# A precise chloroplast genome of *Nelumbo nucifera* (Nelumbonaceae) evaluated with Sanger, Illumina MiSeq, and PacBio RS II sequencing platforms: insight into the plastid evolution of basal eudicots

**DOI:** 10.1186/s12870-014-0289-0

**Published:** 2014-11-19

**Authors:** Zhihua Wu, Songtao Gui, Zhiwu Quan, Lei Pan, Shuzhen Wang, Weidong Ke, Dequan Liang, Yi Ding

**Affiliations:** State Key Laboratory of Hybrid Rice, Department of Genetics, College of Life Sciences, Wuhan University, Wuhan, 430072 Republic of China; BGI-Shenzhen, Shenzhen, 518083 China; College of Life Sciences, Jianghan University, Wuhan, 430056 China; College of Life Sciences, Huanggang Normal University, Huanggang, 438000 Hubei China; Wuhan Vegetable Scientific Research Institute, Wuhan National Field Observation & Research Station for Aquatic Vegetables, Wuhan, 430065 China; Nextomics Biosciences Co., Ltd., Wuhan, 430075 China

**Keywords:** *N. nucifera*, Chloroplast genome sequencing, Basal eudicots, Systematic position, Divergence time, PacBio RS II

## Abstract

**Background:**

The chloroplast genome is important for plant development and plant evolution. *Nelumbo nucifera* is one member of relict plants surviving from the late Cretaceous*.* Recently, a new sequencing platform PacBio RS II, known as ‘SMRT (Single Molecule, Real-Time) sequencing’, has been developed. Using the SMRT sequencing to investigate the chloroplast genome of *N. nucifera* will help to elucidate the plastid evolution of basal eudicots.

**Results:**

The sizes of the *de novo* assembled complete chloroplast genome of *N. nucifera* were 163,307 bp, 163,747 bp and 163,600 bp with average depths of coverage of 7×, 712× and 105× sequenced by Sanger, Illumina MiSeq and PacBio RS II, respectively. The precise chloroplast genome of *N. nucifera* was obtained from PacBio RS II data proofread by Illumina MiSeq reads, with a quadripartite structure containing a large single copy region (91,846 bp) and a small single copy region (19,626 bp) separated by two inverted repeat regions (26,064 bp). The genome contains 113 different genes, including four distinct rRNAs, 30 distinct tRNAs and 79 distinct peptide-coding genes. A phylogenetic analysis of 133 taxa from 56 orders indicated that *Nelumbo* with an age of 177 million years is a sister clade to *Platanus*, which belongs to the basal eudicots. Basal eudicots began to emerge during the early Jurassic with estimated divergence times at 197 million years using MCMCTree. IR expansions/contractions within the basal eudicots seem to have occurred independently.

**Conclusions:**

Because of long reads and lack of bias in coverage of AT-rich regions, PacBio RS II showed a great promise for highly accurate ‘finished’ genomes, especially for a *de novo* assembly of genomes. *N. nucifera* is one member of basal eudicots, however, evolutionary analyses of IR structural variations of *N. nucifera* and other basal eudicots suggested that IR expansions/contractions occurred independently in these basal eudicots or were caused by independent insertions and deletions. The precise chloroplast genome of *N. nucifera* will present new information for structural variation of chloroplast genomes and provide new insight into the evolution of basal eudicots at the primary sequence and structural level.

**Electronic supplementary material:**

The online version of this article (doi:10.1186/s12870-014-0289-0) contains supplementary material, which is available to authorized users.

## Background

The chloroplast genome (cp genome) encodes a set of proteins for photosynthesis and other house-keeping functions that are essential to plant development [1]. Cp genomes are often used for research on plant evolution. Furthermore, cp genomes are predominantly uniparentally inherited [2], have highly conserved gene content and quadripartite organisation, and consist of a large single copy (LSC), a small single copy (SSC) and two inverted copies (IRs). Therefore, cp genome is widely used to trace species history [3-6]. In the past several years, there has been a dramatic increase in the numbers of complete chloroplast genomes from higher plants [7-12]. To date, there have been 437 complete chloroplast genomes of plants deposited in the NCBI database, along with the emergence of next-generation sequencers. These database resources provide information to better understand cp genome evolution in land plants. The ‘living fossil’ *Nelumbo* Adans is a small genus of angiosperms with long evolutionary history. They are perennial aquatic plants that flourished during the middle Albian [13,14]. Now, there are only two surviving species, *Nelumbo nucifera* Gaertn. and *Nelumbo lutea* Willd, respectively. The former is mainly distributed in Asia and northern Australia, and the latter is mainly found in North and South America [15]. *Nelumbo* are economically important aquatic crops with ornamental, edible and medicinal properties. In 1795, Linnaeus placed the *Nelumbo* in *Nymphaea Nelumbo* Linn. In the intervening 200 years, *Nelumbo* has been considered a member of Nymphaeales (water lilies) and was then established as a single family belonging to the Nymphaeales [16].

During the past two decades, DNA sequences have been used to reevaluate the systematic position of *Nelumbo*. The traditional view has been challenged by non-molecular studies [17,18] and *rbcL* sequence data [19]. To date, five different coding genes and several non-coding sequences have been used to reconstruct the relationships of *Nelumbo* [19-25]. Besides the nuclear genome [26], the complete cp genome of *N. nucifera* should be performed to elucidate the genomic evolution of *N. nucifera*. An accurate cp genome map is essential to study the phylogenesis, evolution and resource conservation of *N. nucifera.*

Obtaining an accurate cp genome is a prerequisite to understand its biological function and evolution for higher plants. At the beginning, most of the plant cp genomes were *de novo* assembled from the traditional Sanger sequencing [27-31]. This method is slow, expensive, laborious and low-throughput. More recently, next-generation sequencers such as Illumina, known for being high-throughput and cost-effective, have been used to assemble genomes based on a related reference genome. Because of its short read lengths, it cannot resolve a genome assembly with long repeats or low-GC regions, leading to gaps [32]. Single Molecule, Real-Time sequencing technology (SMRT) is the third-generation sequencing technology developed by Pacific Biosciences (PacBio). The process is as follows: first, DNA-imbedded DNA polymerases are attached to the bottom of 50 nm-wide wells, termed zero-mode waveguides (ZMWs), second, polymerases synthesise DNA using γ-phosphate fluorescently labelled nucleotides in the ZMWs, third, the width of the ZMWs cannot allow light to propagate through the waveguide, but energy can penetrate a short distance and excite the fluorophores incorporated into the growing DNA molecules in the vicinity of the polymerase at the bottom of the well. Compared to Sanger and Illumina platforms, PacBio can generate average read lengths of approximately 3,000 bp, with some reads reaching up to 30 kb with the current PacBio RS II platform. There have been some concerns about accuracy rates and insertion/deletion (indel) events caused by incorporation events or intervals-undetected events, but these can be improved by increased throughput in multiple SMRT cells [33,34]. The optimisation of the assembly method [32,35] and elevation of the accuracy rates make this platform have a great promise for genome sequence finishing. Currently, the PacBio platform is widely applied to *de novo* sequencing for various organisms, including human [36,37], microorganisms [38,39] and plants [40].

In this study, three goals were reached: first, *N. nucifera* (a representative of *Nelumbo*) was selected as the material to evaluate and compare cp genomes , including the accuracy rates and sequence sizes from three types of sequencing platforms, Sanger, Illumina MiSeq, and PacBio RS II. Second, we *de novo* assembled, annotated and analysed the cp genome of *N. nucifera* using PacBio RS II data. Third, to evaluate the systematic position and divergence of *Nelumbo*, as well as other basal eudicots, the cp genome of *N. lutea* was also sequenced by the Sanger platform with an average depth of coverage of 6×. We constructed a large phylogenetic tree that included 133 species from 56 orders (Additional file 1). Finally, we also estimated the divergence time of the basal eudicots, and compared the cp genomic structures to illustrate IR expansions/contractions among these early-diverged eudicots.

## Results and discussion

### *De novo* assembly from Illumina MiSeq and PacBio RS II platforms

**Figure 1:**
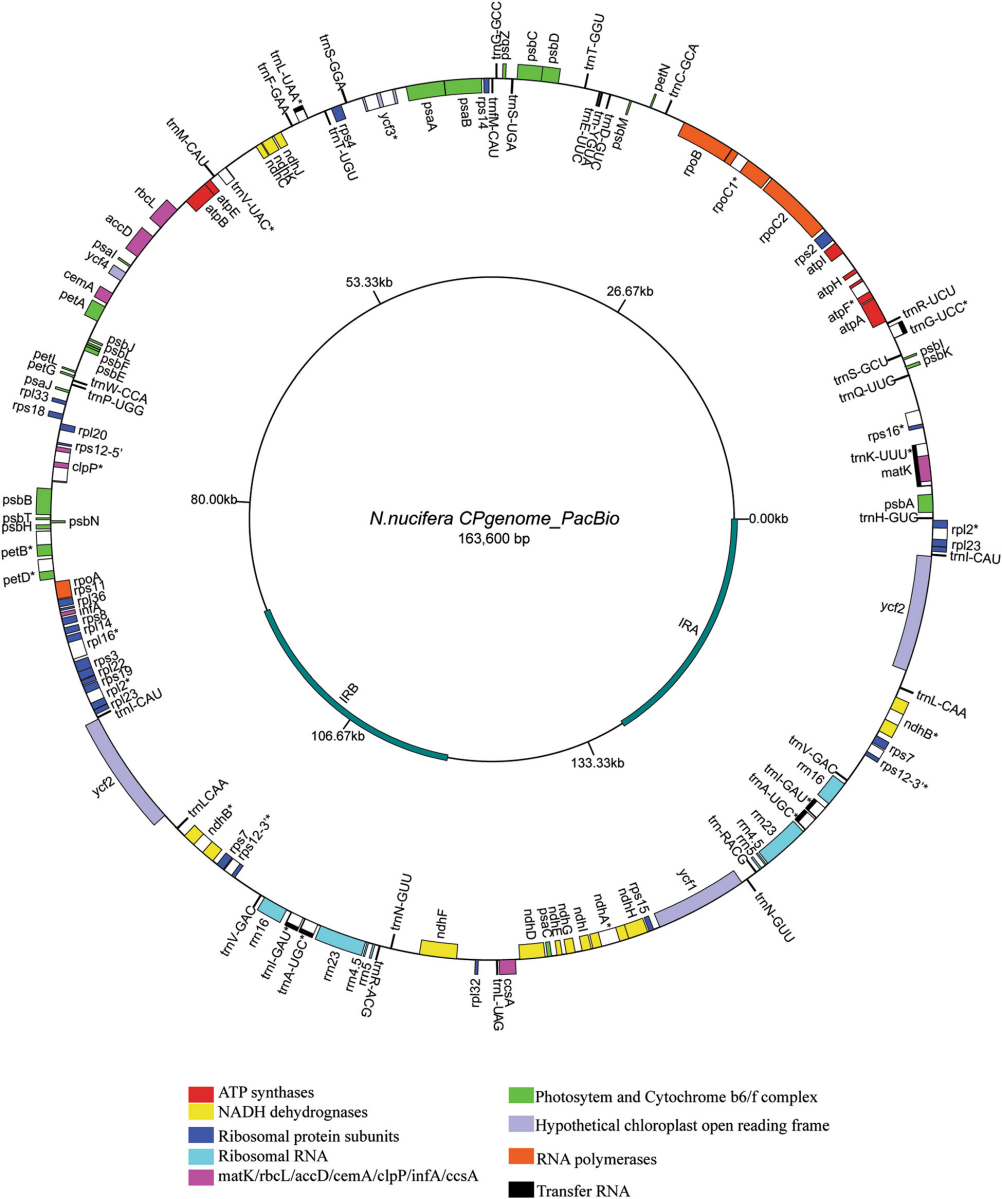
**Gene map of** ***N. nucifera*** **chloroplast genome from PacBio RS II platform.** The inverted repeats are indicated by thick lines. Asterisks indicate genes containing introns. Genes on the outside of the circle are transcribed in a clockwise direction and genes on the inside of the circle are transcribed in a counter-clockwise direction.

Using the HGAP method [32], the PacBio RS II data was *de novo* assembled to one 163,600 bp contig with 105× depth of coverage (Figure 1) using Celera Assembler 7.0. The Illumina MiSeq data were *de novo* assembled to one 163,747 bp contig (Additional file 2: Figure S1) with 712× depth of coverage using Celera Assembler 7.0. The 163,307 bp contig (Additional file 3: Figure S2) for Sanger was assembled with Sequencher software. The sequence gaps marked with NNN from the Illumina MiSeq platform and Sanger technology were filled in using PCR. Data statistics and assembles from Illumina MiSeq and PacBio RS II were summarised in Table 1. Using the Sanger data, we found that the sequence identities among the three sequences were extremely similar, but the lengths of the three contigs from Sanger, Illumina MiSeq and PacBio RS II sequencing platforms were different. Using ClustalW alignment and PCR confirmation, we found a 282 bp deletion in the region of *ndhA* intron using the Sanger platform and a 152 bp insertion in the inverted repeats using the Illumina MiSeq platform (Additional file 2: Figure S1 and Additional file 3: Figure S2). These errors may be caused by low-throughput in the Sanger sequencing reads and short read lengths in the next-generation sequencing methods [41]. Erroneous insertions and deletions caused by sequencing technologies often lead to incorrect analyses of genome features. Additionally, low-throughput techniques and short read lengths are not ideal for reaching certain regions with highly repetitive sequences. However, in some small genomes, such as those of microorganisms, such repeated sequences appear to provide critical insights into the distinctions among bacterial strains [42].

**Table 1:** **Statistics of the** ***N. nucifera*** **chloroplast genome sequencing data from Illumina MiSeq and PacBio RS II**

	**Illumina Miseq**	**PacBio RS II**
Library size(bp)	400	20,000
Number of raw reads	12,164,066	226,904
High quality bases(M)	394	845
Mean read length(bp) (raw-data)	250	4,474
CP average read depth ( error-corrected)	712× (n.a.)	105×
proportion of bases > = Q40	99.99%	99.98%
SC average read depth	493×	83×
IR average read depth	531×	52×
No. of gaps	2	0
No. of contigs	1	1
The total length (bp)	163,747	163,600

Currently, the advance in plastid sequencing is largely promoted by next-generation sequencing technologies. There have been related reports comparing next-generation sequencing platforms for plastid sequencing, such as the GS20 system (454 Life Sciences Corporation) [43] and the Illumina GA II platform [44], and the improved conventional Sanger method [45]. Additionally, the first comparison of next-generation technology (Illumina) with third-generation technology (PacBio) was performed in the last year [40]. However, the comprehensive comparison of the pros and cons of the three representatives of sequencing eras, Sanger, Illumina and PacBio, has not been determined. Especially for the accuracy-challenged PacBio platform, newly developed assembled methods and upgraded chemistry (from C1 to C2) will improve the accuracy rates and throughput [32]. We applied three independent sequencing platforms to evaluate the cp genome of *N. nucifera.* The results confirmed by PCR amplification showed that the *de novo* assembly genome from PacBio RS II platform was the most intact, reaching 100% coverage. Given the sufficient depth (105×), SMRT sequencing by PacBio RS II provides a highly accurate cp genome of *N. nucifera*, as it is highly unlikely that the same error will be randomly observed multiple times [34]. Deep sequencing coverage and additional Illumina library containing large fragments are essential to obtain the accurate structure of genome for Sanger and Illumina Miseq, respectively. Despite small differences among the three *de novo* assembled genomes, the accurate cp genomic structures may have more important roles than the cp genomic sequences in plant development. Meanwhile, the incomplete cp genomic information of *N. nucifera* caused by deletion and insertion from Sanger and Illumina, respectively, cannot reflect a genuine structure of cp genome *in vivo*. Furthermore, the run time of PacBio is very short at only 2 hours [34], which can save considerable time for researchers. Therefore, PacBio RS II platform, characteristics of the long reads and lack of bias in coverage of AT-rich regions, is promising for highly accurate ‘finished’ genomes.

### General features and codon usage of *N. nucifera* cp genome

The final chloroplast circular map of *N. nucifera* from PacBio data corrected with Illumina Miseq data was 163,600 bp. In terms of structure and coding capacity, the cp genome of *N. nucifera* resembles those of eudicots, with minor length variations caused by lineage-specific insertions and deletions. This genome showed the typical quadripartite structure with a large single copy region (LSC, 91,846 bp) and a small single copy region (SSC, 19,626 bp) separated by two copies of an inverted repeat (IR, 26,064 bp) (Figure 1). The cp genome of *N. nucifera* contains the most complete 113 different genes, including four distinct rRNAs (16S, 23S, 4.5S and 5S), 30 distinct tRNAs and 79 distinct peptide-coding genes (including four *ycfs*). Four rRNAs, seven tRNAs and six peptide-coding genes (including *rps12*) are duplicated in the IR region, yielding a total of 130 genes (Table 2).

**Table 2:** **List of genes present in the chloroplast genome of** ***N. nucifera***

	**Group of genes**	**Name of genes**
Protein synthesis and DNA-replication	Ribosomal RNAs (8)	*rrn16*(×2) *rrn23*(×2) *rrn4.5*(×2) *rrn5*(×2)
	Transfer RNAs (37)	trna(ugc)* trnC(gca) trnD(guc) trnE(uuc) trnF(gaa) trnG(gcc) trnL(uaa)* trnL(uag) trnG(ucc)* trnH(gug) trnI(cau)(×2) trnI(gau)*(×2) trnK(uuu)* trnL(caa)(×2) *trnfM(cau) trnM(cau) trnN(guu)*(×2) *trnP(ugg) trnQ(uug) trnR(acg)*(×2) *trnR(ucu) trnS(gcu) trnS(gga) trnS(uga) trnT(ggu) trnT(ugu) trnV(gac)*(×2) trnV(uac)* trnW(cca) trnY(gua)
	Ribosomal proteins small subunit (14)	rps2 rps3 rps4 rps7(×2) rps8 rps11 rps12*(×2)
		rps14 rps15 rps16* rps18 rps19
	Ribosomal proteins large subunit (11)	rpl2 *(×2) rpl14 rpl16* rpl20 rpl22
		*rpl23(×2) rpl32 rpl33 rpl36*
	Subunits of RNA polymerase (4)	rpoA rpoB rpoC1* rpoC2
Photosynthesis	Photosystem I (5)	*psaA psaB psaC psaI psaJ*
	Photosystem II (15)	*psbA psbB psbC psbD psbE psbF psb HpsbI psbJ psbK psbL psbM psbN psbT psbZ*
	Cytochrome b/f complex (6)	petA petB* petD* petG petL petN
	ATP synthase (6)	atpA atpB atpE atpF* atpH atpI
	NADH-dehydrogenase (12)	ndhA*ndhB*(×2) ndhC ndhD ndhE ndhF ndhG ndhH ndhI ndhJ ndhK
	Large subunit of Rubisco (1)	*rbcL*
miscellaneous group	Translation initiation factor IF-1 (1)	*infA*
	Acetyl-CoA carboxylase (1)	*accD*
	Cytochrome c biogenesis (1)	*ccsA*
	Maturase (1)	*matK*
	ATP-dependent protease (1)	clpP*
	Inner membrane protein (1)	*cemA*
Genes of unknown function	Conserved hypothetical chloroplast reading frames (5)	ycf1 ycf2(×2) ycf3*ycf4

Genes with introns are marked with asterisks (*).

The numbers in parentheses represents the number of genes.

Start codon usage of *N. nucifera* was compared to those of eight other basal eudicots (Table 3). In these basal eudicots, ACG, GTG, or ATA appeared to be used as an alternative to ATG as the start codon. Among the changes of start codons, *rpl2* and *rps19* were found in all of the surveyed basal eudicots, but *ndhB* and *ycf2* were only present in *Ranunculus*. Among the 79 distinct chloroplast protein-coding genes of *N. nucifera,* only three genes (*psbL, rpl2* and *rps19*) used an alternative to ATG as the start codon: ACG for *psbL* and *rpl2*, and GTG for *rps19*. An ACG to AUG editing site in the *ndhD*, *psbL* and *rpl2* transcripts is present in most angiosperm plastids [46,47], but we only detected two RNA editing sites (*psbL* and *rpl2*) in the start codon region. Loss of such an editing site in *ndhD* transcripts may be caused by a very slow rate of evolution during the last 160 million years of Nelumbonaceae or back-mutation from C to T in the *ndhD* start codon. This loss of alternative start codons, ACG in *ndhD* may drastically impair the accumulation of the NDH complex in the leaves [48]. Furthermore, we examined codon usage patterns of the 79 distinct chloroplast protein-coding genes in *N. nucifera*. A total of 22,902 codons comprise the 79 different chloroplast protein-coding genes of *N. nucifera*. Overall codon usage in the *N. nucifera* is generally similar to that reported from other genomes, such as *Panax* [22] and *Lotus* [49]. Relative Synonymous Codon Usage (RSCU) analyses suggested that codons from the *N. nucifera* cp genome with the third position nucleotide of A or U were used more frequently than those ending with G or C (Table 4), as observed in most cp genomes of land plants [30]. For example, of the four codons coding for valine, the RSCUs of GUU and GUA were 1.43 and 1.5, but those of GUC and GUG were only 0.49 and 0.58, respectively.

**Table 3:** **Alternative start codon usage in the sequenced basal eudicots**

**Species gene**	***Ranunculus***	***Nandina***	***Nelumbo***	***Platanus***	***Trochodendron***	***Tetracentron***	***Buxus***	***Meliosma***	***Megaleranthis***
*ndhB*	ACG								
*ndhD*	ACG	ACG		ACG	ACG	ACG	ACC	ACG	ACG
*psbC*	GTG	GTG		GTG				GTG	
*psbL*			ACG	ACG	ACG	ACG	ACG	ACG	
*rpl2*	ACG	ACG	ACG	ACG	ATA	ATA	ATA	ACG	ACG
*rps19*	GTG	GTG	GTG	GTG	GTG	GTG	GTG	GTG	GTG
*ycf2*	GTG								
Total No.	6	4	3	5	4	4	4	5	3

**Table 4:** **Relative synonymous codon usage for 79 distinct chloroplast protein-coding genes in** ***N. nucifera***

**Codon**	**Count** ^**1**^	**RSCU** ^**2**^	**Codon**	**Count**	**RSCU**	**Codon**	**Count**	**RSCU**	**Codon**	**Count**	**RSCU**
UUU(F)	784	1.25	UCU(S)	475	1.65	UAU(Y)	658	1.6	UGU(C)	190	1.46
UUC(F)	475	0.75	UCC(S)	284	0.99	UAC(Y)	166	0.4	UGC(C)	70	0.54
UUA(L)	692	1.76	UCA(S)	368	1.28	UAA(*) ^**3**^	36	1.37	UGA(*) ^**3**^	21	0.8
UUG(L)	506	1.29	UCG(S)	148	0.51	UAG(*) ^**3**^	22	0.84	UGG(W)	406	1
CUU(L)	497	1.26	CCU(P)	390	1.61	CAU(H)	427	1.5	CGU(R)	333	1.42
CUC(L)	164	0.42	CCC(P)	177	0.73	CAC(H)	141	0.5	CGC(R)	79	0.34
CUA(L)	331	0.84	CCA(P)	281	1.16	CAA(Q)	605	1.51	CGA(R)	325	1.39
CUG(L)	169	0.43	CCG(P)	123	0.51	CAG(Q)	197	0.49	CGG(R)	106	0.45
AUU(I)	945	1.45	ACU(T)	468	1.58	AAU(N)	822	1.53	AGU(S)	358	1.24
AUC(I)	408	0.63	ACC(T)	224	0.75	AAC(N)	253	0.47	AGC(S)	95	0.33
AUA(I)	605	0.93	ACA(T)	362	1.22	AAA(K)	860	1.48	AGA(R)	426	1.82
AUG(M)	547	1	ACG(T)	133	0.45	AAG(K)	304	0.52	AGG(R)	137	0.58
GUU(V)	455	1.43	GCU(A)	577	1.8	GAU(D)	750	1.59	GGU(G)	531	1.33
GUC(V)	156	0.49	GCC(A)	204	0.64	GAC(D)	196	0.41	GGC(G)	159	0.4
GUA(V)	479	1.5	GCA(A)	348	1.09	GAA(E)	899	1.49	GGA(G)	649	1.62
GUG(V)	185	0.58	GCG(A)	151	0.47	GAG(E)	307	0.51	GGG(G)	263	0.66

^1^Count means the number of codons used in the 79 protein-coding genes.

^2^RSCU represents relative synonymous codon usage.

^3^Codons in bold with an asterisk represent stop codons.

During the evolution of angiosperms, the sizes of the most sequenced cp genomes range from approximately 120 kb to 160 kb in length. However, there are some exceptions for parasitic plants with unique lifestyles, of which the sizes of cp genomes were beyond the scope of 120 kb to 160 kb, such as *Conopholis americana*, with the smallest plastome of 45 kb of land plants [50]. Additionally, the numbers of genes in the cp genomes were present variously in different lineages, such as the losses of *ndh* genes. The events of *ndh* gene losses occurred in most non-photosynthetic plants, such as *Cuscuta reflexa* [51] and the parasitic plants, such as *Epifagus virginiana* [52], and in some non-parasitic, photosynthetic plants, such as *Phalaenopsis aphrodite* [53] and Geraniaceae [54]. The mechanism of the *ndh* gene losses may be explained for that either the genes are transferred to nuclear or they do not participate in the critical life development for the specific lineages [55]. In addition to the *ndh* genes, there were other independent gene losses in different lineages, including *infA*, *rpl*, *rps*, *pet*, *psb* and so on (Additional file 1). For example, the *rpl21* gene loss of the cp genomes in the ancestral clades of gynosperms and angiosperms was compensated by the gene from the mitochondrial genome. The independent loss of *infA* in angiosperms (including almost all Rosaceae) was the result of transfer events from chloroplast to nuclear [56]. The cp genome of *N. nucifera* retained a complete set of genes data, suggesting these genes may be critical to its development.

Alternative start codons of cp genomes widely occurred in land plants, such as pteridophytes [30]. This editing pattern of the initiation codon seems to have occurred independently across the evolution of land plants, which does not correlate with the phylogenetic tree of the plant kingdom. Overall codon usage in the *N. nucifera* cp genome is similar to those of other reported cp genomes [30,57] and mitochondrial genomes [58]. These codon usage patterns may be driven by the composition bias of the high proportion of A/T.

### Phylogenetic and molecular dating analyses of the basal eudicots

**Figure 2:**
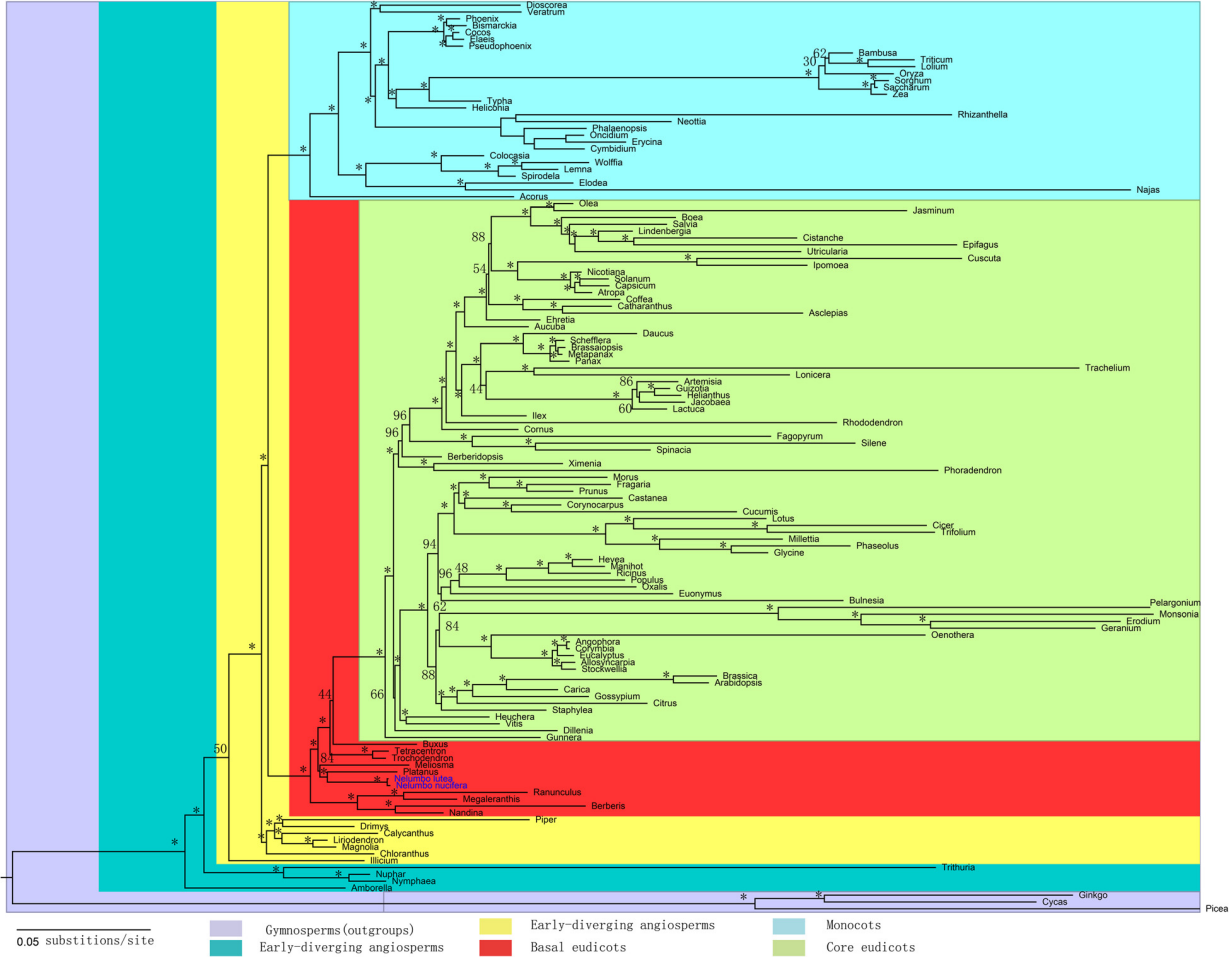
**Phylogenetic tree of the 133 taxa based on 79 chloroplast protein-coding genes.** The ML tree has a -lnL of −1601140.821388 with support values for ML provided at the nodes. Asterisks indicate ML BS =100%. Taxa in blue are the two new genomes sequenced in this study.

Using three data matrices, maximum likelihood (ML) phylogenetic analyses were conducted using 79 protein-coding genes from 56 orders of seed plants. After searching the 56 models with Modeltest 3.7, the general time reversible (GTR) model with rate variations among sites and invariable sites (GTR + G + I) were selected as the best fit for the three data matrices. The phylogenetic trees inferred from the three data matrices showed the same topology. Additionally, the resulting topology, consistent with results from the Angiosperm Phylogeny Group (APG) [59], suggested that the phylogenetic tree was reliable. As sisters to *Meliosma*, *Nelumbo* and *Platanus* form a clade with 100% bootstrap values. This result confirmed that *N. nucifera* is a stem eudicot, supported by the morphological evidence of tricolpate pollen grains [21]. As a result of convergent evolution [60] in the same aquatic environment, a similar morphology has led to the misidentification of *N. nucifera* as a relative of *Nymphaea alba*. A phylogenetic analysis of 133 taxa from 56 orders indicated that *Nelumbo* was the sister clade to *Platanus* which is a genus of tall land trees (Figure 2).

The eudicots comprise the vast majority of the extant angiosperms, with an estimated 200,000 species. The clades can divided into basal eudicots and core eudicots [61,62]. To date, plastid genomes have been completely sequenced for eight basal eudicots, including *Buxus* [63], *Megaleranthis* [64], *Nandina* [43], *Platanus* [43], *Ranunculus* [30], *Trochodendron* [65], *Tetracentron* [65] and *Nelumbo* (in this study). The addition of the un-sampled basal eudicot cp genome of *N. nucifera* will lead to a better understanding of the evolution of basal eudicots.

**Figure 3:**
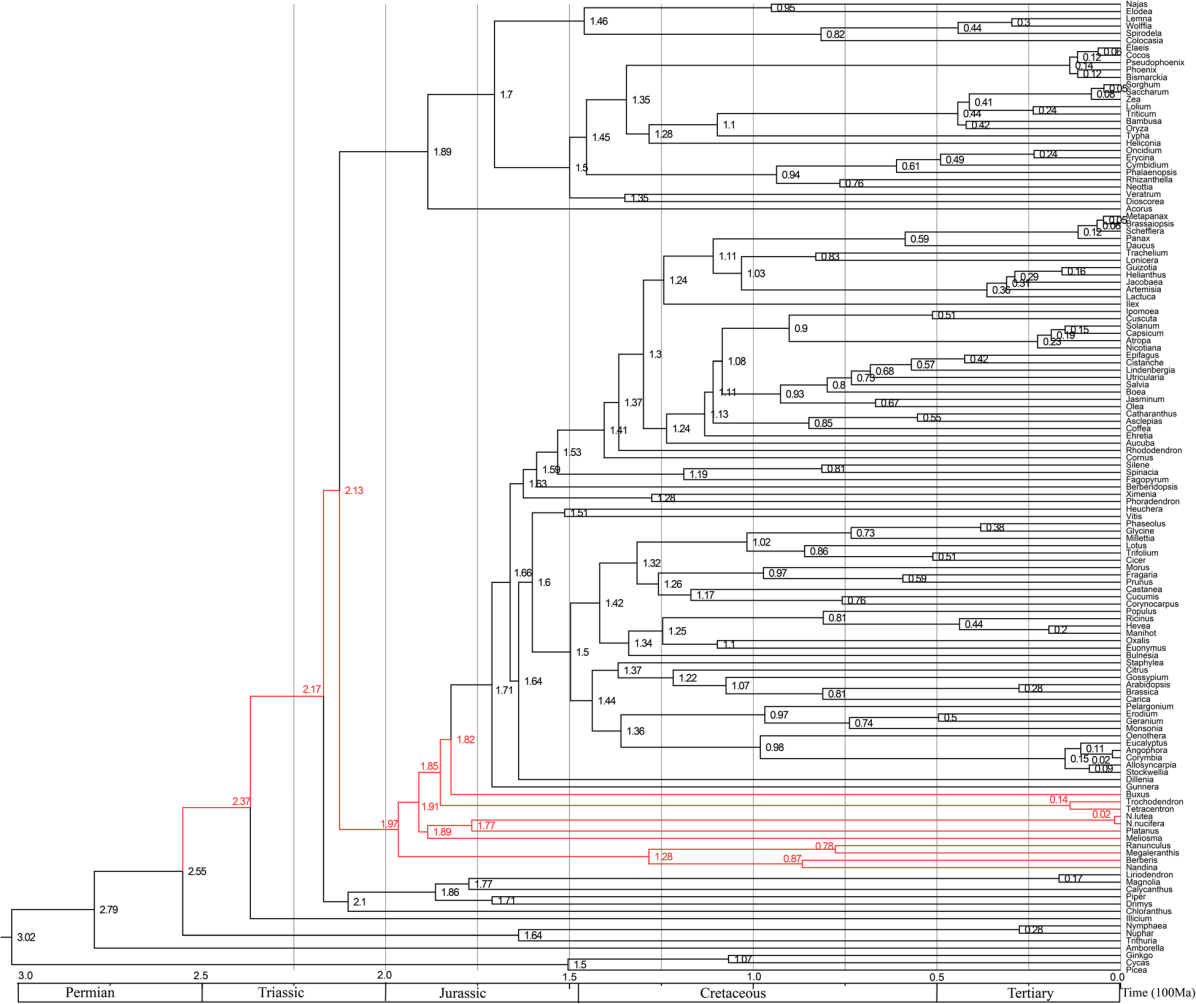
**Posterior estimates of divergence time of 133 taxa on the phylogenetic tree.** The values at the nodes represent mean ages in a 95% highest posterior density (HPD) analysis. Estimations were performed with MCMCTree using the IR (independent rate) model.

In the phylogenetic trees obtained in our study, the analysed basal eudicots, including Ranunculales (*Nandina*, *Berberis*, *Megaleranthis*, *Ranunculus*), Sabiaceae, Proteales (*Platanus*, *Nelumbo*), Trochodendrales (*Trochodendron*, *Tetracentron*) and Buxales (*Buxus*) formed separate clades (Figure 2). To estimate the divergence time in these clades, MCMCTree of PAML4.7 was used with the approximate likelihood calculation method [66]. This analysis dated Ranunculales, Sabiaceae, Proteales, Buxales and Trochodendrales to 197, 189, 189, 185 and 182 million years (Myr) ago, respectively. *Nelumbo* has an age of 177 Myr, and the splitting between the only two extant species, *N. lutea* and *N. nucifera*, is estimated to have occurred approximately 2 Myr ago (Figure 3).

In recent years, along with the released chloroplast data from NCBI, researchers used these cp genomes for plant evolution [67,68]. In our study, we carefully selected different taxa from the NCBI database, of which the cp genomes were potentially published. Additionally, long-branch attraction will mislead to a wrong phylogenetic tree. To avoid long-branch attraction [69], the taxa uniformly distributed in species trees were selected. We controlled the numbers of taxa (no more than 8) in the same order. The saturation of substitution rates of codon sites, especially the third site, affects the topology of phylogenetic tree [28]. In our test, the phylogenetic topology from the matrix containing all three sites of each codon consistent with the results of the other two matrices (1^st^ and 2^nd^ sites, and 3^rd^ site of each codon) verified there was no saturation of substitution rates in our analysed taxa.

Here, 133 taxa uniformly covering 56 orders were adopted to perform the phylogenetic analyses and estimation of divergence time for the basal eudicots. The phylogenetic analyses from three matrices (all three sites, 1^st^ and 2^nd^ sites , and 3^rd^ site of each codon) of 79 chloroplast protein-coding genes supported the phylognesis of *N. nucifera* as a basal eudicot, sister to *Platanus*. Estimations of divergence time showed that *Nelumbo* and *Platanus* began to diverge approximately 177 Myr ago [66]. Additionally, the divergence of the basal eudicots (including *Nelumbo*) from *Nympahea* (the ‘early-diverging’ angiosperm), was approximately 255 Myr ago (Figure 3). The morphological similarity between *Nelumbo* and *Nympahea* caused by convergent evolution is typically contradictory to the similarity of molecular sequences among the three taxa *Nelumbo, Nymphaea* and *Platanus*. Therefore, the phenotypes of these species are determined by the combination of their molecular sequences and living environments.

### The structural evolution within the basal eudicots

**Figure 4:**
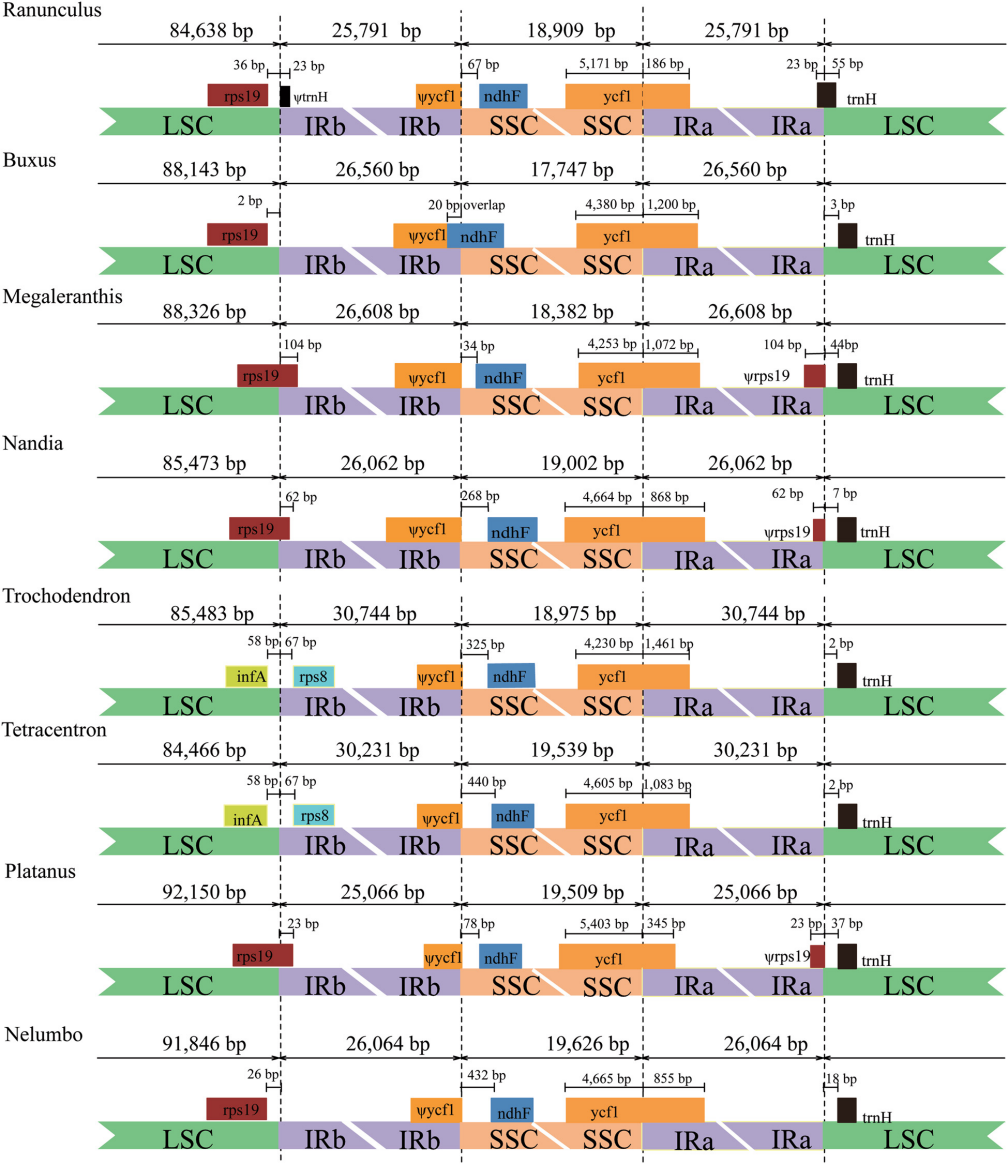
**Comparison of the boundaries of LSC, IR and SSC among eight chloroplast genomes of basal eudicots.**

In angiosperms, frequent contractions and expansions at the junctions of SSC and LSC with IRs contributed to the size variations of cp genomes. Therefore, contractions and expansions of these junctions have been recognised as evolutionary markers for illustrating the relationships among taxa [70]. We were interested in the structural variations of *N. nucifera* and other basal eudicots. The structure of *N. nucifera* cp genome was compared to those of the seven basal eudicots (*Trochodendron*, *Tetracentron*, *Platanus*, *Ranunculus*, *Buxus*, *Megaleranthis*, and *Nandia*). Unlike the other six species, the largest expansions were found in the LSC/IRb boundary of *Trochodendron* and *Tetracentron*, up to 30 kb. The LSC/IRb conjunction of *Trochodendron* and *Tetracentron* expanded into the region between *infA* and *rps8*. However, the junctions of other 6 species appeared to be conserved with only minor expansions (Figure 4). The IRb of *Platanus*, *Megaleranthis* and *Nandia* expanded into the 3′ portion of *rpl19* by 23 bp, 104 bp and 62 bp, respectively. The LSC/IRb boundaries of *Ranunculus*, *Buxus* and *Nelumbo* were located in the intergenic space regions downstream of *rps19* (Figure 4). These data showed that various borders existed in these basal eudicots, even within the same order, such as *Nelumbo* (Proteales) and *Platanus* (Proteales). We speculated that the location of IR/LSC boundaries may not correlate to their positions of phylogenesis.

*N. nucifera* is a member of land plants [71], which flourished during the Cretaceous. When Quaternary glaciations occurred, *N. nucifera* became trapped in water areas in response to environmental stress [72]. Previous reports noted that expansions of IR occurred more progressively in monocots than non-monocot angiosperms, and two hypotheses were proposed to explain IR expansions in the monocots [70]. The IR boundaries of 17 surveyed vascular plants vary among these cp genomes, even between closely related genera of the same family [22]. We wonder whether this clade of basal eudicots maintains the conservative IR boundaries. In this study, expansions and contractions of IR boundaries also varied in these basal eudicots, which was not related to the phylogeny of the lineages. For example, the IR boundaries of *Nelumbo* (Proteales) were more similar to that of *Buxus* (Buxales) than that of its closely related taxa, *Platanus* (Proteales)*.* Despite that fact that each IR of *Nelumbo* was nearly 1 kb longer than that of *Platanus* (Figure 4), the former did not contain the portion of *rps19* as did the latter. We found that variations of IRs were contributed by IR expansion to LSC or by an independent insertion of DNA fragments in IR regions. How the independent insertion occurred is still to be elucidated in future studies.

## Conclusions

We first applied three sequencing platforms to evaluate the cp genome of *N. nucifera.* Using PacBio RS II data, Illumina MiSeq data and Sanger data, we *de novo* assembled, annotated and analysed the cp genome of *N. nucifera*. The precise cp genome of *N. nucifera* is a circular molecule of 163,600 bp with a typical quadripartite structure, containing a LSC region (91,846 bp) and a SSC region (19,626 bp) separated by IR regions (26,064 bp) with a total of 130 genes. The ML trees of 79 combined chloroplast protein-coding genes of 133 taxa confirmed that *N. nucifera* was a member of basal eudicots, sister to *Platanus*. Estimating the divergence time in MCMCTree with an approximate likelihood calculation showed that basal eudicots diverged at 197 Myr, and *Nelumbo* was 177 Myr. The splitting between *N. lutea* and *N. nucifera* was estimated to have occurred approximately 2 Myr. A structural comparison showed that the IR boundaries of basal eudicots occur in various border positions and an independent insertion of IR occurred in *Nelumbo*. This study showed that the PacBio platform will be useful for *de novo* assembly of genomes and the cp genome of *N. nucifera* provided new insight into the evolution of the basal eudicots. We believe that with the appearance of new PacBio sequencing platform, more accurate cp genomes will be obtained to understand the evolution of angiosperms at both the sequence and structural level.

## Methods

### Materials

The materials (*Nelumbo nucifera* Gaertn.) used in the experiment is maintained by Wuhan Vegetable Scientific Research Institute, Wuhan National Field Observation & Research Station for Aquatic Vegetables (30°12′N, 111°20′E).

### Chloroplast genome DNA extraction

High quality DNA was obtained as follows: the unfolded tender leaves of *N. nucifera* were harvested and stored at 4°C in the dark to eliminate starch from the tissue. Chloroplast was isolated using the method of discontinuous sucrose gradient centrifugation, with DNase I digestion [73]. All steps must be conducted at 4°C unless otherwise specified. The chloroplast solutions were gently lysed by adding one-fifth volume of lysis buffer and one-twentieth Proteinase K to a final concentration of 200 μg/ml. The tubes were then gently inverted and mixed once every 15 min during 30-minute water baths at 37°C and then 50°C. After adding cold NH_4_Ac to a final concentration of 0.8 M, the nucleic acids were separately extracted with an equal volume of Tris-saturated phenol/chloroform/isoamyl alcohol (25:24:1) once and chloroform/isoamyl alcohol once (24:1).

Chloroplast genome DNA (cpDNA) was precipitated in two volumes of 100% ethanol overnight at −20°C and centrifuged at 18,000 × g for 20 min. The crude cpDNA was washed twice with 70% ethanol and re-suspended in TE buffer with RNase digestion for 30 min at 37°C. Finally, the values of OD260/280 and OD260/230 were 1.9 and 1.97, respectively.

### Library construction, sequencing and *de novo* assembly

1. Sanger library construction
  The entire chloroplast sequence was amplified using a long-range PCR technique with cpDNA. PCR primers were developed from the alignment of known eudicotyledon chloroplast genomic sequences (Additional file 4). We covered the two chloroplast genomes (*N. lutea* and *N. nucifera*) with PCR products, which showed lengths of between 8 and 15 kb. PCR products were eluted using electrophoresis on low-melting-point agarose gels, followed by column purification (Axgene). The purified products were sheared into random fragments of approximately 1.5 kb and cloned into the pMD18-T vector (TAKARA). The recombinant plasmids were transformed into TOP10 cells and sequenced using an ABI 3730 DNA sequencer.
  Sequence masking and assembly were performed using Sequencher software (Gene Codes Corp., Ann Arbor, MI). Each nucleotide has approximately 7 × coverage in *N. nucifera* and 6 × in *N. lutea*. Gaps and low coverage regions were filled using PCR to make sure each nucleotide was covered by at least three reads.
2. Illumina MiSeq library construction
  Approximately 5 μg cpDNA was used to construct a library containing a 400 bp insert size. Genome DNA sequencing libraries were constructed using TruSeq DNA LT Sample Preparation Kit V2 (Illumina), following the manufacturer’s protocol. The genome DNA was sheared to short fragments using Covaris S220. DNA fragments were adenylated at 3′ ends after end repair. Specific adapters were ligated to both ends of the DNA fragments, and the barcode sequence was included in one of the adaptors. Targeted size DNA fragments were selected by gel-cutting and amplified by 10-cycle PCR using universal primers (Illumina). After purification, quantification, and validation, the validated DNA libraries were sequenced on an Illumina MiSeq Sequencing System following the manufacturer’s standard workflow.
  The raw data were filtered and the obtained clean data were joined by FLASH. The joined reads were aligned to cpBase with bwa-0.7.3a. The aligned reads were then selected for *de novo* assembly with a Celera Assembler 7.0. The final contigs were assembled into one scaffold with SSPACE and lastZ. The two gaps were filled using PCR amplification.
3. PacBio library construction
  A sample of 20 μg pure and high-molecular-weight DNA is required to prepare size- selected approximately 20 kb SMRTbell templates. The cpDNA concentration was measured using both a NanoDrop spectrophotometer and a Qubit fluorometer, and approximately 200 ng of cpDNA was run on a field-inversion gel. The subsequent steps are based on the PacBio Sample Net-Shared Protocol, which is available at http://pacificbiosciences.com/
  The primitive reads from Pacbio RS II were corrected with *SMRT Analysis 2.1*, yielding a total of 9,165 high quality reads, up to 42,623,117 bp. The average length of the reads was 4,651 bp. These reads were assembled using the Celera Assembler 7.0. The reads were aligned to the assembled contigs with bwa-0.7.3a. Eventually, 4 contigs with more than 10x coverage depth were assembled to one contig using the lastZ tool. Finally, to generate the best consensus sequence of the cp genome sequence, Quiver (a new multiread consensus algorithm) was used to correct the error regions [32].

### Genome annotation

The entire chloroplast genome sequences of *N. nucifera* from three platforms were annotated using DOGMA (Dual Organellar GenoMe Annotator) [74]. The genes of *N. lutea* from the Sanger platform were also annotated. For some genes with very short exons, such as *petB* and *petD*, manual annotation was performed. We used tRNAscan-SE to corroborate tRNA boundaries [75]. Physical maps were drawn using GenomeVx [76].

### Codon usage

RSCU (relative synonymous codon usage) [77] of the cp genome of *N. nucifera* was calculated from coding sequences of 79 protein-coding genes. Genes in IR regions were counted only once.

### Phylogenetic analysis

Seventy-nine distinct peptide-coding genes were used for phylogenetic analyses. In addition to the two chloroplast genomes sequenced in the present research, 131 taxa from 56 orders were selected to construct the ML tree. All 79 protein-coding gene sequences were translated into amino acid sequences, which were aligned in ClustalW [77] and manually adjusted. Nucleotide sequences of these genes were aligned by constraining them to the amino acid sequence alignments. The more quickly evolving regions, which are difficult to align, were excluded from the analyses. A FASTA file concatenating 67,862 nucleotides (Additional file 5) was generated, the translated protein-coding sequences of which were seen in Additional file 6. We extracted the first and second sites of codons, and the third site of codons to form two other new matrices. Finally, three matrices were used for phylogenetic analyses. These were established in a DOS platform using our Perl scripts.

The nucleotide file containing 133 concatenated sequences was tested prior to inferring the phylogenetic trees. First, the saturation for substitutions was tested in DAMBE [78] across the three matrices. The results, Iss < Iss.c at p = 0.0000, suggested that there were no saturated sites in these sequences. Second, we used Modeltest 3.7 [79] to determine the most appropriate model of DNA sequence evolution. After searching the 56 models, the general time reversible (GTR) model, with rate variation among sites and invariable sites (GTR + G + I), was selected. To infer phylogenetic trees from three nucleotide matrices, we applied the maximum likelihood method with rapid bootstrapping of 1000 replicates in RAxML-HPC v7.2.8 available on http://www.phylo.org/ [80].

### Estimation of divergence times

The concatenated 79 gene sequences from 133 species and the phylogenetic tree were used for molecular dating analyses. The divergence time was estimated using the Bayesian method implemented in MCMCTree of PAML4.7 [81]. Eight fossil calibrations were incorporated through the previous time (Additional file 7). Using the approximate likelihood calculation method, the gradient g and Hessian H with BASEML using the GTR + Γ5 substitution model were calculated [82]. The independent rate (IR) model [83] for the molecular clock and the GTR + Γ5 model for nucleotide substitutions were set in the mcmctree.ctl control file. To ascertain whether convergence was achieved, two independent MCMC analyses with 10^6^ steps, following a discarded burn-in of 10^5^ steps, were simulated [84,85].

### Availability of supporting data

The precise chloroplast genome of *N. nucifera* sequenced by PacBio RS II and corrected by Illumina MiSeq has been submitted to GenBank (accession KM655836). The chloroplast genomes of *N. nucifera* and *N. lutea* sequenced by Sanger are available on National Center for Biotechnology Information (accession NC_015610 and NC_015605). Other data sets supporting the results of this article are included within the article and its additional files.

## Additional files

Additional file 1
**133 taxa from 56 orders included in phylogenetic analyses with APG III ordinal classification.**
Additional file 2: Figure S1 Gene map of N. nucifera chloroplast genome from the Sanger platform. Additional file 3: Figure S2 Gene map of N. nucifera chloroplast genome from the Illumina MiSeq platform. Additional file 4
**Primers for chloroplast genome amplification in our study.**
Additional file 5
**The DNA sequences alignment of the genes from 133 species used in the phylogenetic analyses.**
Additional file 6
**The amino acid sequences alignment of the proteins from 133 species used in the phylogenetic analyses.**
Additional file 7
**Fossil constraints used in the MCMC analyses.**

## References

[1] Liu J, Qi ZC, Zhao YP, Fu CX, Jenny Xiang QY (2012). Complete cpDNA genome sequence of Smilax china and phylogenetic placement of Liliales-influences of gene partitions and taxon sampling. Mol Phylogenet Evol.

[2] Sodmergen ZQ (2010). Why does biparental plastid inheritance revive in angiosperms?. J Plant Res.

[3] Jansen RK, Cai Z, Raubeson LA, Daniell H, Depamphilis CW, Leebens-Mack J, Muller KF, Guisinger-Bellian M, Haberle RC, Hansen AK, Chumley TW, Lee SB, Peery R, McNeal JR, Kuehl JV, Boore JL (2007). Analysis of 81 genes from 64 plastid genomes resolves relationships in angiosperms and identifies genome-scale evolutionary patterns. Proc Natl Acad Sci U S A.

[4] Moore MJ, Bell CD, Soltis PS, Soltis DE (2007). Using plastid genome-scale data to resolve enigmatic relationships among basal angiosperms. Proc Natl Acad Sci U S A.

[5] Moore MJ, Soltis PS, Bell CD, Burleigh JG, Soltis DE (2010). Phylogenetic analysis of 83 plastid genes further resolves the early diversification of eudicots. Proc Natl Acad Sci U S A.

[6] Olmstead R, Palmer JD (1994). Chloroplast DNA systematics: a review of methods and data analysis. Am J Bot.

[7] Li R, Ma PF, Wen J, Yi TS (2013). Complete sequencing of five araliaceae chloroplast genomes and the phylogenetic implications. PLoS One.

[8] Lin CP, Wu CS, Huang YY, Chaw SM (2012). The complete chloroplast genome of Ginkgo biloba reveals the mechanism of inverted repeat contraction. Genome Biol Evol.

[9] Martin G, Baurens FC, Cardi C, Aury JM, D’Hont A (2013). The complete chloroplast genome of banana (Musa acuminata, Zingiberales): insight into plastid monocotyledon evolution. PLoS One.

[10] Straub SC, Cronn RC, Edwards C, Fishbein M, Liston A (2013). Horizontal transfer of DNA from the mitochondrial to the plastid genome and its subsequent evolution in milkweeds (apocynaceae). Genome Biol Evol.

[11] Yang JB, Yang SX, Li HT, Yang J, Li DZ (2013). Comparative chloroplast genomes of camellia species. PLoS One.

[12] Yi X, Gao L, Wang B, Su YJ, Wang T (2013). The complete chloroplast genome sequence of Cephalotaxus oliveri (Cephalotaxaceae): evolutionary comparison of cephalotaxus chloroplast DNAs and insights into the loss of inverted repeat copies in gymnosperms. Genome Biol Evol.

[13] Crane PR, Herendeen PS (1996). Cretaceous floras containing angiosperm flowers and fruits from eastern North America. Rev Palaeobot Palyno.

[14] Upchurch GR, Crane PR, Drinnan AN (1994). The megaflora from the Quantico locality (Upper Albian), lower cretaceous Potomac group of Virginia. Mem Virginia Mus Nat Hist.

[15] Borsch T, Barthlott W (1994). Classification and distribution of the genus Nelumbo Adans. (Nelumbonaceae). Beitr Biol Pfl.

[16] Cronquist A (1981). An Integrated System of Classification of Flowering Plants.

[17] Ito M (1987). Phylogenetic systematics of the Nymphaeales. Bot Mag Tokyo.

[18] Les D (1988). The origin and affinities of the Ceratophyllaceae. Taxon.

[19] Chase MW, Soltis DE, Olmstead RG, Morgan D, Les DH, Mishler BD, Duvall MR, Price RA, Hills HG, Qiu YL, Kron KA, Rettig JH, Conti E, Palmer JD, Manhart JR, Sysma KJ, Michaels HJ, Kress WJ, Karol KG, Clark WD, Hedren M, Gaut BS, Jansen RK, Kim KJ, Wimpee CF, Smith JF, Furnier GR, Strauss SH, Xiang QY, Plunkett GM (1993). Phylogenetics of seed plants: an analysis of nucleotide sequences from the plastid gene rbcL. Ann Missouri Bot Gard.

[20] Hilu K, Borsch T, Muller K, Soltis DE, Pea S (2003). Inference of angiosperm phylogeny based on matK sequence information. Am J Bot.

[21] Hoot SB, Magallon S, Crane PR (1999). Phylogeny of basal eudicots based on three molecular data sets: atpB, rbcL, and 18 s nuclear ribosomal DNA sequences. Ann Missouri Bot Gard.

[22] Kim KJ, Lee HL (2004). Complete chloroplast genome sequences from Korean ginseng (Panax schinseng Nees) and comparative analysis of sequence evolution among 17 vascular plants. DNA Res.

[23] Savolainen V, Chase MW, Hoot SB, Morton CM, Soltis DE, Bayer C, Fay MF, de Bruijn AY, Sullivan S, Qiu YL (2000). Phylogenetics of flowering plants based upon a combined analysis of plastid atpB and rbcL gene sequences. Syst Biol.

[24] Soltis DE, Soltis PS, Chase MW, Mort ME, Albach DC, Zanis M, Savolainen V, Hahn WH, Hoot SB, Fay MF (2000). Angiosperm phylogeny inferred from 18S rDNA, rbcL, and atpB sequences. Bot J Linn Soc.

[25] Worberg A, Quandt D, Barniske A-M, Löhne C, Hilu KW, Borsch T (2007). Phylogeny of basal eudicots: Insights from non-coding and rapidly evolving DNA. Org Divers Evol.

[26] Wang Y, Fan GY, Liu YM, Sun FM, Shi CC, Liu X, Peng J, Chen WB, Huang XF, Cheng SF, Liu YP, Liang XM, Zhu HL, Bian C, Zhong L, Lv T, Dong HX, Liu WQ, Zhong X, Chen J, Quan ZW, Wang ZH, Tan BZ, Lin CF, Mu F, Xu X, Ding Y, Guo AY, Wang J, Ke WD (2013). The sacred lotus genome provides insights into the evolution of flowering plants. Plant J.

[27] Goremykin VV, Hirsch-Ernst KI, Wolfl S, Hellwig FH (2003). Analysis of the Amborella trichopoda chloroplast genome sequence suggests that amborella is not a basal angiosperm. Mol Biol Evol.

[28] Goremykin VV, Hirsch-Ernst KI, Wolfl S, Hellwig FH (2004). The chloroplast genome of Nymphaea alba: whole-genome analyses and the problem of identifying the most basal angiosperm. Mol Biol Evol.

[29] Kunnimalaiyaan M, Nielsen BL (1997). Fine mapping of replication origins (oriA and oriB) in *Nicotiana tabacum* chloroplast DNA. Nucleic Acids Res.

[30] Raubeson LA, Peery R, Chumley TW, Dziubek C, Fourcade HM, Boore JL, Jansen RK (2007). Comparative chloroplast genomics: analyses including new sequences from the angiosperms Nuphar advena and Ranunculus macranthus. BMC Genomics.

[31] Sato S, Nakamura Y, Kaneko T, Asamizu E, Tabata S (1999). Complete structure of the chloroplast genome of *Arabidopsis thaliana*. DNA Res.

[32] Chin CS, Alexander DH, Marks P, Klammer AA, Drake J, Heiner C, Clum A, Copeland A, Huddleston J, Eichler EE, Turner SW, Korlach J (2013). Nonhybrid, finished microbial genome assemblies from long-read SMRT sequencing data. Nat Methods.

[33] Quail MA, Smith M, Coupland P, Otto TD, Harris SR, Connor TR, Bertoni A, Swerdlow HP, Gu Y (2012). A tale of three next generation sequencing platforms: comparison of Ion Torrent. Pacific Biosciences and Illumina MiSeq sequencers. BMC Genomics.

[34] Roberts RJ, Carneiro MO, Schatz MC (2013). The advantages of SMRT sequencing. Genome Biol.

[35] Koren S, Schatz MC, Walenz BP, Martin J, Howard JT, Ganapathy G, Wang Z, Rasko DA, McCombie WR, Jarvis ED, Phillippy AM (2012). Hybrid error correction and de novo assembly of single-molecule sequencing reads. Nat Biotechnol.

[36] Loomis EW, Eid JS, Peluso P, Yin J, Hickey L, Rank D, McCalmon S, Hagerman RJ, Tassone F, Hagerman PJ (2013). Sequencing the unsequenceable: expanded CGG-repeat alleles of the fragile X gene. Genome Res.

[37] Pugh TJ, Weeraratne SD, Archer TC, Pomeranz Krummel DA, Auclair D, Bochicchio J, Carneiro MO, Carter SL, Cibulskis K, Erlich RL, Greulich H, Lawrence MS, Lennon NJ, McKenna A, Meldrim J, Ramos AH, Ross MG, Russ C, Shefler E, Sivachenko A, Sogoloff B, Stojanov P, Tamayo P, Mesirov JP, Amani V, Teider N, Sengupta S, Francois JP, Northcott PA, Taylor MD (2012). Medulloblastoma exome sequencing uncovers subtype-specific somatic mutations. Nature.

[38] Fichot EB, Norman RS (2013). Microbial phylogenetic profiling with the Pacific Biosciences sequencing platform. Microbiome.

[39] Koren S, Harhay GP, Smith TP, Bono JL, Harhay DM, McVey SD, Radune D, Bergman NH, Phillippy AM (2013). Reducing assembly complexity of microbial genomes with single-molecule sequencing. Genome Biol.

[40] Ferrarini M, Moretto M, Ward JA, Surbanovski N, Stevanovic V, Giongo L, Viola R, Cavalieri D, Velasco R, Cestaro A, Sargent DJ (2013). An evaluation of the PacBio RS platform for sequencing and de novo assembly of a chloroplast genome. BMC Genomics.

[41] Metzker ML (2010). Sequencing technologies - the next generation. Nat Rev Genet.

[42] Ricker N, Qian H, Fulthorpe RR (2012). The limitations of draft assemblies for understanding prokaryotic adaptation and evolution. Genomics.

[43] Moore MJ, Dhingra A, Soltis PS, Shaw R, Farmerie WG, Folta KM, Soltis DE (2006). Rapid and accurate pyrosequencing of angiosperm plastid genomes. BMC Plant Biol.

[44] Straub SC, Fishbein M, Livshultz T, Foster Z, Parks M, Weitemier K, Cronn RC, Liston A (2011). Building a model: developing genomic resources for common milkweed (Asclepias syriaca) with low coverage genome sequencing. BMC Genomics.

[45] Dong W, Xu C, Cheng T, Lin K, Zhou S (2013). Sequencing angiosperm plastid genomes made easy: a complete set of universal primers and a case study on the phylogeny of Saxifragales. Genome Biol Evol.

[46] Hirose T, Sugiura M (1997). Both RNA editing and RNA cleavage are required for translation of tobacco chloroplast ndhD mRNA: a possible regulatory mechanism for the expression of a chloroplast operon consisting of functionally unrelated genes. EMBO J.

[47] Tsudzuki T, Wakasugi T, Sugiura M (2001). Comparative analysis of RNA editing sites in higher plant chloroplasts. J Mol Evol.

[48] Sawai S, Thomason PA, Cox EC (2005). An autoregulatory circuit for long-range self-organization in Dictyostelium cell populations. Nature.

[49] Kato T, Kaneko T, Sato S, Nakamura Y, Tabata S (2000). Complete structure of the chloroplast genome of a legume, Lotus japonicus. DNA Res.

[50] Wicke S, Muller KF, de Pamphilis CW, Quandt D, Wickett NJ, Zhang Y, Renner SS, Schneeweiss GM (2013). Mechanisms of functional and physical genome reduction in photosynthetic and nonphotosynthetic parasitic plants of the broomrape family. Plant Cell.

[51] Funk HT, Berg S, Krupinska K, Maier UG, Krause K (2007). Complete DNA sequences of the plastid genomes of two parasitic flowering plant species, Cuscuta reflexa and Cuscuta gronovii. BMC Plant Biol.

[52] de Pamphilis CW, Palmer JD (1990). Loss of photosynthetic and chlororespiratory genes from the plastid genome of a parasitic flowering plant. Nature.

[53] Chang CC, Lin HC, Lin IP, Chow TY, Chen HH, Chen WH, Cheng CH, Lin CY, Liu SM, Chang CC, Chaw SM (2006). The chloroplast genome of Phalaenopsis aphrodite (Orchidaceae): comparative analysis of evolutionary rate with that of grasses and its phylogenetic implications. Mol Biol Evol.

[54] Weng ML, Blazier JC, Govindu M, Jansen RK (2014). Reconstruction of the ancestral plastid genome in geraniaceae reveals a correlation between genome rearrangements, repeats, and nucleotide substitution rates. Mol Biol Evol.

[55] Wakasugi T, Tsudzuki J, Ito S, Nakashima K, Tsudzuki T, Sugiura M (1994). Loss of all ndh genes as determined by sequencing the entire chloroplast genome of the black pine Pinus thunbergii. Proc Natl Acad Sci U S A.

[56] Millen RS, Olmstead RG, Adams KL, Palmer JD, Lao NT, Heggie L, Kavanagh TA, Hibberd JM, Gray JC, Morden CW, Calie PJ, Jermiin LS, Wolfe KH (2001). Many parallel losses of infA from chloroplast DNA during angiosperm evolution with multiple independent transfers to the nucleus. Plant Cell.

[57] Delannoy E, Fujii S, Des Francs-Small CC, Brundrett M, Small I (2011). Rampant gene loss in the underground orchid *Rhizanthella gardneri* highlights evolutionary constraints on plastid genomes. Mol Biol Evol.

[58] Barth D, Berendonk TU (2011). The mitochondrial genome sequence of the ciliate Paramecium caudatum reveals a shift in nucleotide composition and codon usage within the genus Paramecium. BMC Genomics.

[59] Angiosperm Phylogeny Group (2009). An update of the Angiosperm Phylogeny Group classification for the orders and families of flowering plants: APG III. Bot J Linn Soc.

[60] Kreunen SS, Osborn JM (1999). Pollen and anther development in Nelumbo (Nelumbonaceae). Am J Bot.

[61] Drinnan AN, Crane PR, Hoot SB (1994). Patterns of floral evolution in the early diversification of non-magnoliid dicotyledons (eudicots). Plant Syst Evol.

[62] Soltis PS, Soltis DE, Chase MW (1999). Angiosperm phylogeny inferred from multiple genes as a tool for comparative biology. Nature.

[63] Hansen DR, Dastidar SG, Cai Z, Penaflor C, Kuehl JV, Boore JL, Jansen RK (2007). Phylogenetic and evolutionary implications of complete chloroplast genome sequences of four early-diverging angiosperms: Buxus (Buxaceae), Chloranthus (Chloranthaceae), Dioscorea (Dioscoreaceae), and Illicium (Schisandraceae). Mol Phylogenet Evol.

[64] Kim YK, Park CW, Kim KJ (2009). Complete chloroplast DNA sequence from a Korean endemic genus, Megaleranthis saniculifolia, and its evolutionary implications. Mol Cells.

[65] Sun YX, Moore MJ, Meng AP, Soltis PS, Soltis DE, Li JQ, Wang HC (2013). Complete plastid genome sequencing of Trochodendraceae reveals a significant expansion of the inverted repeat and suggests a Paleogene divergence between the two extant species. PLoS One.

[66] Zhong B, Yonezawa T, Zhong Y, Hasegawa M (2009). Episodic evolution and adaptation of chloroplast genomes in ancestral grasses. PLoS One.

[67] Drew BT, Ruhfel BR, Smith SA, Moore MJ, Briggs BG, Gitzendanner MA, Soltis PS, Soltis DE (2014). Another look at the root of the angiosperms reveals a familiar tale. Syst Biol.

[68] Ruhfel BR, Gitzendanner MA, Soltis PS, Soltis DE, Burleigh JG (2014). From algae to angiosperms-inferring the phylogeny of green plants (Viridiplantae) from 360 plastid genomes. BMC Evol Biol.

[69] Bergsten J (2005). A review of long-branch attraction. Cladistics.

[70] Wang RJ, Cheng CL, Chang CC, Wu CL, Su TM, Chaw SM (2008). Dynamics and evolution of the inverted repeat-large single copy junctions in the chloroplast genomes of monocots. BMC Evol Biol.

[71] Jia RZ, Ming R, Zhu YJ (2013). Genome-wide analysis of Nucleotide-Binding Site (NBS) disease Resistance (R) Genes in Sacred Lotus (Nelumbo nucifera Gaertn.) reveals their transition role during early evolution of land plants. Tropical Plant Biol.

[72] Li JK, Zhou EX, Li DX, Huang SQ (2010). Multiple northern refugia for Asian sacred lotus, an aquatic plant with characteristics of ice-age endurance. Aust J Bot.

[73] Jansen RK, Raubeson LA, Boore JL, de Pamphilis CW, Chumley TW, Haberle RC, Wyman SK, Alverson AJ, Peery R, Herman SJ, Fourcade HM, Kuehl JV, McNeal JR, Leebens-Mack J, Cui LY (2005). Methods for obtaining and analyzing whole chloroplast genome sequences. Method Enzymol.

[74] Wyman SK, Jansen RK, Boore JL (2004). Automatic annotation of organellar genomes with DOGMA. Bioinformatics.

[75] Schattner P, Brooks AN, Lowe TM (2005). The tRNAscan-SE, snoscan and snoGPS web servers for the detection of tRNAs and snoRNAs. Nucleic Acids Res.

[76] Conant GC, Wolfe KH (2008). GenomeVx: simple web-based creation of editable circular chromosome maps. Bioinformatics.

[77] Tamura K, Peterson D, Peterson N, Stecher G, Nei M, Kumar S (2011). MEGA5: molecular evolutionary genetics analysis using maximum likelihood, evolutionary distance, and maximum parsimony methods. Mol Biol Evol.

[78] Xia X, Xie Z (2001). DAMBE: software package for data analysis in molecular biology and evolution. J Hered.

[79] Posada D, Crandall KA (1998). MODELTEST: testing the model of DNA substitution. Bioinformatics.

[80] Stamatakis A, Hoover P, Rougemont J (2008). A rapid bootstrap algorithm for the RAxML Web servers. Syst Biol.

[81] Yang Z (2007). PAML 4: phylogenetic analysis by maximum likelihood. Mol Biol Evol.

[82] dos Reis M, Yang Z (2011). Approximate likelihood calculation on a phylogeny for Bayesian estimation of divergence times. Mol Biol Evol.

[83] Rannala B, Yang Z (2007). Inferring speciation times under an episodic molecular clock. Syst Biol.

[84] Yang Z (2006). Computational Molecular Evolution.

[85] Yang Z, Rannala B (2006). Bayesian estimation of species divergence times under a molecular clock using multiple fossil calibrations with soft bounds. Mol Biol Evol.

