# Supplementary material for: A precise chloroplast genome of Nelumbo nucifera (Nelumbonaceae) evaluated with Sanger, Illumina MiSeq, and PacBio RS II sequencing platforms: insight into the plastid evolution of basal eudicots
Source: BMC Plant Biol. 2014 Nov 19;14:289. doi: 10.1186/s12870-014-0289-0 (PMC4245832; doi:10.1186/s12870-014-0289-0)
Supplement: Additional file 1 — 133 taxa from 56 orders included in phylogenetic analyses with APG III ordinal classification. [file 12870_2014_289_MOESM1_ESM.pdf]

Additional file 1. 133 taxa from 56 orders included in phylogenetic analyses with APG III ordinal classification.

Taxon	APGIII Order	GenBank Accession Number	Genes Not Included
**Gymnosperms(3)**			
Picea abies	Coniferales	NC_021456	*ndhA, ndhB, ndhC, ndhD, ndhE, ndhF, ndhG, ndhH, ndhI, ndhJ, ndhK, rps16*
Cycas taitungensis	Cycadales	NC_009618	
Ginkgo biloba	Ginkgoales	NC_016986	*psbZ, ycf1*
**Basal Angiosperms (7)**			
Amborella trichopoda	Amborellales	NC_005086	*petL, psaI, psbM*
Illicium oligandrum	Austrobaileyales	NC_009600	
Chloranthus spicatus	Chloranthales	NC_009598	
Nuphar advena	Nymphaeales	NC_008788	
Nymphaea alba	Nymphaeales	NC_006050	
Trithuria inconspicua	Nymphaeales	NC_020372	*clpP*
**Magnoliids(5)**			
Drimys granadensis	Canellales	NC_008456	
Calycanthus floridus	Laurales	NC_004993	
Liriodendron tulipifera	Magnoliales	NC_008326	*rpl22*
Magnolia kwangsiensis	Magnoliales	NC_015892	*rpl22*
Piper cenocladum	Piperales	NC_008457	
**Monocots (29)**			
Acorus calamus	Acorales	NC_007407	*accD*
Lemna minor	Alismatales	NC_010109	*infA*
Colocasia esculenta	Alismatales	NC_016753	*infA*
Elodea canadensis	Alismatales	NC_018541	
Najas flexilis	Alismatales	NC_021936	*infA, ndhA, ndhB, ndhC, ndhD,*

			ndhE, ndhF, ndhG, ndhH, ndhI, ndhJ, ndhK, psbH
Spirodela polyrhiza	Alismatales	NC_015891	*infA, rpl20, rps12*
Wolffia australiana	Alismatales	NC_015899	*infA, rps12*
Bismarckia nobilis	Arecales	NC_020366	
Cocos nucifera	Arecales	NC_022417	
Elaeis guineensis	Arecales	NC_017602	
Pseudophoenix vinifera	Arecales	NC_020364	
Phoenix dactylifera	Arecales	NC_013991	
Phalaenopsis aphrodite	Asparagales	NC_007499	*ndhA, ndhB, ndhC, ndhD, ndhE ndhF, ndhG, ndhH, ndhI, ndhJ ndhK, ycf1*
Cymbidium aloifolium	Asparagales	NC_021429	*ndhA, ndhB, ndhC, ndhD, ndhF, ndhG, ndhH, ndhI, ndhK,*
Erycina pusilla	Asparagales	NC_018114	*ndhA, ndhB, ndhC, ndhD,ndhE, ndhF, ndhG, ndhH, ndhI, ndhJ, ndhK, ycf1*
Neottia nidus-avis	Asparagales	NC_016471	*atpA, atpB, atpE, atpF, atpH, atpI, ccsA, cemA, matK, ndhA, ndhB,ndhC, ndhD, ndhE, ndhF, ndhG, ndhH, ndhI, ndhJ, ndhK, petA, petB, petD, petG, petL, petN, psaA, psaB, psaC, psaI, psaJ, psbA, psbB, psbC, psbD, psbE, psbF, psbH, psbI, psbJ, psbK, psbL, psbM, psbN, psbT, psbZ, rbcL, rpoA, rpoB, rpoC1, rpoC2, rps18, ycf3, ycf4*
Oncidium Gower Ramsey	Asparagales	NC_014056	*ndhA, ndhB, ndhC, ndhD,ndhF, ndhG, ndhH, ndhI, ndhJ, ndhK, ycf1*
Rhizanthella gardneri	Asparagales	NC_014874	*atpA, atpB, atpE, atpF, atpH, atpI, ccsA, cemA, matK, ndhA, ndhB,ndhC, ndhD, ndhE, ndhF, ndhG, ndhH, ndhI, ndhJ, ndhK, petA, petB, petD, petG, petL, petN, psaA, psaB, psaC, psaI, psaJ, psbA, psbB, psbC,psbD,*

			psbE, psbF, psbH, psbI, psbJ,psbK, psbL, psbM, psbN, psbT, psbZ,rbcL, rpl22, rpl32, rpl33, rpoA, rpoB,rpoC1, rpoC2, rps12, rps15, rps16,ycf3, ycf4
Dioscorea elephantipes	Dioscoreales	NC_009601	*rps16*
Veratrum patulum	Liliales	NC_022715	*infA, rps16*
Bambusa emeiensis	Poales	NC_015830	*accD, ycf1, ycf2*
Oryza sativa	Poales	NC_001320	*accD, psaJ, ycf1, ycf2*
Lolium perenne	Poales	NC_009950	*accD, ycf1, ycf2*
Saccharum officinarum	Poales	NC_006084	*accD, psbA, ycf1, ycf2*
Sorghum bicolor	Poales	NC_008602	*accD, ycf1, ycf2*
Triticum aestivum	Poales	NC_002762	*accD, ycf1, ycf2*
Typha latifolia	Poales	NC_013823	
Zea mays	Poales	NC_001666	*accD, ycf1, ycf2*
Heliconia collinsiana	Zingiberales	NC_020362	
**Eudicots (90)**			
Brassaiopsis hainla	Apiales	NC_022811	
Daucus carota	Apiales	NC_008325	
Schefflera delavayi	Apiales	NC_022813	
Panax ginseng	Apiales	NC_006290	
Metapanax delavayi	Apiales	NC_022812	
Ilex cornuta Lindl. & Paxton	Aquifoliales	GQ997298-GQ997380	
Guizotia abyssinica	Asterales	NC_010601	
Helianthus annuus	Asterales	NC_007977	
Lactuca sativa	Asterales	NC_007578	*ycf1*
Artemisia frigida	Asterales	NC_020607	
Trachelium caeruleum	Asterales	NC_010442	*accD, clpP, infA, ndhK, rpl23*
Jacobaea vulgaris	Asterales	NC_015543	*rpoC1*

Berberidopsis corallina Hook.f.	Berberidopsidales	EU002158, EU002171, EU002201, EU002274, EU002295, EU002385, EU002466, GQ997932-GQ998004	
Ehretia acuminata R.Br.	Boraginaceae	GQ997215-GQ997297	
Arabidopsis thaliana	Brassicales	NC_000932	*infA*
Brassica napus	Brassicales	NC_016734	*infA*
Carica papaya	Brassicales	NC_010323	*infA*
Buxus microphylla	Buxales	NC_009599	
Fagopyrum esculentum	Caryophyllales	NC_010776	*rpl23*
Spinacia oleracea	Caryophyllales	NC_002202	*rpl23*
Silene conica	Caryophyllales	NC_016729	*infA, matK, rpl23*
Euonymus americanus L	Celastrales	EU002160, EU002170, EU002193, EU002277, EU002321, EU002409, EU002500, GQ998147-GQ998219	
Cornus florida L.	Cornales	EU002157, EU002175, EU002215, EU002276, EU002311, EU002377, EU002491, GQ998074-GQ998146	*psbT*
Staphylea	Crossosomatales	EU002168,	*infA*

colchica Steven		EU002189, EU002261, EU002285, EU002364, EU002453, EU002543, GQ998725-GQ998796	
Cucumis sativus	Cucurbitales	NC_007144	*infA*
Corynocarpus laevigata	Cucurbitales	NC_014807	*infA, ndhD*
Dillenia indica L	Dilleniaceae	GQ997132-GQ997214	
Lonicera japonica Thunb.	Dipsacales	GQ997381-GQ997463	
Rhododendron simsii Planch	Ericales	GQ997782-GQ997859	*accD, clpP, rpl33, ycf1, ycf2*
Cicer arietinum	Fabales	NC_011163	*infA, rpl22, rps16, ycf4*
Glycine max	Fabales	NC_007942	*infA, rpl22, ycf4*
Millettia pinnata	Fabales	NC_016708	*infA, rpl22*
Lotus japonicus	Fabales	NC_002694	*infA, rpl22*
Phaseolus vulgaris	Fabales	NC_009259	*infA, rpl22, rpl33, rps16*
Trifolium subterraneum	Fabales	NC_011828	*accD, infA, ndhB, ndhK, rpl22, rpl32,rps16, rps18, ycf1, ycf4*
Castanea mollissima	Fagales	NC_014674	*infA, rpl22*
Aucuba japonica Thunb.	Garryales	GQ997049-GQ997131	
Coffea arabica	Gentianales	NC_008535	
Asclepias syriaca	Gentianales	NC_022432	*accD, clpP*
Catharanthus roseus	Gentianales	NC_021423	
Erodium texanum	Geraniales	NC_014569	*accD, infA, ycf1, ycf2*
Geranium palmatum	*Geraniales*	NC_014573	*clpP, infA, ycf1, ycf2*
Monsonia speciosa	Geraniales	NC_014582	*accD, clpP, infA, rps12, ycf1, ycf2*
Pelargonium x hortorum	Geraniales	NC_008454	*accD, infA, rpoA*
Gunnera manicata Linden ex Andr é	Gunnerales	EU002162, EU002179, EU002226,	*psbH*

		EU002279, EU002325, EU002413, EU002504, GQ998292-GQ998364	
Boea hygrometrica	Lamiales	NC_016468	*cemA, psbB, psbI, psbZ, rps19*
Salvia miltiorrhiza	Lamiales	NC_020431	
Utricularia gibba	Lamiales	NC_021449	*petD*
Olea woodiana subsp. woodiana	Lamiales	NC_015608	*ccsA, cemA, infA, ndhK, psbZ, rps12*
Jasminum nudiflorum	Lamiales	NC_008407	*accD*
Cistanche deserticola	Lamiales	NC_021111	*accD, atpA, atpB, atpE, atpF, atpH, atpI, ccsA, cemA, ndhA, ndhB, ndhC, ndhD, ndhE, ndhF, ndhG, ndhH, ndhI, ndhJ, ndhK, petA, petB, petD, petG, petL, petN, psaA, psaB, psaC, psaI, psaJ, psbA, psbB, psbC, psbD, psbE, psbF, psbH, psbI, psbJ, psbK, psbL, psbN, psbT, psbZ, rbcL, rpl23, rpoA, rpoB, rpoC1, rpoC2, ycf1, ycf3, ycf4*
Epifagus virginiana	Lamiales	NC_001568	*atpA, atpB, atpE, atpF, atpH, atpI, ccsA, cemA, ndhA, ndhB, ndhC, ndhD, ndhE, ndhF, ndhG, ndhH, ndhI, ndhJ, ndhK, petA, petB, petD, petG, petL, petN, psaA, psaB, psaC, psaI, psaJ, psbA, psbB, psbC, psbD, psbE, psbF, psbH, psbI, psbJ, psbK, psbL, psbM, psbN, psbT, psbZ, rbcL, rpl14, rpl22, rpl23, rpl32, rpoA, rpoB, rpoC1, rpoC2, rps15, rps16, ycf1, ycf3, ycf4*
Lindenbergia philippensis	Lamiales	NC_022859	*accD, matK, ndhF, ndhK, psbF, psbI, rpoC1, rps15, ycf1*
Hevea brasiliensis	Malpighiales	NC_015308	*infA*
Ricinus communis	Malpighiales	NC_016736	*infA*
Manihot	Malpighiales	NC_010433	*infA*

esculenta			
Populus trichocarpa	Malpighiales	NC_009143	*infA, rpl32, rps16, ycf1*
Gossypium hirsutum	Malvales	NC_007944	*infA, rpl22*
Allosyncarpia ternata	Myrtales	NC_022413	*infA, psbL*
Angophora costata	Myrtales	NC_022412	*infA, psbL*
Corymbia eximia	Myrtales	NC_022409	*infA, psbL*
Stockwellia quadrifida	Myrtales	NC_022414	*infA, psbL*
Eucalyptus globulus	Myrtales	NC_008115	*infA, psbL*
Oenothera elata	Myrtales	NC_002693	*infA*
Oxalis latifolia Kunth	Oxalidales	EU002165, EU002186, EU002248, EU002282, EU002350, EU002438, EU002528, GQ998511-GQ998580	*infA, rpl32, rps16*
Platanus occidentalis	Proteales	NC_008335	
Nelumbo lutea	Proteales	NC_015605 (in this study)	
Nelumbo nucifera	Proteales	NC_015610 (in this study)	
Meliosma aff. cuneifolia Franch.	Sabiaceae	GQ997464-GQ997546	
Phoradendron leucarpum (Raf.) Reveal & M.C.Johnst.	Santalales	GQ997713-GQ997781	*infA, ndhA, ndhB, ndhC, ndhD, ndhE, ndhF, ndhG, ndhH, ndhI, ndhJ, ndhK, rpl33, rps15*
Ximenia americana L	Santalales	GQ997860-GQ997931	*All ndh genes*
Heuchera sanguinea Engelm	Saxifragales	EU002163, EU002180, EU002228, EU002280, EU002327,	

		EU002415, EU002506, GQ998365-GQ998437	
Ranunculus macranthus	Ranunculales	NC_008796	*infA*
Berberis bealei	Ranunculales	NC_022457	*psbZ, rpoA*
Nandina domestica	Ranunculales	NC_008336	
Megaleranthis saniculifolia	Ranunculales	NC_012615	
Morus indica	Rosales	NC_008359	*infA*
Fragaria chiloensis	Rosales	NC_019601	*infA*
Prunus persica	Rosales	NC_014697	*infA*
Citrus sinensis	Sapindales	NC_008334	*infA*
Atropa belladonna	Solanales	NC_004561	*infA*
Cuscuta exaltata	Solanales	NC_009963	*infA, ndhA, ndhB, ndhC, ndhD, ndhE, ndhF, ndhG, ndhH, ndhI, ndhJ, ndhK, rpl23, rps16*
Capsicum annuum	Solanales	NC_018552	*infA*
Solanum bulbocastanum	Solanales	NC_007943	
Ipomoea purpurea	Solanales	NC_009808	*infA*
Nicotiana tabacum	Solanales	NC_001879	*infA*
Trochodendron aralioides	Trochodendrales	NC_021426	
Tetracentron sinense	Trochodendrales	NC_021425	
Vitis vinifera	Vitales	NC_007957	
Bulnesia arborea (Jacq.) Engl	Zygophyllales	EU002159, EU002172, EU002205, EU002275, EU002299, EU002388, EU002478, GQ998005-GQ998073	*infA, rps16, ycf1, ycf2*
