# Supplementary figures and images for: A precise chloroplast genome of Nelumbo nucifera (Nelumbonaceae) evaluated with Sanger, Illumina MiSeq, and PacBio RS II sequencing platforms: insight into the plastid evolution of basal eudicots
Source: BMC Plant Biol. 2014 Nov 19;14:289. doi: 10.1186/s12870-014-0289-0 (PMC4245832; doi:10.1186/s12870-014-0289-0)

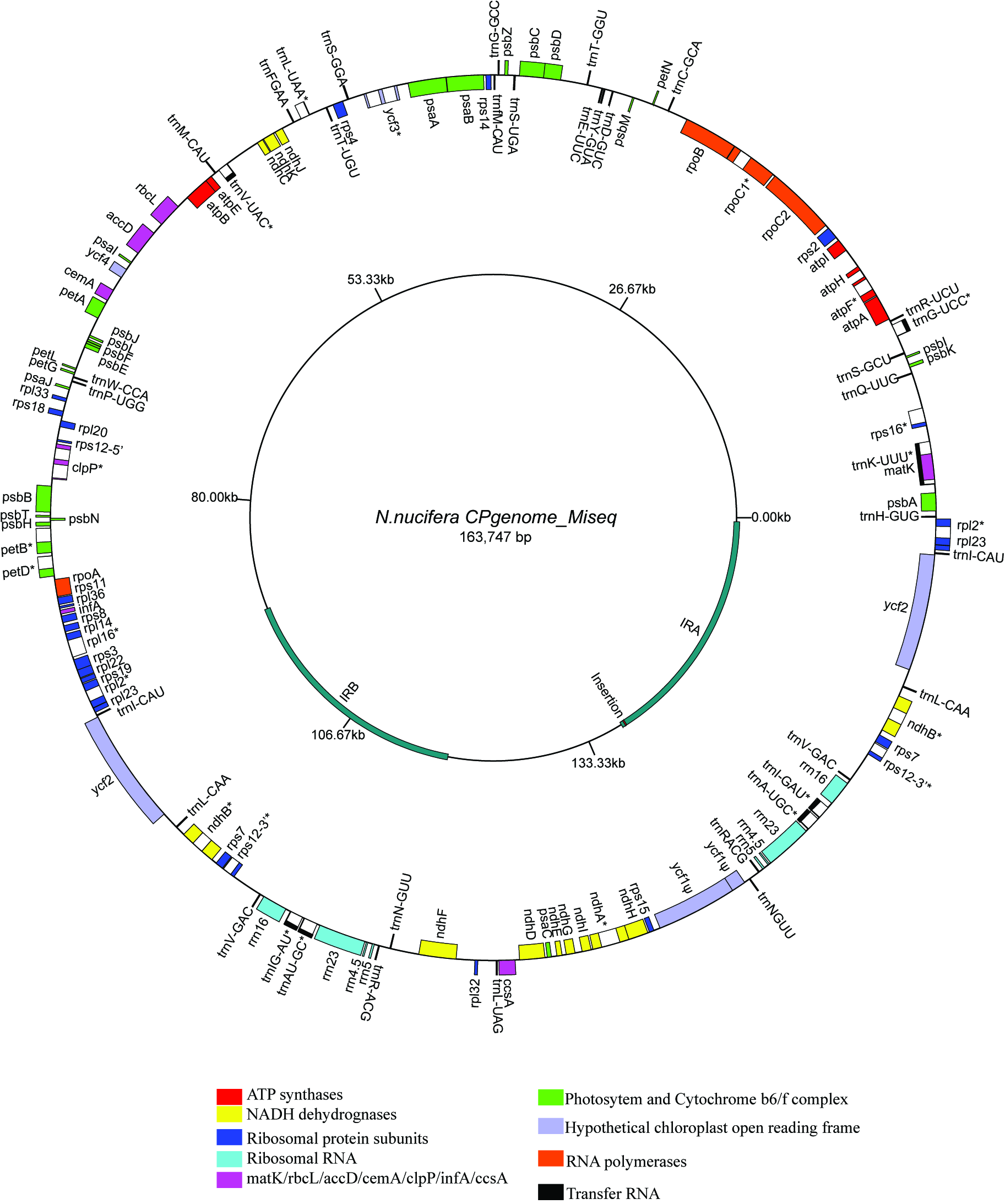

Supplement: Additional file 2: Figure S1 — Gene map of N. nucifera chloroplast genome from the Sanger platform. [file 12870_2014_289_MOESM2_ESM.tiff]

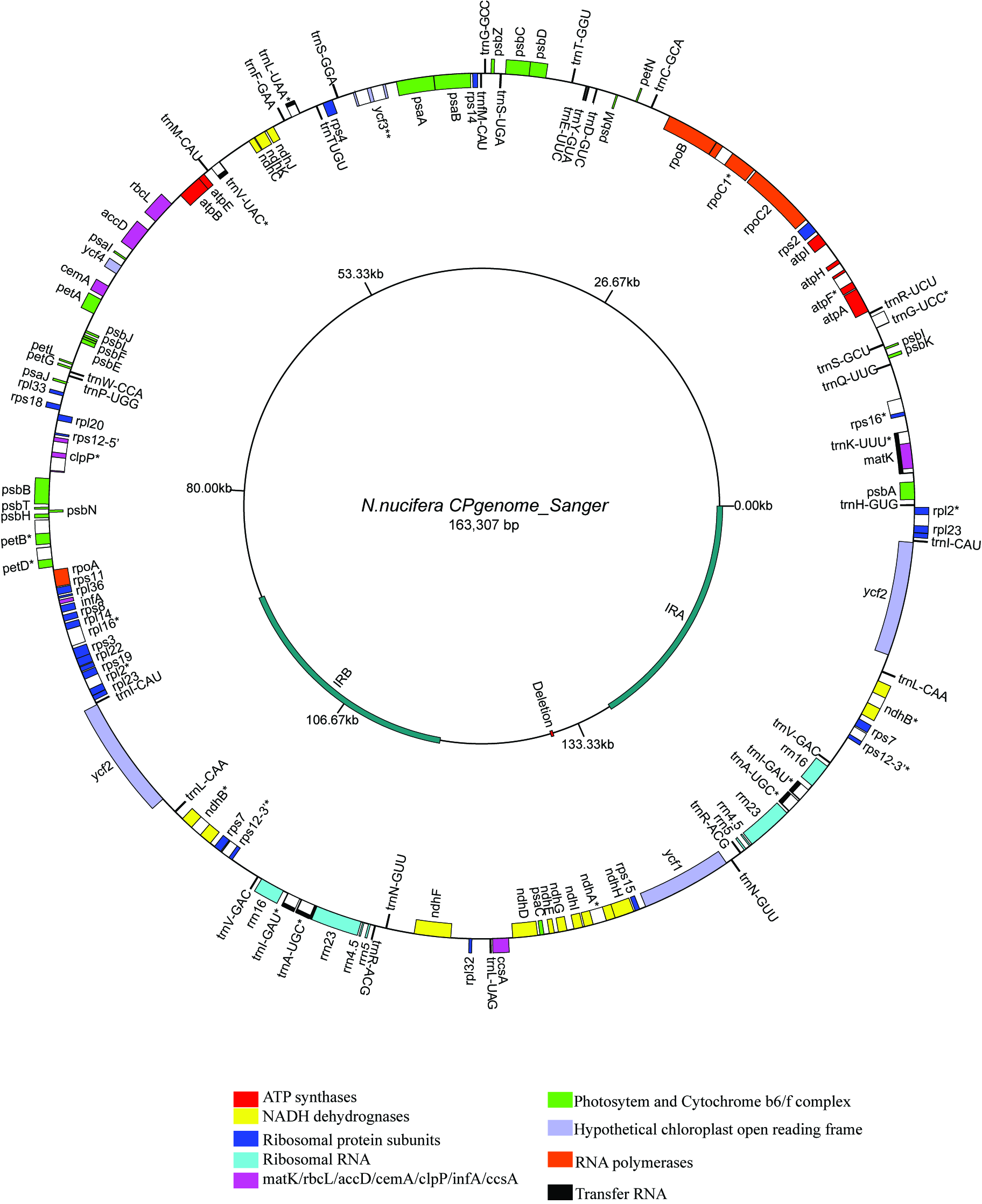

Supplement: Additional file 3: Figure S2 — Gene map of N. nucifera chloroplast genome from the Illumina MiSeq platform. [file 12870_2014_289_MOESM3_ESM.tiff]
