# Supplementary material for: A precise chloroplast genome of Nelumbo nucifera (Nelumbonaceae) evaluated with Sanger, Illumina MiSeq, and PacBio RS II sequencing platforms: insight into the plastid evolution of basal eudicots
Source: BMC Plant Biol. 2014 Nov 19;14:289. doi: 10.1186/s12870-014-0289-0 (PMC4245832; doi:10.1186/s12870-014-0289-0)
Supplement: Additional file 4 — Primers for chloroplast genome amplification in our study. [file 12870_2014_289_MOESM4_ESM.pdf]

Additional file 4. Primers for chloroplast sequence amplification in our study.

**Primer**	**Primer sequence**
Cps1F	GGAAAGGGTAGAAGAATGGGACCTAT
Cps1R	GTACCGAGGGTTCGAATCCCTCTCTT
Cps2F	AAGAGAGGGATTCGAACCCTCGGTAC
Cps2R	GGAACTATTCGTACGTTGTTGAATAG
Cps3F	CTATTCAACAACGTACGAATAGTTCC
Cps3R	ATCAGGCGACACCCGGATTTGAACTG
Cps4F	CAGTTCAAATCCGGGTGTCGCCTGAT
Cps4R	AGAGATGGCCGAGTGGTTGATAGCTC
Cps5F	GAGCTATCAACCACTCGGCCATCTCT
Cps5R	TCCCGTCAGACTTGAACCTAGTTAAA
Cps6F	TTTAACTAGGTTCAAGTCTGACGGGA
Cps6R	CAACCTCGGATCGTTGGTGAGGAACA
Cps7F	TGTTCCTCACCAACGATCCGAGGTTG
Cps7R	GGTGTCCATCCGATGCATCCGCTATG
Cps8F	CATAGCGGATGCATCGGATGGACACC
Cps8R	ACAACGGTATGAACACGATACCAAGG
Cps9F	CCTTGGTATCGTGTTCATACCGTTGT
Cps9R	GCACGTGTCGAATGGATCAGAGAAGG
Cps10F	CCTTCTCTGATCCATTCGACACGTGC
Cps10R	GATTCCTTGGTTCGGACCGAAGAAGC
Cps11F	GCTTCTTCGGTCCGAACCAAGGAATC
Cps11R	CCTCAGTAGCTCAGTGGTAGAGCGGT
Cps12F	ACCGCTCTACCACTGAGCTACTGAGG
Cps12R	CGGCCCTTTGGGAATTCTTCAAGCTC
Cps13F	GAGCTTGAAGAATTCCCAAAGGGCCG
Cps13R	GACATAAGAGATGTCGTTTCTAGTCT
