# Supplementary material for: A precise chloroplast genome of Nelumbo nucifera (Nelumbonaceae) evaluated with Sanger, Illumina MiSeq, and PacBio RS II sequencing platforms: insight into the plastid evolution of basal eudicots
Source: BMC Plant Biol. 2014 Nov 19;14:289. doi: 10.1186/s12870-014-0289-0 (PMC4245832; doi:10.1186/s12870-014-0289-0)
Supplement: Additional file 5 — The DNA sequences alignment of the genes from 133 species used in the phylogenetic analyses. [file 12870_2014_289_MOESM5_ESM.pdf]

Additional file 5. The DNA sequences alignment of the genes from 133 species used in the phylogenetic analyses.

>Aucuba
ATGGAAAGATGGTGGTTTAATTCGATGTTCTTTCTAAGTAAATCAATGGACAGTCTTGG
TCCTATTGAAAATATCAGTGAA------GATCCGACTATAAATGATACGACTAAAAAGATTCA
TAGTTACAGTAATGTTGATAATTTATTCGGTATCAAAGACATTGGGAATTTCATCGCTGA
TGACACTTTTTTAGTTAGGGATAGTAATGGAGACAGTTATTCTATATATTTTGATATTGAA
AATCAGATTTTTGAAATTGACAATGATCAT------TCTTTTCTGAATGAACTAGAAAGTTCT
TTTTATAGTTATCGGAAT---------------AATGGATCTAAGAGTGACGATCCCTACTATGATCG
TTATATGTATGATACTCAATATAGTTGGAATAATCACATTAATAGTTGCATTGATAGTTATC
TTGAG------TCTCAAATCCGTATTGATACTTCCATTGGAAGTGGTAGTGACAATTATGGT--
----TACATTTATAGTTCCATTTGTGGTAGCGAAGGTTCCGGTATACAAACCAGCACGAAT
GGTAGTGATTTAACTATAAGAGAAAGTTCTAATGATCTCGATGTAACTCAAAAATACAG
GCATTTGTGGATTCAATGTGAAAATTGTTATGGATTAAATTATAAGAAATTTTTTAAATC
AAAAATGAATATTTGTGAACAATGTGGATATCATTTGAAAATGAGTAGTTCAGATAGAA
TCGAATTTTCGATCGATCCAGGTACTTGGGATCCTATGGATGAAGACATGGTATCTCTGG
ATCCCATTGAATTTCATTCGGAGGAAGAGCCTTATAAAGATCGTATCGATTCTTATCAAA
GAAAGACAGGATTAACTGAGGCTGTTCAAACAGGTATAGGCCAACTAAACGGTATTCC
CATAGCAATTGGGGTTATGGATTTTCAGTTTATGGGGGGTAGTATGGGATCCGTAGTCGG
GGAGAAAATCACCCGTTTGATTGAGTACGCTACCAATCAATTTCTACCTCTTATTATAGT
GTGTGCTTCCGGGGGGGCACGCATGCAAGAAGGAAGTTTGAGCTTGATGCAAATGGCT
AAAATATCGTCTGCTTTATATGATTATCAATCAAATAAAAAGTTATTCTATGTAGCAATCC
TTACATCTCCTACTACTGGTGGGGTGACAGCTAGTTTTGGTATGTTGGGGGATATCATTA
TTGCTGAACCCAATGCCTACATTGCATTTGCGGGTAAAAGAGTAATTGAACAAACATTG
AATAAAACAGTCCCTGAAGGTTCACAAGCGGCTGAATATTTATTCCAGAAGGGCTTATT
CGATCTAATCGTACCACGTAATCCTTTAAAAGATGTTCTGAGTGAGTTATTGCAGCTCCA
CGCCTTCTTTCCTTTGACT---------------ATGGTAACCATTCGAGCCGACGAAATTAGTAAT
ATTATACGTGAACGTATTGAGCAATATAATAGAGAAGTAAAGATTGTAAATACCGGGAC
CGTACTTCAGGTAGGCGATGGCATTGCTCGTATTCATGGTCTTGATGAAGTAATGGCGG
GCGAATTAGTAGAATTTGAAGAGGGTACAATAGGCATTGCTCTAAATTTGGAATCAAAT
AATGTTGGTGTTGTATTAATGGGTGATGGTTTGATGATACAAGAAGGAAGTTCTGTAAA
AGCAACAGGAAAAATTGCTCAGATCCCAGTGAGTGAGGCCTATTTGGGTCGTGTTATA
AATGCCCTGGCTAAACCTATTGATGGTAGAGGTGAAATTTCAGCTTCTGAATCCCGGTT
AATTGAATCTCCCGCTCCAGGTATTATTTCGCGGCGTTCCGTATATGAGCCTCTTCAAAC
GGGACTTATTGCTATTGATTCGATGATCCCTATAGGACGTGGTCAGCGAGAATTAATTAT
TGGGGACAGACAGACTGGTAAAACAGCAGTAGCCACAGATACGATTCTCAATCAACA
AGGTCAAAATGTAATATGTGTTTATGTAGCTATTGGTCAAAAAGCATCTTCGGTGGCTC
AGGTAGTGACTACTTTCCAGGAAAGGGGAGCCATGGAATACACTATTGTGGTAGCCGA
AGCAGCGGATTCCCCTGCTACATTACAATACCTTGCTCCTTATACAGGAGCAGCTCTGG
CTGAATATTTTATGTACCGTGAACGACACACTTCAATCATTTATGATGATCCCTCCAAAC
AAGCACAAGCTTATCGCCAAATGTCTCTTCTATTACGAAGACCGCCTGGTCGTGAAGCT
TATCCAGGGGATGTTTTTTATTTGCATTCACGCCTTTTGGAAAGAGCCGCTAAATCAAG
TTCTAGTTTAGGTGAAGGAAGTATGACTGCTTTACCAATAGTTGAAACTCAATCGGGAG

ATGTTTCGGCTTATATTCCTACTAATGTAATTTCCATTACGGATGGACAAATATTCTTATC
CGCCGATCTATTCAATGCTGGAATCAGACCTGCTATTAATGTGGGTATCTCCGTTTCCAG
AGTGGGGTCTGCAGCTCAAATTAAGGCCATGAAACAAGTCGCTGGCAAATTAAAATTA
GAACTGGCGCAATTCGCAGAACTAGAAGCCTTTGCACAATTTGCTTCTGATCTCGATAA
AGCTACTCAGAATCAATTGGCAAGAGGTCAACGATTACGTGAATTGCTCAAACAATCC
CAAGCAGCCCCTCTCACGGTGGAAGAACAGATAATGACTATTTATACCGGAACAAATG
GTTATCTTGATTCATTAGAAATTGGACAGGTAAGGAAATTTCTTGTTGAGTTACGTACTT
ACTTAAAAACTAATAAGCCTCAGTTCCAAGAAATCATATCTTCTACCAAGACATTTACC
GAGGAAGCAGAAACCCTTTTGAAAGAAGCTATTCAGGAACAGATGGAACGTTTTATAC
TTCAGGAACAAGCAATGAGAATCAATCCTACTAGTTCTGGTTCTGGGATTTCCACGCTC
GAAAAAAAAAACCTGGGTCGTATCGCCCAAATCATTGGTCCAGTACTGGATGTAGCTT
TTCCGCCGGGCAAGATGCCTAATATTTATAACGCTCTGGTAGTTAAGGGTCGAGATATTG
CCGGTCAACCAATTAATGTGACTTGTGAGGTACAACAATTATTAGGAAACAATCGAGTT
AGAGCTGTAGCTATGAGTGCTACAGATGGTCTAACGAGAGGAATGGAAGTGATTGACA
CGGGAGCTCCTCTAAGTGTTCCGGTTGGTGGATCGACTCTCGGACGAATTTTCAACGT
GCTTGGAGAGCCTGTTGATAATTTAGGTCCTGTAGATACTCGTATAACATCTCCTATTCAT
AGATCTGCGCCCGCCTTTATACAGTTAGATACAAAATTCTCTATTTTTGAAACAGGAATT
AAAGTAGTAGATCTTTTAGCCCCTTATCGCCGTGGAGGAAAAATCGGACTATTCGGGGG
AGCTGGAGTGGGTAAAACAGTACTAATTATGGAATTGATTAACAATATTGCCAAAGCTC
ACGGGGGTGTATCCGTATTTGGCGGAGTGGGTGAACGTACTCGTGAAGGAAATGATCT
TTACATGGAAATGAAAGAATCCGGAGTAATTAATGAACAAAATATTGCAGAATCAAAA
GTGGCTCTAGTTTACGGTCAGATGAATGAACCGCCGGGAGCTCGTATGAGAGTTGGTT
TGACTGCCCTAACCATGGCGGAATATTTCCGCGATGTTAATGAACAAGACGTACTTCTA
TTTATCGACAATATCTTCCGTTTCGTTCAAGCAGGATCCGAAGTATCGGCCTTATTGGGT
AGAATGCCTTCCGCTGTGGGTTATCAACCCACCCTTAGTACCGAAATGGGTACTTTACA
AGAAAGAATTACTTCTACCAAAGAAGGGTCCATAACTTCTATTCAAGCAGTTTATGTAC
CTGCAGACGATTTGACCGACCCTGCTCCTGCTACAACATTTGCACATTTAGATGCTACT
ACCGTACTATCAAGAGGATTGGCTGCCAAAGGTATCTATCCAGCAGTAGATCCTTTAGA
TTCAACGTCAACTATGCTTCAACCCCGGATCGTTGGGGAGGAACATTATGAAACTGCG
CAAAGAGTTAAGGAAACTTTACAACGTTACAAAGAACTTCAGGACATTATAGCTATCCT
TGGGTTGGACGAATTATCCGAAGAGGATCGTTTAACCGTAGCAAGAGCGCGAAAAATT
GAGCGTTTCTTATCACAACCTTTTTTCGTAGCAGAAGTATTTACCGGTTCTCCAGGAAA
ATATGTTGGTCTAGCAGAAACAATTAGAGGGTTTCAATTGATCCTTTCCGGAGAATTAG
ATAGTCTTCCTGAACAGGCCTTTTATTTGGTAGGTAACATCGATGAAGCTACCGCGAAG
GCTATGAACTTAGAAATGGAGAGCAATTTGAAGAAAATGAGCTTAAATCTTTGTGTACT
GACCCCTAATAGAATTGTTTGGGATTCAGAAGTGAAAGAAATCATTTTATCTACTAATAG
TGGTCAAATTGGTGTATTACCAAACCACGCCCCTATTGCCACAGCTGTAGATATAGGTAT
TTTAAGAATACGCCTTAACGACCAATGGGTAACGATGGCTCTGATGGGTGGTTTTGCTA
GAATAGGCAATAATGAGATCACTATTTTAGTAAATGATGCGGAGAAGGGTAGTGACATT
GATCCACAAGAAGCTCGGGAGACTCTTGAAATAGCGGAAGCTAATTTGAGAAAAGCT
GAAGGAAAGAGACAAACAATTGAGGCAAATCTAGCTCTCCGACGAGCTAGGACACGA
GTAGAGTCTATCAACGCGATTTCAATGAAAAATGTAACCGATTCTTTCGTTTCTTTGGG
CCACTGGCCATCCGCCGGGAGTTTCGGGTTTAATACCGATATTTTAGCAACAAATCCAA
TAAATCTAAGTGTAGTGCTTGGTGTATTGATCTTTTTTGGAAAGGGAGTGTTAAGTGATT

TATTAGATAATCGAAAACAGAGGATCTTGAATACTATTCGAAATTCAGAAGAACTGCGT
GAGGGGGCCATTGAACAGTTGGAAAAAGCCCGTGCTCGCTTACGGAAAGTGGAAATG
GAAGCAGATCGGTTTCGAGTGAATGGATACTCTGAGATAGAGCGAGAAAAATTGAATT
TGATTAATTCAACTTATAAGACTTTGGAACAATTAGAAAATTACAAAAACGAAACTATT
CATTTTGAACAACAAAGGGCGATTAATCAAGTCCGACAACGGGTTTTCCAACAAGCGT
TACAAGGGGCTCTAGGAACTCTGAATAGTTGTTTGAACAACGAGTTACATTTACGTACC
ATCAGTGCCAATATTGGCATGTTGGGGGCGATGAAAGAAATAACTGATATGAATCCACT
GATTTCTGCCGCTTCCGTTATTGCTGCTGGATTGGCCGTAGGGCTTGCTTCTATTGGACC
TGGAGTTGGTCAAGGTACTGCTGCGGGTCAAGCTGTAGAAGGTATCGCGAGACAGCCT
GAGGCGGAAGGAAAAATACGAGGTACTTTATTACTTAGTCTAGCTTTTATGGAAGCTTT
AACTATTTATGGACTGGTTGTAGCATTAGCACTTTTATTTGCGAATCCTTTTGTTATGAAT
GTTCTATCATGTTCCATCAATACACTAAAGGGGTTATATGATATATCTGGTGTGGAAGTA
GGCCAACATTTCTATTGGAAAATAGGAGGTTTCCAAGTCCATGGCCAAGTACTTATTAC
TTCTTGGGTTGTAATTGCTATCTTATTAGGTTCAGCCACTATAGCTGTTCGGAACCCACA
AACCATTCCGACTGGGGGTCAGAATTTCTTCGAATATGTCCTTGAATTCATTCGAGATG
TGAGTAAAACTCAAATTGGAGAAGAATATGGTCCTTGGGTTCCTTTTATTGGAACTATG
TTTCTATTTATTTTTGTTTCTAATTGGTCAGGAGCTCTTTTACCTTGGAAAATCATACAAT
TACCTCATGGGGAGTTAGCCGCACCCACGAATGATATAAATACTACTGTTGCTTTGGCTT
TACTCACGTCAGTGGCATATTTCTATGCAGGTCTTACCAAAAAAGGATTAGGTTATTTCG
GGAAATATATTCAACCAACCCCAATCCTTTTACCCATTAACATCTTGGAAGATTTCACAA
AGCCTTTATCACTTAGTTTTCGACTTTTCGGGAATATATTAGCTGATGAATTAGTAGTTGT
TGTTCTTGTTTCTTTAGTACCTTCAGTCATTCCTATACCCGTCATGTTCCTTGGATTATTT
ACAAGCGGTATTCAAGCTCTTATTTTTGCAACCTTAGCCGCGGCTTATATAGGTGAATCT
ATGGAGGGCCATCATATGATATTTTTAACTTTAGAGCATATATTAACTCATATTTCTTTTTC
GATCGTTTCAATTGTAATTACAATTCATTTGATAACCTTATTAGTCGATGAAATCGTAAA
ACTATATGATTCGTCAGAAAAGGGCATGATAGCTATTTTTTTCTGTATAACAGGATTATTA
GCCACTCGTTGGATTTATTCGGGACATTTCCCACTAAGTAATTTATATGAGTCATTAATCT
TCCTTTCATGGAGTTTCTCCATTATTCATATAGTTCCATAT------AAAAAGAAAAATTATTT
AAGTACAATAACTGCGCCAAGTGTTATTTTTACACAAGGCTTTGTTACTTCGGGTCTTTT
AATTGAAATACATCAATCCAGAATATTAGTACCTGCTCTCCAATCCGAGTGGTTAATAAT
GCATGTAAGTATGATGATATTGGGCTATGCAGCTCTTTTATGTGGATCATTATTATCAGTA
GCACTTCTAGTTATTACATTTCGAAAAAACGGAAAGATTTTTTGT------AAAGAAATCGA
ATACATGAATGAAAGAGATAATGTTTTACGAAATACTTCT---------GCTAAGAATTATTAC
AGGTCCCAATTGATTCAACAATTGGATTATTGGAGTTATCGTGTTATTAGTCTAGGGTTT
CTCTTTTTAACCATAGGTATTCTGTCGGGAGCAATATGGGCTAATGAAGCATGGGGATCA
TATTGGAATTGGGACCCAAAGGAAACTTGGGCATTTATTACTTGGATCGTATTCGCGATT
TATTTACATACTCGGACAAATAAAAATTTGCAGGGTGCAAATTCCGCAATTGTGGCGTC
TATAGGCTTTTTTATAATTTGGATATGCTATTTTGGAGTCAATCTATTAGGAATAGGTCTA
CATAGTTATGGTTCATTTACATTAACGATGGCAAAAAAGAAAGCATTCACCCCTCTTTTA
TATCTTGCATCTTTAGTATTTTTGCCCTGGTGGATTTCTCTCTCATTTAATAAAAGTCTGG
AATCTTGGGTTACGAATTGGTGGAATACTAGGCAATCCGAAACTTTTTTGAATGATATTC
AAGAAAAAAGTATTCTAGAAAAATTCATAGAATTAGAGGAACTCCTCTTCTTGGACGA
AATGATCAAGGAATACTCGGAGACACAACTACAAAAGCTTCGTATA---ATCCACAAAGA
AACGATCCAATTAATCAAGATACACAATGAGGATCGTATCCATACGATTTTGCATTTCTC

GACAAATATAATCTGTTTCGTTATTCTAAGCGGTTGTTCTATTTTGGGTAATGAAGAGCT
TGTTATTCTTAACTCTTGGGCTCAGGAATTCCTATATAACTTAAGCGATACAGTCAAAGC
TTTTTCTATTCTTTTATTAACTGATTTATGTATCGGATTCCATTCACCCCATGGTTGGGAA
CTAATGATTGGTTATGTCTACAAAGATTTTGGATTTGTTCATAATGATCAAATTATATCTG
GTCTTGTTTCCACTTTTCCAGTCATTCTAGATACAATTTTTAAATATTGGATTTTCCGTTA
TTTAAATCGTGTATCTCCGTCACTTGTAGTGATTTATCATTCAATGAATGACATGCCTATT
GGTGTTCCAAAAGTACCCTTCCGAAATCCTGGAGAGGAAGATGCATCTTGGGTTGACG
TATACAACCGACTTTATCGAGAAAGATTACTTTTTTTAGGCCAAGAGGTTGATAGCGAG
ATCTCGAATCAACTTATTGGTCTTATGGTATATCTCAGTATCGAAGATGATACCAAAGAT
CTGTATTTGTTTATAAATTCTCCTGGCGGATGGGTAATACCTGGAGTAGCTATTTATGATA
CGATGCAATTTGTGCGACCAGATGTACATACAATATGCATGGGATTAGCTGCTTCAATGG
GATCTTTTATCCTGGTCGGAGGAGAAATTACCAAACGTCTAGCATTCCCTCACGCCAGG
GTAATGATCCATCAACCTGCTAGTTCTTTTTATGAAGCACAAACGGGAGAATTTATCCT
GGAAGCGGAAGAACTGCTGAAACTGCGTGAAAGCCTCACAAGGGTTTATGTACAAAG
AACGGGCAAACCCTTATGGGTTGTATCCGAAGACATGGAAAGAGATGTTTTTATGTCAG
CAACAGAAGCCCAAGCTTATGGAATTGTTGATCTTGTAGCGGTTGAAATGAAAGAACA
AAAATGGATTCATGAAGGTTTAATTACTGAATCGCTTCCCAATGGTATGTTCCGGGTTCG
TTTAGATAATGAAGAAATGATTCTAGGTTATGTTTCAGGAAAGATCCGACGTAGTTTTAT
ACGGATACTGCCAGGAGATAGAGTCAAAATTGAAGTAAGTCGTTATGATTCAACCAGA
GGGCGTATAATTTATCGACTCCGCAACAAGGATTCGAAGGATATGGAGGAATTCCAAAG
ATATTTACAG---GATAGATCTCAGCAACACGACTTCCTATATCCACTTATCTTTCAGGAGT
ATATTTATGCACTTGCTCATGATCATGGTTTAACAGGTTCTGACAATAAATCCAGCTTAC
TAATTGTGAAACGTTTAATTATTCGAATGTATCAACAGAATCATTTGATTATTCCTGTTAA
TGATTCTAACCAAAATCAACTTTTTGGGAAGAATTTGTATTCTCAAATGATATCAGAGGT
ATTTGCAGTCATTGTGGAAATTCCATTTTCTCTACGATTAATAGCTTCCCTGATAGTAAA
ATCTCATAATTTACGATCAATTCATTCAATATTTCCTTTTTTCGAGAACAATTTTTCACAT
CTAAATTATGTGTTAGATATGTTAATACCCCACCCCGTCCATCTGGAAATCTTGGTTCAA
ACCCTTCGCTACTGGGTAAAAGATGCCTCTTCTTTGCATTTATTACGATTCTTTCTCTAC
GAGTATCGTAATTGGAATAGTCTTATTACTAAACCCAGCTCTTCTTTTTCAAAACAAAGA
TTATTCTTTTTCTTATATAATGCTCATGTATGTGAATACGAATCTATCTTCGTCTTTCTCTG
TAACCAATCTTCTTATTTACGATCAACATCTTCTGAAGCCTTTCTTGAGCGAATCTATTT
CTATGGAAAAATAGAATGTCTTGTAGAAGTCTTTGCTAAGGTTTTTAACCTGTGGTTGT
TCAAGGATCCTAACATGCATTATGTTAGGTATCAAGGCAAATCAATTATGGCTTCAAAA
GGTACGTCTCTTTTGATGAATAAATGGAAATATTACCTTATCAATTTTTGGCAATGTCATT
TTTACCTGTGGTCTCACCCAGGAAGGATTTATATAAATCAATTATCCAATCATTCCTTTG
ACTTTATGGGCTATTTTTCAAGTGTGCGACTAAACCCCTCAATGGTACGAAGTCAAATG
CTAGAAAATTCATTTCTAATCGATAATGCTATT------AAGAAGTTCGATACCATTGTTCCA
ATTATTCCTCTGATTGGATCATTGGCTAAAGCGAAATTTTGTAACGTATTAGGGTATCCC
ATTAGTAAGCCGGTTCGGGCCGATTTATCAGATTCTGATATTATTGACCGATTTGGGCGT
ATATGCAGAAATTTTTCTCATTATCATAGCGGATCCTCAAAAAAAAAGAGTTTGTATCGA
ATAAAGTATATACTTCGACTTTCTTGTGCTAGAACTTTGGCCCGGAAACACAAAAGTAG
TGTACGTTTTTTTTTGAAAAGATTAGGCTCGGAATTATTGGAAGAATTCTTTATGGCGGA
AGAACAAGTTCTTCCTTTGACCTTCCCAAGACGGGTTTGGTATTTGGATATTTTTTGTAT
CAACGATTTGGCGAATCATGAAATGATAATTGAT---ATAACAGAAGTACAA---GCTATCA

ATTCTTTTTCCAGATTGGAATCC---TTAAAAGAGATCTATGGGATCATATGGATGCTTGTC
CCTATTTTGACTCTTGTATTAGGAATTACAATAGGCGTACTAGTAATTGTTTGGTTAGAA
AGAGAAATATCTGCAGGGATACAACAACGTATTGGACCTGAATACGCCGGCCCTTTGG
GAATTCTTCAAGCTCTAGCAGATGGTACAAAATTACTTTTCAAAGAAAATATTTTTCCAT
CTAGAGGAGATACTCGTTTATTTAGTATCGGACCATCCATAGCAGTTATATCAATTTTCCT
AAGTTATTCAGTAATTCCTTTTGGATATCGCCTTGTTCTAGCCGATCTCAGTATCGGTGTT
TTTTTATGGATCGCCATTTCAAGTATTGCTCCTGTTGGACTTCTTATGTCAGGATATGGAT
CAAATAATAAGTATTCTTTTTTAGGTGGTCTACGGGCTGCTGCTCAATCAATTAGTTATG
AAATACCATTAACTCTATGTGTGTTATCAATATCTCTACTATCTAACAGTTCAAGTACAGT
TGATATAGTTGAAGCGCAATCAAAATATGGTTTTGGGGGGTGGAATTTATGGCGTCAAC
CTATAGGGTTGATCGTTTTTCTAATTTCTTCCCTAGCCGAGTGTGAGAGATTACCTTTTG
ATTTACCAGAAGCAGAAGAAGAATTAGTAGCAGGTTATCAAACCGAATATTCAGGTATT
AAATTTGGTTTATTTTACATTGCTTCGTATCTGAATCTACTAGTTTCTTCATTATTTGTAAC
AATTCTTTACTTGGGAGGTTGGAATCTTTCTATTCCGTACATATTCGTTCCTGAGCTTTTT
GACATAAATAAAACAGGTAGAGTCTTTGGAACAATAATTGGTATCTTTATTACATTAGCT
AAAACTTATTTGTTCTTGTTCATTCCTATCACAACAAGATGGACTTTACCGAGACTGAG
AATGGACCAACTATTAAATCTTGGATGGAAATTTCTTTTACCTATTTCTCTAGGTAATTTA
TTATTAACAACTTCTTCCCAACTTTTTTCACTGATGATCTGGCATGTACAGAATGAAAAC
TTCATTCTCGATTCTACGAGAATTTTTATGAAAGCCTTTCATTTGCTTCTCTTCGATGGA
AGTTTTATTTTCCCAGAATGTATCCTAATTTTTGGCCTAATTCTTCTTCTGATGATCGATT
CAACCTCTGATCAAAAAGATATACCTTGGTTATATTTCATCTCTTCAACAAGTTTAGTAA
TGAGCATAACGGCCCTATTGTTCCGATGGAGAGAAGAACCTATGATTAGCTTTTCGGGA
AATTTCCAAACGAACAATTTCAACGAAATCTTTCAATTTCTTATTTTACTATGTTCAACT
CTATGTATTCCTCTATCCGTAGAGTACATTGAATGTACAGAAATGGCTATAACAGAGTTT
CTCTTATTCGTATTAACAGCTACTCTAGGAGGAATGTTTTTATGCGGTGCTAACGATTTA
ATAACTATCTTTGTAGCTCCAGAATGTTTCAGTTTATGCTCCTACCTATTATCTGGATATA
CCAAGAAAGATGTACGGTCTAATGAGGCTACTATGAAATATTTACTCATGGGGGGGGCA
AGCTCTTCTATTTTGGTTCATGGTTTCTCTTGGCTATATGGTTCATCCGGGGGAGAGATC
GAGCTTCAAGAAATAGTGAATGGTCTTATCAATACACAAATGTATAACTCCCCAGGAAT
TTCAATTGCGCTCATATTCATCACTGTAGGAATTGGGTTCAAGCTTTCCCCAGCCCCTTC
TCATCAATGGACTCCTGACGTATACGAAGGATCTCCCACTCCAGTCGTTGCTTTTCTTTC
TGTTACTTCGAAAGTAGCTGCTTCAGCTTCAGCCACTCGAATTTTCGATATTCCTTTTTA
TTTCTCATCAAACGAATGGCATCTTCTTCTGGAAATCCTAGCTATTTTTAGCATGATATTG
GGAAATCTCATTGCTATTACTCAAACAAGCATGAAACGTATGCTTGCATATTCGTCCATA
GGTCAAATCGGATATGTAATTATTGGAATAATTGTTGGAGACTCAAATGATGGATATGCA
AGCATGATAACTTATATGCTGTTCTATATCTCCATGAATCTAGGAACTTTTGCTTGCATTG
TATTATTTGGTCTACGTACCGGAACTGATAACATTCGAGATTATGCAGGATTATACACGA
AAGATCCTTTTTTGGCTCTCTCTTTAGCCCTATGTCTCTTATCCCTAGGAGGCCTTCCTC
CACTAGCAGGTTTTTTCGGAAAACTCTATTTATTCTGGTGTGGATGGCAGGCAGGCCTA
TATTTCTTGGTTTTAATAGGACTCCTTACAAGCGTTGTTTCTATCTACTATTATCTAAAAA
TAATCAAGTTATTAATGACTGGACGAAACCAAGAAATAACCCCTCACGTGCGAAATTAT
AGAAGATCTCCTTTAAGATCAAACAATTCCATCGAATTGAGTATGATTGTATGTGTGATA
GCATCTACTATACCAGGAATATCAATGAACCCGATTATTGCAATTGCTCAGGATACCCTT
TTTATGTTTCTGCTTTACGAATATGATATTTTTTGGGCATTTCTAATAATATCAAGTATTGT

TCCTATTTTGGCATTTGTAATTTCCGGAATTTTAGCCCCGATTAGCAAAGGACCAGAGA
AACTCTCTAGTTATGAATCGGGTATAGAACCAATGGGCGATGCTTGGTTACAATTTCGA
ATCCGTTATTATATGTTTGCTCTAGTTTTTGTTGTTTTTGATGTTGAAACGGTTTTTCTTTA
TCCATGGGCAATGAGTTTCGATGTATTGGGTGTATCTGTATTTATAGAAGCTTTCATTTTC
GTGCTTATCTTAATTGTTGGTTCAGTTTATGCATGGCGAAAGGGGGCATTGGAATGGTCT
ACGAATTATTTTCCTTGGTTAACAATAATTGTAGTTTTTCCAATATCTGCGGGTTCCTTAA
TTTTTTTTCTTCCACATAGAGGAAATAGGGTAATTCGGTGGTATACTATTTTTATATGTAT
TTTAGAACTCCTTCTAATGACTTACGTATTCCGTTATCATTTCCAATCGGACGATCCATTA
ATTCAACTGGTGGAGGATTATAAATGGATCAATTTTTTTGATTTCCGTTGGAGATTGGGA
ATTGATGGGTTTTCTATAGGACTTATTTTACTGACGGGATTCATCACCACTTTAGCTACTT
TAGCGGCTTGGCCAGTTACTCGAGATTCTCGATTATTCCATTTCCTGATGTTAGCAATGT
ACAGCGGTCAAATAGGATCATTTTCTTCTCGAGACCTTTTACTTTTTTTCATCATGTGGG
AGTTAGAATTAATTCCCATTTATCTACTTTTAGCCATATGGGGGGGAAAGAAACGTCTGT
ACTCAGCTACAAAATTCATTTTGTACACGGCGGGAGGTTCCGTTTTTCTCTTACTGGGA
GTTCTGGGTGTCGGTTTATATGGTTCTTATGAACCAACATTAAATTTTGAAACATCAGCT
AATCAATCGTATCCTGTGGCCTTGGAAATAATATTCTATATTGGGTTTTTTATTGCTTTTG
CTGTTAAATCGCCGATTATACCCCTACATACATGGTTACCAGATACCCATGGAGAAGCAC
ATTACAGTACTTGTATGCTTTTAGCCGGAATCTTATTAAAAATGGGAGCGTATGGATTGG
TGCGAATCAATATGGAATTATTACCTCACGCTCATTTTCTATTTTCTCCTTGGTTGATAAT
AGTAGGCACAATGCAAATAATTTATGCAGCTTCAACATCTTCTGGCCAACGGAATTTAA
AAAAAAGAATAGCATATTCCTCTGTGTCTCATATGGGTTTCATAATTATAGGAATTGGCT
CTATAACCGAGACAGGACTCAATGGAGCCCTTTTACAAATAATCTCTCATGGATTTATTG
GTGCTGCACTTTTTTTCTTGGCAGGAACGAGTTATGATAGAATACGTCTTGTTTATCTTG
ACGAAATGGGCGGAATAGCTATCCCAATGCCAAAAATATTTACAATGTTCAGTAGCTTT
TCGATGGCCTCCCTTGCATTACCGGGTATGAGTGGTTTTATTGCAGAATTGATAGTATTT
TTTGGAATAATTACCAGCCAAAAATTTCTTTTAATGCCAAAAATACTAATTACTTTTGTA
ATGGCAATTGGAATGATATTAACTCCTATTTATTCATTATCTATGTCACGTCAAATGTTCTA
TGGATACAAGCTATGTAATGCTCCAAACTCTTCTTTTTTTGATTCTGGACCGCGAGAGTT
ATTTCTTTCCCTCTCTATCTTTTTACCCGCAATAGGTATTGGTATATATCCGGATTTCGTTC
TTTCACTATCAGTTGATAAGGTCGAAGTTATTCTATCTCATTTTTTTTATAGAATGATGCT
CGAACATGTACTTGTTTTGAGTGCCTATTTATTTTCTATCGGTATCTATGGATTGATCACA
AGTCGGAATATGGTTAGAGCCCTTATGTGTCTTGAACTTATATTGAATGCAGTTAATCTC
AATTTTGTAACATTTTCTGATTTTTTTGATAGTCGTCAATTAAAGGGAGCCATTTTCTCA
ATTTTTGTTATAGCTATTGCAGCCGCTGAAGCGGCTATTGGCTTGGCTATTGTTTCATCA
ATTTATCGTAACAGAAAATCAACTCGTATCAACCAATCGAATTTGTTGAATAAAATGGA
ACAGACATATCAATATGCATGGATCATACCTTTCGTTCCACTTCCAGTTCCTATATTAATC
GGAGTGGGACTTCTTCTTTTTCCGACGGCAACAAAAAATCTTCGTCGTATGTGGGCTTT
TTATAGTGTTTTATTGTTAAGTATAGTCATGATTTTTTCGATCAATATATCTATTCATCAAA
TAAATAGCAGTTCTATCTATCAATATGTATGGTCTTGGATCATCAATAATGATTTTTCTTTA
GACTTTGGCTACTTGATCGATCCACTTTCTTCTATTATGTCAATATTAATCACTACTGTTG
GAATTATGGTTCTGATTTATAGTGATAATTATATGTCTCACGATCAAGGATATTTGAGATT
TTTTGCTTATATGGGTTTGTTCAGTACTTCCATGTTAGGATTAGTTACTAGTTCTAATTTG
ATACAAATTTATATTTTTTGGGAATTGGTTGGAATATGTTCGTATCTATTAATAGGGTTTT
GGTTCACACGACCTGTTGCGGCAAATGCTTCTCAAAAAGCCTTTGTAACTAATCGTGTA

GGGGATTTTGGTTTATTATTAGGAATTTTAGGTTTTTATTGGATAACAGGTAGTTTTGAAT
TTCGGGATTTATTTGAAATATTCAATAACAATGAAGTCAATTTTTTATTTGTTATTTTATG
CGCCGTTCTATTATTTGCCGGTGCAGTTGCTAAATCTGCACAATTCCCCCTTCATGTATG
GTTACCTGATGCTATGGAAGGGCCTACTCCTATTTCGGCTCTTATACATGCTGCTACTAT
GGTAGCAGCGGGAATTTTTCTTATAGCTCGGCTTATTCCTCTTTTCATAGTCATACCTTAC
ATAATGAATTTAATCTCTTTGATAGGGATAATAACAGTCTTATTAGGAGCTACTTTAGCTC
TTGCTCAAAAAGACATTAAGAGAGGTTTAGCCTATTCTACAATGTCTCAATTGGGTTATA
TGATGTTAGCTCTAGGTATGGGGTCTTATCGAAGTGCTTTATTTCATTTGATTACTCATGC
TTATTCGAAAGCTTTATTGTTTTTAGGATCCGGATCCATTATTCATTCAATGGAAACTATT
GTTGGATATTCTCCAGATAAAAGTCAGAATATGGTTCTTATGGGGGGTTTAACAAAACA
TGTGCCAATTACTAAAACTGCTTTTTTATTAGGTACACTTTCTCTTTGTGGTATTCCACCT
CTTGCTTGTTTTTGGTCCAAAGATGAAATTCTTAATGATACTTGGTTGTATTCGCCGATT
TTCGCAATAATAGCTTGGGCCACAGTGGGATTAACTGCATTTTATATGTTTCGAATCTAT
TTACTTACTTTTGAAGGACATTTAAACGTTAATTTTCAAAATTACAGTGGCAAAAAAAA
TACCTCTTTCTATTCAATATCTCTATGGGGTAAAAGAAGTTCGAACAAAATTAATAAA---
TTAGTAACTTTATTAAAAATGAATTTTTTTTCATTTGGAAATAAGAACAATTCTTCGTATC
CTTATGAATCGGACAATACTATGTTATTTCCTCTACTTGTATTGGTTCTATTTACTTTGTTC
GTTGGAGCTCTAGGAATTCCTTTCGATTTGGATATATTATCCAACTGGTTAACTCCGTCT
ATAAACCTTTTACATCAAAATTTGAAT---GATATAGATTGGTATGAATTTGTAAAAGATGC
AGTTTTTTCAATCAGTATGGCTTTTTTTGGAATATTTATAGCGTCTTTTTTATATAAACCC
GTTTATTCATCTTTACAAAATTTGGACTTACTTAATTCATTTGTTATTCTTTGGGACAAAA
TTATAAATGGCATATATGATTGGTCTTATAATCGTGCTTACATAGATTCTTTTTATACAACA
TCCTTAACTGGGGGAATAAGAAGATTGGCTGAATTAACTCATTTTTTTGATAGACGAAT
AATTGATGGAATTACGAATGGAGTGGGTGTTATGAGTTTCTTTGTAGGAGAAGGTATCA
AATATGTAGGGGGCGGG---CGCATCTCTTCTTATCTTTTCTTGTATTTATCTTATGTATCAA
TCTTTTTATTCATTTTTTCTATGGATTTACCTGGACCAATACATGATTTTCTTTTAGTCTTT
CTGGGATCGGGTCTTATATTAGGAGCTCTAGGAGTGGTATTACTTCCCAATCCAATTTAT
TCTGCCTTTTCGCTGGGATTAGTTCTTGTTTGTATATCCTTATTCTATATTCTATCGAATTC
CCATTTTGTGGCTGCTGCGCAACTCCTTATTTATGTAGGAGCTATAAATGTTTTAATCATT
TTTGCTGTGATGTTCATGAATGGCTCAGAATATTACAAAGATTTTCATCTTTGGACCATT
GGGGACGGGGTTACTTCGATGGTTTGTACAAGTATTTTTGTTTCACTAATGACTACTATT
CCAGATACGTCATGGTACGGGATTATTTGGACTACAAGATCAAACCAAATTGTAGAGCA
AGATTTGATAAGTAATAGTCAACAAATTGGGATTCATTTATCGACAGATTTTTTTCTTCC
ATTTGAACTCATTTCAATAATTCTTTTAGTTGCTTTAATAGGTGCAATTGCTGTAGCGCGT
---ATGACTGCACAAGCTACAAGAAAAGACCTCATGATAGTCAATATGGGTCCTCAACAC
CCATCAATGCATGGTGTTCTTCGACTCATCGTTACTTTAGATGGTGAAGATGTTATTGAC
TGTGAACCAATATTGGGTTATTTACACAGAGGGATGGAGAAAATTGCGGAAAACCGAA
CAATTATACAATATTTACCTTATGTAACACGTTGGGATTATTTAGCTACTATGTTCACAGA
AGCAATAACTGTAAATGCACCAGAGCAATTAGGCAATATCCAAGTACCTAAAAGGGCT
AGCTATATCAGAGTCATTATGTTGGAGTTGAGTCGTATAGCTTCCCATTTGTTATGGCTT
GGTCCTTTTATGGCGGATATTGGCGCACAAACCCCCTTCTTCTATATTTTTCGAGAAAGA
GAATTAATATATGACCTATTCGAAGCTGCCACTGGTATGCGAATGATGCATAATTATTTTC
GTATCGGAGGAGTCGCTGCTGATCTACCTCATGGCTGGATAGATAAATGTTTAGACTTTT
GCGATTATTTTTTAACAAGGATTGCTGAATATCAAAAGCTTATTACACGAAATCCTATTT

TTTTAGAACGAGTTGAAGGAGTGGGCTTTATTAGTGGAGAAGAAGCAATAAATTGGGG
TTTATCAGGACCAATGCTACGAGCTTCCGGAATACAATGGGATCTCCGTAAAGTTGATC
ATTATGAGTGTTACGACGAGTTTGATTGGGAAGTTCAATGGCAAAAAGAGGGAGATTC
ATTAGCTCGTTATTTAGTACGAATCGGTGAAATGACAGAATCCATAAAAATTATTCAACA
GGCCCTGGAAGGAATTCCAGGGGGGCCCTATGAGAATTTAGAAATACGACGCTTTGAC
AAAGTAAGAGATGCCGACTGGAATGATTTTGAATATCGATTCATTAGTAAAAAACCCTC
TCCGACTTTTGAATTGTCGAAGCAAGAACTTTATGTAAGAGTCGAAGCTCCAAAAGGA
GAATTGGGAATTTTTCTGATAGGAGATCAAAGTGTTTTTCCTTGGAGATGGAAAATTCG
CCCCCCGGGTTTTATCAATTTGCAAATTCTTCCTCAGTTAGTTAAAAAAATGAAATTGG
CTGATATTATGACGATACTAGGTAGTATAGATATCATTATGGGAGAAGTTGATCGTATGTT
CTCTATGGTAACTGAGTTCATGAATTATGGTCAACAAACAATACGAGCTGCAAGGTACA
TTGGTCAAGGTTTCATGATTACCTTATCCCATGCGAATCGTTTACCTGTAACTATTCAATA
CCCCTACGAAAAATTGATCACACCCGAACGTTTCCGGGGCCGAATCCACTTTGAATTTG
ATAAATGCATTGCTTGTGAAGTATGTGTTCGTGTATGTCCTATAGATCTACCCGTTGTTGA
TTGGAAATTCGAAACTGATATTCGAAAGAAACGATTGCTTAATTACAGTATTGATTTCG
GGATCTGTATATTTTGTGGTAATTGCGTTGAGTATTGTCCAACAAATTGTTTATCGATGA
CTGAAGAATATGAACTTTCTACTTATGATCGTCACGAATTAAATTATAATCAAATTGCTTT
GGGTCGGTTACCAATGTCAATAATTGACGATTACACAATTCGAACAATTGTAAATTCG---
---ATGCAGGGTCGTTTGTCTGCTTGGCTAGTCAAGCAT
GGGCTAATTCATAGATCTTTGGGCTTTGATTACCAAGGAATAGAAACTTTACAAATAAA
GCCCGAGGATTGGCATTCCATTGCTGTCATTTTATATGTATATGGTTACAATTATCTACGC
TCCCAATGTGCCTATGATGTAGCACCTGGCGGACTGTTAGCTAGTGTGTATCATCTTACG
AGAATAGAGTATGGTGTGGATCAACCGGAAGAGGTATGCATAAAGGTATTTACCCCAA
GGAGGAATCCTAGAATTCCGTCCGTTTTCTGGGTTTGGAAAAGTGTGGATTTTCAAGA
ACGGGAATCTTATGATATGTTGGGAATCTCTTATGATAATCATCCACGTTTGAAACGTAT
CTTAATGCCTGAAAGTTGGATAGGATGGCCCTTACGTAAGGATTATATTGCCCCCAATTT
TTATGAAATACAAGATGCTCAT---
---------------ATGGCGAAAGGGGGCATTGGAATGGTCTTAGCTCCTGAATATTCAGACAAT
AAAAAGAAAAAGGGAAAAAATAAGATTGAGACA---ATTATGAATTCCATTGAGTTTCCT
TTACTTGATCGAACAACCCCAAATTCAGTTATTTCAACTACATTAAATGATCTTTCAAAT
TGGTCAAGACTCTCTAGTTTATGGCCGCTTCTCTATGGTACCAGTTGTTGCTTCATTGAA
TTTGCTTCACTAATAGGCTCACGATTCGACTTTGATCGTTATGGACTAGTACCAAGATCG
AGTCCTAGACAAGCGGACCTAATTTTAACAGCCGGAACAGTAACAATGAAAATGGCTC
CCTCTTTAGTGAGATTATATGAGCAAATGCCTGAACCAAAATATGTTATTGCTATGGGAG
CATGTACAATTACAGGGGGGATGTTCAGTACTGATTCTTATAGTACTGTTCGGGGAGTC
GATAAACTAATTCCTGTGGATGTCTATTTGCCGGGCTGTCCACCTAAACCGGAAGCAGT
TATAGATGCTATAACAAAACTTCGTAAGAAAATATCTCGAGAAATCTATGAAGATAGAAT
TAGGTCTCAACGCGAGAATCGGTGTTTTACTACCAATCACAAGTTTCATGTTGGACGCA
GTATTCATACTGGAAAGTATGATCAAGGATTCCTCTATCAACCGCCATCTATTGCAGAGA
TCTCTCCTGAAACATTTTTCAAATACAAAAGTTCAGTATCTTCCCATAAATTAGTGAATA
TGCAAACTAGAAATACTTTTTCTTGGATAAAGGAACAGATTACTCGATCCATTTCCGTAT
CGCTCATGATATATATCATAACTCGGACATCCATTTCAAGTGCATATCCCATTTTTGCACA
GCAGGGTTATGAAAATCCACGAGAAGCGACTGGGCGTATTGTATGCGCCAATTGCCATT
TAGCTAATAAGCCCGTGGATATTGAGGTTCCACAAGCGGTACTTCCTGATACTGTATTTG

AGGCAGTTGTTCGAATTCCTTATGATATACAAATGAAACAAGTTCTTGCTAATGGGAAA
AAAGGGGGTTTGAATGTGGGGGCTGTTCTTATTTTACCGGAGGGGTTTGAATTAGCTCC
TCCCGATCGTATTTCTCCTGAGATGAAAGAAAAGATAGGCAATCTGTCTTTTCAGGGCT
ATCGCCCCAATAAAAAAAATATTCTTGTGATAGGCCCCGTTCCTGGCCAGAAATATAGT
GAAATCACCTTTCCTATTCTTTCCCCGGACCCCGCTACTAAGAAAGATGTTCACTTCTTA
AAATATCCTATATACGTAGGCGGAAACAGGGGAAGGGGTCAGATTTATCCCGATGGGAG
CAAGAGTAACAATACGGTTTATAATGCTACAGCAGCAGGTATAGTAAGCAAAATCATAC
GAAAAGAAAAGGGGGGATATGAAATAACCATAACGGATGCATCGGATGGACGTCAAGC
GGTTAATATTATCCCTCCAGGACCAGAACTTCTTGTTTCAGAAGGTGAATCTATCAAATT
TGATCAACCATTAACGAGTAATCCTAATGTGGGTGGATTTGGTCAGGGAGATGCAGAAA
TAGTACTTCAAGATCCATTACGTGTCCAAGGCCTTTTGTTCTTCTTGGCATCTGTTATTT
TGGCACAAATCTTTTTGGTTCTTAAAAAGAAACAGTTCGAGAAGGTTCAATTGTCCGA
AATGAATTTCATGAGTAAAGTCTATGATTGGTTCGAAGAACGTCTTGAGATTCAGGCGA
TTGCGGATGATATAACTAGTAAATATGTTCCTCCCCATGTCAACATATTTTATTGTCTGGG
AGGAATTACGCTTACTTGTTTTTTAGTACAAGTAGCTACAGGGTTTGCTATGACTTTTTA
CTATCGTCCGACCGTTACTGAAGCTTTTGCTTCTGTTCAATATATAATGACCGAAGCTAA
CTTTGGTTGGTTAATCCGATCAGTTCATCGATGGTCGGCAAGTATGATGGTACTAATGAT
GATCCTGCATGTATTTCGTGTGTATCTCACCGGTGGATTTAAAAAACCTCGCGAATTGA
CTTGGGTTACAGGTGTGGTTCTGGGTGTATTGACCGCATCTTTTGGCGTAACTGGTTATT
CCTTACCTCGGGACCAAATTGGTTATTGGGCAGTGAAAATTGTAACAGGTGTACCTGA
AGCTATTCCTGTAATAGGATCGCCTTTGGTAGAATTATTGCGTGGAAGTGCTAGTGTGG
GACAATCCACCTTGACTCGTTTTTATAGTTTACACACTTTTGTATTGCCGCTTCTTACTG
CCGTATTTATGTTAATGCACTTTCCAATGATACGTAAACAAGGCATTTCTGGTCCTTTAA
TGGGAGTAACAAAAAAACCTGACTTGAATGATCCTGTATTAAGAGCTAAATTGGCTAA
AGGTATGGGTCATAATTATTACGGAGAGCCCGCATGGCCCAATGATCTTTTATATATTTTT
CCAGTAGTAATTCTTGGCACTATTGCATGTAACGTAGGCTTAGCGGTTCTAGAACCATC
AATGATTGGTGAACCAGCAGATCCATTTGCAACCCCCTTGGAAATATTACCTGAATGGT
ATTTCTTTCCCGTATTTCAAATACTTCGTACAGTACCCAATAAATTGTTGGGTGTTCTTTT
AATGGTTTCAGTACCCGCCGGATTATTAACAGTACCTTTTTTAGAGAATGTTAATAAATT
CCAAAATCCATTTCGCCGTCCAGTAGCGACAACCGTCTTTTTGATTGGTACCGCAGTGG
CCCTTTGGTTGGGTATTGGTGCAACATTGCCTATTGATAAATCCCTAACTTTAGGTCTTT
TTATGATTGAAGTTTTTCTATTTGGAATCGTGTTAGGTCTAATTCCTATTACTTTGGCTGG
ATTATTCGTAACTGCATATTTACAATACAGGCGTGGTGATCAGTTGGACCTTATGCCTAC
TATAACTAGTTATTTTGGTTTTCTACTAGTGTCTTTAACTATAACCTCAGCTCTATTTATTG
GTCTGAGCAAAATACGACTTATTATGGATATAGTAAGTCTCGCTTGGGCTGCTTTAATGG
TAGTCTTTACATTTTCCCTTTCACTCGTAGTGTGGGGAAGAAGTGGGCTCATGATTATTC
GTTCGCCGGAACCAGAAGTCAAAATTTTGGTAGATAGGGATCACATAAAAACGTCTTT
CGAGGAATGGGCCAGACCGGGTCATTTCTCAAGAACAATAGCTAAAGGCCCTGATACT
ACCACTTGGATCTGGAACCTACATGCTGATGCTCACGATTTCGATAGCCATACCAGTGA
TTTGGAGGAGATTTCTCGAAAGGTATTTAGTGCCCATTTCGGTCAACTCTCCATCATCTT
TCTTTGGCTGAGTGGCATGTATTTCCACGGTGCTCGTTTTTCCAATTATGAAGCCTGGCT
AAGCGATCCAACTCACATTGGGCCTAGTGCCCAGGTGGTTTGGCCAATAGTGGGTCAA
GAAATATTGAATGGTGATGTGGGCGGGGGTTTCCGAGGAATACAAATAACCTCTGGTTT
TTTTCAGATTTGGCGAGCATCTGGAATAACTAGTGAATTACAACTCTATTGTACCGCAAT

TGGTGCATTGGTCTTTGCAGCGTTAATGCTTTTTGCTGGTTGGTTTCATTATCATAAAGC
GGCTCCAAAATTGGCTTGGTTTCAAGATGTAGAATCCATGTTGAATCACCATTTAGCGG
GGCTACTAGGACTTGGGTCTCTCGCTTGGGCGGGGCATCAAGTACATGTGTCTTTACCG
ATTAACCAATTTCTAAACGCTGGAGTAGATCCTAAAGAGATACCACTTCCTCATGAATTT
ATCTTGAATCGGGATCTTTTGGCTCAACTTTATCCCAGTTTTGCCGAGGGAGCAACCCC
ATTTTTCACCTTGAATTGGTCAAAATATGCAGAATTTCTTACTTTTCGCGGAGGATTAGA
TCCAGTAACTGGGGGTCTATGGCTAACCGATATTGTACACCATCATTTAGCTATTGCAAT
TCTTTTCCTGATAGCAGGCCACATGTATAGGACCAACTGGGGGATTGGTCATGGCCTAA
AAGATATTTTAGAAGCTCATAAGGGTCCATTTACAGGCCAGGGTCATAAAGGACTATAT
GAGATCCTAACAACGTCATGGCATGCTCAATTATCTCTTAACCTAGCTATGTTAGGCTCT
TTAACCATTGTTGTAGCTCACCATATGTATTCCATGCCCCCTTATCCATATCTAGCTACTG
ACTATGGTACACAATTGTCATTGTTCACACATCACATGTGGATTGGTGGATTTCTCATAG
TTGGTGCTGCTGCGCATGCAGCCATTTTTATGGTAAGAGACTATGATCCAACTACTCGAT
ACAACGATCTATTAGATCGTGTCCTTAGGCATCGTGATGCAATCATATCACATCTCAACT
GGGCATGTATATTTTTAGGCTTTCACAGTTTTGGTTTGTATATTCATAATGATACCATGAG
TGCTTTAGGGCGTCCTCAAGATATGTTTTCAGATACCGCGATACAATTACAACCCGTCTT
TGCTCAATGGATACAAAACACCCATGCTTTAGCACCTGGTGCAACGGCTCCTGGTGCA
ACAACAAGCACCAGTTTAACTTGGGGGGGTGGTGATTTAGTAGCAGTGGGTGGCAAG
GTGGCTTTGTTACCTATTCCATTGGGAACCGCAGATTTTTTGGTACACCACATTCATGCA
TTTACAATTCATGTAACGGTATTGATCCTCCTGAAAGGTGTTCTCTTTGCTCGTAGCTCC
CGTTTGATACCGGATAAAGCAAATCTTGGTTTTCGTTTTCCTTGTGATGGACCTGGAAG
AGGGGGGACATGTCAAGTATCGGCTTGGGATCATGTCTTCTTAGGACTATTCTGGATGT
ACAATGCAATTTCGGTAGTAATATTCCATTTCAGTTGGAAAATGCAGTCAGATGTTTGG
GGCAGTATAAGTGATCAAGGGGTAGTAACTCATATCACGGGAGGCAACTTTGCGCAGA
GTTCTATTACCATTAATGGGTGGCTCCGCGATTTCTTATGGGCACAGGCATCCCAGGTAA
TTCAGTCTTATGGTTCTTCATTATCTGCATACGGCCTGTTTTTCCTAGGTGCTCATTTTGT
ATGGGCTTTTAGTTTAATGTTTCTATTCAGCGGACGTGGTTATTGGCAAGAACTTATTGA
ATCTATCGTTTGGGCTCATAATAAATTAAAAGTTGCTCCTGCTACTCAGCCGAGAGCTTT
GAGCATTGTACAAGGACGTGCTGTAGGAGTAACCCATTACCTTCTGGGTGGAATTGCC
ACAACATGGGCGTTCTTCTTAGCAAGAATTATTGCAGTAGGAATGGCATTAAGATTTCC
AAGGTTTAGCCAAGGCTTAGCTCAGGACCCCACTACTCGTCGTATTTGGTTTGGTATTG
CTACCGCACATGACTTCGAGAGTCATGATGATATTACTGAGGAACGTCTTTATCAGAATA
TTTTTGCTTCTCACTTCGGGCAATTAGCAATAATTTTTCTGTGGACTTCCGGAAATCTGT
TTCATGTGGCTTGGCAAGGAAATTTTGAGGCATGGGTACAGGACCCTTTACATGTAAGA
CCTATTGCTCATGCAATTTGGGATCCCCATTTTGGTCAACCAGCTGTGGAAGCTTTTACT
CGAGGGGGTGCTCTTGGCCCAGTGAATATCGCTTATTCTGGTGTTTATCAGTGGTGGTAT
ACAATCGGTTTACGCACTAATGAGGATCTTTATACTGGAGCTTTTTTTCTATTATTTCTTT
CTGCTATATCCTTAATAGCAAGTTGGTTACACTTACAACCGAAATGGAAACCGAGTGTT
TCGTGGTTCAAAAATGCCGAATCTCGTCTCAATCATCATTTGTCAGGACTCTTCGGCGT
AAGTTCCTTGGCTTGGACAGGGCATTTAGTACATGTCGCTATTCCTGGATCCAGAGGGG
AGTACGTCCGATGGAATAATTTCTTAGATGTATTACCGCACCCCCAAGGATTAGGCCCA
CTTTTTACAGGTCAGTGGAATCTTTATGCTCAAAACCCAGATTCAAGTAGTCATTTATTT
GGCACCTCCCAAGGAGCAGGAACCGCCATTCTTACTCTTCTCGGGGGATTCCATCCAC
AAACGCAAAGTTTATGGCTGACTGATATGGCTCATCATCATTTAGCTATTGCATTTATTTT

TCTCGTTGCTGGTCATATGTATAGAACTAACTTCGGGATTGGGCACAGTATGAACGATCT
TTTAGATGCACATATTCCTCCCGGGGGGAGGTTGGGACGTGGACATAAGGGTCTTTATG
ACACAATCAATAACTCGCTTCATTTTCAATTAGGCCTTGCTCTAGCTTCTTTAGGGGTTA
TTACTTCCTTGGTAGCTCAACACATGTATTCTTTACCTGCTTATGCATTCATAGCACAAG
ACTTTACTACTCAAGCTGCGTTATATACTCATCATCAATATATCGCGGGATTCATCATGAC
GGGAGCTTTTGCTCATGGAGCTATATTCTTCATTAGAGATTACAATCCGGAGCAGAATG
AAGATAATGTATTGGCAAGAATGTTAGACCATAAAGAAGCTATCATATCTCATTTAAGTT
GGGCCAGCCTCTTTCTGGGATTCCATACCTTGGGACTTTATGTTCATAATGATGTCATGC
TTGCCTTTGGTACTCCGGAGAAACAAATTTTGATCGAACCTATATTTGCTCAATGGATAC
AATCTGCTCATGGTAAAACTTCATACGGGTTCGATGTACTTTTATCTTCAACGAGTGGTC
CGGCATTCAATGCCGGTCGAAGCATCTGGTTGCCCGGTTGGTTAAATGCTGTTAATGAG
------AATAGTAATTCACTATTCTTAACAATAGGTCCTGGAGACTTTTTGGTTCATCATGCT
ATTGCTCTGGGTTTACATACAACTACATTGATCTTAGTAAAAGGTGCTTTAGATGCACGT
GGTTCCAAGTTAATGCCAGATAAAAAGGATTTCGGTTATAGTTTTCCGTGCGATGGCCC
AGGACGAGGCGGTACTTGTGATATTTCAGCTTGGGACGCATTTTATTTGGCAGTTTTTT
GGATGTTAAATACCATTGGATGGGTTACTTTTTATTGGCATTGGAAGCACATCACATTAT
GGCAGGGTAACGTTTCGCAGTTTAATGAATCTTCCACTTATTTGATGGGCTGGTTAAGA
GATTATTTATGGTTAAACTCTTCACAACTTATCAATGGATATAACCCTTTTGGTATGAATA
GTTTATCGGTTTGGGCGTGGATGTTCTTATTTGGACATCTTGTTTGGGCTACTGGATTTA
TGTTCTTAATTTCCTGGCGTGGGTATTGGCAGGAATTGATTGAAACTTTAGCATGGGCT
CATGAACGCACACCTTTGGCCAATTTGATTCGCTGGAGAGATAAACCAGTGGCCCTTTC
CATTGTGCAAGCAAGATTGGTTGGATTAGCCCACTTTTCTGTAGGTTATATATTCACTTA
TGCAGCTTTCTTGATTGCCTCTACGTCGGGCAAATTTGGTATGTCACATTCAGTAAAGAT
TTATGATACATGTATAGGGTGTACTCAATGTGTCCGAGCCTGCCCCACTGATGTATTAGA
AATGATACCCTGGGACGGATGTAAAGCTAAACAAATTGCTTCTGCTCCAAGAACAGAG
GATTGTGTCGGTTGTAAGAGATGCGAATCTGCCTGTCCAACGGATTTCTTGAGTGTTCG
AGTTTATTTAGGGAATGAATCAACTCGTAGCATGGGTCTAGGTTATATGACAACTTTCAA
CTTTCCCTCCATTTTTGTGCCTTTAGTAGGCCTAGTATTTCCGGCAATTGCAATGGCTTC
TTTATCTCTTCATGTTCAAAAAAACAAGATTGTTATGCGAGATCTAAAAACATATCTCTC
CGTGGCACCGGTACTAAGTACGCTATGGTTGGGGTCTTTAGCGGGTCTATTGATAGAGA
TTAATCGTTTTTTCCCGGATGCGTTGACATTCCCCTTTTTTTCATTCATGACTGCAATTTT
AGAGAGACGCGAAAGCGAAAGCCTATGGGGTCGTTTCTGTAACTGGATAACCAGCACT
GAAAACCGTCTTTACATTGGATGGTTTGGTGTTTTGATGATCCCTACCTTATTGACCGCA
ACTTCTGTATTTATTATCGCCTTCATTGCTGCCCCTCCAGTAGATATTGATGGTATTCGTG
AACCTGTTTCTGGATCTCTACTTTATGGAAACAATATTATCTCAGGTGCCATTATTCCGA
CTTCTGCAGCTATAGGTTTGCATTTTTACCCAATATGGGAAGCGGCATCCGTTGATGAAT
GGTTATACAACGGCGGTCCTTATGAACTAATTGTTCTACACTTCTTACTTGGTGTAGCTT
GTTACATGGGTCGTGAGTGGGAGCTTAGTTTCCGTCTGGGTATGCGACCTTGGATTGCT
GTTGCATATTCAGCTCCTGTTGCAGCTGCTACCGCTGTTTTCTTGATCTACCCAATTGGT
CAAGGAAGTTTTTCTGATGGTATGCCTCTAGGAATCTCTGGTACTTTCAACTTCATGATT
GTATTCCAGGCTGAGCACAACATCCTTATGCACCCATTTCACATGTTAGGCGTAGCTGG
TGTATTCGGCGGCTCCCTATTCAGTGCTATGCATGGTTCCTTGGTAACTTCTAGTTTGAT
CAGGGAAACCACAGAAAATGAATCTGCTAATGAAGGTTACAGATTCGGTCAAGAGGG
AGAAACTTATAATATCGTAGCCGCTCATGGTTATTTTGGTCGATTGATCTTCCAATATGCT

AGTTTCAACAACTCTCGTTCGTTACATTTCTTCCTAGCTGCTTGGCCTGTAGTAGGTATC
TGGTTTACCGCTTTAGGTATCAGCACTATGGCTTTCAACCTAAATGGTTTCAATTTCAAC
CAATCTGTAGTTGATAGTCAAGGTCGTGTAATTAACACTTGGGCTGATATCATTAACCGT
GCTAACCTTGGTATGGAAGTTATGCATGAACGTAATGCTCATAACTTTCCTCTAGACCTA
GCTGCTATTGAAGCTCCATCTACAAATGGAATGGGTTTGCCTTGGTATCGTGTTCATACC
GTTGTATTGAATGATCCCGGCCGGTTGCTTTCTGTCCATATAATGCATACAGCTCTGGTT
GCTGGTTGGGCTGGTTCGATGGCTCTGTATGAATTAGCAGTTTTTGATCCTTCTGACCCT
GTTCTTGATCCAATGTGGAGACAGGGCATGTTCGTTATACCCTTCATGACCCGTTTAGG
AATAACCAACTCATGGGGCGGTTGGAGTATTACA---GGAGGGGCTATAACGAATCCGGG
TATTTGGAGTTACGAAGGTGTAGCCGGGGCACATATTGTGTTTTCTGGTTTATGCTTCTT
GGCAGCTATCTGGCATTGGGTGTATTGGGATCTAGAAATATTTTGTGATGAACGTACAG
GAAAACCTTCTTTGGATTTGCCCAAAATCTTTGGAATTCATTTATTTCTCTCAGGGGTGG
CTTGCTTTGGTTTTGGTGCATTTCATGTAACAGGTTTGTATGGTCCTGGAATATGGGTGT
CCGATCCTTATGGACTAACAGGAAAAGTACAACCTGTAAATCCAGCGTGGGGCGTGGA
AGGTTTTGATCCTTTTGTTCCGGGAGGAATAGCCTCTCATCATATTGCAGCAGGGACAT
TGGGCATATTAGCGGGTCTATTCCATCTTAGCGTCCGCCCGCCACAACGTTTATACAAA
GGATTACGTATGGGGAATATTGAAACCGTCCTTTCCAGTAGTATCGCTGCTGTCTTTTTT
GCAGCTTTTGTTGTTGCCGGAACTATGTGGTATGGTTCAGCAACTACCCCCATCGAATT
ATTTGGGCCCACTCGTTATCAATGGGATCAGGGGTACTTCCAGCAAGAGATATATCGAA
GAGTTAGTGCTGGGCTAGCTGAAAATCGAAGTTTATCAGAAGTCTGGTCTAAAATTCCT
GAAAAATTAGCTTTTTATGATTACATCGGCAATAATCCGGCAAAAGGGGGATTATTCAG
AGCGGGCTCAATGGATAACGGGGATGGAATAGCGGTCGGATGGTTAGGACACCCTATC
TTTAGAGATAAAGAAGGGCGTGAACTTTTTGTACGTCGTATGCCTACTTTTTTTGAAAC
ATTTCCGGTCGTTTTGGTAGACGGCGACGGAATTGTTAGAGCCGATGTTCCTTTTAGAA
GGGCAGAATCGAAGTATAGTGTCGAACAAGTAGGTGTAACTGTTGAATTCTACGGCGG
TGAACTCAATGGAGTCAGTTATAGTGATCCCGCTACTGTGAAAAAATATGCTAGACGTG
CTCAATTGGGTGAAATTTTTGAATTAGATCGTGCTACTTTGAAATCTGATGGTGTTTTTC
GTAGCAGTCCAAGGGGTTGGTTTACTTTTGGGCATGCTTCATTTGCTTTGCTCTTCTTCT
TCGGACACATTTGGCATGGTGCTAGAACCTTGTTCAGAGATGTTTTTGCTGGTATTGAC
CCAGATTTGGATGCTCAAGTAGAATTTGGAGCATTCCAAAAACTTGGAGATCCAACTA
CAAGACGACAAGTAGTT-----------------------------------GTGGAAACGCTCTTTAATGGAAC
TTTAGCTTTAGCTGGTCGTGACCAAGAAACCACCGGTTTCGCTTGGTGGGCCGGCAAT
GCCCGACTTATCAATTTATCCGGTAAACTACTAGGGGCTCATGTCGCCCATGCCGGATTA
ATCGTATTCTGGGCCGGAGCAATGAACCTATTTGAGGTGGCTCATTTCGTACCAGAAAA
ACCTATGTATGAACAAGGATTAATTTTACTTCCCCACCTAGCTACGCTAGGTTGGGGGGT
AGGTCCTGGGGGGGAAGTTATAGACACCTTTCCATACTTTGTATCTGGAGTACTTCATTT
AATTTCCTCTGCAGTATTGGGATTTGGCGGTATTTATCATGCACTTCTGGGACCTGAGAC
CCTTGAAGAATCTTTTCCATTCTTCGGTTATGTATGGAAAGATAGAAATAAAATGACCA
CAATTTTAGGTATTCACTTAATCTTGTTAGGTATAGGTGCTTTTCTTCTAGTATTCAAGGC
TCTTTATTTTGGGGGCATATATGATACCTGGGCTCCGGGAGGGGGAGATGTAAGAAAAA
TTACTAACTTGACCCTTAGCCCAAGTATTATATTTGGTTATTTACTAAAATCGCCCTTTGG
GGGGGAAGGATGGATTGTTAGTGTGGATGATTTGGAAGATATAATCGGAGGGCATGTAT
GGTTAGGTTCCATTTGTATACTTGGTGGAATCTGGCATATCTTAACCAAACCCTTCGCGT
GGGCTCGACGTGCACTTGTATGGTCTGGAGAGGCTTACTTATCTTATAGTTTAGGGGCT

TTAGCTGTCTTTGGTGTCATTGCTTGTTGTTTTGTCTGGTTCAATAATACCGCTTATCCTA
GTGAGTTTTACGGCCCCACTGGACCAGAAGCTTCTCAAGCTCAAGCATTTACTTTTCTA
GTTAGAGACCAACGTCTTGGGGCTAACGTGGGATCCGCTCAAGGACCTACTGGGTTAG
GTAAGTATCTAATGCGTTCCCCGACTGGAGAGGTCATTTTTGGAGGAGAAACTATGCGT
TTTTGGGATCTGCGTGCTCCCTGGTTAGAACCTCTAAGGGGTCCAAATGGGTTGGACTT
AAGTAGGCTGAAAAAAGATATACAACCTTGGCAAGAACGGCGTTCCGCAGAATATATG
ACTCATGCTCCTTTAGGTTCTTTAAATTCCGTGGGTGGCGTGGCTACCGAAATCAATGC
AGTCAATTATGTCTCTCCTAGAAGTTGGTTAGCTACCTCTCATTTTGTTCTAGGCTTCTT
CCTCTTCGTAGGTCATTTGTGGCACGCGGGAAGGGCTCGTGCAGCTGCAGCAGGATTT
GAAAAAGGAATTGATCGTGATTTTGAACCTGTTCTTTCCATGACCCCTCTTAATATGACT
ATAGCCCTTGGTAAATTTACCAAAGACGAAAAT---TTATTTGATATTATGGATGACTGGTT
ACGGAGGGACCGTTTCGTTTTTGTAGGCTGGTCCGGTCTATTGCTCTTTCCTTGTGCCTA
TTTTGCTTTAGGGGGTTGGTTCACAGGTACAACCTTTGTAACCTCATGGTATACCCATG
GATTGGCTAGTTCCTATTTGGAAGGCTGCAATTTCTTAACTGCCGCAGTCTCTACTCCTG
CTAATAGTTTAGCACATTCTTTGTTGTTACTATGGGGTCCTGAAGCACAAGGAGATTTTA
CTCGTTGGTGTCAATTAGGCGGTCTGTGGATTTTTGTTGCTCTCCATGGCGCTTTCGGA
CTAATAGGTTTCATGTTACGTCAATTCGAGCTTGCTCGATCTGTTCAATTGCGACCTTAT
AATGCAATCGCATTCTCTGCTCCAATTGCTGTTTTTGTTTCTGTATTTCTGATTTATCCAC
TAGGTCAATCTGGTTGGTTCTTTGCGCCTAGTTTTGGTGTAGCAGCTATATTTCGATTCA
TCCTCTTTTTTCAAGGGTTTCATAATTGGACGTTGAACCCATTTCATATGATGGGAGTTG
CCGGTGTATTGGGCGCTGCTTTGCTATGCGCTATTCATGGTGCTACTGTAGAAAATACTT
TATTTGAAGATGGTGATGGCGCAAATACATTCCGTGCTTTTAACCCAACTCAAGCTGAA
GAAACTTATTCAATGGTCACTGCTAACCGCTTTTGGTCCCAAATCTTTGGGGTTGCTTT
TTCCAATAAACGTTGGTTACATTTCTTTATGTTATTTGTACCAGTAACCGGTTTATGGATG
AGTGCTCTTGGAGTAGTCGGTCTGGCCCTGAACCTACGTGCCTATGACTTCGTTTCTCA
GGAAATTCGTGCGGCGGAAGATCCTGAATTTGAGACTTTCTACACCAAAAATATTCTCT
TAAATGAAGGTATTCGTGCTTGGATGGCGGCTCAAGATCAGCCTCATGAAAACCTTATA
TTCCCTGAGGAGGTTCTACCACGTGGAAACGCTCTTATGTCTGGAAGCACAGGAGAAC
GTTCTTTTGCTGATATTATTACCAGTATTCGATACTGGGTCATTCATAGCATTACTATACCT
TCCCTATTCATTGCGGGTTGGTTATTCGTCAGCACCGGTTTAGCTTACGATGTGTTTGGA
AGCCCTCGGCCAAACGAGTATTTCACAGAGAGCCGACAAGGAATTCCATTAATAACTG
GCCGTTTTGATTCTTTGGAACAACTCGATGAATTTAGTAGATCTTTTATGACTATAGATC
GAACCTATCCAATCTTTACAGTGCGATGGTTGGCTGTTCACGGCCTAGCTGTACCTACC
GTTTCTTTTTTGGGGTCAATATCAGCAATGCAGTTCATCCAACGAATGGCTACACAAAC
TGTTGAGAACAGTTCTAGATCTGGT---CCAAGACGAACTACTGTAGGGAGTTTATTGAA
ACCATTGAATTCGGAATATGGTAAAGTAGCTCCTGGATGGGGAACGACTCCTTTGATGG
GTGTTGCAATGGCTCTATTTGCGATATTTCTATCTATTATTTTGGAGATTTATAATTCTTCC
GTTTTATTGGATGGAATTTCAATGAATATGCTTACTCTTAAACTCTTCGTTTACACAGTA
GTGATATTCTTTGTTTCTCTCTTCATCTTTGGATTCTTATCTAATGATCCAGGACGTAATC
CCGGACGTGAAGAAATGGCCGATACTACTGGAAGGATTCCTCTTTGGATAATAGGTACT
GTAACTGGTATTCTTGTGATCGGTTTAATAGGTATTTTCTTTTATGGTTCATATTCCGGAT
TGGGTTCATCTCTGATGCTTAATATCTTTAGTTTGATCTGTATCTGTCTTAATTCTGCCCT
TTAT---AGTAGTTTTTTCTTCACCAAATTGCCCGAGGCATATGCTTTTTTGAATCCAATCG
TAGATGTTATGCCAGTCATACCTCTGTTCTTTTTTCTCTTAGCCTTTGTTTGGCAAGCTG

CGGTAAGTTTTCGAATGACACAATCAAACCCGAACGAACAAAATGTTGAATTGAATCG
TACCAGTCTCTACTGGGGGTTATTACTCATCTTTGTACTTGCTGTTTTATTTTCCAATTAT
TTCTTCAATATGGAAGTCAATATTCTTGCATTTATTGCTACTGCACTGTTCATTCTAGTTC
CTACTGCCTTTCTACTTATCATTTACGTAAAAACAATCAGTCAAAATGATATGGAAACAG
CAACCCTAGTTGCCATCTTTATATCTGGTTTACTTGTAAGTTTTACTGGGTACGCCTTATA
TACCGCTTTTGGGCAACCTTCTCAACAACTAAGAGATCCTTTTGAGGAACATGGGGAC
ATGGAAGCATTGGTTTATACATTCCTCTTAGTCTCGACTCTAGGGATAATTTTTTTCGCTA
TCTTTTTTCGAGAACCGCCTAAAGTTCCAACTAAAAAGATGAAAATGACTCTTGCTTTC
CAATTGGCTGTTTTTGCATTAATTGCTACTTCATCAATCTTATTGATTAGTGTACCCGTTG
TATTTGCTTCTCCTGATGGTTGGTCAAGTAACAAAAATGTTGTATTTTCTGGTACATCAT
TATGGATTGGGTTAGTCTTTCTGGTAGGTATCCTTAATTCTCTCATCTCT--------------------
ATGTCACCACAAACAGAGACTAAAGCAAGTGTTGGATTCAAAGCTGGTGTTAAAGATT
ACAAATTGACTTATTATACTCCTGACTATGAAACCAAAGATACTGATATCTTGGCAGCAT
TCCGAGTAACTCCTCAACCTGGAGTTCCACCTGAAGAAGCAGGGGCTGCAGTAGCTG
CCGAATCTTCTACTGGTACATGGACAACTGTGTGGACCGATGGACTTACCAGCCTTGAT
CGTTACAAAGGGCGATGCTACCACATTGAGCCCGTTGCTGGAGAAGAAAGTCAATTTA
TTGCTTATGTAGCTTACCCTTTAGACCTTTTTGAAGAAGGTTCTGTTACTAACATGTTTA
CTTCCATTGTGGGTAATGTATTTGGGTTCAAAGCCCTGCGCGCTCTACGTCTGGAAGAT
CTGCGAATCCCTGTTGCTTATACTAAAACTTTCCAAGGCCCGCCTCATGGTATCCAAGT
TGAGAGAGATAAATTGAACAAGTATGGTCGTCCCCTGTTGGGATGTACTATTAAACCTA
AATTGGGATTATCTGCTAAAAACTACGGTAGAGCAGTTTATGAATGTCTTCGCGGTGGA
CTTGATTTTACCAAAGATGATGAGAACGTGAACTCCCAACCATTTATGCGTTGGAGAGA
TCGTTTCTTATTTTGTGCCGAAGCAATTTATAAAGCACAGGCTGAAACAGGTGAAATCA
AAGGGCATTACTTGAATGCTACTGCAGGTACATGCGAAGATATGATGAAAAGGGCTGTA
TTTGCTAGAGAGTTGGGAGTTCCTATCGTAATGCATGACTACTTAACAGGGGGATTCAC
TGCAAATACTAGCTTGGCTTTTTATTGCCGAGATAATGGACTACTTCTTCACATCCACCG
CGCAATGCATGCAGTTATTGATAGACAGAAGAATCATGGTATGCACTTCCGTGTACTAG
CTAAAGCGTTACGTATGTCTGGCGGAGATCATATTCACGCTGGTACCGTAGTAGGTAAA
CTTGAAGGGGAAAGAGAAATAACTTTGGGCTTTGTTGATTTACTGCGTGATGATTTTAT
TGAAAAAGATCGAAGTCGCGGTATTTATTTCACTCAAGATTGGGTCTCTCTACCAGGTG
TTCTGCCCGTGGCTTCGGGGGGTATTCACGTTTGGCATATGCCTGCTCTGACCGAGATC
TTTGGAGATGATTCCGTACTACAGTTCGGTGGAGGAACTATAGGACACCCTTGGGGAA
ATGCACCGGGTGCCGTAGCTAATCGAGTAGCTCTAGAAGCATGTGTACAAGCTCGTAAT
GAGGGACGTGATCTTGCTCGTGAGGGTAATGAAATTATCCGTGAGGCTTCCAAATGGA
GTCCTGAACTAGCTGCTGCTTGTGAGGTATGGAAGGAGATCAAATTTGAATTTCCAGC
AATGGATACTTTG------ATGGCGATACATTTATACAAAACTTCTACCCCGAGCACACGCA
ATGGAACCGTAGACAGTCAAGTGAAATCCAATCCACGAAATAATTTGATCTCTGGACA
GCATCGTTGTGGTAAAGGTCGTAATGCCAGAGGAATCATTACCGCAGGGCATAGAGGG
GGAGGTCATAAGCGTCTATACCGTAAAATCGATTTTCGACGGAATGAAAAAGACATATA
TGGTAGAATCGTAACCATAGAATACGACCCTAATCGAAATGCATACATTTGTCTCATACA
CTATGGGGATGGTGAGAAGAGATATATTTTACATCCCAGAGGGGCTATAATTGGAGATA
CCGTTGTTTCTGGTACAGAAGTTCCTATAAAAATGGGAAATGCCCTACCTTTGACCGAT
ATGCCCTTAGGCACGGCCATACATAACATAGAAATCACACTTGGAAAGGGTGGACAATT
AGCTAGAGCAGCGGGTGCTGTAGCGAAACTGATTGCAAAAGAGGGGAAATCGGCCAC

ATTAAAATTACCTTCTGGGGAGGTCCGTTTGATATCCAAAAACTGCTCAGCAACAGTCG
GACAAGTGGGGAATGTTGGAGTGAACCAGAAAAGTTTGGGTAGAGCCGGATCTAAGC
GTTGGCTAGGTAAGCGTCCTGTAGTAAGAGGAGTAGTTATGAACCCTGTAGACCATCCC
CATGGGGGTGGTGAAGGGAAGGCCCCAATTGGTAGAAAAAAACCCACAACCCCTTGG
GGTTATCCTGCACTTGGAAGAAGAAGTAGAAAAAGGAATAAATATAGTGATAATTTGAT
TCTTCGCCGCCGTAGTAAAATGATTCAACCTCAGACCTATTTGAATGTAGCGGATAACA
GCGGGGCCCGCGAATTGATGTGTATTCGAATCATAGGAGCTAGC---AATCGCCGATATGC
TCATATTGGTGACGTTATTGTTGCTGTGATCAAAGAAGCAGTACCCAATATGTCCCTAGA
AAGATCAGAAGTGGTCAGAGCTGTAATTGTACGTACCTGTAAAGAACTCAAACGTGAC
AACGGTATGATAATACGATATGATGACAATGCTGCAGTTGTCATTGATCAAGAAGGAAA
TCCAAAAGGAACTCGAGTTTTTGGTGCGATCGCCCGGGAATTGAGACAGTTAAATTTT
ACTAAAATAGTTTCATTAGCTCCCGAGGTATTAATGCTTAGTCCCAAAAGAACCAGATT
CCGTAAACAACATAGAGGAAGAATGAAGGGAATATCTTATCGAGGTAATCATATTTGTT
TCGGTAAATACGCTCTTCAGGCACTTGAACCTGCTTGGATCACATCTAGACAAATAGAA
GCAGGTCGACGAGCAATGACACGAAATGCACGTCGTGGTGGAAAAATATGGGTACGTA
TATTTCCAGACAAACCTGTTACAATAAGACCCACAGAAACACGTATGGGTTCCGGGAA
AGGATCCCCTGAATATTGGGTAGCTGTTGTTAAACCCGGCCGAATACTTTATGAAATGG
GTGGAGTAACAGAAAATATAGCCAGAAGGGCTATTTCAATAGCAGCATCCAAAATGCCT
ATACGAACTCAATTTATTATTTCGGGAATGACCAGAATTAAACGGGGATATATAGCTCGG
AGACGTAGAACAAAAATTCGTTTATTTGCATCCAGCTTTCGAGGAGCTCATTCAAGGCT
TACTAGAACTATTTCTCAACAGAAAATAAGAGCTTTGGTTTCGGCTCATCGGGATAGGA
ATAAGCAAAAGAGAAATTTTCGTCGTTTGTGGATCACTCGGATAAACGCAGTAATCCGC
GAA---AGGGGAGTATCCTATAGTTATAGTCGATTAATACATGATCTGTACAAGAGACAGT
TGCTTCTTAATCGTAAAATACTTGCACAAATAGCTATATCAAATAGGAATTGTTTTTATAT
GATTTCCAACGAGATCATAAAAGAAGTAGATTGGAAGGAATCCACCGGAATAATTATGC
TAAAGGAAGTATACGCTTTAGGTCAACATATATGTATGTCTGCGGACAAAGCACGAAGA
GTAATTGATCAAATTCGTGGACGTTCCTATGAGGAAACACTTATGATACTAGAACTCAT
GCCTTATCGAGCATGTTATCCCATTTTTAAATTGGTTTATTCGGCAGCAGCAAATGCTAG
TTACAATATGGGTTCTAACGAAGCCGATTTAATCATTAGTAAAGCCGAAGTCAACGAGG
GTACTACCGCGAAGAAATTTAAACCCCGAGCTAGGGGACGTAGTTTTCCGATAAAAAG
ACCTACCTGTCATATAACTATTGTAGTGAAAGATATCTCCTTAGATGGGGGAGTATGGGA
CAAAAAAATGGATGGAATCAAATATGCAGTATTTACAGACAAAAGTATTCGGTTATTGG
GGAAAAAT---CAATATACTTCTAATGTCGAATCAGGATCAACTAGGACAGAAATAAAGC
ATTGGGTCGAACTCTTCTTTGGTGTCAAGGTAATAGCTATGAATAGTCAGCGACTTCCG
GGAAAGGGTAGAAGAATGGGACCTATTATGGGACATACAATGCATTACAGACGTATGAT
CATTACGCTTCAACCGGGTTATTCTATTCCACCTCTTAGAAAGAAAAGAACTATGGCAG
TTCCAAAAAAACGTACTTCTATATCAAAAAAGCATATTCGTAAAAATATTTGGAAAAGG
AAAGGATATTGGGCAGCGTTAAAAGCTTTTTCGTTAGGAAAATCTCTTTCTACCGGGAA
TTCAAAAAGTTTTTTTGTGCAACAAACACAAACAATGGCCAAGGGGAAAGATGTCCG
AGTAACGGTTATTTTGGAATGTACCAGTTGTGCCCGAAACGGTGTTAAT------AAGGTAT
CAAGGGGCATTTCCAGATATATTACTCAAAAGAACCGGCGCAATACGCCAAATCGATTG
GAATTGAGAAAATTCTGTCCCTATTGTTACAAACATACAAGTCATGGGGAGATAAAGAA
AATGAAAATAAGGGCTTCTGTTCGTAAAATTTGTGAAAAATGTCGACTAATCCGTAGGC
GAGGACGAATTAGAGTAATTTGTTCCAACCCGAGACATAAACAAAGACAAGGAATGGT

TCGAGAGAAAGTAACAGTATCTACTCGGACACTGCAGTGGAAGTGTGTTGAATCAAGA
GCAGACAGTAAGCGTCTTTATTATGGGCGCTTTATTCTGTCTCCACTTATGAAAAGTCA
AGCCGACACAATAGGCATTGCGATGCGAAGAGCTTTGCTTGGAGAAATAGAAGGAACA
TGTATCACACGCGCAAAATCTGAG------AAAATACCACACGAATATTCTACCATAGTGGG
TATTCAAGAATCAATACATGAAATTTTAATGAATTTGAAAGAAATTGTATTGAGAAGTAA
TCTATATGGAACTTGTGACACATCTATTTGTGTCAGAGGTCCTGGATATATAACTGCTCA
AGATATCATCTTACCGCCTTATGTAGAAATCGTTGATAAGACACAGCATATAGCTAGCTT
GACAGAACCCATTGATTTGTGTATTGGATTACAAATCGAGAGGAATCGCGGATATCTTAT
AAAAACGCCAAAT---AACTTTGATGGAAGTTAT------CCCATAGACGCTGTATTCATGCCT
GTTCGAAACGTGAATTATAGTATTCATTCTTATGGGAATGGGAATGAAAAACAAGAGAT
ACTTTTTCTCGAAATATGGACAAATGGAAGTTTAACTCCGAAAGAAGCACTTCATGAA
GCCTCCCGAAATTTGATTGATTTATTTATCCCCTTTCTACATATGGAAGAAGAA------AAC
TTACATTTAGAGGACAATCAA---CACACGGTTCCTTTATCCCCTTTTACCTTTCATGATAA
ATTGGCT---AAACTAAGAAAAAACAAAAAC---AAAATAGCATTGAAATCGATTTTTATTG
ATCAATCAGAATTGCCTCCCAGGATCTATAATTGCCTCAAAAGGTCCAATATATATACATT
ATTGGACCTTTTGAATAACAGCCAAGAAGATCTTATGAAAATTGAACAGTTTCGCATAC
AAGATGTAAAACAGATATTGGGCATTCTGGAAAAACAT------TTTGCAATTGACGAAAA
AGAAGTTTTAATGCTCCGGGATGGA---AATGAGGGAATGTCTACAATACCTGGGTTTAA
TCAGATACAATTTGAAGGATTTTGTAGGTTCATTGATCAGGGCTTGACGGAAGAACTTT
CTAAGTTTCCAAAAAATTGAAGATACAGATCAAGAAATAGAATTTCAATTATTTGTGGAA
ACATATCAATTGGTAGAACCGTTGATAAAAGAAAGAGATGCTGTGTATGAATCACTCAC
CTATTCTGCGGAATTATATGTATCCGCGGGATTAATTTGGAAAACCAGTAGGGATATGCA
AGAACAAACAATTTTTATTGGAAACATTCCTCTAATGAATTCCCTGGGAACTTCTATAGT
AAATGGAATATATAGAATTGTGATCAATCAAATATTGCAAAGCCCCGGGATTTATTACCG
GTCAGAATTGGACCATAACGGAATTTCGGTTTATACCGGCACCATAATATCAGATTGGG
GAGGAAGATCAGAATTAGAGATTGATAGAAAAGCAAGGATATGGGCTCGTGTGAGTAG
GAAACAAAAAATATCTATTCTAGTTCTATCATCAGCTATGGGTTCGAATCTAAGAGAAAT
TCTAGAGAATGTTTGCTATCCTGAAATTTTTTTGTCTTTTCTGAATGATAAGGAGAGAAA
AAAGATTGGGTCAAAAGAAAATGCCATTTTGGAGTTTTATCAACAATTTGCTTGTGTAG
GCGGGGATCCGGTCTTTTCTGAATCATTATGTAAGGAATTACAAAAGAAATTCTTTCAA
CAAAGATGTGAATTAGGAAGGATTGGTCGACGAAATATGAACCGGAGACTGAACCTTG
ATATACCCCAGAACAATACATTTTTGTTACCGCGAGATATATTGGCGGCCGCGGATCATT
TGATTGGAATTAAATTTGGAATGGGTACACTTGACGAT------ATGAATCATTTGAAAAATA
AACGTATTCGTTCTGTAGCGGATCTTTTACAAGATCAATTCGGATTGGCTCTGGTTCGTT
TAGAAAATGCGGTTCGAGGAACTATATGCGGAGCAATTAGG---CATAAATTG---ATCCCG
ACCCCTCAGAATTTGGTAACTTCAACTCCA------------TTAACAACTACTTATGAATCTTTT
TTCGGTTTATACCCATTATCTCAAGTTTTGGATCGAACTAATCCATTGACACAAATAGTT
CATGGGAGAAAATTGAGTTATTTGGGTCCTGGAGGACTGACAGGGCGAACTGCTAGTT
TTCGGATACGAGATATCCATCCTAGTCACTATGGGCGTATTTGCCCAATTGATACATCTG
AAGGAATCAATGTTGGACTTATTGGATCCTTAGCAATTCATGCGAGGATTGGTCATTGG
GGG---TCTCTAGAAAGCCCGTTTTAT---------AAAATTTCTGAGAGATCAACAGGG------GT
ACGGATCCTTTATTTATCACCAGGTAGAGATGAATACTATAGGGTAGCGGCAGGAAATT
CTTTGGCCTTGAATCAGGGTATTCAGGAAGAACAGGTTGTTCCAGCTCGATACCGTCA
AGAATTCCTGACTATTGCATGGGAACAGGTTCATCTTCGAAGTATTTTTCCCTTCCAATA

TTTTTCTATTGGTGCTTCTCTCATTCCTTTTATCGAACATAATGATGCGAATCGGGCTTTA
ATGAGTTCTAATATGCAACGTCAAGCAGTTCCCCTTTCTCGGTCTGAGAAATGCATTGT
TGGAACTGGGTTGGAACGCCAAGCGGCTCTAGATTCAGGGGCTCTTGCTATAGCCGAA
CGTGAGGGGAAGATCATTTATACTGATACTGATAAGATCCTTTTATCAGGTAATGGAGA
AACTCTAAGCATTCCATTAGTTATGTATCAACGTTCCAACAAAAATACTTGTATGCATCA
AAAACCCCAGGTTCAGCGGGGTAAATGCATTAAAAAGGGACAAATTTTAGCGGACGGT
GCCGCTACAGTTGGTGGCGAACTCGCTTTAGGGAAAAACGTATTAGTAGCTTATATGCC
ATGGGAAGGTTACAATTTTGAAGATGCAGTACTAATTAGCGAGCGCTTGGTATATGAGG
ATATTTATACTTCTTTTCACATACGGAAATATGAAATTAAGACTCATGTGACAAGCCAAG
GC---CCTGAAAGGGTCACTAACGAAATACCGCATTTAGAAGCCCATTTACTCCGCAATT
TAGACAAAAATGGAATTGTAATGCTGGGATCTTGGGTAGAGACGGGTGATATTTTAGTA
GGTAAATTAACGCCCCAGATGGTGAAAGAA---TCGTCGTATGCCCCGGAAGATAGATTG
TTACGAGCCATACTTGGCATTCAGGTATCTACTTCAAAAGAAACTTGTCTAAAACTACC
TATAGGTGGTAGGGGTCGGGTTATTGATGTGAGATGGATCCAGAAAAAGGGG---GGTTC
TAGTTATAATCCAGAAACGATTCGTGTATATATTTCACAGAAACGGGAAATCAAAGTAG
GCGATAAAGTAGCTGGAAGACATGGAAATAAGGGTATCATTTCCAAAATTTTGCCTAGA
CAAGATATGCCTTATTTGCAAGATGGAAGACCCGTTGATATGGTTTTTAACCCATTAGGA
GTACCTTCACGAATGAATGTAGGACAAATATTTGAATGTTCACTCGGGTTAGCAGGAGG
TCTGCTAGACAGACATTATCGAATAGCACCTTTTGATGAGAGATATGAACAAGAAGCTT
CGAGAAAACTAGTGTTTTCTGAATTATATGAAGCCAGTAAGCAAACAGCGAATCCGTG
GGTATTTGAACCCGAGTATCCAGGAAAAAGCAGAATATTTGATGGAAGAACGGGGGAT
CTTTTTGAACAACCCGTTATAATAGGAAAGCCTTATATCTTGAAATTAATTCATCAAGTT
GATGATAAAATCCATGGACGTTCCAGCGGACATTATGCGCTTGTTACGCAACAACCCCT
TAGAGGAAGGGCCAAGCAAGGGGGACAACGGGTAGGAGAGATGGAGGTTTGGGCTTT
AGAAGGATTTGGTGTTGCTCATATTTTACAAGAGATGCTTACTTATAAATCTGATCATATT
AGAGCTCGCCAGGAAGTACTTGGTACTACGATCATTGGAGGAACAATACCTAATCCCG
AGGATGCTCCAGAATCTTTTCGATTACTCGTTCGAGAACTACGATCTTTGGCTCTGGAA
CTGAATCATTTCCTTGTATCTGAGAAGAACTTCCAGATTAATAAGAAGGAAGCTATGAA
TCAGAATTTTTCTTCTATGATCGATCGGTATAAACATCAACAGCTCCGAATTGGATCAGT
TTCTCCTCAACAAATAAGTGCTTGGGCTACTAAAATCCTGCCTAATGGAGAGATAGTTG
GAGAGGTGACAAAACCATATACTTTTCATTACAAAACCAATAAACCGGAAAAAGATGG
ATTATTTTGTGAAAGAATTTTTGGGCCTATAAAAAGTGGGATTTGTGCTTGTGGAAATTA
TCGAGTAATCGGAAATGAAAAAGAAGACCCGAAATTTTGTGAACAATGCGGAGTCGA
ATTTATTGATTCTCGGATACGAAGGTATCAAATGGGCTACATCAAACTCGCATGCCCAGT
AACCCATGTGTGGTATTTGAAACGTCTTCCTAGTTATATTGCGAATCTTTTAGATAAACC
TCTTAAAGAATTAGAAGGCCTGGTATATTGCGAT------TTTTCTTTTGCTAGGCCC---ATAG
CTAAAAAACCTACTTTCTTACGATTACGAGGTACATTCGAATATGAAATCCAATCCTGG
AAATACAGCATCCCACTTTTTTTTACTACCCAAGGTTTCGATACATTTCGCAATCGAGAA
ATCTCTACTGGAGCAGGTGCTATCCGAGAACAATTAGCCGATCTAGATTTACGAATTATT
ATAGATAATTCATTGGTAGAATGGAAAGAATTAGGGGAAGAG---GGGACCTTAGGGAAT
GAATGGGAAGATCGAAAAGTTGGAAGAAGAAAGGATTTTTTGGTTAGACGTATGGAAT
TGGCTAAGAATTTTATTCGAACAAACATAGAACCAGAATGGATGGTTTTGTGTCTATTA
CCGGTTCTTCCTCCTGAGTTGAGACCAATCATTCAGATAGATGGGGGCAAACTAATGAG
CTCGGATATTAATGAACTCTATAGAAGAGTTATCTATCGGAACAATACTCTTACCGATCT

ATTAACAACAAGTAGATCTACGCCAGGAGAATTAGTAATGTGTCAGGAGAAATTGGTA
CAAGAAGCCGTGGATACCCTTCTTGATAATGGAATCCGCGGACAACCAATGAGGGACG
GTCATAATAAAGTTTACAAGTCGTTTTCAGATGTAATTGAAGGCAAAGAAGGAAGATTT
CGTGAAACTCTGCTCGGTAAACGGGTCGATTATTCAGGGCGTTCCGTCATTGTCGTGGG
CCCTTCACTTTCATTACATCGATGTGGATTGCCTCGCGAAATAGCAATAGAGCTTTTCCA
GACATTTGTAATTCGTGGTCTAATTAGACAACATCTTGCTTCGAACATAGGAGTTGCTA
AGAGTAAAATTCGGGAAAAAGAACCGATTGTATGGGAAATACTTCAAGAAGTTATACA
GGGGCATCCTGTATTGCTGAATAGAGCGCCTACCCTGCATAGATTAGGCATACAGGCAT
TCCAGCCCATTTTAGTGGAAGGGCGTGCTATTTGTTTACATCCATTAGTTTGTAAGGGAT
TCAATGCAGACTTTGATGGGGATCAAATGGCTGTTCATGTACCTTTATCTTTGGAGGCT
CAAGCGGAGGCCCGTTTACTTATGTTTTCTCATATGAATCTTTTGTCTCCAGCCATTGGG
GATCCCATTTCCGTACCAACCCAAGATATGCTTATTGGACTTTATGTATTAACGAGTGGG
AACCGTCGAGGTATTTGTGTAAATAGGTATAATCCCTGGAATCGC---------AGAAACTATC
AAAATGAAAGAAGCGACCATAATAACTATATGGATACG---------GAACCCTTTTTTTGTAA
TTCCTATGATGCAATCGGGGCTTATCGGCAGAAACGAATCAATTTAGATAGTCCTTTGTG
GCTCCGGTGGCGACTAGATCAACGT---GTTATTGCTTCA------AGAGAAGTTCCCATCGA
AGTTCACTATGAATCTTTAGGTACCTATTATGAGATTTATGGACACTATCTAATAGTAAGA
AGTATAAAAAAAGAAATTCTTTTTATATACATTCGAACCACTGTTGGTCATATTTCTCTTT
ATCGAGAAATCGAAGAAGCCATACAGGGGTTTTGTCAGGCCTGTTCATATGGTACC------
------------------ATGGAGGTACTTATGGCAGAACGGGCCAATCTAGTCTTTCACAATAAAGT
GATAGACGGAACTGCCATGAAACGACTTATTAGTAGATTAATAGATCACTTCGGAATGG
CATATACATCACACATCCTGGATCAAGTAAAGACTCTGGGTTTCCAACAAGCTACTGCT
ACATCCATTTCATTAGGAATTGATGATCTTTTAACAATACCTTCTAAGGGATGGCTAGTT
CAAGACGCTGAACAACAAAGTTTGATTTTGGAAAAACACCATCATTATGGGAATGTAC
ACGCAGTAGAAAAATTACGTCAATCCATTGAGATATGGTATGCTACAAGTGAATATTTG
CGACAAGAAATGAATCCTAATTTTAGGATGACTGACCCTTTTAATCCAGTTCATATAATG
TCTTTTTCGGGAGCTAGAGGAAATGCATCTCAGGTACATCAATTAGTCGGTATGAGAGG
ATTAATGTCGGATCCCCAAGGACAAATGATTGATTTACCCATTCAAAGCAATTTACGCG
AAGGACTCTCTTTAACAGAATATATCATTTCTTGCTACGGAGCCCGTAAAGGGGTTGTG
GATACGGCTGTACGAACATCAGATGCTGGATATCTCACGCGCCGACTTGTTGAAGTAGT
TCAACACATTGTTGTACGTCGAACAGATTGTGGCACCGTCCGAGGTATTTCTGTGAGTC
CTCGAAATGGGATGATGCCG---GAAAGGTTTTTTATCCAAACATTAATTGGTCGTGTATT
AGCAGATGATATATATATATGGGTCCACGTTGTATTGCCACTCGAAATCAAGATATTGGGAT
TGGACTTGTCAATCGATTCATAACC------------------TTTCGAGCACAACCAATATCTATTCG
AACTCCCTTTACTTGTAGGAGTACATCTTGGATCTGTCGATTATGTTATGGCCGGAGTCC
TACTCATGGCGACCTGGTTGAATTGGGAGAAGCTGTAGGTATTATTGCAGGTCAATCTA
TTGGAGAACCGGGTACTCAATTAACATTAAGAACTTTTCATACTGGCGGAGTATTCACA
GGGGGTACTGCAGAACATGTGAGAGCCCCTTCTAATGGAAAAATAAAATTCAATGAGG
ATTTGGTTCATCCGACACGTACACGTCACGGACATCCTGCCTTTCTATGTTCTATAGACT
TGTATGTAACTATTGAGAGTGAAGATATTATACATAATGTGAATATTCCACCCAAAAGTT
TTCTTTTAGTTCAAAACGATCAATATGTAGAATCAGAACAAGTGATTGCTGAGATTCGT
GCGGGAACATCCACT---TTAAATTTGAAAGAGAAGGTTCGAAAACATATTTATTCTGAC
TCAGAGGGAGAA---ATGCATTGGAGTACTGATGTGTATCATGTGCCTGAATTTACATATG
GTAATGTTCATCTCTTACCAAAAACAAGTCATTTATGGATATTATTAGGAGGACCGTGCA

GA------TCAAGTCTAGTCTCCCTTCCGCTCCACAAGGATCAAGATCAAATGAACGTTCAT
------TCTCTTTCTGTCAAGCGAAGAGATATTTCTAACCTCTCA------GTAACTAATGATCA
G---GCGAGACACAAATTATTTAGTTCGGATTTTTCTGGTAAA---GAATATAGGATTCATGA
TGAT---TCAGAACAGAATCGAATCATATGTACTGGTCGTTGTAATCTCATATATCCG---GC
CATTCTCCACGAGAATTCT------GATTTATTGGCAAAGAGGCGAAGAAATAGATTCATCA
TTTCACTCCAATCGAGTCAAGAACGCGAGAACGAACTAATGCCCCGTTCAGGTATCTC
GATTGAAATACCC------ATAAATGGTATTTTCCGTCGAAATAGTATTCTTGCCTATTTCGA
CGACCCTCGATATCGAAGAAAGAGTTCGGGAATTACTAAATATGGGACTATAGAAATGC
CCTCAATCGTAAAAAAAGATGAT------TTGATTGAGTATCGAGGA---GTCAAGGAATTTA
GGCCAAAATACATGAAAGTAGATCGATTTTTTTTCATTCCCGAGGAAGTGTATCTCTTAC
CCGGATCTTCTTCCATAATGGTACGGAACAATAGTATCATTGGGGTAGATACACAAATCA
CTTTAAATACAAGAAGCCGAGTAGGCGGGTTGGTCCGAGTGGAGAGAAAAAAAAAAA
GAATTGAACTTAAAATCTTTTCTGGAGATATCCATTTTCCTGGAGAGACAGATAAAATAT
CCCGACATAGTGGCGTTTTGATACCA---CCGGGA---ACGGTAAAAACAAATTTTAAGGA
ATCCAAAAAA------TTGAAAGATTGGATCTATGTCCAACGG------ATCACACCTAGTAAG--
----AAAAAGTTTTTTGTTTTTGTTCGACCTGTCGTCACATATGAAATAAGGGATGGTATA
AATTTAGCAACACTTTTCCCCCCGGATCTGTTGCAGGAAAGGGATAATGTGCAATTTCG
AGTTGTCAATTATATCCTTTATGGAAATGGCAAACCAATTCGGGGAATTTCTGACACAA
GTATTCAATTAGTTCGGACTTGTTTAGTATTGAATTGGGACCAAGACAAAAAA---------A
GTGCCTCTAGCAAAGAAGCCCGCGCTTCCTTTGTTGAAATAAGGACAAATGTTTTGATT
CGAGATTTCCTAAGAATCGACTTAGTGAAATGCCCTATTTCTTATATTGGAAAAAGGAAT
GATCCATCGGGTTCCGGATTGATCTCTGAT---AATGGATCAGATCGCACCAATATCACTC
CCTTTTCTTCCATTTATTCCTAT---------TCCAAGGCAAGGATTCAACAACCCCTTAACCA
AAATCAAGGAACTATTCATACCTTGTTGAATAGA---------AATAAGGAATGCCAATCTTTG
ATAATTTTGTCATCATCCAATTGTTCTCGAATGGGTCCA------TTGAACGATGTAAAATAT
CACAATGTG---------ATAAAAGAATCA---CCCCTAATTCTAATTAGGAATTTGTTTGGCCCT
TTAGGAACA------GCCCTTCAAATTGGGAATTTTGATTCA------TTTTACCATTTA------ATA
ACTCATAATCAGATCTTG---GTAACTAATTATTTGCAACTTGACAAT---------TTAAAACAG
ACTTTTCAAGGACTTAAA---------TATTATTTAATGGATGAAAAT------GGGAAAATTTATAA
TCCTGACCCCTGCAGTAACATCATTTTGAATCCATTCAATTTGAATTGGTATTTTCTCCAT
CACAATTATTGTGAAGAGACATCT---ACAATAATTAGTCTTGGGCAGTTTATTTGTGAAA
ATGTATGTATAGCCAAAAATGGACCACAT---CTAAAA------TCGGGTCAAGTTCTAATTGT
TCAA---GTTGACTCTGTAGTAATACGATCAGCTAAGTCCTATTTAGCCACCCCAGGAGC
AACCGTTCATGGCCATTATGGGGAAATCCTTTATGAAGGAGATACATTAGTTACATTTAT
ATATGAAAAATCGAGATCTGGTGATATAACGCAAGGTCTTCCAAAAGTGGAACAGGTG
TTAGAAGTGCGTTCGATTGATTCAATATCGATGAATCTAGAAAAGAGGGTTGAGGGTTG
GAACGAATGTATAACAAGAATTCTTGGAATTCCTTGGGGATTCTTGATTGGTGCTGAGC
TAACTATAGCGCAAAGTCGTATCTCTTTGGTTAATAAGATCCAAAGAGTTTATCGATCCC
AGGGGGTGCAGATCCATAATAGGCATCTAGAAATTATTGTACGTCAAATAACCTCAAAA
GTTTTGGTTTCAGAA---------GATGGAATGTCTAATGTTTTTTTACCCGGAGAACTAATTG
GATTGTTGCGAGCGGAACGAATGGGGCGCGCTTTGGAAGAAGGGATCTGTTACCGAG
CCGTCTTATTGGGAATAACAAGAGCTTCTCTCAATACTCAAAGTTTCATATCTGAAGCTA
GTTTTCAAGAAACTGCTCGAGTTTTAGCAAAAGCAGCTCTCCGGGGTCGTATCGATTG
GTTAAAAGGCCTGAAAGATAATGTTGTTCTGGGGGGGATGATACCCGTTGGTACTGGAT

TTAAAGGATTA------GTGCATCTTTCAAGGCAACATAACAACATTCCT------TTGGAAACT
AAAAAGAAGAATCTATTCGAGAGGGAAATGAGA------GATATTTTGTTCCATCACAGAA
AATTATTAGATTCTTGCCTTTCAAAGAATTTCCATGATATATCAGAACAACCATTTATAGG
A------------------TTTAATGATTTCATGACAAGAAGATATTGGAACATAAATTTGGAAGAGA
TGATGGAGGCGGGGGTTCATTTTGGCCATGGTACTAGGAAATGGAATCCTAAAATGGCA
TCTTATATCTCTGCAAAACGTAAGGGTATTCATATTACAAATCTTACTAGAACTGCTCGT
TTTTTATCAGAAGCTTGTGATTTGGTTTTTGATGCAGCAAGTAGGGGAAAACAATTCTT
AATTGTTGGTACAAAAAATAAAGTAGCTGATTCAGTAGCACGGGCTGCAATAAAGGCT
CGGTGTCATTATGTTAATAAAAAATGGCTCGGCGGTATGTTAACAAATTGGTCCACTAC
AGAAACGAGACTTCATAAGTTCAGGGACTTGAGAACAGAACAAAAAACAGGGAGAC
TCAACCGTCTTCCGAAAAGAGATGCAGCTATGTTGAAAAGACAATTATCTCGCTTGCA
AACATATCTCGGCGGGATTAAATATATGACAGGGTTACCCGATATTGTAATCATTGTTGAT
CAGCACGAAGAATATACGGCCCTTCGAGAGTGTATCACTTTGGGAATTCCAACAATTTG
TTTACTCGATACAAATTGTGACCCCGATCTCGCAGATATTTCGATTCCAGCGAACGATG
ACGCTATATCTTCAATCCGATTAATTCTTAACAAATTAGTATTCGCAATTTGTGAGGGTC
GTTCT---AGCTATATAAGAAATCCTATGGGACAAAAAATAAATCCACTTGGTTTCAGACT
TGGTACAACCCAAGGTCATCATTCCCTTTGGTTTGCACAACCAAAAAATTATTCTGAAG
GTCTACAAGAAGATCAAAAACTAAGAGATTGTATCAAGAATTATGTACAAAAAAATATG
AAAATACCCTCTGGC---GTCGAGGGAATTGCACGTATAGAAATTCAAAAAAGAATCGAT
CTCATCCAGGTTATAATTTTTATGGGATTTCCAAAGTTATTAATAGAAAGTCGGCCGCGA
------------GGAATCGAAGAATTACAGATGAATCTACAAAAAGAATTTAATTGTGTGAATCG
AAAACTTAACATTGCTATCACAAGAATTGCAAAACCTTACGGAAACCCTAATATTCTTG
CAGAATTTATAGCCGGCCAATTAAAAAATAGAGTTTCCTTTCGAAAAGCAATGAAAAA
AGCTATTGAATTAACTGAACAAGCAGATACAAAAGGAATTCAAATACAAATTGCAGGG
CGTATCGACGGAAAAGAAATTGCACGTGTCGAATGGATCAGAGAAGGTAGGGTTCCCC
TACAAACCATTCGAGCTAAAATAGATTATTGTTCCTATACAGTTCGAACTATTTATGGGG
TATTAGGCATAAAAATTTGGATATTTATAGACCAGGAAATGTCGCGTTATCGAGGACCTC
GTTTCAAAAAAATACGTCGCCTGGGGGCATTACCAGGACTAACTAATAAAAGGCCTAG
AGCCGGAAGTGATCTTAGAAACCAATCGCGTTCCGGGAAA---AAATCTCAATATCGTAT
TCGCTTAGAAGAAAAACAAAAATTGCGTTTTCATTATGGTCTTACAGAACGACAATTAC
TGAAATACGTCCGTATCGCCAGAAAAGCCAAGGGGTCAACAGGTCAAGTTTTACTACA
ATTACTTGAAATGCGTTTGGATAACATCCTTTTTCGATTGGGTATGGCTTCGACTATTCC
CGGAGCCCGTCAATTAGTTAACCATAGACATATTTTAGTTAATGGTCGTATAGTAGATAT
ACCAAGTTATCGCTGCAAACCACGCGATATTATTACAGCGAGGGATGAACAAAAATCC
AGAGCTCTGATTCAAAATTCGCTCGATTCATCCACTCGT---GAGGAATTGCCAAAACAT
TTGACTCTTCACCCATTCCAA------TATAAAGGATTAGTCAATCAAATAATAGATAGTAAA
TGGGTCGGTTTGAAAATAAATGAATTGCTAGTTGTAGAATATTATTCTCGTCAGACTATG
TCACGTCGAGGTACTGCAGAAGAAAAAACTGCAAAATCCGATCCAATTTATCGTAATC
GATTAGTTAACATGTTGGTTAACCGTATTCTGAAACACGGAAAAAAATCATTGGCTTAT
CAAATTATCTATCGAGCCGTGAAAAAGATTCAACAAAAGACAGAAACAAATCCACTAT
CTGTTTTACGTCAAGCAATACGTGGAGTAACTCCCGATATAGCAGTAAAAGCAAGACG
TGTAGGTGGATCGACTCATCAAGTTCCCATTGAAATAGGATCCACACAAGGAAAAGCA
CTTGCCATTCGTTGGTTATTAGCGGCATCCCGAAAACGCCCGGGTCGAAATATGGCTTT
CAAATTAAGTTCCGAATTAGTGGATGCTGCCAAAGGGAGTGGCGATGCCATACGCAAA

AAGGAAGAGACTCATAGAATGGCAGAGGCAAATAGAGCTTTTGCACATTTTCGTATGG
GTAGGGACACTATTGCTGAGATAATAACCTCTATACGAAATGCTGATATGGATAGAAAA
AGAGTGGTTCGAATAGCATCTACTAATATTACCGAAAATATTATTAAAATACTTTTACGA
GAAGGTTTTATCGAAAACGTGAGAAAACATCGAGAAAACAACAAAGATTTTTTGGTTT
TAACCCTGCGACATAGAAGA------AATAAGAAAGGGCCTTAT---------ATCAATTTTTTAAA
TTTAAAACGGATCAGTCGACCTGGTCTACGAATCTATTCTAACTATCAACGAATTTCTAG
AATTTTAGGTGGGATGGGAATTGTCATTCTTTCTACTTCTCGGGGTATAATGACAGACCG
AGAAGCTCGACTAGAAGGAATCGGTGGAGAAATTTTGTGTTATATATGGATGGCAAAA
GCTATACCGAGAATTGGTTCACGTAGAACTGGACGTATTGGTTCACGTAAGAGTGCAC
GTAGAATACCAAAGGGAGTTATTCATGTTCAAGCAAGTTTCAATAATACCATTGTGACT
ATTACCGATGTACGGGGTCGGGTAGTTTCTTGGTCCTCCGCCGGTACTTGTGGATTCAA
GGGTACAAGAAGAGGGACACCATTTGCTGCTCAAACCGCAGCAGGAAATGCTATTCGT
ACAGTAGTAGATCAGGGTATGCAACGAGCAGAAGTCATGATAAAAGGTCCCGGTCTCG
GAAGAGACGCAGCATTACGAGCTATTCGTAGAAGTGGTATACTATTAACTTTCGTACGG
GATGTAACCCCTATGCCACATAATGGCTGTAGACCTCCGAAAAAAAGACGTGTGATGC
CAACTATTAAACAACTTATTAGAAATACAAGACAGCCAATCAGAAATGTCATGAAATCC
CCCGCTCTTCGGGGATGCCCTCAGCGTCGAGGAACATGTACTAGGGTGTATACTATCAC
CCCAAAAAAACCAAACTCTGCCTTACGTAAAGTTGCCAGAGTACGATTAACCTCTGGA
TTTGAAATCACTGCTTATATACCCGGTATTGGCCATAATTCACAAGAACATTCTGTAGTC
TTAGTAAGAGGGGGAAGGGTTAAGGATTTACCCGGTGTGAGATATCACATTGTTCGAG
GAACCCTAGATGCTGTCGGAGTAAAGGATCGTCAACAAGGGCGTTCTAAATATGGGGT
AAAAAAGCCAAAAATGGCAAGGAAAAGTTTGATTCAGAGGGAGAAAAAGAGGAAAA
ATTTGGAACAGAAATATCATTTGATTCGTAGATCTTCAAAAAAAGAAATAAGC---------A
AAGTTCCGTCGTTGAGTGACAAATGGGAAATTTATGGAAAATTACAATCCCCGCCGCG
GAATAGTGCACCTACACGTCTTCATCGGCGTTGTTTTTCGACCGGAAGACCGAGAGCT
AACTATCGAGACTTTGGACTATCTGGACACATACTTCGTGAAATGGTTCACGCATGTTT
GTTGCCAGGGGCAACAAGATCAAGTTGGATGGTAAAAAATTCATTCATTTCAGTTATTT
CACAA------GAAGAAAACACAGAAAACAGGGGGTCCGTTGAATTTCAAGTATTCAGTT
TCACCAATAAGATACGGAGACTTACTTCACATTTGGAATTGCACAAAAAAGATTATTTA
TCTCAGAAGGGTCTACGGAAAATTTTGGGAAAACGCCAACGGCTGCTGACTTATTTGT
CAAAAAAAAATAAAGTACGTTATAAAGAATTAATAGGTCAGTTGAATATTCGAGAGATA
AAAACTCGT------ATGGTAAAACTTCGTTTGAAACGATGTGGTAGAAAGCAACGAGCCG
TCTATCGAATCGTTGCAATTGATGTTCGATCCCGAAGAGAAGGAAGAGATCTTCGGAG
AGTGGGTTTTTATGATCCGATAAAGAATCAAACTTATTTAAACGTTCCTACTATTCTATAT
TTCCTTGAAAAAGGTGCTCAACCTACAGGAACTGTTCAGGATATTTTAAAGAAGGCAG
AGGTTTTTAAGGAACTTCGCCCTAAT------------------ATGGATAAATCCAAGCGACCTTTT
CTGAAATCCAAACGATCTTTTCGTAGGCGTTTGCCCCCGATTCAATCGGGGGATCGAAT
TGATTATAGAAACATGAGTTTAATTAGTCGATTTATTAGTGAACAAGGAAAAATATTATC
TAGACGAGTGAATAGATTGACCTTGAAACAACAACGATTAATTACTATTGCTATAAAAC
AAGCTCGTATTTTATCTTTGTTACCTTTTCTTAATAATGAAAAA------CAATTTGAAAGGG
CCGAGTCGACCGCTAGAACTACTGGTCTTAGAACCAGAAATAAAGTGACACGTTCACT
AAAAAAAAATCCTTTTGTAGCGAATCATTTATTAAGAAAAATTGAGAAACTTAACACA
AAAGCAGACAAAGAAATAATAGTAACTTGGTCCCGGGCATCTACCATTATACCCACAAT
GATAGGCCATACTATTGCTATCCATAATGGAAAGGAACATTTGCCTATTTATATAACAGAT

CGCATGGTAGGCCACAAATTGGGAGAATTTTCACCTACTTTAAATTTCCGAGGACATGC
GAAAAGCGATAATAGATCTCGTCGTATGATTTTGAAATCTTTTCTACTAGGTAATCTAGT
ATCCTTATGCATGAAGATAATCAATTCGGTCGTTGTGGTGGGACTCTATTATGGATTTCT
GACCACATTCTCCATAGGGCCCTCTTATCTCTTCCTTCTCCGAGCTCAGGTTATGGAAG
AAGGAGAAGAAGGAACCGAGAAGAAGGTATCAGCAACAACTGGTTTTATTACGGGAC
AGCTCATGATGTTCATATCGATCTATTATGCACCTCTGCATCTAGCATTGGGTAGACCTCA
TACAATAACTGTCCTAGCTCTACCATATCTTTTGTTTCATTTCTTCTGGAACAATCACAA
ACACTTTTTTGATTATGGATCTACTACCAGAAATTCAATGCGTAATCTCAGCATTCAATG
TGTATTCCTGAATAATCTCATTTTTCAATTATTCAACCATTTCATTTTACCAAGTTCAATG
TTAGCTAGATTAGTCAACATTTATATGTTTCGATGCAACAACAAGATGTTATTTGTAACA
AGTAGTTTTGTTGGTTGGTTAATTGGTCACATTTTATTCATGAAATGGGTTGGATTGGTA
TTAGTCTGGATACGGCAAAATCATTCTATTAGATCGTACATTCGATCTAATAAGTACCTT
GGGTCAGAATTGAGAAATTCTACGGCTCGGATCTTTAGTATTCTTTTATTTATTACCTGT
GTCTACTATTTAGGAAGAATACCGTCACCCATTTTTACTAAGAAACTGAAAGAAACCGA
AGAAGATAAACCTCTTGTGACCCTTCTTTTTGATTATAACCGATGGAATCGCCCATTCCG
TTACATAAAAAATAATAGATTTGAGAAAGCTGTAAGAAATGAAATGTCACAATATTTTTT
TTACACATGTCGAAATGATGGAAAACAAAGAATATCGTTTACATATCCGCTCAGTTTGT
CAACTTTTTGGGAAATGATAGCAAAAAGGATATCCCTGTCTACACTAGAAAAACTCTCC
TCTGATGAACCAGACAGTGATTGGGTTTATACTAACAAACAAAAAAGTAAC---TTGAAC
AACGAGTTTTTAAACAGAATTGAAGCTTTAAACAAGAGATCCCTTCCTCTGGATATACT
CGAAAAAAGGACTAGATTGTGTAATGATGAGACTAAAAAAGAATACTTGCATAAAATG
TATGATCCTTTATTGAACGGACCATACCGTGGAACAATAAAAAAAAGGGTTTCCCCTAT
AGAGCCATTTGGAATAAATCGAATTCATGCTATCCTTTACAAAGAATTTGAACAGAAAT
ACCCTAATGGTAAATCTATTGGAATAAAAGAAATCAGTAAAAAAGTCCCTCGATGGTCA
TACAAATTAATCGATTTGGAACAACAATCA---GGACAGATTCGTTCAAGAAAAGCTAAA
CGCGTAGTTATTTTTACTGATGCTTTGATACGCTATTCGCAACAATCGGATTTTCGTCAA
AACATAATCAAAGGTTCTATGCGCGCTCAAAGGCGTAAAACATTTATTTGGGAACTATT
TCAAGCTAATGTTCATTCCCCCCTTTTTTTGGACAGACTAAATAAAACCTCCCTTTTTTC
TTTTGATATT------TCCGGAATGATGAAAATCTTTTTTCAAAATAGAAAGGAGAAACCAC
GGATAGAGATAGCAGAAGCGTGGGATACCATTCCATTTGCGCAAGTAATAAGAGGTTCT
ATGTTAATAACTCAATCGATTCTTAGAAAATATATTATATTACCTTCATTGATAATAGCTAA
AAATTTGAGCCGTATGTTATTATTGCAACTTCCCGAGTGGTCTGAGGATTTAGAAGAAT
GGAATAAAGAAATGCATGTTAAATGTACCTATAATGGTGTTCAATTATCAGAAACAGAA
TTTCCGAAAAATTGGTTAACAGACGGTATTCAGATAAAAATCCTATTTCCTTTCTGTCTG
AAACCTTGGCACAAATCTAAAAAAGGACAAAAAGATGATTTTTGTTTTTTAACAGTTT
GGGGAATGGAAGCTGAACTTCCTTTTGGTTCTCCTCGAAAACGACCTTCCTTTTTTGA
GCCCATTTTTAAGGAACTGGAAAAAAAAAATTGGAAAATTAAAAAAGAAGTATTTTCTA
GTT---CTAAGAGTTTTAAAAGAAAGAACAAAATTACTTCTAACGGTTTCAAAAGAAATA
AAAAAATGGGTTAGTAAAAGTGTTCTA------TTTCTAAAAAGAATACTAAAAGAACTCT
CAAAAGTAAATGATTCTATAATCAGCAATCAGATAATTCATGAATCATTTAGTCAAATCG
GATCTCCAGATTGGACAAATTATTCACTGACAGAAAAAAAAATGAAGGATCTGGCTGA
TAGAACAAGGACAATCCGAAATCAAATCGAAAGAATTACAAAAGAGAAAAAAAAAAG
TCCTAACAAAACAAGTTATAATGATAAAAGATTAGAATCGCCAAAAAATATTTGGCAAA
TATTCAAAAGAAAAAATGATCGATTCATCTGTAAATTACATTATTTATAAAACTTTTCGT

TGAAAAAATATACATATATATATTTCTATGTATCATTAATATTCCCAGAATCAATACACAAC
TTTTT---CACTTTATTTCATATGACTTATCCTCCTTGTCACAAGCATATGTATTTTACAAAT
TATCACAAATCCAACTTATTAACTTGTATAAATTAAGATCTGTTCTTCAATATACGTCTTT
TTTTCTTAAGACTGAAATAAAGGATTTTTTACACAAGGAATATTTCATTCTGAATTAAG
CCATAAAAAACCTCAAAGTTATGGCATGAATCAATGGAAAAACTGGTTAAGGGGGCGT
TATCAATATGATTTATCTCAGATTAGGTGGGCTAGATTAATACCACAAAAATGGCGAAAT
AGAGTCAATTGGCGCCGTACGGCTCAAAATCAAAATAAATGGGATTCATATGAAAAAG
ACCAA------TTAATTCATTACAAAAAACAA---AATGTCAAAAAATGCTATAAATATGATTT
TTTATCATATAAATCTATTAATGGTAATATTATGTATTCAGATAGAAAATTTTTTGATTGGA
AAATTCTCAATTTTTGTCTTAGATTTTTTGATTGGATGGGAATGAATGAAGAAATACTAA
ATCGTTGGTTCTTCCCAGAATTTGTGCTACTTTATAATTCATATAAAATAAAATCGTGGGT
TATACCAAACAAATTACTTCTTTTACGTTACAAGAGCAACACGGAAGCAGAACTAAAA
TCCTTCCTGAAACGTTATTTGCTTTTTCAATTGAGATGGGACGATGCTTTGAATCAAAG
AATGATCAATAATATCAAAGTATATTGTCTCCTGCTTAGACTTATAAATCCAAGAAAAAT
TACTATATCCTCGATTCAAAGGAGAGAAATGAGTCTGGATATAATGCTGGTTCAGAAGA
ATTTAACTCTTCCAGAATTAATGAAA---GGAGTGTTGATTATCGAACCCCTTCGTCTGTC
CGTAAAAAAATGATGGACAATTTTTTATGTATCAAACCATAGGTATTTCATTGGTTCATAA
GAGTAAGTACAATCATTATGATTTATTTGTTCCTGAAAATATTTTATCAGCTAAACGGCGT
AGAGAATTGAGAATTCTAATTTGTTTTAGATTTATTTGGCCTAATTATCGATTAGAAGATT
TAGCTTGTATGAATCGCTATTGGTTTGATACCAATAATGGGAGTCGTTTCAGTATGTTAA
AGATACATATGTATCCAATGAAAGGACATCAATTCAAATCCTGGATTTTCGAATTGAGA
GAGATATTGAGAGAGATCAAGAATTCTCACTATTTCTTAGATTCATGGACCCAATTCAAT
TCAGTGGGATCTTTCATTCACATTTTTTTCCACCAAGAACGTTTTATAAAACTTTTTGAC
CCCCGAATTTGGAGTATCCTACTTTCA---CGCAATTCA------CAGGGTTCAACAAGCAAT
CGATATTTCACG---------ATCAAGGGT---GTAATACTCTTTGTAGTAGCGGTCCTTATATATC
GT------------ATTAACAATCGAAATATGGTCGAAAGAAAAAATCTCTATTTGATAGGGCTT
CTTCCT---------------ATACCTATGAATTCCATTGGACCCAGAAATGATACATTGGAAGAAT
CCGTT---GGGTCTTCCAATATCAATAGGTTGATTGTTTCG---CTCCTGTATCTTCCAAAAG
GAAAAAATATCTCGGAGAGTTGTTTCCTGAATCCGAAAGAGAGTACTTGGGTTCTCCC
AATAACT------------AAAAAGTTTAGCATGCCTGAATCTAACTGGGGTTCGCGGTGGTGG
AGGAACTGGATCGGAAAAAAGAGG------GATTCTAGTTGTAAGATATCTAAT---------GAA
ACCGTCGCTGGAATTGAGATCTTATTCAAAGAGAAAGATATCAAA---TATCTGGAGTTT
CTTTTTGTATATTATATGGAT------GATCCGATCCGCAAGGACCATGATTGGGAATTGTTTG
ATCGTCTTTCTCTGAGGAAGAGGCGAAATATAATCAACTTG------AATTCGGGACCACTA
TTCGAAATCTTAGTGAAACACTGGATTTGTTATCTCATGTCTGCTTTTCGTGAAAAAATA
CCAATTGAAGTGGAGGGT---TTCTTCAAACAACAAGGG---GCTGGGTCAACTATTCAAT
CAAATGAAATTGAGCACGTTTCCCAT------CTCTTCTCGAGAAACAAGTGGCCTATTTCT
TTGCAAAATTGTGCTCAATTTCATATGTGGCAATTCCGCCAAGATCTCTTCGTT---------A
GTTGGGGGAAG---AATCCGCACGAATCGGATTTTTTGAGGAAC---GTATCGAGAGAGAA
TTTGATTTGGTTAGACAATGTGTGGTTGGTAAACAAGGATCGG---------TTTTTTAGC------
AAGGTACGGAATGTATCGTCAAATATTCAATATGATTCC---------ACAAGA------TCTAGTT
TCGTTCAAGTAACGGATTCTAGCCAATTGAAAGGATCTTCT---GATCAATCCAGAGATCA
TTTGGATTCCATTAGT------------AATGAGGATTCGGAA------------------------------------TAT
CACACATTGATCAATCAAAGAGAGATTCAACAACTAAAAGAAAGATCGATTCTT---TGG

GAT------CCTTCCTTTCTTCAAACGGAACGAACAGAGATAGAATCAGAC------CGATTCCC
CAAA------TGCCTTTCTGGATATTCC------TCAATGTCCCGGCTATTCGCG---GAACGTGAG
AAGCAGATGATTAATCATCTGCTTCCGGAAGAAATCGAAGAATTTCTT------GGGAATCC
TACAAGATCCGTTCGTTCTTTTTTCTCTGATAGATGG------TCAGAACTTCAT---CTGGGT
TCGAATCCTACTGAGAGGTCCACTAGAGATCAG------------AAATTGTTGAAGAAACAAC
AAGATCTTTCT---TTTGTCCCTTCCAGGCGATCGGAA------------AATCAAGAAATGGTTAA
TATATTCAAGATAATTACGTATTTACAAAAT------ACCGTCTCAATTCATCCTATTTCATCA
GATCCGGGATGTGATATGGTTCCG---AAGGATGAACCGGAT---ATGGACAGTTCCAATAA
GATTTCATTCTTGAAAAAAAATCCATTTTTTGATTTA------TTTCATCTATTCCATGACCGG
AACAGGGAAGGATACACGTTA------CACCACGATTTTGAATCAGAA------GAGAGATTTC
AAGAAATGGCAGATCTATTCACTCTATCAATAACCGAGCCG---GATCTGGTGTATCATAA
GGGATTTGCCTTTTCTATTGATTCCTACGGATTGGATCAAAAACAA------TTCTTGAATGA
GGTATTCAACTCCAGGGATGAATCGAAAAAGAAATCT---------TTATTGGTTCTACCTCCT
ATTTTTTAT------GAAGAGAATGAATCTTTTTATCGAAGGATCAGAAAAAAATGGGTCCG
G---ATCTCCTGCGGGAATGATTTGGAAGATCCAAAACCAAAAATAGTGGTATTTGCTAG
CAACAACATAATGGAGGCAGTCAATCAATATAGATTGATCCGAAATCTGATTCAAATCC
AATATAGCACCTATGGG------TGCATAAGAAATGTATTGAATCAATTCTTTTTAATGAAT----
-----AGATCCGATCGCAACTTCGAATATGGA---------ATTCAAAGGGATCAAATAGGAAAG
GATACTCTGAATCATAGAACTATAATGAAATATACGATCAACCAACATTTATCGAATTTG
AAAAAG---AGTCAGAAGAAATGGTTCGATCCT------------CTTATTTCTCGAACCGAGAGA
TCCATGAATCGGGATCCTGATGCATATAGATACAAATGGTCCAATGGGAGCAAGAATTT
CCAGGAACATTTGGAACATTTCGTTTCTGAGCAGAAGAGCCGT---------TTTCAAGTAGT
GTTCGATCGATTACGTATTAATCAATATTCGATTGATTGGTCTGAGGTTATCGACAAAAA
AGATTTGTCTAAGCCACTTCGTTTCTTTTTGTCCAAG---------------------------TCACTTCTT
TTTTTGTCC---------------------AAGTTGTTTTTCTTTTTGTCTAAC---TCACTTCCTTTTTTCT
TTGTGAGTTTCGGGAATATCCCCATTCATAGGTCCGAG---ATCTACATCTAT---GAATTGA
AAGGTCCGAATGATCAACTCTGCAATCAGTTGTTAGAATCAATAGGTCTTCAAATTGTT
CATTTGAAAAAATTGAAACCCTTC------TTATTGGAT---------GATCATGATACTTCC---CAA
AAATCGAAATTCTTGATCAATGGAGGAACAATATCACCCTTTTTGTTCAATAAGATACC
A---AAGTGG---ATGATTGACTCATTCCATACTAGA------AATAATTGCAGGAAATCCTTTG
ATAACACGGATTCCTATTTCTCAATGATATTCCACGATCAAGACAATTGGCTGAATCCCG
TGAAACCATTTCATAGAAGTTCATTGATATCTTCTTTTTATAAAGCAAATCGACTTCGAT
TCTTGAATAATCCACATCACTTCTGCTTCTAT------TGTAACAAAAGATTCCCTTTTTATGT
G------------GAAAGGGCCCGTATCAAT------AATTATGATTTTACGTATGGA---CAATTCCTC
AATATCTTGTTCATTCGCAACAAA---ATATTTTCTTTGTGCGTCGGTAAA------AAAAAAC
ATGCTTTTTGGGGG---AGAGATACTATTTCACCAATCGAGTCACAGGTATCTAACATATT
CATACCTAACGATTTTCCACAAAGTGGT---GACGAAACGTATAACTTGTACAAATCT------
---TTCCATTTTCCAAGTCGATCCGATCCATTCGTTCGT------AGAGCTATTTACTCGATCGC
AGACATTTCTGGAACACCTCTAACAGAGGGACAAATAGTCAATTTTGAAAGAACTTAT
TGTCAACCTCTTTCAGATATGAATCTATCTGATTCAGAAGGGAAGAACTTGCATCAGTA
TCTCAAT------TTCAATTCAAACATGGGTTTGATTCACACTCCATGTTCTGAGAAATATTT
ACCATCCGAAAAG------AGGAAAAAA------------------------CGGAGTCTTTGTCTAAAG------
AAATGCGTTGAGAAAGGGCAGATGTATAGAACCTTTCAACGAGATAGTGCTTTTTCAA
CTCTCTCAAAATGGAATCTATTCCAAACATATATGCCATGGTTCCTTACTTCGACAGGGT

ACAAATATCTAAATTTGATATTTTTAGATACTTTTTCAGACCTATTG------CCGATACTAAG
TAGC---AGTCAAAAATTTGTATCCATTTTTCATGATATTATGCATGGATCAGATATATCATG
GCGAATTCTTCAGAAAAAAATTG-----------------TGTCTTCCACAATGGAATCTG---ATAAAT
GAGATTTCGAGTAAGTGTTTACATAAT------CTTCTTCTGTCCGAAGAAAGGATTCATCG
AAAT------AATGAGTCACCATTGATATCGACACATCTGAGATCGCCAAATGTTCGGGAGT
TC---CTCTATTCAATTCTTTTCCTTCTTCTTGTTGCTGGATAT---CTCGTTCGTACACATCT
TCTCTTTGTTTCCCGG------GCCTCTAGTGAGTTACAGACAGAGTTCGAAAAGGTCAAA
TCTTTGATGATTCCATCATCTATGATTGAGTTGCGAAAACTTCTGGATAGGTATCCTACA
TCTGAACCGAATTCTTTCTGGTTAAAGAATCTCTTTCTAGTTGCTCTGGAACAATTAGG
AGATTCTCTAGAAGAAATATGGGGTTCTGCTTCTGGCGGCAACATGCTATTGGGTGGTG
GTCCCACT---------------TATGGG---GTCAGT---------TCTAAGAAGAAATATTTGAATATCAA
TCTCATCGATCTCATAAGTCTCATA------------CCAAATCCCATCAATCGAATCACTTTTTC
GAGAAATACGAGACATCTAAGTCATACAAGTAAAGAGATCTATTCATTGATAAGAAAAA
GAAAAAACGTGAACGGGGATTGGATTGATGATAAAATAGAATCCTGGGTCGCGAACAG
CGATTCGATTGATGATGAAGAAAGAGAATTTTTGGTTCAGTTCTCC------ACCTTAACGA
CA------GAAAAAAGGATTGATCAAATTTATTGAGTCTGACT---CATAGTGATCATTTA-----
----------TCAAAGAATGACTCTGGTTATCAAATAATTGAACAACCGGGAGCAATTTACTTA
CGATACTTAGTTGACATTCAT------AAAAAGTATCTAATGAATTATGAGTTCAATACATCC
TGTTTAGCAGAAAGACGGATATTCCTTGCTCATTATCAGACAATCACTTAT------TCACAA
ACTTCGTGTGGGGCTAATAGTTTTCATTTCCCATCTCATGGA---------AAACCCTTTTCGC
TCCGCTTAGCCTTATCCCCCTCT---AGGGGTATTTTAGTGATAGGTTCTATAGGAACTGG
ACGATCCTATTTGGTCAAATACCTAGCGACAAACTCCTATGTTCCTTTCATTACGGTATT
TCTGAACAAGTTCCTGGATAACAAGCCTAAAGGTTTTCTTATTGAT---------------GATATC
GAT---ATTGATGATAGTGACGATATTGATGATAGTGAC--
--------------------------GATATCGATCGTGACCTTGATACGGAGCTG---GAACTGCTAACTAG
GATGAATGCGCTAACTATGGATATGATGTCGGAA---------ATAGACCAATTTTATATCACCC
TTCAATTCGAATTAGCAAAAGCAATGTCTCCTTGCATAATATGGATTCCAAACATTCATG
ATCTGGATGTGAATGAGTCGAATTACTTATCCCTCGGTCTATTAGTGAACCATCTCTCCA
GG---------GATTGTGAAAGATGTTCC---ACTAGAAATATTCTTGTTATTGCTTCGACTCATA
TTCCCCAAAAAGTGGATCCCGCTCTAATAGCCCCGAATAAATTAAATACGTGCATTAAG
ATACGAAGGCTTCTTATTCCACAACAACGAAAGCACTTTTTCACTCTTTCATATACTAGG
GGATTTCACTTGGAAAAGAAAATGTTCCATACT------AATGGATTCGGGTCCATAACCAT
GGGTTCCAATGCACGAGATCTTGTAGCACTTACCAATGAGGCCCTATCGATTAGTATTAC
ACAGAAGAAATCAATTATAGACACTAATACAATTAGGTGCGCTCTTCATAGACAAACTT
GGGATTTGCGA---------TCCCAGGTAAGATCGGTTCAGGATCATGGGATCCTTTTCTATCA
GATAGGAAGGGCTGTAGCACAAAATGTACTTCTAAGTAATTGCCCCATAGATCCTATATC
TATCTATATGAAGAAGAAA------TCATGTAACGAAGGGGATTCTTATTTGTACAAATGGTA
CTTCGAACTTGGAACGAGCATGAAGAAATTAACGATACTTCTTTATCTTTTGAGTTGTT
CTGCCGGATCGGTCGCTCAAGATCTTTGGTCTCTACCC------GGACCCGATGAAAAA---A
ATGGGATCACTTCTTATGGACTCGTTGAGAATGATTCT---GATCTAGTTCATGGCCTATTA
GAAGTAGAAGGCGCTCTGGTGGGA------TCTTCACGGACAGAAAAAGATTGCAGTCAG
TTTGATAATGAT------CGAGTGACATTGCTTCTTCGGCCCGAACCAAGGAAT---CCCTTAG
ATATGATGCAAAACGGATCTTGTTCTATCCTTGATCAGAGATTTCTCTATGAAAAATAC---
---GAATCGGAGTTTGAAGAA---GGGGAGGGAGAAGGAGCCCTTGACCCGCAACAG------

------ATAGAGGAGGAT------------TTATTCAATCAC------ATAGTTTGGGCTCCT------AGAATA
TGGCGCCCT---------TGGGGCTTTCTATTTGATTGTATCGAAAGGCCCAATGAATTGGGAT
TTCCC---TATGGGTCCAGGTCATTTCGGGGCAAG---------CGGATCATTTATGATGAAGAG
GATGAGCTTCAAGAGAATGATTCGGAGTTCTTGCAGAGTGGAACCGTGCAGTACCAGA
CACGAGATAGATCTTCCAAAGAACAAGGCCTTTTTCGAATAAGCCAATTCATTTGGGAC
CCTGCGGATCCACTCTTTTTCCTATTCAAA---GATCAGCCCCTTGGCTCTGTGTTTTCAC
ATCGAGAATTATTTGCAGATGAAGAGATGTCAAAGGGGCTTCTTACTTCCCAAACAGAT
CCTCCTACATCTATATATAAACGCTGGTTTATC------AAGAATACGCAAGAAAAGCACTTC
GAATTGTTGATTAATCGTCAGAGATGGCTTAGA---------ACCAATAGTTCATTATCT------A
ATGGATCTTTCCGTTCTAAT------ACTCTATCCGAGAGTTATCAGTATTTATCAAATCTGTT
CCTATCTAAC---GGAAGGCTATTGGATCAAATGACAAAGACATTGTTGAGAAAAAGATG
GCTTTTCCCGGATGAAATGAAAATTGGATTC------ATG---------------------------ATGCCTAGA
TCGCGG---ATAAATGGAAATTTTATTGATAAGACCTTTTCAATTGTAGCCAATATCTTATT
ACGAATAATTCCGACAACTTCAGGAGAAAAAGAGGCATTTACCTATTATAGAGATGGG--
----ATGTCAGCTCAATCCGAAGGAAATTATGCGGAAGCTTTACAGAATTATTATGAAGCT
ATGCGACTAGAAATTGATCCCTATGATCGAAGTTATATACTCTATAATATAGGCCTCATCC
ACACAAGTAACGGAGAACATACGAAAGCTTTGGAATATTATTTTCGAGCACTAGAACG
AAACCCATTCTTACCACAAGCTTTTAATAATATGGCCGTGATCTGTCATTAC---CGGGGA
GAACAGGCCATTCGACAGGGAGATTCTGAAATTGCGGAGGCTTGGTTCGATCAAGCCG
CTGAGTATTGGAAACAGGCTATAGCGCTTACCCCAGGTAATTATATTGAAGCGCATAATT
GGTTGAAGATCACGAGGCGTTTCGAA------ATGAGTTGGCGATCAGAACATATATGGATA
GAACTTATAAGGGGGTCTCGAAAAATAAGTAATTTCTGCTGGGCCTTTATCCTTTTTTTA
GGTTCATTAGGATTCTTATTGGTTGGAACTTCCAGTTATCTTGGTAGAAATTTGATATCTT
TTTTTTCGTCTCAACAAATCATTTTTTTTCCACAAGGGATCGTGATGTCTTTCTATGGGA
TCGCGGGTTTCTTTATTAGTTCCTATTTGTGGTGCACAATTTTGTGGAATGTAGGTAGTG
GTTATGATCGATTCGATAGAAAAGAAGGAATAGTTTGTATTTTTCGTTGGGGATTTCCTG
GAAAGAATCGTCGCATATTCCTCCGATTCCTTATAAAAGATATTCAGTCCGTCAGAATAG
AAGCTAAAGAGAGTATTTATGCTCGTCGTGTCCTTTATATGGACATCAGAGGCCAGGGG
GCCATTCCCTTGACTCGTACTGATGAGAAT---TTGACTCCACGAGAAATTGAACAAAAA
GCTGCTGAATTGGCCTATTTCTTGCGTGTACCAATTGAAGTGTTT
>Berberidopsis
ATGGAAAAACGGTGGTTCAATTCGATGTTGTCTCTAAGTAAATCAATGGACAGTCTTGG
TCCTATTGAAAATACCAGTGGAAGCGAAGATCCGAGTATAAATGATACGGATAAAAACA
TTCATAGTTACAGTAATGTTGATCATTTATTCGTCGTCAGGGACATTCGGAATTTCATCT
CTGATGACACTTTTTTAGTTAGAGATAGTAATGGGGACAGTTATTCCATATATTTTGATAT
TGAAAATCATATTTTTGAGGTTGACAATGATCAT------TCTTTTCTGAGTGAACTAGAAA
GTTATTTTTATAGTTATAGGAATTCTAGTTATCTGAATACTGGATCTAAGAGTGATGATCC
TTACTATAATCGTTACATGTATGATACTAAATATAGTTGGAATAATCACATTAATAGTTGCA
TTGACAGTTATCTTCAA------TCTCAAATCCGTATTGATAGT---------TACGGTAGTGGCAA
TTACAGT------TACATTTATAGTTACATTTGTGGTAGCGAGAGTTTCAGTATAAAAACTAG
CATGAATGGTGGTGATTTAACTATAAGAGAAAGTTCTAATGATCTCGATGTAACTCAAA
AATACAGGCATTTGTGGGTTCAATGCGAAAATTGTTATGGATTAAATTATAAGAAATTTT
TTAAGTCAAAAATGAATATTTGTGAACAATGTGGATACCATTTGAAAATGAGTAGTTCA
GATAGAATCGAACTTTCGATTGATCCAGGTACTTGGGATCCTATGGATGAAGACATGGT

CTCTCTGGATCCCATTGAATTTCATTCGGAGGAGGAACCTTATAAAGATCGTATCGATTC
TTATCAAAGAAAGACAGGATTAACTGAGGCTGTTCAAACAGGCATAGGCCAACTAAAC
GGTATTCCCATAGCAATTGGGGTTATGGATTTTCAGTTTATGGGGGGTAGTATGGGATCC
GTAGTAGGGGAGAAAATCACTCGTTTGATCGAGTATGCTACCAATAAATTTCTACCACT
TATTATAGTGTGTGCTTCCGGAGGGGCACGCATGCAAGAAGGAAGTTTGAGCTTGATG
CAAATGGCTAAAATATCTTCTGCTTTATATGATTATCAATCAAATAAAAAGTTATTCTATG
TATCAATCCTTACATCTCCTACTACTGGTGGGGTGACAGCCAGTTTTGGTATGTTGGGA
GATATCATTATTGCCGAACCCAATGCCTACATTGCATTTGCAGGTAAAAGAGTAATTGAA
CAAACATTGAATAAGACAGTACCTGAAGGTTCACAAGCGGCCGAATATTTATTCCATAA
GGGTTTATTTGATCTAATCGTACCACGTAATCCTTTAAAAGGCGTTCTGAGTGAGTTGTT
TAAGCTCCACGCTTTCTTTCCTTTGAAT---------------ATGGTAACCATTCGAGCCGACGAA
ATTAGTAATATTATCCGTGAACGTATTGAGCAATATAATAGAGAAGTAAAGATTGTAAAT
ACCGGTACCGTACTTCAAGTAGGCGATGGCATTGCTCGTATTCATGGTCTTGATGAAGT
AATGGCAGGTGAATTAGTAGAATTTGAAGAGGGTACAATAGGCATTGCTCTAAATTTGG
AATCAAATAATGTTGGTGTTGTATTAATGGGTGACGGTTTGATGATACAAGAGGGAAGT
TCTGTAAAAGCAACAGGAAGAATTGCTCAGATACCGGTGAGTGAGGCTTATTTGGGTC
GTGTTATAAATGCTCTGGCTAAACCTATTGATGGTAGAGGTGAAATTTCAGCTTCTGAAT
CTCGGTTAATTGAATCTCCCGCTCCGGGTATTATTTCGAGACGTTCCGTATATGAGCCTC
TTCAAACGGGGCTTATTGCTATTGATTCAATGATCCCTATAGGACGTGGTCAGCGAGAA
TTAATTATTGGGGACAGACAGACCGGTAAAACAGCAGTAGCCACAGATACGATTCTCA
ATCAACAAGGGCAAAATGTAATATGTGTTTATGTAGCTATTGGTCAAAAAGCATCTTCT
GTGGCTCAGGTAGTTAATACTTTTCAGGAAAGGGGAGCGATGGAATACACTATTGTGGT
AGCCGAAACGGCGGATTCCCCTGCTACATTACAATACCTCGCTCCTTATACAGGAGCAG
CTCTGGCTGAATATTTTATGTACCGTGAACGACACACTTCAATCATTTATGATGATCTCT
CCAAACAAGCACAAGCTTATCGCCAAATGTCGCTTCTATTACGAAGACCACCCGGTCG
TGAAGCTTATCCAGGAGATGTTTTTTATTTGCATTCACGCCTTTTGGAAAGAGCCGCTA
AATCAAGTTCTCGTTTAGGTGAAGGAAGTATGACTGCTTTACCAATAGTTGAGACTCAA
TCGGGAGATGTTTCGGCTTATATTCCTACTAATGTAATTTCCATTACAGATGGACAAATAT
TCTTATCCGCCGATCTATTCAATGCTGGAATCAGACCGGCTATTAATGTGGGTATTTCCG
TTTCCAGAGTAGGATCTGCGGCTCAAATTAAAGCCATGAAACAAGTAGCTGGCAAATT
AAAATTGGAACTGGCACAATTCGCAGAATTAGAAGCCTTTGCGCAATTCGCTTCTGATC
TCGATAAAGCTACTCAGAATCAATTGGCAAGAGGTCAACGATTACGTGAATTGCTCAA
ACAATCCCAATCAGCCCCTCTCACGGTGGAAGAACAGATAATGACTATTTATACTGGAA
CGAATGGTTATCTTGATTCATTAGAAATTGGACAGGTAAGGAAATTTCTCGTTGAGTTA
CGTACTTACTTAAAAACGAATAAACCTCAGTTCCAAGAAATCATATCTTCTACCAAGAC
ATTCACCGAGGAAGCAGAAGCTCTTTTGAAAGAAGCTATTCAGGAACAAATGGAACG
CTTTCTACTTCAGGAACAAGCAATGAGAATCAATCCTACTACTTCTGGTCCTGGGGTTT
CCACGCTTGAAAAAAAAAAACCTGGGGCGTATCGCTCAAATCATTGGTCCGGTACTGGA
TGTAGCTTTTCCTCCGGGCAAGATGCCTAATATTTACAACGCTCTGGTAGTTAAGGGTC
GAGATACTGTCGGTCAACCAATTAATGTGACTTGTGAGGTACAGCAATTATTAGGAAAT
AATCGAGTTAGGGCTGTAGCTATGAGTGCTACAGATGGTCTAATGAGAGGAATGGAAG
TGATTGACACGGGAGCTCCTTTAAGTGTTCCAGTCGGTGGAGCGACTCTCGGACGAAT
TTTCAACGTGCTTGGAGAGCCTGTTGATAATTTAGGTCCTGTAGATACTCGCACAACAT
CTCCTATTCATAGATCTGCGCCCGCCTTTATACAGTTAGATACAAAATTATCTATTTTTGA

AACAGGAATTAAAGTAGTAGATCTTTTAGCCCCTTATCGCCGTGGAGGAAAAATCGGA
CTATTCGGGGGAGCTGGAGTGGGTAAAACAGTACTCATTATGGAATTGATCAACAACAT
TGCCAAAGCTCATGGAGGTGTATCTGTATTTGGCGGAGTAGGTGAACGTACTCGTGAA
GGAAATGATCTTTACATGGAAATGAAAGAATCTGGAGTGATTAATGAACAAAATATTGC
AGAATCAAAAGTGGCTCTAGTCTACGGTCAGATGAATGAACCGCCGGGAGCTCGTATG
AGAGTTGGTTTGACTGCCCTAACTATGGCGGAATATTTCCGAGATGTTAATGAACAAGA
CGTACTTCTATTTATCGACAATATCTTCCGTTTCGTCCAAGCAGGATCCGAAGTATCTGC
CTTATTAGGTAGAATGCCTTCCGCTGTGGGTTATCAACCCACCCTTAGTACCGAAATGG
GTACTTTACAAGAAAGAATTACTTCTACCAAAGAAGGGTCCATAACTTCTATTCAAGCA
GTTTATGTACCTGCGGACGATTTGACCGACCCTGCTCCTGCCACGACATTTGCACATTT
AGATGCTACTACCGTACTATCAAGAGGATTAGCTGCCAAAGGTATCTATCCAGCAGTAG
ATCCTTTAGATTCAACGTCAACTATGCTCCAACCTCGGATCGTTGGTGAGGAACATTAT
GAAACTGCGCAACAAGTTAAGCAAACTCTACAACGTTACAAAGAACTTCAGGACATTA
TAGCTATCCTTGGGTTGGACGAATTATCCGAAGAGGATCGTTTAACCGTAGCAAGAGCA
CGAAAAATTGAGCGTTTCTTATCACAACCCTTTTTCGTAGCAGAAGTATTTACCGGTTC
TCCAGGGAAATATGTTGGTCTAGCAGAAACAATTAGAGGGTTTCAATTGATCCTTTCCG
GAGAATTAGATGGTCTTCCTGAACAGGCCTTTTATTTGGTAGGTAACATCGATGAAGCT
ACCGCGAAGGCTATGAACTTAGAAATGGAGAACAAATTGAAGAAAATGACCTTAAATC
TTTGTGTACTGACCCCTAATCGAATTGTTTGGGATTCAGAAGTGAAAGAAATCATTTTAT
CTACTAATAGTGGCCAAATTGGCGTATTACCAAATCATGCCCCTATTGCTACAGCTGTAG
ATATAGGTATTTTGAGAATACGCCTTAACGACCAATGGTTAACGATGGCTCTGATGGGC
GGTTTTGCTAGAATAGGCAATAATGAGATCACTGTTTTAGTAAATGATGCGGAGAAGGG
TAGTGACATTGATCCACAAGAAGCTCAGCAAACTCTTGAAATAGCGGAAGCTAACTTG
AGGAAAGCTGAAGGCAAGAGACAAACAATTGAGGCAAATCTAGCTCTCAGACGAGCT
AGGACACGAGTAGAGGCTATCAATGTGATTTCGATGAAAAATGTAACCGATTCTTTCGT
TTCCTTGGGTCACTGGCCATCCGCCGGGAGTTTCGGGTTTAATACCGATATTTTAGCAA
CAAATCCAATAAATCTAAGTGTAGTACTTGGTGTATTGATCTTTTTTGGAAAGGGAGTG
TTAAGTGATTTATTAGATAATCGAAAACAGAGGATCTTGAATACTATTCGAAATTCAGAA
GAACTACGCGAAGGGGCCATTGAACAGCTGGAAAAAGCCCGGGCTCGCTTACGGAAA
GTTGAAATGGAAGCAGATGAGTTTCGAGTGAATGGATACTCTGAGATAGAACGAGAAA
AATTGAATTTGATTAATTCAACTTATAAGACTTTGGAACAATTAGAAAATTACAAAAATG
AAACCATTCATTTTGAAGAACAAAGAGCGATTAATCAAGTCCGACAGCGGGTTTTCCA
ACAAGCCTTACAAGGAGCTCTAGGAACTCTAAATAGTTGTTTGAACAACGAGTTACAT
TTACGTACCATCAGTGCCAATATTGGCATGTTTGGGGCGATGAAAGAAATAACTGATAT
GAATCCACTGATTGCTGCTGCTTCCGTTATTGCTGCTGGATTGGCCGTAGGGCTTGCTT
CTATTGGACCTGGAGTTGGTCAAGGTACTGCTGCGGGCCAAGCTGTAGAAGGTATCGC
GAGACAGCCCGAGGCGGAGGGAAAAATACGAGGTACTTTATTGCTTAGTCTGGCTTTT
ATGGAAGCTTTAACAATTTATGGACTGGTTGTAGCATTAGCGCTTTTATTTGCGAATCCT
TTTGTTATGAATGTTCTATCATGTTCCATCAACACACTAAAAGGGATATACGATATATCCG
GTGTGGAAGTAGGCCAACATTTCTATTGGCAAATAGGAGGTTTCCAAGTCCATGCCCA
AGTACTTATAACTTCTTGGGTTGTAATTGCTATCTTATTAGGTTCAGCCACTATAGCTGTT
CGGAATCCACAAACCATTCCGACTGACGGTCAGAATTTCTTCGAATATGTCCTTGAATT
CATTCGAGACGTGAGCAAAACTCAGATTGGAGAAGAATATGGTCCTTGGGTTCCCTTTA
TTGGAACTCTGTTTCTATTTATTTTTGTTTCTAATTGGTCCGGGGCTCTTTTACCTTGGAA

AATCATACAGTTACCTCATGGGGAGTTAGCCGCACCCACAAATGATATAAATACTACCG
TTGCTTTAGCTTTACTCACGTCAGTGGCATATTTCTATGCGGGTCTTACCAAAAAAGGAT
TAGGTTATTTCGGTAAATACATTCAACCAACTCCAATCCTTTTACCCATTAACATCTTAG
AAGATTTCACAAAACCTTTATCACTTAGTTTTCGACTTTTCGGGAATATATTAGCTGATG
AATTAGTAGTTGTTGTTCTTGTTTCTTTAGTACCTTCAGTGGTTCCTATACCTGTCATGTT
CCTTGGATTATTTACAAGTGGTATTCAAGCTCTTATTTTTGCAACTTTAGCCGCGGCTTAT
ATAGGCGAATCTATGGAGGGTCATCATATGATATTTTCGACTTTAGAGCATATATTAACTC
ATATCTCCTTTTCGATCGTTTCAATTGTAATTACAATTCATTTGATAACCTTATTAGTCGAT
GAAATCATAGGACTATACGATTCGTCAGAAAAGGGCATGATAGCCACTTTTTTCTGTATA
ACAGGATTATTAGTCACTCGTTGGGTTTATTCGGGGCATTTCCCATTAAGTGATTTATATG
AATCATTAATCTTCCTTTCATGGAGTTTCGCCATTATTCATATGGTTCCGTATTTT---AAAT
ATAAAAATCATTTAAGCGCAATAACCGCGCCAAGTGCTATTTTTACCCAAGGCTTTGCT
ACTTCAGGTCTTTTAACTGAAATGCATCAATCCGCAATATTAGTACCTGCTCTCCAATCC
CAGTGGTTAATGATGCACGTAAGTATGATGGTATTGGGCTATGCAGCTCTTTTATGTGGA
TCATTATTATCAGTAGCACTTCTAGTCATTACATTTCGAAAAAACATAAGGATTTTTGGT
AAAAGCAATGAGATCCAATACATGGATGAAAGAAACAATGTTTTACAAAATACTTCTTT
TTTTTCGGTTAGGAATTATTACAGATCCCAATTGATTCAACAATTGGATCGTTGGAGTTA
TCGTGTTATTAGTCTAGGATTTATCTTTTTAACCATAGGTATTCTCTCGGGAGCAGTATGG
GCTAATGAGGCATGGGGATCATATTGGAATTGGGACCCAAAGGAAACTTGGGCATTTAT
TACTTGGACCATATTCGCGATTTATTTACATACTCGAACAAACACAAATTTGCAAGGTGT
AAATTCCGCAATTGTGGCTTCTCTGGGCTTTCTTATAATTTGGATATGCTATTTTGGGGTC
AATCTATTAGGAATAGGACTACATAGTTATGGTTCATTTACATTAACAATGACAAAAAAG
AAAGCATTCACTCCTCTTCTATATCTTGCATCTATAGTATTTTTGCCCTGGTGGATCTCTC
TCTCATTTAATAAAAGTCTGGAATCTTGGGTTACTAATTGGTGGAATACTAGGCAATCCG
AAACTTTTTTGAATGATATTCAAGAAAAAAATATTCTAGAAAAATTCATAGAATTAGAG
GAGCTCCTCCTCTTGGACGAAATGATAAAGGAATACCCAGGGACACATCTACAAAAGC
TTCGTATAGGA---CACAAAGAAACGATCCAATTGATCAAGATGCACAATGAGGATCGTA
TCCATACGATTTTGCATTTCTCGACAAATATAATCTGTTTCGTTATTCTAAGTGGTTATTC
TATTTTGGGTAATGAAGAACTTGTTATTCTTAACTCTTGGGTTCAGGAATTCCTATATAA
CTTAAGCGACACAATAAAAGCTTTTTCTATTCTTTTATTAACCGATTTATGTATCGGATTC
CATTCGCCCCATGGTTGGGAACTAATGATTGGCTCTGTCTACAAAGATTTTGGATTTGCT
CATAACGATCAAATTATATCTGGTCTTGTTTCCACTTTTCCAGTCATTCTAGATACAATTT
TTAAATATTGGATCTTCCGTTATTTAAATCGTGTATCTCCGTCACTTGTAGTGATTTATCAT
TCAATGAATGACATGCCTATTGGTGTTCCAAAAGTACCTTTTCGAAGTCCTGGAGAGGA
AGATGCATCTTGGGTTGACGTATACAACCGACTTTATCGAGAAAGATTACTTTTTTTAGG
CCAAGAGGTTGATAGCGAGATCTCGAATCAACTTATTGGTCTTATGGTATATCTCAGTAT
AGAGGATGATACCAAAAATTTGTATTTGTTTATAAACTCTCCTGGCGGATGGGTAATACC
CGGAGTAGCTATTTATGATACTATGCAATTTGTGCGACCAAATGTACATACAATATGCAT
GGGATTAGCCGCTTCAATGGGATCTTTTATCCTGGCCGGAGGAGAAATTACCAAACGTC
TAGCATTCCCTCACGCCAGGGTAATGATCCATCAACCTGCTAGTTCTTTTTATGAGGCAC
AAACGGGAGAATTTATCCTGGAAGCGGAAGAACTGCTGAGACTGCGCGAAAGCCTCA
CAAGGGTTTATGCACAAAGAACGGGCAAATCCTTATGGGTTGTATCCGAAGACATGGA
AAGGGATGTTTTTATGTCAGCAACAGAAGCCCAAACTCATGGAATTGTTGATCTTGTAG
CGGTTGAAATGAAAGAACAAAAATGGATTCATGAAGGTTTAATTACTGAATCACTTCCC

AATGGTATGTTCCGGGTTCGTTTAGATAATGAAGATCTGATTCTAGGTTATGTTTCAGGA
AGGATCCGACGTAGTTTTATACGGATACTACCAGGAGATAGAGTCAAAATTGAAGTAAG
TCGTTATGATTCAACCAGAGGGCGTATAATTTATAGACTCCGCAACAAGGATTCGAGCG
ATATGGAGGAATTCCAAGGATATTTAGAA---GATAAATCTCGGCAACATGACTTCCTATA
TCCACTTATCTTTCAGGAGTATATTTATGCGCTTGCTCATGATCAT------GCAGGTTATGAC
AATAAATCCAGTTCACTAATTGTGAAACGGTTAATTACTCGAATGTATCAACAGAATCAT
TTGATTATTTCCGCTAATGATTCTAACCAAAATCCTTTTTTTGGGAAGAATTTGTATTCTC
AAATGATATCAGAAGGATTTGCAGTCATTGTGGAAATTCCATTTTCCCTACGATTAGTAT
CTTCCCTAATAGTAAAATCTCATAAATTACGATCAATTCATTCAATATTTCCTTTTTTAGA
GGATAAATTCTCACATTTAAATTATGTGTTAGATATACTAATACCCCACCCTGTCCATCTG
GAAATCTTGGTTCAAACCCTTCGCTACTGGGTGAAAGATGCCTCTTCTTTGCATTTATTA
CGATTATTTCTCCACGAGTATCGTAAT------TGGAATAGTCTTAAATCCATTTCCATTTTTT
CAAAACAAAGATTACTCTTGTTCCTATATAATTCTCATGTATGTGAATACGAATCCGTCT
TTGTTTTTCTACGTAACCAATCTTCTCATTTACTATCAACATCTTCTGGAGCCCTTCTTGA
ACGAATATATTTCTATGGAAAAATAGAACATGTAGAA---GTCTTTGCTAAAGATTTTATCC
TATGGTTGTGCAAGGATCCTTTCATGCATTATGTTAGGTATCAAGGAAAATCAATTCTGG
CTTCAAAGGGGACGCCTCTTCTGATGAATAAATGGAAATATTACCTTGTCAATTTCTGG
CAATGTCATTTTTACGTGTGGGCTCAACCAGGAAGGATCTATATAAACCAATTATCCAAT
CATTCTCTCAACTTTATGGGCTATCTTTCAAGTGTGCGACTAAACCTTTCAGTGGTACG
GAGTCAAATGCTAGAAAATGCATTTCTAATAGATAAGTCCAGT------AAGAAGTTTGATC
CCATAGTTCCAATTATTCCTTTGATTGGATCATTGGCTAAAGCGAAATTTTGTAACGTATT
AGGGCATCCCATTAGTAAGCCGGTCTGGGCCGATTTATCAGATTCTGATATTATCGACCG
ATTTGGACGTATATGCAGAAATCTTTCTCATTATCACAGCGGATCTTCAAAAAAAAAGA
GTTTGTATCGAATAAAGTATATACTTCGACTTTCTTGTGCTAGAACTTTGGCTCGTAAAC
ACAAAAGTACTGTACGTGCTTTTTTGAAAAGATTAGGTTCGGAATTATTGGGAGAATTC
CTTACGGAGGAAGAACAAGTTCTTTCTTTGATCTTCCCAAGACGGATTTGGTATTTGGA
TATTATTTGTATCAATGATCTGGCCAATCATGAAATGATAATTGAT---ACAACAGAAGTAC
AA---GCTATCAATTCTTTTTCTAGATTGGAATCC---TTAAAAGAGGTCTATGGGATCATAT
GGATGTTTGTCCCTATTTTTACTCCTGTATTGGGAATCACAATAGGTGTACTAGTAATTGT
GTGGTTAGAAAGAGAAATATCCGCAGGGATACAACAACGTATTGGACCGGAATACGCC
GGCCCTTTGGGAATTCTTCAAGCTCTAGCAGATGGGACAAAACTCCTTTTCAAAGAGA
ATCTTCTTCCATCTAGAGGAGATACTCGTTTATTCAGTATCGGACCATCCATAGCAGTCG
TATCAATTCTACTAAGTTATTCAGTAATTCCTTTTGGCTATCGCCTTGTTCTAGCTGATCT
CAGTATCGGTGTTTTTTTATGGATCGCCATTTCAAGTATTGCTCCCATTGGACTTCTTATG
TCAGGCTATGGATCAAATAATAAATATTCCTTTTTAGGTGGTCTACGAGCTGCTGCCCAA
TCAATTAGTTATGAAATACCATTAACTCTATGTGTGTTATCAATATCTCTACTATCTAATAG
TTCAAGTACAGTTGATATAGTTGAGGCGCAGTCAAAATATGGTTTTTGGGGGTGGAATT
TGTGGCGTCAACCTATAGGGTTTATCGTTTTTCTAATTTCTTCCCTAGCAGAATGTGAGA
GATTACCTTTTGATTTACCAGAAGCAGAGGAAGAATTAGTAGCAGGTTATCAAACTGAA
TATTCAGGTATCAAATTTGGTTTATTTTACGTTGCTTCCTATCTAAATCTACTAGTTTCTTC
ATTATTTGTAACAGTTCTTTACTTGGGCGGTTGGAATCTTTCTATTCCGTACATATTCGTT
CCTGAGCTTTTTGAAATAAATAAAGTGAATGGAGTCTTTGGAACGACAATTGGCATCTT
TATTACATTAGCTAAAACTTATTTGTTCTTGTTCATTCCTATCACAACAAGATGGACTTTA
CCTAGGCTGAGAATGGACCAACTATTAAATCTTGGATGGAAATTTCTTTTACCTATTTCT

CTAGGTAATCTATTATTAACAACTTCTTCCCAACTCCTTTCACTGATGATCTGGCATGTA
CAGAATGAAAACTTCATTCTCGATTCTACGAGAATTTTTATGAAAGCGTTTCATTTGCTT
CTCTTCGATGGAAGTTTTATTTTCCCAGAATGTATCCTAATTTTTGGCCTAATTCTTCTTC
TGATGATCGATTCAACCTCTGATCAAAAAGATATACCTTGGTTATATTTCATCTCTTCAA
CAAGTTTAGTAATGAGCATAACGGCCCTATTGTTCCGATGGAGAGAAGAACCTATGATT
AGCTTTTCGGGAAATTTCCAAACGAACAATTTCAACGAAATCTTTCAATTTCTTATTTTA
CTATGTTCAACTCTATGTATTCCTCTATCCGTAGAGTACATTGAATGTACAGAAATGGCTA
TAACAGAGTTTCTGTTATTCATATTAACAGCTACTCTAGGAGGAATGTTTTTATGCGGTG
CTAACGATTTAATAACTATCTTTGTAGCTCCAGAATGTTTCAGTTTATGCTCCTACCTATT
ATCTGGATATACCAAGAAAGATGTACGGTCTAATGAGGCTACTACGAAATATTTACTCAT
GGGTGGGGCAAGCTCTTCTATTCTGGTTCATGGTTTCTCTTGGCTATATGGTTCATCCGG
GGGAGAGATCGAGCTTCAAGAAATAGTGAATGGTCTTATCAATACACAAATGTATAACT
CCCCAGGAATTTCAATTGCGCTTATATTCATCACTGTAGGAATTGGGTTCAAGCTTTCCC
CAGCCCCTTCTCATCAATGGACTCCTGACGTATACGAAGGATCTCCCACTCCAGTCGTT
GCTTTTCTTTCTGTTACTTCGAAAGTAGCTGCTTCAGCTTCAGCCACTCGAATTTTCGAT
ATTCCTTTTTATTTCTCATCAAACGAATGGCATCTTCTTCTGGAAATCCTAGCTATTCTTA
GCATGATATTGGGGAATCTCATTGCTATTACTCAAACAAGCATGAAACGTATGCTTGCAT
ATTCGTCCATAGGTCAAATCGGATATGTAATTATTGGAATAATTGTTGGAGACTCAAATG
ATGGATATGCAAGCATGATAACTTATATGCTGTTCTATATCTCCATGAATCTAGGAACTTT
TGCTCGCATTGTATCATTTGGTCTACGTACCGGAACTGATAACATTCGAGATTATGCAGG
ATTATACACGAAAGATCCTTTTTTGGCTCTCTCTTTAGCCCTATGTCTCTTATCCCTAGGA
GGTCTTCCTCCACTAGCAGGTTTTTTCGGAAAACTCCATTTATTCTGGTGTGGATGGCA
GGCAGGCCTATATTTCTTGGTTTCAATAGGACTCCTTACGAGCGTTGTTTCTATCTACTAT
TATCTAAAAATAATCAAGTTATTAATGACTGGACGAAACCAAGAAATAACCCCTCACGT
GCGAAATTATAGAAGATCTCCTTTAAGATCAAACAATTCCATCGAATTGAGTATGATTGT
ATGTGTGATAGCATCTACTATACCAGGAATATCAATGAACCCGATTATTGCAATTGCTCA
GGATACCCTTTTTATGTTTCTGCTTTACGAATATGATATTTTCTGGGCATTTCTAATAATAT
CAAGCGTTATTCCTATTTTGGCATTTCTAATTTCCGGAGTTTTAGCTCCGATTAGCAAAG
GACCAGAGAAACTTTCTAGTTATGAATCGGGTATAGAACCAATGGGCGATGCTTGGTTA
CAATTTAGAATCCGGTATTATATGTTTGCTCTAGTTTTTGTTGTTTTTGATGTTGAAACGG
TTTTTCTTTATCCATGGGCAATGAGTTTCGATGTATTGGGTGTATCTGTATTTATAGAAGC
TTTCATTTTCGTGCTTATCCTAATTGTTGGTTCAGTTTATGCATGGCGAAAAGGAGCATT
GGAATGGTCTACGAATTATTTTCCTTGGTTAACAATAATTGTAGTTTTGCCGATATCTGC
GGGTTCTTTAATTTTCTTTCTCCCTCATAGAGGAAATAAGATAATTAGGTGGTATACTATA
TGTATATGTATACTAGAACTCCTTCTAACGACCTATGCATTCTGTTATCATTTCCAATTCG
ACGATCCATTAATTCAACTGGCGGAGGATTATAAATGGATCGATTTTTTTGATTTCCACT
GGAGATTGGGAATAGATGGACTTTCTATAGGGCCCATTTTACTGACGGGATTTATCACTA
CTTTAGCTACTTTAGCGGCTTGGCCAGTTACTCGAGATTCTCGATTATTTCATTTCCTGAT
GTTAGCAATGTACAGCGGTCAAATAGGATCATTTTCTTCTCGAGACCTTTTACTTTTTTT
CATCATGTGGGAGTTAGAATTAATTCCCGTTTATCTACTTCTATCCATGTGGGGAGGAAA
GAAACGTCTGTACTCAGCTACAAAGTTTATTTTGTACACTGCAGGAGGTTCCGTCTTTC
TCTTAATGGGAGTTCTGGGTGTCGGTTTATATGGTTCCAATGAACCAACATTAAATTTTG
AAACATCAACTAATCAATCATATCCTGTACCATTGGAAATAATATTCTATATTGGATTTCT
TATTGCTTTTGCTGTCAAATCACCGATTATACCTCTACATACATGGTTACCAGATACCCAC

GGAGAAGCACATTACAGTACTTGTATGCTTCTAGCTGGAATCTTATTAAAAATGGGAGC
GTATGGATTGGTTCGGATCAATATGGAATTATTACCTCACGCTCATTCTATATTTTCTCCT
TGGTTAATGATAGTAGGCACAATGCAAATAATCTATGCAGCTTTAACATCTCCTGGTCAA
CGTAATTTAAAAAAAAGAATAGCCTACTCCTCTGTATCTCATATGGGTTTCATAATTATA
GGAATTTGTTCTATAACCGATACGGGACTCAATGGAGCCATTTTACAAATAATCTCTCAT
GGATTTATTGGTGCTGCGCTTTTTTTCTTGGCAGGAATGAGTTATGATAGAATACGTCTT
GTTTATCTCGATGAAATGGGCGGAATAGCTATCCCAATGCCAAAAATATTTACGATGTTC
AGTAGCTTCTCGATGGCTTCCCTTGCATTACCAGGTATGAGTGGTTTTGTTGCAGAATT
GATAGTCTTTTTTGGAATAATTACCAGCCAAAAATATCTTTTAATGCCAAAAATACTAAT
TACTTTTGTAATGGCAATTGGAATGATATTAACGCCTATTTATTCATTATCTATGTCACGC
CAGATGTTCTATGGATACAAGCTATTTAATGCTCCAAACTCTTATTTTTTTGATTCTGGAC
CGCGAGAATTATTTGTTTCGATCTCTATCTTTCTACCCGTAATAGGTATTGGTATTTACCC
GGATTTTGTTCTCTCACTATCAGTTGACAAGGTCGAAGCTATTCTATCTAATTATTTTTAT
AGAATGATGCTTGAACATGTACTTGTTTTGAGTGCCTATTTATTTTCTATCGGTATCTATG
GATTGATCACGAGCCGAAATATGGTTAGAGCCCTTATGTGTCTTGAACTTATATTGAATG
CAGTTAATATGAATTTCGTAACATTTTCTGATTTTTTTGATAGTCGCCAATTAAAAGGAG
ACATTTTCTCAATTTTTGTTATAGCTATTGCAGCCGCTGAAGCAGCTATTGGACCAGCTA
TAGTTTCGTCAATTTATCGTAACAGAAAATCAACTCGTATCAATCAATCTAATTTATTGA
ATAAGATGGAACATACATATCAATATGCATGGATCATACCTTTCGTTCCACTTCCAGTTC
CTATGTTAATAGGAGTGGGACTTCTCCTTTTTCCGACGGCAACAAAAAATCTTCGTCGT
ATGTGGGCTTTTCCTAGTGTTTTATTGTTACTTATAGTTATGATTTTTTCGATCGATCTGTC
TATTCAGCAAATAAATAGCAGTTCTATCTATCAGTATGTATGGTCTTGGATCATCAATAAT
GATTTTTCTTTAGAGTTCGGCTACTTGATCGATCCACTTACTTCTATTATGTCAATATTAA
TCACTACTGTTGGAATTATGGTTCTTATTTATAGTGACAATTATATGTCTCATGATCAAGG
ATATTTGAGATTTTTTGCTTATATGAGTTTTTCCAATACTTCCATGTTGGGATTAGTTACT
AGTTCTAATTTGATACAAATTTATATTTTTTGGGAATTAGTTGGAATGTGTTCGTATCTAT
TAATAGGTTTTTGGTTCACACGACCTATTGCGGCAAATGCTTGTCAAAAAGCCTTTGTA
ACTAATCGTGTAGGGGATTTTGGTTTATTATTAGGAATTTTAGGTTTTTATTGGATAACAG
GTAGTTTCGAATTTCGGGATTTGTTCGAAATATTCAATAACAATGAGGTCAATTTTGTAT
TTGTTACTTTGTGTGCCTCCCTATTATTTGCCGGTGCAGTTGCGAAATCTGCGCAATTCC
CCCTTCATGTATGGTTACCTGATGCCATGGAGGGGCCTACTCCTATTTCGGCCCTTATAC
ATGCTGCTACCATGGTAGCAGCGGGGATTTTTCTTGTAGCTCGGCTTCTTCCTCTTTTCA
TAGTCATACCTTACATAATGAATCTAATATCTTTGATAGGTATAATAACAGTATTATTAGG
AGCTACTTTAGCTCTTGCTCAAAAAGACATTAAGAGAGGTTTAGCCTATTCTACAATGT
CTCAATTGGGTTATATGATGTTAGCTCTAGGTATGGGGTCTTATCGAGCTGCTTTATTTCA
TTTGATTACTCATGCTTATTCGAAAGCATTATTGTTTTTAGGATCTGGATCAGTTATTCAT
TCAATGGAAGCTGTTGTTGGATATTCTCCAGATAAAAGTCAGAATATGGTTCTTATGGG
AGGTTTAACAAAACATGTGCCAATTACAAAAACTGCTTTTTTATTAGGTACACTTTCTCT
TTGTGGTATTCCACCTCTTGCTTGTTTTTGGTCCAAAGATGAAATTCTTAATGATAGTTG
GTTGTATTCACCGATTTTCGCAATAATAGCTTGTTCCACAGCAGGATTAACCGCATTTTA
TATGTTTCGGATCTATTTACTTACTTTTGAAGGGCATTTAAACGTTCATTTTCAAAATTAC
AGTGGCAAAAAAAATAGCCTGTTCTATTCAATATCTCTATGGGGTAAAGAAGGGCCGA
AAACTATTAACAAAAATTTATTAACTTTATTAACAATGAATTTTTTTTCGTTTGGCAATAA
GAAGACTTCCTCCTATCCTCGTGAATCAGGCAATACTATGCTATTCCCTCTGCTTGTATT

GGGCCTATTTACTTTGTTCGTTGGATCCATAGGAATTCCTTTCGGTTTGGATATATTATCC
AAATGGTTAACTCCGTCTATAAACCTTTTACACCAAAATTCGAAT---TCTGTGGATTGGT
ATGAATTTGTGACAAATGCAATTTTTTCAGTCAGTATAGCTTATTTCGGAATATTTATAGC
GTTTTTTTTATATAAACCTGTTTATTCATCTTTACAAAATTTGAACTTAATTAATTCATTTG
CTACTCTTTGGGACAAAATAATAAATGTGCTATATGATTGGTCATATAATCGTGGTTACAT
AGATGCTTTTTATGCAACATCCTTAACTAAGGGTATAAGAGGATTGGCTGAACTAACTC
ATTTTTTTGATAGACGAGTAATTGATGGAATTACGAATGGGTTTGGTGTTACAAGTTTCT
TTGTAGGAGAGGGTATCAAATATGTAGGGGGTGGG---CGCATCTCTTCTTATCTCTTCTT
CTATTTATCTTATATA------------------------ATGGATTTACCCGGACCAATACATGATTTTCTT
TTAGTTTTTCTGGGATCAGGTCTTATATTAGGGGGTCTCGGAGTGGTATTACTTACCAAT
CCAATTTATTCTGCATTTTCATTGGGATTGGTTCTTATTTGTATATCTTTATTCTATATTCTA
TCGAACTCCCATTTTGTAGCTGCTGCACAGCTCCTTATTTACGTAGGAGCTATAAATGTT
TTAATCATATTTGCTGTGATGTTCATGAATGGTTCAGAATATTACAAAGATTTTCATCTTT
GGACCATTGGAGATGGGGTTACTTCGCTGGTTTGTACGAGTATTTTTGTTTCATTAATTA
CTACTATTCCAGATACGTCATGGTACGGGATTATTTGGACTACAAGATCAAACCAGATTA
TAGAACAAGATTTGATAAGTAATAGCCAACAAATTGGGATTCATTTATCAACAGATTTTT
TTCTTCCGTTTGAACTCATTTCGATAATTCTTTTAGTTGCTTTGATAGGGGCAATTGCTG
TGGCTCGTCAGATGACTGTACCAGCTACAAGAAAAGACCTCATGATAGTCAATATGGGT
CCTCACCATCCATCAATGCATGGTGTTCTTCGACTCATCGTTACTCTAGATGGTGAAGAT
GTTATTGACTGTGAACCAATATTGGGTTATTTACACAGAGGGATGGAAAAAATTGCGGA
AAATCGAACAATTATACAATATCTGCCTTATGTAACACGTTGGGATTATTTAGCTACTATG
TTCACAGAAGCAATAACCGTAAATGCACCAGAGCAGTTGGGAAATATTCAGGTCCCTA
AACGGGCCAGCTATATCAGAGTAATTATGTTGGAGTTGAGTCGTATAGCTTCTCATTTGT
TATGGCTTGGCCCTTTTATGGCAGATATTGGTGCACAGACTCCTTTCTTCTATATTTTCAG
AGAAAGAGAATTGGTATATGATCTATTCGAAGCTGCCACCGGTATGAGAATGATGCATA
ATTATTTTCGTATCGGAGGAGTAGCTGCTGATCTACCTCATGGCTGGATAGATAAATGTT
TGGATTTCTGCGATTATTTTTTAACAGGGGTTGCTGAATATCAAAAGCTTATTACACGAA
ATCCCGTTTTTTTAGAACGAGTTGAAGGGGTAGGCATTATTGGCGGAGAGGAAGCAAT
AAATTGGGGTTTATCAGGACCAATGCTACGAGCTTCCGGAATACAATGGGATCTTCGTA
AAGTTGATCATTATGAGTGTTACGACGAATTTGATTGGGAAGTCCAATGGCAAAAAGA
AGGAGATTCATTAGCTCGTTATTTAGTACGAATCAGTGAAATGACGGAATCCATAAAAA
TTATCCAACAGGCTCTGGAAGGAATTCCGGGGGGGCCCTATGAGAATTTAGAAATCCG
GCGCTTTGATAGAGTAAGGGATCCCGAATGGAATGATTTTGAATATCGATTCATTAGTAA
AAAGCCTTCTCCTACTTTTGAATTGTCGAAACAAGAACTTTATGTGAGAGTCGAAGCG
CCAAAGGGGGAATTGGGAATTTTTCTGATAGGAGATCAGAGTGTTTTTCCTTGGAGATG
GAAAATTCGTCCACCGGGTTTTATCAATTTGCAAATTCTTCCTCAGTTAGTTAAAAGAA
TGAAATTGGCTGATATTATGACAATACTAGGTAGCATAGATATCATTATGGGAGAAGTTG
ATCGTATGTTCCCTATGGTAACTGGATTCATGAATTATGGTCAACAAACAGTACGAGCTG
CAAGGTACATTGGTCAAAGTTTCATGGTTACCTTATCCCACGCGAATCGTTTACCTGTA
ACTATTCAATATCCTTATGAAAAATTGATCACATCGGAGCGTTTCCGCGGTCGAATCCAC
TTTGAATTTGATAAATGCATTGCTTGTGAAGTATGTGTTCGTGTATGTCCTATAGATCTAC
CCGTTGTTGATTGGAAATTGGAAACTGATATTCGAAAGAAACGATTGCTTAATTACAGT
ATTGATTTCGGAATCTGTATATTTTGTGGTAATTGTGTTGAGTATTGTCCAACAAATTGTT
TATCAATGACTGAAGAATACGAACTTTCTACTTATGATCGTCACGAATTGAATTATAATC

AAATTGCTTTGGGTCGTTTACCAATGTCAGTAATTGACGATTACACAATTCGAACAATTT
TGAATTCG---ATAAAAAATAGA--------------------------------ATGCAGGGTCGTTTGTCTGCT
TGGCTAGTCAAGCATGGGCTAGTTCATAGATCTTTGGGCTTCGATTACCAAGGAATAGA
GACTTTACAAATAAAGCCCGAGGATTGGCATTCCATTGCTGTCATTTTATATGTATATGG
TTACAATTATCTACGTTCCCAATGTGCCTATGATGTAGCACCAGGCGGACTGTTAGCTAG
TGTGTATCATCTTACGAGAATAGAGTATGGTGTGGATCAACCGGAAGAGGTATGCATAA
AAGTATTTGCCCCAAGGAGGAATCCTAGAATTCCGTCTGTTTTTTGGGTTTGGAAAAGT
GCGGATTTTCAAGAACGTGAATCTTATGATATGTTGGGAATCTCTTATGATAATCATCCA
CGCCTGAAACGTATCTTAATGCCCGAAAGTTGGATAGGATGGCCCTTACGTAAGGATTA
TATTGCCCCCAATTTTTATGAAATACAAGATGCTCATATGGGCAATGAGTTTCGATGTATT
GGGTGTATCTGTATTTATAGAAGCTTTCATTTTCGTGCTTATCCTAATTGTTGGTTCAGTT
TATGCATGGCGAAAAGGAGCATTGGAATGGTCTTAGCTCCTGAATATTCAGACAATAAA
AAGAAAAAAGGAAAAAAAAAGAACATTGAGACAGTTATGAATTCCATTGAGTTTCCCT
TACTTGATCGAACAACCCAAAATTCAGCTATTTTAACTACATCAAATGATCTTTCAAATT
GGTCAAGACTCTCTAGTTTATGGCCGCTTCTCTATGGTACCAGTTGTTGCTTCATTGAAT
TTGCTTCATTAATAGGTTCACGATTCGACTTTGATCGTTATGGACTGGTACCAAGATCGA
GTCCTAGACAAGCGGACCTAATTTTAACAGCTGGCACAGTAACAATGAAAATGGCTCC
TTCTTTAGTGAGATTATATGAGCAAATGCCTGAACCAAAATATGTTATTGCTATGGGAGC
ATGTACAATTACAGGAGGGATGTTCAGTACCGATTCTTATAGTACTGTTCGGGGAGTCG
ATAAGCTAATTCCTGTGGATGTCTATTTGCCGGGCTGTCCACCTAAACCGGAAGCGGTT
ATAGATGCTATAACAAAACTTCGTAAGAAAATATCTCGAGAAATCTATGAAGATAGAATT
AGGTCTCAAGAGGAGAATCGGTGTTTTACTACCAATCACAAGTTTCATGTTGGACGCA
GTACTCATACTGGAAATTATAATCAAGGATTACTCTATCAATCGCCATCTACTTCAGAGA
TCCCTCCTGAAACATTTTTCAAATACAAAAGTTCAGTATCTTCCCACGAATTAGTGAATA
TGCAAACTAGAAATACCTTTTCTTGGATAAAGGAACAGATTACTCGATCTATTTCCGTAT
CGCTCATGATATATATAATAACTCGGACATCCATTTCAAATGCATATCCCATTTTTGCACA
GCAGGGTTATGAAAATCCACGAGAGGCGACTGGGCGTATTGTATGTGCCAATTGCCATT
TAGCTAATAAACCCGTGGATATTGAGGTTCCACAAGCGGTACTTCCTGATACTGTATTTG
AAGCAGTTGTTCGAATTCCTTATGATATGCAACTGAAACAAGTTCTTGCTAATGGTAAA
AAAGGGGCTTTGAATGTAGGGGCTGTTCTTATTTTACCTGAGGGGTTTGAATTAGCCCC
CCCCGATCGTATTTCTCCTGAGATGAAAGAAAAGATAGGCAATCTGTCTTTTCAGAGCT
ATCGCCCCACTAAAAAAAATATTCTTGTGATAGGTCCTGTTCCTGGTCAGAAATATAGT
GAAATCACCTTTCCTATTCTTTCCCCGGACCCCGCTACTAAGAAAGATGTTCACTTCTTA
AAATATCCCATATACGTAGGCGGAAACAGGGGGAGGGGTCAGATTTATCCCGACGGGA
GCAAGAGTAACAATACGGTTTATAATGCTACAGCAGCAGGTATAGTAAGCAAAATCATA
CGAAAAGAAAAGAGAGGATACGAAATAACCATAGCAGATGCATCGGATGGACGTCAA
GTGGTTGATATTATCCCTCCAGGACCAGAACTTCTTGTTTCAGAGGGTGAATCTATCAA
ACTTGATCAACCATTAACGAGTAATCCTAATGTGGGTGGATTTGGTCAGGGAGATGCAG
AAATAGTACTTCAAGATCCATTACGTGTCCAAGGCCTTTTGTTCTTTTTGGCATCTGTTA
TTTTGGCACAAATCTTTTTGGTTCTTAAAAAGAAACAGTTCGAGAAGGTTCAATTGTCC
GAAATGAATTTCATGAGTAAAGTCTATGATTGGTTCGAAGAACGTCTAGAGATTCAGGC
GATTGCAGATGATATAACTAGTAAATACGTTCCTCCTCATGTTAACATATTTTATTGTCTA
GGAGGAATTACGCTTACTTGTTTTTTAGTACAAGTAGCTACGGGGTTTGCTATGACTTTT
TACTATCGTCCGACTGTTACTGAGGCTTTTGCTTCTGTTCAATACATAATGACTGAAGCT

AACTTTGGTTGGTTAATCCGATCAGTTCATCGATGGTCGGCAAGTATGATGGTTCTAATG
ATGATCCTGCACGTATTTCGTGTGTATCTCACCGGTGGATTTAAAAAACCTCGCGAATT
GACTTGGGTTACAGGCGTGGTTCTGGCTGTATTGACCGCGTCTTTTGGTGTAACTGGTT
ATTCCTTACCTCGGGACCAAATTGGTTATTGGGCAGTAAAAATTGTAACAGGCGTACCT
GAAGCTATTCCTGTAATAGGATCGCCTTTGGTAGAGTTATTACGCGGAAGTGCTAGTGT
GGGACAATCCACTTTAACCCGCTTTTATAGTTTACACACTTTTGTATTGCCTCTGCTTAC
TGCCGTATTTATGTTAATGCACTTCCCAATGATACGTAAACAAGGTATTTCTGGTCCTTT
AATGGGAGTAACAAAAAAACCTGACTTGAATGATCCTGTATTAAGAGCTAAATTAGCTA
AAGGGATGGGTCATAATTATTACGGAGAACCCGCATGGCCCAATGATCTTTTATATATTT
TTCCAGTAGTCATTCTAGGTACTATTGCATGTAACGTAGGCTTAGCGGTTCTAGAACCAT
CAATGATTGGTGAACCGGCAGATCCATTTGCAACTCCTTTGGAAATATTACCTGAATGG
TATTTCTTTCCCGTATTTCAAATACTTCGTACAGTACCCAATAAGTTATTGGGTGTTCTTT
TAATGGTTTCAGTACCTGCGGGATTATTAACAGTACCTTTTTTGGAGAATGTTAATAAAT
TCCAAAATCCATTTCGTCGTCCAGTAGCGACAACCGTCTTTTTGGTTGGTACCGCAGTG
GCCCTTTGGTTAGGCATTGGAGCAACATTACCTATTGATAAATCCCTAACTTTAGGTCTT
TTTATGATTGAAGTTTTTCTATTTGGAATCGTGTTAGGTCTAATTCCTATTACTTTGGCTG
GATTATTCGTAACTGCATATTTACAATACAGGCGTGGTGATCAGTTGGACCTTATGCCTA
CTATAACTAGTTATTTCGGTTTTCTACTAGCGGCTTTAACTATAACCTCAGCTCTATTTAT
TGGTCTGAGCAAAATACGACTTATTATGGATATAGTAAGTCTCGCTTGGGCTGCTTTAAT
GGTAGTCTTTACATTTTCTCTTTCACTCGTAGTATGGGGAAGAAGTGGACTCATGATTAT
TCGTTCGCCGGAACCAGAAGTAAAAATTTTGGTAGATAGGGATCCTATAAAAACTTCTT
TCGAGGAATGGGCCAGACCCGGCCATTTCTCAAGAACAATAGCTAAGGGCCCTGATAC
TACCACTTGGATCTGGAACCTACATGCTGATGCTCACGATTTCGATAGCCATACCAGTG
ATTTGGAGGAGATCTCTCGAAAAGTATTTAGTGCCCATTTCGGTCAACTCTCCATCATCT
TTCTTTGGCTGAGTGGCATGTATTTCCACGGTGCTCGTTTTTCCAATTATGAAGCATGGC
TAAGCGATCCTACTCACATTGGACCTAGTGCCCAGGTGGTTTGGCCAATAGTAGGCCAA
GAAATATTGAATGGTGATGTGGGTGGGGGTTTCCGAGGAATACAAATAACCTCTGGTTT
TTTTCAGCTTTGGCGAGCATCTGGAATAACTAGTGAATTACAACTCTATTGTACCGCAAT
TGGTGCATTGGTTTTTGCAGCGTTAATGCTTTTTGCTGGTTGGTTCCATTATCACAAAGC
TGCTCCAAAATTGGCTTGGTTCCAAGATGTAGAATCTATGTTGAATCACCATTTAGCGG
GGCTACTAGGACTTGGGTCTCTTTCTTGGGCGGGGCATCAAGTACATGTATCTTTACCG
ATTAACCAATTTCTAAACGCTGGAGTAGATCCTAAAGAGATACCACTTCCTCATGAATTT
ATCTTGAATCGGGATCTTTTGGCTCAACTTTATCCCAGTTTTGCCGAGGGAGCAACCCC
ATTTTTCACCTTGAATTGGTCAAAATATGCGGAATTTCTTACTTTTCGTGGAGGATTAGA
TCCAGTAACTGGGGGTCTATGGCTGACCGATATTGCGCACCATCATTTAGCTATTGCAAT
TCTTTTCCTGATAGCGGGTCACATGTATAGGACTAATTGGGGCATTGGTCATGGCCTAAA
AGATATTTTAGAGGCTCATAAAGGTCCATTTACAGGCCAGGGCCATAAAGGCCTATATG
AGATACTAACAACGTCATGGCATGCTCAATTATCTCTTAACCTAGCTATGTTAGGCTCTT
TAACCATTGTTGTAGCTCACCATATGTATTCCATGCCTCCTTATCCATATCTAGCTACTGA
CTATGGTACACAACTGTCATTGTTCACACATCACATGTGGATTGGTGGATTTCTCATAGT
TGGTGCTGCTGCGCATGCAGCTATTTTTATGGTAAGAGACTATGATCCAACTACTCGATA
CAACGATCTATTAGATCGTGTCCTTAGGCATCGCGATGCAATCATATCACATCTCAACTG
GGCATGTATATTTCTAGGCTTTCACAGTTTTGGTTTGTATATTCATAATGATACCATGAGT
GCTTTAGGGCGTCCCCAAGATATGTTTTCAGATACCGCTATACAATTACAACCCGTCTTT

GCTCAATGGATACAAAACACCCATGCTTTAGCACCTGGTGCAACGGCTCCTGGTGCAA
CAACAAGTACCAGTTTGACTTGGGGGGGTGGTGATTTAGTAGCAGTGGGTGGCAAGGT
AGCTTTGTTACCCATTCCATTAGGAACCGCAGATTTTTTGGTACATCACATTCATGCATT
TACGATTCATGTGACAGTCTTGATACTCTTGAAGGGTGTTCTATTTGCTCGTAGCTCCCG
TTTGATACCGGATAAAGCAAATCTTGGTTTTCGGTTCCCTTGTGATGGACCTGGAAGAG
GGGGTACGTGTCAAGTATCCGCTTGGGATCATGTCTTCTTAGGGCTATTCTGGATGTACA
ATGCAATTTCGGTAGTAATATTCCATTTCAGTTGGAAAATGCAGTCAGACGTTTGGGGT
AGTATAAGTGATCAAGGGGTGGTAACTCATATCACGGGAGGAAACTTTGCGCAGAGTT
CCATTACTATTAATGGGTGGCTCCGCGATTTCTTATGGGCACAGGCATCTCAGGTAATTC
AGTCTTATGGTTCTTCATTATCTGCATATGGCCTCTTTTTCTTAGGTGCTCATTTTGTATG
GGCTTTTAGTTTAATGTTTCTATTCAGCGGGCGTGGTTATTGGCAAGAACTTATTGAATC
CATCGTTTGGGCTCATAATAAATTAAAAGTTGCTCCTGCTACTCAGCCTAGAGCCTTGA
GCATTGTACAAGGACGTGCTGTAGGAGTAACCCATTACCTTCTGGGTGGAATTGCCAC
AACATGGGCGTTCTTCTTAGCAAGAATTATTGCAGTAGGAATGGCATTAAGATTTCCAA
GGTTTAGCCAAGGCTTAGCTCAGGACCCCACTACTCGTCGTATTTGGTTTGGTATTGCT
ACCGCACATGACTTCGAGAGTCATGATGATATTACTGAGGAACGTCTTTATCAGAATATT
TTTGCTTCTCACTTCGGGCAATTAGCAATAATTTTTCTGTGGACTTCCGGAAATCTGTTT
CATGTAGCTTGGCAAGGAAATTTTGAGTCATGGGTACAGGACCCTTTACACGTAAGAC
CTATTGCTCATGCAATTTGGGATCCTCATTTTGGTCAACCGGCTGTGGAAGCTTTTACTC
GAGGGGGTGCTCTTGGCCCAGTGAATATCGCTTATTCTGGTGTTTATCAGTGGTGGTATA
CAATCGGTTTACGCACTAATGAAGATCTTTATACTGGAGCTCTTTTTCTATTATTTCTTTC
TGCCATATCCTTAATAGCGGGTTGGTTACACCTACAACCGAAATGGAAACCGAGCGTTT
CATGGTTCAAAAATGCCGAATCTCGTCTCAATCATCATTTGTCAGGACTCTTCGGAGTA
AGTTCCTTGGCTTGGACAGGACATTTAGTCCATGTCGCTATTCCTGGAGCCAGGGGGG
AGTACGTTCGATGGAATAATTTCTTAGATGTATTACCGCATCCCCAAGGGTTAGGACCA
CTTTTTACAGGTCAGTGGAATCTTTATGCTCAAAACCCCGATTCAAGTAGTCATTTATTT
GGTACCTCCCAAGGAGCAGGAACTGCCATTCTAACCCTTCTCGGGGGATTCCATCCAC
AAACGCAAAGTTTATGGCTGACTGATATTGCTCATCATCATTTAGCTATTGCATTTATTTT
TCTCATTGCTGGTCATATGTATAGAACTAACTTCGGGATTGGGCACAGTATGAAAGATCT
TTTAGAAGCACATATTCCTCCTGGGGGACGATTGGGACGTGGACATAAGGGTCTTTATG
ACACAATCAATAATTCGATTCATTTTCAATTAGGCCTTGCTCTAGCCTCTTTAGGGGTTA
TTACTTCCTTGGTAGCTCAACACATGTACTCTTTGCCTGCTTATGCGTTCATAGCACAAG
ACTTTACTACTCAAGCTGCGTTATATACTCATCACCAATACATCGCAGGATTCATCATGA
CAGGAGCTTTTGCTCATGGAGCTATATTTTTCATTAGAGATTACAATCCGGAACAGAAT
GAGGATAATGTATTGGCAAGAATGTTAGACCATAAAGAAGCTATCATATCCCATTTAAGT
TGGGCCAGCCTCTTTCTAGGGTTCCATACCTTGGGACTTTATGTTCATAATGATGTCATG
CTTGCCTTTGGTACTCCGGAGAAACAAATCTTGATCGAACCTATATTTGCTCAATGGATA
CAATCTGCTCATGGTAAAACTTCATATGGGTTCGATGTACTTTTATCTTCAACGAATGGC
CCGGCATTCAATGCGGGTCGAAGCATATGGTTGCCTGGCTGGTTAAATGCTGTTAATGA
G------AATAGTAATTCACTATTCTTAACAATAGGTCCTGGAGACTTTTTGGTTCATCATGC
TATTGCTCTAGGTTTACATACAACTACATTGATCTTAGTAAAAGGTGCTTTAGACGCGCG
TGGTTCCAAGTTAATGCCAGATAAAAAAGATTTCGGTTATAGTTTTCCTTGCGATGGTC
CGGGGCGGGGCGGTACTTGTGATATTTCGGCTTGGGACGCATTTTATTTGGCAGTTTTC
TGGATGTTAAATACCATTGGATGGGTTACTTTTTATTGGCATTGGAAGCACATCACATTA

TGGCAGGGTAACGTTTCACAATTTAATGAATCTTCCACTTATTTGATGGGATGGTTAAG
AGATTATCTATGGTTAAACTCTTCACAACTTATCAATGGATATAACCCTTTTGGTATGAAT
AGTTTATCGGTCTGGGCATGGATGTTCTTATTTGGACATCTTGTTTGGGCTACTGGATTT
ATGTTCTTAATTTCCTGGCGTGGATATTGGCAAGAATTGATTGAAACTTTAGCATGGGCT
CATGAACGCACACCTTTGGCCAATTTGATTCGATGGAGAGATAAACCAGTGGCTCTTTC
CATTGTGCAAGCAAGATTGGTTGGATTAGCCCATTTTTCTGTAGGTTATATATTCACTTAT
GCGGCTTTCTTGATTGCCTCTACATCGGGTAAATTTGGTATGTCACATTCAGTAAAGATT
TATGATACATGTATAGGGTGTACTCAATGTGTCCGAGCGTGCCCCACAGATGTATTAGAA
ATGATACCTTGGGACGGATGTAAAGCTAAGCAAATTGCTTCTGCTCCAAGAACAGAGG
ACTGTGTTGGTTGTAAGAGATGTGAATCCGCCTGTCCAACAGATTTCTTGAGTGTTCGA
GTTTATTTATGGCATGAAACAACGCGCAGCATGGGTCTAGCTTATATGACAACTATCAAC
TTTCCCTCTATTTTTGTGCCTTTAGTAGGCCTAGTATTTCCGGCAATTGCAATGGCTTCTT
TATCTCTTCATGTTCAAAAAAACAAGATTGTTATGCGAGATCTAAAAACATATCTCTCCG
TGGCACCGGTACTAAGTACTCTATGGTTCGGGTCTTTAGCAGGTCTATTGATAGAGATC
AATCGTTTTTTCCCAGATGCGTTGACATTCCCTTTTTTTTCATTCATGACTGTAATTTTAG
AGAGACGCGAAAGCGAAAGCCTATGGGGTCGCTTCTGTAACTGGATAACCAGCACTG
AAAACCGTCTTTACATTGGATGGTTTGGTGTTGTGATGATCCCTACCTTATTGACCGCAA
CTTCTGTATTTATTATCGCCTTCATTGCTGCTCCTCCAGTAGATATTGATGGTATTCGTGA
ACCTGTTTCTGGATCTCTACTTTATGGAAACAATATTATCTCTGGTGCCATTATTCCTACT
TCTGCAGCTATAGGTTTGCACTTTTACCCGATATGGGAAGCGGCATCCGTTGATGAATG
GTTATACAATGGTGGTCCTTATGAGCTAATTGTTCTACACTTCTTACTTGGTGTAGCTTG
TTACATGGGTCGTGAGTGGGAACTTAGTTTCCGTCTGGGTATGCGCCCTTGGATTGCTG
TTGCATATTCAGCTCCTGTTGCAGCTGCTACTGCTGTTTTCTTGATCTACCCAATTGGTC
AAGGAAGCTTTTCTGATGGTATGCCTCTAGGAATCTCTGGTACTTTCAACTTCATGATTG
TATTCCAGGCTGAGCACAACATCCTTATGCACCCATTTCACATGTTAGGCGTAGCTGGT
GTATTCGGCGGCTCCCTATTCAGTGCTATGCATGGTTCCTTGGTAACCTCTAGTTTGATC
AGGGAAACCACAGAAAATGAATCTGCTAATGAAGGTTACAGATTCGGTCAAGAGGAA
GAAACTTATAATATCGTAGCTGCTCATGGTTATTTTGGCCGATTGATCTTCCAATATGCTA
GTTTCAACAATTCTCGTTCTTTACATTTCTTCCTAGCTGCTTGGCCTGTAGTAGGTATCT
GGTTCACTGCTTTAGGTATCAGCACCATGGCTTTCAACCTAAATGGTTTCAATTTCAAC
CAATCTGTAGTTGATAGTCAAGGTCGTGTAATTAATACTTGGGCTGATATTATTAACCGT
GCTAACCTTGGTATGGAAGTTATGCATGAACGTAATGCTCATAACTTCCCTCTAGACCTA
GCTGCTATCGAAGCTCCATCTACAAATGGA---GGTTTGCCTTGGTATCGTGTTCATACCG
TCGTATTGAATGATCCCGGTCGGTTGCTTTCTGTCCATATAATGCATACAGCTCTGGTTG
CTGGTTGGGCTGGTTCGATGGCTCTATATGAATTAGCAGTTTTTGATCCCTCTGACCCCG
TTCTTGATCCAATGTGGAGACAAGGTATGTTCGTTATACCCTTCATGACTCGTTTAGGAA
TAACCAATTCATGGGGTGGTTGGAGTATCACA---GGAGGGACTATAACAAATCCGGGTA
TTTGGAGTTACGAAGGTGTGGCCGGGGCACATATTGTATTTTCTGGCTTGTGCTTCTTG
GCAGCTATCTGGCATTGGGTGTATTGGGATCTAGAAATATTTTGTGATGAACGTACAGG
AAAACCTTCTTTGGATTTGCCCAAGATCTTTGGAATTCATTTATTTCTCTCAGGGGTGGC
TTGCTTTGGTTTTGGCGCATTTCATGTAACAGGCTTATATGGTCCTGGAATATGGGTGTC
CGATCCTTATGGACTAACTGGAAAAGTACAATCTGTAAATCCAGCGTGGGGCGTGGAA
GGTTTTGATCCTTTTGTTCCGGGAGGAATAGCCTCTCATCATATTGCGGCAGGGACATT
GGGCATATTAGCGGGCCTATTCCATCTTAGTGTCCGCCCGCCCCAACGTCTATACAAAG

GATTACGTATGGGCAATATTGAAACCGTCCTTTCTAGTAGTATCGCTGCTGTCTTTTTTG
CAGCTTTTGTTGTTGCTGGAACTATGTGGTATGGTTCAGCAACTACCCCGATCGAATTAT
TTGGTCCCACTCGTTATCAATGGGATCAGGGATACTTTCAGCAAGAAATATATCGAAGA
GTTGGTGCTGGGTTATCCGAAAATCAAAGTTTATCAGAAGCTTGGTCTAAAATTCCTGA
AAAATTAGCTTTTTATGATTATATCGGCAATAATCCGGCAAAAGGGGGATTATTCAGAGC
GGGCTCAATGGACAACGGGGATGGAATAGCCGTTGGATGGTTAGGACACCCTATCTTT
AGAGATAAAGAAGGGCGTGAACTTTTTGTACGTCGTATGCCTACTTTTTTTGAAACATT
TCCAGTTGTTTTGGTAGACGGAGACGGAATTGTTAGAGCCGATGTTCCTTTTAGAAGG
GCAGAATCGAAGTATAGTGTCGAACAAGTAGGTGTAACTGTTGAGTTCTATGGTGGCG
AACTCAATGGAGTCAGTTATAGTGAGCCTGCTACTGTGAAAAAATATGCTAGACGCGCT
CAATTGGGTGAAATTTTTGAATTAGATCGTGCTACTTTGAAATCCGATGGTGTTTTTCGT
AGCAGTCCAAGAGGTTGGTTTACTTTTGGACATGCTTCGTTTGCTCTGCTCTTCTTTTTC
GGACACATTTGGCATGGTGCTAGAACCTTGTTCAGAGATGTTTTTGCTGGTATTGACCC
AGATTTGGATGCTCAAGTGGAATTTGGAGCATTCCAAAAACTTGGAGATCCAACTACA
AAAAGACAAGTAGTC------------------------------------GTGGAAACGCTCTTTAATGGAACTT
TAGCTTTAGCTGGTCGTGACCAAGAAACCACCGGTTTCGCTTGGTGGGCCGGGAATGC
CCGACTTATCAATTTATCCGGTAAACTACTCGGGGCTCATGTAGCCCATGCCGGATTAAT
CGTATTCTGGGCCGGAGCAATGAACCTATTTGAAGTGGCTCATTTCGTCCCAGAGAAA
CCCATGTATGAACAAGGATTAATTTTACTTCCCCACCTAGCTACTCTAGGTTGGGGGGTA
GGTCCTGGGGGGGAAGTTATAGACACCTTTCCATACTTTGTATCTGGAGTACTTCACTT
AATTTCCTCTGCAGTATTGGGCTTTGGCGGTATTTATCATGCACTTTTGGGACCTGAGAC
TCTTGAAGAATCTTTTCCATTCTTCGGTTATGTATGGAAAGATAGAAATAAAATGACCAC
AATTTTGGGTATTCACTTAATCTTGTTAGGTATAGGTGCTTTTCTTCTAGTATTCAAGGCT
CTTTATTTTGGGGGCGTATATGATACCTGGGCTCCCGGAGGGGGAGATGTAAGAAAAAT
TACCAACTTGACCCTTAGCCCAAGTGTTATATTTGGTTATTTACTAAAATCGCCCTTTGG
AGGAGAAGGATGGATTGTTAGTGTGGACGATTTGGAAGATATAATCGGAGGACATGTAT
GGTTAGGTTCCATTTGTATACTTGGTGGAATCTGGCATATCTTAACCAAACCCTTTGCAT
GGGCTCGCCGTGCACTTGTATGGTCTGGAGAGGCTTACTTGTCTTATAGTTTAGGCGCT
TTAGCTGTCTTTGGTTTCATTGCGTGTTGCTTTGTCTGGTTCAATAATACCGCTTATCCTA
GTGAGTTTTACGGGCCCACTGGACCAGAAGCTTCTCAAGCTCAAGCATTTACTTTTCTA
GTTAGAGACCAACGTCTTGGGGCTAACGTGGGATCCGCTCAAGGACCTACTGGTTTAG
GTAAATATCTAATGCGTTCCCCGACCGGAGAAGTCATTTTTGGAGGAGAAACTATGCGT
TTTTGGGATCTGCGTGCTCCCTGGTTAGAACCTCTAAGGGGTCCAAATGGTTTGGACTT
GAGTAGGCTGAAAAAAGACATACAACCTTGGCAAGAACGGCGTTCCGCGGAATATATG
ACTCATGCTCCTTTAGGTTCTTTAAATTCTGTGGGTGGCGTAGCTACCGAGATCAATGC
AGTCAATTATGTCTCTCCTAGAAGTTGGTTATCTACCTCTCATTTTGTTCTAGGATTCTTC
CTATTCGTAGGTCATTTGTGGCACGCGGGAAGGGCTCGTGCAGCTGCAGCAGGATTTG
AAAAAGGAATTGATCGTGATTTTGAACCTGTTCTTTCCATGACCCCTCTTAACATGACT
ATAGCCCTTGGTAAATTTACCAAAGACGAAAAT---TTATTTGATATTATGGATGACTGGTT
ACGGAGGGACCGTTTCGTTTTTGTAGGTTGGTCAGGTCTATTGCTCTTTCCTTGTGCTTA
TTTCGCTTTAGGAGGTTGGTTCACAGGTACAACTTTTGTAACTTCATGGTATACCCATGG
ATTGGCCAGTTCCTATTTGGAAGGCTGCAATTTCTTAACCGCCGCAGTTTCTACTCCTG
CTAATAGTTTAGCACATTCTTTGTTGTTACTATGGGGTCCTGAAGCACAAGGAGATTTTA
CTCGTTGGTGTCAATTAGGCGGTCTGTGGACTTTTGTTGCTCTCCATGGCGCTTTCGGA

CTAATAGGTTTCATGTTACGTCAATTCGAACTTGCTCGATCTGTTCAATTGCGACCTTAT
AATGCAATCGCATTCTCTGCTCCAATTGCTGTTTTTGTTTCTGTATTCCTGATTTATCCAT
TAGGTCAGTCTGGTTGGTTCTTTGCACCCAGTTTCGGTGTAGCAGCTATATTTAGATTCA
TTCTCTTTTTTCAAGGGTTTCATAATTGGACATTGAACCCATTTCATATGATGGGAGTTG
CCGGTGTATTGGGCGCTGCTCTGCTATGCGCTATTCATGGTGCTACCGTAGAAAATACTT
TATTTGAAGATGGTGATGGTGCAAATACATTCCGTGCTTTTAACCCAACTCAAGCTGAA
GAAACTTATTCAATGGTCACCGCTAACCGCTTTTGGTCCCAAATCTTTGGGGTTGCTTT
TTCCAATAAACGTTGGTTACATTTCTTTATGTTATTTGTACCAGTAACCGGTTTATGGATG
AGTGCTCTTGGAGTAGTCGGTCTGGCTCTGAACCTACGTGCCTATGACTTCGTTTCCCA
GGAAATCCGTGCAGCGGAAGATCCTGAATTTGAGACTTTCTACACCAAAAATATTCTCT
TAAACGAAGGTATTCGTGCTTGGATGGCGGCTCAAGATCAGCCTCATGAAAACCTTATA
TTCCCTGAGGAGGTTCTACCCCGTGGAAACGCTCTTATGTCTGGAAGCACAGGAGAAC
GTTCTTTTGCTGATATTATTACCAGTATTCGATACTGGGTCATTCATAGCATTACTATACCT
TCCCTATTCATTGCAGGTTGGTTATTCGTCAGCACGGGTTTAGCTTACGATGTGTTTGGG
AGCCCTCGGCCAAACGAGTATTTCACAGAGAGCCGACAAGGAATTCCATTAATAACTG
GCCGTTTTGATTCTTTGGAACAACTCGATGAATTTAGTAGATCCTTTATGACCATAGATC
GAACCTATCCAATTTTTACAGTGCGCTGGTTGGCTGTTCACGGACTAGCTGTACCTACC
GTTTCTTTTTTGGGGTCAATATCAGCAATGCAGTTCATCCAACGAATGGCTACACAAAC
CGTTGAGGGTAGTTCTAGATCTGGT---CCAAGACGAACTATTATAGGGGATTTATTGAAA
CCATTGAATTCGGAATATGGCAAAGTAGCTCCTGGATGGGGAACTACTCCTTTGATGGG
TGTCGCAATGGCTCTATTTGCGATATTCCTATCTATTATTTTGGAGATTTATAATTCTTCCG
TTTTACTGGATGGAATTTTAATGAATATGCTTACTCTCAAACTCTTCGTTTATACAGTAGT
GATCTTTTTTGTTTCTCTCTTCATCTTCGGATTCCTATCTAATGATCCAGGACGTAATCCT
GGACGTGAAGAAATGGCCGATACTACTGGAAGGATTCCTCTTTGGCTAATAGGTACTGT
AACTGGTATTCCTGTGATCGGTTTAATAGGTATTTTCTTTTATGGTTCATATTCCGGATTG
GGTTCATCCCTGATGCTTAATATCTTTAGTTTGATCTGTATCTGTCTTAATTCTGCCCTTTA
T---AGTAATTTTTTCTTCGCCAAATTGCCCGAGGCCTACGCTTTTTTGAATCCAATCGTA
GATTTTATGCCAGTCATACCTGTGCTCTTTTTTCTCTTAGCCTTTGTTTGGCAAGCTGCT
GTAAGTTTTCGAACGACACAATCAAACCCGAACGAACAAAATGTTGAATTGAATCGTA
CCAGTCTCTACTGGGGGTTATTACTCATTTTTGTACTTGCTGTTTTATTTTCCAATTATTT
CTTCAATATGGAAGTAAATATTCTTGCATTTATTGCTACTGCACTGTTCATTCTAGTTCCT
ACTGCTTTTTTACTTATCATCTACGTAAAAACAGTTAGTCAAAATGATATGGAAACAGC
AACCCTAGTCGCCATCTCTATATCTGGTTTACTTGTAAGTTTTACTGGGTACGCCTTATAT
ACCGCTTTTGGGCAACCCTCTCAACAACTAAGAGATCCATTCGAGGAACACGGGGACA
TGGAAGCATTGGTTTATACATTTCTCTTAGTCTCGACTCTAGGGATAATTTTTTTCGCTAT
CTTTTTTCGAGAACCGCCTAAAGTTCCAACTAAAAAGTTGAAAATGACTATTGCTTTCC
AATTGGCTGTTTTTGCATTAATTGCTACTTCATCAATCTTACTGATTAGTGTACCCGTTGT
ATTTGCTTCTCCTGATGGTTGGTCAAGTAACAAAAATGTTGTATTTTCCGGTACATCATT
ATGGATTGGATTAGTCTTTCTGGTGGGTATCCTTAATTCTCTCATCTCT--------------------A
TGTCACCACAAACAGAGACTAAAGCAAGTGCTGGATTCAAAGCTGGTGTTAAAGATTA
CAAATTGACTTATTATACTCCTGAATATGAAACCAAAGATACTGATATCTTGGCAGCATT
CCGAGTAACTCCTCAACCTGGAGTTCCACCTGAGGAAGCAGGGGCCGCGGTAGCTGC
CGAATCTTCTACTGGTACATGGACAACTGTGTGGACCGATGGACTTACCAGCCTTGATC
GTTACAAAGGACGATGCTACCACATCGAGCCCGTTGCTGGAGCAGAAAATCAATTTATT

GCTTATGTAGCTTACCCCTTAGACCTTTTTGAAGAAGGTTCTGTTACTAACATGTTTACT
TCCATTGTGGGTAATGTATTTGGGTTCAAAGCCCTGCGCGCTCTACGTCTGGAGGATCT
GCGAATCCCTCCTGCTTATTCTAAAACTTTCCAAGGCCCGCCTCATGGCATCCAAGTTG
AGAGAGATAAATTGAACAAGTATGGTCGTCCCCTGTTGGGATGTACTATTAAACCTAAA
TTGGGGTTATCTGCTAAGAACTACGGTAGAGCAGTTTATGAATGTCTTCGCGGTGGACT
TGATTTTACCAAAGATGATGAGAACGTGAACTCCCAACCATTTATGCGTTGGAGAGAC
CGTTTCTTATTTTGTGCCGAAGCACTTTATAAAGCACAGGCTGAAACAGGTGAAATCAA
AGGGCATTACTTGAATGCTACTGCAGGTACATGCGAAGATATGATGAAAAGGGCTGTGT
TTGCCAGAGAATTGGGAGTTCCTATCGTAATGCATGACTACTTAACAGGGGGGTTCACT
GCAAATACTACCTTGGCTCATTATTGCCGAGATAATGGCCTACTTCTTCACATCCATCGT
GCAATGCATGCAGTTATTGATAGACAGAAGAATCATGGTATACACTTTCGTGTACTAGCT
AAAGCGTTACGTATGTCTGGTGGAGATCATATTCACTCTGGTACCGTAGTAGGTAAACT
TGAAGGGGAAAGAGACATCACTTTGGGCTTTGTTGATTTACTACGTGATGATTATATTG
AAAAAGACCGAAGTCGAGGTATTTATTTCCCTCAAGATTGGGTCTCTCTACCAGGTGTT
CTGCCCGTGGCTTCGGGAGGTATTCACGTTTGGCATATGCCTGCTCTGACCGAGATCTT
TGGAGATGATTCCGTACTACAGTTCGGTGGAGGAACTTTAGGACACCCTTGGGGAAAT
GCACCGGGTGCCGTAGCTAATCGAGTAGCTCTAGAAGCATGTGTACAAGCTCGTAATG
AGGGACGTGATCTTGCTCGTGAGGGTAATGAAATTATCCGTGAGGCTAGTAACTGGAG
TCCTGAACTAGCTGCTGCTTGTGCGGTATGGAAGGAGATCAAATTTGAATTCGAAGCA
ATGGATATTTTGGATAAGATGGCGATACATTTATACAAAACTTCTACCCCGAGCACACGC
AATGGAGCCGTAGACAGTCAAGTGAAATCCAATCCACGAAATAATTTGATCTATGGACA
GCATCGTTGTGGTAAAGGTCGTAATGCCAGAGGAATCATTACCGCAGGGCATAGAGGG
GGAGGTCATAAGCGTCTATACCGTAAAATCGATTTTCGACGGAATGAAAAAGACATATA
TGGTAGAATCGTAACCATAGAATACGACCCTAATCGAAATGCATACATTTGTCTCATACA
CTATGGGGATGGTGAGAAGAGATATATTTTACATCCCAGAGGGGCTATAATTGGAGATA
CCATTGTTTCTGGTACAGAAGTTCCTATAAAAATGGGAAATGCCCTACCTTTGACCGATA
TGCCCTTAGGCACGGCCATACATAACATAGAAATCACACTTGGAAAGGGTGGACAATT
AGCTAGAGCAGCGGGTGCTGTAGCGAAACTGATTGCAAAAGAGGGGAAATCGGCCAC
ATTAAAATTACCTTCTGGGGAGGTCCGTTTGATATCCAAAAACTGCTCAGCAACAGTCG
GACAAGTGGGGAATGTTGGGGTAAACCAGAAAAGTTTGGGTAGAGCCGGATCTAAGT
GTTGGCTAGGTAAGCGTCCTGTAGTAAGAGGAGTAGTTATGAACCCTGTAGACCATCCC
CATGGGGGTGGTGAAGGGAGGGCCCCAATTGGTAGAAAAAAACCCACAACCCCTTGG
GGTTATCCTGCACTTGGAAGAAGAAGTAGAAAAAGGAATAAATATAGTGATAATTTGAT
TCTTCGTCGCCGTAGTAAAATGATTCAACCTCAGACCCATTTGAATGTAGCGGACAACA
GCGGGGCTCGAGAATTAATGTGTATTCGAATCATAGGAGCTAGT---AATCGCCGATATGC
TCATATTGGTGATGTTATTGTTGCTGTGATCAAAGAAGCCGTACCAAATATGCCCCTAGA
AAGATCAGAAGTGATCAGAGCTGTAATTGTACGTACCTGTAAAGAACTCAAACGCGAC
AACGGTATGATAATACGATATGATGACAATGCTGCAGTTGTCATTGATCAAGAAGGAAA
TCCAAAAGGAACTCGAGTTTTTGGTGCGATCGCCCGAGAATTGAGACAGTTAAATTTT
ACTAAAATAGTTTCATTAGCTCCTGAGGTATTAATGCTTAGTCCAAAAAGAACCAGATT
CCGTAAACAACATAGAGGAAGAATGAAGGGAATATCTTATCGAGGTAATCGTATTTGTT
TCGGTAGATATGCTCTTCAGGCACTTGAACCCGCTTGGATCACATCTAGACAAATAGAA
GCAGGGCGACGCGCAATGACACGAAATGCACGTCGTGGTGGAAAAATATGGGTACGTA
TATTTCCAGACAAACCAGTTACAGTAAGACCTACGGAAACACGTATGGGTTCGGGGAA

AGGGTCCCCCGAATATTGGGTAGCTGTTGTTAAACCGGGTCGAATACTTTATGAAATGG
GCGGAGTAGCAGAAAATATAGCCAGAAAGGCTATTTCAATAGCGGCGTCCAAAATGCC
TATACGAACTCAATTCATTATTTCGGGAATGACCAGAATTAGACGAGGATATATAGCTCG
GAGACGTAGAACAAAACTTCGTTTATTTGCATCAAGCTTTCGAGGGGCTCATTCAAGA
CTTACTCGAACTATTACTCAACAGAAAATAAGAGCTTTGGTTTCGGCCCATCGGGATAG
AGATAGGCAAAAGAGAGATTTTCGTCGTTTGTGGATTACTCGGATAAACGCAGTAATTC
GCGAG---AATAGGGTATCCTATAGTTATAGTAGATTAATACATGATCTGTACAAAAGACA
GTTGCTTCTTAATCGTAAAATACTTGCACAAATAGCTATATCAAATAGGAATTGTCTTTAT
ATGATTTCCAATGAGATCATAAAA--GAAGTATACGCTTT
AGGTCAACATATATCTATGTCTGCTCACAAAGCGCGAAGAGTAATTGATCAGATTCGTG
GACGTTCCTACGAGGAAACACTTATGATACTCGAACTCATGCCTTATCGAGCATGTTATC
CCATTTTTAAATTGGTTTATTCTGCAGCAGCAAATGCTAGTCACAATATGGGTTTCAACG
AAGCAGATTTAGTCATTAGTAAAGCTGAAGTGAACGAGGGTACTACCGTGAAAAAATT
AAAACCCCGAGCTCGAGGACGTAGTTTTCCGATAAAAAGACCTACTTGTCATATAACTA
TTGTATTGAAAGATATATCCTTAGATGAGGGAGTATGGGACAAAAAAATGGATGGAATC
AAATATGCAGTATTTACAGACAAAAGTATTCGGTTATTGGGGAAAAAT---CAATATACTT
CTAATGTCGAATCAGGATCAACTAGGACAGAAATAAAGCATTGGATCGAACTCTTCTTT
GGTGTCAAGGTAATAGCTATGAATAGTCATCGACTCCCGGGAAAGGGTAGAAGAATGG
GACCTATTATGGGACATACAATGCATTACAGACGTATGATCATTACGCTTCAACCGGGTT
ATTCTATTCCACCTCTTAGAAAGAAAAGAACTATGGCAGTTCCAAAAAAACGTACTTCT
CTGTCAAAAAAGCGTATTCGTAAAAATATTTGGAAAAGGAAGGGATATTGGGCAGCGT
TAAAAGCTTTTTCGTTAGCGAAATCTCTTTCTACCGGAAATTCAAAAAGTTTTTTTGTG
CAACAAAACAAA---ATGGCCAAAGGTAAAGATGTCCGAATAACGGTTATTTTGGAATGT
ACCAGTTGT---GCCCGAAAC------GTTAATAAGGAA------GGCATTTCCAGATATATTACTC
AAAAGAATCGACACAATACGCCTAGTCGATTGGAATTGAGAAAATTCTGTCCCTATTGT
TACAAACATACGATTCATGGGGAGATAAAGAAAATGAAAATAAGGGCCTCTGTTCGTA
AAATTTGTGAAAAATGTCGACTGATCCGTAGGCGGGGACGAATTATAGTAATTTGTTCC
AACCCGAGACATAAACAAAGACAAGGAATGGTTCGAGAGAAAGTAACAGTATCTACT
CGGACACTACAGTGGAAATGTGTTGAATCAAGAGCAGACAGTAAGCGTCTTTATTATG
GACGCTTTATTCTGTCTCCACTTATGAAAGGTCAAGCCGACACAATAGGCATTGCGATG
CGAAGAGCTTTGCTTGGAGAAATAGAAGGAACATGTATCACACGTGCAAAATCTGAG--
----AAAATACCACACGAATATTCTACCATAGTAGGTATTCAAGAATCAGTACATGAAATTT
TAATGAATTTGAAAGAAATTGTATTGAGAAGTAATCTATATGGAACTTGCGACGCGTCTA
TTTGTGTCAGGGGTCCTGGATATGTAACTGCTCAAGACATCATCTCACCACCTTCTGTG
GAAATCGTTGATAATACACAGCATATAGCTAGCTTGACGGAACCAATCGATTTGTGTATT
GGATTACAAATCGAGAGGAATCGCGGATATCGTATAAAAACACTAAAT------AACGACG
GAAGTTAT------CCTATAGATGCTGTATTCATGCCTGTTCGAAATGCGAATCATAGTATTCA
TTCTTATGGAAATGGGAATGAAAAACAAGAGATACTTTTTCTCGAAATATGGACAAATG
GAAGTTTAACTCCTAAAGAAGCACTTCATGAAGCCTCCCGGAATTTGATTGATTTATTTA
TTCCTTTTCTACATGCGGAAGAAGAA------AACTTACATTTAGAGGACAATCAA---CACA
AAGCTACTTTCCCCCTTTTTACCTTTCATGATAGATTGGCT---AAACTAAGGAAAAACAA
AAAA------ATAGCGTTGAAATATATTTTTATTGACCAATTAGAATTGCCTCCCAGGATCTA
TAATTGCCTCAAAAGGTCCAATATACATACATTATCGGACCTTTTGAATAACAGTCAAGA
AGATCTTATGAAAATTGAACATTTTCGCATAGAAGATGTAAAACAGATATTGGGCATTCT

AGAAAAGCAT------TTCACAATTGATTTACCGAAAAATAAGATGCTCCGGGATGGAAAT--
-GAGGGAATGTCTACAATACCTGGGTTTAATCAGATACAATTTGAAGGATTTTGTAGGTT
CATTGATCAGGGCTTGACGGAAGAACTTTTTAAGTTTCCAAAAATTGAAGATACAGATC
AAGAAATTGAATTTCAATTATTTGTGGAAAGATATCAATTGGTAGAACCGTTGATAAAA
GAAAGAAATGCTGTGTATGAATCACTCACATATTCTTCTGAATTATATGTATCCGCGGGA
TTAATTTGGAAAACCAGTAGGGATATGCAAGAACAAACCATTTTTATTGGAAACATTCC
TCTAATGAATTCCCTGGGAACTTCTATAGTAAATGGAATATACAGAATTGTGATCAATCA
AATATTGCAAAGCCCCGGTATTTATTACCGGTCAGAATTGGACCATAACGGAATTTCGG
TCTATACCGGCACCATAATATCAGATTGGGGAGGAAGATCAGAATTAGAGATTGATAGA
AAAGCAAGGATATGGGCTCGTGTGAGTAGGAAACAGAAAATATCTATTCTAGTTCTATC
ATCAGCTATGGGTTCGAATCTAAGAGAAATTCTAGAGAATGTTTGCTACCCTGAAATTT
TCTTGTCTTTCCTGAATGATAAGGAGAAAAAAAAAAATTGGGTCAAAAGAAAATGCCAT
TTTGGAGTTTTATCAACAATTTGCTTGTGTAGGCGGGGATCCGGTATTTTCTGAATCCTT
ATGTAAGGAATTACAAAAGAAATTCTTTCAACAAAGGTGTGAATTAGGAAGGATTGGT
CGACGAAATATGAACCGGAGACTGAACCTTGATATACCTCAGAACAATACATTTCTGTT
ACCGCGAGATATATTGGCAGCTGTGGATCATTTGATTGGAATGAAATTTGGAATGGGTA
CACTTGACGAT------ATGAATCATTTGAAAAATAAACGTATTCGTTCTGTAGCGGATCTCT
TACAAGATCAATTCGGATTGGCTCTGGTTCGTTTAGAAAACGTGGTTCGAGGAACTATA
TGTGGAGCAATTAGG---CATAAATTGATA---CCGACTCCTCAGAATTTGGTAACTTCAAC
TCCA------------TTAACAACCACTTATGAATCTTTTTTCGGCTTACACCCATTATCTCAAGT
TTTGGATCGAACTAATCCATTAACACAAATAGTTCATGGGAGAAAATCGAGTTATTTGG
GCCCTGGAGGATTGACAGGGCGAACTGCTAGTTTTCGGATACGAGATATCCATCCTAGT
CACTATGGGCGTATTTGCCCAATTGACACGTCTGAAGGAATCAATGTTGGACTTATTGG
ATCCTTAGCAATTCATGCGAGGATTGGTCATTGGGGG---TCTCTAGAAAGCCCATTTTAT-
--------GAAATTTCT------GAGAGATCAAAAAAGGTACGGATGCTTTATTTATCACCAAGTA
GAGATGAATACTATATGGTAGCGGCAGGAAATTCTTTGGCCTTGAATCAGGGTATTCAG
GAAGAACAGGTTGTTCCAGCTCGATACCGTCAAGAGTTCCTGACTATTGCATGGGAAC
AGGTTCATCTTCGAAGTATCTTTCCCTTCCAATATTTTTCTATTGGAGCTTCCCTCATTCC
TTTTATCGAGCATAATGATGCGAATCGGGCTTTAATGAGTTCTAATATGCAACGTCAAGC
AGTTCCGCTTTCTCGGCCCGAGAAGTGCATTGTTGGAACTGGGTTGGAACGCCAAGCG
GCTCTAGATTCAGGGGTTCTCGCTATAGCCGAACACGAGGGAAAGATCATTTATACCGC
TACTGACAAGATCATTTTATCAGGTAATGGAGATACTCTAAGCATTCCATTACTTATGTAT
CAACGTTCCAACAAAAATACTTGTATGCATCAAAAACCCCAGGTTCGGCGGGGTAAAT
GCATTAAAAAGGGACAGATTTTAGCGGATGGTGCCGCTACAGTTGGCGGCGAACTCGC
TTTGGGGAAAAACGTATTAGTAGCTTATATGCCATGGGAAGGTTACAATTCTGAAGATG
CGGTACTCATTAGCGAGCGTCTAGTATACGGAGATATTTATACTTCTTTTCACATACGTA
AATATGAAATTCAGACTCATGTGACAAGCCAAGGC---CCTGAAAGGGTCACTAAGGAA
ATACCACATTTAGAAGCCCATTTACTCCGCAATTTAGACAAAAATGGAATTGTGATGCT
GGGATCTTGGGTAGAGGCGGGTGATATTTTAGTAGGTAAATTAACGCCCCAGATGGCGA
AAGAA---TCATCGTATGCCCCGGAAGATAGATTATTACGAGCCATACTTGGCATTCAGGT
ATCCACTTCAAAGGAAACTTGTCTAAAACTACCTATAGGTGGTAGGGGTCGAGTTATTG
ATGTGAGATGGATCCCGAAAAAGGGG---GGTTCTAGTTATAATCCAGAAATGATTCGTG
TATATATTTCACAGAAACGTGAAATCAAAGTAGGTGATAAAGTAGCTGGAAGACATGG
AAATAAAGGTATCGTTTCAAAAATTTTGCCTAGACAAGATATGCCTTATTTGCAAGATG

GAAGACCTGTTGATATGGTCTTCAATCCATTAGGAGTACCTTCACGAATGAATGTAGGA
CAGATATTTGAATGTTCGCTCGGGTTAACGGGGGGTCTGCTAGACAGACATTATCGAAT
AGCACCCTTTGATGAGAGATATGAACAAGAGGCTTCGAGAAAACTAGTGTTTTCTGAA
TTATATGAAGCCAGTAAGCAAACAGCGAATCCATGGGTATTTGAACCCGAGTATCCGGG
AAAAAGCAGAATATTTGATGGAAGAACGGGAGATCCTTTTGAACAACCTGTTATAATA
GGAAAGCCTTATATCTTGAAATTAATTCATCAAGTTGATGATAAAATCCATGGACGTTCC
AGTGGACATTATGCACTTGTTACACAACAACCCCTTAGAGGAAGGGCCAAGCAAGGG
GGACAACGGGTAGGAGAAATGGAGGTTTGGGCCCTAGAGGGATTTGGTGTTGCTAATA
TTTTACAAGAGATGCTTACTTATAAATCTGATCATATTAGAGCTCGCCAGGAAGTACTTG
GTACTACGATCATTGGAGGAACAATACCTAATCCTGAGGATGCTCCAGAATCTTTTCGA
TTGCTCGTTCGAGAATTACGATCTTTGGCTCTAGAACTGAATCATTTCCTTGTATCTGAG
AAGAACTTCCAGATTAATAGGAAGGAAGCT---------------------ATGATCGATCGGTATAAA
CATCAACAACTCCGAATTGGATCAGTTTCTCCTCAACAAATAAGTGCTTGGGCCAAAA
AAATCCTACCTAATGGAGAGATAGTTGGAGAGGTGACAAAACCCTATACTTTTCATTAC
AAAACCAATAAACCGGAAAAAGATGGATTATTTTGTGAAAGAATTTTTGGGCCTATAAA
AAGTGGAATTTGTGCTTGTGGAAATTATCGAGTAATCGGAGATGAAAAAGAAGACCCG
AAATTTTGTGAACAATGCGGAGTCGAATTTGTTGATTCTCGGATACGAAGATATCAAAT
GGGCTACATCAAACTGGCATGCCCAGTAACCCATGTGTGGTATTTGAAACGTCTTCCTA
GTTATATCGCGAATCTTTTAGATAAACCTCTTAAAGAATTAGAAGGCCTAGTATACTGCG
AT------TTTTCTTTTGCTAGGCCC---ATAGCTAAAAAACCTACTTTCTTACGATTACGAGGT
TCATTCGAATATGAAATCCAATCCTGGAAATACAGCATCCCACTTTTTTTTACTACCCAA
GGTTTCGATACATTTCGAAATCGAGAAATCTCTACTGGAGCAGGTGCTATCCGAGAACA
ATTAGCCGATCTGGATTTGCGAATTATTATAGATGATTCATTGGTAGAATGGAAAGAATT
AGGGGAAGAA---GGGCCCACAGGAAATGAATGGGAAGATCGAAAAATTGGAAGAAGA
AAGGATTTTTTGGTTAGACGCATGGAATTAGCTAAGCATTTTATTCGAACAAATGTAAA
ACCAGAGTGGATGGTTTTGTGTCTATTACCAGTTCTTCCTCCCGAGTTGAGACCGATCA
TTCAGATAGATGGGGGTAAACTAATGAGCTCGGATATTAATGAACTTTATAGAAGAGTTA
TCTATCGGAACAATACTCTTACCGATCTATTAACAACAAGTAGATCTACACCAGGGGAA
TTAGTAATGTGTCAGGAGAAATTGGTACAAGAAGCCGTGGATACACTTCTTGATAATGG
AATCCGTGGACAACCAATGAGGGACGGTCATAATAAAGTTTACAAGTCGTTTTCAGAT
GTAATTGAAGGCAAAGAGGGAAGATTTCGCGAGACTCTGCTTGGCAAACGGGTCGATT
ATTCAGGGCGTTCCGTCATTGTCGTAGGCCCTTCACTTTCATTACATCGATGTGGATTGC
CTCGCGAAATAGCAATAGAGCTTTTCCAGACATTTGTAATTCGTGGTCTAATTAGACAA
CATCTTGCTTCGAACATAGGAGTTGCTAAGAGTAAAATTCGGGAAAAAGAACCGATTG
TATGGGAAATACTTCAGGAAGTTATGCAGGGGCACCCTGTATTGCTGAATAGAGCACCT
ACTCTGCATAGATTAGGCATACAGGCATTCCAACCCATTTTAGTGGAAGGGCGTGCTAT
TTGTTTACATCCATTAGTTCGTAAGGGATTCAATGCAGACTTTGATGGGGATCAAATGG
CTGTTCATGTACCTTTATCTTTGGAGGCTCAAGCGGAGGCTCGTTTACTTATGTTTTCTC
ATATGAATCTCCTGTCTCCAGCTATTGGGGATCCCATTTCCGTACCAACGCAAGATATGC
TTATTGGACTCTATGTATTAACGAGCGGGAATCGTCGAGGTATTTGTGCAAATAGGTATA
ATCCATGTAATCGCAGAAAC---------TATCAAAATGAAAGAATTGACGATAATAACTATAA
GTATACGAAAGAAAAAGAACCCTTTTTTTGTAATTCCTATGATGCAATTGGAGCTTATCG
GCAGAAAAGAATCAATTTAGATAGTCCTTTGTGGCTCCGGTGGCGACTAGATCAACGC-
--GTTATTGCTTCA------AGAGAAGCTCCCATCGAAGTTCACTATGAATCTTTGGGTACCT

ATCATGAGATTTATGGGCACTATCTAATAGTAAGAAGTGTAAAAAAAGAAATTCTTTTTA
TATACATTCGAACCACTGTTGGTCATATTTCTCTTTATCGAGAAATCGAAGAAGCCATAC
AAGGATTTTGTCGGGCCTGCTCATATGGTACC------------------------ATGGAGGTACTTATG
GCAGAACGGGCCAATCTGGTCTTTCACAATAAAGTGATAGATGGAACTGCCATGAAAC
GACTTATTAGCAGATTAATAGATCACTTCGGAATGGCATATACATCACACATCCTGGATC
AAGTAAAGACTCTGGGTTTCCAGCAAGCCACTGCTACATCCATTTCATTAGGAATTGAT
GATCTTTTAACAATACCTTCTAAAGGATGGCTAGCCCAAGATGCTGAACAACAAAGTTT
GATTTTGGAAAAACACCATCATTATGGGAATGTACACGCGGTAGAAAAATTACGTCAAT
CCATTGAGATATGGTATGCTACAAGTGAATATTTGCGACAAGAAATGAATCCTAATTTTA
GGATGACTGACCCTTTTAATCCAGTCCATATAATGTCTTTTTCGGGAGCTAGAGGAAAT
GCATCTCAGGTACACCAATTAGTAGGTATGAGAGGATTAATGTCGGATCCCCAAGGACA
AATGATTGATTTACCCATTCAAAGCAATTTACGCGAAGGACTCTCTTTAACAGAATATAT
AATTTCTTGCTACGGAGCCCGCAAAGGAGTTGTGGATACTGCTGTACGAACATCAGAT
GCTGGATATCTCACGCGCAGACTTGTTGAAGTAGTTCAACACATTGTTGTACGTAGAAC
AGATTGTGGCACCATCCGAGGTATTTCTGTGAGTCCTCGAAATGGGATGATGCCG---GA
AAGAATTTTTATCCAAACATTAATTGGTCGTGTATTAGCAGACGATATATATATGGGCCC
ACGATGCATTGCCGCTCGAAATCAAGATATTGGGATTGGACTTGTCAATCGATTCATAA
CC------------------TTTCGAGCACAACCAATATCTATTCGAACTCCTTTTACTTGTAGGAGTA
CATCTTGGATCTGTCGATTATGTTATGGACGGAGTCCTACTCATGGCGACCTGGTCGAAT
TGGGAGAAGCTGTAGGTATTATTGCAGGTCAATCTATTGGAGAACCAGGTACTCAACTA
ACATTAAGAACTTTTCATACCGGCGGAGTATTCACAGGGGGTACTGCAGAACATGTACG
AGCCCCTTCTAATGGAAAAATAAAATTCAATGAGGATTTGGTTCATCCCACACGTACAC
GTCATGGGCATCCTGCTTTTCTATGTTATATAGACTTGTATGTAACTATTGAGAGTGAAG
ATATTCTACATAACGTGAATATTCCACCAAAAAGCTTTCTTTTAGTTCAAAATGATCAAT
ATGTAGAATCAGAACAAGTGATTGCTGAGATTCGCGCGGGAACATCCACT---TTGAATT
TTAAAGAGAGGGTTCGAAAACATATTTATTCTGACTCAGAGGGAGAA---ATGCACTGGA
GTACCGATGTGTACCATGCACCTGAATTTACATATGGTAATGTTCATCTCTTACCAAAAA
CAAGTCATTTATGGATATTATCAGGAGGTCCGTGCAGA------TCCAGTGTAGCCCCTTTTT
CGCTCCACAAGGATCAAGATCAAATGAATGTTCAT------TCTCTTTCTGTCGAACGAAGA
TATATTTCTAACCTCTCA------GTGACTAATGATCAA---GTGAGACACAAATTCTTTAGTT
CGTATTTTTCTGGTAAAAAGGAAGGTAGGATTCCTGATTAT---TCAGAACTTAATCGAAT
TATAGGCACTAGTCGTTGTAATCTCATATATCCT---GCCATTCTCTACGAGAATTCT------G
ATTTATTGGCAAAGAGGCGAAAAAATAGATTCATCATCCCATTCCAATCGATTCAAGAA
CGAGAGAAAGAACTAATGCCCCGTTCAGGTATCTCGATTGAAATACCC------ATAAATGG
TATTTTCCGTAGAAATAGTATTCTTGCTTATTTCGATGATCCTCGATACAGAAGAAAGAG
TTCGGGAATTACTAAATATGGGACTATAGAGGTGCATTCAATCGTCAAAAAAGAGGAT--
----TTGATTGAGTATCGAGGA---GTCAAAGAATTTAGGCCAAAATACATGAAAGTAGATC
GATTTTTTTTCATTCCCGAGGAAGTGCATATCTTACCCGGATCTTCTTCCATAATGGTAC
GGAACAATAGTATCATTGGAGTAAATACACAAATCACTTTAAATACAAGAAGCAGAGTA
GGCGGATTGGTCCGAGTGGAGAGAAAAAAAAAAAGGATTGAACTTAAAATCTTTTCT
GGAGATATCCATTTTCCTGGAGAGACAGATAAGATATCCCGACACAGTGGCATCTTGAT
ACCACCAGGAACG------GGAAAAGCAAATTCCAAGGAATCCAAAAAA------TTGAAAAG
TTGGATCTATGTCCAACGG------ATCACACCTACCAAG------AAAAAGTATTTTGTTTTGG
TTCGACCCGTAGTCACATATGAAATAGCAGACGGTATAAATTTAGCAACACTTTTCCCC

CAGGATCTGTTGCAGGAAAGGGATAATGTGCAACTTCGAGTTGTCAATTATATCCTTTAT
GGAAATAGCAAACCAATTCGGAGAATTTCTGACACAAGTATTCAATTAGTTCGGACTTG
TTTAGTATTGAATTGGGACCAAGACAAAAAA---------AGTTCTTCTATCGAAGAGGCCCA
TGCCTCCTTTGTTGAAGTAAAGACAAATGGTTTGATTCGAGATTTCCTAAGAATCGACT
TAGTGAAATCCCCTATTTCGTATACCGGAAAAAGGAATGATCCGTCAGGTTCAGGATTG
ATTTCTGAT---AGTGGATCAGATCGCACCAATATCAATCCGTTTTATTCCATTTATTCCAA
GGCAAGG---ATTCAACAATCACTTAGCCAA------------AATCAAGGAACTATTCGTACGTT
GTTGAATAGA---------AATAAGGAATGCCAATCTTTGATAATTTTGTCATCATCCAATTGTT
CTCGAATGGGTCCA------TTCAACAGTGTAAAATATCACAATGTG---------ATAAAAGAAT
CAGATCCCCTAATTCCAATTAGGAATTTGTTGGGTCCTTTAGGAACA------GCCCTTCAA
ATTGCGAATTTTTATTCA------TTTTACCATTTA------ATAACTCATAATCAGATCTTG---GTA
ACTAACTATTTGCAACTTGACAAT---------TTAAAACAGGCT---CAAGTACTTAAA---------T
ATTATTTAATGGATGAAAAT------GGGAGAATTTATAATCCCGATCCATGCAGTAACATCC
TTTTGAATCCATTCAATTTGAATTGGTATTTTCTCCATCACAATTATTGTGAAGAGACAT
CC---ACAAAAATGAGTCTTGGACAGTTTATTTGTGAAAATGTATGTATAGCCAAAAACG
GACCACAC---CTAAAA------TCGGGTCAAGTTCTAATTGTTCAA---GTTGACTATGTAGTA
ATAAGATTAGCTAAGCCTTATTTGGCCACCCCAGGAGCAACTGTTCATGGCCATTATGG
GGAAATCCTTTACGAAGGAGATACATTAGTTACATTTATATATGAAAAATCGAGATCTGG
TGATATAACGCAGGGTCTTCCAAAAGTGGAACAAGTGTTAGAAGTGCGTTCGATTGAT
TCAATATCGATGAATCTAGAAAAGAGGGTTAAGGGTTGGAACGAACATATAACAAGAA
TTCTTGGAATTCCTTGGGGATTCTTGATCGGTGCTGAGCTAACTATAGTGCAAAGTCGT
ATCTCTTTGGTTAATAAGATCCAAAAGGTTTATCGATCCCAGGGGGTGCAGATCCATAAT
AGGCATATAGAAATTATTGTACGTCAAATAACATCAAAAGTGTTAGTTTCAGAA---------G
ATGGAATGTCTAATGTTTTTTCACCCGGAGAGCTAATTGGATTGTTGCGAGCGGAACGA
ACGGGGCGTGCTTTAGAAGAAGCGATCTGTTACCGAGCCATCTTATTGGGAATAACGA
GAGCATCTTTGAATACTCAAAGTTTCATATCCGAAGCGAGTTTTCAAGAAACTGCTCGA
GTTTTAGCAAAAGCTGCTCTCCGGGGTCGTATCGATTGGTTGAAAGGCCTGAAAGAGA
ACGTTGTTCTCGGGGGGATGATACCCGTTGGTACCGGATTCAAAGGATTA------GTGCAC
CGTTCAAGGCAACATAACAACATT------CTTTTGGAAACCAAAAAGAAGAATCTATTCG
AGGGGGAAATGAGA------GATATTTTGTTCCACCACAGAGAATTATTCGATTCTTGCGTT
TCAAAGAATTTCCATGATACATCAGAACAATCATTTATGGGA------------------TTGAATGTT
AATATGACAAGAAGATATTGGAACATCAATTTGGAAGAGATGATGGAAGCAGGAGTTC
ATTTTGGCCATGGTACTAGGAAATGGAATCCTAGAATGGCACCTTATATCTCTGCAAAG
CGTAAAGGTATTCATATTACAAATCTTACTAGAACTGCCCGTTTTTTATCAGAAGCTTGT
GATTTAGTTTTTGATGCAGCAAGTAGGGGAAAACAATTCTTAATTGTTGGTACCAAAAA
TAAAGCAGCTGATTCAGTAGCATGGGCTGCAATAAGGGCTCGGTGTCATTATGTTAATA
AAAAATGGCTCGGTGGTATGTTAACGAATTGGTCCACTACAGAAACGAGACTTCATAA
GTTCAGGGACTTGAGAACGGAACAAAAGACGGGGAGACTCAACCGTCTTCCGAAAA
GAGATGCAGCTATGTTGAAGAGACAATTATCTCACTTGCAAACATATCTGGGTGGGATT
AAATATATGACGGGGTTGCCCGATATTGTAATCATCGTTGATCAGCAAGAAGAATATACG
GCCCTTCGAGAATGTATCACTTTGGGAATCCCAACGATTTGTTTAATCGATACAAATTGT
GACCCGGATCTCGCAGATATTTCGATTCCAGCGAATGATGACGCTATAGCTTCAATCCGA
TTAATTCTTAACAAATTAGTATTCGCAATTTGTGAGGGTCGTTCT---AGCTATATAAGAAA
TCCTATGGGACAAAAAATAAATCCACTTGGTTTCAGACTTGGTACAACCCAAGGTCATC

ATTCCCTTTGGTTTGCACAACCAAAAAATTATTCCGAGGGTCTACAAGAAGATCAAAA
AATACGGGATTGTATCAAGAATTATGTACAAAAAAATATGAGAATATCCTCTGGT---GTC
GAGGGAATTGCACGTATAGAGATTCAAAAAAGAATTGATCTGATCCAGGTCATAATCTA
TATGGGATTCCCAAAGTTATTAATAGAGGGTAGACCACGA------------GGAATCGAAGAAT
TACAGATGAATATACAAAAAGAATTTAATTGTGTGAACCGAAAACTAAACATTGCTATT
ACAAGAATTGCAAAACCTTACGGACACCCTAATATTCTTGCAGAATTTATAGCCGGACA
ATTAAAGAATAGAGTTTCATTTCGCAAGGCAATGAAAAAAGCTATTGAATTAACTGAAC
AAGCAGATACAAAAGGAATTCAAGTACAAATTGCAGGGCGTATCGACGGAAAAGAAA
TTGCACGTGTCGAATGGATCAGAGAAGGTAGGGTTCCCCTACAAACCATTCGAGCTAA
AATTGATTATTGTTCCTATACAGTTGGAACTATCTATGGGGTATTGGGCATAAAAATTTG
GATATTTATAGACGAGGAAATGTCGCGTTACCGAGGGCCTCGTTTCAAAAAAATACGCC
GTCTGGGGGCTTTACCGGGACTAACTAATAAAAGGCCTAGAGCCGGAAGTGATCTTAG
AAACCAATCGCGTTCCGGGAAA---AGATCTCAATATCGTATTCGTCTAGAAGAAAAACA
AAAATTGCGTTTTCATTATGGTCTTACAGAACGACAATTACTTAAATACGTTCGTATCGC
TGGAAAAGCCAAAGGGTCAACAGGTCAGGTTTTACTACAATTACTTGAAATGCGTTTG
GATAACATCCTTTTTCGATTGGGTATGGCTTCGACTATTCCTGGAGCCCGCCAATTAGTT
AACCATAGACATATTTTAGTTAACGGTCGTATAGTAGATATACCAAGTTATCGCTGCAAA
CCCCGAGATATTATTACAGCGAGGGATGAACAAAAATCCAAAGCTCTGATTCAAAATTA
TCTTGATTCATCCCCCCAT---GAGGAATTGCCAAACCATTTGACTCTTCACCCATTCCAA-
-----TATAAAGGATTAGTCAATCAAATAATAGATAGTAAATGGGTCGGTTTGAAAATAAAT
GAATTGCTAGTCGTAGAATATTATTCCCGTCAGACTATGTCACGTCGAGGTACTGCAGA
AGAAAAACCTGCAAAATCCGATCCAATTTATCGTAATCGATTAGTTAACATGTTGGTTA
ACCGTATTCTGAAACACGGAAAAAAATCATTGGCTTATCAAATTATCTATCGAGCCGTG
AAAAAGATTCAACAAAAGACAGAAACAAATCCACTATCTGTTTTACGTCAAGCAATAC
GTGGAGTAACTCCCGATATAGCAGTAAAAGCAAGACGTGTAGGCGGATCGACTCATCA
AGTTCCCATTGAAATAGGATCCACACAAGGAAAAGCACTTGCCATTCGTTGGTTATTAG
GGGCATCCCGAAAACGTCCGGGTCGAAATATGGCTTTCAAATTAAGTTCCGAATTAGTG
GATGCTGCCAAAGGGAGTGGCGATGCCATACGCAAAAAGGAAGAGACTCATAGAATG
GCAGAGGCAAATAGAGCTTTTGCACATTTTCGTATGGGCAGGGACACTATTGCTGACAT
AATAACCTCTATACGAAATGCTGACATAGATAGAAAAGGAACGGTTCGAATAGCATTTA
CTAACATCACCGAAAACATTGTTAAAATACTTTTACGAGAAGGTTTTATCGAAAACGTG
AGGAAACATCGGGAAAGCAACAAATATTTTTTAGTTTCAACCCTGCGACATAGAAGG---
---AATAGGAAAAGACCATATAGA---------AATATTTTAAATTTAAAACGGATCAGTCGACC
CGGTCTACGAATCTATTCTAACTATCAACGAATTCCTAGAATTTTAGGTGGGATGGGGAT
TGTAATTCTTTCTACTTCTCGAGGTATAATGACAGACCGAGAGGCTCGACTAGAAGGAA
TCGGCGGAGAAATTTTGTGTTATATATGGATGGCAAAACCTATACCAAGAATTGGTTCA
CGTAGGAATGGACGTATTGGTTCACGTAAGAGTGCACGTAGAATACCAAAGGGAGTTA
TTCATGTTCAAGCAAGTTTCAACAATACCATTGTGACTGTTACAGATGTACGGGGTCGG
GTGGTTTCTTGGTCCTCTGCCGGTACTTGTGGATTCAGGGGTACAAGAAGAGGGACAC
CATTTGCTGCTCAAACCGCAGCAGGAAATGCTATTCGTACGGTAGTGGATCAAGGTATG
CAACGAGCAGAAGTCATGATAAAGGGTCCGGGTCTCGGAAGAGATGCAGCATTACGA
GCTATTCGTAGAAGTGGTATACTATTAAGTTTCGTACGGGATGTAACTCCTATGCCACAT
AATGGCTGTAGACCCCCTAAAAAAAGACGTGTGATGCCAACTATTAAACAACTTATTAG
AAACACAAGACAGCCAATCAGAAATGTCACAAAATCCCCCGCTCTTCGGGGATGTCCT

CAGCGCCGAGGAACATGTACTAGGGTGTATACTATCACCCCCAAAAAACCAAACTCTG
CCTTACGTAAAGTTGCCAGAGTACGATTAACCTCTGGATTTGAAATCACTGCTTATATAC
CTGGTATTGGCCATAATTCACAAGAACATTCTGTAGTCTTAGTAAGAGGGGGAAGGGTT
AAGGATTTACCCGGTGTGAGATATCACATTGTTCGAGGAACCCTAGATGCTGTCGGAGT
AAAGGATCGTCAACAAGGGCGTTCTAAATATGGGGTCAAAAAGCCAAAAATGGCAAG
GAAAAGTTTGATTCAGAGGGAGAAGAAGAGGCAAAAATTGGAACAGAAATATCATTT
GATTCGTCGATCCTCAAAAAAAGAAATAAGC---------AAAGTTCCGTCATTGAGTGACA
AATGGGAAATTCATGGAAAGTTACAATCCCCACCACGTAATAGTGCACCTACACGTCTT
CATCGACGTTGTTTTTCGACCGGAAGACCGAGAGCTAACTATCGAGACTTTGGGTTATC
CGGACACATACTTCGTGAAATGGTTCATGCATGTTTGTTGCCAGGGGCAACAAGATCA
AGTTGGATGGTAAAAAATTCATTCATCTCGGTTATTTCGCAAGAAGAA---------AACAGG
------GGGTCTGTTGAATTTCAAGTATTCAGTTTCACCAATAAGATACGGAAACTTACTTC
ACATTTGGAATTGCACAGAAAAGACTATTTATCTCAGAGAGGTCTACGGAAAATTCTGG
GAAAACGTCAACGGCTGCTGGCTTATTTGTCAAAAAAAAATAGAGTACGTTATAAAGA
ATTAATTGGTCAGTTGAATATTCGAGAGCCAAAAACTCGT------ATGGTAAAACTTCGTT
TGAAACGATGTGGTAGAAAGCAACGAGCCGTCTATCGAATCGTTGCAATTGATGTTCG
ATCCCGAAGAGAAGGAAGAGATCTTCGGAAAGTGGGTTTTTATGATCCGATAAAGAAT
CAAACTTATTTAAATGTTCCTGATATTCTATATTTCCTTGAAAAAGGTGCTCAACCTACA
GGAACTGTTCATGATATTTTAAAGAAGGCGGAGGTTTTTAAGAAACTTCGCCTTAATCA
ACCCAAATTAAAT---ATGGATAAATCCAAGCGACCTTTTTTTAAATCCAAGCGATCTTTT
CGTAGGCGTTTGCCCCCGATCCAATCGGGGGATCGAATTGATTATAGAAACATGAGTTT
AATTAGTCGATTTATTAGTGAACAAGGAAAAATATTATCTAGACGAGTGAATAGATTGA
CCTTGAAACAACAACGATTAATTACTATTGCTATAAAACAAGCTCGTATTTTATCTTCGT
TACCTTTTCTTAATAATGAGAAA------CAATTTGAAAGAATCGAGTCGACCCCTAGAACT
ACTGGTCTTAGAACCAGAAATAAAGTGACACGTTCACTAAAAAAAAATCCCTTTGTAG
CAAATCATTTATTAAGAAAAATTGATAAGCTTAACACAAAGGCGGAAAAAGAAATAAT
AGTAACTTGGTCCCGGGCATCTACCATTATACCCACAATGATCGGCCATACTATTGCTAT
CCATAATGGAAAGGAGCATTTGCCTATTTATATAACAGATCGTATGGTAGGCCACAAATT
GGGAGAATTTGCACCTACTCTAAATTTCCGGGGACATGCAAAAAACGATAATAGATCTC
GTCGTATGATTTTTAAATCTTTTCTACTAGGTAATCTAGTATCCTTATGCATGAAGATAAT
CAATTCGGTCGTTGTGGTCGGACTCTATTATGGATTTCTGACCACATTCTCCATAGGGCC
CTCTTATCTCTTCCTTCTCCGAGCTCAGGTTATGGAAGAAGGAGAAGACGGAACCGAG
AAGAAGGTATCAGCAACAACTGGTTTTATTACGGGACAGCTCATGATGTTCATATCGAT
CTATTATGCGCCTCTGCATCTAGCATTGGGTAGACCTCATACAATAACTGTCGTAGCTCT
ACCGTATCTTTTGTTTCATTTCTTCTGGAACAATCACAAACACTTTTTTGATTATGGATCT
ACTACCAGAAATTCAATGCGTAATCTCAGCATTCAATGTGTATTCCTGAATAATCTCATT
TTTCAATTATTCAACCATTTCATTTTACCAAGTTCAATGTTAGCCAGATTAGTCAACATTT
ATATGTTTCGATGCAACAACAAGATGTTATTTGTAACAAGTAGTTTTGTTGGTTGGTTAA
TTGGTCACATTTTATTCATGAAATGGGTTGGATTGGTATTAGTCTGGATACGGCAAAATA
ATTCTATTAGATCTCTTATTCGATCTAATAAGTACCTTGTGTCAGAATTGAGAAATTCTAT
GGCTCGAATCTTTAGTATTCTCTTATTTATTACCTGTGTCTACTATTTAGGCAGAATACCG
TCACCCATTCTTACTAAGAAACTGAAAGAAACCGAAGAAGATAAACCTCTTGTAACTC
TTCTTTTCGACTATAAACGATGGAATCGTCCATTGCGATATATAAAAAATGATAGATTTG
CAAATGCTGTAAGAAATGAAATGTCACAATATTTTTTTATACATGTCGAAGTGATGGA

AAACAAAGAATATCTTTTACGTATCCACCCAGTTTATCAACTTTTGGGGAAATGATACA
AAGAAAGATGTCTTTGTCCACAACAGAAAAACTCTCCTCGGATGAACTGTATAATCATT
GGATTTATACCAATGAACAAAAAAGGAAC---CTAAGCAACGAGTTTATAAAAAGAATTG
CGGCTCTAGACAAGGGATCTCTTTCGATGGATGTACTCGAAAAAAGGATTCGATTGTGT
AATGATGAGACTAAAAAAGAATACTTGCCTAAAATGTATGATCCTTTCTTGAATGGACC
CTATCGTGGAACAATCAAAAAAAATGTTTTCATCTATAGAAACAGTTTGGTTAAATAAGA
TTCATGGTATACTT---CGCGAATTTGAACAGAAAGATCTCAATGATCAATCTATTGGAAT
AAACGAAATGAGTAAAAAAGTCCCTCGATGGTCATACAAATTAATCGATTTGGAACAA
CAAGAGGGGGGAGAGATTCGCTCAAGAAAAGCCAAACGTGTAGTTATTTTTACTGATG
CTTTGATACGTTATTCGCAACAATCGGATTTTCGTCGAGACATAATCAAAGGTTCTATGC
GCGCTCAAAGACGTAAAACAGTTATTTGGGAACTATTTCAAGCAAATGCACATTCCCC
GCTTTTTTTGGACAGAATAGACAAATCCCCCTTTTTTTCTTTTGATATC------TCCGGACT
GATGAAACTCATTTTTAGAAATAGAAAAGAGAAAGCACGGATAGAAATAGCAGAAGTT
TGGGATACCATTCTATTTGCTCAAGTAATAAGAGGTTTCATGTTAGTAACCCAATCGATT
CTTAGAAAATATGTTATATTCCCTTCATTGATAATAGCTAAAAATATCGGTCGTATATTATT
ATTTCAATTTCCCGAATGGTCTGAGGATTTAAAGGGGTGGAATAGAGAAATGCATGTTA
AATGCACTTATAATGGTGTTCAATTATCAGAAACAGAATTTCCGAAAAACTGGTTAACA
GACGGTATTCAGATAAAGATCCTATTTCCTTTTTGTCTGAAACCTTGGCATAGATCTAAG
AAAGGACAAAAAAATGATTTTTGTTTTTTAACAGTTTGGGGAATGGAAGCTGAACTGC
CTTTTGGTTCTCCCAGAAAACGACCTTCCTTTTTTGAACCCATTTTTAAAGAACTCGAA
AAAAAAATTATAAAATTGAAAAAGAAGTGTTTTCTAGTT---CTAAGAGGTTTAAAAGAA
AGAACAAATTTGTTTCTAGAGGTCTCAAAAGAAAAAAAAAAAATGGGTTCTCAAAAGC
GTTCTA------TTTATAAAAAGAATAACAAAAGAACTCTCAAAAGCAAATGATTATATAAT
CAGTAATCGGATGATTCATGAATCGTCCATTCAAATTCGATCTATGGATTGGACAAATTA
TTCACTGACAGAAAAAAAAAATGAAGGATCTAACTGATAGAACAAGCACAATCAGAAA
TCAAATAGAAAGAATTACAAAAGACAAGAAAAAAAAGTCCTAACAAAACAAGTTATGA
TGCTAAAAGATTAGAATCACCAAAAAAATATTTGGCAGGTAGTAAAAAGAAGAAATGCT
CGATTAATCCGTAAATCACATTATTTTTTAAAATTTTTGATTGAAAGGATATACATAGATA
TCTTTCTATGTATCATTAATATTCCCAGAGTCAATGCACAACTTTTT---CACTTTATTTCGT
GTGACTTATCCTCCTTGTCACAAGCATATGTATTTTACAAATTAGCACAAGCCCAAGTTA
TTAACTTGTATAAGTTAAGATCTGTCCTTCAATATACATCTTTTTTTCTTAAGAAAAAAA
GAAAGGATTATTTTACACAAGGAATATTTCATTCCGAATTAAGGCATAAGAAACTTCGG
AATTCTGGAATGAATCAATGGAAAAACTGGTTAAAGGGTCATTATCAATACGATTTATCT
CAGATTAGATGGTCTAGATTAGTGCCACAAAAATGGCGAAATAGAATCAATGAACGTC
GTACGGCTCAAAAT---AACAAATGGGATTCATATGAAAAAGACCAA------TTAATTCATTA
CAAAAAACAAGATAATTTTAAAAAACACTATAGATATGATCTTTTATCATATAAATATATT
AATGATGATATTATGTATCCGAATAGAAAATATTTTGATTGGAGAATTCTCCATTTTTGTC
TTAGACTTTTTGATTGGATGGGAATGAATGAAGAAATACTAAATCGTTGGTTCTTCCCA
GAACTTGTGCTACTTTATAATGCATATAAGATTAAACCATGGGTTATACCAATCAAATTA
CTTCTTTTAAATTCCAAGAGCAACACGGAAGCAGAACTCGATTTCTTCCTGAAAAGATA
TTTGCTTTTTCAATTGAGATGGAACGATCCTTTGAATCAAAGATTGATCAATAATATCAA
AGTATATTGTCTCCTGCTTAGACTAATAAATCCAAGAGAAATTGCTATATCCTCTATTCAA
AGGAGAGAAATGAGTCTGGATATAATGCTTATTCAGAAGAATTTAACTCTTACAGAATT
GATGAAA---GGGATATTGGTTATTGAGCCAGTTCGTCTGTCTGTAAAAAGTGATGGACA

ATTTATTATGTATCAAACCATAGTAATTTCATTGGTTCATAAGAGTAAGCACAATCATTCT
GATTTGCTTGTTCCTGAAAATATTTATCGCCTAGACGTCGTAGAGAATTGAGAATTCTA
ATTTGTTTCAGATTTCTTTGGCCCAATTATCGATTAGAAGATTTAGCTTGTATGAATCGCT
ATTGGTTTGATACCAACAATGGCAGTCGTTTCAGTATGGTAAGGATACATATGTATCCAA
TGAAAGGACATCAATTCAAATCCTGGATTTTCGAATTGAGAGAGATATTGAGAGAGATC
AAGAATTCTCACTATTTCTTAGATTCATGGACCCAATTCAATTCAGTGGGATCTTTCATT
CACATTTTTTTCCACCAAGAACGTTTTATAAAACTCTTTGACCCCCGAATTTGGAGTATC
CTACTTTCA---CGCAATTCA------CAGGGTTCAACAAGCAATCGATATTTC---------ACGAT
CAAGGGTGTAGTACTCTTTGTAGTAGCG---GTCCTTATATATCGT------------ATTAACAATC
GAAATATGGTCGAAAGAAAAAATCTCTATTTGATAGGGCTTCTTCCT---------------ATACC
TATGAATTCGATTGGACCCAGAAATGATACATTGGAAGAATCCTTT---GGGTCTTCCAAT
ATCAATAGGTTGATTGCTTCG---CTCTTGTATCTTCCAAAAGGAAAAAAGATCTCTGAG
AGTTCTTTCCTGGATCCGAAAGAGAGTACTTGGGTTCTCCCAATAACT------------AAAAA
GTGTATCATGCCTGAATCTAACTGGGGTTCGCGGTGGTGGAGGAACTGGATCGGAAAA
AAGAGG------AATTCTAGTTGTAAGATATCTAAT---------GAAACCGTCGCTGGAATTGAG
ATCTCATTCAAAGAGAAAGATATCAAA---TATCTGGAGTTTCTTTTTGTATATTATATGGA
T------GATCCGATCCGCAAGGACCATGATTGGGAATTGTTTGATCGTCTTTCTCTGAGGA
AGAGGCGAAACATAATCAACTTG------AATTCGGGACAGCTATTCGAAATCTTAGTGAA
ACACTGGATTTGTTATCTCATGTCTGCTTTTCGTGAAAAAATACCAATTGAAGTGGAGG
GT---TTCTTCAAACAACAAGGG---GCTGGGTCAACTATTCAATCAAATGATATTGAGCAT
CTTTCCCAT------CTCTTCTCGAGAAACAAGTGGGCTATTTCTTTGCAAAATTGTGCTCAA
TTTCATATGTGGCAATTCCGCCAAGATCTCTTCGTT---------AGTTGGGGGAAG---AATCC
GCACGAATCGGATTTTTTGAGGAAC---GTATCGAGAGAGAATTGGATTTGGTTAGACAA
TGTGTGGTTGGTAAACAAGGATCGG---------TTTTTTAGC------AAGGTACGGAATGTATC
GTCAAATATTCAATATGATTCC---------ACAAGA------TCTAGTTTCGTTCAAGTAACGGAT
TCTAGCCAATTGAAAGGATCTTCT---GATCAATCCAGAGATCATTTGGATTCCATTAGT----
--------AATGAGGATTCGGAA---------------------------------------TATCACACATTGATCAATCA
AAGAGAGATTCAACAACTAAAAGAAAGATCGATTCTT---TGGGAT------CCTTCCTTTCT
TCAAACGGAACGAACAGAGATAGAATCAGAC------CGATTCCCGAAA------TGCCTTTCT
GGATATTCC------TCAATGTCCCGGCTATTCACG---GAACGTGAGAAGCAGATGAATAAT
CATCTGCTTCCGGAAGAAATCGAAGAATTT------CTTGGGAATCCTACAAGATCCATTCG
TTCTTTTTTCTCTGACAGATGG------TCAGAACTTCAT---CTGGGTTCGAATCCTACTGAG
AGGTCCACTAGAGATCAG------------AAATTGTTGAAGAAAGAACAAGATGTTTCT---TTT
GTCCCTTCCAGGCGATCGGAA------------AATAAAGAAATGGTTAATATATTCAAGATAATT
ACGTATTTACAAAAT------ACCGTCTCAATTCATCCTATTTCATCAGATCCGGGATGTGATA
TGGTTCCG---AAGGATGAACCGGAT---ATGGACAGTTCCAATAAGATTTCATTCTTGAAC
AAAAATCCATTTTTTGATTTA------TTTCATCTATTCCATGACCGGAACAGGGGGGGATAC
ACGTTA------CACCACGATTTTGAATCAGAA------GAGAGATTTCAAGAAATGGCAGATC
TATTCACTCTATCAATAACCGAGCCG---GATCTGGTGTATCATAAGGGATTTGCCTTTTCT
ATTGATTCCTACGGATTGGATCAAAAACAA------TTCTTGAATGAGGTATTCAACTCCAG
GGATGAATCGAAAAAGAAATCTTTA---------TTGGTTCTACCTCCTATTTTTTAT------GAA
GAGAATGAATCTTTTTATCGAAGGATCAGAAAAAAATGGGTCCGG---ATCTCCTGCGGG
AATGATTTGGAAGATCCAAAACCAAAAATAGTGGTATTTGCTAGCAACAACATAATGGA
GGCAGTCAATCAATATAGATTGATCCGAAATCTGATTCAAATCCAATATAGCACCTATGG

G------TACATAAGAAATGTATTGAATCGATTCTTTTTAATGAAT---------AGATCCGATCGCA
ACTTCGAATATGGAATTCAA---------AGGGATCAAATAGGAAACGATACTCTGAATCATA
GAACTAGAATGAAATATACGATTAACCAACATTTATCGAATTTGAAAAAG---AGTCAGA
AGAAATGGTTCGATCCT------CTTATTTTTATTTCTCGAACCGAGAGATCCATGAATCGGG
ATCCTGATGCATATAGATACAAATGGTCCAATGGGAGCAAGAATTTCCAGGAACATTTG
GAACATTTCGTTTCTGAGCAGAAGAGCCGT---------TTTCAAGTAGTGTTCGATCGATTA
CGTATTAATCAATATTCGATTGATTGGTCTGAGGTTATCGACAAAAAAGATTTGTCTAAG
TCACTTCGTTTCTTTTTGTCCAAGTCACTTCTT------------------------TTTTTGTCC----------
-----------AAGTTGCTTCTCTTTTTGTCTAACTCACTTCCT---TTTTTCTTTGTGAGTTTCGG
GAATATCCCCATTCATAGGTCCGAG---ATCCACATCTAT---GAATTGAAAGGTCCGAATGA
TCAACTCTGCAGTCAGTTGTTAGAATCAATAGGTCTTCAAATCGTTCATTTGAAAAAAT
GGAAACCCTTC------TTATTGGAT---------GATCATGATACTTCC---CAAAAATCGAAATTCT
TGATCAATGGAGGAACAATATCACCATTTTTGTTCAATAAGATACCA---AAGTGG---ATG
ATTGACTCATTCCATACTAGA------AATAATCGCAGGAAATCCTTTGATAACACGGATTCC
TATTTCTCAATGATATCCCACGATCAAGACAATTGGCTGAATCCCGTGAAACCATTTCAT
AGAAGTTCATTGATATCTTCTTTTTATAAAGCAAATCGACTTCGATTCTTGAATAATCCA
CATCACTTCTGCTTCTAT------TGTAACAAAAGATTCCCTTTT---------TAT---GTGGAAAAG
GCCCGTATCAATAATTATGAT------TTTACGTATGGA---CAATTCCTCAATATCTTGTTCATT
CGCAACAAA---ATATTTTCTTTGTACGTCGGTAAA------AAAAAACATGCTTTTTGGGGG-
--AGAGATACTATTTCACCAATCGAGTCACAGGTATCTAACATATTCATACCTAACGATTT
TCCACAAAGTGGTGACGAAACG---TATAACTTGTACAAATCT---------TTCCATTTTCCAA
TTCGATCCGATCCATTCGTTCGT------AGAGCTATTTACTCGATCGCAGACATTTCTGGAA
CACCTCTAACAGAGGGACAAATAGTCAATTTTGAAAGAACTTATTGTCAACCTCTTTCA
GATATGAATCTATCTGATTCAGAAGGGAAGAACTTGCATCAGTATCTCAAT------TTCAAC
TCAAACATGGGTTTGATTCACACTCCATGTTCTGAGAAATATTTACCATCCGAAAAG-----
-AGGAAAAAA------------------------CGGAGTCTTTGTCTAAAG------AAATGCGTTGAGAAA
GGGCAGATGTATAGAACCTTTCAACGAGATAGTGCTTTTTCAACTCTCTCAAAATGGAA
TCTATTCCAAACATATATGCCATGGTTCCTTACTTCGACAGGGTACAAATATCTAAATTTG
ATATTTTTAGATACTTTTTCAGAC---CTATTGCCGATACTAAGTAGCAGT------CAAAAATT
TGTATCCCTTTTTCATGATATTATGCATGGATCAGATATATCATGGCGAATTCTTCAGAAA
AAATTG------------------TGTCTTCCACAATGGAAT---CTGATAAGTGAGATTTCGAGTAAGT
GTTTACATAAT------CTTCTTCTGTCCGAAGAAATGATTCATCGAAATAATGAGTCA------C
CATTGATATCGACACATCTGAGATCGCCAAATGTTCGGGAGTTC---CTCTATTCAATCCT
TTTCCTTCTTCTTGTTGCTGGATAT---CTCGTTCGTACACATCTTCTCTTTGTTTCCCGA---
---GCCTCTAGTGAGTTACAGACAGAGTTCGAAAAGGTCAAATCTTTGATGATTCCATCA
TACATGATTGAGTTGCGAAAACTTCTGGATAAATATCCTACATCTGAACCGAATTCTTTC
TGGTTAAAGAATCTCTTTCTAGTTGCTCTGGAACAATTAGGAGATTCTCTAGAAGAAAT
ATGGGGTTCTGTTTCTGGCGGCAACATGCTATTGGGTGGTGGTCCC---------------GCTTAT
GGG---GTCAAATCAATACGTTCTAAGAAGAAATATTTGAATATCAATCTCATCGATATCAT
CGATCTCATA---AGTATCATACCAAATCCCATCAATCGAATCACTTTTTCGAGAAATACG
AGACATCTAAGTCATACAAGTAAAGAGATCTATTCATTGATAAGAAAAAGAAAAAAACG
TGAACGGTGATTGGATTGATGATAAAATAGAATCCTGGGTCGCGAACAGTGATTCGATT
GATGATGAAGAAAGAGAATTCTTGGTTCAGTTCTCC------ACCTTAACGACA------GAAA
AAAGGATTGATCAAATTCTATTGAGTCTGACT---CATAGTGATCATTTATCA---------------A

AGAATGACTCTGGTTATCAAATGATTGAACAACCGGGAGCAATTTACTTACGATACTTA
GTTGACATTCAT------AAAAAGTATCTAATGAATTATGAGTTCAATACATCCTGTTTAGCA
GAAAGACGGATATTCCTTGCTCATTATCAGACAATCACTTAT------TCACAAACCTCGTGT
GGGGCTAATAGTTTTCATTTCCCATCTCATGGA---------AAACCCTTTTCGCTCCGCTTAG
CCCTATCCCCCTCT---AGGGGTATTTTAGTGATAGGTTCTATAGGAACTGGACGATCCTAT
TTGGTCAAATACCTAGCGACAAACTCCTATGTTCCTTTCATTACGGTATTTCTGAACAAG
TTCCTGGATAACAAGCCTAAAGGTTTTCTTATTGAT---------------GATATCGATGATATTGA
TGCTAGTGACGATATTGATGCTAGTGACGATATTGATGATAGT-----------------------------------
-------------GACGATATCGATCGTGACCTTGATACGGAGCTGGAGCTGCTAACTATGATGAA
TGCGCTA---------ACTATGGATATGATGCCG---------GAAATAGACCGATTTTATATCACCCT
TCAATTCGAATTAGCAAAAGCAATGTCTCCTTGCATAATATGGATTCCAAACATTCATGA
TCTGGATGTGAATGAGTCGAATTACTTATCCCTCGGCCTATTAGTGAACTATCTCTCC-----
----AGGGATTGTGAAAGATGTTCC---ACTAGAAATATTCTTGTTATTGCTTCGACTCATATT
CCCCAAAAAGTGGATCCCGCTCTAATAGCTCCGAATAAATTAAATACATGCATTAAGATA
CGAAGGCTTCTTATTCCACAACAACGAAAGCACTTTTTCACTCTTTCATATACTAGGGG
ATTTCACTTGGAAAAGAAAATGTTCCATACT------AATGGATTCGGGTCCATAACCATGG
GTTCCAATGCACGAGATCTTGTAGCACTTACCAATGAGGCCCTATCGATTAGTATTACAC
AGAAGAAATCCATTATAGACACTAATATAATTAGATCCGCTCTTCATAGACAAACTTGGG
ATTTGCGA---------TCCCAGGTAAGATCGGTTCAGGATCATGGGATCCTTTTCTATCAGAT
AGGAAGGGCTATTGCACAAAATGTACTTCTAAGTAATTGCCCCATAGATCCTATATCTAT
CTATATGAAGAAGAAA------TCATGTAACGAGGGGGATTCTTATTTGTACAAATGGTACT
TCGAACTTGGAACGAGCATGAAGAAATTAACGATACTTCTTTATCTTTTGAGTTGTTCT
GCCGGATCGGTCGCTCAAGATCTTTGGTCTCTACCC------GGACCCGATGAAAAA---AAT
GGGATCACTTCTTATGGACTCGTTGAGAATGATTCT---GATCTAGTTCATGGCCTATTAGA
AGTAGAAGGCGCTCTGGTGGGA------TCCTCACGAACAGAAAAAGATTGCAGTCAGTT
TGATAATGAT------CGAGTGACATTGCTTCTTCGGCCCGAACCAAGGAAT---CCCTTAGAT
ATGATGCAAAATGGATCTTGTTCTATCGTTGATCAGAGATTTCTCTATGAAAAA------TAC
GAATCGGAGTTTGAAGAA---GGGGAAGGAGAAGGAGCCCTCAACCCGCAA------------C
AGATAGAGGAGGAT------------TTATTCAATCAC------ATAGTTTGGGCTCCT------AGAATAT
GGCGCCCT---------TGGGGCTTTCTATTTGATTGTATCGAAAGGCCCAATGAATTGGGATT
TCCC---TATTGGGCCAGGTCATTTCGGGGCAAG---------CGGATCATTTATGATGAAGAGG
ATGAGCTTCAAGAGAATGATTCGGAGTTCTTGCAGAGTGGAACCGTGCAGTACCAGAC
ACGAGATAGATCTTCCAAAGAACAAGGCTTTTTTCGAATAAGCCAATTCATTTGGGACC
CTGCAGATCCACTCTTTTTCCTATTCAAA---GATCAGCCCTTTGTCTCTGTGTTTTCACAT
CGAGAATTCTTTGCAGATGAAGAGATGTCAAAGGGGCTTCTTACTTCCCAAACAGATC
CTCCTACATCTATATATAAACGCTGGTTTATC------AAGAATACGCAAGAAAAGCACTTCG
AATTGTTGATTAATCACCAGAGATGGCTTAGA---------ACCAATAGTTCATTATCT------AAT
GGATCTTTCCGTTCTAAT------ACTCCATCCGAGAGTTATCAGTATTTATCAAATCTGTTCC
TATCTAAC---GGAACGCTATTGGATCAAATGACAAAGACATTGTTGAGAAAAAGATGGC
TTTTCCCGGATGAAATGAAAATTGGATTC------ATG---------------------------ATGCCTAGATC
GCGG---ATAAATGGAAATTTTATTGATAAGACCTTTTCAATTGTAGCCAATATCTTATTAC
GAATAATTCCGACAACTTCAGGAGAAAAAGAGGCATTTACCTATTATAGAGATGGG------
ATGTCGGCTCAATCCGAAGGAAATTATGCGGAAGCTTTACAGAATTATTATGAAGCTAT
GCGACTAGAAATTGATCCCTATGATCGAAGTTATATACTCTATAACATAGGCCTTATCCA

CACAAGTAACGGAGAACATACAAAAGCTTTGGAATATTATTTTCGGGCACTAGAACGA
AACCCGTTCTTACCACAAGCTTTTAATAATATGGCCGTGATCTGTCATTAC---CGGGGAG
AACAGGCCATTCGACAGGGAGATTCTGAAATTGCAGAGGCTTGGTTCGATCAAGCAGC
TGAGTATTGGAAACAAGCTATAGCGCTTACTCCTGGTAATTATATTGAAGCGCATAATTG
GTTGAAGATCACGAGGCGTTTCGAA------ATGAGTTGGCGATCAGAACGTATATGGATA
GAACTTATAACGGGGTCTCGAAAAACAAGTAATTTCTGCTGGGCCTTTATCCTTTTTTA
GGTTCATTAGGATTCTTATTGGTTGGAACTTCCAGTTATCTTGGTCGGAATTTGATATCTT
TATTTCCGTCTCAGCAAATCATTTTTTTCCACAAGGGATTGTGATGTCTTTCTATGGGA
TCGCAGGTCTCTTTATTAGCTCCTATTTGTGGTGCACAATTTCGTGGAATGTAGGTAGTG
GTTATGATCGATTCGATAGAAAAGAAGGAATAGTGTGTATTTTTCGTTGGGGATTTCCTG
GAAAAAATCGTCGCATCTTCCTCCGATTCCTTATGAAAGATATTCAGTCCATCAGAATAG
AAGTTAAAGAGGGTATTTATGCCCGTCGTGTCCTTTATATGGAAACCAGAGGCCAGGGG
GCCATTCCCTTGACTCGTACTGATGAGAAT---TTGACTCCACGAGAAATTGAACAAAAA
GCTGCTGAATTGGCCTATTTCTTGCGTGTACCAATTGAAGTATTT
>Bulnesia

---ATGAAAAATCACGAAAAT------------------------------
--------------TACCAAGATTCTTATTACAAC---------TCAGATACTGAGTCGAATTGGAATAAT
CACATCAATCGTTGCATTAATAGTTATTTTAAT------TCTCAAATGAAC-----------------------
------------------TACATTTTCAATTATAATGAAAATGAAAGTCAAAGTTCCATAAAAACTA
GCATCAACGAGAGTGAA------ATCAAAAATGAT---------------CAGAAGCCTGATATAAGTC
AATTGTGGGTTCAATGCGAAAATTGTTTTGAATTAAATTATAAAAGATTTTTGAAGTCA
AAGATGAATATTTGTGAACATTGTGGATATCATCTGAAAATGAGTAGTTCAGATAGAATA
GAACTTTCGATTGATCCGGGTACTTGGGATCCTATGGATGAAGAAATGGTATTGGATGA
ACCAGAAGAAGAATCTCGTCTTTCTGATCCATATCCCGATTATATTTCTTCTTATCAAAG
AAAGACAGGATTAACTGAGGCTGTTCAAACGGGTACAGGTCGACTCAATGGTATTCCT
GTAGCAATTGGGGTTATGGATTTTCAGTTTATCGGAGGTAGTATGGGATCCGTAGTCGGT
GAGAAAATTACTCGTTTGATTGACTATGCTATTAATCAACATTTACCTCTTATTATAGTAT
GTGCTTCTGGTGGAGCTCGCATGCAAGAAGGAAGTTTTAGCTTGATGCAAATGGCTAA
AATATCTTCTGTTTTATATAGTTATAAAAAAAAGAAAGGGTTATTTTATGTATCAATTCTT
ACATCTCCTACTACTGGTGGGGTGACAGCGAGTTTTGGTATGTTGGGG---------------------
---ATATCATTATTG
CCGAGCCTCATGCCT------ATATTGCATTTGCGGGTAAAAGAG--------------------------------
-------------ATGGTAACCATTCGAGCTGATGAAATTAGGAATATTATCCGTGAACGTATTGA
GCAATATAATCGAGAAGTCAAAATTGTAAATACCGGTACCGTGCTTCAAGTAGGTGACG
GTATTGCTCGTATTTATGGTCTCGATGAAGTAATGGCAGGGGAATTAGTAGAGTTTGAA
GAGGGTACCGTAGGCATTGCTCTTAATTTAGAATCCAAGAATGTTGGTGTTGTGTTAAT
GAGTGATGGTTTGATGATACAAGAGGGAAGCTCTGTAAAATCAACGGGAAGAATTGCT
CAGATACCTGTGAGTGAGGCTTATTTGGGTCGTGTTCTAAATGCTTTGGCTAAACCTATT
GATGGTAGAGGTGAAATTTCAGCTTCTGAATCTCGGTTAATTGAATCTCCCGCTCCAGG
TATTGTTTCAAGACGTTCTGTATATGAGCCTCTTCAAACAGGACTGATTGCTATTGATTC
AATGATACCTATAGGACGTGGTCAACGAGAATTAATTATTGGGGACAGACAGACCGGTA
AAACAGCAGTGGCTACTGATACGATTCTGAATCAACAAGGAGAAAATGTAATATGCGT

TTATGTAGCTATTGGCCAAAAAGCATCTTCTGTAGCTCAGGTGGTGACTACTTTACAGG
AAAAGGGAGCAATGGAATATACTATTGTAGTAGCAGAACCCGCGGATTCTCCTGCTACA
TTACAATATCTCGCTCCTTATACAGGAGCAGCTTTGGCTGAATATTTTATGTACCGTAAA
CGTCATACTTTAGTGATTTATGATGATCTCTCCAAACAAGCACAGGCTTATCGACAAATG
TCTCTTCTATTACGAAGACCACCTGGACGAGAAGCTTATCCAGGAGATGTTTTTTATTT
GCATTCACGTCTTTTGGAAAGAGCTGCTAAACTAAATTCTAGTTTAGGTGAAGGAAGTA
TGACTGCTTTACCGATAGTTGAGACCCAATCAGGAGATGTTTCAGCTTATATTCCTACTA
ATGTAATTTCCATTACAGATGGGCAAATATTCTTATCTGCCGATCTATTCAATGCTGGCAT
CAGACCTGCTATTGATGTAGGTATTTCCGTTTCCAGAGTAGGATCGGCAGCCCAAATTA
AAGCCATGAAACAAGTAGCTGGTAAATCAAAATTGGACTTAGCGCAATTCACAGAATT
AGAATCATTTGCACAATTTGCTTCTGATCTCGACAAAACTACTCAGAAGCAATTGGCAA
GAGGTCGACGATTACGTGAACTTCTGAAACAATCCCAATCAGATCCACTTCCGGTGGA
AGAACAGATCCTGACTATTTATACCGGAACAAATGGTTATCTTGATTCATTAGAGATTGA
TCAGGTAAAGAAATTTCTTGGTGATTTACGTACTTATTTGAAAACGAATAAACCTAAGT
TCAAAGAAATTATATCTTCTACCAAGACATTTACCAATGAAGCAGAAGCTCTTTTAAAA
GAAGCTATTCAGGAACAGATGGAACGCTTTCTACTTCAGGAACAAGTAATGAGAACGA
ATCCTTCTACTTCAGGTTCTGGGGTTTCCGCACTTGAAAAAAAANNCCTGGGACATATA
GTTCAAATCATTGGTCCCGTATTGGATGTAGCGTTTCCTCCCGGGAAAATGCCTAAGAT
TTACAATGCTCTCGTAGTGAAGGCTGAAAATACTGTTGGTCAAGAAATTAATGTCACTT
GTGAAGTACAGCAATTATTAGGAAATAATCGAGTTAGGGCTGTAGCTATGAGCGCTACA
GATGGTCTAATGAGAGGAATGGAAGTTATTGACACAGGAGCTCCTTTAAGTGTTCCCGT
CGGTGGAGTGACTTTAGGACGAATTTTCAACGTACTTGGGGAACCTGTTGACAATTTA
GGCCCTGTAGATACTAGCACAACATCTCCCATTCATAAGTCTGCGCCTGCCTTTATACAG
TTAGATACAAAATTATCTATTTTTGAAACAGGAATTAAAGTAGTTGATCTTTTAGCCCCT
TATCGTCGGGGAGGAAAGATAGGACTCTTCGGTGGAGCTGGAGTAGGTAAAACAGTAC
TGATTATGGAATTGATTAACAACATTGCCAAAGCTCACGGGGGTGTATCTGTATTTGGC
GGAGTAGGTGAACGTACTCGTGAAGGAAATGATCTTTACATGGAAATGAAAGAATCTG
GAGTCATTAATGAAACAAATCTTTCAGAATCAAAAGTTGCGTTAGTATATGGTCAGATG
AATGAACCACCGGGGGCTCGTATGAGAGTTGGTTTGACTGCCCTAACTATGGCGGAATA
TTTCCGAGACGTTAATAAACAAGACGTGCTTCTATTTATTGACAATATTTTTCGTTTCGT
TCAAGCAGGATCTGAAGTATCTGCTTTATTAGGTAGGATGCCTTCAGCTGTGGGTTATC
AACCTACCCTTAGTACCGAAATGGGTTCTTTACAAGAAAGAATTACTTCTACCAAACAA
GGGTCTATAACTTCTATCCAAGCAGTTTATGTACCTGCGGACGATTTGACCGATCCCGCT
CCTGCTACGACATTTGCACATTTAGATGCTACTACCGTACTATCAAGAGGATTGGCCGC
AAAAGGTATCTATCCAGCAGTTGATCCTTTAGATTCAACATCAACTATGCTTCAACCTCA
AATCGTAGGAGAGGAACATTATGAAACTGCGCAACAAGTTAAGCAAACTTTACAACGT
TATAAAGAACTTCAAGATATTATAGCCATTCTTGGTTTGGACGAATTATCCGAAGAGGAT
CGCTTAACCGTAGCAAGAGCACGAAAAATTGAGCGTTTCTTATCACAACCCTTTTTCGT
AGCAGAAGTATTTACGGGTTCTCCTGGGAAATATGTTGGTCTAGCAGAAACAATTAGGG
GGTTTCAATTGATCCTTTCCGGAGAATTAGATAGTCTTCCTGAACAGGCTTTCTATTTAG
TAGGTAATATTGATGAAGCTACTGCGAAGGCTACGAATTTAGAA---------------------ATGA
CCTTCAATCTTTGTGTACTAACTCGAAATCGAATTGTTTGGGATTCAGAAGTGAAAGAA
ATCATTTTATCTACTAATAGTGGACAAATTGGCATATTACCAAACCATGCACCTATTGCC
ACAGCCGTAGATATAAGTATTTTGAAAATACGTCTTAATGAACAATGGATAACAATGTTT

CTGATGGATGGTTTTGCTAGAATAAGAAATAATGAGGTCACTATTTAGTAAAGGATGC
AGAGAAGGGTAGTGATATTGATCCACAAGAGGCGAAACAAACTCTTGAAATGGCAGA
AGCTGACTTGAAAAAAGCTGAAGGAAAAAGACAAACAATTGAAGCAACTCTAGCTCT
CAAACGAGCTAAGACAAGAGTAGAGGCTATCAATTTTGTAAATATGAATAATTTAACCG
ATTCTTTCATTTCTTTAGGTCATTGGCCGTCCGCCGAGAGCTTCGGGTTTAATACCGATA
TTTTAGCAACGAATCCAATAAATCTAGGTATAGTGATTGGTGTATTGATCTTTTTTGGAA
AGGGAGTGTTAAGTGATTTATTAGATAATCGAAAACAGAGAATCTTGAATACTCTTCGA
AACTCAGAAGAACTGAGTGAAGGGGCCATTGAACAGTTGGAAAAAGCTCGAACCCGT
TTACAGAAAGTTGAAATGGAAGCAGATCAGTTTCGAGTGAATGGATATTCTGAGATAG
AAAAGCAAAAATTGAATTTGATTAATTCAACTTACAAGACTTTGGAACAATTAGAAAAT
TACAAAAATGAAACGATTCGTTTTGAAGAACAAAGAGCAATTAATCAAGTCCGACAAC
GGGTTTTTCAACAAGCCTTACAAGGAGCTTTAGTCACTTTGAATAGTTGTTTGAACAAC
GAGTTACATTTTCGTACTATAAATGCGAATATTGATCTGTTTGGGAAGATGAAAGAAATA
ACTGATATGAATCCACTTATTTCTGCCGCTTCTGTTATTGCTGCTGGATTGGCTGTAGGA
CTTGCTTCAATTGGACCTGGGATTGGTCAAGGGACTGCTGCGGGCCAAGCTGTAGAAG
GTATTGCGAGACAACCCCAAGCGGAGGGAAAAATACGAGGTACTTTATTGCTTAGTTT
GGCTTTTATGGAAGCTTTAACAATTTATGGACTAGTTGTAGCATTAGCTCTTTTATTTGC
GAATCCTTTTGTTATGACTGTTCTATTATGTTCCATGAACACACTCAAAGGGTTATATGAT
CTATCTGGTGTAGAAGTAGGCCAACATTTCTATTGGCAACTAGGAGCTTTCCAAGTGCA
TGGCCAAGTACTTATTACTTCTTGGGTTGTAATTTTTATCTTATTAGGTTCATCCATTATA
GCTGTTCGGAATCCACAAACCATTCCTACTAATGGTCAGAATTTCTTCGAATATGTCCTT
GAATTCATTCGAGATGTCAGTAAAACACAGATTGGAGAAGAATATGGTCCATGGGTTCC
ATTTATTGGAACTATGTTTTTATTTATTTTTGTTTCGAACTGGTCAGGGGCTCTTTTACCT
TGGAAAATCATACAGTTACCTCATGGGGAATTAGCTGCGCCCACAAATGATATAAATACT
ACTGTTGCTCTAGCTTTACTCACGTCAGTAGCATATTTCTATGCTGGTATTAGCAAAAAG
GGATTAAGTTATTTCGGTAAATATATTAAACCAACTCCAATCCTGTTACCCATTAACATCT
TAGAAGATTTCACAAAACCTTTATCACTTAGTTTTCGACTTTTTGGAAATATATTAGCAG
ATGAATTAGTAGTTGTTGTTCTTGTTTCTTTAGTACCTTTGTTGGTTCCGATACCTGTCAT
GTTCCTTGGTTTATTTACAAGTGGTATTCAAGCTCTTATTTTTGCAACTTTAGCTGCGGC
TTATATAGGCGAATCTATGGAGGGTCATCATATGATATTTTTGACTTTAGAACATATATTA
ACTCATATATCCTTTTCAATCATTTCTATTGTAATTATAATTCATTTGATAATTTTATTCATC
GATGAAATTATAGAACTATATGATTCGTCCGAAAAGGGCATGATTGCTATTTTTTTATGCA
TAACGGGATTACTATTTACTCGTTGGATTTCTTTAGGGCATTTTCCATTAAGTGATTTATA
TGAATCCTTAATATGTCTTTCATGGAGTTTGTCCATTGTTCATATAGTTTCCTACTGGAAA
AAACATGCAAATCCTTTCAGTACAATAACTAGGTCAAGCGCTATTTTGACACAAGGATT
TGCTACTTCAGGTCTTTTAACTGGAATGCATCAATCCGTAATCTTAGTACCCGCTCTTCA
ATCTCAATGGTTAATGATGCACGTAAGTATGATGATATTGGGTTATGCAGCTCTTTTATTT
GGATCTTTATTATCAGTAGCTCTCTTAGTTATTCAATTTAGAAAATTCTTCAATATTTTTGA
TAAAAACAAAGAGATTCAATACATGAAAGAAAAAGGAAATATTTTGTTA-----------------
ACCTTAAATTATTACAAATTTCAATTGATTGAACAATTAGATCATTGGAGTTCTCGTATTA
TTAGTTTAGGTTTTATCTTTTTAACCATAGGTATTCTTTCGGGAGCGGTATGGGCTAATG
AAGCATGGGGATCTTATTGGAATTGGGATCCAAAAGAAACTTGGGCCTTTATTACTTGG
CTCATCTTTGCTATTTATTTACATATTAGAACAAATAAAAATTTTGAGAGTATCAATTCTG
CAATTGTGTCTTCTGTAGGTTTTTTTATAATTTGGATATGTTATTTTGGGGTCAATCTATTA

GGAATAGGACTACATAGTTATGGTTCATTTACG------ATGAAAAAAAAGAAAACATCCAT
TTCCTTTATATATCTTGTATCCATGGTATTTTTACCCTGGTGGATCTCTTTCTCATTGAATA
AAAGTCTGAAATCTTGGATTATAAATTGGTGGAATAATAGGCAATCTGAAATTTTTATGA
ATTCTATTCCAGAAAGATATGTTATAGAAAAATTCATAGAATTAGAAGAGCTCTCCCTTT
TGGACGAAATGATAAAGGAAGACCCAGACCCACAAATGCAAAAGCTTCATATTGGAAT
TCACAACGAAACAATCGAATTGATCAACATATACAATGAAGATCGTATCCAAATGATTT
TTGATTTATCCACAAATATAATCGCTTTCATTATTCTAAGTGGTTATTATATTCTAGGTAAT
GAAGAACTTGTTATTCTGAATTCTTGGGTTCAAGAATTCCTATATAATTTAAGCGATACA
ATCAAGGCTTTTGCGATTCTTTTCTTAACTGATTTATGTATAGGATTCCATTCCCCTCATC
TCTGGGAACTCATGATTAGTTCTGTTTACAAAGATTTTGGATTTGATCATAATGATCAAA
TTATATCTTTTCTTGTTTCCACGCTTCCAGTTATTCTGGATACAATTTTGAAATATTGGAT
CTTCCGTTATTTAAATAGTGTATCTCCATCACTTGTAGTGATTTATCATTCATTGAATGAC
ATGCCAGTTGGTGTTCCAAAAGTACCTTTTCTAATTCCTGGAGACGAAGATGCATCTTG
GGTTGACCTATACAACCGACTTTATCGACAAAGATTACTTTTTTTAGGCCAAGAGGTTA
ATGACGAGATTTCAAATCAACTTATTGGTCTTATGGTATATCTAAGTCTCGAAGATTCTA
CCTTAAATCAGTATTTATTCATAAACTCTCCCGGTGGATTAATACTACCCGGAATAGGGA
TTGTTAGTACTATGAGCACGTTGGTACCTGAGGTACAAACTATATGTATGGGATCTGCTA
TTTCAACGGGATGTTTTATTCTGGTCGGAGGAGAAATTACCAAACGTATGGCATTCCCT
CACGCCAGGGTAATGCTCCATCAACCTGCTAGTTCTTTTTTAGAGGCCCCAATGCGAGA
TATGATCCTGGAAGGAGAAGAAATACTCAATCTGTACGAAATTGTCACAACAACTTATG
TAATAAGAACAGGTAAACCTTTATGGGTTATAGTGGAAGACATGAAAAAGGATGTTTTT
ATGTCAGCAGAGGAAGCCAAAAATTATGGGCTTGTTGATCTTATATCGGAAGG
A???
???
??ATGAAGAAATTTCGAGGATATTT
AGAATTACATAGATCTCGGCAATACAAATTTCTATACCCACTTCTTTTTAGGGAGTATATT
TATGTACTTGCTCACGATCATAGTTTCGTAGGCTACGACAATCAATATAGTCTATTGATTG
TAAAACGTTTAATTAATCGAATGTATCAACAGAATGATTGGAGGATTCCCTCCAAGGAT
TCAAAACAAAATTCATTGTTTGGAAAGAATTCTTATTTCCAACAAATATCAGAGGGATT
CGCAGTTATTTTGGAAATTCCATTTTCCCTACGATTCTTATATTCCTTAATAGCAAAATCG
TATAATTTACGATCCATTCATTCCATATTTCCTTTTTTAGAAGACAAATTATCACATTTAAA
TTATGTATTAGATGTACTAATACCCTACCCTATTCATCCGGAAATTTTTATTCAAATTCTTC
GCTACTGCTTGAAAGATGCTTCCGCTTTGTATTTATTACGACTTTTTATTCATGACTATTG
GAATTTTCATAGTCTTATTACCCAATTTATTTCCATCTTCTCAAAACCAAGATTTTCCTTC
TTCCTATATAATTCTTATGTGTGTGAATATGAATCCATTTTTCTTTTTCTCGGTAACCAATT
TTCTCATTTACGATCATCATCTTATGGTCCTTTTATTGAACGAATCCATTTCTATCAAAAA
ACAGAACATCTTCTAAAAGTCTTTGCTAATCTACCTATCTTCTGGTTTTTTAAGGATCCT
TTTCTGCTATATGTTCGATATAAAGAAAAATCCATTTTTGCTTCAAAAGATACGCCGCTT
TGGATTCATAAATGGAAATCTTACTTTGTCAATTTATTTCAATATCATTTTTATGTGTGGC
CTCAACCAGAAAGGATCTATATAAACCAATTATCCAAGCATAACCTCTATTTATTAGGTT
ACTTTTCAAGTGTGCAACTCAATTCTTCATTGGTACGAAGTCAAATGCTAGAAAATTCA
TTTCTAATAGAAAAT------GCTATGAATAAGCTTGATACAATTGTTCCAATGATTCCAATG
ATTCGATCATTGGCTAAAGCCCAATTCTGTAATTCATTAGGTCATCCCATTAGTAAGCCT
AACTGGATTGATTTATCGGATTCGGATATTATTGATCAATTTGCGCGTATATGTAGAAATC

TTTCTCATTATTTTAGCGGATCCTCAAAAAAAAAAGAATTTATATCGCATAAAATATATACT
TAGACTTTCTTGTGTGAAAACTTTGGCTCGTAAGCACAAAAGTACTGTACGCGCTTTGT
TGAAAAAATTAGGTTCAGAATTCTTGGAAGAGTTTTTNATACAGGAAGAACATACTCTT
TCGTTTCTTTTTTCAAGACGGATTTGGTATTTGGATATTATTTCTCGCAATGATCTTTCGA
ATTATCAAATGATCATTAATGCAACA---GAAGTACAA---GATATCAATTCTTTTTCTATATT
TCAATTA---CAAAAAGAATCCTATGAGATTCTATGGGCGCTGATCCCTATTTTGACTCTG
GTATTGGGAATTACAATAGGTGTATTAGTAATTGTGTGGTTAGAAAGAGAAATATCTGCA
GTAATACAACAACGTATTGGACCTGAATACGCAGGTCCTTTTGGAATTCTTCAATCTTTA
GCAGACGGAACAAAATTACTTTTCAAAGAGAATCTTTTTCCATCTAGGGGAGATACTAA
CTTATTCAGTATCGGACCATCCATAGCAGTAATATCAATTTTATTAAGTTATTCAGTAATT
CCTTTTGGATATAATCTTATTTTATCTGATCTAAATATTGGTGTTTTTTTATGGATTGCCGT
TTCAAGCATTGCTCCCATTGGACTTCTTATGTCAGGGTATGGATCCAACAATAAATATTC
CTTTTTAGGTGGTTTAAGAGCTGCTGCTCAATCAATTAGTTATGAAATACCATTAACTCT
TTGTGTCTTATCAATATCTCTACTATCTAACAGTTCAAGTACAGTTGATATAGTTGAAAC
ACAATCAAAATATGGGCTTTGGGGATGGAATTTATGGCGTCAACCTATAGGATTTATCAT
TTTTTTAATTTCTTCTTTGGCAGAATGCGAAAGATTACCCTTTGATTTACCGGAAGCAGA
AGAAGAATTAGTAGCAGGCTACCAAACCGAATATTCAGGTATTAAATTTGGTTTGTTTTA
TGTTGCTTCTTATCTAAATCTATTAGTTTCTTCATTATTTGTAACAGTTCTTTATTTGGGCG
GTTGGAATCTATCTATTCCATACATATTTGTTCCTGAACTTTTTGAAATAAATAGAACGA
GTAGCATTTTTCAAATGACAATTAATATTTTTATTACATTAGCCAAAACATATTTGTTCTT
ATTCATTTCGATCGCAACAAGATGGACTTTACCTAGATTAAGAATGGACCAATTATTAAA
TCTTGGATGGAAATTTCTTTTACCCATTTCTCTCGGGAATCTATTATTAACAACTTCGTTT
CAGCTCCTTTCACTC---ATGAAATCCTTTCATTTG
CTTCTCTTCGATGGAAGTTTGATTTTCCCAGAATGTATCCTAATTTTTGGTCTCATTCTTC
TTCTGATGATCGATTTAACCTCTGATCAAAAAGATATACCTTGGTTATATTTCATCTCTTC
AACAAGTTTAGTAATGAGCATAACGGTCCTATTGTTCCGATGGAGAGAAGAACCTATGA
TTAGCTTTTCGGGAAATTTCCAAACGAACAATTTCAACGAACTTTTTCAATTTCTTATTT
TATTATGTTCAACTTTATGTATTCCTATATCCGTAGAGTACATTGAATGTACAGAAATGGC
TATAACAGAGTTTCTTTTATTCATATTAACAGCTACTCTAGGAGGAATGTTTTTATGCGGT
GCTAACGATTTAATAACTATCTTTGTAGCTCCAGAATGTTTCAGTTTATGCTCCTACCTAT
TATCTGGATATACTAAGAAAGATGTACGGTCTAATGAGGCTACTACGAAATATTTACTCA
TGGGGGGGGCAAGCTCTTCTATTCTGGTTCATGCTTTCTCTTGGTTATATGGTTCATCCG
GGGGAGAGATCGAACTTCAAGAAATAGTGAATGGTCTTATCAATACACAAATGTATAAC
TCCCCAGGAATTTCAATTGCGCTTATATTCATTACTGTAGGAATTGGGTTCAAGCTTTCC
CCAGCCCCTTCTCATCAATGGACTCCTGACGTATACGAAGGATCTCCCACTCCAGTCGT
TGCTTTTCTTTCTGTTACTTCGAAAGTAGCTGCTTCAGCTTCAGCCACTCGAATTTTTGA
TATTCCTTTTTATTTCTCATCAAACGAATGGCATCTTCTTCTGGAAATCCTAGCTATTCTT
AGCATGATATTGGGAAATTTCATTGCTATTACTCAAACAAACATGAAACGCATGCTTGC
ATATTCGTCCATCGGTCAAATCGGATATGTAATTATTGGAATAATTGTTGGAGACTTCAAT
GGTGGATATGCAAGTATGATCACTTATATGCTGTTCTATATCTCTATGAATCTAGGAACTT
TTGCTTGCATTGTATCATTTGGTCTACGCACCGGAACGGATAACATTCGAGATTATGCAG
GATTATACACGAAAGATCCTTTTTTGGCTCTCTCTTTAGCCCTATGTCTCTTATCCCTAGG
AGGTCTTCCTCCACTAGCAGGTTTTTTCGGAAAACTCCATTTATTCTGGTGTGGATGGC
AGGCAGGTCTATATTTATTAGTTTCAATAGGACTCTTTACGAGCGTTGTTTCTATCTACTA

TTATCTAAAAATAATCAAGTTATTCATGACTGGACGAAACCAAGAAATAACCCCTCACG
TGCGAAATTATAGAAGATCGCCTTTCAGATCAAAAAATTCCATCGAATTGAGTATGATT
GTATGTGTGATAGCATCTACTATACCAGGAATATCAATGAACCCGATTATTGAAATTGCT
CAGGATACCCTTTTTATGTTTCTACTTTATGAATATGATCTCTTTTGGGCATTTCTAATAAT
ATCAAGTGTAATTCCTATTTTGGCATTTTTCATTTCTGGAGTTTTAGCCCCGATTAGCAA
AGGTCCAGAGAAACTTTCTAGCTATGAATCGGGCATAGAACCGATGGGTGATGCTTGG
TTACAATTTCGAATCCGTTATTATATGTTTGCTCTAGTTTTTGTTGTTTTTGATGTTGAAA
CGGTTTTTCTTTATCCATGGGCAATGAGTTTTGATATATTAGGTGTATCTGTATTTATAGA
AGCTTTGATTTTCGTGCTTATCTTAATTGTTGGTTTAGTTTATGCGTGGCGAAAGGGGGC
ATTGGAATGGTCTACGAATGATTTTCCTTGGTTAACACTAATTGTTGTTTTTCCAATATTG
GCGGGTTCCTTCATTTTTTTTCTGCCTCAGAGAGGGAATAAAACAATTAGGTGGTATAC
TATATGTGTATGCATCTTAGAGCTTCTTCTGACAACCTATGCATTCTGTTATTATTTTAAAT
TAGATGATCCTTTAATCCAATTAGGAGAGGATTATCAATGGATTCCCTTTTTTGATTTTGA
CTGGAGATTGGGAATCGATGGACTTTCTATAGGACCTATTTTACTGACTGGATTTATTAC
CACTTTAGCTACGTTAGCGGCTTGGCCAATTACTCGAGATTCCAGATTCTTTCATTTTTT
GATGTTAGCAATGTATAGTGGTCAAATAGGATTATTCTCATCTCGAGACATTTTACTTTTT
TTTATCATGTGGGAGTTAGAATTAATTCCTGTTTATTTACTTCTATCCATGTGGGGTGGGA
AAAAACGTCTTTATTCAGCTACAAAGTTTATTTTATATACTGCGGGAGGTTCCATTTTTC
TATTAATAGGAGTTCTGGGTATTGGTTTATATGGTTCCAATCAACCAATATTAAATTTTGA
AACATCAGCTAACCAATCTTATCCTATAGCATTAGAAATTATATTCTATATTGGATTTTTTA
TTGCATTCGCTGTTAAATTACCAATTCTGCCCTTACATACATGGTTGCCAGACACTCACG
GAGAGGCACATTACAGTACTTGTATGCTTCTAGCTGGAATATTATTAAAAATGGGAGCAT
ATGGCTTGGTTCGGATCAATATGGAATTATTACCCCACGCCCATTCTCTATTTTCTCCTTG
TTTGGTGATAATAGGTACATTACAAATAATCTATGCAGCTTCAACATCTCCAGGTCAACG
TAATTTAAAAAAAAGAATAGCATATTCGTCTGTATCTCATATGGGTTTTATAATTATAGGA
ATTGGATCTATCAATGATATGGGACTCAATGGAGTCATTTTACAAATAATTTCACATGGA
TTTATTGGAGCTGCACTTTTTTTCTTAGCAGGAACAAGTTATGATCGGATACGTCTTGTT
TATCTTGACGAAATGGGTGGAATGGCTATACCGATTCCAAAAATATTTACGATGTTCAAT
ATCTTATCAATGGCATCCCTCGCATTACCGGGGATGAGCGGTTTTGTTTCAGAATTGATA
GTCTTTTTTGGAATAATTACGAGCCAAAAATATCTTTTAATGGCAAAAATACTAATTACT
TTTGTCATGGCAATTGGAATGATATTAACTCCTATTTATTCATTATCGATGCTACGTCAAA
TGTTCTATGGATATAAACTATTTAGTACTTCAAATTCGTATTTCTTTGATTCTGGACCACG
AGAATTATTTATTTCTATTTCGATTCTTCTACCTGTAATAAGTATTGGTATTTATCCGGATT
TTATTATCTCCTTATCCGTTGACAAGGTGGAAGCAATTTTATCTAAGTATTTTTATAGAAT
GATGCTAGAACATGTACTTGTTTTGAGTGCTTATTTATTTTCTATAGGTATTTATGGATTAA
TTACGAGTCGGAATATGGTTAGAGCACTTATGTGTCTCGAACTTATGCTTAATGCAGTG
AATATAAATTTTGTAACATTTTCTGACTTTTTTGATAGTCGCCAATTAAAAGGAAATATTT
TCTCAATTTTTGTCATAGCTATTGCAGCTGCTGAAGCTGCTATTGGACTAGCTATTGTTT
CTTCAATTTATCGTAACAGAAAATCAACTTGTATCAATCAATCAAATTTATTGAATAAGA
TGGAACATATGTATCAATATTCATGGATCATACCTTTTCTTCCACTTCCAGTTCCTATTTT
AATAGGAGTTGGGCTTATATTGTTTCCGACAGCTACAAAAAATTTTCGCCGTATATGGGC
TTTTCCTAGTATTTTATTGTTAAGTATAGCCATGATTTTTTCAACTGAGTTGTCTATTCAG
CAAATAAATAGTAATTCTATCTATCAATATGTAGGGTCTTGGACTATCAATAATGATTTTT
CTTTTGAATTAGGCTATTGCATTGATCCACTTACTTGTATTATGTCAATCTTAATAACTAC

TGTTGCAATTCTGGTTCTTTTTTATAGTGACAATTATATGTTCTATGATGAAGGATATTTG
AGATTTTTTGCTTATATAAGTTTTTTCACTACTTCAATGTTAGGATTAGTTACTAGTTCGA
ATTTGATACAAATTTATATTTTTTGGGAATTAGTTGGACTTTGTTCTTATCTATTAATTGGT
TTTTGGTTTACACGACCTAGTGCAGCAAGTGCTTGTCAAAAAGCATTTGTAACTAATCG
TGTAGGGGATTTTGGCTTATTATTAGGAATTTTAGGTTTTTATTGGATAACAGGTAGTTTC
GAATTTCGAGATTTGTTCGAAATATCAAAAAAT---GAGATTAATTCTTTATTTCTTTCTTT
ATGCTCCTTCTTATTATTCACTGGTGCAGTTGCTAAATCTGCCCAATTCCCTCTTCATGTA
TGGTTACCAGACGCTATGGAAGGTCCGACCCCGATCTCAGCTTTGATACATGCGGCTAC
TATGGTAGCTGCGGGAATTTTTCTTGTAGCCCGTCTTCTTCCTCTTTTTATAGTTGTACCT
TATATTATGAATATAATTGCTATTCTAGGCATAATAACACTACTGTTCGGAGCTACTTTAG
CTCTTGCTCAAAAAGATGTTAAGAGAGGTTTAGCTTATTCGACAATGTCTCAATTGGGC
TATATGATGTTAGCTCTAGGTATGGGGTCTTATCGAGCTGGTTTATTTCATTTGATTACTC
ATGCTTATTCTAAAGCATTGTTATTTTTAGGATCCGGATCTATTATTCATTCAATGGAAAG
TCTTGTTGGATATTCTCCGAATAAAAGTCAAAATCTAGTTATCATGGGTGGTTTAAAAAA
GCATGTGCCGATTACAAAAACTGCCTTTTTATTAGGTACACTTTCACTTTGTGGTATTCC
ACCTCTTGCTTGTTTTTGGTCTAAAGATGAAATTCTTTATGATAGTTGGTTATATTCATCG
ATCTTCGCCATAATAGCTTGTTCCACGGCAGGATTGACTGCATTTTATATGTTTCGGATTT
ATTTACTTATTTTCGAAGGACATTTCAACTTTCATTTTCAAAATTACAGTGGAAAAAAA
AGTAGTTCATTCTATTCAATATCGATATGGGGTAAAGAAGGAAAAAAAATTATTAAAAA
CAATAATCTATTTACACCAAATATAAATTTTTTGAAATTCACTAAAAAT---ATTTTTTTGTA
TCCTCGTGAGTCAGATAATACTATGCTATTTCCAATGATTATATTAGTTCTATTTACTTTGT
TTATTGGAGCGATAGGA------TTGGATTTTGATTTATTATCAAAATTGTTAACTCCATCTAT
AAATCTTTTAGAACAAACTTCAAAT---TCTGAGGGTTGG---CAATTTTTGACAAATGCAA
CTTTTTCAGTAAGTATAGCTTTTATTGGAATATTTATAGCGTCTTTTTTATATAATCCCTTT
TATTCGTCTTTAACAAATTTTCATTTAATGAATTTATTTTATATTCTTTGGGACAAAATAAT
CAATTTTATCTATGATTGGTCATATAATCGCGGTTACATAGATTATTTTTACGAAATATTAT
TTATCAAAAGTATAAGAAAATTATCTGAATTCACTCATTTTTTTGATAGACGAATAATTG
ATGGAATTCCAAATGGGTTTGGTATTACTAGTTTTTTTGTGGCAGAAGGTATCCGATATG
TGGGGAGTGGG---CGTATCTCGTCTTATCTTTTATTATATTTATCCTATGTGTTAATTTTTTT
ATTGATATACTACATGGATTTACCAGGACCAATACACGATTTTATTTTAGTTTTTCTGGGA
TCAGGTCTTATATTAGGAAGTTTAGGAGTAGTATTATTTAGCAACTCAATGTATTCTGCTT
TTTCATTAGGATTGGTTCTTTTTTGTATATCTTTATTATATATTTCATCAAACTCCCATTTTG
TAGCTGCAGCACAACTTCTTATTTATGTGGGAGCTATAAATGTTTTAATCATATTTGCTGT
GATGTTCATGAACAATTCGGAATATTCCAAGAATTTCAATATTTTAACCTTTCGGAATGG
AATTACTTCCTTGGTTTGTATAAGTATTTTTTTTTCACTAGTGACTACTATTTTAGATACG
TCATGGTATAGTATTATTTGGACTACTAGATCAAATCATATTATAGAACAAGAGTTAATCA
ATAATAGTCAACAAATTGGGATTCATTTATCAACAGATTTTTTCCTTCCCTTTGAACTCA
TTTCAATTATTCTTTTAGTTTCTTTGATAGGTGCAATTGCTATGGCTCGTCAAATGAATAT
ACCAGCAACAAGAAAAGACTTAATGATAATCAATATGGGTCCCCATCACCCGTCTATGC
ACGGTGTTCTTCGGTTGATAGTTACTCTAGATGGTGAAGATGTTATTGATTGTGAACCCA
TATTGGGTTATTTACACCGAGGGATGGAAAAAATTGCAGAAAACCGAACAATTATACAA
TATTTGCCTTATGTAACCCGTTGGGACTATTTAGCTACTATGTTCACAGAAGCAATAACT
GTAAATGGACCAGAACAATTAGGAAATATTCAAATACCAAAAAGAGCCAGCTATATCA
GAGTCATTATGTTGGAATTAAGTCGTATCGCTTCACATCTTTTATGGCTTGGACCTTTTAT

GGCAGATATTGGTGCACAGACTCCATTTTTCTATATTTTCAGAGAAAGAGAATTAATATA
TGATCTATTCGAAGCTGCCACCGGTATGAGAATGATGCATAATTATTTTCGTATTGGAGG
AGTAGCAGCCGATCTACCTTATGGTTGGATAGATAAATGTTTGGATTTTTGCGATTATTTT
TCAAAAGGGATTTCTGAATATAAAAAACTTATTACCAGAAATCCTATTTTTTTAGAACGA
GTTGAGGGAGTAGGCATTATTGGTGAAGAGGAAGCAATCAATTGGGGTTTATCAGGAC
CAATGTTACGAGCTTCTGGAATAAAATGGGATCTTCGTAAAGTTGATCATTATGAGTCTT
ATGACGAATTTGATTGGGAAGTCCAGTGGCAAAAAGAAGGAGATTCTCTTGCTCGTTA
TTTAGTTCGAATTAGTGAAATGGCTGAATCTATCAAAATTATTCAACAAGCTCTGGAAG
GAATTCCAGGAGGCCCTTATGAGAATTTGGAAACTCGATGTTTTGATAAAAAGAGGGG
TCCAAAGTGGGGTGATTTTGAATATCGATTCATTAGTAAAAAACCTTCTCCTACATTTGA
ATTGCCAAAACAAGAACTTTATGTGAGAGTTGAGGCACCAAAAGGAGAATTAGGAATT
TTTCTGATAGGAGATCAGAGCGGGTTTCCTTGGAGATGGAAAATTCGTCCGCCAGGTTT
TATCAATTTGCAAATTCTTCCTCAATTAGTTAAAAGAATGAAATTGGCTGATATTATGAC
AATACTAGGAAGCATAGATATAATTATGGGGGAGGTTGATCGTATGTTCCCTATGGTAAC
TGGGTTCATGAATTATGGTCAACAAACCGTACGAGCTGCAAGATACATCGGTCAAGGTT
TCATGATTACTTTATCCCACGCAAATCGTTTACCCGTAACTATTCAATATCCTTATGAAAA
ATTAATAACATCAGAACGTTTCCGCGGTCGAATTCATTTTGAATTTGATAAATGTATTGC
TTGTGAAGTATGTGTTCGTGTATGTCCTATTGATCTCCCTGTTGTTGATTGGAAATTCGA
AAATGATATTCGAAAAAAACGATTATTTAATTACAGTATTGATTTTGGAATTTGTATCTTT
TGTGGTAATTGTGTTGAGTATTGCCCGACAAATTGTTTATCTATGACTGAAGAATATGAA
CTTTCTACTTATGATCGTCATGAATTAAATTATAATCAAATTGCTTTGGGTCGTTTACCAA
TACCAGTATTTGATGACTATACAATTCGAACAATTTTGAATTCG--------------------------------
----------------ATGCAGGGTCCTTTGTCTGCTTGGCTATTTAAACATGGTTTAGTTCATAGAT
CTTTGGGTTTCGATTACCAAGGAATCGAAACTTTACAAGTAAAATCCAAAGATTGGTAT
TCCATTGCTGTAATTTTATATGTATATGGTTACAATTATCTACGTTCCCAATGTGCCTATGA
TGTAGCACCCGGTGGGCTTTTAGCTAGTGTTTATCATCTTACAAGAATAACGTATGGTAT
AGATCAACCAGAAGAGGTATGCATCAAAGTATTTGCTCCAAGGGTGGATCCTAAAATTC
CGTCTGTTTTCTGGGTTTGGAAAAGTGCGGATTTTCAAGAACGCGAATCTTACGATATG
TTAGGAATCTTTTATGATAATCATCCACGCTTGAAACGCATCTTAATGCCTGAAAGTTGG
ATAGGATGGCCTTTACGTAAGGATTATATTGCCCCCAATTTTTATGAAATACAAGATGCT
CAT--
---ATGAATTCCATTGAGTTTCCTTTACT
TGATCGAACAACAAAAAATTCAGTTATTTCAACTACATTAAATGATTTTTCCAATTGGTC
AAGACTTTCCAGTTTATGGCCACTTCTGTATGGTACGAGTTGTTGCTTCATTGAATTTGC
TTCATTAATAGGTTCAAGATTCGATTTTGATCGTTATGGATTAGTACCAAGATCGAGTCC
TAGACAAGCAGATCTCATTTTAACAGCCGGCACAGTAACAATGAAAATGGCTCCTTCTT
TAGTAAGATTATATGAACAAATGCCTGAACCAAAATATGTGATTGCTATGGGAGCCTGTA
CAATTACAGGAGGGATGTTCAGTACTGATTCGTATAGTACTGTCCGGGGAGTCGATAAG
CTAATTCCTGTGGATATCTATTTACCGGGTTGTCCGCCTAAACCGGAAGCGGTGATAGAT
GCTATAACAAAACTTCGTAAGAAAATATCTCGAGAAATCTATGAAGATCGAATTCAGTA
TCAA---GAGAATCGGTGTTTTACTACTACTCACAAATTTCATGTCAGACGCAGTACTCAT
ACCGGAAATTATGAACAAGGATTACTTTATCAATCGCCATTTACTTCAGAAATACCTACT
------TTTTTAAAATATAAAAGTTCAGTATCTTCTCACGAATTTCTGAATATGCAAACTAGA
AAGATTTTTTCTTGGATCAATGAAAAGATTACTCAATCCATTTCCATATCGTTTCTATTTA

TAATAATGACTGATGCATCCATTTCAAATGCATATCCCATTTTTGCGCAGCAAGGTTATG
AAAATCCACGAGAAGCTACTGGACGGATTGTATGTGCAAACTGCCATTTAGCGAATAA
ACCTGTAGATATTGAGGTTCCACAAGCGGTACTTCCTGATACTGTATTTGAAGCAGTTAT
TCGAATTCCCTATGATAAGCAACTTAAACAAGTTCTTGCTAATGGTAAAAAGGGGTCTT
TAAATGTAGGAGCTGTTCTTATCTTACCCGAGGGTTTTGAATTAGCTCCCTCGGATCGTA
TTTCGCCCGAGATGAGAGAAAAGATAGGCAATCTATATTTTCAGAGTTATCGTCCCAAT
CAAAAAAATATTCTTGTGATAGGTCCCGTTTCCGGTCAGAAATATAGTGAAATCACTTT
TCCTATCCTTTCCCCGGACCCTTCTACTAATAAAGATGTTTACTTCTTAAAATATCCCATA
TATGTGGGTGGGAACAGGGGAAGAGGTCAGATTTATCCAGATGGGAGTAAGAGTAATA
ATACAGTTTATAATGCTACAGCAGGAGGTATAGTAAGCAAAATTTTACGAAAAGAAAAA
GGGGGGTACGAAATCACCATATCGGATCCATCCAATGGGCGTCAAGTAGTTGATATTATT
CCACCAGGACCAGAACTTCTTGTTTCGGAGGGTGAATCTATCAAACTTGATCAACCATT
AACAAGTAATCCTAATGTGGGTGGATTTGGTCAGGGAGATGCAGAAATAGTACTTCAA
GATCCATTACGTGTTCAAGGCCTTTTGTTCTTCTTTGCATCTGTTATTTTGGCACAAATC
TTTTTGGTTCTTAAAAAGAAACAATTTGAAAAAGTTCAACTATCGGAAATGAATTTTAT
GAGTAAAGTTTATGATTGGTTCGAAGAACGTCTCGAGATTCAGGCCATTGCAGATGATA
TAACTAGTAAATATGTTCCTCCCCATGTTAATATCTTTTATTGTTTAGGAGGAATTACGCT
TACTTGTTTTTTAGTACAAGTAGCTACAGGGTTTGCTATGACTTTTTACTATCGTCCAAC
TGTTACTGACGCTTTTGCTTCGGTTCAATACATAATGACGGAAGCGAACTTTGGTTGGT
TAATCCGATCAGTTCATCGATGGTCGGCAAGTATGATGGTTCTAATGATGATCCTGCACG
TGTTTCGCGTGTATCTTACTGGTGGTTTTAAAAAACCTCGAGAATTAACTTGGGTGACG
GGTGTGATTCTGGCTGTATTGACGGCATCTTTTGGTGTAACTGGTTATTCTTTACCTTGG
GATCAAATTGGTTATTGGGCAGTCAAAATTGTAACAGGCGTACCTGAAGCGATTCCTGT
AATAGGTTCCCCTTTGGTAGAGTTATTACGTGGAAGTGCTAGTGTAGGACAATCCACTT
TAACTCGTTTTTATAGTTTACACACTTTTGTATTACCTCTTCTTACTGCTGTATTTATGTTA
ATGCACTTCCCAATGATACGTAAACAAGGTATTTCTGGTCCTTTAATGGGAGTAACAAA
AAAACCTGACTTGAATGATCCTGTATTAAGAGCTAAATTGGCTAAGGGAATGGGTCATA
ATTACTATGGAGAACCCGCATGGCCAAATGATCTTTTATATATTTTTCCAGTAGTAATTTT
AGGTACTATTGCATGTAACGTTGGTTTAGCGGTTTTGGAACCATCAATGATTGGTGAAC
CGGCAGATCCTTTTGCAACTCCTTTGGAAATCTTACCTGAATGGTATTTTTTTCCTGTAT
TTCAAATACTTCGTACAGTACCCAATAAGTTATTGGGCGTTCTTTTAATGGTTTCGGTAC
CTGCGGGATTATTAATAGTACCTTTTTTGGAAAATGTGAATAAATTCCAAAATCCATTTC
GTCGCCCAGTAGCAACAACTGTTTTTTTGATTGGTACTGTAGTGGCTCTTTGGTTGGGG
ATTGGTGCAACATTGCCTATTGATCAATCGCTTACTTTAGGTCTTTTTATGATTGAAGTTT
TTCTATTTGGAATTGTCTTAGGTCTAATTCCTATTACTTTGGCTGGATTATTTGTAACTGC
ATATTTACAATACAGACGTGGTGATCAGTTGGACTTTATGCCTACTATAACTAGTTATTTT
GGTTTTCTACTAGCAGCTTTAACTATTACTTCAGCTCTATTTATTGGTTTGAGCAAGATA
CGACTTATTATGGATATAATAAGTCTTTCTTGGGCTGCGTTAATGGTAGTCTTTACATTTT
CTCTTTCGCTTGTAGTATGGGGGAGAAGTGGACTCATGATTATTCGTTCACCGGAACAA
GAAGTCAAAATTTTGGTAGATCGGGATCCCGTAAAAACTTCTTTCGAGGAATGGGCTA
AACCTGGCCATTTCTCAAGAACAATAGCTAAAGGGCCAGATACTACCACTTGGATCTG
GAATCTACACGCTGATGCTCACGATTTCGATAGCCATACCAATGATTTGGAGGAAATTT
CTAGAAAAGTATTTAGTGCACATTTTGGTCAACTATCCATCATCTTTCTTTGGCTAAGTG
GCATGTATTTTCACGGTGCTCGGTTTTCAAATTATGAAGCATGGTTAAGCGATCCTACTC

ACATTGGACCTAGTGCGCAGGTAGTTTGGCCAATAGTGGGCCAAGAAATATTGAATGG
TGATGTGGGTGGAGGTTTCCGAGGAATACAAATAACCTCTGGTTTTTTTCAAATGTGGC
GAGCATCTGGAATAACTAATGAATTGCAGCTCTATTGTACAGCAATTGGTGCATTGATTT
TTGCAGCGTTAATGCTTTTTGCCGGTTGGTTCCATTATCACAAAGCTGCTCCAAAATTG
TCTTGGTTTCAAGACGTAGAATCCATGTTGAATCACCATTTAGCAGGGCTACTAGGACT
TGGATCTCTTGGTTGGGCAGGACATCAAATACATGTATCTTTACCAATTAACCAATTTCT
AAACGCTGGAGTAGATCCTAAAGAAATCCCACTTCCTCATGAATTTATCTTGAATCGGG
ATCTTTTGGCTCAACTTTATCCTAGCTTTGCTGAGGGAGCAACTCCATTTTTCACTTTGA
ATTGGTCAAAATATGCGGAATTTCTTACATTTCGTGGAGGATTAGATCCAGTAACTGGG
GGTCTATGGTTGACTGATATTGCACATCATCATTTAGCTATTGCTATTCTTTTCTTAATAG
CGGGTCACATGTATAGGACCAATTGGGGCATTGGTCATGATATCAAAGATCTTTTAGAG
GCTCATAAAGGTCCATTTACAGGCCAGGGACATAAAGGTCTATATGAGATCCTAACAAC
GTCATGGCATGCTCAATTATCTCTTAACCTAGCTATGTTAGGCTCTTTAACTATTATTGTA
GCTCATCATATGTATTCCATGCCCCCTTATCCATATTTAGCTACTGACTATGGTACACAAC
TGTCATTGTTCACACATCACATGTGGATTGGCGGATTTTTGATAGTTGGTGCTGCTGCGC
ATGCAGCTATTTTTATGGTAAGAGATTATGATCCAACTACTCGATACAACGATCTATTAGA
TCGTGTCCTTAGACATCGTGATGCAATCATATCACATCTTAATTGGGTATGTATCTTTCTA
GGGTTTCACAGTTTTGGTTTATATATTCATAATGATACCATGAGTGCTTTAGGGCGCCCT
CAAGATATGTTTTCTGATACTGCTATACAATTACAACCTGTCTTTGCTCAATGGATACAA
AACACACATGCTTTAGCCCCTAGCACAACGGCTCCAGGTGCAACAACAAGCACTAGTT
TGACTTGGGGGGCTAGTAATTTAGTTGCAGTGGGTGGTAAAGTGGCTTTGTTACCTATT
CCATTAGGAACCGCAGATTTTTTGGTCCATCACATTCATGCATTTACAATTCATGTGACG
GTATTGATACTCCTGAAAGGAGTTCTGTTTGCTCGTAGCTCTCGTTTGATACCAGATAAA
GCAAATCTTGGTTTTCGTTTTCCTTGTGATGGACCCGGAAGAGGAGGAACATGTCAAG
TATCCGCTTGGGATCATGTTTTCTTAGGTCTGTTTTGGATGTACAACGCAATTTCAGTAG
TAATATTCCATTTTAGTTGGAAAATGCAGTCAGATGTTTGGGGTAGTATAAGCGATCAAG
GTGTTGTAACTCATATTACCGGAGGAAACTTTGCGCAGAGTTCCATTACGATTAATGGT
TGGCTTCGTGATTTCTTATGGGCGCAGGCGTCCCAGGTAATTCAGTCTTATGGCTCTTCA
TTATCTGCATATGGTCTCTTTTTTCTAGGTGCTCATTTTGTATGGGCTTTCAGTTTAATGT
TTCTATTCAGCGGGCGTGGTTATTGGCAAGAACTTATTGAGTCCATAGTTTGGGCTCATA
ATAAATTAAAAGTTGCTCCTGCTATTCAGCCTAGAGCCTTGAGCATTGTACAGGGACGT
GCTGTAGGGGTAACTCATTACCTTCTGGGTGGAATTGCCACAACATGGGCGTTCTTCTT
AGCAAGAATTATTGCAGTAGGAATGGCATTAAGATTTCCAAGGTTTAGCCAAGGCTTAG
CTCAAGACCCCACTACTCGTCGTATTTGGTTTGGTATTGCTACCGCGCATGACTTCGAA
AGTCATGATGATATTACTGAGGAACGTCTTTATCAGAATATTTTTGCTTCTCATTTCGGA
CAATTAGCAATCATTTTTCTGTGGACTTCCGGAAACCTGTTTCATGTAGCTTGGCAAGG
AAATTTCGAGCAATGGGTACAGGACCCTTTGCACGTAAGACCTATTGCTCATGCAATTT
GGGATCCTCATTTTGGTCAACCGGCTGTAGAAGCCTTTACTAGAGGAGGTGCTCTTGGC
CCAGTCAATATTGCTTATTCTGGCGTTTATCAGTGGTGGTATACAATTGGTTTACGTACTA
ATGAAGATCTTTATACAGGATCCCTTTTTCTATTATTTCTTTCCGCACTATTCTTGATAGC
GGGTTGGTTACATTTACAACCGAAATGGAAACCAAGCGTCTCATGGTTCAAAAATGCC
GAATCTCGTCTAAATCATCATTTGTCAGGACTCTTCGGAGTCAGTTCCTTAGCTTGGAC
AGGACATTTAGTCCATGTTGCTATTCCTGGATCCAGGGGCGAATATGTTCGATGGAATAA
TTTCTTAGATGTATTACCGCATCCCCAAGGGTTAGGCCCTTTTTCACAGGTTCAGTGGA

ATCTTTATGCTCAAAATCCTGATTCGAGTAGTCATTTATTTGGTACCTCCCAAGGAGCAG
GAACTGCCATTCTAACCCTTCTTGGAGGGTTCCATCCACAAACACAAAGTTTATGGCTG
ACTGATATTGCACATCATCATTTAGCTATTGCATTTATTTTTCTTGTTGCTGGTCATATGTA
TAGAACTAACTTCGGGATCGGGCACAGTATCAAAGATCTTTTAGAAACACACATTCCTC
CGGGGGGGAGATTGGGCCGTGGGCATAAGGGTCTTTACGACACAATTAATAATTCGCTT
CATTTTCAATTAGGCCTTGCCCTAGCTTCTTTAGGGGTTATTACTTCTTTGGTAGCTCAA
CACATGTATTCTTTACCTGCTTATGCCTTCATAGCGCAGGATTTTACTACTCAAGCTGCC
CTATATACTCATCACCAATACATCGCAGGATTCATTATGACAGGAGCTTTTGCTCATGGG
GCTATATTTTTTATTAGAGATTACAATCCAGAACAGAATAAGGATAATGTATTGGCAAGA
ATGTTAGATCATAAAGAAGCTATCATATCCCATTTAAGTTGGGCCAGCCTATTTTTAGGA
TTCCACACTCTTGGACTTTATGTTCATAATGATGTCATGCTTGCTTTCGGTACTCCAGAG
AAACAAATCTTGATCGAACCTATCTTTGCCCAATGGATACAATCTGCTCATGGTAAAAC
TTCATATGGTTTCGATGTACTTTTATCTTCAAAAACTAGCCCAGCATTCAACGCGGGTCA
AAGTATATGGTTGCCTGGCTGGTTAAATGCTATTAATGAG------AATAGTAATTCACTATT
CTTAACAATAGGTCCTGGAGATTTTTTGGTTCATCATGCAATTGCCTTAGGATTACATAC
AACTACATTGATTTTAGTAAAAGGTGCTTTAGACGCACGTGGTTCAAAGTTAATGCCAG
ATAAAAAAGATTTTGGTTATAGTTTTCCTTGCGACGGTCCAGGACGGGGTGGGACTTGT
GATATTTCAGCTTGGGACGCATTTTATTTGGCCGTTTTCTGGATGTTAAATACCATTGGA
TGGGTTACTTTTTATTGGCATTGGAAACACATTACATTATGGCAGGGAAACGTTTCACA
GTTTAATGAATCATCCACTTATTTGATGGGATGGTTAAGGGATTATCTATGGTTAAACTC
GTCACAGCTTATCAATGGATATAATCCTTTTGGTATGAATAGTTTATCAGTCTGGGCATG
GATGTTTTTATTTGGGCATCTTGTTTGGGCTACTGGATTTATGTTCTTAATTTCCTGGCGT
GGATATTGGCAGGAATTGATTGAAACTTTAGCATGGGCTCATGAACGTACACCTTTGGC
TAATTTGATTCGATGGAGAGATAAACCAGTAGCTCTTTCCATTGTACAAGCAAGGTTAG
TTGGATTAGCCCACTTTTCTGTTGGTTATATATTTACTTATGCGGCTTTCTTGATTGCCTCT
ACATCGGGCAAATTTGGTATGTCACATTCAGTAAAGATTTATGATACATGTATAGGGTGT
ACTCAATGTGTCAGAGCTTGCCCTACAGATGTATTAGAAATGATACCTTGGGACGGTTG
TAAAGCTAAACAAATTGCTTCTGCTCCAAGAACAGAGGATTGTGTTGGTTGTAAGAGA
TGTGAATCCGCTTGTCCAACAGATTTCTTAAGTGTTCGAGTTTATTTATGGCATGAAACA
ACTCGAAGCATGGCTCTAGCTTATATGACATTTCTAAGCTTTCCCTCTATTTTTGTCCCTT
TAGTGGGTTTAGTATTTCCGGCAATTGCAATGGCTTCTTTATTTCTTTATATTCAAAAAA
ACAAAATTTCGATGCGAGATCTAAAAACATATCTCTCTACGGCACCGGTATTAAGTACT
CTATGGTTTGGATCTTTAGCAGGTCTATTAATAGAAATTAATCGTTTTTTCCCCGATGCGT
TGACATTCCCCTTTTTTTCATTCATGACTGCAATTTTAGAGAGACGCGAAAGCGAAAGC
CTATGGGGTCGCTTCTGTAACTGGATCACCAGCACTGAAAACCGTCTTTACATTGGCTG
GTTCGGTGTTTTGATGATCCCTACTTTATTAACCGCAACTTCTGTATTTATTATCGCCTTC
ATTGCTGCTCCTCCAGTAGATATTGATGGTATTCGTGAACCTGTTTCTGGATCTCTACTTT
ATGGTAACAATATTATTTCTGGTGCCATTATTCCTACTTCTGCAGCTATAGGTTTGCACTT
TTACCCAATCTGGGAAGCAGCGTCTGTTGACGAGTGGTTATACAATGGCGGTCCTTATG
AGTTAATTGTTCTACACTTCTTACTTGGTGTAGCTTGTTACATGGGTCGTGAGTGGGAA
CTTAGTTTCCGTTTAGGTATGCGCCCTTGGATTGCTGTTGCATATTCAGCTCCTGTTGCA
GCTGCAACTGCTGTTTTCTTGATTTATCCAATTGGTCAAGGAAGTTTTTCTGACGGTATG
CCGTTAGGAATCTCTGGTACTTTTAACTTCATGATTGTATTCCAGGCTGAGCACAACATC
CTTATGCACCCATTCCACATGTTAGGCGTAGCTGGTGTATTCGGCGGTTCCCTTTTCAGT

GCTATGCATGGTTCCTTGGTAACTTCTAGTTTGATCAGGGAAACCACAGAAAATGAATC
TGCTAATGCAGGTTATCGATTTGGTCAAGAGGAAGAAACTTATAATATCGTTGCTGCTCA
TGGTTATTTTGGACGATTAATCTTCCAATATGCTAGTTTTAACAATTCTCGTTCTTTACAT
TTCTTCCTAGCTGCTTGGCCTGTAGTAGGTATTTGGTTCACTTCTCTAGGTATCAGCACT
ATGGCTTTTAACTTAAACGGTTTCAATTTCAACCAATCTGTAGTTGATAGTCAAGGTCG
CATCATTAATACCTGGGCTGATATTATTAACCGTGCTAACCTTGGTATGGAAGTTATGCAT
GAACGTAATGCTCACAATTTCCCTCTAGACCTAGCTTCGGTTGAAGCTCCATCTATTAAT
GGAATGGGTTTGCCTTGGTATCGTGTTCATACTGTTGTTTTGAATGATCCTGGTCGTTTG
CTTTCTGTCCATATCATGCATACAGCTCTTGTTGCCGGTTGGGCTGGTTCGATGGCTCTA
TATGAATTAGCAGTTTTTGATCCCTCTGATCCCATTCTTGATCCAATGTGGAGGCAGGGC
ATGTTCGTTATACCTTTCATGACTCGTTTAGGAATAACGAATTCATGGGGTGGCTGGAGT
ATTACA---GGAGGGACTGTAACAAATCCTGGTATTTGGAGTTACGAAGGCGTAGCTGCG
GCACATATTGTGTTTTCTGGCTTGTGTTTTTTGGCAGCTATTTGGCATTGGGTGTATTGG
GATCTAGAAATATTTTGTGATGAACGTACAGGAAAACCTTCTTTGGATTTGCCTAAGAT
CTTTGGAATTCATTTATTTCTTTCAGGATTAGCTTGCTTTGGTTTTGGGGCATTTCATGTA
ACAGGATTGTATGGTCCTGGAATCTGGGTGTCCGATCCTTATGGACTAACTGGGAAGGT
ACAACCTGTAAGTCCAGCTTGGGGTGTGGAAGGTTTTGATCCTTTTGTTCCGGGAGGA
ATAGCCTCTCATCATATTGCAGCAGGGACGCTGGGCATATTAGCGGGATTATTTCATCTT
AGTGTCCGTCCGCCGCAACGTCTATACAAAGGATTACGTATGGGTAATATTGAAACCGT
ACTTTCTAGCAGTATTGCTGCTGTCTTTTTTGCAGCTTTTGTTGTTGCTGGAACTATGTG
GTATGGGTCAGCAACTACCCCGATCGAATTATTTGGTCCTACTCGTTACCAATGGGATCA
GGGATATTTCCAGCAAGAAATCTATCGAAGAGTTAATGCTGCTCTATCCGAGAATCAAA
GTCTATCTGAAGCTTGGTCTCAAATTCCTGAAAAATTAGCCTTTTATGATTATATAGGCA
ATAATCCAGCAAAAGGAGGATTATTTAGAGCAGGCTCAATGGACAATGGGGATGGAAT
AGCTGTTGGGTGGTTAGGGCATCCTATTTTTAGAGATAAAGAAGGACGTGAACTCTTTG
TGCGTCGTATGCCTACTTTTTTTGAAACATTTCCAGTTGTTTTGGTAGATGGAGATGGAA
TTGTTCGCGCCGATGTTCCTTTTAGACGAGCAGAATCTAAATATAGTGTCGAACAAGTA
GGTGTAACAGTTGAGTTTTATGGCGGTGAACTAAATGGAGTTAGTTATAGTGATCCAGC
TACTGTGAAAAAATATGCTAGACGTGCTCAATTGGGTGAAATTTTTGAATTAGATCGTG
CTACTTTGAAATCCGATGGTGTTTTTCGTAGTAGTCCGAGGGGTTGGTTTACTTTTGGA
CATGCTTCATTTGCTCTACTTTTCTTCTTCGGACATATTTGGCATGGTGCTAGAACCTTG
TTCAGAGATGTTTTTGCTGGTATTGACCCGGATTTGGATACTCAAGTGGAATTTGGAGC
ATTCCAAAAAATTGGAGATCCAACTACAAAAAGACAAGTTGTT-----------------------------
-----GTGGAAACGCTCTTTAATGGAACTTTAGCTTTAGCCGGTCGTGACCAAGAAACCAC
TGGGTTCGCTTGGTGGGCCGGGAATGCACGACTTATCAATTTATCCGGTAAACTGTTGG
GAGCTCATGTAGCCCATGCTGGATTAATCGTATTCTGGGCCGGAGCAATGAATCTATTTG
AAGTGGCTCATTTTGTACCAGAGAAGCCTATGTATGAACAAGGATTAATTTTGCTTCCA
CATTTAGCTACTTTAGGTTGGGGAGTAGGTCCTGGCGGGGAAGTGATAGACACGTTTCC
ATACTTTGTATCGGGAGTACTTCATTTAATCTCCTCTGCAGTATTGGGCTTCGGTGGTATT
TATCATGCACTTCTGGGACCCGAGACCCTTGAAGAATCTTTTCCATTCTTCGGTTATGTA
TGGAAAGATAGAAATAAAATGACCACAATTTTGGGTATTCATTTAATTTTATTAGGTCTA
GGTGCTTTTCTTCTAGTATTCAAGGCTCTTTATTTTGGTGGCGTATATGATACCTGGGCTC
CAGGTGGAGGAGATGTAAGAAAAATTAGCAACTTGACTCTTAACCCGAGTGTTATATTT
GGTTATTTATTGAAATCCCCTTTTGGGGGAGAGGGATGGATTGTTAGTGTGGATGATTTA

GAAGATATAATTGGAGGACATGTATGGTTAGGTTCCATTTGTATACTTGGTGGAATCTGG
CATATCTTAACCAAACCTTTCGCATGGGCTCGTCGTGCACTTGTATGGTCTGGAGAAGC
TTACCTATCTTATAGTTTAGGAGCTTTATCTGTTTTCGGTTTCATTGCTTGTTGCTTTGTC
TGGTTTAATAATACTGCTTATCCTAGTGAGTTTTATGGGCCTACTGGACCAGAAGCTTCT
CAAGCTCAAGCATTTACTTTTCTAGTTAGAGACCAACGTCTTGGTGCTAACGTAGGATC
CGCTCAAGGGCCTACCGGTTTAGGTAAATATCTAATGCGCTCTCCGACCGGAGAAATTA
TTTTTGGGGGCGAAACTATGCGTTTTTGGGACCTACGTGCTCCTTGGTTAGAACCTCTA
AGAGGCCCTAACGGTTTGGACTTGAGTAGAATTAAAAAAGATATCCAGCCTTGGCAAG
AACGACGTTCCGCGGAATATATGACTCATGCTCCTTTAGGTTCTTTAAATTCCGTGGGTG
GCGTAGCTACCGAGATCAATGCAGTCAATTATGTTTCTCCTAGAAGTTGGTTAGCTACTT
CTCATTTTGTTCTAGGATTCTTCCTATTTGTAGGTCATTTATGGCACGCAGGAAGGGCTC
GTGCCGCTGCAGCAGGATTTGAAAAAGGAATTGATCGTGATACTGAACCTGTTCTTTTC
ATGACTCCTCTTAACATGACTATAGCCCTTGGTAAATTTACCAAAGACGAAAAT---TTAT
TTGATATTATGGATGACTGGTTACGTAGGGACCGTTTCGTTTTTGTAGGTTGGTCTGGTC
TATTGCTCTTTCCTTGTGCCTATTTTGCTCTAGGAGGTTGGTTTACAGGTACAACCTTTG
TAACTTCATGGTATACTCATGGATTGGCTAGCTCTTATTTGGAAGGTTGCAACTTCTTAA
CCGCTGCAGTTTCTACTCCTGCTAATAGTTTAGCGCATTCTTTGTTGTTACTATGGGGTC
CTGAAGCACAAGGAGATTTTACTCGTTGGTGTCAATTAGGCGGTCTATGGACTTTTGTT
GCTCTACATGGTGCTTTTGGACTAATAGGTTTCATGTTACGTCAATTTGAACTTGCTAGA
TCTGTTCAATTGCGTCCTTATAATGCAATCTCATTTTCTGCTCCAATTGCTGTTTTTGTTT
CTGTATTCCTGATTTATCCCCTAGGTCAGTCTGGTTGGTTCTTTGCACCTAGTTTTGGTG
TAGCAGCTATCTTTCGATTCATCCTTTTTTTCCAAGGGTTTCATAATTGGACATTAAACC
CTTTTCATATGATGGGAGTTGCTGGTGTATTGGGCGCTGCTTTACTATGCGCTATTCATG
GTGCTACCGTAGAAAATACTTTATTTGAGGACGGTGATGGTGCAAACACATTCCGCGCT
TTTAATCCAACTCAAGCTGAAGAAACTTATTCAATGGTTACTGCTAATCGCTTTTGGTCC
CAAATCTTTGGGGTTGCTTTTTCTAATAAACGTTGGTTACATTTCTTTATGTTATTTGTAC
CAGTAACTGGTTTATGGATGAGTGCTCTTGGAGTAGTCGGTCTAGCTTTGAACCTACGT
GCCTATGACTTCGTTTCTCAAGAAATCCGTGCAGCAGAAGATCCTGAATTTGAAACTTT
CTACACAAAAAATATCCTATTAAATGAAGGTATTCGTGCTTGGATGGCGGCTCAAGATC
AGCCTCATGAAAACCTTATATTCCCTGAGGAGGTTCTACCACGTGGAAACGCTCTTATG
TCTGGAAGTACAGGAGAACGTTCTTTTGCTGATATTATTACTAGTATTCGATATTGGGTC
ATTCATAGCATTACTATACCTTCCCTATTCATTGCGGGTTGGCTATTTGTCAGCACGGGTT
TAGCTTATGATGTATTTGGAAGCCCTCGCCCAAACGAATATTTTACAGAGAGCAGACAA
GGAATTCCATTAATAACTGAACGTTTTGATTCTTTGGAACAACTGAATGAATTTAGTAGA
TCTTTTATGACCATAGATCGAACCTATCCAATTTTTACAGTACGATGGTTGGCTGTTCAT
GGACTAGCTGTACCTACCGTTTCTTTTTTGGGATCAATATCAGCAATGCAGTTCATCCAA
CGAATGGCTACACAAACTGCTGAGAGTAATTCTAGATCTAGT---CCAAGACGAACTCCT
GTAGGGAGTTTATTGAAACCATTGAATTCTGAATATGGTAAAGTAGCTCCTGGATGGGG
AACTACTCCTTTGATGGGTGTTGCAATGGCCTTATTCGCGGTATTCCTATCGATTATTTTG
GAGATTTATAATTCTTCCGTTTTACTGGATGGAATTTCAATGAATATGCTTACTTTAAAAC
TATTTGTTTATACGGTAGTGATCTTTTTTGTTTCTCTCTTCATTTTTGGATTCCTATCTAAT
GATCCAGGACGTAATCCTGGACGTGAAGAAATGGCAGATACTACTGGAAGGATTCCTC
TTTGGATAATAGGTACTTTAACTGGTATTCTTTTGATCGGTTTAATAGGTATTTTCTTTTAT
GGTTCATATTCTGGATTGGGTTCATCCCTTATGCTTAATATATTTAGTTTGATCTGTATCTG

GTTTAACTCTGATCTTTATTCAAATCATTTTTTATCGGCAAAATTGCCTGAAGCGTACGC
TTTTTTGAGCCCAATCGTAGATTTTATGCCAGTAATTCCTTTACTCTTTTTTCTATTAGCT
TTTGTTTGGCAAGCTTCTGTAAGTTTTCGAATGACACGATCCAACCCAAATGAACAAAA
TGTCGAATTGAATCGTACCAGTCTTTACTGGGGATTATTACTCATTTTTGTACTTGCTGTT
TTATTTTCCAATTATTTCTTCAATATGGAAGTAAATATTGCCGCATTTATTGCTACTGCATT
ATTCGTTCTAGTTCCTACTGCTTTTTTAATAATAATATACGTAAAAACAGTTAGTCAAAA
C---ATGGAAACAGCAACTCTAGTCGCCATCTCTATATCTGGTTTACTTGTGAGTTTTACT
GGGTATGCCTTATATACTGCTTTTGGGCAGCCCTCTCAACAACTAAGAGATCCGTTCGA
GGAGCACGGGGACATGGAAGCATTGGTTTATACATTTCTTTTAGTCTCGACTCTAGGAA
TAATTTTTTTTGCTATTTTTTTTCGAGAACCGCCTAAAGTTCCAACTAAAAAGATGAAAA
TGACTATTGCTTTCCAGTTAGCTGTTTTTGCATTAATTATTACTTCCTCAATATTATTGATT
AGTGTCCCCGTTGTATTTGCTTCCCCTGAAGGTTGGTCTAGTAACAAAAAGATTGTATT
TTCTGGTACATCATTATGGATTGGATTAGTCTTCCTGGTGGGTATACTTAATTCTCTCATC
TCT--------------------ATGTCACCACAAACAGAGACTAAAGCAAGTGTTGGATTCAAAGCT
GGTGTTAAAGATTATAAATTGACTTATTATACTCCTGAATATGAAACAAAAGATACTGAT
ATTTTGGCAGCATTTCGAGTAACCCCTCAACCTGGAGTTCCGCCCGAAGAAGCAGGGG
CTGCAGTAGCTGCTGAATCTTCTACTGGTACATGGACAACTGTGTGGACTGATGGACTT
ACCAGTCTTGATCGTTACAAAGGACGATGCTACAACATTGAGCCTGTAGCTGGAGAAG
AAAATCAATATATTGCTTATGTAGCTTACCCGTTAGACCTTTTTGAAGAAGGTTCCGTTA
CTAACATGTTTACTTCCATTGTGGGTAATGTCTTTGGGTTCAAAGCCCTGCGTGCTCTAC
GTTTGGAGGATTTGCGAATTCCAAAGTCTTATATTAAAACTTTCGAAGGACCACCTCAT
GGTATCCAAGTTGAGAGAGATAAATTGAATAAGTATGGTCGTCCTTTATTGGGATGTACT
ATTAAACCTAAATTAGGTCTATCCGCGAAGAATTATGGTAGAGCAGTTTATGAATGTCTT
CGAGGTGGACTTGATTTTACCAAAGATGATGAGAACGTTAACTCCCAACCATTTATGCG
TTGGAGAGACCGTTTCCTATTTTGTGCCGAAGCACTTTTTAAAGCACAAGCTGAAACA
GGTGAGATCAAAGGACATTACTTAAATGCTACTGCAGGTACATGCGAAGAAATGATCA
AAAGGGCTGTGTGTGCCAGAGAATTGGGAGCTCCTATCGTAATGCACGATTACTTAAC
AGGTGGATTCACTGCAAATACTAGTTTGGCTCATTATTGCCGAGATAATGGTCTACTTCT
TCACATTCACCGTGCAATGCATGCAGTTATTGATAGACAGAAGAATCATGGTATGCATTT
CCGTGTATTAGCTAAAGCTTTACGCTTGTCCGGAGGAGATCATATTCACGCTGGTACCGT
AGTAGGTAAACTTGAAGGGGAAAGAGAGATTACTTTAGGCTTTGTTGATTTACTACGTG
ATGATTTTGTTGAAAAAGATCGAAGCCGCGGTATTTATTTCACCCAAGATTGGGTCTCT
TTGCCAGGAGTTATACCTGTTGCTTCAGGTGGTATTCACGTTTGGCATATGCCTGCTCTA
ACCGAGATTTTTGGAGATGATTCCGTACTACAATTTGGTGGAGGAACTTTAGGACATCC
TTGGGGTAATGCACCTGGTGCCGTAGCGAATCGAGTAGCTCTAGAAGCGTGTGTACAA
GCTCGTAATGAGGGACGTGATCTTGCTCGTGAAGGGAATACAATTATCCGTGAGGCTAG
CAAATGGAGTCCTGAGTTAGCTGCTGCTTGTGAAGTATGGAAGGAGATCAAATTTGAA
TTCCCTGCAATGGATACTTTA------ATGGCGATACATTTATACAAAACTTCTACCCCGAGA
ACACGCAATGGAGCTTTAGACAGTCAAGTGAAATCCAATCCACGAAAGAATTTGATTT
ATGGACAGCATCGTTGT---AAAGGTCGTAATGCCAGAGGAATCATTACTGTAAGGCATA
GAGGGGGAGGTCATAAGCGTCTATATCGTAAAATAGATTTTCGACGGAAGGAAAAAGA
CATATATGGTAGAATCGTAACCATAGAATACGACCCTAATCGAAATGCATACATTTGTCT
CATCCACTATGGGGATGGTGAGAAGAGATATATTTTACATCCCAGAGGGGCTATAATTG
GAGATACCATTGTATCTGGTACAGAAGTTCCTATCCAAATGGGAAATGCCCTACCTTTG

ACCGAGATGCCCTTAGGCACGGCCATACATAACATCGAAATCACATTTGGAAAGGGTG
GACAATTAGTTAGAGCAGCAGGTGCTGTAGCAAAACTAATTGCAAAAGAGGGGAAAT
CGGCCACATTAAAATTACCTTCTGGGGAGGTCCGTTTAATATCCAAAAATTGCTCAGCA
ACAGTTGGACAAGTGGGTAATGTTGGGGTGAACCAGAAAAGTTTGGATCGAGCCGGA
TCGAAATGTTGGCTAGGTAAGCGTCCTGTAGTCAGAGGAGTAGTTATGAACCCTGTAG
ACCACCCGCACGGGGGTGGGGAAGGAAGAGCGCCAATTGGCAGAAAAAAACCCGCA
ACCCCTTGGGGTTATCCTGCGCTTGGAAGAAGAAGTAGAAAAAGGAATAAATATAGTG
AGAATTTGATTCTTCGTCGACGTAGT---ATGATTCAACCTCAAACCCATTTGAATGTAGC
GGATAACAGCGGGGCCCAAGAATTGATGTGTATTCGAATCATAGGAGCTAGT---AATCG
TCGATATGCCCATATTGGTGATATTATTGTTGCTGTAATAAAAGAAGCAATACCAAACAC
ACCGATCGAAAAATCAGAAGTTATTCGAGCGGTAATTGTGCGTACTTCTAAAGAACTAA
AACGTGACAACGGTATGATAATACGATATGATGACAACGCTGCAGTGATCATTGATCAA
GAGGGAAATCCAAAAGGAACTCGAATTTTTGGTGCAATCCCCCGTGAATTGAGACAAT
TCAATTTCACAAAAATAGTTTCATTAGCACCCGAAGTATTAATGCTTAGTCCAAAAAGA
ACTAGATTCCGTAAACAACATAGAGGAAGATTAAAAGGAAGATCTTATCGAGGGAATC
GTATTTGTTTCGCTAGATATGCTCTTCAAGCACTTGAACCTGCTTGGATAACATCTAGAC
AAATAGAAGCAGGTCGACGAGCAATGACACGAAATATACGCCGTGGTGGAAAAATATG
GGTACGTATCTTTCCAGACAAACCAGTTACAATGAGACCTGCAGAAACGCGTATGGGT
TCGGGAAAAGGATCCCCAGAATATTGGGTCGCTGTCGTTAAACCCGGCAGGATACTTTA
TGAGATGGGAGGAGTATCTGAAAATATAGCCAGAAGAGCTATTTCAATAGCAGCATCCA
AAATGCCCATACGAACTCAATTCATTATTGCAGAAATGACCAGAATTAAACGAGGATAT
ATAGCACATAGACGTAGAGCAAAAATTCGTTTATTTGTATCAGGTTTTCGAGGAGCCCA
TTCAAGAATTACTCGAAGTATTACTCAACAGAGAATAAGAGCTTTGGTTTCATCTGATC
GAGATCGAGATAGAAAAAAGAGAGATTTTCGCCGTTTGTGGATTATTCGGATAAATGCA
GTAATTCGCGAGAAT---ATATTATCTTATAGTTACAGCAAATTTATACACAATATTTACAAG
AAACAGTTGCTTCTTAATCGTAAAATACTTTCACAAATAGCTATATTTAATAGGAATTGT
CTTTATATGATTGCCAATGAAATC--ATAATAAATCAAGCATA
CGCTTTCGGTGACAATATACGTATGTCTGCGAATAAAGCGCGAAGAATAATTGATCAAA
TTCGTGGGCGTTCCTATGAAGAAACACTTATGATACTAGAACTCATGCCTTATCGAGGG
TGTTATCCAATTTTTCAATTAGTTTATTCTGCAGCAGCAAATGCTAGTCATAATCAGGGT
TTAGAGAAAAAAAATTTAGTAATTACTCAAGCTGAAGTAAACGAAAGTACTACGGCAA
AAAAATTCAAACCTAGAGCCCGAGGGCGCAGTTATTCAATAAAAAGACGAACTTGTAA
TATAACTATTGTATTAACAGATGTATCTTTA-------------------------ATAGATGGAATCAAATAT
TCAGTAGTTACAGACAAAGGTATTCGGTTATTGTTGAAAAAT---CAATATACTTTTAATGT
CGAATCAGGATCAACTAGGACAGAAATCAAGCATTGGGTCGAACTCTTCTTTGGTGTC
AAGGTAATCGCTGTGAATAGTCATCGACTCCCGGGAAAGAGTAGAAGAATGGGACCTA
TTATGGGACATACAATGCATTACAAACGTATGATCGTTACGCTTCCACCGGGTTATTATAT
TCCACCTCTTAGAAGAAAAGAACTGATGGCAGTTCCAAAGAAGCGTACTTCTATATCA
AAAAAACGTATTCGTAAGAATATTTGGAAAAAAAAAGGGATATTGGACAGCATTGAAAT
CTTTTTCATTAGCTAAATCGCTTTTTAGCGGAAATTCAAAAAGTTTCTTTGTACAACAGA
TAAAGATAATGGCCAAGGGTAAAGATATCCGTATAACGATAATTTTGGAATGTACTGAAT
GT---GCTCGAAAA------ATTGATAAAAAA------GGTATTTCCAGATATATTACTCAAAAGAA
TCGACACAATACGCCAAATCGATTGGAATTGAAAAAGTTTTGTTCGTATTGTTTCAAAT
ATACGATTCACAAGGAGATCCAAAAAATGAAAATAAGAGCTTCTGTTCGTAAAATTTGT

GAAAAATGTCGACTGATCCGTAGAAGAGGACGAATTATAGTAATTTGTTCCAACCCAA
AACATAAGCAAAGACAAGGAATGGTTCGAGAGAAAATAGAAGTATCTACTCGGACACT
CCAATGGAAGTGTGTGGAATCAAGAGCAGACAGTAAGCGTCTTTATTATGGGCGCTTT
CTTCTTTCACCACTGAAGAAAGGTCAAGCTGATACAATAGGCATTGCAATGCGACGAG
TTTTGCTTGGAGAAATAGAAGGAACAAGTATAACAAGTGCAAAATCTGAG------AACAT
ACCACATGAATATTCTAGCATAGTAGGTGTTGAAGAATCAGTACATGAAATTTTAATGAA
TTTAAAAGAAATTGTATTGAGAAGTCATCTTTATGGAATTTGCGACGCGTCTATTTGTGT
CAAAGGTCCTGGATGTGTAACGGCCCAAGACATCATTTTACCATCTTATGTGGAAATCG
TTGATAACACACAACATATAGCGAACTTAACAGAACCCATTGATTTTTGTATTAGATTAC
AAATAGAAAGGAATCGGGGATATCGTATAAAAACGCCAAATCAATTTCAAGAGGGAAT
TTAT------CCTATAGATGCTGTATTCATGCCTGTTAGAAATGCAAACCATAGTATTCATTCT
TATGGTAATGGTAGTGAAACACAAGAGATACTCTTTCTTGAAATATGGACAAATGGAAG
TTTAACTCCTAAAGAAGCACTTCATGAAGCCTCCCGCAATTTGATTGATTTATTTATTCC
CTTTCTACATGCGGAAGAAGAA------AACTTACATTTAGAAAACAATCAA---AACAAGTA
TACTTTACCCCTTTTTTCTTTTCATGATCAATTCAAT---CAAATAAAAAAAAATCAAAAA-
--GAAATAGAATTCAAATCTATTTTTATTGACCAATTAGAATTGACTCCAAAGATATATAAT
TGTCTCAAAAGGTCTAATATACATACATTATTTGATCTTTTGAATAATCGTAAAGAAGAC
CTGATGAGAATTGAACATTTTCGCATAGAAGACATAAATCATATATTTCACATTTTAGAA
AAGAATTTCGCAACTACAATTAATTTACCGAAAAAGAAAATGCTCATGGATGAAAAT---
GAAGTAATGTCTACAATACCTGGATTGAATCAGATACAATTTGAAGGATTTTGTAAATTC
ATTGATAGGGGTTTAATAGAAGAACTTTATAAATTTCAAAAAATTGAAGATGCAGATCA
AGAAATTGAATTTCAATTATTTGTAGAAACTTATAAATTGGTAGAACCTTCAATAACAGA
ACGAGATGCTATATATCAATCACTCACATATTCTTCTGAATTACATGTATCTGCAGGATTA
ATTTGGAAAACTGTTAGAGGTATGCAAGAACAAACCATTTTTATTGGAAACATTCCCTT
AATGAATTCTCTGGGGACTTTTATAGTCAATGGAATATACAGAATTGTCATCAATCAAAT
ATTACAAAGCCCTGGTATATATTATCGATCAGAATTGGATCATAATGGAATTCCCGTCTAT
ATTGGCACGATAATATCAGATTGGGGAGGAAGATTAGAATTAGAGATTGATAGAAAAGC
AAGGATATGGGCTCGTGTAAGTAGAAAGCAAAAAATATCTATTTTAGTTTTATCATCAGC
TATGGGTTTGAATCTCAGAGAAATTCTGGAAAATGTTTGTTACCCTGAAATGTTCTTGT
CTTTCTTGAATGATAAGGAGAAAAAAAAAAATTGGGTCAAAAGAAAATGCCATTTTGGA
GTTTTATCAACAGTTTGCTTCAGTAGGTGGAGATCCGGTCTTTTCTGAATCCTTATGTAA
AGAATTACAAAAGAAATTCTTTCAACAGAGGTGTGAATTAGGAAAGATTGGTCGACGA
AATATGAATCTAAGATTCAATCTCGATATACCTCAGAACAATACATTTTTATTACCTCGAG
ATATATTGGCAGCTACTGATCATTTGATTGGAATTAAATTTGGAATCGGCACGCTTGACG
AT------ATGAATCATTTGAAAAATAAACGTATTCGATCTGTATCAGATCTTTTACAAGATC
AATTGGGATTGGCTCTAGTTCGTCTAGAAAATGTGATTAAAGGAACTATATCCGGAGCC
ATTCGG---CATAAATTAATA---CCGACTCCTCAAAATTTAGTAACTTCAACTCCA-----------
TTAACAACTACCTATGAATCTTTTTTCGGATTACACCCATTATCTCAAGTTTTTGATCGA
ACGAATCCATTGACACAAATAGTTCATGGTAGAAAATTAAGTTATTTGGGTCCCGGAGG
ATTGACAGCACGAACTGCTAATTTTCGGATACGAGATATTCATCCTAGTCACTATGGGCG
CATTTGCCCAATTGACACGTCGGAAGGAATCAATGTTGGGCTTATTGGATCCTTAGCAA
TTCATGCGAGAATTGGTCATTGGGGA---TCTCTAGAAAGCCCGTTCTAT---------GAAATT
TCTGAACAGTCAAAA------AAAGTACGGATCCTTTCTTTATCACCAAATCGAGATGAATA
TTATATGATAGCAGCAGGAAATTCTTTGGCACTCAATCGGGATATTCAGGAAGAGCAGG

TTGTTCCAGCCCGATACCGTCAAGAATTTTTAACTATTGCATGGGAACGGATTCATCTTC
GAAGTATTTTTCCTTTCCAATATTTCTCGATTGGAGCTTCCCTAATTCCTTTTATCGAGCA
TAATGATGCGAATCGAGCTTTAATGAGTTCGAATATGCAGCGTCAAGCAGTTCCACTTT
CTCAATCTGAAAAATGCATTGTTGGAACTGGGTTAGAACGCCAAGCAGCTCTAGATTC
CGGGGTTCCTGTGATATCCGAACAGGAGGGAAAGATAATTTATAGCGATACTAAAAAG
GTCGTTTTATCGACCAATGGAAATACTTTAAACATTCCACTAATTATGTACCAACGTTCC
AATAAAAATACTTGCATTCATCAAAAAGCTCGGGTTCAATATGGTAAATGGATTAAAAA
GGGACAACTTTTAGCGGATGGTGCTTCTACAGTTGGTGGGGAACTTGCTTTGGGGAAA
AATATATTAGTGGCTTATATGCCATGGGAAGGTTACAATTTTGAAGATGCAGTACTTATT
AGCGAACGTCTAGTATATGAAAATATTTATACTTCTTTTCACATAAGGAAATATGAAATT
CAGACTCATGTGACAAGTCAAGGC---CCTGAAAAGATCACCAAAGAAATACCACATCT
AGAGGCTCATTTACTTCGTAATTTAGACAAAAATGGAATTGTGATGTTGGGATCTTGGG
TAGAGACAGGTGATGTTTTAGTAGGTAAATTAACACCTCAGATGACGAAAGAA---TCCT
CGTATGCTCCGGAAGATAGATTATTACGCGCCATACTCGGCATCCAAGTATCCACTTCAA
AGGAAACTTGTCTAAAATTACCTATAGGTGGTAGGGGTCGAGTTATTGATGTGAGATGG
ATCCAAAAAAAAGGG---GGTTCTAGTTATAATCCAGAAATCATTCGTGTCTATATTTTAC
AGAAACGTGAAATCAAAGTAGGTGATAAAGTAGCTGGAAGACATGGAAATAAGGGTAT
CATTTCAAAAATTTTGACTAGACAGGATATGCCTTATTTACAAGATGGAAGATGTGTTGA
TATGGTCTTCAACCCATTAGGAGTACCCTCACGAATGAATGTAGGACAGATATTTGAAT
GTTCACTCGGGTTAGCCGGAAGTCTCCTAGATAGACATTATCGAATAGCACCTTTTGAT
GAGAGATATGAAAAAGAGGCTTCCAGAAAACTAGTGTTTTCGGAATTATATGAAGCCA
GTAAGCAAACAGCAAATCCATGGGTATTTGAACCAGAGTATCCAGGAAAAAGCAAAAT
ATTTGACGGAAGAACAGGAGATCCTTTTGAACAACCTGTTCTAATAGGAAAGCCTTATA
TCTTGAAATTAATTCATCAAGTGGATGATAAAATACATGGACGCTCCAGTGGACATTATG
CACTTGTTACACAACAACCCCTTAGAGGAAGAGCCAAGCAAGGGGGGCAGCGGGTAG
GAGAAATGGAGGTTTGGGCTCTAGAAGGATTTGGTGTTGCTCATATTTTACAAGAAATG
CTTACTTATAAATCTGATCATATCAAAGCTCGTCAAGAAGTACTTGGTACTACAATAATT
GGAGGAACAATACCGAAACCCCAGGATGCTCCAGAATCGTTTCGATTGCTCGTTCGAG
AACTACGATCTTTGGCTCTAGAACTGAATCATTTCCTTGTATCTGAGAAGAATTTGAAG
ATGACTAGGAAGGAAGCTATGAATAATTATTTTTCTTCTATGATCGATCAATATAAACATC
AACAACTCCGAATTGGATCAGTTTCTCCTCAACAAATAAGTGCTTGGGCTACTAAAATC
CTACCTAATGGAGAAATAGTTGGAGAAGTAACAAAGCCTTATACTTTTCATTACAAAAC
CAATAAACCTGAAAAAGATGGATTATTTTGTGAAAGAATTTTTGGGCCTATAAAAAGTG
GAATTTGTGCTTGTGGAAATTATCGAGTAATCGGAGATGAAAAAGAAAAA---AAATTTT
GTGAACAATGCGGAGTCGAATTTGTAGATTCTCGGGTACGAAGATATCAAATGGGCTAC
ATTCAACTGGCATGCCCAGTAACGCATGTGTGGTATTTGAAACGTCTTCCTAGTTATATC
GCGAATATTTTAGATAAACCTCTTAAAGAATTAGAAGGCCTAGTATACTGCGATTTT------
TCTTTTGCTAGGCCT---GTAATTAAAAAACCAACTTTTTTACGATTACGAGGTTTATTCG
AATATGAAATCCAATCTTGGAAATACAGCATCCCACTTTTTTTTACTACCCAAGGTTTCG
ATACATTTCGAAATCGAGAAATTTCGAGTGGAGCGGGTGCGATTCGAGAACAGTTATCT
GATCTGGATTTGCGAAGTATTATAAATTCTTCATTAGTAGAATGGAAAGAATTGGTAGAA
GAA---GGTCCCACAGGTAATGAGTGGGAGGATAGAAAAATTGGACGAAGAAAAGATTT
TTTGGTTAGACGAATGGAATTAGCTAAGCATTTTCTTCGAACAAACATAGAACCAGAAT
GGATGGTTTTATGTCTATTACCGGTTCTTCCCCCCGAGTTGAGACCTATCATTCAGATTG

ATGGGGGAAGCTAATGAGCTCAGATATTAATGAACTCTATAGAAGAGTTATCTATCGA
AACAATACTCTTAAGGATCTATTGATAACAAGTAGATCTACACCAGGAGAATTAGTAAT
GTGTCAAGAGAAATTGGTACAAGAAGCCGTAGATACACTTCTTGACAATGGAATTCGA
GGACAACCTATGAGGGATAGTCATAATAAGGTTTATAAGTCATTTTCAGATGTCATTGAA
GGCAAAGAGGGAAGATTTCGTGAGACTTTACTTGGCAAACGAGTCGATTATTCAGGAC
GTTCTGTCATTGTTGTAGGTCCCTCACTTTCATTACATCAATGTGGATTGCCTGGCGAAA
TAGCAATAGAACTTTTCCAGACATTTGTAATTCGGGGTCTAATTCGAAAGAATCTTGCT
TCTAACATAGGAGTGGCTAAGAGTCAAATTCGGGAGCAAGAACCCATTATATGGGAAAT
ACTTCAGGAAGTTATGCGGGGTCATCCTGTATTGCTGAATAGAGCACCCACTCTGCATA
GATTAGGAATACAAGCATTCCAGCCTATTTTAGTGGAAGGACGTGCTATTTGTTTACATC
CATTAGTTTGTAAGGGATTCAATGCGGACTTTGATGGTGATCAAATGGCTGTTCATGTAC
CTTTATCTTTGGAGGCTCAAGCAGAGGCTCGTTTACTTATGTTTTCTCATATGAATCTCTT
ATCTCCAGCTATTGGAGATCCGATTTCTGTACCAACCCAAGATATGCTTATGGGTCTTTA
TGTATTAACAAGTGGGAATCGTCGAGGTATTTGTGCAAATAGGTATAATCCATGTAATCG
T---------AAAAATTATAAAAATGAAAAAATTCTAAATAAGAATTGTGAGTACACGAAAGA
A---GAACCTTTTTTTGTTAATTCCTACGATGCAATTGGGGCTTATCAGCAAAAAAGAATT
TATTTAGATAGTCCTTTGTGGCTTAGGTGGAAACTAGATCAACGT---GTTATTGCTTCA---
---AGAGAAACTCCAATAGAAGTTCAGTATGAATCTCTGGGTATCTATCATGAGATTTATG
GAAACTATATAATAGTCAGAGGCATTAAAAAAGAAATTCTTTGGATATATATTCGAACTA
CTGTTGGTCATATTTTTCTTTATCGAGAAATAGAAGAAGCTATACAAGGGTTTTCTCGGG
CTGGTTTATATGGTACC------------------------ATGGAGGTACTTATGGCAGAACGGGCCAAT
CTGATCTATCACAATAAAGCGATAGACGGAACTGCCATTAAACGACTAATTAGCAGATT
CATAGATCATTTCGGAATGGCGTATACATCACACATCCTGGATCAAATAAAGACTCTGG
GTTTCCAGCAAGCTACTTCTGCATCCATTTCATTAGGAATTGATGATCTTTTAACAATAC
CTTCTAAAGGATGGCTCGTCCAAGATGCTGAACAACAAAGTTTCATTTTTGAAAAACA
CCATAATTATGGGAATGTACATGCGGTAGAAAAATTACGCCAATCTATTGAGATATGGTA
TGCTACAAGTGAATATTTGCGACATGAAATGAATCCTAATTTTAGGATTACTGACCCTTT
GAATCCAGTCCATATAATGTCTTTTTCGGGAGCTAGGGGAAATGTATCTCAAGTACATCA
ATTAGTAGGTATGAGAGGCTTAATGTCAGATCCTCAAGGCCAAATGATTGATTTACCCAT
TCAAAGCAATTTACGCGAAGGATTGTCTTTAACAGAATATATTATATCTTGTTATGGAGC
TCGCAAAGGAGTTGTAGATACTGCTGTAAGAACATCAGACGCTGGATATCTGACTCGC
AGGCTTGTTGAAGTAGTTCAACACATTGTTGTACGTAGAACAGATTGTGGCACTATACG
AGGAATTTCTGTGAGTCCTAACAAAGGTATGATGCCA---GAAAGAATTTTTATTCAAAC
ATTAATTGGTCGTGTATTAGCAGATGATATATATATAGGTTCACGATGCATTGCCATTCGA
AATCAAGATATTGGGATTGGACTTGTCAATCGATTCATAACT-----------------TTTCGAAAA
CAATCAATATTGATTCGAACTCCCTTTACTTGTAGAAGTACATCTTGGATCTGTCGATTAT
GTTATGGTCGGAGTCCTACTCACGGTGACCTGGTCGAATTGGGGGAAGCTGTAGGTATT
ATTGCAGGTCAATCTATTGGGGAACCGGGCACTCAACTAACATTAAGAACTTTTCATAC
TGGCGGAGTATTCACAGGAGGTACTGCAGAACATGTAAGAGCCCCTTCTAATGGAAAA
ATACAATTCAATGAAGATTTGGTTCATCCCACACGTACACGTCACGGACATCCTGCTTTT
ATATGTGATATAGATTTGTATGTAACTATTGAAAGTGAAGATATTCTACATAACGTAACTA
TTCCATCAAAAAGTTTTCTTTTAGTTAAAAATAATCAATATGTACAATCAGAACAGGTGA
TTGCTGAGATTCGTGCAAGAACAGATAATTTG---ATTGTGAAAGAGAGAGTTAGAAAA
CATATTTATTCTGACTCAGAGGGAGAA---ATGCACTGGAGTACTGATGTATACCATGCAC

CTGAATGGACATATAGTAATGTCCATCTCTTACCAAAAACAAGCCATTTATGGATATTAT
CAGGAGGTTCGTGTAAA------TCCAGCGTATTACCCCTTTCACTCCATAAGGATCAAGAT
CAATTGTTGAGTTCG------GGTAAAAAAAAAACTATTCTTCCTTATTCTTCAGTGTTT------
AATCCAAGTCGATAT---ATGGGT---
---------------------CATTATGATTTCCCTGCT---ATTCTTCACGAGAATTATGAT------TTATTAG
GAAAAAGGCGAAGAAATAGATTTCTCATTCCATTCCAATTGATGGCAGAACAATATAAC
GAAGAAATGCTGATGCCGCGTCTCTGGATTCAAATACCCCCA------AAAGGTATTTTCTA
TAGAAATAGTATTCTTGCTTATTTTGATGATCCTCAATACAGAAGAAAGAGTTCAGGAAT
TATTAAATATGGGACTATAGGGACACATTCAATTGCCAAAAAAGAGGAT------TTGATCG
AGCATAAA---GGAGTGAAAGAATTTCAGCCAAAATACATGAAAGTAGATCAATTTTTTT
TCATTCCCGAAGAAGTGCATATTTTACCTGAGTCTTCTTCCATAATGGTACGCGACAATA
GTCTTATTGGAGTCGATACACAAATCACTCTCAATACAAGAAGCCGAGTGGGCGGATT
GGTTCGAGTGGAGAAAAAAAAAAAAAAGGATTGAACTTAAAATCTTTTCTGGAGATATC
CATTTTCCTAGCGATACAGATAAGATATCCCGACACAGTGGGATCCTGATCCCGCCA---G
GAATGATAAAAACAATTTCGAAA---GAATCAACAAAA------TTGAAAAATTGGATCTATG
TCCAACGT------ATCATACTTACAAAAACAAAA------TATTTTGTTTTGGTTCGACCTGTAA
TGCCGTATGAAATAACGGATGGTGTCAATTTAGGAGCACTTTTCCTGCAAGATTTGTTC
CAAGAAAAGGATAATCTAAAACTTAAAGTTGTTAATTATATCCTTTATGAAAATGGGAA
A---------CCAATTTCTGACACAAGTATTCAATTAGTTCGGACTTGTTTAATGTTGAGTGTG
GGTCAAGACAAA---------AAAAATTCTTCTATTGAAGAAGCCTACACTTCTTTTATTGGA
GTAACTATCAATGGTTTGATTCGAGATTTCCTAAAAGTTGACTTAATGAAA---CATATTTC
ATATATCAGAAAAAGGAAAGATACTTCAGGTTCAAGATTCATTTCTCATAATAATGCATC
AGATAGTATTAATATTAACCCATTTTCTATTCAGTCTAATTATAAGGAT------AAGGAACAA
ATTCAAAATTTATTGATTCAAAATAACAATACTATTCGTTGTACATTATCGAATCAAACTC
AAAATAAGGAATGCCGATCTTTGATAATTTTGTCACCATCGAATTGTTTTCGTTTTCAAA
TAAAACCTTTCAACAATTTAAAATATCAAAAT---------GTGATAAAAGAATCAGAAAAAG
ATTCTCTCAATTTCAATTCGTTGGGTCCTTTTGGAACA------TCCATTCCAATTGCGAATT
TTTATTCA------TTTGATTATTTCATAACT------TATAATCCAATCTCG---ATCATTAAATATTT
GCAAGTTGACAAT---------TTAAAATGGACTTTTCAAATACTT------CAA---TCGTATTTAAT
TGATGAAAGAGAACAA------ATTTATAATCCTGACTCGTGTAGTAACATTGTTTTGAATG
CCTTTCATTTGAATTGGTATTTTCTCTATTATCAA------------AAATCCACAATAATTAGC---
CTTGGAGAGTTTATTTGTGAAAATGTATCTATAGCTAAAAATGGACCG---------CCTCTAA
AATCGGGTCAAGTTCTAATTGTTCAA---GTTGACTCTGTAATAATTAGATCAGCTAAGCC
TTATTTAGCAACTCCAGGAGCAACTGTTCATGGACATTATGGAAAGATAATTGATGAAG
GAGATACATTAGTAACATTTTTATATGAAAAGTCGAGATCTGGTGATATAACTCAAGGTC
TTCCAAAAGTAGAACAAGTGTTAGAAGTTCGTTCAATTGATTCAATATCAATGAATCTA
GAAAAAAGAATTGAGAGTTGGAACAAAGGTATAGCAAGAGTTCTTGGAATTCCTTGG
AGATTCTTGATTGGTGCTGAGTTAACGATAGCCCAAAGTCGTCTATCTTTAGTTAATAAG
ATACAAAAGGTTTATCGATCACAGGGAGTACAGATTCATAATAGACATATCGAAATTATT
GTACGTCAAATAACATCAAAAGTGTTGGTTTCAGAA---------GATGGAATATCGAATGTT
TTTTTACCCGGAGAGCTAATTGGATTGTTGCGAGCTGAACGAACAGGACGTGCTTTAG
AAGAAACGATATGTTATCGAGCTATTTTATTGGGAATAACGAAAGCCTCTCTGAATACTC
AAAGTTTTCTATCCGAGGCTAGTTTTCAAGAAACTGCTCGAGTTTTAGCAAAATCTGCT
CTCCGCGGTCGTATCGATTGGCTAAAGGGGTTAAAAGAAAACGTTGTTCTAGGGGGAA

TGATACCTGTTGGTACTGGATTCAAAGGGTTAGTGTACTGTTCA------AGGAAACATAAT
AAAATGATT------TGGGAAACAAAAAAAAATCATTTATTTGAGCAAGAA---GTG---AGAG
ATATTTTTTTCCACCATAGAAAATTACTGAATTCT---
--------------------------ATGACAAAACGATATTGGAACATAAATCTAGAAGAGATGATGGAA
GCAGGAGTTCATTTTGGTCATGGTACTCGAAAATGGAATCCTAAAATGGGTCCTTATATT
TCTGCAAAGCGTAAAGGTATTCATATTTTAAATATTACCCGAACGGCTCGTTTTTTATCA
GAAGCTTGTGATTTGGTTTTTGATGCAGCATCGAAAGGAAAACAATTCTTAATTGTTGG
TACTAAAACTAAAGCAGCTGATTTAGTAGCACGTGCTGCAATAAAGAGTCGCTCTCATT
ATGTTAATAAAAAATGGCCCGGGGGTATGTTAACAAATTGGTCCACTACAGAAACAAG
ACTTCATAAGTTCAGAGACTTGAGAATGAAACAAAAGATGGGGAGACTCAATCGTCTT
CCCAAAAGAGATGCAGCTATGTTAAAGAGAAAATTATCCCACTTGCAAACCTATTTGGG
CGGGATTCAATATATGAAGGGATTACCTGATATTGTAATAATCGTTGATCAGCAAGAAGA
ATATACGGCTCTTCGAGAATGTATCACTTTAGGAATTCCAACAATTTGTTTAATCGATAC
AAATTGTGACCCCGATCTTGCAGATATTTCGATTCCAGCCAATGATGATGCTATCGCTTC
AATCCGATTCATTCTTAACAAATTAGTATTCGCAATTTGTGAGGGTCGTTCT---AGCTATA
TACGAAATTTGATGGGGCAAAAAATCAATCCACTCGGTTTTAGACTTGGTATAACTCAA
AGCCATCATTCTATTTGGTTTGCACAACCAAAAAATTATTCCGAAGGTCTACAAGAAGA
TAAAAAAATACGAGACTGTATACAAAATTATATACAAAAAAATATGAAAATATCTTCT---
GGTGTTGAAGGAATTGCATGTATAGAGATTCAAAAGAGAATCGATCTGATTCAAATCAT
AATTTATATGGGATTTCCAAAATTATTAATAAAAGGTAGGTCTCAA------------AAAATCGA
AGAATTACAAACGAATATAAAAAAAGAAATTCATTGTGTGAATCGAAAAATTAACATTG
CTATTAAAAGAATTTCAAATCCTTATGGACACCCTAAGATTCTTGCAGAATTTATAGCCG
GACAACTCAAGAATAGAGTCTCCTTTCGAAAAGCAATGAAAAAAGCTATTGAATTAAG
TGAACAGACAGATACAAAAGGAATTAAAGTACAAATTGCAGGCCGTCTCGATGGAAA
AGAAATTGCACGTGTCGAATGGATCCGAGAAGGTAGGGTTCCTTTACAAACTATTCGA
GCTCCAATTGATTATTGTTCTTATACAGTTCGAACTATCTATGGGGTATTGGGAATAAAA
ATTTGGATATTTATA---------ATGTCGCGTTACCGAGGGCCTCGTTTCAAAAAAATACGCC
GTCTGGGGGCTTTACCGGGACTTACTAATAAAAGGCCTAAAGCCAAAACCATTACTAG
AAATCAATCGCGCTCTGGTAAA---AAATCTCAATATCGTATTCGTTTAGAAGAAAAACA
AAAATTGCGTTTTCATTATGGTCTTACAGAGCGACAATTACTTAAATATGTACGTATCGC
CAGAAAAACCAAGGGATCCACAGGTCAGGTTTTACTACAATTAATTGAAATGCGTTTG
GATAATATCCTGTTTCGATTAGGTATGGCTTCGACTATACCTCAAGCCCGCCAATTTGTT
AACCATAGACACATTTTAGTTAATGATCATATAGTCAATATACCAAGTTATCGCTGCAAA
CCCCGAGATATTATTACAGTGCGTGATAACCAAAAATCTAGAACTCTGGTCCAAAATTA
TCTTGATTCG---CCTAAC---CAGGAATTGGCAAAACATTTGACTCTTCAGCCATTCCAA--
----TATAAAGGATTAGTCAATCAAATAATAGATAGTAAGTGGGTCGGTTTAAGAATAAATG
AATTACTGGTTGTAGAATATTATTCTCGTCAGACTATGTCACGTCGAGGGACTGCAGAA
AAAAAAACTGCAAAATCCGATCCAATTTATCGTAATCGATTAGTTAACATGTTGGTTAA
CCGTATTCTGAAACACGGAAAGAAATCATTGGCTTATCAAATTCTCTATCGAGCCATGA
AAAATATTCAACAAAAGACAGAAAAAAATCCACTATCTGTTTTGCGTCAAGCAATACG
TGGAGTAACTCCCGATATAGCAGTCAAAGCAAGACGTGTAAGCGGATCGACTCATCAA
GTTCCCATTGAAATAGGATCTACACAAGGAAAAGCACTTGCCATTCGTTGGTTATTAGC
GGCATCCCGAAAACGTCCGGGTCGAAATATGGCTTTCAAATTAAGTTCCGAATTAGTGG
ATGCTGCAAAAGGGAGTGGCGATGCTATACGCAAAAAGGAAGAGACTCATAGAATGG

CAGAGGCAAATAGAGCTTTTGCACATTTTGGTATGAGTAAGGACACTATTGCTGACATA
ATAACCTCTATACGAAACGCTGACATGAATAGAAAGGGAACAGTTCGAATAACATATAC
TAATATAACTGAAAATTTTGTGAAAATACTTTTACGAGAAGGTTTTATCAAAAACGTCA
GAAAACATCAAGAAAGGGGCAAATATTTTTTGGTTTTAACTTTACGCTATAAAAGG------
AATACAAAAGGATCCCATAAAACG---ATG------TTGAATTTAAAACGAATTAGTCGGCCT
GGCTTAAGAATATATTCTAATTATCAAAAAATTCCTAAAATTTTAGGCGGGATGGGTATT
TTAATTCTTTCGACTTCACGAGGTATAATGACAGACCGAGAGGCTAGACTAGAAAAAAT
CGGCGGAGAAATTTTGTGTTGTATATGGATGGCAAAACCGATAAAAAGAATTGGTTCAC
GTAGAAATGTACGTATTACTTCACGTAAGAATACGCGCAAAATACCAAAAGGAATTATT
CATGTTCAAGCAAGTTTCAACAATACCATTGTAACTGTTACAGATGTGCGAGGTAGGGT
GATTTCTTGGTCCTCTGCTGGTACTTGTGGATTCAAGGGCACAAGAAGAGGAACACCC
TTTGCTGCTCAAACCGCAGCAGGAAATGCTATTCGAACAGTAGTAGATCAAGGTATGC
AAAGAGCAGAAGTCATGATAAAAGGTCCAGGTCTTGGAAGAGATGCTGCATTAAGAG
CTATTCGTAGAAGTGGTCTATTATTAAGTTTTGTGCGGGATGTAACTCCTATGCCACATA
ATGGATGTAGACCTCCTAAAAAAAGACGTGTGATGCCAACTATTAAACAACTTATTAGA
AACGCAAGACAGCCGATTAGAAATGTGACGAAATCCCCCGCTCTTCGGGGATGTCCTC
AACGTCGAGGAACATGTACTAGGGTGTATACTATCACCCCCAAAAAACCAAACTCTGC
CTTACGTAAAGTTGCCAGAGTACGATTAACCTCTGGATTTGAAATCACTGCTTATATACC
TGGTATTGGCCATAATTTACAAGAACATTCTGTAGTCTTAGTCAGAGGAGGGAGGGTGA
AGGATTTACCCGGTGTGAGATATCACATTGTTCGAGGAACCCTAGATGCTGTCGGAGTA
AAGGATCGTCAACAAGGGCGTTCTAAATATGGGGTCAAAAAGCCAAAAATGGCAAAG
AAAAGTTTGATTCAGAGGGAGAAGAAGAGGCAAAAATTGGAACAAAAATATCATTTG
ACTCGTCGATTTTTAAAAAACGAAAGAAACAAA------GTATCATTGAGTGATAAAGAGA
AATGGGAAATTAAGGGAAAGTTACAATCCCTACCACGTAATAGTGCTCCTACGCGTCTT
CATCGACGCTGTTTTTTGACCGGAAGACCAAGAGGTAATTATCGAGACTTTGGTTTATC
CCGACATATACTTCGTGAAATGTTTCATGCATGTTTGTTGCCAGGGGCAACAAGATCGA
GTTGGATGGTAAAAAATTCATTCATCTCAGCTATTTCACAACAAGAAAAAGAAGAAAA
CAAA------GGATCTGTTGAATTTCAAATCTTAAGTTTCACAAACAGGATACAAAAACTTA
CTTCACATTTAGAATTACACAAAAAGGATTATTCATCTCAAAGAGGTCTACGAAAAATT
CTGGGAAAACGTCAACGACTGCTTTATTATTTGTCAAGGAAAAATCCAATAAGTTATAA
GGAGTTAATTAATCAGCTGGGGATTCGTGAGTTAAAAAAAAATGGT--
-???
???
??
ATGGATAAATCCAAACGACTTTTTCTTAAATCCAAACGATCTTTTCGTAGGCGTTTGCCC
CCGATACAGTCGGGGGATCGAATTGATTATAGAAATCTGAGTTTACTCAATCGATTTCTC
AGTGAACAAGGAAAAATCTTATCTAGACGGGTAAATAGATTGACTTTAAAACAACAAC
GATTAGTTACTATTGCTATAAAACAGGCTCGTATTTTATCTTTGTTACCTTTTATGAATAAT
GAAACA------CATTTTGAAAGAAGGAAATCGACTGCTAGAACTCCTGGGTTTAGAACCA
AAAAGAAAATGACACGT---AAAAAAAAAAATCCTTTTGTAGCACATCCTTTATTAAAAA
AAATAAAGAGACTTAACACAAAATCGGAAAAAGAAATAATAGTAACTTGGTCCCGGGC
ATCCACCATTATACCCATAATGATTGGACATACTATTGCTATACATAATGGAAAGGAACA
CATACCTATTTATATTGAGGATCGTATGGTAGGCCATAAATTGGGAGAATTTGTCTCGAC
TCTCAATTTTAGAGGACATGCGAAAAATGACAGTAGATCTCGTCG

T???
??
??
??
??
??
??
??
??
??
??
??
??
??
??
??
??
??
??
??
??
??
??
??
??
??
??
??
??
??
??
??
??
??
??
??
??
??
??
??
??
??
??
??

??
??
??
??
??
??
??
??
??
??
??
??
??
??
??
??
??
??
??
??
??
??
??
??
??
??
??
??
??
??
??
??
??
??
??
??
??
??
??
??
??
??
??
??

???
???
???
???
???
???
???
???
???
???
???
???
???
???
???
???
???
???
???
???
???
???
???
???
???
???
???
???
???
???
???
???
???
???
???
???
???
???
???
???
???
???
?????????????????????????????????????ATGGCTAGATCTCGT---ATAAATGGAAATTTTATTGA
TAAGACCTTTTCAATTGTAGCCAATATCTTATTACGAATAATTCCGACAACTTCAGGAGA

AAAAGAGGCATTTACTTATTATAGAGATGGG------ATGTCGGCTCAGTCCGAAGGAAATT
ATGCGGAAGCTTTACAAAATTATTATGAAGCTATGCGACTAGAAATTGACCCCTATGATC
GAAGTTATATACTCTATAACATAGGCCTTATCCACACAAGTAATGGAGAACATACAAAA
GCTTTGGAATATTATTTTCGGGCACTCGAACGAAACCCGTTCTTACCACAAGCTTTTAAT
AATATGGCCGTGATCTGTCATTAC---CGGGGAGAACAGGCCATCCGACAAGGAGATTCC
GAGATTGCAGAAGCTTGGTTCGATCAAGCCGCTGAGTATTGGAAACAAGCTATAGCGC
TTACTCCTGGTAATTATATTGAAGCGCAGAATTGGTTAAAGATCACGAGACGTTTCGAA-
-----ATGAGTTTGCGATCAGAATTGATATGGATAGAACTTATAGGGGGGTCTCGAAAAAC
AAGTAATTTTTTTTGGGCTTTTATCCTTTTTTTTGGTTCATTAGGATTCTTATTGGTTGGA
ATTTCCAGCTATCTTGGTAGGAATTTGATATCTTCATTTCCGTCTCAACAAATCCTTTTTT
TTCCACAAGGGATCGTGATGTCTTTCTATGGGATTGCAGGTCTATTTATTAGTTCCTATTT
GTGGTTTACAATTTGGTGGAATGTAGGTAGTGGTTATGATCGATTCGATAAAAAACAAG
GATTAGTTTGTATTTTTCGTTGGGGATTTCCTGGAAAAAATCGTCGTATCTTCCTCAGAT
TTCTTATAAAAGACATTCAATCCATCAGAATAGAAGTGAAAGAGGGTGTTTATGCTCGT
CGTGTACTTTATATGGAAATAAGGGGTCAGGGAGCCATTCCTTTGACACATAGTGATGA
TAAT---TGGACTCCACGAGAAATTGAACAAAAAGCTGCCGAATTAGCCTATTTTTTGCG
TGTACCAATGGAAGTCTTT
>Cornus
ATGGAAAAGTGGTGGTTCAATTCGATGCTGTTTCTGAGTAAATCAACGGGTAGTCTTGG
TCCTATTGAAAATACCAGCGAAAGTGAAGATCCAGATAGAAAT---------AAAAACATTCA
TAGTAATAGTAATGTTGATCATTTATTCAGTGTCAAGGACATTCGGAATTTCATTTCTGAT
GACACTTTTTTAGTTAGGGATAATAATGGGGACAGTTATTCCATATATTTTGATATTGAAA
ATCAGATTTTTGAGATTGACAATGATCAT------TCTTTTCTAAGTGAACTAGAAAGTTCTT
TTTATAGTTATCGGAAT---------------AAGGGATCTAAGAGTAACGATCCCTACTATGATCGT
TACATGTATGATACTCAATATAGTTGGAATAATCACATTAATAGTTGCATTGACAGTTATC
TTCAT------TCTCAAATCTGTATTGATACTTACATTTTAAGTGATAGTGACAATTACAGTGA
TAGTTACATTTATAGTTCCATTTGTGGTAGCGATAGTTCCGGTATAAAAACCAGCATGAA
TGGTAGTGATTTAACTATAAGAGAAAGTTCTAATGATCTCGATGTAACTCAAAAATACA
AGCATTTGTGGGTTCAATGCGAAAATTGTTATGGATTAAATTATAAGAAATTTTTGAAGT
CAAAAATGAATATTTGTGAACAATGTGGATATCATTTGAAAATGAGTAGTTCAGATAGA
ATCGAACTTTCGATTGATCCGGGCACTTGGGATCCTCTGGATGAAGACATGGTCTCTCT
GGATCCCATTGAATTTCATTCGGATGAGGAGCCTTATAAAGATCGTATCGATTCTTATCA
AATAAAAACAGGGTTAACTGAGGCTGTTCAAACAGGCATCGGCCAACTAAACGGTATT
CCCATAGCAATTGGGGTTATGGATTTTCAGTTTATGGGGGGTAGTATGGGATCCGTAGTT
GGGGAGAAAATTACCCGTTTGGTCGAGTATGCTACCAATAAATTTCTACCTCTTATTATA
GTATGTGCTTCGGGAGGGGCACGCATGCAAGAAGGAAGTTTGAGCTTGATGCAAATGG
CTAAAATTTCTTCTGCTTTATATGATTATCAATCAAATAAAAAGTTATTTTATGTATCAATC
CTTACATCGCCTACTACTGGTGGGGTGACAGCCAGTTTTGGTATGTTGGGGGATATCATT
ATTGCCGAACCCAATGCCTACATTGCATTTGCGGGTAAAAGAGTAATTGAACAAACATT
GCATAAAACAGTACCCGAAGGTTCACAAGCGGCCGAATATTTATTCCAGAAGGGCTTAT
TCGATTTAATCGTACCGCGTAATCCTTTAAAGGGCGTTCTGAGTGAATTATTTCAGCTCC
ACGCTTTCTTTCCTTTAAATCAAAATTCAAGCAAGATGGTAACCATTCGAGCCGACGAA
ATTAGTAATATTATCCGTGAACGTATTGAGCAATATAATAGAGAAGTAAAGATTGTAAAT
ACCGGTACTGTACTTCAAGTAGGCGATGGCATTGCTCGTATTCATGGTCTTGATGAAGT

AATGGCAGGTGAATTAGTAGAATTTGAAGAGGGGACAATAGGCATTGCTTTGAATTTG
GAATCAAACAATGTTGGTGTTGTATTAATGGGTGACGGTTTGATGATACAAGAAGGAA
GTTCTGTAAAAGCAACAGGAAGAATTGCTCAGATACCGGTGAGTGAGGCCTATTTGGG
TCGTGTTATAAATGCCCTGGCTAAACCTATTGATGGTAGAGGTGAAATTTCAGCTTCCG
AATCTCGGTTAATTGAATCCCCCGCTCCGGGTATTATTTCGCGACGTTCCGTATATGAGC
CTCTTCAAACGGGGCTTATTGCTATTGATTCGATGATCCCTATAGGACGTGGTCAGCGA
GAATTAATTATTGGGGACAGGCAGACTGGTAAAACAGCAGTAGCCACAGATACGATTC
TCAATCAACAGGGGCAAAATGTAATATGTGTTTATGTAGCTATTGGTCAAAAAGCATCT
TCGGTGGCTCAGGTAGTGACTAACTTCCAGGAAAGGGGGGCGATGGAATACACTATTG
TAGTAGCCGAAACAGCGGATTCCCCTGCTACATTACAATATCTCGCTCCTTATACAGGA
GCGGCTCTGGCTGAATATTTTATGTATCGTGAACGACACACTTCAATCATTTATGATGAT
CTCTCAAAACAAGCGCAAGCTTATCGCCAAATGTCGCTTCTATTACGAAGACCGCCCG
GTCGCGAAGCTTATCCAGGGGATGTTTTTTATTTGCATTCACGCCTTTTGGAAAGAGCC
GCTAAATTAAGTTCTCGTTTAGGTGAAGGAAGTATGACCGCTTTACCAATAGTTGAGAC
TCAATCAGGAGATGTTTCGGCTTATATTCCTACTAATGTAATTTCCATTACTGATGGACA
AATATTTTTATCCTCCGATCTATTTAATGCCGGAATCAGGCCTGCTATTAATGTGGGTATC
TCCGTTTCCAGAGTAGGATCTGCGGCTCAAATTAAAGCCATGAAACAAGTAGCCGGCA
AATTAAAATTGGAACTGGCGCAATTCGCAGAATTAGAAGCCTTTGCACAATTCGCTTCT
GATCTTGATAAAGCCACTCAGAATCAATTGGCAAGAGGTCAACGATTACGTGAATTGCT
CAAACAATCCCAATCAGCCCCTCTCGCGGTGGAAGAGCAGATAATGACTATTTATACCG
GAACAAATGGTTATCTTGATTCATTAGAAATTGGACAGGTAAGGAAATTTCTTGTTGAG
TTACGTACTTACTTAAAAACTAATAAACCTCAGTTCCAAGAAATCATATCTTCTACCAAG
ACATTCACCGAGGAAGCAGAAGCTCTTTTGAAAGAAGCTATTCGGGAACAGATGGAA
CGTTTTATACTTCAGGAACAAGCAATGAGAATAAATCCTACTACTTCTGGTCCTGGAAT
TTCCACGCTTGAAAAAAAAAACCTGGGGCGTATCGCTCAAATCATTGGTCCGGTACTA
GATGTAGCTTTTCCTCCGGGCAAGATGCCTAATATTTATAACGCTTTGGTAGTTAAAGGT
CGAGATACTGCCGGTCAACCAATTAATGTGACTTGTGAGGTACAACAATTATTAGGGAA
CAATCGAGTTAGAGCTGTAGCTATGAGTGCTACGGAGGGTCTAACGAGAGGAATGGAA
GTGGTTGATACGGGAGCTCCTCTAAGTGTTCCGGTTGGTGGGGCGACTCTCGGACGAA
TTTTCAATGTGCTTGGAGAGCCCGTTGATAATTTAGGTCCTGTAGATACTCGCATAACAT
CTCCTATTCATAGATCTGCGCCCGCCTTTATACAGTTAGATACAAAATTATCTATTTTTGA
AACAGGAATTAAAGTAGTAGATCTTTTAGCCCCTTATCGACGTGGAGGAAAAATTGGA
CTGTTCGGAGGAGCTGGAGTGGGTAAAACAGTACTCATTATGGAATTGATTAACAATAT
TGCCAAAGCTCATGGGGGTGTATCCGTATTTGGCGGAGTAGGTGAACGTACTCGCGAA
GGAAATGATCTTTACATGGAAATGAAAGAATCCGGAGTGATTAATGAACAAAATATTGC
GGAATCAAAAGTAGCTCTAGTTTACGGTCAGATGAATGAACCGCCGGGAGCTCGTATG
AGAGTTGGTTTGACTGCCCTAACTATGGCGGAATATTTCCGAGATGTTAATGAACAAGA
CGTACTTCTATTTATTGACAATATCTTCCGTTTCGTCCAAGCAGGATCCGAAGTATCCGC
CTTATTGGGTAGAATGCCTTCCGCTGTGGGGTATCAACCCACCCTTAGTACTGAAATGG
GTTCTTTACAAGAAAGAATTACTTCTACTAAAGAAGGGTCCATAACTTCTATTCAAGCA
GTTTATGTACCTGCAGACGATTTGACCGACCCGGCTCCCGCTACGACATTTGCACATTT
AGATGCCACTACCGTACTATCAAGAGGATTAGCTGCCAAAGGCATCTATCCAGCAGTAG
ATCCTTTAGATTCAACGTCAACTATGCTTCAGCCTCGGATCGTTGGTGAGGAGCATTAT
GAAACTGCGCAAAGAGTTAAGCAAACTTTACAACGTTACAAAGAACTTCAGGACATTA

TAGCTATCCTTGGGTTGGACGAATTATCCGAAGAGGATCGTTTAACCGTAGCAAGAGCA
CGAAAAATTGAGCGTTTCTTATCACAACCTTTTTTCGTAGCAGAAGTATTTACGGGTTC
TCCAGGGAAATATGTTGGTTTAGCAGAAACAATTAGAGGGTTTCAATTGATCCTTTCCG
GAGAATTAGATGGTCTTCCCGAACAGGCCTTTTATTTGGTAGGTAACATCGACGAAGCT
ACCGCGAAGGCTATGAACTTAGAAATGGAGAGTAATTTGAAGAAAATGACCTTAAATC
TTTGTGTACTGACCCCTAATCGAATTGTTTGGGATTCAGAAGTGAAAGAAATCATTTTAT
CTACTAATAGTGGTCAAATTGGCGTATTACCAAATCACGCCCCTATTGCCACAGCTGTAG
ATATAGGTATTTTGAGAATACGCCTTAACGACCAATGGTTAACGATGGCTCTGATGGGC
GGTTTTGCTAGAATAGGCAATAATGAGATCACTGTTTTAGTAAATGATGCGGAGAGGGG
TAGTGACATTGATCCCCAAGAAGCTCAGCAAACTCTTGAAATAGCGGAAGCTAATTTG
AGGAAAGCCGAGGGCAAGAGACAAACAATTGAGGCAAATTTAGCTCTCAGACGAGCT
AGGACACGAGTAGAGGCTATCAATGTAATTTCAATGAAAAATGTAACCGATTCTTTCGT
TTCCTTGGGCCACTGGCCGTTCGCCGGGAGTTTCGGGTTTAATACCGATATTTTAGCAA
CAAATCCAATAAATCTAAGCGTAGTGCTTGGTGTATTGATCTTTTTTGGAAAGGGAGTG
TTAAGTGATTTATTAGATAATCGAAAACAGAGGATCTTGAATACTATTCGAAATTCAGAA
GAACTACGCGGAGGAGCCATTGAACAGCTGGAAAAAGCCCGGGCTCGCTTGCGGAAA
GTGGAAATGGAAGCAGATCAGTTTCGCGTGAATGGATACTCTGAGATAGAGCGAGAAA
AATTGAATTTGATTAATTCAACTTATAAGACTTTGGAACAATTAGAAAATTACAAAAATG
AAACCATTAATTTTGAACAACAAAGGGCAATTAATCAAGTCCGACAACGGGTTTTCCA
ACAAGCCCTGCAAGGGGCTCTAGGAACTCTGAATAGTTGTTTGAATAACGAGTTACAT
TTACGTACCATCAGTGCCAATATTGGCATCTTTGGGGCGATGAAAGAAATAACCGATAT
GAATCCACTGATTTCTGCCGCTTCCGTTATTGCTGCTGGATTGGCTGTAGGGCTTGCTTC
TATTGGACCTGGAGTTGGTCAAGGTACTGCTGCGGGCCAAGCTGTAGAAGGTATCGCG
AGACAGCCCGAGGCGGAGGGAAAAATAAGAGGTACTCTATTGCTTAGTCTGGCTTTTA
TGGAAGCTTTAACAATTTATGGACTAGTTGTAGCATTAGCACTTTTATTTGCAAATCCTT
TTGTTATGAATGTTCTATCATGTTCTATCAACACACTAAAAGGTTTATACGATATATCCGG
TGTGGAAGTAGGCCAACATTTCTATTGGAAAATAGGGGGTTTCCAAGTTCATGGCCAA
GTACTTATTACTTCTTGGGTTGTAATGGCTATCTTATTAGGTTCAGCCACCATAGCTGTTC
GGAACCCACAAACCATTCCGACTGGTGGTCAGAATTTCTTCGAATATGTCCTTGAATTC
ATTCGAGATGTGAGCAAAACTCAAATTGGAGAAGAATATGGTCCTTGGGTTCCCTTTAT
TGGAACTATGTTTCTATTTATTTTTGTTTCTAATTGGTCGGGGGCTCTTTTACCTTGGAAA
ATAATACAATTACCCCATGGGGAGTTAGCCGCACCCACGAATGATATAAATACTACTGTT
GCTTTGGCTTTACTCACGTCAGTGGCATATTTCTATGCAGGTCTTACCAAAAAAGGATT
AGGTTATTTCGGGAAATATATTCAACCAACTCCAATCCTTTTACCCATTAACATCTTAGA
AGATTTCACAAAACCGTTATCACTTAGTTTTCGACTTTTCGGGAATATATTAGCTGATGA
ATTAGTAGTTGTTGTTCTTGTTTCTTTAGTACCTTCAGTGGTTCCTATACCTGTCATGTTC
CTTGGATTATTTACAAGTGGTATTCAAGCTCTTATTTTTGCAACTTTAGCCGCGGCTTATA
TAGGTGAATCCATGGAGGGTCATCATATGATATTTTCAACTTTAGAGCATATATTAACTCA
TATCTCCTTTTCGATCGTTTCAATCGTAATTACAATTCATTTGATAACCTTATTAGTCGAT
GAAATCGTAAGACTATATGATTCGTCAGAAAAGGGCATGATAGCTGCCCTTTTCTGTATA
ACAGGATTATTAGTCACTCGGTGGATTTATTCGGGGCATTTCCCGTTAAGTGATTTATAT
GAATCATTAATTTTTCTTTCATGGAGTTTCTCTATTATTCACATAGTTCCGTAT------AAAA
AGAAAAATCATTTAAGCGCAATAACCGCACCGAGTGCTATTTTTACCCAAGGCTTTGCT
ACTTCAGGCCTTTTAACTGAAATACATCAATCCACAATATTAGTACCCGCTCTCCAATCC

GAGTGGTTAATAATGCACGTAAGTATGATGGTATTGGGCTATGCAGCTCTTTTATGCGGA
TCATTATTATCAGTAGCGCTTCTAGTCATTACATTTCGAAAAGACATAAAGATTTTTTGT--
----AAAGAAATCCAATACATAAATAAAAAAAGCAATGTTTTGGGAAATACTTCT---------G
CTGGGAATTATTACAGGTCCCAATTGATTCAACAATTGGATCATTGGAGTTATCGTGTTA
TTAGTCTAGGGTTTATTTTTTTAACCATAGGGATTCTTTCGGGAGCAGTATGGGCTAATG
AGGCATGGGGATCATATTGGAATTGGGACCCAAAGGAAACTTGGGCATTTATTACTTGG
ACCATATTCGCGATTTATTTACATACTCGAACAAATAAAAATTTGCAGGGTGCAAATTCC
GCAATTGTGGCTTCTATAGGCTTTCTTATAATTTGGATATGCTATTTTGGAGTCAATCTAT
TAGGAATAGGGCTGCATAGTTATGGTTCATTTACATTAACGATGAAAAAAAAAATAAAA
CATTCA---CTCCTCTTCTTTGTATCTATAGTATTTTTGCCCTGGTGGATTTCTCTCTCATTT
AATAAAAGTCTGGAATCTTGGGTTACAAATTGGTGGAATACTAGGCAATCTGAAATTTT
TTTGAATGATATTCAAGAAAAGAGTATTCTAGAAAAATTCATAGAATTAGAAGACCTCC
TCCTCTTGGAGGAAATGATAAAGGGATACTCGAAGATACATCTACAAAAGCTTCATATA
GGAATCCACAAAGAAACGATCCAATTAATCAAGATACACAATGAGGATCGTATCCATAC
GATTTTGCACTTCTCGACAAATATAATCTGTTTCATTATTCTAAGTGGTTACTCTATTCTG
GGTAATGAAGAACTTGTTATTCTTAACTCTTGGACTCAGGAATTCCTATATAACTTAAGC
GACACAATAAAAGCTTTTTCTATTCTTTTATTAACTGATTTATGTATAGGATTCCATTCGC
CCCATGGTTGGGAACTAATGATTGGCTCTGTCTACAAAGATTTTGGATTTGTTCATAACG
ATCAAATTATATCTGGTCTTGTTTCTACTTTTCCAGTCATTCTAGACACAATTTTTAAATA
TTGGATTTTCCGTTATTTAAATCGTGTATCTCCGTCACTTGTAGTGATTTATCATTCAATG
AATGACATGCCTATTGGTGTTCCAAAAGTACCTTTTCGAAGTCCTGGAGAGGAAGACG
CATCTTGGGTTGACGTATACAACCGACTTTATCGAGAAAGATTACTTTTTTTAGGCCAA
GAGGTTGATAGCGAGATCTCGAATCAACTTATTGGTCTTATGGTATATCTCAGTATCGAG
GATGATACCAAAGATCTGTATTTGTTTATAAACTCTCCTGGCGGATGGGTAATACCCGGA
ATAGCTATTTTTGATACTATGCAATTTGTGCAACCAGATGTACATACAATATGCATGGGAA
TGGCTGCTTCAATGGGATCTTTTATCCTGGTCGGGGGAGAAATTACCAAACGTCTAGCA
TTCCCTCACGCCAGGGTAATGATCCATCAACCTGCGAGTGCTTATTATGAGGCACAAAC
GGGAGAATTTATCCTGGAAGCGGAAGAACTGCTGAAACTGCGCGAAACCCTCACAAG
GGTTTATGTACAAAGAACGGGCAAACCCTTATGGGTTGTATCTGAAGACATGGAGAGG
GATGTTTTTATGTCAGCAACAGAAGCCCAAGCTTATGGAATCGTTGATCTTGTAGCGGT
TGAAATGAAAGAACAAAAATGGATTCATGAAGGTTTAATTACCGAATCGCTTCCCAATG
GTATGTTCCGGGTTCGTTTAAATAATGAGGATCTGAGTCTAGGTTATGTTTCGGGCAAGA
TCCGACGTAGTTTTATACGGATACTGCCCGGGGATAGAGTCAAAATTGAAGTAAGTCGT
TATGATTCAACCAGGGGGCGTATAATTTATCGACTCCGCAACAAGGATTCGAAGGATAT
GGAGGAATTCCAAAGATATTTAGAA---GCTAAATCTCGGCAACACGATTTCCTATATCCA
CTTATCTTTCAGGAGTATATTTATACACTTGCTCATGATCGT------GCAGATTATGACAATA
AATCCAGCTTACTAATTGTAAAACGTTTAATTACTCGAATGTATCAACAGAATCATTTGC
TTATTTCCGCTAATGATTCGAACCAAAATCCATTTTGGGGGAAGAATTTGTATTTTCAAA
TGATATCGGAGGGATTTGCAGTCATTGTCGAAATTCCATTTTCTCTACGATTAATATCTTC
CCTCATAGTAAAATCTCAGAATTTACGATCAATTCATTCAATATTTCCTTTTTTAGAGGAC
ACATTTTTACATTTAAATTATGTGTTAGATATACGAATACCCTATCCCGTCCATCTGGAAA
TCTTGGTTCAAATTCTTCGTTACTGGGTAAAAGATGCCTCTTCTTTGCATTTATTACGGT
TCTTTCTACACGAGTATCGTAATCGTAATTGGAATAATTTGAAACTCAGTTCCTCTTTTT
CAAAACGAAGATTATTCTTGTTCCTATATAATTCTCATGTATGTGAATACGAATCCATTTT

TCTCTTTTTCCGTAACCAATCTTCTCACTTACGATCAACATCTTTTGGAGCCCTTCTTGA
ACGAATATATTTCTATGGAAAAATAGAACATGTAGAA---GTCTTTGCTAAGGATTTTATCC
TATGGTTGTTCAAGGATCCTTTCATACATTATGTTAGGTATCAAGGAAAATCAATTCTGG
CTGCAAAGGGGACGCCTCTTTTGATGAATAAATGGAAATATTACTTTGTCAATTTCTGG
AAATGTCATTTTTACGTGTGGTATCAACCAGTAACAATCTATATAAACCAATTATCCAATT
ATTCCCTCGACTTTCTGGGATATCTTTCAAACGTGCGCCCAAACCCCGCAATGGTACGG
AGTCAAATGCTAGAAAATTCGTTTCTAATAGATAATGCGATT------AAAAAGTTCGATAC
CATAGTTCCCATTATTCCTCTGATTGGATCATTAGCTAAAGCGAAATTTTGTAATGTCTTA
GGGCACCCAATTAGTAAGCCGGTATGGGCCGATTTATCAGATTCTGATATTATTGACCGA
TTTGGTCATATATGCAGAAATCTTTCTCATTATTATAGCGGATCCTCAAAAAAAAAGAGT
TTGTATCGAATAAAGTATATACTTCGACTTTCTTGTGCTAGAACTTTAGCTCGTAAACAC
AAAAGTACTGTACGCGCTTTTTTGAAAAGATTAGGCTCGGAATTATTGGAAGAATTCCT
TATGGAGGAGGAACAAGTTCTTTCTTTGACCTTCCCAAGACGGATTTGGTATTTGGATA
TTATTTGTATCAATGATCTGGCCAACCACGAAATGATAATTGAT---ACAACAGAAGTACA
A---GCTATCAATTCTTTTTCCAGATTGGAATCG---TTAAAAGAGGTCTATGAGATCATATG
GATGCTTGTCCCTATTTTGACTCTTGTATTAGGAATCACAATAGGTGTACTAGTAATTGTT
TGGTTAGAAAGAGAAATATCAGCAGGAATACAACAACGTATTGGACCTGAATACGCCG
GCCCTTTGGGAATTCTTCAAGCTCTAGCAGATGGGACAAAATTACTTTTCAAAGAGAA
CCTTCTTCCATCTAGAGGAGATCCTCGTTTATTCAGTATCGGACCATCCATAGCAGTTAT
ATCAATTTTACTAAGTTATTTAGTAATTCCTTTTGGCTATCGCCTTGTTCTAGCCGATCTC
AGTATTGGTGTTTTTTTATGGATTGCTATTTCAAGTATTGCTCCTGTTGGGCTTCTTATGT
CAGGATATGGATCAAATAATAAATATTCCTTTTTAGGTGGTCTGCGAGCTGCTGCTCAAT
CAATTAGTTATGAAATACCATTAACTCTATGTGTGTTATCAATATCTCTACTATCTAACAG
TTCAAGTACAGTTGATATAGTTGAAGCACAGTCAAAATATGGGTTTTGGGGATGGAATT
TGTGGCGTCAACCTATAGGGTTTATCATTTTTCTAATTTCTTCCCTAGCGGAATGTGAAA
GATTACCTTTTGATTTACCAGAAGCGGAAGAGGAGTTAGTAGCGGGTTATCAAACCGA
ATACTCAGGTATCAAATTTGGTTTGTTTTACGTTGCTTCCTATCTAAATCTACTAGTTTCT
TCATTATTTGTAACAGTTCTTTACTTGGGCGGTTGGAATCTTTCTATTCCGTACATACTCG
TTCCTGAGCTTTTTGAAATAAATAAAGCGGGTATAGTCTTTGGAACAATAATTAGCATCT
TTATTACATTAGCTAAAACTTATTTGTTCTTGTTCATTTCTATCACAACAAGATGGACTTT
ACCGAGGCTGAGAATGGACCAACTATTAAATCTTGGATGGAAATTTCTTTTACCTATTTC
TCTAGGTAATCTATTATTAACAACTTCTTCCCAACTTATGTCACTGATGATCTGGCATGTA
CAGAATGAAAACTTCATTCTCGATTCTACGAGAATTTTTATGAAAGCCTTTCATTTGCTT
CTCTTCGATGGAAGTTTTATTTTCCCAGAATGTATCCTAATTTTTGGCCTAATTCTTCTTC
TGATGATCGATTCAACCTCTGATCAAAAAGATATATCTTGGTTATATTTCATCTCTTCAAC
AAGTTTAGTAATGAGCATAACGGCCCTATTGTTCCGATGGAGAGAAGAACCTATGATTA
GCTTTTCGGGAAATTTCCAAACGAACAATTTCAACGAAATCTTTCAATTTCTTATTTTAC
TATGTTCAACTCTATGTATTCCTCTATCCGTAGAGTACATTGAATGTACAGAAATGGCTAT
AACAGAGTTTCTCTTATTCGTATTAACAGCTACTCTAGGAGGAATGTTTTTATGCGGTGC
TAACGATTTAATAACTATCTTTGTAGCCCCAGAATGTTTCAGTTTATGCTCCTACCTATTA
TCTGGATATACCAAGAAAGATGTACGGTCTAATGAGGCTACTATGAAATATTTACTCATG
GGTGGGGCAAGCTCTTCTATTCTGGTTCATGGGTTCTCTTGGCTATATGGTTCATCCGGG
GGAGAGATCGAGCTTCAAGAAATAGTGAATGGTCTTATCAATACACAAATGTATAACTC
CCCAGGAATTTCAATTGCGCTCATATTCATCACTGTAGGAATTGGGTTCAAGCTTTCCCT

AGCCCCTTCTCATCAATGGACTCCTGACGTATACGAAGGATCTCCCACTCCAGTCGTTG
CTTTTCTTTCTGTTACTTCGAAAGTAGCTGCTTCAGCTTCAGCCACTCGAATTTTCGATA
TTCCTTTTTATTTCTCATCAAACGAATGGCATCTTCTTCTGGAAATCCTAGCTATTCTTAG
CATGATATTAGGGAATCTCATTGCTATTACTCAAACAAGCATGAAACGTATGCTTGCATA
TTCGTCCATAGGTCAAATCGGATATGTAATTATTGGAATAATTGTTGGAGACTCAAATGA
TGGATATGCAAGCATGATAACTTATATGCTGTTCTATATCTCCATGAATCTAGGAACTTTT
GCTTGCATTGTATTATTTGGTCTACGTACCGGAACTGATAACATTCGAGATTATGCAGGA
TTATACACGAAAGATCCTTTTTTGGCTCTCTCTTTAGCCCTATGTCTCTTATCCCTAGGA
GGTCTTCCTCCACTAGCAGGTTTTTTCGGAAAACTCTATTTATTCTGGTGTGGATGGCA
GGCAGGCCTATATTTCTTGGTTTCAATAGGACTCCTTACAAGCGTTGTTTCTATCTACTAT
TATCTAAAAATAATCAAGTTATTAATGACTGGGCGAAACCAAGAAATAACCCCTCACGT
GCGAAATTATAGAAGATCTCCTTTAAGATCAAACAATTCCATCGAATTGAGTATGATTGT
ATGTGTGATAGCATCTACTATACCAGGAATATCAATGAACCCGATTATTGCAATTGCTCA
GGATACCCTTTTTATGTTTCTGCTTTACGAATATGATATTTTTTGGGCATTTCTAATAATAT
CAAGTATTATTCCTATCTTGGCATTTTTCATTTCCGGAGTTTTAGCTCCGATTAGCAAAG
GACCAGAGAAACTTTCTAGTTATGAATCGGGTATAGAACCAATGGGCGACGCTTGGTTA
CAATTTAGAATCCGTTATTATATGTTTGCGTTAGTTTTTGTTGTTTTTGATGTTGAAACGG
TTTTTCTTTATCCATGGGCAATGAGTTTCGATGTATTGGGTGTATCCGTATTTATAGAAGC
TTTAATTTTTGTGCTTATCTTAATTGTTGGTTCAATTTATGCATGGCGAAAGGGGGCATT
GGAATGGTCTACGAATGATTTTCCTTGGTTAACAATAATTGTAGTTTTGCCAATAGCCGC
GGGTTCTTTAATTTTCTTTCTTCCCCATAGAGGAAATAGGGTAATTAAGTGGTATACTATT
TGTATATGTATATTAGAGCTCCTTCTAACAACCTATGCATTCTGTTATCATTTCCAATCAG
ACGATCCATTAATCCAACTAGTGGAGGATTATAAATGGATCGATTTTTTTGATTTCCACT
GGCGATTGGGAATAGATGGACTTTCTATAGGACCCATTTTACTGACAGGATTCATTACTA
CTTTAGCTACTTTAGCGGCTTGGCCAGTTACTCGGGATTCTCGATTATTCCATTTCCTGA
TGTTAGCAATGTACAGCGGTCAAATAGGATCATTTTCTTCTCGGGACCTTTTGCTTTTTT
TTATCATGTGGGAGTTAGAATTAATTCCCGTTTATCTACTTTTATCCATGTGGGGAGGAA
AAAAGCGTCTCTATTCAGCTACAAAATTTATTTTGTACACTGCGGGGGGTTCCATTTTTC
TCTTAATGGGAGTTCTGGGTGTCGGTTTATATGGTTCTAATGAACCAACATTAAATTTTG
AAACATCGGCTAATCAATCGTATCCTGTGGTATTGGAAATAATATTCTATATTGGGTTTTT
TATTGCGTTTGCTGTCAAATCACCGATTATACCCCTACATACATGGTTACCAGATACCCAT
GGAGAAGCACATTACAGTACCTGTATGCTTCTAGCTGGAATCTTATTAAAAATGGGAGC
ATATGGATTGGTTCGGATCAATATGGAATTATTACCGCACGCTCATTCTATATTTTCTCCT
TGGTTTATCATAGTAGGTACAACGCAAATAATCTACGCAGCTTCAACATCCCTTGGCCA
ACGTAATTTAAAAAAAAGAATAGCCTATTCTTCTGTATCTCATATGGGTTTCATAATTATA
GGAATAGGCTCTATAACCGATACGGGACTCAATGGAGCCATTTTACAAATAATCTCTCAT
GGATTTATTGGTGCCGCGCTTTTTTTCTTGGCAGGAACGAGTTATGATAGAATACGTCTT
GCTTATCTCGACGAAATGGGCGGAATAGCTATCCTAATGCCAAAAATATTCACAATGTTC
AGTAGCTTTTCGATGGCTTCTCTTGCATTACCGGGCATGAGTGGTTTTGTTGCAGAATT
GATAGTATTTTTTGGAATAATTACCAGCCAAAAGTATCTTTTAATGACAAAAATACTAAT
TACTTTTGTAATGGCAATTGGGATGATATTAACTCCTATTTATTCATTATCTATGTCACGTC
AGATGTTCTATGGATACAAGCTATTTAATGCTCCAAACTCTTATTTTTTTGATTCTGGACC
GCGAGAGTTATTTGTTTCGATCTCTATCTTTTTACCTGTCATAGGTATTGGTATTTACCCC
GATTTTGTTCTTTCACTATCAGTTGACAAGGTCGAACTTATTCTATCTAATTATTTTTCTA

GAATGATGCTCGAACATGTCCTTATTTTGAGTGCCTATTTATTTTCTATCGGTATCTATGG
ATTGATTACAAGTCGAAATATGGTTAGAGCCCTTATGTGTCTTGAACTTATATTGAATGC
AGTTAATATAAATTTTGTAACATTTTCTGATTTTTTTGATAGTCGTCAATTAAAAGGAAC
CATTTTTGCAATTTTTGTTATAGCTATTGCAGCCGCTGAAGCAGCTATTGGACTGGCTAT
TGTTTCGTCAATTTATCGTAATCGAAAATCAACTCGTATCAACCAATCGAATTTGTTGAA
TAAGATGGAACATACATATCAATATGCGTGGATCATACCTTTGATTCCACTTCCAGTTCC
TATGTTAATAGGAGTGGGGCTTCTTCTTTTTCCGACGGCAACAAAAAAATCTCCGTCGTA
TATGGGCTTTTCCTAGTGTTCTATTGTTAAGTATAGTCATGATTTTTTCGATCGATCTGTC
TATTCAGCAAATGAATAGCAGTTCTATCTATCAATATGTATGGTCTTGGATCATCAATAAT
GATTTTTCTTTAGAATTGGGCTACGTAATCGATCCACTTACTTCTATTATGTCAATATTAA
TTACTAGTGTTGGAATTATGGTTCTTATTTATAGTGATAATTATATGTCTCATGATCAAGG
ATATTTGAGATTTTTTGCTTATATGAGTTTTTTCAGTACTTCCATGTTGGGATTAGTTACT
AGTTCAAATTTGATACAAATTTATATTTTTTGGGAATTGGTTGGAATGTGTTCCTATTTAT
TAATAGGATTTTGGTTCACACGACCGGTTGCGGCAAATGCTTGTCAAAAAGCGTTTGTA
ACTAATCGTGTAGGGGATTTTGGTTTATTATTAGGAATTTTAGGTTTTTATTGGATAACAG
GTAGTTTTGAATTTCGGGATTTATTCGAAATAGTAAATAACAATGAGGTCAATTTATCAT
TTATTACTGTGTGTGCCGTTCTAGTATTTGCCGGTGCAGTTGCTAAATCTGCACAATTCC
CCCTTCATGTATGGTTACCTGATGCCATGGAAGGGCCTACTCCTATTTCGGCTCTTATAC
ACGCTGCTACTATGGTAGCGGCAGGAGTTTTTATTGTAGCTCGGCTTCTTCCTCTTTTCA
CAGTCATACCTTACATACTGAATCTAATATCTTTGATAGGTATAATAACAGTATTATTAGG
CGCTACTTTAGCTCTTGCTCAAAAAGACATTAAGAGAAGTTTAGCCTACTCTACAATGT
CTCAGTTGGGTTATATGATGTTAGCTCTAGGTATGGGGTCTTATCGAACTGCTTTATTTCA
TTTGATTACCCATGCATATTCGAAAGCATTATTGTTTTTAGGATCCGGATCTGTTATTCAT
TCAATGGAAACTATTGTTGGATATTCTCCGGATAAAAGTCAGAATATGGTTCTTATGGGG
GGTTTAACAAAACATGTACCAATCACAAAAATTTCTTTTTTATTAGGTACACTTTCTCTT
TGTGGTATTCCACCTCTTGCTTGTTTTTGGTCCAAAGATGAAATTCTTAATGATAGTTGG
TTGTATTCACCAATTTTTGCAATAATAGCTTGCTCTACAGCAGGATTAACCGCATTTTATA
TGTTTCGGATCTATTTACTTACTTTTGAGGGTCATTTAAACGTTCATTTTCAAAATTATAG
CGGCAAAAAAAAATACCCCGTTCTATTCAATATCTCTATGGGGTAAAGTAGGCTCGAAAA
CAATTAACAAA---TTATTAACTTTATTAACAATGAATTTTTTTTCATTTTGCAATAAGAAC
ACTTATTCATATCCTTATGAGTCGGACAATACTATGTTATTTCCTCTGCTTGTATTAGCCCT
ATTCACTTTGTTCGTTGGATCCATAGGAATTCCTTTCGATTTGGATATATTATCCAAATGG
TTAAATCCGGCTATAAACCTTTTACATCAAAATTCGAAT---TTGATGGATTGGTATGAATT
TGTGAAAGATGCAATTTTTTCAGTTAGTATAGCTTATTTTGGAATTTTTATAGCATCTTTT
TTATATAAACCCGTTTATTCATCTTCGCAAAATTTGAACTTAATTAATTCATTTGTTACTC
TTAGGGACAAAATAATAAATGGGATATATGATTGGTCATATAATCGTGGTTACATAGATG
CTTTTTATGCAGCATTTTTAACTGGAGGTATAAGAGGATTGGCTAAATTAATTCATTTTTT
TGATAGACGGATAATTGATGGAATTACGAATGGGGTTGGTATTATGAGTTTCTTTGTAGG
AGAAGGCATCAAATATATAGGAGGGGGG---CGCATTTCTTCTTATCTCTTCTTGTATTCAT
CTTATGTATCAATCTTTTTAGTAATTTAC---ATGGATTTACCTGGACCAATACATGATTTTC
TTTTAGTTTTTCTGGGATTGGGTATTATATTGGGAGGTCTGGGAGTAGTATTACTTAACA
ATACAATTTTTTCCGCCTTTTCATTGGGATTGGTTCTTGTTTGTATCTCCCTATTCTATATT
CTATCTAATTCCCATTTTGTAGCCGCTGCGCAACTTCTTATTTACGTAGGAGCTATAAATA
TTTTAATCATATTTGCTGTGATGTTCATGAATGGTTCAGAATATCACAAAGATTTTTATCT

TTGGACCGTTGGGGATGGGGTTACTTCGATGGTTTGTACAAGTATTTTTGTTTCACTAAT
TACTACTATTCTAGATACGTCATGGTACGGGATTATTTGGACTACAAGATCAAACCAGAT
TCTAGAACAAGATTTGATAAATAATAGTCAACAAATTGGGATTCATTTATCAACAGATTT
TTTTCTTCCATTTGAACTCATTTCAATAATTCTTTTAGTTGCTTTAATAGGCGCAATTGCT
GTAGCCCGTCAAATGACTGCAACAGCTACAAGAAAAGACCTCATGATAGTCAATATGG
GCCCTCAACACCCATCAATGCATGGTGTTCTTCGACTCATCGTTACTCTAGATGGTGAA
GATGTTATTGACTGTGAACCAATATTGGGTTATTTACACAGAGGGATGGAAAAGATTGC
GGAAAACCGAACAATTATACAATATTTGCCTTATGTAACACGTTGGGATTATTTAGCTAC
TATGTTCACAGAAGCAATAACCGTAAATGCACCAGAGCAGTTAGGAAATATTCAAGTAC
CCAAAAGGGCTAGCTATATCAGAGTAATTATGTTGGAATTAAGTCGTATAGCTTCTCATT
TGTTATGGCTTGGCCCCTTTATGGCCGATATTGGTGCGCAGACCCCCTTCTTCTATATTTT
TCGAGAAAGAGAATTAATATATGACCTATTCGAAGCTGCTACCGGTATGCGAATGATGC
ATAATTATTTTCGTATCGGAGGAGTAGCTGCTGATCTACCTCATGGTTGGATAGATAAAT
GTTTGGATTTTTGCGATTATTTTTTAACAGGGGTTGCTGAATATCAAAAGCTTATTACGC
GGAATCCTATTTTTTTAGAACGAGTTGAGGGGGTGGGCATTATTGGAGGCGAAGAGGC
AATAAATTGGGGTTTATCAGGACCAATGCTGCGAGCTTCTGGAATACAATGGGATCTTC
GTAAAGTTGATCATTATGAGTGTTACGATGAATTTGACTGGGAAGTCCAATGGCAAAAA
GAGGGGGATTCATTAGCTCGTTATTTAGTACGAATCGGTGAAATGAAAGAATCCATAAA
AATTATTCAACAGGCTCTAGAAGGAATTCCGGGGGGGCCCTATGAGAATTTAGAAATCC
GACGCTTTGATAAAGTAAGGAATCCCGAATGGAATGATTTTGAATATCGATTTATTAGTA
AAAAACCTTCTCCAACTTTTGAATTGTCGAACCAAGAACTTTATGTAAGAGTCGAAGC
CCCAAAAGGGGAATTGGGAATTTTTCTCATAGGAGATCAGAGTGTTTTTCCTTGGAGAT
GGAAAATTCGCCCACCAGGTTTTATCAATTTGCAAATTCTTCCTCAGTTAGTTAAAAAA
ATGAAATTGGCCGATATTATGACGATCCTAGGTAGCATAGATATCATTATGGGAGAAGTT
GATCGTATGTTCCCTATGGTAACTGGGTTCATGAATTATGGTCAACAAACAATACGGGCT
GCAAGATACATTGGTCAAAGTTTCATGATTACCTTATCCCATGCGAATCGTTTACCCGTA
ACTATTCAATACCCCTATGAAAAATTAATCACATCGGAGCGTTTCCGCGGCCGAATCCA
CTTTGAATTTGATAAATGCATTGCTTGTGAAGTATGTGTTCGCGTATGTCCTATAGATCTA
CCTGTTGTTGATTGGAAATTGGAAACGGATATTCGAAAGAAACGATTGCTTAATTACAG
TATTGATTTCGGAATCTGTATATTTTGTGGTAATTGCGTTGAGTATTGTCCAACAAATTGT
TTATCGATGACTGAAGAATATGAACTTTCTACGTATGATCGTCACGAATTGAATTATAAT
CAAATTGCTTTGGGTCGTTTACCAATATCGGTAATTGACGATTCCACGGTTCGAACAATT
TGGAATTCG---ATGCAGGGTCGTTTGTCTGCTTGGCT
AGTCAAGCATGGGCTAATTCATAGATCTTTAGGCTTTGATTACCGAGGAATAGAGACTT
TACAAATAAAGCCCGAGGATTGGCATTCCATTGCTGTCATTTTATATGTATATGGTTACAA
TTATCTACGCTCCCAATGTGCATATGATGTAGCACCAGGCGGGCTGTTAGCTAGTGTGTA
TCATCTTACGAGAATAGAGTATGGTGTGGATCAACCGGAAGAGGTATGCATAAAAGTAT
TTGCCCCAAGGAGGAATCCTAGAATTCCGTCTGTTTTCTGGGTTTGGAAAAGTGTGGAT
TTTCAAGAACGGGAATCTTATGATATGTTGGGAATCTCTTATGATAATCATCCACGCTTG
AAACGTATCTTAATGCCGGAAAGTTGGATAGGGTGGCCCTTACGTAAGGATTATATTTCC
CCCAATTTTTATGAAATACAAGATGCTCAT---
------------------------------ATGGCGAAAGGGGGCATTGGAATGGTCTTAGCTCCTGAATATTC
AGACAATAAAAAGAAAAAA---------AATACGATTGAGATGGTTATGAATTCTATTGACTT
TCCCTTACTTGATCGAACAACCCCAAATTCTGTTATTTCAACTACATCCAATGATCTTTC

AAATTGGTCAAGACTCTCTAGTTTATGGCCGCTTCTCTATGGTACCAGTTGTTGCTTCAT
TGAATTTGCTTCACTAATAGGCTCACGATTCGACTTTGATCGTTACGGACTGGTACCAA
GATCGAGTCCTAGACAAGCGGACCTGATTTTAACAGCCGGAACAGTAACAATGAAAAT
GGCTCCTTCTTTAGTGAGATTATATGAGCAAATGCCGGAACCAAAATATGTTATTGCTAT
GGGAGCATGTACAATTACAGGGGGAATGTTCAGTACCGATTCTTATAGTACTGTTCGTG
GAGTCGATAAGCTAATTCCTGTGGATGTCTATTTACCGGGCTGTCCACCAAAACCGGAA
GCAGTTATAGATGCTATAACAAAACTTCGTAAGAAAATATCTCAAGAAATCTATGAAGA
TAGAATTAGGTCTCACCAGGAGAATCGGTGTTTTACTACCAATCACAAGTTTCATGTTG
GACGCAGTATTCATACTGGAAATTATAATCAAGGATTACTTTATCAACCGCCATCTACTT
CAGAGATCCTTCCTGAAACATTTTTCAAATACAAAAGTTCAGTATCTTCCCACGAATTA
GTGAATATGCAAACTAGAAATACTTTTTCTTGGATAAAGGAACAGGTTACTCGATCCAT
TTCCGTATCGCTCATGATATATATCATAACTCGGGCATCCATTTCAAGCGCATATCCCATT
TTTGCACAGCAGGGTTATGAAAATCCACGAGAAGCGACGGGGCGTATTGTATGTGCCA
ATTGCCATTTAGCTAATAAGCCCGTGGATATTGAAGTTCCACAAGCGGTACTTCCGGAT
ACTGTATTTGAAGCAGTTGTTCGAATTCCTTATGATATGCAACTGAAGCAAGTTCTTGCT
AATGGTAAAAGGGGGGCTTTGAATGTGGGGGCTGTTCTAATTTTACCTGAGGGGTTTG
AACTAGCGCCTCCCGATCGTATTTCTCCCGAGATGAAAGAAAAGATAGGCAATCTATCT
TTTCAGAGCTATCGCCCCAATAAAAAAAATATTCTTGTGATAGGCCCCGTTCCTGGTCA
GAAATATAGTGAAATCACCTTTCCTATTCTTTCCCCAGACCCCGCTACGAAGAAAGATG
TTCACTTCTTAAAATATCCTATATACGTAGGCGGAAACAGGGGAAGGGGTCAGATTTAT
CCCGACGGGAGCAAGAGTAACAATACAGTTTATAATGCTACAGCAGCGGGTATAGTAA
GCAAAATCGTACGAAAAGGAAAGGGGGGGTATGAAATAACCATAGCAGATGCGTCGG
ATGGACGTCAAGTAGTTGATAGTATCCCTCCAGGGCCCGAACTTCTTGTTTCAGAGGGC
GAATCTATCAAATTTGATCAACCATTAACAAGTAATCCGAATGTGGGCGGATTTGGTCA
GGGAGATGCAGAAATAGTACTTCAAGATCCATTACGTGTCCAAGGTCTTTTGTTCTTCT
TGGCATCTGTTATTTTGGCACAAATATTTTTGGTTCTTAAAAAGAAACAGTTCGAGAAG
GTTCAATTGTCCGAAATGAATTTCATGAGTAAAGTCTATGATTGGTTCGAAGAGCGCCT
CGAGATTCAGGCGATTGCAGATGATATAACTAGTAAATACGTTCCTCCCCATGTCAACAT
ATTTTATTGTTTAGGAGGAATTACGCTTACTTGTTTTTTAGTACAAGTAGCTACGGGGTT
TGCTATGACTTTTTACTATCGTCCGACCGTTACTGAAGCTTTTGCCTCGGTTCAATACAT
AATGACTGAAGCTAATTTTGGTTGGTTAATCCGATCAGTTCATCGATGGTCGGCAAGTA
TGATGGTCCTAATGATGATCCTGCATGTATTTCGTGTGTATCTCACCGGTGGATTTAAAA
AACCTCGCGAATTGACTTGGGTTACGGGCGTGGTTCTGGCCGTATTGACTGCATCTTTT
GGCGTAACTGGTTATTCCTTACCTTGGGACCAAATTGGTTATTGGGCAGTAAAAATTGT
AACGGGTGTGCCTGAAGCGATTCCTGTAATAGGATCGCCTTTGGTAGAATTATTGCGGG
GAAGTGCTAGTGTGGGACAATCCACCTTGACTCGTTTTTATAGTTTACACACTTTTGTAT
TGCCCCTTCTTACTGCCGTATTTATGTTAATGCACTTCCTAATGATACGTAAACAGGGTAT
TTCTGGTCCTTTAATGGGAGTAACAAAAAAACCTGACTTGAATGATCCTGTATTAAGAG
CTAAATTGGCTAAAGGGATGGGTCATAATTATTACGGAGAGCCCGCATGGCCCAATGAT
CTTTTATATATTTTTCCAGTAGTAATTCTAGGTACTATTGCATGTAACGTGGGCTTAGCGG
TTCTAGAACCATCAATGATTGGTGAACCAGCAGATCCATTTGCAACCCCCTTGGAAATA
TTACCCGAATGGTATTTCTTTCCCGTATTTCAAATACTTCGTACAGTACCCAATAAATTGT
TGGGTGTTCTTTTAATGGTTTCAGTACCTGCGGGATTATTAACAGTACCTTTTTTAGAGA
ATGTTAATAAATTCCAAAATCCATTTCGTCGTCCGGTAGCGACAACCGTCTTTTTGATTG

GTACCGCAGTGGCCCTTTGGTTGGGTATTGGTGCAACATTACCTATTGATAAATCCTTAA
CTTTAGGTCTTTTTATGATTGAAGTTTTTCTATTTGGAATCGTGTTAGGTCTAATTCCTAT
TACTTTGGCTGGATTATTTGTAACTGCATATTTACAATACAGACGTGGTGATCAGTTGGA
TCTTATGCCTACTATAACTAGTTATTTCGGTTTTCTACTAGCGGCTTTAACTATAACCTCG
GCTCTATTTATTGGTCTGAGCAAGATACGACTTATTATGGATATAGTAAGTCTCGCTTGG
GCTGCTTTAATGGTAGTCTTTACATTTTCCCTTTCACTCGTAGTATGGGGAAGAAGTGG
ACTCATGATTATTCGTTCGCCGGAACCAGAAGTAAAAATTTTGGTAGATAGGGATCACA
TAAAAACTTCTTTCGAGGAATGGGCCAGACCAGGTCATTTCTCAAGAACACTAGCGAA
AGGCCCTGATACTACCACTTGGATCTGGAACCTACATGCTGATGCTCACGATTTCGATA
GCCATACCAGCGATTTGGAGGAGATCTCTCGAAAAGTATTTAGTGCCCATTTCGGTCAA
CTCTCCATCATCTTTCTTTGGCTGAGTGGTATGTATTTCCACGGTGCTCGTTTTTCCAATT
ATGAAGCATGGCTAAGCGATCCTACTCACATTGGCCCTAGTGCCCAGGTGGTTTGGCCA
ATAGTGGGCCAAGAAATATTGAATGGTGATGTGGGCGGGGGTTTCCGAGGAATACAAA
TAACCTCCGGTTTTTTTCAGATTTGGCGAGCATCTGGAATAACTAGTGAATTACAACTCT
ATTGTACCGCAATTGGTGCATTGGTCTTTGCAGCGTTAATGCTTTTTGCTGGTTGGTTCC
ATTATCATAAAGCTGCTCCAAAATTGGCTTGGTTTCAAGATGTAGAATCTATGTTGAATC
ACCATTTAGCGGGGCTACTTGGACTTGGGTCTCTTTCTTGGGCGGGGCATCAAGTACAC
GTATCTTTGCCGATTAACCAATTTCTAAACGCCGGTGTAGATCCTAAAGAGATACCACTT
CCTCATGAATTTATCTTGAATCGGGATCTTTTGGCTCAACTTTATCCCAGTTTTGCCGAG
GGAGCAACCCCGTTTTTCACCTTGAATTGGTCAAAATATGCGGAATTTCTTACTTTTCG
TGGAGGATTAGATCCAGTTACTGGGGGTCTATGGCTGACCGATATTGCACACCATCATTT
AGCTATTGCAATTCTTTTCCTGATAGCAGGTCACATGTATAAGACCAATTGGGGCATTGG
TCATGGCCTAAAAGATATTTTAGAAGCTCATAAAGGTCCATTTACAGGCCAGGGCCATA
AAGGCCTATATGAGATCCTAACAACGTCATGGCATGCTCAATTATCTCTTAACCTAGCTA
TGTTAGGCTCTTTAACCATTGTTGTAGCTCATCATATGTATTCCATGCCCCCTTATCCATAT
CTAGCTACTGACTATGGTACACAACTGTCATTGTTCACACATCACATGTGGATTGGCGG
ATTTCTCATAGTTGGTGCTGCTGCGCATGCAGCCATTTTTATGGTAAGAGACTATGATCC
AACTACTCGATACAACGATCTATTAGATCGTGTCCTTAGGCATCGCGATGCAATCATATC
ACATCTCAACTGGGCATGTATATTTCTAGGCTTTCACAGTTTTGGGTTGTATATTCATAAT
GATACCATGAGCGCTTTAGGGCGCCCCCAAGATATGTTTTCAGATACCGCTATACAATTA
CAACCCGTCTTTGCTCAATGGATACAAAACACCCATGCTTTAGCACCTGGTTCAACAGC
TCCTGGTGCAACAGCAAGCACCAGTTTAACTTGGGGGGGTGGTGATTTAGTAGCAGTG
GGCGGCAAAGTGGCTTTGTTACCTATTCCATTAGGAACTGCAGATTTTTTGGTACATCA
CATTCATGCATTTACGATTCATGTGACGGTATTGATACTCCTGAAAGGTGTTCTATTTGC
TCGTAGCTCCCGTTTGATACCGGATAAAGCAAATCTTGGTTTTCGTTTTCCTTGTGATGG
ACCTGGAAGAGGGGGGACATGTCAAGTATCGGCTTGGGATCATGTCTTCTTAGGACTAT
TCTGGATGTACAATGCAATTTCGGTAGTAATATTCCATTTCAGCTGGAAAATGCAGTCA
GATGTTTGGGGCAGTATAAGTGATCAAGGGGTAGTAACTCATATTACGGGAGGAAACTT
TGCGCAGAGTTCCATTACTATTAATGGGTGGCTCCGCGATTTCTTATGGGCGCAGGCAT
CTCAGGTAATTCAGTCTTATGGTTCTTCATTATCTGCATATGGCCTTTTTTTCCTAGGTGC
TCATTTTGTATGGGCTTTCAGTTTAATGTTTCTATTCAGTGGACGTGGTTATTGGCAAGA
ACTTATTGAATCCATCGTTTGGGCTCATAATAAATTAAAAGTTGCTCCTGCTACTCAGCC
TAGAGCCTTGAGCATTGTACAAGGACGCGCTGTAGGAGTAACCCATTACCTTCTGGGT
GGAATTGCCACAACATGGGCGTTCTTCTTAGCAAGAATTATTGCAGTAGGAATGGCATT

AAGATTTCCAAGGTTTAGCCAAGGCTTAGCTCAGGACCCCACTACTCGTCGTATTTGGT
TTGGTATTGCTACCGCACATGACTTCGAGAGTCATGATGATATTACTGAGGAACGTCTTT
ATCAGAATATTTTTGCTTCTCACTTCGGTCAATTAGCAATAATTTTTCTGTGGACTTCCG
GAAATCTGTTTCATGTAGCTTGGCAAGGAAATTTTGAGTCATGGGTACAGGACCCTTTA
CATGTAAGACCTATTGCTCATGCAATTTGGGATCCTCATTTTGGTCAACCAGCTGTGGA
AGCTTTTACTCGAGGGGGTGCTCCTGGTCCAGTGAATATCGCTTATTCTGGTGTTTATCA
GTGGTGGTATACAATCGGTTTACGCACTAATGAAGATCTTTATACTGGAGCTCTTTTTCT
ATTATTTCTTTCTGCCATATCCTTAATAGCAGGTTGGTTACACCTACAACCGAAATGGAA
ACCGAGTGTTTCGTGGTTCAAAAATGCGGAATCTCGTCTCAACCATCATTTGTCAGGAC
TCTTCGGAGTAAGTTCTTTGGCTTGGACAGGGCATTTAGTTCATGTCGCTATTCCTGCAT
CCAGAGGGGAGTACGTTCGATGGAATAATTTCTTAGATGTATTACCGCATCCCCAAGGG
TTAGAACCACTTTTTACAGGTCAGTGGAATCTTTATGCGCAAAACCCCGATTCAAGTAG
TCATTTATTTGGTACCTCCCAAGGAGCGGGAACTGCCATTCTAACCCTTCTCGGGGGAT
TCCATCCACAAACGCAAAGTTTATGGCTGACTGATATGGCGCATCATCATTTAGCTATTG
CATTTATTTTTCTCATTGCTGGTCATATGTATAGAACTAACTTCGGGATTGGGCACAGTAT
GAAAGATCTTTTAGATGCACATATTCCTCCGGGGGGGCGACTGGGACGTGGGCATAAG
GGTCTTTATGACACAATCAATAATTCGATTCATTTTCAATTAGGCCTTGCTCTAGCTTCTT
TAGGGGTTGTTACTTCCTTGGTAGCTCAACACATGTACTCTTTACCCCCTTATGCATTCA
TAGCCCAAGATTTTACTACTCAGGCTGCGTTATATACTCATCACCAATACATCGCAGGAT
TCATCATGACAGGGGCTTTTGCTCATGGAGCTATATTTTTCATTAGAGATTACAATCCGG
AACAAAATGAGGATAATGTATTGGCAAGAATGTTAGACCATAAAGAAGCTATCATATCC
CATTTAAGTTGGGCCAGCCTCTTTCTGGGGTTCCATACCTTGGGACTTTATGTTCATAAT
GATGTCATGCTTGCTTTTGGTACTCCGGAGAAACAAATCCTGATCGAACCTATATTTGCT
CAATGGATACAATCTGCCCATGGTAAAACTTCATATGGGTTCGATGTCCTTTTATCTTCA
ACGAATGGCCCGGCATTCAATGCGGGTCGAAGCATATGGTTGCCCGGTTGGTTAAATGC
TGTTAATGAG------AATAGTAATTCACTATTCTTAACAATAGGTCCCGGAGACTTTTTGGT
TCATCATGCTATTGCTCTAGGTTTACATACAACTACATTGATTTTAGTAAAAGGTGCTTTA
GACGCACGTGGTTCCAAGTTAATGCCAGATAAAAAGGATTTCGGTTATAGTTTTCCTTG
CGACGGTCCGGGACGAGGCGGTACTTGTGATATTTCGGCTTGGGACGCATTTTATTTAG
CAGTTTTCTGGATGTTAAATACCATTGGATGGGTTACTTTTTATTGGCATTGGAAACACA
TCACATTATGGCAGGGTAACGTTTCGCAGTTTAATGAATCTTCCACTTATTTGATGGGAT
GGTTAAGAGATTATTTATGGTTAAACTCTTCCCAACTTATCAATGGATATAACCCCTTTG
GTATGAATAGTTTATCGGTCTGGGCGTGGATGTTCTTATTTGGGCATCTTGTTTGGGCTA
CTGGATTTATGTTCTTAATTTCATGGCGTGGATATTGGCAGGAATTGATTGAAACTTTAG
CATGGGCTCATGAACGGACACCTTTGGCCAATTTGATTCGATGGAGAGATAAACCAGT
GGCCCTTTCCATTGTGCAAGCAAGATTGGTTGGATTAGCCCACTTTTCTGTAGGTTATAT
ATTTACGTATGCAGCTTTCTTGATTGCCTCGACATCGGGTAAATTTGGTATGTCACATTC
AGTAAAGATTTATGATACATGTATCGGGTGTACTCAATGTGTCCGAGCTTGCCCCACAG
ATGTATTAGAAATGATACCTTGGGACGGATGTAAAGCGAAGCAAATTGCTTCTGCTCCA
AGAACAGAAGACTGTGTCGGTTGTAAGAGATGTGAATCCGCTTGTCCAACGGATTTCT
TGAGTGTTCGAGTTTATTTATGGCATGAAACAACTCGCAGCATGGGTCTAGCTTATATGG
CAACTTTTGACTTTTCCTCTATTTTGGTACCTGTAGTAGGCATAGTATTTCCGGCAATTG
CAATGGCTTCTTTATCTCTTCATGTTCAAAACAACAAGATTGTTATGCGAGATCTAAAA
ACATATCTCTCCGTGGCGCCGGTATTAAGTACTCTATGGTTTGGGGCTTTAGCAGGCCTA

TTGATAGAGATTAATCGTTTTTCCCGGATGCGTTGACATTCCCCTTTTTTTCATTCATGA
CTGCAATTTTAGAGAGACGCGAAAGCGAAAGCCTATGGGGTCGCTTCTGTAACTGGAT
AACCAGCACTGAAAACCGTCTTTACATTGGATGGTTTGGTGTTGTGATGATCCCTACCT
TATTGACCGCAACTTCTGTATTTATTATCGCCTTCATTGCTGCTCCTCCAGTAGATATTGA
TGGTATTCGTGAACCTGTTTCTGGATCTCTACTTTATGGAAACAATATTATCTCAGGTGC
CATTATTCCTACTTCTGCAGCTATAGGTTTGCACTTTTACCCGATCTGGGAAGCGGCATC
CGTTGATGAATGGTTATACAATGGTGGTCCTTATGAGCTAATTGTTCTACACTTCTTACT
TGGTGTAGCTTGTTACATGGGTCGTGAGTGGGAACTTAGTTTCCGTCTGGGTATGCGCC
CGTGGATTGCTGTTGCATATTCAGCTCCTGTTGCAGCTGCTACTGCTGTTTTCTTGATCT
ACCCAATTGGTCAAGGAAGTTTTTCTGATGGTATGCCTTTAGGAATCTCTGGGACTTTC
AACTTCATGATTGTATTCCAGGCTGAGCACAACATCCTTATGCACCCATTTCACATGTTA
GGCGTAGCTGGTGTATTCGGCGGCTCCCTATTCAGCGCTATGCATGGTTCCTTGGTAACT
TCTAGTTTGATCAGGGAAACCACAGAAAACGAATCTGCTAATGAAGGGTACAGATTCG
GTCAAGAGGAAGAAACTTATAATATCGTAGCTGCTCATGGTTATTTTGGCCGATTAATCT
TCCAATATGCTAGTTTCAACAACTCTCGTTCTTTACATTTCTTCCTAGCTGCTTGGCCTG
TAGTAGGTATCTGGTTCACCGCTTTAGGTATCAGCACTATGGCTTTCAACCTAAATGGTT
TCAATTTCAACCAATCTGTAGTTGATAGTCAAGGCCGTGTAATTAATACTTGGGCTGATA
TCATTAACCGCGCTAACCTTGGTATGGAAGTTATGCACGAACGTAATGCTCATAACTTC
CCTCTAGACCTAGCTGCTATTGAAGCTCCATCTACAAATGGAATGGGTTTACCTTGGTAT
CGTGTTCATACCGTTGTATTGAATGATCCCGGCCGGTTGCTTTCTGTTCATATAATGCATA
CAGCTCTGGTTGCTGGTTGGGCCGGTTCGATGGCTCTGTATGAATTAGCAGTTTTTGAT
CCCTCTGACCCTGTTCTTGATCCAATGTGGAGACAGGGTATGTTCGTTATACCCTTCATG
ACTCGTTTAGGAATAACTAATTCATGGGGCGGTTGGAATATTACA---GGAGGGACTATAA
CCAATCCGGGTATTTGGAGTTATGAAGGTGTGGCCGGGGCACATATTGTGTTTTCTGGT
TTATGCTTCTTGGCAGCTATCTGGCATTGGGTGTATTGGGATCTAGAAATATTTTGCGAT
GAGCGTACAGGAAAACCCTCTTTGGATTTGCCCAAGATCTTTGGAATTCATTTATTTCTT
TCAGGGGTGGCTTGCTTTGGTTTTGGTGCATTTCATGTGACAGGCTTGTACGGTCCTGG
AATATGGGTATCCGATCCTTATGGACTAACGGGAAAAGTACAACCTGTAAATCCAGCGT
GGGGCGTGGAAGGTTTTGATCCTTTTGTTCCGGGAGGAATCGCCTCTCATCATATTGCA
GCGGGGACATTGGGCATATTAGCGGGTCTATTCCATCTTAGCGTCCGCCCGCCTCAACG
TCTATACAAAGGATTGCGTATGGGCAATATTGAAACCGTCCTTTCCAGTAGTATCGCTGC
TGTCTTTTTTGCAGCTTTTGTTGTTGCCGGAACTATGTGGTATGGTTCAGCAACTACCCC
CATCGAATTATTTGGGCCCACTCGTTATCAATGGGATCAGGGGTACTTCCAGCAAGAGA
TATATCGAAGAGTTAGTGCCGGGCTAGCCGAAAATCAAAGTTTATCAGAAGCTTGGTCT
AAAATTCCCGAAAAATTGGCTTTTTATGATTACATCGGCAATAATCCAGCAAAGGGGGG
ATTATTCCGGGCAGGCTCAATGGATAACGGGGATGGAATAGCAGTTGGATGGTTAGGAC
ACCCCGTCTTTAGAGATAAAGAAGGACGTGAACTTTTTGTACGCCGTATGCCTACCTTT
TTTGAAACATTTCCAGTCGTTTTGGTAGACGGAGACGGAATTGTTAGAGCCGATGTTCC
TTTTAGAAGGGCAGAATCGAAGTATAGTGTCGAACAAGTAGGCGTAACTGTTGAGTTC
TATGGTGGCGAACTCAATGGAGTCAGTTATAGTGATCCTGCTACTGTGAAAAAATATGC
TAGACGCGCTCAATTAGGTGAAATTTTTGAATTAGATCGTGCTACTTTGAAATCCGATG
GTGTTTTTCGTAGCAGTCCAAGGGGTTGGTTTACTTTTGGGCATGCTTCATTTGCTCTG
CTCTTCTTCTTCGGCCACATTTGGCATGGTGCTCGAACCTTGTTCAGGGATGTTTTTGCT
GGTATTGATCCAGATTTGGATGCTCAAGTGGAATTTGGAGCATTCCAAAAACTTGGAGA

TCCAACTACACGAAGACAAGTAGTC---------------------------------GTGGAAACGCTCTTT
AATGGAACTTTAGCTTTATCCGGTCGTGACCAAGAAACCACCGGTTTCGCTTGGTGGG
CCGGAAATGCCCGACTTATCAATTTATCCGGTAAACTATTAGGGGCTCATGTAGCCCATG
CCGGATTAATCGTATTCTGGGCCGGAGCAATGAACCTATTTGAGGTGGCTCATTTCGTA
CCAGAGAAGCCTATGTATGAACAAGGATTAATTTTACTTCCCCACCTAGCTACTCTAGG
TTGGGGGGTAGGTCCTGGTGGGGAAGTTATAGACACCTTTCCATACTTTGTATCCGGAG
TACTTCACTTAATTTCCTCTGCAGTATTGGGCTTTGGCGGTATTTATCATGCACTTCTGG
GACCCGAGACTCTTGAAGAATCTTTTCCATTCTTCGGTTATGTATGGAAAGATAGAAAT
AAAATGACTACAATTTTAGGTATTCACTTAATTTTGTTAGGTATAGGTGCTTTTCTTCTAG
TATTCAAGGCTCTTTATTTTGGGGGCATATATGATACCTGGGCTCCCGGGGGGGGAGAT
GTAAGAAAAATTACCAACTTGACCCTTAGCCCAAGTATTATATTTGGTTATTTACTAAAA
TCGCCTTTTGGGGGAGAAGGGTGGATTGTTAGTGTGGACGATTTGGAAGATATAATCGG
GGGACATGTATGGTTAGGTTCCATTTGTATACTTGGTGGAATTTGGCATATCTTAACCAA
ACCCTTCGCATGGGCTCGACGCGCACTTGTCTGGTCGGGAGAGGCTTACTTATCTTATA
GTTTAGGTGCTTTAGCTGTTTTTGGTTTCATTGCTTGTTGCTTTGTCTGGTTCAATAATAC
CGCTTATCCTAGTGAGTTTTACGGACCCACTGGACCAGAAGCTTCTCAAGCTCAAGCAT
TTACTTTTCTAGTTAGAGACCAACGTCTTGGGGCTAACGTGGGATCCGCTCAAGGACCT
ACTGGTTTAGGTAAATATCTAATGCGTTCCCCGACTGGAGAAGTCATTTTTGGAGGAGA
AACCATGCGTTTTTGGGATCTGCGTGCCCCCTGGTTAGAACCTTTAAGGGGTCCAAATG
GGTTGGACTTAAGTAGGCTGAAAAAAGACATACAGCCTTGGCAAGAACGGCGTTCCG
CAGAATATATGACCCATGCTCCTTTAGGCTCTTTAAATTCCGTGGGTGGCGTAGCTACCG
AGATCAATGCAGTCAATTATGTCTCTCCTAGAAGTTGGTTAGCTACCTCTCATTTTGTTT
TGGGATTCTTCTTCTTCGTAGGTCATTTGTGGCACGCAGGAAGGGCTCGTGCAGCTGC
AGCAGGATTTGAAAAAGGAATTGATCGCGATTTTGAACCCGTTCTTTCCATGACCCCTC
TTAATATGACTATAGCCCTTGGTAAATTTACCAAAGACGAAAAT---TTATTTGATATTATG
GATGACTGGTTGCGGAGGGACCGTTTCGTTTTTGTAGGTTGGTCCGGTCTATTACTCTT
TCCTTGTGCCTATTTCGCTTTAGGGGGTTGGTTCACTGGTACAACCTTTGTAACTTCATG
GTATACCCATGGATTGGCCAGTTCCTATTTGGAAGGCTGCAATTTCTTAACTGCCGCAG
TTTCTACTCCTGCTAATAGTTTAGCACATTCTTTGTTGTTACTATGGGGTCCTGAAGCAC
AAGGAGATTTTACTCGTTGGTGTCAATTAGGGGGTCTGTGGACTTTTGTTGCTCTCCAT
GGCGCTTTCGGACTAATAGGTTTCATGTTACGTCAATTCGAACTTGCTCGATCTGTTCAA
TTGCGACCTTATAATGCAATCGCATTCTCTGCTCCAATTGCTGTTTTTGTTTCTGTATTTC
TGATTTATCCACTAGGCCAATCTGGTTGGTTTTTTGCGCCCAGTTTTGGCGTAGCGGCTA
TATTTCGCTTCATCCTTTTTTTTCAAGGGTTTCATAATTGGACGTTGAACCCATTTCATAT
GATGGGAGTTGCCGGTGTATTGGGCGCTGCTTTGCTATGCGCTATTCATGGTGCTACCGT
AGAAAATACTTTATTTGAAGATGGTGATGGTGCAAATACATTCCGCGCTTTTAACCCAA
CTCAAGCTGAAGAAACTTATTCAATGGTCACCGCTAACCGCTTTTGGTCCCAAATCTTT
GGGGTTGCTTTTTCCAATAAACGTTGGTTACATTTCTTTATGTTATTTGTACCAGTAACC
GGTTTATGGATGAGTGCTCTTGGAGTAGTCGGCCTGGCCCTGAACCTACGTGCCTATGA
CTTCGTTTCTCAGGAAATCCGCGCAGCGGAAGATCCTGAATTTGAGACTTTCTACACCA
AAAATATTCTTTTAAACGAAGGTATTCGTGCTTGGATGGCCGCTCAAGATCAGCCTCAT
GAAAACCTTATATTCCCTGAGGAGGTTCTACCCCGTGGAAACGCTCTTATGTCTGGAAG
TACAGGAGAACGCTCTTTTGCTGATATTATTACCAGTATTCGATACTGGGTCATTCATAG
CATTACTATACCTTCCTTATTCATTGCGGGTTGGTTATTCGTCAGCACGGGTTTAGCTTAC

GATGTGTTTGGAAGCCCTCGGCCAAACGAATATTTCACAGAGAGCCGACAAGGAATTC
CATTAATAACTGGCCGTTTTGATCCTTTGGAACAACTCGATGAATTTAATAGATCCTTTA
TGACCATAGATCGAACCTATCCAATTTTTACAGTGCGATGGTTGGCTGTTCACGGACTA
GCTGTACCTACCGTTTCTTTTTTGGGGTCAATATCAGCAATGCAGTTCATCCAACGAATG
GCTACACAAACTGTTGAGGGTAGTTCTAGATCTGGT---CCAAGACGAACTACTGTGGGG
AGTTTATTGAAACCATTGAATTCGGAATATGGTAAAGTAGCTCCTGGATGGGGAACGAC
TCCTTTGATGGGTGTTGCAATGGCTCTATTTGCGGTATTTCTATCTATTATTTTGGAGATT
TATAATTCTTCCGTTTTATTGGATGGAATTTCAATGAATATGCTTACTCTTAAACTCTTTG
TTTACACAGTAGTGATATTCTTTGTTTCTCTTTTCATCTTTGGATTCCTATCTAATGATCC
GGGACGTAATCCTGGACGTGAAGAAATGGCCGATACTACTGGAAGGATTCCTCTTTGG
CTAATAGGTACTGTAACTGGTATTCTTGTAATCGGTTTAATAGGTATTTTCTTTTATGGTT
CATATTCCGGATTGGGTTCATCCCTGATGCTTAATATCTTTAGTTTGATCTGTATCTGTCTT
AATTCTGCCCTTTAT---AGTAGTTTTTTCATCGCCAAATTGCCCGAGTCCTATGCTTTTTT
GAATCCAATCGTGAATGTTATGCCAGTCATACCTGTGCTCTTCTTTCTCTTAGCCTTTGT
TTGGCAAGCTGCTGTAAGTTTTCGAATGACACAATCAAACCCGAACGAACAAAATGTC
GAATTGAATCGTACCAGTCTCTACTGGGGGTTATTACTCATTTTTGTACTTGCTGTTTTAT
TTTCCAATTATTTCTTCAATATGGAAGTAAATATTCTCGCATTTATTGCTACTGCACTGTT
CATTCTAGTTCCTACTGCTTTTCTACTTATCATTTATGTAAAAACAATCAGTCAAAATGAT
ATGGAAACAGCAACCCTAGTCGCCATCTTTATATCTGGTTTACTTGTAAGTTTTACTGGG
TACGCCTTATATACCGCTTTTGGGCAGCCTTCTCAACAACTAAGAGATCCATTCGAGGA
A C A T G G G G A
C??
??????????????????ATGACTATTGCTTTCCAATTGGCTGTTTTTGCATTAATTGCTACTTCCT
CAATCTTATTGATTGGCGTACCCCTTGTATTTGCTTCTCCTGATGGTTGGTCAAGCAACA
AAAATGTTGTATTTTCCGGTACATCATTATGGATTGGATTAGTCTTTCTGGTGGGTATCCT
TAATTCTCTCATCTCT--------------------ATGTCACCACAAACAGAGACTAAAGCAAGTGT
TGGATTCAAAGCTGGTGTTAAAGAGTACAAATTGACTTATTATACTCCTACCTATGAAAC
CAAAGATACTGATATCTTGGCAGCATTCCGGGTAACTCCTCAACCTGGAGTTCCACCTG
AAGAAGCGGGGGCCGCGGTAGCTGCCGAATCTTCTACTGGTACATGGACAACTGTGTG
GACCGATGGACTTACTAGCCTTGATCGTTACAAAGGGCGATGCTACCACATCGAGCCC
GTTGCTGGAGAAGAAAGTCAATTTATTGCTTATGTAGCTTACCCCTTAGACCTTTTTGA
AGAAGGTTCTGTTACTAATATGTTTACTTCCATTGTGGGTAATGTATTTGGGTTCAAAGC
CCTGCGTGCTCTACGTCTGGAAGATCTGCGAATTCCTGTTGCTTATGTTAAAACTTTCC
AAGGCCCGCCTCATGGCATCCAAGTTGAGAGAGATAAATTGAACAAGTACGGTCGTCC
CCTGTTGGGATGTACTATTAAACCTAAATTGGGGTTATCCGCTAAAAACTACGGTAGAG
CGGTTTATGAATGTCTACGCGGTGGACTTGATTTTACCAAAGATGATGAGAACGTGAAC
TCCCAACCATTTATGCGTTGGAGAGACCGTTTCTTATTTTGTGCAGAAGCACTTTATAA
AGCACAGGCTGAAACAGGCGAAATCAAAGGGCATTACTTGAATGCTACTGCAGGTACA
TGCGAAGAAATGATGAAAAGGGCTATATTTGCCAGAGAGTTGGGGGTTCCTATCGTAAT
GCATGACTACTTAACAGGGGGATTCACTGCAAATACTAGTTTGGCTCATTATTGCCGGG
ATAATGGTCTACTTCTTCACATCCACCGTGCAATGCATGCAGTTATTGATAGACAGAAG
AATCATGGTATCCATTTTCGTGTACTAGCTAAAGCGTTACGTATGTCTGGTGGAGACCAT
ATTCACTCTGGTACCGTAGTAGGTAAACTTGAAGGGGAAAGAGAGATCACTTTGGGCT
TTGTTGATTTACTGCGTGATGATTTTGTTGAAAAAGACCGAAGTCGTGGTATTTATTTCA

CTCAAGATTGGGTCTCCCTGCCGGGTGTTCTGCCCGTGGCTTCGGGGGGTATTCACGTT
TGGCATATGCCTGCTCTGACCGAGATCTTTGGGGATGATTCTGTACTACAGTTCGGTGG
AGGAACTTTAGGACACCCTTGGGGAAATGCACCGGGTGCCGTAGCTAATCGAGTAGCT
CTAGAAGCATGTGTACAAGCTCGTAATGAGGGGCGTGATCTTGCTCGTGAGGGTAATG
AAATTATCCGCGAGGCTAGTAAATGGAGTCCTGAACTAGCTGCTGCTTGTGAGGTATGG
AAGGAGATTAAATTTGAATTCGAAGCAATGGATACTTTG------ATGGCGATACATTTATAC
AAAACTTCTACCCCGAGCACACGCAATGGAGCCGTAGACAGTCAAGTGAAATCCAATC
CACGAAAGAATTTGATCTATGGACAGCATCGTTGTGGTAAAGGTCGTAATGCCAGAGG
AATCATTACCGCAGGGCATAGAGGGGGAGGTCATAAGCGTCTATACCGTAAAATCGATT
TTCGACGGAATGAAAAAGACATATATGGTAGAATCGTAACCATAGAATACGACCCTAAT
CGAAATGCATACATTTGTCTCATACACTATGGGGATGGTGAGAAGAGATATATTTTACAT
CCCAGAGGGGCTATAATTGGAGATACCATTGTTTCTGGTACAGAAGTTCCTATAAAAAT
GGGAAATGCCCTACCTTTGACCGATATGCCCTTAGGCACGGCCATACATAACATAGAAA
TCACACTTGGAAAGGGTGGACAATTAGCTAGAGCAGCGGGTGCTGTAGCGAAACTGAT
TGCAAAAGAGGGGAAATCGGCCACATTAAAATTACCTTCTGGGGAGGTCCGTTTGATA
TCCAAAAACTGCTCAGCAACAGTCGGACAAGTGGGAAATGTTGGGGTGAACCAGAAA
AGTTTGGGTAGAGCCGGATCTAAACGTTGGCTAGGTAAGCGTCCTGTAGTAAGAGGAG
TAGTTATGAACCCTGTAGACCATCCCCATGGGGGTGGTGAAGGGAGGGCCCCAATTGG
TAGAAAAAAACCCACAACCCCTTGGGGTTATCCTGCACTTGGAAGAAGAAGTAGAAA
AAGGAATAAATATAGTGATAATTTGATTCTTCGTCGCCGTAGTAAAATGATTCAACCCCA
GACCCATTTGAATGTAGCGGATAACAGCGGGGCTCGAGAATTGATGTGTATTCAAATCA
TAGGAACTAGC---AATCGTCGATACGCTCATATTGGTGACGTTATTGTTGCTGTGATCAA
AGAAGCAGTACCAAACATGCCCCTAGAAAGATCAGAAGTAGTCAGAGCTGTAATTGTG
CGTACCTGTAAAGAACTCAAACGTGACAACGGTATGATAATACGATATGATGACAATGC
TGCAGTTGTCATTGATCAAGAAGGAAATCCAAAAGGAACTCGAGTTTTTGGTGCGATC
GCCCGGGAATTGAGACAGCTAAATTTTACTAAAATAGTTTCATTAGCTCCCGAGGTATT
AATGCTTAGTCCCAAAAGAACCAGATTCCGTAAACAACATAGAGGAAGAATGAAGGG
AATATCTTATCGAGGTAATCATATTTGTTTTGGTAAATATGCTCTTCAGGCACTTGAACCC
GCTTGGATCACATCTAGACAAATAGAAGCCGGACGACGAGCAATGACACGAAATGCAC
GTCGTGGTGGAAAAAATATGGGTACGTATTTTTCCCGACAAACCGGTTACAGTAAGACCT
ACAGAAACGCGTATGGGTTCGGGGAAAGGATCCCCTGAATTTTGGGTAGCTGTTGTTA
AACCGGGTCGAATACTTTATGAAATGAGCGGAGTAACAGAAACTATAGCCAGAAAGGC
TATTTCAATAGCGGCGTCAAAAATGCCTATACGAACTCAATTCATTATTTCGGGAATGAC
CAGAATTAAGCGAGGATATATAGCTCGGAGACGTAGAACAAAAATTCGTTTATTTGCAT
CAAGCTTTCGGGGGGCTCATTCAAGACTTACTCGAACTATTACTCAACAGAAAATAAA
AGCTTTGGTTTCGGCTAATAGGGATAGAGATAGGCAAAAGAGAAATTTTCGTCGTTTGT
GGATCACTCGGATAAACGCAGGAATTCGTGAA---GGGGGGGTCTCTTATAGTTATAGTA
GATTAATACACGATCTGTACAAGAGACAGTTGCTTCTTAATCGTAAAATACTTGCACAA
GTCGCTATATTAAATAGGAATTGTCTTTATATGATTTCCAATGAGATCATAAAA--------------
-------------------ATGCTAAGGAAAGTATACGCTTTTAGTCGATATATATCTATGTCTGCTCAC
AAAGCGCGAAGAGTAATTGATCAAATTCGCGGACGTTCATATGAAGAAACACTTATGAT
ACTAGAACTCATGCCTTATCGAGCATGTTATCCCATTTTTAAATTGGTTTATTCTGCAGC
AGCAAATGCCAGTTACAGTCTGGGTTCCGATGAAACAAATTTAATCATTAGTAAAGCTG
AAGTCAACGAGGGCACTACCATTAAGAAATTAAAACCTAGAGCTAGAGGACGTAGTTA

TCCAATAAAAAGACCTACTTGTCATATAACTATTGTAATAACAGATATATCCATAGAG-----
----------------ATGGATGGAATCAAATATGCAGTAGTTACAGACAAAAGTATTCAGTTATTG
GGGAAAAAT---CAATATACTTCTAATGTCGAATCAGGATCAACTAGGACAGAAATAAAG
CATTGGGTCGAACTCTTCTTTGGTGTCAAGGTAATAGCTATGAATAGTCATCGACTTCC
GGGAAAGGGTAGAAGAATGGGACCTATTATGGGACATACAATGCATTACAGACGTATGA
TCATTACGCTTCAACCGGGTTATTCTATTCCACCTCTTAGAAAGAAAACAACTATGGCA
GTTCCAAAAAAACGTACTTCTATATCAAAAAAGCGTATTCGAAAAAATATGTGGAAAA
AGAAGGGATATTGGGCAGCGCTAAAAGCCTTTTCGTTAGCGAAATCCCTTTCTACCGG
AAATTCAAAAAGTTTTTTGTGCGACAAACAAATAAAATGGCCAAGGGTAAAGATGTC
CGAGTAACGGTAATTTTGGAATGTACCAGTTGTGTCCGAAACGGCGTTAATGTTAATAA
GGCATCAACGGGCATTTCCAGATATATTACTCAAAAGAATCGACACAATACGCCTAATC
GATTGGAATTGCGAAAATTCTGTCCCTATTGTCACAAACATACGATTCATGGGGAGATA
AAGAAAATGAAAATAAGGGCTTCTGTTCGTAAAATTTGTGAAAAATGTCGACTAATCC
GCAGGCGGGGACGAATTATAGTAATTTGTTCCAACCCGAGACACAAACAAAGACAAG
GAATGGTTCGAGAGAAAGTAACAGTATCTACTCGGACACTACAGTGGAAGTGTGTTGA
ATCAAGAGCAGACAGTAAGCGTCTTTATTATGGACGCTTTATTCTGTCTCCACTTATGAA
AGGCCAAGCCGACACAATAGGCATTGCGATGCGAAGAGCTTTGCTTGGAGAAATAGA
AGGAACGTGTATCACACGCGCAAAATCTGAG------AAAATACAACACGAATATTCTACC
ATAGTAGGTATTCAAGAATCAGTACATGAAATTTTAATGAATTTGAAAGAAATTGTATTG
AGAAGTACTTTATATGGAACTTGTGACGCGTCTATTTGTGTCAAGGGTCCCGGATATGT
AACTGCTCAAGATATCATCTCACCGCCTTATGTGGAAATCGTTGATAATACCCAGTATAT
AGCTAGCTTGACGGAACCCATTGATTTGTGTATTGGATTACAAATCGAAAGGAATCGCG
GATATCGTATAAAACCGCCAAAT---AACTTTGACGGAAATTAC------CCGATAGATGCTGT
ATTCATGCCTGTTCGAAATGCGAATCATAGTATTCATTCTTATGGGAATGGGAATGAAAA
GCAAGAGATACTTTTTCTTGAAATATGGACAAATGGAAGTTTAACTCCTAAAGAAGCA
CTTCATGAAGCCTCCCGGAATTTGATTGATTTATTTATTCCTTTTTTACATACGGAAGAA
GAA------AACTTACATTTAGAGGACAATCAA---CACAAGGTTACTTTACCCACTTTTACC
TTTCATGATAAATTAGCT---AAACTAAGGAAAAACAAAAAA---AAAATAGCATTCAAATA
TATTTTTATTGACCAATCAGAATTATCTCCCAGAATCTATAATTGCCTCAAAAGGTCCAAT
ATATATACATTATTGGACCTTTTGAATAACAGTCAAGAAGATCTTATGAAAATGAAAGAT
TTTCGCATAGAAGATGTAAAAGAGATATTGGACATTCTAGAAAAGCGG------TTCGCAA
CCGATTTACTGAAAAATAGGATGCTTCGGGATGGA---AATGAGGGAATGTCTACAATAC
CTGAGTTTAATCAGATACAATTTGAAGGATTTTGTAGGTTCATTGATCGGGGCTTGACG
GAAGAACTTTATAAGTTTCCAAAAATTGAAGATACAGATCAAGAAATTGAATTTCAATT
ATTTGTGGAAACATATCAATTGGTAGAACCGTTGATAAAAGAAAGAGATGCTGTGTATG
AATCACTCACATATTCTTCTGAATTATATGTATCCGCGGGATTAATTTGGAAAACCGGTA
GGGATATGCAAGAACAAACCATTTTTATTGGAAACATTCCTCTAATGAATTCCCTGGGA
ACTTCTATAGTAAATGGAATATACAGAGTTGTAATCAATCAAATATTGCAAAGCCCCGGT
ATTTATTACCGGTCAGAATTGGACCATAATGGAATTTCGGTCTATACCGGCACCATAATA
TCAGATTGGGGGGGAAGATCAGAATTAGAGATTGATAGAAAAGCAAGGATATGGGCTC
GTGTGAGTAGGAAACAAAAAATATCTATTCTAGTTCTATCATCAGCTATGGGTTCGAATC
TAAGAGAAATTTTAGAGAATGTTTGCTATCCTGAAATTTTTTTGTCTTTTCTGAATGATA
AGGAGAAAAAAAAAAATTGGGTCAAAAGAAAATGCCATTTTAGAGTTTTATCAACAATT
TGCTTGTGTAGGCGGGGATCCGGTATTTTCCGACTCCTTATGTAAGGAATTACAAAAAA

AATTCTTTCAACAAAGATGTGAATTAGGGAGGATTGGTCGACGAAATATGAACCGGAG
ACTGAACCTTGATATACCTCAGAACAATACATTTTTGTTACCGCGAGACATATTGGCAG
CCGCGGATCATTTGATTGGAATGAAATTTGGAATGGGTACACTTGACGAT------ATGAAT
CATTTGAAAAATAAACGCATTCGTTCTGTAGCGGATCTTTTACAAGATCAATTCGGATTG
GCTCTGGTTCGTTTAGAAAATGTGGTTCGAGGAACTATATGTGGAGCAATTAGG---CATA
AATTG---ATACCGACTCCTCAAAATTTGGTAACTTCAACTCCA-----------TTAACAACTAC
TTATGAATCTTTTTTCGGCTTACACCCATTATCTCAAGTTTTGGATCGAACTAATCCATTG
ACACAAATAGTTCATGGGAGAAAATTGAGTTATTTGGGCCCCGGGGGATTGACAGGGC
GAACCGCGAGTTTTCGGATACGAGATATCCATCCTAGTCACTATGGGCGTATTTGCCCAA
TTGATACATCTGAAGGAATCAATGTTGGACTTATTGGATCCTTAGCAATTCATGCGAGGA
TTGGTCATTTGGGG---TCTCTCGAAAGCCCGTTTTAT---------GAAATTTCTGAAAGACCA
AAAAAG------GTACGGGTGCTTTATTTATCACCAGGTAGAGATGAATACTATATGGTAGC
GGCAGGAAATTCTTTGGCGTTGAATCAGGGTATTCAGGAAGAACAGGTTGTTCCAGCT
CGATATCGTCAAGAATTCCTGACTATTGCATGGGAACAGGTTCATCTTCGAAGTATTTTT
CCCTTCCAATATTTTTCTATTGGAGCTTCCCTCATTCCTTTTATCGAGCATAATGATGCAA
ATCGGGCTTTAATGAGTTCTAATATGCAACGTCAAGCAGTTCCGCTTTCTCAGTCCGAG
AAGTGCATTGTTGGAACTGGGTTGGAACGCCAAGCGGCTCTAGATTCAGGGGCTCTGG
CTATAGCCGAACACGAGGGAAAGATCGTTTATACCGATACTGACAAGATAGTTTTATCA
GGTAATGGAAATACTATAAGCATTCCATTAGTTATGTATCAACGTTCCAACAAAAATACT
TGTATGCATCAAAAACCCCGGGTTCAGGGGGGTAAATGCATTAAAAGGGGACAAATTT
TAGCGGACGGTGCCGCTACAGTTGGTGGCGAACTCGCTTTGGGGAAAAATATATTAGT
AGCTTATATGCCATGGGAAGGTTACAATTCTGAAGATGCAGTACTCATTAGCGAGCGCT
TGGTATATGAAGATGTTTATACTTCTTTTCACATACGGAAATATGAAGTTCAGACTCATG
TGACAAGCCAAGGT---CCCGAAAGGGTCACTAGCGAAATACCGCATTTAGAAGCCCAT
TTACTCCGCAATTTAGACAAAAATGGAATTGTGATGCTGGGATCTTGGGTAGAGACGG
GGGATATTTTAGTAGGTAAATTAACGCCCCAGATGGTGAAAGAA---TCATCGTATGCCCC
GGAAGATAGATTATTGCGGGCCATACTTGGCATTCAGGTATCTACTTCAAAGGAAACTT
GTTTAAAACTACCTATAGGGGGTAGGGGTCGAGTTATTGATGTGAGATGGATCCAGAAA
AGGGGG---GGTTCTAGTTATAATCCAGAAACGATTCGTGTATATATTTCACAGAAACGTG
AAATCAAAGTAGGCGATAAAGTAGCCGGAAGACATGGAAATAAAGGTATCATTTCAAA
AATTTTGCCTAGACAAGATATGCCTTATTTGCAAGATGGAAGGCCTGTTGATATGGTCTT
CAACCCATTAGGAGTACCTTCAAGAATGAATGTAGGGCAGATATTTGAATGTTCACTCG
GGTTAGCGGGGGGTCTGCTAGACAGACATTATCGAATAGCACCTTTTGATGAGAGATAT
GAACAAGAAGCTTCAAGAAAACTAGTGTTTTCTGAATTATATGAAGCCAGTAAGCAAA
CAGCGAATCCATGGGTATTTGAACCCGAGTATCCAGGAAAAAGCAGAATATTTGATGG
AAGAACGGGGGATCCTTTTGAACAACCTGTTCTAATAGGAAAGCCTTATATCTTGAAAT
TAATTCATCAAGTTGATGATAAAATCCATGGACGTTCCAGTGGCCATTATGCACTTGTTA
CACAACAACCCCTTCGAGGAAGGGCCAAGCAAGGGGGACAGCGGGTAGGAGAAATG
GAGGTTTGGGCTCTAGAGGGATTTGGTGTTGCTCATATTTTACAAGAGATGCTTACTTAT
AAATCTGATCATATTAGAGCTCGCCAAGAAGTACTTGGGACTACGATCGTTGGAGGAA
CAATACCTAATCCTGAGGATGCTCCAGAATCTTTTCGATTGCTCGTTCGAGAACTACGA
TCTTTGGCTCTGGAACTGAATCATTTCCTTGTATCTGAGAAGAACTTACAGATTAATAG
GAAGGAAGCTATGAATCAGAATTTTTCTTCTATGATCGATCGGTATAAACATCAACAAC
TCCGAATTGGATTAGTTTCTCCTCAACAAATAAGCGCTTGGGCCAAAAAAATCCTACCT

AATGGAGAGATAGTTGGAGAGGTAACAAAACCCTACACTTTTCATTACAAAACCAATA
AACCGGAAAAAGATGGATTATTTTGTGAAAGAATTTTTGGGCCTATAAAAAGCGGAATT
TGCGCTTGCGGAAATTATCGAGTAATCGGAGATGAAAAGGAAGACCCGAAATTTTGTG
AACAATGCGGAGTCGAATTTGTTGATTCTCGGATACGCAGATATCAAATGGGCTACATC
AAACTGGCATGCCCAGTAACCCATGTGTGGTATTTGAAACGTCTTCCTAGTTATATTGCG
AATCTTTTAGATAAACCTCTTAAAGAATTAGAAGGCCTAGTATACTGCGAT------TTTTCT
TTTGCTAGGCCC---ATAGCTAAAAAACCCACTTTCTTACGATTACGAGGTTCATTCGAAT
ATGAAATCCAATCTTGGAAATACAGCATCCCGCTTTTTTTACTACCCAAGGCTTCGATA
CATTTCGAAATCGAGAGATCTCTACTGGAGCAGGTGCTATCCGAGAACAATTATCCGAT
CTAGATTTACGAATTATTATAGATTATTCCTTGGTAGAATGGAAAGAATTAGGGGAAGAA
---GGGCTCACAGGGAATGAATGGGAAGATCGAAAAATTGGAAGAAGAAAGGATTTTTT
GGTTAGACGCATGGAATTAGCTAAGCATTTTATTCGAACAAATATAGAACCAGAATGGA
TGGTCTTGTGTCTATTACCAGTTCTTCCTCCCGAGTTGAGACCGATCATTCAGATAGATG
GGGGTAAACTAATGAGCTCGGATATTAATGAACTCTACCGAAGAGTTATCTATCGGAAC
AATACTCTTACCGATCTATTAACAACCAGTAGATCTACGCCAGGAGAATTAGTAATGTGT
CAGGAGAAATTGGTACAAGAAGCCGTGGATACACTTCTTGATAACGGAATTCGCGGAC
AACCAATGAGGGACGGTCATAATAAAGTTTACAAGTCATTTTCAGATGTAATTGAAGGC
AAAGAGGGAAGATTTCGCGAGACTCTGCTTGGTAAACGGGTCGATTATTCGGGGCGTT
CCGTCATTGTCGTAGGCCCTTCACTTTCATTACATCGATGTGGATTGCCTCGCGAAATAG
CAATAGAGCTTTTCCAGACATTTGTAATTCGTGGTCTAATTAAACAACATCTTGCTTCGA
ACATAGGGGTTGCTAAGAGTAAAATTCGGGAAAAAGAACCGATTGTATGGGAAATACT
TCAGGAAGTTATGCAGGGGCATCCTGTATTGCTGAATAGAGCACCTACTCTGCATAGAT
TAGGCATACAGGCATTCCAGCCTATTTTGGTAGAAGGGCGCGCTATTTGTTTACATCCAT
TAGTTCGTAAGGGATTCAATGCCGACTTTGATGGGGATCAAATGGCTGTTCATGTACCC
TTATCTTTGGAGGCTCAAGCGGAGGCCCGTTTACTTATGTTTTCTCATATGAATCTTTTAT
CTCCCGCTATTGGGGATCCCATTTCCGTACCAACCCAAGATATGCTTATTGGACTCTATG
TATTAACGAGCGGGAATCGTCGAGGTATTTGTGTAAATAGGTATAATCCATGTAATCGC--
-------AGAGACTATCAAAATGAAAATATT------------TATAAGTATACGAAA------GAACCCTT
TTTTTGCAATTCCTATGATGCAATTGGAGCTTATCGGCAGAAAAGAATCCATTTAGATAG
TCCTTTGTGGCTCCGATGGCGACTAGATCAACGC---GTTATTGCTTCA------AGAGAAGC
TCCCATCGAAGTTCACTATGAATCTTTGGGTACCTATTATGAGATTTATGAGCACTATCTA
ATAATAAGAAGTATAAAAAAAGAAATTATTTTGATATACATTCGAACCACTGTTGGTCAT
ATTTCTCTTTATCGAGAAATCGAAGAAGCCATACAAGGGTTTTGTCGGGCCTGTTCATA
TGGTACC------------------------ATGGAGGTACTTATGGCAGAACGGGCCAATCTGGTCTATC
ACAATAAAGCGATAGACGGAACTGCCATGAAACGACTTATTAGTAGATTAATAGATCAC
TTCGGAATGGCATATACATCACACATCCTAGATCAAGTAAAGACTCTGGGTTTTCAGCA
AGCCACCGCTACATCCATTTCATTAGGAATTGATGATCTTTTAACAATACCTTCTAAGGG
ATGGCTAGTTCAAGATGCTGAACAACAAAGTTTGATTTTGGAAAAACACCATCATTATG
GGAATGTACACGCGGTAGAAAAATTACGTCAATCCATTGAGATATGGTATGCTACAAGT
GAATATTTGAGACAAGAAATGAATCCCAATTTTAGGATGACTGACCCTTTTAATCCAGT
CCATATAATGTCTTTTTCGGGAGCTAGAGGAAATGCATCTCAGGTACATCAATTAGTAGG
TATGAGGGGATTAATGTCGGATCCCCAAGGACAAATGATTGATTTACCTATTCAAAGTAA
TTTACGTGAAGGACTCTCTTTAACAGAATATATCATTTCTTGCTACGGAGCCCGCAAAG
GAGTTGTGGATACTGCTGTACGAACATCAGATGCTGGATATCTCACGCGCAGGCTTGTT

GAGGTAGTTCAACACATTGTTGTACGTCGAACAGATTGTGGCACCGTTCGAGGTATTTC
TGTGAGTCCTCAAAATGGGATGATACCG---GAAAGAATTTTTATCCAAACATTGATTGGT
CGTGTATTAGCGGACGATATATATATGGGTCCACGATGCATTGCCACTAGAAATCAAGAT
ATTGGAATTGGACTTATCAATAGATTCATAACC------------------TTTCGAGCACAACCAATA
TCTATTCGAACTCCCTTCACTTGTAGGAGTACATCTTGGATCTGTCGATTATGTTATGGC
CGGAGTCCTACTCACGGCGACCTGGTCGAATTGGGGGAAGCTGTAGGTATTATTGCGG
GTCAATCTATTGGAGAACCGGGTACTCAATTAACATTAAGAACTTTTCATACTGGCGGA
GTATTCACAGGGGGTACTGCAGAACATGTACGAGCCCCTTCTAATGGAAAAATAAAATT
CAATGAGGATTTGGTTCATCCGACACGTACACGTCACGGACATCCGGCTTTTCTATGTT
CTATAGACTTGTATGTAACTATTGAGAGTGAAGATATTATACATAATGTGAATATTCCACC
CCAAAGTTTTCTTTTAGTTCAAAATGATCAATATGTAGAATCAGAACAAGTGATTGCTG
AGATTCGCGCAGGAACATCCACT---TTGAATTTTAAAGAGAGGGTTCGAAAACATATTT
ATTCTGACTCAGAGGGAGAA---ATGCACTGGAGTACCGATGTGTATCATGCACCTGAAT
TTACATATGGTAATGTTCAGCTCTTACCAAAAACAAGTCATTTATGGATATTATCTGGGA
GGCCATGCAGG------TCCAGTCTAGCCTCCCTTTCGCTCCACAAGGATCAAGATAAAAT
GAACGCCCAT------TCTCTTTCTGTCAAGCGAAGATATATTTCTAACCTCTCA------GTGAC
TAATGATCAA---GCGAGACACAAATTCTTTAGTTCGGATTTTTCTGGTAAA---CAAGATA
GAATTCCTGATTAT---TCAGAACGTAATCGAATCATATGTACTGGTCATTGTAATCTCACA
TATCCT---GCCATTTTCCACGAGAATTCG------GATTTATTGGCAAAGAGGCGAAGAAATA
GATTCATCATTCCACTCCAATCGATTCAGGAACGCAAGAAAAAAACGAATACCCCGTTC
AGGTATCTCGATTGAAATACCC------ATAAATGGCATTTTCCGTAGAAATAGTATTCTTGC
TTATTTCGATGATCCTCGATACAGAAGAAAAAGTTCGGGAATTACTAAATATGGGACTAT
AGAAGCGCATTCAATCGTCAAAAAAGAAGAT------TTGATTGAGTATCGGGGA---GTCAA
GGAATTTAGGCCAAAATACATGAAAGTAGATCGATTTTTTTTCATTCCCGAGGAAGTGC
ATATCTTACCCGGATCTTCTTCCATAATGGTACGAAACAATAGTCTCATTGGGGTAGATA
CACAAATCGCTTTCAATACAAGAAGCCGAGTAGGCGGGTTGGTGCGAGTGGAGAGAA
AAAAAAAAAGAATTGAACTTAAAATCTTTTCTGGAGATATCCATTTTCCGGGAGAGAC
AGATAAGATATCCCGACACAGCGGCATCTTGATACCA---CCAGGA---ACGGGAAAAAAA
AATTCCACGGAATCAAAAAAA------TTGAAAAATTGGATCTATGTCCAACGG------ATCA
CACCTACCAAG------AAAAAATATTTTGTTTTGGTTCGACCTGTAGTCGCATATGAAATA
ACGGACGGTATAAATTTAGCAACACTTTTCCCCCCGGATCTGTTGCAGGAAAGGGATAA
TGTGCAACTTCGAGTTGTCAATTATATCCTTTATGGAAATGGCAAACCAATTCGGGGAA
TTCCTGACACAAGTATTCAATTAGTTCGGACTTGTTTAGTGTTGAATTGGGGCCAAGAC
AAAAAA---------AGTTCTTATATCGACGAGGCCCATGCTTCTTTTGTTGAAATAAGGACA
AATGGTTTGATTCAAGATTTCCTAAGAATCGACTTAGTGAAGTCCCCTATTTCGTATACC
AGCAAAAGGAATGATCCATCAGGTTCAGGATTGATCTCTGAA---AATGGATCGGATCGC
ACCAATATCAATCCGTTTTCTTCCATTTAT---------------TCCAAGGCAAAGATTCAACAAT
CCCTTAACCAAAATCAAGGAACTATCCATACGTTGTTGAATAGA---------AATAAAGAAT
GCCAATCTTTGATAATTTTGTCATCATCCAATTGTTCTCAAATGGGTCCA------TTCAACG
ATGTAAAATATCACAATGTG---------ATAAAAGAATCA---CCTCTAATTCGAATTAGGAATT
TGTTGGGTCCTTTAGGAACA------GCTCTTCCAATTTATAATTTTGATTCA------TTTTACC
ATTTAAATTTAATAACTCATAATCAACTCTTG---GTAACTAACTATTTGCAACTTGACAAT-
--------TTAAAACAGGCTTTTCAAGTACTTAAA---------TATTATTTAATGGATGAAAAT------
GGGAAAATTTATAATCCCGATCCCTGCAGTAATATCTTTTTGAATCCATTAAATTTGAATT

GGTATTTTCTCCATCACAATTATTGTGAAGAGACATCT---ACAATAATTAGTCTTGGACA
GTTTATTTGCGAAAATGTATGTATAGCCAAAAACGGACCACAC---CTAAAA------TCGGG
TCAAGTTCTAATTGTTCAA---ATTGACTCTTTAGTAATACGATCAGCTAAGCCTTATTTGG
CTACCCCAGGAGCAACTGTTCATGGCCATTATGGTGAAACCCTTTACGAAGGAGATAC
ACTAGTTACATTTATATATGAAAAATCGAGATCTGGTGATATAACGCAGGGTCTTCCAAA
AGTAGAACAGGTGTTAGAAGTACGTTCGATTGATTCAATATCGATGAATCTAGAAAAGA
GGGTTGAGGGTTGGAACGAATGTATAACAAGAATTCTTGGAATTCCTTGGGGATTCTTG
ATTGGTGCTGAGCTAACTATAGCGCAAAGTCGTATCTCTTTGGTTAATAAGATCCAAAG
GGTTTATCGATCCCAGGGGGTGCAGATCCATAATAGGCATATAGAAATTATTGTACGTCA
AATAACATCAAAAGTCTTGGTTTCAGAA---------GATGGAATGTCTAATGTTTTTTCACCC
GGAGAACTAATTGGATTGTTGCGAGCGGAACGTATGGGGCGTGCTTTGGAAGAAGCGA
TCTGTTACCGAGCCATCTTGTTGGGAATAACAAGAGCATCTCTGAATACTCAAAGTTTC
ATATCTGAAGCAAGTTTTCAAGAAACTGCTCGAGTTTTAGCAAAAGCCGCTCTACGGG
GTCGTATCGATTGGTTGAAAGGCCTGAAAGAAAACGTTGTTCTGGGGGGGAGGATACC
CGTAGGTACCGGATTCAAAGGATTA------GCGCACCCCTCAAGGCAACATAACAACATT
CCC------TTGGAAACAAAAAAGAAGAGTCTATTCGAGGGGAAAATGAGA------GATATTT
TGTTCCACCACCGAAAATTATTTGATTCTTGCCTTTCAAAGAATTTCCATGATACATCAG
AACAATCATTTATAGGA------------------TTTAATGATTCCATGACAAGAAGATATTGGAACA
TCAATTTGGAAGAGATGATGGAGTCGGGAGTTCATTTTGGCCATGGTACTAGAAAATG
GAATCCTAGAATGGCACCTTATATCTCTGCCAAGCGTAAAGGTATTCATATTACAAATCT
TACTAGAACTGCTCGTTTTTTATCAGAAGCTTGTGATTTGGTTTTTGATGCAGCAAGTA
AGGGAAAACAATTCTTAATTGTTGGTACCAAAACTAAAGCAGCTGATTCAGTAGCACG
GGCTGCGCTAAAGGCTCGGTGTCATTATGTTAATAAAAAATGGCTCGGTGGTATGTTAA
CAAATTGGGCCACTACAGAAACGAGACTTCATAAGTTCAGGGACTTGAGAACGGAAC
AAAAAACGGGGAAACTCAACCGTCTTCCGAAAAGGGATGCCGCTATGTTGAAAAGAC
AATTATCTCACTTGCAAACGTATCTGGGCGGGATTAAATATATGACGGGGGTACCCGATA
TTGTAATCATTGTTGATCAGCACGAAGAATATACGGCCCTTCGAGAATGTATCACTTTGG
GAATTCCAACAATTTGTTTAATCGATACAAATTGTGACCCGGATCTCGCGGATATTTCGA
TTCCAGCTAACGACGATGCTATAGCTTCAATCCGATTAATTCTTAACAAATTAGTATTCG
CAATTTGTGAGGGGCGTTCT---AGCTATATAAGGAATTCTATGGGACAAAAAATAAATCC
ACTTGGTTTCAGACTTGGTATAACCCAAGGTCATCATTCCCTTTGGTTTGCACAACCAA
AAAATTATTCTGAGGGTCTCCAAGAAGATCAAAAAATACGGGATTGTGTTAAGAATTAT
GTACAAAAAAATATGCGAATATCCTCCGGT---GTTGAGGGAATTGCACGTATCGAGATTC
AAAAAAGAATCGATCTGATCCAGGTCATAATCTATATGGGATTCCCAAAGTTATTAATAG
AAAGCCGGCCGCGA------------GGAATCGAAGAATTGCAGGTGAATCTACAAAAAGAAT
TTAATTGTGTGAACAGAAAACTTAACATTGCTATCACAAGAATTGCAAAACCTTATGGA
AACCCTAACATTCTTGCGGAATTTATAGCCGGACAATTAAAGAATAGAGTTTCATTTCG
CAAAGCAATGAAAAAGGCTATTGAATTAACTGAACAAGAAGATACAAAAGGAATTCA
AGTACAAATTGCAGGGCGTATCGACGGAAAAGAAATTGCGCGTGTCGAATGGGTCAG
AGAAGGTAGGGTTCCGCTACAAACCATTCGAGCGAAAATCGATTATTGTTCTTATACAG
CTCGAACTATCTATGGGGTATTGGGCATCAAAATTTGGATATTTATAGACGAGGAAATGT
CGCGTTACCGAGGACCTCGTTTCAAAAAAATACGCCGCCTGGGGGCTTTACCGGGACT
AACTAATAAAAGGCCTAGAGCCGGAAGCGATCTTAGAAACCAATCGCGTTCCGGTAAA
---AGATCTCAATATCGTATTCGTCTAGAAGAAAAACAAAAATTACGTTTTCATTATGGTC

TTACAGAACGACAATTACTTAAATACGTTCGTATCGCCGGAAAAGCCAAGGGGTCAAC
AGGCCAGGTTTTACTACAATTACTTGAAATGCGTTTGGATAACATCCTTTTTCGATTGGG
CATGGCTTCGACTATTCCGGGAGCCCGCCAATTAGTTAACCATAGACATATTTTAGTTAA
TGGTCGTATAGTAGATATACCAAGTTATCGCTGTAAACCCCAAGATATTATTACGGCGAG
GGATGAACAAAAATCCAGAGCTCTGATTCAAAATTCTCTCGAGTCATCCCCTCAT---GA
GGAATTGCCGAAACATTTGACTGTTCACCCATTCCAA------TATAAAGGATTAGTCAATC
AAATAATAGATAGTAAATGGGTCGGTTTGAAAATAAATGAATTGCTAGTCGTAGAATATT
ATTCCCGTCAGACTATGTCACGTCGAGGTACTGCAGAAGAAAAAACTGCAAAATCTGA
TCCAATTTATCGTAATCGATTAGTTAACATGTTGGTTAACCGTATTCTGAAACACGGAAA
AAAATCATTGGCTTATCAAATTCTCTATCGAGCCGTGAAAAAGATTCAACAAAAGACA
GAAACAAATCCACTATCTGTTTTACGTCAAGCAATACGTGGAGTAACTCCCGATATAGC
AGTAAAAGCAAGACGTGTAGGTGGGTCGACTCATCAAGTTCCCATTGAAATAGGATCC
ACACAAGGAAAAGCACTTGCTATTCGTTGGTTATTAGCGGCATCCCGAAAACGTCCGG
GTCGAAATATGGCTTTCAAATTAAGTTCCGAATTAGTGGATGCTGCCAAAGGGAGTGGC
GATGCCATACGCAAAAAGGAAGAGACTCATAGAATGGCAGAGGCAAATAGAGCTTTTG
CACATTTTCGTATGGGTAGGGACACTATTGCTGACGTAATAACCTCTATACGAAATGCCG
ACATGGATAGAAAAAGAGTGGTTCGAGTACCATCTACTACTATTACCGAAAACATTGTT
AAAATACTTTTACGAGAAGGTTTTATCGAAAACGTAAGGAAACATTGGGAAAGCAACA
AATATTTTTTGGTTTCAACCCTGCGACATAGAAGG------AATAGGAGAAGGCCTTATAGA
---------AATCTTTTAAATTTAAAACGGATCAGTCGACCCGGTTTACGAATCTATTCTAACT
ATCAACGAATTCCTAGAATTTTAGGTGGAATGGGAATTGTAATTCTTTCTACTTCTCGAG
GGATAATGACAGACCGAGAGGCTCGCCAAGAAGGAATCGGCGGAGAAATTTTGTGTTA
TATATGGATGGCAAAAGCTATACCAAGAATTGGTTCACGTAGAAATGCACGTATTGGTT
CACGTAAGAATGTGCGTAGAATACCAAAGGGGGTTATTCATGTTCAAGCAAGTTTCAAT
AATACCATTGTGACTGTTACAGATGTACGGGGTCGGGTAGTTTCTTGGTCCTCTGCCGG
TAGTTGTGGGTTTAGGGGTACAAGACGGGGGACACCTTTTGCTGCTCAAACTGCAGCA
GGAAATGCTATTCGTACAGTCGTGGATCAAGGTATGCAACGAGCGGAAGTCATGATAA
AGGGTCCCGGTCTCGGAAGAGACGCAGCATTACGAGCTATTCGTAGAAGTGGTATACT
ATTAACTTTCGTACGGGATGTAACCCCTATGCCACATAATGGCTGCAGGCCTCCTAAAA
AAAGACGCGTGATGCCAACCATTAAACAACTTATTAGAAATACAAGACAGCCAATCAG
AAATGTCACAAAATCCCCCGCTCTTCGGGGATGCCCTCAGCGTCGAGGAACATGTACT
AGGGTGTATACTATCACCCCAAAAAAACCAAACTCTGCCTTACGTAAAGTTGCCAGAG
TACGATTAACCTCTGGATTTGAAATCACTGCTTATATACCTGGTATTGGCCATAATTCACA
AGAACATTCTGTAGTCTTAGTAAGAGGGGGAAGGGTTAAGGATTTACCCGGTGTGAGA
TATCACATTGTTCGAGGAACCCTAGATGCTGTCGGAGTAAAGGATCGTCAACAAGGGC
GTTCTAAATATGGGGTAAAAAAGCCAAAAATGGCAAGGAAAAGTTTGATTCAGAGGG
AGAAGAAGAGGCAAAAATTGGAACAGAAATATCATTTGATTCGTCGATCCTCAAAAAA
AGAAATAAGC---------AAAGTTCCGTCGTTGAGTGACAAATGGGAAATTCATGGAAAGT
TACAATCCCCCCCACGGAATAGTGCACCTACACGCCTTCATCGACGTTGTTTTTTGACC
GGAAGACCGAGAGCTAACTACCGGGACTTTGGACTATCCGGACACATACTTCGCGAAA
TGGTTCATGCATGTTTGTTGCCAGGGGCAACAAGATCAAGTTGGATGGTAAAAAATCC
ATTCATCTCGGTTATTTCCCAAGAA---------AAAGAAGAAAAGAGGGGGTCCGTTGAATT
TCAAGTATTCGTGTTCACCAATAAGATACAGAGACTTACTTCCCATTTGGAATTGCACA
AAAAAGACTATTTATCTCAGAGGGGTCTGCGGAAAATTCTGGGAAAACGTCAACGGCT

ATTGGCTTATTTGTCAAAAAAAAAATAGAGTACGTTATAAAGAATTAATTAGTCAGTTGG
GTATTCGAGAGACAAAAACTCGT------ATGGTAAAACTTCGTTTGAAACGATGTGGTAG
AAAGCAACGAGCCGTCTATCGAATCGTTGCAATTGATGTTCGATCCCGAAGAGAGGGA
AGAGATCTTCGGAAAGTGGGTTTTTATGATCCGATAAAGAATCAAACTTATTTAAATGT
TCCTGCTATTCTATACTTCCTTGAAAAAGGCGCTCAACCTACAGGAACTGTTCATGATAT
TTTAAAGAAGGCGGAGGTTTTTAAGGAACTTCGTTCTAAT------------------ATGGATAAAT
CCAAGCGACCTTTTCTTAAATCCAAGCGATCTTTTCGTAGGCGTTTGCCCCCGATCCAA
TCGGGGGATCGAATTGATTATAGAAACATGAGTTTAATTAGTCGATTTATTAGTGAACAA
GGAAAAATATTATCTAGACGAGTGAATAGATTAACCTTGAAACAACAACGATTAATTAC
TATTGCTATAAAACAAGCTCGTATTTTATCTTCGTTACCTTTTCTTAATAATGAGAAA------
CAATTTGAAAGAACCGAGTCGACCGCTAGAACTACCGGTCTTAAAACCAGAAATAAAG
TGACACGTTCACTAAAAAAAAACCCTTTTGTAGCAAATCATTTATTAAAAAAAATTGAT
AAGCTTAACACAAAAGCGGAAAAAGAAATAATAGTAACTTGGTCCCGGGCATCTACCA
TTATACCTACAATGATCGGACATACTATTGCTATACATAATGGCAAGGAACATTTGCCTAT
TTATATAACGGATCGTATGGTTGGACACAAATTAGGAGAATTTGCACCTACTTTAAATTT
CCGAGGACATGCAAAAAGCGATAATAGATCTCGTCGTATGATTTTTAAATCTTTTCTACT
AGGTAATCTAGTATCCTTATGCATGAAGATAATCAATTCGGTCGTTGTGGTCGGACTCTA
TTATGGATTTCTGACCACATTCTCCATAGGGCCCTCTTATCTCTTCCTTCTACGAGCTCA
GGCTATGGAAGAAGGAGAAGAAGGAACCGAGAAGAAGGTATCAGCAACAACTGGTTT
TATTACGGGACAGCTCATGATGTTCATATCGATCTATTATGCGCCTCTGCATCTAGCATTG
GGTAGACCTCATACAATAACTGTCCTAGCTCTACCGTATCTTTTGTTTCATTTCTTCTGG
AACAATCACAAACACTTTTTTGATTATGGATCGACTACCAGAAATTCAATGCGTAATCT
CAGCATTCAATGTGTATTCCTGAATAATCTCATTTTTCAATTATTCAACCATTTCATTTTA
CCAAGTTCAATGTTAGCCAGATTAGTCAACATTTATATGTTTCGATGCAACAACAAGAT
GTTATTTGTAACAAGTAGTTTTGTTGGTTGGTTAATTGGTCACATTTTATTCATGAAATG
GGTTGGATTGGTATTAGTCTGGATACGGCAAAATCATTCTATTAGATCGCTTATTAGATC
GAATAAGTACCTTGTGTCAGAATTGAGAAATTCTATGGCTCGGATCTTTAGTATTCTTTT
ATTTATTACCTGTGTCTACTATTTGGGCAGAATACCGTCACCCATTCTTACTAAGAAACT
GAAAGAAACCGAAGAAGATAAATCGCTTGTGATTCTTCTTTTCGACTATAACCGATGGA
ATCGACCATTGCGATATATAAAAAACCAACACTTTGAAAAGGCTGTAAGAAATGAAAT
GTCACAATATTTTTTTTATACATGCCAAAGTGATGGAAAACAAAAAATATCTTTTACGTA
TCCACCCAGTTTATCAACCTTTTGGGAAATGATACAAAAAAAGATGTCCTTGTACACAA
TAGAAAACCCCCCCTCTGACGAACTATACAATAATTGGATTTATACCAACGAAGAAAAA
AGTAAG---TTAAGCAACGAATTTATAAACCGAATCGAGGCTCTAGACGGAGGATCCCTT
TCTTTGAATCTACTTGAAAAAAAAACTAGATTGTGTAATGATGAAACCAGGCAAGAATA
TTTGCCTAAAATGTATGATCCTTTCTTGAACGGATCGTCTCGTGGAACAATCAAAAAAG
GGTTTTCACCTATAGAAAAAATTTGGATAAATAAGATTCACGGTATCCTTTATCGAGAAT
TTGAACAAAAAGATCCTAATGATCAATCTATTGAAATACAAGAAATCATTAAAAAAGTT
CCTCGGTGGTCATACAAATTAATCGATTTGGAACAACAACAGGAAAGACAGATTCGCT
CAAGAAAAGCGAAACGTGTAGTGATTTTTACTAATGCTTTAATACGTTATTCACAACAA
TCGGATTTTCGTCGAAACATAATCAAAGGTTCTATGCGCGCTCAAAGACGTAAAATAGT
TATTGAGGAACTGTTTCAAGCAAATGTGCATTCCCCCCTTTTTTTGGACAGAATAGACA
AATCCCTCCTTTTTTCTTTTGATATC------TCCAGACTAATGAAACTCATTTTGAAAAATC
GAAAGGAGAAAACGCGGATAGAAATAGCAGAAGCCTGGGATACCATTCCGCTTGCTCA

AGTAATAAGAGGTTCCATGTTAGTAACTCAATCGATTTTTAGAAAATATATTATATTACCT
TCATTGATAATAGCTAAAAATATTGGCCGTATTTTATTATTGCAATTTCCCGAGTGGTCTG
AGGATTTAAAAGAATGGGCTGGCGAAATGCATGTTAAATGCACCTATAACGGTGTTCAA
TTATCAGAAACAGAATTTCCGAAAAATTGGTTACTAGATGGTATTCAGATAAAAATCTTA
TTTCCTTTCTGTCTGAAACCTTGGCACAAACCAAAGAAAGTAAAAAAAGCCGATTTTT
GTTTTTTAACAGTTTGGGGAATGGAAACCGACCTTCCTTTTGGTTCTCCTCGAAAACGG
CCTTCTTTTTTTGAACCCATTTTTAAGGAACTCGAAAATCAAATTGGTCAATTGAAAAA
TAAGTCTTTTATAGTT---CTAAGAGTTTTAAAAGAAAGAATCGAATTCTTTCTAAGGGTG
TTAAACGAAACAAAAAAATGGGTTATCAAAAGCATTCTA------TTTATAAAAAGAATACT
AAAAGAACTTTCAAAAGTAAATGATTCTACAATCAACGATCAGAAAATTCATGAATCGT
TTAGTCAAATTCGATCTACGAATTGGACAAATTATTCACTGACAGAAAAAAAAATGAA
AGATCTAACTGATAGAACAAGCACAATAAGAAATCAAATAGAAAGAATTACAAAAGAG
AAAAAAACAAGTCCTAACAAAACAAGTTATAATGTTAAAAGATTAGAATCACCAAAAA
CTATTTGGCAAATGTTAAAAAGAAGAAACGTTCGATTACTCCGTAAATTACATTATTTTA
TAAAATTTTTCATTGAAAAGATATCCATGGATATCTTTCTATATATCATTAATATTCCCAGA
ATCAATACACAACTTTTT---CACTTCATTTCCTGTGACTTACCCTCCTTGTCACAAGCATA
TGTATTTTACAAATTATCCCAAACCCAAGTTATTAACTTGTATAAATTAAGATCTGTTCTT
CAATATACATCTTTTTTTCTTAAGAATACAATAAAGGATTATTTTACACAAGGTATATTTC
ATTTCGAATTAAGCCATAAGAAACTTCGGAATTCTGGAATGAATCAATGGAAAAATTGG
TTAAAAGGTCATTATCAATACGATTTCTCTCGGATTAAATGGTCTAGATTAATACCACAA
AAATGGCGAAATAGAGTCAATCAACGTCGTACAGCTCAAACTAAAAACAAACGGGATT
CATATGAAAAAGACCAA------CTAATTCATTACAAAAAACAA---AATTTTCAAAAAAACT
ATAGATATGATCTTTTATCATATAAATCTATTAATGGTGATATTGGGTATCCGGATAGAAAA
TATTTTGATTGGAAAATTATAAATTTTTGTCTTAGATTTTTAGATTGGATGGGAATGAATG
AAGAAATACTAAATCGTTGGTTCTTCCCAGAATTTTTGTTACTTTATAATATATATAAAAT
AAAACCATGGGTTATACCAAGCAAATTGCTTCTTTTAAACTACAAGAATAACACGGAA
GCAGAACTCCATTTCTTCTTGAACAAGTATTTGCTTTTTCAATTGAGATGGAATGAGAC
TTTGAATCAAAGAATGATCAATAATATCAAAGTATATTGTCTCCTGCTTAGACTGAGAAA
TCCAAAAGAAATTGCTATATTCTCGATTCAAAGAAGAGAAATAAGTCTGGATATAATGC
TGATTCAAAAGAATTTAACTCTTACAGAATTGATGAAA---GGAATATTGATTATCGAACC
CGTTCGTCTGTTTTTAAAAAACGACGGACAATTTATTATGTATCAAACCCTCGGTATTTC
ATTGGTTCATAAGAGTAAGCACAATCATTATGATTTGCTTATTCCTGAAAATATTTTATCA
TCTAGACGTTGTAGAGAGTTGAGAATTCTAATTTGTTTCAGATTCCTTTGGCCCAATTAT
CGATTAGAAGATTTAGCTTGTATGAATCGTTATTGGTTTGATACCAATAATGGAAGTCGT
TTCAGTATGTTAAGGATACATATGTATCCAATGAAAGGACATCAATTCAAATCCTGGATT
TTCGAATTGAGAGAGATATTGAGAGAGATCAAGAATTCTCACTATTTCTTAGATTCATG
GACCCAATTCAATTCAGTGGGATCTTTCATTCACATTTTTTTTCACCAAGAACGTTTTAT
AAAACTCTTTGACCCCCGAATTTGGAGTATCCTACTTTCA---CGCAATTCA------CAGGG
TTCAACAAGCAATCGATATTTCACG---------ATCAAGGGT---GTAATACTCTTTGTAGTAGC
GGTCCTTCTATATCGT------------ATTAACAATCAAAATATGGTCGAAAGAAAAAATCTCTA
TTTGATAGGGCTTCTTCCT---------------ATACCTATGAATTCCATTGGACCCAGAAATGATA
CGTTGGAAGAATCCTTT---TGGTCTTCCAATATCAACAGGTTGATTGTTTCG---CTCCTGT
ATCTTCCAAAAGGAAAAAAGATCTCTGAGAGTTGTTTCCTGGATCCGAAAGAGAGTAC
TTGGGTTCTCCCAAGTACT------------AAAAAGTGTATCATGCCTGAATCTAACTGGGGTT

CGCGGTGGTGGAGGAGCTGGATCGGAAAAAAGAGG------GATTCTAGTTGGAAGATAT
CTAAT---------GAAACCGTCGCTGGAATTGAGATCTTATTTAAAGAGAAAGATATCAAA---
TATCTGGAGTTTTGTTTTGTATATTATATGGAT------GATCCGATCCGCAAGGACCATGATT
GGGAATTGTTTGATCGTCTTTCTCTGAGGAAGAGGCGAAATATAATCAACTTG------AAT
TCGGGACCGCTATTCGAAATCTTAGTGAAACACTGGATTTGTTATCTCATGTCTGCTTTT
CGTGAAAAAATACCAATTGAAGTGGAGGGT---TTCTTCAAACAACAAGGG---GCTGGGT
CAACTATTCAATCAAATGATATTGAGCATGTTTCCCAT------CTCTTCTCGAGAAACAAGT
GGGCTATTTCTTTGCAAAATTGTGCTCAATTTCATATGTGGCAATTCCGCCAAGATCTCC
TCGTT---------AGTTGGGGGAAG---AATCCGCACGAATCGGATTTTTTGAGGAAC---GTAT
CGAGAGAGAATTGGATTTGGTTAGACAATGTGTGGTTGGTAAACAAGGGTCGG---------
TTTTTTAGC------AAGGTACGGAATGTATCGTCAAATATTCAATATGATTCC---------ACAA
GA------TCTAGTTTCGTTCAAGTAACGGATTCTAGCCAATTGAAAGGATCTTCT---GATCA
ATCCAGAGATCATTTGGATTCCATTAGT------------AATGAGGATTCGGAA------------------
------------------TATTACACATTGATCAATCAAAGAGAGATTCAACAACTAAAAGAAAGATC
GATTCTT---TGGGAT------CCTTCCTTTCTTCAAACGGAACGAACAGAGATAGAATCAGA
C------CGATTCCCGAAA------TGCTTTTCTGGATATTCC------TCAATGTCCCGGCTATTCAC
G---GAACGTGAGAAGCAGATGATTAATCATCTGCTTCCGGAAGAAATCGAAGAATTTCT
T------GGGAATCCTACAAGATCCATTCGTTCTTTTTTCTCTGACAGATGG------TCAGAAC
TTCAT---CTGGGTTCGAATCCTACTGAGAGGTCCACTAGAGATCAG------------AAATTGTT
GAAGAAACAACAAGATCTTTCT---TTTGTCCCTTCCAGGCGATCGGAA------------AATAA
AGAAATGGTTAATATATTCAAGATAATTACGTATTTACAAAAT------ACCGTTTCAATTCAT
CCTATTTCATCAGATCCGGGATGTGATATGGTTCCG---AAGGATGAACCGGAT---ATGGA
CAGTTCCAATAAGATTTCATTCTTGAACAAAAATCCATTTTTTGATTTA------TTTCATCTA
TTCCATGACCGGAACAGGGGAGGATACACGTTA------CACCACGATTTTGAATCAGAA---
---GAGAGATTTCAAGAAATGGCAGATCTATTCACTCTATCAATAACCGAGCCG---GATCT
GGTGTATCATAAGGGATTTGCCTTTTCTATTGATTCCTACGGATTGGATCAAAAACAA----
--TTCTTGAATGAGGTATTCAACTCCAGGGATGAATCGAAAAAGAAATCT---------TTATTG
GTTCTACCTCCTATTTTTTAT------GAAGAGAATGAATCTTTTTATCGAAGGATCAGAAAA
AAATGGGTCCGG---ATCTCCTGCAGGAATGATTTGGAAGATCCAAAACCAAAAATAGTG
GTATTTGCTAGCAACAACATAATGGAGGCAGTCAAGCAATATAGATTGATCCGAAATCT
GATTCAAATCCAATATAGCATCTATGGG------TACATAAGAAATGTATTGAATCGATTCTTT
TTAATGAAT---------AGATCCGATCGCAACTTCGAATATGGA---------ATTCAAAGGATCA
AATAAGAAAGGATACTCTGAATCATAGACCTATAATGAAATATACGATCAACCAACATTT
ATCAAATTTGAAAAAG---AGTCAGAAGAAATGGTTCGATCCTCTT------ATTTTTATTTCT
CGAACCGAGAGATCCATGAATCGGGATCCTAATGCATATAGATACAAATGGTCCAATGG
GAGCAAGAATTTCCAGGAACATTTGGAACATTTCGTTTCGGAGCAGAAGAGCCGT------
---TTTCAAGTAGTGTTCGATCGATTACGTATTAATCAATATTCGATTGATTGGTCTGAGGT
TATCGACAAAAAAGATTTGTCTAAGCCACTTCGTTTCTTTTTGTCCAAG------------------
------TCACTTCTTTTTTTGTCC---------------------AAGTTGCTTTTCTTTTTGTCTAAC---TCA
CTTCCTTTTTTCTTTGTGAGTTTCGGGAATATCCCCATTCATAGGTCCGAG---ATCTACGT
CTAT---GAATTGAAAGGTCCGAATGATCAACTCTGCAATCAGTTGTTAGAATCAATAGGT
CTTCAAATCGTTCATTTGAAAAAATGGAAACCCTTC------TTATTGGAT---------GATCATG
ATACTTCC---CAAAAATCGAAATTCTTGATCAATGGAGGAAGAATATCACCATTTTTGTT
CAATAAGATACCA---AAGTGG---ATGATTGACTCATTCCATACTAGA------AATAATCGCA

GGAAATCCTTTGATAACATGGATTCCTATTTCTCAATGATATCCCACGATCAAGACAATT
GGCTGAATCCCGTGAAACCATTTCATAGAAGTTTATTGATATCTTCTTTTTATAAAGCAA
ATCGACTTCGATTCTTGAATAATCCACATCACTTCTGCTTCTAT------TGTAACAAAAGAT
TCCCTTTTTATGTG------------GAAAAGGCCCGTATCAAT------AATTATGATTTTACGTATG
GA---CAATTCCTCAATATCTTGTTCATTCGCAACAAA---ATATTTTCGTTGTGCGTCGGTA
AA------AAAAAACATGCTTTTGGGGGG---AGAGATACTATTTCACCAATCGAGTCACCGG
TTTCTAACATATTCATACCTAATGATTTTCCACAAAGTGGT---GACGAAACGTATAACTTG
TACAAATCT---------TTCCATTTGCCAAGTCGATCCGATCCATTCGTTCGT------AGAGCTAT
TTACTCGATCGCAGACATTTCTGGAACACCTCTAACAGAGGGACAAATAGTCAATTTTG
AAAGAACTTATTGTCAACCTCTTTCAGATATGAATCTATCTGATTCAGAAGGGAAGAAC
TTGCATCAGTATCTCAAT------TTCAATTCAAACATGGGTTTGATTCACACTCCATGTTCT
GAGAAATATTTACCATCCGGAAAG------AGGAAAAAA------------------------CGGAGTCTTT
GGCTAAAG------AAATGCGTTGAGAAAGGACAGATGTATAGAACCTTTCAACGAGATAG
TGCTTTTTCAACTCTCTCAAAATGGAATTTATTCCAAACATATATGCCATGGTTCCTTACT
TCGACAGGGTACAAATATCTAAATTTTATATTTTTAGATACTTTTTCGGACCTATTG------C
CGATACTAAGTAGC---AGTCAAAAATTTGTATCCATTTTTCATGATATTATGCATGGATCA
GATATATCATGGCGAATTCTTCAGAAAAAATTG------------------TGTCTTCCACAATGGAA
TTGG---ATAAGTGAGATTTCGAGTAAGTGTTTACATAAT------CTTCTTCTGTCCGAAGAA
ATGATTCATCGAAAT------AATGAGTCACCATTGATATCGACACATCTGAGATCGCCAAAT
GTTCGGGAGTTC---CTCTATTCAATCCTTTTCCTTCTTCTTGTTGCTGGATAT---CTCGTTC
GTACACATCTTCTCTTTGTTTCCCGG------GCCTCTAGTGAGTTACAGACAGAGTTCGAA
AAGGTCAAATCTTTGATGATTCCATCATCTATGATTGAGTTGCGAAAACTTCTGGATAG
GTATCCTACATCTGAACCGAATTCTTTCTGGTTAAAGAATCTCTTTCTAGTTGCTCTGGA
ACAATTAGGAGATTCTCTAGAAGAAATACGGGGTTCTGCTTCTGGCGGCAACATGCTAT
TGGGTGGTGGTCCCGCT---------------TATGGG---GTCAAATCAATACGTTCTAAGAAGAAA
TATTTGAATATCAATCTCATCGATCTCATAAGTATCATA------------CCAAATCCCATCAATC
GAATCACTTTTTCGAGAAATACGAGACATCTAAGTCATACAAGTAAAGAGATCTATTCA
TTGATAAGAAAAAGAAAAAACGTGAACGGGGATTGGATTGATGATAAAATAGAATCCT
GGGTCGCGAACAGTGATTCGATTGATGATGAAGAAAGAGAATTCTTGGTTCAGTTCTC
C------ACCTTAACGACA------GAAAAAAGGATTGATCAAATTCTATTGAGTCTGACT---CAT
AGTGATCATTTATCA---------ATATCAAAGAATGACTCTGGTTATCAAATGATTGAACAAC
CGGGAGCAATTTACTTACGATACTTAGTTGACATTCAT------AAAAAGTATCTAATGAATT
ATGAGTTCAATACATCCTGTTTAGCAGAAAGACGGATATTCCTTGCTCATTATCAGACAA
TCACTTAT------TCACAAACCTCGTGTGGGGCTAATAGTTTTCATTTCCCGTCTCATGGA--
-------AAACCCTTTTCGCTCCGCTTAGCCTTATCCCCCTCT---AGGGGTATTTTAGTGATAG
GTTCTATAGGAACTGGACGATCCTATTTGGTCAAATCCCTAGCGACAAACTCCTATGTTC
CTTTCATTACGGTATTTCTGAACAAGTTCCTGGATAACAAGCCTAAAGGTTTTCTTATTG
AT---------------GATATCAAT---ATTGAGGATAGTGACGATATTGATGATAGTGACGATATTGA
TGCTAGTGACGATATTGAC--GATATCGATCATGACCTT
GATACGGAGCTG---GAGCTGCTAACTATGATGAATGCGCTAACTATGGATATGATGCCGG
AA---------ATAGACCGATTTTATATCATCCTTCAATTCGAATTAGCAAAAGCAATGTCTCC
TTGCATAATATGGATTCCAAACATTCATGATCTGGATGTGAATGAGTCGAATTACTTATC
CCTCGGTCTATTAGTGAACCATCTCTCCAGG---------GATTGTGAAAGATGTTCC---ACTA
GAAATATTCTTGTTATTGCTTCGACTCATATTCCCCAAAAAGTGGATCCCGCTCTAATAG

CTCCGAATAAATTAAATACGTGCATTAAGATACGAAGGCTTCTTATTCCACAACAACGA
AAGCACTTTTTCACTCTTTCATATACTAGGGGATTTCACTTGGAAAAGAAAATGTTTCAT
ACT------AATGGATTCGGGTCCATAACCATGGGTTCCAATGCACTAGATCTTGTAGCACTT
ACCAATGAGGCTCTATCGATTAGTATTACACAGAAGAAATCAATTATAGACACTAATACA
ATTAGATCCGTTCTTCATAGACAAACTTGGGATTTGCGA---------TCCCAGGTAAGATCGG
TTCAGGATCATGGGATCCTTTTCTATCAGATAGGAAGGGCTGTTGCACAAAATGTATTTC
TAAGTAATTGCCCCATAGATCCTATATCTATCTATATGAAGAAGAAA------TCATGTAACG
AAGGGGATTCTTATTTGTACAAATGGTACTTCGAACTTGGAACGAGCATGAAGAAATTA
ACGATACTTCTTTATCTTTTGAGTTGTTCTGCCGGATCGGTCGCTCAAGATCTTTGGTCT
CTACCC------GGACCCGATGAAAAA---AATGGGATCACTTCTTATGGACTCGTTGAGAAT
GATTCT---GATTTAGTTCATGGCCTATTAGAAGTCGAAGGCGCTCTGGCGGGA------TCCT
CACGGACAGAAAAATATTGCAGTCAGTTTGATAATGAT------CGAGTGACGTTGCTTCTT
CGGCCCGAATCAAGGAAT---CCCTTAGATATGATGAAAAATGGATCTTGTTCTATCCTTG
ATCAGAGATTTCTCTATGAAAAAGAC------GAATCGGAGTTTGAAGAA---GGGGAGGGA
GAAGGAGCCCTCGACCCGCAACAG------------ATAGAGGAGGAT------------TTATTCAATC
AC------ATAGTTTGGGCTCCT------AGAATATGGCGCCCT---------TGGGGCTTTCTATTTGA
TTGTATCGAAAGGCCCAATGAATTGGGATTTCCC---TATTGGTCCAGGTCATTTCGGGGC
AAG---------CGGATCATTTATGATGAAGAGGATGAGCTTCAAGAGAATGATTCGGAGTTC
TTGCAGAGTGGAACCATGCAGTACCAGACACGAGATAGATCTTCCAAAGAACAAGGC
CTTTTTCGAATAAGCCAATTCATTTGGGACCCTGCGGATCCACTCTTTTTCCTATTTAAA-
--GATCGGCCCTTTGTCTCTGTGTTTTCACATCGAGAATTATTTGCAGATGAAGAGATGT
CAAAGGGGCTTCTTACTTCCCAAACAGATCCTCCTACATCTATATATAAACGCTGGTTTA
TC------AAGAATACGCAAGAAAAGCACTTCGAATTGTTGATTAATCGCCAGAGATGGCTT
AGA---------ACCAATAGTTCATTATCT------AATGGATCTTTCCGTTCTAAT------ACTCTATC
CGAGAGTTATCAGTATTTATCAAATCTGTTCCTATCTAAC---GGAACGCTATTGGATCAAA
TGACAAAGACATTGTTGAGAAAAAGATGGCTTTTCCCGGATGAAATGAAAATTGGATT
C------ATG---------------------------ATGCCTAGATCGCGA---ATAAATGGAAATTTTATTGATAA
GACCTTTTCAATTGTAGCCAATATCTTATTACGAATAATTCCGACAACTTCAGGAGAAA
AAGAGGCATTTACTTATTACAGAGATGGG------ATGTCAGCACAATCCGAAGGAAATTAT
GCGGAAGCTTTACAGAATTATTATGAAGCTATGCGACTAGAAATTGATCCCTATGATCGA
AGTTATATACTCTATAACATAGGCCTTATCCACACAAGTAATGGAGAACATACGAAAGC
GTTGGAATATTATTTTCGAGCACTAGAACGAAATCCATTCTTACCACAAGCTTTTAATAA
TATGGCCGTGATCTGTCATTAC---CGGGGAGAACAAGCCATTCGACAGGGAGATTCTGA
AATTGCGGAGGCTTGGTTCGATCAAGCCGCTGAGTATTGGAAACAAGCTATAGCGCTT
ACTCCTGGTAATTATATTGAAGCACATAATTGGTTGAAGATCACGAGGCGTTTCGAA-----
-ATGAGTTGGCGATCAGAACGTATATGGATAGAACTTATAACGGGGTCTCGAAAAACAG
GTAATTTCTGCTGGGCCTTTATCCTTTTTTTAGGTTCATTAGGATTCTTATTGGTTGGAAT
TTCCAGTTATCTTGGTAGAAATTTGATATCTTTATTTCCGTCTCAGCAAATCATTTTTTTT
CCACAAGGGCTCGTGATGTCTTTTTATGGGATTGCGGGTCTCTTTATTAGCTCCTATTTG
TGGTCCACAATTTCGTGGAATGTAGGTAGTGGTTATGATCGATTTGATATAAAAGAAGG
AATAGTATGTATTTTTCGTTGGGGATTTCCTGGAAAAAATCGCCGCATCTTCCTCCGATT
CCTTATAAAAGATATTCAGTCCGTCAGAATAGAAGTTAAAGAGGGTATTTATGCTCGTC
GTGTCCTTTATATGGAAATCAGAGGTCAGGGGGCTATTCCCTTGACTCGTACTGATGAG
AAT---TTGACTCCACGAGAAATTGAACAAAAAGCTGCTGAATTGGCTTATTTCCTGCGT

GTACCGATTGAAGTCTTT
>Dillenia
ATGGAAAAACGGTGGTTCAATTCTATGTTGTCTATAAGTAAATCAATGCACAGCCTTGG
TCCTATTGAAAATACTAGTGGAAGTAAAGACTCGATTATAAATGATACAGATAAAAACA
TTCATAGTTACAGTAATATTGATTATTTATTCAGCCTCAGGGATATTCGGAATTTCATCTC
TGATGACATTTTTGTAATTAAGGATAGTAATGGGGGCAGTTATTCCATCTATTTCGATATT
GAAAATCAGATTTTTGAGATTGACAATGATCAT------TCTTTTCTGAATGAACGAGAAAG
TTCTTTTTATAGTTATCAGAATTCTAGTTATTTGAATAATGGATCTAAGAGGGATGATTCC
TACTATGATCGTTATATATATGATACTCAATCTAGTTGGAATAATCACATTAATAGTTGTAT
TGACAGTTATCTTCAT------TCTCAAATCCGTATTGATAGTTACATTTTAAGTGGTAGTCAC
AATTACAGTGACAGTTACATTTATAGTTACATTTGGAGT------AGTTCCAATATAAGAACT
AATACGAATGATAGTGATTTAACTATAAAAGAAAGTTCGAATGATCTCGATGTAACTCA
AAAATATAAGCATTTGTGGGTTCAATGCGAAAATTGTTATGGATTAAATTATAAGAAATT
TTGTAAGTCAAAAATGAATATTTGCGAACAATGTGGATATCATTTGAAAATGAGTAGTT
CAGATCGAATCGAACTTTCGATTGATCCAGGTACTTGGGATCCTATGGATGAGGATATG
GTTTCTCTGGATCCCATTGAATTTCATTCGGAAGAGGAACCTTATAAAGATCGTATCCAT
TCTTATCAAAAAAAGACAGGATTAAGCGAGGCTGTTCAAACAGGCACAGGTCAATTAA
ACGGTATTCCCATAGCAATTGGGGTTATGGATTTTCAGTTTATGGGGGGTAGTATGGGAT
CCGTAGTAGGCGAGAAAATCACACGTTTGATCGAGTATGCTACCAATAAATTTCTACCT
CTTATTATAGTGTGTGCTTCTGGAGGAGCACGCATGCAAGAAGGAAGTTTGAGCTTGAT
GCAAATGGCTAAAATATCTTCCGCTTTATATGATTATCAATCAAATAAAAAGTTATTTTAT
GTATCAATTCTCACATCTCCTACTACTGGTGGGGTAACAGCCAGTTTTGGTATGTTAGGG
GATATTATTATTGCCGAACCCAACGCCTACATTGCATTTGCGGGTAAAAGAGTAATTGA
GCAAACATTGAATAAGACAGTACCTGAAGGTTCACAAGCAGCTGAATATTTATTCCATA
AAGGCTTATTTGATTCAATCGTACCACGGAATCTTTTAAAAGGTGTTCTGAGTGAGTTA
TTTCAGCTCCACGCTTTCTTTCCTTTGAATCAAAATTCAATCAAGATGGTAACCATTCGA
GCCGACGAAATAAGTAATATTATCCGTGAACGTATTGAACAATATAATAGGGAAGTAAA
GATTGTAAATACCGGTACTGTACTTCAAGTAGGCGACGGCATTGCTCGTATTCACGGTC
TTGATGAAGTAATGGCAGGCGAATTAGTAGAATTTGAAGAGGGTACAATAGGCATTGCT
CTGAATTTGGAATCAAATAATGTTGGTGTTGTATTAATGGGTGACGGTTTAATGATCCAA
GAAGGAAGTTCTGTAAAAGCAACAGGAAAAATTGCTCAAATACCTGTGAGTGAGGCTT
ATTTGGGTCGTGTTATAAATGCTTTGGCTAAACCTATTGATGGTAGAGGTGAAATTTCAG
CTTCTGAATCTCGGTTAATTGAATCTCCCGCTCCAGGGATTATTTCGAGACGTTCTGTAT
ATGAGCCTCTTCAAACGGGGCTTATTGCTATTGATTCGATGATTCCTATAGGACGTGGTC
AGCGAGAATTAATTATTGGGGACAGACAGACCGGTAAAACCGCAGTAGCTACAGATAC
GATTCTCAATCAACAAGGGCAAAATGTAATATGTGTTTATGTAGCTATTGGGCAAAAGG
CATCTTCTGTGGCTCAAGTAGTGACTACTTTCCAGGAAAGGGGAGCGATGGAATACAC
TATTGTGGTAGCCGAAACGGCGGATTCCCCGGCTACATTACAATACCTCGCTCCTTATAC
AGGAGCAGCTCTGGCTGAATATTTTATGTATCGTGAACAACACACTTCAATTATTTATGA
CGATCTCTCCAAACAAGCGCAAGCTTATCGCCAAATGTCTCTGCTATTACGAAGACCAC
CTGGGCGCGAAGCTTATCCAGGAGATGTTTTTTATTTGCATTCACGTCTTTTAGAAAGA
GCCGCTAAATCAAGTTCTCTTTTAGGTGAAGGAAGTATGACTGCTTTACCAATAGTGGA
AACTCAATCAGGAGATGTTTCAGCTTATATTCCCACTAATGTAATTTCCATTACAGATGG
ACAAATATTCTTATCCGCCGATCTATTCAATGCTGGAATCAGACCTGCTATTAATGTAGGT

ATTTCCGTCTCCAGAGTAGGATCCGCAGCTCAAATTAAAGCCATGAAACAAGTAGCTG
GTAAATCAAAATTGGAGCTGGCTCAATTCGCAGAATTAGAAGCCTTTGCACAATTCGC
GTCTGATCTCGATAAAGCTACTCAGAATCAATTGGCAAGAGGTCAACGATTACGTGAAT
TGCTCAAACAATCTCAATCGGCCCCTCTTACGGTGGAAGAACAGATAATGACTATTTAT
ACCGGAACAAACGGTTATCTTGATTCCTTAGAAATTGGACAGGTAAGGAAATTTCTCGT
TGAGTTACGTACTTACTTAAAAACGAATAAACCTCAGTTCCAAGAAATAATATCTTCTA
CTAAGACATTTACCGAGGAAGCAGAAGCTCTTTTGAAAGATGCTATTCAGGAACAGAT
GAAACGCTTTCTACTTCAGGAACAAGCAATGAGAATAAATCCTACTACTTCTGGTCCTG
GGGTTTCCACACTTGAAAAAAAAAGCCTGGGGCGTATCGCTCAAATCATTGGTCCAGT
GCTGGATGTAGCCTTTCCACCGGGAAAGATGCCTAATATTTACAACGCTCTTTTAGTTA
AGGGTCGAGATACTGCCGGTCAACAAATTAATGTGACTTGTGAAGTACAACAATTATTA
GGAAATAATCGAGTTAGAGCTGTAGCTATGAGTGCTACGGATGGTCTAATGAGAGGAAT
GGAAGTGATTGACACAGAAGCTCCTCTAAGTGTTCCAGTCGGTGGAGCGACTCTCGGA
CGAATTTTCAACGTCCTTGGGGAACCTATTGATAATTTAGGTCCTGTAGATACTCGCACA
ACTTCTCCTATTCATAGATCCGCGCCTGCCTTTATACAGTTAGATACAAAATTATCTATTT
TTGAAACAGGAATTAAAGTAGTAGATCTTTTAGCCCCTTATCGTCGTGGAGGAAAAATA
GGTCTATTCGGAGGAGCTGGAGTGGGTAAAACAGTACTCATTATGGAACTGATCAACA
ACATTGCCAAAGCTCATGGGGGTGTATCTGTATTTGGCGGAGTAGGTGAACGTACTCGT
GAAGGAAATGATCTTTACATGGAAATGAAAGAATCTGGAGTGATTAATGAACAAAATAT
TGCAGAATCAAAAGTGGCTCTAGTCTACGGTCAGATGAATGAACCGCCAGGAGCTCGT
ATGAGGGTTGGTTTAACTGCCCTAACTATGGCGGAATATTTTCGAGATGTTAATGAACA
AGACGTACTTCTATTTATCGACAATATCTTCCGTTTCGTCCAAGCAGGATCCGAAGTATC
TGCCTTATTGGGGCGAATGCCTTCCGCTGTGGGTTATCAACCCACTCTTAGTACCGAAA
TGGGTTCTTTACAAGAAAGAATTACTTCTACCAAAAGAGGGTCCATAACTTCTATTCAA
GCAGTTTATGTACCTGCGGACGATTTGACCGATCCTGCTCCTGCCACGACATTTGCACA
TTTAGATGCTACTACCGTACTATCAAGAGGATTAGCTGCCAAAGGTATCTATCCCGCAGT
AGATCCTTTAGATTCAACGTCAACTATGCTCCAACCTCGGATCGTTGGTGAGGAACATT
ATGAAACTGCGCAAAAAGTTAAGCAAACTTTACAACGTTACAAAGAACTTCAGGACAT
TATAGCTATTCTTGGGCTGGACGAATTATCCGAAGAGGATCGTTTAACCGTAGCAAGAG
CACGAAAAATTGAGCGTTTCTTATCACAACCCTTTTTTGTAGCAGAAGTATTCACTGGT
TCCCCAGGGAAATATGTTGGTCTAGCAGAAACAATTAGAGGGTTTCAATTGATCCTTTC
CGGAGAATTAGATGGTCTACCTGAACAAGCCTTTTATTTAGTAGGTAACATCGACGAAG
CTACCGCGAAGGCTATGAACTTAGAAATGGAGAGTAATTTGAAGAAAATGACCTTAAA
TCTTTGTGTACTGACCCCTAATAGAATTGTTTTGGATTCAGGGGTGAAAGAAATCATTTT
ATCTACTAATAGTGGCCAAATTGGCATATTACCAAACCACGCCCCTATTGCCACAGCTGT
AGATATAGGTATTTTGAGAATACGCCTTAACGACCAATGGTTAACAATGGCTCTGATGG
GTGGTTTTGCTAGAATAAGCAATAATGAGATCACTATTTTAGTAAATGATGCGGAGAAA
GGTAGTGACATTGATCCACAAGAAGCTCAGCAAACTCTTGAAATAGCGGAAGCTAATT
TGAGGAAGGCTGAAGGCAAGAGACAAACAATTGAGGCTAATCTAGCTCTCAGACGAG
CTAGGACACGAGTGGAGGCTATTAATGTAATTTCGATGAAAAATGTAACCGATTCTTTC
GTTTCT---GGTCACTGGCCATACGCTGGGAGTTTCGGGTTTAATACCGATATTTTAGCAA
CAAATCCAATAAATCTAAGTGTAGTGATTGGTGTCTTAATCTTTTTTGGAAAGGGAGTG
TTAAGTGATTTATTAGATAATCGAAAACAGAAGATATTGAATAGTATTCGAAATTCAGAA
GAACTGCGCGGGGGGGCTATTGATCAGCTGGAAAAAGCTCGGGCCCGTTTACGGAAA

GTGGAAATGGAAGCGGATCAATTTCGAGTGAATGGATACTCTGAAATAGAACGAGAAA
AGTTGAATTTGATTAATTCAACTTATAAGACTTTAGAACAATTAGAAAATTACAAAAAT
GAAACCATTCAGTTTGAACAACAAAGAGCGCTTAATCAAGTCCGACAACGGGTTTTCC
AACAAGCCTTACAAGGAGCTCTAGGAACGTTGAATAGTTGTTTGAATAACGAGTTACA
TTTACGTACCATCAATGTTAATATTGGTATGTTTGGGGCGATAAAAGAAATAACTGATAT
GAATCCACTGATTTCTGCCGCTTCCGTTATTGCCGCTGGATTGGCTGTAGGGCTTGCTTC
TATTGGGCCTGGAGTTGGTCAAGGTACTGCCGCAGGACAAGCTGTAGAAGGTATCGCG
AGACAGCCCGAGGCGGAGGGAAAAATACGAGGTACTTTATTGCTTAGTTTAGCTTTTAT
GGAAGCTTTAACAATTTATGGATTGGTTGTAGCATTAGCGCTTTTATTTGCAAATCCTTT
TGTTATGAATGTTCTATCATGTTCCATCAACACACTAAAAGTGTTATACGATATATCCGGT
GTGGAAGTAGGCCAACATTTCTATTGGCAAATAGGGGGTTTCCAAGTCCATGCCCAAGT
ACTTATTACTTCGTGGGTTGTAATTGCTATCTTATTAGGTTCGGCCATTATAGCTGTTCGG
AATCCACAAACCATTCCGACTGATGGTCAGAATTTCTTTGAATATGTCCTTGAATTCATT
CGAGACGTGAGCAAAACCCAAATAGGAGAAGAATATGGCCCTTGGGTCCCCTTTATTG
GAACTATGTTTCTATTTATTTTTGTTTCTAATTGGTCAGGGGCTCTTTTACCTTGGAAAAT
CATACAGTTACCTCATGGGGAATTAGCCGCACCCACGAATGATATAAATACTACCGTTGC
TTTAGCTTTACTCACGTCAGTGGCATATTTCTATGCGGGTCTTAGTAAAAAAGGATTAGG
TTATTTCGGTAAATACATTCAACCAACTCCAATCCTTTTACCTATTAATATCTTAGAAGAT
TTCACAAAACCTTTATCGCTCAGTTTTCGACTTTTCGGGAATATATTGGCTGATGAATTA
GTAGTTGTTGTTCTTGTTTCTTTAGTACCTTTGGTAGTTCCTATACCTGTCATGTTCCTTG
GATTATTTACAAGTGGTATTCAAGCTCTTATTTTTGCAACTTTAGCTGCCGCTTATATAGG
CGAATCCATGGAAGGTCATCATATGATATTTTTGACTTTAGAACATATATTAACTCATATC
TCTTTTTCAATCATTTCAATTCTAATTACAATTCATTTTATAACCCTATTATCCGATGAAAC
GGTAGGACTATCTAATTCGAAAAAAGAGGGCATGATAGTGACTTTTTCATGTATAACAG
GATTATTAATTACTCGTTGGATTTATTCACAGCATTTCCCATTAAGTGATTTATATGAATC
AGTAATCTTCCTTTCATGGGCTTTCTCTATTATTCATATGATTCCGTATTTT---ATGCATAA
AAACTATTTAAGCGCAATAACCGCGCCAAGTGCTATTTTTACCCAAGGCTTTGCTACTT
CAGGTTTTTTAACTGAAATACATCAATCAAAAATATTAGTACCCGCTCTACAATCCCAGT
GGTTAATGATGCACGTAAGTATGATGGTATTGGGCTATGCAGCTCTTTTATGTGGATCATT
ATTATCAGTAGCTCTTTTGGTTATTACATTTCGAAAAGACATA---------AGTAAAAGCAAT
CAAATCCAACGCATGAATGAAAGATACAATATTTTACAAAATCCTTCTCTTATTTCTGCG
AGGAATTATTACAGGTCCCAATTGATTCAACAGTTGGATTATTGGAGTTATCGTGTTATT
AGTCTAGGGTTTCTCTTTTTAACCATAGGCATTCTTTCGGGGGCAGTATGGGCTAATGA
GGCATGGGGGTCATATTGGAATTGGGACCCAAAAGAAATTTGGGCATTTATTACTTGGA
CCATATTCGCAATTTATTTACATACTCGAACAAACACAAATTTGAAAGGTGCAAATTCC
GCAATTGTGTCCTTTATGGGCTTTCTTATAATTTGGATATGCTATTTTGGTATCAATCTATT
AGGAATAGGTCTACACAGTTATGGGTCATTTACCTTAATAATGGAACAAAAGAAGCTAT
TCACTCCCTTTTTATATCTTTTATCTATAGTATTCTTGCCCTGGGGGATCTCTCTCTCATTT
AATAAAAGTATGGAATCTTGGGTTATTAATTGGTGGAATACTAGCAAATCTGAAACTTTT
TTGAATGATATTCAAGAAAAGAGTATTCTAGAAAAATTCATAAAGTTAGAGGAACTTTT
TCTCTTGGACGAAATGATAAAGGAATACCCGGAAACACATTTACAAAAGCTTTGTATAG
AAATCTACAAAGAAACGATCCAATTGATCAAGGTATACAATGAAGATCGTATCCATGCA
ATTTTACACTTCTCGACAAATATCATTTCTTTTATTATTCTAAGTGGTTATTCTATTCTGGG
TAATGAAGAACTTGTTATTCTTAACTCTTGGGTTCAGGAATTCCTATATAACTTAAGCGA

CACAATCAAAGTCTTTTCTATTCTTTTATTTACCGATTTATGTATCGGATTTCATTCCCCC
CATGGTTGGGAGCTAATGATTGGTTCTGTTTACAAAGATTTTGGATTTGTTCATAACGAT
CAAATTATATCTGGTCTTGTTTCTACGTTCCCGGTCATTTTAGATACAATTTTCAAATATT
GGATCTTCCGTTATTTAAATCGTGTATCTCCGTCACTTGTAGTGATTTATCATTCAATGAA
TGACATGCCTATTGGTGTTCCAAAAGTACCTTTTCGAAGTCCTGGGGAAGAAGATGCTT
CTTGGGTCGACGTATACAACCGACTTTATCGAGAAAGATTACTTTTTTTAGGCCAAGAG
GTTGATAACGAGATTTCGAATCAACTTATTGGTCTTATGGTATATCTCAGTATAGAGGAT
GATACTAAAGATCTTTATTTGTTTATAAACTCTCCTGGTGGATGGGTAATACCTGGAGTA
GCTATTTATGATACTATACAATTTGTACAACCAGATGTACATACAATATGCATGGGATTAG
CCGCCTCAATGGGATCTTTTATCTTGGTAGGAGGAGAAATTACCAAACGTCTAGCATTC
CCTCACGCCAGGGTAATGATCCATCAACCTGCTAGTTCTTTTTATGAGGCACAAACAGG
AGAGTTTATCCTGGAAGCGGAAGAACTACTGAAACTGCGCGAAACCATCACAAGAGT
TTATGTACAAAGAACGGGCAAACCTTTATGGCTTGTATCCGAAGACATGGAAAGAGAT
GTTTTTATGTCAGCAAGAGAAGCCCAAGCTTATGGAATTATTGATCTTGTAGCGGTTGA
AATGAAAGAAACAAAATGGATTCATGAAGGTTTAATTACCGAATCACTTCCCAATGGTA
TGTTCCGGGTTCGTTTAGATAACGAAGATATGATTCTAGGTTATGTTTCCGGAAGGATCC
GACGTAGTTTTATACGGATATTACCAGGGGATAGAGTAAAAATTGAAATAAGTCGTTAT
GATTCAACCAGAGGGCGTATAATTTATAGACTCCGCAACAAAGATTCGAACGATATGGA
GGAATTCCAAGTATATTTAGAACGAGATATATCTCGGCAACATGACTTTCTATATCCACTT
ATCTTTCAAGAGTATATTTATGCACTTGCTCATGATCATGGA---GTGGGTTATGACAATAA
ATCCAGTTTTCTAATTGTGAAACGTTTAATTACTCGAATGTATCAATGGAATCATTTGAAT
ATTTCCCTTAATGATTCTAACAAAAAGAAATTTGTTGTGAACAATTTAGATTTTCAAATG
ATATTAGAAGGATTTTCAGTCATTGCGGAAATTCCATTTTCGCTCCAATTATTATCTTTCC
TAATAGAAAAATTCCATAATTTACGATCAATTCATTCGATATTTCCTTTTTTAGAGGACAA
ATTCTCACATTTAAATTATGTGTCAGATATACTAATACCCCACCCCGTTCATCTGGAAATC
TTGATTCAAACTCTGCGCTACTGGGTTAAAGATGCCTCTTCCTTGCATTTTTTACGATTT
TTTCTTCATGAGTATCGTAATTGGACTAGTTTTATTACTAAATCCATTTTCTTTTTTTCAA
AACCACGATTTTTTTTCTTCCTATATAATTCTCATGTATATGAATACGAATCCATTTTTGGT
TTTCTCTGTAACCAATCTTCCCATTTACAATCAACATCTTATGGAGCCCTTCTTGGTCGA
ATTTTTTTCTATGAAAAAAGAAAGCATCTTGTAGAAACTCTTGCTAATGATTTTAACTTA
TGGTTGTGCAAAGATCCTTTCATGCATTATGTTAGGCATCAAGAAAAAATTATTCTGGCT
TCAAAGGGGACGGCTCTTCTGATGAATAAATGGAAATATTACTTTGTCAATCTTTGGCA
ATGTAATTTTGATGTGTGGGCTCAACCAGGAAGGATCCATATAAAACAATTATCCAATAA
TTGCTTACATTTTCTAGGCTATCTTTCAAGTGTGCGGCTAAATCCTTCAATGGTATGGAG
TCAAATACTAGAAAATGCATTTCTAATAGATAATACTATT------AAAAAGCTCGATGCCCT
AGTTCCAATTTTTCCGCTGATTGAATCATTGGCTAAGGCCGGGTTTTGTAACGGATTAG
GCCATCCCATTAGTAAGCCGGTATGGGCCGATTCATCAGATTCTGATATTATCGACCGAT
TCGGGCGTATATGTAGAAATCTTTCTCATTATCATAGTGGGTCCTCAAAAAAAAAGAGT
TTGTATCAAATAAAGTATATACTTCGAATTTCTTGTGCTAGGACTTTGGCTCGTAAACAC
AAAAGTACTATACGTGCTTTTTTGAAAAGATTAGGTTCGGAATTATTGGAAGAATTCCTT
ACAGAGGATGAACAAATTATTTCTTTGATCTTACCAGGACAGATTTGGTATTTGGATATT
ATCTATATCGCTGATTTGGCCGATCATTCAATGATAATTAATACAACA---GAAGTACAA---
AATATCCATTTTTTTTCCAGATTGGAATTC---TTAAAAGAGATCTATGAGATCATATGGAT
GCTTGTCCTTCTTTTAACTCTTGTATTGGGACTCACAATAAGTGTACTAGTAATTGTGTG

GTTAGAAAGAGAGATATCCGCAGGGATACAACAACGTATTGGGCCCGAGTATGCCGGG
CCTTTGGGAATTCTTCAAGCTCTAGCAGATGGGATAAAACTACTTTTCAAAGAGAGCCT
TCTTCCATCTAGAGGGGATACTCGTTTATTCAGCATTGGACCATCTATATCAGTCATATCA
ATTCTACTAAGTTATTCAGTAATTCCTTTTGGCTATGGCCTTGTTCTAGCCGATCTTAGTA
TTGGTGTTTTTTTATGGATTTCCTTTTCAAGTATTGCTCCTATTGGGCTTCTTATGTCAGG
ATACGGATCAAATAATAAATATTCCTTTTTAGGTGGTCTACGCGCTGCTGCTCAATCGAT
TAGTTATGAAATACCATTAGCCCTATGTGTGTTATCAATATCTCTACTATCTAACAGTTCA
AGTACAGTTGATATAGTTGAGGCACAGGCAAAATATGGTTTGTGGGGGTGGAATTTTTG
GCGTCAACCTATAGGAGTTCTCGTTTTTCTAATTTCTTCCCTAGCAGAATGTGAGAGATT
GCCTTTTGATTTACCAGAAGCAGAAGAAGAATTAGTAGCAGGTTATCAAACTGAATATT
CAGGTATAAAATTTGCATTATTTTATGTTGCTTCTTATCTAAATCTATTAGTTTCCTCATTA
TTTGTAACAGTTCTTTACTTTGGGGGTTGGGATCTCTCTATTCCGTACATATTCGTTCCTG
AACTTTTTGAAATAAATAAAACGGGCGGGGTCTTTCAAACCACAATTGGTATCTTTATT
ACATTAATTAAAACTTATTTGTTTTTGTTCATTTCTATCACAACAAGATGGACTTTACCG
AGGCTAAGAATAGACCAACTCTTAAATCTTGGATGGAAATTTCTTTTACCTATTTCTCTA
GGTAATTTATTATTAACAACTTCTTTCCAACTCTTTTCACTGATGATCTGGCATGTACAG
AATGAAAACTTCATTCTCGATTCTACGAGAATTTTTATGAAAGCCTTTCATTTGCTTCTC
TTCGATGGAAGTTTGATTTTCCCAGAATGTATCCTAATTTTTGGCCTAATTCTTCTTCTGA
TGATCGATTTAACCTCTGATCAAAAAGATATACCTTGGTTATATTTCATCTCTTCAACAA
GTTTAGTAATGAGTATAACGGCCCTATTGTTCCGATGGAGAGAAGAGCCTATGATTAGC
TTTTCGGGAAATTTCCAAACAAACAATTTCAACGAAATCTTTCAATTTCTTATTTTACTA
TGTTCAACTCTATGTATTCCTCTATCTGTAGAGTACATTGAATGTACAGAAATGGCTATA
ACAGAGTTTCTGTTATTCGTATTAACAGCTACTCTAGGAGGAATGTTTTTATGCGGTGCT
AACGATTTAATAACTATCTTTGTAGCTCCAGAATGCTTCAGTTTATGCTCCTACCTATTAT
CTGGATATACCAAGAAAGATGTACGGTCTAATGAGGCTACTACGAAATATTTACTCATG
GGTGGGGCAAGCTCTTCTATTCTGGTTCATGGTTTCTCTTGGCTATATGGTTCATCCGGG
GGAGAGATCGAGCTTCAAGAAATAGTGAATGGTCTTATCAATACACAAATGTATAACTC
CCCAGGAATTTCAATTGCGCTTATATTCATCACTGTAGGAATTGGGTTCAAGCTTTCCCC
AGCCCCTTCTCATCAATGGACTCCTGACGTATACGAAGGATCTCCCACTCCAGTCGTTG
CTTTTCTTTCTGTTACTTCGAAAGTAGCTGCTTCAGCTTCAGCCACTCGAATTTTGGATA
TTCCTTTTTATTTCTCATCAAACGAATGGCATCTTCTTCTGGAAATCCTAGCTATTCTTAG
CATGATATTGGGGAATCTCATTGCTATTACTCAAACAAGCATGAAACGTATGCTTGCATA
TTCGTCTATAGGTCAAATCGGATATGTAATTATTGGAATAATTGTTGGAGACTCAAATGA
TGGATATGCAAGCATGATAACTTATATGCTGTTCTATATCTCCATGAATCTAGGAACTTTT
GCTTGCATTGTATTATTTGGTCTACGTACCGGGACTGATAACATTCGAGATTACGCAGGA
TTATACACGAAAGATCCTTTTTTGGCTCTCTCTTTAGCCCTATGTCTCTTATCCCTAGGA
GGTCTTCCTCCACTAGCAGGTTTTTTCGGAAAACTCCATTTATTCTGGTGTGGATGGCA
GGCAGGCCTATATTTCTTGGTTTCAATAGGACTCCTTACGAGCGTTCTTTCTATCTACTAT
TATCTAAAAATAATCAAGTTATTAATGACTGGACGAAACCAAGAAATAACCCCTCACGT
GCGAAATTATAGAAGATCGCCTTTAAGATCAAACAATTCCATCGAATTGAGTATGATTGT
ATGTGTGATAGCATCTACTATACCAGGAATATCAATGAACCCGATTATTGCAATTGCTCA
GGATACCCTTTTTATGTTTCTGCTTTACGAATATGATATTTTCTGGACATTTCTAATAATAT
CAAGTTTTATTCCTATTTTAGCATTTATCATTTCCGGAGTTTTAGCTCCGATTAGAAAAG
GACCAGAAAAACTTTCTAGTTATGAATCGGGTATAGAACCAATGGGCGATGCTTGGTTA

CAATTTCGAATACGTTATTATATGTTTGCTTTAGTTTTTGTTGTTTTTGATGTTGAAACTG
TTTTTCTTTATCCATGGGCAATGAGTTTTGATCTATTGGGTGTATCTGTATTTATAGAAGC
TTTAATTTTCGTGCTTATCCTAATTGTTGGTTCAGTTTATGCATGGCGAAAAGGGGCATT
GGAATGGTCTACGAATTTTTTTCCTTGGTTAACAATAATTGTAGTTTTGCCGATATTCGC
GGGTTCTTTAATTTTCTTTCTCCCTCATAGAGGAAATAAGATCTTTAGGTGGTATACTCTA
TGTATATGTTTTTTAGAACTTCTTCTAACGACCTATGTGTTTTGTTATCATTTCCAATTGA
ATGACCCATTAATTCAATTGGCGGAGGATTATAACTGGATCAATTTTATTAATTTTCATTG
GAGATTAGGAATAGATGGACTTTCTATAGGACCTATTTTACTAACGGGATTTATCACTAC
TTTAGCTACTTTAGCGGCCTGGCCAGTTACTCGGGATTCACGATTATTTCATTTCCTTAT
GTTAGCAATGTACAGTGGTCAAATAGGATCATTTTCTTCTCGCGATCTTTTACTTTTTTT
CATCATGTGGGAATTCGAATTAATTCCTGTTTATTTACTTCTATCCATGTGGGGAGGAAA
GAAACGTCTATATTCAGCTACAAAGTTTATTTTGTACACTGCAGGAGGTTCTGTTTTTCT
CTTAATGGGGGTTCTTGGTATTAGTTTATATGGTTTTAATGAACCAATATTTTATTTTGAA
ACATCAGCTAATCAATCGTATCCTCTGGCATTGGAAATACTATTCTATATTGGATTTCTTA
TCGCTTTTGCTGTCAAATTACCCATTATACCCTTACATACATGGTTACCAGATACTCATGG
CGAAGCACATTACAGTACTTGTATGCTTCTAGCTGGAATCTTATTAAAAATGGGAGCATA
TGGATTGGTGCGGATAAATATGGAATTATTATCTCACGCCCATTATATCTTTTCTCCCTGG
TTGGTGATAGTAGGTACAATACAAATAATCTATGCAGCTTCAACATCTCCTGGTCAGCG
GAATTTAAAAAAAAGAATAGCCTATTCTTCCGTATCTCATATGGGTTTCATAATTATCGG
AATCGGTTCAATAACTGATACGGGACTCAATGGAGCCCTTTTACAAATAATATCTCATGG
TTTTATTGGTGCTGCGCTTTTTTTCTTGGCAGGAACAAGTTATGATAGACTACGTCTTGT
TTATCTTGACGAAATGGGTGGAATAGCTATTTCAATGCCAAAAATTTTCACGATGTTCA
GTAGCTTCTCGATGGCCTCCCTTGCATTACCGAGTATGAGTGGTTTTGTTGCAGAATTAA
TAGTCTTTTTTGGAATAATTACCAGCCAAAAATTTTTTTAATGCCAAAAATTTTAATTA
CTTTTATAATGGCAATTGGAATGATATTAACTCCCATTTATTCATTATCTATGTTACGCCAA
ATGTTCTATGGGTACAAACTATTTAATGTCCCCAATTCTTATTTTTTTGATTCTGGACCGC
GAGAGTTATTTGTTTTGATCTCTATCTTTCTCCCTGTAATAGGTATTGGTATTTACCCAGA
TTTTGTTCTTTCACTATCAGTTGACAAGGTTGAGTATATTTTATCTAATTATTTTTATAGAA
TGATACTCGAACATGTGCTTGTTTTGAGTTCCTATTTGTTTTCTATCGGTATCTATGGATT
GATTACGAGCCGAAATATGGTTAGAGCCCTTATATGTCTTGAACTTATACTAAATGCAGT
TAATATAAATTTCATAACATTTTCCGATTTTTTTGATAGTCGCCAGTTGAAAGGAGACAT
TTTTTCAATTTTTGTTATAGCCATTGCAGCCGCTGAAGCAGCTATTGGACCTGCTATTGT
TTCGTCAATTTATCGTAACAGAAAATCAACTCGTATCAATCAATCAAATTTGTTGAATAA
GATGGAACATACATATCGATATGCGTGGATTGTCCCTTTCATTCCACTTACAGTTCCCAT
GTTAATAGGGCTGGGACTTCTCTTTTTTCCAACGGGAACAAAAAATCTTCGTCGTATAT
GGGCTTTTCCAAGTGTTTTATTGTTAAGTATAGCTATGATTTTTTCGACTGATTTATCTAT
TCAACAAATAAATGATAGTTATAGCTATCAATATGTATGGTCTTGGACCATCAATAATGAT
TTTTCTTTAGAGTTTGGGCACTTGATTGACCCACTTACTTCTATTATGTCAATATTAATCA
CTACCGTTGGAATTACGGTTCTTATTTATAGTGACAATTATATGTCTCATGATCAAGGATA
TTTGAGATTTTTTGCTTATATGAGTTTTTCCAATTCTGCCATGTTGGGATTAGTTACTAGT
TCAAATTTAGTACAAATTTATATTTTTTGGGAATTAGTTGGAATGTGTTCGTATTTATTAAT
AGGTTTTTGGTTCACGCGACCTATTGCAGCAAATGCTTGTCAAAAAGCGTTTATAACTA
ATCGTGTAGGGGATTTTGGTTTATTATTAGGAATTTTAGGTCTTTATTGGGTAACGGGTA
GTTTTGAATTTCAGGATTTGTTCGAAATATTCAATAACCGTGAGGTCCATTATTTATTTGT

TACTTTGTGTGCCTTCTTATTATTTCTTGGCGCAGTTGCTAAATCCGCACAATTTCCCCTT
CATGTATGGTTACCTGATGCTATGGAAGGACCTACTCCTATTTCGGCTCTTATACACGCC
GCCACTATGGTAGCAGCCGGAATTTTTCTTGTAGCCCGCCTTTTCCCTCTTTTTATAGCC
TTACCTTACATAATGAATCTAATATCTTTGATAGGGATAATAACAGTACTCTTAGGAGCTA
CTTTAGCTCTTGCTCAAAAAGATATTAAGAAAACTTTAGCCTATTCTACAATGTCTCAAT
TGGGTTATACGATGTTAGCTCTAGGTATGGGGTCTTATCGAGCCGCTTTATTTCATTTGAT
TACTCATGCTTATTCGAAAGCATTATTGTTTTTAGGATCTGGATCCATTATTCATTCAATG
GAAGCTATTGTTGGGTATTCCCCAGAAAAAAGTCAGAATATGGCTCTTATGGGTGGTTT
AGCAAAACATATTCCAATTACAAAAACTGCCTTTTTATTAGGTACACTTTCCCTTTGTGG
CATTCCACCTCTTGCTTGTTTTTGGTCAAAAGATGAAATTCTTAATGATAGTTGGTTGTA
TTCAACAATTTTCGCAATCATGGCCTGCTCTACAGCGGGATTTACCGCATTTTATATGTT
TCGGATTTATTTACTTACTTTTGAAGGGTATTTTAACATTCATTTTCAAAATTATAGTGGA
AAAAAAAACAGCTTGGTCTATTCAATATCTCTATGGGGTAAAGAAGGGACAAAACCTA
TTAAAAAAAACCTATTAACTTTATTAACATTAAATTTTTTTTCATTTGAAAATAAAAACA
CGTTT---TGTCCGCATGAATCGAAAAATACTATGATATTTCCTATACTTCTATTGGTTCTAT
TTACTTTGTTTGTTGGGTTCATAGGAATCCCTTTCCATTTTGATATATTGTCGAAATTAAT
AAATCCATCTATAAACCTTTTACATCAAAATTTGAATAATTTGGAAGATTGGTATGAATTT
ATAACAAATTCAATTCGTTCAGTCAGTATAGCTTATTTAGGGATATTTATAGCGGCTTTTT
TCTATAAGCCCGTTTCGTCATCTCTCCAAAATTTGGACTTAATTAATTTATTTGCTAATTT
GTGGAACAAAATAAAAAATGTAATATATGATTGGTCATATAATCGTGGTTATATAGATGC
TTTTTATGTAGTTTCTTTAACTAAGGGCATAAAAGGATTGGCTGAACTAACTCATTTTTT
TGATAAACAGGTAATTGATGGAATTATAAATGGTGTTGGTATTCTGAATTTCTTTGTAGG
AGAAGTTATCAAATATATAGGAGGAGGC---CGCATTTCTTTTTATCTATTTTTTTATTTATC
TTCTGTATTTATTTTGTTATTAATTTATTACATGGGTTTACCTGGACTAATACACGATTTTC
TTTTAGTCTTTCTGGGATTAGGTCTTATATTAGGAGGGCTAGGGGTGGTATTACTTACCA
ATCCAATTTTTTCTGCCTTTTCGTTGGGATTGGTTCTTGTTTGTATATCCTTATTCTATATT
TCAGCGAACTCCCATTTTGTAGCTGCCGCACAGCTCCTTATTTATGTGGGAGCTATAAAT
GTTTTAATCATATTTGCTGTGATGTTCATGAATGGTTCAGAATATTACAAAGATTTTCATC
TTTGGACCATCGGAGATGGGATTAGTTCGCTGGTTTGTACAAGTATTTTTGTTTCACTAA
TTACTACTATTCCAGATACGTCATGGTACGGTATTATTTGGACTACAAGATCAAACCAGA
TTATAGAACAAGATTTGATAAGTAATGGTCAACAAATTGGGATTCATTTATCAACAGATT
TTGTTCTTCCATTTGAACTTATTTCGATAATTCTTTTAGTTGCTTTAATAGGTGCAATTGC
TGCGGCTCGTCAAATGACTGTATCAGCTACAAAAAAAGACTTCATGATAGTCAATATGG
GCCCTCACCACCCGTCAATGCACGGTGTTCTTCGACTTATTGTTACTCTAGATGGTGAA
GATGTTATTGACTGTGAACCAATATTGGGTTATTTACACAGAGGGATGGAAAAAATTGC
GGAAAACCGAACAATTATACAATATTTGCCTTATGTAACACGTTGGGATTATTTAGCTAC
TATGTTTACAGAAGCAATAACCGTAAATGCACCAGAGCGTTTAGGAAATATTCAAGTAC
CTAAAAGAGCCAGCTATATCAGAGTTATTATGTTGGAGTTGAGTCGTATAGCTTCTCATT
TGTTATGGCTTGGCCCTTTTTTGGCAGATATTGGTGCGCAGACTCCCTTTTTCTATATTTT
CAGAGAAAGAGAATTCGTATATGATTTATTTGAAGCCGCCACCGGTATGAGAATGATGC
ATAATTTTTTTCGTATTGGAGGAGTAGCTGCTGATCTACCTCATGGTTGGATAGATAAAT
GTTTAGATTTCTGCGATTATTTTTTAACGGGGGTTTCCGAATATCAAAAGCTTATTACAC
GAAATCCTATTTTTTTAGAACGGGTTGAAGGAGTAGGAATTATTAATGAGGAGGAAGCA
AAAAATTGGGGCTTATCAGGACCAATGTTACGCGCTTCCGGAATACAATGGGATCTTCG

TAAAGTTGATCATTATGAATGTTACGACGAATTTGACTGGGAAGCAAAATGGCAAAAA
GAAGGAGATTCATTAGCTCGTTATTTAGTACGAATCAGTGAAATGATGGAATCCATAAA
AATTATTCAACAGGCTCTGGAAGGAATTCCGGGGGGACCCTATGAAAATTTAGAAATCC
GACGCTTTGATAGAGTAATAAATTCCGAATGGAATGATTTTGAATATCGATTTATTAGTA
AAAAGCCATCCCCTACTTTTGAATTGTCAAAACAAGAACTTTATGTAAGAGTAGAAGC
CCCAAAGGGGGAATTGGGAATTTTTTTAATAGGAGATCAGAGTGTTTTTCCTTGGAGAT
GGAAAATTCGCCCGCCGGGTTTTATCAATTTGCAAATTCTTCCTCAGTTAGTTAAAAGA
ATGAAATTGGCTGATATTATGACGATATTAGGTAGCATAGATATCATTATGGGAGAGGTT
GATCGTATGTTTCCTATAGTAACCGGGTTCATGAGTTATGGTCAACAAACAATACGAGC
TGCAAGGTACATTGGTCAAAGTTTCATGATTACCTTATCTCACGCCAATCGTTTACCCGT
AACTATTCAATACCCTTATGAAAAATCGATCACATCGGAGCGTTTCCGCGGTCGAATCC
ACTTTGAATTTGATAAATGCATTGCTTGTGAAGTATGTGTTCGTGTATGTCCTATAGATCT
ACCTGTTGTTGATTGGAAATTTGAAACTGATATTCGAAAGAAACGGTTGTTTAATTACA
GTATTGATTTTGGAATCTGTATATTTTGTGGTAATTGCGTTGAGTATTGTCCAACAAATTG
TTTATCGATGACTGAAGAATATGAACTTTCTACTTATGATCGTCACGAATTGAATTATAAT
CAAATTGCTTTGGGTCGTTTACCAATGTCAATAATGAACGATTACACAATTCGAACAAT
TTTAAATTCG---------CAAATAAAAAACAGA------------------------ATGCAAGGCCGTTTGTC
TGCTTGGTTAGTGAAGCATGAACTAGTTCATAGATCTTTGGGTTTCGATTACCAAGGAG
TAGAGACTTTACAAATAAAGCCTGAGGATTGGCATTCCATTGCTGTCATTTTCTATGTAT
ATGGTTACAATTATCTACGTTCCCAATGTGGCTATGATGTAGCACCAGGCGGGCTATTAG
CTAGTGTGTATCATCTTACAAGAATAGAGTATGGTGTAGATCAACCGGAAGAGGTATGC
ATAAAAGTATTTACTTCAAGGGGGAATCCTAGAATTCCGTCTGTTTTCTGGGTTTGGAA
AAGTGCGGATTTTCAAGAACGGGAATCTTATGATATGTTGGGAATCTTTTATGATAATCA
TCCGCGCTTGAAGCGTATCTTAATGCCTGAAAGTTGGATAGGATGGCCCTTGCGTAAGG
ATTATATTGCCCCCAATTTTTATGAAATACAAGATGCTCAT---------------------------------------
---ATGGTCTTAGTTCCTGACTATTC
AGTCAATAAAAAGAAAAAAGGAAAAAAAAAAAATTGAGACAATTATGAATTCCATTGA
GTTTCCCTTACTTGATCGAACAACCCAAAATTCAGTTATTTCAACTACATCAAATGATCT
TTCAAATTGGTCAAGGCTCTCTAGTCTATGGCCACTTCTTTATGGTACCAGTTGTTGCTT
CATTGAATTTGCTTCATTAATAGGCTCACGATTCGACTTTGATCGTTATGGACTGGTACC
GAGATCGAGTCCTAGACAAGCAGACCTAATTTTAACAGCTGGCACAGTAACAATGAAA
ATGGCGCCTTCTTTAGTGAGATTATATGAGCAAATGGCTGAACCAAAATATGTTATTGCT
ATGGGAGCCTGTACAATTACAGGGGGGATGTTCAGTACCGATTCTTATAGTACTGTTCG
GGGAGTCGATAAGCTAATTCCTGTGGATGTCTATTTGCCGGGCTGTCCGCCTAAACCGG
AGGCAGTTATAGATGCTATAACAAAACTTCGTAAAAAAATATCTCGAGAAATATATGAA
GATAGAATTCAGTCT------GAGAATCGGTGTTTTACTACCAATCATAAGTTTCATGTTGGG
CGCAGTATTCATACTGGAAATTATAATAAAGAATTACTCTATCAATCCCCATCTATTTCAA
AGACCCCTCCTGAAACATTTGTCAAATACAAGAGTTCGGGACTTTCTCACGAATTAGT
GAATATGCAAATTATAAATACCTTTTCTTGGATAAAGGAACAGATTACTCGATTCATTTC
TGTATCGCTCATGATATATATAATCACTCGGACATCCATTTCAAATGCATATCCCATTTTC
GCACAGCAGGGTTATGAAAATCCACGAGAAGCGACTGGGCGTATTGTATGTGCTAATT
GCCATTTAGCTAATAAGCCCGTGGATATTGAAGTTCCACAAGCGGTACTTCCTGATACT
GTATTTGAAGCAGTTGTTCGAATTCCTTATGATATGCAACTGAAACAAGTTCTTGCTAAT
GGGAAAAAAGGGGCCTTGAATGTAGGGGCTGTTCTTATTTTACCTGAAGGGTTTGAATT

AGCCCCTCCTGATCGTATTTCTCCTGAGATGAAAGAAAAAATAGGCAATCTGTCTTTTC
AGAGCTATCGCCCGACTAAAAAAAATATTCTTGTAATAGGGCCTGTTCCCGGTCAGAAA
TATAGTGAAATTATCTTTCCTATTCTTTCTCCGGACCCCGCTACTAAGAAAGATGTTCAC
TTCTTAAAATATCCCATATACGTAGGTGGAAACAGGGGAAGGGGTCAGATTTATCCTGA
TGGGAGCAAGAGCAACAATACTGTTTATAATGCTACAGCAGCAGGTATAGTAAGCAAA
ATTATACGAAAAGAAAAGGGCGGATACGAAATAACCATAGCGGATGCATCGGATGGCC
ATCAAGTGGTTGATATTATCCCTCCAGGACCAGAACTTCTTGTTTCAGAGGGTGAATCT
ATCAAACTTGATCAACCATTAACGAGCAATCCTAATGTGGGTGGATTTGGTCAGGGAGA
TGCAGAAATAGTACTTCAAGATCCATTACGTATCCAAGGCCTTTTGTTCTTTTTAGCATC
TATTATTTTGGCACAAATCTTTTTGGTTCTTAAAAAGAAACAGTTTGAAAAGGTTCAAT
TATCCGAAATGAATTTTATGAGTAAAGTCTATGATTGGTTCGAGGAACGTCTTGAGATTC
AGGCGATTGCAGATGATATAACTAGTAAATATGTTCCTCCCCATGTCAACATATTTTATTG
TCTAGGGGGAATTACGCTTACTTGTTTTTTAGTACAAGTAGCTACGGGGTTTGCTATGAC
TTTTTACTATCGTCCGACCGTTACTGAGGCTTTTGCTTCTGTTCAATACATAATGACTGA
AGCTAATTTTGGTTGGTTAATTCGATCAGTTCATCGATGGTCGGCAAGTATGATGGTTCT
AATGATGATTCTGCACGTATTTCGTGTGTATCTCACCGGTGGATTTAAAAAACCCCGTG
AGTTGACTTGGGTTACGGGTGTGGTTCTGGCTGTATTGACCGCCTCTTTTGGTGTAACT
GGGTATTCTTTACCTTGGGACCAAATTGGTTATTGGGCAGTAAAAATTGTAACAGGTGT
CCCTGAAGCTATTCCTGTAATAGGATCACCTTTGGTAGAATTATTACGCGGAAGTGCTAG
TGTGGGACAATCTACTTTGACTCGTTTTTATAGTTTACATACTTTTGTATTACCTCTTCTT
ACGGCCGTATTTATGTTAATGCATTTCCCAATGATACGAAAACAAGGGATTTCTGGTCCT
TTAATGGGAGTAACAAAAAAACCTGACTTGAATGATCCTGTATTAAGAGCTAAATTAGC
TAAAGGAATGGGTCATAATTATTACGGAGAACCCGCATGGCCCAATGATCTTTTATATAT
TTTTCCAGTAGTAATTCTAGGTACTATTGCATGTAATGTGGGCTTAGCAATTCTCGAACC
ATCAATGATTGGTGAACCGGCAGATCCATTTGCAACTCCTTTGGAAATATTACCTGAAT
GGTACTTTTTTCCCGTATTTCAAATACTTCGTACAGTGCCAAATAAGTTATTGGGTGTTC
TTTTAATGGTTTCAGTCCCCGCGGGATTATTAACAGTCCCTTTTTTGGAGAATGTTAATA
AATTCCAAAATCCATTTCGGCGTCCAGTAGCGACAACCATCTTTTTGATTGGCACTGTG
GTGGCCCTTTGGTTGGGTATTGGAGCAACATTACCTATTGATAAATCCTTAACTTTAGGT
CTTTTTATGATTGAAGTTTTTCTTTTTGGAATCGTGTTAGGTCTAATTCCTATTACTTTAG
CTGGGTTATTCGTGACTGCATATTTACAATATAGACGTGGTGACCAATTGGACCTTATGC
CTACTATAACTAGTTATTTCGGTTTTCTACTAGCAGCTTTAACTATAACCTCAGCTCTATT
TATTGGTCTGAGCAAGATACGACTTATTATGGATATAGTAAGTCTTGCTTGGGCTGCTTT
AATGGTAGTCTTTACATTTTCCCTTTCACTCGTAGTATGGGGAAGAAGTGGACTCATGA
TTATTCGTTCACCGGAACCAGAAGTAAAAATTTTGGTAGATAGGGATCCCATAAAAACT
TCTTTTGAGGAATGGGCCAGACCCGGTCATTTCTCAAGAACAATAGCTAAGGGCCCTG
ATACTACTACTTGGATCTGGAACCTTCATGCTGATGCTCACGATTTCGATAGCCATACCA
GTGATTTGGAGGAGATTTCCCGAAAAGTATTTAGTGCCCATTTCGGTCAACTCTCCATC
ATCTTTCTTTGGCTGAGTGGCATGTATTTCCACGGTGCTCGTTTTTCCAATTATGAAGCA
TGGCTAAGCGATCCTACTCACATTGGACCTAGCGCTCAGGTGGTTTGGCCAATAGTGGG
CCAAGAAATATTGAATGGTGATGTGGGCGGGGGTTTCCGAGGAATACAAATAACCTCT
GGTTTTTTTCAGATTTGGCGAGCATCTGGAATAACTAGTGAATTACAACTTTATTGTACT
GCAATTGGTGCATTGATCTTTGCAGCCTTAATGCTTTTTGCTGGTTGGTTTCATTATCAC
AAAGCCGCTCCAAAATTGACTTGGTTCCAAGATGTAGAATCTATGTTGAATCACCATTT

AGCGGGGCTACTGGGACTTGGGTCTCTTTCTTGGGCAGGGCATCAAATACATGTATCTT
TACCGATTAACCAATTTTTAAACGCTGGGGTAGATCCTAAAGAGATCCCACTTCCTCAT
GAATTTATCTTGAATCGGGATCTTTTGGCTCAACTTTATCCCAGTTTTGCCGAGGGAGC
AACCCCTTTTTTCACCTTGAATTGGTCAAAATATGCAGAATTTCTTACTTTTCGTGGAGG
ATTAGACCCAGTAACTGGCGGCCTATGGCTGACCGACATTGCACACCACCATTTAGCTA
TTGCAATTCTTTTCCTGATAGCGGGTCACATGTATAGGACCAATTGGGGCATTGGTCATG
GTCTAAAAGATATTTTAGAGGCTCATAAAGGTCCATTTACGGGCCAAGGCCATAAAGGC
TTATATGAGATCCTAACAACATCATGGCATGCTCAATTATCTCTTAACCTAGCTATGTTAG
GCTCTTTAACCATTGTTGTAGCTCACCATATGTATTCCATGCCCCCTTATCCATATCTAGC
TACTGACTATGGTACACAACTGTCATTGTTTACACATCATATGTGGATTGGTGGATTTCT
TATAGTGGGTGCTGCTGCGCATGCAGCCATTTTTATGGTAAGAGATTATGATCCAACTAC
TCGGTATAACGACCTATTAGATCGTGTCCTTAGGCATCGCGATGCAATCATATCCCATCT
CAACTGGGCATGTATATTTCTAGGCTTTCACAGTTTTGGTTTGTATATTCATAATGATACC
ATGAGCGCTTTAGGGCGTCCCCAAGATATGTTTTCAGATACCGCTATACAATTACAACCT
GTTTTTGCTCAATGGATACAAAACACCCACGCTTTAGCACCTGGTGCTACGGCTCCTGG
TGCAATAACAAGTACCAGTTTGACTTGGGGGGGTGGTGATTTAGTAGCAGTGGGTGGT
AAAGTAGCTTTGTTACCTATTCCATTAGGAACCGCAGATTTTTTGGTACATCACATTCAT
GCATTTACGATTCACGTGACGGTATTGATACTCCTGAAAGGTGTTCTATTTGCTCGCAGC
TCCCGTTTGATACCGGATAAAGCAAATCTTGGTTTTCGTTTCCCTTGTGATGGACCTGG
AAGAGGGGGTACTTGTCAAGTATCCGCTTGGGATCATGTCTTCTTAGGGCTATTCTGGA
TGTACAATGCAATTTCGGTAGTAATATTCCATTTTAGCTGGAAAATGCAGTCAGATGTTT
GGGGTAGTATAAGCGATCAAGGGGTGGTAACTCATATTACGGGAGGGAATTTTGCGCA
GAGTTCTATTACTATTAATGGGTGGCTCCGCGATTTCTTATGGGCACAGGCATCCCAAGT
AATTCAGTCTTATGGTTCTTCATTATCTGCCTATGGCCTCTTTTTCCTAGGTGCTCATTTT
GTATGGGCTTTTAGTTTAATGTTTCTATTTAGCGGTCGTGGTTATTGGCAAGAACTTATT
GAATCCATTGTTTGGGCTCATAATAAATTAAAAGTTGCTCCTGCTACTCAGCCTAGAGC
CTTGAGCATTGTACAAGGGCGCGCTGTAGGAGTAACCCATTACCTTCTGGGTGGAATTG
CCACAACATGGGCATTCTTCTTAGCAAGAATTATTGCAGTAGGAATGGCATTAAGATTT
CCAAGGTTTAGCCAAGGCTTAGCTCAGGACCCCACTACTCGTCGTATTTGGTTTGGTAT
TGCTACCGCACATGACTTCGAGAGTCATGATGATATTACTGAGGAACGTCTTTATCAGA
ATATTTTTGCTTCTCACTTCGGGCAATTAGCAATAATTTTTCTGTGGACGTCCGGAAATC
TGTTTCATGTAGCTTGGCAAGGAAATTTCGAGTCATGGGTACAGGATCCTTTACATGTA
AGACCTATTGCTCATGCAATTTGGGATCCTCATTTTGGTCAACCGGCTGTGGAAGCTTT
TACTCGGGGGGGTGCCCTTGGTCCAGTGAATATAGCTTATTCTGGTGTTTATCAGTGGT
GGTATACAATCGGTTTACGCACTAATGAAGATCTTTATACTGGAGCTCTTTTTTTGTTATT
TCTTTCTGCCATATCTTTAACAGCGGGTTGGTTACACCTACAACCAAAATGGAAACCGA
GCGTTTCGTGGTTCAAAAATGCCGAATCTCGTCTCAATCATCATTTGTCAGGACTATTC
GGGGTAAGTTCCTTGGCTTGGACGGGACATTTAGTCCATGTCGCTATTCCTGGATCCAG
GGGGGAGTACATTCGATGGAATAATTTCTTAGATGTATTACCGCATCCCCAAGGGTTAG
GTCCACTTTTTACAGGTCAGTGGAATCTTTATGCTCAAAACCCCGATTCAAGTAGTCAT
TTATTTGGTACCTCTCAAGGAGCGGGAACTGCCATTCTAACCCTTCTCGGAGGATTCCA
TCCACAAACGCAAAGTTTATGGCTGACTGATATTGCTCATCATCATTTAGCTATTGCATT
TATTTTTCTCGTTGCTGGTCATATGTATAGAACTAATTTCGGGATTGGGCACAGTATGAA
AGATCTTTTAGAAGCGCATATTCCTCCGGGGGGACGCTTGGGACGTGGGCATAAGGGT

CTTTATGACACAATTAATAATTCGATTCATTTTCAATTAGGCCTTGCTCTAGCCTCTTTAG
GGGTTATTACTTCCTTGGTAGCCCAACATATGTACTCTTTACCCGCTTATGCGTTCATAGC
ACAAGACTTTACTACCCAAGCTGCATTATATACTCATCACCAATATATTGCAGGATTCATT
ATGACAGGGGCTTTTGCTCATGGAGCTATATTTTTCATTAGAGATTACAATCCGGAACA
GAATGAGGATAATGTATTGGCAAGAATGTTAGACCATAAAGAAGCGATAATATCCCATTT
AAGTTGGGCCAGCCTCTTTCTGGGGTTTCATACCCTGGGACTTTATGTTCATAATGATGT
CATGCTTGCTTTTGGTACTCCGGAGAAACAAATATTGATCGAACCTATATTTGCCCAATG
GATACAATCTGCTCATGGTAAAACTTCATATGGGTTCGATGTACTTTTATCTTCAACAAC
TGGCCCAGCATTCAATGCGGGTCGAAGTATATGGTTGCCTGGCTGGTTAAATGCAGTTA
ATGAG------AATAGTAATTCATTATTCTTAACAATAGGACCCGGAGACTTCTTGGTTCATC
ATGCTATTGCTCTAGGTTTACATACAACTACATTGATCTTAGTCAAAGGTTCCTTAGATG
CGCGTGGTTCCAAGTTAATGCCAGATAAAAAGGATTTCGGTTATAGTTTTCCTTGCGAT
GGTCCGGGACGGGGTGGTACTTGTGATATTTCGGCTTGGGACGCATTTTATTTGGCAGT
TTTCTGGATGTTAAATACCATTGGATGGGTTACTTTTTATTGGCATTGGAAGCACATCAC
ATTATGGCAGGGTAACGTTTCACAGTTTAATGAATCTTCCACTTATTTGATGGGATGGTT
AAGAGATTATCTATGGTTAAACTCTTCACAACTTATCAACGGATATAACCCTTTTGGTAT
GAATAGTTTATCGGTCTGGGCGTGGATGTTCTTATTTGGACATCTTGTTTGGGCTACTGG
ATTTATGTTCTTAATTTCCTGGCGTGGATATTGGCAGGAATTGATTGAAACTTTAGCATG
GGCTCATGAACGTACACCTTTGGCCAATTTGATTCGATGGAGGGATAAACCTGTGGCTC
TTTCCATTGTGCAAGCAAGATTGGTTGGATTAGTTCACTTTTCTGTAGGTTACATATTCA
CTTATGCAGCTTTCTTGATTGCCTCTACATCGGGCAAATTTGGTATGTCACATTCAGTAA
AGATTTACGATACATGTATAGGGTGTACTCAATGTGTCCGAGCCTGCCCCACAGATGTAT
TAGAAATGATACCTTGGGACGGATGTAAAGCTAAGCAAATTGCTTCTGCTCCAAGAAC
AGAGGATTGTGTTGGTTGTAAGAGATGTGAATCCGCATGCCCAACGGATTTCTTGAGTG
TTCGAGTTTATTTATGGCATGAAACAACTCGTAGCATGGGTCTAGCTTATATGACAACTT
TCAATTTCCCCTCTATTTTTGTGCCTTTAGTGGGTCTAGTATTTCCAACAATTGCAATGG
CTTCTTTATTTCTTCATGTTCAAAAAAACAAGATTGTTATGCGAGATCTAAAAACATATC
TCTCCGTGGCACCGGTACTAAGTACTCTATGGTTCGGGTCTTTAGCAGGTCTATTGATAG
AGATCAATCGTCTATTCCCGGATGCGTTGACATTCCCTTTTTTTTCATTCATGACTGCAA
TTTTAGAGAGACGCGAAAGCGAAAGCCTATGGGGTCGCTTCTGTAACTGGATAACCAG
CACTGAAAACCGTCTTTACATTGGATGGTTTGGTGTTTTGATGATCCCTACCTTATTGAC
CGCAACTTCTGTATTTATTATTGCCTTCATTGCTGCTCCTCCAGTAGATATTGATGGTATT
CGTGAACCTGTTTCTGGATCTTTACTTTATGGAAACAACATTATTTCTGGTGCCATTATT
CCTACTTCTGCAGCTATTGGTTTGCACTTTTACCCGATATGGGAAGCGGCATCCGTTGAT
GAATGGTTATACAATGGTGGTCCTTATGAGTTAATTGTTCTACACTTCTTGCTTGGTGTA
GCTTGTTACATGGGTCGTGAGTGGGAACTTAGTTTCCGTTTGGGTATGCGCCCTTGGAT
TGCTGTTGCATATTCAGCTCCTGTTGCAGCTGCTGCTGCTGTTTTCTTGATCTATCCAAT
TGGTCAAGGAAGCTTTTCTGATGGTATGCCTCTAGGAATTTCTGGTACTTTCAACTTCAT
GATTGTATTCCAGGCTGAGCACAACATCCTTATGCACCCATTTCACATGTTAGGCGTAG
CTGGTGTATTCGGCGGTTCCCTATTCAGTGCTATGCATGGTTCCTTGGTAACTTCTAGTT
TGATCAGGGAAACTACAGAAAATGAATCTGCTAACGCAGGTTACAGATTTGGTCAAGA
GGAAGAAACTTATAATATCGTAGCCGCTCATGGTTATTTTGGCCGATTAATCTTCCAATAT
GCTAGTTTCAACAATTCTCGTTCTTTACATTTCTTCCTAGCTGCTTGGCCTGTAGTAGGT
ATTTGGTTCACTGCTTTAGGTATCAGTACCATGGCTTTCAACTTAAATGGTTTCAATTTC

AACCAATCTGTAGTCGATAGCCAAGGTCGTGTAATTAATACCTGGGCTGATATCATTAAC
CGTGCTAACCTTGGTATGGAAGTTATGCATGAACGTAATGCTCATAACTTCCCTCTAGAC
CTAGCTGCTGTTGAAGCTCCATCAATAAATGGAATGGGTTTGCCTTGGTATCGTGTTCAT
ACCGTCGTCTTAAATGATCCTGGTCGGTTGCTTTCTGTCCATATAATGCATACAGCTCTG
GTTGCTGGTTGGGCCGGTTCGATGGCTCTATATGAATTAGCAGTTTTTGATCCTTCTGAC
CCCGTTCTTGATCCAATGTGGAGACAGGGTATGTTCGTTATACCCTTCATGACTCGTTTA
GGAATAACCAATTCGTGGGGCGGTTGGAGTATCACA---GGGGGAACTATAACGAATCCG
GGTATTTGGAGTTACGAAGGTGTAGCCGGCGCACATATTGTGTTTTCTGGCTTGTGCTT
CTTGGCAGCTATCTGGCATTGGGTGTATTGGGATCTAGAAATTTTTTGTGATGAACGTAC
AGGAAAACCTTCTTTGGATTTACCCAAGATCTTTGGAATTCATTTATTTCTCTCCGGGGT
GGCTTGCTTTGGTTTTGGCGCATTTCATGTAACAGGCTTGTATGGTCCTGGGATATGGGT
GTCCGATCCTTATGGACTAACTGGAAAAGTACAATCTGTAAATCCAGCGTGGGGCGTG
GAAGGGTTTGATCCTTTTGTTCCGGGAGGAATAGCTTCTCATCATATTGCAGCAGGAAC
ATTGGGTATATTAGCGGGGCTATTCCATCTTAGTGTACGGCCCCCCCAACGTCTATACAA
AGGATTACGTATGGGAAATATTGAAACCGTACTCTCTAGTAGTATCGCGGCTGTCTTTTT
TGCAGCTTTTGTTGTTGCTGGAACTATGTGGTATGGTTCAGCAACTACCCCGATCGAAT
TATTTGGACCCACTCGTTATCAATGGGATCAGGGATACTTCCAGCAAGAAATATATCGA
AGAGTTGGCGCAGGCCTAGCCGAAAATCAAAGTTTATCAGAAGCTTGGTCTAAAATTC
CTGAAAAATTAGCTTTTTATGATTACATCGGAAATAATCCGGCAAAAGGGGGATTATTC
AGAGCGGGCTCAATGGACAACGGGGATGGCATAGCTGTTGGATGGTTAGGGCACCCTA
TCTTTAGAGATAAAGGGGGACGTGAACTTTTTGTACGTCGTATGCCGACTTTTTTTGAA
ACATTTCCAGTCGTTTTGGTAGACGGAGATGGAATTGTTAGAGCGGATGTTCCTTTTAG
AAGGGCAGAATCGAAGTATAGTGTCGAACAAGTAGGCGTAACTGTTGAGTTCTATGGT
GGCGAACTCAATGGAGTTAGTTATAGTGATCCCGCTACTGTAAAAAAATATGCTAGACG
TGCTCAATTGGGCGAAATTTTTGAATTAGATCGTGCTACTTTGAAATCCGATGGTGTTTT
TCGTAGCAGCCCAAGGGGCTGGTTTACTTTTGGACATGCTTCATTTGCTCTGCTCTTCT
TCTTCGGACACATTTGGCATGGTTCTAGAACCTTGTTTAGAGATGTTTTTGCTGGTATTG
ATCCAGATTTGGATGCTCAAGTGGAGTTTGGAGCATTCCAAAAAGTTGGGGATCCAAC
TACAAAAAGGCAAGTAGTC--------------------GTGGAAACGCTCTTTAATGGA
ACTTTAGCTTTAGCTGGTCGTGACCAAGAAACCACCGGTTTTGCTTGGTGGGCCGGGA
ATGCCCGACTTATCAATTTATCCGGTAAATTACTCGGGGCTCATGTAGCCCATGCTGGAC
TAATCGTATTCTGGGCCGGAGCAATGAACCTATTTGAGGTGGCTCATTTCGTACCAGAA
AAGCCCATGTATGAACAAGGATTAATTTTACTTCCTCACCTTGCTACTCTAGGTTGGGG
AGTAGGCCCTGGTGGGGAAGTTATAGACACCTTTCCGTACTTTGTATCTGGAGTACTTC
ACTTAATTTCCTCTGCAGTATTAGGCTTTGGCGGTATTTATCATGCACTTCTGGGACCTG
AGACTCTTGAAGAATCTTTTCCATTCTTCGGTTATGTATGGAAAGATAGAAATAAAATG
ACCACAATTTTGGGTATTCACTTAATCTTGTTAGGTCTAGGCGCTTTTCTTCTAGTATTC
AAGGCTCTTTATTTTGGAGGTGTATATGATACCTGGGCTCCCGGGGGGGGAGATGTAAG
AAAAATTACCAACTTGACCCTTAGTCCCAGTGTTATATTTGGTTATTTACTAAAATCCCC
CTTTGGAGGGGAGGGGTGGATTGTTAGTGTGGACGATTTGGAAGATATAATTGGGGGA
CATGTATGGTTAGGTTCCATTTGTATACTTGGTGGAATCTGGCATATCTTAACCAAACCC
TTTGCATGGGCCCGCCGTGCACTTGTATGGTCTGGAGAAGCTTATTTGTCTTATAGTTTA
GGTGCTTTATCCGTCTGTGGTTTTATTGCTTGTTGTTTTGTCTGGTTCAATAATACCGCTT
ATCCTAGTGAGTTTTATGGTCCTACTGGGCCAGAAGCTTCTCAAGCTCAAGCATTTACT

TTTCTAGTCAGAGACCAACGCCTTGGGGCTAACGTGGGATCCGCTCAAGGGCCTACTG
GTTTAGGTAAATATTTAATGCGTTCTCCGACCGGAGAAGTCATTTTTGGAGGAGAAACT
ATGCGTTTTTGGGATCTGCGTGCTCCTTGGTTAGAACCTCTAAGGGGTCCCAATGGTTT
GGACTTGAGTAGACTGAAAAAAGACATACAACCTTGGCAAGAACGGCGTTCCGCAGA
ATATATGACTCATGCTCCTTTGGGCTCTTTAAATTCCGTGGGTGGCGTAGCTACCGAAAT
CAATGCCGTCAATTATGTCTCCCCTAGAAGTTGGTTAGCCACTTCGCATTTTGTTCTAGG
ATTCTTCCTATTCGTAGGCCATTTGTGGCACGCGGGAAGGGCTCGCGCTGCTGCAGCA
GGATTTGAAAAAGGAATTGATCGGGATTTTGAACCTGTTCTTTCCATGACCCCTCTTAA
CATGACTATAGCCCTTGGTAAATTTACCAAAGACGAAAATGATTTATTTGATATTATGGA
TGACTGGTTACGTAGGGACCGTTTCGTTTTTGTAGGTTGGTCCGGTCTATTGCTCTTTCC
TTGTGCTTATTTCGCTTTAGGAGGTTGGTTTACAGGTACAACCTTTGTAACTTCGTGGTA
TACCCATGGATTGGCCAGTTCCTATTTGGAAGGCTGCAATTTCTTAACGGCCGCGGTTT
CGACTCCTGCTAATAGTTTAGCACATTCTTTGTTGTTACTATGGGGACCTGAAGCACAA
GGAGATTTTACTCGTTGGTGTCAATTAGGTGGTCTGTGGACTTTTATTGCTCTCCATGGT
GCTTTCGCCCTAATAGGTTTTATGTTACGTCAATTCGAACTTGCTCGATCTGTTCAATTA
CGACCTTATAATGCAATCGCATTCTCGGGTCCAATTGCAGTTTTTGTTTCTGTATTTTTGA
TTTATCCACTAGGTCAGTCTGGTTGGTTCTTTGCACCTAGTTTTGGTGTAGCAGCTATAT
TTCGATTCATACTCTTTTTCCAAGGGTTTCATAATTGGACGTTGAACCCATTTCATATGAT
GGGAGTTGCCGGTGTATTGGGCGCTGCTCTGCTATGCGCTATTCATGGTGCTACCGTGG
AAAATACTTTATTTGAAGATGGTGATGGTGCAAATACATTCCGTGCTTTTAACCCAACTC
AAGCTGAAGAAACTTATTCAATGGTTACCGCTAACCGCTTTTGGTCCCAAATCTTTGGG
GTTGCTTTTTCCAATAAACGTTGGTTACATTTCTTTATGTTATTTGTACCAGTAACCGGTT
TATGGATGAGTGCTCTTGGAGTAGTCGGTCTGGCTCTGAATCTGCGTGCCTATGACTTC
GTTTCCCAGGAAATCCGTGCAGCGGAAGATCCTGAATTTGAAACTTTCTACACTAAAA
ATATTCTCTTAAACGAAGGTATTCGTGCTTGGATGGCGGCTCAAGATCAGCCTCATGAA
AACCTTATATTCCCTGAGGAGGTTCTACCCCGTGGAAACGCTCTTATGTCTGGAAGCAC
AGGAGAACGTTCTTTTGCTGATATTATTACCAGTATTCGATACTGGGTCATTCATAGCATT
ACTATACCTTCCCTATTTATTGCGGGTTGGTTATTCGTCAGCACAGGTTTAGCTTACGAT
GTGTTTGGAAGCCCTCGGCCAAACGAGTATTTTACAGAGAGCCGACAAGGAATCCCAT
TAATAACTGGCCGTTTTGATCCTTTGGAACAACTCGATGAATTTAGTAGATCTTTTATGA
CTATAGATCGAACCTATCCAATTTTTACAGTGCGATGGTTAGCTGTTCACGGACTAGCTG
TACCTACCGTTTCTTTTTTGGGGTCAATATCAGCAATGCAGTTCATCCAACGAATGGCTA
CACAAAGCGTTGAGGATAGTTCTAGATCTGGT---CCAAGACGAACTATTATAGGAGATTT
ATTGAAACCATTGAATTCGGAATATGGTAAAGTAGCTCCCGGATGGGGAACTACTCCTT
TGATGGGTGTCGCAATGGCTTTATTTGCAGTATTTCTATCTATTATTTTGGAGATTTATAAT
TCTTCTGTTTTATTGGACGGAATTTCAATGAATATGCTTACTCTCAAACTCTTTGTTTAC
ACAGTAGTGATATTTTTTTGTTTCTCTCTTTATCTTTGGATTCCTATCTAATGATCCAGGGC
GTAATCCTGGGCGTGAAGAAATGGCCGATACTACTGGAAGGATTCCTCTTTGGCTAATA
GGTACTGTAACTGGCATTCTTGTGATCGGTTTAATAGGTATTTTCTTTTATGGTTCATATT
CCGGGTTGGGTTCATCCCTGATGTTTAACATCTTTAGTTTGACCTGTATCTGTCTTAATT
CTGTCCTTTATTCGAGTAATTTTTTCCTCTCCAAATTGCCCGAGGCCTATGCTTTTTTGAA
TCCAATCGTAGATGTTATGCCCGTCATACCTCTGCTTTTTTTTCTTTTAGCCTTTGTTTGG
CAAGCCGCTGTAAGTTTTCGAACGACACAATCAAACCCGAACGAACAAAATGTTGAAT
TGAATCGTACCAGTCTCTACTGGGGGTTATTACTCATTTTTGTACTTGCTGTTTTATTTTC

AAATTATTTCTTCAATATGGAAGTAAATATTCTCGCATTTATTGCTACTGCACTGTTCATT
CTAGTCCCTACAGCTTTTTTACTTATCATATACGTAAAAACAGTTAGTCAAAATGATATG
GAAACAGCAACTCTAGTCGCTATCTTTATATCTGGTTTACTTGTAAGTTTTACTGGGTAC
GCCTTATATACCGCTTTTGGGCAACCCTCTCAACAACTAAGAGATCCATTCGAGGAACA
CGGGGACATGGAAGCATTGGTTTATACATTCCTCTTAGTCTCAACTCTAGGAATAATTTT
TTTTGCTATTTTCTTTCGGGAACCACCTAAAGTTCCAACTAAAAAGATAAAAATGACTA
TTGCTTTCCAATTGGCTGTTTTTGCATTAATTGCTACTTCATCGATCTTATTGATTAGCGT
ACCCGTTGTATTTTCTTCTCCTGATGGTTGGTCAAGTAACAAAAATGTTGTATTTTCCGG
TACATCATTATGGATTGGATTAGTCTTTTTGGTGGGTATCCTTAATTCTCTCATCTCT-------
-------------ATGTCACCACAAACAGAGACTAAAGCAAGTGTTGGATTCAAAGCTGGTGTT
AAAGATTACAAATTGACTTATTATACTCCTGACTATGAAACCAAAGATACTGATATCTTG
GCAGCATTTCGAGTAACTCCTCAACCTGGAGTTCCGCCTGAAGAAGCAGGGGCCGCG
GTAGCTGCCGAATCTTCTACTGGTACATGGACAACTGTGTGGACCGATGGACTTACTAG
TCTTGACCGTTACAAAGGACGATGCTACCACATCGAGCCCGTTCCTGGAGAAGAAAGT
CAATTTATTGCTTATGTAGCTTACCCCTTAGACCTTTTTGAAGAAGGTTCTGTTACTAAC
ATGTTTACTTCCATTGTGGGTAATGTATTTGGGTTCAAAGCCCTTCGCGCTCTACGTCTG
GAGGATCTTCGAATCCCTCCTGCTTATACGAAAACTTTCTGGGGCCCGCCTCATGGCAT
CCAAGTTGAGAGAGATAAATTGAACAAGTATGGGCGTCCTCTATTGGGATGTACTATTA
AACCTAAATTGGGGTTATCCGCTAAGAACTATGGTAGAGCAGTTTATGAATGTCTCCGC
GGTGGACTTGATTTTACCAAAGATGATGAGAACGTGAACTCCCAACCATTTATGCGTTG
GAGAGACCGTTTCCTATTTTGTGCCGAAGCAATTTTTAAAGCGCAGGCTGAAACAGGC
GAAATCAAAGGACATTACTTGAATGCTACTGCAGGTACATGTGAAGAAATGATGAAAA
GGGCTGTATTTGCCAGAGAGTTGGGAGTTCCTATCATAATGCATGACTACTTAACAGGG
GGATTCACTGCAAATACTAGCTTGGCTCATTATTGCCGAGATAATGGCCTACTTCTTCAC
ATCCATCGTGCAATGCATGCAGTTATTGATAGACAGAAGAATCATGGTATGCATTTTCGT
GTACTAGCTAAAGCGTTACGTATGTCTGGTGGAGATCATATTCACGCTGGTACCGTAGT
AGGTAAACTTGAAGGGGAAAGAGACATCACTTTGGGCTTTGTTGATTTACTACGTGAT
GATTTTGTTGAAAAAGACCGAAGTCGGGGTATTTTTTTCACTCAAGATTGGGTCTCCCT
AGCAGGCGTTCTGCCCGTGGCTTCGGGGGGTATTCACGTTTGGCATATGCCGGCTCTGA
CCGAGATCTTTGGAGATGATTCCGTACTACAGTTCGGTGGAGGAACTTTAGGGCACCCT
TGGGGAAATGCACCGGGTGCTGTAGCTAATCGAGTAGCTGTAGAAGCATGTGTACAAG
CTCGTAATGAGGGGCGTGATCTTGCTCGTGAGGGTAATGAAATTATCGAGGAGGCTAG
CAAATGGAGTCCTGAACTAGCTGCTGCTTGTGCGATATGGAAGAAAATCAAATTTGAAT
TCGAACCAGTGGATACTATT------ATGGCGATACATTTATACAAAACTTCTACCCCGAGCA
CACGCAATGGAGCCGTAGACAGTCAAGTGAAATACAATCCACGAAATAATTTGATCTAT
GGACAGCATCGTTGTGGTAAAGGTCGTAATGCCAGAGGAATCATTACCGCAGGGCATA
GAGGGGGAGGTCATAAGCGTCTATACCGTAAAATCGATTTTCGACGGAATGAAAAAGA
CATATATGGTAGAATCGTAACCATAGAATACGACCCTAATCGAAATGCATACATTTGTCT
CATACACTATGGGGATGGTGAGAAGAGATATATTTTACATCCCAGAGGGGCTAGAATTG
GAGATACCATTGTTTCTGGTACAGAAGTTCCTATAAAAATGGGAAATGCCCTACCTTTG
ACCGATATGCCCTTAGGCACGGCCATACATAACATAGAAATCACACTTGGAAAGGGTGG
ACAATTAGCTAGAGCAGCGGGTGCTGTAGCGAAACTGATTGCAAAAGAGGGGAAATC
GGCCACATTAAAATTACCTTCTGGGGAGGTCCGTTTGATATCCAAAAACTGCTCAGCAA
CAGTCGGACAAGTGGGGAATGTTGGGGTGAACCAGAAAAGTTTGGGTAGAGCCGGAT

CTAAGTGTTGGCTAGGTAAGCGCCCTGTAGTAAGAGGAGTAGTTATGAACCCTGTAGA
CCATCCCCATGGGGGTGGTGAAGGGAGGGCCCCAATTGGTAGAAAAAAACCCACAAC
CCCTTGGGGCTATCCTGCACTTGGAAGAAGAAGTAGAAAAAGGAATAAATATAGTGAT
AAGTTGATTCTTCGTCGCCGTAGTAAAATGATTCAACCTCAGACCCATTTAAATGTAGC
GGACAACAGCGGGGCTCGAGAATTGATGTGTATTCGAATCATAGGAGCTAGT---AACCG
CCGATATGCTCATATTGGTGACGTTATTGTTGCTGTGATCAAAGAAGCAGTACCAAATAT
GCCTCTAGAAAGATCAGAAGTAATCAGAGCTGTAATTGTACGTACCTGTAAAGAACTC
AAACGCGACAACGGTATGATAATACGATATGATGACAATGCTGCAGTTGTCATTGATCA
AGAGGGAAACCCAAAAGGAACCCGAATTTTTGGTGCGATAGCCCGGGAATTAAGGCA
ATTAAATTTTACTAAAATAGTTTCATTAGCGCCTGAAGTGTTAATGCTTAGTCCAAAAAG
AACAAAATTCCGTAAACAACATAGAGGAAGACTGAAAGGAATATCTTATCGAGGTAAT
CATATTTGTTTCGGTAGATATGCTCTTCAAACACTTGAACCCGCTTGGATCACTTCTAGA
CAAATAGAAGCAGGGCGACGAGCAATGGCACGAAGTGCGCGCCGTGGAGGAAAAATA
TGGGTACGTATATTTCCAGACAAACCAATTACATTAAGACCTACGGAAACACGTATGGG
TTCGGGTAAAGGATCTCCCGAATATTGGGTAGCTGTCGTTAAACCAGGACGAATACTTT
ATGAAATGGGCGGAGTGCCAGAAAATACAGCCAGAAAAGCTATTTCAATAGCGGCGTC
AAAAATGCCTATACGAACTCAATTCATTATTTCGGGAATGACTAGAATTAGACGAGGAT
ATATAGCGCGTAGACGTAGAACAAAAATTCGTTTATTTACATCAAGTTTTCGAGGAGCC
CATTCAAGACTTACTCGAACTATTACTCAACAGAAAATAAGAGCTTTGGTTTCGGCTCA
TCAGGATAGAAGTAGGCAAAAAAGAGATTTTCGTCGTTTGTGGATTACTCGAATAAATG
CAGCAATTCGAGGGAGC------GTATCCTATAGTTATAGTAAATTTATACATGATCTATACA
AGAGTCAATTGCTTCTTAATCGTAAAATACTTGCACAAATAGCTATATCAAATAAGAATT
GTCTTTATATGATTTCCAATGAAATTATCAAA---------------------------------ATGATAAAGGA
AGTATACGCTTTAGGTCATTATATCCGTATGTCGGCTCACAAAGCGCGAAGAGTAATTGA
TCAGATTCGTGGACGTTCCTATGAGGAAACGCTTATGATACTCGAACTCATGCCTTATC
GAGCATGTTATCCCATTTTTAAATTGATTTATTCTGCAGCAGCAAATGCTAGTCACAATA
TGGGCATCAATGAAGCAGATTTAATCATTAGTAAAGCCGAAGTCAATGAAAGCACTACT
ATGAAAAAATTAAAACCTCAGGCTAGAGGCCGCAGTTACCTAATAAAAAGACCTACAT
GTCATATAACCATTGTATTGCAAGATATATCCTTATAT---------------------ATGGATGGAATCA
AATATGCAGTATTTACAGACAAAAGTATTCGGTTATTGGGGAAAAAT---CAATATACTTCT
AATGTCGAATCAGGATCAACTAGGACAGAAATAAAGCATTGGGTCGAACTCTTCTTTG
GTGTCAAGGTAATAGCTATGAATAGTCATCGACTCCCGGGAAAGGATAGAAGAATGGG
ACCCATTATGGGACATACAATGCATTACAGACGTATGATCATTACGCTTCAACCGGGTTA
TTCTATTCCACCTCTTAGAAAGAAAAGAAATATGGCAGTTCCAAAAAAAACGTACTTCTA
TGTCAAAAAAGCATATTCGAAAAAATATTTGGAAAAGGAAGGGTTGTTGGTCAGCATT
AAAAGCCTTTTCTTTAGCGAAATCTCTTTCTACTGGAAATTCAAAAAGTTTTTTTGTCC
GACAAAACAAA---ATGGCCAAAGGGAAAGATGCCCGAGTAACGGTTATTTTGGAATGT
ACTAGTTGC---GTTCGAAAC------GTTAATAAGGAATCAACGGGGATTTTCAGATATATTA
CTCAAAAGAATCGACATAATACGCCTGGTCGATTGGAATTGAGAAAATTCTGTCCCTAT
TGTTACAAACATACAATTCATGGAGAGATAAAGAAAATGAAAATACGTGCCTCTGTTCG
TAAAATTTGTGAAAAATGTCGCCTGATCCGTAGGCGGGGACGAATTATAGTAATTTGTT
CCAACCCGAGACATAAACAAAGACAAGGAATGGTTCGAGAACAAATAAGAGTATCTAT
TCGGACACTGCAGTGGAAGTGTGTTGAATCAAGAATAGACAGTAAGCGTCTTTATTATG
GACGCTTTATTCTATCTCCACTTATGAAAGGGCAAGCCGACACAATCGGCATTGCGATG

CGAAGAGCTTTGCTTGGAGAAATAGAAGGAACATGTATCACGCGTGCAAAATCTGAA--
----AAAATACCACACGAATATTCTACTATGCGAGGTATTCAAGAATCAGTACATGAAATTT
TAATGAATTTAAAAGAAATTGTATTGAGAAGTAATCTGTACGGAACTCGAAACGCGTCC
ATTTGTGCTAGGGGCCCCGGATATGTAACTGCTCAAGATATCATCTTACCCCCTTCTGTA
GAAATCATTGATAATACACAGCATATAGCTAGCCTAACAGAACCAATAAATTTATATATT
GAATTACAAATTGAGAGAAATCGCGGATATCATATAAAAACACCAAATAACTTTCAAGA
CGAAAGTTAT------CCTATAGATGCTGTATTCATGCCTGTTCGAAATGCGAATCATAGTATT
CATTCTTATGGAAATGGGAATGAAAAACAAGAAATCCTTTTTCTCGAAATATGGACAAA
CGGAAGTTTAACTCCTAAAGAAGCGCTTCATGAAGCCTCCCGGAATTTTATTGATTTAT
TTATTCCTTTTATACATGTGGAAAAAGAAAAC------TTGTATTTAGGGGACAATCAA---GA
CAAGGATACTTTTCCCCTTTTTACCTTTCATGATGAATTGGCT---AAACTAAGAAAAAAC
AAAAAA---GAAATAGCATTGAAATATCTTTTTATTGACCAATTAGAATTGCCTCCCAGGA
TCTATAATTGCCTCAAAAGGTCCAATATACACACATTATTGGACCTTTTAAATAACAGTC
AAGAAGATCTTATGAAAATTGAGTATTTTCGCAGAGAAGATGTAAAACAGATATTTGGC
ATTCTAGAAAAGCAT------TTAAATTTTTTTTTAACGAAGAATAAGATGCTCGGGGATGG
AAAT---GAGGTAATGTCTACAATACCTGGGTTTAATCAGATACAATTTGAAGGATTTTGT
AGATTCATTGATCAAGGCTTGGCAGAAGAACTTTCTAAGTTTCCAAAAATTGAAGATAC
AGATCAAGAAATTGAATTTCAATTATTTGTGGAAACATATCAATTGGTAGAACCGTTGAT
AAAAGAAAGAGATGCTGTATATGAATCACTTACATATTCTTCTGAATTATATGTATCCGC
GGGATTAATTTGGAAAACAAATCGAGATATGCAAGAACAAACAATTTTTATTGGAAACA
TTCCTCTAATGAATTCTTTGGGAACTTCTATAATAAATGGCATATACAGAATTGTAATCAA
TCAAATATTACAAAGTCCCGGTATTTATTATCGATCAGAATTGGACCATAAGGGAATTTC
GGTCTATACTGGCGCCATAATATCAGATTGGGGGGGAAGATCAGAATTAGAGATTGATA
GAAAAGCAAGGATATGGGCTCGCGTAAGTAGGAAGCAGAAAATATCTATTCTAGTTTTA
TCATCAGCTATGGGTGCGAATCTAAAAGCAATTCTAGAGAATGTTTACTACCCTGAGAT
TTTCTTGTCTTTCCTGAATGATAAGGAGAAAAAAAAAAATTGGGTCAAAAGAAAATGCC
ATTTTGGAGTTTTATCAACAATTTGCTTGTGTAGGTGGGGATCCGGTATTTTCTGAATCC
TTATGTAAGGAATTACAAAAGAAATTCTTTCAACAAAGATGTGAATTAGGAAGGATTGG
TCGACGAAATATAAACCGGAGACTGAATCTTGATATACCTCAGAATAATACATTTTTATT
ACCCCGAGATATATTAGCAGCTGCGGATCATTTGATTGGACTGAAATTTGGAATGGGTA
CACTTGACGAT------ATGAATCATTTGAAAAATAAACGTATTCGTTCTGTAGCGGATCTCT
TACAAGATCAATTCGGATTGGCTCTGGTTCGTTTAGAAAATTTGGTTCGAGGAACTATA
TGTGGAGCAATTAGG---CATAAATTGCTA---CCGACTCCTCAGAATTTGGTAACTTCAAC
TCCA------------TTAACAACCACTTATGAATCTTTTTTTGGCTTACACCCATTATCTCAAGT
TTTGGATCGAACTAATCCATTGACACAAATAGTTCACGGGAGAAAATCGAGTTATTTGG
GCCCTGGAGGGTTGACGGGGCGAACTGCTAGTTTTCGTATACGAGATATTCATCCTAGT
CACTATGGGCGTATTTGCCCAATTGATACGTCCGAAGGAATCAATGTTGGGCTTATTGGA
TCCTTAGCAATTCATACGAGGATTGGTCATTGGGGG---TCTCTAGAAAGCCCCTTTTATG
ATTAT---GAGATTTCTGAGAAATCAAAAAAA------GTACGGATGCTTTATTTATCACCAAG
TGGAGATGAGTACTATATGGTAGCGGCGGGAAATTCTTTGGCATTAAATCATGGTATTCA
GGAAGAACAGGTTGCTCCAGCTCGATACCGTCAAGAATTCCTGACTATTGCGTGGGAA
CAGGTTCATCTTCGAAGTATTTTTCCCTTCCAATATTTTTCTATTGGGGCTTCCCTCATTC
CTTTTATCGAGCATAATGATGCGAATCGGGCTTTAATGAGTTCTAATATGCAACGTCAAG
CAGTTCCGCTTTCTCGGTCTGAGAAGTGCATTGTTGGAACTGGGTTGGAACGCCAAGT

GACTCTAGATTCAGGGGTTCTCACTATAGCCGAACATGAGGGAAATATAATTTATACCGA
TACTGCCAAGATCCTTTTATCAGGTAATGGAGATATTCGAAGCATTCCATTAGTTATGTAT
CAACGTTCCAACAAAAATACTTGTATGCATCAAAAACCTCAGGTTCAGTGGGGTAAAT
ACATTAAAAAAGGACAAATCTTAGCGGATGGTGCCGCTACAGTTGGTGGTGAACTCGC
TTTGGGGAAAAACGTATTAGTAGCTTATATGCCATGGGAAGGTTACAATTCTGAAGATG
CAGTACTTATTAGCGAGCGTATGGTATATGGAGATGTTTATACTTCTTTTCACATACGGA
AATATGAAATTCAGACTCATGTGACAAGCCAAGGC---CCCGAAAGGATCACTAACGAA
ATACCGCATTTAGAAGCCCATTTACTCCGCAATTTAGACAAAAATGGAATTGTGATGCT
AGGATCTTGGGTAGAGACTGGTGATATTTTAGTAGGTAAATTAACCCCTCAGATGGCAA
AAGAA---TCATCGTATGCCCCGGAAGATAGATTATTACGAGCCATACTTGGCATTCAGGT
ATCCACTTCAAAGGAAACTTGTCTAAAACTACCTATCGGTGGCAGGGGTCGAGTTATTG
ATGTGAGATGGATCCGGAAAAAAGGG---GGGTATAGTTATAATCCCGAAACGATTCGTG
TATATATTTCACAGAAACGTGAAATCAAAGTAGGTGATAAAGTTGCTGGAAGACATGGA
AATAAAGGTATCGTTTCAAAAATTTTGCCTAGGCAAGATATGCCTTATTTTCAAGATGGA
AGCCCCGTCGATATGGTCTTCAACCCATTAGGAGTACCTTCACGAATGAATGTAGGGCA
GATATTTGAATGCTCACTCGGGTTAGCGGGGGGCCTGCTAAACAGACATTATCGAATAT
TACCTTTTGATGAGAGATATGAACAAGAGGCTTCAAGAAAACTCGTGTTTTCTGAATTA
TATAAAGCCAGTAAGCAAACAGCGAATCCATGGATATTTGAACCCGAGTATCCGGGAA
AAAGCAGAATATTTGATGGAAGAACTGGGGATCCTTTTGAACAACCTGTTATAATAGGA
AAGCCTTATATCTTGAAATTAATTCATCAAGTTGATGATAAAATCCATGGACGTTCCAGT
GGGCATTATGCACTTGTTACACAACAACCTCTTAGAGGAAGGGCCAAGCAAGGGGGA
CAACGGGTAGGAGAAATGGAGGTTTGGGCGCTAGAAGGATTTGGGGTTGCTCATATTT
TACAAGAGATGCTTACCTATAAATCTGATCATATTAGAGCTCGCCAGGAAGTGCTTGGT
ACTACGATCATTGGAGGAACAATACCTAATCCCGAGGATGCTCCAGAATCTTTTCGATT
GCTCGTTCGAGAACTACGATCTTTGGCTCTGGAATTGAATCATTTCCTTGTATCTGAGA
AGAACTTCCGGATTAATAGGAAGGAAGCTATGAATCAGAATTTTTCTTCTATGATCGATC
GGTATAAACATCAACAACTCCGAATTGGATCAGTTTCTCCTCAACAAATAAGTGCTTGG
GCCAATAAAACCCTACCTAATGGAGAGATAGTTGGAGAGGTGACAAAACCCTATACTT
TTCATTACAAAACCAATAAACCGGAAAAAGATGGATTATTTTGTGAAAGAATTTTTGGG
CCTATAAAAAGTGGAATTTGTGCTTGTGGAAATTATCGAGTAATCGGGGATGAAAAAG
AAGACCCGAAATTTTGTGAACAATGCGGAGTCGAATTTGTTGATTCTCGGATACGAAG
ATATCAAATGGGCTATATCAAACTGGCATGCCCAGTAACCCACGTGTGGTATTTGAAAC
GCCTTCCTAGTTATATCGCGAATCTTTTAGATAAACCTCTTAAAGAATTAGAAGGCCTAG
TATACTGCGAT------TTTTCTTTTGCTAGGCCC---ATAGCTAAAAAACCTACTTTCTTACGA
TTACGAGGTTCATTCGAATATGAAATCCAATCCTGGAAATACAGCATCCCACTTTTTTTT
ACTACCTCAGGCTTTGAGACATTTCGAAGTCGAGAAATCTCTACTGGAGCAGGGGCTA
TCCGAGAACAATTATCCGATCTGGATTTGCGAATTATTATAAATTATTCATTGGTAGAGT
GGAAAGAATTAGGGGAGGAA---GGGCCCACAGGTAATGAATGGGAAGATCGAAAAAT
TGGAAGAAGAAAGGATTTTTTGGTTAGACGCATGGAATTGGCTAAGCATTTTATTCGAA
CAAATGTAGAACCAGAATGGATGGTTTTGTGTCTATTACCAGTTCTTCCTCCCGAGTTG
AGACCGATCATTCAGATAGATGGGGGAAAACTAATGAGCTCGGATATTAATGAACTCTA
TAGAAGAGTTATCTATCGGAACAATACTCTTACCGATCTATTAACAACAAATAGATCTAC
GCCAGGTGAATTAGTAATGTGTCAGGAGAAATTGGTACAAGAAGCCGTGGATACCCTT
CTTGATAATGGAATCCGCGGACAACCAATGAGGGACAGTCATAATAAGGTTTACAAATC

ATTTTCAGATGTAATTGAAGGCAAAGAGGGAAGATTTCGTGAGACTCTGCTTGGAAAA
CGGGTCGATTATTCGGGTCGTTCTGTCATTGTCGTAGGGCCCTCACTTTCATTACATCGA
TGCGGATTGCCTCGCGAAATAGCAATAGAACTTTTCCAGACATTTGTAATTCGTGATCT
AATTAGACAACATCTTGCTTCAAACATAGGAGTTGCTAAGAGTAAAATTCGGGAAAAA
GAACCGATTGTATGGGAAATACTTCAGGAAGTTATGCAGGGGCATCCCGTATTGCTGAA
TAGAGCACCTACTCTGCATAGATTAGGCATACAAGCATTCCAACCAATTTTAGTGGAGG
GACGCGCTATTTGTTTACATCCATTAGTTTGTAAGGGATTCAACGCAGACTTTGATGGG
GATCAAATGGCCGTTCATGTACCTTTATCTTTGGAGGCTCAAGCGGAAGCTCGTTTACT
TATGTTTTCTCATATGAATCTCTTGTCTCCAGCTATTGGGGATCCCATTTCCATACCAACG
CAAGATATGCTTATTGGACTCTATGTATTAACGAGCGGGAATCGTCGAGGTATTTGTGCA
ACTAGGTATAATCCATGTAATTGCAAAAAC---------TATCAAAATGAAAGAATTGACGATA
TTACCTATAAGTATACGAAA------GAACCCCTTTTTTCGAATTCCTACGATGCAATTGGAG
CTTTTCGTCAGAAAAGAATCAATTTAGATAGTCCTTTGTGGCTTCGTTGGCGACTAGAT
CAACGC---ATTATTGCTTCA------AGAGAAGCTCCCATCGAAGTTCACTATGAATCTTTGG
GTACCTATCATGAGATTTATGGGCATTATTTAATAGTAAGAAGTGTAAAAAAAGAAATTC
TTTGTATATACATTCGAACCACTGTTGGTCATATTTCTCTTTATCGAGAAATCGAAGAAG
CTATACAAGGGTTTTGTCGAGCCTGTTCATATGGTACC------------------------------------ATG
GCAGAATGCGCCAATTTGGTCTTTCACAATAAAGTGATAGATGGAACTGCCATGAAAC
GTCTTATTAGAAGATTAATAGATCACTTCGGAATGGCATATACATCACATATCCTGGATCA
AATAAAGACTCTGGGGTTCCAGCAAGCCACTGCTACATCCATTTCATTAGGAATTGATG
ATCTTTTAACAACACCCTCTAAGGGATGGCTAGTCCAAGATACTGAACAACAAAGTTT
GATTTTGGAAAAACACCATCATTATGGGAATGTACACGCGGTAGAAAAATTACGGCAAT
CCATTGAGATATGGTATGCTACAAGTGAATATTTGCGACAAGAAATGAATCCTAATTTCA
GGATGACTGACCCCTTTAATCCAGTCCATATAATGTCTTTTTCGGGAGCTAGAGGAAAT
GTATCTCAGGTACACCAATTAGTAGGTATGAGAGGATTAATGTCGGATCCCCAAGGACA
AATGATTGATTTGCCCATTCAAAGCAATTTACGCGAGGGACTCTCTTTAACAGAATATAT
AATTTCTTGCTATGGAGCCCGAAAAGGAGTTGTGGATACTGCTGTACGAACATCAGATG
CTGGATATCTCACGCGAAGACTTGTTGAAGTAGTTCAACACATTGTTGTACGTAAAACA
GATTGTGCCACCACTCGAGGTATTTCTGTAAGTCCTCGAAATGGAATGATGCCG---GAA
AGGATTTTTATTCAAACACTAATTGGTCGTGTATTAGCAGACGATATATATATGGGTCCA
CGGTGCATTGCCACTCGAAATCAAGATATTGGGATTGGACTTATCAAGCGATTCATAAA
C------------------CTTCGAGCACAATCAATATATATTAGAACTCCCTTTACTTGTAAGAGTAT
ATCTTGGATCTGTCAATTATGTTATGGTCGGAGTCCTACTCATGGTGATCTGGTCGAATT
GGGAGAAGCTGTCGGTATTATTGCGGGTCAATCTATTGGAGAACCAGGTACTCAACTAA
CATTAAGAACTTTTCATACCGGCGGAGTATTTACAGGGGGTACTGCAGAACATGTACGA
GCCCCCTCTAATGGAAAAATTAAATTCAATGAGGATTTGGTTCATCCCACACGTACACG
TCATGGACATCCTGCTTTTCTATGTTATATAGACTTGTATGTAACCATTGAGAGTGAGGAT
ATTATACATAACGTGAATATTCCGCCAAAAAGTTTTATTTTAGTTCAAAATGATCAATATG
TGGAATCAGAACACGTGATTGCTGAGATTCGCACGGGAACGTCTACT---TTAAATTTTA
AAGAGAGGGTTCGAAAACATATTTATTCTGATTCAGAGGGGGAA---ATGCACTGGAGTA
CTGATGTGTATCATGCACCTGAATTTATATATGGTAATGTTCATCGCTTACCAAAAACAA
GCCATTTATGGATATTATCAGGGGGCCCGTGCAGA------TCTAGTGTAGTCCCTTTTTCCT
TACATAAGGATCAAGATCAAATGAATATTCAT------TCTCTTTCTGTTGAACAAAAATATA
TTTCTAACTCTTCA------TTGACTAACGATAAA---GTGAGACACAAAATCGGTAATTCAG

GGTTTTCTGATAAAAAAGAAGGAGGGATCCCTGATTAT---TCAGAAATTAATCGAATCA
GATGCACTAGTCATTCTAATCTCATATATCCC---GCTATTCTTTATGGGAATTCT------AATT
TATTGGCAAAGAGGCGAAGGAATGGATTCATCATCCCATTCCAATCGATTCAAAAACGA
GAGAAAGAACTAATACCCCCTTCAGGCATCTCGATTGAAATACCC------ATAAATAGTAT
TTTCTGTATAAATAGTATTTATGCTTATTTCGACGATCCTCGATACAGAAGAAGGAGTTC
GGGAATTACTAAATATGGGACTGTAGAAGTGCATTCAATTGTCAAAAAAGAAGAT------T
TGATTGAGTATCGAGGA---GTCAAAGAATTTAGGCCAAAATACAGGAAAGGAGATCGA
TTTTTTTTCATTCCCGAGGAAGTACATATCTTACCTGGATCCTCTTTTATAATGGTACGGA
ACAATAGTATCATTGGAGTAGATACACGAATCACTTTAAATACAAGAAGCCGAGTAGGT
GGGTTGGTCCGAGTTGAGAGAAAAAAAAAAAGTATTGAACTTCAAATATTTTCTGGGG
ATATCCATTTTCCTGGAGAAACAGATAAGATATCCCGACACAGTGGCATCTTGATACCA
CCAGGAACG------CGAAAAACAAATTCTAAGGAATCTAAAAAA------TTGAGAAATTGG
ATCTATGTCCAACGG------ATCGCACTTACCAAG------AAAAAGTATTTTGTTTTGATTCG
GCCTGTAGTCACATATGAAATAGCGGACGGTATAAATTTAGCAACACTTTTTCCCCAAG
ATCCTTCGCGGGAAAGAGATAATGTGAAACTTCGAGTTGTCAATTATATCCTTTATGGA
AATGGAAAACCAATTCGGGGAATTTTTGACACAAGTATTCAATTAGTTCGGACTTGTTT
AATATTGAATTGGGATCAAGACAAAAAA---------AGTTCTTCTATCGGGGAGGCCCATGC
TTCTTTTGTTGAAGTAACGACAAATTGGCTAATTAGAGATTTCCTAAGAATTGATTTAGC
GAAATCCCCCATTTCGTATACCGGAAAAAGGAATGATCCATCAGGTTCAGGATTGATCT
TTGAT---AATGAATCAGATCGC------ATCAATCCGTTTTATTCCATTTATTCTAAGGCAAGG
---ATTCAAAAATCACTTAAACAA------------AATCAAGGAACTATTCGTATATTATTGAATA
GA---------AATAAGGAATGCCAATCTTTGATAATTTTGTCATCATCCAATTGTTCTCGAAT
GGGTCTA------TTCAACAATGTAAAATATTACAATGGG---------ATAAAAGAATCAGATCCC
CTAATTCCAATTAGGAATTTTTTTGGTCCTTTAGGAACA------GCCCTTCAAATTGCGAAT
TTTTATTCG------TTTTACCATTTA------ATAACTCATAATCAGATCTTG---GTAATTAAATAT
TACCAACTTGACAAT---------TTAAAACAGGCTTTTCAACTACTTAAA---------TATTCTTTA
ATGGATGAAAAT------GGTAGAATTTTGAATCCTGATCCATGCAGTAACATCATTTTGAAT
GCATTCAATTTGAATTGGTATTTTTTCCATCACAATTCTTGTGATGCCACATCT---ACAAT
AATTAGTCTTGGACAGTTTATTTGTCAAAATGGATATATAGCCAAAAACAGATCACAC---
CTAAAA------TCGGGTCAAGTTCTAATTGTTCAA---GTGGACTCTTTAGTAATAAGATCAG
CTAAACCTTATTTAGCCACTTCAGGAGCAACTGTTCATGGCCATTATGGGGAAATATTTT
ATGAAGGAGATACATTAATTACATTTATATATGAAAAATCAAGATCTGGTGATATAACGC
AGGGTCTTCCAAAAGTGGAACAAGTTTTAGAAGTGCGTTCAATTGATTCAATATCTATG
AACCTAGAAAAAAGGATTGAGAGTTGGAACGAACGTATAACAAGAATTCTTGGAATTC
CTTGGGGATTCTTGATTGGTGCTGAACTAACTATAGCACAAAGTCGTATCTCGTTGGTT
AATAAGATCCAAAAGGTTTATCGATCCCAAGGGGTGCAAATTCATAATAGACATATAGA
AATTATTGTACGTCAAATAACATCAAAGGTGTTGGTTTCCGAA---------GATGGAATGTCT
AATGTTTTTTCACCTGGAGAATTAATTGGATTGTTGCGAGCGGAACGAACGGGTCGTG
CTTTGGAAGAAGCGATCTGTTATCGAGCCGTCTTATTGGGAATCACGAAAGCATCTCTG
AATACTCAAAGTTTTATATCCGAAGCGAGTTTTCAAGAAACTGCTCGAGTTTTAGCAAA
AGCTGCTCTCCGGGGCCGTATTGATTGGTTGAAAGGCCTGAAAGAGAACGTTGTTCTG
GGGGGGATGATACCCGTTGGTACCGGATTCAAGGGATTA------GTGCACCCTTCAAGGC
AACATAACAATATTCCT------TTGGAAACCAAAAAGAAGAATCTATTCGAGGGGGAAAT
GAGA------GATATTTTATTCCACCACAAAGTCTTGCAT---------------------TTTAAAGAATTT

TCA---ATGACAAGAAGATATTGGAACATCAATTTAGA
AGAGATGATGGAAGCAGGAGTTCATTTTGGTCATGGAACTAAGAAATGGAATCCAAGA
ATGGCACCTTATATCTCTGCAAAGCGTAAAGGTATTCATATTACAAATCTTACCAAAACT
GCTCGTTTTTTATCAGAAGCTTGTGATTTAGTTTTTAATGCAGCAAGTAGGGGAAAACA
ATTCTTAATTGTTGGTACCAAAAATAAAGCAGCTGATTCAGTAGCATGGGCTGCAATAA
GAGCCCGGTGTCATTATGTTAATAAAAAATGGCTCGGTGGTATGTTAACGAATTGGTCC
ACTACAGAAACGAGACTTCATAAGTTCAGGGACTTGAGAACGGAACAAAAGACGGGA
AGACTCAACCGTCTTCCGAAGCGGGATGCGGCTATGTTGAAGAGACAATTGTCTCACT
TGCAAACATATCTGGGTGGGATTAAATATATGACGGGTTTACCCGATATTGTGATCATCG
TTGATCAGCAAGAAGAATATACGGCTCTTCGAGAATGCATTACTTTTGGAATTCCAACA
ATTTGTTTAATTGATACAAATTGTGACCCCGATCTTGCAGATATTTCGATTCCAGCGAAT
GATGACGCTATTGCTTCAATTCGATTAATTCTTAACAAATTAGTCTTCGCAATTTGCGAG
GGGCGTTCT---AGCTATATAAGAAATCCTATGGGACAAAAAATAAATCCACTTGGTTTCA
GACTTGGTACAACCCAAGGTCATCATTCTATTTGGTTTACACAACCAAAAAATTATTCT
GAAGATCTACAAGAAGATCAAAAAATACGGAATTGTATCAAAAATTATGTACAAAAAA
ATATGAGAATATCCTCCGGC---GTAGAGGGAATTGCACATATAGAAATAAAAAAAAAAA
TCGATCTGATCCAGATCATAATCTATATGGGATTTCCAAAATTATTAATAGAAGGTAGAC
CACGA------------GGAATCGAAGAATTAGAAATGAATGTACAAAAAGAATTGAATTGTAT
AAATAAAAAACTCAACATTGCAGTTGCAAGAATTACAAAACCTTATGGGCACCCTAATA
TTCTTGCAGAATTTATAGCCGGTCAATTAAAAAATAGAGTTTCATTTCGCAAGGCAATG
AAAAAAGCTATTGAATTAACTAAACAAGCAGATATAAAAGGAATTCAAATACAAATTGC
AGGGCGTATCGATGGAAAAGAAATTGCACGTGTTGAATGGATCAGAGAAGGTAGGGTT
CCTCTACAAACCATTAGAGCTAAAATTGATTATTGTCCCTATACAGTTCGAACTATTTAC
GGTGTATTAGGCATAAAAATTTGGATATTTGTAGGCAAGGACATGTCGCGTTACCGAGG
GCCTCGTTTCAAAAAAAATACGCCGTCTGGGATCTTTGCCAGGATTAACTAGTAAAAGG
CCTAAAACCCGAAGTGATCTTAGAAACCAATCGCGTTCTGGGAAA---AGATCTCAATAT
CGTATTCGTCTAGAAGAAAAACAAAAATTGCGTTTTCATTATGGTCTTACAGAACGACA
ATTACTTAAATACGTTCGTATCGCCGGAAAAGCAAAAGGGTCAACAGGTCAGGTTTTA
CTACAATTACTTGAAATGCGTTTGGATAACATACTTTTTCGATTGGGCATGACTTCTACT
ATTCCTGGAGCCCGCCAATTAGTTAACCATAGACATATTTTAGTTAATGATCGTATAGTA
GATATACCAAGTTATCGCTGCAAACCTCGAGATATTCTTACTGTGAAGGATGAACACAA
ATCCAGAGCTCTGATTCAAAATTATCTTGATTCATCCCCCCAC---GAGGAATTGCCAAAA
CATTTGACTCTTCATCCATTCCAATAT------AAAGGATTAGTCAATCAAATAATAGATAGTA
AATGTGTCGGTTTGAAAATAAATGAATTGCTAGTCGTAGAATATTATTCCCGTCAGACTA
TGTCACGTCGAGGTACTACAGAAGAAAAAACTGCAAAATCCGATCCAATTTATCGTAAT
CGATTAGTTAACATGTTGGTTAACCGTATTCTGAAACACGGAAAAAAAATCATTGGCTTA
TCAAATTATCTATCGAGCCGTGAAAAAGATTCAACAAAAGACAGAAACAAATCCACTA
TCTGTTTTACGTCAAGCAATACGTGGAGTAACTCCCGATATAGCAGTAAAAGCAAGAC
GTGTAGGCGGATCGACTCATCAAGTTCCCATTGAAATAGGATCCACACAAGGAAAAGC
ACTTGCCATTCGTTGGTTATTAGGGGCATCCCGAAAACGTCCGGGTCGAAATATGGCTT
TCAAATTAAGTTCCGAATTGGTGGATGCTGCCAAAGGGAGTGGCGATGCCATACGCAA
AAAGGAAGAGACTCATAGAATGGCAGAGGCAAATAGAGCTTTTGCACATTTTCGTATG
GGTAGAGACACCATTGCTGACATAATAACCTCTCTACGAAATGCTGACATGAATAGAAA
AGGAACAGTTCGAATACCATCAACTAACCTTACAGAAAACATTGTTAAAATACTTTTAC

GAGAGGGTTTTATCGAAAACGTGAGAAAACATCAGGAAAACAAGAAATCTTTTTTGGT
TTTAACCTTACGACATAAAAGA------AATAGGAAAGGCACATATAGAACT---ATTTTC------
AATTTAAAACGTATCAGTCGACCCGGTCTACGAATCTATTCTAACTATAAACGAATTCCT
AGGATTTTAGGGGGAATGGGGATTTTAATTCTTTCTACTTCGCGAGGTATAATGACAGA
CCGGGAGGCTCGGCTAGAAGGAATCGGTGGAGAAATTTTGTGCTATATATGGATGGCA
AAACTTATACCAAGAATTGGTTCACGTAAAAATGAACGTATTAGTTCGCGTAAGAATGC
ACGTAGAATACCAAAAGGGGTTATTCATGTTCAAGCAAGTTTCAACAACACCATTGTA
ACTGTTACAGACGTACGTGGTCGGGTAGTTTCTTGGTCCTCCGCCGGTACTTGTGGGTT
CAAAGGTACAAGACGAGGGACACCGTTTGCTGCCCAAACCGCAGTAGGAAATGCGAT
TCGTACAGTAGTGGATCAAGGTATGCAACGAGCAGAAGTCATGATAAAAGGCCCTGGT
CTCGGAAGAGATGCAGCATTACGGGCTATTCGTAGAAGTGGTATACTATTAAATTTCGTA
CGGGATGTAACCCCTATGCCGCATAATGGCTGTAGACCCCCTAAAAAAAGACGTGTGAT
GCCAACTATTAAACAACTTATTAGAAACACAAGACAGCCAATCAGAAATGTCACGAAA
TCCCCCGCTCTTCGGGGATGCCCTCAGCGCCGAGGAACATGTACTAGGGTGTATACTAT
CACCCCCAAAAAACCAAACTCTGCCTTACGTAAAGTTGCCAGAGTACGATTAACCTCT
GGATTTGAAATCACTGCTTATATACCTGGTATTGGCCATAATTCACAAGAACATTCTGTA
GTCTTAGTAAGAGGGGGAAGGGTTAAGGATTTACCCGGTGTGAGATATCACATTGTTCG
AGGAACCCTAGATGCTGTCGGAGTAAAGGATCGTCAACAAGGGCGTTCTAAATATGGG
GTCAAAAAGCCAAAAATGGCAAGGAAAAGTTTGATTCAGAGGGAGAAGAAGAGGCA
AAAATTAGAACAAAAATATCATTTGATTCGTCGATCCTCAAAAAAAGAAATAAGC--------
-AAAGTTCCGTTGTTGAGTGACAAATGGGAAATTCATGGAAAGTTACAATCCTCACCAC
GTAATAGTGCACCCACACGTCTTCATCGACGTTGTTTTTCGACCGGAAGACCGAGAGC
TAACTATCGAGACTTTGGACTATCCGGACACATACTTCGTGAAAAGGTTCATGCGTGTT
TGTTGCCAGGGGCAACAAGATCAAGTTGGATGGTAAAAAACTCCTTCATTTCAGTTAA
TTCACACGAA------GAAAAAGAAGAAAAAAAAGGGTCCGTTGAATTTCAAGTATTCAG
TTTCACTAATAAAATACGAAGACTTACTTCACATTTGGAATTGCACAGAAAAGACTATT
TATCTCATAGAGGTTTACGGAAAATTTTGGGAAAGCGCCAACGCTTGTTGGCTTATTTG
TCAAAGAAAAATAAAGTACGTTATAAAAAATTAATTAGTCAGTTAGGTATTCGGGAAAC
AAAAACTCCT------ATGGTAAAACTTCGTTTGAAACGATGTGGTAGAAAGCAACGGGCT
GTCTATCGAATCGTTGCAATTGATGTTCGATCCCGAAGAGAGGGAAGAGATCTTCGAA
AAGTGGGCTTCTATGATCCGATAAAGAATAAAACTTATTTAAATGTTCCTGCTATTCTCT
ATTTCCTTGACAAGGGCGCTCAACCTACAGGAACTGTTTATGATATTTCAAAAAAGGTA
GAAGTTTTTAAGGAGCTTCGCCTTAATGAAACAAAATTCAATTCAATGGATAAATCCAA
GCGACCTTTTCTTAAATCCAAGCGATCTTTTCGTAGGCGTTTGCCCCCGATCCAATCGG
GGGACCAAATTGATTATAGAAATATGAGTTTAATTAGTCGATTTATTAGTGAACAAGGAA
AAATATTATCTAGACGAGTAAATAGATTGACTTTGAAACAACAACGATTTATTACTATTG
CTATAAAACAAGCTCGTATTTTATCTTCGTTACCTTTTCTGAATAATGAAAAA------CAAT
TTGAAAGAACCGAGTCAACCACTAGAACTACTGGTCTTAGAACCCGAAATAAAGTGAC
ACGT---CTAAAAAAAAATCCTTTTGTAGCGAATCATTTATTAAAAAAAATTAATAAACTT
AACACAAAAGCAGAAAAAGAAGTAATAATAAGTTGGTCACGGGCATCTACCATTATAC
CGACAATGATTGGGCATACAATTGCTATTCATAATGGAAAAGAGCATTTACCTATTTATAT
AACGGATCGCATGGTAGGCCATAAATTGGGAGAATTTGTACCTACTCTAAATTTCAGGG
GACATGCGAAAAATGATACTAGATCTCGTCGTATGGTTTTTAAATCTTTTATACTAGGTA
AT------------TGCATGAAGATAATCAATTCGGTCGTTGTGGTCGGACTCTATTATGGGTTTC

TGACCACATTCTCTATAGGGCCCTCTTATCTCTTCCTTCTCCGAGCTCAGGTTATGGAAG
AAGGAGAAGAAGGAACCGAGAAGAAGATATCAGCAACAACTGGTTTTATTACGGGAC
AGCTCATGATGTTCATATCGATCTATTATGCGCCTCTGCATCTAGCATTGGGTAGACCTCA
TACAATAACTATCTTAGCTCTACCGTATCTTTTGTTTCATTTCTTCTGGAACAATCACAA
ACACTTTTTTGATTATGGATCTACTACCAGAAATTCAATGCGTAATCTCAGCATTCAATG
TGTATTCCTGAATAATCTCATTTTTCAATTATTCAACCATTTCATTTTACCAAGTTCAATG
TTAGCCAGATTAGTCAACATTTATATGTTTCGATGCAACAACAAAATGTTATTTGTAACA
AGTAGTTTTGTTGGTTGGTTAATTGGTCACATTTTATTCATGAAATGGGTTGGATTAGTA
TTAGTCTGGATACGGCAAAATCATTCTATTAGATCTCTTATTCGATCTAATAAGTACCTTG
TGTCAGAATTGAGAAATTCTATGGCTCGAATCTTTAGTATTCTCTTATTTATTACCTGTGT
CTACTATTTAGGAAGAATACCGTCACCCATTCTTACT------CTGAAAGAAAAAGAAGATC
CTAAACCTATAGTAACTATTCTTTTCGATTATAAACGATGGAATCGCCCATTTCGATATAT
AAAAAATAATAAATTTGATAAGGCTGTAATAAATAAAATGTCACAATATTTTTTTATACA
TGTCAAAGTGATGGAAAACAAAGAATATCTTTTACCTATTCACTCAGTTTAGCAACTTT
TTTTGAAATGATACAAAAAAAAATTAGTTTGTCCACAACAGAAAAGGGTTTAAAT---TT
TTTATATAATCATTGGGTTTTTATCAACGAAAAAACAAAGAACAATTTAAGTAAAGAGT
TTCTAAATAGAATAAAAAGTTTAAACAAAGGATCC------TTAAATGGATTTGAAAAAAG
AACTAGATTGTGTAATGATGAAACTAAAAATGAGTATTTAACTCAAATATATGATCTTCT
CATAAACGGTCCTTATCGCGGAACAAAAAAAAACTTACAA------TTTGATACAATTTGGA
TAAATAAGATTCATAGTATTCTT---CGAAAATTTGAACAAAAAGATTCTAATGATCAA---A
TTGGAATAAACGAAATCAGTAAAAAAGTCCCTCAACATTCATATAAATTAATTGATTTG
GAACAACAGGAGGGACAACAAATTCGTTCAAGAAAATTTAAACGTGTAGTAATTTTTA
CCGATGCTTTGATACGTTATTCACAACAATCGGATTTTCGTCGAGGCATAATCAAAGGTT
CCATGCGTGCTCAAAGACGTAAAACAATTACTTGGAAATTCTTTCAAGCAAATGTGCAT
TCCCCACTTTTTTTGGACAGAATAGACAAATCCATTTTTTTGTCTTTTGAAATT------TCC
GGACTGATAAAACAAATTTTTATAAATAGGGAGGAGAAAGCTCGGATAAAAATAGCGG
AAGCCTGGGATACCATTTTATTTGCTCAAGTAATAAGAGGCTCTATGTTAGTAATACAAT
CAATTCTTAGAAAATCTATTGTATTACCCTCATTGATAATAGCGAAAAATATCAGCCGGA
TCTTATTATTTCAATTTCCAGAGTGGTCCGAAGATTTAAAGGAGTGGAATAGAGAAATG
CATGTTAAATGCACTTATAATGGGGTTCAATTATCAGAAACAGAATTCCCCAAAAACTG
GTTAACAGACGGTATTCAAATAAAAATCCTATTTCCTTTCTGCCTGAAACCTTGGCACA
AATCTAAGAGAGAAAAAACCAAT---TTTTGTTTTTTAACAGTTTGGGGAATGGAAGCAG
AACTACCCTTTGGCTCACCCCGAAAAAGGCCTTCCTTTTTTGAACCCATTTTTAAAGAA
CTCAAAAATAAAATTCGAAAATTGAAAAAGGAATGTTTTTTTGTT---TTTACAATTTTAA
AAGAAAGAACTAAACAAAAATTTTCATTTTCAAACGAAACAAAAAAAATGGTTTATCAA
AAATGCTCTA------TTTATAAAACAAATAATAAAAGAACTCTCAAAAATAAATGGTTTGA
TAATAAATAATCAAATGATTCAGGAACCATCTATTTCAATTCAATCTATGGATTTGCAAA
ATTATTCACTGACAGAAAAAAAGATAAAGGATCTAACAGATAAAACACGCATAATTAG
AAATCAAATAGAACAAATTACAAAAGACAAGAAAAAAAAATACTAAGACTAAGCAAAA
AAATTCTAATATTAAAGAACCACAAAAAAATACTTGGCAAATAAGAAAAAGAAAAAAT
GCTCAATTAATTCGCAAATTTTATTATTTTATACAATTTTTCATTGAACGAATATATATGGA
TACCCTTCTATGTATCATTAATATTAAT---ATTACAACACAACTTTTTATTCACTTTATTTCA
---GACTTGTCTTCTTTGTCCCAAGCATATGTATTTTACAAATTAGTACAAACACAACTTA
GTAACTTGTATAGTTTAAAACCTATTCTACTATATCTTAAATTTAAACTTAAAAATGAAT

AAAAGATTATTTCATACAAGGAATAATTCATTCCGAATTAAAACAGAACAAATTTCGAA
ATTGTGAAATGACTCAATGGAAAAACTGGTTAACAGGT---TATCAATATAATTTATATCA
GATTAAATTGTCTAAATTAGTACCACAAAAATGGAGAAATAAAATTCATCAATCCTTTAT
GTTTCAAAATAAAAGCAGATGCTATTTATATGAAAAAGACCAA------TTAATTGATTACG
AAAAAGAA------TTTAAAAAACACTATTTGTATGATCTTTTATCATATAAATATATTAAA----
-----------ACCCCG---AAATATTTTGATTGGGGAATTCCCCATTTTTTTTTATTATTTTTGAT
TGGATGGGAATGAATGAAGAAATACTAAATCGTTGGTTCTTTCCAGAATTTGTATTCCTT
TATAACACATATAAGATTAAACAATGGGGTATACCAATAAAATTACTTCTTTTTAATTACA
AAAGCCACACGGAAGCAGAACTTGATTTATTCCTCAAAAAGTATTTACTTTTTCAATTG
AGATGGGACGATTCATTGAATCAAAGAATGATCAATAATGTAAAGGTATATTGTTTCCTG
CTTAGATTAAGAAATTCAAAAGAAATTGCTATATCCTCTATTAAAAAGGAGGAAATGAG
TTTGGATATAATGCTAATTCAGAAAAATGTAAAGTTTACAGAATTGATGCAAAAGGGAA
TATTAATTATCGAACCGATTCGTCTATCTAAAACAACTAATAGACAATTTATTATGTATCA
AACCATACGTATCCCATTGGTTCATAAGAGTAAATACTATGATTTTGATTTGTTTGTCCTT
GAAAATATTTTATCCTCTAGACATCGCAGAGAATTAAGAATTAGAATTTATTTCATATTTC
TTTGGCCTAATTATAGATTAGAGGATTTAGCTTGTATGAATCGCTATTGGTTTGATACCAA
TAATGGCAGTCGTTTTAGCACGTTAAGGGTACATATGTACCCGATGAAAGGACATCAAT
TCAAATCCTGGATTTTTGAATTGAGAGAGATATTGAGAGAGATCAAGAATTCTCACTAT
TTCTTAGATTCATGGACCCAATTCAATTCAGTGGGATCTTTCATTCACATTTTTTTCCAC
CAAGAACGTTTTATAAAACTCTTTGACCCCCGAATTTGGAGTATCCTACTTTCA---CGCA
ATTCA------CAGGGTTCAACAAGCAATCGATATTTC---------ACGATCAAGGGTGTAGTAC
TATTTGTAGTAGCG---GTCCTTATATATCGT------------ATTAACAATCGAAATATGGTCGAA
AGAAAAAATCTCTATTTGATAGGGCTTCTTCCT--------------ATACTTATGAATTCCATTGG
ACCCAGAAATGATACATTGGAAGAATCTTTT---GGGTCTTCCAATATTAATAGGTTGATT
CTTTCG---CTCCTGTATCTTCCAAAAGGAAAAAAGATCTCTGAGAGTTGTTTCCTGGAT
CCGAAAGAGAGTACTTGGGTTCTCCCAAGAACT------------AAAAAGTGTATCGTGCCTG
AATCTAACTGGAGTTCGCGGTGGTGGAGGAACTGGATCGGAAAAAAGAGG------GATT
CTAGTTGTAAGATATCTAAT---------GAAACCGTCGCTGGAATTGAGATCTCATTCAAAGA
GAAAGATATCAAA---TATCTGGAGTTTCTTTTTGTATATTATATGGAT------GATCCGATCC
GCAAGGACCATGATTGGGAATTGTTTGATCGTCTTTCTCCGAGGAAGAGGCGAAACAT
AATCAACTTGAATTTGAATTCGGGACAGCTATTCGAAATCCTAGTGAAACACTGGATTT
GTTATCTCATGTCTGCTTTTCGTGAAAAAATACCAATTGAAGTAGAGGGT---TTCTTCAA
ACAACAAGGA---GCTGGGTCAACTATTCAATCAAATGATATTGAGCATCTTTCCCCT------
CTCTTCTCGAGAAACAAGTGGGCTATTTCTTTGCAAAATTGTGCTCAATTTCATATGTG
GCAATTCCGCCAAAACCCTTTCGTT---------AGTTGGGGGAAG---AATCCGCACGAATCG
GATTTTTTGAGGAAG---GTATCGAGAGAGAATTGGATTTGGTTAGACAATGTGTGGTTG
GTAAACAAGGATCGG---------GTTTTTAGC------AAGGTACGGAATGTATCGTCAAATATTC
AATATGATTCC---------ACAAGA------TCTAGTTTCGTTCAAGTAACGGATTCTAGCCAATT
GAAAGGATCCTCT---GATCAATCCAGAGATCATTCCGATTCCATTAGT------------AATGAG
GATTCGGAA---------------------------------------TATCACACATTGCTCAATCAAAGAGAGATT
CAACAACTAAAAGAAAGATCGATTCTT---TGGGAT------CCTTCCTTTCTTCAAACGGAA
CGAACAGAGATAGAATCAGAC------CGATTCCCGAAA------CGCCTTTCTGGATATTCC----
--TCAATGTCCCGGCTATTCGCG---GAACGTGAGAAGCAGATGAATCATCATCTGCTTCC
GGAAGAAATCGAAGAATTT------CTTGGGAATCCTACAAGATCCATTCGTTCTTTTTTCT

CTGACAGATGG------TCAGAACTTCAT---CTGGGCTCGAATCCTACTGAGAGGTCTACTA
GA------------------AAATTGTTGAAGAAACAACAAGGTCTTTAT---TTTGTCCCTTCCAGGC
GATCGGAA------------AATAAAGAAATGGTTAATATATTCAAGATAATTACGTATTTACAAA
AT------ACCGTCTCAATTCATCCTATTTCATCAGATCCGGGATGTGATATGGTTCCG---AAG
GATGAACCGGAT---ATGGACAGTTCCAATAAGATTTCATTCTTGAACAAAAATCCATTTT
TTGATTTC------TTTCATCTATTCCATGACCAGAACAGGGGGGGATACACGTTA------CAC
CACGATTTTGAATCAGAA------GAGAGATTTCAAGAAATGGCAGATCTATTCACTCTATC
AATAACCGAGCCG---GATCTGGTGTATCATAAGGGATTTGCCTTTTCTATTGATTCCTACG
GATTGGATCAAAAACAA------TTCTTGAATGAGGTATTCAACTCCAGGGATGAATCGAA
AAAGAAATTTTTA---------TTGGTTCTACCTCCTATTTTTTAT------GAAGAGAATGAATCTT
TTTATCGAAGGATCAGAAAAAAAATGGGTCCGG---ATCTCTTGCGGGAATGATTTGGAAG
ATCCAAAACCAAAAAAAGTGGTATTTGCTAGCAACAACATAATGGAGGCAGTCAATCC
ATATAGATTGATCCGAAATCTGATTCAAATCCAATATAGCACCTATGGCTATGGGTACATA
AGAAATGGATTGAATCGATTCTTTTTAATGAAT---------AGATCCGATCGCAACTTCGAAT
ATGGAATTCAA---------AAAGATCAAATAGGAAATGATACTCTGAATCATAGAACTATAAT
GAAATATATGATCAACCAACATTTATCGAATTTGAAAAAG---AGTCAGAAGAAATGGTT
TGATCCT------CTTATTTTTCTTTCTCGAACCGAGAGATCCATGAATCGGGATCCTGATGC
ATATAGATACAAATGGTCCAATGAGAACAAGAATTTCCAGGAACATTTGGAACATTTGG
TTTCGGAGCAGAAAAGTCGT---------TTTCAAGTAGTCTTCGATCGATTACGTATTAATCA
ATATTCGATTGATTGGTCTGAGGTTATCGACAAAAAAGATTTGTCTAAGTCACTTCGTTT
CTTTTTGTCCAAGTCACTTCTT---------------------------TTTTTGTCC---------------------AAGTT
GCTTCTCTTTTTGTCTAACTCACTCCCT---TTTTTCTTTGTGAGTTTCGAGAATATCCCCA
TTCATAGGTCCGAG---ATCCACATCTAT---GAATTGAAAGGTCCGAATGATCAACTCTGC
AATCAGTTGTTAGAATCAATAGGTCTTCAAATCGTTCATTTGAAAAAATGGAAAGCCTT
C------TTATTGGAT---------GATCATGATACTTCC---CAAAAATCGAAATTCTTGATCAATGG
AGGAACAATATCACCATTTTTGTTCAATAAGATACCA---AAGTGG---ATGATTGACTCATT
CCATACTAGA------AATAATCGCGGGAAATCCTTTGATAACACGGATTCCTATTTCTCAAT
GATATCCCACGATCAAGACAGTTGGCTGAATCCCGTGAAACCATTTCATAGAAGTTCAT
TGATATCTTCTTTTTATAAAGCAAATCGACTTCGGTTCTTGAATAATCCACACCACTTCT
TCTTCTAT------TGTAACAAAAGATTCCCTTTT---------TAT---GTGGAAAAGGGCCGTATTG
AGAATTATGAT------TTTACGTATGGA---CAATTCCTCAATATCTTGTTCATTCGCAACAAA
---ATATTTTCTTTGTGCGTCGGTAAA------AAAAAACATGCTTTTTTGGAG---AGAGATAC
TATTTCACCAATCGAGTCACAGGTATCTAACATATTCATACCTAACGATTTTCCACAAAG
TGGTGACGAAAGG---TATAACTTGTACAAATCT---------TTCTATTTTCCAATTCGATCCGA
TCCATTCGTTCGT------AGAGCTATTTTCTCGATCGCAGACATTTTTGGAACACCTCTAAC
AGAGGGACAAATAGTCAATTTTGAAAGAACTTATTGTCAACCTCTTTCAGATATGAATC
TATCTGATTCAGAAGGGAAAAACTTGCATCAGTATCTCAAT------TTCAATTCAAACATG
GGTTTGATTCACACTCCATGTTCTGAGAAATATTTACCATCCGAAAAG------AGGAAAAA
A------------------------CGGAGTCTTTGTCTAAAG------AAATGCGTTGAGAAAGGGCAGATG
TATAGAACCTTTCAACGAGATAGTGCTTTTTCAACTCTCTCAAAATGGAATCTATTCCAA
ACATATATGCCATGGTTCCTTACTTTGACAGGGTACAAATATCTAAATTTTATATTTTTAG
ATACTTTTTCAGAC---CTATTGCCGATACTAAATAGCAGT------CAAAAATTTTTATCTATT
TTTCATGATATTATGCATGGAGCAGATATATCATGGCGAATTCTTCAGAAAAAATTG-------
-----------TGTCTTCCACAATGGAAT---CTGATAAGTGAGATTTCGAGTAAGTGTTTACATAA

T------CTTCTTCTGTCCGAAGAAATGATTCATCAAAAGAATGAGTCA------CCATTGATATC
GACACATCTGAGATCGCCAAATGTTCGGGAGTTC---CTCTATTCAATCCTTTTTCTTCTT
CTTGTTGCTGGATAT---CTCGTTCGTACACATCTTCTCTTTTTTTCCCGA------GCCTCTAG
TGAGTTACAGACAGAGTTCGAAAAGGTCAAATCTTTAATGATTCCATCATACATGATTG
AGTTGCGAAAACTTCTGGATAGGTATCCTACATCTGAACTGAATTCTTTCTGGTTAAAG
AATCTCTTTCTAGTTGCTCTGGAACAATTAGGAGATTCTCTAGAAGAAATACGGGGTTC
TGCTTCTGGCGGCAACATGCTATTGGGTGGTGGTCCC---------------GCTTATGGG---GTCA
AATCAATACGTTCTAAGAAGAAATATTTGAATATCAATCTCATCAATATTATCGATCTCAT
A---AGTATCATACCAAATCCCATCAATCGAATCACTTTTTCGAAAAATACGAGACATATA
AGTCATACAAGTAAAGAGATCTATTCATTGATAAGAAAAAGAAAAAACGTGAACGGTG
ATTGGATTGATGATAAAATAGAATCCTGGGTTGCGAACAGTGATTCGATTGATGATGAA
GAAAGAGAATTCTTGATTCAGTTCTCC------ACCTTAACGACA------GAAAAAAGGATTG
ATCAAATTCTATTGAGTCTGACT---CATAGTGATCATTTATCA---------------AAGAATGACT
CTGGTTATCAAATGATTGAACAACCGGGAGCAATTTACTTACGATACTTAGTTGACATTC
AT------AAAAAGTATCTAATGAATTATGAGTTCAATACATCCTGTTTAGCAGAAAGACGG
ATATTCCTTGCTCATTATCAAACAATCACTTAT------TCACAAACCTTGTGTGGGGCTAAT
AGTTTTCATTTCCCATCTCATGGA---------AAACCCTTTTCGCTCCGCTTAGCCCTATCCC
CCTCT---AGTGGTATTTTAGTGATAGGTTCGATAGGAACTGGGCGATCCTATTTGGTCAA
ATACCTAGCGACAAACTCCTATGTTCCTTTCATTACGGTATTTCTGAACAAGTTCCTGGA
TAACAAGCCTAAAGGTTTTCTTATTGAT---------------GATATCGATGATATTGATGATAGTG
ACGATATTGATGATAGTGACGATATTGATGATAGT--G
ACGATATCGATCGTGACCTTGATACGGAGCTGGAGCTGCTAACTATGATGAATGCGCTA-
--------ACTATGGATATGATGCCG---------GAAATAGACCGATTTTATATCACTCTTCAATTCG
AATTAGCAAAAGCAATGTCTCCTTGCATAATATGGATTCCAAACATTCATGATCTGGATG
TGAATGAGTCGAATTACTTATCCCTCGGTCTATTAGTGAACCATCTCTCC---------AGGGA
TTGTGAAAGATGTTCC---ACTAGAAATATTCTTGTTATTGCTTCGACTCATATTCCCCAAA
AAGTTGATCCCGCTCTAATAACTCCAAATAAGTTAAATACATGCATTAAGATACGAAGG
CTTCTTATTCCACAACAACGAAAGCACTTTTTCACTCTTTCATATACTAGGGGATTTCAC
TTGGAAAAGAAAATGTTCCATACT------AATGGATTTGGATCCATAACCATGGGTTCCAA
TGCACGAGATCTTGTAGCACTTACCAATGAGGCCCTATCGATTAGTATTACACAAAAGA
AATCAATTCTAGACACTAATACAATTAGATCCGCTCTTCATAGACAAACTTGGGATTTGC
GA---------TCCCAGGTAAGATCGGTTCAGGATCATGGGATCCTTTTCTATCAGATAGGAA
GGGCTGTTGCACAAAATGTACTTCTAAGTAATTGCCCCATAGATCCTATATCTATCTATAT
GAAGAAGAAA------TCATGTAACGAAGGGGATTCTTATTTGTACAAATGGTACTTCGAA
CTTGGAACGAGCATGAAGAAATTAACGATACTTCTTTATCTTTTGAGTTGTTCTGCCGG
ATCGGTCGCTCAAGATCTTTGGTCTCTACCC------GGACCCGATGAAAAA---AATGGAAT
CACTTCTTATGGACTCGTTGAGAATGATTCT---GATCTAGTTCATGGGCTATTAGAAGTA
GAAGGCGCTCTGGTGGGA------TCCTCACGGACAGAAAAAGATTGCAGTCAGTTTGATA
ATGAT------CGAGTGACATTGCTTCTTCGGCCCGAACCAAAGAAT---CCCTTAGATATGAT
GCAAAACGGATCTTGTTCTATCGTTGATCAGAGATTTCTCTATGAAAAA------TACGAAT
CGGAGTTTGAAGAA---GGGGAAGGAGAAGGAGCCCTCGACCCGCAA------------CAGAT
AGAGGAGGAT------------TTATTCAATCAC------ATAGTTTGGGCTCCT------AGAATATGGC
GCCCT---------TGGGGCTTTCTATTTGATTGTATCGAAAGGCCCAATGAATTGGGATTTCC
C---TATTGGGCCAGGTCATTTCGGGGCGAG---------CGGATCATTTATGATGAAGAGGATG

AGCTTCAAGAGAATGATTCGGAGTTCTTGCAGAGTGGAAGCATGCAGTACCAGACACG
AGATAGATCTTCCAAAGAACAAGGCTTTTTTCGAATAAGCCAATTCATTTGGGACCCTG
CGGATCCGCTCTTTTTCCTATTCAAA---GATCAGCCCTTTGTCTCTGTGTTTTCACATCG
AGAATTCTTTGCAGATGAAGAGATGTCAAAGGGGCTTCTTACTTCCCAAACAGATCCT
CCTACATCTATATATAAACGCTGGTTTATC------AAGAATACGCAAGAAAAGCACTTCGA
ATTGTTGATTCATCGCCAGAGATGGCTTAGA---------ACCAATAGTTCATTATCT------AATG
GATCTTTCCGTTCTAAT------ACTCTATCCGAGAGTTATCAGTATTTATCAAACCTCTTCCT
ATCTAAC---GGAAGGCTATTGGATCAAATGACAAAGACATTGTTGAGAAAAAGATGGCT
TTTCCCGGATGAAATGCAAATT--ATGCCTAGATCGCGA---
ATAAATGGAAATTTTATTGATAAGACCTTTTCAATTGTAGCCAATATCTTATTACGAATAA
TTCCGACAACCTCAGGAGAAAAAGAGGCATTTACCTATTACAGAGATGGA------ATGTT
AGCTCAATCCGAAGGAAATTATGCGGAAGCTTTACAGAATTATTATGAAGCTATGCGAC
TAGAAATTGATCCCTATGATCGAAGTTATATACTCTATAACATAGGCCTTATCCACACAA
GTAACGGAGAACATACGAAAGCTTTGGAATATTATTTTCGGGCACTAGAACGAAATCCA
TTCTTACCACAAGCTTTAAATAATATGGCCGTGATCTGTCATTAC---CGAGGAGAACAGG
CCATTCGACAGGGAGATTCGGAAATTGCGGAGGCTTGGTTCGATCAAGCCGCTGAGTA
TTGGAAACAAGCTATAGCGCTTACTCCTGGAAATTATATTGAAGCACATAATTGGTTGA
AGATCACAAGGCGTTTTGAA------ATGAGTTGGCGATCAGAACGTATATGGATAGAACTT
ATAGCGGGGTCTCGAAAAAGAAGTAATTTCTGCTGGGCCTTTATCCTATTTTTAGGTTCA
TTAGGATTCTTATTGGTTGGAACTTCCAGTTACCTTGGTAGGAATTTGATATCTTTATTTC
CGTCTCAGCAAATAGTTTTTGTTCCACAAGGGATCGTGATGTCGTTCTATGGGATCGCG
GGTCTCTTTATTAGCTCCTATTTGTGGTGCACAATTTCATGGAATGTAGGTAGTGGTTAT
GATCGATTTGATAGAAAAGAAGGAATAGTGTGTATTTTTCGTTGGGGATTTCCTGGAAA
AAACCGTCGAATCTTCCTCCGATTCTTTATGAAAGATATTCAGTCCATCAGAATAGAAG
TTAAAGAGGGTATTTATGCTCGTCGTGTCCTTTATATGGAAATCCGAGGCCAGGGAGCC
GTTCCTTTGACTCGTACTGATGAGAAT---TTGACTCCACGAGAAATTGAACAAAAAGCT
GCGGAATTGGCTTATTTCTTGCGTGTACCAATTGAAATATTT
>Ehretia
ATGGAAAGATGGTGGTTTAATTCGATGTTGTTTCTAAATAAATCAATGGACAGTCCTGG
TCCTATTGAAAATACCAGTGAGAGTGAAGATCCGAATCTAAAAGATAGGGCTAAAAAC
ATTCATAGTTACAGTAATCTTGATTCTTTATTCGGCGTCAAAGACATTCGGAATTTCATC
TCTGATGACACTTTTTTAGTTAGGGATAGTAATGGAGACAGTTATTCCATATATTTTGATA
TTGAAAATCAGATTTTTGAAATTGACAACGATCAT------TCTTTTCTGAGTGAACTAGAA
AGTTCTTTTTATAGTTATCGGAATTCTAGTTATCTGAATAATAGACATACGAGTGAAGAT
CCGTACTATAATCGTTACATGTATGATACTCAATATAGTTGGAATAATCACATTAATAGTT
GCATTGACAGTTATCTTCAG------TCTCAAATCTGCATTGAGACTTCCATTATAAGTGGTA
GTGATAATTACAGT------TACATTTCTAGGTCCATTTGGGGTAGCGAGGGTTCCAGTATAC
GAACCCGCACGAAGGGTAGTGATTTAACTATAAGAGAAAGCTCTAATGATCTCGATGTA
ACTCAAAAATACAGACATTTGTGGGTTCAATGCGAAAATTGTTATGGATTAAATTATAA
AAAAATTTTGAAATCAAAAATGAATATTTGTGAACAATGTGGATATCATTTGAAAATGA
GTAGTTCAGATAGAATCGAACTTTCGATCGATCCAGGTACTTGGGATCCTATGGATGAA
GACATGGTCTCTTTGGATCCCATTGAATTTCATTCGGAGGAGGAGCCTTATAAAGATCG
TATTGATTCTTATCAAAGAAAGACAGGATTAACTGAGGCTGTTCAAACGGGCATAGGCC
GACTAAACGGTATTCCCCTAGCAATTGGGGTTATGGATTTTCAGTTTATGGGGGGTAGTA

TGGGATCCGTAGTAGGGGAGAAAATAACTCGTTTGATTGAGTACGCTACCAATAAATTT
CTACCTCTTATTATAGTGTGTGCTTCCGGGGGGGCACGTATGCAAGAAGGAAGTTTGAG
CTTGATGCAAATGGCTAAAATATCGTCTGCTTTATATGATTATCAATCAAATAAAAAGTTA
TTTTATGTATCAATCCTTACATCTCCTACTACTGGTGGGGTGACAGCTAGTTTTGGTATGT
TGGGGGATATCATTATTGCCGAACCCAATGCCTACATTGCATTTGCGGGTAAAAGAGTA
ATTGAGCAAACATTAAATAAAACAGTACCGGAAGGTTCACAAGCGGCTGAATATTTATT
CCAGAAGGGCTTATTTGACCTAATCGTACCACGTAATCCTTTAAAAAGCGTTCTTAGTG
AACTATTGAAGCTTCACGCTTTCTTTCCTTTGGATCAAAATTCAATCAAGATGGTAACC
ATTCGAGCCGACGAAATTAGTAATATTATCCGTGAACGTATTGAACAATATAATAGAGAA
GTAAAGATTGTAAATACCGGTACCGTACTTCAAGTAGGCGATGGCATTGCTCGTATTCAT
GGTCTTGATGAAGTAATGGCGGGTGAATTAGTAGAATTTGAAGAGGGTACAATAGGCAT
TGCTCTGAATTTGGAATCAAATAATGTTGGTGTTGTATTAATGGGCGATGGTTTGATGAT
ACAGGAGGGAAGTTCTGTAAAAGCAACAGGAAGAATTGCTCAGATACCAGTGAGTGA
GGCCTATTTGGGTCGTGTTATAAATGCTCTGGCTAAACCTATTGATGGTAGAGGTGAAAT
TTCAGCTTCTGAATCTCGATTAATTGAATCTCCTGCTCCAGGTATTATTTCGCGGCGTTC
CGTATATGAACCTCTTCAAACCGGGCTTATTGCTATTGATTCGATGATCCCTATAGGACG
TGGTCAGCGAGAATTAATTATTGGGGACAGGCAGACCGGTAAAACAGCAGTAGCCACA
GATACGATTCTTAATCAACAAGGTCAAAATGTAATATGCGTTTATGTAGCTATTGGTCAA
AAAGCATCTTCTGTGGCCCAGGTAGTAAATACTTTACAGGAAAGGGGGGCGATGGAAT
ACACTATTGTGGTAGCCGAAACGGCGGATTCCCCTGCTACATTACAATATCTCGCTCCTT
ATACAGGAGCAGCTCTGGCTGAATATTTTATGTACCGTGAACGACACACTTCAATCATTT
ATGATGATCCCTCCAAACAAGCACAAGCTTATCGCCAAATGTCTCTTCTATTACGAAGA
CCCCCTGGCCGCGAAGCTTATCCAGGGGATGTTTTTTATTTGCATTCACGCCTTTTGGA
AAGAGCCGCTAAATCAAGTTCTAGTTTAGGTGAAGGAAGTATGACCGCCTTACCAATA
GTTGAAACTCAATCGGGAGATGTTTCGGCTTATATTCCTACAAATGTAATTTCCATTACT
GATGGACAAATATTCTTATCTGCCGATCTATTCAATGCTGGAATCAGACCCGCTATTAAT
GTGGGTATCTCCGTTTCCAGAGTGGGGTCTGCAGCTCAAATTAAAGCCATGAAACAAG
TAGCTGGTAAATTAAAATTGGAACTGGCGCAATTTGCAGAATTAGAAGCCTTTGCACAA
TTTGCTTCTGATCTCGATAAAGCTACTCAGAATCAATTGGCAAGAGGTCAACGATTACG
TGAATTGCTTAAACAATCCCAAGCAGCTCCTCTCGCGGTGGAAGAACAGATAATGACT
ATTTATACCGGAACAAACGGTTATCTTGATTCATTAGAAATTGGACAGGTAAGGAAATT
TCTTGTTGAGTTACGTACTTACGTAAAAACGAATAAGCCTCAGTTCCAAGAAATCATAT
CTTCTACCAAGACATTTACTGAGGAAGCAGAAGCCCTTTTGAAAGAAGCTATTCAAGA
ACAAATGGACCGTTTTCTACTTCAAGAACAAGCAATGAGAATCAATCCTACAGCTTCT
GGTTCTGGGGTTTCCACGCTTGAAAAAAAAAACCTGGGGCGTATCGTCCAAATCATCG
GTCCGGTACTAGATGTCGCCTTTCCGCCGGGCAAGATGCCTAATATTTATAACGCTCTGG
TAGTTAAAGGTCGAGATACTGTTGGTCAACCAATTAATGTGACTTGTGAGGTACAGCAA
TTATTAGGAAATAATCGAGTTAGAGCTGTAGCTATGAGTGCTACAGATGGTCTAACGAG
GGGAATGGAAGTGGTTGACACAGGAGCTCCTCTAAGTGTTCCGGTTGGTGGAGCGAC
TCTGGGACGAATTTTCAACGTGCTTGGAGAGCCTGTTGATAATTTAGGTCCTGTAGATA
CTCGTACAACATCTCCTATTCATAGACCTGCACCCGCTTTTATACAGTTAGATACAAAAT
TATCTATTTTTGAAACAGGAATTAAAGTAGTAGATCTTTTAGCCCCTTATCGCCGTGGAG
GAAAAATCGGACTATTCGGGGGAGCTGGAGTGGGTAAAACAGTACTCATTATGGAATT
GATTAACAATATTGCCAAAGCTCATGGGGGTGTATCCGTATTTGGCGGAGTGGGTGAAC

GTACTCGCGAAGGAAATGATCTTTACATGGAAATGAAAGAATCTGGAGTTATTAATGAA
GAAAATATTGCAGAATCCAAAGTAGCTCTAGTTTACGGCCAGATGAATGAACCGCCAG
GAGCTCGTATGAGAGTTGGTTTGACTGCCCTAACTATGGCGGAATATTTCCGAGATGTT
AATGAACAAGACGTACTTCTATTTATCGACAATATCTTCCGTTTCGTTCAAGCAGGATCC
GAAGTATCGGCCTTATTGGGTAGAATGCCTTCCGCTGTGGGTTATCAACCCACCCTGAG
TACCGAAATGGGTTCTTTACAAGAGAGAATTACTTCTACCAAAGCGGGGTCCATAACTT
CTATTCAAGCAGTTTATGTACCCGCAGATGATTTGACCGACCCTGCTCCTGCTACGACA
TTTGCACATTTAGATGCTACTACCGTACTATCAAGAGGATTAGCTGCCAAAGGGATCTAT
CCAGCAGTAGATCCTTTAGATTCAACGTCAACCATGCTTCAACCTCGGATCGTTGGTGA
GGAACATTATGAAACTGCGCAAAGAGTGAAGCAAACTTTACAACGTTATAAAGAACTT
CAGGACATTATAGCTATCCTTGGGTTGGACGAATTATCTGAAGAGGATCGTTTAACCGT
AGCAAGAGCGCGAAAAATTGAGCGTTTCTTATCACAACCCTTTTTCGTAGCAGAAGTA
TTTACCGGTTCTCCAGGGAAATATGTTGGTCTAGCAGAAACAATTAGAGGGTTTCAATT
GATCCTTTCCGGAGAATTAGATGGTCTTCCTGAACAGGCCTTTTATTTGGTAGGTAATAT
CGATGAAGCTACCGCGAAGGCTATGAACTTAGAAATGGAGAGCAATTTGAAGAAAATG
ACCTTAAATCTTTGTGTACTGACCCCTAATCGAATTGTTTGGGATTCAGAAGTGAAAGA
AATCATTTTATCTACTAATAGTGGTCAAATTGGCGTATTACCAAATCACGCCCCTATTGCC
ACAGCTGTAGATATAGGTATTTTGAGAATACGCCTTAACGACCAATGGGTAACGATGGC
TCTGATGGGTGGTTTTGCTAGAATAGGTAATAATGAGATCACTGTTTTAGTAAATGATGC
GGAGAAAGCTAGTGACATTGATCCACAAGAAGCTCAGAAAACTCTTGAAATAGCGGA
AGCTAATTTGAGAAAAGCTGAAGGAAAGAGACAAACAATTGAGGCAAATCTAGCTCT
CCGACGAGCTAGGACACGAGTAGAGGCTATCAATGCGATGATTATGAAAAATGTAACC
GATTCTTTCGTTTCT---GGCCACTGGCCATCTGCCGGGAGTTTCGGGTTTAATACCGATA
TTTTAGCAACAAATCCAATAAATCTAAGTGTAGTGATTGGTGTATTGATCTTTTTTGGAA
AGGGAGTGTTAAGTGATTTATTAGATAATCGAAAACAGAGGATTTTGAATACTATTCGA
AATTCCGAAGAACTGCGTGGGGGGGCCATTGAACAGCTGGAAAAAGCCCGGTCTCGC
TTACGGAAAGTGGAAATGGAAGCCGAGCAGTTTCGAGTGAATGGATACTCTGAGATAG
AGCGAGAAAAATTGAATTTGATTAATTCAACTTATAAGACTTTGGAACAATTAGAAAAT
TACAAAAATGAAACTATTCAGTTTGAACAGCAAAGGGCGATTAATCAAGTACGACAAC
GGGTTTTTCAACAAGCCTTACAAGGAGCTCTAGGAACTTTGAATAGTTGTTTGAACAA
CGAGTTACATTTACGCACCATTAGTGCCAATATTGGCATGTTGGGGGCGATGAAAGAAA
TAACTGATATGAATCCACTGATTTCTGCCGCTTCCGTTATTGCTGCTGGATTGGCCGTAG
GGCTTGCTTCTATTGGACCTGGAGTTGGTCAAGGGACTGCTGCGGGTCAAGCTGTAGA
GGGTATCGCGAGACAGCCCGAGGCTGAGGGAAAAATACGAGGTACTCTATTGCTTAGT
CTAGCTTTTATGGAAGCTTTAACAATTTATGGACTGGTTGTAGCATTAGCACTTTTATTT
GCGAATCCTTTTGTTATGAATGTTCTATCATGTTCCATCAACACACTTAAAGGGTTATAC
GATATATCCGGTGTGGAAGTAGGCCAACATTTCTATTGGCAAATAGGGGGTTTCCAAGT
CCATGGTCAAGTACTTATTACTTCTTGGGTTGTAATTGCTATCTTATTAGGTTCAGCCGCT
ATAGCTGTTCGGAACCCACAAACCATTCCGACTGGGGGTCAGAATTTCTTCGAATATGT
TCTTGAATTCATTCGAGATGTGAGTAAAACTCAAATTGGAGAAGAATATGGTCCTTGGG
TTCCTTTTATTGGAACTATGTTTTTATTTATTTTTGTTTCTAATTGGTCAGGAGCTCTTTTA
CCTTGGAAAATCATACAATTACCTCATGGAGAGTTAGCCGCACCCACGAATGATATAAA
TACTACTGTTGCTTTGGCTTTACTCACGTCAGTGGCATATTTCTATGCGGGTCTTAGTAA
AAAGGGATTAGGTTATTTCGGGAAATATATTCAACCAACTCCAATCCTTTTACCCATCAA

CATCTTAGAAGATTTCACAAAGCCTTTATCGCTTAGTTTTCGACTTTTCGGGAATATCTT
AGCTGATGAATTAGTAGTTGTTGTTCTTGTTTCTTTAGTACCTTCAGTCGTTCCTATACCT
GTCATGTTCCTTGGATTATTTACAAGTGGTATTCAAGCTCTTATTTTTGCAACTTTAGCC
GCGGCGTATATAGGTGAATCCATGGAGGGCCATCATATGATATTTTCAACCTTAGAGCAT
ATATTAACTCATATTTCTTTTTCGATCGTTTCAATTGTAATTACAATTCATTTGATAACCTT
TTTAGTTGATAAAACTGTAAAACTATATGATTCATCAGAAAAGGGCATGATAGTTACTTT
TTTCTGTATAACAGGATTATTAGTTACTCGTTGGATTTATTCAGGGCATTTCCCATTAAGT
GATTTATATGAATCATTAATTTTCCTTTCATGGAGTTTCTCTCTTATTCATATAGTTCCGTA
T------AAAAATAAAAATGATTTAAGCACAATAACTGGCCCGAGTGCTATTTTTACTCAAG
GCTTTGCTACTTCAGGTCTTTTAACTGAAATACATCAATCTGAAATATTAGTACCTGCCC
TTCAATCCGAATGGTTAATAATGCACGTAAGTATGATGATATTGGGCTATGCAGCCCTTT
TATGTGGATCATTATTATCAGTAGCACTTCTAGTCATTACATTTCGAAAAAGAAGAAAGA
TTTTTTGT------AAAGAAATCGAATACATGAATGAAAGAAGCAATGTTTTACAAAATACG
TCT---------GCTAAGAATTATTACAGATCCCAATTAATTCACCAATTGGATTATTGGAGTTA
TCGGATTATTAGTCTAGGATTTATCTTTTTAACCGTAGGAATTCTTTCGGGAGCAGTATG
GGCTAACGAAGCATGGGGATCCTATTGGAATTGGGACCCAAAGGAAACTTGGGCATTC
ATTACTTGGATCGTATTCGCGATTTATTTACATACTCGAACAAATATAAATTTGCAGGGG
GCAAATTCCGCAATTGTGGCGTCTATAGGCTTTCTTATAATTTGGATATGCTATTTTGGGG
TCAATCTATTAGGAATAGGGCTACATAGTTATGGTTCATTTGCATTAACAATGGCAAAAA
AGAAAGTATTCACTCCCCTTTTCTATCTTGCATCTATAGTATTTTTGCCCTGGTGGATTTC
TCTCTCATTTAATAAAAGTCTGGAATCCTGGGTTACTAATTGGTGGAATACTGGGCAATC
CGAAATTTTTTTGAATGATATTCAAGAAAAGAGTATTCTAGAAAAATTCATAGAATTAG
AGGAACTCCTCTTCTTGGACGAAATGATCAAAGAATACTCGGAGACACATCTCCAAAA
ACTTCGTATA---ATCCACAAAGAAACGATCCAATTAATTAAGATACACAATGAGGATCGT
ATCCATACAATTTTGCACTTCTCGACAAATCTAATCTGTTTTGTTATTCTAAGCGGTTATT
CTATTTTGGGTAATGAAGAACTTGTTATTCTTAACTCTTGGACTCAGGAATTCCTATATA
ACTTAAGCGACACAGTCAAAGCTTTTTCTATTCTTTTATTAACTGATTTATGTATCGGATT
CCATTCACCACACGGCTGGGAACTAATGATTGGCTCTGTCTACAAAGATTTTGGATTTG
TTCATAATGATCAAATTATATCTGGTCTTGTTTCCACTTTTCCAGTCATTCTCGATACAAT
TTTTAAATATTGGATTTTCCGTTATTTAAATCGCCTATCTCCGTCACTTGTAGTTATTTATC
ATTCAATGAATGACATGCCTATTGGTGTTCCAAAAGTACCTTTCCGGAGTCCTGGAGAG
GAAGATGCATCTTGGGTTGACGTATACAACCGACTTTATCGAGAAAGATTACTTTTTTTA
GGTCAAGAGGTTGATAGCGAGATCTCGAATCAACTTATTGGTCTTATGGTATATCTTAGT
ATTGAGGATGATACCAAGGATCTATATTTGTTTATAAACTCTCCCGGGGGATGGGTAATA
CCTGGAGTAGCTATTTATGATACTATGCAATTTGTGCGACCAGATGTCCATACAATATGC
ATGGGATTAGCCGCTTCAATGGGATCTTTTATCCTGGTCGGAGGAGAAATTACCAAACG
TCTAGCATTCCCTCACGCCAGGGTAATGATCCATCAACCTGCTAGTTCTTTTTATGAGGC
ACAAACGGGAGAATTTATCCTGGAAGCGGAAGAACTGCTGAAACTGCGTGAAACCCT
CACAAGGGTTTATGTACAAAGAACAGGCAAACCATTATGGGTTGTATCCGAAGATATGG
AAAGAGATGTTTTTATGTCAGCAACGGAAGCCCAAGCTTATGGAATTGTTGATCTTGTA
GCGGTTGAAATGAAAGAACAAAAATGGATTCATGAAGGTTTAATTACTGAATCGCTTC
CCAACGGTATGTTCCGGGTTCGTTTAGATAATGAAGATCTGATTCTAGGTTATGTTTCAG
GAAAGATCCGACGTAGTTTTATACGGATACTGCCAGGAGATAGAGTCAAAATTGAAGT
AAGTCGTTATGATTCCACCAGAGGACGTATAATATATCGACTCCGCAACAAGGATTCGA

AAGATATGGAGGAAATTCAAAGATATTTACAG---GATAGATCTCAACAACACGACTGCC
TATATCCACTTATCTTTCAGGAGTATATTTATGCACTTGCTCATGATCATGGTTTACCAGG
TTATGACAATAAATCCAGTTTACTAATTGTGAAACGTTTAATTACTCGAATGTATCAACA
AAATCATTTTTTTATTTCTGTTAATGATTCGAACCAAAATCAATTTTTTGGGAAGAATTT
GTATTCTCAAATGATATCAGAGGGATTTGCATTTATTGTCGAAATGCCGTTTTCTCTACG
ATTAATATCTTCTCTAATATTCAAATCTCATAATTTACGGTCAATTCATTCAATATTTCCTT
TTTTAGAGGACAACTTTTCACATTTAAATTGTGTGTTAGATATACTAATACCCCACCCCG
TTCATCCGGAAATCTTGGTTCAAACTCTTCGCTTTTGGGTAAAAGACGCCTCTTCTTTG
CATTTATTACGATTCTTTCTCCACGAGTATTGGAATAAT------CTTATTACCCCCAGC---TCT
TCTTTTTCAAAACAAAGATTATTCTTCTTCTTATATAATTCTCATGTATGTGAATACGAAT
CCATTTTCGTCTTTCTCCGCAATCAATCTTCTCATTTACGATCAACATCTTTTGGAGCCC
TTCTTGAACGAATATATTTCTATGGAAAAATAGAACGTCTTGTAGAAGTCTTTGCT---GA
TTTTACCCTATGGTTGTTCAAGGATCCGTTCATACATTATGTTAGGTATCAAGGAAAATT
AATTTTGGCTTCAAAGGGGACGCCTCTTTTGATGAATAAATGGAAATATTACCTTGTCA
ATTTTTGGCAATGTTATTTTTACCTGTGGTCGCGCCCAGGAAGAATCTATATAAACCAAT
TATCCAATCATTCCCTTGACTTTATGGGCTATCTTTCAAGTGTGCGACTAAACCCTTCAA
TGGTACGGAGTCAAATGCTAGAAAATGCATTTCTAGTTAATAATGTTATT------AAGAAGT
TTGATACTCTTCTTCCAATTATTCCTCTGATTGGATCATTGGCTAAAGCGAAATTTTGTA
ACATATTAGGGAATCCCATTAGTAAGCCGGTTCGGGCTGATTTATCAGATTCTGATATTAT
TGATCGATTTGGGCGTATATGCAGAAATCTTTCTCATTATCATAGCGGATCTTCGAAAAA
AAAGAGTTTGTATCGAATAAAGTATATACTTCGACTTTCTTGTGCTAAAACTTTGGCTCG
GAAACACAAAAGTAGTGTACGTGCTTTTTTGAAAAGATTCGGCTCGGAATTATTGGAA
GAATTTCTTACATCGGAAGAACAAGTTCTTTCTTTGACCTTCCCAAGAAGGATTTGGTA
TTTGGATATTATTTGTATCAATGATCTGGCTAATCATCAAATGATAATTGAT---ACAACAG
AAATAGAA---GCTATCAATTCTTTTTCCAGATTGGAATCT---TTAAAAGAAATCTATGGGA
TCATATGGATACTTGTCCCTATTTTGATTCTTGTATTAGGAATCACAATCGGCGTTCTAGT
AATTGTTTGGTTAGAAAGAGAAATTTCTGCAGGGATACAACAACGTATTGGCCCCGAAT
ATGCCGGTCCCTTGGGAATTCTTCAAGCCCTAGCAGATGGTACAAAACTACTTTTCAAA
GAGAATCTTCTTCCATCTAGAGGAGATGCTCGTTTATTCAATATCGGACCGTCCATAGCA
GTCATATCAATTTTACTAAGTTATTCAGTAATTCCTTTTGGCTATCACCTTGTTCTAGCTG
ATCTTAGTATTGGCGTTTTTTTATGGATCGCCATTTCAAGTATTGCTCCGGTTGGACTTCT
TATGTCAGGATATGGGTCAAACAATAAATATTCCTTTTTAGGTGGTTTACGGGCTGCTGC
TCAATCAATTAGTTATGAAATACCATTAACTCTATGTGTGTTATCAATATCTCTACTATCTA
ACAGTTCAAGTACAGTTGATATAGTTGAAGCGCAGTCAAAATATGGTTTTTGGGGGTGG
AATTTGTGGCGTCAACCTATAGGGTTTATCATTTTTCTAATTTCTTCTCTAGCCGAGTGT
GAGAGATTACCTTTTGATTTACCAGAAGCAGAAGAAGAATTAGTAGCAGGTTATCAAA
CCGAATATTCCGGTATCAAATTTGGTTTATTTTACGTTGCTTCGTATTTGAATCTACTAGT
TTCTTCATTATTTGTAACAGTTCTTTACTTGGGGGGTTGGAATTTTTCTATTCCGTATATAT
TAGTTCCTGAACTTTTTGACATAAATAAAGCGGGTAAAGTCTTTGGAACAATAATCGGT
ATCTTTATTACATTATCTAAAACTTATTTGTTCTTGTTCATTCCTATTGCAACAAGATGGA
CTTTACCGAGGCTGAGAATGGACCAACTATTAAATCTTGGATGGAAATTTCTTTTACCTA
TTTCTTTAGGTAATCTATTATTAACAACTTCGTCCCAACTTCTTTCACTGATGATCTGGCA
TGTACAGAATGAAAACTTCATTCTCGATTCTACGAGAATTTTTATGAAAGCCTTTCATTT
GCTTCTCTTCGATGGAAGTTTGATTTTCCCAGAATGTATCCTAATTTTTGGCCTAATTCTT

CTTCTGATGATCGATTCAACCTCTGATCAAAAAGATATACCTTGGTTATATTTCATCTCTT
CAACAAGTTTAGTAATGAGCATAACGGCCCTATTGTTCCGATGGAGAGAAGAACCTATG
ATTAGCTTTTCGGGAAATTTCCAAACGAACAATTTCAACGAAATCTTTCAATTTCTTATT
TTACTATGTTCAACTCTATGTATTCCTCTATCCGTAGAGTACATTGAATGTACAGAAATG
GCTATAACAGAGTTTCTCTTATTCGTATTAACAGCTACTCTAGGAGGAATGTTTTTATGC
GGTGCTAACGATTTAATAACTATCTTTGTAGCTCCAGAATGTTTCAGTTTATGCTCCTAC
CTATTATCTGGATATACCAAGAAAGATGTACGGTCTAATGAGGCTACTATGAAATATTTA
CTCATGGGTGGGGCAAGCTCTTCTATTCTGGTTCATGGTTTCTCTTGGCTATATGGTTCA
TCCGGGGGAGAGATCGAGCTTCAAGAAATAGTGAATGGTCTTATCAATACACAAATGTA
TAACTCCCCAGGAATTTCAATTGCGCTCATATTCATCACTGTAGGAATTGGGTTCAAGCT
TTCCCCAGCCCCTTCTCATCAATGGACTCCTGACGTATACGAAGGATCTCCCACTCCAG
TCGTTGCTTTTCTTTCTGTTACTTCGAAAGTAGCTGCTTCAGCTTCAGCCACTCGAATTT
TCGATATTCCTTTTTATTTCTCATCAAACGAATGGCATCTTCTTCTGGAAATCCTAGCTAT
TCTTAGCATGATATTGGGAAATCTCATTGCTATTACTCAAACAAGCATGAAACGTATGCT
TGCATATTCGTCCATAGGTCAAATCGGATATGTAATTATTGGAATAATTGTTGGAGACTC
AAATGATGGATATGCAAGCATGATAACTTATATGCTGTTCTATATCTCCATGAATCTAGGA
ACTTTTGCTTGCATTGTATTATTTGGTCTACGTACCGGAACTGATAACATTCGAGATTAT
GCAGGATTATACACGAAAGATCCTTTTTTGGCTCTCTCTTTAGCCCTATGTCTCTTATCC
CTAGGAGGTCTTCCTCCACTAGCAGGTTTTTTCGGAAAACTCTATTTATTCTGGTGTGG
ATGGCAGGCAGGCCTATATTTCTTGGTTTTAATAGGACTCCTTACAAGCGTTGTTTCTAT
CTACTATTATCTAAAAATAATCAAGTTATTAATGACTGGGCGAAACCAAGAAATAACCC
CTCACGTGCGAAATTATAGAAGATCCCCTTTAAGATCAAACAATTCCATCGAATTGAGT
ATGATTGTATGTGTGATAGCATCTACTATACCAGGAATATCAATGAACCCGATTATTGCAA
TTGCTCAGGATACCCTTTTTATGTTTCTGCTCTACGAATATGATATTTTTTGGGCATTTTT
AATAATATCAAGTCTTATTCCTATTTTGGCATTTTTGATTTCCGGAGTTTTAGCCCCGACT
AGCAAAGGACCAGAGAAACTTTCTAGTTACGAATCGGGTATAGAACCAATGGGTGACG
CTTGGTTACAATTTCGAATCCGTTATTATATGTTTGCTCTAGTTTTTGTTGTTTTTGATGTT
GAAACGGTTTTTCTTTATCCATGGGCAATGAGTTTCGATGTATTGGGTGTATCTGTATTTA
TAGAAGCTTTAATTTTCGTGCTTATCTTAATTGTTGGTTTAGTTTATGCATGGCGAAAGG
GGGCATTGGAATGGTCTACGAATGATTTTCCTTGGTTAACAACAATTGTAGTTTTGCCA
ATATTTGCGGGTTCCCTAATTTTCTTTCTTCCCCATAGAGGGAATAGGGTAATTAGATGG
TATACAATATGTATATGTATTTTGGAACTCCTTCTAACGACCTATGCATTCTGTTATCATTT
CCAATCGGACGATCCATTAATCCAACTAGTAGAGGATTTTAAATGGATCAAATTTTTTGA
TTTCCATTGGAGATTAGGAATAGATGGGCTTTCTATAGGACCCATTTTACTGACGGGATT
CATCACTACTTTAGCTACTTTAGCGGCTCGGCCAATTACTCGGGATTCTCGATTATTCCA
TTTCCTGATGTTAGCAATGTACAGCGGTCAAATAGGATCATTTTCTTCTCGGGACCTTTT
ACTTTTTTTCATCATGTGGGAGTTAGAATTAATTCCTGTTTATTTACTTCTATCCATGTGG
GGAGGAAAGAAACGTCTGTACTCAGCTACAAAATTTATTTTGTACACAGCAGGAGGTT
CCGTTTTTCTCTTAATGGGGGTTCTGGGTATCAGTTTATATGGTTCTAATGAGCCAACAT
TAAATTTTGAAACGTCAGTTAATCAGTCGTATCCTGTGGCTTTAGAAATAATATTCTATAT
TGGATTTTTTATTGCTTTTGCTGTCAAATCGCCGATTATACCCCTACATACATGGTTACCA
GATACCCATGGAGAAGCACATTACAGTACTTGTATGCTTCTAGCGGGAATCTTATTAAA
AATGGGAGCGTATGGGTTAGTTCGTATCAATATGGAACTATTACCTCACGCCCATTCTAT
ATTTTCTCCTTGGTTGGTAATAGTAGGTACCATGCAAATAATCTATGCAGCTTCAACATC

TCTTGGCCAACGCAATTTAAAAAAAAGAATAGCCTATTCCTCTGTATCCCATATGGGTTT
CATAATTATAGGAATTGGTTCTATAACAGATACAGGGCTGAATGGAGCCCTTTTACAAAT
AATCTCTCATGGATTTATTGGTGCTGCACTTTTTTTCTTGGCAGGAACAACGTATGATAG
AATACGTCTTGTTTATCTTGACGAAATGGGCGGAATAGCTATCCCCCTGCCAAAAATATT
CACGATGTTCAGTAGCTTTTCGATGGCTTCCCTTGCATTACCAGGTATGAGTGGTTTTGT
TGCCGAATTGATAGTATTTTTTGGAATAATTACCAGCCAAAAATATCTTTTAATGCCAAA
AATACTAATTACTTTTGTAATGGCAATTGGAATGATATTAACTCCTATTTATTCATTATCTA
TGTTACGCCAGATGTTCTATGGATACAAGCTTTTTAATGCCCCAAACTCTTATTTTTTTGA
TTCTGGACCGCGAGAGTTATTTCTTTCGATCTGTATCTTTTTACCCGTAATAGCAATTGG
TATGTACCCGGATTTCGTTCTTTCACTATCGGTTGACAAGGTCGAAGTTATTCTATCTAA
TTCTTTTTATAGAATGATACTCGAACATGTACTTGTTTTGAGTGCCTATTTATTTTCTATC
GGAATCTATGGATTGATCACAAGTCGAAATATGGTTAGAGCCCTTATGTGCCTTGAACTT
ATATTGAATGCAGTTAATATCAATTTTGTAACATTTTCTGATTTTTTTGATAATCGTCAATT
AAAAGGAGACATTTTCTCAATTTTTGTTATAGCTATTGCAGCCGCTGAAGCAGCTATTG
GACCGGCTATTGTTTCATCAATTTATCGTAATAGAAAATCAACTCGTATCAATCAATCAA
ATTTGTTGAATAAAATGGAACAGACATATCAATATGCGTGGATCATACCTTTGCTTCCGC
TTACAGTTCCTATGTTAATAGGAGTGGGACTTCTTCTTTTTCCGACAGCAACAAAAAAT
CTTCGTCGTATGTGGGCTTTTCAGAGTATTTTATTGTTAAGTATAGTCATGATTTTTTCAA
TCAATTTGTCTATTCAGCAAATAAATAGCAGTTCTATCTATCAAAATGTATGGTCTTGGAT
CATTAATAATGATTTTTCTTTAGAATTCGGCTACTTGATCGATCCACTTACTTCTATTATGT
CAATATTAATCACTACTGTTGGAATTATGGTTCTTATTTATAGTGATAATTATATGTCTCAT
GATCAAGGATATTTGAGATTTTTTGCTTATATGAGTTTTTTCAGTACTTCCATGTTGGGAT
TAGTTACTAGTTCTAATTTGATCCAAATTTATATTTTTTGGGAATTGGTTGGAATGTGTTC
CTATCTATTAATAGGGTTTTGGTTCGCACGACCTGTTGCGGCAAATGCTTGTCAAAAAG
CGTTTGTAACTAATCGTGTAGGGGATTTTGGTTTATTATTAGGAATTTTAGGTTTTTATTG
GATAACTGGTAGTTTCGAATTTCGGGATTTATTCGAAGTATTCAATAATAATGAGGTCAA
TTTATTCTTTGCTACTTTATGTGCCATTCTATTATTTGCCGGTGCAGTTGCTAAATCTGCA
CAATTTCCACTTCATGTATGGTTACCGGATGCCATGGAGGGACCTACTCCTATTTCGGCT
CTTATCCATGCTGCTACTATGGTAGCAGCGGGAATTTTTCTTGTAGCTCGCCTTCTTCCT
CTTTTCATAGTTATACCTTATATAATGAATTTTATCGCATTGATAGGAATAATAACAGTATT
CTTAGGAGCTACTTTAGCTCTTGCTCAAAAAGACATTAAAAGGGGTTTAGCCTATTCCA
CAATGTCTCAGTTGGGTTATATGATGTTAGCTCTAGGTATGGGGTCTTATCGAAATGCTT
TATTTCATTTGATTACTCATGCTTATTCCAAAGCATTATTATTTTTAGGATCCGGATCCGTT
ATTCATTCAATGGAAACTGTTGTTGGCTATTCTCCAGATAAAAGTCAGAATATGGTTCTT
ATGGGAGGTTTAACAAAACATGTACCAATTACCAAAACTGCTTTTTTATTAGGTACACT
TTCTCTTTGTGGTATTCCACCTCTTGCTTGTTTTTGGTCTAAAGATGAAATTCTTAATGAT
AGTTGGTTGTATTCACCGATTGTCGCAATAATAGCTTGGGCCACAGCGGGATTTACCGC
ATTTTATATGTTTCGGATCTATTTACTTACTTTTGAGGGGCATTTAAATGTTCATTTTCAA
AATTACAGTGGCAAACAAAATACCCCCTTCTATTCATTATCTCTGTGGGGTAAAAGGGG
TTCGAAAGGAATTAACAAA---TTAGTAACA---------ATCAATTTTTTTTCATTTAAGAATAA
AAAGCCTTATTCCTATCCTTCGGAATCGGATAATACTATGTTATTTCCCCTACTTATATTG
GGCCTATTTACTTTGTTCGTTGGATCTCTAGGAATTCCTTTC---GTGGATATATTATCCAA
ATGGTTAGCTCCGTCTATAAACCTTTTGCATCAAAAATCGAAT---TCAATGGATTGGTATG
AATTTTTGAAAGATGCTGTTTTTTCAGTCAGTATAGCTTATTTCGGAATATTGATAGCGT

CCTTTTTATATAAACCTATTTATTCTTCTTTACAAAATTTCGACTTAATTAATTCATTTGTT
ATTGTTTGGGACAAAATTCTAAATGGCATATACGATTGGTCATATAATCGTGCTTACATA
GATGCTTTTTATAGAACATCCTTAACGGGAGGGATAAGAGGATTGGCTGAATTAACTCA
TCTTTTTGATCGACGAGTAATTGATGGAATTACGAATGGAGTTGGTGTTATGAGTTTCTT
TGTAGGAGAAGGTATCAAATATGTAGGGGGCGGG---CGTATCTCTTCGTATCTTTTCTTG
TATTTATTTTCTGTATCAATCTTTTTATTAATTTATTACATGGATTTACCTGGACCAATACA
TGATTTTCTTTTAGTCTTTCTGGGATCGGGTCTTATATTAGGAGGTCTAGGAGTAGTATTA
CTTCCCAATCCAATTTATTCTGCCTTCTCGTTGGGGTTGGTTCTTGTCTGTATATCCTTAT
TCTATATTCTATCGAACTCCTATTTTGTAGCTGCCGCACAGCTCCTTATTTACGTCGGAGC
TATAAATGTTTTAATCATTTTTGCTGTGATGTTCATGAATGGTTCAGAATATTACAAAGAT
TTTCATCTTTGGACCGTTGGGGATGGAGTTACTTCGATGGTTTGTACAAGTCTTTTTATT
TCACTAATTACTACTATTCTAGATACGTCATGGTACGGGATTATTTGGACTACAAAATCA
AACCAGATTATAGAGCAAGATTTGATAAGTAATAGTCAACAAATTGGAATTCATTTATCA
ACAGATTTTTTTCTTCCATTTGAATTCATTTCAATAATTCTTTTAGTTGCTTTAATAGGTG
CAATTGCTGTAGCTCGTCAAATGACTGCACCAGTTACAAGAAAAGACCTCATGATAGT
AAATATGGGTCCTCATCACCCATCAATGCATGGTGTTCTTCGACTCATCGTTACTCTAGA
TGGTGAAGATGTTATTGACTGTGAACCAATATTGGGTTATTTACACAGAGGGATGGAGA
AAATTGCGGAAAACCGAACAATTATACAATATTTGCCTTATGTAACACGTTGGGATTATT
TAGCTACTATGTTCACAGAAGCAATAACCGTAAATGGACCCGAACAGTTAGGAAATATT
CAAGTACCTAAAAGGGCTAGCTATATTAGAGTCATTATGTTGGAGTTGAGTCGTATAGCT
TCCCATTTGTTATGGCTAGGCCCTTTTATGGCAGATATTGGGGCACAGACCCCCTTCTTC
TATATTTTTCGAGAAAGAGAACTGATATATGACCTATTCGAAGCTGCCACTGGTATGCGA
ATGATGCATAATTATTTTCGTATTGGAGGAGTAGCTGCTGATCTACCTTATGGCTGGATA
GATAAATGTTTGGATTTTTGCGATTATTTTTTAACAGGGGTTGCTGAATATCAAAAGCTT
ATTACACGGAATCCTATTTTTTTAGAACGAGTTGAAGGCGTAGGCATTATTGGCGGAGA
AGAAGCAAAAAATTGGGGTTTATCAGGACCAATGCTACGAGCTTCCGGAATACAATGG
GATCTTCGTAAAGTTGATCATTATGAGTGTTACGACGAATTTGATTGGGAGGTTCAGTG
GCAAAAAGAGGGGGATTCATTAGCTCGTTATTTAGTACGAATCAGTGAAATGACAGAA
TCCATAAAAATTATTCAGCAGGCTCTGGAAGGAATCCCAGGGGGCCCCTATGAGAATTT
AGAAATCCGACGCTTTGATAAAGTAAAAGATCCCGAATGGAATGATTTTGAATATCGAT
TTATTAGTAAAAAACCTTCTCCGACTTTTGAATTGTCGAAGCAAGAACTTTATGTGAGA
GTCGAAGCCCCAAAAGGAGAATTGGGAATTTTTTTGATAGGAGATCAGAGTGTTTTTC
CTTGGAGATGGAAAATTCGCCCACCGGGTTTTATCAATTTGCAAATTCTTCCTCACTTA
GTTAAAAGAATGAAATTGGCTGATATTATGACAATACTAGGTAGCATAGATATCATTATG
GGAGAAGTTGATCGTATGTTCCCTATGGTAACTGAGTTCATGAATTATGGTCAACAAAC
AATACGAGCAGCCAGGTACATTGGTCAAGGTTTCATGATTACCTTGTCCCACGCGAATC
GTTTACCAGTAACTATTCAATACCCCTATGAAAAATTGATCGCATCGGAACGTTTCCGA
GGCCGAATCCACTTTGAATTTGATAAATGCATTGCTTGCGAAGTATGTGTTCGTGTATGT
CCTATAGATCTACCCGTTGTTGATTGGAAATTGGAAACTGATATTCGAAAGAAACAATT
GCTTAATTACAGTATTGATTTTGGGGTCTGTATATTTTGTGGTAATTGTGTTGAGTATTGT
CCAACAAATTGTTTATCAATGACTGAAGAATATGAACTTTCTACTTATGATCGTCACGAA
TTGAATTATAATCAAATTGCCTTGGGTCGGTTACCGATGTCAGTAATTGACGATTACACA
ATTCGAACAATTTCGAATTCA---ATGCAGGGTCGTTT
GTCTGCTTGGCTAGTCAAGCATGGACTAATTCATAGATCTTTGGGCTTTGATTACCAAG

GAATAGAGACTTTACAAATAAAGCCCGAGGATTGGCATTCCATTGCTGTCATTTTGTAT
GTATATGGTTACAACTATCTACGCTCCCAATGTGCCTATGATGTAGCACCCGGCGGACTG
TTAGCTAGTGTCTATCATCTTACGAGAATAGAGTATGGTGTGGATCAACCAGAAGAGGT
ATGCATAAAAGTATTTGCCTCAAGGAGGAATCCGAGAATTCCGTCCGTTTTCTGGGTTT
GGAAAAGTGTGGATTTTCAAGAACGAGAATCTTATGATATGTTGGGAATCTCTTATGAT
AATCATCCACGCTTGAAACGTATCTTAATGCCTGAAAGTTGGATAGGATGGCCCCTACG
TAAGGATTATATTGCCCCCAATTTTTATGAAATACAAGATGCTCATATGGGCAATGAGTT
TCGATGTATTGGGTGTATCTGTATTTATAGAAGCTTTAATTTTCGTGCTTATCTTAATTGTT
GGTTTAGTTTATGCATGGCGAAAGGGGGCATTGGAATGGTCTTAGCTCCTGAATGTTCA
GACAATAAAAAGAAAAAAGGAAAAAAAAAAGATTGAGACA---GTTATGAATTCCATTGA
GTTTCCTTTACTTGATCGAACAACCCCAAATTCAGTTATTTCAACTACATTAAATGATCT
TTCAAATTGGTCAAGACTCTCTAGTTTATGGCCGCTTCTCTACGGTACCAGTTGTTGCTT
CATTGAATTTGCTTCACTAATAGGCTCGCGATTCGACTTTGATCGTTATGGACTAGTACC
AAGATCGAGTCCTAGACAAGCGGATCTAATTTTAACAGCCGGAACAGTAACAATGAAA
ATGGCCCCCTCTTTAGTGAGATTATATGAACAAATGCCGGAACCAAAATATGTTATTGCT
ATGGGAGCGTGTACAATTACAGGGGGGATGTTCAGTACCGATTCTTATAGTACTGTTCG
GGGGGTCGATAAGCTAATTCCTGTAGATGTCTATTTGCCTGGCTGTCCACCTAAACCGG
AAGCAGTTATAGATGCTATAACAAAACTTCGTAAGAAAATATCTCGAGAAATCTATGAA
GATAGAATTAGGTCTCAACGAGCGAATCGGTGTTTTACTACCACTCACAAGTTTCATGT
TGGACGCAGTATTCATACTGGAAATTATGATCAAAGATTCCTTTATCAACCGTCATCTAC
TTCAGAGATCCCTACTGAAACTTTTTTCAAATACAAAAGTTCAGCATCTTCCCACGAAT
TAGTGAATATGCAAACTAGAAATACTTTTTCTTGGATAAAGGAACAGATTACTCGATCTA
TTTCCGTATCGCTCATGATATATATCATAACTCGGACATACATTTCAAATGCATATCCCATT
TTTGCACAGCAGGGTTATGAAAATCCACGAGAAGCGACTGGGCGTATTGTATGTGCCA
ATTGTCATTTAGCTAATAAGCCCGTGGAGATTGAGGTTCCACAAGCGGTACTTCCTGAT
ACTGTATTTGAAGCAGTTGTTCGAATTCCTTATGATATGCAACTGAAACAAGTTCTTGCT
AATGGTAAAAAAGGGGGTTTGAATGTAGGGGCTGTTCTTATTTTACCGGAAGGGTTTG
AATTAGCTCCTCCCGATCGTATTTCTCCCGAGCTGAAAGAAAAGATAGGCAATTTATCT
TTTCAGAGCTATCGCCCCAATAAAAAAAATATTCTTGTGATAGGCCCCGTCCCTGGTCA
GAAATATAGTGAAATCACCTTTCCTATTCTTTCCCCGGATCCTGCTACTAAGAAGGATGT
TCACTTCCTAAAATATCCTATATACGTAGGCGGGAACAGGGGAAGGGGTCAGATTTATC
CCGACGGGAGCAAGAGTAACAATACAGTTTATAATGCTACAGCAACGGGTATAGTAAG
TAAAATCATACGAAAAGAGAAGGGGGGATATGAAATAACCATAACAGATGCGTCGGAT
GGACGTCAAGTGGTTGATATTATCCCTCCAGGACCAGAACTTCTTGTTTCAGAGGGCG
AATCTATCAAATTTGATCAACCATTAACAAGTAATCCTAATGTGGGCGGATTTGGTCAG
GGAGATGCAGAAATAGTACTTCAAGATCCATTACGTGTTCAAGGCCTTTTGTTCTTCTT
GGCATCTGTTATTTTGGCACAAATCTTTTTGGTTCTTAAAAAGAAACAGTTCGAGAAGG
TTCAATTAGCAGAAATGAATTTCATGAGTAAAGTCTATGATTGGTTCGAAGAACGTCTT
GAGATTCAGGCGATTGCGGATGATATAACTAGTAAATACGTTCCTCCTCACGTCAATATA
TTTTATTGTTTAGGGGGGATTACGCTTACTTGTTTTTTAGTACAAGTAGCTACAGGGTTT
GCTATGACCTTTTACTATCGTCCGACCGTTACTGAGGCTTTTGCTTCTGTTCAATACATA
ATGACTGAAGCTAACTTTGGTTGGTTAATCCGATCAGTTCATCGATGGTCGGCAAGTAT
GATGGTCTTAATGATGATCCTGCATGTGTTTCGTGTGTATCTCACCGGTGGGTTTAAAAA
ACCTCGCGAATTGACTTGGGTTACAGGTGTGGTTTTGGCTGTATTGACCGCATCTTTTG

GCGTAACCGGTTATTCCTTACCTCGGGACCAAATTGGTTATTGGGCAGTTAAAATTGTA
ACAGGTGTACCTGAAGCTATTCCTGTAATAGGATCACCTTTGGTAGAATTATTGCGCGG
AAGTGCTAGTGTGGGACAATCCACTTTGACTCGTTTTTATAGTTTACACACTTTTGTATT
GCCGCTTCTGACTGCCGTATTTATGTTAATGCACTTTCTAATGATACGTAAACAAGGTAT
TTCTGGTCCTTTAATGGGAGTAACAAAAAAACCAGACTTGAATGATCCTGTATTAAGAG
CTAAATTAGCTAAAGGGATGGGTCATAATTATTACGGAGAGCCCGCATGGCCCAATGAT
CTTTTATATATTTTTCCAGTAGTAATTTTAGGTACTATTGCATGTAACGTAGGCTTAGCGG
TTCTAGAACCATCAATGATTGGTGAACCGGCAGATCCATTTGCAACCCCCTTGGAAATA
TTACCTGAATGGTATTTCTTTCCCGTATTTCAAATACTTCGTACAGTGCCCAATAAATTAT
TGGGTGTTCTTTTAATGGTTTCAGTACCCGCGGGATTATTAACAGTACCTTTTTTAGAGA
ATGTTAATAAATTCCAAAATCCATTTCGGCGTCCAGTAGCGACAACCGTTTTTTTGATCG
GTACCGCAGTTGCCCTTTGGTTGGGGATTGGTGCAACATTACCTATTGATAAATCCCTAA
CTTTAGGTCTTTTCATGATTGAAGTTTTTCTATTTGGAATCGTGTTAGGCCTAATTCCTAT
TACTTTGGCTGGATTATTCGTAACTGCATATTTACAATACAGGCGTGGTGATCAATTGGA
CCTTATGCTTACTATAACTAGTTATTTTGGTTTTCTACTAGCGGCTTTAACTATAACCTCA
GCTCTATTTATTGGTCTGAGCAAGATACGACTTATTATGGATATAGTAAGTCTCGCTTGG
GCTGCTTTAATGGTAGTCTTTACATTTTCCCTTTCACTCGTAGTGTGGGGAAGAAGTGG
ACTCATGATTATTCGTTCGCCGGAACCAGAAGTAAAAATTTTGGTAGATAGGGATCCCG
TAAAAACTTCTTTCGAGGAATGGGCCAGACCGGGTCATTTCTCAAGAACAATAGCTAA
AGGGCCTGATACTACCACATGGATCTGGAACCTACATGCTGATGCTCACGATTTCGATA
GCCATACCAGTGATTTGGAGGAGATCTCTCGAAAAGTATTTAGTGCCCATTTCGGTCAA
CTCTCCATCATCTTTCTTTGGCTGAGCGGCATGTATTTCCACGGTGCTCGTTTTTCCAAT
TATGAGGCGTGGCTAAGTGATCCAACTCACATTGGACCTAGCGCCCAGGTGGTTTGGC
CAATAGTGGGCCAAGAAATATTGAATGGTGATGTAGGCGGGGGTTTCCGAGGAATACA
AATAACCTCTGGTTTTTTTCAGATTTGGCGAGCATCTGGAATAACTAGTGAATTACAAC
TCTATTGTACCGCAATTGGTGCATTGGTCTTTGCAGCGTTAATGCTTTTTGCTGGTTGGT
TTCATTATCATAAAGCGGCTCCAAAATTGGCTTGGTTTCAAGATGTAGAATCTATGTTGA
ATCACCATTTAGCGGGGCTACTAGGACTTGGGTCTCTCTCTTGGGCGGGGCATCAAGTT
CATGTATCTTTACCGATTAACCAATTTCTAAACGCTGGAGTAGATCCTAAAGAGATACCA
CTTCCTCATGAATTTATCTTGAATCGGGATCTTTTGGCTCAACTTTATCCCAGTTTTGCT
GAGGGAGCAACCCCACTTTTCACCTTGAATTGGTCAAAATATGCGGAATTTCTTACTTT
TCGTGGAGGATTAGATCCAGTAACTGGGGGTCTATGGCTGACTGATATTGCTCATCATCA
TTTAGCTATTGCAATTCTTTTCCTGATAGCGGGTCACATGTATAGGACCAACTGGGGCAT
TGGTCATGGTATAAAAGATATTTTAGAAGCTCATAAAGGTCCATTTACAGGTCAGGGCC
ATAAAGGCCTATATGAGATCCTAACAACGTCATGGCATGCTCAATTATCTCTTAACCTAG
CTATGTTAGGCTCTTTAACCATTGTTGTAGCTCACCACATGTATTCCATGCCTCCTTATCC
ATATCTAGCTACTGACTATGGTACACAACTGTCATTGTTCACACATCACATGTGGATTGG
TGGATTTCTCATAGTTGGTGCTGCTGCTCATGCAGCCATTTTTATGGTAAGAGACTATGA
TCCAACTACTCGATACAACGATCTATTAGATCGTGTCCTTAGACATCGTGATGCAATCAT
CTCACATCTCAACTGGGCATGTATATTTCTAGGCTTTCACAGTTTTGGTTTGTATATTCAT
AATGATACCATGAGCGCTTTAGGGCGTCCTCAAGATATGTTTTCAGATACCGCTATACAA
TTACAACCCGTTTTTGCTCAATGGATACAAAACACCCATGCTTTAGCACCTGGTGCAAC
GGCTCCTGGTGCAACAGCAAGCACCAGTTTAACTTGGGGGGGTGGTGATTTAGTAGCA
GTGGGTGGCAAAGTGGCTTTGTTACCTATTCCATTAGGAACCGCAGATTTTTTGGTGCA

TCACATTCATGCATTTACGATTCATGTGACGGTATTGATACTCCTGAAAGGTGTTCTATT
TGCTCGTAGCTCCCGTTTGATACCGGATAAAGCAAATCTTGGTTTTCGTTTTCCTTGTGA
TGGACCTGGAAGAGGGGGTACATGTCAAGTATCGGCTTGGGATCATGTCTTCTTAGGA
CTATTCTGGATGTACAATGCAATTTCGGTAGTAATATTCCATTTCAGTTGGAAAATGCAG
TCAGATGTTTGGGGCAGTATAAGCGATCAAGGGGTAGTAACTCATATCACGGGAGGAA
ACTTTGCGCAAAGTTCTATTACTATTAATGGGTGGCTCCGCGATTTCTTATGGGCACAGG
CATCTCAGGTAATTCAGTCTTATGGTTCTTCATTATCTGCATATGGTCTTTTTTTCCTAGG
TGCTCATTTTGTATGGGCTTTTAGTTTAATGTTTCTATTCAGTGGACGTGGTTATTGGCA
AGAACTTATTGAATCCATCGTTTGGGCTCATAATAAATTAAAAGTTGCTCCTGCTACTCA
GCCGAGAGCCTTGAGCATTGTACAAGGACGTGCTGTAGGAGTAACCCATTACCTTCTG
GGTGGAATTGCCACAACATGGGCGTTCTTCTTAGCAAGAATTATTGCAGTAGGAATGGC
ATTAAGATTTCCAAGGTTTAGCCAAGGCTTAGCTCAGGACCCCACTACTCGTCGTATTT
GGTTTGGTATTGCTACCGCACATGACTTCGAGAGTCATGATGATATTACTGAGGAACGT
CTTTATCAGAATATTTTTGCTTCTCACTTCGGTCAATTAGCAATAATTTTTCTGTGGACTT
CCGGAAATCTGTTTCATGTAGCTTGGCAAGGAAATTTTGAGTCATGGGTACAGGACCCT
TTACATGTAAGACCTATTGCTCATGCAATTTGGGATCCTCATTTTGGTCAACCGGCTGTG
GAAGCTTATACTCGAGGGGGTGCTCTTGGCCCAGTGAATATCGCTTATTCTGGTGTTTAT
CAGTGGTGGTATACAATTGGTTTACGCACTAATGAAGATCTTTATACTGGAGCTCTTTTT
CTATTATTTCTTTCTGCCCTATTCTTAATAGCAGGTTGGTTACATCTACAGCCGAAATGGA
AACCGAGCGTTTCGTGGTTCAAAAATGCTGAATCTCGTCTGAATCATCATTTATCAGGA
CTCTTCGGAGTAAGTTCCTTGGCTTGGACAGGACATTTAGTCCATGTCGCTATTCCTGG
ATCCAGAGGGGAGTACGTTCGATGGAATAATTTCTTAGATGTATTACCGCATCCCCAAG
GGTTAGGCCCCCTTTTTACAGGTCAGTGGAATCTTTATGCTCAAAACCCCGATTCGAGT
AGTCATTTATTTGGGACCTCCCAAGGAGCAGGAACTGCCATTCTAACTCTTCTCGGGGG
ATTCCATCCACAAACACAAAGTTTATGGCTGACTGATATTGCTCATCATCATTTAGCTAT
TGCATTTATTTTTCTCGTTGCTGGTCATATGTATAGAACTAATTTTGGGATTGGGCACAGT
ATAAAAGATCTTTTAGATGCACACATTCCTCCGGGGGGACGATTGGGACGTGGGCATAA
GGGTCTTTATGACACAATCAATAATTCGCTTCATTTTCAATTAGGCCTTGCTCTAGCTTC
TTTAGGGGTTATTACTTCCTTGGTAGCTCAACACATGTACTCTTTACCCGCTTATGCATTC
ATAGCACAAGATTTTACTACTCAAGCTGCATTATATACTCATCACCAATATATCGCAGGAT
TCATCATGACAGGAGCTTTTGCTCATGGAGCTATATTTTTCATTAGAGATTATAATCCGG
AGCAAAATGAAGATAATGTATTGGCAAGAATGTTAGAGCATAAAGAAGCTATCATATCT
CATTTAAGTTGGGCCAGTCTCTTCCTGGGATTCCATACCTTGGGACTTTATGTTCATAAT
GATGTCATGCTTGCCTTTGGTACTCCAGAGAAGCAAATCTTGATCGAACCTATATTTGCT
CAATGGATCCAATCTGCTCACGGTAAAACTTCATATGGGTTTGATGTACTTTTATCTTCA
ACGAGCGGCCCGGCATTCAATGCGGGTCGAAGCATCTGGTTGCCCGGTTGGTTAAATG
CTGTTAATGAA------AATAGTAATTCATTATTCTTAACAATAGGTCCTGGAGACTTTTTGG
TTCATCATGCTATTGCTCTGGGTTTACATACAACTACATTGATCTTAGTAAAAGGTGCTTT
AGATGCACGTGGTTCCAAGTTAATGCCAGATAAAAAAGATTTCGGTTATAGTTTTCCGT
GCGATGGTCCGGGCCGAGGCGGTACTTGTGATATTTCGGCATGGGACGCATTTTATTTG
GCAGTTTTTTGGATGTTAAATACTATTGGATGGGTTACTTTTTATTGGCATTGGAAGCAC
ATCACATTATGGCAAGGAAACGTTTCACAGTTTAATGAATCATCCACTTATTTGATGGGC
TGGTTAAGAGATTATTTATGGTTAAACTCTTCACAACTTATCAATGGATATAACCCTTTTG
GTATGAATAGTTTATCGGTCTGGGCATGGATGTTCTTATTTGGACATCTTGTTTGGGCTA

CTGGATTTATGTTCTTAATTTCCTGGCGTGGATATTGGCAGGAATTGATTGAAACTTTAG
CGTGGGCTCATGAACGCACACCTTTGGCCAATTTGATTCGCTGGAGAGATAAACCGGT
GGCCCTTTCCATTGTGCAAGCAAGATTGGTTGGATTGGCCCACTTTTCTGTAGGTTATAT
ATTCACTTATGCAGCTTTCTTGATTGCCTCTACATCGGGCAAATTTGGTATGTCACATTC
AGTAAAGATTTATGATACATGTATAGGATGTACTCAATGTGTCCGGGCCTGCCCTACCGA
TGTATTAGAAATGATACCTTGGGACGGATGTAAAGCGAAGCAAATTGCTTCTGCTCCAA
GAACGGAGGACTGTGTTGGTTGTAAGAGATGTGAATCTGCCTGTCCAACGGATTTCTT
GAGTGTTCGAGTTTATTTATGGCATGAAACAACTCGCAGTATGGGTCTAGCTTATATGAC
AACATTCAACTTTCCCTCTATTTTTGTGCCTTTAGTAGGCCTAGTATTTCCGGCAATTGC
AATGGCTTCTTTATTTCTTTATGTTCAAAAAAACAAGATTGTTATGCGAGATCTAAAAAC
ATATCTCTCCGTGGCACCAGTACTAAGTACGCTATGGTTCGGGGCTTTAGCAGGTCTATT
GATAGAGATTAATCGTTTTTTCCCAGATGCGTTGACATTCCCCTTTTTTTCATTCATGACT
GCAATTTTAGAGAGACGCGAAAGCGAAAGCCTATGGGGTCGCTTCTGTAACTGGATAA
CCAGCACTGAAAACCGTCTTTACATTGGATGGTTTGGTGTTTTGATGATCCCTACCTTAT
TGACCGCAACTTCTGTATTTATTATCGCCTTCATTGCTGCTCCTCCAGTAGATATTGATGG
TATTCGTGAACCTGTTTCTGGATCTCTACTTTACGGAAACAATATTATTTCAGGTGCCAT
TATTCCTACTTCTGCAGCTATAGGTTTGCACTTTTACCCAATATGGGAAGCGGCATCCGT
TGATGAATGGTTATACAACGGTGGTCCTTATGAACTTATTGTTCTACACTTCTTACTTGG
TGTAGCTTGTTACATGGGTCGTGAGTGGGAACTTAGTTTCCGTCTGGGTATGCGACCTT
GGATTGCTGTTGCATATTCAGCTCCTGTTGCAGCTGCTACCGCTGTTTTCTTGATTTACC
CAATTGGTCAAGGAAGTTTTTCTGACGGTATGCCTCTAGGAATCTCTGGTACTTTCAAC
TTTATGATTGTATTCCAGGCTGAGCACAACATCCTTATGCACCCATTTCACATGTTAGGC
GTAGCTGGGGTATTCGGCGGCTCCCTATTTAGTGCTATGCATGGTTCCTTGGTAACTTCT
AGTTTGATCAGGGAAACCACAGAAAACGAATCTGCTAATGCAGGTTACAGATTCGGTC
AAGAGGAAGAAACTTATAATATTGTAGCTGCTCATGGTTATTTTGGCCGATTGATCTTCC
AATATGCTAGTTTCAACAACTCTCGTTCGTTACACTTCTTCCTAGCAGCTTGGCCTGTAG
TAGGTATCTGGTTCACTGCTTTAGGTATCAGCACTATGGCTTTCAACCTAAATGGTTTCA
ATTTCAACCAATCCGTAGTTGATAGTCAAGGCCGTGTAATTAATACTTGGGCTGATATCA
TTAACCGTGCTAACCTTGGTATGGAAGTTATGCATGAACGTAATGCTCATAACTTCCCTC
TAGACTTAGCGGCTATCGAAGCTCCATCTACAAATGGAATGGGTTTGCCTTGGTATCGT
GTTCATACCGTTGTATTGAATGATCCCGGTCGGTTGCTTTCTGTTCATATAATGCATACAG
CTCTGGTTGCTGGTTGGGCCGGTTCTATGGCTCTGTATGAATTAGCAGTTTTTGATCCTT
CTGACCCTGTTCTTGATCCAATGTGGAGACAGGGCATGTTCGTTATACCCTTCATGACT
CGTTTAGGAATAACAAATTCATGGGGCGGTTGGAGTATCACA---GGAGGAACTGTAACG
AATCCGGGTATTTGGAGTTACGAAGGTGTAGCTGGGGCACATATTGTGTTTTCCGGCTT
ATGCTTTTTGGCAGCTATCTGGCATTGGGTGTATTGGGATCTAGAAATATTTTGCGATGA
ACGTACAGGAAAACCTTCTTTGGATTTGCCCAAGATCTTTGGAATTCATTTATTTCTTTC
AGGGGTGGCTTGCTTTGGTTTTGGTGCATTTCATGTAACAGGCTTGTATGGTCCTGGAA
TATGGGTGTCCGACCCTTATGGACTAACCGGAAAAGTACAACCCGTAAATCCAGCGTG
GGGCGTGGAAGGCTTTGATCCTTTTGTTCCGGGAGGAATAGCCTCTCATCATATTGCAG
CAGGGACATTGGGCATATTAGCGGGTCTATTCCATCTTAGCGTCCGCCCGCCACAACGT
CTATACAAAGGGTTGCGTATGGGAAATATTGAAACCGTCCTTTCCAGCAGTATCGCTGC
CGTCTTTTTTGCAGCTTTTGTTGTTGCCGGAACTATGTGGTATGGCTCAGCAACTACCC
CCATCGAATTATTTGGGCCCACTCGTTATCAATGGGATCAGGGGTACTTCCAACAAGAG

ATATATCGAAGAGTGAGTGCTGGGCTAGCAGAAAATCAAAGTTTATCAGAAGCCTGGT
CTAAAATTCCTGAAAAATTAGCTTTTTATGATTACATCGGCAATAATCCGGCAAAAGGA
GGATTATTCAGGGCCGGCTCAATGGATAATGGGGATGGAATAGCGGTCGGATGGTTAGG
ACACCCTATCTTTAGAGATAAAGAAGGGCGTGAACTTTTTGTACGTCGTATGCCTACTT
TTTTTGAAACATTCCCAGTCGTTTTGGTAGACGGCGACGGAATTGTTAGAGCCGATGTT
CCTTTTAGAAGGGCAGAATCAAAGTATAGTGTTGAACAAGTAGGTGTAACTGTTGAATT
CTACGGCGGCGAACTCAACGGAGTCAGTTATAGTGATCCTGCTACTGTGAAAAAATATG
CTAGACGTGCTCAATTGGGTGAAATTTTTGAATTAGATCGTGCTACTTTGAAATCCGAT
GGTGTTTTTCGTAGCAGTCCAAGGGGTTGGTTTACTTTTGGACATGCTTCATTTGCTTT
GCTCTTCTTCTTCGGACACATTTGGCATGGTGCTAGAACCTTGTTCAGAGATGTTTTTG
CTGGTATTGACCCAGATTTGGATGCTCAAGTAGAATTTGGAGCATTCCAGAAACTTGGA
GATCCAACTACAAGAAGACAGGTAGTC------------------------------------GTGGAAACGCTCT
TTAATGGAACTTTAGCTTTAGCTGGCCGTGACCAAGAAACCACCGGTTTCGCTTGGTG
GGCCGGGAATGCCCGACTTATCAATTTATCCGGTAAACTACTAGGGGCTCATGTAGCCC
ATGCTGGATTAATCGTATTCTGGGCCGGAGCAATGAACCTATTTGAAGTGGCTCATTTC
GTACCAGAGAAGCCTATGTATGAACAAGGATTAATTTTACTTCCCCACCTAGCTACTCTA
GGTTGGGGGGTGGGTCCTGGGGGGGAAGTTATAGACACTTTTCCATACTTTGTATCTGG
AGTACTTCATTTAATTTCCTCCGCAGTATTGGGCTTTGGCGGTATTTATCATGCACTTCTG
GGACCTGAGACGCTTGAAGAATCTTTTCCATTCTTTGGTTATGTATGGAAAGATAGAAA
TAAAATGACCACAATTTTAGGTATTCACTTAATCTTGTTAGGTCTAGGTGCTTTTCTTCT
AGTATTCAAGGCTCTTTATTTTGGGGGCGTATATGATACCTGGGCTCCGGGAGGGGGAG
ATGTAAGAAAAATTACCAACTTGACCCTTAGCCCAAATATTATATTTGGTTATTTACTAA
AATCGCCCTTTGGAGGGGAAGGGTGGATTGTTAGTGTGGACGATTTAGAAGATATAATC
GGAGGACATGTATGGTTAGGTTCCATTTGTATACTTGGTGGAATCTGGCATATTTTAACC
AAACCCTTCGCGTGGGCTCGACGCGCATTTGTATGGTCTGGAGAGGCTTACTTATCTTA
TAGTTTAGGGGCTTTAGCCGTGTTTGGTTTCATTGCTTGCTGTTTTGTCTGGTTCAATAA
TACCGCTTATCCTAGTGAGTTTTACGGACCCACTGGACCAGAAGCTTCTCAAGCTCAA
GCATTTACTTTTCTAGTTAGAGACCAACGCCTTGGGGCTAACGTGGGATCCGCTCAAGG
ACCTACTGGTTTAGGTAAATATCTAATGCGTTCCCCGACCGGAGAAGTCATTTTTGGAG
GAGAAACTATGCGTTTTTGGGATCTGCGTGCTCCTTGGTTAGAACCTCTAAGGGGTCCA
AATGGGTTGGACTTGAGTAGGTTGAAAAAAGATATACAACCTTGGCAAGAACGGCGTT
CCGCAGAATATATGACTCATGCTCCTTTAGGTTCTTTAAATTCCGTGGGTGGCGTAGCTA
CCGAGATCAATGCAGTTAATTATGTCTCTCCTAGAAGTTGGTTAGCTACCTCTCATTTTG
TTCTAGGATTCTTCTTCTTCGTAGGTCACTTGTGGCATGCGGGAAGAGCTCGTGCAGCT
GCAGCGGGGTTTGAAAAAGGAATTGATCGTGATTTTGAACCTGTTCTTTCCATGACCCC
TCTTAATATGACTATAGCCCTTGGTAAATTTACCAAAGACGAAAAT---TTATTTGATATTAT
GGATGACTGGTTACGGAGGGACCGTTTCGTTTTTGTAGGCTGGTCCGGTCTATTGCTCT
TTCCTTGTGCCTATTTCGCTTTAGGGGGTTGGTTCACAGGTACAACCTTTGTAACTTCAT
GGTATACCCACGGATTGGCTAGTTCCTATCTGGAAGGCTGCAATTTCTTAACTGCCGCA
GTTTCTACTCCTGCTAATAGTTTAGCACATTCTTTGTTGTTACTATGGGGTCCTGAAGCA
CAAGGAGATTTTACTCGTTGGTGTCAATTAGGCGGTCTGTGGACTTTTGTTGCTCTCCA
TGGCGCTTTCGGACTAATAGGTTTCATGTTACGTCAATTCGAGCTTGCTCGATCTGTTCA
ATTGCGACCTTATAATGCAATTGCATTCTCTGCTCCAATTGCTGTTTTTGTTTCTGTATTC
CTGATTTATCCACTAGGTCAGTCTGGTTGGTTTTTTGCGCCTAGTTTTGGTGTAGCAGCT

ATATTTCGATTCATCCTCTTTTTTCAAGGGTTTCATAATTGGACGTTGAACCCATTTCATA
TGATGGGAGTTGCCGGTGTATTGGGAGCTGCTTTGCTATGCGCTATTCATGGTGCTACC
GTAGAAAATACTTTATTTGAAGATGGTGATGGTGCAAATACATTCCGTGCTTTTAACCCA
ACTCAAGCTGAAGAAACTTATTCAATGGTCACCGCTAACCGCTTTTGGTCCCAAATCTT
TGGGGTTGCTTTTTCCAATAAACGTTGGTTACATTTCTTTATGTTATTTGTACCAGTAAC
CGGTTTATGGATGAGTGCTCTTGGAGTAGTCGGTCTGGCCCTGAACCTACGTGCCTATG
ACTTCGTTTCTCAGGAAATTCGCGCAGCGGAAGATCCTGAATTTGAGACTTTCTACACC
AAAAATATTCTTTTAAACGAAGGTATTCGCGCTTGGATGGCGGCTCAAGATCAGCCTCA
TGAAAATCTTATATTCCCTGAGGAGGTTCTACCACGTGGAAACGCTCTTATGTCTGGAA
GCACAGGAGAACGTTCTTTTGCTGATATTATTACCAGTATTCGATACTGGGTCATTCATA
GCATTACTATACCTTCCCTATTCATTGCGGGTTGGTTATTCGTCAGCACCGGTTTAGCTTA
CGATGTATTTGGAAGCCCTCGGCCAAACGAGTATTTCACAGAGAGCCGACAAGGTATT
CCATTAATAACTGGCCGTTTTGATCCTTTGGAGCAACTCGATGAATTTAGTAGATCGTTT
ATGACTATAGATCGAACCTATCCAATTTTTACCGTACGATGGTTGGCTGTTCACGGCCTA
GCTGTACCTACCGTCTTTTTTTTGGGGTCAATCTCAGCAATGCAGTTCATCCAACGAAT
GGCTACACAAACTGTTGAGAAGAGTTCTAGATCTGGT---CCAAGACGAACTACTGTAG
GGAGTTTATTAAAACCGTTGAATTCGGAGTATGGTAAAGTAGCTCCTGGGTGGGGAAC
GACTCCATTGATGGGGGTTGCAATGGCTCTATTTGCGATATTTCTATCTATTATTTTGGAG
ATTTATAATTCTTCCGTTTTATTGGATGGAATTTCAATGAACATGCTTACTCTCAAACTTT
TCGTTTACACAGTAGTGATATTTTTTGTTTCTCTCTTCATCTTTGGATTCCTATCTAATGAT
CCAGGGCGTAATCCTGGACGTGAAGAAATGGCCGATACTACTGGAAGGATTCCTCTTT
GGATCATAGGTACTGTAACTGGTATTCTTGTGATTGGTTTAATAGGTATTTTCTTTTATGG
TTCATATTCTGGATTGGGTTCATCCCTGATGCTTAATATCTTTAGTTTG------ATCTGTCTT
AATTCTGCCCTTCAT---AGTAGTTTTTTCTTCGGCAAATTGCCCGAGGCCTATGCTTTTTT
GAATCCAATCGTAGATGTTATGCCAGTCATACCTGTGCTTTTTTTTCTCTTAGCCTTTGTT
TGGCAAGCTGCTGTAAGTTTTCGAATGACACAATCAAACCCAAACGAACAAAGTGTTG
AATTGAATCGTACCAGTCTCTACTGGGGGTTATTACTCATTTTTGTACTTGCTGTTTTATT
TTCCAATTATTTCTTCAATATGGAAGTAAATATTCTTGCATTTATTGCTACTGCACTATTCA
TTCTAGTTCCTACCGCTTTTCTACTTATTATTTACGTAAAAACAGTCAGTCAAAATGATAT
GGAAACAGCAACCCTAGTTGCCATCTTTATATCTGGTTTACTTGTAAGTTTTACTGGGTA
CGCCTTATATACCGCTTTTGGGCAACCTTCTCAACAACTAAGAGATCCATTCGAGGAAC
ATGGGGACATGGAAGCATTGGTTTATACATTCCTCTTAGTCTCGACTCTAGGGATAATTT
TTTTCGCTATCTTTTTTCGAGAACCGCCTAAAGTTCCAACTAAAAAGATGAAAATGACT
CTTGCTTTCCAATTGGCTGTTTTTGCATTAATTGCTACTTCATCAATCTTATTGATTAGCG
TACCTGTTGTATTTGCTTCTCCTGATGGTTGGTCAAGTAACAAAAATGTTGTATTTTCTG
GTACATCATTATGGATTGGGTTAGTCTTTCTAGTGGGTATTCTTAATTCTCTTATCTCT-----
---------------ATGTCACCACAAACAGAGACTAAAGCAAGTGTTGGATTCAAAGCTGGTGT
TAAAGAGTACAAATTGACTTATTATACTCCTCAATACCAAACCAAAGATACTGATATCTT
GGCAGCATTCCGAGTAACTCCTCAACCCGGAGTTCCCCCTGAAGAAGCAGGGGCCGC
AGTAGCTGCCGAATCTTCTACTGGTACATGGACAACTGTGTGGACCGATGGACTTACCA
GCCTTGATCGTTACAAAGGGCGATGCTACTACATCGAGCCCGTTCCTGGAGAAAAAGA
GCAATTTATTGCTTATGTAGCTTATCCTTTAGACCTTTTTGAAGAAGGTTCTGTTACTAA
CATGTTTACTTCCATTGTAGGTAATGTATTTGGGTTCAAAGCCCTACGCGCTCTACGTCT
GGAAGATCTGCGAATCCCTCCTGCTTATATTAAAACTTTCCAGGGCCCGCCTCATGGGA

TCCAAGTTGAGAGAGATAAATTGAACAAGTATGGTCGTCCTCTGTTGGGATGTACTATT
AAACCTAAATTGGGATTATCTGCTAAAAACTACGGTAGAGCTGTTTATGAATGTCTTCG
CGGTGGACTTGATTTTACCAAAGATGATGAAAACGTGAACTCCCAACCATTTATGCGTT
GGAGAGATCGTTTCTTATTTTGTGCCGAAGCACTTTATAAAGCACAGGCTGAAACAGG
TGAAATCAAAGGGCATTACTTGAATGCTACTGCAGGTAACTGCGAAGAAATGATGAAA
AGGGCTGTATTTGCTAGAGAATTGGCAGTTCCTATCGTCATGCACGACTACTTAACAGG
GGGATTCACTGCAAATACTACCTTGGCTAGTTATTGCCGAGATAATGGTCTACTTCTTCA
CATCCACCGTGCAATGCATGCAGTTATTGATAGACAGAAGAATCATGGTATACACTTCC
GTGTACTAGCTAAAGCGTTACGTATGTCTGGTGGAGATCATATTCACTCTGGTACCGTAG
TAGGTAAACTTGAAGGGGAAAGAAACATCACTTTGGGCTTTGTTGATTTACTGCGTGAT
GATTATATTGAAAAAGACCGAAGTCGCGGTATCTTTTTCACTCAAGATTGGGTCTCTCT
ACCGGGTGTTCTGCCTGTGGCTTCAGGAGGTATTCACGTTTGGCATATGCCGGCTTTGG
TCGAAATCTTTGGGGATGATTCCGTACTACAGTTCGGTGGAGGAACTTTAGGACATCCT
TGGGGTAATGCGCCAGGTGCCGTAGCTAACCGAGTCTCTCTAGAAGCATGTGTACAAG
CTCGTAATGAAGGGCGTAATCTTGCTACTGAGGGTAATGAAATTGTTCGTGAGGCTAGC
AAATGGAGTCCTGAACTAGCTGCTGCTTGTGAGGTATGGAAGGAGATCAAATTTGAGT
TTGAAGACGTGGATACATTGGATAAGATGGCGATACATTTATACAAAACTTCTACCCCG
AGCACACGCAATGGAACCGTAGACAGTCAAGTGAAATCCAATCCACGAAATAATTTGA
TCTATGGACAGCATCGTTGTGGTAAAGGTCGTAATGCCAGAGGAATCATTACCGCAAG
GCATAGAGGGGGAGGTCATAAGCGTCTATACCGTAAAATCGATTTTCGACGGAATGAA
AAAGACATATATGGTAGAATCGTAACCATAGAATACGACCCTAATCGAAATGCATACATT
TGTCTCATACACTATGGGGATGGTGAGAAGAGATATATTTTACATCCCAGAGGGGCTATA
ATTGGAGATACCATTGTTTCTGGTACAGAAGTTCCTATAAAAATGGGAAATGCCCTACC
TTTGACCGATATGCCCTTAGGCACGGCCATACATAACATAGAAATCACACTTGGAAAGG
GTGGACAATTAGCTAGAGCAGCGGGTGCTGTAGCGAAACTGATTGCAAAAGAGGGGA
AATCGGCCACATTAAAATTACCTTCTGGAGAGGTCCGTTTGATATCCAAAAACTGCTCA
GCAACAGTCGGGCAAGTGGGGAATGTTGGGGTGAACCAGAAAAGTTTGGGTAGAGCC
GGATCTAAGCGTTGGCTAGGTAAGCGTCCTGTAGTAAGAGGAGTAGTTATGAACCCTGT
AGACCATCCCCATGGGGGTGGTGAAGGGAGAGCCCCAATTGGTAGAAAAAAACCCAC
AACCCCTTGGGGTTATCCTGCACTTGGAAGAAGAAGTAGAAAAAGGAAGAAATATAGT
GATAATTTGATTCTTCGTCGTCGTAGTAAAATGATTCAACCTCAGACCCATTTAAATGTA
GCGGATAACAGCGGGGCTCGAGAATTGATGTGTATTCGAATCATAGGAGCTAGC---AAT
CGTCGATATGCTCATATTGGTGACGTTATTGTTGCTGTGATCAAAGAAGCAGTACCAAAT
ATGCCCCTAGAAAGATCAGAAGTAGTGAGAGCTGTAATTGTGCGTACCTGTAAAGAAC
TCAAACGTGACAACGGTATGATAATACGATATGATGACAATGCTGCAGTTGTTATTGATC
AAGAAGGAAATCCAAAAGGAACTCGAATTTTTGGTGCGATCGCTCGGGAATTGAGAC
AATTAAATTTTACTAAAATAGTTTCATTGGCTCCCGAGGTATTAATGCTTAGTCCCAAAA
GAACCAGATTCCGTAAACAACATAGAGGAAGAATGAAGGGAATATCTTATCGAGGTAA
TCATATTTGTTTCGGTAAATATGCTCTTCAGGCACTTGAACCCGCTTGGATCACATCTAG
GCAAATAGAAGCGGGTCGACGAGCAATGACACGAAATGCACGTCGTGGCGGAAAAAT
ATGGGTACGTATTTTTCCAGACAAACCAGTTACACTAAGACCCGCAGAAACCCGTATG
GGTTCGGGGAAAGGATCTCCTGAATATTGGGTAGCTGTTGTTAAACCAGGTCGAATACT
TTATGAAATGAGTGGAGTAACAGAAAATATAGCTAGAAGGGCTATTTCAATAGCAGCAT
CCAAAATGCCTATACAAACTCAATTCATTATTTCGGGAATGACCAGAATTAAACGGGGA

TACGTAGCTCGGAGACGTAGAACAAAAATTCGTTTATTTGCATCAAGCTTTCGAGGGG
CTCATTCAAGACTTACTCGAACTATTACTCAACAGAAAATAAGAGCTTTGGTTTCGGCT
CATCGGGATAGGGATAGGCAAAAGAGAAATTTTCGTCGATTGTGGATCACTCGAATAA
ACGCAGTAATTCGCGAA---AGGGGAGTATCCTATAGTTATAGTAGATTAATACACGATCT
GTACAAGAAACAGTTGCTTCTTAATCGTAAAATACTTGCACAAATAGCTATATCCAATAG
GAATTGTCTTTATATGATTTCCAACGAGATCAGAAAAGAAGTAGATTGGAAAGAATCCA
CCGGAATAATTATGCTAAAGGAAGTATATGCTTTAGGTCAACATATATCCATGTCTGCTG
ACAAAGCACGAAGAGTAATTGATCAAATTCGTGGACGTTCCTACGAGGAAACACTTAT
GATACTAGAGCTCATGCCCTATCGAGCGTGTTATCCAATTTTTAAATTAGTTTATTCAGC
AGCCGCAAATGCTAGTTACAATATGGGTTCCAACGAAGCCAATTTAGTCATTAGTCAAG
CCGAAGTCAACGAGGGTAGCACCGTAAAGAAATTCAAACCTCGAGCTAAAGGGCGTA
GTTATCCGATAAAAAGACCTACCTGTCATATAACTATTGTAATGAAAGATATATCTTTAGA
TGGGGGAATATGGGACAAAAAAATGGATGGAATCAAATATGCAGTATTTACAGACAAA
AGTATTCGGTTATTGGGGAAAAAT---CAATATACTTCTAATGTCGAATCAGGATCAACTA
GGACAGAAATAAAGCATTGGGTCGAACTCTTCTTTGGTGTCAAGGTAATAGCTATGAAT
AGTCATCGACTTCCGGGAAAGGGTAGAAGAATGGGACCTATTATGGGACATACAATGC
ATTACAGACGTATGATCATTACGCTTCAACCGGGTTATTCTATTCCACCTCTTAGAAAGA
AAAGAACTATGGCAGTTCCAAAAAAACGTACTTCTATATCAAAAAAGCGTATTCGTAA
AAATATTTGGAAAAGGAAGGGATATTGGACAGCGTTAAAAGCTTTTTCGTTAGGTAAAT
CTCTTTCTACAGGGAAGTCAAAAAGTTTTGTTGTACGACAAACAAATAAGATGGCCAA
GCGTAAAGATGTCCGAGTAACGGTGATTTTGGAATGTACCAGTTGTGTCCGAAACGGT
GCT------AATAAGATATCAACGGGCATTTCCAGATATATTACTCAAAAGAACCGGCAGAA
TACGCCTAATCGATTAGAATTGAGAAAATTCTGTCGCTATTGTTACAAACATACCATTCA
TGGGGAGATAAAGAAAATGAAAATAAGAGCTTCTGTTCGTAAAATTTGTGAAAAATGT
CGACTAATCCGTAGGCGGGGACGAATTATAGTAATTTGTTCCAACCCGAGACATAAACA
AAGACAAGGAATGGTTCGAGAGAAAGTAATAGTATCTACTCGGACACTACAGTGGAAG
TGTGTTGAATCAAGAACAGATAGTAAACGTCTTTATTATGGACGCTTTATTCTATCTCCA
CTTATGAAAGGTCAAGCCGATACAATAGGCATTGCGATGCGAAGAGCTTTGCTTGGAG
AAATAGAAGGAACATCTATCACACGTGTAAAATCTGAATCTGAGAAAGTCCCACATGA
ATATTCTACCATATCGGGTATTCAAGAATCGGTACATGAAATTTTAATGAATTTGAAAGA
AATTGTATTGAGAAGTAATCTATATGGAACTTGTGACGCGTCTATTTGTGTTAGGGGTCC
TGGATATGTAACTGCTCAAGACATCATCTTACCGCCTTATGTGGAAATTGTTGATAATAC
ACAACATATAGCTAGCTTGACGGAACCAATTGATTTGTGTATTGGATTACAAATCGAGA
GAAATCGAGGATATCTTATAAAAACGCCACAT---AACTTTGATGGAAGTTAT------CCTAT
AGATGCTGTATTCATGCCTGTTCGAAATGCGAATCATAGTATTCATTCTTATGGGAATGG
AAATGAAAAACAAGAGATACTTTTTCTCGAAATATGGACAAATGGAAGTTTAACTCCG
AAAGAAGCACTTCATGAAGCCTCTCGGAATTTGATTGATTTATTTATTCCTTTTTTACATA
TGGAAGAAGAA------AACGTACATTTAGAGGACAATCAA---CACACGGTTCCTTTATCCC
CTTTTATCTTTCATGATAAATTGGCC---AAACTCAGAAAAAAGAAAAAA---AAAATAGCA
TTGAAATCGATTTTTATTGACCAATCAGAATTGCCTCCAAGGATCTATAATTGTCTCAAA
AGGTCCAATATATATACATTATTGGACCTTTTGAATAACAGTCAAGAAGATCTTATGAAA
ATTGAGCATTTTCGCATAGAAGATGTAAAACAGATATTGGGCATTCTAGAAAAACAT-----
-TTCGCAATTGATTTATCAAAAAGTAAGATGCTCGGGGATGGA---AATGAGGGAATGTCT
ACAATACCTGGATTTAATCAGATACAATTTGAAGGATTTTGTAGGTTCATTGATCAGGGT

TTGACAGAAGAACTTTATAAGTTTCCAAAAATTGAAGATACAGATCAAGAAATCGAATT
TCAATTATTTGTGGAAACATATCAATTGGTAGAACCGTTGATAAAGGAAAGAGATGCTG
TGTATGAATCACTCACATATTCTTCTGAATTATATGTATCCGCGGGATTAATTTGGAAAAC
CAGCCGGGATATGCAAGAACAAACCATTTTTATTGGAAATATTCCTCTAATGAATTCCCT
GGGAACTTCTATAGTAAATGGAATATATAGAATTGTGATCAATCAAATATTGCAAAGCCC
CGGTATTTATTACCGGTCAGAATTGGACCATAACGGAATTTCGGTCTATACCGGCACCAT
AATATCAGATTGGGGAGGAAGACCAGAATTAGAGATTGATAGAAAAGCAAGGATATGG
GCTCGTGTGAGTAGGAAACAAAAAATATCTATTCTAGTTCTATCATCAGCTATGGGTTCG
AATCTAAGAGAAATTCTAGAGAATGTTTGCTATCCTGAAATTTTTTTGTCTTTTCTGAAT
GATAAGGAGAGAAAAAAAAATTGGGTCAAAAGAAAATGCCATTTTGGAGTTTTATCAAC
AATTTGCTTGTGTAGGCGGGGATCCGGTATTTTCTGAATCCTTATGTAAGGAATTACAAA
AGAAATTCTTTCAACAAAGATGTGAATTAGGAAGGATTGGTCGACGAAATATGAACCG
AAGACTAAATCTTGATATACCCCAGAACAATACATTTTTGTTACCACGAGATATATTGAC
AGCCGCCGATCATTTGATTGGACTGAAATTTGGAATGGGTACACTTGACGAT------ATGA
ATCATTTGAAAAATAAACGTATTCGTTCTGTAGCCGATCTTTTACAAGATCAATTCGGAT
TGGCTCTGGTTCGTTTAGAAAATGTGGTTCGAGGAACTATATGTGGAGCAATTCGG---C
ATAAATTG---ATACCGACTCCTCAGAATTTGGTAACTTCAACTCCA------------TTAACAAC
TACTTATGAATCCTTTTTCGGGTTACACCCATTATCTCAAGTTTTGGATCGAACTAATCCA
TTGACACAAATAGTTCATGGAAGAAAATTGAGTTATTTGGGACCTGGAGGACTGACAG
GGCGAACTGCTAGTTTTCGTATACGAGATATCCATCCTAGTCACTACGGGCGTATTTGCC
CAATTGACACATCTGAAGGAATCAATGTTGGACTTATTGGATCCTTAGCAATTCATGCG
AGGATTGGTCATTGGGGA---TCTCTAGAAAGCCCGTTTTAT---------GAAATTTCTGAGAG
ATCAACAGGG------GTACGGATGCTTTATTTATCACCAGGTAGAGATGAATACTATATGGT
AGCGGCAGGAAATTCTTTGGCCTTGAATCAGGATATTCAGGAAGAACAGGTTGTTCCA
GCTCGATACCGTCAAGAATTCCTGACTATTGCATGGGAACAGGTTCATCTTCGAAGTAT
TTTTCCCTTTCAATATTTTTCTATTGGAGCTTCCCTCATTCCTTTTATCGAACACAATGAT
GCGAATCGAGCTTTAATGAGTTCTAATATGCAACGTCAAGCAGTTCCTCTTTCTCGGTC
CGAGAAATGCATTGTTGGAACTGGGTTAGAACGACAAGCAGCTCTAGATTCGGGGGCT
CTTGCTATAGCCGAAGGCGAGGGAAAGATCATTTCTACTGATACTGACAAGATCCTTTT
ATCAGGTAATGGAAATACTCTAAGTATTCCATTAGTTATGTATCAACGTTCCAACAAAAA
TACTTGTATGCATCAAAAACCCCAGGTTCGGCGGGGTAAATGCATTAAAAAAGGACAA
ATTTTAGCGGATGGTGCTGCTACGGTTGGCGGCGAACTTGCTTTGGGGAAAAATGTATT
AGTAGCTTATATGCCGTGGGAAGGTTACAATTTTGAAGATGCAGTACTCATTAGCGAGC
GTTTGGTATATGAAGATATTTATACTTCTTTTCATATACGGAAATATGAAATTCAGACTCA
TGTGACAAGCCAAGGC---CCTGAAAGGGTCACTAACGAAATACCGCATTTAGAAGCCC
ATTTACTACGCAATTTAGACAAAAATGGAATTGTGATGCTGGGATCTTGGGTAGAGACG
GGTGATATTTTAGTAGGTAAATTAACGCCCCAGATGGTGAAAGAA---TCGTCGTATGCCC
CGGAAGATAGATTGTTACGAGCCATACTTGGCATTCAGGTATCTACTTCAAAAGAAACT
TGTCTAAAACTACCTATAGGTGGTCGGGGTCGGGTTATTGATGTGAGGTGGATCCAGAA
AAAAGGG---GGTTCTAGTTATAATCCAGAAACGATTCGTGTATATATTTCACAGAAACGT
GAAATCAAAGTAGGCGATAAAGTAGCTGGAAGACACGGAAATAAGGGGATTATTTCCA
AAATTTTGCCTAGACAAGATATGCCTTATTTGCAAGATGGAAGACCCGTTGATATGGTC
TTCAACCCATTAGGAGTCCCTTCACGAATGAATGTAGGACAGATATTTGAATGTTCACT
CGGGTTAGCAGGGAGTCTGCTAGACAGACATTATCGAATAGCACCTTTTGATGAGAGGT

ATGAACAAGAAGCTTCAAGAAAACTAGTGTTTCTGAATTATATGAAGCCAGTAAGCA
AACAGCGAATCCATGGGTATTTGAACCCGAGTATCCAGGAAAAAGCAGAATATTTGAT
GGAAGAACAGGGAATCCTTTTGAACAACCCGTTATAATAGGAAAGCCTTATATCTTGAA
ATTAATTCATCAAGTTGATGATAAAATCCATGGACGTTCCAGTGGGCATTATGCCCTTGT
TACACAACAACCTCTTAGAGGAAGGGCCAAACAAGGGGGACAACGGGTAGGAGAAAT
GGAGGTTTGGGCTCTAGAAGGATTTGGTGTTGCTCATATTTTACAAGAGATGCTTACTTA
TAAATCTGATCATATTAGAGCTCGCCAGGAAGTACTTGGTACTACGATCATTGGAGGAA
CAATACCTAATCCTGAGGATGCTCCAGAATCTTTTCGATTGCTCGTTCGAGAACTACGA
TCTTTAGCTCTGGAACTGAATCATTTCCTTGTATCTGAGAAGAACTTCCAGATTAATAGG
AAGGAAGCTATGAATCAGAATTTTTCTTCTATGATCGATCGGTATAAACATCAACAGCTC
CGAATTGGATTAGTTTCTCCTCAACAAATAAGTGCTTGGGCCACTAAAATCCTGCCTAA
TGGAGAGATAGTTGGAGAGGTGACAAAACCCTATACTTTTCATTACAAAACCAATAAA
CCGGAAAAAGATGGATTATTTTGTGAAAGAATTTTTGGACCTATAAAAAGTGGAATTTG
TGCTTGTGGAAATTATCGAGTAATCGGAGATGAAAAAGAAGACCCGAAATTTTGTGAA
CAATGCGGAGTCGAATTTGTTGATTCTCGGATACGAAGATATCAAATGGGCTACATCAA
ACTCGCATGCCCCGTAACCCATGTGTGGTATTTAAAACGTCTTCCTAGTTATATTGCGAA
TCTTTTAGATAAACCTCTTAAAGAATTAGAAGGCTTAGTATACTGCGAT------TTTTCTTT
TGCTAGGCCC---ATAACTAAAAAACCCACTTTCTTACGATTACGAGGTTTATTCGAATAT
GAAATCCAATCTTGGAAATACAGCATCCCACTTTTTTTTACTACCCAGGGCTTCGATACA
TTTCGCAATCGAGAAATCTCTACTGGTGCAGGTGCTATCCGAGAACAATTAGCCGACCT
AGATTTACAAATTATTATAGATAAATCGTTGGTAGAATGGAAAGAATTAGGGGAAGGA---
GGGCCCACAGGGAATGAATGGGAAGATCGAAAAGTTGGAAGAAGAAAAGATTTTTTG
GTTAGACGCATGGAATTGGCTAAGCATTTTATTCGAACAAATATAGAACCGGAATGGAT
GGTTTTGTGTCTATTACCAGTTCTTCCTCCTGAATTGAGACCGATCATTCAGATAGATGG
GGGTAAACTAATGAGCTCGGATATTAATGAACTCTATAGAAGAGTTATCTATCGAAACA
ATACTCTTACCGATCTATTAACAACAAGTAGATCTACGCCAGGAGAATTAGTAATGTGTC
AGGAGAAATTAGTACAAGAAGCCGTGGATACACTTCTTGATAATGGAATCCGCGGACA
ACCAATGAGGGACGGTCATAATAAAGTTTACAAGTCATTTTCAGATGTAATTGAAGGCA
AAGAGGGAAGATTTCGTGAGACTCTGCTTGGCAAACGGGTTGATTATTCAGGACGTTC
CGTCATTGTCGTGGGTCCTTCACTTTCATTACATCGATGTGGATTGCCTCGCGAAATAGC
AATAGAGCTTTTCCAGACATTTGTAATTCGGGGTCTAATTAGACAACATCTTGCTTCGA
ACATAGGAGTTGCTAAGAGTAAAATTCGGGAAAAAGAACCCATTGTATGGGAAATACT
TCAGGAAGTTATGCAGGGGCATCCTGTATTGCTGAATAGAGCACCCACTCTGCATAGAT
TAGGTATACAGGCATTCCAGCCCGTTTTAGTGGAGGGGCGTGCTATTTGTTTACATCCAT
TAGTTTGTAAGGGATTCAATGCAGATTTTGATGGGGATCAAATGGCTGTTCATGTACCTT
TATCTTTGGAGGCTCAAGCGGAGGCCCGTTTACTTATGTTTTCTCATATGAATCTTTTGT
CTCCAGCTATTGGGGATCCCATTTCCATACCAACTCAAGATATGCTTATTGGACTCTATG
TATTAACGAGCGGAAATCGCCGAGGTATTTGTGTAAATAGGTATAATCCATGTAATCGC--
-------AGAAACTATCAAAATCAAAGAAGTGACAATAATAACTATAAGTATACG---------GAG
CCCTTTTTTTGTAATTCCTATGATGCAATTGGAGCTTATCGGCAGAAACGAATCAATTTA
GATAGTCCTTTGTGGCTCCGGTGGCGACTAGATCAACGC---GTTATTGCTTTA------AGA
GAAACTCCCATCGAAGTTCATTATGAATCTTTAGGTACTTATTATGAGATTTATGGACAC
TATCTAATTGTAAGAAGTATAAAAAAAGAAATTCTTTTTATATACATTCGAACCACTGTT
GGTCATATTTCTCTTTATCGAGAAATCGAAGAAGCCATACAGGGGTTTTCTCAGGCCTG

TTCATATGGTACC------------------------ATGGAGGTACTTATGGCAGAACGGGCCAATCTGG
TCTTTCACAATAAAGTGATAGACGGAACTGCCATGAAACGACTTATTAGTAGATTAATA
GACCACTTCGGAATGGCATATACATCACACATCTTGGATCAAGTAAAAACTCTGGGTTT
TCAACAAGCTACTGCTACATCCATTTCATTAGGAATTGATGATCTTTTAACAATACCTTC
TAAGGGATGGCTAGTACAAGATGCTGAACAACAAAGTTTGATTTTGGAAAAACACCAT
CATTATGGGAATGTACACGCGGTAGAAAAATTACGTCAATCCATTGAAATATGGTATGCT
ACAAGTGAATATTTGCGACAAGAAATGAATCCTAATTTTAGGATGACTGACCCTTTTAA
TCCAGTTCATATGATGTCTTTTTCGGGAGCTAGAGGAAACGCATCTCAGGTACATCAAT
TAGTAGGTATGAGAGGATTAATGTCGGATCCTCAAGGACAAATGATTGATTTACCCATTC
AAAGCAATTTACGTGAAGGGCTCTCTTTAACAGAATATATCATTTCTTGCTACGGAGCC
CGTAAAGGGGTCGTGGATACTGCTGTACGAACATCAGATGCTGGATATCTCACGCGCA
GACTTGTTGAAGTAGTTCAACATATTGTTGTACGTCGAACAGATTGTGGCAGTACCCGG
GGGATTTCTGTGAGTCCTCGGAATGGAATGATGCCG---GAAAGGATTTTTATCCAAACA
TTAATTGGTCGTGTATTAGCAGATGATATATATATGGGCCCGCAATGTATTGCCACTCGCA
ATCAAGACATTGGGATTGGACTTGTAAATCGATTTCTAACT------------------TTTCGAGCA
CAACCAATATCTATTCGAACTCCCTTTACTTGTAGGAGTACATCTTGGATCTGTCGATTA
TGTTATGGCCGGAGTCCTACTCATGGCGATCTGGTTGAATTGGGAGAAGCTGTAGGTAT
TATTGCGGGTCAATCGATTGGAGAACCGGGTACTCAATTAACATTAAGAACTTTTCATA
CCGGTGGAGTATTCACAGGGGGTACTGCAGAACATGTGCGAGCCCCTTCTAATGGAAA
AATCAAATTTAATGAGGATTTGGTTCATCCGACACGTACACGTCATGGACATCCAGCTT
TTCTATGTTCTATAGACTTGTATGTAACTATTGAGAGTGAAGATATTATACATAATGTGAA
TATTCCACCCAAAAGTTTTCTTTTAGTTCAAAATGATCAATATGTAGAATCCGAACAAGT
GATTGCCGAGATTCGCGCGGGAACATCCACT---TTGAATTTTAAAGAGAAGGTTCGAAA
ACATATTTATTCTGACTCAGACGGCGAA---ATCCACTGGAGTACCGATGTGTATCATGCA
CCTGAATTTACATATGGTAATGTTCATCTATTACCAAAAACAAGTCATTTATGGATATTAT
TAGGAGGGCCGTACAGA------TCCAGTCTAGTCTTCCTCTCACTCCACAAGGATCAAGA
TCAAATGAACGCGCAT------TCTCTTTCTGTCAAGGGGGGAGATACTTCTAATCGCTCA---
---GTAACTAATGATCAG---GTGAGACACAAATTCTTTAGTTCGGATTTTTCTGGTAAA---G
AAGATAAGATTCCTGATTAT---TCAGACCTTAATCGAATCATATGTACTGGTTCTTGTAAT
CTCATATATCCG---GCCATTCTCCACGATAATTCT------GATTTATTGTCAAAGAGGCGAAG
AAATAGATTCATCATTCCACTCCAATGGATTCAAGAATGCGAGAACGAACTAATGTCCT
GTTTAGGTATCTCTATTGAAATCCCC------ACAAATGGTATTTTCCGTAGAAATAGTATTCT
TGCTTATTTCGATGATCCTCGATACAGAAGAAAGAGTTCGGGAATTACTAAATACGGGA
CTATAGAAATGCATTCAATCCTCAAAAAAGAGGAT------TTGATTGAATATCGAGGA---GT
CAAGGAATTTAGGCCAAAATACATGAAAGTAGATCGGTTTTTTTTCATTCCCGAGGAAG
TACATATCTTACCCGGATCTTCTTCCATAATGGTACGGAACAATAGTCTCATTGGGGTAG
ATACCCCAATCACTTTAAATATAAGAAGCCGAGTAGGCGGGTTGGTCCGGGTGGAGAA
AAAAAAAAAAAGAATTGAACTTAAAATCTTTTCTGGAGATATCCATTTTCCTGGCGAG
ACAGATAAGATATCCCGACATAGCGGCGTTTTGATACCA------CCAGGAACGGGAAAAA
CAAATTCCAAGGAATCAAAAAAA------TTGAAAAATTGGATCTATGTCCAACGG------AT
CACACCTAGCAAG------AAAAAGTATTTTGTTTTGGTTCGACCTGTCGTCACATATGAAA
TAACGGATGGTATAAATTTATCAACACTTTTCCCTCCGGATCTGTTGCAGGAAAGGGAT
AATGTGCAACTTCGAGTTGTCAATTATATCCTTTATGGAAATGGCAAACCAATTCGAGG
AATTTCTGACACAAGTATTCAATTAGTTCGGACTTGTTTAGTATTGAGTTGGGACCAAG

AAACAAAA---------AGTTCTTCTATCGAAGAAGCCCGCGCTTCCTTTGTTGAAATAAGGA
CAAATGGTTTGATTCGACATTTCTTAAGAATTGACTTAGTGAAATCTCCTATTTCGTATAT
CGGAAAAAGGAATGATCCATTGGATTCACGATTGCTCTCTGAT---AATGGATCAGATTGC
ACCAATATCAATCCGTTTTCTTCCATTTATTCCTAT---------TCCAAAGGAAGAATTCAACA
ATCCCTTAACCAAAATCAAGGAACTATTCATACGTTGTTGAATAGA---------AATAAGGAA
TGCCAATCGTTGATAATTTTGTCATCATCCAATTGTTTTCGAATGGGGCCA------TTCAAT
GATGTAAAATATCACAGTGTG---------ATAAAAGAATCAGATTCCCGAATTCCAATTAGGA
ATTCGTTGGGCCCCTTAGGAACA------GCCCTTCAAATTGCGAATTTTTATTCA------TTTT
ATCATTTA------ATAACTCATAATCAGATCTTG---GTAACTCACTATTTGCAACTTGAC------
AAT---TTAAAACACACCTTTCAAGTAATTAAA---------TATTATTTAATGGATGAAAAT------
GGGAAAATTTATAATCCCGACCCAGGCAGTAATATTATTTTGAATCCATTCCATTTGAAT
TGGTATTTTCTCCATCACAATTGTTGTGAAGAGACATTT---ACAATAATTAGTCTTGGAC
AGTTTATTTGTGAAAATGTATGTATAGCCAAAAACAGACCCCAC---CTAAAA------TCGG
GTCAAGTTATACTTGTTCAA---GTTGACTCTATAGTAATACGATCCGCTAAGCCTTATTTG
GCCACCCCAGGAGCAACTGTTCATGGACATTATGGGGAAATCCTTTACGAAGGAGATA
CATTAGTTACATTTATATATGAAAAATCGAGATCTGGTGATATAACGCAGGGTCTTCCAA
AAGTGGAACAGGTGTTAGAAGTGCGTTCGATCGATTCAATATCGATGAATCTAGAAAA
GAGGGTTGAGGTTTGGAACGAATGTATAACAAGAATTCTTGGAATTCCGTGGGGATTC
TTGATTGGTGCTGAGCTGACTATAGTGCAAAGTCGTATCTCTTTGGTTAATAAGATCCAA
AAGGTTTATCGATCTCAGGGGGTGCAGATTCATAATAGGCATATAGAAATTATTGTACGT
CAAATAACATCAAAGGTTTTGGTTTCAGAA---------GATGGAATGTCTAATGTTTTTTCAC
CCGGAGAGCTGATTGGATTGTTGCGAGCGGAACGAATGGGGCGCGCTTTGGAAGAAG
CGATCTGTTACCGAGCCATCTTATTGGGAATAACAAGAGCATCACTCAATACTCAAAGT
TTCATATCTGAAGCGAGTTTTCAAGAAACTGCTCGGGTTTTAGCAAAAGCAGCTCTCC
GGGGTCGTATCGATTGGTTAAAAGGCCTGAAAGAGAACGTTGTTTTGGGGGGGATGAT
ACCTGTTGGTACCGGATTCAAAGGATTA------GTGCACCTTTCAAAACAACATAACAAG
ATTCCT------TTGGAAACAAAAAAAAAGAATCTATTCGAGGGGGAAATGAGG------GATA
TTTTGTTCCACCACAGGAAATTATTTGATTCTTCCCTTTCAAAGAATTTCCATGATACAT
CAGAA---ATGACAAGAAGATATTGGAACATCAATTTGG
AAGAGATGATGGAGGCGGGAGTTCATTTTGGCCATGGTACTAGGAAATGGAATCCTAA
AATGGCACCTTATATTTCTGCAAAGCGTAAAGGTATTCATATTATAAATCTTACTAGAAC
TGCTCGTTTTTTATCAGAAGCTTGTGATTTGGTTTTTGATGCGGCAAGGAGGGGAAAAC
AATTCTTAATTGTTGGTACCAAAAATAAAGCAGCTGATTCAGTAGCATGGGCTGCAATA
AGGGCCCGGTGTCATTATGTTAATAAAAAATGGCTCGGCGGTATGTTAACGAATTGGTC
CACTACAGAAACGAGACTTCATAAGTTCAGGGACTTGAGAATGGAACAAAAAACGGG
GAGACTCAACCGCCTTCCGAAAAGAGATGCAGCTATGCTGAAAAGACAATTATTTCGC
TTGCAAACATATCTGGGGGGAATTCAATATATGACAGGGTTACCCGATATTGTAATCATC
GTTGATCAGCACGAAGAATATACGGCCCTGCGAGAGTGTATCACTTTGGGAATTCCAAC
AATTTGTTTAATCGATACAAATTGTGACCCCGATCTCGCAGATATTTCGATTCCAGCGAA
TGATGACGCTATATCTTCAATCCGATTGATTCTTAACAAATTAGTATTCGCAATTTGTGAG
GGGCGTTCT---AGCTATATAAGAAATCCTATGGGACAAAAAATAAATCCACTTGGTTTCA
GACTTGGTACAACCCAGGGTCATCATTCCCTTTGGTTTGCCCAACCAAAAAATTATTCT
GAGGGTCTACAAGAAGATCAAAAAATAAGAGATTGTATCAAGAATTATGTACAAAAAA
ATATGAGAATATCCTCTGGG---GTCGAGGGAATTGCACGTATAGAGATTCAAAAAAGAA

TCGATCTGATCCAGGTCATAATCTTTATGGGATTTCCAAAGCTATTAATAGAAAGTCGAC
CGCGA------------GGAATCGAAGAATTACAGACGAATTTACAAAAAGAATTTAATTGTGT
GAGCCGAAAACTTAATATTGCTATCACAAGAATTGCAAAACCTTATGGAAACCCTAATA
TTCTTGCAGAATTTATAGCCGGACAATTAAAGAATAGAGTTTCATTTAGAAAAGCAATG
AAAAAAGCTATTGAATTAACTGAACAAGCAGATACAAAAGGAATTCAAGTACAAATTG
CAGGGCGTATTGACGGAAAAGAAATTGCACGTGTCGAATGGATCAGAGAAGGTAGGG
TTCCCCTACAAACCATTCGAGCTAAAATTGATTATTGTTCCTATACAGTTCGAACTATCTA
TGGGGTATTAGGTATCAAAATTTGGATATTTATAGACGAGGAAATGTCACGTTACCGAG
GACCTCGTTTCAAAAAAATACGCCGGCTGGGGGCTTTACCAGGACTAACTAATAAAAG
GCCTAGAGCCGGAAGTGATCTTAGAAACCAATCGCGTTCCGGGAAA---AAATCTCAATA
TCGTATTCGCCTAGAAGAAAAACAAAAATTGCGTTTTCATTATGGTCTTACAGAACGAC
AATTACTTAAATATGTTCGTATCGCCAGAAAAGCCAAGGGATCAACAGGTCAAGTTTTA
CTACAATTACTTGAAATGCGTTTGGATAACATCCTTTTTCGATTGGGTATGGCTTTGACT
ATTCCCGCAGCCCGTCAATTAGTTAACCATAGACATATTTTAGTTAATGGGCGTATAGTA
GATATACCAAGTTATCGCTGCAAACCCCGAGATATTATTACAGCGAAGGATGAACAAAA
ATCCAGAACTCTGATTCAAAATTCTCTCGATTCATCCCCTCAT---GAGGGATTACCAAAC
CATTTGACCCTTCACCCATTCCAA------TATAAAGGATTAGTCAATCAAATAATAGATAGT
AAATGGGTCGGTTTGAAAATAAATGAATTGCTAGTCGTAGAATATTATTCTCGTCAGACT
ATGTCACGTCGAGGTACTGCAGAAAAAAAAACAGCAAAATCCGATCCAATTTATCGTA
ATCGATTAGTTAACATGTTGGTTAACCGTATTATGAAACACGGAAAAAAATCATTGGCTT
ATCAAATTATCTATCGAGCCGTGAAAAAGATTCAACAAAAGACAGAAACAAATCCACT
ATCTGTTTTACGTCAAGCAATACGTGGAGTAACTCCCGATATAACAGTAAAAGCAAGAC
GTGTAGGTGGATCGACTCATCAAGTTCCCATTGAAATAGGATCCACACAAGGAAAAGC
ACTTGCCATTCGTTGGTTATTAGCGGCATCCCGAAAACGTCCGGGGCGAAATATGGCTT
TCAAATTAAGTTCCGAATTAGTGGATGCTGCCAAAGGGAGTGGCGATGCCATACGCAA
AAAGGAAGAGACTCATAGAATGGCAGAGGCAAATAGAGCTTTTGCACATTTTCGTATG
AGTAGGGACACTATTGCTGAGATAATAACCTCTATACGAAATGCTGATATGGATAGAAA
AAGAGTAGTTCGAATAGCATCTACTAATATTACCGAAAGTATTGTTAAAATACTTTTACG
AGAAGGGTTTATCGAAAACGTGAGAAAACATCAAGAAAACAACAAATCTTTTTTGGTT
TTAACCCTGCGACATAGAAGG------AATAGGAAAAGACCCTAT---------AGAAATATTTTTA
ATTTAAAACGGATCAGTCGACCTGGTCTACGAATCTATTCTAACTATCAACGAATTCCTA
GAATTTTAGGTGGGATGGGGATTGTAATTCTTTCTACTTCTCGAGGTATAATGACAGACC
GAGAGGCTCGAGAACAAAGAATCGGCGGAGAAATCTTGTGTTATATATGGATGGCAAA
AGCTATGCCGAGAATTGGTTCACGTAGGAATGGACGTATTGGTTCGCGCAAGAGTACG
CGTAGAATACCAAAGGGGGTTATTCATGTTCAAGCAAGTTTCAATAATACCATTGTCAC
TGTTACAGATGTACGGGGTCGGGTGGTTTCTTGGGCCTCTGCCGGTACTTGTGGATTCA
AAGGTACTAGAAGGGGGACGCCATTTGCTGCTCAAACCGCAGCAGGAAATGCTATTCG
TACAGTAGTAGATCAAGGTATGCAACGAGCAGAAGTCATGATAAAAGGTCCCGGTCTC
GGAAGAGACGCAGCATTACGAGCTATTCGTAGAAGTGGTATACTATTAACTTTCGTACG
GGATGTAACCCCCATGCCACACAATGGCTGTAGACCTCCGAAAAAAAGACGTGTGATG
CCAACTATTAAACAACTTATTAGAAATACAAGACAGCCAATGAGAAATGTCACGAAATC
CCCCGCTCTTCGGGGATGCCCTCAGCGCCGAGGAACATGTACTAGGGTGTATACTATCA
CCCCCAAAAAACCAAACTCTGCCTTACGTAAAGTTGCCAGAGTACGATTAACCTCTGG
ATTTGAAATCACTGCTTATATACCCGGTATTGGCCATAATTTACAAGAACATTCTGTAGT

CTTAGTAAGAGGGGGAAGGGTTAAGGATTTACCCGGTGTGAGATATCACATTGTTCGA
GGAACCCTAGATGCTGTCGGAGTAAAGGATCGTCAACAAGGGCGTTCTAAATATGGGG
TAAAAAAGCCAAAAATGGCAAGGAAAAGTTTGATTCAGAGGGAGAAGAAGAGGCAA
AAATTGGAACAGAAATATCATTTGATTCGTCGATCCTCAAAAAAAGAAATAAGC---------
AAAGTTCCCTCGTTGAGTGACAAATGGAAATTTATGGAAAGTTACAATCCCCACCAC
GGAATAGTGCACCGACACGCCTTCATCGACGTTGTTTTCGACCGGAAGACCGAGGGC
TAATTATCGAGACTTTGGACTATCCGGACACATACTTCGTGAAATGGTTCATGCATGTTT
GTTGCCGGGAGCAACAAGATCAAGTTGGATGGTAAAAAATTCATTCATTTCAGTTATTT
CTCAA------GAAGAAAACAAAGAAAACAGAGGGTCTGTTGAATTTCAAGTATTCAGTTT
TACCAATAAGATACGAAGACTTACTTCACATTTAGAATTGCACAAAAAAGACTATTTAT
CTCAGAGAGGTTTGCGAAAAATTTTGGGAAAACGTCAACGACTGCTGGCTTATTTGTC
AAAAAAAAATAGAGTACGTTATAAAGAATTAATCGGTCAGTTGGATATTCGAGAGACA
AAAACTCGT------ATGGTAAAACTTCGTTTGAAACGATGTGGTAGAAAGCAACGAGCCG
TCTATCGAATCGTTGCAATTGATGTTCGATCCCGAAGAGAGGGAAAAGATCTTCGGAA
AGTGGGTTTTTATGATCCGATAAAGAATCAAACTTATTTAAACGTTCCTGCTATTCTATAT
TTCCTTGAAAAGGGCGCTCAACCTACAGGAACTGTTCAGGATATTTTAAAGAAGGCGG
AGTTTTTTAAGGAACGTCGCCCTAATCAAACGGAATTCAAT---ATGGATAAATCCAAGC
GACCTTTTCTTAAATCCAAGCGATCTTTTCGTAAGCGTTTGCCCCCGATTCAATCGGGG
GATCGAATTGATTATAGAAACATGAGTTTAATTAGTCGATTTATTAGTGAACAGGGAAA
AATATTATCTAGACGGGTAAATAGATTGACCTTGAAACAACAACGATTAATTACTATTGC
TATAAAACAAGCCCGTATTTTATCTTTGTTACCTTTTCTCAATAATGAGAAA------CAATT
TGAAAGAACCGAGTCGGCCGCTAGAACTACAGGTATTAGAACCAGCAATAACGTGACA
CGTTCACTAAAAAAAAATCCTTTTGTAGCCAATCATTTATTAAAAAAAATTGATAAGCTT
AACACAAAAGCAGAAAAAGAAATAATAGTAACTTGGTCCCGGGCATCTACCATTATAC
CCACAATGATCGGCCATACTATTGCTATCCATAATGGGAAGGAGCATTTGCCTGTTTATAT
AACAGATCGTATGGTAGGCCACAAATTGGGAGAATTCGCACCTACTTTAAATTTCCGAG
GACATGCCAAAACCGATAATAGATCTCGTCGTATGATTTTTCAATCTTTTCTACTAGGTA
ATCTAGTATCCTTATGCATGAAGATAATCAATTCGGTCGTTGTGGTCGGACTCTATTATG
GATTTCTGACCACATTCTCCATAGGGCCCTCTTATCTCTTCCTTCTCCGAGCTCAGGTTA
TGGAAGAAGGA---------ACCGAGAAGAAGGTATCAGCAACCACTGGTTTTATTACGGGA
CAGCTCATGATGTTCATATCGATCTATTATGCGCCTCTGCATCTAGCATTGGGTAGACCTC
ATACAATAACTGTCCTAGCTCTACCATATCTTTTGTTTCATTTCTTCTGGAACAATCACA
AACACTTT---------GGATCTACTACCAGAAATTCAATGCGTAATCTCAGCATTCAATGTGT
ATTCCTGAATAATCTCATTTTTCAATTATTCAACCATTTCATTTTACCAAGTTCAATGTTA
GCCAGATTAGTCAACATTTATATGTTTCGATGCAACAACAAGATGTTATTTGTAACAAGT
AGTTTTCTTGGTTGGTTAATTGGTCACATTTTATTCATGAAATGGCTTGGATTGGTATTA
GTCTGGATACGGCAAAATCATTCTATTAGATCGCTTATTCGATCTAATAAGTACCTTGTGT
CAGAATTGAGAAATTCTATGGCTCGGATCTTTAGTATTCTCTTATTTATTACCTGTGTCTA
CTATTTAGGCAGAATACCGTCACCCATTCTTACTAAGAAACTGAAACAAACCGAAGAA
GATAAACCTCTTGGAACTCTTCTTTTCGATTATAACCGATGGAATCGCCCATTTCGCTAC
ATAAAAAATAATCGATTTGAAAAGGCTGTAAGAAACGAAATGTCACAATACTTTTTTGA
CACATGCCGAAGTGATGGAAAACAAAGAATATCTTTTACATACCCACCAAGTTTGTCAA
CTTTTTTGAAAATGATAAAAAAAAGGATATTCTTGTCAACACTAGAAAAAACCCCCTCT
GATGAACTAGACAACCGTTGGGTTTATACCAACAAAGAAAAAAGTAAT---CTAACCAAC

GAGTTTCTAAATAGAATTGAAGTTCTAGACAGGGAACCCCTTTCTCTAGATATAATTGA
AACAAGGACTAGACTGTGTAATGACAATACTAAAAAAGAATATTTGCCTAAAATGTATG
ATCCTTTCTTGAACGGACCATATCGTGGAACAATCAAAAAATGGTTTTCACCTAAAAAG
AGAGTTGGGATAAATCGGATTCATAGTATCCTTTACCAAGAATTAGTAAATTCAGATCCT
AATGGTCAATCTATTGGAATAAAAGAAATCAGTAAAAAAGCCCCTCGCAGGTCATACA
AATTAATCGATTTGGAACAACAATCA---GGACAAATTCGCTCAAGAAAAGCCAAACGT
GTGGTGATTTTTACTATTGCTTTGATACGCTATTCACAACAATCAGATTTTCGACGAGGC
ATAATCAAAGGTTCTATGCGTGCTCAAAGGCGTAAAACAGTTATTTGGGAGCTGTTTCA
AGCAAATGCGCATTCCCCCCTTTTTTTGGACAGAATAAAAAAATCCCCGCTTTTTTCGT
TTGATATC------TCCCGACTAATCAACCCAATTTTTAGAAATAGAAAGGAGAAAGCACGG
ATACAGGTAGCAGAAGCCTGGGATACCATTCCATTTGCACAAGGAATAAGAGGTTCCAT
GTTAATAACTCAATCAATTCTTAGAAAATATATTCTATTACCTTCCTTGATAATAGCCAAA
AATATTGGCCGTATGTTATTATTTCAACTTCCTGAATGGTCTGAGGATTTACAGGAATGG
AATAGAGAAATGCATGTTAAATGTACCTATAACGGTGTTCAATTATCAGAAACAGAATT
TCCGAAAAATTGGTTCACAGACGGGATTCAGATAAAAATCCTATTTCCTTTCCGTCTGA
AACCTTGGCACAGACCTAAAAAAAAACAAAAAGATGATTTTTGTTTTTTAACAGTTTC
AGGAATGGAAGCTGAACTTCCTTTTGGTTCTCCTCGAAAACGACCTTCCTTTTTTGAGC
CCATTATTAGGAAACTCAAAAAAAAAATTGCAAAATTAAAAAAGAAGTATTTTTTAGCT
---CTAAGGGTTTTAAAAGGAAAAACAAAATTACTTCTAAAAGTTTCAAAAGAAACAAA
AAAATGGGTTATGAAAAACATTCTA------TTTAGAAAAATAATAAGAAACGAACTCTCA
AAAGTTAATGATTCTATAATCAGCAATCAGATAATTCATGAATCATTTAATCAAATTCAAT
CTCCAGATTGGACAAATTCTTCACTGACAGAAAAAAAAATGAAAGATCTGACTGATAG
AATAAGTATCATTAGAAATCAAATAGAAAGAATTACAAAAGAGAAAAAAAAAAGTCTT
ACCAAAAGAAGTTATAATGCTAAAAGATTAGAGTTGCCAAAAAATATTTGGCAAATAGT
AAAAAGAAGAAATCCTCGATTAACCTATAAATTACATTATTTTCTAAAATTTTTCATTGA
AAGAATGTACATAGATATTTTTTTATCTATCATTAATATTCCCAGAATCAATACACAACTT
TTT---CGCTTTATTTCAAGTGATTTATCTTACTTGTCACAAGCATATGTATTTTACAAGTTA
TCACAAACCCAAGTTAGAAATTTGTATAAATTAAGATCTGTTCTTCAATATACATCGTTT
TTTCTTAAGACTGAAATAAAGGATTCTTTTACACTAAGAATATTTCAACCTGAATTAAGG
CATAAGAAACTTAAGAATTATGGAATGAATCATTGGAAAAACTGGTTAAGAGGGCATTA
TCAATACGATTTATCTCAGATTAGATGGTCTAGATTAATACCACAAAAATGGCGAAATAG
AATTAATCAATGTCGTATGACTCAAAAAAAAAAACAAATGGGATTCATATGAAAAAGAC
CAA------TTAATTCATTACAAAAAACAA---AATTTTTCAAAATACTATAAATATGATCTTTT
ATCATATAAATCTCTTAATGACGATTTTTTATATTTCGATAGAAAATATTTGGATTGGAAA
ATCATCAATTTTTATCTTAGACTTTTTGATTGGATGGAAATGAATGAAGAAATACTAAAT
CGTTGGTTCTTCCCAGAATTTGTGCGACTTTATAATGCATATAAAATGAAACCGTGGGTT
ATACCAAGCAAATTACTTCTTTTAAATTACAAAAGTAACACGGAAGCAGAACTAGATAT
CTTCCTGAAACGTTATTTGCTTTTTCAATTGAGATGGGACGATTCTTTGAATCAAAGAAT
GATCAATAATATCAAGGTATATTGTCTCCTGCTTAGACTGATAAATCCAAGAAAAATTAC
TATATCCTCGATTCAACGGAGAGAAATAAGTTTGGATATAATGCTGATTGAAAAGAATTT
AACTCTTACAGAATTGATGAAA---GGCGTATTGATTATCGAACCCCTTCGTCTGTCTGTA
AAAAATGATGGACAATTTATTATGTATCAAACCATAGGTATTTCCTTGGTTCATAAAAGT
AAGCACAATCATTTTAATTTGCTTGTTCCTGAAAATATTTTTTCATCTAGACATCGTAGA
GAGTTGAGAATTCTAATTTGTTTCAGATTTCTTTGGCCTAATTATCGATTAGAAGATTTA

GCTTGTATGAATCGTTATTGGTTTGATACCAATAATGGTAGTTGTTTTAGTATGTTAAGGA
TACATATGTATCCAATGAAAGGACATCAATTCAAATCCTGGATTTTCGAATTGAGAGAG
ATATTGAGAGAGATCAAGAATTCTCACTATTTCTTAGATTCATGGACCCAATTCAATTCA
GTGGGATCTTTCATTCACATTTTTTTCCACCAAGAACGTTTTATAAAACTCTTTGACCCC
CGAATTTGGAGTATCTTACTTTCA---CGCAATTCA------CAGGGTTCAACAAGCAATCGAT
ATTTCACG---------ATCAAGGGT---TTAATACTCTTTGTAGTAGCGGTCCTTATATATCGT----
--------ATTAACAATCGAAATATGGTCGAAAGAAAAAATCTCTATTTGATAGGGCTTCTTC
CT--------------ATACCTATGAATTCCATTGGACCCAGAAATGATACATTGGAAGAATCCGT
T---GGGTCTTCCAATATCAATAGGTTGATTGTTTCG---CTCCTGTATCTTCCAAAAGGAAA
AAAGATCTCTGAGAGTTGTTTCCTGAATCCGAAAGAGAGTACTTGGGAGAACCCAAGT
ACT------------AAAAAGTGTAGCATGCCTGAATCTAACTGGGGTTCGCGGTGGTGGAGGA
ACTGGATCGGAAAAAGGAGG------GATTCTAATTGTAAGATATCTAAT---------GAAACCG
TCGCTGGAATTGAGATCTTATTCAAAGAGAAAGATATCAAA---TATCTGGAGTTTCTTTT
TGTATATTATATGGAT------GATCCGATCCGCAAGGACCATGATTGGGAATTGTTTGATCG
TCTTTCTCTGAGGAAGAGGCGAAATATAATCAACTTG------AATTCGGGACCGCTATTCG
AAATCTTAGTGAAACACTGGATTTCTTATCTCATGTCTGCTTTTCGTGAAAAAATACCAA
TTGAAGTGGAGGGT---TTCTTCAAACAACAAGGG---GCTGGGTCAACTATTCAATCAAA
TGATATTGAGCATGTTTCCCAT------CTCTTCTCGAGAAACAAGTGGGCTTTTTCTTTGCA
AAATTGTGCTCAATTTCATATGTGGCAATTCCGCCAAGATCTCTTCGTT---------AGTTGG
GGGAAG---AATCCGCACGAATCGGATTTTTTGAAGAAC---GTATCGAGAGAGAATTTGA
TTTGGTTAGACAATGTGTGGTTGGTAAATAAGGATCGG---------TTTTTTAGC------AAGGT
ACGGAATGTATCGTCAAATATTCAATATGATTCC---------ACAAGA------TCTAGTTTCGTT
CAAGTAACGGATTCTAGCCAACTGAAAGGATCTTCT---GATCAATCCAGAGATCATTTG
GATTCCATTAGT------------AATGAGGATTCGGAA------------------------------------TATCACA
CATTGATCAATCAAAGAGAGATTCAACAACTAAAAGAAAGATCGATTCTT---TGGGAT---
---CCTTCCTTTCTTCAAACGGAACGAACAGAGATAGAATCAGAC------CGATTCCCGAAA
------TGCCTTTCTGGATATTCC------TCAATGTCCCGGCTATTCACG---GAACGTGAGAAGC
AGATGATTAATCATCTGCTTCCGGAAGAAATCGAAGAATTTCTT------GGGAATCCTACA
AGATCCGTTCGTTCTTTTTTCTCTGATAGATGG------TCAGAACTTCAT---CTGGGTTCGA
ATCCTACTGAGAGGTCCACTAGAGATCAG------------AAATTGCTGAAGAAACAACAAGA
TCTTTCT---TTTGTCCCTTCCAGGCGATCGGAA------------AATAAAGAAATGGTTAATATA
TTCAAGATAATTACGTATTTACAAAAT------ACCGTATCAATTCATCCTATTTCATCAGATC
CGGGGTGTGATATGGTTCCG---AAGGATGAACCGGAT---ATGGACAGTTCCAATAAGATT
TCATTCTTGAACAAAAATCCATTTTTGGATTTA------TTTCATCTATTCCATGACCGGAAC
AGGGGAGGATACACCTTA------CACCACGATTTTGAATCAGAA------GAGAGATTTCAAG
AAATGGCAGATCTATTCACTCTATCAATAACCGAGCCG---GATCTGGTGTATCATAAGGG
ATTTGCCTTTTCTATTGATTCCTACGGATTGGATCAAAAACAA------TTCTTGAATGAGGT
ATTCAACTCCAGGGATGAATCGAAAAAGAAATCT---------TTATTGGTTCTACCTCCTAGT
TTTTAT------GAAGAGAATGAATCTTTTTCTCGAAGGATCAGAAAAAAATGGGTCCGG---
ATCTCCTGCGGGAATGATTTGGAAGATCCAAAACCAGAAATAGTGGTATTTGCTAGCAA
CAACATAATGGAGGCAGTCAATCAATATAGATTGATCCGAAATCTGATTCAAATCCAATA
TAGTACCTATGGG------TACATAAGAAATGTATTGAATCGATTCTTTTTAATGAAT---------A
GATCCGATCGCAACTTCGAATATGGA---------ATTCAAAGGGATCAAATAGGAAAGGATA
CTCTGAATCATAGAACTATAATGAAATATACGATCAACCAACATTTCTCGAATTTGAAAA

AG---AGTCAGAAGAAATGGTTCGATCCTCTT------ATTTTGATTTCTCGAACCGAGAGAT
CCATGAATTGGGATCCTGATGCATATAGATACAAATGGTCCAATGGGAGCAAGAATTTC
CAGGAACATTTGGAACATTTTGTTTCTGAGCAGAAGAGCCGT---------TTTCAAGTAGTG
TTCGATCGATTACGTATTAATCAATATTCGATTGATTGGTCTGAGGTTATCGACAAAAAA
GATTTGTCTAAGCCACTTCGTTTCTTTTTGTCCAAG---------------------------TCACTTCTTT
TTTTGTCC---------------------AAGTTGCTTTTCTTTTTGTCTAAC---TCACTTCCTTTTTTCT
GTGTGAGTTTCGGGAATATCCCCATTCATAGGTCCGAG---ATCTACATCTAT---GAATTGA
AAGGTCCGAATGATCAACTCTCCAATCAGTTGTTAGAATCAATAGGTCTTCAAATTGTT
CATTTGAAAAAATTGAAACCCTTC------TTATTGGAT---------GATCATGATACTTCC---CAA
AAATCGAAATTCTTGATCAATGGAGGAACAATATCACCATTTTTGTTCAATAAGATACCA
---AAGTGG---ATGATTGACTCATTCCATACTAGA------AATAATCGCAGGAAATCCTTTGAT
AACACGGATTCCTATTTCTCAATGATATTCCACGATCAAGACAATTGGCTGAATCCCTTG
AAACCATTTCATAGAAGTTCATTGATATCTTCTTTTTATAAAGCAAATCGACTTCGATTC
TTGAATAATCCACATTACTTCTGCTTCTAT------TGTAACACAAGATTCCCCTTTTCTGTG--
----------GAAAAGGCCCGTATCAAT------AATTATGATTTTACGTATGGA---CAATTCCTCAAT
ATCTTGTTCATTCGCAACAAA---ATATTTTCTTTGTGCGTCGGTAAA------AAAAAACATG
CTTTTGGGGGG---AGAGATACTATTTCACCAATCGAGTCACAGGTATCTAACATATTCAT
ACCTAACGATTTTCCACAAAGTGGT---GACGAAGCGTATAACTTGTACAAATCT---------T
TCCATTTTCCAAGTCGATCCGATCCATTCGTTCGT------AGAGCTATTTACTCGATCGCAG
ACATTTCTGGAACACCTCTAACAGAGGGACAAATAGTCAATTTTGAAAGAACTTATTGT
CAACCTCTTTCAGATATGAATCTATCTGATTCAGAAGGGAAGAACTTGCATCAGTATCT
CAAT------TTCAATTCAAACATGGGTTTGATTCAGACTCCATATTCTGAGAAATATTTACC
ATCCGAAAAG------AGAAAAAAA------------------------CGGAGTCTTTGTCTAAAG------AAA
TGCGTTGAGAAAGGGCAGATGTATAGAACCTTTCAACGAGATAGTGCTTTTTCAACTCT
CTCAAAATGGAATCTATTCCAAACATATATGCCATGGTTCCTTACTTCGACAGGGTACAA
ATATCTAAATTTGCTATTTTTAGATACTTTTTCAGACCTATTG------CCGATACTAAGTAGC-
--AGTCAAAAATTTGTATCCATTTTTCATGATATTATGCATGGATCAGGTATATCATGGCGA
ATTCTTCAGAAAAAATTG------------------TGTCTTCCACAATGGAATCTG---ATAAGTGAG
ATTTCGAGTAAGTGTTTACATAAT------CTTCTTCTGTCCGAAGAAATGATTCATCGAAAT-
-----AATGAGTCACCATTGATATCGACACATCTGAGATCGCCAAAGGTTCGGGAGTTC---C
TCTATTCAATCCTTTTCCTTCTTCTTGTTGCTGGATAT---CTCGTTCGTACACATCTTCTCT
TTGTTTCCCGG------GCCTCTAGTGAGTTACAGACAGAGTTCGAAAAGGTCAAATCTTT
GATGATTCCATCATCTATGATTGAGTTGCGAAAACTTCTGGATAGGTATCCTACATCTGA
ACCGAATTCTTTCTGGTTAAAGAATCTCTTTCTAGTTGCTCTGGAACAATTAGGATATTC
TCTAGAAGAAATACGGGATTCTGCTTCTGGCGGCAACATGCTTGGT---------CCCGCT-----
----------TATGGG---GTCAAATCAATACGTTCTAAGAAGAAATATTTGAATATCAAT---------C
TCATCGATCTCATA------------CCAAATCCCATTAATCGAATCACTTTTTCGAGAAATACGA
GACATCTAAGTCATACAAGTAAAGAGATCTATTCATTGATAAGAAAAAGAAAAAACGT
GAACGGGGATTGGATTGATGATAAAATAGAATCCTGGGTCGCGAACAGTGATTCGATTG
ATGATGAAGAAAGAGAATTCTTGGTTCAGTTCTCC------ACCTTAACGACA------GAAAA
AAGGATTGATCAAATTCTATTGAGTCTGACT---CATAGTGATCATTTA---------------TCAAA
GAATGACTCTGGTTATCAAATGATTGAACAACCGGGAGCAATTTACTTACGATACTTAG
TTGACATTCAT------AAAACGTATCTAATGAATTATGAGTTCAATACATCCTGTTTAGCAG
AAAGACGGATATTCCTTGCTCATTATCAGACAATCACTTAT------TCACAAACTTCGTGTG

GGGCTAATAGTTTTCATTTCCCATCTCATGGA---------AAACCCTTTTCGCTCCGCTTAGC
CTTATCCCCCTCT---AGGGGTATTTTAGTGATAGGTTCTATAGGAACTGGACGATCCTATT
TGGTCAAATACCTAGCGACAAACTCCTATGTTCCTTTCATTACGGTATTTCTGAACAAGT
TCCTGGATAACAAGCCTAAAGGTTTTCTTATTGAT--------------GATATCGAT---ATTGATGA
TAGTGACGATATTGATGCTAGTGACGATATCGATGCTAGTGAC--------------------------------
------------------GATATCGATCGTGACCTTGATACGGAGCTGCTGGAACTGCTAACTAGGA
TGAATGCGCTAACTATGGATATGATGCCGGAA---------ATAGACCGATTTTATATCACCCTT
CAATTCGAATTAGCAAAAGCAACGTCTCCTTGCATAATATGGATTCCAAACATTCATGAT
CTGGATGTGAATGAGTCGAATTACTTATCCCTCGGTCTAGTAGTGAACCATCTATCCAGG
---------GATTGTGAAAGATGTTCC---ACTAGAAATATTCTTGTTATTGCTTCGACTCATATT
CCACAAAAAGTGGATCCCGCTCTAATAGCTCCGAATAAATTAAATACGTGCATTAAGAT
ACGAAGGCTTCTTATTCCACAACAACGAAAGCACTTTTTCACTCTTTCATATACTAGGG
GATTTCACTTGGAAAAGAAAATGTTCCATACT------AACGGATTCGGGTCCATAACCATG
GGTTCCAATGCACGAGATCTTGTAGCACTTACCAATGAGGCCCTATCGATTAGTATTACA
CAGAAGAAATCAATTATAGACACTAATACAATTAGATCCGCTATTCATAGACAAACTTG
GGATTTGCGA---------TCCCAGGTAAGATCGGTTCAGGATCATGGGATCCTTTTCTATCAG
ATAGGAAGGGCTGTAGCACAAAATGTACTTCTAAGTAATTGCCCCATAGATCCTATATCT
ATCTATATGAAGAAGAAA------TCATGTAACGAAGGGGATTCTTATTTGTACAAATGGTA
CTTCGAACTTGGAACGAGCATGAAGAAATTAACGATACTTCTTTATCTTTTGAGTTGTT
CTGCCGGATCGGTCGCTCAAGATCTTTGGTCTCTACCC------GGACCCGATGAAAAA---A
ATGGGATCACTTCTTATGGACTCGTTGAGAATGATTCT---GATCTAGTTCATGGCCTATTA
GAAGTAGAAGGCGCTCTGGTGGGA------TCTTCACGGACAGAAAAAGATTGCAGTCAG
TTTGATAATGAT------CGAGTGACATTGCTTCTTCGGCCCGAACCGAGGAAT---CCCTTAG
ATATGATGCAAAACGGATCTTGTTCTATCCTTGATCAGAGATTTCTCTATGAAAAATAC---
---GAATCGGAGTTTGAAGAA---GGGGAGGGAGAAGGAGCCCTTGACCCGCAACAG------
------ATAGAGGAGTAT------------TTATTCAATCAC------ATAGTTTGGGCTCCT------AGAATA
TGGCGCCCT---------TGGGGCTTTCTATTTGATTGTATCGAAAGGCCCAATGAATTGGGAT
TTCCC---TATTGGTCCAGGTCATTTCGAGGCAAG---------CGGATCATTTATGATGAAGAG
GATGAGCTTCAAGAGAATGATTCGGAGTTCTTGCAGAGTGGAACCGTGCAGTACCAGA
CACGAGATAGATCTTCCAAAGAACAAGGCTTTTTTCGAATAAGCCAATTCATTTGGGAC
CCTGCAGATCCACTCTTTTTCCTATTCAAA---GATCAGCCCCCTGGCTCTGTGTTTTCAC
ATCGAGAATTATTTGCAGATGAAGAGATGTCAAAGGGGCTTCTTACTTCCCAAACGGAT
CCTCCTACATCTATATATAAACGCTGGTTTATC------AAGAATACGCAAGAAAAGCACTTC
GAATTGTTGATTAATCGTCAGAGATGGCTTAGA---------ACCAATAGTTCATTATCT------A
ATGGATCTTTCCGTTCTAAT------ACTCTATCCGAGAGTTATCAGTATTTATCAAATCTCTT
CCTATCTAAC---GGAACGCTATTGGATCAAATGACAAAGACATTGTTGAGAAAGAGATG
GCTTTTCCCGGATGAAATGAAAATTGGATTC------ATG-------------------------ATGCCTAGA
TCGCGG---ATAAATGGAAATTTTATTGATAAGACCTTTTCAATTGTAGCCAATATCTTATT
ACGAATAATTCCGACAACTTCAGGAGAAAAAGAGGCATTTACCTATTACAGAGATGGG-
-----ATGTCAGCTCAATCCGAAGGAAATTATGCGGAAGCTTTACAGAATTATTATGAAGCT
ATGCGACTAGAAATTGATCCTTATGATCGAAGTTATATACTCTATAATATAGGTCTTATCC
ACACAAGTAATGGAGAACATACGAAAGCTTTGGAATATTATTTTCGAGCACTAGAACG
AAACCCATTCTTACCGCAAGCTTTTAATAATATGGCCGTGATCTGTCATTAC---CGAGGA
GAACAGGCCATTCGACAGGGAGATTCTGAAATTGCGGAGGCTTGGTTCGATCAAGCCG

CTGAGTATTGGAAACAGGCTATAGCGCTTACTCCCGGGAATTATATTGAAGCACATAATT
GGTTGAAGATCACGAGGCGTTTCGAA------ATGAGTTGGCGATCAGAACATATATGGATA
GAACTTATAAGGGGGTCTCGAAAAATAAGTAATTTCTGCTGGGCCTTTATCCTTTTTTTA
GGTTCATTAGGATTCTTATTGGTTGGAATTTCCAGTTATCTTGGTAGAAATTTGATATCTT
TTTTTCCGTCTCAGCAAATCGTTTTTTTTCCACAAGGGATCGTGATGTCTTTCTACGGGA
TCGCGGGTCTCTTTATTAGCTCCTATTTGTGGTGCGCAATTTCCTGGAATGTAGGTAGTG
GTTATGATCGATTCGATAGAAAGGAAGGAATAGTGTGTATTTTTCGTTGGGGATTTCCTG
GAAAAAATCGTCGTATATTCCTCCGATTCCTTATAAAAGATATTCAGTCCGTTAGAATAG
AAGTTAAAGAGGGTATTTCTGCTCGTCGTGTCCTTTATATGGACATAAGAGGACGGGGG
GCCATTCCCTTGACTCGTACTGATGAGAAT---TTGACTCCACGAGAAATTGAACAAAAA
GCTGCCGAATTGGCCTATTTCTTGCGTGTACCAATTGAAGTATTT
>Euonymus
ATGGAAAAACGGTGGTTCAATTCGATGTTATCTTTAAGTAAATCAATGGATAGTCTTGG
TCCTATTGAAAATGTTAGTGGACGTGAAGATCCTATTGAAAATGATAGGGATAAAAAAA
TGCATCCTTACAGTAATCTTGATCATTTAGCAGGCGGCAGGGGCTTTCGTAATTTCATTT
CTGATGGAACTTTTTTATTAAAGGATAATAATAGGAACGCTTATTCCGTCCATTTTGATAT
TGAAAATCAAACTTTTGAGATTGACAATGAGCAT------TCGTTTATAAGTGAACTGGAAA
GTTCTTTTTATAGTTATCGAATTTCGAGTTATCTGAATAATGAATCCGATAGTGAAGATAG
TGATCCCCCCTCGGTGGTATATAATACTAAACATAGTTGGAATAATCACATTAATAGTTG
CATTGACACTTATCTTCGTTTTCGTTCTCCAATCGGTAGTGATAGTTATATTTTACGTGGT
GGTGGCAATTACAGTGAAAGTTACATTTATAATTCCATTTGTAGGAACGAGAGTTCTAG
TCTAAGACCTATCACGAATGATATTGATTTAACTCTAAGAGAAAGTTCTAATGATCTCGA
TGTAACTCAAAAATACAGGCATCTCTGGGTTCAGTGCGAAAATTGTTATGGATTAAATT
ATAAGAAATTTTTTAAGTCAAAAATGAATATTTGTGAACAATGTGGATCTCATTTGAAAA
TGAGTAGTTCAGATAGAATTGAACTTTCGATTGATCCGGGCACTTGGAATCCTATGGAT
GAAGACATGGTTTCTGTAGATCCCATTGAATTTCATTCAGAGGAGGAACCTTATAAAGA
TCGTATTGACTCTTATCAAAGAAAGACAGGATTAACCGAGGCTGTTCAAACAGGTACA
GGTCAACTAAATGGTATTCCCGTAGCAATTGGAGTTATGGATTTTATGTTTATGGGGGG
AGTATGGGATCCGTGGTAGGAGAGAAAATTACCCGTTTGATCGAGTATGCTACCAATCA
ATTTTTACCTCTTATTCTAGTGTGTGCTTCTGGAGGAGCACGCATGCAAGAAGGAAGTT
TGAGCTTGATGCAAATGGCTAAAATATCTTCCGCTTTATTTGATTATCAATCAAATAAAA
AGTTATTTTATGTATCAATCCTTACATCTCCTACTACTGGCGGAGTGACAGCAAGTTTTG
GGATGTTGGGAGATATCATTATTGCCGAACCAAATGCCTACATTGCATTTGCGGGTAAA
AGAGTAATTGAGCAAACATTGAATAAGACAGTCCCTGAAGGTTCACAAGCGGCTGAAT
ATTTATTCCATAAGGGCTTATTCGATCAAATCATACCACGTAATCTTTTAAAGGGCGTTC
TGAGTGAGTTATTTCAGTTCCATGCTTTCTTTCCTTTGAATCAAAATTCAATCAAGATGG
TAACCATTCGAGCCGACGAAATTAGTAATATTATCCGTGAACGTATTGAACAATATAATA
GAGAAATAAAGATTGTAAATATCGGTACCGTACTTCAAGTAGGCGACGGCATCGCTCGT
ATTTACGGTCTTGATGAAGTAATGGCGGGTGAATTAGTAGAATTTGAAGAGGGTACAAT
CGGCATTGCTCTTAATTTGGAATCAAATAATGTTGGTGTTGTATTAATGGGTGGTGGTTT
AATGATAGAAGAGGGAAGCTCTGTAAAAGCAACAGGAAGAATTGCTCAGATACCAGT
GAGTGAGGCTTATTTGGGTCGTGTTATAAATGCCCTGGCTAAACCTATTGATGGTCGAG
GCGAAATTTCAGCTTCTGAATCTCGGTTAATTGAATCTCCTGCTCCAGGGATTATTTCGA
GGCGCTCCGTATATGAGTCTCTTCAAACAGGCCTTATTGCTATTGATTCGATGATCCCGA

TAGGACGTGGTCAACGAGAATTAATTATTGGGGACAGACAGACCGGTAAAACCGCGGT
AGCCACAGATACGATTCTCAATCAACAAGGGGAAAATGTAATATGTGTTTATGTAGCTA
TTGGTCAAAAAGCATCCTCTGTGGCTCAGGTGGTGACTACTTTACAGGAAAGAGGAGC
AATGGAATATACTATTGTGGTAGCTGAAATGGCGGATTCTCCAGCTACATTACAGTACCT
CGCCCCTTATACTGGAGCGGCTCTAGCTGAATATTTTATGTACCGTGAACAACACACTTT
AATCATTTATGATGATCTCTCCAAACAAGCACAGGCTTATCGCCAAATGTCTCTTCTATT
ACGAAGACCACCCGGTCGTGAAGCTTATCCAGGAGATGTTTTTTATTTGCATTCACGTC
TTTTAGAAAGAGCCGCTAAATCAAGTTCTAGTTTAGGTGAAGGAAGTATGACTGCTTTA
CCAATAGTTGAGACCCAATCGGGAGATGTTTCAGCTTATATTCCTACTAATGTAATTTCC
ATTACAGATGGACAAATCTTCTTATCCGCCGATCTGTTTAATGCCGGAATCAGGCCTGCT
ATTAATGTGGGTATTTCCGTCTCGAGAGTAGGATCCGCGGCTCAAATTAAAGCGATGAA
ACAAGTCGCGGGCAAATTAAAATTGGAATTGGCTCAATTCGCAGAATTAGAAGCCTTC
GCGCAATTCGCTTCTGATCTCGATAAAGTTACTCAGAATCAATTGGCGAGAGGTCAACG
ATTACGCGAGTTGCTCAAACAATCCCAATCTGCTCCTCTCGCGGTGGAGGACCAGATAA
TGACTATTTATACCGGAACGAATGGCTATCTTGATTCGTTAGAAATTGGACAAGTAAGA
AAATTTCTTGTTGAGTTACGTACTTACTTAAAAACAAATAAACCTCAGTTCCAAGAAAT
CATATCTTTTACCAAGGTATTCACCGAAGAGGCAGAAGCCCTTTTGAAAGAAGCTATTC
AGGAACAGATGAAACGTTTTATACTTCCGGAACAGTTAATGAAAAAAAATCCTACTACT
TCTGGCATGGAGGTTTCCGCCCTTGAAAAAAAAAACCTCGGACGTATTGCTCAAATCA
TTGGTCCAGTACTGGATGTAGCTTTTTCCCCGGGCAAGATGCCTAATATTTACAACGCTC
TAGTGGTTAAGGGTCGAGATACGTCCGGTCACACAATTAATGTGATTTGCGAAGTACAG
CAATTATTAGGAAATAACCAAGTTCGTGCTGTAGCTATGAGTGCCACAGATGGTCTAAT
GAGAGGGATGGACGTGATTGACACGGGAGCTCCTCTAAGTGTTCCAGTCGGCGTAGCG
ACTCTCGGACGAATTTTCAACGTACTCGGTGAGCCTGTTGATAATTTAGGTCCTGTAGA
TACTCGTACAACATCTCTTATTCATAAATCAGCGCCCGCCTTTATACAGTTAGAAACAAA
ATTGTCTATTTTTGAAACAGGAATTAAAGTAGTAGACCTTTTAGCTCCTTATCGCCGTGG
AGGAAAAATCGGACTATTCGGGGGGGCTGGAGTGGGTAAAACGGTCCTAATTATGGAA
TTGATCAACAACATTGCCAAAGCTCATGGAGGTGTATCCGTCTTTGGCGGAGTGGGTG
AACGTACGCGCGAAGGAAATGATCTTTACATGGAAATGAAAGAATCTGGAGTAATTAA
TGAACAAAATATTGCAGAATCAAAAGTGGCTTTAGTTTATGGCCAGATGAATGAACCGC
CAGGAGCTCGTATGAGAGTTGGTTTAACCGCTCTAACTATGGCGGAATATTTCCGAGAT
GTTAATGAACAAGACGTACTTCTATTTATCGACAATATCTTCCGTTTCGTCCAAGCTGGA
TCCGAAGTATCCGCCTTATTGGGGAGAATGCCCTCCGCTGTGGGTTATCAACCCACCCT
TAGTACTGAAATGGGTTCTTTACAAGAAAGAATTACTTCTACCAAAAAAGGGTCCATAA
CTTCTATTCAAGCTGTTTATGTACCGGCGGACGATTTGACTGACCCTGCTCCGGCCACG
ACCTTTGCACACTTAGATGCTACTACTGTACTATCACGAGGACTAGCTGCCAAGGGTAT
CTACCCGGCAGTAGATCCTTTAGATTCAACGTCAACTATGCTACAACCTCGAATTGTTG
GTGAGGAGCATTATGAAACTGCGCAAAGAGTGAAGCAAACTTTACAACGTTACAAAG
AGCTTCAGGACATTATAGCTATCCTGGGGTTGGACGAATTGTCCGAAGAGGACCGCTTA
AACGTAGCAAGAGCACGAAAAATAGAGCGTTTCTTATCACAACCCTTTTTCGTCGCAG
AAGTTTTTACTGGCTCTCCGGGGAAATATGTCGGTCTAGCAGAAACAATTAGAGGGTTT
AAATTGATCCTTTCCGGAGAATTAGATGGTTTTCCTGAGCAGGCCTTTTATTTGGTAGGT
AACATCGATGAAGCTACTACTAAGGCTACGAATTTAGAAATGGAGAACAAATTGAAGA
AAATGACCTTAAATCTTTGTGTACTGACCCCGAATCGAATTATTTGGGATTCAGAAGTG

AAAGAAATCATTTTATCTACTAATAGTGGACAAATTGGCGTATTACCAAATCATGCGCCT
ATTGCCACAGCCATAGATATAGGTATTTTGAGAATACGCCTTAATGACCAATGGTTAACG
ATGGCTTTGATGGGCGGTTTTGCTAGAATAGGAAATAATGAGATCACTGTTTTAGTGAA
TGATGCGGAAAAGAGTATTGACATTGATCCACAAGAAGCTCGTAAAACTCTTGAAATG
GCGGAAGATAACTTGAAGAAAGCCGAAGGTAAGAGGCAAACAATTGAGGCAAATCTC
GCTCTCAGACGAGCTAGGACACGGGTGGAGGCTATCAATGGGATGGCGATGAAAAATG
TAACCGATTCTTTCCTTTTCGTGGGTCACTGGCCATACGCCGGGAGTTTCGGGTTTAAT
ACCGATATTTTAGCAACAAATCCAATAAATCTAAGCGTAGTCCTTGGTGTATTGATTTTT
TTTGGAAAGGGGGTGTTAAGTGATTTATTAGATAATCGAAAACAGAGGATCTTGAATAC
TATTCGAAATTCAGAAGAACTCCGCGAGGGAGCTATTGAACAGCTGGAAAAAGCCTG
GGACCGTTTAAGGAAAGCTGAACTGGAAGCAGATCAGTTTCGAGCGGATGGATATTCT
GAGATAGAACGAGAAAAATTCAATTTGATTAATTCAACTTCAAAGATTTTAGAACAATT
AGAAAATTACAAAAACGAAACCATTCATTTTGAACAAGAAAGAGCAATTAATCAAGTT
CGACAACGGGTTTTCCAACAAGCCCTACAAGAAGCTCTAGGAACTCTGAATAGTTCTT
TGACCGACGAGTTACATTTACGTACTATCAACGCTAATATTGGCATCTTTGGGACGATGA
AAGAAATAACTAATATGAATCCACTGATTTCTGCCGCTTCTGTGATTGCTGCTGGGTTG
GCCGTCGGTCTTGCTTCTATTGGACCTGGGGTTGGTCAAGGGACTGCTGCGGGTCAAG
CTGTAGAAGGCATTGCGAGACAGCCCGAGGCAGAGGGAAAAATACGAGGGACTTTAT
TGCTTAGTCTGGCTTTTATGGAAGCTTTAACAATTTATGGATTAGTTGTAGCATTAGCGC
TTTTATTTGCGAATCCTTTTGTTATGAATGTTCTATTATGCTCCATCAGTACACTCAAAGG
ATTATACGATCTATCTGGTGTGGAAGTAGGTCAACATTTCTATTGGGAAATAGGAGGTTT
CAAAATCCATGGCCAAGTACTTATTACGTCTTGGGTTGTAATTGCTATCTTAGTAGGTTC
AGCCATTGTAGCTGTTCGGAATCCACAAACCATTCCGACTGCCAGTCAGAATTTCTTCG
AGTACGTCCTTGAATTCATTCGAGACGTGAGCAAAACTCAGATTGGAGAAGAATATGG
TCCATGGGTTCCCTTTATTGGAACCATGTTTCTATTTATTTTTGTTTCGAATTGGTCGGGG
GCACTTTTACCTTGGAAACTCATACAGTTACCTCAGGGGGAGTTATCCGCACCTACGAA
TGATATAAATACTACTGTTGCTTTAGCTTTGCTTACATCAGTAGCATATTTCTATGCGGGT
CTTACCAAAAAAGGATTAGGTTATTTCAGTAAATACATTCAACCAACTCCAATCCTTTTG
CCCATTAACGTTTTAGAAGATTTCACAAAACCTTTATCGCTCAGTTTTCGACTTTTTGGA
AATATACTAGCGGATGAATTAGTGGTTGTTGTTCTTGTTTCTTTAGTACCTTTAGTAGTTC
CTATACCTGTCATGTTCCTTGGATTATTTACAAGTGGTATTCAAGCTCTTATTTTTGCAAC
TTTAGCCGCGGCTTATATAGGTGAATCCATGGAGGGTCATCATATGATATTTTCTACTTTA
GAGCATATATTAACTCATATATCCGTTTCGGTTGTTTCCATTGTAATTACAATTCATTTGAT
AACTATATTACTCGATGAAATCGTCGGACTATATGATTCGTCAGAAAAGGGAACGATAG
CCACTTTTTTCTGTATAACTGGATTATTAGTTACTCGTTGTATTTATTTGGGACATTTACC
GTTAAGTAATTTATATGAATCATTAGTCTTTCTTTCATGGAGCTTATTCATTATTCATATAA
TTCCGTATTTTAAAAAACATCAAAAAAATTTTAGCCTAATAACCGCGCCAAGTGCACTT
TTTACCCAAGGCTTTGCTACTTCCGGTTTTTTAACTGAAATGCATCGGTCTGCATTATTA
GTACCGGCTCTACAATCCCAGTGGTTAGTAATGCACGTAAGTATGATGGTATTGGCCTAT
GCGGCCCTTTTATGTGGATCATTATTATCAGTGGCTCTTCTAGTCATTACATTTCGAAAAA
TCATAAGGATTCCTTTTCTTTTTAAAGAGATTCAATACATGAACGGAAGTGGCAGTGTT
TTACGA---ACTTCTTTTCTTTCTTCTAAAAATTATTACAGGTCTCAGTTGATTCACCAATT
AGATTGTTGGAGTTATCATATTATTAGTATAGGATTTATCCTTTTAACTATAGGTATTCTTT
CGGGAGCAGTCTGGGCTAATGAGGCATGGGGATCATATTGGAGTTGGGACCCAAAGGA

AACTTGGGCATTTATTACTTGGGCAATATTCGCAATTTATTTACATACTAGAACACATAA
AAAATGGGAAGGTGTAAATTCCGCAATTGTGGCTTTTATAGGCTTTCTTCTAATTTGGAT
ATGTTATTTAGGGGTTAATCTATTAGGAATAGGGCTGCACAGTTATGGTTCATTTACATTA
ATAATGGCAAAAAAGAAAGCGTTCACTCCCCTTCTATATCTTGCATCTATAGTATTTTTG
CCCTGGTGCGTCTCTCTCTCCTTTCAAAAAAGTCTAGAATCTTGGATTACTAATTGGTG
GAATACTAGAGAATCCGAAACTTTTTTGAATGATATTAAAGAAAAAACTATTCTCGAAA
AATTCATAGAATTAGACGAACTCTTCCTCTTGGAAGAAATGATAAAGGAATACCCGGAA
ACACATTTACAAAAGCTTCGTATCGGAATCCACAAAGAAATGATCAAATTGATCAAGAT
GTACAATGAGGATGGTATCCATACGATTTTCCAATTCTCGACAAATATAATCTATTTCCTT
ATTTTAAGCGGTTATTCTATTCTAGGTAATCAAGAACTTCGTTTTCTTAATTCTTGGGTTC
AAGAATTCCTATATAACTTAAGCGACACAATAAAAGCCTTTTCTATTCTTTTATTAACTG
ATTTATGTATAGGATTTCATTCACCCCACGGTTGGGAACTAATGATTGGTTCTATCTACA
AAGATTTTGGATTTACTCATAATGATCAGATTATATCTGGTCTTGTTTCCACTTTTCCAGT
CATATTAGATACAATTTTTAAATATTGGATCTTCCGCTATTTAAATCGTCTATCTCCCTCAC
TTGTAGTGATTTATCATTCAATGAATGACATGCCTATTGGTGTTCCAAGAATACCCTTTC
GAAATCCTGGGGAAAACGCTTCATCCTGGGTTGACATATACAACCGACTTTATCGAGAA
AGATTGCTTTTTTTAGGCCAAATAATTAATACCCATCTAGCGAATCAACTTATTGCTCTTA
TATTATATCTAACTATGGAGAGTGATACCAAAGATCTTTATTTGTTTATCAACTCTCCAGG
CGGATGGGTAATACCTGGAATAGCTCTTTATGATACTATGCAATTTGTGCGACCAGATGT
ACAGACAATATGCCTGGGATTGGCCGCTTCAATGGGATCTTTTATCCTGGTCGGAGGAA
AAATTACCAAACGTTTAGCATTCCCTCACGCCAGGGTAATGATTCATCAGCCTTTTAGT
GCTTTTTTTATGGCCCAAGTAGGAGAATTTGTCCTGGAAGCGGAAGAACTGCTGAAGC
TGCGCGAAACCATCACAAGGGTTTATGTACAAAGAACGGGCAAACCCTTATGGATGGT
ATCCGAAGACATGGAAAGGGATGCTTTTATGTCAGCAACAGAAGCCCAAGCTCATGGA
ATTGTTGATCGTGTAGCGGTTGAAATGAAAGAACAAAAATGGATTCATGAAGGTTTAAT
TACTGAATCACTTCCAAATGGTATGTTTCGGGTTCGTTTAGATAATGAAGATTTCATTCT
AGGTTATATTTCCGGAAGGATCCGGCGCAGTTTTATACGTATACTACCGGGGGATAGAGT
AAAAATTGAAGTAAGTCGTTATGATTCCACTAGAGGACGTATAATTTATAGACTCCGCA
ACAAAGAT---------AAAGAAAAAATTCCAAAGATATTTAGAACTAGATCCATCTAGGCAGC
ATGACTTCCTATACCCACTTATCTTTAGGGAGTATAGTTATGTGCTTTCCCATGATTATGG
TTTAGTCAGTTATGAAAATAAATCTAGTTTACTAATTGTAAAACGTTTAATTATTCGAATG
GATCAACAAAATCATTTGATTATTTCTACTCATTATTCTAACCAAAATATATTTTTTAAAA
AGAATTTATATTATCAAATGATATCAGAGGGTTTCTCAGTCATTGTGGAAATTCCATTTTC
CCTACGATTCGCACCTTCTTTAATAGTAAAATCTCATAATTTACGATCAATTCATTCAATA
TTTCCTTTTTTAGAGGACAAATTTTCACATTTAACTTATGTATTAGATGTACTAATACCCT
ATCCGATCCATCTGGAAATCCTGGTTCAAATCCTTCGCTGCTGGGTGAAAGATGTTTCT
TCTTTGCATTTATTACGATTTGTTCTCCATGAGTATTGGAATTGGAATAGTCTTCTTACTA
AATCCATTTACATTTTTTCACAACCAAGATTTTTCTTGTTCCTATATAATTCTTATGTGTC
GGAATACGAATCTATCTTTCTTTTTCTACGTAAACAAGATTCTCATTTATGGTCAACATC
CTCTCGGGTCCTTCTTGAGCGAATCCATTTCTATGGAAAAATAGAACATCTTGCAGAAG
TCTTTCCTAGTCAGGTTATCCTTTGGTTGTTGAAGGATCCTTGCACACATTATGTTAGAT
ATCAAGGAAAATGCATTCTGGCTTCAAAAGATATGCCATTTCTGATGAATAAATGGAAA
TTTTACCTTGTTAATTTATTTCAATGTCATTTTTTTGCGGGGTCTCAACCGGAAAGGATC
TATATAAACCAATTATCCAAGCATTCTCTCGACTTTTTAGGCTATCTTTCAAGTGTGCAA

CTAAATTCTTCAGTAGTGCGGAGTCAAATGGTGGACAATGCGTTTCTAATAGATAAC-----
-GCTATGAAGAAACTCGATACAAGAGTTCCACTTATTTCTTTGATTCGATTATTGGCAAA
AGCGAGATTTTGTAACGTATTAGGACATCCCATTAGTAAAACGACCCGGGCTGATTCAT
CGGATTCGGATATTATCGACCGATTTGTGCGTATATGTATAAATCTTTCTCATTATTACAG
TGGATCCTCAAAAAAAAAGAGTTTGTATCGAATAAAATATGTACTTCGACTTTCTTGTG
TTAAAACTTTGGCTCGTAAACACAAAAGTACTGTACGCGCTTTTTTGAAAAGATTAGGT
TCGGAATTATTGGAAGAATTTTTTACAGAGGACGAAGAGATTCTTTCTTTGAGCTTCCC
AAGACGGATTTGGTATTTTGATATTATTTGTATCAACGACCTGGCCCCAGTTCTGATGAT
ACTTGATACACCA---GAAATACAAGATATTCAT---TCTTTTTCCGGATTAGAATCC---TTAA
AAGAGATATATAACATTATATGGATGCTTGCTCCTATTTTAACTCTTGTATTGGGAATAAC
AATAGGGGTACTAGTAATTGTGTGGTTAGAAAGAGAAATAGCCGCAGGAGTACAACAA
CGTATTGGGCCCGAATATGCTGGTCCTTTAGGAGTTCTTCAAGCTCTAGCAGACGGGAT
AAAACTACTTTTTAAAGAGAATCTTCTTCCATCTCGGGGAGATACTCGTTTATTCAGCG
TTGGCCCATCCATAGCAGTCATATCAATTCTACTAAGCTATTCAGTAATTCCTTTTGGCTA
TCACCTAGTTTTAGCTGATCTAAAGATTGGTGTTTTTTTATGGATTGCCATTTCAAGTATT
GCTCCCATCGGACTTCTTATGTCAGGATATGGATCAAATAATAAATATTCTTTTTTAGGTG
GTCTACGAGCCGCTGCTCAATCAATTAGTTATGAAATACCATTAACTCTATGTGTGTTAT
CAATATCTCTACTATCTAATAGTTTAAGTACAGTTGATATAGTTGAGGCACAGTCAAAAT
ATGGTCTTTGGGGGTGGAATTTGTGGCGGCAACCTATAGGGTTTATTGTTTTTCTAATTT
CTTCTCTAGCAGAATGCGAGAGATTGCCCTTTGATTTACCAGAAGCGGAAGAAGAATT
AGTAGCCGGTTATCAAACCGAATATTCGGGTATCAAATTTGGTTTATTTTATGTTGCTTC
CTATCTAAACCTATTAGTTTCGTCATTATTTGTAACAGTTCTTTACTTGGGCGGTTGGAAT
ATTTCTATTCCGCACTTTTTTGTTCCTAAGCTTTTTGAACTAAATAAAGTGAGTGGGGTT
TTTGGAACAACAACGGGTATCTTTATTATATTGGCTAAAACTTATTTGTTCTTGTTCATTT
CTATCACAACAAGATGGACTTTACCTAGACTAAGAATGGATCAACTATTAAATCTTGGC
TGGAAATTTCTTTTACCTATTTCTCTTGGCAATCTATTATTAACAACCTCTTCACAACTCC
TTTCATTGATGATCTGGCATGTACAGAATGAAAACTTCATTCTCGATTCTACGAGAATTT
TTATGAAAGCCTTTCATTTGCTTCTCTTCGATGGAAGTTTGATTTTCCCAGAATGTATCC
TAATTTTTGGCCTAATTCTTCTTCTGATGATCGATTCAACCTCTGATCAAAAAGATATAC
CTTGGTTATATTTCATCTCTTCAACAAGTTTAGTAATGAGCATAACAGCCCTATTGTTCC
GATGGAGAGAAGAACCTATGATTAGCTTTTCGGGAAATTTCCAAACGAACAATTTCAA
CGAAATCTTTCAATTTCTTATTTTACTATGTTCAACTCTATGTATTCCTCTATCCGTAGAGT
ACATTGAATGTACAGAAATGGCTATAACAGAGTTTCTGTTATTCGTATTAACAGCTACTC
TAGGAGGAATGTTTTTATGCGGTGCTAACGATTTAATAACTATCTTTGTAGCTCCAGAAT
GTTTCAGTTTATGCTCCTACCTATTATCTGGATATACCAAGAAAGATGTACGGTCTAATG
AGGCTACTATGAAATATTTACTCATGGGTGGGGCAAGCTCTTCTATTCTGGTTCATGGTT
TCTCTTGGCTCTATGGTTCATCCGGGGGAGAGATCGAGCTTCAAGAAATAGTGAATGGT
CTTATCAATACACAAATGTATAACTCCCCAGGAATTTCAATTGCGCTTATATTCATCACTG
TAGGAATTGGGTTCAAGCTTTCCCTAGTCCCTTCTCATCAATGGACTCCTGACGTATAC
GAAGGATCTCCCACTCCAGTCGTTGCTTTTCTTTCTGTTACTTCGAAAGTCGCTGCTTC
AGCTTCAGCCACTCGAATTTTCGATATTCCTTTTTATTTCTCATCAAACGAATGGCATCT
TCTTCTGGAAATCCTAGCTATTCTTAGCATGATATTGGGGAATCTCATTGCTATTACTCAA
ACAAGCATGAAACGTATGCTTGCATATTCGTCCATAGGTCAAATCGGATATGTAATTATT
GGAATAATTGTTGGAGACTCAAATGGTGGATATGCAAGCATGATAACTTATATGCTGTTC

TATATCTCCATGAATCTAGGAACTTTTGCTTGCATTGTATCATTTGGTCTACGTACCGGA
ACTGATAACATTCGAGATTATGCAGGATTATACACGAAAGATCCTTTTTTGGCTCTCTCT
TTAGCCCTATGTCTCTTATCCCTAGGAGGTCTTCCTCCACTAGCAGGTTTTTTCGGAAAA
CTCCATTTATTCTGGTGTGGATGGCAGGCAGGCCTATATTTCTTGGTTTCAATAGGACTC
CTTACGAGCGTGGTTTCTATATACTATTATCTAAAAATAATCAAGTTATTAATGACTGGAC
GAAACCAAGAAATAACCCCTCACGTGCGAAATTATAGAAGATCCCCTTTAAGATCAAA
CAATTCCATCGAATTGAGTATGATTGTATGTGTGATAGCATCTACTATACCAGGAATATCA
ATGAACCCGATTATGGAAATTGCTCAGGATACCCTTTTTATGGTTCTGCTTTACGAATAC
GATCTTTTCTGGGCATTTCTAATAATCTCAAGTGTTATCCCTGTTTTGGCATTTCTAATTT
CCGGCGTTTTGGCCCCCCTTAACAAAAGGCCAGAGAAGCTTTCGAGTTATGAGTCGGG
TATAGAACCAATGGGCGATGCTTGGTTACAATTTAGAATCCGTTATTACATGTTTGCTCT
CGTTTTTGTTATTTTTGATGTTGAAACGGTTTTTCTTTATCCGTGGGCAATGAGTTTTGA
TGTAGTAGGAGTATCCGTATTTATAGAAGCTTTCATTTTTGTGCTTATCCTAATTGCCGGT
TTAGTTTATGCATGGCGCAAAGGAGCATTAGAATGGTCTACGAATTTTTTTCCTTGGTTA
ACAATCTTTGTAGTCTTTCCGATATCCGCAGGTTCCTTAATTTTCTTTCTGCCTCAGGGA
GGAAATAAAGTAATTAGGTGGTATGCTATATGTATATGTGTATTAGAACTTCTTTTAATGA
CCTACGTATTCTGCTATCGTTTCCAATCGGACGATCTATTAATTCAATTGGCGGAGAATTA
TAAGTGGGTCGATTTTTTTGGTTTTTACTGGAGATTGGGAATAGATGGACTTTCTATAGG
ACCCATTTTACTGACAGGATTTATCACTACTTTAGCTACTTTAGCGGCTTGGCCGGTTAC
TCGAGATTCCAGACTATTCCATTTCTTGATGTTAGCCATGTATAGCGGTCAAATAGGATT
ATTTTCTTCTCGAGATCTTTTACTTTTTTTCATCATGTGGGAGTTAGAATTAATTCCCGTT
TATCTACTTATATTTATGTGGGGGGGAAAGCAACGTTTGTATTCAGCTACAAAGTTTATT
TTATACACCGCAGGGGGTTCTCTTTTTTTATTAATAGGAATTCTAGGTATCTGTTTATATG
GTTCCAACGAGCCAACATTAAATTTTGAAACATCAGCCAATCAACCCTATCCTTTAGCG
CTGGAAATAATATTCTATATTGGATTTCTTATTGCTTTTGCTGTCAAATTACCGATTATACC
TTTACATACATGGTTACCAGATACTCACGGAGAGGCACATTACAGCACTTGTATGCTTCT
AGCCGGAATCTTATTAAAAATGGGAGCATATGGGTTGGTTCGGATCAATATGGAATTATT
ACCCCATGCTCACTCTCTAGTTTCTCCCTGGTTGATAATAATAGGCACAATTCAAATAAT
TTATGCAGCTTCAACATCTCCTAGTCAACGTAATTTAAAAAAAAGAATAGCCTATTCCTC
GGTATCTCATATGGGTTTTATAATTATAGGAATTTGCTCTCTAAGCGATACGGGACTTAAC
GGAGCTCTTTTACAAATAATATCTCATGGATTTATTGGTGCTGCACTTTTTTTCGTGGCA
GGAACGAGTTATGATAGAATACGTCTTCTTTATCTCGATAAAATGGGAGGAATGGCTATC
TCGGTTCCAAAAATATTCACGATGTTCAGTATCTCATCAATGGCTTCTCTTGCATTACCG
GGCATGAGCGGTTTTTTTGCAGAATTGATAATATTTTTTGGAATAATTACTAGCCAAAAA
TTTCTTTTACTGGCAAAAATACTAATTATTTTTGTCATGGCAATTGGAACGATATTAACTC
CTGTTTATTTATTGTCTATGTTACACCAGATGTTCTATGGATACAAGCTATTTAATGCCTC
AAACTCTTCTTTTTTGGATTTTGGACCGCGAGAGTTGTTTATTTCAATCTCTATCCTTCT
ACCTGTAATAGGTATTGGCATTTATCCGGACTTCGTTTTCTCATTATCAGGTGACAAGGT
CGAGGCTATTTTGTATAATTAT---------ATGATGCTAGAACATATACTTGTTTTGAGTGCCTA
TTTATTTTGTATAGGTATTTATGGATTGATCACGAGCCAAAATATGGTTAGAGCCCTTATG
TGTCTTGAACTTCTAATGAATGCAATTAATATAAATTTCGTCACATTTTCTGATTTTTTTG
ATAGTCGCCAATTAAAAGGAACTATTTGCTCAATATTTGTTATAGCTATTGCAGCGGCTG
AAGCAGCTATTGGACCGGCTATTGTTTCCTCAATTTATCGTAACAGAAAATCAACGCGT
ATCAATCAATCTAATTTGTTGAATAAGATGGAACATATATATCAATATTCCGGGATCATCC

CTTTTATTACACTTCCAGTTCCTATGGTAATAATGGGGGGGCTTCTACTTTTTCCGACAG
TAACAAAAAAAAATTCGCCGTATGTTGTCTTTTTCTAGTGTTGTTTTGTTAAGTATAGTCA
TGCTTTTTTCACTAAATCTTCTTCTTCAGCAAATCTATAGCAGTTCTATCTATAAATCAGT
GTGGTCTTGGACTATCAATAATGATTTTTCTTTAGAGTTCGGCTATTTGATCGACCCGCT
GACTTCTATTATGTTAATATTGATCACTACTGTTGGAATCATGGTGCTTATTTATAGCGAC
AATTATATGTCTCATGATCAAGGATATTTGAGATTTTTTGCTTCTATGAGTTTTTTCAATA
CTTCAATGTTGGGTTTAGTCTCGAGTTGTAATTTAATACAAATTTACATTTTTTGGGAATT
GGTTGGGATGTGTTCTTATCTATTAATAGGTTTTTGGTTTACGCGACCTCTTGTGGGTAC
TGCGTGTCAAAAGGCGTTTATAACTAATCGTGTCGGGGATTTTGGTTTATTATTAGGAAT
TTTAGGTTTTTATTGGATAACGGGTAGTTTCGAATTTCGGGATTTGTTCGAAATATTCAA
CAAC---GAGATTGATTTGTTATTTGTTACTTTGTGTTCCTTGCTATTATTTTCCGGTGCAG
TTGCTAAATCCGCGCAGTTTCCCCTTCATGTGTGGTTACCAGATGCCATGGAAGGGCCT
ACTCCTATTTCGGCTCTCATACATGCTGCTACTATGGTAGCAGCAGGCATTTTTCTTGTA
GCTCGCCTTCTTCCTCTTTTCTTAGTCATACCTTACGTTATGAATTTAATAGCCTTAATAG
GTATATTAACAGTACTATTAGGAGCTACTTTAGCTCTTGCTCAAAAAGATATTAAGAGAG
GTTTAGCTTATTCTACAATGTCTCAATTGGGCTATATCATGTTAGCTTTAGGTCTGGGCTC
TTCTCGAGCCGCTTTATTTCATTTGATTACTCACGCTTATTCAAAAGCTTTGTTGTTTTTA
GGATCTGGATCAATTATTCATTCAATGGAGGCTATTGTTGGATATTCTCCCGATAAAAGT
CAGAATATGGTTCTTATGGGCGGTTTAACAAAACATGTTCCAATTACAAAAATTTCTTTT
TTACTAGGTACACTTTCTCTTTGTGGTATTCCTCCCTTTGCATGTTTTTGGTCGAAAGAT
GAAATACTTAATGATAGTTGGTTATATTCACCGATTTTTTCAATAATCGCTTGTTCCGCGG
CTGGATTAACCGCATTTTATATGTTTCGGATCTATTTACTTACTTTTGAAGGTCATTTGAA
TCCTCATTTTCAAGATTACAGTGGAAAAAAAGGTAGCTCTTTCTATTCAATATCTTTATG
GGTAAAGAGGGGCCAAGTCCGATNAAAAAAA---TATTATCTTTATTNAAAAATAAATCT
TTTTTGCAATACTTTAAAT---------CCTTATCCTCATGAATCGAACAATACTATGCTAGCTC
CTATACTTATATTGGCCTTATTTACCTTGTTTAGTGGGGTCATAGGAAAT------GGATTGGA
TATATTATCCAAATTGTTAACTCCACCGATAAACCTGTTAGATCAAAATTCAAAA---TCC
AAGGATTGG---CAATTTTTGACAAATGCAACTTTTCCAGTCAGTATAGCTTTTGGGGGA
ATTTTGTTCGCATCCTTTTTATACAAGCCTGTTTATTCATCTTTACAAAATTTGAACTTCC
TAAATTTAGTTGTCATTCTTAGGGACAAACTAATATATTTAATATATCGTTTGTCATATAAT
CGTGGTTATATAGATGCTTTTTATGCAAAATTTATAACTAGGGGTCTAAGAAGATTGGCA
GAACTAACTCATTTTTTTGATAGACAAATAATCGATAGAATTACAAATGTAATGGGTCTT
ACAAGTTTTTTTCTTGGAGAGGTTATAAAATATGTAGGAGGCGGT---CGCATCTCTTCGT
ATCTCTTATTGTATTTGTTCGCTGTATTCATTTTTTTAGTAATAAAATTGATGGATTTACCT
GGATCAATACATGATTTTCTTTTAGTATTTCTGGGATTGGGCCTTATATTATTAGGTTTAG
GGGTGGTATTGCTTACCAACCCAATTTATTCCGCCTTTTCGTTGGGATTGGTTCTTATTT
GTATAGCCTTATTCTATATTTTATCAAATTCCTATTTTGTAGCTGCCGCACAACTCCTTATT
TACGTAGGAGCCATAAATGTTTTAATCATATTTGCTGTGATGTTCATGAATGGTTCAGAA
TACCACAACGCTTTTTATCTTTGGAACGCTGGAGGCGGGGTTACTTCACTGGTTTGTAC
AAGTATTTTTCTTTTACTAATTACTACTATTCCAGATACGTCATGGTATGGGATTATTTGG
ACTACAAGATCAAACCAGATTATAGAGAAAGATTTGATAACTAATAGTCAACAAATTGG
AATTCGTTTATCAACAGATTTTTTTCTTCCATTTGAACTCATTTCAATAATTCTTTTAGTT
GCGTTAATAGGCGCGATTGCTGCGGCTCGTCAGATGAATGTATCATCTCCAAGACAAGA
CCTCATGATAGTCAATATGGGCCCACACCATCCATCAATGCATGGTGTTCTTCGACTCAT

CGTTACTCTAGATGGTGAAGATGTTATTGACTGTGAACCAATATTAGGTTATTTACACAG
AGGGATGGAAAAAATTGCGGAAAACCGAACAATTATACAATATCTGCCCTATGTAACAC
GTTGGGATTATTTAGCTACGATGTTCACAGAAGCAATAACGGTAAATGGACCAGAACG
GCTGGGAAATATTCAAATACCTAAAAGAGCTAGCCATATCAGAGTAATTATGTTGGAGT
TGAGTCGTATAGCTTCTCATCTGTTATGGCTTGGCCCTTTTATGGCAGATATTGGTGCGC
AAACCCCTTTCTTCTATATTTTCAGAGAAAGGGAATTAGTATATGATCTATTCGAAGCTG
CCACTGGGATGAGAATGATGCATAATTTTTTTCGTATCGGAGGAGTAGTGGCCGATCTA
CCTCATGGCTGGATAGATAAATGCTTGGATTTCTGCGATTATTTTTTAACAAGGGCTGCT
GAATATCAAAACCTTATTACGCGAAATCCTATTTTTTTAGAACGAGTTGAAGGCGTAGG
TATTATTAGTGCAGAGGAAGCAATAAATTGGGGTTTATCAGGACCAATGCTACGAGCTT
CTGGAATACAGTGGGATCTTCGCAAAGTTGATCATTATGAATGTTACAACGAATTTGATT
GGGAAGCTCCGTGGCAAAAAGAAGGCGATTCATTAGCTCGTTATTTAGTCCGAATCAG
TGAAATGAAGGAATCCATAAAAATTATTCAACAGGCTCTGGAAAGAATTCCGGGGGGG
CCCTATGAGAATTTAGAAACCCGATGTTTTGATAGAGAGAGGGATCAAAACTGGAATG
ATTTTGAATATCGATTCATTAGTAAAAAAACCTCTCCTACTTTTGAATTGCCGAAACAAG
AACTTTATGTGAGAGTCGAAGCACCAAAGGGAGAATTGGGAATTTTTCTGATAGGGGA
CCAGAGTGGTTTTCCTTGGAGATGGAAAATTCGTCCACCGGGGTTTATTAATTTGCAAA
TTCTTCCTCAGTTAGTTAAAAGAATGAAATTGGCTGATATTATGACGATACTAGGTAGCA
TCGATATCATTATGGGGGAAGTTGATCGTATGCTCCCTATGGTAACTGGGTTCAGAAATT
ATGGTCAACAAACAATAAGGGCTGTAAGGTACATTGGTCAAAGTTTTATGATTACCTTA
TCCCACGCAAATCGTTTACCCGTAACTATTCAATACCCTTATGAAAAATTAATTACATCG
GAGCGTTTCCGCGGTCGAATCCATTTTGAATTTGATAAATGTATTGCTTGTGAGGTATGT
GTTCGTGTATGTCCTATAGATTTACCCGTTGTTGATTGGCAATTCGAAACGAATATTCGA
AAGAAACGATTGCTTAATTATAGTATTGATTTTGGAATCTGTATATTTTGTGGTAACTGCG
TTGAGTATTGTCCAACAAATTGTTTATCAATGACTGAAGAATATGAGCTTTCTACTTATG
ATCGTCACGAATTGAATTATAATCAAATTGCTTTGGGTCGTTTACCAATATCAGTAATTG
ACGATTATACAATTCGAACGATTTCGACTTTGGAC--AT
GCAAGGTTCTTTGTCTGCTCGGCTAGTCAAACACGGGCTAGTTCATAGATCTTTGGGCT
TCGATTACCAAGGAATAGAAACTTTACAAATAAAGCCCGCGGATTGGCATTCCATTGCT
GTCATTTTATATGTATATGGTTACAATTATCTACGTTCCCAATGTGCCTACGATGTAGCAC
CAGGCGGACTGTTAGCTAGTGTGTATCATCTTACGAGAGTGGCGTATAGTATAGATCAA
CCGGAAGAGGTATGCATAAAAGTATTTGCCCCAAGGAGGAATCCTAGAATTCTGTCTGT
TTTCTGGGTTTGGAAAAGCGCGGATTTTCAAGAAAGGGAGTCTTATGATATGTTCGGAA
TTTCGTATGAGAATCATCCGCGCTTGAAACGCATCTTAATGCCCGAAAGTTGGATAGGG
TGGCCTTTACGTAAGGATTATATTGCCCCCGATTTTTATGAAATACAAGATGCTCAT--------
----
---ATGAATTCCATTGCGTTTCCCTTACTTGATCGA
ACAACCCAA---TCTGTTATTTCAACTACATCAAATGATCTTTCAAATTGGTCAAGACTCT
CCAGTTTATGGCCCCTTCTCTACGGTACGAGTTGTTGCTTCATTGAATTTGCTTCATTAAT
AGGTTCACGCTTCGACTTTGATCGTTATGGACTAGTACCACGATCTAGTCCTAGACAAG
CAGATCTAATTTTAACAGCCGGCACTGTAACAATGAAGATGGCACCCTCTTTAGTGAGA
CTCTATGAACAAATGCCTGAACCTAAATATGTAATTGCCATGGGAGCCTGTACAATTAC
AGGTGGGATGTTCAGTACCGAGTCTTATAGTACTGTTCGGGGAGTCGATAAGCTAATTC
CCGTAGATGTTTATTTGCCTGGTTGCCCACCTAAACCAGAGGCGGTTATAGATGCCATA

ACAAAACTTCGTAAGAAAATATCTCGAGAAATATCTGAAGATCGAATTAGGGCTCAAC
AAGAAAATCGTTGTTTTACTACCAATCACCAGTTTCGCCTTGGGCGCAGTACTCATACT
GGAAATTATGATCAAGGATTACTCTATCAACCACTATCTACGTCAGAGATCCCTACTGAA
ACATTTTTTAAATACACAGCGGGGGTATCTTCCCACGACTTAGTAAATATGCAAACTAG
AAATACCTTTTCTTGGATAAAGGAAGAGATTACTCGATCTTTTTCCGTATCGCTCATTATA
TCTATAATGACTTGGGCATCCATTTCAAATGCATATCCCATTTTTGCACAGCAGGGTTAT
GAAAATCCACGAGAAGCAACTGGACGTATTGTATGCGCCAATTGCCATTTAGCTAACA
AGCCCGTGGATATTGAGGTTCCCCAGGCAGTACTTCCCGATACTGTATTTGAAGCAGTT
ATTCGAATTCCTTATGATAAGCAACTGAAACAAGTTCTTGCTAATGGCAAAAAAGGCGC
CTTGAATGTTGGGGCTGTTCTTATTTTACCTGAGGGATTTGAATTAGCCCCCCCCGATCG
TATTTCTCCTGAGATGAAGGAAAAGATGGGAAATCTGTCTTTTCAGAGCTATCGACCTA
CTAAAAAAAATATTCTTGTGATAGGGCCTGTTCCTGGTCAGAAATATAGTGAAATCACC
TTTCCTATTCTTTCCCCCGACCCCACTACTAAGAAGGACGTTCACTTCCTAAAATATCCC
ATATACGTAGGCGGGAACAGGGGAAGGGGTCAGATTTATCCTGATGGGAGCAAGAGTA
ACAATACAGTTTATAATGCTACAGCAGCGGGTATAGTAAGCAAAATTATACGAAAAGAA
AAGGGGGGGTACGAAATAACCATAACCGATGCATCGGATGGACGCCAAGTAATTGATAT
TATACCTCCAGGACCAGAACTTCTTGTTTCAGAGGGTGAATCTATCAAATTGGATCAGC
CATTTACGAGTAATCCTAACGTGGGTGGATTTGGTCAGGGGGATGCGGAAATAGTACTT
CAAGACCCAGCACGGGTCCAAAGTCTTTTGTTCTTCTTCGCGTCGGTTATTTTGGCACA
AATCTTTTTGGTTCTTAAAAAGAAACAGTTTGAGAAGGTTCAACTGTCCGAAATGAAT
TTCATGAGTAAAGTCTATGATTGGTTCGAAGAACGTCTTGAGATTCAGGCGATTGCAGA
TGATATAACTAGTAAATACGTTCCTCCTCATGTCAACATATTTTATTGTTTAGGAGGAATA
ACGCTTACTTGTTTTTTAGTACAAGTGGCTACGGGGTTTGCTATGACTTTTTACTATAGG
CCAACCGTTACTGAGGCTTTTGCTTCTGTTCAATACATAATGACTGAAGCCAACTTTGG
TTGGTTAATCCGATCAGTTCATCGATGGTCGGCAAGTATGATGGTCCTAATGATGATTCT
GCACGTATTCCGTGTGTATCTCACCGGTGGCTTTAAAAAACCCCGTGAATTGACTTGGA
TTACAGGTGTGGTTCTTGCTGTCTTGACCGCCTCTTTTGGCGTAACCGGTTATTCCTTAC
CTTGGGACCAAATTGGCTATTGGGCAGTCAAAATAGTAACGGGTGTACCGGAAGCTATT
CCTGTAATAGGATCCCCTTTGGTAGAGTTATTGCGCGGAAGTGCTAGTGTGGGCCAATC
CACTTTGACTCGTTTTTATAGTTTACACACTTTTGTATTACCTCTTCTTACTGCCGTATTT
ATGTTAATGCATTTCTTAATGATCCGTAAGCAAGGTATTTCCGGTCCTTTAATGGGAGTA
ACAAAAAAACCTGACTTGAATGATCCTGTATTGCGAGCTAAATTGGCTAAAGGGATGG
GTCATAATTATTACGGAGAACCCGCATGGCCTAATGATCTTTTATATATTTTTCCAGTAGT
CATTCTAGGTACTATTGCATGTAATGTGGGCTTAGCAGTTCTAGAACCTTCAATGGTTGG
GGAACCTGCAGAGCCATTTGCAACTCCTTTGGAAATATTGCCTGAATGGTATTTCTTTC
CCGTATTTCAAATACTTCGTACAGTACCCAATAAGTTATTAGGCGTTCTTTTAATGGTTTC
AGTACCTGCGGGATTATTAACAGTACCTTTTTTGGAGAGTGTTAATAAATTTCAAAATCC
CTTTCGTCGTCCAGTAGCGACAACTGTCTTTTTAATTGGTACTGTAGTGGCCCTTTGGTT
GGGTATTGGAGCAACATTACCTATTGATAAATCCTTAACTTTAGGGCTTTTTATGATTGA
AGTTTTTCTATTTGGAATCGTCTTAGGTCTAATTCCTATTACTTTGGCTGGGTTATTCGTA
ACTGCATATTTACAATATAGACGTGGTGATCAGCTGGGCCTTATGCCTACGATAACTAGT
TATTTCGGTTTTCTACTAGCGGCTTTAACTATAACCTCCGTTCTATTTATTGGTCTGAGTA
AGATACGACTTATTATGGATATAGTAAGTCTCGCTTGGGCTGCTTTAATGGTAGTCTTTA
CATTTTCCCTTTCACTCGTAGTATGGGGAAGAAGTGGACTCATGATTATTCGTTCGCCG

GAACCAGAAGTAAAAATTTTGGTAGATAGGGATCCCATAAAAACTTCGTTCGAGGAAT
GGGCCAGACCCGGCCATTTCTCAAGAACAATAGCCAAGGGACCTGATACTACCACTTG
GATCTGGAACCTACATGCCGATGCTCACGATTTCGATAGCCATACCTCCGATTTGGAGG
AGATCTCTCGAAAAGTATTTAGTGCTCATTTTGGTCAACTCTCCATAATCTTTCTTTGGT
TGAGTGGTATGTATTTCCACGGTGCTCGTTTTTCCAATTATGAAGCATGGCTGAGCGATC
CTACTCACATTGGTCCCAGTGCACAGGTAGTTTGGCCAATAGTGGGCCAAGAAATATTG
AATGGTGATGTGGGCGGGGGTTTTCGAGGAATACAAATAACCTCTGGTTTTTTTCAGAT
TTGGCGAGCATCTGGAATAACTAGTGAATTACAACTCTATTGTACCGCAATTGGTGCGT
TGGTCTTTGCAGCCTTAATGCTTTTTGCTGGTTGGTTCCATTATCACAAAGCTGCTCCAA
AATTGGCTTGGTTCCAAGATGTCGAATCTATGTTGAATCACCATTTAGCGGGGCTATTAG
GACTTGGGTCTCTTTCTTGGGCGGGGCATCAAGTACATGTGTCGTTACCGATTAACCAA
TTTCTAAATGCTGGAGTAGATCCTAAAGAGATCCCCCTTCCTCATGAATTTATCTTGAAT
CGGGATCTTTTGGCTCAACTTTATCCTAGTTTTGCTGAAGGAGCAACTCCTTTTTTCAC
CTTGAATTGGTCAAAATATGCAGAATTTCTTACTTTTCGCGGAGGATTAGATCCAATAAC
TGGGGGTCTTTGGCTGACCGATATTGCACACCATCACTTAGCTATTGCAATTCTTTTCCT
GATAGCGGGTCACATGTATAGAACTAACTGGGGCATTGGTCATGATATAAAAGATATTTT
AGAGGCTCATAAGGGTCCATTTACAGGCCAAGGTCATAAAGGTCTATATGAGATCCTAA
CAACGTCATGGCATGCTCAATTATCTCTTAACCTAGCTATGTTAGGCTCTTTAACCATTG
TTGTAGCTCACCATATGTATTCCATGCCCCCTTATCCATATCTAGCTACTGACTATGGTAC
ACAGCTGTCATTGTTCACACATCACATGTGGATCGGTGGGTTTCTCATAGTTGGTGCTG
CGGCCCATGCAGCCATTTTTATGGTAAGAGACTATGATCCAACTACTCGATACAACGAT
CTATTAGATCGTGTCCTTAGGCATCGCGATGCAATCATATCACATCTCAACTGGGTATGT
ATATTTTTAGGCTTTCACAGTTTTGGTTTATATATTCACAATGATACCATGAGCGCTTTAG
GACGCCCACAAGATATGTTTTCAGATACCGCCATACAATTACAACCTGTCTTTGCTCAAT
GGATACAAAACACCCATGCTTTAGCACCTGGTGCAACGGCCCCAGGTGCAACAACAA
GCACCAGTTTGACTTGGGGAGGTAGTGATTTAGTAGAAGTGGGGGGGAAGGTTGCTTT
GTTACCTATTCCATTAGGTACCGCAGATTTTTTGGTACATCACATTCATGCATTTACGATT
CATGTGACAGTATTGATCCTCCTGAAAGGTGTTTTATTTGCTCGTAGCTCTCGTTTGATA
CCGGATAAAGCAAATCTTGGTTTTCGTTTTCCTTGTGATGGGCCTGGAAGAGGGGGGA
CATGTCAAGTATCTGCTTGGGATCATGTCTTCTTAGGATTATTCTGGATGTACAATGCAA
TTTCGGTAGTAATATTCCATTTCAGTTGGAAAATGCAGTCAGATGTTTGGGGTAGTATAA
GTAATCAAGGGGTGGTAACTCATATCACGGGAGGAAACTTTGCCCAAAGTTCCATTACT
ATTAATGGGTGGCTGCGCGATTTCTTATGGGCACAGGCATCCCAGGTAATTCAGTCTTAT
GGTTCTGCATTATCTGCATATGGCCTTTTTTTCCTAGGTGCTCATTTTGTATGGGCTTTTA
GTTTAATGTTTCTATTCAGCGGACGTGGTTATTGGCAAGAACTTATTGAATCCATCGTTT
GGGCTCATAATAAATTAAAAGTTGCTCCTGCTACCCAGCCTAGAGCCTTGAGCATTGTA
CAAGGACGTGCTGTAGGCGTAACTCATTACCTTCTGGGTGGAATTGCCACAACATGGG
CGTTCTTCTTAGCAAGAATTATTGCAGTAGGAATGGCATTAAGATTTCCAAGGTTTAGC
CAAGGATTAGCTCAGGACCCCACTACTCGTCGTATTTGGTTTGGTATTGCTACCGCACA
TGACTTCGAGAGTCATGATGATATTACCGAGGAACGTCTTTATCAGAATATTTTTGTTTC
TCACTTCGGACAATTAGCAATAATTTTTTTGTGGACTTCCGGAAATCTGTTTCATGTAGC
TTGGCAAGGAAATTTTGAAGCATGGGTACAGGACCCTTTACATGTAAGACCTATTGCTC
ATGCAATTTGGGATCCCCATTTTGGTCAACCGGCTGTGGAAGCTTTTACTCGAGGAGGT
GCTCTTGGCCCAGTCAATATCGCGTATTCCGGTGTTTATCAGTGGTGGTATACCACAGGT

ATACGTACTAATGGGGATCTTTATATTGGAGCTCTTTTTCTATTATTTCTTTCTGCGCTATC
CCTAATAGCGGGTTGGTTCCACCTACAACCGAAATGGAAACCGAGTGTTTCGTGGTTC
AAAAATGCCGAATCTCGTCTCAATCATCATTTGTCAGGACTATTCGGAGTAAGTTCCTT
GGCTTGGACAGGACATTTAGTCCATGTCGCTATTCCGGGATCCAGGGGGCAATACGTTC
GATGGAACAATTTCTTAGATGTAGCACCGCACCCCCAAGGATTAGGCCCCCTTTTTACA
GGGCAGTGGAATCTTTATGCTCAAAACCCCGATTCAAGTAGTCATTTATTTGGTACTTCC
CAAGGAGCTGGAACTGCCATTCTAACCCTTCTCGGGGGTTTCCATCCGCAAACGCAAA
GTTTATGGCTGACCGATATTGCACACCATCACTTAGCTATTGCAATTCTTTTCCTGATAG
CGGGTCACATGTATAGAACTAACTTCGGGATTGGGCACAGTATAAAAGATCTTTTAGAA
GCACATGTTCCGCCGGGAGGTCGATTGGGACGCGGACATAAGGGTCTTTATGACACAA
TCAACAATTCACTTCATTTTCAATTAGGCCTTGCTCTAGCTTCTTTAGGGGTTATTACTTC
CTTGGTAGCTCAACACATGTACTCTTTACCTGCGTATGCGTTTATAGCGCAAGACTTTAC
TACTCAAGCTGCATTATATACTCATCACCAATACATCGCAGGATTCATCATGACAGGAGC
TTTTGCTCATGGAGCTATCTTTTTTATTAGAGATTACAATCCGGAGCAGAATGAGGATAA
TGTATTGGCAAGAATGTTAGACCATAAAGAAGCTATCATATCCCATTTAAGTTGGGCCA
GCCTCTTTCTGGGATTCCATACCTTGGGCCTTTATGTTCATAATGATGTCATGCTTGCTTT
TGGTACTCCGGAGAAACAAATCTTGATTGAACCCATATTTGCCCAATGGATACAATCTG
CACATGGTAAAACTTCATATGGGTTCGATGTACTCTTATCTTCAACGAATAGCCCCGCCT
TCAATGCCGGTCGAAACATATGGTTGCCCGGTTGGTTAAATGCTATTAATGAG------AAT
AGTAATTCACTCTTCTTAACAATAGGGCCTGGAGACTTCTTGGTTCATCATGCTATTGCT
CTAGGTTTACATACAACTACATTGATCTTAGTAAAAGGCGCTTTAGATGCACGTGGTTCT
AAGTTAATGCCAGATAAAAAGGATTTTGGTTATAGTTTTCCGTGCGATGGTCCGGGACG
CGGCGGTACTTGTGATATTTCGGCTTGGGACGCGTTTTATTTGGCAGTTTTTTGGATGTT
AAATACCATTGGATGGGTTACTTTTTATTGGCATTGGAAACACATCACATTATGGCAAGG
GAACGTTTCACAGTTTAACGAATCTTCCACTTATTTGATGGGATGGTTAAGAGATTATCT
ATGGTTAAACTCTTCACAACTTATCAATGGATATAATCCTTTTGGTATGAATAGTTTATCG
GTCTGGGCGTGGATGTTCTTATTTGGACATCTTGTTTGGGCTACCGGATTTATGTTCTTA
ATTTCCTGGCGTGGATATTGGCAAGAATTGATTGAAACTTTAGCATGGGCTCATGAACG
CACACCTTTGGCTAATTTGATTCGATGGAGAGATAAACCAGTGGCTCTTTCCATTGTGC
AAGCAAGATTGGTTGGATTAGCCCACTTTTCTGTAGGTTATATATTCACTTATGCGGCTT
TCTTGATTGCCTCTACATCAGGGAAATTTGGTATGTCGCATTCAGTAAAGATTTATGATA
CATGTATAGGGTGTACCCAATGCGTCCGAGCCTGCCCCACAGATGTATTAGAAATGATA
CCTTGGGACGGGTGTAAAGCTAAGCAAATTGCTTCTGCTCCAAGAACAGAGGACTGC
GTCGGCTGTAAGAGATGTGAATCGGCCTGTCCAACGGATTTTTTGAGTGTTCGAGTTTA
TTTGTGGCATGAAACAACTCGAAGCATGGGTCTAGCTTATATGACAACTTTCAACGTAC
CCTCTATTTTTGTGCCTTTGGTAGGCCTAGTTGTTCCGGCAATTGCAATGGCTTCTTTAT
CTCTTCATGTTCAAAGAAATAAGATTGTTATGCGAGATCTAAAAACATATCTCTCCGCG
GCACCGGTACTAAGTACTTTATGGTTTGGGTCTTTAGCAGGTCTATTGATAGAGATTAAT
CGTTTTTTCCCAGATGCGTTGACATTCCCTTTTTTTTCATTCATGACTGCAATTTTAGAG
AGACGCGAAAGCGAAAGCCTATGGGGTCGCTTCTGTAATTGGATAACCAGCACCGAAA
ACCGTCTTTACATTGGGTGGTTTGGTGTTTTGATGATCCCTACTTTATTGACCGCAACTT
CTGTATTTATTATCGCTTTTATTGCTGCCCCTCCAGTAGATATTGATGGTATTCGTGAACC
TGTTTCTGGATCTCTACTTTATGGAAACAATATTATTTCTGGTGCCATTATTCCTACTTCT
GCAGCGATCGGTTTGCACTTTTACCCAATCTGGGAAGCGGCATCCGTTGATGAATGGTT

ATACAATGGCGGTCCTTATGAGCTAATTGTTTTACACTTCTTACTTGGTGTAGCTTGTTA
CATGGGTCGTGAGTGGGAACTTAGTTTCCGTTTGGGTATGCGGCCTTGGATTGCTGTTG
CATATTCAGCTCCTGTTGCAGCTGCTACTGCTGTTTTCTTGATCTATCCAATAGGCCAAG
GAAGTTTTTCTGATGGTATGCCTTTAGGAATCTCTGGTACTTTCAACTTTATGATTGTATT
CCAAGCTGAACACAATATCCTTATGCATCCATTTCACATGTTAGGCGTAGCTGGTGTATT
CGGCGGTTCCCTATTCAGTGCTATGCATGGTTCCTTGGTAACTTCTAGTTTGATTAGGGA
AACCACCGAAAATGAATCTGCTAATGCAGGTTACAGATTCGGTCAAGAAGAAGAAACT
TATAATATCGTAGCTGCTCATGGTTATTTTGGCCGATTGATCTTCCAATATGCTAGTTTCA
ACAATTCTCGTTCTTTACATTTCTTCCTAGCCGCTTGGCCTGTAGTAGGTATCTGGTTCA
CCGCTTTAGGTATTAGCACTATGGCTTTCAACCTAAATGGTTTCAATTTCAACCAATCTG
TAGTTGATAGTCAAGGCCGTGTAATTAATACTTGGGCTGATATTATTAATCGCGCTAACC
TTGGTATGGAAGTTATGCATGAACGTAATGCTCATAACTTCCCTCTAGACCTAGCTACTG
TCGAAGCTCCATCCACAAATGTAATGGGTTTGCCTTGGTATCGTGTTCATACCGTCGTAT
TGAATGATCCCGGTCGTTTGCTGGCTGTCCATATAATGCATACGGCTCTGGTTGCTGGTT
GGGCTGGTTCGATGGCTCTATATGAATTAGCAGTTTTTGATCCCTCTGACCCTGTTCTCG
ACCCAATGTGGAGACAAGGTATGTTCGTTATACCCTTTATGACTCGTTTAGGAATAACC
GATTCATGGGGCGGTTGGACTATCACA---GGCGGGACTATAACGAATCCGGGTATTTGG
AGTTACGAAGGTGTGGCCGGGGCACATATTGTGTTTTCTGGATTGTGCTTCTTGGCAGC
TATCTGGCATTGGGTGTATTGGGATCTAGAAATATTTACTGATGAACGTACAGGAAAAC
CTTCTTTGGATTTGCCCAAGATCTTCGGAATTCATTTATTTCTCTCAGGAGTGGCTTGCT
TTGGGTTTGGCGCATTCCATGTAACAGGATTGTACGGTCCTGGAATCTGGGTGTCCGAT
CCTTATGGACTAACCGGAAAGGTCCAATCTGTAAATCCAGCGTGGGGTGTGGAAGGTT
TTGATCCTTTTGCTCCGGGAGGAATAGCCTCTCATCATATTGCAGCAGGGACATTGGGC
ATATTAGCAGGACTATTTCATCTTAGTGTCCGTCCGCCACAACGTCTATACAAAGGATTG
CGTATGGGAAATATTGAAACCGTCCTTTCCAGTAGTATTGCTGCTGTCTTTTTTGCGGCT
TTTGTTGTTGCTGGAACTATGTGGTATGGTTCAGCAACTACTCCGATTGAATTATTTGGT
CCCACTCGTTATCAATGGGATCAGGGATATTTCCAGCAAGAAATATATCGAAGAGTTGT
TGCTGGGCTAGCCGAGAATCAAAGTTTATCCGAAGCTTGGTCTAAAATTCCTGAAAAAT
TAGCTTTTTATGATTACATTGGGAATAATCCGGCAAAAGGGGGGTTGTTCAGAGCGGGT
TCGATGGATAACGGGGATGGAATCGCTGTTGGGTGGTTAGGGCATCCTGTCTTTAGAGA
TAAAGAAGGCCGTGAACTTTTTGTGCGTCGTATGCCTACTTTTTTTGAAACATTTCCAG
TTGTTTTGGTAGACGGTGACGGAATTGTTAGAGCCGATGTTCCTTTTCGAAGGGCGGA
ATCGAAGTATAGTGTCGAGCAAGTAGGTGTAACTGTTGAGTTCTATGGCGGCGAACTC
AATGGCGTCAGTTATAGTGATCCCGCTACTGTTAAAAAATATGCTAGACGTGCTCAATT
GGGTGAAATCTTTGAATTAGATCGTGCTACTTTGAAATCCGATGGTGTTTTTCGTAGTAG
TCCAAGGGGTTGGTTTACTTTTGGACATGCTTCATTTGCTCTGCTCTTCTTCTTTGGGCA
CATTTGGCATGGCGCTAGAACCCTGTTCAGAGATGTTTTTGCTGGTATTGATCCAGATTT
GGACGCTCAAGTAGAATTTGGAGCATTCCAAAAACTAGGGGATCCAACTACAAAAAG
ACAAGTAGTT--GTGGAAACGCTCTTTAATGGAACTTTAGCTT
TAGCTGGTCGTGACCAAGAAAGCACCGGGTTCGCTTGGTGGGCCGGGAATGCCCGAC
TTATCAATTTATCTGGTAAACTGCTGGGGGCTCATGTAGCACATGCCGGATTAATCGTAT
TCTGGGCCGGCGCAATGAACTTATTTGAAGTGGCTCATTTCGTACCGGAGAAACCAAT
GTATGAACAAGGATTAATTTTACTTCCCCACCTAGCTACTCTAGGTTGGGGGGTAGGTC
CTGGGGGAGAAGTTATAGATACCTTTCCATACTTTGTATCTGGAGTGCTTCACTTAATTT

CCTCCGCAGTATTAGGCTTTGGCGGTATTTATCATGCACTTCTGGGACCTGAGACCCTT
GAAGAATCTTTTCCATTCTTTGGTTATGTATGGAAAGATAGAAATAAAATGACAACAAT
TTTGGGTATTCACTTAATCTTGTTAGGTCTAGGTGCCTTTCTGTTAGTATTTAAAGCTCTT
TATTTTGGGGGCGTATATGATACCTGGGCCCCGGGGGGGGGAGATGTAAGAAAAATTAC
CAACTTGACTCTTAGTCCAAGTGTTATATTTGGGTATTTACTAAAATCCCCCTTTGGGGG
AGAAGGATGGATTGTTAGTGTGGACGATTTCGAAGATATAATTGGAGGACATGTATGGA
TAGGTTCCATTTGTATACTTGGTGGAATCTGGCATATCTTAACCAAACCTTTTGCATGGG
CTCGCCGTGCGCTTGTATGGTCTGGAGAGGCTTACTTGTCTTATAGTTTAGCTGCTTTAT
CTGTTTTTGGTTTCATTGCTTGTTGCTTTGTCTGGTTCAATAATACCGCTTATCCTAGTGA
GTTTTACGGGCCTACCGGCCCAGAAGCTTCTCAAGCCCAAGCATTTACTTTTCTAGTTA
GAGACCAACGTCTTGGGGCTAACGTGGGATCTGCTCAAGGACCTACCGGTTTAGGTAA
ATATCTAATGCGTTCCCCGACCGGAGAAGTTATTTTTGGAGGTGAAACTATGCGTTTTT
GGGATTTGCGTGCTCCCTGGTTAGAACCTCTAAGGGGTCCCAATGGTTTGGACTTGAGT
AGGCTGAAAAAAGACATACAACCCTGGCAAGAACGACGTTCCGCGGAATATATGACCC
ATGCCCCGTTGGGCTCTTTAAATTCTGTGGGTGGCGTAGCTACCGAGATCAATGCGGTC
AATTATGTTTCTCCTAGAAGTTGGTTAGCTACCTCTCATTTTGTTCTAGGATTCTTCCTTT
TCGTAGGTCATTTATGGCACGCGGGAAGGGCTCGAGCGGCTGCTGCGGGATTTGAAAA
AGGAATTGATCGCGATTTTGAGCCTGTTCTTTCCATGACTCCTCTTAACATGACTATAGC
CCTTGGTAAATTTACCAAAGAAGAAAAGGATTTATTTGATATTATGGATGACTGGTTAA
GGAGGGACCGTTTCGTTTTTGTAGGTTGGTCCGGTCTATTGCTCTTTCCTTGTGCCTATT
TCGCCTTAGGAGGTTGGTTCACAGGTACAACCTTTGTAACTTCATGGTATACCCATGGA
TTAGCCAGTTCCTATTTGGAAGGCTGCAACTTCTTAACCGCTGCAGTTTCTACTCCTGCT
AATAGTTTAGCGCATTCTTTGTTGTTACTATGGGGTCCCGAAGCACAAGGGGATTTTAC
CCGCTGGTGTCAATTGGGTGGTCTGTGGACTTTTGTTGCTCTCCACGGCGCTTTTGGAC
TAATCGGTTTCATGTTACGTCAATTTGAACTTGCTCGATCTGTTCAATTGCGACCTTATA
ATGCAATTTCATTCTCTGCTCCAATCGCTGTTTTTGTTTCGGTATTCTTGATTTATCCACT
AGGTCAGTCTGGTTGGTTTTTTGCACCTAGTTTTGGTGTAGCAGCTATATTTCGATTCAT
CCTCTTTTTCCAAGGATTTCATAATTGGACATTGAACCCATTTCATATGATGGGGGTTGC
TGGTGTATTGGGCGCTGCTCTGCTATGTGCTATTCATGGGGCTACCGTCGAAAATACTTT
ATTTGAAGATGGTGATGGTGCAAATACATTCCGCGCTTTTAACCCAACTCAAGCTGAAG
AAACTTATTCTATGGTTACTGCCAACCGCTTTTGGTCTCAAATCTTTGGGGTTGCTTTTT
CCAATAAACGTTGGTTACATTTCTTTATGTTATTTGTACCAGTAACCGGTTTATGGATGA
GCGCTCTTGGAGTAGTTGGTCTAGCTCTGAACCTACGTGCCTATGACTTCGTTTCTCAG
GAAATCCGTGCAGCGGAAGATCCCGAATTTGAGACTTTTTACACCAAAAATATTCTTTT
AAACGAAGGTATTCGTGCTTGGATGGCGGCTCAAGATCAGCCTCATGAAAACCTTATAT
TCCCTGAGGAGGTTCTACCCCGTGGAAACGCTCTTATGTCTGGAAGCACAGGAGAACG
TTCTTTTGCTGATATTATTACCAGTATTCGATACTGGGTCATTCATAGCATTACTATACCTT
CCCTATTCATTGCGGGTTGGTTATTCGTCAGCACGGGATTAGCTTACGATGTATTTGGAA
GCCCCCGTCCAAACGAGTATTTTACAGAAAGCCGACAAGGAATTCCATTAATAACTGG
CCGTTTTGATCCTTTGGAACAACTTGATAAATTAAGTAGATCTTTTATGACCATAGATCG
AACCTATCCAATTTTTACAGTGCGATGGTTGGCTGTTCACGGACTAGCTGTACCAACAG
TTTTTTTCTTGGGGTCAATATCAGCAATGCAGTTTATCCAACGAATGGTTACACAAAGC
GTTGAGGGTAGTTCTAGA---TCTCGTCCAAAACGAACTGGTGTAGGGGGGTTATTGAA
ACCATTGAATTCGGAATATGGTAAAGTAGCTCCTGGATGGGGAACTACTCCATTGATGG

GAGTCACAATGGCCTTATTTGCAATATTTCTATCTATTATTTAGAGATTTATAATTCTTCC
GTTTTACTAGACGGAATTTCAATGAATATGCTTACTCTCAAACTTTTTGTTTACACGGTA
GTGATATTCTTTGTTTCTCTCTTCATCTTCGGATTCCTATCTAATGATCCAGGACGAAATC
CCGGACGCGAAGAAATGGCCGATACTACTGGAAGGATTCCTCTTTGGATAATAGGTACT
GTAACTGGTATTCTTGTGATTGGTTTAATAGGTATTTTCTTTTATGGTTCATATTCTGGATT
GGGCTCATCCCTGATGCTTAATATCTTTAGTTTGATCTGGATCTGTTTTAATTCTCTCCTT
TATTCAAGCAGTTTTCTCTTCGCTAAATTGCCTGAGGCCTACGCTTTTTTGAAGCCAATC
ATAGATGTTATGCCAGTCATCCCTGTGCTCTTTCTTCTCTTAGCCTTTGTTTGGCAAGCT
GCTGTCAGTTTTCGAACGACACAATCAAACCCGAACGAACAAAATGTTGAATTGAATC
GTACCAGTCTCTACTGGGGGTTATTACTCATTTTTGTACTTGCTGTTTTATTTTCCAATTA
TTTTTTCAATATGGAAGTCAATATTCTCGCATTTATTGCTACTGCACTGTTCATTCTAGTT
CCTACTGCCTTTTTGCTTATAATATACGTAAAAACGATCAGTCAAAGTGATATGGAAATA
GCAACCCTAGTTGCCATCTTTATATCTGGTTTACTTGTAAGTTTTACTGGGTATGCCTTAT
ATACTGCTTTTGGGCAACCTTCTCAACAACTAAGAGATCCATTCGAGGAACACGGGGA
CATGGAAGCATTGGTTTATACATTCCTCTTAGTCTCGACTCTAGGGATCATTTTTTTCGCT
ATCTTTTTTCGAGAACCACCTAAAGTTCCAACTAAAAAGATGAAAATGACTATTGCTTT
CCAATTGGCTGTTTTTGCATTAATTGCTACGTCATTAATCTTACTGATTAGTGTACCCGTT
GTATTTGCTTCTCCTGAGGGTTGGTCGAGTAACAAAAATGTTGTATTTTCGGGGACATC
TTTATGGATTGGATTAGTCTTTCTGGTAGGTATCCTTAACTCTCTCATCTCT------------------
---ATGTCACCACAAACCGAGACTAAAGCGAGTGTTGGATTCAAGGCTGGCGTTAAAGA
TTATAAATTGACTTATTATACTCCTGACTATGAAACCAAAGATACTGATATCTTGGCAGC
ATTCCGAGTAACTCCTCAACCTGGAGTTCCGCCTGAAGAAGCGGGGGCGGCGGTAGCT
GCTGAATCTTCTACTGGTACATGGACAACTGTGTGGACCGATGGGCTTACCAATCTTGA
TCGTTACAAAGGACGATGCTACCACATCGAGCCCGTTGCTGGAGAAGAAAGTCAATTT
ATTGCTTATGTAGCTTATCCTTTAGACCTTTTCGAAGAAGGTTCTGTTACTAACATGTTT
ACTTCCATTGTAGGTAATGTCTTTGGTTTCAAAGCCCTGCGCGCTCTACGTCTGGAGGA
TTTGCGAATCCCCACTGCTTATTCTAAAACTTTCCAAGGCCCGCCGCATGGTATCCAAG
TTGAGAGAGATAAATTGAACAAGTATGGACGCCCTCTATTGGGATGTACTATTAAACCT
AAATTGGGATTATCCGCCAAGAATTATGGTAGAGCAGTTTATGAATGTCTCCGCGGTGG
ACTTGATTTTACCAAAGATGATGAGAACGTAAACTCCCAACCATTTATGCGTTGGAGAG
ACCGTTTCTTATTTTGTGCCGAAGCGCTTTATAAAGCACAGGCTGAAACAGGTGAAATC
AAAGGGCATTACTTGAATGCTACTGCCGGTACATGCGAAGAAATGATGAAAAGGGCTG
TATGTGCTAGAGAACTGGGTGTCCCTATCGTAATGCATGACTACTTAACGGGGGGATTC
ACTGCAAATACTACCTTGGCTCATTATTGCCGAGATAATGGTCTACTTCTTCACATCCAC
CGTGCAATGCATGCAGTTATTGATAGACAGAAAAATCATGGTATACACTTTCGTGTACTA
GCGAAGGCCCTACGTATGTCTGGTGGAGACCATATTCATGCTGGTACCGTAGTAGGTAA
ACTTGAGGGTGAAAGAGATATCACTTTAGGCTTTGTTGATTTACTACGTGATGATTTTAT
TGAAAAAGACCGAAGCCGCGGTATTTATTTCACTCAAGATTGGGTCTCTCTACCGGGTG
TTCTGCCTGTGGCTTCCGGGGGTATTCACGTTTGGCATATGCCTGCTCTGACCGAGATC
TTTGGTGATGATTCCGTACTACAATTCGGTGGAGGAACTTTAGGGCATCCTTGGGGAAA
CGCACCTGGTGCTGTAGCTAATCGAGTAGCTCTAGAAGCATGTGTACAAGCTCGTAATG
AGGGACGTGATCTTGCTCGTGAAGGTAATGAAATTATCCGCGAGGCTAGCAAATGGAG
TCCTGAACTAGCTGCTGCTTGTGAAGTATGGAAGGAGATCAAATTTGAATTCCAAGCA
ATGGATACTTTG------ATGGCGATACATTTATACAAAACTTCTACCCCGAGCACACGCAAT

GGAGCCGTAGACAGTCAAGTGAAATCCAATAAACGAAATAATTTGATCTATGGACAGC
ATCGTTGTGGTAAAGGTCGTAATGCCAGAGGAATCATTACCGCAGGGCATAGAGGGGG
AGGTCATAAGCGTCTATACCGTCAAATCGATTTTCGACGGAAGGAAAAAGACATATATG
GTAGAATCGTAACCATAGAATACGACCCTAATCGAAATGCATACATTTGTCTCATACACT
ATGGGGATGGTGAGAAGAGATATATTTTACATCCCAGAGGGGCTATAATTGGAGATACC
ATTGTTTCTGGTACAGAAGTTCCTATAAAAATGGGAAATGACCTACCTTTGACCGATAT
GCCCTTAGGCACGGCCATACATAACATAGAAATCACACTTGGAAAGGGTGGACAATTA
GCTAGAGCAGCAGGTGCTGTAGCGAAACTGATTGCAAAAGAGGGGAAATCGGCCACA
TTAAAATTACCTTCTGGGGAGGTCCGTTTGATATCCAAAAACTGCTCAGCAACAGTCGG
ACAAGTAGGGAATGTTGGAGTGAACCAGAAAAGTTTGGGTAAAGCCGGATCTAAATGT
TGGCTGGGTAAGCGTCCTATAGTAAGAGGAGTAGTTATGAACCCTGTAGACCATCCCCA
TGGGGGTGGTGAAGGGAGGGCCCCAATTGGTAGAAAAAAACCCGTAACCCCATGGGG
TTATCCTGCACTTGGAAGAAGAAGTAGAAAAAGGAATAAATATAGCGATAATTTGATTC
TTCGTCGCCGTAGTAAAATGATTCAACCTCAGACTCATTTGAATGTGGCGGATAACAGC
GGGGCCCGAGAATTGATGTGTATTCGAATCATAGGAACTAGT---AATCGACGATATGCTG
AAATTGGTGACGTTATTGTTGCTGTGATCAAGGAAGCATTACCAAATACATCCCTAGAA
AAATCAGAAGTGATCAGAGCTGTAATTGTGCGTACTTGTAAAGAATTCAAACGCGACA
ATGGCATGATAATACGATATGATGACAATGCTGCAGTTGTCATTGATCAAGAAGGAAAT
CCGAAAGGAACTCGAATTTTTGGTGCGATCACCCGGGAATTGAGACAGTTAAATTTCA
CTAAAATAGTTTCACTAGCACCCGAGGTATTAATGCTTAGTCCCAAAAGAACCAGATTC
CGTAAACAACATAGAGGAAGAATGAAAGGAATATCTTATCGAGGTAATCATATTTCTTT
CGGTAGATATGCTCTTCAGGCACTTGAACCCGCGTGGATTACATCTAGACAAATAGAAG
CGGGTCGGCGGGCAATGACACGAAATGTCCGCCGCGGTGGAAAAATATGGGTACGTTT
ATTTCCAGACAAACCGGTTACGGTAAGACCTACAGAAACACGTATGGGTTCGGGGAAA
GGATCTCCTGAATATTGGGTAGTTGTCGTGAAACCAGGTAGAATACTTTATGAAATGGA
CGGAGTAGGAGAAAATATAGCTCAAAAAGCTATTTCAATAGCGGCTTCAAAAATGCCTA
TACGAACTCAATTCATTATTTCGGTAATGACCAGAATTAGACGAGGATATATAGCTCGGA
ACCGTAGAACAAAAATGCGTTTATTTGCCTCAAGCTTTCGGGGGGCTCGTTCAAGACTT
ACTCGAACTATTACTCAACAGAAAATACGAGCTTTGGTTTCGGCTCATCGGGATAGAGA
TAGGCAAAAGAGAGATTTTCGTCGTTTGTGGATCACGCGAATAAATGCAGTAATTCGCG
AGAGG---CGGGTATCCTATAGTTATAGTATATTAATGCACAGTTTGTACAAGAGGCAGTT
GCTTCTTAATCGTAAAATACTTGCGCAAATGGCTATATTAAATAAAAAAAGTCTTTATAT
GATTTCCAATGAAATAATAAAA--------------------------------ATGATAAAAAAAGTATACGCA
ATAGGTCAGCATATGTCTATGTCTGCTCACAAAGCAAAAATAGTAATTGATCAGATTCGT
GGACGTTTTTACGAGGAAACACTTAGGATACTCGAACTCATGCCTTTTGAAAGATGTTA
TCCCGTTTTTAAATTGGTTTATTCTGCAGCAGCAAACGCGAGTCACAATATTGAGTCCA
ACAAATCAAACTTAATCATTAGTAAAGCCGAAGTCAACGAAGGTGGAACTCTGAAAAA
ATTTAAACCTAGAGCTCGAGGACGAAGTTATCCGATAAAACGACGCACTTGTCATATAA
CTATTGTATTGAAAGATATATCTTTAGGT---------------------ATGGATGGAATCAAATATGCA
GTATTTACAGACAAAAGTATTCGGTTATTGGGGAAAAAT---CAATATACTTCTAATGTCG
AATCAGGATCAACTAGGACAGAAATAAAGCATTGGGTCGAACTCTTCTTTGGTGTTAA
GGTAATAGCTATGAATAGCCATCGACCCCCGGGAAAGGGTAGAAGAATGGGACCTATT
GTAGGACATACAATGCATTACAGACGTATGATCATTACGCTTCAACCGGGTTATTCTATT
CCACCTCTTAGAAAGAAAAGAACTATGGCAGTTCCAAAAAAGCGCACTTCTATATCAA

AAAAGCGTATTCGTAAAAATAGTTGGAAAAGAAAGGGATATTGGGCAGCGTTGAAAGC
CTTTTCATTAGCGAAATCCCTTGCTACGGGGAATTCAAAAAGTTTTTTTGTGCGA------C
AAATAATGGCCAGGGGTAAAGATACCCGAGTTAAAGTTATTTTGGAATGCACCGGGTGT
---GTTCGAACGGGTGTTGTTAATAAGAAA------GGCATTTCCAGATATATTACTCAAAAA
AATCGACACAATACACCCGGTCGATTGGAATTGAGAAAATTCTGTCCCTATTGTTACAA
ACATAGGATTCATGGGGAGATAAAGAAAATGAAAATAAGAGCCTCTGTTCGTAAAATTT
GTGAAAAATGTCGACTGATCCGTAGACGAGGACGAATTATGGTAATTTGTTCCAACCCT
AGACATAAACAAAGACAGGGAATGGTTCGAGAGAAAGTCAAAGTATCTACTCGAACA
CTACAGTGGAAGTGTGTTGAATCGAGAACAGACAGTAAGCGTCTTTTTTATGGACGCT
TTATTCTGTCTCCACTTATGAAAGGTCAAGCCGACACAATAGGGATTGCAATGCGAAGA
GCTTTGCTTGGAGAAATAGAAGGAACATCTATCACACGTGCAAAATCTGAA------AAAA
TACCACACGAATATTCTACCGTAGTGGGTATTCAAGAAACGGTACATGAACTTTTAATG
AATTTGAAAGAAATTGTATTGAGAGGAAATTTGTATGGAATTCAAAACGGGTCGATTTG
TATCAAGGGTCCGGGATATGTAACTGCTCAAGACCTCGTCTTACCCCCTTCTGTCGAAA
TCGTTGATAATACACAACATATAGCTATACTAACAGAACCAATTTATTTTTGTATTGAATT
ACAAATCGAGAGACATCGAGGATATCATATAAAAACACCCAATAACTTTCAAGAAGGA
AGTTTT------CCTATCGATGCTGTATTCATGCCTGTTCGAAATACAAATTATAGTATTCAGT
CTTATAGAAATGGGAGTGAAAAAGAAGAGATACTTTTTCTCGAAATATGGACAAATGG
AAGTTTAACTCCTAAAGAAGCACTTCATGAAGCCTCTCGGAATTTGATTGATTTATTCAT
TCCTTTTCTACATGCGGAAGAAGAA------AACTTACATTTAGAAAACGACTGG---TACAA
GGTTACTTTACCCCTTTTTACCTTTCATGATAGATTGACT---AAACTAAAGAAAAATCAA
AAA---GAAATGGCATTGAAATATATTTTTATTGATCAATTAGAATTGACTTCTAGGACCTA
TAATTGCCTCAAAAGGTCTAATATACATACATTATTTGACCTTTTAAATAAGAGTCACGA
ATACCTTATGAAAATGGAACATTTTCACATTGAAGACGTGAAACATATATTGAACATTCT
AGAAAAAAAAAAA---TTCGCAACTGATTTACCAAAGAATAAAATGCTGGGGGATGGAA
AT---GAGGGAACGTCTACAATACCTGGGTTTAATCAGATACAATTTGAAGGGTTTTGTAG
GTTTATTGATCATGGCTTAACCGAAGAACTTTATAAGTTTCCAAAAATTGAAGATACAG
AGCAAGAAATTGAATTCCAATTATTTGCGGAAACATATCAATTAGTGGAACCATTGATA
AAAGAAAGAGATGCTGTATATGAAGCAATCACATATTCTTCTGAATTATATGTATCTGCG
AGATTAATTTGGAAAACCAGTAGGGATATACAAAAACAAACCCTTTTTTTTGGAAACAT
TCCTCTAATGAATTCCCTGGGAACCTCTATAGTAAATGGAATATACAGAACTGCGATCAA
TCAAATCTTGCAAAGCCCCGGTATCTATTATCGGTCCGAAGCGGACCATAACGGAATTT
CGGTCTATACCGGCAGCATAATATCAGATTGGGGAGGAAGATTCGAATTAGAGATTGAT
AGAAAAGCAAGGATATGGGCTCGTGTAAGTAGGAAACAGAAAATCTCTATTCTAGTTC
TATCATCAGCTATGGGTTTGAATCTAAGGGAAATTTTAGAAAATGTTTGCTACCCTGAGA
TTTTCTTGTCTTTCCTAAATGAGAAGGAGAAAAAAAAAAATAGGGTCAAAGGAAACTGC
TATTTTGGAGTTTTATCAACAATTTACGTGTGTAGGCGGAGATCCAGTATTTTCTGAATC
TCTATGTAAGGAATTACAAAAAAAAATTCTTTCAACAAAGATGTGAATTAGGAAGGATTG
GTCGACGAAATATGAACCAGAGATTGAATCTTGATCTACCTCCGACTAATACATTTTTGT
TACCGAGAGATATATTGGCGGCTGCGGATTATTTGATTGGAATGAAATTTGGAATGGGC
ACGCTTGATGAT------ATGAATCATTTAAAAAATAAGCGTATTCGTTCGGTTGCGGATCTC
TTACAAGATCAATTTGGATTGGCTTTGGTTCGTTTAGAAAATATGGTTCGAGGCACTATA
TGTGGAGCAATTAGA---CATAAATTGATA---CCGACTCCTCAGAGTTTGGTACCTTCAAC
TCCA------------TTAACAACTACTTATGAATCTTTTTTCGGGTTACACCCATTATCTCAAGT

TTTAGATCGAACTAATCCATTGACACAAACAGTTCATGGGAGAAAGTTGAGTTATTTGG
GACCTGGAGGATTGACAGGGCGAACTGCTAATTTTCGGATACGAGATATCCATCCTAGT
CACTATGGACGCATTTGTCCAATTGACACGTCTGAAGGAATCAACGTTGGGCTTATTGG
ATCCTTAGCAATTCATGCGAAAATTGGTCATTGTGGC---TCTCTAGAAAGTCCGTTTTAT-
--------GAAATCTTTGAAGAATCAAAATCAAAAAAAAACCGGATGTTTTATTTATCACCAA
ATAGAGATGAATACTGTATGATAGCAGCAGGAAATTCTTTGGCACTGAGTCGAGGTATT
CAGGAAGAACAGGTTGTTCCAGCTCGATACCGTCAAGAATTCCTGACTATTGCATGGG
AACAGGTTCATCTTCGCAGTATTTTTCCCTTCCAATATTTTTCTATTGGAGCTTCCCTTAT
TCCTTTTATCGAGCATAATGACGCGAATCGAGCTTTAATGAGTTCTAATATGCAACGTCA
AGCAGTTCCGCTTTCTCAGTCCGAGAAATGCATTGTTGGAACTGGAGCGGAACGACAG
GTGGCTCTAGATTCAGGAGTTTCTGTTATAGCCGAACACGAGGGAAGGATCATTTATAC
CGATACTGACAAAATTTTTTTATTAGGCAATGAGGATATTTTCAATATTCCATTAGTTGTG
TATCAACGTTCTAACAAAAATACTTGTATGCATCAAAAAGGTCGGGTTCAGCGGGATAA
GTGCATTAAAAAGGGACAAATTTTAGGAGATGGTGCCGCTACAGTCGGTGGCGAACTC
ACTTTGGGTAAAAACATATTAGTAGCTTATATGCCGTGGGAGGGTTACAATTCTGAAGA
TGCGGTACTCATTAGCGAGCGTATGATATATGGAGATATTTATACTTCTTTTCACATACGG
AAATATGAAATTCAGGCTCATGTGACAAGTCAAGGT---TCCGAAAGGATCACTAACGAA
ATACCGCATCTAGAAGCCCATTTACTCCGAAATTTAGACAAAAATGGAATTGTGGCGCT
GGGATCGTGGGTAGAGACGGGCGATATTTTAGTAGGTAAATTAACGCCTCAGATGGCA
AAAGAG---TCATCGTATGCCCCTGAAGATAGGTTATTACGAGCCATACTTGGCATTCAGG
TGTCCAGTTCAAAGGAAACTTGTCTAAAACTCCCTACAGGTGGTAGGGGCCGAGTTAT
TGATGTTAGATGGGTCCAGAAAAAGGGG---GGTTCGGATTCTAATCCAGAAACCATTCG
TGTATATATTTTACAGAAACGTGAAATCAAAGTAGGCGATAAAGTAGCTGGAAGACATG
GAAATAAAGGTATCGTTTCCAAAATTTTGCCTAGACAAGATATGCCTTATTTGCAAGATG
GAGGAACTGTTGATATGGTCTTCAACCCATTAGGAGTACCTTCCCGAATGAATGTAGGA
CAGATATTTGAATGTTCGCTGGGGTTAGCGGGAGATCTTCTAGATAGACATTATCGAATA
GCGCCTTTTGATGAGAGATATGAACAAGAGGCTTCGAGAAAATTAGTGTTTTCTGAATT
ATATGAAGCCAGCAAGCAAACAGCCAATCCATGGGTCTTTGAACCTGAGTATCCCGGA
AAAAGCAGAATATTTGATGGAAGAACAGGAAATCCCTTTGAACAGCCTGTTATAATAG
GAAAGCCTTATATCTTGAAATTAATTCATCAGGTTGATGATAAAATACATGGACGCTCCA
GTGGGCATTATGCACTTGTTACACAACAACCCCTTAGAGGAAGGGCCAAGCAAGGAG
GACAACGTATAGGAGAAATGGAGGTTTGGGCTCTAGAGGGCTTTGGTGTTTCTCATATT
CTACAAGAGATGCTTACTTATAAATCTGATCATATTAAAGCTCGTCAAGAAGTACTTGGT
ACTACGATCGTTGGAGGAACAATACCTAAACCCGAGGATGCTCCAGAATCTTTTCGATT
GCTCGTTCGAGAACTACGATCTTTAGCTTTGGAACTGAATCATTTCTTTGTGTCTGAGA
AGAACTTCCAGATTAATAGGAAGGAAGCT--------------------ATGATCGATCGGTATAAAC
ATCAACAACTACGAATTGGATCAGTTTCTCCTCAACAAATAAGTGCTTGGGCCAATAAA
ATCCTACCTAACGGAGAGCTTGTTGGAGAGGTGACAAAACCCTATACTTTTCATTACAA
GACCAATAAACCTGAAAAAGAGGGATTATTCTGTGAAAGGATTTTTGGTCCTATAAAA
AGTGGAATTTGTGCTTGTGGAAATTATCGAGGAATCGGAGATGAAAAAGAAGACCTAA
ATTTTTGTGAACAATGCGGAGTCGAATTTGTTAATTCTCGGATACGCAGATATCAAATGG
GCTACATCAAATTGGCATGCCCAGTAACCCATGTGTGGTATTTGAAACGTCTTCCTAGT
TTTATCGCCAATCTTTTAGATAAATCTATTAAAGAATTGGAAGGCCTAGTATACTGCGAT--
----TTTTCTTTTGCTAGGCCC---ATAGCAAAAAAACCTACTTTCTTACGATTACGGGGTTC

ATTCGAATATGAAATCCAATCCTGGAAATACAGCATCCCACTTTTTTTTACTACCCAAGG
TTTCGATACATTTCGAAATCGAGAAATTTCTACTGGAGCAGGTGTTATCCGAGACCAAT
TAGCCGATCTGGATTTGCGAATTATTATAGAGTATTCGTTGGTCGAATGGAACGAATTAG
GGGAAGAA---AGACCCATGGATAATGAATGGGAAGATCGAAAGGTTGTTCGAAGAAAG
GATTTTTTGGTTAGACGTATGGAATTAGCTAAGCATTTCTTCGAACAAATATAGAACCA
GAATGGATGGTTTTATGTCTATTACCGGTTCTTCCCCCCGAGTTGAGACCGATCATTCAG
ATAGATGGGGGTAAATTAATGAGCTCAGATATTAATGAGCTCTATAGAAGAGTTATCTAT
CGAAACAATACTCTTGCCGATCTATTAACAACAAGTCGATCTACGCCAGGGGAATTAGT
AATGTGTCAAGAGAAATTGGTACAAGAAGCTGTCGATACACTTCTTGATAATGGCATCC
GCGGCCAACCAATGAGAGATGGTCATAATAAGGTTTACAAGTCGTTTTCAGATGTAATT
GAAGGCAAAGAAGGAAGATTTCGTGAGACTCTGCTTGGCAAACGGGTTGATTATTCGG
GACGTTCTGTCATTGTCGTAGGCCCCTCACTGCCATTACATCGATGTGGGTTGCCCCGG
GAAATAGCAATAGAGCTTTTCCAAACATTTGTAATTCGTGGTCTAATTAGACAACATCTT
GCTTCGAACATAGGAGTTGCTAAGAGTAAAATTAGGGAAAAAGAACCGATTGTATGGG
AAATACTTCAGGAAGTTATGCAGGGGCATCCTGTATTGCTGAATAGAGCTCCTACTCTG
CATAGATTAGGCGTACAGGCATTCCAACCAATTTTAGTGGAAGGACGTGCTATTTGTTT
ACACCCATTAGTTTGTAAAGGATTCAATGCAGACTTTGATGGGGATCAGATGGCTGTTC
ATGTGCCTTTATCATTAGAGGCTCAAGCGGAGGCTCGTTTACTTATGTTTTCTCATACGA
ATCTCCTGTCTCCGGCTATCGTCGATCCCATTGCCTTACCGACCCAAGATATGCTTATTG
GGCTCTATGTATTAACGAATGGGGATCGACGAGGTATTTGTGCAAATAGGTATAATCCAT
GCAATCGTAGAAAT---------TTTCAAAATGAAAGAATTCCCTATAATAACTATAGGGATAC
GAAAAAAAAAGTTATTTTTTTTTGTAATTCCTATCATGCAATTGGAGCTTATCGGCAGAA
AAGAATCGATTTAGATAGTTCTTTGTGGCTCCGATGGCAATTAGATCAACGC---GTTATT
GCTTCA------AGAGAAGCTCCGATCGAAGTTCACTATGAATCTTTGGGTACCTATCATGA
GATTTATGGACACTATCTAATAGTACGAAGTATCAAAAGAGAAATTCTTTGTATATACCT
TCGGACCACCGTTGGTCATATTTCTCTTTATCGAGAAATCGAAGAGGCTATACAAGGGT
TTTGTCGGGCCTTCTCGTATGGTATC------------------------ACGGAGGTACTTATGGCCGAA
GGGGCCAATCTGGTCTATCACAATAAAGCGATAGATGGAACTGTCATTAAACGGCTTAT
TAGCAGATTAATAGATCACTTCGGAATGGCGTATACATCACATATCCTGGATCAAGTAAA
GACTCTAGGTTTCCACCAAGCCACCGCTACATCCATTTCATTAGGAGTTGATGATCTTTT
AACAATACCTTCTAAGGGATGGCTAGTCCAAGACGCTGAACAACAAAGTTTTCTTTTG
GAAAAACAGCATCATTATGGGAATGTACACGCGGTAGAAAAATTACGTCAATCTATTGA
GATATGGTATGCTACAAGCGAATATTTGCGCCAAGAAATGAATCCTAATTTTAGAATGAC
TGAACCTGGTAATCCAGTCCATATAATGTCTTTTTCGGGAGCTAGAGGAAATGCATCTC
AAGTACACCAATTAGTAGGTATGAGAGGATTAATGTCGGATCCACAAGGACAAATGATT
GATTTACCCATTCAAAGCAATTTACGCGAAGGACTTTCTTTAACAGAATATATAATTTCT
TGCTATGGAGCCCGCAAAGGAGTTGTGGATACTGCTGTACGAACATCGGATGCTGGATA
TCTTACGCGCAGACTTGTTGAAGTAGTTCAACACATTGTTGTACGTAGAACAGATTGTG
GCACCAGCCGAGGAATCTCTGTGAGTCCTCAAAATGAAATAATGTCG---GAAAGACTTT
TTATACAAACATTAATTGGTCGTGTATTAGCAGATGATATCTATATGGGTTCCCGATGCAT
TGCCGTTAGAAATCAAGATATTGGGGTTTGGCTTGTCAATCGATTCATAACC--------------
---TTTCAAACGCAATTCATATCCCTTCGAACCCCCTTTACTTGTAGGAGTATGTCTTGGA
TCTGTCGCTTATGTTATGGCCGAAGTCCTACTCATGGCGACCTGGTTGAATTAGGAGAA
GCTGTAGGTATTATTGCGGGTCAATCTATTGGAGAGCCGGGTACTCAACTAACATTACG

AACTTTTCATACCGGCGGAGTATTCACAGGGGGTACTGCAGAACATGTACGAGCCCCT
TCTAACGGGAAAATCAAATTTAATGAGGATTTGGTTCATCCCACACGTACACGCCATGG
GCATCCTGCTTTTCTATGTTATATGGACTTGTATGTAACTATTGAAAGTGAAGATATTCTA
TATAACGTGACTATTCCACAAAAAAGTTTTCTTTTAGTTCAAAATGATCAATATGTAGAA
TCAGAACAAGTGATTGCCGAGATTCGCGCGGGAACATACACTTTG---AATTTTAAAGAA
AGGGTCCGAAAGCATATCTATTCCGACTCCGAAGGAGAA---ATCCACTGGAGTACTGAT
GTGTACCATACACCTGAATTTGCATATAGTAATGTCCACCTCTTACCAAGAACAAGCCAT
TTATGGATATTAAAAGGAAGTTCGTGCAGA------TACCGTGTAGTCTCTTTTTCAATACAT
AAGGATCAAGATCAAGTTCATTCTCTT------TCTGTTTCTGCCGAAGGA---AGATATATTT
CGAGCTTT---TCAGGAAATAATGATCAA---GTTAGATCCAGATTCTTTAGTTCGGATCTTT
CCGGTAAA---CAAAGTAAGATTACTGAATAT---TCAGAGCATAATCGAATCATATGTACTG
GTCATTGTAATCTCATATATCCCCCA---ATTCTACATGAGAGTTATGAT------TTATTGGCAA
AGAGGCGAAGAAATAGATTCATCATTCCATTCCAATCGATTCCAGAACGGGAGAAAGA
ACTAATGTCCCCTGCCGATATCTCGATTGAAATACCCATAAAT------GGTATTTTCCGTCG
AAAAAGTATTTTTGCTTATTTCGATGATCTTCAATACAGAAGAAACAGTTCAGGAATTA
CTAAATATGGGACTATAGGAGTGCATTCAATCCTCAAAAAAGAGGAT------TTGATTGAA
TATCGA---GGAGTCAAAGAATTTAAGCCAAAATACATGAAAATAGATAGATTTTTTTTCA
TTCCCGAGGAAGTACATATTTTACCTGAATCTTCTTCCATAATGGTACGGAACAATAGTA
TCATTAGAGTGGATACACGAATTGCTTTAAATACAAGAAGCCGAGTAGGCGGCTTGGTC
CGAGTGGAGAAAAAAAAAAAAGAGATTGAACTTAAAATATTTTCTGGAGATATCCATT
TTCCTGGAGAGACAGATAAGATCTCCCGACACAGCGGGATCTTGATACCACCAGGAAC
GATAAAAAAA------AATTCGAAGGAATCAAAAAAT------TTGCAAAATTGGATCTATGTCC
AACGG------ATTACACCTACTAAGAAAAAG------TATTTTATTTTTGTTCGGCCCGTAATCA
TATATGAAATAGCAGACGGTCTAAATTTATCAACACTTTTCCCTCAAGATCTGTTGCAGG
AAAGGGAAAACCTGCAACTTCGAGTTGTTAATTATATCCTTTATGGATATGGTAAACCT
GTTTTGGGAATTTCTGACCCAAGTATTCAATTAATTCGTACTTGTTTATCGCTGGATTGG
GATCAAGAAAAA---------AAAAGTTCTTCTATTGAGGAGGCTCATGCTTCTTTTATTGAA
GTAAGTACAAGAGGTCTGATTCGATATTTCTTACGAATTGACTTAGTGAAATCCCATAGT
TTGTATAGCCGAAAAAGGAAGGATTTCTCAAGTTCTGGATTCCTCTAT---GATAATGCGT
CAGAGCGTGCCAATATCAATCCATTTTAT---------CTCAAGGCAAGA---------------ATTCAA
CAATCACTTAGACAAAATCAAGGAACTATTAGTACGTTGTTGAATAGA---------AATAAG
GAATACCAATCTTTGATAATTTTATCATCACCTAATTGTTTTCGAATGGGTCCA------TTCA
ACAATGTAAAATATCACAAT---------GTGATAAAAGAAAGA---CCTATAATTTTAATTAGGA
ATTACTTGGGCCCTTTAGGA------ACAGCTCTTCAAATTGCGAATTATTCTTCATATTCAT
TTTACCATTTTATA------ACTCATAATCAGATCTCG---GTAACTAAATATTTGCAACTTGAG
CTTGACAAT---TTAAAAAAGAACGTTCAAGTAATTCAAATTAAA---TATTATTTAATGAAT
GAAAATGGGAGA------GTTTCTAAGCCCGATCCATGCAGTAACATCATTTTGAATCAATT
CAATTTGAATTGGCATTTTCTCTATCATGATTATTGTGAAGAAAGATCCCCAATAATTAG
C---TTGGGGCAGCTGATTTGTGAAAATCTATGTATAGCCAAAAATGGACCA---------CACC
TAAAATCGGGTCAAGTTATAATTGTTCAA---GTCGACTCGGTAGTAATAAGATCAGCTAA
GCCTTATTTGACCACCCCAGGAGCAACTCTTCATGGCCATTATGGAGAAATCCTTTCCG
AAGGCGATACGTTAGTTACATTTATATATGAAAAATCAAGATCTGGTGATATAACGCAGG
GTCTGCCAAAAGTGGAACAAGTGTTAGAAGTGCGCTCTATTGATTCAATATCGAGCAA
CCTAGAAAAGAGAGTTGAAGGTTGGAACGCGTGTATAACACGAATTCTGGGGATTCCT

TGGACATTCTTGATTGGTGGTGAGCTAACTATAGTGCAAAGTCGTATCTCTTTGGTTAAT
AAGATCCAAAAGGTTTATCGATCCCAGGGGGTGCAGATCCATAACAGACATATAGAAAT
TATTGTACGTCAAATAACATCAAAAGTGTTGGTTTCCGAA---------GAGGGAATGTCTAA
TGTTTTTTTACCTGGAGAACTGATTGGATTGTTGCGAGCGGAACGAACAGGACGTGCT
TTGGAAGAAGCGCTCTGTTACCGAGCCATATTATTGGGAATAACGAGAGCATCTCTTAA
TACTCAAAGTTTCATATCTGAAGCGAGTTTTCAAGAAACTGCTCGAGTTTTAGCAAAA
GCTGCTCTCAAGAGTCGTATCGATTGGTTGAAAGGTTTGAAAGAGAACGTTGTTCTAG
GGGGTATGATACCTGTTGGTACCGGATTCAAAGGATTTGTGCGTTTTTCG------CAGCAA
CATAATAACATTTCTTTGAAAACTAAAAAGACGAAT------TTATTCAAGGGTGAAGTGAG
A------GATATTTTGTTCTACCACAGAGAATTTTTTGATTCGTGCATTTCAAAGAATTTTCA
TGATCCAGCGGAACAACCTTTTATAGGATTTAATCATTCC------------------ATGACAAGAA
GATATTGGAACATAAATTTGAAAGAGATGGTGGAAGCCGGAGTTCACTTTGGTCATGGT
ACTAGGAAATGGAATCCGAGAATGGCACCTTATATCTCTGAAAAGCGTAAAGGTATTCA
TATTACAAATCTTACTAAAACTGCTCGGTTTTTATCAGAAGCTTGTGATTTAGTTTTTGA
TGCAGCAAGTAAGGGAAAACAATTCTTAATTGTTGGTACCAAAACTAAAGCAGCTGAT
TCAGTAGCACGAGCTGCAATACGGGCTCGGTGTCATTATGTTAATAAAAAATGGCTCGG
TGGTATTTTAACAAATTGGTCCACTACAGAAACGAGACTTCATAAGTTCCGAGACTTTA
GAATGGAACAAAAGACGGGGGGATTCAACCGTCTTCCGAAAAGAGATGCGGCTGTGT
TGAAGAGACAGTTATCCCACTTGCAAACATATTTGGGCGGGATTAAATATATGATGGGG
TTACCCGATATTGTAATAATCGTTGATCAGCAAAAAGAATATACAGCTCTTCGAGAATGT
ATCATTTTGGGAATTCCAACGATTTGTTTAATTGATACAAATTGTGACCCGGATCTCGCA
GATATTTCGATTCCAACAAACGATGACGCTATAGCTTCAATCCGATTAATTCTTAACAAA
TTAGTATTTGCAATTTGTGAGGGACGTTCT---AGCTATATACGAAATCCTATGGGACAAA
AAATAAATCCACTTGGTTTCAGACTTGGCACAAACCAAAGTCATCATTCTCTTTGGTTT
GTGCAACCAACAAATTATTCAGAAGGTCTGCAAGAAGATCAAAAAATACGAAACTTTA
TCAAAAACTATGTACAACAAAACATAAAAATATCTTCCGGT---GTTGAGGGAATTGCAC
GTATAGAGATTCAAAAAAGAATTGATCTGATTCAGGTCCTAATATATATAGGATTCCCGA
AGTTATTACTGGAGAGTAGG------------ACTCGAAGAATCGAAGAATTACAGAGGAAGTT
ACAAAAAGAATTAAATTGTATGAACCGAAAACTAAACATTGCTATTACAAGGATTGCAA
ATCCTTATGGACATCCTACTATTCTTGCAGAATTTCTAGCCGGACAATTAAAAAGCCGA
GTTTCATGTCGTAAAGCAATGAAAAAAGCTATTGAATTAGCTGAACAGGCGAATACAA
AAGGAATTCAAGTACAAATTGCAGGGCGTATCAACGGAAAAGAAATTGCACGTGTTGA
ATGGATCCGAGAAGGTAGGGTTCCTCTACAAACAATTCGAGCTAAAATCGATTTTTGTT
CCTATACTGCTCAAACGCTTTATGGGGTATTGGGAATCAAAATTTGGATATTTGGAGACG
AGGAAATGTCACGTTACCGAGGGCCTCGTTTCAAAAAAATACGCCGGCTGGGGGTTTT
ACCGGGACTAACTAGTAAAAGGCCCCGAGTCGGAAACGATCTTAGAAATCAATCGCGC
TCCGGTAAA---AAATCTCAATATCGTATTCGTTTAGAAGAAAAGCAAAAATTGCGTTTTC
ATTATGGTCTTACAGAACGACAATTACTTAAATACGTTCGTATCGCCGGAAAAGCAAAA
GGTTCAACAGGTCAAATTTTACTACAATTACTTGAAATGCGTTTGGATAACATCCTTTTT
CGATTGGGTATGGCTTCGACCGTTCCGCAAGCCCGCCAATTAGTTAACCATAGACATAT
TTTAGTTAATGGTCGTATAGTGGATATACCAAGTTATCGCTGCAAACCGCGCGATATTATT
ACAGCAAAGGATGAACAAAAATCTAGAGCTCTGATTCAAAATTATCTTGATTCATTCCC
CCAT---GAGGAAGTGCCAAAACATTTGACTCTTCACCCAATCCAA------TATAAAGGATT
AGTTAATCAAATAATCGATAGTAAATCGATCGGTTTAAAAATAAATGAATTGCTAATCGT

AGAATATTATTCTCGTCAGGCTATGTCACGTCGAGGTACTGCAGAAGAAAAAACTGCA
AAATCCGATCCAATTTATCGTAATCGATTAGTTAATATGTTGGTTAACCGTATTCTGAAAC
ACGGAAAAAAATCATTGGCTTATCAAATTCTCTATCGAGCCATGAAAAAGATTCAACAA
AAGACAGAAACAAATCCACTATCTGTTTTACGTCAAGCAATACGTGGAGTAACTCCCG
ATATAGCAGTAAAAGCAAGACGTGTAGGCGGATCGACTCATCAAGTTCCCATTGAAATA
GGATCCACACAAGGAAAAGCACTTGCCATTCGTTGGTTATTAGGGGCATCCCGAAAAC
GTCCGGGTCGAAATATGGCTTTCAAATTAAGTTCTGAATTAGTAGATGCTGCCAAAGGG
AGTGGCGATGCCATACGCAAAAAGGAAGAGACTCATAGAATGGCAGAGGCAAATAGA
GCTTTTGCACATTTTCGTATGGGTAGGGATACTCTTGCTGACATAATAAACTCTCTACGA
AATGCTGCTATGAATAGAAAAGGAACGATTCGAATACCGTTTACTAATATCACCAAAAA
CATTATTAAAATACTTTTACGAGAAGGTTTTATTGAAAACGCCAGGAAACATCGCGAAA
GCGACAAATATTTTTTGGTTTTAACCCTACGACATAGA---------------AAAGGGAGAACTA
GT---------------TTAAATTTAAAAAGAATAAGTCGACCGGGTCTACGAATCTATTCTAACTA
TCAACAAATTCCTAGAATTTTAGGCGGAATGGGGATTGTAATACTGTCTACTTCTCGAG
GTATAATGACAGACCGAGAGGCTCGACTAGAAGGAATCGGAGGAGAAATTTTGTGTTA
TATATGGATGGCCAAAACTATAACAAAAACCAGTTCTCGTAGGAATGTACGTATTGGCT
CACGTAAGAATACACGTAGAATACCAAAAGGAGTTATTCATGTTCAAGCAAGTTTCAA
CAATACCATTGTGACTGTGACGGATGTACGGGGTCGGGTTATTTCTTGGTCCTCCGCCG
GCACTTGTGGATTCAAGGGTACACGAAGAGGGACGCCGTTTGCTGCCCAAACCGCAG
CAGGAACCGCTATTCGTGCAGTAGTGGATCAAGGGATGCAACGAGCAGAAGTCATGAT
AAAGGGTCCTGGCCTCGGACGAGATGCAGCATTAAGAGCTATTCGTAGAAGTGGTATA
CTGTTAAGTTTCGTACGAGATGTAACTCCTATGCCACATAATGGCTGCAGGCCACCTAA
AAAAAGACGCGTCATGCCAACTCTTAAACAACTTATTAGGAATACAAGGCAGCCAATA
AAAAATGTCACGAAATCCCCCGCTCTTAGGGGATGTCCTCAGCGCCGAGGAACATGTA
TTCGGGTGTATACTATCACCCCCAAAAAACCAAACTCTGCCTTACGTAAAGTTGCCAGA
GTACGATTAACCTCTGGATTTGAAATCACTGCTTATATACCTGGCATTGGCCATAATTCA
CAAGAACATTCTGTAGTCTTAGTAAGAGGGGGAAGGGTTAAGGATTTACCCGGTGTGA
GATATCATATTGTTCGAGGAACCCTGGATGCTGTCGGAGTAAAGGATCGTCAACAAGG
GCGTTCTAAATATGGGGTCAAAAAGCCAAAAATGGCAAGGAAAGGTTTGATTCAAAG
GGAAAAGACGAGGCAAAAATTGGAACAAAAATATCATTTGATTCGTAGATCCTCAAGA
AAAGAAATAAGC---------AAAGTTCTGTCATTGAGTGATCAATGGGAAATTCATGGAAAG
TTGCAGTCCCCACCGCGGAATAGTGCAGCGACACGTCTTCATCGACGTTGTTTTTCGAC
TGGAAGACCAAGAGCTAACTATCGAGACTTTGGGTTATCCGGACACATACTTCGTGAA
ATGGTTCATGCAGGTTTGTTGCCAGGGGCAACAAGATCAAGTTGGATGGTCAAAAATG
CATTCATCTCAGTTATTTCACAAAACGAAAAAGAAAAAAACAAAAACAAGGGATCCGT
TGAATTGCAAGTATTCGTTTTTACTAATAAGATCCGAAGAATTACTTCACATTTGGAATT
GCATAGAAAAGACTATTTATCTCAGAGAGGCCTCCGGAAAATTATAGGAAAACGCCAA
CGGCTGCTGTCTTATTTGTCAAAGAAAAATGAAGTACGTTATAAACAATTAATTAGTCA
GTTGGATATTCGGGAACCAAAAACACGT------ATGCTCAAACTTCGTTTGAGACGGTGT
GGTCGAAAGCAACGAACTATTTATAGAATCGTTGCAATTGATGTTCGATCGCGAAGAGA
AGGAAAACCTCTTCGTAAGGTTGGTTTTTATGATCCAATAAATAATCATACCTATTTAAA
TTTTCCTGACATTCTATATTTCCTTGAAAAGGGTGCTCAACCTACAGAAACAGTTCAGG
ACATTTCAAAGAAATCGGGGGTTTTTACACAATTCTGCCTTAATCAA---AGCAAATTCA
ACATGCATAAATTCAAGCGACTTTTTCATAAATCCAAGCGTTCTTTGCGTAGGCGTTTG

CCCCCGATCAAATCGGGGGATCGAATTGCTTATAGAAACATGAGTTTAATTAGTCGATTT
ATTAGTGAACAAGGAAAAATATTATCTAAACGGGTAAATCGATTGACCTTAAAACAACA
ACGATTAATTACTATTGCTATCAAACAAGCTCGTATTTTATCTTTGTTACCTTTTCTTAAT
AATGAAAAA------CAATTTGAAAAGGGCGAGTCGACTGCTAGAACTGCTGGTCTTAGA
ACCCGAAATCAAGTGGCACGT---CTAAAAAAAAATCCTTTTGTAGCGAATCATTTATTG
GGAAAAATAAATAAGCTTAACACAAAGGCGGAAAAAGAAATAATAGTAACTTGGTCCC
GGGCATCCACCATTATACCCACAATGATCGGACATACCATTGCTATCCACAATGGAAAG
GAACATTTGCCTCTTTATATAACAGATCGGATGGTAGGACATAAATTGGGAGAATTTGC
ACCTACTCTCAATTTCCGGGGACATGCAAAAAATGATAATAGATCTCGTCGTATGATTTT
CAAATCTTTTATACTAGGTAATCTAGTATCCTTATCCCTGAAGATAATCAATTCGGTCGTT
GTGGTCGGACTCTACTATGGATTTCTGACCACATTTTCCATAGGACCCTCTTATCTCTTC
CTTCTCCGAGCTCGGGTTATGGAAGAAGGAGAAGAAGGAACCGAGAAGAAGGTATCA
GCAACAACAGGGTTTATTACGGGACAGCTCATGATGTTCATATCGATCTATTATGCGCCT
CTGTATCTAGCATTTGGTAGACCTCATACAATAACTGTCCTAGCCCTACCGTATCTTTTGT
TTCATTTCTTCTGGAACAATCACAAACACTTTTTTGATTATGGATCTACTACCACAAATT
CAATGCGTAATCTTAGCATTCAATGTGTATTCCTGAATAATCTCATTTTTCAATTATTCAA
CCATTTCATTTTACCAAGTTCAATGTTAGTCAGATTAGTCAACATTTATATGTTTCGATGC
AACAACAAGATGTTATTTGTAACAAGTAGTTTTGTTGGTTGGTTAATTGGTCACATTTTA
TTCATGAAATGGGTTGGGTTCGTATTAGTCTGGATACAGCAAAAAAATTCTATTAGATCT
---ATTCGATCTAATAAGTACCTTGTGTCAGAATTGAGAAATTCTATGGCTCGAATCTTGA
GTATTCTCTTATTTATTACCTGTGTCTACTATTTAGGCAGAATACCGTCACCCCTTTTTAC
TAAGAAACTGAAAGAAACCGAAGAAGATAAAACTCTTGTAACTAGACTTTTTGACTAT
AAACGGTGGAATCGCCCATTTCGATATATAAAAAACAACGGAAGAGAAAAGGCTGTAA
GAAATGAAATGTCACAATTTTTTTTTTATACATGCCTAAGTGATGGAAAAGAAAAAATT
CCTTTTACATACCTGCCGAGTTTGTCCATTTTTTTCGACCTAATAGAAAGAAAGATGCCC
ATATTCACAACAGAAAAAAATGACCTCTCCAAAATTATATAATCGTTGGAGTTATATGAAT
GACCAAAAAAAGAATAAACTAAATAATGAATTTCTAAATAGAGTAGAAACTCTAGATTT
ACATTTTTTAGCTTTTACTTTACTCGAAAAAAGAACTAGATTGTGTAAA------AATACAA
AAGAATACTTACCTAAAATCTATGATCCTTTATTGAATGGTCCCTCACGCAGAAAGATCA
AAAAGTTGTTTTTATTCCCACCCTGGATAACGATAAATAGAATTCACGGTCTCCTT--------
-TTTGACCAAAAACGGGGAGAGGATAGAAAAATTGGAATAAATGAAATAAGTAAAAAA
GTTCCTCGCTGGTCATACAAATTAATCGAGTTAGAACAACAAGAAAGAGAAGAAATTC
GTTCAAGAAAATCCAAACGTGTAATAATTTTTACAGAGGCTTTAATACGTTATTCACAA
CAAGCAGACTTTCGCCGTGACATAATAAAAGGCTCCATGCGTCCTCAAAGACGTAAAA
CAATTACTTGGGAACTCTTTCAACCAAATATACATTCCCCCCTTTTTTTGGACCGACTAG
ACAAACCCCTTTTGTTTTCTTTTGATATC------TCGCAGATGATGAAGATAATGTTTATCA
ATAGGGAGGAAAAAGCACGGGTAGAAATAGCAGAAGCCTGGGATAACATTCTATTTGC
TCACTTAATAAGAGGCTCGTTGCTAGTAATCCAATCCATTCTTAGAAAATATATTTTCTTA
CCTTTATTGATAATAGCTAAAAATACCATCCGTACACTATTATTTCAATTTCCAGAATGGT
CTGAGGATTTAAAAGATTGGAATAAAGAAATGCATGTTAAATGCACCTATAATGGAGTT
CAATTATCCGAAAAAGAATTTCCGAAAAACTGGTTAACAGAGGGTATTCAGATAAAAA
TCCTATTCCCTTTTCGCCTGAAACCTTGGCACAGAGTTAAGAAAGGGAAAAAAAAGAA
TTTTTGCTTTTTAACAGTTTTTGGAATGGAAACTGAATTTCCTTTTGGTTCTACCAAAAG
AAGACTTTTTGATTTTAAACCCATTTGTAAAAAACTCGAAGAAAAGGTTAGAAAGTTT

ACAAAAAAGAGTTTTCTATTT---ATAAGGATTTTAAAAAAAAGACCAAAGTTAAATTTC
ACAAAATCAAAAAAAAGTAAAAAAAGAGTTATCAAAAAAAGTCCATTTTTTTTTTTA
AAGAACAAATAAAAGAACTAATC---------------ATAACTAATAAAAAGACGATTCATGAAT
CCTCGATTCAAATTATACCTATGGATTGGACAAATTCTTCACTGGCCGAAGCAAGAATG
CAAGATTTGACTAATAAAAAAAGGACAATCATAAAT---------CAAATAAAAAAAAAAAA
AGAGAAGAGTCCTAACAAAATAACTTATGGTACGAAAAGATTCGGATCATTAAAAAAG-
--------ATACTAAAAAGAAGAAATGCTCGAGTAGTCCGCAAATTCCATTTTTTAAAAAAA
AAAAATATTGAGAAAATATACATAGATATCTTTCTATCTATCATTCAAATTCTCAGAATCA
ATGCAGAACTTTTT---------ATTAAC---GACTTATCTTCTTTGTCACAAGCATATGTATTTTA
CAAATTATCACAAACCAAAAAGCTTAACTTATATAAGTTAAGATCAGCTCTTCAATATAC
AACTATTTTTCTTAAAAATAAAATAAAGGGTTATTTTGTACAGGGAATATTTCATTCCCA
ATTAAGACATAAAAAACCGATT---TCCACCATGAATCAATGGAAAAACTGGTTAAAAGC
CGGTTATCAATACGATTTATCTCAGATAAGATGGTCTAGATTAGTACCACAAAAATGGCG
AACTATTGTTAATCAAGGATGTATAGCTGAAAATAAAAGCGATTCATATGAAAAATCATA
TGAAAAAGACCGATTAATTCCGTACGAAAAAAAA---AATTTTAAAAAACACTATAGATA
TGATCTTTTAGCATATAAATTTATTAATGATGAGATTGTGGATCCGGCTAGAAAATATTTT
GATTGGAGAATTCTCCGTTTTTGTATTAGACTTTTTGATTGGATGGAAATGAATGAAAG
AATATTAAGTCATTGGTTCTTCCCAGAAATTTTGATACTTTACAATGCATATAAGAAAAC
CCCCGTATCCATACCAATCAAATTACTTCTTTTTGATTACAAAAGTTCTACGGAAGCGGA
GCTTGATTTCCTCCTAAAAAGGTATTTCTGTTTTCAATTACGATGGAATGCTTCTGTAAA
TCAAAGAGTACTCAATAATATAAAAGTATTTTGTCTCCTGCTTAGACTGATAAATCCAAA
CGAAATTGCTATATCCTCTATTCAAAGGAGAGAACTGAGTCTCGATATTTTACTAATTCA
GAACAATTTGAGTCTTACAGAATTTTTGAAAAAAGGAATATTGATTATCGAACCAGTTC
GTCTGGCTGTAAAAAATGTCGGACAGTTTCTTATGTACCAAACCATAAATATTTCATTGG
GTCATAAGAGTAAGCACAATCTTGATGATTTGTTTGTTCCTGAAAATATCTTTTTATCTC
AACGTCGTAGAGAATTCGGAATTCTAATTTCTTTACAGGTTTTTTGGACCAATTATCGAT
TAGAAGATTTAGCTTGTATGAATCGTTGTTGGTTTGATACCAATAATGGAAGTCGTTTCA
ATATGGTAAGAATACATATGTATCCAATGAAAGGACATCAATTCCAATCCTGGATTTTCG
AATTGAGAGAGATATTGAGAGAGATCAAGAATTCTCACTATTTCTTAGATTCATGGACC
CAATTCAATTCAGTGGGATCTTTCATTCACATTTTTTTCCATCAAGAACGTTTTATAAAA
CTCTTGGACTCCCGAATTTTGAGTATCCTACTTTCA---CGCAATTCA------CAGGGTTCAA
CAGGCAATCGATATTTC---------ACGATCAAGGGTGTAGTCTTATTTGTAGTAGCG---GTCT
TTATATATCGT------------ATTAACAATAGAAAGATGGTCGAAAGAAAAAATCTTTATTTGA
CAGGGCTTCTTCCA--------------ATACCTATGAATTCCATTGGACCCAGAAATGAGAAATT
GGAAGAATCTTTT---GGGTCTTCCAATATCAATAGGTTGATTCTTTCG---CTCCTGTATCT
TCCAAAAGGAAAAAAGATCTCTGAGAGCGGTTTCCTGGATCCGAAAGAGAGTACTTG
GGTTCTCCCAATAACT------------AAAAAGTGTATCATGCCTGAATCTAACTGGAGTTCGC
GGTGGTGGAGGAACTGGATCGGAAAAAAGAGG------GATTCTAGTTGTAAGATATCTAA
T---------GAAACCGTCGCTGGAATTGAGATCTCATTCAAAGAGAAAGATCTCAAA---TATC
TGGAGTTTCTTTTTGTATATTATATGGAT------GATCCGATCCGCAAGGACCAGGATTGGG
AATTGTTTGATCGTCTTTCTCCGAGGAAGAGGCGAAACATAATCAACTTG------AATTCG
GGACGGCTATTCGAAATTTTAGTGAAAGACTGGATTTGTTATCTGATGTTTGCTTTTCGT
GAAAAAATACCAATTGAAGTGGAGGGT---TTCTTCAAACAACAAGGA---GCTGGGTCA
ACTATTCAATCAAATGAGATTGAGCATGTTTCCCAT------CTCTTCTCGAGAAAGAAGTG

GGCTATTTCTTTGAAAAATTGTGCTCAATTTCATATGTGGCAATTCCGCCAAGATCTCTT
CGTT---------AGTTGGGGAAAG---AATCCGCACGAATCGGATTTTTTGAGGAAC---ATATC
GAGAGAGAATTGGATTTGGTTATACAATGTGTGGTTGGTAAACAAGGATCGG---------TT
TTTTAGC------AAGGTACGGAATGTATCGTCAAATATTCAATATGATTCC---------ACAAGA-
-----TCTAGTTTCCTTCAAGTAAGGGATTCTAGCCAATTGAAAGGATCTTCT---GATCAATC
CAGAGATCATTTCGATTCCATTAGT------------AATGAGGATTCGGAA--------------------------
------------TATCACACATTGATCAATCAAAGAGAGATTCAACAACTAAAAGAAAGATCGA
TTCTT---TGGGAT------CCTTCCTTTCTTCAAACGGAACTAACAGAGATAGAATCAGAC----
--CGATTCCCTAAA------TGCCTTTCTGGATATTCC------TCAATGTCCCGGCTATTCGCG---
GAACGTGAGAAGGAGATGAATAATCATCTGCTTCCGGAAGAAATCGAAGAATTT------C
TTGGGAATCCTACAAGATCCATTCGTTCTTTTTTCTCTGACAGATGG------TCAGAACTT
CAT---CTGGGTTCGAATCCTACTGAGAGGTCCACCAGAGATCGG------------AAATTGTTG
AAGAAAGAACAAGATGTTTCT---TTTGTCCCTTCCAGGCGATCGGAA------------AATAAA
GAAATAGTTAATATATTCAAGATCATTACGTATTTACAAAAT------ACCGTCTCAATTCATC
CTATTTTATCAGATCCGGGATGTGATAGGGTTCCG---AAGGATGAACTGGAT---ATGGAC
AGTTCCAATAAGATTTCATTCTTGAACAAAAATCCATTTTTGGATTTA------TTTCATCTAT
TCCATGACCGGAACAGGGGGGGATACACCTTA------CACCACGATTTTGAATCAGAA-----
-GAGAGATTTCAAGAAATGGCGGATCTATTCACTCTATCAATAACCGAGCCG---GATCTG
GTGTATCATAAGGGATTTACCTTTTCTATTGATTCCTACGGATTGGATCAAAAACAA------
TTCTTGAATGAGGTATTGGAT---------GAATCGAAAAAGAAATCTTTA---------TTGGTTCT
ACCTCCTATTTTTTAT------GAAGAGAATGAATCTTTTTATCGAAGGATCAGAAAAAAATG
GGTTCGG---ATCTCCTGCGGGAATGATTTGGAAGATCCAAAAGCAAAAAGAGTGGTATT
TGCTAGCAACAACATAATGGAGGCAGTTAATCAATATAGATTGATCCGAAATCTGATTC
AAATCCAATATAGCACCTATGGG------TACATAAGAAATGTATTGAATCGATTCTTTTTAAT
GAAT---------AGACCCGATCGCAACTTCGAATATGTAATTCAA---------AGGGATCAAATAG
GAAATGATACTCTGAATCATAGAACTATAATGAAATATACGATCAACCAACATTTATCGA
ATTTGAAAAAG---AGTCAGAAGAAATGGTTCGACCCT------CTTATTTGGATTTCTCGAA
CCGAGAGATCCATGAATCGGGATCCTAATGCATATAGATACAAATGGTCCAATGGGAAC
AAGAATTTCCAGGAACATTTGGAACATTTCATTTCTGAGCCGAAGAGCCATTTT---CATT
TTCAAGTAGTCTTCGATCGATTACGTATTAATCAATATTCGATTGATTGGTCTGAGGTTAT
CGACAAAAAAAGATTTGTCTAAGTCACTTCGTTTCTTTTTGTCCAAGTTACTTCTT----------
-----------------TTTTTGTCC--------------------AAGTTTCTTCTCTTTTTGTCTAACTCACTTCC
T---TTTTTCTTTGTGAGTTTCGGGAATATCCCCATTCATAGGTCCGAG---ATCCACATCTAT
---GAATTGAAAGGTCCGAATAATCAACTCTGCAATCAGTTGTTAGAATCAATAGGTCTTC
AAATCGTTCATTTGAAAAAATGGAAACCCTTC------TTATTGGAT---------GATCATGATAC
TTCC---CAAAAATCGAAATTCTTGATCAATGGAGGAACAATATCACCATTTTTGTTCAAT
AAGATACCA---AAGTGG---ATGATTGACTCATTCCATACTAGA------AATAATCGCGGGAA
ATCTTTTGATAAGACGGATTCCTATTTCTCAATGATATCCCACGATCAAGACAATTGGCC
GAATCCCGTGAAACCATTTCATATAAGTTCATTGATATCTTCTTTTTATAAAGCAAATCG
ACTTCGATTCGTGAAGAATCCACATCACTTCTACTTCTAT------TGTAACAAAAGATTCCC
TTTT---------TAT---GTGGAAAAGGCCCGTATCAATAATTATGAT------TTTACGTATGGA---C
AATTCCTCAATATCTTGTTCATTCGCAACAAA---ATATTTTCTTTGTACGCCGGTAAA------
AAAAAACATGCTTTTTTGGAG---AGAGATACTATTTCACCAATCGAGTCACAGGTATCT
AACATATTCATACCTAACGATTTTTCAGAAAGTGGTGACGAAAGG---TATAACTTGTACA

AATCT---------TTCCATTTTCCAATTCGATCCGATCCATTCGTTCGT------AGAGCTATTTAC
TCGATCGCAGACATTTCTGGAACGCCTCTCACAGAGGAACAAATAGTCAATTTTGAAA
AAACTTATTGTCAACCTCTTTCAGATATGAATCTATCTGATTCAGAAAGGAAGAACTTG
CATCAGTATCTCAAT------TTCAATTCAAACATGGGTTTGATTCACACTCCATGTTCTGAG
AAATATTTACCATCCGAAAAG------AGGAAAAAA------------------------CGGAGTCTTTGTC
TAAAG------AAATGCGTTGAGAAAGGGCAGATGTATAGAACCTTTCAACGAGATAGTGC
TTTTTCAACTCTCTCAAAATGGAATCTATTCAAAACATATATGCCATGGTTCCTTACTTC
GACAGGTTACAAATATCTAAATTGGAGATTTTTAGATACTTTTTCAGAC---CTATTGCCGA
TACTAAGTAGCAGTAGC---CAAAAATTTGTATCCATTTTTCGGGATATTATGCATGGATCA
GATATATCATGGCGAATTCTTCAGAAAAAATGG------------------GGTCTTCCACAATGGAA
T---CTGATAAGTGATATTTCGAGTAAGTGTTTACATAAT------CTTCTTTTGTCCGAAGAAA
TGGTTCATCGAAATAATGAGTCA------CCATTGATATCGACACATCTGAGATCGCCAAAT
GTTCGGGAGTTG---CTCTATTCAATCCTTTTCCTTCTTCTTGTTGCTGGATAT---CTTGTTC
GTACACATCTTCTCGTTGTTTCCCGA------GCCTATAGTGAGTTACAGACAGAGTTCGAA
AAGGTCAAATCTTTGATGATTCCATCATACATGATTGAGTTGCGAAAACTTCTGGATAG
GTAT---------GAACTGAATTCTTTCTGGTTAAAGAATCTCTTTCTAGTTGCTCTGGAACAA
TTAGGAGATTCTCTAGAAGAAATACGGGGTTCTGCTTCTGGCGGCAACATGCTATGGGG
TGGTGGTCCC---------------GCTTATGGG---GTCAAATCAATACGTTCTAAGAAGAAATATT
TGAATATCAAT---------CTCATCGATCTCATA---AGTATCATACCAAATCCCATCAATCGAAT
CACTTTTTCGAGAAATACGAGACATCTAAGTCATACAAGTAAAGAGATCTATTCATTGAT
AAGAAAAAGAAAAAACGTGAACGGTGATTGGATTGATGATAAAATCGAATCCTTGGTC
GCGAACAGCGATTCGATTGATGATAAAGAAAGAGAATTCTTGGTTCAGTTCTCC------A
CCTTAACGACA------GAAAAAAGGATTGATCAAATTCTATTGAGTCTGACT---CATAGTG
ATCATTTATCA---------------AAGAATGACTCTGGTTATCAAATGATTGAACAACCAGGAG
CAATTTACTTACGATACTTAGTTGACATTCAT------AAAAAGTATCTAATGCATTATGAATT
CAATACGTCCTGTTTAGCAGAAAGACGGCTATTCCTTGCTCATTATCAGACAATCACTTA
T------TCACAAATCTCGTGTGGGGCTAATAGTTTTCATTTCCCATCTCATGGA---------AAA
CCCTTTTCGCTCCGCTTAGCCCTATCCCCCCCT---AGGGCTATTTTAGTGATAGGTTCTAT
AGGAACTGGACGATCCTATTTGGTCAAATACCTAGCGGAAAACTCCTATGTTCCTTTCA
TTACAGTATTTCTGAACAAGTTCCTGGATAACAAGCCTAAGGGTTTTCTTATTGAT--------
-------GATAGTGACGATATTGATCTGAGTGACGATATTGACCGT-----------------------------------
--GACCTTGATACGGAGCTGGAGCTTCTAACTAGGA
TGAATGCGCTA---------ACTATGGATATGATGCTT---------GAAATAGACCGATTTTATATCA
CCCTTCAATTCGAATTAGCAAAAGCAATGTCTCCTTGCATAATATGGATTCCAAACATTC
ATGATCTGAACGTGAATGAGTCGAATTACTTATCCCTCGGTCTATTAGTGAACTATCTCT
CC---------AGGGATTGTGAAAGATGTTCC---ACTAGAGATATTCTTGTTATTGCTTCGACTC
ATATTCCCAAAAAAGTGGATCCCGCTCTAATAGCTCCGAATAAATTAAATACATGCATTA
AGATACGAAGGCTTCTTATTCCACAACAACGAAAGCACTTTTTCACTCTTTCCTATACT
CGGGGATTTCACTTGGAAAAGAAAATGTTCCATACT------AATGGATTCGGGTCCATAAC
TATGGGTTCCAAGGTACGAGATCTTGTAGCACTTACCAATGAGGCCCTATCGATTAGTAT
TACACAGAAGAAATCAATTATAGACACTAATATAATTAGATCTGCTCTTCATAGACAAAC
TTGGGATTTGCGA---------TCCCAGGTAAGATCGGTTCAGGATCATGGGATCCTTTTTTAT
CAGATAGGAAGGGCTGTTGCACAAAACGTACTTCTAAGTAATTGTACCATGGATCCTAT
ATCTATCTATATGAAGAAGAAA------TCATGTAACGAAGGGGATTCCTATTTGTACAAATG

GTACTTCGAACTTGGAACGAGCATGAAGAAATTAACGATACTTCTTTATCTTTTGAGTT
GTTCTGCCGGATCGGTCGCTCAAGACCTTTGGTCTCTACCC------GGACCCGATGAAAA
A---AATGGGATCACTTATTATGGACTCGTTGAGAATGATTCT---GATCTAGTTCATGGCCT
ATTAGAAGTAGAAGGCGCTCTGGTGGGA------TCCTCACGGACAGAAAAAGATTGCAG
TCAATTTGATAATGAT------CGAGTGACATTGCTTCTTCGGCCCGAACCAAGGAAT---CCC
TTAGATATGATGCAAAATGGATCTTGTTCTATCGTTGATCAGAGATTTCTCTATGAAAAA-
-----TACGAATCGGAGTTTGAAGAA---GTGGAAGGAGAAGGAGTCCTCGACCCGCAA----
--------CAGAGAGAGGAGGAT-----------TTATTCAATCAC------ATAGTTTGGGCTCCT------A
GAATATGGCGCTAT---------TGGGGCTTTCTATTTGCTTGTATCGAAAGGCCCAATGAATT
GGGATTTCCC---TATTGGGCCAGGTCATTTCGGGGTAAG---------CGGATCATTTATGATG
AAGAGGATGAGCTTCAAGAGAATGATTCGGAGTTCTTGCAGAGTGGAACCATGCAGTA
CCAGATACGAGATAGATCTTCTAAGGAACAAGGCTTTTTTCGAATAAGCCAATTCATTT
GGGACCCTGCCGATCCACTCTTTTTCCTATTCAAA---GATCAGCCCTTTGTCTCTGTGTT
TTCACATCGAGAATTCTTTGCAGATGAAGAGATGTCAAAGGGGCTTCTTACTTCCCAGG
GGGATCCCCCCACATCTATATATAAACGCTGGTTTATC------AAGAATACGCAAGAAAAG
CACTTCGAATTGTTGATTCATCGCCAGAGATGGCTTAGA---------ACCAATAGTTCATTAT
CT------AATGGATTTTTCCGTTCTAAT------ACTCTATCCGAGAGTTATCAGTATTTATCAAA
TCTGTTCCTATCTAAC---GGAACGCTATTGGATCAAATGACAAAGACAGTGTTGAGAAA
AAGATGGCTTTTCCCGGATGAAATGAAAATTGGATTC------ATG------------------------ATG
CCGAGATCTCGG---ATAAATGGAAATTTTATTGATAAGACCTTTTCAATTGTAGCCAATAT
CTTATTACGAATAATTCCGACAACTTCAGGAGAAAAAGAGGCATTTACCTATTACAGAG
ATGGG------ATGTCGGCTCAATCCGAAGGAAATTATGCGGAAGCTTTACAGAATTATTAT
GAAGCTATGCGACTAGAAATTGATCCCTATGATCGAAGTTATATACTCTATAACATAGGA
CTTATTCACACAAGTAACGGAGAACATACGAAAGCTTTGGAATATTATTTTCGAGCACT
AGAACGAAACCCGTTTTTACCGCAAGCTTTTAATAATATGGCCGTGATCTGTCATTAC---
CGTGGAGAACAGGCCATTCGGCAGGGAGATTCTGAAATTGCGGAGGCTTGGTTCGATC
AAGCCGCCGAATATTGGAAACAAGCTATAGCGCTTACTCCTGGAAATTATATTGAAGCA
CAGAATTGGTTGAAAATCACAAGACGTTTTGAA------ATGAGTTGTCGGTCAGAACGTA
TATGGATAGAATTTATAGCAGGGTCTCGAAAAACAAGTAATTTATGCTGGGCCTTTGTC
CTTTTTTTAGGTTCATTAGGGTTCTTATTGGTTGGAACTTCTAGTTATCTTGGCCAGAATT
TGATATCTTTATTCCCCTCTCAGCAAATAATTTTTTTTCCACAAGGGATCGTGATGTCTTT
CTATGGGATTGCAGGTCTCTTTATTGGCTCCTATTTGTGGTGCACAATTTCGTGGAATGT
GGGTAGTGGTTACGATCTATTCGATAAAAAGGAAGGAATAGTGTGTATTTTTCGCTGGG
GATTTCCTGGAAAAAATCGTCGTATCTTCCTCCGATTCTTTATGAAAGATATTCAATCCC
TCAGAATAGAAGTGAAAGAGGGTATTTATGCTCGTCGTGTCCTTTATATGGAAATCAGA
GGTCAGGGGGCTATTCCCTTGACTCGTACTGATGAGAAT---TTGACTCCACGAGAAATT
GAGCAAAAAGCGGGTGAATTGGCCTATTTCTTGCGTGTACCCATTGAAGTCTTT
>Gunnera
ATGGAAAAATGTTGGTTCAATTCCATGTTGTCTCTAAGTAAATCAATGGACAGTCTTGG
TCCTATTGAAAATACTAGTGGAAGTGAAGACCCGACTATAAATGATACAGATAAAAACA
TTCATAATTCCAGTAATGTTGATCTTTTATTCAGTGTCAGGGACATTAAAAATTTAATATC
TGATGACACTTTTTTAGTTAGGGATAGTAATGGGGACAGTTATTCCATATATTTTGATATG
GAAAATCATATTTTTGAGGTTGACAATGATCAT------TCTTTTCTGAGTGAACTAGAAAG
TTCTTCCTATAGTTATAGGAATTCTAGTTATCTGACTAATGGATCTCAGAGTGACGATCC

CTACTATGATCGTTATATGTATGATACTAAATATAGTTGGAATAATCACATTATTAGTTGCA
TTGACGTTTATCTTCGT------TCTCAAATACGTATTGATAGTTCGACTTTAAGTGGTAGTG
ACAATTACAGTTACATTTACATTTATAGTTATATTTGTGGGAGTGAGAGTTCAAGTATAA
GAACTAGCACGAATGGTAGTGATTTAATTATAAGAGAAAGTTCTAATGATCTTGATGTA
ACTCAAAAATACAGGCATTTGTGGATTCAATGCGAAAATTGTTATGGATTAAATTATAAG
AAATTTTTTAAGTCAAAAATGAATATTTGTGAACAATGTGGATATCATTTGAAAATGAGT
AGTTCAGAGAGAATCGAACTTTCGATTGATCCAGGTACTTGGGATCCTATGGATGAAGA
CATGGTCTCTCTGGATCCTATTGAATTTCATTCAGAAGAGGAACCTTATAAAGATCGTAT
CGATTCTTATCAAATAACGACAGGATTAACTGAGGCTGTTCAAACAGGCATAGGTCAAC
TAAACGGTATTCCCATAGCAATTGGGGTTATGGATTTTCAGTTTATGGGAGGTAGTATGG
GATCCGTAGTAGGCGAGAAAATAACCCGTTTGATCGAATATGCTACCAAAAGATTTCTA
CCTCTTATTATAGTATGTGCTTCTGGAGGAGCACGCATGCAAGAAGGAAGTTTGAGCTT
GATGCAAATGGCTAAAATATCTTCCGCTTTATATGATTATCAATTAAATAAAAAGTTATTC
TATATAGCAATCCTTACATCTCCTACTACTGGTGGGGTGACAGCTAGTTTTGGCATGTTG
GGGGATATCATTATTGCCGAACCCAATGCCTACATTGCATTTGCGGGTAAAAGAGTAATT
GAACAAACATTGAATAAGACAGTACCTGAAGGTTCACAAGCGGCTGAATATTTATTCCA
TAAGGGCTTATTCGATCCAATCGTACCACGTAATCCTTTAAAAGGCGTTCTGAGTGAGT
TATTTCAACTCCACGCTTTCTTTCCTTTGAATCAAAATTCAATCAAGATGGTAACCCTTC
AAGCCGACGAAATTAGTAATATTATCCGTGAACGTATTGAGCAATATACTATAGAAGTAA
AGATTGTAAATACCGGTACCGTACTTCAAGTAGGCGATGGCATTGCTCGTATTCATGGTC
TTGATGAAGTAATGGCAGGTGAATTAGTAGAATTTGAAGAGGGTACAATAGGCATTGCT
CTGAATTTGGAATCAAATAATGTTGGTGTTGTATTAATGGGTGACGGTTTGATGATACAA
GAGGGAAGTTCTGTAAAGGCAACAGGAAGAATTGCTCAGATACCAGTGAGTGAGGCC
TATTTGGGTCGTGTTATAAATGCCCTGGCTAAACCTATTGATGGTAGAGGTGAAATTTCA
GCTTCTGAATCTCGTTTAATTGAATCTCCGGCCCCAGGTATTATTTCGAGACGATCCGTA
TATGAGCCCCTTCAAACGGGTCTTATTGCTATTGATTCAATGATTCCTATAGGACGCGGT
CAGCGAGAATTAATTATTGGGGACAGACAGACTGGTAAAACAGCAGTAGCCACAGAC
ACGATTCTCAATCAGCAAGGACAAAATGTAATATGTGTTTATGTAGCTATTGGTCAAAA
AGCATCTTCTGTGGCTCAGGTAGTGACTACTTTCCAGGAAAGGGGAGCGATGGACTAC
ACTATTGTGGTAGCCGAAATGGCGGATTCCCCTGCTACATTACAATATCTTGCTCCTTAT
ACAGGAGCAGCTTTGGCTGAATATTTTATGTATCGTGAACGACATACTTCAATCATTTAT
GATGATCCCTCTAAACAAGCACAAGCTTATCGTCAAATGTCTCTTCTATTAAGAAGACC
GCCTGGTCGCGAAGCTTATCCGGGAGATGTTTTTTATTTGCATTCACGTCTTTTGGAAA
GAGCCGCTAAATCAAGTTCTCGTTTAGGTGAAGGAAGTATGACCGCTTTACCAATAGTT
GAGACTCAATCGGGAGATGTTTCGGCTTATATTCCTACTAATGTGATTTCCATTACAGAT
GGCCAAATATTCTTATCCGGGGATCTATTCAATGCTGGCATCAGACCTGCTATTAATGTG
GGTATTTCCGTCTCCAGAGTCGGATCTGCAGCTCAAATTAAAGCCATGAAAAAAGTAG
CTGGCAAATTAAAATTGGAACTGGCGCAATTCGCAGAATTAGAAGCTTTTGCACAATTC
GCTTCTGATCTCGATAAAGCTACTCAGAATCAATTGGCAAGAGGTCAACGATTACGTGA
ATTGCTCAAACAATCCCAATCATCCCCTCTCACGGTGGAAGAACAGATAATGACTATTT
ATACTGGAACAAATGGCTATCTTGATTCATTAGAAATTGGGCAGGTAAGTAAATTTCTC
GTTGAGTTACGTACTTACTTAAAAACCAATAAACCTCAGTTCCAAGAAATCATATCTTCT
ACCAAGATATTAACCGAAGAAGCAGAAGACCTTTTGAAAGAAGCTATTCAGGAACAGA
TGGAACGCTTTCTACTTCAG---------ATGAGAATTAATCCTACTACTTCTGGTCCTGGGGT

TTCCACACTTGAAAAAAAAAAACCTGGGGCGTATCGCTCAAATCATTGGTCCGGTACTG
GATGTAGCTTTTCCCCCGGGCAAGATGCCTAATATTTACAACGCTCTCGTAGTTAAGGGT
AGAGATACTGTTGGTCAACAAATTAATGTGACTTGTGAGGTACAGCAATTATTAGGAAA
TAATAGAGTTAGGGCTGTAGCTATGAGTGCTACAGACGGTCTAACGAGAGGAATGGAA
GTGATTGACACGGGAGCTCCTCTAAGTGTTCCAGTCGGTGGAGCGACTCTCGGACGAA
TTTTCAACGTACTTGGAGAACCTGTTGATAATTTAGGTCCTGTAGAGACTCGCACAACC
TCTCCTATTCATAGATCTGCGCCCGCCTTTATACAGTTAGATACAAAATTATCTATTTTG
AAACAGGAATTAAAGTAGTAGATCTTTTAGCCCCTTATCGCCGTGGAGGAAAAATCGG
GCTATTCGGGGGAGCTGGAGTGGGTAAAACAGTACTCATTATGGAACTAATCAACAAC
ATTGCCAAAGCTCATGGGGGTGTATCCGTATTTGGCGGAGTAGGTGAACGTACTCGTGA
AGGAAATGATCTTTACATGGAAATGAAAGAATCCGGAGTGATTAATGAACAAAATATTG
CAGAATCAAAAGTAGCTCTAGTCTACGGTCAGATGAATGAACCGCCGGGAGCTCGTAT
GAGAGTTGGTTTGACTGCCCTTACTATGGCGGAATATTTCCGAGATGTTAATGAACAAG
ACGTACTTCTATTTATCGACAATATCTTCCGTTTCGTCCAAGCAGGATCCGAAGTATCCG
CCTTATTGGGCAGAATGCCTTCCGCTGTGGGTTATCAACCCACCCTTAGTACCGAAATG
GGTTCTTTACAAGAAAGAATTACTTCTACCAAAGAAGGGTCCATAACTTCTATTCAAGC
AGTTTATGTACCTGCGGACGATTTGACCGACCCCGCTCCTGCCACAACATTTGCACATT
TAGATGCTACTACTGTATTATCAAGAGGATTAGCTGCCAAAGGTATCTATCCAGCAGTAG
ATCCCTTAGATTCAACGTCAACTATGCTCCAACCTCGGATCGTTGGTGAGGAACATTAT
GAAATCGCGCAAAGAGTTAAGCAAACCTTACAACGTTACAAAGAACTTCAGGACATTA
TAGCTATCCTTGGGTTGGACGAATTATCCGAAGAGGATCGTTTAACCGTAGCAAGAGCA
CGAAAAATTGAGCGTTTCTTATCACAACCCTTTTTCGTAGCAGAAGTATTTACGGGTTC
TCCAGGGAAATATGTTGGTCTAACAGAAACAATTAGAGGGTTTCAATTGATCCTTTCCG
GAGAATTAGATAGTCTTCCTGAACAAGCCTTTTATTTGGTAGGTAATATCGATGAAGCTA
CCGCGAAGGCTATAAACTTAGAATTAGAAATGGAGGAGAGCAAAATGACCTTAAATCT
TTGTGTACTGACTCCTAATAAAATTGTTTGGGATTCAGAAGTGAAAGAAATTATTTTATC
TACTAATAGTGGTCAAATTGGCGTATTACCAAACCACGCCCCTATTGCCACAGCTGTAG
ATATAGGTATTTTAAGAATACGTCTTAATGACCAATGGTTAACGATGGCTCTGATGGGTG
GTTTTGCTAGAATAGGCAATAATGAAATCACTGTTTTAGTAAATGATGCGGAGAAGGGT
AGTGACATTGATCCACAAGAAGCTCAGCAAACTCTTGAAATAGCGGAAGCTAACTTGA
GGAAAGCCGAAGGCAAGAGACAAACAATTGAGGCGAATCTAGCTCTCAGACGAGCTA
GGACACGAGTAGAGGCTCTCAATGTAATTTCGATGAAAAATGTAACCGATTCTTTCGTT
TCTTTGGGTCACTGGCCATCCGCCGGGAGTTTCGGGTTTAATACCGATATTTTAGCAAC
AAATCCAATAAATCTAAGTGTAGTGCTTGGTGTTTTGATCTTTTTTGGAAAGGGAGTGT
TAAGTGATTTATTAGATAATCGAAAACAGAGGATCTTGAATACTATTCGAAATTCAGAA
GAACTACGTGGGGGAGCCATTGAACAGCTGGAAAAAGCCCAGGCCCGTTTACGTAAA
GTGGAAATAGAAGCAGACCAGTTTCGAGTGAATGGATACTCTGAGATAGAACGAGAA
AAGTTGAATTTGATTAATTCAACTTATAAGACTTTGGAACAATTAGAAAATTACAAAAA
CGAAACCATTCATTTTGAACAACAAAGAGCGATTAATCAAGTCCGACAACGGGTTTTC
CAACAAGCCTTACAAGGCGCTCTAGGAACTCTAAATAGTCGTTTGAACAACGAGTTAC
ATTTACGTACCATCAGTGCTAATATTGGCGTGTTTGGGGCGATGAAAGAGATAACTGATA
TGAATCCACTGATTTCTGCCGCTTCCGTTATTGCTGCTGGATTGGCCGTAGGGCTTGCTT
CTATTGGACCTGGAGTTGGCCAAGGTACTGCTGCGGGCCAAGCTGTAGAAGGTATCGC
GAGACAACCCGAGGCGGAGGGAAAAATACGAGGTACTTTATTGCTTAGTCTGGCTTTT

ATGGAAGCTTTAACAATTTATGGATTGGTTGTAGCATTAGCGCTTTTATTTGCAAATCCC
TTTGTTATGAATGTTCTATCATGTTCCATCAACACACTAAATGTGTTATACGATATATCCG
GTGTAGAAGTAGGCCAACATTTCTATTGGCAAATAGGAGGTTTTCAAGTCCATGCCCAA
GTACTTATTACTTCTTGGGTTGTAATTGCTATCTTATTAGGTTCAGCTACTATAGCTGTTC
GAAATCCTCAAACTATTCCAACTGACGGTCAGAATTTCTTCGAATATGTTCTTGAATTCA
TTCGAGACGTGAGCAAAACTCAGATTGGAGAAGAATATGGTCCTTGGGTTCCCTTTATT
GGAACTATGTTTCTATTTATTTTTGTTTCTAATTGGTCAGGGGCTCTTTTACCTTGGAAAA
TCATACAGTTACCTCATGGGGAGTTAGCCGCACCCACTAATGATATAAATACTACTGTTG
CTTTAGCTTTACTCACGTCAGTAGCATATTTCTATGCGGGTCTTACAAAAAAAGGATTAG
GTTATTTCGGTAAATACATTCAACCAACTCCAATCCTTTTACCCATTAACATCTTAGAAG
ATTTTACAAAGCCTTTATCACTTAGTTTTCGACTTTTCGGGAATATATTAGCTGATGAATT
AGTAGTTATTGTTCTTGTTTCTTTAGTACCTTCAGTGGTTCCTATACCTGTCATGTTCCTT
GGATTATTTACAAGTGGTATTCAAGCTCTTATTTTTGCAACTTTAGCCGCGGCTTATATAG
GCGAATCCATGGAGGGTCATCATATGATATTTTTGACTTTAGAGCATATATTAACTCATAT
ATCCTTTTCGATCGTTTCAATTGTAATTACTATTTATTTAATAACCTTATTAGTCAATGGAA
TCGTAGAACTATATGATTCATCAGAAAAAGGCATGATAGCTACTTTTTTTGTATAACAG
GGTTATTAGTAACCCGTTGGATTTTTTCGGGACATTTCCCATTAAGTGATTTATATGAATC
ATTAATCTTCCTTTCCTGGAGTTTCTCCATTATTAATATGGTTCCGTAT------AAATATCAA
AAAACTTTAAGCACAATAACAGCACCAAGTGCTATTTTTACTCAAGGCTTTGCTACTTC
AGGTCTTTTAACTGAAATGCATCAATCCACAATATTAGTACCTGCTCTACAATCCCAATG
GTTAATGATGCACGTAAGTATGATGGTATTGAGCTATGCAGCTCTTTTATGTGGATCATTA
TTATCAGTAGCTCTTCTAGTCATTACATTTCGAAAAGACATAATGATTTTTTGT------AAA
GAGACCCAATACATGAATGAAGGAAACAATATTTTACGAAACACTTCT---------GCTAGA
AATTATTACAGGTCCCAATTAATTCAACAATTGGATCATTGGAGTTATCGTGTTATTAGTA
TAGGGTTTATCTTTTTAACCATAGGTATTCTTTCAGGAGCAGTATGGGCTAATGAAGCGT
GGGGATCATATTGGAATTGGGACCCAAAGGAAACTTGGGCATTTATTACTTGGACCATC
TTTGCGATTTATTTACATACTCGAACAAATAAAAAGTTTCAAGGTCCAAATTCCGCAATT
GTGGCTTCTATGGGCTTTCTTATAATTTGGATATGCTATTTTGGGGTCAATCTGTTAGGAA
TAGGGCTACATAGTTATGGTTCATTTATATTAACAATGGCAAAAAAGAAAGCATTCACTC
CCCTTCTATATCTTGCATCTATAGTATTTTTGCCCTGGTGGATTTCTCTCTCATTTAGTAAA
AGTCTGGAATCTTGGGTTACTAATTGGTGGAATACGAGTAAATCCGAATTTTTTTTGAAT
GATATTCAAGAAAAGAGCATTCTAGAAAAATTTATAGAAATAGAGGAACTCTTCCTCTT
GGACGAAATGATAAAGGAATACCCGGAGACACATCTACAAAAGCTTCATATA---ATACA
TAAAGAAACGATCCAATTGATCAAAATGCACAATGAGGATCGTATCCATACGATTTTGC
ATTTCTCGACCAATATAATCTGTTTCGTTATTCTAAGCGGTTATTCTATTCTGGTTAATGA
CGAACTTGTTATTCTTAACTCTTGGGTTCAGGAATTCCTATATAACTTAAGTGACACAAT
AAAAGCTTTTTCTATTCTTTTCTTAACTGATTTTTGTATTGGATTCCATTCACCCCCTGGT
TGGGAACTAATAATTGGCTCTGTCTCCAAAGATTTTGGATTTTATCATAATGATCAAATC
ATATCTGGTCTTGTTTGCACTTTTCCAGTCATTCTAGATACAATTTTTAAATATTTGATCT
TCGATTATTTAAGTCGTGTATCTCCATCACTTGTAGTGATTTATCATTCAATGAATGACAT
GCCTATTGGTGTTCCACGAGTACCCCATCGAATTCCTGGAGAGGAAGATGCATATTGGG
TTGACATATACAACCGACTTTATAGAAACAGAATAATGTTTTTAGGCCAAGAGGTTAGT
AGCGAGATCTCGAATCAACTTATTTCTCTTTTGGTATATCTCACTATAGAAGAAGATCGC
AAAGATGTTTTTCTGTTTATAAACTCTCCTGGCGGGTGGGTAGTACCCGGAGTAGCTCT

TTATGATACTATGCGATTTGTGCAACCAGAAATACATACAATATGCATGGGAATGGCCGC
TTCAATGGGATCTTTTCTCCTGGCCGGAGGAGAAATTACCAAACGTATAGCATTCCCTC
ACGCCAGGGTAATGATCCATCAACCTGCTAGTTCTTTTTATGAGACACGAACGGGAGAA
TTTATCCTGGAATCGGAAGAACTGCTGAAACTGCGCGAAATCATCACAAAGGTTTATGT
ACAAAGAACGGGCAAACCCTTCTGGGTTATATCTGAAGACATGGAAAGGGATGTTTTT
ATGTCAGCAACCGAAGCCCAAGCTTATGGACTTATTGATATTATACTGATTCAAATGAAA
GAACAAAAAATGGATTCATGAAGGTTTAATTACTGAATCACTTCCCAATGGTATGTTCCG
GGTTCGTTTAGATAATGAAGATCTGATTCTAGGTTATGTTTCAGGAAGGATCCGACGTA
GTTTTATACGGATACTACCAGGAGATAGAGTCAAAATTGAAGTAAGTCGTTATGATTCA
ACCAGAGGGCGTATAATTTATAGACTACGTAACAAAGATTCGGGTGATATGGAGGAATT
CAAAATATATTTAGAA---GATAGATTTCGGCAACATTACTTCCTATATCCACTTATTTTTCA
AGAGTATATTTATGCACTTGCTCATGATCATGGTTTATTTGGTTATGACAATAAATACAGT
TCACTAATTGTAAAACGTTTAATTACTCGAATGTATCACCAGAAGTATTTGATTAGTTCG
GCTAATGATTCTAACCAAAATCCATTTTTTGGGAATAATTTGTATTCTCAACTGATATCAG
AGGGATTAGCGGTTATTGTGGAAATTCCATTTTCGCTAAGATTAATATCTTATCGAATAG
CCAAATATCATAATTTACGATCAATTCATTCAATATTTCCTTTTTTAGAGGACAAATTCTC
ACATTTAAATTATGTGTCAGATATACTAATACCCCACCCCATCCATCTGGAAATCTTGGTT
CAAACCCTTCGCTACTGGGTGAAAGATGCTCCTTCTTTGCATTTAGTACGATTTTTTCTA
CACGAGTTTCGTAATTGGAATAGTCTTATTACTAATAAATCCATTTACTTTTTTTCACAAA
GATTATTCTTATTACTATATAATTCTCATGTATGTGAATACGAATACATGTTCATTTTTCTC
CGTAATCAATCTTATCATTTACGATCAACATCTTCTGAGGTCCTTCTTGAGCGAATATATT
TTTATGGAAAAATAGAACAGCTTGTAGGAGTCTTTATTAATGATTTTATCTTATGTATGTT
CAAGGATCCCTTCATGCATTATGTTAGGTATCAAGGAAAATCCATTTTGGCTTCAAAGG
GAACGATCCTTATGATGAATAAATGGAAATATTACTTTATAAATTTCTGGCAATGTCATTT
TTACGTGTGGTCTCAACCGGGAAGGATCTATATAAACCAATTATACAATCATCCCCTAGA
CTTGCTGGGCTATCTTTCAAGTGTGCGACTAAGTCCTTCAGTGTTACGGAGTCAAATGT
TAGAAAATTCATTTATAATAGATACTGCTATT------AAGAAGTTTGATACTATAATTCCAAT
TATTACTTTAATTGGATCATTGGCTAAAGCTAAATTTTGTAATGTATTAGGGCATCCCATT
AGTAAGCCAGCTTGGGCCGATTCATCAGATTCTGATATTATCGACCGATTTGGGCGGATA
TGCAGAAATCTTTCTCATTATCACAGTGGATCCTCAAAAAAAAATAATTTGTATCGAATA
AAGTATATACTTCGCCTTTCTTGTGCTAAAACTTTAGCTCGTAAACATAAAAGTACTGTA
CGTGCTTTTTTAAAAAGATTAGGTTCGGAATTATTGGAAGAATTCTTTACGGAGGAAGA
ACACGTTCTTTCTTTGATCTTACCAAGACGTATTTGGTATTTGGATATTATTTGTATCAAT
GATCTGGTCATTCATGAAATGATAATTGATACAACA---GAAGTACAA---GCTATCAATTCT
TTTTCCAGATTGGAAAAC---TTAATAGAGCTTTATGGAATCATATGGATGCTTGTCCCTAT
TTTTACTCCTGTATTGGGAATCACAATAGGTGTACTAGTAATTGTGTGGTTAGAAAGAG
AAATATCCGCAGGGATACAACAACGTATTGGACCTGAATACGCCGGTCCTTTGGGAATT
CTTCAAGCTATAGCAGATGGAACAAAACTACTTTTCAAAGAGGATCTTCTTCCATCTAG
AGGAGATACTCGCTTATTCAGTATTGGACCATCTATAGCAGTTATATCAATTCTACTAAGT
TATTCAGTAATTCCTTTTAGCTATCACCTTGTTCTAGCCGATCTCAGTATTGGTGTTTTTT
TATGGATAGCCATTTCCAGTATTGCTCCCATTGGACTTCTTATATCAGGATATGGATCGAA
TAATAAATATTCCTTTTTAGGTGGTCTACGAGCTGCTGCTCAATCCATTAGTTATGAAATA
CCATTAACTCTATGTGTGTTATCAATATCTCTACTATCTAACAGTTCAAGTACAGTTGATA
TAGTTGAGGCGCAGTCAAAATATGGGTTTTGGGGATGGAATTTGTGGCGTCAACCTATA

GGATTTATCGTTTTTATAATTTCTTCACTAGCAGAATGTGAGAGATTACCTTTTGATTTAC
CAGAAGCAGAGGAAGAATTAGTAGCAGGTTATCAAACCGAATATTCAGGTATAAAATTT
GGTTTGTTTTACGTTGCTTCCTATCTAAATCTATTAGTTTCTTCATTATTTGTAACAGTTCT
TTATTTGGGCGGTTGGAATCTCTCTATTCCATACATATGGATTTCTGAGTTTTTTGAAATA
AATAAAGTGGGTGGAGTCTTTATAACGACAATTGGTATCTTTATTACATTAGCTAAAACT
TATTTGTTCTTGTTCATTTCTATCACAACAAGATGGACTTTACCTAGGCTGAGAATGGAT
CAACTATTAAATCTTGGGTGGAAATTTCTTTTACCGATTTCACTAGGTAATCTATTATTAA
CAACTTCTTTCCAACTTCTTTCACTGATGATCTGGCATGTACAGAATGAAAACTTCATTC
TCGATTCTACGAGAATTTTTATGAAAGCGTTTCATTTGCTTCTCTTCGATGGAAGTTTTA
TTTTCCCAGAATGTATCCTAATTTTTGGCCTAATTCTTCTTCTGATGATCGATTCAACCTC
TGATCAAAAAGATATACCTTGGTTATATTTCATCTCTTCAACAAGTTTAGTAATGAGCAT
AACTGCCCTATTGTTCCGATGGAGAGAAGAACCTATGATTAGCTTTTCGGGAAATTTCC
AAACGAACAATTTCAACGAAATCTTTCAATTTCTTATTTTACTATGTTCAACTCTATGTAT
TCCTCTATCCGTAGAGTACATTGAATGTACAGAAATGGCTATAACAGAGTTTCTGTTATT
CGTATTAACAGCTACTCTAGGAGGAATGTTTTTATGCGGTGCTAACGATTTAATAACTAT
CTTTGTAGCTCCAGAATGTTTCAGTTTATGCTCCTACCTATTATCTGGATATACCAAGAA
AGATGTACGGTCTAATGAGGCTACTACGAAATATTTACTCATGGGTGGGGCAAGCTCTT
CTATTCTGGTTCATGGTTTCTCTTGGCTATATGGTTCATCCGGGGGAGAGATCGAGCTTC
AAGAAATAGTGAATGGTCTTATCAATACACAAATGTATAACTCCCCAGGAATTTCAATT
GCGCTTATATTCATCACTGTAGGAATTGGGTTCAAGCTTTCCCCAGCCCCTTCTCATCAA
TGGACTCCTGACGTATACGAAGGATCTCCCACTCCAGTCGTTGCTTTTCTTTCTGTTACT
TCGAAAGTAGCTGCTTCAGCTTCAGCCACTCGAATTTTCGATATTCCTTTTTATTTCTCA
TCAAACGAATGGCATCTTCTTCTGGAAATCCTAGCTATTCTTAGCATGATATTGGGGAAT
CTAATTGCTATTACTCAAACAAGCATGAAACGTATGCTTGCATATTCGTCCATAGGTCAA
ATCGGATATGTAATTATTGGAATAATTGTTGGAGACTCAAATGATGGATATGCAAGCATG
ATAACTTATATGCTGTTCTATATCTCCATGAATCTAGGAACTTTTGCTTGCATTGTATCATT
TGGTCTACGTACCGGAACTGATAACATTCGAGATTATGCAGGATTATACACGAAAGATC
CTTTTTTGGCTCTCTCTTTAGCCCTATGTCTCTTATCCCTAGGAGGTCTTCCTCCACTAGC
AGGTTTTTTCGGAAAACTCCATTTATTCTGGTGTGGATGGCAGGCAGGCCTATATTTCTT
GGTTTCAATAGGACTCCTTACGAGCGTTGTTTCTATCTACTATTATCTAAAAATAATCAA
GTTATTAATGACTGGACGAAACCAAGAAATAACCCCTCACGTGCGAAATTATAGAAGAT
CCCCTTTAAGATCAAACAATTCCATCGAATTGAGTATGATTGTATGTGTGATAGCATCTA
CTATACCAGGAATATCAATGAACCCGATTATTGCAATTGCTCAGGATACCCTTTTTATGTT
TCTGCTTTATGAATATGATATTTTCTGGGCATTTCTAATAATATCAAGTATTATTCCTATTT
TGGCATTTCTAATTTCCGGAGTTTTAGCTCCGATTAGCAAAGGGCCAGAAAAACTTTCT
AGTTATGAATCAGGTATAGAACCAATGGGTGATGCTTGGTTACAATTTCGAATCCGTTAT
TATATGTTTGCTCTAGTTTTTGTTGTTTTTGATGTTGAAACGGTTTTTCTTTATCCATGGG
CAATGAGTTTAGATGTATTGGGTGTATCAGTATTTATAGAAGCCTTCATTTTTGTGCTTAT
CCTAATTGTTGGTTCAGTTTATGCATGGCGAAAAGGAGCGTTGGAATGGTCTACGAATC
ATTTTCCTTGGTTAACAATAATTGTAGTTTTTCCCATATCTGCGGGTTCCTTAATTTTATTT
CTTCCTCAGAGAGGAAATAAAGTAATAAGATGGTATACTTTATGTATTTGTATACTAGAA
CTACTTCTAACTACCTATGCATTCTGTTATCATTTCCAATTGGACGATCCATTAATGCAAC
TGTCAGAGGACTATAAATGGATAAATTTTTTTGATTTTCACTGGAGATTAGGAATCGATG
GACTTTCTATAGGACCCATTTTACTGACGGGATTCATCACTACTTTAGCTACTTTGTCGG

CTTGGCCAGTTACTCGAGATCCCCGATTATTCTATTTCCTGATGTTAGCAATGTACAGCG
GTCAAATAGGATCATTTTCTTCTCAAGACCTTTTACTTTTTTTCATCATGTGGGAGTTAG
AATTAATTCCCGTTTATCTACTTCTATCCATGTGGGGAGGAAAGAAACGCCTATACTCAG
CTACAAAGTTTATTTTGTACACTGCAGGAGGTTCCATTTTTCTATTAATGGGAGTTCTGG
GGATTGGTTTATATGGTTCCAATAAACCAACATTAAATTTTGAAACATCAGCTAATCAAT
CGTATCCTGTGGCATTGGAAATAATATTCTATATTGGATTTCTTATTGCTTTTGCTGTAAA
ATTACCTATTATACCCCTACATACATGGTTACCGGACACACATGGAGAAGCACATTACAG
TACTTGTATGCTTCTAGCCGGAATCTTATTAAAAATGGGGGCATATGGATTGGTTCGGAT
CAATATGGAATTATTACCCCATGCTCATTCTATATTTTCTCCCTGGTTGATGATAGTAGGC
ACAGTGCAAATAATCTATGCAGCTTCAACATCTCCGGGTCAACGTAATTTAAAAAAAAG
AATAGCCTATTCCTCTGTATCTCATATGGGTTTTCTAATTATAGGAATTGGATCTCTAACC
GACACGGGACTCAATGGAGCCATTTTACAAATAATCTCTCATGGATTTATTGGTGCTGC
ACTTTTTTTCTTATCAGGAACGAGTTATGATAGAATACGTCTTGTTTATCTTGACGAAAT
GGGCGGAATAGCTATCGAAATGCCAAAAATATTCACGATGTTCAGTAGCTTCTCGATGG
CTTCCCTTGCATTACCGGGCATGAGTGGTTTTGTTGCAGAATTGATAGTATTTTTTGGAA
TAATTACCAGCCAAAAATATCTTTTAATTCCAAAAATACTAATTACTTTTGTAATGGCAAT
TGGAATGATATTAACTCCTATTTATTCATTATCTATGTCACGACAGATGTTCTATGGATAC
AAGCTATTTAATGCTCCAAATTCTTATTTTTTTGATTTTGGACCACGAGAGTTATTTGTTT
CGATCTTTATCTTTCTACCCGTAATAGGTATTGGTATTTACCCGGATTTCGTTCTCTCACT
ATCGGTTGACAAGGTTGAAGCTATTCTATCTAATTATTTTTATAGAATGATGCTCGAACA
TGTACTTGTTTTGAGTGCCTATTTATTTTCTATCGGTATTTATGGATTGATCACAAGCCGA
AATATGGTTAGAGCTCTTATGTGTCTTGAACTTATACTGAATGCAGTTAATATAAATTTGG
TAACATTTTCTGATTTTTTTGATAGTCGCCAATTAAAAGGAGATATTTTTTCCATTTTTGT
TATAGCTATTGCAGCCGCTGAAGCAGCTATTGGACCAGCTATTGTTTCGTCAATTTATCG
TAACAGAAAATCAATTCGTATCAATCAATCGAATTTGTTGAATAAAATGGAACATACATA
TCAATATGCATGGATCATACCCTTCATTCCACTCCCAGTTCCTATGTTAATAGGAGTGGG
GCTTCTACTTTTTCCGACGTCAACAAAAAATCTTCGACGTATGTGGGCTTTTACTAGTG
TTTTATTGTTAAGTATAGTTTTGATTTTTTCGATCGATCTGTCTATTCAGCAAATAAATAG
TAGTTCCATCAATCAATATGTATGGTCTTGGACTATTAATAATGATTTTTCTTTAGAGTTC
GGGCATTTCATCGATCCACTTACTTCTATTATGTTACTATTAATCACTACTGTTGGAATTA
TGGTTCTTATTTATAGTGATAATTATATGTCTCATGATCAAGGATATTTGAGATTTTTTGCT
TATATGAGTTTTTCCAATACTTCCATGTTGGGATTAGTTACTAGTTCTAATTTGATACAAA
TTTATATTTTTTGGGAACTAGTTGGAATGTGTTCCTACCTATTAATAGGTTTTTGGTTTAC
ACGACCTATTGCAGCAAATGCTTGTCAAAAAGCGTTTGTAACTAATCGTGTAGGAGATT
TTGGTTTATTATTAGGAATTTTAGGTATTTATTGTATAACGGGTAGTTTTGAATTTCGAGA
TTTTTTCAAAATTTTTAATAACAATGAGGTCAATTTTTTATTTATTACTTTTTGTGCCTCC
CTATTATTTGCCGGTGCAGTTGCTAAATCCGCACAATTCCCCCTTCATGTCTGGTTACCT
GATGCCATGGAGGGACCTACTCCTATTTCGGCTCTTATACATGCTGCTACTATGGTAGCA
GCGGGAATTTTTCTTGTAGCTCGGCTTCTTCCTCTTTTCATACTTATACCTTACATAATGA
ATCTAATATCTTTAATAGGTATAATAACACTACTATTAGGAGCTACTTTAGCTCTTGCTCA
AAAAGACATTAAAAGGGGTTTAGCCTATTCGACAATGTCTCAATTGGGTTATACGATGT
TAGCTCTAGGTATGGGGTCTTATCAAGCTGCTTTATTTCATTTGATTACTCATGCTTATTC
GAAAGCATTATTGTTTTTAGGATCCGGGTCAATTATTCATTCAATGGAAGCTATTGTTGG
ATATTCTCCAGATAAAAGTCAGAATATGGTTCTTATGGGTGGTTTAACAAAACATGTGCC

AATTACAAAAATTGCTTTTTTATTAGGTACACTTTCTCTTTGTGGTATTCCACCTCTTGCT
TGTTTTTGGTCAAAAGATGAAATTCTTAATGATAGTTGGTTGTATTCACCTAGTTTTGCA
CTAATAGCTTATTCCACAGCAGGATTAACTGCATTTTATATGTTTCGGATATATTTACTTA
CTTTTGAAGGACATTTTAATGTTAATTTAAAAAATTACAGTGGAAAAAAAAATAACTCG
TTATATTCAATTTCTATATGGGGTAAAGAAGGGTCTAAAACAATTCAAAAAAAATTATTA
ACTTTATTAACACGGAATTTTTTTTCGTTTGGCAATAGGGGCACTTACTTGTATCCACAT
GAATCGGACAATACAATGCTATTTCCTCTGCTTTTATTGGTCTTATTTACTTTGTTCATTG
GATTCATAGGAATTCCTTTCTATAATAATATATTATTGAAATGGTTAACTCCGTCTATAAG
CCTTTTACATAAAAATTTGGAG---TCCTCCGATTGGTATGAATTTGTGACCAATTCAATTT
TTTCGGTAAGTATAGCTTATTTAGGAATATTTATAGCATCCCTTTTATATAGGCCTGTTTAT
TCATCTTTAAAAAATTTGAACTTAATAAATTCATTTGCTATTTTTTGGGACAAAATAATA
AATGGGGTATATAATTGGTCATATAATCGTGGTTATATAGATGCTTTTTATACAACATATTT
AATAGGGGGTATAAGAGGATTAGCTGAACTAACAAATTTTTTTGATAGAAGAATAATTG
ATGGAATTACTAATGGAGTTGGTATTACAAGTTTCTTTGTAGGAGAGGGGATCAAATATA
TAGGGGGTGGA---CGTATCTCTTCTTATCTCTTCTTGTATTTATCTTATGTATCAATATTTTT
ATTAATT---TACATGGATTTACCCGGACCAATACATGATTTTCTTTTAGTCTTTCTGGGAT
CAGGTCTTATATTAGGAGGTCTAGGAGTGGTATTATTTACCAATCCAATTTATTCTGCCTT
TTCATTGGGGTTGGTTCTTGTTTGTATATCCTTATTATATATTCCATCGAACTCACATTTTG
TAGCTGCTGCACAACTTCTTATTTACGTAGGAGCTATAAATGTTTTAATCATATTTGCTGT
GATGTTCATGAATGGTTCAGAATATTACAAAGATTTTCATTTTTGGACCGTTGGAGATGG
GGTTACTTCACTGGTTTGTACAAGTATTTTTGTTTCACTAATTACTACTATTTCAGATACA
TCGTGGTACGGAATTATTTGGACGACAAGATCAAACCAGATTATAGAACAAGATTTAAT
AAGTAATATTCAACAAATTGGGATTCATTTATTAACAGATTTTTTTCTTCCATTTGAACTC
ATTTCAATAATTCTTTTAGTTGCTTTGATAGGTGCAATTTCTGTGGCTCGTCAGATGACT
GTACCAGCTACAAGAAAAGACCTCATGATAATCAATATGGGCCCTCAACACCCATCAAT
GCACGGTGTTCTTCGACTCATCGTTACTCTAGATGGTGAAGATGTTATTGACTGCGAAC
CAATATTGGGTTATTTACACAGAGGTATGGAAAAAATTGCGGAAAACCGAACAATTATA
CAATATCTGCCTTATGTAACACGTTGGGATTATTTAGCTACTATGTTCACAGAAGCAATA
ACTGTAAATGCACCAGAGCAGTTGGGAAATATTCAAGTACCTAAAAGAGCCAGCTATAT
CAGAGTAATTATGTTGGAGTTGAGTCGTATAGCCTCTCATTTGTTATGGCTTGGCCCTTT
TATGGCAGATATTGGTGCACAGACTCCTTTCTTCTATATTTTCAGAGAAAGAGAATTGAT
ATATGATCTATTCGAAGCTGCCACCGGTATGAGAATGATGCATAATTATTTCCGTATCGG
AGGAGTAGCTGCTGATCTACCTCATGGCTGGATAGATAAATGTTTGGATTTCTGTGATTA
TTTTTTAACAGGGGTTGCTGAATATCAAAAGCTTATTACGCGGAATCCCATTTTTTTAGA
ACGAGTTGAAGGAGTAGGCATTATTGGTGGAGAGGAAGCAATAAATTGGGGTTTATCA
GGACCAATGTTACGAGCTTCTGGAATCCAATGGGATCTTCGTAAAGTTGATCATTATGA
GTCTTATGACGAATTTGACTGGGAAGTACAATGGCAAAAAGAAGGAGATTCATTAGCT
CGTTATTTGGTACGAATTGGTGAAATGAAAGAATCCATAAAAATTATTCAACAGGCTCT
GGAAGGAATTCCGGGAGGGCCCTATGAGAATTTAGAAATCCGGCGCTTTGATAGAGCA
AGGGATTCAGAATGGAATGAGTTTGAATATCGATTCATTAGTAAAAAGCCTTCTCCTAC
TTTTGAATTGTCGAAACAAGAACTTTATGTGAGAGTCGAAGCCCCAAAAGGTGAATTG
GGAATTTTTATGATAGGGGATCAAAGTGTTTTTCCTTGGAGATGGAAAATTCGCCCACC
AGGTTTTATAAATTTGCAAATTCTCCCCCAGTTAGTTAAAAGAATGAAATTGGCCGATAT
TATGACAATACTAGGTAGCATAGATATCATTATGGGAGAAGTTGATCGTATGTTCCCTAT

GGTAACTGGGTTCATGAATTATGGTCAACAAACAGTACGAGCTGCAAGGTACATTGGT
CAAAGTTTCATGATTACCCTATCCCACGCGAATCGTTTACCTGTAACTATTCAATATCCTT
ATGAAAAATTGATTACATCGGAACGTTTCCGTGGTCGAATCCACTTTGAATTTGATAAA
TGCATTGCTTGTGAAGTGTGTGTTCGTGTATGTCCTATCGATCTACCCGTTGTTGATTGG
AAATTGGAAACTAATATTCGAAAGAAACAATTGCTTAATTACAGTATTGATTTTGGAATC
TGTATATTTTGTGGTAATTGCGTTGAGTATTGTCCAACAAATTGTTTATCAATGACTGAA
GAATATGAACTTTCTACTTATGATCGTCACGAATTGAATTATAATCAAATTGCTTTAGGTC
GTTTACCAATATCAGTAATTGACGATTACACAATTCGAACAATTTTAAATTCG---ATCAA
AAATGGA----------------------------------ATGCAGGGTCGTTTGTCTGCTTGGCTAGTCAAGCA
TGGGCTAGTTCATAGATCTTTGGGCTTCGATTACCAAGGAATAGAGACTTTACAAATAA
AGCCCGAGGATTGGCATTCCGTTGCTGTAATTTTATATGTATATGGTTACAATTATCTACG
TTCCCAATGTGCTTATGATGTAGCACCAGGCGGACTGTTAGCTAGTGTGTATCATATTAC
TAGAATACAGTATGGTGCGGATCAACCAGAAGAGGTATGTATAAAAGTATTTGCCCCAA
GGAGGAATCCTAGAATTCCGTCTGTTTTCTGGATTTGGAAAAGTGCGGATTTTCAAGAA
CGTGAATCCTATGATATGTTGGGAATCTCTTATGATAATCATCCACGCCTGAAACGTATC
TTAATGCCTGAAAGTTGGATAGGATGGCCCTTACGTAAGGATTATATTGCCCCGAATTTT
TATGAAATACAAGATGCTCATATGGGCAATGAGTTTAGATGTATTGGGTGTATCAGTATT
TATAGAAGCCTTCATTTTTGTGCTTATCCTAATTGTTGGTTCAGT------TTATGCATGGCGA
AAAGGAGCGTTGGAATGGTCTTAGCTCCTGAATATTCAGACAATAAAAAAGTAAAAAA
AAATAACATTGAGACAGTTATGAATTCCATTGAGTTTCCGTTACTTGATCGAACAACCC
AAAATTCCGTTATTTCAACTACATCAAATGATCTTTCAAATTGGTCAAGACTCTCCAGTT
TATGGCCGCTTCTCTATGGTACTAGTTGTTGCTTCATTGAATTTGCTTCATTAATAGGCTC
ACGGTTCGACTTTGATCGTTATGGGCTGGTACCAAGATCTAGTCCTAGGCAAGCGGACC
TAATTTTAACAGCTGGCACAGTAACAATGAAAATGGCTCCTTCTTTAGTGAGATTATATG
AGCAAATGCCTGAACCAAAATATGTTATTGCTATGGGAGCATGTACGATTACAGGTGGG
ATGTTCAGTACCGATTCTTATAGTACTGTTCGGGGGGTAGATAAGCTAATTCCAGTGGAT
GTATATTTGCCAGGCTGTCCACCTAAACCGGAAGCAGTTATAGATGCTATAACAAAACT
TCGTAAAAAAATATCTCGAGAAATATATGAAGATAGAATTAGGTCTCAACAGGATAATC
GATGTTTTACTACTAACCACAAGTTTCATGTTGAACGCAGTACTCATACTGGAAATTATG
ATCAAGGATTACTCTATCAATCACCATCTACTTCAGAAATCACTTCTGAAACATTTTTAA
AATATAAAAGCTCAGTATCTTCACACGAATTAGTGAATATGCAAACTAAAAATACCTTTT
CTTGGATAAAGGAACAGATTACTAGATCCATTTCCGTATTGTTTATGATATATATAATAAC
TCGGACATCCATTTCAAATGCATATCCCATTTTTGCACAGCAGGGTTATGAAAATCCAC
GAGAAGCGACTGGACGTATTGTATGTGCAAATTGCCATTTAGCTAATAAACCCGTGGAT
ATTGAGGTCCCACAAGCGGTACTTCCTGATACTGTATTTGAAGCAGTTGTTCGAATTCC
TTATGATATGCAACTTAAACAGGTTCTTGCTAATGGTAAAAGAGGGGCTTTGAATGTGG
GGGCTGTTCTTATTTTACCTGAGGGGTTTGAATTAGCTCCGCCCGATCGTATTTCTCCCG
AGATGAAAGAAAAGATCGGCAAGCTGTCTTTTCAGAGCTATCGCCCCACTAAAAAAAA
TATTCTTGTGATAGGTCCTGTTCCTGGTCAGAAATATAGTGAAATTACCTTTCCTATTCTT
TCCCCAGACCCCGCTACTAAGAAGGATGTTAACTTTTTAAAATATCCCATATATGTAGGC
GGAAACAGGGGAAGGGGACAGATTTATCCCGACGGGAGCAAGAGTAACAATACAGTT
TATAATGCTACAGCAGCAGGTATAGTAAGCAAAATCACACGAAAAGAAAAAGGGGGAT
ACGAAATAACCATAGCGGATGCGTCGGATGGGCGTCAAGTGGTTGATATTATACCTCCA
GGACCAGAACTTCTTGTTTCAGAAGGTGAATCTATCAAACTTGATCAACCATTAACGA

GTAATCCTAATGTGGGTGGATTTGGTCAGGGAGATGCAGAAATAGTACTTCAGGATCCA
TTACGAGTCCAAGGCCTTTTGTTCTTCTTGGCATCTGTTATTTTGGCACAAATCTTTTTG
GTTCTTAAAAAGAAACAGTTTGAGAAGGTTCAATTGTCCGAAATGAATTTCATGAGTA
AAGTCTATGATTGGTTCGAAGAACGCCTCGAGATTCAGGCGATTGCAGATGATATAACT
AGTAAATATGTTCCTCCGCATGTCAACATATTTTATTGTTTAGGAGGAATTACGCTTACTT
GTTTTTTAGTACAAGTAGCTACGGGGTTTGCTATGACTTTTTACTATCGTCCGACCGTTA
CTGAGGCTTTTGCTTCTGTTCAATACATAATGACTGAAGCTAATTTTGGTTGGTTAATCC
GATCAGTTCATCGATGGTCGGCAAGTATGATGGTCCTAATGATGATACTACACGTATTTC
GTGTGTATCTCACCGGTGGATTTAAAAAACCTCGCGAATTGACTTGGGTTACAGGTGTG
GTTCTAGCTGTATTGACTGCATCTTTTGGTGTAACTGGTTATTCCTTACCTTGGGACCAA
ATTGGATATTGGGCAGTCAAAATTGTAACAGGCGTACCTGAAGCTATTCCGGTAATAGG
ATCACCTGTGGTAGAGTTATTACGTGGAAGTGCTAGTGTGGGACAATCCACTTTGACTC
GTTTTTATAGTTTACACACTTTTGTATTACCTCTTCTTACTGCCGTATTTATGTTAATGCAC
TTCTCAATGATACGTAAGCAAGGTATTTCTGGTCCTTTAATGGGAGTAACAAAAAAACC
TGACTTGAATGATCCTGTATTAAGGGCAAAATTGGCTAAAGGGATGGGTCATAATTATTA
CGGAGAACCCGCATGGCCCAATGATCTTTTATATATTTTTCCAGTAGTAATTTTAGGTACT
ATTGCATGTAATGTAGGTTTAGCGGTTCTAGAACCATCAATGATTGGTGAACCAGCAGA
TCCGTTTGCAACTCCTTTGGAAATATTACCCGAATGGTATTTCTTTCCTGTATTTCAAATA
CTTCGTACAGTACCTAATAAGTTATTGGGTGTTCTTTTAATGGTTTCAGTACCTGCGGGA
TTATTAACAGTACCTTTTTTAGAGAATGTTAATAAATTCCAAAATCCATTTCGTCGTCCA
GTAGCTACAACCGTCTTTTTGATTGGTACCGCAGCGGCTCTTTGGTTGGGTATTGGAGC
AACATTACCTATTGATAAATCCCTAACTTTAGGTCTTTTTATGATTGAAGTTTTTCTATTT
GGAATCGTGTTAGGTCTAATTCCTATTACTTTGGCTGGATTATTTGTAACTGCATATTTAC
AATATAGACGTGGTGATCAGTTGGACCTTATGCCTACTATAACTAGTTATTTCGGTTTTCT
ACTAGCGGCTTCAACTATAACCTCAGCTCTATTTATTGGTCTAAGCAAGATACGACTTAT
TATGGATATAGTAAGTCTCGCTTGGGCTGCTTTAATGGTAGTCTTTACATTTTCCCTTTCA
CTCGTAGTGTGGGGAAGAAGTGGACTTATGATTATTCGTTCGCCGGAACCAGAAGTAA
AAATTTTGGTGGATAGGGATCCCGTAAAAACTTCTTTCGAGGAATGGGCCAGACCCGG
TCATTTCTCAAGAACAATAGCTAAGGGCCCTGATACTACCACTTGGATCTGGAACCTAC
ATGCTGATGCTCACGATTTCGATAGCCATACCAGTGATTTGGAGGAGATCTCCCGAAAA
GTATTTAGTGCTCATTTCGGTCAACTCTCCATCATCTTTCTTTGGCTGAGTGGCATGTATT
TCCACGGTGCTCGTTTTTCCAATTATGAAGCATGGCTAAGTGATCCTACTCACATTGGA
CCTAGTGCCCAGGTGGTTTGGCCAATAGTGGGCCAAGAAATATTGAATGGTGATGTAG
GCGGGGGTTTCCGAGGAATACAAATAACCTCTGGTTTTTTTCAGATTTGGCGAGCATCT
GGAATAACTAGTGAATTACAACTTTATTGTACCGCAATTGGTGCATTGGTCTTTGCAGCC
TTAATGCTTTTTGCTGGTTGGTTCCATTATCACAAAGCTGCTCCAAAATTGGCTTGGTTT
CAAGATGTGGAATCTATGTTGAATCACCATTTAGCGGGGTTACTTGGACTTGGTTCTCTT
TCTTGGGCGGGACATCAAGTACATGTATCTTTACCGATTAACCAATTTCTAAATGCTGGA
GTAGATCCTAAAGAGATCCCACTTCCTCATGAATTTATCTTGAATCGGGATCTTTTGGCT
CAACTTTATCCCAGTTTTGCCGAGGGAGCAACCCCATTTTTCACCTTGAATTGGTCAAA
ATATGCAGAATTTCTGACTTTTCGTGGAGGATTAGATCCAGTAACTGGGGGTCTATGGC
TGACCGATATTGCACATCATCATTTAGCTATTGCAATTCTTTTCCTGATAGCGGGTCACAT
GTATAGGACCAACTGGGGCATTGGTCATGGTCTAAAAGATATTTTAGAGGCTCATAAAG
GTCCATTTACAGGTCAGGGCCATAAAGGTTTATATGAGATCCTAACAACATCATGGCAC

GCTCAATTATCTCTTAACCTAGCTATGTTAGGTTCCTTAACCATTGTTGTAGCTCACCATA
TGTATTCCATGCCCCCTTATCCATATTTAGCTACTGACTATGGTACACAACTGTCATTGTT
TACACATCACATGTGGATTGGTGGATTTCTTATAGTTGGTGCTGCTGCGCATGCAGCTAT
TTTTATGGTAAGAGACTATGATCCAACTACTAGATACAACGATCTATTAGATCGTGTCCT
TAGGCATCGTGATGCAATCATATCACATCTCAACTGGGCATGTATATTTCTAGGCTTTCA
CAGTTTTGGTTTGTATATTCATAATGATACCATGAGCGCTTTAGGGCGTCCCCAAGATAT
GTTTTCAGATACCGCTATACAATTACAACCCGTCTTTGCTCAATGGATACAAAACACCCA
TGCTTTAGCACCCGGTGCAACAGCTCCTGGTGCAACAACAAGCACCAGCTTGACTTGG
GGGGGTGGTGATTTAATAGCAGTAGGTGGCAAAGTAGCTTTATTACCTATTCCATTAGG
AACCGCAGATTTTTTGGTACATCACATTCATGCATTTACGATTCATGTGACGGTATTGAT
ACTTCTGAAAGGTGTTCTATTTGCCCGTAGCTCCCGTTTGATACCGGATAAAGCAAATC
TTGGTTTTCGTTTTCCTTGTGACGGGCCTGGAAGAGGGGGGACATGTCAAGTATCCGC
TTGGGATCATGTATTCTTAGGGCTATTCTGGATGTACAATGCAATTTCGGTAGTAATATTT
CATTTCAGTTGGAAAATGCAGTCAGATGTTTGGGGTAGTATAAGTGATCAAGGGATAGT
AACTCATATCACGGGAGGAAACTTTGCACAGAGTTCCATTACTATTAATGGGTGGCTCC
GCGATTTCTTATGGGCACAGGCATCCCAGGTAATTCAGTCTTATGGTTCTTCATTATCTG
CGTATGGTCTTTTTTTCCTAGGTGCTCATTTTGTATGGGCTTTCAGTTTAATGTTTCTATT
CAGCGGGCGTGGTTATTGGCAAGAACTTATTGAATCCATCGTTTGGGCTCATAATAAATT
AAAAGTTGCTCCTGCTACTCAGCCTAGAGCCTTGAGCATTGTACAAGGACGTGCTGTA
GGAGTAACTCATTACCTTCTGGGTGGAATTGCCACAACATGGGCATTCTTCTTAGCAAG
AATTATTGCAGTAGGAATGGCATTAAGATTTCCAAGGTTTAGCCAAGGCTTAGCTCAGG
ACCCCACTACTCGTCGTATTTGGTTTGGTATTGCTACTGCACATGACTTCGAGAGTCATG
ATGATATTACTGAGGAACGTCTTTATCAGAATATTTTTGCTTCTCACTTCGGGCAATTAG
CAATAATTTTTCTGTGGACTTCCGGAAATCTGTTTCATGTAGCTTGGCAAGGAAATTTT
GAGTCATGGGTACAGGACCCTTTACATGTAAGACCTATTGCTCATGCAATTTGGGATCC
TCATTTTGGTCAACCAGCTGTGGAAGCTTTTACTCGAGGGGGTGCTCTTGGCCCAGTG
AATATCGCTTATTCTGGTGTTTATCAGTGGTGGTATACAATCGGCTTACGCAATAATGAA
GATCTTTATACTGGAGCTCTTTTTCTGTTATTTCTTTCTGCCATATCCTTAATAGGGGGTT
GGTTACACCTACAACCGAAATGGAAACCGAGTGTTTCGTGGTTCAAAAATGCCGAATC
TCGTCTCAATCATCATTTGTCAGGACTCTTCGGAGTAAGTTCCTTGGCTTGGACAGGAC
ATTTAGTCCATGTCGCTATTCCTGCATCCAGGGGAGAATATGTTCGATGGAATAATTTCT
TAAATGTATTACCGCATCCCCAAGGGTTAGGACCACTTTTTACAGGTCAGTGGAATCTT
TATGCTCAAAACCCCGATTCAGGTAGTCATTTATTTAGTACCTCCCAAGGAGCAGGAAC
TGCCATTCTAACCCTTCTCGGGGGATTCCATCCACAAACGCAAAGTTTATGGCTGACTG
ATATTGCTCATCATCATTTAGCTATTGCATTTATTTTTCTCGTTGCTGGTCATATGTATAGA
ACTAATTTCGGGATTGGGCACAGTATGAAAGATCTTTTAGAAGCACATATTCCTCCTGG
GGGGCGATTGGGACGTGGGCATAAGGGTCTTTATGACACAATCAATAATTCAATTCATT
TTCAATTAGGCCTTGCTCTAGCCTCTTTAGGGGTTATTACTTCGTTGGTAGCTCAACATA
TGTACTCTTTACCTGCTTATGCGTTCATAGCACAAGACTTTACTACTCAAGCTGCGTTAT
ATACTCATCACCAATACATCGCAGGATTCATCATGACCGGAGCTTTTGCTCATGGAGCTA
TATTTTTCATTAGAGATTACAATCCGGAACAGAATGAGGATAATGTATTGGCAAGAATGT
TAGACCATAAAGAAGCTATCAAATCACATTTAAGTTGGGCCAGCCTATTTCTGGGGTTC
CATACCTTGGGACTTTATGTTCATAATGATGTCATGCTTGCTTTTGGTACTCCGGAGAAA
CAAATCTTAATCGAACCTATATTTGCCCAATGGATACAATCTGCTCACGGTAAAACTTCA

TATGGGTTCGATGTACTTTTATCTTCAACGAATGGCCCGGCATTCAATGCGGGTCGAAG
TATATGGTTGCCTGGCTGGTTAAATGCCATTAATGAG------AATAGTAATTCACTATTCTTA
ACAATAGGTCCTGGAGACTTCTTGGTTCATCATGCTATTGCGCTAGGTTTACATACAACT
ACATTGATCTTAGTAAAAGGCGCTTTAGATGCGCGTGGTTCCAAGTTAATGCCAGATAA
AAAGGATTTTGGTTATAGTTTTCCTTGCGATGGTCCGGGACGAGGTGGTACTTGTGATA
TTTCGGCTTGGGACGCATTTTATTTGGCAGTTTTTTGGATGTTAAATACCATTGGATGGG
TTACCTTTTATTGGCATTGGAAACACATTACATTATGGCAAGGTAACGTTTCACAGTTTA
ATGAATCTTCAACTTATTTGATGGGATGGTTAAGAGATTATCTATGGTTAAACTCTTCAC
AACTTATCAATGGATATAACCCTTTTGGTATGAATAGTTTATCGGTCTGGGCGTGGATGT
TCTTATTTGGACATCTTGTTTGGGCTACTGGATTTATGTTCTTAATTTCCTGGCGTGGATA
TTGGCAAGAATTGATTGAAACTTTAGCATGGGCTCATGAACGCACACCTTTGGCCAATT
TGATTCGATGGAGAGATAAACCAGTGGCTCTTTCCATTGTGCAAGCAAGATTGGTTGGA
TTAGCCCACTTTTCTGTAGGTTATATATTCACTTATGCGGCTTTCTTGATTGCCTCTACAT
CGGGCAAATTTGGTATGTCACATTCAGTAAAGATTTATGATACATGTATAGGGTGTACTC
AATGTGTCCGAGCCTGCCCCACAGATGTATTAGAAATGATACCTTGGGACGGATGCAAA
GCTAAGCAAATTGCTTCTGCTCCAAGAACAGAGGACTGTGTTGGTTGTAAAAGATGTG
AATCTGCCTGTCCAACAGATTTCTTGAGTGTTCGAGTTTATTTATGGCATGAAACAACT
CGTAGCATGGGTCTAGCTTATATGATATTTCTCAACTTACCCTCTATTTTTGTGCCTTTAG
TGGGCCTAGTATTTCCGGCAATTGCAATGGCTTCTTTATCTATTCATATTCAAAAAAACA
AGATTGTTATGCAAGATCTAAAAACATATCTCTCCGTGGCACCGGTACTAAGTACTCTAT
GGTTCGGGTCTTTAGCGGGTCTATTGATAGAGATCAATCGTTTATTCCCGGATGCGTTGA
CATTCCCTTTTTTTTCATTCATGACTGCAATTTTAGAGAGACGCGAAAGCGAAAGCCTA
TGGGGTCGCTTCTGTAATTGGATAACCAGCACTGAAAACCGTCTTTACATTGGATGGTT
TGGTGTTTTGATGATCCCTACCTTATTGACCGCAACTTCTGTATTTATTATCGCCTTCATT
GCTGCTCCTCCAGTAGATATTGATGGTATTCGTGAACCTGTTTCTGGATCTCTACTTTAT
GGAAACAATATTATTTCTGGTGCTATTATTCCTACTTCTGCAGCTATAGGTTTGCACTTTT
ACCCGATATGGGAAGCGGCATCCGTTGATGAATGGTTATACAATGGTGGTCCTTATGAA
CTAATTGTTCTACACTTCTTACTTGGTGTAGCTTGTTACATGGGTCGTGAGTGGGAACTT
AGTTTCCGTCTGGGTATGCGCCCTTGGATTGCTGTTGCATATTCAGCTCCTGTTGCAGCT
GCTACTGCTGTTTTCTTGATCTACCCAATTGGTCAAGGAAGCTTTTCTGATGGTATGCCT
CTAGGAATCTCTGGTACTTTCAACTTCATGATTGTATTCCAGGCTGAGCACAACATCCTT
ATGCACCCATTTCACATGTTAGGCGTAGCTGGTGTATTCGGCGGCTCCCTATTCAGTGCT
ATGCATGGTTCCTTGGTAACCTCTAGTTTGATCAGGGAAACCACAGAAAATGAATCTGC
TAATGAAGGTTACAGATTCGGTCAAGAGGAAGAAACTTATAATATCGTAGCTGCTCATG
GTTATTTTGGCCGATTGATCTTCCAATATGCTAGTTTCAATAATTCTCGTTCTTTACATTT
CTTCTTAGCTGCTTGGCCTGTAGTAGGTATCTGGTTTACCGCTTTAGGTATCAGTACCAT
GGCTTTCAACCTAAATGGTTTCAATTTCAACCAATCTGTAGTTGATAGTCAAGGTCGTG
TAATTAATACCTGGGCTGATATCATTAACCGTGCTAACCTTGGTATGGAAGTTATGCATG
AACGTAATGCTCATAACTTCCCTCTAGACCTAGCTGCTGTCGAAGCTCCATCCACAAAT
GGA---GGTTTGCCTTGGTATCGTGTTCATACCGTCGTATTGAATGATCCCGGTCGGTTGC
TTTCTGTCCATATAATGCATACAGCTCTGGTTGCTGGTTGGGCCGGCTCGATGACTCTAT
ATGAATTAGCAGTTTTTGATCCCTCTGACCCCGTTCTTGATCCAATGTGGAGACAGGGT
ATGTTTGTTATCCCCTTCATGACTCGTTTAGGAATAACCAATTCATGGGGAGGTTGGAGT
ATCACA---GGAGGGGCTATAACGAATCCGGGTATTTGGAGTTATGAAGGCGTGGCCGGG

GCACATATTATGTTTTCTGGTTTGTGCTTTTTGGCAGCTATCTGGCATTGGGTGTATTGG
GATCTAGAAATATTTTCTGATGAACGTACAGGAAAACCTTCTTTGGATTTGCCAAAGAT
CTTTGGAATTCATTTATTTCTCTCCGGGGTGGCTTGTTTTGGTTTTGGCGCATTTCATGT
AACAGGCTTGTATGGTCCCGGAATATGGGTGTCCGATCCTTATGGACTAACTGGAAAAG
TACAACCTGTAAATCCAGCGTGGGGCGTGGAAGGTTTTGATCCCTTTGTTCCGGGAGG
AATAGCCTCTCATCATATTGCAGCAGGTACATTGGGCATATTAGCAGGCCTATTTCATCTT
AGTGTCCGTCCGCCCCAACGTCTATACAAAGGATTACGTATGGGCAATATTGAAACCGT
CCTTTCCAGTAGTATCGCTGCTGTCTTTTTTGCAGCTTTTGTAGTTGCGGGAACTATGTG
GTATGGTTCAGCAACTACCCCGATCGAATTATTTGGTCCCACTCGTTATCAATGGGATCA
GGGATACTTCCAGCAAGAAATATATCGAAGAATTGGTGCTGGGCTAGCCGAAAATCAA
AGTTTATCAGAAGTTTGGTCTAAAATTCCCGAAAAATTAGCTTTTTATGATTACATCGGC
AATAATCCGGCAAAAGGGGGATTATTTAGAGCGGGCTCAATGGACAACGGGGATGGAA
TAGCTATTGGATGGTTAGGACACCCTATCTTTAGAGATAAAGAAGGGCGTGAACTTTTT
GTACGTCGTATGCCTACTTTTTTTGAAACATTTCCAGTCGTTTTGGTAGACGGAGACGG
AATTGTTAGAGCCGATGTTCCTTTTAGAAGGGCAGAATCGAAGTATAGTGTCGAACAA
GTAGGTGTAACTGTTGAGTTCTATGGTGGTGAACTCAATGGAGTCAGTTATAGTGATCC
TGCTACTGTGAAAAAATATGCTAGGCGTGCTCAATTGGGTGAAATTTTTGAATTAGATC
GTGCTACTTTGAAATCCGATGGTGTTTTTCGTAGCAGTCCGAGGGGTTGGTTTACTTTT
GGGCATGCTTCGTTTGCTCTGCTCTTCTTCTTCGGACACATTTGGCATGGTGCTAGAAC
CTTGTTCAGAGATGTTTTTGCTGGTATTGATCCAGATTTGGATACTCAAGTGGAATTTGG
AGCATTCCAAAAACTTGGAGATCCAACTACAAAAAGACAAGTAGTC------------------------
------------GTGGAAACGCTCTTTAATGGAACTTTAGCTTTAGCTGGTCGTGACCAAGAAA
CCACCGGTTTCGCTTGGTGGGCCGGGAATGCCCGACTTATCAATTTATCCGGTAAATTA
CTCGGGGCTCATGTAGCCCATGCCGGATTAATCGTATTCTGGGCCGGAGCAATGAACCT
ATTTGAAGTGGCTCATTTCGTACCAGAGAAACCCATGTATGAACAAGGATTAATTTTAC
TTCCCCATCTAGCTACTCTAGGTTGGGGGGTAGGTCCTGGTGGGGAAGTTATAGACACC
TTTCCATACTTTGTGTCTGGAGTACTTCACTTAATTTCCTCTGCAGTATTGGGCTTTGGC
GGTATTTATCATGCACTTCTAGGACCTGAGACTCTTGAAGAATCTTTTCCATTCTTCGGT
TATGTATGGAAAGATAGAAATAAAATGACCACAATTTTGGGGATTCACTTAATCTTGTTA
GGTATCGGTGCTTTTCTTTTAGTATTTAAGGCTCTTTTTTTTGGGGGCGTATATGATACCT
GGGCTCCTGGAGGGGGAGATGTAAGAAAAATTACTAACTTGACCCTTAGCCCAAGTGT
TATATTTGGTTATTTACTAAAATCTCCCTTTGGAGGAGAAGGATGGATTGTTAGTGTGGA
CGATTTGGAAGATATAATTGGAGGACATGTATGGTTAGGTTCCATTTGTATACTTGGTGG
AATCTGGCATATCTTAACCAAACCCTTTGCATGGGCTCGCCGCGCACTTGTATGGTCTG
GAGAGGCTTACTTGTCTTATAGTTTAGGTGCTTTATCTGTCTTTGGTTTCATTGCTTGTTG
CTTTGTATGGTTCAACAATACCGCTTATCCTAGTGAGTTTTATGGGCCGACTGGACCAG
AAGCTTCTCAAGCTCAAGCATTTACTTTTCTAGTTAGAGACCAACGTCTTGGGGCTAAC
GTGGGGTCCGCTCAAGGACCTACTGGTTTAGGTAAATATCTAATGCGTTCCCCGACTGG
AGAAGTCATTTTTGGAGGAGAAACTATGCGTTTTTGGGATCTGCGTGCTCCTTGGTTAG
AACCTCTAAGGGGTCCCAATGGTTTGGACTTGAGTAGGCTGAAAAAAGACATACAACC
TTGGCAAGAACGTCGTTCCGCGGAATATATGACTCATGCTCCTTTGGGTTCTTTAAATTC
CGTGGGTGGCGTAGCTACCGAGATCAATGCAGTCAATTATGTTTCTCCTAGAAGTTGGT
TAGCTACCTCTCATTTTGTTCTAGGATTCTTCCTATTCGTAGGCCATTTGTGGCATGCGG
GAAGGGCTCGTGCAGCTGCAGCAGGATTTGAAAAAGGAATTGATCGTGATTTTGAACC

TGTTCTTTCCATGACCCCTCTTAACATGACTATAGCCCTTGGTAGATTTACCAAAGACGA
AAAAGATTTATTTGATACTATGGATGACTGGTTACGAAGGGACCGTTTCGTTTTTGTAG
GTTGGTCCGGCCTATTACTCTTTCCTTGTGCCTATTTTGCTTTAGGAGGTTGGTTCACAG
GTACAACCTTTGTAACTTCGTGGTATACCCATGGATTGGCTAGTTCCTATTTGGAAGGAT
GCAATTTCTTAACCGCCGCAGTTTCTACTCCTGCTAATAGTTTAGCACATTCTTTGTTGC
TACTATGGGGTCCTGAAGCACAAGGAGATTTTACTCGTTGGTGTCAATTAGGTGGTCTG
TGGACGTTTGTTGCTCTCCATGGGGCTTTCGGACTAATAGGTTTCATGTTACGTCAATTT
GAACTTGCTCGATCTGTTCAATTGCGACCTTATAATGCAATCGCATTCTCTGGTCCAATT
GCTGTTTTTGTTTCTGTATTCCTGATTTATCCACTAGGTCAGTCTGGTTGGTTCTTTGCA
CCTAGTTTTGGTGTAGCAGCTATATTTCGATTCATCCTCTTTTTCCAAGGGTTTCATAATT
GGACGTTGAACCCATTTCATATGATGGGAGTTGCCGGTGTATTGGGCGCTGCTCTGCTA
TGCGCTATTCACGGTGCTACCGTAGAAAATACTTTATTTGAGGATGGTGATGGTGCAAA
TACATTCCGTGCTTTTAACCCAACTCAAGCTGAAGAAACTTATTCAATGGTCACCGCTA
ACCGCTTTTGGTCCCAAATCTTTGGGGTTGCTTTTTCCAATAAACGTTGGTTACATTTCT
TTATGTTATTTGTACCAGTAACTGGTTTATGGATGAGTGCTCTTGGAGTAGTCGGTCTGG
CTCTGAACCTACGTGCCTATGACTTCGTTTCCCAAGAAATCCGTGCAGCGGAAGATCCT
GAATTTGAGACTTTCTACACCAAAAATATTCTCTTAAACGAAGGTATTCGTGCTTGGAT
GGCGGCTCAGGATCAGCCTCATGAAAACCTTATATTCCCTGAGGAGGTTCTACCCCGTG
GAAACGCTCTTATGTCTGGAAACACAGGAGAACGTTCTTTTGCTGATATTATTACCAGT
ATTCGATACTGGGTCATTCATAGCATTACTATACCTTCCCTATTTATTGCGGGTTGGTTAT
TCGTCAGCACGGGTTTAGCTTATGATGTATTTGGAAGCCCTCGGCCAAACGAATATTTT
ACAGAGAACCGACAAGGAATTCCATTAATAACCGGCCGTTTTGATCCTTTGGAACAAC
TCGATGAATTTAGTAGATCCTTTATGACCATAGATAGAACCTTTCCAATTTTTACAGTGC
GATGGTTGGCTGTTCACGGACTAGCTGTACCTACCGTTTCTTTTTTGGGGTCAATATCA
G C A A T G C A G T T C A T C C A A C G
A???
??
???ATGCTTACTCTCAAACTCTTCGTTTACAC
AGTAGTTATATTCTTTGTTTCTCTCTTCATCTTCGGATTCCTATCTAATGACCCGGGGCGT
AATCCTGGACGGGAAGAAATGGCCGACACTACTGGAAGGATTCCTCTTTGGCTAATAG
GTACTGTAACTGGTATTCCTGTGATTGGTTTAATAGGTATTTTCTTTTATGGTTCATATTC
CGGATTGGGTTCATCCCTGATGCTTAATATCTTAAGTTTGATCTGTATCTGTCTTAATTCT
ACCCTTTATTCGAGTAGTTTTTTCTTAGGCAAATTGCCCGAGGCCTACGCTTTTTTGAAT
CCAATCGTAGATTTTATGCCAGTTATCCCTCTGCTCTTTTTTCTCTTAGCCTTTGTTTGGC
AAGCTGCTGTAAGTTTTCGAACGACACAATCAAACCCGAACGAACAAAGTGTTGAATT
GAATCGTACCAGTCTCTACTGGGGGTTATTACTAATTTTTGTACTTGCTGTTTTATTTTCC
AATTATTTCTTCAATATGGAAGTAAATATTCTCGCATTTATTGCTACTGCACTGTTCATTC
TAGTTCCTACTGCTTTTTTACTTATCATATATGTAAAAACAGTCAGTCAAAATGATATGG
AAACAGCAACCCTAGTCGCCATCTCTATATCTGGTTTACTTGTAAGTTTTACTGGGTACG
CCTTATATACTGCTTTTGGGCAACCCTCTCAACAACTAAGAGATCCATTTGAGGAACAC
GGGGACATGGAAGCATTGGTTTATACATTCCTTTTGGTCTCGACTCTAGGGATAATTTTT
TTCGCTATCTTTTTTTCGAGAACCACCTAAAGTTCCAACTAAAAAGATAAAAATGACTAT
TGCTTTTCAATTGGCTGTTTTTGCATTAATTGCTACTTCATTAATCTTACTGATTAGTGTA
CCCGTTGTATTTGCTTCTCCTGATGGTTGGTCAAGTAACAAAAATGTTGTATTTTCCGGT

ACATCATTATGGATTGGATTAGTCTTTCTAGTGGGTATCCTTAATTCTCTCATTTCT---------
------------ATGTCACCACAAACAGAGACTAAAGCAGGTGTTGGATTCAAAGCTGGTGTTA
AAGATTACAAATTGACTTATTATACTCCTGACTATGAAACCAAAGATACTGATATCTTGG
CAGCATTCCGAATGACTCCTCAACCCGGAGTTCCACCTGAGGAAGCAGGGGCCGCGGT
AGCTGCCGAATCTTCTACTGGTACATGGACAACTGTGTGGACTGATGGACTTACCAGCC
TTGATCGTTACAAAGGACGATGCTACGGCATCGAGCCCGTTGCTGGAGAAGAAAATCA
ATATATTGCTTATGTAGCTTACCCTTTAGACCTTTTTGAAGAAGGTTCTGTTACTAACATG
TTTACTTCCATTGTGGGGAATGTATTTGGGTTCAAAGCCCTACGCGCTCTTCGTCTGGA
GGATCTGCGAATCCCTCCTGCTTATGTTAAAACCTTCCAAGGCCCACCTCATGGCATCC
AAGTTGAGAGAGATAAATTGAACAAGTATGGTCGTCCCCTATTGGGATGTACTATTAAA
CCTAAATTGGGTTTATCTGCTAAGAACTACGGCAGAGCAGTTTATGAATGTCTTCGCGG
TGGACTTGATTTTACCAAAGATGATGAGAACGTGAACTCCCAACCATTTATGCGTTGGA
GAGACCGTTTCTTATTTTGTGCCGAAGCAATTTATAAAGCACAGGCTGAAACAGGTGA
AATCAAAGGACATTACTTGAATGCTACTGCAGGTACATGTGAAGAAATGATCAAAAGG
GCGGTATTTGCCAGAGAATTGGGGGTTCCTATCGTAATGCATGACTACTTAACAGGGGG
ATTCACCGCAAATACTAGCTTGGCTTTTTATTGCCGAGATAATGGCCTACTTCTTCACAT
CCACCGTGCAATGCATGCAGTTATTGATAGACAGAAGAATCATGGTATACACTTTCGTG
TACTAGCTAAAGCGTTACGTATGTCCGGTGGAGATCATATTCACTCTGGTACCGTAGTAG
GTAAACTTGAAGGGGAAAGAGAAATCACTTTGGGTTTTGTTGATTTACTACGTGATGAT
TTTATTGAAAAAGACCGAAGTCGGGGTATTTATTTCACTCAAGATTGGGTCTCTTTACC
AGGTGTTTTACCCGTGGCTTCGGGGGGTATTCACGTTTGGCATATGCCCGCTCTGACCG
AGATCTTTGGGGATGATTCTGTACTACAGTTCGGTGGAGGAACTTTAGGACACCCTTGG
GGAAATGCACCGGGTGCCGTAGCTAATCGAGTAGCTCTAGAAGCATGTGTACAAGCTC
GTAATGAGGGGCGTGATCTTGCTCGTGAGGGTAATGAAATTATCCGTGAGGCTTCCAAA
TGGAGTCCTGAACTAGCTGCTGCTTGTGAGGTATGGAAGGAGATCAAATTTGAATTCG
AAGCAATGGATACTTTG------ATGGCGATACATTTATACAAAACTTCTACCCCAAGCACA
CGCAATGGAGCCGTAGACAGTCAAGTGAAATCCAATCCACGAAATAATTTGATCTATGG
ACAGAATCGTTGTGGTAAAGGTCGTAATGCCAGAGGAATCATTACCGCAAGGCATAGA
GGGGGAGGTCATAAGCGTCTATACCGTAAAATCGATTTTCGACGGAATGCAAAAGACAT
ATATGGTAGGATCGTAACCATAGAATACGACCCTAATCGAAATGCATACATTTGTCTCAT
ACACTATGGGGATGGTGAGAAGAGATATATTTTACATCCCAGAGGGGCTATAATTGGAG
ATACCATTGTTTCTGGTACAGAAGTTCCTATAAAAATGGGAAATGCCCTACCTTTGACC
GATATGCCCTTAGGCACGGCCATACATAACATAGAAATCACACTTGGAAAGGGTGGAC
AATTAGCTAGAGCAGCGGGTGCTGTAGCGAAACTGATTGCAAAAGAGGGGAAATCGG
CCAAATTAAAATTACCTTCTGGGGAGGTACGTTTGATATCCAAAAACTGCTCAGCAACA
GTCGGACAGGTGGGGAATGTTGGGGTGAACCAGAAAAATTTGGGTAGAGCCGGATCT
AAGTGTTGGCTAGGTAAGCGTCCTGTAGTAAGAGGAGTAGTTATGAACCCTGTAGACC
ATCCCCATGGAGGTGGTGAAGGGAGGGCCCCGATTGGTCGAAAAAAACCCACAACCC
CTTGGGGTTATCCTGCACTTGGAAGAAGAAGTAGAAAAAGGAATAAATATAGTGATAAT
TTGATTGTTCATCGCCGTAGTAAAATGATTCAACCTCAGACCCATTTGAATGTAGCGGA
CAACAGCGGAGCTCGAGAATTGATGTGTATTCGAATCATAGGAGCTAGT---AATCGGCG
ATATGCTCATATTGGTGACGTTATTGTTGCTGTGATCAAAGAAGCAATACCAAATATGCC
TTTACAAAGATCAGAAGTGATCAGAGCTGTAATTGTACGTACCTGTAAAGAACTCAAA
CGTGACAACGGTATGATAATACGATATGATGACAATGCTGCAGTTGTCATTGATCAAGA

AGGAAATCCAAAAGGAACTCGAGTTTTTGGTGCGATCGCTCGGGAATTGAGACAGTTA
AATTTTACTAAAATAGTTTCATTAGCTCCCGAGGTATTAATGCTTAGTCCCAAAAGAACC
AGATTCCGTAAACAACATAGAGGAAGAATGAAGGGAATATCTTATCGAGGTAATCGTAT
TTGTTTCGGTAGATATGCTCTTCAGGCACTTGAACCCACTTGGATCACATCTAGACAAA
TTGAAGCGGGTCGACGAGCAATTACACGACATGCACGTCGTGGTGGAAAAATATGGGT
ACGTATATTTCCCGACAAACCCATTACAGTAAGACCCGCGGAAACACGTATGGGTTCGG
GTAAAGGATCTCCCGAATATTGGGTAGCTGTCGTTAAACCAGGTCGAATACTTTATGAA
ATGGGCGGAGTAACAGAAAATGCAGCCAGAGAAGCTATTTCACTAGCGGCGTCCAAAA
TGCCTATACGAACTCAATTCATTATTTCAGGAATGACCAGAATTAGACGAGGATATATAG
CTCGGAGACGTAGAACAAAAATTCGTTTATTTGTATCAAGCTTTCGAGGGGCTCATTCA
AGACTTACTCGAACTATTACTCAACAGAAAATAAGAGCTTTGGTTTCGGCTCATCGGGA
TAGAGATAAGCAAAAGAGAAATTTTCGTCGTTTGTGGATCACTCGGATAAATGCAGTAA
TTCGCGAT---AACAGGGTATCCTATAGTTATAGTAGATTAATAAACGATCTGTACAAGAG
GCGGTTGCTTCTTAATCGTAAAATACTTGCACAAATAGCTATATCAAATAGGAATTGTCT
TCATATGATTTCCAATGAGATCATAAAAATAAAA------------------------------ATGATTAATGT
ATATGCTTTAGGTCAACATATATGCATTTCTGCTAACAAAGCAAGAAGAGTAATTGATCA
GATTCGTGGACGTTCCTACGAGGAAACACTTATGATACTAGAACTCATGCCTTATCGAG
CATCTTATCCCATTTTTAAATTGATTTATTCTGCAGTAGCAAATGCTACTCACAATAGGG
GTTTCAACGAAGCAAATTTATTCATTAGTAAAATGGAAGTCAACGAAGGTACTACCGTG
AAAAAATTAAAACCTCGAGCTCGAGGACGTAGTTATCCGATAAAAAGATCCACTTGTC
ATATAAAAATTGTATTAACAGATATATCCTTATATGGGGGGGTATGGGACAAAAAAATGG
ATGGAATCAAATATTCAGTATTTACAGACAAAAGTATTCGGTTATTGGTGAAAAAT---CA
ATATACTTCTAATGTCGAATCAGGATCAACTAGGACAGAAATAAAGCATTGGGTCGAAC
TCTTCTTTGGCGTCAAGGTAATAGCTATGAATAGTCATCGACTCCCGGGAAAGGGTAGA
AGAATGGGACCTATTATGGGACATACAATGCATTACAGACGTATGATCATTACGCTTCAA
CCGGGTTATTCTATTCCACCTGTTAGAAAGAAAAGAACTATGGCAGTTCCAAAAAAAC
GTACTTCTCTGTCAAAAAAGCGTATTCGTAAAAATATTTGGAAAAAGAAGGGCTATTGG
GCAGCGTTAAAAGCTTTTTCGTTAGCGAAATCTCTTTCTAGCGGAAATTCTAAAAGTTT
TTTT------CTACGAAAAAAAATGAGT------AAAGATGTCCGAGTAACAGTTATGTTAGAAT
GTATCAGTTGTGTCCAAAACGGT------GGTGATAAGGAATCAAAGGGCGTTTCCAGATAT
ATTACTGAAAAGAATCGACACAATACACCAGGTCGATTGGAGTTGAAAAAATTCTGTC
CCTTTTGTTCCAAACAGACGATTCATGTGGAGATAAAGAAAATGAAAATAAGGGCCTC
CGTTCGTAAAATTTGTGAAAAATGTCGACTGATCCGTAGGCGGGGACGAATTATAGTAA
TTTGTTCCAACCCGAGACATAAACAAAGACAAGGAATGGTTCGAGAGAAAATAACAGT
ATCTACTCGGACACTACAGTGGAAGTGTGTTGAATCAAGAACAGACAGTAAGCGTCTT
TATTATGGACGCTTTATTATGTCTCCACTTATGAAAGGTCAAGCCGACACAATAGGCATT
GCAATGCGAAGAGCTTTGCTCGGAGAAATAGAAGGAACATGTATCACACGTGCAAAAT
CTGAG------AAAATACCACATGAATATTCTACCATAGTAGGTATTCAAGAATCAGTACATG
AAATTTTAATGAATTTGAAAGAAATTATATTGAAAAGTAATATATATGGAACTCGCGACG
CGTCTATTTGCGTCAGAGGTCCTGGATATGTAACTGCTCAAGACATTATCTCACCGCCTT
CTGTGGAAATTGTTGATAATACACAGCATATAGCTAGCCTGACGGAACCAATTGATTTG
TGTATTCAATTACAAATTGAAAAGAATCGCGGATATCGTATAAAAACGCCAAATACCTAT
CAAGATGGAAGTTAT------CCTATAGATGCTGTATTCATGCCTGTTCGAAATGCGAATCAT
AGTATTCATTCTTATGGGAATGGGAAGGAGAAACAAGAAATACTTTTTCTCGAAATCTG

GACAAATGGAAGTTTAACTCCTAAAGAAGCACTTCATGAAGCCTCCCGGAATTTGATT
GATTTATTTATTCCTTTTCTACATGCGGAAGAAGAA------AACTTACATTTAGAGGACAAT
CAA---TACAAGGTTACTTTACCCTTTTTTACCTTTCATGATAGATTGGCT---AAACTAAGG
AAAAATAAAAAA---GAAATAGCATTGAAATCAATTTTTATAGACCAATCAGAATTGCCTT
CCAGGACCTATAATTGCCTCAAAAGTTCCAATATACATACATTATTGGACCTTTTGAATA
ACAGTCAAGAAGATCTTATGAAAATTAAGCATTTTAGCATAGAAGATGTAAAACAGATA
TTGGACATTCTAGAAAAGCAA--------------------TTTCACAAT---ATGCTCCGGGATGGAAA
T---GAGGAAATGTCTACAATACCTGGATTTAGTCAGATACAATTTGAAGGATTTTGTAGA
TTCATTGATCAGGGCTTGACGGAAGAACTTTATAAGTTTCCAAAAATTGAAGATACAGA
TCAAGAAATTGAATTTCAATTATTTGTGGAAACATATCAATTGGTCGAACCGTTGATAA
AAGAAAGAGATGCTGTGTATGAATCAATCACATATTCTTCTGAATTATATGTATCCGCGG
GATTAATTTGGAAAACCAGTAGGGATATGCAAGAACAAACAATTTTTCTTGGAAACATT
CCTATAATGAATTCCCTGGGAACTTCTATAGTAAATGGAATATACAGAATTGTGATCAAT
CAAATATTGCAAAGCCCCGGTATTTATTACCGGTCAGAATTGGACCATAACGGAATTTC
GGTATATACCGGCACTATAATATCAGATTGGGGGGGAAGATTAGAATTAGAGATTGATAG
AAAAGCAAGGATATGGGCTCGTGTGAGTAGGAAACAGAAAATATCTATTCTAGTTCTAT
CATCAGCTATGGGGTCGAATCTAAGAGAAATTCTAGATAATGCTTGCTATCCTGAAATTT
TCTTGTCTTTCCTGAATGATAAGGAGAAAAAAAAGATTGGGTCAAAAGAAAATGCCAT
TTTGGAGTTTTATCAACAATTTTCTTGTGTAGGCGGGGATCCGGTATTTTCTGAATCCTT
ATGTAAGGAATTACAAAAGAAATTCTTTCAGCAAAGATGTGAATTAGGAAGGATTGGT
CGACGAAATATGAACCAGAGACTAAATCTTGATATACCTCAGAACAATACATTTTTGTT
ACCGCGAGATATATTGGCAGCTGCGGATCATTTGATTGGAATGAGATTTGGAATGGGTA
CACTTGACGAT------ATGAATCATTTAAAAAATAAACGTATTCGTTCTGTAGCGGATCTCT
TACAAGATCAACTCGGATTAGCTCTGGCTCGTTTAGAAAATGTGGTTCGCGGAACTATA
TGTGGAGCAATTAGG---CATAAATTGATA---CCGACTCCTCAGAATTTGGTAACTTCAAC
TCCA------------TTAACAACCACTTATGAATCTTTTTTCGGCTTACACCCATTATCGCAAGT
TTTGGATCGAACTAATCCATTGACACAAATAGTTCATGGGAGAAAGTTGAGTTATTTGG
GCCCTGGAGGATTGACGGGTCGAACTGCTAGTTTTCGGATACGAGATATCCATCCTAGT
CACTATGGACGTATTTGCCCAATTGACACGTCTGAAGGAATCAATGTTGGACTTATTGG
ATCCTTAGCAATTCATGCGCGGATTGGTCATTGGGGG---TCTCTAGAAAGTCCATTTTAT-
--------GAAATTTCTGAGAGATCAAAAAAG------GTACGGATGCTTTATTTATCACCAAGTA
GAGATGAATACTATATGGTAGCGGCGGGAAATTCTTTGGCGTTGAATCAAGGTATTCAG
GAAGAACAGGTTGTTCCCGCTCGATACCGTCAAGAATTCCTGACTATTGCATGGGAAC
AGGTTCATCTTCGAAGTATTTTTCCCTTCCAATATTTTTCTATCGGAGCTTCCCTCATTCC
TTTTATCGAGCATAATGATGCGAATCGGGCTTTAATGAGTTCTAATATGCAGCGTCAAGC
AGTTCCGCTTTCTCGTTCCGAGAAGTGCATTGTTGGAACTGGGTTGGAACGCCAAGCG
GCTCTAGATTCAGGGGTTTCCGCTATAGCGGAACACGAGGGAAAGATCATTTATACCGA
TACTGACAAGATCATTTTATCAGGTAATGGGGATACTCTAAGCATTCCATTAGTTATGTA
CGAACGTTCCAACAAAAATACTTGTATGCATCAAAAACCCCAGGTTCAGCGGGGTAAC
TGCATTAAAAAGGGACAAATTTTAGCGGATGGTGCCGCTACAGTTGGCGGCGAACTTA
CTTTGGGAAAAAACTTATTAGTAGCTTATATGCCATGGGAAGGTTACAATTCTGAAGAT
GCGGTACTCATTAGCGAGCGTCTGGTCTATGGCGATATCTATACTTCTTTTCACATACGG
AAATATGAAATTCAGACTCATGTTACAAGTCAAGGA---CCCGAAAGGATTACTAATGAA
ATACCGCATTTAGAAGCCTATTTACTCCGCAATTTAGACAAAAATGGAATTGTGATGCTG

GGATCTTGGGTAGAGACGGGTGATATTTTAGTAGGTAAATTAACACCTCAGATGGCGAA
AGAA---TCATCATATGCCCCGGAAGATAGATTATTACGAGCCATACTTGGCATTCAAGTA
TCCACTTCAAAGGAAACTTGTCTAAAACTACCTATAGGCGGTAGGGGCCGAGTTATTGA
TGTGAGATGGATACAGAAAAAAAGG---GGTTCTAGTTATAATCCAGAAATGATTCGTGT
ATATATTTCACAGAAACGTGAAATCAAAGTAGGTGATAAAGTAGCTGGAAGACATGGA
AATAAAGGTATCATTTCAAAAATTTTGCCTAGGCAAGATATGCCTTATTTGCAAGATGGA
AGACCTGTTGATATGGTCTTCAATCCATTAGGAGTACCTTCACGAATGAATGTCGGACA
GATATTTGAATGCTCACTCGGGTTAGCGGGGGGTCTGTTAGACAGACATTATAGAATAG
CACCTTTTGACGAGAGATATGAACAAGAGGCTTCTAGAAAACTAGTGTTTTCTGAATTA
TATGAAGCCAGTAAGCAAACAGCGAATCCATGGGTATTTGAACCCGAGTATCCGGGAA
AAAGCAGAATATTTGATGGAAGAACGGGAGATCCTTTTGAACAACCTGTTATAATAGG
AAAGTCTTATATCTTGAAATTAATTCATCAAGTTGATGATAAAATTCATGGACGTTCCAG
TGGACATTATGCACTTGTTACACAACAACCTCTTAGAGGAAGGGCCAAGCAAGGGGGA
CAACGGGTAGGAGAAATGGAGGTTTGGGCTTTAGAGGGATTTGGTGTTGCTCATATTTT
ACAAGAGATGCTTACTTATAAATCTGATCATATTAGAGCTCGCCAGGAAGTACTTGGTA
CTACGATCATCGGAGGAACAATACCTAACCCTGAGGATGCTCCAGAATCTTTTCGGTTG
CTTGTTCGAGAACTACGATCTTTGGCTTTGGAACTGAATCATTTCCTTGTATCTGAGAA
GAACTTCCAGATTAATAGGAAGGAAGCT---------------------ATGATCGATCAATATAAACAT
CAACAACTCCGAATTGGATCAGTTTCTCCTCAACAAATAAGTGCTTGGGCTAATAAAAT
CCTACCTAATGGAGAGATAGTTGGAGAGGTGACAAAACCCTATACTTTTCATTACAAAA
CTAATAAGCCGGAAAAAGATGGATTATTTTGTGAAAGAATTTTCGGTCCTATAAAAAGT
GGAATTTGTGCTTGTGGAAATTATCGAGTAATCGGAGATAAAAAAGAAAACCCGAAAT
TTTGTGAACAATGTGGAGTCGAATTTGTTGATTCTCGGATACGAAGATATCAAATGGGC
TACATCAAATTGGCATGCCCAGTAACTCATGTGTGGTATTTGAAACGGCTTCCTAGTTAT
ATCGCGAATCTTTTAGATAAACCTCTTAAAGAATTAGAAGGCCTAGTATACTGCGAT------
TTTTCTTTTGCTAGGCCC---ATAGCTAAAAAACCTACTTTCTTACGATTACGGGGTTCATT
CGAATATGAAATCCAATCCTGGAAATACAGCATCCCACTTTTTTTTACTACTCAAGGCTT
CGATACATTTCGAAATCGAGAAATCTCTACTGGAGCAGGTGCTATCCGAGAACAATTAT
CCGATCTGGATTTGCGAATTATTATAGATTATTCATTGGTAGAATGGAAAGAATTAGGGG
AAGAA---GGGACCACAGGTAATGAATGGGAAGATCGCAAAATTGGAAGAAGAAAGGA
TTTTTTGGTTAGACGCATGGAATTAGCTAAGCATTTTATTCGAACAAATGTAGAACCAG
AACGGATGGTTTTGTGTCTATTACCAGTTCTTCCTCCCGAGTTGAGACCGATCATTCAG
ATAGATGGAGGTAAACTAATGAGCTCGGATATTAATGAACTCTATAGACGAGTTATCTAT
CGGAATAATACTCTTACCGATCTATTAACAACAAGTAGATCTACGCCAGGGGAATTAGT
AATGTGTCAGGAGAAATTGGTACAAGAAGCCGTGGATACACTTCTCGATAATGGAATC
CGCGGACAACCAATGAGGGATGGTCATAATAAAGTTTACAAGTCGTTTTCAGATGTAAT
TGAAGGCAAAGAGGGAAGATTTCGTGAGACTCTGCTTGGCAAACGGGTAGATTATTCG
GGGCGTTCCGTCATTGTTGTAGGCCCTTCACTTTCATTACATCGATGTGGATTGCCTCGC
GAAATAGCAATAGAGCTTTTCCAGACATTTGTAATTCGTGGTCTAATTAGACAACACGT
TGCTTCGAACATAGGGGTTGCTAAGAGTAAAATTCGTGAAAAAGAACCAATTGTCTGG
GAAATACTTCAGGAAGTTATGCAGGGGCATCCTGTATTGCTGAATAGAGCACCTACTCT
GCATAGATTAGGCATACAGGCATTCCAACCAATTTTAGTGGAAGGACGCGCTATTTGTT
TACATCCATTAGTTCGTAAGGGATTCAATGCAGACTTTGATGGGGATCAAATGGCTGTT
CATGTACCTTTATCTTTGGAAGCTCAAGCGGAGGCTCGTTTACTTATGTTTTCTCATATG

AATCTCCTGTCTCCAGCTATTGGGGATCCCATTTCTGTACCAACTCAAGATATGCTTATT
GGACTCTATGTATTAACGAGCGGGAATCGTCGAGGTATTTGTGCAAATAGGTATAATCCA
TGTAATCGCAGAAAC---------TATAAAAATAAAAGAATTGCCGATAATAAG------TATACGA
AAGAAAAGGAACCCTTTTTTTGTAATTCCTATGATGCAATTGGAGCTTATCGGCAGAAA
ATAATAAATTTAGATAGTCCTTTGTGGCTCCGGTGGCGACTAGATCAACGC---GTTATTG
CTTCA------AGAGAAGTTCCTATCGAAGTTCACTATGAATCCTTGGGTACCTACCATGAG
ATTTATGGGCACTATCTAATAGTAAGAAGCGTAAAAAAAGAAATTCTTTGTATATACATT
AGAACCACTGTTGGTCATATTTCCTTTTATCGAGAAATCGAAGAAGCCATACAAGGGTT
TTGTCGGGCCTGCTCATATGGTATC------------------------ATGGAGGTACTTATGGCAGAAC
GGGCCAATCTGGTCTTTCACAATAAAGTGATAGATGGAACTGCCATGAAACGACTTATT
AGCAGATTAATAGATCACTTTGGAATGGCATATACATCACACATCCTGGATCAAGTAAA
GACTCTGGGTTTCCAGCAAGCCACTGCTACATCCATTTCATTAGGAATTGATGATCTTTT
AACAATACCTTCTAAGGGATGGCTAGTCCAAGATGCTGAACAACAAAGTTTTATTTTGG
AAAAAAACCATCAGTATGGAAATGTACACGCGGTAGAAAAATTACGTCAATCCATTGA
GATATGGTATGCTACAAGTGAATATTTGCGACAAGAAATGAATCCTAATTTTAGGATGAC
TGACCCTTTTAATCCAGTCCATATAATGTCTTTTTCGGGAGCTAGAGGAAATGCATCTCA
AGTACACCAATTAGTAGGTATGAGGGGATTAATGTCGGATCCCCAAGGACAAATGATTG
ATTTACCTATTCAAAGCAATTTACGCGAAGGGCTCTCTTTAACGGAATATATCATTTCTT
GTTACGGAGCCCGCAAAGGAGTTGTGGATACTGCTGTACGAACATCAGATGCTGGTTA
TCTCACGCGCCGACTTGTTGAAGTAGTTCAACACATTGTTGTACGTAGAACAGATTGTG
GCACCGTCCGCGGTATTTCTGTGAGTCCTCGAAATGGTATGATGCCG---GAAAGAATTT
TTATACAAACATTAATTGGTCGTGTATTAGCAGACGATATATATATAGGTCCACGATGCAT
TGCCACTCGAAATCAAGATATTGGGATTGGACTTGTCAATCGATTCATAACC---------------
---TTTCGAACACAACCAATATCTATTCGAACCCCTTTTACTTGTAGGAGTACATCTTGGA
TCTGTCGATTATGTTATGGTCGGAGTCCTACTCATGGCGACCTCGTCGAATTGGGAGAA
GCTGTAGGTATTATTGCGGGTCAATCTATTGGCGAACCAGGTACTCAACTAACATTAAG
AACTTTTCATACCGGCGGCGTATTCACAGGGGGTACTGCAGAACATGTACGAGCACCTT
ATAATGGAAAAATAAAATTCAATGAGGATTTGGTTCATCCCACACGTACACGTCATGGG
CATCCTGCTTTTCTATGTTATATAGACTTGTATGTAACTATTGAGAGTGAAGATATTATAC
ATAACGTGAATATTCCACCAAAAAGTTTTCTTTTAGTTCAAAATGATCAATATGTAGAAT
CAGAACAAGTGATTGCTGAGATTCGTGCGGGAACATCCACT---TTGAATTTTAAAGAGA
GGGTTCGAAAACATATTTATTCTGACTCAGAGGGAGAA---ATGCACTGGAGTACCGATG
TGTACCATGTACCTGAATATACATATGGTAATGTTCACCTCTTACCAAAAACAAGTCATT
TATGGATATTATCAGGGGGACCGTGCAGA------TCTAGTGTAGTCTCTTTTTCACTCCACA
AGGATCAAGATCAAATGAACGTTCAT------TCTCTTTCTGTCGAACAAAGATATATTTCTA
ACTTCTCG------GCGATTAATAATCAA---GTGAGACACAAATTGTTTAGTTCCGATCTTTA
TGGTAAAAAAGAAGGTAGTATTCCTGATTAT---TCAGAACTTAATCGAATCATATGCACT
GGTCATTGTAATTTCATATATCCT---GCCATTCTCCACGAGAATTTT------TATTTATTGGCA
AAGAGACGAAGAAATAGATTCATCATTCCATTCCAATCGATTCAAGAACGAGAGAAAG
AACTAATGCCCCGTTCAGGTATCTCAATTGAAATACCC------ATAAATGGTATTTTCCGTA
GAAATAGTATTCTTGCTTATTTCGATGATTTTAGATACAGAAGAACTGGTTCGGGAATTA
CTAAATATGGGACTATAGAGATGCATTCAATCATCAAAAAAGAGGAT------TTGATTGAG
TATCGAGGG---GTCAAAGAATTTAGGCCAAAATACATCAAAGTAGATCGGTTTTTTTTCA
TTCCCGAGGAAGTACATATCTTACCCGGATCTTCTTACGTAATGGTACGGAACAATAGTA

TCATTGGAGTAGATACACAAATCACTTTTAATACAAGAAGCCGAGTAGGCGGATTGGTC
CGAGTGGAGAAAAAAAAAAAAAAGTATTGAACTTAAAATATTTTCTGGAAATATCCATTT
TCCTGGCGAGACAGATAAGATATCCCGACACAGTGGGATCTTGATCCCACCAGGAACG-
-----GGAAAAACAAATTCCAAGGAATCCAAAGAA------TTGAAAAATTGGATCTATGTCC
AACGG------ATTACACCTACCAAG------AAAAAATATTTTGTTTTGGTTCGACCTGTAATC
ACATATGAAATAACAGACGGTATAAATTTAGCAACACTTTTCCCCCAGGATCTGTTGCA
GGAAAGGGATAATGTGCAATTTCGAGTTGTCAATTATATCCTTTATGGAAATGGCAACC
AAATTCGGGGAATTTCTGACACAAGTATTCAATTAGTTCGGACTTGTTTAGTATTGAATT
GGGACCAAGACAAAAAG---------AACTCTTCTATCGAAGAGGCTCCTGCTTCTTTTGTTG
AAGTAAGGACAAATGGTCTGATTCGAGATTTCCTAAGAATAGACTTAGTGAAATCCCCT
ATTTCGTATACCGGAAAAAGGAATGATCCGGCAGGTTCAGGGTTGATCTCTGAT---AAT
GGATCAGATCGCACCAATATCAATCCCTTTTATTCCATTTATTCCAAGGGAAGG---ATTC
AACAATCACTTAGCCAA------AATAAAAATCAAGGAACTATTCGTACGTTGTTGAATAGA
---------AATAAGGAATGCCAATTTTTTCTAATTTTGTCATCATCCAATTGCTCCCGAATGG
GTCCA------TTTAACGGTGTAAAATATAAGAATGTG---------ATAAAAGAATCAGATCCCCT
AATTCCAATTAGGGATTCGTTGGGTCCTTTAGGAACA------GCCCTTAAAATTGCGAATT
TTTATTCA------TTTTACCATTTA------ATAACCCATAATCACATCTTG---GTAACGAAATATT
TACAACTTGACAAT---------TTAAAACAAACTTTTCAAGTACTTACA---------TATTATTTAA
TGGATGAAAAT------GGGAGGATTTATAATCCTGATCTATGCAGTAACATTATTTTGAATA
CATTCAATTTGAATTGGTATTTTCTTCATCACAATTTTCGTGAAGAGACATCC---ACAATA
ATTAGTCTTGGACAGTTTATTTGTGAAAATGTATGTATAGCCAAAAATGGACCACAC---C
TAAAA------TCGGGTCAAGTTATAATTGTTCAA---GTTGGCTCTGTAGTAATAAGATCAGC
TAAGCCTTATTTGGCCACGCCAGGAACAACTGTTCATGGCCATTATGGAGAAATACTTT
ATGAAGGAGATACATTAGTTACATTTATATATGAAAAATCGAGATCTGGTGACATAACGC
AGGGTCTTCCAAAAGTAGAACAAGTGTTAGAAGTACGTTCGATTGATTCAATATCGCTA
AACCTAGAAAAAAGGATTGAGGGTTGGAACAACCGTATAACAAGAATTCTTGGAATTC
CGTGGGGATTCTTAATTGGTGCTGAGCTAACTATAGTGCAAAGTCGTATCTCTTTGGTTA
ATAAGATACAAAAGGTTTATCGATCCCAAGGGGTGCAGATCCACAATAGGCATATAGAA
ATTATTGTACGTCAAATAACATCAAAAGTATTGGTTTCAGAA---------GATGGAATGTCTA
ATGTTTTTTCACCCGGAGAACTAATTGGATTGTTGCGAGCGGAACGAACGGGGCGTGC
TTTGGAAGAAGCGATCTGTTACCGAGCCATATTATTGGGAATAACGAGGGCATCTCTGA
ATACTCAAAGTTTCATATCCGAAGCTAGTTTTCAAGAAACTGCTCGAGTTTTAGCAAAA
GCTGCTCTCCGGGGTCGTGTCGATTGGTTGAAAGGCCTGAAAGAGAACGTTGTCCTGG
GGGGTATGATACCGGTTGGTACTGGGTTCAAAGGATTA------GTGTAC-----------TATAAC
AACATTCCT------TTGGAACCCAAAAAGAAGAATCTATTTGAGGGGGAAATGAGA------G
ATATTTTGTTCCACCACAGAGAATTATTTGATTCTTTCATTTTAAAGAATTTACATGATAC
ATCAGAACAATCATTTATGGGA-----------------TTTAATGATTGCATGACAAAAAGATATTG
GAACATAAATTTGGAAGAGATGATGGAAGCAGGAGTTCATTTTGGTCATGGTACTAGA
AAATGGAATCCTAGAATGGCACCTTATATCTCTGCAAAGCGTAAAGGTATTCATATTACA
AATCTTACTAGAACTGCTCGTTTTTTATCAGAAGCTTGTGATTTAGTTTTTGATGCAGCA
AGTAGGGGAAAACAATTCTTAATTGTTGGTACCAAAAATAAAGCAGCTGATTCAGTAG
CGTGGGCTGCAATAAGGGCTCGGTGTCATTATGTTAATAAAAAATGGCTCGGTGGTATG
TCAACGAATTGGTCCACTACAGAAACGAGACTTCATAAGTTCAGGGACTTAAGAGCGG
AACAAAAGACGGGTAGACTCAACCGTCTTCAGAAAAGAGATGCAGCTGTGTTGAAGA

GACAATTATCTCACTTAGAAACATATCTGGGTGGGATTAAATATATGACGTGGTTACCTG
ATATTGTAATCATCGTTGATCAGCAAGAAGAATATACGGCTCTTCGAGAATGTGTCACTT
TGGGAATTCCCACGATTTGTTTAATTGATACAAATTGTGACCCGGATATCGCAGATATTT
CGATTCCAGCGAATGATGACGCTATAGCTTCAATCCGATTAATTCTTAACAAATTAGTAT
TCGCAATTTGTGAGGGTCGTTCTAGC---TATATAAGAAATCCTATGGGACAAAAAATAAA
TCCACTTGGTTTCAGACTTGGTACAACTCAAGGTCATCATTCCCTTTGGTTTGCACAAC
CCAAAAATTATTCAGAGGGTCTACAAGAAGATCAAAAAATACGTAATTGTATCAAGAAT
TATATTCAAAAAAATATTAAAATATCCTCTGGT---GTCGAGGGAATTGCCCGTATAGAGAT
TAAAAAAAGAATCGATCTAATCCAGGTCATAATCTATATGGGATTCCCAAAATTATTAAT
AGAAGGTAGACCACAA------------GGAGTCGAAGAATTACAGATCAATGTACAAAAAGA
ATTTAATTCTGTAAACAAAAAACTCAACATTGCTATTACAAGAATTGAAAAACCTTACG
GAAACCCCAATATTCTTGCAGAATTTATAGCCGGACAATTAAAAAATAGGGTTTCATTTC
GCAAGGCAATAAAAAAAGCTATTGAATTAACTGAACAAGCAGATACAAAAGGAATTCA
AGTACAAATTGCAGGACGTATCGACGGAAAAGAGATTGCACGTGTCGAATGGATCAGA
GAAGGTAGGGTTCCCCTACAAACCATTCGAGCAAAAATTGATTATTGTTCCTATACAGT
TCGAACTATCTATGGGGTATTAGGCATCAAAATTTGGATATTTGTAGACGAAGAAATGTC
GCGTTACCGAGGGCCTCGTTTCAAAAAAATACGCCGTCTGGGAGCTTTACCGGGACTA
ACTAGTAAAAGGCCTAGAGCCGGAAGTGATCTTAGAAACCAATCACGTTCCGTGAAA--
-AGATCTCAATATCGGATTCGTCTAGAAGAAAAACAAAAATTGCGTTTTCATTATGGTCT
TACAGAACGACAATTACTTAAATATGTTCGTATCGCCGGAAAAGCCAAAGGGTCAACA
GGTCAGGTTTTATTACAATTACTTGAAATGCGTTTGGATAACATCCTTTTTCGATTGGGT
ATGGCTTCGACTATTCCTGGAGCCCGCCAATTAGTTAACCATAGACATATTTTAGTGAAT
GGTCGTATAGTGGATATACCAAGTTATCGCTGCAAACCCCGAGATATTATTACGGCGAG
GGATGAAGAAAAATCCAGAGCTCTGATTCAAAATTTTCTTGATTCATCCACACAT---GC
GGAATTACCAAAACATTTGACTCGTCACCCATTTCAA------TATAAAGGGTTAGTCAATC
AAATAATAGATAGTAAATGGGTCAGTTTGAAAATAAATGAGTTGTTAGTCGTAGAATATT
ATTCCCGTCAGATTATGTCACGTCGAGGTACTGCAGAAGAAAAAACTGCAAAATCCGA
TCCAATTTATCGTAATCGATTAGTTAACATGTTGGTTAACCGTATTCTGAAACACGGAAA
AAAATCATTGGCTTATCAAATTATCTATCGAGCCATGAAAAAGATTCAACAAAAGACAG
AAACAAATCCACTATCTGTTTTACGTCAAGCAATACGTGGAGTAACTCCCGATATAGCA
GTAAAAGCAAGACGTGTGGGCGGATCGACTCATCAAGTTCCCATTGAAATAGGATCCA
CACAAGGAAAAGCACTTGCCATTCGTTGGTTATTAGGGGCATCCCGAAAACGTCCGGG
TCGAAATATGGCTTTCAAATTAAGTTCCGAATTAGTGGATGCTGCCAAAGGGAGTGGCG
ATGCCATACGCAAAAAGGAAGAGACTCATAGAATGGCAGAGGCAAATAGAGCTTTTGC
ACATTTTCGTATGGGTAAGGACACTATTGCTAACATAATAACCTCTATACGAAATGCTGA
CATGGATAGAAAAGGAACGGTTCGAATAGCATTTACTAACATCACCGAAAACTTTGTTA
AAATACTTTTACAAGAAGGTTTTATCGAAAACGTGAGGAAACATCAGGAAAGAAACA
AATATTTCTTAGTTTTAACCCTGCGACATAGAAGG------AATAGGAAAGGGCCATATAGA
ACT---ATTTTAAAT------TTAAAACGGATCAGCCGACCCGGTCTACGAATCTATTCTAACTA
TCAACGAATTCCTAGAATTTTAGGTGGAATGGGAATTGTAATTCTTTCTACTTCTCGAGG
TATAATGACAGACCGAGAGGCTCGACTAGAAAGAATCGGCGGAGAAATTTTGTGTTATA
TATGGATGGCAAAACCTATACCAAGAATTGGTTCACGTAGGAATGGACGTATTGGTTCA
CGTAAGAATGCACGTAGAATACCAAAGGGAGTTATTCATGTTCAAGCAAGTTTTAACAA
TACCATTGTTACTGTTACAGATATACGGGGTCGGGTGGTTTCTTGGTCCTCCGCTGGTAC

TTGTGGATTCAGCGGTACAAGAAGAGGGACGCCATTTGCTGCTCAAACCGCAGCAGG
AAATGCTATTCGTACAGTAGTGGATCAGGGTATGCAACGAGCAGAAGTCATGATAAAG
GGTCCTGGTCTCGGAAGAGATGCAGCATTACGAGCTATTCGTAGAAGTGGTATACTATT
AAGTTTCGTACGAGATGTAACCCCTATGCCACATAATGGCTGTAGACCTCCTAAAAAAA
GACGTGTTATGCCAACTATTAAACAACTTATTAGAAACACAAGACAGCCAATCAGAAAT
GTTACGAAATCCCCCGCGCTTCGGGGATGTCCTCAGCGCCGAGGAACATGCACTAGGG
TGTATACTATCACCCCCAAAAAACCAAACTCTGCCTTACGTAAAGTTGCCAGAGTACGA
TTAACCTCTGGATTTGAAATCACTGCTTATATACCTGGTATTGGCCATAATTCACAAGAA
CATTCTGTAGTCTTAGTAAGAGGGGGAAGGGTTAAGGATTTACCCGGTGTGAGATATCA
CATTGTTCGAGGAACCCTAGATGCTGTCGGAGTAAAGGATCGTCAACAAGGGCGTTCT
AAATATGGGGTCAAAAAGCCAAAAATGGCAAGGAAAAGTTTGATTCAGAGGGAGAAG
AAGAGACAAAAATTGGAACAGAAATATCATTTGATTCGTCGATCCTCAAAAAAAGAAA
TAAGC---------AAAGTTCAGTCGTTGAATGACAAATGGGAAATTCATGGAAAGTTACAAT
CCCCACCACGTAATAGTGCACCTACACGTCTTCATCGACGTTGTTTTCAACCGGAAGA
CCGAGAGCTAACTATCGAGATTTTGAGCTATCCGGACATATACTCCGTGAAATGGTTGA
TGCATGTTTGTTGCCAGGGGCAACAAGATCAAGTTGGATGGCAAAAAATTCATTCATCT
CAGTTATTTCGCAAAAAGAAAAAGAAAAA------AACACGGGGTCTATTGAATTTCAAGT
ATTAAGTTTCACCAATAAAATACGGAGACTTACTTCACATTTGAAATTACACAGAAAAG
ATTATTTATCTCAGAGAGGTCTACGAAAAATTTTGGGAAAACGTCAACGGATGCTGACT
TATTTGTCAAAGAAAAATAGAGTACGTTATAAAGAATTAATTGATCAGTTGGATATTCGA
GATACAAAAACTCGT------ATGGTAAAACTTCGTTTGAAACGATGTGGTAGAAAGCAAC
GAGCCGTCTATCGAATTGTTGCAATTGATGTTCGATCCCGAAGAGAAGGAAGAGATCTT
CAGAAAGTGGGTTTTTATGATCCGATAAAGAATCAAACTTATTTAAATGTTCCTGCTATT
CTATATTTCCTTGAAAAAGGTGCTCAACCTACAGGAACTGTTTATGATATTTCAAAGAA
GGCAGAGGTTTTTAAGGAACTTCGCGTTAATCAAACGAAATGTAAT---ATGGATAAATC
CAAGCGACCCTTTCTTAAATCCAAGCGATCTTTTCGTAGGCGTTTGCCCCCGATCCAAT
CGGGGGATCGAATTGATTATAGAAACATGAGTTTAATTAGTCGATTTATTAGTGAACAA
GGAAAAATATTATCTAGACGGGTGAATAGGTTGACCTTGAAACAACAACGATTAATTAC
TATTGCTATAAAACAAGCTCGTATTTTATCTTCATTACCTTTTCTTAACAATGAAAAA------
CAATTTGAAAGAATCGAGTCGACTGCTAGAACTACTGGTCTTAGAACCAGAAATAAAG
TGACACGTTCACTAAAAAAAAACCCTTTCGTAGCAAATCAGTTATTAAGAAAAATTGAT
AAGCTTAACACAAAGGCAGAAAAAGAAATAATAGTAACTTGGTCCCGGACATCTACCA
TTATACCCACAATGATCGGCCATACTATTGCTATCCATAATGGAAAAGAACATTTACCTAT
TTATATAACAGATCGTATGGTAGGCCACAAATTGGGAGAATTTGCCCCTACTCTAAATTT
CCGGGGACATGCCAAAAACGATAATAAATCTCGTCGTATGATTTTGAAATCTTTTCTACT
AAGTAATCTAGTATCCTTATGCATGAAGATAATCAATTCGGTCGTTGTGGTCGGACTCTA
TTATGGATTTCTGACCACATTCTCCATAGGGCCCTCTTATCTCTTACTTCTCCGAGCTCA
GGTTATGGAAGAAGGA---------ACCGAGAATAAGGTATCAGCCACAACTGGTTTTATTAC
GGGACAGCTCATGATGTTCATATCGATCTATTATGCGCCTCTGCATCTAGCCTTGGGTAG
ACCTCATACAATAACTGTATTAGCTCTACCGTATCTTTTGTTTCATTTCTTCTCGAACAAT
CACAAAAACTTTTTTTATTATGGATCTACTAGCAGAAATTCAATGCGTAATTTCAGCATT
CAATGTGTATTCCTGAATAATCTAATTATTCAATTATTCAACCTTTTTCTTTTACCAAGTT
CAATGTTAGCCAGATTAGTCAACATTTATATGTTTCGATGCAACAACAAGATGTTATTTG
TAATAAGTAGTTTTGTTGGTTGGTTAATTGGTCACATTTTATTCATGAAATGGGTTGGAT

TGGTATTAGTCTGGATACGGCAAAATAGATCT------------ATTAGATCTAATAAGTACCTTG
TGTCAGAATTGAGAAAT------TCTCGAATCGTTAGTATTCTCTTATTTATTACCTGTGTCTA
CTATTTAGGCAGAATACCGTCACCCATTTTTACTAAAAAACTGAAAGAAACCGAAGAA
GATAAACCTCTTGTAACTCTTCTTTTCGACTATAAAAGGTGGAATCGTCCATTAAGATAT
ATAAGA---TATGAGTTTGAAAATACTTTAAAAAATGAAATGTCACAATATTTTTTTATAC
ATGTAGAAGTAATGGAAAAGAAAGAATATCTTTTATGTATCCACCCAGTTTGTCAAATTT
TTTTGAAATGATACAAAAAAAGATGTTTTTGTACACAAAAGAAACATTTTTATCTGATG
AACGATATAATCATTGGGTTTATACCAATGAAAAAAAAAGGAACAACTTAAGCAACGA
ATTTATAAATAGAATAAAAGCTTTAGACAAAAGACCCATTTTTCTGGATATATTCGAAAA
AATAACTAGA------TTGAATAATAATAAAAAAAAATACTTACCTAAAACATATGACCCCTT
CTTAGCT---CCTTATCGTGGAACAATAAACAAATTGTCTTCACCTATAGAGATAGCTTCG
ATTAATAAGATTTATAGTATACTTTACCAAGAATTTGAACAGAAAGATATATCAAATATA--
-------------GAAATCAGTAAAAAAGTTCCTTTATGGACACACAAATTCATCGAGTTTGAAC
AAATCGAGAGAGAAGAAATTCGTTCAAGAAAAGTCAAACATGCAGCTTATGTTAGTGA
TAATGTGATAGATTTTGAGACAGAATCGGATTTTCGCCGGGACATAATTAAAGGATCCA
TGCGCGCTCAAAGACGTAAAACGGTTATTTTTAAACTGTTTCAATCAAATGCACATTCC
CCTCTTTTTTTGGACAGA---ACAGACCCCCTTTTTTATTTTGATAGCTTTGGACTGACGA
AACTAATTTCT---------AGAAATAAAGAAAGCAACGAACAGAAGATAAAAATTGAAGAG
TGGGATAACATTATCTTTGGTCACGTAATAAGGAGTTGCATATTATTAATCCAATCGATTC
TTAGAAAATATATGATATTTCCCTTATTGATAATAATTAAAAATATCGGCCGTATATTATTA
TTTCAAGTTGCCGAATGGGGTGAGGATTTTGCAGATTTGAAAAAAGAAATACATGTTAT
TTCCAATAAGAGTGGCGTTGAATTATCAGAAAAAGAAGTTAGGATAACTTCTTTTTTAA
ACGGTATTCAAATAAAAATCCTATTTCCTTTCTGTTTGAAACCTTGGCATAAATATAAGG
ATGAAAATACAACAAAAAAGAAAAAAAATAAAGTCAAAAAAATGACAGCTGAACGGC
CTTTTGGTTTTCCCCAACTCGAGCCTTCATTTTTTGGACCCATTTTTAACGAACTAAAA--
---AAAAAAATTTTTCAATATATTTTAATGGTCTCAAA
CAAAATCAAAAAATGGGTTATCAACGGCATTCTATTTATAAAAATAATAATAATGAAAGG
ACTTTCACAAGTAAACCCAATTGATTCTATAATCAGTAATCAAATGATTAATTACCCG-----
-------TACATTCAAATTCTATCTACAGATCAGAAAAATTATTTACTGACAGAAAAACAAAT
AAAGGATTCGGCTGAGATAATAAGAACAATAAAAAAT-----------------------------AAAATA
GAAAGAAAAGACAAGAAAATAGAATCACCAAAAAATATTTGGCAGATATTAAAA------A
ATGTTCGAGTAATACATAAATCACATTATTTTTTAAAATTATTCATTGAAAAGATATACAT
AGATATCCTTATATCTATTATTAATTCTATAATAGATATTCCCAGAATTAAT--------------CAT
GACTTATCCTACTTATCGCAAGCATATGTATTTTACAAATTATCACAGAACCAAGTTTCT
AACTTAAGC---------TTTGTCCTTCAATATACATCTTTTTTTTCTTAAAAATGAAATAAAAGA
TTATTTAACACACGGAATATTTCATTCCGAGTTACAGCATAATAAAATTCATAATTATGAA
ATGAATCAATGGAAAAACTGGTTAAGGGTTCATTATCAATATGATTTCTCTCTGATTAGG
TGGTCTAGATTAGTAATACAAAAAAGGCAAAATAAAGTAAACCAATGTCGTACAGTTC
AAAATAAAAACAAATGGGATTCATATGAAAAAGATCGA------TTAATTCATTATGAAAAA
CAA---AATTTGAAAAAACACTATATATATAATCTTTTATCATATAAATATCTTAATATAAAT
CTGGATAGACCGGATAGAAAATATTTTGGTTTTGGAATTCTAAATTTTTGTCTTAGA------
------------------------------------TTCTTACCA---GAATTTAGGCAACTTTATAATGCATATAAGAT
TAAACCATGGGTCCTACCAATCAAAGTACTTTTTTTTGATAAGCATATAAACATAGAAAC
AGAACCCGATTTTTTACTGAAAAAGTATTTGCTTTTTCAATTGGCATGGTAT---------------

--ATATTCAATAATGTCAAGGCATATTATATCCTGTTTAGGGCGATAGAGGCAAGAGAAAT
TTCTATAGCCTATATTAAAAACGGACAAGTGAGTCTGGATCTAATGCGG---ATTCATGAT
CATTTAACTCTTAATGAATTGGTGAAAAGGCTATTGTTGATTGACCCGATTCGTCTGTCT
ATAAACAATGATAGACAAATTATTATGTATCAAACCATAGGTATTGCATTGGTTCATAAA
AATAAGCACAATAATTATGATTTTCTTGTTCCTGAAAATATTTTATCATCTAGACGTCGTA
GAGAATTGAGA------ATTCTAAGTGTATTTCTTTGGCCCAATTATCGATTAGAAGATTTAG
CTTGTATGAATCGTTATTGGTTTAATACCAATAATGGCAGTCGTTTCAGTATGTTAAGGAT
ACATATGTATCCAATGAAAGGACATCAATTCAAATTCTGGATTTTCGAATTGAGAGAGAT
ATTGAGAGAGATCAAGAATTCTCACTCTTTCTTAAATTCATGGACCCAATTCAATTCAG
TGGGATCTTTCATTCACATTTTTTTCCACCAAGAACGTTTTATAAAACTCTTTGACCCCC
GAATTTTGAGTATCCTACTTTCA---CGCAATTCA------CAGGGTTCAACAAGCAATCGATA
TTTCACG---------ATCAAGGGTGTACTACTATTTGTAGTAGCG---GTCCTTATATATCGT------
------ATTAACAATCGAAATATGGTCGAAAGAAAAAATCTCTATTTGATAGGGCTTCTTCCT
---------------ATATCTATGAATTCCATTGGACCCAGAAATGATACATTGGAAGAATCTTTT---
GGGTCTTCCAATATCAATAGGTTGATTGTTTCG---CTCCTGTATCTTCCAAAAGTAAAAA
ATATTTCTGAGAGTTGTTTCCTGGATCCGAAAGAGAGTACTTTGGTTCTCCCAATAACT-
-----------AAAAAGTGTATCATGCCTGAATCTAACTGGGGTTCGCGGTGGTGGAGGAACT
GGATCGGAAAAAAGAGG------GATTCTAGTTGTAAGATATCTAAT---------GAAACCGTCG
CTGGAATTGAGATCTCATTCAAAGAGAAAGATATCAAA---TATCTGGAGTTTATTTTTGT
ATATTATATGGAT------GATCCGATCCGCAAGGACCATGATTGGGAATTGTTTGATCGTCT
TTCTCCGAGGAAGAGGCGAAACATAATCAACTTG------AATTCGGGAGAGTTATTCGAA
ATCTTAGTGAAACACTGGATTTGTTATCTCATGTCTGCTTTTCGTGAAAAAATACCAATT
GAAGTGGAGGGT---TTCTTCAAACAACAAGGA---GCTGGGTCAACTATTCAATCAAATG
ATATTGAGCATGTTTCCCAT------CTCTTCTCGAGAAACAAGTGGGCTATTTCTTTGCAAA
ATTGTGCTCAATTTCATATGTGGCAATTCCGCCAAGATCTCTTCGTT---------AGTTGGGG
GAAG---AATCCGCACGAATCGGATTTTTTGAGGAAC---GTATCGAGAGAGAATTGGATT
TGGTTAGACAATGCGTGGTTGGTAAACAAGGATCGG---------TTTTTTAGC------AAGGTA
CGGAATGTATCGTCAAATATTCAATATGATTCC---------ACAAGA------TCTAGTTTCGTTC
AAGTAACGGATTCTAGCCAATTGAAAGGATCTTCT---GATCAATCCAGAGATCATTTCGA
TTCCATTAGT------------AATGAGGATTCGGAA------------------------------------TATCACACA
TTGATCAATCAAAGAGAA---------CTAAAAGAAAGATCGATTCTT---TGGGAT------CTTTC
CTTTCTTCAAACGGAACGAACAGAGATAGAATCAGAC------CGATTCCCGAAA------TGC
CTTTCTGGATATTCC------TCAAGGTCCCGGCTATTCGCG---GAACGTGAGAAGCAGATG
AATAATCATCTGCTTCCGGAAGAAATCGAAGAATTTCTT------GGGAATCCTACAAGATC
CATTCGTTCTTTTTTCTCTGACAGATGG------TCAGAACTTCAT---CTGGGTTCGAATCCT
ACTGAGAGGTCCACTAGAGATCAG------------AAATTGTTGAAGAAACAACAAGATGTTT
CT---TTTGTCCCTTCCAGGCGATCGGAA------------AATAAAGAAATGGTTAATATATTCAA
GATAATTACGTATTTACAAAAT------ACCGCCTCAATTCATCCTATTTCATCAGATCCGGGA
TCTGGTATGGTTCCG---AAGGATGAACCGGAT---ATGGGCAGTTCCAATAAGATTTCATT
CTTGAACAAAAATCCATTTTTTTATTTA------TTTCATCTATTCCATGACCGGAACAGGGG
GGGATACACGTTA------GACCAAGATTTTGAATCAGAA------GAGAGATTTCAAGAAATG
GCAGATCTATTCACTCTATCAATAACCGAGCCG---GATCTGTTGTATCATAAGGGGTTTG
CCTTTTCTATTGATTCTTACGGATTGGATCAAAAACAA------TTCTTGAATGAGGTATTCA
ACTCCAGGGATGAATCGAAAAATAAATCTTTA---------TTGGTTCTACCTCCTATTTTTTAT

------GAAGAGAATGAATCTTTTTATCGAAGGATCAGAAAAGAATGGGTCCGG---ATCTCC
TGCAGGAATGATTTGGAAGATCCAAAACAAAAAATAGTGGTATTTGCTAGCAACAACA
TAATGGAGGCAGTCAATCAATATAGATTGATCCGAAATCTGATTCAAATCCAATATAGCA
CCTATGGG------TACATAAGAAATGTATTGAATCGATTCTTTTTAATGAAT---------AGATCC
GATCGCAACTTCGAATATGGAATTCAA---------AGGGATCAAATAGGAAATGATACTCTG
AATCATAGAACTATAATGAAATATACGATCAACCAACATTTATCGAATTTGAAAAAG---A
GTCAGAAGAAATGGTTCGATCCT------CTTATTTTTATTTCTCGAACCGAGAGATCCATGA
ATCGGGATCCTGATGCATATAGATACAAATGGTCCAATGGGAGCAAGAATTTACAGGAA
CATTTGGAACATTTCGTTTCTGAGCAGAAGAGCCGT---------TTTCAAGTAGTGTTCGATC
GATTACGTATTAATCAATATTCGATTGATTGGTCTGAGGTTATCGACAAAAAAGATTTGT
CTAAGTCACTTCGTTTCTTTTTGTCCAAGTTG---
-------------CTTTTCTTTTTGTCTAAC---TCACTTCCTTTTTTCTTTGTGAGTTTCGGGAATA
TCCCCATTCATAGGTCCGAG---ATCCATATCTAT---GAATTGAAAGGTCCGAATGATCAAC
TCTGCAATCAGTTGTTAGAATCAATAAGTCTTCAAATCGTTCATTTGAAAAAATTGAAA
CCCTTC------TTATTGGAT---------GATCATGATACTTCC---CAAAAATCTAAATTCTTGATCA
ATGGAGGAACAATATCACCATTTTTGTTCAATAAGATACCA---AAGTGG---ATGATTGAC
TCATTCCATACTAGA------AATAATCGCAGGAAATCCTTTGATAACACGGATTCCTATTTA
TCAATGATATCCCACGATCAAGACAATTGGCTGAATCCCGTGAAACCATTTCATAGAAG
TTCATTGATATCTTCTTTTTATAAAGCAAATCGACTTCGATTCTTGAATAATCCACATCAC
TTCTGCTTCTAT------TGTAACAAAAGATTCCCTTTTTATGTG------------GAAAAGGCCCGT
ATCAATAATTATGAT------TTTACGTATGGA---CAATTCCTCAATATCTTGTTCATTCGCAAC
AAA---ATATTTTCTTTGTGCGTCGGTAAA------AAAAAACATGCTTTTTTGGAG---AGAGA
TACTATTTCACCAATCGAGTCACAGGTATCTAGCATATTCATACCTAACGATTTTCCACA
AAGTGGTGACGAAACG---TATAACTTGTACAAATCT---------TTCCATTTTCCAATTCGATC
CGATCCATTCGTTCGT------AGAGCTATTTACTCGATCGCAGACATTTCTGGAACACCTCT
AACAGAGGGACAAATAGTCAATTTTGAAAGAACTTATTGTCAACCTCTTTCAGATATGA
ATCTATCTGATTCAGAAAGGAAGAACTTGCATCAGCATTTCAAT------TTCAATTCAAAC
ATGGGTTTGATTCACACTCCATGTTCTGAGAAATATTTACCA------TCCGAAAAGAGGGA
AAAA------------------------CGGAGTCTTTGTCTAAAT------AAATGCGTTGAGAAAGGGCAG
ATGTATAGAACCTTTCAACGAGATAGTGCTTTTTCAACTCTCTCAAAATGGAATCTATTC
CAAACATATATGCCATGGTTCCTTACTTCGACAGGGTACAAATATCTAAATTTGATATTTT
TAGATACTTTTTCAGACCTATTGCCG---ATACTAAGTAGAAGTATC---AAAAAATTTGTAT
CCATTTTTCATGATATTATGCATGGATCGGATATATTATGGCGAATTCTTCAGGAAAAATT
G-----------------TGTCTTCCAGAATGGAATCCG---ATAAGTAAGATTTTGAGTAAGTGTTTA
CATAAGCTTAAGCTTCTTCTGCTCGAAGAAATGATTCATCGAAATAATGAGTCA------CC
ATTGATATCGACACATCTGAGACCGCCAAATGTTCGGGAGTTC---CTTTATTCAATCCTT
TTCCTTCTTCTTGTTGCTGGATAT---CTCGTTTGGACACATCTTATCTTTGTTTCCATTTCC
GCAGCCTCTAGTGAGTTACAGACAGAGTTCGAAAAGGTCAAATCTTTGATGATTCCAT
CATACATGATTGAGTTGCGAAAACTTCTGGATAGGTATCCTACATCTGAACTGAATTCTT
TCTGGTTAAAGAATCTCTTTCTAGTTGCTCTGGAACAATTAGGAGATTCTCTAGAAGAA
ATATGGGGTTCTGCTTTTGGCGGCAACATGCTATTGGGTGGTGGTCCT--------------ACTT
ATGGG---GTCAAATCAATACGTTCTAAGAAGAAATATTTGAATATCAATCTCATCGATATC
ATCGATCTCATA---AGTATCATACCAAATCCCATCAATCGAATCACTTTTTCGAGAAATAC
GAGACATCTAAGTCATACAAGTAAAGAGCTCTATTCATTGATAAGAAAAAGAAAAAAG

GTGAAAGGTGCTTGGATTTATGATAAAATAGAATCCTGGGTCGCGAACAGTGATTCGAT
TGATGATGAAGAAAGAGAATTCTTGGTTCAGTTCTCC------ACCTTAACGACA------GAA
AAAAGGATTGATCAAATTCTATTGAGTATGACT---CAGAGTGATCATTTATCA--------------
AAGAATGACTCTGGTTATCAAATGATTGAACAACCGGGAGCAATTTACTTACGATACTT
AGTTGACATTCAT------AAAAAGTATCTAATGAATTATGAGTTCAATACATCCTGTTTAGC
AGAAAGACGGATATTCCTTGCTCATTATCAGACAATCACTTAT------TCACAAACCTCGT
GTGGGGCTAATAGTTTTAATTTCCCATCTCATGGA---------AAACCCTTTTCGCTCCGCTT
AGCCCTATCCCCCTCT---AGGGGTATTTAGTGATAGGTTCTATAGGAACTGGACGATCC
TATTTGGTCAAATACCTAGCGACAAACTCCTATGTTCCTTTCATTACGGTATTTCTGAAC
AAGTTCCTGAATAACAAGCTTAAAGGTTTTATTTTTGATGATAGTGATAGTGACGATATT
GATGATATTGATGATAGTGACGATATTGATGCTAGTGACGATATTGATGCTAGTGACGATA
TTGAT-----------------------------------GATAATGACGATATCGATCGTGACCTTGATCCGGAGC
TGGAGCTGCTAATGGATGCGCTAACTATA---ACTATGGATATGATGCCG---------GAAATAG
ACCTATTTTATATCACCCTTCAATTCGAATTAGCAAAAGCAATGTCTCCTTGCATAATATG
GATTCCAAACATTCATGATCTGGATGTGAATGAGTCGAATTACTTATCCCTCGGTCTATT
AGTGAACTATCTCTCC---------AGGGATTGTGAAAGATGTTCC---ACTAGAAATATTCTTG
TTATTGCTTCGACTCATATTCCCCAAAAAGTGGATCCCGCTCTAATAGCTCCGAATAAAT
TAAATACATGCATTAAGATACGAAGGCTTCTTATTCCACAACAACGAAAGCACTTTTTC
ATTCTTTCATATACTAGGGGATTTCACTTGGAAAAGAAAATGTTCCATACT------AATGGA
TTCGGGTCCATAACCATGGGTTCCAATGCACGATATCTTGCAGTACTTACCAATGAGGC
CCTATCAATTAGTATTACACAGAAGAAATCAATTATAGACACTAATACAATTAGATCCGC
TCTTCATAGACAAACTTGGGATTTGCGA---------TCCCAGGTAAGATCGGCTCAGGATCA
TGGGATCCTTTTCTATCAGATAGGAAGGGCTGTTGCACAAAATGTACTTCTAAGTAATT
GCCTCATAGATCCTATATCTATCTATATAAAGAAGAAA------TCATGTAACGAAGGGGATT
CTTATTTGTACAAATGGTACTTCGAACTTGGAACGAGCATGAAGAAATTAACGATACTT
CTTTATCTTTTGAGTTGTTCTGCCGGATCGGTCGCTCAAGATCTTTGGTCTCTACCC------
GGACCCGATGAAAAA---AATGGTATCACTTCTTATGGACTCGTTGAGAATGATTCT---GA
TCTAGTTCATGGCCTATTAGAAGTAGAAGGCGCTCTGGTGGGA------TCCTCACGGACA
GAAAAAGATTGCAGTCAGTTTGATAATGAT------CGAGTGACATTGGTTCTTCGGCCCGA
ACCAAGGAAT---CCCTTAGATATGATGCAAAATGGATCTTGTTCTATCGTTGATCAGAGA
TTTATCTATGAAAAA------TACGAATCGGAGTTTGAAGAA---GGGGAAGGAGAAGGAGC
CCTCGACCCGCAA------------CAGATAGAGGAGGAT------------TTATTCAATCAC------ATAG
TTTGGGCTCCT------AGAATATGGCGCCCT---------TGGGGCTTTCTATTTGATTGTATCGAA
AGGCCCAATGAATTGGGATTCCCC---TATTGGGCCAGGTCATTTCGGGGCAAG---------C
GGATCATTTATGATGAAGAGGATGAGCTTCAAGAGAATGATTCGGAGTTCTTGCAGAGT
GGAACCATGCAGTACCAGACACTAGATAGATCTTCCAAAGAACAAGGCTTTTTTCGAA
TAAGCCAATTCATTTGGGACCCTGCAGATTCACTCTTTTTCCTATTCAAA---GATCAGCC
CTTTGTCTCTGTGTTTTCACATCGAGAATTCTTTGCAGATGAAGAGATGTCAAAGGGGT
TTCTTACTTCCCAAACAGATCCTCCTACATCTATATATAAACGCTGGTTCATC------AAGA
ATACGCAAGAAAAGCACTTCGAATTGTTGATTTATCGCCAGAGATGGCTTAGAACC------
---AATAGTTCATTATCT------AATGGAGCTTTCCGTTCTAAT------ACTCCATCCGAGAGTTA
TCAGTATTTATCAAATCTGTTCCTATCTAAC---GGAACGCTATTGGATCAAATGACAAAG
ACATTGTTGAGAAAAAGATGGCTTTTCCCGGATGAAATGAAAATTGGATTC------ATG----
----------------------ATGCCTAGATCGCGG---ATAAATGGAAATTTTATTGATAAGACCTTTTC

AATTGTAGCCAATATCTTATTACGAATAATTCCGACAACTTCAGGAGAAAAAGAGGCAT
TTACCTATTACAGAGATGGG------ATGTCAGCTCAATCCGAAGGAAATTATGCGGAAGCT
TTACAGAATTATTATGAAGCTATGCGACTAGAAATTGATCCCTATGATCGAAGTTATATAC
TCTATAACATAGGACTTATCCACACAAGTAACGGAGAACATACGAAAGCTTTGGAATAT
TATTTTCGGGCACTAGAACGAAACCCATTCTTACCGCAAGCTTTTAATAATATGGCCGTG
ATCTGTCATTAC---CGGGGAGAACAAGCCATTCGACAAGGAGATTCTGAAATTGCGGA
GGCTTGGTTCGATCAAGCCGCTGAGTATTGGAAACAAGCTATAGCGCTTACTCCTGGTA
ATTATATTGAAGCGCATAATTGGTTGAAGATCACAAGGCGTTTCGAA------ATGAATTGG
CGATCAGAACGTATATGGATAGAGCTTATAACGGGATCTCGAAAAATAAGTAATTTCTG
CTGGGCCTTTATCCTTTTTTTAGGTTCATTAGGATTCTTATTGGTTGGAACTTCCAGTTAT
CTTGGTAGGAATTTGATATATTTATTTCCGTCTCAGCAAATACTTTTTTTTCCACAAGGGA
TCGTGATGTCTTTCTATGGGATCGCAGGCCTCTTCATTAGCTCCTATTTGTGGTGCACAA
TTTCGTGGAATATAGGTAGTGGTTATGATCGATTCGATAGAAAAGAAGGAATAGTATGTA
TTTTTCGGTGGGGATTTCCTGGAAAAAATCGTCGCATCTTCCTTCGATTCCTTATGAAA
GATATTCAGTCTATCAGAATAGAAGTTAAAGAGGGTATCTATGCTCGTCGTGTCCTTTAT
ATGGAAATCAGGGGCCAGGGAGCCATTCCCTTGACTCCTACTGATGAGAAT---TTTACT
CCACGAGAAATTGAAAAAAAAGCTGCTGAATTAGCCTATTTTTTGCGTGTACCAATTGA
AGTTTTT
>Heuchera
ATGGAAAAACGGCGGTTCAATTCGATGTTGTCTCTAAGTAAATCAATGGACAGTCTGG
GTCCTATTGAAAATACCTGTGGTAGTGAAGATCCGATTATAACCGATACGTATAAAAAAA
TTCCTCGTTACAATAATATTGATCATTTATTTGGCGTTCGGGACATTCGGAATTTCATCTC
TGATGACACTTTTTTAGTTAAGGATAGTAATGGGAACAGTTATTCCATATATTTTGATATT
GAAAATCAGATTTTTGAGATTGATAATGATCAT------TCTTTTCTGAGTGAAGTAGAAAG
TTCTTTTTATAGTTATCGGAATTCTAGTTATCTGAATCATGTATCTAAGGGTGACGATCCC
CATTATGATCGTTACATGTATGATACTAAATATAGTTGGAATAATCATATTAATAGTTGCAT
TGACAGTTATCTTCGT------TCTCAAATCCATATTGATAGTTACATTTTAAGTAATAGTGAT
AATTACGGT------TACATTTATAGTTACATTTGTGGTAACAAGAATTCCAGTATAAGAACT
AGCACGAATGGTAGTGATTTAACTAAA------AGTTCTAATGATCTCGAGGTAACTCAAAA
ATACAGACATTTGTGGGTTCAATGCGAAAATTGTTATGGATTAAATTATAAGAAATTTTT
TAAATCAAAAATGCATATTTGTGAACAATGTGGATATCATTTGAAAATGAGTAGTTCAGA
TAGAATCGAACTTTCGATTGATCCAGGTACTTGGGATCCTCTGGATGAAGACATGGTCT
CTCTAGATCCCATTGAATTTCATTCGGAGGAGGAACCTTATAAAGAGCGTATTGATTCTT
ATCAAAGAAAGACAGGATTAACTGAGGCTGTTCAAACAGGCACAGGTCAACTAAACG
GTATTCCTATAGCAATTGGGGTTATGGATTTTCAGTTTATGGGGGGTAGTATGGGATCTG
TAGTAGGCGAGAAAATCACCCGTTTGGTCGAGTATGCTACCAATAAATTTCTACCTCTTA
TTCTGGTATGTGCTTCCGGAGGAGCACGCATGCAAGAAGGAAGTTTGAGCTTGATGCA
AATGGCTAAAATATCTTCTGCTTTATATGATTATCAATCAAATAAAAAGTTATTCTATGTA
TCAATCCTTACATCTCCTACTACTGGTGGGGTGACAGCTAGTTTTGGTATGTTGGGGGAT
ATCATTATTGCCGAACCCAATGCCTACATTGCATTTGCGGGTAAAAGAGTAATTGAACA
AACATTGAATAAGACAGTACCTGAAGGTTCACAAGCGGCTGAATATTTATTCCATAAGG
GCTTATTCGATCCAATCGTACCACGTAATCTTTTAAAAGGCGTTCTGAATGAGTTATTTC
AGCTCCACGCTTTCTTTCCTTTGAAAAAAAATGCAATCAAGATGGTAACCATTCGAGCT
GATGAAATTAGTAATATTATCCGGGAACGTATTGAGCAATATAATAGAGAAGTAAAGATT

GTAAATACCGGTACCGTACTTCAAGTAGGCGATGGCATTGCTCGTATTCATGGCCTTGAT
GAAGTAATGGCAGGTGAATTAGTAGAATTTGAAGAGGGTACAATAGGCATTGCTTTGAA
TTTGGAATCCAATAATGTTGGTGTTGTATTAATGGGTGATGGTTTGATGATACAAGAAGG
AAGTTCTGTAAAAGCAACAGGAAGAATTGCTCAGATACCTGTCAGTGAGGCTTATTTG
GGTCGTGTTATAAATGCTCTGGCTAAACCTATTGATGGTAGAGGTGAAATTCCAGCTTC
TGAATCTCGGTTAATTGAATCTCCTGCTCCAGGTATTATTTCGAGACGTTCCGTATATGA
GCCTCTTCAAACAGGGCTTATCGCTATTGATGCGATGATACCTATAGGACGTGGTCAGC
GAGAATTAATTATTGGGGACAGACAGACCGGTAAAACAGCAGTAGCCACAGATACGAT
TCTCAATCAACAAGGACAAAATGTAATATGTGTTTATGTAGCTATTGGTCAAAAGGCAT
CTTCTGTGGCTCAGGTAGTGACTAATTTCCAGGAAAGGGGAGCAATGGAATACACTATT
GTGGTAGCCGAAACAGCGGATTCCCCTGCTACATTACAATACCTCGCTCCTTATACAGG
AGCAGCTCTGGCTGAATATTTTATGTACCGTGAACGACACACTTCAATCATTTATGATGA
TCTCTCCAAACAAGCACAAGCTTATCGCCAAATGTCTCTTCTATTACGAAGACCCCCCG
GTCGCGAAGCTTATCCAGGAGATGTTTTTTATTTGCATTCACGCCTTTTGGAAAGAGCC
GCTAAATCAAGTTCTCGTTTAGGTGAAGGAAGTATGACCGCTTTACCAATAGTTGAGAC
CCAATCGGGAGATGTTTCGGCTTATATTCCTACTAATGTAATTTCTATTACAGATGGACA
GATATTCTTATCCGCCGATCTATTCAATTCTGGAATCAGACCTGCTATTAATGTGGGTATT
TCCGTCTCTAGAGTAGGATCCGCAGCTCAAATTAAAGCCATGAAACAGGTAGCTGGCA
AATCAAAATTGGAACTGGCCCAATTTGCAGAATTAGAAGCCTTTGCACAATTTGCTTCG
GATCTTGATAAAGCTACTCAGAATCAATTGGCAAGAGGTCAACGATTACGTGAATTGCT
TAAGCAATCCCAATCAGCCCCTCTCACAGTAGAAGAACAAATAATGACTATTTATACCG
GAACAAATGGTTATCTTGATTCATTAGAGATTGGACAGGTAAGGAAATTTCTCGTTGAG
TTACGTACTTACTTAAAAACGAATAAACCTCAGTTTCAAGAAATCATATCTTCTACCAA
GACATTCACCGAGGAAGCAGAAGCCCTTTTGAAAGAAGCTATTCAGGAACAGATGGA
ACGTTTTCTACTTCAGGAACAAGCAATGAGAATCAATCCTACTACTTCTGATCCTGGGG
TTTCCACGCTTGAAAAAAAAAACCTGGGGCGTATCGTTCAAATTATTGGTCCGGTACTG
GATGTAGCTTTTCCCCCGGGCAAGATGCCTAATATTTACAACGCTCTGGTAGTTAAGGG
TCGAGATACTGTCGGTCAAGAAATTAATGTAACTTGTGAGGTACAGCAATTATTAGGAA
ATAATCGAGTTAGAGCTGTAGCTATGAGTGCTACAGATGGTCTAAGGAGAGGAATGGA
CGTGATTGACACGGGAGCTGCTCTAAGTGTTCCAGTCGGTGGAGCGACTCTCGGACGA
ATTTTCAACGTACTTGGAGAACCTGTTGATAATTTAGGTCCTGTAGATACTCGCACAAC
ATCTCCTATTCATAGATCTGCGCCCGCCTTTATACAGTTAGATACAAAATTATCTATTTTT
GAAACAGGAATTAAAGTAGTAGATCTTTTAGCCCCTTATCGTCGTGGAGGAAAAATCG
GACTATTTGGGGGAGCTGGAGTGGGTAAAACGGTACTCATTATGGAATTGATCAACAA
CATTGCCAAAGCTCATGGGGGTGTATCTGTATTTGGCGGAGTAGGTGAACGTACTCGTG
AAGGAAATGATCTTTACATGGAAATGAAAGAATCTGGAGTGATTAATGAACAAAATATT
GCAGAATCAAAAGTAGCTCTAGTCTACGGCCAGATGAATGAACCGCCGGGAGCTCGTA
TGAGAGTTGGTTTGACTGCCCTAACTATGGCGGAATATTTCCGAGATGTTAATGAACAA
GACGTACTTCTATTTATCGACAATATCTTCCGTTTCGTCCAAGCAGGATCCGAAGTATCT
GCCTTATTGGGTAGGATGCCTTCCGCCGTGGGTTATCAACCCACCCTCAGTACCGAAAT
GGGTACTTTACAAGAAAGAATTACTTCTACCAAAGAGGGTTCCATAACTTCTATTCAAG
CAGTTTATGTACCTGCGGACGATTTGACCGACCCTGCTCCTGCTACGACATTTGCACAT
TTAGATGCTACTACCGTACTGTCAAGAGGATTAGCTGCCAAAGGCATCTATCCAGCAGT
AGATCCTTTAGATTCAACGTCAACTATGCTCCAACCTCGGATCGTTGGTGAGGAACATT

ATGAAATTGCACAAAGAGTTAAGCAAACTTTACAACGTTACAAAGAACTTCAGGACAT
TATAGCTATCCTTGGGTTGGACGAATTATCCGAAGAGGATCGTTTAACCGTAGCAAGAG
CACGAAAAATTGAGCGTTTCCTATCACAACCTTTTTTCGTAGCAGAAGTTTTTACTGGT
TCTCCGGGGAAATATGTTGGTCTAGCAGAAACAATTCGAGGGTTTCAATTAATCCTTTC
GGGGGAATTAGATAGTCTTCCTGAACAAGCCTTTTATTTGGTAGGTAACATCGATGAAG
TTACCGCGAAGGCTATGAACTTAGAAATGGAGAGCAATTTGAAGAAAATGACCTTAAA
TCTTTGTGTACTGACCCCTAATCGAATTGTTTGGGATTCAGAAGTGAAAGAAATCATTT
TATCTACCAATAGTGGACAAATTGGCGTATTACCAAATCATGCCCCTATTGCCACAGCTG
TAGATATAGGTATTTTGAGAATACGCCTTAACGACCAATGGTTAACGATGGCTCTGATGG
GTGGTTTTGCTAGAATAGGTAATAATGAGATCACTATTTTAGTAAATGATGCGGAGAAG
GGTAGTGACATTGATCCCCAAGAAGCTCAACAAACTCTTGAAATAGCGGAAACTAACT
TGAAGAAAGCTGAAGGCAAGAGACAAACAATTGAGGCAAATCTAGCTCTCAGACGAG
CTAGGACACGAGTAGAGGCTATCAATGTGATTTCAATGAAAAATGTAACCGATTCTTTC
GTTTCCTTGGGTCACTGGCCATCCGCCGGGAGTTTCGGGTTTAATACCGATATTTTAGC
AACAAATCTAATAAATCTAAGTGTAGTGCTCGGTGTATTGATCTTTTTTGGAAAGGGAG
TGTTAAGTGATTTATTAGATAATCGAAAACAGAGGATTTTGAATACTATTCGAAATTCAG
AAGAATTACGTGGGGGGGCCATTGAACAGCTGGAAAAAGCCCGGGCCCGCTTACGGA
AAGTCGAAATGGAAGCAGATCAGTTTCGAGTGAATGGATACTCTGAGATAGAACGCGA
AAAATTGAATTTGATTAATTCAACTTATAAGACTTTGGAACAATTAGAAAATTACAAAA
ATGAAACCATTAATTTTGAACAACAAAGAGCGATTAATCAAGTCCGACAACGGGTTTT
CCAACAAGCTTTACAAGGAGCTCTAGGGACTTTGAATAGTTGTTTGAACAACGAGTTA
CATTTACGTACCATTAGTGCTAATATTGGTATGTTTGGGGCGATGAAAGAAATAACTGAT
ATGAATCCATTGATTTCTGCCGCTTCCGTTATTGCTGCTGGATTGGCCGTAGGGCTTGCT
TCTATTGGACCTGGAGTTGGTCAAGGTACTGCTGCGGGCCAAGCTGTAGAAGGTATTG
CGAGACAGCCTGAGGCAGAAGGAAAAATACGCGGTACTTTATTACTTAGTCTAGCTTTT
ATGGAAGCTTTAACAATTTATGGACTGGTTGTAGCATTAGCGCTTTTATTTGCAAATCCT
TTTGTTATGAATGTTCTATCATGTTCCATCACCCTAAAT---GGGTTATACGATATATCCGGT
GTGGAAGTAGGCCAACATTTCTATTGGCAAATAGGAGGTTTACAAGTCCATGCCCAAGT
ACTTATTACCTCTTGGGTTGTAATTGCTATCTTATTAGGTTCTGCCACTATAGCTGTTCGG
AATCCACAAACCATTCCGACTGACGGTCAGAATTTCTTCGAATATGTTCTTGAATTCATT
CGCGACGTGAGCAGAACTCAGATTGGAGAAGAATATGGTACTTGGGTTCCCTTTATTG
GAACGATGTTTCTATTTATCTTTGTTTCTAATTGGTCAGGGGCTCTTTTACCTTGGAAAA
TCATACAGTTACCCCATGGGGAGTTAGCCGCACCCACGAATGATATAAATACTACGGTT
GCTTTAGCTTTACTCACGTCAGTGGCATATTTTTATGCGGGTCTTTCCAAAAAAGGATTA
GGTTATTTCGGTAAATACATTCAACCAACTCCAATCCTTTTACCCATTAACATCTTAGAA
GATTTCACAAAACCTTTATCACTTAGTTTTCGACTTTTCGGGAATATATTAGCTGATGAA
TTAGTAGTTGTTGTTCTTGTTTCTTTAGTACCTTTAGTGGTTCCTATACCGGTTATGTTCC
TTGGATTATTTACAAGTGGTATTCAAGCTCTTATTTTTGCAACTTTAGCTGCGGCTTATAT
AGGAGAATCTATGGAGGGTCATCATATGATATTTTCGACTTTCGAACATATATTAACTCAT
ATATCCTTTTCTATTGTTTCAATTTTAATTACAATTTATTTCATAACCTTATTAGTGGATGA
AATCGTAGGACTAGATGATTCGTCAGAAAAGGGCATGATAGCGACCTTTTTCTGTATAA
CAGGATTATTAATCGCGCGGTGGATTTATTCACGACATTTTCCATTAAGTGATTTATATGA
ATCATTAATCTTCCTTTCATGGAGTTTCTCCATTATTCATATGGTTCCGTATTTT---AAACA
TAAAAATCATTTAAGCGCAATAACCGCGCCAAGTGCGATTTTTACCCAAGGCTTTGCTA

CTTCGGGTCTTTTAACGGAAATGCATCAATCCGCAATATTAGTACCTGCTCTACAATCCC
AATGGTTAATGATGCATGTAAGTATGATGGTATTGGGCTATGCAGCTCTTTTATGTGGAT
CATTATTATCAGTAGCTCTTTTAGTCATTACATTTCGAAAAGACATAAGGATTTTCGGTA
AAAGCAATGAGATCCAATACATAAATAAAAGAAGCAATGTTTTACGAACGACTTCTTTT
CTTTCTGATAGGAATTATTACAAGTATCAATTAATTCAACAATTGGATCATTGGGGTTATC
GTGTTATTAGTCTAGGGTTTATCTTTTTAACCATAGGTATTCTTTCAGGAGCAGTATGGG
CTAATGAGGCATGGGGATCATATTGGAATTGGGACCCAAAAGAAACTTGGGCATTTATT
ACTTGGACCATATTTGCGATTTATTTACATACGCGAACAAATACAAATTTGCAAGGTGCA
AATTCTGCAATTGTGGCTTCGATGGGGTTTCTTATAATTTGGATATGTTATTTTGGGGTCA
ATCTATTAGGAATAGGGCTACATAGTTATGGTTCATTTAACATC---ATGGCAAAAAAAAA
AGCATTCACTCCCCTTCTATATCTTGCATCTATAGTATTTTTGCCCTGGTGGATCTCTCTC
TTATTTAATAAAAGTCTGGAATCCTGGGTTACAAATTGGTGGAATACTAGGCAATCTGA
AACTTTTTTGAATGATATTCAAGAAAAGAGTATTCTCGAAAAAATTCATAGAATTAGAGG
AACTCTTCCTGTTGAACGAAATGATAAAGGAATATCCAGAGACACATCTACAAAAGCTT
AGTCTAGGA---CACAAAGAAACGATCCAATTGATCAAGATGCACAATGAGGATCGTATC
CATACGATTTTACACTTCTCGACAAATCTAATCTGTTTCGTTATTCTAAGTGGTTATTCTA
TTCTGGGTAATGAAGAACTTGTTATTCTTAACTCTTGGGTTCAGGAATTCTTATATAACT
TAAGCGATACAATAAAAGCTTTTTCTATTCTTTTATTAACTGATTTGTGTATCGGATTCCA
TTCGCCCCATGGTTGGGAACTAATGCTTGGCTCTGTCTACAAAGATTTTGGATTTGCTC
ATAACGATCAAATTATATCCGGTCTTGTTTCCACTTTTCCAGTCATTCTAGATACAATTTT
AAAATATTGGATCTTCCGTTATTTAAATCGTGTATCTCCGTCACTTGTAGTGATTTATCAT
TCAATGAATGACATGCCTATTGGTGTTCCAAAAGTACCTTTTCGAAGTCCTGGAGAGGA
AGATGCATCTTGGGTTGACGTATACAACCGACTTTATCGAGAAAGATTGCTTTTTTTAG
GCCAAGAGGTTGATAGTGAGATCTCTAATCAACTTATTGGTCTTATGGTATATCTCAGTA
TAGAGGATGATACCAAAGATCTGTATTTGTTTATAAACTCTCCCGGAGGATGGGTAATAC
CCGGAGTAGCTATTTATGATACTATGCAATTTGTACGACCAGATGTACATACAATATGCAT
GGGGTTGGCCGCTTCAATGGGATCCTTTATCCTGGTCGGAGGAGAAATTACCAAACGT
CTAGCATTCCCTCACGCCAGGGTAATGATCCATCAACCCGCTAGCTCTTTTTATGAGGC
ACAAACGGGAGAATTTATCCTGGAAGCGGAAGAACTACTGAAACTACGCGAAACCAT
CACAAGGGTTTATGTACAAAGAACGGGCAAACCCTTATGGGTTGTATCCGAAGATATGG
AAAGGGATGTTTTTATGTCAGCAACAGAAGCCCAAGCTCATGGAATTGTTGATCTTGTA
GCGGTTGAAATGAAAGAACAAAAATGGGTTCATGAAGGTTTAATTACTGAATCACTTC
CCAATGGTATGTTCCGGGTTCGTTTAGATAATGAAGATCTGATTCTAGGTTATGTTTCGG
GAAGGATCCGACGTAGTTTTATACGGATACTACCAGGAGATAGAGTCAAAATTGAAGTA
AGTCGTTATGATTCAACCCGAGGGCGTATAATTTATAGACTCCGCAACAAGGATTCGAA
CGATATGGGGGAATTTCAAGGATATTTAGAA---GATAAATTTCGGCAACATGACTTCCTA
TATCCACTTATCTTTCAGGAGTATATTTATGCACTTGCTCATGATCATGTTTTATTTGGTTA
TGACAATAAATCCAGTTCACTAATTGTGAAACGTTTAATTACTCGAATGTGTCAACAGA
ATCATTTTATTATTTCTGCTAATGATTCGAACCAAAATCAATTTTTTGGGAAGAATTTATA
TTCTCAAATGATATCAGAGGGATTTGCAGTCATTGTGGAAATTCCGTTTTCCCTACGATT
TGTATCTTCCTTATTAGTAAAATCTCATAATTTACGATCAATTCATTCAATATTTCCTTTTT
TAGAAGACAATTTTTTACATTTAAATTATGTGTCAGATATACTAATACCCCACCCCATCCA
TCTTGAAATATTGGTTCAAACTCTTCGCTACTGGGTGAAAGATGCCTCTTCATTGCATTT
ATTACGATTCTTTCTCTATGAGTATCAGAATTGGAATAGTCTTATTACTAAGAAATCAATT

TCTATTGTTTCACAAAGATTATTCTTATTTTATATAATTCTTATGTATGTGAATACGAATC
TATCTTCATTTTTCTTTGTAACCAATCTTCTCATTTACGATCAACATCTTCTGGAACTCTT
TTTGAGCGAATACATTTCTATGGAAAAATAAAACATGTTGTAGAAGTCTTTTCTAATGAT
TTTATCCTATGGTTGTTCAAAGATCCGTTCATGCATTATGTTAGGTATCAAGGAAAATCA
ATTCTGGCATCAAAAGGTACCCCCCTTCTGCTGAATAAATGGAAATATTACCTTGTCAAT
TTATGGCAATGTAATTTTTACGTATGGTCTCAACCAGGAAGGATCCATATAAACCAATTA
TCCAATCATTCCCTCGACTTTCTGGGCTATCTTTCGAGTGTGCAACTAAATCCTTCAGTA
GTGCGGAGTCAAATGCTAGAAAATTCATTGATAATGGATAATGCTATT------AAGAAGTT
GGATATCATAGTTCCAATTATTCCTTTAATTAGATCATTGGCTAAAGCTAAATTCTGTAAC
GTAGTAGGGGATCCCATTAGTAAGCCGGCCTGGGCCGATTCATCAGATTCTGATATTATC
GACAGATTTGTGCGCATATGCAGAAATATTTCTCATTATCACAGCGGATCCTCAAAAAA
AAAGAGTTTGTATCGAATAAAGTATATACTTCGACTTTCTTGTGCTCGAACTTTGGCTCG
TAAACACAAAAGTACTGTACGTGCTTTTTTGAAAAGATTAGGTTCGGAATTATTAGAAG
AATTCCTTACGGAGGACGAACAAGTGCTTTCTTTGATCTTTCCAAGACGGGTTTGGTAT
TTGGATATTATTTGTATCAATGATTTGGTCAATCATGAAATGATAATTGAT---ACAACAGA
AGTACAA---GATATCAATTCTTTTTCCCGATTGGAATCC---TTAAAAGAGGTCTATGGGAT
CATATGGATGCTTATCCCTATTTTGACTCTTGTATTAGGAATCACAATAGGTGTACTAGTA
ATTGTGTGGTTAGAAAGAGAAATATCAGCAGGGATACAACAACGTATTGGACCTGAAT
ACGCCGGCCCGTTGGGAATTCTCCAAGCTTTAGCAGATGGGACAAAACTACTTTTCAA
AGAAAATATTCTTCCATCTAGAGGAGATACTGGTTTATTCAGTATCGGACCATCCATAGC
GGTCATATCAATTCTACTAAGTTATTCAGTAATTCCTTTTGGTTATCACCTTGTTCTAGCC
GATCTCAATATCGGTGTTTTTTTATGGATCGCCATTTCAAGTATTGCTCCCATTGGACTTC
TTATGTCAGGATATGGATCAAATAATAAATATTCCTTTTTAGGTGGTTTACGAGCTGCTG
CTCAATCGATTAGTTATGAAATACCGTTAACTCTATGTGTGTTATCAATATCTCTACTATCT
AACAGTTTGAGTACAGTTGATATAGTTGAGGCACAGTCAAAATATGGGTTTTGGGGGT
GGAATTTGTGGCGTCAACCTATAGGGTTTATCGTTTTTCTAATTTCCTCCCTAGCAGAAT
GTGAGAGATTACCTTTTGATTTACCAGAAGCAGAGGAAGAATTAGTAGCAGGTTATCA
AACCGAATATTCAGGTATCAAATTTGGTTTATTTTATGTTGCTTCCTATCTAAATCTACTA
GTTTCTTCATTCTTTGTAACAGTTCTTTACTTGGGTGGGTGGAATCTCTCTATTCCGTAC
CTATTTGTTCCTGAACTTTTTGAAATAAATAAAGCGAGTGGAGTTTTTGGAACGACACT
TGGTCTCTTTATTACATTAGCTAAAACATATTTGTTCTTGTTCATTCCTATAACAACAAGA
TGGACTTTACCTAGGCTAAGAATGGACCAATTATTAAATCTTGGGTGGAAATTTCTTTTA
CCTATTTCTCTAGGTAATCTATTATTAACAACTTCTTCCCAACTCCTTTCACTGATGATCT
GGCATGTACAGAATGAAAACTTCATTCTCGATTCTACGAGAATTTTTATGAAAGCCTTT
CATTTGCTTCTCTTCGATGGAAGTTTTATTTTCCCAGAATGTATCCTAATTTTTGGTCTAA
TTCTTCTTCTGATGATCGATTCAACCTCTGATCAAAAAGATATACCTTGGTTATATTTCAT
CTCTTCAACAAGTTTAGTAATGAGCATAACGGCCCTATTGTTCCGATGGAGAGAAGAAC
CTATGATTAGTTTTTCGGGAAATTTCCAAACGAACAATTTCAACGAAATCTTTCAATTTC
TTATTTTACTATGTTCAACTCTATGTATTCCTCTATCCGTAGAGTACATTGAATGTACAGA
AATGGCTATAACAGAGTTTCTGTTATTCGTATTAACAGCTACTCTAGGAGGAATGTTTTT
ATGCGGTGCTAACGATTTAATAACTATCTTTGTAGCTCCAGAATGTTTCAGTTTATGCTC
CTACCTATTATCTGGATATACCAAGAAAGATGTACGGTCTAATGAGGCTACTACGAAATA
TTTACTCATGGGTGGGGCAAGCTCTTCTATTCTGGTTCATGGTTTCTCTTGGCTATATGG
TTCATCCGGGGGAGAGATGGAGCTTCAAGAAATAGTGAATGGTCTTATCAATACACAAA

TGTATAACTCCCCAGGAATTTCAATTGCGCTTATATTCATCACTGTAGGAATTGGGTTCA
AGCTTTCCCCAGCCCCTTCTCATCAATGGACTCCTGACGTATACGAAGGATCTCCCACT
CCAGTCGTTGCTTTTCTTTCTGTTACTTCGAAAGTAGCTGCTTCAGCTTCAGCCACTCG
AATTTTCGATATCCCTTTTTATTTCTCATCAAACGAATGGCATCTTCTTCTGGAAATCCTA
GCTATTCTTAGCATGATATTGGGGAATCTCATTGCTATTACTCAAACAAGCATGAAACGT
ATGCTTGCATATTCGTCCATAGGTCAAATCGGATATGTAATTATTGGAATAATTGTTGGAG
ACTCGAATGGTGGATATGCAAGCATGATAACTTATATGCTGTTCTATATCTCCATGAATCT
AGGAACTTTTGCTTGCATTGTATCATTTGGTCTACGTACCGGAACTGATAACATTCGAGA
TTATGCAGGATTATACACGAAAGATCCTTTTTTGGCTCTCTCTTTAGCCCTATGTCTCTTA
TCCCTAGGAGGTCTTCCTCCACTAGCAGGTTTTTTCGGAAAACTCCATTTATTCTGGTG
TGGATGGCAGGCAGGCCTATATTTCTTGGTTTCAATAGGACTCCTTACGAGCGTTGTTT
CTATCTACTATTATCTAAAAATAATCAAGTTATTAATGACTGGACGAAACCAAGAAATAA
CCCCTCACGTGCGAAATTATAGAAGATCCCCTTTAAGATCAAACAATTCCATTGAATTG
AGTATGATTGTATGTGTGATAGCATCTACTATACCAGGAATATCAATGAACCCGATTATTG
CAATTGCTCAGGATACCCTTTTTATGTTTCTGCTTTACGAATATGATATTTTCTGGGCTTT
TCTAATAATATCAAGTCTGATTCCTATTTTGGCATTTCTAATTTCCGGAGTTTTAGCTCCG
ATTAGCAAAGGACCAGAGAAACTGTCGAGTTATGAATCGGGTATCGAACCAATGGGCG
ATGCTTGGGTACAATTTCGAATCCGTTATTATATGTTTGCTCTAGTTTTTGTTGTTTTTGA
TGTTGAAACGGTTTTTCTTTATCCATGGGCAATGAGTTTCGATGTATTGGGTGTATCCGC
ATTTATAGAAGCTTTAATTTTCGTGCTTATCCTAATTGTTGGTTCAGTTTATGCATGGCGA
AAGGGAGCATTGGAATGGTCTACGAATTATTTTCCTTGGTTAACAATAATTGTAGTTTTG
CCGATATTTGCGGGCTCCTTAATTTTCTTTCTCCCCCATAGAGGAAATAAGATAATTAGG
TGGTATACTATATGTATATGCATCTTGGAGCTCCTTCTAACGACCTATGCATTCGGTTATC
ATTTTCAATTGGATGATCCATTAATCCAATTTTCGGAGGATTATAAATGGATCAATTTTTT
TGATTTTTATTGGAGATTGGGAATAGATGGACTTTCTATAGGACCTATTTTACTGACAGG
ATTTATCACCACTTTAGCTACTTTAGCGGGTCGGCCAGTTACTCGAGATTCCCGATTATT
CCATTTCTTGATGTTAGCAATGTACAGTGGTCAAATAGGATCATTTTCTTCTCGAGACCT
TTTACTTTTTTTCATCATGTGGGAGTTAGAATTAATTCCTGTTTATCTACTCCTATCTATGT
GGGGAGGGAAGAAACGTCTGTACTCAGCTACAAAGTTTATTTTGTACACTGCAGGAGG
TTCTATTTTTCTCTTAATGGGAGTGCTGGGTATCGGTTTATATGGTTCCAATGAACCAAC
ATTAAATTTTGAAACATTAGCTAATCAGTCGTATCCTATAGCATTGGAAATACTATTCTAT
CTTGTATTTCTTATTGCTTTTGCTGTCAAATCGCCGATTATACCTCTACATACATGGTTAC
CAGACACCCATGGAGAAGCACATTACAGTACTTGTATGCTTCTAGCTGGAATCTTATTA
AAAATGGGAGCGTATGGGTTGATTCGGATCAATATGGAATTATTACCCCGTGCTCATTCT
ATATTTTCTCCCTGGTTGTTGATAGTAGGCACAATACAAATCATCTATGCAGCTTCAACA
TCTTCCGGCCAACGTAATTTAAAAAAAAGAATAGCCTATTCCTCTGTATCTCATATGGGT
TTCATAATTATCGGAATTAGCTCTCTAACTGATACAGGACTCAATGGAGCCATTTTACAA
ATAATCTCTCATGGATTTATTGGCGCTGCACTTTTTTTCTTGGCAGGAACCAGTTATGAT
AGAATACGCCTTGTTTATCTCGACGAAATGGGTGGAATAGCTATTCCAATGCCAAAAAT
ATTCACAATGTTCAGTATCTTATCGATGGCTTCCCTTGCATTACCGGGCATGAGTGGTTT
TGTTGCAGAATTGCTAGTATTTTTTGGAATAATTACCAGCCAAAAGTATCTTTTAATGCC
AAAAATAATAATTACTTGTGTAATGGCAATTGGAATGATATTAACTCCTATTTATTCATTA
TCTATGTCACGCCAGATGTTCTATGGATACAAGCTATTTAATGCCCCAAACTCTTATTTTT
TTGATTCTGGACCGCGAGAGTTATTTGTTTCGATTTCTATCTTTCTACCGGTAATAGGGA

TTGGTATTTACCCGGATTTCGTTCTTTCATTATCAGTTGACAAGGTCGAAACTATTCTATC
TAATTTTTTTTATAGAATGATGCTCGAACATGTACTTATTTTGAGTGCCTATTTATTTTCTA
TCGGTATCTATGGATTGATCACGAGTCGAAATATGGTTCGAGCCCTTATGTGTCTTGAAC
TTATACTGAATGCAGTTAATATAAATTTCGTAACATTTTCGGATTTTTTTGATAATCGCCA
ACTAAGAGGAGACATTTTCTCAATTTTTGTTATAGCTATTGCAGCCGCTGAAGCAGCTAT
TGGACTAGCAATTGTTTCGTCAATTTATCGTAACAGAAACTCTATTCGTATCAACCAATC
GAATTTGTTGAATAAAATGGAACATACATATCCATATGCATGGATCATACCTTTCGTTCC
ACTTCCTGTTCCTGTCTTAATAGGAGTGGGACTTCTCCTTTTTCCGACGGCAACCAAGA
ATCTTCGTCGTATGTGGGCTTTTCCTAGTGTTTTATTATTAAGTATAGTTATGATTTTTTCG
GCGGATCTGTCTATTCAGCAAATAAATAGCAATTCCATATATCAATATGTATGGTCTTGGA
CTATCAATAATGATTTTTCTTTAGAGTTCGGCCACCTGATCGACCCACTTACTTCTATTAT
GTTAATATTAATCACTACTGTTGGAATTATGGTTCTTATTTATAGTGATAATTATATGTCTC
ATGATCAAGGATATTTAAGATTTTTTGCTTATATGAGTTTTTTCAATACTTCCATGTTGGG
ATTAGTTACTAGTTCTAATTTGATACAAATTTATATTTTTTGGGAATTAGTTGGAATGTGT
TCATATCTATTAATAGGTTTTTGGTTCACAAGACCTATTGCGGCAAATGCTTGTCAAAAA
GCTTTTGTAACTAATCGTATAGGGGATTTTGGTTTATTCTTAGGAATTTTAGGTCTTTATT
GGATAACAGGTAGTTTTGAATTTAGAGATTTGTTCGAAATATTCAATAACAATGAGTTAA
ATTTATTATTTGTTACTTTGTGTGCTTTTCTATTATTTTCCGGTGCAGTTGCTAAATCCGC
GCAATTTCCCCTTCATGTATGGTTACCCGATGCCATGGAGGGGCCTACTCCTATTTCGGC
TCTTATCCATGCTGCTACCATGGTAGCAGCGGGAATTTTTCTTGTCGCTCGGCTTCTTCC
TCTTTTCATAGTTATACCTTACATAATGAATCTAATATCTTTGATAAGTATAATAACAGTAC
TATTAGGAGCTACTTTAGCTCTTGCTCAAAAAGATATTAAGAGAAGTTTAGCCTATTCTA
CAATGTCTCAATTGGGTTATACGATGTTAGCTTTAGGCATGGGGTCGTATCGAGCTGCTT
TATTTCATTTAATTACCCATGCTTATTCGAAAGCATTATTGTTTTTAGGATCTGGATCAATT
ATTCATTCAATGGAAGCTATTGTCGGATATTCTCCAGATAAAAGTCAGAATATGGTTCTT
ATGGGTGGTTTAACAAAACATGTGCCAATTACAAAAACAGCTTTTTTTTTAGGTACACT
TTCTCTTTGTGGTATTCCACCACTTGCTTGTTTTTGGTCCAAAGATGAAATTCTTAATGA
TACTTGGTTATATTCACCAATTTTCGCAATAATAGCTTGTTCCACAGCTGGGTTAACTGC
ATTTTATATGTTTCGGATTTATTTACTTACTTTTGAAGGGCATTTAAACGTTCATTTTCAA
AATTACAGTGGAAAAAAAACGAGCTCGTTCTATTCAATATCTCTATGGGGTAAAGAAGG
TCCAAAAACGACTAAAACAAAATTATTTACTTTATTAACAATGAATTCTTTTTCGACTTG
GAATAAGAAAACTTTTTCATATCCCCATGAATCGGATAATACTATGCTATTCTCTCTGCTT
GTATTGGTCCTATTTACTTTGTTCGTTGGAGCCATAGGAATTCCTTTCGATTTAGATATAT
TATCTAAATGGTTAACCCCGTCTATAAACCTTTTACATAAAAATTTGACT---TCTGTGGAT
TGGTATGCATTTGTGACAAATGCAATTTTTTCAGTCAGTATAGCGTATTTCGGAATACTT
ATAGCGTCCCTTTTATATAAGCCTGTTTATTCATCTTTACAAAATTTGAACTTAATTAATT
CATTTGTTATTTTTTGGGACAAAATAATATATGTGATATATGGTTGGTCATATAATCGTGG
TTACATAGATGCTTTTTATGCAACATCTTTCACTAAGGGTATAAGAGGGTTAGCTGAATT
AACTCATTTTTTTGATAGACGAGTAATTGATGGAATTACGAATGGCGTTGGTATTACGAG
TTTCTTTGTAGCAGAAGGCATCAAATATGTAGGGGGTGGC---CGCATCTCTTCTTATCTT
TTCTTGTATTTATCTTATGTATCAATATTTTTATTAAGTTACTACATGGATTTACCTGGACC
AATACATGATTTTCTTTTAGTCTTTCTGGGATCGGCTCTTATATTAGGAGGTCTAGGAGT
GGTATTACTTACCAATCCAATTTATTCTGCATTTTCGTTGGGATTGGTTCTTGTTTGTATA
TCTTTATTCTATATTTCATCAAACTCCCATTTTGTAGCTGCTGCACAGCTCCTTATTTACG

TGGGAGCTATAAATGTTTTAATCATATTTGCTGTGATGTTTATGAATGGTTCAGGATCTTA
CAAAGATTTTACTCTTTGGACCGTTGGGGATGGGGTTACTTCGGTGGTTTGTACAAGTA
TTTTTGTTTCACTAATTACTATTATTCCAGATACGTCATGGTATGGTATTATTTGGACTAC
AAGATCAAACCAGATTATAGAGCAAGATTTGATAAGTAATAGTCAACAAATTGGGATTC
ATTTATCAACAGATTTTTTTCTTCCATTTGAACTCATTTCCATAATTCTTTTAGTTGCTTT
GATAGGTGCAATTACTGTGGCTCGTCAGATGAATGTACCAGCGACAAGAAAAGACCTC
ATGATAGTAAATATGGGTCCTCACCACCCATCAATGCATGGTGTTCTTCGACTCATTATT
ACTCTAGATGGTGAAGATGTTATTGACTGTGAACCAATATTGGGTTATTTACACAGAGG
AATGGAAAAAATTGCGGAAAACCGAACAATTATACAATATCTGCCTTATGTAACACGTT
GGGATTATTTAGCTACTATGTTCACAGAAGCAATAACCGTAAATGCACCAGAGCAATTA
GGAAATATTCAAGTACCGAAAAGAGCCAGCTATATCAGAGTAATTATGTTGGAGTTGAG
TCGTATAGCTTCTCATTTGTTATGGCTTGGTCCTTTTATGGCAGATATTGGTGCACAGAC
CCCGTTCTTCTATATTTTCAAAGAAAGAGAATTAGTATATGATTTATTCGAAGCTGCCAC
TGGTATGAGAATGATGCATAATTATTTTCGTATCGGAGGAGTGGCTGTTGATCTACCTCA
TGGCTGGATAGATAAATGTTTGGATTTCTGTGATTATTTTTTAACAGGGATTGCTGAATAT
CAAAAACTTATTACACGAAATCCTATTTTTTTAGAACGAGTTGAGGGAGTAGGCATTATT
AGCGGAGAAGAAGCAATAAATTGGGGTTTATCAGGACCAATGCTACGAGCTTCCGGAA
TACAATGGGATCTTCGTAAAGTTGATCATTATGAGTGTTACGACGAATTTGATTGGGAG
GTCCAATGGCAAAAAGAAGGAGATTCACTAGCTCGTTATTTAGTACGAATCAGTGAAA
TGACGGAATCTATAAAAATTATTCAACAGGCTCTGGAAGGAATTCCAGGGGGGCCCTAT
GAGAATTTAGAAATCCGATCCTTTGATAGAGTAAGGGATCCCGAATGGAATGATTTTGA
ATATAGATTCATTAGTAAAAAGCCTTCGCCCACTTTTGAATTATCGAAACAAGAACTTTA
TGTGAGAGTCGAAGCACCAAAGGGAGAGTTGGGAATTTTTTTGATAGGAGATCAAAGT
GTTTTTCCTTGGCGATGGAAAATCCGACCGCCGGGTTTTATCAATTTGCAAATTCTTCCT
CAGTTAGTTAAAAGAATGAAATTGGCTGATATTATGACGATACTAGGTAGTATAGATATC
ATTATGGGAGAAGTTGATCGTATGTTCCCTATGGTAACCGGGTTCATGAATTATGGTCAA
CAAACAGTACGAGCTGCAAGGTACATTGGTCAAAGTTTCATGATTACCTTATCCCACGC
AAATCGTTTACCTGTAACTATTCAATATCCTTATGAAAAATTGATCACATCGGAGCGTTT
CCGCGGTCGAATCCACTTTGAATTTGATAAATGCATTGCTTGTGAAGTATGTGTTCGTGT
ATGTCCTATAGATTTACCTGTTGTCGATTGGAAATTAGAAACTGATATTCGAAAGAAAC
GATTGCTTAATTACAGTATTGATTTCGGAATCTGTATATTTTGTGGTAATTGCGTTGAGTA
TTGTCCAACAAATTGTTTATCAATGACTGAAGAATATGAACTTTCTACTTATGATCGTCA
CGAATTGAATTATAATCAAATCGCTTTGGGTCGTTTACCAATGCCAGTAATTGATGATTA
CACAATTCGAACAATTTTGAATTCG---ATAAAAAATGGA---------------------------------ATGC
AGGGTCGTTTGTCTGCTTGGCTAGTAAAGCATGGGCTAGTTCATAGATCTTTAGGCTTT
GATTACCAAGGAATAGAGACTTTACAAATAAAGCCGGAGGATTGGCATTCCATTGCTGT
AATTTTATATGTATATGGTTACAATTATCTACGTTCCCAATGTGCCTATGATGTAGCACCA
GGTGGACTGTTAGCTAGCGTGTATCATCTTACGAGAATAGAGTATGCTGTAGATCAACC
GGAAGAGGTATGCATAAAAGTATTTGCCCCAAGGAGGAATCCTAGAATTCCGTCTGTTT
TCTGGGTTTGGAAAAGTGCGGATTTTCAAGAACGGGAATCTTATGATATGTTGGGAATC
TCTTATGATAATCATCCACGCCTGAAACGTATCTTAATGCCTGAAAGTTGGATAGGATGG
CCCTTGCGTAAGGATTATATTGCCCCCAATTTTTATGAAATACAAGATGCTCAT-------------
--ATGGCGAAAGGGAGCATTGG
AATGGTCTTAGCTCCTGAATATTCAGACAATAAAAAGAAAAAAGGAACAAAAAAGAA

GATTGAGACAGTTATGAATTCCATCGAGTTTCCCTTACTCGATCGAACAACCCAAAATT
CAGTCATTTCAACTACATCAAATGATCTTTCAAATTGGTCAAGACTTTCCAGTTTATGGC
CGCTTCTCTATGGTACCAGTTGTTGCTTCATTGAATTTGCTTCATTAATAGGCTCACGAT
TCGACTTTGATCGTTATGGACTGGTACCAAGATCCAGTCCTAGACAAGCGGACCTAATT
TTAACAGCTGGTACAGTAACAATGAAAATGGCCCCTTCTTTAGTGAGATTATACGAACA
AATGCCTGAACCAAAATATGTTATTGCTATGGGAGCATGTACAATTACAGGGGGGATGT
TCAGTACCGATTCTTATAGTACTGTTCGAGGAGTCGATAAGCTAATTCCTGTGGATGTCT
ATTTGCCAGGCTGTCCACCTAAACCGGAGGCAGTTATAGATGCTATAACAAAACTGCGT
AAGAAAATATCTCGAGAAATCTATGAGGATAGAATTAGGTCTCAACGAGGGAATCGGT
GTTTTACTACCAATCACAAGTTTCATGTTGGACGCAGTACTCATACTGGAAATTATGATC
AAGGATTACTCTATCAATCGCCGTCTACTCCAGAGATCCCCCCGGAAACATTTTTCAAA
TACAAAAGTTCAGTATCTTCTCACGAATTAGTGAATATGCAAACTAGAAATACCTTTTCT
TGGACAAAGGAACAGATTACTCGATCCATTTCCGTATCGCTCATGATATATATAATCACT
CGGACATACATTTCAAATGCATATCCCATTTTTGCACAACAGGGTTATGAAAATCCACG
AGAAGCGACTGGGCGTATTGTATGTGCCAATTGCCATTTAGCTAGTAAGCCCGTGGATA
TTGAGGTTCCACAAACGGTACTTCCTGATACTGTATTTGAAGCAGTTGTTCGAATTCCT
TATGATATGCAACTGAAACAAGTTCTTGCTAATGGTAAAAAGGGAGGTTTGAATGTGG
GGGCTGTCCTTATTTTACCCGAGGGGTTTGAATTAGCCCCCCCCGATCGTATTTCTCCCG
AGATGAAAGAAAAGATAGGCAATCTGTCTTTTCAGAGCTATCGCCCCACAAAAAAAAA
TATTCTTGTGATAGGTCCTGTTCCTGGTCAGAAATATAGTGAAATAACCTTTCCTATTCTT
TCTCCCGACCCCGCTAGTAAGAAAGATGTTCACTTTTTAAAATATCCCATATATGTAGGC
GGGAACAGGGGACGGGGCCAGATTTATCCCGACGGGAGTAAGAGTAACAATACAGTTT
ATAATGCTACAGCAGCAGGTATAGTAAACAAAATTATACGAAAAGAAAAAGGGGGGTA
CGAAATAACTATCGCGGATCCATCGGATGGACGTCAAGTGGTTGATATTATCCCTCCAG
GGCCAGAACTTCTTGTTTCAGAGGGTGAATCTATCAAACTTGATCAACCATTAACGAGT
AATCCTAATGTGGGTGGATTTGGTCAGGGAGATGCCGAAATAGTACTTCAAGATCCATT
ACGTGTCCAAGGCCTTTTGTTCTTCTTGGCATCTGTTATTTTGGCACAAATCTTTTTGGT
TCTTAAAAAGAAACAGTTTGAGAAGGTTCAATTGTCTGAAATGAATTTCATGAGTAAA
GTATATGATTGGTTCGAAGAACGCCTCGAGATTCAGGCGATTGCAGATGATATAACTAG
TAAATACGTTCCTCCTCATGTCAACATCTTTTATTGTCTAGGAGGAATTACGCTTACTTG
TTTTTTAGTACAAGTAGCTACGGGGTTTGCTATGACTTTTTACTATCGTCCGACCGTTAC
TGAGGCTTTTGCTTCTGTTCAATACATAATGACTGAAGCTAACTTTGGTTGGTTAATCCG
ATCAGTTCATCGATGGTCGGCAAGTATGATGGTCCTAATGATGATCCTGCACGTATTTCG
TGTGTATCTCACCGGTGGATTTAAAAAACCTCGCGAATTGACTTGGGTTACAGGTGTGG
TTCTGGGTGTATTGACCGCATCTTTTGGTGTAACTGGTTATTCCTTACCTTGGGACCAAA
TTGGTTATTGGGCAGTAAAAATTGTAACAGGCGTACCTGACGCTATTCCTGTAATAGGA
TCGCCTTTGGTAGAGTTATTACGCGGAAGTGCTAGTGTGGGACAATCCACTTTGACTCG
TTTTTATAGTTTACATACTTTTGTATTACCCCTTCTTACTGCTGTATTTATGTTAATGCACT
TCCCAATGATACGTAAGCAAGGTATTTCTGGTCCTTTAATGGGAGTAACAAAAAAACCT
GACTTAAATGATCCTGTATTAAGAGCTAAATTGGCTAAAGGTATGGGCCATAATTATTAC
GGAGAGCCCGCATGGCCCAATGATCTTTTATATATTTTTCCAGTAGTTATTTTAGGTACTA
TTGCATGTAACGTAGGCTTAGCAGTTCTAGAACCGTCAATGATTGGTGAACCAGCAGAT
CCATTTGCAACTCCTTTGGAAATATTACCTGAATGGTATTTCTTTCCCGTATTTCAAATAC
TTCGTACAGTACCCAATAAGTTATTGGGTGTTCTTTTAATGGTTTCAGTACCTGCGGGAT

TATTAACAGTACCCTTTTTGGAGAATGTTAATAAATTCCAAAATCCATTTCGGCGTCCAG
TAGCAACAACCGTCTTTTTGATTGGTACTGCGGTGGCCCTTTGGTTGGGTATTGGAGCA
ACATTACCTATTGATAAATCCCTAACTTTAGGTCTTTTTATGATTGAAGTTTTTCTATTTG
GAATCGTATTAGGTTTAATTCCTATTACTTTGGCTGGATTATTCGTAACTGCATATTTACA
ATACAGACGTGGTGATCAGTTGGACCTTATGCCTACTATAACTAGTTATTTCGGTTTTCT
ACTAGCGGCTTCAACTATAACCTCAGCTTTATTTATTGGTCTAAGCAAGATACGACTTAT
TATGGATATAGTAAGTCTCGCTTGGGCTGCTTTAATGGTAGTCTTTACATTTTCCCTTTCA
CTCGTAGTATGGGGAAGAAGTGGACTCATGATTATTCGTTCGCCGGAACCAGAAGTAA
AAATTTTGGTAGATAGGGATCCCATAAAAACTTCTTTCGAGGAATGGCCAGACCCGGT
CATTTCTCAAGAACAATAGCTAAGGGCCCTGAGACTACCACTTGGATCTGGAACCTACA
TGCTGATGCTCACGATTTCGATAGCCATACCAGTGATTTGGAGGAGATTTCTCGAAAAG
TATTTAGTGCTCATTTCGGTCAACTCTCCATCATCTTTCTTTGGCTGAGTGGCATGTATTT
CCACGGTGCTCGTTTTTCCAATTATGAAGCATGGCTAAGCGATCCTACTCATATTGGACC
TAGTGCCCAGGTGGTTTGGCCAATAGTGGGCCAAGAAATATTGAATGGTGACGTGGGT
GGGGGTTTCCGAGGAATACAAATAACCTCTGGTTTTTTTCAAATTTGGCGAGCATCCGG
AATAACTAGTGAATTACAACTCTATTGTACCGCAATTGGTGCATTGGTCTTTGCAGCGTT
AATGCTTTTTGCTGGTTGGTTCCATTATCACAAAGCTGCTCCAAAATTGGCTTGGTTCC
AAGACGTAGAATCTATGTTGAATCACCATTTAGCGGGGTTACTAGGACTTGGGTCTCTT
TCTTGGGCGGGGCATCAAGTACATGTATCTTTACCGATTAACCAATTTCTAAACGCTGG
AGTAGATCCTAAAGAGATCCCACTTCCTCATGAATTTATCTTGAATCGGGATCTTTTGGC
TCAACTTTATCCCAGTTTTGCTGAGGGAGCAACCCCGTTTTTCACCTTGAATTGGTCAA
AATATGCGGAATTCCTTACTTTTCGTGGAGGATTAGATCCAGTAACTGGGGGTCTATGG
CTGACCGATATTGCACACCACCATTTAGCTATTGCAATTCTTTTCCTGATAGCAGGTCAC
ATGTATAGGACCAACTGGGGCATTGGTCATGGTCTAAAAGATATTTTAGAGGCTCATAA
GGGTCCATTTACAGGCCAGGGCCATAAAGGACTATATGAGATCCTAACAACGTCATGGC
ATGCTCAATTATCTCTTAACCTAGCTATGTTAGGCTCTTTAACCATTGTTGTAGCTCACCA
TATGTATTCCATGCCCCCTTATCCATATCTAGCTACTGACTATGGTACAGTACTGTCATTG
TTCACGCATCACATGTGGATTGGTGGATTTCTCATAGTGGGTGCTGCTGCGCATGCAGC
CATTTTTATGGTAAGAGACTATGATCCAACTACTCGATACAACGATCTATTAGATCGTGT
CCTTAGACATCGCGATGCAATCATATCGCATCTCAACTGGGCATGTATATTTTTAGGCTTT
CACAGTTTTGGTTTGTATATTCATAATGATACCATGAGTGCGTTAGGGCGTCCTCAAGAT
ATGTTTTCAGATACCGCTATACAATTGCAACCTGTCTTTGCTCAATGGATACAAAACACC
CATGCTTTAGCGCCTGGTGCAACGGCTCCTGGTGCAACAACAAGCACCAGTTTGACTT
GGGGGGGTGGTGATTTAGTAGCAGTAGGTGGCAAAGTAGCTTTGTTACCTATTCCATTA
GGAACCGCAGATTTTTTGGTACATCACATTCATGCATTTACTATTCATGTGACGGTCTTG
ATACTCCTGAAAGGTGTTCTATTTGCTCGTAGCTCGCGTTTGATACCGGATAAAGCAAA
TCTCGGCTTTCGCTTCCCTTGTGATGGACCTGGAAGAGGGGGTACATGTCAAGTATCTG
CTTGGGATCATGTCTTCTTAGGGCTATTCTGGATGTACAATGCTATTTCGGTAGTCATATT
CCATTTCAGTTGGAAAATGCAGTCAGATGTTTGGGGTAGTATAAGTGATCAAGGGGTAG
TAACTCATATTACGGGAGGAAACTTTGCGCAGAGTTCCATTACTATTAATGGATGGCTCC
GCGATTTCTTATGGGCACAAGCATCCCAAGTAATTCAGTCTTATGGTTCTTCATTATCTG
CATATGGACTTTTTTTCCTAGGTGCTCATTTTGTATGGGCTTTTAGTTTAATGTTTCTATT
CAGCGGGCGTGGTTACTGGCAAGAACTTATTGAATCCATCGTTTGGGCTCATAATAAAT
TAAAAGTTGCTCCTGCTACTCAGCCTAGAGCCTTGAGCATTGTACAAGGACGTGCTGTA

GGAGTAACCCATTACCTTCTGGGTGGAATTGCCACAACATGGGCGTTCTTCTTAGCAAG
AATTATTGCAGTAGGAATGGCATTAAGATTTCCAAGGTTTAGCCAAGGCTTAGCTCAGG
ACCCCACTACTCGTCGTATTTGGTTTGGTATTGCTACCGCACATGACTTCGAGAGTCAT
GATGATATTACTGAGGAACGTCTTTATCAGAATATTTTTGCTTCCCACTTCGGGCAATTA
GCAATAATTTTTCTGTGGACTTCCGGAAATCTGTTTCATGTAGCTTGGCAAGGAAATTT
TGAGTCGTGGGTACAGGATCCTTTACACGTAAGACCTATTGCTCATGCAATTTGGGATC
CTCATTTTGGTCAACCTGCTGTGGAAGCTTTTACTCGAGGGGGTGCTCTTGGCCCAGTG
AATATCGCTTATTCTGGTGTTTATCAGTGGTGGTATACAATCGGTTTACGTACTAATGAA
GATCTTTATACTGGAGCTCTTTTTCTATTATTTCTTTCTGCCGTATCCTTAATAGCGGGTT
GGTTACACTTACAACCGAAATGGAAACCGAGCGTTTCCTGGTTCAAAAATGCTGAATC
TCGTCTCAATCATCATTTGTCAGGACTTTTCGGAGTAAGTTCCTTGGCTTGGACAGGGC
ATTTAGTCCATGTTGCTATTCCTGGATCTAGGGGGGAGTACGTTCGCTGGAATAATTTCT
TAGATGTATTACCGCATCCCCAAGGGTTAGGCCCACTTTTTACAGGTCAGTGGAATCTTT
ATGCTCAAAACCCTGATTCAAGTAGTCATTTATTTGGTACTTCTCAAGGAGCAGGAACT
GCCATTCTAACCCTTCTCGGGGGATTTCATCCACAAACGCAAAGTTTATGGTTGACTGA
TATGGCTCATCATCATTTAGCTATTGCATTTATTTTTCTCGTTGCTGGTCATATGTATAGAA
CTAACTTCGGGATTGGGCATAGTATGAAAGATCTGTTAGAAGCACATGTTCCTCCGGGG
GGACGATTGGGGCGTGGACATAAAGGTCTTTATGATACAATCAATAATTCGATTCATTTT
CAATTAGGTCTTGCTCTAGCCTCTTTAGGGGTTGTTACTTCTTTGGTAGCTCAACACATG
TACTCTTTACCTGCTTATGCGTTCATAGCACAAGACTTTACGACTCAAGCTGCGTTATAT
ACTCATCACCAATACATCGCAGGATTCATCATGACAGGAGCTTTTGCTCATGGAGCTATA
TTTTTCATTAGAGATTACAATCCGGAACAGAATGAGGATAATGTATTGGCAAGAATGTT
AGACCATAAAGAAGCTATCATATCCCATTTAAGCTGGGCCAGCCTCTTTCTGGGGTTCC
ATACCTTAGGACTTTATGTTCATAATGATGTCATGCTTGCTTTCGGTACTCCGGAAAAAC
AAATCTTGATCGAACCTATATTTGCCCAATGGATACAATCTGCTCATGGTAAAACTTCGT
ATGGGTTCGATGTACTTTTATCTTCAACGAATGGCCCAGCATTCAATGCTGGTCGAAGC
ATATGGTTGCCTGGCTGGTTAAATGCTATTAATGAG------AATAGTAATTCACTATTCTTAA
CAATAGGTCCTGGCGATTTCTTGGTTCATCATGCTATTGCTCTAGGTTTACATACAACCA
CATTGATCTTAGTAAAGGGTGCTTTAGATGCACGTGGTTCTAAGTTAATGCCAGATAAA
AAGGATTTCGGTTATAGTTTTCCTTGCGATGGTCCGGGACGAGGTGGTACTTGTGATAT
TTCGGCTTGGGACGCTTTTTATTTGGCAGTTTTCTGGATGTTAAATACCATTGGATGGGT
TACTTTTTATTGGCATTGGAAGCACATCACATTATGGCAGGGTAACGTTTCACAATTTAA
TGAATCTTCCACTTATCTGATGGGATGGTTAAGAGATTATCTATGGTTAAACTCTTCACA
ACTTATCAATGGATATAACCCTTTTGGTATGAATAGTTTATCGGTCTGGGCGTGGATGTT
CTTATTTGGACATCTTGTTTGGGCTACTGGATTTATGTTCTTAATTTCCTGGCGCGGATAT
TGGCAGGAATTGATTGAAACTTTAGCATGGGCTCATGAACGCACACCTTTGGCCAATTT
GATTCGATGGCGAGATAAACCAGTGGCCCTTTCTATTGTGCAAGCAAGATTGGTTGGAT
TAGCCCACTTTTCTGTAGGTTATATATTTACTTATGCGGCTTTCTTGATTGCGTCTACATC
GGGCAAATTTGGTATGTCACACTCAGTAAAGATTTATGATACATGTATAGGGTGTACTCA
ATGTGTTCGAGCCTGCCCCACAGATGTATTAGAAATGATACCTTGGGATGGATGTAAAG
CTAAACAAATTGCTTCTGCTCCAAGAACAGAAGACTGTGTCGGTTGTAAGAGATGTGA
ATCCGCCTGTCCAACGGATTTCTTGAGTGTTCGAGTTTATTTATGGCATGAAACAACTC
GCAGCATGGGTCTAGCTTATATGACAACTTTCAACTTACCCTCTATTTTTGTGCCTTTAG
TGGGCCTAGTATTTCCGGCAATTGCAATGGCTTCTTTATCTCTTCATGTTCAAAGAAACA

GGATTTTTATGCGAGATCTAAAAACATATCTTTCCGTGGCACCGGTACTAAGTACTCTAT
GGTTCGGGGCTTTAGCAGGTTTATTGATAGAGATCAATCGTTTATTCCCGGATGCGTTGA
CATTCCCTTTTTTTTCATTTATGACTGCAATTTTAGAGAGACGCGAAAGTGAAAGCCTAT
GGGGTCGCTTCTGTAACTGGATAACCAGCACCGAAAACCGTCTTTACATTGGATGGTTT
GGCGTTTTGATGATCCCTACCTTATTGACCGCAACTTCTGTATTTATTATCGCCTTCATTG
CTGCTCCTCCAGTAGATATTGATGGTATTCGTGAACCTGTTTCTGGATCTCTACTTTACG
GAAACAATATTATTTCTGGTGCCATTATTCCTACTTCTGCAGCTATAGGTTTGCACTTTTA
CCCAATCTGGGAAGCAGCATCCGTTGATGAATGGTTATACAATGGTGGTCCTTATGAAC
TAATTGTTCTACACTTCTTACTTGGTGTAGCTTGTTACATGGGTCGTGAGTGGGAACTTA
GTTTCCGTCTGGGTATGCGTCCTTGGATTGCTGTTGCATATTCAGCTCCCGTTGCAGCTG
CGACTGCTGTTTTCTTGATCTACCCAATTGGTCAAGGAAGCTTTTCTGATGGTATGCCTC
TAGGAATCTCTGGTACTTTCAACTTCATGATTGTATTCCAGGCTGAGCACAACATCCTTA
TGCACCCATTTCACATGTTAGGCGTAGCTGGTGTATTCGGCGGCTCCCTATTCAGTGCTA
TGCATGGTTCCTTGGTAACCTCTAGTTTGATCAGGGAAACCACAGAAAATGAATCTGCT
AATGAAGGTTACAGATTCGGGCAAGAGGAAGAAACTTATAATATCGTAGCTGCTCATGG
TTATTTTGGCCGATTGATCTTCCAATATGCTAGTTTCAATAATTCTCGTTCTTTACATTTCT
TCCTAGCCGCTTGGCCTGTAGTAGGTATCTGGTTTACTGCTTTAGGTATCAGCACCATGG
CTTTCAACCTAAATGGTTTCAATTTCAACCAATCTGTAGTTGATAGTCAAGGTCGTGTAA
TTAATACCTGGGCTGATATTATTAACCGTGCTAACCTTGGTATGGAAGTTATGCATGAAC
GTAACGCTCATAACTTCCCTCTAGACCTAGCTGCTGTCGAAGCTCCGTCTACAAATGGA
ATGGGTTTGCCTTGGTATCGTGTTCATACTGTTGTATTGAATGATCCCGGTCGGTTGCTT
GCTGTCCATATAATGCATACAGCTCTCGTTGCTGGTTGGGCCGGTTCGATGGCTCTATAT
GAATTAGCAGTTTTTGATCCTTCTGATCCCGTTCTTGATCCAATGTGGAGACAAGGTAT
GTTCGTTATACCCTTCATGACTCGTTTAGGAATAACCAATTCATGGGGCGGTTGGAGTAT
CACA---GGAGGTACCATAACGAATCCAGGCATTTGGAGTTACGAAGGTGTGGCCGGAG
CGCATATTGTGTTTTCTGGCTTGTGCTTCTTGGCAGCTATTTGGCATTGGGTGTATTGGG
ATCTAGAAATATTTAGTGATGAACGTACAGGAAAACCTTCTTTGGATTTGCCCAAGATT
TTTGGAATTCATTTATTTCTTTCAGGGGTGGCTTGCTTTGGTTTTGGCGCATTTCATGTA
ACAGGGTTGTATGGTCCTGGAATATGGGTGTCCGATCCTTATGGACTAACTGGAAAAGT
ACAATCTGTAAATCCAGCGTGGGGTGTGGAAGGTTTTGATCCTTTTGTTCCGGGAGGA
ATAGCCTCTCATCATATTGCAGCGGGGACGTTGGGCATATTAGCGGGCCTATTCCATCTT
AGTGTTCGTCCGCCTCAACGTCTATACAAAGGATTACGTATGGGCAATATTGAAACCGT
ACTTTCCAGTAGTATCGCTGCTGTCTTTTTTGCTGCTTTTGTTGTTGCTGGAACTATGTG
GTATGGTTCAGCAACTACCCCGATCGAATTATTTGGTCCCACTCGTTATCAATGGGATCA
GGGATACTTCCAGCAAGAAATATATCGAAGAATTAGTGCTGGACTAGCCGAAAATCAA
AGTTTATCAGAAGCTTGGTCTAAAATTCCCGAAAAACTAGCTTTTTATGATTACATTGGC
AATAATCCGGCAAAAGGGGGATTATTCAGAGCGGGCTCAATGGACAACGGGGATGGAA
TAGCTGTTGGATGGTTAGGACACCCTGTCTTTAGAGATAAAGAAGGGCGTGAACTTTTT
GTACGCCGTATGCCTACCTTTTTTGAAACATTTCCAGTTGTTTTGGTAGATGGAGACGG
AATTGTTAGAGCTGATGTTCCTTTTAGAAGGGCAGAATCAAAGTATAGTGTCGAACAA
GTAGGTGTAACTGTTGAGTTCTATGGCGGCGAACTTAACGGAGTTAGTTATAGTGATCC
GGCTACTGTTAAAAAATATGCTAGACGCGCTCAATTGGGTGAAATTTTTGAATTAGATC
GTGCTACTTTGAAATCCGATGGTGTTTTTCGTAGCAGCCCAAGGGGTTGGTTTACTTTT
GGACATGCTTCATTTGCTTTGCTCTTCTTCTTCGGACACATTTGGCATGGTGCTAGAACC

TTGTTCAGAGATGTTTTTGCTGGTATTGACCCAGATTTGGATGCTCAAGTGGAATTTGG
AGCATTCCAAAAACTTGGAGATCCAACTACAAGAAGACAAGTAGTC-----------------------
------------GTGGAAACGCTCTTTAATGGAACTTTAGCTTTAGCTGGTCGTGACCAAGAAA
CCACCGGTTTCGCTTGGTGGGCCGGGAATGCCCGACTTATCAATTTATCTGGTAAACTA
CTTGGGGCTCATGTAGCCCATGCCGGATTAATCGTATTCTGGGCTGGAGCAATGAACCT
ATTTGAAGTGGCTCATTTCGTACCAGAGAAGCCCATGTATGAACAAGGATTAATTTTAC
TCCCCCACCTAGCTACGCTCGGTTGGGGGGTAGGTCCTGGTGGGGAAGTTATAGACAC
CTTTCCCTACTTTGTATCTGGAGTACTTCACTTAATTTCCTCTGCAGTATTGGGCTTTGG
CGGTATTTATCATGCACTTCTGGGACCTGAGACTCTTGAAGAATCTTTTCCATTCTTCGG
TTATGTATGGAAAGATAGAAATAAAATGACCACAATTTTGGGTATTCACTTAATCTTGTT
AGGTCTAGGTGCTTTTCTTCTAGTATTCAAGGCTCTTTATTTTGGAGGCATATATGATACC
TGGGCTCCGGGGGGGGGGGATGTAAGAAAAATTACCAACTTGACCCTTAGCCCAAGTG
TTATATTTGGTTATTTACTAAAATCTCCTTTTGGGGGAGAAGGATGGATTGTTAGTGTGG
ATGATTTGGAAGATATAATTGGAGGACATGTATGGTTAGGTTCCATTTGTATACTTGGTG
GAATCTGGCATATCTTAACCAAACCCTTTGCATGGGCTCGCCGTGCACTTGTATGGTCT
GGAGAGGCTTACTTGTCTTATAGTTTAGCTGCTTTATCTATCTTTGGTTTCATTGCTTGTT
GCTTTGTGTGGTTCAATAATACCGCTTATCCTAGTGAGTTTTACGGGCCCACTGGACCA
GAAGCTTCTCAAGCTCAAGCATTTACTTTTCTAGTTAGAGACCAACGTCTCGGGGCTAA
TGTGGGATCGGCTCAAGGACCGACAGGTTTAGGTAAATATCTAATGCGTTCCCCAACTG
GAGAAGTCATTTTTGGAGGAGAAACTATGCGTTTTTGGGATCTTCGTGCTCCCTGGTTA
GAACCTCTAAGGGGTCCCAACGGTTTGGACTTGAGTAGGCTGAAAAAAGACATACAA
CCTTGGCAAGAACGGCGTTCCGCGGAATATATGACTCATGCTCCTTTAGGTTCTTTAAA
TTCCGTGGGTGGCGTAGCTACAGAAATCAATGCAGTCAATTATGTCTCTCCTAGAAGTT
GGTTATCTACCTCTCATTTTGTTCTAGGATTCTTCCTATTCGTAGGTCATTTGTGGCACGC
AGGAAGGGCTCGTGCAGCTGCAGCAGGATTTGAAAAAGGAATTGATCGTGATTTTGAA
CCTGTTCTTTCCATGACCCCTCTTAACATGACTATAGCCCTTGGTAAATATACCAAAGAC
GAAAATGATTTATTTGATATTATGGATGACTGGTTACGGAGAGACCGTTTCGTTTTTGTA
GGTTGGTCCGGTCTATTGCTCTTTCCTTGTGCGTATTTCGCTTTAGGGGGTTGGTTCACA
GGTACAACCTTTGTAACTTCATGGTATACCCATGGATTGGCCAGTTCCTATTTGGAAGGC
TGTAATTTCTTAACCGCCGCAGTTTCAACCCCTGCTAATAGTTTAGCACATTCTTTGTTG
TTACTATGGGGTCCTGAAGCCCAAGGAGATTTTACTCGTTGGTGTCAATTAGGCGGTCT
GTGGACTTTTGTTGCTCTCCATGGCGCTTTCGGACTAATAGGTTTCATGTTACGTCAATT
CGAACTTGCTCGATCTGTTCAATTGCGACCTTATAATGCAATTGCATTTTCTGGTCCAAT
TGCTGTTTTTGTTTCTGTATTCCTGATTTATCCACTAGGTCAGTCTGGTTGGTTCTTTGCG
CCTAGTTTTGGTGTAGCAGCTATATTTCGATTCATCCTCTTTTTCCAAGGGTTTCATAATT
GGACGTTGAACCCATTTCATATGATGGGAGTTGCCGGTGTATTGGGCGCTGCTCTGCTA
TGCGCTATTCATGGTGCTACTGTAGAAAATACTTTATTTGAAGATGGTGATGGTGCAAAT
ACATTCCGTGCTTTTAACCCAACTCAAGCTGAAGAAACTTATTCAATGGTCACCGCTAA
CCGCTTTTGGTCCCAAATCTTTGGGGTTGCTTTTTCCAATAAACGTTGGTTACATTTCTT
TATGTTATTTGTACCAGTAACCGGTTTATGGATGAGTGCTCTTGGAGTAGTCGGTCTGGC
TCTGAACCTACGTGCCTATGACTTCGTTTCCCAGGAAATCCGTGCAGCGGAAGATCCTG
AATTTGAGACTTTCTATACCAAAAATATTTTATTAAACGAAGGTATTCGTGCTTGGATGG
CGGCTCAAGATCAGCCTCATGAAAACCTTATATTCCCTGAGGAGGTTCTACCACGTGGA
AACGCTCTTATGTCTGGAAGCACAGGAGAACGTTCTTTTGCTGATATTATCACTAGTATT

CGATACTGGGTCATTCATAGCATTACTATACCCTCCCTATTCATTGCGGGTTGGTTATTCG
TCAGCACGGGTTTAGCTTACGATGTGTTTGGAAGCCCTCGGCCAAACGAGTATTTTACA
GAGAGCCGACAAGGAATTCCATTAATAACTGGCCGTTTTGATTCTTTGGAACAACTCGA
TGAATTTAGTAGATCCTTTATGACCATAGATCGAACCTATCCAATTTTTACAGTACGATG
GTTGGCTATTCACGGACTAGCTGTACCTACCGTTTCTTTTTTGGGGTCAATATCAGCAAT
GCAGTTCATCCAACGAATGGCTACACAAACCGTTGAGAGTAGTTCCAGATCTGGT---CC
AAGACAAACTAATGTAGGGAATTTATTGAAACCATTGAATTCGGAATATGGTAAAGTAG
CTCCTGGATGGGGAACTACTCCTTTGATGGGTGTCGCAATGGCTCTATTTGCGATATTCC
TATCTATTATTTTGGAGATTTATAACTCTTCTGTTTTACTGGACGGAATTTCAATGAATAT
GCTTACTCTCAAACTCTTCGTTTACACAGTAGTGATATTCTTTGTTTCTCTCTTCATCTTC
GGATTCCTATCTAATGATCCAGGACGTAATCCTGGACGTGAAGAAATGGCCGATACTAC
TGGAAGGATTCCTCTTTGGCTAATAGGTACTGTAGCTGGTATTCTTGTGATCGGTTTAAT
AGGTATTTTCTTTTATGGTTCATATTCAGGATTGGGTTCATCCCTGATGCTTAATATTTTTA
GTTTGATCGGTATCTGTCTTAATTCTGCCCTTTAT---AGTAGTTTTTTCTTTGCCAAATTAC
CCGAGGCCTACGCTTTTTTAAATCCAATTGTAGATTTTATGCCAGTAATACCTTTGCTGT
TTTTTCTCTTAGCCTTTGTTTGGCAAGCTGCTGTAAGTTTTCGAACGACACAATCAAAC
CCGAACGAACAAAGTGTTGAATTGAATCGTACCAGTCTCTACTGGGGGTTATTACTCAT
TTTTGTACTTGCTGTTTTATTTTCCAATTATTTTTTCAATATGGAAGTAAATATTCTTGCAT
TTATTGCTACTGCACTGTTCATTCTAGTTCCTACTGCTTTTTTACTTATCATATACGTAAA
AACAGTCAGTCAAAATAATATGGAAACAGCAACCCTAGTCGCCATCTCTATATCTGGTT
TACTTGTAAGTTTTACTGGGTACGCCTTATATACCGCTTTTGGGCAACCCTCTCAACAAC
TAAGAGATCCATTCGAGGAACATGGGGATATGGAAGCATTGGTTTATACATTCCTCTTAG
TCTCGACTCTAGGGATAATTTTTTTCGCTATCTTTTTTCGAGAACCGCCTAAAGTTCCAA
CTAAAAAG------ATGACTATTGCTTTCCAATTGGCTGTTTTTGCATTAATTGCTACTTCAT
CAATCTTATTGATTAGTGTACCCGTTGTATTTGCTTCTCCTGACGGTTGGTCAAGTAAGA
AAAATGTTGTATTTTCTGGTACATCATTATGGATTGGATTAGTCTTTCTGGTAGGTATCCT
TAATTCTCTCATCTCT---------------------ATGTCACCACAAACAGAGACTAAAGCAAGTGT
TGGATTCAAAGCTGGTGTTAAAGATTACAAATTGACTTATTATACTCCTGATTATGAAAC
CAAAGATACTGATATCTTGGCAGCATTCCGAGTAACTCCTCAACCTGGAGTTCCACCTG
AGGAAGCGGGGGCAGCGGTAGCTGCTGAATCTTCTACTGGTACATGGACAACTGTGTG
GACCGATGGACTTACCAACCTTGATCGTTACAAAGGACGATGCTATCACATCGAGCCCG
TTGCTGGAGAAGAAAGTCAATTTATTGCTTATGTAGCTTACCCCTTAGACCTTTTTGAA
GAAGGTTCTGTTACTAACATGTTTACTTCCATTGTGGGTAATGTATTTGGGTTCAAAGCC
CTGCGCGCTCTACGTCTAGAGGATCTGCGAATCCCTCCTGCTTATGTTAAAACTTTTCA
AGGCCCGCCTCATGGCATCCAAGTTGAGAGAGATAAGTTGAACAAGTATGGTCGTCCC
CTATTGGGATGTACTATTAAACCTAAATTGGGGTTATCTGCTAAGAACTACGGTAGAGC
AGTTTATGAATGTCTCCGTGGTGGACTTGACTTTACCAAAGATGATGAAAACGTGAACT
CCCAACCATTTATGCGTTGGAGAGACCGTTTCTTATTTTGTGCCGAAGCACTTTATAAA
GCACAAGCTGAAACAGGTGAAATCAAAGGGCATTACTTGAATGCTACTGCAGGTACAT
GCGAAGAAATGATCAAAAGGGCAGTATTTGCCAGAGAATTGGGAGTTCCTATCGTAAT
GCACGATTACTTAACAGGGGGATTCACTGCAAATACTAGCTTATCTTTTTATTGCCGAGA
TAATGGCCTACTTCTTCACATCCATCGTGCAATGCATGCAGTTATTGATAGACAGAAGAA
TCATGGTATACACTTTCGTGTACTAGCTAAAGCGTTACGTATGTCTGGTGGAGATCATAT
TCACTCTGGTACCGTAGTAGGTAAACTTGAAGGGGAAAGAGAGATCACTTTGGGCTTT

GTTGATTTACTACGTGATGATTTTATTGAAAAAGATCGAAGTCGCGGTATTTATTTCACT
CAAGATTGGGTCTCTCTACCAGGTGTTCTGCCCGTGGCTTCAGGGGGTATTCACGTTTG
GCATATGCCTGCTCTGACCGAGATCTTTGGAGATGATTCTGTACTACAGTTCGGTGGAG
GAACTTTAGGACACCCTTGGGGAAATGCACCGGGTGCCGTAGCTAATCGAGTTGCTCT
AGAAGCATGTGTACAAGCTCGTAATGAAGGACGTGATCTTGCTCGTGAGGGTAATGAA
ATTATCCGTGAGGCTTGCAAATGGAGTCCTGAACTAGCTGCTGCTTGTGAAGTATGGAA
GGAGATCAAATTTGAATTCGAGGCAATGGATACTTTG------ATGGCGATACATTTATACA
AAACTTCTACCCCGAGCACACGCAACGGAGCCGTAGACAGTCAAGTGAAATCCAATC
CACGAAATAATTTGATCTATGGACAGCATCATTGTGGTAAAGGTCGTAATGCCAGAGGA
ATCATTACCGCAGGGCATAGAGGGGGAGGTCATAAGCGTCTATACCGTAAAATCGATTT
TCGACGGAATAAAAAGGACATATATGGTAGAATCGTAACCATAGAATACGACCCTAATC
GAAATGCATACATTTGTCTCATACACTATGGGGATGGTGAGAAGAGATATATTTTACATC
CCAGAGGGGCTATAATCGGGGATACCATTGTTTCTGGTACAGAAGTTCCTATAAAAATG
GGAAATGCCCTACCTTTGACCGATATGCCCTTAGGCACGGCCATACATAACATAGAAAT
CACACTTGGAAAGGGTGGACAATTAGCTAGAGCAGCGGGTGCTGTAGCGAAACTGATT
GCAAAAGAGGGGAAATCGGCCACATTAAAATTACCTTCTGGGGAGGTCCGTTTGATAT
CCAAAAACTGCTCAGCAACAGTCGGACAAGTGGGGAATGTTGGGGTGAACCAGAAA
AGTTTGGGTAGAGCCGGATCTAAGCGTTGGCTAGGTAAGCGTCCTGTAGTAAGAGGAG
TAGTTATGAACCCTGTAGACCATCCCCATGGGGGTGGTGAAGGGAGGGCCCCAATTGG
TAGAAAAAGACCCACAACCCCTTGGGGTTATCCTGCACTTGGAAGAAGAAGTAGAAA
AAAGAATAAATATAGTGATAATTTGATTCTTCGTCGCCGTAGTAAAATGATTCAACCCCA
GACCTATTTGAATGTAGCGGACAACAGCGGGGCCCGAGAATTGATGTGTATTCGAATCA
TAGGAGCTAGT---AATCGCCGATATGCTCATATTGGTGATGTTATTGTTGCTGTGATCAAA
GAAGCAGTACCAAATATGCCTCTCAAAAGATCAGAAGTGATCAGAGCTGTAATTGTAC
GTACTTGTAAAGAACTCAAACGTGACAATGGTATGATAATACGATATGATGACAATGCT
GCAGTTGTCATTGATCAAGAAGGAAATCCAAAAGGAACTCGAGTTTTTGGTGCGATCG
CCCGAGAATTGAGACGGTTAAATTTTACAAAAATAGTTTCATTAGCCCCTGAAGTATTA
ATGCTTAGTCCCAAAAGAACCAGATTCCGTAAACAACATAGAGGAAGAATGAAGGGAA
TATCTTATCGAGGTAATCATATTTGTTTCGGTAGATATGCTCTTCAGGCACTTGAACCCG
CTTGGATCACATCTAGACAAATAGAAGCAGGGCGACGAGCAATGACACGAAATGCAC
GCCGTGGGGGAAAAATATGGGTACGTATATTTCCAGACAAACCCGTTACAGTAAGACC
TGCGGAAACACGTATGGGGTCGGGTAAAGGATCTCCCGAATATTGGGTAGCTGTCGTTA
AACCAGGTAGAATACTTTATGAAATGGGCGGAGTAACAGAAAATATAGCCAGAAAGGC
TATTTCAATAGCAGCATCCAAAATGCCTATACGAACTCAATTCATTATTTCG---ATGACCA
GAATTAGACGAGGATATGTAGCTCGTAAACGTAGAACAAAAATTCGTTTATTTGCATCA
AGCTTTCGGGGGGCTCATTCAAGACTTACTCGAACTATTACTCAACAGAAAATAAGAG
CTTTGGTTTCTGCTCATCGGGATAGAGATAGGCAAAAGAGAGATTTTCGTCGTTTGTGG
ATCACTCGGATAAATGCAGTAATTCGCGGGAGT---AAGGTATCCTATAGTTATAGTCGATT
AATACACGATCTGTACAAGAGGAAATTGCTTCTTAATCGTAAAATACTTGCACAAATAG
CTATATTAAATAGGAATTGTCTTTATATGATTTCCAATGAAATCATAAGAGAAGGGGATT
GTAAGGAATCCACCGGAAAAATTATGTTGAAGGAAGTATACGCTTTAGGTCAACATATA
TGTATGTCTGCTCACAAAGCGCGAAGAGTAGTTGATCAGATTCGTGGACGTTCCTACG
AGGAAACACTTATGATACTAGAACTCATGCCTTATCGAGCATGTTATCCCATTTTTAAAT
TGGTTTATTCTGCAGCAGCAAATGCTAGTCACAATATGGGTTTCAATGAAGCAGATTTA

ATCATTAGTAAAGCCGAAGTCAATGAGGGTACTACCGTGAAAAAATTAAAACCTCGAG
CTAGAGGACGTGGTTATCCGATAAAAAGGTCCACCTGCCATATAACTATTGTATTGAAA
AATACATCCTTA------------------------ATGGATGGAATCAAATATGCAGTATTTACAGACAAA
AGTATTCGGTTATTGAGGAAAAAT---CAATATACTTCTAATGTCGAATCAGGATCAACTA
GGACAGAAATAAAGCATTGGGTCGAACTCTTCTTTGGTGTCAAGGTAATAGCTATGAAT
AGTCATCGACTCCCGGGAAAGGGTAGAAGAATGGGACCTATTATGGGGCATACAATGC
ATTACAGACGTATGATCATTACGCTTCAACCGGGTTATTCTATTCCACCTCTTAGAAAGA
AAAGAACTATGGCAGTTCCAAAAAAACGTACTTCTATGTCAAAAAAGCGTATTCGTAA
AAATTATTGGAAAAAGAAGGGATATTGGGCAGCGTTAAAAGCTTTTTCATTATCGAAAT
CTCTTTCAACCGGAAATTCAAAAAGTTTTTTT------------CCGACAATGGCCAAGAGTAA
AGATGTCCGAGTAACGGTTATTTTGGAATGTACCAGTTGTGTGCGAAATGGTGTGAAT---
---AAAGAATCCATGGGCATTTCCAGATATATTACTCAAAAGAATCGACACAATACGCCTA
GTCGATTGGAATTGAGAAAATTCTGTCCCTATTGTTACAAACATACGATTCATGGGGAG
ATAAAGAAAATGAAAATAAGGGCCTCTGTTCGTAAAATTTGTGAAAAATGTCGACTGA
TCCGTAGGCGGGGACGAATTATAGTAATCTGTTCCAACCCAAGACATAAACAAAGACA
AGGAATGGTTCGAGAGAAAGTAACAGTATCTACTCGGACACTAGAGTGGAAGTGTGTT
GAATCAAGAGCAGACAGTAAGCGTCTTTATTATGGACGCTTTATTTTGTCTCCACTTATG
AAAGGTCAAGCCGACACAATAGGCATTGCGATGCGAAGAGCTTTGCTTGGAGAAATAG
AAGGAACATGTATCACACGTGCAAAATCTGAG------AAAATACCACATGAATATTCTACC
ATAGTAGGTATTCAAGAATCAGTACATGAAATTTTAATGAATTTGAAAGAAGTTGTATTG
AGAAGTAATCTGTATGGAACTCGCAACGCGTCTATTTGTGTCAGTGGTCCTGGATATGT
AACTGCTCAAGACATCATCTCACCGCCTTCTGTGGAAATCGTTGATAATACACAGCATA
TAGCTAGCCTGACTGAACCAATTGACTTGTGTATTGGATTACAAATCGAAAAGAATCGC
GGATATCGTATAAAAACGCCAAAT------AACGACGGAAGTTAT------CCTATAGATGCTGTA
TTCATGCCTGTTCGAAATGCGAATCATAGTATTCATTCTTATGGGAATGGGAATGAAAAA
CAAGAGATACTTTTTCTCGAAATATGGACAAATGGAAGTTTAACTCCTAAAGAAGCACT
TCATGAAGCCTCTCGGAATTTGATTGATTTATTTATTCCCTTTCTACATGCGGAAGAAGA
A------AACTTACATTTAGAGGACAATCAA---CACAAGGTTACTTTACCCCTTTTTACTTTT
CATGATAGATTGGCT---AAACTAAGGAAAAACAAAAAA------ATAGCATTAAAATATATTT
TTATTGACCAATCAGAATTGCCTCCCAGGATCTATACTTGCCTCAAAAGGTCCAATATAC
ATACATTATTGGACCTTTTGAATAACAGTCAAGAAGATCTTATGAAAATAGAACATTTTC
GCATAGAAGATGTAAAAGAGATATTGGGCTTTTTAGAAAAGCAT------TTCGAAATTGAT
TTACCGAAGAATAAGATGCTCCGGAATGGAAAT---GAGGGAATGTCTACAATACCTGGG
TTTAATCAGATACAATTTGAAGGATTTTGTAGGTTTATTGATCAGGGCTTGACGGAAGA
ACTTTATAAGTTTCCAAAAATTGAAGATACAGATCAAGAAATTGAATTTCAATTATTTGT
GGAAACATATCAATTGGTTGAACCGTTGATAAAAGAAAGAGATGCTGTGTATGAATCAC
TCACATATTCTTCTGAATTATATGTATCTGCGGGATTAATTTGGAAAACCAGTAGGGATAT
GCAAGAACAAACAATTTTTATTGGCAACATTCCTCTAATGAATTCCCTGGGAACTTCTAT
AGTAAATGGAATATACAGAATTGTGATCAATCAAATATTGCAAAGCCCCGGAATTTATTA
CCGGTCAGAATTGGACCATAACGGAATTTCGGTCTATACGGGCACCATAATATCAGATT
GGGGAGGAAGATTAGAATTAGAGATTGATAGAAAAGCACGGATATGGGCTCGTGTGAG
TAGGAAACAGAAAATATCTATTCTAGTTCTATCATCAGCTATGGGGTCGAATCTAAGAG
AAATTCTAGAGAATGTTTGCTACCCGGAAATTTTTTTGTCGTTCCTGAATGATAAGGAG
AAAAAAAAAATTGGGTCAAAAGAAAATGCCATTTTGGAGTTTTATCAACAATTTGCTT

GTGTAGGCGGGGATCCAGTATTTTCTGAATCCGTCTGTAAGGAATTACAAAAGAAATTC
TTTCAACAAAGGTGTGAATTAGGAAGGGTTGGTCGACGAAATATGAATCGGAGACTTA
ATCTTGATATACCTCAGAACAATACATTTTTGTTACCGCGAGATATATTGGCAGCTGCGG
ATCATTTGATTGGAATGAAATTTGGAATGGGTACACTTGACGAT------ATGAATCATTTGA
AAAATAAACGTATTCGTTCTGTAGCGGATCTCTTACAAGATCAATTCGGATTGGCTCTG
GTTCGTTTAGAAAATGTGGTTCGAGGAACTATAGGCGGAGCAATTAGG---CATAAATTG
ATA---CCGACTCCTCAGAATTTGGTAACTTCAACTCCA------------TTAACAACCACTTATG
AATCCTTTTTCGGCTTACACCCATTATCTCAAGTTTTGGATCGAACTAATCCATTGACAC
AAATAGTTCATGCGAGAAAATTGAGTTATTTGGGCCCTGGAGGATTGACAGGGCGAAC
TGCTAGTTTTCGGATACGAGATATCCATTCTAGTCACTATGGACGCATTTGCCCAATTGA
CACGTCTGAAGGAATCAATGTTGGACTTATTGGATCTTTAGCAATTCATGCGCGAATTG
GTCATTGGGGA---TCTCTAGAAAGCCCTTTTTAT---------GAAATTTCTGAGTCTGAGAGA
TCAAAAAAGGTACGGATGCTTTATTTATCACCAAGTAGAGATGAATACTATATGATAGCG
GCAGGAAATTCTTTGGCGCTGAATCAGGGTATTCAGGAGGAACAGGTTGTTCCAGCTC
GATACCGTCAAGAATTCCTGAGTATTGCATGGGAACAGGTTCATCTTCGAAGTATTTTT
CCCTTCCAATATTTTTCTATTGGAGCTTCCCTCATTCCTTTTATCGAGCATAATGATGCGA
ATCGGGCTTTAATGAGTTCTAATATGCAACGTCAAGCAGTTCCGCTTTCTCGGTCCGAA
AAGTGCATTGTTGGAACTGGGTTGGAACGACAAGCAGCTCTAGATTCAGGAGTTCCCG
CTATAGCCGAACACGAGGGAAAGATTATTTATACTGATACTGACAAGATCATTTTATCAG
GTAATGGAAATACTCTAAGCATTCCATTAATTATGTATGAACGTTCCAACAAAAATACTT
GTATGCATCAAAAACCCCAGGTCCATCGGGGTAAATGTATTAAAAAAGGACAAATTTTA
GCGGATGGTGCCGCTATCGTTGGCGGCGAACTCGCTTTGGGTAAAAACGTATTAGTAGC
TTATATGCCATGGGAAGGTTACAATTCTGAAGATGCAGTACTTATTAGCGAGCGTCTGGT
ATATGGAGATATTTATACTTCTTTTCACATACGGAAATATGAAATTCAGACTCATGTGAC
AAGCCAAGGC---CCTGAAAGGATCACTAATGAAATACCGCATTTAGAAGCCCATTTACT
CCGTAATTTAGACAAAAATGGAATTGTGAGGCTTGGATCTTGGGTAGAGACGGGCGAT
ATTTTAGTAGGTAAATTAACGCCTCAGATGGCGAAAGAA---TCATCGTATGCCCCCGAA
GATAGATTATTACGAGCCATATTGGGCATTCAGATATCCACTTCAAAGGAAACTTGTCTA
AAACTGCCTATAGGCGGTAAGGGTCGAGTTATTGATGTGAGATGGATCCAGAAAAAGG
GG---GGTTCTAGTTATAATCCAGAAACGATTCGTGTATATATTTCACAGAAACGTGAAAT
CAAAGTAGGTGATAAAGTAGCTGGTAGACATGGAAATAAAGGCATCATTTCCAAAATTT
TGCCTAGACAGGATATGCCTTATTTGCAAGATGGAAGACCTGTGGATATGGTCTTCAAC
CCATTAGGAGTACCTTCACGAATGAATGTAGGACAGATATTTGAATGCTCGCTGGGGTT
AGCGGGGAGTCTGCTAGACAGGCATTATCGAATAGCACCTTTTGATGAGAGATATGAGC
AAGAGGCTTCGAGAAAACTAGTGTTTTCTGAATTATATGAAGCTAGTAAGCAAACAGC
AAATCCATGGGTATTTGAACCCGAATATCCGGGAAAAAGTAGAATATTTGATGGAAGAA
CGGGAGATCCTTTTGAACAACCCGTTATAATAGGAAAGCCTTATATCTTGAAATTAATTC
ATCAAGTTGATGATAAAATCCATGGACGTTCCAGTGGACATTATGCACTTGTTACACAA
CAACCCCTGAGAGGAAGGGCCAAGCAAGGGGGACAGCGGGTAGGAGAAATGGAGGT
TTGGGCTCTAGAGGGATTTGGCGTTGCTCATATTTTGCAAGAGATGCTTACTTATAAATC
TGATCATATTAGAGCTCGTCAGGAAGTACTTGGTACTACGATCATTGGAGGAACAATAC
CTAACCCCGAGGATGCTCCCGAATCTTTTCGATTGCTCGTTCGAGAATTACGATCTTTG
GCTCTGGAACTGAATCATTTTCTTGTATCTGAAAAAAATTTCCAGATTAATAGGAAGGA
AGCTATGAATCAGAATTTTTCTTCTATGATCGATCGGTATAAACATCAACAACTCCGAAT

TGGATTAGTTTCTCCTCAACAAATAAGTGCTTGGGCCAATAAAATCCTACCTAATGGAG
AAATAGTTGGAGAGGTGACAAAACCCTATACTTTTCATTACAAAACCAATAAACCGGA
AAAAGATGGATTATTTTGTGAAAGAATTTTTGGGCCTATAAAAAGTGGAATTTGTGCTT
GTGGAAATTATCGAGTAATCGGAGATGAAAAAGAAGACCCGAAATTTTGTGAACAATG
CGGAGTCGAATTTGTTGATTCTCGGGTACGAAGATATCAAATGGGCTATATCAAACTGG
CATGTCCAGTAACCCATGTGTGGTATTTGAAACGTCTTCCTAGTTATATCGCGAATCTTT
TAGATAAACCTCTGAAAGAATTAGAGGGCCTAGTATACTGCGATTTT------TCTTTTGCTA
GGCCC---GTAGCCAAAAAACCCACTTTCTTACGATTACGAGGTTTATTCGAATATGAAAT
CCAATCCTGGAAATACAGCATCCCACTTTTTTTTACAACCCAGGGCTTCGATACATTTCG
AAATAGAGAAATCTCTACCGGAGCAGGTGCTATCCGAGAACAATTAGCCGATCTGGATT
TGCGACTTATTATAGATTCTTCATTGGTAGAATGGAAAGAATTAGGGGAAGAA---GGTC
CCACAGGTAATGAATGGGAAGATCGAAAAATTGGAAGAAGAAAAGATTTTTTGGTTAG
ACGCATGGAATTAGCTAAGCATTTTATTCGAACAAATATAGAACCAGAATGGATGGTTT
TGTGTCTATTACCAGTTCTTCCTCCCGAGTTGAGACCGATCATTCAGATCGATGGGGGT
AAACTAATGAGTTCGGATATTAATGAACTCTATAGACGAGTTATCTATCGGAACAATACT
CTTAACGATCTATTAACAACAAGTAGATCTACACCAGGGGAATTAGTAATGTGTCAGGA
GAAATTGGTACAAGAAGCCGTGGATACGCTTCTTGATAATGGAATCCGCGGACAACCA
ATGAGGGACGGTCATAATAAGGTTTACAAGTCGTTTTCGGATGTAATTGAAGGCAAAG
AGGGAAGATTTCGTGAGACTCTGCTTGGCAAACGGGTTGATTATTCGGGGCGTTCCGT
CATTGTCGTAGGCCCTTCACTTTCATTACATCGATGTGGATTGCCTCGCGAAATAGCAAT
AGAGCTTTTCCAGACATTTGTAATTCGTGGTCTAATTAGACAACATCTTGCTTCGAACAT
AGGAGTTGCTAAGAGTAAAATCCGGGAAAAAGAACCGATTGTATGGGAAATACTTCAG
GAAGTTATGCAGGGGCATCCTGTATTGCTCAATAGAGCACCTACTCTGCATAGATTAGG
CATACAGGCATTCCAACCAGTTTTAGTGGAAGGACGCGCTATTTGTTTACATCCATTAGT
TCGTAAGGGATTTAATGCAGACTTTGATGGGGATCAAATGGCTGTTCATGTACCTTTATC
TTTGGAGGCTCAAGCGGAGGCTCGCTTACTTATGTTTTCTCATATGAATCTCTTGTCTCC
AGCTATTGGGGATCCCATTTCCGTACCAACTCAAGATATGCTTATTGGACTCTATGTATTA
ACGAGGGGGAATCGCCGAGGTATTTGTGCAAATAGGTATAATCCACGTAATTGC---------
ATAAATTATCAAAATGAAAGAATTCGCACTAATAACTATAAGTATACGAAAGAA---GAAC
CCTTTTTTTGTAATTCGTATGATGCAATTGGAGCTTATCGGCAGAAAAGAATCAATTTAG
ATAGTCCTTTGTGGCTCCGGTGGCGACTAGATCAACGC---GTTATTGCTTCA------AGAG
AAGCTCCCGTCGAAGTGCATTATGAATCTTTGGGTACTTATCATGAGATTTATGGGCACT
ATCTAATAGTAAGAAGTGTAAAAAAAGAAATTATTTGTATATACATTCGAACCACCGTTG
GTCATATTTCTCTTTATCGAGAAATCGAAGAAGCTATACAAGGGTTTTGTCGAGCCTGT
TCATATCGTACC------------------------ATGGAGGTACTTATGGCAGAACGGGCCAATCTGGT
CTTTCACAATAAAGTGATAGATGGAACTGCCATGAAACGACTTATTAGCAGATTAATAG
ATCACTTCGGAATGGCATATACATCACACATCCTGGATCAAGTAAAGACTCTAGGTTTC
CAGCAAGCCACTGCTACATCTATTTCATTAGGAATTGATGATCTTTTAACAATACCTTCT
AAAGGATGGCTAGTCCAAGACGCTGAACAACAAAGTTTGATTTTGGAAAAACACCATC
ATTATGGGAATGTACACGCAGTAGAAAAATTACGCCAATCTATTGAGATATGGTATGCTA
CAAGTGAATATTTGCGACAAGAAATGAATCCTAATTTTAGGATGACTGATCCCTTTAATC
CAGTCCATATGATGTCTTTTTCGGGAGCGAGAGGAAATGCATCTCAGGTACACCAATTA
GTAGGTATGAGAGGATTAATGTCGGATCCCCAAGGACAAATGATTGATTTACCCATCCA
AAGCAATTTACGCGAAGGACTCTCTTTAACAGAATATATCATTTCTTGCTACGGAGCCC

GCAAAGGAGTTGTGGATACTGCTGTACGAACATCAGATGCTGGATATCTCACGCGCAG
ACTTGTTGAAGTAGTTCAACACATTGTTGTACGTAGAACAGATTGTGGCACCTCCCGA
GGTATTTCTGTGAGTCCTCAAAATGGGATGATGCCG---GAAAGAATTTTCATCCAAACA
TTAATTGGTCGTGTATTAGCGGACGATATATACATGGGTTCACGATGCATTGCCATTCGA
AATCAAGATATTGGGATTGGACTTGTCAATCGATTCATAACC------------------TTTCGAGC
ACAACCAATATCTATTAGAACTCCCTTTACTTGTAGGAGTACATCTTGGATCTGTCGATT
ATGTTATGGTCGGAGTCCTACTCATGGTGACCTGGTCGAATTGGGGGAAGCTGTAGGTA
TTATTGCGGGTCAATCTATTGGAGAACCAGGTACTCAACTAACATTAAGAACTTTTCAT
ACTGGTGGAGTATTCACGGGGGGTACTGCAGAACATGTACGAGCCCCTTCTAATGGAA
AAATAAAATTCAATGAGGATTTGGTTCATCCCACACGTACACGTCATGGACATCCTGCT
TTTCTATGTTCTATAGACTTGTATGTAACTATTGAGAGTGAAGATATTATACATAATGTGA
ATATTCCACCAAAAAGTTTTATTTTAGTTAAAAATGATCAATATGTAGAATCAGAACAAG
TGATTGCTGAGATTCGTGCGGGAACATCTACT---TTTAATTTTAAAGAGAGGGTTCGAA
AACATATTTATTCTGATTCACAGGGAGAA---ATGCACTGGAGTACCGATGTGTACCATGC
ACCTGAATTTACATATGGTAATGTTCATCTCTTACCAAAAACCAGTCATTTATGGATATTA
TCAGGAAGTTCTTGCAGA------TCCAATGTAGTCCCCTTTTCGCTCCACAAAGATCAAGA
TCAACTGAACGTTCAT------TCTCTTTCTGTCGAACGAAAATATATTTCTAACTTCTCA-----
-GTGACTAATGATCAA---GTGAGACATAAACTCTTTAGTTCGGATCGTTCTGGTAAA------
---AGGATTCCTGATTAT---TCAGAACTTAATCGAATCATATGTACTGATCATTGTAATTTCA
TATATCCG---GCCATTCTCCACGAGAATTAT------GATTTATTGTCAAAGAGGCGAAAAAA
TAGATTAATCATTCCATTCCAATCGATTCAAGAACGAGAGAAAGAACAAATGCCCCATT
CAGGTATCTCGATTGAAATACCC------ATAAATGGCATTTTCCGTAAAAATAGTATTATGG
CTTATTTCGATGACCCCCGATACAGAAGAAACAGTTCGGGAATTACTAAATATGGTACTA
TAGAGGTGCATTCAATCGTCAAAAAAGAGGAT------TTGATTGAGTATCGAGGA---GGCA
AAGAGTTTAAGCCAAGATACATGCAAGTAGATAGATTTTTTTTCATTCCCGAGGAAGTG
CATATTTTGCCCGGATCTTCTTCCATAATGGTGCGGAACAATAGTATCATTGGAGTATCT
ACACGAATCACTTTAAATAAAAGAAGCCGCGTAGGCGGATTGGTCCGAATGGAGAGAA
AAAAAAGGGGAATTGAACTTAAAATCTTTTCTGGAGATATCTATTTTGCTGGAGAGACA
GATAAAATAGCCCGACACAGTGGCATCTTAATACCACCAGGAACG------GGAAAAACAA
ATTCCAAGGAATCAAAAAAA------TTGAAAAATTGGCTCTATGTCCAACGG------ATCAC
ACCTACCAAG------AAAAAGTATTTTGTTTTGGTTCGACCTGTAGTCACATACGAAATAG
CGGACGGTATAAATTTAGCAACACTTTTCCCCGAGGATCTGTTGCAGGAAAGGGATAAT
ATGAAACTTCGAGTTGTCAATTATATCCTTTATGGAAACGGAAAACCAATTCGGGGAAT
TTCTGACACAAGTATTCAATTAGTTCGTACTTGTTTAGTATTGAATTGGGACCAAGACA
AAAAA---------AGTTCTTCTGTCGGAGAGGCCCATGCCTCCTTTGTTGAAGTAAGTACAA
CTGGTCTGATTCGAGATTTCCTAAGAATTGACGTAGTGAAATCCCCTATTTCGTATACCG
GAAAAAGGAACAGTCCATCAAGTTCAGGATTGGTCTATGAT---AATGGATCAGATCGCA
CCAATATCAATCCGTTTTAT---------TCCAAGGCAAAG---ATTCAACAATCACTTAGCCAA-
-----------AATCAAGGAACTATTCGTACCTTGTTCAATAGA---------AATAAGGAATTCCAAT
CTTTGATAATTTTATCATCATCCAATTGTTCTCGAATGGGTCCA------TTCAAC---------GAT
CACAACGTG---------ATAAAAGAATCAGACTTCCTAATTCCAATTAGGAATTCGTTGGGT
CCTTTAGGAACA------GCCCTTCAAATTGAAAATTTTTATTCA------TTTTACGATTTA------
ATAACTCGTAATCAGATCTTG---GTAACTAAATATTTGCAACTTGAGAAT---------TTAAAA
CAGATTTTTCAAGTACTTAAA---------TATTTTTTAATGGATGAAAAT------GGGAGGATTTA

TAATCCGGATCCGTGCAGTACCATCATTTTGAATCCATTCAATTTGAATTGGTACTTTCT
CCATCATAATTATTGTGAAGAAACAACC---ACAAAAGTTAGTCTCGGACAGTTTATTTGC
GAAAATGTATGTATAGCCAAAAACGGACCACAC---CTAAAA------TCGGGCCAAGTTCTA
ATTGTTCAA---GTTGACTCTATAGTAATAAGATCGGCTAAGACTTATTTGGCCACTCCAG
GAGCAACCGTTCGCGGCCATTATGGGGAAATCCTTTACGAAGGGGATACATTAGTTACA
TTTATATATGAAAAATCGAGATCTGGTGATATAACGCAGGGTCTTCCAAAGGTGGAACA
GGTGTTAGAAGTGCGTTCGATTGATTCAATATCGATAAACCTAGAAAAGAGAGTTGAG
GGTTGGAATGAGCGTATAACAAGAATTCTTGGAATTCCCTGGGGATTCTTGATTGGTGC
TGAGCTAACTATAGTGCAAAGTCGTATCTCTTTGGTTAATAAAATCCAAAAGGTTTATCG
ATCCCAGGGGGTGCAGATTCATAATAGGCATATAGAAATTATTGTACGTCAAATAACATC
AAAAGTGTTGGTTTCAGAA---------GATGGAATGTCTAATGTTTTTTCACCCGGAGAACT
AATTGGATTGTTGCGAGCGGAACGAACGGGTCGTGCTTTGGAAGAAGTGATCTGTTAT
CGAGCCATTTTATTGGGAATAACTAGAGCATCTCTGAATACTCAAAGTTTCATATCCGAA
GCGAGTTTTCAAGAAACCGCTCGAGTTTTAGCAAAAGCAGCTCTCCGTGGTCGTATCG
ATTGGCTGAAGGGCCTGAAAGAGAACGTTGTTCTGGGAGGGATGATACCCGTTGGTAC
CGGATTCAAAAGATTA------GTGCACCATTCAAGGCAACATAAGAACATT------TCTTTGG
AAACCAAAAAGAAAAATTTATTCGAGGGGGAAATGAGA------GATATTTTGTTCCACCA
CAGAGAATTATTTAATTTTTGCATTTCAAAGAATTTCCATGATACATCAGAACAATATGG
GATT---------------------------------ATGACAAGAAGATATTGGAACATTAATTTGGAAGAGAT
GATGGAAGCGGGAGTTCATTTTGGTCATGGTACTAGGAAGTGGAATCCTAGAATGGCC
CCTTATATCTCTGCAAAGCGTAAAGGTATTCATATTACAAATCTTACTAGAACTGCTCGT
TTTTTATCAGAAGCTTGTGATTTAGTTTTTGATGCAGCAAGTAGGGGAAAACAATTCTT
AATTGTTGGGACAAAAAATAAAGCAGCTGATTCAGTAGCACGGGCTGCAATAAAGGCT
CGATGTCATTATGTTAATAAAAAATGGCTCGGTGGTATGTTAACAAATTGGTCCACTACA
GAAACGAGACTTCATAAGTTCAGGGACTTGAGAACGGAACAAAAGACGGGGGGGCTC
AATCGTCTTCCGAAAAGAGATGCAGCTATGTTGAAGAGACAACTATCTCACTTGCAAA
CATATCTGGGTGGGATTAAATATATGACGGGAGTCCCCGATATTGTAATCATCGTTGATC
AGCAAGAAGAATATACGGCTCTTCGAGAATGTATCACTTTGGGAATTCCAACGATTTGT
TTAATCGATACAAATTGTGACCCCGATCTCGCAGATATTTCGATTCCAGCGAATGATGAC
GCTATAGCTTCAATCCGATTAATTCTTAACAAACTAGTATTTGCGATTTGTGAGGGTCGT
TCT---AGCTATATAAGAAATCCTATGGGACAAAAAATAAATCCACTTGGTTTCAGACTTG
GTACAACCCAAGGTCATCATTCCCTTTGGTTTGCACAACCAAAAAATTATTCTGAGGGT
CTACAAGAAGATCAAAAAATACGGGATTGTATCAAGAATTATGTACAAAAAAATATGAG
AATATCCTCTGGT---GTCGAGGGAATTGCACGAATAGAAATTCAAAAAAGAATCGATCT
GATTCAGGTCATAATTTATATGGGATTCCCAAAGTTATTAATAGAAGGTAGACCACGA----
--------AGAATTGAAGACTTACAGATGAATGTACAAAAAGAATTAAATTATGTGAACCGA
AAACTCAACATTGCTATTACAAGAATTGCAAAACCTTACGGAAACCCTAATATTCTTGC
AGAATTTATAGCCGGACAATTAAAAAATAGAGTTTCATTTCGCAAGGCAATGAAAAAA
GCTATTGAATTAACTGAACAGGCAGATACAAAAGGAATTCAAGTACAAATTGCAGGAC
GTATCGACGGAAAAGAAATTGCACGTGTCGAATGGATCAGAGAAGGGAGGGTTCCCCT
ACAAACCATTCGAGCTAAAATTGATTATTGTTCCTATACAGTTCGAACTATATATGGGGT
ATTAGGCATCAAAATTTGGATATTTGTAGACGAG---ATGTCGCGTTACCGAGGGCCTCGT
TTCAAAAAAATACGCCGTCTGGGGGCTTTACCGGGACTAACTAGTAAAAGGCCTAGAG
CCGGAAGTGATCTTAGAAACCAATCGCGTTCCGGTAAA---AGATCTCAATATCGTATTCG

TCTAGAAGAAAAACAAAAATTGCGTTTTCATTATGGTCTTACAGAACGACAATTACTTA
AATATGTCCGTATCGCCGGAAAAGCCAAAGGTTCAACAGGTCAGGTTTTACTACAATTA
CTTGAAATGCGTTTGGATAACATCCTTTTTCGATTGGGTATGGCGTCGACTATTCCTGGA
GCCCGCCAATTAGTTAACCATAGACATATTTTAGTTAATGGTCGTATAGTAGATATACCA
AGTTATCGCTGCAAACCACGAGATATTATTACAGCGAAGGATGAACAAAAATCCAGAA
CTCTGATTCAAAATTATCTTGATTCATCCCCTCAT---GAGGAATTGCCAAAATATTTGACT
CTTCACCCGCTCCAATAT------AAAGGATTAGTCAATCAAATAATAGATAATAAATGGGTC
GGTTTGAAAATAAATGAATTGCTAGTCGTAGAATATTATTCCCGTCAAACTATGTCACGT
CGAGGTACTGCAGAAGAAAAAACTGCAAAATCCGATCCAATTTATCGTAATCGATTAGT
TAACATGTTGGTTAACCGTATTCTGAAACACGGAAAAAAATCATTGGCTTATCAAATTAT
CTATCGAGCCGTGAAAAAGATTCAACAAAAGACAGAAACAAATCCACTATCTGTTTTA
CGTCAAGCAATACGTGGAGTAACTCCCGATATAGCAGTAAAAGCAAGACGTGTAGGCG
GATCGACTCATCAAGTTCCCATTGAAATAGGATCCACACAAGGAAAAGCACTTGCCAT
TCGTTGGTTATTATGGGCATCCCGAAAACGTCCGGGTCGAAATATGGCTTTCAAATTAA
GTTCCGAATTAGTGGATGCTGCCAAAGGGAGTGGCGATGCCATACGCAAAAAAGAAG
AGACTCATAAAATGGCAGAGGCAAATAGAGCTTTTGCACATTTTCGTATGGGTAGGGA
CACTATTGCTGAGATAATAACCTCTATACGAAATGCTGACATGCATAGAAAAGGAACGG
TTCGAATAGCATCTACTAACATCACTGAAAACATTGTGAAAATACTTTTACGAGAAGGT
TTTATAGAAAACGTGAGAAAGCATCGGGAAAACAACAAAGATTTTTTGGTGTTAACCC
TACAACATAGAAGG------AATAGGAAAGGACCATATAAAACT---ATT------TTAAATTTAAA
ACGGATCAGTCGACCTGGTCTACGAATCTATTCTAACTATCAAAGAATTCCTAGAATTTT
GGGTGGTGTGGGGATTGTAATCCTTTCTACTTCTCGAGGTATAATGACAGACCGAGAGG
CTCGACTAGAAGGAATCGGTGGAGAAATTTTGTGTTATATATGGATGGCAAAACCTATA
CCAAGAATTGGTTCACGTAGGAATGGACGTATTGGTTCACGTAAGAGTGCACGTAGAC
TACCAAAGGGAGTTATTCATGTTCAAGCAAGTTTCAACAATACCATTGTGACTGTTACA
GATGTACGGGGTCGGGTGGTTTCTTGGTCCTCTGCCGGTACTTGTGGATTCAGGGGTAC
AAGAAGAGGGACACCATTTGCTGCTCAAACAGCAGCAGGAAACGCTATTCGTACAGT
AGTGGATCAAGGTATGCAACGAGCAGAAGTAATGATAAAAGGTCCTGGTCTCGGAAGA
GATGCAGCATTACGGGCTATTCGCAGAAGTGGTATACTATTAAGTTTTGTACGGGACGT
AACCCCTATGCCACATAATGGCTGTAGACCTCCTAAAAAAAGACGTGTGATGCCAACC
ATTAAACAACTTATTAGAAACACAAGACAGCCAATAAGAAATGTCACGAAATCCCCCG
CTCTTCGGGGATGTCCTCAGCGACGAGGAACATGTACTAGGGTGTATACTATCACCCCC
AAAAAACCAAACTCTGCCTTACGTAAAGTTGCCAGAGTACGATTAACCTCTGGATTTG
AAATCACTGCTTATATACCTGGTATTGGCCATAATTCACAAGAACATTCTGTAGTCTTAG
TAAGAGGGGGAAGGGTTAAGGATTTACCCGGTGTGAGATATCACATTGTTCGAGGAAC
CCTAGATGCTGTCGGAGTAAAGGATCGTCAACAAGGGCGTTCTAAATATGGGGTCAAA
AAGCCAAAAATGGCACGGAAAAGCTTGATTCAGAGGGAAAAGAAGAGGCAAAAATT
GGAACAGAAATATCATTTGATTCGTCGATCCTCAAAAAAAGAAATAAGC---------AAAGT
TACGTCGTTGAGTGACAAATGGAAATTCATGGAAAGTTACAATCCCCACCACGTAATA
GTGCGCCTACACGTCTTCATCGACGTTGTTTTTCGACCGGAAGGCCGAGAGCTAACTAT
CGAGACTTTGGGCTATCCGGACACATACTTCGTGAAATGGTTCATGCATGTTTGTTGCC
AGGGGCAACAAGATCAAGTTGGATGGTAAAAAATTCATTCATTTCAGTTTTTTTGCAAG
AA---GAA---AAAGAGGAAAACCGGGGGTCTGTTGAATTTCAAGTATTCAGTTTCACCAA
TAAGATACGAAGGCTCACTTCGCATTTGGAATTGCACAGAAAAGACTATTTATCTCAGA

GAGGTCTACGTAAAATTCTGGGAAAACGTCAACGACTACTGGCCTATTTGTCAAAAAA
AAATAGAGTACGTTATAAAGAATTAATTGGTCGGTTAGATATTCGGGAACTAAAAACTC
AC------ATGGTAAAACTTCGTTTGAAACGATGTGGTAGAAAGCAACGAGCCGTCTATCG
AATCGTTGCAATTGATGTTCGATCCCGAAGAGAAGGAAGAGATCTTCAAAAAGTTGGT
TTTTATGATCCGATAAAGAATCAAACTTATTCAAATGTTCCTGCTATTCTATATTTCCTTG
CAAAAGGCGCTCAACCTACAGGAACTGTTCATGATATTTTAAAGAGGGCAGAGGTTTT
TAACGAATTTCGCTTTAATCAAACGAAATTCAAT---ATGGATAAATCCAAGCGACCTTTT
CTTAAATCCAAGCGATCTTTTCGTAGGCGTTTGCCCCCGATCCAATCGGGGGATCGAAT
TGATTATAGAAATATGAGTTTAATTAGTCGGTTTATTAGTGAACAAGGAAAAATATTATCT
AGACGAGTGAATAGATTGACCTTGAAACAACAACGATTAATTACTATTGCTATAAAACA
AGCTCGTATTTTATCTTTGTTACCTTTTCTTAATAACGAGAAA------CAGTTTGAAAGAAC
CGAGTCGACCGCTAGAACTACTGGTTTTCGAACCAGAAATAAAGTGGCACGT---CTAA
AAAAAAATCCTTTTGTAGCGAATAATTTATTAAAAAAAATTACTAAGCTTAACACAAAG
TCGGAAAAAGAAATAATAGTAACTTGGTCCCGAGCATCCACCATTATACCCACAATGAT
AGGACATACTATTGCTATCCATAATGGAAAAGAGCATTTGCCTATTTATATAACAGATCG
TATGGTAGGCCACAAATTGGGAGAATTTGCACCTACTCTAAATTTCCGGGGACATGCGA
AAAATGATAATAGATCTCGTCGTATGATTTTGAAATCTTTTCTACTAGGTAATCTAGTATC
CTTATGCATGAAGATAATAAATTCGGTCGTTGTGGTCGGACTCTATTATGGATTTCTGAC
CACATTCTCCATAGGGCCCTCTTATCTCTTCCTTCTCCGAGCTCAGGTTATGGAAGAAG
GAGAAGAAGGAACCCAGAAGAAGGTATCAGCAACAACTGGTTTTATTACGGGACAGC
TCATGATGTTCATATCGATCTATTATGCGCCTCTGCATCTAGCATTGGGTAGACCTCATAC
AATAACTGTCCTAGCTCTACCGTATCTTTTGTTTCATTTCTTCTGGAACAATCACAAACA
CTTTTTTGATTATGGATCTACTACCAGAAATTCAATGCGTAATCTCAGCATTCAATGTGTA
TTCCTGAATAATCTCATTTTTCAATTATTCAACTATTTCATTTTACCAAGTTCAATGTTAG
CCAGATTAGTCAACATTTATATGTTTCGATGCAACAACAAGATGTTATTCGTAACAAGTA
GTTTTGTTGGTTGGTTAATTGGTCACATTTTATTCATGAAATGGGTTGGATTGGTATTAG
TCTGGATACGGCAAAATAATTCTATTAGATCTCTTATTCGATCTAATAAGTACCTTGTGTC
AGAAGTGAGAAATTCTATGGCTCGAATCTTTAGTATTCTCTTATTTATTACCTGTGTCTAC
TATTTAGGCAGAATACCGTCACCCACTGTTACTAAGAAACTGAAAGAAACCGAAGAAG
ATAAACCTCTGGTGACCCGTCTTTTCGACTATAAACGATGGACTCGTCCATTGCGGTATA
TAAAAAATGATCGATTTGAAAATGCTGTAAGAAATGAAATGTCACAATATTTTTTTACA
CATGTCGAAGTGATGGAAAACAAAGAATATCTTTTACGTCTCCACCTAGTTTGTCAACT
TTTTTAGAAATGATACAAAGAAAGATGTCTTTGTACACAACGGAAAACCCTTCCTCTTA
TGAACTGTATAATCAGTGGGTTTATACCAATGAACAAAACGAAAAC---CTAAGCAACGA
GTTTCTAAGTAGAATAGAGGCTCTAGACAAGGGATCTCTTCCTCTGGATGTACTCGAAA
AAAAGACTAGATTATGTAATGATGAAACTAAAAAAGAATACTTACCTAAAAAATATGAT
CCTTTCTTGAACGGGCCTTATCGTGGAACAATCAAATTTATTTTTCCACCTATCGGAACT
CTTTGGCTAAATAAGATCCACGGTATCCTTGATCGAGAATTTCAACAAAAAGATACTAA
TGATCAAGGAATAGGAATAAACGAAATCACTAAAAAAATCCCTCGATGGTCATACAAAT
TAATCGATTTAGAACAACAAGAGAGA---CAGATTCGTTCAAGAAAAGCTAAACGTGTA
GTCATTTTTACTGATGCTTTAATACGTTATTCTCAACAATCGGATTTTCGTCGAGACATAA
TAAAAGGCTCCATGCGCGCTCAAAGACGTAAACTAGCTATTTGGGAACTGTTTCAAGC
AAATGTACATTCCCCACTTTTTTTGGACAGAATAGACAACCCCCCCCTTTTTTCATTTGA
TATC------TCCGAACTGATGAAACTAATTTTCATAACTATAGAAGAGAAAGTGCGAATAG

AAATAGCCGAAGCCTGGGATACCATTATATTTGCTCAAGTAATAAGAGGTTTAGTCTTAG
TAACACAATCAATTCTTAGAAAATCTATTATATTACCGTCATTAATAATAGCTAAAAATAT
TGGTCGTATGCTATTATTTCAATTTCCCGAGTGGTCCGAGGATTTAAAGGATTGGAAGA
GGGAAATGCATGTTAAATGCACCTATAATGGTGTTCAATTATCAGAAACAGAATTTCCG
AAAAACTGGTTAACAGATGGTATTCAAATAAAGATACTATTTCCTTTCTGTCTGAAACC
TTGGCACAGATCTAAGAAAGGGAAAAAAGATGATTTTTGTTTTTTAACAGTTTGGGGA
ATGGAAGCTGAACTGCCTTTTGGTTCTCCCCGAAAAAGACCCTCTTTTTTTGAACCCGT
TTTTCAAGAACTCGGAAAAAAAAATTCTAAAATGGAAAAGGAATTGTTTTCTAGCT---CT
AAGAGTTTTTAAAGAAAGAACAAATTTCTTTCTAATAGTCCCAAAAGAAATAAAAAAA
TGGATTATCAAAAAAAATTCTC------CTTATACAAAGAATAATAAAAGAACTATCAAAACT
AAATGATTCAATAATCAGCAATAGTATGATTTATGAATCGTCCAGTCAAATTCAATCTATC
GATTGGACAAATTATTCACTGACAGAAAAAAAAATCAAAGATCTAACTGATAGAACAA
GCCTAATCAGAACTCAAATAGGAAAAATTACAAAAGAAAAGAAAACCAGTGCTAACA
AGAAAAGTCATGATGCTAAAAGATTAGAATCTGCCCCAATTTTTTGGCAGATGTTAAAA
AGAATAAATATTCGATTAATCCGTAAATCATATTTTTTTATAAAATTTTTCGTTGAAAGAA
TATATATAGATATCTTCTTATGTATAATTAATATTCCTCGGATCAATACACAACTTTTT---CA
CTTTATA------AACTTATCCTCTTTGTCACAAGCATATGTATTTTACAAATTATCACAAACC
CAAGTTATTAGCTTGGATAAGTTAAGATCTATCCTTCAATATACATCTCTTTTTCTTAAAA
ATGAAATAAAGGATTATTTTGCACAAGGAATATTGCATTCCGAATTAAAACATAAGAAA
CTTCGGAATTCTGGAATGAATCAATGGAAAAGTTGGTTAAGGGGTCATTATCAATATAA
TTTCTCAAAGATTAGATGGTCTAGATTAGTACCACAAAAATGGCGAAATAGAGTTAATC
AAGGTTGGAGGGCCCAAAAT---AACAAATGGGATTCGTATGAAAAAGACCAA------GTA
ATTCATTACGAAAAA---GAAAATTTTAAAAAACACTATAGGTATGATCTCTTATCATCTA
AATCTATTAATGATGGTATTATGGATCCGGATAGAAAATATTTTGATTGGAAAATTCTCCA
CTTTTGTCTTAAA---TTTGATTGGATGGGAATGAATGAAGAAATACTAAATCGTTGGTTC
TTCCCGGAATTTGCGCTACTTTATAATTCATATAAAATTCAACCCTGGGCCATACCCATC
AAATTACTTCTTTTTAATTATAAAAGCAATACAGAAGCAGAACTCGATTTATTTCTAAAA
AAGTATTTGCTTTTTCAATTGAGATGGGATGATTCTTTAAATCAAAGAATGATCAATAAT
ATCAAGGTATATTGTCTCCTGCTTAGACTGATAAACCCAAGAGAAATTGCTATATCCTCT
ATTGAACGGGGAGAAATGAGTCTGGATATAATGCTGATTCAGAAAGATTTAACTCTTAC
AGAATTGATGAAA---GGGATATTTATTATCGAACCGGTTCGTCTGTCTGTCAAAAATGAT
GGACAATTTATTATGTATCAAACCATAAGTATTTCATTGGTTCATAAGAGTAAAGACAAT
CATTATGCTTTGCTCGTTCCTGAAAATATTTTATCACCTAGACGTCGTAGAGAGTTTAGA
ATTCTAATTTGTTTCAGATTTCTTTGGCCCACTTATCGATTAGAAGATTTAGCTTGTATGA
ATCGCTATTGGTTTGATACCACTAATGGCAGTCGTTTCGGTATGGTAAGGATACATATGT
ATCCGATGAAAGGACATCAATTCAAATCCTGGATTTTCGAATTGAGAGAGATATTGAGA
GAGATCAAGAATTCTCACTATTTCTTAGATTCATGGACCCAATTCAATTCAGTGGGATCT
TTCATTCACATTTTTTTCCACCAAGAACGTTTTATAAAACTCTTTGACCCCCGAATTTGG
AGTATCCTACTTTCA---CGCAATTCA------CAGGGTTCAACAAGCAATCGATATTTC--------
-ACGATCAAGGGTGTAGTACTATTTGTAGTAGCG---GTCCTTATATATCGT------------ATTA
ACAATCGAAATATGGTCGAAAGCAAAAATCTCTATTTGACAGGGCTTCTTCCT-------------
--ATACCTATGAATTCCATTGGACCCAGAAATGATACATTGGAAGAATCTTTT---GGGTCT
TCCAATATCAATAGGTTGATTGTTTCG---CTCCTGTATCTTCCAAAAGGAAAAAAGATCT
CTGAGAGTTGTTTCCTGGATCCGAAAGAGAGTACTTGGGTTCTCCCAATAACT------------

AAAAAGTGTATCATGTCTGAATCTAACTGGGGTTGGCGGTGGTGGAGGAACTGGATCG
GAAAAAAGAGG------GATTCTAGTTGTAAGATATCTAAT---------GAAACCGTCGCTGGAA
TTGAGATCTCATTCAAAGAGAAAGATATCAAA---TATCTGGAGTTTCTTTTTGTATATTAT
ATGGAT------GATCCGATCCGCAAGGACCATGATTGGGAATTGTTTGATCGTCTTTCTCCG
AGGAAGAGGCGAAACATAATCAACTTG------AATTCGGGACAGCTATTCGAAATCTTAG
TGAAACACTGGATTTGTTATCTCATGTCTGCTTTTCGTGAAAAAATACCAATTGAAGTG
GAGGGT---TTCTTCAAACAACAAGGA---GCTGGGTCAACTATTCAATCAAATGATATTGA
GCATGTTTCCCAT------CTCTTCTCGAGAAACAAGTGGGCTATTTCTTTGCAAAATTGTG
CTCAATTTCATATGTGGCAATTCCGCCAAGATCTCTTCGTT---------AGTTGGGGGAAG---
AATCCGCACGAATCGGATTTTTTGAGGAAC---GTATCGAGAGAGAATTGGATTTGGTTA
GACAATGTGTGGTTGGTAAACAAGGATCGG---------TTTTTTAGC------AAGGTACGGAAT
GTATCGTCAAATATTCAATATGATTCC---------ACAAGA------TCTAGTTTCGTTCAAGTAA
CGAATTCTAGCCAATTGAAAGGATCTTCT---GATCAATCCAGAGATCATTTCGATTCCATT
AGT------------AATGAGGATTCGGAA------------------------------------TATCACACATTGATCA
ATCAAAGAGAGATTCAACAACTAAAAGAAAGATCGATTCTT---TGGGAT------CCTTCCT
TTCTTCAAACGGAACGAACAGAGATAGAATCAGAC------CGATTCCCGAAA------TGCCT
TTCTGGATATTCC------TCAATGTCCCGGCTATTCACG---GAACGTGAGAAGCAGATGAAT
AATCATCTGCTTCCGGAAGAAATCGAAGAATTT------ATTGGGAATCCTACAAGATCCAT
TCGTTCTTTTTTCGCTGACAGATGG------TCAGAACTTCAT---CTGGGTTCGAATCCTACT
GAGGGGTCCACTAGAGATCAG------------AAATTGTTGAAGAAAGAACAATATCTTTCT---
TTTGTCCCTTCCAGGCGATCGGAA------------AATAAAGAAATGGTTAATATATTCAAGAT
AATTACGTATTTACAAAAT------ACCGTCTCAATTCATCCTATTTCATCAGATCCGGGATGT
GATATGGTTCCG---AAGGATGAACCGGAT---ATGGACAGTTCCAATAAGATTTCAGTCTT
GAACAAAAATCCATTTTTTGATTTA------TTTCATCTATTCCATGACCGGAACAGGGGGG
GATACACGTTA------CACCACGATTTTGAATCAGAA------GAGAGATTTCAAGAAATGGC
AGATCTATTCACTCTATCAATAACCGAGCCG---GATCTGGTGTATCATAAGGGATTTGCC
TTTTCTATTGATTCCTACGGATTGGATCAAAAACAA------TTCTTGAATGAGGTATTCAAC
TCCAGGGATGAATCGAAAAAGAAATCTTTA---------TTGGTTCTACCTCCTATTTTTTAT----
--GAAGAGAATGAATCTTTTTATCGAAGGATCAGAAAAAAAATGGGTCCGG---ATCTCCTG
CGGGAATGATTTGGAAGATCCAAAACCAAAAATAGTGGTATTTGCTAGCAACAACATA
ATGGAGGCAGTCAATCAATATAGATTGATCCAAAATCTGATTCAAATCCAATAT------TAT
GGG------TACATAAGAAATGTATTGAATCGATTCTTTTTAATGAAT---------AGATCCGATCA
CAACTTCGAATATGGAATTCAA---------AGGGATCAAATAGGAAATGATACTCTGAATCAT
AGAACTATAATGAAATATAGGATCAACCAACATTTATCGAATTTGAAAAAG---AGTCAG
AAGAAATGGTTCGATCCT------CTTATTTTTATTTCTCGAACCGAGAGATCCACGAATCG
GGATCTTAATGCATATAGATACAAATGGTCCAATGGGAGCAAGAATTTCCAGGAACATT
TGGAACATTTCGTTTCTGAGCAGAAGAGCCGT---------TTTCAAGTAGTGTTCGATCGATT
ACGTATTAATCAATATTCGATTGATTGGTCTGAGGTTATCGACAAAAAAGATTTGTCTAA
GTCACTTCGTTTCTTTTTGTCCAAGTCACTTCTT------------------------TTTTTGTCC--------
-------------AAGTTGCTTCTCTTTTTGTCTAACTCACTTCCT---TTTTTCTTTGTGAGTTTCG
GGAATATCCCCATTCATAGGTCCGAG---ATCCACATCTAT---GAATTGAAAGGTCCGAAT
GATCAACTCTGCAATCAGTTGTTAGAATCAATAGGTCTTCAAATCGTTCATTTGAAAAA
ATTGAAACCCTTC------TTATTGGAT---------GATCATGATACTTCC---CAAAAATCGAAATT
CTTGATCAATGGAGGAACAATATCACCATTTTTGTTCAATAAGATACCA---AAGTGG---A

TGATTGACTCATTCCATACTAAAACTAAAAATAATCGCAGGAAATCCTTTGATAACACG
GATTCCTATTTCTCAATGATATCCCACGATCAAGACAATTGGCTGAATCCCGTGAAACCA
TTTCATGGAAGTTCATTGATATCTTCTTTTTATAAAGCAAATCGACTTCGATTCTTGAATA
ATCCACATCACTTCTGCTTCTAT------TGTAACAAAAGATTCCCTTTT---------TAT---GTGG
AAAAGGCCCGTATCAATAATTATGAT------TTTACGTATGGA---CAATTCCTCAATATCTTG
TTCATTCGCAACAAA---ATATTTTCTTTGTGCGGCAGTCAA------AAAAAATATGCTTTTT
GGGAG---AGAGATACTATTTCACCAATCGAGTCACAGGTATCTAACATATTCATACCTAA
CGATTTTCCACAAAGTGGTGACGAAACG---TATAACTTGTCCAAATCT---------TTCTATTT
TCCAATTCGATCCGATCCATTCGTTCGT------AGAGCTATTTATTCGATCGCAGACATTTC
TGGAACACCTCTAACAGAGGGACAAATAGTCAATTTTGAAAGAACTTATTGTCAACCT
CTTTCAGATATGAATCTATCTGATTCAGAAGGGAAGAACTTGCATCAGTATCTCAAT------
TTCAATTCAAACATGGGTTTGATTCACACTCCATGTTCTGAGAAAGATTTACCATCCGA
AAAG------AGGAAAAAA------------------------CGGAGTCTTTGTCTAAAG------AAATGCGTT
GAGAAAGGGCAGATGTATAGAACCTTTCAACGAGATAGTGCTTTTTCAACTCTCTCAA
AATGGAATCTATTCCAAACATATATGCCATGGTTCCTTACTTCGACAGGGTACAAATATC
TAAATTTGATATTTTTAGATACTTTTTCAGAC---TTATTGCCGATACTAAGTAGCAGT------
CAAAAATTTGTATCCATTTTTCATGATATTATGTATGGATCAGATATATCATGGCGAGTTC
TTCAGAAAAAATTG------------------TGTCTTCCACAACGGAAT---CTGATAAGTGATATTTC
GAGAAAGTGTTTACATAAT------CTTCTTCTGTCCGAAGAAATGATTCATCGAAATAATG
AGTCA------CCATTGATATCGACACATCTGAGATCGCCAAATGTTCGGGAGTTC---CTCTA
TTCAATCCTTTTCCTTCTTCTTGTTGCTGGATAT---CTCGTTTGTACACATCTTCTCTTTGT
TTCCCGA------GCCTCTAGTGAGTTACAGACAGAGTTCGAAAAGGTCAAATCTTTGATG
ATTCCATCATACATGATTGAGTTGCGAAAACTTCTGGATAGGTATCCTACATCTGAACTG
AATTCTTTCTGGTTAAAGAATCTCTTTCTAGTTGCTCTGGAACAATTAGGAGATTCTCTA
GAAGAAATACGGGGTTCTGCTTCTGGCGGCAACATGCTATGGGGTGGTGGTCCC---------
------GCTTATGGG---GTCAAATCAATACGTTCTAAGAAGAAATATTTGAATATCAAT---------
CTCATCGATCTCATA---AGTATCATACCAAATCCCATCAATCGAATCACTTTTTCGAGAAA
TACGAGACATCTAAGTCATACAAGTAAAGAGATCTATTCATTGATAAGAAAAAGAAAA
AACGTGAACGGTGATTGGATTGATGATAAAATAGAATCCTGGGTCGCGAACACTGATTC
GATTGATGATGAAGAAAGAGAATTCTTGGTTCAGTTCTCC------ACCTTAACGACA------
GAAAAAAGGATTGATCAAATTCTATTGAGTATGACT---CATAGTGATCATTTATCTAGTGA
TCATTTATCAAAGAATGACTCTGGTTATCAAATGATTGAACAACCGGGAGCAATTTACT
TACGATACTTAGTTGACATTCAT------AAAAAGTGTCTAATGAATTATGAGTTCAATACAT
CCTGTTTAGCAGAAAGACGGATATTCCTTGCTCATTATCAGACAATCACTTAT------TCAC
CG------TGTGGGGCTAATAGTTTTCATTTCCCATCTCATGGA---------AAACCCTTTTCGCT
CCGCTTAGCCCTATCCCCCTCT---AGGGGTATTTTAGTGATAGGTTCTATAGGAACTGGA
CGATCCTATTTGGTCAAATACCTAGCGACAAACTCCTATGTTCCTTTCATTACGGTATTT
CTGAACAAGTTCCTGGATAACAAGCCTAAAGGTTTTCTTATTGAT---------------GATATCG
AT-----------------GATATTGATGCTAGTGACGATATTGATGATAGT--------------------------------
---------------GACGATATCGATCGTGACCTTGATACGGAGCTGGAGCTGCTAACTATGATG
AATGCACTA---------ACTATGGATATGATGCCG---------GAAATAGACCGATTTTATATCACC
CTTCAATTCGAATTAGCAAAAGCAATGTCTCCTTGCATAATATGGATTCCAAACATTCAT
GATCTGGATGTGAATGAGTCGAATTACTTATCCCTCGGTCTATTAGTGAACTATCTCTCC-
--------AGGGATTGTGAAAGATGTTCC---ACTAGAAATATTCTAGTTATTGCTTCGACTCAT

ATTCCCCAAAAAGTGGATCCCGCTCTAATAGCTCCGAATAAATTAAATACATGCATTAAG
ATACGAAGGCTTCTTATTCCACAACAACGAAAGCACTTTTTCACTCTTTCATATACTAGG
GGATTTCACTTGGAAAAGAAAATGTTCCATACT------AATAGATTCGGGTCCATAAGCAT
GGGTTCCAATGCACGAGATCTTGTAGCACTTACCAATGAGGCCCTATCGATTAGTATTAC
ACAGAAGAAATCAATTATAGACACTAATACAATTAGATCCGCTCTTCATAGACAAACTT
GGGATTTGCGA---------TCCCAGGTAAGATCGGTTCAGGATCACGGGATCCTTTTCTATC
AGATAGGAAGGGCTGTTGCACAAAATGTACTTCTAAGTAATTGCCCCATAGATCCTATAT
CTATCTATATGAAGAAGAAA------TCATGTAACGAAGGGGATTCTTATTTGTACAAATGGT
ACTTCGAACTTGGAACGAGCATGAAGAAATTAACGATACTTCTTTATCTTTTGAGTTGT
TCTGCCGGATCGGTCGCTCAAGACCTTTGGTCTCTACCC------CGACCCGATGAAAAA---
AATGGGATCACTTCTTATGGACTCGTTGAGAATGATTCT---GATCTAGTTCATGGCCTATT
AGAAGTAGAAGGCGCTCTGGTGGGA------TCCTCACGGACAGAAAAAGATTGCAGTCA
GTTTGATAATGAT------CGAGTGACATTGTTTCTTCGGCCCGAACCAAGGAAT---CCCTTA
GATATGATCCAAAATGGATCTTGTTCTATCGTTGATCAGAGATTTCTCTATGAAAAA------
TACGAATCGGAGTTTGAAGAA---------GGAGAAGGAGCCCTCGACCCGCAA------------CA
GATAGAAGAGGAT------------TTATTCAATCAC------ATAGTTTGGGCTCCT------AGAATATG
GCGCCCT---------TGGGGCTTTCTATTTGATTGTATCGAAAGGCCCAATGAATTGGGATTT
CCC---TATTGGGCCAGGTCATTTCGGGGCAAG---------CGGATCATTTATGATGAAGAGGA
TGAGCTTCAAGAGAATGATTCGGAGTTCTTGCAGAGTGGAACCGTGCAGTACCAGACA
CGAGAGAGATCTTCCAAAGAACAAGGCTTTTTTCGAATAAGCCAATTCATTTGGGACC
CTGCAGATCCACTCTTTTTCCTATTCAAA---GATCAGCCCCCTGTCTCTGTGTTTTCACA
TCGAGAATTCTTTGCAGATGAAGAGATGTCAAAGGGGCTTCTTACTTCCCAAACAGAT
CCTCCTACATCTATATATAAACGCTGGTTTATC------AAGAATACGCAAGAAAAGCACTTC
GAATTGTTGATTCATCGCCAGAGATGGCTTAGA---------ACCAATAGTTCATTATCT------A
ATGGATCTTTCCGTTCTAAT------ACTCTATCCGAGAGTTATCAGTATTTATCAAATCTGTT
CCTATCTAAC---GGAACGCTATTGGATCAAATGACAAAGACATTGTTGAGAAAAAGATG
GCTTTTCCCGGATGAAATTAAAATTGGATTC------ATG---------------------------ATGCCTAGA
TCGCGG---ATAAATGGAAATTTTATTGATAAGACCTTTTCAGTTGTAGCCAATATCTTATT
ACAAATAATTCCGACAACTTCAGGAGAAAAAGAGGCATTTACCTATTACAGAGATGGG-
-----ATGTCAGCTCAATCCGAAGGAAATTATGCGGAAGCTTTACAGAATTATTATGAAGCT
ATGCGACTAGAAATTGATCCCTATGATCGAAGTTATATACTCTATAACATAGGCCTTATCC
ACACAAGTAACGGAGAACATACGAAAGCTTTGGAATATTATTTTCGGGCACTAGAACG
AAATCCGTTCTTACCACAAGCTTTTAATAATATGGCCGTGATCTGTCATTAC---CGGGGA
GAACAGGCCATTCGACAGGGAGATTCGGAAATTGCGGAGGCTTGGTTTGATCAAGCCG
CTGAGTATTGGAAACAAGCTATAGCACTTACTCCTGGTAATTATATTGAAGCGCATAATT
GGTTGAAGATCACGAGGCGTTTCGAA------ATGAGTTGGCGATCAGAACATATATGGATA
GAACTTATAACGGGGTCTCGAAAAATAAGTAATTTCTGCTGGGCCTTTATTCTTTTTTTA
GGTTCATTAGGATTCTTATTGGTTGGAACTTCCAGTTATCTTGGTAGGAATTTGATATCTT
TATTTCCGTCTCAGCAAATAATTTTTTTTCCACAAGGAATCGTGATGTCTTTCTATGGCA
TCGCGGGTCTCTTTATTAGCTCCTATTTGTGGTGCACAATTTCGTGGAATGTAGGTAGTG
GTTATGATAGATTCGATAGAAAAGAAGGAGTTGTGTGTATTTTTCGTTGGGGATTTCCT
GGAAAAAATCGTCGCATCTTCCTTCGATTCCTTATGAAAGATATTCAGTCCATCAGAATA
GAAGTTAAAGAGGGTATTTCTGCCCGTCGTGTCCTTTATATGGACATCAGAGGCCAGGG
GGCCATTCCCTTGACTCGTACTGATGAGAAT---TTGACTCCACGAGAAATTGAACAAAA

AGCTGCCGAATTGGCCTATTTCTTGCGTGTACCAATTGAAGTATTT
>Ilex
ATGGAAAGATGCCGGTTCAATTCGATGTTGTTTCTAAGTAAATCAATGGACAGTCTTGG
GCCTATTGAAAATACCAGTGAAAGTGAAGAGCCAAATATAAATGATCCGGATAAAAAC
ATTTATAGTTCCAGTAATGTTGATCATTTATTCAGCATCAAGGACATTCGGAATTTGATCT
CTGATGACACTTTTTTAGTTAGGGATAGTAATGGGGACAGCTATTCCATATATTTTGATAT
TGAAAATCAGATTTTTGAAATTAACAATGATCAT------TCTTTTCTGAGTGAACTAGAAA
GTTCTTTGTATAGTTATCGGAATTCTAGTTATCTGAATAATGGATCTAAGAGTGACGATC
CCTACTATGATCGTTACATGTATGATACTCAATATAGTTGGAATAATCACATTAATAGTTG
CATTGATAGTTATCTTCAG------TCTCAAATCTGTATTGATACTTACATTGTAAGTGGGAG
TGACAATTACAGT------TACATTTATAGTTCTATTTGTGGTAACGAGGGTTCC------AGTAC
TAGCACGAATGGTAGTGATTTAACTATAAGAGAAAGTTCTAATGATCTCGATGTAACTC
AAAAATACAGGCATTTGTGGGTTCAATGCGAAAATTGTTATGGATTAAATTATAAAAAA
TTTTTTAAATCAAAAATGAATATTTGTGAACAATGTGGATATCATTTGAAAATGAGTAGT
TCAGATAGAATCGAACTTTCGATTGATCCAGGTACTTGGGATCCTATGGATGAAGACAT
GGTCTCTCTGGATCCCATTGAATTTCATTCGGAGGAGGAGCCTTATAAGGATCGTATTGA
TTCTTATCAAAGAAAGACAGGATTAACTGAGGCTGTTCAAACAGGCATAGGTCAACTA
AACGGTATTCCCGTAGCACTTGGGGTTATGGATTTTCAGTTTATGGGGGGTAGTATGGG
ATCCGTAGTCGGGGAGAAAATCACACGTTTGATTGAGTACGCTACTAAGAAATTTCTAC
CTCTTATTATAGTGTGTGCTTCCGGGGGGGCACGCATGCAAGAAGGAAGTTTGAGCTT
GATGCAAATGGCTAAAATCTCTTCTGCTTTATATGATTATCAATCAAATAAAAAGTTATTC
TATGTATCAATCCTTACATCTCCTACTACTGGTGGGGTGACAGCGAGTTTTGGTATGTTG
GGGGATATCATTATTGCTGAACCCAATGCCTACATTGCATTTGCAGGTAAAAGAGTAATT
GAACAAACATTAAATAAAACAGTACCTGAAGGTTCACAAGCGGCTGAATATTTATTCCA
GAAGGGCTTATTCGATCTAATCGTACCACGTAATCTTTTAAAAAGCGTTCTAAGTGAATT
ATTTCAGCTCCACGCTTTCTTTCCTTTGAATCAAAATTCAATCAAGATGGTAACCATTCG
AGCCGACGAAATTAGTAATATTATCCGTGAACGTATTGAACAATATAATAGAGAAGTAA
AGATTGTAAATACCGGTACCGTACTTCAAGTAGGTGATGGAATTGCTCGTATTCATGGTC
TTGATGAAGTAATGGCAGGTGAATTAGTAGAATTTGAAGAGGGTACCATAGGCATTGCT
CTGAATTTGGAATCAAATAATGTTGGTGTTGTATTAATGGGTGATGGTTTGATGATCCAA
GAAGGAAGTTCTGTAAAAGCAACAGGAAAAATTGCTCAGATACCAGTGAGTGAGGCC
TATTTGGGTCGCGTTATAAATGCCCTGGCTAAACCTATTGATGGTAGAGGTGAAATTTCA
GCTTCTGAATCTCGGTTAATTGAATCTCCCGCTCCCGGTATTATTTCGCGCCGTTCCGTAT
ATGAGCCTCTTCAAACGGGGCTTATTGCTATTGATTCGATGATCCCTATAGGACGCGGTC
AGCGAGAATTAATTATTGGGGACCGGCAGACCGGTAAAACAGCAGTAGCCACGGATAC
GATTCTCAATCAACAAGGTCAAAATGTAATATGTGTTTATGTAGCTATTGGTCAAAAAG
CATCTTCTGTGGCTCAGGTAGTGACTACTTTCCAGGAAAGGGGAGCGATGGACTACAC
TATTGTGGTAGCCGAAACAGCGGATTCCCCTGCTACATTACAATACCTCGCTCCTTATAC
AGGAGCGTCTTTGGCTGAATATTTTATGTACCGTAAAGAACACACTTTAATCATTTATGA
TGATCCCTCCAAACAAGCACAAGCTTATCGCCAAATGTCTCTTCTATTACGAAGACCGC
CTGGTCGCGAAGCTTATCCAGGGGATGTTTTTTATTTGCATTCACGCCTTTTGGAAAGA
GCCGCTAAATCAAGTTCTCATTTAGGTGAAGGAAGTATGACCGCTTTACCAATAGTTGA
AACTCAATCGGGAGATGTTTCGGCTTATATTCCTACTAATGTAATTTCCATTACTGATGG
ACAAATATTCTTATCCGCCGATCTATTCAATTCTGGAATCAGACCCGCTATTAATGTGGG

TATCTCGGTTTCCAGAGTGGGGTCTGCAGCTCAAATTAAAGCCATGAAACAAGTAGCT
GGCAAATTAAAATTGGAACTGGCGCAATTCGCAGAATTAGAAGCCTTTGCACAATTTTC
TTCTGATCTCGATAAAGCTACTCAGAATCAATTGGCAAGAGGTCAACGATTACGTGAAT
TGCTCAAACAATCCCAAGCAGCCCCTCTCACGGTGGAAGAACAGATAATGACTATTTAT
ACCGGAACAAATGGTTATCTTGATTCATTAGAAATTGGACAGGTAAGGAAATTTCTTGT
TGAGTTACGTACTTACTTAAAAAGTAATAAACCTCAGTTCCAAGAAATCATATCTTCTAC
TAAGACATTCACTGAGGAAGCAGAAGCCCTTTTGAAAGAAGCTATTCAGGAACAGCT
GGAACGTTTTATGCTTCAGGAACAAGCAATGAGAAACAATCCTACTACGTCTGGTTCT
GGGGTTTCCACGCTTGAAAAAAAAAACCTGGGGCGTATTGCCCAAATCATTGGTCCGG
TACTAGATGTAGCTTTTGCGCCGGGCAAGATGCCTAATATTTATAACGCTCTGGTAGTTA
AGGGTCGAGATACTGCCGGTCAACCAATTAATGTGACTTGTGAGGTACAACAATTATTA
GGAAACAATCGAGTTAGAGCTGTAGCTATGAGTGCTACAGATGGTTTAACGAGAGGAA
TGGAAGTGATTGATACGGGAGCTCCACTAAGTGTTCCGGTCGGGGGAGCAACTCTCGG
ACGAATTTTTAACGTGCTTGGAGAACCCGTTGATAATTTAGGTCCTGTAGATACTCGCA
CAACATCTCCTATTCATAGATCCGCGCCCGCCTTTATACAGTTAGATACAAAATTATCGAT
TTTTGAAACCGGAATTAAAGTAGTAGATCTTTTAGCCCCTTATCGCCGTGGAGGAAAAA
TCGGACTATTCGGGGGGGCTGGAGTGGGTAAAACAGTACTCATTATGGAATTGATTAAC
AATATTGCCAAAGCTCATGGGGGTGTATCCGTATTTGGCGGAGTAGGTGAACGTACTCG
TGAAGGAAATGATCTTTACATGGAAATGAAAGAATCTGGGGTTATTAATGAACAAAATA
TTGCCGAATCAAAAGTGGCTCTAGTTTACGGTCAGATGAATGAACCTCCGGGAGCTCG
TATGAGAGTTGGTTTGACTGCCCTAACTATGGCGGAATATTTCCGAGATGTTAATGAAC
AAGACGTACTTCTATTTATCGACAATATCTTCCGTTTCGTCCAAGCAGGATCCGAAGTAT
CTGCCTTATTGGGTAGAATGCCTTCCGCGGTGGGTTATCAACCCACCCTTAGTACCGAA
ATGGGTACTTTACAAGAAAGAATCACTTCTACCAAAGAAGGTTCCATAACTTCTATTCA
AGCAGTTTATGTACCTGCAGATGATTTGACCGACCCTGCCCCTGCTACGACATTTGCAC
ATTTAGATGCTACTACCGTACTATCAAGAGGATTAGCTGCCAAAGGTATCTATCCAGCAG
TAGATCCTTTAGATTCAACGTCAACTATGCTACAACCTCGGATCGTTGGTGAGGAACAT
TATGAAACTGCGCAAGAGGTTAAGCAAACTTTGCAACGTTACAAAGAACTTCAGGACA
TTATAGCTATCCTTGGGTTGGACGAATTATCCGAAGAGGATCGTTTAACCGTAGCAAGA
GCGCGAAAAATTGAGCGTTTCTTATCACAACCTTTTTTCGTAGCAGAAGTATTTACCGG
TTCTCCAGGGAAATACGTTGGTCTAGCAGAAACAATTAGAGGGTTTCAATTGATCCTTT
CCGGAGAATTAGATGGTCTTCCTGAACAGGCCTTTTATTTGGTAGGTAACATTGATGAA
GCTACCGCGAAGGCTATGAACTTAGAAATGGAGAGCAATTTGAAGAAAATGATCTTAA
ATCTTTGTGTACTGACCCCTAATCGAATTGTTTGGGATTCAGAAGTAAAAGAAATCATT
TTATCTACTAATAGTGGCCAAATTGGCGTATTACCAAACCACGCCCCTATTGCAACAGCT
GTAGATATAGGTATTTTGAGAATACGCCTTAACGACCAATGGTTAACGATGGCTCTGATG
GGTGGTTTTGCTAGAATAGGTAATAATGAGATTACTGTTTTAGTAAATGATGCGGAGAA
GAGCAGTGACATTGATCCACAAGAAGCTCAGCAAACTCTTGAAATAGCGGAAGCTAAT
TTGAGAAAAGCTGAAGGAAAGCGACAAACAATTGAGGCAAATCTAGCTCTCCGACGA
GCTAGGACACGAGTAGAGGCCATCAATGCAATTTCGATGAAAAATGTAACCGATTCTTT
CGTTTCTTTGGGCCACTGGCCATCCGCCGGGAGTTTCGGGTTTAATACCGATATTTTATC
AACAAATCCAATAAATCTAAGTGTAGTGCTTGGTGTATTGATCTTTTTTGGAAAGGGAG
TGTTAAGTGATTTATTAGATAATCGAAAACAGAGGATCTTGAATACTATTCGAAATTCAG
AAGAATTACGTGGAAGAGCCATTGAACAGCTGGAAAAAGCCCGGGCTCGCTTACGGA

AAGTGGAAATGGAAGCAGAGCAGTTTCGAGTGAATGGATACTCTGAGATAGAGCGAG
AAAAATTGAATTTGATAAATTCAACTTATAAGACTTTGGAACAATTAGAGAATTACAAA
AATGAAACTATTCATTTTGAACAACAAAGGGTGATTAATGAAGTCCGACAACGGGTTT
TCCAACAAGCCTTACAAGGAGCTCTAGGAACTCTGAATAGTTGTTTGAACAACGAGTT
ACATTTACGTACCATCAGTGCCAATATTAGCATGTTGGGGGCGATGAAAGAAATAACTG
ATATGAATCCACTGATTTCTGCCGCTTCCGTTATTGCTGCTGGATTGGCTGTAGGGCTTG
CTTCTATTGGACCTGGAGTTGGCCAAGGTACTGCTGCGGGCCAAGCTGTAGAAGGTAT
TGCGAGACAGCCCGAGGCGGAGGGAAAAATACGAGGTACTTTATTGCTTAGTCTAGCT
TTTATGGAAGCTTTAACAATTTATGGACTGGTTGTAGCATTAGCACTTTTATTTGCGAAT
CCGTTTGTTATGAATGTTCTAGCATGTTCCATCAACACACTAAAAGGGTTATACGATATA
TCCGGTGTGGAAGTAGGCCAACATTTCTATTGGAAAATAGGGGGTTTCCAAGTCCATG
GCCAAGTACTTATTACTTCTTGGGTTGTAATTGCTATCTTATTAGGTTCAGCTACTATAGC
TGTTCGGAACCCACAAACCATTCCGACCGGCGGTCAGAATTTCTTCGAGTATGTCCTTG
AATTCATTCGAGATGTAAGTAAAACCCAAATTGGAGAAGAATATGGTCCTTGGGTTCCT
TTTATTGGAACTATGTTTCTATTTATTTTTGTTTCTAATTGGTCAGGAGCTCTTTTACCTT
GGAAAATCATACAATTACCTCATGGGGAGTTAGCCGCACCCACAAATGACATAAATACT
ACTGTTGCTTTGGCTTTACTCACGTCAGTGGCATATTTCTATGCGGGTCTTAGCAAAAA
AGGATTAGGTTATTTTGGGAAATATATTCAACCAACTCCAATCCTTTTACCCATTAACAT
CTTAGAAGATTTCACAAAGCCTTTATCACTTAGTTTTCGACTTTTCGGGAATATATTAGC
TGATGAATTAGTAGTTGTTGTGCTTGTTTCTTTAGTACCTTTAGTGATTCCTATACCTGTC
ATGTTCCTTGGATTATTTACAAGTGGTATTCAAGCTCTTATTTTTGCAACTTTAGCCGCG
GCTTATATAGGTGAATCCATGGAGGGCCATCATATGATATTTTTAACTTTAGAGCATATAT
TAACTCATATTTCTTTTTCGATGGTTTCAATTGTAATTACAATTCATTTGATAACCTTATTT
TTCGATGAAATCGTAAAACTATATGATTCATCAGAAAAAGGCATGATAACTACTTTTTTC
TGTATCACAGGATTATTAGTCACTCGTTGGATTTATTCGGGACATTTCCCCCTAAGTAAT
TTATATGAATCATTAATATTTCTTTCATGGAGTTTCTCCATTATTCATATAGTTCCGTAT------
AAAAAAAAAAAACCATTTAAGCACAATAACTGCACCAAGTGCTATTTTTACCCAAGGCT
TTGCTACTTCAGGTATTTTAACTGAAATACATCAATCCGAAATATTGGTACCCGCTCTCC
AATCTGAATGGTTAATAATGCACGTAAGTATGATGATATTGGGCTATGCAGCTCTTTTATG
TGGATCATTATTATCAGTAGCACTTCTAATCATTACATTTCGAAAGAACAGAAAAATTCT
TTGT------AAAAAAATCCAATACATGAATGAAAGAAACGATGTTTTACAAAATACTTCT---
------GATAAGAATTATTACAGGTCCCAGTTGATTCAACAATTGGGTTATTGGAGTTATCGT
GTTATTAGTCTAGGGTTTATCTTTTTAACCATCGGTATTCTTTCGGGAGCAGTATGGGCT
AATGAAGCATGGGGATCATATTGGAATTGGGACCCAAAGGAAACTTGGGCATTTGTTA
CTTGGATCGTATTCGCAATTTATTTACATACTCGAACAAATAAAAATTTGCAGGATACAA
ATTCCGCAATTGTGGCGTCTATAGGCTTTCTTATAATTTGGATATGCTATTTTGGGGTCAA
TCTATTAGGAATAGGTCTACATAGTTATGGCTCATTTATGTTAACGATGGCAAAAAAGAA
AGCATTCACTCCTCTTTTATATCTTGCATCTATAGTATGTTTGCCCTGGTGGATTTCTCTC
TCATTTAATAAAAGTCTGGAATCTTGGGTCACTAATTGGTGGAATACTGGGCAATCCGA
AACCTTTTTGAATGATATTCAAGAAAAGAGTATTCTAGAAAAATTCATAGAATTAGAGG
AACTCCTCCTCTTGGACGAAATGATCAAGGAATACTCAGAGACCCATCTACAAAAACT
TCGTATA---ATCCACAAAGAAACGATCCAATTAATCAAGATACACAACGAAGATCGTATC
CATACGATTTTGCACTTCTCGACAAATATAATCTGTTTCGTTATTTTAAGCGGTTATTCTA
TTTGGGGTAATGAAGAACTTGTTATTCTTAACTCTTGGGCCCAGGAATTCCTATATAACT

TAAGCGACACACTAAAAGCTTTTTCTATTCTTTTATTAACCGATTTATGTATCGGATTCCA
TTCGCCCCATGGTTGGGAACTAATGATTGGATCTGTCTACAAAGATTTTGGATTTGTTCA
TAATGATCAAATTATATCTGGTCTTGTTTCTACTTTTCCAGTCATTCTAGATACAATTTTA
AAATATTGGATTTTCCGTGATTTAAATCGTGTATCTCCGTCGCTTGTAGTGATTTATCATT
CAATGAATGACATGCCTATTGGTGTTCCAAAAGTACCTTTTCGAAGTCCTGGAGAGGA
AGATGCATCTTGGGTTGACGTATACAACCGACTTTATCGAGAAAGATTACTTTTTTTAGG
CCAAGAGGTTGATAGTGAAATATCGAATCAGCTTATTGGTCTTATGGTATATCTCAGTAT
CGAGGATGATACCAAAGATCTGTATTTGTTTATAAACTCTCCTGGCGGATGGGTAATACC
CGGAGTAGCTATTTATGATACTATGCAATTTGTGCGACCAGATGTACATACAATATGCAT
GGGATTAGCCGCTTCAATGGGATCTTTTATCCTAGTCGGAGGAGAAATTACCAAACGTC
TAGCATTCCCTCACGCCAGGGTAATGATCCATCAACCTGCTAGTTCTTTTTATGAGGCAC
AAACGGGAGAATTTATCCTGGAAGCGGAAGAACTGCTGAAACTGCGTGAAACCCTCA
CAAGGGTTTATGTACAAAGAACGGGTAAACCTTTATGGGTTGTATCCGAAGACATGGA
AAGAGATGTTTTTATGTCAGCAACAGAAGCCCAAGCTTATGGAATTGTTGATCTTGTAG
CGGTTGCAATGAAAGAACAAAAATGGATTCATGAAGGTTTAATTACTGAATCGCTTCCC
AATGGTATGTTCCGGGTCCGTTTAGATAATGAAGATCTGATTCTCGGTTATGTTTCAGGA
AAGATCCGACGTAGTTTTATACGGATACTGCCGGGAGATAGAGTCAAAATTGAAGTAA
GTCGTTATGATTCAACCCGAGGGCGTATAATTTATCGACTCCGCAACAAGGATTCGAAG
GATATGGAGGAATTCCAAAGATATTTCGAG---GATAGATCTCAACAACACGACTTTCTAT
ATCCACTTATCTTTCAGGAGTATATTTATGCACTTGCTCATGATCATGGTTTAGCAGGTTA
TGACAATAAATCCAGCTTACTAATTGTGAAACGTTTAATTACTCGAATGTATCAACAGAA
TCATTTGATTCTTTCGACTAATGATTCTAACCAAAATAAAATTTTTGGGAAGAATTTGTA
TTCTCAAATGATATCAGAGGGATTTGCAGTCATTGTGGAAATTCCATTTTCTGTACGATT
AATATCTTCCCTAATAATAAAATCTCATAATTTACGATCAATTCATTCAATATTTCCTTTTT
TAGAGGATAAATTTTTACATTTAAATTATGTGTTAGATATACTAATACCCCACCCCGTCCA
TCTGGAAATCTTGGTTCAAACTCTTCGTTACTGGGTAAAAGATACTTCTTCTTTGCATTT
ATTACGATTCTTTCTCTACGAGGATCGTAATTGGAATAGTCTTATTACTAAACCCGGTTC
TTCTTTTTCAAAACAAAGATTCTTTTTCTTCTTATATAATTCTCATGTATGTGAATACGAA
TCTATCTTCGTTTTTCTCCGTAATCAATCTTCTCATTTACGATCAACATCTTCTGGAGTTC
TTCTTGAACGAATATATTTCTATGGAAAAATAGAATATCTTGTAGAAGTCTTTGCTAAGG
CTTTTAACCTATGGTTGTTCAAGGATCCTTTCATACATTATGTTAGGTATCAAGAAAAAT
CAATTCTGGCTTCAAAAGGGACGTCTCTTTTGATGAATAAATGGAAACATTACCTTGTC
AATTTCTGGCAATGTTATTTTTACCTGTGGTCTCAACCAGGAAGAATCCATATAAACCAA
TTTTCCAATTATTATCTTGACTTTATGGGCTATTTTTCAAGTGTGCGACTAAATCCTTTAA
TGGTACGGAATCAAATGCTAGAAAATGCATTTCTAATCGATAATGCTATT------AAGAAGT
TCGATATCAGTGTTCCAATTATTCCTCTGATTGGATCGTTGGCGAAAGCGAAATTTTGTA
ACGGATTAGGGCATCCTATTAGTAAGCCGGTTCGGGCCGATTTATCAGATTCTGATATTA
TTGATCGATTTGGGCGTATATACAGAAATCTTTTTCATTATCATAGCGGATCCTCAAAAA
AAAAGAGTTTGTATCGAATAAAGTATATACTTCGATTTTCTTGTATTAGAACTTTGGCTC
GTAAACACAAAAGTACTGTACGTACTTTTTTGAAAAGATTAGGATCGGAATTATTGGAA
GAATTCTTTAAGGCGGAAGACCAAGTTCTTTCTTTGACCTTTCCAAAACGGATTTGGTA
TTTGGATATTATTTGTATCAATGATTTGGTTAATCATGAAATGATAATTGAT---ACAACAG
AAGTACAA---GCTATCAATTCTTTTTCCAGATTGGAATCC---TTACAAGAGGTCTATGGG
ATCATATGGATGCTTGTCTATATTTTGACTACTGTATTAGGAATCACAATAGGTGTACTAG

TAATTGTTTGGTTAGAAAGAGAAATATCTGCAGGGATACAACAACGTATTGGACCTGAA
TACGCCGGACCTTTAGGAATTCTTCAAGCTCTAGCAGATGGTACAAAATTACTTTTCAA
AGAGAATCTTCTTCCATCTAGAGGAGATAATCGTTTATTCAGTATCGGACCATCTATAGC
AGTCATCTCAATTCTACTAAGTTATTCAGTAATTCCTTTTGGATATCATCTTGTTCTAGCT
GATCTAAGTATTGGTGTTTTTTTATGGATTGCCATTTCCAGTATTGCTCCCGTTGGACTTC
TTATGTCAGGATATGGATCAAATAATAAATATTCCTTTTTAGGTGGTCTCCGAGCTGCTG
CTCAATCAATTAGTTATGAAATACCATTAACTCTATGTGTGTTATCAATATCTCTACTATCT
AACAGTTCAAGTACAGTTGATATAGTTGAAGCGCAATCAAAATATGGTTTTTGGGGATG
GAATTTGTGGCGTCAACCTATAGGGTTTATCATTTTTCTAATTTCTTCCCTAGCCGAGTG
TGAAAGATTGCCCTTTGATTTGCCAGAAGCAGAAGAAGAATTAGTAGCAGGTTATCAA
ACCGAATATTCAGGTATCAAATTTGGTTTATTTTACGTTGCTTCATATCTGAATCTACTGC
TTTCTTCATTATTTGTAACAGTTCTTTACTTGGGGGGTTGGAATCTTTCTATTCCGTACAT
ATTTGTTCCTGAGCTATTTGAAATAAATAAAGCAGGTAGAGTCTTTGGAACAATAATTG
GTATCTTTATTACATTAGCTAAAACTTATTTGTTCTTGTTCATTTCTATCACAACAAGATG
GACTTTACCGAGACTGAGAATGGACCAACTATTAAATCTTGGGTGGAAATTTCTTTTAC
CTATTTCTTTAGGTAATCTATTATTAACAATTTCTTCCCAACTTCTTTCACTGATGATCTG
GCATGTACAGAATGAAAACTTCATTCTCGATTCTACGAGAATTTTTATGAAAGCCTTTCA
TTTGCTTCTCTTCGATGGAAGTTTTATTTTCCCAGAATGTATCCTAATTTTTGGCCTAATT
CTTCTTCTGATGATCGATTCAACCTCTGATCAAAAAGATATACCTTGGTTATATTTCATCT
CTTCAACAAGTTTAGTAATGAGCATAACGGCCCTATTGTTCCGATGGAGAGAAGAACCT
ATGATTAGCTTTTCGGGAAATTTCCAAACGAACAATTTCAACGAAATCTTTCAATTTCTT
ATTTTACTATGTTCAACTCTATGTATTCCTCTATCCGTAGAGTACATTGAATGTACAGAAA
TGGCTATAACAGAGTTTCTCTTATTCGTATTAACAGCGACTCTAGGAGGAATGTTTTTAT
GTGGTGCTAACGATTTAATAACTATCTTTGTAGCTCTAGAATGTTTCAGTTTATGCTCCTA
CCTATTATCTGGATATACCAAGAAAGATGTACGGTCTAATGAGGCTACTATGAAATATTT
ACTCATGGGTGGGGCAAGCTCTTCTATTCTGGTTCATGGTTTCTCTTGGCTATATGGTTC
ATCCGGGGGAGAGATCGAGCTTCAAGAAATAGTGAACGGTCTTATCAATACACAAATG
TATAACTCCCCAGGAATTTCAATTGCGCTCATATTCATTACTGTAGGAATTGGGTTCAAG
CTTTCCCCAGCCCCTTCTCATCAATGGACTCCTGACGTATACGAAGGATCTCCCACTCC
AGTCGTTGCTTTTCTTTCTGTTACTTCGAAAGTAGCTGCTTCAGCTTCAGCCACTCGAA
TTTTCGATATTCCTTTTTATTTCTCATCAAACGAATGGCATCTTCTTCTGGAAATCCTAGC
TATTCTTAGCATGATATTGGGAAATCTCATTGCTATTACTCAAACAAGCATGAAACGTAT
GCTTGCATATTCGTCCATAGGTCAAATCGGATATGTAATTATTGGAATAATTGTTGGAGA
CTCAAATGATGGATATGCAAGCATGATAACTTATATGCTGTTCTATATCTCCATGAATCTA
GGAACTTTTGCTTGCATTGTATTATTTGGTCTACGTACCGGAACTGATAACATTCGAGAT
TATGCAGGATTATACACGAAAGATCCTTTTTTGGCTCTCTCTTTAGCCCTATGTCTCTTAT
CCCTAGGAGGTCTTCCTCCACTAGCAGGTTTTTTCGGAAAACTCTATTTATTCTGGTGT
GGATGGCAGGCAGGCCTATATTTCTTGGTTTTAATAGGACTCCTTACAAGCGTTGTTTCT
ATCTACTATTATCTAAAAATAATCAAGTTATTAATGACTGGACGAAACCAAGAAATAACC
CCTCACGTGCGAAATTATAGAAGATCTCCTTTAAGATCAAACAATTCCATCGAATTGAG
TATGATTGTATGTGTGATAGCATCTACTATACCAGGAATATCAATGAACCCGATTATTGCC
ATTGCTCAGGATACCCTTTTTATGTTTCTGCTTTACGAATATGATATTTTTTGGACATTTCT
AATAATATCAAGTCTTATTCCTATTTTGGCATTTTTCATTTCCGGAGTTTTAGCCCCAATT
AGCAAAGGACCAGAGAAACTCTCTAGTTATGAATCGGGTATAGAACCAATGGGCGACG

CTTGGTTACAATTTCGAATTCGTTATTATATGTTTGCTTTAGTTTTTGTCGTTTTTGATGTT
GAAACGGTTTTTCTTTATCCATGGGCAATGAGTTTCGATGTATTGGGTGTATCTGTATTTA
TAGAAGCTTTAATTTTCGTGCTTATTTTAATTGTTGGTTCAGTTTATGCATGGCGAAAGG
GGGCATTGGAATGGTCTACGAATTATTTTCCTTGGTTAACAATAATTGTAGTTTTGCCAA
TATCTGCAGGTTCCTTAATTTTCTTTCTTCCCCATAAAGGAAATAGGGTCATTCGGTGGT
ATACTATATTTATATCTATTTTAGAACTCCTTCTGACGACCTATGCATTCTGTTATCATTTC
CAATTGGACGATCCATTAATCCAACTAGTGGACGATTATAAATGGATCAATTTTTTTGAC
TTCCATTGGAGATTAGGAATAGATGGACTTTCTATCGGGCCCATTTTACTAACGGGATTC
ATTACTACTTTAGCTACTTTAGCGGCTCGGCCAGTTACTCGAGATTCTCGATTATTCCATT
TCTTGATGTTGGCAATGTACAGTGGTCAAATAGGACCATTTTCTTCTCGGGACCTTTTAC
TTTTTTTCATCATGTGGGAGTTAGAATTAATTCCCGTTTATCTGCTTCTATCTAGGTGGGG
AGGAAAGAAACGTCTGTACTCAGCTACAAAATTTATTTTGTACACAGCGGGAGGTTCC
ATTTTTCTCTTAATGGGAGTTCTGGGTATCGGTTTATATGGTTCTAATGAACCAACATTA
AATTTTGAAACATCAGCTAATCAGTCGTATCCTGTGGCCTTGGAAATAATATTTTATATTG
GATTTTTTATTGCTTTTGCTGTCAAATCACCGATTATACCCCTACATACATGGTTACCAGA
TACCCACGGAGAAGCACATTACAGTACTTGTATGCTCCTAGCCGGAATCTTATTAAAAA
TGGGAGCATATGGGTTGATTCGGATCAATATGGAATTATTACCTCACGCTCATTCTATATT
TTCTCCTTGGTTGATAATAGTAGGCACAATACAAATAATCTATGCAGCTTTAACATCTCC
TGGCCAACGTAATTTAAAAAAAAGAATAGCCTATTCTTCTGTATCTCATATGGGTTTCAT
CCTTATAGGAATTGGTTCTATAACCGCTACAGGCCTCAATGGGGCCATTTTACAAATAAT
CTCTCATGGATTTATTGGTGCTGCACTTTTTTTCTTGGCAGGAACAAGTTATGATAGAAT
ACGTCTTGTTTATCTCGACGCAATGGGCGGAATAGCTATCCCAATGCCAAAAATATTCA
CGATGTTCAGTAGCTTTTCGATGGCTTCCCTTGCATTACCGGGTATGAGTGGTTTTGTTG
CAGAATTGATAGTCTTTTTTGGAATAATTACCAGCCAAAAATATCTTTTAATGCCAAAAA
TACTAATTACTCTTGTAATGGCAATTGGAATGATATTAACTCCTATTTATTCATTATCTATG
TCACGCCAGATGTTCTATGGATACAAGTTATTTAATGCTCCAAACTCTTATTTTTTTGATT
CTGGACCGCGAGAGTTATTTGTTTCGATCTCTATCTTTCTACCCGTAATAGGTATTGGTAT
GTACCCGGATTTCGTTCTTTCACTATCAGTTGACAAAGTCGCAGTTATTCTATCTAATTAT
TTTTATAGAATGATACTCGAACATGTACTTGTTTTGAGTGCCTATTTATTTTCTGTCGGTC
TCTATGGATTGATCACAAGTCGAAATATGGTTAGGGCCCTTATGTGTCTTGAACTTATAT
TGAATGCAATTAATATGAATTTTGTAACATTTTCCTATTTTTTTGATAGTCGGCAATTAAA
AGGAGATATTTTTTCAATTTTTGTTATAGCTATTGCAGCCGCTGAAGCAGCTATTGGACT
GGCTATTGTTTCATCAATTTATCGTAACAGAAAATCAACTTGTATCAACCAATCGAATTT
GTTAAATAAGATGGAACAGACATATCAATATGCATGGATAATACCCTTCGTTCCACTTCC
AGTTCCTATGTTAATAGGGGTGGGACTTCTTCTTTTTCCCACGGCAACAAAAAATCTTC
GTCGTATGTGGTCCTTTCATAGTATTTTATTGTTAAGTATAGTCATGATTTTTTCGATTGAT
CTGTCTATTCAGCAAATAAATAGCAGTTCTATCTATCAATATGTATGGTCTTGGATCATCA
CTAATGATTTTTCTTTAGAATTCGGCTACTTAATTGATCCACTTACTTCTATTATGTCAATA
TTAATCACTACTGTTGGAATTATGGTTCTTATTTATAGTGATAATTATATGTCTCATGATCA
AGGATATGTAAGATTTTTTGCTTATATGAGTTTTTTCAGTACTTCCATGTTGGGATTAGTT
ACTAGTTCTAATTTGATACAAATTTATATTTTTTGGGAATTGGTTGGAATATGTTCGTATC
TATTAATAGGGTTTTGGTTCACACGACCTGTTGCGGCAAATGCTTGTCAAAAAGCCTTT
GTAACTAATCGTGTCGGGGATTTTGGTTTATTATTAGGAATTTTAGGTTTTTATTGGATAA
CAGGTAGTTTTGAATTTAGGGATTTATTCGAAATATTCAATAACAATGAGGTCAATTTTT

TATTTGTTACTTTGTGTGGCGTTCTATTATTTGCCGGTGCAGTTGCTAAATCTGCACAATT
CCCCCTTCATGTATGGTTAGCTGATGCCATGGAAGGACCTACTCCTATTTCGGCTCTTAT
ACATGCTGCTACTATGGTAGCAGCGGGAATTTTTCTTGTCGCGCGGCTTTTTCCCCTTTT
CATAGTCATACCTTCCCTAATGAATTTAATCTCTTTGGTAGGAATAATAACAGTATTTTTA
GGAGCTACTTTAGCTCTTGCTCAAAAAGATATTAAGAGAGGTTTAGCCTATTCCACAAT
GTCTCAATTAGGTTATATGATGTTAGCTCTAGGTATGGGATCTTATCAAAGTGCTTTATTT
CATTTGATTACTCATGCTTATTCGAAAGCCTTATTGTTTTTAGGATCTGGATCCGTTATTC
ATTCAATGGAAACTCTTGTTGGATATTCTCCAGATAAAAGTCAGAATATGGTTCTTATGG
GGGGTTTAACAAAACATGTGCCAATTACCAAAAATGCTTTTTTATTAGGTACACTTTCC
CTTTGTGGTATTCCGCCTCTTGCTTGTTTTTGGTCCAAAGATGAAATTCTTCATGATAGT
TGGTTGTATTCACCGCTTTTCGCAATAATAGCGTGGTCCACAGCGGGATTAACCGCATT
TTATATGTTTCGGATCTATTTACTTACTTTTGAAGGGCATTTAAACGTTCATTTTCAAAAT
TATAGTGGAAAAAAAAATACTCCATTCTATTCAATATCTCTATGGGGAAAGGGGGATTC
GAGAGGGATTAACACA---TTATTAAGTTTATTACGAATGAATTTGAAAACATTTGGCAAT
AAAAAGACTTATTTGTATCCTTATGAATCGGACAATACTATGTTATTCCCTCTACTTCTAT
TGGTTCTATTTACTTTGTTCGTTGGATTCCTAGGAATTCCTTTCGATTTGGATATATTATCC
AAATGGTTAACTCCTTCTATAAACCTTTTACACGAAAATTCGAAT---TCGACAAGTTGGT
ATGAATTTGTCAAAGATGTTGGTTTTTCAGTGAGTATAGCTTATTTAGGAATATTTATAGC
GTCCTTTTTATATAAACCTCTTTATTCATCTTTACAAAATTTGGACTTAATTAATTCATTTG
TTACTCTTTGGGACAAAATAATAAATGTCATATATAGCTGGTCATATAATCGTGGTTACAT
AGATGCTTTTTATGCAAGATCCTTAACTGGGGGAATAAGGGGATTGGCTGAATTAACTC
ATTTTTTTGATAGACGAGTAGTTGATGGAATTACAAATGGAGTTGGTGTTATGAGTTTCT
TTGTAGGAGAAGGTATCAAATCTGTAGGGGGCGGG---CGCATCTCTTCTTATCTTTTCTT
GTATTTATCTTATGTATCAATCTTTTTATTCATTTACTCTATGGATTTACCTGGACCAATAC
ATGATTTTCTTTTAGTCTTTCTGGGATCGGGTCTTATATTAGGAGGTTTAGGAGTAGTATT
ACTTCCCAATCCAATTTATTCTGCCTTTTCATTGGGATTGGTTCTTGTTTGTATATCCTTAT
TCTATATTCTATCGAACTCCCATTTTGTAGCTGCTGCGCAGCTCCTTATTTATGTAGGAGC
TATAAATGTTTTAATCATTTTTGCTGTAATGTTTATGAACGGTTCAGAATATTACAAAGAT
TTTCATCTTTGGACTGTTGGGGATGGGGTTACTTCGATAGTTTGTACAAGTCTTTTTGTT
TCACTAATGACTACTATTCCAGATACGTCATGGCACGGGATTATTTGGACTACAAAATCA
AATCAGATTATAGAGCAAGATTTGATAAGTAATAGTCAACAAATTGGGATTCATTTATCA
ACAGATTTTTTTCTTCCATTTGAACTCATTTCAATAATTCTTTTAGTTGCTTTAATAGGTG
CAATTGCTGTAGCTCGTCAAATGACTGCACCAGCTACAAGAAAAGACCTCATGATAGT
CAATATGGGTCCTCACCACCCATCAATGCATGGTGTTCTTCGACTCATCGTTACTCTAGA
TGGTGAAGATGTTATTGACTGCGAACCAATATTGGGTTATTTACACAGAGGAATGGAAA
AAATTGCAGAAAACCGAACAATTATACAATATTTGCCTTATGTAACACGTTGGGATTATT
TAGCTACTATGTTCACAGAAGCAATAACTGTAAATGCACCAGAACAGTTAGGCAATATT
CAAGTACCTAAAAGGGCGAGCTACATCAGAGTCATTATGTTGGAGTTGAGTCGTATAGC
TTCGCATTTGTTATGGCTTGGCCCTTTTATGGCAGATATTGGGGCACAGACCCCCTTCTT
CTATATTTTTCGAGAACGAGAATTGATATATGACCTATTCGAAGCTGCCACCGGTATGCG
AATGATGCATAATTATTTTCGTCTCGGAGGAGTAGCTGCTGATCTACCTCATGGATGGAT
AGATAAATGTTTAGATTTTTGCGATTATTTTTTAACAGGGGTTGCTGAATATCAAAAGCT
TATTACACAGAATCCTATTTTTTTAGAACGAGTTGAAGGAGTAGGCATTATTGGCGGAG
AAGAAGCAATAAATTGGGGTTTATCAGGACCAATGCTACGAGCTTCCGGAATACAATG

GGATCTTCGTAAAGTTGATCATTATGAGTGTTACGACGAATTTGATTGGGAAGTACAAT
GGCAAAAAGAAGGGGATTCGTTAGCTCGTTATTTAGTACGAATCAGCGAAATGACAGA
ATCTATAAAAATTATTCAACAGGCTCTAGAAGGAATTCCAGGGGGGCCCTATGAGAATT
TAGAAATCCGACGCTTTGATAGAGTAAGGGATCCCGAATGGAATGATTTTGATTATCGA
TTCATTAGTAAGAAACCTTCTCCGACTTTTGAATTATCACAGCAAGAACTTTATGTAAG
AGTCGAAACCCCAAAAGGAGAATTGGGAATTTTTCTGATAGGAGATCAGAGTGTTTTT
CCCTGGAGATGGAAAATTCGCCCACCCGGTTTTATCAATTTGCAAATTCTTCCTCAGTT
AGTTAAAAAAATGAAATTGGCTGATATTATGACGATACTAGGTAGCATAGATATCATTAT
GGGAGAAGTTGATCGTATGTTCCCTATGGTAACTGAGTTCATAAATTATGGTCAACAAA
CAGTACGAGCTGCAAGGTACATTGGTCAAGGTTTCACGATTACCTTATCCCACGCGAAT
CGTTTACCTGTAACTATTCAATACCCCTATGAAAAATTGATAATATCAGAACGTTTCCGC
GGCCGAATCCACTTTGAATTTGATAAATGCATTGCTTGTGAAGTATGTGTTCGTGTATGT
CCTATAGATCTACCCGTTGTTGATTGGAAATTGGAAACTGATATTCGAAAGAAACGATT
GCTGAATTACAGTATTGATTTCGGAATCTGTATATTTTGTGGTAATTGCGTTGAGTATTGT
CCAACAAATTGTTTATCAATGACTGAAGAATATGAACTTTCTACATATGATCGTCACGAA
TTGAATTATAATCAAATTGCTTTGGGTCGATTACCAATGTCAGTAATTGACGATTACACA
ATTCGAACAGTTTTGAATTCG---ATGCAGGGTCGTTT
GTCTGCTTGGCTAGTCAAGTATGGGCTAATTCATAGATTTTTGGGCTTTGATTACCAAGG
AATAGAGACTTTACAAATAAAACCCGAGGATTGGCCTTCCATTGCTGTCATTTTATATGT
ATATGGTTACAATTATCTACGCTCCCAATGTGCCTATGATGTAGCACCAGGCGGACTCTT
AGCTAGTGTGTATCATCTTACGAGAATAGGGTATGGTGTGGATCAACCAGAAGAGGTAT
GCATAAAAGTATTTGCCCCAAGGAGGAATCCTAGAATTCCGTCTGTTTTCTGGGTTTGG
AAAAGTGTGGATTTTCAAGAACGGGAATCTTATGATATGTTGGGAATTTCTTATGATAAT
CATCCACGCTTGAAACGTATCTTAATGCCTGAAAGTTGGATAGGATGGCCCTTACGTAA
GGATTATATTGCCCCCAATTTTTATGAAATACAAGATGCTCAT------------------------------------
----
----------------------------ATGAATTCCATTCAATTTCCCTTACTTGAGCGAACAACCCAAAATT
CAGTTATTTCAACTACATCAAATGATCTTTCAAATTGGTCAAGACTCTCTAGTTTATGGC
CGCTTCTCTATGGTACCAGTTGTTGCTTCATTGAATTTGCTTCACTAATAGGCTCACGAT
TCGACTTTGATCGTTATGGACTAGTACCAAGATCGAGTCCTAGACAAGCAGACCTAATT
TTAACAGCCGGAACAGTAACAATGAAAATGGCTCCTTCTTTAGTGAGATTATATGAGCA
AATGCCTGAACCAAAATATGTTATTGCTATGGGAGCATGTACAATTACAGGGGGGATGT
TCAGTACCGATTCTTATAGTACTGTTCGGGGAGTCGATAAGCTAATTCCTGTGGATGTCT
ATTTGCCGGGCTGTCCCCCTAAACCGGAAGCAGTTATAGATGCTATAACAAAACTTCGT
AAGAAAATATCTCGAGAAATCTCTGAAGATAGAATTAGGTCTCAACGGGAGAATCGTT
GTTTTACTACCAATCACAAGTTTCATGTTGGGCGTAGTATTCATACGGGAAATTATGATC
GAGGATTTCTTTATCAACCGCCATCTACTTCAGAGATCCCTCCGGAAACATTTTTCAAAT
ACAAAAGTTCAGTATCTTCCCAAGAATTGGTGAATATGCAAGCTAGAAATACTTTTTCT
TGGATAAAGGAACAGATTACTCGATCCATTTCCGTATCGCTCATGATATATATAATAACTC
GGACATCCATTTCAAGTGCATATCCCATTTTTGCACAGCAAGGTTATGAAAATCCACGA
GAAGCGACTGGGCGTATTGTATGTGCAAATTGTCATTTAGCTAATAAGCCCGTCGATATT
GAGGTTCCACAAGCGGTACTTCCTGATACTGTATTTGAAGCAGTTGTTCGAATTCCTTA
TGATATGCAACTGAAACAAATTCTTGCTAATGGTAAAAAGGGGGCTTTGAATGTAGGG
GCTGTTCTTATTTTACCGGAGGGGTTTGAATTAGCTCCTCCCGATCGTATTTCTCCCGAG

ATGAAAGAAAAGATAGGCAATCTATCTTTTCAGAGCTATCGCCCCAATAAAAAAAATAT
TCTTGTGATAGGCCCTGTTCCTGGTCAGAAATATAGTGAAATTATCTTTCCTATTCTTTCC
CCGGACCCCGCTACTAAGAAAGATGTTCACTTCTTAAAATATCCTATATACGTAGGCGG
AAACAGGGGAAGGGGGCAGATTTATCCTGACGGGAGCAAGAGTAACAATACAGTTTAT
AATGCTACAGCAGCAGGTATAGTAAGCAGAATCATACGAAAAGAAAAAGGAGGGTAC
GAAATAACCATAACGGATGCATCGGATGGACGTCAAGTGGTTGATATTATCCCTCCAGG
ACCAGAACTTCTTGTTTCAGAAGGCGAATCTATCAAATTTGATCAACCATTAACAAGTA
ATCCTAATGTGGGGGGATTTGGTCAGGGAGATGCAGAAATAGTACTCCAAGATCCATTA
CGTGTCCAAGGCCTTTTGTTCTTCTTGGCATCTGTTATTTTGGCACAAATCTTTTTGGTT
CTTAAAAAGAAACAGTTCGAGAAGGTTCAATTGTCCGAAATGAATTTCATGAGTAAAG
TCTATGATTGGTTCGAAGAACGTCTCGAAATTCAGGCGATTGCGGATGATATAACTAGT
AAATACGTGCCTCCTCATGTCAACATATTTTATTGTCTAGGAGGAATTACGCTTACTTGT
TTTTTAGTTCAAGTGGCTACGGGATTTGCTATGACTTTTTACTATCGTCCAACCGTTACT
GAGGCTTTTGCTTCTGTTCAATATATAATGACTGAAGCTAATTTTGGTTGGTTAATCCGA
TCAGTTCATCGATGGTCGGCAAGTATGATGGTCCTAATGATGATCCTGCATGTATTTCGT
GTGTATCTCACCGGTGGATTTAAAAAACCTCGCGAATTGACTTGGGTTACAGGTGTGGT
TCTGGGTGTATTGACCGCATCTTTTGGCGTAACTGGTTATTCCTTACCTCGGGACCAAAT
TGGTTATTGGGCAGTGAAAATTGTAACAGGTGTACCTGAAGCTATTCCTGTAATAGGAT
CGCCTTTGGTAGAATTATTGCGCGGAAGTGCTAGTGTGGGACAATCTACTTTGACTCGT
TTTTATAGTTTACACACTTTTGTATTACCGCTTCTTACTGCCGTATTTATGTTAATGCATTT
CCCAATGATACGTAAACAAGGTATTTCTGGTCCTTTAATGGGAGTAACAAAAAAACCTG
ACTTGAATGATCCTGTATTAAGAGCTAAATTGGCTAAAGGTATGGGTCATAATTATTACG
GAGAACCCGCATGGCCCAACGATCTTTTATATATTTTTCCAGTAGTAATTCTAGGTACTA
TTGCATGTAACGTAGGCTTAGCGGTTCTAGAACCATCAATGATTGGTGAACCGGCAGAT
CCATTTGCAACCCCTTTGGAAATATTACCCGAATGGTATTTTTTTCCCGTATTTCAAATAC
TTCGTACAGTACCCAATAAATTATTGGGTGTTCTTTTAATGGTTTCAGTACCCACGGGAT
TATTAACAGTACCTTTTTTAGAGAATGTTAATAAATTCCAAAATCCATTTCGTCGTCCAG
TAGCAACAACCGTCTTTTTGGTTGGTACTGTAGTAGCCCTTTGGTTGGGTATTGGTGCA
ACATTACCTATTGATAAATCCCTAACTTTAGGTCTTTTTATGATTGAAGTTTTTCTATTTG
GAATCGTGTTAGGTCTAATTCCTATTACTTTGGCTGGATTATTCGTAACTGCATATTTACA
ATACAGGCGTGGTGATCAGTTGGACCTTATGCCTACTATAACTAGTTATTTCGGTTTTCT
ACTAGCGGCTTTAACTATAACCTCAGCTCTATTTATTGGGTTGAGCAGGATACGACTTAT
TATGGATATAGTAAGTCTCGCTTGGGCTGCTTTAATGGTAGTCTTTACATTTTCCCTTTCA
CTTGTAGTATGGGGAAGAAGTGGACTCATGATTATTCGTTCGCCGGAACCAGAAGTAA
AAATTTTGGTAGATAGGGATCACATAAAAACTTCTTTTGAGGAATGGGCCAGACCGGG
TCATTTCTCAAGAACAATAGCTAAAGGCCCTGATACTACCACTTGGATCTGGAACCTAC
ATGCTGATGCTCACGATTTCGATAGCCATACCAGTGATTTGGAGGAGATCTCTCGAAAA
GTATTTAGTGCACATTTCGGTCAACTCTCCATTATCTTTCTTTGGCTGAGTGGCATGTATT
TCCACGGTGCTCGTTTTTCCAATTATGAAGCATGGCTAAGCGATCCGACTCACATTGGA
CCTAGTGCCCAAGTGGTTTGGCCAATAGTGGGCCAAGAAATATTGAATGGTGATGTGG
GCGGGGGTTTCCGAGGAATACAAATAACCTCTGGTTTTTTTCAGCTTTGGCGAGCATCT
GGAATAACTAGTGAATTACAACTTTATTGTACCGCAATTGGTGCATTGGTCTTTGCAGC
GTTAATGCTTTTTGCTGGTTGGTTTCATTATCATAAAGCTGCTCCAAAATTGGCTTGGTT
TCAAGATGTAGAATCTATGTTGAATCACCATTTAGCGGGGCTACTAGGACTTGGGTCTC

TCTCTTGGGCGGGGCATCAAGTACATGTATCTTTACCGATTAACCAATTTCTAAACGCTG
GAGTAGATCCTAAAGAGATACCACTTCCTCATGAATTTATCTTGAATCGGGATCTTTTGG
CTCAACTTTATCCCAGTTTTGCTGAGGGAGCAACCCCGTTTTTCACCTTGAATTGGTCA
AAATATGCAGACTTTCTTACTTTTCGTGGAGGATTAGATCCAGTAACTGGGGGTCTATG
GCTGACCGATATAGCACACCATCATTTAGCTATTGCAATTCTTTTCCTGATAGCAGGTCA
CATGTATAGAACCAACTGGGGTATTGGTCATGGCCTAAAAGATATTTTAGAAGCTCATA
AAGGTCCATTTACAGGTCAGGGCCATAAAGGCCTGTATGAGATCCTAACAACGTCATG
GCATGCTCAATTATCTCTTAATCTAGCTATGTTAGGCTCTTTAACCATTGTTGTAGCTCAC
CATATGTATTCCATGCCCCCTTATCCATATCTAGCTACTGACTATGGTACCCAACTGTCAT
TGTTCACACATCACATGTGGATTGGTGGATTTCTCATAGTTGGTGCTGCTGCGCATGCA
GCTATTTTTATGGTAAGAGACTATGATCCAACTACTCGATACAACGATCTATTAGATCGT
GTCCTTCGGCATCGCGATGCAATCATATCACATCTCAACTGGGCATGTATATTTCTAGGC
TTTCACAGTTTTGGTTTGTATATTCATAATGATACCATGAGTGCTTTAGGGCGTCCTCAA
GATATGTTTTCAGATACCGCTATACAATTACAACCTGTCTTTGCTCAATGGATACAAAAC
ACCCATGCTTTAGCACCTAGTGCAACCGCTCCTGGTGCAACAACAAGTACCAGTTTAA
CTTGGGGGGGTGGTGATTTAGTAGCAGTGGGCGGCAAAGTGGCTTTGTTACCTATTCCA
TTAGGAACCGCAGATTTTTTGGTCCATCACATTCATGCATTTACGATTCATGTAACGGTA
TTGATACTCCTAAAAGGTGTTCTATTTGCTCGCAGCTCCCGTTTGATACCGGATAAAGC
AAATCTTGGTTTTCGTTTTCCTTGTGATGGACCTGGAAGAGGGGGGACATGTCAAGTAT
CAGCTTGGGATCATGTCTTCTTAGGCCTATTTTGGATGTACAATGCAATTTCGGTAGTAA
TATTCCATTTCAGTTGGAAAATGCAGTCAGATGTTTGGGGCAGTATAAGTGATCAAGGG
ATAGTAACTCATATCACGGGAGGAAACTTTGCGCAGAGTTCCATTACTATTAATGGGTG
GCTCCGTGATTTCTTATGGGCACAAGCATCCCAGGTAATTCAGTCTTATGGTTCTTCATT
ATCTGCATATGGCCTTTTTTTCCTAGGTGCTCATTTTGTCTGGGCTTTTAGTTTAATGTTT
CTATTCAGTGGACGTGGTTATTGGCAAGAACTTATTGAATCCATCGTTTGGGCTCATAAT
AAATTAAAAGTTGCTCCTGCTACTCAGCCGAGAGCCTTGAGCATTATACAAGGACGTG
CTGTAGGAGTAACCCATTACCTTCTGGGTGGAATTGCCACAACATGGGCATTCTTCTTA
GCAAGAATTATTGCAGTAGGAATGGCATTAAGATTTCCAAGGTTTAGCCAAGGCTTAGC
TCAGGACCCCACTACTCGTCGTATTTGGTTTGGTATTGCTACCGCACATGATTTCGAAA
GTCATGATGATATTACTGAGGAACGTCTTTATCAGAATATTTTTGCTTCTCACTTTGGAC
AATTAGCAATAATTTTTCTGTGGACTTCCGGAAATCTGTTTCATGTAGCTTGGCAAGGA
AATTTTGAGTCATGGGTACAGGACCCTTTACATGTAAGACCTATTGCTCATGCAATTTGG
GATCCTCATTTTGGTCAACCGGCTGTGGAAGCTTTTACTCGAGGGGGTGCTCTTGGCCC
AGTGAATATCGCTTATTCTGGTGTTTATCAGTGGTGGTATACAATCGGTTTACGCACTAA
TGAAGATCTTTATACTGGAGCTCTTTTTCTATTATTTCTTTCTGCCGTATCCTTAATAGCA
GGCTGGTTACACTTACAACCAAAATGGAAACCGAGCGTTTCGTGGTTCAAAAATGCCG
AATCTCGTCTTAATCATCATTTGTCAGGACTCTTCGGCGTAAGTTCCTTGGCTTGGACA
GGGCATTTGGTGCATGTCGCTATTCCTGGATCCAGAGGGGAGTCCGTTCGATGGAATAA
TTTCTTAGATGTATTACCGCATCCCCAAGGGTTAGGCCCGCTTTTTACAGGTCAGTGGA
ATCTTTATGCTCAAAATCCGGATTCAAGTAGTCATTTATTTGGTACCTCCCAAGGAGCAG
GAACTGCCATTCTAACCCTTCTCGGGGGATTCCATCCACAAACGCAAAGTTTATGGCTG
ACTGATATTGCTCATCATCATTTAGCTATTGCATTTATTTTTCTCATTGCTGGTCATATGTA
TAGAACTAACTTCGGGATTGGGCACAGTATGAAAGATCTTTTAGATGCGCATATTCCTC
CGGGGGGGGGATTGGGACGTGGGCATAAGGGTCTTTATGACACAATCAATAATTCGATT

CATTTTCAATTAGGCCTTGCTCTAGCTTCTTTAGGGGTTATTACTTCCTTGGTAGCTCAA
CACATGTACTCTTTACCTGCTTATGCATTCATCGCACAAGACTTTACTACTCAAGCTGCG
TTATATACTCATCACCAATACATCGCGGGATTCATCATGACAGGAGCTTTTGCTCATGGA
GCTATATTTTTCATTAGAGATTACAATCCGGAACAAAATGAGGATAATGTATTGGCAAGA
ATGTTAGACCATAAAGAAGCTATCATATCTCATTTAAGTTGGGCCAGCCTCTTTCTAGGG
TTCCATACCTTGGGACTTTATGTTCATAATGATGTCATGCTTGCCTTTGGTACTCCGGAG
AAACAAATCTTGATCGAACCTATATTTGCTCAATGGATACAATCTGCTCATGGTAAAACT
TCATATGGGTTCGATGTACTTTTATCTTCAACGAATGGACCGGCATTCAACGCGGGTCG
AAGCATATGGTTGCCCGGTTGGTTAAATGCTGTTAATGAG------AATAGTAATTCACTATT
CTTAACAATAGGTCCTGGAGACTTTTTGGTTCATCATGCTATTGCTCTGGGTTTACATAC
AACTACATTGATCTTAGTAAAAGGTGCTTTAGATGCACGTGGTTCCAAGTTAATGCCAG
ATAAAAAGGATTTCGGTTATAGTTTTCCGTGCGATGGCCCGGGACGAGGCGGTACTTGT
GATATTTCGGCTTGGGACGCATTTTATTTGGCAGTTTTTTGGATGTTAAATACCATTGGA
TGGGTTACTTTTTATTGGCATTGGAAGCACATTACATTATGGCAGGGTAACGTTTCACAG
TTTAATGAATCTTCCACTTATTTGATGGGCTGGTTACGAGATTATTTATGGTTAAACTCTT
CACAACTTATCAATGGATATAACCCTTTTGGTATGAATAGTTTATCGGTCTGGGCGTGGA
TGTTCTTATTTGGACATCTTGTTTGGGCTACTGGATTTATGTTCTTAATTTCCTGGCGTGG
ATATTGGCAGGAATTGATTGAAACTTTAGCATGGGCTCATGAACGCACACCTTTGGCCA
ATTTGATTCGCTGGAGAGATAAACCAGTGGCCCTTTCCATTGTTCAAGCAAGATTGGTT
GGATTAGCTCACTTTTCTGTAGGTTATATATTCACTTATGCAGCTTTCTTGATTGCCTCTA
CATCAGGCAAATTTGGTATGTCACATTCAGTAAAAATTTATGATACATGTATAGGGTGTA
CTCAATGTGTCCGCGCCTGCCCCACCGATGTATTAGAAATGATACCTTGGGACGGCTGT
AAAGCTAAACAAATTGCTTCCGCTCCAAGAACAGAGGACTGTGTCGGTTGTAAGAGAT
GTGAATCTGCCTGCCCAACGGATTTCTTGAGTGTTAGAGTTTATTTAGGGAATGAAACA
ACTCGTAGCATGGGTCTAGCTTATATGATAACTCTCAACCTTCCCTCTATTTTTGTGCCTT
TAATAGGCCTAGTATTTCCGGCAATTGCAATGGCTTCTTTATTTCTTCATGTTCAAAAAA
ACAAGATTGTGATGCGAGATCTAAAAACATATCTCTCCGTGGCACCGGTAATAAGTACT
CTATGGTTCGGGTCTTTAGCAGGTCTATTGATAGAGATTAATCGTTTTTTCCCGGATGCG
TTGACATTCCCCTTTTTTTCATTCATGACTGCAATTTTAGAGAGACGCGAAAGCGAAAG
CCTATGGGGTCGCTTCTGTAACTGGATAACCAGCACTGAAAACCGTCTTTACATTGGAT
GGTTTGGTGTTTTGATGATCCCTACCTTATTGACCGCAACTTCTGTATTTATTATCGCCTT
CATTGCTGCTCCTCCAGTAGATATTGATGGTATTCGTGAACCTGTTTCTGGATCTCTACT
TTATGGAAACAATATTATCTCAGGTGCCATTATTCCTACTTCTGCAGCTATCGGTTTGCA
CTTTTACCCAATCTGGGAAGCGGCATCCGTTGATGAATGGTTATACAATGGTGGTCCTTA
TGAACTAATTGTTCTACACTTCTTACTTGGTGTAGCTTGTTACATGGGTCGTGAGTGGG
AACTTAGTTTCCGTCTGGGTATGCGACCTTGGATTGCTGTTGCATATTCAGCTCCTGTTG
CAGCTGCTGCTGCTGTTTTCTTGATCTACCCAATTGGTCAAGGAAGTTTTTCTGATGGTA
TGCCGCTAGGAATCTCTGGTACTTTCAACTTTATGATTGTATTCCAGGCTGAGCACAAC
ATCCTTATGCACCCATTTCACATGTTAGGCGTGGCTGGTGTATTCGGCGGCTCCCTATTC
AGTGCTATGCATGGTTCCTTGGTAACTTCTAGTTTGATCAGGGAAACCACAGAAAATGA
ATCTGCTAATGCAGGTTACAGATTCGGTCAAGAGGAAGAAACTTATAATATCGTAGCCG
CTCATGGTTATTTTGGCCGATTGATTTTCCAATATGCTAGTTTCAACAACTCTCGTTCTTT
ACATTTCTTCCTAGCTGCTTGGCCTGTAGTAGGTATCTGGTTCACTGCTTTAGGTATCAG
CACTATGGCTTTCAACCTAAATGGTTTCAATTTCAACCAATCTGTAGTTGATAGTCAAG

GCCGTGTAATTAATACTTGGGCTGATATTATTAACCGTGCTAACCTTGGTATGGAAGTTA
TGCATGAACGTAACGCTCATAACTTCCCTCTAGATCTAGCTGCTATCGAAGCTCCATCTA
CAAATGGAATGGGTTTGCCTTGGTATCGTGTTCATACCGTTGTATTGAATGATCCCGGTC
GGTTACTGTCCGTCCATATAATGCATACAGCTCTGGTTGCTGGTTGGGCCGGTTCGATG
GCTCTGTATGAATTAGCAGTTTTTGATCCCTCTGACCCTGTTCTTGATCCAATGTGGAGA
CAGGGTATGTTCGTTATACCCTTCATGACTCGTTTAGGAATAACCAATTCATGGGGCGGT
TGGAGTATCACA---GGAGGGACTGTAACGAATCCGGGTATTTGGAGTTACGAAGGTGT
GGCAGGGGCACATATTGTGTTTTCTGGCTTGTGCTTCTTGGCAGCTATTTGGCATTGGG
TGTATTGGGATCTCGAAATATTTTGTGATGAACGTACAGGAAAACCTTCTTTAGATTTGC
CCAAGATCTTTGGAATTCATTTATTTCTTGCAGGGGTAGCTTGCTTTGGTTTTGGTGCAT
TTCATGTAACAGGCTTGTATGGTCCTGGAATATGGGTGTCCGATCCTTATGGACTAACGG
GAAAAGTACAATCTGTAAATCCAGCGTGGGGCGTGGAAGGTTTTGATCCTTTTGTTCC
GGGAGGAATAGCCTCTCATCATATTGCAGCAGGGACATTGGGCATATTAGCCGGTCTAT
TCCATCTTAGCGTCCGCCCGCCCCAACGTCTATACAAAGGATTGCGTATGGGCAATATT
GAAACCGTCCTTTCCAGTAGTATCGCTGCTGTCTTTTTTGCAGCTTTTGTTGTTGCCGG
AACTATGTGGTATGGTTCAGCAACTACCCCAATCGAGTTATTTGGGCCTACTCGTTATCA
ATGGGATCAGGGGTACTTCCAGCAAGAGATATATCGAAGAGTTAGTGCTGGGCTAGCC
GAAAATCAAAGTTTATCAGAAGCTTGGTCTAAAATTCCTGAAAAATTAGCTTTTTATGA
TTACATCGGCAATAATCCGGCAAAAGGGGGATTATTCAGGGCAGGCTCAATGGATAAC
GGAGATGGAATAGCGGTTGGATGGTTAGGACACCCTATCTTTAGAGATAAAGAAGGCC
GTGAACTTTTTGTACGCCGTATGCCTACTTTTTTTGAAACATTTCCGGTTGTTTTAATAG
ACGGCGACGGAATTGTTAGAGCCGATGTTCCTTTTAGAAGGGCAGAATCGAAGTATAG
TGTCGAACAAGTAGGTGTAACTGTTGAGTTCTACGGTGGCGAACTCAATGGAGTCAGT
TATAGTGATCCTGCTACTGTGAAAAAATATGCTAGACGTGCTCAATTGGGTGAAATTTTT
GAATTAGATCGTGCTACTTTGAAATCCGATGGTGTTTTTCGTAGCAGTCCAAGGGGTTG
GTTTACTTTTGGACATGCTTCATTTGCTTTGCTCTTCTTCTTCGGACATATTTGGCATGGT
GCTAGAACCTTGTTCAGAGATGTTTTTGCTGGTATTGACCCAGATTTGGATGCTCAAGT
AGAATTCGGAGCATTCCAAAAACTTGGAGATCCAACTACAAGAAGACAAGTAGTT------
------------------------------GTGGAAACGCTCTTTAATGGAACTTTAGCTTTAGCTGGTCGTGA
CCAAGAAACCACCGGTTTCGCTTGGTGGGCCGGAAATGCCCGACTTATCAATTTATCCG
GTAAACTACTAGGGGCTCATGTAGCCCATGCCGGATTAATCGTATTCTGGGCCGGAGCA
ATGAACCTATTTGAAGTGGCTCATTTTGTACCAGAGAAGCCTATGTATGAACAAGGATT
AATTTTACTTCCCCACCTAGCTACTCTAGGTTGGGGGGTAGGTCCTGGTGGGGAAGTTA
TAGACACCTTTCCATACTTTGTATCTGGAGTACTTCATTTAATTTCCTCTGCAGTATTGGG
CTTTGGCGGTATTTATCATGCACTTCTGGGACCTGAGACGCTTGAAGAATCTTTTCCATT
CTTCGGTTATGTATGGAAAGATAGAAATAAAATGACCACAATTTTAGGTATTCACTTAAT
CTTGTTAGGTATAGGTGCTTTTCTTCTAGTATTCAAGGCCCTTTATTTTGGGGGCGTATAT
GATACCTGGGCTCCGGGAGGGGGAGATGTAAGAAAAATTACCAACTTGACCCTTAGCC
CAAGTATTATATTTGGTTATTTACTAAAATCCCCCTTTGGAGGGGAAGGATGGATTGTTA
GTGTAGACGATTTGGAAGATATAATCGGAGGACATGTATGGTTAGGTTCCATTTGTATAC
TTGGTGGAATCTGGCATATCTTAACCAAACCCTTCGCATGGGCTCGACGCGCACTTGTA
TGGTCTGGAGAGGCTTACTTATCTTATAGTTTAGGGGCTATATCTGTCTTTGGTTTCATTG
CTTGTTGTTTTGTCTGGTTCAATAATACTGCTTATCCTAGTGAGTTTTACGGACCCACTG
GACCAGAAGCTTCTCAAGCTCAAGCATTTACTTTTCTAGTTAGAGACCAACGTCTTGG

GGCTAACGTGGGATCCGCTCAAGGACCTACCGGTTTAGGTAAATATTTAATGCGTTCCC
CCACCGGAGAAGTCATTTTTGGAGGAGAAACTATGCGTTTTTGGGATCTGCGTGCTCC
GTGGTTAGAACCTCTAAGGGGTCCAAATGGGTTGGACTTGAGTAGGTTGAAAAAAGA
CATACAACCTTGGCAAGAACGGCGTTCTGCAGAATATATGACTCATGCTCCTTTAGGTT
CGTTAAATTCCGTGGGTGGCGTAGCTACTGAGATCAATGCAGTCAATTATGTCTCTCCTA
GAAGTTGGTTAGCTACTTCTCATTTTGTTCTAGGATTCTTCTTCTTCGTAGGTCATTTGT
GGCACGCGGGAAGAGCTCGTGCGGCTGCAGCAGGATTTGAAAAAGGAATTGATCGTG
ATTTTGAACCTGTTCTTTCCATGACTCCTCTTAATATGACTATAGCCCTTGGTAAATTTAC
CAAAGACGAAAAT---TTATTTGATATTATGGATGACTGGTTACGGAGGGACCGTTTCGTT
TTTGTAGGCTGGTCCGGTCTATTGCTCTTTCCTTGTGCCTATTTCGCTTTAGGGGGTTGG
TTCACAGGTACAACCTTTGTAACTTCATGGTATACTCACGGATTGGCCAGTTCCTATTTG
GAAGGCTGTAATTTCTTAACTGCCGCCGTTTCTACTCCTGCTAATAGTTTAGCACATTCT
TTATTGTTACTATGGGGTCCTGAAGCCCAAGGAGATTTTACTCGTTGGTGTCAATTAGG
CGGTCTATGGACTTTTGTTGCTCTCCATGGCGCTTTTGGACTAATAGGTTTTATGTTACG
TCAATTCGAGCTTGCTCGATCTGTTCAATTGCGACCTTATAATGCAATCGCATTCTCTGC
TCCAATTGCTGTTTTTGTTTCTGTATTCCTGATTTATCCACTAGGTCAGTCTGGTTGGTTC
TTTGCGCCTAGTTTTGGTGTAGCAGCTATATTTCGATTCATCCTCTTTTTTCAAGGATTTC
ATAATTGGACGTTAAACCCATTTCATATGATGGGAGTTGCTGGTGTATTAGGCGCTGCTT
TGCTATGCGCTATTCATGGTGCTACCGTAGAAAATACTTTATTTGAAGATGGTGATGGTG
CAAATACATTCCGTGCTTTTAACCCAACTCAAGCTGAAGAAACTTATTCAATGGTCACC
GCTAACCGCTTTTGGTCCCAAATCTTTGGGGTTGCTTTTTCCAATAAACGTTGGTTACAT
TTCTTTATGTTATTTGTACCAGTAACCGGTTTATGGATGAGTGCTCTTGGAGTAGTCGGT
CTGGCCCTGAACCTACGTGCCTATGACTTCGTTTCTCAGGAAATTCGCGCAGCGGAAG
ATCCTGAATTTGAGACTTTCTACACCAAAAATATTCTCTTAAACGAAGGTATCCGTGCTT
GGATGGCGGCTCAAGATCAGCCTCATGAAAACCTTATATTCCCTGAGGAGGTTCTACCA
CGTGGAAACGCTCTTATGTCTGGAAGCACAGGAGAACGTTCTTTTGCTGATATTATTAC
CAGTATTCGCTACTGGGTTATTCATAGCATTACTATACCTTCCTTATTCATTGCGGGTTGG
TTATTCGTCAGCACCGGTTTAGCTTATGATGTGTTTGGAAGCCCTCGGCCAAACGAGTA
TTTCACAGAGAGCCGACAAGGAATTCCATTAATAACTGGGCGTTTTGATTCTTTGGAAC
AACTTGATGAATTTAGTAGATCTTTTATGACCATAGATCGAACCTATCCAATTTTTACAG
TGCGATGGTTGGCTGTTCACGGCCTAGCTGTACCTACCGTTTCTTTTTTGGGATCAATAT
CAGCAATGCAGTTCATCCAACGAATGGCTACACAAACTGTTGAGAACGCTTCTAGATC
TGGT---CCAAGACGAACTACTGTAGGGAGTTTATTGAAACCATTGAATTCGGAATATGGT
AAAGTAGCTCCTGGATGGGGAACAACTCCTTTGATGGGTGTCGCAATGGCTTTATTTGC
GATATTTCTATCTATTATTTTGGAGATTTATAATTCTTCCGTCTTATTGGATGGAATTTCAA
TGCAAATGCTTACTCTCAAACTCTTCGTTTACACAGTAGTGATATTCTTTGTTTCTCTCT
TCATCTTTGGATTCCTATCTAATGATCCAGGACGTAATCCTGGACGTGAAGAAATGGCC
GATACGACTGGAAGGATTCCTCTTTGGATAATAGGTACTGTAACTGGTATTCTTGTGATC
GGTTTACTAGGTATTTTCTTTTATGGTTCATATTCCGGATTGGGTTCATCCCTGATGCTTA
ATATCTTTAGTTTAATCTGTATCTGTCTTAATTCTACTCTTTATTCGAGTAGTCCTTTCTTG
GCCAAATTGCCCGAGGCCTATGCTTTTTTGAATCCAATCGTAGATATTATGCCAGTCATA
CCTCTACTCTTTTTTCTCTTAGCCTTTGTTTGGCAAGCTGCTGTAAGTTTTCGAATGACA
CAATCAAACCCGAACGAACAAAGTGTTGAATTGAATCGTACTAGCCTCTACTGGGGAT
TATTACTCATTTTTGTACTTGCTGTTTTATTTTCCAATTATTTCTTCAATATGGAAGTAAAT

ATTCTTGCATTTATTGCTACTGCACTGTTCATTCTAGTTCCTACTGCTTTTCTACTTATCAT
TTACGTAAAAACAGTTAGTCAAAATGATATGGAAACAGCAACTCTAGTCACCATCTTTA
TATCTGGTTTACTTGTAAGTTTTACTGGGTACGCCTTATATACCGCTTTTGGGCAACCTT
CTCAACAACTAAGAGATCCGTTCGAGGAACATGGGGACATGGAAGCATTGGTTTATAC
ATTCCTTTTAGTCTCGACTCTAGGGATAATTTTTTTCGCTATCTTTTTTCGCGAACCGCCT
AAAGTTCCAACTAAAAAGATGAAAATGACTCTTGCTTTCCAATTGGCTGTTTTTGCATT
AATTGCTACTTCATCAATCTTATTGATTAGTGTACCCGTTGTATTTGCTTCTCCTGATGGT
TGGTCTAGTAACAAAAATGTTGTATTTTCTGGTACATCATTATGGATTGGATTAGTCTTTC
TGGTGGGTATCCTTAATTCTCTCATCTCT--------------------ATGTCACCACAAACAGAAAC
TAAAGCAAGTGTTGGATTCAAAGCTGGTGTTAAAGATTACAAATTGAATTATTATACTC
CTGACTATGTCACCAAAGATACTGATATCTTGGCAGCATTCCGAGTATCTCCTCAACCTG
GAGTTCCACCTGAAGAAGCAGGGGCCGCGGTAGCTGCTGAATCTTCTACTGGTACATG
GACAACTGTGTGGACTGATGGACTTACCAGCCTTGATCGTTACAAAGGGCGATGCTAC
CAAATCGAGCCCGTTGCTGGAGAAGAAAATCAATTTATTGCTTATGTAGCTTACCCATT
AGATCTTTTTGAAGAAGGTTCTGTTACTAATATGTTTACTTCCATTGTGGGTAATGTATTT
GGGTTCAAAGCCCTGCGTGCTCTACGTCTGGAAGATTTGCGAATCCCTCCTGCTTATAC
TAAAACTTTCCAAGGCCCGCCTCATGGCATCCAAGTTGAGAGAGATAAATTGAACAAG
TATGGTCGTCCCCTGTTGGGATGTACTATTAAACCGAAATTGGGATTATCTGCTAAAAAC
TACGGTAGAGCAGTTTATGAATGTCTACGCGGTGGACTTGATTTTACCAAAGATGATGA
GAATGTGAACTCCCAACCATTTATGCGTTGGAGAGATCGTTTCTTATTTTGTACCGAAG
CACTTTTTAAAGCACAGGCTGAAACAGGTGAAATCAAAGGGCATTACTTGAATGCTAC
TGCAGGTACATGCGAAGAAATGATGAAAAGGGCTGTATTTGCCAGAGAATTGGGAGTT
CCTATCGTAATGCATGACTACTTAACAGGGGGATTTACTGCAAATACTACCTTAGCTCAT
TATTGCCGAGATAATGGCCTACTTCTTCACATCCACCGCGCAATGCATGCAGTTATTGAT
AGACAGAAGAATCATGGGATGCACTTTCGTGTACTAGCTAAAGGGTTACGTATGTCTGG
TGGAGATCATATTCACGCCGGTACCGTAGTAGGTAAACTTGAAGGGGAAAGGGAGATC
ACTTTGGGATTTGTTGATTTATTGCGTGATGATTTTATTGAAAAAGATCGAAGTCGCGGT
ATTTATTTCACTCAAGATTGGGTCTCTCTACCAGGTGTTCTGCCCGTGGCTTCGGGAGG
TATTCACGTTTGGCATATGCCTGCTCTGACCGAGATCTTTGGGGATGATTCCGTACTACA
GTTCGGTGGAGGAACTTTAGGCCACCCTTGGGGAAATGCACCGGGTGCCGTAGCTAAT
CGAGTAGCTCTAGAAGCATGTGTAAAAGCTCGTAATGAGGGGCGTGATCTTGCTCGTG
AGGGTAATGAAATTATCCGTGAGGCTAGCAAATGGAGTCCTGAACTAGCTGCTGCTTGT
GAGATCTGGAAGGAGATCAAATTTGAATTTGAGGCAATGGATACTTTG------ATGGCGAT
ACATTTATACAAAACTTCTACCCCGAGCACACGCAATGGAGTCGTAGACAGTCAAGTG
AAATCCAATCCACGAAATCATTTAATCTATGGACAGCATCGTTGTGGTAAAGGTCGTAA
TGCCAGAGGAATCATTACCGCAGGGCATAGAGGGGGAGGTCATAAGCGTCTATACCGT
AAAATCGATTTTCGACGGAATGAAAAAGACATATATGGTAGAATCGTAACCATAGAATA
CGACCCTAATCGAAATGCATACATTTGTCTCATACACTATGGGGATGGTGAGAAGAGAT
ATATTTTACATCCCAGAGGGGCTATAATTGGAGATACCATTGTTTCTGGTACAGAAGTTC
CTATAAAAATGGGAAATGCCCTACCTTTGACCGATATGCCCTTAGGCACGGCCATACATA
ACATAGAAATCACACTTGGAAAGGGTGGACAATTAGCTAGAGCAGCGGGTGCTGTAGC
GAAACTGATTGCAAAAGAGGGGAAATCGGCCACATTAAAATTACCTTCTGGGGAGGTC
CGTTTGATATCCAAAAACTGCTCAGCAACAGTCGGACAAGTGGGGAATGTTGGGGTGA
ACCAGAAAAGTTTGGGTAGAGCCGGATCTAAGCGTTGGCTAGGTAAGCGTCCTGTAGT

AAGAGGAGTAGTTATGAACCCTGTAGACCATCCCCATGGGGGTGGTGAAGGGAGGGC
CCCAATTGGTAGAAAAAAACCCACAACCCCTTGGGGTTATCCTGCACTTGGAAGAAGA
AGTAGAAAAAGGAATAAATATAGTGATAATTTGATTCTTCGTCGCCGTAGTAAAATGATT
CAACCTCAGACCCATTTGAATGTAGCGGATAACAGCGGGGCTCGAGAATTGATGTGTAT
TCGAATCATAGGAGCTAGC---AATCGTCGATATGCTCATATTGGTGACATTATTGTTGCTG
TGATCAAAGAAGCAGTGCCAAATATGCCCTTAGAAAAATCAGAAGTGGTCAGAGCTGT
AATTGTACGTACCTGTAAAGAACTCAAACGTGACAACGGTATGATAATACGATATGATG
ACAATGCTGCAGTTGTCATTGATCAAGAAGGAAATCCAAAAGGAACTCGAGTTTTTGG
TGCGATTGCACGGGAATTGAGACAGTTAAATTTTACTAAAATAGTTTCATTAGCTCCCG
AGGTATTAATGCTTAGTCCCAAAAGAACCAGATTCCGCAAACAACATAGAGGAAGAAT
GAAGGGAATATCTTATCGAGGTAATCATATTTGTTTCGGTAAATATGCTCTTCAAGCACT
TGAACCTGCTTGGATCACATCTAGACAAATTGAAGCAGGTCGACGAGCAATGACACGA
AATGCACGTCGTGGTGGAAAAATATGGGTACGGATATTTCCAGACAAACCAGTTACAGT
AAGACCCACAGAAACACGTATGGGTTCGGGGAAAGGATCCCCTGAATATTGGGTAGCT
GTTGTTAAACCAGGTCGAATACTTTATGAAATGGGTGGAGTAACAGAAAATATAGCCAG
AAAGGCTATTTCAATAGCAGCGTCCAAAATGCCTATACGAACTCAATTCATTATTTCGGG
AATGACCAGAATTAGACGGGGATATATAGCTCGGAGACGTAGAACAAAAATTCGTTTAT
TTGCATCAAGCTTTCGAGGGGCTCATTCAAGACTTACTCGAACTATTACTCAACAAAAA
ATAAGAGCTTTAGTTTCGGCTCATCGGGATAGGAATAGGCAAAAGAGAAATTTTCGTCG
TTTGTGGATCACTCGAATAAATGCAGTAATTCGCGAA---AGAGGGGTATCCTATAGTTAT
AGTAGATTAATCCATGATCTGTACAAGAGACAGTTGCTTCTTAATCGTAAAATACTTGCA
CAAATAGCTATATCAAATAGGAATTGTATTTATATGATTTCCAATGAGATCATAAAAGAA
GTAGATTGGAAGGAATCCACCGGAAGAATTATGTTAAAAGAAGTATACGCTTTAGGTCA
ACATATATCTATGTCTGCTCACAAAGCGCGAAGAGTAATTGATCAAATTCGTGGACGTT
CCTACGAAGAAACACTTATGATACTAGAACTCATGCCTTATCGAGCATGTTATCCCATTT
TTAAATTGGTTTATTCTGCAGCAGCAAATGCTAGTTACAATATGGATTCCAACGAAGCA
AATTTATTTATTAGTAAAGCCGAAGTCAACGAGGGTACTACCGTAAAGAAATTAAAACC
TCGAGCTCGAGGGCATAGTTATCCGATAAAAAGACCTACTTGCCGTATAACTATCGTAG
TGAAGGATATATCCTTAGATGGGGGAGTATGGGACAAAAAAATGGATGGAATCAAATAT
GCAGTATTTACAGACAAAAGTATTCGGTTATTGGGGAAAAAT---CAATATACTTCTAATG
TCGAATCAGGATCAACTAGGACAGAAATAAAGCATTGGGTCGAACTCTTCTTTGGTGT
CAAGGTAATAGCTATGAATAGTCATCGACTTCCGGGAAAGGGTAGAAGAATGGGACCT
ATTATGGGACATACAATGCATTACAGACGTATGATCATTACGCTTCAACCGGGTTATTCT
ATTCCACCTCTTAGAAAGAAAAGAACTATGGCAGTTCCAAAAAAACGTACTTCTATATC
AAAAAAGCGTATTCGTAAAAATATTTGGAAACGGAAGGGATATTGGGCAGCGTTAAAA
GCTTTTTCGTTAGGGAAATCTCTTTTGACCGGAAATTCAAAAAGTTTTTTTGTGCGACA
AACAAATAAGATGGCCAAGGGTAAAGATGTCCGAGTAACAGTTATTTTGGAATGTACC
AGTTGTGTCCGAAACAGTGTTAAT------AAGGGATCAACGGGCATTTCCAGATATATTAC
TCAAAAGAACCGGCACAATACGCCTAATCGATTGGAATTGAGAAAATTCTGTCCCTATT
GTTACAAACATACGATTCATGGGGAGATAAAGAAAATGAAAATAAGAGCTTCTGTTCGT
AAAATTTGTGAAAAATGTCGATTAATCCGTAGGCGGGGACGAATTATAGTAATTTGTTC
CAACTCGAGACATAAACAAAGACAAGGAATGGTTCGAGAGAAAGTAACAGTATCTAC
TCGGACACTACAGTGGAAGTGTGTTGAATCAAGAGCAGACAGTAAGCGTCTTTATTAT
GGACGTTTTATTTTGTCGCCACTTATGAAAGGTCAAGCTGACACAATAGGCATTGCGAT

GCGAAGAGCTTTGCTTGGAGAAATAGAAGGAACATGTATCACACGCGCAAAATTTGAG
------AAAATACCACACGAATATTCTACCATAGTGGGTATTCAAGAATCAGTACATGAAAT
TTTAATGAATTTGAAAGAAATTGTATTGAGAAGTAATCTATATGGAACTTGCGACGCATC
TATTTGTGTCAGGGGTCCTGGATATGTAACCACTCAAGATATCATCTTACCGCCTTATGT
CGAAATCGTTGATAATACACAGCATATAGCTAGTTTGACGGAACCAATTGATTTGTGTAT
TGGATTACAAATCGAGAGGAATCGTGGATATCTTATAAAAACGCCAAAT---AACTTTGAC
GGAAGTTAT------CCTATAGATGCTGTATTCATGCCTGTTCGAAATGCGAATCATAGTATTC
ATTCTTATCGGAATGGGAATGAGAAACAAGAGATACTTTTTCTCGAAATATGGACAAAT
GGAAGTTTAACTCCTAAAGAAGCACTTCATGAAGCCTCCCGGAATTTGATTGATTTATT
TATTCCCTTTTTACATATGGAAGAAGAA------AACTTACATTTAGAGGACAATCAA---CAC
ACGATTCCTTTACCCCCTTTTAACTTTCATGATAAATTGGCT---AAACTAAGAAAAAACA
AAAAA---AAAATAGCATTGAAATCAATTTTTATTGACCAATTAGAATTGCCTCCCAGGAT
CTATAATTGCCTCAAAAGGTCCAATATATATACATTATTGGACCTTTTGAATAATGTTCAA
GAAGATCTTATGAAAATTGAACATTTTCGCATAGAAGATGTAAAACAGATATTGGGGGT
TCTAGAAAAGCAT------TTCGCAATTGATTTACCAAAAAATAAGATGCTCCGGGATGGA--
-AATGAGGGAATGTCTACAATACCTGGGTTTAATCAGATACAATTTGAAGGATTTTGTAG
GTTCATTGATCAGGGTTTGACGGAAGAACTTTATAAGTTTCCAAAAATTGAAGATACCG
ATCAAGAAATTGAATTTCAATTATTTGTGGAAACATACCAATTGGTCGAACCATTGATAA
AAGAAAGAGATGCTGTGTATGAATCACTCACATATTCTTCTGAATTATATGTATCCGCGG
GATTAATTTGGAAAACCAGTAGGGATATGCAAGACCAAACAATTTTTATTGGAAATATT
CCTCTAATGAATTCTCTGGGAACTTCTATAATAAATGGAATATATAGAATTGTGATCAATC
AAATATTGCAAAGCCCGGGCATTTATTACAAGTCAGAATTGGACCATAACGGAATTTCG
GTCTATACCGGCACCATAATATCAGATTGGGGAGGAAAATCAGAATTAGAGATTGATAA
AAAAGCAAGGATATGGGCTCGTGTGAGTAGGAAACAAAAAATATCTATTCTAGTTCTAT
CATCAGCTATGGGGTCGAATCTAAGAGAAATTCTAGAGAATGTTTGTTATCCTGAAATT
TTTTTGTCTTTTCTGAATGATAAGGAGAAAAAAAAAATTGGGTCAAAAGAAAATGCCA
TTTTGGAGTTTTATCAACAATTTGCTTGTGTAGGTGGGGATCCAGTATTTTCGGAATCCT
TATGTAAAGAATTACAAAAGAAATTCTTTCAACAAAGATGTGAATTAGGAAGGATTGGT
CGACGAAATATGAACCGGAGACTGAATCTTGATATACCCCAGAACAATACATTTTTGTT
ACCACGAGATATATTGGCAGCCGCGGATCATTTGATTGGAATGAAATTTGGAATGGGTA
CACTTGACGAT------ATGAATCATTTGAAAAATAAACGTATTCGTTCTGTAGCGGATCTTT
TACAAGATCAATTCGGATTGGCTCTGGTTCGTTTAGAAAATGTAGTTCGAGGAACTATA
TGTGGAGCAATTAGG---CATAAATTT---ATACCGACTCCTCAGAATTTGGTAACTTCAAC
TCCA------------TTAACAACTACTTATGAATCTTTTTTCGGTTTACACCCATTATCTCAAGT
TTTGGATCGAACTAATCCATTGACACAAATAGTTCATGGGAGAAAATTGAGTTATTTGG
GACCTGGAGGATTGACAGGGCGAACTGCTAGTTTTCGAATAAGAGATATCCATCCTAGT
CACTATGGACGTATTTGCCCAATTGACACATCTGAAGGAATTAATGTTGGACTTATTGGA
TCCTTAGCAATTCATGCGAGGATTGGTCATTGGGGG---TCTCTAGAAAGCCCCTTTTAT---
------GAAATTTCTGAGAGATCAAAAGGG------GTACGGGTGCTTTATTTATCACCCCATAG
AGATGAATACTATATGGTAGCGGCAGGAAATTCTTTGGCCTTGAATCAGGGTATTCCGG
AAGAACAGGTTGTTCCAGCTCGATATCGTCAAGAATTCCTGACTATTGCATGGGAACA
GGTTCATCTTCGAAGTATTTTTCCCTTCCAATATTTTTCTATTGGAGCTTCCCTCATTCCT
TTTATCGAGCATAATGATGCGAATCGGGCTTTAATGAGTTCTAATATGCAACGTCAAGCC
GTTCCTCTTTCTCGGTCCGAGAAGTGCATTGTTGGAACTGGGTTGGAACGCCAAGCGG

CTCTAGATTCAGGGGCTCTTGCTATATCCGAACACGAGGGAAAGATCATTTATACCGAT
ACTGACAAGATCTTTTTATCGGGTAATGGAGATACTTTAAGCATTCCATTAGTTATGTATC
AACGTTCCAACAAAAATACTTGTATGCATCAAAAAACCCAGGTTCGGCGGGGTAAATG
CATTAAAAAGGGACAAATTTTAGCGGATGGCACCGCTACAGTTGGTGGCGAACTCGCT
TTGGGGAAAAACGTATTAGTAGCTTATATGCCATGGGAAGGTTACAATTCTGAAGATGC
AGTACTCATTAGCGAGCGCTTGGTATATGAAGATATTTATACTTCTTTTCACATACGGAA
ATATGAAATTCAGACTCATGTGACAAGCCAAGGC---CCCGAAAAGGTCACTAATGAAAT
ACCGCATTTAGAAGCCCATTTACTCCGCAATTTAGACAAAAATGGAATTGTGATGCTGG
GATCTTGGGTAGAGACGGGTGATATTTTAGTAGGTAAATTAACGCCCCAAATGGTGAAA
GAA---TCTTCGTATGCCCCGGAACATAGATTGTTACGAGCCATACTTGGCATTCAGGTAT
CTACTTCCAAAGAAACTTGTCTAAAACTACCTATAGGTGGTAGGGGTCGGGTTATTGAT
GTAAGATGGATTCAGAAAAAAGGG---GGTTCTAGTTATAATCCAGAAACGATTTGTGTA
TATATTTCACAGAAACGTGAAATCAAAGTAGGGGATAAAGTAGCTGGAAGACATGGAA
ATAAAGGTATCATTTCAAAAATTTTGCCTAGACAAGATATGCCTTATTTGCAAGATGGAA
GACCCGTTGATATGATCTTCAACCCATTAGGAGTACCTTCACGAATGAATGTAGGGCAG
ATATTTGAATGTTCACTCGGGTTAGCGGGGGGTCTGCTCGACAGACATTATCGAATAGC
GCCTTTTGATGAGAGATATGAACAAGAAGCTTCGAGAAAACTAGTGTTTTCTGAATTAT
ATGAAGCCAGTACGCAAACAGCGAATCCGTGGGTATTTGAACCCGAGTATCCAGGAAA
AAGCAGAATATTTGATGGAAGAACGGGGGATCCTTTTGAACAACCTGTTATAATAGGA
AAGCCTTATATCTTGAAATTAATTCATCAAGTTGATGATAAAATCCATGGACGTTCCAGT
GGACATTATGCACTTGTTACACAACAACCCCTTAGAGGAAGAGCCAAGCAAGGGGGA
CAACGGGTAGGAGAAATGGAGGTTTGGGCTCTAGAGGGATTTGGCGTTGCTCATATTTT
ACAAGAGATGCTTACTTATAAATCTGATCATATTAGAGCTCGCCAGGAAGTACTTGGTA
CTACGATCATTGGAGGAACAATACCTAATCCTGAGGATGCTCCAGAATCTTTTCGATTG
CTTGTTCGGGAACTACGATCTTTGGCTCTGGAACTGAATCATTTCCTTGTATCTGAGAA
GAACTTCCAGATTACTAGGAAGGAAGCTATGAATCAGAATTTTTCTTCTATGATCGATC
GGTATAAACATCAACAACTCCGAATTGGATCAGTTTCTCCTCAACAAATAAGTGCTTGG
GCCACTAAAATCTTGCCTAATGGAGAGATAGTTGGAGAGGTGACAAAACCCTATACTTT
TCATTACAAAACCAATAAACCAGAAAAAGATGGATTATTTTGTGAAAGAATTTTTGGCC
CTATAAAAAGTGGAATTTGTGCTTGTGGAAATTATCGAGTAATCGGAGATGAAAAAGA
AGACTCGAAATTTTGTGAACAATGCGGAGTCGAATTTGTTGATTCTCGGATACGAAGAT
ATCAAATGGGCTACATCAAACTCGCATGCCCAGTAACCCATGTGTGGTATTTGAAACGT
CTTCCTAGTTATATTGCGAATCTTTTAGATAAACCTCTTAAAGAATTAGAAGGCCTAGTA
TACTGCGATCCCAATTTTTCTTTTGCTAGGCCC---GTAGCTAAAAAACCCACTTTCTTAC
GATTACGAGGTTTATTCGAATATGAAATCCAATCCTGGAAATACAGCATCCCACTTTTTT
TTACTACCCAAGGCTTCGATACATTTCGAAATCGGGAGATCTCTACTGGAGCCGGTGCT
ATCCGAGAACAACTAGCCGATCTAGATTTACGAATTATTATAGATTATTCATTAGTAGAAT
GGAAGGAATTAGGGGAAGAA---GGGTTCACAGGGAATGAATGGGAAGATCGAAAAGT
TGGAAGAAGAAAGGATTTTTTGGTTAGACGCATGGAATTAGCTAAGCATTTTATTCGAA
CAAATATAGAACCAGAACGGATGGTTTTGTGTCTATTACCAGTTCTTCCTCCCGAGTTG
AGACCGATTATTCAGATAGATGGGGGTAAACTAATGAGTTCAGATATTAATGAACTCTAT
AGAAGAGTTATCTATCGGAACAATACTCTTACCGATCTATTAACAACAAGTAGATCTAC
GCCAGGAGAATTAGTAATGTGTCAGGAGAAATTGGTACAAGAAGCCGTGGATACACTT
CTTGATAATGGAATCCGCGGACAACCAATGAGGGACGGTCATAATAAAATTTACAAGTC

GTTTTCAGATGTAATTGAAGGCAAAGAGGGAAGATTTCGCGAGACTCTGCTCGGCAAA
CGGGTCGATTATTCAGGGCGTTCTGTCATTGTCGTGGGCCCTTCACTTTCATTACACCGA
TGTGGATTACCTCGGGAAATAGCAATAGAGCTTTTCCAGACATTTGTCATTCGTGGTCT
AATTAGACAACATCTTGCTTCGAATATAGGAGTTGCTAAGAGTAAAATTCGGGAAAAA
GAACCGCTTGTATGGGAAATACTTCAAGACGTTATGCAGGGACATCCTGTATTGCTGAA
TAGAGCACCTACTCTGCATAGATTAGGCATACAGGCATTCCAGCCCGTTTTAGTGGAAG
GGCGCGCTATTTGTTTACATCCATTAGTTTGTAAGGGATTCAATGCAGATTTTGACGGGG
ATCAAATGGCTGTTCATGTACCTTTATCTTTGGAGGCTCAAGCGGAGGCCCGTTTACTTA
TGTTTTCTCATATGAATCTTGTGTCTCCAGCTATTGGGGATCCCATTTCCGTACCAACTC
AAGATATGCTTATTGGACTTTATGTATTAACGAGCGGGAATCGTCGAGGTATTTGTGTAA
ATAGGTATAATCCATGTAATCTT---------AGAAACTATCAAAATGAAAGAATTTACGATAAT
AACTATAAGTATACAAAA------GAACCTTTTTTTTGTAATTCCTATGATGCAATTGGAGCT
TATCGACAGAAACAAATTAATTTAGATAGTCCTTTGTGGCTCCGGTGGCGACTAGATCA
ACGC---GTTATTGCTTCA------AGAGAAGTTCCCATCGAAGTTCATTATGAATCTTTGGGT
ACCTATTATGAGATTTATGGGCACTATCTAATAGTAAGAAGTATAAAAAAAGAAATTCTT
TTTATATACATTCGAACCACTGTTGGTCATATTTCTCTTTATCGAGAAATCGAAGAAGCC
ATACAGGGGTTTTGTCAAGCCTGTTCATATGGTCCC------------------------ATGGAGGTACT
TATGGCAGAACGGGCCAATCTGGTCTTTCACAATAAAGTGATAGACGGAACTGCCATG
AAACGACTTATTAGTAGATTAATAGATCACTTCGGAATGGCATATACATCACATATCCTG
GATCAAGTAAAGACTCTGGGTTTTCAACAAGCTACTGCTACATCCATTTCATTAGGAAT
TGATGATCTTTTAACAATACCTTCTAAGGGATGGCTAGTTCAAGATGCTGAACAACAAA
GTTTAATTTTGGAAAAACACCATCATTATGGGAATGTACACGCGGTAGAAAAATTACGT
CAATCCATTGAGATATGGTATGCTACAAGTGAATATTTGCGACAAGAAATGAATCCTAAT
TTTAGGATGACTGACCCTTTTAATCCAGTCCATATAATGTCTTTTTCGGGAGCTAGAGGA
AATGCATCTCAGGTACATCAATTAGTAGGTATGAGAGGATTAATGTCGGATCCTCAGGG
ACAAATGATTGATTTACCCATTCAAAGCAATTTACGCGAAGGACTCTCTTTAACAGAAT
ATATTATTTCTTGCTACGGAGCCCGTAAAGGGGTTGTGGATACTGCTGTACGAACATCA
GATGCTGGATATCTTACGCGCAGACTTGTTGAAGTAGTTCAACACATTGTTGTACGTCG
AACAGATTGTGGCACCGTCCGAGGTATTTCTGTGAGTCCTCGAAATGGGATGATGCCG-
--GAAAGGATTTTTATCCAAACATTAATTGGGCGTGTATTAGCAGATGATATCTATATGGG
TGCACGATGCGTTGCCACTCGAAATCAAGATATTGGGATTGGACTTGTCAATCGATTCA
TAACC------------------TTTCGAGCACAACCAATACCTATTCGAACTCCCTTTACTTGTAGG
AGTACATCTTGGATTTGTCGATTATGTTATGGCCGGAGTCCTACTCATGCCGACCTGGTC
GAATTGGGAGAAGCTGTAGGTATTATTGCAGGTCAATCTATTGGAGAACCGGGTACTCA
ATTAACATTAAGAACTTTTCATACCGGCGGAGTATTCACAGGGGGTACTGCAGAACAG
GTGCGAGCCCCTTCTAATGGAAAAATAAAATTCAATGAGGATTTGGTTCAGCCGACAC
GTACACGTCATGGACATCCTGCCTTTCTGTGTTCTATAGACTTGTATGTAACTATTGAGA
GTGAAGATATTCTACATAATGTGAATATTCCACCCAAAAGTTTTCTATTAGTTCAAAACG
ATCAATATGTAGAATCAGAACAAGTGATTGCTGAGATTCGCGCGGGAACATCCACT---T
TGAATTTTAAAGAGAGGGTTCGAAAACATATTTATTCTGACTCAGAGGGAGAA---ATGC
ACTGGAGTACCGATGTGTATCATGCACCTGAATTTACATATGGTAATGTTCATCTCTTAC
CAAAAACCAGTCATTTATGGATATTATTAGGACAGCCATGCAGA------TCTAGTCTAGTCT
CCCCTTCGCTCCACAAGGATCAAGATCAAATGAATGCTTAT------TCTCTTTCTGTCAAG
CGAAGATATATTTCTAACCTCTCA------GTAACTAATAATCAA---GTGAGACACAAATTCT

TTAGGTCGGATTTTTCTGGTAAA---GAAGATAGGATTCCTGATTAT---TCAGAACTCAATC
GAATCATATGCACTGGTCGTTGTAATCTCATCTATCCG---GCCATTCTTCCCGAAAATTCT
------GATTTATTGGCAAAGAGCCGAAGAAATCGATTCATTATTCCACTCCAATCGATTCA
AGAACGCGAGAACGAACTAATGCCCCGTTCAGATATCTCGATTGAAATACCC------ATAA
ATGGCATTTTCCGCAGAAAGAGTATTCTTGCTTATTTCGATGATCCTCGATACAGAAGA
AAGAGTTTGGGAATTACTAAATATGGGACTATAGAAGTGCATTCAATCGTCAAAAAAGA
GGAT------TTGATTGAGTATCGAGGA---GTTAAGGAAGTTAGGCCAAAATACATGAAAGT
AGATCGATTTTTTTTCATTCCCGAGGAAGTGCATATTTTACCCGGATCTTCTTCCATAATG
GTACGGAACAATAGTATCATTGGGGTAGATACACAAATCACTTTAAATACAAGAAGCCG
AGTAGGCGGATTGGTCCGAGTGGAGAGAAAAAAAAAAAAGAATTGAACTGAAAATCTT
TTCGGGGGATATCCATTTTCCTGGAGAGACAGATAAGATATCCCGACATAGTGGCGTCT
TGATCCCG---CCAGGA---ACGGGAAAAACAAATTCCAAGGAATCAAAAAAA------TTGA
AAAATTGGATCTATGTCCAACGG------ATCACACCTATCAAG------AAAAAGTATTTTGTT
TTGGTTCGACCTGTAGTCACATATGAAATAACGGACGGTATAAATTTAGCAACACTTTT
CCCCCTCGATCTGTTGCAGGAAAGGGATAATGTGCAACTTCGAGTTATCAATTATATCCT
TTATGGAAATGGCAAACCAATTCGGGGAATTTCTGACACAAGTATTCAATTAGTTCGGA
CTTGTTTAGTATTGAATTGGGACCAAGACAAAAAA---------AGTTCTTCTATCGAAGAGG
CCCGCGCTTCCTTTGTTGAAATAAAGACAAAAGGTTTGATTCGAGATTTCCTAAGAATC
GACTTAGTGAAATCCTCTATTTCGTATACCAGAAAAAGGAATGATCCATCGGGGGCAGG
ATTGATCTCTGAT---AATGGCTCAGATCGCACCAATATCAATCCGTTTTCTTTTATTTATAC
CTAT---------TCCAAGGCAAGGATTCAAGAATCCCTTAACCAAAATCAAGGAACTATTCAT
ACGGTATTGAATAAA---------AATAAGGAATGCCAATCCTTGATAATTTTGTCATCATCCA
ATTGTTCTCGAATGGGCCCA------TTCAACGATGTAAAATATCACAATATG---------ATAAA
AGAATCA---CCCCCAATTCCAATTAGGAATTCGTTGGGATCTTTAGGAACA------GCCTTT
CAAATCGCGAATTTTTATTCA------TTTTACCATTTA------ATAACTTATAATCCGATTTTG---
GTAAATAACTATTTCCAACTTGACAAT---------TTAAAACAGACTTTTCAAGTACTTAAA--
-------TATTATTTAATGGATGAAAAT------GGAAAATTTTATAATCCCGATCCACGCAGTAAT
ATCATTTTGAATCCATTCAATTTGAATTGGTATTTTCTCCATCACAATTATTGTGAAGAGA
CATCT---ACAATAATTAGTCTTGGACAGTTTATTTGTGAAAATGTATGTATAGCCAAAAA
CGGACCCCAC---CTAAAA------TCGGGTCAAGTTCTAATTGTTCAA---GGCGACTCTGTA
GTAATACGATCAGCTAAGCCTTATTTGGCCACCCCAGGAGCAACTGTTAATGGCCATTA
TGGGGAAATCCTTTACGAAGGAGATATATTAGTTACATTTATATATGAAAAATCGAGATC
TGGTGATATAACACAGGGTCTTCCAAAAGTGGAACAGGTGTTAGAGGTGCGTTCGATT
AATTCAATAGCAATGAATCTCGAAAAGAGGGTTGAGGGTTGGAACGAATGTATAACAG
GAATTCTTGGAATTCCTTGGGGATTCTTGATTGGTGCTGAGCTAACTATAGTGCAAAGT
CGTATATCTTTGGTTAATAAGATCCAAAAGGTTTATCGATCTCAGGGGGTGCAGATCCAT
AATAGGCATATAGAAATTATTGTACGTCAAATAACCTCAAAAGTATTGGTTTCAGAA------
---GATGGAATGTCTAATGTTTTTTCACCCGGAGAACTAATTGGATTGTTACGAGCGGAA
CGAATGGGGCGTGCTTTGGAAGAAGCGATCTGTTACCGAGCCGTCTTATTGGGAATAA
CAAGAGCATCTCTGAATACTCAAAGTTTTATATCTGAAGCGAGTTTTCAAGAAACTGCT
CGAGTTTTAGCAAAAGCAGCTCTCGGGGGTCGTATCGATTGGTTGAAGGGCCTGAAAG
AGAACGTTGTTCTGGGGGGGATGATACCCGTTGGTACCGGATTCAAAGGATTA------GT
GCACCCTTCAAGGCAACATAACAACATTCCC------CTGGAAACCAAAAAGAAGAATCTA
TTTGAGGGGGAAATGAGA------GATATTTTGTTCCACCACAGAAAATTATTGGATTCTTG

CCTTTCAAAGAATTTCCATGATATATCAAACAATCATTTA------------------------------------AT
GACAAGAAGATATTGGAACATCAATTTGGAAGAGATGATGGAGGCAGGAGTTCATTTT
GGCCATGGTACTAGGAAATGGAATCCTAAAATGGCACCTTATATCTCTGCAAAGCGTAA
AGGTATTCATATTACAAATCTTACTAGAACTGCTCGTTTTTTATCAGAAGCTTGTGATTT
GGTTTTTGATGCAGCAAGTATGGGAAAAAAATTCTTAATTGTTGGCACTAAAAATAAAG
CAGCTGATTCAGTAGCATGGGCTGCAATAAGGGCTCGTTGTCATTATGTTAATAAAAAA
TGGCTCGGCGGTATGTTAACGAATTGGTCCACTACAGAAACGAGACTTCATAAGTTTAG
AGACTTGAGAACGGAACAAAAAACAGGGAGACTCAACCGTCTTCCGAAACGAGATG
CAGCTATGTTGAAAAGACAATTAGCTCACCTGCAAACATATCTGGGCGGAATTAAATAT
ATGACAGGTTTACCCGATATTGTAATCATCGTTGATCAGCACGAAGAATATACAGCCCTT
CGAGAATGTATCACTTTAGGAATTCCAACAATTTGTTTAATCGATACAAATTGTGACCCC
GATCTTGCAGATATTTCGATTCCAGCGAACGATGACGCTATATCTTCAATCCGATTAATT
CTTAACAAATTAGTATTTGCAATTTGTGAGGGTCGTTCT---AGCTATATAAGAAATACGAT
GGGACAAAAAATAAATCCACTTGGTTTCAGACTTGGTACAACCCAAGGTCATCATTCC
CTTTGGTTTGCACCACCAAAAAATTATTCCGAAGGTCTACAAGAAGATCAAAAAATAA
GAGATTGTATCAAGAATTATGTACAAAAAAATAGCAAAATATCCTCTGGC---GTCGAGG
GAATTGCACGTATAGAGATTCAAAAAAGAATCGATCTGATCCAGGTCATAATCTATATGG
GATTCCCAAAGTTTTTAATAGAAAGTCGACCGCGA------------GGGATCGAAGAATTACA
GATGAATCTACAAAAAGAATTTAATGGTGTGAACCGAAAACTTAATATTGCTATCACAA
GAATTGCAAAACCTTATGGAAACCCTAATATTCTTGCAGAATTTATAGCCGGCCAATTAA
AAAATAGAGTTTCATTTCGCAAAGCAATGAAAAAAGCTATTGAATTAACCGAACAAGC
AGATACAAAAGGAATTCAAGTACAAATTGCAGGGCGTATCGACGGAAAAGAAATTGCA
CGTGTCGAATGGATCAGAGAAGGTAGGGTTCCCCTACAAACCATTCGTGCTAAAATTG
ATTATTGTTCCTATACAGTTCGAACTATCTATGGGGTCTTAGGCATCAAAATTTGGATATT
TATAGACGAAGAAATGTCACGTTACCGAGGACCTCGTTTCAAAAAAATACGCCGCCTG
GGAGCTTTACCAGGACTAACTAATAAAAGGCCTAGAGCCGGAAATGATCTTAGAAACC
AATCGCGTTCCGGTAAA---AAATCTCAATATCGGATTCGCCTAGAAGAAAAACAAAAAT
TGCGTTTTCATTATGGTCTTACAGAACGACAATTACTTAAATACGTTCGTATCGCCGGAA
AAGCCAAGGGGTCAACAGGTCAGGTTTTACTACAATTACTTGAAATGCGTTTGGATAA
CATCCTTTTTCGATTGGGTATGGCTCCGACTATTCCCGGAGCCCGCCAATTAGTTAATCA
TAGACATATTTTAGTTAATGGTCGTATAGTAGATATACCAAGTTATCGCTGCAAACCCCG
AGATATTATTACGGCGAGGGATGAACAAAAATCCATAGTTCTGGTTCAAAATTCTCTCG
ATTCATCCCCTCAT---GAGGAATTGCCAAAACATTTGACTCTTCACCCATTCCAA------TA
TAAAGGATTAGTCAATCAAATAATAGATAGTAAATGGGTCGGTTTGAAAATAAATGAAT
TGTTAGTCGTAGAATATTATTCGCGTCAGACTATGTCACGTCGAGGTACTGCAGAAGAA
AAAACTGCAAAATCCGATCCAATTTATCGTAATCGATTAGTTAACATGTTGGTTAACCGT
ATTCTGAAACACGGAAAAAAATCATTGGCTTATCAAATTGTCTATCGAGCCGTGAAAA
AGATTCAACAAAAGACAGAAACAAATCCACTATCTGTTTTACGTCAAGCAATACGTGG
AGTAACTCCCGATATAGCAGTAAAAGCAAGACGTGTAGGTGGATCGACTCATCAAGTT
CCCATTGAAATAGGATCCACACAAGGAAAAGCACTTGCCATTCGTTGGTTATTAGCAGC
ATCCCGAAAACGTCCGGGTCGAAATATGGCTTTCAAATTAAGTTCCGAATTAGTGGATG
CTGCCAAAGGGAGTGGCGATGCCATACGCAAAAAGGAAGAGACTCATAGAATGGCAG
AGGCAAATAGAGCTTTTGCACATTTTCGTATGGGTAGGGACACTATTGCTGACATAATA
ACCTCTATACGAAATGCTGATATGGATAGAAAAAGAGTGGTTCGAATAGCATCTACTAAT

ATCACTGAAAATCTGGTTAAAATACTTTTACGAGAAGGTTTTATCGAAAACGTGAGAA
AACCTCGAGAAAAAAACAAATATTTTTTGGTTTTAACCCTGCGACATAGAAGG------AAT
AGGAAAGGGCCCTAT---------AGAAATATTTTAAATTTAAAACGGATCAGTCGCCCTGGT
CTACGAATCTATTCTAACTATCAACGAATTCCTAGAATTTTAGGCGGGATAGGGATTGTA
ATTCTGTCTACTTCTCGAGGTATAATGACAGACCGAGAAGCTCGACTAGAAAAAATCG
GCGGAGAAATTTTGTGTTATATATGGATGGCAAAAGCTATACCGAGAGTTGGTTCACGT
AGGAATGGACGTATTGGTTCACGTAAGAGTGCACGTAGAATACCAAAGGGAGTTATTC
ATGTTCAAGCAAGTTTTAATAATACCATTGTGACTGTTACAGATGTAAGAGGTCGGGTG
GTTTCTTGGTCCTCGGCCGGTACTTGTGGATTCAGGGGTACAAGAAGAGGGACACCGT
TTGCTGCTCAAACCGCAGCAGGAAATGCTATTCGTGCAGTAGTGGACCAAGGTATGCA
ACGAGCAGAAGTCATGATAAAAGGCCCCGGTCTCGGAAGAGACGCAGCATTACGAGC
TATTCGTAGAAGTGGTATACTATTAACTTTCGTACGGGATGTAACCCCTATGCCGCATAA
TGGCTGTAGACCTCCTAAAAAAAGACGCGTGATGCCAACTATTAAACAACTTATTAGA
AATACAAGACAGCCAATCCGAAATGTCACGAAATCCCCCGCTCTTGGGGGATGCCCTC
AGCGTCGAGGAACATGTACTAGGGTGTATACTATCACCCCAAAAAAACCAAACTCTGC
CTTACGTAAAGTTGCCAGAGTACGATTAACCTCTGGATTTGAAATCACTGCTTATATACC
CGGTATTGGCCATAATTCACAAGAACATTCTGTAGTCTTAGTAAGAGGGGGAAGGGTTA
AGGATTTACCCGGTGTGAGATATCACATTGTTCGAGGAACCCTAGATGCTGTCGGAGTA
AAGGATCGTCAACAAGGGCGTTCTAAATATGGGGTAAAAAAGCCAAAAATGGCAAGG
AAAAGTTTGATTCAGAGGGAGAAAAAGAGGCAAAAATTGGAACAGAAATATCATTTG
ATTCGTCGATCCTCAAAAAAAGAAATAAGC---------AAAGTTCCGTTATTGAGTGACAAA
TGGAAAGTTTATGGAAAGTTACAATCCCCACCGCGGAATAGTGCACCTACACGCCTTCA
TCGACGTTGTTTTTCGACCGGAAGACCGCGAGCTAACTATCGAGACTTTGGGCTATCCG
GACACATACTTCGTGAAATGGTTCATGCATGTTTGTTGCCAGGGGCAACAAGATCAAG
TTGGATGGTCAAAAATTCATTCATTTCCGTTACTTCACAA------GAAGAAAAGAAAGAA
AACAGGGGGTCTGTTGAATTTCAAGTAGTCAGTTTTACCAATAAGATACGGAGACTTAC
TTCACATTTGGAATTGCACAAAAAAGACTATTTATCTCAGAGAGGTCTACGGAAAATTC
TGGGAAAACGTCAACGGCTATTGGCTTATTTGTCAAAAAAAAAATAGAGTACGTTATAAA
GAAATAATTGGTCAGTTGGATATTCGAGAGATAAAAACTCGT------ATGGTAAAACTTCG
TTTGAAACGATGTGGTAGAAAGCAACGAGCCGTCTATCGAATCGTTGCAATTGATGTTC
GATCCCGAAGAGAAGGAAGAGATCTTCGGAAAGTGGGTTTTTATGATCCGATAAAGAA
TCAAACTTATTTAAATGTTCCTGCTATTCTATATTTCCTTGAAAAGGGTGCTCAACCTAC
AGGAACTGTTCAGGATATTTTAAAGAAGGCGGAGGTTTTTAAAGAACTTCGCTCTAATC
AAACGAAATTCAAT---ATGGATAAATCCAAGCGACCTTTTTTTAAATCCAAGCGATCTTT
TCGTAGGCGTTTGCCCCCGATTCAATCGGGGGATCGAATTGATTATAGAAACATGAGTT
TAATTAGTCGATTTATTAGTGAACAAGGAAAAATATTATCTAGGCGAGTGAATAGATTGA
CCTTGAAACAACAACGATTAATTACTACTGCTATAAAACAAGCTCGTATTTTATCTTCGT
TACCTTTTCTTAATAATGAGAAA------CAATTTGAAAGAACCGAGTCGACGGCTAGAACT
ACTGGTCTTAGAACTAGAAATAAAGTGACACGTTCACTAAAAAAAAAATCCTTTTGTAG
CAAACCATTTATTAAGAAAAATTGAGAAGCTTAACACAAAAGCAGAAAAAGAAATAAT
AGTAACTTGGTCCCGGGCATCTACCATTATACCCACAATGATCGGCCATACTATTGCTAT
CTATAATGGAAAGGAACATTTGCCTATTTATATAACAGACCGTATGGTAGGCCACAAATT
GGGAGAATTTGCACCTACTTTACATTTCCGAGGACATGCGAAAAGCGATAATAGATCTC
GTCGTATGATTTTTAAATCTTTTCTACTAGGTAATCTAGTACCCTTATGTATGAAGATAAT

AAATTCGGTCGTTGTGGTCGGACTCTATTATGGATTTCTGACCACATTCTCCATAGGGCC
CTCTTATCTCTTCCTTCTCCGAGCTCAGATTATGGAAGAAGGA---------ACCGAGAAGAA
GGTATCAGCAACAACTGGTTTTATTACGGGACAGCTCATGATGTTCATATCGATCTATTA
TGCGCCTCTGCATCTAGCATTGGGTAGACCTCATACAATAACTGTCCTAGCTCTACCGTA
TCTTTTGTTTCATTTCTTCTGGAACAATCACAAACACTTTTTTGATTATGGATCTACTACC
AGAAATTCAATGCGTAATCTCAGCGTTCAATGTGTATTCCTGAATAATCTCATTTTTCAA
TTATTCAACCATTTCATTTTACCAAGTTCAATGTTAGCCAGATTAGTCAACATTTATATGT
TTCGATGCAACAACAAGATGTTATTTGTAACAAGTAGTTTTGTTGGTTGGTTAATTGGT
CACATTTTATTCATGAAATGGGTTGGATTGGTATTAGTCTGGATACGGCAAAATCATTCT
ATTAGATCGCTTATTCGATCTAATAAGTACCTTGTGTCAGAATTGAGAAATTCTATGGCT
CGGATCTTTAGTATTCTCTTATTTATTACCTGTGTCTACTATTTAGGGAGAATACCGTCAC
CCATTCTTACTAAGAAACTGAAAGAAACTGAAGAAGATAAACCTCTTGTGACTCTTCT
TTTCGATTATAACCGATGGAATCGCCCATTTCGATACATAAAAAATAAGCGATTTGAAAA
GAATGTAAGAAATGAAACATCACAATATTTTTTTTATACATGCCAAAATGATGGAAAAC
AAAGAATATCTTTTACATATCCACTCAGTTTGTCAACTTTTTGGGAAATGATACAAAGA
AGGATGTCCCTGTCCACATTAGGAAAAATCGCCTCTGATGAACTGTACACTCATTGGAT
TTATACCAACAAACAACAAAATAAC---ATAAACAATGAGTTTATAAATAGAATTGAGACT
CTCGACATGGGATCTCTTTTTCTGGATATACTCGAAAAACGGACTAGATTGTGTAATGAT
GAGACTAAAAAAGACTACTTGCCTAAAATGTATGATCCTTTCTTGAATGGGCCATATCG
TGGAACAATCAAAAAATGGTTTTCACCTATAGAGACAATTGGAATAAATAAGATTCATG
GTATTCTTTGCCGAGAATTTGAACAGAAAGACCCTAATGATCAATCTATTGGAATAAAC
GAAATCAGTAAAAAAATCCCTCGATGGTCATACAAATTAATAGATTTGGAACAACAGTC
A---GGACAGATTCGCTCAAGAAAAGCCAAACGGGTAGTCATTTTTACTAGTGCTTTGAT
ACGCTATTCACAACAATCGGATTTTCGTCGAAATATAATCAAAGGTTCGATGCGTAGTC
AAATGCGGAAAATAGTTATTTGGGAACTGTTTCAAGCAAATGTGCATTCCCCCCTTTTT
TTGGACAGAATAAACAAATCCCCCCTTTTTTCGTTTGATATC------TCCGGACTAATGAA
ACTAATTTTTAGAAATAGAAAGGAAAAAGCACGGATAGAAATAGCAGAAGCCTGGGAT
ACCATTCCATTTGCACAAGTAATAAGAGGCTCTATGTTAATAACTCAATCGATTCTTAGA
AAATATATTATTTTACCTTTATTGATAGTAGCTAAAAATATGGGTCGTATGTTATTATTGCA
ACTTCCTGAATGGTCTAAGGATTTACAGGAATGGAATAGAGAAATGCATGTTAAATGTA
CCTATAATGGTGTTCAATTATCAGAAACCGAATTTCCGAAAAATTGGTTAACAGACGGT
ATTCAGATAAAAATCCTATTTCCTCTCTGTCTGAAACCTTGGCACAGATCTAAGATAAA
AAAAAAAGATGATTTTTGTTTTTTAACGGTTTGGGGAATGGAAGCTGAACTTCCTTTTG
GTTCTCCCCGAAAAAGACCTTCTTTTTTTGAGCCCATTTTTAAAGAACTCAAAAAAAA
ATTAAAAAAAATTGAAAAAGAAATATTTTCTAGTT---TTAAGAGTTTTAAAGGGAAGAAC
AAACTTTTTTCTAACAGTCTCAAAAGAAACAAAAAAATTGGGTCATCAAAAGTGTTCTA-
-----TTTATAAAAAGAATAAGAAAAGTACTTTCAAAAGTAAATGATTCTATAATCAACAAT
CAGATAATTCATGAATCGTTTAATCAAAGTCGATCTACAGATTGGACAAATTATTCCCTG
ACAGAAAAAAAACGGAAGGATCTGACTGATAGAACACGCACAATTAGAAATCAAATA
GAAAAAATTACAAACGACAAAAAAAAAAAATTCTAACAAAACAACTTATAATGCCAAA
AGACTAGAATCACTAAAAAATATTTGGCAAATATTAAAAAGAAGAAATGCTCGATTAAT
CAGTAAATTACATTATTTTATAAAAATTTTCATTGAAAGAATCTACATAGATGTCTTTCGA
TGTATCATTAATATTCCCCAAATCAATATACAACTTTTT---CACTTTATTTCGTGTGACTTA
TCCTCCTTGTCACAAGCATATGTATTTTACAATTTATCCCAAACCCACTTGGAT---------AA

ATTAAGATCTGTTCTTCAATATACATCTTTTTTTCTTAAGACTGGGATAAAGGATTCTTTT
ACACAAGGAATAATTCATTCTGAATTAAGATATAAGAAATTTCGGAGTTATGGAGCGAA
TCAATGGAAAAACTGGTTAAGGGGTCATTATCAATACGATTTATCTCAGATTAGATGGTC
TAGATTAATCCCACAAAAATGGCGAAATAGAGTCAATCAATGTCGTACAGCTCAAAAG
AAAAAGAAATGGAATTCATATGAAAAAAACCAA------TTACTTTATTACAAAAAACAA---
AATTTTCAAAAATACTTTAAATATGATCTTTTATCATATCAATCTATTAATGGTGATATTAT
GTATCCAGATAGAAAATATTTTGATTGGAAAATTCTCAAGTTTTGTCTTAGATTTTTTGA
TTGGATGGGAATGAATGAAGAAATACTAAATTGTTGGTTCTTCCCCGAATTTGTGCTAC
TTTATAATGCATATAAAATGAAACCGTGGATTATACCAAGCAAATTACTTCTTTTAAATTA
CAAGAGCAACACGGAAGCAGAACTAAATTTCTTCCTGAAACGATATTTGCTTTTTCAAT
TGAGATGGGACGATGCTTTGAATCAAAGAATGATCAATAATATTAAGGTATATTGTCTCC
TGCTTAGACTGAGAAACCCAAGAAAAATTACTATATCCTCTATTCAAAGAAGAGAAATG
AGTCTGAATATAATGCTGATTCAGAAGAATTTACCTCTTACAGAATTGATGAAA---GGGA
TATTGATTATCGAATCGGTTCGTCTGTCTGTAAAAAATGATGGACAATTTATTATGTATCA
AACCATAGGTATTTCATTGGTTCATAAGAGTAAACGCAATCATTCGAATTTACTTGTTCC
TGAAAATATTTTATCATCTAGACGTCGTAGAGAATTGAGAATTTTAATTTGTTTCAGATT
TCTTTGGCCGAATTATCGATTAGAAGATTTAGCTTGTATGAATCGCTATTGGTTTGATAC
GAATAATGGCAGTCGTATCAATATGTTAAGACTACGTATGTATCCAATGAAAGGACATCA
ATTCAAATCCTGGATTTTCGAATTGAGAGAGATATTGAGAGAGATCAAGAATTCTCACT
ATTTCTTAGATTCATGGACCCAATTCAATTCAGTTGGATCTTTCATTCACATTTTTTTCCA
CCAAGAACGTTTTATAAAACTTTTTGACCCCCGCATTTGGAGTATCCTACTTTCA---CGC
AATTCA------CAGGGTTCAACAAGCAATCGATATTTCACG---------ATCAAGGGT---GTAAT
ACTCTTTGTATTAGCGGTCCTTATATATCGT------------ATTAACAATCGAAAGATGGTCGA
AAGAAAAAATCTCTATTTGATAGGGCTTCTTCCT---------------ATACCTATGAATTCCATTG
GACCCAGAAATGATACATTGGAAGAATCCGTT---GGGTCTTCCAATATCAATAGGTTGAT
TGTTTCG---CTCCTGTATCTTCCAAAAGGAAAAAAGATCTCTGAGAGTTGTTTTCTGAA
TCCGAAAGAGAGTACTTGGGTTCTCCCAATAACT------------AAAAAGTGTAGCATGCCT
GAATCTAACTGGGGTTCGCGGTGGTGGAGAAACTGGATCGGAAAAAAGAGG------GAT
TCTAGTTGTAAGATATCTAAT---------GAAACCGTCGCTGGAATTGAGATCTTATTCAAAG
AGAAAGATATCAAA---TATCTGGAGTTTCTTTTTGTATATTATATGGAT------GATCCGATC
CGCAAGAACCATGATTGGGAATTGTTTGATCGTCTTTCTCTGAGAAAGAGGCGAAATAT
AATCAACTTG------AATTCGGGACCGCTATTCGAAATCTTAGTGAAACACTGGATTTGTT
ATCTCATGTCTGCTTTTCGTGAAAAAAATACCAATTGAAGTAGAGGGT---TTCTTCAAACA
ACAAGGG---GCTGGGTCAACTATTCAATCAAATGATATTGAGCATGTTTCCCAT------CTC
TTCTCGAGAAACAAGTGGGCTATTTCTTTGCAAAATTGTGCTCAATTTCATATGTGGCA
ATTCCGCCAAGATCTCTTCGTT---------AGTTGGGGGAAG---AATCCGCCCCAATCAGATT
TTTTGAGGAAC---GTATCGAGAGAGAATTTGATTTGGTTAGACAATGTGTGGTTGGTAA
ACAAGGATCGG---------TTTTTTAGC------AAGGTACAGAATGTATCGTCAAATATTCAATA
TGATTCC---------ACAAGA------TCTAGTTTCGTTCAAGTAACGGATTCTAGCCAATTGAA
AGGATCTTCT---GATCAATCCAGAGATCATTTGGATTCCATTAGT------------AATGAGGAT
TCGGAC---------------------------------------TATCACACATTGATCAATCAAAGAGAGATTCAA
CAACTAAAAGAAAGATCGATTCTT---TGGGAT------CCTTCCTTTCTTCAAACGGAACGA
ACAGAGATAGAATCAGAC------CGATTCCCGAAA------TGCCTTTCTGGATATTCC------TC
AATGTCCCGGCTATTCACG---GAACGTGAGAAGCAGATGATTAATCATCTGTTTCCGGA

AGAAATCGAAGAATTTCTT------GGGAATCCTACAAGATCCGTTCGTTCTTTTTTCTCTG
ACAGATGG------TCAGAACTTCAT---CTGGGTTCGAATCCTACTGAGAGGTCCACTAGAG
ATCAG------------AAATTGTTGAAGAAACAACAAGATCTTTCT---TTTGTCCCTTCCAGGC
GATCGGAA------------AATAAAGAAATGGTTAATATATTCAAGATAATTACGTATTTACAAA
AT------ACCGTCTCAATTCATCCTATTTCATCAGATCCGGGATGTGATATGGTTCCG---AAG
GATGAACCGGAT---ATGGACAGTTCCAATAAGATTTCATTCTTGAACAAAAATCCATTTT
TTGATTTA------TTTCATCTATTCCATGACCGGAACAGGGGAGGATACACGTTA------CAC
CACGATTTTGAATCAGAA------GAGAGATTTCAAGAAATGGCAGATTTATTCACTCTATC
AATAACCGAGCCG---GATCTGGTGTATCATAAGGGATTTGCCTTTTCTATTGATTCCTACG
GATTGGATCAAAAACAA------TTCTTGAATGAGGTATTCAACTCCAGGGATGAATCGAA
AAAGAAATCT---------TTATTGGTTCTACCTCCTATTTTTTAT------GAAGAGAATGAATCTT
TTTATCGAAGGATCAGAAAAAAAATGGGTCCGG---ATTTCCTGCGGGAATGATTTGGAAG
ATTCAAAACCAAAAATAGTGGTATTTGCTAGCAACAACATAATGGAGGCAGTCAATCAA
TATAGATTGATCCGAAATCTGATTCAAATCCAATATAGCACCTATGGG------TACATAAGA
AATGTATTGAATCGATTCTTTTTAATGAAT---------AGATCCGATCGCAACTTCGAATATGG
A---------ATTCAAAGGGATCAAATAGGAAAGGATACTATGAATCATAGAACTATAATGAAA
TATACGATCAACCAACATTTATCGAATTTGAAAAAG---AATCAGAAGAAATGGTTCGAT
CCTCTT------ATTTTTATTTCTCGAACCGAGAGATCCATGAATCGGGATCCTAATGCATATA
GATACAAATGGTCCAATGGGAGCAAGAATTTCCAGGAACATTTGGAACATTTCGTTTCT
GAGCAGAAGAGCCGT---------TTTCAAGTAGTGTTCGATCGATTACGTATTAATCAATATT
CGATTGATTGGTCTGAGGTTATCGACAAAAAAGATTTGTCTAAGCCACTTCGTTTCTTT
TTGTCCAAG---------------------------TCACTTCTTTTTTTGTCC---------------------AAGTTGCTT
TTCTTTTTGTCTAAC---TCACTTCCTTTTTTCTTTGTGAGTTTCGGGAATATCCCCATTCAT
AGGTCCGAG---ATCTACATCTAT---GAATTGAAAGGTCCGAATGATCAACTCTGCAATCA
GTTGTTAGAATCAATAGGTCTTCAAATCGTTCATTTGAAAAAATGGAAACCCTTC------T
TATTGGAT---------GATCATGATACTTCC---CAAAAATCGAAATTCTTGATCAATGGAGGAA
CAATATCACCATTTTTGTTCAATAAGATACCA---AAGTGG---ATGATTGACTCATTCCATA
CTAGA------AATAATCGCAGGAAATCCTTTGATAACACGGATTCCTATTTCTCAATGATAT
TCCACGATCAAGACAATTGGCTGAATCCCGTGAAACCATTTCATAGAAGTTCATTGATA
TCTTCTTTTTATAAAGCAAATCGACTTCGATTCTTGAATAATCCACATCACTTCTGCTTCT
AT------TGTAACAAAAGATTCCCTTTTTATGTG------------GAAAAGGCCCGTATCAAT------
AATTATGATTTTACGTATGGA---CAATTCCTCAATATCTTGTTCATTCGCAACAAA---ATAT
TTTCTTTGTGCGTCGGTAAA------AAAAAACATGCTTTTTGGGGG---AGAGATACTATTTC
ACCAATCGAGTCACAGGTATCTAACATATTCATACCTAACGATTTTCCACAAAGTGGT---
GACGAAACGTATAACTTGTACAAATCT---------TTCCATTTTCCAAGTCGATCCGATCCAT
TCGTTCGT------AGAGCTATTTACTCGATCGCAGACATTTCTGGAACACCTCTAACAGAG
GGACAAATAGTCAATTTTGAAAGAACTTATTGTCAACCTCTTTCAGATATGAATCTATCT
GATTCAGAAGGGAAGAACTTGCATCAATATCTCAAT------TTCAATTCAACCATGGGTTT
GATTCACACTCCATGTTCTGAGAAATATTTACCATCCGAAAAG------AGGAAAAAA-------
-----------------CGGAGTCTTTGTCTAAAG------AAATGCGTTGAGAAAGGGCAGATGTATAG
AACCTTTCAACGAGATAGTGCTTTTTCAACTCTCTCAAAATGGAATCTATTCCAAACATA
TATGCCATGGTTCCTTACTTCGACAGGGTACAAATATCTAAATTTGATATTTTTAGATACT
TTTTCAGACCTATTG------CCGATACTAAGTAGC---AGTCAAAAATTTGTATCCATTTTTCA
TGATATTATGCATGGATCAGATACATCATGGCGAATTCTTCAGAAAAAATGG---------------

--TGTCTTCCACAATGGAATCTG---ATAAGTGAGATTTCGAGTAAGTGTTTACATAAT------
CTTCTTCTGTCCGAAGAAATGATTCATCGAAAT------AATGAGTCACCATTGATATCGACA
CATCTGAGATCTCCAAATGTTCGGGAGTTC---CTCTATTCAATCCTTTTCCTTCTTCTTGT
TGCTGGATAT---CTCGTTCGTACACATCTTCTCTTTGTTTCCCGG------GCCTCTAGTGAGT
TACAGACAGAGTTCGAAAAGGTCAAATCTTTGATGATTCCATCATCTATGATTGACTTA
CGAAAACTTCTGGATAGGTATCCTACATCTGAACCGAATTCTTTCTGGTTAAAGAATCTA
TTTCTAGTTGCTCTGGAACAATTAGGAGATTCTTTAGAAGAAATACGGGGTTCTGCTTC
TGGCGGCAACATGCTATTGGGTGGTGGTCCCGCT---------------TATGGG---GTCAAATCAA
TACGTTCTAAGAAGAAATATTTGAATATCAATCTCATCGATCTCATAAGTATCATA----------
--CCAAATCCCATCAATCGAATCACTTTTTCGAGAAATACAAGACATCTAAGTCATACAA
GTAAAGAGATCTATTCATTGATAAGAAAAAGAAAAAACGTGAACGGGGATTGGATTGA
TGATAAAATAGAATCCTGGGTCGCGAACAGTGATTCGATTGATGATGAAGAAAGAGAA
TTCTTGGTTCAGTTCTCC------ACCTTAACGACA------GAAAAAAGGATTGATCAAATTCT
ATTGAGTCTGACT---CATAGTGATCATTTA---------------TCAAAGAATGACTCTGGTTATCA
AATGATTGAACAACCGGGAGCAATTTACTTACGATACTTAGTTGACATTCAT------AAAA
AGTATCTAATGAATTATGAGTTCAATACATCCTGTTTAGCAGAAAGACGGATATTCCTTG
CTCATTATCAGACAATCACTTAT------TCACAAACCTCGCGTGGGGCTAATAGTTTTCATT
TCCCATCTCATGGA---------AAACCCTTTTCGCTCCGCTTAGCCTTATCCCCCTCT---AGGG
GTATTTTAGTGATAGGTTCTATAGGAACTGGACGATCCTATTTGGTCAAATACCTAGCGA
CAAACTCCTATGTTCCTTTCATTACGGTATTTCTGAACAAGTTCCTGGATAACAAGCCTA
AAGGTTTTCTTATTGAT---------------GATATCGAT---ATTGATGATAGTGACGATATTGATGA
TAGTGACGATATTGATGCTAGTGACAATATTGATGCT------------------------------------AGTGA
CGATATCGATCGTGACCTTGATACGGAGCTG---GAACTGCTAACTATGATGAATGCGCTA
ACTATGGATATGATGCCGGAA---------ATAGACCGATTTTATATCACCCTTCAATTCGAATT
AGCAAAAGCAATGTCTCCTTGCATAATATGGATTCCAAACATTCATGATCTGGATGTGA
ATGAGTCGAATTACTTATCCCTCGGTCTATTAGTGAACCATCTCTCCAGG---------GATTG
TGAAAGATATTCC---ACTAGAAATATTCTTGTTATTGCTTCAACTCATATTCCCCAAAAAG
TGGATCCCGCTCTAATAGCTCCGAATAAATTAAATACGTGCATTAAGATACGAAGGCTTC
TTATTCCACAACAACGAAAGCACTTTTTCACTCTTTCATATACTAGGGGATTTCACTTGG
AAAAGAAAATGTTCCATACT------AATGGATTCGGGTCCATAACCATGGGTTCCAATGCA
CGAGATCTTGTAGCACTTACCAACGAGGCTCTATCGATTAGTATTACACAGAAGAAATC
AATTATAGACACTAATACAATTAGATCCGCTCTTCATAGACAAACTTGGGATTTGCGA----
-----TCCCAGGTAAGATCGGTTCAGGATCATGGGATCCTTTTCTATCAGATAGGAAGGGCT
GTAGCACAAAATGTACTTCTAAGTAATTGCCCCATAGATCCTATATCTATCTATATGAAGA
AGAAA------TCATGTAACGAAGGAGATTCTTATTTGTACAAATGGTACTTCGAACTTGGA
ACGAGCATGAAGAAATTAACGAGACTTCTTTATCTTTTGAGTTGTTCTGCCGGATCGGT
CGCTCAAGATCTTTGGTCTCTACCC------GGACCCGATGAAAAA---AATGGGATCACTTC
TTATGGACTTGTTGAGAATGATTCT---GATCTAGTTCATGGCCTATTAGAAGTAGAAGGC
GCTCTGGTGGGA------TCCTCACGGACAGAAAAAGATTGCAGTCAGTTTTATAATGAT----
--CGAGTGACATTGCTTCTTCGGCCCGAACCAAGGAAT---CCCTTAGATATGATGCAAAA
TGGATCTTGTTCTATCCTTGATCAGAGATTTCTCTATGAAAAATAC------GAATCGGAGTT
TGAAGAA---GGGGAAGGAGAAGGAGCCCTCGACCCGCACCAG------------ATAGAGGAG
GAT------------TTATTCAATCAC------ATAGTTTGGGCTCCT------AGAATATGGCGCCCT-------
--TGGGGCTTTCTATTTGATTGTATCGAAAGGCCCAATGAATTGGGATTTCCC---TATTGG

TCCAGGTCATTTCGGGGCAAG---------CGGATCATTTATGATGAAGAGGATGAGCTTCAA
GAGAATGATTCGGAGTTCTTGCAGAGTGGAACCATGCAGTACCAGACACGAGATAGAT
CTTCCAAAGAACAAGGCCTTTTTCGAATAAGCCAATTCATTTGGGACCCTGCAGATCCA
CTCTTTTTCCTATTCAAA---GATCAGCCCTTTGTCTCTGTGTTTTCACATCGAGAATTATT
TGCAGATGAAGAGATGTCAAAGGGGCTTCTTACTTCCCAAACAGATCCTCCTACATCTA
TATATAAACGCTGGTTTATC------AAGAATACGCAAGAAAAGCACTTCAAATTGTTGATT
AATCGCCAGAGATGGCTTAGA---------ACCAATAGTTCATTATCT------AATGGATCTTTCC
GTTCTAAT------ACTCTATCCGAGAGTTATCAGTATTTATCAAATCTGTTCCTATCTAAC---
GGAACGCTATTGGATCAAATGACAAAGACATTGTTGAGAAAAAGATGGCTTTTCCCGG
ATGAAATGAAAATTGGATTC------ATG---------------------------ATGCCTAGATCGCGG---ATA
AATGGAAATTTTATTGATAAGACCTTTTCAATTGTAGCCAATATCTTATTACGAATAATTC
CGACAACTTCAGGAGAAAAAGAGGCATTTACATATTATAGAGATGGA------ATGTCAGC
TCAATCGGAAGGAAATTATGCGGAAGCTTTACAGAATTATTATGAAGCTATGCGACTAG
AAATTGATCCCTATGATCGAAGTTATATACTCTATAACATAGGCCTTATCCACACAAGTA
ACGGAGAACATACGAAAGCTTTGGAATATTATTTTCGAGCACTAGAGCGAAACCCATTC
TTACCACAAGCTTTTAATAATATGGCCGTGATCTGTCATTAC---CGGGGAGAACAGGCCA
TTCGACAGGGAGATTCTGAAATTGCAGAGGCTTGGTTCGACCAAGCCGCTGAGTATTG
GAAACAGGCTATAGCGCTTACTCCTGGGAATTATATTGAAGCGCATAATTGGTTGAAGA
TCACAGGGCGTTTCGAA------ATGAGTTGGCGATCAGAACATATATGGATAGAACCTATA
ATGGGGTCTCGAAAAGCAAGTAATTTCTGGTGGGCCATTATCCTTTTTTTAGGTTCATTA
GGATTCTTATTGGTTGGAACTTCTAGTTATCTTGGTAGAAATTTGATATCTTTATTTCCGT
CTCAGCAAATTCTTTTTTTTCCACAAGGGATCGTGATGTCTTTCTATGGGATCGCCGGTC
TTTTTATTAGCTCCTATTTGTGGTGCACAATTTCGTGGAATGTAGGTAGTGGTTATGATC
GATTCGATCGAAAAGAAGGAATAGTGTGTATTTTTCGTTGGGGATTTCCTGGAAAAAAT
CGTCGCATCTTCCTCCGATTCCTTATAAAAGATATTCAGTCTGTCAGAATAGAAGTTAAA
GAGGGTATTTATGCTCGTCGTGTCCTTTATATGGACATCAGAGGCCAGGGGGCCATCCC
CTTGACTCGTACTGATGAGAAT---TTTACTCCACGAGAAATTGAGCAAAAAGCTGCTGA
ATTAGCATATTTCTTGCGTGTCCCAATTGAAGTATTT
>Lonicera
CTGAAAAAATTGTGGTTCCATTTGATGTTATTGGAAAGCGATCCTGAGGAGGGTAACGA
TCCGTTCCATGACGAGGAGGGTAACGATAACCCGCATGACTCTGAAAGCGATCCTAGG
TTACCGGCATACTCTGGTGCTCCTGATCTTCTTGAGAAGCGCCTTCTAATATATTTTCCG
AAGGATGATCCTAACGCTACAAGAAAGAAAAATCCCAAAGGCCTTTGTTATTATTTTAG
GCATTGGGGAAGTTATCTTGATAGGGATAGGGATAGGGGGAAGAATAGGGAGAAGGAG
AAGCATATTGCTATGGTTCGCGCTCGACTATATCTATATAAAGATAAGGGCCACACTACA
GACGATCCCAGTTATGAGCGCTTTATAGTTGATCCTAAATATTTTCCGAAGGATAACGAT
AGGGAGAAGATAGACAACTTTTTTAACGAGGCTTCCCATATAGTAAAGAGCTTCTCTAT
AGTAGAGAGATATCCGAGCTATGGAGGTGATTATGGGGGTGATTCTGACTCTGGGGACT
ATGGAAGTGATGAAAGTGATCCGGATTATGGAAGTGATCCGTCCCTTCAAAAAAAAAG
AAATAGGAAGAATTACACTCACAAATACAAGCATTTGTGGCTTATGTGCGATGAGTGTT
ATACATTAAATTATAGGAGATTTTTGAAAGAAAGATTGTATATTTGTGAAGGATGTGGAT
TTCATTTGAAAATGAATAGTTCAGAGAGACTCAAACTTTTGATCGATCCGGATACTTGG
GATCCTATGAATGAAAAGATGGCCTCTCTGGATCCCATTGAATTTCATTCGGAGGAGGA
TCCTTATATAGATCGTGTCAATTCTTATCGAAAAAAGACAGGATTAACTGAGGCTATTCA

AACCGGCCTATGCCAATTAAATGGTATTCCCACAGCAATGGGGGTTATGGAGTTTGGGT
TTATGGGGGGTAGTATGGGATCCGTAGTCGGGGAGAAAATAACCCGTTTGGTTGAGTAT
GCCACTAATCAAGCTCTACCTCTTATTATAGTGTGTGCTTCCGGGGGGGCACGCATGCA
AGAAGGAAGTTTGAGCTTGATGCAAATGGCTAAAATATCTTCTTCTTTATATGATTTTCA
AACAAAGAAAAAGTTATTCTATGTATCAATCCTTACATCTCCTACTACCGGTGGGGTGA
CAGCCAGTTTTGGTATGTTGGGGGATGTCATTGTTGCCGAACCCGATGCCTATATTGCAT
TTGCGGGTAAAAGGGTAATTGAACTAACATTGAATAAAGAAGTACCTGAAGGTTCACA
AGAGACGGAATATTTATTCGAGAAGGGGTTATTCGATCTAGTCATACCACGTAAGAATTT
AAAAAGTATTCTGAATAAGTTATTTGGGTCCCACGGTTTCTTTCCTTTTACTTTGAATCA
AAATCACATGGTAACCATTCGAGCCGACGAAATTAGTAATATTATCCGTCAACGTATTGA
GCAATATAATAGAGAAGTAAAGATTGTAAATACCGGTACCGTACTTCAAGTAGGCGATG
GCATTGCTCGTATTCATGGTCTTGATGAAGTAATGGCGGGTGAATTGGTAGAATTTGGA
GAGGGTACAATAGGCATTGCTCTGAATTTGGAATCCAATAACGTTGGTGTTGTTTTAAT
GGGCGGTGGTTTGATGATACAAGAAGGAAGTTCTGTAAAAGCAACAGGAAGAATTGC
TCAGATACCAGTGAGTGAGGCCTATTTGGGTCGTGTTATAAATGCTCTGGCTAAACCTA
TTGATGGTAGAGGTGAAATTGCATCTTCGGAATATCGGTTAATTGAATCTCCTGCTCCAG
GTATTATTTCGCGACGTTCCGTATATGAACCTCTTCAAACGGGACTTATTGCTATTGATTC
GATGATCCCTATAGGGCGTGGTCAGCGAGAATTAATTATTGGGGACAGACAAACCGGTA
AAACAGCAGTAGCAACAGATACGATTCTTAACCAACAAGGCCAAAATGTAATATGCGT
TTATGTAGCTATTGGTCAAAAAGCATCTTCTGTGGCTCAAGTAGTGACCACTTTCCGAG
AAAAGAGGGCGATGGAATACACTATTGTGGTAGCAGAAACGGCGGATTCCCCTGCTAC
ATTACAATACCTAGCTCCCTATACAGGAGCATCTCTGGCTGAATATTTTATGTATCGTGA
ACGACACACTTCAATTATTTATGATGATCTCTCCAAACAAGCACAAGCTTATCGCCAAA
TGTCTCTTCTATTACGAAGACCACCCGGCCGCGAAGCTTATCCAGGGGATGTTTTTTATT
TACATTCACGCCTTTTGGAAAGAGCTGCTAAATTAAGTTCTCAGTTAGGTGAAGGAAGT
ATGACTGCTTTACCAATAGTCGAAACTCAATCGGGAGATGTTTCGGCTTATATTCCGACT
AATGTTATTTCGATTACTGATGGACAAATATTCTTATCCGCCGACCTATTCAATGCTGGAA
TCAGACCCGCTATTAATGTCGGTCTCTCCGTTTCCAGAGTTGGATCTGCGGCTCAAATT
AAAGCCATGAAACAAGTAGCTGGCAAATTAAAATTAGACCTGGCGCAATTCACAGAAT
TAGAAGTCTTTGCACAATTTTCTTCTGATCTTGAGAAAACGACTCAGAATCAATTGGCA
AGAGGTCGACGATTACGCGAATTGCTCAAACAATCCCAAGCAAACCCTCTCGCGGTGG
AAGAACAGATAATGACTATCTATACCGGAACAAATGGTTATCTTGATTCATTCGAAGTTG
GACAGGTAAGGGGTTTTCTTGACAAGCTACGTGAGTACTTAAAAAATAATAAACCGCA
GTTTCAAGAAATCATATCTTCTACCAAGACTTTCACCGAAGAAGCAGAAGCCCTTTTG
AAAGAAGCTATTCAGGAACAGACGGAGCGTTTTATACTTCAGGAAGAAGTAATGAGAA
TCGATCCGACTACTTCCGGTTCTGGGGTTTCCACGCTTGAAAAAAAAAACTTGGGGCG
TATCGTCCAAATCATTGGTCCGGTACTAGATGTGGTTTTTCCGCCAGGCAAGATGCCTA
ATATTTATAACGCTCTGGTAGTTAAGGGTCGAGATACTGCCGGTCAACCAATTAATGTGA
CTTGTGAGGTACAGCAATTATTAGGAAACAATCGAGTTAGGGCTGTAGCTATGAGTGCT
ACAGACGGTCTAACGAGAGGAATGGAAGTGATTGATACGGGAGCTCCTCTAAGTGTTC
CGGTCGGTGGAGCGACTCTTGGACGAATTTTCAACGTGCTTGGAGAGCCTGTTGATAA
TTTAGGTCCTGTAGATACTCGCACAACATCCCCTATTCATAGATCTGCGCCCGCCTTTAT
AGAGTTAGATACGAAATTATCTATTTTTGAAACCGGAATTAAAGTAGTAGATCTTTTAGC
CCCCTATCGCCGTGGAGGAAAAATAGGACTATTCGGGGGGGCTGGAGTGGGCAAAAC

GGTACTCATTATGGAATTGATTAACAATATTGCCAAAGCTCATGGGGGTGTATCTGTATT
TGGGGGAGTAGGTGAACGTACTCGTGAAGGAAATGATCTTTACAAGGAAATGAAAGA
ATCTGGGGTGATTAATGAACAAAATATTGCAGAATCAAAAGTGGCTCTAGTTTACGGTC
AGATGAATGAACCACCGGGAGCCCGTATGAGGGTTGGTTTGACTGCCCTAACTATGGC
GGAATATTTCCGAGATGTTAATAAACAAAACGTACTTCTATTTATCGACAATATCTTCCG
TTTCGTCCAAGCAGGATCTGAAGTATCTGCTTTATTGGGTAGAATGCCTTCCGCTGTGG
GTTATCAACCTACCCTTAGTACCGAAATGGGTACTTTACAAGAAAGAATTACTTCTACC
AAGAAGGGGTCCATAACTTCTATTCAAGCGGTTTATGTCCCTGCAGACGATTTGACCGA
CCCTGCTCCTGCTACGACATTTGCACATTTAGATGCTACTACCGTACTATCAAGAGGATT
GGCCGCCAAGGGTATCTATCCAGCAGTAGATCCTTTAGATTCAACGTCAACTATGCTAC
AACCTGGGATCGTTGGTAAGAACCATTATGAAACAGCGCAAAGGGTTAAGCAAACTTT
ACAACGTTACAAAGAACTTCAGGACATTATAGCTATCCTTGGGTTGGACGAATTATCCG
AAGAGGATCGTTTAACCGTAGCAAGAGCACGAAAAATTGAGCGTTTCTTATCACAACC
TTTTTTTGTAGCAGAAGTATTTACCGGTTCTCCAGGGAAATATGTTGGTCTAACAGAAA
CAATTAGAGGGTTTCAATTGATCCTTTCCGGAGAATTAGATGTTCTTCCCGAACAGGCC
TTTTATTTGGTAGGTACCATCGATGAAGCTACCGCGAAGGCTATGAACTTAGAAATGGA
GAGCAATTTAAAGAAAATGACCTTAAATCTTTGTGTACTGACCCCTAATCGAATTGTTT
GGGATTCAGAAGTGAAAGAAATCATTTTATCTACTAATAGTGGCCAAATTGGCGTATTA
CCAAATCACGCCCCTATTGCCACAGCTGTCGATATAGGTATTTTGAGAATACGCCTTAAC
GACCAATGGGTAACGATGGCTCTGATGGGTGGTTTTGCTAGAATAGGTAATAATGAGAT
CACTGTTTTAGTAAATAATGCGGAAAAGAGTAGTGACATTGATCCACACGAAGCTCAG
CAAACTCTTGAAATAGCGGAAGCTGGTTTGAAAAAAGCTGAAGGAAAAAGACAAACA
ATTGAGGCAAATCTAGCCCTCCGACGAGCTAGGACACGAGTAGAGACTATCAGGGCGA
TTTCAATGAAAAATGTAACCGATTCTTTCGTTTCTTTGGGTCACTGGCCATCTGCCGGG
AGTTTCGGGTTTAATACCGATATTTTAGCAACAAATCCAATAAATCTAAGTGTAGTGCTT
GGGGTATTGATCTTTTTTGGAAAGGGAGTGTTAAGTGATTTATTAGATAATCGAAAACA
GAGGATTCTGAATGCTATTCGAAATTCAGAAGAACTGCGTGGGGGGGCCATTGAACGG
CTAGAAAAAGCCCGGGCTCACTTACGGAAAGTGGAAATGGAAGCAGATCAGTATCGA
GTGAATGGATACTCTGAGATAGAACGAGAAAGGTTGATTTTGATTAATTCAACTTATAA
GACTTTGGAACAACTAGAAAATGACAACAACGAAACTATTCATTTTGAACAACAAAGG
GCAATTAATCAAGTCCGACAACGGGTTTTCCAACAAGCCTTACAAGGAGCTCTAGGAA
CTTTGAATAGTTGTTTGAACAACGAGTTGCATTTACGTATCATCAGTGCCAATATTGACA
TATTGGGGGCGATGAACGAAATAACCGATATGAATCCACTGATTTCTGCCGCTTCCGTTA
TTGCTGCTGGATTGGCTGTAGGGCTTGCTTCTATTGGACCTGGAGTTGGTCAAGGAACT
GCTGCGGGCCAAGCTGTAGAAGGTATCGCGAGACAGCCCGAGGCAGAGGGAAAAATA
CGAGGTACTTTATTGCTTAGTCTAGCCTTTATGGAAGCTTTAACTATTTATGGCTTGGTT
GTAGCATTAGCACTTTTATTTGCGAATCCTTTTGTTATGAATGTTCTATCATGTTCCATCA
ACACACTAAAGGGGTTATACGATATATCCGGTGTAGAAGTAGGCCAACATTTCTATTGG
AAAATAGGTGGTTTCCAAGTCCACGGCCAAGTACTTATTACTTCTTGGGTTGTAATTGC
TATCTTATTAGGTTCAGCCACTATAGCTGTTCGGAACCCACAAACCATTCCGACCGGCA
GTCAGAATTTCTTCGAATATGTCCTTGAATTCATTCGAGATGTGAGTAAAACTCAAATT
GGAGAAGAATATGGTCCTTGGGTTCCTTTTATTGGAACTATGTTTCTATTTATTTTTGTTT
CTAATTGGTCAGGAGCTCTTTTGCCTTGGAAAATCATACAATTACCTCACGGGGAGTTA
GCCGCACCCACGAATGATATAAATACTACTGTTGCTTTAGCTCTACTCACGTCAGTGGC

ATATTTCTATGCGGGTCTTAGCAAAAGGGGATTGGGTTATTTTGGGAAATATATTCAACC
AACTCCAATCCTTTTACCTATTAACATATTAGAAGATTTCACAAAGCCTTTATCACTCAG
TTTTCGACTTTTCGGAAATATATTAGCTGATGAATTGGTAGTTGTTGTTCTTGTTTCTTTA
GTACCTTCAGTGGTTCCTATACCTGTCATGTTCCTTGGATTATTTACAAGTGGTATTCAA
GCTCTTATTTTTGCAACTTTAGCCGCGGCTTATATAGGTGAATCCATGGAGGGTCATCAT
ATGAGATTTTTAACTTTAGAGCATATATTAACTCATATTTCCTTTTCAACCGTTTCAATTG
TAATTACAATTCATTTGATAACCTTATTAGCCGATAAAATTAGAAAGCTATATGATTCGTC
CGCAAAGGGCATGATAGCGGCTTTTTTATGTATAACGGGATTATTAGTCACTCGTTGGGT
TTATTCAGGACATTTCCCATTAAGTGATTTATATGAATCATTAATCTTCCTTTCGTGGAGT
TTTTCAGTTTTTCATATAGTTCCGTAT------AAAAAGAAACAAAATTTAAGCACAATAACT
GGGTCAAGTGTTATTTTTACCCAAGGCTTTGCTACTTCAGGTATTTTAGATGAAATACAT
CAATCCACAATATTAGTACCTGCTCTCCAATCCGAGTGGTTAATAATGCACGTAAGTATG
ATGATATTGGGCTATGCAGCTCTTTTATGTGGATCATTATTATCAGTAGCACTTCTAGTCA
TTACATTTCGAAAAAACAGAAAGATTTTTTGT------AAAGAAATCCAATACATGAATGAA
AGGGGGAATGTTTTACGAAAGACTTCT---------GCTAAGAATTATTACAGGTGTCAATTGA
TTCAACAGTTGGATTATTGGAGTTATCGTATAATTAGTCTAGGGTTTCTCTTTTTAACCAT
AGGTATTCTTTCAGGAGCAGTATGGGCTAATGAAGCATGGGGATCATATTGGAATTGGG
ACCCAAAGGAAACTTGGGCATTTATTACTTGGACCGTATTCGCGATTTATTTACATACTC
GAACAAATAAAAAGCTACGGGGTACAAATTCTGCAATTGTGGCGTCTATAGGATTTCTT
ATAATTTGGATATGCTATTTTGGGGTCAATCTATTAGGAATAGGACTACATAGTTATGGTT
CATTTAATTTAGGTATGGCAAAAAAGAAAACGTTCACTCCTCTTTTATATCTCGCATCTC
TTGTCTTTTTGCCCTGGTGGATTTCTCTCTCAGTTAATAAAAGTTTGGAATCTTGGGTTA
CTAATTGGTGGAATACTAGGCAATCCGAAATCTTTTTGAATGATAGTCAAGAAAAGAGT
ATTCTAGAAAAATTCATAGAATTAGAGGAACTCGCCCTCTTGGACGAAATGATCAAGG
AATACTCGGAGACACATCTACAAAACCTTCGTATA---ATCCACAAAGAAACGATCCAAT
TAATCAAGATACACAACGAAGATCATATCCGTATGATTTTGCACTTCTCGACAAATATAA
TCTGTTTCATTATTCTAAGCGGTTATTCTATTTTGGGTACTGAAGAACTTGTTATTCTTAA
CTCTTGGGCTCAGGAATTCCTATATAACTTAAGTGACACAATAAAAGCTTTTTCTATTCT
TTTAGTAGTCGATTTTTGGGTCGGATTCCATTCGCCCCATGTTTGGGAACTAATGATTGG
CTCTGTCTACAAAGATTTTGGATTTGTTCATAATGATCAAATTCTATCTGTTCTTGTTTCC
ACTATTCCAGTCATTCTAGATACCCTTTTTAAATATTGGCTTTTCTTTTATTTAAATCGCGT
ATCTCCGTCACTTGTAGTGATTTATCATTCAATGAATGACATGCCTGTAGGTGTTCCAAA
AATACCCTTTCAAATTTCTGGAGACGAAGAAGCGTCTTGGGTTGACGTATACAACGGA
CTTTATCGAAGGGGATTCCTTTTTATAGTCCAAGAGATTGATACCGAACTCTCAAATCA
AATTGTAGGTCTTATGGTATTTCTCAGTTTGGAGGGTGAGGTAAACGATCTGTATTTCTT
TATATCCTCTCTCGGCGGATCCATGGTAGATGGATTCGCTATTTTTGATATGCTGGTATAT
TTGCCACCAACGATGCATACATTGAGTATCGGAGAATGCGCCTCAATGGCATCTTTGAT
CGTGGCCGGAGGAACATTTACCCAACGTATAGCATTCCCTCACAGTAGGATAATGATCC
ATCAACCTAATACTTTTCCTTTTGAGACACCAACGGGAGAAACTTTCCTCGAAATAAAG
GAATTCTGTCGCCTGCGTGCGTTGGTCGGAGAGGCTTATGCAAAAATAACGGGCCAAC
CCCTTTGGGTTGTAGAGATAGACCTGGAAAGAGATATTTTTATGTCAGCACAAGAAGCC
AAAGATTATGGAATTGTTGATCTTGTAGCGAGTGGCCAAAAAGACCCAAAACGTATTCA
TGAGGGTTTAATTACGGAATCACTTCCAAATGGTATGTTCCGAGTTCATTTAGATAATGG
AGATCTGATTATAGGTTATATTTCAGGAAAGATCCGACGTAGTTTTATACGGATACTTCC

AGGCGATAAAGTCCAAGTTGAAGTAAGTCGTTATGATTCAACCAGAGGGCGTATAATTT
ATCGGCTTCGCAACAAGGACTCGAAAGATATGGAGGAATTCAAAAGAGATTTCGAG---
GCTGGGTCTCAACAACATTACTTCTTATATCCACTTATCTTTCAGGAGTATATTTATGCAC
TTGCTTATGATCATGGTTTAGTAGGGTATGACAATAAATCTAGCTTACTATTTGTGAAAC
GTTTAATTACTCGAATGTATCAAAAGAATCATTTGATTTTTTCTGTTAAGGATTCTAACC
AAAATGTATTTTTGGGGAAGAATTTGTATTCTCAAATGATATCAGAGGGGTTTGCAGTC
ATTGTAGAAATTCCATTTTCTCTACGCTTTTTATCTTCCCTAAGAGTCAAATCTCAGAAT
TTACGATCCATTCATTCAGTATTTCCTTTTTTAGAGGACAAATTTTCACATTTAAACTATG
TATTAGATATACTAATACCTTACTCAGCCCATTTGGAAATTCTGGTTCAAACTCTTCGCTA
CTGGGTAAAAGATGCCCCCTCTTTGCATTTATTACGATTCTTTCTCCACGAGTATCCTAA
TTTGACTAGTCTTATTATTAAAACCGGTTCTTCTTTTTCAAAACAAAGATTATTTTTCTTC
CTATATAATTCTCATGTATGTGAATACGAATTCATCTTCGTCTTTCTCCGTAACCAATCTT
CTCATTTACGATCAACATCTTCTGGAGCCCTTCTTGAACGAATATATTTTTATGAAAAAA
TAGAACATCTTGTAGAAGTCTTTGCTAAGGATTTTAATCTATGGTTGTTCAAGGATCCTT
TCATGCATTATGTTAGGTATCAAGGAAAGTCAATTTTCGCTTCAAAGGGGACCTTTCTTT
TGATGAATAAATGGAAATATTACTTTGTACGTTTCTGGCAATGTCATTTTTACCAGTGGT
TTCAACCAGAAAGGATTTCTATAAACCAATTATCCAAACATTCGCTCGACCTTCTAGGTT
ATCTTTCAAGTGTGCGGCTAAACCCCTTAATGGCGCGCAGTCAAATGCTGGAAAATGC
ATTTCTAATCGATAATGCTGGT------AAGAAGTTCGATACCATTGTTCCAATTAGTCCTCT
GATTGGATCATTGGCTAAAGCGAAATTTTGTAACGTATTAGGGCATCCTGTTAGTAAGG
TGCTTTGGGCCGATTTATCAGATTCTGAGATTATTGACCGATTTGGGCGGATATGCAGAA
ATCTTTCTCATTATCATAGCGGATCTTCACAAAAAAAGAGTTTGGATCGAATAAAGTATA
TACTTCGACTTTCTTGTGCTAGAACTTTGGCTCGTAAACACAAAAGTACTGTACGTGCT
TTTTTGAAAAGATCAGGCTCGGAATTCTTAGAAGACTTCTTTACGGCGGAAGAACAGG
TTCTTTTCTTGACTTTACCAAGAACGATTTGGTATTTGGATATTATTTGTATCAATGATTT
GGCCAATCATGAAATGATAATTGAT---ACAACAGAAGTACAA---GCTATCAATTCTTTTTC
GAGATTGGAATCC---TTAAAAGAAGTCGATGGGATCATATGGATGCTTGTTCCTATTTTC
AGTCTTGTATTAGGAATCACAATAGGTGTACTAGTAATTGTTTGGTTAGAAAGAGAAAT
ATCTGCAGGGATACAACAACGTATTGGACCCGAATACGCCGGTCCTTTTGGAATTCTTC
AAGCTCTAGCAGATGGTACAAAATTACTTTTCAAAGAGAATCTTCTGCCATCTCGAGGA
GATATTCGTTTATTCAGTATCGGACCATCCATCGCAGTCATATCAATTCTACTAAGTTATT
TAGTAATTCCTTTTGGCTATCATCTTGTTCTAGCTGATCTCAGTATCGGTGTTTTTTTATG
GATTGCAATTTCAAGTATTGCTCCCATTGGACTTCTTATGTCAGGATATGGATCAAATAAT
AAATATTCTTTTTTAGGCGGTTTACGAGCTGCTGCTCAATCAATTAGTTATGAAATCCCA
TTAACTCTATGTGTGTTATCAATATCTCTACTATCTAACAGTTCAAGTACAGTTGATGTAG
TTGAAGCCCAATCAAAATATGGTTTTTGGGGATGGAATTTGTGGCGTCAACCTATAGGG
TTTATCGTTTTTCTAATTTCTTCCCTAGCCGAGTGTGAGAGATTACCTTTTGATTTACCAG
AAGCAGAAGAAGAATTGGTAGCAGGTTATCAAACCGAATATTCAGGTATCAAATTTGG
TTTATTTTATGTTGCTTCGTATTTGAATCTCTTAGTTTCTTCATTATTTGTAACAGTTCTTT
ACTTGGGGGGTTGGAATCTTTCCATTCCGTACATATTAGTTCCTCCGCTTTTTGAAATAA
ATAAAGAGAGTCGAGTCTTTGGAACAATAATGGGTATCTTTATTACATTAGCTAAGACTT
ATTTGTTCTTGTTCATTCCTATCATAACAAGGTGGACTTTGCCGAGGTTAAGAATGGAC
CAACTATTAAATCTCGGGTGGAAATTTCTTTTACCTATTTCTCTAGGTAATCTATTATTAA
CAACCTCTTCCCAACTTCTTTCACTGATGATCTGGCATATACAGAATGAAAACTTCATTC

TCGATTCTACGAGAATTTTTATGAAAGCCTTTCATTTGCTTCTCTTCGATGGAAGTTTGA
TTTTCCCAGAATGTATCCTAATTTTTGGCCTAATTCTTCTTCTGATGATCGATTCAACCTC
TGATCAAAAAGATATACCTTGGTTATATTTCATCTCTTCAACAAGTTTAGTAATGAGCAT
AACGGCCCTCTTGTTCCGATGGCGAGAAGAACCTATGATTAGCTTTTCGGGAAATTTCC
AAACGAACAATTTCAACGAAATCTTTCAATTTCTTATTTTACTATGTTCAACTCTATGTAT
TCCTCTATCCGTAGAGTACATTGAATGTACAGAAATGGCTATAACAGAGTTTCTCTTATT
CGTATTAACAGCTACTCTAGGAGGAATGTTTTTATGCGGTGCTAACGATTTAATAACTAT
CTTTGTAGCTCCAGAATGTTTCAGTTTATGCTCCTACCTATTATCTGGATATACTAAGAAA
GATGTACGGTCTAATGAGGCTACTATGAAATATTTACTCATGGGTGGGGCAAGCTCTTCT
ATTCTGGTTCATGGTTTCTCTTGGCTATATGGTTTATCCGGGGGAGAGATCGAGCTTCAA
GAAATAGTGAATGGTCTTATCAATACACAAATGTATAACTCCCCAGGAATTTCAATTGCG
CTCATATTCATCACTGTAGGAATTGGGTTCAAGCTTTCCCCAGCCCCTTCTCATCAATGG
ACTCCTGACGTATACGAAGGATCTCCCACTCCAGTCGTTGCTTTTCTTTCTGTTACTTCG
AAAGTAGCTGCTTCAGCTTCAGCCACTCGAATTTTTGATATTCCTTTTTATTTCTCATCA
AATGAATGGCATCTTCTTCTGGAAATCCTAGCTATTCTTAGCATGATATTGGGGAATCTC
ATTGCTATTACTCAAACAAGCATGAAACGTATGCTTGCATATTCGTCCATAGGTCAAATC
GGATATGTAATTATTGGAATAATTGTTGGAGACTCAAATGATGGATATGCAAGCATGATA
ACTTATATGCTGTTCTATATCTCCATGAATCTAGGAACTTTTGCTTGCATTGTATTATTTGG
TCTACGTACCGGAACTGATAACATTCGAGATTATGCAGGATTATACACGAAAGATCCTTT
TTTGGCTCTCTCTTTAGCCCTATGTCTATTATCCCTAGGAGGTCTTCCTCCACTAGCAGG
TTTTTTCGGAAAACTCTATTTATTCTGGTGTGGATGGCAGGCAGGCCTATATTTCTTGGT
TTTAATAGGACTCCTTACAAGCGTTGTTTCTATCTACTATTATCTAAAAATAATCAAGTTA
TTAATGACTGGACGAAACCAAGAAATAACTCCTCACGTGCGAAATTATAGAAGATCTC
CTTTCAGATCAAAGAATTCCATCGAATTGAGTATGATTGTATGTGTGATAGCATCTACTAT
ACCAGGAATATCAATGAACCCGATTATTGCAATTGCTCAGGATACCCTTTTTATGTTTCT
GCTTTACGAATATGATATTTTTTGGACATTTCTAATAATCTCAAGTCTTATTCCTATTTTGG
CATTTTTCATTTCCGGAGTTTTAGCCCCGATTAGCAAAGGGCCGGAAAAACTTTCTAGT
TATGAATCGGGTATAGAACCAATGGGTGACGCTTGGGTACAATTTAGAATCCGCTATTAT
ATGTTTGCCCTAGTTTTTGTTGTTTTTGATGTTGAAACGGTTTTTCTTTATCCATGGGCA
ATGAGTTTCGATGTATTGGGTGTATCTGTATTTATAGAAGCTTTCATTTTCGTGCTTATCT
TAATTGTTGGTTCAGTTTATGCATGGCGAAAGGGGGCATTGGAATGGTCTACGAATGAT
TTTCCTTGGTTAACAATAATTGTAGTTTTGCCAATATCTGCGGGTTCCTTAATTTGCTTTC
TTCCCCATAGAGGGAATAAGGTAATTAGGTGGTATACCATATGTATATGTGTTTTAGAAC
TTCTTCTAACAACTTATGCATTCTGTTATCATTTCCAATCGGACGATCCATTAATCCAATT
AGTGGAGGACTATAAATGGATCCATTTTTTTGATTTCCATTGGAGATTAGGAATAGACG
GACTTTCTATAGGACTCATTTTACTAACGGGATTCATCACTACTTTAGCTACTTTAGCGG
CTCGGCCAGTTACTCGAGATTCTCGATTCTTCCATTTCCTGATGTTAGCAATGTACAGCG
GTCAAATAGGATCATTTTCTGCTCGGGACCTTTTACTTTTTTTCATCATGTGGGAGTTAG
AACTCATTCCGGTTTATCTCCTTCTATCTATGTGGGGAGGAAAGAAACGTCTGTACTCG
GCTACAAAATTTATTTTGTACACGGCGGGGGGTTCGGTTTTTCTCTTAATGGGAGTTCTT
GGTGTCGGTTTATATGGTTCGAATGAACCAACATTAAATTTTGAAACATCAGTTAATCA
GCGGTATCCTGTGGCCTTGGAAATAATATTCTATATTGGATTTTTTATTGCTTTTGCTGTA
AAATCGCCGATTATACCCCTACATACATGGTTACCAGATACCCATGGAGAAGCACATTAC
AGTACTTGTATGCTTCTAGCCGGAATCTTATTAAAAATGGGAGCGTATGGATTAGTTCGG

ATTAATATGGAATTATTACATCATGCTCATTCTATATTTTCTCCTTGGTTAGTGATAGTAGG
CACAATACAAATAATCTATGCAGCTTCAACATCTCCCGGCCAACGTAATTTAAAAAAAA
GAATAGCCTATTCCTCTGTATCTCATATGGGTTTCATACTTATAGGAATTGGTTCTATAAC
CGATACGGGACTCAATGGAGCCATTTTACAAATAATATCTCATGGATTTATTGGTGCTGC
ACTTTTTTTCTTGGCAGGAACAGGTTATGATAGAATACGTCTTGTTTATCTTGACGAAAT
GGGCGGAATAGCTATTCAAATGCCAAAAATCTTCACGATGTTCAGTACCTTTTCAATGG
CCTCCCTTGCATTACCCGGTATGAGTGGTTTTGCTGGAGAATTCATAGTCTTTTTTGGCA
TAATTACCAGCCAAAAATATCTTTTAATGCCAAAAATACTCATTACTTTTGTAATGGGAA
TTGGAATGATATTAACCCCCATTTATTCATTAGCTATGTCACGCCAAATGTTCTATGGATA
TAAGTTATTTAATGCTCCAAATTCTTATGTTTTTGATTCTGGACCGCGAGAGTTATTTGTT
TCGATTTCTATCTTTCTACCCATATTAGGTATTGGTATGTACCCCGATTTTGTTCTTTCACT
ATCAGTTGATAAGGTCGAAGTTATTCTATCCAATTTTTTTTATAGAATGATGCTCGAACAT
GTACTTGTTTTGAGTGCCTATTTATTTTCTGTCGGTCTCTATGGATTGATCACGAGTCGA
AATATGGTTAGGGCCCTTATGTGTCTTGAACTTATATTGAATGCAGTTAATATCAATTTTG
TAATATTTTCTGATTTTTTTGATAGTCGCCAATTAAAAGGAGCCATTTTTTCCATTTTTGT
TATAGCTATTGCGGCCGCTGAAGCAGCTATTGGACTAGCTATTGTTTCATCAATTTATCGT
AATAGAAAATCAACTCGTATTAATCAATCGAATTTGTTGAATAAGATGGAACAGACATC
TCAATATGCATGGATAATACCCTTCTTGCCACTTCCAGTTCCTATGCTAATAGGACTGGG
ACTTCTTCTTTTTCCGACAGCAACAAAAAATTTTCGTCGTATGTGGGCTTTTCAGAGTG
TTTTATTATTAAGTATAGTCATGATTTTTTCGATGAATCTATCTATTCAGCAACTAAATAGC
AGTTCTATCTATCAATATGTATGGTCTTGGATCATCAATAATGATTTTTCTTTTGAATTAG
GCTACTTGATTGATCCACTTACTACTATTATGTCAATATTAATCACTACTGTTGGAATTAT
GGTTCTTGTTTATAGTGATAATTATATGTCTCATGATCAAGGATATTTAAGATTTTTTGCTT
ATATGAGTTTTTTCAGTAGTTCTATGTTGGGATTAGTTACTAGTTCTAATTTGATACAAAT
TTATATTTTTTGGGAATTAGTTGGAATGTGTTCGTATCTATTGATAGGGTTTTGGTTCACA
CGACCTGTTGCAGCAAATGCTTGTCAAAAAGCGTTTGTAACTAATCGTGTTGGGGATTT
TGGTTTATTATTAGGAATTTTAGGTTTTTATTGGCTAACGGGCAGTTTCGAATTTAGGGA
TTTATTCGAAATATTCAATAACAATGAGGTCCATTTTTTATTTGTTATTTTGTGTGCCGTT
CTTTTTTTTGCAGGCGCAGTTGCTAAATCTGCACAATTTCCCCTCCATGTATGGTTACCC
GATGCCATGGAGGGCCCTACTCCTATTTCGGCTCTTATACATGCTGCTACTATGGTAGCA
GCGGGAGTTTTTCTTGTAGCTCGGCTTCTTCCTCTTTTCATAATCATACCTTACATAATGA
ATTTAATCTCTTTACTAGGGATAATAACAGTATTATTAGGGGCTACTTTAGCTCTCGCTCA
AAAAGATATTAAGAGGGGTTTAGCCTATTCCACAATGTCTCAATTGGGATATATGATGTT
AGCTCTAGGTATGGGGTCTTATCGCAGTGCTTTATTTCATTTAATTACTCATGCTTTTTCG
AAAGCATTATTGTTTTTAGGATCTGGATCCATTATTCATGCAATGGAAACTATTGTTGGAT
ATTCGCCAAATAAAAGTCAGAATATGGCTCTTATGGGAGGTTTAACAAAACATGTGCCG
ATTACCAAAACCGCTTTTTTATTAGGTACACTTTCTCTTTGTGGTATTCCACCCCTTGCTT
GTTTTTGGTCCAAGGATGAAATTCTTAATGATAGTTGGTTGTATTCACCGATTTTCGCAA
TAATAGCTTGGTCTACAGCGGCATTAACCGCATTTTATATGTTTCGGATCTATTTACTTAC
TTTTGAAGGACACTTAAACGCTAATTTTCAAAATTACAGTGGAAAAAAAAATAAACCC
TTCGATTCAATATCTCTATGGGGTAAAGAAGGTTCGAAAAGGATTAAAAAA---TTATTAA
CTTTATTAACAATAAATTTTTTTTCATTGGGGAATAAGAAGTCTTATTTATATCCTTATGA
ATCGGATAATACTATGTTATTCCCAATACTTGTATTGGGTCTATTTACTTTGTTCGTTGGAT
TCCTAGGAATTCCTTTCAATTTAGATATATTATCAAAATGGTTAACCCCATATATAAACCT

TTTACATCCAAATTTGAAT---TCAATGGATTGGTATGAATTTGCGAAAGATGCAGTTTTTT
CAGTCAGTATAGCCTATTTCGGAATAGTTATAGCGTCCCTTTTATATAAACCTGTTTATTC
ATCTTTTCAAAATTTGTACTTAATTAATTCATTTGTTATTCTTTCGGACAAAATCATAAAT
GGTCTATATGATTGGTCATATAATCGTGGTTACATAGATGCTTTTTATGCAACATCCTTAA
CCGGGGGTATAAGAGGATTGGCGGAATTTACTCATTTTTTTGATAGACGAGTAATTGAT
GGAATTACGAATGGAGTTGGTATTATGAGTTTCTTTGTAGGAGAGGGTATCAAATCTGT
TGGGGGCGGG---CGTATCTCTTCTTATCTTTTCTTGTATTTATCGTATGTATCCATTTTTTT
ATTAATTTACTACATGGATTTACCTGGACCAATACATGATTTTCTTTTAGTCTTTCTGGGA
TTGGGTTTTATATTAGGAGGTCTAGGAGTGGTGTTACTTCCCAATCCAATTTATTCTGCC
TTTTCGCTGGGATTGGTTCTTGTTTGTATATCCTTATTCTATATTCTATCGAATTCCTATTT
TGTCGCTGCTGCGCAGCTCCTTATTTACGTAGGGGCCATAAATGTTTTAATCATTTTTGC
TGTGATGTTCATGAAGGGTTCAGAATATTACAAGGATTTTCATCTTTTGGCCGTTGGGG
ATGGGCTTACTGCGATGGTTTGTACAAGTCTTTTTGTTTCACTAATTACTACTATTCCAG
ATACGTCGTGGTACGGGATTCTTTGGACTACAAAATCAAGCCAAATTATAGAGCAAGAT
TTGATAAGTAATAGTCAACAAATTGGGATTCATTTATCAACAGATTTTTTTCTTCCGTTT
GAACTCATTTCAATAATTCTTTTAGTTTCGTTAATAGGTGCAATTGCTGTAGCTCGTCAA
ATGACTACGCCAGTTACAAGAAAAGACCTCATGATAGTCAATATGGGTCCTCACCACCC
ATCAATGCATGGTGTTCTTCGACTCATTGTTACTCTAGATGGAGAAGATGTTATTGACTG
TGAGCCAGTATTGGGTTATTTACATAGAGGTATGGAAAAAATTGCGGAAAACCGAACA
ATTATACAATATTTGCCTTATGTAACACGTTGGGATTATTTAGCTACTATGTTCACCGAAG
CAATAACTGTAAATGCACCGGAGCAGTTAGGCAATATTCAAGTACCTAAAAGGGCCAG
CTATATCAGAGTCATTATGCTGGAGTTAAGTCGTATAGCTTCGCATTTGTTATGGCTTGG
CCCTTTTATGGCAGATATTGGGGCACAGACCCCTTTCTTCTATATTTTTCGAGAAAGAGA
ATTGATATATGACCTATTCGAAGCTGCCACCGGTATGCGAATGATGCATAATTTTTTTCGT
ATCGGAGGGGTAGCTGCTGATTTACCTCATGGATGGATAGATAAATGTTTGGATTTTTGC
GATTATTTTTTAACAGAGGTTGCTGAATATCAAAATCTTATTACACGCAATCCTATTTTTT
TAAAACGAGTTGAAGGAGTAGGCATTATTGGCGGAAAGGAGGCAATAAATTGGGGTTT
ATCGGGACCAATGCTACGAGCTTCCGGAATACAATGGGATCTTCGTAAAGTTGATCAGT
ATGAGTGTTACGACGAGTTCGATTGGGAAGTCCAATGGCAAAATGAGGGGGATTCATT
AGCTCGTTATTTAGTACGAATCAATGAAATGGCAGAATCCATAAAAATGATTCAACAGG
CTCTAGAAGGAATTCCGGGGGGGCCCTATGAGAATTTAGAAATCCGACGTTTTGATCCA
CTAAGAGATCCGAAATGGAATGATTTTGACTATCGATTTATTAGTAAAAAACCTTCTCCG
ACTTTTGAATTGTCGAAGCAAGAACTTTATGTGAGAGTTGAAGCCCCAAAAGGAGAAT
TGGGAATTTTTCTGATAGGGGATAAGAATGTTTTTCCTTGGAGATGGAAAATCCGACCA
CCGGGTTTTATCAATTTGCAAATTCTTCCTCAGCTAGTTAAAAGAATGAAATTGGCTGAT
ATTATGACGATATTAGGTAGCATAGATATCATTATGGGAGAAGTTGATCGTATGTTCTCTA
TGATAACTGAGTTAGTGAATTATGGTCAACAAACAGTACGAGCTGCCAGATACATTGGT
CAAAGTTTCACGATTACTTTATCGCATGCGAATCGTTTACCCGTAACTATTCAATACCCC
TACGAAAAATTGATCACATCAGAGCGTTTCCGCGGCCGAATCCATTTTGAATTTGATAA
GTGCATTGCTTGTGAAGTATGTGTTCGCGTATGTCCTATAGATCTACCCATTGTTGATTG
GAAATTGGAAACGGATATTCGAAAGAAACGATTGCTTAATTACAGTATTGATTTTGGAA
TCTGTATATTTTGTGGTAATTGCATTGAGTATTGTCCAACAAATTGTTTATCAATGACTGA
AGAATATGAACTTTCTGTGTATGATCGTCACGAATTGAATTATAATCAAATTGCTTTGGG
TCGGTTACCAATGTCAGTAATCGACGATTACACAATTCGAACAATTTTGAATTTG---------

---------------------ATGCAGGGTCATTTGTCTACTTGGCTAGTCAAGCATGGG
CTAATTCATCGATCTTTGGGCTTTGATTACCAAGGAACGGAGACTTTACAAATAAAACC
CGAGGATTGGCATTCCATTGCTGTCATTTTATATGTATATGGTTACAATTATCTACGCTCC
CAATGTGCCTATGATGTAGCACCTGGCGGACTGTTAGCAAGTGTGTATCATCTTACGAG
AATAGAGTATGGCGTGGATCAACCAGAAGAGGTATGCATAAAAGTATTTGCCCCGAGG
AGGAATCCTAGAATTCCGTCTGTTTTCTGGGTTTGGAAAAGTGTAGATTTTCAAGAACG
GGAATCTTATGATATGTTGGGAATCTCTTATGATAATCATCCACGTTTGAAACGTATCTTA
ATGCCTGAAAATTGGATAGGATGGCCTTTACGTAAGGATTATATTGCCCCCAATTTTTAT
GAAATACAAGATGCTCAT--
-----------------ATGGTCTTAGCTCCTGAATATTCAGACAATAAACAGAAAAATG
AAAAAAATAAGATTGACACAGTTATGAATTCCATTGAGTTTCCCTTACTTGATCAAACA
ACCCAAAATTCAGTTATTTCAACTACATTAAATGATCTTTCAAATTGGTCAAGACTCTCT
AGTTTATGGCCGCTTCTCTATGGTACCAGTTGTTGCTTCATTGAATTTGCTTCACTAATA
GGCTCACGATTCGACTTTGATCGTTATGGACTAGTACCAAGATCAAGTCCTAGACAAGC
GGACCTAATTTTAACAGCCGGAACGGTAACAATGAAAATGGCTCCTTCTTTAGTGAGAT
TATATGAGCAAATGCCTGAACCAAAATATGTTATTGCTATGGGGGCATGTACAATTACAG
GGGGGATGTTCAGTACCGATTCTTATAGTACTGTTCGGGGAGTCGATAAGCTAATTCCG
GTGGATGTCTATTTGCCGGGCTGTCCGCCTAAACCGGAAGCGGTTATCGATGCTATAAC
AAAACTTCGTAAGAAAATATCTCGAGAAATCTATGAAGATAGAATTAGGTATCAACAGG
AAAATCGGTGTTTTACTACCAATCACAAGTTTCAGGTTTGGCGTAGTAGTCATACTGGA
AATTATGATCAAGGATTCCTTTATCAAGTGCCACCTACTTCAGAGATCCCT---GAAACAT
TTTTCAAATACAAAGGTTCAGTATCTTCCCACGAATTGGTGAATATGCAAACCAGAAAT
ATTTTTTCTTGGATAAAGGAACAGAGTACTCGATCCATTTCCGTATTGCTCATGATATATA
TCATAACTCGGACATCCATTTCAAGCGCATATCCCATTTTTGCACAGCAGGGTTATGAA
AATCCACGCGAAGCGACGGGGCGTATTGTATGTGCCAATTGCCATTTAGCTAATAAGCC
CGTGGATATTGAGGTTCCACAAGCGATACTTCCTGATACTGTATTTGAAGCGGTTGTTC
GAATTCCTTATGATATACAACTGAAACAGGTTCTTGCTAATGGTAAAAAGGGGGCTTTG
AATGTGGGTGCTGTTCTTATTTTACCGGAGGGGTTTGAATTAGCCCCTCCCGATCGTATT
TCTCCCGAGATGAAAGAAAAGATAGGCAATCTGTCTTTTCAGAGCTACCGCCCCAATA
AAAAAAATATTCTTGTGATAGGCCCTGTTCCTGGTCAGAAATATAGTGAAATCACCTTT
CCTATCCTTTCCCCGGACCCAGTTACTAAGAAAGATGTTCACTTCTTAAAATATCCTATA
TATGTAGGCGGAAACAGGGGAAGGGGTCAGATTTATCCCGACGGGAGCAAAAGTAAC
AATACAGTTTATAATGCTACAGCAGCGGGTATAGTAAACAAAATTATACGAAAAGAAAA
AGGGGGGTACGAAATAACTATAGCGGATACATCGGATGGACATCAAGTGGTTGATATTA
TCCCTCCAGGGCCAGAACTTCTTGTTTCAGAAGGCGAATCTATCAAATTTGATCAACCG
TTGACAAGTAATCCTAATGTGGGCGGATTTGGTCAGGGAGATGCAGAAATAGTACTTCA
AGATCCATTACGTGTCCAAGGCCTTTTGTTCTTCTTGGCATCTGTTATTTTGGCACAAAT
CTTTTTGGTTCTTAAAAAGAAACAGTTCGAGAAGGTTCAATTGTCCGAAATGAATTTCA
TGAGTAAAGTTTATGATTGGTTTGAAGAACGTCTCGAGATTCAGGCAATTGCGGATGAT
ATAACTAGTAAATATGTTCCTCCCCATGTCAACATATTTTATTGTCTAGGAGGAATTACGC
TTACTTGTTTTTTGGTACAAGTGGCTACGGGGTTTGCTATGACTTTTTACTATCGTCCGA
CCGTTACTGACGCTTTTGCTTCTGTTCAATACATAATGACTGAAGCTAATTTTGGTTGGT
TAATCCGATCTGTTCATCGATGGTCGGCAAGTATGATGGTTCTAATGATGATCTTACACG
TATTTCGCGTGTATCTCACCGGTGGATTTAAAAAACCTCGAGAATTGACTTGGGTTACT

GGGGTGGTTCTGGCTGTATTGACCGCATCTTTTGGCGTAACCGGTTATTCCTTACCTTGG
GACCAAATTGGTTATTGGGCAGTGAAAATTGTAACAGGTGTCCCTGAAGCTATTCCTGT
AATAGGACCTCCTTTGGTAGAATTATTGCGCGGGAGTGCTAGTGTGGGACAATCCACTT
TGACTCGGTTTTATAGTTTACACACTTTTGTATTACCACTTCTTACTGCGGTATTTATGTT
AATGCACTTTCCGATGATACGTAAACAAGGCATTTCTGGTCCTTTAATGGGAGTAACAA
AAAAACCTGACCTGAATGATCCTGTATTAAGAGCGAAATTGGCTAAAGGTATGGGTCAT
AATTATTACGGAGAGCCCGCGTGGCCTAATGATCTTTTATATATTTTTCCTGTAGTCATTC
TAGGTACTATTGCATGTAACGTAGGCTTAGCGGTTCTAGAACCATCAATGATTGGTGAA
CCAGCAGATCCATTTGCAACCCCTTTAGAAATATTACCCGAATGGTATTTCTTTCCCGTA
TTTCAAATACTTCGTACAGTACCCAATAAATTATTGGGTGTTCTTTTAATGGTTTCAGTA
CCCGCGGGATTATTAACAGTACCTTTTTTAGAGAATGTTAATAAATTTCAAAATCCATTT
CGTCGTCCAGTAGCGACAACGGTCTTTTTGATTGGTACCGCAGTGGCCCTTTGGTTGG
GTATTGGTGCAACATTACCTATTGATAAATCCCTAACTTTAGGTCTTTTTATGATTGAAGT
TTTTCTATTTGGAATCGTGTTAGGTCTAATTCCTATTACTTTGGCTGGATTATTCGTAACT
GCATATTTACAATACAGGCGTGGCGATCAGTTGGACCTTATGCCTACTATAACTAGTTAT
TTCGGTTTTCTACTAGCGGCTTTAACTATAGCCTCAGTTCTTTTTATTGGTCTGAGCAAG
ATACGACTTATTATGGATATAGTAAGTCTTGCTTGGGCTGCTTTAATGGTAGTCTTTACAT
TTTCCCTTTCACTCGTAGTATGGGGAAGAAGTGGGCTCATGATTATTCGTTCGCCGGAA
CCAGAAGTCAAAATTTTGGTAGATAGGGATCACATAAAAACTTCTTTCGAGGAATGGG
CCAAACCCGGTCATTTCTCAAGAACAATAGCTAAAGGCCCTGATACTACCACTTGGATC
TGGAACCTACATGCTGATGCTCACGATTTCGATAGCCATACCGGTGATTTGGAGGAGAT
CTCTCGAAAAGTATTTAGTGCCCATTTCGGTCAACTCTCCATCATCTTTCTTTGGCTGAG
TGGCATGTATTTCCACGGTGCTCGTTTTTCCAATTATGAAGCATGGCTAAGTGATCCGAC
TCACATTGGGCCGAGTGCCCAGGTGGTTTGGCCAATAGTGGGCCAAGAGATATTGAAC
GGTGATGTGGGCGGGGGCTTCCGAGGAATACAAATAACCTCGGGTTTCTTTCAGCTTT
GGCGAGCATCTGGAATAACTAGCGAATTACAACTCTATTGTACCGCAATTGGTGCATTG
ATCTTTGCAGCGTTAATGCTTTTTGCTGGTTGGTTTCATTATCATAAAGCTGCTCCAAAA
TTGGCTTGGTTTCAAGATGTAGAATCGATGTTGAATCACCATTTAGCGGGGTTACTAGG
ACTTGGGTCTCTCTCTTGGGCGGGGCATCAAGTCCATGTATCTTTACCGATTAACCAATT
TCTAAATGCTGGGGTAGATCCTAAAGAGATACCACTGCCTCATGAATTTATCTTGAATCG
GGATCTTTTGGCTCAACTTTATCCCAGTTTTGCCGAGGGAGCAACCCCTTTTTTCACCT
TGAATTGGTCAAAATACGCAGACTTTCTTACTTTTCGTGGAGGATTAGATCCAGTAACT
GGAGGTCTATGGCTGACCGATATTGCACACCATCATTTAGCTATTGCAATTCTTTTCCTG
ATAGCAGGTCATATGTATCGGACCAACTGGGGCATTGGTCATGGCCTAAAAGATATTTTA
GAAGCTCATAAGGGTCCATTTACAGGCCAGGGCCATAAAGGCCTATATGAAATCCTAAC
AACGTCATGGCATGCTCAATTATCTCTTAACCTAGCGATGCTAGGCTCTTTAACCATTGT
TGTAGCTCATCATATGTATTCCATGCCCCCTTATCCATATCTAGCTACTGACTATGGTACA
CAACTGTCATTGTTTACACATCACATGTGGATTGGTGGATTTCTCATAGTTGGGGCTGCT
GCGCATGCAGCCATTTTTATGGTAAGAGACTATGATCCAACTACTCGATATAACGATCTA
TTAGATCGTGTCCTTAGGCATCGCGATGCTATCATATCACATCTCAACTGGGCATGTATAT
TTCTAGGTTTTCACAGTTTTGGTTTGTATATTCATAATGATACTATGAGCGCTTTAGGGCG
CCCTCAAGATATGTTTTCAGATACCGCTATTCAATTACAACCCGTCTTTGCTCAATGGAT
CCAAAACACCCATGCTTTAGCACCCGGTGCAACGGCTCCTGGTGCAACAACAAGCAC
CAGTTTAACTTGGGGGGGCGGTGATTTAGTAGCAGTGGGTGGCAAAGTGGCTTTGTTA

CCTATTCCATTAGGAACCGCAGATTTTTTGGTACATCACATTCATGCATTTACGATTCATG
TGACCGTATTGATACTCCTGAAAGGTGTTCTATTTGCTCGTAGTTCCCGTTTGATACCGG
ATAAAGCAAATCTTGGTTTTCGTTTTCCTTGTGATGGACCTGGAAGAGGAGGGACGTG
TCAAGTATCGGCTTGGGATCATGTCTTCTTAGGACTGTTCTGGATGTACAATGCAATTTC
AGTAGTAATTTTCCATTTCAGTTGGAAAATGCAGTCAGATGTTTGGGGCAGTATAAGTG
ATCAAGGGGTAGTAACTCATATCACGGGAGGAAATTTTGCGCAGAGTTCCATTACTATT
AATGGGTGGCTTCGCGATTTCTTATGGGCACAAGCATCCCAGGTAATTCAGTCTTATGG
TTCTTCATTATCTGCATATGGCCTTTTTTTCCTAGGTGCTCATTTTGTATGGGCTTTTAGTT
TAATGTTTCTATTCAGTGGACGTGGTTATTGGCAAGAACTTATCGAATCCATCGTTTGGG
CTCATAATAAATTAAAAGTTGCTCCTGCTACTCAGCCAAGAGCCTTAAGCATTGTACAA
GGACGCGCTGTAGGAGTAACCCATTACCTTCTGGGTGGAATTGCCACAACGTGGGCGT
TCTTCTTAGCAAGAATTATTGCAGTAGGAATGGCATTAAGATTTCCAAGGTTTAGCCAA
GGCTTAGCCCAGGACCCCACTACTCGTCGTATTTGGTTTGGTATTGCTACCGCACATGA
CTTCGAGAGTCATGATGATATTACTGAGGAACGTCTTTATCAGAATATTTTTGCTTCTCA
CTTCGGGCAATTAGCAATAATTTTTCTGTGGACTTCCGGAAATCTGTTTCATGTAGCTTG
GCAAGGGAATTTTGAGTCATGGGTACAGGACCCTTTACATGTAAGACCTATTGCTCATG
CAATTTGGGATCCTCATTTTGGTCAACCGGCTGTAGAAGCTTTTACTCGAGGAGGTGCT
CTTGGCCCAGTGAATATCGCCTATTCCGGTGTTTATCAGTGGTGGTATACAATAGGTTTA
CGCACTAATGAAGATCTTTATACTGGCGCCCTTTTTCTATTATTTCTTTCTGCCATATCCTT
AATAGCAGGTTGGTTACACCTACAACCGAAATGGAAACCAAGCGTTTCATGGTTCAAA
AATGCGGAATCTCGTCTCAATCATCATTTGTCAGGACTCTTCGGCGTAAGTTCCTTGGC
TTGGACAGGACATTTAGTACATGTCGCTATTCCTGGATCCAGGGGGCAGTACGTTCGAT
GGAATAATTTCTTAGATGTAGTACCACATCCCCAAGGCTTAGGCCCGCTTTTTACAGGT
CAGTGGAATCTTTATGCTCAAAATCCCGATTCAACTAGTCATTTATTTGGTACCTCCCAA
GGAGCAGGAACTGCTATTCTAACCCTTCTCGGGGGATTTCATCCACAAACGCAAAGTTT
ATGGCTGACTGATATGGCTCATCATCATTTAGCTATTGCATTTATTTTTCTCGTTGCAGGG
CATATGTATAGAACTAACTTCGGGATTGGGCACAGTATGAAAGATCTTTTAGATGTGCAT
CTTCCCCCCGGGGGGCGATTGGGACGTGGGCATAGGGGTCTTTATGACACAATCAATAA
TTCGATTCATTTTCAATTAGGCCTTGCCCTAGCTTCTTTAGGGGTTATTACTTCCTTGGTA
GCTCAACACATGTACTCTTTACCTGCTTATGCATTCATAGCACAAGACTTTACTACTCAA
GCTGCGTTATATACTCATCACCAATACATCGCAGGATTCATCATGACAGGAGCTTTTGCT
CATGGAGCTATATTTTTCATTAGAGATTACAATCCGGAACAGAATGAGGATAATGTATTG
GCAAGAATGTTAGACCATAAAGAAGCTATCATATCTCATTTAAGTTGGGCCAGCCTCTT
TCTGGGGTTCCATACTTTGGGACTTTATGTTCATAATGATGTCATGCTTGCCTTTGGTAC
TCCGGAGAAACAAATCTTGATCGAACCTATATTTGCTCAATGGATACAATCTGCTCATG
GTAAAACTTCCTATGGGTTCGATGTACTTTTATCTTCAACGAACGGCCCGGCATTCAAT
GCTGGTCGAAGCATATGGTTGCCGGGTTGGTTAAATGCTATTAATGAG------AATAGTAA
TTCACTATTCTTAACAATAGGTCCCGGAGACTTTTTGGTTCATCATGCTATTGCTCTGGG
TTTACATACAACTACATTGATCTTAGTAAAAGGTGCTTTAGATGCACGCGGTTCCAAGTT
AATGCCAGATAAAAAAGATTTCGGTTATAGTTTTCCGTGCGATGGCCCGGGGCGAGGC
GGTACTTGTGATATTTCGGCTTGGGACGCATTTTATTTGGCAGTTTTTTGGATGTTAAAT
ACTATTGGATGGGTTACTTTTTATTGGCATTGGAAGCATATCACATTATGGCAGGGTAAC
GTTTCGCAGTTTAATGAATCTTCCACTTATTTGATGGGCTGGTTAAGAGATTATTTATGG
TTAAACTCTTCACAACTTATCAATGGATATAACCCCTTTGGTATGAATAGTTTATCAGTCT

GGGCGTGGATGTTCTTATTTGGACATCTTGTTTGGGCTACTGGATTTATGTTCTTAATTTC
CTGGCGCGGATATTGGCAGGAATTGATTGAAACTTTAGCATGGGCTCATGAGCGTACAC
CTTTGGCCAATTTGATTCGTTGGAGAGATAAACCGGTGGCCCTTTCTATTGTACAAGCA
CGATTGGTTGGATTAGCCCACTTTTCTGTAGGTTATATATTCACTTATGCAGCTTTCTTGA
TTGCCTCTACATCAGGCAAATTTGGTATGTCACATTCAGTAAAAATTTATGATACATGTAT
AGGATGTACTCAATGTGTCCGAGCCTGCCCCACCGATGTATTAGAAATGATACCTTGGG
ATGGATGTAAAGCTAAACAAATTGCTTCCGCTCCAAGAACAGAGGACTGTGTCGGTTG
TAAGAGATGCGAATCTGCCTGTCCAACAGATTTCTTGAGTGTTCGAGTTTATTTATGGCA
TGAAACAACTCGCAGCATGGGTCTAGCTTATATGACAACTTTCAACTTTCCCTCTGTTT
TTGTGCCTTTGGTGGGCCTAGTATTTCCGGCAATTGCAATGGCTTCTTTATCTCTTCATG
TTCAAAAAAATAAGATTGTTATGCGAGATCTAAAAACATATCTCTCCGCAGCACCGGTA
CTAAGTACTTTATGGTTCGGGTCTTTAGCAGGTCTATTGATAGAGATTAATCGTTTTTTCC
CGGATGCGTTGACATTCCCCTTTTTTTCATTCATGACTGCAATTTTAGAGAGACGCGAA
AGCGAAAGCCTATGGGGTCGCTTCTGTAACTGGATAACTAGCACTGAAAACCGTCTTT
ACATTGGATGGTTTGGTGTTTTGATGATCCCTACCCTGTTGACCGCAACTTCTGTATTTA
TTATCGCCTTCATTGCTGCTCCTCCAGTAGATATTGATGGGATTCGTGAACCTGTTTCTG
GATCTCTACTTTATGGAAACAATATTATCTCGGGTGCCATTATTCCTACTTCTGCAGCTAT
AGGTTTGCACTTTTACCCAATATGGGAAGCGGCATCGGTTGATGAATGGTTATACAATG
GTGGTCCTTATGAACTAATTGTTCTACACTTCTTACTAGGTGTAGCTTGTTACATGGGTC
GTGAGTGGGAGCTTAGTTTCCGTCTGGGTATGCGACCTTGGATTGCTGTTGCATATTCA
GCTCCTGTTGCAGCCGCAACTGCTGTTTTCTTGATCTACCCAATTGGTCAAGGAAGTTT
TTCTGACGGTATGCCTCTAGGAATCTCTGGTACTTTCAACTTCATGATTGTATTCCAGGC
TGAGCACAACATTCTTATGCACCCATTTCACATGTTAGGCGTAGCTGGGGTATTCGGCG
GCTCCCTATTCAGTGCTATGCATGGTTCCTTGGTAACCTCTAGTTTGATCAGGGAAACC
ACAGAAAATGAATCTGCTAATGAAGGTTATAGATTCGGTCAAGAAGAAGAAACTTATAA
TATTGTAGCCGCCCATGGTTATTTTGGCCGATTGATCTTCCAATATGCTAGTTTCAACAA
CTCTCGTTCTTTACATTTCTTCCTAGCCGCTTGGCCTGTAGTAGGTATCTGGTTCACTGC
TTTAGGTATCAGCACTATGGCTTTCAACCTAAATGGTTTTAATTTCAACCAATCTGTAGT
TGATAGTCAAGGCCGTGTAATTAATACTTGGGCTGATATCATTAATCGTGCTAACCTTGG
TATGGAAGTTATGCATGAACGTAATGCTCACAACTTCCCTCTAGACCTAGCCGCTCTTG
AAGCTCCATCTACAAATGGAATGGGTTTGCCTTGGTATCGTGTTCATACCGTTGTATTGA
ATGATCCCGGTCGACTGCTTTCTGTCCATATAATGCATACAGCTCTGGTTGCGGGTTGGG
CCGGTTCTATGGCTCTGTATGAATTAGCAGTTTTTGATCCCTCTGACCCCGTTCTTGATC
CAATGTGGAGACAGGGTATGTTCGTTATACCATTCATGACTCGTTTAGGAATAACCAATT
CATGGGGTGGTTGGGGTATCACA---GGGGGGACTATAACGAATCCAGGTATTTGGAGTT
ACGAAGGTGTGGCCGGGGCACATATTGTGTTTTCTGGCTTGTGCTTCTTGGCAGCTATC
TGGCATTGGGTATATTGGGATCTAGAAATATTTTGTGATGAACGTACAGGAAAACCTTCT
TTGGATTTACCCAAAATCTTTGGAATTCATTTATTTCTCGCAGGAGTGGCGTGCTTTGGT
TTTGGTGCATTTCATGTAACAGGCTTGTATGGTCCTGGAATATGGGTGTCCGATCCTTAT
GGACTGACGGGAAAAGTGCAATCTGTAAATCCAGCCTGGGGCGTGGAAGGTTTTGATC
CTTTTGTTCCGGGAGGAATAGCCTCTCATCATATTGCAGCAGGGGCATTGGGCATATTG
GCGGGCCTATTCCATCTTAGCGTTCGCCCACCCCAACGCCTATACAAAGGATTGCGTAT
GGGCAATATTGAAACCGTCCTTTCCAGTAGTATTGCTGCTGTCTTTTTTGCAGCTTTTGT
TGTTGCCGGAACTATGTGGTATGGTTCAGCAACTACTCCGATCGAATTGTTTGGGCCTA

CTCGTTATCAATGGGATCAGGGGTACTTTCAGCAAGAGATATACCGAAGGGTTAGTGCT
GGACTAGCCGAAAATCAAAGTTTATCAGAAGCTTGGTCTAAAATTCCCGAAAAATTGG
CTTTTTATGATTACATCGGCAATAATCCGGCAAAAGGGGGATTATTCAGGGCGGGCTCA
ATGGATAACGGGGATGGAATAGCCGTTGGATGGTTAGGGCATCCTATCTTTAGAGATAA
AGAAGGACGCGAACTTTTTGTACGTCGTATGCCTACTTTTTTTGAAACATTTCCGGTCG
TTTTGGTAGACGGCGACGGAATTGTTAGAGCCGATGTTCCTTTTCGAAGGGCCGAATC
GAAGTATAGTGTCGAACAAGTAGGTGTAACTGTTGAGTTCTACGGTGGCGAACTCAAC
GGAGTCAGTTATAGCGATCCTGCTACTGTGAAAAAATATGCTAGACGTGCTCAATTGGG
TGAAATTTTTGAGTTAGATCGTGCTACTTTGAAATCCGATGGTGTTTTTCGTAGCAGTCC
GAGAGGTTGGTTTACTTTTGGACATGCTTCATTTGCTTTGCTCTTTTTCTTCGGACACAT
TTGGCATGGTGCTAGAACCTTGTTCAGAGATGTTTTTGCTGGTATTGACCCAGATTTGG
ATGCTCAAGTAGAATTTGGAGCATTCCAAAAACTTGGAGATCCAACTACAAGAAGACA
GGTAGTC------------------------------------GTGGAAACGCTCTTTAATGGAACTTTAGCTTTAG
CTGGTCGTGACCAAGAAACCACTGGTTTCGCTTGGTGGGCCGGGAATGCCCGGCTTAT
AAATTTATCCGGTAAACTACTAGGGGCTCATGTAGCCCATGCCGGATTAATCGTATTCTG
GGCCGGAGCAATGAACCTATTTGAAGTGGCTCATTTCGTACCAGAGAAGCCTATGTATG
AACAAGGATTAATTTTACTCCCCCACCTAGCTACTCTAGGTTGGGGGGTAGGTCCCGGC
GGGGAAGTTATAGACACTTTTCCATACTTTGTATCTGGAGTACTTCATTTAATCTCCTCT
GCAGTATTGGGCTTTGGCGGTATTTATCATGCACTTCTAGGACCTGAGACGCTTGAAGA
ATCTTTTCCATTCTTCGGTTATGTATGGAAAGATAGAAATAAAATGACTACAATTTTAGG
TATTCACTTAATCTTGTTAGGTATAGGTGCTTTTCTTTTAGTATTCAAGGCTCTTTATTTT
GGGGGCGTATATGATACCTGGGCGCCGGGAGGGGGAGATGTAAGAAAAATTACCAACT
TGACCCTTAGTCCAAGTATTATATTCAGTTATTTACTAAAATCGCCCTTTGGAGGGGAAG
GATGGATTGTTAGTGTAGACGATTTGGAAGATATAATCGGAGGACATGTATGGGTAGGT
TCCATTTGTATACTTGGCGGAATCTGGCATATCTTAACCAAACCCTTCGCATGGGCTCGA
CGCGCACTTGTATGGTCCGGGGAGGCTTACTTATCTTATAGTTTAGCGGCTTTATCTGTC
TTTGGATTTATTGCTTGTTGTTTTGTATGGTTCAATAATACCGCCTATCCTAGTGAGTTTT
ACGGACCTACTGGACCCGAAGCTTCTCAAGCTCAAGCATTTACGTTTCTAGTTAGAGAT
CAACGTCTTGGAGCTAACGTCGGATCCGCTCAAGGACCTACCGGTTTAGGTAAATATTT
AATGCGTTCCCCGACCGGAGAAGTCATTTTTGGAGGAGAAACTATGCGTTTTTGGGAT
CTGCGCGCCCCCTGGTTAGAACCATTAAGGGGTCCAAATGGATTGGACTTGAGTAGGC
TGAAAAAAGATATACAACCTTGGCAAGAACGGCGTTCCGCAGAATATATGACTCATGCT
CCTTTAGGTTCTTTAAATTCCGTGGGCGGCGTAGCTACCGAGATCAATGCAGTCAATTAT
GTCTCTCCTAGAAGTTGGTTAGCTACTTCTCATTTTGTTCTAGGATTCTTCTTGTTTGTA
GGTCATTTGTGGCATGCGGGAAGGGCTCGTGCAGCTGCAGCAGGGTTTGAAAAAGGA
ATTGATCGTGATTTTGAACCTGTTCTTTCCATGACCCCTCTAAATATGACTATAGCCCTT
GGTAAATTTACCAAAGACGAAAATGATTTATTTGATATTATGGATGACTGGTTACGGAG
AGACCGTTTCGTTTTTGTAGGCTGGTCCGGTCTATTGCTCTTTCCTTGTGCCTATTTCGC
TGTAGGGGGTTGGTTCACAGGTACAACCTTTGTAACTTCATGGTATACCCACGGATTGG
CCAGTTCCTATTTGGAAGGCTGCAATTTCTTAACTGCCGCAGTTTCGACTCCTGCTAAT
AGTTTAGCACATTCTTTGTTGTTACTATGGGGTCCTGAAGCACAAGGAGATTTTACTCG
TTGGTGTCAATTGGGCGGTCTGTGGACTTTTGTTGCTCTCCATGGTGCTTTCGGACTAA
TAGGTTTCATGTTACGTCAATTCGAACTTGCTCGATCTGTTCAATTGCGACCTTATAATG
CAATCGCATTCTCTGGCCCAATTGCTGTTTTTGTTTCTGTATTCCTGATTTATCCACTAGG

TCAGTCTGGTTGGTTCTTTGCACCTAGTTTTGGTGTAGCGGCTATATTCCGATTCATCCT
CTTTTTTCAAGGATTTCATAATTGGACGTTGAACCCATTTCATATGATGGGAGTTGCCGG
TGTATTGGGCGCTGCTTTGCTATGCGCTATTCATGGTGCTACCGTAGAAAATACTTTATTT
GAAGATGGTGATGGTGCAAATACATTCCGTGCGTTTAACCCAACCCAAGCTGAAGAAA
CTTATTCAATGGTCACCGCTAACCGCTTTTGGTCCCAAATCTTTGGGGTTGCTTTTTCCA
ATAAACGTTGGTTACATTTCTTTATGTTATTTGTACCAGTAACCGGTTTATGGATGAGTG
CTCTTGGAGTAGTCGGTCTGGCTCTGAACCTGCGTGCCTATGACTTCGTTTCTCAGGAA
ATTCGTGCGGCTGAAGATCCTGAATTTGAGACTTTCTACACCAAAAATATTCTCTTAAA
CGAAGGGATTCGTGCTTGGATGGCGGCTCAAGATCAGCCTCATGAAAACCTTATATTCC
CTGAGGAGGTTCTACCCCGTGGAAACGCTCTTATGTCTGGAAGCACAGGAGAACGTTC
TTTTGCTGATATTATTACCAGTATTCGCTACTGGGTCATTCATAGCATTACTATACCCTCC
CTATTCATTGCGGGTTGGTTATTCGTCAGCACCGGCTTAGCTTACGATGTGTTTGGAAG
CCCTCGGCCAAACGAGTATTTCGCAGAGAACCGACAAGGAATTCCATTAATAACTGGC
CGTTTTGATCCTTTGGAACAACTCGACGAATTTAGTAGATCTTTTATGACTATCGATCGA
ACCTATCCAATTTTTACAGTGCGATGGTTGGCTGTTCACGGCCTAGCTGTACCTACTGTT
TCTTTTTTGGGGTCAATCTCAGCAATGCAGTTCATCCAACGAATGGCTACACAAACTGT
TGAGAGTGGTTCTAGATCTGGT---CCAAGACGAACTAATGTAGGGAGTTTATTGAAACC
ATTGAATTCGGAATACGGAAAAGTAGCCCCTGGATGGGGAACGACTCCGTTGATGGGA
GTTGCAATGGCTCTATTTGCGATATTTCTATCTATTATTTTGGAGATTTATAATTCTTCCGT
TTTATTGGATGGAATTTCAATGAATATGCTTACTCTTAAACTTTTCGTTTACACAGTAGT
GATATTCTTTGTTTCTCTGTTCATCTTTGGATTTCTATCTAATGATCCAGGACGTAATCCT
GGACGTGAAGAAATGGCCAATACTACTGGAAGGATTCCTCTTTGGATAATAGGTACTGT
AGCTGGGATTCTTGTGATCGGTTTAATAGGTATTTTCTTTTATGGTTCATATTCCGGATTG
GGTTCATCGCTGATGCTTAATATCTTTAGTTTGATCTATATCTGTCTTAATTCTACCCTTTA
TTCGACTAGTCTTTTCTTCGCCAAATTGCCCGAGGCCTATGCTTTTTTGAATCCTATCGT
AGATGTTATGCCAGTCATACCTTTGTTCTTTTTTCTCTTAGCCTTTGTTTGGCAAGCTGC
TGTAAGTTTTCGAACGACACAATCAAACCCAAACGAACAAAATGTTGAATTGAATCGT
ACCAGCCTTTATTGGGGATTATTACTCATTTTTGTACTTGCTGTTTTATTTTCCAATTATTT
CTTCAATATGGAAGTAAATATTCTTGCATTTATTGCTACTGCACTGTTCATTCTAGTCCCT
ACTGCGTTTCTACTTATCATTTACGTAAAAACCATAAGTCAAAATGATATGGAAGCAGC
AACCCTACTCGCCATCTTTATATCTGGTTTACTTGTAAGTTTTACTGGGTACGCCTTATAT
ACCGCTTTTGGGCAACCTTCTCAACACCTAAGAGATCCATTCGAGGAACATGGGGACA
TGGAAGCATTGGTTTATACATTCCTCTTAGTCTCGACTCTAGGGATCATTTTTTTCGCTAT
CTTTTTTAGAGAACCACCCAAAGTCCCAACGAAAAAA------ATGACTCTTGCTTTCCAA
TTGGCCGTTTTTGCATTAATTGCTACTTCATCAATCTTATTAATTAGCGTACCCGTTGTAT
TTGCTTCTCCTGATGGTTGGTCAAGTAACAAAAATGTTGTATTTTCCGGTACATCATTAT
GGATTGGATTAGTCTTTCTGGTAGGTATCCTTAATTCTCTCATCTCT--------------------ATG
TCACCACAAACAGAGACTAAAGCAAGTGTTGGATTCAAAGCGGGTGTTAAAGATTACA
AATTGACTTATTATACTCCTGACTATGAAACCAAAGATACTGATATCTTGGCAGCATTCC
GAGTAACTCCTCAACCCGGAGTTCCGCCTGAAGAAGCGGGGGCCGCGGTAGCTGCTG
AATCTTCAACCGGTACATGGACAACTGTGTGGACCGATGGACTTACCAGCCTTGATCGT
TACAAAGGGCGATGCTACCACATCGAGCCCGTTGCTGGAGAAGAAAATCAATTTATTG
CTTATGTAGCTTACCCATTAGACCTTTTTGAAGAAGGTTCTGTTACTAACATGTTTACTT
CTATTGTGGGTAATGTATTTGGGTTCAAAGCCCTGCGCGCTCTACGTCTGGAAGATCTG

CGAATCCCTGTCTCTTATGTTAAAACTTTCCAAGGCCCGCCTCATGGTATCCAAGTTGA
GAGAGATAAATTGAACAAGTACGGCCGCCCCCTGTTGGGATGTACTATTAAACCTAAAT
TGGGGTTATCTGCTAAAAACTACGGTAGGGCGGTTTATGAATGTCTACGTGGTGGACTT
GATTTTACCAAAGATGATGAGAACGTGAACTCCCAACCATTTATGCGTTGGAGAGATCG
TTTCTTATTTTGTGCCGAAGCCCTTTTTAAAGCACAAGTTGAAACAGGTGAAATCAAA
GGGCATTACTTGAATGCTACTGCAGGTACATGTGAAGAAATGATCAAAAGGGCTGTATT
TGCCAGAGAATTGGGAGTTCCTATCGTAATGCACGACTACTTAACAGGGGGATTCACTG
CAAATACCAGCCTGGCTCACTATTGCCGAGATAATGGCCTACTTCTTCACATCCACCGT
GCAATGCATGCAGTTATTGATAGACAGAAGAATCATGGTATACACTTTCGTGTACTAGCT
AAAGCGTTACGTATGTCTGGTGGAGATCATATTCACTCCGGTACCGTAGTAGGTAAACT
TGAAGGGGAAAGAGAGATCACTTTGGGCTTTGTTGATTTACTGCGCGATGATTTTATTG
AAAAAGATCGAAGTCGCGGTATTTATTTCACTCAAGATTGGGTCTCTCTACCGGGTGTT
CTGCCCGTGGCTTCGGGGGGTATTCACGTTTGGCATATGCCCGCTTTGACCGAGATCTT
TGGAGATGATTCCGTCCTACAGTTCGGCGGAGGAACTTTAGGACACCCTTGGGGAAAT
GCACCTGGTGCCGTAGCTAATCGAGTCGCTCTAGAAGCATGTGTACAAGCTCGTAATGA
GGGGCGTGATCTTGCTCGTGACGGTAATGAAATTATCCGTAGGGCTAGCAAATGGAGTC
CTGAACTAGCTGCTGCTTGTGAGGTATGGAAGGAGATCAAATTTGAATTTCAAGCAAT
GGATACTTTG------ATGGCAATACATTTATACAAAACTTCTACCCCGAGCACACGCAATG
GGACCGTAAACGGTCAAGTAAAACCCAACCCACGAAATAATTTGATCTATGGACAGCA
TCGTTGTGGTAAAGGTCGTAATGCCAGAGGAATCATTACCGCAGGGCATAGAGGGGGA
GGTCATAAGCGTCTATACCGTAAAATTGATTTTCGACGGAATGAAAAAAACATATATGG
CAGAATCGTAACCATAGAATACGACCCTAATCGAAATGCATCCATTTGTCTCATACACTA
TGGGGATGGTGAGAAGAGATACATTTTACATCCCAGAGGGGCTATAATTGGAGATACCA
TTGTTTCTGGTACAGAAGTTCCTATAAAAATGGGAAATGCCCTACCTTTGACCAATATG
CCCTTAGGCACGGCCATACATAACATAGAAATGACACTTGGAAAGGGTGGACAATTAG
CTAGAGCGGCGGGTGCTGTAGCAAAACTGATTTCAAAAGAGGGGAAATCGGCCACATT
AAAATTACCTTCTGGGGAGGTCCGTTTGATATCCAAAAACTGCTCAGCAACAGTCGGA
CAAGTGGGGAATGTTAGGGTGAACCAGAAAAGTTTGGGTAGAGCCGGATCTAAGCGT
TGGCTAGGTAAGCGTCCTGTAGTAAGGGGAGTAGTTATGAACCCTGTAGACCATCCCCA
CGGGGGTGGGGAGGGGAAGGCCCCAATTGGTAGAAAAAAACCCACAACCCCTTGGG
GTTATCCTGCACTTGGAAAAAGAAGTCGAAAACGGAATAAATATAGTGATAATTTGATT
CTTCGTCGCCGTAATAAAATGATTCAACCTCAGACCTATTTGAATGTAGCGGATAACAG
CGGGGCTCGAGAATTGATGTGTATTCGAATCATAGGAGCTAGC---AATCGCCGATATGCT
CATATTGGTGACGTTATTGTTGCTGTGATTAAAGAAGCAGTGCCAAAGATGCCCCTAGA
AAGATCAGAAGTGGTCAGAGCTGTAATTGTACGTACTTGTAAAGAACTCAAACGTGAC
AGCGGTATGATAATACGATATGATGACAATGCTGCAGTCCTCATTGATCAAGAAGGAAA
TCCAAAGGGAACTCGGGTTTTTGGTGCGATTGCCGGGGAGTTGAGACAGTTAAATTTT
ACTAAAATAGTTTCATTAGCTCCCGAGGTATTAATGCTTAGTCCTAAAAGAACTAGATTC
CGTAAACAACATAGAGGAAGAATGAAGGGAATATCTTATCGAGGTAATCACATTTGTTT
CGGTAAATATGCTCTTCAGGCACTTGAACCCACTTGGATCACATCGAGACAAATAGAA
GCAGGTCGACGAGCAATGACACGAAATGCACGTCGTGGTGGAAAAATATGGGTACGTA
TATTTCCAGACAAACCGGTTACAGTAAGATCCGCAGAAACACGTATGGGTTCGGGGAA
AGGATCCCCCGAATATTGGGTAGCTGTTGTTAAACCGGGCCGCATCCTTTATGAAATGG
GTGGAGTAACAGAAAATATAGCCAGAAAGGCTATTTCGATAGCAGCGTCCAAAATGCC

TATACGGGCTCAATTCATTATTGCGGGAATGACCAGAACTAAACGGGGATCTATAGCTC
GGAGACGTAGAACAAAAGTGCGTTCCTTTGTATCAAGCTTTCAAGGGGCTCATTCAAG
ACTTACTCGAACAATGAGTCAACAGAAAATAAGAGCTTTGGATTCAGCACATCGGGAT
AGGGATAGGCAAAAGAGAAACTTTCGTCGTTTGTGGATTACTCGAATAAACGCAGTAA
TGCGCGAA---AGGGGGGTATCCTATAGTTATAGTAGATTAATACACGATCTGTACAAGAG
ACAGTTGCTTCTTAATCGTAAAATACTTGCACAAATAGCTATATCAAATAGGAATTCCAT
TTCTATTATTTCCAACGAAATCGTAAAAGAAGTCGATTGGAAAGAATCCGCAGGAATAA
TTATGTTCGAGGACGTATATGCTTTAGGTCAACATATAGCTATGTCTGCTCACAAAGCGC
GAAGAGTAATTGATCAAATTCGTGGACGTTCCTACGAAGAAACACTTATGATACTAGAA
CTCATGCCTTATCGAGCATGTTATCCAATTTTTAAATTGGTTTATTCTGCAGCAGCAAAT
GCTAGTTACATTCTGGGTTCCAATGAAGCAAATTTAATCATTAGTAAAGCTGAAGTCAA
CGAGGGTACTGCCGTGAAGAAATTAAAACCTCGAGCTCGAGGACGTAGTTATCCGATA
AAAAGACCTACCTGCCATATAACTATTATAGTGAAAGATATGTCCTTACCTGGGGGAGTA
TGGGACAAAAAAATGGATGGAATCAAATATGCAGTATTTACAGACAAAAGTATTCGGTT
ATTGTCGAAAAAT---CAATATACTTCTAATGTTGAATCAGGATTAACTAGGACAGAAATA
AAGCATTGGGGCGAACTCTTCTTTGGTGTCAAGATAATAGCTATGAATAGTCATCGACT
TCAGGGCAAGGGTAGAAGAATGGGACCTAGTATGGGACAAACAATGCATTACAGACGT
ATGATCATTACGCTTCAACCGGGTTATTCTATTCCACCTCTTAGAAAGAACTTAAATATG
GCAGTTCCAAAAAAACGTACTTCTATATCAAAAAAACGTATTCATAAAAATATTTGGAA
GGGGAAGGGATATTGGTTCGCGTTAAAAGCTTTGTCATTGGGGAAATCTCTTTCGACCG
GGAATTCAAAAAGTTTTTTTGTGCGACAAACAAATAAGATGGCTAAGAGTAAAGATGT
CCGAATAACGGTTATTTTGGAATGTACCAGTTGTGTTCGAAATGGTGTTAATGTTGATAA
GGTATCAACGGGCATTTCCAGATATATGACTCAAAAGAACCAGCACAATACGCCCAATC
GATTGGAATTGAGAAAATTCTGTCCCTATTGTTACAAACATACGATTCATGGGGAGATA
AAGAAAATGAAAATAAGAGCTTCTGTTCGTAAAATTTGTGATAAATGTCGACTAATCCG
TAGGCGGGGACGAATTATAGTAATTTGTTCCAACCCAAGACATAAACAACGACAAGGA
ATGGTTCAAGAGAAAGTAACAGTATCTACTCGGACACTAGAGTGGAAGTGCGTTGAAT
CAAGAGCAGACAGTAAGCGTCTTTATTATGGACGCTTTATTCTGTCTCCACTTATGAAA
GGTCAAGCTGACACAATAGGCATTGCGATGCGAAGAGCTTTGCTTGGAGAAATAGAAG
GAACATGCATCACACGCGCAAAATCTGAG------AAAATACCACATGAATATTCTACCATA
GTGGGTATTCAAGAATCAGTACATGAAATTTTAATGAATTTGAAAGAAATTGTATTGAG
AAGTAATCTATATGGAACCTGCGACGCGTCCATTTGTGTCAGGGGTCCCGGAAATGTAA
CTGCTCAAGATATCATCTCACCGCCCTATGTCGAAATCATTGATAATACACAGCATATAG
TTAGCTTGACGGAACCAATTGATTTTTGTATTGGATTACAAATCGAGAGGAATCGTGGA
TATCTTATAAAAACGCCAAAT---CATTTTGATGGAAGTTAT------CCCATAGATGCTGTATT
CATGCCTGTTCGAAATGCGAATCATAGTATTCATTCTTATGGGAATGAGAATGAGAAAC
AAGAAATACTCTTTCTCGAAATATGGACAAACGGAAGTTTAACTCCTAAAGAAGCACT
TCATGAAGCCTCTCGGAATTTGATTGATTTATTTATTCCTTTTTTACATACAGAAGAAGA
A------AATTTACATTTAGAGGACAATCAA---CACACGGTTCCTTTAACCCCCTTTACCTTT
CATGAGAAATTGGCT---AAACTAAGAAAAAAAAAA------------------------

------------------------------ATGCTCCAGGATGGAAAT---GAGGGAATG
TATACAATACCCGGGTTGAATCAGATACAATTTGAAGGATTTTGTAGGTTCATTGATCAG
GGCTTGATGGAAGAACTTTCTAAGTTTCCAAAAATAGAAGATACGGATCAAAAAGCGG

AATTTCAATTATTTATGGAAACATATCAATTTGTAGAACCATTGATAAAAGAAAGAGATG
CTGTGTATAAATTACTCACATATTCTTCTGAATTATATGTATCCGCAGGACTAATTTGGAA
AACCCGCAGGGATATGCAAGAACAAACAATTTTCATTGGAAACATTCCTCTAATGAATT
CTCTGGGAACTTCTATAGTAAAGGGAATATATAGAATTGTGATCAATCAAATATTGCAAA
GCCCGGGTATTTATTACCGGTCAGAATTGGACCATAATGGAATTGAGGCCTATACCGGC
ACCATAATATCAGATTGGGGAGGAAGATCAGGATTAGAGATTGATAGAAAAGCAAGGA
TATGGGCTCGCGTGAGTAGGAAACAAAAAATATCTATCCTAGTTTTATCATCAGCTATGG
GTTCGAATCTACGAGAAATTCTGGAGAATGTTTGCTATCCCGAAATATTTTTGTCTTTTC
TGAATGCTAAGGAGAAAAAAAAAAATTGGGTCAAAAGAAAATGCTATTTTGGAGTTTTA
TCAACAATTTGCTTGTGTAGGCGGAGATCCGGTATTTTCTGAATCCTTATCTAAGGAATT
ACAAAAGAAATTCTTTCAACAAAAATGTGAATTAGGAAGGATTGGGCGGCGAAATATG
AATCGGAGACTGAACCTTGATATACCCCAGAACAATACATTTTTGTTACCACGAGATATA
TTGGCAGCCGCGGATCATTTGATTGGAATGAAATTTGGAATGGGTCCACTTGACGAT----
--ATGAATCATTTGAAAAATAAACGTATTCGTTCTGTAGCAGATCTTTTACAAGATCAATT
CGGATTGGCTCTGTCTCGTTTAGAAAATGTGATTCGAGGAACTATATCTGGAGCACTTA
GA---CATAAATTGATA---CCGACTCCTCAGAATTTGGTAACTTCAACTCCA------------TTA
ACAACTACTTATGAATCTTTTTTCAGTTTACACCCATTATCTCAAGTTTTGGATCGAACT
AATCCATTAACACAAATAGTTCATGGTAGAAAATTGAGTTATTTGGGCCCTGGAGGATT
GACAGGGCGAACTGCTAGTTTTCGAATACGAGATATCCATCCTAGTTACTATGGACGTAT
TTGCCCAATTGACACATCTGAAGGAATAAATGTTGGACTTATTGGATCCTTAGCAATTCA
TGCGAGGATTGGTCGTTGGGGG---TCTCTAGAAAGCCCATTTTAT---------GAAATTTCTG
AGAAACCGAAAGGG------GCACGGATGCTTTATTTATCACCAGGTCGAGATGAATACTCT
ATGGTAGCTGCAGGCAATTCTTTGGCCTTGAATCAGAGTATTCAGGAGGAACAGGTTG
TTCCAGCTCGATACCGTCAAGAATTCCTGACTGTTGCATGGGAACAAGTTCATCTTCGA
AGTATTTTTTCCTTCCAATATTTTTCTATTGGAGCTTCCCTCATTCCTTTTATCGAGCATAA
TGATGCGAATCGGGCTTTAATGAGTTCTAATATGCAACGTCAAGCAGTTCCTCTTTCTC
GGTCCGAGAAGTGCATTGTTGGAACTGGGTTGGAACGCCAAGCAGCTCTGGATTCGG
GGGCTCTTGCTATAGCCAAACACGAGGGAAAGATCAGTTATATCGATACGGACAAGATA
CTTTTATCAAGTAATAGAGATACTCTAAGCATT---------ATGGGTCGGGGTTCCAACAAAA
AGACTTGTATGCATCAAAAACCCCAGGTTCGGCGGGTTAAATGCATTAAAAAGGGACA
AATTTTAGGGTATGGCGCCGCTACGGTTGGTGGTGAACTCGCTTTGGGGAAAAACGTA
TTAGTAGCTTATATGCCATGGGAAGGTTACAATTCTGAAGATGCAGTACTCATTAGCGA
GCGCTTGGTATATGAAGATATTTATACTTCTTTTCACATACGGAAATATGAAACTAAGATT
TGTGTGACAAGCCAAGGC---CCCGAAAGGGTCACTAATGAAATACCGCATTTAGAAGC
CCATTTACTCCGCAATTTAGACAAAAGGGGAATTGTAATGCTGGGATCTTGGGTAGAGG
CGGGTGATATTTTAGTAGGTAAATTAACGCCCAAAATTGTCAAAGAA---TCGTCGTCTG
CCCCCGAAGCTAGATTGTTACGAGCCATACTCGGCATTCAGGCAGCTACTTCAAAGGA
AACTTGTCTAAAACTACCTATAGGTGGTAGGGGTCGGGTTATTGATGTGAGATGGATCC
AGCAAAATGAGGTTTCTGGTTCTAAT---CCAGAAAGGATTTGTGTATCTATTTTACAGAA
ACGTGAAATCAAAGTAGGCGATAAAGTAGCTGGAAGACATGGAAATAAGGGTATCATT
TCCAAAATTTTGCCTAGACAAGATATGCCTTATTTGCAAGATGGAAGACCTGTTGATAT
GGTCTTCAACCCATTAGGAGTCCCTTCACGAATGAATGTAGGACAGATATTTGAATGTT
CACTCGGGTTAGCGGGGGATCTGCTAAACAGACATTATCGAATAGCGCCTTTTGATGAG
AGATATGAACAAGAAGCTTCGAGAAAACTTGTGTTTTCTGAATTATATGAAGCCAGTAA

GCAAACAGCAAATCCATGGGTATTTGAACCCGAGTATCCAGGAAAAAGCAGAATATTT
GATGGACGAACGGGGGATCCTTTTGAACAACCTGTTATAATAGGAAAGCCTTACATCTT
GAAATTAATTCATCAAGTTGATGATAAAATCCATGGACGTTCCAGCGGACAGTATGCCG
TTGTTACACAACAAGCCGTTAGAGGAAGGTCCAGGGGAGGGGGACAACGGGTAGGAG
AAATGGAGGTTTGGGCTCTAGAGGGATTTGGCGTTGCTCATATTTTACAAGAGATGCTT
ACTTATAAATCTGATCATATGAGAGCCCGACAGGGAATACTTGGTACTACACTCATCGG
AGGAACAATACCTAATCCTGAGGGTGCTCCAGAATCTTTTCGATTGCTCGTTCGAGAAC
TACGATCTTTGGCTCTGGAATTGAATCATTTCCTTGTATCTGAGAAGAATTTCCAGATTC
ATAGGGAGGAAGCT---------------------ATGATCGATCGGTATAAACATCAACAACTTCGAA
TTGGATTGGTTTCTCCTCAACAAATAAGTACTTGGGCCAACAAAATCCTGCCCAATGGA
GAGATCGTTGGAGAGGTGACAAAATCCTCTACTTTTCATTACAAAACCAAAAAACCGC
AAAAAGGTGGATTATTTTGTGAAAGAATTTTTGGGCCTATCAAAAGTGGAATTTGTGCT
TGTGGAAATTATCGAGTAATCGGAGATGCAAAAGACGACCCGAAATTTTGTGAACAAT
GCGGAGTCGAATTTGTTGATTCTCGGATACGAAGATATCAAATGGGCTACATCAAACTC
GCATGCCCAGTAACTCATGTGTGGTATTTGAAACGTCTTCCTAGTTATATTGCGAATCTT
TTAGATAAACCTCTTAAAGAATTAGAAGGCCTAGTATATTGCGAT------TTTTCTTTTGCT
AGGCCC---ATAACCAAAAAACCGACTTTCTTACGATTACGAGGTTCATTCGAATATGAA
ATCCAATCTTGGAAATCTATCATCCCACCCTTTTTTACTAGCCAAGGCTTCGATACATTT
CGAAATCGAGAGATCTCTACTGGCGCAGGTGCTATCCGAGAACAATTAGCCGATCTAGA
TTTACGAATTATTGTAGATTATTCGTTGGTAGAATGGAAAGAGTTGGGGGAAGAA---GG
GCTTACAGGGAATGAATGGGAAGATCGAAAAGTTGGAAGAAGAAAACATTTTTTGGCT
AGACGCATGGAATTAGCTAAGCATTTTATTCGAACAAATATAGAACCAGAATGGATGGT
TTTGTGTCTATTACCAGTTCTTCCTCCTGAGTTGAGACCGCTTTTTCGGACAGATAGTAG
TAGGCTAGTGAGGTCGGATATTAATGAACTCTATAGACGAGTTATCTATCGGAACAATAC
TCTTACTGATCTATTAACAACAAGTCGATTTGCGCCGGGAGAATTAGTAATGTGTCAGG
AGAAATTGGTACAAGAAGCCGTGGATTCACTTCTTGATAATGGAATAGGCGGGCAACC
AACGAAGGACGGTTCTAATCAAGTTTACAAGTCGTTTTCAGATAGAATTGCAGGTAAA
GAGGGAAGATTTCGTGAGACTCTGCTTGGCAAACGAGTCGATTATTCAGGGCGTTCCG
TTATTGTCGTGGGCCCTTCACTTTCATTACATCGATGTGGATTGCCTCGCGAAATAGCAA
TAGAACTTTTCCAGACATTTGTAATTCGTGGTCTAATTAGACAGCATCTTGCTTCGAACA
TACGAGTTGCCAAGAGTCAAATTCGGGAAAAAGAACCGATTGTATGGGAAATACTTCA
GGAAGTTATGCAGGGGCATCCGGTATTGCTGAATAGAGCGCCCACTCTGCATAGATTAG
GCATACAAGCATTTCAGCCCGTTTTAGTGGAAGGGCGTGCTATTTGTTTACATCCGTTA
GTTTGTAAAGGATTCAATGCAGACTTTGACGGGGATCAAATGGCTGTTCATGTGCCTTT
ATCTTTAGAGGCTCAAGCAGAGGCACGTTTACTTATGTTTTCTCATCTGAATCTTTTGTC
TCCAGCTATTGGGAATCCCATTTCCGTACCAACCCAAGATATGCTTATTGGACTCTATGT
CTTAACGAGGGGGAATCGTCGAGGTGTTTGTGTAAATAGGTATAATCCATGGAATTGC---
------AGAAACTATCAAAATGAAAGAATTTTCGATAATAACTATAAGTATACGAAA------GA
ACCCCTTTTTTCTAATTCCTATGATGCAATTGGGGCTTATCGGCAGAAACGAATCAATTT
AGATAGTCCTTTGTGGCTACGGTGGCGATTAGACCAACGC---GTTATTGCTACA------AG
AGAAGCTCCCATCGAAGTGCACTATGAATCTTTGGGTACCTATTATGAGATTTATGGACA
CTATCTAATAGTAAGAAGTATAAAAAAAGAAATTCTTTATATATATATTCGAACCACTGTT
GGTCATATTTTTCTTTATCGAGAAATCGAAGAAGCCATACAAGGGTTTTGTCAGGCCTG
CTCA---------------------------------ATGGAGGTACTTATGGCAGAACGGGCCAATCTGGTCTT

TCACAATAAAGTGATAGACGGAACTGCCATCAAACGACTTATTAGTCGATTAATAGATC
ATTTCGGAATGGCATATACATCACACATCCTGGATCAAGTAAAGACTCTGGGTTTTCAA
CAAGCTACCGCTACATCCATTTCATTAGGAATTGATGATCTTTTAACAATACCTTCTAAG
AGATGGCTAGTTCAAGATGCTGAACAACAAAGTTTGATTTTGGAAAAACACCATCATTA
TGGGAATGTACACGCGGTAGAAAAATTACGTCAATCCATTGAGATCTGGTATGCTACAA
GTGAATATTTGCGACAAGAAATGAATCCTAATTTTAGGATGACTGACCCTTTTAATCCA
GTTCATATAATGTCTTTTTCGGGAGCTAGGGGAAATGCATCTCAGGTACATCAATTAGTA
GGTATGAGAGGATTAATGTCGGACCCCCAAGGACAAATGATTGATTTACCCATTCAAAG
CAATTTACGCGAAGGGCTCTCTTTAACAGAATATATAATTTCTTGTTACGGAGCCCGCA
AGGGGGTTGTGGATACTGCTGTACGAACCTCGGATGCTGGATATCTCACGCGAAGACT
TGTTGAAGTAGTTCAACACATTGTTGTACGTCGAACAGATTGTGGGACCGCCCAAGGT
ATTTCTGTGAGTCCTCGAAATGGGACGATGGCG---GAAAGTATTTTGATCCAAACATTA
ATAGGTCGTGTATTAGCGGATGATATATATATGGGTCCACGATGCATTGCCGCCAGAAAT
CAAGATATTGGAATTGGGCTTGTCAACCGATTCATAACC------------------TTTCGAGCACA
ACCAATATCTATACGAACTCCCTTTACTTGTAGGAGTACATCTTGGATCTGTCGATTATGT
TATGGCCGGAGTCCTACTCATGGTGACCTGGTCGAGTTGGGAGAAGCTGTCGGTATTAT
TGCAGGCCAATCTATTGGAGAACCGGGTACTCAATTAACATTAAGAACTTTTCATACCG
GTGGAGTATTCACGGGGGGTACTGCAGAATATGTACGAGCCCCCTCTAATGGAAAAATC
AAATTCAATGAGGATTTGATTCATCCAACACGTACACGTCACGGGTATCCTGCTTTTCTA
TGTTCGATAGACTTGTATGTAACTATTGAAAGTGAAGATATTATACATAATGTGAATATTC
CACCCAAAAGTTTTCTTTTAGTTCAAAACGAGCAATATGTAGAATCAGAACAAGTGATT
GCTGAGATTCGCGCAGGAACATCCACT---TTGAATTTTAAGGAGAAGGTTCGAAAACAT
ATTTATTCTGACTCAGGGGGAGAA---ATGCACTGGAATACCGATGTGTATCATGCACCCG
AATTTACATATGGTAATGTTCATCTCTTACCAAAAACAAGTCATTTATGGGTATTATTAGG
ACAGCCATGCAGA------TCTAGTCTAGTCTCCCTTTCGAGCCACAAGGATCAAGATCAA
ATGAACGCTCAT------TCTCTTTCTGTCAAGCGAAAATATATTTCTAACCCTTCC------GTA
ACTAATGATCAA---GCGAGACACAAATTCTTTAGTTCGGATTTTTATGGTCAA---GAAGA
TAGGATTCCTGATTAT---TCAGAACTTACTCGAATCATATGTATTGGTCGTTGTAATCTCAT
ATATCCG---GCCATTCTCCACGAGAATTCT------GATTTATTGGCAAAAAGGCGAAGAAAT
AGATTCATCATTCCACTCCAATCGATTCAAGAAGGCGAGAACGAACTAATGCTCTCTTC
AGGTATCACAATTGAAATGCCC------ATAAATGGTATTTTCCGTAGAAATAGTATTCTTGC
TTATTTCGACGATCCTCAATACAGAAGAAAGGGTTCGGGAATTACTAAATATGGGATTC
TAGAGGTGGATTCAATCCTCAAAAAAGAGGAT------TTGGATGAGTATCGAGAA---GTCG
AGGAATTTAGTCCAAAAAATATGAAAGTAGATCGATTCTTTTTCATTCCCGAGGAAGTG
CATATCTTATCCGAATCTTCTTCCCTAATGGTACAGAACAATAGTATTATTGGGGTAGATA
CACAAATGACTTTAACTACAAGAAGCCGAGCAGCCGGGTTGGTACGAGTGGAGAGAA
AAAAAAACAGAATTGAACTTAAAATCTTTTCTGGAGATATCCATTTTCCTGGAGAGACA
GATAAGATATCCCGACATAGTGGCGTCTTGATACCACAAGGTATG------GGAAAAAAAAA
TTCCAAG------AAATCC------ATGAAAGATTGGATCTATGTCCAACGG------ATCACACCTA
CCCCC------AAGAAGTATTTTGTTTTGGTTCGACCTGTAGTCACATATGAAATAACGGAC
GGTATAAATTTAGCA---CTTTTCCCCCCAGATCTGTTGCAGGAAAGGGATAATGTGCAA
CTTCGAATTGTCAATTATATCCTTTATGGAAATGGCAAATCAATTCGGGGAATTTCTGAC
ACAAGTATTCAATTAGTTCGGATTTGTTTAGTATTGAATTGGGACCAAGACAAAAAG----
-----AGTTCGTTGGTCGAAGAGGGGCGTCCTTCCTTTGTTGAAATAAGGACAAATGGTTT

GATTCGAGATTTTCTAAGAATCGATTTAGCGAAATCCCCTATTTTGTATACCGGAAAAAG
GAATGATACATCGGGTTCAGGATTGATCTCCGAG---AATGGATCAGATCGCACCAATATC
AATCCATTTTCTTCCATTTTTTCCTAT---------TCCGGGGCAAAGATTCAAGAATCCGTTAA
CCAAAATCAAGGAACTATTCATACATTGTTGAATAGA---------AATAAGGAATGCCAATCT
TTGATAATTTTTTCATCATCCAATTGTTCTCGAATAGGTTCA------TGCAACGATGTAAAA
TATGACAATGTG---------ATAAAAGAATTA---CCCCTAATA---------AATTCGTTGGGCCCTT
TAGGAACA------GCCCTTCCAATTGAAAATTTTTATTTA------TTTTCCCATTTA------ATAA
CTCATAATCAGATCTTG---GTAACGAACTATTTGCAACTTGACAATTTAAAACAGATAAA
ACAGACCTTTCAAGTACTTAAC---------TATTATTTCATCGACGAAAAT------GCCAAAATT
TATAATTCTGATCCATGCAGGAACATCATTTTGAATCCATTCAAGTTAAATTGGTATTTGA
TACCTCACAATTATTTTGAAAAAACATCT---ACAATAATGAGTCTTGGGCAGTTTATTTG
TGAAAATGGATGTATAGCCAAAAACAGACCCCAC---CTAAAA------TCGGGTCAAGTTCT
AATTGTTCAA---GTTGACTCTGTAGTAATACGATCAGCTAAGCCGTATTTAGCCATTCCC
GGAGCAACTGTTCATGGCCATTATGGGAGAATCCTTTATGCAGGAGATACGTTAGTTAC
ATTTATATATGAAAAATCGAGATCAGGTGATATAACGCAGGGTCTTCCAAAAGTAGAAC
AGGTGTTCGAAGTTCGTTCGATTGATTCAATATCGATGAATCTCGAACAGAGGGTTGAG
GGTTGGAACGAATGTATAACAAGAATTCTTGGAATTCCTTGGGGATTCTTGATTGGTGC
TGAGCTAACTATAGTGCAAAGTCGTATCTCTTTGGTTAATAAGATCCAAAAAGTTTATCG
ATCCCAGGGGGTGCAGATCCATAATAGACATATAGAAATTATTGTACGTCAAATAACATC
AAAAGTGTTGGTTTCAGAA---------GATGGAATGTCAAATGTTTTTTTACCCGGAGAACT
AATTGGATTGTTGCGAGCGGAACGAACGGGGCGCGCTTTGGAAGAAGCGATCTGTTAC
CGAGCCATCTTATTGGGAATAACAAGAGCATCTCTGAATACTCAAAGTTTCATATCCGA
AGCGAGTTTTCAAGAAACTGCTCGAGTTTTAGCAAAAGCAGCTCTCCGGGGTCGTATC
GATTGGTTGAAAGGCTTGAAAGCAAACGTTGTTCTGGGCGGAATGATACCAGTTGGTA
CGGGATTCAAAGGCTTA------GTGCATCCTTCAAGGAAACATAACAACATTCCC------TTG
GAAACCAAAAAGAAGAATCTATTCGAGGGGGAAATGAGA------GATATTTTGTTCCACC
ACAAAAATGTATTTGATTCCTGCTTTTCACAAAATTTCTATGATACACCGGAGCAATCAT
TTATAACA------------------TTTAATGATTCCATGACCAGAAGGTATTGGAACATCAATTTGG
AAGAGATGATGGAGGCAGGAGTTCATTTTGGCCATGGTATTAGGAAATGGAATCCTAA
AATGGCACCTTATATTTCTGCAAAGCGTAAAGGTAATCATATTACAAATCTTACTAGAAC
TGCTCGTTTTTTATCAGAAGCTTGTAATTTAGTTTTTGATGCAGCAAGTAGGAGAAAAC
AATTCTTAATTGTTGGCACTAAAAAAAAAGCAGCTGATTCAGTAGCACGGGCTGCAAT
AAGGGCTCGGTGTCATTATGTTAATAAAAAATGGCCTGGCGGTATGTTAACGAATTGGT
CCACTACAGAAATGAGACTTCATAAGTTTAGAGACTTGAGAACGCAACAAAAAACAG
GGAGACTTAACCGTCTTCCGAAAAGAGATGCGGCTATGTTGAAAAGGAGATTATCTCA
CTTGCAAACATATTTGGGTGGGATTAAATATATGACGGGGTTACCCGATATTGTAATTGT
CGTTGATCAGCACGAAGAATATACGGCCCTTCGAGAATGTATCACTTTGGGAATTCCAA
CAATTTGTTTAATCGATACAAATTGTGACCCCGATCTCGCCGATCTTCCGATTCCAGCGA
ACGATGACGCTATATCTTCAATCCGATTAATTCTTAACAAATTAGTATTCGCAATTTGTGA
GGGCCGTTCT---AGCTATCTAAGAAATCCGATGGGACAAAAAATAAATCCACTTGGTTT
CAGACTTGGTACAACTCAAAGTCATCATTCCCTTTGGTTTGCGCAACCAAAAAATTATT
CTGAAGGTCTACAAGAAGATCAAAAAATAAGAAATTCTATCAAAAATTATATACAAAAA
AATATGAGAATAGCTTCCGGC---GTCGAGGGAATTGGACGTATAGAGATTCAAAAAAGT
ATCGATCTGATACAGGTCATAATCTATATGGGATTCTCAAAGTTATTAATAAAAAGTCGG

ATGCGAGAAATTAGGAATTTACAAGAAGAATTACAGATGAATCTACAAAAAGAATTTCA
TTGGGTGAACCGAAAACTTAACATTGCTATCACAGAAATTGAAAAACCTTATGGAAAC
CCTAATATTCTGGCAGAATTTATAGCCAACCAATTAAAGAATAGAGTTTCAGTTCGCAA
AGCAATGAAAAAGGTTATTGAATTAACTGAAAAAGAAGGTACAAAAGGAATTCAAGT
ACAAATAGCAGGGCGTATCAATGGAAAAGATATTGCACGTGTCGTATGGATCAGAGAA
GGTAGGGTTCCTCTACAAACCATTCGAGCTAAAATTGATTATTGCTCCTATACAGTTCGA
ACTATCCATGGGGTATTAGGCATCAAAATTTGGATATTTATAGACGGGGAAATGTCGCGT
TACCGAGGACCTCGTTTTAAAAAAATACGCCGCCTGGGGGCTTTGCCGGGACTAACTA
AAAAGGGGCCTATAGGT-----------------CAATCGCGTTCCGTGAAA---AAATCTCAATATC
GTATTCGCCTTGAAGAAAAACAAAAATTGCGTTTTCATTATGGTCTTACAGAACGGCAA
TTACTTAAATATGTTCGTATCGCCGGAAAAGCCAAGGGGTCAACAGGTCAGGTTTTACT
ACAATTACTTGAAATGCGATTGGATAACATCCTTTTTCGATTGGGTATGGCTCCGACTAT
TCCCGGAGCTCGCCAATTAGTTAACCATAGACATATTTTAGTCAACGGCCGTATCGTAGA
TATACCAAGTTATCGCTGCAAACCCCGAGATATTATTACGGCGCGGGATGAACAAAACT
CTAGAGCTCTGATTCAAAATTCTCTCAATTCATCCCCTCATAAAAATAAATTGCCAAAAC
ATTTGACTCTTCAGCCATTCCAA------TATAAAGGATTAGTCAATCAAATAATAGATAGTA
AATGGGTCGGTTTGAAAATAAATGAATTGCTAGTCGTAGAATATTATTCTCGTCAGACTA
TGTCACGTAGAGGTACTGCAGAAAAAAAAACTGCAAAATACGATCCAATTTATCGTAAT
CGATTAGTTAACATGTTGGTTAACCGTATTCTGAAACACGGAAAAAAAGCATTGGCTTA
TAAAATTCTCTATCGAGCCATAAAAAAGATTCACCAAGAGACAAAAAGAAATCCACTA
TCTGTTTTACGAGAAGCAATACGTAAAGTAACTCCCAAGATAGCAGTAAAAGCAAGAC
GTGTAAGTGGATCGACTCGTCAAGTTCCCATTGAAATAGGATCCAAAAAAGGAAAAGT
ACTTGCCATTCGTTGGTTATTAGCGGCATCCCGAAAACGTCCGGGTCAAGATATGGCTT
TCAAATTAAGTTCCGAATTCATGGATGCTTCCAAAGGGAGTGGCGATGCCATACGCAA
AAAGGAAGAGACTCGTAGAATGGCAGAAGCAAATAGAACTTTTGCACATTTTCGTATG
GGTAGGGATACTATTGCTGACATAATAACCTCTATAAGAAATGCTGATATGGATAGAAAA
AGAGTGGTTCGAATAGCATCTACCAATATCACTGAAAATATTGTTAAAATACTTTTACGA
GAAGGTTTTATCGAAAATGCGAGAAAACATCGAGAAAACAGCAAATCTTTTTTGGTTT
TAACCCTACAACATAGGAGG------AATAGGCAAAAACCTTATAGA---AAGAGAAACGGT
TTAAATTTAAAACGGATCAGTCGACCCGGTCTACGAATCTATTCTAACTATCAACGAATT
CCTAGAATTTTAGGCGGGATGGGGGTTGTAATTCTTTCTACTTCTCGAGGTATAATGACA
GACCGAGAGGCTCGACTAGAAAGAATCGGCGGAGAAATTTTGTGTTATATATGGATGG
CAAAAGCTATACCGAGAATTGGTTCACATAGGAAGGGACGTTTTGGTTCACGTAAGAG
TACACGCCGAATACCAAAGGGAGTTATTCATGTTCAAGCAAGTTTCCATAATACCATTG
TGACTGTTACAGATGTACGGGGTCAAGTAGTTTCTTGGTCCTCTGCCGGTACTTGTGGA
TTCAGAGGTAGAAGAAGAGGGACACCGTTTGCTGCTCAAACCATAGCAGGAAATGCG
ATTCGTACAGTAGTAGATCAAGGTATGAAACGAGCCAACGTCATGATAAAGGGTCCTG
GTCTCGGAAGAGACGCAGCATTGCGAGCTATTCGCAGAAGCGGCATACTATTAACTTTC
ATACGTGATGTAACCCCCCTCCCACATAATGGCTGTAGACCTCCGAAAAGAAGACGTG
TGATGCCAACTATTAAACAACTTATTAGAAATACAAGACAGCCAATCAGAAATGTCACA
AAAGCCCCCGCTCTTCGGGGATGCCCTCAGCGTCGAGGAACATGTACTAGGGTGTATA
CTATCACCCCCAAAAAACCAAACTCTGCCTTACGTAAAGTTGCCAGAGTACGATTAAC
CTCTGGATTTGAAATCACTGCTTATATACCCGGTATTGGCCATAATTCAAAAGAACATTC
TGTAGTCTTAGTAAGAGGGGGAAGGGTTAAGGATTTACCCGGTGTGAGATATCACATTG

TTCGAGGAACCCTAGATGCTGACAAAGTAGAGGGTCGTCAACAAGGGCGTTCTAAATA
TGGGGTAAAAAAGCCAAAAATGGCAAGAAAAAGTTTGATTCAGAGGGAGAAGAAGA
GGCAAAAATTGGAACAGAAATATCATTTGATTCGTCGATCCTCAAAAAAAGAAATAAG
A---------AAAGTTCCGTCGTTGAGTGACAAACGGGAAATTTATGGAAAGTTACAATCCCC
GCCGCGGAATAGTGCACCTACACGCCTTCATCGACGTTGTTTTTTGACCGGAAGGCCG
AGAGCTAACTATCGAGACTTTGGACTATCCGGTCACATACTTCGTGAAATGGTTCATGC
ATGTTTGTTGCCAGGGACGACAAGATCCAGTTGGATGCTAAAAGATGCATTCGTTTCAA
TTATTTTGCAA------GAGGAAAACAAAGAAAACAGGGGGTCCGCTGAATTTCAAGTAGT
TAATTTCACCAATAAGATACGGAAACTTACTTTACATTTGAAATTGCACAAAAAGGACT
ATTCGTCTCAGAGAGGCCTGCTAAAAATTCTGGGAAAACGTCAACGGCTGCTGGCTTA
TTTGTCAAACAAAAACAGAATACGT---ATGGTAA
AACTTCGTTTGAAACGATGTGGTAGAAAGCAACGAGCTGTCTATCGAATTGTTGCAATT
GATGTTCGATGCCGAAGAGAGGGAAGAGCTCTTCGGAAAGTGGGTTTTTATGATCCGA
TAAAGAATCAAACTTATTTAAACGTTCCTGCTATTCTCTATTTCCTTGAAAAAGGTGCTC
AGCCTACAGGAACTGTTGGGGATATTTTAAGGAAGGCGGAGGCTTTTAAGGAACTTCG
CCCTAAGCAAACGCAATTAAGAAAAATGGATAAATCCAAGCGACCCTTTCTGAAATCC
AAGCGACCCTTTCGGAAACGTTTACCCCCAATCCAACCGGGGGATCAAATTGAGTATA
GAAACATGAGTTTAATTAGTCGATTTATTAGTGACCAAGGAAAAATATTACCTCGACGG
GTGAAAAGATTAACCTTGAAACAACAACGATTAATTACTCTTGCTATAAAACAAGCTCG
TATTTTATCTTCGTTACCCTTTCTTAATAATAGGAAACTG---AAGACGAAAAACAAAAAA
AATAAGCGAAAGAAACGCCTTAAGAATAAATTTCATCCGGTGACACATTCATTCAAAA
AAAATCCTTTTGTAGCAAATAATTTACTAAGAAAAATGGATAAGCTTAACACAAAAGAA
GAAAAAGAAATAATAATAACTTGGTCCCGGGCATCTACCATTATACCCACAATGGTCGG
TCATACTATTGCTATCCATAATGGAAAGGAGCATCTGCCTATTTATATCACAGATCGTATG
GTAGGCCACAAATTGGGAGAGTTTGCACCTACTTTAAATTTCCGAGGACACGCGAAAA
GCGATAATAGATCGCGTCGTATGACTTTACCATTTTTTCAACTAGCGAATCTCGTATCCTT
ATGCATGAAAATAATAAATTCGGCCGTTGTGGTCGGGCTCTATTATGGATTTCTGACCAC
ATTCTGCGTAGGGTCCTCTTATCTCTTCCTTCTACGAGCTCATGTTATAGAAGAAGCAAG
A---------GAAAAGAAGATATCAGCAACAACGGGTTTTCTTATGGGACAGCTCATAATGTT
CATATCGATCTATTATGTACCTCTGCATCTAGCATTGGGTAGACCTCATACAATAACTGTC
CTAGTTTGCCCGTATCTTTGGTTTCACGTCCGTTGGAATAATGACAAAAAAAAAAAATT
TTTTGCTTCTACTACCAGAGATTCAATGCGTAATCTCAGCACTCAAGGGCTATTCCTAAA
TCATCTCGTTTTTCAATCACTCAACCCTTACTTTTTACCAAGTGCTATGTTAGCAAGACT
AGTCAACGTTTACATGTTTCGATGCAACAACAAGATTTTATTTGTAACAAGTAGTTTTG
TTGGTTGGTTAATTGGTTTCATTTTATTAATGAAA------------TTACTGGTCTGGATACAGC
ACAACCGTTTTATTAGATTGTACGTTCGATTTAATAAGTACCTTATGTCAGAATTTACGTA
CCGTATATTGCGAACCTTTAGTATTATCTTATTTATTACCTGTGTCTACTATTTAGGCAGAA
TGCCTTCACCCCTTCTTACTAAGAAACTGGAAAAATTTGAATTTGTTAAACTTCCTGTG
ATCCTTCTTTTTGATTATAAAGATTGGAATCGACCATTACGATACATAAAAAACAGGGAT
TTTAAAGGGGCTGTAAAAAGCGAAACGGCACAATATTTTTTTGATACATGCCGAAGTGA
TGGAAAACAAAGAATCTCTTTTACATATCCGCCCAGTTTGTCAACTTTTTTGGAAATGA
TACAAAGACGAATAGACGAGTTCACGCTCCCCCCC------TATGATGAACTGTATAATCAT
TGGGTTTATACAAAGACCAAAAAAAAGAACAAGATAAAAAACGAATTACTTAATAGAA
TTAAGGCTCTAGACATGGGATTTCTTTCTCGGGATATACTTGAAAAAAGAACTAGATTG

GTTAAGGACCGGCCTAATCAAAAATTATTGTCTCAAGAGTATGATCCTTTTTTGAACGG
ACCATATCGTGGAACACCCCAACAGCGGTTTTCACCT---AAGACAGTTAAAATGAATAA
GATTCATGGTATCCTTCAGAAAGAATTTGAACAGAAAAATTTGAGGGTCCCTGGAATTG
GAATCAAAGAAATCGATAAAAAAGTCCCTCGATGGTCATCCAAATTAATCGAAATGGA
AGCAGAATTTCTCGACCATCTTTGCTCAAGAGCACTGAAAGAAATAGTGCTATCTAAA
GAGATTTACCTAGATATATTGAAAGAGCCAGATTTTGAGCGAGACCTAATCATGAGTTC
TACACGCGCTCAAAGGCGGAAAGTTATTATTTTGAAACTGCTTCACCCAAAGCCGCAG
TCCCCGCTTTTTTGGGGCAGAATCAAAAGTGTTCTCGGTTATTTTTTTACTCAT-----------
CCAATAAAATTTATTTTTATAAATAAGGAAGAGCTAATGCGGATACAAGTGGAGAGCGC
ATGGGAAAACCTGCCATATGGACCGGGAATACGAAGTTCCTTATTACTAATGCAATCGT
TTCTTAGAAAAAATATAAGTCTCCCTTTACTCATAATAGCTAAAAATATTGGCCGTTTGT
TATTTTTGCAAGTTCCTGAGTGGGCTGAGGATTTTCAGGAATTGAATAGAGAAAGGCAT
TTTCCATGCACCCATCTTGGTGTTGGACTAAACGATCCAGAGGTTTTGGCCCATTGGGC
GGAAGAAGGTTTTGACATAAAAATCCTATATCCTTTCCGCTTGAAACCTTGGCACAGAT
CTAAGAAGGAAGTGGAAGATGATTTTGGTTTTTTAACCATTTTGGGAATGGAAACTGA
ATATCCTTTCGGTTCTCCCCGAAAAAGAACTTCCTTTTTTAAACCCATTTTTAAGAAACT
AGGAAAACAA--ATAAAAAAA
AAGAAGGCTTTTGAAGATTTTAAAGTTCTAGGG---GTTTTCAAAAAAGTAATATCAGAA
TTCTCAAAGAGAAAT------ACAATCAACAATCAGATAATTGAAGAATCGTTTACTCAAAT
TCGATCTATAGATCGGGCAAATAAGAATCTAACGGAACAAGAAATGAAGAATCTAACT
GATAGAACAAGCACAATCAAAAATAAAATACAAAGACTTACAAAAGATAAAAAAAAA-
-------------------ACAAATTCTAATGTAGAATCACAACCACTAATTTGGCAAATATTAAAAC
GAAGAAACGCTCGATTAATCAGCCAATTCCATTATTTTCTCAAAATTTTTATTGAAAAAA
TATACATAGATATCTTTCCACGTATCATTAATATTACCAGAATCAATACACAACCTTTA-----
------------GACTTATCTTCCTTGTCGCAAAGAGATGTATTTTTCAAATTATCACAACCCCA
AGCTATTAACTTGTCTAAGTTAAGATCTGCTCTTCAATATACATCTTTTTTTCTTAAGACT
GCAATAAAGGATTCTTTTATACAGGGAATATTTCATTCCGAATTAAAGCATAAGAAACCT
CGA------AGTTATGATCAATGGAAAAACTGGTTAAGAGGTCAT---CAATACAACTTATCTC
CGATTAGATGGTCTAGATTAATACCACAAAAATGGCGAAATAGAGTCAATCAACGTTGT
ACGGCTGAAAATAAACAAAAATGGAGTTCAGATGAAAATGAAAAAGACCTATTATTTC
ATTACACAAATCCA---AATTTTCAAAAATACTATAGATATGGTCTTTTATCATATAAATCTC
TTAATGCTAATATTATTGATCCAGATCGAAAATATTTTGATTGGAAAATGCAAAATTTTGC
TCTAAACTTTTTTGATTGGATAAAAATAGATAAAGAAAGACTAAGGAACTGGTTCTTCC
CAGAACATGTGCTACTTTATACTGCATATAAAGTGAAACCGTGGATTATACCAAATCCAC
TTTTTCTTGGAAATGGCGAAGCTTTTAGAGGAAGAATAAGAACCCCCGATTTAGTTCAG
TATTGGCTTTTTCAATTGAAATGGCACAATTATTTTGGGGAAACAATACCCGGACTAATG
AAACAATTTTCAGCCCAGCTAGAGTGGCTAAATTACCAAAAATGGGTGATAACCTATCT
GAACAATAAACTATTCAGTATAAATCAACATCTCATTCACTATGAAACTACACTAGAGAT
GGGTGAGCTGGGGGAGGTAGTGATTCTCGAATCGAATCGTCTGTATCTAGGAACTTATA
GCCAAATTATTATGTATCAGACCATACGCATTTCATTGGTTGATCAAAGTAAGCAAAATC
ATTATGATTTTCTTGTTCCCGAAACTATTTTATCATCTAGACGTCGTAGAGAGTTGAGAA
TTCTAATTTCTTTCAAATTTCTTTGGCCCAATTATCGATTAGAAGATTTAGCTTGTATGAA
TCGTTATTGGTTTGATACCAATAATGGGAGTCGTTTCAGCATGTTAAGGATATATATGTAT
CCAATGATCCGATATTCATTCCAACCTTTGATTTTCGAATTAAGAGAGATATTGAGAGAG

ATCAAGAATTCTCACCATTTATTAGATTCATGGACCCAATTCAATTCAGTAGCATCTTTC
ATTCACATTTTTTTCCACCAAGAACGTTTTATAAAACTTTTTGACCCCCGAATTTGGAGT
ATTCTACTTTCA---CGCAATTCA------CAGGGTTCAACAAGCAGTCGATATTTCAGG-------
--ATCAAGGGTGTAATACTCTTTGGAGTAGCG---GTCCTTCTATATCGT------------ATTAAC
AGTCGAAATATGGTCGAAAGAAAAAATCTCTATTTGATAGGGCTTCTTCCT---------------A
TACCTATGAATTCCATTGGGCCCAGAAACGATACATTGGAAGAATCCGTT---GGGTCTTC
CAATATCAATAGGTTGATTGTTTCG---CTCCTGTATCTTCCAAAAGGAAAAAAGATCTCT
GAGAGTTGTTTCCTGAATCCGAAAGAGAGTACTTGGGTTCTCCCAATAACT------------A
AAAAGTGTAGCATGCCTGAATCTAACTGGGGTTCGCGGTGGTGGAGGAACTGGATCGG
AAAAAAGAGG--

--GATTCT--
--------AGCCAATTGAAAGGATCTTCT---GATCAATCCAGAGATCCTTTGGATTCCGTTAGT
------------AATGAGGATTCGGAA--TATCACCCATTGATCAATC
AAAGAGAGATTCAACAACTAAAAGAAAGATCGATTCTT---TGGGAT------CCTTCCTTTC
TTCAAACGGAACGAACAGAGATAGAATCAGAC------CGATTCCCGAAA------TGCCTTTC
TGGATATTCC------TCAATGTCCCGGCTATTCACG---GAACGTGAGAAGCAGATGATTAAT
CATCTGCTTCCGGAAGAAATCGAAGAATTTCTT------GGGAATCCTACAAGATCCGTTCG
TTCTTTTTTCTCTGACAGATGG------TCAGAACTTCAT---CTGGGTTCGAATCCTACTGAG
AGGTCCACTAGAGATCAG------------AAATTGTTGAAGAAACAAGAAGATCTTTCT---TTT
GTCCCTTCCAGGCGATCGGAA------------AATAAAGAAATGGTTAATATATTCAAGATAATT
ACATATTTACAAAAT------ACCGTCTCAATTCATCCTATTTCATCCGATCCGGGATGTGATA
TGGTTCCG---AAGGATGAACCGGAT---ATGGACAGTTCCAATAAGATTTCATTCTTGAAC
AAAAATCCATTTTTGGATTTA------TTTCATCTATTCCATGACCGGAACAGGGGAGGATA
CACGTTA------CACCACGATTTTGAATCAGAA------GAGAGATTTCCAGAAATGGCGGAT
TTATTTACTCTATCAATAACCGAGCCG---GATCTGGTGTATCATAGGGGATTTGCTTTTTC
TATTGATTCCTACGGATTGGATCAAAAACAA------TTCTTGAATGAGGTATTCAACTCCA
GGGATGAATCGAAAAAGAAATCTTTA---------TTGGTTCTACCTCCTATTTTTTAT------GA
AGAGAATGAATCTTTTTATCGAAGGATCAGAAAAAAATGGGTCATG---ATCTCCTGCGG
GAATGATTTGGAAGATCCAAAACCAAAAAGAGTGGTATTTGCTAGCAACAACATAATG
GAGGCAGTCACTCAATCTAGATTGATCGGAAATCTGATTCAAATCCAATATAGCACCTAT
GGG------TACATAAGAAATGTATGGAATCGATTCTTTTTAATGAAT---------AGATCCGATC
GCAACTTCGAATATGGAATTCAA---------GGGGATCAAATAGGAAAGGATACTCTGAATC
ATAGAACTATAATGAAATATACGATCAACCAACATTTATCGAATTTGAAAAAG---AGTCA
GAAGAGATGGTTCGATCCT------CTTATTTTGATTTCTCGAACCGAGAGATCCATGAATC
GGGATCCTGATGCATATAGATACAAATGGTCCAATGGGAGCAAGAATTTCCAGGAACAT
---------TTCGTTTCTGAGCAGAAGAGCCGT---------TTTCAAGTAGTGTTCGATCGATTACG
TATTAATCAATATTCGATTGATTGGTCTGAGGTTATCGACAAAAAAGATTTGTCTAAGCC
ACTTCGTTTCTTTTTGTCTAAGCCACTTCGTTTCTTTTGGTCCAAGTCACTTCGTTTCTT
TTTGTCCAAGTCACCTCTTTTTTTGTCTAAGTTGCTTTTCTTTTTGTCTAAC---TCACTTC
CTTTTTTCTTTGTGAGTTTCGGGAATATCCCCATTCATAGGTCCGAG---ATCTACATCTAT-

--GAATTGAAAGGTCCGAATGATCAACTCTGCAATCAGTTGTTAGAATCAATAGGTCTTC
AAATCGTTCATTTGAAAAAATGGAAACCCTTC------TTATTGGAT---------GATCATGATAC
TTCC---CAAAAATCGAAATTCTTGATCAACGGAGGAACAACCTCGCCATTTTTGTTCAA
TAAGATACCA---AAGTGG---ATGATTGACTCATTTCATACTAGA------AAGAATCGCAGGA
AATCCTTTGATAACCCGGATTCCTATTTCTCAATGATATTCCACGATCAAGACAATTGGC
TAAATCCCGTGAAACCATTTCATAGAAGTTCATTGATATCTGCTTTTTATAAAGCAAATC
GACTTCGATTCTTGAATAATCCACACCACTTCTGCTTCTAT------TGTAACAAAAGATTCC
CTTTTTATGTG------------GAAAAGGCCCGTATCAATAATTATGAT------TTTACGTATGGA---
CAATTCCTCAATATCTTGTTCATTCGCAACAAA---ATATTTTCTTTGTGCGTCGGTAAA----
--AAAAAACATGCTTTTTGGGGG---AGAGGTACTATTTCACCAATCGAGTCACAGGTATC
TAACATATTCATACCTAATGATTTTCCACAAAGTGGTGACGAGACA---TATAACTTATAC
AAATCT---------TTCCATTTTCCAAGTCGACCCGATCCATTCGTTCGT------AGAGCTATTTA
CTCGATCGCAGACATTTCTGGAACACCTCTAACAGAGGGACAAATGGTCCATTTTGAA
AGAACTTATTGTCAACCTCTTTCAGATATGAATCTATCTGATTCAGAAGGGAAGAACTT
GCATCAGTAT------------TTCAATTCAAACATGGGTTTGATTCACACTCCATGTTCTGAGAA
ATATTTACCATCCGAAAAG------AGGAAAAAA------------------------CGGAGTCTTTGTCTA
AAG------AAATGCGTTAAGAAAGGGCAGATGTATAGAACCTTTCAACGAGATAGTTCTT
TTTCAACTCTCTCCAAATGGAATCTATTCCAAACATATATGCCATGGTTCTTTACTTCGA
CAGGGTACAAATATCTAAATTTGATATTGTTAGATATTTTTTCAGACCTATTGCCGATACT
AAGTAGGAGTCAA---------AAATTTGTATCCAATATTCATATTATGCGTCGATTAGGT---ATA
TCATGGCGAATTCTTCAGAGAAAATGGGGT------------------CTTCAA---TGGAATTGG---AT
AAGTGAGATTTCGAGTAAGTGTTTACAT------CAGCTTCTTCTGTCCGAAGAAATGATTC
ATCGAAATAATGAGTCA------CCATTGATATCGACACATCTGAGATCGCCAAATGTTCGG
GAGTTC---CTCTATTCAATCCTTTTCCTTCTTCTTGTTGCTGGATAT---CTCGTTCCTACAC
ATCTTCTCTTTGTTTCCCGG------GCCTCTAGTGAGTTACAGACAGAGTTCGAAAAGGTC
AAATCTTTGATGATTCCATCATCTATGATTGAGTTGCAAGAACTTCTGGAGAGTGATCCT
CCATCTGAACCAAATGATCCTATATTCAAGCATCCCCTTCAAGCTAGGCTGGAACAATT
AGCAGATTTTCTAGTATGGAAGGTTTTTTTTTCTGTCGGCGACATGCTATTTAATTTGTCT
---ATCGATCTCGTCGATATCGATATCATCGATCTC
ATAAGTAACATA------------CCAAATCCCAACGATCAAATCACTTTTTCGAGAAATACGAG
ACATCTAAGTCATACAAGTAAAGAGATCTATTCATTGATAAGAAAAGTGAAGGTTGGGA
ACGGGGATTGGGCTGATGATCAAGTAGAATCCTGGGTCGCAAACAGTGATTACGTTGA
TGATGAAGAAAGAGAATTCTTGGTTCAGTTCTCCACC---TTAACGACAGAAAAA---AGG
ATTGAGATTGATCAAATTCTATGGAGTCTGACT---CATAGTGATCATTTCTCA---------------
AAGAATGACTCTGGTTATCAAATGATTGAACAACCGGGAGAAATTTACTTACGATACTT
AGTTGACATTCAT------AAAAAGTATCTAATGAATTATGAGTTCAATACATCCTGTTTAGC
GGAAAGACGGATATTCCTTGCTCATTATCAGACAATCACTTAT------TCACAAACCCCGT
GTGGGGCTAATAGTTTTGATTTCCCATCGCCTGGA---------AAACTCTTTTCGCTCCGCTT
AGCCTTATCCCCCGCT---AGGGGTATTTTAGTGATAGGTTCTATAGGAACTGGACGATCC
TATTTGGTCAAATACCTAGCGACAAACTCTTATGTTCCTTTCATTACGGTATTGTATGAG
GAAGAGCTTGATAATGATCCTTCTAATTTTGCTGCTTATAAGGATGTTGAGATGTTTTTT
GAGGTTATTGATGATATTGATGATAGTAAGATTGATGAGAGTGATATTGATGAGAGTCAA
ATTGATGAG------------------------------------AGTCCGATTGATGATAGGACGATTGATAGGCA
GATGGATCATAGGCTGGACGGGATAACCGCATGGGATGCGTTAGGTATGGAGGTGTTGT

CGTTAAGAAACCACTATTATATCAACCTTCAATTCGAATTAGCAAAAGCAATGTCTCCTT
GCATAATATGGATGCCAAACATTCATGATCTGCGTGTGAAGGAGTCGGATTGCTTATCCC
TCGGTCTATTAGTGAACCATCTCTCCGGG---------GATTGTGAAAGATGTTCC---ACTAGA
AATATTCTTGTTATTGCTTCGACTCATATTCCCCAAAAGGTGGATCCCGCTGTAATAGCT
CCGAATAAATTAACTACGTGCATTAAGATACGAAAGCTTCTTATTCCACACCAACGAAA
GCACTTTCTCACTCTTTCATATACTAGGGGATTTCGCTTGGAAAATCAAATGTTTCATAC
T------AATAGATTCGGGTACATAACCATGGGTTCCAATCCACAAGATCTTGCAGCACTTA
CCAATGAGGCCCTATCGATTAGTATTATACAGAAGAAATCAATTATAGACACTAATACAA
TTCGATTCGCTCTTCATAGACAAACTTGGGATTTGCGA---------TCCCAGGTAAGATCGG
CTCAGGATCGGGGGATCCTTTTCTATCAGATAGGAAGGGCTGTAGCACAAAATGTACTT
CTAAGTAATTGCCTCATAGATCCTATATCTATCTATCTCAAGAAGAAA------TCATGTAAC
GAAGGGGATTCTTATTTGTACAAATGGTACTTCGAACTTGGAACGAGCATGAAGAAATT
AACGATACTTCTTTATCTTTTGAGTTGTTCTGCCGGATCGGTCGCTCAAGATCTTTGGTC
TCTACCC------GGACCCGATGAAAAA---ACTGGGATCGCTTATTATAGACCCGCTGAGAA
TGATTCT---GATCTAGTTCATGGCCTATTAGAAGTAGAAGGCGCTCTGGTGGGA------GA
CTCACGGACAGAAAAGGATTGCAGTCAGTTTGATAATGAT------CGAGTGACATTGCTTC
TTCGGCCCGAACCAAGGAAT---CCCTTAGATATGATGCAAATTGGATATTTTTCTATCCTT
GATGTGGGATTTCTCTATCAAAGA------TCCGAATTGGAGTTTGAAGAC---------GGGGAA
GGAGCCCTCGACCTGGAA---------------------CAGGAGTTC------TTCACTAAC------------ATA
GTTTGGGCTCCT------AGAATATGGAGCCCT---------TGGGACTTTCTATTGGATTGTATCG
ACATTCCAACTGAATTGGGATTTCCCTTCTATGGGTCCGATGAAAAGTATGCTGGA---GA
TAAGGAAGAGTATTATGATGAAGAGGAAGAGAATGATGAAGAAGAGGATGAGCTTCA
AGAGAATGATTCGGAGTTCTTGCAGACACGAGATATATCTTACAAAAAAGAAAGCCTT
TTTCGACTAAGCCGATTCATTTGGAACCCTGGAGATCCACTCTTTGGGCTATTCGTA---G
ATGAGTCCTCTGTCTCTGTGTTTTTACATCGAGAATTATTTGCAGATGAAGCACAGAGC
CTTTTGACTTCCGTGACTTCCGAAGAACCTTCTAGATCTATGATGAAAGGCTGGTTTAT
CGAC---AATAAGGCGCAAGAAAAGCACTTCGAATTCTTGCTGAATTGGCATAGATGGTT
TAGAACC---------CCTAGTTCATTATCT------AATGGATCTTTCCGTTCTAAT------ACTCTAT
TCGAGAGTTATCAGTATTTATCAAATCTGTTCCTATCTAAC---GAAACGCTATTGGATCAA
ATGAGAAATACATTGCTGAGAAAAAGATGGCTTTTCCCGGATGAAAATTGGATTCATGG
A------ACAGGAGAAAGATTTAAAGATATG------ATGCCCAGATCGCGG---ATAAATGGAAA
TTTTATTGATAAGACCTTTTCGATTGTAGCCAATATCTTATTACGAATAATTCCGACAACT
TCAGGAGAAAAAGAGGCATTTACCTATTACAGAGATGGA------ATGTCAGCTCAATCTG
AAGGAAATTTTGCGGAAGCCTTACAGAATTATTATGAAGCTATGCGACTCGAAATTGAT
CCCTACGATCGAAGTTATATACTCTATAACATAGGACTTATCCATACAAGTAACGGAGAA
CATACGAAAGCTTTGGAATATTACTTTCGAGCGCTAGAACGAAACCCATTCTTACCACA
AGCTTTTAATAATATGGCCGTGATCTGTCATTAC---CGAGGAGAACAGGCCATTCGACA
GGGAGATTCTGAAATTGCGGAGGCTTGGTTCGATCAAGCCGCTGAGTATTGGAAACGG
GCTATAGCGCTTACTCCCGGTAATTATATTGAAGCGCATAATTGGTTGAAGATCACCAGG
CGTTTCGAA------ATGAGTTGTCGATCAGAACATATATGGATAGAACCTATAACGGGGTC
TCGAAAAACAAGTAATTTCTGCTGGGCCATTATCCTTTTTTTAGGTTCATTAGGATTCTT
ATTGGTTGGAACTTCCAGTTATTTTGGTAGAAATTTCACATCTTTATTTCCGTCTCAGCA
AATCGTTTTTTTTCCACAAGGGCTCGTGATGTCTTTCTATGGGATCGCGGGTCTCTTTAT
TAGTTCCTATTTGTGGTGCACAATTTCGTGGAATGTAGGTAGTGGTTATGATCGATTCGA

TAGAAAAGAAGGAATAGTGTGTATTTTTCGTTGGGGATTTCCTGGAAAAAATCGTCGCA
TCTGCCTCCGATTCTTTATAAAGGATATTCAGTCCATCAGAATAGAAGTTAAAGAGGGT
CTTTATGCTTGTCGTGTCCTTTATATGGACATCAGAGGTCAGGGGGCCATTCCTTTGACT
CGTACTGATGAGAAT---TTGACTCCACGGGAAATTGAACAAAAAGCTGCTGAATTGGCC
TATTTCTTGCGTGTGCCAATTGAAGTATTT
>Meliosma
ATGGAAAAATGGTGGTTCAATTCGATATTGTCTCTAAGTAAATCAATGGGCAGTCTTGG
TCCTATTAGAAAGACCAATGGAAGGGAAAACCCCTTTATAAATGATACGGAGAAAAATA
TTCCTAGTTGTAGTAATGTTGATCATTTATTCGGTGTCAGGAACATTCGGAGTTTCATCT
TTGATGATACTCTTTTAGTTAGGGATAGTAATGGTTATAGTTATTCGATATATTTTGATATT
GAAAATCAGCTTTTTGAGATTGACAATGAT------CATTCTTTTCTGAGTGAACTAGAAAA
AGAACTTTCTAGTTATCTGAATTCTAATTATCTGAATACTGGGTCTAAGAGTGACAATCA
CTACTATGATCGTTACATGTATGATACTAAATATAGTTGGAATAATCATATTAATAGTTGCA
TTGACAGTTATCTTTGTTCTGAAATCAGT------ATTGATAGTTACATTTCAAATGGTAGCG
ACAATTACAGTGACAGTTACATTTATAGTTACATTTGTAGTGACAGTAGAAGTTCTATAA
GA---------AATGACAGTGAT---AATAAAAGAGGAAGATAT---GATCTCGAGATAAATAAAA
GATACAGGACTTTGTGGGTTCAATGCGAAAATTGTTATGGATTAAATTATAAGAAATTGT
TTCGGTCAAAAATGAATATTTGTGAACAATGTGGATATCATTTGAAAATGAGTAGTTCA
GATAGAATCGAACTTTCGATTGATCCGGGCACTTGGGATCCTATGGATGAAAACATGGT
CTCTACGGACCCGATTGAATTTCATTCGGAGGCAGACCCTTATAAGGATCGTATCGATTT
TTATCAAAGAAAGACAGGGTTAACTGAGGCTGTTCAAACAGGCATAGGTCAACTAAAT
GGTATTCCCGTCGCAATGGGGGTTATGGATTTTAAGTTCATGGGGGGTAGTATGGGATC
CGTAGTAGGCGAGAAAATCACCCGTTTGATCGAGTATGCTACTCATAGATCTCTACCTC
TTATTATAGTGTGTGCTTCCGGAGGAGCACGTATGCAAGAAGGAAGTTTGAGCTTGATG
CAAATGGCTAAAATATCTTCCGCTTCATATAATTATCAATCAAATAAAAAGTTATTCTATG
TATCAATCCTTACATCTCCTACAACCGGCGGGGTGACAGCTAGTTTTGGTATGTTGGGA
GATATCATTATTGCCGAACCCAATGCCTACATTGCATTTGCGGGTAAAAGAGTAATTGAA
CAAACATTGAATAAGACAGTACCTGAGGGTTCACAAGCGGCTGAGTATTCATTCCATAA
GGGCTTATTCGATTCAATCGTACCCCGTAATCTTTTAAAAGGCGTTCTTAGTGAGTTATT
TTATCTCCACGGTTTCTTTCCCTTGAATCAAAATTCAATCAAGATGGTAACCCTTCGAGC
CGACGAAATTAGTAATATTATCCGTGAACGTATTGAGCAATATAATAGAGAAGTAAAGAT
TGTGAATACTGGCACCGTACTTCAAGTAGGCGACGGCATTGCTCGTATTCATGGTCTTG
ATGAAGTAATGGCAGGTGAATTAGTAGAATTTGAAGAGGGTACAATTGGCATTGCTCTG
AATTTGGAATCAAATAATGTTGGTGTTGTATTAATGGGTGACGGTTTGATGATACAAGA
AGGAAGTTCTGTAAAAGCAACAGGAAGAATTGCTCAGATACCGGTGGGCGACGCTTAT
TTGGGTCGTGTTATAAATGCCCTGGCTAAACCTATTGATGGTAGAGGTGAAATTTCAGC
TTCTGAATCTCGGTTAATTGAATCGCCCGCCCCAGGTATCATTTCGAGACGTTCCGTATA
TGAGCCTCTTCAAACAGGGCTTATTGCCATTGATTCGATGATCCCTATAGGACGTGGTC
AGCGAGAATTAATTATTGGGGACAGGCAAACCGGTAAAACAGCAGTAGCCACAGATAC
TATTCTCAATCAAAAAGGGCAAAATGTAATATGTGTTTATGTAGCTATTGGTCAAAAAG
CATCTTCTGTGGCTCAGGTAGTTACTACTTTCCAGGAAAGGGGGGCGATGGAATACACT
ATTGTGGTAGCCGAAACGGCAGACTCGCCCGCTACATTACAATACCTCGCTCCTTATAC
AGGAGCGGCTCTGGCTGAATATTTTATGTATCGTGAACGACATACTTCAATCATTTATGA
TGATCTCTCCAAACAAGCACAAGCTTATCGACAAATGTCTCTTCTATTACGAAGACCGC

CCGGTCGCGAAGCTTATCCAGGAGATGTTTTTATTTGCATTCACGCCTTTTGGAAAGA
GCCGCTAAATCAAGTTCGCGTTTAGGTGAAGGAAGTATGACCGCTTTACCAATAGTTGA
GACTCAATCGGGAGACGTTTCGGCTTATATTCCTACTAATGTAATTTCCATTACAGATGG
ACAAATATTCTTATCCGCCGATCTATTCAATGCCGGAATCCGGCCTGCTATTAATGTGGG
TATTTCTGTCTCCAGAGTAGGATCTGCAGCTCAAATTAAAGCCATGAAACAAGTAGCTG
GCAAATCAAAATTAGAATTGGCTCAATTCGCAGAGTTAGAAGCCTTTGCACAATTTGCT
TCTGATCTTGATAAAGCTACTCAGAATCAATTGGCAAGAGGTCAACGATTACGTGAATT
GCTCAAACAATCCCAATCAGCCCCTCTCACGGTGGAAGAACAGATATTGACTATTTATA
CTGGAACGAATGGATATCTTGATTCGTTAGAAATTGGACAAGTAAAGAAATTTCTCGTT
CAGTTACGTAACTACTTAAAAACGAATAAACCTCAGTTCCAAGAAATCATATCTTCGAC
CAAGACATTCACCGAGGAAGCAGAAACCCTTTTGAAGGAAGCTATTCCGGAACAGAT
AGAACGCTTTCTACTTCAGGAACAAACAATGAGAATCAATCCTACTACTTCTGGTCCTG
GAGTTCCCGCACTTGAAGAAAAAAACCTGGGGCGTATCGCTCAAATCATTGGTCCGGT
ACTGGATGTAGCTTTTCCCCCGGGCAAAATGCCTAATATTTACAACGCTTTGGTAGTTA
AGGGTCGAGATACTGTCGGCCAGCAAATTAATGTGACTTGTGAGGTACAGCAATTATTA
GGAAATAATCGAGTTAGAGCTGTAGCTATGAGTGCTACAGACGGTCTGATGAGAGGAA
TGGACGTGATTGACACGGGAGCTCCTCTAAGTGTTCCAGTCGGTGGAGCAACTCTCGG
ACGAATTTTCAACGTTCTTGGAGAGCCTGTTGATAATTTAGGTCCTGTAGATACTCGCA
CAACATCCCCTATTCATAGATCCGCACCTGCCTTTATACAGTTAGATACCAAATTATCTAT
TTTTGAAACAGGAATTAAAGTAGTGGATCTTTTAGCTCCTTATCGCCGTGGAGGAAAAA
TCGGACTATTCGGGGGAGCTGGAGTGGGTAAAACAGTACTCATCATGGAATTGATCAA
CAACATTGCCAAAGCTCATGGAGGTGTATCTGTATTTGGCGGAGTAGGCGAACGTACTC
GTGAAGGAAATGATCTTTACATGGAAATGAAAGAATCTGGAGTGATTAATGAACAAAA
TATTGCAGAATCAAAAGTAGCTCTAGTCTATGGTCAGATGAATGAACCGCCGGGAGCTC
GTATGAGAGTTGGTTTGACTGCCCTAACCATGGCGGAGTATTTCCGGGATGTTAATGAA
CAAGACGTACTTTTATTTATCGACAATATCTTCCGTTTCGTCCAAGCAGGATCCGAAGTA
TCTGCCTTATTAGGCAGAATGCCTTCCGCTGTGGGTTATCAACCTACCCTTAGTACAGA
AATGGGCTCTTTGCAAGAAAGAATTACTTCTACCAAAGAAGGATCCATAACTTCTATTC
AAGCAGTTTATGTACCTGCGGACGATTTGACCGACCCTGCTCCTGCCACGACATTTGCA
CATTTAGATGCTACTACCGTACTATCAAGAGGATTAGCTGCTAAAGGTATCTATCCAGCA
GTAGATCCTTTAGATTCAACGTCAACTATGCTCCAACCTCGGATTGTTGGTGAGGAACA
TTATGAAACTGCGCAAAGAGTTAAGCAAACTTTACAACGTTACAAAGAACTTCAGGAC
ATTATAGCTATCCTTGGGTTGGACGAATTATCTGAAGAGGATCGTTTAACCGTAGCAAG
AGCGCGAAAAATTGAGCGTTTCTTATCACAACCCTTCTTCGTAGCAGAAGTATTTACCG
GTTCTCCAGGGAAATATGTTGGTCTAGCAGAAACAATTAGAGGATTTCAACTGATCCTT
TCCGGAGAATTAGATGGTCTTCCTGAGCAGGCCTTTTATTTGGTAGGTAACATCGATGA
AGCTACCGCGAAGGCTATGAACTTAGAAGTGGAGAGCAATTTGAAGAAAATGACCTTA
AATCTTTGTGTACTGACTCCTAATCGAATTGTTTGGGATTCAGAAGTGAAAGAAATCAT
TTTATCTACTAATAGTGGCCAAATTGGCGTATTACCAAATCACGCCCCTATTGCCACAGC
TGTAGATATAGGTATTTTGAGAATACGCCTTAATGACCAATGGTTAACGATGGCTCTGAT
GGCGGCTTCGCTAGAATAGGCAATAATGAGATCACCATTTTAGTAAATGATGCGGAGA
AGGGTAGTGACATTGATCCGCAAGAAGCTCAGCGAACTCTTGAAATAGCTGAAGCTAA
CTTGAGTAGAGCTGAAGGCAAAAGACAAACAATTGAGGCGAATCTAGCTCTCAGACG
AGCTAGGACGCGAGTAGAGGCTATCAATGTCATTTCGATGAAAAATGTAACCGATTCTT

TCGTTTCCTTGGGTTACTGGCCATCCGCCGGGAGTTTCGGGTTTAATACCGATATTTTAG
CAACAAATCCAATAAATCTAAGTGTAGTGCTTGGTGTATTGATCTTTTTTGGAAAGGGA
GTGTTAAGCGATTTATTAGATAATCGAAAACAGAGGATCTTGAGTACTATTCGAAGTTC
AGAAGAACTACGTGAAGGGGCCATTCAACAGCTGGAAAAAGCCCGGGCCCGCTTGCG
GAAAGTAGAAATGGAAGCAGATGAGTTTCGAGTGAATGGATATTCTGAGATAGAGCGA
GAAAAATTGAATTTGATTAATTCAACTTATAAGAATTTGGAACAATTAGAAAATTACAA
AAATGAAACCATTCATTTTGAACAACAAAGAGCGATTAATCAAGTCCGACAACGAGTT
TTTCAACAAGCCTTACAAGGAGCTCTAGGAACTCTGAATAGTTGTTTGAACAGCGAGT
TACATTTACGCACCATCAATGCTAATATTGGCATGTTTGGGGCGATGAAAGAAATAACT
GATATGAATCCACTGATTTCTGCCGCTTCCGTTATTGCTGCTGGATTAGCCGTAGGGCTT
GCTTCTATTGGACCTGGAGTTGGTCAAGGTACTGCTGCGGGCCAAGCCGTAGAAGGTA
TCGCGAGACAACCAGAGGCAGAGGGTAAAATACGAGGTACTTTATTACTTAGTCTGGC
TTTTATGGAAGCTTTAACAATTTATGGACTGGTCGTGGCATTAGCGCTTTTATTTGCGAA
TCCCTTTGTTATGAATGTTCTACCATGTTCCATCAACACACTAAAAGAATTCTATGATATA
TCCGGTGTGGAAGTAGGCCAGCATTTCTATTGGCAAATAGGAGGTTTCCAAGTCCATGC
CCAAGTACTTATTACTTCTTGGTTCGTAATTGCTATCTTATTAGGTTCAGCCACTATAGCT
GTTCGGAATCCACAAACCATTCCTACCGACGGTCAGAATTTCTTCGAATATGTCCTTGA
ATTCATTCGAGACTTAAGCAAAACTCAGATTGGAGAAGAATATGGTCCTTGGGTTCCCT
TTATTGGAACTATGTTCTTATTTATTTTTGTTTCTAACTGGTCAGGGGCTCTTTTACCTTG
GAAAATCATACAGTTACCTCATGGGGAGTTAGCCGCACCCACGAATGATATAAATACTA
CTGTTGCTTTAGCTTTACCCACGTCAGTGGCATATTTCTATGCGGGTCTTACCAAAAAA
GGATTAGGTTATTTCGGTAAATACATTCAACCTACTCCAATACTTTTACCAATTAACATCC
TAGAAGATTTCACAAAACCTTTATCACTTAGTTTTCGACTTTTCGGGAATATATTGGCTG
ATGAATTAGTAGTTGTTGTTCTTGTTTCTTTAGTACCTTTAGTAGTTCCTATACCTGTCAT
GTTCCTTGGATTATTTACAAGTGGTATTCAAGCTCTTATTTTTGCAACTTTAGCCGCGGC
TTATATAGGCGAATCCATGGAGGGTCATCATATGATATTTTTGACTTTAGAACATATATTA
ACTTATATCTCTTTTTCGATCGTTTCAATTGTAATTACGATTCATTTGATGACCTTATTAGT
CGATGAAATCATAGGACTATATGATTCGTCAGAAAAAGGCATGATAGTTACTTTTTTTT
TATAACAGGATTATTAGTTACTCGTTGGATTTATTCGGGTCATTTCCCATTAAGTGATTTA
TATGAATCATTAATGTTCCTTTCATGGAGTTTTTCCATTATTCATATGGTTACGAAAATAC
GGAACCATCAAAACCATTTAAGCGCAATAACCGCGCCAAGTGCTATTTTTACCCAAGG
CTTTGTCATTTCGGGTCTTTTAACTGGAATGCATCAATCCGCCATATTAGTACCCGCTCT
CCAATCCCAGTGGTTAATGATGCACGTAACTATGATGTTATTGAGCTATGCAGCTCTTTT
ATGTGGATCATTATTATCAGTAGCTCTTCTAGTCATTACATTTCGAAAAAACATAGAGAT
TGTTGGTAAAAGCAATAAGATCCAATCCATGAATGAAAGAAATACTCTTTTACAAAACA
CTTCTTTTCTTTCCTTTAGAAATTATCACAGGTATCAATTGATTCAGCAATTGGATCATTG
GAGTTATCGTGTGATTAGTCTCGGATTTATCTTTTTAACCATAGGTATTCTTTCAGGAGC
AGTATGGGCTAATGAGGCATGGGGTTCGTATTGGAATTGGGACCCTAAGGAAACTTGG
GCATTTATTACTTGGACCATATTCGCGATTTATTTCCATATTCGAACAAATAACAATTTGC
AAGGTGCGAATTCTGCAATTGTGGCTTCTATGGGATTTCTTATAATTTGGATATGCTATTT
TGGAGTCAATCTATTAGGAATAGGACTACATAGTTATGGTTCATTCATATTAACAATGCC
AAAAAAGAAAGCATTCACTCCCCCCCCATATCTTGCATCTATATTATTTTTGCCCTGGTG
GATCTCTCTTTCATTTAATAAAAGTCTGGAATCTTGGGTTACTAATTGGTGGAATACTAG
ACAATCTGAAACTTTTTTGAATGATATTCAAGAAAAGTGCGTTCTAGAAAAATTTATAG

AATTAGAAGAACTATTCCTGTTGGACGAAATGATAAAGGAATACCCGGAGACACATATA
CAAAAGCTTCGTATAGGAATCCACAAAGAAACGATACAATTGGTCAAGATGTACAATG
AGGATCATATCCATGCCATTTTGGACTTCTCGACAAATATAATATGTTTCGCTATTCTAAG
TGGTTATTCTATTCTGGGTAATGAAGAACTTGTCAGTCTGAATTCTTGGGTTCAGGAATT
CCTATATAACTTAAGCGACACAATAAAAGCTTTTTCTATTCTTTTATTAACTGATTTATGC
ATCGGATTCCACTCGCCCCATGGTTGGGAACTAATGATTGGTTCTGTCTACAAAGATTT
TGGATTTTCTCATAACGATCAAATTATATCTGGTCTTGTTTCCACTTTTCCAGTCATTCTA
GATACAATTTTTAAATATTGGATCTTCCGTTATTTAAATCGTGTATCTCCGTCACTTGTAG
TGATTTATCATTCAATGAATGACATGCCTATTGGTGTTCCAAAAGTACCTTTTCGGAGTC
CTGGAGAGGAAGATGCAATTTGGGTTGACGTATACAACCGACTTCATCGAGAAAGATT
ACTTTTTTTAGGCCAAGGGGTTGAAAGCGAGATCTCGAATCAACTTATTGGTCTCATGG
TATATCTCAGTATAGAGGATGATACCAGGGATCTATATTTGTTTATAAACTCTCCCGGTGG
ATGGGTAATACCCGGAATAGCAATTTATGATACTATGCAATTTGTGCCACCAGATGTACA
TACAATATGCATGGGATTAGCCGCTTCAATGGGCTCTTTTATCCTGGTCGGAGGAGAAA
TTACCAAACGTCTAGCATTCCCTCACGCCAGGGTAATGATCCACCAACCTGCTAGTTCT
TTTTATGAGGCACAAACAGGAGAATTTATCCTAGAAGCGGAAGAACTACTGAAACTTC
GCGAAACCCTCACAAGGGTTTATGTACAAAGAACGGGCAACCCCCTATGGGTTGTATC
CGAAGACATGGAAAGAGATGTTTTTATGTCAGCAACAGAAGCCCAAGCTCATGGAATT
GTTGATCTTGTAGCGGTCGAAATGAAAGAACAAAAATGGATTCATGAAGGTTTAATTAC
TGAATCACTTCCCAATGGTATGTTCCGGGTTCGTTTAGATAATGAAGATCTGATTCTAGG
TTATGTTTCAGGAAGGATCCGACGTAGTTTTATACGGATACTACCAGGAGATAGAGTCA
AAATCGAAGTAAGTCGTTATGATTCAACCAGAGGGCGTATAATTTATAGACTCCGCAAC
AAAGAAGCGAACGATATGGAGGAATTACAAGAATATTTAGAAATAGATGGCTCTTGGC
AACAAGACTTTCTATATCCACTTCTATTTCAGGAGTATATTTATGCACTTGCTCATGATCA
TGGTTTAGAAAATTTCGGT---AAATCCAGTTTACTGATTGTGAAACGTTTAATTACTCAA
ATGTATCAACAGAATCATTTGAGTATTCCGGCTAATGATTCGAACCAAAATCAATTAGTT
GTCAAGAATTTGGATTCTCAAATGATATCAGAGGGATTTTCAGTCATTGTGGAAATTCC
ATTTTTGCTGCGATTAGTATCTTCCCTAATAGAAAAATCTCATAATTTACGATCAATTCAT
TCAATATTTCCTTTTCTAGAGGACAAATTCTCACATTTAAATTATGTGTCACATATACTAA
TACCCCATCCCATACACTGGGAAGTCATGGTTCAAAACCTTCGCTGCTGGGTACAAGAT
GCACCTTCTTTGCATTTATTGCGATTCTTTCTCCACGAATATCATAATTCGAATAGTCTTA
TTACTAAATCGATTTCCATTTTGAAAAAACAAAGATTATTCTGTTTTCTGTATAATTCTTA
TATATATGAATGCGAATCCATAGTCGTTTTTATCCGTAAAAAATCTTCTCATTTACGATCA
ACATCTTCTGGAGCCCTTCTTGAGCGAGCACATTTCTATGGAAAAATGGAACATCTTGT
AGTAGTGTTTCGTAATGATTTTAACCTATGGTTTTTCAAGGAGCCTTTTATCCATTATGCC
AGATATCAAGGAAAAGCAATTCTGGCTTCAAAGGGCACTCCGCTTCTAATGAAGAAAT
GGAAATATCACCTTTTAAATTTATGGCAATGTCATTTTTTACTTATGGTCTCAACCGAGCA
GGATCCATATAAACCGATTATCCAATCATTCCCTTCAATTTCTGGGCTATCTTTCAAGTGT
ACGACTAAATCCTTCAGTGGTTAGGAGTCAACTGCTAGAAACTTCATTTATAATAGATAT
TGCTATT------AAGAAATTCGATACCATAATTCCAATTATTCCTCTGATCGGATCATTGGCT
AAAGCTAAAATTTGTAACGTATCAGGACATCCTATTAGTAAGCCGGTCTGGGCCGATTC
ATCCGATTCTGATATTATCGATCGATTTGGGTGGATATGCAGAAATCTTTCTCATTATCAC
AGTGGATCCTCAAAAAAAAATGGTTTGTATCGAATAAAGTATATACTTCGACTTTCGTG
TGCTAAAACTTTGGCTCGTAAACACAAAAGTACTGTACGCGCTTTTTTGAAACGATTAG

GTTCGGAATTATTGGAAGAATTCCTTACGAAGGAAGAACAAGTTCTTTCTTTGATCTTC
CCAAGACGGATTTGGTATTTGGATATTATTCGTATCAATGATCTGGCTAATTATGAAATGA
TAATTGATACGACA---GAAGTACAA---GCTATCAATCCTTTTTCCAGATCGGAA---TCCTT
AAAAGAGGTCTATGAGCTCATATGGCTATTTGTTCCTATTTTTACTCCTGTATTGGGAAT
CACAATAGGGGTACTAGTAATTGTATGGTTAGAAAGACAAATCTCCGCAGGGATACAAC
AACGTATTGGACCTGAATATGCTGGCCCTTTGGGAATTCTTCAAGCTCTAGCAGATGGG
ACCAAACTACTTTTCAAAGAGGATCTTCTCCCATCTAGAGGAGATAGTCGTTTATTCAG
TATTGGACCATCTATAGCGGTCATATCAATTCTATTAAGTTATTCAGTAATTCCGTTTGGC
TATCGCCTTGTTTTAGCCGATCTCAGTATAGGTGTTTTTTTATGGATCGCCATTTCAAGCA
TTGCTCCTATTGGACTTCTTATGTCAGGATATGGATCGAATAATAAATATTCCTTTTCAGG
TGGTCTACGAGCTGCTGCTCAATCGATTAGTTATGAAATACCATTAACTCTATGTGTGTT
ATCAATATCTCTACTATCTAACAGTTCAAGTACAGTTGATATAGTTGAGGCGCAGTCAAA
ATATGGTTTTTGGGGGTGGAATTTGTGGCGTCAACCTATAGGGTTTATCGTTTTTCTAAT
TTCTTCCCTGGCGGAATGTGAGAGATTACCTTTTGATTTACCAGAAGCGGAGGAAGAA
TTAGTAGCAGGTTATCAAACCGAATATTCAAGTATCAAATTTGGTTTATTTTACGTTGCT
TCCTATCTAAATCTACTAGTTTCTTCATTATTTGTAACAGTTCTTTATTTGGGCGGTTGGA
ATTTCTCTATTCCGTACATATTCATTCCTGAACTGTTAAGAATAAAT---------GGAGTCTTT
GGAACGACAATTGGTATCTTTATTACATTAGCTAAAGCTTATATGTTCCTGTTCATTTCTA
TCACAACAAGATGGACTTTACCTAGGATGAGAATGGACCAACTATTAAATCTTGGCTGG
AAATTTCTTTTACCTATTTCTCTAGGTAATCTATTATTGACAACTTCCTTCCAACTCTTTT
CACTCATGATCTGGCATGTACAGAATGAAAACTTCATTCTCGATTCTACGAGAATTTTTA
TGAAAGCGTTTCATTTGTTTCTCTTCCATGGAAGTTTCATTTTCCCAGAATGTATCCTAA
TTTTTGGCCTAATTCTTCTTCTGATGATCGATTCAACCTCTGATCAAAAAGATATACCTT
GGTTATATTTCATCTCTTCAACCAGTTTAGTAATGAGCATAACGGCCCTATTGTTCCGAT
GGAGAGAAGAACCTATGATTAGCTTTTCGGGAAATTTCCAAACGAACAGTTTCAACGA
AATCTTTCAATTTCTTATTTTACTATGTTCAACTCTATGTATTCCTCTATCCGTAGAGTACA
TTGAATGTACAGAAATGGCTATAACAGAGTTTCTGTTATTCGTATTAACAGCTACTCTAG
GAGGAATGTTTTTATGTGGTGCTAACGATTTAATAACTATCTTTGTAGCTCCAGAATGTT
TCAGTTTATGCTCCTACCTATTATCTGGATATACCAAGAGAGATGTACGGTCTAATGAGG
CTACCACGAAATATTTACTCATGGGTGGGGCAAGCTCTTCTATTCTGGTTCATGGTTTCT
CTTGGCTATATGGTTCATCCGGGGGAGAGATCGAGCTTCAAGAAATAGTGAATGGTCTT
ATCAATACACAAATGTATAACTCCCCAGGAATTTCAATTGCGCTTATATTCATCACTGTA
GGAATTGGGTTCAAGCTTTCCCCAGCCCCTTCTCATCAATGGACTCCTGACGTATACGA
AGGATCTCCCACTCCAGTCGTTGCTTTTCTTTCTGTTACTTCGAAAGTAGCTGCTTCAG
CTTCAGCCACTCGAATTTTCGATATTCCTTTTTATTTCTCATCAAACGAATGGCATCTTCT
TCTGGAAATCCTAGCTATTCTTAGCATGATATTGGGGAATCTCATTGCTATTACTCAAAC
AAGCATGAAACGTATGCTTGCATATTCGTCCATAGGTCAAATCGGATATGTAATTATTGG
AATAATTGTTGGAGACTCAAATGATGGATATGCAAGCATGATAACTTATATGCTGTTCTAT
ATCTCCATGAATCTAGGAACTTTTGCTCGCATTGTATCATTTGGTCTACGGACCGGAACT
GATAACATTCGAGATTATGCAGGATTATACACGAAAGATCCTTTTTTGGCTCTCTCTTTA
GCCCTATGTCTCTTATCCCTAGGAGGTCTTCCTCCACTAGCAGGTTTTTTCGGAAAACT
CCATCTATTCTGGTGTGGATGGCAGGCAGGCCTATATTTCTTGGTTTCAATAGGACTCCT
TACGAGCGTTGTTTCTATCTACTATTATCTAAAAATAATCAAGTTATTAATGACTGGACG
AAACCAAGAAATAACCCCTCACGTGCGAAATTATAGAAGATCTCCTTTAAGATCAAAC

AATTCCATCGAATTTAGTATGATTGTATGTGTGATAGCATCTACTATACCAGGAATATCAA
TGAACCCCATTATTGCAATTGCTCAGGATACCCTTTTTATGTTTCTGCTTCACGAATATGA
TATTTTCTGGGCATTTCTAATAATATCAAGTATTATTCCTATTTTGGCATTTCTAATTTCTG
GAGTTTTGGCCCCGATTAGCGCAGGACCCGAGAAACTTTCTAGTTATGAATCGGGTATA
GAACCAATGGGCGATGCTTGGTTACAATTTCGAATCCGTTATTATCTGTTTGCTCTAGTT
TTTGTTGTTTTTGATGTTGAAACGGTTTTTCTTTATCCATGGGCGATGAGTTTCGATGTA
TTGGGTGTATCTGTATTTATAGAGGCTTTAATTTTTGTGCTTATCCCAATTGTTGGTTCAG
TTTATGCATGGCGAAAAGGAGCATTGGAATGGTCTACGAATTATTTTCCTTGGTTAACTA
TAATTGTAGTTTTGCCGATATCTGCGGGTTCTTTAATTTTCTTTCTCCCTTATAGAGGAAA
TAAGGTGATTCGATGGTATACTATTTGTATATGTTTATTAGAACTCCTTTTAACGACCTAT
GCATTTTGTTATCATTTTCAATTGGACGATCCATTAATCCAACTCGAGGAGGAGTATAAA
TGGATCAATTTTTTTGATTTTCACTGGAGATTAGGAATCGATGGACTTTCCATAGGACCC
ATTTTACTGACGGGATTCATCACAACTTTAGCCACTTTAGCGGCTTGGCCAGTTACTCG
AGATTCCCGATTGTTCCATTTCCTGATGTTAGCAATGTACAGCGGTCAAATAGGATCATT
TTCTTCTCGAGACCTTTTACTTTTTTTCATCATGTGGGAGTTAGAATTAATTCCTGTTTAC
CTACTTCTATCCATGTGGGGAGGGAAGAAACGTCTCTACTCAGCTACAAAGTTTATTTT
GTACACTGCAGGGGGTTCTATTTTTCTCTTAATGGGAGTTTCGGGTATGGGTTTATATGG
TTCCAATGAACCAACATTAAATTTTGAAACATCAGCTAGTCAATCATATCCTGTGGCATT
GGAAATAATATTCTATTTTGGATTTCTTATTGCTTATGCTGTCAAATCACCGATTATACCC
CTACATACATGGTTACCAGATACCCATGGCGAAGCACATTACAGTACATGTATGCTTTTA
GCCGGAATCTTATTAAAAATGGGAGCGTATGGATTGGTTCGGATCAATATGGAATTATTA
CCCCACGCCCATTCTATATTTTCTCCCTGGTTGATGATAATAGGCACAATTCAAATAATTT
ATGCAGCTTCAACATCTCCCGGTCAACGCAATTTAAAAAAGAGAATAGCCTATTCCTCC
GTATCTCATATGGGTTTCACAATTATAGGAATTGGTTCCATAACCGATACGGGACTCAAT
GGAGCCATTTTACAAATAATCTCTCATGGATTTATTGGTGCTGCACTTTTTTTCTTGGCA
GGAACGAGTTACGATCGAATACGTCTTGTTTATCTCGACGAAATGGGAGGAATAGCTAT
CCCAATGCCAAAAATATTTATGATGTTCAGTAGCTTCTCGATGGCCTCTCTTGCATTGCC
GGGAATGAGTGGTTTTGTTGCAGAATTGATAGTCTTTTTTGGAATAATTACCAGCCAAA
AATATCTTGTACTTCCAAAAATAGTAATTATTTTTGTAATGGCAGTTGGAATGATATTAAC
CCCTATTTATTCATTATCTATGTCACGTCAGATGTTCTATGGATACAAGCTATTCAATGCA
CCAAACTATTCTTTTTTTGATTCTGGACCACGAGAACTGTTTGTTTCGATCTCTATCTTT
CTACCCGTAATAGGTATTGGTATTTATCCAGATTTCGTTCTTTCACTGTCAGTTGACAAG
GTAGAAGCTATTTTATCTAATTACTTTTATAGAATGATGCTCGAACATGTGCTTGTTTTGA
GTGCCTATTTATTTTCTATCGGTATCTATGGATTGATTACGAGCCGACATATGGTTAGAGC
GCTTATGTGTCTTGAGCTTATACTGAATGCAGTTAATATCAATTTCGTAACATTTTCTGAT
TTTTTTGATAGTCGCCAATTAAAAGGAGACATTTTCTCGATTTTTGTTATAGCTATTGCC
GCCGCTGAAGCAGCTATTGGGCCAGCTATTGTTTCGTCAATTTATCGTAACAGAAAGTC
AACTCGTATCAATCAATCGAATTTGTTGAATAAAATGGAGCAGACATATCAATATGCATG
GATCATACCTTTCGTTCCACTTCCAGTTCCTATGTTAATAGGAGTGGGACTTCTCCTTCT
TCCGAAGGCAACAAAAAATCTTCGTCGTATATGGGCTTTTCCTAGTGTTTTATTGCTAAG
TGTAGTTATGATACTTGCGGCCGATCTGTCTATTCAGCAAATAAATGGCAGTTACATCTA
TCAATATGTATGGTCTTGGACCATCAATAATGATTTTTCCTTAGAGTTTGGCTACTTGATC
GATCCACTTACTTCTATTATGTCAATATTAATTACTACTGTTGGAATCCTGGTTCTTATTTA
TAGTGACAATTATATGTCTCATGATCAAGGATATTTGAGATTTTTTGCTTATATGAGTTTT

TCCAATACTTCCATGTTGGGATTAGTTACTAGTTCCAATTTGATACAAATTTATATCTTTT
GGGAATTAGTTGGAATGTGTTCGTATCTATTAATAGGTTTTTGGTTCACACGACCAATTG
CAGCAAATGCTTGTCAAAAAGCGTTTGTAACTAATCGTGTAGGGGATTTTGGTTTATTAT
TAGGAATCTTAGGTCTTTATTGGATAACGGGTAGTTTTGAATTTCGGGATTTGTTCGAAA
TATTCAATAACAATGAGGTCAATTCTTTATTTGTTACTTTGTGTGCCTCTCTATTATTCGT
CGGTGCAGTTGCTAAATCTGCACAATTCCCCCTTCATGTATGGTTACCTGATGCCATGGA
GGGGCCTACTCCCATTTCGGCTCTTATCCATGCTGCTACTATGGTAGCAGCGGGAATTTT
TCTTGTAGCTAGGCTTCTTCCTCTTTTCACAGCCATACCTTACATAATGTATCTAATTTCT
TTGATAGGCATAATAACAATACTTTTAGGAGCTACTTTGGCTCTTGCTCAAAAAGACATT
AAGAGAGGTTTGGCTTATTCGACAATGTCGCAATTGGGTTATATTATGTTAGCTCTAGGT
ATGGGGTCTTATCAAGCTGCTTTATTTCATTTGATCACTCATGCCTATTCGAAAGCATTAT
TGTTTTTAGGGTCCGGATCAATTATTCATTCAATGGAAACCATTGTTGGATATTCTCCAG
ATAAAAGTCAGAACATTGTTCTTATGGGTGGTTTAACAAAATATGTGCCAATTACAAAA
ACTTCGTTTTTATTAGGTACACTTTCTCTTTGTGGTATTCCACCTCTTGCTTGTTTTTGGT
CCAAAGACGAAATTCTTAATGATAGTTGGTTGTATTCACCCATTTTCGCGATAATAGCTT
GGGTTACAGCAGGATTAACTGGATTTTATATGTTTCGCATGTATTTACTTACTTTTGAGG
GGCAGTTAAACGTTTATTTTCAAAATTACAGTGGCAAAAAAAACATATCGGGCTATTCA
ATATCTATATGGGGTAAAGAAGGACCGAAACCAATTCAAAAAATC---------------------TTT
CTTTTTGCGGTTGGAAATAAAGAAACTTCTTCGTATCCCCATGAATCGGACAATACTAT
GCTATTGCCTTTGCTTGTATTGGCCCTATTTACTTTGGTCGTTGGATCCATAGGAATTCCT
TTTGATTTTGATATATTATCGAAATGGTTAACTCCCTCAATAAACCTTTTACATCCAAATT
CGAAT---TCTTTTGATTGGTATGAATTTGTGACAAATGGCATTTTTTCAGTCAGTATCGCC
TATTTCGGAATAGTTATAGCGTCTCTTTTATATACGCCTGTATATTCATCTTTCCAGAATTT
CGATGTAATCAATTCATTTGTGATTCTATGGGACAAAATAATAAATGTGATATATAATTGG
TCATATAATCGTGGTTATATAGACGTTTTTTATGCAACAACCTTAACTAGAGGGATAAGA
GGATTAGCTGAACTAACTTATTTTTTTGATAGACGAGTAATTGATGGAATTACGAATGGA
GTTGGTATTACAAGTTTTTTTGTAGGAGAAGGAATCAAATATTTTGGGGGTGGG---AGA
ATCTCTTCTTATCTCTTCTTGTATTTATCTTATGTATCCATCTTTTTATTAATTTATTACATG
GATTTGCCTGGACCAATACATGATTTTCTTTTAGTATTTCTGGGATCAGGTCTTATATTAG
GAGGTCTAGGAGTGGTATTACTTACCAATCCCATTTTTTCTGCCTTTTCATTGGGATTGG
TTCTTGTTTGTATATCCTTATTCCATATTCCATCGAACTCCCATTTTGTAGCTGCTGCACA
GCTCCTTATTTACGTGGGGGCTATCAATGTCTTAATCCTATTTGCTGTGATGTTCATGAGT
GGTTCAGAATATTACAATGATTTCCAGCTTTGGACCGTTGGCGATGGGGTTACTTCACT
GGTTTGTACAAGCATTTTTGTTTCACTAATTACTACTATCCCAGATACGTCATGGTACGG
GATTATTTGGACTACAAGATCAAACCAGATTATAGAGCAGGACTTGATAAATAATAGTC
AACAAATTGGGATTCATTTATCAACAGATTTTTTTCTTCCATTTGAACTCATTTCGATAAT
TCTTTTAGTTGCTTTGATAGGTGCAATTTCTATGGCTCGTCAGATGACTGTACCTGCTAT
AAAAAAAGACCTTATGATAGTCAATATGGGTCCTCAGCACCCATCAATGCATGGTGTTC
TTCGACTCGTCGTTACTCTAGATGGTGAAGATGTTATTGACTGTGAACCCATATTAGGTT
ATTTACACAGAGGGATGGAAAAAATTGCGGAAAACCGAACAATTATACAATATCTCCCT
TATGTAACACGTTGGGATTATTTAGCTACTATGTTCACAGAGGCAATAACAGTAAATGCA
CCAGAACAGTTGGGAAATATTCAAGTTCCTAAAAGAGCCAGCTATATCAGAGTAATTAT
GCTGGAGCTGAGTCGTATAGCTTCTCATTTGTTATGGCTTGGCCCTTTTATGGCGGATAT
CGGTGCACAGACTCCTTTCTTCTATATTTTCCGAGAGAGAGAATTGATATATGATCTATT

CGAAGCTGCCACAGGTATGCGAATGATGCATAATTATTTCCGTATCGGAGGAGTAGCTG
CTGATTTACCTCATGGCTGGATAGATAAATGTTTGGATTTCTGCGATTATTTTTTAACAGG
GGTTACTGAATATCAAAACCTTATTCGGCGGAATCCTATTTTTTTGGAACGAGTTGAAG
GGGTAGGCATTATTGGTAGAGAGGAAGCAATAAATTGGGGTTTATCAGGACCAATGCTA
CGAGCTTCCGGAATCCAATGGGATCTTCGTAAAGTTGATCATTATGAGTGTTACGACGA
ATTTGATTGGGAAGTCCAATGGCAAAAAGAAGGAGATTCATTAGCTCGTTATTTAGTAC
GAATCAGTGAAATGACGGAATCCATCAAAATTATTCAACAGGCTCTGGAAGGAATTCC
GGGGGGGCCCTACGAGAATTTAGAAGTCCGCCGTTTTGATAGAGCAAAGGATTCCGAA
TGGAATGATTTTGAATATCGATTCATTAGTAAAAAGCCTTCTCCTACTTTTGAATTATCG
AAACAAGAACTTTATGTGAGAGTGGAAGCCCCAAAGGGGGAATTGGGAATTTTTTTGA
TAGGGGATCAGAGTGTTTTTCCCTGGAGATGCAAAATTCGTCCACCGGGTTTTATCAAT
TTGCAAATTCTTCCTCAGCTAGTTAAAAGAATGAAATTGGCTGATATTATGACGATACTA
GGTAGTATAGATATCATTATGGGAGAAGTTGATCGTATGTTCCCTATGTTAACCGGGTTG
ATGAATTATGGTCAACAAACAGTACGAGCTGCAAGGTACATCGGTCAAAGTTTCATGAT
TACCTTATCCCACACGAATCGTTTACCTATAACTATTCAATATCCTTATGAAAAATTGATC
ACATCAGAGCGTTTCCGCGGTCGAATCCATTTTGAATTTGATAAATGCATTGCTTGTGA
AGTATGTGTTCGTGTATGCCCTATAGATCTACCCGTTGTTGATTGGAGATTGGAAACTGA
TATTCGAAAGAAACGATTACTTAATTACAGTATTGATTTTGGAATCTGTATATTTTGTGGT
AACTGCGTCGAGTATTGTCCAACAAACTGTTTATCAATGACTGAAGAATATGAACTTTC
GACTTATGATCGTCACGAATTGAATTATAATCAAATTGCTTTGGGTCGGTTACCAATGTC
AATAATTGGAGATTATACAATTCGAACAATTATGAATTCGAAAATAACCACAGGCAACC
CCCTTGATTCAAAAACGATTACCAATTACATGCAGGGTCGTTTGTCTGCTTGGCTAGTC
AAGCATGGGCTAGTTCATAGATCTTTAGGCTTCGATTACCAAGGAATAGAGACTTTACA
AATAAAGCCCGAGGATTGGCACTCCATTGCTGTCATTTCATATGTATATGGTTACAATTAT
CTACGTTCCCTGTGTGCCTATGATGTAGCACCAGGCGGACTGTTAGCTAGTGTGTATCAT
CTTACGAGAATACAGTCTGGTGTGGAGCAACCAGAAGAGGTATGCATAAAAATATTTG
CCCCAAGGAGGAATCCTAGAATTCCATCTGTTTTCTGGATTTGGAAAAGTGCAGATTTT
CAAGAACGGGAATCTTATGATATGTTTGGAATCTCTTATGATAATCATCCACGCCTGAAA
CGTATCTTGATGCCTGAAAGTTGGATCGGATGGCCCTTACGTAAGGATTATATTGCCCCC
AATTTCTATGAAATACAAGATGCTCATATGGGCGATGAGTTTCGATGTATTGGGTGTATC
TGTATTTATAGAGGCTTTAATTTTTGTGCTTATCCCAATTGTTGGTTCAGTTTATGCATGG
CGAAAAGGAGCATTGGAATGGTCTTAGCTCCTAAATATTCAGACAATAAAAAGCAAAA
AGAAGAAAAAAATGACATTGAGACAGTTATGAATTCCATTGAGTTTCCGTTACTTGAG
CGAACAACCCAAAATTCAGTTATTTCAACTACATCGAATGATCTTTCAAATTGGTCAAG
ACTCTCCAGTTTATGGCCCCTTCTCTATGGTACCAGTTGTTGCTTCATTGAATTTGCTTC
ATTAATAGGCTCTCGATTCGACTTTGATCGTTATGGACTGGTACCAAGATCGAGTCCTAG
GCAAGCCGACCTCATTTTAACAGCCGGCACAGTAACAATGAAAATGGCTCCTTCTTTA
GTGAGATTATATGAGCAAATGCCTGAACCAAAATATGTCATTGCTATGGGAGCCTGTACT
ATTACAGGAGGAATGTTCAGTACCGATTCTTATAGTACTGTTCGGGGAGTCGATAAGCT
AATTCCTGTGGATGTCTATTTGCCGGGTTGCCCACCTAAACCGGAGGCAGTTATAGATG
CTATAACAAAACTTCGTAAGAAGGTATCTCGAGAAATCTATGAAGATAGAATTGGGTCT
CAACAGGAAAATCGATGTTTTACTACCAATCACAAGTTTCATGTTGGGCGCAGTACTCA
TACTGGAAATTATGATCAAGAATTACTCTATCAATCGCCATCTACTTCAGAGATCCCTTC
TGAAACATTTTTCAAATACAAAAGTGGAACATCTTCCCACGAATTAGTGAATATGCAAA

ATAGAAATACCTTTTCTTGGATAAAGGAACAGATGATTCGATCTATTTCCGTATCGATCA
TGATAAATGTAATAACTCAGACATCCATTTCAAATGCGTATCCCATTTTTGCACAGCAGG
GTTATGAAAATCCACGAGAAGCGACTGGTCGTATTGTATGTGCCAATTGCCATTTAGCT
AATAAGCCCGTGGATATTGAAGTTCCACAAGCAGTACTTCCTGATACTGTATTTGAAGC
AGTTGTTAGAATCCCTTATGATATGCAACTGAAACAAGTTCTTGCTAATGGCAAAAAGG
GGGCTTTGAATGTAGGAGCTGTTCTTATTTTACCTGAGGGATTCGAATTAGCTCCCCCA
GATCGTATTTCTCCCAAGATGAAAGAAAAGCTGGGCAATCTATCTTTTCAGAGTTATCG
CCCAACTAAAAAAAATATTCTTGTGATAGGTCCTGTTCCTGGTCAGAAATATAGTGAAA
TCACCTTTCCTATTCTTTCCCCGGACCCCGCTACTAAGAAAGACGTTCACTTCTTAAAAT
ATCCCATATACGTAGGCGGGAACAGGGGAAGGGGTCAGATTTATCCCGATGGGAGCAA
GAGTAACAATACCGTCTATAATGCTACAGCAGCCGGTATAGTAAGCAAAGTTGTACGTA
AAGAAAAGGGGGGATATGAAATAAACATAGCGGATGCATCGGATGGACACCAAGTGGT
TGATATTATACCTCCAGGACCGGAACTTCTTGTTTCAGAGGGCGAATCGATCAAGCTTG
ATCAACCATTAACGAGTAATCCCAATGTGGGTGGATTTGGTCAGGGAGATGCAGAAATA
GTACTTCAAGATCCATTACGTGTCCAAGGTCTTTTATTTTTCTTGGCGTCTGTTATTCTG
GCACAAATTTTTTTGGTTCTTAAAAAGAAACAATTTGAGAAGGTTCAATTGTCCGAAAT
GAATTTCATGAGTAAAGTATATGATTGGTTCGAAGAACGTCTCGAGATTCAGGCGATTG
CAGATGATATAACTAGTAAATATGTCCCTCCCCATGTCAACATATTTTATTGTCTAGGAGG
GATTACGCTTACTTGTTTTTTAGTACAAGTAGCTACGGGGTTTGCTATGACTTTTTATTAT
CGTCCGACCGTTACCGAGGCTTTTGCCTCTGTTCAATATATAATGACTGAAGCGAACTT
TGGTTGGTTAATCCGATCAGTTCATCGATGGTCGGCAAGTATGATGGTCCTAATGATGAT
CCTGCACGTATTTCGTGTGTATCTCACCGGCGGATTTAAAAAACCTCGCGAATTGACTT
GGGTTACGGGTGTGGTTCTGGCTGTATTGACCGCATCTTTTGGTGTAACTGGTTATTCCT
TACCTCGGGACCAAATTGGTTATTGGGCAGTAAAAATCGTGACAGGCGTACCTGAAGC
TATTCCTGTAATAGGATCACCTTTGGTAGAGTTATTACGTGGAAGTGCTAGTGTGGGTCA
ATCCACTTTGACTCGTTTTTATAGTTTACACACTTTTGTATTACCTCTTCTTACTGCCGTA
TTTATGTTAATGCACTTCCCAATGATACGTAAGCAAGGTATTTCTGGTCCTTTAATGGGA
GTAACAAAGAAACCTGACTTGAATGATCCTGTATTAAGAGCAAAATTGGCTAAAGGGA
TGGGTCATAATTATTACGGAGAACCCGCATGGCCCAATGATCTTTTATATATTTTTCCAGT
AGTAATTCTAGGTACGATTGCATGTAACGTAGGCTTAGCGGTTCTAGAACCGTCAATGA
TTGGTGAACCAGCAGATCCATTTGCAACTCCTTTGGAAATATTACCCGAATGGTACTTC
TTTCCCGTATTTCAAATACTCCGTACAGTACCCAATAAGTTATTGGGTGTTCTTTTAATG
GTTTCAGTACCTGCGGGATTATTGACAGTACCCTTTTTGGAGAATGTTAATAAATTCCAA
AACCCATTTCGTCGTCCAGTAGCCACAACTGTCTTTTTGATCGGTACCGTAGTAGCTCT
TTGGTTGGGTATTGGAGCAACATTACCTATTGAAAAATCCCTAACTTTAGGTCTTTTTAT
GATTGAAGTTTTTCTATTTGGAATTGTGTTAGGTCTAATTCCTATTACTTTGGCTGGATTA
TTCGTAACTGCATATTTACAATACAGACGCGGTGATCAGTTGGACCTTATGCCTACTATA
ACTAGTTATTTCGGTTTTCTACTGGCGGCTTCGACTATAACCCCGGCTCTATTTATTGGTC
TGAGCAAGATACGACTTATTATGGATATAGTAAATCTCGCTTGGGCTGCTTTAATGGTAG
TCTTTACATTTTCCCTTTCACTCGTAGTATGGGGAAGAAGTGGACTCATGATTATTCGTT
CGCCGGAATCAGAAATGAAAATTTTGGTGGATAGGGATCCCGTCAAAACTTCATTCGA
GGAATGGGCCAGACCCGGTCATTTCTCAAGAACAATAGCTAAGGGCCCTGATACTACC
ACTTGGATCTGGAACCTACATGCCGATGCTCACGATTTCGATAGCCATACCAGTGATTT
GGAGGAGATCTCTCGAAAAGTATTTAGTGCCCATTTCGGTCAACTCTCCATCATCTTTAT

TTGGCTGAGTGGCATGTATTTCCATGGTGCCCGTTTTTCCAATTATGAAGCATGGCTAAG
CGACCCTACTCACATTGGACCCAGCGCTCAGGTGGTTTGGCCAATAGTGGGCCAAGAA
ATATTGAATGGTGATGTGGGCGGGGGTTTCCGAGGGATACAAATAACCTCTGGTTTTTT
TCAGCTTTGGCGAGCATCTGGAATAACGAATGAATTACAACTCTATTGTACCGCAATTG
GTGCATTGGTCTTTGCAGCGTTAATGCTTTTTGCTGGTTGGTTCCATTATCACAAAGCA
GCTCCGAAATTGGCCTGGTTCCAAGATGTAGAATCTATGTTGAATCACCACTTAGCGGG
GTTACTAGGACTTGGTTCTCTTGGTTGGGCGGGACACCAAGTACATGTATCTTTACCGA
TTAACCAATTTCTAGACGCTGGAGTAGATCCTAAAGAGATCCCACTTCCTCATGAATTTA
TCTTGAATCGGGATCTTTTGGCTCAACTTTATCCCAGTTTTGCCGAGGGAGCAACCCCA
TTTTTCACCTTGAATTGGTCAAAATATGCGGAATTTCTTACTTTTCGTGGAGGATTAGAT
CCAGTAACAGGGGGTCTATGGCTGACCGATATTGCACACCATCATTTAGCTATTGCAGT
TCTTTTTCTCGTTGCTGGTCATATGTATAGAACTAACTGGGGCATTGGTCATGGCCTGAA
AGATATTTTAGAGGCTCATAAGGGTCCATTTACAGGTCAGGGACATAAAGGCCTCTATG
AGATCCTAACAACGTCATGGCATGCTCAATTATCTCTTAACCTAGCTATGTTGGGCTCTT
TAACCATTGTTGTAGCTCACCATATGTATTCCATGCCCCCTTATCCATATCTAGCTACTGA
CTATGGTACACAACTTTCATTATTCACACATCACATGTGGATCGGTGGATTTCTCATAGT
TGGTGCTGCTGCACATGCAGCCATTTTTATGGTAAGAGATTATGATCCAACTACTCGATA
CAACGATCTATTAGACCGTGTCCTTAGGCACCGCGATGCGATCATATCACATCTCAACT
GGGCATGTATATTTCTAGGCTTTCACAGTTTTGGTTTGTATATTCATAATGATACTATGAG
CGCTTTAGGGCGTCCCCAAGATATGTTTTCAGATACCGCTATCCAATTACAACCCGTCTT
TGCTCAATGGGTACAAAACACCCACGCTTTAGCACCTGGTGCAACAGCTCCTGGTGCA
ACAGCAAGTACCAGCTTGACTTGGGGGGGTGGTGATTTAGTAGCAGTAGGCGGCAAA
GTAGCTTTGTTACCTATTCCATTAGGAACCGCAGATTTTTTGGTGCATCACATTCATGCA
TTTACGATCCATGTGACGGTATTGATACTACTGAAAGGTGTTCTATTTGCTCGTAGCTCC
CGTTTGATACCTGATAAAGCAAATCTTGGGTTTCGTTTCCCTTGTGACGGACCTGGAAG
AGGGGGGACATGTCAAGTATCCGCCTGGGATCATGTCTTCTTAGGTCTATTCTGGATGT
ACAATTCCATTTCGGTAGTAATATTCCATTTCAGTTGGAAAATGCAGTCGGATGTTTGGG
GTAGTATAAGTGATCAAGGGGTGGTAACTCATATCACGGGAGGAAACTTTGCGCAGAG
TTCCATTACTATTAATGGGTGGCTCCGTGATTTTTTATGGGCACAGGCATCCCAGGTAAT
TCAGTCTTATGGTTCTTCATTATCCGCGTATGGCCTTTTTTTTCTAGGTGCTCATTTTGTC
TGGGCTTTCAGTTTAATGTTTCTATTCAGCGGCCGTGGTTATTGGCAAGAACTTATTGAA
TCCATCGTTTGGGCTCATAACAAATTAAAAGTTGCTCCTGCTACTCAGCCTAGAGCCTT
GAGCATTGTACAAGGACGTGCTGTAGGAGTAACCCATTACCTTCTGGGTGGAATTGCC
ACAACATGGGCGTTCTTCTTAGCAAGAATTATTGCAGTAGGAATGGCATTACGATTTCC
AAGGTTTAGCCAAGGCTTAGCTCAGGACCCCACTACTCGTCGTATTTGGTTTGGTATTG
CTACCGCGCATGACTTCGAGAGTCATGATGATATTACTGAGGAACGTCTTTATCAGAATA
TTTTTGCTTCTCACTTCGGACAGTTAGCAGTAATTTTTCTGTGGACTTCCGGAAATCTGT
TTCATGTAGCTTGGCAAGGAAATTTTGAGTCATGGGTACAAGACCCTTTACATGTCAGA
CCTATTGCTCATGCAATTTGGGATCCTCATTTTGGTCAACCGGCTGTGGAAGCCTTTACT
CGAGGGGGTGCTCTCGGCCCAGTGAATATCGCGTATTCTGGTGTTTATCAGTGGTGGTA
TACAATCGGCTTACGCACTAATGAAGATCTTTATACTGGAGCTCTTTTTCTATTGTTTCTT
TCTGCCATATCCTTAATAGCGGGTTGGTTACACCTACAACCGAAATGGAAACCCAGTGT
CTCCTGGTTCAAAAATGCCGAATCCCGTCTCAATCACCATTTGTCAGGACTCTTCGGAG
TAAGTTCCTTGGCTTGGACAGGGCATTTAGTTCATGTCGCTATTCCCGGATCCAGAGGG

CAGTCTGTTCGATGGAATAATTTCTTAGATGTATTACCGCATCCCCAAGGGTTAGGCCCG
CTTTTTACAGGTCAATGGAATATTTATGCCCAAAACCCCGATTCCGGTAGTCATTTATTT
GGTACCTCCCAAGGAGCGGGAACTGCCATTCTAACCCTTCTTGGGGGATTCCATCCAC
AAACGCAAAGTTTATGGCTGACCGATATGGCACACCATCATTTAGCTATTGCAGTTCTTT
TTCTCGTTGCTGGTCATATGTATAGAACTAACTTCGGGATTGGGCACAGTATGAAAGAT
CTTTTAGAAGCACATATTCCTCCGGGGGGGCGATTGGGACGTGGACATAAAGGTCTTTA
TGACACAATCAATAATTCGATTCATTTTCAATTAGGCCTTGCTCTAGCCTCTTTAGGGGT
TATTACTTCCTTGGTAGCTCAACACATGTACTCTTTACCTGCTTATGCATTCATTGCACA
GGACTTTACTACTCAAGCTGCATTATATACTCATCACCAATACATCGCAGGATTCATCAT
GACAGGAGCCTTTGCTCATGGAGCTATATTCTTCATTAGAGATTACAATCCGGAGCAGA
ATGAGGATAATGTATTGGCAAGAATGTTAGACCATAAAGAAGCTATCAAATCCCATTTA
AGTTGGGCCAGCCTCTTTCTGGGTTTCCATACCTTAGGACTTTATGTTCATAACGACGTC
ATGCTCGCTTTTGGTACTCCGGAGAAACAAATCTTGATCGAACCCATATTTGCCCAATG
GATACAATCTGCTCATGGCAAGACTTCATATGGGTTCGATGTACTTTTATCTTCAACGAC
TGGCCCAGCATTCAACGCGGGTCGAAGCATATGGTTGCCCGGTTGGTTAAATGCTGTTA
ATGAG------AATAGTAATTCACTATTCTTAACAATAGGTCCTGGAGACTTCTTGGTTCATC
ATGCTATTGCTCTAGGTTTACATACAACTACATTGATCTTAGTAAAAGGCGCTTTAGATG
CGCGTGGTTCCAAGTTAATGCCAGATAAAAAGGATTTCGGTTATAGTTTTCCTTGCGAC
GGTCCGGGACGGGGTGGTACTTGTGATATTTCGGCTTGGGACGCATTTTATTTGGCAGT
TTTCTGGATGTTAAATACCATTGGATGGGTTACTTTTTATTGGCATTGGAAGCACATCAC
ATTATGGCAGGGTAACGTTTCACAGTTTAATGAATCCTCCACTTATTTGATGGGATGGTT
AAGAGATTATCTATGGTTAAACTCTTCACAACTTATCAATGGATATAACCCTTTTGGTAT
GAATAGTTTATCGGTCTGGGCGTGGATGTTCTTATTTGGACATCTTGTTTGGGCTACTGG
ATTTATGTTCTTAATTTCCTGGCGCGGATATTGGCAGGAATTGATTGAAACTTTAGCATG
GGCTCATGAACGAACACCTTTGGCTAATTTGATTCGATGGAGAGATAAACCTGTGGCTC
TTTCCATTGTACAAGCAAGATTGGTTGGGTTAGCCCATTTTTCTGTAGGTTATATATTCA
CTTATGCAGCCTTCTTGATTGCCTCTACATCAGGCAAATTTGGTATGTCACACTCAGTAA
AGATTTATGATACATGTATAGGGTGTACTCAATGTGTACGAGCCTGCCCCACAGATGTAT
TAGAAATGATACCTTGGGACGGATGTAAAGCTAAGCAAATTGCTTCCGCTCCAAGAAC
AGAGGACTGTGTAGGTTGTAAGAGATGTGAATCTGCCTGTCCAACAGATTTCTTGAGT
GTCCGGGTTTATTTATGGCATGAGACAACTCGCAGCATGGGTCTAGCTTATATGATAACT
CTCAACTTACCCTCTATTTTTGTTCCTTTAGTGGGCCTAGTATTTCCGGCAATTGCAATG
GTTTCTTTATTTCTTCATGTTCAAAAAAACAAGATTGTCATGCGAGATATAAAAACATAT
CTCTCTGTGGCACCTGTGCTAAGTACTCTATGGTTCGGGGCTTTAGCAGGTCTATTGATA
GAGATCAATCGTTTATTCCCGGATGCGTTGGCATTCCCTTTTTTT------ATGACTGCAATT
TTAGAGAGACGCGAAAGCGAAAGCCTATGGGGTCGCTTCTGTAACTGGATAACCAGCA
CTGAAAACCGTCTTTACATTGGATGGTTCGGTGTTTTGATGATCCCTACCTTATTGACCG
CAACCTCTGTATTTATTATCGCCTTCATTGCTGCTCCTCCAGTAGATATTGATGGTATTCG
TGAACCTGTTTCCGGGTCTCTACTTTATGGAAACAATATTATTTCGGGTGCCATTATTCCT
ACTTCTGCGGCTATAGGTTTGCACTTTTACCCGATATGGGAAGCGGCATCCGTTGATGA
ATGGTTATACAATGGTGGTCCTTATGAACTAATTGTTCTACACTTCTTACTTGGTGTAGC
TTGTTACATGGGTCGTGAGTGGGAACTTAGTTTCCGTCTGGGTATGCGCCCTTGGATTG
CTGTTGCATATTCAGCTCCTGTTGCAGCTGCTGCTGCTGTTTTCTTGATCTACCCAATTG
GTCAAGGAAGCTTCTCTGATGGTATGCCCCTAGGAATCTCTGGTACTTTCAACTTCATG

ATTGTATTCCAGGCTGAGCACAACATCCTTATGCACCCATTTCACATGTTAGGTGTAGCT
GGTGTATTCGGCGGCTCCCTATTCAGTGCTATGCATGGTTCCTTGGTAACCTCTAGTTTG
ATCAGGGAAACCACTGAAAATGAATCTGCTAACGAAGGTTACAGATTTGGTCAAGAGG
AAGAAACTTATAATATCGTAGCTGCTCATGGTTATTTTGGCCGATTGATCTTCCAATATGC
TAGTTTCAACAATTCTCGTTCTTTACATTTCTTTTTAGCTGCTTGGCCTGTAGTAGGTATC
TGGTTCACTGCTTTAGGTATCAGTACCATGGCTTTCAACCTAAACGGTTTCAATTTCAA
CCAATCCGTAGTTGACAGTCAAGGTCGTGTAATTAACACTTGGGCTGATATCATCAACC
GTGCTAACCTTGGTATGGAAGTTATGCATGAACGTAATGCTCACAACTTCCCTCTAGAC
CTAGCTGCTGTTGAAGCTCCATCTACAAATGGAATGGGTTTGCCTTGGTATCGTGTTCAT
ACCGTCGTATTGAATGATCCCGGTCGGTTGCTTTCTGTCCATATAATGCATACAGCTCTA
GTTTCTGGTTGGGCCGGTTCGATGGCTCTATACGAATTAGCAGTTTTTGATCCCTCTGAC
CCCGTTCTTGATCCAATGTGGAGACAGGGTATGTTCGTTATACCCTTCATGACTCGTTTA
GGAATAACCAATTCATGGGGTGGTTGGAGTATTACA---GGAGGGACTATAACGAATCCG
GGTATTTGGAGTTACGAAGGTGTGGCCGGGGCACATATTGTGTTTTCTGGTTTGTGCTT
CTTGGCAGCTATCTGGCATTGGGTATATTGGGATCTAGAAATTTTCTGTGATGAACGTAC
AGGAAAACCTTCTTTGGATTTGCCCAAGATCTTTGGAATTCATTTATTTCTCTCAGGGGT
GGCTTGCTTCGGGTTTGGCGCATTTCATGTAACAGGCTTGTATGGTCCTGGAATATGGG
TATCCGATCCTTATGGACTAACTGGAAAAGTACAATCTGTAAATCCCGCGTGGGGCGTG
GAAGGTTTTGATCCTTTTGTTCCGGGAGGAATAGCCTCTCATCATATTGCAGCAGGGAC
ATTAGGCATATTAGCGGGTCTATTCCATCTTAGTGTCCGCCCGCCCCAACGTCTATACAA
AGGATTACGTATGGGCAATATTGAAACTGTCCTTTCCAGTAGTATCGCTGCTGTCTTTTT
TGCAGCTTTTGTTGTTGCTGGAACTATGTGGTATGGTTCAGCAACTACCCCGATCGAAT
TATTTGGTCCCACTCGTTATCAATGGGATCAAGGCTACTTCCAGCAAGAAATATATCGAC
GAGTTGGTACTGGGCTAGCCGAAAATCAAAGTTTATCAGAAGCTTGGTCTAAAATTCC
CGAAAAATTAGCTTTTTATGATTACATCGGCAATAATCCAGCAAAGGGGGGATTATTCA
GAGCGGGCTCAATGGACAACGGGGATGGAATAGCTGTTGGATGGTTAGGACACCCCAT
CTTTAGAGATAAAGAGGGGCGTGAGCTTTTTGTACGTCGTATGCCTACTTTTTTTGAAA
CATTTCCAGTAGTTTTAGTAGACGGAGACGGAATTGTGAGAGCCGATGTTCCTTTTAGA
AGGGCAGAATCGAAATATAGTGTCGAACAAGTAGGTGTAACTGTTGAGTTCTATGGTG
GCGAACTCAATGGAGTCAGTTATAGTGATCCTGCTACTGTGAAAAAATATGCTAGACGT
GCTCAATTGGGTGAAATTTTTGAATTAGATCGTGCTACTTTGAAATCGGATGGTGTTTTT
CGTAGCAGTCCAAGGGGTTGGTTTACTTTTGGACATGCCTCGTTTGCCTTGCTCTTCTT
TTTCGGACACATTTGGCATGGTGCTAGAACCTTGTTCAGAGATGTTTTTGCTGGTATTG
ACCCAGATTTGGATGCTCAAGTGGAATTTGGAGCATTCCAAAAACTTGGAGATCCAAC
TACAAAGAGACAAGTAGTC-----------------------------------GTGGAAACGCTCTTTAATGGA
ACTTTAGCTTTAGCTGGTCGTGACCAAGAAACCACTGGTTTCGCTTGGTGGGCCGGGA
ATGCCCGACTTATCAATTTATCCGGTAAACTACTCGGAGCTCACGTAGCCCATGCCGGA
TTAATCGTATTCTGGGCCGGAGCAATGAACCTATTTGAAGTGGCTCATTTCGTACCAGA
GAAGCCCATGTATGAACAAGGATTGATTTTACTTCCCCATCTAGCTACTCTAGGCTGGG
GGGTAGGTCCTGGTGGGGAAGTTATAGACACCTTTCCATACTTTGTATCTGGAGTACTT
CACTTAATTTCCTCTGCAGTATTGGGCTTTGGCGGTATTTATCATGCACTTCTAGGACCT
GAGACTCTTGAAGAATCTTTTCCATTCTTCGGTTATGTATGGAAAGATAGAAATAAAAT
GACTACAATTTTGGGTATTCACTTAATCTTGTTAGGTATAGGTGCTTTTCTTCTAGTACTC
AAGGCTCTTTATTTTGGGGGTGTATATGATACCTGGGCTCCCGGTGGGGGAGATGTAAG

AAAAATTACCAACTTGACCCTTAGCCCAAGTGTTATATTTGGTTATTTACTAAAATCGCC
CTTTGGGGGAGAAGGGTGGATTGTTAGTGTGGACGATTTAGAAGATATAATTGGAGGA
CATGTATGGTTAGGTTCCATTTGTATACTTGGTGGAATCTGGCATATCTTAACCAAACCT
TTTGCATGGGCTCGCCGCGCACTTGTATGGTCTGGAGAGGCTTACTTGTCTTATAGTTTA
GGTGCTTTAGCTGTCTTTGGTTTCATCGCTTGTTGCTTTGTCTGGTTCAATAATACCGCT
TATCCTAGTGAGTTTTATGGGCCCACCGGGCCAGAAGCTTCTCAAGCTCAAGCATTTAC
TTTTCTAGTTAGAGACCAACGTCTTGGGGCTAACGTGGGATCCGCTCAAGGACCCACT
GGTTTAGGTAAATATCTAATGCGTTCCCCGACTGGAGAGGTCATTTTTGGAGGAGAAAC
TATGCGTTTTTGGGATCTCCGTGCTCCTTGGTTGGAACCTCTAAGGGGTCCCAATGGTT
TGGACTTAAGTAGGCTGAAAAAAGACATACAACCTTGGCAAGAACGGCGTTCCGCGG
AATATATGACTCATGCTCCTTTGGGTTCTTTAAATTCCGTGGGTGGCGTAGCTACTGAGA
TCAATGCAGTCAATTATGTCTCTCCTAGAAGTTGGTTAGCTACCTCTCATTTTGTTCTAG
GATTCTTCCTATTCGTGGGTCATTTGTGGCATGCGGGAAGGGCCCGTGCAGCTGCAGCA
GGATTTGAAAAAGGAATTGATCGTGATTTTGAACCTGTTCTTTCCATGACCCCTCTTAA
CATGACTATAGCCCTTGGTAGATTTACCAAAGACGAAAATGATTTATTTGATATTATGGA
TGACTGGTTACGGAGAGACCGTTTTGTTTTTGTAGGTTGGTCCGGCCTATTGCTATTTCC
TTGTGCCTATTTCGCTTTAGGAGGTTGGTTCACCGGTACAACCTTTGTAACTTCATGGTA
TACCCATGGATTGGCTAGTTCCTATTTGGAAGGCTGCAATTTCTTAACCGCCGCAGTTTC
TACTCCTGCTAATAGTTTAGCACATTCTTTGTTGCTACTATGGGGTCCTGAAGCACAAG
GCGATTTTACTCGTTGGTGTCAGTTAGGCGGTCTGTGGACTTTTGTTGCTCTCCACGGC
GCTTTCGGACTAATAGGTTTCATGTTACGTCAATTCGAACTTGCTCGATCTGTTCAATTG
CGACCTTATAATGCAATCGCATTCTCTGCTCCAATTGCTGTTTTTGTTTCTGTATTCCTGA
TTTATCCACTAGGTCAGTCTGGTTGGTTCTTTGCACCTAGTTTTGGTGTAGCAGCTATAT
TTCGATTCATCCTCTTCTTCCAAGGGTTTCATAATTGGACGTTGAACCCATTTCATATGA
TGGGAGTTGCCGGCGTATTGGGCGCTGCTCTGCTATGCGCTATTCATGGTGCTACCGTA
GAAAACACTTTATTCGAAGATGGTGACGGTGCAAATACATTCCGTGCTTTTAACCCAAC
TCAAGCCGAAGAGACTTATTCAATGGTCACCGCTAACCGCTTTTGGTCCCAAATCTTTG
GGGTTGCTTTTTCCAATAAACGTTGGTTACATTTCTTTATGTTATTTGTACCAGTAACCG
GTTTATGGATGAGTGCTCTTGGAGTAGTCGGTCTAGCTCTGAACCTACGGGCCTATGAC
TTTGTTTCCCAGGAAATCCGTGCAGCAGAAGATCCTGAATTTGAGACTTTCTACACCAA
AAATATTCTCTTAAACGAAGGTATTCGTGCTTGGATGGCGGCTCAGGATCAGCCTCATG
AAAACCTTATATTCCCTGAGGAGGTTCTACCCCGTGGAAACGCTCTTATGTCTGGAAGC
ACGGGAGAACGTTCTTTTGCTGATATTATTACCAGTATTCGATACTGGGTCATTCATAGC
ATTACGATACCTTCCCTATTCATTGCGGGTTGGTTATTCGTCAGCACGGGTTTAGCTTAC
GATGTGTTTGGAAGCCCTCGACCGAACGAGTATTTTACAGAGAGCCGACAAGGAATTC
CATTAATAACTGGCCGTTTTGATCCTTTGGCACAACTCGATGAATTTAGTAGATCTTTTA
TGACCATAGATCGAACCTATCCAATTTTTACAGTGCGATGGTTGGCTGTTCACGGACTA
GCTGTACCTACCGTTTCTTTTTTGGGGTCAATATCAGCAATGCAGTTCATCCAACGAATG
GCTACACAAACCGTTGAGGGTAGTTCTAGA---TCTGGTCCAAGACAAACTGGTGTAGG
GTCTTTATTGAAACCATTGAATTCGGAATATGGTAAAGTAGCCCCTGGATGGGGAACTA
CCCCTTTGATGGGTGTCGCAATGGCTCTATTTGCGATATTCTTATCTATTATTTTGGAGAT
TTATAATTCTTCCGTTTTACTGGATGGAATTTCAATGAGTATGCTTACTCTCAAACTCGTC
GTTTACACAGTAGTGATATTCTTTGTTTCTCTCTTCATCTTCGGATTCCTATCTAATGATC
CAGGGCGTAATCCTGGGCGTGAGGAAATGGCCGATACTACTGGAAGGATTCCTCTTTG

GCTAATAGGTACTGTAACTGGTATTCCTGTGATCGGTTTAATAGGTATTTTCTTTTACGGT
TCATATTCCGGATTGGGTTCATCCCTGATGCTTAATATCTTAAGTTTAATCTGGATCTGTC
TTAATTCTGCCCTTCATTCGAGTAGTTTTTTATTCGCCAAATTGCCTGAGGCTTACGCCT
TTTTCAATCCAATCGTAGATGTTATGCCAGTCATACCTGTGCTCTTTTTTCTCTTAGCCTT
TGTTTGGCAAGCTGCTGTAAGTTTTCGAACGACACAATCAAACCCGAACGAACAAAAT
GTTGAATTGAATCGTACCAGTCTCTACTGGGGGTTACTACTCATTTTCGTACTTGCTGTT
TTATTTTCAAATTATTTCTTCAATATGGAAGTAAATATTCTCGCATTTATTGCTACTGCACT
GTTCATTCTAGTTCCTACTGCTTTTTTACTTATCATCTACGTAAAAACAGTCAGTCAAAA
TGATATGGAAACAGCAACCCTAGTCGCCATATTTATATCTGGTTTACTTGTCAGTTTCAC
CGGGTACGCCTTATATACCGCTTTTGGGCAACCCTCTCAACAACTAAGAGATCCATTCG
AGGAACACGGGGACATGGAAGCATTGGTTTATACATTCCTCTTAGTCTCGACTCTAGGG
ATAATTTTTTTCGCTATCTTTTTTCGCGAACCACCTAAAGTTCCGACTAAAAAGATGAA
AATGACTATTGCTTTTCAATTGGCTGTTTTTGCATTAATTGCTACTTCATCAATTTTACTG
ATTAGTGTACCCGTTGTATTTGCTTCTCCTGATGGTTGGTCAAGTAACAAAAATGTTGTA
TTTTCTGGTACATCATTATGGATTGGATTAGTCTTTCTGGTGGGTATTCTTAATTCTCTCA
TCTCT---------------------ATGTCACCACAAACAGAGACTAAAGCAAGTGTTGGATTTAAAG
CTGGTGTTAAAGATTACAAATTGACTTATTATACTCCTGAATATGAAACCAAAGATACTG
ATATCTTGGCAGCATTCCGAGTAACTCCTCAACCTGGAGTTCCACCTGAGGAAGCAGG
GGCCGCAGTAGCTGCCGAATCTTCTACTGGTACATGGACAACTGTGTGGACCGATGGA
CTTACCAGCCTTGATCGTTACAAAGGACGATGCTACCACATCGAGCCCGTTGCTGGAG
AAGAAAGTCAATTTATTGCTTATGTAGCCTACCCTTTAGACCTTTTTGAAGAAGGTTCT
GTTACTAACATGTTTACTTCCATTGTGGGTAATGTATTTGGGTTCAAAGCCCTACGCGCT
CTACGTCTGGAGGATCTGCGAATCCCTGTTGCTTATGTTAAAACTTTCCAAGGCCCGCC
TCATGGCATCCAAGTTGAGAGAGATAAATTGAACAAGTATGGTCGTCCCCTATTGGGAT
GTACTATTAAACCAAAATTGGGGTTATCCGCTAAGAACTACGGTAGAGCTGTTTATGAA
TGTCTCCGAGGTGGGCTTGATTTTACCAAGGATGATGAGAACGTGAACTCCCAGCCAT
TTATGCGTTGGAGAGACCGTTTCTTATTTTGTGCCGAAGCACTTTTTAAAGCACAGACT
GAAACAGGCGAAATCAAAGGACATTACTTGAATGCTACTGCAGGTACATGCGAAGAAA
TGATGAAAAGGGCTGTATTTGCCAGAGAATTGGGAGTTCCTATCGTAATGCATGACTAC
TTAACGGGGGGATTTACTGCAAATACTACCTTGGCTCATTATTGCCGAGATAATGGCCTA
CTTCTGCACATCCATCGCGCAATGCATGCAGTTATTGATAGACAAAAGAATCATGGTAT
GCACTTTCGTGTACTAGCTAAAGCGTTACGTATGTCTGGTGGAGATCATATTCATGCTGG
TACCGTAGTAGGTAAACTTGAAGGTGAAAGAGAAATCACCTTGGGCTTTGTGGATTTA
CTACGCGATGATTTTATTGAAAAAGACCGAAGTCGCGGTATTTATTTCACTCAGGATTG
GGTTTCTCTACCGGGTGTTCTGCCCGTGGCTTCAGGGGGTATTCACGTTTGGCATATGC
CTGCTCTGACCGAGATCTTTGGGGATGATTCCGTACTACAGTTCGGTGGAGGAACTTTA
GGACATCCTTGGGGAAATGCACCGGGTGCCGTAGCTAATCGAGTAGCCCTAGAAGCGT
GTGTACAAGCTCGTAATGAAGGACGTGATCTTGCTCGTGAAGGTAATGAAATTATCCGT
GAGGCCAGCAAATGGAGCCCTGAACTAGCTGCTGCTTGTGAGGTATGGAAGGAGATC
AAATTCGAATTCGAAGCAATGGATACTTTG------ACGGCGATACATTTATACAAAACTTCT
ACCCCGAGCACACGCAATGGAGCCGTAGACAGTCAAGCGAAATCCAATCCACGAAAT
AATTTGATCTATGGACAGCATCGTTGTGGTCAAGGTCGTAATGCCAGAGGAATCATTAC
CGCAGGGCATAGAGGGGGGGGTCATAAGCGTCTATACCGTAAAATCGATTTTCGACGG
AATGAAAAAGACATACCTGGTAGAATCGTAACCATAGAATACGACCCTAATCGAAATGC

ATACATTTGTCTCATACACTATGGGGATGGTGAGAAGAGATATATTTTACATCCCAGAGG
GGCTATAATTGGAGATACCATTGTTTCTGGTACAGAAGTTCCTATAAAAATTGGAAATGC
CCTACCTTTGACCGATATGCCCTTAGGCACGGCCATACATAACATAGAAATCACACTTG
GAAAGGGTGGACAATTAGCTAGAGCAGCGGGTGCTGTAGCGAAACTGATTGCAAAAG
AGGGTAAATCGGCCACATTAAAATTACCTTCTGGGGAGGTCCGTTTGATATCCAAAAAC
TGCTCAGCAACAGTCGGACAAGTGGGTAATGTTGGGGTGAACCAGAAAAGTTTGGGT
AGAGCCGGATCTAAGTGTTGGCTAGGTAAGCGTCCTGTAGTAAGAGGAGTAGTTATGA
ACCCTGTAGACCATCCCCATGGGGGTGGTGAAGGGAGGGCCCCAATTGGTAGAAAAA
AACCCACAACCCCTTGGGGTTATCCTGCGCTTGGAAGAAGAAGTAGAAAAAGGAATA
AATATAGTGATAGTTTGATTCTTCGTCGCCGTAGTAAAATGATTCAACCTCAGACTCATT
TGAATGTAGCGGACAACAGTGGGGCTCGAGAATTGATGTGTATTCGAATCATAGGAGCT
AGT---AATCGCCGATATGCTCATATTGGTGACGTTATTGTTGCTGTAATCAAAGAAGCAG
TGCCCAATATGCCTCTAGAAAGATCAGAAGTGATCAGAGCTGTAATTGTACGTACGTGT
AAAGAACTCAAACGTGACAACGGTATGATAATACGATATGATGACAATGCTGCAGTTGT
CATTGATCAAGAAGGAAATCCAAAAGGAACTCGAGTTTTTGGTGCGGTTGCTCGGGAA
TTGAGACAGTTGAATTTTACTAAAATAGTTTCATTAGCTCCCGAGGTATTAATGCTTAGT
CCCAAAAGAACCAGATTTCGTAAACAACATAGAGGAAGAATGAAGGGAAAATCGTATC
GAGGCAGTCGTATTTGTTTCGGTAGATACGCTCTTCAGGCACTTGAACCCGCTTGGATC
ACATCTAGACAAATAGAAGCAGGGCGACGAGCAATGACACGATATGCACGTCGTGGTG
GAAAAATATGGATACGCATATTTCCAGACAAACCCGTTACATTAAGACCTACGGAAACA
CGTATGGGTTCGGGGAAAGGATCTCCTGAATATTGGGTATCTGTCGTTAAACCGGGTCG
AATACTTTATGAAATGGGCGGAGTATCAGAAATTATAGCCAGAGAAGCTATTTCAATAG
CAGCATCAAAAATGCCTATACGAACTCAATTCGTTATTGCGGGAATGACTAGAATTAGA
CGAGGATATATAGCTCGGAGACGTAGAACAAAAATTCGTTTATTTTCATCAACCTTTCG
CGGGGCTCATTCAAGGCTTACTCGAACTATTACTCAACAGAAAATGAGAGCTTTGGTTT
CGGCTCATCGGGATAGGGGTAGGCAAAAGAGAGATTTTCGTCGTTTGTGGATCACTCG
GATAAATGCAGTAATTCGTGAGAAT------GTATTCTATAGTTATAGTAGATTAATACATGAT
CTGTATAAGGGGCAATTGTTTCTTAATCGTAAAATACTTGCACAAATAGCTATATCAAAT
AGGAATTGTCTTTACATGATTTTTAATGAGATCATCAAA---------------------------------ATGAT
AAAGGAAGTATACGCTTTAGGTCAACATATATGTATGTCTGCTCACAAAGCACGAAGAG
TTATTGATCAAATTCGTGGGCGCTCCTACGAAGAAACACTTATGATACTAGAGCTCATG
CCTTATCGAGCGTGTTATTCCATTTTTAAATTGATTTATTCTGCAGCAGCAAATGCTAGTC
ACAATATGGGTTTCAACGAAGCAGATTCAATCATTAGTAAAGTCGAAGTCAACGAGGG
TACTACCATGAAAAGATTAAAACCTCGAGCTCGAGGACGTAGTTTTCCGATAAAAAGA
CCCACCTGTCATATAACTATTGTATTGAAAGACATATCCTTAGATGGGGTGATATGGGAC
AAAAAAATGGATGGAATCAAATATGCAGTATTTACAGACAAAAGTATTCGGTTATTGGT
GGCGAACAATCAATATACTTCTAATGTCGAATCAGGATCAACTAGGACAGAAATAAAGC
ATTGGGTCGAACTCTTCTTTGGTGTCAAGGTAATATCTATGAATAGTCATCGACTCCCGG
GAAAGGGTAGAAGAATGGGACCTATTATGGGGCATACAATGCATTACAGACGTATGATC
ATTACGCTTCAACCGGGTTATTCTATTCCACCTCTTAGAAAGAAAAGAACTATGGCAGT
TCCAAAGAAACGTACTTCTATCTCAAAAAAGCGCATTCGTAAAAATATTTGGAAAAGA
AAGGGATATTGGGCAGCGGTAAAAGCTTTTTCGTTAGCGAAATCTCTTTCTACCGGGAA
TTCAAACAGTTTTTTTGTGCGG------------ATGGCCAAGGGTAAAGATGTCAGAGTCACA
GTTATTTTGGAATGTACCAGTTGTGTCCGAAACGGTGTTAAT------AAGGAATCACCGGG

CATTTCCAGATATATTACTCAAAAGAATCGACACAATACGCCTAGTCGATTGGAATTGA
GAAAATTCTGTCCCTATTGTTACAAGCATACAATTCATGGGGAGATAAAGAAAATGAAA
ATAAGGGCCTCTGTTCGTAAAATTTGTGAAAAATGTCGACTGATCCGCAGGCGGGGAC
GAATTTTACTAATTTGTTCCAACCCGAGACATAAACAAAGACAAGGAATGGTTCGAGA
AGAAGTAGCAGTATCTACTCGGACACTACAGTGGAAGTGTGTTGAATCAAGAGCAGAC
AGTAAGCGTCTTTATTATGGACGATTTATTATGTCTCCGCTTATGAAAGGTCAAGCCGAT
ACGATAGGCATCGCGATGCGAAGGGCTTTGCTTGGAGAAATAGAGGGAACATGTATCA
CACGCGCTAAATCTGAG------ACGATACCGCATGAATATTCTACCATAGTAGGTATTGAA
GAATCAGTACATGAAATTTTAATGAATTTGAAAGAAATTGTATTAAGAAGTAATCTGTAT
GGAACTCGCGACGCATCTATTTGCGTCAGAGGTCCTGGATACGTAACTGCTCAAGACAT
CATCTCACCGTCTTCTGTGGAAATAGTTGATACTACACAGCATATAGCGAGCCTGACGG
AACCAATTAATTTGTGTATTGGATTACAAATCGAGAGGAATCACGGATATCGTATGAAA
ACACCAAAGAACTCTCAAGATGGAAGTTAT------CCTATAGATGCTGTATTCATGCCTGTT
CGAAATGCGAATCATAGTATTCATTCTTATGGGAATGGGAATGAGAAACAAGAGATACT
TTTTCTCGAAATATGGACGAATGGAAGTTTAACTCCTAAAGAAGCACTTCACGAAGCC
TCCCGGAATTTGATTGATTTATTTATTCCTTTTCTACATACGGAAGAAGAG------GACATA
ATTATAGAGGACAATCAA---AACAGGTTTACTGTACCTTTTTTTACCTTTCATGATAGATT
GGCT---AAACTACGGAAAAACAAAAAA------ATAGCATTGAAATTCATTTTTATTGACCA
ATCAGAATTGCCTCCTAGGACCTATAATTGCCTCAAAAGATCCAATATACATACATTATT
GGACCTTTTGAATAAGAGTCAAGAAGATCTTATGAGAATTGAACATTTTCGGATAGAAG
ATGTAAAACACATATTGGGCATTCTACAAAAGCAT------TTCGAAATTAATTTACCGAAG
AATAAGATGTTCTGGGATGGAAAT---GAGGGAATGTCTACAATACCGGGGTTTAGTCAG
ATACAATTTGAGGGATTTTGTAAGTTCATTGATCAGGGCTTGACGGAAGAACTTTATAA
GTTTCCAAAAAATTGAAGATCCGGATCAAGAAATTGAATTTCAATTATTTGTGGAAACAT
ATCAATTGGTAGAACCCTTTATAAAAGAAAGAGATGCTGTGTATGAATCACTTACATATT
CTTCTGAATTATATGTACCTGCGGGATTAATTTGGAAAACCGGTAGGGATATGCAAGAA
CAAACCATTTTTATTGGAAACATTCCGCTAATGAATTCTCTGGGAACCTCTATAGTAAAT
GGAATATACCGAATTTTGATCAATCAAATATTGCAAAGCCCCGGTATTTATTACCGTTCA
GAATTGGACCATAACGGAATTTCGGTCTATACTGGCACCATAATATCAGATTGGGGAGG
AAGATCAGAATTAGAGATTGATAGAAAAGCAAGGATATGGGCTCGTGTGAGTAGGAAA
CAAAAAATATCGATTCTAGTTCTATCATCAGCTATGGGTTCGAATCTAAAAGAAATTCTA
GATAATGTTTGCTACCCTGAAATTTTCTTGTCTTTCCTGAATGATAAGGAGAAAAAAAA
GATTGGGTCAAAAGAAAATGCCATTTTGGAGTTTTATCAACAATTTGCTTGTGTAGGCG
GGGATCCGGTATTTTCTGAGTCCTTATGTAAAGAATTACAAAAGAAATTTTTTCAACAA
AGATGTGAATTAGGAAGGATTGGTCGCCGCAATATGAACCGGAGACTGAATCTTGATAT
ATCTCAGAACAATACATTTTTGTTACCGCGAGATGTATTGGCCGCTGGGGATCAGTTGA
TCGGAATGAAATTTGGAATGGGTACACTTGACGAT------ATGAATCACTTGAAAAATAAA
CGTATTCGTTCTGTAGCGGATCTGTTACAAGATCAATTCGGATTGGCTCTGGTTCGTTTA
GAAAATGTGGTTCGAGGAACTATATGTGGAGCAATTAGG---CATAAATGGATA---CCGAC
TCCTCATAATTTGGTAACTTCAACTCCA------------TTAACAACCACTTATGAATCATTTTT
CGGCTTACACCCCTTATCTCAAGTTTTGGATCAAACTAATCCATTGACACAAATAGTTCA
TGGGCGAAAATTGAGTTATTTGGGTCCTGGGGGGTTGACAGGGAGAACGGCTAGTTTT
CGGATCCGAGATATCCATCCTAGTCACTATGGACGTATTTGCCCAATTGACACGTCTGA
AGGAATCAATGTTGGACTTATTGGATCCTTAGCGATTCATGCGAGGATTGGTCATTGGG

GG---TCTTTAGAAAGCCCATTTTAT---------GAAATATCTGAGAGATCA------AAAAAGGTA
CGGATGGTTTATCTATCACCAAGTAGAGATGAATACTATATCGTAGCGGCAGGAAATTCT
TTGGCGTTGAATCAGAATGTCCAGGAAGAAGAGGTTGTTCCAGCTCGATACCGTCAAG
AATTCCTGACTATTGCATGGGAACAGGTTCATCTTCGAAGCATTTTTCCCTTCCAATATT
TTTCTATCGGAGCTTCCCTCATTCCTTTTATCGAGCATAATGATGCGAATCGGGCTTTAAT
GAGTTCTAATATGCAACGTCAAGCAGTTCCGCTTTCTCGGTCCGAGAAGTGCATTGTTG
GAACTGGGTTGGAACGCCAAGCGGCTCTAGATTCGGGGGTTTCCGCCATAGCCGAACA
CGAGGGAAAGATCATTTATACCGATATTGACAAGATTATTTTATCAGGCAATGGAGACA
CTCTAAAGATTCCATTGGTTATGTATCAACGTTCCAACAAAAATACTTGTATGCATCAAA
AACCCCAGGTTCCGCGGGGTAAATGCATGAAAAAGGGCCAAATTTTAGCGGACGGTG
CCGCTACAGTTGGTGGTGAACTCGCTTTGGGAAAAAAACGTATTAGTAGCTTATATGCCA
TGGGAAGGCTACAATTCTGAAGACGCGGTACTCATTAGCGAGCGTCTGGTATATGGAGA
TATTTATACTTCTTTTCACATACGGAAATATGAAATTCAGACTCATGTGACAAACCAAGG
A---CCTGAAAGAATCACTAATGAAATACCGCATTTAGAAGTCCATTTACTCCGCAACTTA
GACAGAAATGGAATTGTGATGTTAGGATCTTGGGTAGAAACGGGTGATATTTTAGTAGG
TAAATTAACTCCTCAGATGGCGAAAGAA---TCATCCTATGCTCCCGAAGATAGATTATTA
CGAGCCATACTTGGCATTCAGGTATCTACTTCAAAGGAAACGTGTCTAAAACTACCTAT
AGGTGGCAGGGGTCGAGTTATTGATGTGAGATGGATCCAGAAAAAGGGG---GGTTCCA
GTTATAATCCAGAAACAATTCGTGTATATATTTCACAGAAACGTGAAATCAAAGTAGGT
GATAAAGTAGCTGGAAGACATGGGAATAAAGGTATCATTTCCAAAATTTTGCCTAGACA
AGATATGCCTTATTTGCAAGATGGAACACCTGTTGATATGGTCTTCAACCCATTAGGAGT
ACCTTCACGAATGAATGTAGGACAGATATTTGAATGTTCCCTCGGGTTAGCGGGGGATC
TGCTAGACAGACATTATCGAATAGCACCCTTTGATGAGAGATATGAACAAGAGGCTTCG
AGAAAACTAGTGTTTTCTGAATTATATGAAGCCAGTAAGCAAACAGCGAATCCATGGGT
ATTTGAACCCGAGTATCCGGGAAAAGGCATAATATTTGATGGAAGAACGGGAGATCCTT
TTGAACAACCAGTTATAATAGGAAAGTCCTATATCCTGAAATTAATTCATCAAGTTGATG
ATAAAATCCACGGACGTTCCAGTGGACATTATGCGCTTGTTACCCAACAACCCCTTAGG
GGAAGGGCTAAGCAAGGGGGACAACGGGTAGGAGAAATGGAAGTTTGGGCCCTAGA
GGGATTTGGTGTTGCTCATATTTCACAAGAGATGCTCACTTATAAATCGGATCATATTAG
AGCTCGTCAGGAAGTACTTGGTACTACAATCATTGGAGGAACAATACCTAACCCTGCA
GATGCTCCAGAATCTTTTCGATTGCTCGTTCGAGAACTACGATCTTTGGCTCTGGAACT
GAATCATTTTCTTGTATCTGAGAAGAACTTCCAGATTAATAGGAAGGAAGCT--------------
------ATGATCGACCGGTATAAACATCAACAACTTCGAATTGGATTAGTTTCTCCTAAACA
AATAAGTGCTTGGGCCAGTAAAACCCTACCTAATGGAGAGATAGTTGGAGAGGTGACA
AAACCCTATACTTTTCATTACAAAACCAATAAACCGGAGAAAGATGGATTGTTTTGTGA
AAGAATTTTTGGGCCTATAAAAAGTGGAATTTGTGCTTGTGGAAATTATCGAGTAATCG
GAGATGAAAAAGAAGACCCGAAATTTTGTGAACAATGTGGAGTCGAATTTGTTGATTC
TCGGATACGAAGATATCAAATGGGATACATCAAATTGGCATGTCTAGTAACTCATGTGTG
GTATTTGAAACGTCTTCCTAGTTATATCGCGAATCTTTTAGATAAACCTCTTAAAGAATT
AGAAGGTTTAGTATACTGCGAT------TTTGCTTTTGCTAGGCCC---GTCGCTAAAAAACCT
ACTTTCTTACGATTACGAGGTTCATTCGAATATGAAATCCAATCCCGGAAATACAGCATC
CCTCTTTTTTTTACTACCCAAGGCTTCAATACATTTCGAAATCGAGAAATCTCTACTGGA
GCAGGTGCTATCAAAGAACAATTAGCCGATCTGGATTTGCGAATTATTACAGATTATTCA
TTGGTAGAATGGAAAGAATTAGGGGAAGAA---GGGCCCACAGGTAATGAATGGGAAGA

TAGAAAAATTGGAAGAAGAAAGGATTTTTTGGTTAGACGCATGGAATTAGCTAAGCAT
TTTATTCGAACAAATGTAGAACCAGAACGGATGGTTTTGTGCTTATTGCCAGTTCTTCC
TCCCGAGTTGAGACCGATCATTCAGATAGATGGAGGTAAACTGATGAGTTCGGATATTA
ATGAACTCTATAGAAGAGTTATCTATCGAAACAATACTCTTACCGATCTATTAACAACAA
GTAGATCTACGCCAGGGGAATTAGTAATGTGTCAGGAGAAATTGGTACAAGAAGCAGT
GGATACACTTCTTGATAATGGGATCCGGGGACAACCAATGAGGGACGGCTATAATAAAG
TTTACAAGTCGTTTTCAGATGTAATTGAAGGCAAAGAGGGAAGATTTCGCGAGACTCT
ACTTGGTAAACGGGTCGATTATTCGGGGCGTTCCGTCATTGTCGTGGGCCCTTCGCTTT
CATTACATCGATGTGGATTGCCTCGCGAAATCGCAATAGAACTTTTCCAAACATTTGTAA
TTCGTGGTCTAATCAGACAACATGTTGCGCCCAATATAGGAGTTGCTAAAAGTAAAATT
CGGGAAAAAGAACCAATTGTATGGGAAATCCTTCAAGAAGTTATGCAGGGGCATCCTG
TATTGCTGAATAGAGCACCCACTCTGCATAGATTAGGGATACAGGCATTCCAACCCATTT
TAGTGGAAGGACGTGCTATTTGTTTACATCCATTAGTTTGTAAGGGATTCAATGCAGAC
TTTGATGGGGATCAAATGGCTGTTCATGTACCCTTATCTTTGGAGGCTCAAGCGGAGGC
TCGTTTACTTATGTTTTCTCATATGAATCTCTTGTCTCCAGCTATTGGAGATCCCATTTCC
GTACCAACTCAAGATATGCTTATTGGACTCTATGTATTAACGATCGGGAATCGTCGAGGT
ATTTGTGCAAATAGGTATAATCCATGTAATCACAGAAAC---------TATCAAAATAAAAGAA
TTGACGATAATAACGAGAAGTATACGAAAGAAAAAGAACCCTATTTTTGTAGTTCCTAT
GATGTACTTGGAGCTTATCAGCAGAAACGAATCACTTTAGATAGTCCTTTGTGGTTCCG
ATGGCGACTAGATCAACGC---GTCATTGCATCA------AGAGAAGTTCCCGTCGAAGTTCA
ATATGAATCGTTGGGTACCTATCATGAGATTTATGGTCACTATCTAATAGTAAGAAGTGT
AAAAAAAGAAATCCTTTGTATATACATTCGAACCACCGTTGGTCATATTTCTTTTTATCG
AGAAATAGAAGAAGCTATACAAGGGTTTTGGCGTGCCTGCTCATACACGGTACCTAAG
CTAAGTAATTATATGATAATGGAGGCACTTATGGCAGAACGGGCCGATCTGGTCTTTCA
CAATAAAGTAATAGATGGAACTGCCATGAAACGACTTATTAGCAGATTAATAGATCACT
TCGGAATGGCATATACATCACACATCCTGGATCAAATAAAGACTCTGGGTTTCCAGCAA
GCCACTGCTACATCCATTTCATTAGGAATTGATGATCTTTTAACAATACCTTCTAAGGGA
TGGCTAGTCCAAGATGCTGAACAACAAAGTTTGATTTTGGAAAAACACCATCATTATG
GGAATGTACACGCGGTAGAAAAATTACGTCAATCCATTGAGATATGGTATGCTACAAGT
GAATATTTGCGCCAAGAAATGAATCCTAATTTTAGGATGACTGACCCTTTTAATCCAGTC
CATATAATGTCTTTTTCGGGAGCTCGAGGAAATGCATCTCAGGTACACCAATTGGTAGG
TATGAGAGGCTTAATGTCGGATCCCCAAGGGCAAATGATTGATTTGCCCATTCAAAGCA
ATTTACGTGAAGGACTCTCTTTAACGGAATATATTATTTCTTGCTATGGAGCCCGCAAAG
GAGTTGTGGATACTGCTGTACGAACATCAGATGCTGGATACCTCACGCGCAGACTTGTT
GAAGTAGTTCAACACATTGTTGTACGTAGAACAGATTGTGGTACCATCCGAGGGATTTC
TGTGAGTCCTCGAAATGGGATGATGACC---GAAAGAATTTTTATCCAAACATTAATTGGT
CGTGTATTAGCAGACGATATATATATGGGTCCACGATGCATTGCTATTCGAAATCAAGATA
TTGGGATTGGACTTGTCAATCGATTCATAACC------------------TTTCGAGCACAACCAATA
TCTATTCGAACCCCCTTTACTTGCAGGAGTACATCTTGGATCTGTCGATTATGTTATGGT
CGGAGTCCCACTCATGGCGATCTGGTCGAATTGGGAGAAGCAGTAGGTATTATTGCGG
GTCAATCTATTGGGGAACCGGGTACTCAACTAACATTAAGAACTTTTCATACTGGTGGA
GTATTCACAGGGGGTACTGCAGAACATGTACGAGCTCCTTCTAATGGAAAAATAAAATT
TAATGAGGATTTGGTTCATCCCACACGTACACGTCATGGACATCCTGCTTTTCTATGTTA
TATAGACTTGTATGTAACTATTGAGAGTCAGGATATTATACATAATGTGAATATTCCGCCA

AAAAGTTTTCTTTTAGTTCAAAATGATCAATATGTAGAATCAGAACAAGTGATTGCTGA
GATTCGCGCGGGAACATCCACTTTG---AATTTTAAAGAGAGGGTTCGAAAACATATTTA
TTCTGACTCAGAGGGAGAA---ATGCACTGGAGTACCGATGTGTACCATGCACCTGAATA
TACATATGGTAATGTTCATCTCTTACCAAAAACAAGTCATTTATGGATATTATCAGGAGG
TCCGTGCAGA------TCTAGTATAGCCCCTTTTTCGCTCCACAAGGATCAAGATCAAATCA
ACCTTCAT------TCTCTTTCTGTCGAACGAAGATCTATTTCTAAC------CTCTCAGTGACTA
ATGATCAA---GTGAGACACAAATTGTTTAGTTCGGATCTTTCGGGG------AGGGGTAGGA
TTCTTGATTAT---TCCGGACCTGATCGAATCATATGCAATGATCATTGTAATTTCAGATAC
CCTACC---ACTCTCCACGAGAATTCTGAT------TTATTGGCAAAGAGGCGAAGAAATAGA
TTCATCATTCCATTCCAATCTAATCAAGAACGAGAGAAAGAACTAATGCTCTGTTCAGG
TATCTCGATTGAAATACCCATAAAT------GGTCTTTTCCGTAGAAATAGTATTCTTGCTTAT
TTCGATGATCCCCGATACAGAAGAAACAGTTCGGGAATTACTAAATATGGGACTATAGA
AGTGCATTCAATTCTCAAAAAAGAGGAT------TTGATTGAGTATCGAAGA------AAAGAA
TTTAGGCCAAAATACATGAAAGTAGATCGATTCTTTTTCATTCCTGAGGAAGTGCATATC
TTACCCGGATCTTCTTCCATAATGGTACAGAACAATAGTATCATTGGAGTAGATACACGA
ATAACTTTAAATACAAGAAGCCGAGTAGGTGGATTGGTCCGAGTGGAGAGAAAAAAA
AAAAAGATTGAATTGAAAATCTTTTCTGGAGATATCCATTTTCCTGGAGAGACAGATAA
GATATCCCGGCACAGTGGCATCTTGATACCACCGGGAACAAAAAAAAAA------AACTCT
AAGGAATCAAAA------AAATGGAAAAATTGGATCTATGTCCAACGG------ATCACACCTA
CTAAGAAAAAG------TATTTTGTTTTGGTTCGACCCGTAGTCACATATGAAATAGCAGAC
GGGATAAATTTAGCAACGCTTTTCCCCCAGGATCTGCTGCAGGAAAGGGATAATGTGC
AACTTAGAGTTGTCAATTATATCCTTTATGGAAACGGCAAACCAATTCGAGGAATTTCT
CACACAAGTATTCAATTAGTTCGAACTTGTTTAGTATTGAATTGGGACCAAGACAAAAA
A---------GATTCTTCTATAGAAGAGGTTCATGCTTCCTGTGTCGAAGTAAGGGCAATTGAT
CTGATTCGAGATTTCATAAGAATGGACTTAGTTAAGCCCCCTATTTCATATACCGTAAAA
AGGAATGATCCGGCAGGTTCAGGATTGATTCCCGAT---AATGGATCAGATCGCACCAATA
TCAATCCTTTTTAT---------TCCAAGGCAAGG---------------ATTCAGCAATCACTTAGCCAA
CATCAAGGAACTATTCGTACGTTGTTGAATAGA---------AATAAGGAATGCCACTCTTTGA
TAATTTTGTCATCATCCAATTGTTTTCGA------ATGGGCCTATTCAACGGTTTGAAATATCA
TAAT---------GTGACAAAAGAATCAGATTCCCTAATTCCAATTCGGAATTCGTTGGGGCCC
TTGGGGACT------GTCCCTAAAATTGCGAATTTTGATTTA------TCTTATCATTTAATAACG-
-----CATAATCAGATCTTG---AGAAATAAATATTTGCTACTTGACAAT---------TTTAAAAAG
ATTTTCCAAGTACTTAAA---------TATTATTTAATGGATGAAAATGGGGGA------ATTAATAA
TCCCGATCCATCCAGTAACATCATTTTGAATCCAGTCAATTTGAATTGGTATTTTCTCCAT
CACGATTATTGTGAAGAGACACCCATAATAATTAGC---CTTGGACAATTTATTTGTGAAA
ATGTATGTATATACAAAAAGAGACCA---------CACCTAAAATCTGGTCAAGTTCTAATTGT
CCAT---GTTGACTCTTTAGTAATAAGATCAGCTAAGCCCTATTTGGCCACTACAGGAGCA
ACGGTTCATGGCCATTATGGGGAAATCCTGTACGAAGGAGATACATTAGTTACATTTATA
TATGAAAAATCGAGATCTGGTGATATAACGCAGGGTCTTCCAAAGGTGGAACAAGTGT
TAGAAGTGCGTTCGATTGATTCAATATCGATGAATCTAGAAAAGAGGGTTGAAGGTTGG
AACGAACATATAACAAGAATTCTTGGAATTCCTTGGGGATTCTTGATTGGTGCTGAGCT
AACTATAGCGCAAAGTCGTATCTCTTTAGTTAATAAGATCCAAACGGTTTATCGATCCCA
GGGGGTGCAGATTCATAATAGGCATATAGAAATTATTGTACGTCAAATAACATCAAAAG
TCTTGGTTTCAGAA---------GATGGAATGTCTAATGTTTTTTCACCCGGAGAACTAATTGG

ATTGTTGCGAGCGGAACGAACGGGGCGTACTTTGGAAGAAGCGATCTGTTACCGAGCC
GTCTTATTGGGAATAACGAGGGCATCTCTGAATACTCAAAGTTTCATATCCGAGGCGAG
TTTTCAAGAAACTGCTCGAGTTTTAGCAAGAGCCGCTCTACGAGGTCGTATCGATTGGT
TGAAAGGCCTGAAAGAGAACGTTGTTCTGGGGGGGATGATACCCGTTGGTACCGGATT
CAAAGGATTGGTGCACTGTTCA------AGGCAACACAAGAACATTCCT------TTGGAAATA
AAAACGAAGAATCTATTCGGGGGGGAAATGAGA------GATATTTTGTTCCACCACAGAG
AATTATTTGATTCTTGCATCCAAAAAAATTTCCATGATACATCAGAACAA--------------------
----------------------ATGACAAGAAGATATTGGAACATCAATTTGGAAGAGATGATGGAAGC
GGGAGTTCATTTTGGTCATGGTACTAGGAAATGGAATCCTAGAATGGCACCTTACATCT
CTGCAAAGCGTAAAGGTATTCATATTACAAATCTTACTAGAACTGCTCGTTTTTTATCAG
AAGCTTGTGATTTAGTTTTTGATGCAGCAAGTAGGGAAAAACAATTCTTAATAGTTGGT
ACCAAAAATAAAGCGGCTGATTCAGTAGCATCGGCTGCAATAAGGGCTCGGTGTCATTA
TGTTAATAAAAAATGGCTCGGTGGTATGTCAACGAATTGGTCCACTACAGAAACGAGA
CTTCATAAGTTCAGGGACTTGAGAGTGGAACAAAAGACGGGGAGACTCAATCGTCTTC
CGAAAAGAGATGCAGCAATGTTGAAGAGACAATTATCGCACTTGCAAACATATCTGGG
CGGGATCAAATATATGACGGGGTTACCCGATATTGTAATCATCGTTGATCAGCAAGAAG
AATATACGGCTCTTCGAGAATGTGTCACTTTGGGAATTCCAACGATTTGCTTAATCGATA
CAAATTGTGACCCGGATCTCGCAGATATTTCGATTCCAGCGAATGATGACGCTATAGCTT
CAATCCGATTAATTCTTAACAAATTAGTATCCGCAATTTGTGAGGGTCGTTCTAGC---TAT
AGAAGAAATCGTATGGGACAAAAAATAAATCCACTTGGTTTCAGACTTGGTACAACTC
AAGGTCATCATTCTGTTTGGTTTGCACAACCAAAAAAGTATTCTGAGGGTCTCGAGGA
AGATCAAAAAATACGAGATTGTATCAAGAATTATGTACAAAAAAATATGAGAATATCCT
CCGGT---GTCGAGGGAATTGCACGTATAGAGATTCAAAAAAGAATCGATCTCATCCAGG
TCATAATCTATATAGGATTCCCAAAGTTGTTAATAGAGGGTAGACCACGA------------GGA
GTCGAAGAATTACAGATCAATGTACAAAAAAAGTTTCATTCTGTGAACCGAAAACTCA
ACATTGCTATCACAAGAATTGCAAAACCTTATGGACAACCTAATATTCTTGCAGAATTTA
TAGCTGGACAATTAAAGAATAGAGTTTCATTTCGAAAGGCGATGAAAAAGGCTATTGA
ATTAACTGAACAAGCAGATACAAAAGGAATTCAAGTGCAAATTGCAGGGCGTATCGAC
GGAAAAGAAATTGCACGTGTCGAATGGATTAGAGAAGGTCGAGTTCCCCTACAAACCA
TTCGAGCTAAAATTGATTATTGTTCCTATACAGTTCGAACTATCTATGGGGTATTGGGCAT
CAAAATTTGGATATTTGTAGACGAGGAAATGTCTCGTTACCGAGGACCTCGTTTTAAAA
AAATACGCCGTCTAGGGGCTTTACCGGGACTAACTAGTAAAAGGCCTAGAGCTGGAAG
TGATCTTAGAAACCAATCGCGTTCCGGGAAA---AGGTCGCAATATCGTATTCGTCTAGA
AGAAAAACAAAAATTGCGTTTTCATTATGGTCTGACAGAGCGGCAATTACTTAAATACG
TTCGTATCGCTGGAAAAGCCAAAGGATCAACAGGTAAAGTTTTACTACAATTACTTGAA
ATGCGTTTGGATAACATCCTTTTTCGATTGGGTATGTCTTCGACCATTCCTGGAGCCCGG
CAATTAGTTAACCATAGACATATTTTAGTTAATGGTCGTATAGTAGATATACCAAGTTATC
GTTGCAACCCCCGAGATCTTATTACGGCGAAGGATGAACAAAAATCCAGAGCTCTGAT
TCAAAATTATCTTGATTCATCCCCCCAT---GAGGAATTGCCAAAACATTTGACTCTTCAC
TCATTCCAATAT------AAAGGATTAGTCAATCAAATAATAGATAGTAAATGGGTTGGTTTG
AAAATAAATGAGTTGCTAGTCGTAGAATATTATTCCCGTCAGACTATGTCACGTCGAGGT
ACTGCAGAAGAAAAAACTGCAAAATCCGATCCAATTTATCGTAATCGATTAGTTAACAT
GTTGGTTAACCGTATTCTGAAACACGGAAAAAAATCATTGGCTTATCAAATTCTCTATC
GAGCCGTGAAAAAGATTCAACAAAAGACAGAAACAAATCCACTATCTGTTTTACGTCA

AGCAATACGTGGAGTAACTCCCGATATAGCAGTAAAAGCAAGACGTGTAGGCGGATCG
ACTCATCAAGTTCCCATTGAAATAGGATCTACACAAGGAAAAGCACTTGCCATTCGTTG
GTTATTAGGGGCATCCCGAAAACGTCCGGGTCGAAATATGGCTTTCAAATTAAGTTCCG
AATTAGTGGATGCTGCCAAAGGGAGTGGCGATGCCATACGCAAAAAGGAAGAGACTC
ATAGAATGGCAGAGGCAAATAGAGCTTTTGCACATTTTCGTATGGGTAGGGACACTATT
TCCGACATAATAACTTCTATACGAAATGCTGACATGGAAAAAAAAGGAACGGTTCGAAT
AGCATTTACTAATATCACCGAAAACATTGTTAAAATACTTCTACGAGAAGGTTTTATCGA
AAACGTGAGGAAACATCGGGAAAGCAACAAATATTTCTTGGTTTCAACCCTGCGACAT
AGAAGG------AATAGGAAAGGACCATATAGA---------ACTATTTTA------AAACGTATCAGC
CGACCCGGTCTACGAATCTTTTCCAACTATCAACGAATTCCTAGGATTTTAGGTGGAAT
GGGGATTGTAATTCTTTCTACTTCTCGAGGTATAATGACAGACCGAGAGGCTCGACTAG
AAGGAATCGGAGGAGAAATTTTGTGTTATATATGGATGGCAAAACCTATACTCAGAATT
GGTTCACGTAGGACTGGACGTATTGGTTCACGTAAAAATGGACGTAGAATCCCAAAGG
GAGTTATTCATGTTCAAGCAAGTTTCAACAATACCATTGTGACTGTTACAGATGTACGG
GGTCGGGTGATTTCTTGGTCCTCCGCCGGTACTTGTGGATTCAGGGGCACAAGAAGAG
GGACACCGTTTGCTGCTCAAACCGCAGCAGGAAATGCTATTCGTACAGTAATGGAGCA
GGGAATGCAACGGGCAGAAGTCATGATAAAGGGTCCTGGGCTCGGAAGAGATGCAGC
ATTACGAGCCATTCGTAGAAGTGGTATACTATTAAGTTTCGTACGGGACGTAACCCCTAT
GCCACATAATGGATGTAGACCTCCTAAAAAACGACGTGTGATGCCAACTATTAAACAAC
TTATTAGAAACACAAGACAGCCAATCCGAAATGTCACGAAATCCCCCGCTCTTCGGGG
ATGTCCTCAGCGCCGAGGAACATGTACTAGGGTGTATACTATCACCCCCAAAAAACCA
AACTCTGCCTTACGTAAAGTTGCCAGAGTACGATTAACCTCTGGATTTGAAATCACTGC
TTATATACCTGGTATTGGCCATAATTCACAAGAACATTCTGTAGTCTTAGTAAGAGGAGG
GAGGGTTAAGGATTTACCCGGTGTGAGATATCACATTGTTCGAGGAACCCTAGATGCTG
TCGGAGTAAAGGATCGTCAACAAGGGCGTTCTAAATATGGGGTCAAAAAGCCAAAAAT
GGCAAGGAAAAGTTTGATTCAGAGGGAGAAGAAGAGACAGAAATTGGAAGAGAAAT
ATCATTTGATTCGTCGATCCTCAAAAAAAGAAATAAGT---------AAAGTTCCATCGTTGA
GGGACAAATGGGAAATTCATGGAAAATTACAATCCCCACCGCGTAATAGTGCACTTAC
ACGTCTTCATCGACGTTGTTTTTTGACCGGAAGACCGAGAGCTAACTATCGAGACTTTG
GGTTATCCGGACACCTACTTCGTGAAATGGTTCATGCATGTTTGTTGCCGGGGGCAACA
AGATCAAGTTGGATGGTCAAAAATACATTCATCTCAGTTATTTCGCAAGAC------GAAAA
AGTAGCGAGCAGCGGGTCTGTTGAATTTCAAGTATTCCGTTTCACCAATAAGATACGGA
GACTTACTTCACATTTGAAGTTGCACAGAAAAGACTATTTATCTCAGAGAGGTCTACGG
AAAATTCTGGGAAAACGTCAACGGCTCCTGGCTTATTTTTCAAAGAAAAATAGAGTAC
GTTATAAAGAATTAATTTTTCAATTGGATATTCGGGAGCCAAAAACTCGT------ATGGTAA
AACTTCGTTTGAAACGATGTGGTAGAAAGCAACGAGTCATCTATCGAATCGTTGCAATT
GATGTTCGATCCCGAAGAGAAGGAAGAGATCTTCGGAAAGTGGGTTTTTACGATCCGA
TAAAGAATCAAACTTATTTAAATGTTCCCGCTATTCTATATTTCCTTGAAAAGGGCGCTC
AGCCTACAGGAACTGTTCATGATATTTCAAAGAGGGCAGAGGTATTTAAGGAACTCCG
TCTTAATCAAACGAAATCAAATTACATGGATAAATTCAAGCGACCTTTTCTTAAATCCA
AGCGATCTTTTCGTAGGCGTTTGCCCCCAATTGGATCGGGGGATCGAATTGACTATAGA
AATATGAGTTTAATTAGTCGATTTATTAGTGAACAAGGAAAAATATTATCTAGACGAGTG
AATAGATTGACCTTGAAACAACAACGATTAATTACTATTGCTATAAAACAAGCTCGTATT
TTATCTTCGTTACCTTTTCTTAATAATGAGAAA------CAATTTGAGAGAACTGAGTCGAC

CCCTAGAACTGCGGGTCCTAGAACCAGAAATAAAGTGACACGTTCCCTAAAAAAAAAT
CCTTTTGTAGCTAATCATTTATTAGGAAAAATTGAGAAGCTCAACATGAGAGCGGAAAA
AGAGATAATACTAACTTGGTCCCGGGCATCTACCATTATACCCACAATGATCGGCCATAC
AATTGCTATTCATAATGGAAAAGAACATTTGCCTGTTTATATAACAGATCGTATGGTAGG
TCACAAATTGGGAGAATTTGCACCTACTCTTAATTTCCGGGGACATGCGAGAAACGATA
ATAGCTCTCGTCGTATGATTTTGAAATCTTTTCTACTAGGCAATCTATTATCCTTATGCAT
GAAGATAATAAATTCGGTCGTTGTGGTCGGACTCTATTATGGATTTCTGACCACATTCTT
CATAGGGCCCTCTTATCTCTTCCTTCTCCGAGCTCGGGTTATGGAAGAAGGA---------AC
CGAGAAGGAGGTATCAGCAACAACTGGTTTTATTACAGGACAGCTCATGATGTTCATAT
CGATCTATTATGCGCCTCTGCATCTAGCATTGGGTCGACCTCATACAATAACTGTCCTAG
TTCTACCGTATCTTTTGTTTCATTTCTTCTGGAACAATCACAAACACTTTTTTGATTATGG
ATCTACTACCAGAAATTCAATGCGTAATCTCAGCATTCAATGTGTATTCCTGAATAATCT
CATTTTTCAATTATTCAACCATTTCATTTTACCAAGTTCAACGTTAGCCAGATTAGTAAA
CATTTATATGTTTCGATGCAACAACAAGATGTTATTTGTAACAAGTAGTTTTGTTGGTTG
GTTAATTGGTCACATTTTATTCATGAAATGGGTTGGATTGGTATTATTCTGGATACGACA
AAATCATTCTATTAGATCT------GTACTTATTAAGTACCTTGTGTCAGCATTGAGAAATTCT
ACGGCTCGAATCTTTAGTATTCTCTTATTTATCACCTGTGTCTACTATTTAGGCAGAATAC
CGTCACCTATTGTCACTAAAAAACTGAAAGAAACCGAAGAAGACAAACCTCTTGTGA
CTCTTCTTTTTGACTATAAACGATGGAATCGTCCATTGCGATATATAAAAAACAATCGAT
TTGAAAATGCTGTAAGAAATGAAATGTCACAATATTTTTTTGATACATGCCGAAGTGAT
GGAAAACAAAGAATATCTTTTACATATCCACCCAGTTTGTCAACTTTTTGGGAAATGAT
ACAAAGAAAGATGTCTTTGTGCACGACAGAAAAACTCTCC---GAAGAACTGTATAATC
ATTGGATTTATACCAATGAACAAAAAAGGAACAGCTTGAAGAACGAATTTTTGAATCG
AATGAAAGCACTAAACAAGGGATTT------CGGGATGTACTCGAAAAACGAACTCGACTG
TGTAATGATGAAACTGAACAAAAATGCTTTCCTAAAATGTACGATCCTTTTTTGAACGG
A------CGCTATCGCGTAACAACCAACTATTCACATGATTACAAGGTCTGGATAAATAAAA
TTCATGGTATGCTTTACGACACATTTGAACAGAAAAATGTGAATGATCAA---ATTGAAAT
AAAAGAAATTCGTAAAAAGGTCCCTCGATGGTCATACAAATTGATTGATTTGGAAATAC
AGAAGAGAAAAGGGATTCGTTCAAGAAAAGCCAAACGTGTAGTCATTTTTACTGATGC
TTTGATACGTTATTCGCAACAATCGGATTTTCGTCGACATTTCATCAAAGGATCGATACG
CGCTCAAAGATGTAAAACAGTTCCTTGGAAACTGTTTCAACGAAATGTGCATTCCCCG
CTTTTTTTGGACCGAATAGACAAACCTTTTTTTTTCTCTTTTGATATC------TCCGGAACG
ATGAAGCTAATTTTTAGTAATAAAGATAAAAATGAGCGGATAACAATAGCAGAAACCTG
GGATACTTTTATATTTGCTCAAGTAATAAGGGGTTCCATGTTAGTAACCCAATCGATTCTT
AGGAAATATATTATATTGCCGTCATTGATAATAGCTAAAAATATCGGCCGTATGCTATTAT
TTCAATTCCCCGAATGGTACGAGGATTTGACGGAGTGGAGTAGAGAAATGCATGTTAA
ATGCACCTATAATGGTGTTCAATTATCAGAAATAGAATTTCCGAAAAATTGGTTAACAG
ACGGTATTCAGATAAAAATCCTATTTCCTTTCTGTCTGAAACCTTGGCACAAATCTAAG
GAAAAAAAAGAAGATAATTTTTGCTTTTTAACAGTCTGGGGAATGGAAGCCGAACTAC
CTTTTGGTTCTCCCCGCAAACGGCCTTCCTTTTTTGAACCCATTTATAAAGAACTGGAA
AAAAAAAATTTTAAAGTAAAA---AAAAAATGTTTTGTAGTTCTAAGAGTTTTAAAAGAA
AGAACAAAATGGTTTCTAAAGGTCTCAAAAGAAAAAAAAAGATGGGTTAGTAAAAAA
GTTCTA------TTTATAAAAACAAAAACAAATGAACTTGTAAAAGGAAAT---TCTCTAGTC
AGTAATGATATTTTTCATGAATCGTCGATTGGAATTAGATCCATAGATTGGACAAATTATT

CACTGACAGAAACAAAAATGAAGGATCTGGCTGATAGGACAAGAACAATCAGAAATC
AAATAGAAAGAATCACAAAAGACAACAAAAAAAGTCCTAATGAAACAAGTTGGGATG
ATAAAAGATCAAAATCGCCGAAAAATATTTGGCAGATATTAAAAAGAAGAAATGCCCG
ATTAATGCGTAAATGGCATTATTTTTTGAAATTTTTCTTTGAAAGGATATCTATGTATATCC
TTCTATGTATTATTAATATTCCCAGAATCAATACAACATTTTTCATTCACTTTATTTCGTGT
GACCTAGCCGCCTTATCACAAGCCTATGTATTTTACAAATTATCACAAACCAAAATTCTT
AACAAGTATCACTTGAGATCTGTACTTAAATATACATCTCTTTTTCTTAAGGATAGAATA
AAGTATTTTTTTAGACAAGGAATATTTCATTCCGAATCAGAGCATAAAAAAACTTCGAAA
TTCTGGAATGAATGAATGGAAAACCTGGTTAAGGGGTCATTATCAATACGATTTATCTC
AGACTAGATGGTCTAGATTAGTACCGAAAAAATGGCGAACTAGAATCAATCAATGTCAT
ACGGTTCAAAATAAAAACAGATTTGCTTCCTATGAAAAAGACCAA------TTAATTCATTA
CGAGAAACAAGAAAAATTTAAAAAACAGTACAGATATGGTCTTTTATCACATAAATATA
TTAATGATTCTATTATTGATCCAGATAGAAAATATTTTGATTGGAGAATTCTAAATTTTTTT
ATTAGCAATTTCAATTGGATGGGAATGAATGAAGAAATACTAAATCGTTTGTTCTTGCC
AGAATTTTTGCTATTTTATGATGCATATAAGATGAAACCATGGGTCATACCAACCAAATT
ACTTTTTTTTAATTTCAAGAACAACACAAAAGCAGAATTAAATTTCTTCCTGAAAAGAT
ATTTTCTTTTTCAATTGAGATGGGGTGATCCTTTGAATCAAAGAATAATCAATAATATAA
AGGTATATTGTTTCCTACTTAGACTGATAAATCCAAAGGAAATTGCTATATCCTCGATTC
AAAGAGGAGAAATGGATCTGGATGTAATGCTAAGTCAGAAGGATCTATCTCTTAAAGA
ATTACTAAAAAAAGGGATATTTCTTATCGAACCAATTCGTCTGTCTATAAAATGGGATGG
ACAGTTTATTATGTATAAAACCATAGGTATTTCATTGGTCCATAAGAGTAAGCACAATCA
TTATGATTTGCTTGTTCCTGAAAATATTTTATCATCTAGATGTCGTAGAGAATTGAGAATT
CTAATTTGTTTCAGATTTCTTTGGCCCAATTATCGATTAGAAGATTTAGCTTGTATGAATC
GCTATTGGTTTGATACCACTAATGGTAGTCGTTTCAGTATGTCAAGGATACATATGTATCC
AATGAAACGACATCAATTCCAATCCTGGATCTTCGAATTGAGAGAGATATTGAGAGAGA
TCAAGAATTCTCACTATTTCTTAGATTCATGGACCCGATTCGATTCATTGGGATCTTTCA
CTCCCATTTTCTTCCACCAAGAGCGTTTTATGAAACTCTTTGACCCCCGAATTTGGAGT
CTCCTACTTTCA---CGCGATTCA------CGGGGGTCAAAAAGCAATCGATATTTCACG-------
--ATCAAAGGTGTAGGACTGCTTGTAGTAGCG---GTCCTTATATAT------------CGTATTAATA
ACCGAAATAGGGTC------AAAAATCTCTATTTGACGGGGCTTCTT---------------CCTATACC
TATGAATTCCATTGGACCCAGAAATGAGACATTGGAAGAATCTTTT---GGGTCTTCCAAT
ATCAATAGGTTGATTGTTTCG---CTCCTCTATCTTCCAAAAGGGAAAAAGATCTCTGAG
AGTTGTTTCATGGATCCGAAAGAGAGTACTTGGGTTCTCCCAATAACT-----------AAAAA
GTGTATCATGCCTGAATCTAACTGGGGTTCGCGGTGGTGGAGGAACCGGATCAGAAAA
AAGAGG------GATTCTAGTTGTAAGATATCT---------AATGAAACCGTAGCTGGAATTGAG
ATCTCATTCAAAGAGAAAGATAGCAAA---TATCTGGAGTTTCTTTTTGTATCCTATACGG
AT------GATCCGATCCGCAAGGACCATGACTGGGAATTGTTTGATCGTCTTTCTCCGAGG
AAGAAGCGAAACATAATCAACTTG------AATTCGGGACAGCTATTCGAAATCTTAGTGA
AACACTGGATTTGTTATCTCATGTCTGCTTTTCGTGAAAAAAGACCAATTGAAGTGGAA
GGT---TTCTTCAAACAACAAGGA---GCTGAGTCAACTATTCAATCAAATGATATTGAGCA
TGTTTCCCAT------CTCTTCTCGAGAAACAAGTGGGGTATTTCTTTGCAAAATTGTGCTC
AATTTCATATGTGGCAATTCCGCCAAGATCTCTTCGTT---------AGTTGGGGGAAG---AAT
CCGCACGAATCAGATTTTTTGAGGAAC---GTATCGAGAGAGAATTGGATTTGGTTAGAC
AATGTGTGGTTGGTAAACAAGGATCGG---------TTTTTTAGC------AAGGTGCGGAATGTA

TCGTCAAATATTCAATATGATTCC---------ACAAGA------TCTATTTTCGTTCAAGTAACGG
ATTCTAGCCAATTGAAAGGATCTTCT---GATCAATCCAGAGATCATTTCGATTCCATTAGT
------------AATGAGGATTCGGAA--------------------------------------TATCACACATTGATCAATC
AAAGAGAGATTCAACAACTAAAAGAAAGATCGATTCTT---GGGGAT------CCTTCCTTTC
TTCAAACGGAACGAACAGAGATAGAATCAGAC------CGATTCCCGAAA------TGCCTTTC
TGGATATTCC------TCAATGTCCCGGCTATTCACG---GAACGTGAGAAGCAGATGAATAAT
CATCTGCTTCCGGAAGAAATCGAAGAATTTATT------GGGAATCCTACAAGATCAATTCG
TTCTTTTTTCTCTGACAGATGG------TCAGAACTGCAT---CTGGGTTCGAATCCTACTGAG
AGGTCCACTAGAGATCAG------------AAGTTGTTGAAGAAACAACAAGATGTTTCT---TTT
GTCCCTTCCAGGCGATCGGAA------------AAGAAAGAAATGGTTGATATATTCAAGATAAT
TACGTATTTACAAAAT------ACCGTCTCAATTCATCCTATTTCATCAGATCCGGGATGTGAT
ATGGTTCCG---AAGGATGAACCGGAT---ATGGACACTTCCAATAAGATTTCATTCTTGAA
CAAAAATCCATTTTTTGAT------TTATTTCATCTATTCCATGACCGGAACAGGGGGGGATA
CACGTTA------CACCACGATTTTGAATCAGAA------GAGAGATTTCAAGAAATGGCAGAT
CTATTCACTCTATCAATAACCGAGCCG---GATCTGGTGTATCATAAGGGATTTGCCTTTTC
TATTGATTCCTACGGATTGGATCAAAAAAAA------TTCTTGAATGAGGTATTCAACTCCA
GGGATGAATCGAAAAAGAAATCTTTA---------TTGGTTCTACCTCCTATTTTTTAT------GA
AGAGAATGAATCTTTTTATCGAAGGATCAGAAAAAAAATCGGTCCGG---ATCTCCTGCGG
GAATGATTTGGAAGATCCAAAACCAAAAATAGTGGTATTTGCTAGTAACAACATAATGG
AGGCAGTCAATCAATCTAGATTGATCCGAAATCTGATTCAAATCCAATATAGCACCTATG
GG------TACATAAGAAATGTATCGAATCGATTCTTT---------TTAATGAATAGATCCGATCGC
AACTTCGAATATGGAATTCAA---------AGGGATCAAATAGGAAATGATACTCTGAATCATA
GAACTATAATGAAATATATGATCAACCAACATTTCTCGAATTTGAAAAAG---AGTCAGA
AGAAATGGTTCGATCCT------------CTTATTTCTCGAACCGAGAGATCCATGAATCGGGAT
CCTGATGCATATAGATACAAATGGTCCAATGGGAGCAAGAATTTCCAGGAACATTTGGA
ACATTTCGTTTCTGAGCAGAAGAGC---------CGTTTTCAAGTAGTGTTCGATCGATTACGT
ATTAATCAATATTCGATTGATTGGTTCGAGGTTATCGACAAACAAGATTTGTCTAAGTCA
CTTCGTTTCTTTTTGTCCAAGTCA---------------------------CTTCTCTTTTTGTCC----------------
-----AAGTCACTTCTCTTTTTGTCCAAGAAGTCACTTCCTTTTTTCTTTGTGAGTTTCGGG
AATATCCCCATTCATAGGTCCGAG---ATCCACATCTAT---GAATTGAAAGGTCCGAATGAT
CAACTCTGCAATCAGTTGTTAGAATCAATAGGTGTTCAAATCGTTCATTTGAATAAATTG
AAACCCTTCTTATTGTTATTGGAT---------GATCATGATACTTCC---CAAAAATCGAAATTC
TGGATCAATGGAGGAACAATATCACCATTTTTGTTCAATAAGATACCA---AAGTGG---AT
GATTGACTCATTCCATACT------AGAAAGAATCGCAGGAAATCCTTTGATAACACGGATT
CCTATTTCTCAATGATATCCCACGATCGAGACAATTGGCTGAATCCCGTGAAACCATTTC
ATAGAAGTTCATTGATCTCTTCTTTTTATAAAGCAAATCGACTTCGATTCTTGAATAATC
CACATCACTTCTGGTTCTAT------TGTAACAAAAGATTCCCTTTTTATGTG------------GAAA
AGGCCCGTATCAAGAATTCTGAT------CTTACGTAT---GGACAATTCCTCAATATCTTGTTC
ATTCGCAAC------------AAAATGTGCGTCGGTAAA------AAAAAACATGCTTTTTTGGAG---
AGAGATACTATTTCACCCATCGAGTCACAGGTATCTAACATATCCATACCTAACGATTTT
CCACAAAGTGGTGACGAAACG---TATAACTTGTACAAATCT---------TTCCATTTTCCAAT
TCGATCCGATCCATTCGTTCGT------AGA---------GCTATCGCAGACATTTCTGGAACACCT
CTAACAGAGGGACAAATAGTCAATTTTGAAAGAACTTATTGTCAACCTCTTTCAGATAT
GAATCTATCTGATTCAGAAGGGAAGAACTTGCATCAGTATCTCAAT------TTCAATTCAA

ACATGGGTTTGATTCACACTCCATGTTCTGAGAAATATTTACCA------TCCGAAAAGAGG
AAAAAA----------------------CGGAGTCTTTGTCTAAAG------AAATGCGTTGAGAAACGGC
AGATGTACAGAACCTTTCAACGAGATAGTGCTTTTTCAAATCTCTCAAAATGGAATCTG
TTCCAAACATATATGCCATGGTTCCTTACTTCGACAGGGTGCAAATATCTAAATTTCACC
CTTTTAGATACTTTTTCAGGCCTATTG------CCGATACTAAATAGCAGT---CAAAAATTTGT
ATCCATTTTTCATGATATTATGCATGGATCAGATATATCATGGCCAATTCCTCAGAAAAAA
CGG------------------GGTCTTCCACAATGGAATCTG---ATAAGTGAGATTTCGAATAAGTGT
TTACATAAT------CTTCTTCTGTCCGAAGAAATGATTCATCGAAATAATGAGTCACCCGTT
CCATTGATATGGACACATCTGAGATCACCAAATGCTCGGAAGTTC---CTCTATTCAATCC
TTTTCCTTCTTCTTGTTGCTGGATAT---CTCGTTCGTCCACATCTTCTCTTTGTTTCCCGA-
-----GCCTCTAGTGAGTTACAGACAGAGTTCGAAAAGATCAAATCTTTGATGATTCCATC
ATACATGATTGAGTTGCGAAAACTTCTGGATAGGTATCCTACATCTGAACTGAATTCTTT
CTGGTTAAAGAATCTCTTTCTAGTTGCTCTGGAACAATTAGGAGATTCTCTAGAAGAAA
TACGGGGTTCTGCTTCTGGCGGCAACATGCTATTGGGTGGTGGTCCC---------------GCTTA
TGGG---GTCAAATCAATACGTTCTAAGAAGAAATATTTGAATATCAATCTCATCGATCTC
ATCGATCTCATA---AGTATCATACCAAATCCCATCAATCGAATCACTTTTTCGAGAAATAC
GAGACATCTAAGTCGTACAAGTAAAGAGATCTATTCATTGATAAGAAAAAGAAAAAAC
GTGAACGGTGATTGGATTGATGATAAAATAGAATCCTGGGTCGCGAACAGTGATTCGAT
TGATGATGAAGAAAGAGAATTCTTGGTTCAGTTCTCC------ACCTTAACGACA------GAA
AAAAGGATTGATCAAATTCTATTGAGTCTGACT---CAGAGTGATCATTTATCA---------------
AAGAATGACTCTGGTTATCAAATGATTGAACAGCCGGGATCAATTTACTTACGATACTT
AGTTGACATTCAT------AAAAAGTATCTAATGAATTATGAGTTCAATAGATCCTGTTTAGC
AGAAAGACGGATATTCCTTGCTCATTATCAGACAATCACTTAT------TCACAAACCTCGT
GTGGGACTAATAGTTTTCATTTCCCATCTCATGGA---------AAACCCTTTTCGCTCCGCTT
AGCCCTATCCCCCTCT---AGGGGTATTTTAGTGATAGGTTCTATAGGAACTGGACGATCA
TATTTGGTCAAATACCTAGCGACAAACTCCTATGTTCCTTTCATTACGGTATTTCCGAAC
AAGTTCCTGGATGACAAACCTAAAGGTTATCCTATTGATGAT---------ATTGATGATAGTG
ATATTGATGATCGTGACGATATTGAT--
---GATAGTGACGATATCGATGATGACCTTGATACGGAGCTGCTAACTATGACGAATGCGC
TA---------ACTATGTATATGACG---------CCGAAAATAGACCGATTTGATATCACCCTTCAAT
TCGAATTAGCAAAAGCAATGTCTCCTTGCATAATATGGATTCCAAACATTCATGATCTGT
ATGTGAATGAGTCGAATTACTTATCCCTCGGTCTATTAGTGAACTATCTCTCC---------GG
GGGTTGTGAAAGATGTTCC---ACTAGAAATATTCTTGTTATTGCTTCGACTCATATTCCCC
AAAAAGTGGATCCCGCTCTAATAGCTCCGAATAAATTAAATACATGCATTAAGATACGA
AGGCTTCTTATTCCACAACAACGAAAGCACTTTTTCACTCTTTCATATACTAGGGGCTTT
CGCTTGGAAAAGAAAATGTTCCATACT------AATGGATTCGGGTCCATAACCATGGGTTC
CAATGCACGAGATCTTGTAGCACTTACCAATGAGGCCCTCTCAATTAGTATTACACAGA
AGAAATCAATTATAGACACTAATACAATTAGATCCGCTCTTCATAGACAAACTTGGGATT
TGCGA---------TCCCAGGTAAGATCGGTTCAGGATCATGGGATCCTTTTCTATCAGATAGG
AAGGGCTGTTGCACAAAATGTACTTCTAAGTAATTGCCCCATAGATCCTATATCTATCTAT
ATGAAGAAGAAA------TCATGTAAGGAAGGGGATTCTTATTTGTACAAATGGTACTTCGA
ACTTGGAACGAGCATGAAGAAATTAACGAGACTTCTTTATCTTTTGAGTTGTTCTGCCG
GATCGGTCGCTCAAGATCTTTGGTCTCTACCC------GGACCCGATGAAAAA---AATTGGA
TCACTTCTTATGGATTCGTTGAGAATGATTCT---GATCTAGTTCATGGCCTATTAGAAGTA

GAAGGCGCTCTGGTGGGA------TCCTCACGGACAGAAAAAGATTGCAGTCAGTTTGATA
ATGAT------CGAGTGACATTGCTTCTTCGGTCCGAACCAAGGAAT---CCCTTAGATATGAT
CCAAAATGGATCTTGTTCTATCGTTGATCAGAGATTTCTATATGAAAAA------TACAAATC
GGAGTTTGAAGAA---GGGGAA------GGAGCCCTCGACCCGCAA------------CAGATAGAG
GAGGAT------------TTATTCAATCAC------ATAGTTTGGGCTCCT------AGAATATGGCGCCCT
---------TGTGGCAATCTATTTGATTGTATCGAAAGGCCCAATGAATTGGGATTTCCC---TAT
TGGGCCAGGTCATTTCGG---------GGCAAGCGGATCATTTATCATAAAGAGGATGAGCTT
CAAGAGAATGATTCGAAGTTCTTGCAGAGTGGAACCATGCAGTACCAGACACGAGATA
GATCTTCCAAAGAACAAGGCTTTTTTCGAATAAGCCAATTCATTTGGGACCCTGCAGAT
CCATTCTTTTTCCTATTCAAA---GATCAGCCCTTTGTCTCTGTGTTTTCACGTCGAGAATT
CTTTGCAGATGAAGAGATGTCAAAGGGGCTTCTTACTTCCCAAACAAATCCTCCTACAT
CTATATATAAACGCTGGTTCATC------AAGAATACGCAAGAAAAGCACTTCGAATTGTTG
ATTCATCGCCAGAGATGGCTTAGAACC---------AATAGCTCATTATCT------AATGGATCTTT
CCGTTCTAAT------ACTCCATCCGAGAGTTATCAGTATTTATCAAATCTGTTCCTATCTAAT-
--GGAACACTATTGGATCAAATGATAAAGACATTGTTGAGAAAGAGATGGCTTTTCCCG
GATGAAATGAAACATTTGATT------CATGTAACAGGAGAAAGATTTCCCATTCCTATGCCT
AGATCGCGG---ATAAATGGAAATTTTATTGATAAGACCTCTTCAATTGTAGCCAATATCTT
ATTACGAATAATTCCGACAAGTTCAGGAGAAAAAGAGGCATTTACCTATTACAGAGATG
GG------ATGTCAGCTCAATCCGAAGGAAATTATGCGGAAGCTTTACAGAATTATTATGAA
GCTACGCGACTAGAAATTGATCCCTATGATCGAAGTTATATACTCTATAATATAGGCCTTA
TTCACACAAGCAATGGAGAACATACGAAAGCTTTGGAATATTATTTCCGGGCACTAGA
ACGAAATCCATTCTTACCGCAAGCTTTTAATAATATGGCCGTGATCTGTCATTAC---CGG
GGAGAACAGGCCATTCGACAGGGAGATTCTGAAATTGCGGAGGCTTGGTCCGATCAA
GCTGCTGAGTATTGGAAACAAGCTATAGCGCTTACCCCAGGTAATTATATTGAAGCACA
TAATTGGTTGAAGATCACGAGGCGGTTCGAA------ATGAACTGGCGATCAGAACGTATAT
GGATAGAACTTATAACGGGGTCTCGCAAAATAAGTAATTTCTGCTGGGCCTTTATCCTTT
TTTTAGGTTCACTAGGATTCTTATTGGTTGGAATTTCCAGTTATCTTGGTAGGAATTTGAT
ATCCTTATTTCCATCTCAGCAAATAATTTTTTTTCCACAAGGGCTCGTGATGTCTTTCTAT
GGGATCGCAGGTCTGTTCATTAGCTCCTATTTGTGGTGCACAATTTCGTGGAATGTGGG
TAGTGGTTATGATCGATTCGATAGAAAAGGAGGAATAGTGTGTATTTTTCGTTGGGGATT
TCCTGGAATAAATCGTCGCATCTTCCTTCGATTCCTTATGAGAGATATCCAGTCGATCAG
AATAGAAGTTAAAGAAGGTCTTTATCCTCGTCGTGTCCTTTATATGGAAATCAGAGGAC
AGGGAGCCATTCCCCTGACTCGTACTGATGAGAAT---TTGACTCCACGAGAAATTGAAC
AAAAAGCTGCTGAATTGGCCTATTTCTTGCGCGTACCCATTGAAGTATTT
>Arabidopsis
ATGGAAAAATCGTGGTTCAATTTTATGTTTTCTCTAAGTAAAGCAATGGATAGT---TTTC
CTGGTGAAAAGACTACTATAAGTCAAGACCGTTTTATATATGATATGGATAAAAACTTTT
ATGGTTCCAATAATGTTGATCTTTTAGTTAGCTCCAAGGACATTCGCAATTTCATATCGG
ATGACACCTTTTTTGTTAGGGATAGTAATAAGAATAGTTATTCTATATTTTTTGATAAAAA
AAAAAAAATTTTTGAGATTGACAATGAT---------------TTTAGTGACCTAGAAAAATTTTTT
TATAGTTATTGTAGTTCTAGTTATCTAAATAATAGATCTAAAGGTGACAAC---CACTATGA
TCCTTACATTAAGGATACTAAATATAATTGTACTAATCACATTAATAGTTGCATTGATTCTT
ATTTTCGT------TCTTACATCTGTATTGATAATAACTTTTTAATCGATAGTAATAATTTTAAT
GAAAGTTACATTTATAATTTCATTTGTAGTAGTAAAAATTACAAGATAAGAACTAATAGG

AATCGTAGTAATTTAATAAGT---------TCTAAG---TTCGATATAACTCAAAACTACAATCAA
TTGTGGATTCAATGCGACAATTGTTATGGATTAATGTATAAG---------AAAGTCAAAATGA
ATGTTTGTGAACAATGTGGACATTATTTGAAAATGAGTAGTTCAGAAAGAATCGAGCTT
TCGATTGATCCGGGTACTTGGAATCCTATGGATGAAGACATGGTCTCTGCGGATCCCATT
AAATTTCATTCGAAGGAGGAACCTTATAAAAACCGTATTGACTCTGCGCAAAAAACTA
CAGGATTGACTGACGCTGTTCAAACAGGTACAGGTCAACTAAACGGTATTCCGGTAGC
CCTTGGGGTTATGGATTTTCGGTTTATGGGGGGTAGTATGGGATCCGTAGTAGGCGAAA
AAATAACTCGTTTGATCGAGTATGCTACCAATCAATGTTTACCTCTTATTTTAGTGTGTTC
TTCCGGAGGAGCACGAATGCAAGAAGGAAGTTTAAGTTTGATGCAAATGGCTAAAATT
TCTTCGGTTTTATGTGATTATCAATCAAGTAAAAAGTTATTCTATATATCAATTCTTACATC
TCCTACTACCGGTGGAGTGACAGCTAGTTTTGGTATGTTGGGGGATATCATTATTGCCGA
ACCCTATGCCTATATTGCATTTGCGGGTAAAAGAGTAATTGAACAAACATTGAAAAAAG
CCGTGCCTGAAGGTTCACAAGCGGCTGAATCTTTATTACGTAAGGGCTTATTGGATGCA
ATTGTACCACGTAATCTTTTAAAAGGTGTTCTGAGCGAGTTATTTCAGCTCCATGCTTTT
TTTCCTTTGAACACAAAT---------ATGGTAACCATTAGAGCCGACGAAATTAGTAATATTA
TCCGTGAACGTATTGAGCAATATAATAGAGAAGTAACGATTGTAAATACCGGTACCGTA
CTTCAAGTGGGCGATGGCATCGCTCGTATTTATGGTCTTGATGAAGTAATGGCAGGTGA
ATTAGTAGAATTTGAGGAGGGTACTATAGGTATTGCCCTTAATTTAGAATCCAATAATGT
TGGTGTTGTATTAATGGGTGACGGTTTGATGATCCAAGAAGGAAGTTCAGTCAAAGCT
ACGGGAAAAATTGCTCAGATACCGGTGAGTGAGGCTTATTTGGGGCGTGTTATAAACG
CCTTGGCTAACCCTATTGATGGTCGAGGTAAGATTTCCGCTTCTGAATCTCGGTTAATTG
AATCTCCTGCCCCAGGTATTATTTCGAGACGTTCTGTATATGAGCCTCTTCAAACAGGA
CTTATTGCTATTGATTCCATGATCCCTATAGGACGCGGCCAGCGAGAATTAATTATTGGT
GACAGACAGACCGGTAAAACAGCAGTAGCCACAGATACAATTCTCAATCAACAAGGC
CAAAATGTAATATGTGTTTATGTAGCTATTGGTCAAAAAGCTTCTTCCGTGGCTCAGGTA
GTGACCAGTTTACAGGAACGAGGGGCAATGGAATACACTATTGTGGTAGCTGAAACGG
CCGATTCCCCAGCTACGTTACAATACCTCGCGCCTTATACAGGAGCAGCCTTGGCTGAA
TATTTTATGTACCGTGAACAACACACTTTAATAATTTATGATGATCTTTCCAAACAAGCA
CAAGCTTATCGACAAATGTCTCTTCTATTACGAAGACCGCCGGGTCGTGAAGCTTATCC
AGGAGATGTTTTTTATTTACATTCACGTCTTTTAGAAAGAGCCGCTAAATTAAGCTCTCA
ATTAGGTGAAGGGAGTATGACTGCCTTACCAATCGTCGAGACCCAGTCAGGAGATGTT
TCAGCTTATATTCCTACTAATGTAATTTCCATTACAGATGGACAAATATTCTTATCCGCCG
ATCTTTTTAATGCTGGAATCAGACCTGCTATTAATGTAGGGATTTCTGTCTCGAGAGTAG
GATCCGCCGCTCAAATTAAAGCTATGAAACAGGTAGCTGGAAAATTAAAATTGGAATT
GGCTCAATTCGCTGAATTAGAAGCCTTTTCCCAATTTTCTTCTGATCTCGATAAAGCTAC
TCAGAATCAATTGGCAAGAGGTCAACGATTGCGTGAGTTACTGAAACAATCCCAATCA
GCCCCCCTCACAGTGGAAGAACAGATAATGACCATTTATACCGGAACAAATGGTTATCT
GGATGGATTAGAAATTGGACAAGTAAGAAAATTTCTCGTTCAATTACGCACTTACTTAA
AAACGAATAAACCTCAGTTTCAAGAAATCATAGCCTCTACCAAGACATTAACCGCTGA
AGCAGAAAGCTTTTTGAAAGAAGGTATTCAAGAGCAACTAGAACGTTTCCTACTTCAG
GAGAAAGTAATGAGAACAAATCCTACTACTTCAAATCCAGAGGTTTCGATACGTGAAA
AAAAAAACCTGGGACGTATCGCCCAAATCATTGGTCCGGTACTGGATGTAGCCTTCCCC
CCGGGCAAAATGCCTAATATTTACAATGCTCTGGTGGTTAAGGGTCGAGATACTCTTGG
TCAAGAAATTAATGTGACTTGTGAAGTACAGCAATTATTAGGAAATAATCGAGTTAGAG

CTGTAGCTATGAGTGCGACAGAGGGTTTAAAGAGAGGAATGGACGTGGTTGATATGGG
AAATCCTCTAAGTGTTCCAGTCGGCGGAGCGACTCTAGGACGAATTTTCAACGTGCTT
GGGGAACCCGTTGATAATTTAGGTCCTGTCGATACTCGCACAACATCTCCTATCCATAAA
TCCGCGCCTGCTTTTATAGAATTAGATACAAAATTATCGATTTTTGAAACAGGAATTAAA
GTAGTAGATCTTTTGGCCCCTTATCGTCGTGGGGGAAAAATTGGACTATTCGGTGGGGC
TGGCGTGGGTAAAACAGTACTAATTATGGAATTGATCAACAACATTGCTAAAGCTCATG
GTGGTGTATCCGTATTTGGTGGAGTAGGCGAACGGACTCGTGAAGGAAATGATCTTTAC
ATGGAAATGAAAGAATCTGGAGTCATTAATGAACAAAATCTTGCGGAATCCAAAGTGG
CCCTAGTTTATGGTCAGATGAATGAACCGCCAGGAGCTCGTATGAGAGTTGGTCTGACT
GCCTTAACTATGGCAGAATATTTCCGAGATGTTAATGAGCAAGACGTACTTCTATTTATC
GACAATATCTTCCGTTTCGTACAAGCAGGATCCGAGGTATCCGCCTTATTGGGTAGAAT
GCCTTCTGCTGTGGGTTATCAACCCACCCTTAGTACCGAAATGGGTACTTTACAAGAAA
GAATTACTTCTACGAAAAAGGGGTCCATAACCTCTATTCAAGCAGTTTATGTACCTGCA
GATGATTTGACTGACCCCGCACCTGCCACCACATTTGCACATTTAGATGCGACTACCGT
ACTATCAAGAGGATTAGCTGCCAAAGGTATCTATCCAGCGGTAGATCCTTTAGATTCAA
CGTCAACTATGCTACAACCTCGAATCGTTGGCGAGGAACATTATGAAACTGCGCAACA
AGTAAAACAAACTTTACAACGTTACAAGGAGCTTCAGGACATTATAGCTATCCTGGGGT
TGGATGAATTATCCGAAGAGGATCGCTTAACCGTAGCAAGAGCACGAAAAATTGAGCG
TTTCTTATCACAACCCTTTTTCGTAGCAGAAGTATTTACAGGTTCTCCGGGAAAATATGT
TGGTCTAGCGGAAACAATTAGAGGGTTTAATTTGATCCTTTCCGGAGAATTTGATTCTC
TTCCCGAACAGGCCTTTTACTTAGTGGGTAACATCGATGAAGCTACTGCGAAGGCTACG
AACTTAGAAATGGAGAGTAAATTGAAGAAAATGACCTTAAATCTTTGTGTACTGACTCC
GAATCGAATTGTTTGGGATTCAGAAGTAAAAGAAATCATTTTATCTACTAATAGTGGAC
AAATTGGTGTATTAGCAAACCACGCGCCGATTGCCACAGCTGTTGATATAGGTATTTTG
AAAATACGCCTTGCTAACCAATGGTTAACGATGGCTCTGATGGGCGGTTTTGCTAGAAT
AGGCAATAATGAAATCACTATTTTAGTAAATGATGCAGAGAAGAATAGTGACATTGATC
CACAAGAAGCTCAGCAAACTCTTGAAATAGCAGAAGCTAACTTGAGAAAAGCTGAAG
GTAAGAGACAGACAATTGAGGCTAATCTAGCTCTCAGACGAGCTCGGACACGAGTCG
AGGCTCTCAATACGATT---ATGAAAAATTTAACCGATTCTTTCGTTTACTTGGGTCACTG
GCCATCCGCCGGGAGTTTCGGATTTAATACCGATATTTTAGCAACAAATCCAATAAATCT
AAGTGTAGTTTTCGGTGTATTGATCTTTTTTGGAAAGGGAGTGTTAAATGATTTATTAGA
TAACCGAAAGCAGAGGATATTAAATACTATTCGAAATTCAGAAGAACTGCGTGAAGGA
GCTATTCAACAATTAGAAAATGCCCGGGCGCGCTTGCGTAACGTAGAAACGGAAGCGG
ATAAGTTTCGCGTGAATGGATACTCTGAAATCGAACGAGAAAAATTGAATTTGATTAAT
TCAACTTATAAAACTTTGAAACAATTAGAAAATTACAAAAATGAAACCATTCTTTTTGA
GCAACAAAGAACAATTAATCAAGTCCGCGAACGGGTTTTCCAACAAGCTTTACAAGG
AGCTATAGGAACCCTAAATAGTTGTTTGAGTAATGAGTTACATTTACGTACTATTAATGC
AAATATTGGGATGTTTGGTACGATGAAAGAAATAACTGATATGAATCCACTGGTTTCTG
CTGCTTCGGTTATTGCTGCTGGGTTGGCTGTTGGGCTTGCTTCTATTGGACCTGGAGTC
GGTCAAGGTACAGCTGCGGGTCAAGCTGTCGAAGGTATCGCGAGACAACCTGAGGCA
GAAGGAAAAATACGAGGTACTTTATTGCTTAGTTTGGCTTTTATGGAAGCTTTAACAAT
TTATGGCCTGGTTGTAGCATTAGCGCTTTTATTTGCGAATCCTTTTGTTATGAATGTTTTA
TCATGTTCCATCAACACACTAATAAAATTATATGAGATATCTGGTGTAGAAGTAGGCCAA
CATTTCTATTGGCAAATAGGGGGTTTCCAGGTCCATGCCCAAGTTCTTATTACTTCTTGG

GTTGTAATTGCTATCTTATTAGGTTCCGCAGTTCTAGCGATTCGCAATCCACAAACAATT
CCAACTGACGGCCAAAATTTCTTTGAATTTGTCCTTGAATTCATTCGAGACGTGAGTAA
AACCCAGATTGGAGAAGAATATGGTCCATGGGTTCCCTTTATTGGAACCCTGTTTTTATT
TATTTTTGTTTCTAACTGGTCAGGAGCCCTTTTACCGTGGAAAATTATCCAGTTACCTCA
AGGGGAGTTAGCAGCACCAACGAATGATATAAATACGACGGTTGCTTTAGCTTTACTCA
CATCAGTAGCCTATTTTTATGCGGGGCTTAGCAAAAAAGGATTAGGGTATTTCAGTAAAT
ACATTCAACCAACCCCAATTCTTTTACCCATTAACATCTTAGAAGATTTTACAAAACCCC
TATCACTTAGTTTTCGACTTTTCGGAAATATATTAGCCGATGAATTAGTCGTTGTTGTTCT
TGTTTCTTTAGTACCTTTAGTGGTTCCTATACCTGTTATGTTCCTTGGATTATTTACAAGC
GGGATTCAAGCTCTCATTTTTGCCACTTTAGCTGCGGCTTATATAGGTGAGTCTATGGAA
GGTCATCATATGATTTTTTCAATTTTAGAGCATATATTAACTCATATATCTTTTTCGGTCGT
TTCAATTGTGCTGACAATTTATTTTTTAACTTTATTAGTTAATGAAATCATAGGATTTTTT
GATTCATCAGATAAAGGAATCATAATTACGTTTTTTGGTATAACAGGATTATTATTAACTC
GTTGGATTTATTCAGGACATTTTCCATTAAGTAATTTATATGAATCATTAATTTTTCTTTCG
TGGGCTTTTTCAATTATTCATATGGTTTCCTATTTTAATAAACAACAAAATAAGTTAAAC
ACAATAACTGCGCCAAGTGTTATTTTTATTCAGGGTTTTGCTACTTCAGGTCTTTTAAAC
AAAATGCCTCAGTCTGCAATATTAGTACCAGCTCTCCAGTCCCAGTGGTTAATGATGCA
CGTAAGTATGATGATATTAGGCTATGGCGCTCTGTTATGCGGATCATTATTATCAATAGCT
CTTCTAGTGATTACATTTCGCAAAGTCGGACCTACTTTTTGGAAAAAGAATGTACTTTA
CTACATAAATGAAAGAAATTCTATTTTACTAAACATTAATTTTAGTTTTTCTAGAAATTAT
TATAGGTATCAACTGATTCAACAATTAGATTTTTGGAGTTTTCGTATTATTAGTCTTGGAT
TTATCTTTTTAACCGTCGGCATTCTTTCAGGAGCTGTCTGGGCTAATGAGACATGGGGT
TCATATTGGAACTGGGACCCAAAAGAAACTTGGGCATTTATTACTTGGACTATATTCGC
AATTTATTTACATATTAAAACAAATAGGAATGTTAGGGGTATAAATTCTGCAATTGTGGC
TTTGATCGGTTTTATTTTAATTTGGATATGCTATTTTGGCGTCAATCTTTTAGGAATAGGT
TTACATAGTTATGGTTCATTTACATCGAATATGGCAAAAAAGAAAGCATTCATTCCTTTT
TTTTATTTTCTATCTATAGTCTTTTTGCCCTGGTTGATCTCTCTCTGCTGTAATAAAAGTT
TGAAAACTTGGATTACTAATTGGTGGAATACTAGACAATGCGAAACTTTTTTGAATGAT
ATTCAAGAAAAAAGTTTTCTAGAAAAATTCATACAATTAGAGGAACTATTCCAGCTCGA
TGAAATGATAAAGGAATACCCAGAAACCAATTTACAACAATTTCGTCTAGGA---CACAA
AGAAACGATCCAATTCATCAAGATACACAATGAGTATAATATCCATACAATCTTGCACTT
CTCGACAAATCTAATATCTTTCGTTATTCTAAGTGGTTATTCCTTTTGGGGTAAGGAAAA
GCTTTTTATTCTGAATTCTTGGGTTCAAGAATTCCTATATAATTTAAGTGATACAATTAAA
GCTTTTTCGATTCTTTTATTAACTGATTTATGTATCGGATTCCATTCGCCTCACGGTTGGG
AACTAATGATTGGTTATATTTACAAAGATTTTGGGTTTGCTCATTATGAGCAAATTTTATC
TGGTCTAGTTTCTACCTTTCCAGTAATTCTTGATACAATTTTTAAATATTGGATTTTTCGT
TATTTAAATCGTGTATCTCCGTCACTTGTAGTGATTTATCATGCAATAAACGACATGCCTA
TTGGCGTTCCAAAAGTACCTTTTCGAAGTCCTGGAGAAGGAGATACATCTTGGGTTGA
CATATACAACCGACTTTATCGAGAAAGATTATTTTTTTTAGGCCAAGAGGTTGATACCGA
AATCTCGAATCAACTTATTAGTCTTATGATATATCTCAGTATAGAAAAGGATACCAAAGA
TCTTTATTTGTTTATAAACTCTCCTGGTGGATGGGTAATATCTGGAATGGCTATTTATGAT
ACTATGCAATTTGTGCGACCCGATGTACAGACAATATGCATGGGATTGGCCGCTTCAAT
AGCATCCTTTATCCTAGTCGGAGGAGCAATTACCAAACGTATAGCATTCCCTCACGCT---
GTAATGATCCATCAACCCGCTAGTTCGTTTTATGAGGCACAAACGGGAGAATTTATCTT

GGAAGCGGAAGAATTACTTAAACTTCGCGAAACCATCACAAGGGTTTATGTACAAAGA
ACGGGCAAACCTATATGGGTTATATCCGAAGACATGGAACGGGATGTTTTTATGTCAGC
A A C A G A A G C C C A A G C T C A T G G A A T T G T T G A T C T T G T A G C G G T T C A
A??
??
???ATGGATAAATTTCAAGGATATTT
AGAGTTCGATGGGGCTCGGCAACAGAGTTTTCTATATCCACTTTTTTTTCGGGAGTATAT
TTATGTACTTGCTTATGATCATGGTTTAGCGGATTATGACAAAAAATATAGTTCACTAATT
ACGAAACGCTTAATTTTGCGAATGTATGAACAGAATCGTTTGATTATTCCCACTAAGGA
TGTGAACCAAAATTCCTTTTTGGGGAGTCTTTTCTATTATCAAATGATATCTGTTTTATTT
GCAGTGATTGTCGAAATTCCATTTTCCCTAAGATTAGGATCCTCTTTTTTAAAAAAATCT
TATAATTTACAATCAATTCATTCAATATTTCCCTTTTTAGAAGACAAATTAGGACATTTTA
ATTATGTGTTAGATGTACTAATACCTTACCCCATCCATCTAGAAATCTTGGTTCAAACCCT
ACGTTACCGGGTAAAAGATGCCTCTTCTTTGCATTTTTTTCGGTTCTGTTTATACGAGTA
TTGT------AATTGGAAGAATTTTAAA---------------TCAATTCCAAGATTTTTCTTGTTCTTAT
ATAATTCTCATGTATGTGAATACGAATCCATCTTTTTTTTTCTACGCAAGCGGTCTTCGCA
TTTACGATCGACATCTTATGAAGTCCTTTTTGAGCGAATTGTATTCTATGGAAAAATACA
CCATTTTTTCAAAGTTTTTGTTAATCCGGCAATCCTAGGGTTGCTCAAGGATCCTTTCAT
ACATTATGTTAGATATCACGGAAGATGCATTCTGGCAACAAAGGATACGCCGCTTCTGA
TGAATAAATGGAAATATTATTTTGTTAATTTATGGCAATGTTATTTTTCGGTATGGTTTCA
ATCGCAAAAGGTCAATATAAATCAATTATCTAAAGATAATTTAGAGTTTCTGGGTTATCT
GTCAAGTTTGCGATTAAACCCTTTAGTGGTACGTAGTCAAATGCTAGAAAACTCATTTC
TAATAGATAAT------GTTAGAATCAAATTGGATAGCAAAATTCCAATTTCTTCTATTATTGG
ATCGTTGGCTAAAGATAAATTTTGTAATGTATTAGGGCATCCCATTAGTAAAGCAACCTG
GACGGATTCATCAGATTCTGATATTCTCAACCGATTTGTGCGGATATGCAGAAATATTTC
TCATTATTACAGCGGATCTTCAAAAAAAAAGAATTTGTATCGAATAAAATATATACTTCG
TCTTTGTTGTGTTAAAACTTTGGCTCGTAAACACAAAAGTACTGTACGCACTTTTTTAA
AAAGGTTGGGCTCTGGTTTATTGGAAGAATTCCTTACGGGGGAAGACCAAGTTCTTTC
TTTAATCTTCCCAAGACGAATTTGGTATTTGGATATTCTTTATCTTAATGATTTGGTCAAT
CATGAAATGATAATTTATGCAACA---GCAGTCCAAACTATAAAT---TCTTTTGTTAAATTG
GAATCT---TTAAAAGAGGTCTATGGACTCATATGGATATTTGTCCCTATATTTTCTCTTGTA
TTGGGAATCATAACAGGTGTACTAGTAATTGTGTGGTTAGAAAGAGAAATATCTGCAGG
GATACAACAACGTATTGGACCTGAATACGCCGGCCCGTTGGGAATTCTTCAAGCTCTAG
CCGATGGGACAAAACTACTTTTCAAAGAAAATCTTCGTCCATCTAGAGGAAATACTCCT
TTATTTAGTATTGGACCATCTATAGCAGTTATCTCTATTTTACTAAGTTATTCAGTAATTCC
CTTTAGCAATCACCTTGTTTTAGCGGATCTCAATATCGGTATTTTTTTATGGATTGCCATC
TCAAGTATTGCTCCTATTGGACTTCTTATGTCAGGATATGGATCAAATAATAAATATTCTT
TTTTAGGTGGTCTGCGAGCTGCTGCCCAATCGATTAGTTATGAAATACCATTAACTCTAT
GTGTTTTATCAATATCTCTACTATCTAACAGTTTAAGTACAGTTGATATAGTTGAGGCAC
AATCAAAATATGGTTTTTGGGGATGGAATTTGTGGCGTCAACCTATAGGTTTTATCATTT
TTCTAATTTCTTCCCTAGCAGAATGCGAGAGGTTACCGTTTGATTTACCAGAAGCGGAA
GAAGAATTAATAGCAGGTTATCAAACTGAATATTCAGGTATAAAATTTGGTTTATTTTAC
GTTGCTTCTTATCTAAATCTATTAATTTCCTCATTATTTGTAACAGTTCTATACTTAGGCG
GTTGGAATATTTCTATTCCGTATATATCTATTCTGGAGCTATTTCAAAGG---------GATCAA

ATTTTTGGAACAACAATTGGTATCTTTATTACATTAGCTAAAACTTATTTGTTTTTGTTCG
TTTCTATCGCAACAAGATGGACTTTACCTAGGCTAAGAATGGATCAACTATTAAATCTTG
GATGGAAATTTCTTTTACCTATTTCCCTTGGTAATCTATTATTAACCACTTCTTTCCAACT
CTTTTCACTCATGATCTGGCATGTACAGAATGAAAATTTCATTCTCGATTCTACGAGAAT
TTTTATGAAAGCCTTTCATTTGCTTCTCTTCGATGGAAGTTTTATTTTCCCAGAATGTATC
CTAATTTTTGGCCTAATTCTTCTTCTGATGATCGATTCAACCTCTGATCAAAAAGATATA
CCTTGGTTATATTTCATCTCTTCAACAAGTTTCGTAATGAGCATAACGGCCCTATTGTTC
CGATGGAGAGAAGAACCTATGATTAGCTTTTCAGGAAATTTCCAAACGAACAATTTCA
ACGAAATCTTTCAATTTCTTATTTTACTATGTTCAACTCTCTGTATTCCTCTATCCGTAGA
GTACATTGAATGTACAGAAATGGCTATAACAGAGTTTCTGTTATTCATATTAACAGCTAC
TCTAGGAGGAATGTTTTTATGTGGTGCTAACGATTTAATAACTATCTTTGTAGCTCCAGA
ATGTTTCAGTTTATGCTCCTACCTATTATCTGGATATACCAAGAAAGATATACGATCTAAT
GAGGCTACTATGAAATATTTACTCATGGGTGGGGCAAGCTCTTCTATTCTGGTTCATGGT
TTCTCTTGGCTATATGGTTCATCCGGGGGAGAGATTGAGCTTCAAGAAATAGTGAATGG
TCTTATCAATACACAAATGTATAACTCCCCAGGAATTTCAATTGCGCTTATATTCATCACT
GTAGGAATTGGGTTCAAGCTTTCCCTAGCCCCTTCTCATCAATGGACTCCTGACGTATA
CGAAGGATCTCCCACTCCAGTCGTTGCTTTTCTTTCTGTTACTTCGAAAGTAGCTGCTT
CAGCTTCAGCCACTCGAATTTTCGATATTCCTTTTTATTTCTCATCAAATGAATGGCATCT
TCTTCTGGAAATCCTAGCTATTCTTAGCATGATATTCGGGAATCTCATTGCTATTACTCAA
ACAAGCATGAAACGTATGCTTGCATATTCGTCCATAGGTCAAATCGGATATGTAATTATT
GGAATAATTGTTGGAGACTCAAATGGTGGATATGCGAGCATGATAACTTATATGCTGTTC
TATATCGCCATGAATCTAGGAACTTTTGCTTGCATTATATTATTTGGTCTACGTACCGGAA
CTGATAACATTCGAGATTATGCAGGATTATACACAAAAGATCCTTTTTTGGCTCTCTCTT
TAGCTCTATGTCTCTTATCCCTAGGAGGTCTTCCTCCACTAGCAGGTTTTTTTGGAAAAC
TCCATTTATTCTGGTGTGGATGGCAGGCAGGCCTATATTTCTTGGTTTCAATAGGACTCC
TTACGAGCGTTCTTTCTATCTACTATTATCTAAAAATAATCAAGTTATTAATGACTGGACG
AAACCAAGAAATAACCCCTCACATGCGAAATTATAGAATATCCCCTTTAAGATCAAACA
ATTCCATCGAATTGAGTATGATTGTATGTGTGATAGCATCTACTATACCAGGAATATCAAT
GAACCCGATTATTGCGATTGCTCAGGATACCCTTTTTATGTTTCTGCTTTACGAATATGAT
ATTTTCTGGGCATTTTTACTAATATCAAGTGCTATTCCTGTTTTGGCATTTCTAATTTCCG
GAGTTTTATCTCCAATTAGGAAGGGGCCGGAGAAACTTTCTAGTTATGAATCAGGTATA
GAACCGATCGGGGATGCTTGGTTACAATTTAGAATCCGTTATTATATGTTTGCTCTAGTT
TTTGTTGTTTTTGATGTTGAAACTGTTTTTCTGTATCCGTGGGCAATGAGTTTCGATGTA
CTGGGGGTATCTGCTTTTATAGAAGCTTTCATTTTCGTGCTTATCCTAATTCTTGGTTTAG
TTTATGCATGGCGAAAAGGAGCATTGGAATGGTCTACGAATGATTTTCCTTGGTTAACA
ATAATTGTTGTTTTTCCAATATCTGCTGGTTCATTAATGTTATTTCTCCCGCATAGGGGAA
ATAAAGTCAATAAATGGTATACTATATGCATTTGCATCTTAGAACTTCTTCTAACGACCTA
CGCTTTTTGTTATAATTTTAAAATGGACGATCCATTAATTCAACTGTCCGAAGATTATAAA
TGGATCGATTTTTTTGATTTTTACTGGAGAATGGGAATAGATGGACTTTCTATAGGAACT
ATTTTACTGACGGGATTTATTACTACTTTAGCCACTTTAGCGGCTTTTCCAGTTACTCGG
GATTCCCGATTTTTTCATTTCCTGATGTTAGCAATGTACAGCGGTCAAATAGGATCATTT
TCTTCTCGGGATCTTCTACTTTTTTTCATCATGTGGGAATTAGAATTAATTCCCGTTTATC
TACTTTTATCCATGTGGGGTGGAAAGAAACGTCTGTATTCGGCTACAAAATTTATTTTGT
ACACGGCAGGAAGTTCTATTTTTTTATTAATAGGAGTTTTAGGTATAAGTTTATATGGTTC

GAACGAACCAACATTAAATTTAGAACTCTTAGCTAATAAATCCTATCCTGTTACACTTGA
AATACTATTTTATATTGGATTTCTTATTGCTTTTGCCGTCAAATCACCGATTATACCTTTAC
ATACTTGGTTACCTGACACCCACGGTGAGGCACATTACAGTACCTGTATGCTTCTCGCT
GGAATCTTATTAAAAATGGGAGCCTATGGGTTGGTTCGAATCAATATGGAATTATTACCC
CACGCTCATTCTATGTTTTCTCCTTGGTTGTTGGTAGTCGGTACAATCCAAATAATTTATG
CAGCTTCAACATCTCCCGGTCAACGTAATTTAAAAAAGAGAATAGCCTATTCTTCTGTA
TCTCATATGGGTTTTATAATTATAGGTATTAGTTCTATAACGGATCCTGGACTTAATGGAG
CTATTTTACAAATAATTTCTCATGGATTTATTGGCGCTGCACTTTTTTTCTTGGCAGGAA
CGAGTTATGATAGAATTAGGCTTGTTTATCTTGATGAAATGGGTGGAATGGCTATCTCCA
TTCCAAAAATATTTACAATGTTCACTATTTTATCGATGGCTTCCCTTGCATTGCCGGGCAT
GAGTGGTTTTATTGCAGAATTCATTGTTTTTTTTGGAATAATTACCAGCCAAAAATATTT
CTTAATTTCCAAAATTTTCATTATTTTTGTAATGGCAATTGGAATGATATTAACTCCTATAT
ATTTATTATCTATGTTACGCCAAATGTTCTATGGATACAAGTTAATTAATATCAAAAACTT
TTCTTTTTTTGATTCTGGACCCCGAGAGTTATTTCTTTCAATCTCTATTCTTTTACCCATA
ATTGGTATTGGGATTTATCCTGATTTTGTGCTCTCATTAGCAAGTGACAAGGTCGAATCC
ATTTTATCTAATTATTTTTATGGAATGATACTCGAACATGTACTTGTTTTGAGTGCCTATTT
ATTTTTGATTGGTCTTTATGGATTGATCACAAGTCGAAATATGGTTAGGGCTCTTATGTG
TCTTGAACTTATACTCAATGCAGTTAATATGAATTTCGTAACATTTTCTGATTTTTTTGAT
AATTCCCAACTAAAAGGGGAAATTTTCTGCATTTTTGTTATAGCAATTGCAGCCGCTGA
AGCAGCTATTGGATTAGCTATAGTCTCGTCAATTTATCGTAACAGAAAATCAATTCGCAT
AAACCAATCGACCTTATTAAATAAGATGGAACATACATATCAATATTCATGGATCATCCC
TTTCATTCCACTTCCAGTACCTATTTTACTAGGAGTTGGACTTCTCCTTTTTCCGACAGC
AACAAAAAACCTTCGCCGCATGTGGACTTTTCTTAGTATTTTTTTGTTAAGTATAGTTAT
GATCTTTTCAATCTATCTATCTATTCAACAAATTTTTCTAAGTTGCATTCATCAAAATGTA
TGGTCTTGGACCATAAATAATGAATTTTCTTTTGAGTTCGGTTACTTTATCGATCCACTTA
CTTCTATTATGTCAATATTAATTACAACTGTTGGGATTTTGGTTCTGATTTATAGTGACAA
TTATATGTCGCATGATCAAGGATACCTGAGATTTTTTGCTTATATGGGTTTTTTTAATACT
TCAATGTTAGGATTAGTTACTAGTTCTAATTTGATCCAAGTTTATTTTTTTTGGGAATTAG
TTGGAATGTGTTCGTATTTATTAATAGGTTTTTGGTTCACACGACCTATTGCAGCGAATG
CTTGTCAAAAAGCTTTTGTAACTAATCGTGTAGGGGATTTTGGTTTATTATTAGGAATTT
TAGGTCTTTATTGGATAACTGGCAGTTTTGAATTTCAGGATTTGTTCGAAATATTCAATA
AT---AGAGTAAATCTCTTATTCCTTACTTTGTGTGCATTTCTATTATTTGTAGGTCCTATTG
CTAAATCCGCACAATTTCCTCTTCATGTATGGTTACCTGATGCCATGGAGGGCCCTACTC
CCATTTCGGCTCTTATACATGCTGCTACTATGGTAGCGGCGGGTATTTTTCTTGTAGCTC
GTCTTCTTCCTCTTTTTATCGTTATCCCTTCTATAATGTATATAATATCTTTGATAGGTATAA
TAACAGTACTCTTAGGAGCCACTTTAGCTCTTGCTCAAAAAGACATTAAGCGGGGTTTA
GCCTATTCTACAATGTCTCAACTCGGTTATATGATGTTAGCTCTAGGTATGGGGTCTTATC
GATCTGCTTTATTTCATTTGATTACTCATGCTTATTCGAAAGCTTTGTTGTTTTTAGGATC
TGGATCCATTATTCATTCAATGGAAGCTATAGTTGGATATTCTCCCGATAAAAGTCAGAA
TATGATTCTTATGGGTGGTTTGACAAAACATGTCCCGATTACAAAAACGGCCTTTTTAAT
AGGAACACTCTCTCTTTGTGGTATCCCCCCCTTGCTTGTTTTTGGTCGAAAGATGAAA
TTCTTAATGATAGTTTGTTATTTTCGCCAATTTTTGCAATAATAGCTTGTTCAACAGCGG
GATTAACCGCATTTTATATGTTTCGGATTTATTTACTTACTTTTGAAGGACATTTAAACAC
TTATTTTCTAAATTACAGTGGAAAAAAAAGTGGCTCCTTCTATTCACTCTCTTTATGGGG

TAAAGAAGAAGAAAAAAAACTTAATAAA---TTAGTACCATTATTAACAATGAATTTTTTT
TGCGGACTTAATACAAGAACTTTCTATTATCCGCATGAATCAGACAATACTATTCTATTTC
CTATGCTTATTTTGGTTTTATTTACTTTGTTTATTGGAGCCATAGGA------TTCGACTTTGA
TATATTATCAAAATTTTTCACGCCGTCAATAAACCTTTTGCATAAAAATTCACAA---TTTG
TAGATTGG---GAATTTTTGAGAAATGCAACTTTTTCGGTCAGTATAGCCTTTTTTGGAAT
ATTTATAGCATACTGTTTATATAAGCCTTTTTATTCATCTTTATTAAATTTAACCTTACTTAA
TTCATTTCAAATTCATTGGGAAAAACTAATAAATTTTGTATATAATTGGTCATATAATCGT
GGTTACATAGATTCTTTTTTTAAAACATCTTTAATTGAAAGTATAAGAAGATTAGCAAAA
CAAACGACTTTTTTTGATAAACGAATCATTGATGGAATTACAAATGGAGTAGGTATTAC
AAGTTTCTTTGTAGGAGAAGTAACAAAATATATAGGTGGAAGT---CGCATCTCTTCTTAT
CTGTTCTTATATTTGTCCTATGTATTGATTTTTTTAATGATCCTCTTTATGGATTTGCCTGG
ACCAATACATGATTTTCTTTTAGTTTTTCTGGGATCCGGTCTTCTAGTAGGGGGTCTGGG
AGTGGTATTACTTCCCAACCCAATATTTTCAGCCTTTTCCTTAGGATTTGTTCTTGTTTGT
ATATCTTTATTGTATATTCTATCAAATTCCCATTTTGTAGCTGCTGCACAACTCCTTATTTA
TGTGGGGGCCATAAATGTTTTAATCATATTTGCTGTGATGTTCATGAATGATTCAGAATAT
TCCACAGATTTCAATCTGTGGACCATTGGGAATGGGATTACTTCGTTGGTTTGTACAAC
TATTCTTTTTTTATTAATGTCTACTATTCTCGATACGTCATGGTACGGGGTTATTTGGACT
ACAAAATTAAACCAGATTCTAGAGCAAGATTTAATAAGTAATAGTCAACAAATAGGAAT
TCATTTATCAACAGATTTTTTTCTGCCATTTGAACTCATTTCAATAATTCTTTTAGTTGCT
TTGATAGGTGCAATTTCTGTGGCTCGTCAAATGAAGAGACCAGTTACAGGAAAAGATC
TTATGATAGTCAATATGGGACCTCACCACCCATCCATGCATGGTGTTCTTCGCTTAATTG
TTACTCTAGATGGTGAGGATGTTGTTGACTGTGAACCCATATTAGGTTATTTACACAGAG
GAATGGAAAAAATTGCAGAAAACCGAGCAATTATACAATATTTACCGTATGTAACGCGA
TGGGATTATTTAGCTACTATGTTTACAGAAGCAATAACAGTAAATGGACCAGAACAATT
GGGAAATATTCAAGTTCCTAAAAGGGCCAGCTATATCAGAGTAATTATGCTGGAATTGA
GTCGTATAGCTTCTCATCTGTTATGGCTCGGCCCTTTTATGGCAGATATTGGTGCACAGA
CTCCCTTTTTCTATATTTTCAGAGAACGAGAATTTGTATATGATCTATTCGAAGCTGCCA
CCGGTATGAGAATGATGCATAATTTTTTTCGTATTGGAGGAATAGCGGCTGATTTACCTT
ATGGTTGGATAGATAAATGCTTGGATTTTTGTGATTATTTTTTAACAGAGGTTGTTGAATA
TCAAAAACTCATTACACGAAATCCTATTTTTTTAGAACGGGTTGAAGGAGTTGGGATTA
TTGGTGGGGAAGAAGCAATAAATTGGGGTTTATCCGGACCAATGCTACGCGCATCCGG
AATACCATGGGATCTTCGTAAAATTGATCGTTATGAGTCTTACGATGAATTTGAATGGGA
AATTCAATGGCAAAAACAAGGAGATTCATTAGCTCGTTATTTAGTACGACTTAGCGAAA
TGACAGAATCCATAAAAATTATTCAACAGGCTCTGGAAGGACTTCCAGGGGGTCCCTAT
GAGAATTTAGAAAGCAGGGGCTTTGATAGAAAAAGGAATCCAGAATGGAATGATTTTG
AATATCGATTCATTAGTAAAAAACCTTCTCCTACTTTTGAATTATCAAAACAAGAACTTT
ATGTAAGAGTTGAAGCTCCAAAAGGGGAGTTGGGAATTTTTCTCATAGGAGATCAAAG
CGGTTTTCCTTGGAGATGGAAAATACGACCACCGGGTTTTATTAATTTGCAAATTCTTCC
TGAACTAGTTAAAAGAATGAAATTGGCTGATATTATGACGATACTCGGTAGCATAGATAT
AATTATGGGAGAAGTTGATCGTATGCTTCCTATGATAACCGGGTTCATGAATTATGGTCA
ACAAACCCTACGAGCTGCAAGGTATATTGGTCAAGGTTTCATGATTACCTTATCCCACA
CAAATCGTTTACCTGTAACTATTCAATATCCCTATGAAAAATTAATAACATCGGAACGTT
TCCGCGGTCGAATCCATTTTGAATTTGATAAATGCATTGCTTGTGAAGTATGTGTTCGAG
TATGTCCTATAGATTTGCCTGTTGTTGATTGGAAATTGGAAACTAATATTCGAAAAAAAC

GATTGCTTAATTACAGTATTGATTTTGGAATTTGTATATTTTGTGGTAATTGTGTTGAGTA
TTGTCCAACAAATTGTTTGTCAATGACTGAAGAATATGAATTTTCCACTTATGATCGTCA
CGAATTGAATTATAATCAAATAGCTTTGGGTCGTTTACCAATGTCAGTAATTGACGATTA
CACTATTCGAACAATTTGGAATTCA------GGGGTAAACCCTTTAATT-------------------------AT
GCAGGGCACTTTGTCCGTTTGGCTAGCCAAACGCGGGCTGGTTCATAGATCGTTGGGC
TTCGATTATCAAGGAATAGAGACTTTACAAATAAAGCCCGAAGATTGGCATTCTATTGC
TGTAATTTTATATGTATATGGTTACAATTATTTACGTTCGCAATGTGCCTATGATGTGGCA
CCAGGTGGCCTCTTAGCCAGCGTGTATCATCTTACGAGAATAGAATATGGTGTTAATCA
AGCGGAAGAAGTCTGCATAAAAGTATTTACTCACAGGAGTAATCCTAGAATTCCATCCG
TTTTCTGGGTTTGGAAAAGTACGGATTTTCAAGAACGGGAATCTTATGATATGTTAGGA
ATCACTTATGATAGCCATCCACGACTGAAACGGATCCTAATGCCCGAGAGTTGGATAGG
GTGGCCTTTACGTAAGGATTATATTGCCCCCAATTTTTATGAAATACAAGATGCTTAT------
----
--ATGAATTCCATTAAGTTTCCCATACTTGATCG
AACAACAAAAAATTCCGTTATTTCAACTACGTTAAATGATCTTTCAAATTGGTCAAGAC
TTTCCAGCCTATGGCCGCTTCTTTATGGTACCAGTTGTTGTTTCATTGAATTTGCCTCATT
AATAGGCTCCCGATTTGACTTTGATCGTTATGGGTTAGTACCAAGATCGAGTCCTAGAC
AGGCGGACCTTATTTTAACAGCAGGTACAGTAACAATGAAAATGGCTCCTTCTTTAGTG
CGATTATATGAACAAATGCCTGAACCGAAGTATGTTATTGCTATGGGAGCCTGTACAATT
ACAGGGGGGATGTTCAGTACCGATTCTTATAGTACTGTTCGAGGAGTTGATAAGCTAAT
TCCTGTAGATGTCTATTTGCCGGGTTGTCCACCTAAACCAGAGGCTGTTATAGACGCTAT
AACAAAGCTTCGTAAGAAAATAGCTAGAGAAATCTATAAGGATCGAATTAGACCTCAA
CAGGGTAATCGGTGTTTTACTACCAATCACAAGTTTTTTGTTGTACGCAGTCCGCATATT
GGAAATTATGATCAAGAACTACTCTATCCACCATCATCTACTTCAGAGATCTCT---GAAA
CATTTTTTAAATACAAAAGTCCAGTATCGTCCCACGAATTAGTGAATATGCAAACTAGA
AATACCTTTTCTTGGATAAGGGAAGAGATTACTCGCTCCATATCTGTCTCACTCATAATAT
ATATAATAACTTGGGCATCCATTTCAAGTGCATATCCGATTTTTGCCCAGCAGAATTATG
AAAATCCACGAGAGGCGACTGGGCGTATTGTATGTGCCAATTGCCATTTAGCTAATAAG
CCCGTGGATATTGAGGTTCCACAAACGGTACTTCCTGATACTGTATTTGAAGCAGTTGT
TAAAATTCCTTATGATATGCAACTAAAACAAGTTCTAGCTAATGGTAAAAAAGGGGCTT
TGAATGTGGGAGCTGTTCTTATTTTACCGGAGGGTTTTGAATTAGCCCCCCCGATCGTA
TTTCACCCGAGATGAAAGAAAAGATAGGAAATCTTTCTTTTCAGAATTATCGTCCCAAT
AAAAAAAATATTCTTGTGATAGGTCCTGTTCCTGGTCAAAAATATAGTGAAATAACCTT
TCCTATTCTTGCTCCAGACCCTGCTACTAATAAAGATGTTCACTTCTTAAAATATCCTATA
TATGTAGGTGGAAACAGGGGAAGGGGTCAGATTTATCCTGATGGTAGCAAAAGTAACA
ATACAGTTTATAATGCTACGGCAGGAGGGATAATAAGTAAAATTTTACGAAAAGAAAAG
GGGGGATACGAAATAACCATAGTGGATGCATCGAATGGACGCGAAGTTATTGATATTAT
CCCTCGAGGCCTAGAACTTCTTGTTTCAGAGGGCGAATCCATTAAACTCGATCAACCAT
TAACGAGTAATCCTAATGTGGGTGGATTTGGTCAGGGGGATGCGGAAATAGTACTTCAA
GATCCATTACGTGTCCAAGGTCTTTTGTTCTTCTTAGGATCGGTTGTTTTGGCACAAATC
TTTTTGGTTCTTAAAAAGAAACAGTTTGAGAAGGTTCAATTATCCGAAATGAATTTTAT
GAGTAAAGTTTATGATTGGTTCGAAGAACGTCTTGAGATTCAGGCGATTGCAGATGATA
TAACTAGTAAATATGTTCCTCCGCATGTCAACATATTTTATTGTCTAGGCGGAATTACCCT
TACTTGTTTTTTAGTACAAGTAGCTACGGGATTTGCTATGACTTTTTATTACCGTCCAAC

CGTTACTGAAGCTTTTGCTTCTGTTCAATATATAATGACTGAAGCTAACTTTGGTTGGTT
AATCCGATCAGTTCATCGATGGTCGGCAAGTATGATGGTCCTAATGATGATCCTGCACGT
ATTTCGTGTATACCTCACCGGTGGTTTTAAAAAACCTCGCGAATTAACTTGGGTTACTG
GTGTGGTTCTGGGTGTATTGACCGCATCTTTTGGTGTAACAGGTTATTCTTTACCTTGGG
ATCAAATTGGTTATTGGGCGGTCAAAATTGTAACAGGTGTACCTGACGCTATTCCGGTA
ATAGGATCACCTCTTGTAGAATTATTACGCGGAAGTGCTAGTGTTGGACAATCCACTTT
GACTCGTTTTTATAGTTTACACACTTTTGTATTACCTCTTCTTACGGCCGTCTTTATGTTA
ATGCATTTCCTAATGATACGTAAGCAAGGTATTTCTGGTCCCTTAATGGGAGTAACAAA
AAAACCAGATTTGAATGATCCTGTATTACGAGCTAAATTAGCTAAAGGTATGGGTCACA
ATTATTACGGGGAACCCGCATGGCCCAACGACCTTTTATATATTTTTCCAGTAGTTATTCT
TGGTACCATTGCCTGTAACGTAGGCTTAGCGGTTTTAGAACCATCAATGATTGGTGAAC
CTGCGGATCCTTTTGCAACTCCTTTGGAAATATTACCTGAATGGTATTTCTTTCCTGTATT
TCAAATACTTCGTACAGTGCCTAACAAATTATTGGGTGTTCTTTTAATGGTTTCAGTACC
AGCGGGATTATTAACCGTACCCTTTTTGGAAAATGTTAATAAGTTTCAAAATCCATTTCG
TCGTCCAGTAGCGACAACCGTCTTTTTGATTGGCACCGCGGCGGCCCTGTGGTTAGGTA
TTGGAGCAACATTACCGATTGATAAATCTCTAACTTTAGGTCTTTTTATGATTGAAGTTT
TTTTATTTGGAATCGTCTTAGGTCTAATTCCTATTACTTTGGCTGGATTATTCGTAACTGC
TTATTTACAATACAGACGTGGTGATCAGTTGGACTTTATGCCTACTATAACTAGTTATTTC
GGTTTTCTACTAGCAGCTTTAACTATAACCTCAGTTCTGTTTATTGGTCTAAGCAAAATA
CGACTTATTATGGATATAGTAAGTCTCGCATGGGCTGCTTTAATGGTAGTTTTTACATTTT
CCCTCTCTCTCGTAGTGTGGGGAAGAAGTGGACTCATGATTATTCGTTCGCCGGAACCA
GAAGTCAAAATTTTGGTAGATAGGGATCCCATAAAAACTTCTTTCGAGGAATGGGCTAA
ACCCGGTCATTTCTCAAGAACAATAGCTAAGGGACCTGATACTACCACTTGGATCTGGA
ACCTACATGCTGATGCTCACGATTTTGATAGTCATACCAGTGATTTGGAGGAAATCTCTC
GAAAAGTATTTAGTGCCCATTTCGGCCAACTCTCTATCATCTTTCTTTGGCTGAGTGGCA
TGTATTTCCATGGTGCTCGTTTTTCCAATTATGAAGCATGGCTGAGTGATCCTACTCACA
TTGGACCTAGTGCTCAGGTGGTTTGGCCAATAGTGGGCCAAGAAATCCTGAATGGAGA
TGTGGGCGGAGGCTTCCGAGGAATACAAATAACCTCAGGCTTTTTTCAGATTTGGCGA
GCATCCGGAATAACTAGTGAATTACAACTTTATTGTACCGCAATTGGCGCATTGGTCTTC
GCAGCCTTAATGCTTTTTGCTGGTTGGTTCCATTATCACAAAGCAGCTCCAAAATTGGC
TTGGTTCCAAGATGTAGAATCTATGTTGAATCACCATTTAGCAGGGCTACTAGGACTTG
GGTCCCTTTCTTGGGCAGGACATCAAGTACATGTATCTTTACCGATTAACCAATTTCTAA
ACGCTGGAGTAGATCCTAAAGAAATACCGCTTCCTCATGAATTTATCTTGAATCGGGAT
CTTTTGGCTCAACTTTATCCAAGTTTTGCTGAAGGAGCAACTCCCTTTTTTACCTTAAAT
TGGTCAAAATACTCGGAATTTCTTACTTTTCGTGGCGGATTAGATCCAGTGACTGGGGG
TCTATGGTTAACCGATATAGCACATCATCATTTAGCTATCGCAATTCTTTTTCTAATCGCG
GGTCATATGTATAGGACCAACTGGGGTATTGGTCATGGTATAAAAGATATTTTAGAGGCT
CATAAAGGTCCATTTACAGGCCAAGGCCATAAAGGCCTATATGAAATTCTAACAACATC
ATGGCATGCTCAATTATCTCTTAACCTAGCTATGTTAGGCTCTTTAACCATTATTGTAGCT
CACCATATGTATTCCATGCCCCCTTATCCATATCTAGCTACTGACTATGCTACACAACTGT
CATTGTTCACACATCACATGTGGATTGGTGGATTTCTCATAGTTGGTGCTGCTGCGCATG
CAGCCATTTTTATGGTAAGAGACTATGATCCAACTAATCGATATAACGATTTATTAGATCG
TGTCCTGAGGCATCGCGATGCAATCATATCACATCTCAACTGGGTATGTATATTTCTAGG
CTTCCACAGTTTTGGTTTGTATATTCATAATGATACCATGAGTGCTTTAGGGCGTCCACA

AGATATGTTTTCAGATACTGCTATACAATTACAACCAGTCTTTGCTCAATGGATACAAAA
TACCCATGCTTTAGCACCTGGTGTAACAGCCCCTGGTGAAACAGCGAGCACCAGTTTG
ACTTGGGGGGGCGGTGAGTTAGTAGCAGTGGGTGGCAAAGTAGCTTTGTTACCTATTC
CATTAGGAACGGCAGATTTTTTGGTACATCATATTCATGCATTTACAATTCATGTGACGG
TATTGATACTGTTGAAAGGTGTTCTATTTGCTCGTAGCTCGCGTTTAATACCAGATAAAG
CAAATCTTGGTTTTCGTTTCCCTTGTGATGGGCCGGGAAGAGGAGGAACATGTCAAGT
ATCTGCTTGGGATCATGTCTTCTTAGGACTATTCTGGATGTACAATGCTATTTCCGTAGTA
ATATTCCATTTCAGTTGGAAAATGCAGTCAGATGTTTGGGGTAGTATAAGTGATCAAGG
GGTGGTAACTCATATTACTGGAGGAAACTTTGCACAGAGTTCCATTACTATTAATGGGT
GGCTCCGCGATTTCTTATGGGCACAAGCATCTCAGGTAATTCAGTCTTATGGTTCTTCGT
TATCTGCATATGGTCTTTTTTTCCTAGGTGCTCATTTTGTATGGGCTTTCAGTTTAATGTT
TCTATTCAGCGGGCGTGGTTATTGGCAAGAACTTATTGAATCCATTGTTTGGGCTCATAA
TAAATTAAAAGTTGCTCCTGCTACTCAGCCTAGAGCCTTGAGCATTATACAAGGACGTG
CTGTAGGAGTAACCCATTACCTTCTGGGTGGAATTGCCACAACATGGGCGTTCTTCTTA
GCAAGAATTATTGCAGTAGGAATGGCATTAAGATTTCCAAGGTTTAGCCAAGGCTTAGC
TCAGGACCCCACTACTCGTCGTATTTGGTTTGGTATTGCTACCGCACATGACTTCGAGA
GTCATGATGATATTACTGAAGAACGTCTTTATCAGAATATTTTTGCTTCTCATTTCGGGC
AATTAGCAATAATTTTTCTGTGGACTTCCGGAAATTTGTTTCATGTAGCTTGGCAAGGA
AATTTTGAGACATGGGTACAAGACCCTTTACATGTAAGACCGATTGCTCATGCTATTTG
GGATCCTCATTTTGGTCAACCGGCTGTGGAAGCGTTTACTCGAGGAGGTGCTCTTGGC
CCGGTGAATATAGCTTATTCTGGTGTTTATCAGTGGTGGTATACAATCGGTTTACGTACT
AATGAAGATCTTTATACTGGAGCTCTTTTTCTATTATTTCTTTCTGCCCTATCCTTAATAG
GGGGTTGGTTACACCTACAACCAAAATGGAAACCAAGAGTTTCGTGGTTCAAAAATGC
TGAATCTCGTCTGAATCATCATTTGTCAGGACTATTCGGGGTAAGCTCATTGGCTTGGA
CAGGTCATTTAGTCCATGTCGCTATTCCTGCATCCAGGGGGGAATATGTTCGATGGAATA
ATTTCTTAAATGTATTACCGCATCCCCAAGGGTTAGGCCCACTTTTTACGGGTCAGTGG
AATCTTTATGCTCAAAACCCCGACTCGAGTAGTCATTTATTTGGTACCTCCCAAGGATCC
GGAACTGCCATTCTAACCCTTCTTGGGGGATTCCATCCACAAACGCAAAGTTTATGGCT
AACCGATATGGCACATCATCATTTAGCTATCGCAATTCTTTTCCTCATTGCGGGTCATATG
TATAGAACGAACTTTGGAATCGGACACAGTATAAAAGATCTTTTAGAAGCACATATTCC
TCCGGGAGGACGATTGGGGCGTGGGCATAAGGGTCTTTATGACACAATCAATAATTCGA
TTCATTTTCAATTAGGCCTTGCTCTAGCCTCCTTAGGAGTTATTACTTCCTTGGTAGCTC
AACACATGTACTCTTTACCTGCTTATGCGTTCATAGCGCAAGATTTTACGACTCAAGCTG
CGTTATATACCCATCACCAATATATTGCAGGATTCATCATGACAGGAGCTTTTGCTCATG
GAGCTATCTTTTTTATTAGAGATTACAATCCAGAACAGAATGAGGATAACGTATTAGCAA
GAATGTTAGACCATAAAGAAGCTATCATATCCCATTTAAGTTGGGCCAGCCTCTTTCTAG
GGTTCCATACTTTGGGACTTTATGTTCATAACGACGTCATGCTTGCTTTTGGTACTCCCG
AAAAACAAATCTTGATCGAACCTATATTTGCCCAATGGATACAATCTGCTCATGGGAAA
ACTTCATATGGATTTGATGTACTTTTATCTTCGACAAGTGGCCCAGCATTTAATGCGGGT
CGCAGCATATGGTTGCCTGGCTGGTTAAATGCTATTAATGAG------AATAGTAATTCATTA
TTCTTAACAATAGGTCCTGGAGATTTCTTGGTTCATCATGCTATTGCTTTAGGTTTACATA
CAACTACATTGATCTTAGTAAAAGGTGCTTTAGATGCACGTGGTTCCAAGTTAATGCCA
GATAAAAAGGATTTCGGGTATAGTTTTCCTTGCGATGGTCCGGGACGAGGTGGTACTTG
TGATATTTCGGCTTGGGACGCATTTTATTTGGCAGTTTTCTGGATGTTAAATACTATTGGA

TGGGTTACTTTTTATTGGCATTGGAAACACATCACATTATGGCAGGGTAACGTTTCACA
GTTTAATGAATCTTCCACTTATTTGATGGGATGGTTAAGAGATTATCTATGGTTAAACTCT
TCACAACTTATCAATGGATATAACCCGTTTGGTATGAATAGTTTATCAGTCTGGGCATGG
ATGTTCTTATTTGGGCATCTTGTTTGGGCTACAGGATTTATGTTCTTAATTTCCTGGCGTG
GTTATTGGCAGGAATTGATTGAAACTTTAGCGTGGGCTCATGAACGCACACCTTTGGCA
AATTTGATTCGCTGGAAAGATAAACCAGTAGCTCTTTCAATCGTGCAAGCAAGATTGGT
TGGATTAGCTCACTTTTCTGTAGGTTATATATTCACTTATGCGGCTTTCTTGATTGCCTCC
ACATCGGGCAAATTCGGTATGTCACATTCAGTAAAAATTTATGATACTTGTATAGGATGT
ACTCAATGTGTCCGAGCATGCCCTACAGACGTATTAGAAATGATACCTTGGGATGGATG
TAAAGCTAAGCAAATAGCTTCTGCTCCAAGAACCGAGGACTGTGTTGGTTGTAAGAGA
TGTGAATCCGCCTGTCCAACGGATTTTTTGAGCGTTCGAGTTTATTTATGGCATGAAAC
AACTCGAAGTATGGGTCTAGCTTATATGACAACTTTCAACTTACCCTCTATTTTTGTGCC
TTTAGTAGGCCTAGTCTTTCCGGCAATTGCAATGGCTTCTTTATTTCTTCATATTCAAAA
AAATAAGATTTTTATGCGAGATCTAAAAACATATCTTTCCGTAGCACCGGTACTAAGTAC
TCTATGGTTCGGTTCGTTAGCAGGTTTATTAATAGAGATTAATCGTTTATTTCCAGATGCA
TTAACATTTCCCTTTTTTTCATTCATGACTGCAATTTTAGAGAGACGCGAAAGCGAAAG
CCTATGGGGTCGCTTCTGTAACTGGATAACTAGCACTGAAAACCGTCTTTACATTGGAT
GGTTTGGTGTTTTGATGATCCCTACCTTATTGACCGCAACTTCTGTTTTTATTATCGCATT
CATTGCTGCTCCTCCAGTAGATATTGATGGTATTCGTGAACCTGTTTCTGGATCTCTTCT
TTACGGAAACAATATTATTTCCGGTGCCATTATTCCTACTTCTGCAGCTATTGGATTGCAT
TTTTACCCAATCTGGGAAGCTGCATCCGTTGATGAATGGCTATACAACGGCGGTCCTTA
TGAACTAATTGTTCTACACTTTTTACTTGGTGTAGCTTGTTATATGGGTCGTGAGTGGGA
ACTTAGTTTCCGTCTGGGTATGCGTCCTTGGATTGCTGTTGCATATTCAGCTCCTGTTGC
AGCTGCGACTGCTGTTTTCTTGATCTATCCAATTGGTCAGGGAAGTTTTTCTGATGGTAT
GCCTCTAGGAATCTCTGGTACTTTCAACTTTATGATTGTATTCCAGGCTGAGCACAACAT
TCTTATGCACCCATTTCACATGTTAGGTGTAGCTGGTGTATTCGGCGGCTCCCTTTTTAG
TGCTATGCATGGTTCCTTGGTAACTTCTAGTTTGATCAGGGAAACCACAGAAAATGAAT
CTGCTAATGAAGGTTACAGATTCGGGCAAGAAGAAGAAACTTACAACATTGTAGCTGC
TCACGGTTATTTTGGCCGATTGATTTTCCAATATGCTAGTTTCAACAATTCTCGTTCTTTA
CATTTCTTCTTAGCGGCTTGGCCGGTAGTAGGTATTTGGTTTACTGCTTTAGGTATTAGT
ACTATGGCTTTCAACCTAAATGGTTTCAATTTCAACCAATCAGTAGTTGATAGTCAAGG
ACGTGTTATTAATACTTGGGCTGATATTATTAACCGTGCTAACCTTGGTATGGAAGTTAT
GCATGAACGTAATGCTCACAACTTCCCTCTAGACCTAGCTGCTGTTGAGGCTCCATCTA
CAAATGGAATGGGTTTGCCTTGGTATCGTGTTCATACTGTTGTATTGAATGATCCCGGTC
GTTTACTTGCTGTTCATATAATGCATACTGCTCTAGTTGCTGGTTGGGCTGGTTCGATGG
CTCTATATGAATTAGCAGTTTTTGATCCCTCCGACCCCGTTCTTGATCCAATGTGGAGAC
AAGGTATGTTCGTTATACCTTTCATGACTCGTTTAGGAATAACCAATTCTTGGGGCGGTT
GGAATATTACA---GGGGGGACTATAACGAATCCGGGTCTTTGGAGTTACGAAGGGGTAG
CCGGAGCACATATAGTGTTTTCTGGTTTGTGCTTCTTGGCAGCTATTTGGCATTGGGTAT
ATTGGGATCTAGAAATTTTTTGTGATGAACGTACAGGAAAACCTTCTTTGGATTTGCCC
AAGATTTTTGGAATTCATTTATTTCTTTCAGGAGTGGCTTGCTTCGGTTTTGGCGCATTT
CATGTAACAGGATTATATGGTCCTGGAATATGGGTATCCGACCCTTATGGACTAACCGGA
AAGGTACAACCCGTAAACCCGGCGTGGGGCGTGGAGGGTTTTGACCCTTTTGTTCCGG
GAGGAATAGCCTCTCATCATATTGCAGCAGGGACGTTGGGTATATTAGCGGGCCTATTC

CATCTTAGTGTTCGTCCGCCTCAACGTCTATACAAAGGATTACGTATGGGCAATATTGAA
ACCGTCCTTTCCAGTAGTATTGCTGCTGTCTTTTTTGCAGCTTTTGTTGTTGCTGGAACT
ATGTGGTATGGTTCTGCAACTACTCCCATCGAATTATTTGGTCCTACTCGTTATCAATGG
GATCAGGGATACTTTCAACAAGAAATATATCGAAGAGTTAGTGCTGGACTGGCTGAAA
ATCAAAGTTTATCAGAAGCTTGGGCGAAAATTCCTGAAAAATTAGCTTTTTATGATTATA
TTGGTAATAATCCAGCAAAAGGGGGGTTATTCCGAGCGGGTTCAATGGACAATGGGGA
TGGAATAGCTGTTGGATGGTTAGGCCACCCCGTCTTTAGAAATAAAGAAGGGCGTGAA
CTTTTTGTACGCCGCATGCCTACTTTTTTTGAAACTTTTCCGGTAGTTTTGGTAGACGGA
GACGGAATTGTTAGAGCTGACGTCCCGTTTAGAAGGGCAGAATCAAAATATAGTGTCG
AACAAGTAGGTGTAACTGTTGAGTTTTATGGTGGTGAACTCAATGGAGTGAGTTATAGT
GATCCCGCAACTGTGAAAAAATATGCTAGACGGGCTCAATTGGGTGAGATTTTTGAATT
AGATCGTGCGACTTTGAAATCTGATGGTGTTTTTCGTAGCAGCCCAAGAGGTTGGTTTA
CGTTTGGACATGCTTCGTTTGCTCTACTTTTCTTCTTTGGACACATTTGGCATGGTGCTA
GAACCCTCTTCAGAGATGTTTTTGCTGGTATTGATCCAGATTTGGATGCTCAAGTGGAA
TTCGGGGCATTCCAAAAACTTGGAGATCCAACTACAAAACGACAAGCAGTCATGAAA
ACCTTATATTCCCTGAGGAGGTTCTACCACGTGGAAACGCTCTTTAATGGAACTTTAGC
TTTAGCTGGTCGTGACCAAGAAACCACCGGTTTCGCTTGGTGGGCCGGGAATGCCCGA
CTTATCAATTTATCTGGTAAACTTTTGGGAGCTCATGTAGCCCATGCCGGATTAATCGTA
TTCTGGGCCGGAGCAATGAACTTATTTGAAGTGGCCCATTTTGTACCTGAAAAGCCCAT
GTATGAACAAGGATTAATTTTACTTCCCCACCTAGCCACTTTAGGATGGGGGGTAGGTC
CTGGGGGAGAAGTTATAGACACCTTTCCGTACTTTGTATCTGGAGTACTTCACTTAATTT
CTTCTGCAGTTTTGGGCTTTGGCGGTATTTATCATGCACTTCTGGGACCCGAAACTCTT
GAAGAATCTTTTCCCTTTTTCGGTTATGTATGGAAAGATAGAAATAAAATGACCACCATT
TTGGGTATTCACTTAATTTTGTTAGGTGTAGGTGCCTTTCTTCTAGTATTCAAGGCTCTCT
ATTTTGGGGGCGTATATGATACCTGGGCTCCGGGAGGGGGGGATGTAAGAAAAATTAC
AAACTTGACTCTTAGCCCAAGTGTTATATTTGGTTATTTACTAAAATCTCCCTTTGGGGG
AGAAGGATGGATTGTTAGTGTGGACGATTTGGAAGATATAATTGGAGGACATGTATGGT
TAGGTTCCATTTGTATATTTGGTGGAATCTGGCATATCTTAACCAAGCCTTTTGCATGGG
CTCGCCGCGCACTTGTATGGTCTGGGGAGGCTTACTTGTCTTATAGTTTAGCTGCTTTAT
CTGTTTGTGGTTTCATTGCTTGTTGTTTTGTCTGGTTTAATAATACCGCTTACCCTAGTGA
GTTTTACGGACCTACGGGGCCAGAAGCTTCTCAAGCTCAAGCATTTACTTTTCTAGTTA
GAGACCAACGTCTTGGAGCTAACGTGGGGTCTGCTCAAGGACCTACAGGTTTAGGTAA
ATACTTAATGCGTTCTCCGACTGGAGAAGTTATTTTTGGAGGAGAAACAATGCGTTTTT
GGGATCTGCGTGCTCCCTGGTTAGAACCTTTAAGGGGTCCTAACGGTTTGGACTTGAGT
AGGTTGAAAAAAGACATACAACCTTGGCAAGAACGACGTTCGGCAGAATATATGACTC
ATGCTCCTTTAGGTTCGTTAAATTCTGTAGGGGGCGTAGCTACTGAGATCAATGCAGTC
AATTATGTCTCTCCGAGAAGTTGGTTATCTACCTCTCATTTTGTTCTAGGATTTTTCCTAT
TCGTGGGTCATTTATGGCACGCGGGAAGGGCTCGGGCAGCGGCAGCGGGATTTGAAA
AAGGAATTGATCGTGATTTTGAACCTGTTCTTTCCATGACTCCTCTTAACATGACTATAG
CCCTTGGTAAATTTACCAAAGACGAAAAAGATTTATTTGATATTATGGATGACTGGTTAC
GGAGGGACCGCTTCGTTTTTGTAGGTTGGTCTGGTCTATTGCTCTTTCCTTGTGCCTATT
TCGCTTTAGGGGGTTGGTTCACAGGTACAACCTTTGTAACTTCATGGTATACTCATGGAT
TGGCCAGTTCCTATTTAGAAGGTTGCAATTTTTTAACCGCTGCAGTTTCTACTCCTGCTA
ATAGTTTAGCGCATTCTTTGTTGTTACTGTGGGGTCCTGAAGCACAAGGAGATTTTACT

CGTTGGTGTCAATTAGGTGGTCTATGGGCTTTTGTTGCTCTCCACGGCGCTTTCGCATTA
ATAGGTTTTATGTTACGTCAATTTGAACTTGCTCGATCTGTTCAATTGCGACCTTATAATG
CAATCGCATTCTCTGGTCCCATTGCTGTTTTTGTTTCTGTCTTCCTAATTTATCCACTAGG
TCAATCTGGTTGGTTCTTTGCGCCTAGTTTTGGTGTAGCGGCTATATTTCGATTCATCCT
CTTTTTCCAAGGGTTTCATAATTGGACATTGAACCCATTTCATATGATGGGAGTCGCCGG
TGTACTGGGCGCGGCTCTGCTATGCGCTATTCATGGTGCTACTGTAGAAAATACTTTATT
TGAAGATGGTGATGGTGCAAATACATTCCGTGCTTTTAACCCAACTCAAGCCGAAGAA
ACTTATTCAATGGTCACCGCTAACCGCTTTTGGTCACAAATCTTTGGGGTTGCTTTTTCC
AATAAACGTTGGTTACATTTCTTTATGTTATTTGTACCGGTAACTGGTTTATGGATGAGT
GCTCTTGGAGTAGTCGGTTTAGCTTTGAACCTACGTGCCTATGACTTCGTTTCCCAGGA
AATCCGTGCAGCGGAAGATCCAGAATTTGAGACTTTCTATACTAAAAATATTCTTTTAA
ACGAAGGTATTCGTGCTTGGATGGCGGCTCAAGATCAGCCTCATGAAAACCTTATATTC
CCTGAGGAGGTTCTACCACGTGGAAACGCTCTTATGTCTGGAAGCACAGGAGAACGTT
CTTTTGCTGATATTATTACCAGTATTCGATACTGGGTCATTCATAGCATTACTATACCTTCC
CTATTCATTGCGGGCTGGTTATTCGTCAGCACCGGTTTAGCTTACGATGTGTTCGGGAG
CCCTCGTCCAAACGAGTATTTTACAGAGAGCCGACAAGGCATTCCATTAATAACAGGC
CGTTTTGATCCTTTGGAACAACTCGATGAATTTAGTAGATCTTTTATGACTATAGATAGG
ACCTATCCAATTTTTACAGTGCGCTGGTTGGCTGTTCATGGACTAGCTGTACCTACCGTT
TCTTTTTTGGGGTCAATATCAGCAATGCAGTTCATCCAACGAATGGCTACACAAACTGT
TGAAGATAGTTCTAGATCTGGT---CCAAGAAGCACTACTGTAGGGAAGTTATTGAAACC
ATTGAATTCCGAATATGGTAAAGTAGCTCCCGGATGGGGAACGACCCCTTTGATGGGTG
TTGCAATGGCTCTATTTGCGGTATTCCTATCTATTATTTTGGAGATTTATAATTCTTCTGTT
CTACTGGATGGAATTTCAGTGAATATGCTTACTCTCAAACTTTTTGTATACACTGTAGTT
ATATTCTTTGTTTCTCTCTTCATATTTGGATTCCTATCTAATGATCCAGGACGTAATCCGG
GACGTGAAGAAATGGCTGATACTACTGGAAGGATTCCTCTTTGGGTAATAGGTACTGTA
GCTGGTATTCTTGTGATCGGTTTAATAGGTATTTTCTTTTATGGTTCATATTCAGGATTAG
GTTCATCCCTGATGCTTAATATATTTAATTTGATCTGTATTTTTTTTAATTCTACCCTTTTTT
CAAGCACTTTTTTAGTCGCCAAATTGCCAGAGGCCTACGCCTTTTTGAATCCAATCGTA
GATGTTATGCCCGTAATACCTCTTTTCTTTCTTCTCTTAGCCTTTGTTTGGCAAGCCGCT
GTAAGTTTTCGAATGACACAATCAAATCCGAACGAACAAAGTGTTGAATTAAATCGTA
CCAGTCTCTATTGGGGGTTATTACTCATTTTTGTACTTGCTGTTTTATTTTCGAATTATTTC
TTCAATATGGAAGTAAATATTCTTGCATTTATTGCTACTGCACTCTTCATTCTCGTTCCTA
CTGCTTTTTTTGCTTATAATTTACGTAAAAACCGTTAGTCAAAATGATATGGAAACAGCA
ACCCTAGTCGCCATCTTTATATCTGGGTTACTTGTAAGTTTTACTGGGTATGCTCTATATA
CTGCCTTTGGGCAACCCTCTCAACAACTAAGAGATCCATTCGAGGAACACGGGGACAT
GGAAGCATTGGTTTATACATTTCTCTTAGTATCCACTTTAGGGATAATTTTTTTCGCTATT
TTTTTTCGGGAACCACCTAAAATTTCAACTAAAAAA------ATGACTATTGCTTTCCAATT
GGCAGTTTTTGCATTAATTATTACTTCATCAATCTTACTGATTAGTGTACCCGTTGTATTT
GCGTCTCCTGATGGTTGGTCGAGTAACAAAAATGTTGTATTTTCTGGTACATCTTTATGG
ATTGGATTAGTCTTCTTGGTGGGTATCCTTAATTCTCTTATCTCT--------------------ATGTC
ACCACAAACAGAGACTAAAGCAAGTGTTGGGTTCAAAGCTGGTGTTAAAGAGTATAA
ATTGACTTACTATACTCCTGAATATGAAACCAAGGATACTGATATCTTGGCAGCATTCCG
AGTAACTCCTCAACCTGGAGTTCCACCTGAAGAAGCAGGGGCTGCGGTAGCTGCTGA
ATCTTCTACTGGTACATGGACAACTGTGTGGACCGATGGGCTTACCAGCCTTGATCGTT

ACAAAGGACGATGCTACCACATCGAGCCCGTTCCAGGAGAAGAAACTCAATTTATTGC
GTATGTAGCTTATCCCTTAGACCTTTTTGAAGAAGGTTCGGTTACTAACATGTTTACCTC
GATTGTGGGTAATGTATTTGGGTTCAAAGCCCTGGCTGCTCTACGTCTAGAGGATCTGC
GAATCCCTCCTGCTTATACTAAAACTTTCCAAGGACCACCTCATGGTATCCAAGTTGAA
AGAGATAAATTGAACAAGTATGGACGTCCCCTATTAGGATGTACTATTAAACCAAAATT
GGGGTTATCCGCGAAAAACTATGGTAGAGCAGTTTATGAATGTCTACGTGGTGGACTTG
ATTTTACCAAAGATGATGAGAATGTGAACTCCCAACCATTTATGCGTTGGAGAGACCGT
TTCTTATTTTGTGCCGAAGCTATTTATAAATCACAGGCTGAAACAGGTGAAATCAAAGG
GCATTATTTGAATGCTACTGCGGGTACATGCGAAGAAATGATCAAAAGAGCTGTATTTG
CCAGAGAATTGGGAGTTCCTATCGTAATGCATGACTACTTAACAGGGGGATTCACCGCA
AATACTAGTTTGTCTCATTATTGCCGAGATAATGGCCTACTTCTTCACATCCACCGTGCA
ATGCACGCTGTTATTGATAGACAGAAGAATCATGGTATGCACTTCCGTGTACTAGCTAA
AGCTTTACGTCTATCTGGTGGAGATCATATTCACGCGGGTACAGTAGTAGGTAAACTTG
AAGGAGACAGGGAGTCAACTTTGGGCTTTGTTGATTTACTGCGCGATGATTATGTTGAA
AAAGATCGAAGCCGCGGTATCTTTTTCACTCAAGATTGGGTCTCACTACCTGGTGTTCT
GCCTGTGGCTTCAGGGGGTATTCACGTTTGGCATATGCCTGCTTTGACCGAGATCTTTG
GAGATGATTCTGTACTACAATTCGGTGGAGGAACTTTAGGCCACCCTTGGGGAAATGC
ACCGGGTGCCGTAGCCAACCGAGTAGCTCTGGAAGCATGTGTACAAGCTCGTAATGAG
GGACGTGATCTTGCAGTCGAGGGTAATGAAATTATCCGTGAAGCTTGCAAATGGAGTC
CTGAACTAGCTGCTGCTTGTGAAGTATGGAAAGAGATCACATTTAACTTCCCAACCATC
GATAAATTAGATGGCATGGCGATACATTTATACAAAACTTCTACCCCGAGCACACGCAA
TGGAGCCGTAGACAGTCAAGTGAAATCCAATCCACGAAATAATTTGATCTGTGGGCAG
CATCATTGTGGTAAAGGTCGTAATGCCAGAGGAATAATTACCGCAAGGCATAGAGGGG
GAGGTCATAAGCGTCTATACCGTAAAATAGATTTTCGACGAAATGCAAAAGACATATAT
GGTAGAATCGTAACCATAGAATACGACCCTAATCGAAATGCATACATTTGTCTCATACAC
TATGGGGATGGTGAGAAGAGATATATTTTACATCCCAGAGGGGCTATAATTGGAGATAC
CATTGTTTCTGGTACAGAAGTTCCTATAAAAATGGGAAATGCCCTACCTTTGACCGATAT
GCCCTTAGGCACGGCCATACATAATATAGAAATCACACTTGGAAGGGGTGGACAATTAG
CTAGAGCAGCGGGTGCTGTAGCGAAACTGATTGCAAAAGAGGGGAAATCGGCCACAT
TAAAATTACCTTCTGGAGAGGTCCGTTTGATATCCAAAAACTGCTCAGCAACAGTCGG
ACAAGTGGGAAATGTTGGGGTAAACCAGAAAAGTTTGGGTAGAGCCGGATCGAAATG
TTGGCTAGGTAAACGTCCTGTAGTAAGAGGAGTAGTTATGAACCCTGTCGACCATCCCC
ATGGAGGTGGTGAAGGGAGGGCTCCAATTGGTAGAAAAAAACCCGTAACCCCCTGGG
GTTATCCTGCACTTGGAAGAAGAACTAGAAAAAGGAAAAAATATAGTGAGACTTTGAT
TCTTCGTCGCCGTAGTAAAATGATTCAACCACAAACCTATTTGAATGTAGCAGACAACA
GCGGGGCTAGAGAATTGATGTGTATTCGAATAATAGGAGCTAGT---AATCGCCGATATGC
TCATATTGGTGACGTTATTGTTGCTGTAATCAAAGAAGCAATACCAAATACTCCTCTAGA
AAGATCAGAAGTGATTAGAGCTGTAATTGTACGTACTTGTAAAGAACTCAAACGTAAC
AATGGGACGATAATACGATATGATGACAATGCCGCAGTTGTCATTGATCAAGAAGGAAA
TCCAAAAGGAACTCGAGTTTTTGGGGCGATCCCACGGGAATTGAGACAATTAAACTTT
ACTAAAATAGTTTCATTAGCTCCTGAGGTATTAATGCTTAGTCCAAAAAGAACCAGATT
TCGTAAACAACATCGAGGAAGACTAAAAGGAATATCTTCTCGTGGGAATCGTATTTGTT
TTGGCAGATATGCTCTTCAAACACTTGAACCCGCTTGGATCACATCTAGACAAATAGAA
GCAGGGCGACGCGCAATGACACGAAATGTACGACGTGGTGGAAAAATTTGGGTACGTA

TATTTCCAGACAAACCAGTTACAGTAAGACCTGCGGAAACGCGTATGGGTTCTGGGAA
AGGATCCCCTGAGTATTGGGTAGCTGTGGTTAAACCAGGTAAAATCCTTTATGAAATGG
GTGGTGTACCCGAAAATATAGCAAGAAAAGCTATTTCAATAGCGGCATCAAAAATGCCT
ATAAAAACCCAATTCATTATTTCTGAAATGACTAGAATTAAACGCGGATATATAGCTCGG
AGGCGTAGAACAAAACTTCGTTTATTTGCATCAAGCTTTCGAGGGGCTCATTCACGGCT
TACACGAACTATGACTCAACAGAGAATAAGAGCTTTAGTTTCGGCTCATCGGGATAGG
GGTAAAAGAAAAAGAGATTTTCGCCGTTTATGGATCACTCGAATAAATGCCGTAATTCA
CGAAATG---GGGGTATTCTATAGTTATAATGAATTCATACACAATCTATACAAGAAGCAAT
TACTTCTTAATCGGAAAATACTTGCACAAATAGCTCTATTAAATAGGAGTTGTCTTTATA
CAATTTCGAATGACATAAAAAAA------------------------------ATGATAAAGGAAGTATATG
CTTTAGGGCAATATATATCTATGTCTGCCCATAAAGCACGGAGAGTAATTGATCAAATAC
GTGGACGTTCCTATGAAGAAGCACTTATGATATTAGAACTTATGCCTTATCGAGGATGTT
ATCCCATTTTTAAATTGGTTTATTCTGCTGCAGCGAACGCTAGTCACAATAAGGGTTTCA
AAGAAACCAATTTAGTCATTAGTAAAGCTGAGGTGAACCAAGGGAATACTGTGAAAAA
ATTAAAACCTCGTGCCCGAGGACGAAGTTACCCAATAAAACGATCCACTTGTCATATAA
CTATCGTATTGGAAGATATATCCTTCTATGGAGGCCTATGGGACAAAAAAATGGATGGAA
TCAAATATGCAGTATTTACAGACAAAAGTATTCGGTTATTGGGGAAAAAT---CAATATAC
TTTTAATGTCGAATCAGGATCAACTAGGACAGAAATAAAGCATTGGGTCGAACTCTTCT
TTGGTGTCAAGGTAATAGCTATGAATAGTCATCGACTCCCCGGAAAGGTTAAAAGAATG
GGACCTATTCTGGGACATACAATGCATTACAGACGTATGATCATTACGCTTCAACCGGG
TTATTCTATTCCACCTCTTAGAAAGAAAAGAACTATGGCAGTTCCAAAAAAACGTACTT
CTATCTCGAAAAAGCGTATTCGTAAAAAAATTTGGAAAAGGAAGGGATATTGGACATC
GTTGAAAGCTTTTTCATTAGGGAAATCACTTTCTACAGGTAATTCAAAAAGTTTTTTTGT
ACAGCAAAATAAA---ATGGCCAAGGGTAAAGATGTTCGAGTAACAATTATTTTGGAATG
TACCAGTTGTGTTCGAAATGATATT------AAGAAAGAAGCCGCGGGAATTTCCAGATATA
TTACTCAAAAGAATCGGCATAACACCCCTAGTCGATTGGAATTGAGAAAATTCTGTCCC
TATTGTTATAAACATACAATTCACGGGGAAATCAAGAAAATGAAAATAAGGGCTTCCGT
TCGTAAAATTTGTGAAAAGTGTCGACTAATCCGCAGGAGGGGACGAATTATAGTAATTT
GTTCCAACCCGAGGCATAAACAAAGACAAGGAATGGTTCGAGAGAAAGTCAAAGTAT
CTACTCGGACACTACAGTGGAAGTGTGTTGAATCAAAAAGAGACAGTAAGCGTCTTTA
TTATGGACGCTTTATTCTGTCTCCACTTATGAAAGGCCAAGCCGACACAATAGGCATTG
CGATGCGAAGAGCTTTACTTGGCGAAATAGAAGGAACATGTATTACACGTGCAAAATC
TGAG------AACATACCACATGACTATTCTAACATAGCAGGTATTCAAGAATCAGTACATG
AAATTTTAATGAATTTGAACGAGATTGTATTAAGAAGTAATCTATATGGAACGCGCAAC
GCGCTTATTTGTGTCCAAGGTCCTGGATATATAACTGCTCGAGACATTATTTTACCGCCC
GCTGTGGAAATCATTGATAATACACAGCATATAGCTACCTTAACAGAACCAATAGATTTG
TGTATTGAATTAAAAATTGAGAGGAATCGCGGATATAGTCTAAAAATGTCAAATAACTT
TGAA---GACAGA---AGTTATCCTATCGATGCTGTATTCATGCCTGTTGAAAATGCCAATCA
TAGTATTCATTCTTATGGGAATGGGAATGAAAAACAAGAGATTCTTTTTCTAGAAATATG
GACAAATGGAAGTTTAACTCCTAAAGAAGCACTTCATCAAGCCTCCCGGAATTTGATTA
ATTTATTTATTCCTTTTCTACATGTAGAAGAAGAA------ACGTTCTATTTAGAGAACAATC
AA---CATCAAGTTACTTTACCCTTTTTTCCTTTTCATAATCGATTAGTT---AACCTAAGAA
AAAAAAAAAAAAAAGAACTAGCCTTTCAATATATTTTTATTGACCAATTAGAATTGCCT
CCCAGAATCTACAATTGTCTCAAAAAATCCAATATACATACATTATTGGACCTTTTGAAT

AACAGTCAAGAAGACCTTATCAAAATTGAACATTTTCACGTAGAAGATGTAAAAAAGA
TATTAGACATTCTAGAAAAAAAAA------------------------------ATGCTTGGGATGAAAAA--
-GAGGGAACATCTGCAATACCCGGATTTAATCAGATACAATTTGAAGGGTTTTATCGGTT
TATTGATCAGGGTTTAATAGAAGAACTTGCTAAGTTTCCAAAAATTGAAGATATAGATC
ACGAAATTGAATTTCAATTATTTGTGGAAACATATCAATTGGTAGAACCTCTGATAAAA
GAACGAGATGCTGTCTATGAATCACTTACATATTCTTCTGAATTATATGTATCCGCGGGAT
TAATTTGGAAAACCAGTAGGAATATGCAAGAACAAAGAATTTTTATTGGAAACATTCCT
TTAATGAATTCCCTTGGAACTTCTATAGTAAACGGAATTTACCGAATTGTGATCAATCAA
ATATTGCAAAGTCCCGGTATCTATTACCAGTCAGAATTGGATCATAATGGGATTTCGGTC
TATACCGGCACCATAATATCAGATTGGGGAGGCAGGTTAGAATTAGAGATTGATAAAAA
AGCAAGAATATGGGCTCGGGTGAGTAGGAAACAAAAAATATCTATTCTAGTTCTATCAT
CAGCTATGGGTTTGAATCTACGAGAAATTCTAGAGAATGTTTGCTACCCTGAAATTTTC
TTATCTTTCTTGACCGATAAGGAGAAAAAAAAAAATAGGATCAAAAGAAAATGCTATTTT
GGAGTTTTATCAACAATTTTCTTGTGTAGGTGGGGATCCTATATTTTCTGAATCCTTATGT
AAGGAATTACAAAAAAAAATTCTTTCACCAAAGGTGTGAATTAGGAAGGATTGGTCGCC
GAAATATTAATTGGAGACTGAATCTTAATATACCTCAGAACAATATATTTTTGTTACCAC
GAGATGTGTTAGCAGCTGCCGATCATTTGATTGGGATGAAATTTGGAATGGGTACACTT
GATGAT------ATGAATCATTTGAAAAATAAACGTATTCGCTCTGTAGCGGATCTTTTACAA
GACCAGCTCGGGTTGGCTCTGGCTCGTTTAGAAAATGTAGTTAAGGGAACTATCTCCG
GAGCAATTAGG---CATAAATTGATA---CCTACTCCTCAGAATTTGGTAACTTCAACTCCG-
-----------TTAACAACTACTTATGAATCCTTTTTCGGATTACACCCATTATCTCAAGTTTTGG
ATCGAACAAATCCATTGACACAAATCGTTCATGGGAGAAAATTGAGTTATTTGGGCCCT
GGCGGATTAACAGGGCGAACTGCTAATTTTCGGATACGAGATATCCATCCTAGTCACTA
TGGGCGTATTTGTCCCATTGACACGTCTGAAGGAATCAATGTTGGACTTATTGGATCTTT
ATCAATTCATGCCAGGATTGGTGATTGGGGG---TCGTTAGAAAGTCCATTTTAT---------G
AACTCTTTGAGAAATCAAAAAAAGCACGGATACGGATGCTTTTTTTATCACCAAGTCA
AGATGAATATTATATGATAGCGGCAGGAAATTCTTTGGCTCTTAATCGGGGCATTCAAGA
AGAACAGGCTGTTCCAGCTCGATACCGCCAAGAATTTTTGACTATCGCATGGGAAGAG
GTTCATCTTCGAAGCATTTTCCCTTTCCAATATTTTTCCATTGGAGCTTCTCTAATTCCTT
TTATCGAACATAATGATGCGAATCGAGCTTTAATGAGTTCTAATATGCAACGTCAAGCA
GTTCCACTTTCTCGGTCCGAAAAGTGCATTGTTGGAACTGGGTTGGAACGCCAAGTGG
CTTTAGATTCAGGGGTTCCCGCTATAGCCGAACACGAGGGAAAAATCCTTTATACTGAC
ACTGAGAAGATCGTTTTTTCGGGCAATGGTGATACTCTAAGCATTCCATTAATTATGTAT
CAACGCTCAAACAAAAATACTTGTATGCATCAAAAACCTCAGGTTCGTCGGGGTAAAT
GCATTAAAAAGGGACAAATTTTAGCGGATGGTGCTGCTACAGTTGGTGGGGAACTCGC
TTTGGGGAAAAATATATTAGTGGCTTATATGCCATGGGAAGGATACAATTTTGAAGATGC
GGTACTCATTAGTGAGTGTCTAGTATATGGTGATATTTATACTTCTTTCCACATACGGAAA
TATGAAATTCAGACGCATGTGACAACCCAAGGT---CCTGAAAGGATCACTAAAGAAATA
CCGCATCTAGAAGGTCGTTTACTCCGAAATTTAGACAAAAATGGAATTGTGATGCTAGG
ATCGTGGGTTGAAACAGGTGATATTTTAGTAGGTAAATTAACACCTCAAGTGGCGAAA
GAA---TCCTCCTATGCTCCGGAAGATAGGTTATTACGAGCTATACTTGGCATTCAGGTAT
CGACTTCAAAAGAAACTTGTTTAAAATTGCCTATAGGTGGTAGAGGTCGAGTTATTGAT
GTGAGGTGGGTTCAGAAAAAGGGT---GGTTCAAGTTATAACCCAGAAATAATTCGTGTA
TATATTTCACAGAAACGTGAAATAAAAGTAGGTGATAAAGTAGCTGGAAGACATGGAA

ATAAAGGTATCATTTCAAAAATTTTGCCTAGACAGGATATGCCTTATTTGCAAGACGGG
AGACCCGTGGATATGGTCTTCAACCCATTAGGAGTACCCTCACGCATGAATGTAGGACA
GATATTTGAATGCTCGCTTGGGTTAGCGGGAAGTCTGCTAGATAGACATTATCGAATAG
CCCCTTTTGATGAGAGATATGAACAAGAGGCTTCGAGAAAACTGGTGTTTTCAGAATTA
TATGAAGCCAGTAAGCAAACAGCGAATCCATGGGTATTTGAACCCGAGTATCCAGGAA
AAAGCCGCATTTTTGATGGAAGAACAGGAGATCCTTTTGAACAGCCTGTGATAATAGG
AAAGCCCTATATCTTGAAATTAATTCATCAGGTTGATGATAAAATACACGGACGTTCTAG
TGGACATTATGCACTTGTTACACAACAACCCCTTAGAGGCCGTTCCAAGCAGGGGGGG
CAGCGGGTAGGCGAAATGGAGGTTTGGGCTCTGGAGGGGTTTGGTGTTGCTCATATTTT
ACAAGAGATGCTTACTTATAAATCTGATCATATTAGAGCTCGCCAAGAAGTACTTGGTA
CCACTATCATTGGAGGAACAATACCTAAACCAGAAGATGCTCCGGAATCTTTTCGATTA
CTTGTTCGAGAACTACGATCTTTGGCTCTGGAACTGAATCATTTCCTTGTATCTGAGAA
GAATTTCCAGATTAATAGGAAGGAAGTT---------------------ATGATCGATCGGTATAAACAT
CAACAACTCCGAATTGGCTTAGTTTCTCCTCAGCAAATAAGTGCTTGGGCCACTAAAAT
AATACCTAATGGAGAGATAGTTGGAGAGGTGACAAAACCCTATACTTTTCATTACAAAA
CCAATAAACCGGAAAAAGATGGATTATTTTGTGAAAGGATTTTTGGACCTATAAAGAGT
GGAATTTGCGCTTGTGGAAATTATCGAGTGATCGGAGATGAAAAAGAAGACCCAAAGT
TTTGTGAACAATGTGGAGTTGAATTTGTTGATTCTCGGATACGAAGATATCAAATGGGA
TACATAAAACTGACATGTCCTGTAACTCATGTATGGTATTTGAAACGTCTTCCTAGTTAT
ATTGCGAATCTTTTAGATAAACCTCTTAAAGAATTAGAAGGCCTAGTATACTGCGAT------
TTTTCTTTTGCTAGGCCC---ATAACTAAAAAACCTACTTTCTTACGATTACGAGGTTCATT
TGAATATGAAATTCAATCCTGGAAATACAGCATCCCACTTTTTTTACTACTCAAGGTTT
CGATATATTTCGAAATCGAGAAATTTCTACTGGGGCGGGTGCTATCCGAGAACAATTAG
CCGATTTAGATTTGCGAATTATTATAGAAAATTCGTTGGTAGAATGGAAACAATTAGGA
GAAGAA---GGGCCTACGGGGAATGAATGGGAAGATCGAAAAATTGTAAGAAGAAAAG
ATTTTTTAGTTAGACGTATGGAATTAGCTAAGCATTTTATTCGAACAAATATAGAACCCG
AATGGATGGTTTTATGTCTCTTACCGGTTCTTCCTCCCGAGTTGAGACCCATCATTCAGA
TAGAAGGGGGTAAACTAATGAGTTCAGATATTAATGAACTCTATAGAAGAGTTATCTATC
GGAACAATACTCTTACCGATCTATTAACAACAAGTAGATCTACACCGGGGGAATTAGTA
ATGTGTCAGGAAAAATTGGTACAAGAAGCCGTGGATACACTTCTTGATAATGGAATCCG
TGGACAACCCATGAGGGATGGTCATAATAAGGTTTACAAGTCATTTTCAGATGTAATTG
AAGGCAAAGAGGGAAGATTTCGCGAGACTCTGCTTGGGAAACGGGTCGATTATTCGG
GGCGCTCGGTGATTGTCGTTGGACCTTCACTTTCATTACATCGCTGTGGATTGCCTCGC
GAAATAGCAATAGAGCTCTTCCAGACATTTGTAATTCGTGGTTTAATTAGACAACACCT
GGCTTCGAACATAGGAGTTGCTAAGAGTCAAATTCGTGAAAAAAAGCCGATTGTCTGG
GAAATCCTTCAAGAAGTCATGCAGGGGCATCCCGTATTACTGAATAGAGCACCTACCCT
ACATAGATTAGGCATACAGTCATTCCAACCTATTTTAGTGGAAGGACGCACTATTTGTTT
ACATCCATTAGTTTGTAAGGGGTTCAATGCAGACTTTGATGGGGATCAAATGGCTGTTC
ATGTGCCTTTATCTTTAGAGGCTCAAGCAGAGGCTCGTTTACTTATGTTTTCTCATATGA
ATCTCTTATCTCCAGCTATTGGAGATCCCATTTCGGTACCGACTCAAGATATGCTTATTG
GACTCTATGTATTAACGAGCGGCACTCGTCGAGGTATTTGTGCAAACAGATATAATCCAT
GTAATCGAAAAAAC---------TATCAAAATGAAAGAATTTACGAAACAAACTATAAGTATA
CG------AAAGAACCCTTTTTTTGCAATTCCTATGATGCAATTGGAGCTTATCGACAGAAA
AAAATCAATTTAGATAGTCCTTTGTGGCTTCGGTGGCAATTAGATCAACGC---GTTATTG

CTTCA------AGAGAAGTTCCTATCGAAGTTCACTATGAATCTTTTGGTAACTATCATGAGA
TTTATGCACACTATCTAATAGTAAGAAGTGTAAAAAAAGAAAATTTTTGTATATATATTC
GAACCACAGTTGGTCATATTTCTTTTTATCGAGAAATCGAGGAAGCTATACAAGGTTTT
TCTCAAGCCTGTTCATATGATACC------------------------------------ATGGCGGAACGGGCCAA
TCTGGTATTTCACAATAAAGTGATAGATGGAACTGCTATTAAACGACTTATTAGCCGATT
AATAGATCACTTCGGGATGGCATATACATCACACATCCTAGATCAAGTAAAGACTCTGG
GTTTCCAGCAAGCCACTGCTACATCCATTTCATTAGGAATTGATGATCTTTTAACGATAC
CTTCTAAGGGCTGGCTTGTCCAAGATGCTGAACAACAAAGTTGGATTTTGGAAAAACA
CCATCATTATGGGAATGTACATGCGGTAGAAAAATTACGCCAATCTATTGAGATATGGTA
TGCTACAAGTGAATATTTGCGACAAGAAATGAATCCTAATTTTAGGATGACGGACCCCT
TCAATCCAGTCCATATGATGTCTTTTTCGGGAGCTAGGGGAAATGCATCTCAAGTACAT
CAATTAGTAGGTATGAGAGGATTAATGTCGGATCCCCAAGGACAAATGATTGATTTACC
TATTCAAAGCAATTTACGCGAAGGACTGTCTTTAACAGAATATATTATTTCTTGCTATGG
AGCCCGTAAAGGAGTTGTAGATACTGCTGTACGCACATCAGATGCTGGATATCTTACGC
GTCGACTTGTTGAAGTAGTTCAACATATTGTTGTACGTAGAACAGATTGTGGCACTATC
CGAGGGATTTCGGTGAGTCCTAAAAATCGGATGATGTCA---GAAAGAATTTTTATCCAA
ACATTAATTGGTCGTGTCTTAGCAGACGATATATATATAGGTTCCCGATGTGTCGCCTTTC
GAAATCAAGATCTTGGGATTGGACTTGTCAATCGATTAATAACC------------------TTTGGA
ACACAATCAATATCCATTCGAACTCCCTTTACTTGTCGGAGTACATCTTGGATCTGTCGA
TTATGTTATGGTCGGAGTCCCACTCATGGTGACCTCGTTGAATTGGGGGAAGCTGTAGG
TATTATCGCGGGTCAATCGATCGGCGAACCGGGGACTCAACTAACATTAAGAACTTTTC
ATACCGGCGGAGTATTTACAGGAGGTACTGCCGAACATGTACGAGCCCCTTATAATGGA
AAAATCAAATTCAATGAGGATTTGGTTCATCCTACACGTACACGTCACGGGCATCCTGC
CTTTCTATGTTATATAGACTTGTCTGTAATTATTGAGAGCGAAGATATTATACATAGCGTG
ACTATTCCACCAAAAAGTTTTCTTTTAGTTCAAAATGATCAATATGTGGAATCAGAACA
AGTGATTGCTGAGATTCGCGAGGGAACATACACTTTT---CATTTTAAAGAGAGGGTTAG
AAAATATATTTATTCTGACTCAGAGGGCGAA---ATGCACTGGAGTACTGATGTGTCCCAT
GCACCCGAATTTACATATAGTAATGTCCACCTCTTACCAAAAACAAGTCATTTATGGATA
TTATCCGGAGGTTCATGTGGA------TCTAGTCTAATTCGTTTTTCGATCCATAAAGATCAA
GATCAAATGAATATACCC------TTTCTTTCCGCCGAAAGA---AAATCTATTTCT------CTCTC
AGTGAATAATGATCAA---GTGAGCCAAAAATTTTTTAGTTCCGATTTTGCCGATCCAAA
A---TTAGGGATTTACGATTAT---TCAGAATTGAATGGAAACTTAGGTACTAGTCATTATAA
TTTAATATATTCTGCT---ATTTTTCATGAGAACTCGGAT------TTATTAGCAAAAAGACGAA
GAAATAGATTTCTCATTCCATTCCAATCGATTCAAGAGCAAGAGAAAGAATTCATACCG
CAG---GGTATCTCGGTTGAAATACCCATAAAT------GGTATTTTCCGTAGAAATAGTATTTT
TGCTTTTTTTGATGATCCTAGATACCGAAGAAAGAGTTCCGGAATTCTTAAATATGGGA
CTCTAAAGGCGGATTCAATCATCCAAAAAGAGGAT------ATGATTGAGTATCGA---GGAG
TCCAAAAAATTAAGACAAAATACATGAAAGTAGATCGCTTTTTTTTCATTCCTGAGGAA
GTGCATATTTTACCCGAATCCTCTGCCATAATGGTACAGAACTATAGTATCATTGGAGTG
GATACACGACTCACTTTAAATATAAGAAGCCAAGTCGGCGGGTTGATCCGAGTAGAGA
AAAAAAAAAAAAGGATTGAACTCAAAATATTTTCAGGGGATATCCATTTTCCGGACAA
GACAGATAAAATATCCCGACACAGTGGCATCTTGATACCGCCAGGAAGAGGAAAAAAA
------AACTCGAAGGAATCCAAAAAA------TTTAAAAATTGGATTTATGTCCAACGG------A
TTACACCAACCAAGAAAAAG------TTTTTTGTTTTGGTGCGGCCCGTAGCCACCTATGA

GATAGCGGACAGTATAAATTTGGCAACACTCTTCCCACAAGATCTCTTTCGGGAAAAG
GATAATATTCAACTTCGAGTTTTCAACTATATCCTTTATGGAAATGGCAAACCAACTCGA
GGAATTTCTGACACAAGTATTCAATTAGTTCGAACTTGTTTAGTCTTGAATTGGGAC-----
-------------AAAAATTCTTCTCTCGAGGAGGTCCGTGCTTTCTTTGTTGAAGTAAGTACAA
AGGGTTTGATTCAAGATTTCATAAGAATTGGCTTAGTGAAATCCCATATTTCATATATAA
GAAAAAGGAATAATTCGCCAGATTCGGGATTGATCTCT------------GCAGATCACATGAAT-
-----CCGTTTTATTCGATTTCGCCCAAGTCTGGCATT------------CTTCAACAATCACTTAGA
CAAAATCACGGAACTATTCGCATGTTCTTAAATCGA---------AATAAGGAGTCCCAATCTT
TGTTAATTTTATCATCATCTAATTGTTTTAGAATGGGTCCA------TTTAATCATGTAAAACA
TCACAAT---------GTGATAAACCAATCAAATACTCTAATTACAATTAAAAATTCGTCGGGC
CCCTTAGGAACA------GCTACCCCAATTTCAAATTTTTATTCG------TTTTTGCCTTTACTA
ACT------TATAATCAGATCTCT---TTAATTAAATATTTTCAACTTGATAAC---------TTAAAAT
ATATTTTTCAAAAAATT------AAC---TCTTATTTAATCGATGAAAACGGAATA------ATTTTA
AATCTCGACCCATATAGTAACGTTGTTTTGAATCCATTCAAATTGAATTGGTATTTTCTTC
ATCAAAATTATTGTGAGGAAACGTCCACAATAATTAGT---CTTGGACAATTTTTTTGTGA
AAATGTATGTATAGCTAAAAAAGAACCG---------CACCTAAAATCGGGTCAAGTTTTAAT
TGTTCAA---AGGGATTCCGCAGTAATAAGATCCGCTAAGCCCTATTTGGCTACTCCGGG
AGCAAAAGTTCATGGGCATTACAGCGAAATTCTTTACGAAGGGGATACATTAGTTACAT
TTATATATGAAAAATCGAGATCCGGTGATATAACCCAAGGTCTTCCAAAAGTAGAACAA
GTTTTAGAAGTCCGCTCGATTGATTCAATATCGTTGAACTTAGAAAAGCGGATTAAGGG
TTGGAACAAGTGTATAACAAGAATTCTTGGAATTCCTTGGGGATTCTTGATTGGTGCTG
AGTTAACTATAGTGCAAAGTCGTATTTCTTTGGTTAATAAGATTCAAAAGGTTTATCGAT
CCCAGGGGGTGCAGATTCATAATAGGCATATCGAAATTATTGTACGTCAAATAACATCA
AAAGTTTTGGTTTCCGAA---------GAGGGAATGTCTAATGTTTTTTACCTGGAGAACTTA
TTGGATTATTACGAGCAGAACGAACGGGGCGTGCTTTAGAAGAAGCAATCTGTTATCG
AGCCGTTTTATTAGGAATAACTCGAGCATCTTTGAATACTCAAAGTTTTATATCCGAAGC
AAGTTTTCAAGAAACTGCTCGAGTTTTAGCAAAAGCTGCTCTTCGGGGTCGTATCGATT
GGTTGAAAGGCCTGAAAGAAAATGTTGTTCTAGGGGGGGTGATCCCCGCCGGGACCG
GATTCAAAGGATTGGTGCATTGTTCA------CGGCAACATACCAACATTATTTGGAAAAA
AAAACAAAGAATTTAGCTTTATTCGAGGGAGATATGAGA------GATATTTTATTCTACCAC
AGGGAATTTTGTGACTCTTCTATTTCCAAATCTGACTTTTCTAGGATT------------------------
----------------------ATGACAAAAAGATATTGGAACATCGATTTGGAAGAGATGATGAGAGC
AGGAGTTCATTTTGGACATGGTACGAGGAAATGGAATCCTAGAATGGCACCTTATATTT
CTGCAAAGCGTAAAGGTATTCATATTATAAATCTGACTAGAACTGCTCGTTTTTTATCAG
AAGCTTGTGATTTAGTTTTTGATGCGGCAAGTAGGGGAAAACAATTCTTAATTGTTGGG
ACAAAAAATAAAGCAGCTGATTTAGTGTCGCGGGCTGCAATACGGGCTCGGTGTCATT
ATGTTAATAAAAAATGGCTCGGCGGCATGTTAACAAATTGGTCCACTACCGAAAAAAG
ACTTCATAAGTTTAGGGACTTGAGAACTGAACAAAAGACAGAGGGATTCAACCGTCTT
CCGAAAAGGGATGCAGCTGTGTTGAAGAGACAATTATCTCGCTTGGAAACATATCTAG
GCGGGATTAAATATATGACGGGATTGCCTGATATTGTAATCATCCTCGATCAGCAAGAAG
AATATACGGCTCTTAGAGAATGTATCACTTTGGGAATTCCAACCATTTCTTTAATCGATA
CAAATTGTAATCCCGATCTCGCGGATATTTCTATTCCCGCAAATGATGACGCTATAGCTT
CAATTCGATTCATTCTTAACAAATTAGTATTCGCAATTTGTGAGGGCCGTTCTAGC---TAT
ATACAAAATTCTATGGGACAAAAAATAAATCCACTTGGTTTCAGACTTGGTACAACCCA

AAGTCATCATTCTCTTTGGTTTGCACAACCAAAAAAGTATTCTGAAGGTTTAGAAGAA
GATAAAAAAATACGAGACTGTATTAAAAATTATGTCCAAAAAAATATAAGAATATCCTCT
GGT---ATGGAGGGAATTGCACGTATTGAAATTCAAAAAAGAATCGATCTCATCCAGATC
ATAATCTATATGGGATTTCCTAAATTATTAATTGAAGATAAA------------CCCCGAAGAGTC
GAAGAATTACAGATGAATGTTCAAAAAGAACTAAATTGTGTCAATAGAAAACTCAACA
TTGCTATTACCAGAATTTCCAATCCGTATGGGGATCCTAATATTCTTGCAGAATTTATCGC
TGGACAATTAAAAAATCGCGTTTCTTTTCGAAAAGCAATGAAAAAAGCTATTGAATTAA
CTGAACAGGCGAATACAAAAGGAATTCAAGTACAAATTGCAGGACGTATCGACGGAA
AAGAAATTGCACGTGTTGAATGGATCAGAGAAGGCAGAGTTCCTTTACAAACAATTGA
AGCTAAAATTGATTATTGTTCCTATACAGTTCGAACTATTTATGGGGTTTTAGGAATAAA
AATTTGGATATTCGTAGACGAAGAAATGTCACGTTACCGAGGGCCTCGTTTTAAAAAAA
TACGCCGTCTGGGGGCTTTACCGGGACTAACTAGTAAAAGGCCTAAGGCAGGAAGCGA
TCTTAGAAACCAATCACGCTCCGTAAAA---AAATCTCAATATCGTATTCGTTTAGAAGAA
AAACAAAAATTGCGTTTTCATTATGGTCTTACAGAACACCAATTACTTAAATATGTGCGT
ATAGCCGGAAAAGCCAAGGGGTCAACAGGTCAAGTTTTACTACAATTACTTGAAATGC
GTTTGGATAACATCCTTTTTCGATTGGGTATGGCTTTGACTATTCCTCAAGCGCGCCAAT
TAGTTAATCATGGACATATTTTAGTTAATGGTCGTATAGTTGATATACCAAGTTATCGCTG
CAAACCCCGAGATATTATTACAGTGAAGGATGAACAAAACTCTAGAACTTTGGTTCAA
AATCTTCTTGATTCATCTGCCCCT---GAGGAATTGCCAAACCATCTGACTCTTCACACAT
TCCAA------TATGAAGGGTTAGTCAATCAAATAATAGATAGGAAATGCGTCGGTTTGAAA
ATAAATGAATTGCTTGTCGTAGAATATTACTCTCGACAGACTATGTCACGCCGAGGTACT
GCAGAAGAAAAAACTGCAAAATCCGATCCAATTTATCGTAATCGATTAGTTAACATGTT
GGTTAACCGTATTCTGAAACACGGAAAAAAATCATTGGCTTATCAAATTATCTATCGAG
CCTTGAAAAAGATTCAACAAAAGACAGAAACAAATCCACTATCTGTTTTACGTCAAGC
AATACGTGGAGTAACTCCCGATATAGCAGTAAAAGCAAGACGTGTAGGCGGGTCAACT
CATCAAGTTCCCATTGAAATAGGATCCACGCAAGGAAAAGCACTTGCCATTCGTTGGTT
ATTAGGGGCATCCCGAAAACGTCCGGGTCGAAATATGGCTTTCAAATTAAGTTCCGAAT
TAGTGGATGCTGCCAAAGGGAGTGGCGATGCCATACGCAAAAAGGAAGAGACTCATA
GAATGGCAGAGGCAAATAGAGCTTTTGCACATTTTCGTATGGGGAAAGACACCATTGC
TGATATAATAACCTCTATACGAAATGCTGACATGAATAGAAAAGGAACGGTTCGGATAG
GATCGACTAACATCACGGAAAGTATTGTTAAAATCCTTTTACGAGAGGGTTTTATCGAA
AACGTAAGGAAACATCGCGAAAACAACCAATATTTTTTGATTTTAACCCTAAGACATAG
ACGA------AATAAGAAAGAATCCTACAAA---------ACGATTTTAAATTTAAAGAGAATAAG
TCGACCGGGTCTACGAATCTATTCTAACTCTCAACGAATTCCACGAATTTTAGGCGGAA
TAGGAATTGTAATCCTTTCGACTTCTCAAGGTATAATGACAGACCGAGAAGCTCGACTA
AAAAGAATCGGCGGAGAAATTTTGTGTTATATATGGATGGCAAAACCTATATTAAGAAT
TGGTTCGCGTAAAAATACACGTAGTGGTTCACGTAAAAATGTACGTAGAATACCAAAG
GGAGTTATTCATGTTCAAGCAAGTTTCAACAATACCATTGTAACCGTTACAGATGTACG
GGGTCGGGTGATTTCTTGGTCCTCCGCAGGTACTTGTGGATTCCGGGGTACAAGAAGA
GGAACACCCTTTGCTGCCCAAACCGCAGCAGGAAATGCTATTCGAGCAGTAGTGGATC
AAGGTATGCAACGAGCTGAAGTAAGGATAAAAGGCCCTGGACTCGGAAGAGATGCGG
CATTACGAGCTATTCGTAGAAGCGGTATACTTTTAAGTTTCGTACGAGATGTAACCCCTA
TGCCACATAATGGTTGTAGACCCCCTAAAAAAAGACGTGTAATGCCAACCATTAAACA
ACTTATTAGAAATACAAGACAGCCAATCCGAAACGTCACGAAATCCCCAGCGCTTCGG

GGATGCCCTCAGCGACGAGGAACATGTACTCGGGTGTATACTATCACCCCCAAAAAAC
CAAACTCTGCTTTACGTAAAGTTGCCAGAGTACGATTAACCTCGGGATTTGAAATCACT
GCTTATATACCTGGTATTGGCCATAATTTACAAGAACATTCTGTAGTCTTAGTAAGAGGG
GGAAGGGTTAAGGATTTACCCGGTGTGAGATATCACATTGTTCGAGGAACCCTAGATGC
TGTCGGAGTAAAGGATCGTCAACAAGGGCGTTCTAAATATGGGGTCAAAAAGCCAAA
AATGGCAAAGAAAAGTTTGATTTATAGGGAGAAGAAGAGGCAAAAATTGGAAAAAAA
ATATCATTTGATTCGTCGATCCTCAAAAAAGGAAATAAGT---------AAGATTCCGTCGCTA
AGTGAGAAATGGAAAATTCATGGAAAATTACAATCCCCACCGCGTAATAGTGCACCTA
CACGTCTTCATCGACGTTGCTTTTCGACCGGAAGACCGAGAGCTAACTATCGAGACTT
TGGACTATCTGGACACATCCTTCGGGAAATGGTTCAGGCATGTTTGTTGCCAGGGGCA
ACAAGATCAAGCTGGATGATAAAAAATATAGTCATTTCA------TTTGAAGAA---CAA---A
AAGAAGAAAGCAGGGGATCCGTTGAATTTCAAGTATTTAGTTTCACTAATAAGATACGA
AGACTTACTTCACATTTGGAATTGCACAGAAAAGATTATTTATCTCAGCGGGGTCTACG
AAAAATTCTGGGAAAACGTCAACGACTGCTGGCTTATTTGTCAAAAAAAAAATAGAGTA
CGTTATAAAGAATTAATTAATCAGTTGAATATTCGGGAATTAAAAACTCGT------ATGGTA
AAACTTCGTTTAAAACGATGTGGTAGAAAGCAACGAGCCGTTTATCGAATCCTTGCAAT
TGATGTTCGATACCGAAGAGAAGGAAGAGATCTTAGTAAGGTGGGTTTTTATGATCCCA
TAACTAATCAAACTTTTTTAAACCTTTCTGCTATTCTCGATTTTCTTAAAAAAGGAGCTC
AACCAACAAGAACAGCTCATGATATTTCAAAGAAGGCTGGGATTTTTACGGAA-----------
------------------ATGAATAAATCTAAGCGACTTTTTACTAAATCCAAGCGATCTTTTCGTAG
GCGTTTGCCCCCGATCCAATCGGGGGATCGAATTGATTATAGAAACATGAGTTTAATTA
GTCGATTTATTAGTGAACAAGGAAAAATATTATCTAGACGGGTGAATAGAGTGACTTTA
AAACAACAACGATTAATCACTATTGCTATAAAACAAGCTCGTATTTTATCTTTGTTACCT
TTTCTTAATAATCAG------AAACAATTTGAAAGAAGTGAGTCGACTCCTAGAACTACTAG
TCTTAGAACCAGAAAAAAAGTGACACGTTCACTAAAAAAAAACCCTTTTGTAGCAAA
GCATTTATTAAGAAAAATAGAGAAGCTTAATACAAAGGCGGAAAAAGAAATCATAATA
ACTTGGTCCCGGGCATCCACCATTATACCCACAATGATTGGCCATACGATCGCTATTCAT
AATGGAAGGGAACACTTACCCGTTTATATAATAGATCTTATGGTAGGACATAAATTAGGA
GAATTTTCACCTACTATAAATTTTCGAGGACATGCCAAAAATGATAATAGATCTCGTCGT
ATGGTTTTTCAATCTTTTATACTAGGTAATCTAGTATCCTTATGCATGAAGATAATAAATT
CGGTCGTTGTGGTCGGACTCTATTATGGATTTCTGACCACATTTTCCATAGGGCCCTCTT
ATCTCTTCCTTCTCCGAGCTCGGGTTATGGACGAAGGAGAAGAAGGAACCGAGAAGA
AAGTATCAGCAACAACAGGTTTTATTGCGGGACAGCTCATGATGTTCATATCGATCTATT
ATGCGCCTCTGCATTTAGCATTGGGTAGACCTCATACAATAACTGTCCTAGCTCTACCGT
ATCTTTTGTTTCATTTCTTCTGGAACAATCACAAACACTTTTTTGATTATGGATCTACTAC
CAGAAATGAAATGCGTAATCTTCGCATTCAATGTGTATTCCTGAATAATCTCATTTTTCA
ATTATTCAACCATTTCATTTTACCAAGTTCAATGTTAGCCAGATTAGTCAACATTTATATG
TTTCGATGCAACAACAAGATGTTATTTGTAACAAGTAGTTTTGTTGGTTGGTTAATTGGT
CACATTTTATTCATGAAATGGGTTGGATTGGTATTAGTCTGGATACAGCAAAATAATTCT
ATTAGGTCT---ATTAGATCTAATAAGTTCCTTGTGTCAGAATTGAGAAATTCTATGGCTCG
AATCTTTAGTATTCTCTTATTTATTACCTGTGTCTACTATTTAGGCAGAATACCATCACCC
ATTTTTACTAAGAAACTTAAAGGAACCGAAGAAGCTAAACCATTTGTAACTCTCGTTTT
CGATTATAAAAGATGGAATCGACCAAACCGATATATAAAAAATGATAAAATTGAAAATAT
TGTAAGAAATGAAATGTCACAATATTTTTTTTATACATGCCAAAGTGATGGAAAAGAAC

GAATATCTTTTACATATCCCCCCAACCTTTCCACCTTTTTTGAAATGATCCAAAAAAGGA
TACCTTCATTTACAAAAGAAAAAAAAAACCTTTGACCAAGTTTCTACTTATTGGAGTTTG
ATCCATGAAGAAAAAAAGGGAAAACTTAAAAAAAGAATTTTTAAATAGAATTGAAGCTT
TAGAT---------TCTGTTGAAAATATACTGGAAAAAACGACTCGCTTTTGTTATAACGAAGC
TAAAAAAGAATATTTACCTAAAATTTATGATCCTTTTTTGCATGGAATCTCTCGGGGAAG
AATCAAAAAATTACCTCCATTCTTAGGAGGATCCTGGATAAACAAGATTCATGGTCTAC
TTTATAAAAAATTTGAGCAAACAGATAGCAACGATCAAGAT------TTCCCTGAAATCAAT
AAAAAAGTTCCTCGATGGTCATACAAATTAATAGAGTTGGAAGAATTGGAAGGCGAAG
GAATTCGTTCAAGAAAAGCAAAACGTGTAGTGGTTTTTACTGATGCTTTGATCCGTTAT
TCGCAACAATCTGATTTTCGTCGAGAGATAATTAAAGGATCCATGCGTTCCCAAAGGCG
TAAAACTGTTATTTGGGAATTTTTTCAAGCAAAAGTACATTCCCCCCTTTTTTTTGATAG
AATAGATAAA------CTTTTTTTTTTTTCGTTTGATATATGGGGGCTAAAAAAAAAAATTAT
TAAAAATAGAGAAGAAACAAGACGGATAGAAATTGCAGAAACTTGGGATAGCTTCCTA
TTTGCTCAAATAATAAGAGGTTCTCTCTTAGTAACTCAATCAATTCTTAGAAAATATATTA
TATTACCTTTATTGATAATAATTAAAAACAGTGTCCGTATGTTATTATTTCAATTTCCCGA
GTGGTCCCAGGATTTAAAGGATTGGAAACGTGAAATGCATGTTAAATGTACTTATAATG
GGGTTCAACTATCCGAAACAGAATTTCCAAGAAACTGGTTAACGGATGGTATTCAGATA
AAAATCCTATTTCCTTTTTATCTTAAACCTTGGCATAAATCTAAAAAAGGAGAAAAAAA
TGATTTTTGTTTCTTAACAGTTTGGGGAATGGAAACCGAACTACCGTTTGGTTCTGCCC
AAAGAAAGCCTTCTTTTTTTGAACCTATTTCTAAAGAATTAAAAAAAAGAATCAAAAA
ATTAAAAAAAAAAGTCTTTTGTGGTT---TTAAAGATTTTCAAAGAAAGAGCACCCATTTT
CCTAAAAGTCGCAAAAGAAACTAAAAACTGGATTCTCAAAAACTTTATT------TTTATAA
AAGGA------------ATTTCAAAACGAAATGATTCAATAATAAGTAATCAGATGATTCATGAA
CTA---GTTCAAAATAAATCCTTGGAGTGGACAAAT---TCACTTAGCGAAAAAAAAAATCA
AAAATTTGATTGATAGAAAAAAGACAATCAGAAATCAAATAGAAGAAATTTCAAAAGA
AAAACAAAACAGTTGTACCAAACTGCGTTATGATTCTAAAATAATTGAGTCATCAAAA
AAAATTTGGCAGACATTCAAA------AATACCCGATTAATTCGTAAATCCATTTTTTTTTTT
AAATTTTGCATCGAACAAATGTCTATAGCTATTTTTCTAGGTATCATTAATATTCCAAGAA
TTACTACACAACTT------------------TATGACCTATGCTCTTTATCACAAGCATATGTATTTTA
CAAATTATCACAAATT---GTTAGTAACTTTTGTAAATTAAAGGCTGTTCTTGAATATACAT
CCTTTTTTGTTAAGAATAAAATAAAGGTTTTTTTTGAACACGGAATCTTTCAT---GAATT
GAAAAATAAAACCTTTTTG---TCCGAAGTAAATCAATGGAAAAACTGGTTACGAAGTC
AGTATCAATACAATTTACCCCAGATTTCATGGGCTAGATTAGTAACCCAAAATTGGAAA
AATAAAATAAATAAAGATTCTCTAGTTCTAAACCCAACCAAAGAGGATTCATATGAAAA
AAAGAAA------TTTGATAATTATAAAAAACAA---AATGTAAAAAAAGATTCTATATATAAT
CTTTTTTGCTATAAATCTATTCATTATAATATTCGGGGTCGGCATAGAAAATATTTGGATT
GGAGAATTCTTAACTTTTGGTTTACATTTTTTGATTGGATGGGAATGAATGAAGAAATAC
TAAATCAGTTTTTCTTTCCGGAATTTTTCTTATTTTCTAGTACATATAAAATGAAACCATG
GGTCATACCAATCAAATTACTTCTTTTAAATAGTAAT------ACAGAAGCGGAACTTGATTT
ATTTCTGACAAGATATTCGCGTTTTCAATTGCGATGGAAT---------AATCAAAAAATTCTC
AATAATGTAAAAGTATACTGTCTCTTGGTTAGACTAAACAATCCAAACGAAATAGCGGT
ATCTTCTATTGAAAGAGGAGAGATGAGCCTAGACATTCTAATGATTGAGAAAAATTTCA
CTTTTGCAAAATTAATGAAAAAAGGAATATTGATTATTGAACCTGTGCGTTTGTCTGTAC
AAAACGATGGACAACTTATTATATATAGAACCATAGGTATTTCATTGGTTCATAAAAATA

AACACAATTATGAT---TTCTTTGTCCCTGAAAAAATTCTATCCCCTAAACGACGTAGAGA
ATTTCGAATTCTAATTTGTTTCACATTTCTTTGGCCCAATTTTAAATTAGAAGATTTAGCT
TGTATGAATCGCTATTGGTTTAATACTACTAACGGAAATCATTTCAGTATGATAAGAATA
CGCATGTATACGATGAAAGGACATCAATTCAAATCCTGGATTTTCGAATTGAGAGAAAT
AGTGAGAGAGATCAAGAATGCTCACTATTTCTTAGATTCATGGACCCAATTCAATTCAG
TGGGATCTTTCATTCATATTTTTTTCCACCAAGAACGTTTTAGAAAACTCTTGGACCCTC
GGATTTTTAGTATCCTACTTTTG---CGCAATTCA------CAGGGTTCAACAAGCAATCGATA
TTTC---------ACGATCAAGGGTGTAGTACTATTTGTAGTAGCG---GCCCTTCTATATCGT-----
-------ATTAACAATCGAAATATGGTCGAAAGCAAAAATCTCTATTTGAAAGGGCTTCTTCC
T--------------ATACCTATGAATTCCATTGGACCCAGAAATGATACATCGGAAGAATCTTTT-
--GGGTCTTGCAATATCAATAGGTTGATTGTTTCG---CTCCTGTATCTTACAAAAGGAAAA
AAGATCTCTGAGAGCTGTTTCCGGGATCCGAAAGAGAGTACTTGGGTTCTCCCAATAA
CT------------CAAAAGTGTATCATGCCTGAATCTAACTGGAGTTCGCGGTGGTGGAGGAA
CTGGATCGGAAAAAAGAGG------GGTTTTTGTTGTAAGATATCTAAT---------GAAACCGT
CGCTGGAATTGATATCTCATTTAAAGAGAAAGATATCAAA---TATCTGGAGTTTCTTTTT
GTATATTATATGGAT------GATCCGATCCGCAAGGGCCATGATTGGGAATTGTTTGATCGT
CTTTCTCCGAGTAAGAGGCGAAACATAATCAACTTG------AATTCGGGACAGCTATTCGA
AATCTTAGTGAAAGACTGGATTTGTTATCTCATGTTTGCTTTTCGTGAAAAAATACCAAT
TGAAGTGGAGGGT---TTCTGCAAACAACAAGGA---GCTGGGTCAACTATTCAATCAAAT
GATATTGAGCATGTTTCCCAT------CTCTTCTCGAGAAACAAGTGGGCTATTTCTTTGCAA
AATTGTGCTCAATTTCATATGTGGCAATTCCACCAAGATCTCTTCGTT---------AGTTGGG
GGAAG---AATCCGCACGAATCGGATTTTTTTAGGAAA---ATATCGAGAGAGAATTGGATT
TGGTTAGACAATGTGTGGTTGGTAAACAAGGATAGA---------TTTTTTAGC------AAGGTA
CGAAACGTATCGTCAAATATTCAATATGATTCT---------ACAAGA------TCTAGTTTCGTTC
AAGTAACGGATTCTAGCCAATTGAACGGATCTTCT---GATCAATTCATAGATCCTTTCGA
TTCCATTAGT------------AATGAGGATTCGGAATATCAC---------------------------------TATCAC
ACATTGATCAATCAAAGAGAGATTCAACAACTAAAAGAAAGATCGATTCTT---TTGGAT-
-----CCTTCCTTTATTCAAACGGAAGGAAGAGAGATAGAATCAGAC------CGATTCCCTAA
A------TACCTTTCTGGCTATTCC------TCAATGCCCCGGCTATTCACG---GAACGTGAGAA
GCGAATGAATAATCATCTGCTTCCGGAAGAAAGCGAAGAATTT------CTTGGGAATCCTA
CAAGAGCCATTCGTTCTTTTTTCTCTGACAGATGG------TCAGAACTTCAT---CTGGGTTC
GAATCCTACTGAGAGGTCCACTAGGGATCAG------------AAATTGTTGAAGAAAGAACAA
GATGTTTCT---TTTGTCCCTTCCAGGCGATCGGAA------------AATAAAGAAATAGTTAATA
TATTCAAGATAATTACGTATTTACAAAAT------ACCGTCTCAATTCATCCTATTTCATCAGA
TCTGGGATGTGATATGGTTCCG---AAGGATGAACTGGAT---ATGGACAGTTCCAATAAGA
TTTCATTCTTGAACAAAAATCCATTTTTTGATTTA------TTTCATCTATTCCATGAACGGA
AGAGGGGGGGATACACGTTA------CGCCAC------GAGTCAGAA------GAGAGATTTCAAG
AAATGGCAGATCTATTCACTCTATCAATAACCGAGCCG---GATCTGGTGTATCATAAGGG
ATTTGCCTTTTCTATTGATTCCTACGGATTGGATCAAAGACAA------TTCTTGAAGGAGG
TTTTCAACTTCAGGGATGAATCAAAAAAGAAATCTTTA---------TTGGTTCTACCTCCTAT
TTTTTAT------GAAGAAAATGAATCTTTTTATCGAAGGCTCAGAAAAAATTTGGGTCCGG---
ATCTCCTGCGGGAATTATTTGGAAGATCAAAAA------AGAGTGGTATTTGCTAGCAACAA
CATAATGGAGGCAGTCAATCAATATAGATTGATCCGAAATATGATTCAAATCCAATTCCA
ATATAGTCCCTATGGGTACATAAGAAATGTATTGAATCGATTCTTTTTAATGAAG---------A

GACCTGATCGCAACTTCGAATATGGAATTCAA---------AGGGATCTAATAGGAAATGATA
CTCTGAATCATAGAACTATAATGAAAGATACGATCAACCAACATTTATCGAATTTGAAA
AAG---AGTCAGAAGAAATGGTTCGATCCT------CTTATTTTTCTTTCTCAAACCGAGAGAT
CCATAAATCGGGATCCTAATGCATATAGATACAAATGGTCCAATGGGAGCAAGAATTTC
CAGGAGCATTTGGAACATTTCGTTTCTGAGCGGAAGAGCCGT---------TTTCAAGTAGTG
TTCGATCAATTATGTATTAATCAATATTCGATTGATTGGTCTGAGGTTATTGATAAAAAAG
ATTTGTCTAAGTCACTTCGTTTCTTTTTGTCTAAGTTACTTCGTTTCTTTTTGTCCAAG---
TTACTTCTTTTTTTGTCC---------------------AAGTTACTTCTCTTTTTGTCTAACTCACTTCC
T---TTTTTCTTTGTGAGTTTCGAGAATATCCCTATTCATAGGTCTGAG---ATCCACATCTAT-
--GAATTGAAAGGTCCGAACGATCAACTCTGCAATCAGTTGTTAGAATCAATAGGTCTTC
AAATCGTTCATTTGAAAAAGTTGAAACCCTTT------TTATTGGAT---------GATCATAATACT
TCT---CAAAAATCGAAATTCTTGATCAATGGAGGAACAATATCACCATTTTTGTTCAATA
AAATACCA---AAGTGG---ATGATTGACTCATTCCATACTAGA------AAGAATCGCAGGAAA
TCTTTTGATAACACGGATTCATATTTCTCAATCGTATCCCACGATCAAGACAATTGGCTG
AATCCCGTGAAACCATTTCAGAGAAGTTCATTGATATCTTCTTTTTCTAAAGCAAATCG
ACTTCGATTCTTGAATAATCCACATCACTTCTGTTTCTAT------TGTAACAAAAGATTCCC
TTTT---------TAT---GTGGAAAAGGCCCGTCTCAATAATTCTGATTTTACGTTTACGTATGG
A---CAATTCCTCACTATCTTGTTCATTCACAACAAA---ACATTTTCTTCGTGCGGTGGTA
AA------AAAAAACATGCTTTTTTGGAG---AGAGATACTATTTCACCTATCGAGTCACAGG
TATCTAACATATTCATATCTAACGATTTTCCACAAAGTGGTGACGAAAGG---TATAACTT
GTACAAATCT---------TTCCATTTTCCAATTCGATCCGATCCATTAGTTCGT------AGAGCTA
TTTACTCGATTGCAGACATTTCTGGAACACCTCTAATAGAGGGACAAAGAGTCAATTTT
GAAAGAACGTATTGTCAAACTCTTTCAGATATGAATCTATCCGATTCAGAAGAGAAGAG
CTTGCATCAGTATCTCAAT------TTCAATTCAAACATGGGTTTGATTCACACTCCATGTTC
TGAGAAATATTTACAG---------------AGGAAAAAA------------------------CGGAGTCTTTGCCT
AAAA------AAATGCGTTGACAAAGGGCAGATGGATAGAACCTTTCAACGAGATAGTGCT
TTTTCAACTCTCTCAAAATGGAATCTATTCCAAACATATATGCCATGGTTCTTTACTTCG
ACAGGGTACAAATATCTAAATTTGATATTTTTAGATACTTTTTCAGAC---CTATTGCGGAT
ACTAAGTAGCAGT------CAAAAATTTGTATCCATTTTTCATGATATTATGCATGGATTAGAT
ATATCATGGCGAATTCTTCAGAAAAAATTG------------------TGTCTTCCACAAAGGAAT---C
TGATAAGTGAGATTTCGAGTAAGTCTTTACATAAT------CTTCTTCTGTCCGAAGAAATGA
TTCATCGAAATAATGAGTCA------TCATTGATATCGACACATCTGAGATCGCCAAATGTTC
GTGAGGTC---CTCTATTCAATCCTTTTCCTTCTTCTTGTTGCTGGATAT---ATCGTTCGTAC
ACATCTTCTCTTTGTTTCCCGA------GCCTATAGTGAGTTACAGACAGAGTTCGAAAAGA
TCAAATCTTTGATGATTCCATCATACATGATTGAGTTGCGAAAACTTCTGGATAGGTATC
CTACATCTGAACTGAATTCTTTCTGGTTAAAGAATCTTTTTCTAGTTGCTCTGGAACAAT
TAGGAGATTGTCTAGAAGAAATACGGGGTTCT------GGCGGCAACATGCTATGGGGTGG
TGATCCC---------------GCTTATGGG---GTCAAATCAATACGTTCTAAGAAGAAAGATTTGA
AAATAAATTTCATCGATATCATCGATCTCATA---AGTATCATACCAAATCCCATCAATCGA
ATCACTTTTTCGAGAAATACGAGACATCTAAGTCATACAAGTAAAGAGATCTATTCATT
GATAAGAAAAAGAAAAAACGTGAGCGGTGATTGGATTGATGATAAAATAGAATCCTGG
GTCGCGAACAGTGATTCGATTGATGATAAAGAAAGAGAATTCTTGGTTCAGTTCTCC----
--ACCTTAAGGGCA------GAAAAAAGGATTGATCAAATTCTATTGAGTCTGACT---CATAG
TGATCATTTATCA---------------AAGAATGACTCTGGTTATCAAATGATTGAACAACCGGGA

ACAATTTACTTACGATACTTAGTTGACATTCAT------AAAAAGTATCTAATGAATTATGAG
TTCAATACATCCTGTTTAGCAGAAAGACGGATATTCCTTGCTCATTATCAGACAATCACT
TAT------TCACAAACTTCGTGTGGGGCTAATAGTTTTCATTTCCCGTCTCATGGA---------A
AACCCTTTTCGCTCCGCTTAGCCCTATCCCCCTCT---AGGAGTATTTTAGTGATAGGTTCT
ATAGGAACTGGACGATCCTATTTGGTCAAATACCTAGCGACAAACTCCTATGTTCCTTTC
ATTACAGTATTTCTGAACAAGTTCCTGGATAACAAGCCGAAAGGTTTTTTATTGAT------
---------GATATCGAT------GATGATAGTGACGATATTGATGCTAGTAAC------------------------------
-------------------------------------GATATTGATCGTGAACTTGATACGGAGCTGGAGCTTCTAA
CTATGATGAATGCGCTA---------ACTATGGATATGATGTTG---------GAAATAGACCGATTTT
ATATCACCCTTCAATTCGAATTAGCAAAAGCAATGTCTCCTTGCATAATATGGATTCCAA
ACATTCATGATCTTGATGTGAATGAGTCGAGTTACTTAGCCCTCGGTCTATTGGTGAACT
CTCTCTCC---------AGGGATTGTGAAAGATGTTCC---ACTAGAAATATTCTTGTTATTGCTT
CGACTCATATTCCCCAAAAAGTGGATCCCGCTCTAATAGCCCCGAATAAATTAAATACAT
GCATTAAAATACGAAGGCTTCTTATTCCACAACAACGAAAGCACTTTTTCACTCTTTCC
TATACTAGGGGATTTCACTTGGAAAAGAAAATGTTCCATACT------AATGGATTCGAGTC
TATAACCATGGGTTCCAGTGCACGAGATCTTGTAGCACTTACCAATGAGGCCCTATCAA
TTAGTATTACACAGAAGAAATCAATTATAGACACTAATACAATTAGATCTGCTCTTCATA
GACAAACTTGGGATTTGCGA---------TCCCAGGTAAGATCGGTTCAGGATCATGGGATCC
TTTTCTATCAGATAGGAAGGGCTGTTGCACAAAATGTACTTATAAGTAATTGCCCCATAG
ATCCTATATCTATCTATATGAAGAAGAAA------TCATGTAACGAAGGGGATTCTTATTTGT
ACAAATGGTACTTCGAGCTTGGAACGAGCATGAAGAAATTCACGATACTTCTTTATCTT
TTGAGTTGTTCTGCCGGATCGGTCGCTCAAGACCTTTGGTCTCTACCC------GTACCCGA
TGAAAAA---AATAGGATCACTTCTTATGGATTCGTTGAGAATGATTCG---GATCTAGTTCA
TGGCCTATTAGAAGTGCAAGGCGCTTTGGTGGGA------TCCTCACGGACAGAAAAAGAT
TGCAGTCAGTTTGATAATGAT------CGAGTGACATTGCTTTTTCGCTCCGAACCAAGGGA
T---CCCTTATATATGATGCAAGATGGATCTTGTTCTATCGTTGATCAGAGATTTCTCTATGA
AAAA------TACGAATCGGAGTTTGAAGAA---GGGGAAGGAGAAGGAGTCCTCGACCCG
CAA------------CAGATAGAGGAGGAT------------TTATTCAATCAC------ATAGTTTGGGCTCC
T------AGAATATGGCGCCCT---------CGGGGCTTTCTATTTGATTGTATCGAAAGGCCTAAT
GAATTGGGATTTCCC---TATTCGGCCGGGTCATTTCGGGGCAAG---------CGGATCATTTA
TGATGAAAAGTATGAGCTTCAAGAGAATGATTCGGAGTTCTTGCAGAGCGGAACCATG
CAGTACCAGAGACGAGATAGGTCTTCCAAAGAACAAGGCTTTTTTAGAATAAGCCAAT
TCATTTGGGACCCCGCAGATCCACTCTTTTTCCTATTCAAA---GATCAACCCTTTGTCTC
TGTGTTTTCACATCGAGAATTCTTTGCAGATGAAGAGATGTCAAAGGGGCTTCTTACTT
CCCAAACGGATCCTCCTACATCTATATATAAACGCTGGTTTATC------AAGAATACGCAAG
AAAAGCACTTCGAATTGTTGATTCAGCGCCAGAGATGGCTTAGA---------ACCAATAGTT
CATTATCT------AATGGATTTTTCCGTTCTAAT------ACTCGATCCGAGAGTTATCAGTATTT
ATCAAATCTGTTCATATCTAAC---GGAACGCTATTGGATCGAATGACAAAGACATTGTTG
AAAAAAAGATGGCTTTTCTCGGATGAAATGAAAATAGGATTC------ATG---------------------
-----ATGCCAAGATCGCGT---ATAAATGGAAATTTTATTGATAAAACCTTTACAATTGTAGC
CGATATCTTATTACGAGTCATTCCGACAACTTCCGGAGAAAAAGAGGCATTTACTTATTA
CAGAGATGGT------ATGTCGGCTCAATCTGAAGGAAATTATGCGGAAGCATTACAGAATT
ATTATGAAGCTATGCGACTAGAAATTGACCCCTATGATCGAAGTTATATACTCTATAATAT
AGGCCTTATCCATACAAGTAATGGGGAACATACCAAAGCTTTAGAATATTATTTTCGGGC

ATTAGAACGAAACCCCTTTTTACCACAAGCTTTTAATAATATGGCTGTGATCTGTCATTA
CCGT---GGAGAACAGGCCATTCAACAAGGAGATTCTGAAATGGCGGAGGCTTGGTTCG
CTCAAGCCGCTGAGTATTGGAAACAGGCTATAACGCTTACTCCTGGTAATTATATTGAA
GCACAGAATTGGTTGACGATCACGAGGCGCTTCGAA------ATGAGTTGGCGATCAGAAT
CTATATGGATAGAATTTATAACGGGGTCTCGAAAAACAAGTAATTTCTGCTGGGCCTTTA
TCCTATTTTTAGGTTCATTAGGATTCTTATTGGTTGGAACTTCCAGTTATCTTGGTAGAAA
TGTTATATCGTTATTTCCGTCCCAGCAAATCATTTTTTTTCCACAAGGGATTGTGATGTCT
TTCTATGGGATCGCAGGTCTCTTTATTAGTTGCTATTTGTGGTGCACTATTTTGTGGAATG
TGGGTAGTGGTTATGATCTTTTCGACCGAAAAGAAGGAATAGTGCGTATTTTTCGTTGG
GGATTTCCTGGAAAAAGTCGTCGCATCTTTTTACGATTCTTTATGAAAGATATTCAGTCC
ATCAGAATAGAAGTTAAAGAGGGTGTTTCTGCCCGGCGTGTCCTTTATATGGAAATTCG
AGGTCAAGGGGCTATTCCTTTAATTCGTACTGATGAGAAT---TTTACTACACGAGAAATT
GAGCAAAAAGCTGCTGAATTGGCTTACTTCTTGCGTGTACCAATTGAAGTATTT
>Oryza
???
???
???
???
???
???
???
???
???
???
???
???
???
???
???
???
????ATGGCAACCCTTCGAGTCGACGAAATTCATAAAATTCTCCGCGAACGTATTGAACA
ATATAATAGAAAAGTAGGGATTGAGAATATCGGTCGCGTAGTTCAAGTGGGGGATGGGA
TTGCTCGTATTATAGGTCTTGGTGAAATAATGTCAGGCGAATTAGTCGAATTTGCAGAA
GGGACTAGGGGTATTGCTCTGAATTTGGAATCCAAAAATGTTGGGATTGTATTAATGGG
CGATGGGTTGATGATACAAGAGGGCAGTTTTGTAAAAGCAACAGGAAGAATTGCTCAG
ATACCCGTGAGCGAGGCTTACTTGGGTCGTGTTATAAATGCTCTGGCTAAACCTATTGAT
GGGAGAGGCGAAATTGTAGCTTCGGAATCTCGCTTAATTGAATCTCCTGCTCCGGGTAT
AATTTCCAGGCGTTCTGTATATGAACCCCTTCAAACGGGGCTTATTGCTATCGATTCGAT
GATTCCTATTGGGCGCGGTCAGCGAGAGTTAATTATTGGGGACAGACAAACCGGCAAA
ACAGCAGTAGCTACAGATACAATTCTCAATCAAAAAGGGCAAGATGTAATATGTGTTTA
TGTAGCTATCGGTCAAAGAGCATCCTCCGTAGCTCAAGTAGTAACTACTTTCCATGAAG
AGGGGGCCATGGAATACACTATTGTAGTAGCTGAAATGGCGGATTCCCCTGCTACATTA
CAATACCTCGCTCCTTATACGGGAGCAGCCCTGGCTGAGTATTTTATGTACCGCGAACG
GCATACTTTAATAATTTATGATGATCTCTCCAAACAGGCACAAGCTTATCGCCAAATGTC

CCTTCTATTAAGAAGACCCCCCGGCCGCGAAGCTTACCCAGGGGATGTTTTTATTTGC
ATTCACGCCTTTTAGAAAGAGCCGCTAAATTAAATTCTCTTTTAGGGGAAGGAAGTATG
ACTGCTTTACCAATAGTTGAGACTCAATCTGGAGACGTTTCCGCCTATATTCCTACTAAT
GTAATCTCCATTACAGATGGACAAATATTCTTATCCGCAGATCTATTCAATGCCGGAATT
CGGCCTGCTATTAATGTGGGTATTTCCGTTTCCAGAGTAGGATCCGCGGCTCAAATTAA
AGCCATGAAACAAGTAGCTGGCAAATCAAAATTGGAATTAGCTCAATTCGCGGAGTTA
CAAGCCTTTGCACAATTCGCCTCTGCTCTCGATAAAACAAGTCAGAATCAATTGGCAA
GGGGCCGACGATTACGAGAATTGCTTAAACAATCCCAAGCAAATCCTCTTCCAGTGGA
AGAGCAGATAGCTACTATTTATATCGGAACAAGAGGATATCTTGATTCCTTAGAAATTGG
ACAGGTAAAGAAATTTCTGGATGAGTTACGTAAACACCTAAAAGATACTAAACCTCAA
TTCCAAGAAATTATATCTTCTAGCAAGACATTCACCGAGGAAGCGGAAATCCTTTTGAA
GGAAGCTATTCAGGAACAACTCGAACGGTTTTCCCTTCAGGAACAAACAATGAGAAC
CAATCCTACTACTTCTCGTCCCGGGGTTTCTACAATTGAAGAAAAAAGTACAGGGCGTA
TCGATCAAATTATTGGACCCGTGCTGGATGTCACTTTTCCCCGGGGCAAGTTACCTTATA
TTTATAATGCTTTGGTAGTCAAGAGTCGAGACACTGACGGTAAGCAAATTAATGTAACT
TGTGAGGTACAACAATTATTAGGAAATAATCGAGTTAGAGCTGTAGCTATGAGTGCTAC
AGATGGGTTGATGAGAGGAATGGAAGTGATTGACACGGGAGCTCCTCTCAGTGTTCCT
GTCGGTGGAGCTACTCTTGGACGAATTTTCAACGTTCTTGGGGAGCCTGTTGACAATTT
GGGTCCTGTAGATACTAGTGCAACATTCCCTATTCATAGATCCGCGCCCGCCTTTATCGA
GTTAGATACGAAATTATCCATCTTTGAAACTGGTATTAAGGTGGTCGATCTTTTAGCTCC
TTATCGGCGTGGAGGAAAAATCGGACTATTTGGGGGAGCTGGAGTAGGTAAAACAGTA
CTCATCATGGAATTAATCAACAATATTGCTAAAGCTCACGGGGGCGTATCCGTATTTGGC
GGAGTAGGGGAACGGACTCGTGAAGGAAATGATCTTTATATGGAAATGAAGGAATCTG
GAGTAATTAATGAAAAAAATCTTGAGGAATCAAAGGTAGCTCTAGTCTATGGCCAAATG
AATGAACCGCCAGGAGCTCGTATGAGAGTTGGTTTGACTGCCCTAACTATGGCAGAATA
TTTCCGAGATGTTAATAAGCAAGACGTGCTTCTATTCATCGATAATATCTTTCGTTTTGTT
CAAGCAGGATCGGAGGTATCTGCCTTATTAGGGAGAATGCCCTCTGCAGTGGGTTATCA
ACCTACTCTTAGTACAGAAATGGGTTCTTTGCAAGAAAGAATTACTTCTACTAAAAAGG
GATCTATAACTTCGATCCAAGCGGTTTATGTACCTGCGGACGATTTGACCGACCCTGCT
CCTGCTACAACATTTGCACATTTGGATGCTACTACCGTACTTTCCAGAGGATTAGCTTCC
AAAGGGATTTATCCTGCAGTAGATCCTTTAGATTCAACCTCAACTATGTTACAACCTCG
GATCGTTGGCAACGAACATTATGAAACTGCGCAAAGAGTTAAGCAAACTTTACAACGT
TACAAAGAACTTCAGGACATTATCGCAATTCTTGGGTTGGATGAATTATCGGAGGAGGA
TCGTTTAACTGTAGCAAGAGCACGAAAAATTGAGCGCTTCTTATCACAACCGTTTTTTG
TGGCAGAAGTTTTTACCGGTTCTCCGGGAAAGTATGTTGGTCTTGCAGAAACTATTAGG
GGATTTCAACTAATCCTTTCCGGAGAATTAGACGGCCTACCCGAACAGGCTTTTTATTT
GGTGGGTAACATCGATGAAGCTAGCACGAAAGCTATAAACTTAGAAGAGGAGAACAA
ATTGAAGAAAATGAAATTAAATCTTTATGTACTGACTCCTAAGCGAATTATTTGGGATTG
TGAAGTGAAAGAAATCATTTTATCTACTAATAGTGGCCAAATTGGCGTATTACCAAACC
ACGCCCCCATTAACACAGCTGTAGATATGGGTCCCTTGAGAATACGCCTCAACGATCAA
TGGTTAACGGCGGTTCTGTGGAGCGGTTTTGCCAGAATAGTTAATAATGAGATCATCAT
TTTAGGAAATGATGCGGAACTGGGTAGTGACATTGATCCGGAAGAAGCTCAACAGGCA
CTTGAAATAGCCGAAGCTAACGTGAGTAGAGCTGAGGGTACGAAAGAATTGGTTGAA
GCGAAGGTAGCTCTCAGACGAGCTAGGATACGAGTCGAGGCTGTTAATTGGATTCCCA

TGAAAAATGTAACCCATTCTTTCGTTTTTTTAGCTCACTGGCCATCCGCTGGGAGTTTC
GGGCTTAATACCGATATTTTAGCAACAAATCTAATAAATCTAACTGTAGTGGTTGGTGTA
TTGATTTATTTTGGAAAGGGAGTGTTAAAAGATTTATTAGATAATCGAAAACAGAGGAT
CTTGAGTACTATTCGAAATTCGGAAGAATTGCGTAGAGGAACCATTGAGCAGCTCGAA
AAAGCTCGAATTCGATTACAGAAAGTCGAACTAGAAGCGGATGAGTATCGAATGAATG
GATACTCTGAGATAGAACGAGAAAAAGCAAATTTGATTAATGCCACTTCTATTAGTTTG
GAACAATTAGAAAAGTCTAAAAACGAAACCCTTTATTTTGAAAAACAAAGGGCGATGA
ATCAGGTCCGACAACGGGTTTTCCAACAAGCCGTACAAGGAGCTCTAGGAACTCTGAA
TAGTTGTTTGAATACCGAGTTACATTTCCGTACGATTCGTGCTAATATTAGCATTCTCGG
GGCCATGGAA------------ATGAATCCACTAATTGCTGCTGCTTCCGTTATTGCTGCTGGATT
GGCCGTAGGTCTTGCTTCTATTGGGCCTGGAGTTGGTCAAGGTACTGCTGCAGGACAA
GCTGTAGAAGGTATTGCGAGACAGCCAGAAGCAGAAGGTAAAATACGCGGTACTTTAT
TGCTTAGTCTAGCTTTTATGGAAGCTTTAACAATTTATGGACTAGTTGTGGCACTGGCGC
TTTTATTTGCGAACCCTTTTGTTATGAATATTATACCGTGTTCCATTAAAACACTCAAGG
GGTTATACGATATATCGGGCGTAGAAGTAGGCCAACACTTCTATTGGCAAATAGGAGGT
TTCCAAATTCATGCCCAAGTACTTATCACTTCTTGGGTCGTAATTACTATCTTGCTAGGT
TCAGTTATCATAGCTGTTCGCAATCCACAAACCATCCCAACCGATGGTCAGAATTTCTT
CGAATATGTCCTTGAGTTTATTCGAGACTTAAGCAAAACTCAGATTGGAGAAGAATACG
GTCCCTGGGTTCCCTTTATTGGAACTATGTTCCTTTTTATTTTTGTTTCGAACTGGTCGG
GTGCTCTTTTACCTTGGAAAATTATACAGTTACCCCATGGAGAATTAGCAGCGCCCACG
AATGATATAAATACTACTGTTGCTTTAGCTTTACTCACGTCAGCGGCATATTTTTATGCGG
GTCTTAGCAAAAAAGGATTGAGTTATTTCGAGAAATATATTAAACCAACTCCAATCCTT
TTACCAATTAACATATTAGAAGATTTCACAAAACCATTATCGCTTAGTTTTCGACTTTTC
GGGAATATATTGGCGGATGAATTAGTCGTTGTTGTTCTTGTTTCTTTAGTCCCCTTAGTA
GTTCCTATACCGGTCATGTTTCTTGGATTATTTACAAGCGGTATTCAAGCTCTTATTTTTG
CAACGTTAGCCGCAGCCTATATAGGTGAATCCATGGAGGGTCATCATATGCTATTTGCAA
CTTTAGAACATATACTAACTCATATTTCTTTCTCAACAATTTCAATTGTGATTACGATTCA
TTTAATAACCTTATTAGTTCGTGAACTTGGGGGATTGCGTGATTCGTCAGAAAAAGGAA
TGATAGCCACTTTTTTCTGTATAACAGGATTCTTAGTTTCTCGTTGGGCTTCTTCGGGAC
ATTTTCCATTAAGTAATTTATATGAGTCATTGATCTTCCTTTCATGGGCTCTGTATATTCTT
CATATGATTCCTAAGATACAGAACTCTAAAAATGATTTAAGCACAATAACTACGCCGAG
TACTATTTTAACGCAAGGCTTTGCCACGTCGGGTCTTTTAACTGAAATGCATCAATCCA
CAATACTAGTACCTGCTCTACAATCTCAGTGGTTAATGATGCATGTCAGTATGATGTTAC
TAAGCTATGCGACTCTTTTGTGCGGATCCTTATTATCCGCCGCTCTTCTAATGATTAGATT
TCGAAAGAATTTAGATTTCTTTTCGAAAAAGAAGGAGATTGAATATTTCTATGCAAAAA
GAAGTGCTTTAAAAAGCACCTTTTTTCCTTTATTTCCAAATTATTACAAATATCAATTAAT
TGAGCGTTTGGATTCTTGGAGTTATCGTGTCATTAGTCTAGGGTTTACCCTTTTAACCAT
AGGTATTCTTTGTGGAGCAGTATGGGCTAATGAGGCGTGGGGATCCTACTGGAATTGGG
ATCCTAAAGAAACTTGGGCATTTATTACTTGGACCATATTTGCAATTTATTTACATAGTAG
AACAAATCCAAATTGGAAGGGTACGAAGTCAGCATTTGTAGCTTCCATAGGATTTCTTA
TAATTTGGATCTGTTATTTTGGTATCAATCTATTAGGAATAGGTTTACATAGTTATGGTTCA
TTTACATTACCCATGAAAAAAAAGAAAGCATTGCCTTCTTTCCTATATCTTGTATTTATC
GTACTTTTGCCTTGGGGAGTCTCTTTCTCTTTTAACAAATGTCTGGAACTTTGGATTAAG
AATTGGTGGAATACCAGGCAATCCCAAACTCTCTTAACTGCTATTCAAGAGAAAAGAG

TTCTAGAAAGATTCATGGAATTAGAAGACCTTTTTATCTTGGACGAAATGATAAAAGAG
AAACCGAATACACATGTACAAAACCCCCCTATAGGAATACGCAAGGAAATAATACAATT
GGCCAAAATAGATAATGAGGGTCATCTCCATATCATTTTGCATTTCTCGACAAATATAAT
CTGTTTGGCTATTCTAAGTGGTTCTTTTTTTCTGGGTAAAGAGGAACTTGTTATTTTGAA
TTCTTGGGTTCAGGAATTCTTCTATAACTTAAATGACTCAGTAAAAGCTTTTTTTATTCT
TTTAGTTACTGATTTTTTTGTTGGATTTCACTCCACCCGCGGTTGGGAACTACTAATTCG
TTGGGTCTATAACGATCTTGGATGGGTTCCTAACGAGCTAATTTTCACTATTTTTGTTTGT
AGTTTTCCTGTGATTCTAGATACATGTTTGAAATTTTGGGTCTTTTTTTGTTTAAACCGC
CTATCTCCTTCGCTTGTAGTCATTTATCATTCAATTAGTGAAATGCCCATTGGTGTTCCAA
AAGTACCTTACCGGATTCCCGGAGATGAAGAAGCGACTTGGGTTGACTTATACAATGTT
ATGTATCGAGAAAGGACACTTTTTTTAGGTCAAGAGATTCGTTGCGAGGTCACGAATCA
TATTACAGGTCTCATGGTATATCTCAGTATAGAAGATGGAAGTAGCGATATTTTTTTGTTT
ATAAACTCCCCCGGCGGTTGGTTAATCTCAGGAATGGCGATTTTTGATACGATGCAAAC
GGTAACACCAAATATATATACAACATGCCTAGGAATAGCCGCGTCCATGCCGTCCTTCAT
TCTGCTTGGAGGAGAACCCACCAAGCGTATAGCATTCCCCCACGCGAGGATTATGCTTC
ACCAACCTGCTAGTGCTTATTATCGGGCAAGAACACCAGAATTTTTACTAGAAGTGGAA
GAGTTACACAAAGTTCGCGAAATGATCACAAGGGTTTATGCACTAAGAACAGGCAAGC
CTTTTTGGGTTGTATCCGAAGACATGGAAAGGGATGTTTTTATGTCAGCAGACGAAGCC
AAAGCTTATGGACTTGTCGATATTGTAGGGGATGAACCGAGAGAAGCAAAAATTACTT
TCGAGGGTTTAGTTATGGAAGCCCTACCCAACGGAATGTTCCGCGTTCGCCTAGAGAAT
GACACCATCATCCTGGGCTATATTTCAGGAAAGATCCGGTCTAGTTCTATACGAATACTG
ATGGGGGATAGGGTCAAAATTGAAGTAAGTCGTTATGATTCAAGCAAGGGGCGTATAAT
TTATAGACTTCCCCATAAGGATTCGAAGGATATGGAAAAATTCGAGGGTTATTCAGAAA
AACTTAAATTTCCTAGACAATATTTCGTCTACCCACTACTCTTTCAGGAGTACATTTATG
TGTTTGCCCATGATTATGGATTAATTGGTTCTAATAACAAATTTAGTTCACTACTTGTGA
AACGGTTAATGATTCGAATGTATCAGCAGAATTTTTGGATTAATTTGGTTAATCATCCTA
ACCAAGATCGATTATTGGATTCTGAGTTTTATTCTCAGATTCTATCTGAGGGGTTTGCGA
TCGTTGTAGAAATCCCATTCTCGCTACGAGAACAGTCTTGTCCGATACCAAAGTTTCAG
AATTTACGATCTATTCATTCAATATTTCCTTTTTTAGAAGACAAATTTTTGCACTTACATT
ATCTAGCACATATAGAAATACCCTATCCTATCCATTTGGATATCTTGCTTCAACTCCTTCA
ATACCGGATCCAAGATGTTCCATCCTTGCATTTATTGCGATTCTTTCTCAACTACTACTC
GAATTGGAATAGTTTTATTACTAAATCCATTTTTATTTTGAAAAAAAAAAGACTATTTCG
GTTCCTATATAACTCTTATGTATCAGAATATGAATTTTTCTTGTTGTTTCTTCGTAAACAA
TCTTCTTGCTTGCGATTAACTTCTTCCGGAACCTTTCTGGAACGAATCATCTTTTCTAGG
AAGATGGAACAT------TTAATGTACCCTGCTCGGAAAACCATATGGTTCGTTATGGATCCC
CTTATGCATTATGTTCGATATCAAGGAAAGGCAATTCTTGCATCAAAAGGAACTCTTCTT
TTGAAGAAGAAATGGAAATGTTACCTTGTCCGTTTGTGGCAATATTCTTTCTCTTTTTGG
ACTCAACCGCAAAGGATCCATCTAAACCAATTAGAAAACTCTTGCTTCGATTTTCTGGG
GTACTTTTCAAGTGTACCAATAAATTCTTTGTTAGTAAGGAATCAAATGCTGGAGAATT
CATTTCTAATAGATACTCAAATG------AAAAAATTCGATACCAAAGTCCCTGTTACTCCTC
TCATTGGATCTTTAGCAAAAGCCCAATTTTGTACTGGATCGGGGCATCCTATTAGTAAAC
CAATTTGGACCGATTTATCGGATTGGGATATTCTTGATCGGTTTGGTCGGATATGTAGAA
ATCTTTTTCATTATCACAGTGGATCTTCAAAAAAGAAGACTTTGTATCGACTAAAGTATA
TACTTCGACTTTCATGCGCTAGAACTTTAGCTCGTAAACATAAAAGCACGGTACGAGCT

TTTATGCAATGGTTGGGTTCGGTATTTTTAGAAGAATTTTTTACGGAAGAAGAGCAAGT
TTTTTCTTTGATGTTCGCCAAACGTATTTGGTATTTGGATATTCTCCGTATCAATGATCTG
GTGAATCCTCTTATGATAATAGACAGGGTA---CAGGTAGAA---GCTATCAATTCTTTTTCG
AACTTGGAA---TTATTAAAAGAAGTCTATGGACTGATATGGATTCTA---CCCATTTTGACC
CTCTTACTGGGAATCACAATAGAAGTACTCGTAATTGTGTGGTTAGAAAGAGAAATATC
CGCATCGATACAACAACGTATTGGTCCTGAATATGCTGGCCCCCTGGGACTGCTTCAAG
CTATAGCAGATGGAACTAAGCTACTTTTTAAAGAGGATATCTTGCCATCCCGAGGGGAT
ATTCCCTTATTTAGCATTGGACCGTCTATAGCAGTCATATCAATTTTATTAAGTTTTTTAG
TTATTCCTTTGGGATATCGCTTTGTTTTAGCGGATCTTAGTATTGGTGTTTTTTTATGGATT
GCCATTTCAAGTATTGCTCCTATTGGTCTTCTTATGGCAGGATATAGCTCAAATAATAAAT
ATTCTTTTTCAGGTGGTCTACGAGCTGCCGCTCAATCTATTAGTTATGAAATCCCATTAA
CTTTTTGTGTACTAGCAATATCTCTACTATCCAACAGTTCAAGTACAGTTGATATAGTTG
AAGCACAGTCAAAATATGGTTTTTTTGGATGGAATCTTTGGCGTCAGCCTATAGGTTTT
CTGGTTTTTCTAATTTCTTCTTTGGCGGAATGTGAAAGATTACCCTTTGATTTACCAGAA
GCGGAGGAAGAATTAGTAGCAGGTTATCAAACCGAATATTCTGGTATCAAATATGGTTT
ATTTTATCTTGTTTCTTACCTAAATTTATTAGTTTCCTCTTTATTTGTAACAGTTCTCTACT
TAGGCGGGTGGAATTTGTCTATTCCCTATATATCCTTTTTTGGATTTTTCCAAATGAATAA
AATGGTTGGAATTTTAGAAATGACAATGAGTATCTTTATTACATTAACTAAAGCTTATTTA
TTTCTCTTCATTTCTATCACAATAAGATGGACTTTACCCAGGATGAGAATGGATCAGTTA
TTAAATCTTGGATGGAAATTTCTTTTACCTATTTCCCTGGGCAATCTCTTATTAACAACTT
CTTCCCAACTTGTTTCACTAATGATCTGGCATGTACAGAATGAAAACTTCATTCTCGATT
CTACGAGAATTTTTATGAAAGCGTTTCATTTGCTTCTCTTCCAGGGAAGTTTCATTTTCC
CAGAATGTATCCTAATTTTTGGCCTAATTCTTCTTCTGATGATCGATTTAACCTCTGATCA
AAAAGATAGACCTTGGTTCTATTTCATCTCTTCAACAAGTTTAGTAATAAGCATAACGG
CCCTATTGTTCCGATGGAGAGAAGAACCTATAATTAGCTTTTCGGGAAATTTCCAAACG
AACAATTTCAACGAAATCTTTCAATTTCTTATTTTATTATGTTCAACTTTATGTATTCCTCT
ATCCGTAGAGTACATTGAATGTACAGAAATGGCTATAACAGAGTTTCTGTTATTCGTATT
AACAGCTACTCTAGGGGGAATGTTTTTATGTGGTGCTAACGATTTAATAACTATCTTTGT
AGCTCCAGAATGTTTCAGTTTATGTTCCTACCTATTGTCTGGATATACCAAGAGAGATCT
ACGGTCTAATGAGGCTACTATGAAATATTTACTCATGGGTGGGGCAAGCTCTTCTATTCT
GGTTCATGGTTTCTCTTGGCTATATGGTTCATCTGGGGGGGAGATCGAGCTTCAAGAAA
TTGTGAACGGTCTTATCAATACACAAATGTATAACTCCCCAGGAATTTCAATTGCGCTTA
TATCCATCACTGTAGGACTTGGGTTCAAGCTTTCCCCAGCCCCTTTTCATCAATGGACT
CCTGACGTCTACGAAGGATCCCCCACTCCAGTCGTTGCTTTTCTTTCTGTTACTTCGAA
AGTTGCTGCTTCAGCTTCAGCCACGCGAATTCTCGATATTCCTTTTTATTTCTCATCAAA
CGAATGGCATCTTCTTCTGGAAATCCTAGCTATTCTTAGCATGATATTGGGGAATCTCCT
TGCTATTACTCAAACAAGCATGAAACGTATGCTTGCATATTCGTCCATAGGGCAAATCG
GATATGTAATTATTGGAATAATTGTTGGAGACTCAAATGATGGATATGCAAGCATGATAA
CTTATATGCTTTTCTATATCTCCATGAATCTAGGAACTTTTGCTTGCATTGTATTATTTGGT
CTACGTACCGGAACTGATAACATTCGAGATTATGCAGGATTATACACGAAAGATCCTTTT
TTGGCTCTCTCTTTAGCCCTATGTCTCTTATCCCTAGGAGGCCTTCCTCCACTAGCAGGT
TTCTTCGGAAAACTCTATCTATTCTGGTGTGGATGGCAAGCAGGCCTATATTTCTTGGTT
TCAATAGGACTCCTTACGAGCGTTCTTTCTATCTACTATTATCTAAAAATAGTCAAGTTAT
TAATGACTGGACGAAACCAAGAAATAACCCCTTATGTGCGAAATTATAGAAGATCCCCT

TTAAGATCAAACAATTCCATCGAATTGAGTATGACTGTATGTGTGATAGCATCTACTATA
CCAGGAATATCAATGAACCCCATTCTTGCAATTGCTCAGGATACCCTCTTTATGTTTCTG
CTTCACGAATATGATATTTTTTGGGCATTTCTAATAATAGCAAGCCTTATTCCTATTTTAG
CATTTTGGATTTCAGCACTTTTAGCTCCGGTTCGTGAAGGACCAGAGAAGCTTTCTAGT
TATGAATCGGGTATAGAACCCATGGGGGGGGCTTGGTTACAATTCCGAATACGCTATTA
CATGTTTGCGCTAGTTTTTGTTGTTTTTGATGTGGAAACGGTCTTTCTCTACCCTTGGGC
AATGAGTTTCGACGTATTGGGTATATCCGTTTTTATCGAAGCTTTCATTTTCGTGCTTATC
CTAGTTGTTGGTTTAGTTTATGCATGGCGAAAAGGAGCCTTGGAATGGTCTATGAGTTC
TTTTCCTTGGTTAACAATACTTGTTGTTTTGCCGATATTTGCGGGTTCATTAATTTTCTTT
TTACCTCATAGGGGAAACAAAATCGTTAGGTGGTATACTATGTCTATTTGTTTATTAGAA
TTCCTTCTAATGACTTATGCATTCTGTTATCATTTCCAATTGGAGGATCCCTTAATCCAAT
TAAAAGAGGATTCTAAATGGATAGATGTCTTCAATTTCCACTGGAGATTGGGAATCGAT
GGACTTTCATTAGGATCTATTTTATTGACAGGATTTATGACTACTTTAGCTACTTTAGCAG
CTTGGCCGGTTACCCGGAATTCCCGATTATTCTATTTCCTGATGCTAGCAATGTATAGCG
GTCAAATAGGATTATTTTCTTCGCGAGACCTTTTACTTTTTTTTATCATGTGGGAGTTAG
AATTAATTCCTGTTTACTTACTTTTATCCATGTGGGGGGGGAAGAGGCGTCTCTATTCAG
CTACAAAGTTTATTTTGTATACTGCAGGTGGTTCCATTTTTTTCTTAATCGGAGTTCTAG
GTATGGGCTTATACGGTTCCAACGAACCAAGATTAGATTTGGAAAGATTAATTAATCAAT
CATACCCTGCAACATTGGAAATACTATTTTATTTTGGCTTCCTTATTGCTTATGCTGTCAA
ATTGCCGATTATACCCCTACATACGTGGTTACCAGATACCCATGGGGAAGCGCATTACAG
TACATGTATGCTTTTAGCGGGAATCCTATTAAAGATGGGAGCATACGGATTGATTCGGAT
CAATATGGAATTGTTACCTCATGCTCATTATCTATTTTCCCCTTGGTTAGTAATAATAGGA
GCGATGCAAATAATCTATGCAGCTTCAACTTCTCTTGGCCAACGCAATTTCAAAAAAAG
AATAGCCTACTCCTCCGTCTCTCACATGGGTTTCATTATTATAGGAATTGGTTCCATAAC
CAACATTGGACTCAATGGAGCTATTTTACAAATATTATCCCATGGATTTATTGGGGCTAC
ACTTTTTTTCTTAGCGGGAACGGCTTGTGATAGAATGCGCCTTGTTTATCTCGAAGAAC
TGGGAGGGGCTTCTATCCCAATGCCAAAAATTTTTACCATGTTTAGTAGCTTTTCAATGG
CTTCTCTTGCCTTACCAGGAATGAGTGGTTTTGTTGCGGAATTAGTAGTATTTTTTGGAC
TAATTACTAGTCCAAAATTTCTGTTAATGCCAAAAATGCTAATTACTTTTGTAATGGCAA
TTGGAATGATATTAACTCCTATTTATTTATTATCTATGTTACGACAGATGTTCTATGGATAC
AAGCTATTTCATGTTCCAAACGAAAATTTTGAGGATTCCGGCCCGCGAGAACTCTTTCT
TTTAATCTGTATCTTTTTACCAGTAATAGGAATCGGTATTTATCCAGATTTTGTTCTCTCG
CTATCCGTTGACAGGGTAGAGGCTCTGTTATCCAATTATTATCCTAAAATGATGTTTGAG
CATGTACTTTTTTTGAGTGTCTATTTATTTTCGATTGGTATCTATGGATTGATCACAAGCC
GAAACATGGTTAGAGCTCTAATATGCCTTGAACTTATACTGAATTCAATTAATCTAAATC
TCGTAACATTTTCTGATCTATTTGATAGCCGCCAATTAAAAGGAGACATTTTCGCAATTT
TTGTTATAGCCCTTGCGGCTGCTGAAGCAGCTATTGGATTATCCATTCTTTCTTCCATCCA
TCGTAATAGGAAATCAACTCGTATCAATCAATCTAATTTTTTGAATAATATGGAACATACA
TATCAATATGCATGGGTAATCCCTCTTCTCCCACTTCCAGTTATTATGTCAATGGGGTTTG
GACTTTTTCTTGTTCCAACAGCAACAAAAAAATCTTCGTCGCATATGGGCTTTTCCTAGT
GTTTTACTTTTAAGTATAGCTATGGTATTCTCAGTTCACCTGTCTATTCAACAAATAAATG
GAAGTTCTATCTATCAATATCTATGGTCTTGGACCGTCAATAATGATTTTTCCTTAGAATT
TGGATACTTAATCGACCCGCTTACTTCTATTATGTTAATACTAATTACTACTGTAGGAATC
CTGGTTCTTATTTATAGTGATGATTATATGTCTCACGATGAGGGATATTTGAGATTTTTTG

TTTATATAAGTTTTTTCAATACTTCCATGTTGGGATTGGTTACTAGTTCCAATTTGATACA
AATTTATTTTTTTTGGGAGCTTGTGGGAATGTGTTCCTATTTATTGATAGGCTTTTGGTTT
ACACGGCCAATTGCAGCGAGTGCTTGTCAAAAAGCTTTTGTAACTAATCGTGTAGGGG
ATTTTGGTCTGTTATTAGGAATTCTAGGTTTTTTTTGGATAACAGGTAGTTTAGAGTTTC
GGGATTTGTTTAAAATAGCTAATAACAATGAGATTAACTCCTTGCTTACTATTTTGTGTG
CTTTTTTATTATTCCTTGGTGCGGTTGCGAAATCGGCACAATTCCCTCTTCACGTATGGT
TACCCGATGCTATGGAAGGACCCACCCCCATTTCAGCTCTTATACACGCAACAACTATG
GTTGCTGCGGGGATTTTTCTTATAGCTCGACTTCTTCCTCTTTTCATATCCCTACCTTTGA
TAATGAGTTTCATTTCTTTAATAGGTACACTAACACTTTTCTTAGGAGCCACTTTAGCTC
TTGCTCAGAGAGATATTAAAAGAAGCTTAGCCTATTCTACAATGTCTCAATTGGGTTATA
TGATGTTAGCTCTAGGTATAGGTTCTTATCAAGCTGCTTTATTCCATTTGATCACTCATGC
TTATTCGAAAGCTTTATTATTCTTGGGATCCGGATCTGTTATTCATTCAATGGAACCTCTT
GTTGGATATTCACCAGATAAAAGTCAGAATATGGTTCTTATGGGTGGTTTAAGAAAATA
CATTCCAATTACAAGAACTTGTTTTTTATGGGGTACCCTTTCTCTTTGTGGTATTCCACC
TCTTGCTTGCTTCTGGTCCAAAGATGAAATCCTTAGTAATAGTTGGTTATATTCACCCTT
TTTTGGAATAATAGCTTCTTTTACTGCAGGATTAACTGCGTTTTATATGTTTCGGATATAT
TTACTTACTTTTGATGGGTATTTGCGTGTTCATTTTCAAAATTACAGTAGTACTAAAGAG
GATTCGTTGTATTCAATATCGTTATGGGGAAAAAGGATATCTAAAGGAGTCAATAGA---G
ATTTCGTTTTATCAACAGCGAAGTTTTTTTCAACTAAAAAC------ACTTTTGTCTATCCTC
ATGAACCGGGAAATACTATGCTATTTCCTCTTCTTATATTACTGCTTTGTACTTTGTTTATT
GGATCTATAGGAATCCATTTTGAATTAACCATATTATCAAAGTGGCTAACTCCCTCAATC
AACTTTTTCCAAGAAAGTTCTAATTCGTCCATAAATTCATATGAATTTATCACTAATGCA
ATTTCTTCTGTAAGTCTAGCTATTTTTGGTCTATTCATAGGCATATATGTTTTATGGATCTG
CTTACTCTTTTTTTCAGAATTTGATTTAATAAATTCCTTTGTATATTTTTTCCATCAACTAA
AAAAAAAGATATATAGTTGGTCATATAATCGCGGTTATATAGATATTTTCTATACTAGGAC
CTTTACCTTGGGTATAAGAGGATTAACCGAACTAACGCAGTTTTTCGACAAGGGTGTCA
TTGATGGAATTACCAATGGAGTAGGTCTTGCTAGTTTTTGTATAGGAGAAGAAATTAAAT
AT---GTAGGGGGAGGTCGAATTTCGTCTTATTTATTCTTTTTTTTATGTTATGTATCTGTGT
TCTTATTCTTTTTTCTTATGGATTTACCTGGGCCAATACATGAAATTCTTGTGCTATTTGG
GGGATTTGTTCTTCTACTAGGGGGTCTAGGGGTGGTATTACTTACCAACCCAACTTTTTC
TGCTTTTTCGCTAGGATTAGTTCTTGTTTGTATATCCTTATTCTATATTTTATTGAATTCCT
ACTTTGTAGCTGTGGCACAACTTCTTATTTATGTGGGAGCTATAAATGTCTTGATCATATT
TGCCGTAATGTTCGTAAATGGCTCAGAATGGTCTAAAGATAAGAATTTTTGGACTATTG
GAGATGGGTTCACTTCACTCGTTTGTATAACTATTCCTTTTTCACTAATGACTACTATCCC
AGATACGTCATGGTATGGAATTCTTTGGACTACAAGATCAAACCAAATAGTAGAACAGG
GTCTCATAAATAACGTTCAACAAATTGGGATTCATTTAGCAACCGATTTTTATCTTCCAT
TTGAACTCATTTCCATAATTCTTCTAGTTTCTTTAATAGGTGCAATTACTATGGCTCGGCA
AATGAGTCTACCGCTTACAAGAAAAGATCTCATGATAGTCAATATGGGCCCTCAGCACC
CATCAATGCATGGTGTTCTTCGACTGATCGTTACTCTCGATGGTGAAGATGTTATTGATT
GTGAACCCATATTAGGCTATTTACACAGAGGAATGGAAAAAATCGCGGAAAACCGAAC
TATTATACAATACTTACCTTATGTAACACGTTGGGATTATTTAGCTACTATGTTTACAGAA
GCAATAACGGTAAATGCACCAGAATTCTTGGAGAATATTCAAATACCCCAAAGAGCCA
GCTATATTAGGGTAATTATGTTAGAATTGAGCCGTATAGCTTCTCACTTGTTATGGCTTGG
ACCTTTTATGGCAGATCTCGGTGCGCAGACTCCTTTTTTTTATATTTTTAGAGAGAGAGA

ATTAATATATGATCTATTTGAAGCTGCTACAGGTATGCGAATGATGCATAATTACTTTCGC
ATCGGAGGAGTTGCTGCCGATCTGCCTTATGGATGGATCGATAAATGTTTAGATTTCTGT
GATTATTTTTTACGAGGAGTTATTGAATATCAACAACTTATTACACAGAATCCCATTTTTT
TGGAACGAGTTGAGGGAGTTGGTTTTATTAGCGGAGAAGAAGCTGTAAATTGGGGCTT
ATCGGGCCCCATGTTACGAGCTTCTGGAATACAATGGGATCTTCGTAAAGTTGATCTTT
ACGAGTCTTACAATCAATTCGATTGGAAAGTCCAATGGCAAAAAGAAGGGGATTCATT
AGCACGCTATTTAGTACGAATCGGTGAAATGAGGGAATCCATCAAAATTATTCAACAGG
CTGTAGAAAAAATTCCTGGGGGCCCTTATGAGAATTTAGAAGTCCGACGC---AAGAAA
GCAAAGAATTCCGAATGGAATGATTTTGAATATCGATTTCTTGGTAAAAAACCTTCACC
CAATTTTGAATTGTCAAAACAAGAGCTTTATGCAAGAGTGGAAGCCCCAAAAGGTGAA
TTAGGAATTTATCTGGTAGGAGATGATAGTCTTTTCCCCTGGAGATGGAAAATTCGTCC
ACCCGGTTTTATTAATTTGCAAATTCTTCCTCAGCTAGTCAAAAAAATGAAATTGGCTG
ATATCATGACGATATTAGGTAGTATAGATATCATTATGGGAGAAGTTGATCGTATGTTCCC
TATGGTAACTGGGTTCATG------GGTCAACAAACAATACGCGCAGCAAGGTACATTGGT
CAAAGTTTCATAATTACCTTATCCCACACAAATCGTTTACCTATAACGATTCACTACCCC
TATGAAAAATCAATTACATCGGAGCGTTTCCGGGGGCGAATCCACTTTGAATTTGATAA
ATGTATTGCTTGTGAAGTATGTGTTCGCGTATGCCCTATAGATCTACCTCTTGTGGATTG
GAGATTTGAAAAGGATATTAAAAGGAAACAATTGCTTAATTATAGTATTGATTTCGGAG
TTTGTATATTTTGTGGTAATTGTGTTGAGTACTGTCCGACAAACTGTTTATCAATGACTG
AAGAATATGAACTTTCTACTTATGATCGTCATGAATTGAATTACAATCAAATTGCTTTGA
GTCGGTTACCAATCTCCATAATGGGAGATTACACAATTCAAACAATTAGGAATTCAAAA
ATAGACGAAGAAAAATCTTGGAATTCAAGAACGATTACTGATTACCAGCAGGGTTGGT
TATCTAATTGGCTAGTCAAACATGAGGTGGTTCATAGATCTTTGGGCTTCGATCATCGAG
GAATAGAGACTTTGCAAATAAAAGCAGAGGATTGGGATTCCATTGCTGTCATTTTATAT
GTATATGGTTACAATTATTTACGTTCCCAATGTGCTTATGACGTTGCACCCGGTGGATCT
TTAGCTAGCGTGTATCATCTTACGAGAATACAGTATGGTATAGATAACCCAGAAGAAGTA
TGCATAAAAGTCTTTGCCCAAAAGGATAATCCTAGAATCCCATCTGTCTTCTGGATTTG
GAGAAGTTCCGATTTTCAAGAACGCGAATCTTTTGATATGGTGGGAATCTCTTATGATAA
TCATCCACGCCTTAAACGTATCCTAATGCCTGAAAGTTGGATAGGCTGGCCCTTACGTA
AGGACTATATAACCCCCAATTTCTATGAAATACAAGATGCTCAT---------------------------------
----
-------------------------------ATGAGTTTGATTGAGTTTCCCCTACTTGACCAAAGAAGTTCCAA
TTCCGTTATTTCAACTACACTAAAAGATCTTTCGAATTGGTCAAGACTCTCTAGTTTATG
GCCCCTTCTATATGGTACCAGTTGTTGTTTCATTGAATTTGCTTCATTAATAGGCTCACGA
TTCGACTTTGATCGTTATGGATTGGTACCAAGATCAAGTCCTAGGCAAGCGGACCTAAT
TTTAACAGCCGGTACAGTAACAATGAAAATGGCTCCCTCTTTAGTGAGATTATATGAGC
AAATGCCTGAACCAAAATACGTCATTGCTATGGGAGCTTGTACTATTACTGGTGGAATG
TTCAGTACGGATTCCTATAGTACTGTTCGGGGAGTTGATAAGTTAATTCCTGTGGACGTC
TACTTGCCGGGTTGCCCACCTAAACCAGAGGCTGTTATAGATGCCCTAACAAAACTTCG
TAAGAAGATATCGCGAGAAATAGTTGAGGATCGAACTCTATCTCAA---AAAAATCGATG
TTTTACTACCAGCCACAAGCTTTATGTTCGGCGCAGTACTAATACTGGAACTTATGAGC
AAGAATTGCTCTATCAATCACCATCTACTTTAGACATATCTACT------TTTTTCAAATCCAA
AAGTCCAGTATCTTCCTACAAATTAGTGAATATGGAAAATAGAAATACTTTTTCTTGGGT
AAAGGAACAGATGACTCGATCGATTTCTGTATCGATCATGATATACGTAATAACTCGGAC

ATCTATTTCAAATGCATATCCCATTTTTGCGCAGCAAGGTTATGAAAACCCACGAGAAG
CAACTGGACGAATTGTATGTGCCAATTGCCATTTAGCGAATAAGCCTGTGGATATTGAA
GTTCCCCAAGCAGTGCTTCCCGATACTGTATTTGAAGCAGTTCTTCGAATTCCTTATGAT
ATGCAACTGAAACAAGTTCTTGCTAATGGGAAAAAGGGAGGGTTGAATGTGGGTGCT
GTTCTTATTTTGCCCGAGGGATTCGAATTAGCGCCGCCCGACCGTATTTCTCCTGAGTTG
AAAGAAAAGATAGGAAATCTCTCTTTTCAGAGTTATCGTCCCAATAAAAAAAATATTCT
TGTGATAGGCCCTGTTCCCGGTAAGAAATATAGTGAAATCGTCTTTCCCATTCTTTCCCC
CGACCCTGCTATGAAGAAAGACGTTCATTTCTTAAAATATCCCATATATGTGGGGGGAA
ACCGAGGAAGGGGACAGATCTATCCTGATGGTAGCAAGAGTAACAATACGGTCTATAA
TGCAACGTCAACAGGTGTAGTAAGAAAAATACTGCGTAAAGAAAAGGGGGGATATGA
AATATCCATAGTCGATGCATCGGATGGACGCCAAGTGATTGATCTTATACCTCCCGGTCC
AGAACTTCTTGTTTCAGAGGGGGAATCGATCAAGCTTGATCAACCATTAACAAGTAATC
CTAATGTGGGAGGGTTTGGTCAGGGGGATGCAGAAATAGTGCTTCAGGATCCATTACG
CGTTCAAGGCCTTTTGTTCTTCTTCGCATCCGTTATTTTGGCACAAGTTTTTTTGGTTCT
CAAAAAGAAACAGTTTGAAAAGGTTCAATTGTACGAAATGAATTTCATGAGTAAAGTA
TATGATTGGTTTGAGGAACGTCTTGAGATTCAGGCAATTGCAGATGATATAACTAGTAA
ATATGTTCCTCCTCATGTCAACATATTTTATTGTTTAGGGGGAATTACACTTACTTGTTTT
CTAGTACAAGTTGCTACCGGTTTTGCTATGACTTTTTACTATCGCCCAACCGTTACAGAG
GCTTTTTCCTCGGTTCAATACATAATGACCGAGGCCAACTTTGGTTGGTTAATCCGATCA
GTTCATCGATGGTCAGCAAGTATGATGGTTCTAATGATGATCCTGCACGTATTTCGTGTG
TATCTCACAGGTGGGTTTAAAAAACCCCGCGAATTAACTTGGGTCACTGGTGTGGTTTT
AGCTGTATTAACTGCATCGTTTGGTGTAACTGGTTATTCTTTACCTTGGGATCAAATTGG
TTATTGGGCAGTCAAAATTGTGACAGGTGTGCCTGACGCGATTCCGGTAATAGGATCAC
CTTTAGTGGAGTTATTACGTGGAAGTGCTAGTGTGGGTCAATCCACTTTGACTCGTTTTT
ATAGTTTACATACCTTTGTACTTCCTCTGCTTACTGCCGTATTTATGTTAATGCACTTTCT
AATGATACGTAAGCAAGGTATTTCGGGCCCTTTAATGGGAGTAACAAAGAAACCTGAC
TTAAACGATCCTGTATTAAGAGCAAAATTAGCTAAAGGGATGGGACATAATTATTACGG
GGAACCCGCGTGGCCCAACGATCTTTTATATATTTTTCCAGTAGTAATTCTAGGTACTAT
TGCATGTAATGTAGGTTTAGCGGTTCTCGAGCCGTCAATGATTGGTGAACCGGCGGATC
CGTTTGCAACTCCTCTGGAAATATTACCCGAGTGGTACTTCTTTCCCGTGTTTCAAATAC
TCCGTACAGTACCTAATAAGTTATTGGGCGTTCTCTTAATGGTTTCTGTGCCGACGGGCT
TATTGACAGTACCTTTTCTAGAGAATGTCAATAAATTCCAAAATCCATTTCGTCGCCCAG
TAGCTACAACCGTTTTTTTAATCGGTACTGCAGTAGCTCTTTGGTTAGGTATTGGAGCAA
CATTACCCATTGAAAAATCCTTAACTTTAGGTCTTTTTATGATTGAAGTTTTTCTATTTGG
AATCGTCTTAGGCCTAATTCCTATTACTTTAGCGGGATTATTCGTGACTGCGTATTTGCAA
TACAGACGTGGGGATCAGTTGGATCTTATGCTTACTATAACTAGTTATTTCGGTTTTCTAT
TGGCTGCTTTAACTCTAACCCTAGCTCTATTTATTGGCTTGAACAAGATACGTCTTATTAT
GGATATAGTAAGTCTCGCTTGGGCTGCTTTAATGGTAGTCTTTACATTTTCTCTTTCACTA
GTAGTATGGGGGAGGAGTGGACTCATGATGATTCGTTCGCCGGAACCAGAAGTAAAAA
TTGTTGTGGATAGGGATCCTGTAAAAACATCTTTTGAGGAATGGGCCAGACCCGGCCAT
TTCTCAAGAACACTAGCTAAGGGCCCTGATACTACCACTTGGATCTGGAACCTACATGC
TGATGCTCACGATTTCGATAGTCATACAGGTGATTTGGAGGAGATCTCTCGAAAAGTCT
TTAGTGCTCATTTCGGTCAACTCTCCATTATCTTTCTTTGGTTGAGTGGCATGTACTTCC
ATGGTGCCCGTTTTTCCAATTATGAAGCATGGCTAAGTGATCCTACTCACATTGGACCC

AGTGCTCAGGTAGTTTGGCCAATCGTAGGGCAAGAAATATTGAATGGTGATGTAGGCG
GGGGTTTCCGAGGAATCCAAATATCCTCCGGCTTTTTTCAGATTTGGCGAGCATCCGGA
ATAACTAGTGAATTACAACTCTATTGTACCGCAATCGGTGCATTGATTTTTGCATCGTTA
ATGCTTTTTGCTGGGTGGTTCCATTATCACAAAGCCGCTCCCAAATTAGCCTGGAGTCA
TGACGTAGAATCCATGTTGAATCATCACTTAGCAGGGTTATTAGGACTTGGGTCTCTTTC
TTGGGCGGGGCACCAAATCCATGTATCTTTACCGATTAACCAATTTCTTGACGCTGGGG
TTGATCCTAAGGAGATACCACTTCCTCATGAATTTATCTTGAATCGCGACCTTTTGGCTC
AACTTTATCCTAGTTTTGCCGAACGAGCAACCCCTTTTTTCACCTTGAATTGGTCCAAAT
ACGCAGAATTTCTTAGTTTTCGCGGAGGGCTAGATCCAATAACCGGCGGTCTATGGTTG
AGCGATATTGCGCACCATCATTTAGCTATTGCTATTCTTTTCCTGATCGCAGGTCATATGT
ATAGGACCAACTGGGGTATTGGTCATGGCCTGAAAGATATTTTGGAGGCTCATAAAGGC
CCATTTACAGGACAGCGCCATAAAGGTCTCTATGAAATCCTAACAACGTCATGGCATGC
TCAATTATCTCTTAACCTAGCTATGCTAGGCTCTACAACCATTGTTGTAGCTCATCATATG
TACTCTATGCCCCCCTATCCATACCTAGCTACTGACTATGGTACACAACTTTCCTTGTTC
ACACACCACATGTGGATTGGCGGATTTCTAATAGTTGGTGCTGCTGCACATGCAGCCAT
TTTTATGGTAAGAGACTATGATCCAACGACTCGATACAACGATCTATTAGATCGTGTCCT
TAGACACCGTGATGCAATCATATCCCACCTTAACTGGGTATGTATATTTCTAGGTTTTCA
CAGTTTTGGTTTATACATTCATAATGATACCATGAGTGCTTTAGGTCGTCCCCAAGATAT
GTTTTCGGATACCGCCATACAATTACAACCGATCTTTGCTCAATGGGTACAAAATCTCCA
TGCTGGCGCGCCTAGGCTAACAGCTCCTGGTGCAACAACAAGTACCAGCTTAACGTGG
GGAGGTGGCGAGTTAGTAGCAGTAGGCGGCAAAGTTGCTTTGTTACCTATTCCATTAGG
AACCGCAGATTTTTTAGTCCATCACATTCACGCATTTACAATCCATGTGACTGTATTAAT
ACTTTTGAAAGGTGTTTTATTTGCTCGCAGTTCCCGTTTGATACCCGATAAAGCAAATCT
TGGTTTTCGCTTCCCTTGCGATGGGCCTGGGCGAGGGGGAACATGTCAAGTATCCGCC
TGGGATCATGTTTTCTTAGGTCTATTCTGGATGTACAATTCCATTTCGGTAGTCATTTTCC
ATTTCAGTTGGAAAATGCAGTCGGATGTTTGGGGTACTATAAGTGATCAAGGGGTGGTA
ACTCATATCACAGGGGGAAACTTTGCACAGAGTTCCATTACGATTAATGGGTGGCTCCG
AGATTTCTTGTGGGCACAGGCATCCCAAGTAATTCAGTCTTATGGTTCTTCATTATCCGC
ATATGGTCTTTTTTTCTTAGGTGCTCATTTTGTCTGGGCCTTCAGTTTAATGTTTTTATTC
AGCGGCCGTGGTTATTGGCAAGAACTGATTGAATCTATCGTTTGGGCTCATAACAAATT
AAAAGTTGCTCCTGCTACTCAGCCTAGAGCCTTGAGCATTATACAAGGACGTGCTGTAG
GAGTAACCCATTACCTTCTGGGTGGAATTGCCACAACATGGGCATTCTTCTTAGCGAGA
ATTATTGCAGTAGGAATGGAATTAAGATTTCCCAGGTTTAGCCAAGGCTTAGCTCAGGA
CCCCACTACTCGTCGTATTTGGTTTGGTATTGCTACCGCACATGATTTCGAAAGTCATGA
TGATATTACTGAGGAACGTCTTTATCAGAACATTTTTGCTTCTCACTTTGGGCAATTAGC
AATAATCTTTCTATGGACGTCCGGAAATCTGTTTCATGTCGCTTGGCAAGGAAATTTTG
AATCATGGATACAGGATCCTTTACACGTAAGACCTATTGCTCATGCGATTTGGGATCCTC
ATTTTGGTCAACCCGCTGTGGAAGCCTTTACTCGAGGAGGCGCCGCCGGTCCAGTGAA
TATCGCCTATTCTGGGGTTTATCAGTGGTGGTATACAATTGGATTACGGACCAATGAAGA
TCTTTATACAGGAGCTCTTTTTCTATTATTTCTTTCTACCCTATCCTTAATAGGGGGTTGG
TTACATCTACAACCCAAATGGAAACCAAGCCTTTCGTGGTTCAAAAACGCGGAATCTC
GTCTCAATCATCATTTGTCAGGACTTTTCGGGGTAAGTTCTTTGGCTTGGACAGGACAT
TTAGTTCATGTCGCTATTCCAGCATCCAGGGGAGAGTACGTTCGATGGAATAATTTCTTA
GATGTATTACCCTATCCACAGGGGTTGGGACCCCTTCTGACGGGTCAGTGGAATCTTTA

TGCCCAAAACCCCGATTCGAGTAATCATTTATTTGGTACCACTCAAGGAGCGGGAACTG
CTATTCTAACTCTTCTTGGGGGATTCCATCCACAAACACAAAGTTTGTGGCTGACCGAT
ATTGCTCATCATCATTTAGCTATTGCATTTATTTTTCTCATTGCCGGTCATATGTATCGAAC
TAACTTCGGAATTGGGCACAGTATCAAAGATCTTTTAGAAGCACATACTCCTCCGGGGG
GTCGATTAGGGCGTGGGCATAAGGGCCTTTACGACACAATCAATAATTCGATTCATTTT
CAATTAGGTCTTGCTCTAGCTTCTTTAGGGGTTATTACTTCCTTAGTAGCTCAACATATGT
ACTCTTTACCTTCTTATGCATTCATAGCACAAGACTTTACTACTCAAGCAGCTTTATATAC
TCATCACCAATACATTGCAGGGTTCATCATGACAGGGGCTTTTGCTCATGGAGCTATTTT
TTTCATTAGAGATTACAATCCGGAACAGAATGAGGATAATGTATTGGCAAGAATGTTAG
ACCATAAAGAAGCTATCATATCTCATTTAAGTTGGGCTAGTCTCTTCCTAGGATTCCATA
CTTTGGGCCTTTATGTTCATAATGACGTCATGCTTGCTTTTGGTACTCCAGAAAAGCAAA
TCTTGATCGAACCTATATTTGCTCAATGGATACAATCGGCTCATGGTAAGACGACATATG
GGTTCGATATACTCTTATCTTCAACGAGCGGTCCCGCTTTCAATGCGGGTCGAACCCTAT
GGTTACCCGGATGGTTGAATGCTGTTAACGAG------AATAGTAATTCGCTTTTCTTAACA
ATAGGACCTGGAGATTTCTTGGTTCATCATGCTATTGCTTTAGGTTTGCATACAACTACA
TTGATTTAGTAAAGGGTGCTTTAGATGCACGCGGTTCCAAATTAATGCCGGATAAAAA
AGATTTTGGATATAGTTTTCCTTGCGACGGCCCTGGTCGCGGCGGTACTTGTGATATTTC
TGCTTGGGACGCGTTTTATTTGGCAGTTTTCTGGATGTTAAATACCATTGGATGGGTTAC
TTTTTATTGGCATTGGAAACACATCACATTATGGCAGGGCAACGTTTCACAATTTAATGA
ATCCTCCACTTATTTGATGGGATGGTTAAGAGATTACCTATGGTTAAACTCTTCACAACT
TATCAATGGATATAATCCTTTTGGGATGAATAGTTTATCGGTATGGGCGTGGATGTTCTTA
TTTGGACATCTTGTTTGGGCGACTGGATTTATGTTCTTAATTTCCTGGCGTGGATATTGG
CAGGAATTAATTGAGACTTTAGCATGGGCTCATGAACGGACACCTTTGGCTAATTTAAT
TCGCTGGAGAGATAAGCCCGTGGCTCTTTCCATTGTGCAAGCAAGATTGGTGGGATTA
GCTCACTTTTCCGTGGGTTATATATTCACTTATGCCGCTTTCTTGATTGCCTCAACATCAG
GCAAGTTTGGTATGTCACATTCCGTAAAAATTTATGATACATGTATAGGATGCACTCAAT
GTGTACGAGCTTGTCCAACAGATGTATTAGAAATGATACCTTGGGATGGATGTAAAGCC
AAGCAAATTGCTTCCGCGCCAAGAACCGAAGATTGTGTGGGTTGTAAGAGATGCGAAT
CCGCCTGCCCAACAGATTTTTTAAGTGTCCGCGTTTATTTAGGACCTGAGACAACCCGC
AGCATGGCTCTATCTTATATGATGGATTTTAACTTACCCTCTATTTTCGTGCCTTTAGTAG
GCTTAGTATTTCCGGCAATTGCAATGGCTTCTTTATTTCTTTATGTGCAGAAAAATAAGA
T T G T
C??
???ATGACTGCAATTTTAGAGAGACGCGAAA
GTACAAGCCTGTGGGGTCGCTTCTGCAACTGGATAACTAGCACCGAAAACCGTCTTTA
CATCGGATGGTTCGGTGTTTTGATGATCCCTACCTTATTGACCGCAACTTCTGTATTTATT
ATCGCCTTCATCGCTGCCCCTCCAGTAGATATTGATGGTATTCGTGAGCCTGTTTCTGGT
TCTTTACTTTACGGAAACAATATTATCTCTGGTGCCATTATTCCTACTTCTGCGGCGATCG
GATTGCACTTTTACCCAATTTGGGAAGCTGCATCTGTTGATGAGTGGTTATACAATGGT
GGTCCTTATGAGCTAATTGTTCTACACTTCTTACTTGGTGTAGCTTGTTATATGGGTCGT
GAGTGGGAACTGAGTTTCCGTCTGGGTATGCGTCCTTGGATTGCTGTTGCATATTCAGC
TCCTGTTGCAGCTGCTACTGCTGTTTTCTTGATTTACCCTATTGGTCAAGGAAGTTTCTC
TGATGGTATGCCTTTAGGAATATCTGGTACTTTCAACTTTATGATTGTATTCCAGGCAGA
GCATAACATCCTTATGCATCCATTTCACATGTTAGGTGTAGCTGGTGTATTCGGCGGTTC

CCTATTCAGTGCTATGCATGGTTCCTTGGTAACCTCTAGTTTGATCAGGGAAACCACTG
AAAACGAATCTGCTAATGAGGGTTACAGATTTGGTCAAGAGGAAGAAACTTATAATATT
GTGGCCGCTCATGGTTATTTTGGCCGATTAATCTTCCAATATGCTAGTTTTAACAACTCT
CGTTCTTTACACTTCTTCTTGGCTGCTTGGCCTGTAGTAGGGATTTGGTTCACTGCTTTA
GGTATTAGTACTATGGCTTTCAATCTAAACGGATTCAATTTCAACCAATCTGTAGTTGAT
AGCCAAGGTCGCGTTATTAATACTTGGGCTGATATCATCAACCGTGCTAATCTTGGTATG
GAAGTAATGCACGAACGTAATGCTCACAACTTCCCTCTAGACCTAGCTGCTCTTGAAGT
TCCATCTCTTAATGGAATGGGTTTGCCTTGGTATCGTGTTCATACTGTCGTATTGAATGAT
CCGGGTCGATTGCTTTCGGTGCATATAATGCACACAGCTCTAGTTTCTGGTTGGGCTGG
CTCGATGGCTTTATACGAATTAGCGGTTTTTGATCCCTCTGATCCTGTTCTGGATCCAAT
GTGGAGACAAGGTATGTTCGTCATTCCTTTCATGACTCGTTTAGGAATAACCAATTCGT
GGGGTGGTTGGAGTATTTCA---GGAGGAACTGTAACGAATCCGGGTATTTGGAGTTATG
AAGGCGTGGCAGGGGCGCATATTGTGTTTTCTGGCTTGTGTTTCTTGGCAGCTATCTGG
CATTGGGTATATTGGGACCTCGAAATATTCTGTGATGAGCGGACGGGAAAACCTTCTTT
GGATTTGCCCAAGATCTTTGGAATTCATTTATTTCTTGCAGGGGTGGCTTGCTTTGGCTT
TGGCGCATTTCATGTAACTGGTTTGTATGGTCCTGGGATATGGGTGTCTGATCCTTATGG
ACTAACTGGAAAAGTACAAGCTGTAAATCCGGCGTGGGGTGCAGAAGGTTTTGATCCT
TTTGTTCCGGGGGGAATAGCTTCTCATCATATTGCTGCGGGTACATTGGGCATATTAGCG
GGCCTATTCCATCTTAGTGTCCGTCCACCTCAACGTCTATACAAAGGATTACGTATGGGC
AATATTGAAACTGTACTTTCCAGTAGTATCGCTGCTGTTTTTTTTGCAGCTTTCGTAGTT
GCCGGAACTATGTGGTATGGGTCAGCAACTACCCCAATTGAATTGTTTGGGCCTACTCG
TTATCAGTGGGATCAGGGATACTTTCAGCAAGAAATATATCGAAGAGTTAGTGATGGGT
TAGCCGAAAATCTCAGTTTATCAGAAGCTTGGTCTAAAATTCCCGAAAAATTAGCCTTT
TATGATTATATTGGTAATAATCCGGCAAAGGGGGGATTATTCAGAGCAGGCTCAATGGA
CAATGGGGATGGAATAGCTGTTGGATGGTTAGGACATCCTATCTTTAGAGATAAAGAAG
GGCGCGAACTTTTTGTACGCCGTATGCCTACTTTTTTTGAAACATTTCCGGTTGTTTTGG
TAGATGAAGAGGGAATTGTGAGAGCGGACGTTCCTTTTAGAAGAGCAGAATCCAAATA
TAGTGTTGAACAAGTAGGCGTAACGGTGGAGTTCTATGGTGGCGAACTTAATGGAGTA
AGTTATTCTGATCCTGCTACTGTAAAAAAATATGCGAGGCGTTCCCAATTAGGGGAAAT
TTTTGAATTAGATCGGGCTACTTTGAAATCAGATGGTGTTTTTCGCAGCAGTCCAAGGG
GTTGGTTCACTTTTGGTCATGCTACCTTTGCTTTGCTCTTCTTTTTCGGACACATTTGGC
ATGGGGCTAGAACCTTGTTCGCAGATGTTTTTGCTGGTATTGATCCAGACTTAGATGCT
CAAGTGGAATTTGGAGCAATCCAAAAACGTGGAGATCCAACTACAGGGAGACAGCCA
GTCATGAAAATCTTATATTCCCTGAGGAGGTTCTACCACGTGGAAACGCTCTTTAATGG
AACTTTTGTTTTAGCTGGTCGTGACCAAGAAACCACCGGTTTTGCTTGGTGGGCCGGG
AATGCCAGACTTATCAATTTGTCGGGTAAACTACTTGGAGCTCACGTAGCCCATGCAGG
ATTAATCGTATTCTGGGCCGGAGCAATGAACCTATTTGAAGTGGCCCATTTCGTACCAG
AAAAACCCATGTATGAACAAGGGTTGATTTTACTTCCGCACTTAGCTACTCTAGGTTGG
GGAGTAGGGCCCGGGGGAGAAGTTCTAGATACTTTTCCGTACTTTGTATCTGGAGTACT
TCATCTAATTTCCTCCGCAGTCTTAGGCTTCGGTGGCATTTATCACGCGCTTCTGGGACC
GGAGACTCTTGAAGAATCTTTTCCATTCTTTGGTTATGTGTGGAAAGATAGAAATAAAA
TGACTACAATTTTGGGTATTCACCTAATTTTGTTAGGTATAGGTGCTTTTCTTCTAGTACT
CAAAGCTCTTTATTTTGGCGGTATATATGATACCTGGGCTCCTGGGGGAGGAGATGTAA
GAAAAATTACCAATTTGACCCTTAGCCCCGGTGTTATATTTGGTTATTTACTAAAATCCC

CTTTTGGGGGAGAGGGTTGGATTGTTAGTGTGGATGATTTAGAAGATATAATTGGGGGA
CATGTATGGTTGGGTTTCATTTGTGTATTTGGCGGAATTTGGCATATCTTAACCAAACCC
TTCGCATGGGCTCGCCGTGCATTTGTATGGTCTGGAGAAGCTTACTTGTCTTATAGTTTA
GGCGCTTTATCTGTCTTTGGTTTTATCGCTTGTTGTTTTGTCTGGTTCAATAATACAGCTT
ATCCGAGTGAGTTTTATGGACCTACTGGGCCAGAAGCTTCTCAAGCTCAAGCATTTACT
TTTCTAGTTAGAGATCAGCGTCTTGGAGCTAATGTGGGATCTGCCCAAGGACCCACAG
GTTTAGGTAAATATCTAATGCGTTCCCCAACGGGAGAGGTTATTTTTGGAGGGGAAACT
ATGCGTTTTTGGGACCTTCGTGCTCCATGGTTAGAACCTCTAAGGGGCCCCAACGGTTT
GGACTTGAGTAGGTTGAAAAAAGACATACAACCTTGGCAAGAACGACGTTCGGCAGA
ATATATGACCCATGCTCCTTTAGGCTCTTTAAATTCTGTGGGTGGCGTAGCTACCGAGAT
CAATGCAGTTAATTATGTTTCTCCTAGAAGTTGGTTAGCGACTTCCCATTTTGTTCTAGG
ATTCTTCTTTTTTGTGGGCCATTTGTGGCATGCAGGAAGAGCCCGGGCTGCTGCAGCA
GGATTTGAAAAGGGAATCGATCGTGATTTGGAGCCTGTTCTTTACATGACCCCTCTTAA
CATGACTATAGCCCTTGGTAGAGTTACTAAAGAAGAAAATGATTTATTTGATATTATGGA
CGACTGGTTACGAAGGGACCGTTTTGTTTTTGTAGGATGGTCTGGCCTATTGCTTTTTC
CTTGTGCTTATTTCGCTTTAGGAGGTTGGTTTACAGGGACAACTTTTGTAACTTCTTGGT
ATACCCATGGATTGGCGAGTTCCTATTTGGAAGGTTGCAATTTCTTAACCGCAGCAGTT
TCCACCCCTGCCAATAGTTTAGCACACTCTTTGTTGCTACTATGGGGCCCGGAAGCACA
AGGGGATTTTACTCGTTGGTGTCAATTAGGTGGTCTGTGGACTTTTGTTGCTCTCCATG
GGGCTTTTGCACTAATAGGTTTCATGTTACGTCAATTTGAACTTGCTCGGTCTGTTCAAT
TGCGGCCTTATAATGCAATTTCATTCTCTGGCCCAATCGCTGTTTTTGTTTCCGTATTCCT
GATTTATCCACTGGGGCAATCCGGTTGGTTCTTTGCGCCGAGTTTTGGCGTAGCAGCGA
TATTTCGATTCATCCTCTTCTTCCAAGGATTTCATAATTGGACGTTGAACCCATTTCATAT
GATGGGAGTTGCCGGAGTATTAGGCGCGGCTCTGCTATGCGCTATTCATGGGGCAACCG
TGGAAAACACTCTATTTGAGGACGGTGATGGTGCAAATACCTTCCGCGCTTTTAACCCA
ACTCAAGCTGAAGAAACTTATTCAATGGTCACCGCTAATCGCTTTTGGTCCCAAATCTT
TGGTGTTGCTTTTTCCAATAAACGTTGGTTACATTTCTTTATGCTATTTGTACCGGTCACC
GGTTTATGGATGAGTGCTATTGGCGTAGTCGGCCTGGCTCTGAACCTACGTGCCTATGA
CTTCGTTTCCCAGGAAATCCGTGCAGCGGAAGATCCTGAATTTGAGACTTTCTACACCA
AAAATATTCTTTTAAACGAGGGTATTCGTGCGTGGATGGCAGCTCAGGATCAGCCTCAT
GAAAATCTTATATTCCCTGAGGAGGTTCTACCACGTGGAAACGCTCTTATGTCTGGAAG
CACGGGAGAACGTTCTTTTGCTGATATTATTACCAGTATTCGATACTGGGTTATTCATAG
CATTACTATACCTTCCCTATTCATTGCGGGTTGGTTATTTGTCAGTACGGGTTTAGCTTAT
GACGTGTTTGGAAGTCCTCGGCCAAACGAGTATTTCACAGAAAGCCGACAAGGAATTC
CGTTAATAACCGACCGTTTTGATTCTTTAGAACAACTCGATGAATTTAGTAGATCCTTTA
TGACCATAGATCGAACCTATCCTATTTTTACAGTGCGATGGCTGGCTGTTCACGGACTA
GCTGTACCTACTGTTTTTTTCTTGGGATCAATATCAGCAATGCAGTTCATCCAACGAATG
GCTACACAAACCGTTGAAGATAGTTCTAGACCTGGA---CCAAGACAAACTCGCGTAGG
TAATTTATTGAAACCCTTGAATTCGGAATATGGGAAAGTAGCTCCGGGTTGGGGGACTA
CTCCTTTTATGGGGGTCGCAATGGCTTTATTCGCGGTATTCCTATCTATTATTTTAGAAAT
TTATAATTCTTCCGTTTTACTGGACGGAATTTTAATGAATATGCTTACTCTCAAACTTTTT
GTTTATACAGTAGTGATATTCTTTGTTTCCCTCTTTATCTTTGGATTCTTATCTAATGATCC
AGGACGTAATCCTGGGCGTGACGAGATGGCCGATACTACTGGAAGAATTCCTCTTTGG
CTGATAGGTACTGTAACCGGTATTGCTGTGATTGGTTTAATAGGTGTTTTCTTTTACGGT

TCATATTCTGGATTGGGTTCATCTCTAATGCCTAATATACTTAGTTTAACCTGTATTTGTTT
TAATTCTGTTATTTATCCGACTAGTTTTTTCTTCGCCAAATTGCCCGAAGCTTATGCCATT
TTCAATCCAATCGTGGATTTTATGCCTGTCATACCTGTACTCTTTTTTCTATTAGCCTTTG
TTTGGCAAGCTGCTGTAAGTTTTCGAATGACACAATCAAACCCGAATGAACAAAATGT
TGAATTGAATCGTACCAGTCTATACTGGGGTTTATTACTCATTTTTGTACTTGCTGTTTTA
TTTTCCAATTACTTCTTCAATATGGAAGTCAATATTCTCGCATTTATTGCTACTGCATTGT
TCATTCTAGTTCCTACTGCCTTTTTACTTATTATTTATGTAAAAACAGTCAGCCAAAATGA
TATGGAAACAGCAACTTTAGTCGCCATCTCCATATCTGGTTTACTTGTAAGCTTTACTGG
GTATGCCTTATATACCGCGTTTGGGCAACCCTCTCAACAATTAAGAGATCCATTCGAAG
AACACGGGGACATGGAAGCATTGGTTTATACGTTCCTTTTAGTTTCGACTTTAGGGATA
ATTTTTTTCGCTATCTTCTTCCGAGAACCACCTAAGGTTCCGACTAAAAAAGTGAAAAT
GACTATTGCTTTCCAATTAGCTGTTTTTGCATTAATTGTGACTTCCTCAGTCTTAGTCATT
AGTGTACCCCTTGTATTTGCTTCTCCTGATGGTTGGTCAAACAATAAAAACGTTGTATTT
TCCGGTACATCATTATGGATTGGACTGGTCTTTCTGGTAGCTATTCTAAATTCTCTCATTT
CT--------------------ATGTCACCACAAACAGAAACTAAAGCAAGTGTTGGATTTAAAGCTG
GTGTTAAGGATTATAAATTGACTTACTACACCCCGGAGTACGAAACCAAGGACACTGAT
ATCTTGGCAGCATTCCGAGTAACTCCTCAGCCGGGGGTTCCGCCCGAAGAAGCAGGGG
CTGCAGTAGCTGCCGAATCTTCTACTGGTACATGGACAACTGTTTGGACTGATGGACTT
ACCAGTCTTGATCGTTACAAAGGCCGATGCTATCACATCGAGCCCGTTGTTGGGGAGG
ATAATCAATATATCGCTTATGTAGCTTATCCATTAGACCTATTTGAAGAGGGTTCTGTTAC
TAACATGTTTACTTCCATTGTGGGTAACGTATTTGGTTTCAAAGCCCTACGCGCTCTACG
TCTGGAGGATCTGCGAATTCCCCCTACTTATTCAAAAACTTTCCAAGGTCCGCCTCATG
GTATCCAAGTTGAAAGGGATAAGTTGAACAAATACGGTCGTCCTTTATTGGGATGTACT
ATTAAACCAAAATTGGGATTATCTGCAAAAAATTATGGTAGAGCATGTTATGAGTGTCTA
CGCGGTGGACTTGATTTTACCAAAGATGATGAAAACGTAAACTCACAACCATTTATGCG
TTGGAGGGACCGTTTTGTCTTTTGTGCCGAAGCTATTTATAAATCACAGGCCGAAACCG
GTGAAATTAAGGGGCATTACTTGAATGCGACTGCAGGTACATGCGAAGAAATGATTAA
AAGAGCTGTATTTGCGAGGGAATTAGGGGTTCCTATTGTAATGCATGACTACTTAACCG
GGGGGTTCACCGCAAATACTAGTTTGGCTCATTATTGCCGCGACAACGGCCTACTTCTT
CACATTCACCGAGCAATGCATGCAGTTATTGATAGACAGAAAAATCATGGTATGCATTT
CCGTGTATTAGCTAAAGCATTGCGTATGTCTGGGGGAGATCATATCCACGCTGGTACAGT
AGTAGGTAAGTTAGAAGGGGAACGCGAAATGACTTTAGGTTTTGTTGATTTATTGCGCG
ATGATTTTATTGAAAAAGATCGTGCTCGCGGTATCTTTTTCACTCAGGACTGGGTATCCA
TGCCAGGTGTTATACCGGTGGCTTCAGGGGGTATTCATGTTTGGCATATGCCAGCTCTG
ACCGAAATCTTTGGAGATGATTCTGTATTGCAATTTGGTGGAGGAACTTTAGGACATCC
TTGGGGTAATGCACCTGGTGCAGCAGCTAATCGGGTGGCTTTAGAAGCCTGTGTACAA
GCTCGTAACGAAGGGCGCGATCTTGCTCGTGAAGGTAATGAAATTATCCGATCAGCTTG
CAAATGGAGTCCTGAACTAGCCGCAGCTTGTGAAATATGGAAAGCGATCAAATTCGAG
TTCGAGCCGGTAGATAAACTAGATAGCACGGCGAAACATTTATACAAAACACCTATCCC
GAGCACACGCAAGGGAACCATAGATAGGCAAGTGAAATCCAATCCACGAAATAATTTG
ATCCATGGACGGCACCGTTGTGGTAAAGGTCGTAATTCCAGAGGAATCATTACCGCAA
GGCATAGAGGGGGAGGTCATAAGCGCCTATACCGTAAAATCGATTTTCGACGGAATCA
AAAAGACATATCTGGTAGAATCGTAACCATAGAATACGACCCTAATCGAAATGCGTACA
TTTGTCTCATACACTATGGGGATGGTGAGAAGGGATATATTTTACATCCCAGAGGGGCTA

TAATTGGAGATACTATTGTTTCTGGTACAAAAGTTCCTATATCAATGGGAAATGCCCTAC
CTTTGACCGATATGCCCTTAGGCACGGCCATACATAACATAGAAATCACACGTGGAAGG
GGTGGGCAATTAGCTAGAGCAGCAGGTGCTGTAGCGAAACTGATTGCAAAAGAAGGT
AAATCGGCCACTTTAAGATTACCATCTGGGGAGGTCCGTTTGGTATCCCAAAACTGCTT
AGCAACAGTCGGACAAGTGGGTAATGTTGGGGTGAACCAAAAAAGTTTGGGTAGAGC
CGGGTCTAAGTGTTGGCTAGGTAAACGCCCCGTAGTAAGAGGGGTAGTTATGAACCCT
GTGGACCACCCCCATGGGGGCGGTGAAGGGAAAGCCCCCATTGGTAGAAAAAAACCC
ACAACCCCTTGGGGTTATCCTGCGCTTGGAAGAAGAACTAGGAAAAGGAAAAAATATA
GTGATAGTTTTATTCTTCGTCGCCGTAAG---ATGATTCAACCTCAGACCCTTTTAAATGTA
GCAGATAACAGTGGAGCTCGAAAATTGATGTGTATTCGAGTCATAGGAGCTGCTAGTAA
TCAGCGATATGCTCGTATTGGTGATGTTATTGTTGCTGTAATCAAAGACGCAGTGCCCC
AAATGCCTCTAGAAAGATCCGAAGTAATTCGAGCTGTAATTGTACGTACATGTAAAGAG
TTCAAATGCGAAGACGGTATAATAATACGCTATGACGACAATGCAGCGGTTATCATTGAT
CAAAAAGGAAATCCAAAAGGAACTCGCGTTTTTGGCGCGATCGCCGAGGAATTGAGA
GAATTGAATTTTACTAAAATAGTTTCATTAGCTCCTGAAGTATTAATGCTTAGTCCCAAA
AGAACCAGATTTCGTAAACAACATAGAGGAAGAATGAAGGGAAAATCCTACCGAGGC
AATTGTATTTGTTTTGGTAGATATGCTCTTCAAGCACTTGAACCCACTTGGATTACGGCG
AGGCAGATAGAAGCAGGACGAAGAGCAATGACACGATATGCACGTCGTGGTGGAAAA
ATCTGGGTACGTATATTTCCCGACAAACCGGTTACAATAAGACCGACCGAAACACGTAT
GGGTTCGGGAAAGGGATCCCCCGAATATTGGGTAGCCGTTGTTAAACCAGGTCGAATA
CTTTATGAAATGGGCGGAGTATCCGAAACTGTAGCTAGACGAGCTATTTCCATAGCTGC
CAGCAAAATGCCCATACGAAGTCAATTTCTTCGGTTAGAAATGACCAGAGTTCCGCGA
GGATATATAGCTCGGAGACGACGGGCAAAAATGCGTTCATTTGCCTCAAACTTTAGAG
GGGCTCATTTAAGACTTAATCGAATGATTACTCAACAGGTAAGAAGAGCGTTTGTTTCC
TCTCATCGAGATAGAGTCAGGCAAAAGAGGGATTTTCGGCGTTTGTGGATCAGTCGGA
TAAACGCAGCAACGCGGATACAT------GTATTCGATAATTATAGTAAATTAATACACAATC
TTTACAAGAAAGAATTGATTCTTAATCGTAAAATCCTTGCACAAGTAGCTGTATTAAATT
CAAATAATCTTTACACGATTTCCAATAAAATAAAGATTATCAAT---------------------------AG
GATAAAGCGGGTTTTCAAAGCACAAAAACGCATCCACATGTCCGTTTTCAAAGTACAA
AGAGTTCTGGATGAGATTCGTTGGCGTTACTACGAAGAAACTGTTATGATATTGAACCT
CATGCCTTATCGAGCATCTTATCCCATCTTAAAGTTGGTTTATTCGGCAGCAGCAAATGC
TACTCATTATAGGGATTTCGACAAAGCAAATTTATTCATCACTAAAGCCGAAGTCAGTA
GGAGTACTATTATGAATAAATTCAGACCTCGGGCTCGAGGACGTAGTTCTCCCATAAAA
AAAACCATGTGTCATATAACAATTGTACTAAAT---GTAAAGAAATCG---------------------AT
GGATGGAATCAAATACGCAGTATTTACAGAAAAAAGTCTTCGTTTATTGGGAAAGAAT--
-CAATATACTTTTAATGTCGAATCGGGATTCACTAAGACAGAAATAAAGCATTGGGTCGA
ACTCTTCTTTGGTGTTAAGGTAGTAGCTGTGAATAGCCATCGACTACCCGGAAAGGGTA
GAAGAATGGGCCCTATTCTAGGACATACAATGCATTACAGACGTATGATCATTACCCTTC
AACCGGGTTATTCTATTCCACTTCTAGATAGAGAAAAAAACATGGCAGTTCCAAAAAA
ACGTACTTCGATGTCAAAAAAGCGTATTCGTAAAAATCTTTGGAAGAAAAAGACTTATT
TTTCCATAGTACAATCTTATTCTTTAGCAAAATCATTTTCTGGCGTCAAACCAAAGGGTT
TTTCTCGGCAACAAACAAATAATATGGCCAAGGGGAAAGATGTTAGAATCAGAGTTATT
TTGCAATGTGTGAGTTGTGTTCGAAAA------GGG------GAGGAGTCGGCAGGGATTTCTA
GATATAGTACTCAAAAGAATCGCCACAATACACCCGGACAATTAGAATTAAGAAAATTT

TGTCGTTATTGTCGTAAGCATACGATTCATGCCGAAATAAAGAAAATGAAAATAAGAGC
TTCTGTTCGTAAAATTTGTACAAAATGTCGACTGATTCGTAGGCGTGGACGAATTAGAG
TCATTTGTTCCAATCCGAAGCATAAACAAAGACAGGGGATGGTTCGAGAGGAGGTAGC
AGGATCCACTCAAACACTACAGTGGAAGTGTGTTGAATCAAGAGTAGATAGTAAGCGT
CTTTATTATGGTCGTTTCATTCTGTCCCCGCTTAGAAAAGGTCAAGCGGATACCGTTGGT
ATTGCCTTGCGAAGAGCTTTACTTGGAGAAACAGAAGGAACATGTATCACACATGCAA
AATTTGGG------AGCGTGCCGCACGAATATTCTACAATAGCAGGTATTGAAGAATCGGTA
CAAGAAATTTTACTAAATTTGAAAGAAATTGTATTGAGAAGTAATCTCTATGGAGTTAG
AACCGCATCAATTTGCGTCAAAGGTCCTAGATACATAACTGCCCAAGATATTATCTTACC
GCCTTCCGTAGAAATCGTTGATACGGCACAACCTATAGCTAACTTGACAGAACCCACG
GATTTCCGTATTGAGTTACGGATCAAGAGAGATCGCGGATATCACACGGAAGTCAGAA
AGAACACTCAAGATGGAAGTTAT------CCTATAGATGCTGTATCCATGCCTGTTCGAAAT
GTGAATTATAGTATTTTTGCTTGTGGGAATGGAAATGCAAAATATGAGATACTTTTTCTA
GAAATATGGACCAATGGAAGTTTAACCCCTAAGGAAGCGCTTTATGAGGCTTCTCGTAA
TTTGATTGATTTATTTCTTCCTTTTCTTCACACGGAGGAAGAG------GGTACTCGTTTCCA
AGAAAATAAA---AACAGGTTTACTTCCCCCCTTTTAAGCTTTCAAAAAAGATTAACT---A
ATCTAAAGAAAAACAAAAAA---AGAATTCCATTGAATTGTATTTTTATTGATCAATTAGA
ACTGCCTTCTAGAACGTATAATTGTCTCAAAAGGGCCAATATCCATACACTATTGGACCT
TTTGAGTAAGACGGAAGAAGATCTGATGAGAATTGACAGTTTTCGTATGCAAGATGGA
AAACAGATATGGGACACTCTAGAGAAGCAT------CTCCCAATGGATTTACCTAAGAATAA
GATGCTCCGGAATGGAAAT---GAGGGAATGTCCACAATACCCGGATTTAGTCAGATCCA
ATTCGAGGGATTTTGTAGGTTCATTAATCAAGGCTTGGCAGAAGAACTTGAGAAGTTTC
CAACAATTAAAGATCCAGATCACGAAATTTCATTTCAATTATTTGCGAAAGGATATCAAT
TGCTAGAACCTTCGATAAAAGAAAGGGATGCTGTGTATGAATCACTCACCTATTCTTCC
GAATTATACGTATCCGCGCGATTAATTTTTGGTTTC------GATGTGCAAAAGCAAACCATT
TCTATTGGAAACATTCCTATAATGAATTCCTTAGGAACCTTTATAATAAATGGAATATACC
GAATTGTGATCAATCAAATATTGCTAAGTCCTGGTATTTACTACCGCTCGGAATTAGACC
ATAAAGGAATTTCTATCTACACCGGGACTATAATATCAGATTGGGGAGGAAGATCGGAA
TTAGCAATTGATAAAAAAGAAAGGATATGGGCTCGCGTGAGTAGAAAACAAAAGATAT
CTATTCTAGTTCTATCATCAGCTATGGGTTCAAATCTAAAAGAAATTCTAGATAATGTTTC
CTACCCTGAAATTTTCTTGTCTTTCCCTAATGCTAAGGAGAAGAAGAGGATTGAGTCAA
AAGAAAAAGCTATTTTGGAGTTTTATCAACAATTTGCTTGTGTAGGTGGGGACCTGGTA
TTTTCGGAATCCTTATGTGAGGAATTACAAAAGAAATTTTTTCAACAAAAATGTGAATT
AGGAAGGATTGGTCGACGAAATATGAATCGAAGACTGAATCTTGATATACCTCAGAAC
AGCACCTTCTTGTTACCGCGAGATGTATTGGCCGCTACGGATCATTTGATTGGAATGAA
ATTTGAAACGGGTATACTTGACGATGACGATATGAATCACTTGAAAAATAAACGTATTC
GTTCGGTTGCGGATCTGTTACAAGATCAATTCGGACTGGCTCTTGGTCGTTTACAACAT
GCGGTTCAAAAAACTATCCGTAGAGTATTCATACGTCAATCAAAA------CCGACTCCACA
AACTTTGGTAACTCCAACTTCAACCTCGATTTTATTAATAACTACTTACGAGACCTTCTT
TGGTACATATCCCTTATCTCAAGTTTTTGATCAAACCAATCCATTGACACAAACGGTTCA
TGGGCGAAAAGTGAGTTGTTTGGGTCCTGGAGGATTGACGGGGAGAACTGCAAGTTT
TCGGAGCCGAGATATCCATCCGAGTCACTATGGGCGTATTTGTCCAATTGACACGTCCG
AAGGAATCAACGTTGGACTTACTGGATCTTTAGCTATTCATGCGAGAATTGATCATTGG
TGGGGATCCGTAGAGAGTCCTTTTTATGAAATATCTGAGAAAGCAAAGAAAAAAAAAG

AG------AGACAGGTGGTTTATTTATCACCAAATAGAGATGAGTATTATATGATAGCAGCA
GGAAATTCTTTGTCCTTGAATCGGGGTATTCAGGAAGAACAGGTTGTTCCAGCTAGATA
CCGTCAAGAATTCCTGACTATTGCATGGGAACAGATTCATGTTAGAAGTATTTTTCCTTT
CCAATATTTTTCTATTGGGGGTTCTCTCATTCCTTTTATTGAGCATAATGATGCGAATCGA
GCTTTAATGAGTTCTAATATGCAGCGCCAAGCAGTTCCGCTTTCTCGGTCCGAGAAGTG
CATTGTTGGAACTGGATTGGAACGCCAAACAGCTCTAGATTCGAGGGTTTCTGTTATAG
CCGAACGCGAGGGAAAGATCATTTCTACTAATAGTCACAAGATCCTTTTATCAAGTAGT
GGGAAGACTATAAGTATTCCTTTAGTTACCCATCGGCGCTCTAACAAAAATACTTGTATG
CACCAAAAACCTCGGGTTCCGCGGGGTAAATCCATTAAAAAAGGACAAATTTTAGCGG
AGGGGGCTGCTACGGTTGGTGGGGAACTTGCTTTAGGAAAAAACGTATTAGTAGCTTAT
ATGCCATGGGAAGGTTACAATTTTGAAGACGCAGTATTAATTAGCGAACGTTTGGTATA
TGAGGATATTTATACTTCTTTTCACATCCGAAAATATGAAATTCAGACGGATACAACAAG
CCAAGGCTCCGCTGAAAAAATCACTAAAGAAATACCACATCTAGAAGAACATTTACTC
CGCAATTTGGACAGAAATGGAGTTGTGAAGTTGGGGTCCTGGGTAGAAACAGGCGATA
TTTTAGTAGGTAAATTAACGCCTCAGATAGCGAGCGAA---TCGTCCTATATCGCGGAAGC
TGGATTATTACGGGCCATATTTGGTCTTGAGGTATCCACTTCAAAAGAAACTTCTCTCA
AACTACCGATAGGTGGAAGAGGACGCGTTATCGATGTGAAATGGATCCAGAGG----------
-----------GATCCCATAATGGTTCGTGTATATATTTTACAAAAACGCGAAATCAAAGTTGGG
GATAAAGTAGCCGGAAGACACGGGAATAAGGGGATCATTTCCAAAATTTTGCCTAGGC
AAGATATGCCCTATTTGCAAGATGGAACGCCTGTTGATATGGTTTTCAATCCCTTAGGAG
TACCCTCCCGAATGAATGTGGGACAAATATTTGAAAGCTCGCTCGGATTAGCAGGGGAT
CTGCTAAAGAAACATTATAGAATAGCACCCTTTGATGAGAGATATGAGCAAGAGGCTTC
AAGAAAACTTGTGTTTTCAGAATTATATGAAGCCAGTAAACAAACAAAAAATCCGTGG
GTATTTGAACCCGAGTACCCGGGAAAAAGCAGAATATTTGATGGAAGAACAGGAGACC
CCTTCGAACAGCCTGTTCTAATAGGGAAGTCCTATATCTTAAAATTAATTCATCAAGTTG
ATGAGAAAATCCACGGACGCTCTACTGGGCCCTATTCACTTGTTACACAACAACCCGTT
AGAGGAAGAGCCAAGCAAGGGGGACAACGAATAGGAGAAATGGAAGTTTGGGCTTT
AGAAGGATTTGGTGTTGCTCATATTTTACAAGAGATACTTACTTATAAATCTGATCATCTT
ATAGCTCGCCAAGAAATACTTAACGCTACGATCTGGGGAAAACGAGTACCTAATCACG
AGGATCCTCCAGAATCTTTTCGAGTGCTTGTTCGAGAACTACGATCTTTGGCTCTAGAA
CTGAACCATTTCCTTGTATCTCAGAAGAACTTCCAGGTTAATAGGGAAGAAGTT----------
-----------ATGATTGACCAATATAAACATCAACAACTTCAAATTGGACTCGTTTCCCCTCAA
CAAATAAAGGCTTGGGCTAACAAAACACTACCTAATGGGGAAGTCGTTGGCGAAGTCA
CAAGGCCCTCCACTTTTCATTATAAAACCGATAAACCAGAAAAAGATGGATTGTTTTGC
GAAAGAATCTTTGGACCCATAAAAAGCAGAATTTGTGCTTGTGGAAATTCTCGAGCGA
GCGGAGCTGAAAACGAAGACGAAAGATTTTGCCAAAAATGCGGAGTAGAATTTGTTG
ATTCTCGGATACGAAGATATCAAATGGGATACATCAAACTCGCATGTCCCGTGACTCAT
GTGTGGTATTTAAAAGGTCTTCCTAGTTATATCGCGAATCTTTTAGATAAACCCCTTAAG
AAATTGGAGGGCCTAGTATATGGCGAT------TTCTCTTTTGCTAGGCCC---AGTGCTAAAA
AACCAACTTTCTTACGATTACGAGGTTTATTCGAGGATGAAATTTCATCCTGTAACCATA
GCATTTCCCCCTTTTTTTCTACCCCAGGCTTTACAACATTTCGAAATCGGGAAATTGCG
ACAGGAGCAGGTGCTATTAGAGAACAATTAGCAGATTTGGATTTGCGAATTATTTTAGA
GAATTCCTCGGTCGAATGGAAGGAATTAGAAGACGAG---GGGTATAGTGGAGATGAAT
GGGAAGATAGAAAAAGACGAATAAGAAAAGTTTTTTTGATTAGACGCATGCAATTGGC

GAAACATTTTATTCAAACAAATGTAGAACCAGAATGGATGGTTTTGTGCTTATTACCAG
TTCTTCCTCCCGAATTAAGACCCATTGTTTATAGGTCTGGGGATAAAGTAGTGACTTCG
GATATTAATGAACTTTATAAGAGAGTTATCCGTCGGAACAACAATCTTGCCTATCTATTA
AAAAGAAGTGAATTAGCGCCAGCAGATTTAGTAATGTGCCAGGAAAAATTGGTACAAG
AAGCCGTGGATACACTTCTTGATAGTGGGTCCCGCGGGCAACCAACGAGGGATGGTCA
CAATAAAGTATACAAATCACTTTCAGATGTAATTGAAGGTAAAGAGGGGAGGTTTCGC
GAAACTCTGCTTGGGAAACGGGTCGATTACTCGGGGCGTTCTGTCATTGTTGTGGGTC
CTTCACTTTCATTACATCAATGTGGGTTACCTCTAGAGATAGCAATAAAGCTTTTTCAGC
TATTTGTAATTCGCGATTTAATCACGAAACGTGCTACTTCTAATGTTAGGATTGCTAAAA
GGAAAATTTGGGAAAAGGAACCCATTGTATGGGAAATACTTCAAGAAGTTATGAGGGG
ACATCCTGTACTGTTGAATAGAGCACCTACCCTGCATAGATTAGGCATACAGGCTTTCC
AACCCACTTTAGTAGAGGGGCGTACTATTTGTTTACACCCATTAGTGTGTAAAGGTTTC
AATGCAGACTTTGATGGGGATCAAATGGCTGTTCATCTACCTTTATCCTTGGAAGCTCA
GGCGGAAGCTCGTTTACTTATGTTTTCTCATATGAATCTCCTATCTCCCGCTATTGGGGAT
CCTATTTGCGTACCAACCCAAGACATGCTTATCGGACTTTATGTATTAACGATTGGAAAC
CGTCGAGGTATTTGTGCAAATAGATATAATAGTTGCGGAAAC---------------TATCCAAACC
AAAAAGTAAATTACAATAAT---AAGTATACGAAAGATAAAGAATCTCTTTTTTCTAGTTC
CTATGATGCACTGGGAGCTTATAGACAGAAACAAATCTGTTTAGACAGTCCCTTGTGGC
TCCGATGGAAACTAGATCAACGC---GTCATTGGGTTA------AGAGAAGTTCCGATTGAAG
TTCAATATGAATCTTTGGGGACTTATCGTGAGATTTATGCCCACTATCTAGTAGTGGGAA
ATAGAAAAAAGGAAATCCGTTCTATATACATTCGCACCACTCTTGGTCATATTTCTTTTT
ATAGAGAAATAGAGGAAGCCATACAAGGATTTAGTCAGGCCTATTCATACACTATC-------
------------------------------ATGGCGGAACGGGCCAATCTGGTCTTTCAGAATAAAGAGATAGA
TGGAACTGCTATGAAACGACTTATTAGCAGATTAATAGATCATTTCGGAATGGGATATAC
ATCCCATATACTGGATCAAATAAAAACGCTGGGCTTCCATCAAGCCACTACTACATCGAT
TTCATTAGGAATCGAGGATCTTTTAACAATACCCTCTAAGGGATGGTTAGTCCAAGATG
CGGAACAACAGAGTTTTCTTTTGGAAAAACACTATTATTATGGGGCTGTACACGCGGTA
GAAAAATTACGCCAATCCGTTGAAATCTGGTATGCTACAAGTGAATATTTGAAACACGA
AATGAATTCGAATTTTCGGATAACAGATCCTTCTAATCCAGTCTATCTAATGTCTTTTTCA
GGAGCTAGAGGAAATGCATCGCAGGTACACCAATTAGTAGGTATGCGAGGATTAATGG
CGGATCCTCAAGGACAAATGATTGATTTACCTATTCAAAGCAATTTACGCGAGGGACTT
TCTTTGACAGAATATATAATTTCCTGCTACGGAGCCCGCAAAGGGGTTGTAGATACTGC
TGTACGAACGGCGGATGCTGGATATCTTACACGTAGACTTGTTGAAGTAGTTCAACATA
TTATTGTGCGTAGAAGAGATTGTGGTACTATCCAAGCTATTTCTGTGAGTCCTCAAAATG
GGATGACGGAA------AAACTTTTTGTACAAACATTAATTGGTCGTGTATTAGCAAACGAT
ATATATATCGGTTCACGGTGCATTGCCACTCGAAATCAAGATATTGGAATTGGGTTAGTC
AATCGATTCATAACCACCTTTCGAGCACAGCCATTTCGAGCACAACCAATATATATTAGA
ACCCCCTTTACTTGCCGGAGCACATCTTGGATCTGTCAATTATGTTATGGTCGGAGTTCC
ACTCATGGCGATCTGGTCGAATTGGGGGAAGCTGTAGGTGTTATTGCGGGTCAATCTAT
TGGGGAGCCAGGGACTCAACTAACATTAAGAACTTTTCATACTGGTGGGGTATTCACA
GGGGGTACTGCCGACCTTGTACGATCCCCTTCAAATGGAAAAATCCAATTCAATGGGG
ATTTGGTTCACCCCACACGTACCCGTCATGGGCAGCCTGCTTTTCTATGTTATATAGACT
TGCATATAACTATTCAGAGTCAGGATATTCTACATAGTGTGACTATTCCTTCAAAAAGCT
TGATTCTAGTGCAAAATGATCAATATGTAGAATCCGAACAAGTAATTGCGGAGATTCGT

GCCGGAACGTCCGCTTTG------TTTAAAGAAAAGGTACAAAAACATATTTATTCCGAATC
AGATGGGGAA---ATGCACTGGAGTACCGATGTTTATCATGCGCCCGAATATCAATATGGT
AATCTTCGTCGATTACCAAAAACAAGCCATTTATGGATATTGTCAGTAAGTATGTGCAG
A------TCTAGTATAGCTTCTTTTTCGCTCCACAAGGATCAAGATCAAATGAATACTTATTC
CTTTTCTGTTGACGGAAGG------TATATCTTTGGCCTCTCG------ATGGCTGAT---GATGAG
GTAAGACATAGACTGTTGGATACTTTTGGT------------GATAGGGAAATTCTTGATTATTCA
---ACGCCGGATCGAATCATGTCCAATGGTCATTGGAATTTTGTCTATCCTTCT---ATTCTT
CAAAATAAT------TTTGATTTGTTAGCGAAAAAGCGAAGAAATAGGTTCGCCATTCCATT
ACAGTATCATCAAGAACAAGAGAAAGAACCAATATCCTGTTTTGGGATTTCGATTGAAA
TACCCTTTATG------GGTGTTTTACGTAGAAATACTATAGTTGCTTATTTTGACGACCCAC
GATACAAAAAAGAT------GGAATTGTTAAATTTAGAAACCCGGAGGAAGATTCCGTCTTA
AAA------AAGGGGGTTTTGATTGAGCATCGAGGA---ACAAAAGAATTTAGTCTAAAATA
C---GAAGTAGATCGGTTTTTTTTCATTCTCCAAGAACTGCATATCTTGCCGAGATCCTCA
TCCCTAAAGGTACTTGACAATAGTATTATTGGAGTGGATACACAACTCACAAAAAATAC
AAGAAGTCGGCTGGGTGGATTGGTTCGAGTGAAGAGAAAAAAAAGCCATACGGAACT
AAAAATCTTTTCTGGAGATATTCATTTTCCTGAAGAGGCGGATAAGATATTAGGT------GG
CAGTTTGATACCACTAGAAAGAGAAAAAAAA------GATTCGAAGGAA---TCAAAA---AA
AAGGGAAAATTGGGTCTATGTTCAGTGGAAAAAAATTCTCAAGAGCAAGGAAAAG-----
-TATTTTGTTTTGGTTCGACCTGCAGTCGCGTATGAAATGAACGAAGGGAGAAATTTAG
CAACACTTTTCCCACAGGATCTCTTCGAGGAAGAGGGTAATCTCCAACTTCGACTTGT
CAATTTTATTTCTCATGAAAATAGCAAGTTAACTCAAAGAATTTATCATACGAAT---CAAT
TCGTTCGAACTTGCTTAGTATTGAATTGGGAACAAGAAGAAAAAGAGGAGGCTCGT----
-----------------GCTTCCCTTGTTGAGATAAGAGCAAATGGTCTGATTCGCGATTTCCTAAG
AATTGGGTTAATCAAATCCACTATTTCGTATACACGAAAAAGGTATGATAGCAGAAGTG
CAGGACTGATTCTCCAT---------TTAGATCGCACCAATACCAATTCCTTTTATTCCAAGGC
GAAGATTCAATCA---------------CTTAGCCAACATCAAGAAGCTATTGGT------------ACCTT
GTTGAATCGAAATAAA---------GAATACCAATCTTTGATGGTTTTGTCGGCATCCAACTGT
TCTCGAATTGGT------TTTTTTAAGAATTCAAAAAATCCC---------AATGGGGTAAAAGAAT
CGAATCCTAGAATTCCTATTCCAAATTTTTCGGGACTCTTAGGTACTATTGCACCT---AGT
ATATCGAATTTTTCTTCATCT---TACTATTTACTAACG---------TATAATCAGATCCTGTTAA
AA---AAGCATTTGCTCCTTGAC---------AATTTGAAACAAAACTTCAAAGTACTTCAAGG
ACTTAAACACTCTTTAATAAATGAAAATCAAAGG------ACTTCAAATTTCGAT------AGTA
ACATCATGTTGGATCCATTCCAGTTAAATTGGCACTTTCTCCCTCATGATTCTTGGGAAG
AGACATCAGCAAAAATTCAC---CTTGGACAATTTATTTGCGAAAATGTATGTCTATTTAA
ATCGCACATA------------AAAAAATCAGGTCAAATTTTCATTGTAAAT---ATAGATTCCTTT
GTTATAAGAGCAGCTAAGCCTTATTTGGCCACTACAGGAGCAACTGTTCATGGTCATTA
TGGAGAAATCCTTTACAAAGGGGATAGGTTAGTTACGTTTATATACGAAAAAGCGAGAT
CTAGTGACATAACGCAAGGTCTTCCAAAAGTAGAACAAATCTTCGAAGCGCGTTCAAT
TGATTCACTATCGCCGAATCTCGAAAGGAGAATTGAGGATTGGAATGAGCGTATACCAA
GAATTCTTGGGGGTCCTTGGGGATTCTTGATTGGAGCTGAGTTAACCATAGCCCAAAGT
CGTATCTCTTTGGTTAATAAGATCCAAAAGGTTTATCGATCCCAAGGGGTACAGATCCAT
AATAGACATATAGAGATTATTATACGCCAAGTAACATCAAAAGTGCGGGTTTCCGAA-----
----GATGGAATGTCTAATGTTTTTTCACCTGGGGAATTAATTGGACTATTGCGAGCAGAG
CGAGCAGGGCGGGCTTTGGATGAATCGATCTATTATCGGGCAATCTTATTGGGAATAAC

AAGGGTTTCCCTGAATACCCAAAGTTTCATATCTGAAGCAAGTTTTCAAGAAACTGCTC
GAGTTTTAGCAAAAGCTGCCTTACGAGGTCGTATTGATTGGTTGAAAGGCCTGAAAGA
AAACGTAGTTCTGGGGGGGATTATACCTGTTGGTACCGGATTCCAAAAATTTGTGCACC
GTTACCCA------CAAAACAAGAACCTTTAT------TTCGAAATTCAAAAAAAAAAACTATTT
GCGTCGGAAATG------AGAGATATTTTGTTTCTCCATACAGAATTAGTTTCTTCTGATTCT
GACGTAACAAAC---------TCAGAATCACCATTTACCCCATTTATA------------------------ATGA
CAAGAAGATATTGGAACATCAATTTGAAAGAGATGATAGAAGCGGGAGTTCATTTTGG
TCATGGTATTAAGAAATGGAATCCTAAAATGGCCCCTTACATCTCGGCAAAGCGTAAAG
GTACTCATATTACAAATCTCGCTAGAACCACCCGTTTTTTATCAGAAGCTTGTGATTTAG
TTTTTGATGCAGCAAGTCAGGGAAAAAGCTTCTTAATTGTTGGTACCAAAAAAAGAGC
AGCGGATTTAGTAGCATCAGCTGCAATAAGGGCTCGTTGTCATTATGTTAATAAAAAGT
GGTTCAGTGGTATGTTAACGAATTGGTCGATTACTAAAACTAGACTTTCTCAATTTAGA
GACTTAAGAGCAGAAGAAAAGATGGAAAAATTCCACCATCTCCCAAAAAGAGATGTG
GCAATCTTGAAGAGAAAATTATCTACCTTGCAAAGATATCTCGGCGGGATCAAATATAT
GACGAGGTTGCCTGACATTGTGATCGTCCTCGATCAGCAAAAAGAGTATATAGCTCTTC
GGGAATGTGCCATTTTGGGGATTCCTACTATTTCTTTAGCCGATACAAATTGTGACCCAG
ATCTCGCGAATATATCGATTCCAGCCAACGATGACACTATGACTTCAATTCGATTGATTC
TTAACAAATTAGTATTTGCAATTTGTGAGGGCCGTTCTCTC---TATATAAGAAATCATATG
GGACAAAAAATAAATCCACTTGGTTTCAGACTTGGTACAACCCAAAATCACCATTCCTT
TTGGTTCGCACAACCAAAAAATTATTCTGAAGGTCTACAGGAAGATAAAAAAATACGG
AATTGTATCAAGAACTATATACAAAAGAATAGGAAAAAGGGCTCGAATAAAATAGAAG
CAATTACACATATAGAAATTCAAAAAGAAATCGATACAATCCACGTCATAATCCATATTG
GATTCCCCAAT---TTATTAAAGAAAAAAGGA---------------GCAATCGAAGAATTAGAGAA
AGATCTACAAAAAGAAGTTAATTCTGTAAACCAGAGACTTAATATTGGGATCGAAAAA
GTGAAAGAACCTTATAGACAACCTAACATTCTTGCAGAATATATAGCTTTCCAATTAAA
AAATAGAGTTTCATTCCGAAAGGCAATGAAAAAAGCCATTGAATTAACTAAAAAAACA
GATATAAAGGGAGTAAAAGTAAAAATTGCAGGCCGTCTCGCGGGAAAAGAAATTGCG
CGTGCCGAGTGCATCAAAAAAGGTAGACTTCCCCTCCAAACAATTCGCGCTAAAATTG
ATTATTGCTGCTATCCAATTCGAACTATCTATGGAGTATTAGGAGTTAAAATTTGGATATT
CGTAGACGAAGAAATGTCCCGTTATCGAGGACCTCGTTTTAAAAAAATACGCCGTCTG
GGAGCTTTACCAGGACTCACTAGAAAAACACCTAAATCCGGAAGTAATCTGAAAAAG
AAATTCCATTCTGGGAAA---AAAGAACAATATCGTATTCGTCTTCAAGAAAAACAGAAA
TTGCGTTTTCATTATGGTCTGACAGAACGACAATTACTTAGATATGTACATATCGCTGGA
AAAGCAAAAAGTTCAACAGGTCAGGTTTTACTACAATTACTTGAAATGCGTTTGGATAA
TATCCTTTTTCGATTAGGTATGGCTTCAACCATTCCCGAGGCCCGGCAATTAGTTAACCA
TAGACATATTTTAGTTAATGGTCGTATAGTCGATATACCAAGTTTTCGTTGCAAACCCCG
AGATATTATTACTACGAAGGATAACCAAAGATCAAAACGTCTGGTTCAAAATTCTATTG
CTTCATCCGACCCGAAA------TTGCCAAAGCATTTGACGATTGACACA------TTGCAATAT
AAAGGACTAGTAAAAAAAATCCTGGATAGGAAGTGGGTCGGTCTCAAAATAAATGAGT
TGTTAGTTGTAGAATATTACTCTCGTCAAACTATGTCACGTCGAGGTACTGCAGAAAAA
AGAACTGCAAAATCCGATCCAATTTTTCGTAATCGATTAGTTAACATGGTGGTTAACCGT
ATTATGAAAGACGGAAAAAAATCATTGGCTTATCAAATTCTCTATCGAGCCGTGAAAAA
GATTCAACAAAAGACAGAAACAAATCCACTATTGGTTTTACGTCAAGCAATACGTAGA
GTAACTCCCAATATAGGAGTAAAAACAAGACGTAAAAAAGGATCGACGCGGAAAGTT

CCGATTGAAATAGGATCTAAACAAGGAAGAGCACTTGCCATTCGTTGGTTATTAGAAGC
ATCCCAAAAGCGTCCGGGTCGAAATATGGCTTTCAAATTAAGTTCCGAATTAGTAGATG
CTGCCAAAGGGGGTGGGGGTGCCATACGCAAAAAGGAAGCGACTCATAGAATGGCAG
AGGCAAATAGAGCTCTTGCACATTTTCGTATGGGCAAGGACACTATTGCTGATTTACTA
ACCTCTATAAGAAACGCGGACATGAATAAAAAAGGAACTGTTCGGGTAGTATCTACAA
ATATTACCGAAAACATTGTTAAAATACTTCTACGAGAGGGTTTTATTGAAAGTGTTCGG
AAACATCAGGAAAGTAACAGATATTTCTTGGTTTCAACTTTGCGACATCAAAAGAGAA
AGACTAGAAAGGGAATATATAGAACT---------AGAACCTTTTTAAAGCGTATCAGCCGAC
CTGGCTTACGAATTTATGCCAACTATCAAGGAATTCCTAAGGTTTTGGGCGGAATGGGA
ATTGCTATTCTTTCTACTTCTCGAGGTATAATGACAGATCGAGAAGCTCGACTAAACAG
AATTGGGGGAGAAGTCTTATGTTATATATGGATGACAAAAGCTATACCAAAAATTGGTT
CACGTAGGAAAGTACGTATTGGTTTGCGTAAGAGTGCACGTAGAATAACAAAAGGAGT
TATTCATGTTCAAGCTAGTTTCAACAATACTATTATAACTGTTACAGATCCGCAAGGTCG
GGTGGTTTTCTGGTCCTCCGCGGGTACTTGTGGATTCAAAAGCTCAAGAAAAGCATCA
CCCTATGCTGGTCAAAGAACAGCTGTAGATGCTATTCGTACAGTGGGT---------TTGCAA
CGAGCAGAAGTTATGGTAAAGGGTGCTGGTAGTGGAAGAGATGCCGCATTACGAGCCA
TTGCTAAAAGTGGTGTACGATTAAGTTGTATACGCGATGTAACACCTATGCCGCATAATG
GGTGTCGACCTCCTAAAAAAAGACGTCTGATGCCAACGGTTAAACAACTTATTAGAAA
CGCAAGACAGCCAATACGAAATGCTAGAAAATCGGCCGCGCTTAAGGGATGTCCTCAG
CGTCGAGGAACATGTGCTAGGGTGTATACTATCAACCCCAAAAAACCCAACTCTGCCTT
ACGTAAAGTTGCCAGAGTACGATTAACCTCTGGATTTGAAATCACTGCTTATATACCTG
GTATTGGCCATAATTTACAAGAACATTCTGTAGTATTAGTAAGAGGAGGAAGGGTTAAG
GATTTACCCGGTGTGAGATATCGCATTATTCGAGGAGCCCTAGATGCTGTCGCAGTAAA
GAATCGTCAACAAGGGCGTTCTAAATATGGGGTCAAAAAGCCAAAAATGGCAAAAAA
AAGTTTGATTCAGAGAGAGAGGAAGCGGCAGAAATTAGAACAGAAATATCATTTGATT
CGTCGATCTTCAAAAAAAAAGATAAGAAGCAAAGTTTACCCTTTGAGTTTGAGTGAAA
AAACGAAAATGCGAGAAAAATTGCAATCCCTGCCACGTAATAGTGCACCGACACGCCT
TCATCGACGTTGTTTTTTGACCGGAAGACCTAGAGCTAACTATCGAGACTTTGGGCTAT
CCGGACACATACTTCGAGAAATGGTTTATGCATGTTTGTTACCGGGTGCAACAAGATCC
AGTTGGATGAAAAAGAGAAAAATTTTTGGATTTATGGTTAAAGAAGAAAAAGAAGAA
AAC------TGGGGTTCTGTTGAATTTCAAGTATTCAGTTTCACCAATAAGATACGGAGACT
TGCTTCACATTTGGAATTACACAAAAAAGATTTTTCATCGGAAAGAGGTCTCCGAAGA
CTTTTGGGAAAACGTCAACGTTTGCTGGCTTATTTGGCAAAGAAAAATAGAGTACGTTA
TAAGAAATTAATCAGTCAGTTGGATATTAGGGAGCGG--------------ATGTTAAAACTTCGT
TTAAAACGATGTGGTAGAAAGCAA--------------------------------CGT-----------------------------
------TTTTATGATCCGATAAAGAATCAAACTTGTTTAAATGTTCCAGCTATTCTCTATTTCC
TTGAAAAAGGTGCTCAACCTACAAGAACCGTTTCTGATATTTTAAGGAAGGCGGAATT
CTTTAAAGAAAAAGAAAGAACTTTGAGT------------TTTCGTAAATCCAAGCAAACTTTT
CGTAAATTCAAACAACCTTTTCGCAAAAGGCGTCCTCGGATTGGCCCGGGGGATCGAA
TTGATTATAGAAACATGAGTTTAATTAATCGATTTATTAGTGAACAAGGAAAAATATTAT
CGAGACGAATAAATAGATTAACCTTGAAACAACAACGATTAATTACTCTTGCTATAAAA
CAGGCTCGTATTTTATCTTTCTTACCATTTCGTAACTATGAGAATGAGAAACAATTTCAA-
--GCCCAGTCAATTTCAATAATTACAGGTCCTAGACCCAGAAAAAATGTGACACGAAAA-
--AAAACGAATCCTTTTGTAGCTCATCATTTATTGGCAAAAATAGAAAAGGTCAATATGA

AGGAGGAGAAAGAAACAATAGTAACGTGGTCCCGGGCATCTAGCATTCTACCCGCAAT
GGTTGGCCATACAATCGCGATTCATAATGGAAAGGAACATATACCTATTTACATAACAAA
TCCTATGGTAGGTCGCAAATTGGGGGAATTCGTACCAACTCGGCATTTCACGAGTTATG
C A A G A A A G G A T A C T A A A T C T C G T C G
T??
???
???
???
???
???
???
???
???
???
???
???
???
???
???
???
???
???
???
???
???
???
???
???
???
???
???
???
???
???
???
???
???
???
???
???
???
???
???

??
??
??
??
??
??
??
??
??
??
??
??
??
??
??
??
??
??
??
??
??
??
??
??
??
??
??
??
??
??
??
??
??
??
??
??
??
??
??
??
??
??
??
??

??
??
??
??
??
??
??
??
??
??
??
??
??
??
??
??
??
??
??
??
??
??
??
??
??
??
??
??
??
??
??
??
??
??
??
??
??
??
??
??
??
??
??
??

??
??
???????????????????????ATGCCTAGATCCCGT---ATAAATGGAAATTTCATTG
ATAAGACCTTCTCAATTGTAGCCAATATTTTATTGCGAATAATTCCGACAACCTCCGGGG
AAAAAAGGGCATTTACTTATTATAGAGATGGG------ATGTTGGCTCAATCCGAAGGAAAT
TATGCGGAAGCTTTGCAAAATTATTATGAAGCTACGCGACTAGAAAGTGATCCCTATGA
TCGAAGTTATATACTCTATAACATAGGCCTTATACACACAAGCAATGGAGAGCATACAA
AGGCTTTGGAATATTATTTCCGGGCACTAGAACGAAACCCCTTCTTACCACAAGCTTTT
AATAATATGGCCGTGATCTGTCATTAC------GGAGAACAGGCCATTCTACAGGGTGATTC
AGAAATTGCAGAAGCTTGGTTTGATCAAGCTGCTGAGTATTGGAAACAAGCTATAGGC
CTTACTCCGGGAAATTATATTGAAGCACAGAACTGGTTGAAGATTACGAAGCGCTTTGA
ATTTGAAATGAATTGGCGATCAGAACACATATGGATAGAACTTCTAAAAGGTTCTCGAA
AAAGAGGTAATTTTTTCTGGGCCTGTATTCTTTTTCTAGGTTCACTAGGATTCTTAGCGG
TTGGGGCTTCCAGTTATCTTGGTAAGAATATTATATCTGTACTTCCATCTCAACAAATTCT
TTTTTTTCCACAGGGGGTCGTGATGTCTTTCTACGGAATCGCAGGCCTATTCATTAGCGC
TTACTTGTGGTGCACTATTTTGTGGAATGTAGGCAGTGGTTATGACCGATTCGATAGAA
AAGAGGGAGTAGTGTGCATTTTTCGTTGGGGATTCCCTGGAATAAAACGTCGCGTCTTT
CTTCGATTCCTTATGCGGGATATCCAATCAATTAGAATTCAGGTTAAAGAAGGTCTTTTT
CCTCGTCGTATCCTTTATATGGAAATCCGGGGCCAGGGGGCCATTCCCTTGACTCGTAC
TGATGAGAAGTTTTTTACTCCACGAGAAATTGAACAAAAAGCTGCCGAATTGGCTTATT
TCTTGCGCATACCAATGGAAGTATTT
>Epifagus
ATGGAAAGATGGCGGTTTAGTCTGATG------ATAAAGAAATCATTGGATAGTGTTAGTTC
TATTGAAAATACTATCGAAAATAAAGATTCTAATATAAAAGGTAGGGCTCAAAACATTC
GTAGTTACAATAATATCATTTATTTATTTGGCGTCGAATACAACCGAAATTTAATACCAAA
TGATACTTTTTTATTTAGGGATAATAAGGGAGATAGTTTTTAT---TCTTTTAATATAGAAAA
TCATATTTTTGAAATTGATATCAAT------TTTTCTTGTCTGAGTGAACTAGAAATAGAATTT
TATAGTTATCATGGTTTTGATTATTTTAATAATGGATCC---ATTGAAGATTACTTATACAAT-
-----ATGTATGATACTCCGTCA------AATAATCACATTACTAGTTGTATTGAAAGTTATCTTC
AG------TCTCAAATATGCGTCAATTCCATTATAAGTGATAGTAGT---AGTTAT------------ATT
TTTGGATGCTTT---GATAGCAGGAGGTCCGGTATACGAAACCGTGCGCAGAGTAGTTAT
TTAACTATTAGAGAAAGCTTTAATAATCTTGATTCAACTAAAAAATACAAGCATTTATGG
GTTCAATGCGAAAATTGTTATGGATTAAATTACAAAAAATTTTTGAAATCAAAAATTAAT
CTTTGTGAACAATGTGGATATCATTTTAAAATGAGTAGTTCAGAAAGAATAGAAGTTTT
GGTTGACCCCGATACTTGGTATCCTATGGATGAAGACATGTCCTCTCTGGATCCCATTGA
ATTTCATTCGGAGGAGGAA---TATAAAGATCGTATTTATTCTTATCAAAAAAGGACGGGA
TTAACCGAGGCTGTTCAAACAGGTTTAGGTCAACTAAATGGTATTCCCATAGCAATTGG
AGTTATGGATTTTCAGTTTATGGGGGGTAGTATGGGATCGGTAGTCGGTGAGAAAATAA
CCCGTTTGATTGAGTACGCTACCAATAAAATTTTACCTCTTATTATAGTGTGTGCTTCGG
GGGGGGCTCGCATGCAAGAAGGAAGTTTGAGCTTAATGCAAATGGCTAAAATATCGTC
TGCCTTATTTGATTATCAATCAAATAAAAAATTATTGTATGTATCAATCCTTACATCGCCC
ACTACCGGTGGGGTAACAGCTAGTTTTGGTATGTTGGGAGATATCATTATTGCCGAACC
AAATTCCTACATTGCATTTGCGGGTAAAAGAGTAATTGAGCAAACATTGCATAAAATAG
TACCCGAAGGTTCACAAGCGGCTGAATATTTATTCCAGAAGGGCTTATTTGACCTAATA

ATACCGCGTAATCTTTTAAAAAGTGTTCTAGGTGAGTTATTTAAGCTCCACGCTTTCTTT
C C T T T G A A T - - - - - - - - - - - - - -
-???
??
??
??
??
??
??
??
??
??
??
??
??
??
??
??
??
??
??
??
??
??
??
??
??
??
??
??
??
??
??
??
??
??
??
??
??
??
??
??
??

??
??
??
??
??
??
??
??
??
??
??
??
??
??
??
??
??
??
??
??
??
??
??
??
??
??
??
??
??
??
??
???ATGCCTATTGGTGT
TCCAAAAGTACCTTTCCAAATTCCTGGAGAGGAAGATGCATCTTGGGTTGACGTATACA
ACCGACTTTATCGCGAAAGATTACTTTTTTTAGGCCAAGAAGTTGATAGTGATATCTCTA
ATCAACTTATTGGTCTTATGGTATATCTCAGTATCGAGGATGATACTAAAGAGATTTATTT
ATTTATAAACTCTCCAGGTGGGTGGGTAATACCTGGAGTAGCTATTTATGATACTATGCA
ATTTGTTCGCCCAGAGGTCCATACAATATGCATGGGATTGGCCGCCTCAATGGGATCTTT
TCTCCTGGTTGGAGGAGAAATTACCAAACGTTTAGCATTCCCTCACGCTAGGGTAATGA
TCCATCAACCCGCAAGTTATTTTTTTGGGGCGCAAACGGGAGAATTTATCCTGGAAGCT
GAAGAATTGCTGAAACTTCGCGAAACCCTCACAAGAGTTTATGTACAAAGGACAGGC
AAACCATTATGGGTTATATCGGAAGACATGGAAAGAGACGTTTTTATGTCAGCAAAAG
ACGCCCAGGCTTATGGAATTGTTGATCTTGTAGCAGTTGAAATGAAAGAACAAAAATG
GATCCATGAAGGTTTAATTACTGAATCACTTACCAATGGCATGTTCTGGGTTAGATTAGA
TAATAAAGATCTTATTATAGGTTATGTTTCAGGAAATATCCGACATAGTTTTATACGGATC

CTGCCTGGGGATAAAGTAAAAATAGAAGTAAGTCGTTATAATTCCACCAGAGGGCGTAT
AATTTATCGACTCCGAAACAAGTATTATAAAGAT--
--AT
GTATAAACAGAACCTTTTTATTATTTCTTCTAACGATTCTAAATACAATTTTTTG---TTGA
AAAAATTGTATTATGAAATTATATTGGAGGGACTTACTTTTATTTTGGAATTTTTTTTTTTT
ATACGATTAATATCTTATCTAATAGAAAAATTAAATAATTTACGATCAATTCTTTCAATATT
TCCTTTTTTAGAAGACAATTTTTCAAATTTAAAATTTATATTAGATATACTAATACCCCGA
AAAGTCCATGCGGAAATCTTAGTTCAAACTCTTCGATATTGGATAAAAGATGCCACTTA
TTTGCATTTATTACGATTTTGGCTCAACAAT------AATTGGAATAGTATTATTAATGATGGT-
-----TATTTTTACCCTAATAAAATATTATTATTATTATATAATTCTTATGTATGTAAATATGAAT
CCATTTTTGTCTTTCTGCGTAACCAATCTTATCATTTTCGATCAACACCCTTTATAATGCT
TCTTGAACGAATCTATTTTTATTTTAAAATAGAACGTCTGGTGAATCCCTTTTTAAAGGA
TTTTAACTTGTGGTTGATCAAGGAACCTTGCCTCCATTATTTTAGGTATCGAAGACAATA
CATTTTAGCTTCAAAAGGTACGTCTCTCTTCCTTAATAAATGGAAATGCTACGTTATTAC
TTTTTGGCAATGGTATTTTTCGCTGTGGTTTTTTTCAAGAAGTATTTATATAAAAAAATTA
TTAAATAATTCTTTTGAAATTTTAACCTATCATTCAAGCTTGAATATAAACCCTTCATTTG
TACTGAGTCAAATTTTAGAAACTCCATTTATAAGCAATAATACTATT------AAAAAAGTTA
ATATCCTTGTCCCAATTATTCTTTCGATTTCA------GATAAAGAGAAATTTTTTAACGTATT
AGGGCATCCCACTAGTAACCTGTTTCGGGTTGATTTATCAGATTATAATATTATTGGCCGA
TTGGAATGTATATATAGAAATCTTTATCATTATCTTAGTGGATCTTCAAAAAAAAAATAGTT
TATATTTAATAAAGTATATACTTCTACTTTCTTGCGTTAGAACTTTGGCTCGGAAACACA
AAAGTACTGTACGTACTTTTGCGAAAAGATTGTGCTTAGAATTATTGGAAGGATTTTTTA
TATCGGAAGAAGACATTCTTTTTCTGAAGTTCCAAAAACAATTTTGGTATTTAAATATTT
A T T C T A T A - - - - - - A A T T A T C T G G C C - -
-??
???
???
???
???
???
???
???
???
???
???
???
???
???
???
???
???
???
???
???

??
??
??
??
??
??
??
??
??
??
??
??
??
??
??
??
??
??
??
??
??
??
??
??
??
??
??
??
??
??
??
??
??
??
??
??
??
??
??
??
??
??
??
??
??
??
??
??

??
??
??
??
??
??
??
??
??
??
??
??
??
??
??
??
??
??
??
??
??
??
??
??
??
??
??
??
??
??
??
??
??
??
??
??
??
??
??
??
??
??
??
??

???
???
???
???
???
???
???
???
???
???
???
???
???
???
???
???
???
???
???
???
???
???
???
???
???
???
???
???
???
???
???
???
???
???
???
???
???
???
???
???
???
???
???
???
???
???

??
??
??
??
??
??
??
??
??
??
??
??
??
??
??
??
??
??
??
??
??
??
??
??
??
??
??
??
??
??
??
??
??
??
??
??
??
??
??
??
??
??
??
??

???
???
???
???
???
???
???
???
???
???
???
???
???
???
???
???
???
???
???
???
???
???
???
???
???
???
???
???
???
???
???
???
???
???
???
???
???
???
???
???
???
???
???
???
???
???
???
???
???

??
??
??
??
??
??
??
??
??
??
??
??
??
??
??
??
??
??
??
??
??
??
??
??
??
??
??
??
??
??
??
??
??
??
??
??
??
??
??
??
??
??
??
??

???
???
???
???
???
???
???
???????????????ATGGCGATACATTTATACAAAACTTCTACCCCGAGCACACGCAATGGA
ACCGTATACAGTCAAGTGAAATCCAATCCACGAAAAAATTTGATCTATGGACAGCATCA
TTGTGGTAAAGGTCGTAATGTCAGAGGAATCATTACCACAAGGCATAGAGGGGGAGGT
CATAAGCGTCTATACCGTAAAATAAGTTTTATACGGAATGAAAAATACATATATGGTAGA
ATCATAACCATAGAATACGACCCTAATAGAAATGCATACATTTGTCTCATACACTATGGG
GATGGTGATAAGAGATATATTTTACATCCCAGAGGGGCTATAATTGGAGATACCCTTGTT
TCTGGTACAGAAGTACCTATAATAATAGGAAATGCCCTACCTTTGACCGATATGCCCTTA
GGCACGGCCATACATAACATAGAAATCACACTTGGAAAGGGTGGACAATTAGTTAGAG
CAGCGGGTGCTGTAGCGAAACTTATTGCAAAAGAGGGGAAATTGGCCACATTAAAATT
ACCTTCTGGGGAGGTCCGTTTGATATCCAAAAACTGCTCAGCAACAGTCGGACAGGTG
GGGAATGTTGGGGTTAATAAGAAAAGTTTGGGTAGAGCCGGATCAAAGCGTTGGCTAG
GTAAGCGTCCTGTAGTAAGAGGAGTAGTTATGAACCCTATAGACCACCCCCATGGGGGT
GGTGAGGGTAGAGCCCCTATTGGTAGAAAAAAACCCACAACCCCCTGGGGTTATCCTG
CACTCGGAAGAAGAAGTAGAAAAATTAATAAATATAGTGATAATTTTATTGTTCGTCGC
C G T A G T A A
G??
???
???
???
??????????????ATGCTTAGTCCACAAAAAACTAGATTTCGTAAACAACATAGAGGAAGA
ATGAAGGGAATATCTTATCGAGGTAATAATATTTGTTTTGGTAAATATGGTCTTAAAGCA
CTTGAACCCGCTTGGATCACACCCAGACAAATTGAGGCAGGTCGACGCGCAATAACAC
GAAAATTCCGTCGTGGTGGAAAAATATGGGTCCGCGTATTTCCAGATAAACCAGTTACA
GTAAGATCATCTGAAACACGTATGGGTTCAGGGAAAGGATCCCATAAATATTTTGTAGC
TGTTGTTAAACCGGGACTAATACTATATGAAATAGGTGGAGTAACTGAAAATATAGCTA
AGCGGGCTATTTTAATAGCAGCATCAAAAATGCCAATGCAAACTCAATTTATTATTTCTG
GAATGACCAGAATTAAACGTGGATATATAGCGCGAAGACGTATAAAAAAATTTCGCTTA
TTTGCATCAAGTTTTCTAAATGCCCACTCAAGACTTACTCGAACTATTACTCAACAGAA
AATAAGAGCTTTGGTTTCATCTGATAGGGATAGGAATAATAAAAAAAGAAAATTTCGTA
GTTTGTGGATCACTCGGATAAACGCAGTAATTAGAGAAGAG---GGGGTATCTTATAGTTA
TAAAAATTTTATATATGCTCAATACAAGATACAGTTGCTTATTAATCGTAAAATACTAGCC
CAAATAGCTATATTAAATAGAAATTTTTTTTATATGATTTTCAATGAAATCAGAAAAGAG
G C A G A T T T A A A A G A A T A C A T C A G A A T A A A
T??
???
???
???

??
??
??
??
???-----
-????????????ATGGCCAAGAGTAAAGATGCGCGAGTAGCGGTCATTTTAGAATGTACTAG
TTGTATCCGAAACAGT------GTAAATAAGGTACTTACGGGTATTTCCCGATATATTACTCA
AAAGAACCGTCGCAATACGCCTAATCGATTAGAATTGAGAAAATTCTGTCCCTATTGTT
ATAAACATATGATTCACGGGGAAATAAAGAAAATGAAAATAAGAGCTTCCATTCGTAAA
ATTTGTGAAAAGTGTAGGTTAATCTGTAGGCGTAGGCGCATTATAGTAATTTGTTCCAAC
C C G A G A C A T A A A C A A C G G C A A G G
A???
??
??
??
??
??
??
??
??
??
??
??
??
??
??
??
??
??
??
??
??
??
??
??
??
??
??
??
??
??
??
??
??

???
???
???
???
???
???
???
???
???
???
???
???
???
???
???
???
???
???
???
???
???
???
???
???
???
???
???
???
???
???
???
???
???
???
???
???
???
???
???
???
???
???
???
???

???
???
???
???
???
???
???
???
???
???
???
???
???
???
???
???
???
???
???
???
???
???
???
???
???
???
???
???
???
???
???
???
???
???
???
???
???
???
???
???
???
???
???
???
???
???

??ATGACAAGAAGATAT
TGGAATATAGACTTGGAAGAGATGATAGGGGCAGGAGTTTCTTTTGGGCATGGTACTAG
AAAATGGAATCCAAAAATGGCACCTTATATCTCTGTAAAGCATAAAGGTATTCATTTTAC
AAATCTTACGAAAACTGCTCGTTTTTTATCTGAAGCCTGTGATTTGGTTTTTTATGCAGC
AAGTAGGGGAAAACAATTCTTGATTGTTGATACAAAAAATAAAGCAGCTGATTCAGCA
GCATGGGCTGCAATAAAGGCCCGGTGTCATTGTGTTAATAAAAAATGGCCCGGAGGGA
TGTTAACGAATTGGTCCACTACAGAAACGCGACTTCATAAGTTAAGGGACTTGATAATG
GAACAAAAAGCAGGAAGACTCAACCGCCTTAAAAAAAAGGATGAGGCTGCCGTGAA
AAGACAATTAGCTCGATTGCAAACATATCTAGGTGGTATAAAATATATGACAAGGTTACC
AGACATCGTAATCATCGTTGATCAGCACGAAGAATATAAGGCTCTGCACGAGTGTATCA
CCATAGGAATTCCTACAATTGGTTTAATCGATACAAATTGTGATCCCGATCTAGCAGATA
TTTCTATTCCAGCGAATGATGACGCTATTTCTTCAATACGTTTAATTCTTAACAAATTGGT
ATTCGCTATTTGTGAGGGCCGTTCC---GGCTATATAATAAAAAATATGGGACAAAAAATA
AATCCACTTGGTTTTAGGCTTGGTACAACCCAAAGTCATCATTCCTTTTGGTTTGCACA
ACCAAAAAATTATTATAAGGGTATACAAGAAGATCAAAAAATAAGAGATTTTATAAAAA
ATTATGTAAAAAATAATATTATAATATCCCCTGAC---ACCGAAGGAATTGCCTATATAGAG
ATTCAAAAAAGAATCGATTTTTTAAAAATAATGATTTTTATAGGATTCAAA---TTTTTAAT
AGAAAATAGACAACTAGGAATT------ATTAAAGAAGCATTACATATAGATTTAAAAAAA
ACTTTCATTATGTTAATAGAAAACTTATAATTGATATCATAAGAATTACAAAACCTTATAG
AAACCCTAATATTCTTGCAGAATTTATAGCCGATCAATTAAAGAATAGAGTTTCTTTTAG
AAAAACAATGAAAAAAGCTATTGAATTAACGGAAAGCGAAGATACAAAAGGAATTCA
AGTACAAATTTCAGGGCGTATCGACGGAAAAGAAATTGCACGTGTTGAATGGATCAGA
GAGGGCAGAGTCCCCCTACAAACTATTCAAGCTAAAATAAATTATTGTTCCTATATGGTT
AGGACTACCCACGGGGTATTAGGCATAAAAATTTGGATTTTTATAGAAAAGGAAATGTC
ACGTTACCGGGGACCTAGTTTAAAAAAAATACGACGCCTGGGGGCTTTACCAGGACTA
ACTAATAAAAGGTCTAAAGCCGAAAACGATTTTATAAAAAAATTGCGTTCTGATAAA---
AAATCTCAATATCGTATTCGCCTAGAAGAAAAACAAAAATTACGTTTTAATTATGGTCTT
AGAGAACGACAATTACGTAAATATTTTAGTATCGCAATAAAAACCAGGGGGTCAACCG
GTAAAGTTTTAATGCAATTACTTGAAATGCGCTTGGATAACATCATTTTTAGATTGGGTA
TGGCTTCGACTATTCCTGCGGCCCGCCAATTAGTTAACCATAGACATGTTTTAGTAAATG
GTCGTATAGTAGATATACCAAGTTATCGCTGCAAATCCCGAGATATTATTATGGCAAGGG
ATGAACAACAATCCAACACTTTTATTAATAATTGTATCAATTATTCCACACATCGTATGG
AAGCACCAAATCACTTAACTCTTCTTCACCCA---------TTCAAAGGATTAGTCAATCAAAT
AATAGATAGTAAATGGGTCGGTTTTAAAATAAATGAATTGCTAGTCGTAGAATATTATTT
TCGTAAAACTATGTCACGTCGAGGTACTGCAGAAGAAAAAACAGCAAAACCCGATCC
AATTTATTGGAATCGATTAGTTAACATGTTGGTTAACCGTATTCTGAAACACGGAAAAA
AATCATTGGCTTATCAAATAATCTATCGAGCCTTGAAAAAAATTCAACAAAAGACAGAA
AAAAATCCACTATATGTTTTACGTCAAGCAATACGTGGAGTAACTCCCGATATAGCAGT
AAAAGCAAGACGTGTAGGGGGATCGACTCATCAAGTTCCCATTGAAATAGGATCCACA
CAAGGAAAAGCACTTGCCGTTCGTTGGTTATTAGTGGCATCAAAAAAAAGGCCCGGTC
AAAATATGGCTTTCAAATTAAGTTCCGAATTAGTAGATGCTGCAAAAGGGAGTGGCGAT
GCCATACGCAAAAAGGAAGAGACTCATAAAATGGCAGAGGCAAGTAGAGCTTTTGCA
CATTTGCGTATGGGTAGAGACACTATTTTGGAAATAATAAACTCTATACGAAATGCTGAT
AGGGGTAGAAAAAGAGTTGTTCGCATAACATCTACAAATATTACCGAAAATTTTGTTAA

AATACTTTTTATAGAAGGTTTTATTGAAAACGCAAGAAAACATAGAGAAAAAAACAAA
TATTATTTTACTTTAACCCTACGACATAGAAGG------AATAGTAAAAGACCTTAT---------AT
AAATATTTTAAATTTAAAACGAATAAGTCGGCCCGGTCTACGAATCTATTCAAACTCTCA
ACAAATTCCTTTAATTTTAGGCGGAATAGGAATTGTAATTCTTTATACCTCTAGGGGTAT
AATGACAGATAGGGAGGCTCGACTAAAAGGAATCGGCGGAGAATTGTTGTGTTATATAT
GGATGGCAAAAGCTATACCAATAACTGGTTCACGTAGGAATGTACATGTTGGTTCACGT
AAGAGTTCATTTAGAATACAAAAGGGAGTTATTCATGTACAAACAAGTTTCAATAATAC
CATTGTTGCAGTTACAGATATAAAGGGTCGGGTTGTTTCCTGGTCCTCGGCTGGTACTT
GTGGATTTAAGGGTACGAGAAGAGGGACATCCTTTGCTGCCCAAATTGCAGCAACAAA
TGCTATTCGT------ATAGTCCAAGGTATGCAACGAGCAGAAGTAATGATAAAAGGTCCCG
GTATAGGAAGAGACGCAGTATTACGAGCTATTCGTGGAAGTGGTGTACTATTAACTTTT
GTACGGGATGTAACCCCTATGCCACATAATGGCTGTAGACCACCTAAAAAAAGACGTG
TGATGCCAACTATAAAACAACTTATTAGAAAAAAAAGACAGCCAAACCTAAATGTAAC
TAAATCTCCCGCCCTACGGGGATGTCCTCAGCGTCGAGGAACTTGTACTAGGGTGTATA
CTATCACCCCAAAAAAACCAAACTCTGCCTTACGTAAAGTTGCCAGAGTACGATTAAC
CTCTGGAATTGAAATAACTGCTTATATACCCGGTATTGGCCATAATTCACAAGAACATTC
TTCAGTCTTAGTAAGAGGGGGAAGGGTTAAGGATTTACCCGGTGTAAGATATCACATTA
TTCGAGGAACCCTAGATGCTGTCGGAGTAAAGGATCGTCAACAAGGGCGTTCTAAATA
TGGGGTAAAAATAAAAAATATGGCAAGAAAAAGTTTGATTCAGAGGGAAAAGGGCAG
GCTAAAGTTGGAAAATAAATATCATTTTATTCGTCGATCCTCAAAAAATGAAATAAGT----
-----AAAGTTCCATCATTGAGTGACAAATGGGAAATTTATGGAAAGTTAGAATCCCCACC
ACGTAATAGTGCACCTACGCGCCTTCGTCGTCGTTGTTTTTATACCGGAAGGCCAAGAG
CTAACTACCGAGACTTTGGACTATGCGGACACATACTTCGCGAAATGGTTAATGCATGT
TTGTTGCCAGGAGCAACAAGATCAAGTTG
G???
??
??
??
??
??
?????????????????ATGTATAAATTTAAG--------------------CGATCTTTTCGTAGGCGTTTGTC
CCCGATTGGGTCGGGGAATCTAATTTATTATAGAAACATGAGTTTAATTAGTCGATTTATT
AGTGAACAAGGAAAAATATTATCTAGACGAGTTAATAGATTGACCTTGAAACAACAAC
GATTAATTACTATTGCTATAAAACAAGCTCGTATTTTATCTTTATTACCTTTTATAAATAAT
GAAAAA------CAATTTGAAAGAATCGAGTCCATCACCAGAGTTAAGGGT---TTTATTAAA
AAA---GTGATACATTCACTTAAAAAAAATCTTTTTGTAGCCAATCATCTACGGGCGAAA
ATTAATAAGCTTAATAATAAAAAAAAAAAAAGAAATAATAGTAACGTGGTCCCGCGCATC
TACTATTATACCCATAATGATCGGGCATATGATTTCTATTCATAATGGTAAAGAACATTTG
CCTATTTATATAACAGATCATATGGTAGGTCACAAATTAGGAGAATTTGTACCTACTTTAA
ATTTCCGAGGACACGCAAAAAGTGATAATAGGTCACGTCG
T???
??
??
??

??
??
??
??
??
??
??
??
??
??
??
??
??
??
??
??
??
??
??
??
??
??
??
??
??
??
??
??
??
??
??
??
??
??
??
??
??
??
???????????????????????ATGAAAGAACATCCATACAAATCCTGGATTTTAGAATTG----------
--AGAGAGATAAAGAATTCTCACTATTTCTTAGATTCATGGACTAAATTCAATTCAGTAGG
GTCTTACATTAACATTTTTTCCCACCAAGAACGTTTTATAAAACTATTTGACCCCCGAAT
TTTGAGTATCCTACTTTCA---CGCAATTCA------CAGGGTTCAACAAGCAATAGATATTTC
ACG---------ATCAAGGGTGTAATACTCTTTGTAGTAGCG---GTACTTATATATCGT------------
ATTAACAATCGAAATATGGTCGAAATAAAAAATATATATTGGAGGGGGCTTCTTCCT------

---------ATACCTATGAATTCCATTGGACCCAGAAATGATACATTGGAAGAATTGGTC---GG
GTCTTACAATATAAATAGGTTTATTGTTTCG---CTCCTCTATCTTACAAAAGGGAAAAATA
TCTCTGAGAGTTTTTTCCTGAATCTGAAAGAGAGTACTTTGGTTCTCCCAATAACT-------
-----AAAAAGTGTAGCATGCCTGAATCTAACTGGGGTTCACGGTGGTGGAGGAACTGGA
CCGGAAAAAAATAGG------GATTATAGTTGCAAGATATCTAAT---------GAAACCGTCGCTG
GAATTGAGATCTTATTCAAAGAGAAAGATAAAAAA---TATCTTGAGTTTATTTTTTTTTAT
TATATGGAT------GATCCAATTCGCAAGGACCGTGATTGGGAATTGTTTGATCGTCTTTCT
CCGAGTAAGAGGCTAAATAAAATCAACTTT------TATTCGGGACCGCTATTCGAAATCTT
AGTGAAACGCCGGATTTATTATCTCATGTCTGCTTTTCGTGAAAAAATACCAATTGAAG
TAGTGAAGGGTTTCTTCAAACAACAAAAG---GTTGGGTCAACTATTCAATCAAATGATA
TTGAGCATGTTTCTCAT------TTCTTCTCGAGAAATAAGCGGGCTATTTCTTTGAAAAATA
GTGCTCAATTTAATATGTGGCAATTTCGCCAGGATCTCTTAGTT---------AGTTGGGGGGA
G---AATCCGCACGAATCGGATTTTTTGAGGAAC---GTTTCGAGAGCGAATTGGATTTGGT
TAAACAATGTGTGGTTGGTAAACAAGTATCGG---------TTTTGTAGA------AAGGTACGGA
ATGTATCGTCAAATATTAAATATAAATAT---GATTCCACAAGATCAAGATCCAGTTTCGTT
CAAGTAACGGATTCTAGCCAACTGAAAGGATCTTAT---TATAAATCTAGCGGTCATTTTTA
TTCCGTTATTAGT---------AATGAGGATTCGGAA------------------------------------TATCACAC
ATTGATCAATCAAAGAGAGATTAAACCACTAAAA---------TCTATTTTTTTTGAT------CCT
TCCTTTCTTCAAACGGAAGCAACAGAGATAGAATCAGAC------CAATTACAGAAA------C
GCCCTTCTGGATATTCC------------TCAACGCTATTCACG---GAACATGAGAAGCAGATGA
TTAATCATATGCTTCCGGAAGAAATCGAAGAATTTATTGGG------AATCCTACAAGATTG
GTTCATTCTTTTTTATCTGATAGATGG------TCAGAACTTCAT---CTGGGTTCGAATCCTAC
TGAGAGGTCCACTAGAGATCAT------------AAATTGTTGAAGAAACAACAAGATCTTTCT--
-TTTGTCCCTTCCAGGCGATCGGAA------------AATAAAGAACTGGTTAATATATTAAAGAT
AATTACGTATTTAAAAAAT------ACTGTTTCTATTCATCCTATTTCATCAGATCCGGGGTGT
GATGGGGTTCTG---AAGGATGAACCGGAT---ATGGACAGTTCCAATAAGATTTCAGTCTT
TAACAAAAATACTTTTATTTATTTA------TTTCATCTATTCCATGACTGGAACAGGGTAGG
ATACACCTTAAACCTACACCACGATTTTGAATTAGAA------GAGAGATTTCAAGAAAAG
GCAGATCTATTCACTCTATCAATAACCGAGCCA---GATCTGGTGTATCACAAGGGATTTT
CCTTTTCTATTTAT---------ATGGATCAAAAACAA--

------------AAAATGGTGGTATTTGCTAGCAACAACATAATGGAGGCAGTCAATCAATCTAG
ATTTATACGAAATATGATCAAAATCCAATATAGTACCTATGGA------TACATAAGAAATGTA
TTACACCGATTCTTTTTAATGAAT---------AGATCCGATCACAACTTAGAATATGAAATTAA
A---------AGGGATCAAATAGGAAAGGACACTCTGAATCATAGAACTATAATTAAATATATG
ATCAACCAACATTTATCGAATTTTAAAAAG---AGTCAGAATAAATGGTTCAATCCT------A
TTCTCTTTTTTTTCTCGAACCGAGAGATCCGTAAATCGGAATCCTGATGCATATAGATACA
AAAGGTCCAACGGGAGCAATAATTTTTTGGAACATCTGGAACATTTCGTTTCTGAGCA
GAAGAGCCATTTT---AAATTTAAAATAGTGTTTGATCTAATACGTTTTAATCAATATTCGA
TTGATTGGTCTGCGTTTATCGACACAAAAGATTTGTCTAAGCCACTTCGTTTCTTTTTAT
CCAAG--TTGCTTTTCTTTTTGTCTAA
C---TCACTTCCTTTTTTCTGTGTGAGTTTCGGTAATATCCCCATTCATAGGTCCGAG---AT
CTACATCTAT---GAATTGAAAGATCCGAATGATCAACTCTGTAATCAGTTTTTAGAACCA
ATAGATCTTAAAATTGTTCATTTAAAAAAAAGGAAACCCTTC------TTATTGGGT---------T

ATCATGGTACTTCC---CGCAAATTGAAATTATTGATCACTGGAGGAAGA------CCTTTTTT
ATTCAATAAGATACCA---AGGTGT---ATGATTGACTCATTCCATACTATA------AATAATCG
CAGTAAATCCTTTGATAACACGGATTCCTATTTATCAATGATATTCCACAATAAAGACAA
TTGGCTTAATCTCGTGAAACCATTTCATAGAAGTTCATTGATATCTTATTTTTATAAAGCA
AATCGACTTCAATTCTTGAATAATCCACATAACTTCTGCTTCTAT------TGTAACACAAGA
TTACCCTTTTACGTA------------GAAAAGGCCCATATCCATAATTATTAT------TTTACGTATG
GA---CAATTCCTCAATATCTTGTTTATTCGCAACAAA---ATATTTTCTTTGTGCGTCGATA
AA------AAAAAACATGCTTTTTGGGGGGGGAGAGATACTATTTCACCAATAGAGTCACA
GGTATCTAAAATATTCATACCTAAAAATTTTCCACAAAGTGGTGATGAAACA---TATAAC
TTGTCCCAACCT---------TTCCATTTTCCAAGCCGATATGACCCATTCGTTCGT---------------
--TTGATCGCAAACATTTATGGAACACCTCTAACAGAGGGACAAATAGTAAATTTAGGAA
GAACTTATTGTCAACCTCTTTCAGATATGAATCTATCTGATTCAGAAGGGAAGAACTTT
CATCAGTATCTCAATTTC------AATTCAAACATGGGTTTGATTCACACCCCATGTTCTGAT
AAATATTTACCATCTGAAAAG------AGGAAAAAA------------------------CGTAGTCTTTGTAT
AAATAAATATAAATGCGTTGAGAAAGGGCAGATGTATAGAACCTTTCAACGAAAGGTT
GCTTTTTCAACTCTCTCAAAATGGAATCTATTCCAAACATATATGCCATGGTTCCTTACT
TCGGCAGGGTACAAATATATAAATTTGATATTTTTAGATACTTTTTCGGAACTATTGTCGA
TACTAAGTAGCAGT---------AAAAAATTTGTATCCATTTTTAATAATATTATGCATGGATCA
GGTATATCATGGCGAATTATTAATAAAAAAACGG------------------TGTCTTCCA------------CTG
ATAAGTGAGATTTCGAGTAAGTGTTTACATAAT------CTTCTTCTGTCCGAAGAAACGATT
CGTCAAAATAATGAATCA------CCATTAATATCGACACATCTCAGATCGCCAAATGTGCG
GGAGTTC---CTCTATTCAATCCTTTTCCTTCTTCTTGTTGTTGGATAT---CTCGTTCGTACA
CATCTTCTCTTTGTTTCCCGG------GCCTCTAGCGAGTTACAGACAGAGTTTAAAAGGGT
CAAATCATTGATGATTCCATCATCTATGATTGAGTTGCGGAAACTTCTGAATAGATATCC
TACA---GCATCTAATTCTTTCTGGTTAAAGAATCTATTTATAGTTGCTATGGAACAATTAG
TATATTCTCTAGAAGAAATACGGGCTTCTGGCGGCAAC---------------CTACTTGGTCCCGC
T---------------TATGGGGTCAAATCAATATGTTCT---AAGAATAAATATTTTAATATCAAT-------
-----------CTCATC---GATCTCATACCCAATCCCATCAATAGAATCATTTTTTCGAGAAATAT
GAGACATCTAAGTCATACAAGTAAAGAGATCTATTCATTGATAAGAAAAAGAAAAAAC
GTGAATGGGGATTGGATTGATGATATAATAGAATCCTGGGTCGCGAACAGTGATTCGAT
TGATGATGAAGAAAGAGAATTCTTGGTTCAGTTCTCC------GCCTTAACGACA------GAA
AAAAGGATTTATCAAATTCTATTGAGTCTGACT---CATAGTGATCATTTATCA---------------
AAAAACGACTCTGGTTATAAAATGATTGAACAACCGGGAGCAATTTACTTGCGATACTT
AGTTGACATTCAT------AAAAAGTATCTATTGAATTATGAGTGCAATACATCCTGTTTAGT
AGAAAGACGGGTATTCCTTGCTCATTCTCAGACAATCACTTAT------TCACAAACTTCTC
GTGGGACTAATACTTTGCATTTCCCATCTCAAGGA---------AAACCCTTTTCGATCAGCTT
AGCCTTATCCCCCTCT---AAGGGGATTTAGTGATAGGTTCTATAGGAACTGGACGATCC
TTTTTGGTAAAATACCTAGCGACAAACTCCTATGTTCCTTTCATTACGGTATTTCTGAAC
AAGTTCCTGGATAACAAGCCTAAAGGTTTTCTTGTTGAT---------------GATAATGAC---AA
TGATTCTAGTGACGATATTTATGCTAGTGACGATATTAATAGT-----------------------------------
------------------------------GACCTTGATACGGAGCTGGAACTTATAACTATGATGAATGCGCT
A---------ACTATGGATATGATGTTG---------GAACTAGACCGATTTTTTACCACCCTTCAATT
AGAATTAGCAAAAGCAATGTCTCCTTGGATAATATGGATTCCAAACATTCATGATCTGGA
TGTGAATGAGTCGAATTACTTATCCTTCGGTCTATTAGTGAACCATCTCTCT----------------

-GAAAGATGTTCC---ACTAATAATATTATTGTTATTGCTTCAACTCATATTCCCAAAAAAG
TGGATCCCGCTCTACTAGCTCCTAATAAATTAAATACGTGCATTAAGATACGAAGGCTTC
TTATTCCACAACAACGAAAGCACTTTTGCACTCTTTCATATACTAGGGGATTTCACTTG
GAAAATAAAATTTTCCATACT------AATGGATTCGGTTCCATAACCATGGGTTCCAGTGC
AAGAGATCTTGTAGCACTTACCAATGAGGCCCTATCGATTAGTATTACACAGAATAAATC
AATTCTAGACACTAATACAATTAGATCCGCTCTTCATAGACAAACTTGGGATTTGCGA---
------TCCGGGGTAAGATCGTTTCAGGATAATGGGATCCTTTCTTATCAGATAGGAAGGGC
TATAACACAAAATGTACTTCTAAGTAATTGTCCCATAGATCCTATATCTATCTATATGAAG
AAGAAATCATGTACATGTAACGGGGGGGATTATTATTTTTACAAATGGTACTTTGGACTT
GGAACGAGCATGAAGAAATTAACGATACTTCTTTATCTTTTGAGTTGTTCTGCCGGATC
GGTCGCTCAAGATCTTTGGTCTCTACCC------GGACCCGCTGAAAAA---AATGGGATCAC
TTCTTATGGACTCGTTGAGAATGATTCG---GATCTAGTTCGTGGCCTATTAGAAGTAGAA
GGCGCTCTGGTGGGA------TCTTCACGGACAGAAAAAGATTGCAGTCCGTTTGATAATG
ATCGAGTTATATTTACATTGATTCTTCGGCCCGAACCGGGTAAT---CCCTTAGATATTATTA
AAAAGGGATCTTGTTCTATCTTTGATCATAGATTTATCTATGAAAAA------TACGAATCGG
AGTTTGAAGAA---GGGTATGGGGAAGGAGCCCTTGACCCGCAACAG------------ATAGAG
GAGGAT------------TTATTCAATCAC------ATAGTTTGGGCTCCT------AGAATATGGCGCCCT
---------TGGGGCTTTATATTTTATTGTATCGAAAGACCCAATGAATTGGGATTTCCT---TATT
GGTCCAGGTCATTTAGA---------GGCAAGCGGATCGTTTATGATAAAGATGGTGAGCTTC
AAGAGAATGATTCGGAGTTATTGAAGAGTGGAACCGTGCAGTACCAGACACGAGATAG
ATCTTCCAAAGAACAAGGCCTTTTAAAAATAAACCAATTCATTTGGGATCCTGCAGATC
CTCTCTTTTTCCTATTAAAA---GATCAGCCCCCTGGCTCTGTGTTTTCACATCGAAGATT
TTTTGCAGATGAGGAGATGTCAAAAGGTCTTCTTACTTCCCAAAAGGAT---CCTACATCT
ATATATAAACGCTGGTTTATC------AAGAATACACAAGAACAGCACTTCGAATTGTTGATT
AATCGTCAGAGATGGCTTAGAACC---------AAAAGTTCATTATCTAAATCTAATGGATCTT
TTCGTTCTAAT------ACTCTATTCGAGAGTTATCAGTATTTATCAACTCTGTTCCTATCTAAT
---GGAACGCTATTTGATAAAATGACAAAGACATTGTTAATAAAAAGATGGCTTTTCCCG
G A T G A A A T G C A A A T G -
-???
??
??
??
??
??
??
??
??
??
??
??
??????????
>Zea
??
??

???
???
???
???
???
???
???
???
???
???
???
???
???
???
????ATGGCAACCCTTCGAGTCGACGAAATTAATAAAATTCTCCGCGAACGTATTGAACA
ATATAATAGGAAAGTAGGGATTGAGAATATCGGTCGCGTAGTGCAAGTGGGGGATGGG
ATTGCTCGTATTATAGGTCTTGGTGAAATAATGTCAGGTGAATTAGTCGAATTTGCAGAA
GGGACGAGAGGTATTGCTCTGAATTTGGAATCTAAAAATGTTGGGATTGTATTAATGGG
CGATGGGTTGATGATACAAGAGGGAAGTTTTGTAAAAGCAACAGGAAGAATTGCTCAG
ATACCCGTGAGCGAGGCTTACTTGGGTCGTGTTATAAATGCTCTCGCTAAACCTATTGAT
GGGAGAGGCGAAATTGTCGCTTCAGAATCTCGCTTAATTGAATCTCCTGCTCCGGGTAT
AATTTCTAGGCGTTCCGTATATGAACCCCTTCAAACAGGGCTTATTGCTATCGATTCGAT
GATCCCCATAGGGCGCGGTCAGCGAGAGTTAATTATTGGGGACAGACAGACTGGCAAA
ACAGCAGTAGCCACAGATACAATTCTCAATCAAAAAGGTCAAGATGTAATATGTGTTTA
TGTAGCTATCGGTCAAAGAGCATCCTCCGTGGCTCAAGTAGTAACTACTTTCCACGAAG
AGGGGGCCATGGAATACACTATTGTAGTAGCTGAAATGGCGGATTCACCCGCTACATTA
CAATATCTCGCTCCTTATACGGGAGCAGCCCTGGCTGAGTATTTTATGTACCGCGAACG
GCATACCTTAATAATTTATGATGATCTCTCCAAACAGGCACAAGCTTATCGCCAAATGTC
CCTTCTATTAAGAAGACCTCCCGGCCGCGAAGCTTATCTAGGGGATGTTTTTTATTTGCA
TTCACGCCTTTTAGAAAGAGCCGCTAAATTAAATTCTCTTTTAGGCGAAGGGAGTATGA
CCGCTTTACCAATAGTGGAGACTCAATCTGGAGACGTTTCCGCCTATATTCCTACTAATG
TAATTTCAATTACAGATGGACAAATATTCTTATCCGCGGATCTATTCAATGCCGGAATTC
GACCTGCTATTAATGTGGGTATTTCAGTTTCCAGAGTAGGATCCGCAGCTCAAATTAAA
GCCATGAAACAAGTAGCTGGCAAATCAAAATTGGAACTAGCTCAATTCGCAGAATTAC
AAGCCTTTGCACAATTCGCCTCCGCTCTGGATAAAACAAGTCAGAATCAATTGGCAAG
GGGTCGACGATTACGGGAATTGCTTAAACAATCCCAATCAAACCCTCTCCCAGTGGAA
GAGCAGGTAGCTACTATTTATACCGGAACGAGAGGATATCTTGATTCGTTAGAAATTGA
ACAGGTAAAGAAATTTCTGGATGAGTTACGTAAACACCTAAAAGATACTAAACCTCAA
TTCCAAGAAATTATATCTTCTAGTAAGACATTCACCGAGCAAGCAGAAACCCTTTTGAA
GGAAGCTATTCAGGAACAGCTTGAACGGTTTTCCCTTCAGGAACAAACAATGAGAACC
AATCCTACTACTTCGCGTCCCGGGATTTCCACAATTGAAGAAAAAAGCGTAGGGCGTAT
TGATCAAATTATTGGACCCGTGCTGGATATCACTTTTCCCCCGGGCAAGTTGCCTTATAT
TTATAATGCTTTGATAGTCAAGAGTCGAGACACTGCCGATAAGCAAATTAATGTGACTT
GTGAGGTACAACAATTATTAGGAAATAATCGAGTTAGAGCTGTGGCTATGAGTGCTACA

GAGGGGTTGATGAGAGGAATGGAAGTGATTGACACGGGAACTCCTCTCAGTGTTCCA
GTCGGTGGAGCTACTCTCGGACGAATTTTTAACGTTCTTGGGGAGCCTATTGACAATTT
GGGTCCTGTGGATACTAGTGCAACATTTCCTATTCATAGATCTGCGCCTGCCTTTATCGA
GTTAGATACGAAATTATCTATCTTTGAAACAGGTATTAAGGTGGTCGATCTTTTAGCTCC
CTATCGACGTGGAGGAAAAATCGGACTGTTTGGGGGGGCAGGAGTAGGTAAAACAGT
ACTCATCATGGAATTAATCAATAACATTGCTAAAGCTCATGGAGGCGTATCCGTATTTGG
CGGAGTAGGGGAACGGACTCGTGAAGGAAATGATCTTTATATGGAAATGAAGGAATCC
GGAGTAATTAATGAAAAAAATATTGAGGAATCAAAGGTAGCTCTAGTCTATGGCCAAAT
GAATGAACCGCCGGGAGCTCGTATGAGAGTTGGTTTAACTGCCCTAACTATGGCAGAAT
ATTTCCGAGATGTTAATAAGCAAGACGTGCTTTTATTCATCGATAATATCTTTCGTTTTGT
TCAAGCAGGATCGGAAGTATCCGCCTTATTAGGCAGAATGCCCTCCGCAGTGGGTTATC
AACCTACCCTTAGTACAGAAATGGGTTCTTTGCAAGAAAGAATTACTTCTACCAAAAA
GGGATCTATAACTTCGATCCAAGCAGTTTATGTACCTGCAGACGATTTGACCGACCCTG
CTCCTGCCACAACATTTGCACATTTGGACGCTACTACCGTACTTTCCAGAGGATTAGCT
TCCAAGGGTATTTATCCCGCAGTGGATCCTTTAGATTCAACCTCAACTATGTTACAGCCT
CGGATTGTTGGCAACGAACATTATGAAACTGCGCAAAGAGTTAAGGAAACTTTACAAC
GTTACAAAGAACTTCAGGACATTATCGCAATTCTTGGATTGGATGAATTATCGGAGGAG
GATCGTTTAACTGTAGCAAGAGCACGAAAAATCGAGCGGTTCTTATCACAACCGTTCTT
TGTGGCAGAAGTTTTTACCGGTTCTCCAGGAAAGTATGTTGGTCTTGCAGAAACAATTA
GGGGATTTCAACTAATCCTTTCCGGAGAATTAGACGGCCTACCCGAACAGGCTTTTTAT
TTAGTGGGGAACATCGATGAAGCTAGCACGAAAGCTATAAACTTAGAAGAGGAGAGC
AAATTGAAGAAAATGAAATTAAATCTTTATGTACTGACTCCTAAACGAATTATTTGGGAT
TGTGAAGTGAAAGAAATCATTTTATCTACTAATAGTGGCCAAATTGGTGTATTACCAAA
CCACGCCCCCATTAACACAGCTGTAGATATGGGTCCTTTGAGAATACGCCTGAACGACC
AATGGTTAACGGCGGTTCTGTGGAGTGGTTTTGCAAGAATAGTTAATAATGAGATCATC
ATTTTAGGAAATGATGCAGAACTGGGTAGTGACATTGATCCAGAAGAAGCTCAACAGG
CACTTGAAATAGCCGAAGCTAACTTGAGTAAAGCTGAAGGTACGAAAGAATTGGTTGA
AGCGAAGCTAGCTCTCAGACGAGCTAGGATACGAGTCGAGGCTGTCAATTGGATTCCC
ATGAAAAATGTAACCCATTCTTTTGTTTTTTTAGCTCACTGGCCATTCGCTGGGAGTTTC
GGGCTTAATACCGATATTTTAGCAACAAATCTAATAAATCTAACTGTAGTGGTTGGTGTA
TTGATTTTTTTTGGAAAGGGAGTGTTAAAAGATTTATTAGATAATCGAAAACAGAGGAT
TTTAAGTACTATTCGAAATTCGGAAGAATTGCGTAAAGGGACCCTTGAACAACTCGAA
AAAGCTCGTATTCGATTACAGAAAGTCGAACTGGAAGCAGATGAGTATCGAATGAATG
GATACTCTGAAATAGAACGAGAAAAAGAAAATTTGATTAATGCTACTTCTATTAGTTTG
GAACAATTAGAAAAGTCTAAAAACGAAACCCTTTATTTTGAAAAACAAAGGGCGATGA
ATCAGGTCCGACAACAGGGTTTCCAACAAGCTGTACAAGGAGCTCTAGGAACTCTGAA
TAGTTGTTTGAATACCGAGTTACATTTCCGTACGATTCGTGCTAATATTGGCATTCTCGG
GGCCATAGAATGGAAGAGA---ATGAATCCACTAATTGCTGCTGCTTCCGTTATTGCTGCT
GGATTGGCCGTAGGGCTTGCTTCTATTGGGCCCGGAGTTGGTCAAGGTACTGCTGCAG
GACAAGCTGTAGAAGGTATTGCGAGACAGCCAGAAGCAGAAGGTAAAATAAGAGGTA
CTTTATTGCTTAGTCTAGCTTTTATGGAAGCTTTAACAATTTATGGACTGGTTGTGGCAC
TAGCGCTTTTATTTGCGAACCCTTTTGTTATGAATATTACACCATGTTCCATTAAAACACT
CAAGGGGTTATATGATATATCGGGTGTAGAAGTAGGGCAACACTTCTATTGGCAAATAG
GAGGTTTCCAAATTCATGCCCAAGTACTCATCACTTCTTGGGTCGTAATTACTATTTTGC

TAGGTTCAGTTATCATAGCTGTTCGGAATCCACAAACCATCCCGACCGATGGTCAGAAT
TTCTTCGAATATGTCCTTGAGTTTATTCGAGACTTGAGCAAAACTCAGATTGGAGAAGA
ATATGGTCCCTGGGTTCCCTTTATTGGAACTATGTTCCTTTTTATTTTTGTTTCGAATTGG
TCAGGTGCTCTTTTACCTTGGAAAATTATAGAGTTACCCCATGGGGAATTAGCAGCGCC
CACGAATGATATAAATACTACTGTTGCTTTAGCTTTACTAACATCAGCGGCATATTTTTAT
GCGGGTCTTAGCAAAAAAGGATTGAGTTATTTCGAAAAATATATTAAACCAACCCCAAT
CCTTTTACCTATTAACATCCTAGAAGATTTCACAAAACCATTATCACTTAGTTTTCGACT
TTTCGGAAATATATTGGCGGATGAATTAGTCGTTGTTGTTCTTGTTTCTTTAGTCCCCTTA
GTAGTCCCTATACCGGTCATGTTTCTTGGATTATTTACAAGCGGTATTCAAGCTCTTATTT
TTGCAACGTTAGCCGCAGCCTATATAGGTGAATCCATGGAAGGTCATCATATGCTATTTG
CAACTTTAGAACATATACTAACTCATATCTCTTTCTCAACCATTTCAATTGTGATTACGAT
TCATTTGATAACCTTATTAGTTCGTGAACTTAGGGGATTACGTGATTCGTCAGAAAAAG
GAATGATAGCTACTTTTTTCTCTATAACAGGATTTTTAGTCTCTCGTTGGGTTTCTTCGG
GACATTTTCCATTAAGTAATTTATATGAGTCATTGATCTTCCTTTCATGGACTCTGTATATT
CTTCATACTATTCCTAAGATACAGAACTCGAAAAATGATTTAAGCACAATAACTACGCC
AAGTACTATTTTAACGCAAGGCTTTGCCACGTCGGGTCTTTTAACTGAAATGCATCAAT
CCACAATACTAGTACCTGCTCTACAATCTCAGTGGTTAATGATGCATGTCAGTATGATGT
TACTAAGCTATGCAACTCTTTTGTGTGGATCCTTATTATCCGCCGCTCTTCTAATCATTAG
ATTTCGAAAGAATTTCGATTTCTTTTCA---CTAAAGGAGATTGAATATTTATATGCAAAA
AGAAGTGCTTTAAAAAACACCTCTTTTCCCGTATTTCCAAATTATTACAAATATCAATTA
ACTGAGCGTTTGGATTCTTGGAGTTATCGTGTCATTAGTCTAGGGTTTACTCTTTTAACC
GTGGGTATTCTTTGTGGAGCAGTATGGGCTAATGAGGCATGGGGATCCTATTGGAATTG
GGATCCTAAGGAAACTTGGGCATTTATTACCTGGACCATATTTGCAATATATTTACATAGT
AGAAAAAATCCAAATTGGAAGGGTACGAATTCCGCACTTGTAGCTTCGATAGGATTTCT
TATAATTTGGATCTGCTATTTTGGTATCAATCTGTTAGGAATAGGTTTACATAGTTACGGT
TCATTTACATTACCAATGAAAAAAAAGAAAGCATTGCCTTCTTTCTTATATCTTGTATTTA
TCGTACTTTTGCCCTGGGGAGTCTCTTTCTCTTTTAACAAATGTCTGGAACTTTGGATTA
AGAATTGGTGGAATACCAGGCAATCCGAAACTTTCTTAACTGATATTCAAGAGAAAAG
GATTCTAGAAGGATTCATAGAATTAGAGGAACTTTTTCTCTTGGACGAAATGATAAAAG
AGAAACCGAAGACACATGTACAAAAACTTCCTATAGGAATACACAAGGAAATAATACA
ATTGGCCAAAATAGATAATGAGGATCATCTCCATATCATTTTGCATTTCTCAACAAATATA
ATCTGTTTGGCTATTCTAAGTGGTTCTTTTTTTCTGGGTAAAGAAGAACTTGTCATTTTG
AATTCTTGGGTTCAGGAATTTTTCTATAACTTAAATGATTCAATAAAAGCTTTTTTTATTC
TTTTAGTTACTGATTTTTTTGTTGGATTTCACTCTACCCGCGGTTGGGAACTACTAATTC
GTTGGGTCTACAACAATCTTGGATGGGCTCCTAACGAGCTAATTTTCACTATTTTTGTTT
GTAGTTTTCCTGTGATTCTAGACACGTGTTTGAAATTTTGGGTCTTTTTTTGTTTAAACC
GTCTATCTCCTTCACTTGTAGTCATTTATCATTCAATTAGTGAAATGCCCATTGGTGTTCC
AAAAGTACCTTACCGGATTCCCGGAGATGAAGAAGCGACTTGGGTTGACTTATACAAT
GTTATGTATCGAGAAAGGACACTTTTTTTAGGTCAAGAGATTCGTTGCGAGATCACAAA
TCATATTACAGGTCTGATGGTATATCTCAGTATAGAAGATGGAAATAGCGATATTTTTTTG
TTTATAAATTCCCTTGGCGGTTGGCTAATCTCAGGAATGGCGATTTTTGATACGATGCAA
ACGGTGACACCTGATATATATACAATATGCCTCGGAATAGCCGCGTCCATGGCCTCCTTC
ATTCTGCTTGGAGGAGAACCCACCAAGCGTATAGCATTCCCTCACGCTAGGATTATGCT
TCACCAACCTGCTAGTGCTTATTATCGGGCAAGGACACCAGAATTTTTACTAGAAGTGG

AAGAGTTACACAAAGTTCGCGAAATGATCACAAGGGTTTATGCACTAAGAACAGGCAA
GCCTTTTTGGGTTGTATCCGAAGACATGGAAAGGGATGTTTTTATGTCAGCAGACGAA
GCCAAAGCTTATGGACTTGTCGATATTGTAGGGGATGAACCGAGAGAAGCAAAAGTCA
CTTTCGAAGGTTTAGTTACGGAAGCCCTACCCAACGGAATGTTCCGCGTTCGCCTAGA
GAATGACACCATCATCCTGGGCTATATTTCAGGAAAGATCCGGTCTAGTTCTATACGAAT
ACTGATGGGGGATAGGGTCAAAATTGAAATAAGTCGTTATGATTCCAGCAAAGGTCGTA
TAATTTATAGACTTCCCCATAAGGATTCGAAGGATATGGAAAAATTCGAAGGGTATTCA
GAAAAACAGAAATCTCGTCAACACTACTTCGTCTACCCACTTCTCTTTCAGGAATATAT
TTATGCATTTGCTCATGATTATGGATTATTTGGTTGTAATAACAAATTTAGTTCACTACTT
GTGAACCGTTTAATTATCCGAATGTATCAGCAGAATTTTTTAATTAATTCGGTTAATTATC
CTAACCAAGATCGATTGTTGGATTCTGAGTTTTATTCTCAGATTCTATCTGAGGGGTTTG
CGATCGTTGTAGAAATCCCACTCTCGCTAGGGCAACTATCTTGTCCGATACCAAATTTTC
AAAATTTACAATCTATTCATTCAATATTTCCCTTTTTAGAAGACAAATTTTTGCATTTACA
TTATCTATCACATATAAAAATACCCTATCCTATCCATTTAGAAATCCTGGTTCAACTCCTT
GAATACCGGAGTCAAGATGTTCCCTCTTTGCATTTATTGCGATTCTTTCTCTACTATTATT
CGAATTGGAATAGTTTTATTACTAAATCCATTTTTCTTTTGAAAAAAAAAAGACTATTTC
GATTCCTATATAACTCTTATGTATCAGAATATGAATTTTTCTTGTTGTTTCTTCATAAACAA
TCTTCTTGCTTACGATTAACATCTTCTGGAACCTTTCTGGAACGAATCATCTTTTCTGGG
AAGATGGAACAT------GTAATGTACCCGGGGCGGAAAACCATATGGTTCTTTATGGATCC
TCTTATGCATTATGTTCGATATCAAGGAAAGGCAATTCTTGCATCAAAAGGCACTCTTCT
TTTGAAGAAGAAATGGAAATCTTACCTTGTCAATTTCTCGCAATATTTTTTCTCTTTTTG
GACTCAACCACAAAGGATTCGTCTAAACCAATTAACAAACTCTTGCTTCGATTTTCTGG
GGTACCTTTCAAGTGTACCAATAAATACTTTGTTAGTAAGGAATCAAATGCTGGAGAAT
TCTTTTCTAATAGATACTCGAATG------AAAAAATTCGATACCACAGTCCTTGCAACTCC
CCTTGTCGGATCCTTATCAAAAGCTCAATTTTGTACTGGATCGGGGCATCCTATTAGTAA
ACCCGTTTGGACTGATTTATCAGATTGGGATATTCTTGATCGTTTTGGTCGGATATGTAG
AAATATTTTTCATTATCATAGTGGATCTTCGAAAAAACAGACTTTGTATCGACTAAAATA
TATACTTCGACTTTCATGTGCTAGAACTTTAGCTCGTAAACATAAAAGTACGGTACGAA
CTTTTATGCAACGATTGGGTTCGGTATTTTTAGAAGAATTTTTTACGGAAGAAGAGCAA
GTTTTTTCTTTGATGTTCACCAAATGTATTTGGTATTTGGATATTATCCGTATCAATGACC
TGGTGAATCCTCTTATGATAATAGATAGGGTA---GAGGTAGAA---ACTATCAATTCTTTTT
CGAAATCGGAA---TTATTTAAAGAAATCTACGGACTTATATGGATTCTA---CCCATTTTTG
CCCTCCTACTGGGAATCACAATAGAAGTACTCGTAATTGTGTGGTTAGAAAGAGAAATA
TCCGCATCGATACAACAACGTATTGGTCCTGAATATGCTGGCCCCCTGGGACTGCTTCA
AGCTATAGCAGATGGAACTAAACTACTTTTAAAAGAGGATATCCTCCCATCCCGAGGAG
ATATTCCTTTATTTAGCATTGGTCCCTCTATAGCAGTCATATCCATTTTATTAAGTTTTTTA
GTTATCCCTTTAGGATATCGTTTTGTTTTAGCTGATCTTAGTATTGGTGTTTTTTTATGGAT
TGCGATTTCAAGTATTGCTCCTATTGGTCTTCTCATGGCAGGATATAGCTCAAATAATAA
ATATTCTTTTTCAGGCGGTCTACGAGCGGCTGCTCAATCTATTAGTTATGAAATACCATT
AACTTTTTGTGTACTAGCAATATCTCTACTATCTAATAGTTCAAGTACAGTTGATATAGTT
GAAGCACAGTCCAAATATGGTTTTTTTGGATGGAATCTTTGGCGTCAGCCTATAGGTTT
TCTAGTTTTTCTAATTTCTTCTTTGGCAGAATGTGAAAGATTACCCTTTGATTTACCAGA
AGCAGAAGAAGAATTAGTAGCAGGTTATCAAACCGAATATTCTGGTATTAAATATGGTT
TATTTTATCTTGTTTCTTACCTAAATTTATTAGTTTCCTCTTTATTTGTAACTGTTCTATACT

TAGGCGGGTGGAATTTCTCTATTCCCTATATATCCTTTTTTGGATTTTTCCAAATGAATAA
AATAATTGGAATTTTGGAAATGGTAATAGGTATCTTTATTACATTAACTAAAGCTTATTTA
TTTCTCTTCATTTCTATCACAATAAGATGGACTTTACCCAGGATGAGAATGGATCAGTTA
TTAAATCTTGGATGGAAATTTCTTTTACCTATTTCTCTGGGCAATCTCTTATTAACAACTT
CTTCCCAACTAGTTTCACTAATGATCTGGCATGTACAGAATGAAAACTTCATTCTCGATT
CTACGAGAATTTTTATGAAAGCGTTTCATTTGCTTCTCTTCCATGGAAGTTTCATTTTCC
CAGAATGTATCCTAATTTTTGGCCTAATTCTTCTTCTGATGATCGATTTAACCTCTGATCA
AAAAGATAGACCTTGGTTCTATTTCATCTCTTCAACAAGTTTAGTAATAAGCATAACGG
CCCTATTGTTCCGATGGAGAGAAGAACCTATAATTAGCTTTTCGGGAAATTTCCAAACG
AACAATTTCAACGAAATCTTTCAATTTCTTATTTTATTATGTTCAACTTTATGTATTCCTCT
ATCCGTAGAGTACATTGAATGTACAGAAATGGCTATAACAGAGTTTCTGTTATTCGTATT
AACAGCTACTCTAGGGGGAATGTTTTTATGTGGTGCTAACGATTTAATAACTATCTTTGT
AGCTCCAGAATGTTTCAGTTTATGTTCCTACCTATTGTCTGGATATACCAAGAGAGATCT
ACGGTCTAATGAGGCTACTATGAAATATTTACTCATGGGTGGGGCAAGCTCTTCTATTCT
GGTTCATGGTTTCTCTTGGCTATATGGTTCATCTGGGGGGGAGATCGAGCTTCAAGAAA
TTGTGAATGGTCTTATCAATACACAAATGTATAACTCCCCAGGAATTTCAATTGCGCTTA
TATTCATCACTGTAGGACTTGGGTTCAAGCTTTCCCCAGCCCCTTTTCATCAATGGACTC
CTGACGTCTACGAAGGATCCCCCACTCCAGTCGTTGCTTTTCTTTCTGTTACTTCGAAA
GTAGCTGCTTCAGCTTTAGCCACGCGAATTCTCGATATTCCTTTTTATTTCTCATCAAAC
GAATGGCATCTTCTTCTGGAAATCCTAGCTATTCTTAGCATGATATTGGGGAATCTCCTT
GCTATTACTCAAACAAGCATGAAACGTATGCTTGCATATTCGTCCATAGGGCAAATCGG
ATATGTAATTATTGGAATAATTGTTGGAGACTCAAATGATGGATATGCAAGCATGATAAC
TTATATGCTGTTCTATATCTCCATGAATCTAGGAACTTTTGCTTGCATTGTATTATTTGGTC
TACGTACCGGAACTGATAACATTCGAGATTATGCAGGATTATACACGAAAGATCCTTTTT
TGGCTCTCTCTTTAGCCCTATGTCTCTTATCCCTAGGAGGCCTTCCTCCACTAGCAGGTT
TCTTCGGAAAACTCTATCTATTCTGGTGTGGATGGCAAGCAGGCCTATATTTCTTGGTTT
CAATAGGACTCCTTACGAGCGTTCTTTCTATCTACTATTATCTAAAAATAATCAAGTTATT
AATGACTGGACGAAACCAAGAAATAACCCCTTATGTGCGAAATTATAGAAGATCCCCTT
TAAGATCAAACAATTCCATCGAATTGAGTATGACTGTATGTGTGATAGCATCTACTATAC
CAGGAATATCAATGAACCCCATTCTTGCAATTGCTCAGGATACCCTCTTTATGTTTCTGC
TTCACGAATATGATATTTTTTGGACATTTCTAATAATAGCAAGCCTTATTCCTATTTTGGTA
TTTTGGATTTCAGGACTTTTAGCCCCGGTTAGTGAAGGACCAGAGAAGCTTTCTAGTTA
TGAATCGGGTATAGAACCCATGGGGGGGGCTTGGTTACAATTCCGAATACGCTATTACA
TGTTTGCGCTAGTTTTTGTTGTTTTTGATGTGGAAACAGTCTTTCTCTACCCTTGGGCAA
TGAGTTTCGACGTATTGGGTGTATCCGTTTTTATCGAAGCTTTCATTTTTGTGCTTATCCT
AGTTGTTGGTTTAGTTTATGCATGGCGAAAAGGAGCCTTGGAATGGTCTATGAGTTATT
TTCCTTGGTTAACAATACTTGTTGTTTTGCCGATATTTGCAGGTTCATTAATTTTCTTTTT
ACCTCATAAAGGAAATAAAATCGTTAGGTGGTATACTATAGCTATTTGTTTATTAGAATTC
CTTCTAATGACTTATGCATTCTGTTATCATTTCCAATTGGAGGATCCTTTAATCCAATTAA
AGGAGGATTCTAAATGGATAGATGTTTTCGATTTCCACTGGAGATTGGGAATCGATCCA
CTTTCATTAGGATCTATTTTATTGACAGGATTTATCACTACTTTAGCTACTTTAGCGGCTT
GGCCGGTTACTCGGAATTCGCAATTATTCTATTTCCTGATGCTAGCAATGTATAGCGGTC
AAATAGGATTATTTTCTTCACGAGACCTTTTACTTTTTTTTATCATGTGGGAGTTAGAATT
AATTCCTGTTTACTTACTTTTATCCATGTGGGGGGGAAAGAGGCGTCTGTATTCAGCTAC

CAAGTTTATTTTGTATACTGCAGGCGGTTCCATTTTTTTCTTAATTGGAGTTCTGGGTATG
GGATTATATGGTTCCAATGAACCCGGATTAGATTTAGAAAGATTGATTAATCAATCATAC
CCTACAACATGGGAAATACTACTGTATTTTGGCTTCCTTATTGCTTATGCTGTCAAATTG
CCGATTATACCTTTACATACGTGGTTACCATATACCCATGGGGAAGCGCATTACAGTACA
TGTATGCTTTTAGCCGGAATTCTATTAAAGATGGGAGCATACGGATTGATTCGGGTCAAT
ATGGAATTGTTACCGCATGCTCATTATCTATTTTCCCCTTGGTTGGTAATAATAGGAGCG
GTGCAAATAATCTATGCAGCTTCAACTTCTCTTGGTCAACGAAATTTCAAAAAAAGAAT
AGCCTACTCCTCCGTATCTCACATGGGTTTCATAATTATAGGAATTGGTTCCATAACCAA
CATTGGACTAAATGGAGCTATTTTACAAATATTATCTCATGGATTTATCGGTGCTACACTT
TTTTTCTTGGCGGGAACGGCTTGTGATAGAATGCGTCTTGTTTATCTCGAAGAACTGGG
GGGAATATCTATCCCAATGCCAAAAATTTTTACCATGTTTAGTAGCTTTTCAATGGCTTC
TCTTGCCTTGCCGGGAATGAGCGGTTTTGTTGCAGAATTAGTAGTATTTTTTGGACTAAT
TACTAGTCCTAAATTTATGTTAATGCCAAAAATGCTAATTACTTTTGTAATGGCAATAGG
AATGATATTAACTCCTATTTATTTATTATCTATGTTACGCCAGATGTTCTATGGATACAAGC
TATTTCATGTTCCAAACAAAAATTTTGTAGATTCTGGACCACGAGAACTCTTTCTTTTAA
TTTGTATCTTTTTACCAGTAATAGGAATTGGTATTTATCCAGATTTGGTTCTCTCGCTATC
CGTTGATAGGGTAGAGGTTCTATTATCCAATTATTATACTAAAATGATGTTTGAGCGAGT
ACTTTTTTTGAGTGTCTATTTATTTTCGATTGGTATCTATGGATTGATCACAAGCCGAAA
CATGGTTAGAGCTCTAATATGTCTTGAACTTATACTGAATTCAATTAATCTAAATCTCGTA
ACATTTTCTGATCTATTTGATAGTCGCCAATTAAAAGGAGACATTTTCGCAATTTTTGTT
ATAGCCCTTGCGGCTGCTGAAGCAGCTATTGGACTATCCATTCTTTCTTCCATCCATCGT
AACAGGAAATCAACTCGTATCAATCAATCCAATTTTTTGAATAATATGGAACATACATAT
CAATATGCCTGGGTAATTCCTCTTCTCCCACTTCCAGTTATTATGTCAATGGGATTTGGA
CTTTTTCTTATTCCTACAGCAACAAAAAATCTTCGTCGGATATGGGCTTTTCCTAGTATT
TTACTCTTAAGTATAGCTATGGTATTCTCACTTCACCTGTCTATTCAACAAATAAATGGA
AGTTCTATCTATCAATATCTATGGTCTTGGACCATCAATAATGATTTTTCCTTAGAATTTG
GATACTTGGTCGACCCCCTTACGTCTATTATGTTAATACTAATTACTACTGTAGGAATCTT
AGTTCTTATTTATAGTGACGATTATATGTCTCACGATGAAGGATATTTGAGATTTTTTGTT
TATATAAGTTTTTTTAATACTTCCATGTTAGGATTGGTTACTAGTTCCAATTTGATACAAA
TTTATTTTTTTTGGGAACTTGTGGGAATGTGTTCCTATTTATTGATAGGTTTTTGGTTTAC
GCGGCCAATTGCAGCGAGTGCTTGTCAAAAAGCTTTTGTAACTAATCGTGTAGGGGAT
TTTGGTCTGTTATTAGGAATTTTAGGTTTTTTTTGGATAACGGGTAGTTTGGAGTTTCGG
GATTTGTTCAAAATAGCTAATAACAATGGGATTAATTCCTTACTTACTACTTTGTGTGCTT
TTTTATTATTCCTTGGTGCAGTTGCAAAATCTGCACAATTTCCTCTTCACGTATGGTTAC
CTGATGCTATGGAAGGACCCACTCCTATTTCGGCTCTTATACACGCAGCAACTATGGTT
GCTGCGGGGATTTTTCTTCTAGCTAGACTTCTTCCTCTTTTCATATCCCTACCCTGGATAA
TGAGTTTCATTTCTTTAATAGGTACAATAACACTCTTCTTAGGAGCCACTTTAGCTCTTG
CTCAGAGAGATATTAAAAGAAGCTTAGCCTATTCTACAATGTCTCAATTGGGTTATATGA
TGTTAGCTCTAGGTATAGGTTCTTATCAAGCTGCTTTATTCCATTTGATCACTCATGCTTA
TTCGAAAGCTTTATTGTTCTTAGGATCCGGATCCGTTATTCATTCAATGGAACCTCTTGT
TGGATATTCACCAGATAAAAGTCAGAATATGGTTCTTATGGGTGGTTTAAGAAAATACG
TTCCAATTACAAGAACTACTTTTTTATGTGGTACACTTTCTCTTTGTGGTATTCCACCTCT
TGCTTGCTTCTGGTCCAAAGATGAAATCCTTAGTAATAGTTGGTTGTATTCACCCTTTTT
TGGAATAATAGCTTCTTTTACTGCAGGATTAACTGCATTTTATATGTTTCGGATATATTTA

CTTACTTTTGATGGGTATTTGCGTGTTCATTTTCAAAATTACAGTAGTACTAAAGAAGGT
TCGTTGTATTCAATATCCTTATGGGGAAAAAGTATATCCAAAGGAGTCAATAGG---GATT
TTGTTTTATCAACAATGAAGTTTTTTTCAGCTAAAAAT------ACTTTTGTCTATCCTCATG
AAACGGGAAATACTATGCTATTTCCTCTTCTTATATTACTACTTTTTACTTTGTTCATTGG
ATCCATAGGAATCCATTTTGAGTTAACCATATTATCAAAGTGGCTAACTCCTTCAATAAA
CTTGTTCCAGGAAAATTCTAATTCTTCCATAAATTCATATGAATTTCTCACTAATGCAATT
TCTTCTGTAAGTTTAGCAATTTTTGGTCTATTCATAGCATATATCTTTTATGGATCTGCTTA
TTCTTTTTTTCAGAATTTGAATTTTCAAAATTCCCTTGTAAGCTTTTTGGATGAAGTAAA
AAAAAAGATATACAGCTGGTCATATAATCGTGGTTATATAGATTTTTTCTATACTAGGGTT
TTTATCCTAGGTATAAGAAAGTTAGCCGAACTAACGCATTTTTTTGATAAAGGTGTCATT
GATGGAATTACCAATGGAGTAGGTCTTGCTGGTTTTTGTATAGGAGAAGAAATCAAATA
T---GTAGGGGGAGGGCGAATATCGTCTTATCTATTCTTTTTTTTATGTTATGTATCCTTGTT
CTTATTCTTTATTCCAATGGATTTACCTGGACCAATACATGAGATTCTTGTGCTATTTGGG
GGATTTGGTCTTCTACTAGGAGGTCTAGGAGTAGTATTACTTACCAACCCAATTTATTCT
GCCTTTTCGCTGGGATTAGTTCTTGTTTGTATATCCTTATTCTATTTTTTATTAAATTCCTA
CTTTGTAGCTGTCGCACAACTTCTTATTTATGTGGGAGCCATAAATGTCTTGATCATATTT
GCTGTAATGTTTGTAAACGGCTCAGAGTGGTCTAAAGATAAGAATTATTGGACTATTGG
AGATGGGTTTACTTTACTCCTTTGTATAACTATTCCTTTTTCACTAATGACTACTATCCCA
GATACGTCGTGGTATGGAATTCTTTGGACTACAAGATCAAACCAAATAGTAGAACAGG
GTCTCATAAATAACGTTCAACAAATTGGGATTCATTTAGCAACCGATTTTTATCTTCCGT
TTGAACTCATTTCCCTAATTCTTCTAGTTTCTTTAATAGGTGCAATTACTATGGCTCGACA
AATGAGTCTATCGCTTAAAAGAAAAGATCTCATGATAGTCAATATGGGCCCTCAACACC
CATCAATGCATGGTGTTCTTCGACTGATTGTTACTCTCGATGGTGAAGATGTTATTGATT
GCGAACCCATATTAGGGTATTTACACAGAGGAATGGAAAAAATCGCGGAAAACAGAAG
TATTATACAATACTTGCCTTATGTAACACGATGGGATTATTTAGCTACTATGTTTACAGAA
GCAATAACGGTAAATGCACCAGAATTCTTAGAGAATATTCAAATACCCAAAAGAGCCA
GCTATATTAGAGTAATTATGTTAGAATTGAGCCGTATAGCTTCTCATTTGTTATGGCTTGG
ACCTTTTATGGCGGATCTCGGGGCGCAGACTCCCTTTTTCTACATTTTTAGAGAGAGAG
AATTGATATATGATCTATTTGAAGCTGTCACAGGTATGCGAATGATGCATAATTACTTTCG
CATCGGAGGAGTCGCTGCCGATCTCCCTTATGGATGGATGGATAAATGTTTAGATTTCTG
TGATTATTTTTTACAAGGAGTTGTTGAATATCAAGAACTTATTACACAGAATCCCATTTT
TTTAGAACGAGTTGAAGGAGTCGGTTTTATTAGCGGAGAAGAAGCTGTAAATTGGGGC
TTATCGGGACCAATGTTACGAGCTTCTGGAATACAATGGGATCTTCGTAAAATTGATCCT
TATGAGTCTTACAATCAATTCGATTGGAAAGTCCAATGGCAAAAAGAAGGAGATTCGTT
AGCTCGCTATTTAGTACGAGTCGGTGAAATGAGGGAATCCATAAAAATTATTCAACAGG
CTGTAGAGAAAATTCCTGGAGGACCTTATGAGAATTTAGAAGCCCGACGC---AAGAAA
GCAAAGAATCCCGAATGGAATGATTTTGAATATCGATTTCTTGGTAAAAAACCTTCGCC
CAATTTTGAATTATCAAAGCAAGAGCTTTATGTAAGAGTAGAAGCTCCAAAAGGCGAA
TTAGGAATTTATCTGGTAGGAGATGATAGTCTTTTCCCCTGGAGATGGAAAATTCGTCC
ACCGGGTTTTATTAATTTGCAAATTCTTCCTCAGCTAGTTAAAAAAATGAAATTGGCTGA
TATCATGACAATATTAGGTAGTATAGATATCATTATGGGGGAAGTTGATCGTATGTTCCCT
ATGCTAACTGGGTTCATTAGTTATGGTCAACAAACAATACGCGCCGCAAGATACATTGG
TCAAAGTTTCATAATTACCTTATCCCACACAAATCGTTTACCTATAACGATTCACTACCC
TTATGAAAAATCAATTACATCGGAGCGTTTCCGGGGGCGAATACACTTTGAATTTGATA

AATGCATTGCTTGTGAAGTATGTGTTCGCGTATGCCCGATAGATCTACCCCTTGTGGATT
GGAGATTTGAAAAGGATATTAAAAGAAAACAATTGCTTAATTATAGTATTGATTTCGGA
GTTTGTATATTTTGCGGTAACTGTGTTGAATACTGTCCCACAAATTGTTTATCAATGACT
GAAGAATATGAACTTTCTACTTATGATCGTCATGAATTGAATTACAATCAAATTGCTTTG
AGTCGGTTACCAATCTCCATAATGGGAGATTACACAATTCAAACAATTAGGAATTCGAA
AATAGACGAAGAAAAATCTTGGAATTCAAGAACGATTACAGATTACCAGCAGGGTTGG
TTATCTAATTGGCTAGTTAAACATGACGTGGTTCATAGGTCTTTGGGCTTCGATCATCGA
GGAGTAGAGACTTTACAAATAAAAGCGGGGGATTGGGATTCCATTGCTGTCATTTTATA
TGTATATGGTTACAATTATTTACGTTCCCAATGTGCTTATGACGTAGCACCCGGTGGATC
TTTAGCTAGCGTGTATCATCTTACGAGAATACAGTATGGTATAGATAACCCAGAAGAGGT
ATGCATAAAAGTCTTTGCCCAAAAGGATAATCCTAGAATCCCGTCCGTTTTCTGGGTTT
GGAGAAGTGCCGATTTTCAAGAACGCGAATCTTATGATATGGTGGGAATTTCTTATGAT
AATCATCCACGTCTTAAACGTATCTTAATGCCTGAAAGTTGGATAGGCTGGCCCTTACGT
AAGGACTATATAACCCCCAATTTCTATGAAATACAAGATGCTCAT------------------------------
----
-------------------------------ATGAGTTTGATTGAGTTTCCGTTACTCGACCAAACAAGTTCCA
ATTCCGTTATTTCAACTACACCAAACGATCTTTCAAATTGGTCAAGACTCTCAAGTTTAT
GGCCCCTTCTATATGGTACCAGTTGTTGTTTCATTGAATTTGCTTCATTAATAGGCTCAC
GATTCGACTTTGATCGTTATGGATTGGTACCCAGATCAAGTCCTAGACAAGCGGACCTA
ATTTTAACAGCTGGTACGGTAACAATGAAAATGGCTCCATCTTTAGTGCGATTATATGAG
CAAATGCCTGAACCGAAATACGTCATTGCTATGGGAGCCTGTACTATTACAGGGGGCAT
GTTCAGTACAGATTCCTATAGTACTGTTCGAGGAGTTGATAAGTTAATTCCTGTGGACG
TCTACCTGCCGGGTTGCCCGCCTAAACCAGAGGCTGTTATAGATGCCCTAACAAAACTT
CGTAAGAAGATAGCGCGAGAAATAATTGAGGATCGAACTCTATGTCAAAGTAAAAATA
GATCTTTTACTACCCGTCACAAACTTTATGTTCGGCGCAGTACTCACACTGGAACTTAC
GAACAAGAATTGCTCTATCAATCACCATCTACTTTAGATATATCTACT------TTTTTCAAAT
CCAAAAGTTCAGTATCTTCCTACAAATTAGTGAATATGGAAAATAGAAAAACTTTTTCT
TGGTTAAAGGAACAGATGATTCGATCGATTTCTGTATCGATCATGATATACGTAATAACT
CGCACATCTATTTCAAACGCATATCCCATTTTTGCGCAGCAGGGTTATGAAAACCCGCG
GGAAGCAACTGGACGAATTGTATGTGCCAATTGCCATTTAGCTAATAAGCCTGTGGATA
TTGAAGTTCCCCAAGCAGTGCTTCCTGATACTGTATTTGAAGCAGTTCTTCGAATTCCTT
ATGATATGCAGCTGAAACAAGTTCTTGCTAATGGTAAAAAGGGAGGGTTGAATGTGGG
TGCTGTTCTTATTTTGCCCGAGGGATTCGAATTAGCGCCGCCCGACCGTATTTCTCCTGA
GTTGAAAGAAAAGATAGGAAATCTCTCTTTTCAGAGTTATCGTCCCAATAAAAAAAATA
TTCTTGTGATAGGCCCTGTTCCCGGTAAGAAATATAGTGAAATTGTCTTTCCCATTCTTT
CCCCCGATCCCGCTACAAAAAAAGACGTTCATTTCTTAAAATATCCCATATATGTAGGGG
GAAACCGAGGAAGGGGACAGATCTATCCTGACGGTAGCAAGAGTAACAATACGGTCTA
TAATGCTACGTCAACAGGTATAGTAAAAAAAATACTACGTAAAGAAAAGGGGGGATAT
GAAATATCCATAGTGGATGCATCGGATGGGCGCCAAGTGATTGATATTATACCTCCCGGG
CCAGAACTTCTTTTTTCAGAGGGGGAATCGATCAAGCTTGATCAACCATTAACAAGCA
ATCCTAATGTAGGAGGGTTTGGTCAGGGGGATGCAGAAATAGTGCTTCAGGATCCATTG
CGCGTCCAAGGCCTTTTGTTTTTCTTCGCATCTGTTATTTTGGCACAAGTTTTTTTGGTT
CTCAAAAAGAAACAGTTTGAAAAGGTTCAATTGTACGAAATGAATTTCCTCAATAAAG
TATATGATTGGTTTGAAGAACGTCTTGAGATTCAGGCAATTGCAGATGATATAACTAGTA

AATATGTTCCTCCTCATGTCAACATATTTATTGTTTAGGGGGAATTACACTTACTTGTTT
TCTAGTACAAGTCGCTACCGGTTTTGCTATGACTTTTTACTACCGACCGACCGTTACAG
AGGCTTTTTCCTCCGTTCAATATATAATGACGGAGGCCAACTTTGGTTGGTTAATCCGAT
CGGTTCATCGATGGTCAGCAAGTATGATGGTTCTAATGATGATCCTGCACGTATTTCGTG
TGTATCTCACAGGTGGATTTAAAAAACCCCGTGAATTAACTTGGGTCACAGGCGTGGTT
TTGGCTGTATTGACTGCATCATTTGGTGTAACTGGTTATTCTTTGCCTTGGGATCAAATT
GGTTATTGGGCAGTCAAAATTGTTACAGGTGTACCCGAAGCTATTCCGGTAATAGGGTC
CCCTTTAGTGGAATTATTACGTGGAAGTGCTAGTGTGGGCCAATCCACTTTGACTCGTT
TTTATAGTTTACATACCTTTGTACTACCTCTGCTTACTGCCGTATTTATGTTAATGCACTTT
CCAATGATACGTAAGCAAGGTATTTCGGGTCCGTTAATGGGAGTAACAAAGAAACCTG
ACTTAAATGATCCTGTATTAAGAGCAAAATTAGCTAAAGGGATGGGACATAATTATTATG
GGGAACCCGCGTGGCCCAACGATCTTTTATACATTTTTCCAGTAGTAATTCTAGGTACTA
TTGCGTGTAATGTAGGTTTAGCGGTTCTAGAGCCGTCAATGATTGGTGAACCGGCGGAT
CCGTTTGCAACTCCTTTGGAAATATTACCCGAGTGGTACTTCTTTCCCGTATTTCAAATA
CTCCGTACAGTACCCAATAAGTTATTGGGCGTTCTCTTAATGGTTTCTGTGCCAACGGG
CTTATTGACAGTACCCTTTCTAGAGAATGTCAATAAATTCCAAAATCCATTTCGTCGCCC
AGTAGCTACGACCGTTTTTTTAATCGGTACTGCAGTAGCTCTTTGGTTAGGTATTGGAGC
AACATTACCCATTGATAAATCCTTAACTTTAGGTCTTTTTATGATTGAAGTTTTTCTATTT
GGAATCGTCTTAGGCCTAATTCCTATTACTTTAGCGGGATTATTCGTGACTGCGTATTTG
CAATACAGGCGTGGGGATCAGTTGGATCTTATGCTTACTATAACTAGTTATTTCGGTTTT
CTACTGGCTGCTTTAACTATAACCCCAGCTCTATTTATTAGCTTGAACAAGATACGTCTT
ATTATGGATATAGTAAGTCTCACTTGGGCTGCTTTAATGGTAGTCTTTACATTTTCTCTTT
CGCTAGTAGTATGGGGGAGGAGTGGACTCATGATTATTCGTTCGTCGGAACCAGAAGTT
AAAATTGCTGTGGATAGGGATCCTATAAAAACATCTTTTGAGGAATGGGCCAGACCCG
GGCATTTCTCAAGAACAATAGCTAAGGGCCCCGATACTACCACTTGGATCTGGAACCTA
CATGCTGATGCTCACGATTTCGATAGTCATACCGGTGATTTGGAGGAGATCTCCCGAAA
AGTCTTTAGTGCTCATTTCGGCCAACTCTCCATTATCTTTCTTTGGTTGAGTGGCATGTA
CTTCCACGGTGCCCGTTTTTCCAATTATGAAGCATGGCTAAGCGATCCTACTCACATTG
GACCCAGTGCTCAGGTAGTTTGGCCAATAGTAGGACAAGAAATTTTGAATGGTGATGT
AGGCGGGGGTTTCCGAGGAATCCAAATAACCTCTGGTTTTTTTCAGATTTGGCGAGCAT
CCGGAATAACTAGTGAATTACAACTCTATTGTACCGCAATTGGTGCATTGATTTTTGCAT
CGTTAATGCTTTTTGCCGGTTGGTTCCATTATCACAAAGCCGCTCCCAAATTGGCCTGG
TTCCAAGATGTAGAATCCATGTTGAATCACCACTTAGCGGGATTATTAGGACTTGGGTC
TCTTTCTTGGGCGGGACACCAAATCCATGTATCTTTACCGATTAACCAATTTCTCGACGC
TGGGGTTGATCCTAAAGAGATACCACTTCCTCATGAATTTATCTTGAATCGCGACCTTTT
GGCTCAACTTTATCCTAGTTTTGCCGAAGGAGCAACCCCTTTTTTCACCTTGAATTGGT
CCAAATATGCGGAATTTCTTAGTTTTCGCGGAGGACTAGATCCAATAACCGGTGGTCTA
TGGTTGAGCGATATTGCACACCATCATTTAGCTATTGCTATTCTTTTCCTGATCGCTGGTC
ATATGTATAGGACCAATTGGGGTATTGGTCATGGACTTAAAGATATTTTGGAGGCTCATA
AGGGCCCATTTACAGGACAAGGCCATAAAGGTCTCTATGAAATCCTAACAACGTCATG
GCATGCTCAATTATCTCTTAACCTAGCTATGCTAGGCTCTACAACTATTGTTGTAGCTCAT
CATATGTACTCTATGCCCCCCTATCCATACCTAGCTACTGACTACGGTACACAACTTTCC
TTGTTCACACACCACATGTGGATTGGCGGATTTCTAATAGTTGGTGCTGCTGCACATGC
AGCCATTTTTATGGTAAGAGACTATGATCCAACTACTCGATACAACGATCTATTAGATCG

CGTCCTTAGACACCGCGATGCAATCATATCCCACCTTAACTGGGTATGTATATTTCTGGG
TTTTCACAGTTTTGGCTTGTACATTCATAATGATACCATGAGTGCTTTAGGCCGTCCCCA
AGATATGTTTTCGGATGCCGCCATACAATTACAACCCATCTTTGCTCAATGGATACAAAA
TATCCATGCTGGCGCGCCTGGCGTAACAGCTCCTGGTGCAACAACAAGTACCAGCTTA
ACGTGGGGAGGCGGCGAGTTAGTAGCTATAGGCGGCAAAGTAGCTTTGTTACCTATTCC
ATTAGGAACCGCAGATTTTTTAGTCCATCACATTCACGCATTTACCATCCATGTGACTGT
ATTAATACTTTTGAAAGGTGTTTTATTTGCTCGCAGTTCCCGTCTGATACCTGATAAAGC
AAATCTTGGCTTTCGCTTCCCTTGCGACGGACCTGGGCGAGGGGGAACATGTCAAGTA
TCCGCCTGGGATCATGTTTTCTTAGGTCTATTCTGGATGTACAATTCTATTTCGGTAGTCA
TTTTCCATTTCAGTTGGAAAATGCAGTCGGATGTTTGGGGTACTATAAGTGATCAAGGG
ATAGTAACTCATATCACAGGGGGAAACTTTGCGCAGAGTTCCATTACGATTAATGGTTG
GCTTCGAGATTTCTTGTGGGCACAGGCATCCCAAGTAATTCAGTCTTATGGTTCTTCATT
ATCTGCATATGGTCTTTTTTTCTTAGGCGCTCATTTTGTCTGGGCTTTCAGTTTAATGTTT
TTATTCAGCGGCCGTGGTTATTGGCAAGAACTCATTGAATCTATCGTTTGGGCTCATAAC
AAATTAAAAGTTGCTCCTGCTACTCAGCCTAGAGCCTTGAGCATTATACAAGGACGTGC
TGTAGGAGTAACCCATTACCTTCTGGGTGGAATTGCCACAACATGGGCATTCTTCTTAG
CGAGAATTATTGCAGTAGGAATGGAATTAAGATTTCCCAGGTTTAGCCAAGGCTTAGCT
CAGGACCCCACTACTCGTCGTATTTGGTTTGGTATTGCTACCGCACATGATTTCGAAAG
TCATGATGATATTACTGAGGAACGTCTTTATCAGAACATTTTTGCTTCTCACTTTGGGCA
GTTAGCAATAATCTTTCTATGGACGTCCGGAAATCTGTTTCATGTAGCTTGGCAAGGAA
ATTTTGAATCATGGATACAGGATCCTTTACACGTAAGACCTATTGCTCATGCCATTTGGG
ATCCTCATTTTGGGCAACCCGCTGTGGAAGCCTTTACTCGAGGAGGTGCTGCCGGTCC
AGTGAATATCGCTTATTCTGGGGTTTATCAGTGGTGGTATACAATTGGATTGCGCACCAA
TGAAGATCTTTATACTGGAGCTCTTTTTCTATTATTTCTTTCTACGCTATCCTTAATAGGG
GGTTGGTTACATCTACAACCCAAATGGAAGCCAAGCCTTTCGTGGTTCAAAAACGCCG
AATCTCGTCTGAATCATCATTTGTCAGGACTTTTCGGAGTAAGTTCTTTGGCTTGGACA
GGACATTTAGTTCATGTTGCTATTCCCGGATCCAGGGGGGAGTACGTTCGATGGAATAA
TTTCTTAGATGTATTACCCTATCCCCAGGGGTTGGGTCCCCTTCTGACGGGTCAGTGGA
ATCTTTATGCCCAAAATCCTGATTCGAGTAATCATTTATTTGGTACCACTCAAGGAGCGG
GAACTGCCATTCTGACCCTTCTTGGGGGATTCCATCCACAAACACAAAGTTTGTGGCT
GACCGATATTGCTCATCATCATTTAGCTATTGCATTTATTTTTCTCATTGCCGGTCATATGT
ATCGAACTAACTTCGGAATTGGGCACAGTATCAAAGATCTTTTAGAAGCACATACTCCT
CCGGGGGGTCGATTAGGACGTGGGCATAAAGGCCTTTATGATACAATCAATAATTCGAT
TCATTTTCAATTAGGCCTTGCTCTAGCTTCCTTAGGGGTTATTACTTCCTTAGTAGCTCA
ACATATGTACTCTTTACCTGCTTATGCATTCATAGCACAAGACTTTACTACTCAAGCTGC
TTTATATACTCATCACCAATACATTGCAGGGTTCATCATGACAGGGGCTTTTGCTCATGG
AGCTATTTTTTTCATTAGGGATTACAATCCGGAACAGAATGAAGATAATGTATTGGCAAG
AATGTTAGACCATAAGGAAGCTATCATATCTCATTTAAGTTGGGCTAGCCTCTTCCTAGG
ATTCCATACCTTGGGCCCTTATGTTCATAACGACGTTATGCTTGCTTTTGGTACTCCAGA
AAAGCAAATCTTGATTGAACCTATATTTGCCCAATGGATACAATCTGCTCATGGTAAGA
CGACATATGGGTTCGATATACTCTTATCTTCAACGAATGGCCCCACTTTCAATGCAGGTC
GAAACATATGGTTGCCCGGATGGTTGAATGCTGTTAATGAG------AATAGTAATTCGCTT
TTCTTAACAATAGGACCTGGGGATTTCTTGGTTCATCATGCTATTGCTCTAGGTTTGCAT
ACAACTACATTGATTTTAGTAAAGGGTGCTTTAGATGCACGCGGTTCCAAATTAATGCC

GGATAAAAAGGATTTCGGGTATAGTTTTCCTTGCGACGGCCCAGGGCGCGGCGGTACT
TGTGATATTTCTGCTTGGGACGCGTTTTATTTGGCAGTTTTCTGGATGTTAAATACCATT
GGATGGGTTACTTTTTATTGGCATTGGAAACACATTACATTATGGCAGGGCAACGTTTC
ACAATTTAATGAATCCTCCACTTATTTGATGGGATGGTTAAGAGATTACCTATGGTTAAA
CTCTTCACAACTTATTAATGGATATAATCCTTTTGGGATGAATAGTTTATCAGTATGGGCT
TGGATGTTCTTATTTGGACATCTTGTTTGGGCTACAGGATTTATGTTCTTAATTTCCTGGC
GTGGATATTGGCAGGAATTAATTGAGACTTTAGCATGGGCTCATGAACGGACACCTTTG
GCTAATTTAATTCGCTGGAGAGATAAGCCCGTGGCTCTTTCCGTTGTGCAAGCAAGATT
GGTCGGATTAGCCCACTTTTCCGTGGGTTATATATTCACTTATGCAGCTTTCTTGATTGCC
TCAACATCAGGCAAGTTCGGTATGTCACATTCTGTAAAAATTTATGATACATGTATAGGA
TGCACTCATTGTGTACGAGCTTGCCCAACAGATGTATTAGAAATGATACCTTGGGATGG
ATGTAAAGCCAAGCAAATTGCTTCCGCGCCGAGAACCGAAGATTGTGTGGGTTGTAAG
AGATGCGAATCCGCCTGTCCAACGGATTTTTTAAGTGTCCGCGTTTATTTAGGGCCTGA
AACAACCCGCAGCATGGCTCTATCTTATATGACAGATTTTAACTTACCCTCTATTTTCGT
ACCTTTAGTAGGCTTAGTATTTCCGGCAATTGCAATGACTTCCTTATTTCTTTATGTGCA
GAAAAATAAGATTGTCATGCGGGATATAAAAACATATCTCTCTGTAGCACCCGTGCTAA
GTACTCTATGGTTTGGGGCTTTAGCAGGTTTATTGATAGAAATCAATCGTTTATTCCCAG
ATGCTTTGTCATTCCCTTTTTTT------ATGACTGCAATTTTAGAGAGACGCGAAAGTACA
AGCCTGTGGGGTCGCTTCTGCAACTGGATAACTAGCACCGAAAACCGTCTTTACATTG
GATGGTTCGGTGTTTTGATGATCCCTACCTTATTGACCGCAACTTCCGTATTTATTATCGC
CTTCATCGCTGCTCCTCCAGTAGATATTGATGGTATTCGTGAGCCTGTTTCTGGTTCTTT
ACTTTATGGAAACAATATTATCTCTGGTGCTATTATTCCTACTTCTGCGGCGATAGGGTTG
CATTTTTACCCAATTTGGGAAGCTGCATCTGTTGATGAATGGTTATACAATGGCGGTCCT
TATGAGCTAATTGTTCTACACTTCTTACTTGGTGTAGCTTGTTATATGGGTCGTGAGTGG
GAACTTAGTTTCCGTCTGGGTATGCGCCCTTGGATTGCTGTTGCATATTCAGCTCCTGTT
GCAGCTGCTACTGCTGTTTTCTTGATTTACCCTATTGGTCAAGGAAGTTTCTCTGATGGT
ATGCCTTTAGGAATATCTGGTACTTTCAACTTTATGATTGTATTCCAGGCAGAGCACAAC
ATCCTTATGCATCCATTTCACATGTTAGGTGTAGCTGGTGTATTCGGCGGTTCCCTATTCA
GTGCTATGCATGGTTCCCTTGTAACCTCTAGTTTGATCAGGGAAACCACTGAAAATGAG
TCTGCTAATGAGGGTTACAAATTTGGTCAGGAAGAAGAGACTTATAATATTGTGGCTGC
TCACGGTTATTTTGGTCGATTAATCTTCCAATATGCTAGTTTCAACAATTCTCGTTCTTTA
CACTTCTTCTTGGCTGCTTGGCCTGTAGTAGGGATCTGGTTCACTGCTTTAGGTATTAGT
ACTATGGCATTCAACCTAAATGGTTTCAATTTCAACCAATCTGTAGTTGATAGCCAAGG
TCGCGTTATTAATACTTGGGCTGATATCATCAACCGTGCTAATCTTGGTATGGAGGTAAT
GCACGAACGTAATGCTCACAACTTCCCTCTAGACCTAGCTGCTCTTGAAGTTCCTTACC
TTAATGGAATGGGTTTGCCTTGGTATCGTGTTCATACTGTCGTATTGAATGATCCGGGTC
GATTGCTTTCGGTGCATATAATGCACACAGCTCTAGTTTCTGGTTGGGCTGGTTCGATG
GCTTTATACGAATTAGCGGTTTTTGATCCCTCTGATCCTGTTCTGGATCCAATGTGGAGA
CAAGGTATGTTCGTCATCCCCTTCATGACTCGTTTAGGAATAACGAATTCGTGGGGTGG
TTGGAGTATTTCA---GGAGGAACTGTAACGAATCCGGGTATTTGGAGTTATGAAGGTGT
GGCAGGTGCGCATATTGTGTTTTCTGGCTTGTGTTTCTTGGCAGCTATCTGGCATTGGGT
ATATTGGGACCTAGAAATATTCTGTGATGAGCGGACGGGAAAACCTTCTTTGGATTTGC
CCAAGATCTTTGGAATTCATTTATTTCTTGCAGGGGTGGCTTGTTTTGGCTTTGGTGCAT
TTCATGTAACGGGTTTATATGGCCCTGGGATATGGGTGTCCGATCCTTACGGACTCACTG

GAAAAGTACAAGCTGTAAATCCTGCGTGGGGTGCAGAAGGTTTTGATCCTTTCGTTCC
GGGAGGAATAGCTTCGCATCATATTGCTGCGGGTACTTTGGGCATATTAGCGGGCCTATT
CCATCTAAGTGTCCGTCCGCCTCAACGTCTATACAAAGGGTTACGTATGGGCAATATTG
AAACTGTACTTTCCAGTAGTATCGCTGCTGTTTTTTTTGCTGCTTTCGTAGTTGCCGGAA
CTATGTGGTATGGATCAGCAACTACCCCAATCGAATTATTTGGGCCTACTCGTTATCAGT
GGGATCAGGGATACTTTCAGCAAGAAATATATCGAAGAGTTAGCGATGGGTTAGCCGA
AAATCTTAGTTTATCAGAAGCTTGGTCTAAAATTCCCGAAAAATTAGCCTTTTATGATTA
TATTGGTAATAATCCGGCAAAGGGGGGATTATTCAGAGCAGGCTCAATGGACAATGGG
GATGGCATAGCTGTTGGATGGTTAGGACATCCCGTCTTTAGAGATAAAGAAGGACGCG
AGCTTTTTGTACGTCGTATGCCTACTTTTTTTGAAACATTTCCGGTCGTTTTGGTAGATG
AAGAGGGAATTGTGAGAGCGGACGTTCCTTTTAGAAGAGCAGAATCCAAATATAGTGT
TGAACAAGTAGGCGTAACGGTGGAGTTCTATGGTGGCGAACTTAATGGAGTAAGTTAT
TCTGATCCTGCTACTGTAAAAAAATATGCGCGGCGTGCTCAATTAGGGGAAATTTTTGA
ATTAGATCGAGCTACTTTGAAATCAGATGGTGTTTTTCGCAGCAGTCCAAGGGGTTGGT
TCACTTTTGGTCATGCTACCTTTGCTTTGCTCTTCTTTTTCGGACACATTTGGCATGGCG
CTCGAACCTTGTTCCGAGATGTTTTTGCTGGTATTGATCCAGACTTGGATGCTCAAGTG
GAATTTGGAACATTCCAAAAAGTTGGAGATCCAACTACAAGGAGACAGGCAGCCATG
AAAATCTTATATTCCCTGAGGAGGTTCTACCACGTGGAAACGCTCTTTAATGGAACTTT
CGTTTTAGCTGGTCGTGACCAAGAAACCACCGGCTTTCCTTGGTGGGCTGGGAATGCC
AGACTTATCAATTTGTCCGGTAAACTACTTGGAGCTCATGTAGCCCATGCCGGATTAATC
GTATTCTGGGCCGGAGCAATGAACCTATTTGAAGTGGCCCATTTCGTACCAGAAAAGC
CCATGTATGAACAAGGGTTGATTTTACTTCCGCACTTAGCTACTCTAGGTTGGGGAGTA
GGGTCGGGGGGAGAAGTTCTAGATACTTTTCCTTACTTTGTATCCGGAGTACTTCACTT
AATTTCCTCCGCAGTCTTAGGCTTCGGTGGCATTTATCACGCGCTTCTGGGACCCGAGA
CTCTTGAAGAATCTTTTCCATTCTTTGGTTATGTATGGCAAGATAGAAATAAAATGACTA
CACTTTTGGGTATTCACTTAATTTTGTTAGGTCTAGGTGCTTTTCTTCTAGTACTCAAGG
CTCTTTATTTTGGCGGCGTATATGATACCTGGGCCCCGGGGGGGGGGGATGTAAGAAAA
ATTACCAATTTGACCCTTAGCCCCGGTGTTATATTTGGTTATTTACTAAAATCCCCTTTTG
GGGGAGAAGGATGGATTGTTAGTGTGGATGATTTAGAAGATATAATTGGGGGACATGTA
TGGTTGGGTTCCATTTGTGTACTTGGCGGAATTTGGCATATCTTAACCAAACCCTTTGCA
TGGGCTCGCCGTGCATTTGTATGGTCTGGCGAAGCTTACTTGTCTTATAGTTTAGGTGCT
TTATCTGTTTTTGGTTTTATCGCTTGTTGTTTTGTCTGGTTCAATAATACGGCTTATCCAA
GTGAGTTTTACGGGCCCACTGGACCAGAAGCTTCTCAAGCTCAAGCATTTACTTTTCTA
GTTAGAGACCAGCGTCTTGGAGCTAATGTAGGGTCTGCCCAAGGACCGACGGGTTTAG
GTAAATATCTAATGCGTTCCCCAACGGGAGAGGTTATTTTTGGAGGGGAAACTATGCGT
TTTTGGGACCTTCGTGCTCCGTGGTTAGAACCTCTAAGGGGCCCCAACGGTTTGGACT
TGAGTAGGTTGAAGAAAGACGGCCAACCTTGGCAAGAACGACGTTCAGGAGAATATA
TGACCCATGCTCCTTTAGGCTCTTTAAATTCCGTGGGTGGCGTAGCTACCGAGATCAAT
GCTGTTAATTATGTCTCCCCTAGAAGTTGGTTAGCGACCTCCCATTTTGTTCTAGGATTC
TTCTTTTTTGTGGGCCATTTGTGGCATGCAGGAAGAGCCCGAGCTGCTGCAGCAGGCT
TTGAAAAGGGAATTGATCGCGATTTGGAACCTGTTGTTTACATGACCCCTCTTAACATG
ACTATAGCCGTTGGTAGAGTTACTAAAGAAGAAAACGATCTATTCCCCCTTATCGACGA
CTGGTTACGAAGGGACCGCTTCGTTTTTGTAGGATGGTCCGGCTTATTGCTCTTTCCTT
GTGCTTATTTCGCTTTAGGGGGTTGGTTTACAGGGACTACTTTTGTAACTTCTTGGTATA

CCCATGGATTGGCTAGTTCCTATTTGGAAGGTTGCAATTTCTTAACCGCGGCGGTTTCC
ACACCTGCCAATAGTTTAGCACACTCTTTGTTGCTACTATGGGGACCGGAAGCACAGG
GAGATTTTACTCGTTGGTCTCAATTAGGTGGTCTTTGGACTTTTCTCGCTCTCCATGGAG
CTTTTGCACTAATAGGTTTCATGCTACGTCAATTTGAACTTGCTCGGCCTGTTCAATTGC
GGCCTTATAATGCAATTTCATTCTCTGGTCCAATCGCTGTTTTTCTTTCCGTATTCCTTAT
TTATCCACTGGGACAATCTGGTTGGTTCTTCTCGCCGAGTTTTGGCGTAGCAGCTATATT
TCGCTTCATCCTTTTCTTCCAAGGATTTCATAATTGGACGTTGAAACCATTTCATATGAT
GGGAGTTGCCGGAGTATTAGGCGCAGTTCTGCTATGCGCTATCCACGGGGCTACCGTAG
AAAACACTCTATTCGAAGATGGCGATGGTGCAAATACCTTCCGTGCTTTTAACCCAACT
CAAGCTGAAGAAACTTATTCAATGGTCACTGCTAACCGCTTTTGGTCCCAAATCTTTGG
TGTTGCTTTTTCCAATAAACGTTGGTTACATTTCTTTATGCTATTTGTACCCGTCACCGGT
TTATGGATGAGTGCTATTGGCGTAGTTGGTCTGGCTCTGAACCTACGTGCCTATGACTTC
GTTTCCCAGGAAATCCGTGCAGCGGAAGATCCTGAATTTGAGACTTTCTACACCAAAA
ATATTCTTTTAAACGAGGGTATTCGTGCGTGGATGGCAGCTCAGGATCAGCCTCATGAA
AATCTTATATTCCCTGAGGAGGTTCTACCACGTGGAAACGCTCTTATGTCTGGAAGCAC
GGGAGAACGTTCTTTTGCTGATATTATTACCAGTATTCGATACTGGGTTATTCATAGCATT
ACTATACCTTCCCTATTCATTGCGGGTTGGTTATTTGTCAGTACGGGTTTAGCTTATGAC
GTGTTTGGAAGTCCTCGACCAAACGAGTATTTCACGGAAAGCCGACAAGGAATTCCAT
TAATAACCGACCGTTTTGATTCTTTAGAACAACTAGATGAATTTAGTAGATCCTTTATGA
CCATAGATCGAACCTATCCTATTTTTACAGTGCGATGGCTGGCTGTTCACGGATTAGCTG
TACCCACGGTTTTTTTCTTGGGATCAATATCAGCAATGCAGTTCATCCAACGAATGGCTA
CACAAACCGTTGAAGATAGTTCTAGACCTAAG---CCAAAACGGACTGGCGCAGGTAGT
TTATTGAAACCCTTGAATTCGGAATATGGGAAAGTTGCTCCGGGTTGGGGGACTACTCC
TTTTATGGGGGTCGCAATGGCTTTATTCGCGATATTCCTATCTATAATTTTAGAAATTTATA
ATTCTTCTGTTTTACTGGACGGAATTTTAACGAATATGCTTACTCTCAAACTTTTTGTTTA
TACAGTAGTGATATTCTTTGTTTCCCTCTTTATCTTTGGATTCTTATCTAATGACCCAGGA
CGTAATCCTGGGCGTGAGGAGATGGCCGATACTACTGGAAGAATTCCTCTTTGGCTGAT
AGGTACTGTAACTGGTATTCTTGTGATTGGTTTAATAGGTTTTTTCTTTTACGGTTCGTAT
TCTGGATTGGGTTCATCTCTAATGCCTAATATACTTAGTTTAACCTGTATCTGTTTTAATT
CTGTTCTTTGTCCGACTAGCTTTTTCTTCGCCAAATTACCCGAAGCTTATGCCATTTTCA
ACCCAATCGTGGATGTTATGCCTGTCATACCTGTACTCTTTTTTCTATTAGCGTTTGTTTG
GCAAGCTGCTGTAAGTTTTCGAATGACACAATCAAACCCGAATGAACAAAATGTTGAA
TTGAATCGTACCAGTCTATACTGGGGTTTATTACTCATTTTTGTACTTGCTGTTTTATTTT
CCAATTACTTCTTCAATATGGAAGTAAATATTCTCGCATTTATTGCTACAGCATTGTTCAT
TCTAGTTCCTACTGCCTTTTTACTTATTATTTATGTAAAAACAGCCAGTCAAAATGATATG
GAAACAGCAACTTTAGTCGCCATCTCCATATCTGGTTTACTTGTAAGCTTTACTGGGTAT
GCCTTATATACCGCGTTTGGGCAACCCTCTCAACAATTAAGAGATCCATTCGAAGAACA
TGGGGACATGGAAGCATTGGTTTATACGTTCCTTTTAGTTTCGACTTTAGGGATAATTTT
TTTCGCTATCTTCTTCCGAGAACCACCTAAGGTTCCGACTAAGAAA------ATGAATATTG
CTTTCCAATTAGCTGTTTTTGCATTAATTGCGACTTCCTCAGTTTTAGTAATTAGAGGAC
ACCTTGTATTTGCTTCTCCTGATGGTTGGTCAAATAATAAAAACGTTGTATTTTCCGGTA
CATCATTATGGATTGGATTAGTCTTTCTGGTAGCTATTCTGAATTCTCTTATTTCT-----------
----------ATGTCACCACAAACAGAAACTAAAGCAAGTGTTGGATTTAAAGCTGGTGTTAA
GGATTATAAATTGACTTACTACACCCCGGAGTACGAAACCAAGGATACTGATATCTTGG

CAGCATTCCGAGTAACTCCTCAGCTCGGGGTTCCGCCTGAAGAAGCAGGAGCTGCAGT
AGCTGCGGAATCTTCTACTGGTACATGGACAACTGTTTGGACTGATGGACTTACCAGTC
TTGATCGTTACAAAGGACGATGCTATCACATCGAGCCCGTTCCTGGGGACCCAGATCAA
TATATCTGTTATGTAGCTTATCCATTAGACCTATTTGAAGAGGGTTCTGTTACTAACATGT
TTACTTCCATTGTGGGTAACGTATTTGGTTTCAAAGCCTTACGCGCTCTACGTTTGGAG
GATCTACGAATTCCCCCTGCTTATTCAAAAACTTTCCAAGGTCCGCCTCACGGTATCCA
AGTTGAAAGGGATAAGTTGAACAAGTACGGTCGTCCTTTATTGGGATGTACTATTAAAC
CAAAATTGGGATTATCCGCAAAAAATTACGGTAGAGCGTGTTATGAGTGTCTACGCGGT
GGACTTGATTTTACCAAAGATGATGAAAACGTAAACTCACAACCATTTATGCGCTGGAG
AGACCGTTTCGTCTTTTGTGCCGAAGCAATTTATAAAGCACAAGCCGAAACTGGTGAA
ATCAAGGGGCATTACTTGAATGCGACTGCAGGTACATGCGAAGAAATGATTAAGAGAG
CTGTATTTGCAAGGGAATTAGGGGTTCCTATTGTAATGCATGACTACTTAACAGGAGGA
TTCACCGCAAATACTACTTTGTCTCATTATTGCCGCGACAACGGCCTACTTCTTCACATT
CACCGAGCAATGCATGCAGTTATTGATAGACAGAAAAATCATGGTATGCATTTCCGTGT
ATTAGCTAAAGCATTGCGTATGTCGGGGGGAGATCATATCCACTCCGGTACAGTAGTAG
GTAAGTTAGAAGGGGAACGCGAAATAACTTTAGGTTTTGTTGATTTATTGCGCGATGAT
TTTATTGAAAAAGATCGTTCTCGCGGTATCTTTTTCACTCAGGACTGGGTATCCATGCCA
GGTGTTATACCGGTGGCTTCTGGGGGTATTCATGTTTGGCATATGCCAGCTCTGACCGA
AATCTTTGGAGATGATTCCGTATTACAATTTGGTGGAGGAACTTTAGGACATCCTTGGG
GAAATGCACCTGGTGCAGCAGCTAATCGTGTGGCTTTAGAAGCCTGTGTACAAGCTCG
TAACGAAGGGCGCGATCTTGCTCGTGAAGGTAATGAAATTATCAAAGCAGCTTGCAAA
TGGAGTGCTGAACTAGCCGCAGCTTGTGAAATATGGAAGGAGATCAAATTTGATTTCA
AAGCGATGGATACCATA------ACGGCGAAACATTTATACAAAACACCTATCCCGAGCACA
CGCAAGGGAACCGTAGACAGGCAAGTGAAATCCAATCCACGAAATAAATTGATCCATG
GACGGCACCGTTGTGGTAAAGGTCGTAATGCCAGAGGAATCATTACCGCAAGGCATAG
AGGGGGAGGTCATAAGCGCCTATACCGTAAAATCGATTTTCGACGGAATCAAAAAGAC
ATATCTGGTAGAATCATAACCATAGAATACGACCCTAATCGAAATGCATACATTTGTCTC
ATACACTATGGGGATGGTGAGAAGAGATATATTTTACATCCCAGAGGGGCTATAATTGG
AGATACTATTGTTTCTGGTACAAAAGTTCCTATATCAATGGGAAATGCCCTACCTTTGAC
CGATATGCCCTTAGGCACGGCCATACATAACATAGAAATCACACGTGGAAGGGGTGGG
CAATTAGCTAGAGCAGCAGGTGCTGTAGCGAAACTGATTGCAAAAGAGGGTAAATTGG
CCACTTTAAGATTACCATCTGGGGAGGTCCGTTTGGTATCCCAAAACTGCTTAGCAACA
GTCGGACAAGTGGGTAATGTTGGGGTGAACCAAAAAAGTTTGGGTAGAGCCGGATCT
AAGTGTTGGCTAGGTAAACGCCCCGTAGTAAGAGGGGTAGTTATGAACCCTGTGGACC
ACCCCCATGGGGGTGGTGAAGGGAAAGCCCCCATTGGTAGAAAAAAACCCACAACCC
CTTGGGGTTATCCTGCGCTTGGAAGAAGAACTAGGAAAAGGAAAAAATATAGTGATAG
TTTTATTCTTCGTCGCCGTAAG---ATGATTCAACCTCAGACCCTTTTAAATGTAGCAGAT
AACAGTGGAGCTCGAAAATTGATGTGTATTCGAGTCATAGGAGCCGCTGGTAATCAGC
GATATGCTCGTATTGGTGATGTTATTATTGCTGTAATCAAAGACGCAGTGCCCCAAATGC
CTCTAGAAAGATCCGAAGTAATTCGAGCTGTAATTGTACGTACACGTAAAGAATTCAAA
GGCGACGACGGTATAATAATACGCTATGATGACAATGCAGCAGTTATCATTGATCAAAA
AGGAAATCCAAAAGGAACTCGAGTTTTTGGCGCGGTTGCCGAAGAATTAAGAGAATT
GAATTTGACTAAAATAGTTTCATTAGCTCCTGAAGTATTAATGCTTAGTCCCAAAAGAA
CTAGATTTCGTAAACAACATAGAGGAAGAATGAAGGGAAAATCCTGCCGAGGCAATCA

TATTTGTTTTGGTAGATATGCTCTTCAAGTACTTGAACCCGCTTGGATCACCGCGAGAC
AGATAGAAGCAGGACGAAGAGCAATGACACGATATGCACGTCGTGGTGGAAAAATATG
GGTGCGTATATTTCCCGACAAACCGGTTACAATAAGACCCACAGAAACACGTATGGGC
TCGGGAAAGGGATCCCCCGAATATTGGGTAGCCGTTGTTAAACCAGGTCGAATACTTTA
TGAAATGAGCGGAGTATCCGAAACTGTAGCTAGAGCAGCTATCTCTATAGCTGCCAGTA
AAATGCCCATACGAAGTCAATTTCTTCGATTAGAGATGACCAGAGTTCCGCGAGGATAT
ATAGCTCGGAGACGACGAACAAAAATGCGTTCATTTGCCTCAAACTTTAGGGGGGCTC
ATTTAAGACTTAATCGAGTGATTACTCAACAAGTAAGAAGAGCTTTTGTTTCTTCTCAT
CGAGATAGAGGCAGGCAAAAGAGGGATTTTCGTCGTTTGTGGATCACTCGGATAAACG
CAGCAACACGGGTATAT------GTATTCAATAGTTATAGTAAATTAATACACAATCTGTCCA
AGAAGGAATTGATTCTTAATCGTAAAATGCTTGCACAAGTAGCTGTATCAAATCCAAAT
AATCTTTACACGATTTCCAATAAAATAAGGATCATCAAT---------------------------AGGATAA
AGCGGGTTTTCAAAGCACAAAAACGCATACATATGTCTGTTTTCAAAGCACAAAGAGT
TCTAGATGAGATTCGCTGGCGTTACTACGAGGAAACCGTTATGATACTGAATCTCATGC
CTTATCGAGCATCGTATCCCATCTTAAAGTTGGTTTATTCGGCAGCAGCAAATGCTACTC
ATTATAGGGATTTCGACAAAACGAATTTATTCATCACTAAAGCCGAAGTCAGTAGGAGT
ACTATTATGAAAAAATTCAGACCTCGAGCTCGAGGACGTAGTTATTCTATAAAAAAAAC
GATGTGTAATATAACAATTGTACTAAAT---GTAAAGAAATCA---------------------ATGGATGG
AATCAAATACGCAGTATTTACAGAAAAGAGTCTTCGTTTATTGGGAAAGAAT---CAATAT
ACTTTTAATGTCGAATCGGGATTCACTAAGACAGAAATAAAGCATTGGGTCGAACTCTT
CTTTGGTGTTAAGGTAGTAGCTGTGAATAGCCATCGACTACCCGGAAAGGGTAGAAGA
ATGGGACCTATTCTGGGACATACAATGCATTACAGACGTATGATCATTACCCTTCAACCG
GGTTATTCTATTCCACTTCTAGATAGAGAAACGAACATGGCAGTTCCAAAAAAACGTAC
TTCGATGTCAAAAAAGCGTATTCGTAAAAATCTTTGGAAAAAAAAGACTTATTTTTCCA
TAGTACAATCTTATTCTTTAGCAAAATCATTTTCTAGGGGCAAACCAAAAGGTTTTTCTG
GGCAACAAGCAAACAAAATGGCCAAGGGCAAAGATGTTAGAATCCGAGTTATTTTGG
AATGTATTAGTTGTGTTCGAAAG------GGT------AAGGAATCGACGGGGATTTCTAGATAT
AGTACTCAAAAGAATCGCCACAATACGCCCGGACAATTAGAATTAAGAAAATTTTGTC
GTTATTGTCGCAAGCATACCACTCATAATGAAATAAAGAAAATGAAAATAAGAGCTTCC
GTTCGTAAAATTTGTACAAAATGTCGACTGATTCGCAGGCGTGGGCGAATTAGAGTTAT
TTGTTCCAATCCGAAGCATAAACAAAGACAGGGGATGGTTCGAGAAGAGATAACAGG
ATCCACTCAAACACTAGAGTGGAAGTGTGTTGAATCAAGAGTAGATAGTAAACGTCTT
TATTATGGTCGTTTCATTCTGTCTCCGCTTAGAAAAGGTCAAGCGGATACTGTCGGTATT
GCCTTGCGAAGAGCTTTACTTGGAGAAATAGAAGGAACATGTATCACACGCGCCAAAT
TTGGG------AACGTGCCACATGAATATTCCACAATAGTAGGTATTGAAGAATCCATACAA
GAAATTTTACTAAATTTGAAAGAAATTGTATTGAGAAGTAATCTCTATGGAGTTAGAGA
CGCATCAATTTGCGTCAAAGGTCCTAGATACATAACCGCTCAAGATATAATCTTACCGCC
TTCCGTAGAAATCGTTGATACGACACAACCTATAGCTAACTTGAGAGAACCCGTTGATT
TCTGTATTGAGTTACAGATCAAGAGAGATCGCGCATATCATACGGAACTCAGAAAGAA
CTCTCAAGATGGAAGTTAT------CCTATAGATGCTGTATTCATGCCTGTTCGAAATGTGAA
TTATAGTATTTTTTCTTGTGGGAATGGAAATGAAAAACACGAGATACTTTTTTTAGAAAT
ATGGACGAATGGAAGTTTAACTCCTAAAGAAGCGCTTTATGAAGCTTCTCGTAATTTGA
TTGATTTATTTCTTCCTTTTCTACACACAGAGGAAGAG------GGCACTAGTTTCGAAGAA
AATAAA---AACAGGTTAACTCCACCCCTTTTAACCTTTCAAAAAAGATTCACT---AATCT

AAAGAAAAACAAAAAA---GGAATTCCATTGAATTGTATTTTTATTGATCAATTAGAATTG
CCTTCTAGAACGTATAATTGTCTCAAAAGGGCCAATATACATACACTATTGGACCTTTTG
AGTAAGACTGAAGAAGATCTTATGCAAATTAATAGTTTTCGTATGGAAGATGGAAAACT
GATATGGGACACTCTAGAGAAGCAT------CTCCCAATTGATTTACCTAAGAACAAGATGC
TCCGGAATGGAAAT---GAGGGAATGTCCACAATACCCGGATTTAGTCAGATCCAATTCG
AGGGATTTTGTAGATTCATTAATCAAGGCTTGGCGGAAGAACTTGAGAAGTTTCCCAC
AATTAAAGATCCAGATCACGAAATTGCATTTCAATTATTTGCGAAAGGATATCAATTGCT
AGAACCCTCGATAAAAGAAAGGAATGCTGTGTATGAATCACTCACCTATTCTTCCGAAT
TATATGTATCCGCGAGATTAATTTTTGGTTTC------GATGTGCAAAAGGAAACCATTTCTA
TTGGAAACATTCCTATAATGAATTCCTTAGGAACCTTTATAATAAATGGAATATACCGAA
TTGTGATCAATCAAATATTGCTAAGTCCTGGTATTTACTACCGCTCGGAATTAGACCATA
AGGGAATTTCTATATATACCGGGACTATAATATCAGATTGGGGAGGAAGGTCGGAATTA
GCAATTGATAAAAAAGAAAGGATATGGGCTCGCGTGAGTAGAAAACAAAAGATATCTA
TTTTAGTTCTATCATCAGCTATGGGTTCGAATCTAAGAGAAATTTTAGATAATGTTTCCTA
CCCCGAAATTTTCTTGTCTTTCCCGAATGCTAAGGAGAAGAAGAGGATTGAGTCAAAA
GAAAAAGCTATTTTGGAGTTTTATCAACAATTTGCTTGTGTAGGTGGGGACCTGGTATT
TTCGGAGTCCTTATGCGAGGAATTACAAAAGAAATTTTTTCAACAAAAAATGTGAATTAG
GAAGAGTTGGTCGACGAAATATGAATCGGAGACTGAATCTTGATATCCCTCAGAACAAT
ACATTCTTGTTACCACGAGATGTATTGGCCGCTACGGATCATTTGATTGGAATGAAATTT
GGAACGGGTATACTTGACGATGACGATATGAATCACTTGAAAAATAAACGTATTCGTTC
GGTTGCAGATCTGTTACAAGATCAATTCGGACTGGCTCTTGGCCGTTTACAACATGCGG
TTCAAAAAACTATCCGTAGAGTATTCATACGTCAATCGAAA------CCGACTCCACAAACT
TTGGTAACTCCAACTTCAACTTCGATTTTATTAATAACTACTTATGAGACCTTTTTTGGC
ACATACCCCTTAGCTCAAGTTTTTGATCAAACCAATCCATTGACACAAACGGTTCATGG
GCGAAAAGTGAGTTGTTTGGGTCCAGGAGGATTGACGGGGAGAACTGCAAGTTTTCG
GAGCCGAGATATTCATCCGAGTCACTATGGGCGTATTTGTCCAATTGACACGTCCGAAG
GAATCAATGTTGGACTTACCGGATCGTTAGCTATTCATGCGAGAATTGATCACTGGTGG
GGATCTATAGAGAGTCCGTTTTATGAAATATCTGAGAAAGCAAAAGAAAAAAAAGAG--
----AGACAGGTAGTTTATTTATCACCAAATAGAGATGAATATTATATGATAGCAGCAGGAA
ATTCTTTGTCCTTGAATCAGGGTATTCAGGAAGAACAGGTTGTTCCAGCTAGATACCGT
CAAGAATTCCTGACTATTGCATGGGAACAGATTCATGTTAGAAGTATTTTTCCTTTCCAA
TATTTTTCTATTGGAGGTTCTCTCATTCCTTTTATTGAGCATAATGATGCGAATCGGGCTT
TAATGAGTTCTAATATGCAGCGCCAAGCAGTTCCACTTTCTCGGTCCGAGAAGTGCATT
GTTGGAACTGGATTGGAACGCCAAACAGCTCTAGATTCGAGGGTTTCCGTTATAGCCC
AACGCGAGGGAAAGATCATTTCTAGTGATAGTCACAAGATCCTTTTATCAAGTAGTGGG
AAGACCATAAGTATTCCTTTAGTTGCCCATCGGCGCTCTAACAAAAATACTTGTATGCA
CCAAAAACCTCGGGTTCCGCGGGGTAAATCCATTAAAAAAGGGCAAATTTTAGCGGAG
GGAGCAGCTACAGTTGGCGGGGAACTTGCTTTAGGAAAAAACGTTTTAGTAGCTTATA
TGCCCTGGGAGGGTTACAATTTTGAAGACGCAGTACTAATTAGTGAACGTTTGGTATAT
GAGGATATTTATACTTCTTTTCACATCCGAAAATATGAAATTCAGACGGATACGACAAGC
CAAGGCTCCGCTGAAAAAATCACTAAACAAATACCACATCTAGAAGAACATTTACTTC
GCAATTTGGACAGAAATGGAGTTGTAAGGTTGGGATCCTGGGTAGAAACTGGCGATAT
TTTAGTAGGTAAATTAACGCCTCAGATAGCGAGCGAA---TCGTCCTATATCGCGGAAGCT
GGATTATTACGGGCTATTTTTGGTCTTGAGGTATCCACTTCAAAAGAAACTTCTCTCAA

ACTACCTATAGGTGGAAGAGGGCGCGTTATCGATGTGAAATGGATCCAGAGG-------------
--------GACCCCATAATGGTTCGTGTATATATTTTACAGAAACGTGAAATCAAAGTTGGGG
ATAAAGTAGCTGGAAGACACGGGAATAAGGGGATCATTTCCAAAATTTTGCCTAGGCA
AGATATGCCCTATTTGCAAGATGGAGCACCTGTTGATATGGTCTTCAATCCCTTAGGAGT
ACCCTCACGAATGAATGTGGGACAAATATTTGAAAGCTCGCTCGGATTAGCAGGGGAT
CTGCTAAAGAAACATTATAGAATAGCACCCTTTGATGAGAGATATGAGCAAGAGGCTTC
AAGAAAACTTGTGTTTTCGGAATTATATGAAGCCAGTAAACAAACAAAAAATCCATGG
GTATTTGAACCCGAGTACCCGGGAAAAAGCAGAATATTTGATGGAAGAACAGGAGATC
CCTTCGAACAGCCTGTTCTAATAGGGAAGTCCTATATCTTAAAATTAATTCATCAAGTTG
ATGAGAAAATTCATGGACGTTCTACTGGGCCCTACTCACTTGTTACCCAACAACCCGTT
AGAGGAAGAGCCAAGCAAGGGGGACAACGAATAGGAGAAATGGAAGTTTGGGCTTT
AGAAGGATTTGGTGTTGCTCATATTTTACAAGAGATACTTACTTATAAATCTGATCATCTT
ATAGCTCGCCAAGAAATACTTAATGCTACGATCTGGGGAAAAAGAATGCCTAATCACGA
GGATCCGCCAGAATCTTTTCGAGTCCTCGTTCGAGAACTACGATCTTTGGCTCTAGAAC
TGAACCATTTCCTTGTATCTGAGAAGAACTTCCAGGTTAATAGGGAGGATGTT-------------
--------ATGATTGACCAATATAAACATAAACAACTTCAAATTGGACTCGTTTCCCCTCAAC
AAATAAAGGCTTGGGCTAAAAAAATCCTACCTAATGGGGAAGTCGTTGGCGAAGTCAC
AAGGCCCTCCACTTTTCATTATAAAACCGATAAACCAGAAAAAGATGGATTGTTTTGCG
AAAGAATCTTTGGACCCATAAAAAGCGGAATTTGTGCTTGTGGAAACTCTCGAGCGAG
CGTACGTGAAAACGAAGACGAAAGATTTTGCCAAAAATGCGGGGTAGAATTTGTTGAT
TCTCGGATACGAAGATATCAAATGGGATACATCAAACTAGCATGTCCAGTGACTCATGT
GTGGTATTTGAAAGGTCTTCCTAGTTATATTGCGAATCTTTTAGATAAACCCCTTAAGAA
ATTGGAGGGCCTAGTATATGGTGAT------TTCTCTTTTGCTAGGCCC---AGCGCTAAAAAA
CCTACTTTCTTACGATTACGGGGTTTATTCGAAGATGAAATTTCATCCTGTAACCACAGT
ATTTCTCCCTTTTTTTCTACCCCAGGCTTTGCAACATTTCGAAATCGGGAAATTGCAAC
AGGAGCAGGCGCTATTAGAGAACAATTAGCAGATTTAGATTTGCGAATTATTATAGAGA
ATTCTTTGGTTGAATGGAAGGAATTAGAAGACGAG---GGGTATAGTGGAGATGAATGGG
AAGATAGAAAAAGACGAATAAGAAAAGTTTTTTTGATTAGACGCATGCAATTGGCGAA
ACATTTTATTCAAACAAATGTAGAACCAGAATGGATGGTTTTGTGCTTATTACCGGTTCT
TCCTCCCGAATTGAGACCCATTGTTTATAGATCTGGGGATAAAGTAGTGACTTCAGATAT
TAATGAACTTTATAAGAGAGTTATCCGTCGGAACAACAACCTTGCCTATCTATTAAAAA
GAAGTGAATTAGCACCAGCAGATTTAGTAATGTGCCAGGAAAAATTGGTACAAGAAGC
CGTGGATACACTTCTTGATAGTGGGTCCCGCGGGCAACCGACGAGGGATGGTCACAAT
AAAGTATACAAATCACTTTCAGATGTAATTGAGGGTAAAGAGGGGAGGTTTCGCGAGA
CTCTGCTTGGGAAACGGGTCGATTACTCGGGGCGTTCTGTCATTGTTGTGGGTCCTTCG
CTTTCATTACATCAATGTGGATTACCTCTAGAGATAGCAATAAAGCTTTTTCAGCTATTT
GTAATTCGCGATTTAATCACGAAACGTGCTACTTCTAATGTCAGGATTGCTAAAAGGAA
AATTTGGGAAAAGGAACCCATTGTATGGGAAATACTTCAAGAAGTTATGCGGGGACAT
CCTGTACTGTTGAACAGAGCACCTACCCTGCATAGATTAGGCATACAGGCCTTCCAACC
CACTTTAGTAGAGGGACGTACTATTTGTTTACATCCATTAGTGTGTAAGGGTTTCAATGC
GGACTTTGATGGGGATCAAATGGCTGTTCACCTACCTTTATCCTTGGAAGCTCAGGCGG
AAGCCCGTTTACTTATGTTTTCTCATATGAATCTCCTGTCTCCCGCTATTGGAGATCCTAT
TTGCGTGCCAACCCAAGACATGCTTATCGGACTTTATGTATTAACGATTGGAAACCGTC
TAGGTATTTGTGCAAATAGATATAATAGTTGCGGAAAC---------------TCTCCAAATAAAAA

AGTAAACTACAATAATTATAAGTATACGAAAGATAAAGAACCCCATTTTTCTAGTTCCTA
TGATGCACTGGGAGCTTATAGACAAAAACGAATCGGTTTAAACAGTCCCTTGTGGCTC
CGATGGAAACTAGATCAACGC---ATCGTTGGGTCA------AGAGAAGTTCCGATTGAAGTT
CAATATGAATCTTTTGGGACTTATCATGAGATTTATGCCCACTATCTAGTAGTGGGAAAT
AGAAAAAAAGAAATCCGTTCTATATACATTCGAACCACTCTTGGTCATATTTCTTTTTAT
AGAGAAATAGAGGAAGCCATACAAGGATTTAGTCGGGCCTATTCATACACTATC----------
--------------------------ATGGCAGAACGGGCCAACCTGGTCTTTCATAATAAAGAGATAGAC
GGAACTGCTATGAAACGGCTTATTAGCAGATTAATAGATCATTTCGGAATGGGATATACA
TCCCATATACTGGATCAAATAAAGACTCTGGGCTTCCATCAAGCCACTACTACATCGATT
TCTTTAGGAATCGAGGATCTTTTAACAATACCCTCTAAAGGATGGTTAGTCCAAGACGC
GGAACAACAGAGTTTTCTTTTGGAGAAACACTATTATTATGGAGCTATACACGCGGTAG
AAAAATTACGCCAATCCGTTGAGATATGGTATGCTACAAGTGAATATTTGAAACAAGAA
ATGAATTCGAATTTTCGAATAACGGATCCTTCTAATCCAGTCTATCTAATGTCTTTTTCAG
GAGCCAGAGGAAATGCATCTCAGGTACACCAATTAGTAGGTATGAGAGGGTTAATGGC
GGATCCTCAAGGACAAATGATTCATTTACCTATTCAAAGCAATTTACGCGAGGGACTTT
CTTTGACAGAATATATAATTTCCTGCTACGGAGCCCGAAAAGGGGTTGTAGATACTGCT
GTACGAACAGCGGATGCTGGCTATCTTACACGTAGACTTGTTGAAGTAGTTCAACATAT
TATTGTGCGTAGAAGAGATTGTGGTACTATCCAAGGTATTTCTGTGAGTCCTCAAAATG
GGATGACGGAA------AAACTTTTTGTCCAAACACTAATTGGTCGTGTATTAGCAGACGAT
ATATATATTGGTTCACGATGCATTGCTTCTCGAAATCAAGATATTGGAATTGGATTAGTCA
ATCGATTCATAACTGCCTTTCGAGCACAACCGTTTCGAGCACAACCAATATATATTAGAA
CCCCCTTTACTTGCCGGAGCACATCTTGGATCTGTCAATTATGTTATGGGCGGAGTCCC
ACTCATGGGGATCTGGTCGAATTGGGGGAAGCTGTAGGTATTATTGCGGGTCAATCAAT
TGGGGAGCCGGGGACTCAATTAACATTAAGAACTTTTCATACTGGTGGAGTATTCACAG
GGGGTACTGCCGACCTTATACGATCCCCCTCGAATGGAAAAATCCAATTCAATGAGGAT
TTGGTTCACCCCACACGTACCCGTCATGGGCAGCCTGCTTTTCTATGCTATATAGACTTG
CATGTAACTATTCAGAGTCAGGATATTCTACATAGTGTGAATATTCCGTTAAAAAGCTTG
ATTCTAGTGCAAAATGATCAATATGTAGAATCTGAACAAGTAATTGCGGAGATTCGTGC
CGGAACGTCCACTTTG------TTTAAAGAAAAGGTACAAAAGCATATTTATTCCGAATCAG
ACGGGGAA---ATGCACTGGAGTACTGATGTTTACCATGCGCCCGAATATCAATATGGTAA
TCTTCGTCGATTACCAAAAACAAGCCATTTATGGATATTGTCAGTAAGTATGTGCAGA---
---TCCAGTATAGCATCTTTTTCGCTGCACAAGGATCAAGATCAAATGAATACTTATTCTTT
TTCTGTTGATGGAAGA------TATATCTTTGACTTCTCA------ATGGCTAAT---GATCAAGTA
AGCCATAGACTGTTGGATACTTTTGGT------------GATAGAGAAATTCTTGATTATTTA---A
CGCCAGATCGAATCGTGTTCAACGGTCATTGGAATTGTTTCTATCCTTCC---ATTCTTCA
AGATAAT------TCAGATTTGTTGGCGAAAAAGCGAAGAAATAGGCTCGTCGTTCCATTAC
AATATCATCAAGAACAAGAAAAAGAGCGAATATCCTGTTTGGGTATTTCAATGGAAATA
CCCTTTATG------GGTGTTTTACGTAGAAATACTATTTTTGCTTATTTTGACGATCCACGAT
ACAGAAAAGAT------GGAATTGTTAAATTTAGAAGCCCGGAGGAAGATTCTATCTTAAA
A------AAAGAGGGTTTTATTGAGCATCGAGGA---ACAAAAGAATTTAGTCTAAAATAC---
GAAGTAGATCGGTTTTTTTTCATTCTTCAAGAACTGCATATCTTGCCGAGATCCTCATCC
CTAAAGGTACTTGACAATAGTATTATTGGAGTCGATACACAACTCACAAAAAATACAAG
AAGTCGACTAGGTGGATTGGTCCGAGTGAAGAGAAAAAAAAGCCATACAGAACTCAA
AATCTTTTCCGGAGATATTCATTTTCCTGAAGAGGCGGATAAGATATTAGGT------GGCAG

TTTGATACCACCAGAAAGAGAAAAAAAA------GATTCTAAGGAA---TCAAAA---AAAAG
GAAAAATTGGGTTTATGTTCAACGGAAAAAATTTCTCAAAAGCAAGGAAAAG------TAT
TTTGTTTCGGTTCGACCTGCAGTCGCATATGAAATGGACGAAGGGATAAATTTAGCAAC
ACTTTTCCCGCAGGATCTCCTGCAAGAAGAGGATAATCTCCAACTTCGACTTGTCAATT
TTATTTCTCATGAAAATAGCAAGTTAACTCAAAGAATTTATCACACGAAT---CAATTCGT
TCGAACTTGCTTAGTAGTGAATTGGGAACAAGAAGAAAAAGAGGGAGCTCGT----------
----------GCTTCTCTTGTTGAGGTAAAAACAAATGATCTAATTCGCGATTTCCTAAGAATT
GAGTTAGTCAAGTCTACTATTTTGTATACACGAAGAAGGTATGATAGGACAAGTGTAGG
ATTGATTCCCAAT---------TTAGATCGCAACAATACCAATTCCTTTTATTCCAAGGCGAAG
ATTCAATCA---------------CTTAGCCAACATCAAGAAGTTATTGGC------------ACCTTGTTGA
ATCGAAATAAA---------GAATATCCATCTTTGATGATTTTGTTGGCATCCAACTGTTCTCG
AATTGGT------TTATTCAAGAATTCTAAATATCCC---------AATGCGGTAAAAGAATCGAAT
CCTAGAATTCCTATT---GATATTTTTGGGCTCTTAGGCGTTATTGTACCT---AGTATATCGA
ATTTTTCTTCATCT---TACTATTTATTAACG---------CATAATCAGATCTTGTTAAAA---AAAT
ACTTGTTCCTTGAC---------AATTTGAAACAAACCTTACAAGTACTTCAAGGACTTAAAT
ACTCTTTAATAGACGAAAATAAAAGG------ATTTCGAATTTTGAC------AGTAACATCATG
TTGGAGCCATTCCATTTGAATTGGCACTTTCTCCATCATGACTCATGGGAAGAGACATT
GGCAATAATTCAC---CTTGGACAATTTATTTGTGAAAATTTATGTCTATTTAAATTACACA
TA------------AAAAAATCTGGTCAAATTTTCATTGTTAAT---ATGGACTCCTTTGTTCTAAG
AGCAGCTAAGCCTTATTTGGCCACTATAGGAGCAACTGTTCATGGTCATTATGGAAAAA
TCCTTTACAAAGGAGATAGGTTAGTTACGTTTATATATGAAAAATCGAGATCCAGTGATA
TAACGCAAGGTCTTCCAAAAGTAGAACAAATCTTCGAAGCGCGTTCAATTGATTCACTA
TCGCCGAATCTCGAAAGGAGAATTGAGGATTGGAATGAACGTATACCAAGAATTCTTG
GGGTTCCCTGGGGATTCTTGATTGGAGCTGAGCTAACCATAGCCCAAAGTCGTATCTCT
TTGGTTAATAAGATCCAAAAGGTTTATCGATCCCAAGGGGTACAGATCCATAATAGACAT
ATAGAGATTATTATACGCCAAGTAACATCAAAAGTAAGGGTTTCCGAA---------GATGGA
ATGTCTAATGTTTTTTTACCTGGGGAATTAATAGGACTATTGCGAGCGGAACGAGCAGG
GCGGGCTTTAGATGAATCGATCTATTATCGGGCAATCTTATTGGGAATAACAAGGGCTTC
CCTGAATACCCAAAGTTTCATATCTGAAGCAAGTTTTCAAGAAACTGCTCGAGTTTTAG
CAAAAGCTGCCCTACGAGGTCGTATTGATTGGTTGAAAGGTCTGAAAGAAAACGTAGT
TCTGGGGGGGATTATACCTGTTGGTACCGGATTCCAAAAATTTGTGCATCGTTCCCCA---
---CAAGACAAGAACCTTTAT------TTAGAAATTCAAAAAAAAAATCTATTCGCGTCGGAA
ATG------AGAGATATTTTGTTTCTCCATACAGAATTAGTTTCTTCTGATTCTGACGTAACA
AAC---------TCAGAGACCCCGTTTACCCCTATTTATACGATT-----------------ATGACAAGAA
GATATTGGAACATCAATTTGAAAGAGATGATAGAAGCGGGAGTTCATTTTGGTCATGGT
ATTAAGAAATGGAATCCCAAAATGGCCCCTTACATCTCGGCAAAGCGTAAAGGTACTCA
TATTACAAATCTTGCTAGAACAGCTCGTTTTTTATCAGAAGCTTGTGATTTAGTTTTTGA
TGCAGCAAGTCAGGGAAAAAGCTTCTTAATTGTTGGTACCAAAAAAAGAGCAGCGGA
TTTAGTAGCATCAGCTGCAATAAGGTCTCGTTGTCATTATGTTAATAAAAAGTGGTTCAG
CGGTATGTTAACGAATTGGTCGATTACCAAAACTAGACTTTCTCAATTTAGAGACTTAA
GAGCAGAAGAAAAAAATGGGAAAATTCCACCATCTCCCAAAAAGAGATGCGGCAATCT
TAAAGAGAAAATTATCTACCTTGCAAAGATATCTCGGCGGGATCAAATATATGACGAGG
TTGCCTGACATTGTGATCGTCCTTGATCAGCAAAAAGAGTATATAGCTCTTCAGGAATG
TGCCATTTTGGGGATTCCTACTATTTCTTTAGTCGATACAAATTGTGACCCAGATCTTGC

GAATATATCGATTCCTGCCAACGATGACACTATGACTTCAATTCGATTAATTCTTAACAA
ATTAGTATTTGCAATTTCTGAGGGCCGTTCTCTC---TATATAAGAAATCGTATGGGACAA
AAAATAAATCCACTCGGTTTCAGACTTGGTACAACCCAAAATCACCATTCCTTTTGGTT
CGCACAACCAAAAAATTATTCTGAAGGTCTACAGGAAGATAAAAAAATACGGAATTGT
ATCAAGAACTATATACAAAAGAATAGGAAAAAAGGCTCGAATAAAATGGAATCAATTA
CACATATAGAAATTCAAAAAGAAATCGATACAATCCACGTCATAATCCATATTGGATTCC
CCAAC---TTATTAAAGAAAAAGGGA---------------GCAATCGAAGAATTAGAGAAAGATCT
CCAAAAGGAAGTTAATTCTGTAAACCAGAGACTTAATATTGCTATCGAAAAGGTTAAA
GAACCTTATAGACAACCTAATATTCTTGCGGAATATATAGCTTTCCAATTAAAAAATAGA
GTTTCATTCCGAAAAGCAATGAAAAAAGCTATTGAATTAACTAAAAAAGCGGATATAA
AGGGAATAAAAATCCAAATTGCGGGTCGTCTAGCAGGAAAAGAAATTGCACGTGCCG
AATGCATCAAAAAGGGTAGACTACCCCTCCAAACAATTCGCGCTAAAATTGATTATTGC
TGCTATCCAATTCGAACTATCTATGGAGTATTAGGTGTCAAAATTTGGATATTCGTAGAA
GAAGAAATGTCCCGTTATCGAGGGCCTCGTTTAAAAAAAATACGCCGTCTGGGAGCTT
TACCAGGACTCACTAGAAAAACGCCTAAATCCGGAAGTAATCAGAAAAAGAAATTCCA
TTCTGGGAAA---AAAGAGCAATATCGTATTCGTCTTCAAGAAAAACAGAAATTGCGTTT
TCATTATGGTCTGACAGAACGACAATTACTTAGATATGTACATATCGCTGGAAAAGCAA
AAAGATCCACAGGTCAGGTTTTACTACAATTACTTGAAATGCGTTTGGATAATATCCTTT
TTCGATTGGGTATGGCTTCAACCATTCCTGGGGCCCGACAATTAGTTAACCATAGACATA
TTTTAGTTAATGGTCGTATAGTTGATATACCAAGCTTTCGTTGCAAACCCCGAGATATTAT
TACTACGAAGGATAACCAACGATCAAAACGTTTGGTTCAAAATTATATTGCTTCATCCG
ATCCGAAA------TTACCAAAGCATTTGACGGTTGACACA------TTGCAATATAAAGGACTA
GTAAAAAAAATTCTAGATAGGAAGTGGGTCGGTCTCAAAATAAATGAGTTGTTAGTTGT
AGAATATTATTCTCGCCAGACTATGTCACGTCGAGGTACTGCAGAAAAAAGAACCGCA
AAATCCGATCCAATTTTTCGTAATCGATTAGTTAACATGGTGGTTAACCGTATTATGAAA
GACGGAAAAAAATCATTGGCTTATCAAATTCTCTATCGAGCCGTGAAAAAGATTCAAC
AAAAGACAGAAACAAATCCACTATTGGTTTTACGTCAAGCAATACGTAGAGTAACTCC
CAATATAGGAGTAAAAACAAGACGTAAAAAAGGATCGACGCGGAAAGTTCCGATTGA
AATAGGATCTAAACAAGGAAGAGCACTTGCCATTCGTTGGTTATTAGAAGCATCCCAA
AAGCGTCCGGGTCGAAATATGGCTTTCAAATTAAGTTCCGAATTAGTAGATGCTGCCAA
AGGGAGTGGGGGTGCCATACGCAAAAAGGAAGCGACTCATAGAATGGCAGAGGCAAA
TAGAGCTCTTGCACATTTTCGTATGGGCAAGGACACTATTGCTGATTTACTAACCTCTAT
AAGAAACGCAGACATGAATAAAAAAGGAACTGTTCGAGTAGTATCCACAAATATTACC
GAAAACATTGTTAAAATACTTCTACGAGAGGGTTTTATTGAAAGTGTTCGGAAACATCA
GGAAAGTAACAGATATTTCTTGGTTTCAACTTTGCGACATCAAAGGAGAAAGACTAGA
AAGGGAATATATAGAACT---------AGAACCTTTTTAAAGCGTATCAGCCGACCTGGCTTAC
GAATTTATGCCAACTATCAAGGAATTCCTAAGGTTTTGGGCGGAATGGGAATTGCTATT
CTTTCTACTTCTCGAGGTATAATGACAGATCGAGAAGCTCGACTAAACAGAATTGGGG
GAGAAGTTTTATGTTATATATGGATGACAAAAGCTATACCAAAAATAGGTTCACGTAAG
AAAGTGCGTATTGGTTTGCGTAAGAGTGCACGTAGAATAACAAAAGGGATTATTCATGT
TCAAGCTAGTTTCAACAATACCATTATAACCGTTACAGACCCACAAGGTCGGGTCGTTT
TCTGGTCCTCCGCAGGTACTTGTGGATTCAAAAGCTCAAGAAAAGCATCACCCTATGC
TGGTCAAAGAACAGCAGTAGATGCTATTCGTACAGTGGGT---------TTGCAACGAGCAG
AAGTTATGGTAAAAGGTGCTGGTAGCGGAAGAGATGCCGCATTACGAGCCATTGCTAA

AAGTGGTGTACGGTTAAGTTGTATACGCGATGTAACACCTATGCCGCATAATGGATGTC
GACCTCCTAAAAAAAGACGTCTGATGCCAACGGTTAAACAACTTATTAGAAATGCAAG
ACAGCCAATACGAAATGCTAGAAAATCGGCCGCGCTTAAGGGATGTCCTCAGCGTCGA
GGAACATGTGCTAGGGTGTATACTATCAACCCCAAAAAACCCAACTCTGCCTTACGTAA
AGTTGCCAGAGTACGATTAACCTCTGGATTTGAAATCACTGCTTATATACCTGGTATTGG
CCATAATTTACAAGAACATTCTGTAGTATTAGTAAGAGGAGGAAGGGTTAAGGATTTAC
CCGGTGTGAGATATCGCATTATTCGAGGAACCCTAGATGCTGTCGCAGTAAAGAATCGT
CAACAAGGGCGTTCTAAATATGGGGCCAAAAAGCCAAAAATGGCAAAAAAAAGTTTG
ATTCAGAGGGAGAAGAAGCGGCAGAAATTAGAACAAAAATATCATTTGATTCGTCGAT
CCTCAAAAAAAAAGATAAGAAGCAAAGTTTCCCCTTTGAGTTTGAGCGAAAAAACAA
AAATGCAAGAAAAATTGCAATCCCTACCACGTAATAGTGCACCTACACGCCTTCATCGA
CGTTGTTTTTTAACCGGAAGACCTAGAGCTAACTATCGAGACTTTGGGCTATCCGGACA
CATACTTCGAGAAATGGTTTATGCATGTTTGTTACCGGGTGCAACAAGATCCAGTTGG---
------------------------ATGGTTAAAGAAGAAAAACAAGAAAAC------AGGGGTTCTGTTGAA
TTTCAAGTATTCAGTTTCACCAATAAGATACGGAGACTTGCTTCACATTTAGAATTACAC
AAAAAAGATTTTTCATCGGAAAGAGGTCTACGAAGACTTTTGGGAAAACGTCAACGTT
TGCTGGCTTATTTGGCAAAGAAAAATAGAGTACGTTATAAGAAATTAATAAGTCAGTTG
GATATTCGGGAGAAG---------------ATGGTAAAACTTCGTTTAAAACGATGTGGTAGAAAG
CAACAAGCTATCTATCGAATCGTTGCAATTGATGTTCGATCTCGAAGAGAAGGAAGAG
ATCTTCGAAAAGTAGGTTTTTATGATCCGATAAAGAATCAAACTTGTTTAAATGTTCCA
GCTATTCTCTATTTCCTTGAAAAGGGTGCTCAACCTACAAGAACAGTTTATGATATTTTA
AGGAAGGCAGAATTCTTTAAAGATAAAGAAAGAACTTTGAGT------------TTTCATAAATC
TAAGCAACCTTTTCGTAAATTCAAACAACCTTTTCGTAAAAGGCGTTCTCGAATAGGCC
CGGGGGATCGAATTGATTATAGAAACATGAGTTTAATTAATCGATTTATTAGTGAACAAG
GAAAAATATTATCTAGACGAATAAATAGATTAACCTTGAAACAACAACGATTAATTACTC
TTGCTATAAAACAGGCTCGTATTTTATCTTTCTTACCATTTCGTAACTATGAGAATGAGA
AGCAATTTCAA---GCCCAGGCAATTTCAATAATTACTGGTCCTAGACACAGAAAAAATG
TGACACGAAAA---AAAACGAATCCTTTTGTAGCTCGTCATTTATTGGCAAAAATCGAAA
AGGTCAATATGAAGGAGGAGAAAGAAATAATAGTAACGTGGTCCCGGGCATCTAGCAT
TCTACCCGCAATGGTTGGCCATACAATCGCGATTCATAATGGAAAAGAACATATACCTAT
TTACATAACAAATCCTATGGTAGGTCGCAAATTGGGGGAATTCGTGCCTACTCGGCATT
T C A C G A G T T A T A C A A G A A A G G A T A C T A A A T C T C G T C G
T???
??
??
??
??
??
??
??
??
??
??
??

???
???
???
???
???
???
???
???
???
???
???
???
???
???
???
???
???
???
???
???
???
???
???
???
???
???
???
???
???
???
???
???
???
???
???
???
???
???
???
???
???
???
???
???

???
???
???
???
???
???
???
???
???
???
???
???
???
???
???
???
???
???
???
???
???
???
???
???
???
???
???
???
???
???
???
???
???
???
???
???
???
???
???
???
???
???
???
???

???
???
???
???
???
???
???
???
???
???
???
???
???
???
???
???
???
???
???
???
???
???
???
???
???
???
???
???
???
???
????????????????????????????????????ATGCCTAGATCCCGT---ATAAATGGAAATTTCATTG
ATAAGACCTCCTCGATTGTAGCCAATATTTTATTGCAAATAATTCCGACAACCTCCGGGG
AAAAAAGGGCATTTACTTATTATAGAGATGGG------ATGTTGGCTCAATCCGAAGGAAAT
TATGCGGAAGCTTTGCAGAATTATTATGAAGCTACGCGACTAGAAATTGATCCCTATGAT
CGAAGTTATATACTATATAACATAGGCCTTATACACACAAGCAATGGAGAGCATACAAA
GGCTTTGGAATATTATTTCCGGGCGCTAGAACGAAACCCCTTCTTACCGCAAGCTTTTA
ATAATATGGCCGTGATCTGTCATTAC---CGAGGAGAACAGGCCATTCTACAGGGCGATTC
GGAAATTGCGGAAGCTTGGTTTGATCAAGCTGCTGAGTATTGGAAACAAGCTATAGCG
CTTACTCCAGGAAATTATATTGAAGCACAGAACTGGTTAAAGATTACGAAGCGCTTTGA
ATTTGAAATGAATTGGCGATCAGAACACATATGGATAGAACTTCTAAAAGGTTCTCGAA
AAAGAGGTAATTTTTTCTGGGCCTGTATTCTTTTTCTAGGTTCATTAGGATTCTTAGCGG
TTGGGGCTTCCAGTTATCTTGGTAAGAATATGATATCTGTACTTCCATCTCAACAAATTC
TTTTTTTTCCACAGGGGGTCGTGATGTCTTTCTACGGAATCGCGGGCCTATTCATTAGCT
CCTATTTGTGGTGCACTATTTTATGGAATGTAGGCAGTGGTTATGACCGATTCGATAGAA

AAGAGGGAATAGTGTGCATTTTTCGTTGGGGATTCCCTGGAATAAAACGTCGCATCTTC
CTTCAATTCCTTGTGCGGGATATCCAATCAATTAGAATTCAGGTTAAAGAGGGTCTTTAT
CCTCGTCGTATCCTTTATATGGAAATCCGGGGCCAGGGGGTCATTCCCTTGACTCGTACT
GATGAGAAGTTTTTTACTCCACGAGAAATTGAACAAAAAGCTGCCGAATTGGCCTATT
TCTTGCGTGTACCAATTGAAGTATTT
>Nicotiana
ATGGAAAGATGGTGGTTTAATTCGATGTTGTTTCTAAATAAATCAATGGGCAGTCTTGG
TCCTATTGAAAATACCAATGAA------GATCCAAATCGAAAA------GTGAAAAACATTCATA
GTTGCAGTAATGTTGATTATTTATTCGGCGTTAAAGACATTCGGAATTTCATCTCTGATG
ACACTTTTTTAGTTAGTGATAGGAATGGAGACAGTTATTCCATCTATTTTGATATTGAAA
ATCATATTTTTGAGATTGACAACGATCAT------TCTTTTCTGAGTGAACTAGAAAGTTCTT
TTTATAGTTATCGAAACTCGAATTATCGGAATAATGGATTTAGGGGCGAAGATCCCTACT
ATAATTCTTACATGTATGATACTCAATATAGTTGGAATAATCACATTAATAGTTGCATTGAT
AGTTATCTTCAG------TCTCAAATCTGTATAGATACTTCCATTATAAGTGGTAGTGAGAAT
TACGGT------TACATTTATAGGGCCGTTTGTGGTAACGAGGGTTCCAGTAGACGAACTCG
CACGAAGGGCAGTGATTTAACTATAAGAGAAAGTTCTAATGATCTCGAGGTAACTCAA
AAATACAGGCATTTGTGGGTTCAATGCGAAAATTGTTATGGATTAAATTATAAGAAATTT
TTGAAATCAAAAATGAATATTTGTGAACAATGTGGATATCATTTGAAAATGAGTAGTTC
AGATAGAATTGAACTTTTGATCGATCCGGGTACTTGGGATCCTATGGATGAAGACATGG
TCTCTCTAGATCCCATTGAATTTCATTCGGAGGAGGAGCCTTATAAAGATCGTATTGATT
CTTATCAAAGAAAGACAGGATTAACCGAGGCTGTTCAAACAGGCATAGGCCAACTAAA
CGGCATTCCCGTAGCAATTGGGGTTATGGATTTTCAGTTTATGGGGGGTAGTATGGGATC
CGTAGTCGGAGAGAAAATCACCCGTTTGATTGAATACGCTGCCAATCAAATTTTACCCC
TTATTATAGTGTGTGCTTCTGGGGGGGCGCGCATGCAGGAAGGAAGTTTGAGCTTGATG
CAAATGGCTAAAATATCGTCTGCTTTATATGATTATCAATTAAATAAAAAGTTATTTTATG
TATCAATCCTTACATCTCCGACAACTGGTGGAGTGACAGCTAGTTTTGGTATGTTGGGG
GATATCATTATTGCCGAACCCAACGCCTACATTGCATTTGCAGGTAAAAGAGTAATTGA
ACAAACATTGAATAAAACAGTACCCGAAGGTTCACAAGCAGCTGAATACTTATTCCAG
AAGGGTTTATTCGACCTAATTGTACCACGTAATCTTTTAAAAAGCGTTCTGAGTGAGTT
ATTTAAGCTCCACGCCTTTTTTCCTTTGAATCAAAAGTCAAGCAAAATGGTAACCATTC
GAGCTGACGAAATTAGTAATATTATCCGTGAACGTATTGAACAATATAATAGAGAAGTA
AAGATTGTAAATACCGGTACCGTACTTCAAGTAGGCGACGGCATTGCTCGTATTCACGG
TCTTGATGAAGTAATGGCGGGTGAATTAGTCGAATTTGAAGAGGGTACAATAGGCATTG
CTCTGAATTTGGAATCAAATAATGTTGGTGTTGTATTAATGGGCGATGGTTTATTGATAC
AAGAAGGAAGTTCTGTAAAAGCAACGGGAAGAATTGCTCAGATACCCGTGAGTGAGG
CTTATTTGGGTCGTGTTATAAATGCCCTGGCTAAACCTATTGATGGTAGAGGTGAAATTT
CAGCTTCTGAATTTCGATTAATCGAATCTGCCGCCCCGGGTATTATTTCGCGCCGTTCCG
TATATGAGCCTCTTCAAACCGGGCTTATTGCTATTGATTCGATGATCCCTATAGGACGTG
GTCAGCGAGAATTAATTATTGGGGACAGACAGACCGGTAAAACAGCAGTAGCCACAGA
TACGATCCTCAATCAACAAGGTCAAAATGTAATATGTGTTTATGTAGCTATTGGGCAAA
AAGCATCTTCTGTGGCCCAGGTCGTAACTACTTTACAGGAAAGGGGAGCGATGGAATA
CACTATTGTGGTAGCCGAAACGGCAGATTCCCCTGCTACATTACAATACCTTGCTCCTTA
TACAGGAGCAGCTCTGGCTGAATATTTTATGTATCGTGAACGACACACTTTAATCATTTA
TGATGATCCCTCCAAACAAGCGCAAGCTTATCGCCAAATGTCTCTTCTATTACGAAGAC

CGCCCGGTCGTGAAGCTTATCCAGGAGATGTTTTTATTTGCATTCACGCCTTTTGGAA
AGAGCCGCTAAATTAAGTTCTAGTTTAGGTGAAGGAAGTATGACCGCCTTACCAATAGT
TGAAACCCAATCGGGAGATGTTTCGGCTTATATTCCTACTAATGTAATTTCCATTACTGA
TGGACAAATCTTCTTATCCGCCGACCTATTCAATTCTGGAATCAGACCTGCTATTAATGT
GGGTATCTCCGTTTCCAGAGTGGGGTCCGCAGCTCAAATAAAAGCCATGAAACAAGTA
GCTGGTAAATTAAAATTAGAACTAGCACAATTCGCAGAATTAGAAGCCTTTGCACAATT
TGCTTCTGATCTCGATAAAGCTACTCAGAATCAATTGGCAAGAGGTCAACGATTACGTG
AATTGCTTAAACAATCCCAATCAGCTCCTCTCACGGTAGAAGAGCAGATAATGACTATT
TATACCGGAACAAACGGCTATCTTGATTCATTAGAAGTTGGACAGGTAAGGAAATTTCT
TGTTGAGCTACGTACTTACTTAAAAACTAATAAACCTCAGTTCCAAGAAATCATATCTTC
TACCAAGACATTTACCGAGGAAGCAGAAGCCCTTTTGAAAGAAGCTATTCAGGAACAA
ATGGACCGTTTTATACTTCAGGAACAAGCAATGAGAATCAATCCTACTACTTCTGGTTC
TGGGGTTTCCACGCTTGAAAAAAAAAACCCGGGGCGTGTCGTCCAAATCATCGGTCCG
GTACTAGATGTAGCCTTTCCCCCGGGCAAGATGCCGAATATTTATAACGCTCTGGTAGTT
CAAGGTCGAGATAGTGTTGGTCAACCAATTAATGTGGCTTGTGAGGTACAGCAATTATT
AGGAAATAATCGAGTTAGGGCTGTAGCTATGAGTGCTACAGAGGGTCTAACGAGAGGA
ATGGAAGTGATTGACACAGGAGCTCCTATAAGTGTTCCGGTCGGGGGAGCGACTCTGG
GACGAATTTTTAACGTGCTCGGAGAACCTGTTGATAATTTAGGGCCTGTAGATACTAGT
ACAACGTCTCCTATTCATAGATCTGCGCCCGCCTTTATACAGTTGGATACAAAATTATCT
ATTTTTGAAACAGGAATTAAAGTAGTAGATCTTTTAGCCCCTTATCGCCGTGGAGGAAA
AATCGGACTATTCGGGGGAGCTGGAGTGGGTAAAACAGTACTTATTATGGAATTGATTA
ACAATATTGCTAAAGCTCACGGGGGCGTATCCGTATTTGGCGGAGTGGGTGAACGTACT
CGGGAAGGAAATGATCTTTACATGGAAATGAAAGAATCTGGAGTAATTAATGAAGAAA
ATATTGCAGAATCAAAAGTAGCCCTAGTTTATGGTCAGATGAATGAACCGCCGGGAGCT
CGTATGAGAGTTGGTTTGACTGCCCTAACTATGGCGGAATATTTCCGAGATGTTAATGA
GCAAGACGTACTTCTTTTTATTGACAATATCTTCCGTTTCGTCCAAGCAGGATCCGAAG
TATCGGCCTTATTGGGTAGAATGCCTTCCGCTGTGGGTTATCAACCCACCCTGAGTACC
GAAATGGGTTCTTTACAAGAAAGAATTACTTCTACCAAAGAAGGGTCCATAACCTCTAT
TCAAGCAGTTTATGTACCCGCAGACGATTTGACCGACCCTGCTCCTGCTACGACATTTG
CACATTTAGATGCTACTACCGTACTATCAAGAGGATTGGCTGCCAAAGGTATTTATCCAG
CAGTAGATCCTTTAGATTCAACGTCAACCATGCTTCAACCTCGGATCGTTGGTGAGGAA
CATTACGAAACCGCCCAAAGAGTTAAGCAAACTTTACAACGTTACAAAGAACTTCAGG
ACATTATAGCTATCCTTGGATTGGACGAATTATCCGAAGAGGATCGTTTACTCGTAGCAA
GAGCGCGAAAAATTGAGCGTTTCTTATCACAACCCTTTTTCGTAGCAGAAGTATTTACC
GGTTCTCCAGGGAAATATGTTGGTCTAGCAGAAACAATTCGAGGATTTCAATTGATCCT
TTCCGGAGAATTAGATGGTCTTCCTGAACAGGCCTTTTATTTGGTAGGTAATATCGATGA
AGCTACCGCGAAGGCTATGAACTTAGAAATGGAGAGCAATTTGAAGAAAATGACCTTA
AATCTTAGTGTACTGACCCCTAATCGAATTGTTTGGGATTCAGAAGTGGAAGAAATTGT
TTTATCTACTAATAGCGGTCAAATTGGCATATTACCAAATCACGCCCCTATTGCCACAGC
TGTAGATATAGGGATTTTGAGAATACGTCTTAACGACCAATGGTTAACGATGGCTCTGAT
GGGTGGTTTTGCTAGAATAGGCAATAATGAGATCACTGTTTTAGTAAATGATGCGGAGA
AGGGTAGTGACATTGATCCACAAGAAGCTCAGCAAACTCTTGAACTAGCGGAAGCTAA
TGTGAAAAAGGCTGAAGGAAGGAGACAAAAAATTGAGGCAAATCTAGCTCTCCGACG
AGCTAGAACACGGGTGGAGGCTATCAATCCGATTTCAATGAAAAACGTAACCGATTCT

TTCGTTTCT---GGCCACTGGCCATCTGCCGGGAGTTTCGGGTTTAATACCGATATTTTAG
CAACAAATCCAATAAATCTAAGTGTAGTGCTTGGTGTATTGATTTTTTTTGGAAAGGGA
GTGTTAAGTGATTTATTAGATAATCGAAAACAGAGGATCTTGAATACTATTCGAAATTCA
GAAGAACTGCGTGGAGGGGCTATTGAACAACTCGAAAAAGCTCGTTCTCGCTTACGG
AAAGTAGAAAGCGAAGCCGAGCAGTTTCGAGTGAATGGATACTCTGAAATAGAACGA
GAAAAATTGAATTTGATTAATTCAACTTATAAGACTTTGGAACAATTAGAAAATTACAA
AAACGAAACGATTCAGTTTGAACAGCAAAGGGCGATTAATCAAGTCCGACAACGGGT
TTTCCAACAAGCCTTACGAGGAGCTCTAGGAACTCTGAATAGTTGTTTGAACAACGAG
TTACATTTACGTACCATTAGTGCCAATATTGGCATGTTGGGAACAATGAAAGAAATAAC
TGATATGAATCCACTGATTTCTGCCGCTTCCGTTATTGCTGCTGGATTGGCCGTAGGGCT
TGCTTCTATTGGACCCGGAGTTGGTCAAGGGACTGCTGCGGGTCAAGCTGTAGAGGGT
ATCGCGAGACAGCCTGAGGCAGAGGGAAAAATACGAGGTACGCTATTGCTTAGTCTAG
CTTTTATGGAAGCTTTAACAATTTATGGACTGGTTGTAGCATTAGCACTTTTATTTGCGA
ATCCTTTTGTTATGAATGTTCTATCATGTTCCATCAATACTCTAAAGGGGTTATACGATAT
ATCCGGTGTGGAAGTAGGCCAACATTTCTATTGGCAAATAGGGGGTTTCCAAGTACATG
GCCAAGTACTTATTACTTCTTGGGTTGTAATTGCTATCTTATTAGGTTCAGCCACTATAGC
TGTTCGGAACCCACAAACCATTCCGACCGGGGGTCAGAATTTCTTCGAATATGTTCTTG
AATTTATTCGAGATGTGAGTAAAACTCAAATTGGCGAAGAATATGGGCCCTGGGTTCCT
TTTATTGGCACTATGTTTTTATTTATTTTTGTTTCTAATTGGTCAGGAGCTCTTTTACCTTG
GAAAATCATACAATTACCTCATGGGGAGTTAGCCGCACCCACGAATGATATAAATACTA
CTGTTGCTTTGGCTTTACTCACATCAGTGGCATATTTCTATGCGGGTCTTACAAAAAAA
GGATTAGGTTATTTCGGAAAATATATTCAACCAACCCCAATCCTTTTACCCATTAACATC
TTAGAAGATTTCACAAAACCTTTATCACTTAGTTTTCGACTTTTCGGGAATATCTTAGCT
GATGAATTAGTAGTTGTTGTTCTTGTTTCTTTAGTACCTTTAGTAGTTCCTATACCTGTCA
TGCTCCTTGGATTATTTACAAGTGGTATTCAAGCTCTTATTTTTGCAACTTTAGCCGCGG
CTTATATAGGTGAATCCATGGAGGGCCATCATATGATATTTTCAACCTTAGAGCATATATT
AACTCACATTTCCTTTTCGATCGTTTCAATTGTAATTACAATTCATTTGATAACCTTTTTA
GTCGATGAAATCGTAAAACTATACGATTCATCAGAAAAGGGCATAATAGTTACTTTTTTC
TGTATAACAGGATTATTAGTTACTCGTTGGATTTCTTCTGGACATTTCCCACTAAGCGAT
TTATATGAATCATTAATTTTCCTTTCATGGAGTTTCTCCCTTATTCATATAATTCCGTAT-----
-AAAAATGTTTTAATTTTAAGTAAAATAACTGGCCCTAGTGCTATTTTTACCCAAGGCTT
TGCTACGTCAGGTATTTTAACTGAAATACACCAATCTGTAATATTAGTACCTGCTCTTCA
ATCCGAGTGGTTAATAATGCACGTAAGTATGATGATATTGGGCTATGCAGCTCTTTTATG
TGGATCATTATTATCAGTAGCACTTCTAGTGATTACATTTCGAAAAAACAGACAGCTTTT
TTAT------AAGGAAAATGTTTTACAAAATACTTCTTTT------------------------------GCTAAAAA
TTATTACAGGTCCCAATTGATTCAACAATTGGATTATTGGAGTTATCGGGTTATTAGTTTA
GGATTTACTTTTTTAACCATAGGAATCCTTTCGGGAGCGGTATGGGCTAATGAAGCGTG
GGGGTCGTATTGGAATTGGGACCCAAAAGAAACTTGGGCATTTATTACTTGGATCGTAT
TTGCAATTTATTTACATACTCGAACAAATAGAAATTTGCGGGGTGCAAATTCTGCAATTG
TAGCGTCTATAGGCTTTCTTATAATTTGGATATGCTATTTTGGGGTCAATCTTTTAGGAAT
AGGGTTACATAGTTATGGTTCTTTTCCATCAACAATGGCAAAAAAGAAAGCATTCACTC
CTCTTTTCTATCTTGCATCTATAGTATTTTTGCCCTGGTGGATTTCTTTCTCAGTTAATAAA
TGTCTGGAATCTTGGGTTACCAATTGGTGGAATACTGGGCAATCCGAAATTTTTTTGAAT
AATATTCAAGAAAAGAGTCTTCTAGAAAAATTCATAGAATTAGAGGAACTCCTCTTCTT

GGACGAAATGATCAAGGAATACTCGGAAACACATCTCGAAGAGTTTGGGATA---ATCCA
TAAAGAAACGATCCAATTAATCAAGATACAAAATGAGAATCGTATCCATACGATTTTGC
ACTTCTCGACAAATATCATCTGTTTTATTATTCTAAGCGGGTATTCAATTTTGGGTAATGA
AAAACTTGTTATTCTTAACTCTTGGGCTCAGGAATTCCTATATAACTTAAGTGACACAGT
AAAAGCTTTTTCTATTCTTTTATTAACTGATTTATGTATCGGATTCCATTCACCCCACGGT
TGGGAATTAATGATTGGCTCTATCTATAAAGATTTTGGATTTGTTCATAATGATCAAATCA
TATCTGGTCTTGTTTCCACCTTTCCAGTCATTCTCGATACAATTTTTAAATATTGGATTTT
CCGTTATTTAAATCGTCTGTCTCCGTCACTTGTAGTTATTTATCATTCAATGAATGACATG
CCTATTGGTGTTCCAAAAGTCCCTTTCCGAAGTCCTGGAGAGGAAGATGCATCTTGGG
TTGACGTATACAACCGACTTTATCGAGAAAGATTACTTTTTTTAGGCCAAGAGGTTGAT
AGCGAGATTTCGAATCAACTTATTGGTCTTATGGTATATCTCAGTATCGAGGATGAGACC
AAAGATCTGTATTTGTTTATAAACTCTCCTGGGGGCTGGGTAATACCTGGGGTGGCTATT
TATGATACTATGCAATTTGTGCGACCAGATGTCCATACAATATGCATGGGATTAGCCGCT
TCAATGGGATCTTTTATCCTGGTCGGAGGAGAAATTACCAAACGTCTAGCATTCCCTCA
CGCTAGGGTAATGATCCATCAACCTGCTAGTTCTTTTTATGAGGCACAAACAGGCGAAT
TTGTCCTGGAAGCGGAAGAACTGCTGAAACTGCGTGAAACCCTCACAAGGGTTTATGT
ACAAAGAACGGGGAAACCCTTATGGGTTGTATCCGAAGATATGGAAAGAGATGTTTTT
ATGTCAGCAACAGAAGCCCAAGCTTATGGAATTGTTGATCTTGTAGCGGTTGA
A???
??
??????????????????????????????????????ATGGAAGAAATCCAAAGATATT
TACAG---GATAGATCGCAACAACACAACTTCCTATATCCACTTATCTTTCAGGAGTATATT
TATGCACTTGCTCATGATCATGGTTTACCAGGTTATAACAATAAATTAAGTTTCCTAATTG
TGAAACGTTTAATTACTCGAATGTATCAACAGAATCATTTTCTTATTTCTACTAATGATTC
TAACAAAAATTCATTTTTGGGGAAGAGTTTGTATTCTCAAATGATATCAGAGGGATTTG
CGTTTATTGTGGAAATTCCGTTTTCTCTACGATTAATATCTTCTTTAATTTTTAAATCTTAT
AATTTACGATCAATTCATTCAACATTTCCTTTTTTAGAGGACAATTTTTCACATCTAAATT
ATGTATTAGATATACTAATACCCTACCCTGTTCATCTGGAAATCTTGGTTCAAACTCTTCG
CTATTGGGTAAAAGATGCCTCTTCTTTACATTTATTACGATTCTTTCTCCATGAATTTTGG
AATTTGAATAGTCTTATTACTCCCGGT---TACTCCTTTTCAAAACAAAGATTCTTCTTCTT
CTTATATAATTCTTATGTATATGAATGCGAATCCACTTTCGTCTTTCTACGGAACCAATCT
TCTCATTTACGATCAACATCTTTTGGAGCCCTTCTTGAACGAATATATTTCTATGGAAAA
ATAGAACGTCTTGTAGAAGTCTTTGCT---GATTTTACCCTATGGTTATTCAAGGATCCTTT
CATGCATTATGTTAGGTATCAAGGAAAATCCATTCTGGCTTCAAAAGGGACGTTTCTTTT
GATGAATAAATGGAAATTTTACCTTGTCAATTTTTGGCAATGTCATTGTTCTCTGTGCTT
TCACACAGGAAGGATCCATATAAACCAATTATCCAATCATTCCCGTGACTTTATGGGCTA
TCTTTCAAGTGTGCGACTAAATCCTTCAATGGTACGTAGTCAAATGTTAGAAAATTCAT
TTCTAATCAATAATGCAATT------AAGAAGTTCGATACCCTTGTTCCAATTATTCCTTTGAT
TGGATCATTAGCTAAAGCAAACTTTTGTACCGTATTAGGGCATCCCATTAGTAAACCGGT
TTGGTCCGATTTATCAGATTCTGATATTATTGACCGATTTGGGCGTATATGCAGAAATCTT
TTTCATTATTATAGCGGATCTTCCAAAAAAAAGACTTTATATCGAATAAAGTATATACTTC
GACTTTCTTGTGCTAGAACTTTAGCTCGGAAACACAAAAGTACTGTACGCACTTTTTTG
AAAAGATCGGGCTCGGAATTATTGGAAGAATTCTTAACGTCGGAAGAACAAGTTCTTT
CTTTGACCTTCCCACGACGGATTTGGTATTTGGATATTTTTTGTATCAATGATCTGGCGA

ATTATCAAATGATAATTGAT---ACAACAGAAATAGAA---ACTATCAATTCTTTTTCCAAAT
TGGAATCC---TTAAAAGAAGTCTATGGGATCATATGGATGCTTTTCCCTATTTTGACTCTT
GTATTAGGAATCACAATAGGTGTACTAGTAATTGTTTGGTTAGAAAGAGAAATATCTGC
AGGAATACAACAACGTATCGGACCTGAATATGCTGGCCCTTTAGGAATTCTTCAAGCTC
TAGCAGATGGGACAAAACTACTTTTGAAAGAGAACCTTATTCCATCTACAGGAGATACT
CGTTTATTCAGTATCGGACCATCCATAGCAGTAATATCCATCTTTCTAAGTTATTCAGTAA
TTCCTTTTGGTGATCACCTTGTTCTAGCCGATCTTAGTATTGGTGTTTTTTTCTGGATTGC
CATTTCAAGTATTGCTCCCGTTGGACTTCTTATGTCGGGATATGGATCAAATAATAAATAT
TCTTTTTTAGGTGGTCTACGGGCAGCTGCTCAATCAATTAGTTATGAAATACCATTAGCT
CTATGTGTGTTATCAATATCTCTACTATCTAACAGTTTAAGTACAGTTGATATAGTGGAAG
CGCAGTCAAAATATGGCTTTTGGGGGTGGAATTTGTGGCGTCAACCCATCGGGTTTATC
GTTTTTCTAATTTCTTCTCTAGCCGAGTGTGAAAGATTACCTTTTGATTTACCAGAAGCA
GAAGAAGAATTAGTAGCAGGGTATCAAACCGAATATTCAGGTATCAAATTTGGTTTATT
TTACATTGCTTCATATCTGAATCTACTAGTTTCTTCATTATTTGTAACAGTTCTTTATTTGG
GAGGTTGGAATCTTTCTATTCCGTACATATTTGTTCCTGAGCTATTTGGCATAAATAAAA
GGGGTAAAGTCTTTGGAACACTAATTGGTATCTTTATCACATTAGCCAAAACTTATTTGT
TTTTGTTCATTCCTATTGCAACAAGATGGACTTTACCGAGGCTGAGAATGGACCAACTA
TTAAATCTTGGGTGGAAATTTCTTTTACCTATTTCTCTAGGTAATCTATTATTGACAACCT
CGTCCCAACTTCTTTCACTGATGATCTGGCATGTACAGAATGAAAACTTCATTCTCGATT
CTACGAGAATTTTTATGAAAGCCTTTCATTTGCTTCTCTTCGATGGAAGTTTGATTTTCC
CAGAATGTATCCTAATTTTTGGCCTAATTCTTCTTCTGATGATCGATTCAACCTCTGATCA
AAAAGATATACCTTGGTTATATTTCATCTCTTCAACAAGTTTAGTAATGAGCATAACGGC
CCTATTGTTCCGATGGAGAGAAGAACCTATGATTAGCTTTTCGGGAAATTTCCAAACGA
ACAATTTCAACGAAATCTTTCAATTTCTTATTTTACTATGTTCAACTCTATGTATTCCTCT
ATCCGTAGAGTACATTGAATGTACAGAAATGGCTATAACAGAGTTTCTCTTATTCGTATT
AACAGCTACTCTAGGGGGAATGTTTTTATGCGGTGCTAACGATTTAATAACTATCTTTGT
AGCCCCAGAATGTTTCAGTTTATGCTCCTACCTATTATCTGGATATACCAAGAAAGATGT
ACGGTCTAATGAGGCTACTATGAAATATTTACTCATGGGTGGGGCAAGCTCTTCTATTCT
GGTTCATGGTTTCTCTTGGCTATATGGTTCATCCGGGGGAGAGATTGAGCTTCAAGAAA
TAGTAAACGGTCTTATCAATACACAAATGTATAACTCCCCAGGAATTTCAATTGCGCTCA
TATTCATTACCGTAGGAATTGGGTTCAAGCTTTCCCCAGCCCCTTCTCATCAATGGACTC
CTGACGTATACGAAGGATCTCCCACTCCAGTCGTTGCTTTTCTTTCTGTTACTTCGAAA
GTAGCTGCTTCAGCTTCAGCCACTCGAATTTTCGATATTCCTTTTTATTTCTCATCAAAC
GAATGGCATCTTCTTCTGGAAATCCTAGCTATTCTTAGCATGATATTGGGAAATCTCATT
GCTATTACTCAAACAAGCATGAAACGTATGCTTGCATATTCGTCCATAGGCCAAATCGG
ATATGTAATTATTGGAATAATTGTTGGAGACTCAAATGATGGATATGCAAGCATGATAAC
TTATATGCTGTTCTATATCTCCATGAATCTAGGAACTTTTGCTTGCATTGTATTATTTGGTC
TACGTACCGGAACTGATAACATTCGAGATTATGCAGGATTATACACAAAAGATCCTTTTT
TGGCTCTCTCTTTAGCCCTATGTCTCTTATCCCTAGGAGGTCTTCCTCCACTAGCAGGTT
TTTTCGGAAAACTCTATTTATTCTGGTGTGGATGGCAGGCAGGCCTATATTTCTTGGTTT
TAATAGGACTCCTTACAAGCGTTGTTTCTATCTACTATTATCTAAAAATAATAAAGTTATT
AATGACTGGACGAAACCAAGAAATAACCCCTCACGTGCGAAATTATAGAAGATCCCCT
TTAAGATCAAACAATTCCATCGAATTGAGTATGATTGTATGTGTGATAGCATCTACTATAC
CAGGAATATCAATGAACCCAATTATTGCAATTGCTCAGGATAGCCTTTTTATGTTTCTGC

TTTACGAATATGATTTTTTTTGGGCATTTCTAATAATATCTATCCTTGTTCCTATTTTGGCA
TTTTTAATTTCCGGAGTGTTAGCCCCGATTAGCAAAGGGCCGGAGAAACTTTCTACTTA
TGAGTCGGGTATAGAACCAATGGGCGATGCTTGGTTACAATTTCGAATCCGTTATTATAT
GTTTGCTCTAGTTTTTGTTGTTTTTGATGTTGAAACGGTTTTTCTTTATCCATGGGCAAT
GAGTTTCGACGTATTGGGTGTATCTGTATTTATAGAAGCTTTCATTTTCGTGCTTATCTTA
ATTATTGGTTTAGTTTATGCATGGCGAAAGGGGGCATTGGAATGGTCTACGAATTATTTT
CCTTGGTTAACAATAATTGTAGTTTTTCCAATATTTGCGGGTTCCTTAATTTTCTTTCTTC
CCCATAAAGGAAATAGGGTAATTAGGTGGTATACGATATGTATATGTATTTTAGAACTCC
TTCTAACGACTTATGCATTTTGTTATCATTTCCAATCGGATGATCCATTAATCCAACTAGT
GGAGGATTATAAATGGATCAATTTTTTTGATTTCCATTGGAGATTAGGAATAGATGGACT
TTCTATAGGACCCATTTTATTAACAGGATTTATCACTACTTTAGCTACTTTAGCGGCTTGG
CCAGTTACTCGAGATTCTCGATTATTCCATTTTCTCATGTTAGCAATGTACAGTGGTCAA
ATTGGATCATTTTCGTCTCGGGACCTTTTACTTTTTTTCATCATGTGGGAGTTAGAATTAA
TTCCTGTTTATCTACTTCTATGCATGTGGGGAGGAAAGAAACGTCTGTACTCAGCTACA
AAATTTATTTTGTACACGGCGGGGGGTTCTGTTTTTCTCTTAATGGGAGTTTTGGGTCTT
GCTTTATATGGTTCTAATGAACCAACATTAAATTTTGAAACATCAGTTAATCAGTCATAT
CCTGTGGTTTTAGAAATAATATTCTATATTGGATTTTTTATTGCTTTTGCTGTCAAATCGC
CCATTATCCCCCTACACACATGGTTACCAGATACCCATGGAGAAGCACATTACAGTACT
TGTATGCTTCTAGCCGGAATTTTATTAAAAATGGGAGCGTATGGATTAATTCGAATCAAT
ATGGAATTATTACCTCATGCCCATTCTATATTTTCTCCTTGGTTGATGATAATAGGTACAAT
ACAAATAATCTATGCAGCTTTAACATCTCTTGGCCAACGGAATTTAAAAAAAAAGAATAG
CCTATTCCTCTGTCTCTCATATGGGTTTCATAATTATAGGAATTAGTTCTCTAACCGACAC
GGGACTTAATGGAGCCCTTTTACAAATAATATCTCATGGATTTATTGGTGCTGCACTTTT
TTTCTTGGCGGGAACGACTTATGATAGAATCCGCCTTGTTTATCTTGACGAAATGGGCG
GAATAGCTATTCCAATGCCAAAAATGTTCACGATGTTCAGTAGCTTTTCGATGGCTTCCC
TTGCATTACCAGGTATGAGTGGTTTTGTTGCCGAATTGATAGTATTTTTTGGAATAATTA
CCGGCCAAAAATATCTTTTAATTCCAAAAATACTAATTACTTTTGTAATGGCAATTGGAA
TGATATTAACTCCTATTTATTCATTATCTATGTCACGCCAGATGTTCTATGGATACAAGCTA
TTTAATGCCCCGAAGGATTCTTTTTTTGATTCTGGACCGCGAGAGTTATTTCTTTCGATC
TCCATCTTTTTACCCGTAATAGGTATTGGTATATACCCGGATTTCGTTCTTTCATTAGCAG
TTGACAAGGTCGAAGTTATTCTATCTAATTTTTTTTATAGAATGATTCTCGAACATGTACT
TGTTTTGAGTGCCTATTTATTTTCTATCGGGATCTATGGATTGATCACAAGTCGAAATATG
GTTAGAGCCCTTATGTGTCTTGAACTTATATTGAATGCGGTTAATATAAATTTTGTAACAT
TTTCTGATTTTTTTGATAATCGTCAATTAAAAGGAGACATTTTCTCAATTTTTGTTATAGC
TATTGCAGCCGCTGAAGCAGCCATTGGACTGGCTATTGTTTCATCAATTTACCGTAACA
GAAAATCAACTCGTATCAACCAATCAAATTTGTTGAATAATATGGAACAGACATATGAAT
ATGCGTGGATCATACCTTTCATTCCACTTCCAGTCCCTATGTTAATAGGAGCGGGACTTT
TTCTTTTTCCAACGGCAACAAAAAGTTTTCGCCGTATGTGGGCTTTTCAGAGTGTTTTA
TTGTTAAGCATAGTCATGGTTTTTTCAATCTACCTGTCTATTCAGCAAATAAATAGCAGT
TCTTTTTATCAATATGTATGGTCTTGGATCATCAATAATGATTTTTCTTTAGACTTCGGATA
CTTGATCGACCCACTTACTTCTATTATGTCAATATTAATCACTACGGTTGGAATTATGGTT
CTTATTTATAGTGATAATTATATGGCTCATGATCAAGGCTATTTGAGATTTTTTGCTTATAT
GAGTTTTTTCAGTACTTCCATGTTGGGATTAGTTACTAGTTCGAATTTGATACAAATTTAT
ATTTTTTGGGAATTGGTTGGGCTGTGTTCCTATCTATTAATAGGATTTTGGTTTACACGA

CCTGTTGCGGCAAATGCTTGTCAAAAAGCGTTTGTAACTAATCGTGTAGGGGATTTTGG
TTTATTATTAGGAATTTTAGGTTTTTATTGGATAACAGGGAGTTTCGAATTTAGGGATTTA
TTCGAAATATTCAATAACAATGAAGTCGATTTTTTATTTGTTACTTTATGTGCTGTTCTCT
TATTTGCCGGTGCAGTTGCTAAATCCGCCCAATTCCCCCTTCATGTATGGTTACCTGATG
CCATGGAGGGGCCTACTCCCATTTCGGCTCTTATACATGCTGCTACTATGGTAGCGGCG
GGAATTTTTCTTGTAGCTCGGCTTCTTCCTCTTTTCAGAGTTATACCTTACATAATGTATT
TGATCTCGGTTATAGGAATAATAACAGTATTATTAGGAGCTACTTTAGCTCTTGCTCAAA
AAGACATTAAGAGAGGTTTAGCCTATTCCACAATGTCTCAATTGGGTTATATGATGTTAG
CTCTTGGTATGGGGTCTTATCGAAGCGCTTTATTTCATTTGATTACTCATGCTTATTCCAA
AGCATTATTATTTTTAGGATCCGGATCCATTATTCATTCAATGGAAACTATTGTTGGATAT
TCTCCAGCTAAAAGTCAGAATATGGGTCTTATGGGAGGTTTAAGAAAACATGTACCAAT
TAGCAAAATCACATTTTTATTAGGTACACTTTCTCTTTGTGGTATTCCACCTCTTGCTTGT
TTTTGGTCCAAAGATGAAATTCTTAATGATAGTTGGTTGTATTCGCCAATTTTCGCAATA
ATAGCTTGGGCCACGGCGGGATTAACCGCATTTTATATGTTTCGGATCTATTTACTTACTT
TTGAAGGGCATTTAAACGCTCATTTTCCAAATTATGGTGGGAAACAAAAAACCCCCTTC
TATTCAATATCTCTATGGGGTAAA---------AACGGAGTTAAGAAA---TTATTAACT---------A
TGAATTTTTTTGCATTTGAGCATAAAGCGGTTTATTCCTATCCTTATGAATCGGACAATAC
TATGCTATTCCCAATATTTGTATTAGGACTCTTTACTTTGTTTGTTGGATCTATAGGAATTC
CTTTCAATTTGGATATATTATCGAAATGGTTAGCTCCATCTATAAATCTTTTGCATCAAAA
GTCGAAT---TCGATGGATTGGAATGAATTTTTAAAGGATGCAGTTCTTTCAGTCAGTATA
GCTTATTTCGGAATATTTATAGCATCCTTTTTATATAAACCCATTTATTCTTCTTTAAAAAA
TTTTGAGTTAATTAATTCTTTTGTTATTCTGTGGGACAAAATTATAAATGGCATATATGAT
TGGTCATATAATCGTGCTTACATAGATGCTTTTTATACAAGATTCTTAGTTGGTGGGATAA
GAGGATTAGCTGAATTTACTCATTTTTTTGATCGACGAGTAATTGATGGGATGACTAACG
GGGTTGGGGTTATTAGTTTCATTGTAGGAGAAGGTATCAAATATATAGGGGGCGGA---CG
CATCTCTTCTTATCTTTTCTTGTATTTAGCTTATGTTTCAATCTTTTTATTAGTTTATTACAT
GGATTTATCTGAACCAATACATGATTTTCTTTTAGTCTTTCTAGGGTCGGGTCTTATATTA
GGGGGTCTAGGAGTGGTATTACTTCCCAATCCAATTTATTCTGCCTTTTCCTTGGGATTG
GTTCTTGTTTGTACATCGTTATTCTATATTCTATCTAACTCCTATTTTGTAGCTGCTGCGCA
GCTACTTATTTACGTAGGAGCTATAAATGTTTTAATCATTTTTGCTGTGATGTTCATGAAT
GGTTCAGAATATTACAAAGATTTTCATCTTTGGACCGTAGGAGATGGAATTACTTCGAT
GGTTTGTATAAGTCTTTTTATTTCACTAATTACTACTATTTCAGATACGTCATGGTACGGG
ATTATTTGGACTACAAGATCAAACCAGATTATAGAGCAAGATTTTATAAGTAATAGTCAA
CAAATTGGAATTCATTTATCAACAGATTTTTTTCTTCCATTTGAACTGATTTCAATAATCC
TTTTAGTTGCTTTAATAGGTGCAATTGCTGTAGCTCGTCAAATGACTGCACCAACTACA
AGAAAAGACCTCATGATAGTCAATATGGGGCCTCAGCACCCATCAATGCACGGTGTTCT
TCGACTCATCGTTACTCTAGATGGTGAAGATGTTGTCGACTGCGAACCAATATTGGGTT
ATTTACATAGAGGGATGGAGAAAATTGCGGAAAACCGAACAATTATACAATATTTGCCT
TATGTAACACGTTGGGATTATTTAGCTACTATGTTCACAGAAGCAATAACCATAAATGGA
CCCGAACAATTAGGCAATATTCAAGTACCTAAAAGGGCTAGCTATATCAGAGTCATTAT
GTTGGAGTTGAGTCGGATAGCTTCTCATTTGTTATGGCTAGGCCCTTTTATGGCGGATAT
TGGTGCGCAGACCCCTTTCTTCTATATTTTTCGAGAAAGAGAATTGATATATGACCTCTT
CGAAGCTGCCACCGGTATGCGAATGATGCATAATTATTTTCGTATCGGGGGAGTGGCTG
CCGATCTACCCTATGGCTGGATAGATAAATGTTTGGATTTTTGCGATTATTTTTAACAGG

GGTTGCTGAGTATCAAAAACTTATTACCCGGAATCCTATTTTTTTAGAACGAGTTGAAG
GCGTAGGCATTATTGGGGGAGACGAAGCATTAAATTGGGGGTTATCGGGACCAATGCTA
CGAGCTTCCGGAATAGAATGGGATCTTCGTAAAGTTGATCATTATGAGTCTTACGACGA
ATTTGATTGGCAGGTTCAATGGCAACGAGAAGGGGATTCATTAGCTCGTTATTTAGTAC
GAATCGGTGAAATGACAGAATCCATAAAGATTATTCAACAGGCTCTGGAAGGAATTCC
AGGAGGGCCTTACGAAAATTTAGAAATCCGACGTTTTGACAGATTAAAAGATCCTGAA
TGGAATGATTTTGAATATCGGTTTATTAGTAAAAAACCTTCTCCAACTTTTGAATTGTCG
AAACAAGAACTTTATGTGAGAGTTGAAGCCCCAAAAGGAGAATTGGGAATTTTTCTCA
TAGGAGATCAGAGCGTTTTTCCTTGGAGATGGAAAATTCGCCCACCAGGTTTTATCAAT
TTGCAAATTCTTCCTCAGTTAGTTAAAAGAATGAAATTGGCTGATATTATGACAATACTA
GGTAGCATAGATATCATTATGGGAGAAGTTGATCGTATGCTCCCTATGATAACTGAATTC
ATAAATTATGGTCAACAAACAATACGAGCCGCCAGATACATCGGCCAAGGTTTCATGAT
TACCCTGTCCCACGCAAATCGTTTACCTGTAACTATTCAATACCCCTACGAAAAATTGAT
CACATCGGAACGTTTCCGAGGCCGAATCCACTTTGAATTTGATAAATGCATTGCTTGTG
AAGTATGTGTGCGTGTATGTCCTATAGATTTACCCGTTGTTGATTGGAAGTTGGAAACTG
ATATTCGAAAGAAACGATTGCTTAATTACAGTATTGATTTTGGAATCTGTATATTTTGTGG
TAATTGCGTTGAGTATTGTCCAACAAATTGTTTATCAATGACCGAAGAATATGAACTTTC
TACTTATGATCGTCACGAATTGAATTATAATCAAATCGCTTTGGGTCGCTTACCAATGTC
AGTAATTGATGATTACACAATTCGAACAATTTCGAATTTA--
--------ATGCAGGGTCGTTTGTCTGCTTGGCTAGTCAAGCATGGGCTAATTCATAGATCTTT
GGGCTTTGATTACCAAGGAATAGAGACTTTACAAATAAAGCCCGAGGATTGGCATTCCA
TTGCTGTCATTTTTTATGTATATGGGTACAACTATCTCCGCTCTCAATGTGCCTATGATGT
AGCGCCTGGCGGGCTGTTAGCTAGTGTGTATCATCTTACGAGAATAGAGGATGGTGTGG
ATCAACCAGAGGAGGTATGCATAAAAGTATTTGCCTCCAGGAGGAATCCTAGAATTCCG
TCTGTTTTCTGGGTTTGGAAAAGTGTGGATTTTCAAGAACGAGAATCATATGATATGTT
GGGAATCTCTTATGATAATCATCCACGCTTGAAACGTATCTTAATGCCTGAAAGTTGGAT
AGGATGGCCCTTACGTAAGGATTATATTGCCCCCAATTTTTATGAAATACAAGATGCTCA
T--ATG
GTCTTAGCTCCTGAATATTCAGACAATAAAAAGAAAAACGGAAAAAATAAGATTGAGA
CA---GTTATGAATTCCATTCAGTTTCCTTTACTTGATCGAACAACCCAAAATTCAGTTATT
TCAACTACATTAAATGATCTTTCAAATTGGTCAAGACTCTCTAGTTTATGGCCGCTTCTC
TATGGTACCAGTTGTTGCTTCATTGAATTTGCTTCACTAATAGGCTCACGCTTCGACTTT
GATCGTTATGGACTAGTACCAAGATCGAGTCCTAGACAAGCGGATCTAATTTTAACAGC
CGGAACAGTAACAATGAAAATGGCCCCCTCTTTAGTGAGATTATATGAGCAAATGCCTG
AACCAAAATATGTTATTGCTATGGGAGCCTGTACAATTACAGGCGGGATGTTCAGTACC
GATTCTTATAGTACTGTTCGGGGAGTCGATAAGCTAATTCCTGTAGATGTATATTTGCCA
GGTTGCCCACCTAAACCGGAAGCGGTTATAGATGCTATAACAAAACTTCGTAAGAAAAT
ATCTCGAGAACTCTATGAAGATCGAATTAGGTCTCAACGAGCGAATCGGTGTTTTACTA
CCAATCACAAGTTTCATGTTCAACACAGTATTCATACGGGAAATTATGATCAAAGAGTT
CTTTATCAACCCCCATCTACTTCAGAGATCCCTACGGAAATCTTTTTCAAATACAAAAAT
TCAGTATCTTCCCCCGAATTAGTGAATATGCAAACTAGAAATGCTTTTTCTTGGCTAAAG
AAACAGATTACTCGATCTATTTCCGTATCGCTCATGATATATATCTTAACTCGGACATCCA
TTTCAAGTGCATATCCCATTTTTGCACAGCAGGGTTATGAAAATCCACGAGAAGCGACT
GGGCGTATTGTATGTGCCAATTGCCATTTAGCTAATAAGCCCGTGGAGATTGAGGTTCC

ACAAGCGGTACTTCCTGATACTGTATTTGAAGCAGTTGTTCGAATTCCTTATGATATGCA
ACTGAAACAGGTTCTTGCTAATGGTAAAAGGGGGGGGTTGAACGTGGGGGCTGTTCTT
ATTTTACCGGAGGGGTTTGAATTAGCTCCTCCCGATCGTATTTCTCCCGAGATGAAAGA
AAAGATTGGCAATTTGTCTTTTCAGAGCTATCGCCCCAATAAAAAAAAATATTCTTGTGAT
AGGCCCTGTCCCTGGTCAAAAATATAGTGAAATAACCTTCCCTATTCTTTCCCCGGACC
CTGCTACTAAGAAGGATGTTCACTTCTTAAAATATCCTATATACGTAGGCGGGAACAGG
GGAAGGGGTCAGATTTATCCCGACGGCAGCAAGAGTAACAATACTGTTTATAATGCTAC
AGCAGCAGGTATAGTAAGCAAAATCATACGAAAAGAAAAGGGTGGGTATGAGATAACC
ATAACGGATGCGTCGGATGGACGTCAAGTGGTTGATATTATCCCTCCCGGACCAGAACT
TCTTGTTTCCGAGGGCGAATCTATCAAATTTGATCAACCATTAACGAGTAATCCTAATGT
AGGCGGATTTGGTCAGGGAGATGCAGAAATAGTACTTCAAGATCCATTACGTGTCCAA
GGACTTTTGTTCTTCTTGGCATCTGTTATTTTGGCACAAATCTTTTTGGTTCTTAAAAAG
AAACAGTTCGAGAAGGTTCAATTGGCCGAAATGAATTTCATGAGTAAAGTATATGATTG
GTTCGAAGAACGTCTCGAGATTCAAGCAATTGCGGATGATATAACTAGTAAATACGTTC
CTCCCCACGTCAATATATTTTATTGTTTAGGGGGAATTACGCTTACTTGTTTTTTAGTACA
AGTAGCTACTGGGTTTGCTATGACTTTTTACTATCGTCCGACCGTTACTGAGGCTTTTGC
TTCTGTTCAATACATAATGACTGAAGCCAACTTTGGTTGGTTAATCCGATCAGTTCATCG
ATGGTCGGCAAGTATGATGGTCCTAATGATGATCCTGCATGTATTTCGTGTGTATCTCAC
CGGCGGATTTAAAAAACCTCGCGAATTGACTTGGGTTACAGGTGTGGTTCTGGCTGTAT
TAACCGCATCTTTTGGCGTAACTGGTTATTCCTTACCTTGGGACCAAGTCGGTTATTGGG
CAGTGAAAATAGTAACAGGTGTCCCTGACGCTATTCCTGTAATAGGATCACCCTTGGTC
GAATTATTGCGCGGAAGCGCTAGTGTGGGACAATCTACTTTGACCCGTTTTTATAGTTTA
CACACTTTTGTATTGCCGCTTCTTACTGCCGTATTTATGTTAATGCACTTTCCAATGATAC
GTAAACAAGGTATTTCTGGGCCTTTAATGGGAGTAACAAAAAAACCTGACTTGAATGA
TCCTGTATTAAGAGCTAAATTGGCTAAAGGTATGGGTCATAATTATTATGGAGAGCCCGC
ATGGCCCAATGATCTTTTATATATTTTTCCAGTAGTAATTCTAGGTACTATTGCATGTAAT
GTAGGCTTAGCCGTTTTAGAACCATCAATGATTGGTGAACCGGCAGATCCATTTGCAAC
CCCTTTGGAAATATTACCTGAATGGTATTTCTTTCCTGTATTTCAAATACTTCGTACAGTG
CCCAATAAATTATTGGGGGTTCTTTTAATGGTTTCAGTACCTGCGGGATTATTAACAGTA
CCTTTTTTAGAGAATGTTAATAAATTCCAAAATCCATTTCGCCGTCCAGTAGCGACGAC
TGTCTTTTTGATTGGTACCGCAGTCGCCCTTTGGTTGGGCATTGGTGCAACATTACCTAT
TGATAAATCCCTAACTTTAGGTCTTTTTATGATTGAAGTTTTTCTATTTGGAATCGTGTTA
GGTCTAATTCCTATTACTTTGGCTGGATTATTCGTAACTGCATATTTACAATATAGGCGTG
GTGATCAGTTGGACCTTATGCTTACTATAACTAGTTATTTCGGTTTTCTATTAGCGGCTTT
AACTATAACCTCAGCTCTATTTATTGGTCTGAGCAAGATACGACTTATTATGGATATAGTA
AGTCTTGCTTGGGCTGCTTTAATGGTAGTCTTTACTTTTTCCCTTTCACTCGTAGTGTGG
GGAAGAAGTGGACTCATGATTATTCGTTCGCCGGAACCAGAAGTTAAAATTTTGGTAG
ATAGGGATCCCGTAAAAACTTCGTTCGAGGAATGGGCCAGACCGGGTCATTTCTCAAG
AACAATAGCTAAAGGGCCTGATACTACCACTTGGATCTGGAACCTACATGCTGATGCTC
ACGATTTCGATAGCCATACCAGTGATTTGGAGGAGATCTCTCGAAAAGTATTTAGTGCA
CATTTCGGTCAACTCTCCATCATCTTTCTTTGGCTGAGCGGCATGTATTTCCACGGTGCT
CGTTTTTCTAATTATGAAGCGTGGCTAAGTGATCCAACTCACATTGGGCCTAGTGCCCA
GGTGGTTTGGCCAATAGTGGGCCAAGAAATATTAAATGGTGACGTAGGCGGGGGTTTC
CGAGGAATACAAATAACCTCTGGTTTTTTTCAGATTTGGCGAGCATCTGGAATAACTAG

TGAATTACAACTCTATTGTACAGCAATTGGGGCATTGGTCTTTGCAGCGTTAATGCTTTT
TGCTGGTTGGTTTCATTATCATAAAGCGGCGCCAAAATTGGCTTGGTTTCAAGATGTAG
AATCTATGCTGAATCACCATTTGGCAGGGCTACTAGGACTTGGGTCTCTCTCTTGGGCG
GGGCATCAAGTACATGTATCTTTACCGATTAACCAATTTCTAAACGCTGGAGTAGATCCT
AAAGAGATACCACTTCCTCATGAATTTATCTTGAATCGAGATCTTTTGGCTCAACTTTAT
CCCAGTTTTGCCGAGGGAGCAACCCCATTTTTCACCTTGAATTGGTCAAAATATGCGGA
CTTTCTTACTTTTCGTGGAGGATTAGATCCAGTAACTGGGGGTCTGTGGCTGACTGATA
TTGCCCATCACCATTTAGCTATTGCAATTCTTTTCCTGATAGCGGGTCACATGTATAGGA
CCAACTGGGGTATTGGTCATGGACTAAAAGATATTTTAGAAGCTCATAAAGGTCCATTT
ACAGGTCAGGGCCATAAAGGCCTATATGAGATCCTAACAACGTCATGGCATGCTCAATT
ATCTCTTAACTTAGCTATGTTAGGCTCTTTAACCATTGTTGTAGCTCACCATATGTATTCC
ATGCCCCCTTATCCGTATCTAGCTACTGACTATGGTACACAACTGTCATTGTTCACACAT
CACATGTGGATTGGTGGATTTCTCATAGTTGGTGCTGCCGCGCATGCAGCCATTTTTATG
GTAAGAGATTATGATCCAACTACTCGGTACAACGATCTATTAGATCGTGTCCTTAGACAT
CGTGATGCAATCATATCCCATCTCAACTGGGCATGTATATTTCTAGGCTTTCACAGTTTT
GGTTTGTATATTCATAATGATACCATGAGCGCTTTAGGGCGTCCTCAAGATATGTTTTCA
GATACCGCTATACAATTACAACCCGTTTTTGCTCAATGGATACAAAACACCCATGCTTTA
GCACCTGGTGCAACGGCTCCTGGTGCAACAGCAAGTACCAGTTTAACTTGGGGGGGT
GGTGATTTAGTGGCAGTGGGTGGCAAGGTGGCTTTGTTGCCTATTCCATTAGGAACCGC
GGATTTCTTGGTACATCACATTCATGCATTTACGATTCATGTGACGGTATTGATACTCTTG
AAAGGTGTTCTATTTGCTCGCAGTTCCCGTTTGATACCGGATAAAGCAAATCTTGGTTT
TCGTTTTCCTTGTGATGGACCTGGAAGAGGGGGTACATGTCAAGTATCGGCCTGGGATC
ATGTCTTCTTAGGACTATTCTGGATGTACAATGCAATTTCGGTAGTAATATTCCATTTCAG
TTGGAAAATGCAGTCAGATGTTTGGGGCAGTGTAAGTGATCAAGGGGTAGTAACTCAT
ATCACGGGAGGAAACTTTGCGCAAAGTTCTATTACTATTAATGGGTGGCTCCGCGATTT
CTTATGGGCACAGGCATCCCAGGTAATTCAGTCTTATGGTTCTTCATTATCTGCATATGG
CCTTTTTTTCCTAGGTGCTCATTTTGTCTGGGCTTTTAGTTTAATGTTTCTATTCAGTGGA
CGTGGTTATTGGCAAGAACTTATTGAATCCATCGTTTGGGCTCATAATAAATTAAAAGTT
GCTCCTGCTACTCAGCCGAGAGCCTTGAGCATTATACAAGGACGTGCTGTAGGAGTAA
CCCATTACCTTCTGGGTGGAATTGCCACAACATGGGCATTCTTCTTAGCAAGAATTATTG
CAGTAGGAATGGCATTACGATTTCCAAGGTTTAGCCAAGGCTTAGCTCAGGACCCCACT
ACTCGTCGTATTTGGTTTGGTATTGCTACCGCACATGACTTCGAGAGTCATGATGATATT
ACTGAGGAACGTCTTTATCAGAATATTTTTGCTTCTCACTTTGGTCAATTAGCAATAATT
TTTCTGTGGACTTCCGGAAATCTGTTTCATGTAGCTTGGCAAGGAAATTTTGAGTCGTG
GGTACAGGACCCTTTACATGTAAGACCTATTGCTCATGCAATTTGGGATCCTCATTTTGG
TCAACCGGCCGTGGAAGCTTTTACTCGAGGGGGTGCTCTTGGCCCAGTGAATATCGCTT
ATTCTGGTGTTTATCAGTGGTGGTATACAATCGGTTTACGCACTAATGAAGATCTTTATA
CTGGTGCTCTTTTTCTATTATTTCTTTCTGCCATATCCTTAATAGCAGGTTGGTTACACCT
ACAACCGAAATGGAAACCGAGCGTTTCCTGGTTCAAAAATGCCGAATCTCGTCTGAAT
CATCATTTGTCAGGACTCTTTGGCGTAAGTTCCTTGGCTTGGACAGGGCATTTAGTTCA
TGTTGCTATTCCTGCATCCAGAGGGGAGTACGTTCGGTGGAATAATTTCTTAGATGTATT
ACCGCATCCCCAAGGGTTAGGCCCACTTTTTACAGGTCAATGGAATCTTTATGCTCAAA
ACCCCGATTCAAGTAGTCATTTATTTGGTACCGCCCAAGGGGCGGGAACTGCCATTCTA
ACTCTTCTCGGGGGATTCCATCCACAAACGCAAAGTTTATGGCTGACTGATATTGCCCA

TCACCATTTAGCTATTGCATTTATTTTTCTCGTTGCTGGTCATATGTATAGAACCAATTTC
GGGATTGGGCACAGTATGAAAGACCTTTTAGATGCACATATTCCCCCGGGGGGACGATT
GGGGCGTGGACATAAGGGTCTTTATGACACAATCAATAATTCGCTTCATTTTCAATTAG
GCCTTGCTCTAGCTTCTTTAGGGGTTATTACTTCTTTGGTAGCTCAACACATGTACTCTT
TACCTGCTTATGCATTCATAGCACAAGACTTTACTACTCAAGCTGCATTATATACCCACC
ACCAATATATCGCAGGATTCATCATGACAGGAGCTTTTGCTCATGGAGCTATATTTTTCA
TTAGAGATTACAATCCGGAGCAAAATGAAGATAATGTATTGGCAAGAATGTTAGAGCAT
AAAGAAGCTATCATATCTCATTTAAGTTGGGCCAGCCTCTTTCTGGGATTCCATACCCTG
GGACTTTATGTTCATAATGATGTCATGCTTGCCTTTGGCACTCCGGAGAAGCAAATCTT
GATTGAACCTATATTTGCTCAATGGATACAATCCGCTCATGGTAAAACTTCATATGGGTT
CGATGTACTTTTATCTTCAACGAGTGGTCCAGCATTCAATGCGGGTCGAAGCATCTGGT
TGCCGGGTTGGTTAAATGCTGTTAATGAA------AATAGTAATTCATTATTTTTAACAATAG
GTCCTGGAGACTTTTTGGTTCATCATGCTATTGCTCTTGGTTTACATACAACTACATTGA
TCTTAGTAAAAGGTGCTTTAGATGCACGTGGTTCCAAGTTAATGCCAGATAAAAAGGAT
TTCGGTTATAGTTTTCCGTGCGATGGCCCAGGACGAGGCGGTACTTGTGATATTTCGGC
ATGGGACGCGTTTTATTTGGCAGTTTTTTGGATGTTAAATACTATTGGATGGGTTACTTT
TTATTGGCATTGGAAGCACATCACATTATGGCAGGGTAACGTTTCACAGTTTAATGAAT
CTTCCACTTATTTGATGGGCTGGTTAAGGGATTATTTATGGTTAAACTCTTCACAACTTA
TCAACGGATATAATCCTTTTGGTATGAATAGTTTATCGGTTTGGGCATGGATGTTCTTATT
TGGACATCTTGTTTGGGCTACTGGATTTATGTTCTTAATTTCTTGGCGTGGATATTGGCA
GGAATTGATTGAAACTTTAGCATGGGCTCATGAACGCACACCTTTGGCCAATTTGATTC
GCTGGAGAGATAAACCAGTGGCCCTTTCCATTGTACAAGCAAGATTGGTTGGATTAGCT
CACTTTTCTGTAGGTTATATATTCACTTATGCGGCTTTCTTGATTGCCTCTACGTCGGGCA
AATTTGGTATGTCACATTCAGTAAAGATTTATGATACGTGTATAGGATGTACTCAATGTG
TCCGAGCCTGCCCCACCGATGTATTAGAAATGATACCTTGGGACGGTTGTAAGGCTAAA
CAAATTGCTTCTGCTCCACGAACAGAGGACTGTGTTGGTTGTAAGAGATGTGAATCCG
CCTGTCCAACAGATTTCTTGAGTGTCCGAGTTTATTTATGGCATGAAACAACTCGCAGT
ATGGGTCTAGCTTATATGACAAATTTGAACCTTCCATCTATTTTTGTGCCGTTAGTAGGC
CTAGTCTTTCCGGCAATTGCAATGGCTTCTTTATTTCTTCATGTTCAAAAAAATAAGATT
GTTATGCGAGATCTAAAAACATATCTCTCTGTGGCACCAGTACTAAGTACGCTATGGTTC
GGGGCTTTAGCAGGTCTATTGATAGAGATTAATCGTTTTTTCCCGGATGCGTTGACATTC
CCCTTTTTTTCATTCATGACTGCAATTTTAGAGAGACGCGAAAGCGAAAGCCTATGGGG
TCGCTTCTGTAACTGGATAACTAGCACTGAAAACCGTCTTTACATTGGATGGTTTGGTG
TTTTGATGATCCCTACCTTATTGACGGCAACTTCTGTATTTATTATTGCCTTCATTGCTGC
TCCTCCAGTAGACATTGATGGTATTCGTGAACCTGTTTCAGGGTCTCTACTTTACGGAA
ACAATATTATTTCCGGTGCCATTATTCCTACTTCTGCAGCTATAGGTTTACATTTTTACCC
AATCTGGGAAGCGGCATCCGTTGATGAATGGTTATACAACGGTGGTCCTTATGAACTAA
TTGTTCTACACTTCTTACTTGGCGTAGCTTGTTACATGGGTCGTGAGTGGGAGCTTAGT
TTCCGTCTGGGTATGCGACCTTGGATTGCTGTTGCATATTCAGCTCCTGTTGCAGCTGCT
ACCGCAGTTTTCTTGATCTACCCAATTGGTCAAGGAAGTTTTTCTGATGGTATGCCTCTA
GGAATCTCTGGTACTTTCAATTTCATGATTGTATTCCAGGCTGAGCACAACATCCTTATG
CACCCATTTCACATGTTAGGCGTAGCTGGTGTATTCGGCGGCTCCCTATTCAGTGCTATG
CATGGTTCCTTGGTAACTTCTAGTTTGATCAGGGAAACCACAGAAAATGAATCTGCTAA
TGAAGGTTACAGATTCGGTCAAGAGGAAGAAACTTATAACATCGTAGCCGCTCATGGTT

ATTTTGGCCGATTGATCTTCCAATATGCTAGTTTCAACAACTCTCGTTCGTTACACTTCT
TCCTAGCTGCTTGGCCTGTAGTAGGTATCTGGTTTACCGCTTTAGGTATCAGCACTATGG
CTTTCAACCTAAATGGTTTCAATTTCAACCAATCTGTAGTTGACAGTCAAGGCCGTGTA
ATTAATACTTGGGCTGATATCATTAACCGTGCTAACCTTGGTATGGAAGTTATGCATGAA
CGTAATGCTCACAACTTCCCTCTAGACCTAGCTGCTATCGAAGCTCCATCTACAAATGG
AATGGGTTTGCCTTGGTATCGTGTTCATACCGTTGTATTGAATGATCCCGGTCGGTTGCT
TTCTGTTCATATAATGCATACAGCTCTGGTTGCTGGTTGGGCCGGTTCGATGGCTCTGTA
TGAATTAGCGGTTTTTGATCCTTCTGATCCTGTTCTTGATCCAATGTGGAGACAGGGTAT
GTTCGTTATACCCTTCATGACTCGTTTAGGAATAACCAATTCATGGGGCGGTTGGAGTAT
CACA---GGGGGGACTGTAACGAATCCGGGTATTTGGAGTTACGAAGGTGTAGCTGGAG
CACATATTGTGTTTTCTGGCTTATGCTTTTTGGCAGCTATCTGGCATTGGGTGTATTGGG
ATCTAGAAATATTTTGTGATGAACGTACAGGAAAACCTTCTTTGGATTTGCCAAAGATC
TTTGGAATTCATTTATTTCTCTCAGGGGTGGCTTGCTTTGGTTTTGGTGCATTTCATGTA
ACAGGCTTGTATGGTCCCGGAATATGGGTGTCCGACCCTTATGGACTAACGGGAAAAGT
ACAACCTGTAAATCCAGCGTGGGGCGTGGAAGGTTTTGATCCTTTTGTTCCAGGAGGA
ATAGCCTCTCATCATATTGCAGCAGGAACATTGGGCATATTAGCGGGCCTATTCCATCTT
AGCGTCCGTCCGCCACAACGTCTATACAAAGGATTGCGTATGGGAAATATTGAAACCGT
CCTTTCCAGTAGTATCGCTGCTGTCTTTTTTGCAGCTTTTGTTGTTGCCGGAACTATGTG
GTATGGTTCGGCAACAACCCCGATTGAATTATTTGGGCCCACTCGTTACCAATGGGATC
AGGGGTACTTCCAGCAAGAAATATATCGAAGAGTTAGTGCTGGGCTAGCAGAAAATCA
AAGTTTATCAGAAGCCTGGTCTAAAATTCCTGAAAAATTAGCTTTTTATGATTACATCGG
CAATAATCCGGCAAAAGGGGGATTATTCAGAGCGGGCTCAATGGATAACGGGGATGGA
ATAGCGGTTGGATGGTTAGGACACCCTATCTTTAGAGATAAAGAAGGCCGTGAACTTTT
TGTACGTCGTATGCCTACTTTTTTTGAAACATTTCCGGTCGTTTTGGTAGATGGCGATGG
AATTGTTAGAGCCGATGTTCCTTTTAGAAGGGCAGAATCGAAGTATAGTGTTGAACAA
GTAGGTGTAACTGTTGAGTTCTACGGCGGTGAACTCAACGGCGTCAGTTATAGTGATCC
TGCTACTGTGAAAAAATATGCTAGACGTGCTCAATTGGGTGAAATTTTTGAATTAGATC
GTGCTACTTTGAAATCCGATGGTGTTTTTCGTAGCAGTCCAAGGGGTTGGTTTACTTTT
GGGCATGCTTCGTTTGCTTTGCTCTTCTTCTTCGGACACATTTGGCATGGTGCTAGAAC
CTTGTTCAGAGATGTTTTTGCTGGTATTGACCCAGATTTAGATGCTCAAGTCGAATTTG
GAGCATTCCAAAAACTTGGAGATCCAACTACAAAAAGACAGGCAGCC--------------------
--------------GTGGAAACGCTCTTTAATGGAACTTTAGCCTTAGCTGGTCGTGACCAAGAA
ACCACTGGTTTCGCTTGGTGGGCCGGGAATGCCCGACTTATCAATTTATCCGGTAAACT
ACTAGGGGCTCATGTAGCCCATGCTGGATTAATCGTATTCTGGGCCGGAGCAATGAACC
TATTTGAAGTGGCCCATTTCGTACCAGAGAAGCCTATGTATGAACAAGGATTAATTTTAC
TTCCCCACCTAGCTACTCTAGGTTGGGGGGTAGGCCCTGGGGGAGAAGTTATAGACAC
CTTTCCATACTTTGTATCTGGAGTACTTCATTTAATTTCTTCTGCAGTATTGGGCTTTGGC
GGCATTTATCATGCACTTCTGGGACCTGAGACACTTGAAGAATCTTTTCCCTTCTTTGG
TTATGTCTGGAAAGATCGAAATAAAATGACCACAATTTTAGGTATTCACTTAATCTTGTT
AGGTCTAGGTGCTTTTCTTCTAGTATTCAAGGCTCTTTATTTTGGGGGCGTATATGATAC
CTGGGCTCCGGGAGGGGGAGATGTAAGAAAAATTACCAACTTGACCCTTAGCCCGAGT
ATCATATTTGGTTATTTACTAAAATCCCCTTTTGGAGGGGAAGGATGGATTGTTAGTGTG
GACGATTTAGAAGATATAATCGGAGGACATGTATGGTTAGGTTCCATTTGTATACTTGGT
GGAATCTGGCATATCTTAACCAAACCCTTCGCATGGGCTCGACGCGCACTTGTATGGTC

TGGAGAGGCTTACTTATCTTATAGTTTAGGGGCTTTATCCGTCTTTGGTTTCATTGCTTGT
TGTTTTGTCTGGTTCAATAATACCGCTTATCCTAGTGAATTTTACGGACCTACTGGACCA
GAAGCTTCTCAAGCTCAAGCATTTACTTTTCTAGTTAGAGACCAACGTCTTGGGGCTAA
CGTGGGATCCGCTCAAGGACCTACTGGTTTAGGTAAATATCTAATGCGTTCCCCGACTG
GAGAAGTCATTTTTGGAGGAGAAACTATGCGTTTTTGGGATCTGCGTGCTCCATGGTTA
GAGCCTCTAAGGGGTCCAAATGGGTTAGACTTGAGTAGGTTGAAAAAAGACATACAAC
CTTGGCAGGAACGGCGTTCCGCAGAATATATGACTCATGCTCCTTTAGGTTCTTTAAATT
CCGTGGGTGGTGTAGCTACCGAGATCAATGCAGTCAATTATGTCTCTCCTAGAAGTTGG
TTAGCTACCTCTCATTTTGTTCTAGGATTCTTCTTCTTCGTAGGTCATTTGTGGCACGCG
GGAAGGGCTCGTGCAGCTGCAGCAGGATTTGAAAAAGGAATTGATCGTGACTTTGAA
CCTGTTCTTTCCATGACCCCTCTTAATATGACTATAGCCCTTGGTAAGTTTACCAAAGAC
GAAAATGATTTATTTGATATTATGGATGACTGGTTACGGAGGGACCGTTTCGTTTTTGTA
GGCTGGTCCGGTCTATTGCTCTTTCCTTGTGCCTATTTCGCTGTAGGGGGTTGGTTCACA
GGTACAACCTTTGTAACTTCATGGTATACCCATGGATTGGCCAGTTCTTATTTGGAAGGC
TGCAATTTCTTAACTGCCGCGGTTTCTACTCCTGCTAATAGTTTAGCACATTCGTTGTTG
TTACTATGGGGTCCTGAAGCACAAGGAGATTTTACTCGTTGGTGTCAATTGGGGGGTCT
GTGGACTTTTGTTGCTCTCCATGGAGCTTTTGGCCTAATAGGTTTCATGTTACGTCAATT
CGAGCTTGCTCGATCTGTTCAATTGAGACCTTATAATGCAATCGCATTCTCTGGTCCAAT
TGCTGTTTTTGTTTCTGTATTTCTGATTTATCCACTGGGTCAGTCTGGTTGGTTCTTTGC
ACCTAGTTTTGGTGTAGCAGCTATATTTCGATTCATCCTCTTTTTTCAAGGGTTTCATAAT
TGGACGTTGAACCCATTTCATATGATGGGAGTTGCCGGTGTATTGGGCGCTGCTTTGCT
ATGCGCCATTCATGGTGCTACCGTAGAAAATACTTTATTTGAAGACGGTGATGGTGCAA
ATACATTCCGTGCTTTTAACCCAACTCAAGCCGAAGAAACTTATTCAATGGTCACCGCT
AACCGCTTTTGGTCCCAAATCTTTGGGGTTGCTTTTTCCAATAAACGTTGGTTACATTTC
TTTATGTTATTTGTACCAGTAACCGGTTTATGGATGAGTGCTCTTGGAGTAGTCGGTCTA
GCCCTGAACCTACGTGCCTATGACTTCGTTTCTCAGGAAATTCGCGCAGCGGAAGATC
CTGAATTTGAGACTTTCTACACCAAAAATATTCTCTTAAACGAAGGTATTCGCGCTTGG
ATGGCGGCTCAAGATCAGCCTCATGAAAACCTTATATTCCCTGAGGAGGTTCTACCACG
TGGAAACGCTCTTATGTCTGGAAGCACAGGAGAACGTTCGTTTGCTGATATTATTACCA
GTATTCGATACTGGGTCATTCATAGCATTACTATACCTTCCCTATTCATTGCGGGTTGGTT
ATTTGTCAGCACCGGTTTAGCTTACGATGTGTTTGGAAGCCCTCGGCCAAACGAGTATT
TTACAGAGAGCCGACAAGGAATTCCATTAATAACTGGCCGTTTTGATCCTTTGGAACAA
CTCGATGAATTTAGTAGATCGTTTATGACTATAGATCGAACCTATCCAATTTTTACAGTAC
GATGGTTGGCTGTTCACGGCCTAGCTGTACCTACCGTCTTTTTTTTGGGATCAATATCAG
CAATGCAGTTCATCCAACGAATGGCTACACAAACTGTTGAAAACAGTTCT---AGATCTG
GTCCAAGACGAACTGCGGTAGGAGATTTATTAAAACCATTGAATTCGGAATATGGTAAA
GTAGCTCCTGGGTGGGGAACTACTCCTTTGATGGGTGTCGCAATGGCCTTATTTGCGGT
ATTTCTATCTATTATTTTGGAGATTTATAATTCTTCCGTTTTATTGGATGGAATTTCAATGA
ATATGCTTACTCTCAAACTCTTCGTTTACACAGTAGTGATATTTTTTGTTTCTCTCTTCAT
CTTTGGATTCCTATCTAATGATCCCGGACGTAATCCTGGACGTGAAGAAATGGCCGATA
CTACTGGAAGGATTCCTCTTTGGATAATAGGTACTGTAGCTGGTATTCTTGTAATCGGTT
TAATAGGTATTTTCTTTTATGGTTCATATTCCGGATTGGGTTCATCCCTCATGCTTAATACC
TTTAGTTTGATCGGTATCTGTCTTAATTCGACCCTTTTTTCGAGTAGTTTTTTCTTCGGCA
AATTGCCCGAGGCCTATGCTTTTTTGAATCCAATCGTAGATATTATGCCAGTCATACCTC

TGTTTTTTTTCTCTTAGCCTTTGTTTGGCAAGCTGCTGTAAGTTTTCGAACGACACAAT
CAAACCCGAACGAACAAAATGTTGAATTGAATCGTACCAGTCTCTACTGGGGGTTATTA
CTCATTTTTGTACTTGCTGTTTTATTTTCCAATTATTTCTTCAATATGGAAGTAAATATTCT
TGCATTTATTGCTACTGCACTATTTATTCTAGTTCCTACCGCTTTTCTACTTATCATTTACG
TAAAAACAGTCAGTCAAAACGATATGGAAACAGCAACCCTAGTCGCCATCTTTATATCT
GGTTTACTTGTAAGTTTTACTGGGTACGCCTTATATACCGCTTTTGGGCAACCTTCTCAA
CAACTAAGAGATCCATTCGAGGAACATGGAGACATGGAAGCATTGGTTTATACATTCCT
CTTAGTCTCGACTCTAGGGATAATTTTTTTCGCTATCTTTTTTCGAGAACCGCCTAAAGT
TCCAACTAAAAAGAAC---ATGACTCTTGCTTTCCAATTGGCTGTTTTTGCATTAATTGCT
ACTTCATTAATCTTATTGATTAGCGTACCCGTTGTATTTGCTTCTCCTGATGGCTGGTCAA
GTAACAAAAATGTTGTATTTTCTGGTACATCCTTATGGATTGGATTAGTCTTTCTGGTGG
GTATCCTTAATTCTCTCATCTCT--------------------ATGTCACCACAAACAGAGACTAAAGC
AAGTGTTGGATTCAAAGCTGGTGTTAAAGAGTACAAATTGACTTATTATACTCCTGAGT
ACCAAACCAAGGATACTGATATATTGGCAGCATTCCGAGTAACTCCTCAACCTGGAGTT
CCACCTGAAGAAGCAGGGGCCGCGGTAGCTGCCGAATCTTCTACTGGTACATGGACAA
CTGTATGGACCGATGGACTTACCAGCCTTGATCGTTACAAAGGGCGATGCTACCGCATC
GAGCGTGTTGTTGGAGAAAAAGATCAATATATTGCTTATGTAGCTTACCCTTTAGACCTT
TTTGAAGAAGGTTCTGTTACCAACATGTTTACTTCCATTGTAGGTAACGTATTTGGGTTC
AAAGCCCTGCGCGCTCTACGTCTGGAAGATCTGCGAATCCCTCCTGCTTATGTTAAAAC
TTTCCAAGGTCCGCCTCATGGGATCCAAGTTGAAAGAGATAAATTGAACAAGTATGGT
CGTCCCCTGTTGGGATGTACTATTAAACCTAAATTGGGGTTATCTGCTAAAAACTACGGT
AGAGCTGTTTATGAATGTCTTCGCGGTGGACTTGATTTTACCAAAGATGATGAGAACGT
GAACTCACAACCATTTATGCGTTGGAGAGATCGTTTCTTATTTTGTGCCGAAGCACTTTA
TAAAGCACAGGCTGAAACAGGTGAAATCAAAGGGCATTACTTGAATGCTACTGCAGGT
ACATGCGAAGAAATGATCAAAAGAGCTGTATTTGCTAGAGAATTGGGCGTTCCGATCGT
AATGCATGACTACTTAACGGGGGGATTCACCGCAAATACTAGCTTGGCTCATTATTGCC
GAGATAATGGTCTACTTCTTCACATCCACCGTGCAATGCATGCGGTTATTGATAGACAG
AAGAATCATGGTATCCACTTCCGGGTATTAGCAAAAGCGTTACGTATGTCTGGTGGAGA
TCATATTCACTCTGGTACCGTAGTAGGTAAACTTGAAGGTGAAAGAGACATAACTTTGG
GCTTTGTTGATTTACTGCGTGATGATTTTGTTGAACAAGATCGAAGTCGCGGTATTTATT
TCACTCAAGATTGGGTCTCTTTACCAGGTGTTCTACCCGTGGCTTCAGGAGGTATTCAC
GTTTGGCATATGCCTGCTCTGACCGAGATCTTTGGGGATGATTCCGTACTACAGTTCGG
TGGAGGAACTTTAGGACATCCTTGGGGTAATGCGCCAGGTGCCGTAGCTAATCGAGTA
GCTCTAGAAGCATGTGTAAAAGCTCGTAATGAAGGACGTGATCTTGCTCAGGAAGGTA
ATGAAATTATTCGCGAGGCTTGCAAATGGAGCCCGGAACTAGCTGCTGCTTGTGAAGTA
TGGAAAGAGATCGTATTTAATTTTGCAGCAGTGGACGTTTTGGATAAGATGGCGATACA
TTTATACAAAACTTCTACCCCGAGCACACGCAATGGAACCGTAGACAGTCAAGTGAAA
TCCAATCCACGAAATAATTTGATCTATGGACAGCATCATTGTGGTAAAGGTCGTAATGC
CAGAGGAATCATTACCGCAAGGCATAGAGGGGGAGGTCATAAGCGTCTATACCGTAAA
ATCGATTTTCGACGGAATGAAAAAGACATATATGGTAGAATCGTAACCATAGAATACGA
CCCTAATCGAAATGCATACATTTGTCTCATACACTATGGGGATGGTGAGAAGAGATATAT
TTTACATCCCAGAGGGGCTATAATTGGAGATACCATTGTTTCTGGTACAGAAGTTCCTAT
AAAAATGGGAAATGCCCTACCTTTGACCGATATGCCCTTAGGCACGGCCATACATAACA
TAGAAATCACACTTGGAAAGGGTGGACAATTAGCTAGAGCAGCGGGTGCTGTAGCGA

AACTGATTGCAAAAGAGGGGAAATCGGCCACATTAAAATTACCTTCTGGGGAGGTCCG
TTTGATATCCAAAAACTGCTCAGCAACAGTCGGACAAGTGGGGAATGTTGGGGTGAAC
CAGAAAAGTTTGGGTAGAGCCGGATCTAAGCGTTGGCTAGGTAAGCGTCCTGTAGTAA
GAGGAGTAGTTATGAACCCTGTAGACCATCCCCATGGGGGTGGTGAAGGGAGAGCCCC
AATTGGTAGAAAAAAACCCACAACCCCTTGGGGTTATCCTGCACTTGGAAGAAGAAGT
AGAAAAAGGAATAAATATAGTGATAATTTGATTCTTCGTCGCCGTAGTAAAATGATTCA
ACCTCAGACCCATTTAAATGTAGCGGATAACAGCGGGGCTCGAGAATTGATGTGTATTC
GAATCATAGGAGCTAGC---AATCGTCGATATGCTCATATTGGTGACGTTATTGTTGCTGT
GATCAAAGAAGCCGTACCAAATATGCCCCTAGAAAGATCAGAAGTAGTCAGAGCTGTA
ATTGTGCGGACCTGTAAAGAACTCAAACGTGACAACGGGATGATAATACGATATGATG
ACAATGCTGCAGTTGTTATTGATCAAGAAGGAAATCCAAAAGGAACTCGAATTTTTGG
TGCAATCGCCCGGGAATTGAGAGAATTAAATTTTACTAAAATAGTTTCATTAGCTCCCG
AGGTATTAATGCTTAGTCCCAAAAGAACCAGATTCCGTAAACAACATAGAGGAAGAAT
GAAGGGAATATCTCATCGAGGTAATCATATTTCTTTCGGTAAATATGCTCTTCAGGCACT
TGAACCTGCTTGGATTACATCTAGACAAATAGAAGCAGGCCGACGAGCAATGACACGA
AATGCACGTCGTGGTGGAAAAATATGGGTACGTATATTTCCAGACAAACCAGTTACACT
AAGACCCGCAGAAACACGTATGGGTTCAGGAAAAGGATCCCCTGAATATTGGGTAGCT
GTTGTTAAACCGGGTCGAATACTTTATGAAATGGGTGGAGTAACAGAAAATATAGCCAG
AAGGGCTATTTCACTAGCAGCATCTAAAATGCCTATACGAACTCAATTCATTATTTCG---A
TGACCAGAATTAAACGGGGATATATAGCTCGGAGACGTAGAACAAAAATTCGTTTATTT
GCATCAAGCTTTCGGGGGGCTCATTCAAGGCTTACTCGAACTATTACTCAACAGAAAAT
AAGAGCTTTAGTTTCGGCTCATCGGGATAGGGATAGGAAAAAGAGAGATTTTCGTCGT
TTGTGGATCACTCGGATAAACGCAGTAATTCGCGAA---AGGGGAGTATCCTATAGTTATA
GTAGATTAATACACGATCTGTACAAGAGACAGTTGCTTCTTAACCGTAAAATACTTGCA
CAAATAGCTATATCAAATAGGAATTGTCTTTATATGATTTCGAACGAAATCATAAAGGAA
GTAGATTGGAAAGAATCCACCAGAATAATTATGCTAAAGGAAGTATATGCTTTAGGTGA
ACATATATCTATGTCTGCTGACAAAGCACGAAGAGTAATTAATCAAATTCGCGGCCGTT
CCTATGAGGAAACGCTTATGATACTAGAACTCATGCCCTATCGAGCATGTTATCCCATTT
TGAAATTGATTTATTCTGCAGCAGCAAATGCTAGTTACAATATGGGTTCCAGCGAAGCC
AATTTAGTCATTAGTAAAGCCGAAGTCAATGGAGGTACTACTGTGAAGAAATTGAAAC
CTCGAGCTCGAGGACGTAGTTTTCCAATAAAAAGATCGACCTGTCATATAACTATTGTA
ATGAAAGATATATCTTTAGATGGGGGATTATGGGACAAAAAAATGGATGGAATCAAATA
TGCAGTATTTACAGACAAAAGTATTCGGTTATTGGGGAAAAAT---CAATATACTTCTAAT
GTCGAATCAGGATCAACTAGGACAGAAATAAAGCATTGGGTCGAACTCTTCTTTGGTG
TCAAGGTAATAGCTATGAATAGTCATCGACTTCCGGGAAAGAGTAGAAGAATGGGACC
TATTATGGGACATACAATGCATTACAGACGTATGATCATTACGCTTCAACCGGGTTATTCT
ATTCCACCTCTTAGAAAGAAAAGAACTATGGCAGTTCCAAAAAAACGCACTTCGACAT
CAAAAAAGCGTATTCGTAAAAATATTTGGAAAAGGAAGGGATATTCGATCGCATTAAAG
GCTTTTTCATTAGCGAAATCTCTTTCTACCGGGAATTCAAAAAGTTTTTTTGTACGCCAA
ACAAAAATAATGGCCAAGGGGAAAGATGTCCGAGTAACGGTGATTTTGGAATGTACTA
GTTGTGTCCGAAACAGTGTTGAT------AAGGTATCAAGAGGTATTTCCAGATATATTACTC
AAAAGAACCGGCACAATACGCCTAATCGATTAGAATTGAAAAAATTCTGTCCCTATTGT
TACAAACATACGATTCATGGGGAGATAAAGAAAATGAAAATAAGAGCTTCCGTTCGTA
AAATTTGTGAAAAATGTCGACTAATCCGTAGGCGGGGACGAATTATAGTAATTTGTTCC

AACCCGAGACATAAACAAAGACAGGGAATGGTTCGAGAGAAAGTAACAGTATCTACT
CGGACACTACAGTGGAAGTGTGTTGAATCAAGAACAGACAGTAAACGCCTTTATTATG
GACGCTTTATTCTGTCTCCACTTATGAAAGGCCAAGCCGACACAATAGGCATTGCGATG
CGAAGAGCTTTACTTGGAGAAATAGAAGGAACATGTATCACACGTGTAAAATCTGAG---
---AAAGTCCCACATGAATATTCTACCATAACGGGCATTCAAGAATCGGTACATGAAATTT
TAATGAATTTGAAAGAAATTATATTAAGAAGTAATCTATATGGAACTTCTGACGCATCTA
TTTGTGTCAAGGGTCCTGGATCTGTAACTGCCCAAGATATCATCTTGCCGCCTTATGTAG
AAATCGTTGATAATACACAACATATAGCCAGTTTAACGGAACCAATTGATTTTTGTATTG
GATTACAAATAGAGAGAAATCGTGGATATCTTATAAAAACGCCACAT---AACTTTGATGG
AAGTTAT------CCTATAGATGCTGTATTCATGCCTGTTCGAAACGCGAATCATAGTATTCAT
TCTTATGGGAATGGGAATGAAAAACAAGAGATACTTTTTCTCGAAATATGGACAAACG
GAAGTTTAACTCCGAAAGAAGCACTTCATGAAGCATCCCGAAATTTAATTGATTTATTT
ATTCCCTTTTTACATATGGAGGAAGAC------AACTTATATTTACAGGACAATCAA---CACA
CGGTTCCTTTATCCCCTTTTACCTTTCATGATAAATTGGCT---AAACTCATAAAAAACAA
AAAA---AAAATAGCATTGAAATCGATTTTTATTGACCAATCAGAATTGCCTTCCAGGATC
TATAATTGCCTCAAAATGTCCAATATATATACATTGTTGGACCTTTTGAATAACAGCCAA
GAAGATCTTATGAAAATTGAACATTTTCGTAGCGAAGATGTAAAACGGATATTGGGCAT
TTTAGAAAAATAT------TTCGTAATTGATTTAGCAAAAAATAAGATGCTCGGGGATGGA---
AATGAGGGAATATCTACAATACCTGGATTTAATCAGATACAATTTGAAGGATTTTGTAGG
TTCATTGATCAAGGTTTGACGGAAGAACTTTATAAGTTTCCAAAAATTGAAGATACAGA
TCAAGAAATTGAATTTCAATTATTTGTGGAAACATATCAATTGGTCGAACCCTTGATAAA
GGAAAGAGATGCTGTGTATGAATCACTCACATATTCTTCTGAATTATATGTATCCGCGGG
ATTAATTTGGAAAAACAGTAGGGATATGCAAGAACAAACAATTTTTATCGGAAACATTC
CTCTAATGAATTCCCTGGGAACTTCTATAGTCAATGGAATATATAGAATTGTGATCAATC
AAATATTGCAAAGTCCCGGTATTTATTACCGATCAGAATTGGACCATAACGGAATTTCGG
TCTATACCGGCACCATAATATCAGATTGGGGAGGAAGATCAGAATTAGAAATTGATAGA
AAAGCAAGGATATGGGCTCGTGTAAGTAGGAAACAAAAAATATCTATTCTAGTTCTATC
ATCAGCTATGGGTTTGAATCTAAGAGAAATTCTAGAGAATGTTTGCTATCCTGAAATTTT
TTTGTCTTTTCTGAGTGATAAGGAGAGAAAAAAAAATTGGGTCAAAAGAAAATGCCATT
TTGGAGTTTTATCAACAATTTGCTTGTGTAGGTGGCGATCCGGTATTTTCTGAATCCTTA
TGTAAGGAATTACAAAAGAAATTCTTTCAACAAAGATGTGAATTAGGAAGGATTGGTC
GACGAAATATGAACCGAAGACTGAACCTTGATATACCCCAGAACAATACATTTTTGTTA
CCACGAGATATATTGGCAGCCGCCGATCATTTGATTGGGCTGAAATTTGGAATGGGTGC
ACTTGACGAT------ATGAATCATTTGAAAAATAAACGTATTCGTTCTGTAGCAGATCTTTT
ACAAGATCAATTCGGATTGGCTCTGGTTCGTTTAGAAAATGTGGTTCGGGGGACTATAT
GTGGAGCAATTCGG---CATAAATTG---ATACCGACACCTCAGAATTTGGTAACCTCAACT
CCA------------TTAACAACTACTTATGAATCCTTTTTCGGTTTACACCCATTATCTCAAGTT
TTGGATCGAACTAATCCATTGACACAAATAGTTCATGGGAGAAAATTAAGTTATTTGGG
CCCTGGAGGACTGACAGGGCGCACTGCTAGTTTTCGGATACGAGATATCCATCCTAGTC
ACTATGGACGTATTTGCCCAATTGACACATCTGAAGGAATCAATGTTGGACTTATTGGAT
CCTTAGCAATTCATGCGAGGATTGGTCATTGGGGA---TCTCTAGAAAGCCCTTTTTAT-----
----GAAATTTCTGAGAGGTCAACCGGG------GTACGGATGCTTTATTTATCACCAGGTAGA
GATGAATACTATATGGTAGCGGCAGGAAATTCTTTAGCCTTAAATCAGGATATTCAGGAA
GAACAGGTTGTTCCAGCTCGATACCGTCAAGAATTCTTGACTATTGCATGGGAACAGG

TTCATCTTCGAAGTATTTTTCCTTTTCAATATTTTTCTATTGGAGCTTCCCTCATTCCTTTT
ATCGAACATAATGATGCGAATCGAGCTTTAATGAGTTCTAATATGCAACGTCAAGCAGT
TCCTCTTTCTCGCTCCGAGAAATGCATTGTTGGAACTGGGTTGGAACGACAAGCAGCT
CTAGATTCGGGGGCTCTTGCTATAGCCGAACGCGAGGGAAGGGTCGTTTATACCAATAC
TGACAAGATTCTTTTAGCAGGTAATGGAGATATTCTAAGCATTCCATTAGTTATATATCAA
CGTTCCAATAAAAATACTTGTATGCATCAAAAACTCCAGGTTCCTCGGGGTAAATGCAT
TAAAAAGGGACAAATTTTAGCGGATGGTGCTGCTACGGTTGGTGGCGAACTTGCTTTG
GGGAAAAACGTATTAGTAGCTTATATGCCGTGGGAGGGTTACAATTCTGAAGATGCAGT
ACTTATTAGCGAGCGTTTGGTATATGAAGATATTTATACTTCTTTTCACATACGGAAATAT
GAAATTCAGACTCATGTGACAAGCCAAGGC---CCTGAAAAAGTAACTAATGAAATACC
GCATTTAGAAGCCCATTTACTCCGCAATTTAGATAAAAATGGAATTGTGATGCTGGGAT
CTTGGGTAGAGACAGGTGATATTTTAGTAGGTAAATTAACACCCCAGGTCGTGAAAGA
A---TCGTCGTATGCCCCGGAAGATAGATTGTTACGAGCTATACTTGGTATTCAGGTATCT
ACTTCAAAAGAAACTTGTCTAAAACTACCTATAGGTGGCAGGGGTCGGGTTATTGATGT
GAGGTGGATCCAGAAGAGGGGT---GGTTCTAGTTATAATCCCGAAACGATTCGTGTATAT
ATTTTACAGAAACGTGAAATCAAAGTAGGCGATAAAGTAGCTGGAAGACACGGAAATA
AAGGTATCATTTCCAAAATTTTGCCTAGACAAGATATGCCTTATTTACAAGATGGAAGAT
CCGTTGATATGGTCTTTAACCCATTAGGAGTACCTTCACGAATGAATGTAGGACAGATAT
TTGAATGTTCACTAGGGTTAGCAGGGAGTCTGCTAGACAGACATTATCGAATAGCACCT
TTTGATGAGAGATATGAACAAGAAGCTTCGAGAAAACTTGTGTTTTCTGAATTATATGA
AGCCAGTAAGCAAACAGCGAATCCATGGGTATTTGAACCCGAATATCCAGGAAAAAGC
AGAATATTTGATGGAAGGACGGGGAATCCTTTTGAACAACCCGTTATAATAGGAAAGC
CTTATATCTTGAAATTAATTCATCAAGTTGATGATAAAATCCATGGGCGCTCCAGTGGAC
ATTATGCGCTTGTTACACAACAACCCCTTAGAGGAAGAGCCAAACAGGGGGGACAGC
GGGTAGGAGAAATGGAGGTTTGGGCTCTAGAAGGGTTTGGGGTTGCTCATATTTTACA
AGAGATGCTTACTTATAAATCGGATCATATTAGAGCTCGCCAGGAAGTACTTGGTACTAC
GATCATTGGGGGAACAATACCTAATCCCGAAGATGCTCCAGAATCTTTTCGATTGCTCG
TTCGAGAACTACGATCTTTAGCTCTGGAACTGAATCATTTCCTTGTATCTGAGAAGAAC
TTCCAGATTAATAGGAAGGAAGCTATGAATAATAATTTTTCTTCTATGATCGATCGATATA
AACATCAACAGCTCCGAATTGGATCAGTTTCTCCTCAACAAATAAGTGCTTGGGCCACT
AAAATCCTGCCTAATGGAGAGATAGTTGGAGAGGTAACAAAACCCTATACTTTTCATTA
CAAAACTAACAAACCGGAAAAAGATGGATTATTTGTGAAAGAATTTTTGGTCCTATAA
AAAGCGGAATTTGTGCTTGTGGAAATTATCGAGTAATCGGAGATGAAAAAGAAGACCC
GAAATTTTGTGAACAATGCGGGGTCGAATTTGTTGATTCTCGGATACGAAGGTATCAAA
TGGGCTATATCAAACTCGCATGCCCAGTAACCCATGTGTGGTATTTAAAACGTCTTCCTA
GTTATATTGCGAATCTTTTAGATAAACCTCTTAAAGAATTAGAAGGCCTAGTATACTGCG
AT------TTTTCTTTTGCTAGGCCC---ATAACTAAAAAGCCCACTTTCTTACGATTACGAGG
TTTATTCGAATATGAAATCCAATCTTGGAAATACAGCATCCCACTTTTTTTTACTACCCA
GGGTTTCGATACATTTCGCAATCGAGAAATCTCTACTGGCGCGGGTGCTATCCGAGAAC
AATTAGCCGATCTAGATTTACGAATTATTATAGAGAATTCGTTGGTAGAATGGGAAGAAT
TGGGGGAAGAA---GGGCACACAGGGAATGAATGGGAAGATCGAAAAGTTGGAAGAAG
AAAGGACTTTTTGGTTAGACGCGTGGAATTGGCTAAGCATTTTATTCGAACAAATATAG
AGCCAGAATGGATGGTTTTGTGTCTATTACCAGTTCTTCCTCCTGAGTTGAGACCGATC
ATTCAGATAGATGGGGGTAAACTAATGAGCTCAGATATTAATGAACTCTATAGAAGAGT

TATCTATCGGAACAATACCCTTACCGATCTATTAACAACAAGTAGATCTACGCCAGGAG
AATTAGTAATGTGTCAGGAGAAATTAGTACAAGAAGCCGTGGATACACTTCTTGATAAT
GGAATCCGGGGACAACCAATGAGGGACGGTCATAATAAAGTTTACAAGTCATTTTCTG
ATGTAATTGAAGGCAAAGAGGGAAGATTTCGTGAGACTCTGCTTGGCAAACGAGTCGA
TTATTCAGGACGTTCTGTCATTGTCGTGGGTCCTTCACTTTCATTACATCGATGTGGATT
GCCTCGTGAAATAGCAATAGAACTTTTCCAGACATTTGTAATTCGTGGTCTAATTAGAC
AACATCTTGCTTCGAACATAGGAGTTGCTAAGAGTAAAATTCGAGAAAAAGAGCCGAT
TGTTTGGGAAATCCTTCAAGAAGTTATGCAGGGACATCCTGTATTGCTGAATAGAGCAC
CCACTTTGCATAGATTAGGCATACAGGCATTCCAGCCCGTTTTAGTGGAGGGGCGCGCT
ATTTGTTTACATCCATTAGTTTGCAAGGGATTCAATGCTGATTTTGATGGAGATCAAATG
GCTGTTCATGTACCTTTATCCTTGGAGGCTCAAGTAGAGGCCCGTTTACTTATGTTTTCT
CATATGAATCTTTTGTCTCCGGCTATTGGGGATCCCATTTCCGTACCAACGCAAGATATG
CTTATTGGACTCTATGTATTAACGAGCGGAAATCATCGAGGTATTTGTGTAAATAGATAT
AATCCATGTAATCGC---------AGAAACTATCAAAATCAAAAAAGAAGTGACAATAGTTAT
AAGTATACG---------GAACCGTTTTTTTCTAATTCCTATGATGCAATTGGGGCTTATCGGC
AGAAACGAATCAATTTAGATAGTCCTTTGTGGCTCCGGTGGCGACTAGATCAACGC---G
TTATTGCTTCA------AGAGAAACTCCCATCGAAGTTCACTATGAATCTTTAGGTACTTTTT
ATGAGATTTATGGACACTATCTAATAGTAAGAAGTCTAAAAAAACAAATCCTTTTTATAT
ACATTCGAACCACTGTTGGTCATATTGCTCTTTATCGAGAAATCGAAGAAGCTATACAG
GGGTTTTCTCGGGCCTATTCATCTGGTACC------------------------------------ATGGCAGAACG
GGCCAATCTGGTCTTTCACAATAAAGCGATAAACGGAACTGCCATGAAACGACTTATTA
GTAGATTAATAGATCACTTCGGAATGGCATATACATCACATATCCTGGATCAAGTAAAAA
CTCTGGGGTTCCAACAAGCTACTGCTACATCCATTTCATTAGGAATTGATGATCTTTTAA
CAATACCTTCTAAGGGATGGCTAGTTCAAGATGCTGAACAACAAAGTTTGATTTTGGAA
AAACACCATCATTATGGGAATGTACACGCGGTAGAAAAATTACGTCAATCCATTGAAAT
ATGGTATGCTACAAGTGAATATTTGCGACAAGAAATGAATCCTAATTTTAGGATGACTG
ACCCTTTTAATCCAGTTCATATAATGTCTTTCTCGGGAGCTAGAGGAAATGCATCTCAGG
TACATCAATTAGTAGGTATGAGAGGATTAATGTCAGATCCTCAAGGACAAATGATTGATT
TACCTATTCAAAGCAATTTACGCGAAGGACTCTCTTTAACAGAATACATCATTTCTTGCT
ACGGAGCCCGTAAAGGAGTTGTGGATACTGCTGTACGAACATCAGACGCTGGATATCT
CACTCGCAGACTTGTTGAAGTAGTTCAACACATTGTTGTACGTCGAACGGATTGTGGC
ACCGCCCGGGGTATTTCTGTGAGTCCTCGGAATGGGATGATGCCG---GAAAGGATTTTT
ATCCAAACATTAATTGGTCGTGTATTAGCAGATGATATATACATGGGTCCGCGATGTATTG
CCACTAGAAATCAAGACATTGGCATTGGACTTGTAAATCGATTCATAACC------------------
TTTCGGGCACAACCAATCTCTATTCGAACTCCCTTTACTTGTAGGAGTACATCTTGGATT
TGTCGATTATGTTATGGCCGGAGTCCTACTCATGGCGACCTGGTTGAATTGGGGGAAGC
TGTAGGTATTATTGCAGGTCAATCGATTGGAGAACCGGGTACTCAATTAACATTAAGAA
CTTTTCATACCGGAGGAGTATTCACGGGGGGTACTGCAGAACATGTGCGAGCCCCATCT
AATGGAAAAATCAAATTCAATGAGGATTTGGTTCATCCGACACGTACACGTCATGGGCA
TCCCGCCTTTCTATGTTCTATAGACTTGTATGTAACTATTGAGAGTGAAGATATTCTACAT
AATGTGAATATTCCACCCAAAAGTTTGCTTTTAGTTCAAAACGATCAATATGTAGAATC
AGAACAAGTAATTGCTGAGATTCGCGCAGGAATATCCACT---TTGAATTTTAAAGAGAA
GGTTCGAAAACATATTTATTCTGATTCAGACGGAGAA---ATGCACTGGAGTACCGATGT
CTATCATGCACCCGAATTTACATACGGTAATGTTCATCTATTACCAAAAACAAGCCATTT

ATGGATATTATTAGGAAGGCCGTGCAGG------TCCAGTCTAGTCTACCTTTCGATCCACA
AGGATCAGGATCAAATGAATGCGCAT------TTTCTTTCTGGCAAGCGAAGATATACTTCT
AACCTCTCA------GTAACCAACGATCAG---GCGAGGCAGAAATTATTTAGTTCGGATTTT
TCTGGTAAA---GAAGATAGGATTCCTGATTAT---TCAGACCTTAATCGAATTATATGTGCT
GGTCAGTATAATCTCGTATATTCG---CCTATTCTCCACGAGAATTCT------GATTTATTGTC
AAAAAGGCGAAGAAATAAATTCATCATTCCACTACACTCGATTCAAGAACTCGAGAAT
GAACTAATGCCCTGTTCAGGTATCTCGATTGAAATCCCC------GTAAATGGTATTTTCCGT
CGAAATAGTATTCTTGCTTATTTCGATGATCCTCGATACAGAAGAAAGAGTTCGGGCATT
ATTAAATATGGGACTGTAGAAACGCATTCAGTCATCAAAAAAGAGGAT------TTGCTTGA
GTATCGAGGA---GTCAAGGAATTTAGGCCAAAATACATGAAAGTAGATCGATTTTTTTC
ATTCCTGAAGAGGTGCATATCTTGCCCGGATCTTCTTCCATAATGGTACGGAACAATAGT
ATCGTTGGGGTAGATACACAAATCACCTTAAATCTAAGAAGCCGAGTCGGTGGGTTGG
TCCGGGTGGAGAGAAAAAAAAAAACGAATTGAACTTAAAATCTTTTCTGGAGATATCCA
TTTTCCTGGAGAGACAGATAAGATATCCCGACATACCGGCGTTTTGATACCA---CCAGG
A---ACAGGAAAAAGAAATTCCAAGGAATCCAAAAAA------GTGAAAAATTGGATCTATG
TCCAACGG------ATTACACCTAGCAAG------AAAAAGTTTTTTGTTTTAGTTCGACCTGTC
GTCACATATGAAATAACGGACGGTATAAATTTAGCAACCCTTTTTCCACCGGATCCATTG
CAGGAAAGGGATAATGTGCAACTTCGAATTGTCAATTATATCCTTTATGGAAATGGCAA
ACCGATTCGAGGAATTTCTGACACAAGTATTCAATTAGTTCGGACTTGTTTAGTATTAAA
TTGGAACCAAGACAAAAAA---------AGTTCTTCTTGCGAAGAAGCCCGTGCTTCTTTTG
TTGAAATAAGGACAAATGGTTTGATTCGACATTTCCTAAGAATCAACTTAGTGAAATCC
CCTATTTCGTATATCGGAAAAAGGAATGATCCGTCGGGGTCAGGATTGCTCTCTGAT---A
ATGGATCAGATTGTACCAATATCAACCCCTTTTCTTCCATTTATTCCTAT---------TCCAAG
GCAAAAATTCAACAATCTATTAACCAACCTCAAGGAACTATTCATACGTTGTTAAATAG
A---------AATAAGGAATGTCAGTCGTTGATAATTTTGTCAGCAGCCAATTGTTCTCGAATG
GGGCCA------TTCAAGGATGTAAAATATCACAGTGTG---------ATAAAAAAATCA---CCCCT
AATTCCAATTAGGAATTCATTGGGCCCTTTAGGAACA------TCCCTTCCAATTGAGAATT
TTTATTCA------TCTTACCATTTA------ATAACTCATAATCAGATCTTA---GTAACTAACTATT
TGCAACTTGACAAT---------TTAAAACAGACTTTTCAAGTGATTAAATTTAAA---TATTATT
TAATGGATGAAAAT------GGAAAAATTTTTAATCCCGATCCGTGTCGTAACATTATTTTAA
ATCCATTCAATTTGAATTGGTATTTTCTCCATCACAATTATTGTGAAGAGACATCT---AAA
ATAATTAGTCTTGGACAGTTTATTTGTGAAAATGTATGTATAGCCAAAAATGGACCGCCC
---CTCAAA------TCGGGTCAAGTTATACTTGTTCAA---GTTGATTCGATAGTGATACGATC
AGCTAAGCCTTATTTGGCCACCCCCGGAGCAACTGTTCATGGCCATTATGGGGAAACCC
TTTACGAAGGAGATACATTAGTTACATTTATATATGAAAAATCGAGATCTGGTGATATAA
CACAAGGTCTTCCAAAAGTAGAACAGGTGTTAGAAGTGCGTTCGGTTGATTCAATATC
CATGAATCTAGAAAAGAGGATTGAGGGTTGGAACAAATGTATAACAAGAATTCTTGGA
ATTCCTTGGGGATTCTTGATTGGTGCTGAGCTAACTATAGCGCAAAGCCGAATCTCTTT
GGTTAATAAAATCCAACAGGTTTATCGCTCCCAGGGGGTGCAGATTCATAATAGGCATC
TAGAAATTATTGTACGTCAAATAACATCAAAAGTGTTGGTTTCAGAA---------GATGGAA
TGTCTAATGTTTTTTCACCCGGAGAACTTATTGGATTGTTGCGAGCAGAACGAATGGGG
CGCGCTTTGGAAGAAGCGATCTGTTACCGAGTCGTCTTATTGGGAATAACAAGAGCAT
CTCTCAATACTCAAAGTTTCATATCTGAAGCGAGTTTTCAAGAAACTGCTCGAGTTTTA
GCAAAAGCGGCTCTCCGGGGTCGTATCGATTGGTTGAAAGGCCTGAAAGAGAACGTT

GTTTTGGGGGGGGTGATACCCGTTGGTACCGGATTCAAGGGATTA------GTGCACCCTTC
AAAACAACATAACAACATTCCT------TTGGAAACAAAAAAAAAGAATCTATTCGAGGGG
GAGATGAGA------GATATTTTGTTCCACCACAAAAAATTATTTGATTCTTGTCTTTCAAA
AAATTTCCACGATATACCCGAACAATCGTTTATAGGA------------------TTTAATGATTCCAT
GACAAGAAGATATTGGAACATAAATTTGGAAGAGATGATGGAGGCAGGAGTTCATTTT
GGTCATGGTACTAGGAAATGGAATCCTAAAATGGCGCCTTATATTTCTGCAAAGCGTAA
GGGTATTCATATTACAAATCTTACTAGAACTGCTCGTTTTTTATCAGAAGCTTGTGATTTA
GTTTTTGACGCAGCAAGTAGGGGAAAACAATTCTTAATTGTTGGTACCAAAAATAAAG
CAGCTGATTCAGTAGAGTGGGCTGCAATAAGGGCCCGGTGTCATTATGTTAATAAAAAA
TGGCTTGGCGGTATGTTAACGAATTGGTCCACTACCGAAACAAGACTTCATAAGTTCAG
GGACTTGAGAATGGAACAAAAAACAGGGAGACTCAACCGTCTCCCGAAAAGAGATGC
TGCTATGTTGAAAAGACAATTATCTCGCTTGCAAACATATCTGGGCGGGATTAAATATAT
GACAGGGGTACCCGATATTGTAATCATCGTTGATCAGCACGAAGAATATACGGCCCTGC
GAGAGTGTATTACTTTAGGAATTCCAACAATTTGTTTAACCGATACAAATTGTGACCCC
GATCTCGCAGATATTTCAATTCCAGCGAATGATGACGCCATATCCTCAATCCGATTAATT
CTTAACAAATTAGTATTCGCAATTTGTGAGGGTCGTTCT---AGCTATATAAGAAATCCTAT
GGGACAAAAAATAAATCCACTTGGTTTCAGACTGGGTACAACCCAAGGTCATCATTCC
CTTTGGTTTTCACAACCAAAAAATTATTCTGAAGGTTTACAAGAAGATCAAAAAATAA
GAGATTGTATCAAGAATTATGTACAAAAGAATATGAGAACGTCCTCTGGC---GTCGAGG
GAATTGCACGTATAGAGATTCAAAAAAGAATCGATCTGATCCAGGTCATAATCTTTATG
GGATTCCCAAAATTATTAATAGAAAGTCGACCGCGC------------GGAATCGAAGAATTACA
AACGACCTTACAAAAAGAATTTCATTGTGTAAACCGAAAACTGAACATTGCTGTCACA
AGAATTGCAAAACCTTATGGAAACCCTAATATTCTTGCAGAATTTATAGCTGGACAATTA
AAGAATAGAGTTTCCTTTCGAAAAGCAATGAAAAAGGCTATTGAATTAACAGAACAAG
CAGATACAAAAGGAATTCAAATACAAATTGCGGGGCGTATCGACGGAAAAGAAATTGC
ACGTGTCGAATGGATCAGAGAAGGTAGGGTTCCCCTACAAACGATTCGAGCGAAAATT
GATTATTGCTCTTATACAGTTCGAACTATCTATGGAGTATTGGGCATCAAAATTTGGATAT
TTCTAGACGAGGAAATGTCGCGTTACCGAGGACCTCGTTTCAAAAAAATACGCCGCCT
GGGGGCTTTACCAGGACTAACTAATAAAAAGCCTAGGAACGGAAGTGATCTTAGAAAC
CAATCGCGTTCCGGGAAA---AAATCTCAATATCGTATTCGTCTAGAAGAAAAACAAAAA
TTGCGTTTTCATTATGGTCTTACAGAACGACAATTACTTAAATACGTTCGTATCGCCAGA
AAAGCCAAGGGGTCAACAGGTCAAGTTTTACTACAATTACTTGAAATGCGTTTGGATA
ACATCCTTTTTCGATTGGGTATGGCTTCAACTATTCCCGCAGCCCGTCAATTAGTTAACC
ATCGACATATTTTAGTTAATGGTCGTATAGTAGATATACCGAGTTATCGCTGCAAACCCC
GAGATATTATTACGGCAAAGGATGAACAAAAATCCAGAGCTCTGATTCAAATTTCTCTC
GATTCATCCCCTCAT---GAGGAATTACCAAACCATTTGACCCTTCACCCATTCCAA------T
ATAAAGGATTAGTCAATCAAATAATAGATAGTAAATGGGTCGGTTTGAAAATAAATGAA
TTGCTAGTCGTAGAATATTATTCTCGTCAGACTATGTCACGTCGAGGTACTGCAGAAAA
AAAAACAGCAAAATCCGATCCAATTTATCGTAATCGATTAGTTAACATGTTGGTTAACC
GTATTCTGAAACACGGAAAAAAAATCATTGGCTTATCAAATTATCTATCGAGCCGTGAAA
AAGATTCAACAAAAGACAGAAACAAATCCACTATCCGTTTTACGTCAAGCAATACGTG
GAGTAACTCCCGATATAACAGTAAAAGCAAGACGTGTAGGTGGATCGACTCATCAAGT
TCCCATTGAAATAGGATCCACACAAGGAAAAGCACTTGCCATTCGTTGGTTATTAGCGG
CATCCCGAAAACGTCCGGGTCGAAATATGGCTTTCAAATTAAGTTCCGAATTAGTGGAT

GCTGCCAAAGGGAGTGGCGATGCCATACGCAAAAAGGAAGAGACTCATAGAATGGCA
GAGGCAAATAGAGCTTTTGCACATTTTCGTATGGGTAGGGACACTATTGCTGAGATAAT
AACTTCTATACGAAATGCTGATATGGATCGAAAAAGGGTGGTTCGAATAGCATCTACTA
ATATTACCGAAAATATTGTTCAAATACTTTTACGAGAAGGTTTTATCGAAAACGTGAGA
AAACATCGAGAAAAAAACAAATATTTTTTGGTTTTAACCCTGCGACATAGAAGG------A
ATAGGAAAAGACCCTAT---------AGAAATATTTTAAATTTAAAACGAATCAGTCGACCTG
GTCTACGAATCTATTCTAATTATCAACGAATTCCGCGAATTTTAGGTGGAATGGGGATTG
TAATTCTTTCTACTTCTCGAGGTATAATGACAGACCGAGAGGCTCGACTAGAAGGAATA
GGTGGAGAGATTTTGTGTTATATCTGGATGGCAAAAGCTATACCGAAAATTAGTTCGCG
TAGGAATGGACGTATTGGTTCACGTAAGGGTGCACGTAGAATACCAAAGGGAGTTATT
CATGTTCAAGCAAGTTTCAATAATACCATTGTCACTGTTACAGATGTACGGGGTCGAGT
AGTTTCTTGGTCCTCCGCCGGTACTTCTGGATTCAAAGGTACGAGAAGAGGAACACCG
TTTGCTGCTCAAACCGCAGCAGCAAACGCTATCCGTACAGTAGTGGATCAAGGTATGC
AACGAGCAGAAGTCATGATAAAAGGTCCCGGTCTCGGAAGAGATGCAGCATTACGAG
CTATTCGTCGAAGTGGTATACTATTAACTTTCGTACGGGATGTAACTCCTATGCCACATA
ATGGCTGTAGACCTCCGAAAAAAAGACGTGTGATGCCAACTATTAAACAACTTATTAG
AAATACAAGACAGCCAATCAGAAATGTCACGAAATCCCCCGCTCTTCGGGGCTGTCCT
CAGCGTCGAGGAACATGTACTAGGGTGTATACTATCACCCCAAAAAAACCAAACTCTG
CCTTACGTAAAGTTGCCAGAGTACGATTAACCTCTGGATTTGAAATCACTGCTTATATAC
CCGGTATTGGCCATAATTTACAAGAACATTCTGTAGTCTTAGTAAGAGGGGGAAGGGTT
AAGGATTTACCCGGTGTGAGATATCACATTGTTCGAGGAACCCTAGATGCTGTCGGAGT
AAAGGATCGTCAACAAGGGCGTTCTAAATATGGGGTAAAAAAGCCAAAAATGGCAAG
GAAAAGTTTGATTCAGAGGGAGAAGAAGAGGCAAAAATTGGAACAGAAATATCATTC
GATTCGTCGATCCTCAAAGAAAGAAATAAGC---------AAGGTTCCGTCGTTGAGTGACA
AATGGGAAATTTATGGAAAGTTACAATCCCCACCACGGAATAGTGCACCTACACGCCTT
CATCGACGTTGTTTTTTGACCGGAAGGCCGAGAGCTAACTATCGAGACTTTGGACTATC
CGGACACATACTTCGTGAAATGGTTCATGCATGTTTGTTGCCAGGAGCAACAAGATCA
AGTTGGATGGTAAAAAATTCTGTCATTTCAGTTATTTCTCAAGAA------GAAAAG---------
AGAGGATCTGTTGAATTTCAAGTATTCAATTTCACCAATAAGATACGGAGACTTACTTC
ACATTTAGAATTGCACAAAAAAGACTATTTATCTCAGAGAGGTTTGAAGAAAATTTTGG
GAAAACGTCAACGACTCCTAGCTTATTTGTCAAAAAAAAATAGAGTACGTTATAAAGA
ATTAATTAATCAGTTGGACATTCGAGAGACAAAAACTCGT------ATGGTAAAACTTCGTT
TGAAACGATGTGGTAGAAAGCAACGAGCCGTCTATCGAATCGTTGCAATTGATGTTCG
ATCCCGAAGAGAAGGAAAAGATCTTCGGAAAGTGGGTTTTTATGATCCGATAAAGAAT
CAAACTTATTTAAATGTTCCTGCTATTTTATATTTCCTTGAAAAAGGGGCTCAACCTACA
GGAACTGTTCAGGATATTTTAAAGAAGGCGGAGGTTTTTAAGGAACTTCGTCCTAATCA
ATCC-----------ATGGATAAATCCAAGCGACCTTTTCTTAAATTCAAGCGATCTTTTCGTAG
GCGTTTGCCCCCGATTCAATCGGGGGATCGAATTGATTATAGAAACATGAGTTTAATTA
GTCGATTTATTAGTGAACAAGGAAAAATATTATCAAGACGAGTGAATAGATTGACCTTG
AAACAACAACGATTAATTACTCTTGCTATAAAACAAGCTCGTATTTTATCTTTGTTACCC
TTTCTCAATAATGAGAAA------CAATTTGAAAGAACCGAGTCGACCGCTAGAACTACTG
GTTTTAAAGCCCGAAATAAAGTGACACGTTCACTAAAAAAAAATCCCTTTGTAGCCAA
TCATTTATTAAAAAAAAATTGATAAGCTTAACACAAAAGCAGAAAAAGAAATAATAGTAA
CTTGGTCCCGGGCATCTACCATTATACCCACAATGATCGGTCATACGATTGCTATCCATA

ATGGAAAAGAGCATTTGCCTATTTATATAACGGATAGTATGGTAGGCCACAAATTGGGA
GAATTTGCACCTACATTAAATTTTAGAGGACATGCAAAAAGCGATAATAGATCTCGTCG
TATGATTTTTCAATCTTTTCTACTAGGTAATCTAGTATCCTTATGCATGAAGATAATCAATT
CGGTCGTTGTGGTCGGACTCTATTATGGATTTCTGACCACATTCTCCATAGGGCCCTCTT
ATCTCTTCCTTCTCCGAGCTCTGGTTATGGAAGAAGGA---------ACCGAGAAGAAGGTAT
CAGCAACAACTGGTTTTATTACGGGGCAGCTCATGATGTTCATATCGATCTATTATGCGC
CTCTGCATCTAGCATTGGGTAGACCTCATACAATAACTGTCCTAGCTCTACCATATCTTTT
GTTTCATTTCTTCTGGAACAATCACAAACACTTTTTTGATTATGGATCTACTACCAGAAA
TTCAATGCGTAATCTCAGCATTCAATGTGTATTCCTGAATAATCTCATTTTTCAATTATTC
AACCATTTCATTTTACCAAGTTCAATGTTAGCCAGATTAGTCAACATTTATCTCTTTCGA
TGCAACAGCAAGATCTTATTTGTAACAAGTGGTTTTGTTGGTTGGTTAATTGGTCACAT
TTTATTCATGAAATGGCTTGGATTGGTATTAGTCTGGATACGGCAAAATCATTCTATTAGA
TCGTACATTCGATCTAATAAGTACCTTGTGTTAGAATTGAGAAATTCTATGGCTCGGATC
TTTAGTATTCTCTTATTTATTACCTGTGTCTACTATTTAGGCAGAATACCCTCACCCATTC
TTACTAAGAAACTAAAAGAAACTGAAGAAGATCAGCCTCTTGTGAGTCTTCTTTTCGA
TTATAATCGATGGAATCGACCATTTCGCTACATAAAGAATAATCGATTTGAACAGGCCAT
AAGAACGGAAATGTCACAATATTTTTTTAACACATGTAAAAGTGATGGAAAACAAAGA
ATATCTTTTACATACCCGCCAAGTTTATCAACTTTTTGGAAAATGATAAAAAGAAGGATA
CCCCTATTGTCACTCCAAAAAACACTCCCTAATGAACTGGACAATCAGTGGATTTCTAC
CAACAAAGAAAAAAGTAAT---CTGAATAAGGAATTTTTAAATCGACTTGAAGTTCTAGA
CAAGGAATCTTTTTCTCTCGATATACTTGAAACAAGGACTAGATTGTGTAATGATGATAC
TAAAAAAGAATACGTGCCTAAAATGTATGATCCTTTGTTAAATGGACCATATCGAGGAA
CAATCAAAAAAAAGTTTTCACCTAAAGAGAGGGTTAGAATAAATAGGATTCATACTATC
TTTTACCAAGAATTGGAACAAAAAGCTCCTAATGGGACATCGATTGGAATAAAAGAAA
TCAGTAAAAAAATACCTCGCTGGTCACATAAATTAATCGAATTGGAACAACAATCG---G
GTCAAATTCGTTCAAGAAAAGCCAAACGTGTAGTGATTTTTACTGCCGCTTTAATACGC
TATTCGCAACAACCAGATTTTCGGCGAGGTATAATCAAAGGCTCTATGCGGGCTCAAAG
GCGCAAAACAGTTATTTGGAAACTCTTTCAAGCAAATGTGCATTCCCCCCTTTTTCTTG
ACAGAATAACCCCCCCCTTTCTTTTTTCTTTTGATATC------TCTGGACTGATTAAACCAA
TTTTTCGAAATAGAAAGGAAAAAGCACGGATAGAGATAGCAGAAGCCTGGGATACCAT
TCCTTTTGCGCAAATAATAAGAGGTTACATGTTAATAACTCAATCAATTCTTCGAAAATA
TATTGTATTACCTTCATTGATAATAGCCAAAAATCTCGGGCGTATGTTAGTCTTGCAACTT
CCTGAATGGTCTGAAGATTTGCAGGAATGGAATAGAGAAATGCATATTAAATGTACTTAT
AATGGTGTTCAATTATCAGAAACAGAATTTCCAAAAAATTGGTTGAAAGATGGCATTCA
GATCAAAATATTATTTCCTTTTTGTCTGAAACCTTGGCATATATCTAAAAAAAAACAAAA
AGATGATTTTTGTTTTTTAACAGTTTGGGGAATGGAAGCTGAACTTCCCTTTGGTTCTC
CCCGAAAACGCCCTTCTTTTTTTGAGCCCATTTTTAAGGAACTCGAAAAAAAAATTGG
AAAATTCAAAAAGAAATATTTTATAACT---CTAAAAGTTTTCAAAGGAAAAATAAAATTA
TTTCGAAGAATTTCAAAAGAAACCAAAAAATGGCTTATCAAAAGTAGTCTA------TTTAT
AAAAAAAATGAAAAAAGAACTTTCAAAAGTAAATGATTCTCTAATGAGTAATCAGATA
ATTAACGAATCATTTAGTCAAATTGAATCTGGAAATTGGCCAAATTCTTCACTGATAGA
AAGCAAAATGAAGGATTTGACTGATAGAACAAGTACAATCAAAAATCAAATAGAAAGA
ATTACAAAAGAGAAAAAGAAAAGTCCTAATAAA---ACAAATAATATTAAAAAATTCGAA
TCGCCAAAAAAGATTTTCCAAATATTAAAAAGAAGAAATACTCGATTAATATGGAAATT

CCATTATTTTCTAAAATTATTCATTCAAAGATTATACATCGATCTATTTTTATCTATCATTAA
TATTCCCAGAATTAATACACAACTCTTT------TTTATTTCGTTTGATTTATCCTACTTATCAC
AAGCATATGTATTTTACAAATTATCACAACCCCAAGTTATTAATTTGTCTAAATTAAGATC
TGTTCTTCAATATACGTCTTTTTTCCTTAAGACTAAAATAAAGGACTATTTTACACTAGG
AATATTTCATTCTGAATTAAAACATAAGAAACTTCAGAGTTATAGAATCAATCAATGGAA
AAACTGGTTAAGACGGCATTATCAATACGATTTATCTCAGATTAGATGGTCTAGATTAAT
GCCACAAAAATGGCGAAATAGGGTTAATCAAGGTTGTATGGCTCAAAATCGAAACAAA
TGGAATTCATATGAAAAAGACCAA------TTAATTCATTACAAAAAAGAA---AATTTTCAA
AAATGTTATAGATATGATCTTTTAGCATATAAATCGATTAATGGCAATATTCTATATCTCGA
TAGAAAATATTTTGATTGGAAAATTATCCATTTTTCTCTTAGATTTTTTGATTGGATGGGA
ATGAATGAAAAAATATTAAATCGTTGGTTCTTCCCAGAATTTGTACCACTTTATAATGTAT
ATAAAATAAAACCGTGGATTATACCAAGCAAATTACTTCTTTTAAATTACAAAAGCAAC
ACGGAAGCAGAACTAGATTTGTTCTTGAAACGTTATTTGCTTTTTCAATTGAGATGGAA
CGATGCTTTGAATCAAAGAATGATCGAGAATATCAAGGTATATTGTCTCCTGCTTCGACT
GATAAATCCAAGCAAAATTGCTATATCGTCAATTCAAAGGAGAGAAATGAGTTTGGATA
TAATGCTGATTCAGAAGAATTTAACTCTTACAGAATTGATGAAG---GGGATATTAATTAT
CGAACCTATTCGGTTGTCTGTAAAAAAATAATGGACAATTTATTATGTATCAAACCATAGG
TATTTCATTGGTTCATAAAAGTAAACACAATCATTTTGATTTGCTTGTTCCTGAAAATATT
TTATGGTCTAGACGTCGTAGAGAATTGAGAATTCGAAGTTTGTTTAGATTTCTTTGGCCT
AATTATCGATTAGAAGATTTAGCTTGTATGAATCGTTATTGGTTTGATACCAATAATGGTA
GTCGTTTCAGTATCTTAAGGATACATATGTATCCAATGAGAGGACATCAATTCAAATCCT
GGATTTTCGAATTGAGAGAGATATTGAGAGAGATCAAGAATTCTCACCATTTCTTAGAT
TCATGGACCCAATTCAATTCAGCGGGATCCTTCATTCACATTTTTTTCCACCAAGAACG
TTTTCTAAAACTCTTTGACCCCCGAATTTGGAGTATCCTACTTTCA---CGCAATTCA------
CAGGGTTCAACAAGCAATCGATATTTCACG---------ATCAAGGGT---GTAATACTCTTTGT
AGTAGCGGTCCTTATATATCGT------------ATTAACAATCGAAATATGGTCGAAAGAAAAA
ATCTCTATTTGATAGGGCTTCTTCCT---------------ATACCTATGAATTCCATTGGACCCAGA
AATGATACATTGGAAGAATCCGTT---GGGTCTTCCAATATCAATAGGTTGATTGTTTCG---
CTCCTGTATCTTCCCAAAGGAAAAAAGATCTCTGAGAGTTGTTTCCTGAATCCGAAAG
AGAGTACTTGGGTTCTCCCAATAACT-----------AAAAAGTGTAGCATGCCTGAATCTAAC
TGGGGTTCGCGTTGGTGGAGGAACTGGATCGGAAAAAAGAGG------GATTCTAGTTGTA
AGATATCTAAT---------GAAACCGTCGCTGGAATTGAGATCTTATTCAAAGAGAAAGATCT
CAAA---TATCTGGAGTTTCTTTTTGTATATTATATGGAT------GATCCGATCCGCAAGGACC
ATGATTGGGAATTGTTTGATCGTCTTTCTCTGAGGAAGAGTCGAAATAGAATCAACTTG
------AATTCGGGACCGCTATTCGAAATCTTAGTGAAACACTGGATTTCTTATCTCATGTCT
GCTTTTCGTGAAAAAATACCAATTGAAGTGGAGGGT---TTCTTCAAACAACAAGGG---G
CTGGGTCAACTATTCAATCAAATGATATTGAGCATGTTTCCCAT------CTCTTCTCGAGAA
ACAAGTGGGCTATTTCTTTGCAAAACTGTGCTCAATTTCATATGTGGCAATTCCGCCAA
GATCTCTTCGTT---------AGTTGGGGGAAG---AATCCGCCCGAATCGGATTTTTTGAGGAA
C---GTATCGAGAGAGAATTGGATTTGGTTAGACAATGTGTGGTTGGTAAACAAGGATCG
G---------TTTTTTAGC------AAGGTACAGAATGTATCGTCAAATATTCAATATGATTCC--------
-ACAAGA------TCTAGTTTCGTTCAAGTAACGGATTCTAGCCAACTGAAAGGATCTTCT---
GATCAATCCAGAGATCATTTGGATTCCATTAGT------------AATGAGGATTCGGAA-----------
----------------------------TATCACACATTGATTAATCAAAGAGAGATTCAACAACGAAAAGA

AAGATCGATTCTT---TGGGAT------CCTTCCTTTCTTCAAACGGAACGAAAAGAGATAGA
ATCAGGC------CGATTCCCGAAA------TGCCTTTCTGGATATTCC------TCAATGTCCCGGC
TATTCACG---GAACGTGAGAAGCAGATGATTAATCATCTGTTTCCGGAAGAAATCGAAG
AATTTCTT------GGGAATCCTACAAGATCCGTTCGTTCTTTTTTCTCTGATAGATGG------T
CAGAACTTCAT---CTGGGTTCGAATCCTACTGAGAGGTCCACTAGGGATCAG------------A
AATTGTTGAAGAAACAACAAGATCTTTCT---TTTGTCCCTTCCAAGCGATCGGAA---------
---AATAAAGAAATGGTTAATATATTCAAGATAATTACGTATTTACAAAAT------ACCGTCTC
AATTCATCCTATTTCATCAGATCCGGGATGTGATATGGTTCCG---AAGGATGAACCGGAT-
--ATGGACAGTTCCAATAAGATTTCATTCTTGAACAAAAATCCATTTTTTGATTTA------TT
TCATCTATTCCATGACCGGAACAGGGGAGGATACACGTTA------CACTACGATTTTGAAT
CAGAA------GAGAGATTTCAAGAAATGGCAGATCTATTCACTCTATCAATAACCGAGCCG
---GATCTGGTGTATCATAAGGGATTTGCCTTTTCTATTGATTCCTGCGGATTGGATCAAAA
ACAA------TTCTTGAATGAGGCCAGGGATGAA---------TCGAAAAAGAAATCT---------TTA
TTGGTTCTACCTCCTATTTTTTAT------GAAGAGAATGAATCTTTTTCTCGAAGGATCAGA
AAAAAATGGGTCCGG---ATCTCCTGCGGGAATGATTTGGAAGATCCAAAACCAAAAAT
AGTGGTATTTGCTAGCAACAACATAATGGAGGCAGTCACTCAATATAGATTGATCCGAA
ATCTGATTCAAATCCAATATAGTACCTATGGG------TACATAAGAAATGTATTGAATCGATT
CTTTTTAATGAAT---------AGATCCGATCGCAACTTCGAATATGGA---------ATTCAAAGGG
ATCAAATAGGAAAGGATACTCTGAATCATAGAACTATAATGAAATATACGATCAACCAAT
ATTTATCGAATTTGAAAAAG---AGTCAGAAGAAATGGTTCGAGCCTCTT------ATTTTGAT
TTCTCGAACCGAGAGATCCATGAATCGGGATCCTGATGCATATAGATACAAATGGTCCA
ATGGGAGCAAGAATTTCCAGGAACATTTGGAACAGTCCGTTTCGGAGCAGAAGAGCC
GT---------TTTCAAGTAGTGTTCGATCGATTACGTATTAATCAATATTCGATTGATTGGTCT
GAGGTTATCGACAAAAAAGATTTGTCTAAGCCACTTCGTTTCTTTTTGTCCAAG----------
-----------------TCACTTCTTTTTTTGTCC--------------------AAGTTGCTTTTCTTTTTGTCTAA
C---TCACTTCCTTTTTTCTGTGTGAGTTTCGGAAATATCCCCATTCATAGGTCCGAG---AT
CTACATCTAT---GAATTGAAAGGTCCGAATGATCAACTCTGCAATCAGTTGTTAGAATCA
ATAGGTCTTCAAATTGTTCATTTGAAAAAATGGAAACCCTTC------TTATTGGAC---------G
ATCATGATACTTCC---CAAAAATCGAAATTCTTGATCAATGGAGGAACAATATCACCATT
TTTGTTCAATAAGATACCA---AAGTGG---ATGATTGACTCATTCCATACTAGA------AATAA
TCGCAGGAAATCCTTTGATAACCCGGATTCCTATTTCTCAATGATATTCCACGATCAAGA
CAATTGGCTGAATCCCGTGAAACCATTTCATAGAAGTTCATTGATATCTTCTTTTTATAA
AGCAAATCGACTTCGATTCTTGAATAATCCACATCACTTCTGCTTCTAT------TGGAACAC
AAGATTCCCCTTTTCTGTG------------GAAAAGGCCCGTATCAAT------AATTCTGATTTTAC
GTATGGA---CAATTCCTCAATATCTTGTTCATTCGCAACAAA---ATATTTTCTTTGTGCGT
CGGTAAA------AAAAAACATGCTTTTTGGGGG---AGAGATACTATTTCACCAATCGAGTC
ACAGGTATCTAACATATTCATACCTAACGATTTTCCACAAAGTGGT---GACGAAACGTAT
AACTTGTACAAATCT---------TTCCATTTTCCAAGTCGATCCGATCCATTCGTTCGT------A
GAGCTATTTACTCGATCGCAGACATTTCTGGAACACCTCTAACAGAGGGGCAAATAGTC
AATTTTGAAAGAACTTATTGTCAACCTCTTTCAGATATGAATCTATCTGATTCAGAAGGG
AAGAACTTGCATCAGTATCTCAAT------TTCAATTCAAACATGGGTTTGATTCACACTCCA
TGTTCTGAGAAAGATTTATCATCCGAAAAG------AGGAAAAAA------------------------CGGA
GTCTTTGTCTAAAG------AAATGCGTTGAGAAAGGGCAGATGTATAGAACCTTTCAACG
AGATAGTGCTTTTTCAACTCTCTCAAAATGGAATCTATTCCAAACATATATGCCATGGTT

CCTTACTTCGACAGGGTACAAATATCTAAATTTGATATTTTTAGATACTTTTTCAGACCTA
TTG------CCAATACTAAGTAGC---AGTCAAAAATTTGTACCCATTTTTCATGATATTATGCA
TGGATCAGGTATATCATGGCGAATTCTTCAGAAAAAATTG------------------TGTCTTCCAC
AATGGAATCTG---ATAAGTGAGATCTCGAGTAAGTGTTTACATAAT------CTTCTTCTGTC
CGAAGAAATGATTCATCGAAAT------AATGAGTCACCATTGATATCGACACATCTGAGAT
CGCCAAATGCTCGGGAGTTC---CTCTATTCAATCCTTTTCCTTCTTCTTGTTGCTGGATAT
---CTCGTTCGTACACATCTTCTCTTTGTTTCCCGG------GCCTCTAGTGAGTTACAGACAG
AGTTCGAAAAGGTCAAATCTTTGATGATTCCATCATCTATGATTGAGTTGCGAAAACTT
CTGGATAGGTATCCTACATCTGAACCGAATTCTTTCTGGTTAAAGAATCTCTTTCTAGTT
GCTCTGGAACAATTAGGAGATTCTCTAGAAGAAATACGGGGTTCTGCTTCTGGCGGCA
ACATGCTTGGT---------CCCGCT---------------TATGGG---GTCAAATCAATACGTTCTAAGAA
GAAAGATTGGAATATCAATCTCATCGAGATCATCGATCTCATA------------CCAAATCCCAT
CAATCGAATCACTTTTTCGAGAAATACGAGACATCTAAGTCATACAAGTAAAGAGATCT
ATTCATTGATAAGAAAAAGAAAAAACGTGAACGGGGATTGGATTGATGATAAAATAGA
ATCCTGGGTCGCGAACAGTGATTCGATTGATGATGAAGAAAGAGAATTCTTGGTTCAG
TTCTCC------ACCTTAACGACA------GAAAATAGGATTGATCAAATTCTATTGAGTCTGAC
T---CATAGTGATCGTTTA---------------TCAAAGAATGACTCTGGTTATCAAATGATTGAACA
ACCGGGAGCAATTTACTTACGATACTTAGTTGACATTCAT------AAAAAGCATCTAATGA
ATTATGAGTTCAATCCATCCTGTTTAGCAGAAAGACGGATATTCCTTGCTCATTATCAGA
CAATCACTTAT------TCACAAACTTCGTGTGGGGAAAATAGTTTTCATTTCCCATCTCATG
GA---------AAACCCTTTTCGCTCCGCTTAGCCTTATCCCCCTCT---AGGGGTATTTTAGTGA
TAGGTTCTATAGGAACTGGACGATCCTATTTGGTCAAATACCTAGCGACAAACTCCTAT
GTTCCTTTCATTACGGTATTTCTGAACAAGTTCCTGGATAACAAGCCTAAAGGTTTTCTT
CTTGAT---------------GAGATCGAT---ATTGATGATAGTGACGATATTGATGATAGTGACAAT
CTTGATGCTAGTGAC--GATATCGATCGTGACCT
TGATACGGAGCTG---AAACTGCTAACTAGGATGAATGGGCTAACTATGGATATGATGCC
GGAA---------ATAGACCGATTTTATATCACCCTTCAATTCGAATTAGCAAAAGCAATGTCT
CCTTGCATAATATGGATTCCAAACATTCATGATCTGGATGTGAATGAGTCGAATGACTTA
GCCCTCGGTCTATTAGTGAACCATCTCTCCAGG---------GATTGTGAAAGATGTTCT---AC
TAGAAATATTCTTGTTATTGCTTCGACTCATATTCCCCAAAAAGTGGATCCCGCTCTAAT
AGCTCCGAATAAATTAAATACGTGCATTAAGATACGAAGGCTTCTTCTTCCACAACAAC
GAAAGCACTTTTTCACTCTTTCATATACTAGGGGATTTCACTTGGAAAAGAAAATGTTC
CATACT------AACGGATTCGGGTCCATAACCATGGGTTCCAATGCACGAGATCTTGTAGC
ACTTACCAATGAGGTCCTATCGATTAGTATTACACAGAAGAAATCAATTATAGACACTAA
TACAATTAGATCCGCTCTTCATAGACAAACTTGGGATTTGCGA---------TCCCAGGTAAGA
TCGGTTCAGGATCATGGGATCCTTTTCTATCAGATAGGAAGGGCTGTAGCACAAAATGT
ACTTCTAAGTAATTGCCCCATAGATCCTATATCTATCTATATGAAGAAGAAA------TCATGT
AACGAAGGGGATTCTTATTTGTACAAATGGTACTTCGAGCTTGGAACGAGCATGAAGA
GATTAACGATACTTCTTTATCTTTTGAGTTGTTCTGCCGGATCGGTCGCTCAAGATCTTT
GGTCTTTATCC------GGACCCGATGAAAAA---AATGGGATCACTTCTTATGGACTCGTTG
AGAATGATTCT---GATCTAGTTCATGGCCTATTAGAAGTAGAAGGCGCTCTGGTGGGA---
---TCTTCACGGACAGAAAAAGATTGCAGTCAGTTTGATAATGAT------CGAGTGACATTG
CTTCTTCGGCCCGAACCGAGGAAT---CCCTTAGATATGATGCAAAACGGCTCTTGTTCTA
TCCTTGATCAGAGATTTCTCTATGAAAAATAT------GAATCGGAGTTTGAAGAA---GGGG

AGGGAGAAGGAGCCCTTGACCCGCAG-------------------GAGGAT-----------TTATTCAATC
AC------ATAGTTTGGGCTCCT------AGAATATGGCGCCCT---------TGGGGCTTTCTATTTGA
TTGTATCGAAAGGCCCAATGAATTGGGATTTCCC---TATTGGTCCAGGTCATTTCGGGGC
AAG---------CGGATCATTTATGATGAAGAGGATGAGCTTCAAGAGAATGATTCGGAGTTC
TTGCAGAGTGGAACCATGCAGTACCAGACACGAGATAGATCTTCCAAAGAACAAGGC
CTTTTTCGAATAAGCCAATTCATTTGGGACCCTGCAGATCCACTCTTTTTCCTATTCAAA
---GATCAGCCCCCTGGCTCTGTGTTTTCACATCGAGAATTATTTGCAGATGAAGAGATGT
CAAAGGGGCTTCTTACTTCCCAAACAGACCCTCCTACATCTATATATAAACGCTGGTTTA
TC------AAGAATACGCAAGAAAAGCACTTCGAATTGTTGATTAATCGTCAGAGATGGCTT
AGA---------ACCAACAGTTCATTATCT------AATGGATCTTTCCGTTCTAAT------ACTCTATC
CGAGAGTTATCAGTATTTATCAAATCTGTTCCTATCTAAC---GGAACGCTATTGGATCAAA
TGACAAAGACATTGTTGAGAAAAAGATGGCTTTTCCCGGATGAAATGAAAATTGGATT
C------ATG---------------------------ATGCCTAGATCACGG---ATAAATGGAAATTTTATTGATAA
GACCTTTTCAATTGTAGCCAATATCTTATTACGAGTAATTCCGACAACTTCAGGAGAAA
AAGAGGCATTTACCTATTACAGAGATGGG------ATGTCGGCTCAATCCGAAGGAAATTAC
GCGGAAGCTTTGCAGAATTATTATGAAGCTATGCGACTAGAAATTGATCCCTATGATCG
AAGTTATATACTCTATAATATAGGGCTTATCCACACAAGTAATGGAGAACATACGAAAGC
TTTGGAATATTATTTTCGAGCACTAGAACGAAACCCATTCTTACCACAAGCTTTTAATAA
TATGGCCGTGATCTGTCATTAC---CGGGGAGAACAGGCCATTCAACAGGGAGATTCTGA
AATTGCAGAGGCTTGGTTCGATCAAGCCGCTGAGTATTGGAAACAGGCTATAGCGCTT
ACTCCCGGTAATTATATTGAAGCGCATAATTGGTTGAAGATCACGAGACGTTTCGAA-----
-ATGACTTGGCGATCAGAACATATATGGATAGAACTTATAACGGGGTCTCGAAAAATAA
GTAATTTCTGCTGGGCCTTTATCCTTTTTTTAGGTTCATTAGGCTTCTTATTAGTTGGAAC
TTCCAGTTATCTTGGTAGAAATTTGATATCTTTTTTTCCGCCTCAGCAAATCATTTTTTTT
CCACAAGGACTCGTGATGTCTTTCTACGGAATTGCGGGTCTCTTTATTAGCTCTTATTTG
TGGTGCACAATTTCCTGGAATGTAGGTAGTGGTTATGATCGATTCGATAGAAAGGAAGG
AATAGTCTGTATTTTTCGTTGGGGATTTCCGGGAAAAAATCGTCGCATATTCCTCCGATT
CCTTATAAAAGATATTCAGTCCGTTAGAATAGAAGTTAAAGAGGGTATTTCTGCTCGTC
GTGTTCTTTATATGGACATCCGAGGCCAGGGGTCCATTCCCTTGACTCGTACTGATGAG
AAT---TTGACTCCACGAGAAATTGAACAAAAGGCTGCTGAATTAGCCTATTTCTTGCGT
GTACCAATTGAAGTATTT
>Spinacia
ATGAAAAAATGGTGGTTCAATTCGATGTTGTCTTTAAGTAAATCAATGGACAGTCTTGG
TCCTATTGAAAATACCAGTACAAAGGAAGATCCGAGTCTAAATGATCCAGAAAAACAA
ATTCATAGTTACAGTAATGTTAATCATTTAGTTCGTGGCATTGACATTCAGAATTTCATCT
CGGATGATACTTTTTTACTTATAGATACTAAAGGGGACAGTTATTCCATATATTTTGATAT
TGAAAATCAAATTTTTGAGATTGACAACGATCATTCT------TTTCTGAGTAAACTGCAAA
GTTCTTTTTCAAATTATTGGAATTCAAGTTATCTGAACTCTAGATCTAAGAATGGCGATA
CTTATAATGGTCATTCGCTGTATTATACTAACGATAGTTGGAATAATCACATTAATAATTG
CATTGACAGTTATCTTCAT------TCTCAAATCCGTAGTGACAGTTCCATCCTAAGTGGTAA
TGACAGTTACATTTTGAGTTACATTTTTAAT-----------AATAGGAGTGAAAGTTTCAGTAA
AAGAAGTAATGGCAGTAATTTAACTAGAAGAGAAAGTTCT---AATCTTGATGTAACTCA
AAAATATAGACATTTGTGGGTTCAATGCGAAAGTTGTTATGCATTAAATTATAAGAAATT
GCTGAAGTCAAAAATGGGTATTTGTGAACAATGTGGATATCATTTGAAAATAAGTAGTT

CAGATAGAATCGAACTTCTGATTGATCCCGGTACTTGGAATCCAATGGATGACGATATG
GTCTCTATGGATCCTATCGGATTTCATTCGGAGGAGGAAGCTTATAAAGATCGTATTGAT
TCTTATCAAATAAAGACGGGTTTAACTGAAGCTGTTCAAACCGGTATAGGTCAACTAAA
CGGTATTCCTGTAGCAATTGGGGTTATGGATTTTCAGTTTATGGGAGGTAGTATGGGATC
TGTAGTAGGGGAAAAAATAACCCGGTTGATTGAGTATGCTTCCAATAAATTCATACCCC
TTATTATAGTATGTGCTTCCGGAGGGGCACGTATGCAAGAAGGAAGCTTGAGCTTAATG
CAAATGGCAAAAATCTCGTCTGTTTTATATGATTATCAATCAAATAAAAAGTTATTTTATG
TATCAATCCTTACATCTCCTACTACTGGTGGGGTGACAGCCAGCTTTGGTATGTTGGGG
GATATCATTATTGCCGAACCTAACGCCTACATTGCATTTGCGGGTAAAAGAGTAATTGA
ACAAACATTGAATAAGACAGTACCTGAAGGTTCACAAGCAGCCGAATTTTTATTCCATA
AGGGTTTATTTGATCCAATCGTACCGCGTAATCTTTTAAAAGGCGTTCTAAGCGAGTTAT
TTGAGCTGCATGCTTTTTTTCCTTTGAATCAAAATAAAGGCGAGATGGCAACCATTCGA
GCAGATGAAATTAGCAAAATTATCCGTGAACGTATTGAAGGATATAATCGAGAAGTAAA
GGTTGTAAATACCGGTACAGTGCTTCAAGTGGGTGACGGCATTGCTCGTATTCACGGTC
TTGATGAAGTAATGGCAGGTGAATTAGTAGAATTTGAAGAGGGAACAATAGGTATTGCT
CTGAATTTGGAATCAAATAATGTTGGTGTTGTATTAATGGGTGACGGGTTGATGATACA
AGAGGGAAGTTCTGTAAAAGCAACAGGAAGAATTGCTCAGATACCCGTGAGTGAGGC
TTATTTAGGGCGTGTTATAAATGCCCTAGCTAAACCTATTGATGGTAGGGGTGAAATTAC
AGCTTCTGAATCTCGGTTAATTGAATCTCCCGCTCCTGGTATTATGTCCAGACGTTCCGT
GTATGAGCCTCTTCAAACCGGACTTATTGCTATTGATGCAATGATCCCTGTAGGGCGTG
GTCAGCGAGAATTAATTATCGGGGACAGACAAACTGGTAAAACAGCAGTAGCCACAGA
TACGATTCTCAATCAACAAGGACAAAATGTAATATGTGTTTATGTAGCTATTGGACAAA
AAGCATCTTCTGTGGCGCAGGTAGTTACTAACTTCCAGGAAAGGGGAGCAATGGAATA
CACTATTGTGGTAGCCGAAACAGCGGATTCCCCCGCTACATTACAGTATCTCGCTCCTTA
TACCGGAGCGGCGCTGGCGGAATATTTTATGTACCGTGAGCGACACACTTTAATCATTT
ATGATGATCTTTCCAAACAAGCACAAGCTTATCGACAAATGTCTCTTCTATTACGAAGA
CCACCTGGACGTGAAGCTTATCCGGGAGATGTCTTTTATTTGCATTCACGCCTTTTGGA
AAGAGCCGCTAAATTAAGTTCTCTTTTAGGTGAAGGAAGTATGACCGCTTTACCAATAG
TCGAGACCCAAGCAGGAGATGTTTCGGCTTATATTCCTACTAATGTAATTTCCATTACAG
ATGGACAAATATTCTTATCCGCCGATCTATTCAATGCTGGAATCAGACCGGCTATTAATG
TGGGTATTTCTGTTTCTCGGGTAGGATCTGCGGCTCAAATTAAAGCCATGAAAAAAGTA
GCTGGTAAATTAAAATTGGAACTGGCGCAATTTGCAGAATTAGAAGCCTTTGCACAATT
TGCTTCTGATCTTGATAAAGCTACTCAGAATCAATTGGCAAGAGGGCAACGATTACGCG
AATTGCTTAAACAACCCCAATCCGCCCCTCTCACGGTAGAAGAACAGGTAATGACTATC
TATACCGGAACGAATGGTTATCTTGATTCATTAGAACTTGATCAAGTAAGAAAATATCTT
GTTGAATTACGTACTTATGTAAAAACGAATAAACCGGAGTTCCAAGAAATTATATCTTCT
ACCAAGACATTTACTGAGGAAGCAGAAGCCCTTTTAAAAGAAGCTATTCAAGAACAGA
TGGAACGCTTTCTACTTCAGGAACAAGCAATGAGAATCAATCCTACTACTTCTGATCCT
GGGGTTTCCACACTTGAAAAAAAAAACCTGGGGCGTATCGCTCAAATTATTGGTCCGG
TACTGGACGTAGCCTTTCCTCCAGGCAAGATGCCCAATATTTACAACGCTCTAATAGTTA
AAGGTCGAGATACTGCTGGTCAACCAATGAATGTGACTTGTGAAGTACAACAATTATTA
GGAAATAATAGAGTTAGAGCTGTAGCTATGAGTGCTACAGATGGTCTAACGCGAGGAAT
GGAAGTTATCGACACAGGAGCTCCTTTAAGCGTTCCAGTCGGTGGAGCGACTCTTGGA
CGAATTTTTAACGTGCTTGGGGAGCCTGTTGATAATTTAGGTCCAGTAGATACACGCAC

AACATCTCCTATTCATAGATCGGCGCCCGCCTTTACACAGTTAGATACAAAATTATCTAT
TTTTGAAACAGGAATTAAAGTGGTAGATCTTTTAGCCCCTTATCGCCGTGGAGGAAAAA
TCGGACTATTTGGGGGAGCGGGAGTGGGTAAAACAGTACTCATTATGGAATTGATTAAC
AATATTGCCAAAGCTCATGGGGGCGTATCCGTATTTGGCGGAGTAGGTGAACGTACTCG
TGAAGGAAATGATCTTTACATGGAAATGAAAGAATCCGGAGTTATCAATGAACAAAATA
TCGCAGAATCAAAGGTGGCTTTAGTTTATGGGCAAATGAATGAACCGCCAGGAGCGCG
TATGAGAGTTGGGTTGACTGCCCTAACTATGGCGGAATATTTCCGAGATGTTAATGAAC
AAGACGTACTTCTATTTATTGACAATATCTTCCGTTTCGTCCAAGCAGGATCTGAAGTAT
CTGCCTTATTGGGTCGAATGCCTTCCGCTGTGGGTTATCAACCTACTCTTAGTACCGAAA
TGGGCTCGTTACAGGAAAGAATTACTTCTACAAAAGAAGGGTCCATAACTTCGATTCA
AGCAGTTTATGTACCTGCGGACGATTTGACCGATCCCGCTCCTGCTACGACATTTGCAC
ATTTAGATGCTACTACCGTACTATCAAGAGGATTGGCCGCCAAAGGGATCTATCCAGCA
GTAGATCCGCTAGATTCAACGTCAACTATGCTCCAACCTAGGATCGTTGGCGAGGAACA
TTATGAAATTGCGCAAAGAGTTAAGGAAACCTTACAACGTTACAAAGAACTTCAGGAC
ATTATAGCTATCCTTGGTTTGGACGAATTATCCGAAGAAGATCGTTTAACTGTAGCAAG
AGCACGAAAAATTGAGCGTTTCTTATCACAACCCTTTTTCGTAGCAGAAGTATTTACAG
GCTCTCCAGGAAAATATGTTGGTCTAGCAGAAACAATTAGAGGGTTTCAATTGATCCTT
TCCGGAGAATTAGACAGTCTTCCTGAACAGGCTTTTTATTTGGTAGGTAACATCGACGA
AGCTACCGCGAAAGCTATGAACTTAGAAATGGAGAGCAAATTAAAGAAAATGACCTTA
AATCTTTGTGTACTGACTCCGAATCGAAGTATTTGGAATTCAGAAGTAAAAGAAATCAT
TTTATCTACTAATAGTGGCCAAATTGGCGTATTACCAAACCATGCCCCTACTGCCACAGC
GGTAGATATAGGAATTTTAAGAATACGCCTTAACGACCAATGGTTAACATTGGCTCTGAT
GGGCGGTTTTGCTAGAATAGGCAATAATGAGATTACTATTTTAGTAAATGATGCTGAGA
GGGGTAGTGACATTGATCCCCAAGAAGCCCAACAAACTCTTGAAATAGCAGAAGCTAA
CTTGAGGAAAGCGGAAGGAAAGAGACAAAAAATTGAAGCAAATTTAGCTCTCAGACG
AGCTAGGACACGAGTCGAGGCTAGCAATACGATTTCTATGAAAAATGTAACCGATTCTT
TCGTTTTCTTGGGTCACTGGCCATCCGCCGGGAGTTTCGGGTTTAATACCGATATTTTAG
CAACAAATCTAATAAATCTCAGTGTAGTGCTTGGTGTATTGATCTTTTTTGGAAAGGGA
GTGTTAAGTGATTTATTAGATAATCGAAAACAGAGGATCTTGAATACTATTCGAAATTCA
GAAGAACTACGCGGAAAGGCCATTGAACAGCTGGAAAAAGCCCGGGCTCGCTTAAAG
AAAGTCGAAATGGACGCGGATCAGTTTCGAGTGAATGGATATTCTGAAATAGAACGAG
AAAAGATGAATTTGATTAATTCAACTTATAAAACTTTAGAACAATTTGAAAATTACAAA
AACGAAACCATTCAGTTTGAACAACAAAAAGCGATTAATCAAGTCCGACAGCGGGTTT
TCCAACAAGCCTTACAAGGAGCTCTAGGAACTCTGAATAGTTGTTTGAACAATGAGTT
ACATTTACGGACCATCAATGCTAATATTGGCATGTTTGGTGCGATGAACGAAATAACTG
ATATGAATCCACTGATTGCTGCCGCTTCCGTTATTGCTGCTGGATTGGCTGTAGGGCTTG
CTTCTATTGGACCTGGAGTTGGTCAAGGTACTGCTGCGGGACAAGCTGTAGAAGGTAT
TGCGAGACAGCCCGAAGCAGAAGGAAAAATACGAGGTACTTTATTACTTAGTTTAGCG
TTTATGGAAGCTTTAACAATTTATGGATTGGTTGTAGCATTAGCGCTTTTATTTGCGAATC
CTTTTGTTATGAATGTTCTATCATATTCAATCAACCCGCTAAAG---TTATATGCTATATCGG
GTGTGGAAGTAGGTCAACATTTTTATTGGCAAATAGGAGGTTTCCAAATCCATGGCCAG
GTCCTTATAACTTCTTGGGTTGTAATTGCTATCTTATTAGGTTCAGCTGCTATAGCTGTTC
GGAGTCCGCAAACAATTCCGACTGGTGGTCAAAATTTTTTTGAATATGTCCTTGAATTC
ATCCGAGACGTGAGCAAAACTCAAATTGGCGAAGAATATCGCCCGTGGGTTCCCTTTA

TTGGGACTATGTTTCTATTTATTTTTGTTTCTAATTGGTCAGGGGCTCTTTTACCTTGGAA
AATCATACAGTTACCTCATGGGGAGTTAGCCGCACCCACGAACGATATAAATACTACTG
TTGCTTTAGCTTTACTCACGTCAGTAGCCTATTTCTATGCAGGTCTTACAAAAAAAGGAT
TAGGTTATTTTGGTAAATACATTCAACCAACTCCAATTCTTTTACCCATTAACATCTTAGA
AGATTTCACAAAACCGCTATCACTTAGTTTTCGACTTTTCGGAAATATATTAGCGGATGA
ATTAGTAGTTGTTGTTCTTGTTTCTTTAGTACCTTTAGTGGTTCCTATACCTGTCATGTTT
CTTGGCTTATTTACAAGTGGTATTCAGGCTCTTATTTTTGCAACTTTAGCCGCAGCTTATA
TAGGCGAATCCCTGGAAGGTCATCATATGATATTTTCTACTTTAGAGCATATTTTAACTCA
TATTTCTTTTTCAACGGTTTCCGTTGTAATTACACTCCATTTGATAACTTTATTAGTCAAT
GAAATAGTGGGACTATATAATTCATTAGAAAAAGGCATGCTAGTTACTTTTTTCTGTATA
ACAGGATTATTAGTTACTCGTTGGGTTTATTGGAAGCATTTCCCATTAAGTGATCTATATG
AATCATTAATCTTCCTTTCCTGGAGTTTCTACCTTATTCATATGATTCCATCT------TTTTTA
AAAAAAAAAAATTCTTTAATAACTGCACCAAGTGCTATTTTTACCCAAGGATTTGCTAC
ATCGGGCCTATTAACTGAAATGCATCAATCCGGAATATTAGTACCCGCCCTACAATCACA
GTGGTTAATGATGCATGTAAGTATGATGGTATTGGGTTATGCAGCTCTTTTAGGCGGATC
ATTATTATCTGTAACACTTCTAATTATTATATTTCAAAAAGATATACAAGTTTTTGATAAA
CGAAAAGAGATTCAATACATAAATGAAAAAAGCAATATAGTACAA---GCTTCGCCCTCT
TATGTTCGAAATTATTACAGGTCCCAATTGATTGAACAACTGGATCATTGGAGTTATCGG
GTTATTAGTCTAGGATTTATCTTTTTAACCATAGGTATTCTTTCAGGGGCAGTATGGGCC
AATGAGGCATGGGGGTCATATTGGAACTGGGACCCAAAGGAAACTTGGGCGTTTATTA
CTTGGACCATATTTGCAATTTATTTACATATTCGAACAAATAAAAATTTGCGGGGTGCAA
ATTCTGCAATTGTGGCTTTTATAGGCTTTCTTATAATTTGGATATGCTATTTTGGGGTTAAT
CTATTAGGAATAGGGCTACATAGTTATGGTTCATTTACATTAACGATGGAAAAAAAGAA
AGTATTTATTCCTTTTCTATATCTTATATCTATAGTATTTTTACCCTGGTGGATCTATCTATC
ATTTCAAAAAAGTCTGGAATCTTGGGTTACTACTTGGTGGAATACTAAGCAATCTGAAA
CTTTTTTGAATGATATTCAAGAAAAAAAGCTTCTCGAAAAATTCATCGAATTAGAGGAG
CTCCGCTTGTTGGACGAAATGATAAAGGAATATCCAGAAACACAGTTACAAAAACTCG
GTATAGGAATCCACAATGAAACGATTCAATTGATCAAGATGCACAATGAGGATTGTATC
CATATGATTTTGCACTTCTCGACAAATTTAATCTGTTTCCTTATTCTAGGCGGTTATTCCA
TTCTGGGAAATAAAGAACTTATTCTTCTTAATTCTTGGGTTCAGGAATTCCTATATAACTT
AAGCGACACAATAAAAGCTTTTTCTATTCTTTTAGTAACCGATTTATGTATCGGATTTCA
TTCACCCCAGGGTTGGGAACTACTAATTGAATCTATCTACAAAGATTTTGGATTTGCCG
ATAACGATCAAATTATATCGAGTCTTGTTTCCACTTTTCCAGTCATTCTAGATACAATTTT
GAAATATTGGATATTCCGTTCTTTAAATCGTGTATCCCCATCGCTTGTAGTGATTTATCAT
TCAATGAATGACATGCCTATTGGTGTTCCAAAAGTTCCCTTTCGAAGTCCTGGAGAGGA
AGATGCATCTTGGGTTGACGTATACAACCGACTTTATCGAGAAAGATTACTTTTTTTAGG
CCAAGAAGTTGATAGCGAGATCTCGAATCAACTTATTGGTCTTATGGTATATCTCAGTAT
CGAAGATGATACCAAAGATTTGTATTTGTTTATAAATTCTCCTGGCGGATGGGTAATACC
AGGAGTAGCTATTTATGATACCATGCAATTTGTGCGACCAGATGTACATACAATATGCAT
GGGGTTAGCTGCTTCAATGGGATCTTTTATCCTGGTCGGAGGAGAAATTACCAAACGTT
TAGCATTCCCTCACGCTAGGGTAATGATTCATCAACCCGCAAGCTCTTTCTATGAAGCC
CAAACGGGAGAGTTTCTCCTGGAAGCGGAAGAACTTTTGAAATTGCGCGAAACCCTC
ACAAAGGTTTATGGACAAAGAACGGGCAAACCCTTATGGGTTGTATCCGAAGATATGG
AAAGGGATGTTTTTATGTCAGCAACAGAAGCCCAAGCTCATGGAATTATTGATCTTGTC

GCGGTCGAAATGAAAGAACAAAAATGGATTCATGAAGGTTTGATTACCGAATCACTTC
CTAATGGTATGTTCTGGGTTCGTTTAGATAATGAAGATCCGATTCTGGGTTATGTTTCAG
GACGGATACGACGTAGTTCTATACGAATTCTACCGGGAGATAGAGTTAAGATTGAAGTA
AGCCGTTATGATTCAACTAGAGGTCGTATAATTTATAGACTGCGCAACAAGGATTCGAA
CGATATGGAAGAATTCCAAAGACATATAGAA---GACAGGTCTTGGCAACATAATTTTTA
TATCCACTTATCTTTCAGGAATATATTTATGCATTTGCATATGATCAT------TCAGGCAACA
GTAAA---TACAGTTTACTGCTTGTAAAACGTTTAATTACTCGAATGTATCAACAGAATCA
TTTGATTCTTTCTGCTAATGATTCGAATCAAAATGTAATTTTGGGGAAGAATTTGTATTC
ACAAATGATAACAGAAGGGTTTGCCGTCATTGTGGAAGTTCCATTTTCCCTACTATTAAT
ATCTTCCCTAATAGTAAAATCTCATAATTTGCGATCAATTCATTCAATATTTCCCTTTTTA
GAGGACAAATTATTACATTTAAATTATGTGTTAGATATATTAATAACTTACCCCGCCCATC
TCGAAATCTTGGTTCAAACTCTTCGCTACTGGGTGAAAGATGCCTCTTCTTTGCATTTAT
TACGATTTTTTCTTTATGAGTATCGTAAT------TTGAATAGTCTTGAATTGATTTCCTTTTT
GAAAAAACAAAGATTATTCTTATTCCTATATAATTTGCATGTATGTGAATACGAATCCCTT
TTTGTTTTTCTCTGCAACCAATCCTCTTATTTACGACCAACATCTTTTGGAGCCCTTATT
GAACGAATCTATTTCTGCGGAAAGCTAAAATATGTAAAA---GTTTTTACTAAGGATTTTA
TCCTATGGATTTTCAGAGAACCTTTCCCACATTATGTTAGGTATCAAGGAAAATCAATTC
TGGCTTCAAAAGGGACATCTCTTCTGATGCATAAATGGAAATATTACCTTATCTATTTCG
GGCAATGTCATTTTTCTGTGTGGTCTCAACCAAAAAGACTCTATATCAATCGATTATCAA
ACCATTCCCTAGACTTTATGGGTTTTCTTTCAAGGGTGCGACTCAATTCTTCAGTAATAC
GGAGTCAAATGTTAAAAAATTCATTTCTAATAGAGAATATTAGT------AAGAAGTTCGAT
ACCGTAGTTCCAATTATTCCTTTGGTTGGGTCATTGGCTAAAGCGAAATTTTGTAATGTA
TTAGGGCATCCCATTAGTAAGTCGGTCTGGACCGATTTATCAGATTCTGATATTCTTGAT
CGATTTGGACGTATATGTAGAAATATTTCTCATTATTATAGCGGCTCTTCAAGAAAAAAA
AGTTTGTATCGAATAAAGTATATACTTCGACTTTCTTGTGCTAGAACTTTGTCTCGTAAA
CACAAAAGTACTGTACGTGCTTTTTTGAAAAGATTAGGTTCAGAATTTTTAGAAGAATT
CTTTACAGAGGAAGAAAAAGCTCTTTCTTTGATCTTACCAAGACGTATTTGGTATTTGG
ATATTATTTGTATTCATAATTTGGCCAATGATGAAATGATAATTGATACAACAACAACAA
AAGTACAAGCTATCAATTCTTTTTCCAGATTGGAA---TTCTTAAAAGAGGTTTATGAGAC
TATATGGATGCTTTTCCCTATTTTGATTCTCGTATTGGGAATTACAATAGGTGTACTAGTA
ATTGTATGGTTAGAAAGAGAAATATCCGCAAGTATACAACAACGTATTGGACCTGAATA
TGCTGGTCCTTTGGGGATTCTTCAAGCTTTAGCGGACGGAACAAAACTGCTTTTTAAA
GAAAACCTTCTTCCATCTAGGGGGGATACATATTTATTTAGTATTGGACCTTCTATAGCA
GTCATATCGATTCTACTAGGTTACTTAATAATTCCTTTTGGGTCTCGCCTTGTTCTAGCCG
ATCTCAGTATTGGTGTTTTTTTATGGATCGCTGTTTCAAGCATTGCTCCGATTGGACTTC
TTATGTCAGGATATGGATCAAATAATAAATATTCCTTTTTAGGTGGTCTACGGGCTGCTG
CCCAATCAATTAGTTATGAAATACCATTAACTCTATGTGTGTTATCCATATCTCTACTATCT
AATAGTTCAAGTACAGTTGATATAGTTGAGGCGCAGTCAAAATATGGTTTTTGGGGATG
GAATTTGTGGCGGCAACCTATAGGATTTATTGTTTTTATAATTTCTTCTCTAGCAGAATGC
GAGAGATTGCCTTTTGATTTACCAGAAGCAGAAGAAGAATTAGTAGCAGGTTATCAAA
CCGAATATTCCGGTATTAAATTTGGTTTATTTTATGTTGCGTCTTATTTAAATCTACTAATC
TCTTCATTATTTGTAACCGTTCTTTACTTGGGTGGTTGGAATCTCTCTATTCCATACATAT
TCATCTCGGAGTTTTTTGAAATAAATAAAATAGATGGAGTCTTTGGAACAACAATTGGT
ATTTTCATTACATTAGCTAAAACTTTTTTGTTCTTGTTCATTCCTATCACAACAAGATGG

ACTTTACCTAGACTGAGAATGGACCAATTATTAAATCTTGGATGGAAATTTCTTTTACCT
ATTTCTTTAGGTAATCTATTATTAACAACTTCTTCCCAACTCTTTTCATTAATGATCTGGC
ATGTACAGAATGAAAACTTCATTCTCGATTCTACGAGAATTTTTATGAAAGCCTTTCATT
TGCTTCTCTTCGATGGAAGTTTGATTTTTCCAGAATGTATCCTAATTTTTGGCCTAATTCT
TCTTCTGATGATCGATTCAACCTCTGATCAAAAAGATATACCTTGGTTATATTTCATCTCT
TCAACAAGTTTAGTAATGAGCATAACAGCCCTGTTGTTCCGATGGAGAGAAGAACCTA
TGATTAGCTTTTCAGGAAATTTCCAAACGAACAATTTCAACGAAATCTTTCAATTTCTTA
TTTTACTATGTTCAACTCTATGTATTCCTCTATCCGTAGAGTACATTGAATGTACAGAAAT
GGCTCTAACAGAGTTTCTGTTATTCATATTAACAGCTACTCTAGGAGGAATGTTTTTATG
CGGTGCTAACGATTTAATAACTATCTTTGTAGCTCCAGAATGTTTCAGTTTATGCTCCTA
CCTATTATCTGGATATACCAAGAAAGATGTACGGTCTAATGAGGCTACTACGAAATATTT
ACTCATGGGTGGGGCAAGCTCTTCTATTCTGGTTCATGGTTTCTCTTGGCTATATGGTTC
ATCCGGTGGAGAGATCGAGCTTCAAGAAATAGTGAATGGTCTTATCAATACACAAATGT
ATAACTCCCCGGGAATTTCAATTGCGCTTATATTCATCACTGTAGGAATTGGGTTCAAGC
TTTCCCCAGCCCCTTCTCATCAATGGACTCCTGACGTATACGAAGGATCTCCCACTCCA
GTCGTTGCTTTTCTTTCTGTTACTTCGAAAGTAGCTGCTTCAGCTTCAGCCACTCGAAT
TTTCGATATTCCCTTTTATTTCTCATCAAACGAATGGCATCTTCTTCTGGAAATCCTAGCT
ATTCTTAGCATGATATTGGGGAATCTCATTGCTATTACTCAAACAAGCATGAAACGTATG
CTTGCATATTCGTCCATAGGTCAAATCGGATATGTAATTATTGGAATAATTGTTGGAGAC
TCAAATGATGGATATGCAAGCATGATAACTTATATGCTGTTCTATATCTCCATGAATCTAG
GAACTTTTGCTTGCATTGTATTATTTGGTCTACGTACCGGAACTGATAACATTCGAGATT
ATGCAGGATTATACACGAAAGATCCTTTTTTGGCTCTCTCTTTAGCCCTATGCCTCTTATC
CCTGGGGGGTCTTCCTCCACTAGCAGGTTTTTTCGGAAAAATCTATTTATTCTGGTGTG
GATGGCAGGCAGGCCTATATTTCTTGGTTTTAATAGGACTCCTTACGAGCGTTCTTTCTA
TCTACTATTATCTAAAAATAATCAAGTTATTAATGACTGGACGAAACCAAGAAATAACCC
CTCACGTGCGAAATTATAGAAGATCCCCTTTAAGATCAAAGAATTCCATCGAATTCAGT
ATGATTGTATGTGTGATAGCATCTACTATACCAGGAATATCAATGAACCCGATTATTACAA
TTGCTCAGGATACCCTTTTTATGTTTCTACTTTATGAATATGATATTTTCTGGGCATTTTTA
ATAATATCAAGTGTTATTCCTATTTTAGCATTTCTATTTTCCGGAATTTTAGCACCGATTA
GCAAAGGACCCGAGAAACTTTCTAGTTACGAATCGGGTATAGAACCTATGGGGGATGC
TTGGTTACAATTTCGAATCCGTTATTATATGTTTGCTCTAGTTTTTGTTGTTTTTGATGTT
GAAACGGTTTTTCTTTATCCATGGGCAATGAGTTTCGATATATTGGGTGTATCCGTATTTA
TAGAAGCTTTAATTTTCGTGCTTATCCTAATTGTTGGTTTAGTTTATGCATGGCGAAAAG
GAGCATTGGAATGGTCTACGAATTCTTTTCCCTGGTTAACAACAATTGTAGTTTTGCCCA
TATTTGCCGGTTCTTTAATTTTCTTATTTCCTCATAGAGGAAATAAGGTTATTAGGTGGTA
TACTATATGTATTTCTATGATAGAGCTCCTTCTAATGACCTATGTGTTTTTTTATCATTTTC
AACCGGATGATCCATTAATCCAACTGGTTGAAGATTATAAATGGATCAACTTTTTTGATT
TCCATTGGAGATTAGGAATCGATGGACTTTCGATAGGACCCATTTTACTAACGGGATTCA
TCACTACTTTAGCTACTCTAGCGGCTTGGCCTGTTACTCGAAATTCGCAATTATTTCATT
TCTTGATGTTAGCGATGTATAGTGCTCAAATAGGATTATTTTCGTCCCGAGATCTTTTACT
TTTTTTCATAATGTGGGAATTAGAATTAATTCCTGTTTATCTACTTTTATCCATGTGGGGG
GGAAAGAAACGTCTCTACTCCGCTACTAAATTTATTTTGTATACTGCGGGGGGTTCTATT
TTTCTATTAATGGGAGTTCTAGGTGTTGGTTTATATGGTTCCAATGAACCAACATTAAAT
TTAGAAACATTAGTTAATCAATCGTATCCTGTGGCATTAGAAATAATATTCTATATTGGAT

TTTTTATTGCTTTTGCTGTCAAATTACCGATTATACCCTTACATACATGGTTACCAGATAC
CCACGGAGAAGCGCATTACAGTACTTGTATGCTGCTAGCCGGAATTTTATTAAAAATGG
GAGCATATGGATTGGTTCGGATCAATATGGAATTATTACCTCATGCTCATTCGATATTTTC
TCCTTGGTTGATGATAATAGGCACAATGCAAATAATCTATGCAGCTTCAACATCTCCCGG
GCAACGTAATTTAAAAAAAAGAATAGCCTATTCCTCCGTATCTCACATGGGTTTCATAAT
TATAGGAATTAGTTCTATAACCGATACGGGCCTTAATGGGGCCATTTTACAAATAATTTC
TCATGGATTTATTGGTGCTGCACTTTTTTTCTTAGCGGGAACTAGTTACGATAGAATACG
CCTTGTTTATCTCGACGAAATGGGCGGAATCGCGATTCCAATGCCAAAAATATTCACAC
TCTTCAGTAGCTTTTCAATGGCATCTCTTGCATTACCCGGCATGAGTGGTTTCATTGCCG
AATTAATAGTATTTTTTGGACTAATTACTAGCCAAAAATATCTTTTAATACCAAAACTACT
AATTACTTTTGGAATGGCAATTGGAATGATATTAACTCCTATTTATTTATTATCTATGTCAC
GTCAAATGTTCTATGGATATAAATTATTTAACATTTCAAACTCTTCTTTTTTTGATTCTGG
TCCGCGAGAGTTGTTTGTTTCAACTTCTATCTTTTTACCAGTAATAGGTATTGGGGTTTA
CCCGGATTTAGTTCTCTCACTATCAGTTGAGAAAGTGGAAGCTATTTTATCCAATTATTT
TTATAGAATGATACTCGAGCATGTACTTGTTTTGAGTGCTTTTTTATTTTCTATCGGGATT
TATGGATTAGTCACGAGTCGAAATTTAGTTCGCGCTCTTATGTGCCTTGAACTTATATTG
AATGCAGTTAATCTAAACTTTGTAACATTTTCTGATTTTTTTGATAGTCGCCAATTAAAG
GGAAACATTTTCTCCATTTTTGTTATAGCCATTGCAGCCGCTGAAGCAGCTATTGGACC
GGCTATTGTTTCTTCAATTTATCGTAACAGAAAATCCATTCGTATCAATCAATCGAATTTA
TTGAATAAAATGGAACATATATACCAATATGCATGGATCATACCTTTCCTTCCACTTCCG
GTTCCCTTGTTAATAGGAGCTGGACTTTTATTTTTTCCGACGGCAACAAAAAATCTTCG
GCGTATATGGGCTTTTTCTAGTATTTCGTTGTTAAGTATAGTTATGATTTTTTCGATGAAG
CTGGCTATTCAACAAATAAATAGTAATTCTATTTATCAATATCTATGGTCTTGGACCATTA
ATAATGACTTTTCTTTAGAATTTGGCTACTTGATGGACCCACTTACTTCTATTATGTCAAT
GTTAATTACTACTGTTGCAATTCTGGTTCTTATTTATAGTGATAATTATATGTCTCATGATC
AAGGATATTTGAGATTTTTTGCGTATATGAGTTTTTTCAATACTTCCATGTTGGGATTAGT
TACTAGTTCTAATTTGATACAAATTTATATTTTTTGGGAATTAGTGGGAATGTGTTCCTAT
CTATTAATAGGTTTTTGGTTTACACGACCTATTGCCGCAAATGCTTGTCAAAAAGCGTTT
GTGACTAATCGTGTAGGAGATTTTGGCTTATTATTAGGAATTTTAGGTCTTTATTGGATAA
CAGGTAGTTTCGAGTTTCGGGATTTGTTTGAAATATTCAATAAT---GAGGTCAATTCCTTA
TTTTGTATTTTATGTGCTTTCTTGTTATTTGCCGGTGCAGTTGCTAAATCAGCTCAATTTC
CCCTTCATGTATGGTTACCTGATGCTATGGAGGGGCCTACTCCTATTTCAGCTCTCATCC
ATGCTGCTACCATGGTAGCGGCGGGGATTTTTCTAGTTGCTCGACTCCTGCCCCTTTTTG
TAGTTATACCTTACATAATGTATGTAATATCTTTTATAGGTATAATAACAGTACTATTAGGA
GCGACCTTAGCTCTTGCTCAAAAAGACATTAAGAGGAGTTTAGCTTATTATACAATGTC
TCAATTGGGTTATATGATGTTAGCTCTAGGTATGGGTTCTTATCGAACGGCTTTATTTCAT
TTAATTACTCATGCTTATTCAAAAGCATTATTGTTTTTAGCATCTGGATCTCTTATTCATTC
AATGGGAACTATTGTTGGATATTCTCCGGATAAAAGTCAGAATATGGTTCTTATGGGGG
GGTTAACAAAACATGTACCAATTACAAAAACTTCTTTTTTAATAGGTACACTTTCTCTTT
GTGGTATTCCGCCTCTTGCTTGTTTTTGGTCCAAAGATGAAATTCTTAATGATAGTTGGG
TGTATTCGCCGATTTTCGCAATAATCGCTTATTTCACAGCCGGATTAACCGCATTTTATAT
GTTTAGAATCTATTTACTTACATTTGAAGGGCATTTAAACTTTTTTTGTAAAAATTACAG
TGGAAAAAAAAGTAGTTCTTTTTATTCAATATCTTTATGGGGCAAAAAAGAATTGAAAA
CGATTAATCAAAAATTATTAAACTTATTAACAATGAATTTTTTTTCGTTTGAAAATAAGA

ATATTTCTTTATATCCTTATGAATCGGACAATACTATGTTATTTCCTCTGATTATATTGATCA
TGTTTACTTTATTCGTTGGATTCATAGGAATCCCATTTGATTTGGATATATTAACTAAATG
GTTAACTCCATCTATAAATCTTTTACATTCCAATTCAGAAAATTTTGTGGATTGGTATGAA
TTTGTGATAAATGCAATTTTTTCAATTAGTATAGCTTTTTTTGGAATATTTATAGCCTTCTT
TTTTTATAAACCTATTTATTCATCCTTAAAAAATTTTGACTTAATTAATTCATTTGATATTT
TGGGGGACAATATAATAACTATTATATACAATTGGTCTGCTAACCGTGGTTATATAGATGC
TTTTTATTCAACATTCTTAATTAAGGGTATAAGAAGTTTGTCTGAACTAGTTTCTTTTTTT
GATAGACGAATAATTGATGGAATTCCTAATGGTTTTGGTGTTACAAGTTTTTTTGTAGGT
GAAGGTATCAAATATGTAGGAGGTGGC---CGAATCTCTTCGTATCTTTTTTGGTATTTATT
ATATGTATCCATTTTTTTATTCATTTTTACTATGGATTTACCTGGACCAATACATGATTTTC
TTTTAGTCTTTCTGGGGTCGGGTCTTATATTAGGGGCTCTAGGAGTGGTATTATTTACCA
ATCCTATTTTTTCTGCCTTTTCATTGGGATTGGTTCTTGTGTGTATATCTTTATTCTATATT
CTAGCAAACTCCCACTTTGTAGCTTCTGCACAACTCCTTATTTATGTGGGAGCTATAAAT
GTATTAATCATATTTTCTGTGATGTTCATGAGTGGTCCAGAATATGACAAAAAGTTTCAG
CTGTGGACTGTTGGGGACGGGGTTACTTCATTGGTTTGTATAAGTCTTTTTGTTTCATTA
ATTAGTACTATTCTAAATACATCTTGGTATGGGATTATTTGGACTACAAAATCAAACCAG
ATTCTCGAACAAGATTTAATAAACGCTAGTCAACAAATTGGAATTCATTTATCAACCGAT
TTTTTTCTTCCATTTGAACTCATTTCAATAATTCTTTTAGTTTCTTTAATAGGTGCAATTG
CTGTGGCTCGTCAAATGGCTGTACCAACTACCAGAAAAGACCTCATGATAGTCAATATG
GGCCCTCACCATCCATCAATGCACGGTGTTCTCCGACTCATCGTTACTTTAGACGGTGA
AGATGTTATTGACTGTGAACCGATAGTGGGTTATTTACACAGAGGTATGGAAAAAATTG
CGGAAAATCGAACAATTATACAATATCTGCCTTATGTAACACGTTGGGATTATTTAGCTA
CTATGTTTACAGAAGCAATAACTGTAAATGGACCAGAACAATTGGGAAATATTCAAGTA
CCTAAAAGAGCTAGCTATATCAGAGTAATTATGTTGGAGTTAAGTCGGATAGCTTCGCAT
TTATTATGGCTCGGCCCTTTTATGGCCGATATTGGCGCGCAGACCCCTTTTTTCTATATTT
TAAGAGAAAGGGAATTAATATATGATTTATTCGAAGCTGCTACCGGTATGAGAATGATGC
ATAATTATTTTCGTATTGGAGGAGTAGCTGCCGATCTTCCTTATGGATGGATAGATAAATG
TTTAGATTTTTGTGATTATTTTTTAATAGGGCTTACTGAATACCAAAAACTTATTACGCGA
AATCCTATTTTTTTAGAACGAGTTGAGAACGTCGGCATTATTGGTGGAGAAGAAGCAAT
AAATTGGGGTTTATCAGGCCCAATGCTACGCGCTTCCGGAATACAATGGGACCTGCGTA
AAGTTGATCATTATGAGTGTTACGACGAATTTGATTGGGAAGTCCAATGGCAAAAAGA
AGGAGATTCATTAGCTCGTTATTTAATACGAATCGGTGAAATGGCCGAATCTGTAAAAA
TTATACAACAAGCTCTAGAAGGAATTCCAGGGGGTCCCTATGAGAATTTAGAAATTCGA
CGCTTTAATAGAATAAAATATCCTGAATGGAATGATTTTGAATATCGATTCATTAGTAAA
AAGCCATCTCCTGCTTTTGAATTGTCGAAACAAGAGCTTTATGTAAGAGTCGAAGCCC
CAAAGGGAGAATTAGGAATATTTTTGATAGGAGATCAGAGTGTTTTTCCTTGGAGATGG
AAAATTCGTCCCCCGGGTTTTATCAATTTGCAAATTCTTCCTCAGTTAGTTAAAAAAATG
AAATTGGCTGATATTATGACGATACTAGGTAGCATAGATATCATTATGGGAGAAGTTGAT
CGTATGTTCCCTATGGTAACTGGGTTCATCAATTATGGTCAACAAACAATACGCGCGGC
AAGGTACATTGGTCAAAGTTTTATGATTACCCTATCCCATGCTAACCGCTTACCTGTAAC
TATTCAATATCCTTATGAAAAATTAATAACATCGGAGCGTTTTCGTGGTCGAATTCACTT
TGAATTCGATAAATGCATTGCTTGTGAAGTATGTGTTCGTGCATGTCCTATAGATTTGCC
CGTTGTTGATTGGAAATTGGAAACGGATATTCGAAAGAAACGGTTGCTTAATTACAGCA
TTGATTTCGGAATTTGTATATTTTGTGGTAATTGTGTTGAGTATTGTCCAACCAATTGTTT

ATCAATGACTGAAGAATATGAGCTTTCTACTTATGATCGTCACGAATTAAATTATAATCA
AATTGCTCTGGGTCGTTTACCAATATCAATAACCGATGATTACACAATTCGAACAATTTT
GAATTCG-------------------------ATGCAGGGTCGTTTGTCTGCTTGGCTAG
TCAAACACGGGTTAGTTCATAGATCCTTGGGTTTTGATTACCAAGGAATAGAGACTTTA
CAAATAAAGCCCGAAGATTGGCATTCCATTGCTGTTATTTTATATGTATATGGTTACAATT
ATTTACGTTCTCAATGTGCTTATGATGTAGCACCGGGCGGGCTGTTAGCTAGTGTGTATC
ATCTTACGAGAATAGAGTACGGCGTGGATCAACCGGAAGAGGTATGCATAAAAGTATTT
GCTCCAAGGCGGAATCCTAGAATTCCATCCGTTTTCTGGGTTTGGAAAAGCGCGGATTT
TCAAGAACGGGAATCTTATGATATGTTTGGAATCTCTTATGATAATCATCCACGCCTGAA
ACGTATTTTAATGCCCGAAAGTTGGATAGGATGGCCCTTACGTAAGGATTATATTGTCCC
GAATTTTTATGAAATACAAGATGCTTAT--------------------------------
---A
TGAATTCCATTGAGTTTCCACTGCTTGATCAAATAGCCCAAAATTCTGTTATTTCAACTA
CATCAAACGATCTTTCAAATTGGTCAAGACTCTCAAGTTTATGGCCGCTTCTCTACGGT
ACCAGCTGTTGCTTCATTGAATTTGCTTCATTAATAGGCTCACGATTTGACTTTGATCGT
TATGGACTCGTACCAAGAGCGAGCCCTAGACAAGCGGACCTAATTTTAACAGCCGGTA
CCGTAACAATGAAAATGGCGCCTTCCTTATTGAGATTATATGAGCAAATGCCTGAACCA
AAATATGTTATTGCTATGGGAGCATGTACAATTACAGGGGGGATGTTCAGTACCGATTCT
TATAGTACTGTTCGAGGAGTTGATAAATTAATTCCCGTGGATGTCTATTTGCCGGGCTGC
CCACCTAAACCCGAAGCAGTTATAGATGCTATAACAAAACTTCGTAAGAAAATATCTCG
GGAAATCTATGAAGATAGAATTAAGTCT------AAGAATCGATGTTTTACTATCAATCACAA
GTTTCGTGTTGGGCGGAGTATTCATACCGGAAATTATGATCAAGCATTATTATATAAATCT
CCATCGACTTCAGAGATTCCTCCTGAAACATTTTTCAAATACAAAAACGCAGCATCTTC
CCGCGAATTAGTGAATATGCAAACTATAAATACCTTTTCTTGGATAAAAGAACAGATTAC
TCGATCCATTTCCATATCACTTATATTATATATAATAACTCGGTCATCCATTGCGAATGCCT
ATCCCATTTTCGCACAGCAAGGTTATGAAAATCCACGAGAGGCGACGGGACGTATTGTA
TGTGCCAATTGCCATTTAGCTAATAAGCCCGTGGATATTGAGGTTCCACAAGCGGTCCT
TCCAGATACTGTATTTGAAGCAGTTGTTCGAATTCCTTATGATATGCAATTAAAACAAGT
TTTAGCTAACGGTAAAAAGGGTGGCTTGAATGTGGGGGCTGTTCTTATTTTACCTGAGG
GATTTGAATTAGCCCCACCCGATCGTATTTCTCCAGAGATGAAAGAAAAAATGGGGAAT
CTTTCTTTTCAGAGCTATCGCCCCAATAAACAAAATATTCTTGTGATAGGTCCTGTTCCT
GGGCAAAAATATAGTGAAATTACCTTTCCTATTCTTGCCCCGGACCCTGCCACTAAAAA
AGACGTTCACTTCTTAAAATATCCGATATATGTAGGTGGTAACAGGGGAAGGGGTCAAA
TTTATCCTGACGGAAGCAAGAGTAATAATACAGTTTATAATTCCACAGCAACAGGTATA
GTAAAGAAAATTGTACGAAAAGAAAAGGGCGGATACGAAATAAACATAGCGGATGCCT
CGGATGGACGTGAAGTGGTTGATATTATCCCTCGAGGACCAGAGCTTCTTGTTTCCGAG
GGTGAATCTATCAAACTTGATCAACCATTAACGAGTAATCCTAATGTGGGTGGGTTTGG
TCAGGGAGACGCAGAAGTAGTACTTCAAGACCCACTGCGCATACAAGGTCTTTTGTTT
TTCTTTGCGTCTGTTATTTTAGCACAAATCTTTTTGGTTCTTAAAAAGAAACAATTCGAG
AAGGTTCAATTGTCCGAAATGAATTTCATGAGTAAAGTCTATGATTGGTTCGAAGAACG
TCTCGAGATTCAGGCGATTGCGGATGATATAACTAGTAAATATGTTCCTCCTCATGTCAA
CATATTTTATTGTCTAGGCGGAATTACGCTTACTTGTTTTTTAGTACAAGTAGCTACAGG
GTTTGCGATGACTTTTTACTATCGTCCAACCGTTACTGATGCTTTTGCTTCTGTTCAATAT
ATAATGACTGAAGTCAACTTTGGTTGGTTAATACGATCCGTTCATCGGTGGTCGGCAAG

TATGATGGTTCTAATGATGATCCTGCATGTATTTCGTGTGTATCTTACCGGTGGGTTTAAA
AAACCTCGAGAATTGACTTGGGTTACAGGCGTGGTTCTGGGTGTATTGACCGCGTCTTT
TGGAGTAACTGGTTATTCCTTACCTTGGGACCAAATTGGCTATTGGGCAGTAAAAATTG
TAACAGGCGTACCGGATGCTATTCCTGTAATAGGATCGCCTTTGGTAGAGTTATTACGTG
GAAGTGCTAGTGTGGGACAATCTACTTTGACTCGTTTTTATAGTTTACACACTTTTGTAT
TGCCTCTTCTTACTGCCGTATTTATGTTAATGCACTTTCTAATGATACGTAAACAAGGTAT
TTCTGGTCCCTTAATGGGAGTAACAAAAAAACCTGACTTGAATGATCCTGTATTAAGGG
CTAAATTAGCGAAAGGTATGGGGCATAATTATTATGGGGAGCCCGCGTGGCCTAATGAT
CTTTTATATATTTTTCCTGTAGTAATTTTAGGTACTATTGCGTGTAACGTAGGCTTAGCAG
TTTTAGAACCATCAATGATTGGCGAGCCGGCGGATCCATTTGCAACTCCTTTGGAAATA
TTACCTGAATGGTATTTTTTTCCTGTATTTCAAATACTTCGTACAGTACCAAATAAGTTAT
TAGGCGTTCTTTTAATGGCTTCAGTACCTGCGGGATTATTAACAGTTCCTTTTTTAGAAA
ATGTTAATAAATTCCAAAATCCATTTCGTCGTCCAGTAGCTACAACTGTCTTTTTGGTTG
GTACCGTAGTGGCCCTTTGGTTAGGTATTGGAGCAACATTACCTATTGATAAATCCCTAA
CTTTAGGCCTTTTTATGATTGAAGTTTTTCTATTTGGAATCGTCTTAGGTCTAATTCCTAT
TACTTTGGCTGGATTATTTGTAACTGCATATTTACAATATAGACGTGGTGATCAGTTGGAT
CTTATGTCTACTCTAACTAGTTATTTCGGTTTTTTACTAGCGGCTTTAACTATAACTTCCG
CTTTATTTATAGGTCTAAACAAGATACGACTTATTATGGATATTGTAAGTCTCGCTTGGGC
TGCTTTAATGGTAGTCTTTACATTTTCTCTTTCACTTGTAGTATGGGGAAGAAGTGGACT
CATGATTATTCGTTCGCCGGAACCAGAAGTAAAAATTTTGGTAGATAGGGATCCCGTAA
AAACTTCGTTTGAGGCATGGGCCAAACCTGGTCATTTTTCAAGAACAATAGCTAAGGG
CCCTGAAACTACTACATGGATCTGGAACCTACATGCTGATGCTCACGATTTCGATAGCC
ATACCAGCGATTTGGAGGAGATCTCCCGAAAAATTTTTAGTGCCCATTTCGGTCAACTC
TCCATCATCTTTCTTTGGCTGAGTGGTATGTATTTCCACGGTGCCCGTTTTTCCAATTATG
AAGCATGGCTAAGCGATCCTACTCACATTGGACCTAGTGCCCAAGTAGTTTGGCCGATA
GTGGGTCAAGAAATATTGAATGGTGATGTAGGGGGAGGTTTCCGAGGAATACAAATAA
CCTCTGGGTTTTTTCAAATTTGGCGAGCATCTGGAATAACTAGTGAATTACAACTTTATT
GTACCGCAATTGGTGCATTGGTCTTTGCAGCATTAATGCTTTTTGCTGGTTGGTTCCATT
ATCACAAAGCGGCTCCAAAATTAGCTTGGTTCCAAGATGTGGAATCTATGTTGAATCAC
CATTTAGCAGGACTACTAGGACTTGGGTCTCTTTCTTGGGCGGGTCATCAAATCCATGT
ATCTTTACCGATTAACCAATTTCTAAATGCTGGAGTAGATCCTAAAGAGATACCGCTTCC
TCATGAATTAATCTTGAATCGGGATCTTTTGGCTCAACTTTATCCCAGTTTTGCCGAAGG
AGCAACCCCGTTTTTCACTTTGAATTGGTCAAAATATGCGGACTTTCTTACTTTTCGTG
GAGGGTTAGACCCAGTAACTGGAGGTCTGTGGCTGACCGATACTGCACACCATCATTT
AGCTATTGCAATTCTTTTCCTGATAGCGGGTCATATGTATAGAACGAACTGGGGCATTGG
GCATGGTCTAAAAGATATTTTAGAGGCTCATAAAGGTCCATTTACGGGCCAGGGACATA
AAGGCTTATATGAGATCTTAACAACGTCATGGCATGCTCAATTAGCTCTTAACCTAGCTA
TGTTAGGTTCTTTAACCATCGTTGTAGCTCATCATATGTATGCGATGCCCCCTTATCCATA
TCTAGCTACTGACTATGGTACCCAACTTTCATTGTTTACACATCACATGTGGATTGGTGG
ATTTCTCATAGTTGGTGCTGCTGCGCATGCGGCCATTTTTATGGTAAGAGACTATGATCC
AACTACTCGATACAACGACCTATTAGATCGGGTTCTTAGACATCGCGATGCAATCATATC
ACATCTTAACTGGGCATGTATATTTCTAGGATTTCACAGTTTTGGTTTATATATTCATAAT
GATACCATGAGTGCTTTAGGGCGTCCTCAAGATATGTTTTCAGATACCGCTATACAATTA
CAACCTGTCTTCGCTCAATGGATACAAAACACCCATGCTTTAGCGCCTAGTGCAACGGC

TCCGGGTGCAACAGCAAGCACCAGTTTGACTTGGGGAGGTAGTGATTTAGTAGCAGTG
GGTGGAAAAGTAGCCTTGTTACCCATTCCACTAGGGACCGCAGATTTTTTAGTCCACCA
CATTCATGCTTTTACAATTCATGTGACGGTACTAATACTTCTAAAAGGCGTTCTATTTGC
TCGTAGCTCTCGTTTGATACCGGATAAAGCAAATCTTGGGTTTCGCTTCCCTTGTGATG
GACCTGGAAGAGGGGGAACATGCCAAGTATCCGCGTGGGATCATGTATTCTTAGGGCT
ATTCTGGATGTACAACTCAATTTCGGTAGTAATATTTCATTTCAGTTGGAAAATGCAGTC
AGATGTTTGGGGTAGTATAAGCGATCAAGGGGTGGTAACTCATATCACTGGAGGAAAC
TTTGCGCAAAGTTCTATTACTATTAATGGGTGGCTACGTGATTTCTTATGGGCGCAAGCA
TCCCAGGTAATTCAGTCTTACGGTTCTTCATTATCTGCATATGGTCTCTTTTTCCTAGGTG
CTCATTTTGTATGGGCTTTTAGTTTAATGTTTTTATTTAGCGGGCGTGGTTATTGGCAAG
AACTTATTGAATCCATCGTTTGGGCTCATAATAAATTAAAAGTTGCTCCTGCTACTCAGC
CTAGAGCCTTGAGCATTGTACAAGGACGTGCTGTAGGAGTAACCCATTACCTTCTGGGT
GGAATTGCCACAACATGGGCGTTCTTCTTAGCAAGAATTATTGCAGTAGGAATGGCATT
AAGATTTCCAAGGTTTAGCCAAGGCTTAGCTCAGGACCCCACTACTCGTCGTATTTGGT
TTGGTATTGCTACCGCACATGACTTCGAGAGTCATGATGATATTACTGAAGAACGTCTTT
ATCAGAATATTTTTGCTTCTCACTTTGGGCAATTAGCAATCATTTTTCTGTGGACTTCCG
GAAATCTGTTTCATGTAGCTTGGCAAGGAAATTTTGAGTCATGGGTACAGGACCCTTTA
CACGTAAGACCTATTGCTCATGCAATTTGGGATCCTCATTTTGGTCAACCTGCTGTAGA
AGCTTTTACTCGGGGAGGTGCTCTTGGCCCAGTGAATATCGCTTATTCTGGTGTTTATCA
GTGGTGGTATACAATAGGTTTACGCACTAATGAAGATCTTTATACTGGAGCTCTTTTTCT
ATTATTTCTTTCTGTTATATCTTTACTAGGGGGTTGGTTACACCTACAACCAAAATGGAA
ACCGAGCGTTTCGTGGTTCAAAAATGCCGAATCCCGTCTCAACCATCACTTGTCAGGA
CTCTTTGGAGTAAGTTCCTTGGCTTGGACAGGACATTTAGTTCATGTCGCTATTCCCGG
ATCCAGAGGAGAGTACGTTCGATGGAATAATTTCTTAGACGTATTACCACATCCTCAAG
GATTAGGTCCCCTTTTTACAGGTCAGTGGAATCTTTATGCTCAAAACCCCGATTCAAGC
AGTCATTTATTTGGTACCTCCCAAGGGGCGGGAACTGCCATCCTAACCCTTCTTGGGGG
ATTTCATCCACAAACGCAAAGTTTATGGCTAACTGATATGGCTCATCATCATTTAGCTAT
TGCATTTGTTTTTCTTGTTGCGGGTCATATGTATAGAACGAACTTTGGGATTGGGCACAG
TATGAAAGATCTTTTAGAAGCGCATATTCCCCCAGGGGGACGATTGGGACGCGGACATA
AAGGTCTTTATGACACAATCAACAATTCGCTTCATTTTCAATTAGGACTTGCCCTAGCGT
CTTTAGGTGTTATAACTTCCTTGGTAGCCCAACACATGTACTCTTTACCTGCTTATGCGT
TCATAGCCCAAGATTTTACGACTCAAGCTGCGTTATATACACATCACCAATACATCGCAG
GATTCATTATGACAGGAGCTTTTGCTCATGGAGCTATATTTTTCATTCGAGATTACAATC
CGGAACAGAATGAAGATAATGTATTGGCAAGAATGTTAGACCATAAAGAAGCTATTATA
TCCCATTTAAGTTGGGCCAGTCTCTTTTTAGGGTTCCATACCTTGGGTCTTTATGTTCAT
AATGATGTCATGCTTGCTTTTGGTACTCCAGAAAAACAAATTTTGATCGAACCCATATTT
GCTCAATGGATACAATCTGCTCATGGGAAAACTTCATATGGGTTTGATGTACTTTTATCC
TCAACAAGTGGACCGGCATTCAATGCGGGTCGAAGTATATGGTTGCCTGGCTGGTTAAA
TGCTGTTAATGAG------AATAGTAATTCCCTATTCTTAACAATAGGGCCTGGAGACTTTTT
GGTTCATCATGCTATTGCTCTAGGTTTACATACAACTACATTGATCTTAGTAAAAGGTGC
TTTAGATGCACGCGGTTCAAAGTTAATGCCAGATAAAAAGGATTTCGGTTATAGTTTTC
CTTGCGATGGTCCAGGACGAGGCGGTACTTGTGATATTTCGGCTTGGGACGCATTTTAT
TTGGCGGTTTTCTGGATGTTAAATACCATTGGGTGGGTTACCTTTTATTGGCATTGGAAA
CACATCACATTATGGCAGGGTAATGTTTCACAATTTAATGAATCTTCCACTTATTTGATG

GGATGGTTAAGAGATTATCTATGGTTAAACTCTTCACAACTTATCAATGGATATAATCCAT
TTGGTATGAATAGTTTATCGGTTTGGGCCTGGATGTTCTTATTTGGACATCTTGTTTGGG
CTACGGGATTTATGTTCTTAATTTCGTGGCGTGGATATTGGCAGGAGTTGATTGAAACTT
TAGCATGGGCTCATGAACGCACACCTTTGGCCAATTTGATTCGATGGAGAGATAAACCG
GTAGCTCTTTCCATTGTACAAGCAAGATTAGTTGGATTAGCCCACTTCTCCGTAGGTTAT
ATATTCACATATGCGGCTTTCTTGATTGCCTCTACATCGGGCAAATTCGGTATGTCACATT
CAGTTAAGATTTATGATACATGTATAGGATGTACTCAATGTGTTCGAGCTTGCCCCACAG
ATGTATTAGAAATGATACCTTGGGATGGATGTAAAGCTAAACAAATAGCTTCTGCTCCA
AGAACAGAGGATTGTGTTGGTTGTAAGAGATGTGAATCCGCTTGTCCAACGGATTTTTT
AAGCGTTCGGGTTTATTTATGGCATGAAACAACCCGCAGTATGGGTCTAGGGTAT---------
ATGAACTTTCCCTCTATTTTTGTGCCTTTAGTAGGCCTAGTATTTCCGGCAATTGCAATG
GCTTCTTTATTTCTTTATGTTCAAAAAAATAAGATTGTTATGCGAGATTTCAAAACATATC
TTTCTGTGGCACCGGTACTAAGCACTTTATGGTTTGGGTCTTTAGCTGGTCTATTAATAG
AAATCAATCGTTTTTTCCCAGATGCGTTGACATTCCCCTTTTTTTCATTCATGACTGCAA
TTTTAGAGAGACGCGAAAGCGAAAGCCTATGGGGTCGCTTCTGTAATTGGATAACCAG
CACTGAAAACCGTCTTTACATTGGATGGTTTGGTGTTTTGATGATCCCTACCTTATTGAC
TGCAACTTCTGTATTTATTATAGCCTTCATTGCTGCTCCTCCAGTAGATATTGATGGTATT
CGTGAACCTGTTTCCGGATCTCTACTTTACGGAAATAATATTATTTCGGGTGCCATTATTC
CTACTTCTGCAGCTATAGGGTTGCACTTTTATCCAATCTGGGAAGCGGCATCGGTTGAT
GAGTGGTTATACAATGGTGGTCCTTATGAACTAATTGTTCTACACTTCTTACTTGGTGTA
GCTTGTTATATGGGTCGTGAGTGGGAACTTAGTTTCCGTCTAGGTATGCGCCCTTGGATT
GCTGTTGCATATTCCGCTCCAGTTGCAGCGGCTACTGCTGTTTTCTTGATCTACCCAATC
GGTCAAGGAAGCTTTTCTGATGGTATGCCTCTAGGAATCTCCGGTACTTTCAACTTTAT
GATTGTATTCCAGGCTGAGCACAACATCCTTATGCACCCATTTCACATGTTAGGTGTAGC
TGGTGTATTCGGCGGCTCCCTATTTAGTGCTATGCATGGTTCCTTGGTAACTTCTAGTTT
GATCAGGGAAACCACAGAAAATGAATCTGCTAATGAAGGTTACAGATTCGGTCAAGAG
GAAGAAACTTATAATATCGTAGCTGCTCATGGTTATTTTGGTCGATTGATCTTCCAATATG
CTAGTTTCAACAACTCTCGTTCTTTACACTTCTTCTTAGCTGCTTGGCCTGTAGTAGGTA
TTTGGTTTACTGCTTTAGGTATTAGTACTATGGCTTTCAACTTAAATGGTTTCAATTTCAA
CCAATCTGTAGTTGATAGTCAAGGTCGTGTAATTAATACTTGGGCTGATATCATTAACCG
TGCTAACCTTGGTATGGAAGTTATGCATGAACGTAATGCTCATAACTTCCCTCTAGACCT
AGCTGCTATTGAAGCTCCATCTACAAATGGAATGGGTTTGCCTTGGTATCGTGTTCATAC
TGTTGTATTGAATGATCCCGGCCGGTTGATTTCTGTCCATATCATGCATACAGCTCTGGT
TGCTGGTTGGGCCGGTTCGATGGCTCTATATGAATTAGCTGTTTTTGATCCCTCTGACCC
CGTTCTTGATCCAATGTGGAGACAGGGTATGTTCGTTATACCTTTCATGACTCGTTTAGG
AATAACCAATTCGTGGGGCGGTTGGAGTATTACA---GGGGGTACTATAACGGATCCTGG
CATTTGGAGTTACGAAGGTGTGGCCGGAGCGCATATTATGTTTTCGGGCTTGTGCTTTT
TGGCCGCTATTTGGCATTGGGTATATTGGGATTTAGAAATATTTTCGGATGAACGTACAG
GAAAACCTTCTTTGGATTTGCCTAAGATTTTTGGAATTCATTTATTTCTTTCCGGGGTGG
CTTGTTTTGGTTTTGGTGCATTTCATGTAACAGGCTTGTACGGCCCGGAATATGGGTAT
CCGATCCTTATGGACTAACTGGAAAAGTACAACCTGTAAGTCCAGCATGGGGCGTGGA
AGGTTTTGATCCTTTTGTTCCAGGAGGAATAGCCTCTCATCATATTGCAGCAGGAACATT
AGGTATATTAGCAGGTCTATTCCATCTTAGTGTCCGTCCGCCTCAACGTCTATACAAAGG
ATTACGTATGGGCAACATTGAAACCGTTCTTTCTAGTAGTATCGCTGCTGTCTTTTTTGC

AGCTTTTGTTGTTGCGGGAACTATGTGGTATGGTTCCGCAACTACCCCGATCGAATTATT
TGGCCCCACTCGTTATCAATGGGATCAGGGATACTTTCAGCAAGAAATATACCGACGAG
TAAGTGCTGGGCTAGCCGAAAATCAAAGTTTCTCAGAAGCTTGGTCAAAAATTCCCGA
GAAATTGGCTTTTTATGATTACATTGGAAATAATCCGGCGAAAGGGGGATTATTTAGAG
CAGGCTCAATGGACAACGGCGATGGAATAGCTGTTGGATGGTTAGGACACCCTATTTTT
AGAGATAAAGAAGGACGTGAACTCTTTGTACGACGTATGCCTACTTTTTTTGAAACCTT
TCCGGTCGTTTTGATAGATGGGGACGGAATTGTTCGAGCTGATGTTCCTTTTAGAAGAG
CAGAATCTAAGTATAGCGTTGAACAAGTAGGTGTAACTGTTGAGTTTTACGGCGGCGA
ACTAAACGGAGTTAGTTATAGCGATCCTGCTACTGTCAAAAAATATGCTAGACGTGCTC
AATTAGGTGAAATTTTCGAATTAGACCGTGCTACTTTGAAATCAGATGGTGTTTTTCGC
AGTAGTCCGAGGGGTTGGTTTACTTTTGGACATGCTTCCTTTGCCCTACTCTTCTTCTTC
GGACACATTTGGCATGGGTCTAGAACCTTGTTCAGAGATGTTTTTGCTGGGATTGACCC
CGATTTGGATGTTCAAGTAGAATTTGGCGCATTCCAAAAAATTGGAGATCCAACTACAA
GAAGACAAGGAGTTATGAAAACCTTATATTCCCTGAGGAGGTTCTACCCCGTGGAAAC
GCTCTTTAATGGAACTTTAACTTTAGCCGGCCGTGACCAAGAAACCACCGGTTTCGCTT
GGTGGGCCGGGAATGCTCGACTTATCAATTTATCTGGTAAACTACTTGGAGCTCATGTA
GCCCATGCCGGATTAATCGTATTCTGGGCCGGAGCAATGAACTTATTTGAAGTGGCTCA
TTTCGTACCAGAGAAGCCTATGTATGAACAAGGATTAATTTTACTTCCCCACCTAGCTAC
TCTAGGTTGGGGGGTAGGCCCTGGCGGGGAAGTTATAGACACTTTTCCATACTTTGTAT
CGGGAGTACTTCACTTAATTTCGTCGGCCGTATTGGGCTTTGGTGGTATTTATCATGCAC
TTTTGGGCCCTGAAACTCTTGAAGAATCTTTTCCATTCTTCGGTTATGTGTGGAAAGAT
AGAAATAAAATGACCACAATTTTGGGCATTCACTTAATCTTGTTAGGTATAGGCGCTTTT
CTTCTAGTATTCAAGGCTCTTTATTTTGGGGGCGTATATGATACATGGGCTCCCGGAGGT
GGAGATGTAAGAAAAATTACCAACGTCACCCTTAGTCCAAGTATTATATTTGGTTGTTTA
CTAAAATCGCCTTTTGGAGGAGAAGGGTGGATTGTTAGTGTAGACGATTTGGAAGATAT
AATAGGGGGGCATGTATGGATAGGTGTCATTTGTATACTTGGTGGAATCTGGCATATTTT
AACAAAACCTTTTGCATGGGCTCGCCGCGCACTTGTCTGGTCTGGCGAGGCTTATTTGT
CTTATAGTTTGGCTGCTTTATCTGTCTTTGGTTTCATTGCTTGTTGTTTTGTCTGGTTCAA
TAATACTGCTTATCCTAGTGAGTTTTACGGGCCCACTGGACCAGAAGCTTCTCAAGCTC
AAGCATTTACTTTTTTAGTTAGAGACCAACGTCTTGGGGCTAACGTGGGATCCGCTCAA
GGACCTACTGGTTTAGGTAAGTATCTAATGCGTTCTCCGACTGGAGAAGTTATTTTTGG
GGGCGAAACTATGAGGTTTTGGGATTTGCGCGCTCCGTGGTTAGAACCCCTAAGAGGT
CCAAACGGTTTGGACTTGAGTCGCCTGAAAAAAGATATACAACCTTGGCAAGAACGA
CGTTCAGCAGAATATATGACTCATGCTCCTTTAGGTTCTTTAAATTCTGTGGGTGGGGTG
GCTACCGAGATCAATGCAGTCAATTATGTCTCTCCTAGAAGTTGGTTATCTACTTCTCAT
TTTGTTCTAGGATTCTTCCTATTCGTAGGGCATTTGTGGCATGCGGGAAGGGCTCGTGC
AGCTGCAGCAGGATTTGAAAAAGGAATTGATCGTGATTTTGAACCTGTTCTTTCGATGA
CCCCTCTTAATATGACTATAGCCGTTGGTAAATTTACCAAAGACGAAAAAGATTTATTTG
ATAGTATGGATGACTGGTTACGGAGGGACCGTTTTGTTTTTGTAGGTTGGTCTGGTCTAT
TGCTCTTTCCTTGTGCTTATTTCGCCTTAGGGGGTTGGTTTACGGGTACAACCTTTGTAA
CTTCATGGTATACCCATGGATTGGCCAGTTCTTATTTGGAAGGCTGCAATTTCTTAACCG
CAGCAGTTTCTACTCCTGCTAATAGTTTAGCCCACTCTTTATTGTTACTGTGGGGTCCTG
AAGCACAAGGGGATTTTACTCGTTGGTGTCAATTAGGTGGCCTGTGGGCTTTTGTTGCT
CTTCATGGTGCTTTCGCACTAATAGGTTTTATGTTACGTCAATTCGAACTTGCTCGGTCT

GTTCAATTGCGCCCTTATAATGCAATCGCATTCTCTGGTCCAATTGCTGTTTTTGTTTCT
GTATTCCTGATTTATCCACTAGGTCAATCTGGTTGGTTCTTTGCGCCTAGTTTTGGTGTA
GCAGCTATATTCCGATTCATTCTCTTTTTTCAAGGGTTTCATAATTGGACGCTGAACCCA
TTTCATATGATGGGAGTTGCCGGTGTATTGGGCGCCGCTCTGCTATGCGCTATTCATGGT
GCTACCGTTGAAAATACTTTATTTGAAGATGGTGATGGGGCAAATACATTCCGGGCTTTT
AACCCAACCCAAGCTGAAGAAACTTATTCAATGGTCACCGCTAACCGCTTTTGGTCCC
AAATCTTTGGGGTTGCTTTTTCCAATAAACGTTGGTTACATTTCTTTATGTTATTTGTACC
AGTAACCGGTTTATGGATGAGTGCTCTTGGAGTAGTCGGTCTGGCTTTGAACCTACGTG
CCTATGACTTCGTTTCCCAGGAAATCCGTGCAGCTGAAGATCCTGAATTTGAAACTTTT
TACACCAAAAATATTCTCTTAAACGAGGGTATCCGTGCTTGGATGGCGGCTCAAGATCA
GCCTCATGAAAACCTTATATTCCCTGAGGAGGTTCTACCCCGTGGAAACGCTCTTATGT
CTGGAAGCACAGGAGAACGTTCTTTTGCTGATATTATTACCAGTATTCGATACTGGGTTA
TTCATAGCATTACTATACCTTCCCTATTCATTGCGGGTTGGTTATTCGTCAGCACAGGTTT
AGCTTACGATGTGTTTGGAAGCCCTCGTCCAAACGAATATTTCACAGAGAGCCGACAA
GGAATTCCATTAATAACTGGCCGTTTTGACTCTTTGGAACAACTTGATGAATTTAGTAGA
TCCTTTATGACCATAGATCGAACCTATCCAATTTTTACAGTGCGCTGGCTGGCTATTCAC
GGACTGGCTGTACCTACCGTTTCTTTTTTGGGGTCAATATCCGCAATGCAGTTCATCCA
ACGAATGGCTACACAAACCGTTGAGAGTAGTTCTAGA---TCTCGTCCAAAACCTACTAC
TGTAGGGGCTTTATTGAAACCGTTGAATTCGGAATATGGTAAAGTAGCTCCTGGGTGGG
GAACTACTCCTTTGATGGGAGTTGCAATGGCTTTATTTGCAGTATTCTTATCTATTATTTT
GGAAATTTATAATTCGTCCGTTTTACTGGATGGAATTTCCATGAATATGCTTACTCTCAA
ACTCTTTGTTTACACAGTAGTAATATTCTTTGTTTCTCTCTTCATTTTCGGATTCCTATCT
AATGACCCAGGACGTAATCCTGGACGCGAAGAAATGGCCGATACTACTGGAAGAATTC
CTCTTTGGATAATAGGGACTGTAGCTGGTATTCTTGTGATCGGTTTAGTAGGTATTTTCTT
TTATGGTTCATATTCTGGATTAGGCTCATCGCTGATGCTTAATATCTTTAGTTTGATCTGT--
----CTTAATTCTGCCCTTTATTCGAGTAGTTTTTTCTTCGGCAAATTACCTGAGGCCTACG
CTTTTTTGAGTCCAATCGTAGATTTTATGCCAGTCATACCTTTATTCTTTTTTCTATTAGC
CTTTGTTTGGCAAGCTGCTGTAAGTTTTCGAACGACACAATCAAACCCGAACGAACAA
AATGTTGAATTGAATCGTACCAGTCTTTATTGGGGGCTATTACTCATTTTTGTACTTGCT
GTTTTATTTTCGAATTATTTTTTCAATATGGAAGTAAATATTCTCGCATTTATTGCTACTGC
ACTATTCATTCTAGTTCCTACTGCTTTTTTACTTATTATCTATGTAAAAACGGTTAGCCAA
AATGATATGGAAACAGCAACCCTAGTCGCCATCTTTATATCTGGTTTACTTGTAAGTTTT
ACTGGGTATGCTTTATATACCGCTTTTGGGCAACCCTCTCAACAATTAAGAGATCCCTTC
GAGGAACACGGAGACATGGAAGCATTGGTTTATACATTTCTATTAGTCTCTACTTTAGG
GATTATTTTTTTTGCGATCTTTTTTCGGGAACCTCCTAAAATTTCAACTAAAAAA------AT
GACTATAGCTTTCCAATTGGCTGTTTTTGCATTAATTGCCACTTCATCAATCTTACTGATT
AGCGTACCCGTTGTATTTGCTTCTCCCGACGGTTGGTCAAGTAACAAAAATATTGTATTT
TCCGGTACATCATTATGGCTTGGATTAGTCTTTCTGGTGGGTATCCTTAATTCTCTCATCT
CT---------------------ATGTCACCACAAACAGAGACTAAAGCAAGTGTTGGATTTAAAGCTG
GTGTTAAAGATTACAAATTGACTTATTATACTCCTGAGTATGAAACCCTAGATACTGATA
TCTTGGCAGCATTCCGAGTAAGTCCTCAACCTGGAGTTCCACCCGAAGAAGCAGGGGC
TGCAGTAGCTGCTGAATCTTCTACTGGTACATGGACAACTGTATGGACCGACGGACTTA
CCAACCTTGATCGTTACAAAGGACGATGCTACCACATCGAGCCCGTTGCTGGAGAAGA
AAATCAATATATTTGTTATGTAGCGTATCCTTTAGACCTTTTTGAAGAAGGTTCTGTTACT

AACATGTTTACTTCCATTGTGGGTAACGTATTTGGGTTCAAAGCCTTGCGTGCTCTACG
TTTGGAAGATTTGCGAATCCCTGTTGCTTATGTAAAAACTTTCCAAGGCCCGCCTCACG
GTATCCAAGTTGAGAGAGATAAATTGAACAAGTATGGTCGTCCCCTATTGGGATGCACC
ATTAAACCTAAATTAGGTTTATCCGCTAAAAACTATGGTCGCGCAGTTTATGAATGTCTT
CGCGGTGGACTTGATTTTACCAAAGATGATGAAAACGTGAACTCCCAGCCGTTTATGC
GTTGGAGAGACCGTTTCCTATTTTGTGCCGAAGCTCTTTATAAAGCACAAGCCGAAAC
AGGCGAAATCAAAGGGCATTACTTGAATGCTACCGCGGGTACATGCGAAGATATGATG
AAAAGGGCTGTATTTGCCAGAGAATTGGGCGTTCCTATTGTAATGCATGACTACTTAAC
AGGGGGATTCACTGCAAATACTACCTTGTCTCATTATTGCCGAGATAATGGTCTACTTCT
TCACATCCACCGTGCAATGCACGCAGTTATTGATAGGCAGAAGAATCATGGTATGCACT
TCCGTGTACTAGCGAAAGCGTTACGTCTATCTGGTGGAGATCATATTCACTCTGGTACC
GTAGTAGGTAAGCTTGAAGGAGAAAGAGATATTACTTTAGGCTTTGTTGATTTACTACG
TGATGATTATACTGAAAAAGACCGAAGTCGCGGTATTTATTTCACTCAATCTTGGGTTTC
CACACCAGGTGTTCTGCCTGTTGCTTCAGGCGGTATTCACGTTTGGCATATGCCTGCTC
TAACCGAGATCTTTGGGGATGATTCTGTACTACAGTTTGGTGGAGGAACTTTAGGACAC
CCTTGGGGGAATGCACCAGGTGCTGTAGCAAACCGAGTAGCTCTAGAAGCATGTGTAC
AAGCTCGTAATGAGGGACGTGATCTTGCTCGCGAAGGTAATACAATTATTCGCGAGGCT
ACCAAATGGAGTCCTGAACTAGCTGCTGCTTGTGAAGTATGGAAGGAAATCAAATTTG
AATTCCCAGCAATGGATACAGTC------ATGGCGATACATTTATACAAAACTTCTACCTCGA
GCACACGCAATGGAGCCGTT------CAAGTGAAATCCAATCCACGAAATAATTTGATCTCT
GGACAGCGTCGTTGTGGTAAAGGTCGTAATGCCAGAGGAATCATTACCGCAAGGCATA
GAGGGGGAGGTCATAAGCGTCTATACCGTAAAATAGATTTTAGACGGAATGAAAAAGA
CATATATGGTAAAATCGTAACTATAGAATACGACCCTAATCGAAATGCATACATTTGTCT
CATACACTATGGGGATGGCGAGAAGAGATATATTTTACATCCCAGAGGGGCTATAATTG
GAGATACCATTGTTTCTGGTACAGAAGTTCCTATAAAAATGGGAAATGCCCTACCTTTG
ACCGATATGCCCTTAGGCACGGCCATACATAACATAGAAATCACACTTGGAAGGGGTGG
ACAATTAGCTAGAGCAGCGGGTGCTGTAGCGAAACTGATTGCAAAAGAGGGGAAATC
GGCCACATTAAAATTACCTTCTGGGGAGGTCCGTTTGATATCCAAAAACTGCTCAGCAA
CAGTCGGACAAGTGGGGAATGTTGGAGTGAACCAGAAAAGGTTGGGTAGAGCCGGAT
CTAAGCGTTGGCTAGGTAAGCGTCCTGTAGTAAGAGGAGTAGTTATGAACCCTGTAGA
CCATCCCCACGGGGGTGGTGAAGGGAGGGCCCCAATTGGTAGAAAAAGCCCTACAAC
CCCTTGGGGTTATCCTGCACTTGGAAGAAGAAGTAGAAAAAGGAATAAATATAGTGAT
AATTTTATTATTCGTCGACGTAGTAAAATGATTCAACCTCAGACCCATTTGAATGTAGCA
GACAACAGCGGGGCTCGAGAATTGATGTGTATTCGAATCATAGGAGCTAGC---AACCGT
CGATATGCTCGTATTGGTGACGTTATTGTTGCTGTGATCAAAGAAGCAATCCCCAACAC
GCCCTTAGAAAGATCAGAAGTGATCAGAGCTGTAGTTGTACGTACTTGTAAAGAACTA
AAACGCGACAACGGTATGATAATACGATATGATGACAATGCTGCAGTTATCATTGATCA
AGAAGGAAATCCAAAAGGAACTCGAATTTTTGGTGCAATTGCCCGGGAATTGCGACA
AAAA---TTTGCGAAAATAGTTTCATTAGCTCCTGAGGTATTAATGCTTAGTCCAAAAAG
AACTAGATTCCGTAAACAACATAGAGGAAGAATGAAGGGAATATCTTATCGAGGGAAT
CGTATTTGTTTCGGAAGATATGCTCTTCAGGCACTTGAACCTGCTTGGATCACGTCTAG
GCAAATAGAAGCAGGGCGGCGAGCAATGACACGAAATGCGCGTCGCGGTGGAAAAAT
ATGGGTACGTATATTTCCAGACAAACCAGTTACAGTAAGACCCGCGGAAACCCGTATG
GGTTCGGGGAAAGGATCTCCCGAATATTGGGTAGCTGTTGTCAAACCAGGTCGAATAC

TTTATGAAATAAGCGGAGTAGCCGAAAATATAGCCCGAAGGGCTGTCGCAATAGCGGC
ATCGAAAATGCCTATACGAACTCAATTCATTATTTCAGGAATGACTAGAGTTAAAAGAG
GATATATAGCTCGAAGGCGCAGAAAAAAAATTCGTTTTTTTGCATCAAGCTTTCGAGGG
GCTCATTCAAGACTTACTCGAACTATTGCTCAACAGAAAATAAGAGCTTTGGTTTCGGC
TCATCGGGATAGAGATAGGCAAAAGAGAGATTTTCGTCGTTTATGGATCACTCGAATAA
ACGCAGCAATTCGCGAA---AGGGGGGTATACTATAATTATAGTAAATTTATACACGATCT
GTACAAGAGACAGTTGCTTCTTAATCGTAAAATACTTGCACAAATAGCTATATTAAATCC
AAATTGTATTTATATGATTTATAATGAGATCATAAAAAAAGAAGATTGCAAAAAAATACCT
CGAAATTATTTTTTTTAAAGAGATAACTACAAGAGGTTACTCGATATCTATGTCTGTTGA
CAAAGCCCGAAGAGTAATTGATCAGATCCGTGGGCGTTCCTACGCCGAAACACTTATG
ATATTAGAACTCATGCCCTATCGAGCATGTTATCCCATTTTTAAATTGATTTATTCTGCAG
CAGCAAATGCTAGTCATAATAAACAATTTAATAAAGCAAATTTAATTATTAGTAAAGCCG
AAGTGAATAAGGGCATTACCTTAAAAAAAGTAAAACCGCGAGCTCGAGGACGTAGTTA
TATGATAAAAAGACCTACTTGTCATATAACTATTGTATTAAGAGATATAACCCACTTTTAT
G A G G T A A T G - - - - - - - -
-???
??
??
????????????????ATGGCAGTTCCAAAAAAGCGTACTTCGATTTACAAAAAACGTATTCGT
AAAAATATTTGGAAAAAAAAAGGGATATTGGGCGGCGTTAAAAGCCTTTTCACTAGCAA
AATCTCTTTCTACCGGGAATTCAAAAAGTTTTTTTGTACGCAAAATAAGTAATATGGCTA
AGGGTAAAGATGTCCGAGTAAAAGTTATTTTGGAATGTACTGGTTGT---GTTCGAAAG---
---GTTAATAAGGGA------GGCGTTTCTAGATATATTACTCAAAAGAATCGCCACAATACGC
CTAGTCGGTTGGAATTGCGAAAATTCTGTCCCTATTGTTACAAGCATACAATTCATGGA
GAGATAAAGAAAATGAAAATAAGAGCTTCTGTTCGTCCAATTTGTGAAAAATGTCGAC
TGATCCGCAGACGGGGACGAATTATAGTAATTTGTTCTAACCCAAAACATAAACAACGA
CAAGGAATGGTTCGAGAGAAAATAAGAGTATCTACTCAGACACTTCAGTGGAAGTGTG
TTGAATCAAGAACAGATAGTAAATGTCTTCATTATGGACGCTTTATTCTCTCTCCACTTA
TGAAGGGTCAAGCTGATACAATAGGCATTGCGATGCGAAGAGCGTTACTTGGAGAAAT
AGAAGGAACATGTATCACACGTGCAAAATCTGAA------AAAATACCACACGAATACTCT
ACCATATTAGGTATTCAAGAATCAGTACACGAAATTTTAATGAATTTGAAAGAAATAGTA
TTGAGAAGTAATCTATATGGAACTTGTGAGGCGTCTATTTGTGTTAGGGGCCCCAGAGG
TGTAACTGCTCAAGATATCATCTTGCCACCTTATGTAGAAATAGTTGACAATACGCAGCA
TATCGCCAGCTTGACGGAACCAATTGATTTGTGTATTGGATTACAACTCGAGAGAAATC
GGGGATATCATATAAAAGCGCCAAATAACTTTCAAGACGGAAGTTTT------CCTATAGAT
GCTCTATTCATGCCTGTTCGGAACGTGAATCATAGTATTCATTCTTATGGAAATGGGAAT
GAAAAACAAGAGATACTTTTTCTCGAAATATGGACAAATGGTAGTTTAACTCCGAAAG
AAGCACTTTATGAAGCCTCTCGGAATTTAATTGATTTATTAATTCCTTTTCTACATGCGG
AAGAAAAC---------GTAAATTTAGAGGACAATCAA---CACAAAGTTTCTTTACCCCTTTTT
ACCTTTCATAATAGATTGGCT---GAAATAAGGAAAAACAAAAAA---AAAATAGCATTGA
AATTCATTTTTATTGACCAATTAGAATTGCCACCTAGGATCTACAATTGCCTAAAAAAAT
CCAATATACACACATTATTGGACCTTTTGAATAACAGTCAAGAAGATCTTATTAAAATGA
AGCATTTTCGCATAGAAGACGTAAAACAAATATTTGGCACTCTAGAAAAGCAT------TTC
GTAATTGATTTAAAAAATAAAAGAATGCTACGGGATGGAAAT---GAGGGAATGTCTACA

ATCCCTGGGTTTAATCAGATACAATTTGAAGGATTTTGGAGGTTCATTGATCAGGGCTTA
ACGGAAGAACTTTCTAAGTTTCCAAAAATGGAAGATACAGACCAAGAAATTGAATTTC
AATTATTTGTGGAAACATATCAATTAGCAGAACCCTTGATAAAAGAAAAAGATGCTGTG
TATGAATCACTTACATATTCTTCTGAATTATATGTGTCCGCGGGATTAATTTGGAAAACCC
GTAGGGAGATGCAAGAACAAACAATTTTGATTGGAAATATTCCTCTAATGAATTCTCTA
GGAACTTTTATAGTAAATGGAATATACAGAATTGTGATCAATCAAATATTGCAAAGCCCG
GGTATTTATTACCGATCCGAATTAGACCATAACGGAATTTCGGTCTATACCGGCACCATA
ATATCAGATTGGGGAGGAAGATCAGAATTAGAGATTGATAGAAAAGCACGGATATGGG
CTCGGGTGAGTAGGAAACAGAAAATATCTATTTTAGTTCTATCATCAGCTATGGGTTCA
AATCTAAGAGAAATTCTGGATAATGTTTGCTACCCGGAAATTTTCTTGTCTTTCCTGAAT
GATAAGGAGAAAAAAAAAAATTGGGTCAAAAGAAAATGCCATTTTGGAGTTTTATCAAC
AATTTGCTTGTGTGGGTGGGGATCCGGTATTTTCGGAATCCTTATGTAAGGACTTGCAA
AAAAAATTCTTTCAACAAAGATGCGAATTAGGAAGGATTGGTCGACGAAATATGAACC
GGCGACTGAACCTTGATATACCCGAAAATAATACGTTTTTGTTACCGCGGGATATATTGG
CAGCTGCGGATCATTTGATTGGAATGAAATTTGGAATGGGTACACTTGATGAT------ATG
AATCATTTGAAACATAAACGTATTAGGTCTGTCGCAGATCTCTTACAAGATCAATTCGGA
TTGGCTCTAGTTCGTTTAGAAAACGTGGTTCGAGGAACTATAAGTGGAGCAATTCGG---
CATAAATTAATA---CCGACTCCTCAGAATTTGGTAACTTCAACTCCA------------TTAACAA
CGACTTTTGAATCTTTTTTTGGATTACACCCATTATCTCAAGTTTTGGATCGAACTAATC
CATTGACACAAATAGTTCATGGGAGAAAATTGAGTTATTTGGGTCCTGGGGGATTGACA
GGGCGAACCGCTAGTTTTCGGATACGAGATATCCATCCTAGTCACTACGGGCGTATTTG
CCCAATTGACACATCTGAAGGAATCAATGTTGGGCTTATTGGGTCCTTAGCAATTCATG
CGAGAATTGGTCCTTGGGGG---TCTCTAGAAAGCCCTTATTAT---------GAAATTTCTGAG
AGATCAAAA------AGGGTACAGATGCTTTATTTATCGCCAAGTAGGGATGAATACTATATG
TTAGCGTCCGGAAATTCTTTGGCCCTGAATCAAGGTATTCAGGAAGAACAGGTTGTTC
CAGCTCGATATCGTCAAGAATTCCTGACTATTGCGTGGGAACAGGTTCATTTTCGAAGT
ATCTTTTCCTTCCAATATTTTTCTATTGGAGCTTCTCTCATTCCTTTTATCGAGCACAATG
ATGCAAATCGAGCTTTAATGAGTTCTAATATGCAACGTCAAGCAGTTCCGCTTTCTCAG
TCCGAGAAGTGCATTGTTGGAACGGGGTTGGAACGCCAAGTGGCCCTAGATTCGGGG
GTTCTCGCTATAGCCGAACATGAGGGAAAGATCATTTATACCAACACTGACAAGATTGT
TTTATTAGGGAATGGGAATACTGTAAGCATTCCATTAGTTATGTATCAACGTTCCAACAA
AAATACTTGTATGCATCAAAAACCTCAGATTCCGCGGGGGAAATGTGTTAAAAAGGGG
CAAATTTTAGCAGATGGTGCCGCTACAGTTGGTGGTGAGCTAGCTTTGGGAAAAAACG
TATTAGTAGCTTATATGCCGTGGGAAGGTTACAATTTTGAAGATGCGGTACTCATTAGTG
AGCGTCTTGTGTATGAAGATATTTATACTTCTTTTCACATACGGAAATATGAAATTCAAA
CTTATGTGACAAGTCAAGGT---CCTGAAAAGGTCACTAGCGAAATACCACATTTAGAAG
CTCATTTACTCCGCAATTTAGACAAAAATGGAATTGTGAGGTTGGGATCATGGGTAGAA
ACAGGTGATATTTTGGTAGGTAAATTAACGCCTCAGATGGCAAAAGAA---TCTTCGTAT
GCCCCCGAAGATAGATTATTACGAGCTATACTTGGCATTCAGGTATCCACATCAAAGGA
AACTTGTCTAAAACTACCTATAGGTGGTAGGGGCCGAGTTATTGATGTGCGATGGATCC
AGAAAAAGGGG---GGTTCCAGTTATAATCCAGAAACGATTCATGTATATATTTCACAAA
AACGTGAAATCAAAGTAGGCGATAAAGTAGCTGGAAGACATGGAAATAAGGGTATCAT
TTCCAGAATTTTGCTTAGACAAGATATGCCATATTTGCAAGATGGAAGACCTGTTGATAT
GATCTTTAACCCACTAGGAGTACCTTCACGAATGAATGTAGGACAGATATTTGAATGTT

CGCTCGGGTTAGCGGGGAGTCTGCTAGATAGACATTATCGAATAGCACCTTTTGATGAG
AGATATGAGCAAGAGGCTTCGAGAAAACTGGTGTTTTCTGAATTATATGAAGCCAGTA
AGCAAACAGCGAATCCGTGGGTATTTGAACCCGAGTATCCGGGAAAGAGTCGAATATT
TGATGGAAGAACGGGAGATCCTTTTGAACAACCCGTTATAATAGGAAATCCTTATATATT
GAAATTAATTCATCAAGTTGATGATAAAATTCATGGACGTTCTAGTGGACATTATGCACT
TGTTACACAACAACCCCTTCGAGGAAGAGCCAAGCAAGGAGGACAGCGGGTAGGAG
AAATGGAGGTTTGGGCTCTAGAAGGATTTGGTGTTGCTCATATTTTACAAGAGATGCTT
ACTTATAAATCAGATCATATTAAAGCTCGCCAGGAAGTACTTGGTACTACGATCATTGGG
GGAACAATACCTAACCCTGAAGATGCTCCAGAATCTTTTCGACTGCTCGTCCGAGAACT
ACGATCTTTGGCTCTGGAACTGAATCATTTCCTTGTATCTGAGAGGAATTTCCAGATTAA
TAGGATGGAAGCT---------------------ATGATCGATCAGTATAAACATCAACAACTCCGAATT
GGATCAGTTTCTCCTCAACAAATAAGTGCTTGGGCCACAAAAATCCTACCTAATGGAG
AGATAGTTGGAGAGGTGACAAAACCTTATACTTTTCATTACAAAACCAATAAACCAGA
AAAAGATGGATTATTTTGCGAAAGAATTTTTGGGCCTATAAAAAGTGGAATTTGTGCTT
GTGGAAATTATCGAGTAATCGGAGATGAAAAAGAAGACCCAAAATTTTGTGAACAATG
CGGAGTCGAATTTGTTGATTCTCGAATACGAAGGTATCAAATGGGCTATATTAAATTGGC
ATGCCCGGTAACTCACGTGTGGTATTTGAAACGTCTTCCTAGTTATATCGCGAATTTTTT
AGATAAACCTCTTAAAGAATTAGAGGGCCTAGTATACTGCGATTTT------TCATTTGCTAG
GCCT---ATAGCGAAAAAACCTACTTTTTTACGATTACGCGGTTTATTCGAATATGAAATC
CAATCCTGGAAATACAGCATCCCCCTTTTTTTTACTACCCAAGGTTTTGATACATTTCGA
AATAGAGAAATATCTACTGGAGCAGGTGCTATTCGAGAACAATTAGCCGATTTGGATTT
GCGTACTATTATAGATTATTCATTCGCAGAATGGAAAGAGTTAGGGGAAGAA---GGGTCT
ACAGGAAATGAATGGGAAGACCGAAAAGTTGGAAGAAGAAAGGATTTTTTAGTTAGA
CGCATGGAATTAGTTAAGCATTTTATTCGAACAAATATAGAACCAGAATGGATGGTTTTG
TGTCTATTACCTGTTCTTCCTCCCGAGTTGAGACCGATCATTCAGATAGATGGAGGTAAA
TTAATGAGTTCGGATATTAATGAACTTTATAGAAGAGTCATCTATCGGAACAATACTCTT
ACTGATCTATTATCAACAAGTAGATCTACGCCGGGAGAATTAGTAATGTGTCAGGAGAA
ATTAGTACAAGAAGCCGTAGATACACTTCTTGATAATGGAATCCGTGGCCAGCCAATGA
GGGATGGTCATAATAAAGTTTACAAGTCATTTTCAGATGTAATTGAGGGTAAAGAGGGA
AGATTTCGTGAGACTTTACTTGGTAAACGGGTCGATTATTCCGGACGTTCCGTCATTGT
CGTAGGCCCTTCACTTTCATTACACCGATGTGGATTGCCTCGTGAAATTGCAATAGAGC
TCTTCCAGACATTTGTAATTCGTGGTCTAATTAGGCAACATCTTGCTTCGAACATAGGA
GTTGCTAAGCGTAAAATTCGGGAAAAAGAGCCGATTGTATGGAAAATACTGCAGGAAG
TTATGCAGGGGCATCCTGTATTATTAAATAGAGCACCTACTCTTCATAGATTAGGTATACA
GGCGTTCCAACCTATTTTAGTGGAAGGGCGCGCTATTTGTTTACATCCGTTAGTTTGTAA
AGGATTCAACGCAGACTTTGATGGGGATCAAATGGCTGTTCATGTACCTTTATCTTTGG
AGGCTCAAGCAGAGGCCCGTTTACTTATGTTTTCTCATATGAATCTTTTGTCTCCAGCTA
TTGGCGATCCCATTTCCGTACCAACCCAAGATATGCTTATTGGACTCTATATATTAACGA
GCGGGAATCGTCGAGGTATTTGTGCAAATAGATATAATCCATGGAATCAC---------AAAA
CCTATCAAAATGAAAGAATTGATGATACTAATTATAAGTCTATGAAA------GAACCCTTTT
TTTGTAATTTCTATGATGCAATTGGAGCTTATCGTCAGAAAAGAATTCATTTAGATAGCC
CTTTGTGGCTCCGGTGGCAACTAGATCAACGC---ATTATTGCTTCA------AAAGAAGCTC
CCATTGAAGTTCACTATGAATCTTTGGGCACCTATCATGAGATTTATGCGCACTATCTAAT
AATAAGAAGTGTAAAAAAAGAAATTATTGATATATATATTCGAACTACTGTTGGTCATAT

TTCTCTTTATCGAGAAATTGAAGAAGCTATACAAGGATTTTATCAAGCCTGCTCA---------
----------------------------------ATGGCAGAACGGGCCAATTTGGTCTTTCACAATAAAGCGAT
AGATGGAACTGCCATGAAACGACTTATTAGTAGATTAATAGATCATTTTGGAATGGCATA
TACGTCACACATCCTAGATCAACTAAAGACTCTGGGTTTCCAGCAAGCCACTGCTACAT
CCATTTCATTAGGAATTGATGATCTTTTAACAATACCTTCTAAAGGATGGCTAGTCCAAG
ATGCTGAACAGCAAAGTTTGATTTTGGAAAAACACCACCATTATGGGAATGTACACGC
GGTAGAAAAATTACGTCAATCCATTGAGATATGGTATTCTACAAGTGAATATTTGCGACA
AGAAATGAATCCCAATTTTAGGATGACTGACCCTTATAATCCAGTCCATATAATGTCTTT
TTCGGGAGCTAGAGGAAATGTGTCTCAGGTACATCAATTAGTAGGTATGAGAGGATTAA
TGTCGGATCCACAAGGACAAATGATTGATTTACCTATTCAAAGCAATTTACGGGAAGGA
CTCTCTTTAACAGAATATATAATTTCTTGTTACGGAGCCCGCAAAGGGGTTGTGGATACT
GCTGTACGAACATCCGATGCTGGATATCTCACGCGCAGGCTTGTCGAAGTAGTTCAACA
TATTGTTGTGCGTAGAAGAGATTGTGGCACCATTCGAGGTATTTCTGTAAGTCCTCAAA
ACAGTACGATGCCG---GAAAGAATTTTGATCCAAACATTAATTGGTCGTGTATTAGCAG
ACGATATATATATGGGTTCACGATGCATTGCCACCCGAAATCAAGATATCGGGGTTGGAC
TTGTCAATCGATTCATAACG------------------CTTCGAACCCAACTAATATCCATCCGAACTC
CTTTTACTTGTAGGAGTGCGTCTTGGATCTGTCGATTATGTTATGGCCGGAGTCCTACTC
ACGGCGGCCTGGTTGAATTGGGAGAAGCTGTAGGTATTATTGCAGGTCAATCCATTGGG
GAACCTGGTACTCAACTAACATTAAGAACTTTTCATACGGGTGGAGTATTCACAGGAG
GGACTGCAGAACATGTACGAGCCCCTTCTAATGGAAAAATCCAATTCAATGAGGATTT
GGTTCATCCCACACGTACACGTCATGGACACCCTGCCTTTCTATGTTATATCGACTTGTA
TGTCACTATTGAGAGTGACGATATTCTACATAATGTGAATATTCCGCCAAAAAGTTTTCT
TTTAGTTCAAAATGATCAATATGTTGAATCTGAACAAGTGATTGCTGAAATTCGCGCAG
GGACATCCACCTTG---AATTTTAAGGAAAGGGTTCGAAAACATATTTATTCTGATTCAGA
AGGAGAA---ATGCACTGGAGTACTGATGTGTACCATGCACCCGAATTTACATATGGTAAT
GTTCATCTCTTACCAAAAACAAGTCATTTATGGGTATTATCAGGAAAGCCGTACAGA-----
-TCGAGTGTAGTCCCCTTTTCGCTCTCCAAGGATCAAGATCAAATGAACACTCAT------TC
TCTTTCTTTCGAACAAATATATATTTCTAACCCCTCA------GTCACTAACGATCAA---GTAA
AAGATAAATTATCGGACTCT---TTTAGTAAAAAA---GAGGATAGGATTACGGATTAT---TC
AGAACTTAATCGAATAGGT---------CATTGTAATCTCATCTATCCT------------GCAAAAAATT
TGGAT------TTATTGGCAAAGAAACGAAGAAATAGATTTATTATTCCATTTCAAGGAAGT
CAAGAACGAAAAAAAGAATTAATGTCCCTTTCCGGTATCTCGATTGAAATACCCATAAA
T------GGTATTTTCCGTAAAAATAGTATTTTTGCTTATTTCGATGATCCTCGATACCGAAGA
AAGAGTTCGGGAATTACAAAATATGGGACTATAGAGATGCATTCAATCGTTAAAAAAGA
GGAT------TTGATTGAATATCGA---GGAGTCAAAGAATTTCGACCAAAATACATGAAAGT
CGATCGGTTTTTTTTCATTCCTGAAGAAGTTCATATCTTAGCCGGATCTTCGTCCATAAT
GGTACGCAACAATAGTATCATTGGAGTAGATACGTGGATCACTTTAAATACAAGAAGCC
GAATAGGCGGGGTGGTCCGAGTGGAGAGGAAAAAAAAAAAAGATTGAACTTACCATTT
TTTCGGGAGATATCCATTTTCCCGGGGAGACAGATAAGATATCCCGACACAGTGGCATT
TTGATACCACCAAGCAGGAAA------------AATTCCAAGGACTCAAAAAAT------TTAAAAA
AATGGATCTATGTCCAACGG------ATCACCCCTACTAAGAAAAAA------TATTTTGTTTTG
GTTCGGCCGGTAGTCCCATATGAAATAACGGACGGTATCAATTTAGCAACACTTTTCCC
TCAGGATTTGTTGCAGGAAAGGGATAATGTGCAACTTCGAGTTGTCAATTATATCCTTTA
TGGAAATGGTAAAGTCACTCGAGGAATTTCTGACACAAGTATTCAATTAGTACGTACTT

GTTTAGTATTGAATTGGAATCAAGATAAA---------AAAGGTTCTTCTATCGAAGAGGCCC
GCGGTTCCTTTGTTGAAGTAAGAACAAATGGTATGATTCAAGATTTCTTAAAGGTCAAT
TTAGTGAAACCCGCTATTTCATATATCAGTAAAAGAAATGATCCATCA---TCCGAAAAAA
AGGAGGGA---------TCAGATCATACCAATATGAATCCATTTTATTCCATTTATATTTATCCA
AAGACA---------AAACTTCAAAAATCATTTAACCAAAACCAAGGAACTGTCCGTACGTT
GTTGGGTATA---------AACAAAGAATGTCAATTTTTTTTAATTTTGTCATCATCCAATTGTT
TTCGAATAGGTCCA---------------TTCAAGGGT------------------GTCAAATATCCCGATCCCCT
AATTCCAATTAGGAATTCATTTGGTCCTTTAGGAACA------GCCCTTCAAATTGCTAATTT
TTTTTCA------TTTTACTATTTAATAACT------CATAATCAGATCTTG---GTAACTAATTATTT
GCAACTTGACAAT---------TTAAAACAAACCTTTCAACCATTTAAATTTCAA---TATTATTT
AATGGATGAAAATGGAAGA------ATTTATAATCCCGATCCATGCAGTAACATCATTTTTAA
TCCATTCAAGTTGAATTGGTATTTTCTTCATTATCATTTTTGTGAAGAGACATCCACCAA
AATTGAT---CTTGGACAATTTGTTTGTGAAAATGTATGTATAACCAAAAAAGGAACG------
---CACCTAAAATCGGGTCAAGTTCTAATTGTTCAA---TTTGACTCTGTAGTAATAAGATC
GGCTAAGCCTTATTTGGCTACTCCAGGAGCAACACTTCATGGTCATTATGGAGAAATCA
TTTATGAAGGGGATACATTAGTTACATTTATATATGAAAAATCGAGGTCTGGTGACATAA
CGCAAGGTCTTCCAAAAGTGGAACAAGTCTTAGAAGTTCGTTCGATTGATTCAATATCC
ATCAATCTAGAAAAGAGGATTGATAGTTGGAACGAACGTATAACAAGAATTCTTGGAA
GTCCTTGGGGATTCTTGATTGGGGCTGAGCTAACTATAGCGCAAAGTCGTATTTCTTTG
GTTAACAAGATCCAAAAGGTTTATCGATCACAAGGGGTGCAGATCCATAATAGACATAT
AGAAATTATTGTACGTCAAATAACATCAAAAGTCTTGGTTTCAGAA---------GATGGAAT
GTCTAATGTTTTTTTGCCCGGAGAACTAATTGGATTGTTTCGGGCGGAACGAACGGGA
CGCGCTTTGGAAGAAGCGATATGTTACCGCGCTACCTTATTGGGAATAACGAGAGCATC
TCTGAATACTCAAAGTTTTATCTCCGAAGCGAGTTTTCAAGAAACTGCTCGAGTTTTAG
CAAAAGCAGCTCTACGGGGCCGTATTGATTGGTTGAAAGGACTAAAAGAAAACGTTGT
TCTGGGGGGGATGATACCTGTTGGTACCGGATTCAAAGGATTCGTACACCATTCA------A
GTCAACATAAG------GACATTCCTTTGAAAACAAAAAAGCAGAATTTATTCGAGGGAGA
AATGGGA------GATATTTTGTTTTACCACAGAGAATTATTTGAGTCTTGTCTTTCAAAGAA
T--ATGACAAGAAGATATTGGAACATCAAT
TTGGAAGAGATGATGGAAGCGGGAGTTCATTTTGGTCACGGTACTCGGAAATGGAATC
CTCGAATGTCGCCTTATATCTCTGCAAAATGTAAAGGTATTCATATTATAAATCTTACTAG
AACTGCTCGTTTTTTATCAGAGGCTTGTGATTTAGTTTTTGATGCATCAAGTAGAGGAA
AACAATTTTTAATTGTTGGGACAAAAAATAAAGCAGCTGATTCAGTAGCACGGGCTGC
AATAAGGGCTCGCTGTCATTATGTTAATAAAAAGTGGCTTGGGGGTATGTTAACGAATT
GGTCCACGACAGAAACGAGACTTCACAAATTCAGGGACTTGAGAATGGAACAAACGG
CAGGGAGACTCGCTCGTCTTCCAAAGAGAGATGCGGCGGTGGTGAAGAGACAATTAT
CTCACTTGCAAACATATTTGGGTGGGATCAAATATATGACAGGGTTACCGGATATTGTAA
TCATCGTTGATCAACAGGAAGAATATACGGCCCTTCGAGAATGTATTACTTTGGGAATT
CCAACTATTTGTTTAATTGATACAAATTGTAACCCGGATCTCGCAGATATTTCGATTCCG
GCAAATGATGACGCTATAGCTTCAATTCGATTAATTCTTACCAAATTAGTATTTGCAATTT
GTGAAGGCCGCTCT---AGCTATATAAGAAATCCTATGGGACAAAAAATAAATCCACTTG
GTTTCCGACTTGGTACAACACAAAGTCATTATTCCCTTTGGTTTTCACAACCAAAAAAC
TATGCAGAAGGTCTACAAGAAGATCAAAAAATAAGGGATTGTATAAAAAATTATGTACA
AAAAAATACAAAAACATCCTCGGGT---GTCGAAGGAATTGCACGTATTGAAATTCAAAA

AAGAATCGATCTGATACAGGTGATAATCCATATGGGATTCCCAAAATTATTAATAGAAAA
TAGACCACAA------------GGGGTCGAAGACCTAAAAATAAATGTACAAAAAGAATTGAAT
TGCGTGAACCGAAAACTCAACATCGCTATTACAAGAATTGCAAAACCTTACGGGGACC
CTAATATTCTTGCAGAATTTATAGCCGGACAACTAAAGAGTAGGGTATCATTTCGAAAA
GCAATGAAAAAAGCTATCGAATTAACCGAACAAGCTGATACAAAAGGAATTCAAATCC
AAATTGCCGGCCGTATCGATGGAAAAGAAATTGCACGTATCGAATGGATAAGAGAAGG
TAGGGTTCCCCTACAAACGATTCGCGCAAAAATCGATTATTGTGCCTATACCGTTCGAA
CAATCTATGGCGTATTGGGGATAAAAATTTGGATATTTATGGGCGAGGAAATGTCGCGTT
ACCGAGGGCCTCGTTTCAAAAAAATACGCCGTCTAGGAGCTTTACCTGGACTAACTAAT
AAAAGGCCTAGAGCCGGAAGTGATCTTAGAAACCAATCACGTTCCGGGAAA---AGATC
TCAATATCGTATTCGTCTAGAAGAAAAACAAAAATTGCGTTTTCATTATGGTATTACAGA
ACGACAATTACTTAAATACGTTCGTATCGCCAGAAAAGCCAAAGGGTCAACAGGTCAG
GTTTTACTACAATTACTTGAAATGCGTTTGGATAATATCCTTTTTCGATTGGGCATGGCTC
CGACTATTCCTGGAGCCCGCCAATTAGTTAACCATAGACATATTTTAGTTAACGGCCGCA
TAGTAGATATACCAAGTTATCGTTGCAAACCCCAAGATACTATTATGGCGAGGGATGAA
CAAAAATCCATAGCTCTAATTCAAAATTCTCTTGATTTATCCCCCCGC---GAGGAATTAC
CCAAACATTTGACTCTTAACCCATTCCCA------TATAAAGGATTAGTTAATCAAATAATAG
ATAGTAAGTGGGTCGGTTTAAAAATAAATGAATTACTAGTGGTAGAATATTATTCTCGTC
AGACGATGTCACGTCGAGGTACTGTAGAAGAAAAAACTGCAAAATCCGATCCAATTTA
TCGTAATCGATTAGTTAACATGTTGGTTAACCGTATTCTGAAACACGGAAAAAAATCAT
TGGCTTATCAAATTCTCTATCGAGCCGTGAAAAAGATTCAACAAAAGACAGAAACAAA
TCCACTATCTGTTTTACGTCAAGCAATACGTGGAGTAACTCCCGATATAGCAGTAAAAG
CAAGACGTGTAGGCGGATCGACTCATCAAGTTCCCATTGAAATAGGATCCACACAAGG
AAAAGCGCTTGCCATTCGTTGGTTATTAGGGGCAGCCCGAAAACGTCCGGGTCGAAAT
ATGGCTTTCAAATTAAGTTCCGAATTAGTGGATGCTGCCAAAGGGAGTGGCGATGCCGT
ACGCAAAAAGGAAGAGACTCATAGAATGGCAGAGGCAAATAGAGCTTTTGCACATTTT
CGTATGGGTAAGGACACTATTGCTGATATAATAACCTGTATACGAAATGCTGACATGAAT
AGAAAAGGAACGGTTCGAATAGTATCTACTAACATCACCGAAAACATTGTTAAAATACT
TTTACGAGAAGGCTTTATCGAAAATGCGAGAAAACATCAAGAAAGAAACAAATATTTT
TTGGTTTTAACTCTGCGACATAGAAGA------AACAAGAAAGGGCCTTATCTAAAT---------
ACTTTCCATTTAAAAAGAGTCAGTCGACCTGGTCTACGAATCTATTCTAATTATCAACGA
ATTCCTAGAATTTTAGGTGGAATGGGGATTGCAATTCTTTCTACCTCGAGAGGTATAATG
ACAGATCGGGAGGCTCGACTAGAAGGAATTGGCGGAGAAATTTTATGTTATATATGGAT
GGCAAAACCTATACCAAAAATTGGTTCACGTAGAAATGGACGTATTAGTTCGCGTAAA
AGTGCACGTAAAATACCAAAGGGTGTTATTCATGTTCAAGCAAGTTTTAATAATACCAT
TGTAACTGTTACAGATGTACGAGGTCGAGTCGTTTCTTGGGCTTCTGCCGGTACTTGTG
GATTCAGGGGTACAAAAAGAGGAACACCATTTGCGGCTCAAACCGCAGCGGGAAATG
CTATTCGTACGGTGGTGGAACAAGGTATGCAACGAGCAGAAGTCATGATAAAAGGTCC
TGGTCTCGGAAGGGATGCAGCATTACGGGCTATTCGTAGAAGCGGTATACTATTAAGTT
TCGTGCGAGACGTAACCCCTATGCCGCATAATGGCTGTAGGCCTCCTAAAAAAAGACG
CGTCATGCCAACCATTAAACAACTTATTAGAAACACAAGACAGCCAATCAGAAATGTC
ACGAAATCCCCCGCGCTTCGGGGATGCCCTCAGCGCCGAGGAACATGTACTAGGGTGT
ATACTATCACCCCAAAAAAACCAAACTCTGCCTTACGTAAAGTTGCCAGAGTACGATTA
ACCTCTGGATTTGAAATCACTGCTTATATACCTGGTATTGGCCATAATTTACAAGAACAT

TCTGTAGTCTTAGTAAGAGGGGGAAGGGTTAAGGATTTACCCGGTGTGAGATATCACAT
TGTTCGAGGAACCCTAGATGCTGTCGGAGTAAAGGATCGTCAACAAGGGCGTTCTAAA
TATGGGGTCAAAAAGCCAAAAATGGCAAGGAAAAGTTTGATTCAGCGGGAGAAGAAG
AGAAGAAATTTAGAACAAAAATATCATTTGATTCGTCGATCCTCAAAACAAGAAATACG
C---------AAAGTAACATCGTTGAGTGATAAATGGGAAATTCATGGAAAGTTACAATCTCC
ACCACGTAATAGTGCACCTGCGCGTCTTCATCGACGTTGTTTTTGACCGGAAGACCG
AGAGCTAACATACGAGACTTTGGGTTATCTGGACATATACTTCGTGAAATGGTTCACAC
ATGTTTGTTGCCAGGGGCAACAAGATCAAGTTGGATGAAAAAAAATTCATTCATATCCG
TAATTTCGGATGAA------AAAAAAGAAGAAAACAAGGGTTCTGTTGAATTTCAAGTATT
CTGTTTTACCAATAAGATAAGAAGACTTACTTTACATTTGGAATTGCACAAAAAAGACT
ATTCATCTCAGCGGGGTCTACGAAAAACTCTGGGAAAACGTCAACGACTGCTGGCTTA
TTTGTTAAAGATAAATGGAGTACGTTATAAAGAATTAATCAGTAAATTGAACATTCGAG
AACTAAAAACACGT------ATGGTAAAACTTCGTTTGAAACGATGTGGTAGAAAGCAACG
AGCCGTCTATCGAATCGTTGCAATTGATGTTCGGTCCCGAAGAGAGGGAAGAGATTTG
CAGAAAGTGGGTTTTTATGATCCGATAAAAAGTCAAACTTATTTAAATGTTCCTGCTATT
CTAGATTTTCTTGAAAAGGGTGCTCAACCTACAGAAACTGTCTATGATATTTTAAAGAG
GGCAGAGGTTTTTAAAGAATTTCGACTTAATCAAACGAAGTTCAAT---ATGGATAAATC
CAAGCGACCCTTTATTAAATCCAAGCGCTCTTTTCGTAGGCGTTTGCCCCCGATCCAAT
CGGGGGATCGAATTGATTATAGAAACATGAGTTTAATTAGTCGGTTTATTAGTGAACAA
GGAAAAATTTTATCTAGGCGAGTGAATAGATTGACCTTGAAACAACAACGATTAATTAC
TAGTGCTATAAAACAAGCTCGTATTTTATCTTTGTTACCTTTTCTTAATAATGAGAAA-----
-CAGTTTGAAAGAACCGAGTCGACTACTAGAACTGCTAATTTTAGAACTAAAAATAAAG
TGACACGTTCACTAAAAAAAAATCCTTTTGTAGCGAATCATTTATTAAGAAAAATCGAG
AAGCTTAACAAAAAGGCGGAAAAAGAAATAATAGTAACTTGGTCACGGGCATCTACCA
TTATACCCACAATGATTGGCCATACGATTGCTATCCATAATGGAAGAGAACACTTGCCCA
TTTATATAACAGATCGTATGGTAGGTCATAAATTAGGCGAATTTGCACCAACTCTCAATT
TCCGGGGACATGCAAAAAACGATAATAAGTCTCGTCGTATGATTTTTCAATCTTTTCTAC
TAGGTAATCTAGTATCCTTATGCATGAAGATAATCAATTCGGTCGTTGTGGTCGGACTCT
ATTATGGATTTCTGACCACATTCTCCATAGGGCCCTCTTATCTCTTCCTTCTCCGAGCTC
AGGTTATGGAAGAAGGAGAAGAAGGAACCGAGAAGAAGGTATCAGGAACAACAGGT
TTTATTATGGGACAGCTCATGATGTTCATATCGATCTATTATACGCCTCTGCATCTAGCAT
TGGGTAGACCTCATACAATAACTGTCCTAGCTCTACCCTATCTTTTGTTTCATTTCTTCTG
GAACAATCACAAACACTTTTTTGATTATGGATCCACTAGCAGAAATTCAATGCGTAATC
TCAGCATTCAATGTGTATTCCTGAATAATCTCATTTTTCAATTATTCAACTATTTCATTTTA
CCAAGTTCAATGTTAGCCAGATTAGTGAACATTTATATGTTTCGATGCAACAACAAGAT
GTTATTTGTAACAAGTAGTTTTGTTGGTTGGTTAATTGGTCACATTTTATTCATGAAATG
GGTTGGATTGGTATTAGTCTGGATACAGCAAAATAATTCTATTAGATCTTACCTTGTGTC
A-----------------GAATTGAGAAATTCTATGGCTCGAATCTTTAGTATTCTCTTCTTTATTACC
TGTGTCTACTATTTAGGCAGAATGCCGTCACCTATTTTTACT------CTGAAACAAGAAGA
AGATCCTAAACCCCTTATTTTTCTTCTTTTCGACTATAAACGTTGGAATCGTCCAATGCG
ATATATAAAAAACAATCGGTTTGAAAATGCGGTAAGAAATGAAATGTCACAATATTTTTT
TTATACATGTCAAAATGATGGAAAACAAAGAATTTCTTTTACATATCCACCTAGTTTATC
AATTTTCTGGGAAATGATACAAAGAAAGATTTCTTTGGCTACAACAGAAAAATTCTTAG
ATGATGAACTATACAATTATTGGATTTATACGAATGAACAAAAAAAAAAC---TTAAGCAA

TGAATTTGCTAATAGAATTACAGTTCTAGACAAAGGACTT------ATAGATGTACTTGATAA
AAAAACTAGATTATGT------AAAAGTAAAAATGAATATTTACAAAAAGACCATGATCCTT
TATTAAATGGATCCTATCGTGGTATAAAAAAAACTCTTTTACCTTTTATAGATGAAATTTT
TATAAATAAAATTCATAGTGTTCTGTACCAGGAATTTGAATATAAAGATTCCTTTACTCA
A---ATTGGAATAAAAGAAATAAGTAAAAAGGTTCCTCATTGGTCATACCAACTAATTGA
ATCCGAACAAGAGGAAATGGAACAAATTCGCTCAAGGAGCGGAAAAGAAGTAGTTTT
TTTTACGGATGTTTTGACACGTTATCCACAAGAATCTGATTTTCGTCGAGATATAATCAA
AGGTTCCATGCGTAGTCAAAGACGTAAAATAGTTATTTGGGAATTGTTTCAAGCAAATA
TACATTCCCCACTTTTTTTAGACAGAACGAATAAATCCTCTTTTTTTTCTATTACTTTT-----
-TCCAGATTAATTAAACGAATTTTTAAAAATAAACAAGAACAAATACGACTAGACATAG
CCGAAACTTGGGATACTATTCCAGGTGCTCAAATAATAAGAAGTTTGATATTACTAACTC
AATCAATTCTTAGAAAATACATTCTATTGCCTTTATTGATAACAGGAAAAAAATATCGGTC
GTATATTATTATTCCAACTTCCTGAGTGGTCGGACGATTTCAAGGAGTGGACTAGTGAA
ATGCATATTAAATGTACCTATAATGGTGTTCAATTATCAGAAAAAGAATTTCCTAAAAAC
TGGTTAACAGATGGTATGCAGATAAAGATATTATCTCCTTTCTGTCTAAAACCTTGGCAT
AAATCTATGCAAAATCAAATAGATGCTTTTTGCTTTTTAACAGTTGTGGGATTAGAAACT
GACATCCCTTTTGGTCCTCCCCGAAAACGGCCTTCTTTTTTTCAACCTATTTTTAAACAA
CTCGACAAAAAAATTGAAAAACTTATAAAAGGAAACTTCCAAGTT---CGAAAAAGACT
AAAAGAA------AAATTTTTTCTAAAGTTACAAAATGAAACAAACAATTGGATTATTGAAA
TCTTTCCT------TTTTTTAAAAAAATAATACGAAAAATGTCAACCGTAAATGATTCAAGAA
TCAAAAATCATATGATTCATGAATCATCCGTTCAAATTCGATTCTTGAATCAGACAAATT
CTTCAGTGACCGAAAAAAAAATGAAAGATTTGGCTAATAGAACAAGGATAATTAAAAA
TAAAATAGAAAAAATTTCAAACGACAAACTAAAA---------------------------AAAACTCGTTA
TGGTACGAAAAATTTGGGTCAGATATTAAAAAGAAGAAATGCTCGATTAATCCGTAATT
CAAATTATATTTTGAAATTTTTTAGGGAAAGGATATACGGAGATATATTTCTATATATCATT
AATATTCCCAAAATTAATACACAACTTTTTATTCAGTTTATTTCA---GATTTTTCTTTTTTG
TCACAAGCCTATGTATTTTACAAATTATCCCAAGCCAAAATTCTTAACTTATATAAGTTA
AGATTAGTCCTTCAGTATATATCTTTATTTCTTAAAAATGAAATAAAAGATTTTTTTACTC
AAGGAATAACTAATTCCGAGTTAAAGACTAAAAAACTTCCGAATTCCGGAATGAACCA
ATGGAAAAACTGGTTAAAATTGAAATATCAATACAATTTATCTCAGCTAAAATGGTCTC
GATTAGTACCACAAAAATGGCGAAATAGAGTAACTGAACATTGTGAGGTTGAAAAT-----
----ACAAATTTATATCAAAACGAAGAA------TTAATTAATTCCAAAAAGCAT------TTTCAA
AAGAACTATAGATATGATGTTTTATCATATAAATTTTTTAAT---------------GATAAAAATAAA
TATTTGGATTTTAAAATTCTCGATTTTTCTCTTCGACTTTTTGATTGGATGGGAATGAATG
AAAAAATACTGAGCCGTTGGTTTTTCTCCGAATTTTTTTCATTTTATAATGCATATAAAAT
GAAACCGTGGGTTATACCAATTAATTTGCTTTTTTCAAATCCTAAGAGTGTTATAGAAGC
AGAATTCGATTTATTCCTAAAAAGATATTTGCTTTTTCAATTGAAATGGGCCGATTCTTT
GAATGAAAAACTGATGGATAATATCCAAGTATATTGTCTCGTGCTTCGATTGATCAATCC
AATAGAAATTCTTATATCCTCTATAGAAAGAAAAGAACTGAGTATGGATATAATGCTAGA
TCGGAAAGATTTTAATTGTCCAAACTGGAAACAAAAAAGAGTATTGATTATTGAACCGA
TTCGTCTTTCTATAAGGGGCGATGGACAATTTCTTCTGTATCAAACCATAGGTATTTCAT
TGGTTCATAAGAGTAAACACAACAATTTTGATTTGCTTGCTCCTGAAAATCTTTTATCGC
CTAGGCGTCGTAGAGAATTAAGAATTCTACTTTGTTTG---TTTCTTTGGCCCAATTATCGA
TTAGAAGATTTAGCTTGTATGAACCGTTTTTGGTTTGATACTAATAACGGAAGTCGTTTT

AGTATATTAAGAATACATATGTATCCAATGAAAGGACATCAATTCAAATCCTGGATTTTC
GAATTGAGAGAGATATTGAGAGAGATTAAGAATTCTCACTATTTCTTAGATTCATGGAC
CCAATTCAATTCAGTGGGATCTTTCATTCACATTTTTTTCTACCAAGAACGTTTTTTAAA
ACTCTTTGACCCCCGAATTTGGAGTATCCTACTTTCA---CCCAATTCA------CAGGGTTCA
ACAAGCAATCGATATTTC---------ACGATCAAGGGTGTAGTACTCTTTGTGGTAGTG---GT
CCTTATATATCGT------------ATTACCAATCGAAATATGGTCGAAAGAAAAAATCTCTATTT
GATAGGGCTTTTTCCT---------------ATACCTATGAATTCCATTGGACCCAGAAATGATACAT
TGGAAAAATCCTTT---GGGTCTTCCAATATCAATAGGCTGATTGTTTCA---CTCCTCTATC
TTCCAAAAGGAAAAAAGATTTCTGAGAGTTATTTCCTGGATCCGAAAGAGAGTACTTG
GTTTCTTCCAATAACT------------AAAAAGTGTATCATGCCTGAATCTAACCGAGGTTCGC
GGTGGTGGAGGAACTGGATCGGAAAAAGGAGG------GATTCTAGTTGTAAGATATCTAA
T---------GAAACCGTCGCTGGAATTGAGATCTCATTCAAAGAGAAAGATATCCAA---TATC
TGGAGTTTCCCTTTGTATATTATATGGAC------GATCCGATCCGCAAGGACCATGATTGGG
AATTGTTTGATTGTCTTTCTCTT--

----------------TTTTTGAGGAAC---GTATCGAGAGAGAATTGGATTTGGCTAGACAATGTGC
GGTTGGTAAATAAGGATCGG---------TTTTTTAGC------AAGGTACGGAATGTATCGTCAAA
TATTCAATATGATTTC---------ACAAGA------TCTAGTTTTGTTCAAGTAACGGATTCTAGC
CAATTGAAAGAATCTTCT---GATCAATCTAGAGATCGTTCGAATTCTATTAGT------------AA
TGCGGATTCGGAA---------------------------------------TATCACACATTAATCAATAAAAGAGA
GATTCAACAACTAAAAGAAAGATCGATTCTT---CGGGAT------CCTTCCTTTCTTCAAACG
GAAGGAACAGAGATAGAATCAGAC------CGATTCCCGAAA------TGCCTTTCTGGATATT
CC------TCAATGCCTCGGCTATTCACG---GCACGTGAGAAGCAGATGATTATTCATCTGCT
TCCGGAAGAAATCGAACAACTT------CTTGAGAATCCTACAAGATCTATTCGTTCTTTTT
TCTCTGGCAGATGG------TCAGAACTTCAT---CTGGGTTCAAATCCTACTGAGAGGTCCA
CTAGAGATCCG------------CAATTGTTGAAGAAACAACAAGATGTTTCT---TTTGCCCCTT
CCAGGCAATCGGAA------------AATAAAGAAATGGTTAATATATTCAAGATAATTAAGTATT
TACAAAAT------ACCGTCTCAATTCATCCTATTTCATCAGATCCGGGATGTGATATGGTTC
CG---AAGGATGAACTGGAT---ATGGACAGTTCCGATAAGATTTCATTCTTGAACAAAAAT
TCATTTTTTGACTTA------TTTCATCTATTCCATGACCGAAACAGGGGGGGATACGCATTA
------CACCACGATTTTGAATCCGAA------GAGAAATTTCAAGAAATGGCAGATCTATTCAC
TCTATCAATAACCGATCCG---GATCTGGTGTATCATAGGGGATTTTCCTTTTCTATTGATT
CCTGCGGATTGGATCAAAAACAA------TTCTTGAATGAGGTATTCAACTCCAGGGATGA
ATCAAAAAAGAAATCTTTA---------TTGGTTCTATCTCCTATTTTTTAT------GAAGAGAATG
AATCTTTTTATCGAAGGATCCGAAAAAAAGGGGTTCGA---ATCTCC---------------------------
---AGA
AATGTATTGAATCGATTCTTTTTAATAAAT---------AGATCCGATCGCAGCTTCGAATATGG
AATTCAA---------AGGGATCAAATAGGAAACGATACTTTGAATCATAGAACTATAAGGAA
ATATATGATCAACCAAGATTTCTCGAATTTGAAAAAG---AGTCAGAAGAAATGGTTCGA
TCCT------CTTATTTTTCTTTCTCGAACCGAGAGATTCATGAATCGGGATCCTGATGCATAT
AGATACAAATGGTTCAATGGGAGCAAGAATTTCCAGGAACATTTGGAACATTTCGTTTC
TGAGCAGAAGAGCCGT---------TTTCAAGTAGTGTTCGATCAATTACGTATTAATCAATAT
TCGATTGATTGGTCTGAGGTTATTGACAAAAAAGATTTGTCTAAGTCACTTCGTTTCTTT

TTGTCCAAGTCACTTCGT------------------------TTCTTTTTGTCC---------------------AAGTTGC
TTCTCTTTTTGTCTAACTCACTTCCT---TTTTTCTTTGTGAGTTTCGGGAATATCCCCATTA
ATAGGTCCGAG---ATCCGCATTTAT---GAATTGAAAGGTCCGAATGATCAACTCTACAAT
CCGTTGGTAGAATCAATAGGTCTTCAAATCGTTCATTTGAAAAAATGGAAAGCCTTC----
--TTATTGGAT---------GATCATGATACTTTC---CAAAAATCGAAATTCTTGATCAATGGAGG
AACAATATCACCATTTTTGTTCAATAAGATACCA---AAGTGG---ATGATTGACTCATTCCA
TACTAGA------AATAATAGCGGGAAATCCTTTGATAACACGGATTCCTATTTCTCAATGAT
ATCCCACGATCAAAACAATTGGCTGAATCCCGTAAAACCATTTCATAGAAGTTCATTGA
TATCTTCTTTTTATAAAGCAAATCAACTTCGATTCTTGAATAATCCACATCACTTCTGCTT
CTAT------TGTAACAAAAGATTCCCTTTT---------TAT---ATGGAAAAGGCCCGTATCAATAA
TTCTGAT------TTTACGTATAGA---CAATTCCTCAATATCTTGTTCATTCACAACAAA---CTA
TTTTCTTTGTGTGTCGGTAAA------AAAAAACATGCTTTTTTGGAG---AGAGATACTATTT
CACCAATCGAGTCACAGGTATCTAACATATTCCTACCTAACGATTTTCCA--------------------
--ATTCGATCCGATCTATTAGTTCGT------AGAACTATTTACT
CGATCGCAGACATTTCTGGAACACCTCTAACAGAGGGACAACTAGTCCATTTTGAAAG
AACTTATTGTCAACCTCTTTCCGATATGAATCTATCTGATTCAGAAAAGAAGAACTTGCA
TCAGTATCTCAAT------TTCAATTCAAACATGGGTTTTATTTACACTCCATGTTCTGAGAA
ATATTTACTATCGGAAAAG------AGGAAAAAA------------------------CGGAGTCTTTGTCTAA
AG------AAATGCGTTGAGAAAGGGCAGATGTATAGAACCTTTCAACGAGATAGTGCTTT
TTCAACTCTCTCAAAATGGAATCTATTCCAAACATATATGCCATGGTTCCTTACTTCGAC
AGGGTACAAATATCTAAATTTTCTATTTTTAGATACTTTTTCAGAC---TTATTGCCGATACT
AAGTAGCAGT------CAAAAATTTTTATCTATTCTTCATGATATTATGCACGGATCAGGTATA
TCATGGCGAATTCTTCAGAAAAAATTG------------------TGTCTTCCACCATGGAAT---CTGA
TAAGTGAGATTTCGAGTAAGTGTTTACATAAT------CTTCTTCTGCCCGAAGAAATGATTC
ATCGAAATAATGAGTCA------CCATTGATATGGACACATCTGGCATCGCCAAATGTTCGG
GAGTTC---TTCTATTCAATCCTTTTCCTTCTTTTTGTTGCTGGATAT---CTCGTTCGTACCC
ATCTTCTCTTTGTTTTCCGA------GCCTCTAGTGAGTTACAGACAGAGTTCGAAAGGGTA
AAATCTTTGATGATTCCATCATACATGATTGAGTTGCGAAAACTTCTGGATAGGTATCCT
ACATCGGAACCGAATTCCTTCTGGTTAAAGAATCTCTTTTTGGTTGCTTTAGAACAATT
AGGAGATTCCCTAGAAGAAATAAGGGGTTCTGCTTCTGGCGACAACATGCTATTGGGT
GGTGGTCCCGGTCCC---------GCTTATGGG---TTCAAATCAATACGTTCTAAGAAAAAATA
TTTGAATATTAATCTCATCGATATCCTCGATCTCATA---AGTATCATACCAAATCCCATCAA
TCGAATCACTTTTTCGAGAAATACGAGACATCTAAGTCATACAAGTAAAGAGATCTATT
CATTGATAAGAAAAAGAAAAAGGGTGAACGGTGATTGGATTGATGATAAAATAGAATC
CTGGGTCGCAAGCAGTGATTCGATTGATGATGAAGAAAGAGAATTCTTGGTTCAGTTC
TCC------ACTTTAACGACA------GAAAAAAGGATTGATCAAATTCTATTGAGTCTGACT---
CATAGTGATCATTTATCA---------------AAGAATGACTCTGGTTATCAACTGATTGAACAAC
CGGGAGCAATTTACTTACGATACTTAGTTGACATTCAT------AAAAAGTATCTAATGAATT
ATGAGTTCAATACATCTTGTTTAGCAGAAAGACGAGTATTCCTTGCTCATTATCAGACAA
TCACTTAT------TCACAAACCTCGTGTGGGGCTAATAGTTTTCATTTCCCATCTCATGGA--
-------AAACCCTTTTCGCTCCGCTTAGCCCTATCCCCCTCT---AGAGGTATTTTAGTGATAG
GTTCTATAGGAACTGGACGATCCTATTTGGTCAAATACCTAGCGACAAACTCCTATGTTC
CTTTTATTACGGTATTTCTGAACAAGTTCCTGGATAACAAGCCTAAAGGTTCTCTTATTG
AT---------------GCTAGT------------------GACGATATCGATCGT--

---GACCTTGATACGGAGCTGGAGCTGCTAACTATGATGA
ATGCGCTA---------ACTATGGATATGATGCCG---------GAAATAGACCAATTTTCTATCACCC
TTCAATTCGAATTAGCAAAAGCAATGTCTCCTTGCATAATATGGATTCCAAACATTCATG
ATCTGGATGTGAATGAGTCGAATTACTTATCCCTCGGTCTATTCGTGAACTATCTCTCC---
------AGGGATTGTGAAAGAGGGTCT---ACTAGAAATATTCTTGTTATTGCTTCGACTCATA
TTCCTCAAAAAGTGGATCCCGCTCTAATAGCTCCGAATCAATTAAATACATGCATTAAGA
TACGAAGGCTTCGTATTCCACAACAACGAAAGCACTTTTTCACTCTTTCATATACTAGG
GGATTTCACTTGGAAAAGAAAATGTTCCATACT------AATGGATTCGGGTCCATAACCAT
GGGTTCCAATGTACGAGATCTTGTAGCATTTATCAACGAGGCCCTATCGATTAGTATTAC
ACAGAAGAAATCCATTATAGACACTAATACAATTCGATCCGCTCTTCATAGACAAACTT
GGGATTTGCGA---------TCCCAGGTAAGATCGGTTCAGGATCATGGGATCCTTTTCTATCA
GATAGGAAGGGCTGTTGCACAAAATGTACTTCTAAGTAATTGCCCGATAGATCCTATATC
TACCTATATGAAGAAGAAA------TCATGTAACGAAGGGGATTCTTATTTGTACAAATGGT
ACTTCGAACTTGGAACGAGCATGAAGAAATTAACGATACTTCTTTATCTTTTGAGTTGT
TCTGCCGGATCGGTCGCTCAAGATCTTTGGTCTCTACCC------GGACCCGATGAAAAA---
AACGGGATCACTTCTTATGGACTCGTTGAGAATGATTCT---TATCTAGTTCATGGCCTATT
AGAAGTAGAAGGCGCTCTGGTGGGA------TCCTCACGG------ATAGAAAAAGCTTGCAG
TCAGAATGAT------CGAGTGACATTGTTTCTTCGGCCCGAACTAAGGAAT---CCCTTAGAT
ATGATGCAAAATGGATCTTGTTCTATCCTTGATCATAGATTTCTCTATGAAAAA------TAC
GAATCGGAATTGGAAGAA---------GGGGAAGGAGCCCTCGATCCGCAA------------CAGAT
AGAGGAGGAT------------TTATTCAATCAC------ATAGTTTGGGCTCCT------AGAATATGGA
ACCCC---------TGGGGCTTTCTATTTGATTGTATCGAAAGGCCTAATGAATTGGGATTTCC
C---TATTGGGCCAGGTCATTTCGGGGCAAG---------CGGAGCATTTATGATAAAGAGGATG
AGCTTCAAGAGAATGATTCGGAGTTCTTGCAGAGTGGAACCATGCAGTACCAGACACG
AGATAGATCTTCCAAAGAACAGGGCTTTTTTCGAATAAGCCAATTCATTTGGGACCCTG
CAGATCCACTCTTTTTCCTATTCAAA---GATCAGCCCTTTGTCTCTGTGTTTTCACATCG
AGAATTCTTTGCAGATGAAGAGATATCAAAGGGGCTTCTTACTTCCCAAATGAATCCTC
CTATATCTATATTTCAACGCTGGTTTATC------AAGAATACGCAAGAAAAGCACTTTGAAT
TGTTGATTAATCGCCAAAGATGGCTTAGA---------ACCAACAGTTCATTATCG------AATGG
ATCTTTCCGTTCTAAT------ACTCTATCCGAGAGTTATCAGTATTTATCAAATCTGTTCCTA
TCTAAC---GGAACACTATTGGATCAAATGACAAAGACATTGTTGAGAAAAAGATGGCTT
TTCCCGGATGAAATGAAAATTGGATTC------ATGCAAGAGGAGAAAGATTTTCCATTCCT
TATGTCTAGA------------AATGGGAATTTTATTGATAAGACCTTTTCAGTTGTGGCCAATAT
CTTATTACAAATAATTCCGACAACTTCAGGAGAAAAAGAGGCATTTACTTATTACAGAG
ATGGG------ATGTCAGCTCAATCTGAAGGAAATTATGCGGAAGCTTTACAGAATTATTAT
GAAGCTATGCGACTAGAAATCGATCCCTACGATCGAAGTTATATACTCTATAATATAGGC
CTTATTCACACAAGTAATGGAGAACATACGAAAGCTTTAGAATATTATTTTAGGGCACTA
GAACGAAACCCATTCTTACCACAAGCTTTTAATAATATGGCAGTGATCTGTCATTAC---C
GGGGAGAACAAGCCATTCGACAGGGAGATTCTGAAATTGCGGAGGCTTGGTTCGATC
AAGCCGCTGAGTATTGGAAACAGGCTCTAACGCTTACTCCTGGGAATTATATTGAAGCG
CATAATTGGTTGAAGATCACGGGGCGTTTCGAA------ATGAATTGGCGATCAGAACGTAT
ATGGGTAGAATTTATAACGGGATCTCGAAAAATAAGTAATTTCTGCTGGGCCTTTATACT
TTTTTTAGGTTCATCAGGATTTTTATTGGTTGGAATTTCCAGCTATCTTGGTAAGAATTTT
ATATCTTTATTTCCCCCCCAGCAAATTCTTTTTTTTCCACAAGGGCTGGTGATGTCTTTCT

ATGGGATTGCAGGCCTCTTTATTAGCGCCTACTTGTGGTGTGCAATTTCATGGAATGTAG
GTAGTGGTTATGATCGATTCGATAGAAAAGAAGGAATAGTCTGTATTTTTCGTTGGGGA
TTCCCTGGAAAAAATCGTCGCATCTTCTTCCGATATCTTATAAAAGATATTCAGTCTATTA
GAATAGAACTTAAAGAGGGTATTTATACTCGTCGTGTCCTTTATCTGGAAATCAGAGGT
CAAGGAGCCATTCCTTTGACTCGTACTGATGAGAAT---TTGACTCCACGAGAAATGGAA
CAAAAAGCGGCTGAATTGGCCTATTTCTTGCGTGTACCAATTGAAGTATTT
>Oenothera
ATGAAGCCTACCAAGGGGCCTAAAAAGCCTAACAATAAGGAAGACCTTGAAGGGCCT
AAACCTAACAAGTCTAACGAGGAAGCGGAAGAGCTT------GGGGATAATAAGGAAGAC
CTTGAAGGGAAGTCTAACGAGGAA---GCGGAAGAGCTTGGGGATAAGCAGAAGGATA
AGAAAGATGGGATTTTCGTACTCAATTATGACGATGAGTATGAGGAAGACCTTTATGAC
GATGAGTATGAGGAAGACCTTGAATATGACGATGAGTATGAGGAAGACCTTGAATATGA
TGAGGAATATGACTATGAGGAAGACCTTGAAGGG---GATAATAAGCCTCATAAGGAAGG
GGATAATAAG---------CCTCATAAGGAAGACCTTGAAGGGGATAATAAGCCTCATAAGGA
AGACCTTGAAGGGGATAAGCAGAAGGAAGAAGAGGAGAAGCAGAAGGAAGAGGAG
AAGCTGAAGGACTGTCCCCACTGGTTTCACCAG---ATTTACCCTAAGCATTGGGGC---G
ATCGCAAGTCGGTTCCCGCTAAGGAGGTTCCCGCTCAGTCTACCAAGCGGGATACCGA
TCCGGAGGCGTCTCTAAAATCGAACTATGCGCATTTATGGGTTCACTGCAAACTTTGTT
CTGGATTCAATTATAAGAAAATTTTGAAGTCCAAAAACAATGTTTGTGAACAATGTGGA
TCTCATTTGAAAATGCATAGTTCAGATCGAATCGACCTTATGCTTGATCCAAAGACTTG
GGCTCCTATGCATGAAGGCCTGCTTTCTCTAGATCCTATTGAATTTCATTCGGAGAAAG
ACCCTTATAAAGATCGGGTTGCTTCTTATAAAAGAAAGACAGGATTATCGGAAGCTATT
CAAACGGGCAGAGGTTACCTAAAACGTATTCACCTAGGAATTGGACTTATGGATTTTCA
GTTTATGGGGGGTAGTATGGGATCCGTAGTCGGCGAGAGAATCACCCGTTTGGTCGAGT
ATGCTACCAATCGAGTTTTACCTCTTATTCTAGTGTGTGCTTCCGGAGGGGCACGTATGC
AAGAAGGGAGTTTGAGCTTGATGCAAATGGCTAAAATATCTTCTGCTTTATCTGATTATC
AATTCAATAGAACGGTATTCTATGTAGCTCTCCTTACATCTCCCACTACGGGTGGTGTGA
CGGCTAGCTTTGGGATGTTGGGGGATATCATTCTTGCAGAACCTAATGCCTACATCGCAT
TCGCAGGTAAAAGAGTAATTGAACAAACATTGAATATTGAAGTACCTGAGGGTTCACA
AACGGCTGAATATTTATTCGATAAGGGCTTATTCGATCAAATTGTACCACGCAATCCTTT
AAAGGGTTCTTTGAGTGAGTTATTTAATTTTCACGGTTTTGTTACTTTGAATTCTCAAGT
AATTAATATGGCAACCATTAGAGCCGACGAAATTAGTAATATTATCCGTGAACGGATTGA
GCAATATAATAGAGAAGTAAAGATTGTAAATACTGGTACCGTACTTCAAGTTGGTGACG
GCATTGCTCGTATTTATGGTCTTGATGAAGTAATGGCAGGCGAATTAGTAGAATTTGAA
GAGGGTACAATCGGCATTGCTCTTAATTTGGAATCAAAAAATGTTGGTGTTGTATTAAT
GGGTGACGGTTTACTGATACAAGAGGGAAGTTCTGTAAAAGCAACAGGAAGAATTGC
TCAGATACCCGTGAGTGAGGCTTATTTGGGTCGTGTTATAAATGCCCTGGCAAAACCTA
TTGATGGTCGAGGTGAAATTTCTTCTTCTGAGTCTCGGTTAATTGAATCTCCCGCTCCG
GGTATTATTTCGAGACGTTCCGTATATGAGCCTCTTCAAACAGGACTTATTGCTATTGAT
GCAATGATCCCTATAGGACGTGGCCAGCGAGAATTAATTATTGGAGACAGACAGACTG
GTAAAACAGCAGTAGCCACGGATACGATTCTCAATCAACAAGGGAACAATGTGATATG
TGTTTATGTAGCTATTGGTCAAAAAGCCTCTTCTGTGGCTCAGGTAGTGAATGCTCTCC
AAGAAAGGGGAGCTATGGAATACACTATTGTGGTAGCGGAAGCGGCCGATTCCCCAGC
TACATTACAATACCTCGCCCCTTATACAGGAGCAGCTCTGGCTGAATATTTTATGTACCG

TGAACGACACACTTTAATCATTTATGATGATCCCTCCAAACAAGCACAGGCTTATCGCC
AAATGTCTCTTCTATTACGAAGACCTCCCGGTCGCGAAGCTTATCCGGGAGATGTTTTT
TATTTGCATTCACGCCTTTTGGAAAGAGCCGCTAAATTAAGTTCTCGTTTAGGTGAAGG
AAGTATGACTGCCTTACCAATAGTTGAGACTCAATCGGGAGATGTTTCGGCTTATATTCC
TACTAATGTAATTTCCATTACAGACGGACAAATCTTCTTATCGGCCGATCTATTCAATGC
TGGAATCAGACCCGCGATTAATGTGGGCATTTCTGTCTCCAGAGTAGGATCCGCAGCTC
AAATTAAAGCCATGAAACAAGTAGCTGGAAAATTAAAATTGGAATTGGCGCAATTCGC
AGAATTAGAAGCCTTTGCACAATTCTCTTCTGATCTCGATAAAGCTACCCAGAATCAAT
TGGCAAGAGGTCAACGATTACGTGAGTTGCTTAAACAATCTCAAGCAAAACCTCTCAC
CGTGGCAGAACAGATACTGACTATTTATACTGGGACGAATGGTTATCTTGATTCATTCGA
AATTGCACAGGTAAGGAAATTTCTTGATGAGCTACGTGATTACGTAAAAACGAGGAAA
CCGCAGTTCGAAGAAATCATATCTTCTACTAAGATATTCACCGAGGAAGCACAAGCCCT
TTTGAAAGACGCTATTCAGGAACAGAAGGAACTCTTTCTAGTTCAGGAA------ATGAGA
ATCAATCCTACTACTTCAGGTCCTGGAGTTTCCACACTTGAAAAAAAAAAATCGGGCC
GTATCGCTCAAATCATTGGTCCAGTACTGGATGTAACCTTTCCCCCGGGGAAGATGCCT
AATATTTATAACGCTCTGGTCGTTAAAGGTCGAGATACTGGCGGCCAAGAAATTAATGT
CACTTGTGAAGTACAGCAATTATTAGGAAATAATCGTGTTAGAGCTGTCGCCATGAGTG
CTACAGATGGTCTAACGAGAGGAATGGAAGTGATTGACACGGGAGCTCCTCTAAGTGT
TCCAGTCGGCGGAGCGACTCTAGGACGAATTTTCAACGTGCTCGGAGAGCCTGTTGAT
GAATTAGGACCCGTAGATACTCGTACAACATCTCCTATTCATAGATCCGCGCCCGCCTTT
ATACAGTTAGATACAAAATTATCTATTTTTGAAACAGGAATTAAAGTAGTCGATCTTTTA
GCCCCTTATCGCCGTGGAGGAAAAATAGGCCTATTCGGGGGGGCTGGGGTAGGTAAAA
CAGTCCTCATTATGGAATTGATCAACAACATTGCTAAAGCTCACGGGGGTGTATCCGTA
TTTGGCGGAGTAGGTGAACGTACTCGTGAAGGAAATGATCTTTACATGGAAATGAAGG
AATCCGGCGTCATTAACGAACAAAATATTGCAGAATCGAAGGTGGCTCTAGTCTATGGT
CAGATGAATGAACCCCCAGGAGCTCGTATGAGAGTTGGTTTGACTGCCCTAACGATGG
CGGAATATTTCCGAGATGTTAATAAACAAAATGTACTTCTATTTATCGACAACATCTTCC
GTTTCGTCCAAGCAGGATCCGAAGTATCCGCCTTATTGGGCCGAATGCCTTCCGCTGTG
GGTTATCAACCCACCCTTAGTACCGAAATGGGTTCTTTACAAGAAAGAATTACTTCTAC
TAAAGCGGGATCCATAACTTCTATTCAAGCAGTTTATGTACCGGCGGACGATTTGACCG
ACCCTGCTCCTGCCACGACCTTTGCACATTTAGATGCGACGACCGTCCTATCAAGAGGA
TTGGCTGCCAAAGGTATCTATCCAGCAGTGGATCCTTTAGATTCAACGTCAACTATGCT
CCAACCTCGAATCGTTGGCGACGAACATTATGAAACTGCACAAAGAGTAAAGGAAAC
CTTACAACGTTACAAAGAACTTCAGGACATTATATCTATCCTTGGGTTGGATGAATTATC
CGAAGAGGATCGCTTAACTGTAGCACGAGCACGAAAAATTGAGCGTTTCTTATCACAA
CCCTTTTTCGTAGCAGAAGTATTTACAGGTTCCCCGGGGAAATACGTTGGTCTAGCAGA
AACCATTAGAGGGTTTAAATTGATCCTTTCCGGAGAATTAGATGGTCTTCCTGAACAAG
CCTTTTATTTGGTCGGTACCATCGATGAAGCTACCGCGAAGGCTGCGAACTTAGAAATG
GAGAGTGATTTGAAGAAAATGACCTTAAATCTTTGTGTATTAACCCCTAATCGAATTGT
TTGGGATTCAGAAGTGAAAGAAATCATTTTATCTACTAATAGTGGACAAATTGGCGTATT
ACCAAATCACGCACCTATTGCCACAGCTGTAGATATAGGTATTTTAAGAATACGCCAAA
ACGGACAGTGGTTAACGATGGCTCTGATGGGGGGTTTTGCTAGAATCGGCAATAATGA
GATCACTATTTTAGTAAATGACGCGGAAAAGGGTAGTGACATTGATCCACAAGAAGCC
CAGGAAACTCTTGGACTAGCGGAAGCTAACTTTAGGAAAGCGGAAGGCAAGAGACAA

ACAATTGAAGCAAATCTGGCTCTCAGACGAGCTAGGACACGCGTTGAGGCTATCAATG
TGATTTCAATGAAAAATGTAACCGATTCTTTCGTTTCCTTGGTTCACTGGCCATCCGCCG
GGAGTTTCGGGTTTAATACCGATATTTTAGCAACAAATCCAATAAATCTAAGCGTAGTGC
TTGGTGTATTGATCTTTTTTGGAAAGGGAGTGTTAAGTGATTTATTAGATAATCGAAAAC
AGAGGATCTTGAATACTATTCGTAATTCAGAAGAATTACGCGAGGGGGCCATCGAACA
GCTGGAAAAAGCCCGGGCCCGTTTACAGGACGTGCAAATCGAAGCGGAAGGGTATCG
AGCGTATGGGTACTTTGGGATAGACGAGCAAAGGCACGAGTCGATTAATTCAACTTATA
AGACCTTGGAACAATTAGAAAATAACAAAAACGAAAGCATTCATTTTGAACAACAAA
GGGCGATTAATCAAGTCCGACAGCAGATTTTCCAACAAGCCTTACAAGGAGCTTTAGG
AACTCTGAATAGTTGTTTGAACAACGAGTTACATTTACGGACCATCAGTGCTAATATTG
GCCTGTTTGGGTCGATGAAAGAACTCACTGATATGAATCCACTGATTTCTGCCGCTTCC
GTTATTGCTGCTGGATTAGCTGTCGGGCTTGCTTCTATTGGACCTGGAATTGGTCAAGG
TACTGCTGCGGGACAAGCTGTAGAAGGTATCGCGAGACAGCCCGAGGCAGAGGGAAA
AATACGAGGTACTTTATTGCTTAGTCTGGCTTTTATGGAAGCGTTAACAATTTATGGATT
GGTTGTAGCCTTAGCACTTTTATTTGCGAATCCCTTTGTAATGGATGTTCTATCATGTTCC
AACAACACACTAAAAGGGTTATACGACATATCTGGTGTGGAAGTAGGCCAACATTTCTA
TTGGCAAATAGGAGGTTTTCAAGTCCATGGCCAAGTACTTATTACCTCTTGGGTTGTAA
TTGCTATCTTATTAGGTTCAGCCAGTATAGCTGTTCGGAATCCACAAACAATTCCAAATG
ACAGTCAGAATTTCTTCGAATATATCCTTGAATTCATTCGAGATGTTAGTAAAACCCAGA
TTGGAGAAGAATATGGTCCATGGGTTCCTTTTATTGGAACTATGTTTCTATTTATTTTTGT
TTCTAATTGGTCAGGAGCTCTTTTACCGTGGAAACTCGTAGAGTTACCTCACGGGGAGT
TAGCTGCGCCCACCAATGATATAAATACTACTGTTGCTTTAGCTTTACTCACGTCAGTAG
CCTATTTCTATGCGGGTCTTAGCAAAAAGGGGTTAGGTTATTTCAGTAAATACATACAAC
CAACCCCAATCCTTTTACCCATTAACATCTTAGAAGATTTCACAAAACCTTTATCCCTTA
GTTTTCGACTTTTCGGAAATATATTAGCCGATGAATTAGTAGTTGTTGTTCTTGTTTCTTT
AGTACCTTCAGTCGTTCCTATACCTGTCATGTTCCTGGGATTATTTACGAGTGGTATTCA
AGCTCTTATTTTTGCAACGTTAGCTGCGGCTTATATAGGCGAATCCATGGAGGGTCATCA
TATGATATTTTATACTCTAGAACATATATTAACTCATATATCCTTTTCGCTCGTTTCAATTG
GAATTACAATTTTTTTGATAACCTTATCAGTC---GAAATCATAGGACTATATGATTCGTCA
GAAAAAGGCGTGATAGGAACTTTTTTATGTATAACAGGATTATTAGTCACTCGTTGGGC
GTATTCGGGACATTTTCCATTAAGTAATTTATATGAATCATTACTTTTTCTGTCATGGAGT
TTCGCGATTATTCATATGTTTCCATATTTTAAAAAACAGAAAAGTTATGTAAGGACAATA
ACCTCGTCAAGTACTATTTTTACCCAGGGCCTTGTTACTTCAGGTCTTTTAAGTGAAATG
CAGCAATCAGAAATATTAGTACCTGCGCTCCAATCCCAATGGTTAATGATGCACGTAAG
TATGATGGTATTGGGCTATGCAGCTCTTTTGTGTGGATCATTATTATCAGTAGCTCTCTTA
GTCATTACATTTCGAAAAGCTCTAAGAATTTTTAGTAAAAAGAAAGAGATCCAATATAG
GAATGAACCAAGCAATGTTTTACTA------TCTTTTATTTCTTCTAAAAACTATTACAGGGC
TCAACTGATTCAACAATTAGATCGTTGGAGTTCTCGTATTATTAGTCTAGGATTTATCTTT
TTAACCATAGGTATTCTTTCTGGAGCAGTATGGGCTAATGAGGCGTGGGGATCCTATTG
GAATTGGGATCCAAAAGAAACTTGGGCATTTATTACTTGGACCATGTTTGCGATTTATTT
ACATACTCGAACAAATCCAAATTTTCAAAGTGTAAATTCCGCAATTGTGGCTTTTCTGG
GCTTTATTATAATTTGGATATGCTATTTCGGGGTCAATTTATTAGGAATAGGCTTACATAG
TTATGGTTCATTTAATTTACAT--ATAGTCTTTTTTCCCTG
GTGGATCTCCCTCTTATTTAATAAAGGTCTTGAATCTTGGGTTACTAATTGGTGGAATAC

TACGCACTCGGAAACTTTTTTGACTGATATGCAAGAAAAGAGTATTCTAGACAAATTCA
TAGAATTAGAAGAACTCTTACTCTTAGACGAAATGATAAACGAATACCCGGAAACACAT
CTCCAAACACTTCGTATAGGAATCCACAAAGAAATGGTCCGCTTGATTAAGATGCGCA
ACGAGGATCATATCCATACAATTTTGCACTTATCGACAAATATAATCTGTTTCATTATTTT
CCGCGGTTATTCTATTCTGGGTAATAAAGAACTTCTCATTCTTAACTCTTGGATGCAAGA
ATTCCTATATAACTTAAGTGACACAATAAAAGCTTTTTCGATTCTTTTATTAACTGATTTT
TGTATCGGATTCCACTCGCCCCATGGTTGGGAACTAATGATTGCTTATGTCTACAAAGAT
TTTGGATTTGCTCAGAACGATCAAATTATATCTGGTCTTGTTTCCACTTTTCCAGTCATT
CTAGATACAATTTTTAAATATTGGATTTTTCGTTATTTAAATCGTGTATCACCCTCACTTG
TAGTGATTTATGATTCAATGAATGACATGCCGATTGGTATGCCTAAAATCCCTTTCCTGC
TTGACGGAGAAGACGACGCGACTTGGGTTGACCTGTATAACGTACTTTATCGAACCAG
ATCCATCTTTTTAGGCGACGCAATTCACTTCGAGGTCGCGAATCACATTGCTGGGCTGA
TGATATTTCTCACCATACAGGATGCGACTCAAAATCTTTATTTCTTTATAAACTCTCCCG
GCGGGCTGGCAGTAGCCGGATTACTCATTTATGATACTATGCAATACGTAACACCTCCTG
TGTATACACTAGGCCTGGGGGTACTCGCCTCAATGGCATCCTTTCTACTGGTCGGCGGA
GAAACGTCCAAACGCCTAATGGGCCCTAACGGTAGGGTAATGATACATCAGCCCGAGG
ATTATACTCACAAAGACCAATCGCTAGAGGTACAACTGGACTCAGGGGAAGTAGAAGA
CATTCGCAAAATGGTCATAAGGGTTTATCTAGAAAGAACAAGGCTACCCCGGGAGGTT
CTAAACGACCATTTGGAAAGAAATTATTTTATGACAGCAACCGAAGCCAAATATTATGG
A A T T G T T G A T G A T A T A G G G A T T C A
A??
??
??ATGGAGGAATTCCCGGGATATT
TCGAACTAGATAGATCTCGACAACACGACTTCCTATACCCCCTAATTTTTCGGGAGTCTA
TTTATGCACTTGCTCATGATCATGGTTTAGTTGATTATGACAAAAAATATAGTTTAATAAT
TGTGAAACGTTTAATTACTCGAATGTATCAACGGAATCATTTGATTATTTCGGCTAATGG
TTCTGTTCAAAATCCATTTTGGGGCCAGAATTTGTATTCGAAAATTCTATCAGAAGGGT
TTGCAGTCATTGTGGAAATTCCATTTTCGCTACGAGTTTTATCTTCCTTTATAGCAAAAT
CTCCTACTTTACGATCAATTCATTCAATATTTCCTTTTTTAGAGGACCAATTTTCACATTT
AGATTATTTGTCACATGTACTAATACCCTATCCCATCCATTTGGAAATCGCGGTTCAAAC
CCTTCGCTACTGGGTGAAGGATGCCTCTTCTTTGCATTTATTACGTATCTTTCTACACGA
GTATTGGAAT------AGTTTTAGTACTAAGCATATTACCCTTTTTTTAAAATCAAGATTCTTC
TTGTTCCTCTATAATTCTTATGTATGTGAATACGAATCTATCTTCCTTTTTATCCGCAATCA
ATCTTCGCATTTCCAGTCAACATCTTCTGGAGTCTTTTTTGAGCGAATCCTTTTCTATGT
AAAAATAGACCATCTTGTTGAAGTTTTTGTTGGGTTTCTGATCCGATCCTTCTTCAAGG
ATCCGAATATGCATTATGTTAGATATCAAGGAAAATCTATTCTAGCTTCAAAGGATACAC
CTCTTTTGATGAATAAATGGAAATATTACCTTGTCAATTTATGGCAATATCATTTTTCGGT
CTGGTCTCAACCCGGAAGGATCAACATAAACCAATTAGGTAAGTATTCTCTTGACTTTT
TGGGATATTTTTCAAACGTGCAACTAAAGTCTTCAGTGGTACGAAATCAAACGCTAGA
GAATTCATTTCTAATAAATAAT------GCTATGAAGAAGCTCGAAACAACAGTTCCAATTTT
GCCTCTGATTGGATCGTTGTCTCGAGCGAAATTTTGTAATGCATTAGGACATCCCATTAG
TAAGCCGACCCGGAATGATTCATCCGATTCTGATATTATCGACCGATTTGTGCGTATATG
CAGAAATCTTTCTCATTATCACAGTGGGTCTTCAAAAAAAAAAGAGTTTATATCGAATAA
AGTATATACTTCGACTTTCTTGTGTTAAAACTTTGGCCCGTAAACATAAAAGTAGTGTAC

GCGCTTTTTTGAAAAGATTAGGTTCGGAATTAGGGGACGAATTCCTTACAGAGGAAGG
AGTTGTTCTTGCTGTGATCTTCCCA---CGAATTTGGTATTTGGATATTCCTTGTATCAATG
ATCGGGTAGGCGACGCGATGATAATTGATACAACC---GCAGTACAA---GATATGAATTCT
TTTTCCAGATTGCAATCC---TTGAAAGAGGTCTCTGGAATCATATGGATGCTTGTACCTA
TTTTGAGTCTTGTATTGGGGATTACTCTAGGTGTACTCGTAATAGTGTGGTTAGAAAGG
GAAATATCCGCCGGAATACAACAACGTATTGGGCCTGAATACGCAGGGCCTATGGGAAT
TCTTCAAGCTTTAGCTGATGGAATAAAACTCATTTTCAAAGAGAATCTTCTCCCGTCTC
GAGGAGATACTCGTTTATTCAGCATAGGACCATCCATAGCAGTTATATCCATTTTACTAA
GTTATTCAGTAATTCCTTTTAGCTCTCACCTTGTCTTATCTGATCTCAATATCGGTGTTTT
TTTGTGGATTGCCGTTTCCAGTATTGCTCCCATCGGACTTCTTATGTCAGGATATGGATC
AAATAATAAATATTCTTTTTTAGGTGGTCTACGAGCCGCTGCTCAATCAATTAGTTATGA
AATACCATTAACTCTTTGTCTGTTATCAATATCTCTACTATCTAACAGTTCAAGTACAGTT
GATATAGTTGAAGCCCAATCAAAATATGGTTTGTGGGGGTGGAATCTGTGGCGCCAACC
TATAGGGTTTCTTGTTTTTCTAATTTCTTCCCTAGCAGAATGTGAAAGATTACCCTTTGAT
TTACCAGAAGCAGAAGAAGAATTAGTAGCAGGTTATCAAACCGAATATTCAGGTATCA
AATTTGGTTTATTTTACGTTGCTTCCTATCTAAATCTATTAGTTTCTTCGTTATTTGTAACA
GTTCTTTACTTGGGCGGTTGGAATATCTCTATCTCGTACATATTCGTTCCTGGGCTCTTT--
----GAAAAAGTGGGTAGAGTTTTTGGAACGACAATTGGTATTTTTACTACATTAGCTAAA
ACTTATTTGTTTTTGTTCATTTCTATCACAACAAGATGGACTTTACCTAGGCTAAGAATG
GACCAATTATTAAATCTTGGATGGAAATTTCTTTTACCCATTTCTCTCGGTAATCTATTAT
TAACAACTTCTTCCCAACTTCTTTCACTGATGATCTGGCATGTACAGAATGAAAACCTC
ATTCTCGATTCTACGAGAATTTTTATGAAAGCCTTTCATTTGCCTCTCTTCGATGGAAGT
TTTATTTTCCCAGAAGGTATCCTCATTTTTGGCCTAATTCTTCTTCTGATGATCGATTCAA
CCTCTGATCAAACAGATATACCTTGGTTCTATTTCATCTCTTCAATAAGTTTAGTAATGA
GCATAACGGCCCTATTGTTCCGATGGAGAGAAGAACCTAGGATTCTCTTTTCAGGAAAT
TTCCAAACGAACAATTTCAACGAAATCTTTCAATTTCTTATTTTACTATGTTCAACTCTA
TGCATTCCTCTATCCGTAGAGTACATTGAATGTACAGAAATGGCTATAACAGAGTTTCTG
TTATTCGTATTAACCGCGACCCTAGGAGGAATGTTTTTATGCGGTGCTAACGATTTAATA
ACTATCTTTGTAGCTCCAGAATGTTTCAGTTTATGCTCCTACCTATTATCTGGATATACCA
AGAAAGATGTACGGTCTAATGAGGCTACTATGAAATATTTACTCATGGGGGGGGCAAGC
TCTTCTATTCTGGTTCATGGTTTCTCTTGGCTATATGGTTCATCCGGGGGGGAGATCGAG
CTTCAAGAAATAGTGAATGGTCTTATCAATACACAAATGTATAACTCCCCAGGAATTTC
AATTGCGCTTATATTCATCACTGTAGGAATTGGGTTCAAGCTTTCCCCAGCCCCTTCTCA
TCAATGGACTCCTGACGTATACGAAGGATCTCCCACTCCAGTTGTTGCTTTTCTTTCTGT
TACTTCGAAAGTAGCTGCTTCAGCTTCAGCCACTCGAATTTTCGATATTCCTTTTTATTT
CTCATCAAACGAATGGCATCCTCTTCTGGAAATTCTAGCTATTCTTAGCATGATATTGGG
GAATCTCATTGCTATTACTCAAACAAGCATGAAACGTATGCTTGCATATTCGTCCATAGG
TCAAATCGGATATGTAATTATTGGAATAATTGTTGGAGACGCAAACGGTGGATATGCGA
GCATGATAACTTATATGCTGTTCTATATCTCCATGAATCTAGGAACTTTTGCTTGCATTGT
ATTATTTGGTCTACGTACCGGAACTGATAACATTCGAGATTATGCAGGATTATACACGAA
AGATCCTTTTTTGGCTCTCTCTTTAGCCCTATGTCTCTTATCCCTAGGAGGTCTTCCTCC
ACTAGCAGGTTTTTTCGGAAAACTCCATTTATTCTGGTGTGGGTGGCAGGCAGGCCTAT
ACTTCTTGGTTTCAATAGGACTCTTTACGAGCGTTGTTTCTATCTACTATTATCTAAAAAT
AATCAAGTTATTAATGACTGGACGAAAGCAAGAAATAACACCTCACGTGCGAAATTATA

GAAGATCCCCTTTAAGATCAAACAATTCCATCGAATTGAGTATGATTGTATGTGTAATAG
CATCCACTATACCAGGAATATCAATGAACCCGATTATTGCAATTGCTCAGGATACCCTTT
TTATGTTTCTGCTTTACGAATATGATATTTTCTGGGCATTTCTAATAATATCAAGTGTTATT
CCTATTTTGGCATTTCGAATTTCCGGACTTTTAGCCCCGACTAGCATAGGGCCAGAGAA
ACTTTCGAGTTATGAATCGGGTATAGAACCAATGGGCGATGCTTGGTTACAATTTCGAAT
ACGTTATTATATGTTTGCTTTAGTTTTTGTTGTTTTTGATGTTGAAACCATTTTTCTTTATC
CATGGGCACTGAGTTTCGATATATTGGGGGTATCCGTATTTATAGAAGCTCTCATTTTCG
TGCTTATCTTAGTTCTTGGTTTGGTTTATGCATGGCGAAAAGGAGCGTTGGAATGGTCT
ACGAATTCTTTTCCTTGGTTAACAATAATTGTAGTTTTTCCCATATTGACGGGTTCTTTAA
TTTTCTTGCTACCTCATAGAGGTAATAAGGTCATGAAATGGTATACTTTATGTATATGTAT
TTTAGAGCTTCTTTTAACAACCTATACATTCTGTTATCATTTCCAACTGGACGATCCATTA
ACCCAACTAACAGAGAATTATAAATGGATCCATTTTTTTGATTTTTATTGGAGATTAGGA
ATAGATGGGCTTTCTATAGGACCTATTTTATTGACGGGATTTATTACCACTTTAGCAACTT
TAGCGGCCTGGCCAGTTACTCGAGATGCCCAATTGTTCCATTTTTTGATGTTAGCAATGT
ACAGCGGTCAAATAGGATCATTTTCCTCTAGAGACCTTTTACTTTTTTTCCTAATGTGGG
AGTTCGAATTAATTCCCGTTTATCTACTTCTATCGATGTGGGGGGAAAGAAACGTCTCT
ATTCAGCTACAAAGTTCATTTTGTACACGGCGGGTGGGTCCATTTTTCTATTAATAGGAG
TTCTGGGTATTGGTTTATATGGTTCCAATGAACCAACACTCAATTTTGAAACATTAGCGA
ATCAGTCGTATCCTGTGGCACTCGAAGTAATATTCTATGTGGGATTTCTTATTGCTTTTGC
GGTCAAATTGCCGATTATACCCTTCCATACATGGTTACCAGATACACATGGGGAAGCAC
ATTATAGTACGTGTATGCTTCTAGCTGGAATCTTATTAAAAATGGGAGCTTATGGGTTGG
TTCGAATCAATATGGAATTATTACCTCATGCTCATTGCCTATTTTCTCCCGGGTTGATTAT
AGTAGGTGCAATACAAATAATCTATGCAGCTTCAACATCTCCTGGTCAACTTAATTTAAA
AAAAAGAATAGCCTATTCCTCTATATCTCATATGGGTTTCATAATTATTGGAATTGGATCT
CTAAGCGATACGGGACTTAATGGAGCCATTTTACAAATAATCTCTCATGGCTTTATTGGG
GCGGCTCTTTTTTTCTTGGCCGGAACGAGTTATGATAGAATACGCCTTCTTTATCTCGAT
GAAATGGGTGGAATGGCTATCCCCCTTCCAAAATTATTTACGATGCTCAGTATCTTATCG
ATGGCTTCGCTTGCATTACCGGGCCTGAGCGGTTTTGTTGCCGAATTATTAGTATTCTTT
GGCATAATTACCAGTCAAAAGTATCTTTTAATGCCAAAAATACTAATTGCTTTTTTAATG
GCAATTGGAATGATATTAACTCCTATTTATTCATTATCTATGTTACGCCAAATGTTCTACG
GATACAAGCTATTTAATGTTCCAAACTATTATTTCTTTGATTCTGGTCCGCGAGAGTTATT
TGTTTCGATCTCTCTCCTTCTACCAATAATTGGGATTGGTATTTATCCGGATTTCGTTCTG
TCATTATCGGTTGAAAAAGTAGAAGCTATTATATCTCATTTTTTTTTTTCGATGATACTCG
AACATGTACTTGTTTTGAGTGCCTATTTATTTTCTATAGGTATTTATGGATTGATCACGAG
TCGAAATATGGTTCGGGCTCTTATGTGCCTTGAACTTATACTGAATTCGGTTAATCTTAA
TTTCGTAACATTTTCGGATTTTTTTGATAGTCGCCAATTAAAAGGAGATATTTTTTCCATT
TTTATTATAGCTATTGCAGCCGCCGAAGCCGCTATTGGGTTGGCTATTGTTTCGTCAATTT
ATCGTAACAGAAAATCAATTCGTATCAATCAATCCAATTTATTGAATAAAATGGAATATA
CATATCAATATTCATGGATTATACCTTTCATTCCACTACCAGTTCCTATTCTAATAGGAAT
GGGACTTCTACTTTTTCCGACAGCAACAAAAAATCATCGTCGTGTGTGGTCTTTTCCTA
GCATTTTACTGTTAAGCATGGTTATGCTTCTTTCCGTCTATCTCTCTATTCAACAAATAAA
TAGAAGTTTTATCTATCAATATGTATGGTCTTGGACCATTAATAATGATTTTTCTTTAGAG
TTCGGTCACTTAATCGATCCACTTGCTTCTATTATGTTAATATTAATTACTACTGTTGGAA
TTTTGGTTCTTTTTTATAGTGATAATTATATGTCTCATGATCAAGGATATTTAAGATTTTTT

GCTTATCTGAGTTTTTCAATACCTCAATGTTAGGATTAGTTACTAGTTCTAATTTGATAC
AAATTTATATTTTTTGGGAATTAGTTGGAATGTGTTCTTACCTATTAATAGGTTTTTGGTT
CACGCGACCTATTGCAGCAACTGCTTGTCAAAAAGCTTTTGTAACTAATCGTGTAGGG
GATTTTGGTTTATTATTAGGAATTTTAGGTCTTTATTGGATAACGGGTAGTTTTGAATTTA
GGGATTTGTTCGAAATAGTGAATAAC---CAAGTTCATTTTTTATTTGTTACTTTGTGTTCC
TTTCTCTTATTTGCTGGTGCAGTTGCTAAATCCGCGCAATTCCCCCTTCATGTATGGTTA
CCTGATGCCATGGAAGGGCCCACTCCTATTTCCGCTCTTATCCATGCCGCTACTATGGTA
GCCGCGGGCATTTTTCTTGTAGCTCGGCTTCTTCCTCTTTTTGTAATCACACCTTACATA
ATGAATTTGATATCTTTGATAGGTATAATAACAGTACTATTAGGAGCTACTTTAGCCCTTG
CTCAAAAAGATATTAAAAGAAGTTTAGCTTATTCTACAATGTCTCAACTGGGTTATATGA
TGCTAGCTCTAGGTATGGGTTCTTATCGAGCTGCTTTATTTCATTTGATTACTCATGCTTA
TTCAAAAGCATTGTTGTTTTTAGGCTCCGGATCCATTATTCATTCAATGGAATCCATTGT
TGGCTATTCTCCAGATAAAAGCCAGAATATGGTTCTTATGGGCGGTTTAAAAAAGCATG
TACCAATCACAAAAACGGCTTTTTTGGTGGGTACGCTTTCTCTTTGTGGTATTCCACCT
CTTGCTTGTTTTTGGTCCAAAGATGAAATTCTTAATGATAGTTGGTTGTATTCGCCAATT
TTCGCAATAATAGCTTGTTCCACAGCAGGATTCACTGCATTTTATATGTTTCGTGTTTATT
TACTTACTTTTGATGGACATTTAAACGTTCATTTTCAAAATTATAGTGGTCAAAAAAGTA
GCTCCGTCTATTCAATATCTTTATGGGGAAAACAAGTACCAAAAAGGATTCAAAAT---TT
ATTAAATTTATTGACAATGAATTTTTTTTGGCCGAATAGAAAG---ACTTTTTCTTATCCCC
ATGAGTCGGACAATACTATGCTATTTTCCCTCTTTGTATTAGTGCTCTTTACTTTGTTTGT
TGCAGCCATAGGA------TTTGATTGCGATATATTATCAAAATTGTTAAATCCGTCTATAAA
CCTTTTACATCAAAATTCAAAT---TTTACGGATTGG---GAATTTGTGACCAATGCAAGTTT
TTCCGTCAGTATAGCCCTTTTGGGGATATTTATAGCGACTTTCTTATATAAGCCTATTTATT
CATCTTTACAAAATTTCAACTTACTTAATTCATTTTATGTTATGTGGGACAAAATCCAAA
ATTGGATATATGATTGGTCATATAATCGTGGTTATATAGATTCTTTTTATACAATATCCTTA
ACTGGGGGTATAAGAGGATTGGCTGAACTAAGTCATTTTTTTGATAGACGCGTAATTGA
TGGAATTCTAAACGGATTCGGCCTTACGAGTTTCTTTTTAGGAGAAAGTCTCAAATATT
TCGGGGGCGGA---CGTATCTCTTCGTATCTCTTATTGTATTCCATTTTTATATTCATTTTTTT
ATTAATGGATTCTATGGATTTGCCTGGACCAATACATGATTTTCTTTTAGTTTTTCTGGGA
TCGGGTCTTATAGTAGGAGGTCTGGGGGTGGTCTTACTTACCAATCCCATTTTTTCTGCC
TTTTCGTTGGGATTGGTTCTTGTTTGCATATCCTTATTTTTTAGTTTATCAAACTCCTATTT
TGTAGCGGCTGCGCAGCTACTTATTTACGTGGGAGCTATAAATGTTTTAATCCTATTTGC
TGTGATGTTCATGAATGGTTCAGAATATTCCAAAGATTTGACTCTTTGGACTGTTGGTGA
TGGGATTACGTCGTTGGTTTGTACAAGTATTTTTATTTCACTAATTACTACTATTCTAGAT
ACGTCTTGGTACGGGATTATTTGGACGACAAAATCAAATCAGATTATAGAGCAAGATTT
GATAGGTAATAGTCAACAAATTGGAATTCATTTATCAACTGATTTTTTTCTTCCATTTGA
GCTGATTTCAATAATACTTTTAGTTTCTTTGATAGGTGCAATTGCCGTTGCTCGTCAAAT
GAATGTAACAACGACAAGAAAAGACCTCATGATAGTCAATATGGGACCTCACCACCCA
TCAATGCATGGTGTTCTTCGGCTCATCCTTACTCTAGACGGTGAAGATGTTATTGATTGT
GAGCCAATATTGGGTTATTTACACCGAGGGATGGAAAAAATTGCGGAAAACCGAACTG
TTATACAATATCTGCCTTATGTAACACGGTGGGATTATTTAGCGACTATGTTCACAGAAG
CAATAACCATAAATGGACCCGAACAGTTGGGGAATATTCAAGTACCTAAAAGAGCAAG
TTATATCAGAATAATTATGTTAGAGTTGAGTCGTATAGCTTCTCATCTCTTATGGCTTGGC
CCTTTTATGGCGGATATTGGTGCACAGACTCCCTTTTTCTACATTTTCAGAGAAAGAGA

ATTAGTATATGATCTATTTGAAGCTGCCACCGGTATGAGAATGATGCATAATTATTTTCGT
ATCGGAGGAGTGGCGGCCGATCTACCGTATGGATGGATAGATAAATGTTTGGATTTCTG
TGATTATTTTTTAACAGCAGTTTCGGAATATCAAAAACTTATTACGCGGAATCCTATTTTT
TTAGAACGAGTTGAGGGGGTGGGCATTATTGGCGGGGAAGAAGCAATAAATTGGGGTT
TATCAGGACCAATGCTCCGAGCTTCCGGAATAGAATGGGATCTTCGTAAAGTTGATCGT
TATGAATGTTACGGCGAGTTGGATTGGGAAATCCGGTGGCAAAAAGAAGGGGATTCAT
TAGCTCGTTATTTAGTCCGAATGAGTGAAATGACGGAATCTATCAAAATTATTCAGCAG
GCTCTGGAAGGAATTCCGGGGGGGCCTTATGAAAATTTGGAAATACGATGCTTTGATAG
AGAAAAGGATCCAGAATGGGACGGTTTTGAATATCGATTCATTAGTAAAAAACCTTCTC
CCACTTTTGAATTGCCGAAGCAAGAACTTTATGTACGCGTTGAAGCTCCAAAAGGAGA
ATTGGGAATTTTTTTAATAGGAGATCAAAGTGGTTTTCCTTGGAGATGGAAAATCCGCC
CGCCTGGTTTTATCAATTTGCAAATTCTTCCTCAGTTAGTTAAAAGAATGAAATTGGCTG
ATATTATGACGATACTAGGTAGCATAGATATCATTATGGGAGAAGTAGATCGTATGTTCCC
TATGGTAACTGGGTTCATGAATTATGGTCAACAAACAGTACGAGCTGCAAGGTACATTG
GTCAGGGGTTCATGATTACCTTATCCCACGCAAATCGTTTACCCGTAACTATTCAATATC
CTTATGAAAAATTAATTACATCGGAACGTTTCCGCGGTCGAATCCATTTTGAATTTGATA
AATGCATTGCTTGTGAAGTATGTGTTCGTGTATGTCCTATAGACCTGCCCGTTGTTGATT
GGAAATTGGAAACCGGTGTTCGAAAGAAACGATTGCTTAATTACAGTATTGATTTCGGA
GTTTGTATATTTTGTGGTAACTGCGTTGAGTATTGTCCAACAAACTGTTTATCAATGACG
GAAGAATATGAACTTTCTACTTATGATCGTCACGAATTGAATTATAATCAAATCGCTTTG
GGTCGTTTACCAATGTCAGTAATCGACGATTATACAATTCGGACAATTTTGAATTCA------
---ATGCAGGGTCGTTTGTCTGCTTGGCTAGTCAAGCACG
GGCTAGTTCATAGATCTTTGGGCTTCGATTACCAAGGAATAGAAACTTTACAAATAAAG
CCCGAGGAGTGGCATTCCATTGCTGTCATTTTATATGTATATGGTTACAATTATCTACGTT
CCCAATGTGCTTATGATGTAGCACCCGGTGGACTGTTAGCTAGTGTGTATCATCTTACGA
GAATAGAGTATGGTGTGGATCAAGCGGAGGAGGTATGCATTAAAGTATTTGCTCCAAGG
AATAATCCTAGAATTCCGTCCGTTTTCTGGGTTTGGAAAAGCGCGGATTTTCAAGAACG
AGAGTCTTATGATATGTTGGGAATCCGTTATGATAATCATCCACGACTGAAACGTATCTT
AATGCCGGAAAGTTGGATAGGATGGCCCTTACGTAAGGATTATATTGCCCCCAATTTTTA
TGAAATACAAGATGCTCAT--
--ATGAATTCCA
TAGAATTTACCTTACTTGCTCGAAGAACGCAAAATTCAGTTATTTCAACTACATCAAAT
GATCTTTCAAATTGGTCAAGGCTCTCGAGTTTATGGCCGCTTCTTTATGGTACCAGCTGT
TGCTTCATTGAATTTGCTTCATTAATAGGCTCACGATTCGACTTTGATCGTTATGGACTG
GTACCAAGATCGAGTCCTAGACAGGCGGACCTCATTTTAACAGCTGGCACAGTAACAA
TGAAAATGGCTCCTTCTTTAGTGAGATTATATGAACAAATGCCCGAACCAAAATATGTTA
TTGCTATGGGAGCGTGTACAATTACCGGGGGTATGTTCAGTACCGATTCTTATAGTACTG
TTCGTGGAGTAGACAAGCTAATTCCTGTAGATGTCTATTTGCCAGGCTGCCCGCCTAAA
CCGGAGGCTATTATAGATGCTATAACAAAACTTCGTAAGAAAATATCTAGAGAAATCTAT
GAAGATCGAATTAGGTCTCAAGAGGAGAATCGATGTTTTACTACCAATCACAAGTTTCA
TGTTGGACCGAGTATGCATACTGGAAATTATGATCCAGGATTACTTTATCAATTACCATCT
ACTTCAGAGATCGCTTCTGAAACATTTTTCAAATACAAAAGTTCAGTATCTGCCCACGA
ATTAGTGAAT------ATGAAAAATACCTTTTCTTGGATAAAGAAAGAGATTACTCGATCTAT
TTCCCTATCACTGATGATATATATCATAACTCGGACATCCATTTCAAACGCATATCCCATT

TTTGCACAACAGGGGTATGAAAATCCACGAGAAGCGACTGGACGTATTGTATGTGCCA
ATTGCCATTTAGCTAATAAGCCCGTGGATATTGAGGTTCCCCAAGCGGTACTGCCGGAT
ACTGTATTTGAAGCAGTTGTTCGAATTCCTTATGATAGGCAAGTAAAACAAGTTCTTGC
TAATGGTAAAAAAGGGGGTTTGAATGTGGGCGCTGTTCTTATTTTACCCGAGGGTTTTG
AATTAGCCCCCCCCGCTCGTATTTCACCCGAGATGAAAGAAAGGATAGGCAATCCGTCT
TTTCAGAGCTATCGCCCCACTAAAAAAAATATTCTTGTTATAGGTCCCGTTCCCGGCCA
GAAATATAGTGAAATAACCTTTCCTATTCTTTCTCCGGACCCCGCTACTAATAAAGATGT
TCACTTCTTAAAATACCCCATATATGTCGGCGGGAATAGAGGAAGGGGACAGATTTATC
CCGACGGGAGCAAGAGTAACAATACAGTTTATAATGCGACAGCCGCTGGGATAGTAAG
TAAAATAATACGAAAAGAAAAGGGGGGGTATGAGATAACCATAACGGATGCCTCGGAT
GGGCGTCAAGTGGTTGATATTATCCCCTCAGGACCCGAACTTCTTGTTTCAGAGGGCG
AATCTATCAAACTTGATCAGCCATTAACAAGTAATCCTAATGTGGGTGGTTTTGGGCAA
GGGGATGCAGAAGTAGTACTTCAAGATCCATTACGTGTCCAAGGCCTTTTGTTCTTCTT
GGCATCTGTTATTTTGGCACAAATCTTTTTGGTTCTTAAGAAGAAACAGTTTGAGAAGG
TTCAATTGTCTGAAATGAATTTCATGAGTAAAGTCTATGATTGGTTCGAAGAACGTCTC
GAGATTCAGGCGATTGCGGATGATATAACTAGTAAATATGTTCCTCCTCATGTCAACATA
TTTTATTGTCTGGGGGGAATTACGCTTACTTGTTTTTTAGTACAAGTAGCTACGGGATTT
GCTATGACTTTTTACTACCGCCCAACAGTCACTGAGGCTTTTGCTTCTGTTCAATACATA
ATGACTGAAGCTAACTTTGGTTGGTTAATCCGCTCCGTTCATCGCTGGTCGGCAAGTAT
GATGGTCCTAATGATGATCTTGCACGTATTTCGTGTCTATCTCACAGGTGGTTTTAAAAA
ACCTCGCGAATTAACTTGGGTCACAGGCGTGGTTCTAGCTGTATTGACTGCATCGTTTG
GTGTAACTGGGTATTCCTTACCTTGGGACCAAATTGGTTATTGGGCAGTCAAAATTGTA
ACAGGCGTGCCGGACGCTATTCCTGTAATAGGATCTCCCTTGGTAGAGTTATTACGAGG
AAGTGCTAGTGTGGGACAATCCACTTTGACTCGTTTTTATAGTTTACACACTTTTGTATT
ACCTCTTCTTACGGCCGTATTTATGTTAATGCATTTCCTAATGATACGTAAGCAAGGGAT
TTCTGGTCCTTTAATGGGAGTAACAAAAAAACCTGACTTGAATGATCCTGTATTAAGAG
CTAAATTGGCTAAGGGGATGGGTCATAATTATTACGGCGAACCCGCATGGCCCAACGAC
CTTTTATATATTTTTCCAGTAGTAATTCTGGGTACGATTGCGTGTAACGTGGGCTTAGCG
ATTCTGGAACCATCAATGCTTGGTGAACCCGCGGATCCCTTTGCAACTCCTTTGGAAAT
ATTACCTGAATGGTATTTCTTTCCCGTATTTCAAATACTTCGTACAGTACCCAATAAGTTA
TTGGGCGTTCTTTTAATGGTTTCAGTACCTTCCGGATTATTAACAGTACCTTTTTTGGAG
AATGTTAATAAATTTCAAAATCCATTCCGTCGTCCAGTAGCGACAACGGTCTTTTTGATT
GGGACTGTAGTAGCCCTTTGGTTGGGTATTGGAGCAACATTACCTATCGATAAATCCCTA
ACCTTAGGTCTTTTTATGATTGAAGTTTTTCTATTTGGAATTGTCTTAGGTCTAATTCCTA
TTACTTTAGCTGGATTATTTGTAACTGCATATTTACAATACAGGCGTGGCGATCAGCTGG
ACCTTATGCTTACTATAACTAGTTTTTTCGGTTTTCTACTAGCGGCTTTAACTATAACCTC
AGTTTTATTTATTGGTCTGACCAAGATACGACTTATTATGGATATAGTCAGTCTTGCTTGG
GCTGCTTTAATGGTAGTCTTTACATTTTCCCTTTCACTGGTAGTATGGGGAAGGAGTGG
GCTCATGATTATTCGTTCGCCGGAACCAGAAGTCAAAATTTTGGTAGATAGGGATCCCA
TAAAAACGTCGTTCGAGGAATGGGCTAAACCGGGCCATTTCTCAAGAACAATAGCTAA
GGGCCCTGAGACTACCACTTGGATCTGGAACCTACATGCTGATGCTCACGATTTCGATA
GCCATACCAGCGATTTGGAGGAGATCTCTCGAAAAGTATTTAGTGCCCATTTTGGGCAA
CTTTCCATTATCTTTCTGTGGCTGAGTGGCATGTATTTCCACGGTGCTCGTTTTTCCAATT
ATGAAGCATGGCTAAGCGACCCTACTCACATTGGACCTAGTGCTCAGGTGGTTTGGCC

AATAGTGGGCCAAGAAATATTGAATGGCGATGTGGGCGGGGGTTTCCGAGGAATACAA
ATAACCTCTGGGTTTTTTCAGCTTTGGAGAGCGTCTGGAATAACTAGTGAATTACAACT
CTATTGTACCGCAATAGGCGCATTAGTCTTTGCAGCCTTAATGCTTTTTGCTGGTTGGTT
CCATTATCACAAAGCGGCTCCAAAATTGGCCTGGTTCCAAGATGTCGAATCTATGTTGA
ATCACCATTTAGCAGGGCTACTAGGACTGGGGTCTCTTTCTTGGGCGGGGCATCAAGTA
CATGTATCTTTACCAATTAACCAATTTCTAAACGCGGGAGTAGATCCTAAAGAGATCCC
ACTTCCTCATGAATTTATCTTGAATCGGGATCTTTTGGCTCAACTTTATCCGAGTTTTGC
CGAGGGAGCAACTCCATTTTTTACCTTGAATTGGTCAAAATATGCGGAATTTCTGACTT
TTCGCGGAGGATTAGATCCAGTAACTGGGGGTCTATGGCTGACTGATATTGCTCATCAT
CATTTGGCTATTGCAATTCTTTTCTTGATAGCGGGTCACATGTATAGGACCAACTGGGGC
ATTGGTCATGGTCTAAAAGATATTTTAGAGGCTCATAAAGGTCCATTTACAGGCCAGGG
CCATAAAGGCCTATATGAGATCCTAACAACGTCATGGCATGCTCAATTATCTCTTAACCT
CGCTATGTTAGGCTCTTTAACGATTGTTGTAGCTCACCATATGTATTCCATGCCCCCTTAT
CCATATCTAGCTACTGACTATGGTACCCAACTGTCATTGTTCACACATCACATGTGGATT
GGTGGATTTCTCATAGTTGGTGCTGCCGCGCATGCTGCTATTTTTATGGTAAGAGATTAT
GATCCAACTACTCGATACAACGATCTATTAGATCGTGTCCTTAGGCATCGCGATGCAATC
ATATCACATCTCAATTGGGTATGTATTTTTCTAGGCTTTCACAGTTTTGGTTTGTATATTC
ATAACGATACCATGAGCGCTTTAGGACGCCCTCAAGATATGTTTTCTGATACCGCTATAC
AATTACAACCCGTCTTTGCTCAATGGATACAAAACACCCATGCATTAGCGCCCGGTGCA
ACGGCCCCTGGTGCAACAACAAGCACCAGTTTGGCTTGGGGAGGCGGTGATTTAGTA
GCAGTGGGGGGCAAGGTTGCTTTGTTACCGATTCCCTTAGGAACCGCAGATTTTTTGGT
CCATCACATTCATGCATTTACGATTCATGTAACTGTATTGATACTCTTGAAGGGTGTTCTA
TTTGCTCGTAGCTCTCGTTTGATACCGGATAAAGCAAATCTTGGCTTTCGTTTCCCTTGT
GATGGACCGGGACGGGGAGGGACATGTCAAGTATCCGCTTGGGATCATGTCTTCTTAG
GGCTATTCTGGATGTACAATGCAATTTCGGTAGTCATATTTCATTTCAGTTGGAAAATGC
AGTCAGACGTTTGGGGTAGTATAAGTGATCAAGGAGTGGTAACTCATATAACGGGAGG
AAACTTTGCCCAGAGTTCCATCACTATTAATGGTTGGCTCCGCGATTTCTTATGGGCAC
AGGCATCCCAAGTAATTCAGTCTTATGGTTCTTCATTATCTGCATATGGCCTTTTTTTCCT
AGGTGCTCATTTTGTATGGGCTTTTAGTTTAATGTTTCTATTCAGCGGACGTGGTTATTG
GCAAGAACTTATTGAATCCATCGTTTGGGCTCATAATAAATTAAAAGTTGCTCCTGCTAC
CCAGCCTAGAGCCTTGAGCATTGTACAAGGACGTACTGTAGGAGTAACCCATTACCTTC
TGGGTGGAATTGCCACAACATGGGCATTCTTCTTAGCAAGAATTATTGCAGTAGGAATG
GCATTAAGATTTCCAAGGTTTAGCCAAGGCTTAGCTCAGGACCCCACTACTCGTCGTAT
TTGGTTTGGTATTGCAACCGCGCATGACTTTGAGAGTCATGATGATATTACTGAGGAAC
GTCTTTATCAGAATATTTTTGCTTCTCACTTCGGGCAATTAGCAATCATTTTTCTGTGGA
CTTCCGGAAATCTCTTTCATGTAGCTTGGCAAGGAAATTTTGAGGCATGGGTACAGGAC
CCTTTACATGTAAGACCTATTGCTCATGCAATTTGGGATCCTCATTTTGGTCAACCGGCT
GTGGAAGCTTTTACTCGAGGGGGTGCTCTCGGCCCAGTGAATATCGCTTATTCTGGTGT
TTATCAGTGGTGGTATACAATTGGTTTACGTACTAACGAAGATCTTTATACAGGAGCGCT
TTTTCTATTATTTCTTTCTGCTATATCCTTAATAGCGGGTTGGTTACACCTACAACCGAAA
TGGAAACCGAGCGTTTCATGGTTCAAAAATGCCGAATCTCGTCTCAACCATCATTTGTC
GGGGCTATTCGGTGTAAGTTCTTTGGCTTGGACAGGGCATTTAGTACATGTCGCTATTCC
AGGATCCAGGGGGGAATCCGTTCGATGGAATAATTTCTTAGATGTATTACCGCATCCCG
AAGGGTTAGGACCCCTTTTTACAGGCCAGTGGAATCTTTATGCCCAAAACCCCGATTCA

AGTAGTCATTTATTTGGTACTTCCCAAGGATCGGGAACTGCCATTCTCACCCTTCTAGGT
GGATTCCATCCACAAACGCAAAGTTTATGGTTGACTGATATGGCTCATCATCATTTGGCT
ATTGCATTTCTTTTTCTCATTGCTGGTCATATGTATAGAACCAACTTCGGGATTGGGCAC
AGTATAAAAGATCTTTTAGAAGCGCATACCCCTCCGGGGGGGCGATTGGGGCGTGGAC
ATAAGGGTCTTTATGACACAATCAATAATTCGATTCATTTTCAATTAGGCCTTGCCCTCG
CCTCTTTAGGTGTTATTACTTCCTTGGTAGCCCAACACATGTACTCTTTGCCTGCTTATG
CGTTCATAGCACAAGACTTTACTACTCAAGCTGCGTTATATACTCATCACCAATACATTG
CAGGATTCATCATGACGGGGGCTTTTGCGCATGGGGCTATATTTTTCATTAGAGATTACA
ATCCGGAACAGAATGAGGATAATGTATTGGCAAGAATGTTAGACCATAAAGAAGCTATT
ATATCCCATTTAAGTTGGGCCAGCCTCTTTTTGGGGTTCCATACTTTGGGACTTTATGTT
CATAATGATGTCATGCTTGCTTTTGGTACTCCGGAGAAACAAATATTGATCGAACCTATA
TTTGCCCAATGGATACAATCTGCTCATGGTAAAACCTCGTATGGTTTCGATGTACTTTTA
TCTTCAACGAATAGCCCGGCATTTAATGCGGGTCGAAGCATATGGTTGCCCGGTTGGTT
AAATGCTATTAATGAG------AATAGTAATTCACTATTCTTAACAATAGGTCCTGGAGACTT
CTTGGTTCATCATGCGATTGCTCTAGGGTTACATACCACTACATTGATCTTAGTAAAAGG
TGCTTTAGATGCACGCGGTTCCAAATTAATGCCAGATAAAAAGGATTTCGGTTATAGTTT
TCCTTGCGATGGTCCGGGACGAGGTGGTACTTGTGATATTTCCGCTTGGGACGCATTTT
ATTTGGCAGTTTTCTGGATGTTAAATACCATTGGATGGGTTACTTTTTATTGGCATTGGA
AACACATCACATTATGGCAAGGTAACGTTTCACAGTTTAATGAATCTTCCACTTATTTAA
TGGGATGGTTAAGAGATTATCTTTGGTTAAACTCTTCACAACTTATCAATGGATATAATC
CTTTTGGTATGAATAGTTTATCAGTCTGGGCCTGGATGTTCTTATTTGGACATCTTGTTTG
GGCTACGGGATTTATGTTCTTAATTTCCTGGCGTGGATATTGGCAGGAATTGATTGAAAC
TTTAGCATGGGCTCACGAACGGACGCCCTTGGCTAATTTGATTCGGTGGAGAGATAAA
CCGGTGGCTCTTTCCATTGTGCAAGCAAGATTGGTTGGATTAGCCCACTTTTCTGTAGG
TTATATATTCACTTATGCGGCTTTCTTGATTGCTTCGACATCAGGCAAATTTGGTATGTCG
CATTCAGTAAAGATTTATGATACCTGTATAGGATGTACTCAATGTGTACGTGCCTGCCCG
ACGGACGTATTAGAAATGATCCCTTGGGATGGGTGTAAAGCCAAGCAAATTGCTTCTGC
TCCAAGAACAGAGGACTGTGTTGGTTGTAAGAGATGTGAATCGGCCTGTCCAACGGAT
TTTTTGAGCGTTCGAGTTTATTTATGGCATGAAACAACTCGAAGTATGGGTCTAGCTTAT
ATGACA------GACTTCCCCTCTATTTTTGTGCCTTTAGTGGGCCTAGTATTTCCGGCAATT
GCAATGGCTTCTTTATCTCTTCATGTTGAAAACAACAAGATTGTTATGCGAGATCTAAA
AACATATCTTTCCGTCGCACCGGTACTAAGTGCTCTCTGGTTCGGGGCTTTAGCGGGTC
TATTGATAGAGATCAATCGTTTCTTCCCGGATGCCTTGACATTCCCCTTTTTTTCC---ATG
ACTGCAATTTTAGAGAGACGCGAAAGCGAAAGCCTATGGGGTCGCTTCTGTAACTGGA
TAACCAGCACCGAAAACCGTCTTTACATCGGATGGTTTGGTGTTTTGATGATCCCTACC
CTATTGACCGCAACTTCTGTATTTATTATCGCCTTCATTGCTGCTCCTCCAGTAGATATTG
ATGGTATTCGTGAACCTGTTTCTGGATCTCTTCTTTATGGAAACAATATTATTTCGGGTG
CCATTATTCCTACTTCTGCAGCTATTGGTTTGCATTTTTACCCAATATGGGAAGCAGCAT
CTGTTGATGAATGGTTATACAACGGTGGTCCTTATGAGCTAATTGTTCTACACTTCTTAC
TTGGTGTAGCTTGTTACATGGGCCGTGAGTGGGAACTTAGTTTCCGTCTGGGTATGCGC
CCTTGGATTGCTGTTGCGTATTCAGCTCCTGTTGCAGCTGCTACTGCTGTTTTCTTGATC
TATCCAATTGGTCAAGGAAGTTTTTCTGATGGTATGCCTCTAGGAATTTCTGGTACTTTC
AACTTCATGATTGTATTCCAGGCGGAGCACAACATCCTTATGCACCCATTCCACATGTTA
GGCGTAGCTGGTGTATTCGGCGGCTCCCTATTCAGTGCTATGCATGGTTCCTTGGTAACC

TCTAGTTTGATCAGGGAAACCACAGAAAATGAATCTGCTAATGAAGGTTACAGATTCG
GTCAAGAGGAAGAAACTTATAATATCGTAGCTGCTCACGGTTATTTTGGCCGATTGATC
TTCCAATACGCGAGTTTCAACAACTCTCGTTCTTTACATTTCTTCTTAGCTGCTTGGCCT
GTAGTAGGTATCTGGTTCACTGCTTTAGGTATTAGCACCATGGCTTTCAACCTAAATGGT
TTCAATTTCAACCAATCTGTAGTTGATAGTCAAGGTCGTGTAATTAATACCTGGGCGGAT
ATTATTAACCGTGCTAACCTTGGTATGGAAGTTATGCACGAGCGTAACGCCCATAACTTC
CCTCTAGACCTAGCTGCTGTTGAAGCTCCATCTACAAATGGAATGGGTTTGCCTTGGTA
TCGTGTGCATACCGTCGTATTGAATGATCCTGGCCGTTTGCTGGCTGTCCATATAATGCA
TACAGCTTTGGTTGCTGGTTGGGCCGGTTCGATGGCTTTATATGAATTAGCCGTTTTTGA
TCCCTCTGACCCCGTTCTTGATCCAATGTGGAGACAAGGTATGTTCGTTATACCCTTCAT
GACTCGTTTAGGAATAACTAATTCATGGGGTGGTTGGAGTATCACA---GGGGGAACTGT
AACGAATCCGGGTATTTGGAGTTACGAAGGTGTGGCCGGCTCACATATTCTGTTTTCTG
GCTTGTGCTTCTTGGCAGCTATCTGGCATTGGGTGTATTGGGATTTAGCAATATTTTCTG
ATGAACGCACAGGAAAACCTTCTTTAGATTTGCCCAAGATCTTTGGAATTCATTTATTTC
TTTCAGGGCTAGCTTGCTTTGGTTTTGGCGCATTTCATGTAACAGGGTTGTATGGTCCTG
GAATCTGGGTGTCCGATCCTTATGGACTAACTGGAGAGGTACAACCTGTAAATCCAGCA
TGGGGCGTAGAAGGTTTTGATCCTTTTGTTCCAGGAGGAATAGCCTCTCATCATATTGC
AGCGGGGACTTTAGGTATATTGGCGGGTCTATTTCATCTTAGTGTCCGTCCGCCCCAAC
GTCTCTACAAGGGATTGCGTATGGGCAATATTGAAACCGTCCTTTCCAGTAGTATTGCT
GCTGTCTTTTTTGCAGCTTTTGTTGTTGCTGGAACTATGTGGTATGGTTCAGCAACTACC
CCTATTGAATTGTTTGGTCCCACCCGTTATCAATGGGATCAGGGATACTTCCAGCAAGA
AATATATCGAAGAGTTGGCGCGGGGCTAGCCAAAAATCAAAGCTTATCAGAAGCGTGG
TCTAAAATTCCTGAAAAATTGGCTTTTTATGATTACATCGGGAATAATCCTGCAAAAGG
GGGATTATTCAGAGCGGGTTCAATGGACAGCGGGGATGGAATAGCCGTTGGGTGGTTA
GGACATCCTATCTTTAGAGATAAAGAGGGGCGTGAACTTTTTGTACGTCGTATGCCTAC
TTTTTTTGAAACATTTCCGGTTGTTTTGGTAGACGGAGACGGAATTGTTAGAGCCGACG
TTCCTTTTAGAAGGGCAGAATCGAAGTATAGTGTGGAACAAGTAGGTGTAACCATCGA
GTTCTATGGTGGCGAACTCAATGGAGTCAGTTATAGTGACCCTGCTACTGTTAAAAAAT
ATGCTAGACGCGCTCAATTAGGTGAAATTTTTGAATTAGATCGCGCTACTTTGAAATCG
GATGGTGTTTTTCGTAGCAGTCCGAGAGGCTGGTTTACTTTTGGGCATGCTTCGTTTGC
TCTGCTCTTCTTCTTCGGGCACATTTGGCACGGTGCTAGAACCCTCTTCAGAGATGTTT
TTGCTGGTATTGACCCGGATTTGGATACGCAAGTGGAATTTGGAGCATTCCAAAAACTT
GGAGATCCAACTACAAGAAGACAAGCAGTCATGAAAACCTTATATTCCCTGAGGAGGT
TCTACCCCGTGGAAACGCTCTTTAATGGAACTTTAGCTTTAGCTGGTCGTGACCAAGAA
ACTACCGGTTTCGCTTGGTGGGCCGGGAATGCACGACTTATCAATTTATCCGGTAAACT
TTTGGGGGCTCATGTAGCCCATGCCGGATTAATCGTATTCTGGGCCGGAGCAATGAACC
TATTTGAAGTGGCTCATTTTGTACCAGAGAAGCCCATGTATGAACAAGGATTAATTTTAC
TTCCGCACTTAGCCACTCTAGGTTGGGGAGTAGGTCCTGGTGGGGAAGTTATAGACAC
CTTTCCATACTTTGTATCTGGAGTACTTCACTTAATTTCCTCGGCAGTATTGGGCTTTGG
CGGTATTTATCATGCACTTCTGGGCCCTGAGACTCTTGAAGAATCTTTTCCATTCTTTGG
TTATGTATGGAAAGATAGAAATAAAATGACCACAATTTTAGGTATTCACTTAATCTTGTT
AGGTTTAGGTGCTTTTCTTCTAGTATTCAAGGCTCTTTATTTTGGTGGCGTATATGATACC
TGGGCTCCGGGAGGGGGAGATGTACGAAAAATTACTAACTTGACCCTTAGCCCAAGTA
TTCTATTTGGTTATTTACTAAAATCGCCCTTTGGCGGAGAAGGATGGATTGTGAGTGTG

GATGATTTGGAAGATATAATTGGAGGGCATGTATGGTTAGGTTCCATTTGTATACTTGGC
GGAATCTGGCATATCTTAACCAAACCTTTTGCGTGGGCTCGTCGCGCACTTGTATGGTC
TGGGGAGGCTTACCTGTCTTATAGTTTAGCTGCTTTATCCGTTTTTGGTTTCATTGCTTGT
TGCTTTGTCTGGTTCAATAATACCGCCTATCCTAGTGAATTTTACGGACCGACTGGACCA
GAAGCTTCTCAAGCTCAGGCATTTACTTTTCTAGTTAGAGACCAACGCCTTGGGGCTAA
CGTGGGATCCGCTCAAGGACCTACAGGTTTAGGTAAATATCTAATGCGTTCCCCGACTG
GAGAAGTCATTTTTGGAGGGGAAACTATGCGTTTTTGGGATCTGCGTGCTCCCTGGTTA
GAACCTCTAAGGGGGCCAAATGGGTTGGACTTGAGTAGGCTGAAAAAAGACATACAG
CCTTGGCAAGAACGGCGTTCCGCAGAATATATGACTCATGCTCCTTTAGGTTCTTTAAA
TTCGGTTGGTGGCGTTGCTACCGAGATCAATGCAGTCAATTATGTCTCTCCTAGAAGTT
GGTTAGCTACCTCTCATTTTGTTCTAGGATTCTTCCTATTCGTAGGGCATTTATGGCACG
CGGGAAGGGCTCGTGCAGCTGCAGCAGGATTTGAAAAAGGAATTGATCGTGATTTTGA
ACCTGCTCTTTCCATGACCCCTCTTAACATGACTATAGCCCTTGGTAAATTTACCAAAGA
CGAAAAAGATTTATTTGATATTATGGATGACTGGTTACGTAGGGACCGTTTTGTTTTTGT
AGGTTGGTCCGGTCTATTGCTCTTTCCTTGTGCCTATTTCGCTTTAGGGGGTTGGTTTAC
AGGTACAACCTTTGTAACTTCATGGTATACCCATGGATTGGCCAGCTCCTATTTGGAAG
GATGCAACTTCTTAACCGCCGCAGTTTCGACCCCTGCTAATAGTTTAGCGCATTCTTTGT
TGTTACTATGGGGTCCGGAAGCGCAGGGGGATTTTACTCGTTGGTGTCAATTGGGCGGT
CTGTGGACTTTTGTTGCTCTCCACGGCGCTTTCGCACTAATAGGTTTCATGTTACGTCAA
TTTGAAATTGCTCGATCTGTTCAATTGCGACCTTATAATGCAATCGCATTCTCTGGGCCA
ATTGCTGTTTTTGTTTCTGTATTCCTGATTTATCCACTCGGGCAGTCTGGTTGGTTCTTTG
CGCCTAGTTTTGGTGTAGCAGCTATATTTCGCTTCATCCTCTTTTTCCAAGGGTTTCATAA
TTGGACATTGAACCCGTTTCATATGATGGGAGTTGCTGGTGTATTGGGCGCTGCTCTACT
ATGTGCTATTCATGGTGCTACTGTAGAAAATACTTTATTTGAAGATGGTGATGGTGCAAA
TACATTCCGTGCTTTTAACCCAACTCAAGCTGAAGAAACTTATTCAATGGTCACCGCTA
ATCGCTTTTGGTCCCAAATCTTTGGGGTTGCTTTTTCCAATAAACGTTGGTTACATTTCT
TTATGTTATTTGTACCAGTCACCGGTTTATGGATGAGTGCTCTTGGAGTCGTCGGCCTGG
CTCTGAACCTACGCGCCTATGACTTCGTTTCCCAGGAGATCCGTGCCGCGGAAGATCCT
GAATTTGAAACTTTCTACACCAAAAATATTCTCTTAAACGAAGGTATTCGTGCTTGGAT
GGCGGCTCAAGATCAGCCTCATGAAAACCTTATATTCCCTGAGGAGGTTCTACCCCGTG
GAAACGCTCTTATGTCTGGAAGCACAGGAGAACGTTCTTTTGCTGATATTATTACCAGT
ATTCGATACTGGGTTATTCATAGCATTACTATACCCTCCCTATTCATTGCGGGTTGGTTAT
TCGTCAGCACGGGTTTAGCTTACGATGTGTTTGGAAGCCCTCGTCCAAACGAATATTTT
ACAGAGAGCCGACAAGGAATTCCATTAATAACTGGCCGTTTTGACTCTTTGGAACAAC
TCGACGAATTTAGTAGATCTTTTATGACTATAGATCGAACCTATCCAATTTTTACAGTGC
GATGGTTGGCTGTTCATGGACTAGCTGTACCTACCGTTTCTTTTTTGGGGTCAATATCAG
CAATGCAGTTCATCCAACGAATGGCTACACAAACTGCTGAGGAGAGTTCTAGA---GCTA
GACCAAAAAAAAACAGGTTTAGGGGGGTTATTGAAACCATTAAATTCAGAATATGGTAA
AGTAGCTCCTGGGTGGGGAACTACCCCTTTGATGGGTCTTGCAATGGCTCTATTTGCGG
TATTCTTATCTATTATTTTGGAAATTTATAATTCTTCCGTTTTATTGGACGGAATTTCAATG
AATATGCTTACTCTCAAACTCTTTGTTTACACAGTGGTGATATTCTTTGTTTCTCTCTTCA
TCTTCGGATTCCTATCTAACGATCCAGGACGTAATCCTGGACGTGAAGAAATGGCCGAT
ACTACTGGAAGGATTCCTCTTTGGATAATAGGTACTGTAGCCGGTATTCTTGTGATCGGT
TTAATAGGTATTTTCTTTTATGGTGCATACTCCGGGTTAGGCTCATCTCTGATGCTTAATA

TCTTTAGTTTG------ATCTGTCTTAATTCCGATCTTTATTCGAGTCGTTTTTCTTAGCCAA
ATTACCCGAGGCCTACGCTTTTTTGAATCCAATCGTAGATGTTATGCCAGTAATACCCCT
TTTCTTTTTGCTCTTAGCCTTTGTTTGGCAAGCTGCTGTAAGTTTTCGAATGACACAATC
AAACCCGAACGAACAAGATGTTGAATTGAATCGTACCAGTCTCTACTGGGGGTTATTAC
TCATTTTTGTACTTGCTGTTTTATTTTCCAATTATTTCTTCAATATGGAAGTAAATATTCTC
GCATTTATTGCTACTGCACTTTTTATTCTAGTTCCTACTGCGTTTTTACTTATAATATACGT
AAAAACTGTCAGTCAAAGTGATATGGAAACAGCAACCCTAGTCGCCATCTCTATATCTG
GTTTACTTGTAAGTTTTACGGGGTATGCCTTATATACTGCTTTTGGGCAACCCTCTCAAC
AATTAAGAGATCCGTTCGAGGAACACGGGGACATGGAAGCATTGGTTTATACATTCCTC
TTAGTCTCCACTCTAGGGATCATTTTTTTCGCTATCTTTTTTCGAGAACCGCCTAAAATT
CAAACTAAAAAGACGAAAATGACTATTGCTTTCCAATTGGCTGTTTTTGCATTAATTGC
TACTTCATCACTTTTACTGATTAGTGTACCCGTTGTATTTGCTTCTCCCGAGGGTTGGTC
AAGTAACAAAAATGTTGTATTTTCCGGCACATCATTGTGGATTGGATTAGTCTTTCTGGT
GGGTATCCTTAATTCTCTCATCTCT---------------------ATGTCACCACAAACAGAGACTAAA
GCAAGTGTTGGATTCAAAGCCGGCGTTAAAGATTATAAACTGACTTATTATACTCCTGA
ATACGAAACCAAAGATACTGATATCTTGGCAGCATTCAGAGTAACTCCTCAACCTGGAG
TTCCGCCTGAGGAAGCAGGGGCTGCAGTAGCTGCCGAATCTTCTACTGGTACCTGGAC
AACTGTGTGGACCGATGGGCTTACCAGCCTTGATCGTTATAAAGGAAGATGCTACCACA
TCGAGCCTGTTGCGGGAGAAGAGAATCAATATATATGTTATGTAGCTTACCCCTTAGACC
TTTTTGAAGAGGGTTCTGTTACTAATATGTTTACTTCCATTGTGGGTAATGTATTTGGGTT
CAAAGCCCTGCGTGCTCTACGTCTGGAGGATCTGAGAATCCCTACTGCATATGTTAAAA
CTTTCCAAGGCCCGCCTCACGGCATCCAAGTTGAGAGAGATAAGTTGAACAAGTATGG
CCGTCCCCTATTGGGATGTACCATTAAACCGAAATTAGGGTTATCCGCTAAGAACTACG
GTAGAGCTGTTTATGAATGTCTTCGTGGTGGACTTGATTTTACCAAGGATGATGAAAAC
GTAAACTCACAACCATTTATGCGTTGGAGAGACCGTTTCTTATTTTGTGCCGAAGCAAT
TTATAAATCACAGGCTGAAACTGGTGAAATCAAAGGGCATTACTTGAATGCTACTGCGG
GTACATGCGAAGAAATGATGAAAAGGGCTATATTTGCCAGAGAATTGGGAGTTCCCATC
GTAATGCATGACTACTTAACAGGGGGATTCACTGCAAATACTAGCTTGGCTCATTATTGC
CGAGATAACGGTCTACTTCTTCACATCCACCGTGCAATGCATGCAGTTATTGATAGACA
GAAAAATCATGGTATACACTTTCGTGTACTAGCTAAAGCCTTACGTATGTCTGGTGGAG
ATCATATTCACTCAGGTACCGTAGTAGGTAAACTTGAAGGAGAAAGAGATATTACTTTA
GGCTTTGTTGATTTACTACGTGATGATTTTATTGAAAAAGACCGAAGCCGCGGTATTTAT
TTCACTCAAGATTGGGTCTCTCTACCTGGTGTTTTGCCTGTGGCTTCAGGGGGTATTCA
CGTGTGGCATATGCCTGCTCTGACCGAAATCTTTGGAGATGATTCCGTACTACAATTCG
GTGGTGGAACTTTAGGACACCCTTGGGGAAATGCACCGGGTGCTGTAGCGAATCGAGT
AGCTCTAGAAGCATGCGTACAAGCCCGTAATGAGGGGCGTGATCTTGCTCGTGAGGGT
AATGAAATTATACGGGAGGCTTGCAAATGGAGTCCGGAACTAGCTGCTGCTTGTGAAG
TATGGAAAGAGATCAAATTTGAATTCCAAGCAATGGATACTTTG------ATGGCGATACAT
TTATACAAAACTTCTACCCCGAGCACACGCAATGGAGCCGTAGACAGTCAAGTGAAAT
CCAATACACGAAAAAAATTTGATCTATGGACAGCCTCGTTGTAGTAAAGGTCGTAATGCC
AGAGGAATCATTACCGCAGGGCATAGAGGGGGAGGTCATAAGCGTCTATACCGTAAAA
TCGATTTTCGACGGAATGAAAGAGACATATATGGTAGAATCGTAAGCATAGAATACGAC
CCTAATCGAAATGCATCCATTTGTCTCATACACTATGGGGATGGTGAGAAGAGATATATT
TTACATCCCAGAGGGGCCATAATTGGAGATACCATTGTTTCTGGTACAGAAGTTCCTATA

AAAATGGGAAATGCCCTACCTTTGACCAAGATGCCCTTAGGCACGGCCCTACATAACAT
AGAAATCACACTTGGAAAGGGTGGACAATTAGCTAGAGCAGCGGGTGCTGTAGCGAA
ACTGATTGCAAAAGAGGGGAAATCGGCCACATTAAAATTACCTTCTGGGGAGGTCCGT
TTGATATCCAACAACTGCTCAGCAACAGTCGGACAAGTGGGGAATGTTGGGGTGAACC
AGAAAAGTTTGGGTAGAGCCGGATCGAAACGTTGGCTAGGTAAGCGTCCTGTAGTAAG
AGGGGTAGTTATGAACCCCGTAGACCATCCCCATGGGGGTGGTGAAGGGAGGGCCCCA
ATTGGTAGAAAAAAACCCGCAACCCCTTGGGGTTATCCGGCACTTGGAAGAAGAAGTA
GAAAAAGGAATAAATATAGTGAGAATTTGATTCTTCGTCGCCGTAGTAAAATGATTCAA
CCTCAAACCCGTTTGAATGTCGCGGATAACAGTGGGGCTCGAGAATTAATGTGTATTCG
AATCATCGGAGCTAGT---AATCGTCGATATGCTCATATCGGTGACATTATTGTTGCTGTGA
TCAAGGAAGCATTACCAAGCACCTCCCTAGAAAGATCAGAAGTGGTCAGAGCTGTAAT
TGTACGTACTTGTAAAGAACTTAAATGTGACGACGGTATCATAATACGATATGATGACAA
TGCTGCTGTTGTCATTGATCAAGAAGGAAATCCAAAAGGAACCCGAGTTTTTGGTGCG
ATTGCCCACGAATTGAGAGAGTTAAGTTTCACTAAAATAGTTTCATTAGCACCTGAGGT
ATTAATGCTTAGTCCCAAAAGAACCAGATTTCGTAAACAACATAGAGGAAGAATGAGA
GGGATATCTTATCGCGGCAATCGTATTTGTTTCGGTAAATATGCTCTGCAGGCACTTGAA
CCGGCGTGGATCACATCTAGACAAATAGAAGCAGGGCGTCGAGCAATGACGCGAAAT
GTACGACGTGGTGGAAAAACATGGGTACGTATATTTCCAGACAAACCCGTTACGTTAA
GAGCGGCCGAAACACGTATGGGTTCGGGTAAAGGAAACCCCGAATATTGGGTAGCTGT
CGTCAAACCGGGTAGAATACTTTATGAAATGGGTGGAGTCGCAGAAAATATAGCCAGA
AAGGCGATTTCTATAGCAGCCTCGAAAATGCCTATACGAACTCAATTCATTATTTCAGGA
ATGACCAGAATTAGACGGGGATATATAGCTCGTAGACGTAGAACAAAAACGCGTTTCTT
TGCATCAAGCTGGCGGGGGGCTCGTGGAAACCTGACTCGAGCAATCATTCAACAGAG
AATAAGAGCTTGGTTTTCGTCTCATCGAGATAGAACTAGACAAAAGAGAGATTTTCGTC
GTTTGTGGATCACTCGAATAAATGCAGCAATTCGCGAGAAT------AGATCCTCT------AGT
AAATTAATACACAATCTGTACAAGAGGCAGTTGTTTCTTAATCGTAAAATGCTTGCCCA
ACTAGCTATATTAAATAGGAATTGTCTTTATATGATTTCCAATCAGATCCTAAAA---GAAG
TAGATTGGCAAGAATCCGCTACAATAATGAAAAAAGAAGTCTATGCTTTAGGGCAATAT
ATATCTATGTCTGCTCCCAAAGCGCGAAGGGTGATTGATCAGATTCGTGGGCGGTCCTA
CGAAGAAACCCTTATGCTACTAGCACTTATGCCTTATCGAGCGTGTGATCCCATTTTAAA
ATTAGTGAATTCTGCGGCCGCAAATGCTAGGCACAATATGTCTTTCAATGAAGCAACTT
TAGTTATTAGTAAAGCAGAAGTCAACGAAGGCACTACTGTGAAAAAATTAAAACCGCG
AGCTCGAGGACGGAGTTATCCAATAAGAAGACCCACTTGCCATATAAGAATTGTATTGC
AAGATACGTCTTTCGAT---------------------ATGGATGGAATCAAATATGCAGTATTTACAGA
CAAAGGTGTTCGGTTATTGGGGAAAAAT---CAATATACTTCTAATGTCGAATCAGGATCA
ACTAGGACAGAAATAAAGCATTGGGTCGAACTCTTCTTTGGTGTCAAGGTACAAGCTA
TGAATAGTCATCGACTCCCGGGAAAGGGTAGAAGAATGGGACCTATTATGGGACATAC
AATGCATTACAGACGTATGATCATTACGCTTCAACCGGGTTATTCTATTCCACCTCTTAG
AAAGAAAAGAACTATGGCAGTTCCAAAAAAACGCACTTCTATATCAAAAAAACGTATT
CGTAAAACTATTTGGAAAAAAAAAGGCATATTGGGCATCCTTGAAAGCTTTTTCGTTAGC
GAAGTCTCTTTCTACTGGAAATTCTAAAAGTTTTTTCTACTCGAAATTC------ATGGCCA
GGGGTAAAGATGCCCGTGTAACGGTTATCTTGGAATGTACCAATTGTGTTCGAGGGGGT
------GTTACTAAGGAATCAACGGGTATTTCCAGATATATTACTGAAAAGAATCGACACAA
TACGCCTGGTCAATTGGAATTGAAAAAATTCTGTCCCTATTGTTACAAACAGACAATTC

ACGGGGAGATAAAGAAAATGAAAATACGGGCATCCGTTCGTAAAATTTGTACAAAATG
TCGACTGATCCGTAGGAGAGGACGAATTATAGTAATTTGTTCCAATCCGAGACATAAAC
AAAGACAAGGCATGGTTCGAGAGAAAGTAAGAGTATCTACTCGGACACTCCAGTGGA
AGTGTGTTGAATCAAGAGTAGACAGTAAGCGTCTTTATTATGGACGCTTTATTCTTTCCC
CCCTTATGAAAGGGCAGGCCGATACAATAGGCATTGCGATGCGAAGAGCTTTACTTGG
GGAAATAGAAGGGACATGTATCACCCGCGCCAAATTTGAA------AAAATACCCCATGAA
TATTCTACCATAGTAGGTATTCAAGAATCAGTACATGAAATTTTAATGAATTTGAAAGAA
ATTGTATTGAGAAGTAATTTGTATGGAACTTGTAACGCGTCTATTTGTGTCAAGGGTCCC
GGGTGTGTAACTGCTCAAGACATCCTATTACCACCGTCTGTGGAAATCATTGATAACAC
ACAGCATATAGCTAGCCTAATGGAACCGATTCATTTGTGTATTGGATTACAAATTGAGAG
GAATCGAGGATATCATATAAAATCACCAAATAACTTTCAAGACGGAAGTTAT------CCTAT
AGATGCTGTATTTATGCCTGTTCGAAATGCGAATCATAGTATTCATTCTTATGGGAATCA
AAATGAAAAACAAGAGATACTTTTTCTCGAAATATGGACAAATGGAAGTTTAACTCCTA
AAGAAGCACTTCACGAAGCCTCCCGGAATTTAATTGATTTATTTCTTCCCTTTCTGCATG
CGGAAGAAGAA------AACTTACATTTAGGAAAGAATAAG---TACAATGTGACTTTCCCCC
TTTTTACTTTTCATAAAAAAATTAGCTAAG---CTAAGGAAACCGAAAAAC---GAAATAGCA
TTGAAATCTATTTTTATTGACCAATTAGAATTGCCCCCCAGGATCTATAATTCCCTCAAA
GGGTCCAAGATCCATACATTATCGGACCTTTTGAATAAGAGTCAAGAAGACCTTATGAA
AATGAAACATTTTCGTATAGAAGATGTAAAACAGATCTTGGACATTCTAGAAATAGAAA
AAGCATTTCATAACCGATTTAACGAAGAAAACATGCTCGGGGATGGAAAT---GGGGGAA
TGTGTACAATACCTGGTTTTAATCAGATACAATTTGAAGGATTTTGTAGATTCATTGATC
AGGGCTTAACGGAAGAACTTTCGAAATTTCCAAAAATTGAAGACACGGATCAAGAAAT
TGAATTTCAATTATTTGTGGAAACATATCACTTGGTAGAACCTTTGATAAAAGAACGAG
ATGCTGTATATGAATTACTCACATATTCTTCTGAATTATATATATCCGCGGGATTAATTTGG
AAAAGCAGTAGGGATATGCAAAAACAAACCATTTTTATTGGAAACATTCCTTTAATGAA
TTCCCTGGGAACTTCTATAGTAAATGGAATATACAGAATTGTGATCAATCAAATATTACA
AAGTCCCGGTATCTATTATCGGTCAGAATTGGACCATAACGGAATTTCGGTCTATACCGG
GACGATAATATCAGATTGGGGGGGGAGGTTAGAATTAGAGATTGATAGAAAAGCAAGG
ATATGGGCTCGTGTGAGTAGGAAACAGAAAATATCTATTCTAGTTCTATCATCAGCTATG
GGTTCGAATCTAAGAGAAATTCTAGAGAATGTTTGCTATCCTGAGATGTTCTTGTCTTTC
CTGAATGAGAAAGAGAAAAAAACAATTGGATCAAAAGAAAATGCCATTTTGGAGTTTT
ATCAACAATTTTCTTGTGTAGGCGGGGATCCTGTATTTTCGGAATCCTTATGTAAGGAAT
TACACACGAAATTCTTTCAACAAAGATGTGAATTAGGAAGGATTGGTCGACAAAATAT
GAATCGGAGGCTTAATCTTGATATACCTCAGCACAATACATTTTTGTTACCGCGAGATAT
ATTAGCAGCTGCGGATCATTTGATTGGAATGAAATTCGGAATGGGTACACTTGACGAT---
---ACGAATCATTTGAAAAATAAACGTATTCGTTCTGTAGCCGATCTCTTACAAGATCAAT
TCGGATTGGCTCTGGTTCGTTTAGAAAGTGTGGTTAGACGAACTATATGTGGAGCAATT
AGA---CATAAATTGATA---CCGACTCCTCATAATTTGGTAACTTCAACTCCA------------TTA
ACAACCACTTATGAATCTTTTTTCGGATTACACCCATTATCTCAAGTTTTGGATCGAACG
AATCCATTGACACAAATAGTTCATGGGAGAAAATTGAGTTATTTGGGCCCTGGAGGATT
AACAGCGCGAACTGCTAGTTTTCGGATGCGAGATATTCACCCCAGTTACTATGGACGTA
TTTGCCCAATTGACACGTCTGAAGGAATCAATGTTGGACTTATTGGATCCTTAGCAATT
CATGCGAGAATTGGTCGGGGTGGG---TCTCTAGAAAGCCCATTTTAT---------GAAATCTC
TGAGAGATCA------AACAAAGGACGAATGCTGTATTTCTCACCAAGTCGAGATGAATAC

TATATGGTAGCGGCGGGAAATTCTTTGGCGCTGAATCGGGGTATTCAAGAAGAACAGG
TTGTTCCAGCTCGATACCGTCAAGAATTCCTGACTCTTGCATGGGACCAGGTTCATCTT
CGAAGTATTTTTCCTTTTCAATATTTTTCTATTGGAGCTTCGCTCATTCCTTTTATCGAAC
ATAATGATGCGAATCGAGCTTTAATGAGTTCGAATATGCAACGTCAAGCAGTTCCACTT
TCTCAGTCCGAGAGGTGCATTGTTGGAACTGGGTTGGAACGCCAAGCGTCTCTAGATT
CCGGGGTTACCGCTATAGCCGAACGCGAGGGAAAGATCATTTATACCAATAGCGATAAG
ATCATTTTATCGGGCAGTGGAGATACTCTAAGCATTCCATTGGTTATGTATCAACGTTCC
AACAAAAATACTTGGATGCATCAAAAACCCCAGGTTCCGCGGGGTAAATGCATTAAAA
AAGGACAAATTTTAGCGGACGGTGCGGCTACAGTTGGTGGCGAACTCGCTTTAGGTAA
AAACGTCTTAGTAGCTTATATGCCGTGGGAAGGTTACAATTTTGAAGATGCGGTACTCA
TTAGCGAGCGTCTGGTATATGAAGATATTTATACTTCGTTTCACATACGGAAATATGAAA
TGCAGACTCATGTAACAAGCCAAGGT---CCCGAAAGGATCACTAATGAAATACCGCATC
TAGAAGCCCATTTACTCCGCAATTTAGACAAAAATGGAATTGTGATGATGGGATCTTGG
GTGGAGGCGGGCGAGATTTTAGTAGGTAAATTAACGCCTCAGATGGCGGAAGAAGAGT
CGTCGTATGCCCCAGAGGAGAGATTATTACGAGCCATACTTGGCATTCAGATATCCACTT
CAAAGGAGACTTGCCTAAAACTACCGATAGGTGGTAGGGGTCGAGTTATTGATGTGAG
ATGGATCCAGAAAAGGGGG---GGTTCTAGTTATAATCCAGAAACTATTCGTGTATATATT
TTACAGAAACGCGAAATCAAAGTAGGGGATAAGGTAGCCGGAAGACATGGAAATAAA
GGTATCATTTCCAAAATTTTACCTAGACAAGATATGCCTTATTTGCAAGATGGAAGGCCT
GTTGATATGGTCTTCAATCCATTAGGAGTCCCCTCCCGAATGAATGTAGGACAGATATTT
GAATGCTCGCTCGGATTCGCGGGGGATCTGCTAGAGAGACATTATCGAATAGCACCTTT
TGATGAGAGATATGAGCAAGAGGCCTCAAGAAAACTCGTGTTTTCTGAATTATACGAA
GCCAGTAAGCAAACAGCCAATCCATGGGTATTTGAACCTGAGTATCCGGGAAAAAGCA
GACTATTTGATGGAAGAACGGGGGATCCCTTTGAACAACCTGTTATAATAGGAAAGCCT
TATATCTTGAAATTAATTCATCAAGTTGATGATAAAATACACGGACGTTCCAGTGGACAT
TATGCACGTGTTACACAACAACCCCTTAGAGGACGGGCCAAACAGGGGGGTCAGCGG
GTAGGAGAAATGGAGGTTTGGGCTCTCGAGGGGTTTGGTGTTGCTCATCTTTTACAAG
AGATGCTTACTTATAAATCGGATCATATTAGAGCTCGTCAAGGAGTCCTTGGTACTACGA
TCCTTGGAGGAACAATACCTAAACCCGAGGATGCTCCAGAATCTTTTCGACTGCTCGTT
CGAGAACTACGATCTTTGGCTCTGGAACTGAATCATTTCCTTGTATCTGAAAAGAACTT
CCAGATTAATAGGAAGGAAGCT--------------------ATGATCGATCGGTATAAACATCAACA
ACTCCGAATTGGATCAGTTTCTCCTCAACAAATAAGTACTTGGGCCAATAAAATTCTCC
CTAATGGAGAAATAGTCGGAGAGGTGACAAAACCCTATACTTTTCATTACAAAACCAAT
AAACCGGAAAGGGATGGATTATTTTGTGAAAGAATTTTTGGTCCTATAAAAAGTGGAAT
TTGTGCTTGTGGAACTTATCGAGTAATCGGAGATAAAAAAGAAGACCCGAACTTTTGT
GAACAATGCGGAGTCGAATTTGTTGATTCTCGGATACGAAGATATCAAATGGGATACAT
CAAACTGGCATGCCCAGCAACCCATGTGTGGTATTTGAAACGCCTTCCTAGTTATATCG
CGAATCTTTTAGATAAACCTCTTAAAGAATTAGAAGGCCTAGTATACTGCGAT------TTTT
CTTTTGCCAGGCCC---GTAGCTAAAAAACCAACTTTCTTACGATTACGAGGGTTATTCG
AATATGAAATCCAATCCTGGAAATACAGCATCCCGCTTTTTTTTACTACTCAAGGCTTCG
ATACATTTAGAAATCGAGAAATTTCTACCGGAGCGGGTGCTATCCGCGAACAATTAGCC
GGTCTCGATTTGCGAGTTATTATAGATTATTCGTTGGTAGAGTGGAAGGAATTAGGGGA
AGAA---AGGTCGACGGGTAATGAATGGGAAGATCGCAAAATTGGACGAAGAAAGCAG
TTTTTGGTTAGACGCGTGGAATTAGCTAAGCATTTTATTCGAACAAATATAGAACCAGA

ATGGATGGTTTATGTCTCCTACCAGTTCTTCCTCCCGAACTACGACCCATCATTCAGAT
GGATGGGGGTAAGCTAATGAGCTCGGATATTAATGAACTCTATCGAAGAGTTATCTATCG
GAACAATATCCTTGCCGATCTATTAACAACAAGTAGATCTACACCAGGGGACCTTGTAA
TGGGTCAAGAGAAATTGGTACAAGAAGCCGTAGATACACTTCTTGATAATGGAATCCGT
AGCCGCCCGGTGAGGGATGGTCAGAATAAGGTTTACAAGTCGTTTTCAGATGTAATTG
AAGGCAAAGAGGGAAGATTTCGTGAGACTCTGCTTGGCAAACGGGTCGATTATTCGGG
TCGTTCTGTCATTGTCGTAGGACCTACGCTTCCATTACATCGCTGTGGATTGCCTCGGGA
AATAGCAATAGAGCTTTTCCAGACATTTTTAATTCGTGGGCTCATTAGACAACATCTTGC
TTCGGACATAGTAGGTGCTAAGAGTCAAATTAGGGAAAAAGAGCCGATTGTCTGGGAA
ATACTGCAGCAAGTTATGCAGGGGCATCCCGTATTGCTGAATAGAGCGCCTACTCTGCA
TAGACTAGGCATACAGGCATTTCAACCCATTTTAGTGGAAGGGCGTGCTATTTGTTTAC
ATCCATTAGTTTGTAAGGGATTCAATGCAGACTTTGATGGGGATCAAATGGCTGTTCAT
GTACCTTTATCTTTGGAGGCTCAGACGGAGGCTCGTTTACTGATGTTTTCTCATATGAAT
CTCTTGTCTCCTGCGATGGGGGATCCCATTTCCGTGCCAACCCAAGATATGCTTATTGGT
CTATATATATTAACGAGTGGGAATCCCCGAGGCATTTGTACAAATAGGTATAATCCCTGG
AATCGCAGCAAC---------TACCAAAATGAAAGAATTTCTGATAATAAC------TGGAAAAAA
AAA---GAACCTTTTTTTTGTAATTCTTATGATGCAATTGGAGCTTATCGACAGAAAAGAA
TCCATTTAGATAGTCCTTTGTGGCTTCGGTGGCGACTAGATCAACGC---GTTATTGCTTC
A------AGAGAAGTTCCCATCGAAGTTCAGTATGAATCCTTGGGGACCTATCATGAGATTT
ATGGGCACTATATAATAGTAAGAAGTGTAAAAACAGAAATTCTTTGGATGTACATTCGA
ACAACTGTCGGTCATATTTCTCTTTTTCGAGAAATGGAAGAAGCTATACAAGGGTTTTG
TCGAGCTCGCTGGTATCTCAGT------------------------------------ATGGACGAAGGAGTCAAC
CTAGTCTTTCACAAGAAAGTAATAGATGGAACTGCCATTAAAAGACTTATTAGCCGATT
AATAGATCACTTCGGAATGGCACATACATCACACATTCTAGATCAAGTAAAGACTCTGG
GTTTCCAGCAAGCCACTGCTACATCCATTTCATTAGGGATTGATGATCTTTTAACGATAC
CTTCTAAGGGTTGGTTAGTACAAGATGCTGAGCAACAAAGTTTGAGTTTGGAAAAACA
CCACCATTACGGGAATGTACACGCAGTAGAAAAGTTACGCCAATCCATTGAAGTATGGT
ATGCTACAAGTGAATATTTGCGACAAGAAATGAATCCTAATTTTAGGATGACCGACCCC
TTTAATCCAGTCCACATAATGTCTTTTTCGGGAGCCAGAGGAAATGCATCTCAAGTACA
CCAATTAGTAGGTATGAGAGGATTAATGTCGGATCCTCAAGGACAAATGATTGATTTAC
CTATTCAAAGCAATTTACGCGAGGGACTGTCGTTAACAGAATATATCATTTCTTGCTACG
GAGCCCGCAAAGGAGTTGTGGATACTGCTGTACGAACATCCGATGCTGGATATCTTACG
CGCAGGCTTGTTGAAGTAGTTCAACACATTGTTGTACGTAGAACAGATTGTGGAACCC
TCCGCGGGATTTCTGTGAGTCCTCGGAGG------ATGCCG---GAAAGAATTTTTATTCAAA
CATTAATTGGTCGTGTATTAGCAGACGATATATATATAGGCTCACGGTGTATTGCCATTAG
AAATCAAGATATTGGAATTGGACTTGTCAATCGATTCATAACC------------------TTTCGAAT
CCAACCAATATCCATCCGAACCCCCTTTACTTGTAGAAGTACATCTTGGATCTGTCGATT
ATGTTATGGTAGGAGTCCTACCCATGGTGACCTGGTCGAATTGGGAGAAGCTGTAGGTA
TTATTGCGGGTCAATCTATTGGAGAACCGGGTACTCAACTAACATTAAGAACTTTTCAT
ACTGGCGGAGTATTCACAGGTGGTACTGCAGAACATGTACGGGCCCCCTCGAATGGAA
AAATAAAATTCAATGAGGCTTTGGTTCATCCCGCACGTACGCGTCATGGGCATCCTGCC
CTTCTCTGTTCTATGGACTTGGATGTAACTATTGAGAGTGAAGATATTCTACATAACCTG
ACTATTCCGCCAAAAAGTTTTCTTTTGGTTCAAAATAATCAATATGTAGAATCAGAACA
AGTCATTGCCGAGATTTGCGCGGGAACATCCACTTTC---CATTTTAAAGAAAGGGTTCG

AAAACATATTTATTCGGACTCTGAGGGAGAA---ATGCACTGGAGTACCGATGTGTACCA
TGCACCCGAATTTACATATAGCAATGTCCATCTTTTACCAAAAACAAGTCATTTATGGAT
ATTATCAGGAGGTTCGTGCAGA------TCCCGCGGAGCTCCGTTTTCACTCCACAAGGAT
CAAGATCAAATGAATCCTCGT------TCGACAGAAAGAGAACGA---AGATATCTTTCGAGT
CTC---TCAGCTAATAATGATCAA---ATCAGATACAAATTCTTTAGTTCTTCTTTTTCTGGTA
AAAAAGACGATAGGAGTCCTGGTTAT---TCAGAAATGAATCGAATCATATGTACTCTTCA
TTGTAATCTCATATATCCTTCG---ATTCTACGTGAGAATTCTGAT------TTATTGGCAAAAA
GGAGAAGAAATCGACTTGTCATTCCAGTCCAATCGAGTCAAGAACGAGAGAAAGAAC
TAATACCCCATTCAGGTATTTCGATTGAACTGCCCATAAAT------GGTATTTTCCGGAAAA
AGAGTATTCTTGCTTTTTTTGATGATCCTCGATACAGAACAAAGAGTTCGGGAATTACT
CAATATGAGACTATGGGGATGCACTCCATCGTTAAAAAAGAGGGT------TTGGTTGATTA
TCGA---GGAATCAACGAATTTAAGCCAAAATACATGACAATAGATCGATTTTTTTTCATT
CCCGAAGAAGTACATATTTTACCTGAGTCTTCTTCGATAATGGTACGGAATAATAGTCTG
ATTGGAGTAGATACACGAATCGCTTTAAATACAAGAAGCAGAGCGGGCGGATTAGTCC
GAGTGGAGAGAAAAAAAAAGGGGGATTGCACTTCAAATCTTTTCTGGAACTATCCATTT
TCCTGGAGAGACAGATAAGATATCCTGGGACAGTGGCATCTTGATACCACCAGGAACA
GGAAAAAGA------AATTCTAAGGAATCCAAAAAA------TGGAAAAATGGGATCTATGTCC
AAAGG------ATCACACCTACTAAGAAAAAG------CATTTTGTTTTATTTCGGCCTGTAGTG
ACATATGAAATAGCGGACGGTCTCAATTTAGCAAGACTCTTCCCCCCGGATCTGTGCCA
AGAAAAGGATAATATGCAACTTCAAATTGTCAATTATATTGTTTACGGAAATGGCAAAC
CAATTCGGGAAATTTCTGACACAAGTATTCAATTAGTTCGGACTTGGTTCATTTTGAATT
GGGACCAAGACAAA---------AAAAGTGCTTCTGCCGAGGCGGCCCACGCTTCCTTTGTC
GAAGTAAGGGCAAAAGGTCTGATTCGAGATTTCTTAAGAATCGACTTAGTGAAATCCC
CTATTTTGGATCCGAGAAAAAGGAATGATCCGTCAGGCTCAGGATTGATCTCT---GATAA
TGTCTCAGATCACACTAATATCAATCCCTTTTAT---------TCCAAGCCAAAG---------------AT
GAAACAATCGCCTAGGCAAAATCACGGAACTATTCGGACCTTGTTAAATCAA---------AA
TAAGGAATGCCCGTCTTTGATGATTTTGTCCGCATCTAATTGTTTTCGAATGGGTCCA----
--TTCAATGATGTAAAATCCCAGAAT---------GTGATAAAAGAATCA---GCTATAATTCAAAT
TAGGAATTCAATTGGCCCTTTAGGAACA------GCCCTTCAAGTTGTGAACTTT------GATT
CATTTTACTATTTTATAACT------CATAATCAGGTCTTG---CTAACTAAATATTTGCAAGTTG
AAAAT---------TTAAAACAGACTTTTCAAGTACTT------CAA---TATTATTTAATGGATGAAA
GCGGGAGG------ATTTATAATCCGGATCCCCGCAGTAACATCGTTTTGAATTCATTCAATT
TGAGTTGGTATTTTCTGCCTCACAATTCTTGTGAAGAAATATCTACAATAGTTAGT---CTT
GGACAGTTTATTTGTGAAAATGGATGTATAGCCAAAAATGGACCA---------TACCTAAGA
TCGGGTCAAGTTTTGATTGTTCAA---CTTGACTCTGTAGTAATAAGATCTGCTAAGCCTT
ATTTGGCCACTCCAGGAGCAACTGTTCATGGACATTATGGAGAAATCCTTTATGACGGA
GATACAGTAGTGACATTTCTATATGAAAAATCGAGATCCGGTGATATAACGCAGGGTCT
TCCAAAAGTGGAACAGGTGTTAGAAGTGCGTTCAGTTGATTCAATATCGGTGAACCTA
GAAAAAAGAGTTGAGAATTGGAACGAGCATATAACAAGAATTCTTGGATTTCCTTGGG
GATTCTTGATTGGGGCTGAGCTAACTATAGTGCAAAGTCGGATCTCTTTGGTTAATAAG
ATCCAAAAGGTTTATCGATCCCAGGGGGTCCAAATCCATAATAGGCACATCGAAATTAT
TGTACGCCAAATAACATCCAAAGTGTTGGTTTCAGAG---------GATGGAATGTCTAATGT
TTTTTTACCTAGAGAACTAATTGGATTGTTGCGAGCGGAACGGACGGGGCGCGCTTTG
GAAGAATCGATCTGTTACAAGGCCTTCCTATTGGGAATAACAAGAACATCTTTGAATAC

TCAAAGTTTCATATCCGAAGCGAGTTTTCAAGAAACTGCTCGAGTTTTAGCTAAAGCG
GCTCTCCGGGGTCGTATTGATTGGTTGAAAGGCCTAAAAGAGAACGTTGTTATAGGCG
GGATGATACCCGTTGGGACCGGATTCAAGGGCTTAGTACACTGTTCC------AAGCAACAT
AAAAGCATTCCC---------AAGAATAAGCAC------TTTTTCGAGGGGGAGATCAGA------GAT
ATTTTGTTCCACCACAGAGAATTATTTGATTCTTGCATTTCAAAGAATTTCCACGATACA
CCAGAACAATCATTTAGAGTATTTAATGATTCC------------------ATGACAAGAAGATATTG
GAACATCAATTTAGAAGAGATGATGGAAGCAGGAGTTCATTTTGGTCATGGTATTAAAA
AATGGAATCCTAGGATGGCGCCTTATATTTATGCCAATCGTAAAGGTATTCATATTACAAA
TCTTACTAAAACCGCGCGTTTTTTAGCAGAAGCTTGTGATTTAGTTTTTGATGCAGCAA
GCCGGGGGGGGCAGTTTTTAATTGTTGGTACCAAAAAGCAAGCAGCTGCTTTAGTAGC
ACGGGCTGCAATAAAGGCTCGGTGTCATTATGTTAATAAAAAGTGGCTTGGTGGTATGT
TAACGAATTGGTCCACTACAGAAACGAGACTTCATCAGTTCAGGGACTTGAGAACGGA
ACAAAAGACAGGGAGACTTAACCGTCTTCCAAAAAGAGACGCGGCTATATTGAAGAG
ACAATTATCTCACTTGCAAACATATCTGGGTGGGATTAAATATATGACCGGTTTGCCCGA
TATTCTAATTATCCTTGATCAGCAAGAAGAATATACGGCTCTTCGAGAATGTATTACTTT
GGGAATTCCAACAATTTGTTTAATCGACACCGATTGTGACCCCGATCTTGCAGATCTTC
CGATTCCAGCAAATGATGACGCTATGGCTTCAATCCGATTAATTCTTAACAAATTAGTAT
TCGCGATTTGTGAGGGTCGTTCT---AGCTCTATACGAAATCCTATGGCCCAAAAAATAAA
TCCACTCGGTTTCAGACTTGGTACAACTCAAAGTCATCATTCGATTTGGTTTGCACAAC
CAAAAGATTATTCTGACGGTCTACAAGAAGATCAAAAAATACGAGATTGTATCCAAAAT
TATATACAAAAAAATATGCAAATATCCTCGGATGGTGTCGAGGGGATTGCCCGAATAGA
GATTCGAAAAAGAATTGATCTGATTCAAGTCAAAATCTATATGGGATTCCCAAAGTTATT
AATAGAAGATGGGACACGA------------AGAATCGAAGAATTACAGAGGATTGTACAAAA
AGAAATCAATTCTGTGAACCGAAAAATCAACATTACTATTACAAAAATTACAAAACCCT
ACGGAGACCCCAATATTCTGGCAGAATTTATAGCCGGACAATTAAATAATAGAGTTTCC
TTTCGCAAAGCAATCCAAAAGGCTATTGAATTAGCCGAACAGGCGGATACAAAAGGAA
TTCGAGTACAAATTGGAGGGCGCATCAACGGAGGAGAGATAGCACGTGTCCTATGGAT
GAAAGAAGGTCGGGTTCCTCTACAAACCATTCGAGCTAAAATTGATTATTGTGTCTATA
CAGTCCGAACTATCCATGGGCTATTAGGCATCAAAATTTGGATATTTAGAGAGGAT---AT
GTCGCGTTACCGAGGGCCTCGTTTCAAAAAAATACGCCGTCTGGGGGCTTTACCCGGA
CTAACCAGTAAAAGGCCTAGAGCCGGAAGTGATCCGAGAAACCAAGAACTTTCTGGG
AAC---AAATCTCAATATCGTATTCGTTTAGAAGAAAAACAAAAGTTGCGTTTTCATTATG
GTCTTACAGAACGACAATTGCTTAAATACGTTCGTATCGCCGGAAAAGCCAAAGGGCC
AACAGGTCAGGTTTTACTGCAATTACTTGAAATGCGTTTGGATAACATCCTTTTTCGATT
GGGTATGGCTTCGACCATTCCTCAAGCCCGCCAATTAGTTAACCATAGACATATTTTAGT
TAATGGTCGTATAGTGGATATACCAAGTTATCGTTGCAAACCGCGAGATATTATTACGGC
GAAGGATGATCAAAAATCGAAAGCTATGATTCAAAATTCTCTTGAATCACCCCCCCAT---
GAGGAAGTGCCAAAACATTTGACTTTTCACCCATTCCAA------TATAAAGGATTAGTCAA
TCAAATAATCGATAGTGAATGGGTCGGTTTGAAAATAAATGAATTGCTAGTCGTAGAAT
ATTATTCTCGTCAGACTATGTCACGTCGAGGTACTGCAGAAGAAAAAACTGCAAAATC
CGATCCAATTTATCGTAATCGATTAGTTAACATGTTGATTAACCGTATTCTGAAACACGG
AAAAAAATCATTGGCTTATCAAATTCTCTATCGAGCCATGAAAAAGATTCAACAAAAG
ACAGAAACAAATCCACTATCCGTTTTACGTCAAGCAATACGTAGAGTAACTCCCGATAT
AGCAGTAAAAGCAAGACGTGCAAGCGGATCGACTCATCCAGTTCCCATTGAAATAGGA

TCCACACAAGGAAGAGCACTTGCCATTCGTTGGTTATTAGGGGCATCCCGAAAACGTC
CGGGTCGAAATATGGCTTTCAAATTAAGTTCCGAATTGGTGGATGCTACCAAAGGGAG
GGGCGGTGCCATACGCAAAAGGGAAGAGACTCATAGAATGGCAGAGGCAAATAGAGC
TTTTGCGCATTTTCGTATGGGTAGGGACACTATTGCTGACATAATAACCTCTATACGAAA
TGTTGACATGAATAGAAAAGGGACGGTTCGAATAGAATCTACTAACATGGCCGAAAAG
ATTGTTAAAATACTTTTACGGGAGGGTTTTATCGAAAACGTGCGAAAACACCAGGAAA
ACAAGAAATCTTTTTTGGTTTTAACCCTACGACATAGAAGG------AATAGGAAAGGACC
CTCTAGAACT---TTTTTAAATTTAAATTTAAAACGGATTAGTCGACCTGGCCTCCGAGTC
TATTCTAACTATCAAAAAATTCCTAGAATTTTAGGCGGGATGGGGATTGTAATTCTTTCT
ACTTCTCGAGGGATAATGACAGACCGAGAGGCTAGACTAGAAGGAATCGGCGGAGAA
ATTTTGTGTTATATATGGATGGCAAAATCTATACCAAGCGCTGGTTTACGT------TTACGTT
TACGTTTACGTAAGAGTACGCGTAAAATCCCAAAGGGGGTTATTCATGTTCAAGCGAGT
TTCCACAATACTATTGTGACTGTTACAGATGTGCGGGGGCGGGTAATTTCTTGGTCCTC
CGCCGGTACTTGTGGATTCAAGAGTACAAGAAAAGGGACGCCATTTGCTGCTCAAACC
GCAGCAGGAGATGCTATTCGGCCAGTAGTGGATCAAGGTATGCAACGAGCAGAAGTCA
GGATAAAAGGTCCCGGTCTCGGAAGAGATGCGGCATTACGAGCGATTCGTAGAAGTGG
TATACGATTAAGTTGTATACGTGATGTAACCCCTCTGCCACATAATGGCTGTAGGCCCCC
GAAAAAAAGGCGTGTGATGCCAACTATTAAACAACTTATTAGAAACACAAGACAGCCA
ATCAGAAATGTCACGAAATCGCCCGCCCTGCGGGGATGTCCTCAGCGCCGAGGAACAT
GTACTAGGGTGTATACTATCAACCCCAAAAAACCAAACTCTGCCTTACGTAAAGTTGCC
AGAGTACGATTAACCTCTGGATTTGAAATCACTGCTTATATACCTGGTATTGGCCATAAT
TCACAAGAACATTCTGTAGTCTTAGTAAGAGGGGGAAGGGTTAAGGATTTACCCGGTG
TGAGATATCACATTGTTCGAGGAACCCTAGATGCTGTCGGAGTAAAGGATCGTCAACA
AGGGCGTTCTAAATATGGGGTCAAAAGGCCAAAAATGGCAAGAAAAGGTTTGATTCA
GAGGGAGAAGAAGAGGGAAAAATTGGAACAAAAATATCGTTTGATTCGTCGATCCTCA
AAAAAAGAAATAAGC---------ACAGCTCCGTCATTAAGTGAAAAATGGAAAATTCATGG
AAAGTTACAATCCTCACCACGTAATAGTGCACCTACACGTCTTCATCGACGTTGTTTTT
CGACCGGAAGACCGAGAGCTAACTATCGAGACTTTAGGCTTTCCGGACACATACTTCG
TGAAATGGTTCATGCATGTTTGTTGCCAGGGGCAACAAGATCAAGTTGGATGGTAAAA
AAGGCATTCATTTCCGTTATTTCACAA--------------GAAGAAAACAGGGGGTCTGTTGAA
TTTCAAGTAGTTAGCTTCACCAATAAGATACGAAGACTTACTTCCCATTTGGAATTCCA
CAGAAAAGACTTTTTATCCCAGAGGGGTCTACGTAAAATCCTGGGAAAACGACAACGC
CTGCTGTCTTATTTGTCAAAGAAAGACAAAGTCCGTTATACAGAATTAATTAGTCAGCT
AGATATCCGAGAATTAACAACTCGT------ATGGTAAAACTTCGTTTGAAACGATGTGGTA
AAAAGCAACGAGCCGTTTATCGAATCGTTGCAATTGATGTGCGATCCCGCAGAGAAGG
AAGAGATCTTTGGAAAGTGGGTTTTTATGATCCGATAAATAATAAAACCTATTTAGATAT
TCCAGGTATTCTATATTTTCTTGAAAAAGGCGCTCAACCCACAGGAACTGTTTATGATAT
TTTAAAGAAAGCGGGGGTTTTTACAGAATTTAGTCTTAATCAAATGGAAGTCAAT---ATG
GATAAATCCAAGCGACTCTTTCTTAAATCTAAGCGATCTTTTCGTAGGCGTTTGCCCCC
GATCCAATCGGGGGATCGAATTGATTATAGAAACATCAGTTTAATTAGTCGATTTATTAG
TCAACAAGGAAAAATTTTATCGAGACGGGTAAATAGATTGACTTTAAAACAACAAAGA
TTAATTACTATTGCTATAAAACAAGCTCGTATTTTATCTTTATTACCTTTTCGTCCGAAGG
CGCAA------CGGTTTAAAAGAAGCCAGTCGACCGCCCGAACTGTTGGTCTTAGAACCA
GAAATAAAATGAAACGTTCACTCAAAAACAACCCTTTTGTAGCGAATCCTTTATTAAGA

AAAATGGAGAAGCTAAACAGAAGGGAGGACAAAATACTTATAAGAACTTGGTCTAGG
GCGTCTACGATTATACTTACAATGATCGGCCATACTATTGCTATTCATAATGGAAAAGAG
CATTTGCCTATTTATATAACAGATTATATGGTAGGCCATAAATTGGGAGAATTTGCACCTA
CTATCAATTTTCACGAACATGCGAAAAATGATAATAAATCTCGTCGT--------------------AT
GGTTAATCTCGTATACGTATGCATGAAGATAAACAATTCCGTGGTTATGGTCGGACTCTA
TTATGGATTTATTAGCGCATTTTCCATCGGGTCCTCTTACCTGTTCCTTCTCCGACCACG
GTTTTTGAACGACGATCCAGACGCAATCGAAAAGAAGGCATCAGAAACTGCAGGTTTT
TTTACAGGACAACTTTTGATATTCATATCAATCCTTTATGGGCCTCTGCATCTAGCGTTAG
GTAGACCTCATACAATCCTTTTACTACTTGCACCGTATTTTTTCTTTCATTACTTATTCAG
CAATAGCGGTCAATGGCCGCGTTTTGCT---TTGCTCACTAAGTCAATGCGGAATCGTAG
GTTTCAATTGGTATTCCTGAATAATTTGCTTTTTCAATTATTCAGCCTTAGCTTGTTGGGA
AGGCCAATGTTAACCCGATTAAGCTACATTTATATCTTTCGATGCAACAATAAGATGTTA
TTTGTCCTAAGTAGCTTTGTTGGTTGGTTAATTGGTCATATTTTAGTCCTGAAATGGGCT
GGATTGGTATTTGTTTGGCTACTTCAGGTTATTAGATCGAAGACAAAACAGGTTGAATC
GTCTTCGCTAATAAGCGGCCTAAAAATTAACTTTTATCACCTCTTTAGTATTATCTTATTT
GCTATCTTTCTTTTATATTTAGATCAAAGAAAACTATCGAACGAAACCAAGAAACTTGA
AGAAGCTCAGAAAGACATCCCTATTATCACTTCTCTTTTTGACCCGCAAAAACCACTCC
GTCCATTACGATATATAAAAACGGCTGGCGTTGAAAAGGCTGTCAAAAATGAGATGTC
ACAATATTTTTTTATGCATGCCGAAGCGATGGAAAACAAAGAATATGTTTTACATATCC
ACCCAGTTTGGCAACTTTCTGGGAAATGATACAAAGAAAAATGGCTTCGAGGTTCCCA
TATGCTAAAGCTGCTCCTGGGAGTTATAGACAGTGGATTTCTCGCAATAAAAAAAAAA
AGAACAGCCTGAGTACAGAATTTCAAAATCGAATTAAAACTTTAGATAAAAAAAAAAG
CCTGCAAAATGTCCTCGAAACAAGGAAAAGGTTGTGTAATAATAAAACTAAAAAAGAA
TACTTGCCTGAAATAGCCGATCCTTTTTTGACCGGAGCGCTTCGTGGAAAGTTAAAGAA
TAATATTACAATGAGGATGAAAAACCCGGTAAATAAGCTTCACCTACTTTTTGTGAAAA
CGTTAGTAAATAGAGAAAAGTATTCATTCATTGAACTAGAAGAAGAAATCAATAAAAA
AGTTCCTCGATGGTCATACCAATTAATCGAGTTGGAACAACTGGAGGGAGCCGAGATT
CGAATACTCCCATTCAAACGTGTAGCGGTGTTTACTGAGGCGATACGTTTTTTAGGCCA
TATGTCCGATTTTCGTCGAGGCCTAATCAAGGGATCCGCGCGTCAGGACAGACGTAAA
GCATACGTTTGTCGCACAACTCAAGTAAATGCACGCTCCCCGCTTTTCGCTTTGGGCTT
GGTTAACTTAGCCGTGCAGGTGAAATTCTTTTATGAAACCCGGATAAAAGGGGAAATA
GTGGATGACGTCGAGGATGATATACGAGAGAATGTAACGGAAATGTGGGAGAACATTG
ACTATGGTCAAGTCATAAGAACTTTCATATTATTACTGCATATTTTTCTTAGAAAAAAGG
TTGTATTCCCTGCATTCATAATAGGGAAAAATATAGCCCGTATGTTATTATTGCAAGCTAC
CGAGTGGAAAATAGACTTCGCCCGTTTGAAGAGAGAAAGGTATGCTATATGCACCTATA
ATGGTATGAAAGTCTCAGAAAAAATCTTCCCACCAGACTGGGCCGATGACGGTATTCA
GATACTGGTCACCAACCCTTTTTACCTTAAACCTTGGTACAGATCTAAGCCGAAGAAAG
AACCTTGGTACAGATTTAAGACCCGATTCATTAAAGATCCGAAGAAAGAAAAAGGTCC
GAAGAAAGAAAAACCTTGGTACAGACGATTCTTTTTTCAAAAAGATCCGAAGAAAGA
CCGAGGGGTTCGTTTTTTTGGGATCTTAACCGGTGATCTAATAACCCCGGATTGGGGAG
TCAATCCCATTAGAAACGAACTCAAAAAGAAAATTCGCCAATTTGAAAAGAAACATTC
TATAATTCTAAGCAAACGTTTTCGAAACGTCTTAAAAAAAATGGATAGATAATCTTAAAA
ACGAATTTAGAATGAGTAGGAATTTGCAAGGTCCAGTTAGAGCTTTGAAAGAT---TCAT
TACCAGAAGAAAAAGTGGCGGATCCATCGGATCTGGATAATGAACTAAGAGCAGAGAT

AGATAAAGTTACAAAAGAAAGAAAGAAA------------------TCTCCTGATAAACTCGTACAA
GCAAAAAAAAAATATTTTAAAGAAATTAGAAAGAATACACAAATTTTATTTTCTAAGAAT
CCGATATGTTCTACTATTTTTCATTAAAAGGATATCCAAAAGGATATACCTCAATGCCATT
TCTATTCGCAAGATCCATCCACAACGTTTTATTTTTAAAGAGTCGTCGGACTTAGCCTCT
ATGTCCCAAGCATATGTATTTTACAAATTATACCAAACCCAAACCCAACTTTTAGATAAG
TTAAGATATGTTCTTCAATATACATCCCGTTTTCTTAAGAAAGAACTAAAGGATTATTTT
GCACAAGAACTCTTTCATTCGAAATTAAAACATAAAAATAGTTTG---TCCGGCAAAAAT
CAATGGAAAAACTGGTTAAAGGCTCAGCATCAATATAGCGTATCTCCCATTATATGGAAT
AGCTTAAGCCCCCAAAAATGGCGAACTAAAGTCAATCAAGAACGTATGGACGAAAATA
CAAATAAAAGGTATTCTAATGAAAAACGAAAACAATTCTTTGAAGCAAATTCATTAGAC
---GATATTAAGAACTCTATAAAATATGACCTTTTTTCCTATCAATCTATTAATACCGCGAC
AAATAGTACCGCGGCAACCAGTATTAAGCCTGGGGTTTTTTGGCAAAATTCTCAAGAA
AATGATCCTATTGAAGATGGAAGGCAAAATGTTATTCAACAAAATCTTATCGATTTTTTT
CTTGAAAAAAAAAAGGATACAAACCAAGAATTGCACGCGCAAGCGAAAGCGAGAATT
TGGGATCAAGTACAAGAAAACAAAATTCCTTACACAGCCTTTGGATCAGACATCCGTA
ATTTTCAAGTGATATTGCCGGAGGATGATGAGGAGGACCGATTGGACGAACTCAAATT
GAATGCCTACGAATTGAGTAGACAGAAAATTACAGATAATTTGCTAAGCTCTATCAAAA
GGGAAAGACTGAAAATGGAGTTTTCGACAAGAAACAATAGTCTTGCGACCATTATGCT
TACCCACGGA---ATATTCAGTATCGAACCACTTCGTATATCTAGACAAAATCAGGACGCA
TCATTGATCTATCAACTGATAAAGATTTCCTTGGTTGAGCAGCTTGATCCCAGTGATTCT
GATTTGTTTGTTCCCGAAACTATTTTATCCACCAAACGGCGTAGAGAATTGCGAATTCT
AATTTCTTTCCGTTTTCTTTGGCCTAATTATCGATTAGAAGATTTAGCTTGTATGAATCGA
TATTGGTTTAATGCGCAGAATGGGAGCCGTTTCAGTATGCTAAGGATACGTATGTATCCG
GTGAATCTGAATCCATTCAGATTCTGGGTTTTTGAATTGAGAGAGATATTGAGAGAGAT
CAAGAATTCT------CCATTCAAT------------------TCAGTGGGATCTTTCATTCACATTTTTGT
CCACCAAGAACGTTTTCTAAAACTCTTGGACCCCCGAATTTGGAGTGTCCTGCGTTCA
CAGGGTTCA--ACAGGTGTAGTACTATTTTTGGTAG
CG---GTCCTTATATATCGT------------ATTAACAATCGAAATATGATCGAAAGAAAAAATATC
TATTTGACAGGGCTTCTTCCT--------------ATACCTACGAATTTCGCTGGGCCCAGAAATG
AGACATTGGAAGAATCTTTT---CTGTCTTCCAATATCAATAGGTTGATTGTTTCG---CTCC
TGCATCTTCCAAAAGGAAAAAGGCTCTCTGAGAGTTGTTTCCTGGATCCGAAAGAGAG
TACTCGGGTTCTCCCAATAACTAAG--TGGAGGAA
CTGGATCGGAAAAAGGAGG---

---------------GATTCTAGCCAACTGAAAGGATCTTCT---GATCAATCCAGAGATCATTTCGA
TTCCATTGGC------------ACGGAGGATTCGGAA--------------------------------------TATCACAC
ATTGATCAATCAAAGAGAGATTCAACAACGAAAAGAAAGATCGAGTCTTTTGGAT-------
--CCTTCCTTTCTTCAAACGGAACGAACAGAGATAGAATCAGAC------CGATTCTCTAAA-
-----GGCCTTTCTGGATCTTCC------TCAAAGTCCCGGCTATTCACG---GAAGGTGAGAAG
GAGATGAATAATCATCTCCCTCCGGAAGAAATCGAAGAATTTCTT------GGGAATCCTAC

AAGATCCATTCTTTCTTTTTTCTCTGACGAATGG------TCAGAACTTCAT---CTGGGCTCG
AATCCTACTGAGAGGTCCACTGTGGATCAG------------AAATTGTTGAAGAAAGAACAAG
AGGTTTCT---TTTGCCCCTTTCCGGCGATCGGAA------------ACTAAAGAAATAGTGAATCT
ATTCAAGACAATGGCGTATTTACAAAAG------ACCGTCTCAATTCATCCTATTTCATCAGA
TCCGGGATGTGATATGGTTCCG---AAGGATGAACTGGAT---TCA-------------------------------
---GAAGAGAGAT
TTCAAGAAATGGCAGATCTATTCACTCTATCAATAACCGAGCCG---GATCTGGTGTATCA
TAAGGGATTTGCCTTTTCTATTGATTCTTCCGTATTGGATCAAAAACAA------TTCTTGGC
TGAGGCC---------AGGGATGAATCGAAAAAGAAATCTTTA---------TTGGTTCTACCTCCT
GTTTTTTAC------CAAGAGAATGAATCTTTTTATCGAAGGATCAGAAAAAGGGGG---GTC
CAGATCTCCTGCGGGAATGATTTGGAAGATCCAAAACCAAAAATAGTGGTATTTGCTAG
CAACAACATCGTGGAGGCAGTCAATCAATATAGA---ATCCGAAATCTGATTCAAATCCA
ATATAGCACCCATGGG------TACATAAGAAATGTATTGAATCGATTCTTTTTAATGAAT------
---CGATCCGATCGCAACTTCGAATATGGAATTCAAAGG---------GATCAAATAGGAAATGA
TACTCTGAATCATAGAACTTTTATGAAATATACGATCAACCAACATTTATCGAATTTGAA
AAAG---AGTCAAAAGAAAGGGTCCGATCCT------CTTATTTTGATTTCT------GAGAGATC
CGTGAATCGGGATCCTAATGCATATAGATACAAATGGTCCAAGGGGAGCAAGAATTTCC
AGGAACATTTGGAACATTTCGTTTCTGAGCAGAAGAGCCGT---------TTTCAAGTGGTCT
TCGATCGATATCGAATTAACCAATATTCGAGTGATCGGTCTGAGGTTAGCGACAAAAAA
GATTTGGCTAAGTTCCGTTCTTTCGTTTTCTCCAAG--
----------------------TTACTTCTTTTTTTGTCTAAC---TCACTTCCTTTTTTCTTTGTGAGTTTC
GGGAATCCCCCCATTCAGAGGTCCGAG---ATCCGCGTCTCT---GAATTGAAAGGTCCGA
ATGATCGACTCTGCAATCAGTTCTTAGAATCAATAGGTCTTCAACTCGTTTATTTGAAAA
AATTGAAACCATTC------TTATTGGAT---------GATCATGAGACTTCC---CAAAAATCGAAA
TTATTGTTCAAT------------------------------AAGAAACCA---GAGGGG---ATGATTGACTCATT
CCATACTAGA------AATAATCGCGGGAAATCTTTGGAT---------TCCTATTTCTCAATGATAT
CCCACGATCAAGACAATTGGTTGAATCCCGTGAAACCATTTCATAGAAGTTCATTGATC
TCTTCTTTTTATAAAGCAAATCGACTTCGATTCTTGAATAATCCACATGACTTCGGCTTC
TTT------TGTAACAAAAGATTCCCCTTTTATGTGGAT---------------------ATCAAGAATTTGG
AT------TTTACGTATGGA---CAATTCCTCAATATCTTGTTCATTCGCAACACA---AAATTTT
CTTTGTGCGGCGACAAA------AAAAAACATGCTTTTTTGGAG---AGAGATACTATTTCAT
CAATCGAGTCACAGGTATCTAACCTATTCAAG------GATTTTCCACAAAGTGGTGACGAA
AGG---TATAACTTTTACAAATAT---------TTCCACCTTGCAATGCGATCTGATCCATTAGTT
CGT------AGAGCTATTTACTCGATCGCAGACATTTCTGGAACACCTCTAACAGAGGGAC
AGAGAGTCAATTTTGAAAGAACTTATTGTCAACCTCTTTCAGATATGAATCTATCTGATT
CAGAAGGGAAGAACTTGTATCAGTATCTCAAT------TTCAATTCAAACATGGGTTTGATT
TAT---------TCTGAGAAATGTTTCTCA------TCCGAAAAGAGGAAAAAGAAAAAA-----------
----CCCGAAAAGAGGAAAGAGAAA------AAACCCGAAAAGAGGAAAGAGAAAAAACC
CGAA---------AAGAGGAAAGAG------------------AAAAAACCCGAAAAGAGGAAAGAGAA
AAAACCCGAAAAGAGGAAAGAGAAAAAACCCGAA---AAGAGGAAAGAGAAAAAAC
CCGAAAAGAGGAAAGAGAGCCTTACCTCGACAGGGAGTCCAAAATTTGTATCCATTTG
TTATGCTATATCTGATCCATGCGGAATATCATGGCGAATTCTTCAGAAAAAATTGTGTCT
TTTACAA---------------------------TGGATAAGTGCGATTTCGAATAAGTGTTTCCATAAG------
CTTCTTCTGTCTGAAGAAAGTATTCATCGAAATAATGAGTCACCATCGATGACAGATCT

GAGATGGCCA---------AATCTTGGGGCGTTC---CTCTATTCAATCCTTTTCCTTCTTTTTGT
TGCTGGACAT---CTCGTTTTTTCACACCTTCTCTTTTTATCCCAA------GACTTTAGTGAGT
TACAGAGAGATTTCGCAAGGGCCCAATCTTTGATGATTCCATCATACATCGTGGAGTTG
CGAGAACTTCTGGATATGTACCCCGCACCTCGTTCT------------TTCAAGAAGCTCTTTCT
AGCTGCTCGGGAAAAATTAGTAAAT---------------------TATCTACGATGGGGTGGTGGACG
CAAA---TCTTTTCTTATCCAT---------CTC
TTCGAGCTCCTCAATATCACACCAAATCCCATCGATCGA---ATCGCTTTTTTGAAAAATA
CGAGACATCTAAGTCATACAAGTAAAGAGCTCTATTCATTGATAACAGAGCTAGGGAAC
GGTCCTTGTTGTTTGATTGACGATAAAATAGAATCCTGGATCTCGAACTGTGATGCGAT
TGAGGATAAAGAAAGAGAATTCTTGGTTCCGTTTTGCAAC------TTCACGAGA------GAA
ACAAGGATTGATCAAATTCTATTGAGTTTGACT---CATAGTGATCATTTATCA---------------
AACAATGACTCTGCCTCTCAAATGAGTGAAGAACCGGGAGCATTTTACTTACGACACT
TAGTTGACATTCAT------AAAAAGGGTCTAATGAATTATGAGTGCAATACATCCTGTTTAG
CAGAAAGACGGATATTCCTTGCTCATTATCAGACAATCACT------------TATTCACCGTGC
GGGGATAATCGTTCTCATTTCCCATCTCATGGA---------AAAACCTTTTCGCTCCGCTTAC
CCCTACACCCCTCT---AGGGCTACTTTAGTGATAGGTTCTATAGGAAGTGGACGATCCTA
TTTGGTCAAATCCCTAGCGACAAACTCCTATGTTCCTCTCATTACAGTAGTTCTGAACA
AGTTCCTGAAGAACTGGACGCCTCAAGGTTTTGATATTCAC---GAGAGTGGCGTTTATG
AT---TATGAGGATCGTGACAGTGACGATTATGATGAGCCTGGT-----------------------------------
------------------------------GCCAGTGACGATTATGAGGAT---CGTGATATGGAAGATTTTGTTG
ATAGCGAGATGACGGAGTGGCTAACTAAGACGAATGTGCCACTTGTGTATCAGTTGTTT
TATATCACCCTTCAATTCGAATTAGCAAAAGCAATGTCTCCTTGCATACTATGGATTCCA
AACATTCATGATCTGGATGCGAAAGAGTCGGATTACTTATCCCTCGGTCTATTAGTGAA
CCATCTCTCCAGG---------GATTGTGGAAGACGTTCCACTAAAAATGAGATTCTTGTTATT
GCTTCGACTCATATTCCCCAAAAAGTGGATCCCTCTCTAATAGGTCCGGATGGATTAAGT
ACATGCATTAAGACACGAAGGCTTCTTGTTCCACAACAACAACAGTGCCTTTTCACCC
TTTCATATACTAGGGGATTTCACTTGGAAAATAAAATGTTCCATACTCATACTAATGAATT
CGAGTCCACAATCTTGGGTCCCAGTGTACCAGATCTTGTAGCACTTACCAACGAGGCC
CTATCGATTAGTATTACACAGAAGAAATCAATTATAGACACTACTACAATTAGATATGCT
CTTCATAGAAAAACTTGGGATTTGGAAGCCGAC---AGAAACCTAAGCCCGGCTAAGGA
GCATGGGACCCTTTTCTATCAGGTAGGAAGGGCTTTTGCACACACTGTACTTCTAAGGA
ATTGCCCCATAGATCCTATATCTATCTATATCAAGAAGAAC------TTATGTGAAGCAGGGG
ATTCTTCTTTGTACAAATGGTACTTCGAACTTGGAACGAGCATGAAGAAATTAACGATA
CTTCTTTATCTTTTGACTTGTTCTGCCGGATCGATCGCTCAAGACCTTTTGTCTCCCCCC
GGA---------CCCGATGAACAAAATTTGATCACTTCTTATGGACTCGTTGAGAACGATTCT--
-GATCTAGTTCATGGCCTATTAGAGTTAGAAGGCGCTCTGGTGGGATCCTCGGGAGAAG
GGACAGAAAAAGATTCCAGTCAGTTTGATAATGAT------CGAGTGACATTGCTTCTTCGG
CCCAAACCAAGGAAT---CCCTTAGATATTCAGAGACTTATCTATCAACATCAAAAATAC--
-GAATCGGAGTTGGAAGAA---------GACGACGACGACGACGAG------GACGTCTTCGCCC
CGCAAAAGATGTTG---------------------GAGGAT------------TTATTCAGTGAA------CTAGTTT
GGTCTCCT------AGAATATGGCACCCT---------TGGGACTTTATATTGGATTGTGACGAAGA
GGATGAGCGGCTTGAAGCTCAA---CAAGAGGATGAGCTGCTTGAAGAGGAACACGAG
GAAGAGGAAGAGGAAGAGGAAGAGGATGAGCTTCAAGAGAATGATTCGGAGTTCTTC
CGGAGTGAAACCCAGCAGCCCCAGGCACGAGATGGATTTTCCGAAGAAGAAGGATGT

TTTCGAATAAGCCAATTCATGTGGGTCCCTGGAGATCCACTCTCTTTCCTATACAAA---G
ATACGCCCTTTGTCGAAGTGTTGTCATATCCAGAGGAAGCTACA------GAGATTTCAAAG
GAGCTTCTTCGT---CTTCTTAATGCCCCTAAACGCGCACGTCAACGCTGGTGGACC------
AAGAAGAAGCAAGACAAGCACTACGAACTCGTGCTTGATCGCCAGAGATGGCTTATAA
CC---------AAGAGTTCATTATCTAAATCTAATGGATTTTTCCGTTCTAAT------ACTCCATCT
GAGAGTTATCAGTATTTATCAAATCTGTTCCTATCTAACAGA---AGGCTATTGGATCAAAT
CACAAAGACATTCTTTAGAAAAAAATGGCTTTTCCCGGATGAAATGAAAATCGGATTC-
-----ATGGAACAG---------------------ATGCCTAGATCAAGG---ATAAATGGAAATTTTATTGAT
AAGACCTTTTCAATTGTAGCCAATATCTTATTACGAATAATTCCGACAACTTCAGGGGA
AAAAGAGGCATTTACCTATTACAGAGATGGG------ATGTCGGCTCAGTCAGAAGGAAAT
TATGCGGAAGCTTTACAGAATTATTATGAAGCTATGCGACTAGAAATTGATCCTTATGAT
CGAAGCTATATACTCTATAACATAGGCCTTATCCACACAAGTAATGGAGAACACACGAA
AGCTTTGGAATATTATTTTCGGGCACTAGAACGAAACCCGTTCTTACCCCAAGCTTTTA
ATAATATGGCTGTGATCTGTCATTAC---CGGGGAGAACAGGCCATTCGACAGGGAGATT
CTGAAATTGCGGAGGCTTGGTTCGATCAAGCCGCTGAGTATTGGAAACAAGCTATAGC
GCTTACGCCCGGGAATTATATTGAAGCGCAAAATTGGTTGAAGATCACGAGACGTTTCG
AA------ATGATTTGGCGATCAGAACATATATGGATAGAACTTATAGTGGGGTCTCGAAAA
ATAAGTAATTTCTGCTGGGCCTTTCTCCTTTTTTTAGGTTCATTAGGATTCGTATTGGTTG
GAACTTCCAGTTATCTCGGTAAGAATTTGATATCTTTAGTTTCCTCTCAGCAAATCCATT
TTTTTCCACAGGGGCTCGTGATGTCTTTCTACGGAATCGCGGGTCTCTTTATTAGTTCTT
ATTTGTGGTGCACAATTTCTTGGAATGTGGGTAGTGGCTATGATCGATTCGATCGAAAA
GAAGGAATAGTGTGTATTTTTCGTTGGGGATTCCCGGGAAAAAATCGTCGTATCTTCCT
ACGATTCCGTATGAAAGATATTCAATCCATCAGAATAGAAGTTAAAGAGGGTATTTATGC
GCGTCGTGTCCTTTATATGGAAATAAAAGGGCAGGGGGCCATTCCCTTGACGCGTAGTG
ATGATAAT---TTGACTCTGCGAGAAATTGAGCAAAAAGCCGCCGAATTGGCCTATTTCTT
GCGCGTACCCATTGAAGTTTTT
>Lotus
ATGGAAAAATGGTGGTTAAATTCTATGTTGCGTTTAAGTAAATCAACGGATAGGTTCGG
TCCTATTGAAAATACCAGTGTAAGTGAAGACCCGATTTTAACTGATATGGATAAAAACA
TTCATAGTTACAACAATGTTGATTATTTAGTAGGTGTGGAGAACATCCGGAATTTCCTAT
CTAATAAAAGTTTTTTAGTTAGGGATAGTAAGAGGAACAGTTATTCCATATATTTTGCTAT
TGAAAATCAAATTTTGGAGATTGATAATGATCAT------TTGTTTTTAAGTGAACTAGAAAG
TTTTTTTTCTAGGTATACGAATTCTATCTATTTGAATAACAGCTCTAAAGGGGATGGTGAT
---------CATTACATGTATGATACTGAATCTAGTTGGAATAATAACATTAATAGTTTCATTGA
AAATTATATTCGT------TCTCAAATTTGTATTGATAATTACATTTTAGGTGATGGTGACAAA
TACAATGAT---TACTTTTATAGTTATATTTGTAGTAGGGAGAGGACTAGCATAATAACTAG
CACAAATGATAGTGATTTCACTAAAATAGAAGGTTCTAATGATCTCGATGAGACTCAAA
AATACAAACATTTGTGGATTGAATGCGAAAATTGTTATGGATTAAATTATAAGAAAATTT
TGAAATCAAAAATGAATATTTGTGAACACTGTGGATATCATTTGAAAATGAGCAGTTCA
GATAGAATCGAACTTTCGATTGATCCGGGTACTTGGAATCCTATGGATGAAGACATGAT
TTCTGTGGATCCCATTGAATTTCATTCCGAAGAGGAACCTTATAAAGATCGTATTGATTC
TTATCAAAAAACGACAGGATTAACCGAGGCTGTTCAAACAGGTACGGGTCATTTAAAT
GGTATTCCTGTAGCAATTGGGATTATGGATTTTGAGTTTATGGGAGGTAGTATGGGATCC
GTAGTAGGTGAGAAAATCACCCGGTTGGTCGAATATGCTACCAACCAACTTTTACCTCT

TATTGTAGTATGTGCATCTGGAGGAGCACGTATGCAAGAAGGAAGTTTGAGTTTGATGC
AAATGGCTAAAATATCTTCTGCTTTATATGATTATCAACTAAATAAAAAGTTATTCTATGT
ATCAATCCTTACATCTCCCACTACTGGTGGGGTAACAGCTAGTTTTGGCATGTTGGGGG
ATATCATTATTGCCGAACCAAATGCTTACATTGCATTTGCGGGTAAAAGAGTAATTGAAC
AAACCTTGAATAAGGCAGTACCCGAAGGTTCACAGGCGGCAGAATATTTATTCCATAAG
GGCTTGTTTGATTCAATCGTACCACGTAATCCTTTAAAAGGGATTCTAAGTGAGTTATTT
CAGCTCCACGCTTTCTTTCCTTTGATTCAAAATGAAAAAGAAATGGTAACTATTCGTGC
AGATGAAATTAGTAAAATTATCCGTGAACGGATTGAGCAATATAATACCGAAATTAAAAT
TGTAAATACAGGTACTGTACTTCAAGTAGGCGATGGCATTGCTCGTATTTATGGTCTTGA
TGAAGTAATGGCTGGTGAATTGGTGGAATTTGAAGAGGGTACCATAGGCATTGCTTTGA
ATTTGGAATCCAAAAACGTTGGTGTTGTATTAATGGGCGATGGTTTGATGATACAAGAG
GGAAGTTCGGTAAAAGCAACAGGAAGAATTGCTCAGATACCTGTAAGTGAGGGTTATT
TGGGTCGTGTTATAAATGCTCTGGCTAAACCTATTGACGGTCGGGGAGAAATTTCATCT
TCAGAATCTCGGTTAATCGAATCCCCCGCTCCCGGTATTATTTCGAGACGTTCTGTATAT
GAGCCTCTTCAAACAGGACTTATTGCTATTGATTCGATGATCCCTATAGGGCGCGGCCA
GCGAGAATTAATTATTGGGGACAGACAAACAGGTAAAACAGCAGTAGCCACAGATACG
ATTCTCAATCAACAAGGACAAAATGTAATATGTGTTTATGTAGCTGTTGGTCAAAAAGC
ATCGTCTGTAGCTCAAGTGGTGAATACTTTACAAGAAAGAGGAGCGATGGAATACACT
ATTGTAGTAGCTGAAACGGCAGATTCTCCAGCTACATTGCAATATCTCGCTCCTTATACA
GGAGCAGCTCTCGCTGAATTTTTTATGTACCGTGAACGCCACACTTTAATCATTTATGAT
GATCTCTCCAAACAAGCGCAGGCTTATCGCCAAATGTCTCTTCTATTACGAAGACCACC
CGGTCGCGAAGCTTATCCGGGAGATGTTTTTTATTTGCATTCGCGTCTTTTGGAACGAG
CTGCTAAATTAAGTTCTCAGTTAGGTGAAGGGAGTATGACTGCTTTACCAATAGTTGAG
ACCCAATCAGGAGATGTTTCAGCTTATATTCCTACTAATGTAATTTCCATTACGGATGGC
CAAATATTTTTATCTGCCGATCTATTCAACGCTGGGATCAGACCCGCGATTAATGTGGGT
ATTTCTGTTTCCAGAGTTGGATCGGCGGCTCAAATTAAAGCCATGAAACAAGTAGCCG
GTAAATTAAAATTGGAATTGGCACAATTCGCAGAATTAGAAGCTTTTGCACAATTCGCT
TCTGATCTTGATAAAGCTACTCAAAATCAATTGGCAAGAGGTCAACGATTACGCGAGTT
GCTTAAACAATCCCAATCAGCCCCTCTCACTGTGGAAGAACAGGTAATAACTATTTATA
CCGGAACTAATGGTTATCTTGATTCATTAGAAATTAGACAGGTACGGAAATTTCTCGTTG
AGTTACGTGCTTACTTAAAAACGAATAAACCTCAATTCAACGAAATCATATCTTCTACC
AAGACATTCACTGGGGAAGCAGAAGCTCTTTTGAAAGAAGCTATTCAGGAACAGATG
GAACTCTTTTTACTCCAAGAACAAGTAATGATAATAAATCCTACCACTTCTGATCCTGA
AGTTTCTGCTCTTGAAAAAAAAAAATACGGGACGTATCGCCCAAATAATTGGTCCGGTAC
TAGATGTAACTTTTCCCCCAGGGAAGATGCCCAATATTTACAACGCTCTAATAGTTAAA
GGTCGAGATACTGTTGGTCAACAAATTAACGTGACTTGTGAAGTACAGCAATTATTAGG
AAATAATCGCGTTAGAGCTGTAGCTATGAGTGCTACGGATGGTCTAACGAGAGGAATG
GAAGTTATTGACACAGGAGCTGCTCTAAGTGTTCCGGTGGGTGGAGTAACTCTAGGAC
GAATTTTCAACGTACTTGGCGAGCCTATTGATAATTTGGGTCCCGTAGATACTCGTACAA
CATCTCCTATTCACAGATCCGCGCCTGCCTTTATACAATTAGATACAAAATTATCCATTTT
TGAAACAGGAATTAAAGTAGTAGATCTTTTAGCTCCTTATCGCCGTGGAGGAAAAATAG
GACTTTTCGGTGGAGCTGGAGTGGGTAAAACAGTACTCATTATGGAATTAATCAATAAC
ATTGCCAAAGCTCATGGGGGTGTGTCCGTATTTGGCGGAGTAGGTGAGCGTACTCGTG
AGGGAAACGATCTTTACATGGAAATGAAAGAATCCGGAGTAATTAATGAAAAAAATAT

TGCAGAATCAAAAGTAGCTCTAGTCTACGGTCAAATGAATGAACCGCCTGGAGCTCGT
ATGAGAGTTGGGTTAACTGCCCTAACTATGGCGGAATATTTTCGAGATGTGAATGAACA
AGACGTACTTCTATTTATCGACAATATCTTCCGTTTCGTCCAAGCAGGATCCGAAGTATC
CGCTTTATTAGGTCGAATGCCTTCCGCTGTGGGTTATCAACCTACCCTTAGTACCGAAAT
GGGTTCTTTACAAGAAAGAATTACTTCTACCAAAGAAGGGTCCATAACTTCTATTCAAG
CAGTTTATGTACCCGCAGACGATTTGACTGATCCCGCCCCCGCCACGACATTTGCACAT
TTAGATGCTACTACTGTACTATCAAGAGGATTAGCTGCCAAAGGTATCTATCCAGCAGTA
GATCCTTTAGATTCAACGTCAACTATGCTCCAACCCCGAATCGTTGGTGAAGAACATTA
CGAAACTGCGCAAAGAGTTAAACAAACTTTACAACGTTACAAAGAACTTCAGGATATT
ATAGCTATCCTTGGGTTGGATGAATTATCTGAAGAGGACCGTTTGACCGTAGCAAGAGC
ACGAAAAAATTGAGCGTTTCTTATCACAACCCTTTTTTGTAGCAGAAGTATTTACCGGTT
CACCGGGGAAATATGTTGGTCTAGCAGAAACAATTAGAGGGTTTAAATTGATTCTTTCT
GGTGAATTAGATAGTCTTCCCGAACAAGCCTTTTATTTGGTGGGTAATATCGATGAAGCT
ACCGTGAAGGCTACGAATTTAGAAAAGGAGAGCAAATTGAAGAAAATGACTTTAAATC
TTTGTGTACTGACCCCAAATCGAATTGTTTGGGATTCCGACGTGAAGGAAATTATTTTAT
CTACAAATAGTGGACAAGTTGGAATATTACCCAATCACGCGCCTCTGGCTATGGCTTTA
GATATAGGTATTTTGAGAATACGCCTTAACGACCAATGGTTAACTATGGCTCTGATGGGC
GGTTTTGCTCGAATAGGCAATAATGAAATCACTGTTTTAGTAAATGATGCGGAAAAAGG
AAGTGACATTGATCCACAAGAAGCACAGCAAACTCTTGAAATAGCAGAAGCTAATTTA
AAGGAGGCTAAGGGCAAGAGACAAACAATTGAGGCAAATCTAGCTCTCAGACGAGCT
AGAACACGAGTAGAGTCTATCAATATGATTTCAATGAAAAATATAACCGATCCTTTCCCT
TGCTTGGGTTACTGGCCATCTGCCGGAAGTTTCGGATTTAATACCGATATTTTAGCAACA
AATCCAATAAATCTAAGTATAGTGATAGGTGTATTGATTTTTTTTGGAAAGGGAGTGTTA
AGTGATTTATTAGATAATCGAAAACAGAGAATCTTGAGAACTATTCGAAATTCAGAAGA
ACTACGGGAAGGGGCGATTGAACAGCTGGAAAAAGCCCAGGCCCGCTTAAAGAAAGT
AGAAACAGAAGCAGATCGGTTTAGAGTGAATGGCTACTCTGAGATAGAACGAGAAAA
ATTGAATTTAATTAATTCAATTTATACAACTTTAGAACAATTAGAAAATTACAAAAATGA
AACCATTCATTTTGAACAACAAAGGGCGATTAATCAAGTCCGACAGCGGGTTTTACAA
CAAGCCTTACAAGGAGCTCTAGGAACTCTGAATAGTTGCTTAAATAATGAGTTACATTT
CCGTACCATCGCCGCTAATATTGGTATGTTTGGGGCGATGAAAGAAAAAAATAATATGA
ATCCAATAATTTCTGCCGCTTCCGTTATTGCTGCTGGGTTGGCCGTTGGGCTTGCTTCTA
TTGGACCTGGGGTTGGTCAAGGCACTGCTGCAGGTCAAGCTGTAGAAGGAATTGCGC
GACAACCAGAGGCAGAGGGAAAAATACGAGGTACTTTATTACTTAGTCTGGCTTTTATG
GAAGCTTTAACTATTTATGGGCTGGTTGTAGCATTAGCACTTTTATTTGCAAATCCCTTT
GTTATGAATGTTCTACTATGTTCCATCAACACATTCAAAAGATTATACGATATTTCTGCCG
TGGAAGTAGGTCAACATTTCTACTGGCAAATGGGAGATTTCCAAGTGCATGCCCAAGT
ACTTATTACTTCGTGGGTTGTGATTGCTATCTTATTAGGTTCAACCATTCTAGTTGTTCGA
AATCCACAAACCATTCCCAATTTTGGTCAAAATTTCTTTGAATATGTCCTTGAATTTATT
CGAGACGTAAGCAAAACTCAAATTGGAGAAGAATATGGTCCTTGGGTTCCATTTATCG
GAACTCTATTTCTATTTATTTTTGTTTCGAATTGGTCAGGGGCTCTTTTGCCTTGGAAAA
TCATACAGTTACCTCATGGGGAGTTAGCTGCACCCACAAATGATATAAATACTACTGTTG
CATTAGCTTTACTTACGTCAGTAGCATATTTCTATGCGGGGATTTCAAAAAAAGGATTAG
CTTATTTTGGTAAATATATACAACCTACTCCAATTCTTTTACCGATTAACATCTTAGAGGA
TTTTACAAAACCTTTATCGCTTAGTTTTCGACTTTTCGGAAATATATTAGCTGACGAATT

AGTAGTAGTTGTTCTTGTTTCTTTAGTACCTTTAGTGGTTCCTATACCTGTTATGTTCCTT
GGATTATTTACAAGTGGTATTCAAGCTCTTATTTTTGCTACTTTAGCCGCAGCTTATATAG
GTGAATCCATGGAAGGTCATCATATGATATTTTCAACTTTAGAGCATATATTAACTCATAT
ATCGTTTTCGGTCGTATCAATTGTAATTTCAATTCATTTGATAACCTTATTTGTCAACCAA
ATTGTAGGATTCTATGATTCGTCCAAAAAAGGCATGATAATAACTTTTTTGTGTATAACA
GGATTATTAATTACTCGTTGGTTTTTTTCAGGCCATTTACCATTTAGTGATTTATATGAATC
CTTAATCTTTCTTTCATGGGGTTTTTCGATTTTTTATATGGTTCCGCGTTTTAAAAAACAA
AAAAACGATTTAAGTACAATAATTGCACCTAGTGTTATTTTTACCCAAGGCTTTGCTACT
TCGGGTCTTTTAACCGAAATGCATCAATCTGTAATATTAGTACCCGCTTTACAATCCCAT
TGGTTAATGATGCACGTAAGTATGATGATCTTAGGCTATGCAGCTCTTTTATGTGGATCA
CTATTATCAGTAGCTATTTTAGTCATTACATTTCAAGAATTAATACCAATTTTGGGTAAAA
GCAAAGAAATCAAATACATAAATATGAATGAAAATGTTTTACGAAAAACTTCTTTTTCA
TCTTATAGAAATTATTACAGGTATCAATTTATTCAACAATTGGATCGTTGGGGTTATCGTA
TTATTAGTCTAGGTTTTATCTTTTTAACGATCGGTATTCTTTCAGGAGCAGTATGGGCTAA
CGAGGCATGGGGATCTTATTGGAATTGGGATCCCAAGGAAACTTGGGCCTTTATTACTT
GGACAATATTCGCGATTTATTTACATAGTAGAAAAAATAAAAAACTGGAAGGTTTAAAT
TCTTCAATAGTGGCCTCTATAGGCTTTCTTATAATTTGGATATGTTATTTTGGGGTCAATC
TATTAGGAATAGGACTACATAATTATGGTTCGTTTACATCTAATATGGCAAAAAAGAAAG
CATCTATTCCCTTTCTATCTCTCACATCTATAGTCTTTTTGCCCTGGTGCATCTCTTTTACA
TGTAAGAAAGGTATGGAATATTGGGTTACTAATTGGTGGAATACTAAGCAATCCGAAAT
TTTCTTGAATATCATTCAAGAAAAGAGCATTCTAAAAAAATTCATGGAATTAGAGGAAC
TGTTCTTCTTGGATGAACTGCTAAAGGAATATTCGGAAACACGTCTTCAAATCCTCCGT
ACCGGA---CACAAAGAAACAATTCAATTGATCAAGACGCACAACGAAGATCGTATCTAT
ACAATTTTGCACTTCTCGACAAATATAATCTATTTCATTATTCTAAGTGGTTATTCTATTCT
AGGTAATCAAGAACTTATTATTCTTAACTCTTGGGTTCAAGAATTCCTATATAACTTAAG
TGACACAATAAAAGCTTTTTCTATTCTTTTAGTAACTGATTTATGTATAGGGTTCCATTCG
ACTCATGGTTGGGAACTACTGATTGGGTCCGTCTATAAAGATTTTGGATTTATTCAGAAT
GATCAAATTATATCTGGTCTTGTTTCTACTTTTCCAGTGATTTTAGATACAATTTTAAAAT
ATTGGATTTTCCGTTATTTAAATCGTGTATCTCCTTCACTTGTAGTGATTTATCATTCAAT
GAATGACATGCCCATTGGTGTTCCAAAAGTGCCCTTTCGAAGTCCTGGAGAGGAAGAT
GCATCTTGGGTTGACATATAC---CGACTTTATAGAGAAAGACTGCTTTTTTTAGGCCAGG
AAGTTAATAGTGAAATATCAAATCAACTTATTGGTCTTATGGTATATCTCAGTATAGAGG
ATGATAAGAAAGATTTGTATCTGTTTATTAATTCTCCGGGAGGATGGGTAATCCCCGGAA
TAGCTATTTATGATACTATGCAATTTGTACAACCGGATGTACAGACAGTATGCATGGGAT
TAGCCGCTTCAATGGGATCTTTTGTTTTGGCAGGCGGAAAAATTACCAAACGTCTAGCA
TTCCCTCACGCT---GTAATGATCCATCAACCTGCTAGTTCTTTTTATGAGGCACAAACGG
GAGAATTTATCCTGGAAGCGGAAGAACTCCTGAAACTACGCGAAACTATCACAAGGGT
TTATGTACAAAGAACGGGCAAACCTTTATGGCTTGTATCAGAAGACATGGAAAGGGAT
GTTTTTATGTCAGCAGCAGAAGCCCAAGCCTATGGAATTGTTGATCTTGTAGCGGTTGA
A???
???
???ATGGAGGAATATCAGTTATATTT
GGAACTGGATAGATCTCGTCAACAGGACTTCCTATACCCACTTGTTTTTCACGAATATAT
TTACGGACTCGCTTATAGTCATGATTTATTTGGTTATGATAATAAATATAGTTTACTAATTG

TAAAACGTTTAATTACTCGAATGTATCAACAGAATCATTTGATTATTTCGGCTAATGATTC
TAACAAAAATCGATTTTTGAGGAAGAATTTTTATTCTCAAATAATATCAGAGGGTTTTGC
CATCATCGTGGAAATTCTGTTTTCTTTACAATTAAGCTCGTCCTTAATCATAAAATCTTAT
AATCTGCGATCAATTCATTCTATTTTTCCCTTTTTCGAGGATAAAGTTACATATTTAAATT
ATATATCAGATATACGAGTCCCTTATCCTATCCATCTGGAAATCTTAGTCCAAATCCTTCG
ATACTGGGTGAAAGATGCCCCTTTGTTTCATTTATTAAGACTGTTTCTTTATGATTATTGT
AATTGGAATAGTATTTTTATTAAATCGATTTATACTTTTTCAAAACCAAGATTTTTCTTCT
TCCTATATAATTTTTATGTATGTGAATATGAATCTATCTTTTTTTTTCTACGTACCAAATCT
TTTCATTTACGATTAAAATCTTTTAGAGTTTTTTTTGAGCGAATCTTTTTCTATGCAAAA
AAAGGACATCTTGTAGAAGTATTTGTTAAGTTTTCTACCTTAACATTCTTTAAGGATCCT
TTCATCCATTATGTTAGATATCAGGGAAAATCCATTCTGGCTTCCAAGAATTTGCCTATTT
TAATGAATAAATGGAAATACTATTTTATCCATTTATGGCAATGTTATTTTGATGTTTGGTC
TCAACCGCGAACAATCCGTATAAACCAATTATCCGAATATTCATTTCACTTTTTGGGCTA
TTTTTTAAAGGTGGGGCTGAAGCATTCAGTAGTACGGGGTCAAATGCTGCAAAATGGA
TTTCTAATCAAAATT------ATTATAAAAAAGCTTGATATAATAGTTCCAATTATTCCAATAA
TTAGATTATTGGCTAAATCGAAATTTTGTAATGTATTAGGGAATCCCCTTAGTAAGCCGT
CCTGGGCCGACTTATCCGATTTTGAGATTATTGCCCGATTTTTGCGGATATGCAGAAATC
TTTCTCATTATTACAATGGATCCTCAAAAAAAAAGAGTTTGTATCGAATAAAATATATAC
TTCGGCTTTCATGTATTAAAACTTTGGCCTGTAAACACAAAAGTACCGTACGCGCTTTT
TCGAAAAGATTAGGTTCGGAATTATTGGAAGAATTCTTTACAGAGGAAGAAGAGATTC
TTTCTTTGATCTTTCCAACACGGATTTGGTATTTGGATATTCTTTTTAGCAACGATCTAAT
CAATCATGACATGATAATTGATACAACA---GAAGTCCAAGATATCAAT---TCTTTTTCCAG
ATTGGAATCT---TTTAAAGAGGTCTATGGAGTTCTATGGGTACTCGCCCCTATTTTAATTA
TTGTATTAGGAATCACAATAAGTGTATTAGCAATTGTATGGTTAGAAAGAGAAATATCCG
CGGGGATGCAACAACGTATTGGACCTGAATACGCCGGTCCTTTTGGCGTTCTTCAAGCT
CTAGCAGACGGAACAAAACTACTTTTCAAAGAGAATCTTATTCCATCTAGAGGGGATAT
TCGTTTATTCAGTATCGGACCATCCATATCAGTTATCTCAATTCTAATAACCTATTTAGTA
ATTCCTTTTAGCTATAATTTTGTTTTATCTGATTTGAATATCGGTGTTTTTTTATGGATTGC
TATTTCGAGTATTGCTCCCATTGGACTTCTTATGTCAGGATATGGTTCAAATAATAAATAT
TCTTTTTTGGGTGGTCTGCGAGCTGCTGCTCAATCAATTAGTTATGAAATACCATTAACT
TTATGTGTATTATCAATATCTCTACTATCGAACAGTTTAAGTACAGTTGATATAGTTGATG
CACAATCAAAATATGGGTTTTGGGGATGGAATTTGTGGCGTCAACCTATGGGTTTCGTC
GTTTTTCTAATTTCTTCCCTAGCAGAATGTGAAAGATTACCCTTTGATTTGCCAGAAGCG
GAAGAAGAATTAGTAGCGGGTTATCAAACAGAATATTCGGGTATAAAATTTGGTTTATTT
TATGTTGCTTCCTATTTAAACCTGTTAATTTCTTCATTATTTGTAACAGTTCTTTACTTGG
GTGGTTCGAATATCTCTATTCCGTACATATCCCTTTTTGAGTTTTTTGAAATAAATAAAGA
ATATGGAGTTTTTGGAACTACAATTGGTATCTTTATTACACTAGCTAAAACTTATTTGTTC
TTATTCGTTTCTATCATAACAAGATGGACTTTGCCTAGGCTAAGAATGGATCAATTATTA
AATCTTGGCTGGAAGTTTCTTTTACCTATTTCTCTTGGTAATCTATTATTAACAACTTCGT
CTCAACTGTTTTCACTCATGATCTGACATGTACAGAATGAAAACCTCATTCTCGATTCTA
CGAGAATTTTTATGAAAGCCTTTCATTTGCTTCTCTTCGATGGAAGTTTTATTTTCCCAG
AATGTATCCTAATTTTTGGTCTAATTCTTCTTCTGATGATCGATTCAACCTCTGATCAAAA
AGATATATCTTGGTTCTATTTTATCTCTTCAACAAGTTTAGTAATGAGCATTGTGGTTCTA
TTGTTCCGATGGAGAGAAGAACCTATGATTAGCTTTTCGGGAAATTTCCAAACGAACA

ATTTCAACGAAATCTTTCAATTTCTTATTTTACTATCTTCAACTCTATGTATTCCTCTATCC
GTAGAGTACATTGAATGTACAGAAATGGCTATAACAGAGTTTCTGTTATTCGTATTAACA
GCTACTCTAGGAGGAATGTTTTTATGCAGCGCTAACGATTTAATAACTATCTTTGTAGCT
CCAGAATGTTTCAGTTTATGCTCCTATCTACTATCTGGATATACCAAGAAAGATGTACGG
TCTAATGAGGCTACTATGAAATATTTACTCATGGGTGGGGCAAGCTCTTCTATTCTGGTT
CATGGTTTCTCTTGGCTATACGGTTCATCCGGGGGAGAGATCGAGCTTCAAGAAATAGT
GAATGGTCTTATCAATACACAAATGTATAACTCCCCGGGAATTTCAATTGCGCTTATATT
CATCACTGTAGGAATTGGGTTCAAGCTCTCCCTAGCCCCTTCTCATCAATGGACTCCTG
ACGTATACGAAGGATCTCCCACTCCAGTCGTTGCTTTTCTTTCTGTTACTTCGAAAGTA
GCTGCTTCAGCTTTAGCCACTCGAATTTTCGATATTCCCTTTTATTTCTCATCAAACGAA
TGGCATCTTCTTCTGGAAATCCTAGCTATTCTTAGCATGATATTAGGGAATCTCATTGCTA
TTACCCAAACAAGCATGAAACGTATGCTTGCGTATTCGTCCATAGGTCAAATCGGATAT
GTAATTATTGGAATAATTGTTGGAGACTCAAATGGTGGATATGCAAGCATGATAACTTAT
ATGCTGTTCTATATCTCCATGAATCTAGGAACTTTTGCTTGCATTGTATCATTTGGTCTAC
GTACCGGAACTGATAACATTCGAGATTATGCAGGATTATACACGAAAGATCCTTTTTTG
GCTCTCTCTTTAGCACTATGTCTCTTATCCTTAGGAGGTCTTCCTCCGCTAGCAGGTTTT
TTCGGAAAACTTTATTTATTCTGGTGTGGATGGCAGGCAGGCCTATATTTCTTGGTTTCA
ATAGGACTTCTTATGAGCGTTGTTTCTATCTACTATTATCTAAAAATAATCAAGTTATTAA
TGACTGGACGAAACCAAGAAATAACCCCTCACGTGCGAAATTATAAAAGGTCCCCTTT
AAGATCAAACAATTCCATCGAATTGAGTATGATTGTATGTGTGATAGCATCTACTATATC
AGGAATATCAATGAACCCGATTATTGAAATTGCTCAGGATACCCTTTTTATGTTTTTGCT
TTATGAATATGATATTTTTTGGGCATTTCTAATAATATCGATCTTTATTCCGATTTTGGCAT
TTACAATTTCTGGCTTTTTGGCCCCGATTAACAAAGGGCCAGAGAAACTTTCTAGTTAT
GAATCGGGAATAGAACCAATGGGAGATGCTTGGTTACAATTTCAAATCCGTTATTATATG
TTTGCTCTAGTTTTTGTTGTTTTTGATGTTGAAACGGTTTTTCTTTATCCATGGGCAATG
AGTTTTGATGTATTGGGGATATCCGTATTTATAGAAGCTTTGATCTTTGTGCTTATCCTAA
TTGTTGGTTCAGTTTACGCGTGGCGAAAGGGGGCTTTAGAATGGTCTACGAATTATTTT
CCTTGGTTAACATTAGTTGTAATTTTGCCAATAGCCGGCGGTTCCTTAATTTTCTTATTTC
CTCATAGAGGAAATAAGGTAATTAGGTGGTATACTGTTTGTATTTGTTTGATAGATCTCC
TTTTAACAACCTATGCGTTTTGTTATCATTTCCAATTGGATGATCCGCTAATTCAACTGA
CGGAGAGTTATAAATGGATCAATTTTTTTGATTTTTACTGGAGATTCGGAATAGATGGAC
TCTCTATAGGACCCATTTTACTGACGGGATTTATCACCACTTTAGCTACTTTAGCGGCTC
AGCCGATTACTCGAGAATGCAAATTATTCTATTTCCTGATGTTAGCAATGTATAGCGGTC
AAATAGGACCATTTTCTTCTCGAGACATTTTACTTTTCTTCATCATGTGGGAATTGGAAT
TAATTCCCGTTTATCTACTTTTAGCCATGTGGGGGGGAAAGAAACGTTTGTATTCAGCTA
CAAAATTTATTTTGTATACTGCGGGAAGTTCTGTTTTTTTATTAATGGCAACTCTAGGTAT
CGGTTTATACGGTTCTAATGAACCAACACTCAATTTTGAAACATTAAATAATCAATCATA
TCCCTTGGCGTTGGAAATAATCGTATATATAGGATTTCTTATTGCGTTTGCTGTCAAATCA
CCGATTATACCCTTACATACATGGTTACCGGATACACACGGAGAGGCCCATTACAGCAC
TTGTATGCTTTTAGCCGGAATCTTATTAAAAATGGGAGCATATGGGTTGGTTCGAATTAA
TATGGAATTATTTTCCCACGCTCATTCTATATTTTGCCCTTGGTTAATGATATTAGGTTCCA
TTCAAATAATCTATGCCGCTTCAGCATCTCTTGGTCAACGTAATTTAAAAAAAAGAATA
GCTTATTCCTCAGTATCTCATATGGGTTTCCTAATTATAGGAATTGGTTCTATAAGCGATA
CGGGGCTCAACGGGGCCATTTTACAAATAATATCTCATGGATTTATTGGCGCTGCGCTTT

TTTTCTTGTCAGGAACTAGTTATGATAGACTACGTCTTCTTTATCTTGACGAAATGGGTG
GAATGGCTCTGCCAATGCCAAAAATATTCACGGTTTTCACTATCTTATCGATGGCCTCTC
TTGCATTGCCGGGCATGAGTGGTTTTTTTGCAGAGTTGATAGTTTTTTGGGGAATAATTA
CCAGCCAAAAATATTTTTTAATAATGAAAATACTAATTACTTTTGTAACAGCAATTGGAA
TGATATTAACTCCTATTTATTCATTATCTATCTTACGGCAGATGTTCTATGGATACAAGTTT
TTTAATACGCCAAACTCATATTTTTTTGATTCTGGACCGAGAGAATTATTTATTTCGATTT
CTATCCTTATACCAATAATAGGTATTGGTATTTATCCAGATTTCATTTTCTCATTCTCGGTT
GACAAGGTTGAAGCTGTTCTATCTCATTTT---------ATGATGCTCGAACATGTACTTGTTT
TGAGTGCTTATCTATTTTCTATCGGTATCTATGGATTAATCACGAGTCGAAATATGGTTCG
AGCCCTTATGTGTCTTGAACTTATATTGAATGCAGTAAATATGAATCTCGTAACCTTTTCT
GATTTTTTTGATAATCGCCAATTAAAGGGAAATATTTTTTCAATTTTTGTTATAGCTATTG
CAGCCGCTGAAGCAGCTATCGGACCGGCGATTGTTTCCTCAATTTCGCGTAACAGAAA
ATCAATTCGTATCAATCAATCGAATTTGTTGAATAAAATGGAATATACACATCAATCTTCA
TGGATCATACCCTTCACCCCACTTCCCGTTCCTATATTAATAGGAGTAGGACTTCTACTT
TTTCCTACGGCAACAAAAAAATCTTCGCCGTATGTGGGCTTTTCCTAGTATTTTTTTGTTA
ATTATAGTTATGATTTTTTCTGTAGATCTATCTATTCATCAAATCAAGAACAGTTCTATCTA
TCAATATGTATGGTCTTGGACTATAAATACCGATCTTTCTTTAGAGTTTGGTTACTTGATC
GATTCACTTACCTCTATTATGTCAATATTAATTACTACTGTTGGCATTCTGGTTCTTATTTA
TAGTGATAATTATATGTCTCATGATCAAGGGTATTTGAGATTTTTTGCTTATTTGAGTTTT
TTTAATACTTCAATGTTAGGATTAGTTACTAGTTCGAATTTGATACAAGTTTACATTTTTT
GGGAATTGGTTGGAATGTGTTCTTATCTATTAATAGGTTTTTGGTTCACACGTCCTATTG
CGGCAAATGCTTGTCAAAAAGCGTTTGTAACTAATCGTGTAGGCGATTTTGGTTTATTAT
TAGGAATTTTGGGTCTTTATTGGATTACAGGTAGTTTGGAATTTCGGGATTTATTCCAAA
TAATCAACAAC---GAGGTTAATATTTTTTTTATTACTTTGTGTGCCCTCTTGTTATTTTGTG
GCTCGGTTGCAAAATCTGCCCAATTCCCCCTTCATGTATGGTTACCAGATGCTATGGAA
GGTCCCACCCCTATTTCTGCTCTTATACATGCTGCCACCATGGTAGCAGCAGGAATTTTC
CTTGTAGCTCGACTTCTTCCCTTTTTCATAGCCATACCTTCTATAATGAACGGAATAGCT
TTCATAGGTATAATAACAGTAGTATTAGGGGCCACTTTAGCAATTGCTCAAAAAGATATT
AAGAAAAATTTGGCCTATTCTACCATGTCTCAATTGGGTTATATGATGTTAGCTTTAGGT
ATGGGCTCTTATCGAATTGCGTTATTTCATTTGATTACTCATGCTTATTCAAAAGCATTGT
TGTTTTTAGGGTCCGGATCCATTATTCATTCAATGGAAGCTATTGTTGGGTATTCTCCAA
ATAAAAGTCAAAATATGCTTCTTATGGGTGGTTTAACAAAACATGTGCCAATTACAAAA
ACTGCCTTTTTAATAGGTACGCTTTCTCTTTGTGGTATTCCGCCTTTCGCCTGTTTTTGGT
CCAAGGATGAGATTCTTAATGATAGTTGGTTATACTCACCAATTTTTGCAATAATAGCTT
GTTCAACAGCAGGATTAACCGCATTTTATATGTTTCGGATCTATTTACTTGTTTTTGAAG
GATATTTCAATGTTCATTTTCAAAATTTCAATGGAAAGAAAAATAGTTCATTCTATTCAA
TATCTTTATGGGGTAAA---GAAGGAAAAAAAAAAATTAAAAAATTTATTATCTTTATTAAC
AATGAATTTGTTTCGCGGTACTAAAAAGTTTCCTTGTTTTTATCCTCATGAATCTGACAA
TACTATGTTATTTTCTATGCTTGTCTTAGTACTATTTACTTTCTTCGTCGGAGCCATAGGA-
-----TTTGATTTGGATATATTATCCAAATTGTTAAGTCCGTCAATAGACCTTTTACACCAAA
ATTCAAAC---TCTGTAGATTGG---GAATTTTTTACAAATGCAACTTTTTCGGTGAGTATAG
CTTTTTTCGGAATCTTTATAGCGTCTTTTTTCTATAAACCAGTTTATTCGACTTTACAAAA
TTTGAATTTATTAAATTTATTCGAAATTTTAGCTGATACAATAATAAATGGCATATATGATT
GGTCATATAATCGCGGCTATATAGATGGATTTTATGAAATATCTCTTATTGCCTCTGTAAG

AAAATTAGCGAAACTAAATTCTTTTTTTGATAGACAGGTAATTGATGGAATTCCAAATG
CAGTTGGTATTACAAGTTTTCTTATAGGGGAGGCTTTTAAATATGTAGGAAGTGGG---CG
AATTTCTTCGTATATCTTATTTTTTGTATTTTTTGTATTATTATTTTTAATAATTTTTTATATG
GATTTGCCCAAAACGCTACATGATTTTCTTTTAGTATTTCTGGGATCGGGTCTTATATTAG
GAAGTCTAGGAGTAGTATTACTTACCAACCCAATCTTTTCTGCTTTTTCGTTGGGACTAA
CTCTTGTTTGTATATCTTTATTGTATATTTTATCAAACTCCCATTTTGTAGCTGCATCACAG
TTACTTATTTACGTGGGAGCTATAAATGTTTTAATCATATTTGGTGTGATGTTCATGAATG
GTTCAGAATATTACCAAGATTTTCGTCTTTGGACCGTTGGCGATGGAATGACTTTGATG
GTTTGTACAAGTATTTTTATTTCACTAATAACTACTATTCCAGATACATCATGGTACGGGA
TTATTTGGACTACAAGATCCAATCAGATTATAGAACAAGATTTGATAAGTACTAGTCAAC
AAATTGGAATTCATTTATCAACAGATTTTTTTCTTCCATTTGAACTCATTTCAATAATTCT
TTTAGCTGCTTTAATAGGTGCAATTGTCGTAGCTCGCCAGATGAATGTCCCAGCTACAA
GAAAAGACCTCATGATAGTAAATATGGGGCCTCACCATCCATCAATGCATGGTGTTCTT
CGACTTATTGTTACTCTGGATGGTGAAGATGTTATTGATTGTGAACCAATATTGGGTTAT
TTACACAGAGGAATGGAAAAAATTGCGGAAAATCGAACAATTATACAATATCTGCCTTA
TGTAACACGTTGGGATTATTTAGCCACTATGTTCACAGAAGCAATAACCGTAAATGGAC
CGGAACAGTTAGGAAATATTCAAGTGCCTAAAAGAGCCAGCTATATCCGAGTAATTATG
TTAGAGTTGAGTCGTATAGCTTCTCACCTACTATGGTTGGGACCTTTTATGGCAGATATT
GGTGCACAAACTCCTTTCTTCTATATTTTTAGAGAAAGGGAATTCATATATGATCTATTC
GAAGCTGCGACAGGTATGAGAATGATGCATAATTTTTTTCGTATTGGAGGAGTAGCGGC
CGATCTACCTCACGGTTGGATAGATAAATGTTTTGATTTCTGCAATTATTTTTTCACAAG
GGTTGTTGAATATCAAAAACTTATTACGCGCAATCCTATTTTTTTGGAAAGGGTTGAGG
GGGTGGGTGTTGTTGGTGGAGAGGAAGTAATAAATTGGGGTTTATCAGGACCAATGCT
TCGGGCTTCCGGAATACAATGGGATCTACGTCAAGTTGATAATTATGAGTGTTATGAAG
AATTTGATTGGGAAGTTCAATGGCAAAAAGAGGGAGATTCATTGGCTCGTTATTTAGTT
AGAATTGGTGAAATGATGGAATCCATAAAAATTATTCAACAGGCTTTGGAGGGGATTCC
CGGCGGCCCCTATGAAAATTTAGAAATCCGATGTTTTGGTAGAGAAAAAGAACCAGAA
TGGAATGATTTTGAATATCGATTCATTGGTAAAAAACCGTCTCCAACTTTCGAATTGCC
AAAACAAGAACTTTATGTAAGAGTTGAGGCCCCCAAAGGAGAATTGGGGATTTTTTTA
ATAGGAGATCAAAATGGTTTTCCTTGGAGATGGAAAATTCGTCCACCGGGTTTTATCAA
TTTGCAAATTCTTCCTCAATTAGTTAAAAGAATGAAATTGGCTGATATTATGACAATACT
AGGTAGCATAGATATCATTATGGGAGAAGTTGATCGTATGTTCCTTATGGTAACTGGGTT
CATGAATTATAGTCAACAAACAGTTCGAGCTGCAAGATACATTGGTCAAAGTTTCATGA
TTACCTTATCTCACGCAAATCGTTTACCGGTAACTATTCAATACCCTTATGAAAAATTAAT
AACATCGGAACGTTTCCGGGGTCGAATCCATTTCGAATTTGATAAATGCATTGCTTGTG
AAGTATGTGTTCGTGTATGTCCTATAGATTTACCCGTTGTGGATTGGAAACTAGAAACTA
ATATTCGAAAAAAACGATTGCTAAATTACAGTATTGATTTCGGAATCTGTATTTTTTGTG
GTAACTGTATTGAGTATTGTCCAACAAATTGTTTATCAATGACGGAAGAATATGAACTTT
CGGCTTATGATCGTCATGAATTGAATTATAATCAAATTGCTTTAGGCCGTTTACCCATGTC
AGTAATTGATGATTATACAATTCGAACAATTCAAATAAAA--
---------ATGCAAGGTCGTTTGTCTGCTTGGCTAGTCAAACATGGACTAGTTCATAGATCTT
TGGGTTTCGATTACCAAGGAATAGAGACTTTACAAATAAAGCCTGAGGATTGGCATTCC
ATTGCTGTTATTTTATATGTATATGGTTATAATTATCTACGTTCCCAATGTGCCTATGATGTA
TCACCGGGAGGACTGTTAGCTAGTGTGTATCATCTTACGCGAATAGAGTGTGGTATATAT

CAACCAGAAGAGGTATGCATTAAAATATTTGTTCCAAGGAACAATCCCCGAATTCCTTC
TATTTTCTGGGTTTGGAAAAGTGCGGATTTTCAAGAAAGGGAATCATATGATATGTTAG
GAATCTCGTATGATAATCATCCTCGTCTGAAACGTATTTTAATGCCCGAAAGCTGGATAG
GGTGGCCTTTGCGTAAAGATTATATCACCCCCAATTTTTATGAAATACAAGATGCTCAC--
----
------------------------------ATGAATTCCATTGAGTTTCCCTTAATTGAT
CGAACAACCCAAAATTCAGTTATTTCAACTACATTAAATGATCTTTCAAATTGGTCAAG
ATTATCCAGTTTATGGCCGCTTCTCTATGGTACCAGTTGTTGCTTCATTGAATTTGCTTCA
TTAATAGGATCACGATTCGACTTTGATCGTTATGGATTGGTACCGCGATCTAGTCCCAGG
CAAGCAGACCTAATTTTAACAGCGGGCACGGTAACCATGAAAATGGCTCCTTCTTTAGT
TCGATTATATGAACAAATGCCCGAACCAAAATATGTTATTGCTATGGGAGCCTGCACAAT
TACAGGAGGGATGTTCAGTACAGATTCTTATAGTACTGTTCGGGGGGTAGATAAACTAA
TTCCTGTGGATGTTTATTTGCCGGGCTGTCCACCGAAACCGGAGTCCATTCTAGATGCT
ATAACAAAACTTCGTAAGAAAATATCTCGAGAAATCCATGAAGATCAACTGAGTTCTCA
A---GAGAATAGGTGTTTTACTACCAACCACAAATTTTATGTTGAACGCAGTACTCATACC
GGCAATTATGATCAAGTATTATTCCATCAACCACCGTCTACTTCAGAGATATCCACA------
TTTTTTAGATATCAAAAAGTTCAGTATCCTAACGAAATAGTGAATATGCAAACTAGAAAT
GCTTTTTCTTATATAAAGGAAGAGATTACTCGATCCATTTCCGTATTGCTCGTGATATATA
TAATAATTCGAGCACCCATTTCAAATGCATATCCGATTTTTGCCCAACAGGGTTATGAAA
ATCCGCGAGAAGCAACTGGCCGTATTGTATGTGCCAATTGCCATTTAGCCAATAAGCCC
GTGGATATTGAGGTCCCACAAACGGTACTTCCCGATACTGTATTTGAAGCAGTTGTTCG
AATTCCTTATGATATGCAAGTAAAACAAGTTCTTGCTAATGGTAAAAGGGGCGCTTTAA
ATGTAGGGGCTGTTCTTATTTTACCGGAGGGTTTTGAATTGGCCCCCACCGATCGTATTT
CGCCCGAGATTAAAGAAAAGATGGGCAATCTGTCTTTTCAAAGCTACCGTCCCACGAA
AAAAAATATTCTTGTGGTAGGCCCTGTTCCTGGTCAGAAATATAGTGAGATTACCTTTCC
GATTCTTTCCCCGGATCCCGCTACTAATAGAGATGTTAATTTCTTAAAATATCCTATATAC
GTAGGCGGCAACAGGGGAAGGGGTCAGATTTATCCCGACGGGAGTAAGAGTAATAATA
ATGTTTATAATGCTACAACGAGCGGTATAATAAACAAAATTATACGAAAAGATAAGGGG
GGGTACGAAATAACGATAGTGGATGCATCGGATGGACGTGAAGTAATTGATATTATACC
TCCAGGACCAGAACTTCTTGTTTCAGAGGGTGAATCCATCAAACTTGATCAACCTTTAA
CAAGTAATCCAAATGTGGGTGGATTTGGTCAGGGAGATGCAGAAATAGTTCTTCAAGA
TCCGTTACGTGTCCAAGGTCTCTTGTTCTTCTTGGCATCTATTATTTTCGCACAAATCTTT
TTGGTTCTTAAAAAGAAACAATTTGAGAAGGTTCAATTGTCCGAAATGAATTTTATGAG
TAAAGTCTATGATTGGTTCGAAGAACGTCTTGAGATTCAGGCGATTGCCGATGATATAA
CTAGTAAGTATGTTCCTCCTCATGTCAACATATTTTATTGTTTAGGGGGAATTACACTTAC
TTGTTTTTTAGTCCAAGTAGCCACGGGGTTTGCTATGACTTTTTATTATCGTCCTACAGT
CACTGAAGCTTTTGCTTCTGTTCAATATATAATGACTGAGGCGAATTTTGGTTGGTTAAT
CCGATCGGTTCATCGATGGTCGGCGAGTATGATGGTCTTAATGATGATCCTGCACGTATT
TCGCGTCTATCTTACTGGTGGTTTTAAAAAACCTCGCGAATTGACTTGGGTTACTGGTG
TGGTTTTGGGTGTATTGACCGCATCTTTTGGTGTAACTGGTTATTCATTACCTTGGGACC
AAATTGGTTATTGGGCAGTCAAAATTGTAACAGGCGTACCTGAAGCTATTCCTGTAATA
GGATCGTCGGTGGTAGAGTTATTGCGTGGAAGTGCTAGTGTGGGACAATCTACCCTGA
CTCGTTTTTATAGTTTACATACTTTTGTATTACCTCTCCTTACTGCCGTATTTATGTTAATG
CACTTTTCAATGATACGTAAGCAAGGCATTTCTGGCCCTTTAATGGGAGTAACAAAAAA

ACCTGACTTGAATGATCCTGTATTAAGAGCTAAATTGGCTAAAGGAATGGGTCATAATTA
TTATGGAGAACCCGCATGGCCCAACGATCTTTTATATATTTTTCCAGTAGTGATTCTGGG
TACTATTGCATGTAACGTAGGCTTAGCAGTTCTAGAACCATCAATGATTGGGGAACCCG
CGGATCCATTTGCAACCCCTTTGGAAATATTGCCCGAATGGTATTTCTTTCCTGTATTTC
AAATACTTCGTACAGTGCCAAATAAGTTATTGGGTGTTCTTTTAATGGTTTCAGTACCCG
CAGGATTAGTAACAGTACCCTTTTTGGAAAATGTTAATAAATTCCAAAATCCATTTCGCC
GTCCAGTAGCAACAACCGTTTTTTTAATTGGTACCGCAGTGTCTCTTTGGTTGGGTATT
GGAGCAACATTACCCATTGAGAAATCCCTTACTTTAGGTCTTTTTATGATTGAAGTTTTT
CTATTTGGAATCGTGTTAGGTCTAATTCCTATTACTTTGGCTGGATTATTTGTAACTGCAT
ATTTACAATACCGACGTGGTGATCAGTTGGACCTTATGCCCACTATAACTAGTTATTTTG
GTTTTCTACTAGCGGTTTTAACTATAACCTCTGGTCTATTTATCAGTCTGAGGAAGCTAC
GACTTATTATGGATATAGTAAGTCTTGCCTGGGCTGCTTTAATGGTAGTTTTTACATTTTC
CCTTTCCCTCGTAGTATGGGGAAGAAGTGGACTTATGATTATTCGTTCGCCGGAACCAG
AAGTCAAAATTTTGGTGGATAGGGATCCCATAAAAACTTCTTTCGAGGAATGGGCAAA
ACCCGGCCATTTCTCAAGAACAATAGCTAAGGGACCTGATACTACTACTTGGATCTGGA
ACCTACATGCTGATGCTCATGATTTCGATAGCCATACTAGTGATTTGGAGGATATTTCCC
GAAAAATATTTAGTGCCCATTTCGGGCAACTCTCCATCATCTTTCTTTGGCTGAGTGGCA
TGTATTTCCACGGTGCTCGTTTTTCCAATTATGAAGCATGGCTAAGCGATCCTACTCACA
TTAGACCCAGTGCTCAGGTGGTTTGGCCAATAGTGGGCCAAGAAATATTGAATGGTGAT
GTGGGCGGGGGTTTCCGAGGAATACAAATAACCTCGGGTTTTTTTCAGATTTGGCGCG
CCTCTGGAATAACTAGTGAATTACAACTCTATTGTACCGCAATTGGTGGGTTGGTCTTTG
CAGCCTTAATGCTTTTTGCTGGGTGGTTTCATTATCACAAAGCTGCTCCAAAATTGGCT
TGGTTTCAAGATGTGGAATCCATGTTGAATCACCATTTGGCAGGTCTACTAGGACTGGG
GTCTCTTTCTTGGGCGGGGCATCAAGTACATGTATCTTTACCGATTAACCAATTTCTAAA
TGCTGGAGTAGATCCCAAAGAGATTCCACTTCCTCATGAATTTATCTTAAACCGGGATC
TTTTGGCTCAACTTTATCCAAGTTTTGCCGAGGGAGCAACCCCATTTTTCACTTTGAAT
TGGGCAAAGTACGCGGATTTTCTTACTTTTCGCGGAGGATTGGATCCAGTAACTGGTGG
TCTGTGGCTGACCGATATTGCACATCATCATTTAGCTATTGCCATTCTTTTCTTGATAGCG
GGTCACATGTATAGGACCAACTGGGGTATCGGTCATGGTATAAAAGATATTTTAGAGGC
TCATAAAGGTCCATTTACAGGACAGGGGCATAAAGGTCTATATGAGATTCTAACAACTT
CATGGCATGCTCAATTATCTATTAACCTAGCTATGTTAGGCTCTTTGACCATTATTGTAGC
TCACCATATGTATTCTATGCCCCCTTATCCATACCTAGCGACTGACTATGGTACACAATTG
TCATTGTTCACGCATCACATGTGGATTGGTGGATTTCTCATAGTTGGTGCTGCTGCACAT
GCAGCCATTTTTATGGTAAGAGACTATGATCCAACGACTCGATACAACGATCTATTAGAT
CGTGTCCTTAGGCATCGCGATGCAATCATATCACATCTAAACTGGGTGTGTATATTTTTA
GGCTTTCATAGTTTTGGTTTGTATATTCATAATGATACCATGAGTGCGTTAGGGCGTCCC
CAAGATATGTTTTCAGATACAGCTATACAATTGCAACCCATTTTTGCTCAATGGATACAA
AATACCCACGCTTTAGCACCGGGCATAACGGCCCCTGGTGCAACAACAGGTACCAGTT
TGACTTGGGGGGGTGGTGATTTAGTGGCAGTAGGCAACAAAGTCGCTTTATTACCTATT
CCTTTAGGAACTGCGGATTTTTTGGTCCATCATATTCATGCATTTACAATTCATGTGACG
GTATTGATACTCCTAAAAGGTGTTCTATTTGCTCGTAGCTCAAGATTGATACCAGATAAA
GCAAATCTTGGTTTTCGTTTCCCTTGTGATGGACCAGGAAGAGGGGGAACATGCCAAG
TATCCGCTTGGGATCATGTCTTCTTAGGACTATTTTGGATGTACAATGCAATTTCAGTAG
TAATATTCCATTTCAGTTGGAAAATGCAGTCAGATGTTTGGGGGAGTATAAGCGATCAA

GGGGTAGTAACTCATATCACAGGAGGAAACTTTGCGCAAAGTTCCATTACTATTAATGG
GTGGCTCCGCGATTTCTTATGGGCACAGGCATCTCAGGTAATTCAGTCTTATGGTTCGTC
ATTATCTGCATATGGTCTTTTTTTCTTGGGTGCCCACTTTGTATGGGCTTTTAGTTTAATG
TTTCTATTCAGCGGGCGTGGTTATTGGCAAGAACTTATTGAATCCATCGTTTGGGCTCAT
AATAAATTAAAAGTTGCTCCTGCTACTCAGCCGAGAGCCTTGAGCATTGTACAGGGCC
GGGCTGTAGGAGTAACACATTACCTTCTGGGTGGAATTGCCACAACGTGGGCATTCTTC
TTAGCAAGAATTATTGCAGTAGGAATGGCATTAAGATTTCCAAGGTTTAGCCAAGGTTT
AGCTCAGGACCCCACTACTCGTCGTATTTGGTTTGGTATTGCTACCGCACATGACTTTG
AGAGTCATGATGATATTACTGAGGAACGTCTTTATCAGAATATTTTTGCTTCTCATTTCG
GACAATTAGCAATAATTTTTCTGTGGACTTCCGGGAATCTGTTTCATGTAGCTTGGCAA
GGAAATTTTGAGGCATGGGTACAGGATCCTTTACATGTAAGACCTATTGCTCATGCAAT
TTGGGATCCTCATTTTGGTCAACCGGCTGTAGAAGCTTTTACTCGAGGGGGTGCTCTTG
GTCCAGTGAATATAGCCTATTCTGGTGTTTATCAGTGGTGGTATACTATTGGTTTACGTAC
TAATGGAGATCTTTATAGTGGAGCTCTTTTTCTATTATTTCTTTCTGCCATATCTTTACTAG
CGGGCTGGTTACACCTACAACCGAAATGGAAACCTAGCGTTTCATGGTTTAAAAATGC
CGAATCCCGCCTCAATCATCATTTGTCAGGACTCTTTGGAGTTAGTTCCTTGGCTTGGA
CAGGACATTTAGTCCATGTCGCTATTCCGGGATCTAGGGGAGAATACGTTCGATGGAAT
AATTTTTTAAGTATATTGCCTCACCCCCAAGGATTAGGTCCACTTTTTACAGGTCAGTGG
AATCTTTATGCTCAAAACCCCGATTCCAGTAGTCATTTATTTGGTACCTCCCAAGGGGCT
GGAACTGCTATTTTAACACTTCTCGGGGGATTCCATCCACAAACGCAAAGTTTATGGCT
AACCGATATGGCACACCATCATTTAGCTATTGCCATTCTGTTTCTACTTGCCGGTCATATG
TATAGAACTAACTTCGGTATTGGGCACAGTATAAAGGATCTTTTAGAAGCACATATTCCT
CCCGGGGGTAGATTAGGGCGTGGACATAAGGGTCTTTATGACACAATCAATAATTCAAT
TCATTTTCAATTAGGCCTTGCCTTAGCCTCTTTAGGGGTGATTACTTCTTTGGTAGCTCA
ACACATGTATTCTTTACCCGCTTATGCGTTTATAGCGCAAGACTATACTACTCAAGCTGC
GTTATATACTCATCATCAATACATCGCAGGATTCATTATGACAGGGGCTTTTGCTCACGG
AGCTATCTTTTTTATTCGAGATTACAATCCTGAGCAGAATGAAGATAATGTATTGGCAAG
AATGTTAGATCACAAGGAAGCTATCATATCCCATTTAAGTTGGGCCAGTCTATTTCTGGG
GTTCCATACTTTGGGACTTTATGTCCATAATGATGTTATGCTTGCTTTTGGTACTCCGGA
GAAACAAATCTTGATTGAACCCATATTTGCCCAATGGATACAATCTGCTCATGGGAAAA
CTTCATATGGGTTCGATGTACTTTTATCTTCAACGAATAGTCCGGCGTTCAATGCGGGGC
GAAGCATATGGTTGCCTGGTTGGTTAAACGCTATTAATGAG------AATAGTAATTCTCTAT
TCTTAACAATAGGACCCGGCGACTTCTTGGTTCATCATGCTATTGCTCTTGGTTTACATA
CAACTACATTGATCTTAGTAAAAGGTGCTTTGGATGCGCGTGGTTCCAAATTAATGCCG
GATAAAAAAGATTTTGGTTATAGTTTTCCTTGCGATGGTCCGGGACGGGGCGGTACTTG
TGATATTTCGGCCTGGGACGCTTTTTATTTGGCAGTTTTCTGGATGTTAAATACGATTGG
ATGGGTTACTTTTTATTGGCATTGGAAGCACATTACACTGTGGCAGGGTAATGTTTCAC
AGTTTAATGAATCTTCGACCTATTTAATGGGATGGTTAAGAGATTATCTATGGTTAAACT
CTTCACAACTTATCAATGGATATAACCCTTTTGGTATGAATAGTTTATCAGTCTGGGCGT
GGATGTTTTTATTTGGACATCTTGTTTGGGCTACTGGATTTATGTTCTTAATTTCCTGGCG
CGGATATTGGCAGGAATTAATTGAAACTTTAGCATGGGCTCATGAACGCACACCTTTGG
CTAATTTGATTCGATGGAGAGATAAGCCAGTTGCTCTTTCCATTGTGCAAGCAAGGTTG
GTTGGATTAGCCCACTTTTCGGTAGGTTATATATTTACTTATGCGGCTTTCTTGATTGCCT
CTACATCGGGTAAATTTGGTATGTCACATTCAGTAAAAATTTATGATACATGTATAGGAT

GTACTCAATGTGTCCGAGCCTGTCCTACAGATGTATTAGAAATGATACCTTGGGACGGA
TGTAAAGCTAAGCAAATAGCTTCTGCCCCAAGAACAGAAGACTGTGTTGGTTGTAAAA
GGTGCGAATCCGCTTGTCCGACGGATTTCTTAAGTGTTAGAGTTTATTTATGGCATGAA
ACAACTCGAAGTATGGGTCTAGCTTATATGACA------AACTTACCTTCTATTTTTGTGCCT
TTAGTGGGCCTAGTATTTCCGGCAATGGCAATGGCTTCTTTATTTCTTTATCTTCAAAAA
AACAAGATTTTTATGCGAGATCTAAAAACATATCTCTCCGTGGCACCAGTGGTAAGTAC
TCTATGGTTCGCGGCTTTAGCAGGTCTCTTGATAGAGATCAATCGTTTATTCCCGGATGC
ACTAATATTCCCCTTTTTTTCATTCATGACTGCAATTTTAGAGAGACGCGAGAGCGAAA
ACCTATGGGGTCGCTTCTGTAACTGGATAACTAGCACTGAAAACCGTCTTTACATTGGA
TGGTTTGGTGTTTTGATGATCCCTACCTTATTGACCGCAACTTCTGTATTTATTATCGCTT
TCATTGCTGCCCCTCCGGTAGATATTGATGGTATTCGTGAACCTGTTTCTGGATCTCTAC
TTTACGGAAACAATATCATTTCTGGTGCCATTATTCCTACTTCTGCGGCTATCGGTTTGC
ACTTTTACCCGATCTGGGAAGCAGCATCTGTTGATGAATGGTTATACAATGGCGGTCCT
TATGAACTAATTGTTCTACATTTCTTACTTGGTGTAGCTTGTTACATGGGTCGTGAGTGG
GAACTTAGTTTTCGTCTAGGTATGCGCCCTTGGATTGCTGTTGCATATTCAGCTCCTGTT
GCAGCCGCTACTGCTGTTTTCTTGATCTATCCGATTGGTCAAGGAAGCTTTTCTGACGG
TATGCCTCTAGGAATCTCTGGTACTTTCAACTTTATGATAGTATTTCAGGCTGAGCATAA
CATCCTTATGCATCCATTTCACATGTTAGGTGTAGCTGGTGTATTCGGCGGCTCCCTATTT
AGTGCTATGCACGGTTCCTTGGTAACTTCTAGTTTGATCAGGGAAACCACAGAAAATG
AATCTGCTAATGAAGGTTACAGATTCGGTCAAGAGGAAGAAACCTATAATATTGTAGCT
GCTCACGGTTATTTTGGCCGATTGATCTTCCAATATGCAAGTTTCAACAACTCTCGTTCT
TTACACTTCTTCTTAGCTGCTTGGCCTGTTGTAGGTATCTGGTTTACTGCGTTAGGTATC
AGCACTATGGCTTTCAATTTGAATGGTTTCAATTTCAACCAATCTGTAGTTGATAGTCAA
GGTCGTGTAATTAACACCTGGGCTGATATTATTAACCGAGCTAACCTTGGTATGGAAGTT
ATGCATGAACGTAATGCTCATAACTTCCCTCTAGACCTAGCTGCGGTCGAGGCTCCATC
TATAAATGGAATGGGTTTGCCTTGGTATCGTGTTCATACCGTTGTATTAAATGATCCCGG
CCGTTTGCTTGCTGTCCATATAATGCATACAGCTCTGGTTGCTGGTTGGGCTGGTTCAAT
GGCTCTATATGAATTAGCAGTTTTTGATCCTTCTGATCCCGTTCTTGACCCAATGTGGAG
ACAGGGTATGTTCGTTATACCCTTCATGACTCGTTTAGGAATAACCAATTCGTGGGGCG
GTTGGAATATTACA---GGGGGAACTATAACGAATCCAGGTATTTGGAGTTATGAAGGTG
TGGCCGGGGCGCATATTGTGTTTTCGGGCTTGTGCTTTTTGGCCGCTATCTGGCATTGG
GTCTATTGGGATTTAGAAATCTTTTCTGATGAACGTACTGGAAAACCTTCTTTAGATTTG
CCAAAAATCTTTGGAATTCATTTATTTCTTGCAGGGTTGGCTTGTTTTGGGTTTGGCGCA
TTTCATGTAACTGGATTGTATGGTCCTGGAATATGGGTGTCCGATCCTTATGGACTAACC
GGAAGGGTACAACCCGTAAATCCCGCATGGGGTGTGGAAGGTTTTGATCCCTTTGTTC
CGGGAGGCGTAGCCTCTCATCATATTGCAGCAGGGACATTGGGTATATTAGCGGGCCTT
TTCCATCTTAGTGTGCGCCCACCCCAACGTCTATATAAAGGATTACGTATGGGAAATATT
GAAACCGTCCTTTCCAGTAGTATCGCTGCTGTCTTTTTTGCAGCTTTTGTTGTTGCTGG
AACTATGTGGTATGGTTCAGCAACTACCCCGATTGAATTATTTGGTCCCACTCGTTATCA
ATGGGATCAGGGATACTTCCAGCAAGAAATATATCGAAGAGTTGGTGCTGGGCTAGCT
GAAAATCAAAGTTTCTCAGAAGCTTGGTCTAAAATTCCCGAAAAATTGGCTTTTTATGA
TTACATCGGCAATAATCCGGCAAAAGGAGGTTTGTTTAGAGCGGGTTCAATGGACAAT
GGAGATGGAATAGCCGTCGGTTGGTTAGGACATCCTATCTTTAGGGATAAAGAGGGGC
GTGAACTTTTTGTACGTCGTATGCCTACCTTTTTTGAAACATTTCCGGTTGTTTTGGTAG

ACGGAGACGGAATTGTTAGAGCCGATGTTCCTTTTCGAAGGGCAGAATCGAAATATAG
TGTCGAACAAGTAGGCGTAACTGTTGAATTCTATGGTGGCGAACTCAATGGAGTCAGT
TATAGTGATCCCGCTACTGTCAAAAAATATGCCAGACGTGCTCAATTGGGTGAAATTTT
TGAATTAGATCGTGCTACTTTGAAATCTGATGGTGTTTTTCGTAGCAGTCCGAGGGGTT
GGTTTACTTTTGGACATGCTTCGTTTGCTCTGCTGTTCTTTTTCGGGCACATTTGGCATG
GTTCTAGAACCTTGTTTAGAGATGTTTTTGCCGGTATCGACCCAGATTTAGATTCTCAAG
TCGAATTTGGAGCATTCCAAAAACTTGGAGATCCAACCACAAGAAGACAGGTAGTCAT
GAAAACCTTATATTCCCTGAGGAGGTTCTACCACGTGGAAACGCTCTTTAATGGAACTT
TAGCTTTAACCGGTCGTGACCAAGAAACTACCGGTTTTGCTTGGTGGGCCGGGAATGC
CCGACTTATCAATTTATCTGGTAAACTTCTGGGGGCCCATGTAGCCCATGCTGGATTAAT
AGTATTCTGGGCCGGAGCAATGAACCTATTTGAAGTGGCTCATTTTGTACCCGAGAAGC
CCATGTATGAACAAGGCTTAATTTTACTTCCCCATCTAGCTACTCTAGGTTGGGGGGTCG
GTCCTGGGGGAGAGGTTATAGACACCTTCCCATACTTTGTTTCTGGAGTACTTCACTTA
ATTTCCTCTGCGGTATTAGGATTTGGCGGTATTTATCATGCACTTCTGGGACCTGAGACT
CTTGAAGAATCTTTTCCATTTTTCGGTTATGTATGGAAAGATAGAAATAAAATGACTACC
ATTTTGGGTATTCACTTAATCTTGTTAGGTATAGGGGCTTTTCTTCTAGTATTCAAGGCTT
CTTATTTTGGGGGTATATATGATACTTGGGCTCCCGGGGGAGGAGATGTAAGAAAAATT
ACCAATTTGACCCTTAGTCCAAGTATTATATTTGGTTATTTACTAAAATCACCTTTTGGGG
GAGAGGGGTGGATTGTTAGTGTGGACGATTTGGAAGACATAATTGGGGGACATGTATG
GTTGGGTTCCATTTGTATACTTGGTGGAATCTGGCATATTTTAACAAAACCCTTTGCATG
GGCTCGGCGTGCACTAGTATGGTCTGGAGAAGCTTACTTGTCTTATAGTTTAGCAGCTT
TATCCGTTTTTGGTTTTATTGCTTGTTGCTTTGTCTGGTTTAATAATACCGCTTATCCTAGT
GAGTTTTATGGACCCACTGGACCAGAAGCTTCTCAAGCTCAAGCATTTACTTTTCTAGT
TAGAGACCAACGTCTTGGGGCTAATGTAGGATCCGCTCAAGGGCCTACCGGTTTAGGT
AAATACCTAATGCGTTCTCCGACCGGAGAAGTCATTTTTGGAGGAGAAACTATGCGGTT
TTGGGATCTTCGTGCTCCTTGGTTGGAACCTCTAAGGGGTCCAAATGGTTTGGACTTGA
GTAGACTCAAAAAAGACATACAGCCTTGGCAAGAACGTCGTTCCGCGGAATATATGAC
TCACGCTCCTTTAGGTTCCTTAAATTCCGTGGGGGGTGTAGCTACGGAGATCAATGCAG
TCAATTACGTTTCTCCTAGAAGTTGGTTAGCTACTTCTCACTTTGTTCTAGGATTCTTTCT
ATTCGTGGGTCATTTATGGCACGCTGGAAGAGCTCGCGCAGCTGCCGCGGGATTTGAA
AAAGGAATTGATCGAGATTTTGAACCTGTTCTTTCCATGACTCCACTTAATATGACTATA
GCTCTTGGTAAATTTACCAAAGATGAAAATGATTTATTTGATATTATGGATGACTGGTTA
CGTCGGGACCGTTTCGTTTTTGTGGGTTGGTCCGGTCTATTGCTCTTCCCTTGTGCCTAT
TTCGCCTTGGGGGGTTGGTTCACAGGTACCACCTTTGTAACTTCATGGTATACTCATGG
ATTGGCAAGTTCCTATTTGGAGGGCTGTAATTTCTTAACCGCTGCAGTTTCGACCCCTG
CTAATAGTTTAGCACACTCTTTGTTGTTACTATGGGGTCCTGAAGCACAGGGAGATTTT
ACCCGTTGGTGTCAATTAGGAGGTCTGTGGACTTTTGTTGCTCTCCACGGTGCTTTCGG
ATTAATAGGTTTCATGTTACGTCAATTTGAACTCGCTCGATCTGTTCAATTACGGCCTTAT
AATGCAATTGCATTTTCTGGTCCAATTGCTGTTTTTGTTTCTGTATTCCTGATTTATCCAC
TAGGTCAGTCTGGTTGGTTCTTTGCACCTAGTTTTGGTGTAGCAGCTATATTTCGATTCA
TCCTCTTTTTCCAAGGGTTTCATAATTGGACATTGAACCCATTTCATATGATGGGAGTTG
CAGGTGTATTGGGCGCTGCCCTACTATGCGCTATTCATGGTGCTACCGTGGAAAATACAT
TATTTGAAGATGGTGATGGCGCAAATACATTCCGTGCTTTTAACCCAACCCAAGCTGAA
GAAACTTATTCGATGGTCACCGCTAACCGTTTTTGGTCTCAAATCTTTGGGGTTGCTTTT

TCCAATAAACGTTGGTTACATTTCTTTATGTTATTTGTACCAGTAACTGGTTTGTGGATG
AGTGCTCTTGGAGTAGTCGGTCTGGCCCTGAACCTACGTGCCTATGATTTCGTTTCTCA
AGAAATACGCGCAGCGGAAGATCCTGAATTTGAGACTTTCTACACAAAAAATATTCTCT
TAAACGAAGGTATTCGTGCGTGGATGGCAGCTCAAGATCAGCCTCATGAAAACCTTATA
TTCCCTGAGGAGGTTCTACCACGTGGAAACGCTCTTATGTCTGGAAGCACAGGAGAAC
GTTCTTTCGCTGATATTATTACCAGTATTCGATACTGGATTATTCATAGCATTACTATACCC
TCCCTATTCATCGCGGGTTGGTTATTTGTCAGCACGGGTTTAGCTTACGATGTATTTGGA
AGTCCCCGTCCAAACGAGTATTTTACAGAGAGCCGACAAGGAATTCCTTTAATAACAG
GCCGTTTTGATCCTTTGGAACAACTCGATGAATTTAGTAGATCTTTTATGACCATAGATA
GGACCTTTCCAATTTTTACAGTACGATGGTTAGCTGTTCACGGACTAGCGGTACCCACC
GTTTCTTTTTTGGGATCAATATCAGCAATGCAGTTCATCCAACGAATGGCTACACAAAC
CGTTGAGGATAGTTCTAGA---GCTCGTCCAAGACAAACTTCTGTAGGGAGTTTATTGAA
ACCGTTGAATTCAGAATATGGTAAAGTCGCTCCGGGGTGGGGAACTACTCCTTTGATGG
GTATTGCAATGGCTCTATTTGCAATATTTCTATCTATTATTTTGGAAATTTATAATTCTTCT
ATTTTACTAGACGGAATTTCTATGAATATGCTTACTTTAAAATTATTTGTTTACACAGTAG
TGATATTCTTTGTTTCGCTTTTCATCTTTGGATTCTTATCTAACGACCCAGGACGTAATCC
AGGGCGTGAAGAGATGGCCGATACTACTGGAAGGATTCCTCTTTGGATAATAGGTACTG
TAACTGGTATTACTGTGATCGGTTTAATTGGTATTTTTTTTTATGGTTCGTATTCCGGATT
GGGATCATCCCTGATGATTAATATCGTTAGCTTGGTCTGTATTTATATTAACTCTGTCCCT
TATTCAAGTATTTTTTTCTTGGATAAATTGCCCGAGGCCTACGCTTTTTTGAATCCAATT
GTAGATATTATGCCAGTAATACCCCTATTCTTTTTTCTTTTAGCTTTTGTTTGGCAAGCCG
CTGTAAGTTTTCGAACGACACAATCAAACCCAAATGAACAAAGTGTTGAATTGAATCG
TACCAGTCTTTACTGGGGGTTATTACTCATTTTTGTGCTTGCTGTTTTATTTTCAAATTAT
TTCTTCAATATGGAAGTAAATATTCTCGCATTTATTGCTACTGCGCTCTTCATTCTAGTTC
CTACAGCCTTTTTACTAATAATTTACGTAAAAACGGTAAGTCAAAGTGACATGGAAACA
GCAACCCTAGTTGCCATCTCTATATCTGGTTTACTTGTAAGTTTTACCGGGTATGCCTTAT
ATACCGCTTTTGGGCAACCCTCGCAACAACTAAGAGATCCATTCGAAGAACATGGGGA
CATGGAAGCATTGGTTTATACATTCCTCTTAGTCTCGACTTTAGGAATCATTTTTTTCGCT
ATCTTTTTTAGAGAACCTCCCAAAGTTCCAACTAAAAAA------ATGACTATCGCTTTCCA
ATTAGCTGTTTTTGCATTAATTGCTACTTCATCCATTTTATTGATTAGTGTACCTGTTGTAT
TTGCTTCTCCTGATGGTTGGTCGAGTAATAAAAATGTTGTATTTTCTGGTACATCATTATG
GATTGCATTAGTATTCCTAGTGGGTATTCTTAATTCTCTCATCTCC---------------------ATGT
CACCACAAACAGAAACTAAAGCAAGTGTTGGGTTCAAAGCTGGTGTTAAAGATTATAA
ATTGACTTATTATACTCCTGACTATGAAACCAAAGATACTGATATCTTAGCAGCATTCCG
AGTAACTCCTCAACCTGGAGTTCCACCTGAAGAAGCAGGTGCTGCGGTAGCTGCCGAA
TCTTCTACTGGTACATGGACAACCGTGTGGACCGATGGGCTAACCAGTCTTGATCGTTA
CAAAGGACGATGCTACCACATCGAGCCTGTTCCTGGAGAAGAAAGTCAATTCATTGCT
TATGTAGCTTATCCTTTAGACCTTTTTGAAGAAGGTTCTGTTACTAACATGTTTACTTCCA
TTGTAGGTAATGTATTTGGGTTCAAGGCTCTTCGCGCGCTACGTCTGGAGGATTTGCGA
ATTCCTAATGCTTATGTTAAAACGTTCCAAGGTCCTCCTCACGGAATCCAAGTGGAAAG
AGATAAATTGAACAAGTATGGTCGTCCTCTATTGGGATGTACTATTAAACCTAAATTGGG
GTTATCCGCTAAGAATTACGGTAGAGCAGTTTATGAATGTCTTCGCGGGGGACTTGATT
TTACTAAAGATGATGAAAATGTGAACTCTCAACCATTTATGCGTTGGAGAGACCGTTTC
TTATTTTGTGCTGAAGCGCTTTTTAAAGCACAGGAAGAAACAGGTGAAATCAAAGGGC

ATTACTTGAATGCTACTGCCGGTACATGCGAAGAAATGATGAAAAGAGCGGTATTTGCC
AGAGAATTGGGCGTTCCTATCGTAATGCACGATTATTTAACTGGGGGATTCACTGCAAA
TACTACCTTGGCTCACTATTGCCGGGATAATGGTCTACTTCTTCATATCCACCGTGCAAT
GCACGCAGTTATCGATAGACAGAAAAATCATGGTATGCACTTCCGTGTACTAGCTAAAG
CGTTACGTATGTCTGGTGGAGATCATATTCACGCCGGTACTGTAGTAGGTAAACTTGAA
GGGGAAAGGGAAATCACTTTAGGTTTTGTTGATTTACTACGTGATGATTTTGTTGAAAA
AGATCGAAGTCGCGGTATTTATTTCACTCAGGATTGGGTTTCTTTACCAGGTGTTCTACC
TGTTGCTTCGGGAGGTATTCACGTTTGGCATATGCCTGCTCTGACCGAAATCTTTGGAG
ATGATTCTGTACTCCAATTCGGTGGAGGAACTTTAGGACACCCTTGGGGAAATGCACCT
GGTGCTGTTGCTAATCGAGTAGCTCTTGAAGCGTGTGTACAAGCTCGAAACGAGGGAC
GTGATCTTGCTCGTGAGGGTAATGAAATTATCCGTGAGGCGTCCAAATGGAGTCCTGAA
TTAGCTGCTGCTTGTGAAATATGGAAGGAGATCAAATTTGAATTCCAAGCAATGGATAC
TTTG------ATGGCGATACATTTATACAAAACTTCTACCCCGAGCACACGCAATCGAGCCG
TAGACAGTCAAGTGAAATCCAATCCACGAAATCGTTTGATCTATGGACTACATTGTTGT
AGTAAAGGCCGTAATGCCAGAGGAATCATTACCGCAGGGCATAGGGGGGGGGTCATA
AGCGTCTATACCGTAAAATCGATTTTCGACGGAATGAAAAAAACATATATGGGAAAATC
GTAACCATAGAATACGACCCTAATCGAAATGCATACATTTGTCTCATACACTATGGGGAT
GGTGAGAAAAGATATATTTTACACCCCAGAGGGGCTATAATTGGAGATACCATTGTTTC
TGGTACAGAAGTTCCTATAAAAATGGGAAATGCCCTACCTTTGACCGATATGCCCTTAG
GCACGGCCATACATAACATAGAAATCACATTCGGAAAGGGTGGAAAATTAGCTAGAGC
AGCAGGTGCTGTAGCGAAACTGATTGCAAAAGAGGGGAAATCGGCAACATTAAAATTA
CCTTCTGGGGAGGTCCGTTTGATATCCAAAAACTGCTCAGCAACAGTCGGACAAGTAG
GAAATGTTGGGGTAAACCAGAAAAGTTTGGGCAGGGCCGGATCGAAATGTTGGCTAG
GTAAGCGTCCTGTAGTAAGAGGAGTGGTTATGAACCCTGTAGACCACCCCCATGGGGG
GGGTGAAGGGAGGGCCCCCATTGGTAGAAAAAAACCCGTAACTCCTTGGGGTTATCCT
GCACTTGGAAGAAGAAGTAGAAAAAGGAAGAAATATAGTGATAATTTGATTCTTCGTC
GCCGTACTAAAATGATTCAACCTCAAACCCATTTAAATGTAGCCGATAATAGCGGAGCC
CGCAAATTGATGTGTATTCGAATCATAGGAGCTAGT---AATCGACGATACGCTTATATTGG
TGACATTGTTGTTGCTGTAATCAAAGAAGCAGTACCGAATACACCCTTAGAAAGATCA
GAAGTGATCAGAGCTGTAATTGTACGTACTTGTAAAGAACTCAAACGTAGCAATGGTAT
AATAATACAGTATGATGATAATGCTGCAGTTGTCATTGATCAAGAAGGAAATCCAAAAG
GAACTCGAATTTTTTGCGCAATCGCCCGGGAATTGAGACAGTTCAATTTCACTAAAATA
GTTTCATTAGCGCCTGAGATATTAATGCTTAGTCCAAAAAGAACCAGATTCCGTAAACA
ACATAGAGGAAGAATGAAAGGAATATCCCATCGAGGTAATCAAATTTGCTTTGGAAGAT
ATGCTCTTCAGGCACTTGAACCAGCTTGGATAACATCTAGACAAATAGAAGCGGGGCG
GAGGGCAATGTCACGAAATGTGCGCCGAGGTGGACAAATATGGGTACGCATATTTCCG
GATAAACCTGTTACAGTAAGACCTACCGAAACGCGTATGGGTTCGGGAAAGGGATCTC
CCGAATATTGGGTAGCCGTAGTTAAACCCGGAAAAATAGTTTATGAAATGGGCGGAGTC
GCAGAAAATATAGCCAGAAAAGCTATTTCAATAGCAGCATCCAAAATGCCTATACGAAC
TCAATTCATTATTTCAGGAATGACCAGAATTAAACGAGGATATATAGCGCGAAAACGGA
GGACAAAAATTCGTTTATTTACATCAAGCTTTCGCGGAGCTCATTCAAGACTTACTCGA
ACTATTTCGCAACAAAAAATAAAAGCTTTGGTTTCGGCTCATCGGGACAGAAATCGGA
AAAAAAGAGAATTTCGAGGTTTATGGATCAGTCGAATAAATGCAGGAATTGGTGATAA
CAAAAATATATACTATAGTTATAGTAATTTTATGTATAATCTGTACAAGAAGCAATTGCTT

CTTAATCGTAAAATAGTTGCACAAATAGCTATATTCAAAGGGAATTGTCTTTTTATGATT
G C C A A C G A G A T C A T A A C A -
-???
??
??
??
???????????ATGAATGGAATCAAATATGCAGTATTTACAGACAAAAGTATTCGGTTATTGG
GGAAAAAT---CAATATACTTCTAATGTCGAATCGGGATCAACTAGGACAGAAATAAAGC
ATTGGGTCGAACTCTTCTTTGGTGTCAAGGTAAAAGCTATGAATAGTCATCGACTCCCG
GCAAAGGGTAGAAAAGTGCGACTTATTATGGGACATACAATGCATTACAGACGTATGAT
CATTACGCTTCAACCGGGTTATTCTATTCCACCTCTTAGAAAGAAAAGAACTATGGCAG
TTCCAAAAAAAGCGGACTTCTATATCAAAAAAACTCATTCGTAAGAATTTTTGGAAAAA
GAGGGGATATTGGACAGCATTGAAAGCGTTTTCATTAGCGCAATCCATTTTTACGGGAA
ATTCTAAAAGTTTTTTT------AAA---------ATGGCCAAGGGTAAAGATATCCGAATAATTGT
TATTTTGGAATGTACCGGTTGT---GATCAAAAG------GTTAATAAGGAA------GGTATTTCC
AGATATATTACTCAAAAGAATCGACACAATACGCCCAGTCGATTAGAATTAATAAAATTC
TGTCCTTGTTGTCGCAAACATATGATTCACGCAGAGATAAAGAAAATGAAAATAGGAG
CGTCTGTTCGTAAGATTTGTGAAAAATGTCGATTGATCCGAAGGCGAGGTCGGATTATA
GTAATTTGTTCCAACCCAAGACATAAACAAAGACAGGGAATGGTTCGAGAGAAAGTA
AGAGTATCTACTCGGACACTACAGTGGAAGTGTGTTGAATCAAGAATAGACAGTAAGC
GACTTTATTATGGACGCTTTATTCTGTCTCCACTTATGAAAGGCCAAGCCGATACAATAG
GCATTGCAATGCGAAGAATTTTGCTCGGAGAAATGGAGGGAACATGTATCACACGTGC
AAAATCTGAG------AAAATATCACATGAATATTCTACCATAGTAGGTATTCAAGAACCAGT
ACATGAGATTTTAATGAATTTGAAAGAAATTGTATTGAAAAGTAATCTGTATGGAATTCG
CGATGCGTCTATTTGTTTCAAAGGTCCCGGATATGTAACTGCTCAAAACATTATTTTACC
ACCTTCGGTGGTAATCGTTGATAATACACAACATATAGCTAATGTAACAGAACCAATTCA
GTTGTGTATTGGATTACAAATTGAGAGGGATCGTGGATATCGTATAAACACACTAAAAA
ATTTTCAAGATGGAAGTTAT------AATATAGATGCTATATTCATGCCTGTTCGAAATGCAA
ATCATAGTATTCATTCTTACGTGAATGGTAATGAAAAACAAGAGATCCTCTTTCTCGAAA
TATGGACAAATGGAAGTCTAACTCCTAAGGAAGCGCTTTATGAAGCCTCCCGAAATTTG
ATTGATTTATTTATTCCTTTTTTACATGCAGAAGAAGAA------AAGTTAAATTTTGAAAAC
AATGAA---CATAAAGTTACTTTACCCCTTTTTACTTGTCATGATCTATTGGCTAAAAAACT
AAAGAAAAAAAAAAAAA---GAAATAGCATTCAAATCCATTTTCATTGACCAATTAGAATT
GCCTCCCAAGATCTATAATTGTCTCAAAAAGTCCAATATACATACCTTATTGGAACTTTT
GACTAAGAGTAAGGAAGACCTTATGAAAATTGAACATTTTCGCGTAGAAGATGTAAAA
CACATATTAGACATTTTAGAAATTGAAAAGCATTTTCCA---------------------ATGCTTGGGG
TTGGAAAT---GAGGGAATGTCTACACTACCGGGGTTGAATCAAATCCAATTTGAAGGGT
TTTGTAGGTTCATTGATAGAGGCTTGACAGAAGAACTTTTTAAGTTTCCTAAAATTGAA
GATACAGATCAAGAAATTGAATTTCAATTATTTGTGGAAACTTATCAATTAGTAGAACCT
TCTATAAAAGAAAAAGATGCCGTATATGAATCACTTACATATTCTTCGGAATTATATGTAT
CCGCGGGATTAATTTGGAAAAACAGTAAGAATATCCAAGAACAAACAATTTTTATTGGA
AACATTCCTCTAATGAATTCCCTGGGAACTTCTATAGTAAATGGAATATATAGAATTGTA
ATCAATCAAATATTGCAAAGCCCCGGTATCTATTACCAGTCAGAATTGGACCATAAGGG
AATTTCGGTCTATACCGGCACAATAATATCAGATTGGGGGGGAAGATTAGAATTGGAGA

TTGATAGAAAAGCAAGGATATGGGCTCGCGTGAGTAGAAAACAGAAAATATCTATTCTA
GTTCTATCATCAGCTATGGGTTCGAATTTAAACGAAATTCTCGAAAATGTTTGCTACCCT
GAAATTTTCTTGTCTTTCCTGAATGATAAGGAAGAAAAGAAAATCGGGTCAAAAGAAA
GTGCCATTTTGGAGTTTTATCGACAATTTGCTTGTGTGGGCGGAGATCCAGTATTTCCTG
AATCCTTATGTAGGGAATTACAAAAAAAATTTTTTCAACAAAGATGTGAATTAGGCGAG
ATTGGTCGACGAAATATGAATCGAAGACTGAATCTTGATATACCTCAGAACAATACATT
TTTGTTACCTCGGGATATATTGACAGCTGCGGATCATTTGATTGGAATGAAATTTCGAAT
GGGTACGCTTGATGAT------ATGAATCATTTGAAAAATAAACGTATTCGTTCGGTAGCAG
ATCTCTTACAAGATCAATTTGGATTGGCCCTGGTTCGTTTAGAAAATATGGTTAGAGGA
ACCATATGTGGAGCAATTAGA---TATAAATTGATA---CCGACTCCTCAGAATTTGGTGACT
TCAACGCCA------------TTAACAACCACTTATGAATCTTTTTTCGGCTTACATCCATTATCT
CAAGTTTTGGATCGAACCAATCCATTGACCCAAATAGTTCATGGGAGAAAATTGAGTTA
TTTGGGTCCTGGAGGATTGACGGGGCGAACTGCTAGTTTTCGAATACGAGATATCCATT
CTAGTCACTATGGACGCATTTGTCCAATTGACACCTCTGAGGGAATCAATGTTGGACTT
ATTGGATCCCTCGCAATTCATGCGAGGATTGGTCGTTGGGGG---TCCATAGAAAGTCCA
TTTTTT---------GAAATTTCTGAGAGATCAAAAAGA------ATACACATGCTTTATTTATCAC
CCAGTAGAGATGAATACTATATGATAGCGACAGGAAATTATTTGGCATTGAATCAGGGC
AATCAGGAGGAACAGATTGTTCCAGCTCGATATCGTCAAGAATTTCTGACTATTGCATG
GGAACAGGTTCATCTTCGAAGTATTTTCTCCTTCCAATATTTTTCTATTGGTGCCTCCCTT
ATTCCTTTTATTGAGCATAATGATGCGAATCGAGCTTTAATGAGTTCAAACATGCAACGT
CAAGCAGTTCCACTTTCTCAGTCTGAAAAGTGCATTGTTGGAACTGGATTGGAACGAC
AAGTGGCCTTAGATTCGGGAACTCTCGCTATAGCCGAACACGAGGGAAAGATCATTTAT
AAAGATACAAACAAGATTGTTTTATTTGGAAGTGGAGAGACTCTAAGCGTTCCATTAGT
TATATATCGACGTTCCAACAAAAATACTTGCATGCATCAAAAATCCCAGGTTCAGAGAG
GTAAATGCATTAAAAGGGGACAAATTTTAGCAGATGGTGCTGCTACAGTTGGCGGTGA
ACTCTCTCTGGGAAAAAACGTATTAGTAGCTTATATGCCATGGGAAGGATACAATTCTG
AGGATGCTGTACTCATTAGTGATCGTCTGGTATATGAAGATATTTATACTTCGTTTCATAT
ACGGAAATATGAAATTCAGACTCATGTGACAAGCAATGGT---CCTGAAAGAATCACTAA
TAAGATTCCACACTTAGAAGTCCATTTACTACGAAATTTAGACAAAAATGGACTTGTGA
TCCTGGGATCTTGGGTAGAAGCTGGCGATATTTTAGTGGGTAAATTAACGCCTCAAATG
GCGAAAGAA---TCCTCGTATGCCCCGGAAGATAGATTATTACGAGCTATACTTGGGATTC
AGGTATCTACCTCAAAGGAAACTTGTCTAAAATTACCTATAGGTGGTAGGGGCCGCGTT
ATTGATGTGAGATGGCTCCACAAAAAGGGG---GGTTCCGGTTATAATCCAGAAACTATT
CATATATATATATTACAGAAACGTGAAATCAAAGTAGGTGATAAAGTGGCCGGAAGACA
TGGAAATAAAGGCATTGTTTCCAAGATTTTGGCTAGACAGGATATGCCTTATTTGCAAG
ATGGAAGACCTGTTGATATGGTCTTCAACCCATTAGGGGTACCTTCACGAATGAATGTA
GGACAGATATTTGAATGCTCACTCGGATTAGCGGGGGATGTGCTAGATAGACATTATCG
AATAGCACCCTTTGATGAGAGATATGAACAAGAGGCTTCTAGAAAACTTGTGTTTTCTG
AATTATATCAAGCCAGTAAACAAACATCGAATCCATGGATATTTGAACCCGAGTATCCC
GGAAAAAGCAGAATATTTGATGGAAGAACAGGGACTCCTTTTGAACAGCCTGTTATAA
TTGGAAATCCTTATATCTTGAAATTAATCCACCAAGTTGATGATAAAATACATGGACGTT
CCAGTGGACATTATGCACTTGTTACTCAACAACCTCTTAAAGGAAGGGCCAAGCAAGG
AGGACAGCGGGTAGGTGAAATGGAAGTTTGGGCTCTTGAGGGATTCGGTGTTGCCCAT
ATTTTACAAGAGATGCTTACTTATAAATCTGATCATATTAAAGCTCGTCAAGAAGTACTA

GGGACTACGATTATTGGAGGAACAATATCTAAACCCGTAGATGCTCCAGAATCTTTTCG
ATTGCTCGTTAGAGAACTACGATCTTTGGCTCTGGAACTGAATCATTTCCTTGTCTCTG
AGAAGAACTTCCGGATTCATAGGAAGGAAGTT---------------------ATGATTGATCAGTATA
AACATCAACAACTCCGAATTGGATCAGTTTCTCCTCAACAAATAAGTGCTTGGGCAAC
AAAGATCCTACCTAATGGAGAGATAGTTGGAGAGGTAACAAAACCCTATACTTTTCATT
ACAAAACCAATAAGCCCGAAAAAGATGGATTATTTTGTGAAAGAATTTTTGGACCTATC
AAAAGTGGGATTTGTACTTGTGGAAATTATCGAGTAATTGGAGATAAAAAAGACGAAC
CCAAATTTTGTGAACAATGCGGGGTAGAATTTGTGGATTCTCGGATACGTAGATATCAA
ATGGGCTATATAAAACTAGCATGCCCGGTAACCCATGTGTGGTATTTGAAGCGTCTTCCT
AGTTATATCGCGAATCTTTTAGATAAACCTCTTAAAGAATTAGAAGGTCTAGTATATTGC
GATTTT------TCGTTTGCTAGACCC---GTAGTTAAAAAACCTACTTTCTTACGATTACGAG
GTTTATTCGAATATGAAATCCAATCCTGGAAATACAGCATTCCACTCTTTTTCGCTACCC
ACGGTTTCGATACATTTCGAAATCGCGAAATATCTAGCGGCGCGGGTGCTATCCGAGAA
CAATTAGTCGATCTGGATTTGAGAATTGTTATTGATTCTTCATTGGTAGAGTGGAAAGAA
TTAGGGGAAGAG---AGATCCACGCACAATGAATGGGAAGACCGAAAGGTTGGAAGAA
GAAAGAATTTTTTGGTTCGGCGCATGGAATTAGCTAAACATTTTATCCGAACAAATATA
GAACCGGAATGGATGGTTTTATGTCTCTTACCAGTTCTTCCTCCCGAATTGAGACCTATT
ATTCAAATAGATGGGGGTAAACTAATGAGTTCGGATATTAATGAACTCTATAGAAGAGTT
ATTTATCGGAACAATACTCTTATCGATCTATTGACAACAAGTAGATCTACGCCGGGAGAA
TTAGTAATGTGTCAAGAAAAAATTGGTACAAGAAGCCGTCGATACACTTCTTGATAATGG
AATCCGGGGACAGCCAATGAAGGACGGTCATAATAAGGTTTACAAATCATTTTCAGATA
TAATTGAAGGGAAAGAGGGAAGATTTCGCGAGACTCTGCTTGGCAAACGAGTTGATTA
TTCGGGGCGTTCTGTCATTGTTGTAGGTCCATCACTTTCATTACATCGATGTGGATTGCC
CCGTGAAATAGCAATAGAGCTTTTCCAGACATTTCTAATTCGTAGTTTAATTCGAAAAC
ACTTTGCTTCGAACATAGGGGTTGCTAAGAGTAAAATTCGGGAAAAAGAGCCGATTGT
ATGGGAAATACTTCAAGAAGTTATGCGGGGACATCCTATATTGCTGAATAGAGCACCCA
CTTTGCATAGATTAGGGATACAAGCCTTCCAACCTATTTTAGTGGAAGGACGTGCTATTT
GTTTACATCCATTAGTGTGTAAGGGGTTCAATGCAGACTTTGATGGGGATCAAATGGCT
ATTCATGTCCCTTTATCTTTGGAGGCTCAAGCAGAAGCTCGTTTACTTATGTTTTCTCAT
ACGAACCTTTTGTCTCCGGCTATTGGGGATCCCATTGCCGTACCAACTCAAGATATGCTT
ATTGGACTTTACATATTAACGAGCGGAAATCGTCGAGGTATTTGTGCAAATAGGTATAAT
CGAAGTAATTGG---------AAAAATTCTAAAAATGAAAAAATTCGCGATAAG------AAATAT
ATGAAAAAA---GAACCTTTTTTTTCTAATTCCTATGATGCAATTGGAGCTTTTCGACAGA
AAAAAATCAATTTTGATAGCCCTTTTTGGCTCCGGTGGCGATTAGATAAATGC---ATTAT
GTCGTCA------AGAGAGGCTCCTATCGAAGTTCACTATGAATCTTTGGGTACCTATCATG
ATATTTATGAACATTATTTAGTAATAAGAAGTATAAAAAAACAAATTTGTTGCATATACAT
TCGAACTACTATTGGTCATATTTCTTTTTATAGAGAAATCGAAGAAGCTATACAAGGCTT
TTGTCGAGGCTATTCGTATGGTATC------------------------------------ATGGCAGAACGGGCCA
ATCTAGTCTTTCATAATAAAGTAATAGGTGGAACTGCCATAAAACGAATTATTAGTAGAT
TAATAGATCATTTCGGAATGGCGTATACATCACATATTCTGGATCAAGTAAAGACTCTTG
GGTTCCGTCAAGCTACTACGACATCTATTTCATTAGGAATTGATGACCTTTTAACAATAC
CTTCTAAGGGATGGTTAGTCCAAGATGCTGAACAACAAAGTTTGATTTTGGAAAAACA
TCATCATTATGGGAATGTACATGCGGTAGAAAAATTACGTCAATCCATTGAGATATGGTA
TGCTACAAGTGAATATTTGCGACAAGAAATGAATCCCAATTTTAGTATGACTGACCCCT

TTAATCCAGTCCATATAATGTCTTTTTCGGGAGCTAGGGGAAATGCATCTCAAGTGCAC
CAATTAGTAGGTATGAGAGGATTAATGTCGGATCCCCAGGGCCAAATGATTGATTTACC
CATTCAAAGCAATTTACGTGAAGGACTTTCTTTAACAGAATATATTATTTCTTGTTATGG
AGCCCGCAAGGGGGTTGTGGATACTGCTGTACGAACATCAGATGCTGGATATCTTACAC
GGAGACTTGTTGAAGTGGTTCAACACATTGTTGTACGCCGAACAGATTGCGGTACCGT
TCGAGGGATTTCTGTAAATACTCGGAATAGAATGATGTCA---GAAAGAATTTTGATACA
AACATTAATTGGTCGTGTATTAGCAGACGATATATATATAGGGTCGCGATGCATTGTCGTT
AGAAATCAAGATATTGGAATTGGACTTATCAATCGATTCATAAAC------------------TTTCAA
ACACAACCAATATTTATCCGAACCCCCTTTACCTGTAGGAATACGTCTTGGATCTGTCGA
TTATGTTACGGGCGGAGTCCTATTCATGGAAACCTGGTCGAATTGGGAGAAGCTGTAG
GTATTATAGCCGGCCAATCTATTGGAGAACCTGGTACTCAATTAACATTAAGAACTTTTC
ATACTGGCGGAGTCTTCACGGGTGGTACCGCCGAATATGTGCGATCCCCCTCGAATGGT
AAAATAAAATTCAATGAGAATTCGGCTTACCCTACACGTACACGTCATGGACATCCTGC
CTTTTTATGTTATATAGACTTATATGTAACTATTGAAAGTAACGATATTATGCATAACGTAA
TTATTCCACCCAAAAGTTTTTTATTAGTTCAAAATGATCAATATGTAAAATCTGAACAAA
TAATTGCCGAGATCCGTGCAGGAACATATACTTTA---AATTTGAAAGAAAAGGTTCGAA
AACATATTTTTTCTGATTCGGAAGGAGAA---ATGCATTGGAGTACAAATATATATCACGT
TTCTGAGTTTGCATATAGTAATGTACATATCTTACCAAAAACAAGCCATTTGTGGATATTA
TCGGGAAATTCACACAAG------TCTGATACAGTTTCTTTGTCACTTCTTAAGGATCAAGA
TCAAATGAGCACTCAT------TCCCTTCCTACTGCAAAAAGAAATACTTCGAACTTTTTA---
---GTAAGTAATAATCAA---GTACGA--
--------CTATGTCCGGATCATTGTCATTTTATGCATCCTACT---ATTTCCCCCGATACTTCGAA
T------TTATTAGCAAAAAAGCGAAGAAATAGATTCATTATTCCATTTCTATTCATTCGAGA
ACGAAACAACGAGCTAATGCCC------GATATCTCGGTTGAAATACCTATC------GATGGTA
TTATTCATAAAAATAGTATTCTTGCTTATTTCGATGATCCTCAATACAGAACACAGAGTT
CGGGAATTGCGAAATATAAAACTATAGGAATTCATTCCATTTTTCAAAAAGAAGAT------
TTAATTGAGTATCGA---GGAATAAGGGAATTTAAACCAAAATACATCAAAGTAGATCGAT
TTTTTTTCATTCCCCAAGAAGTGCATATTTTATCCGAATCTTCTTCCATAATGGTCCGGA
ACAATAGTATCATTGGAGTAAATACACCAATCACTTTAAATAAAAAAAGTCGAGTAGGC
GGATTAGTTCGAGTAGAAAAAAACAAAAAAAAAATTGAATTAAAAATCTTTTCTGGAG
ATATCCATTTTCCGGGAGAGATAGATAAGATATCTCAACACAGTGCCATCTTGATACCAC
CT---GAAATGGTAAAAAAAAAAAAATTCTAAGGAATCAAAAAAA------AAGACAAACTGG
AGATATATACAATGG------ATTACAACTACCAAGAAAAAA------TATTTTGTTTTGGTTCGA
CCTGTAATTCTATATGATATAGCGGACAGTATCAATTTAGTAAAACTTTTTCCCCAAGATC
TGTTCAAGGAATGGGATAATCTCGAACTTAAAGTTCTAAATTTTATCCTTTATGGAAATG
GGAAATCAATTCGTGGAATTTTGGACACAAGTATTCAATTAGTTCGAACTTGCTTAGTC
TTGAATTGGAATGAAGACGAA---------AAAAGTTCTTCTATTGAGGAGGCCCTCGCTTCT
TTTGTTGAAGTAAGTACAAATGGTTTGATTCGATATTTCTTAAGAATTGACTTAGTTAAA
---CATATTTCATATATCCGAAAAAGAAATGATCCGTCTAGTTCAGGGTTGATTTCATAC---
AATGAGTCGGATCGTATCAATATCAATCCATTT---------TTTTCTATTTAC---------AAGGAA
AATATTCAACAATCACTTAGCCAAAAGCAC---ACTATTCGTATGTTGTTGAAT---------AG
AAATAAGGAAAACCGATCTTTTATAATTTTGTCATCATCTAATTGTTTT------CAAATGGG
ACCATTCAACAATGTAAAATATCACAAT---------GGGATTAAAGAAGAAAATCATAAAATT
CCAATTAAAATTTCGTTGGGCCCCTTAGGAGTA------GCCCCGCAAATTGCAAATTTTTT

TTCA------TTTTACCATTTAATAACT------CATAATAAAATCTCATCGATAAAGAAAAATTT
GCAACTTAACAAA---------TTCAAGGAAACTTTTCAAGTAATT------AAA---TATTATTTAA
TGGACGAAAATGAGAGA------ATTTATAAGCCCGATCTATACAATAACATCATTTTAAAC
CCATTCCATTTGAATTGGGATTTTATCCATCCTAAT-----------ACGTTTCCAATAATTAGT---
CTTGGGCAATTTATTTGTGAAAATGTATGTATAGTTCAAAATGGACCA---------AACCTAA
AATCGGGTCAAGTTATAACTGTTCAA---ATGGATTTTGTAGGAATAAGATTGGCTAACCC
TTATTTGGCAACCCCGGGAGCAACTATTCATGGACATTATGGAGAAATGCTTTATGAAG
GAGATATATTAGTTACATTTATATATGAAAAATCGAGATCTGGTGATATAACACAAGGTC
TTCCAAAAGTAGAACAGGTATTAGAAGTACGTTCGATTGATTCAATATCAATGAACCTA
GAAAAGAGAATTGATGCTTGGAACGAACGTATAACAGGAATTCTGGGCATTCCTTGGA
GATTCTTGATTGGTGCTGAGCTAACTATAGCTCAAAGTCGTATTTCTTTGGTGAATAAAA
TCCAAAGAGTTTATCGATCCCAGGGAGTGCACATCCATAATAGACATATCGAAATTATTG
TTCGTCAAATAACATCAAAAGTCTTGGTGTCAGAA---------GATGGAATGTCGAATGTAT
TTTCACCGGGGGAACTAATTGGATTGTTACGAGCTCAACGAACAGGGCGCGCCTTGGA
AGAATCTATTTGTTACCGAACTCTATTATTGGGAATAACAAAAACATCTCTGAATACTCA
AAGTTTCATATCCGAAGCAAGTTTTCAAGAAACTGCTAGAGTTTTAGCAAAAGCCGCT
CTCCGAGGTCGTATCGATTGGTTGAAAGGTCTAAAAGAAAACCTTGTTTTAGGGGGGA
TAATACCCGTCGGTACCGGATTCAAAAAAATAGGGGATCGTTCAAGCGCAAGACAAGA
TACCAAGATTACC------TTGGAAACAATAAAA--------------------------------------ATTATTCGA
GGTAGAAAT--ATGAC
AAAAAGATATTGGAACATAACTTTTGAAGAAATGATGGAAGCGGGGGTTCATTTTGGC
CACGGTACTAGGAAATGGAATCCTAAAATGGCACCTTATATATCTATGAAGCGCAAGGG
TATTCATATTATAAACCTTACCCGAACTGCTCGTTTTTTGTCAGAAGCTTGTGATTTAGTT
TTTGATGCAGCAAGCAGGGGAAAACGATTCTTAATTGTTGGTACAAAAAAAAAAGCA
GCCGATTTGGTAGCCCGGGCTGCAATAAGAGCCCGATGTCATTATGTTAATAAAAAATG
GCTCGGAGGTATGTTAACGAATTGGTATACTACAGAAACGAGACTTCGTAAGTTCAGG
GAATTGAGAACGGAACAAAAGACGGGGAAACTCAACTGTCTTCCAAAAAGAGATGCC
GCTATCTTGAAGAGACAATTATCGCACTTGGAAACATATCTGGGCGGCATTAAATATATG
ACAGGGTTACCTGATATTGTAATAATCGTTGATCAACAAGAAGAATATACGGCTCTTCG
CGAATGTATTACTTTAGGAATTCCAACGATTTGTTTAATCGATACAAATTGTGACCCCGA
TCTCGCGGATATTTCGATTCCAGCTAATGACGATGCTATAGCTTCAATCCGATTAATTCTT
AACAAATTAGTATTTGCAATTTGTGAGGGTCATTCTAGC---TATATACGAAATTTTATGGG
ACAAAAAATAAATCCGCTTGGTTTCAGACTTGGTACAACTCAAAGTCATGATTCTATTT
GGTTTGCACAACCAACAAATTATTCCGAGAATTTAAAAGAAGATAAAAAAATAAGAGA
TTGTATCAAGACTTATATACAAAAAACTATAAAAATATCTTCG---GGTGTCGAGGGAATT
GGACGGATAAAGATTCAAAAAAGAATCGATCTGATTCAAGTCATAATCTATATGGGATT
TCCAAAGTTATTAATAGACGGTAAGCCTCGA-----------AGAATCGAAGAATTACAGATAA
ATGTGCAAAAAAAAAATGAATTATGTGAATAGAAAACTCAACATAGCTATTACAAGAATT
GCAAATGCTTATAGAGATCCTAATATTCTTGCAGAATTCATAGCCGGACAATTAAAAAAT
AGAGTTTCGTTTCGTAAAGCAATGAAAAAAGCTATTGAGTTAACTGAACAGGCGGGGA
CAAAAGGAGTTCAAGTACAAATTGCGGGACGTATCGACGGAAAAGAAATTGCACGCG
TAGAATGGATCCGAGAAGGTAGGGTTCCCCTACAAACCATTCGAGCTAAAATTGATTAT
TGTTCCTATACAGTTCGAACTATTTATGGCGTATTAGGGATCAAAGTTTGGATATTTTTAA
ACAAAGAAATGTCACGTTACCGAGGGCCTCGTTTCAAAAAGATACGCCGTCTGGGGGC

TTTACCGGGACTAACCAGTAAAAGGCCTACAGTCGGAAGCGAACTTAGAAATCAATCG
CGCTCCACTAAA---AAATCTCAATATCGTATTCGTTTAGAAGAAAAACAAAAATTGCGC
TTTCATTACGGTCTTACGGAACGACAATTGCTTAAATACGTTCGTATCGCTGGAAAAGC
AAAAGGGTCAACTGGTCAGGTTTTACTACAATTACTTGAAATGCGTTTGGATAACATAC
TTTTTCGATTAGGTATGGCTTCAACTATTCCTCAAGCCAGACAATTAGTTAACCATAGAC
ATGTTTTCGTTAATGGTCATATAGTAGATATACCAAGTTATCGTTGCAAACCACAAGATA
TTATTACAGCGAAGGATAACAAAAAATCTAAAACTCTGATTCAAAATTCTCTTGAATCA
GCCCCCCGC---GAGGAATTGCCAACCCATTTGACTCTTCAGCCATTCCAA------TATAAA
GGATTAGTAAATCAAATAATAGATAGTAAATGGGTTGGTTTGAAAATTAATGAATTGCTA
GTTGTAGAATATTATTCTCGTCAGACTATGTCACGGCGAGGTACTGCAGAAAAAAAAAC
CGCAAAATCCGATCCAATTTATCGTAATCGATTGGTTAACATGTTGGTTAACCGTATTCT
GAAACACGGAAAAAAATCATTGGCTTATCAAATTATCTATCGAGCTATGAAAAAGATTC
AACAAAAGACAGAAACAAATCCACTATCTGTTTTACGTCAAGCAATACGTGGAGTAAC
TCCCGATATAGCAGTAAAAGCAAGACGCGTAGGCGGATCGACTCATCAAGTTCCCATT
GAAATAGGATCCACACAAGGAAAAGCACTTGCTATTCGTTGGTTATTAGGGGCATCCCG
AAAACGTCCGGGTCGGAATATGGCTTTCAAATTAAGTTCCGAATTAGTGGATGCTGCCA
AAGGGAGTGGCGATGCCATACGGAAAAAGGAAGAGACTCATAGAATGGCAGAGGCAA
ATAGAGCTTTTGCACATTTTCGTATGGGTAAGGACACCATCGCTGACATAATAACCTCTA
TACGCAATGCTGACATGAATAGAAAAAGAACTGTTCAAATACCATTTACTAACATCACC
GAAAACACTGTAAAAATACTTTTACGAGAGGGTTTTATTGAAAACGTGAGAAAACATC
GGGAAAGCGACAAATCTTTTTTAGTTTTAACTCTACGATATAGAAGA------AATACGAAA
GGATCATATAAAACT---TTT------TTAAATTTAAAACGGATCAGTACACCCGGTCTACGAA
TCTATTATAACTATCAAAAAATTCCTAGAATTTTAGGAGGTATGGGGATTGTAATTCTTTC
TACTTCGAGGGGTATAATGACAGATCGAGAGGCTCGATTAGAAAAAATCGGCGGAGAA
GTTTTATGTTATATATGGATGGCAAAACCTATACCAAAGATTGGTTCACGTAAAAATGCA
CGTAGTGGTTCT---AAGCATCTTCGTAAAATACCAAAGGGGATTATTCATGTTCAAGCTA
GTTTTAACAATACCATTGTAACTGTTACAGATGTACGAGGTAGGGTGATTTCTTGGTCCT
CCGCCGGTACTTGTGGATTCAAGGGTACACGAAGGGGAACACCATTTGCCGCTCAAAC
CGCAGCAGGAAATGCTATTCGAACAGTAGCGGATCAAGGTATGCAACGAGCAGAAGTC
ATGATAAAAGGTCCCGGTCTCGGAAGAGATGCAGCATTAAGAGCTATTCGTAGAAGCG
GTATATTATTAAACTTTATACGGGATGTAACTCCTATGCCACATAATGGATGTAGGTCACC
TAAAAAAAGACGTGTAATGCCAACTATGAAACAACTTATTAGAAAGACAAGACAGCCA
ATCCGAAATGTCACCAAATCCCCCGCTCTTCGGGGATGCCCTCAGCGCCGAGGAACTT
GTACTAGGGTGTATACTATCACCCCTAAAAAACCAAACTCTGCCTTACGTAAAGTTGCC
AGAGTACGCTTAACCTCTGGATTTGAAATCACTGCTTATATACCCGGTATTGGCCATAAT
TTACAAGAACATTCTGTAGTCTTAGTAAGAGGGGGAAGGGTTAAGGATTTACCCGGTG
TGAGATATCACATTGTTCGAGGAACCCTGGATGCTGTCGGAGTAAAAGATCGTCAACA
AGGGCGTTCTAAATATGGGGCCAAAAAGCCAAAAATGGCAAGGAAAAGTTTGATTCA
GAGGGAGAAAAAGAGGCAAAAATTAGAACAAAAATATCATTTGATTCGCCGATCCTCA
AAAAAAGAAATAAGC---------AAAGTTCCGTCGTTAAGTGAGAAATGGAAAATTCATGG
AAAGTTAGAATCACTACCGCGTAATAGTGCACCCACACGTCTTCATCGACGTTGTTTTT
CAACCGGAAGACCGCGAGCTAATTATCGAGACTTTGGATTATCTGGACACACACTTCGT
GAAATGGTTCATGAATGTTTGTTGCCCGGTGCAACAAGATCAAGTTGGATGGTAAAAA
AATCATTTATACCGGTTATTTCACAAGAA---AAAAAAGAAGAA---AACCCCGGATCGGT

TGAATTTCAAGTATTTAATTTCACCAACAAGATACGAAGACTTACTTCACATTTTGAATT
GCACCGAAAAGATTATTTATCTCAAAGAGGATTACGTAAAATTCTCGGGAAACGGCAA
CGATTACTTTCTTATTTGTCAAAGAAAAATAAAATACGTTATAAAAAATTAATAAATCTG
TTGGATATTCGAGAGTCAAAAATTCGT------ATGGTAAAACTTCGTTTGAAACGATGTGG
TAAAAAGCAACGAGCTGTTTATCGAATCGTTGCAATAGATGTTCGATCTCGAAGAGAG
GGACGAGATCTTCGGAAAGTGGGTTTTTATGATCCAATAAAAAATCAAACCTATTTAAA
TATTCCCGTTATTCTAAATTTCCTTGAGCAAGGTGCTCAACCTACAGGAACTGTTCAGG
ATATTTCAAAAAAGGCAGGTTTCTTTATGGACCTT--------------------------ATGGATAAATC
TAAGCGACTCTTTCTGAAATCCAAGCGATTTTTTCGTAGGCGTTTGCCCCCGATCCAAT
CGGGGGATCGAATTGATTATAAAAACATGAGTTTAATTAGTCGATTTATTAGTGAACAG
GGAAAAATTTTATCTAGACGTGTGAATAGATTGACCTTAAAACAACAACGATTAATTAC
AATTGCTATAAAACAAGCTCGTATTTTATCTTCGTTACCTTTTATTAATAATGAAAAAAA
A---CAATTTGAAAAAAGCGAGACAGCCACTAGAACTACTACTGTTTTTAAAACAAAAA
AAGTGACACGT---CTAACAAAAAATCCTTTTGTAGCGAATCGTTTATTAAGAAAAATAA
ATAATCTTAACACAAAATCAGAAAAAGAAATAATAATAACTTGGTCCAGAACATCTACC
ATTATACCCGCAATGATTGGCCATACCATTGCTATTCACAATGGAAAAGAGCATTTACCG
ATTTATGTAACGGATCGTATGGTAGGCCATAAATTGGGAGAATTTTCTCCTACTCTAAAC
TTCCGGGGACACGCAAAAAATGATAATAGATCTCGGCGTATGATTTTTCAATCTTTTATA
CTGGATAATCTAGTATCCTTATGCCTCAAGATAATTAATTCGGTCATTGTGGTCGGACTCT
ATTATGGATTTATGACCACATTCTCCACAGGGCCCTCTTATCTATTCCTTCTTCGAGCTCA
CGTTATGGAAGAAGGG---------ACCGAGAAGAAAATATCAGCAACAACTGGGTTTATTA
CGGGACAGCTCGTGATGTTCATATCGATCTATTATGCGCCTTTGCATATAGCATTAGATAG
ACCTCATACAATAACTGTCATAACTCTACCGTATCTTTTATTATATTTCCTCGGCAATAAT
CAAAAAAACTTTTTGAATTATGTATACAAGAATCAAAATTCAATACGTCATTTTAGTATT
CAAAGAATATTCTTTCAGAATCTTTTTTTTCAGTTATTAAACCCCTTTTTTTTACCAAGTT
CAATCTTAATGAGATTAGCGAACATTTATATCTTTCAATCAAACAATAAAGTCTTATTTTT
AACAAGTAGTTTTGTTGGTTGGTTAATTGGTCATGTTTTTTTCATGAAATGGATTGGATT
AATGTTAGTCTGGATACAGGAAAAAAACAATTCTATAAAA---ATTCGATCTAATAAGGG
GGTTCTGGCAAAATTTAGAAAGTCT------CAAATTTTTCTTATTTTTTTTTTTATTACCTGT
TTATATTATTTAGGAAGAATACCGCCCATTTATTTTTTTAAAATGTCAGAAATTGAAGAA
AAGCTTAAACCTCTTGTAACTATTCTTTTCGATTATAATCGATGGAATCGTCCATTGCGAT
ATATAAAAAATGATCGATTTGAAAATGTTGTAAGAAATGAAATTTCACAATTTTTTTTTT
TTACATGTCAAAGTGATGGAAAAGAACGAATATCTTTCACATATCCACCTAATTTGTCG
ACTTTTCAGAAAATGATGGAAATGAAAATTTCTCTCTTCACAAGA---GATATAATCTACG
AAGAATTGTCTAATTCTTGGAGATCCACTAATGAAGAAAAAAAAAAAAAAACTAACCAA
TGAATTTTTAAATAGGGTAGAAGTTTTAGATAAGGAATCT------GTGGATATATTCGAAAA
CCGAATTCGATTGTGTAATGATGAAAAAAAAACAAAAATACTTAACTAAAGAATATGATC
CTTTTTTGAATGGACCCTGTCGTGGACAAATCCAAAAATGGTTTTCACCC------TCTCTT
TTTATAAATAAGATTCATGGTATCCTTTATCCAAAATTTGAACAGAAAGATCTGAATGAT
AATTGTATTGGAATAAACGAAATCGTGAAAAAACTTCCCCGATGGTCATACAACCTAAT
CGAGTTGGAACAACTGGAGGGAAAACAAATTCGTTCCAGAAAAGCTAAACGTGTAGT
ACTTTTGACCAAAGCTTTAATACGTTATTCACAGCAACCAGATTTTCGGCGAGACATAA
TAAAAGGATCTATACGCGCTCAAAGACGTAAAACAGTTACTTGTAAACTCTTTCAAAG
AAGTGTGGATTCCCCTCTTTTTTTGGAAAAAATGGAAAAAACCTCCTTCTTTTGTTTTG

ATATTTTAGATTCAAGTAAAATCTTTTTTATGTTCAAAAATAAAGAGGAAAAAAGACGT
ATAGAAATAGGAGAAGCTTGGGATAGCATTATATTTGCTCAAGTAATTAGAGGTTGTTTA
TTAATAACCCAATCGATTCTTAGAAAATATATTCTATTACCTTCATTGATAATAACAAAAA
ATATCGTTCGTATACTATTATTTCAATTTCCCGAATGGTCAGAAGATTTTAGGGATTGGCA
GAGAGAAATGTATATTAAATGCACCTATAATGGGGTTCAATTATCCGAAACAGAATTTCC
AAAAAAATGGCTAACAGACGGTATTCAGATAAAGATCTTATTTCCCTTTCGTCTGAAAC
CTTGGCACAGATCTAAAAAAGTAAAAAAAAAAGAACTTTTGCTTTTTAACAATTTTTGG
AATGGAAGTTGAATTGCCCTTTTCTGGTTATCCCCGAAATTTTTCATTTTTTGATCCCATT
TTAAAAGAACTCAAAAAAAAAA-----------------AAAAAATTGAAAAATATTCTAAAAATTG
TCAATGAAAGAACAAAAAACTTTATAACCACCTTAAAAGAAACATCAAAACGGATCAT
CCAAAGTATTCTTAAAAAAGTTCTATTCCTAAACAAAAAAATAAAAAAA------CTC--------
-ATCACTAAGAATATAATTTATGAATCAACCATTCTAATTCAAGCTATTAATAAGACAAAT
TGTTCATTGACAGAAAAAAAAAATAAAGGCCATAAATGCTAAGACAAAGAAAATCATAA
AGAAAATAGAAAGGATGACAAAA---GAAAATAAGAATTCTAAGAAAACAAGTTCTAAT
ACTAAAGGATTAGAATTAGAAAAAAAGATTTTGCAAATATTACAAAGAAGAAATGTTC
AATTAACCCATAAATTGTATTCCTTTTTTAAATTTTTACTGAATTTTATGAAAACTGATATA
TTTCTTTGTATCGTTAGTGTTCCTAGGATCAATGTACAATTTTTT--------------------------GC
TTTGTCCCAGGCATATGTATTTTTTAAAATATCCCAAATCCAA------AACAAGTATAAATT
CAAATATGTTTTTGACTATAGATCGTTTTTTATTAAGGATGAAATAAAAGATTATTTTATA
CAAGGAATAATTCATTCCAAATTAAGACATAAGAACTCTCCC---TCTTTAAAGAATCAAT
GGACAAATTGGTTAAAGGTTCATTATCAATATGATTTATCTCAGAATAGATGGTCTCGAT
TAGTACAAAAAAACCTGAAAAATAGAATAAATAAACACCGTTTGGATCAAAATAAAAC
CAAATGTGATTCATATAAAAAAACCCAA------TTAATTGTTTCCAAAAACAAA---AAAAT
AAAAAAACAATCTAGATATGATCTTTTGTTATATAAATTTATTAATGATGCTATTCGTTATC
GGGATAGAAAGTATTTTGATTGGGTGGTAATGGAT-----------------------GTAAAAATACAA
AATAATTCTATATCGAATTCGTTTTTTTTTAAATTCCTGAGATTTTATGATGCATATAGGAA
TAAACCGTGGATCATACCAATCAAATTCCTTTTTTTACATTACAAGAATAAGATAGAGGC
AGAAGTTAATTTATTACTAAGAAAGTATTTGAATTTTCATTTGAACTGGAAGGGTTCCTT
AAATAAAAGAGTAATAAATAATGTCAAAGTATATTGTCTACTGATTAGATTGAAAAATAT
AAAACAAATAGCTATATCCTCTATTCAAAGGGGAGAACTAAGTCTAGACATTATGATGA
TTCAAAATGAGAAGGATTCCACTCTTACGGGATTCAGAGGAATTTTTATTATCGAACCA
GTCCGCCTGTCTAGAAAAAACAATGAACAATTTTTTATGTATGAAACCGCGCGCCTCCT
ATTAATTCATAAGAGTAAACGCAAACATTATGCTTTAGTTGTTATTGAAAATATTTTATCG
GCTAGACGTCGTAGAGAATTGAGAATTCTAATTTGTTTC---ATTCTTTGGCCGAATTATC
GATTAGAAGATTTAGCTTGTATAAATCGCTATTGGTTTGATACCTATAATGGAAGCCGTT
TCAGTATAGTAAGGATACATATGTATCCAATGAAAGGACATCAATTCAAATCCTGGATTT
TCGAATTGAGAGAGATATTGAAAGAGATCAAGAATTCTCGCTATTTCTTATATTCATGGA
CCCAATTCAATTCAACGGGATCTTTCATTCACATTTTTTTCCATCAAGAGAGTTTTATAA
AACTCTTGGACTCACGAATTTGGAGTATCCTACTTTCA---GGCAATTCA------CAGGGTT
CAACAAGCAATCGATATTTT---------ACGATCAAGGATGTAGTACTTTTTGTAGTAGCG---A
TCCTTCTATATCGT------------ATTAACAATCGAAAAATGGTCGAAAGAAAAAATCTCTATT
TGACGGGGCTTCTTCCT---------------ATACCTATGAATTCCATTGGACCCAGAAATGATAC
ACTGGAAGAATCTTTT---GAGTCTTCCAATATCAATAGGTTGATTGTTCCG---CTCCTGTA
TCTTCCAAAAGAAAAAAAGATCTCTGAGAGCTCTTTCCTGGATCCGAAAGAGAGTACC

CAGGTTCTCCCAATAACT------------AAAAAGTGTATCATGCCTGAATCTAACTGGGGTTC
GCGGTGGTGGAGGGACTGGATCAGAAAAAAGAGG------GATTCTAGTTGTAAGATATCT
AAT---------GAAACCGTCGCTGGAATTGAGATCTCATTCAAAGAGAAAGATATCAAA---TA
TCTAGAGTTTCCTTTTGTATATTATATGGAT------GATCCGATCCGCAAGGACCATGATTGG
GAATTGTTTGATCGTCTTTCTCCGAGGAAGAGGCGAAACATAATCAACTTG------AATTC
GGGACAGCTATTCGAAATCTTAGTGAAAGACTGGATTTCTTATCTCATGTTTGCTTTTCG
TGAAAAAAAACCAATTGAAGTGGAGGGT---TTCTTCAAACAACAAGGG---GCTGGGTC
AACTATTCAATCAAATGATATTGAGCATGTTTCCCAT------CTCTTCTTGAGGAACAAGCG
GGCTATTTCTTTGCAAAATTGTGCTCAATTTCATATGTGGCAATTCCGCCAAGATCTCTT
CGTT---------AGTTGGGGGAAGAAGCAGCCGCACGAATCGGATTTTTTAAGGAAC---ATA
TCGAGAGAGAATTGGATTTGGTTAGACAATGTGTGGTTGGTAAACAAGGATCGG---------
TTTTTTAGC------AAGATACGGAATGTATCATCAAATATTCAATATGATTCC---------ACGAG
A------TCTAGTTTTATTCAAGTAACGGATTCTAGCCAATTGAAAGGATCTTCT---GATCAA
TCCAGGGATCATTTCGATTCCATTAGG------------AATGAGGATTCGAAA----------------------
----------------TATGACACATTGATCAATCAAAGAGAGATTCAACAACTAAAAGAAAGATC
GATTCTTTGTTGGGAT------CCTTCCTTTCTTCAAACGGAACGAACAGACATAGAATCGG
AGCGATTCCGATTCCCTAAA------AGCCTTTCTGGATATTCC------TCAATGTGCCGACTAT
TCATG---CAACGTGAGAAGCAGATGAATAATCATCTGCTTCCGGAAGAAATCGAAGAAT
TT------CTTGGGAACCCTACAAGAGCCGATCGTTTTTTTTTCTCTGACAGATGG------TCA
GAACTTCAT---CTAGGTTCGAATCCTACTGAGAGGTCCACTAGAGATCAG------------AAA
TTGTTGAAGAAAGAACAAGATGTTTCT---TTTGTTCTTTCCAAGCGATCGGAA------------
AAGAAAGAAATAGTGAATATATTCAAGATAATTCTGTATTTACAAAAT------ACCGTCTCA
ATTCATCCTATTTCATCAGATCCGGTATGTGATATGGTTCCG---AAGGATGAACCG---------
GACAGTTCCAATAAGATGTCATTCTTGAACAAAAAT---------GGTTTA------TTTCATCTAT
TCCATGACCGGAACAGGGGGGTATACACGTTA------CACCACGATTTTGAATCAGAA-----
-GAGATATTTCAAGAAATGGCAGATCTATTCAATCTATCAATAACTGAGCCG---GATCTG
GTGTATCATAAGGGATTTGCCTTTTCTATTGATTCCTCCGGATTGGATCAAAAACAA------
TTTTTGAATGAGGTATTCAACTCAAGGGATGAATCGAAAAAGAAATCTTTA---------TTG
GTTCTACCTACTCTTTTTTAT------GAAGAGAATGAATCTTTTTATCGAAGGATCATAAAA
AAATGGGTCCAG---ACCTCTTGCGGGAATGATTTGGAAGATCCAAAACCAAAAATAGT
GGTATTTACTAGCAACAACATAATGGAGGCAGTCAATCAATATAGATTGATCCGAAATCT
TATTCCAATCCAATATATCACCTATGGGTATGGGTACATAAGAAATGTATTGAATCGATTC
ATTCAAAAGAAT---------CGATTCGATCGCAACTTCGAATATCGAATTCAA---------AGGTA
TCAAATAGAGAATGATACTCTGAATCATAGAACTAGAATGAAATACACGATCAACCAAC
ATTTCTCAAATTTAAAAAAAAAAGAGTCAGAAGAAATGGTTCGATTCT------CTTATTTTG
ATTTCTCGAACCGAGAGATCCATGAATCGGGATCCTAATGCATATAGATACAAATGGTCC
AATGGGAACAAGAATTTCCAGGAACATTTGGACCATTTCATTTCTGAGCAGAATAGCC
GT---------TTTCAAGTAGTGTTCGATCGATTACGTATTAATCAATATTCGATTGATTGGTCT
GAGGTTATCGACAAAAAAGATTTGTCTAAGTCACTTTGTTTCTTTTTGTACAAGTTTCTT
CTC---------------------------TTTTTTTCC---------------------AAGTTTCTTCTCTTTTTGTCTAAA
TCACTTCCT---TTTTTCTTTGTGAGTTTCGGGAGTATCCCCATTCATAGGTCCGAG---ATC
CACATCTAT---GAATTGAAAAGTCCGAATCATCCACTCTGTAATCAGCTGTTCGAATCAA
TAGGTCTTCAAATCGTTCATTTGAAAAAATGGAAACCCTTC------TTATTAGAT---------GA
CCATGATACTTCC---CAAAAATCGAGATTCTTGATCAATGGAGGAACAATATCACCATTT

TTGTTCAATAAGATACCT---AAGTGG---ATGATTGACTCTTTCGATACTATA------AAGAAT
CGTAGAAAATTTTTTGATAACACGGATTCCTATTTCTCAATGATATCCCACGATGAAGAT
AATTGGCTGAATCCCGTGAAACCATTTCATAGAAGTTCATTGATATCCTCTTTTTATAAA
GCAAATCGACTTCGATTCTTGAATAATCGATATCACTTCTGCTTCTAT------TGTAACAAA
AGATTGCCTTTT---------TAT---GTGGAAAAGGCCTGTATCAATAATTATGAT------TTTACGT
ATGGA---CAATTCCTCAATATCTTGTTCATTCGCAACAAA---AGATTTTCTTTGTGCGGCG
GTAAA------AAAAAACATGCTTTTTTGGAG---AGAGATACTATTTCACCAATCGAGTCAC
GGGTATTTAACATATTGATACTTAACGATTTTCCGCAAAGTGGTGACGAAGGG---TATAA
CTTGTACAAATCT---------TTCCATTTTCCAATTCGATCCGATCCATTCGTTCAT------AGAG
CTATTTACTCGATCGCAGACATTTCTGTAACACCTCTAACAGAGGGGCAAATAGTCAAT
TTTGAAAGAACTTATTGTCAACCTCTTTCAGATATGAATCTACCCGATTCAGAAGGGAA
GAACTTGCATCAGTATCTCAAA------TTCAATTCAAACATGGGTTTGATTCATATTCCATG
TTCTGAGAAATATTTACCATCCGAAAAT------AGGAAAAAA------------------------GGGATTC
CTTGTCTAAAA------AAATGCCTTGAGAAAGGGCAGATGTATAGAACCTTTCAACGAGA
TAGTGTTTTTTCAACTCTATCAAAATGGAATCTATTCCAAACATATATACCTTGGTTCCTT
ACTTCGACAGGGTACAAATATCTAAATTTCATATTTTTAGATACTTTTTCAGAC---CTATT
GCCGATACTAAGTAGCAGC------CAAAAATTTGTATCCATTTTTCATGATATTATGCATAGA
TCAGATATATCATGGCGAATTCTTCAGAAAAAAATGG------------------TGTCTTTCACAATG
GAAT---CTGATAAGTGAGATTTCGAGTAAGTGTTTCCATAAT------CTTCTTCTGTCCGAA
GAAATTATTCATCGAAATAATGAGTCA------CCATTGATATCGACACATCTGAGATCGCTA
AATGTTCGGGAGTTC---CTCTATTCAATCCTTTTCCTTCTTCTTGTTGCTGGATAT---CTCG
TTCGTACACATCTTCTCTTTGTTTCTCGA------GTCTATAGTGAGTTACAGACAGAGTTCG
AAAAGGTCAAATCTTTGATGATTCCATCATACATGATTGAGTTGCGAAAACTTCTGGAT
AGGTATCCTACATCTGAACAGAATTCTTTCTGGTTAAAGAATCTCTTTCTAGTTACTCTG
GAACAATTAGGAAATTCTCTAGAAGAAATACGAAGTTCTGCTTCTGGCGGCAACATGC
TATGGGGTGGTGGTTCC---------------GCTTATGGG---GTCAAATCAATACGTTCTAAGAAG
AAATATTTGAATCTC---------------ATCGATCTCATA---AGTATCATACCAAATCCCATCAATC
GAATCGCTTTTTCGAGAAATATGAGACATCTAAGTCATACAAGTAAAGCGATCTATTCAT
TGATAAGAAAAATAAAAAACGTGAATGGTGATTGGATTGATGATAAAATAGAATCCTGG
GTCTCGAACAGTGATTCGATTGATGATAAAGAAAGAGAATTCTTGGTTCAGTTCTCT-----
-ACCTTAACGACA------GAGAAAAGGATTGATCAAATTCTATTGAGTCTGACT---CACAGT
GATCATTTATCA---------------AAGAATAACTCTGGTTATCAAATGATTGAACAACCGGGAA
CAATTTACTTACGATACTTAGTTGACATTCAG------AAAAAGTATCTAATGAATTATGAGT
TCAATACATCCTGTTTAGTAGAAAAACGGATATTCCTTGCTCATTATCAGACAATCACTT
AT------TCACAAACCCTGTGTGGGACTAATAGTTTTCATTTCTCGTCTCATGGG---------AA
ACCCTTTTCGCTCCGCTTAGCCCTATCCCCCCCT---AGGGGTCTTTTAGTGATAGGTTCTA
TAGGAACTGGACGATCCTATTTGGTCAAATACCTAGCTGCAAACTCCTATGTTCCTTTCA
TTAGAGTATTTCTGAACAAGTTCCTGGATAACAAGCCTAAGGGTTTTCTTATTGAT--------
-------GATAGTGACGATAGTCACGATATTGACGATATTGATGATAGTAATGATAGTGACGAT
---ATCGACCGTGACCTTGATACGGAGCTGGAG
TTGCTAACTATGATGAATGCGCTA---------ACTATGGATATGATGCCG---------GAAATAGAC
CGATTTTATATCACCCTTCAATTCGAATTAGCAAAAGCAATGTCTCCTTGCATAATATGG
ATTCCAAACATTCATGATCTGGATGTGAATGAGTCGAATTCCTTATCCCTCGGTCTATTA
GCGAACCATCTCTCC---------AGGGATTGTGAAAGATGTTCC---ACTAGAAATATTCTTGT

TATTGCTTCGACTCATATTCCCCAAAAAGTAGATCCCGCTCTAATAGCTCCGAATAAATT
AAATACATGCATTAAGATACGAAGGCTTCTTATTCCACAACAACGAAAGCACTTTTTTA
CTCTTTCATATACTAGGAGATTTCACTTCGAAAAGAAGATGTTCCATACT------AATGGAT
TCGGGTCCATAACTCTGGGTTCCAATGTACAAGATCTTGTAGCACTTACCAATGAGGCC
CTATCAATTAGTATTATACAAAAGAAATCCATTATAGACACTAATCTAATTAGATCTGCTC
TTCATAAACAAACTTGGGATTTGCGA---------TCTCAGGTAAGATCGGTTCAGGATCATG
GGATCCTTTTCTATCAGATAGGAAGGGCTGTTTCACAAAATGTACTTCTAAGTAATTGCT
CCATAGATCCTATATCTATCTATATGAAGAAGAAA------TCATGTAATGAGGGGGATTCTT
ATTTGTACAAATGGTATTTCGAACTTGGAACGAGCATGAAGAATTTAACGATACTTCTTT
ATCTTTTGAGTTGTTCTGCCGGAGCGGTCGCTCAAGACCTTTGGTCTCTACCC------GGA
CCTGATGAAAAA---AATGGGATCACTTCTTATGGACTCGTTGAGAATGATTCT---GATCT
AGTTCATGGCCTATTAGAAGTAGAAGGCGCTCTGGTGGGA------TCTTCACGGACAGAA
AAAGATTGCAGTCAGTTTGATAATGAT------CGGGTGACATTACTTCTTCGGCCCGAACC
AAGTAAT---CCCTTAAATATGATTCAAAATGGATCTTGTTCTATCGTTGATCAGAGATTTC
TCTATGAAAAA------TATGAATCGGAGTTTGAAGAA---------GGGGAAGGAGTCCTCGAC
CTGCAA------------CAGATAGAGGAGGAT------------TTATTCAATCAC------ATAGTTTGGGC
TCCT------AGAATATGGTGCCCA---------TGGGGCTTTCTATTTGATTGTATCGAAAGGCCC
AATGAATTGGGATTTCCC---TATTGGGCCAGGTCATTTCGGGGCAAG---------CGAATCAT
TTATGATGAAGAGGATGAGCTTCAAGAGAATGATTCGGAGTTCTTGCAGGGTGGAACC
ATGCAGTACCAGACACAAGATAGATCTTCCAAAGAACAAGGCTTTTTTCGAATAAGCC
AATTCATTTGGGACCCCACGGATCCACTCTTTTTCCTATTCAAA---GATCAGCCTTTTGT
CTCTGTGTTTTCACATCGAGAATTCTTTGCAGATGAAGAGATATCAAGGGGACTTCTTA
CTTTCCAAACAGATCTTCCGACATCTATATATAAACGCTGGTTTATC------AAGAATACGC
AAGAAAAGCATTTCGAATTGTTGATTCATCGCCAGAGATGGCTTAGA---------ACCAATA
ATTCATTATCT------AATGGATTTTTCCGTTCTAAT------ACTCTATCCGAGAGTTATCAATAT
TTATCAAATATGTTCCTATCTAAC---GGAACACTATTGGATCAAATGACAAAGACATTGT
TGAGAAAAAGATGGCTTTTCCCGGATGAGATGGTTGTTGCTATC------TGCTCCAATAAC
GAATCATTGGTT------ATGCCTAGATCTCGG---ATAAATGGAAATTTTATTGATAAGACCTT
TTCAGTTGTAGCCAATATCTTATTACAAATAATACCCACAACCTCGGGAGAAAAAGAGG
CATTCACTTATTATAGAGATGGT------ATGTCAGCTCAATCTGAAGGAAATTATGCGGAAG
CTTTACAGAATTATTATGAGGCTATGCGACTGGAAGTTGATCCCTATGATCGAAGTTATA
TACTTTATAACATAGGCCTTATCCACACAAGTAACGGAGAACATACGAAAGCTCTGGAA
TATTATTTTCGGGCACTCGAACGAAATCCGTTCTTACCTCAAGCTTTTAACAATATGGCC
GTGATCTGTCATTAC---CGGGGAGAACAGGCCATTAGACAGGGAGATTCTGAAATTGCC
GAATCTTGGTTCGATCAAGCTGCTGAATATTGGAAACAAGCTATAGCCCTTACCCCCGG
TAATTATATTGAAGCACAGAATTGGTTAAAAATTACAAGGCGTTTTGAA------ATGACTT
GGATATCAGAAGATACGTGGGTAGATTTTGTAACGGGGTGTCGAAACCCCCGTGATTTC
TTCTGGGCATTTATCACTCTTTTAGGTTCAGTAGGGTTTTTATTGGTTGCAGCTTCCAGT
TATCTTCATAAGAATTTTTTATCTTTCATTTCGTCCGAGCTAATTCGTTTTTTGCCACAAG
GGGCTACGATAACTGTTTATGGGACCGCGGGTCTATTTGTTAGTTTGTTTTTGTGGCTTA
CCATTTTTTGGAATGTGGGCAGTGGTTATGATCTTTTCGATAAAAAAAGAGGAATAGTG
TGCATTTTTCGTTACGGATTTCCTGGAAAAAATCGTCGTATTTTTATCCGAGTCCTTATC
AACGATATTCAGTCCCTCATAATACAAAGTAAAGAGGGTAGGTATCTTCGCAGTGTCCT
TTATATGCAAACCAGAGAACAGGGGGCCATTCCCCTAACTGCTGTTGACGATTCT---TG

GAATCCACCAAAAATTGCACAAAAAGCTGGAGATTTGTCCAAGTTATTGCGTGTGCCA
ATTGAAATATTT
>Triticum
???
???
???
???
???
???
???
???
???
???
???
???
???
???
???
???
????ATGGCAACCCTTCGAGTCGACGAAATTCATAAAATTCTCCGCGAACGTATTGAACA
ATATAATAGGAAAGTAGGGATTGAGAATATAGGTCGCGTAGTTCAAGTGGGGGATGGGA
TTGCTCGTATTATAGGTCTTGGTGAAATAATGTCAGGTGAATTAGTCGAATTTGCAGAA
GGTACTAGGGGTATTGCTCTGAATTTGGAATCCAAAAATGTTGGGATTGTATTAATGGG
CGATGGGTTGATGATACAAGAGGGAAGTTTTGTAAAAGCAACAGGAAGAATTGCTCAG
ATACCCGTGAGTGAGGCTTACTTGGGTCGTGTTGTAAATGCTCTGGCTAAACCTATTGA
TGGGAAAGGCGAAATTATAGCTTCCGAATCTCGCTTAATTGAATCTCCTGCTCCAAGTAT
AATTTCCAGGCGTTCCGTATACGAACCTCTTCAAACAGGGCTTATTGCTATCGATTCGAT
GATCCCTATAGGGCGCGGTCAGCGAGAGTTAATTATTGGGGACAGACAGACTGGCAAA
ACAGCAGTAGCCACAGATACAATTCTCAATCAAAAAGGGCAAGGTGTAATATGTGTTTA
TGTAGCTATCGGTCAAAGAGCATCCTCCGTAGCTCAAGTAGTAACTACTTTCCATGAGG
AGGGGGCCATGGAATACACTATTGTAGTAGCTGAAATGGCGGATTCACCTGCTACATTA
CAATACCTCGCTCCTTATACGGGAGCAGCCCTGGCTGAGTATTTTATGTACCGCGAACG
GCATACTTTAATAATTTATGATGATCTCTCCAAACAGGCACAAGCTTATCGCCAAATGTC
CCTTCTATTAAGAAGACCTCCCGGCCGTGAGGCTTATCCAGGGGATGTTTTTTATTTGCA
TTCACGCCTTTTAGAAAGAGCCGCTAAATTAAATTCTCTTTTAGGCGAAGGAAGTATGA
CCGCTTTACCAATAGTTGAGACTCAATCTGGAGACGTTTCCGCCTATATTCCTACTAATG
TAATCTCCATTACAGATGGACAAATATTCTTATCTGCGGATCTATTCAATGCCGGAATTC
GACCCGCTATTAATGTGGGTATTTCTGTTTCCAGAGTAGGATCCGCGGCTCAAATTAAA
GCCATGAAACAAGTAGCTGGCAAATCAAAATTGGAACTAGCTCAATTCGCAGAGTTAC
AAGCCTTTGCACAATTCGCCTCTGCTCTCGATAAAACAAGTCAGAATCAATTGGCAAG
GGGTCGACGATTAAGGGAATTGCTTAAACAATCCCAGGCAAATCCTCTCCCAGTGGAA
GAGCAGATAGCTACTATTTATACCGGAACAAGAGGATATCTTGATTCGTTAGAAATTGA
ACAGGTAAATAAATTTCTGGATGAGTTACGTAAACATCTAAAAGATACTAAACCTCAAT
TCCAAGAAATTATATCTTCTAGCAAGACATTCACCGAGCAAGCGGAAATCCTTTTGAAG

GAAGCTATTCAGGAACAGCTGGAACGGTTTTCTCTTCAG---------ATGAGAACCAATCCT
ACTACTTCTCCTCCCGGGGCTTCCACAATTGAAGAAAAAAGTACAGGTCGTATCGATC
AAATTATTGGACCCGTGCTGGATGTCACTTTTCCCCCAGGCAAGTTACCTTATATTTATA
ACGCTTTGGTAGTCCAGAGTAGAGACACTGACGATAAGCAAATTAATGTGACTTGTGA
GGTACAACAATTATTAGGAAATAATCGAGTTAGAGCTGTAGCTATGAGTGCTACGGACG
GGTTGATGAGAGGAATGGAAGTGATTGACACGGGAGCTCCTCTCAGTGTTCCGGTCGG
TGGAGCTACTCTTGGACGAATTTTCAACGTTCTTGGGGAGCCTGTTGACAATTTGGGTC
CTGTAGATAGTAGTGCAACGTTCCCTATTCATAGATCTGCGCCTGCCTTTATCGAGTTAG
ATACGAAATTATCCATCTTTGAAACAGGTATTAAGGTCGTCGATCTTTTAGCTCCTTATC
GACGTGGAGGAAAAATAGGACTATTTGGGGGGGCTGGAGTAGGTAAAACAGTACTGAT
CATGGAATTAATCAATAACATTGCTAAAGCTCATGGGGGCGTATCCGTATTCGGTGGAGT
AGGGGAACGGACTCGTGAAGGAAATGATCTTTATATGGAAATGAAGGAATCCGGAGTA
ATTAATGAAAAAAATATTGAGGAATCAAAGGTAGCTCTAGTCTATGGCCAAATGAATGA
ACCACCGGGAGCTCGTATGAGAGTTGGTTTAACTGCCCTAACTATGGCGGAATATTTCC
GAGATGTTAATAAGCAAGACGTGCTTTTATTTATCGATAATATCTTTCGTTTTGTTCAAG
CAGGATCAGAGGTATCCGCTTTATTAGGGAGAATGCCCTCCGCAGTGGGTTATCAACCT
ACTCTTAGTACAGAAATGGGTTCTTTGCAAGAAAGAATTGCTTCTACTAAAAAGGGAT
CTATAACTTCGATTCAAGCAGTTTATGTACCTGCAGACGATTTGACCGACCCTGCCCCT
GCCACAACATTTGCACATTTGGATGCTACTACCGTACTTTCCAGAGGATTAGCTTCCAA
GGGTATTTATCCAGCAGTAGATCCTTTAGATTCAACCTCGACTATGTTACAGCCTCGGAT
CGTTGGCAACGAACATTATGAAACTGCGCAAAGAGTTAAGGAAACTTTACAACGTTAC
AAAGAACTTCAGGACATTATTGCAATTCTTGGCTTGGATGAATTATCGGAAGAGGATCG
TTTAACTGTAGCAAGAGCAAGAAAAATTGAGCGTTTCTTATCACAACCGTTCTTTGTGG
CAGAAGTTTTTACTGGTTCTCCAGGAAAGTATGTTGCTCTTGCGGAAACTATTAGGGGA
TTTCAACTAATCCTTTCCGGAGAATTAGACGGCCTACCTGAACAGGCTTTTTATTTGGT
GGGTAACATCGATGAAGCTAGCACGAAAGCTATAACCTTAGAAGAGGAGAACAAATCG
CAGAAAATGAAATTAAATCTTTATGTACTGACTCCTAAGCGAATTATTTGGGATTGTGAG
GTGAAAGAAATCATTTTATCCACTAATAGTGGCCAAATTGGCGTATTACCAAACCACGC
CCCCATTAACACAGCAGTAGATATGGGTCCTTTGAGAATACGCCTCAACGACCAATGGT
TAACAGCGGTTCTGTGGAGCGGTTTTGCGAGAATAGTTAATAATGAGATCATCATTTTA
GGAAATGATGCGGAACTGGGTAGTGATATTGATCCGGAAGAAGCTCAAAAGGCACTTG
AAATAGCCGAAGCTAACTTGAGTAAAGCTGAGGGTACGAAAGATTTGGTTGAAGCGA
AGCTCGCTCTCAGACGAGCTAGGATACGAATCGAGGCTGTCAATTGGATTCCCATGAA
AAATGTAACCCATTCTTTCGTTTTTTTAGCTCACTGGCCATCCGCTGGCAGTTTCGGGCT
TAATACCGATATTTTAGCAACAAATCTAATAAATCTAACTGTAGTGGTTGGTGTTTTGAT
TTTTTTTGGAAAGGGAGTGTTAAAAGATTTATTAGATAATCGAAAACAGAGGATCTTGA
GTACTATTCGAAATTCAGAAGAATTGCGTAGAGGGACCATTGAGCAACTCGAAAAAGC
TCGGATTCGATTACAGAAAGTCGAACTAGAAGCGGATGAGTATCGAATGAATGGATACT
CTGAGATAGAACGAGAAAAAGCAAATTTGATTAATGCTACTTCTATTAGTTTGGAACAA
TTGGAAAAGTCTAAAAACGAAACCCTTTATTTTGAAAAACAAAGGGCAATGAATCAGG
TCCGACAGCGGGTTTTCCAACAGGCCGTACAAGGAGCTCTAGGAACTCTGAATAGTTG
TTTGAATACCGAGTTACATTTCCGTACGATTCGTGCTAATATTGGCATTCTCGGGTCCCT
GGAATGGAAGAGA---ATGAATCCACTAATTGCTGCTGCTTCTGTTATTGCTGCTGGATTG
GCCGTAGGGCTTGCTTCTATTGGACCTGGAGTTGGTCAAGGTACTGCTGCAGGACAAG

CTGTAGAAGGTATTGCGAGACAGCCAGAAGCAGAAGGTAAAATACGAGGTACTTTATT
GCTTAGTCTAGCTTTTATGGAAGCTTTAACAATTTATGGACTAGTTGTGGCACTAGCGCT
TTTATTTGCGAACCCTTTTGTTATGAATATTATACCTTGTTCCATTAAAACACTCAAGGG
GTTATACGATATATCGGGTGTAGAAGTAGGCCAACACTTCTATTGGCAAATAGGAGGTT
TCCAAATTCATGCCCAAGTACTCATCACTTCTTGGGTCGTAATTACTATCTTGCTAGGTT
CAGTTGTCATAGCTGTTCGGAATCCACAAACCATCCCGACCGACGGTCAGAATTTTTTT
GAATATGTCCTTGAGTTTATTCGAGACTTGAGCAAAACTCAGATTGGAGAAGAATATGG
TCCCTGGGTTCCCTTTATTGGAACTATGTTCCTTTTTATTTTTGTTTCGAATTGGTCGGGT
GCTCTTTTACCTTGGAAAATTATAGAGTTACCCCATGGGGAATTAGCAGCGCCCACGAA
TGATATAAATACTACTGTTGCTTTAGCTTTACTCACGTCAGCGGCATATTTTTATGCTGGT
CTTAGCAAAAAAGGATTGAGCTATTTCGAGAAATATATTAAACCAACTCCAATCCTTTT
ACCAATTAACATCCTAGAAGATTTCACAAAACCATTATCACTTAGCTTTCGACTTTTCGG
GAATATATTGGCGGATGAATTAGTCGTTGTTGTTCTTGTTTCTTTAGTCCCCTTAGTAATC
CCTATACCGGTCATGTTTCTTGGATTATTTACAAGCGGTATTCAAGCTCTTATTTTTGCAA
CATTAGCCGCAGCCTATATAGGTGAATCCATGGAGGGTCATCATATGCTATTTGCAACTT
TAGAACATATACTAACTCATATCTCTTTCTCAACGATTTCAATTGTGATTACGATTCATTT
GATAACCTTATTAGTTCGTGAACTTGGAGGATTACGTGATTCGTCAGAAAAAGGAATGA
TAGTTACTTTTTTCTCTATAACAGGATTCTTAGTTTCTCGTTGGGCTTCCTCGGGACATTT
TCCATTAAGTAATTTATATGAGTCATTGATCTCCCTTTCATGGGCCCTGTATATTCTTCATA
CCATTCCTAAAATACAGAACTCTAAAAATGATTTAAGCACAATAACTACGCCAAGTACT
ATTTTAACGCAAGGCTTTGCCACATCGGGTCTTTTAACTGAAATGCATCAATCCACGAT
ACTAGTACCTGCTCTACAATCTCAGTGGTTAATGATGCATGTCAGTATGATGTTATTAAG
CTATGCAACTCTTTTGGGCGGACCCCCATTATCCGCCGCTATTCTAATCATTAGATTTCG
AAATAATTTCCATTTCTTTTCTAAAAAGAAAGATATTAAATATTTTTATGCAAAAAGAAG
TGCTTTAAAAAGAACCTCTGTCCCTTCATTTCCAAATTATTACAAATATCAATTAACGGA
GCGTTTGGATTCTTGGAGTTATCGTGTCATTAGCCTAGGATTTACCCTTTTAACCGGAGG
GATTCTTGGTGGAGCAGTATGGGCTAATGAGGCCTGGGGAGCCTATTGGAATTGGGATC
CCAAGGAAACTTGGGCATTTATTACTTGGACCATATTCGCAATTTATTTACATAGTAGAA
CACATCCAAATTGGAAGGGTACGAATTCCGCACTTATAGCTTCGATAGGATTTCTTATAA
TTTGGATTTGTTATTTTGGTATAAATCTATTAGGAATAGGCTTACATAGTTATGGTTCGTTT
ACATTAACAATGAAAAAAAAGAAAGCATTGCCTTCTTTACTATATCTTGTATTTATCGTA
CTTTTGCCTTGGGGGGTCTCTTCCTCATTTAACAAATGTCTGGAACTTTGGATTAAGAA
TTGGTGGAATACCAGGCAATCCGAAACTCTCTTAACTGATATTCAAGAGAAAAGGATTC
TAGAAAGATTCATAGAATTAGAAGAACTTTCTCTCTTGGACGAGATGATAAAAGGGAA
ACTAAAGACACATGTACAAAAACCTCCTACAGGAATACACAAGGAAATAATACAATGG
GTCAAAATAAATAATGAGGATCATCTCCATACCATTTTGCATTTCTCGACAAATATAATCT
GTTTGGCTATTCTAAGTGGTTCTTTTTTTCTGGGTAAAGAGGAACTTGTCATTTTGAATT
CTTGGGTTCAGGAATTCTTCTATAATTTAAATGACTCAATAAAAGCTTTTTTTATTCTTTT
AGTTACTGATTTTTTTGTTGGATTTCACTCCACCCGTGGTTGGGAACTAGTAATTCGTTG
GGTCTACAACGATTTTGGATGGGCTCCTAACGAGCTTATTTTCACTATTTTTGTTTGTAG
TTTTCCAGTAATTCTAGATACATGTTTGAAATTTTGGGTCTTTTTTTGTTTAAACCGTCTA
TCTCCTTCGCTTGTAGTCATTTATCATTCAATTAGTGAAATGCCCATTGGTGTTCCAAAA
GTACCTTACCGGATTCCTGGAGATGAAGAAGCGACTTGGGTTGACTTATACAATGTTAT
GTATCGAGAAAGGACACTTTTTTTAGGTCAAGAGATTCGTTGCGAGATCACGAATCATA

TTACAGGTCTCATGGTATATCTGAGTATAGAAGATGGAATTAGTGATATTTTTTTGTTTAT
AAACTCCCCCGGCGGGTGGCTAATCTCAGGAATGGCTATTTTTGATACGATGCAAACGG
TGACACCAGATATATATACAATATGCCTCGGAATAGCCGCCTCCATGGCATCCTTCATTC
TGCTTGGAGGAGAACCCACCAAGCGTATAGCATTCCCTCACGCGAGGATTATGCTTCAC
CAACCTGCTAGTGCTTATTATCGGGCAAGGACACCAGAATTTTTACTAGAAGTAGAAGA
GTTACACAAAGTTCGCGAAATGATCACAAGGGTTTATGCAGTAAGAACAGGCAAGCCT
TTTTGGGTTGTATCCGAAGACATGGAAAGGGATGTTTTTATGTCAGCAGACGAAGCCA
AAGCTTATGGACTTGTCGATATTGTAGGGGATGAACCGAGAGAAGCAAAAGTAACTTT
CGAAGGTTTAGTTACGGAAGCCCTACCCAATGGAATGTTCCGCGTTCGGCTAGAGAAT
GACACCATCATCCTAGGCTATATTTCAGGAAAGATACGGTCTAGTTCTATACGAATACTG
ATGGGGGATAGGGTCAAAATTGAAGTAAGTCGTTATGATTCAAGCAAGGGACGTATAAT
TTATAGACTTCCCCATAAGGATTCGAAGGATATGGAAAAATTCGAAGGGTATTCAGAAA
AACATAAATCTCGTCAACAATACTTCGTCTACCCACTTCTCTTTCAGGAATATATTTATG
CATTTGCCCCTGATTATGGATTAGTTGGTTGTAATAACAAATTTAGTTCACTACTTGTGA
AACGTTTAATTATTCGAATGTATCAGCAGAATTTTTTGGATAACTCGGTTAATCATCCTA
ACCAAGATCGATTATTGGATTCTGAGTTTTATTCTCAGATTCTATCTGAAGGGTTTGCGA
TCGTTGTGGAAATCCCATTCTCGCTACGGGAATTATTTTGTCCGATACCAAAGTTTCAG
AATTTACGCTCTATTCATTCAATATTTCCCTTTTTTGAAGACAAATTTTTGCATTTGGATT
ATCTATCACATATAGAAATACCCTATCCTATCCATTTGGAAATCCTGGTTCAACTCCTTCA
ATACCGTATCCAAGATGTTCCATCCTTGCATTTATTGCGATTTCTTCTCAACTACTATTCG
AATTGGAATAGTTTTATTACTAAATCCATTTTTATTTTCAAAAAAAAAAGACTATTTCGA
TTCCTATATAACTCTTATGTATCAGAATATGAATTTTTTTTGTTGTTTCTTCGTAAACAATC
TTCTTGCTTACCATTAGCATCTTCTGGAACTTTTCTGGAACGAATCCACTTTTCTAGGAA
GATGGAACAT------ATAATGTACCCTGGTCGGAAAACCCTATGGTTCTTTATGGATCCTCT
TATGCATTATGTTCGATATCAAGGAAAGGCAATTCTTGCATCAAAAGGCACTTTTCTTTT
GAAGAAGAAATGGAAATGCTACCTTATCAATTTATGGCAATATTATTTCTGTTTTTGGAC
TCAGCCGCGAAGAATCCATATAAACCAATTAGCAAACTCTTGCTTCGATTTTATGGGGT
ACCTTTCAAGTGTACCAAAAAGTTCTTTGTTAGTAAGGAATCAAATGCTGGAGAATTCA
TTTCTAATAGATACTCGAATG------AAAAAATTCGATACCATAGTCCACGCTACTCTCCTC
ATAGGATACTTATCAAAAGCTCAATTTTGTACTGGATCGGGGCATCCTATTAGTAAACCC
ATTTGGACAGATTTATCAGATTGGGATATTCTTGATCGGTTTGGTCGGATATGTAGAAAT
CTTTTTCATTATCACAGTGGATCTTCAAAAAAGAAGACTTTGTATCGACTAAAGTATATA
CTTCGACTTTCATGCGCTAGAACTTTAGGTCCTAAACATAAAAGCACGGTACGAGCTTT
TATGCAATGGTTGGGTTCGGTATTTTTAGAAGAATTTTTTAGGGAAGAAGAGCAAGTTT
TTTCTTTGATGTTCGCCAAACGTATTTGGTATTTGGATATTCTCCGTATCAATGATCTGGT
GAATCCTCTTATGATAATAGATAGGGTA---GAGGTAGAA---ACTATCAATTCTTTTTGGAA
ATTGGAA---TTATTAAAAGAAGTCTATGGACTGATATCGATTCTA---CCCATTTTGACCCT
CCTTTTGGGAATAACAATAGAGGTACTCGTAATTGTGTGGTTAGAAAGAGAAATATCTG
CGTCGATACAACAACGTATTGGTCCTGAATATGCTGGCCCCCTGGGCCTGCTTCAAGCT
ATAGCAGATGGGACTAAGCTACTTTTTAAAGAAGATATTCTGCCATCCCGAGGAGATAT
TTCTTTATTTAGCATTGGACCCTCTATAGCAGTCATATCAGTTTTATTAAGTTTTTTAGTTA
TCCCTTTGGGATATCATTTTGTTTTAGCCGATCTTAGTATTGGTGTTTTTTTATGGATTGC
CATTTCAAGTATAGCTCCTATTGGTCTTCTTATGGCAGGGTATAGCTCAAATAATAAATAT
TCTTTTTCAGGCGGTCTACGAGCTGCTGCTCAATCCATTAGTTATGAAATACCATTAACT

TTTTGTGTGCTAGCAATATCTCTACTATCTAACAGTTCAAGTACAGTTGATATAGTTGAA
GCACAGTCAAAATACGGTTTTTTTGGATGGAATATTTGGCGTCAGCCTATAGGTTTTCTG
GTTTTTTTAATTTCTTCTTTGGCAGAATGTGAAAGATTACCCTTTGATTTACCAGAAGCG
GAGGAAGAATTAGTAGCAGGTTACCAAACTGAATATTCCGGTATCAAATATGGTTTATTT
TATCTTGTTTCTTACCTAAATTTATTAGTTTCTTCTTTATTTGTAACAGTTCTCTACTTGGG
CGGGTGGAATTTATCTATTCCCTATATATCCTTTTTTGATTTTTTCCAAATGAATAAAGCA
GTTGGAATTTTGGAAATGACAATGGGTATATTTATTACATTAACTAAAGCTTATTTATTTC
TCTTCATTTCTATCACAATAAGATGGACTTTACCGAGGATGAGAATGGATCAGTTATTAA
ATCTTGGATGGAAATTTCTTTTACCTATTTCCCTGGGCAATCTCTTATTAACAACCTCTTC
TCAACTTGTTTCACTAATGATCTGGCATGTACAGAATGAAAACTTCATTCTCGATTCTAC
GAGTATTTTTATGAAGGCGTTTCATTTGCTTCTCTTCAATGGAAGTTTCATTTTCCCAGA
GTGTATCCTAATTTTTGGCCTAATTCTTCTTCTGATGTTCGATTCAACCTCTGTTCAAAA
AGATAGACCTTGGTTCTATTTCATCTCTTCAACATGTTTAGTAATAAGCATAACGGCCCT
ATTGTTCCGATGGAGAGAAGAACCTATAATTAGCTTTTCGGGAAATTTCCAAACGAACA
ATTTCAACGAAATCTTTCAATTTCTCATTTTATTATGTTCAACTTTATGTATTCCTCTATCC
GTAGAGTACATTGAATGTACAGAAATGGCTATAACAGAGTTTCTGTTATTCGTATTAACA
GCTACTCTAGGGGGAATGTTTTTATGTGGTGCTAACGATTTAATAACTATCTTTGTAGCT
CCAGAATGTTTCAGTTTATGTTCCTACCTATTGTCTGGATATACCGAGAGAGATCTACGG
TCTAATGGGGCTTCTTTGTAATATTTACTCATGGGTGGGGCAGGCTCTTCTATTCTGGTT
CATGGTTTCTCTTGGCTATATGGTTCATCTGGGGGGGAGATCGAGCTTCAAGAAATTGT
GAACGGTCTTATAAATACACAAATGTATAACTCCCCAGGAATTTCACTTGCGCTTATATC
CATCACTGTAGGACTTGGGTTTAAGCTTTCCCCAGCCCCTTTTCATCAATGGACTCCTG
ACGTCTACGAAGGATCCCTACTCCACTTCGTTGCTTTTCATTCTATTACTTCGAAAGTAG
CTGCTTCAGCCTCAGCCACGCGAATTCTCGAATTCTCTCTTTATTTCTCATCAAACGAAT
GGCATCTTCTTCTGGAAATCCTAGCTATTCTTAGCATGATTTTGGGAAATCTCCTTGCTA
TTACTCAAACAAGCATGAAACGTATGCTTGCATATTCGTCCATAGGGCAAATCGGATAT
GTAATTATTGGAATAATTGTTGGAGACTCAAATGATGGATATGCAAGCATGATAACTTAT
ATGCTGTTCTATATCTCCATGAATCTAGGAACTTTTGCTTGCATTGTATTATTTGGTCTAC
GTACCGGAACTGATAACATTCGAGATTATGCAGGATTATACACGAAAGATCCTTTTTTG
GCTCTCTCTTTAGCCCTATGTCTCTTATCCCTAGGAGGCCTTCCTCCACTAGCAGGTTTC
TTCGGAAAACTCTATCTATTCTGGTGTGGATGGCAAGCAGGCCTATATTTCTTGGTTTCA
ATAGGACTCCTTACGAGCGTTCTTTCTATCTACTATTATCTAAAAATAATCAAGTTATTAA
TGACTGGACGAAACCAAGAAATAACCCCTTATGTGCGAAATTATAGAAGATCCCCTTTA
AGATCAAACAATTCCATCGAATTGAGTATGACTGTATGTGTGATAGCATCTACTATACCA
GGAATATCAATGAACCCCATTCTTGCAATTGCTCAGGATACCCTCTTTATGTTTCTGCTT
CACGAATATGATATTTTTTGGACATTTCTAATAATAGCAAGCCTTATTCCTATTTTGGCAT
TTTCGATTTCAGGACTTTTAGCCCCGGTTAGTGAAGGACCAGAGAAGCTTTCTAGTTAT
GAATCGGGTATAGAACCCATGGGAGGGGCTTGGGTACAATTCCGAATACGCTATTATAT
GTTTGCGCTCGTTTTTGTTGTTTTTGATGTGGAAACCGTCTTTCTATACCCTTGGGCAAT
GAGTTTCGACGTATTGGGTGTATCCGTTTTTATCGAAGCTTTGATTTTCGTGCTTATCTTA
GTTGTTGGTTTAGTTTATGCATGGCGAAAAGGAGCCTTGGAATGGTCTATGAGTTATTTT
CCTTGGTTAACAATACTTGTTGTTTTGCCGATATTTGCAGGTTCATTAATTTTCTTTTTAC
CTCATAAGGGAAACAAAGTCGTTAGGTGGTATACTATATCTATTTGTTTATTAGAATTCC
TTCTAATGACTTATGCGTTCTGTTATCATTTCCAACTGGAGGATCCCTTAATCCAATTAA

AGGAGGATTATAAATGGATAGATGTCTTCGATTTCCACTGGAGATTGGGAATCGATGGA
CTTTCATTAGGATCTATTTTATTGACAGGATTTATCACTACTTTAGCTACTTTAGCAGCTT
GGCCAATTACACGGAATTCGCGATTATTCTATTTCCTGATGCTCGCAATGTATAGTGGTC
AAATAGGATTATTTTCTTCGCGAGACCTTTTACTTTTTTTTATCATGTGGGAGTTAGAATT
AATTCCTGTTTACTTACTTTTATCCATGTGGGGGGGGAAGAGGCGTCTATATTCAGCTAC
AAAGTTTATTTTGTATACTGCAGGCGGTTCCATTTTTTTCTTAATCGGAGTTCTGGGTAT
GGGCTTATATGGTTCCAACGAACCAGGATTAGATTTGGAAAGATTAATTAATCAATCATA
CCCTGCAACCTTGGAAATACTTTTATATTTTGGCTTCCTTATTGCTTATGCTGTCAAATTG
CCGATTATACCCCTACATACGTGGTTACCAGATACCCATGGGGAAGCACATTATAGTACA
TGTATGCTTTTAGCGGGAATATTATTAAAGATGGGAGCATATGGATTGATTCGGATCAAC
ATGGAATTGTTACCTCATGCTCATTATCTATTTTCGCCCTGGTTAGTAATAATAGGAGCGA
TCCAAATAATCTATGCAGCTTCAACTTCTCTTGGTCAACGAAATTTCAAAAAAAGAATA
GCTTATTCCTCTGTATCTCACATGGGTTTCATAATTATAGGAATTGGTTCCATAACCAACA
TTGGACTCAATGGAGCTATTTTACAAATATTATCCCATGGATTTATTGGTGCTACACTTTT
TTTCTTGGCAGGAACCGCTTCTGATAGAATGCGTCTTGTTTATCTCGAAGAACTGGGGG
GAATATCTATCCCAATGCCTAAAATTTTTACCATGTTTAGTAGCTTTTCAATGGCTTCTCT
TGCCTTACCAGGAATGAGTGGTTTTGTCGCAGAATTAGTAGTATTTTTTGGACTAATTAC
TAGTCCAAAATTTCTGTTAATGCCAAAAACACTAATTACTTTTGTAATGGCAATTGGAAT
GATATTAACTCCTATTTATTTATTATCTATGTTACGCCAGATGTTCTATGGATACAAGCTAT
TTAATGTTCCAAACGCAAATTTTGTGGATTCTGGACCACGAGAACTCTTTATTTTAATCT
GTATCTTTTTACCAGTAATAGGAATTGGTATTTATCCAGATTTTGTTCTCTCCCTATCCGT
TGACAGGGTAGAGGCTCTCTTATCCAATTATTATCCTAAAATGATGTTTGAGCATGTACT
TTTTTTGAGTGTCTATTTATTTTCGATTGGTATCTATGGATTGATCACAAGCCGAAACATG
GTTAGAGCTCTAATATGTCTTGAACTTATACTGAATTCAATTAATCTAAATCTCGTAACAT
TTTCTGATCTATTTGATAGTCGCCAATTAAAAGGAGATATTTTCGCAATTTTTGTTATAGC
CCTTGCGGCTGCTGAAGCCGCTATTGGACTATCCATTCTTTCTTCCATCCATCGTAATAG
GAAATCCACTCGTATCAATCAATCTAATTTATTGAATAATATGGAACATACATATCAATAT
GCATGGGTAATCCCTCTTCTCCCACTTCCAGTTATTATGTCAATGGGATTTGGCCTTATTC
TTATTCCGACAGCAACAAAAAATCTTCGTCGCATATGGGCTTTTCCTAGTGTTTTACTCT
TAAGTATAGCTATGGTATTCTCAGTTCAACTGTCTATTCAACAAATAAATGGAAGTTCGA
TCTATCAATATCTATGGTCTTGGACCGTCAATAATGATTTTTCTTTAGAATTTGGATACTT
GATTGACCCACTTACTTCTATTATGTTAATACTAATTACTACTGTAGGAATCCTGGTTCTT
ATTTATAGTGATGGTTATATGTCTCACGATGAAGGATATTTGAGATTTTTTGTTTATATAA
GTTTTTTCAATACTTCCATGTTGGGATTGGTTACTAGCTCCAATTTGATACAAATTTATTT
TTTTTGGGAACTCGTGGGAATGTGTTCTTATTTATTGATAGGCTTTTGGTTTACACGACC
AATCGCAGCGAGTGCTTGTCAAAAAGCTTTTGTAACTAATCGTGTAGGGGATTTTGGTC
TATTATTAGGAATTTTAGGTTTTTTTTGGATAACAGGTAGTTTAGAGTTTAGGGATTTGTT
CAAAATAGCTAATAACAATGGAGTTAACTCTTTACTTACTACTTTGTGTGCTTTTTTATTA
TTCCTTGGTGCAGTTGCGAAATCCGCACAATTCCCTCTTCACGTATGGTTACCCGATGCT
ATGGAAGGACCCACTCCTATTTCAGCTCTTATACAAGCAGCAACTATGGTTGCTGCGGG
AATTTTTCTTCTAGCTCGACTTCTTCCTCTTTTCATATCTTTACCTTTGATAATGAGTTTTA
TTTCTTTAGTAGGTACAATAACACTATTCTTAGGAGCTACTTTAGCTCTTGCTCAGAGAG
ATATTAAAAGAAGCTTAGCCTATTCTACAATGTCTCAATTGGGTTATATGATGTTAGCTCT
AGGTATAGGTTCTTATCAAGCTGCTTTATTCCATTTGATCACTCATGCTTATTCAAAAGCT

TTATTGTTCTTGGGATCCGGATCCGTTATTCATTCAATGGAACCTCTTGTTGGATATTCAC
CAGATAAAAGTCAAAATATGGTTCTTATGGGCGGTTTAAGAAAATACATTCCAATCACA
AGAACTACTTTTTTATGGGGTACACTTTCTCTTTGTGGTATTCCACCTCTTGCTTGCTTC
TGGTCCAAAGATGAAATCCTTAGTAATAGTTGGTTGTATTCGCCCTTTTTTGGAATAATA
GCCTCCTTTACTGCAGGATTAACTGCCTTTTATATGTTTCGGATATATTTACTTACATTTG
ATGGGTATTTGCGTGTTCATTTTCAAAATTACAGTAGCACTAAAGAGAGTTCCTTGTATT
CAATATCCTTATGGGGAAAAAGGATACCCAAAGGAGTGAATAGG---GATTTCGTTTTATC
AACAACGAAGTTTTTTTCAGCTAAAAAC------ACTTTTGCCTATCCGCATGAAACGGGA
AATACTATGTTATTTCCTCTTCTTATATTACTACTTTTCACTTTGTTCATTGGATTCATAGG
AATCTCTTTTGAGTTAACCATATTATCAAAGTGGTTAACTCCCTCAATAAACTTTACTCA
GGAAAGTTCTAATTCTTTTGTAAATTCATATGAATTTATTACTAATGCAATTTCTTCTGTA
ACTCTAGCTATCTTTGGTTTATTCATAGCATATATCTTCTATGGATCCGCTTATTCTTTTTT
TCAGAATTTGGATTTAATAAACTCTTTTTACGAATTTTTAGATCAAGTAAAAAAAAATAT
ATACAGTTGGTCATATAATCGTGGTTATATAGATATTTTCTATACTAGGGTCTTTACCCTC
GGTATAAGAGGGTTAACCGAACTAACGGAGTTTTTTGATAAGGGTGTTATTTATGGAAT
TACCAATGGAGTGGGTCTTCCTAGTTTTTGTATAGGAGAAGAAATTAAATAT---GTAGGG
GGAGGTCGAATCTCGTCTTATTTATTCTTTTTTTTATGTTATGTATCTTTGTTTTTATTCTT
TTTTCTTATGGATTTACCTGGACCAATACATGAGATTCTTATGCTATTTGGGGGATTTGTT
CTTCTACTAGGGGGTCTAGGAGTAGTATTACTTACCAACCCCATTTATTCTGCCTTTTCG
CTGGGATTAGTTCTTGTTTGTATATCCTTATTCTATTTTTTATTAAATTCCTACTTTGTAGC
TGTCGCACAACTTCTTATTTATGTGGGAGCCATAAATGTCTTGATCATATTCGCTGTAAT
GTTCGTAAATGGCTCAGAATGGTCTAAAGATAAGAATTATTGGACTATTGGAGATGGGT
TCACTTCACTCGTTTGTATAACTATTGTTTTTTCACTAATGACTACTATCCCAGATACGTC
GTGGTATGGAATTCTTTGGACTACAAGATCAAACCAAATAGTAGAACAGGGTCTCATAA
ATAATGTTCAACAAATTGGAATTCATTTAGCAACCGATTTTTATCTTCCGTTTGAACTCA
TTTCCATAATTCTTCTAGTTTCTTTAATAGGTGCAATTACTATGGCTCGGCAAATGAGTCT
ACCGCTTACAAGAAAAGATCTCATGATAGTCAATATGGGCCCTCAACACCCATCAATGC
ATGGTGTTCTTCGACTGATCGTTACTCTCGATGGTGAGGATGTTATTGATTGTGAACCCA
TATTAGGCTATTTACACAGAGGAATGGAAAAAATCGCGGAAAACCGAACTATTATACAA
TACTTACCTTATGTAACAAGGTGGGATTATTTAGCTACTATGTTTACAGAAGCAATAACA
GTAAATGCACCAGAATTCTTGGAGAATATTCAAATACCACAAAGAGCCAGCTATATTAG
GGTAATTATGTTAGAATTAAGCCGGATAGCTTCTCACTTGCTATGGCTTGGACCTTTTAT
GGCGGATCTCGGCGCACAGACTCCTTTTTTCTATATTTTTAGAGAGAGAGAATTAATATA
TGATCTATTTGAAGCTGCTACAGGTATGCGAATGATGCACAATTACTTTCGCATCGGAG
GAGTAGCCGCCGATCTACCTTATGGATGGATCGAGAAATGTTTAGATTTCTGTGATTATT
TTTTACGCGGAGTTGTTGAATATCAACAACTTATTACACAGAATCCAATTTTTTTAGAGC
GAGTTGAAGGAGTAGGTTTTATTAGCGGAGAAGAAGCAGTAAATTGGGGCTTATCGGG
ACCGATGTTACGAGCTTCTGGAATACAATGGGATCTTCGTAAAGTAGATCCTTATGAGT
CTTACAACCAATTTGATTGGAAAGTCCAATGGCAAAAAGAAGGGGATTCGTTAGCTCG
CTATTTAGTACGAGTTGGTGAAATGAGGGAATCCATCAAAATAATTCAACAAGCTGTAG
AAAAAATTCCTGGAGGACCTTATGAGAATTTAGAAGTCCGACGC---AAGAAAGAAAAG
AATTCTGAATGGAATGATTTTGAGTATAAATTTCTTGGTAAAAAACCTTCGCCCAATTTT
GAATTGTCAAGACAAGAGCTTTATGTAAGAGTAGAAGCCCCAAAAGGTGAATTAGGGA
TTTATCTAGTAGGAGATGATAGCCTTTTCCCCTGGAGATGGAAAATCCGTCCACCCGGG

TTTATTAATTTGCAAATTCTTCCTCAGCTAGTTAAAAAAATGAAATTGGCTGATATCATG
ACGATATTAGGTAGTATAGATATCATTATGGGGGAAGTTGATCGTATGTTCCCTATGGTAA
CTGGGTTCATGAGTTATGGTCAACAAACAATACGTGCTACAAGGTACATAGGTCAAAGT
TTCATAACTACCTTATCCCACACAAATCGTTTACCTATAACGATTCACTACCCTTATGAA
AAATCAATTACACCAGAGCGTTTCCGAGGGCGAATCCACTTTGAATTTGATAAATGTAT
TGCTTGTGAAGTATGTGTTCGCGTATGTCCGATAGATCTACCCGTTGTGGATTGGAGATT
TGAAAAGGATATTAAAAGAAAACAATTGCTTAATTATAGTATTGATTTCGGAGTTTGTAT
ATTTTGTGGCAATTGTGTTGAGTACTGTCCAACAAGCTGTTTATCAATGACTGAAGAAT
ATGAACTTTCTACCTATGATCGTCATGAATTGAATTACAATCAAATTGCTTTAAGTCGGT
TACCAATCTCCATAATGGGAGATTACACAATTCAAACAATTAGGAATTCGAAAATAAAC
AAAGAAAAATCTTCGAATTCAAGAACGATTACTGATTACCAGCAGGGTTGGTTATCTAA
TTGGCTAGTCAAACATGAGGTGGTTCATAGATCTTTGGGCTTTGATCACCGAGGAATAG
AGACTTTACAAATAAAAGCAGGGGATTGGGATTCCATTGCTGTCATTTTATATGTATATG
GTTACAATTATTTACGCTCCCAATGTGCTTATGATGTAGCACCCGGTGGATCTTTAGCTA
GCGTGTATCATCTTACGAGAATACAATATGGTATAGATAATCCAGAAGAAGTCTGCATAA
AAGTCTTTGCCCAAAAGGATAATCCTAGAATTCCGTCTGTCTTCTGGATTTGGAGAAGT
GCCGATTTTCAAGAACGCGAATCTTATGATATGGTGGGAATCTCTTATGATAATCATCCG
CGCCTTAAACGTATCCTAATGCCTGAAAGTTGGATAGGCTGGCCCTTACGTAAGGATTAT
ATAACCCCCAATTTTTATGAAATACAAGATGCTCAT---
---ATGGTCTTAACT---GAATATTTAGAC---A
AAAAAAAA---GAAGGAAAAGATTCTATTGAGACAGTTATGAATTTGATTGAGTTTCCCT
TACTTGACCAAACAAGTTCCAATTCTGTTATTTCAACTACACCAAATGATCTTTCGAATT
GGTCAAGACTCTCTAGTTTATGGCCCCTTCTATACGGTACCAGTTGTTGTTTCATTGAAT
TTGCTTCATTAATAGGCTCACGATTCGACTTTGATCGTTATGGATTGGTACCAAGATCAA
GTCCTAGGCAAGCAGACCTAATTTTAACAGCCGGTACGGTAACAATGAAAATGGCTCC
CTCTTTAGTGAGATTATACGAGCAAATGCCTGAACCAAAATACGTCATTGCTATGGGAG
CCTGTACTATTACAGGGGGAATGTTCAGTACGGATTCCTATAGTACTGTTCGGGGAGTT
GATAAGTTAATTCCTGTGGATGTCTACTTGCCGGGCTGCCCACCTAAACCGGAGGCAGT
TATAGATGCCCTAACAAAACTTCGTAAGAAGATATCGCGAGAAATAGTTGAGGATCGAA
CTCTATCTCAA---AAAAATAGATGTTTTACTACCAGTCACAAGCTTTATGTTAGGCGCAG
TACTCATACAGGAACTTATGAGCAAGAATTGCTCTATCAATCACCATCTACATTAGATAT
ATCTACT------TTTTTCAAATCCAAAAGTTCAGTACCTTCCTACAAATTAGTGAATATGGA
AAATAGAAATACTTTTTCTTGGGTAAAGGAACAGATAACTCGATCGATTTCTGTATCGAT
CATGATATACGTAATAACTCGGACATCTATTTCAAATGCATATCCCATTTTTGCGCAGCA
GGGTTATGAAAACCCACGAGAAGCAACTGGACGAATTGTATGTGCCAATTGCCATTTA
GCTAGCAAGCCCGTCGATATTGAAGTTCCCCAAGCTGTGCTTCCCGATACTGTATTTGA
AGCAGTTCTTCGAATTCCTTATGATATGCAATTGAAACAAGTTCTTGCTAATGGAAAAA
AGGGAGGGTTGAATGTAGGTGCTGTTCTTATTTTGCCCGAGGGATTCGAATTAGCGCCG
CCCGATCGTATTTCCCCTGAGTTAAAAGAAAAGATAGGAAATCTTGCTTTTCAGAGTTA
TCGTCCCGATAAAAAAAACATTCTTGTGATAGGCCCTGTTCCCGGTAAGAAATATAGTG
AAATTGTCTTTCCCATTCTTTCCCCTGATCCTGCTACGAAGAAAGATGCTCATTTCTTAA
AATATCCCATATATGTAGGGGGAAACCGAGGAAGAGGACAGATCTATCCTGATGGTAGC
AAGAGTAACAATACAGTCTATAATGCTACGTCAACAGGTATAGTAAGAAAAATACTACG
TAAAGAAAAAGGGGGGTATGAAATATCCATAGTTGATGCATCAGATGGACGCCAAGTG

ATTGATATTATACCTCCCGGACCAGAACTTCTTGTTTCAGAGGGGGAATCCATCAAGCT
TGATCAACCATTAACAAGCAATCCTAATGTGGGAGGTTTTGGTCAGGGGGACGCAGAA
ATCGTGCTTCAGGATCCATTACGTGTTCAAGGCCTTTTGAACGTCCTCGAATCCGTTATT
TTGGCACAAGTTTTTTGGTTCTCATCAACGAAACAGTTTGAAAAGGTTCAATTGTACG
AAATGAATTTCATGAGTAAAGTATATGATTGGTTTGAGGAACGTCTTGAGATTCAGGCA
ATTGCAGATGATATAACTAGTAAATATGTTCCTCCCCATGTCAACATATTTTATTGTTTAG
GGGGAATTACACTTACTTGTTTTCTAGTACAAGTCGCTACCGGTTTTGCTATGACTTTTT
ACTACCGACCAACCGTTACAGAGGCTTTTTCCTCGGTTCAATACATAATGACCGAGGCC
AACTTTGGTTGGTTAATCCGATCCGTTCATCGATGGTCAGCAAGTATGATGGTTTTAATG
ATGATCCTGCATGTATTTCGTGTGTATCTCACAGGGGGATTTAAAAAACCCCGTGAATTA
ACTTGGGTCACAGGTGTGGTTTTGGCTGTTTTGACTGCATCATTTGGTGTAACTGGTTA
TTCTTTGCCTTGGGATCAAATTGGCTATTGGGCAGTCAAAATTGTGACAGGTGTACCTG
ACGCCATTCCGGTAATAGGATCGCCTTTAGTGGAGTTATTACGCGGAAGTGCTAGTGTG
GGCCAATCCACTTTGACTCGTTTTTATAGTTTACATACCTTTGTACTGCCTCTGCTTACT
GCCGTATTTATGTTAATGCACTTTCCAATGATACGTAAGCAAGGTATTTCGGGTCCTTTA
ATGGGAGTAACAAAGAAACCAGACTTAAATGATCCTGTATTAAGAGCTAAATTAGCTAA
AGGGATGGGACATAATTATTACGGGGAACCCGCATGGCCCAACGATCTTTTATATATTTT
TCCAGTAGTAATTCTAGGTACTATTGCATGTAATGTAGGTTTAGCGGTTCTCGAGCCATC
AATGATTGGTGAACCGGCAGATCCGTTTGCAACTCCTCTGGAAATATTACCCGAGTGGT
ACTTCTTTCCCGTGTTTCAAATACTCCGTACAGTACCCAATAAGTTATTGGGCGTTCTCT
TAATGGTTTCTGTGCCAACAGGCTTATTGACAGTACCTTTTCTAGAGAATGTCAATAAAT
TTCAAAATCCATTTCGCCGCCCAGTAGCTACGACCGTTTTTTTAATCGGTACTGTAGTAG
CTCTTTGGTTAGGTATTGGAGCAACATTACCCATTGATAAATCATTAACTTTAGGTCTTT
TTATGATTGAAGTTTTTCTATTTGGAATCGTCTTAGGCCTAATTCCTATTACTTTAGCGGG
ATTATTCGTGACTGCGTATTTGCAATACAGGCGTGGGGATCAGTTGGATCTTATGCTTAC
TTTAACTAGTTATTTTGGTTTTCTACTGGCTGCTTTAACTATAACCCCAGCTCTATTTATT
GGCTTGAACAAGATACGCCTTATTATGGATATAGTAAGTCTCGCTTGGGCTGCTTTAATG
GTAGTCTTTACATTTTCTCTTTCACTAGTAGTATGGGGAAGGAGTGGACTCATGATTATT
CGTTCGCCGGAACCAGAAGTAAAAATTGTTGTGGATAGGGATCCTGTAAAAACATCTT
TTGAGGAATGGGCCAGACCCGGCCATTTCTCAAGAACACTAGCTAAGGGCCCTGATAC
TACCACTTGGATCTGGAACCTACATGCTGATGCTCACGATTTCGATAGTCATACTGGTGA
TTTGGAGGAGATTTCTCGAAAAGTCTTTAGTGCTCATTTCGGGCAACTTTCCATTATCTT
TCTTTGGTTGAGTGGCATGTACTTTCATGGCGCCCGTTTTTCCAATTATGAAGCATGGCT
AAGTGATCCTACTCACATTGGACCCAGTGCTCAGGTAGTTTGGCCTATAGTAGGGCAAG
AAATATTGAATGGTGATGTAGGCGGGGGTTTCCGAGGAATCCAAATAACCTCTGGTTTT
TTTCAGCTTTGGCGAGCATCTGGAATAACTAGTGAATTACAACTCTATTGTACTGCAATT
GGTGCATTGATTTTTGCAGCGTTAATGCTTTTTGCTGGTTGGTTCCATTATCACAAAGCC
GCTCCCAAATTGGCCTGGTTCCAAGATGTAGAATCCATGTTGAATCACCACTTAGCGGG
ATTATTAGGACTTGGGTCTCTTTCTTGGGCGGGGCACCAAATTCATGTATCTTTACCAAT
TAACCAATTTCTTGACGCTGGGGTGGATCCTAAAGAGATACCACTTCCTCATGAATTTAT
CTTGAATCGGGACCTTTTGGCTCAACTTTATCCTAGTTTTGCCGAAGGAGCAACCCCTT
TTTTCACTTTAAATTGGTCCAAATACGCAGAATTTCTGACTTTTCGCGGAGGACTAGAT
CCAGTAACCGGTGGTCTCTGGCTGACCGATATTGCGCACCATCATTTAGCTATTGCTATT
CTTTTCCTAATCGCAGGTCATATGTATAGGACCAATTGGGGTATTGGCCATGGACTTAAA

GATATTTTGGAGGCTCACAAGGGCCCATTTACAGGACAAGGCCATAAGGGTCTTTATGA
AATCTTAACAACGTCATGGCATGCTCAATTATCTCTTAACCTAGCTATGCTAGGCTCTAC
AACCATTGTTGTAGCTCATCATATGTATTCTATGCCTCCCTATCCATACCTAGCTACTGAC
TATGGTACACAACTTTCCTTGTTCACACACCACATGTGGATTGGCGGATTTCTAATAGTC
GGTGCTGCTGCACATGCAGCAATTTTTATGGTAAGAGACTATGATCCAACTACTCGATA
CAACGATCTATTAGATCGCGTCCTTAGACACCGCGATGCAATCATATCCCACCTTAACTG
GGTATGTATATTTCTAGGTTTTCACAGTTTTGGCTTGTACATTCATAATGATACCATGAGT
GCTTTAGGCCGTCCACAAGATATGTTTTCGGATACTGCCATACAATTACAACCTATCTTT
GCTCAATGGGTACAAAATATCCATGCTACTGCGCCTGGCGTAACAGCTCCTGGTGCAAC
AACAAGTACTAGCTTAACGTGGGGAGGCGGCGAGTTAGTAGCAGTAGGTGGCAAAGT
GGCTTTGTTACCTATTCCATTAGGAACCGCAGATTTTTTAGTCCATCACATTCATGCATTT
ACCATACATGTGACTGTATTAATACTTTTGAAAGGTGTTTTATTTGCTCGGAGTTCCCGT
TTGATACCCGATAAAGCAAATCTAGGTTTTCGCTTTCCTTGCGATGGGCCTGGCCGAGG
GGGAACATGTCAAGTATCCGCCTGGGATCATGTTTTCTTAGGTTTATTCTGGATGTACAA
TGCAATTTCGGTAGTCATTTTCCATTTCAGTTGGAAAATGCAGTCGGATGTTTGGGGTA
CTATAAGTGATCAAGGGGTGGTAACTCATATTACAGGGGGAAACTTTGCACAGAGTTCC
ATTACGATTAATGGGTGGCTTCGAGATTTCTTGTGGGCACAGGCATCGCAAGTCATTCA
GTCTTATGGTTCTTCATTATCTGCATATGGTCTTTTTTTCTTAGGTGCTCATTTTGTCTGG
GCCTTCAGTTTAATGTTTTTATTCAGCGGCCGTGGTTATTGGCAAGAACTCATTGAATCT
ATCGTTTGGGCTCATAACAAATTAAAAGTTGCTCCTGCTACTCAGCCTAGAGCCTTGAG
CATTATACAAGGACGCGCTGTAGGAGTAACCCATTACCTTCTGGGTGGAATTGCCACGA
CATGGGCATTCTTCTTAGCGAGAATTATTGCAGTAGGAATGGAATTAAGATTTCCCAGG
TTTAGCCAAGGCTTAGCTCAGGACCCCACTACTCGTCGTATTTGGTTTGGTATTGCTACC
GCACATGATTTCGAAAGTCATGATGATATTACTGAAGAACGTCTTTATCAGAACATTTTT
GCTTCTCACTTTGGGCAATTAGCAATAATCTTTCTATGGACGTCCGGAAATCTGTTTCAT
GTAGCTTGGCAAGGAAATTTTGAATCATGGATACAGGATCCTTTACACGTAAGACCTAT
TGCTCATGCGATTTGGGATCCTCATTTTGGTCAACCCGCTGTGGAAGCCTTTACTCGAG
GAGGTGCTGCTGGTCCAGTGAATATTGCTTATTCTGGAGTTTATCAGTGGTGGTATACAA
TAGGATTACGCACCAATGAGGATCTTTATACTGGAGCTCTTTTTCTATTATTTCTTTCTAC
GCTGTCCTTAATAGCGGGTTGGTTACATCTACAACCCAAATGGAAACCAAGCCTTTCGT
GGTTCAAAAACGCGGAATCTCGTCTCAATCATCATTTGTCAGGACTTTTCGGGGTAAGT
TCTTTGGCTTGGACAGGACATTTAGTTCATGTTGCTATTCCCGCATCCAGGGGGGAGTA
CGTTCGATGGAATAATTTCTTAGATGTATTACCCTATCCCCAGGGGTTGGGACCCCTTTT
GACGGGTCAATGGAATCTTTATGCCCAAAACCCTGATTCGAGTAATCATTTATTTGGTAC
CGCTCAAGGAGCCGGAACTGCCATTCTAACTCTTCTTGGGGGATTCCATCCACAAACA
CAAAGTTTGTGGCTGACCGATATGGCTCACCATCATTTAGCTATTGCATTTATTTTTCTCA
TTGCCGGTCACATGTATCGAACTAACTTCGGAATTGGGCACAGTATTAAAGATCTTTTA
GAAGCGCATACTCCTCCGGGGGGTCGATTAGGGCGTGGGCATAAGGGCCTTTATGACA
CAATCAACAATTCGATTCATTTTCAGTTAGGTCTTGCTCTAGCTTCTTTAGGGGTTATTA
CTTCCTTAGTAGCTCAACATATGTACTCTTTACCTCCTTATGCATTCATAGCACAAGACTT
TACTACTCAAGCTGCTTTATATACTCATCACCAATATATTGCAGGGTTCATCATGACAGG
GGCTTTTGCTCATGGAGCTATTTTTTTCATTAGGGATTACAATCCGGAACAGAATGAGG
ATAATGTATTGGCAAGAATGTTAGACCATAAAGAAGCTATCATATCTCATTTAAGTTGGG
CTAGCCTCTTTCTAGGATTCCATACCTTGGGCCTTTATGTTCATAACGACGTCATGCTTG

CTTTTGGTACTCCAGAAAAGCAAATCTTGATCGAACCTATATTTGCCCAATGGATACAAT
CTGCTCATGGCAAGACGACATATGGGTTCGATATACTCTTATCTTCAACGAATGGCCCC
GCTTTCAATGCGGGTCGAAGCCTATGGTTGCCCGGATGGTTGAATGCTGTTAATGAG----
--AATAGTAATTCGCTTTTCTTAACAATAGGACCTGGGGATTTCTTGGTTCATCATGCTAT
TGCTCTAGGTTTGCATACAACTACATTGATTTTAGTAAAGGGCGCTTTAGATGCACGCG
GTTCCAAATTAATGCCGGATAAAAAGGATTTTGGATATAGTTTTCCTTGTGACGGCCCA
GGGCGCGGCGGTACTTGTGATATTTCTGCTTGGGACGCATTTTATTTGGCAGTTTTCTGG
ATGTTAAATACCATTGGGTGGGTTACTTTTTATTGGCATTGGAAACATATCACATTATGG
CAGGGCAACGTTTCACAATTTAATGAATCCTCCACTTATTTGATGGGATGGTTAAGAGA
TTACCTATGGTTAAACTCTTCACAACTTATCAATGGATATAATCCTTTTGGGATGAATAGT
TTATCGGTATGGGCGTGGATGTTCTTATTTGGACATCTTGTTTGGGCTACTGGATTTATGT
TCTTAATTTCCTGGCGGGGGTATTGGCAGGAATTAATTGAGACTTTAGCATGGGCTCAT
GAACGCACACCTTTAGCTAATTTAATTCGCTGGAGAGATAAGCCCGTGGCTCTTTCCAT
TGTGCAAGCAAGATTGGTTGGATTAGCTCACTTTTCCGTGGGTTATATATTCACTTATGC
AGCTTTCTTGATTGCCTCAACATCAGGCAAGTTTGGTATGTCACATTCCGTAAAAATTTA
CGATACATGTATAGGATGCACTCAATGTGTACGAGCTTGTCCAACAGATGTATTAGAAAT
GATACCCTGGGATGGATGTAAAGCCAAGCAAATTGCTTCCGCGCCGAGAACCGAAGAT
TGTGTGGGTTGTAAGAGATGTGAATCTGCCTGCCCGACAGATTTTTTAAGTGTCCGCGT
TTATTTAGGGCCTGAAACAACACGTAGCATGGCTCTATCTTATATGACGGATTTAAACTT
ACCTTCTATTTTCGTGCCTTTAGTAGGCTTAGTATTTCCGGCAATTGCAATGACTTCTTTA
TTTCTTTATGTGCAAAAAAATAAGATTGTCATGCGGGATATAAAAACATATCTCTCTGTA
GCACCCGTGCTAAGTACTCTATGGTTTGGTGCTTTAGCAGGTTTATTGATAGAAATTAAT
CGTTTATTCCCAGATGCTTTGTCATTCCCTTTTTTT------ATGACTGCAATTTTAGAGAGA
CGCGAAAGTACAAGCCTGTGGGGTCGCTTCTGCAACTGGATAACTAGCACTGAAAATC
GTCTTTACATCGGATGGTTCGGTGTTTTGATGATCCCTACCTTATTGACCGCAACTTCTG
TATTTATTATCGCCTTCATCGCTGCCCCTCCAGTAGATATTGATGGTATTCGTGAGCCTGT
TTCTGGTTCTTTACTTTATGGAAACAATATTATCTCTGGTGCTATTATCCCTACTTCTGCG
GCGATCGGATTGCACTTTTACCCAATTTGGGAAGCTGCATCTGTTGATGAGTGGTTATA
CAATGGTGGTCCTTATGAGCTAATTGTTCTACACTTCTTACTTGGTGTAGCTTGTTATATG
GGTCGTGAGTGGGAACTTAGTTTCCGTCTGGGTATGCGTCCTTGGATTGCTGTTGCATA
TTCAGCTCCTGTTGCAGCTGCCACTGCTGTTTTCTTGATTTACCCTATTGGTCAAGGAA
GCTTTTCTGATGGTATGCCTTTAGGAATCTCTGGTACTTTCAACTTTATGATTGTATTCCA
GGCAGAGCATAACATCCTTATGCATCCATTCCACATGTTAGGTGTAGCTGGTGTATTCGG
CGGTTCCCTATTCAGTGCTATGCATGGTTCCTTGGTAACCTCTAGTTTGATCAGGGAAA
CTACTGAAAATGAATCTGCTAATGAGGGTTACAAATTTGGTCAAGAGGAAGAAACTTAT
AATATTGTGGCTGCTCATGGTTATTTTGGCCGATTAATCTTCCAATATGCTAGTTTCAACA
ACTCTCGTTCTTTACACTTCTTCTTGGCTGCTTGGCCTGTAGTAGGAATCTGGTTCACTG
CTTTAGGTATTAGTACTATGGCTTTCAACCTAAATGGTTTCAATTTCAACCAATCTGTAG
TTGATAGTCAAGGTCGCGTTATTAATACTTGGGCTGATATCATCAACCGTGCTAACCTTG
GTATGGAAGTAATGCACGAACGTAATGCTCACAACTTCCCTCTAGACTTAGCTGCTGTT
GAAGTTCCATCTATTAATGGAATGGGTTTGCCTTGGTATCGTGTTCATACTGTCGTATTG
AATGATCCGGGTCGATTGCTTGCGGTGCATATAATGCACACAGCTCTAGTTTCTGGTTG
GGCTGGCTCAATGGCTTTATACGAATTAGCAGTTTTTGATCCCTCTGATCCTGTTCTGGA
TCCAATGTGGAGACAAGGTATGTTCGTAATTCCCTTCATGACTCGTTTAGGAATAACGG

ATTCGTGGGGTGGTTGGAGTATTTCA---GGAGGAACTGTAACAAATCCGGGTATTTGGA
GTTATGAAGGTGTGGCAGGTACGCATATTGTGTTTTCTGGCTTGTGTTTCTTGGCAGCG
ATCTGGCATTGGGTATATTGGGACCTAGAAATATTCTCTGATGAGCGGACGGAAAACC
CTCTTTGGATTTGCCCAAGATCTTTGGAATTCATTTATTTCTTGCAGGGGTGGCTTGCTT
TGGCTTTGGGGCATTTCATGTAACGGGTTTGTATGGTCCTGGGATATGGGTATCCGATCC
TTATGGACTAACTGGAAAAGTACAAGCTGTAAATCCAGCGTGGGGTGCAGAAGGTTTT
GATCCTTTTGTTCCGGGGGGAATAGCTTCTCATCATATTGCTGCGGGTACATTGGGTATA
TTAGCGGGCTTATTCCATCTTAGTGTCCGTCCGCCTCAACGTCTATATAAAGGATTACGT
ATGGGCAATATTGAAACTGTACTTTCCAGTAGTATCGCTGCTGTTTTTTTTGCAGCTTTC
GTAGTTGCTGGAACTATGTGGTATGGGTCAGCAACGACCCCAATCGAATTATTTGGGCC
TACTCGTTATCAGTGGGATCAGGGATACTTTCAGCAAGAAATATATCGAAGAGTTAGCA
ATGGTTTAGCCGAAAATCTTAGTTTATCAGAAGCTTGGTCTAAAATTCCCGAAAAATTA
GCCTTTTATGATTATATTGGTAATAATCCGGCAAAAGGGGGATTATTCAGAGCAGGCTCA
ATGGACAATGGGGATGGAATAGCTGTTGGATGGTTAGGACATCCCGTCTTTAGAGATAA
AGAAGGACGTGAGCTTTTTGTACGCCGTATGCCTACTTTTTTTGAAACATTTCCGGTTG
TTTTGGTAGATGAGGAGGGAATTGTTAGAGCAGACGTTCCTTTTAGAAGAGCAGAATC
CAAATATAGTGTTGAACAAGTAGGCGTAACGGTGGAGTTCTATGGTGGCGAACTTAATG
GAGTAAGTTATTCTGATCCTGCTACTGTAAAAAAATATGCGAGGCGTTCTCAATTAGGG
GAAATTTTTGAATTAGACCGGGCTACTTTGAAATCTGATGGTGTTTTTCGCAGCAGTCC
AAGGGGTTGGTTCACTTTTGGTCATGCTACCTTTGCTTTGCTCTTCTTTTTCGGACACAT
TTGGCATGGCGCTAGAACATTGTTCCGAGATGTTTTTGCTGGTATTGATCCAGATTTGGA
TGCTCAAGTGGAATTTGGAACATTCCAAAAAGTGGGAGATCCAACTACAAGGAAACA
GGCAGCCATGAAAATCTTATATTCCCTGAGGAGGTTCTACCACGTGGAAACGCTCTTTA
ATGGAACTTTCGTTTTAGCTGGTCGTGACCAAGAAACCACCGGCTTTGCTTGGTGGGC
TGGGAATGCCAGACTTATCAATTTGTCCGGTAAACTACTTGGAGCTCATGTAGCCCATG
CCGGATTAATCGTATTCTGGGCCGGAGCAATGAACCTATTTGAAGTGGCCCATTTCGTA
CCAGAAAAGCCCATGTATGGACAAGGGTTGATTTTACTTCCACACTTAGCTACTCTAGG
TTGGGGAGTAGGGCCAGGGGGAGAAGTTCTAGATACTCTTCCGTACTTTGTATCTGGCG
TACTTCACCTAATTTCCTCCGCAGTCTTAGGCTTCGGTGGCATTTATCACGCGCTTCTGG
GACCCGAGACTCTTGAGGAATCTTTTCCATTCTTTGGTTATGTCTGGAAAGATAGAAAT
AAAATGACTACAATTTTGGGTACCCACTTAATTTTGTTAGGCCTAGGTGCTTCTCTTCTA
GTACTCAAGGCTCTTTATTTTGCCGGTGTATATGATACCTGGGCCCCTGGGGGAGGAGA
TGTAAGAAAAATTACCAATTTGACCCTTAGCCCTAGTGTTATATTTGGTTATTTCCTAAA
ACCTCCTTTTGGTGGAGAAGGGTGGATTGTTAGTGTAGATGATTTAGAAGATATAATTG
GTGGGCATGTATGGTTGGGTTTTATTTGTGTACTTGGCGGAATTTGGCATATTTTAACCA
AACCCTTCGCATGGGCTCGCCGTGCATTTGTATGGTGTGGAGAAGCTTACTTGTCTTAT
AGTTTAGCCGCTTTATCTGTCTTTGGTTTTATCGCTTGTTGTTTTGTATGGTTCAATAATA
CAGCTTATCCGAGTGAGTTTTATGGACCCACCGGGCCAGAAGCTTCCCAAGCTCAAGC
ATTTACTTTTCTAGTTAGAGACCAGCGTCTTGGAGCTAATGTGGGATCCGCCCAAGGAC
CCACAGGTTTAGGTAAATATCTAATGCGTTCCCCAACTGGGGAGGTTATCTTTGGAGGG
GAAACTATGCGTTTTTGGGACCTTCGTGCTCCATGGTTAGAACCTCTAAGGGGCCCCAA
CGGTTTGGACTTGAGTAGGTTGAAAAAAGACATACAACCTTGGCAAGAACGACGCTC
AGCAGAATATATGACCCACGCTCCTTTAGGCTCTTTAAATTCCGTGGGTGGCGTAGCTA
CCGAGATCAATGCAGTTAATTATGTTTCTCCTAGAAGTTGGTTAGCGTCTTCCCATTTTG

TTCTAGGATTCTTCTTTTTTGTGGGCCATTTTTGGCATGCAGGAAGAGCCCGGGCTGCT
GCAGCAGGTTTTGAAAAGGGAATCGATCGTGATTTGGAACCTGTTCTTTACATGAACC
CTCTTAACATGACTATAGCCCTTGGTAGAATTCCTAAAGAAGAAAATGATCTATTTGATA
CTATGGATGACTGGTTACGAAGGGACCGTTTCGTTTTTGTAGGATGGTCTGGCCTATTG
CTCTTTCCTTGTGCTTATTTCGCTTTAGGGGGTGTGTTTACAGGGACAACTTTTGTAACT
TCTTGGTATACCCATGGAGTGGCTAGTTCCTATTTGGAAGGTTGTAATTTCTTAACCGCA
GCAGTTTCTACCCCTGCCAATAGTTTAGCACACTCTTTGTTGCTACTATGGGGGCCCGA
AGCACAAGGAGATTTTACTCGTTGGTGTCAATTAGGCGGTCTATGGACTTTTGTAGCTC
TCCACGGGGCTTTTGCACTAATAGGTTTCATGTTACGCCAATTTGAACTTGCTCGGTCT
GTTCAATTGCGGCCTTATAATGCAATCTCATTCTCTGGTCCAATTGCTGTTTTTGTTTCTG
TATTCCTTATTTATCCACTGGGGCAATCTGGTTGGTTCTTTGCGCCGAGTTTTGGCGTAG
CAGCGATATTTCGATTCATCCTTTTCTTTCAAGGATTTCATAATTGGACGTTGAACCCAT
TTCATATGATGGGAGTTGCCGGAGTATTAGGTGCGGCTCTGCTATGCGCTATTCATGGAG
CGACCGTAGAAAACACTCTATTTGAGGACGGTGATGGTGCAAATACCTTCCGTGCTTTT
AACCCAACTCAAGCTGAAGAAACTTATTCAATGGTCACTGCTAACCGCTTTTGGTCCC
AAATCTTTGGTGTTGCTTTTTCCAATAAACGTTGGTTACATTTCTTTATGCTATTTGTACC
CGTCACCGGTTTATGGATGAGTGCTATTGGCGTAGTTGGCTTGGCTCTGAACTTACGTG
CCTATGACTTTGTTTCCCAGGAAATCCGTGCAGCGGAAGATCCTGAATTTGAGACTTTC
TACACCAAAAATATTCTTTTAAACGAGGGTATTCGTGCGTGGATGGCAGCTCAGGATCA
GCCTCATGAAAATCTTATATTCCCTGAGGAGGTTCTACCACGTGGAAACGCTCTTATGT
CTGGAAGCACGGGAGAACGTTCTTTTGCTGATATTATTACCAGTATTCGATACTGGGTTA
TTCATAGCATTACTATACCTTCCCTATTCATTGCGGGATGGTTATTTGTCAGTACGGGTTT
AGCTTATGACGTGTTTGGAAGTCTTAGGCCAAACGAGTATTTCACGGAAAGCCGACAA
GGAATTCCGTTAATAACCGACCGTTTTGATTCTTTAGAACAACTCGATGAATTTAGTAGA
TCCTTTATGACCATAGATCGAACCTATCCTATTTTCACAGTGCGATGGCTGGCTATTCAC
GGACTAGCTGTACCTACTGTTTCCTTCTTGGGATCAATATCAGCAATGCAGTTCATCCAA
CGAATGGCTACACAAACCGTTGAAGATAGTTCTAAACCTAGG---CCAAAACAAACTGG
TGCAGGTAGTTTACTGAAACCCTTGAATTCGGAATATGGGAAAGTCGCCCCGGGTTGG
GGGACTACTCCTTTTATGGGGGTCGCAATGGCTTTATTCGCTATATTCCTATCTATCATTT
TAGAAATTTATAATTCTTCCGTTTTACTGGACGGAATTTTAACCAATATGCTTACTCTCAA
ACTTTTTGTTTATACAGTAGTGATATTCTTTGTTTCACTCTTTATCTTTGGATTCTTATCTA
ATGACCCAGGACGGAATCCTGGACGCGAGGAGATGTCCGATACTACTGGAAGAATTCC
TCTTTGTCTGATAGGTACTGTAGCCGGTATTGCTGTGATTGGTTTAGTAGGTGTTTTCTT
TTATGGTTCATATTCTGGATTGGGTTCATCTCTAATGCCTAATATACTTAGTTTAACCTGTA
TTTGTTTTAATTCTGTTATTTATCCGACTAGTTTTTTCTTCGCCAAATTGCCCGAAGCTTA
TGCCATTTTCAATCCAATCGTGGATTTTATGCCTGTCATACCTCTATTCTTTTTTCTATTAG
CCTTTGTTTGGCAAGCTGCTGTAAGTTTTCGAATGACACAATCAAACCCGAATGAACA
AAATGTTGAATTGAATCGTACCAGTCTATACTGGGGTTTATTACTCATTTTTGTACCTGC
TGTTTTATTTGCCAATTACTTCTTCAATATGGAAGTCAATATTCTCGCATTTATTGCTACT
GCACTGTTCATTCTAGTTCCTACTTCTTTTTTACTTATTATTTATGTAAAAACAGTCAGCC
AAAATAATATGGAAACAGCAACTTTAGTCGCCATCTCCATATCTGGTTTACTTGTAAGCT
TTACTGGGTATGCCTTATATACCGCGTTTGGGCAACCCTCTCAACAATTAAGAGATCCAT
TCGAAGAACACGGAGACATGGAAGCATTGGTTTATACGTTCCTTTTAGTTTCGACTTTA
GGGATAATTTTTTTCGCTATCTTCTTCCGAGAACCACCTAAGGTTCCAACTAAAAGAAT

AAAAATGACTATTGCTTTCCAATTAGCTGTTTTTGCATTAATTGCGACTTCCTCAGTCTT
AGTAATTAGTGTACCCCTTGTATTTGCTTCTCCTGATGGTTGGTCAAATAATAAAAATGT
TGTATTTTCTGGTACATCATTATGGATTGGACTAGTCTTTCTCGTAGCTATTCTGAATTCT
CTCATTTCT--------------------ATGTCACCACAAACAGAAACTAAAGCAGGTGTTGGATTT
AAAGCTGGTGTTAAAGATTATAAATTGACTTACTACACCCCAGAGTATGAAACTAAGGA
TACTGATATCTTGGCAGCATTCCGAGTAAGTCCTCAGCCTGGGGTTCCGCCCGAAGAA
GCAGGGGCTGCAGTAGCTGCCGAATCTTCTACTGGTACATGGACAACTGTTTGGACTG
ATGGACTTACCAGTCTTGATCGTTACAAAGGACGATGCTATCACATCGAGCCTGTTGCT
GGGGAAGACAGCCAATGGATCTGTTATGTAGCTTATCCATTAGACCTATTTGAAGAGGG
TTCCGTTACTAACATGTTTACTTCCATTGTAGGTAACGTATTTGGTTTCAAAGCCCTACG
TGCTCTACGTTTGGAGGATCTACGAATTCCCCCTACTTATTCAAAAACTTTCCAAGGCC
CGCCTCATGGTATCCAAGTTGAAAGAGATAAGTTGAACAAGTATGGTCGTCCTTTATTG
GGATGTACTATTAAACCAAAATTGGGATTATCCGCAAAAAATTATGGTAGAGCGTGTTAT
GAGTGTCTACGTGGTGGACTTGATTTTACCAAAGATGATGAAAACGTAAACTCACAAC
CATTTATGCGCTGGAGAGACCGTTTTGTCTTTTGTGCCGAAGCTATTTATAAATCACAGG
CCGAAACCGGTGAAATCAAGGGGCATTACTTGAATGCGACTGCGGGTACATGTGAAGA
AATGATTAAGAGAGCTGTATTTGCAAGAGAATTAGGGGTTCCTATTGTAATGCATGACT
ACTTAACTGGGGGATTCACCGCAAATACTACTTTGGCTCATTATTGCCGCGACAATGGC
CTACTTCTTCACATTCACCGTGCAATGCATGCAGTTATTGATAGACAGAAAAATCATGGT
ATGCATTTCCGTGTATTAGCTAAAGCATTGCGTATGTCTGGGGGAGATCATATCCACTCC
GGTACAGTAGTAGGTAAGTTAGAAGGGGAACGCGAAATGACTTTAGGTTTTGTTGATTT
ATTGCGCGATGATTTTATTGAAAAAGATCGTGCTCGCGGTATCTTTTTCACTCAGGACT
GGGTATCCATGCCAGGTGTTATACCGGTAGCTTCAGGTGGTATTCATGTTTGGCATATGC
CAGCTCTGACCGAAATCTTTGGGGACGATTCTGTATTACAATTTGGTGGAGGAACTTTA
GGACATCCTTGGGGAAATGCACCTGGTGCAGCAGCTAATCGAGTGGCTTTAGAAGCCT
GTGTACAAGCTCGTAACGAAGGGCGCGATCTTGCTCGCGAAGGTAATGAAATTATCCG
AGCAGCTTGCAAATGGAGTCCTGAACTAGCCGCAGCTTGTGAAGTATGGAAGGCGATC
AAATTCGAGTTCGAGCCGGTAGATACTATTGATAAGACGGCGAAACATTTATACAAAAC
ACCTATCCCGAGCACACGCAAGGGAACCTTAGACAGGCAAGTGAAATCCAATCCACG
AAATAATTTGATCCATGGACGGCACCGTTGTGGTAAAGGTCGTAATTCCAGAGGAATCA
TTACCGCAAGCATAGAGGGGGAGGTCATAAAGCGCCTATACCGTAAAATAGATTTTCGA
CGGAATCAAAAAGACATATCTGGTAGAATCGTAACCATAGAATCCGACCCTAATCGAAA
TGCATACATTTGTCTCATACACTATGGGGATGGTGAGAAGAGATATATTTTACATCCCAG
AGGGGCTATAATTGGAGATACTATTGTTTCTGGTACAAAAGTTCCTATATCAATGGGAAA
TGCCCTACCTTTGACCGATATGCCCTTAGGCACGGTCATGCATAACAAAGAAAACACAC
GAGGAAGGGGTGGGCAATTAGCTAGAGCAGCAGGTGCTGTAGCGAAACACAAAGCAA
AAGAGGGTAAAATGGCCACTTTAAGATTACCATCCGGGGAGGGCCGTATAGTAACCCA
AAACAGCCTAGCAACAGTCGGACAAGAGGGTAAAGTAGGGGTGAACCAAAAAAGTTT
GGGTAGAGCCGGATCTAAGTGTTGGCTAGGTAAACGCCCCGTAGTAAGAGGGGTAGTT
ATGAACCCTGTGGACCACCCCCATGGGGGCGGTGAAGGGAAAGCTCCCATTGGTAGA
AAAAAACCCACAACCCCTTGGGGTTATCCTGCGCTTGGAAGAAGAACTAGGAAAAGG
AAAAAATATAGCGATAGTTTTATTCTTCGTCGCCGTAAG---ATGATTCAACCTCAGACCC
TTTTAAATGTAGCCGATAATAGTGGAGCTCGAAAATTGATGTGTATTCGAGTCATAGGA
GCTGCTGGTAATCAGCGATATGCTCGTATTGGTGATGTTATTGTTGCTGTAATCAAAGAC

GCATTGCCCCAAATGCCTCTAGAAAGATCCGAAGTAATTCGAGCTGTAATTGTACGTAC
ATGTAAAGAGTTCAAATGCGAAGACGGTATAATAATCCGCTATGATGACAATGCTGCGG
TTATAATTGATCAAAAAGGCAATCCAAAAGGAACTCGAGTTTTTGGCGCGATTGCCGA
GGAATTGAGAGGATTGAATTTTACCAAAATAGTTTCATTAGCTCCTGAAGTATTAATGCT
TAGTCCCAAAAGAACCAGATTTCGCAAACAACATAGAGGAAGAATGAAAGGAAAATC
CTGCCGAGGCAATCGTATTTGTTTTGGTAGATATGCTCTTCAAGCACTTGAACCCGCTT
GGATCACGGCGAGACAGATAGAAGCAGGACGAAGAGCAATAACACGATATGCACGTC
GTGGTGGAAAAATATGGGTACGTATATTTCCCGACAAACCGGTTACACTAAGACCCACA
GAAACACGTATGGGCTCGGGAAAGGGGTCCCCCGAATATTGGGTATCCGTTGTTAAAC
CTGGTCGTATACTTTATGAAATGGGGGGAGTATCTGAAACTGTAGCTAGAGCAGCTATC
TCCATAGCTGCCAGTAAAATGCCCATACGAAGTCAATTTATTCGATTAGAGATGACCAG
AGTTCCGCGAGGATATATAGCCCGGAGACGACGAACAAAAATGCGTTCATTTGCCTCA
AACTTTAGAGGGGCTCATTTAAGACTTAATCGAATGATTACCCAACAGGTAAAAAGAG
CTTTTGTTTCCTCTCATCGAGATAGAGGTAGGCAAAAGAGGGATTTTCGTCGTTTGTGG
ATCACTCGGATAAACGCAGCAACGCGGGTATAT------GTATTCGATAGTTATAGTAAATTA
ATACACAACCTGTACAAGAAGAAATTGATTCTTAATCGTAAAATGCTTGCACAAGTAGC
TGTATCAAATCCAAATAATCTTTACACGATTTCCAATAAAATAAAGATCATCAAT-----------
----------------AGGATAAAGAGAGTTTTCAAAGCACAAAAACGTATCCCTATGTCTGTTTTC
AAAGCACAAAGAGTTCTTGACGAGATTCGCTGGCGTTACTACGAGGAAACTGTTATGA
TACTGAACCTCATGCCCTATCGAGCATCTTATCCCATCTTAAAGTTGGTTTATTCGGCAG
CCGCAAATGCTACTCATTATAGGGATTTCGACAAAGCGAATTTATTTATTACTAAAGCCG
AAGTCAGTAGGAGTACTATTATGAAGAAATTCAGACCTCGAGCTCGAGGACGTAGTTT
CCCCATAAAAAAAAGCATGTGTCATATAACAATTGTACTAAAT---GTAAAGAAATCT------
---------------ATGGATGGAATCAAATACGCAGTATTTACAGAAAAAAGTCTTCGTTTATTGG
GAAAGAAT---CAATATACTTTTAATGTCGAATCGGGATTCACTAAGACAGAAATAAAGCA
TTGGGTCGAACTCTTCTTTGGTGTTAAGGTAGTAGCTGTGAATAGCCATCGACTACCCG
GAAAGGGTAGAAGAATGGGCCCTATTCTAGGACATACAATGCATTACAGACGTATGATC
ATTACCCTTCAACCGGGTTATTCTATTCCACTTCTAGATAGAGAAAAAAACATGGCAGT
TCCAAAAAAACGTACTTCAATGTCAAAAAAGCGTATTCGTAAAAATATTTGGAAGAAA
AAGACTTATTTTTCCATAGTACAATCTTATTCTTTAGTAAAATCATTTTCGAGCGGTAAA
CCAAAGGGTTTTTCTGGGCAACAAACAAATAATATGGCCAAGGGAAAAGATGTTAGAA
TCAGAGTTATTTTGGAATGCATCAGTTGTGTTCGAAAA------GGT------GAGGAATCGACG
GGGATTTCTAGATATAGTACTCAAAAGAATCGCCACAATACACCCGGACAATTAGAATT
CAAAAAATTTTGTCGTTATTGTCGCAAGCATACGACTCATCACGAAATAAAGAAAATGA
AAATAAGAGCTTCCGTTCGTAAAATTTGTACAAAATGTCGACTGATTCGTAGGCGTGGA
CGAATTAGAGTCATTTGCTCTAATCCGAAGCATAAACAAAGACAGGGGATGGTTCGAG
AGGAGGTAGCAGGATCCACTCAAACACTACAGTGGAAGTGTGTTGAATCAAGAGTAG
ATAGTAAGCGCCTTTATTATGGTCGTTTCATTCTGTCCCCGCTTAGAAAAGGTCAAGCG
GACACCGTCGGTATTGCCTTGCGAAGAGCTTTACTTGGAGAAATAGAAGGAACATGTA
TCACACGTGCAAAATTTGGG------AGCGTGCCGCACGAATATTCTACAATAGCAGGTATT
GAAGAATCCGTACAAGAAATTTTACTAAATTTGAAAGAAATTGTATTGAGAAGTAATCT
CTATGGAGTTAGAGACGCATCCATTTGCGTCAAAGGTCCTAGATACATAACTGCTCAAG
ATATCATCTTACCGCCTTCCGTAGAAATCGTTGATACGGCACAACCTATAGCTAACTTGA
CAGAGCCCATTGATTTCTGTATTGATTTACAGATCAAGAGAGATCGTGGATATCAGACA

GAACTCAGAAAGAACTATCAAGATGGAAGTTAT------CCTATAGATGCTGTATCCATGCC
TGTTCGAAATGTGAATTATAGTATTTTTTCTTGTGGGAATGGAAATGAAAAACACGAGA
TACTTTTTCTAGAAATATGGACTAATGGAAGTCTAACCCCTAAGGAAGCACTTTATGAG
GCTTCTCGTAATTTGATTGATTTATTTCTTCCTTTTCTACACGCGGAGGAAGAG------GGC
GCTAGTTTCGAAGAAAATAAA---AACAGGTTTACTCCACCCCTTTTTACTTTTCAAAAA
AGATTAACT---AATCTAAAGAAAAACAAAAAA---GGAATTCCATTGAATTGTATTTTTAT
TGATCAATTAGAATTGACTTCTAGAACGTATAATTGTCTAAAAAGGGCCAATATACATAC
ACTATTGGACCTTTTGAGTAAGACTGAAGAAGATCTTCTGAGAATTGACAGTTTTCGTA
TGGAAGATAGAAAACATATATGGGACACTCTAGAGAAGCAT------CTCCCAATTGATTTA
CTTAAGAATAAAATGCTCCGGAATGGAAAC---GAGGGAATGTCCACAATACCCGGATTT
AGTCAGATCCAATTCGAGGGATTTTGTAGGTTCATTAATCAAGCCTTGGCAGAAGAACT
TGACAAGTTTCCAACAATTAAAGATCCAGATCACGAAATTGCATTTCAATTATTTGCGA
AAGGATATCAATTGCTAGAACCCTCGATAAAAGAAAGAGATGCTGTGTATGAATCACTC
ACCTATTCTTCCGAATTATATGTATCTGCGAGATTAATTTTTGGTTTC------GATGTGCAAA
AGCAAACCATTTCTATCGGAAACATTCCTATAATGAATTCCTTAGGAACCTTTATTATAA
ATGGAATATACCGAATTGTGATCAATCAAATATTGCTAAGTCCTGGTATTTACTACCGCT
CGGAATTAGATCATAAGGGAATTTCTATCTACACTGGGACTATAATATCAGATTGGGGAG
GGAGATCGGAATTAGCAATTGATAAAAAAGAAAGGATATGGGCTCGCGTGAGTAGAAA
ACAAAAGATATCTATTCTAGTTCTATCATCAGCTATGGGTTCGAATCTAAGAGAAATTCT
AGATAATGTTTCCTACCCTGAAATTTTCTTGTCTTTCCCGAATGCTAAGGAGAAGAAGA
GGATTGAGTCAAAAGAAAAAGCTATTTTGGAGTTTTATCAACAATTTGCTTGTGTAGGT
GGGGACCTGGTATTTTCGGAGTCCTTATGCGAGGAATTACAAAAGAAATTTTTTCAACA
AAAATGTGAATTAGGAAGGATTGGTCGACGAAATATGAATCGGAGACTTAATCTTGATA
TACCTCAGAACAATACATTCTTGTTACCACGAGATGTATTGGCCGCTACGGATCATTTGA
TTGGAATGAAATTTGGAACGGGTATACTTGACGATGACGATATGAATCACTTGAAAAAT
AAACGTATTCGTTCGGTTGCGGATCTGTTACAAGATCAATTCGGATTGGCTCTTGGTCG
TTTACAACATGCAGTTCAAAAAACTATTCGTAGAGTATTCATACGTCAATCGAAA------C
CGACTCCCCAAACTTTGGTAACTCCAACTTCAACTTCAATTTTATTAATAACTACTTATG
AGACCTTTTTTGGCACATACCCGTTATCTCAAGTTTTTGATCAAACGAATCCATTGACA
CAAACTGTTCATGGGCGAAAAGTGAGTTGTTTAGGTCCTGGAGGGTTGACGGGGAGA
ACCGCAAGTTTTCGGAGCCGAGATATTCATCCGAGTCACTATGGGCGTATTTGTCCAAT
CGACACGTCTGAAGGAATCAATGTTGGACTTACTGGATCCTTAGCAATTCATGCGAGAA
TTGATCACTTGTGGGGATCCATAGAGAGTCCGTTTTATGAAATATCTGAGAAAGCAAAA
GAAAAAAAAGAG------AGACAGGTGGTTTATCTATCACCAAATAGAGATGAATATTATAT
GATAGCAGCAGGAAATTCTTTGTCCTTGAATCAAGGTATTCAGGAAGAACAGGTTGTT
CCGGCTAGATACCGTCAAGAATTCTTGACTATTGCATGGGAACAGATTCATGTTAGAAG
TATTTTTCCTTTCCAATATTTTTCTATTGGGGGTTCTCTCATTCCTTTTATTGAGCACAAT
GATGCGAATCGGGCTTTAATGAGTTCTAATATGCAGCGCCAAGCAGTTCCGCTTTCTCG
GTCCGAGAAATGCATTGTTGGAACTGGATTGGAACGCCAAACAGCTCTAGATTCGAGG
GTTTCCATTATAGCCGAACGCGAGGGAAAGATCATTTCTACTGATAGTCATAAGATACTT
TTATCAAGTAGTGGGAAGACTATAAGTATTCCTTTAGTTAACCACCGTCGCTCTAACAA
AAATACTTGTATGCACCAAAAACCTCGGGTTCCACGGGGTAAATCCATTAAAAAAGGA
CAAATTTTAGCAGAAGGAGCTGCTACAGTTGGGGGCGAACTTGCTTTAGGAAAAAAC
GTATTAGTAGCTTATATGCCATGGGAAGGTTACAATTTTGAAGACGCAGTACTAATTAGC

GAACGTTTGGTATATGAAGATATTTATACCTCTTTTCACATCCGGAAATATGAAATTCAG
ACGGATACGACAAGCCAGGGCTCCGCTGAAAAAATCACTAAAGAAATACCACATCTAG
AAGAACATTTACTCCGCAATTTGGACAGAAATGGAGTTGTTAGGTTGGGATCCTGGGT
AGAAACTGGTGATATTTTAGTAGGTAAATTAACGCCTCAGATAGCGAGCGAA---TCATC
TTATATCGCGGAAGCTGGATTATTACGGGCCATATTTGGCCTTGAGGTTTCCACTTCAAA
AGAAACTTCTCTGAAATTACCTATAGGTGGAAGAGGGCGCGTTATCGATGTGAAATGGA
TCCAAAGG---------------------GACCCGATAATGGTTCGTGTATATATTTTACAGAAACGTGA
AATCAAAGTTGGGGATAAAGTAGCCGGAAGACATGGGAATAAAGGGATCATTTCCAAA
ATTTTGCCTAGGCAAGATATGCCCTATTTGCAAGATGGAACACCCGTTGATATGGTCTTC
AATCCCTTAGGAGTACCCTCCCGAATGAATGTGGGACAAATATTTGAAAGCTCACTCGG
ATTAGCGGGGGATCTGCTAAAGAAACATTATAGAATAGCACCCTTTGATGAGAGATATG
AGCAAGAGGCTTCAAGAAAACTTGTGTTTTCAGAATTATATGAAGCCAGTAAAGAAAC
AAAAAATCCATGGGTATTTGAACCCGAGTACCCGGGAAAAAGCAGAATATTTGATGGA
AGAACAGGCGACCCCTTTGAGCAACCTGTTCTAATAGGGAAGTCCTATATCTTAAAATT
AATTCATCAAGTTGATGAGAAAATTCATGGGCGTTCTACTGGGCCCTACTCACTTGTTA
CACAACAACCGGTTAGAGGAAGAGCCAAGCAAGGGGGACAACGAGTAGGAGAAATG
GAAGTTTGGGCTTTAGAAGGATTTGGTGTTGCTCATATTTTACAAGAGATACTTACTTAT
AAATCTGACCATCTTATAGCTCGCCAAGAAATACTTAATGCTACGATCTGGGGAAAAAG
AATACCTAATCATGAGGATCCTCCAGAATCTTTTCGAGTGCTCGTTCGAGAACTACGAT
CTTTGGCTCTAGAACTGAATCATTTCCTTGTATCTGAAAAGAACTTCCAGGTTAATAGG
GAGGAAGTT---------------------ATGATTGACCAATATAAACATCAACAACTTCAAATTGGA
CTCGTTTCCCCTCAACAAATAAAGGCTTGGGCTAACAAAAACCTACCTAATGGAGAAG
TCGTTGGCGAAGTCACAAGGCCCTCTACTTTTCATTATAAAACCGATAAACCAGAAAA
AGATGGATTGTTTTGCGAAAGAATCTTTGGACCCATAAAAAGCGGGATTTGCGCTTGTG
GCAATTCTCGAGCGAGCGGAGCTGAAAACGAAGACGAAAGATTTTGCCAAAAATGCG
GGGTAGAATTTGTGGATTCTCGGATACGAAGATATCAAATGGGATACATCAAACTCGCA
TGTCCCGTGACTCATGTGTGGTATTTGAAAGGTCTTCCTAGTTATATCGCGAATCTTTTA
GATAAACCTCTTAAGAAGTTGGAGGGCCTAGTATACGGCGAT------TTCTCTTTTGCTAG
GCCC---AGCACTAAAAAACCCACTTTTTTACGATTACGAGGTTTATTCGAAGAGGAAAT
TGCATCCTGTAACCACAGCATTTCTCCCTTTTTTTCTACCCCAGGCTTTGCAACATTTCG
AAATCGGGAAATTGCGACAGGAGCAGGTGCTATTAGAGAACAATTAGCAGATTTGGAT
TTGCGAATTATTATAGAGAATTCTTTGGTCGAATGGAAGGAATTAGAAGACGAG---GGG
TATAGTGGAGATGAGTGGGAAGATAGAAAAAGACGAATAAGAAAAGTTTTTTTGATTA
GACGCATGCAATTGGCGAAACATTTTATTCAAACAAATGTAGAACCAGAATGGATGGTT
TTGTGCTTATTACCGGTTCTTCCTCCCGAATTGAGACCTATTGTTTATAGGTCTGGAGAT
AAAGTAGTGACTTCAGACATTAATGAACTTTATAAGAGAGTTATCCGTCGGAACAACAA
TCTTGCCTATCTATTAAAAAGAAGTGAATTAGCGCCAGCAGATTTAGTAATGTGCCAGG
AAAAATTGGTACAAGAAGCCGTGGATACACTTCTTGATAGTGGGTCCCGCGGGCAACC
GACGAGGGATGGTCACAATAAAGTATACAAATCACTTTCAGATGTAATTGAAGGTAAA
GAGGGAAGGTTTCGCGAGACTCTGCTTGGGAAACGGGTCGATTACTCGGGGCGTTCTG
TCATTGTTGTGGGTCCTTCGCTTTCATTACATCAATGTGGATTACCTCTAGAGATAGCAA
TAAAGCTTTTTCAGCTATTTGTAATTCGCGATTTAATCACGAAACGCGCCACTTCTAATG
TAAGGATTGCTAAAAGGAAAATTTGGGAAAAAGAACCCATTGTATGGGAAATACTTCA
AGAAGTTATGCGGGGACATCCTGTACTGTTAAATAGAGCACCTACCCTGCATAGATTAG

GCATACAGGCCTTTCAACCCACTTTAGTAGAGGGGCGTACTATTTCTTTACACCCATTAG
TGTGTAAGGGTTTCAATGCGGACTTTGATGGAGATCAAATGGCTGTTCATCTACCTTTAT
CCTTGGAAGCTCAGGCAGAAGCCCGTTTACTTATGTTTTCTCATATGAATCTCTTATCTC
CCGCTATTGGGGATCCTATTTGCGTACCAACCCAAGACATGCTTATCGGGCTTTATGTAT
TAACGATTGGAAAGCGTCGAGGTATTTGTGCAAATAGATATAATAGTTGCAGAAAC------
---------TATCCAAATCTAAAAGTAAATTACAATAATTCTAAATATAGGAAAGATAAAGAAC
CCCATTTTTCTAGTTCTTATGATGCACTGGGAGCTTATAGACAAAAACTAATCAGTTTAG
ATAGTCCCTTGTGGCTACGATGGAACCTGGATCAACGC---GTCATTGGGTCA------AGAG
AAGTTCCGATTGAAGTTCAATATGAATCTTTGGGGACTTATCATGAGATTTATGCCCACT
ATCTAATAATGGGAAATAGAAAAAAAGAAATTCGTTCGATATACATTCGAACCACTCTT
GGTCATATTTCTTTTTATAGAGAAATAGAGGAAGCCATACAAGGATTTAGTCAGGCCTAT
TCATACACTACC-----------------------------------ATGGCGGAACGGGCCAATCTGGTCTTTCA
TAATAAAGAGATAGACGGAACTGGTATGAAACGACTTATTAGCAGATTAATAGATCATT
TTGGAATGGGATATACATCCCATATACTGGATCAACTAAAGACTCTGGGCTTCCATCAAG
CCACTACTACATCGATTTCGTTAGGAATCGAGGATCTTTTAACAATACCCTCTAAGGGAT
GGTTAGTCCAAGACGCGGAACAACAGAGTTTTCTTTTGGAGAAACACTATTATTATGG
GGCTGTACACGCGGTAGAAAAATTACGCCAATCTGTTGAGATATGGTATGCGACAAGTG
AATATTTGAAACAAGAAATGAATTCCAATTTTCGGATAACGGATCCTTCTAATCCAGTCT
ATCTAATGTCTTTTTCAGGAGCCAGAGGAAATGCATCTCAAGTACACCAATTAGTAGGT
ATGAGAGGATTAATGTCGGACCCTCAAGGACAAATGATTGATTTACCTATTCAAAGCAA
TTTACGCGAGGGACTTTCTTTGACAGAATATATAATTTCCTGCTATGGAGCCCGCAAAG
GGGTTGTAGATACTGCTGTGCGAACAGCAGATGCTGGATATCTTACACGTAGACTTGTT
GAAGTAGTTCAACATATTATTGTGCGTAGAAGAGATTGTGGTACTATCCGAGGTATTTCT
GTAAGTCCTCAAAATGGGATGACGGAA------AAACTTTTTGTCCAAACACTAATTGGTC
GTGTATTAGCAGACGATATATATATCGGTTCACGATGCATTGCCGCGCGAAATCAAGATA
TTGGAATTGGATTAGTCAATCGATTCATAACTGCCTTTCGAGCACAACCATTTCGAGCA
CAACCAATATATATTAGAACCCCCTTTACTTGCCGAAGCACTTCTTGGATCTGTCAATTA
TGCTATGGCCGGAGTCCTACTCATAGTGATCTGGTCGAATTGGGAGAAGCCGTAGGTAT
TATTGCAGGTCAATCAATTGGGGAGCCAGGGACTCAACTAACATTAAGAACTTTTCATA
CTGGCGGAGTATTCACAGGGGGTACTGCCGACCTTGTACGATCCCCTTCGAATGGAAA
AATAAAATTCAATGAGAATTTGGTTCACCCCACACGTACCCGTCATGGGCAGCCTGCTT
TTCTATGTTATATAGACTTGCATGTAACTATTCAGAGTCAAGATATTCTATATAGTGTGAA
TATTCCCTCAAAAAGCTTGATTCTAGTGCAAAATGATCAATATGTAAAATCCGAACAAG
TAATTGCGGAGATTCGTGCCGGAACGTCCACTTTA------TTTAAAGAAAGGGTACAAAA
GCATATTTATTCTGAATCAGACGGCGAA---ATGCACTGGAGTACTGATGTTTACCATGCG
CCCGAATATCAATATGGTAATCTTCGCCGATTACCAAAAACAAGCCATTTATGGATATTG
TCAGTAAGTATGTGCAGA------TCCAGTATAGCGTCTTTTTCACTCCACAAGGATCAAGA
TCAAATGAATACTTAT--
--------GGT------------GATAGGGAAATTCTTGATTATTCA---ACGTCGGATCGAATCATGTCC
AATGGCCATTGGAATTTTATCTATCCTTCT---ATTTTTCAAGATAAT------TCAGATTTGTTG
GCGAAAAAGCGAAGAAATAGGTTCGTCATTCCATTACAATATCATCAAGAACAAGAGA
AAGAACTAATATCCTGTTTTGGGATTTCGATTGAAATCCCCCTTATG------GGTGTTTTAC
GTAGAAATACTATTTTTGCTTATTTTGACGATCCCCGATACAGAAAAGAT------GGAATTG
TTAAATTTAGAAGCCCAGAGGAAGATTCCATCTTAAAA------AAAGAGGGTTTGATTGA

GCATCGAGGA---ACAAAAGAATTTAGTCTAAAATAC---GAAGTAGATCGGTTTTTTTTCA
TTCTTCAAGAACTTCATATCTTGCCGAGATCTTCATCCCTAAAGATACTTGACAATAGTA
TTATTGGAGTGGATACACAACTCACAAAAAATACAAGAAGTCGACTAGGCGGACTGGT
CCGAGTGAAGAGAAAAAAAAGCCATACGGAACTCAAAATATTTTCCGGAGATATTCAT
TTTCCTGAAGAGGCGGATAAGATATTAGGT------GGCTGTTTGATACCGCCAGAAAGACA
AAAAAAA------GATTCTAAGGAA---TCAAAA---AAAAGGAAAAATTGGGTCTATGTTCA
ACGGAAAAAAATTCTCAAGAGCAAGGAAAAG------TATTTTGTTTCCGTTCGCCCTACA
GTGGCATATGAAATGGACGAAGGAAGAAATTTAGCAACACTTTTCCCGCAGGATCTCT
TGCAAGAAGAAAATAATCTCCAAATTCGACTTGTCAATTTTATTTCTCATGAAAATAGC
AAGTTAACTCAAAGAATTTATCACACAAAT---CAATTTGTTAGAACTTGCTTAGTAGTGA
ATTGGGAACAAGAAGAAAAAGAAAAGGCTGGT---------------------GCTTCCCTTGTTGAG
GTAAGAGCAAATGATCTTATTCGCGATTTCCTAAGAATTGAGTTAGTCAAGTCCACTATT
TCGTATACACGAAAAAGGTATGATAGGACAAGTGCAGGACCGATTCCCCAT---------TTA
GATCGCGCCAATATCAATTCTTTTTATTCCAAGGCGAAGATTGAATCA---------------CTTA
GCCAACATCCAGAAGCTATTGGC------------ACTTTGTTGAATCGAAATAAA---------GAAT
ACCACTCTTTGATGATTTTGTCGGCATCCAACTGTTCTCGAATTGGT------TTATTCAAGA
ATTCAAAACATCCC---------AATGCGATAAAAGAATGGAATCCTAGAATTCCTATT---GAA
ATTTTTGGGCCCTTAGGCGCTATTGTAGCT---AGTATATCGCATTTTTCTTCATCT---TACTA
TTTACTAACG---------CATAATAAAATCCTGTTAAAA---AAATATTTGTTCGTTGAC---------
AATTTAAAACAAACCTTCCAAGTACTTCAAGAACTTAAATACTCTTTAATAGATGAAAA
TAAAAGG------ATTTCTAATTTCGAT------AGTAACATAATGTTGGATCCATTCCTTTTGAA
TTGTCACTTTGTCCATCATGATTCTTGGGAAGAGACATTGGCAATAATTCAC---CTTGGA
CAATTTATTTGTGAAAATGTATGTCTATTTAAATCGCACATA------------AAAAAATCCGGT
CAAATTTTCATTGTAAAT---ATGAATTCCTTTGTTATAAGAGCAGCTAAACCTTATTTGGC
CACTACAGGAGCAACTGTTAATGGTCATTATGGAGAAATCCTTTACAAGGGAGATAGGT
TAGTTACGTTTATATATGAAAAATCGAGATCTAGTGACATAACGCAAGGTCTTCCAAAA
GTGGAACAAATCTTTGAAGCGCGTTCAATTGATTCATTATCCCCGAATCTCGAAAGGAG
AATTGAGGATTGGAATGAGCGTATACCAAGAATTCTTGGGGTCCCTTGGGGATTCTTGA
TTGGAGCTGAGCTAACCATAGCCCAAAGTCGTATCTCTTTGGTTAATAAAATCCAAAAG
GTTTATCGATCCCAAGGGGTACAGATCCATAATAGACATATAGAGATTATTATACGCCAA
GTAACATCAAAAGTGCGGGTTTCCGAA---------GATGGAATGTCTAATGTTTTTTCGCCT
GGGGAATTAATCGGACTATTGCGAGCGGAACGAGCAGGGCGAGCTTTGGATGAATCGA
TCTATTATCGGGCAATCTTATTGGGAATAACAAGGGCTTCCCTGAATACCCAAAGTTTCA
TATCTGAAGCAAGTTTTCAAGAAACTGCTCGAGTTTTAGCAAAAGCTGCCCTACGAGG
TCGCATTGATTGGTTGAAAGGCTTGAAAGAAAACGTAGTTCTGGGGGGGATTATACCT
GTTGGTACCGGATTCCAAAAATTTGTGCATCGTTCCCCA------CAAGACAAGAACCTTTA
T------TTCGAAATAAAAAAAAAAAAATCTATTCGCGTCGGAAATG------AGAGATTTTTTGT
TTCTCCATACAGAATTAGTTTCTTCAGATTCTGACGTAACAAAC------------------------------
------------------------------ATGACAAGAAGATATTGGAACATTAATTTGAAAGAGATGATAGA
AGCAGGAGTTCATTTTGGTCATGGTATTAAGAAATGGAATCCTAAAATGGCCCCTTACA
TTTCGGCAAAGCGTAAAGGTACTCATATTATAAATCTCGCTAGAACGGCTCGTTTTTTAT
CAGAAGCTTGTGATTTAGTTTTTGATGCAGCAAGTCAGGGAAAAAGTTTCTTAATTGTT
GGTACCAAAAAAAGAGCAACAGATTTAGTAGCATCAGCTGCAATAAGGGCTCGTTGTC
ATTATGTTAATAAAAAGTGGTTCAGTGGTATGTTAACGAATTGGTCGATTACGAAAACT

AGACTTTCTCAATTTAGAGACTTAAGAGCAGAAGAAAAAATGGAAAATTCCACCATC
TCCCAAAAAGAGATGTGGCAATCTTGAAGAGAAAATTATCTACCTTGCAAAGGTATCTC
GGCGGGATCAAATATATGACGAGATTGCCAGACATTGTGATCGTCCTTGATCAGCAAAA
AGAGTATATAGCTCTTCGGGAATGTGCCATTTTGGGGATTCCTACTATTTCTTTAGTCGAT
ACAAATTGTGACCCGGATCTCGCGAATATATCGATTCCAGCCAACGATGACACTATGAC
TTCAATTCGATTGATTCTTAACAAATTAGTATTTTCAATTTGTGAGGGCCGTTCTCTC---T
ATATAAGAAATCGTATGGGACAAAAAATAAATCCACTCGGTTTCAGACTTGGTACAACC
CAAAAACACCATTCCTTTTGGTTCGCACAACCAAAAAATTATTCGGAAGGTCTACAGG
AAGATAAAAAAATAAGGGATTGTATCAAGAACTATATACAAAAGAATAGGAAAAAGGG
CTCGAATAAAATAGAATCAATTACACATATAGAAATCCAAAAAGAAATCGATACGATCC
ACGTCATAATCCATATTGGATTCCCAAAT---TTATTAAAGAAAAAAGGA---------------GCA
ATCGAAGAATTAGAGAAAGATCTACAAAAGGAAATTAACTCTGTAAACCAGAGATTTA
ATATTTCTATCGAAAAAGTTAAAGAACCTTATAGACAGCCTAACATTCTTGCAGAATATA
TAGCTTTCCAATTAAAAAATAGAGTTTCATTCCGAAAGGCAATGAAAAAAGCCATTGAA
TTAACTAAAAAAGCAGATATAAGGGGAGTAAAAGTAAAAATTGCGGGTCGTCTCGGAG
GAAAAGAAATTGCACGCGCCGAATCCATTAAAAAGGGTAGACTTCCCCTCCAAACAAT
TCGCGCTAAAATTGATTATTGCTGCTATCCAATTCGGACTATCTATGGAGTATTAGGTGTA
AAAATTTGGATATTCGTAGACGAAGAAATGTCCCGTTATCGAGGACCTCGTTTAAAAAA
AATACGCCGTCTGGGAGCTTTACCAGGACTCACTAGAAAAACACCTAAATCCGGAAGT
AATCTTAAAAAAAAAATTCAATTCTGGGAAA---AAGGAGCAATATCGTATTCGTCTTCAA
GAAAAACAGAAATTGCGTTTTCATTATGGTCTGACAGAACGACAATTACTTAGATATGT
ACATATCGCTGGAAAAGCAAAAAGGTCAACAGGCCAGGTTTTACTACAATTACTTGAA
ATGCGTTTGGATAATATCCTTTTTCGATTGGGTATGGCTTCAACCATTCCTGGGGCCCGC
CAATTAGTCAACCATAGACATATTTTAGTTAATGGTCGTATAGTCAATATACCAAGTTTTC
GTTGCAAACCCAGAGATATTATTACTACGAAAGATAACCAAAGATCAAAAGGTCTGGTT
CAAAATTATATTGCTTCATCGGACCCGAAA------TTGCCAAAGCATTTGGCGATTGACAC
G------TTGGAATATAAAGGACTAGTAAATAAAATCCTAGATAGGAAGTGGGTCGGTCTCA
AAATAAATGAGTTGTTAGTTGTAGAATATTACTCTCGTCAGACTATGTCACGTCGAGGTA
CTGCAGAAAAAAGAACTGCAAAATCCGATCCAATTTTTCGTAATCGATTAGTTAACATG
GTGGTTAACCGTATTATGAAAGACGGAAAAAAATCATTGGCTTATCAAATTCTCTATCG
AGCCGTGAAAAAGATTCAACAAAAGACAGAAACAAATCCACTATTGGTTTTACGTCAA
GCAATACGTAGAGTAACTCCCAATATAGGAGTAAAAACAAGACGTAAAAAAGGATCGA
CGCGGAAAGTTCCGATTGAAATAGGATCTAAACAAGGAAGAGCACTTGCCATTCGTTG
GTTATTAGAAGCATCCCAAAAGCGTCCGGGTCGAAATATGGCTTTCAAATTAAGTTCCG
AATTAGTAGATGCTGCCAAAGGGGGTGGGGGTGCCATACGCAAAAAGGAAGCGACTC
ATAGAATGGCAGAGGCAAATAGAGCTCTTGCACATTTTCGTATGGGCAAGGACACTATT
GCTGATTTACTAACCTCTATAAGAAATGCGGACATGAATAAAAAAGGAACAGTTCGAGT
AGTATCTACAAATATTACCGAAAACATTGTGAAAATACTTCTACGAGAGGGTTTTATTGA
AAGTGTTCGGAAACATCAGGAACGTAACAGATATTTCTTGGTTTCAACTTTGCGACATC
AAAAGAGAAAGACTAGAAAAGGAATATATAGAACA---------AGAACCTTTTTAAAGCGT
ATCAGCCGACCCGGCTTACGAATTTATACCAACTATCAAGGAATTCCTAAAGTTTTGGG
TGGAATGGGAATTGCTATTCTTTCTACTTCTCGAGGAATAATGACAGATCGAGAAGCTC
GACTAAACAGAATTGGGGGAGAAGTCTTATGTTATATATGGATGGCAAAAGCTATACCA
AAAATTGGTTCACGTAAGAAAGTGCGTATTGGTTTACGTAAGAGTGCACGTAGAATAA

CAAAAGGGGTTATTCATGTTCAAGCTAGTTTCAACAATACCATTATAACTGTTACAGAC
CCACAAGGTCGGGTGGTTTTTTGGTCCTCGGCCGGTACTTGTGGATTCAAAAGCTCAA
GAAAAGCATCACCCTATGCTGGTCAAAGAACAGCAGTAGATGCTATTCGTACAGTAGG
T---------TTGCAACGAGCAGAAGTTATGGTAAAGGGCGCTGGTAGTGGAAGAGATGCCG
CATTACGAGCCATTGCTAAAAGTGGTGTGCGATTAAGTTGTATACGCGATGTAACACCT
ATGCCGCATAATGGATGTCGACCGCCTAAAAAAAGACGTCTGATGCCGACGGTTAAAC
AACTTATTAGAAACGCAAGACAGCCAATACGAAATGCTCGAAAAACGGCCGCGCTTAA
AGGATGCCCTCAGCGTCGAGGAACATGTGCTAGGGTGTAT------AACCCCAAAAAACCC
AACTCTGCCTTACGTAAAGTTGCCAGAGTACGATTAACCTCTGGATTTGAAATCACTGC
TTATATACCTGGTATTGGCCATAATTTACAAGAACATTCTGTAGTATTAGTAAGAGGAGG
AAGGGTTAAGGATTTACCCGGTGTGAGATATCGCATTATTCGAGGAACCCTAGATGCTG
TCGCAGTAAAGAATCGTCAACAAGGGCGTTTACAATATGGGGTCAAAAAGCCAAAAAT
GGCAAAAAAAAGTTTGATTCAGAGGGAGAAGAAGCGGCAGAAATTAGAACAGAAATA
TCATTTGATTCGTCAATCTTTAAAAAAAAAGATAAGAAGCAAAGTTTCTCCCTTGAGTT
TGAGTGAAAAAACTAAAATGCGAGAAAAATTGCAATCCCTACCGCGTAATAGTGCACC
TACACGCCTTCATCGACGTTGTTTTTTGACCGGAAGACCTAGAGCTAACTATCGACATT
TTGGGCTATCCGGACACGTACTTCGAGAAATGGTTTATGAATGTTTGTTACCGGGTGCA
ACAAGATCCAGTTGGATGAAAAAGAGAAAAATTTTTGGATTTATGGTTAAAGAAGAAA
AAGAAGAAAAC------AGGGGTTCTGTTGAATTTCAAGTATTCAGTTTCACCAATAAGAT
ACGGAGACTTGCTTCACATTTGGAATTACACAAAAAAGATTTTTCATCGGAAAGAGGT
CTACGAAGACTTTTGGGAAAACGTCGACGTTTGCTGGCTTATTTGGCAAAGAAAAATA
GAGTACGTTATAAGAAATTAATCGGTCAGTTGAATATTCGGGAGCAG---------------ATGTT
AAAACTTCGTTTAAAACGATGTGGTAGAAAGCAACAAGCTGTCTATCGAATCGTTGCA
ATTGATGTTCGATCTCGAAGAGAAGGAAGAGATCTTCGAAAAGTAGGTTTTTATGATCC
GATAAAGAATCAAACTTGTTTAAATGTTCCAGCTATTCTCTATTTCCTTGAAAAAGGTG
CTCAACCTACAAGAACTGTTTATGATATTTTAAGGAAGGCGGAATTCTTTAAAGAAAAA
GAAAGCACTTTGAGT------------TTTAATAAATCCAAGCAACCCTTTCGTAAATCCAAGCA
AACTTTTCGTAAAAGGCGTCCTCGGATTGGCCCGGGAGATCGAATTGATTATAGAAACA
TGAGTTTAATTAATAGATTTATTAGTGAACAAGGAAAAATATTATCGAGACGAATAAATA
GATTAACCTTGAAACAACAACGATTAATTACTCTTGCTATAAAACAGGCTCGTATTTTAT
CTTTCTTACCGTTTCGTAACTATGAGAACGAAAAGCAATTTCAA---GCCCAGTCAATTTC
AATAATTACAGGTTCTAGACCCAGAAAAAATGTGACACGAAAA---AAAACGAATCCTT
TTGTAGCTCATCATTTATTGGCAAAGATAGAAAAGGTCAATATGAAGGAGGAGAAAGA
AACAATAGTAACGTGGTCCCGGGCATCTAGCATTCTACCCACAATGGTTGGCCATACAA
TCGCGATTCATAATGGAAAGGAACATATACCTATTTACATAACAAATCCTATGGTAGGTC
GCAAATTGGGGGAATTCGTGCCTACTCGGCATTTCACGAGTTATGCAAGAAAGGATACT
A A A T C T C G T C G
T???
??
??
??
??
??
??

??
??
??
??
??
??
??
??
??
??
??
??
??
??
??
??
??
??
??
??
??
??
??
??
??
??
??
??
??
??
??
??
??
??
??
??
??
??
??
??
??
??
??
??

??
??
??
??
??
??
??
??
??
??
??
??
??
??
??
??
??
??
??
??
??
??
??
??
??
??
??
??
??
??
??
??
??
??
??
??
??
??
??
??
??
??
??
??

??
??
??
??
??
??
??
??
??
??
??
??
??
??
??
??
??
??
??
??
??
??
??
??
??
??
??
??
??
??
??
??
??
??
??
????????????????????????????????????ATGCCTAGATCCCGT---GTAAATGGAAATTTCATTG
ATAAGACCTCCTCAATTGTAGCCAATATTTTATTGCGAATAATTCCGACAACCTCAGGAG
AAAAAAAGGCATTTACTTATTATAGAGATGGG------ATGTTGGCTCAATCCGAAGGAAAT
TATGCGGAAGCTTTGCAAAATTATTATGAAGCTACGCGACTAGAAATCGATCCCTATGAT
CGAAGTTATATACTCTATAACATAGGCCTTATACACACAAGCAATGGAGAGCATACAAA
GGCTTTGGAATATTATTTCCGGGCACTAGAACGAAACCCCTTCTTACCGCAAGCTTTTA
ATAATATGGCCGTGATCTGTCATTAC---CGAGGAGAACAGGCCATTCTACAGGGTGATTC
GGAAATTGCGGAAGCTTGGTTTGATCAAGCTGCTGAATATTGGAAACAAGCTATAGCG
CTTACTCCGGGAAATTATATTGAAGCACAGAACTGGTTGAAGATTACGAAGCGCTTTGA

ATTTGAAATGAATTGGCGATCAGAACACATATGGGTAGAACTTCTAAAAGGTTCTCGAA
AAAGGGGTAATTTTTTCTGGGCCTGTATTCTTTTTCTAGGTTCACTAGGATTCTTATCGG
TTGGGATTTCCAGTTATCTTGGTAAGAATATTATATCTATACTTCCATCTCAAGAAATTCT
TTTTTTTCCGCAGGGGGTCGTGATGTCTTTCTACGGAATCGCAGGCCTATTCATTAGCTC
CTACTTGTGGTGTACTATTTTGTGGAATGTAGGTAGTGGTTATGACCGATTCGATAGAAA
AGAGGGAATAGTGTGCATTTTTCGTTGGGGATTCCCTGGAATAAAACGTCGCGTCTTCC
TTCGATTCCTTATGCGGGATATCCAATCAATTAGAATTCAGGTTAAAGAGGGTCTTTATC
CTCGTCGTATTCTTTATATGGAAATCCGGGGCCAGGGGATCATTCCCTTGACTCGTACTG
ATGATAAATTTTTTACTCCACGAGAAATTGAACAAAAAGCTGCCGAATTGGCCTATTTC
TTGCGCGTACCAATTGAAGTATTT
>Atropa
ATGGAAAGATGGTGGTTTAATTCGATGTTGTTTCTAAATAAATCAATGGGCAGTCTTGG
TCCTATTGAAAATACCAGTGAA------GATCCAAATCGAAAA------GTGAAAAACATTCAT--
-TGCAGTAATGTTGATTATTTATTCGGCGTTAAAGACATTCGGAATTTCATCTCTGATGAC
ACTTTTTTAGTTAGTGATAGGAATGGAGACAGTTATTCCATCTATTTTGATATTGAAAAT
CAGATTTTTGAGATTGACAACGATCAT------TCTTTTCTGAGTGAACTAGAAAGTTCTTTT
TATAGTTATCGAAACTCGAGTTATCTGAATAATGGATTTAGGGGCGAAGATCCCTACTAT
AATTCTTACATGTATGATACTCAATATAGTTGGAATAATCACATTAATAGTTGCATTGATA
ATTATCTTCAG------TCTCAAATCTGTATAGATACTTCTATTATAAGTGGTAGTGAGAATTA
CGGT------TACATTTATAGGACCATTTGTGGTAATGAGGGTTCCAGTAAACGAACTCGCA
CGAAGGGCAGTGATTTAACTATAAGAGAAAGTTCTAATGATCTCGAGGTAACTCAAAA
ATACAGGCATTTGTGGGTTCAATGCGAAAATTGTTATGGATTAAATTATAAGAAATTTTT
GAAATCCAAAATGAATATTTGTGAACAATGTGGATATCATTTGAAAATGAGTAGTTCGG
ATAGAATTGAACTTTTGATTGATGCGGGTACTTGGGATCCTATGGATGAAGACATGGTC
TCTCTGGATCCCATTGAATTTCATTCGGAGGAGGAGCCTTATAAAGATCGTATTGATTCT
TATCAAAGAAAGACAGGATTAACCGAGGCTGTTCAAACAGGCATAGGCCAACTAAAC
GGCATTCCCGTAGCAATTGGGGTTATGGATTTTCAGTTTATGGGGGGTAGTATGGGATCC
GTAGTCGGAGAGAAAATCACCCGTTTGATTGAACACGCTGCCAATCAAATTTTACCTCT
TATTATAGTGTGTGCTTCCGGTGGGGCGCGCATGCAGGAAGGAAGTTTGAGCTTGATGC
AAATGGCTAAAATATCGTCTGCTTTATATGATTATCAATTAAATAAAAAATTATTTTATGTA
TCAATCCTTACATCTCCGACAACTGGTGGAGTGACAGCTAGTTTTGGTATGTTGGGGGA
TATCATTATTGCCGAACCCAATGCCTACATTGCATTTGCAGGTAAAAGAGTAATTGAAC
AAACATTGAATAAAACAGTACCCGAAGGTTCACAAGCAGCTGAATACTTATTCCAGAA
GGGTTTATTCGACCAAATTGTACCACGTAATCTTTTAAAAAGCGTTCTGAGTGAATTATT
TAAGCTCCACGCCTTTTTTCCTTTGAATCAAAAGTCAAGCAAAATGGTAACCATTCGAG
CTGACGAAATTAGTAATATTATCCGTGAACGTATTGAACAATATAATAGAGAAGTAAAGA
TTGTAAATACCGGTACCGTACTTCAAGTAGGCGATGGCATTGCTCGTATTCACGGTCTT
GATGAAGTAATGGCGGGTGAATTAGTAGAATTTGAAGAGGGTACAATAGGCATTGCTCT
GAATTTGGAATCAAATAATGTTGGTGTTGTATTAATGGGCGATGGTTTGTTGATACAAGA
AGGAAGTTCTGTAAAAGCAACGGGAAGAATTGCTCAGATACCGGTGAGTGAGGCTTAT
TTGGGTCGTGTTGTAAATGCCCTGGCTAAACCTATTGATGGCAGAGGTGAAATTTCAGC
TTCTGAATTTCGATTAATTGAATCTGCCGCCCCAGGTATTATTTCGCGCCGTTCCGTATAT
GAGCCTCTTCAAACCGGGCTTATTGCTATTGATTCGATGATCCCTATAGGGCGTGGTCA
GCGAGAATTAATTATTGGGGACAGACAGACCGGTAAAACAGCAGTAGCCACAGATACG

ATTCTCAATCAACAAGGGCAAAACGTAATATGTGTTTATGTAGCTATTGGGCAAAAAGC
ATCTTCTGTGGCCCAGGTAGTAACTACTTTACAGGAAAGGGGAGCGATGGAATACACTA
TTGTGGTAGCCGAAACGGCAGATTCCCCTGCTACATTACAATACCTTGCTCCTTATACA
GGAGCATCTCTGGCTGAATATTTTATGTATCGTGAACGACACACTTTAATCATTTATGAT
GATCTCTCCAAACAAGCGCAAGCTTATCGCCAAATGTCTCTTCTATTACGAAGACCGCC
CGGTCGTGAAGCTTATCCAGGAGATGTTTTTTATTTGCATTCACGCCTTTTGGAAAGAG
CCGCTAAATTAAGTTCTAGTTTAGGTGAAGGAAGTATGACCGCCTTACCAATAGTTGAA
ACTCAATCGGGAGATGTTTCGGCTTATATTCCTACTAATGTAATTTCCATTACTGATGGA
CAAATCTTCTTATCCGCCGACCTATTCAATTCTGGAATCAGACCCGCTATTAATGTGGGT
ATCTCCGTTTCCAGAGTGGGGTCCGCAGCTCAAATAAAAGCCATGAAACAAGTAGCTG
GTAAATTAAAATTAGAACTAGCACAATTCGCAGAATTAGAAGCCTTTGCACAATTTGCT
TCTGATCTCGATAAAGCTACTCAAAATCAATTGGCAAGAGGTCAACGATTACGTGAATT
GCTTAAACAATCCCAATCAGCTCCTCTCACGGTAGAAGAGCAGATAATGACTATTTATA
CCGGAACAAACGGCTATCTTGATTCATTAGAAGTTGGACAGGTAAGGAAATTTCTTGTT
GAGCTACGTACTTACTTAAAAACTAATAAACCTCAGTTCCAGGAAATCATATCTTCTACC
AAGACATTTACCGAGGAAGCAGAAGCCCTTTTGAAAGAAGCTATTCAGGAACAAATG
AAACGTTTTATACTTCAGGAACAAGCAATGAGAATCAATCCTACTACTTCTGGTTCTGG
GGTTTCCACGCTTGAAAAAAAAAACCTGGGGCGTGTCGTCCAAATCATCGGTCCGGTA
CTAGATGTAGCCTTTCCCCCGGGCAAGATGCCGAATATTTATAACGCTCTGGTAGTTCA
AGGTCGAGATAGTGTTGGTCAACCAATTAATGTGGCTTGTGAGGTACAGCAATTATTAG
GAAATAATCGAGTTAGGGCTGTAGCTATGAGTGCTACAGACGGTCTAACGAGAGGAAT
GGAAGTGATTGACAAAGGAGCTCCTATAAGTGTTCCGGTCGGGGGAGCGACTCTGGG
ACGAATTTTTAACGTGCTCGGAGAGCCTGTTGATAATTTAGGGCCTGTAGATACTAGTA
CAATGTCTCCTATTCATAGATCTGCGCCCGCCTTTATACAGTTAGATACAAAATTATCTAT
TTTTGAAACAGGAATTAAAGTAGTAGATCTTTTAGCCCCTTATCGCCGTGGAGGAAAAA
TCGGACTATTCGGGGGAGCTGGAGTGGGTAAAACAGTACTCATTATGGAATTGATTAAC
AATATTGCTAAAGCTCACGGGGGCGTATCCGTATTTGGCGGAGTGGGTGAACGTACTCG
GGAAGGAAATGATCTTTACATGGAAATGAAAGAATCTGGGGTGATTAATGAAGAAAAT
ATTGCAGAATCAAAAGTGGCCCTAGTTTACGGTCAGATGAATGAACCGCCGGGAGCTC
GTATGAGAGTTGGTTTGACTGCCCTAACTATGGCGGAATATTTCCGAGATGTTAATGAG
CAAGACGTACTTCTATTTATTGACAATATCTTCCGTTTCGTCCAAGCAGGATCCGAAGTA
TCGGCCTTATTGGGTAGAATGCCTTCCGCTGTGGGTTATCAACCGACCCTGAGTACCGA
AATGGGTTCTTTACAAGAAAGAATTACTTCTACCAAAGAAGGGTCCATAACCTCTATTC
AAGCCGTTTATGTACCCGCAGACGATTTGACCGACCCTGCTCCTGCTACGACATTTGCA
CATTTAGATGCTACTACCGTACTATCAAGAGGATTGGCTGCCAAAGGTATTTATCCAGCA
GTAGATCCTTTAGATTCAACGTCAACCATGCTTCAACCTCGGATCGTTGGTGAGGAACA
TTACGAAACCGCCCAAAGAGTTAAGCAAACTTTACAACGTTACAAAGAACTTCAGGA
CATTATAGCTATCCTTGGATTGGACGAATTATCCGAAGAGGATCGTTTACTCGTAGCAAG
AGCGCGAAAAATTGAGCGTTTCTTATCACAACCCTTTTTCGTAGCAGAAGTATTTACCG
GTTCTCCAGGGAAATATGTTGGTCTAGCAGAAACAATTCGAGGATTTCAATTGATCCTT
TCCGGAGAATTAGATGGTCTTCCTGAACAGGCCTTTTATTTGGTAGGTAATATCGATGAA
GCTACCGCGAAGGCTATGAACTTAGAAATGGAGAGCAATTTGAAGAAAATGACCTTAA
ATCTTAGTGTACTGACCCCTAATCGAATTGTTTGGGATTCAGAAGTGGAAGAAATCGTT
TTATCTACTAATAGTGGTCAAATTGGCATATTATCAAATCACGCCCCTATTGCCACAGCT

GTAGATATAGGGATTTTGAGAATACGCCTTAACGACCAATGGTTAACGATGGCTCTGAT
GGGTGGTTTTGCTAGAATAGGCAATAATGAAATCACTGTTTTAGTAAATGATGCGGAGA
AGGGTAGTGACATTGATCCACAAGAAGCTCAGCAAACTCTTGAAATAGCGGAAGCTAA
TGTGAAAAAGGCTGAAGGAAGGAGACAAAAAATTGAGGCAAATCTAGCTCTCCGACG
AGCTAGAACACGGGTGGAGGCTATCAATCCGATTTCAATGAAAAACGTAACCGATTCT
TTCGTTTCT---GGCCACTGGCCATCTGCCGGGAGTTTCGGGTTTAATACCGATATTTTAG
CAACAAATCCAATAAATCTAAGTGTAGTCCTTGGTGTATTGATTTTTTTTGGAAAGGGA
GTGTTAAGTGATTTATTAGATAATCGAAAACAGAGGATCTTGAATACTATTCGAAATTCA
GAAGAACTGCGTGAAGGGGCTATTGAACAACTCGAAAAAGCTCGTTCTCGCTTACGG
AAAGTAGAAACCGAAGCCGAGCAGTTTCGAGTGAATGGATACTCCGAGATAGAACGA
GAAAAATTGAATTTGATTAATTCAACTTATAAGACTTTGGAACAATTAGAAAATTACAA
AAACGAAACGATTCAGTTTGAACAACAAAGGGCGATTAATCAAGTCCGACAACGGGT
TTTCCAACAAGCCTTACGAGGAGCTCTAGGAACTCTGAATAGTTGTTTGAACAACGAG
TTACATTTACGTACCATTAGTGCCAATATTGGCATGTTGGGAACAATGAAAGAAATAAC
TGATATGAATCCACTGGTTTTTGCCGCTTCCGTTATTGCTGCTGGATTGGCCGTAGGGCT
TGCTTCTATTGGACCTGGAGTTGGTCAAGGGACTGCTGCGGGTCAAGCTGTAGAGGGT
ATCGCGAGACAGCCTGAGGCAGAGGGAAAAATACGAGGTACGCTATTGCTTAGTCTAG
CTTTTATGGAAGCTTTAACAATTTATGGACTGGTTGTAGCATTAGCACTTTTATTTGCAA
ATCCTTTTGTTATGAATGTTCTATCATGTTCCATCAATACTCTAAAGGGGTTATACGATAT
ATCCGGTGTGGAAGTAGGCCAACATTTCTATTGGCAAATAGGGGGTTTCCAAGTACATG
GCCAAGTACTTATTACTTCTTGGGTTGTAATTGCTATCTTATTAGGTTCAGCCACTATAGC
TGTTCGGAACCCACAAACCATTCCGACCGGGGGTCAGAATTTCTTCGAATATGTTCTTG
AATTCATTCGAGATGTGAGTAAAACTCAAATTGGAGAAGAATATGGGCCCTGGGTTCCT
TTTATTGGCACTATGTTTTTATTTATTTTTGTTTCTAATTGGTCAGGAGCTCTTTTACCTTG
GAAAATCATACAATTACCTCATGGGGAGTTAGCCGCACCCACGAATGATATAAATACTA
CTGTTGCTTTGGCTTTACTCACATCAGTGGCATATTTCTATGCGGGTCTTACAAAAAAA
GGATTAGGTTATTTCGGAAAATATATTCAACCAACCCCAATCCTTTTACCCATTAACATC
TTAGAAGATTTCACAAAACCTTTATCACTTAGTTTTCGACTTTTCGGGAATATCTTAGCT
GATGAATTAGTAGTTGTTGTTCTTGTTTCTTTAGTACCTTTAGTAGTTCCTATACCTGTCA
TGCTCCTTGGATTATTTACAAGTGGTATTCAAGCTCTTATTTTTGCAACTTTAGCCGCGG
CTTATATAGGTGAATCCATGGAAGGCCATCATATGATATTTTCAACCTTAGAGCATATATT
AACTCACATTTCCTTTTCGATCGTTTCAATTGTAATTACAATTCATTTGATAACCTTTTTA
GTCGATGAAATCGTAAAACTATACGATTCGTCAGAAAAGGGCATAATAGTTACTTTTTT
CTGTATAACAGGATTATTAGTTACTCGTTGGATTTCTTCGGGACATTTCCCACTAAGCGA
TTTATATGAATCATTAATTTTCCTTTCATGGAGTTTCTCCCTTATTCATATAATTCCGTAT---
---AACAATGTTTTAATTTTAAGTAAAATAACTGGCCCAAGTGCTATTTTTACCCAAGGCT
TTGCTACTTCAGGTATTTTAACTGAAATACACCAATCTGGAATATTAGTACCTGCTCTTC
AATCCGAGTGGTTAATAATGCACGTAAGTATGATGATATTGGGCTATGCAGCCCTTTTAT
GTGGATCGTTATTATCAGTAGCACTTCTAGTGATTACATTTCGAAAAAACAGAAAGCTT
TTTTAT------AAGGAAAATGTTTTAGAAAATACTTCT--------------------------------GCTAAAA
ATTATTACAGGTCCCAATTGATTCAACAATTGGATTATTGGAGTTATCGGGTTATTAGTTT
AGGATTTACTTTTTTAACCATAGGAATCCTTTCGGGAGCGGTATGGGCTAATGAAGCGT
GGGGGTCGTATTGGAATTGGGACCCAAAAGAAACTTGGGCATTTATTACTTGGATCGTA
TTTGCAATTTATTTACATACTCGAACAAATATAAATTTACGGGGTGCAAATTCTGCAATTA

TAGCAACTATAGGTTTTCTTATAATTTGGATATGCTATTTTGGGGTCAATCTATTAGGAAT
AGGGTTACATAGTTATGGTTCTTTTACATCAACAATGGCAAAAAAGAAAGCATTCACTC
CTCTTTTCTATCTTGCATCTATAGTATTTTTGCCCTGGTGGATTTCTTTCTCAGTTAATAAA
TGTCTGGAATCTTGGGTTACTAATTGGTGGAATACTGGGCAATCCGAAATTTTTTTGAAT
AATATTCAAGAAAAGAGTCTTCTAGAAAAATTCATAGAATTAGAGGAACTCCTCCTCTT
GGACGAAATGATCAAGGAATACTCGGAAACACATCTCGAAGAGTTTGGGATA---ATCCA
TAAAGAAACGATCCAATTAATCAAGATACAAAATGAGAATCGTATCCATACGATTTTGC
ACTTCTCGACAAATATAATCTGTTTTATTATTCTAAGCGGGTATTCAATTTTGGGTAATGA
AAAACTTGTTATTCTTAACTCTTGGGCTCAGGAATTTCTATATAACTTAAGTGACACAGT
AAAAGCTTTTTCTATTCTTTTATTAACTGATTTATGTATTGGATTCCATTCACCCCACGGT
TGGGAATTAATGATTGGCTCTATCTATAAAGATTTTGGATTTGTTCATAATGATCAAATTA
TATCTGGTCTTGTTTCCACCTTTCCAGTCATTCTTGATACAATTTTTAAATATTGGATTTT
CCGTTATTTAAATCGTCTGTCTCCGTCACTTGTAGTTATTTATCATTCAATGAATGACATG
CCTATTGGTGTTCCAAAAGTTCCTTTCCGAAGTCCTGGAGAGGAAGATGCATCTTGGGT
TGACGTATACAACCGACTTTATCGAGAAAGATTACTTTTTTTAGGCCAAGGGATTAATA
GCGAGATTTCGAATCAACTTATTGGTCTTATGGTATATCTCAGTATCGAGGATGAGACCA
AAGAGCTGTATTTGTTTATAAACTCTCCTGGGGGCTGGGTAATACCTGGGATCGCTATTT
ATGATACTATGCAATTTGTGCGACCAGATGTCCATACAGTATGCATGGGATTAGCCGCTT
CAATGGGATCTTTTATCCTGGTCGGAGGAGAAATTACCAAACGTCTAGCATTCCCTCAC
GCTAGGGTAATGATGCATCAACCTGCTAGTGGTTATTATGAGGCACAAACAGGCGAATT
TGTCCTGGAAGCGGAAGAACTGTTGAAACTGCGTGAAACCCTCACAAGGGTTTATGTA
CAAAGAACGGGCAAACCCTTATGGGTTGTATCCGAAGATATGGAAAAAGATGTTTTTAT
GTCAGCAACAGAAGCCCAAGCTTATGGAATTGTTGATCTTGTAGCGGTTGA
A???
??
??????????????????????????????????????ATGGAAGAAATCCAAAGATATT
TACAG---GATAGCTCGCAACAACACAACTTCCTATATCCACTTATCTTTCAGGAGTATATT
TATGCACTTGCTCATGATCATGGTTTACCAGGTTATAACAATCAATTTAGTTTACTAATTG
TGAAACGTTTAATTACTCGAATGTATCAACAGAATCATTTTCTTATTTCTACTAATGATTC
TAACAAAAATACATTTTTGGGGAAGAGTTTGTATTCTCAAATGATATCAGAGGGATTTG
CATTTATTGTGGAAATTCCATTTTCTCTACGATTAATATCTTCTTTAATTTTAAAATCTCAT
AATTTACGATCAATTCATTCAACATTTCCTTTTTTAGAGGACAATTTTTCACATCTAAATT
ATGTATTAGATATACTAATACCCTATCCCGTTCATCTGGAAATCTTGGTTCAAACTCTTCG
CTATTGGGTAAAAGATGCCTCTTCTTTACATTTATTACGATTCTTTCTCCACGAATATTGG
AATTTGAATAGTCTTATTACTCCCGGT---TACTCCTTTTCAAAAAAAAGATTCTTCTTTTT
CTTATATAATTCTTATGTATATGAATGCGAATCCACTTTCGTCTTTCTACGGAACCAATCT
TCTCATTTACGACCAACATCTTTTGGAGCCCTTCTTGAACGAATATATTTCTATGGAAAA
ATAGAACGTCTTGTAGAAGTCTTTGCT---GATTTGACCCTATGGTTATTCAAGGATCCTTT
CATGCATTATGTTAGGTATCAGGGAAAATCAATTCTGGCTTCAAAAGGGACGTTTCTTT
TGATTAATAAATGGAAATTTTACCTTGTCAATTTTTGGCAATGTCATTTTTCTCTGTGTTT
TCACACAGGAAGGATCCATATAAACCAATTATCCAACCATTCCCGTGACTTTATGGGCTA
TCTTTCAAGTGTGCGACTAAATCCTTCAATGGTACGTAGTCAAATGTTAGAAAATTCAT
TTATAATCAATAATGCAATT------AAGAAGTTCGATACCCTTGTTCCAATTATTCCTTTGAT
TGGATCATTAGCTAAAGAAAACTTTTGTACCGTATTAGGGCATCCCATTAGTAAACCGG

TTTGGTCCGATTTATCAGATTCTGATATTATTGACCGATTTGGGCGTATATGCAGAAATCT
TTTTCATTATTATAGCGGATCTTCCAAAAAAAAGACTTTATATCGAATAAAGTATATACTT
CGACTTTCTTGTGCTAGAACTTTAGCTCGGAAACACAAAAGTACTGTACGCACTTTTTT
GAAAAGATCGGGCTCGGAATTATTGGAAGAATTCTTAACGTCGGAAGAACAAGTTCTT
TCTTTGACCTTCCCACGACGGATTTGGTATTTGGATATTTTTTGTATCAATGATCTGGCG
AATTATCAAATGATAATTGAT---ACAACAGAAATAGAG---ACTATCAATTCTTTTTCCAAA
TTGGAATCC---TTAAAAGAAGTCTATGGAATCATATGGATGCTTGTCCCTATTTTGACTCT
TGTATTAGGAATCACAATAGGTGTACTAGTAATTGTTTGGTTAGAAAGAGAAATATCTGC
AGGAATACAACAACGTATCGGACCTGAATATGCCGGCCCTTTAGGAATTCTTCAAGCTC
TAGCAGATGGGACAAAACTACTTTTTAAAGAGAACCTTATTCCATCTACAGGAGATCCT
CGTTTATTCAGTATCGGACCATCCATAGCAGTAATATCTATCTTTCTAAGTTATTCAGTAA
TTCCTTTTGGTGATCACCTTGTTCTAGCCGATCTTAGTATTGGTGTTTTTTTATGGATTGC
CATTTCAAGTGTTGCTCCCGTTGGACTTCTTATGTCAGGATATGGATCAAATAATAAATA
TTCCTTTTTAGGTGGTCTACGGGCAGCTGCCCAATCAATTAGTTATGAAATACCATTAGC
TCTATGTGTGTTATCAATATCTCTACTATCTAACAGTTCAAGTACAGTTGATATAGTGGAA
GCGCAGTCAAAATATGGTTTTTTAGGGTGGAATTTGTGGCGTCAACCCATCGGGTTTAT
CGTTTTTCTAATTTCTTCTCTAGCCGAGTGTGAAAGATTACCTTTTGATTTACCAGAAGC
AGAAGAAGAATTAGTAGCAGGGTATCAAACCGAATATTCAGGTATCAAATTTGGTTTAT
TTTACATTGCTTCATATCTGAATCTACTAGTTTCTTCATTATTTGTAACAGTTCTTTACTTG
GGAGGTTGGAATCTTTCTATTCCGTACATATTTGTTCCTGAGTTATTTGGCATAAATAAA
AGGGGTAAAGTCTTTGGAACATTAATTGGTATCTTTATTACATTAGCCAAAACTTATTTG
TTTTTGTTCATTCCTATTGCAACAAGATGGACTTTACCGAGGCTGAGAATGGACCAACT
ATTAAATCTTGGATGGAAATTTCTTTTACCTATTTCTCTAGGTAATCTATTATTGACAACT
TCGTCCCAACTTCTTTCATTGATGATCTGGCATGTACAGAATGAAAACTTCATTCTCGAT
TCTACGAGAATTTTTATGAAAGCCTTTCATTTGCTTCTCTTCGATGGAAGTTTGATTTTC
CCAGAATGTATCCTAATTTTTGGCCTAATTCTTCTTCTGATGATCGATTCAACCTCTGATC
AAAAAGATATACCTTGGTTATATTTCATCTCTTCAACAAGTTTAGTAATGAGCATAACGG
CTCTATTGTTCCGATGGAGAGAAGAACCTATGATTAGCTTTTCGGGAAATTTCCAAACG
AACAATTTCAACGAAATCTTTCAATTTCTTATTTTACTATGTTCAACTCTATGTATTCCTC
TATCCGTAGAGTACATTGAATGTACAGAAATGGCTATAACAGAGTTTCTCTTATTCGTAT
TAACAGCTACTCTAGGGGGAATGTTTTTATGCGGTGCTAACGATTTAATAACTATCTTTG
TAGCCCCAGAATGTTTCAGTTTATGCTCCTACCTATTATCTGGATATACCAAGAAAGATG
TACGGTCTAATGAGGCTACTATGAAATATTTACTCATGGGTGGGGCAAGCTCTTCTATTC
TGGTTCATGGTTTCTCTTGGCTATATGGTTCATCCGGGGGAGAGATTGAGCTTCAAGAA
ATAGTAAACGGTCTTATCAATACACAAATGTATAACTCCCCAGGAATTTCAATTGCGCTC
ATATTCATTACCGTAGGAATTGGGTTCAAGCTTTCCCCAGCCCCTTCTCATCAATGGACT
CCTGACGTATACGAAGGATCTCCCACTCCAGTCGTTGCTTTTCTTTCTGTTACTTCGAA
AGTAGCTGCTTCAGCTTCAGCCACTCGAATTTTCGATATTCCTTTTTATTTCTCATCAAA
CGAATGGCATCTTCTTCTGGAAATCCTAGCTATTCTTAGCATGATATTGGGAAATCTCAT
TGCTATTACTCAAACAAGCATGAAACGTATGCTTGCATATTCGTCCATAGGCCAAATCG
GATATGTAATTATTGGAATAATTGTTGGAGACTCAAATGATGGATATGCAAGCATGATAA
CTTATATGCTGTTCTATATCTCCATGAATCTAGGAACTTTTGCTTGCATTGTATTATTTGGT
CTACGTACCGGAACTGATAACATTCGAGATTATGCAGGATTATACACAAAAGATCCTTTT
TTGGCTCTCTCTTTAGCCCTATGTCTCTTATCCCTAGGAGGTCTTCCTCCACTAGCAGGT

TTTTTCGGAAAACTCTATTTATTCTGGTGTGGATGGCAGGCAGGCCTATATTTCTTGGTT
TTAATAGGACTCCTTACAAGCGTTGTTTCTATCTACTATTATCTAAAAATAATCAAGTTAT
TAATGACTGGACGAAACCAAGAAATAACCCCTCACGTGCGAAATTATAGAAGATCCCC
TTTAAGATCAAACAATTCCATCGAATTGAGTATGATTGTATGTGTGATAGCATCTACTATA
CCAGGAATATCAATGAACCCGATTATTGCAATTGCTCAGGATAGCCTTTTTATGTTTCTG
CTTTACGAATATGATTTTTTTTGGGCATTTCTAATAATATCAATCCTTGTTCCTATTTTGGC
ATTTTTCATTTCCGGAGTGTTAGCCCCAATTAGCAAAGGGCCGGAGAAACTTTCTACTT
ATGAGTCGGGTATAGAACCAATGGGCGATGCTTGGTTACAATTTAGAATCCGTTATTATA
TGTTTGCTCTAGTTTTTGTTGTTTTTGATGTTGAAACGGTTTTTCTTTATCCATGGGCAAT
GAGTTTCGACGTATTGGGTGTATCTGTATTTATAGAAGCTTTCATTTTCGTGCTGATCTTA
ATTATTGGTTTAGTTTATGCATGGCGAAAGGGGGCATTGGAATGGTCTACGAATTATTTT
CCTTGGTTAACAATAATTGTCGTTTTTCCTATATTTGCGGGTTCCTTAATTTTCTTTCTTC
CCCATAAAGGAAATAGGGTAATTAGGTGGTATACAATTTGTATATGTATTTTAGAACTCC
TTCTAACGACTTATGCATTTTGTTATCATTTCCAATTGGATGATCCATTAATCCAACTAGT
GGAGGATTATAAATGGATCGATTTTTTTGATTTCCATTGGAGATTAGGAATAGATGGACT
TTCTATAGGACCCATTTTATTAACAGGATTTATCACTACTTTAGCGACTTTAGCGGCTTG
GCCAATTACTCGAGATTCTCGATTATTCCATTTTATCATGTTAGCAATGTACAGTGGTCA
AATTGGATCATTTTCATCCCGGGACCTTTTACTTTTTTTCATCATGTGGGAGTTAGAATT
AATTCCTGTTTATCTACTTCTAGCCATGTGGGGAGGAAAGAAACGTCTGTACTCAGCTA
CAAAATTTATTTTGTACACGGCAGGGGGTTCTGTTTTTCTATTAATGGGAGTTTTGGGTG
TTGCTTTATATGGTTCTAATGAACCAACATTAAATTTTGAAACATTAGTTAATCAGTCATA
TCCTGTGGTTTTAGAAATAATCTTCTATATTGGATTTTTTATTGCTTTTGCTGTCAAATTG
CCCATTATCCCTCTACACACATGGTTACCAGATACCCATGGAGAAGCACATTACAGTAC
TTGTATGCTTCTAGCCGGAATCTTATTAAAAATGGGAGCGTATGGATTAATTCGAATCAA
TATGGAATTACTACCTCACGCCCATTCTATATTTTCTCCTTGGTTGATGATAATAGGTACA
ATACAAATAATCTATGCAGCTTCAACATCTCTTGGCCAACGGAATTTAAAAAAAAGAAT
AGCCTATTCCTCTGTCTCTCATATGGGTTTCATAATTATAGGAATTAGTTCTCTAACCGAT
ACGGGACTTAATGGAGCCCTTTTACAAATAATCTCTCATGGATTTATTGGTGCTGCACTT
TTTTTCTTGGCGGGAACGACTTATGATAGAATCCGCCTTGTTTATCTTGACGAAATGGG
CGGAATAGCTATTCCAATGCCAAAAATGTTCACGATGTTCAGTAGCTTTTCGATGGCTT
CCCTTGCATTACCAGGTATGAGTGGTTTTGTTGCCGAATTGATAGTATTTTTTGGAATAA
TTACCGGCCAAAAATATGTTTTAATTCCAAAAATACTAATTACTTTTGTAATGGCAATTG
GAATGATATTAACTCCTATTTATTCATTATCTATGTCACGCCAGATGTTCTATGGATACAA
GTTATTTAATGCCCCGAAGGATTCCTTTTTTGATTCTGGACCGCGAGAGTTATTTCTTTC
GATTTCCATCTTTTTACCCGTAATAGGTATTGGTATATACCCGGATTTCGTTCTTTCATTA
GCAGTTGATAAGGTCGAAGTTATTCTATCTAATTTTTTTTATAGAATGATTCTCGAACATG
TACTTGTTTTGAGTGCCTATTTATTTTCTATCGGTATCTACGGATTGATCACAAGTCGAAA
TATGGTTAGAGCCCTTATGTGTCTTGAACTTATATTGAATGCGGTTAATATCAATTTTGTA
ACATTTTCTGATTTTTTTGATAATCGTCAATTAAAAGGAGACATTTTCTCAATTTTTGTTA
TAGCTATTGCAGCCGCTGAAGCAGCCATTGGACTGGCTATTGTTTCATCAATTTACCGTA
ACAGAAAATCAACTCGTATCAACCAATCTAATTTGTTGAATAATATGGAACAGACATAT
GAATATGCGTGGATCATACCTTTCATTCCACTTCCAGTCCCTATGTTAATAGGAGCGGGA
CTTATTCTTTTTCCAACGGCAACAAAAAGTTTTCGCCGTATGTGGGCTTTTCAGAGTGT
TTTATTGTTAAGCATAGTCATGATTTTTTCAATCTACCTGTCTATTCAGCAAATAAATAGC

AGTTCTTTTTATCAATATGTATGGTCTTGGATCATCAATAATGATTTTTCTTTAGACTTCG
GATACTTGATCGACCCACTTACTTCTATTATGTCAATATTAATCACTACTGTTGGAATTAT
GGTTCTTATTTATAGTGATAATTATATGGCTCATGATCAAGGCTATTTGAGATTTTTTGCTT
ATATGAGTTTTTTCAGTACTTCCATGTTGGGATTAGTTACTAGTTCTAATTTGATACAAAT
TTATATTTTTTGGGAATTGGTTGGGCTGTGTTCCTATCTATTAATAGGATTTTGGTTTACA
CGACCTGTTGCGGCAAATGCTTGTCAAAAAGCGTTTGTAACTAATCGTGTAGGGGATTT
TGGTTTATTATTAGGAATTTTAGGTTTTTATTGGATAACAGGAAGTTTCGAATTTAGGGA
TTTATTCGAAATATTCAATAACAATGAGGTCAATTTTTTATTTGTTACTTTATGCGCTGTT
CTCTTATTTGCCGGTGCAGTTGCTAAATCCGCCCAATTCCCCCTTCATGTATGGTTACCT
GATGCCATGGAGGGACCTACTCCTATTTCGGCTCTTATACATGCTGCTACTATGGTAGCG
GCGGGAATTTTTCTTGTAGCTCGGCTTCTTCCTCTTTTCAGAGTTATACCTTATATAATGT
ATTTGATCTCGGTTATAGGAATAATAACAGTATTATTAGGAGCTACTTTAGCTCTTGCTCA
AAAAGACATTAAGAGAGGTTTAGCCTATTCCACAATGTCTCAATTGGGTTATATGATGTT
AGCTCTAGGTATGGGGTCTTATCGAAGCGCTTTATTTCATTTGATTACTCATGCTTATTCC
AAAGCATTATTATTTTTAGGATCCGGATCCATTATTCATTCAATGGAAACTATTGTTGGAT
ATTCTCCAGCTAAAAGCCAGAATATGGGTCTTATGGGAGGTTTAAGAAAACATGTACCA
ATTACCCAAATCACATTTTTATTAGGTACACTTTCTCTTTGTGGTATTCCACCTCTTGCTT
GTTTTTGGTCCAAAGATGAAATTCTTAATGATAGTTGGTTGTATTCGCCAATTTTCGCAA
TAATAGCTTGGGCCACGGCGGGATTAACCGCATTTTATATGTTTCGGATCTATTTACTTAC
TTTTGAAGGACATTTAAACGTTCATTTTCAAAATTATGGTGGGAAACAAAAAACCCCTT
TCTATTCAATATCTCTATGGGGTAAA---------AAGGGAGTTAAGAAA---TTATTAACA-------
--ATGAATTTTTTTTCATTTGAGCATAAAACGGTTTATTCCTATCCTTATGAATCGGACAAT
ACTATGCTATTCCCAATATTTGTATTAGGACTCTTTACTTTGTTTGTTGGATCTATAGGAA
TTCCTTTCAATTTGGATATATTATCGAAATGGTTAGCTCCATCTATAAATCTTTTGCATCA
AAAGTCGAAT---TCGATGGATTGGAATGAATTTTTAAAGGATGCAGTCCTTTCCGTCAGT
ATAGCTTATTTCGGAATATTTTTAGCATCCTTTTTATATAAACCTATTTATTCTTCTTTAAA
AAATTTTGAGTTAATTAATTCTTTTGTTATTCTGTGGGACAAAATTATCAATGGCATATAT
GATTGGTCATATAATCGTGCTTACATAGATGCTTTTTATACAAGATTCTTTGTTGGTGGGA
TAAGAGGATTAGCTGAATTTATTCATTTTTTTGATCGACGAGTAATTGATGGGATGACTA
ACGGGGTTGGGGTTATTAGTTTCATTGTAGGAGAAGGTATCAAATATATAGGGGGCGGA
---CGCATCTCTTCTTATCTTTTCTTATATTTAGCTTATGTTTCAGTCTTTTTATTAGTTTATT
ACATGGATTTATCTGAACCAATACATGATTTTCTTTTAGTCTTTCTAGGATCGGGTCTTAT
ATTAGGGGGTCTAGGGGTGGTATTACTTCCCAATCCAATTTATTCTGCCTTTTCCTTGGG
ATTGGTTCTTGTTTGTACATCGTTATTCTATATTCTATCTAACTCCTATTTTGTAGCTGCTG
CGCAGCTACTTATTTACGTAGGAGCTATAAATGTTTTAATCATTTTTGCTGTGATGTTCAT
GAATGGTTCAGAATATTACAAAGATTTTCATCTTTGGACCGTAGGAGATGGAATTACTT
CGATGGTTTGTATAAGTCTTTTTATTTCACTAATTACTACTATTTCAGATACTTCATGGTA
CGGGATTATTTGGACTACAAGATCAAACCAGATTATAGAGCAAGATTTTCTAAGTAATA
GTCAACAAATTGGAATTCATTTATCAACAGATTTTTTTCTTCCATTTGAACTAATTTCAAT
AATCCTTTTAGATGCTTTAATAGGTGCAATTGCTGTAGCTCGTCAAATGACTACACCAAC
TACAAGAAAAGACCTCATGATAGTCAATATGGGGCCTCAGCACCCATCAATGCACGGC
GTTCTTCGACTCATCGTTACTCTAGATGGTGAAGATGTTGTCGACTGCGAACCAATATT
GGGTTATTTACATAGAGGGATGGAGAAAATTGCGGAAAATCGAACAATTATCCAATATT
TGCCTTATGTAACACGTTGGGATTATTTAGCTACTATGTTCACAGAAGCAATAACCATAA

ATGGACCCGAACAATTAGGCAATATTCAAGTACCTAAAAGAGCTAGCTATATCAGAGTC
ATTATGTTGGAGTTGAGTCGGATAGCTTCTCATTTGTTATGGCTCGGTCCTTTTATGGCG
GATATTGGTGCGCAGACCCCTTTCTTCTATATTTTTCGAGAAAGAGAATTAATATATGAC
CTCTTCGAAGCTGCCACCGGTATGCGAATGATGCATAATTATTTTCGTATCGGAGGGGT
GGCTGCCGATCTACCCTATGGATGGATAGATAAATGTTTGGATTTTTGCGATTATTTTTA
ACGGGGATTGCTGAGTATCAAAAACTTATTACCCGGAATCCTATTTTTTTAGAACGAGT
TGAAGGCGTAGGCATTATTGGGGGAGACGAAGCATTAAATTGGGGTTTATCGGGACCA
ATGCTACGAGCTTCCGGAATAGAATGGGATCTTCGTAAAGTTGATCATTATGAGTCTTAC
GACGAATTTGATTGGCAGGTTCAATGGCAACGAGAAGGGGATTCATTAGCTCGTTATTT
AGTACGAATCGGTGAAATGACCGAATCCATAAAGATTATTCAACAGGCTCTGGAAGGA
ATTCCAGGAGGGCCTTACGAAAATTTAGAAATACGACGTTTTGACAGATTAAAAGATCC
TGAATGGAATGATTTTGAATATCGGTTTATTAGTAAAAAACCTTCTCCAACTTTTGAATT
GTCGAAACAAGAACTTTATGTGAGAGTTGAAGCCCCAAAAGGAGAATTGGGAATTTTT
CTCATAGGAGATCAGAGCGTTTTTCCTTGGAGATGGAAAATTCGCCCACCAGGTTTTAT
CAATTTGCAAATTCTTCCTCAGTTAGTTAAAAGAATGAAATTGGCTGATATTATGACAAT
ACTAGGTAGCATAGATATCATTATGGGAGAAGTTGATCGTATGCTCCCTATGATAACTGA
ATTCATAAATTATGGTCAACAAACAATACGAGCCGCCAGATATATCGGCCAAGGTTTCAT
GATTACCCTGTCCCACGCAAATCGTTTACCTGTAACTATTCAATACCCCTACGAAAAATT
GATCACATCGGAACGTTTCCGGGGCCGAATACACTTTGAATTTGATAAATGTATTGCTT
GTGAAGTATGTGTGCGTGTATGTCCTATAGATTTACCCGTTGTTGATTGGAAGTTGGAA
ACCGATATTCGAAAGAAACGGTTGCTTAATTACAGTATTGATTTTGGAATCTGTATATTT
TGTGGTAATTGCGTTGAGTATTGCCCAACAAATTGTTTATCAATGACCGAAGAATATGA
ACTTTCTACTTATGATCGTCACGAATTGAATTATAATCAAATCGCTTTGGGTCGCTTACC
AATGTCAGTAATTGATGATTACACAATTCGAACAATTTCGAATTA---------------------------
-------------------ATGCAGGGTCGTTTGTCTGCTTGGCTAGTCAAGCATGGGCTAATTCATA
GATCTTTGGGCTTTGATTACCAAGGAATAGAGACTTTACAAATAAAGCCCGAGGATTGG
CATTCCATTGCTGTCATTTTTTATGTATATGGGTACAATTATCTCCGCTCTCAATGTGCCTA
TGATGTAGCGCCTGGCGGGCTGTTAGCTAGTGTGTATCATCTTACGAGAATAGAGGATG
GTGTGGATCAACCAGAAGAGGTATGCATAAAAGTATTTGCCTCCAGGAGGAATCCTAG
AATTCCGTCTGTTTTCTGGGTTTGGAAAAGTGTGGATTTTCAAGAACGAGAATCATATG
ATATGTTGGGAATCTCTTATGATAATCATCCACGCTTGAAACGTATCTTAATGCCTGAAA
GTTGGATAGGATGGCCCTTACGTAAGGATTATATTGCCCCCAATTTTTATGAAATACAAG
ATGCTCAT--
--------ATGGTCTTAGCTCCTGAATATTCAGACAATAAAAAGAAAAACGGAAAAAATAAG
ATTGAGACA---GTTATGAATTCCATTCAGTTTCCTTTACTTGATCGAACAGCCCAAAATT
CAGTTATTTCAACTACATTAAATGATCTTTCAAATTGGTCAAGACTCTCTAGTTTATGGC
CGCTTCTCTATGGTACCAGTTGTTGCTTCATTGAATTTGCTTCACTAATAGGCTCACGCT
TCGACTTTGATCGTTATGGACTAGTACCAAGATCGAGTCCTAGACAAGCGGATCTAATT
TTAACAGCCGGAACAGTAACAATGAAAATGGCCCCCTCTTTAGTGAGATTATATGAGCA
AATGCCTGAACCAAAATATGTGATTGCTATGGGAGCCTGTACAATTACAGGCGGGATGT
TCAGTACCGATTCTTATAGTACTGTTCGGGGAGTCGATAAGCTAATTCCTGTAGATGTCT
ATTTGCCAGGTTGCCCACCTAAACCGGAAGCAGTTATAGATGCTATAACAAAGCTTCGT
AAGAAAATATCTCGAGAACTCTATGAAGATCGAATTAGGTCTCAACGAGTGAATCGGT
GTTTTACTACCAATCACAAGTTTCATGTTCGACGCAGTATTCATACGGGAAATTATGATC

AAAGAGTTCTTTATCAACCACCATCTACTTCAGAGATCCCTACGGAAATCTTTTTCAAAT
ACAAAAATTCAGTATCTTCCCCCGAATTAGTGAATATGCAAACTAGAAATGCTTTTTCTT
GGCTAAAGAAACAGATTACTCGATCTATTTCCGTATCGCTCATGATATATATCTTAACTCG
GACATCCATTTCAAGTGCATATCCCATTTTTGCACAGCAGGGTTATGAAAATCCACGAG
AAGCGACTGGGCGTATTGTATGTGCCAATTGCCATTTAGCTAATAAGCCCGTGGAGATT
GAGGTTCCACAAGCGGTACTTCCTGATACTGTATTTGAAGCAGTTGTTCGAATTCCTTA
TGATATGCAACTGAAACAGGTTCTTGCTAATGGTAAAAAGGGGGGGTTGAATGTGGGG
GCTGTTCTTATTTTACCGGAGGGTTTTGAATTAGCTCCGTCCGATCGTATTTCTCCCGAG
ATGAAAGAAAAGATTGGCAATTTGTCTTTTCAGAGCTATCGCCCCAATAAAAAAAATAT
TCTTGTGATAGGCCCTGTCCCTGGTAAAAAATATAGTGAAATAACCTTCCCTATTCTTTC
CCCGGACCCTGCTACTAAGAAGGATGTTCACTTCTTAAAATATCCTATATACGTAGGCGG
GAACAGGGGAAGGGGTCAGATTTATCCCGACGGCAGCAAGAGTAACAATACAGTTTAT
AATGCTACAGCAGCAGGTATAGTAAGCAAAATCATACGAAAAGAAAAAGGGGGATATG
AGATAACCATAACGGATGCGTCGGAGGGACGGCAAGTGGTTGATATTATCCCTCCCGG
ACCAGAACTTCTTGTTTCCGAGGGCGAATCTATCAAATTTGATCAACCATTAACGAGTA
ATCCTAATGTAGGCGGATTTGGTCAGGGAGATGCAGAAATAGTACTTCAAGATCCATTA
CGTGTCCAAGGACTTTTGTTCTTCTTGGCATCTGTTATTTTGGCACAAATCTTTTTGGTT
CTTAAAAAGAAACAGTTCGAGAAGGTTCAATTGGCCGAAATGAATTTCATGAGTAAAG
TATATGATTGGTTCGAAGAACGTCTCGAGATTCAAGCAATTGCGGATGATATAACTAGTA
AATATGTTCCTCCCCACGTCAATATATTTTATTGTTTAGGGGGAATTACGCTTACTTGTTT
TTTAGTACAAGTAGCTACTGGGTTTGCTATGACTTTTTACTATCGTCCGACCGTTACTGA
GGCTTTTGCTTCTGTTCAATACATAATGACTGAAGCCAACTTTGGTTGGTTAATCCGATC
AGTTCATCGATGGTCGGCAAGTATGATGGTCCTAATGATGATCCTGCATGTATTTCGTGT
GTATCTCACCGGCGGATTTAAAAAACCTCGCGAATTGACTTGGGTTACCGGTGTGGTTC
TGGCTGTATTAACCGCATCTTTTGGCGTAACTGGTTATTCCTTACCTTGGGACCAAATTG
GTTATTGGGCAGTGAAAATAGTAACAGGTGTCCCTGACGCTATTCCTGTAATAGGATCA
CCTTTGGTCGAATTATTGCGCGGAAGCGCTAGTGTGGGACAATCTACTTTGACTCGTTT
TTATAGTTTACACACTTTTGTATTGCCGCTTCTTACTGCCGTATTTATGTTAATGCACTTT
CCAATGATACGTAAACAAGGTATTTCTGGGCCTTTAATGGGAGTAACAAAAAAACCTG
ACTTGAATGATCCTGTATTAAGAGCTAAATTGGCTAAAGGTATGGGTCATAATTATTATG
GAGAGCCCGCATGGCCTAATGATCTTTTATATATTTTTCCAGTAGTAATTCTAGGTACTAT
TGCATGTAATGTAGGCTTAGCCGTTTTAGAACCATCAATGATTGGTGAACCGGCAGATC
CGTTTGCAACCCCTTTGGAAATATTACCTGAATGGTATTTCTTTCCTGTATTTCAAATACT
TCGTACAGTGCCCAATAAATTATTAGGGGTTCTTTTAATGGTTTCAGTACCTGCGGGATT
ATTAACAGTACCTTTTTTGGAGAATGTTAATAAATTCCAAAATCCATTTCGCCGTCCAGT
AGCGACGACTGTCTTTTTGATTGGTACCGCAGTCGCCCTTTGGTTGGGCATTGGTGCAA
CATTACCTATTGATAAATCCCTAACTTTAGGTCTTTTTATGATTGAAGTTTTTCTATTTGG
AATCGTGTTAGGTCTAATTCCTATTACTTTGGCTGGATTATTCGTAACTGCATATTTACAA
TATAGGCGTGGTGATCAGTTGGACCTTATGCTTACTATAACTAGTTATTTCGGTTTTCTAT
TAGCGGCTTTAACTATAACCTCAGCTCTATTTATTGGTCTGAGCAAGATACGACTTATTA
TGGATATAGTAAGTCTTGCTTGGGCTGCTTTAATGGTAGTCTTTACTTTTTCCCTTTCACT
CGTAGTGTGGGGAAGGAGTGGACTCATGATTATTCGTTCGCCGGAACCAGAAGTTAAA
ATTTTGGTAGATAGGGATCCCGTAAAAACGTCTTTCGAGGAATGGGCCAGACCGGGTC
ATTTCTCAAGAACAATAGCTAAAGGGCCTGATACTACCACTTGGATTTGGAACCTACAT

GCTGATGCTCACGATTTCGATAGCCATACCAGTGATTTGGAGGAGATCTCTCGAAAAGT
ATTTAGTGCACATTTCGGTCAACTCTCCATCATCTTTCTTTGGCTGAGCGGCATGTATTT
CCACGGTGCTCGTTTTTCTAATTATGAAGCGTGGCTAAGTGATCCAACTCACATTGGGC
CTAGTGCCCAGGTGGTTTGGCCAATAGTGGGCCAAGAAATATTAAATGGTGACGTACG
CGGAGGTTTCCGAGGAATACAAATAACCTCTGGTTTTTTTCAGATTTGGCGAGCATCTG
GAATAACTAGTGAATTACAACTCTATTGTACAGCAATTGGGGCATTGGTCTTTGCAGCG
TTAATGCTTTTTGCTGGTTGGTTTCATTATCATAAAGCGGCGCCAAAATTGGCTTGGTTT
CAAGATGTAGAATCTATGCTGAATCACCATTTGGCAGGGCTACTAGGGCTTGGGTCTCT
CTCTTGGGCGGGGCATCAAGTACATGTATCTTTACCGATTAACCAATTTCTAAACGCTG
GAGTAGATCCTAAAGAGATACCACTTCCTCATGAATATATCTTGAATCGAGATCTTTTGG
CTCAACTTTATCCCAGTTTTGCCGAGGGAGCAACCCCATTTTTCACCTTGAATTGGTCA
AAATATGCGGACTTTCTTACTTTTCGTGGAGGATTAGATCCAGTAACTGGGGGTCTGTG
GCTAACTGATATTGCCCATCACCATTTAGCTATTGCAATTCTTTTCCTGATAGCGGGTCA
CATGTATAGGACCAACTGGGGTATTGGTCATGGACTAAAAGATATTTTAGAAGCTCATA
AAGGTCCATTTACAGGTCAGGGCCATAAAGGCCTATATGAGATCCTAACAACGTCATGG
CATGCTCAATTATCTCTTAACTTAGCTATGTTAGGCTCTTTAACCATTGTTGTAGCTCATC
ATATGTATTCCATGCCCCCTTATCCGTATCTAGCTACTGACTATGGTACACAACTGTCATT
GTTCACACATCACATGTGGATTGGTGGATTTCTCATAGTTGGTGCTGCCGCGCATGCAG
CCATTTTTATGGTAAGAGATTATGATCCAACTACTCGGTACAACGATCTATTAGATCGTG
TCCTTAGGCATCGTGATGCAATCATATCCCATCTCAACTGGGCATGTATATTTCTAGGCTT
TCACAGTTTTGGTTTGTATATTCATAATGATACCATGAGCGCTTTAGGGCGTCCTCAAGA
TATGTTTTCAGATACCGCTATACAATTACAACCCGTTTTTGCTCAATGGATACAAAACAC
CCATGCTTTAGCACCTGGTGCAACGGCTCCTGGTGCAACAGCAAGCACCAGTTTAACT
TGGGGGGGTGGTGATTTAGTGGCAGTGGGTGGCAAGGTGGCTTTGTTGCCTATTCCATT
AGGAACCGCCGATTTCTTGGTACATCACATTCATGCATTTACGATTCATGTGACGGTATT
GATACTCTTGAAAGGTGTTCTATTTGCTCGCAGTTCCCGTTTGATACCGGATAAAGCAA
ATCTTGGTTTTCGTTTTCCTTGTGATGGACCTGAAAGAGGGGGTACATGTCAAGTATCG
GCCTGGGATCATGTCTTCTTAGGACTATTCTGGATGTACAATGCAATTTCGGTAGTAATA
TTCCATTTCAGTTGGAAAATGCAGTCAGATGTTTGGGGCAGTGTAAGTGATCAAGGGG
TAGTAACTCATATCACGGGAGGAAACTTTGCGCAGAGTTCTATTACTATTAATGGGTGG
CTCCGCGATTTCTTATGGGCACAGGCATCCCAGGTAATTCAGTCTTATGGTTCTTCATTA
TCTGCATATGGCCTTTTTTTCCTAGGTGCTCATTTTGTCTGGGCTTTTAGTTTAATGTTTC
TATTCAGCGGACGTGGTTATTGGCAAGAACTTATTGAATCCATCGTTTGGGCTCATAATA
AATTAAAAGTTGCTCCTGCTACTCAGCCGAGAGCCTTGAGCATTATACAAGGACGTGCT
GTAGGAGTAACCCATTACCTTCTGGGTGGAATTGCCACAACATGGGCATTCTTCTTAGC
AAGAATTATTACAGTAGGAATGGCATTACGATTTCCAAGGTTTAGCCAAGGCTTAGCTC
AGGACCCCACTACTCGTCGTATTTGGTTTGGTATTGCTACCGCACATGACTTCGAGAGT
CATGATGATATTACTGAGGAACGTCTTTATCAGAATATTTTTGCTTCTCACTTCGGTCAA
TTAGCAATAATTTTTCTGTGGACTTCCGGAAATCTGTTTCATGTAGCTTGGCAAGGAAA
TTTTGAGTCGTGGGTACAGGACCCTTTACATGTAAGACCTATTGCTCATGCAATTTGGG
ATCCTCATTTTGGTCAACCGGCCGTGGAAGCTTTTACTCGAGGGGGTGCTCTTGGCCCA
GTGAATATCGCTTATTCTGGTGTTTATCAGTGGTGGTATACAATCGGTTTACGCACTAAC
GAAGATCTTTATACTGGCGCTCTTTTTCTATTATTTCTTTCTGCCATATCCTTAATAGCAG
GTTGGTTACACCTACAACCGAAATGGAAACCGAGCGTTTCCTGGTTCAAAAATGCCGA

ATCTCGTCTGAATCATCATTTGTCAGGACTCTTTGGCGTAAGTTCCTTGGCTTGGACAG
GGCATTTAGTCCATGTTGCTATTCCTGCATCCAGAGGTGAGTCCGTTCGGTGGAATAATT
TCTTAGATGTATTACCGCATCCCCAAGGGTTAGGCCCACTTTTTACAGGTCAATGGAATC
TTTATGCTCAAAACCCCGATTCAAGTAGTCATTTATTTGGTACCGCCGAAGGGGCGGGA
ACTGCCATTCTAACTCTTCTCGGGGGATTCCATCCACAAACGCAAAGTTTATGGCTGAC
TGATATTGCCCATCACCATTTAGCTATTGCATTTATTTTTCTCGTTGCTGGTCATATGTATA
GAACCAATTTCGGGATTGGGCACAGTATGAAAGATCTTTTAGATGCACATATTCCTCCG
GGGGGACGATTGGGGCGTGGACATAAGGGTCTTTACGACACAATCAACAATTCGCTTC
ATTTTCAATTAGGCCTTGCTCTAGCTTCTTTAGGGGTTATTACTTCTTTGGTAGCTCAAC
ACATGTACTCTTTACCTGCTTATGCATTCATAGCACAAGACTTTACTACTCAAGCTGCAT
TATATACCCACCACCAATATATCGCAGGATTCATCATGACAGGAGCTTTTGCTCATGGAG
CTATATTGGTCATTAGAGATTACAATCCGGAGCAAAATGAAGATAATGTATTGGCAAGA
ATGTTAGATCATAAAGAAGCTATCATATCTCATTTAAGTTGGGCCAGCCTCTTTCTGGGA
TTCCATACCCTGGGACTTTATGTTCATAATGATGTCATGCTTGCCTTTGGCACTCCGGAG
AAGCAAATCTTGATTGAACCTATATTTGCTCAATGGATACAATCCGCTCATGGTAAAACT
TCATATGGGTTCGATGTACTTTTATCTTCAACGAGTGGCCCAGCATTCAATGCGGGTCG
AAGCATCTGGTTGCCGGGTTGGTTAAATGCTGTTAATGAA------AATAGTAATTCATTATT
CTTAACAATAGGTCCTGGAGACTTTTTGGTTCATCATGCTATTGCTCTTGGTTTACATAC
AACTACATTGATCTTAGTAAAAGGTGCTTTAGATGCACGTGGTTCCAAGTTAATGCCAG
ATAAAAAGGATTTCGGTTATAGTTTTCCGTGCGATGGCCCAGGACGAGGCGGTACTTGT
GATATTTCGGCATGGGACGCGTTTTATTTGGCAGTTTTTTGGATGTTAAATACTATTGGAT
GGGTTACTTTTTATTGGCATTGGAAGCACATCACATTATGGCAGGGTAACGTTTCACAG
TTTAATGAATCTTCCACTTATTTGATGGGCTGGTTAAGGGATTATTTATGGTTAAACTCTT
CACAACTTATCAATGGATATAACCCTTTTGGTATGAATAGTTTATCGGTTTGGGCATGGA
TGTTCTTATTTGGACATCTTGTTTGGGCTACTGGATTTATGTTCTTAATTTCTTGGCGTGG
ATATTGGCAGGAATTGATTGAAACTTTAGCATGGGCTCATGAACGCACACCTTTGGCCA
ATTTGATTCGTTGGAGAGATAAACCAGTGGCCCTTTCCATTGTACAAGCAAGATTGGTT
GGATTAGCTCACTTTTCTGTAGGTTATATATTCACTTATGCGGCTTTCTTGATTGCCTCTA
CGTCGGGCAAATTTGGTATGTCACATTCAGTAAAGATTTATGATACGTGTATAGGATGTA
CTCAATGTGTCCGAGCCTGCCCCACCGATGTATTAGAAATGATACCTTGGGACGGTTGT
AAGGCTAAACAAATTGCTTCCGCTCCACGAACAGAGGACTGTGTTGGTTGTAAGAGAT
GTGAATCCGCCTGTCCAACTGATTTCTTGAGTGTACGAGTTTATTTATGGCATGAAACA
ACTCGCAGTATGGGTCTAGCTTATATGACAAATTTGAACCTTCCATCTATTTTTGTGCCG
TTAGTAGGCCTAGTCTTTCCGGCAATTGCAATGGCTTCTTTATTTCTTCATGTTCAAAAA
AACAAGATTGTTATGCGAGATCTAAAAACATATCTCTCTGTGGCACCAGTACTAAGTAC
GCTATGGTTCGTGGCTTTAGCAGGTCTATTGATAGAGATTAATCGTTTTTTCCCGGATGC
GTTGACATTCCCCTTTTTT------ATGACTGCAATTTTAGAGAGACGCGAAAGCGAAAGCC
TATGGGGTCGCTTCTGTAACTGGATAACTAGCACTGAAAACCGTCTTTACATTGGATGG
TTTGGTGTTTTGATGATCCCTACCTTATTGACGGCAACTTCTGTATTTATTATTGCCTTCA
TTGCTGCTCCTCCAGTAGACATTGATGGAATTCGTGAACCTGTTTCAGGGTCTCTACTTT
ACGGAAACAATATTATTTCCGGTGCCATTATTCCTACTTCTGCAGCTATAGGTTTACATTT
TTACCCAATCTGGGAAGCGGCATCCGTTGATGAATGGTTATACAACGGTGGTCCTTATG
AACTAATTGTTCTACACTTCTTACTTGGCGTAGCTTGTTACATGGGTCGTGAGTGGGAG
CTTAGTTTCCGTCTGGGTATGCGACCTTGGATTGCTGTTGCATATTCAGCTCCTGTTGCA

GCTGCTACCGCAGTTTTCTTGATCTACCCAATCGGTCAAGGAAGTTTTTCTGATGGTAT
GCCTCTAGGAATCTCTGGTACTTTCAATTTCATGATTGTATTCCAGGCTGAGCACAACAT
CCTTATGCACCCATTTCACATGTTAGGCGTAGCTGGTGTATTCGGCGGCTCCCTATTCAG
TGCTATGCATGGTTCCTTGGTAACCTCTAGTTTGATCAGGGAAACCACAGAAAATGAAT
CTGCTAATGAAGGTTACAGATTCGGTCAAGAGGAAGAAACTTATAATATCGTAGCCGCT
CATGGTTATTTTGGCCGATTGATCTTCCAATATGCTAGTTTCAACAACTCTCGTTCGTTA
CACTTCTTCCTAGCTGCTTGGCCTGTAGTAGGTATCTGGTTTACCGCTTTAGGTATCAGC
ACTATGGCTTTCAACCTAAATGGTTTCAATTTCAACCAATCTGTAGTTGACAGTCAAGG
TCGTGTAATTAACACTTGGGCTGATATCATTAACCGTGCTAACCTTGGTATGGAAGTTAT
GCATGAACGTAATGCTCATAACTTCCCTCTAGACCTAGCTGCTATCGAAGCTCCATCTAC
AAATGGAATGGGTTTGCCTTGGTATCGTGTTCATACCGTTGTATTGAATGATCCCGGTCG
GTTGCTTTCTGTTCATATAATGCATACAGCTCTGGTTGCTGGTTGGGCCGGTTCGATGGC
TCTGTATGAATTAGCGGTTTTTGATCCTTCTGATCCTGTTCTTGATCCAATGTGGAGACA
GGGTATGTTCGTTATACCCTTCATGACTCGTTTAGGAATAACCAATTCATGGGGCGGTTG
GAGTATCACA---GGGGGGACTGTAACGAATCCGGGTATTTGGAGTTACGAAGGTGTAGC
TGGAGCACATATTGTGTTTTCTGGCTTATGCTTTTTGGCAGCTATCTGGCATTGGGTGTA
TTGGGATCTAGAAATATTTTGTGATGAACGTACAGGAAAACCCTCTTTGGATTTGCCAA
AGATCTTTGGAATTCATTTATTTCTCTCAGGGGTGGCTTGCTTTGGTTTTGGTGCATTTC
ATGTAACAGGCTTGTATGGTCCCGGAATATGGGTGTCCGATCCTTATGGACTAACGGGA
AAAGTACAACCTGTAAATCCAGCGTGGGGCGTGGAAGGTTTTGATCCTTTTGTTCCAG
GAGGAATAGCCTCTCATCATATTGCAGCAGGAACATTGGGCATATTAGCGGGCCTATTC
CATCTTAGCGTCCGTCCGCCACAACGTCTATACAAAGGATTGCGTATGGGAAATATTGA
AACCGTCCTTTCCAGTAGTATCGCTGCTGTCTTTTTTGCAGCTTTTGTTGTTGCCGGAA
CTATGTGGTATGGTTCGGCAACTACCCCGATTGAATTATTTGGGCCCACTCGTTATCAAT
GGGATCAGGGGTACTTCCAGCAAGAAATATATCGAAGAGTTAGTGCTGGGCTAGCAGA
AAATCAAAGTTTATCAGAAGCCTGGTCTAAAATTCCTGAAAAATTAGCTTTTTATGATTA
CATCGGCAATAATCCGGCAAAAGGAGGATTATTCAGAGCGGGTTCAATGGATAACGGG
GATGGAATAGCGGTTGGATGGTTAGGACACCCTATCTTTAGAGATAAAGAAGGGCGTG
AACTTTTTGTACGTCGTATGCCTACCTTTTTTGAAACATTCCCGGTCGTTTTGGTAGACG
GCGATGGAATTGTTAGAGCCGATGTTCCTTTTAGAAGGGCAGAATCGAAGTATAGTGTT
GAACAAGTAGGTGTAACTGTTGAGTTCTACGGCGGTGAACTCAACGGCGTCAGTTATA
GTGATCCTGCTACTGTGAAAAAATATGCTAGACGTGCTCAATTGGGTGAAATTTTTGAA
TTAGATCGTGCTACTTTGAAATCCGATGGTGTTTTTCGTAGCAGTCCAAGGGGTTGGTT
TACTTTTGGGCATGCTTCGTTTGCTTTGCTCTTCTTCTTCGGACACATTTGGCATGGTGC
TAGAACCTTGTTCAGAGATGTTTTTGCTGGTATTGACCCAGATTTAGATGCTCAAGTCG
AATTTGGAGCATTCCAAAAACTTGGAGATCCAACTACAAAAAGACAGGCAGCCATGA
AAACCTTATATTCCCTGAGGAGGTTCTACCACGTGGAAACGCTCTTTAATGGAACTTTA
GCCTTAGCTGGTCGTGACCAAGAAACCACCGGTTTCGCTTGGTGGGCCGGGAATGCCC
GACTTATCAATTTATCCGGTAAACTACTAGGGGCTCATGTAGCCCATGCTGGATTAATCG
TATTCTGGGCCGGAGCAATGAACCTATTTGAAGTGGCCCATTTCGTACCAGAGAAGCCT
ATGTATGAACAAGGATTGATTTTACTTCCCCACCTAGCTACTCTAGGTTGGGGGGTAGG
CCCTGGGGGAGAAGTTATAGACACCTTTCCATACTTTGTATCTGGAGTACTTCATTTAAT
TTCTTCTGCAGTATTGGGCTTTGGCGGCATTTATCATGCACTTCTGGGACCTGAGACAC
TTGAAGAATCTTTTCCCTTCTTTGGTTATGTCTGGAAAGATCGAAATAAAATGACCACA

ATTTTAGGTATTCACTTAATCTTGTTAGGTATAGGTGCTTTTCTTCTAGTATTCAAGGCTC
TTTATTTTGGGGGTGTATATGATACCTGGGCTCCAGGAGGGGGAGATGTAAGAAAAATT
ACCAACTTGACCCTTAGCCCGAGTATCATATTTGGTTATTTACTAAAATCCCCTTTTGGA
GGGGAAGGCTGGATTGTTAGTGTAGACGATTTAGAAGATATAATTGGAGGACATGTATG
GTTAGGTTCCATTTGTATACTTGGTGGAATCTGGCATATCTTAACCAAACCCTTCGCATG
GGCTCGACGCGCACTTGTATGGTCTGGAGAGGCTTACTTATCTTATAGTTTAGGGGCTTT
AGCCGTCTTTGGTTTCATTGCTTGTTGTTTTGTCTGGTTCAATAATACCGCTTATCCTAGT
GAATTTTACGGACCTACTGGACCAGAAGCTTCTCAAGCTCAAGCATTTACTTTTCTAGT
TAGAGACCAACGTCTTGGGGCTAACGTGGGATCCGCTCAAGGACCTACTGGTTTAGGT
AAATATCTAATGCGTTCCCCGACCGGAGAAGTCATTTTTGGAGGAGAAACTATGCGTTT
TTGGGATCTGCGTGCTCCATGGTTAGAGCCTCTAAGGGGTCCAAATGGGTTAGACTTGA
GTAGGTTGAAAAAAGACATACAACCTTGGCAGGAACGGCGTTCCGCGGAATATATGAC
TCATGCTCCTTTAGGTTCTTTAAATTCCGTGGGTGGTGTAGCTACCGAGATCAATGCAG
TCAATTATGTCTCTCCTAGAAGTTGGTTAGCTACCTCTCATTTTGTTTTAGGATTCTTCTT
CTTCGTAGGTCATTTGTGGCACGCGGGAAGGGCTCGTGCAGCTGCAGCAGGATTTGAA
AAAGGAATTGATCGTGACTTTGAACCTGTTCTTTCCATGACCCCTCTTAATATGACTATA
GCCCTTGATAAGTTTACCAAAGACGAAAATGATTTATTTGATATTATGGATGACTGGTTA
CGGAGGGACCGTTTCGTTTTTGTAGGCTGGTCCGGTCTATTGCTCTTTCCTTGTGCCTAT
TTTGCTGTAGGGGGTTGGTTCACAGGTACAACCTTTGTAACTTCATGGTATACCCATGG
ATTGGCCAGTTCTTATTTGGAAGGCTGCAATTTCTTAACTGCCGCAGTTTCTACTCCTGC
TAATAGTTTAGCACATTCGTTGTTGTTACTATGGGGTCCTGAAGCACAAGGAGATTTTA
CTCGTTGGTGTCAATTGGGGGGTCTGTGGACTTTTGTTGCTCTCCATGGAGCTTTTGGC
CTAATAGGTTTCATGTTACGTCAATTCGAGCTTGCTCGATCCGTTCAATTGAGACCTTAT
AATGCAATCGCATTCTCTGGTCCAATTGCTGTTTTTGTTTCTGTATTTCTGATTTATCCAC
TAGGTCAGTCTGGTTGGTTCTTTGCACCTAGTTTTGGTGTAGCAGCTATATTTCGATTCA
TCCTATTTTTTCAAGGGTTTCATAATTGGACCTTGAACCCCTTTCATATGATGGGAGTTG
CCGGTGTATTGGGCGCTGCTTTGCTATGCGCCATTCATGGTGCTACCGTAGAAAATACTT
TATTTGAAGACGGTGATGGTGCAAATACATTCCGTGCTTTTAACCCAACTCAAGCCGAA
GAAACTTATTCAATGGTCACCGCTAACCGCTTTTGGTCCCAAATCTTTGGGGTTGCTTT
TTCCAATAAACGTTGGTTACATTTCTTTATGTTATTTGTACCAGTAACCGGTTTATGGATG
AGTGCTCTTGGAGTAGTCGGTCTAGCCCTGAACCTACGTGCCTATGACTTCGTTTCTCA
GGAAATTCGCGCAGCGGAAGATCCTGAATTTGAGACTTTCTACACCAAAAATATTCTCT
TAAACGAAGGTATTCGCGCTTGGATGGCGGCTCAAGATCAGCCTCATGAAAACCTTATA
TTCCCTGAGGAGGTTCTACCACGTGGAAACGCTCTTATGTCTGGAAGCACAGGAGAAC
GTTCGTTTGCTGATATTATTACCAGTATTCGATACTGGGTCATTCATAGCATTACTATACC
TTCCCTATTCATTGCGGGTTGGTTATTTGTCAGCACTGGTTTAGCTTATGATGTGTTTGG
AAGCCCTCGGCCAAACGAGTATTTTACAGAGAGCCGACAAGGAATTCCATTAATAACT
GGCCGTTTTGATTCTTTGGAACAACTCGATGAATTTAGTAGATCGTTTATGACTATAGAT
CGAACCTATCCAATTTTTACAGTACGATGGTTGGCTGTTCACGGCCTAGCTGTACCTAC
CGTCTTTTTTTTGGGATCAATATCAGCAATGCAGTTCATCCAACGAATGGCTACACAAA
CTGTTGAGAACAGTTCT---AGATCTGGTCCAAGACGAACTGCAGTAGGAGATTTATTAA
AACCATTGAATTCGGAATATGGTAAAGTAGCTCCTGGATGGGGAACTACTCCTTTGATG
GGTGTCGCAATGGCCTTATTTGCGGTATTTCTATCTATTATTTTGGAGATTTATAATTCTTC
CGTTTTATTGGATGGAATTTCAATGAATATGCTTACTCTCAAACTCTTCGTTTACACAGT

AGTGATATTTTTTGTTTCTCTCTTCATCTTTGGATTCCTATCTAATGATCCCGGACGTAAT
CCTGGACGTGAAGAAATGGCCGATACTACTGGAAGGATTCCTCTTTGGATAATAGGTAC
TGTAGCTGGTATTCTTGTAATCGGTTTAATAGGTATTTTCTTTTATGGTTCATATTCCGGAT
TGGGTTCATCCCTGATGCTTAATACCTTTAGTTTGATCGGTATCTGTCTTAATTCGACCCT
TTATTCGAGTAGTTTTTTCTTCGGCAAATTGCCCGAGGCCTATGCTTTTTTGAATCCAAT
CGTAGATATTATGCCAGTCATACCTCTGTTCTTTTTTCTCTTAGCCTTTGTTTGGCAAGCT
GCTGTAAGTTTTCGAACGACACAATCAAACCCGAACGAACAAAGTGTTGAATTGAATC
GTACCAGTCTCTACTGGGGGTTATTACTCATTTTTGTACTTGCTGTTTTATTTTCCAATTA
TTTCTTCAATATGGAAGTAAATATTCTTGCATTTATTGCTACTGCACTATTTATTCTAGTTC
CTACCGCTTTTCTACTTATCATTTACGTAAAAACAGTCAGTCAAAACGATATGGAAACA
GCAACCCTAGTCGCCATCTTTATATCTGGTTTACTTGTAAGTTTTACTGGGTACGCCTTAT
ATACCGCTTTTGGGCAACCTTCTCAACAACTAAGAGATCCATTCGAGGAACATGGAGA
CATGGAAGCATTGGTTTATACATTCCTCTTAGTCTCGACTCTAGGGATAATTTTTTTCGCT
ATCTTTTTTCGAGAACCGCCTAAACTTCCAACTAAAAAGAAC---ATGACTCTTGCTTTC
CAATTGGCTGTTTTTGCATTAATTGCTACTTCATTAATCTTATTGATTAGCGTACCCGTTG
TATTTGCTTCTCCTGATGGCTGGTCAAGTAACAAAAATGTTGTATTTTCTGGTACATCCT
TATGGATTGGATTAGTCTTTCTGGTGGGTATCCTTAATTCTCTCATCTCT--------------------
ATGTCACCACAAACAGAGACTAAAGCAAGTGTTGGATTCAAAGCTGGTGTTAAAGAGT
ACAAATTGACTTATTATACTCCTGAGTACCAAACCAAGGATACTGATATATTGGCAGCAT
TCCGAGTAACTCCTCAACCTGGAGTTCCACCTGAAGAAGCAGGGGCCGCGGTAGCTG
CCGAATCTTCTACTGGTACATGGACAACTGTATGGACCGATGGACTTACCAGCCTTGAT
CGTTACAAAGGGCGATGCTACCGCATCGAGCGTGTTGTTGGAGAAAAAGATCAATATAT
TGCTTATGTAGCTTACCCTTTAGACCTTTTTGAAGAAGGTTCTGTTACCAACATGTTTAC
TTCCATTGTAGGTAATGTATTTGGGTTCAAAGCCCTGCGCGCTCTACGTCTGGAAGATC
TGCGAATCCCTCCTGCTTATGTTAAAACTTTCCAAGGTCCGCCTCATGGGATCCAAGTT
GAAAGAGATAAATTAAACAAGTATGGTCGTCCCCTGTTGGGATGTACTATTAAACCTAA
ATTGGGGTTATCTGCTAAAAACTACGGTAGAGCTGTTTATGAATGTCTTCGCGGTGGAC
TTGATTTTACCAAAGATGATGAGAACGTGAACTCACAACCATTTATGCGTTGGAGAGAT
CGTTTCTTATTTTGTGCCGAAGCACTTTATAAAGCACAGGCTGAAACAGGTGAAATCAA
AGGGCATTACTTGAATGCTACTGCAGGTACATGCGAAGAAATGATCAAAAGAGCTGTA
TTTGCTAGAGAATTGGGTGTTCCGATCGTAATGCATGACTACTTAACGGGGGGATTCAC
CGCAAATACTAGCTTGGCTCATTATTGCCGAGATAATGGTCTACTTCTTCACATCCACCG
TGCAATGCATGCGGTTATTGATAGACAGAAGAATCATGGTATCCACTTCCGGGTATTAGC
AAAAGCGTTACGTATGTCTGGTGGAGATCATATTCACTCTGGTACCGTAGTAGGTAAAC
TTGAAGGTGAAAGAGACATAACTTTGGGCTTTGTTGATTTACTGCGTGATGATTTTGTT
GAACAAGATCGAAGTCGCGGTATTTATTTCACTCAAGATTGGGTCTCTTTACCAGGTGT
TCTACCCGTGGCTTCAGGAGGTATTCACGTTTGGCATATGCCTGCTCTAACCGAGATCT
TTGGGGATGATTCCGTACTACAGTTCGGTGGAGGAACTTTAGGACACCCTTGGGGTAAT
CCGCCAGGTGCCGTAGCTAACCGAGTAGCTCTAGAAGCATGTGTAAAAGCTCGTAATG
AAGGACGTGATCTTGCTCAGGAAGGTAATGAGATTATTCGCGAGGCTTGCAAATGGAG
CCCGGAACTAGCTGCTGCTTGTGAGGTATGGAAAGAGATCGTATTTAATTTTGCAGCTG
TGGACGTTTTGGATAAGATGGCGATACATTTATACAAAACTTCTACCCCGAGCACACGC
AATGGAACCGTAGATAGTCAAGTGAAATCCAATCCACGAAATAATTTGATCTATGGACA
GCGTCGTTGTGGTAAAGGTCGTAATGCCAGAGGAATCATTACCGCAAGGCATAGAGGG

GGAGGTCATAAGCGTCTATACCGTAAAATCGATTTTCGACGGAATGAAAAAGACATATA
TGGTAGAATCGTAACCATAGAATACGACCCTAATCGAAATGCATACATTTGTCTCATACA
CTATGGGGATGGTGAGAAGAGATATATTTTACATCCCAGAGGGGCTATAATTGGAGATA
CCATTGTTTCTGGTACAGAAGTTCCTATAAAAATGGGAAATGCCCTACCTTTGACCGATA
TGCCCTTAGGCACGGCCATACATAACATAGAAATCACACTTGGAAAGGGTGGACAATT
AGCTAGAGCAGCGGGTGCTGTACCGAAACTGATTGCAAAAGAGGGGAAATCGGCCAC
ATTAAAATTACCTTCTGGGGAGGTCCGTTTGATATCCAAAAACTGCTCAGCAACAGTTG
GACAAGTGGGGAATGTTGGGGTGAACCAAAAAAGTTTGGGTAGAGCCGGATCTAAAC
GTTGGCTAGGTAAGCGTCCTGTAGTAAGAGGAGTAGTTATGAACCCTGTAAACCATCCC
CATGGGGGTGGTGAAGGGAGAGCCCCAATTGGTAGAAAAAAACCCACAACCCCTTGG
GGTTATCCTGCACTTGGAAGAAGAAGTAGAAAAAGGAATAAATATAGTGATAATTTGAT
TCTTCGTCGCCGTAGTAAAATGATTCAACCTCAGACCCATTTAAATGTAGCGGATAACA
GCGGGGCTCGAGAATTAATGTGTATTCGAATCATAGGAGCTAGC---AATCGTCGATATGC
TCATATTGGTGACGTTATTGTTGCTGTGATCAAAGAAGCAGTACCAAATATGCCCCTAG
AAAGATCAGAAATAGTCAGAGCTGTAATTGTGCGTACCTGTAAAGAACTCAAACGTGA
CAACGGGATGATAATACGATATGATGACAATGCTGCAGTTGTTATTGATCAAGAAGGAA
ATCCAAAAGGAACTCGAATTTTTGGTGCAATCGCCCGGGAATTGAGAGAATTAAATTTT
ACTAAAATAGTTTCATTAGCTCCCGAGGTATTAATGCTTAGTCCCAAAAGAACCAGATT
CCGTAAACAACATAGAGGAAGAATGAAGGGAATATCTTATCGAGGTAATCATATTTCTT
TCGGTAAATATGCTCTTCAGGCACTTGAACCTGCTTGGATTACATCTAGACAAATAGAA
GCAGGCCGACGAGCAATGACACGAAATGCACGTCGTGGTGGAAAAATATGGGTACGTA
TATTTCCAGACAAACCAGTTACACTAAGACCCGCAGAAACACGTATGGGTTCAGGAAA
AGGATCCCCTGAATATTGGGTAGCTGTTGTTAAACCGGGTCGAATACTTTATGAAATGG
GTGGAGTAACAGAAAATATAGCCAGAAGGGCTATTTCACTAGCAGCATCCAAAATGCC
TATACGAACTCAATTCATTATTTCA---ATGACCAGAATTAAACGGGGATATATAGCTCGG
AGACGTAGAACAAAAATTCGTTTATTTGCATCAAGCTTTCGAGGGGCTCATTCAAGGCT
TACTCGAACTATTACTCAACAGAAAATAAGAGCTTTAGTTTCGGCTCATCGGGATAGGG
ATAGGAAAAAGAGAGATTTTCGTCGTTTGTGGATCACTCGGATAAACGCAGTAATTCGC
GAA---AGGGGAGTATCCTATAGTTATAGTAGATTAATACACGATCTGTACAAGAGACAGT
TGCTTCTTAACCGTAAAATACTTGCACAAATAGCTATATCAAATAGGAATTGTCTTTATAT
GATTTCGAACGAAATCATAAAGGAAGTAGATTGGAAAGAATCCACCAGAATAATTATGC
TAAAGGAAGTATATGCTTTAGGTGAACATATATCTATGTCTGCTGACAAAGCACGAAGA
GTAATTGATCAAATTCGCGGACGTTCCTATGAGGAAACGCTTATGATACTAGAACTCAT
GCCCTATCGAGCATGTTATCCCATTTTGAAATTGGTTTATTCTGCAGCAGCAAATGCTAG
TTATAATATGGGTTCCAGCGAAGCCAATTTAGTCATTAGTAAAGCCGAAGTCAATGGGG
GTACGACTGTGAAGAAATTGAAACCTCGAGCTCGAGGACGTAGTTTTCCGATAAAAAG
ATCGACCTGTCATATAACTATTGTAATGAAAGATATATCTTTAAATGGGGGATTATGGGA
CAAAAAAATGGATGGAATCAAATATGCAGTATTTACAGACAAAAGTATTCGGTTATTGG
GGAAAAAT---CAATATACTTCTAATGTCGAATCAGGATCAACTAGGACAGAAATAAAGC
ATTGGGTCGAACTCTTCTTTGGTGTCAAGGTAATAGCTATGAATAGTCATCGACTTCCG
GGAAAGAGTAGAAGAATGGGACCTATTATGGGACATACAATGCATTACAGACGTATGAT
CATTACGCTTCAACCGGGTTATTCTATTCCACCTCTTAGAAAGAAAAGAACTATGGCAG
TTCCAAAAAAACGCACTTCGACATCAAAAAAGCGTATCCGTAAAAATATTTGGAAAAG
GAAGGGATATTGGGTAGCATTAAAGGCTTTTTCATTAGCGAAATCTCTTTCTACCGGGA

ATTCAAAAAGTTTTTTTGTACGCCAAACAAAAATAATGGCCAAGGGGAAAGATGTCCG
AGTAACGGTGATTTTGGAATGTACTAGTTGTGTCCGAAACAGTGTTGAT------AAGGTAT
CAAGAGGTATTTCCAGATATATTACTCAAAAGAACCGGCACAATACGCCTAATCGATTA
GAATTGAAAAAATTCTGTCCCTATTGTTACAAACATACGATTCATGGGGAAATAAAGAA
AATGAAAATAAGAGCTTCCGTTCGTAAAATTTGTGAAAAATGTCGACTAATCCGTAGGC
GGGGACGAATTATAGTAATTTGTTCCAACCCGAGACATAAACAAAGACAGGGGATGGT
TCGAGAGAAAGTAACAGTATCTACTCGGACACTACAGTGGAAGTGTGTTGAATCAAGA
ACAGACAGTAAACGCCTTTATTATGGACGCTTTATTCTGTCTCCACTTATGAAAGGACA
AGCCGACACAATAGGCATTGCGATGCGAAGAGCTTTACTTGGAGAAATAGAAGGAACA
TGTATCACACGTGTAAAATTTGAG------AAAGTACCACATGAATATTCTACCATAACGGG
CATTCAAGAATCGGTACATGAAATTATAATGAATTTGAAAGAAATTGTATTGAGAAGTA
ATCTATATGGAACTTCTGAGGCGTCTGTTTGTGTCAAGGGTCCTGGATATGTAACTGCTC
AAGATATCATCTTGCCGCCTTATGTAGAAATCGTTGATAATACACAACATATAGCTAGTTT
AACGGAACCAATTGATTTTTGTATTGGATTACAAATAGAGAGAAATCGTGGATATCTTAT
AAAAACGCCACAA---AACTTTGATGGAAGTTAT------CCTATAGATGCTGTATTCATGCCT
GTTCGAAACGCGAATCATAGTATTCATTCTTATGGGAATGGGAATGAAAAACAAGAGAT
ACTTTTTCTTGAAATATGGACAAACGGAAGTTTAACTCCGAAAGAAGCACTTCATGAA
GCATCCCGAAATTTAATTGATTTATTTATTCCCTTTTTACATATGGAGGAAGAC------AAC
TTATATTTACAGGACAATCAA---CACACGGTTCCTTTATCCCCTTTTACCTTTCATGATAA
ATTGGCT---AAACTCATAAAAAACAACAAA---AAAATAGCATTGAAATCGATTTTTATTG
ACCAATCAGAATTGCCTTCCAGGATCTATAATTGCCTCAAAATGTCCAATATATATACATT
ATTGGACCTTTTGAATAACAGCCAAGAAGATCTTATGAAAATTGAACATTTTCGTAGCG
AAGATATAAAACAGATATTGGGCATTTTAGAAAAATAT------TTCGTAATTGATTTAGCAA
AAAATAAGATGCTCGGGGATGGA---AATGAGGGAATATCTACAATACCTGGATTTAATCA
GATACAATTTGAAGGATTTTGTAGGTTCATTGATCAAGGTTTGACGGAAGAACTTTATA
AGTTTCCAAAAATTGAAGATACAGATCAAGAAATTGAATTCCAATTATTTGTGGAAACA
TATCAATTGGTCGAACCCTTGATAAAGGAAAGAGATGCTGTGTATGAATCACTCACATA
TTCTTCTGAATTATATGTATCCGCGGGATTAATTTGGAAAAACAGTAGGGATATGCAAGA
ACAAACAATTTTTATCGGAAACATTCCTCTAATGAATTCCCTGGGAACTTCTATAGTCAA
TGGAATATATAGAATTGTGATCAATCAAATATTGCAAAGTCCCGGTATTTATTACCGATCA
GAATTGGACCATAACGGAATTTCGGTCTATACCGGCACCATAATATCAGATTGGGGAGG
AAGATCAGAATTAGAAATTGATAGAAAAGCAAGGATATGGGCTCGTGTAAGTAGGAAA
CAAAAAATATCTATTCTAGTTCTATCATCAGCTATGGGTTTGAATCTAAGAGAAATTTTA
GAGAATGTTTGCTATCCTGAAATTTTTTTGTCTTTTCTGAGTGATAAGGAGAGAAAAAA
AATTGGGTCAAAAGAAAATGCCATTTTGGAGTTTTATCAACAATTTGCTTGTGTAAGCG
GCGATCCGGTATTTTCTGAATCCTTATGTAAGGAATTACAAAAGAAATTCTTTCAACAA
AGATGTGAATTAGGAAGGATTGGTCGACGAAATATGAACCGAAGACTTAACCTTGATAT
ACCCCAGAACAATACATTTTTGTTACCACGAGATATATTGGCAGCCGCCGATCATTTGAT
TGGGCTGAAATTTGGAATGGGTGCACTTGACGAT------ATGAATCATTTGAAAAATAAGC
GTATTCGTTCTGTAGCAGATCTTTTACAAGATCAATTCGGATTGGCTCTGGTTCGTTTAG
AAAATGTGGTTCGGGGGACTATATGTGGAGCAATTCGG---CATAAATTG---ATACCGACA
CCTCAGAATTTGGTAACCTCAACTCCA------------TTAACAACTACTTATGAATCCTTTTTC
GGTTTACACCCATTATCTCAAGTTTTGGATCGAACTAATCCATTGACACAAATAGTTCAT
GGGAGAAAATTAAGTTATTTGGGCCCTGGAGGACTGACAGGGCGCACTGCTAGTTTTC

GGATACGAGATATCCATCCTAGTCACTATGGACGTATTTGCCCAATTGACACATCTGAAG
GAATCAATGTTGGACTTATTGGATCCTTAGCAATTCATGCGAGGATTGGTCATTGGGGA-
--TCTCTAGAAAGCCCCTTTTAT---------GAAATTTCTGAGAGGTCAACCGGG------GTACG
GATGCTTTATTTATCACCAGGTAGAGATGAATACTATATGGTAGCGGCAGGAAATTCTTT
AGCCTTAAATCGGGATATTCAGGAAGAACAGGTTGTTCCAGCTCGATACCGTCAAGAA
TTCTTGACTATTGCATGGGAACAGGTTCATCTTCGAAGTATTTTTCCTTTTCAATATTTTT
CTATTGGAGCTTCCCTCATTCCTTTTATCGAACATAATGATGCGAATCGAGCTTTAATGA
GTTCTAATATGCAACGTCAAGCAGTTCCTCTTTCTCGCTCCGAGAAATGCATTGTTGGA
ACTGGGTTGGAACGACAAGCAGCTCTAGATTCGGGGGCTCTTGCTATAGCCGAACGCG
AGGGAAGGGTCGTTTATACCAATACTGACAAGATTCTTTTAGCAGGTAATGGAGATATT
CTAAGCATTCCATTAGTTATATATCAACGTTCCAATAAAAATACTTGTATGCATCAAAAA
CTCCAGGTTCCTCGGGGTAAATGCATTAAAAAGGGACAAATTTTAGCGGATGGTGCTG
CTACGGTTGGTGGCGAACTTGCTTTGGGGAAAAACGTATTAGTAGCTTATATGCCGTGG
GAGGGTTACAATTCTGAGGATGCAGTACTTATTAGCGAGCGTTTGGTATATGAAGATATT
TATACTTCTTTCCACATACGGAAATATGAAATTCAGACTCATGTGACAAGCCAAGGC---C
CTGAAAAAGTAACTAATGAAATACCGCATTTAGAAGCCCATTTACTCCGCAATTTAGAC
AAAAATGGAATTGTGATGCTGGGATCTTGGGTAGAGACGGGTGATATTTTAGTAGGTAA
ATTAACACCCCAGGTCGTGAAAGAA---TCGTCGTATGCCCCGGAAGATAGATTGTTACG
AGCTATACTTGGTATTCAGGTATCTACTTCAAAAGAAACTTGTTTAAAATTACCTATAGG
TGGTAGGGGTCGGGTTATTGATGTGAGGTGGATCCAGAAAAGGGGT---GGTTCTAGTTA
TAATCCCGAAACGATTCGTGTATATATTTCACAGAAACGTGAAATCAAAGTAGGCGATA
AGGTGGCTGGAAGACACGGAAATAAAGGTATCATTTCCAAAATTTTGCCTAGACAAGA
TATGCCTTATTTACAAGATGGAAGATCCGTTGATATGGTCTTTAACCCATTAGGAGTACC
TTCACGAATGAATGTAGGACAGATATTTGAATGTTCACTAGGGTTAGCAGGGAGTCTGC
TAGACAGACATTATCGAATAGCACCTTTTGATGAGAGATATGAACAAGAAGCTTCGAG
AAAACTAGTGTTTTCTGAATTATATGAAGCCAGTAAGCAAACAGCAAATCCATGGGTAT
TTGAACCCGAATATCCAGGAAAAAGCAGAATATTTGATGGAAGGACGGGGAATCCTTT
TGAACAACCCGTTATAATAGGAAAGCCTTATATCTTGAAATTAATTCATCAAGTTGATGA
TAAAATCCATGGGCGTTCCAGTGGACATTATGCGCTTGTTACACAACAACCCCTTAGAG
GAAGAGCCAAACAGGGGGGACAGCGGGTAGGAGAAATGGAGGTTTGGGCTCTAGAA
GGGTTTGGGGTTGCTCATATTTTACAAGAGATGCTTACTTATAAATCGGATCATATTAGA
GCTCGCCAGGAAGTACTTGGTACTACGATCATTGGGGGAACAATACCTAATCCCGAAGA
TGCTCCAGAATCTTTTCGATTGCTCGTTCGAGAACTACGATCTTTAGCTCTGGAACTGA
ATCATTTCCTTGTATCTGAGAAGAACTTCCAGATTAATAGGAAGGAAGCT------------------
--ATGATCGATCGATATAAACATCAACAGCTCCGAATTGGATCAGTTTCTCCTCAACAAAT
AAGTGCTTGGGCCACTAAAATCCTGCCTAATGGAGAGATAGTTGGAGAGGTAACAAAA
CCCTATACTTTTCATTACAAAACTAATAAACCGGAAAAAGATGGATTATTTTGTGAAAG
AATTTTTGGTCCTATAAAAAGCGGAATTTGTGCTTGTGGAAATTATCGAGTAATCGGAG
ATGAAAAAGAAGACCCGAAATTTTGTGAACAATGCGGAGTCGAATTTGTTGATTCTCG
GATACGAAGGTATCAAATGGGCTATATCAAACTCGCATGCCCAGTAACCCATGTGTGGT
ATTTAAAACGTCTTCCTAGTTATATTGCGAATCTTTTAGATAAACCTCTTAAAGAATTAG
AAGGCCTAGTATACTGCGAT------TTTTCTTTTGCTAGGCCC---ATAACTAAAAAACCCAC
TTTCTTACGATTACGAGGTTTATTTGAATATGAAATCCAATCTTGGAAATACAGCATCCC
GCTTTTTTTTACTACCCAGGGTTTCGATACATTTCGAAATCGAGAAATCTCTACTGGCGC

AGGTGCTATCCGAGAACAATTAGCCGATCTAGATTTACGAATTATTATAGAGAATTCATT
GGTAGAATGGGAAGAATTGGGGGAAGAA---GGGCACACAGGGAATGAATGGGAAGAT
CGAAAAGTTGGAAGAAGAAAGGACTTTTTGGTTAGACGCGTGGAATTGGCTAAGCATT
TTATTCGAACAAATATAGAGCCAGAATGGATGGTTTTGTGTCTATTACCAGTTCTTCCTC
CTGAGTTGAGACCGATCATTCAGATAGATGGGGGTAAACTAATGAGCTCAGATATTAAT
GAACTCTATAGAAGAGTTATCTATCGGAACAATACCCTTACCGATCTATTAACAACAAGT
AGATCTACGCCAGGAGAATTAGTAATGTGTCAGGAGAAATTAGTACAAGAAGCCGTGG
ATACACTTCTTGATAATGGAATCCGGGGACAACCAATGAGGGACGGTCATAATAAAGTT
TACAAGTCATTTTCTGATGTAATTGAAGGCAAAGAGGGAAGATTTCGTGAGACTCTGC
TTGGCAAACGAGTCGATTATTCAGGACGTTCTGTCATTGTCGTGGGTCCTTCACTTTCA
TTACATCGATGTGGATTGCCTCGTGAAATAGCAATAGAACTTTTCCAGACATTTGTAATT
CGTGGTCTAATTAGACAACATCTTGCTTCGAACATAGGAGTTGCTAAGAGTAAAATTAG
AGAAAAAGAGCCGATTGTATGGGAAATCCTTCAAGAAGTTATGCAGGGACATCCTGTA
TTGCTGAATAGAGCACCCACTCTGCATAGATTAGGCATACAGGCATTCCAGCCCGTTTT
AGTGGAGGGGCGCGCTATTTGTTTACATCCATTAGTTTGCAAGGGATTCAATGCCGATT
TTGATGGAGATCAAATGGCTGTTCATGTACCCTTATCCTTGGAGGCTCAAGTGGAGGCC
CGTTTACTTATGTTTTCTCATATGAATCTTTTGTCTCCGGCTATTGGGGATCCCATTTCCG
TACCAACGCAAGATATGCTTATTGGACTCTATGTATTAACGAGCGGAAATCGTCGAGGT
ATTTGTGTAAATAGATATAATCCATGTAATCGC---------AGAAACTATCAAAATCAAAAAA
GAAGTGACAATAGTTATAAGTATACG---------GAACCCTTTTTTTCGAATTCCTATGATGC
AATTGGAGCTTATCGGCAGAAACGAATCAATTTAGATAGTCCTTTGTGGCTCCGGTGGC
GACTAGATCAACGC---GTTATTGCTTCA------AGAGAAACTCCCATCGAAGTTCACTATG
AATCTTTAGGTACTTTTTATGAGATTTATGGACACTATCTAATAGTAAGAAGTCTAAAAA
AACAAATCCTTTTTATATACATTAGAACCACTGTTGGTCATATTGCTCTTTATCGAGAAAT
TGAAGAAGCTATACAGGGGTTTTCTCGGGCCTATTCATATGGTACC---------------------------
---------ATGGCAGAACGGGCCAATCTGGTCTTTCACAATAAAGCGATAGACGGAACTGC
CATGAAACGCCTTATTAGTAGATTAATAGAGCACTTCGGAATGGCATATACATCACATAT
CCTGGATCAAGTAAAAACTCTGGGGTTTCAACAAGCTACTGCTACATCCATTTCATTAG
GAATTGATGATCTTTTAACAATACCTTCTAAGGGATGGCTAGTTCAAGATGCTGAACAA
CAAAGTTTGATTTTGGAAAAACACCATCATTATGGGAATGTACACGCGGTAGAAAAATT
ACGTCAATCCATTGAAATATGGTATGCTACAAGTGAATATTTGCGACAAGAAATGAATC
CTAATTTTAGGATGACTGACCCTTTTAATCCAGTTCATATAATGTCTTTCTCGGGAGCTA
GAGGAAATGCATCTCAGGTACATCAATTAGTAGGTATGAGAGGATTAATGTCGGATCCT
CAAGGACAAATGATTGATTTACCTATTCAAAGCAATTTGCGCGAAGGACTCTCTTTAAC
AGAATACATCATTTCTTGCTACGGAGCCCGTAAAGGAGTTGTGGATACTGCTGTACGAA
CATCAGACGCTGGATATCTCACTCGCAGACTTGTTGAAGTAGTTCAACACATTGTTGTA
CGTCGAACGGATTGTGGCACCGCCCGGGGTATTTCTGTGAGTCCTCGGAATGGGATGA
TGCCG---GAAAGGATTTTTAGCCAAACATTAATTGGTCGTGTATTAGCAGATGATATATA
CATGGGTCCGCGATGTATTGCCACTAGAAATCAAGACATTGGCATTGGACTTGTAAATA
GATTCATAACC------------------TTTCGGGCACAACCAATCTCTATTCGAACTCCCTTTACTT
GTAGGAGTACATCTTGGATTTGTCGATTATGTTATGGCCGGAGTCCTACTCATGGCGACC
TGGTTGAATTGGGGGAAGCCGTAGGTATTATTGCAGGTCAATCGATTGGAGAACCGGG
TACTCAATTAACATTAAGAACTTTTCATACCGGAGGAGTATTCACGGGGGGTACTGCAG
AACATGTGCGAGCCCCATCTAATGGAAAAATAAAATTCAATGAGGATTTGGTTCATCCG

ACACGTACACGTCATGGGCATCCCGCCTTTCTATGTTCTATAGACTTGTATGTAACTATT
GAGAGTGAAGATATTCTACATAATGTGAATATTCCACCCAAAAGTTTGCTTTTAGTTCAA
AACGATCAATATGTAGAATCAGAACAAGTAATTGCTGAGATTCGCGCAGGAATATCCAC
T---TTGAATTTTAAAGAGAAGGTTCGAAAACATATTTATTCTGATTCAGACGGAGAA---A
TGCACTGGAGTACCGATGTCTATCATGCACCCGAATTTACATATGGTAATGTTCATCTATT
ACCAAAAACAAGTCATTTATGGATATTATTAGGAGGGCCGTGCAAG------TCCAGTCTAG
TCTACCTTTCGATCCACAAGGATCAGGATCAAATGAATGCGCAT------TCTCTTTCTGGC
AAGCAAAGATATACTTCTAACCTCTCA------GTAACCAATGATCAG---GCGAGGCAGAAA
TTATTTAGTTCGGGTTTTTCTGGTAAA---GAGGATAGGATTCCTGATTAT---TCAGACCTT
AATCGAATCATATGTACTGGTCAGTATAATCTCGTATATTCG---CCTATTCTCCATGAGAAT
TCT------GATTTATTGTCAAAAAGGCGAAGAAATAAATTCATCATTCCACTACACTCGATT
CAAGAACTCGAGAATGAACTAATGCCCTGTTCAGGTATCTCGATTGAAATACCC------GT
AAATGGTATTTTCCGTCGAAATAGTATTCTTGCTTATTTCGATGATCCTCGATACAGAAG
AAAGAGTTCGGGCATTATTAAATATGGGACTATAGAAACGCATTCAGTCATCAAAAAAG
AGGAT------TTGATTGAGTATCGAGGA---GTCAAGGAATTTAGGCCAAAATACATGAAAG
TAGATCGATTTTTTTTCATTCCTGAAGAGGTGCATATCTTGCCCGGATCTTCTTCCATAAT
GGTACGGAATAATAGTATCGTTGGAGTAGATACACAAATCACCTTAAATCTAAGAAGCC
GAGTCGGTGGGTTGGTTCGGGTGGAGAGAAAAAAAAAACGAATTGAACTTAAAATCT
TTTCTGGAGATATCCATTTTCCTGGCGAGACAGATAAGATATCCAGACATACCGGCCTTT
TGATACCA---CCAGGA---ACAGGAAAAATAAATTCCAAGGAATCCAAAAAA------GTGA
AAAAGTGGATCTATGTCCAACGG------ATTACACCTAGTAAG------AAAAAGTTTTTTGTT
TTAGTTCGACCTGTCGTCACATATGAAATAACGGACGGTATAAATTTTGAAACCCTTTTT
CCACCGGATCCATTGCAGGAAAGGGATAATGTGCAACTTCGAATTGTCAATTATATCCT
TTATGGAAATGGCAAACCGATTCGAGGAATTTCTGACACAAGTATTCAATTAGTTCGGA
CTTGTTTAGTATTGAATTGGAACCAAGACAAAAAA---------AGTTCTTCTTGCGAAGAAG
CCCGTGCTTCTTTTGTTGAAATAAGGACAAATGGTTTGATTCGACATTTCCTAAGAATC
AACTTAGTTAAATCCCCTATTTCGTATATCGGAAAAAGGAATGATCCGTCGGGGTCAGG
ATTGCTCTCTGAT---AATGGATCAGATTGTACCAATATCAACCCCTTTTCTTCCATTTATTC
CTAT---------TCCAAGGCAAAAATTCAACAATCTCTTAACCAACCTCAAGGAACTATTCAT
ACGTTGTTGAATAGA---------AATAAGGAATGTCAGTCGTTGATAATTTTGTCATCAGCCA
ATTGTTCTCGAATGGGG---------------ACATTAAAATATCACAGTGTG---------ATAAAAGAC
TCA---CCCCTAATTCCAATTAGGAATTCATTGGGCCCTTTAGGAACA------TGCCTTCCAA
TTGAGAATTTTTATTCA------TCTTACCGTTTA------ATAACTCATAATCAGATCTTA---GTA
ACTAACTATTTGCAACTTGACAAT---------TTAAAACAGACCTTTCAAGTGATTAAATTTA
AA---TATTATTTAATGGATGAAAAT------GGAAAAATTTTTAATCCTGATCCATGTCGTAAC
ATTATTTTAAATCCATTCAATTTGAATTGGTATTTTCTCCATCACAATTATTGTGAAGAGA
CATCT---AAAATAATTAGTCTTGGACAGTTTATTTGTGAAAATGTATGTATAGCCAAAAAT
GGACCGCCC---CTCAAA------TCGGGTCAAGTTATACTTGTTCAA---GTTGACTCGATAGT
GATACGATCAGCTAAGCCTTATTTGGCCACCCCCGGAGCAACTGTTCATGGCCATTATG
GGGAAACCCTTTACGAAGGAGATACATTAGTTACATTTATATATGAAAAATCGAGATCTG
GTGATATAACACAGGGTCTTCCAAAAGTAGAACAGGTGTTAGAAGTGCGTTCGATTGA
TTCAATATCCATGAATCTAGAAAAGAGGGTTGAGGGTTGGAACAAATGTATAACAAGA
ATTCTTGGAATTCCTTGGGGATTCTTGATTGGTGCTGAGCTAACTATAGCGCAAAGCCG
AATCTCTTTGGTTAATAAAATCCAACAGGTTTATCGCTCCCAGGGGGTGCAGATTCATA

ATAGACATATAGAAATTATTGTACGTCAAATAACATCAAAAGTGTTGGTTTCAGAA-------
--GATGGAATGTCTAATGTTTTTTCACCCGGAGAACTTATTGGATTGTTGCGAGCAGAAC
GAATGGGGCGCGCTTTGGAAGAAGCGATCTGTTACCGAGTCGTCTTATTGGGAATAAC
AAGAGCATCTCTCAATACTCAAAGTTTCATATCTGAAGCGAGTTTTCAAGAAACTGCTC
GAGTTTTAGCAAAAGCGGCTCTCCGGGGTCGTATCGATTGGTTGAAAGGCCTGAAAGA
GAACGTTGTTTTGGGGGGGGTGATACCCGTTGGTACCGGATTCAAGGGATTA------GTC
CACCCTTCAAAACAACATAACAACATTCCT------TTGGAAACAAAAAAAAAGAATCTAT
TCGAGGGGGAGATGAGA------GATATTTTGTTCCACCACAGAAAATTATTTGATTCTTGT
CTTTCAAAAAAATTCCACGACATACCCGAACAATCATTTATAGGA------------------TTTAA
TGATTCCATGACAAGAAGATATTGGAACATAAATTTGGAAGAGATGATGGAGGCAGGA
GTTCATTTTGGTCATGGTACTAGGAAATGGAATCCTAAAATGGCGCCTTATATCTCTGCA
AAGCGTAAGGGTATTCATATTACAAATCTTACTAGAACTGCTCGTTTTTTATCAGAAGCT
TGTGATTTAGTTTTTGACGCAGCAAGTAGGGGAAAACAATTCTTAATTGTTGGTACCAA
AAATAAAGCAGCTGATTCAGTAGAGTGGGCTGCAATAAGGGCCCGGTGTCATTATGTTA
ATAAAAAATGGCTTGGCGGTATGTTAACGAATTGGTCCACTACAGAAACAAGACTTCAT
AAGTTCAGGGACTTGAGAATGGAACAAAAAACAGGGAGACTCAACCGTCTCCCGAAA
AGAGATGCAGCTATGTTGAAAAGACAATTATCTCGCTTACAAACATATCTGGGCGGGAT
TAAATATATGACAGGGGTACCCGATATTGTAATCATCGTTGATCAGCACGAAGAATATAC
GGCCCTGCGAGAATGTATTACTTTAGGAATTCCAACAATTTGTTTAACCGATACAAATTG
TGACCCCGATCTCGCAGATATTTCAATTCCAGCGAATGATGACGCCATATCCTCAATCCG
ATTAATTCTTAACAAATTAGTATTCGCAATTTGTGAGGGTCGTTCT---AGCTATATCAGAA
ATCCTATGGGACAAAAAATAAATCCACTTGGTTTCAGACTGGGTACAACCCAAGGTCAT
CATTCCCTTTGGTTTTCACAACCAAAAAATTATTCTGAAGGTTTACAAGAAGATCAAAA
AATAAGAGATTGTATCAAGAATTATGTACAAAAGAATATGAGAACGTCCTCTGGC---GT
CGAGGGAATTGCACGTATAGAGATTCAAAAAAGAATCGATCTGATCCAGGTCATAATCT
TTATGGGATTCCCAAAATTATTAATAGAAAGTCGACCGCGC------------GGAATCGAAGAA
TTACAAATGACCTTACAAAAAGAATTAAATTGTGTAAACCGAAAACTGAACATTGCTG
TCACAAGAATTGCAAAACCTTACGGAAACCCTAATATTCTTGCAGAATTTATAGCTGGA
CAATTAAAGAATAGAGTTTCATTTCGAAAAGCAATGAAAAAGGCTATTGAATTAACAG
AACAAGCAGATACAAAAGGAATTCAAATACAAATTGCGGGGCGTATCGACGGAAAAG
AAATTGCACGTGTCGAATGGATCAGAGAAGGTAGGGTTCCCCTACAAACGATTCGAGC
TAAAATTGATTATTGCGCTTATACAGTTCGAACTATCTATGGAGTATTGGGCATCAAAAT
TTGGATATTTCTAGACGAGGAAATGTCGCGTTACCGAGGACCTCGTTTCAAAAAAATAC
GCCGCCTGGGGGCTTTACCAGGACTAACTAATAAAAAGCCTAGGACCGGAAGTGATCT
TAGAAACCAATCGCGTTCCGGGAAA---AAATCTCAATATCGTATTCGTCTAGAAGAAAA
ACAAAAATTGCGTTTTCATTATGGTCTTACAGAACGACAATTACTTAAATACGTTCGTAT
CGCCAGAAAAGCCAAGGGGTCAACAGGTCAAGTTTTACTACAATTACTTGAAATGCGT
TTGGATAACATCCTTTTTCGATTGGGTATGGCTTCAACTATTCCCGCAGCCCGTCAATTA
GTTAATCATCGACATATTTTAGTTAATGGTCGTATAGTAGATATACCGAGTTATCGCTGCA
AACCCCGAGATATTATTACGGCAAAGGATGAACAAAAATCCAGAGCTCTGATTCAAATT
TCTCTCGATTCATCCCCTCAT---GAGGAATTACCAAATCATTTGACCCTTCACCCATTCCA
A------TATAAAGGATTAGTCAATCAAATAATAGATAGTAAATGGGTCGGTTTGAAAATAA
ATGAATTGCTCGTCGTAGAATATTATTCTCGTCAGACTATGTCACGTCGAGGTACTGCAG
AAAAAAAAACAGCAAAATCCGATCCAATTTATCGTAATCGATTAGTTAACATGTTGGTT

AACCGTATTCTGAAACACGGAAAAAAATCATTGGCTTATCAAATTATCTATCGAGCCGT
GAAAAAGATTCAACAAAAGACAGAAACAAATCCACTATCCGTTTTACGTCAAGCAATA
CGTGGAGTAACTCCCGATATAACAGTAAAAGCAAGACGTGTAGGTGGATCGACTCATC
AAGTTCCCATTGAAATAGGATCCACACAAGGAAAAGCACTTGCCATTCGTTGGTTATTA
GCGGCATCCCGAAAACGTCCGGGTCGAAATATGGCTTTCAAATTAAGTTCCGAATTAGT
GGATGCTGCCAAAGGGAGTGGCGATGCCATACGCAAAAAGGAAGAGACTCATAGAAT
GGCAGAGGCAAATAGAGCTTTTGCACATTTTCGTATGGGTAGGGACACTATTGCTGAGA
TAATAACTTCTATACGAAATGCCGATATGGATCGAAAAAGGGTGGTTCGAATAGCATCT
ACTAATATTACCGAAAATATTGTTCAAATACTTTTACGAGAGGGTTTTATCGAAAACGTG
AGAAAACATCAAGAAAACAACAAATATTTTTTGGTTTTAACCCTGCGACATAGAAGG---
---AATAAGAAAAGACCCTAT---------AGAAATATTTTAAATTTAAAACGAATCAGTCGACC
TGGTCTACGAATCTATTCTAATTATCAACGAATTCCTCGAATTTTAGGTGGAATGGGGAT
TGTAATTCTTTCTACTTCTCGAGGTATAATGACAGACCGAGAGGCTCGACTAGAAGGAA
TAGGTGGAGAAATTTTGTGTTATATCTGGATGGCAAAAGCTATACCGAAAATTAGTTCG
CGTAGGAATGGACGTATTGGTTCACGTAAGGGTGCACGTAGAATACCAAAGGGAGTTA
TTCATGTTCAAGCAAGTTTCAATAATACCATTGTCACTGTTACAGATGTACGGGGTCGA
GTAGTTTCTTGGTCCTCCGCTGGTACTTCTGGATTCAAAGGTACGAGAAGAGGAACAC
CGTTTGCTGCTCAAACCGCAGCAGCAAACGCTATCCGTACAGTAGTGGATCAAGGTAT
GCAACGAGCAGAAGTCATGATAAAAGGTCCCGGTCTCGGAAGAGATGCAGCATTACG
AGCTATTCGTCGAAGTGGTATACTATTAACTTTCGTACGGGATGTAACTCCTATGCCACA
TAATGGCTGTAGACCTCCGAAAAAAAGACGTGTGATGCCAACTATTAACCAACTTATTA
GAAATACAAGACAGCCAATCAGAAATGTCACGAAATCCCCCGCTCTTCGGGGGTGCCC
TCAGCGTCGAGGAACATGTACTAGGGTGTATACTATCACCCCAAAAAAACCAAACTCT
GCCTTACGTAAAGTTGCCAGAGTACGATTAACCTCTGGATTTGAAATCACTGCTTATATA
CCCGGTATTGGCCATAATTTACAAGAACATTCTGTAGTCTTAGTAAGAGGGGGAAGGGT
TAAGGATTTACCCGGTGTGAGATATCACATTGTTCGAGGAACCCTAGATGCTGTCGGAG
TAAAGGATCGTCAACAAGGGCGTTCTAAATATGGGGTAAAAAAGCCAAAAATGGCAA
AGAAAAGTTTGATTCAGAGGGAGAAGAAGAGGCAAAAATTGGAACAGAAATATCATT
CGATTCGTCGATCCTCAAAAAAAGAAATAAGC---------AAGGTTCCGTCGTTGAGTGAC
AAATGGGAAATTTATGGAAAGTTACAATCCCTACCACGGAATAGTGCACCTACACGCCT
TCATCGACGTTGTTTTTTGACCGGAAGGCCGAGAGCTAACTATCGAGACTTTGGACTAT
CCGGACATATACTTCGTGAAATGGTTCATGCATGTTTGTTGCCAGGAGCAACAAGATCA
AGTTGGATGGTAAAAAATTCTGTCATTTCAATTATTTTTCAAGAA------GAAAAG---------
AGAGGATCTGTTGAATTTCAAGTATTCAATTTCACCAATAAGATACGGAGACTTACTTC
ACATTTAGAATTGCACAAAAAAGATTATTTATCTCAGAGAGGTTTGAAGAAAATTTTGG
GAAAACGTCAACGGCTGCTAGCTTATTTGGCAAAAAAAAATAGAGTACGTTATAAAGA
ATTAATTAATCAGTTGGACATTCGAGAGACAAAAACTCGT------ATGGTAAAACTTCGTT
TGAAACGATGTGGTAGAAAGCAACGAGCCGTCTATCGAATCGTTGCAATTGATGTTCG
ATCCCGAAGAGAAGGAAAAGATCTTCGTAAAGTGGGTTTTTATGATCCGATAAAGAAT
CAAACTTATTTAAATGTTCCTGCTATTTTATATTTCCTTGAAAAAGGGGCTCAACCTACA
GGAACTGTTCAGGATATTTTAAAGAAGGCGGAGGTTTTTAAGGAACTTCGTCCTAATCA
ACCTAAATTCAAT---ATGGATAAATCCAAGCGACCTTTTCTTAAATTCAAGCGATCTTTT
CGTAGGCGTTTGCCCCCGATTCAATCGGGGGATCGAATTGATTATAGAAACATGAGTTT
AATTAGTCGATTTATTAGTGAACAAGGAAAAATATTATCAAGACGAGTGAATAGATTGA

CCTTGAAACAACAACGATTAATTACTCTTGCTATAAAACAAGCTCGTATTTTATCTTTGT
TACCTTTTCTCAATAATGAGAAA------CAATTTGAAAGAACCGAGTCGACCGCTAGAAC
TACTGGTTTTAAAGCCCGAAATAAAGTGACACGTTCACTAAAAAAAAATCCCTTTGTA
GCCAATCATTTATTAAAAAAAATTGATAAGCTTAACACAAAAGCAGAAAAAGAAATAAT
AGTAACTTGGTCCCGGGCATCTACCATTATACCCACAATGATCGGTCATACTATTGCTAT
CCATAATGGAAAAGAGCATTTGCCTATTTATATAACGGATAGTATGGTAGGCCACAAATT
GGGAGAATTTGCACCTACATTAAATTTTAGAGGACATGCAAAAAGCGATAATAGATCTC
GTCGTATGATTTTTCAATCTTTTCTACTAGGTAATCTAGTATCCTTATGCATGAAGATAAT
CAATTCGGTCGTTGTGGTCGGACTCTATTATGGATTTCTGACCACATTCTCCATAGGGCC
CTCTTATCTCTTCCTTCTCCGAGCTCTGGTTATGGAAGAAGGA---------ACCGAGAAGAA
GGTATCAGCAACAGCTGGTTTTATTACGGGACAGCTCATGATGTTCATATCGATCTATTA
TGCGCCTCTGCATCTAGCATTGGGTAGACCTCATACAATAACTGTCCTAGCTCTACCATA
TCTTTTGTTTCATTTCTTCTGGAACAATCACAAACACTTTTTTGATTATGGATCTACTACC
AGAAATTCAATGCGTAATCTCAGCATTCAATGTGTATTCCTGAATAATCTCATTTTTCAAT
TATTCAACCATTTCATTTTACCAAGTTCAATGTTAGCCAGATTAGTCAACATTTATCTCTT
TCGATGCAACAACAAGATCTTATTTGTAACAAGTGGTTTTGTTGGTTGGTTAATTGGTC
ACATTTTATTCATGAAATGGCTTGGATTGGTATTAGTCTGGATACGGCAAAATCATTCTAT
TAGACCGTACATTCGATCTAATAAGTACCTTGTGTTAGAATTGAGAAATTCTATGGCTCG
GATCTTTAGTATTCTCTTATTTATTACCTGTGTCTACTATTTAGGCAGAATACCCTCACCC
ATTCTTACTAAGAAACTGAAAGAAACTGAAGAAGATAAACCTCTTGTGAGTCTTCTTTT
CAATTATAATCGATGGAATCGACCATTTCGCTACATAAAGAATAATCGATTTGAACAAGC
CGTAAGAACTGAAATGTCACAATATTTTTTTGACACATGTAAAAGTGATGGAAAACAA
AGAATATCTTTTACATACCCGCCAAGTTTATCAACTTTTTGGAAAATGATAAAAGGAAG
GCTACCCTTATTGTCACTCCAAAAAACCCTCCCTAATGAACTGGACAATCAGTGGGTTT
CTACCAACAAAGAAAAAAGTAAT---CTGAATAAGGAATTTTTAAATCGACTAGAAGTTC
TAGACAAGGAATCTCTTTCTCTGGATATACTCGAAACAAGGACTAGATTGAGTAATGAT
GATACTAAAAAAGAATACGTGCCTAAAATGTATGATCCTTTGTTAAATGGACCATATCGA
GGAACAATCAAAAAAGGGTTTTCACCTAAAGAGAGAGTTATACTAAATAGGATTCATA
CTATCTTTTACCAAGAATTTGAACAAAAAGATCCTAATGGGACATCGATTGGAATAAAA
GAAATCAGTAAAAAAATACCTCGCTGGTCACATAAATTAATCGAATTGGAACAACAATT
G---GGTCAAATTCGTTCAAGAAAAGCCAGACGTGTAGTGATTTTTACTACCGCTTTAAT
AAGCTATTCGCAACAATCAGATTTTCGGCGAGGTATAATTAAAGGCTCTATGCGGGCTC
AAAGGCGCAAAACAGTTATTTGGAAACTATTTCAAGCAAATGTGCATTCCCCCTTTTTT
CTCGACAGAATAACCCCCCCCCGTCTTTTTTCTTTTGATATC------TCTGAACTGATTAAA
CCAATTTTTCGAAATAAAAAGGAAAAAGCACGGATAGCTATAGCCGAAGCCTGGGATA
GCATTCTTTTTGCGCAAATAATAAGAGGTTCCATGTTAATAACTCAATCAATTCTTCGAA
AATATATTCTATTACCTTCATTGATAATAGCCAAAAATCTCGGGCGTATGTTATTCTTGCA
ACTTCCTGAATGGTCTGAAGATTTGGAGGAATGGAATAGAGAAATGCATATTAAATGTA
CTTATAATGGTGTTCAATTATCAGAAACAGAATTTCCAAAAAATTGGTTGAGGGATGGG
ATTCAGATCAAAATTTTATTTCCTTTTTGTCTGAAACCTTGGCATATATCTAAAAAAAAA
CAAAAAGATGATTTTTGTTTTTTAACAGTTTGGGGAATGGAAGCTGAACTTCCCTTTGG
TTCTCCACGAAGTCGCCCTTCCTTTTTTGAGCCCATTTTTAAGGAACTCGAAAAAAAA
AATGGAAAATTAAAAAAGAAATATTTTATAACT---CTAAAAGTTTTAAAAGGAAAAACA
AAATTATTTAGAAGAATTTCAAAAGAAACTAAAAAATGGCTTATCAAAAGTATTCTA-----

-TTTCTAAAAAAAAATAAGAAAAGAACTTTCAAAAATAAAGGATTCTCTAATTAGTAATC
AGATAATTAACGAATCATTTAGTCAAATTGAATCTGTAAATTGGCCAAATTCTTCACTGA
TAGAAACCAAAATGAAGGATTTGACTGATAGAACAAGTACAATAAAAAATCAAATAGA
AAGAATTACAAAAGAGAAAAAGAAAAGTCGTAATAAA---ACAAATAATATTAAAAAAT
TCGAATCGACAAAAAATATTTTTCAAATATTACAAAGAAGAAATACTCGATTAATATGG
AAATTCCATTATTTTATAAAATTATTCATTCAAAGATTATACATCAATCTATTTTTATCTATC
ATTAATATTCCCAGAATTAATACACAACTCTTT------TTTATTTCGTTTGATTTATCCTACTT
GTCACAAGCATATGTATTTTACAAATTATCACAAACCCAAGTTATTAATTTGTCTAAATTT
AGATCTGTTCTTCAATATACGTCTTTTTTCCTTAAGACTAAAATAAAGGACTATTTTACA
CTAGGAATATTTCATTCTGAATTAAAACATAAGAAACTTCAGAGTTATAGAATCAATCAA
TGGAAAAACTGGTTAAGACGGCATTATCAATACGATTTATCTCAGATTAGATGGTCTAGA
TTAATGCCACAAAAATGGCGAAATAGGGTTAATCAAAGTTGTATGGCTCAAAATAAAA
ACAAATGGAATTCATATGAAAACGACCAA------TTAATTCATTACAAAAAAGAA---AATT
TTCAAAAATGTTATAGATATGATCTTTTATCATATAAATCAATTAATGGCAATATTCTATAT
CTCGATAGAAAATATTTTGATTGGAAAATTATCCATTTTTCTCTTAGATTTTTTGATTGGA
TGGGAATGAATGAAAAAATATTTAATCGTTGGTTCTTCCCAGAATTTGTACTACTTTATA
ATGTATATAAAATAAAACCTTGGATTATACCAAGCAAATTACTTCTTTTAAATTACAAAA
GCAACACGGAAGCAGAACTTGATTTGTTCTTGAAACGTTATTTGCTTTTTCAATTGAAA
TGGAACGATGATTTGAATCAAAGAATGATCGAGAATATCAAGGTGTATTGTCTCCTGCT
TCGACTGATAAATCCAACCAAAATTAGTATATCGTCAGTTCAAAGGGGAGAAATGAGTT
TGGATATAATGCTGATTCAGGGGAATTTAACTCTTACAGAATTGATGAAG---GGAATATT
GATTATCGAACCTATTCGGTTGTCTGTAAAAAATAATGGACAATTTATTATGTATCAAAC
CATAGGTATTTCATTGGTTCATAAAAGTAAACACAATCATTTTGATTTACTTGTTCCTGA
AAATATTTTATGGTCTAGACGTCGTAGAGAATTGAGAATTCGAAGTTTTTTTAGGTTTCT
TTGGCCTAATTATCGATTAGAAGATTTAGCTTGTATGAATCGTTATTGGTTTGATAACAAT
AATGGTAGCCGTTTCAGTATCTTAAGGATACATATGTATCCAATGAGAGGACATCAATTC
AAATCCTGGATTTTAGAATTGAGAGAGATATTGAGAGAGATCAAGAATTCTCACCATTT
CTTAGATTCATGGACCCAATTCACTTCAGTGGGATCCTTCATTCACATTTTTTTCCACCA
AGAACGTTTTCTAAAACTCTTTGACCCCCGAATTTGGAGTATCCTACTTTCA---CGCAAT
TCA------CAGGGTTCAACAAGCAATCGATATTTCACG---------ATCAAGGGT---GTAATACT
CTTTGTAGTAGCGGTCCTTATATATCGT------------ATTAACAATCGAAATATGGTCGAAAG
AAAAAATCTCTATTTGATAGGGCTTCTTCCT---------------ATACCTATGAATTCCATTGGAC
CCAGAAATGATACATTGGAAGAATCCGTT---GGGTCTTCCAATATCAATAGGTTGATTGT
TTCG---CTCCTGTATCTTCCCAAAGGAAAAAAGATCTCTGAGAGTTGTTTCCTGAATCC
GAAAGAGAGTACTTGGGTTCTCCCAATAACT------------AAAAAGTGTAGCATGCCTGAA
TCTAACTGGGGTTCGCGTTGGTGGAGGAACTGGATCGGAAAAAAGAGG------GATTCTA
GTTGTAAGATATCTAAT---------GAAACCGTCGCTGGAATTGAGATCTTATTCAAAGAGAA
AGATCTCAAA---TATCTGGAGTTTCTTTTTGTATATTATATGGAT------GATCCGATCCGCA
AGGACCATGATTGGGAATTGTTTGATCGTCTTTCTCTGAGGAAGAGTCGAAATAGAATC
AACTTG------AATTCGGGACCGCTATTCGAAATCTTAGTGAAACACTGGATTTCTTATCT
CATGTCTGCTTTTCGTGAAAAAATACCAATTGAAGTGGAGGGT---TTCTTCAAACAACA
AGGG---GCTGGGTCAACTATTCAATCAAATGATATTGAGCATGTTTCCCAT------CTCTTC
TCGAGAAACAAGTGGGCTATTTCTTTGCAAAACTGTGCTCAATTTCATATGTGGCAATT
CCGCCAAGATCTCTTCGTT---------AGTTGGGGGAAG---AATCCGCCCGAATCGGATTTTT

TGAGGAAC---GTATCGAGAGAGAATTGGATTTGGTTAGACAATGTGTGGTTGGTAAACA
AGGATCGG---------TTTTTTAGC------AAGGTACAGAATGTATCGTCAAATATTCAATATGA
TTCC---------ACAAGA------TCTAGTTTCGTTCAAGTAACGGATTCTAGCCAACTGAAAGG
ATCTTCT---GATCAATCCAGAGATCATTTGGATTCCATTAGT------------AATGAGGATTCGG
AA---------------------------------------TATCACACATTGATTAATCAAAGAGAGATTCAACAAC
GAAAAGAAAGATCGATTCTT---TGGGAT------CCTTCCTTTCTTCAAATGGAACGAAAAG
AGATAGAATCAGGC------CGATTCCCGAAA------TGCCTTTCTGGATATTCC------TCAATG
TCCCGGCTATTCACG---GAACGTGAGAAGCAGATGATTAATCATCTGTTTCCGGAAGAA
ATCGAAGAATTTCTT------GGGAATCCTACAAGATCCGTTCGTTCTTTTTTCTCTGATAGA
TGG------TCAGAACTTCAT---CTGGGTTCGAATCCTACTGAGAGGTCCACTAGAGATCAG-
-----------AAATTGTTGAAGAAACAACAAGATCTTTCT---TTTGTCCCTTCCAGGCGATCGG
AA------------AATAAAGAAATGGTTAATATATTCAAGATAATTACGTATTTACAAAAT------A
CCGTCTCAATTCATCCTATTTCATCAGATCCGGGATGTGATATGGTTCCG---AAGGATGA
ACCGGAT---ATGGACAGTTCCAATAAGATTTCATTCTTGAACAAAAATCCATTTTTTGAT
TTA------TTTCATCTATTCCATGACCGGAACAGGGGAGGATACACGTTA------CACTACGA
TTTTGAATCAGAA------GAGAGATTTCAAGAAATGGCAGATCTATTCACTCTATCAATAA
CCGAGCCG---GATCTGGTGTATCATAAGGGATTTGCCTTTTCTATTGATTCCTGCGGATT
GGATCAAAAACAA------TTCTTGAATGAGGCCAGGGATGAA---------TCGAAAAAGAAAT
CT---------TTATTGGTTCTACCTCCTATTTTTTAT------GAAGAGAATGAATCTTTTTCTCGA
AGGATCAGAAAAAAATGGGTCCGG---ATCTCCTGCGGGAATGATTTGGAAGATCCAAA
ACCAAAAATAGTGGTATTTGCTAGCAACAACATAATGGAGGCAGTCACTCAATATAGAT
TGATCCGAAATCTGATTCAAATCCAATATAGTACCTATGGT------TACATAAGAAATGTATT
GAATCGATTCTTTTTAATGAAT---------AGATCCGATCGCAACTTCGAATATGGA---------AT
TCAAAGGGATCAAATAGGAAAGGATACTCTGAATCATAGAACTATAATGAAATATACGA
TCAACCAATATTTATCGAATTTGAAAAAG---AGTCAGAAGAAATGGTTCGAGCCTCTT---
---ATTTTGATTTCTCGAACCGAGAGATCCATGAATCGGGATCCTGATGCATATAGATACA
AATGGTCCAATGGGAGCAAGAATTTCCAGGAACATTTGGAACAGTCCGTTTCGGAGCA
GAAGAGCCGT---------TTTCAAGTAGTGTTCGATCGATTACGTATTAATCAATATTCGATTG
ATTGGTCTGAGGTTATCGACAAAAAAGATTTGTCTAAGCCACTTCGTTTCTTTTTGTCTA
AGCCACTTCGTTTCTTTTTGTCCAAG---TCACTTCTTTTTTTGTCC---------------------AAGT
TGCTTTTCTTTTTGTCTAAC---TCACTTCCTTTTTTCTGTGTGAGTTTCGGAAATATCCCC
ATTCATAGGTCCGAG---ATCTACATCTAT---GAATTGAAAGGTCCGAATGATCAACTCTGC
AATCAGTTGTTAGAATCAATAGGTCTTCAAATTGTTCATTTGAAAAAATTGAAACCCTT
C------TTATTGGAC---------GATCATGATACTTCC---CAAAAATCTAAATTCTTGATCAATGG
AGGAACAATATCACCATTTTTGTTCAATAAGATACCA---AAGTGG---ATGATTGACTCATT
CCATACTAGA------AATAATCGCAGGAAATCCTTTGATAACCCGGATTCCAATTTCTCAAT
GATATTCCACGATCAAGACAATTGGCTGAATCCCGTGAAACCATTTCATAGAAGTTCAT
TGATATCTTCTTTTTATAAAGCAAATCGACTTCGATTCTTGAATAATCCACATCACTTCTG
CTTCTAT------TGGAACACAAGATTCCCCTTTTCTGTG------------GAAAAGGCCCGTATCA
AT------AATTCTTATTTTACGTATGGA---CAATTCCTCAATATCTTGTTCATTCGCAACAAA-
--ATATTTTCTTTGTGCGTCGGTAAC------AAAAAACATGCTTTTTGGGGG---AGAGATACT
ATTTCACCAATCGAGTCACAGGTATCTAACATATTCATACCTAACGATTTTCCACAAAGT
GGT---GACGAAACGTATAACTTGTACAAATCT---------TTCCATTTTCCAAGTCGATCCGAT
CCATTCGTTCGT------AGAGCTATTTACTCGATCGCAGACATTTCTGGAACACCTCTAAC

AGAGGGGCAAATAGTCAATTTTGAAAGAACTTATTGTCAACCTCTTTCAGATATGAATC
TATCTGATTCAGAAGGGAAGAACTTGCATCAGTATCTCAAT------TTCAATTCAAACATG
GGTTTGATTCACACTCCATGTTCTGAGAAAGATTTATCATCCGAAAAG------AGGAAAA
AA------------------------CGGAGTCTTTGTCTAAAG------AAATGCGTTGAGAAAGGGCAGAT
GTATAGAACCTTTCAACGAGATAGTGCTTTTTCAACTCTCTCAAAATGGAATCTATTCCA
AACATATATGCCATGGTTCCTTACTTCGACAGGGTACAAATATCTAAATTTGATATTTTTA
GATACTTTTTCAGACCTATTG------CCAATACTAAGTAGC---AGTCAAAAATTTGTATCCA
TTTTTCCTGATATTATGCATGGATCAGGTATATCATGGCGAATTCTTCAGAAAAAATTG---
---------------TGTCTTCCACAATGGAATCTG---ATAAGTGAGATAAGTAGTAAGTGTTTACA
TAAT------CTTCTTCTGTCCGAAGAAATGATTCATCGAAAT------AATGAGTCACCATTGAT
ATCGACACATCTGAGATCGCCAAATGCTCGGGAGTTC---CTCTATTCAATCCTTTTCCTT
CTTCTTGTTGCTGGATAT---CTCGTTCGTACACATCTTCTCTTTGTTTCCCGG------GCCTC
TAGTGAGTTACAGACAGAGTTCGAAAAGGTCAAATCTTTGATGATTCCATCATCTATGA
TTGAGTTGCGAAAACTTCTGGATAGGTATCCTACATCTGAACCGAATTCTTTCTGGTTA
AAGAATCTCTTTCTAGTTGCTCTGGAACAATTAGGAGATTCTCTAGAAGAAATACGGGG
TTCTGCTTCTGGCGGCAACATGCTTGGT---------CCCGCT---------------TATGGG---GTCAAA
TCAATACGTTCTAAGAAGAAAGATTGGAATATCAATCTCATCGAGATCATCGATCTCATA
------------CCAAATCCCATCAATCGAATCACTTTTTCGAGAAATACGAGACATCTAAGTCA
TACAAGTAAAGAGATCTATTCATTGATAAGAAAAAGAAAAAACGTGAACGGGGATTGG
ATTGATGATAAAATAGAATCCTGGGTCGCGAACAGTGATTCGATTGCTGATGAAGAAAG
AGAATTCTTGGTTCAGTTCTCC------ACCTTAACGACA------GAAAATAGGATTGATCAAA
TTCTATTGAGTCTGACT---CATAGTGATCATTTA---------------TCAAAGAATGACTCTGGTT
ATCAAATGATTGAACAACCGGGAGCAATTTACTTACGATACTTAGTTGACATTCAT------A
AAAAGCATCTAATGAATTATGAGTTCAATCCATCCTGTTTAGCAGAAAGACGGATATTC
CTTGCTCATTATCAGACAATCACTTAT------TCACAAACTTCGTGTGGGGAAAATAGTTTT
CATTTCCCATCTCATGGA---------AAACCCTTTTCGCTCCGCTTAGCCTTATCCCCCTCT---
AGGGGTATTTTAGTGATAGGTTCTATAGGAACTGGACGATCCTATTTGGTCAAATACCTA
GCGACAAACTCCTATGTTCCTTTCATTACGGTATTTCTGAACAAGTTCCTGGATAACAA
GCCTAAAGGTTTTCTTCTTGAT---------------GAGATCGAT---ATTGATGATAGTGACGATAT
TGATGATAGTGACAATCTTGATGCTAGTGAC--G
ATATCGGTCGTGACCTTGATACGGAGCTG---GAACTGCTAACTAGGATGAATGGGCTAA
CTATGGATATGATGCCGGAA---------ATAGACCGATTTTATATCACCCTTCAATTCGAATTA
GCAAAAGCAATGTCTCCTTGCATAATATGGATTCCAAACATTCATGATCTGGATGTGAAT
GAGTCGAATGACTTATCCCTCGGTCTATTAGTGAACCATCTCTCCAGG---------GATTGTG
AAAGATGTTCT---ACTAGAAATATTCTTGTTATTGCTTCGACTCATATTCCCCAAAAAGT
GGATCCCGCTCTAATAGCTCCGAATAAATTAAATACGTGCATTAAGATACGAAGGCTTCT
TATTCCACAACAACGAAAGCACTTTTTCACTCTTTCATATACTAGGGGATTTCACTTGG
AAAAGAAAATGTTCCATACT------AACGGATTCGGGTCCATAACCATGGGTTCCAATGCA
CGAGATCTTGTAGCACTTACCAATGAGGTCCTATCGATTAGTATTACACAGAAGAAATC
AATTATAGACACTAATACAATTAGATCCGCTCTTCATAGACAAACTTGGGATTTGCGA----
-----TCCCAGGTAAGATCGGTTCAGGATCATGGGATCCTTTTCTATCAGATAGGAAGGGCT
GTAGCACAAAATGTACTTCTAAGTAATTGCCCCATAGATCCTATATCTATCTATATGAAGA
AGAAA------TCATGTAACGAAGGGGATTCTTATTTGTACAAATGGTACTTCGAGCTTGGA
ACGAGCATGAAGAGATTAACGATACTTCTTTATCTTTTGAGTTGTTCTGCCGGATCGGT

CGCTCAAGATCTTTGGTCTTTATCC------GGACCCGATGAAAAA---AATGGGATCACTTC
TTATGGACTCGTTGAGAATGATTCT---GATCTAGTTCATGGCCTATTAGAAGTAGAAGGC
GCTCTGGTGGGA------TCTTCACGGACAGAAAAAGATTGCAGTCAGTTTGATAATGAT---
---CGAGTGACATTGCTTCTTCGGCCCGAACCGAGGAAT---CCCTTAGATATGATGCAAAA
GGGCTCTTGTTCTATCCTTGATCAGAGATTTCTCTATGAAAAATAT------GAATCGGAGTT
TGAAGAA---GGGGAGGGAGAAGGAGCCCTTGACCCGCAG---------------------GAGGAT----
--------TTATTCAATCAC------ATAGTTTGGGCTCCT------AGAATATGGCGCCCT---------TGG
GGCTTTCTATTTGATTGTATCGAAAGGCCCAATGAATTGGGATTTCCC---TATTGGTCCA
GGTCATTTCGGGGCAAG---------CGGATCATTTATGATGAAGAGGATGAGCTTCAAGAGA
ATGATTCGGGGTTCTTGCAGAGTGGAACCATGCAGTACCAGACACGAGATAGATCTTC
CAAAGAACAAGGCCTTTTTCGAATAAGCCAATTCATTTGGGACCCTGCAGATCCACTCT
TTTTCCTATTCAAA---GATCAGCCCCCTGGCTCTGTGTTTTCACATCGAGAATTATTTGC
AGATGAAGAGATGTCAAAGGGGCTTCTTACTTCCCAAACAGATCCTCCTACATCTATAT
ATAAACGCTGGTTTATC------AAGAATACGCAAGAAAAGCACTTCGAATTGTTGATTAAT
CGTCAGAGATGGCTTAGA---------ACCAATAGTTCATTATCT------AATGGATCTTTCCGTT
CTAAT------ACTCTATCCGAGAGTTATCAGTATTTATCAAATCTGTTCCTATCTAAC---GGA
ACGCTATTGGATCAAATGACAAAGACATTGTTGAGAAAAAGATGGCTTTTCCCGGATG
AAATGAAAATTGGATTC------ATG---------------------------ATGCCTAGATCACGG---ATAAAT
GGAAATTTTATTGATCAGACCTTTTCAATTGTAGCCAATATCTTATTACGAGTAATTCCG
ACAACTTCAGGAGAAAAAGAGGCATTTACCTATTACAGAGATGGG------ATGTCGGCTC
AATCCGAAGGAAATTACGCGGAAGCTTTGCAGAATTATTATGAAGCTATGCGACTAGAA
ATTGATCCCTATGATCGAAGTTATATACTCTATAATATAGGGCTTATCCACACAAGTAATG
GAGAACATACGAAAGCTTTGGAATATTATTTTCGAGCACTAGAACGAAACCCATTCTTA
CCACAAGCTTTTAATAATATGGCCGTGATCTGTCATTAC---CGGGGAGAACAGGCCATTC
AACAGGGAGATTCTGAAATTGCGGAGGCTTGGTTTGATCAAGCCGCTGAGTATTGGAA
ACAGGCTATAGCGCTTACTCCCGGTAATTATATTGAAGCGCATAATTGGTTGAAGATCAC
GAGACGTTTCGAA------ATGACTTGGCGATCAGAACATATATGGATAGAACTTATAACGG
GGTCTCGAAAAATAAGTAATTTCTGCTGGGCCTTTATCCTTTTTTTAGGTTCATTAGGCT
TCTTATTAGTTGGAACTTCCAGTTATCTTGGTAGAAATTTGATATCTTTTTTTCCGACTCA
GCAAATCGTTTTTTTTCCACAAGGAATCGTGATGTCTTTCTACGGAATTGCGGGTCTCT
TTATTAGCTCTTATTTGTGGTGCACAATTTCCTGGAATGTAGGTAGTGGTTATGATCGATT
CGATAGAAAGGAAGGAATAGTCTGTATTTTTCGTTGGGGATTTCCGGGAAAAAATCGTC
GCATATTCCTCCGATTCCTTATAAAAGATATTCAGTCCGTTAGAATAGAAGTTAAAGAGG
GTATTTCTGCTCGTCGTGTTCTTTATATGGACATCCGAGGCCAGGGGTCCATTCCCTTGA
CCCGTACTGATGAGAAT---TTGACTCCACGAGAAATTGAACAAAAGGCTGCTGAATTAG
CCTATTTCTTGCGTGTACCAATTGAAGTATTT
>Calycanthus
ATGGGAAAATGGTGGTTCAAT---------TCGCTAAGTAAATCAATGGACAGTCTTGGTCTTA
TTGGAAATACCAGTGGAAGTGAAGACCCCACTCTAAATGATACGAATAAAAACATTCCT
---TGCAATAATGTTGATCATTTTTTCAGTGTCAGGGACATTTGGAGTTTCATCTCTGATG
GCACTTTTTTAGTTAGGGATAATAATGGTAACTGTTATTCCGTATATTTGGATATTGAAAA
TCAGGTTTTTGAGATTGACAATGAT------AGTTCTTTTCTGAGTGAACTAGAAAGTTCTT
TTTCGAGTTATATGAAT---AGTTATCTGAATAGTGGGTCTAAGAGTGACAATCGCTACTAT
GATCGCTACACGTATGATATTAAATATAGTTGGAATAATCACATTAATAGTTGCATTGATA

GTTATCTTCGTTCTGAAATAAAC------ATTGATAGTTACATTTCAAGTGGTAGCGACAATT
ACCGTGACAGTTACATTTATAGTTACATTTGTAGTAAGAGTGGGAGTTCTATAAGAACT
GGCGGTAATAGCAGTGATTTCAATAGAAGAGGAAGATCT---GATTTCGATCTAAATAAA
AAATACAGACATTTATGGGTTCAATGCGAAAATTGTTATGGATTGAATTATAAAAAATTT
GTGAGTTTCAAAATGCATATTTGTGAACAATGTGGATATCATTTGAAAATGAGTAGTTC
AGAGAGAATAGAACTTTCGATTGATTCGGGTACTTGGGATCCTATGGATGAAGACATGG
TCTCTATGGACCCTATTCAATTTCATTCGGAAGAGGAGCCTTATAGAGATCGTATCGATT
CGTATCAAAGAAAGACAGGTTTAACTGAGGCTGTTCAAACGGGCATAGGTCAACTAAA
TGGTATTCCCGTAGCAATTGGGGTTATGGATTTTCAGTTTATGGGGGGTAGTATGGGATC
CGTAGTAGGTGAGAAAATCACCCGTTTGATCGAGTATGCTACTAATAGATCTCTGCCTG
TTATTATGGTGTGTGCTTCTGGAGGAGCGCGCATGCAAGAAGGAAGTTTGAGTTTGAT
GCAAATGGCTAAAATATCTTCCGCTTCATATGATTATCAATTAAATAAAAAGTTATTCTAT
GTATCAATCCTTACATCTCCTACAACCGGCGGAGTAACCGCCAGTTTTGGTATGTTGGG
AGATATCATTATTGCTGAACCCAATGCTTACATTGCGTTTGCGGGTAAAAGAGTAATTGA
ACAAACATTGAATAAGACAGTACCCGATGGTTCACAAGCGGCTGAGTATTCATTCCATA
AGGGCTTATTTGATCCAATCGTACCACGTAATCCTTTAAAAGGTGTTCTGAATGAGTTAT
TTCGGCTACACGGTTTCTTTCCCTTGAATCAAAATTCAAGTAGAATGGTAACCATTCGA
GCCGACGAAATTAGTAATATTATCCGTGAACGTATTGAACAATACAATAGAGAAGTCAA
GATTGTGAATACTGGCACAGTACTTCAAGTAGGCGACGGCATTGCTCGTATTCATGGTC
TTGATGAAGTAATGGCAGGGGAATTGGTAGAATTTGAAGAGGGTACAATAGGCATTGC
TCTTAATTTGGAATCAAATAATGTTGGCGTTGTATTAATGGGCGACGGTTTGACGATACA
AGAGGGAAGTTCTGTAAAAGCAACAGGAAGAATTGCTCAGATACCAGTTAGTGAGGC
TTATTTAGGTCGTGTTATAAATGCTCTGGCTAAACCTATTGATGGTAGAGGTGAAATTTC
GGCTTCGGAATCCCGGTTAATTGAATCTCCTGCTCCAGGTATTATTTCGAGACGTTCCGT
ATATGAGCCTCTTCAAACGGGACTTATTGCTATTGATTCGATGATCCCTATAGGACGCGG
TCAGCGAGAATTAATTATTGGGGACAGACAGACCGGGAAAGCAGCAGTAGCCACAGAT
ACTATTCTCAATCAAAAGGGGCAAAATGTGGTATGTGTTTATGTGGCTATTGGTCAAAA
AGCATCTTCCGTGGCCCAAGTAGTGACCACTTTCCAGGAACGAGGGGCAATGGAATAC
ACTATTGTGGTAGCCGAAACGGCGGATTCGCCTGCTACATTACAATACCTTGCTCCTTAT
ACAGGAGCGGCTCTGGCTGAATATTTTATGTACCGTGAACGACATACTTCAATAATTTAT
GATGATCTCTCCAAACAAGCACAAGCTTATCGTCAAATGTCTCTTCTATTAAGAAGACC
GCCCGGTCGCGAAGCTTATCCAGGAGATGTTTTTTATTTGCATTCACGCCTTTTGGAAA
GAGCCGCTAAATTAAGTTCTCCTTTAGGTGAAGGAAGTATGACTGCTTTACCAATAGTT
GAGACTCAATCTGGGGACGTTTCCGCTTATATTCCTACTAATGTTATTTCCATTACAGAT
GGACAAATATTCTTATCCGCCGATCTATTCAATGCCGGAATCCGTCCTGCCATTAATGTG
GGTATTTCTGTCTCGAGAGTAGGATCCGCGGCTCAAATTAAAGCCATGAAACAAGTAG
CTGGCAAATCAAAATTGGAACTGGCCCAATTCGCAGAGTTAGAGGCCTTTGCACAATT
TGCCTCCGATCTAGATAAAGCCACTCAGAATCAATTGGCAAGAGGTCAACGATTACGC
GAGTTGCTTAAACAATCCCAAGCAGCCCCTCTCACCGTGGAAGAACAGATAGTTACTA
TTTATACCGGAGCGAATGGGTATCTTGATCCGTTAGAAATTGGCCAGGTAAAGAAATTT
CTCGTTCAGTTACGTACCTACTTAAAAACAAATAAACCTCAGCTCCAAGAAATCATATC
TTCTACCAAGACATTCACCGAGGAAGTGGAAGCCCTTTTGAAGGAAGCTATTCCAGAA
CAGATAGAACTCTTTCTACTTCAGGAACAAACAATGAGAATCAATCCTACTACTTCTGG
TCCTGGGGTTTCCACACTTGAAGAAAAAAACCTGGGGCGTATTGCTCAAATCATTGGT

CCAGTACTGGATGTAGCTTTTCCCCCGGGCAAGATGCCTAATATTTACAACGCCTTGGT
AGTTAAGGGTCGAGATACTGTCGGTCAACAAATTAATGTGACTTGTGAAGTCCAACAA
TTATTAGGAAATAATCGAGTTAGAGCTGTAGCTATGAGTGCTACAGATGGTCTGATGAG
AGGAATGGAAGTGATTGACACAGGAGCTCCTCTAAGCGTTCCCGTCGGTGGAGCTACG
CTTGGACGAATTTTCAACGTTCTTGGAGAGCCTGTTGATAATTTAGGTCCTGTAGATAC
TCGCACAACATCTCCTATTCATAGATCTGCGCCTGCTTTTATCCAGTTAGATACCAAATTA
TCAATCTTTGAAACAGGGATTAAAGTGGTGGATCTTTTAGCTCCTTATCGTCGTGGAGG
AAAAATCGGACTATTCGGAGGAGCCGGGGTGGGTAAAACAGTACTCATCATGGAATTA
ATCAACAACATTGCCAAGGCTCATGGAGGTGTATCTGTATTTGGCGGAGTAGGCGAAC
GTACTCGTGAAGGAAATGATCTTTACATGGAAATGAAAGAATCTGGAGTGATTAATGAA
CAAAATATTGCAGAATCAAAAGTAGCTCTAGTCCATGGCCAGATGAATGAACCGCCGG
GAGCTCGTATGAGAGTTGGTTTGACTGCCCTAACCATGGCGGAATATTTCCGGGATGTT
AATGAACAAGACGTACTTCTATTTATCGACAATATCTTCCGTTTCGTCCAAGCAGGATC
AGAAGTATCCGCCTTATTAGGTAGAATGCCTTCCGCTGTGGGTTATCAACCTACCCTTAG
TACAGAAATGGGTTCTTTGCAAGAAAGAATTACTTCTACCAAAGAGGGGTCTATAACTT
CCATTCAAGCAGTTTATGTACCTGCGGACGATTTGACCGACCCTGCTCCTGCTACGACA
TTTGCACATTTAGATGCTACTACCGTACTATCAAGAGGATTAGCTGCCAAGGGTATCTAT
CCAGCAGTAGATCCTTTAGATTCAACGTCAACTATGCTCCAACCTAGGATCGTTGGTGA
GGAACATTATGAAATTGCGCAAAGAGTTAAGCAAACTTCACAACGTTACAAAGAACTT
CAGGACATTATAGCTATCCTTGGGTTGGACGAATTATCCGAAGAGGATCGTTTAACAGT
AGCAAGAGCACGAAAAATTGAGCGTTTCTTATCACAACCGTTCTTCGTAGCAGAAGTA
TTTACTGGTTCCCCAGGGAAATACGTTGGTCTTGCGGAAACAATTAGGGGGTTTCAACT
GATCCTTTCGGGAGAATTAGATGGTCTTCCCGAACAGGCCTTTTATTTGGTGGGTAACA
TCGATGAAGCTACCGCGAAGGCTATGAACTTAGAAGTAGAGAGCAAATTGAAGAAAAT
GACCCTAAATCTTTGTGTACTGACTCCTAATCGAATTATTTGGGATTCAGAAGTGAAAG
AAATCATTTTATCTACTAATAGTGGCCAAATTGGCGTATTACCCAATCACGCCCCTATTG
CCACAGCTGTAGATATAGGTATTTTGAGAATACGCCTCAACGGCCAATGGTTAATGATG
GCTGTAATGGGCGGTTTCGCTAGAATAGGCAATAACGAGATCACCATTTTAGTAAATGA
TGCAGAGAAGGGTAGTGACATTGATCCACAAGAAGCTCAGCGAACTCTTGAAATAGCT
GAAGCTAACTTGAGTAAAGCTGAAGGCAAGAGACAAGTCATTGAGGCGAATCTAGCT
CTTAGACGAGCTAGAACACGAGTAGAGGCTATCAATGTTATT---ATGAAAAATGTAACC
GATTCTTTCGTTTCCTTGGGTCACTGGCCATTCGCTGGGAGTTTCGGGTTTAATACCGAT
ATTTTAGCAACAAATCTAATAAATCTAAGTGTAGTGCTTGGTGTATTGATCTTTTTTGGA
AAGGGAGTGTTAAGTGATTTATTAGATAATCGAAAACAGAGGATCTGGAGTACTATTCG
AAATTCAGACGAACTACGTGAGGTAGCCATTGAGCAGCTCGAAAAAGCCCGGGCTCG
CTTACGGAAAGTGGAAAGAGAAGCAGATGAGTTTCGAGTGAATGGATACTCTGAGATA
GAACGAGAAAAATGGAATTTGATTAATGCCACTTATGAGAATTTGGAACGATTAGAAAA
TTACAAAAATGAAACCATTCATTTTGAACAACAAAGAGCAATTAATCAGGTCCGGCAA
CGAGTTTTCCAACAAGCCTTACAAGGAGCTCTAGGAACTCTGAATAGTCGTTCGAACA
GCGAGTTACATTTACGCACTATCAGCGCTAATATTGGCATGCTCGGGGCCATGAAAGAA
ATAACTGATATGAATCCACTGATTTCTGCCGCTTCCGTTATTGCTGCTGGATTGGCTGTA
GGGCTTGCTTCTATTGGGCCTGGAGTTGGTCAAGGTACTGCTGCAGGCCAAGCCGTAG
AAGGTATCGCAAGACAACCAGAAGCAGAGGGTAAAATACGAGGTACTTTATTGCTCAG
TCTGGCTTTTATGGAAGCTTTAACAATTTACGGACTGGTCGTGGCATTAGCGCTTTTATT

TGCGAATCCTTTTGTTATGAATGTTCTACCCTGTTCTATCAACACACTAAAAGGGTTATA
CGATATATCCGAAGTGGAAGTAGGTCAACATTTCTATTGGCAAATAGGGGGTTTCCAAG
TCCATGCCCAAGTACTTATCACTTCTTGGGTTGTAATTGCTATTTTATTAGGCTCGGCCG
CTATAGCTGTTCGTAATCCACAAACCATTCCAACCGACGGTCAGAATTTCTTCGAATAT
GTCCTTGAATTCATTCGAGACTTGACCAAAACTCAGATTGGAGAAGAATATGGTCCTTG
GGTTCCCTTTATTGGAACTATGTTCCTATTTATTTTTGTTTCTAACTGGTCGGGTGCTCTT
TTACCCCGGAAAATCATACAGTTACCGCATGGAGAGTTAGCCGCACCCACGAATGATAT
AAATACTACTGTTGCTTTAGCTTTACTTACGTCAGTGGCATATTTCTATGCGGGTCTTAC
CAAAAAAGGATTGGGTTATTTTGGTAAATACATTCAACCAACTCCAATACTTTTACCAAT
TAACATCCTAGAAGATTTCACAAAACCTTTATCACTTAGTTTTCGACTTTTCGGGAATAT
ATTAGCTGATGAATTAGTAGTTGTTGTTCTTGTTTCTTTAGTACCTTTGGTGGTTCCTATA
CCTGTCATGTTCCTTGGATTATTCACAAGCGGGATTCAAGCTCTTATTTTTGCAACTTTA
GCCGCGGCCTATATAGGTGAATCCATGGAAGGTCATCATATGATATTTTCAACTTTAGAA
CATATCTTAACTCATATCTCTTTTTCTATCATTTCAATTGTCATTACGATTCATTTGCTGAA
CTTATTAGTCCATGATACCGTAGGACTATGTGATTCGTCAGAAAAAGGCATGATGGCTA
CTTTTTTCTGTATAACAGGATTATTAGTTACTCGTTGGATTTATTCTAGGCATTTTCCGTT
AAGTGATTTATACGAATCCTTGATGTTCCTTTCATGGAGTTTCTCTATTATTCATATGGTT
CCATATTTTAGGAACCATAAAAATTCTTTAAGCGCAATAACCGCGCCAAGTGCTATTTTT
ACCCAAGGCTTTGCCACTTCAGGTCTTTTAACTGAAATGCATCAATCTGCAATATTAGT
ACCCGCTCTACAATCCCAGTGGTTAATGATGCACGTAAGTATGATGTTATTGGGTTATGC
ATCTCTTTTATGTGGATCGTTATTATCAGTAGCTCTTTTAGTCATTACATTTCGAAAAACC
TTAGGTATTCCTGGTAAAAACAATGAGATACGCTACTTCAATGAAAAAAGAAGTGTTTT
AAAAAAAACATCTTTTTTTTCATTTAGAAATTATCACAGGTATCAATTGACTCAGCGATT
GGATCATTGTAGTTATCGTATCATTAGTCTAGGGTTTACTTTTTCAACCATAGGTATTCTT
TCGGGAGCAGTATGGGCTAATGAGGCATGGGGGTCTTATTGGAATTGGGACCCCAAGG
AAACTTGGGCATTTATTACTTGGACCATATTCGCAATTTATTTACATACTAGAACAAATC
AGAGATTTCAAGATGTAGGTCCGGCAATTGTGGCTTCTATGGGATTTCTTATAATTTGGA
TATGCTATTTTGGGGTCAATCTATTAGGAATAGGACTACATAGTTATGGTTCATTCGCATT
AACAGTGCCAAAAAAA---GCATTGACTCCCTTCCCATATCTTGCATCTATAGTTTTTTTG
CCCTGGTGGATCTCTCTCTCATTTACCAAAAGTCTGGAACCTTGGGTTACTAATTGGTG
GAATACCGGGCAATCCAAAACTTTTTTGAATGATATTCAAGAGAAGAACGTTCTGGAA
AGATTCATAGAATTAGAACAACTATTCCTGTTGGACGAAATGATAAAAGAGTATCCCGA
GACACAGATACAAAAGTTTCATATAGGAATCCACAAAGAGACGCTCCAATTGGTCAAA
ATGCACAATGAAGATCATATCCATATCGTTTTGCATTTCTCGACAAATATAATCTGTTTTG
CTATTCTAAGTGGTTATTATTTTCTGGGTAATGAAGAACTTGTTATTCTTAATTCTTGGGT
TCAGGAATTCCTCTATAACTTAAGCGACACAATAAAGGCTTTTTCGATTCTTTTAGTAAC
TGATTTATGTATCGGATTCCACTCACCCCGTGGTTGGGAACTAATGATTGGTTCGGTCTA
CAAAGATTTTGGATTTGCTCATAACGATCAAATTATATCTGGTCTTGTTTCCACTTTTCC
AGTTATTCTAGATACAATTTTGAAATATTGGATCTTCCATTATTTAAATCGTGTATCCCCTT
CACTTGTAGTGATTTATCATTCAATGAATGAAATGCCCATTGGTGTTCCAAAAGTACCTT
TTCGGAGTCCTGGAGAGGAAGACGCGGTTTGGGTTGACGTATACAACCGACTTCATCG
AGAAAGATTACTTTTTTTAGGCCAAGAAGTTGATAGCGAGATCTCGAATCAACTTGTTG
GTCTCATGGTATATCTCACCATAGAGGATGATACCAAGGATCTTTATTTGTTTATAAACTC
TCCTGGCGGATGGGTAATCCCAGGAATAGCTATTTATGACACTATGCAATTTGTGTCACC

CGATGTGCATACAATATGCATGGGATTAGCCGCTTCAATGGGATCTTTCATCCTGGTCGG
AGGAGAAATTACTAAACGTCTAGCATTCCCTCACGCTAGGGTTATGATCCACCAACCTG
CTAGTTCTTTTTATGAGGCACCCACAGGAGAATTTATCCTGGAAGCGGAAGAACTACTG
AAACTCCGCGAAACCCTCACAAGGGTTTATGTACAAAGAACGGGCAATCCTTTGTGGG
TTGTATCCGAAGACATGGAAAGGGATGTTTTTATGTCAGCAACAGAAGCCCAAGCTCA
CGGCATTGTTGATCTTGTAGCGATCGAAATGAAAGAACAAAAATTGATTCATGAAGGTT
TAATTACTGAATCACTTCCCAACGGTATGTTCCGGGTTCGTCTAGATAATGAGGATCTGA
TTCTAGGTTATGTTTCGGGGAGGATCCGACGGAGTTTTATACGGATACTACCAGGAGAT
AGAGTCAAAATCGAAGTAAGTAGTTATGATTCAACTAGGGGCCGTATAATTTATAGACT
TCGCAACAAAGATTCGAATGATATGGAGGAATTACAAGGATATTTAGAAATAGATGGAT
TTCGGCAACACCACTTCCTATATCCGCTTCTACTTCAGGAATATATCTACGCACTTGCTC
ATGATCATGGTTTATTGAGTCATGACAAT---TCCAGTTCACTAATTGTGAAACGCTTAATT
ACTCGAATGCATCAACAGAATCATTTTATTATTTCGGTTAATGATTCTAACCAAAAAGGA
TTCGTTGGGAAGAATTTTCATTCTCAAAAGATATCGGAAGGTTTTGCCGTCATTGTGGA
AATTCCATTTTCACTGCAATTAGTATCTTCTCTAATAGCAAAATTTCAT---TCACGATCTAT
TCATTCAATATTTCCCTTTTTCGAGGACAAATTATCACATTTAAATCATGTGTCAGATATA
CTAATACCCTACCCCATCCATCTGGAAATCTTGGTTCAAACCCTTCGCTGCTGGATACA
GGATGCCCCCTCTTTGCATTTATTGCGATTCTTTCTCCATGAGTATTGGAATTCAAATAGT
CTCATTACTAAATCCATTTCCTTTTTTTCAAAACAAAGATTATTCTTGTTCCTATATAATT
CGCATGTATATGAATGCGAATCCGTATTCATTTTTCTCCGTAAACAATCTTCCCATTTACG
ATCTACATCTTTTGGATCTTTTCTTGAGCGAACACATTTCTATGGAAAAATAGAGCAT---
GTAGTAGTGCTTGGGAATCCGAAG---TTATGGTTGTTCAAAGATCCTTTCGTGCATTATG
TCAGATATCAAGGAAAATCCATTCTAGCTTCAAGGGGGACTCAGTTTCTGATAAAGAAA
TGGAAATATCACCTTGTCAACTTCTGGCAATGTCATTTTTACTTGTGGTCTCAACCAGA
CAGGATTCATCTAAACCAACTATGCAATCATTCCTTCTATTTTCTGGGTTATCTTTCAAGT
GTGCAACTAAACTCTTCGGTGGTAAGGAGTCAAATGCTAGAAAATGCATTTCTAATGGA
TACTGCTATT------AAGAAATTCGAGACCATAGTCCCAATTATTCCTCTGATTGGATCATT
GGCTAAAGCAAAATTTTGTAATGGATCAGGGCATCCCATTAGTAAGCCGTTTCGGACCG
ATTTGTCGGATTCTGAGATTATCAATCGATTTGGTCGGATATGCAAAAATCTTTCTCATTA
TCACAGCGGATCCTCAAAAAAACAGAGTTTGTATCGAATAAAGTTTATACTTCGACTTT
CGTGTGCTAGAACTTTGTCTCGTAAACATAAAAGTACGGTACGCGCTTTTTTGAAAAGA
TTAGGTTCGGAATTATTGGAAGAATTCCTTACGGAGGAAGAACAAGTTCTTTCTTTGCT
ATTCCCAAGACGGATTTGGTATTTGGATATTATCTGTATCAATGATCTAGCCAATCATGAA
ATGATAATTGATACGACA---GAAGTGCAA---GCTATCAATTCTTTTTCCAGATCGGAA---T
CCTTAAAAGAGGTCTATGACCTCTTATGGTTGCTTGTCCCTATTTTTACTCCTGTATTGG
GAATCACAATAGGTGTACTCGTGATTGTGTGGTTAGAAAGAGAAATATCCGCAGGGATA
CAACAACGTATTGGGCCTGAATATGCCGGCCCCTTGGGAATTCTTCAAGCTCTAGCAGA
TGGAACCAAACTACTTTTCAAAGAGGATCTTCTACCATCTAGAGGAGATATTCGTTTATT
CAGTATTGGGCCGTCTATAGCGGTTATATCAATTCTACTAAGTTATTCAGTAATTCCTTTT
GGCTATCGCCTTGTTCTAGCCGATCTCGGTATAGGCGTTTTTTTATGGATCGCTATTTCCA
GTATTGCTCCTATTGGACTTCTTATGTCAGGATATGGGTCGAATAATAAATATTCCTTTTC
AGGTGGTCTACGGGCTGCTGCTCAATCTATTAGTTATGAAATACCATTAACTCCGTGTGT
GTTATCAATATCTCTACTATCTAACAGTTCAAGTACAGTTGATATAGTTGAGGCACAGTC
AAAATATGGATTTTGGGGGTGGAATCTGTGGCGTCAACCTATAGGGTTTATAGTTTTTCT

AATTTCTTCCCTAGCAGAATGTGAGAGATTACCCTTTGATTTACCAGAAGCAGAGGAGG
AATTAGTAGCAGGTTATCAAACCGAATATTCAGGTATCAAATTTGGTTTATTTTACGTTG
CTTCTTATCTAAATCTACTAGTTTCTTCATTATTTGTAACGGTTCTTTACTTGGGTGGGTG
GAATCTTCCTATTCCGTACATATTCATTCCTGAACCTTTCGGAATAAATAAGACGGACGG
AGTCTTTGGAACGACAATTGATATCCTTATTACATTAGCTAAATCCTATTTGTTCCTGTTC
GTTCCTATCATAACAAGATGGACTTTACCTAGGATGAGAATGGACCAACTATTAAATCTT
GGATGGAAATTTCTTTTACCTATTTCTCTAGGTAATCTATTATTAATAACTTCTTTCCAAC
TCGTTTCGTTGATGATCTGGCATGTACAGAATGAAAACTTCATTCTCGATTCTACGAGA
ATTTTTATGAAAGCGTTTCATTTGCTTCTCTTCCATGGAAGTTTCATTTTCCCAGAATGTA
TCCTCATTTTTGGCCTAATTCTTCTTCTGATGATCGATTCAACCTCTGATCAAAAAGATAT
ACCTTGGTTATATTTCATCTCTTCAACAAGTTTAGTAATGAGCATAACGGCCCTATTGTT
CCGATGGAGAGAAGAACCTATGATTAGCTTTTCGGGAAATTTCCAAACGAACAATTTC
AACGAAATCTTTCAATTTCTTATTTTACTATGTTCAACTCTATGTATTCCTCTATCCGTAG
AGTACATTGAATGTACAGAAATGGCTATAACAGAGTTTCTGTTATTCGTATTAACAGCTA
CTCTAGGAGGAATGTTTTTATGTGGTGCTAACGATTTAATAACTATCTTTGTAGCTCCAG
AATCTTTCAGTTTATGCTCCTACCTATTATCTGGATATACCAAGAGAGATGTACGGTCTAA
TGAGGCTACTACGAAATATTTACTCATGGGTGGGGCAAGCTCTTCTATTCTGGTTCATGG
TTTCTCTTGGCTATATGGTTCATCCGGGGGAGAGATCGAGCTTCAAGAAATAGTGAATG
GTCTTATCAATACACAAATGTATAACTCCCCAGGAATTTCCATTGCGCTTATATCCATCAC
TGTAGGAATTGGGTTCAAGCTTTCCCCAGCCCCTTCTCATCAATGGACTCCTGACGTAT
ACGAAGGATCTCCCACTCCAGTCGTTGCTTTTCTTTCTGTTACTTCGAAAGTAGCTGCT
TCAGCTTCAGCCACTCGAATTTTCGATATTCCTTTTTATTTCTCATCAAACGAATGGCAT
CTTCTTCTGGAAATCCTAGCTATTCTTAGCATGATATTGGGGAATCTCATTGCTATTACTC
AAACAAGCATGAAACGTATGCTTGCATATTCGTCCATAGGTCAAATCGGATATGTAATTA
TTGGAATAATTGTTGGAGACTCAAATGATGGATATGCAAGCATGATAACTTATATGCTGT
TCTATATCTCCATGAATCTAGGAACTTTTGCTCGCATTGTCTCATTTGGTCTACGTACCG
GAACTGATAACATTCGAGATTATGCAGGATTATACACGAAAGATCCTTTTTTGGCTCTCT
CTTCAGCCCTATGTCTCTTATCCCTAGGAGGTCTTCCTCCACTAGCAGGTTTTTTCGGAA
AACTCCATCTATTCTGGTGTGGATGGCAGGCAGGCCTATATTTCTTGGTTTCAATAGGAC
TCCTTACGAGCGTTGTTTCTATCTACTATTATCTAAAAATAATCAAGTTATTAATGACTGG
ACGAAACCAAGAAATAACCCCTCACGTGCGAAATTATAGAAGATCCCTTTTAAGATCA
AACAATTCCATCGAATTGAGTATGATTGTATGTGTGATAGCATCTACTATACCAGGAATAT
CAATGAATCCAATTATTGCAATTGCTCAGGATACCCTCTTTATGTTTCTGCTTCACGAATA
TGATATTTTCTGGGCATTTCTAATAATATCAAGTGTCATTCCTATTTTGGCATTTCTAATTT
CCGGAGTTTTAGCCCCGATTCGTGAAGGACCAGAGAAGCTCTCTAGTTATGAATCAGG
TATAGAACCAATGGGGGATGCTTGGTTACAATTCCGAATCCGCTATTACATGTTTGCTCT
AGTTTTTGTTGTTTTTGATGTTGAAACGGTCTTTCTTTATCCATGGGCAATGAGTTTCGA
TGTACTGGGTGTATCTGTATTTATAGAAGCTTTAATTTTCGTGCTTATCCCAATTGTTGGT
TCAGTTTATGCATGGCGAAAGGGAGCGTTGGAATGGTCTACGAGTTATTTTCCTTGGTT
AACAGTAATTGTTGTTTTACCGATATCCGCGGGTTCATCAATTTTCTTTGTCCCTTATAGA
GGGAATAAAGTGATTCGATGGTATACTATTTGTATATGCTTATTAGAAATCCTTCTAACGA
CCTACGCATTCTGTTATCATTTCCAATCGGACGATCCATTAATCCAATTGGAGGAGGCTT
ATAAATGGATACATATTTTTGATTTTCATTGGAGACCGGGAATCGATGGACTTTCCATAG
GACCTATTTTACTGACGGGATTCATCACTACTTTAGCCACTTTAGCGGCTCGGCCAGTTA

CTAGAGATTCGCGATTGTTCCATTTCCTGATGTTAGCAATGTACAGTGGTCAAGTAGGA
TCATTTTCTTCTAGAGACCTTTTACTTTTTTTCCTCATGTGGGAGTTAGAATTAATTCCTG
TTTACCTACTTCTATCCATGTGGGGGGGGAAAAAACGTATGTACTCGGCTACAAAGTTT
ATTTTGTACACAGCGGGGGGTTCCATTTTTCTCTTAATGGGAGTTTCGGGCATGGGTTTA
TATGGCTCCAATGAACCAACATTAAATTTTGAAACATTAGCTAATCAATCGTATCCTTTG
GGATTGGAAATAATATTCTATTTTGGCTTCCTTATTGCTTATGCTGTCAAATCGCCGATTA
TACCCCTACATACATGGTTACCAGATACCCATGGAGAAGCACATTACAGTACATGTATGC
TTCTAGCCGGAATCTTATTAAAAATGGGAGCATACGGATTAGTTCGGATCAATATGGAAT
TATTACCCCATGCTCATTCTATATTTTCTCCCTGGTTGGTCATAGTGGGAGCAATTCAAAT
AATCTATGCAGCTTCAACTTCTTTCGGTCAACGCAATTTCAAAAAGAGAATAGCCTATT
CCTCTGTATCTCATATGGGTTTCACACTTATAGGGATTGGTTCCATAACTGATACGGGAC
TTAATGGGGCCATTTTACAAATAATCTCTCATGGATTTATTGGTGCTGCACTTTTTTTCTT
GGCAGGAACAAGTTACGATAGAATACGTCTTGTTTATCTCGACGAAATGGGAGGAATA
GCTATCCCAATGCCAAAAATATTTACCATGTTCAGTAGCTTCTCGATGGCTTCTCTTGCA
TTGCCGGGAATGAGTGGTTTTGTTGCGGAATTGGTAGTATTTTTTGGAATAATTACCAG
CTCGAAATATCTTTTAATGCCAAAAATAGTAATTTCTTTTGTAATGGCAATTGGAATGATA
TTAACTCCTATTTATTCATTATCTATGTTACGTCGGATGTTCTATGGATATAAGCTATTCAA
CGTCCCAGACTTTTATTTTTTGGATTCTGGACCACGAGAACTATTTGTTTCGATCTGTAT
CTTTCTACCTGTAGTAGGGATTGGTATTTATCCCGATTTCGTTCTCTCCCTATCAGTTGAT
AGGGTAGAGGCTATTCTATCTAATTATTTTCATAAAATGATGACCGAGTATGTACTTATTT
TGAGTGCCTATTTATTTTCGATCGGTATTTATGGACTGATCACAAGCCGAAACATGGTTA
GAGCACTTATGTGTCTTGAGCTTATACTGAATGCGGTTAATATGAATCTCGTAACGTTTT
CTGATTTATTTGATAGTCGCCAATTAAAAGGAGATATTTTCTCGATTTTTGTTATAGCTAT
TGCAGCCGCTGAAGCAGCTATTGGGCCGGCTATTGTTTCATCGATCCATCGTAACCGAA
AATCAACTCGTATCAATCAATCAAATTTGTTGAATAAAATGGAACATACATATCAACATG
CATGGATCATACCTTTCGCTCCACTTCCATTTACTATGTCAATAGGATTGGGACTTCTGC
TTGTTCCGACCGCAACAAAAAATCTTCGTCGTATGTGGACTTTTCCTAGTGTTTCATTG
CTAAGTATAGCAATGGTTTTTTCGGTCAATCTGTCTATTCAACAGATAAATGGCAGTTCT
ATCTATCAACATCTATGGTCTTGGACTATCAATAATGATTTTTCTTTCGAGTTCGGCCACT
TGATCGACCCGCTTACTTCTATTATGTCAATACTAATCACTACTGTTGGAATCATGGTTCT
TATTTATAGTGACAATTATATGTCTCATGATCAAGGATATTTGCGATTTTTTGCTTATATGA
GTTTTTCCAATACTTCAATGTTGGGATTAGTTACTAGTTCCAATTTGATACAAATTTATAT
TTTTTGGGAACTAGTGGGAATGTGTTCGTATTTATTAATAGGTTTTTGGTTCACACGACC
CACTGCAGCAAATGCTTGTCAAAAAGCGTTTGTAACTAATCGTGTAGGCGATTTCGGGT
TATTATTAGGAATTTTGGGTTTTTATTGGATAACTGGTAGTTTCGAATTTCGGGATTTGTT
CGAAATCTTTACTAACAATGGAGTCAATTCTTTATTTGCTACTCTGTGTGCCTTCCTATTA
TTCGTCGGTGCAGTTGCTAAATCCGCACAATTTCCTCTTCATGTATGGTTACCCGATGCC
ATGGAGGGACCTACTCCTATTTCGGCTCTTATACATGCTGCTACTATGGTAGCAGCAGGA
ATTTTTCTTGTCGCTCGGCTTCTTCCGCTTTTCACAGTTATACCTTACATAATGAATCTCA
TTTCTTTGATAGGTGTAATAACATTACTATTAGGAGCTACTTTAGCTCTTGCTCAAAGAG
ACATTAAGAGAAGTTTAGCCTATTCTACAATGTCTCAACTGGGCTATATTATGCTAGCCC
CAGGGATAGGTTCTTATCGAGCTGCTTTATTCCATTTGATTACTCATGCCTATTCGAAAG
CATTATTGTTTTTAGGATCCGGATCCATTATTCATTCAATGGAACCCATTGTTGGCTATTC
TCCAGATAAAAGTCAGAACGTGGTTCTTATGGGTGGTTTAACAAAATATCTGCCAATTA

CAAAAACTACTTTTTTATTAGGTACACTTTCTCTTTGTGGTATTCCACCTCTTGCTTGTTT
TTGGTCTAAAGATGAAATTCTTAATGATAGTTGGTTGTATTCACCAATTTTCGCGATAAT
AGCTTGCTCTACAGCAGGGTTAACTGCATTTTATATGGTTCGGGTGTATTTACTTACTTT
TGATGGGCATTTACACGTTCATTTTCAAAATTACAGTGGGACTCGAAATAGCTCATTTTA
TTCAATATCTATATGGGGGAAAGAAGGAACAAAATTCGTTAATGGA---AATTTGCCTTTA
TCAACACTGAAAAAAAATTTCCAATAAAGAA------ACTTTTGTGTATCCCCATGAATCGGA
CAATACAATGCTATTGCCTCTGCTTGGATTGGTCCTATTTACTTTGTTTGTTGGATCCGTA
GGAATTCCTTTCGAATTTGATATATTATCGAAATGGTTAAATCCATCAATAAACCTTTTAC
ATCCAAATTCGAACGTTTCTGTGGATTGGTATGAATTTGGAACAAATGCAATTTATTCAG
TTAGTATAGCCTGTTTCGGAATATTCATAGCATCTCTTTTCTATGGATCTATTTATTCATCT
TTTCAGAATTTGGATTTCATCAATTCATTTGTTATTTTCTTGGATAGAATAATAAATGTGA
TATACAATTGGTCATATAATCGGGGTTACATAGATGTTTTTTATACAACATGTTTTACTAA
AGGTATAAGAGGATTGGCCGAACTAACTTATCTTTTGGATAGACGACTAATTGACGGAA
TTGCTAATGGTATTGGTATGGCAAGTTTTTTTGTAGGAGAAGGGATCAAATAT---GTGGG
GGGGGGGCGAATATCATCTTATCTCTTCGTGTATTTATCTTATGTATCAATCTTGTTATTAA
TTTACTATATGGATTTACCTGGACCAATACATGATATTCTTTTAGTATTTCTAGGATTAGGT
CTTATATTGGGAGGTCTGGGAGTGGTATTACTTACCAATCCAATTTATTCTGCCTTTTCGT
TGGGATTGGTTCTTGTTTGTATATCCTTATTTCATATTCCATCTAACTCCTATTTTGTAGCT
GCTGCACAGCTCCTTATTTATGTGGGAGCGGTAAATGTTTTAATCGTATTTGCTGTGATG
TTCATGAATGGTTCAGAATATTCCAACGATTTCCATCTTTGGACCGTTGGAGATGGAGT
CACCTCATTGGTTTGTACAAGTATTCTTTTTTCACTAATTACTACTATTTGGAATACGTCA
TGGTACGGGATTATTTGGACTACAAGATCAAACCAGATTATAGAGCAGGACCTAACAA
GTAACGTTCAACAAATTGGTATTCGTTTAGCAACTGATTTTTATCTTCCATTTGAACTCA
TTTCTATAATTCTTTTAGCTGCTTTGATAGGTGCAATTGCTATGGCTCGTCAGATGAATGT
ACCGGCTACAAGAAAAGACCTCATGATAGTCAATATGGGTCCTCACCACCCCTCAATGC
ATGGTGTTCTTCGACTCATCGTTACTCTAGATGGTGAAGATGTTATTGACTGCGAACCT
GTATTGGGTTATTTACACAGAGGAATGGAAAAAATTGCAGAAAACCGAACAATTATAC
AATATCTGCCTTATGTAACACGTTGGGATTATTTAGCCACTATGTTCACAGAAGCAATAA
CAGTAAATGCACCAGAACAATTAGGAAATATTCAAGTACCTAAAAGAGCTAGCTATATC
AGAGTAATCATGCTGGAGCTGAGTCGTATAGCTTCTCATTTGTTATGGCTTGGACCTTTT
ATGGCGGATATCGGTGCACAGACTCCTTTCTTCTATATTTTCAGAGAAAGGGAATTGCTA
TATGATCTATTTGAAGCTGCCACAGGTATGCGAATGATGCATAATTATTTCCGTATCGGA
GGAGTAGCTGCTGATCTACCTCATGGCTGGATAGATAAATGTTTAGATTTCTGCGATTAT
TTTTTAACGGGAGTTGTTGAATATCAAAAGCTTATTACGCGGAATCCCATTTTTTTGGAA
CGAGTTGAAGGGGTGGGCATTATTGGTGGGGAGGAAGCAATAAATTGGGGTTTATCGG
GACCAATGCTACGAGCTTCCGGAATCCAATGGGATCTTCGTAAAGTGGATCATTATGAG
TGTTACGACGAATTCGATTGGCAAGTGCAATGGCAAAAAGAAGGAGACTCATTAGCTC
GTTATTTAGTACGAATCAGTGAAATGACAGAATCTATAAAAATTCTTCAACAAGCTCTG
GAAGGAATTCCGGGAGGGCCCTATGAGAATTTAGAAGGTCGACGCTTTGATAGAGAAA
GTGATTCCGAATGGAATGATTTTGACTATCGATTCATTAGTAAAAAGCCTTCTCCCACTT
TTGAATTGTCGAAACAAGAACTTTATGTGAGAGTAGAAGCTCCAAAGGGAGAATTGGG
AATTTTTCTGATAGGAGATAATAGTGTTTTTCCCTGGAGATGGAAAATTCGTCCGCCAG
GTTTCATCAATTTGCAAATTCTTCCTCACCTAGTTAAAAGAATGAAATTGGCTGATATCA
TGACGATACTAGGTAGTATAGATATCATTATGGGGGAAGTTGATCGTATGTTCCCTATGGT

AACTGGGTTCATGAATTATGGTCAACAAACGGTACGAGCCGCAAGGTACATTGGTCAA
AGTTTCATGATTACTTTATCTCACGCGAATCGTTTACCTGTAACTATTCAATATCCTTATG
AAAAATCGATCACATCAGAGCGTTTTCGTGGTAGAATCCACTTTGAATTTGATAAATGC
ATTGCTTGTGAAGTATGTGTTCGGGTATGCCCCATAGATTTACCCGTTGTTCATTGGAGA
TTGGAAACAGATATTAGAAAGAAACGATTGCTTAATTATAGTATTGATTTCGGAATCTGT
ATATTTTGTGGGAACTGTGTCGAGTATTGTCCAACAAACTGTTTATCAATGACTGAAGA
ATATGAACTTTCTGCTTATAATCGTCACGAATTGAATTATAATCAAATTGCTTTGGGTCG
GTTACCGATGTCAGTAATTGAAGATTACACAATTCGAACAACTAGGAATTCTAAAATAG
CCATGGATAAACCTCTTAATGCAAGAACGGTTACAAATTTCATGCAGGGTCGTTTATCT
GCTTGGCTAGTCAAGCACGGGCTAGTTCATAGATCTTTGGGCTTCGATTACCAAGGAAT
AGAGACTTTACAAATAAAGCCCGAGGATTGGTACTCCATTGCTGTCATTTCATATGTATA
TGGTTATAATTATCTACGTTTCCAGTGTGCCTATGATGTAGCGCCTGGCGGATTGTTAGC
TAGTGTGTATTATCTTACAAGAATACAGTATGGTGTGGATCAACCCGAAGAGGTCTGCA
TAAAAGTATTTGCCCCAAGGAGGAATCCTAAAATCCCGTCTGTTTTCTGGATTTGGAAA
AGTGCGGATTTTCAAGAACGAGAATCTTATGATATGTTGGGGATCTCTTATGAAAATCAT
CCACGCCTGAAACGTATCTTGATGCCCGAAAGTTGGATAGGATGGCCTCTACGTAAGGA
TTATATTGCCCCCAATTTCTATGAAATACAAGATGCTCAT---

---------------------ATGAATTCGACTGAGTTTCCATTACTTGACCGAACAACCCAAAATTCC
GTTATTTCAACTACATTGAATGATCTTTCGAATTGGTCAAGACTTTCTAGTTTATGGCCG
CTTCTTTATGGTACGAGTTGTTGCTTCATTGAGTTTGCTTCATTAATAGGCTCGCGATTC
GATTTTGATCGTTATGGACTAGTACCAAGATCGAGTCCTAGGCAAGCGGACCTAATTTT
AACAGCTGGCACAGTAACAATGAAAATGGCCCCTTCTTTAGTGAGATTATATGAGCAAA
TGCCTGAACCAAAATATGTCATTGCTATGGGGGCCTGTACTATTACAGGAGGGATGTTC
AGTACCGATTCTTATAGTACCGTTCGAGGAGTCGATAAGCTAATTCCTGTGGATGTCTAT
TTGCCGGGCTGCCCACCTAAACCAGAGGCAGTTATAGATGCTATAACGAAACTTCGTAA
GAAAGTATCTCGAGAAATCTATCAAGATAGAATTGGGTCTCAACAGGAAAATAGATGTT
TTACTACCAATCACAAGTTTCATATTGAACGCAGTACTCATACTGGAAATTACGGTCAA
GGATTACTCTACCAATCTCCA---ACTTCAGAAATA---TCTGAAACATTTTTCAAATACAA
AAGTTCAGTATCTTCCCACGAATTAATGAATATGCAAAATAGAAATACCTTTTCTTGGGT
AAAGGAAGAGATGACTCGATTCATTTCCGTATCGATCATGATATATGTAATAACTCGGAC
ATCTATTTCAAACGCATATCCCATTTTTGCGCAGCAGGGTTATGAAAATCCACGAGAAG
CAACTGGGCGTATTGTATGTGCCAATTGCCATTTAGCTAATAAGCCCGTGGATATTGAGG
TTCCACAAGCCGTGCTTCCTGATACTGTATTTGAAGCAGTTGTTCGAATCCCTTATGATA
TGCAACTGAAACAAGTTCTTGCTAATGGTAAAAAGGGGGCTTTGAATGTGGGGGCTGT
CCTTATTTTACCCGAGGGATTCGAATTAGCTCCCCCCGATCGTATTTCTCCCGAGCTGAA
AGAAAAGATGGGCAATCTGTCTTTTCAGAGTTATCGCCCCAATAAAAGAAATATTCTTG
TGGTAGGTCCTGTTCCTGGTCAGAAATATAGTGAAATTGTCTTTCCCATTCTTTCCCCGG
ACCCTGCTACTAAGAAAGACGTTCACTTCTTAAAATATCCCATATATGTAGGCGGGAAC
AGAGGAAGGGGTCAGATTTATCCTGATGGGAGCAAGAGTAACAATACAGTCTATAATG
CTACAGCAGCAGGTATAGTAAGCAGAATAGTACGTAAAGAAAAGGGGGGATATGAAAT
AAGCATAGCCGATGCATCGGATGGACGCCAAGTGGTTGATATTATACCTCCAGGACCAG
AACTTCTTGTTTCAGAGGGGGAATCCATCAAGCTTGATCAGCCATTAACAAGTAATCCT
AACGTGGGTGGATTTGGTCAGGGAGATGCAGAAATAGTACTTCAAGATCCATTACGTG

TCCAAGGTTTGTTGTTTTTCTTGGCATCTGTTATTCTAGCACAAATCTTTTTGGTTCTTA
AAAAGAAACAGTTTGAGAAGGTTCAATTGTCCGAAATGAATTTCATGAGTAAAGTATAT
GATTGGTTCGAGGAGCGTCTCGAGATTCAGGCGATTGCAGATGATATAACTAGTAAATA
TGTTCCTCCTCATGTTAACATATTTCATTGTCTAGGAGGGATCACACTTACTTGTTTTTA
GTACAAGTAGCTACGGGTTTTGCTATGACTTTTTACTACCGTCCGACTGTTACAGAGGC
TTTTGCCTCTGTTCAATACATAATGACTGAAGCCAACTTTGGTTGGTTAATCCGATCGGT
TCATCGATGGTCAGCAAGTATGATGGTCCTAATGATGATCCTACACGTATTTCGTGTGTA
TCTCACAGGTGGATTTAAAAAACCTCGCGAATTGACTTGGGTTACGGGTGTAGTTCTG
GCTGTATTGACTGCATCTTTTGGTGTAACTGGTTATTCCTTACCTTGGGACCAAATTGGT
TATTGGGCGGTAAAAATCGTGACAGGTGTACCTGAAGCTATTCCTGTAATAGGATCACC
CTTGGTAGAGTTATTACGTGGAAGTGCTAGTGTGGGCCAATCCACTTTGACCCGTTTTT
ATAGTTTACACACTTTTGTATTACCTCTTCTTACTGCCGTATTTATGTTAATGCACTTCCC
AATGATACGTAAGCAAGGTATTTCAGGTCCTTTAATGGGAGTAACAAAGAAACCTGAC
TTAAATGATCCTGTATTAAGAGCTAAATTGGCTAAAGGAATGGGGCATAATTATTACGGA
GAACCCGCATGGCCCAATGATCTTTTATATATTTCTCCAGTAGTCATTTTAGGTACTATTG
CATGTAACGTAGGCCTAGCGGTTCTAGAGCCGTCAATGATTGGCGAACCAGCGGATCC
ATTTGCAACTCCTTTGGAAATATTACCTGAATGGTACTTCTTTCCCGTATTTCAAATTCTC
CGTACAGTACCTAATAAGTTATTGGGTGTTCTCTTAATGGTTTTAGTACCAACGGGATTA
TTGACAGTACCATTTTTGGAGAATGTTAATAAATTCCAAAATCCATTTCGTCGTCCAGTA
GCTACAACCGTCTTTTTGATTGGTACCGCGGTAGCCCTTTGGTTAGGTATTGGAGCAAC
ATTACCTATTGATAAATCTTTAACTTTAGGCCTTTTTATGATTGAAGTTTTTCTATTTGGA
ATCGTGTTAGGCCTAATTCCTATTACTTTGGCCGGATTATTCGTAACTGCATATTTACAAT
ACAGACGCGGTGATCAGTTGGACCTTATGCCTACTATAACTAGTTATTTCGGTTTTCTAC
TGGCTGCTTCAACTATAACCCCAGCTCTATTGATTGGTCTGAACAAGATACGACTTATTA
TGGATATAGTAAGTCTCGCTTGGGCTGCTTTAATGGTAGTTTTTACATTTTCCCTTTCACT
CGTAGTATGGGGAAGGAGTGGACTCATGATTATTCGTTCGCCGGAATCAGAAGTGAAG
ATTATGGTGGATAGGGATCCCATAAAAACTTCTTTCGAGGAATGGGCCAGACCCGGCCA
TTTCTCAAGAACAATAGCTAAGGGCCCTGATACTACTACTTGGATCTGGAACCTTCATG
CTGATGCTCACGATTTTGATAGCCATACCAGTGATTTGGAGGAGATCTCTCGAAAAGTA
TTTAGTGCTCATTTCGGTCAACTCTCCATCATCTTTCTTTGGCTGAGTGGCATGTACTTC
CATGGCGCCCGTTTTTCCAATTATGAAGCATGGCTAAGCGATCCTACTCACATTGGACCT
AGCGCCCAGGTGGTTTGGCCAATAGTGGGTCAAGAAATATTGAATGGTGATGTGGGTG
GGGGTTTCCGAGGAATACAAATAACCTCTGGTTTTTTTCAGCTTTGGCGAGCATCTGGA
ATAACTAATGAATTACAACTCTATTGTACCGCAATTGGTGCATTGGTCTTTGCAGCGTTA
ATGCTTTTTGCCGGTTGGTTCCATTATCACAAAGCTGCTCCAAAATTGGCTTGGTTCCA
AGATGTGGAATCTATGTTGAATCACCACTTAGCAGGGTTACTGGGGCTTGGGTCTCTTT
CTTGGGCGGGACACCAAGTCCATGTATCTTTACCGATTAACCAATTTCTAGACGCTGGA
GTGGATCCTAAAGAGATACCACTTCCTCATGAATTTATCTTGAATCGGGATCTTTTGGCT
CAACTTTATCCCAGTTTTGCCGAGGGAGCAACCCCATTTTTCACCTTGAATTGGTCAAA
ATACGCGGACTTTCTTAGTTTTCGTGGGGGATTAGATCCAGTAACGGGTGGCCTATGGC
TGACCGATATTGCACACCATCATTTAGCTATTGCAATTCTTTTCCTGGTAGCTGGTCACA
TGTATAAGACCAACTGGGGCATTGGTCATAGTCTGAAAGATATTTTAGAGGCGCATAAA
GGCCCATTTACAGGCCAGGGCCATAAAGGCCTCTATGAGATCCTAACAACGTCATGGCA
TGCTCAATTATCTCTTAACCTAGCTATGTTAGGCTCTTCAACCATTGTTGTAGCTCACCAT

ATGTATTCCATGCCCCCTTATCCATACCTAGCTATTGACTATGGTACACAACTTTCATTGT
TCACACATCATATGTGGATCGGTGGATTTCTCATAGTTGGTGCTGCTGCACATGCAGCCA
TTTTTATGGTAAGAGACTATGATCCAACTACTCGATACAACGATCTATTAGATCGTGTCC
TTAGACACCGGGATGCAATCATATCACACCTGAACTGGGCATGTATATTTCTAGGCTTTC
ACAGTTTTGGCTTGTATATTCATAATGATACCATGAGCGCTTTAGGGCGTCCCCAAGATA
TGTTTTCAGATACCGCTATCCAATTACAACCCATCTTTGCTCAATGGGTACAAAACACC
CATGCTTTAGCACCTAGTGCAACGGCCCCTGGTGCAACAGCAAGCACCAGTTTGACTT
GGGGGGGTGGTGATTTAGTAGCAGTAGGCGGCAAAGTCGCTTTGTTACCTATTCCATTA
GGAACCGCAGATTTTTTGGTACATCACATTCATGCATTTACGATCCATGTGACCGTATTG
ATACTATTGAAAGGTGTTCTATTTGCTCGTAGCTCACGTTTGATACCTGATAAAGCAAAT
CTTGGTTTTCGTTTCCCTTGTGATGGACCTGGAAGAGGGGGGACATGTCAAGTATCTGC
CTGGGATCATGTCTTCTTAGGTCTATTCTGGATGTACAATGCAATTTCTGTAGTCATATTC
CATTTCAGTTGGAAAATGCAGTCAGATGTTTGGGGTAGTATAAGTGATCAAGGAGTGGT
AACTCATATCACAGGAGGAAATTTTGCACAGAGTTCCATTACTATTAATGGATGGCTTC
GGGATTTTTTATGGGCACAGGCATCTCAGGTAATTCAGTCTTATGGTTCTTCATTATCGG
CATATGGCCTTTTTTTCCTAGGTGCTCATTTTGTCTGGGCTTTCAGTTTAATGTTTCTATT
CAGCGGCCGTGGTTATTGGCAAGAACTCATTGAATCCATCGTTTGGGCTCATAACAAAT
TAAAAGTTGCTCCTGCTACTCAGCCTAGAGCCTTGAGCATTGTACAAGGACGTGCTGTA
GGAGTAACCCATTACCTTCTGGGTGGAATTGCCACAACATGGGCATTCTTCTTAGCAAG
AATTATTGCAGTAGGAATGGCATTAAGATTTCCAAGGTTTAGCCAAGGCTTAGCTCAGG
ACCCCACTACTCGTCGTATTTGGTTTGGTATTGCTACCGCACATGATTTCGAGAGTCATG
ATGATATTACTGAGGAACGTCTTTATCAGAACATTTTTGCTTCTCACTTCGGGCAGTTAG
CAATAATCTTTCTGTGGACTTCCGGAAATCTGTTTCATGTAGCTTGGCAAGGAAATTTT
GAGTCATGGGTACAGGACCCTTTACATGTAAGACCTATTGCTCATGCAATTTGGGATCC
TCATTTTGGCCAACCAGCTGTAGAAGCCTTTACTCGAGGAGGTGCTCTCGGCCCGGTG
AATATCGCTTATTCCGGTGTTTATCAGTGGTGGTATACAATCGGCTTACGCACCAATGAA
GATCTTTATACTGGAGCTCTTTTTCTATTATTTCTTTCTGCCATATCCTTAATTGCGGGTTG
GTTACACCTACAACCCAAATGGAAACCAAGCGTTTCGTGGTTCAAAAATGCCGAATCT
CGTCTCAATCATCATTTGTCAGGACTCTTCGGAGTAAGTTCCTTAGCTTGGACAGGGCA
TTTAGTTCATGTCGCTATTCCCGGAGCAAGGGGGGAGTATGTCAGATGGAATAATTTCTT
AGATGTATTACCACATCCCCAAGGGTTGGCCCCACTTTTTACAGGTCAGTGGAATCTTT
ATGCCCAAAACCCCGATTCCAGTAGTCATTTATTTGGTACCTCCCAAGGGGCAGGAACT
GCCATTCTAACTCTTCTCGGGGGATTCCATCCACAAACGCAAAGTTTATGGCTGACCGA
TATTGCTCATCATCATTTAGCTATTGCATTTATTTTTCTCGTTGCTGGTCATATGTATAGAA
CTAACTTCGGGATTGGGCACAGTATGAAAGATCTTTTAGAAGCACATATTCCCCCAGGG
GGTCGATTGGGACGTGGGCATAAGGGTCTTTATGACACAATCAATAATTCGATTCACTT
TCAATTAGGCCTTGCTCTAGCCTCTTTAGGGGTTATTACTTCCTTGGTAGCTCAACACAT
GTACTCTTTACCTGCTTATGCATTCATAGCACAAGACTTTACTACTCAAGCTGCGTTATAT
ACCCATCACCAATACATTGCAGGGTTCATCATGACAGGAGCCTTTGCTCATGGAGCTAT
ATTCTTCATTCGAGATTACAATCCGGAGCAGAATGAGGATAATGTATTGGCAAGAATGT
TAGACCATAAAGAAGCTATCATATCTCATTTAAGTTGGGCCAGCCTGTTTCTGGGGTTCC
ATACCTTAGGACTCTATGTTCATAACGACGTCATGCTCGCTTTTGGTACTCCGGAGAAA
CAAATCTTGATCGAACCCATATTTGCCCAATGGATACAATCCGCTCATGGTAAGACTTCA
TATGGGTTCGATGTACTTTTATCTTCAACGAATGGACCGGCATTCAATGCTGGTCGAAGT

ATATGGTTACCGGGCTGGTTGAACGCCGTTAATGAG------AATAGTAATTCATTATTCTTA
ACAATAGGTCCTGGAGACTTCTTGGTTCATCATGCTATTGCTCTAGGTTTGCATACAACT
ACATTGATCTTAGTAAAGGGTGCTTTAGATGCACGTGGTTCTAAGTTAATGCCAGATAA
AAAAGATTTCGGTTATAGTTTTCCTTGCGACGGTCCTGGACGAGGTGGTACTTGTGATA
TTTCGGCCTGGGACGCATTTTATTTGGCCGTTTTCTGGATGTTAAATACTATTGGATGGG
TTACTTTTTATTGGCATTGGAAGCACATCACATTATGGCAGGGTAACGTTTCACAATTTA
ACGAATCCTCCACTTATTTGATGGGATGGTTAAGAGATTATCTATGGTTAAACTCTTCAC
AACTTATCAATGGATATAACCCTTTTGGTACGAATAGTTTATCGGTCTGGGCGTGGATGT
TCCTTTTTGGGCATCTTGTTTGGGCTACTGGATTTATGTTCTTAATTTCCTGGCGTGGATA
TTGGCAGGAATTGATTGAAACTTTAGCATGGGCTCATGAACGCACGCCTTTGGCTAATT
TGATTCGATGGAGGGATAAACCAGTGGCTCTTTCCATTGTGCAAGCAAGATTGGTTGGA
TTAGCTCACTTTTCTGTAGGTTATATATTCACTTATGCAGCTTTCCTGATTGCCTCTACAT
CAGGCAAATTTGGTATGTCACATTCAGTAAAGATTTATGATACATGTATAGGGTGTACTC
AATGTGTACGAGCCTGCCCCACAGATGTATTGGAAATGATACCTTGGGACGGATGTAAA
GCTAAGCAAATTGCTTCTGCTCCAAGAACAGAGGACTGTGTGGGTTGTAAGAGATGTG
AATCTGCTTGTCCAACGGATTTCTTGAGTGTTCGGGTTTATTTATGGCATGAGACAACC
CGAAGCATGGGTCTAGCTTATATGACAGATCTCAATTTACCCTCTATTTTTGTGCCTTTA
GTGGGCCTAATGTTTCCAGCAATTGCAATGGCTTCTTTATCTCTTCATATTCAAAAAAAT
AAGATTATTATGCGAGATATAAAAACATATCTCTCCACGGCACCTGTGCTAGCTACTCTA
TGGTTCGGGTCTTTAGCAGGTCTATTGATAGAGATCAATCGTTTATTCCCAGATGCGTTG
ACATTCCCTTTTTTTTCATTCATGACTGCAATTTTAGAGAGACGCGAAAGCACAAGCCT
ATGGGGTCGCTTCTGTAACTGGATAACCAGCACTGAAAACCGTCTTTATATTGGATGGT
TCGGTGTTTTGATGATCCCTACCTTATTGACCGCAACTTCTGTATTTATTATTGCCTTCAT
TGCTGCTCCTCCAGTAGATATTGATGGTATTCGTGAACCTGTTTCTGGATCTCTACTTTAT
GGAAACAATATTATCTCTGGTGCCATTATTCCTACTTCTGCAGCTATAGGTTTGCATTTTT
ACCCGATCTGGGAAGCAGCATCCGTTGATGAATGGTTATACAACGGTGGTCCTTATGAG
CTAATTGTTCTACACTTTTTACTTGGTGTAGCTTGTTACATGGGTCGTGAGTGGGAACTT
AGTTTCCGTCTGGGTATGCGTCCTTGGATTGCTGTTGCATATTCAGCTCCTGTTGCAGCA
GCTACTGCTGTTTTCTTGATCTACCCTATCGGTCAAGGAAGTTTTTCTGATGGTATGCCT
CTAGGAATATCTGGTACTTTCAACTTTATGATTGTATTCCAGGCTGAGCACAACATCCTT
ATGCATCCATTTCACATGTTAGGCGTAGCTGGTGTATTCGGCGGCTCCCTATTCAGTGCT
ATGCATGGTTCCTTGGTAACCTCTAGTTTAATCAGGGAAACCACTGAAAATGAATCTGC
TAATGCAGGTTACAGATTCGGTCAAGAGGAAGAAACTTACAATATAGTAGCCGCTCATG
GTTATTTTGGCCGATTGATCTTCCAATATGCTAGTTTCAACAACTCTCGTTCTTTACACTT
CTTCCTAGCTGCTTGGCCTGTAGTAGGTATTTGGTTCACCGCTTTAGGTATCAGCACCAT
GGCTTTCAACCTAAATGGGTTCAATTTTAACCAATCTGTAGTTGACAGTCAAGGTCGTG
TTATTAACACTTGGGCTGATATCATCAATCGCGCTAACCTTGGTATGGAAGTTATGCATG
AACGTAATGCTCACAACTTCCCTCTAGACCTAGCTGCTGTTGAAGTTCCGTCTACAAAT
GGAATGGGTTTGCCTTGGTATCGTGTTCATACCGTCGTATTGAATGATCCTGGTCGGTTG
CTTTCTGTCCATATAATGCATACAGCTCTAGTTTCTGGTTGGGCCGGTTCGATGGCTCTA
TACGAATTAGCAGTTTTTGATCCCTCTGACCCTGTTCTTGATCCAATGTGGAGACAAGG
TATGTTCGTTATACCCTTCATGACTCGTTTAGGAATAACCAATTCATGGGGTGGTTGGAG
TATCACA---GGAGGAACTATAACGAATCCGGGTATTTGGAGTTACGAAGGTGTGGCCGG
GGCACATATTGTGTTTTCTGGCTTGTGCTTCTTAGCAGCTATCTGGCATTGGGTATATTG

GGACCTAGAAATATTCTGTGATGAACGTACAGGAAAACCCTCTTTGGATTTGCCCAAGA
TCTTTGGAATTCATTTATTTCTCTCAGGGGTGGCTTGCTTTGGGTTTGGCGCATTTCATG
TAACAGGTTTGTATGGTCCCGGAATATGGGTGTCCGACCCTTATGGCCTAACCGGAAAA
GTACAACCTGTAAATCCGGCGTGGGGCGCGGAAGGTTTTGATCCTTTTGTTCCGGGAG
GAATAGCCTCTCATCATATTGCAGCAGGTACATTGGGCATATTAGCGGGTCTATTCCATC
TTAGTGTCCGCCCACCCCAACGTCTATACAAAGGATTACGTATGGGCAATATTGAAACT
GTCCTTTCTAGTAGTATCGCTGCTGTCTTTTTTGCAGCTTTCGTTGTTGCTGGAACTATG
TGGTATGGCTCAGCAACTACCCCGATCGAATTATTTGGTCCCACTCGTTATCAGTGGGAT
CAGGGATACTTCCAGCAAGAAATATATCGAAGAGTTGGCGCCGGACTAGCCGAAAATC
TAAGTTTATCGGAAGCTTGGTCTAAAATTCCCGAAAAATTAGCTTTTTATGATTACATCG
GCAATAATCCAGCGAAGGGTGGATTATTCAGAGCAGGCTCAATGGACAACGGGGATGG
AATAGCTGTTGGATGGTTAGGACACCCCGTTTTTAGAGATAAAGAAGGGCATGAGCTTT
TTGTGCGTCGTATGCCTACTTTTTTTGAAACATTTCCGGTAGTTTTGGTAGACGGAGAC
GGAATTGTGAGAGCCGATGTTCCTTTTCGAAGGGCGGAATCAAAGTATAGTGTCGAAC
AAGTGGGTGTAACTGTTGAGTTCTATGGCGGCGAACTCAATGGAGTCAGCTATAGCGA
TCCTGCTACTGTGAAAAAATATGCTAGACGTGCCCAATTGGGTGAAATTTTTGAATTAG
ATCGTGCTACTTTGAAATCCGATGGTGTTTTTCGGAGCAGTCCAAGGGGTTGGTTCACT
TTTGGACATGCTACGTTTGCTTTGCTCTTCTTTTTCGGACACATTTGGCACGGCGCTAG
AACCTTGTTCAGAGATGTTTTTGCTGGTATTGACCCAGATTTGGATGCTCAAGTGGAAT
TTGGAGCATTCCAAAAACTTGGAGATCCAACTACAAGAAGACAAGTAGTCATGAAAA
CCTTATATTCCCTGAGGAGGTTCTACCCCGTGGAAACGCTCTTTAATGGAACTTTAGCTT
TAGCCGGTCGTGACCAAGAAACCACTGGGTTCGCTTGGTGGGCCGGGAATGCCCGAC
TTATCAATTTGTCCGGTAAACTACTCGGGGCTCACGTAGCCCATGCCGGATTAATCGTAT
TCTGGGCCGGAGCAATGAACCTATTTGAAGTGGCTCATTTCGTACCAGAGAAACCCAT
GTATGAACAAGGATTGATTTTACTTCCCCATCTAGCTACTTTAGGTTGGGGGGTAGGTC
CGGGTGGAGAAGTTATAGACACCTTTCCATACTTTGTATCTGGAGTACTTCACTTAATTT
CCTCTGCAGTCTTAGGCTTTGGCGGTATTTATCATGCCCTTCTTGGACCTGAGACTCTTG
AAGAATCCTTTCCATTCTTCGGTTATGTATGGAAAGATAGAAATAAAATGACCACAATTT
TGGGTATTCACTTAATCTTGTTAGGTATAGGTGCTTTTCTTCTAGTACTCAAGGCTCTTTA
TTTTGGGGGTGTATATGATACCTGGGCTCCAGGGGGGGGAGATGTAAGAAAAATTACC
AATTTGACCCTTAGCCCAGGTGTTATATTTGGTTATTTACTAAAATCTCCCTTTGGGGGA
GAAGGATGGATTGTTAGTGTGGACGATTTGGAAGATATAATTGGAGGACATGTATGGTT
AGGTTCCATTTGTATACTTGGTGGAATCTGGCATATTTTAACCAAACCCTTTGCGTGGGC
TCGCCGTGCATTTGTATGGTCTGGAGAGGCCTATTTGTCTTATAGTTTAGGTGCCTTATC
TGTCTTTGGTTTCATCGCTTGTTGTTTTGTTTGGTTCAATAATACCGCTTATCCTAGTGAG
TTTTATGGGCCCACTGGGCCAGAGGCTTCTCAAGCTCAAGCATTTACTTTTCTGGTTAG
AGACCAACGTCTTGGGGCTAACGTGGGGTCCGCTCAAGGACCTACTGGTTTAGGTAAA
TATCTAATGCGTTCCCCGACCGGAGAGGTCATTTTTGGAGGAGAAACTATGCGTTTTTG
GGATCTCCGTGCTCCTTGGTTGGAACCTCTAAGGGGTCCCAATGGTTTGGACTTGAGTA
GGTTGAAAAAAGACATACAACCTTGGCAAGAACGACGTTCCGCGGAATATATGACTCA
TGCTCCTTTAGGTTCTTTAAATTCCGTGGGTGGCGTAGCTACCGAGATCAATGCGGTCA
ATTATGTCTCTCCTAGAAGTTGGTTAGCTACCTCTCATTTTGTTCTAGGATTCTTCCTATT
CGTGGGCCATTTGTGGCATGCGGGAAGGGCTCGTGCGGCTGCAGCCGGATTTGAAAA
AGGAATCGATCGTGATTTTGAACCTGTTCTTTCCATGACCCCTCTTAACATGACTATAGC

CCTTGGTAGATTTACCAAAGAAGAAAATGATTTATTTGATATTATGGATGACTGGTTAAG
GAGGGACCGTTTCGTTTTTGTAGGTTGGTCCGGTTTATTGCTCTTTCCTTGTGCTTATTT
CGCTTTAGGGGGTTGGTTCACAGGTACAACCTTTGTAACTTCATGGTATACCCATGGAT
TGGCCAGTTCCTATTTGGAAGGCTGCAATTTCTTAACCGCTGCAGTTTCTACTCCTGCT
AATAGTTTAGCACATTCGTTGTTGCTACTATGGGGACCTGAAGCACAAGGAGATTTTAC
TCGTTGGTGTCAATTAGGGGGTCTGTGGACTTTTGTTGCTCTCCATGGGGCTTTCGGAC
TAATCGGTTTCATGTTACGTCAATTTGAACTTGCTCGATCTGTTCAATTGCGACCTTACA
ATGCAATCGCATTCTCTGCTCCAATTGCTGTTTTTGTTTCTGTATTCCTAATTTATCCACT
AGGTCAGTCTGGTTGGTTCTTTGCGCCTAGTTTTGGTGTAGCAGCTATATTTCGATTCAT
CCTCTTCTTCCAAGGGTTTCATAATTGGACGTTGAACCCATTTCATATGATGGGGGTTGC
CGGAGTATTGGGCGCTGCTCTGCTATGCGCTATTCATGGTGCTACTGTAGAAAATACTTT
ATTCGAGGATGGTGACGGTGCAAATACATTCCGTGCTTTTAACCCAACTCAAGCTGAA
GAGACTTATTCGATGGTCACTGCTAACCGCTTTTGGTCCCAAATCTTTGGGGTTGCTTT
TTCCAATAAACGTTGGTTACATTTCTTTATGTTATTTGTACCGGTAACCGGTTTATGGATG
AGCGCTATTGGAGTAGTCGGTCTGGCTCTGAACCTACGTGCCTATGACTTCGTTTCCCA
GGAAATCCGCGCGGCGGAAGATCCTGAATTTGAGACTTTCTACACCAAAAATATTCTCT
TAAACGAAGGTATTCGTGCTTGGATGGCGGCTCAGGATCAGCCTCATGAAAACCTTATA
TTCCCTGAGGAGGTTCTACCCCGTGGAAACGCTCTTATGTCTGGAAGCACGGGAGAAC
GTTCTTTTGCTGATATTATTACCAGTATTCGATATTGGGTTATTCATAGTATTACTATACCT
TCCTTATTTATTGCGGGTTGGTTATTCGTCAGCACAGGTTTAGCTTACGATGTGTTTGGG
AGCCCTCGGCCAAACGAGTATTTCACAGAGAGCCGGCAAGGGATTCCATTAATAACTG
GCCGTTTTGATCCTTTGGCACAACTCGATGAATTTAGTAGATCCTTTATGACCATAGATC
GGACCTATCCAATTTTTACAGTGCGATGGTTAGCTGTTCACGGACTAGCTGTACCTACC
GTTTCTTTTTTGGGGTCAATATCAGCAATGCAGTTCATCCAACGAATGGCTACACAAAC
TGTTGAGGGTAGTTCTAGATCTGGG---CCAAGACGGACTCTTACAGGGGATTTATTGAA
ACCATTGAATTCGGAATATGGTAAAGTGGCTCCTGGATGGGGAACCACCCCATTTATGG
GTGTCGCAATGGCTTTATTTGCCATATTCCTATCTATTATTTTGGAGATTTATAATTCTTCC
GTTTTGTTGGATGGAATTTCAACGAGTATGCTTACTCTCAAACTCTTCGTTTACACAGTA
GTGATATTCTTTGTTTCTCTCTTCATCTTCGGATTCCTATCTAATGATCCAGGACGTAACC
CTGGGCGTGAGGAAATGGCCGATACTACTGGAAGGATTCCTCTTTGGCTTATAGGTACT
GTAACTGGTATTCCTGTGATCGGTTCAATAGGTATTTTCTTTTACGGTTCATATTCCGGAT
TGGGATCATCCCTAATGCTTAATATCTTTAGTTTGATCTGT------CTTAATTCTGCCCTTCA
TTCGAGTAGCTTTTTCTTTGCCAAATTGCCCGAAGCTTATGCTTTTTTCAATCCAATCGT
AGATGTTATGCCAGTCATACCTGTGCTCTTTTTTCTTTTAGCCCTTGTTTGGCAAGCTGC
TGTAAGTTTTCGCACGACACAATCAAACCCGAACGAACAAAGTGTTGAATTGAATCGT
ACCAGTCTCTACTGGGGGTTATTACTCATTTTTGTACTTGCTGTTTTATTTTCCAATTATT
TCTTCAATATGGAAGTCAATATTCTCGCATTTATTGCTACTGCGCTGTTCATTCTAGTTCC
TACTGCCTTTTTACTTATCATCTACGTAAAAACAGTCAGTCAAAGTGATATGGAAACAG
CAACCTTAGTCGCCATCTCCATATCTGGTTCACTTGTAAGCTTTACGGGGTACGCCCTAT
ATACCGCTTTTGGGCAACCCTCTCAACAACTAAGAGATCCATTTGAGGAACACGGGGA
CATGGAAGCATTGGTTTATACATTCCTGTTAGTCTCGACTTTAGGGATAATTTTTTTCGCT
ATCTTTTTTCGAGAACCGCCTAAGGTCCCGACTAAAAAGATGAAAATGATTATTGCTTT
CCAATTGGCTGTTTTTGCATTAATTGCTACTTCATCAATCTTATTGATTAGTGTACCTGTT
GTATTTGCTTCTCCCGATGGTTGGTCAAATAATAAAAATGTTGTATTTTCTGGTACATCAT

TATGGATTGGATTAGTATTTCTGGTAGCTATCCTTAATTCTCTCATCTCT--------------------A
TGTCACCAAAAACAGAGACTAAAGCAGGTGTTGGATTCAAAGCTGGTGTTAAAGATTA
CAAATTGACTTATTATACTCCTGACTATGAAACCAAAGATACTGATATTTTGGCAGCATT
CCGAGTAACTCCTCAACCTGGAGTTCCAGCGGAGGAAGCAGGGGCTGCGGTAGCTGC
CGAATCTTCTACTGGTACATGGACAACTGTGTGGACCGATGGACTTACCAGCCTTGATC
GTTACAAAGGACGATGCTACCACATCGAGCCCGTTGCTGGGGAGGAAAGTCAATTTAT
TGCCTATGTAGCTTATCCTTTAGACCTTTTTGAAGAAGGTTCTGTTACTAACATGTTTAC
TTCTATTGTGGGTAATGTATTTGGGTTCAAAGCCCTACGAGCTCTACGTCTAGAGGATCT
GCGAATTCCTCCTGCTTATACCAAAACTTTCCTAGGCCCGCCCCATGGCATCCAAGTTG
AACGGGATAAATTGAACAAGTATGGTCGTCCCCTATTGGGATGTACTATTAAACCAAAA
TTGGGGTTATCCGCCAAGAACTACGGTAGGGCGGTTTATGAATGTCTCCGCGGTGGACT
TGATTTTACCAAGGATGATGAGAACGTGAACTCCCAACCATTTATGCGTTGGAGAGAC
CGTTTCTTATTTTGTGCCGAAGCCATTTTTAAATCGCAGGCCGAAACAGGTGAAATCAA
AGGACATTATTTGAATGCTACTGCAGGTACATGCGAAGAAATGATCAAAAGGGCCGTG
TTTGCTAGAGAATTGGGAGTTCCTATCGTAATGCATGACTATTTAACAGGGGGATTCACT
GCAAATACTAGCTTGGCTCATTATTGCCGGGACAACGGCCTACTTCTTCACATCCATCG
CGCAATGCATGCAGTTATTGATAGACAGAAAAATCATGGTATGCACTTTCGTGTACTAG
CTAAAGCGTTACGTATGTCTGGTGGAGATCATATTCACGCTGGTACCGTAGTAGGTAAA
CTGGAAGGGGAACGGGAGATCACTTTGGGTTTTGTTGATTTACTACGTGATGATTTTAT
TGAAAAAGACCGAAGTCGCGGCATTTATTTCACTCAAGATTGGGTCTCTATGCCAGGT
GTTCTGCCCGTGGCTTCAGGGGGTATTCACGTTTGGCATATGCCTGCCCTGACCGAGAT
CTTTGGGGATGATTCCGTACTACAGTTCGGTGGAGGAACTTTAGGACACCCTTGGGGA
AACGCGCCTGGTGCAGTAGCTAATCGGGTGGCTTTAGAAGCGTGTGTACAAGCTCGTA
ATGAGGGACGTAATCTTGCTCGTGAAGGTAATGAAATTATCCGTGAAGCTAGCAAATGG
AGCCCTGAACTAGCCGCTGCTTGTGAGGTATGGAAGGAGATCAAATTCGAATTCGAGG
CAATGGATACCTTG------ATGGCGATACATTTATACAAAACTTCTACCCCGAGCACACGC
AATGGAGCCGTAGACGGTCAAGTGAAATCCAATCCACGAAACAATTTGATCTATGGAC
AGCATCATTGTAGTAAGGGTCGTAATGCTAGAGGAATCATTACCGCAAGGCATAGAGGG
GGAGGTCATAAGCGTCTATACCGTAAAATCGATTTTCGACGGAATGAAAAAGACATATC
TGGTAGAATCGTAACCATAGAATACGACCCTAATCGAAATGCATACATTTGTCTCATACA
CTATGGGGATGGTGAGAAAAGATATATTTTACATCCCAGAGGGGCTATAATTGGAGATA
CCATTGTTTCTGGTACAGAAGTTCCTATATCAATGGGAAATGCCCTACCTTTGACCGACA
TGCCCTTAGGCACGGCCATACATAACATAGAAATCACACTTGGAAAGGGTGGACAATT
AGCTAGAGCCGCAGGTGCTGTAGCGAAACTGATTGCAAAAGAGGGTAAATCGGCCAC
ATTAAGATTACCATCTGGGGAGGTCCGTTTGATATCCAAAAACTGCTCAGCAACAGTCG
GACAAGTGGGTAATGTTGGGGTGAACCAGAAAAGTTTGGGTAGAGCCGGATCTAAGC
GTTGGCTAGGTAAGCGTCCTGTAGTAAGAGGAGTAGTTATGAACCCTGTAGACCATCCC
CATGGGGGTGGTGAAGGGAGAGCCCCGATTGGTAGAAAAAAACCCACAACCCCTTGG
GGTTATCCTGCGCTTGGAAGAAGAAGTAGGAAAAGGAATAAATATAGTGATAGTTTTAT
TCTTCGTCGCCGTAAA---ATGATTCAACCTCAGACCCATTTGAATGTAGCGGACAACAG
CGGGGCTCGAGAATTGATGTGCATTCGAATCATAGGGGCTAGT---AATCGCCGATATGCT
CATATCGGTGACGTTATTGTTGCTGTAATCAAAGAAGCAGTGCCCAATATGCCTCTCGA
AAGATCAGAAGTTATTCGAGCTGTAATTGTACGTACATGTAAAGAACTCAAACGCGAC
AACGGTATGATAATACGATATGATGACAATGCGGCAGTTGTCATTGATCAAGAAGGAAA

TCCAAAAGGAACTCGAGTTTTTGGAGCGATTGCTCGGGAATTGAGAAAGTTAAATTTC
ACTAAAATAGTCTCATTAGCCCCTGAAGTCTTAATGCTTAGTCCCAAAAGAACCAGATT
TCGTAAACAACATAGAGGAAGAATGAAGGGAATATCTTATCGAGGCAATCATATTTGTT
TCGGTAGATACGCTCTTCAAGCACTTGAACCCTCTTGGATCACATCTAGACAAATAGAA
GCAGGGCGACGAGCAATGACACGATATGCGCGCCGTGGCGGAAAAATATGGGTACGTA
TATTTCCCGACAAACCCGTTACAGTAAGACCTACGGAAACACGTATGGGTTCGGGGAA
AGGATCCCCCGAATATTGGGTATCTGTCGTTAAACCAGGTCGAATACTTTATGAAATGG
GCGGAGTATCCGAAACTGTAGCCAGAGCGGCTATTTCAATAGCTGCGTGCAAAATGCCT
ATACGAACTCAATTCATTATCGCGAGAATGACCAGAATTAGACGAGGATATATAGCTCG
GCGACGTCGAACAAAAATTCGTTTATTTGCATCAACCTTTCGAGGGGCTCATTCAAGAC
TTACTCGAACTATTACTCAACAGAAAATGAGAGCTTTGGTTTCCACTCAGCGGGATAGA
GGCAGGCGAAAGAGAGATTTTCGTCGTTTGTGGATCACTCGCATAAATGCAGTAACTC
GTGAGAAT------GTATCCTATAGTTATAGTAGATTAATACATGATCTGTACAAGAAGCGGT
TGCTTCTTAATCGTAAAATACTTGCACAAATAGCTATATCAAATAGGAATTGTCTCGACA
CAATTTCCAATGAGATTCTCAAA---------------------------------AAAATGAAGCAAGTACAAG
CTTTAGCTCAACATATATCTATGTCTGCTCACAAAGCACGAAGAGTCGTTGATCAGATT
CGTGGACGTTCCTATGCGGAAACACTTATGCTACTGGAACTCATGCCTTATCGAGCATC
TTATCCCATTTTTAAACTGGTTTATTCTGCAGCAGCAAATGCTAGTCACAATAAGAGTTT
TAACGAAGCTGATTCATTCATTAGTAAAGCCGAAGTCAATGGGGGTACTATCGTGAAAA
AGTTTAAACCTCGGGCTAAAGGACGTAGTTACCCGATAAAAAGACCCACCTGTCATATA
ACTATTGTATTGAAAGATAGATCTAAAAAG---------------------ATGGATGGAATCAAATATG
CAGTCTTTACAGAAAAGAGTATTCGGTTATTGGGGAACAATCAA---TATACTTCTAATGT
CGAATCAGGATCAACTAGGACAGAAATAAAGCATTGGGTCGAACTCTTCTTTGGTGTC
AAGGTAATAGCTATGAATAGTCATCGACTCCCGGGAAAGGGTAGAAGAATGGGA---------
---CATACAATGCATTACAGACGTATGATCATTACGCTTCAACCGGGTTATTCTATTCCACC
TCTTATAGAGAAAAGAACTATGGCGGTTCCAAAGAAACGTACTTCTGTGTCAAAAAAG
CATATTCGTAGAAATATTTGGAAGAGAAAGGGATATCGGGCCGCGTCAAAAGCTTTGTC
TTTAGCTAAATCGATTTCTACCGGACATTCAAAGAGTTTTTTTGTGCGACAAACAAGTA
ATATGGCCAAGGGTAAAGATGTCAGAGTCATAATTATTTTGGAATGTACCGGTTGTGCC
CGAAAT------GGT------AAAGAATCGAGGGGCATTTCCAGATATATTACTCAAAAAAATCG
ACACAATACACCTAGTCGATTGGAATTGAAAAAATTCTGTTCTTATTGTTACAAGCATAC
GATTCATGCGGAGATAAAGAAAATGAAAATAAGGGCCTCCGTTCGTAAAATTTGTGAA
AAATGTCGACTAATCCGTAGGCGGGGACGAATTATAGTAATTTGTTCCAATCCGAGACA
TAAACAGAGACAAGGGATGGTTCGAGAGGAAGTGGCAGTATCCACTCGGACACTACA
GTGGAAGTGTGTTGAATCAAGAACAGACAGTAATCGTCTTTATTATGGACGTTTCGTTT
TGTCCCCGCTTATGAAAGGTCAAGCGGATACGATAGGTATCGCGATGAGAAAGGCTTTA
CTTGGGGAAATAGAAGGAACATGTATAACACGCGCAAAATCTGAG------AAGGTACCGC
ATGAATATTCTACAATAGTTGGTATTGAAGAATCAGTACATGAAATTTTAATGAATTTGA
AAGAAATTGTATTAAAAAGTAATCTGTATGGAACTCGTGACGCATCCATTTGCGTCAGG
GGTCCTAAATACGTAACTGCTCAAGATATCATCTCACCGCCTTCTGTGGAAATAGTTGAT
ACTACACAACATATAGCTAGCCTGACGGAGCCAATTAATTTGTGTATTGAATTACAAATC
GAGAGGGATCGCGGATATCGTATGAAAGCCCCAAATAACTATCAAGATGGAAGTTAT----
--CCTATAGATGCCGTATCCATGCCTGTTCGAAATGCGAATCATAGTATTCATTCTTATGGG
AGTGGGAATGAGAAACAAGAGATACTTTTTCTTGAAATATGGACAAATGGAAGTTTAA

CTCCTAAAGAAGCACTTCACGAAGCTTCTCGTAATTTGATTGATTTATTTATTCCTTTTCT
ACACGCGGAAGAACAGGACATTCATTTGAATTTAGAGGACAATCAA---AACAGGTTTA
CTGTATCCCCTTTTACCCTTCATGATAGATTGGGTAAT---ATAAGGAAAAACAAAAAA---
GAAATTGCATTGAAATGTATTTTTATTGACCAATCAGAATTGCCCACTAGGACCTATAAT
TGTCTGAAAAGGTCCAATATACATACATTATTGGACCTTTTGAATAACAGTCAAGAAGA
TCTTATGAGAATTGAACATTTTCGCATAGAAGATATAAAACAAATATTGGACATTCTACA
GAAGCAT------TTCGGAATTGATTTACCTAAGAATAAGACGTTTCGGGATGGG---AATGA
GGGAATGCCTACAATACCAGGATTTAGTCAGATACAATTTGAGGGATTTTGTAGGTTCA
TTGACCAGGGCTTGACGGAAGAACTTCATAAGTTTCCAAAAATTGAAGATACAGATCA
AGAAATTGAATTTCAATTATTTGTGGAAACATATCAATTGGTAGAACCCTTGATAAAAG
AAAGAGATGCTGTGTATGAATCACTCACATATTCTTCTGAATTATATGTATCCGCGGGAT
TAATTTGGAAAACCGGTCGAGATATGCAAGAACAAACCATATTTATTGGAAACATTCCT
CTAATGAATTCCCTGGGAAACTCTATAGTAAGCGGAATATACAGAATTGTAATCAATCAA
ATATTGCAAAGCCCCGGTATTTATTACCGTTCAGAATTGGACCATAACGGAATTTCTGTA
TATACCGGCACCATAATATCAGATTGGGGGGGAAGACCAGAATTAGAGATTGATAGAAA
AGCAAGGATATGGGCCCGTGTGAGTAGGAAACAAAAAATATCTATTCTAGTTCCATCAT
CAGCTATGGGTTCGAATCTAAGAGAAATTCTAGATAATGTTTGCTACCCTGAAATTTTCT
TGTCTTTCCCGAATGATAAGGAGAAAAAAAAAGATTGGTTCAAGGGAAAATGCCATTTT
GGAGTTTTATAAACAATTTGCTTGTGTAGGCGGGGATCCGGTATTTTCTGAGTCCTTATG
TAAGGAATTACAAAAGAAATTTTTTCAACAAAGATGTGAATTAGGCAGGATTGGTCGA
CGAAATATGAACCGGAGACTGAATATTGATATACCTCAGAACAATACATTTTTGTTGCCA
CGAGACGTATTGGCTGTTGTGGATCATTTGATCGGAATGAAATTTGGAATGGGCACTCT
TGACGAT------ATGAATCACTTGAAAAATAAACGTATTCGTTCTGTAGCAGATCTGTTAC
AGGATCAATTTGGATTGGCTCTGGTTCGTTTAGAAAATGCGGTTCGAGGAACTATATGT
GGAGCAATCAGG---CATAAATTGATC---CCGACCCCTCATAATTTGGTAACTTCAACTCC
A------------TTAACAACCACTTATGAATCGTTTTTCGGCTTACACCCCTTATCTCAAGTTTT
GGATCGAACTAATCCATTGACACAAATAGTTCATGGGCGAAAATCGAGTTATTTAGGCC
CCGGAGGATTAACGGGGCGAACTGCTAGTTTTCGGATACGAGATATCCATCCTAGTCAC
TATGGACGTATTTGCCCAATTGACACGTCCGAGGGAATCAATGTTGGACTTATCGGATC
CTTAGCTATTCATGCGAGGATTGGTCATTGGGGG---TCTATAGAGAGTCCGTTTTAT-------
--GAAATATCTGAGAAATCCAAA------------GAGATCGTTTATTTACCACCAAGTAGAGACG
AATATTACATGGTAGCGGCAGGAAATTCTTTGGCCTTAAATCGGGGTATTCAGGAAGAA
CAGGTTGTTCCAGCTAGATACCGTCAAGAATTCCTGACTATTGCATGGGAACAGATTCA
TCTTCGAAGCATTTTTCCCTTCCAATATTTTTCTATTGGAGCTTCCCTCATTCCTTTTATC
GAGCATAATGATGCAAATCGGGCTTTAATGAGTTCTAATATGCAGCGTCAAGCAGTTCC
GCTTTCTCGGTCCGAGAAGTGCATTGTTGGAACTGGGTTGGAAGGCCAAGCAGCTCTA
GATTCGGGGGTTTCGGCTATAGCAGAACACGAGGGAAAGATCATTTATACGGATACTGA
CAAGATCGTTTTATCAGGTAATAGAGACACTATAAGCATTCCATTGGTTATGTATCAACG
TTCTAACAAAAATACTTGTATGCATCAAAAACCTCGAGTTCCGCGGGGTAAATGCATTA
AAAAGGGGCAAATTTTAGCGGACGGAGCGGCTACAGTTGGTGGCGAACTCGCTTTGG
GAAAAAACGTATTAGTAGCTCATATGCCATGGGAAGGCTACAATTCTGAAGACGCGGT
ACTCATTAGCGAACGTCTGATATATGGGGATATTTATACTTCTTTTCACATACGGAAATAT
GAAATTCAGACTCATGTGACAAGCCAAGGT---CCTGAAAGAATCACTAATGAAATACCA
CATTTAGAAGCCCATTTACTCCGCAATTTAGACAAAAATGGAATTGTGATGCTGGGATC

TTGGATAGAAAGAGGTGATATTTTAGTGGGTAAATTAACGCCTCAGGCGGCGAAAGAA-
--TCGTCGTATGCCCCGGAAGATAGATTATTACGAGCCATACTTGGCATTCAGGTATCCAC
TGCAAAGGAAACTTGTCTAAAACTACCTATAGGCGGAAGGGGTCGAGTTATTGATGTG
AGATGGATCCAGAAAAAAGGG---GGTTCCAGTTATAATCCAGAAACGATTCGTGTATCT
ATTTTACAGAAACGTGAAATCAAAGTGGGTGATAAAGTAGCCGGAAGACATGGAATA
AGGGCATCGTTTCCAAAATTTTGCCTAGACAAGATATGCCTTATTTGCAAGATGGAACG
CCAGTTGATATGGTCTTCAACCCATTAGGAGTACCTTCGCGAATGAATGTGGGACAAAT
GTTTGAATGCTCGCTCGGATTGGCGGGGTACCTGCTGGACAAACATTATAGAATAGCAC
CCTTTGATGAGAGATATGAGCAAGAGGCTTCGAGAAAACTAGTGTTTCCTGAATTATAT
TCAGCCAGTAAGCAAACAGTAAATCCTTGGGTATTTGAACCCGAGTATCCAGGAAAAA
GCAGAATATTTGATGGAAGAACGGGAGATCCTTTCGAACAACCTGTTATAATAGGAAA
GTCCTATATCCTGAAATTAATTCATCAAGTTGATGATAAAATCCACGGACGTTCCAGTGG
ACATTATGCACTTGTTACACAACAACCTCTTAGAGGAAGGGCCAAGCAAGGGGGACA
ACGAGTAGGAGAAATGGAAGTTTGGGCTCTAGAGGGATTTGGTGTTGCTCATATTTCAC
AAGAAATGCTTACTTATAAATCGGATCATATTAGGGCTCGCCAGGAAGTACTTGGTGCT
ACGATCATTGGAGGAACAATACCTAAACCTGAGGATGCTCCTGAATCTTTTCGGTTGCT
CGTTCGAGAACTACGATCTTTGGCTCTGGAACTGAATCATTTCCTTGTATCTGAGAAGA
ACTTCCAGATTAATAGGAAGGAAGCT---------------------ATGATCGACCGGTATAAACATC
AACAACTTCGAATTGGATCAGTTTCTCCTCAACAAATACGTGCTTGGGCCAACAAAAC
CCTACCTAATGGAGAGATAATTGGAGAGGTGACAAAACCCTATACTTTTCATTACAAAA
CCAATAAACCGGAAAAAGATGGATTGTTTTGTGAAAGAATCTTTGGACCTATAAAAAG
TGGAATTTGTGCTTGTGGAAATTATCGAGTGATCGGAGATGAAAAAGAAGACCCGAAA
TTTTGTGAACAATGCGGAGTCGAGTTTGTTGATTCTCGGATACGAAGATATCAAATGGG
ATACATCAAACTGGCATGTCCAGTGACTCATGTGTGGTATTTGAAACGTCTTCCTAGTTA
TATCGCGAATCTTTTAGATAAACCCCTTAAGGAGTTCGAAGGCCTAGTATACTGCGAT----
--TTTTCTTTTGCTAGGCCC---ATAGCTAAAAAACCTACTTTCTTGCGATTACGAGGTTCA
TTCGAATCTGAAATCCAATCCCGGAAATACAGCATCCCACTTTTTTTACTACCCAGGG
CTTCGATACATTTCGAAATCGAGAAATCTCTACTGGAGCAAGTGCTATCAGAGAACAAT
TAGCTGATCCGGATTTGCGAATTATTACAGATCATTCATTGGTAGAATGGAAAGAATTAG
GGGAAGAAGGGTCCACT---GGGAATGAATGGGAAGATAGAAAAATTGGAAGAAGAAA
GGATTTTTTGGTTAGACGCATAGAATTAGCTAAGCATTTTATTCGAACAAATGTAGAACC
AGAACGGATGGTTTTGTGCCTATTACCAGTTCTTCCTCCCGAGTTGAGACCAATCATTC
AGATAGATGGGGGTAAACTAATGAGTTCGGATATTAATGAACTCTATCGAAGAGTTATCT
ATCGGAACAATACTCTTACCGATCCATTAACAACAAGTACATCTACGCCGGGGGAATCA
GTAATGTGTCAGGAGAAATTGGTACAAGAAGCTGTGGATACCCTTCTTGATAATGGGAT
CCGTGGACAACCAATGAGGGACGGTCATAATAAAGTTTACAAGTCCTTTTCAGATGTAA
TTGAAGGCAAAGAAGGAAGATTTCGTGAAACTCTACTTGGTAAACGGGTCGATTACTC
AGGGCGTTCCGTCATTGTCGTGGGCCCTTCACTTTCATTGCATCGATGTGGATTACCTC
GAGAAATAGCAATAGAACTTTTCCAAACATTTGTAATTCGTGGTTTAATCAGACAACAT
GTTGCTTCCAACATAGGAATTGCGAAAAGTAAAATTCGGGAAAAAGAACCAATTGTAT
GGGAAATACTTCAAGAAGTTATGCAAGGGCATCCTGTATTGCTAAATAGAGCGCCCACC
CTGCATAGATTAGGCATACAGGCGTTCCAACCCATTTTAGTAGAGGGACGTGCTATTTG
TTTACATCCATTAGTTCGTAAGGGATTCAATGCAGACTTTGATGGGGATCAAATGGCTG
TTCATGTACCTTTATCTTTGGAAGCTCAAGCGGAGGCTCGTTTACTTATGTTTTCTCATAT

GAATCTCTTGTCTCCGACTATTGGAGATCCCATTTCGGTACCAACTCAAGATATGCTTAT
TGGACTCTATGTATTAACGATCGGGAATCGTCGAGGTATTTGTGCAAATAGGTATAATCC
ATGGAATCGC------GGAAAC---TCTCAAAATGAAAGAGTTAACGATACT---TATATGTATA
CTAAG---AAAGAGCCCTATTTTTGTAGTTCCTCTGATGCACTCGGAGCTTATCGGCAGA
AACGAATCAATTTAGATAGCCCTTTGTGGCTACGGTGGCGACTGGATCAACGT---GTCA
TTGCCTCA------AGAGAAGTTCCTATCGAAGTTCAATATGAATCTTTGGGTACCTATCATG
AAATTTATGGGCACTATCTAATAGTAAGAAGTATAAAAAAAGAAATCCTTTGTATATACA
TTCGAACCACTGTCGGTCATATTTCTTTTTATCGAGAAATAGAAGAAGCCATACAGGGG
TTTTGTCGGGCCTACTCA--ATGGCAGAACGGGCCGAT
CTGGTCTTTCACAATAAGGTGATAGATGGGGCTGCCATGAAACGACTTATTAGCAGATT
AATAGATCACTTCGGAATGGCATATACATCGCACATCCTGGATCAAGTAAAGACTCTGG
GTTTCCAGCAAGCCACCACTACATCCATTTCATTAGGAATTGATGATCTTTTAACAATAC
CTTCTAAGGGGTGGCTAGTCCAAGATGCTGAACAACAAAGTTTGATTTTAGAAAAGCA
CCATCATTATGGGAATGTACACGCAGTAGAAAAATTACGCCAATCCATTGAGATATGGTA
TGCTACAAGTGAATATTTGAGACAAGAAATGCATCCTAATTTTAGGATGACTGATCCTT
CTAATCCAGTCCATATAATGTCCTTTTCGGGAGCTCGAGGAAATGCATCTCAGGTACAC
CAATTAGTAGGTATGAGAGGATTAATGTCGGACCCCCAAGGACAAATGATTGATTTACC
CATTCAAAGCAATTTACGCGAAGGACTTTCTTTAACAGAATATATCATTTCCTGCTATGG
AGCCCGCAAGGGAGTTGTGGATACTGCTGTACGAACATCAGATGCTGGATACCTCACG
CGTAGACTTGTTGAAGTAGTTCAACACATTGTTGTACGTAGAACAGATTGTGGCACTAT
CCGGGGCATTTCCGTGAGTCCTCGAAAT------GGGATGACGGAAAGAATTTGGATCCAA
ACACTAATTGGTCGTGTATTAGCATACGATATATATATGGGTCCACGCTGCATTGCCGCC
CGAAATCAAGATATTGGGATTGGACTTGTCAATCGATTCATAACC------------------TTTCG
AGCACAACCAATATATATTCGAACCCCCTTTCTTTGTAGGAGTACATCTTGGATATGTCG
ATTATGTTATGGTCGGAGTCCCGCTCACGGCGACCTAGTCGAATTGGGAGAAGCAGTA
GGTATTATTGCGGGTCAATCAATCGGCGAACCGGGGACTCAACTAACATTAAGAACTTT
TCATACCGGTGGAGTATTCACAGGTGGTACTGCCGAACATGTACGAGCTCCTTTTAATG
GAAAAATTCAATTCAACGAGGATTTGGTTCACCCCACACGTACACGTCATGGGCATCCT
GCTTTTCTATGTTATACAGACCTGTATGTAACTATTGAGAGTCACGATATTCTACATAATG
TGAATATTCCACAAAAAAGTTTTCTCTTAGTTCAAAATGATCAATATGTAGAATCAGAA
CAAGTGATTGCCGAGATTCGCGCTGGAACATCCACTTTC---AAAGTTAAAGAAAAAGT
TCGAAAACATATTTATTCTGACTCAGAGGGAGAA---ATGCACTGGAGTACCGATGTGTA
CCATGCACCTGAATATACATACGGTAATGTTCATCTCTTACCAAAAACAAGTCATTTATG
GATATTATCAGGAGGTCTG------TGCAGATCCAGTATAGTGCCCTTTTCGCTCCACAAGG
ATCAAGATCAAATGAATGTTCAT------TCTCTTTCTGTGGAACAGAGATATATTTCTGACC
TCTCA------GTAACTAACGTTCGA---GTGAGACACAAATTGTTTAGTTCGGATTCTTCCG
GTAAA---GGGGGGAGGGCTCCTGATTATTCA---GGACCTGATCGAATCATATCCAATGGT
CATTGGAATTTCATATATCCTGCC---ATTCTCCACGAGAATTCTGAT------TTATTGGCTAA
GAGGCGAAGAAATAGATTCATTATTCCATTCCAATCTGATCAAGAACGAGAGAAAGAA
CTAATGCCCCGTTCCGGTATCTCGATTGAAATACCCATAAAT------GGTATTTTACGCAGA
AATAGTATTCTTGCTTATTTCGACGATCCCCGATACAGAAGAAGCAGTTCGGGAATTACT
AAATATGGGACTATAGGGGTGGATTCAATCGTCAAAAAAGAGGAT------TTGATTGAGTA
TCGAAGA---GCAAAAGAATTTAGGCCAAAATACATGAAAGTAGATCGCTTTTTTTTCAT
TCCCGAAGAAGTGCATATCTTACCCGCATCTTCGCCCGTAATGGTACGGAATAATAGTAT

CATTGGAGTAAATACACGAATAGCTTTAAATACAAGAAGCCGAGTAGGTGGATTGGTCC
GAGTGGAGAGAAAAAAAAAAAAAGGATTGAACTGAAAATATTTTCTGGGGATATCCATTT
TCCTGGAGAGACAGATAAGATATCTCGGCACAGCGGCATCTTGATACCACCAGGAACG
GGAAAAAAA------AATGTTAAGGAATCCAAA------AAATGGAAAAATTGGATTTATGTCC
AACGG------ATTACACCTACTAAGAAAAAG------TATTTTGTTTTGGTTCGACCCGTAGTC
ACATATGAAATAGCTGATGGGATCAATTTAGAAACACTTTTCCCCCAGGATCCCTTGCA
GGAAAGGGATAATGTGCAACTACGAGTTGTCAATTATATCCTTTACGGAAATGGCAAAC
CAATTCGAGGAATTTCTCACACAAGTCTTCAATTAGTTCGAACTTGTTTAGTATTGAATT
GGGACCAAGACCGAAATGGT------------TCTATAGAAGAGGTTCATGCTTCCTTTGTTGAA
GTAAGGGCAAATGGGCTGATTCGAGATTTCCTAAAAATTGATTTAGTGAAGTCCCCCAT
TTTGTATAGCGGAAAAAGAAATGATACGACAAGTTCAGGATTCATCCCCAAT---AATGG
ATCAGATCGTACCAATATCAATCCTTTTTATTTCAAGGCGAGG---------------------------ATT
CAATCACTTACCCAACATCAAGGAACTATTCGTACGTTGCTGAATAGA---------AATAAG
GAATGCCAATCTTTTCTAATTTTGTCATCATCCGATTGTTCTCGAATTGGTTCA------TTC
AATGGTTCGAAATCTCACAAAGTGACA---------AAAGAATCAGATCCCACGATTCCCATT
AGGAATTCGTTGGGTCCCTTAGGGACT------GTACCCAAAATTGCGAATTTTGATTCATCT
------TACTATTTAATAACT------CATAATCAGATCTTGTTAAAT---AAATATTTGTCACTTGAC
AAT---------TTAAAACAGACTTTCCAAGTACTTAAATATTAT---------TTAATGGATGAAAAT
GGGAGA------ATTTATAATCCCGATCCACGCAGTAACATCATTTTTAATCCATTCGATTTG
AATTGGTGCTTTCTCCCTCATGATTATTGTGAGGAGACGTCCACCATAATTAAC---CCCG
GGCAGTTTATTTGTGAAAATGTATGTATATCCAAATACGGACCG---------CACATAAAATC
GGGTCAAATTCTAATTGTTCGT---GTTGACTCCTTAGTAATAAGATCAGCTAAGCCTCAT
TTGGCCACTCCGGGAGCAACCGTTCATGGCCATTGTGGAGAAATCCTTTACGAAGGAG
ATACATTAGTTACATTTATATATGAAAAATCGAGATCGGGTGATATAACACAAGGTCTTC
CAAAAGTGGAACAGGTGTTAGAAGCGCGTTCGATTGATTCAATATCGATGAACCTAGA
AAAGAGGGTTGAAGGTTGGAACGAACGTATAACAAAAATTCTTGGGATTCCTTGGGGA
TTCTTGATTGGTGCTGAGCTAACCATAGCGCAAAGTCGTATTTCTTTGGTTAATAAGATC
CAAAAGGTTTATCGGTCCCAGGGGGTGCAGATCCATAATAGGCATATAGAGATTATTGT
ACGTCAAATAACATCAAGAGTGTTGGTTTCAGAA---------GATGGAATGTCTAATGTTTT
TTCACCCGGAGAACTAATCGGATTGTTGCGAGCGGAACGAACCGGGCGTGCTTTGGAA
GAAGCGATCTGTTACCGAGCTATCTTATTGGGAATAACGAGGGCATCTTTGAATACTCA
AAGTTTCATATCCGAAGCGAGTTTTCAAGAAACCGCTCGAGTTTTAGCAAAAGCCGCT
CTACGAGGTCGTATTGATTGGTTGAAAGGCCTGAAAGAGAACGTTGTTCTGGGGGGGA
TGATGCCTGTTGGTACCGGATTCAAAGGATTAGTGCGCCGTTCAAGG------CAACACAA
CAATATTCCT------TTGGAAATCAAAAAGAAAAATCTATTCGAGGGGGAAATGAGA------
GATATTTTGTTCCACCACAGAGAATTATTTGATTCTTGCATCCCAAACAATTTCCCCGAT
ACA---ATGACAAGAAGATATTGGCACATTAATTTG
GAAGAGATGATGGAAGCAGGAGTTCATTTTGGTCATGGTACTAGGAAATGGAATCCTA
GAATGGCACCTTACATCTCCGCAAAGCGTAAAGGTATTCATATTACAAATCTTACTAGA
ACTGCTCGTTTTTTGTCAGAAGCCTGTGATTTAGTTTTTGATGCAGCAAGTATAGGAAA
ACACTTCTTAATAGTTGGTACCAAAAAGAAAGCAGCGGATTCAGTAGCATCAGCTGCA
ATAAGGGCTCGGTGTCATTATGTTAATAAAAAATGGCTCGGTGGTATGTCAACGAATTG
GTCCACTACAGAAACGAGACTTCATAAGTTCAGGGACTTGAGAGCCGAACAAAAAGC
GGGGAAACTCAACCGTTTACCGAAAAGAGATGCGGCAATGTTGAAGAGACAATTATCT

CACTTGCAAACATATTTGGGCGGAATTAAATATATGACGGGGTTACCCGATATTGTAATC
ATCATCGATCAGCAAGAAGAATATACGGCTCTTCGAGAATGTCTCACTTTGGGGATTCC
GACAATTTGTTTAATCGATACAAATTGTGACCCGGATCTCGCGGATATTTCGATTCCAGC
GAACGATGATGCTATAGCTTCAATCCGATTGATTCTTAACAAATTAGTATCCGCGATTTG
TGAGGGCCGTTCTAGC---TATATAAGAAATTGTATGGGGCAAAAAATAAATCCACTCGG
TTTCCGACTTGGT---GAACAAAGTCATCATTCCCTTTGGTTCGCACAACCAAAAAGTTA
TTGCAGAGGTCTACAGGAAGATGAAAAAATACGGGATTGTATCAAGATATATGTACAAA
AAAATATGAGAATATCCTCAGGT---TTCGAAGGAATTGCGCATATAGAGATTCAAAAAA
AAATGGATCTGATCCAGGTCATAATCTCTATTGGATTCCCAAATTTTTTAATAGAAGGT---
---GGAACCCGA------GGAATCGAAGAATTACAGATGAATGTACAAAAGAGGTTTCATTCT
GCGAACCGGAGACTTAACATTGCTATTACAAGAGTTGCAAAACCTTATGGACAACCTA
ATATTCTTGCAGAATATATAGCTCGACAATTAAAGAATAGAGTTTCCTTTCGAAAGGCAA
TGAAAAAAGTTATTGAATTAACTGAACAAGCGGACACAAAAGGAATTCAAGTGCAAA
TTGCAGGGCGTATCGACGGAAAAGAAATTGCACGTGTCGAATGGATCAGAGAAGGCA
GGGTTCCCCTACAAACCATTCGAGCTAAAATTGATCATTGTTCCTATACAGTTCGAACC
ATATATGGGGTATTGGGCATAAAAATTTGGATATTTGTAGACGAACAAATGTCTCGTTAC
CGAGGGCCTCGTTTCAAAAAAAATACGCCGTCTGGGGGCTTTACCGGGACTAACTAGTA
AAAGACCTAGATCCGGAAGTGATCTTAGAAACCAATCGCGTTCTGGTAAA---AAATCTC
AATATCGTATTCGTCTAGAAGAAAAACAAAAATTGCGCTTTCATTATGGTCTGACAGAG
CGACAATTGCTTAGATATGTTCGTATTGCCGGAAAAGCCAAAGGGTCAACTGGTCAGG
TTTTACTACAACTACTTGAGATGCGTTTGGATAACATCCTTTTTCGACTGGGTATGGCTT
CGACCATTCCTGGAGCCAGGCAATTAGTTAACCATAGACATATTTTAGTTAATGGTCGTA
TAGTAGATATCCCAAGTTATCGCTGCAAACCCCGAGATATTATTACTACGAGGGATGAAC
AAAGATCCAGAGCTCTGATTCAAAATTATCTTGATTCACCCTCCCAC---GAGGAATTGC
CAAAACATTTGACTTTTCACCCATCCCAATAT------AAAGGATTGGTAAATCAAATAATAG
ATAGTAAGTGGGTCGGTTTAAAAATCAATGAGTTGCTAGTCGTAGAATATTATTCCCGTC
AGACTATGTCACGTCGAGGTACTGCAGAAGAAAAAACTGCAAAATCCGATCCAATTTA
TCGTAATCGATTAGTTAACATGTTGGTTAACCGTATTCTGAAACACGGAAAAAAAATCAT
TGGCTTATCAAATTGTCTATCAAGCCGTGAAAAAGATTCAACAAAAGACAGAAACAAA
TCCACTATCTGTTTTACGTCAAGCAATATGTAGAGTAACTCCCGATATAGCAGTAAAAGC
AAGACGTGTAGGTGGATCGACTCATCAAGTTCCTATTGAAATAGGATCTACACAAGGA
AAAGCACTTGCCATTCGTTGGTTATTAGGGGCATCCCGAAAACGTCCGGGTCGAAATAT
GGCTTTCAAATTAAGTTCCGAATTAGTGGATGCTGCCAAAGGGAGTGGCGATGCCATAC
GCAAAAAGGAAGAGACTCATAGAATGGCAGAGGCAAATAGAGCTTTTGCACATTTTCG
TATGGGTAGGGACACTATTGCCGATATACTAACTTCTATAAGAAATGCTAACATGGATAA
AAAAGGAACAGTTCGAATAGCATCTACTAATATAACCGAAAACATTGTTAAAATACTTC
TAAGGGAAGGGTTTATTGAAAATGTTAGGAAACATCAGGAAAACAACAAATATTTCTT
GGTTTCAACCCTGCAACATAGAAGG------AATAGGAAAGGAAGATATAGA---------ACTAT
TTTA------AAGCGTATCAGTCGACCTGGTCTACGAATCTATTCCAATTATCAACGAATTCC
TAGGATTTTAGGTGGAATGGGGATCGTAATTCTTTCTACTTCTCAGGGTATAATGACAGA
TCGAGAAGCTCGACTAGAAGGAATTGGAGGAGAAATTTTGTGTTATATATGGATGACA
AAATCTATACCAAGAATTGGTTCACGGAGGGGTGGGCGTATTGCTTCACGTAAGAATGC
ACGTAGAATACCAAAAGGAGTTATTCATGTTCAAGCGAGTTTCAACAATACCATTGTGA
CTGTTACAGATGTAACAGGTCGGGTGGTTTCTTGGTCCTCCGCAGGTACTTGTGGATTC

AGGGGCACAAGAAGAGGGACACCATTTGCTGCTCAAACCGCAGCAGCAAACGCTATT
CGTACAGTAATCGATCAGGGTATGCAACGAGCAGAAGTCATGATAAAGGGCCCCGGGC
TCGGAAGAGATGCAGCATTACGAGCCATTCGTAGAAGTGGTATACTGTTAAGTTTCGTA
CGTGACGTAACCCCTATGCCACATAATGGATGTAGGCCTCCTAAAAAAAGACGTGTGAT
GCCAACTATTAAACAACTTATTAGAAACACAAGACAGCCAATCCGAAATGTCACGAAA
TCCCCTGCTCTTCGAGGATGTCCTCAGCGTCGAGGAACATGTACTAGGGTGTATACTAT
CACCCCCAAAAAACCAAACTCTGCCTTACGTAAAGTTGCCAGAGTACGATTAACCTCT
GGATTTGAAATCACTGCTTATATACCTGGTATTGGCCATAATTCACAAGAACATTCTGTA
GTCTTAGTAAGAGGAGGAAGGGTTAAGGATTTACCCGGTGTGAGATATCACATTGTTCG
AGGAACCCTAGATGCTGTCGGAGTAAAGGATCGTCAACAAGGGCGTTCTAAATATGGG
GTCAAAAAGCCAAAAATGGCAAGGAAAGGTTTGATTCAGAGGGAGAGAAAGAGGCA
AAAATTGGAACAGAAATATCATTTGATTCGTCGATCCCCGAAAAAAGAAATAGGC-------
--AAAGTTTCATCGTTGAGTGACAAGTGGAAAATTCATGGAAAATTGCAATCCCCACCA
CGTAATAGTGCCCCTACACGTCTTCATCGACGTTGCTTTTTGACCGGAAGACCTAGAGC
TAACTATCGAGACTTTGGACTATCCGGACACATACTTCGAGAAAAGGTTCATGCATGTT
TGTTGCCGGGGGCAACAAGATCAAGTTGGATGGTCAAAAATTCATTCATCTCGGTTATT
CCGCAAGAAGAAAAAAACAAA------------GGATCTGTTGAATTTCAAGTATTCAGTTTCA
CCAATAAGATACGGAAACTTACTTCACATTTGGAATTGCACAGAAAAGACTATTCATCT
CAGATAGGCCTGCGGAGAATTCTGGGAAAACGTCAACGGCTGCTGGCTTATTTGTCAA
AGAAAAATAGAGTACGTTATAAAGAATTAATCGATCAGTTAGGTATTCGGGAGCCAAAA
ACTCGT------ATGGTAAAACTTCGTTTGAAACGATGTGGTAGAAAGCAACGAGCCATCT
ATCGAATCGTTGCAATTGATGTTCGATCCCGAAGAGAAGGAAGAGATCTTCGTAAAGT
GGGTTTTTATGATCCGATAAATAACCAAACTTATTTAAATGTTCCTCTTATTCTATATTTCC
TTGAAAAGGGCGCTCAACCTACAGGAACTGTTTATGATATTTTAAAGAAGGCAGAGGT
ATTTAAAGAACTTCAAATTAATCAAAAGAAATCAAAT---ATGGATAAATCCAAGCGACC
TTTTCGTAAATCCAAGAGATCTTTTCGTAGGCGTTTGCCCCCAATTGGATCGGGGGATC
GAATTGATTATAGAAACATGAGTTTAATTAGTCAATTTATTAGTGAACAAGGAAAAATAT
TATCTAGACGAGTGAATAGATTGACCTTGAAACAACAACGATTAATTACTATTGCTATAA
AACAAGCTCGTATTTTATCTTCGTTACCCTTTCTTAATAATGAGAAA------CAGTTTGAGA
GAACCGAATCGATCCCTAGAACTACCGGTCCTAGAACCAGAAAA---GTGACACGTTCA
CTAAAAAAAAATCCTTTTGTAGCTAATCATTTATTGGCAAAAATTGAGAAGCTCAACAT
GAGAGAGGAAAAAGAGACAATAGTAACTTGGTCCCGGGCATCTACCATTATACCCACA
ATGATCGGCCATACAATCGCTATTCATAATGGAAAGGAACATTTACCTATTTTTATAACA
GATCGCATGGTGGGTCACAAATTGGGAGAATTCGCACCTACTCTTACTTTCCGGGGAC
ACGCGAGAACCGATACTAGATCTCGCCGTATGATTTTGAAATCTTTTCTACTAGGTAATC
TATTATCCTTATGCATGAAGATAATAAATTCGGTCGTTGTGGTCGGACTCTATTATGGATT
TCTGACCACATTCTCCATAGGGCCCTCTTATCTTTTCCTTCTCCGAGCTCGGGTTATGGA
AGAAGGA---------ACCGAGAAGGAGGTATCAGCAACAACTGGTTTTATTACAGGACAGC
TCATGATCTTCATATCGATCTATTATGCGCCTCTGCATCTGGCAATGGGTAGACCTTATAC
AATAACTGTACTAGGTCTACCATATCTTTTGTTTCATTTCTTCTGGAAGAATCACAAACA
TTTTTTTGATTATGGATCAACTAACCTAAATTCAATGCGTAATTTCAGCATTCAATCCATA
TTCTTGAATAATTTCATTTTTCAATTATTGAACCATTTCGTTTTACCAAGTTCAACGTTAA
TCAGATTGGTCAACATTTCTATGTTTCGATGCAACAACAAAACGCTATTTGTAATAAGTA
GTTTTGTTGGTTGGTTAATTGGTCACATTTTATTCATGAAATGGGTTGGATTGATATTATT

CTGGATACAGCGAAATCGTTCAATTAGATCC---ATTAGGTCTAATAAGTACCTTGTGTCA
GAATTGAGAAATTTTTTGTCTCGAATGGTTAATATTTCTTATTTATCACCTGCGTATACT
ATTTAGGAAGAATACCTTCGCCTATCCTGACTAAGAAACTGAAAGAAGCCGAAGAAGA
CAAACCTATTGTAACTCTTCTTTTCGACTATAAACGATGGGATCGTCCATTGCGATATATA
AAAAATGATCGTTTTGCAAATGCTGTAAGAAATGAAATGTCACAATATTTTTTTCACAA
ATGTCCAGTTGATGGAAAACAAAAAATATCTTTTACATATCCACCTAGTTTGTCAATTTT
TTTTGAAATGATAGAAAAACATCTTTCTTTGTGTACGACAGAAAAACTATCCCCGGAAG
ATCTGTATAATCATTGGGTTTATACCAATGAAAAAAAAAGATGCAACTTGAACAATGAG
TTCATAAATAGAATAGAAGCTCTAAAC------AAGCTTTCTATGCATGTGCTCGAAAAAAG
GACCAGATTATACAATGATAAGAATGAACAAGAATGCTTGCCTAGAATGTACGATCCTT
TTTTGAACGGACCATACCGTGGAACAATCAAAAAAGTGTATTCACACCTAAGAGTGGT
TTGGATAAATAAGATTGATGATATCCTTTACCAAGAATTTGAAGAGAAAGATACACCAA
ATTATTCTATTGGAATAGAAGAAATTGGTAAAAAGGTTCCTCGATGGTCATACAAATTG
ACCGATTTGCAACAGCAGGAAAGGGAAGCGATTCGTTCAAGAAAATCCAAACGCGTG
GTAATTTATACTGATGCTTTGATACGTTATTCGCAACAATCGGATTTTCGTCGGGATCTAA
TCAAAGGATCTAGGCGTGCTCAAAGACGTAAAACAGTTACTTGGGAAATATTTCAAGC
AAATGTGCATTCCCCACTTTTTTTGGACAGAATAGACAAAACATTTTTTTTTTCTTTTGA
TATC------TCCGGAACGATTAATCTAGCTTTTAGAAATAAAGAGGAGAATGAACGGATAG
CAATAGCAGAAACTTGGGATACTATTATATTTGCTCAAGCAATAAGAGGTATTATGTTAG
TAACCCAATCGATTCTTAGAAAATACATCGTATTGCCTTCATTGATAATAGCTAAAAATC
TTGTCCGTATGTTATTATTTCAATTCCCCGAGTGGTATGAGGATTTGAAGGAGTGGAATA
GAGAAATGCATGTTAAATGTACCTATAATGGTGTTCAATTATCAGAAACAGAATTTCCA
AAAGACTGGTTAACAGACGGTATTCAGATAAAGATCCTATTTCCTTTCTGTCTGAAACC
TTGGCACAGATCTAAGAAAGGAAAAAAAGACAATTTTTGTTTTTTAACAGTCTGGGGA
ATGGAAACCGAACTACCTTTTGGTTCTTCCAGAAAACGACCTTCCTTTTTTGAACCCAT
TTTAAAAGAACTCGAAAAAAAAAATTAGAAAAGCGAAA---AAAAAATATTTTTTAGTTCT
AAGCGTTTTA---------AAAGAATGGTTTCGAAAGGTCTCCAAAGAAAAAACAAGATGGA
TTAGAAAAATAGTTTTA------TTTATAAAAAAAATAATCAAAGAGTTTGCAAAAGTAAAC
---ACCATAATCAGTAATAAGATTCTTAGTGAACCGACCATTCGAATTAGATCCATGGATT
GGACAAATTATTCACTGACAGAAAAAAAAATGAAAGATCTGGCCGACAGGACAACCA
CAATAAGAAATCAAATAGAAAGGATCACAAAAGAAAAAAGAACAAGTCCTAACGAGA
CAAGTTGTGATGATAAAAGATCTGAATCACAGAAACATATTTGGCAGATATCAAAAAGA
AGAAGTGCCTGGTTCATACGTAAATGGCGCTATTTTATTAAAATTTTCAGTGAAAGAATA
TACATAGATATCTTTCTATGCGCCATTAATATTCCCAGGGTCAATGCACAACTTTTC---AA
ACTAATTTCGTGGGATCTATCTTCCTTGTCCCAAGCATATTTATTTTACAAATTATCACAA
ACCCAAGTTATTAACAAGTATCGCTTGAAATCTGTACTTCAATAT---GATCTTTTTCTTAG
GGATAGAATAAGGGATTATTTGACACGAGGAATATTTGATTCCAAG------TTTAAGAAAC
TTCCGAATTCTGGAATGGGTGAGTGGAAAAACTGGTTAAGAGGTCATTATCAATACAAT
TTATCTCAGACTAGATGGTCTAGATTAGTATCAAAAAAATGGCGAGATAGAGTCAATCA
ACATCGTACGATTCAAAATAAAAAA---AGGGATTCCTATGAAAAA------GACCAATTCAT
TCATTACGAGAAACAAGAAAAATTTCAAAAACACTACAGATATGATCTTTTTTCATATA
AATATATTAATAAATATATTATTGATAGGGATAAAAAATATTTGGAATGGAAAATTCTCAA
TTTTTGTCTTAGATTTTTTGGCCGGATGGGAATGAATCAAGGAATGCTATATCGTTGGTT
CTTCTCAGAATTTGTGCCACTTTATGATGCATATAAGATTAAACCATGGATCATACCAAT

CAAATTACTTCTTTTCAATTCCAAGAGCAACAAGGGAGCAGAACTACAGTTCTTCTTG
AAAAGATATTTGCTTTTTCAATTAAGATGGGATGATCCTTTGAATCAAAGAATGATGAAT
AATGTCAAGGTATATTGTCTCCTGCTTAGACTTATAAATCCAAGGGAAATTGCTATATCC
TCTATTCAAAGAGGAGAAATGCGCCTGGATGTAATGTTGATTCAGAAAGATCTAACTCT
TACAGAATTGATAAAAAGGGGAATATTGATTATCGAACCGGTCCGTCTGTCTATAAAAT
GGGATGGGCAATTTATTATGTATCAAACCATAGGCATTTCATTGGTCCATAAGAATAAGC
ACAATGATTATGATTTGCTTGTTCCTGAAAATATTCTATCTCCTAGACGTCGCAGAGAAT
TGAGAATTCTAATCTCCTTCAGATTCCTTTGGCCTAATTATCGATTAGAAGATTTAGCTT
GTATTAATCGCTACTGGTTTGATACCAATAATGGCAGTCGTTTCAGTATGTTAAGGATAC
ATATGTATCCAATGAAACGACATCAATTCAAATCCTGGATCTTCGAATTG------------AGA
GAGATCAAGAATTCTCACTATTTCTTAGATTCATGGACCAAATTCGATTCAGTGGGATCT
TTCACTCACATTTTTTTCCACCAAGAACGTTTTATGAAACTCTTTGACCCCCGAATTTG
GAGTATCCTACTTTCA---CGTGATTCA------CAGGGTTCAACAAGCAATCGATATTTC------
---ACGATCAAAGGTGTAGTACTGCTTGTAGTAGTG---GTCCTTATA------------TATCGTATT
AACAATCGAAATATGGTCGAAAGAAAAAATCTCTATTTGATGGGGCTTCTT---------------C
CTATACCTATGAATTCCATTGGACCCAGAAATGATACATTGGAAGAATCTTTT---TGGTCT
TCCAATCTCAATAGGTTGATTGTTTCG---CTCCTGTATCTTCCAAAAGGGAAAAAGATCT
CTGAGAGTTGTTTCATGGATCCGAAAGAGAGTACTTGGGTTCTCCCAATAACT------------
AAAAAGTGTATCATGCCTGAATCTAACTGGGGTTCGCGGTGGTGGAGGAACCGGATCG
GAAAAAAGAGG------GATTCTAGTTGTAAGATATCT---------AATGAAACCGTAGCTGGAA
TTGAGATCTCATTCAAAGAGAAAGATATCAAA---TATCTGGAGTTTCTTTTTGTATCCTAT
ACGGAT------GATCCGATCCGCAAGGACCATGATTGGGAATTCTTTGATCGTCTTTCTCC
GAGGAAGAAGCGAAACATAATCAACTTG------AATTCGGGACAGCTATTCGAAATCTTA
GTGAAACACTTGATTTGTTATCTCATGTCTGCTTTTCGTGAAAAAAGACCAATTGAAGT
GGAGGGT---TTCTTCAAACAACAAGGA---GCTGAGGCAACTATTCAATCAAATGATATT
GAGCATGTTTCCCAT------CTCTTCTCGAGAAACAAGCGGGGTATTTCTTTGCAAAATTG
TGCTCAATTTCATATGTGGCAATTCCGCCAAGATCTCTTCGTT---------AGTTGGGGGAAG
---AATCGGCACGAATTGGATTTTTTGAGGAAC---GTATCGAGAGAGAATTTGATTTGGTT
AGACAATGTGTGGTTGGTAAACAAGGATCGG---------TTTTTTAGC------AAGGTACGGAA
TGTATCGTCAAATATTCAATATGATTCC---------ACAAGA------TCTATTTTCGTTCAAGTAA
CGGATTCTAGCCAATTGAAAGGATCTTCT---GATCAATCCAGAGATCCTTTCGATTCCAT
TAGT------------AATGAGGATTCGGAA------------------------------------TATCACACATTGATC
AATCAAACAGAGATTCAGCAACTAAAAGAAAGATCGATTCTT---TGGGGT------CCTTCC
TTTCTTCAAACGGAACGAACAGAGATAGAATCAGAT------CGACTCTTGAAA------TGCC
TTTCTGGATATTCC------TCAATGTCCCGGCTATTCACG---GAACGTGAGAAGCAGATGA
ATAATCATCTGCTTCCGGAAGAAATCGAAGAATTTCTT------GGGAATCCTACAAGATCA
ATTCGTTCTTTTTTCTCTGATAGATGG------TCAGAACTTCAT---CTGGGTTCGAATCCTAC
TGAGAGGTCTACTAGAGATCAT------------AAATTGTTGAAGAAACAACAAGATGTTTCT--
-TTTGTCCCTTCCAGGCGATCGGAA------------AATCAAGAAATAGTTGATATATTCAAGAT
AATTACATATTTACAAAAT------ACCGTCTCAATTCATCCTATTTCATCAGATCCGGGATGT
GATATGGTTCCG---AAGGATGAACCGGAT---ATGGACAGTTCCAATAAGATTTCATTCTT
GAACAAAAAATCCATTTTTGGAT------TTATTTCATCTATTCCATGACCGGAACAAAGGGG
GATACAGGTTA------CACCACGATTTTGAATCAGAA------GAGAGATTTCAAGAAATGGC
AGATCTATTCACTCTATCAATAACCGAGCCG---GGTCTGGTGGATCATAAGGGATTTGCC

TTTTCTATCGATTCCTACGGATTGGATCAAAAAAAA------TTCTTGAATGAGGTATTCAAC
TCCAGGGATGAATCGAAAAAGAAATCTTTA---------TTGGTTCTACCTCCTATTTTTTAT----
--GAAGAGAATGAATCTTTTTATCGAAGGATCAGAAAAAAAATCGGTCCGG---ATCTCCTG
CGGGAATGATTTGGAAGATCCAAAACCAAAAATAGTGGTATTTGCTAACAACAACATA
ATGGAGGCGGTCAATCAATATAGATTGATCCGAAATCTGATGCAAATCCAATATAGCAC
CTATGGG------TACATAAGAAATGTATCGAATCGATTCTTTTTAATG---------AATAGATCCG
ATCGCAACTTCGAATATGGAATTCAA---------AGGGATCAAATAGGAAATGATACTCTGA
ATCATCTAACTATAATAAAATATACGATCAACCAACATTTATCGAATTTGAAAAAG---AGT
CAGAAGAAATGGTTCGAT------------CCTCTTATTTCTCGAACCGAGAGATCCATGAATCG
GGATCCTGATGCATATAGATACAAATGGTCCAATGGGAGCAAGAATTTCCAGGAACATT
TGGAACATTTCGTTTCGGAACAGAAGCAC---------CGGTTTCAAGTAGTGTTCGATCGAT
TACGTATTAATCAATATTCGATTGATTGGTCCGAGGTTATCGACAAACAAGATTTGTCTA
AGTCACTTCGTTTCTTTTTGTCCAAGTCA---------------------------CTTCTCTTTTTGTCC-----
----------------AAGTCACTT---CTCTTTTTGTCTAAGTCACTTCCTTTTTTCTTTGTGAGTATC
GGGAATATCCCCATTCATAGGTCCGAG---ATCCACATCTAT---GAATTGAAAGGTCCGAA
TGATCAACTCTGCAATCAGTTGTTAGAATCAATAGGTGTTCAAATCGTTCATTTGAATAA
ATTGAAACCC------TTCTTATTGGAT---------GATCATGATACTTCC---CAAAGATCGAAATT
CTTGATCAATGGAGGAACAATATCACCC---------AATAAGATACCA---AAGTGG---ATGAT
TGACTCATTCCATACT------AGAAATAATCGCAGGAAATCCTTTGAGAACACGGATTCCT
ATTTCTCAATGATATCCCACGATCGAGACAATTGGCTGAATCCCGTGAAACCATTTCATA
GAAGTTCATTAATATCTTCTTTTTATAAAGCAAATAGACTTCGATTCTTGAATCATCCACA
TCACTTCTGGTTCTAT------TGTAACAAAGGATTCCCTTTTTATGTG------------GAAAAGAC
CCGTATCAATAATTATGAT------CTTACATAT---GGACAATTCCTCAATATCTTGTTCATTCG
CAACAAA---ATATTTTCTTTGTGCGTCGGTAAA------AAAAAACATGTTTTTTTGGAG---A
GAGATACTATTTCACCAATCGAGTCACAGGTATCTGACATATTCATACCTAACGATTTTC
CACAAAGTGGTGACGAAACG---TATAACTTGTACAAATCT---------TTCCATTTTCCAATT
CGATCCGATCCATTCGTTCGT------AGAGCTATTTACTCGATCGCAGACATTTCTGGAACA
CCTCTAACAGAGGAACAAATAGTCAATTTTGAAAGAACTTATTGTCAGCCTCTTTCAGA
TATGAATCTATCTGATTCAGAAGGGAAAAACTTGCATCAGTATCTCAGT------TTCAATTC
AAACATGGGTTTGATTCACACTCCATGTTCTGAGAAATATTTACCA------TCAGGAAAGA
GGAAAAAA------------------------CGGAGTCTTTGTCTAAAG------AAATGCGTTGAGAAAC
GGCAGATGGATAGAACCTTTCAACGAGATAGTGCTTTTTCAAATCTCTCAAAATGGAAT
CTGTTCCAAACATATATGCCATGGTTCCTTACTTCGACAGGGTGCAAATATCTAAATTTG
ACCCTTTTAGATACTTTTTCAGACCCATTG------CCGATACTAAGTAGCAGT---CAAAAAT
TTGTATCCATTTTTCATGATATTATGCATGGATCAGATATATCATGGCCAATTCCTCAGAA
AAAATTGTGG------------GCGATTCTTCCACAATGGAATCTG---ATAAGTGAGAGTTCGAG
TAAGTGTTTACAGAAT------CTTCTTCTGTCCGAAGAAATGATTCATCGAAATAATGAGT
CACCCGTTCCATTGATATGGACACATCTGAGATCACCAAATGCTTGGGAGTTC---CTCTA
TTCAATCCTTTTCCTTTTTCTTGTTGCTGGATAT---CTCGTTCGTACACATCTTCTCTTTGT
TTCCCGA------GCCTCTAGTGAGTTACAGACAGGGTTAGAAAGGATCAAATCTTTGATGA
TTCCATCATACATGATTGAGTTGCGAAAACTTCTGGATAGGTATCCTACATCTGAACCGA
ATTCTTTCTGGTTAAAGAATCTCTTTCTAGTTGCTCTGGAACAATTAGGAGATTCTCTGG
AAAAAATACGGGGTTCTGCTTCTGGCGGCAACATGCTATTGGGTGGTGGTCCC-----------
----GCTTATGGG---GTCAAATCAATACGTTCTAAGAAGAAATATTTGAATATCAAT---------C

TCATCGATCTCATC---AGTATCATACCCAATCCCATCAATCGAATCACTTTTTCGAGAAAT
ACGAGACATCTAAGTCGTACAAGTAAAGAGATCTATTCATTGATAAGAAAAAGAAAAA
ATGTGAACGGTGATTGGATTGATGATAAAATAGAATCCTGGGTCGCGAACAGTGATTCG
ATTGATGATGAAGAAAGAGAATTCTTGGTTCAGTTCTCC------ACCTTAACGACG------G
AAAAAGGGATTGATCAAATTCTATTGAGTCTGACT---CATAGTGATCATTTATCA-----------
----AAGAATGACTCTGGTTATCAAATGATTGAACAACCGGGATCAATTTACTTACGATAC
TTAGTTGACATTCAT------AAAAAGTATCTAATGAATTATGAGTTCAATAGATCCTGTTTA
GCAGAAAGACGGATATTCCTTGCTCATTATCAGACAATCACTTAT------TCACAAACCTC
GTGTGGGGCTAATAGTTCTCATTTCCCATCTCATGGA---------AAACCCTTTTCGCTCCGC
TTAGCCCTATCCCCTTCT---AGGGGTATTTAGTGATAGGTTCTATAGGAACTGGACGATC
CTATTTGGTCAAATACCTAGCGACAAACTCCTATGTTCCTTTCATTACGGTATTTCCGAA
CAAGTTTCTGGATGACAAGCCTAAAGGTTATCGTATTGAT---------------GATATCGAT---AT
TGATGATAGTGACGATATCGAT------------ATTGATGATAGTGACGATATT-----------------------
------------------------------GATGATGACCTTGATACGGAGCTGCTAACTATGACGAATGTGCT
A---------ACTATGTATATGACG---------CCGAAAATAGACCGATTTGATATCACCCTTCAATT
CGAATTAGCAAAAGCAATGTCCCCTTGCATAATATGGATTCCAAACATTCATGATCTGTA
TGTGAATGAGTCGAATTACTTATCCCTCGGTCTATTAGTGAACTATCTCTCC---------AGA
GATTGTGAAAGATGTTCC---ACTAGAAATATTCTTGTTATTGCTTCGACTCATATTCCCCA
AAAAGTGGATCCCGCTCTAATAGCTCCGAATAAATCAAATACATGCATTAAGATACGAA
GGCTTCTTATTCCACAACAACGAAAGCACTTTTTCATTCTTTCATATACTAGGGGATTTC
ACTTGGAAAAGAAAATGTTCCATACT------AACGGATTCGGGTCCATAACCATGGGTTCC
AATGCACGAGATCTTGTAGCACTTACCAATGAGGCCCTATCAATTAGTATTACACAGAA
GAAATCCATTATAGACACTAATACAATTAGATCAGCTCTTCATAGACAAACTTGGGATTT
GCGA---------TCCCAGGTAAGATCGGTTCAGGATCATGGGATCCTTTTCTATCAGATAGGA
AGGGCTGTTGCACAAAATGTACTTCTAAGTAATTGCCCCATAGATCCTATATCTATCTATA
TGAAGAAGAAA------TCATGTAAGGAAGGGGATTCTTATTTGTACAAATGGTACTTCGA
GCTTGGAACGAGCATGAAGAAATTAACGATACTTCTTTATCTTTTGAGTTGTTCTGCCG
GATCGGTCGCTCAAGATCTTTGGTCTCCACCC------GGACCCGATGAAAAA---AATTGGA
TCACTTCTTATGGATTCGTTGAGAATGATTCT---GATCTAGTTCATGGC---TTAGAAGTAG
AAGGCGCTCTGTTGGGA------TCCTCACGGACAGAAAAAGATTGCAGTCAGTTTGATAA
TGAT------CGAGTGACATTGTTTCTTCGGTCCGAACCAAGGAAT---CCGTTAGATATGATG
CAAAATGGATCTTGTTCTATCGTTGATCAGAGATTTCTCTATGAAAAA------TATGAATCG
GAGTTTGAAGAA---GGGGAAAGAGAAGGAGCCCTCGACCCGCAA------------CAGATAG
AGGAGGAT------------TTATTCAATCAC------ATAGTTTGGGCTCCT------AGAATATGGCGCC
CT---------TGTGGCAATCTATTTGATTGTATCGAAAGGACCAATGAATTGGGATTTCCC---T
ATTGGGCCAGGTCATTTCGG---------GGCAAGCGGATCATTTATCATAAAGAGGATGAGC
TTCAAGAGAATGATTCGGAGTTCTTGCAGAGTGGAACCATGCAGTACCAGACACGAGA
TAGATCTTCCAAAGAACAAGGCTTTTTTCGAATAAGCCAATTCATTTGGGACCCTGCAG
ATCCATTCTTTTTCCTATTCAAA---GATCAGCCCTTTGTCTCTGTGTTTTCACGCCGAGAA
TTCTTTGCAGATGAAGAGATGTCAAAAGGGCTTATTACTTCCCAAACAAATCCTCCTAC
ATCTATATATAAACGCTGGTTCATC------AAGAATACGCAAGAAAAGCACTTCGAATTGT
TGATTCATCGCCAGAGATGGCTTAGAACC---------AATAGTTCATTATCT------AATGGATC
TTTCCGTTCTAAT------ACTCCATCCGAGAGTTATCAGTATTTATCAAATCTGTTCCTATCT
AAC---GGAACGCTATTGGACCAAATAGCAAAGGCATTGTTGAGAAAGAGATGGCTTTT

CCCGGATGAAATGAAACATTTGATT------CATGTAACAGGAGAAAGATTTCCCATTCCTA
TGCCTAGATCTCGG---ATAAATGGAAATTTTATTGATAAGACCTCTTCAATTGTAGCCAAT
ATCTTATTACGAATAATTCCGACAACTTCAGGAGAAAAAGAGGCATTTACCTATTACAG
AGATGGG------ATGTCAGCCCAATCCGAAGGAAATTATGCGGAAGCTTTACAAAATTATT
ATGAAGCTACGCGACTAGAAATTGATCCCTATGATCGAAGTTATATACTCTATAACATAG
GCCTTATCCACACAAGCAACGGAGAACATACGAAGGCTTTGGAATATTATTTCCGGGCA
ATAGAACGAAATCCATTCTTACCACAAGCTTTTAATAATATGGCCGTGATCTGTCATTAC-
--CGGGGAGAACAGGCCATTCGACAAGGAGATTCTGAAATTGCAGAGGCTTGGTCCGA
TCAAGCTGCTGAATATTGGAAACAAGCTATAGCGCTTACTCCGGGTAATTATATTGAAG
CACATAATTGGTTGAAGATCGCAAGGCGGTTCGAA------ATGAACTGGCGATCAGAACG
TATATGGATAGAACTTATAACGGGGTCTCGAAAAACAAGTAATTTCTGCTGGGCCTGTA
TCCTTTTTTTAGGTTCACTAGGATTCTTATTGGTTGGAACTTCGAGTTATCTTGGTAGGA
ATCTGATATCCCTATTCCCATCTCAGCAAATCATTTTTTTTCCCCAAGGGATCGTGATGTC
TTTCTATGGGATCGCGGGTCTGTTCATTAGTTCCTATTTGTGGTGCACAATTTCGTGGAA
TGTAGGTAGTGGTTATGATCGATTCGATAGAAAAGAAGGAATAGTGTGTATTTTTCGTT
GGGGATTTCCTGGAATAAATCGTCGCATCTTCCTTCGATTTCTTATGAGAGATATCCAGT
CCATCAGAATGGAAGTTAAAGGGGGTCTTTATTCTCGACGTGTCCTTTATATGGAAATC
AGAGGCCAGGGTGCCATTCCCTTGACTCGTACTGATGAGAAT---TTTACTCCACGAGAA
ATTGAACAAAAAGCTGCTGAATTAGCCTATTTTTTGCGCGTGCCAATTGAATTGAAA
>Amborella
ATGGAAAAATGTGGGTTCAATTCGATGTTGTCTCTAAGTAAATCGATGGACAGTCTTGG
TCTTATTGGAAATACCGGTGCAGGTGGAGGCCCTTTGATCTATGATGACACTTTTAAGC
TTAAG---TATTTTGATATTGACGAGATTGACGATGATAGTGACTCTCTGAGTGATATTGA
AAATCAGAATCAGATCTTCTTTGATATTTACAATGATAGTGACTCTCTGGAACTAGATAG
TGAAAATAAGAACTTTGAGATTCACAATGAG------AGTGACTTTATGAGTAAACTAGAA
AGTTCTTTTTTTAGTTATCTCACTTATAGTAATCTCAATAGTGGATCTAAGAGTTACAACC
CCTACTATGATCCTTACATGCATGACACTAGATATAGTTGGAATAATAACATTAATAGTTG
CATTGACAGTTATATTCGTTGTGAAATCGAT------TCC---------ATTTCAAGTGGTAGCGAC
AATTGCAGTGACAGTTATATTTATAGTTACATCTGTAGTGACAATGGGAGTTCTATAAGA
ACTAGAACTAGTGGCAGTTCTTACCATATAAGAGGAAGATCT---AATTTCGAGAAAAAT
AAAAAATTAAAAAAATTATGGGTTCAATGCGAGAATTGTTATGCATTGAATTATAATAAA
TTATTTAGGTCTAAAATGAATGTTTGTGAACAATGTGGATATCACTTGAAAATGAGTAGT
TCAGATAGAATCGAACTCTCGATTGATCCAGGCACTTGGCATCCTATGGATGAAGATAT
GGTCTCCACGGATCCTATTGAATTTCATTCGGAGGAGGAACCTTATAGAGATCGCATCG
ATTCTTATCAAAGACAAGCAGGTTTAACCGATGCTGTTCAAACAGGCATAGGTCAACTA
GAAGGTATTCCGATAGCAATTGGAGTTATGGATTTTCAGTTCATGGGAGGTAGTATGGG
ATCCGTAGTAGGCGAGAAAATAACTCGTTTGATCGAGTATGCTATTGATAGATCTCTCCC
AGTTGTTATTGTGTGTGCTTCTGGAGGAGCACGCATGCAAGAAGGAGGTTTGAGCTTG
ATGCAAATGGCTAAAATATCTTCCGCTTCATATAATTATCAATCCAATAAAAAGTTATTCT
ATGTATCAATCCTTACATCTCCTACAACCGGTGGAGTGACTGCGAGTTTCGGTATGTTG
GGAGATATCATTATTGCCGAACCCAATGCCTACGTAGCATTTGCAGGCAAAAGAGTAAT
TGAAGAAACATTAAATAAGAAAATACCTGATGGTTTGCAAGTAGCTGAGTATTCATTCC
ATAAGGGCTTATTCGATTCAATCGTACCACGTAATCCTTTAAAAGGTGTTCCAAGTGAG
TTATTTCAGCTCCACGGTTTCTTTTCTTCTACTTTCCATCCCCTTAAAATGGTAACCATTC

GAGCTGACGAAATTAGTAATATTATCCGTGAACGGATTGAGCAATATAATAGAGAAGTA
AAGATTGTCAATACTGGCACTGTACTTCAAGTGGGCGACGGCATTGCTCGTATTCATGG
TCTTGATGAAGTAATGGCGGGCGAATTAGTAGAATTTGAAGAGAGTACAATAGGCATTG
CTCCAAATTTGGAATCCAATAATGTTGGTGTTGTATTAATGGGTGATGGTTTAATGATAC
AAGAGGGCAGTTCTGTAAAAGCAACAGGAAGAATTGCTCAGATACCAGTGAGTGAGG
CTTATTTAGGTCGTGTTATAAATGCTCTGGCTAAACCTATTGATGGGAGAGGTGAAATTT
CATCTTCCGAATCTCGGTTAATTGAATCCCCCGCACCAGGTATTATTTCGAGACGTTCCG
TATATGAACCTCTTCAAACAGGTCTTATTGCTATTGATTCCATGATTCCTATAGGACGCG
GTCAGCGAGAATTAATTATTGGGGACAGACAGACCGGTAAAACAGCCGTAGCTACAGA
TACTATTCTGAATCAAAAGGGGCAAAATGTAATATGTGTTTATGTAGCTATTGGTCAAAA
AGCATCTTCCGTGGCTCAGGTAGTGACTACTTTCCAGGAACAAGGAGCAATGGAATAC
ACTATTGTGGTAGCTGAAACGGCGGATTCACCTGCTACATTACAATACCTCGCTCCTTAT
ACAGGAGCAGCCTTGGCTGAATATTTTATGTACCGTGAACGACATACTTTAATAATTCAT
GATGATCCCTCTAAACAAGCACAAGCTTATCGCCAAATGTCTCTTCTATTAAGAAGACC
GCCTGGTCGCGAAGCTTATCCAGGAGATGTTTTTTATTTGCATTCCCGACTTTTGGAAA
GAGCCGCTAAATCAAGTTCTCGTTTAGGCGAAGGAAGTATGACTGCTTTACCAATAGTT
GAGACTCAATCTGGGGACGTTTCGGCTTATATTCCCACTAATGTAATTTCCATTACAGAT
GGACAAATCTTCTTATCTGCCGATCTATTCAATGCTGGAATCCGGCCTGCTATTAATGTG
GGTATTTCCGTTTCCAGAGTAGGATCCGCAGCTCAAATTAAAGCTATGAAACAAGTAGC
TGGCAAATCAAAATTGGAACTGGCTCAATTTGCAGAGTTAGAAGCCTTTGCACAATTC
GCTTCTGATCTCGATAAAGCTACTCAGAATCAATTGGCAAGAGGTCAACGATTGCGCG
AGTTGCTCAAACAATCCCAAGCAGCCCCTCTCGCGGTGGAAGAACAGGTAGTTACTAT
TTATACAGGAGCGAATGGGTATCTTGATCCATTAGAAATTGGACAGGTGAAGAAATTTC
TCGTTCAGTTACGTACTTATTTAAGAACAAATAAGCCTCAGTTCCAAGAAATCATATCTT
CTACCCGGACGTTCACCGAGGAAGCAGAAGCCATTCTGAAAGAAGCTATTCAGGAAC
AGATAGAACTGTTTCTATTTCAGGAACAAACAATGAGAATCAATCCTACTACTTCTGGT
TCTGGTGCTTCTACACTTGCGGAAAAAAGCGTGGGACGTATCGCTCAAATCATTGGTC
CGGTACTGGATGTAGCTTTTCCCCCGGGCAAGATGCCCAATATTTACAACTCTTTGGTA
GTTAAGGGTCGAGATACTGCCGGTCAGCAAATTAATGTGACTTGTGAGGTACAACAATT
ATTAGGAAATAATCGAGTTAGAACGGTAGCTATGAGTGCTACAGATGGTCTAATGAGAG
GAATGGAAGTGATTGACACGGGAGCTCCTCTAAGTGTTCCGGTTGGCGAAGCTACTCT
CGGACGAATTTTCAACGTTCTTGGAGAACCAGTTGATAATTTAGGTCCTGTAGATACTC
GCACAACATCTCCTATTCATAGATCTGCGCCTGCCTTTATACAGTTAGATACCAAATTATC
AATCTTTGAAACAGGCATTAAAGTGGTGGATCTTTTAGCTCCTTATCGTCGTGGAGGAA
AAATCGGGCTATTCGGGGGAGCCGGAGTGGGTAAAACAGTACTCATCATGGAATTGAT
CAATAACATTGCCAAAGCTCACGGAGGTGTATCTGTATTTGGCGGAGTAGGAGAACGT
ACTCGTGAAGGAAATGATCTTTACATGGAAATGAAAGAATCCGGAGTGATTAATGAAC
AAAATATTGCAGAATCAAAAGTCGCTCTAGTCTATGGTCAGATGAATGAACCGCCGGG
AGCTCGGATGAGAGTTGGTTTAACTGCCCTAACAATGGCGGAATATTTCCGTGATGTTA
ATGAGCAAGACGTACTTCTATTTATTGACAATATCTCTCGCTTCGTTCAAGCAGGATCAG
AAGTCTCTGCCTTATTAGGCAGAATGCCTTCTGCCGTGGGTTATCAACCTACCCTTAGTA
CAGAAATGGGTTCTTTGCAAGAAAGAATTACTTCTACTAAAGAGGGATCCATAACTTCC
ATTCAAGCAGTTTATGTACCTGCAGACGATTTGACCGACCCTGCTCCTGCCACGACATT
TGCACATTTAGATGCTACTACCGTACTATCAAGAGGATTAGCCGCCAAAGGTATCTATCC

AGCAGTAGATCCTTTAGATTCAACGTCAACTATGCTCCAACCTCGGATCGTTGGCGAGG
AACATTACGAAACTGCGCAAAGAGTTAAGCAAACTTTACAACGTTACAAAGAACTTCA
AGACATTATAGCTATTCTTGGACCAGATGAATTATCCGAAGAGGATCGTTTAACCGTAG
CAAGAGCACGAAAAATTGAGCGTTTCTTATCACAACCCTTCTTCGTAGCAGAGGTATTT
ACTGGTTCTCCAGGAAAATATGTTGGTCTCGCAGAAACAATTAGGGGGTTTCAACTGA
TCCTTTCCGGAGAATTAGATGGTCTTCCCGAACAGGCTTTTTATCTGGTAGGTAACATC
GATGAAGCTACCGCGAAGGCTATTAACTTAGAAGTAGAGAGCAAATTAAAGAAAATGA
CCCTAAATCTTTGTGTACTGACTCCTAATCGAATCATTTGGGATTCAGAAGTGAAAGAA
ATTATTTTATCCACTAATAGTGGCCAAATTGGTGTATTACCAAATCACGCACCTATTGCA
ACGGCTGTAGATATAGGTATTCTCAAAATACGCCTCCAGGAGCAATGGTTAACAATGGC
TCTGATGGGTGGTTTCGCCAGAATAGGCAATAATGAAATAACCATATTAGTAAATGATGC
GGAGAAGGGTAGTGACATTGATCCGCAAGAAGCTCAGCAAACTCTTGAAATAGCTGA
AGCTAACCTGAGTAGAGCTGAGGGCAAGAGACAAACAATTGAGGCGAATTTAGCTCTT
AGACGAGCTAGGACACGAGTAGAGGCTATCAATGCTATTTCGATGAAAAATGTAACCG
ACTCTTTCGTTTCCTTGGGCCACTGGCCATCTGCCGGAAGTTTCGGATTTAATACCGATA
TTTTTGCAACAAATCCAATAAATCTAAGTGTGGTGCTTGGTGTGTTGATTTTTTTCGGA
AAGGGAGTGTTAAGTGATTTATTAGATAATCGAAAACAGAGAATCTTGAGTACGATTCG
CAATTCAGAAGAACTACGTGGGGGAGCCATTGAGCAGCTTGAAAAAGCCCGGGCTCG
CTTACGGAAAGTGGAAATTGAAGCAGATGAGTTTCGAGTGAATGGATACTCTGAGATA
GAACGAGAAAAAAGTAATTTGATTAATGCTGCTTATGAAAATTTAGAACGATTAGAAAA
TTACAAAAATGAAAGCATTCATTTTGAACAACAAAGAGCGATGAATCAGGTCCGACAG
CGAGTTTTCCAACAAGCCTTGCAAGGAGCTCTAGAAACTCTGAATAGTTATTTGAACA
GCGAGTTACATTTACGCACCATCAGTGCTAATATTGGCATGCTCGGGACCATGAAAAAT
ATAACTGATATGAATCCCCTGATTTCTGCTGCTTCCGTTATTGCTGCTGGATTGGCCGTA
GGACTTGCTTCTATTGGACCCGGAGTCGGTCAAGGTACTGCTGCGGGTCAAGCCGTAG
AGGGTATCGCGAGACAACCAGAAGCAGAGGGTAAAATACGAGGTACTTTATTGCTTAG
CCTGGCTTTTATGGAAGCTTTAACAATTTACGGACTGGTCGTGGCATTAGCGCTTTTATT
TGCGAATCCCTTTGTTATGAGTGTTCTACCATGTTCCATCAACACACTAAAAGGGATATA
CGATCTATCCGGTGTGGAAGTAGGCCAACATTTCTATTGGCAAATTGGGGGTTTCCAAG
TCCATGCCCAAGTACTTATAACTTCTTGGGTCGTAATTGCTATCTTATTAGGTTCAGCCA
CTATAGTTGTTCGGAATCCACAAACCATTCCGACCGACGGTCAGAATTTCTTCGAATAT
GTGCTTGAATTCATTCGAGACTTGACCAAAACTCAGATTGGAGAAGAATATGGTCCCT
GGGTTCCCTTTATTGGAACTATGTTCCTATTTATTTTTGTTTCTAACTGGTCCGGCGCTCT
TTTACCTCGGAAAATCATAGAGTTACCTCAGGGGGAGTTAGCTGCACCTACAAATGATA
TAAATACTACTGTTGCTTTAGCTTTACCCACGTCAGTGGCATATTTCTATGCAGGTATTA
GCAAGAAAGGATTGGGTTATTTCGGTAAATATATTCAACCAACTCCCATACTTTTACCAA
TTAATATCTTAGAAGATTTCACAAAACCTTTATCACTTAGTTTTCGACTTTTTGGGAATAT
ATTGGCTGATGAATTAGTAGTTGTTGTTCTTGTTTCTTTAGTCCCTTCATTGGTTCCTATA
CCTGTCATGTTCCTCGGATTATTCACAAGCGGTATTCAAGCTTTGATTTTTGCAACTTTA
GCCGCGGCTTATATAGGCGAATCCATGGAGGGTCATCATATGATATTCGCGACTTTAGAA
CATATATTAACTCACATCTCTTTTTCAATAATTTCAATTGTCATTACGACTCATTTGATGA
CCTTCGCCCGT---GAAATCGCAGGACTATCTGATTTGTCAGAAAAAGGCATGATAGCTG
TTTTTTTGCGTATAACAGGATTATTAGTCACTCGTTGGATTTATTCAGGACATTTACCATT
AAGTAATTTATATGAATCATTAATTTTTCTTTCATGGGGTTTCTCTCTTATTCATATGATTT

CTAAAATACAGAACCATAAGAATTTTTTAAGTTCAATAACCGCGCCAAGTGCTATTTTA
ACCCAAGGCTTTGTCACTTCGGGTCTTTTAACTGAAATGCATCAATCCGCAATACTAGT
ACCTGCTCTACAATCCCATTGGTTAATAATGCACGTAAGTATGATGTTGTTGAGCTATGC
AGCTCTCTTATGTGGATCCTTATTATCAATAGCTCTTCTAGTCATTACATTTAGAAAAAAG
CTAGATATTATTTTC---------GAGATCCAATATTTGAATGAAAAAAGAAGTATTTTGCAAA
ACACTTCT---TTTTCATTTAGAAATTACCACAAATATCAACTTACTCAGCAATTAGATCAT
TGGAGTTATCGTGTTATTGGTATAGGATTTACCCTTTTAACTTTAGGTATTCTTTCAGGGG
CGGTATGGGCTAATGAGGCATGGGGATCCTATTGGAATTGGGACCCCAAGGAGACTTG
GGCTTTTATTACTTGGACTATATCTGCGATTTATTTACATACTAGAACAAATAAAAACTTG
CAAAGTGCGAATTCAGCAATTGTGGCTTCGATAGGATTTCTTATCATTTGGATATGCTAT
TTTGGGGTCAATCTATTAGGAATAGGCCTACATAGCTATGGTTCATTCGCATTAAGGGTG
TCCAAAAAA---GCATTGACTTCCCTGCCATATCTTGCATCTATAGTTTTTCTGCCCTGGG
GAATTTCTCTATCATTTAATAAAAGTCTGGAACCTTGGGTTACAAATTGGTGGAATACCA
GCCAATACGAAACTCTTTTGAATGAAATTCAAGAAAATAACGTTCTTGAAAGATTCATA
GAATTAGAAGAACTATTCTTGTTGGACGAAATGATAAGGGATTATCCGGAGACACATAT
ACAAAAGCTTCGTATAGGAATCCACAAAGAAACGATAGAATTGGTGAAAATGTACAAT
CAAGATTATATCCATATAATTTCGCATTTCTCGACAAATATAATGAGTTTCGCTATTCTAA
GTGCTTATTCTATTCTGGGTAATGAAGAACTTGTCACTCTTAATTCTTGGGTTCAGGAAT
TCTTCTATAACTTAAGTGACACAATAAAAGCTTTTTCTATTCTTTTACTAACCGATTCATG
TATCGGATTTCATTCGCCCCATGGTTGGGAACTCATGATTGGTTCGGTCTACAAAGATTT
TGGATTTAGTCATAACGATCAAATTATATCCGGTCTCGTTTCCACTTTTCCGGTTATTCTA
GATACTATTTTGAAATATTGGATCTTCCATCATTTAAATCGTATATCTCCTTCACTTGTAGT
TATTTATCATTCAATGAATGAAATGCCCGTTGGTGTTCCAAAAGTACCTTTTCGGAGTCC
TGGAGAGGAAGACGCAACTTGGGTTGACATATACAACCGACTTCATCGAGAAAGATTA
CTTTTTTTAGGCCAAGAGGTTGAAGGTGAGATATCGAATCAAATTGTTGGTCTCATGGT
ATATCTCAGTATAGAGGATGATACCAGGGATCTCTATTTGTTTATAAACTCTCCCGGCGG
ATGGGTAATACCAGGAATAGCTATTTATGACGCTATGCAATTTGTACCGCCAGACGTACA
TACGATATGCATGGGATTAGCCGCTTCAATGGGATCTTTCGTCCTGGCCGGAGGAGAAA
TTACCAAACGTCTAGCATTCCCTCACGCTAGGGTTATGATCCACCAACCCGCTAGTTCT
TTTTATGAGGCACCAGCAGGAGAATTTATCCTGGAAGCGGGGGAGCTACTGAAACTTC
GCGAAACCATCACAAGCGTTTATGTACAAAGAACAGGCAACCCTTTATGGGTTGTATCC
GAAGACATGGAAAGGGATGTTTTTATGTCAGCAACAGAAGCCCAAGCTCATGGCATTG
TTGATCTTATAGCAGTCGAAATGAAAGAACAAAAATTAATTCATGAAGGTTTAATTACT
GAATCACTTCCCAATGGCATGTTCCAAGTTCGCTTAGATAATGGAGACCTGGTTCTAGG
TTATGTTTCAGGAAGGATCCGACGTAGTTTTATACGGATATTACCAGGCGATAGAGTGA
AAATTGAAGTAAGTCGTTATGATTCAACCAGGGGACGTATTATTTATAGGCTTCGCAAC
AAAGATTCGAATGATATGGAGGAATTACGAGGATATTTAGAAATAGATAGACCCCATCA
ACAACGCTTCCTATATCCGCTTCTATTCCAGGAGTCTATCTATGCACTTGCTCATAATCAT
GGTTTATTAGGTCATGACAAG---TCCAGTTCACTAAATGTAAAACGTTTAATTATTCAAA
TGTATCAACAGAATCATTTCATCATTTCATTTAATGATTCTAACCAAAATAGATTTCTTGG
GAGGAATTCTTATTTTCAAATGGTATCAGAGGGTTTTGCAGTAATTGTGGAAATTCCATT
CTCGATGCGATTAGTATCTTCCCTAGGATCTGAATCTCAT---TTCCAATCTATCCATTCAAT
ATTTCCGTTTCTAGAGGACAAACTTTCATATTTAAATTATGTGTTAGATATACTAATACCC
CACCCAATCCATCTGGAAATCTTGGTTCAAAGCCTTCGCCAATGGATACGAGACCTTCC

TTCTTTGCATTTATTGCGGTTTTTTCTCCACGAACATCAGAATTGGAATAGTTTCATTACT
AAATGCAGTTCTTTTTTTTCAAGACAAAGATTGTTTTTGTTCCTGTACAATTTTCATGTA
TATCAATTTGAATCCCTTTTCGTTTTCCTACGTAAACAATTTTTTCATTTACGGTCGATAT
CTTTTGGATCTTTTCTTGAACGAACACATTTCTATGGAAAAATAGAAAAT---GTAGTATC
CATGCGTAATCAGAATATTTTATGGTTGTTCAAGGACCTTTTCATGCATTATGTTAGATAT
AAAGGAAAATCAATTATGGCTTCAAGGGGTACTTATCTCCTTATGAATAAATGGAAATC
TCACCTTGTCAATTTCTGGCAATTCCGTTTTTACTTCTGGTCTCAACCGGGCAGAATCCA
TATAAATGAATTATCCAATCATTCCTTCTATTTCCCGGGCTATCTTTCAGGTCTACGATTA
AATCCCTCGATGGTAAGGAGTGAAATGCTAGAGAATTCATTTATGATAGATGCTGTTATC
------AAGAGATTCGATACAGTAGTCCCAACTATTTTTCTTATTGGATCCTTGGCTAAAGTA
AAATTATGTAACGTATCAGGGCATCCTATTAGTAAGTCGGTCTGGGCCGATTCGTCAGAT
TCTGATATTCTCGATCAATTTGGGCGGATATGCAGAAATCTCTCTCATTATCACAGTGGG
TCCTACAAAAAACACAGTTTGTGTCGAATAAAATATATACTTCGACTTTCGTGTGCTAG
AACTTTGGCTCGTAAACATAAAAGCACGGTACGCGCAATTTTGAAAAGATTAGGTTCG
GAATTCTTGGACGAATTCCTTACGGAGGAACAAGAGATTCTTTCCTTGATTTTTCCAAA
ACGAATTTGGTATTTGGATATTATCCGTATTCATAGTCTGGCCAATCAT---ATGATAATTGA
TACGACA---GAAGTACAA---GCTATCAATTCTTTTTCCAGATTGGAA---TCGTCAAAAGA
GGTCTATGGTCTGATATGGCTATTTATCCCTATTTTTACTCCTGTATCAGGGATCACAATA
GGGGTATTGGTTATTGTGTGGCTAGAAAGAGAAATATCTGCAGGGATACAACAACGCAT
TGGACCTGAATACGCTGGTCCTTTGGGGATTCTTCAAGCTATAGCAGATGGGACCAAAT
TACTTTTCAAAGAGGATCTTCTCCCATCTAGAGGAGATATTCGTTTATTCAGTATCGGGC
CTTCTGTAGTGGTCATATCAATTTTACTAAGTCATTTGGTAATTCCTTTCGGTTATCGCCT
TGTTCTCGCCGATCTAAGTATAGGTGTTTCTCTATGGATCGCAATTTCCAGTATTGCTCCT
ATTGGACTTCTTATGTCAGGATATGCATCAAATAATAAATATTCTTTTTCAGGTGGTCTAC
GAGCTGCTGCTCAATCTATTAGTTATGAAATACCGTTAACTCTGTGTGTGTTATCAATATC
TCTACTATCTAACAGTTCAAGTACGGTTGATATAGTGGAGGCACAGTATAAATATGGTTT
TTGGGGGTGGAATCTGTGGCGTCAACCTATAGGGTTTCTAGCTTTTCTAATTTCTTCCTT
AGCGGAATGTGAGAGGTTGCCCTTTGATTTACCAGAAGCGGAGGAAGAATTAGTAGCG
GGTTATCAAACCGAATATTCAGGTATTAAATTTGGTTTATTTTATCTTGCTTCTTACCTAA
ATCTACTAGTTTCTTCATTATTTGTAACGGTTCTTTATTTGGGTGGGTGGAATCTTTCTAT
TCCATACATAGCAATTCCTGAGCTTTTCAGAATAAATAGAATTGGTGGAGTTTTTGGAA
CAACAATTAGTATCTTTTTTACATTAGCTAAAGCTTATTTGTTCCTGTTCATTCCTATCAC
AACAAGATGGACTTTACCTAGGATGAGAATGGACCAACTATTAAATCTTGGTTGGAAAT
TTCTTTTACCTATTGCTCTAGGTAATCTATTACTGACAACTTCTTCCCAACTTTTTTCGCT
GATGATCTGGCATGTACAGAATGAAAACTTCATTCTCGATTCTACGAGAATTTTTATGAA
AGCCTTTCATTTGCTTCTCTTCCATGGAAGTTTCATTTTCCCAGAATGTATCCTAATTTTT
GGCCTAATTCTTCTTCTAATGATCGATTCAACCTCTGATCAAAAAGATAGACCTTGGTTC
TATTTCATCTCTTCAACAAGTTTAGTAATGAGCATAACGGCCCTATTGTTCCGATGGAGA
GAAGAACCTATGATTAGCTTTTCGGGGAATTTCCAAACGAACAATTTCAACGAAATCTT
TCAATTTCTTATTTTACTATGTTCAACTCTATGTATTCCTCTATCAGTAGAGTACATTGAAT
GTACAGAAATGGCTATAACAGAGTTTCTGTTATTCGTATTAACAGCTACTCTAGGAGGA
ATGTTTTTATGTGGTGCTAACGATTTAATAACTATCTTTGTAGCTCCAGAATGTTTCAGTT
TATGCTCCTACCTATTATCTGGATATACCAAGAGAGATGTACGGTCTAATGAGGCTACTA
CGAAATATTTACTCATGGGTGGGGCAAGCTCTTCTATTCTGGTTCATGGTTTCTCTTGGC

TATACGGCTCATCCGGGGGAGAGATCGAGCTTCAAGAAATAGTGAATGGTCTTATCAAT
ACACAAATGTATAACTCCCCGGGAATTTCGATTGCGCTTCTATCCATCACTGTAGGAATT
GGGTTCAAGCTTTCCCCAGCCCCTTTTCATCAATGGACTCCTGACGTATACGAAGGATC
TCCCACTCCAGTCGTTGCTTTTCTTTCTGTTACTTCGAAAGTAGCTGCTTCAGCTTTAGC
CACTCGAATTTTCGATATTCCTTTCTATTTCTCATCGAACGAATGGCATCTTCTTCTTGAA
ATCCTAGCTATTCTTAGCATGATATTGGGGAATCTCATTGCTATTACTCAAACAAGCATG
AAACGTATGCTTGCATATTCGTCCATAGGTCAAATCGGATATGTAATTATTGGAATAATTG
TTGGAGACTCAAATGATGGATATGCAAGCATGATAACTTATATGCTGTTCTATATCTCCAT
GAATCTAGGAACTTTTGCTCGCATTGTATCATTTGGTCCACGTACCGGAACTGATAACAT
TCGGGATTATGCAGGATTATACACGAAAGATCCTTTTTTGGCTCTCTCTTCAGCCCTATG
TCTCCTATCCCTAGGAGGTATTCCTCCATTAGCAGGTTTTTTCGGAAAACTCCATCTATT
CTGGTGTGGATGGCAGGCGGGCCTATATTTCTTGGTTTCGATAGGACTCCTTACGAGCG
TTGTTTCTATCTACTATTATCTAAAAATAATCAAGTTATTAATGACTGGACGAAACAAAG
AAATAACCCCTCACGTGCGAAATTATAGAAGATCTCCTTTAAGATCAAACAATTCCATC
GAATTGAGTATGATTGTATGTGTAATAGCATCTACTATACCAGGAATATCAATGAACCCA
ATTATTGCAATTGCTCAGGATACCTTCTTTATGTTTCTGCTTCACGAATATGATATTTTCT
GGGCATTTCTAATGATATCAAGTGTTATTCCTATTTTGGCATTTCTAATTTCTGGAGTTTT
AGCCCCGATTAGTCAAGGACCAGAAAAGGTCTCTAGTTATGAATCAGGTATAGAACCA
ATGGGGGATGCTTGGATACAATTCCGAATCCGCTATTACATGTTTGCCCTAGTTTTTGTT
GTTTTTGATGTCGAAACCGTTTTTCTTTATCCATGGGCCATGAGTTTCGATGTATTGGGC
GTATCCGTATTTATAGAAGCTTTAATTTTCGTGCTTATCCCAATCGTTGGTTCAGTTTATG
CATGGCGAAAAGGAGCATTGGAATGGTCTACGAATAATTTTCCTTGGCTAACAGCGATT
CTTCTTTTGCCCATATCCGCGGGTTCTTCAATTCTGTTTATACCTCAGAGAGGAAATAAA
GCGGTTCGGTGGTATACTATATGTATATGCTTATTAGAACTTCTCCTAATGACCTATGTAT
TTTATTATAATTTCCAATTGGACGATCCATTAATCCAATTGGAAGAGGATTGTAACTGGA
TAAATCTTTTTGATTTTCACTGGAGACTAGGAATTGATGGACTTTCCATAGGTCCCATTT
TATTGACGGGATTCATCACTACTTTAGCTACTTCAGCGGCTTGGCCAGTTACTCGAAATT
CGCGATTATTTCATTTCCTGATGTTAGCAATGTATAGCGGTCAAATAGGATCATTTTCTTC
TCGGGACCTTTTACTTTTTTTCATCATGTGGGAGTTAGAATTAATTCCTGTTTACCTACTT
TTATCGATGTGGGGAGGAAAAAAACGTCTGTATTCAGCTACAAAATTCATTTTGTACAC
CGCAGGAGGTTCCATTTTTCTCTTAATAGGAGTTCTGGGTATGGGCCTATATAGTTCCAA
TCAACCCATGTTGAATTTTGAAACATCAGCCAATCAATCGTATCCCGTGGGATTGGAAA
TCCTATTCTATTTCGGCTTCCTTATTGCTTATGCTGCCAAACCACCGATTATACCCCTACA
TACATGGTTACCAGATACTCATGGAGAGGCGCATTACAGTACATGTATGCTTCTGGCTG
GAATCTTATTAAAAATGGGAGCATATGGGTTGGTTCGAATCAATATGGAATTATTACCCC
ATGCTCATTCTCTATTTTCTCCTTGGTTGGTAATAGTGGGAACCCTTCAAATAATCTATGC
AGCTTCAACTTCTCTTGGCCAACGCAATTTAAAAAAGAGAATAGCCTATTCCTCCGTAT
CTCATATGGGTTCCACAATCATAGGAATTGGTTCCATGACCGATACGGCACTCAATGGA
GCCATTTTACAAATAATTTCTCATGGATTTATTGGTGCTGCACTTTTTTTCTTGGCAGGA
ACAAGTTACGATAGAATACGGTTTCTTTATCTCGACGAAATGGGAGGAATAGCTATACC
AATGCCAAAAATATTTACTATGTTCAGTATCTTTTCGATGGCCTCTCTCGCATCGCCAGG
AATGAGTGGTTTTGTTGCAGAATTTTTGGTCTTTTTGGGAATAATTACCAGCCCAAAATA
TCTTTTCATCCCCAAAATACTAATTACTGTTGTAATGGCAATTGGAATGATATTAACTCCC
ATTTATTTATTATCTATGTCACGCCAGATGTTCTATGGATACAAACTATTCAATGTTCCAA

ATTCTTACTTTGTAGATTCTGGACCGCGAGAAATATTTATTTTAATCTCTATCCTTCTACC
TGTAATGGGTATTGGTATTTATCCTGATTTCGTTCTTTCACTATCAGTTGATAAGGTAGAA
GCTATTTTAGCAAATTACTTTAATGGAATGATGTTCGAGTATGCACTTGTTTTAAGTAGTT
ATTTATTTTCTATGGGTATCTATGGATTAATCACAAGTCGAAACATGGTTAGAGCACTTAT
GTGTCTTGAGCTTATACTGAATGCTGTTAATATGAATCTCGTAACATTTTCTGATTTATTT
GATAGTCGACAATTAAAGGGAGACATTTTCTCGATCTTCGTTATAGCTATTGCAGCCGCT
GAAGCAGCTATTGGCCCAGCTATTGTTTCATCAATCCATCGTAACAGAAAATCCACTCG
TATCAATCAATCAAATTTGTTGAAGAAAATGGAACGTACATATCAATATGCATGGATCAT
ACCCTTCCTTACGCTTGCAGTTCCTATTTTGATAGGATTGGGACTCCTGCTTGTTCCCCC
GGCAACAAAAAGTATTCGTCGGATATGGGCTTTTCCCAGTGTTTTATTGTTAAGTATAGT
TATGGTTTTTTCTACCAATTTGTCTATTCAGCAAATAAATGGTAATTCGATTTATCAATAT
CTATGGTCCTGGACCATCAATAGTGATTCTTCCTTAGAGTTCGGCTATTTGATCGATTCA
CTTACTTCTATTATGTCAATATTAATTGCTACTGTTGGAATGATGGTTCTTATCTATAGTGA
TAATTATATGTCTCATGATAAGGGATATTTAAGATTTTTTGCTTATATGAGTTTTTTCAATA
CTTCAATGTTGGGGTTAGTTATTAGTCCCAATTTGATACAAATCTATATTTTCTGGGAACT
TGTGGGAATGTGTTCGTATCTATCAATAGGTTTTTGGTTTACTCGACCAAGTGCAGCAA
ATGCTTGTCAAAAAGCGTTTGTAACTAATCGTGTAGGGGATTTTGGTTTATTATTAGGAA
TCTTAGGTCTTTATTGGATAGCGGGTAGTTTCGAATTTCGGGATTTGTTTGATATATTCAA
TGATAATGTGTTGAATTCCTCTTTTGCTATTCTGTGCGCCTCCTTATTATTCCTGGGTGCA
GTTGCTAAATCCGCACAATTCCCCCTTCATGTATGGTTACCTGATGCCATGGAGGGACC
AACTCCTATTTCGGCTCTTATACATGCTGCTACTATGGTAGCAGCAGGAATTTTTCTTGT
AGCTCGGCTTCTTCCTCTTTTGACAGTTATACCCTACATAATGAATCTTATTTCTTTGATA
GGTGTAATAACAGTACTATTAGGAGCCACTTTGGCTCTTGCTCAAAAGGACATTAAAAG
AAGTTTAGCTTATTCTACGATGTCTCAACTGGGTTATATTATGTTGGCTTCAGGTATAGGT
TCTTATCGAGCCGCTTTATTCCATTCAATCACTCATGCCTATTCGAAAGCATTATTGTTTT
TAGGATCTGGATCAATTATTCATTCTATGGAACCTATTCTTGGGTATTCTCCAGATAAAA
GCCAGAACATGGTTTTTATGGGTGGTTTAAGTAAATATGTGCCGATAACAAAGGCTACT
TTTTTGTTAGGTACGCTCTCCCTTTGTGGTATTCCACCCCTTGCTTGTTTCTGGTCCAAA
GATGAAATTCTTAATAATAGTTGGTTGTATTCGCCAATTTTCGCGATAATAGCTTCTTCTA
CAACAGGATTAACTGCATTTTATATGTTTCGTATGTATTTACTTACTTTTGAAGGGCATTT
ACGCGTTCATTTTAAA---------GGTGATACAAATAGTTCCTTGTATTCAATATCTATATGGG
GGAAAGAAGGACCAGAAGTTTTTAGCAGA---AATTTGATTTTATTATCAATGAATGTTTC
TTCTAAAAAAGAA------ACCTTTCCCTATCCCCACGAATCGGACAATACTATGTTATTCCC
CTTACTTTTATTGGCCATATTGACTTTGTTCGTTGGGTCCGTAGGAATTCGTTTCGACTT
TGACGTGTTATCAAAATGGTTAATTCCATCAATGGACCTTTTTCATGAGTATTTGAATCC
TTCGGCGGATTGGTATGAATTTGCACAAAATGCAATTTATTCAGTCAGTATAGCCTTTTT
TGGAATATTAATAGCCAATCTTTTATATGGGTCTGTTCATTCATCTTTCCAGAATTTGGAC
TTAATCAACTCATTTGCAATCTTTTCGGACCAAGCACTAAATGTCATATACAATTGGTCA
TATAATCGCGGTTACATAGATCTTTATTATGCAACAATCTTAACTAGAGGTATACGAGGAT
TAGCTGAATCCACTCATTTTTTTGATCAACGGATAATTGATGGAGTCACGAATGCAGTT
GGTATTACAAATTTCTTTGTAGGAGAAGCGATCAAATAT---ATGGCGGGTGGAAGAATCT
CTTCTTACCTCTTTTTTTCGTTATCCTCTTTATCAATAGCCCTTATTTTAGTATATATGGATT
TACTTGGACCAATACATGATATTCTTTTGGTATCTTTGGGCTCAGGTATTATATTAGGAAG
TTTAGGAGTAGTATTACTTACCAATCCTATTTATTCTGCCTTTTCATCGGGATTGGTTCTT

GTTCGTATATCCTTATTCCATATTCTATCAAACTCCTATTTTGTAGCTGCTGCACAGCTCC
TTATTTACGTAGGAGCCATAAATGTCTTAATCATATTTGCTGTGATGTTCATGAATGGTTC
AGAATATTACAATAATTTCCATCTTTGGACCGTTGGAGATGGGATCTCTTCAGTAGTTTG
TACAAGTATTCTTTTTTCACTAATTGCTACTATTCTAGATACGTCATGGTACGGGATTATT
TGGACTACAAGATCAAATCAGATCATAGAGCAGGACCTTACAAGTAACGTACAACAAA
TTGGGATTCATTTATCAACTGATTTTTTTCTTCCATTTGAACTCATTTCTATAATTCTTTTA
GTTGCCTTGGTAGGGGCAATTGCTATGGCTCGTGCGATGACTGTACCAGCTACAAGAA
ACGACCTCATGATAGTCAATATGGGTCCTCACCATCCATCAATGCATGGTGTTCTTCGAC
TCATAGTTACTCTAGACGGTGAAGACGTTATTGACTGTGAACCCATATTAGGTTATTTAC
ACAGAGGGATGGAAAAAATTGCAGAAAATCGAACAATCATACAATATCTTCCTTATGTA
ACACGTTGGGATTATTTAGCTACTATGTTCACAGAGGCAATAACAGTGAATGCACCGGA
AGAGTTGGGAAATATTCAAGTACCTAGAAGAGCCAGCTATATCAGAGTAATTATGTTGG
AGCTCAGTCGTATAGCTTCTCATTTGTTATGGCTTGGACCTTTTATGGCTGATATCGGTG
CACAGACTCCTTTCTTCTATATTTTCAGAGAGAGAGAATTGCTATATGATCTATTTGAAA
CTGCCACAGGAATGCGAATGATGCATAATTTTTTCCGTATCGGAGGAGTAGCTGCTGAT
TTACCTCATGGCTGGATAGATAAATGTTTGGATTTCTGTGATTATTTCTTACCAGAAGTT
GCTGAATATCAAAAACTTATTACACGTAATCCCATCTTTTTGGAACGAGTTGAAGGAGT
GGGCTTTATTGCTGAAGAGGAAGCAATAAATTGGGGTTTATCAGGACCAATGTTACGA
GCTTCCGGAATACCATGGGATCTTCGTAAAGTCGATCATTATGAGTGTTACGATGAATTT
GATTGGGAAGTCCAATGGCAAAAAGAAGGAGACTCATTAGCTCGTTATTTAGTACGAA
TCAGTGAAATGATAGAATCCGTCAAAATTATTCAACAGGCTCTAGAAGGAATTCCGGG
GGGGCCCTATGAAAATTTAGAAGCCCGACGCTTTGATAGAGTAAGAAATATAGAATGG
AATGATTTTGAATATCGATTTATTAGTAAAAAACCTTCTCCCACTTTTGAATTGTCGAAA
CAAGAACTCTATGTGAGAGTAGAAGCTCCAAAAGGGGAATTAGGGATTTTTCTAATAG
GAGATAATAGTGTTTTTCCCTGGAGATGGAAAATTCGTCCACCCGGTCTTATCAATTTGC
AAATTCTTCCTCAGCTAGTTAAAAGAATGAAATTGGCTGATATCATGACGATACTAGGT
AGTATAGATATCATTATGGGAGAAGTTGATCGTATGTTCCCTATGGTGACTGGGTTCATG
AATTATGGTCAACAAGCAGTGCGAGCTGCAAGATATATTGGTCAAAGTTTCATGATTAC
CTTATCTCACGCGAATCGTTTACCTGTAACCATTCAGTATCCTTATGAAAAATCGATCAC
ATCAGAGCGTTTCCGGGGGCGAATCCACTTTGAGTTCGATAAATGCATTGCTTGTGAAG
TATGCGTTCGTGTATGCCCGATAGATCTCCCCGTTGTTGATTGGAGATTCGAAACAGATA
TTAGAAAAAAACGATTGCTTAATTATAGTATTGATTTTGGAATCTGTATATTTTGTGGTAA
CTGCGTCGAGTATTGCCCCACAAACTGTTTATCCATGACTGAAGAATATGAACTTTCCA
CCTATGATCGTCACGAATTGAATTATAATCAAATTGCTTTGGGTCGGTTACCAATACCTG
TAGTTGGAGATTACACAATTCAAACAATTATGAATTCGAAAATAGCTATGAATAAACCC
CTTGATTCAAGAACAATTACCAATTACATGCAGGGTCGTTTATCTGCTTGGCTAGTCAA
GCATGAGCTAGTTCATAGATCTTTGGGCTTCGATTACCAAGGAATAGAAACTTTACAAA
TTAAATCCGAAGATTGGTACTCCATTGCTGTCATTTCATATGTATATGGTTACAATTACCT
ACGTTCTCAGTGTGCTTATGATGTAGCACCAGGAGGATTATTAGCTAGTGTGTATCATCT
TACGAGAATACAATATGGTGTGGATCAACCAGAAGAGGTATGTATAAAAGTATTTGCCC
AAAGGAGGAATCCTAGAATCCCGTCTGTTTTCTGGATCTGGAAAAGTTCTGATTTTCAA
GAACGGGAATCTTATGATATGTTGGGAATCTATTATGATAACCACCCACGCCTGAAACGT
ATCTTGATGCCTGAAAGTTGGATTGGTTGGCCCCTACGCAAGGATTATATTGCCCCTAAT
TTCTATGAGATACAGGATGCTCAT---

---ATGAA
TTCTATGGAGTTTCCGTTACTTGACCGAACAACCCCAAATTCAGTTATTTCAACTACAC
CGAATGATCTTTCGAATTGGTCAAGACTCTCCAGTTTATGGCCGCTTCTATATGGTACCA
GTTGCTGCTTCATTGAATTTGCTTCATTAATTGGCTCGCGATTCGACTTTGATCGTTATG
GACTGGTACCAAGATCGAGTCCTAGGCAAGCCGACCTTATTTTAACAGCCGGCACAGT
GACAATGAAAATGGCTCCTTCTTTAGTAAGATTATATGAACAAATGCCTGAACCAAAAT
ATGTCATTGCTATGGGAGCCTGTACTATTACAGGAGGAATGTTCAGTACCGATTCTTATA
GTACCGTTCGAGGAGTCGATAAGTTAATTCCTGTGGATGTCTATTTGCCGGGTTGCCCA
CCTAAACCAGAAGCAATTATAGACGCTATAACGAAACTTCGTAAGAAGGTATCTCGAG
AAATCTATGAAGATAGAATCGCGTCCCAACAGGAAGATCGATGTTTTACTACCAATCAC
AAGTTTCGGGTTGGGCGCAGTATTCATACCGGAAATTACGATCAAGAG---------CTCTAC
CAATCGACTTCAGAGATACTTTCTGAAACACTTTTCAAATACAAGAGTTCACTATCTTC
TCACGAATTAGTGAATATGCAAAATAGAAATACTTTTTCTTGGGTAAAGGAACAGATGA
CTCGATCCATTTTTGTATCGATGATGATATATATAATAACTCGGGCATCTATTTCAAATGCA
TATCCTATTTTTGCGCAGCAGGGTTATGAAAATCCGCGAGAAGCAACTGGACGTATTGT
ATGTGCCAATTGTCATTTAGCTAATAAGCCTGTGGATATTGAGGTTCCACAAGCTGTGCT
TCCTGATACTGTATTTGAAGCAGTTGTTAGAATCCCCTATGATATGCAACTGAAACAAGT
TCTTGCTAATGGTAAAAAGGGAGGTTTGAATGTAGGAGCTGTTCTCATTTTACCCGATG
GATTTGAATTAGCTCCTCCTGATCGTATTTCTCCCGCGATGAAAGAAAAGATAGGTAATT
TATCTTTCCAGAGTTATCGCCCCACTAAAAAAAATATTCTTGTGATAGGTCCTGTTCCTG
GTCAGAAATATAATGAAATTCTCTTTCCAATCCTTTCCCCGGATCCCGCTACTAAGAAA
GAAGTTCACTTCTTGAAATATCCCATATATGTGGGTGGGAATAGAGGAAGGGGTCAGAT
TTATCCCGATGGGAGCAAGAGTAACAATACAGTTTATAATGCCTCAGCATCAGGGATAA
TAAGTAGAATAATACGTAAAGAAAAGGGTGGATATGAAATAACCATAGCTAATGCATTG
GATGGGCGTCAAGTGGTTGATATTATACCTCCAGGACCGGAACTTCTTGTTTCCGAAGG
TGAATCTATCAAACTTGATCAACCATTAACGAGTAATCCCAATGTGGGTGGATTTGGTC
AGGGGGATGCAGAAATAGTACTTCAAGATCCATTACGTATCCAAGGTCTTTTTCTCTTTT
TGGCATCTGTTATTCTGGCACAAATCTTTTTGGTTCTTAAAAAGAAACAGTTTGAGAAG
GTTCAATTGGCTGAAATGAATTTCATGAGTAAAGTATATGATTGGTTCGAGGAGCGTCT
CGAGATTCAGGCGATTGCGGATGATATAACTAGTAAATATGTTCCTCCTCATGTCAATATA
TTTCATTGTCTAGGGGGAATCACACTTACTTGTTTTTTAGTACAAGTAGCTACGGGTTTT
GCTATGACTTTTTACTATCGTCCAACTGTTACAGAGGCTTTTGCCTCTGTTCAATATATAA
TGACTGAAGTAAACTTTGGTTGGTTAATCCGATCAGTTCATCGATGGTCGGCAAGTATG
ATGGTTCTAATGATGATCCTGCACGTATTTCGTGTTTATCTCACAGGCGGATTTAAGAAA
CCTCGCGAATTAACTTGGGTTACGGGTGTGGTTCTGGCTGTATTGACTGCATCTTTTGG
TGTAACTGGTTATTCCTTACCTCGGGACCAAATTGGTTATTGGGCAGTCAAAATCGTGA
CCGGCGTGCCTGAGGCTATCCCTATAATAGGATCGCCCTTGGTAGAGTTATTACGTGGA
AGTGCTAGTGTGGGCCAATCTACCTTGACCCGTTTTTATAGTTTACACACTTTTGTATTA
CCTCTTCTTACTGCCGTATTTATGTTAATGCATTTTCCAATGATACGTAAGCAAGGTATTT
CGGGTCCTTTAATGGGAGTAACAAAGAAACCTGACTTGAATGATCCTGTATTAAGAGCT
AAATTGGCTAAAGGGATGGGACATAATTATTACGGAGAACCCGCATGGCCTAATGATCT
TTTATATATCTTTCCAGTAGTAATTTTAGGTACTATTGCATGTAACGTGGGTCTAGCTGTT
CTAGAACCTTCAATGATTGGTGAACCAGCAGATCCATTTGCAACTCCTTTGGAAATATT
ACCCGAATGGTACTTTTTTCCCGTATTTCAAATACTTCGTACAGTACCTAATAAGTTATT

GGGTGTCCTTTTAATGGTTTCGGTACCAACGGGATTATTGACAGTACCTTTTTTGGAGA
ATGTTAATAAATTCCAAAATCCATTTCGCCGTCCAGTAGCTACAACAGTCTTTTTGTTCG
GTACCGCACTAAGTCTTTGGTTAGGTATTGGAGCAACATTACCTATTGATAAATCCCTAA
CTTTAGGTCTTTTTATGATTGAAGTTTTTCCATTTGGAATCGTGTTAGGTCTAATTCCTAT
TACTTTGGCCGGATTATTTGTAACTGCATATTTACAATACAGACGCGGGGATCAGTTGGA
T C T
T???
??????ATGGATATAGTAAGTCTCGCTTGGGCTGCTTTAATGGTAGTTTTTACATTTTCCCTT
TCACTAGTAGTATGGGGAAGAAGTGGACTCATGATTATTCGTTCGCCGGAATCAGAAGT
GAAGATTATGGTGGATAGGGATCCCATAAAAACTTCTTTCGAGGAATGGGCCAGACCC
GGCCATTTCTCAAGGACAATAGCTAAAGGCCCCAATACTACCACTTGGATCTGGAACCT
ACATGCTGATGCTCACGATTTCGATAGCCATACCAGTGATTTGGAGGAAATCTCTCGAA
AAGTATTTAGTGCTCATTTCGGTCAACTATCCATCATCTTTCTTTGGTTGAGTGGTATGTA
CTTCCATGGTGCCCGTTTTTCCAATTATGAAGCATGGTTGAGCGATCCTACTCACATCGC
ACCCAGTGCCCAGGTGGTTTGGCCAATAGTTGGTCAAGAAATCCTGAATGGCGATGTA
GGTGGAGGTTTCCGAGGAATACAAATAACCTCTGGTTTTTTTCAGATTTGGCGAGCATC
TGGAATAACTAGTGAATTACAACTCTATTGTACCGCAATTGGTGCTTTGGTCTTTGCAGC
GTTAATGCTTTTTGCCGGTTGGTTCCATTATCACAAGGCTGCTCCAAAATTGGCTTGGTT
CCAAGATGTAGAATCCATGTTGAACCACCACTTAGCAGGGTTACTAGGACTTGGGTCTC
TTTCTTGGGCGGGACACCAAGTACATGTATCTTTACCCATTAACCAATTTCTAGACGCG
GGAGTGGATCCTAAAGAGATACCACTTCCTCATGAATTTATCTTGAATCGGGATCTTTTG
GCTCAACTTTATCCCAGTTTTGCCGAGGGATCAACCCCATTTTTTACCTTGAATTGGTCA
AAATACGCGGAATTTCTTACTTTTCGCGGAGGATTAGATCCAGTAACAGGGGGTCTATG
GCTGACCGATATTGCACACCATCATTTGGCTATTGCAATTATTTTCCTGATAGCTGGTCA
CATGTATAGGACCAACTGGGGAATTGGTCATGGCCTAAAAGAGATTTTAGAGGCTCATA
AAGGTCCATTTACAGGCGAGGGTCATAAAGGTCTCTATGAGATCCTAACAACGTCATGG
CATGCTCAATTAGCTCTTAACCTAGCTATGTTAGGCTCTTTAACCATTGTTGTAGCTCAC
CATATGTATTCTATGCCCCCCTATCCATACCTAGCTATTGACTATGGTACACAACTCTCGT
TGTTCACACATCACATGTGGATCGGTGGATTTCTCATAGTTGGCGCTGCTGCACATGCA
GCTATTTTTATGGTAAGAGACTATGACCCAACTACTCGATACAACGACCTTTTAGATCGT
GTCCTTAGGCACCGTGATGCAATCATATCACACCTCAACTGGGCATGTATATTTCTAGGC
TTTCATAGTTTTGGCCTGTATATTCATAATGATACCATGAGCGCTTTGGGGCGTCCTCAA
GATATGTTTTCAGATACCGCTATACAATTACAACCCATCTTTGCTCAATGGATACAAAAC
ACTCATGCTTTAGCACCTGGTGCAACAGCTCCTGGTGCAACAACAAGTACCAGTTTGA
CTTGGGGAGGTGGTGATTTAGTAGCAGTAGGTGGAAAAGTAGCTTTGTTACCTATTCCA
CTAGGAACCGCAGATTTTTTGGTACACCACATTCATGCATTTACGATCCATGTGACCGTA
TTGATACTACTGAAAGGCGTTCTATTTGCTCGTAGCTCCCGTTTGATACCTGATAAAGCA
AATCTGGGTTTTCGTTTCCCTTGTGACGGACCCGGAAGAGGGGGAACATGTCAAGTAT
CCGCCTGGGATCATGTCTTCTTAGGTCTATTCTGGATGTACAATGCGATTTCGGTAGTAA
TATTCCATTTCAGTTGGAAAATGCAGTCGGATGTTTGGGGTAGTATAAGCAATCAAGGG
GTGGTAACTCATATCACGGGAGGAAACTTTGCGCAGAGTTCCATTACTATTAACGGATG
GCTCCGGGATTTTCTATGGGCACAGGCCTCTCAGGTGATCCAGTCTTATGGTTCTTCATT
ATCCGCATATGGCCTGTTTTTCCTAGGTGCTCATTTTGTTTGGGCCTTCAGTTTAATGTTT
CTATTCAGCGGCCGCGGTTATTGGCAAGAACTTATTGAATCCATCGTTTGGGCTCATAA

CAAATTAAAAGTTGCTCCTGCTACTCAGCCTAGAGCCTTGAGCATTGTCCAAGGACGT
GCTGTAGGAGTAACCCATTACCTTCTGGGTGGAATTGCCACAACATGGGCATTCTTCTT
AGCAAGAATTATTGCAGTAGGAATGGCATTAAGATTTCCAAGGTTTAGCCAAGGCTTAG
CTCAGGACCCCACTACTCGTCGTATTTGGTTTGGTATTGCCACCGCACATGACTTCGAG
AGTCATGATGATATCACTGAGGAAGGTCTTTATCAGAATATTTTTGCTTCTCACTTCGGG
CAGTTAGCAATAATCTTCCTGTGGACTTCCGGGAATCTGTTTCATGTGGCTTGGCAAGG
AAATTTTGAGTCATGGGTACAGGACCCTTTACATGTAAGACCTATTGCTCATGCAATTTG
GGATCCTCATTTTGGTCAACCAGCTGTAGAAGCCTTTACTCGAGGGGGTGCTCTCGGTC
CGGTGAATATCGCTTATTCCGGTCTCTATCAGTGGTGGTATACAATCGGCTTGCGCACCA
ATGAAGATCTTTATACTGGAGCTCTTTTTCTATTATTTCTTTCTGCGATATCTTTAATAGCG
GGTTGGTTACATCTACAACCCAAATGGAAGCCAAGCGTTTCGTGGTTCAAAAATGCTG
AATCTCGTCTTAATCATCATTTGTCAGGACTGTTCGGAGTAAGTTCCTTGGCTTGGACA
GGACATTTAGTTCATGTCGCTATTCCCGGATCCAGGGGGGAATACGTCAGATGGAATAA
TTTCCTAGACGTATTACCATCTCCTCAAGGCTTGGGGCCACTTTTTACAGGACAGTGGA
ATCTTTATGCCCAAAACCCCGATTCCAGTGGTCATTTATTCGGTACCTCCCAAGGAGCG
GGAACTGCCATTCTAACCCTTCTCGGGGGATTCCATCCTCAAACTCAAAGCTTATGGCT
GACCGATATTGCTCATCATCATTTAGCTATTGCATTTGTTTTTCTCGTTGCTGGTCATATG
TATAGAACTAACTTCGGGATCGGTCATAGTATAAAAGATCTTTTAGAAGCACATACTCCT
CCGGGGGGTCGATTGGGACGGGCACATAAAGGTCTTTATGACACAATCAATAATTCCAT
TCATTTTCAATTAGGTCTTGCTCTAGCCTCTTTGGGGGTTATTACTTCCTTGGTAGCTCA
ACACATGTATTCTTTACCTCCTTATGCATTCATAGCACAAGACTTTACTACTCAAGCTGC
GTTATATACTCATCACCAATACATCGCAGGCTTCATCATGACAGGGGCCTTTGCTCATGG
AGCTATATTCTTCATTAGAGATTACAATCCGGAACAGAACGAGGATAATGTATTGGCAA
GAATGTTAGACCATAAAGAAGCTATAAAATCTCATTTAAGTTGGGCCAGCTTGTTCCTG
GGTTTCCATACTTTGGGACTCTATGTTCATAACGACGTCATGCTTGCTTTTGGTACTCCA
GAAAAACAAATCTTGATCGAACCTATATTTGCCCAATGGATACAATCCGCTCATGGTAA
GACTTCATATGGATTTGATGTACTCTTATCTTCAACGAACGGCCCAGCATTCAATGCTGG
TCGAAGCTTATGGTTACCTGGCTGGTTGAATGCTATTAATGAG------AATGGTAATTCACT
ATTCCTAACAATAGGTCCTGGAGACTTCTTGGTTCATCATGCTATTGCTCTAGGTTTGCA
TACAACTACATTGATCTTAGTAAAAGGTGCTTTAGATGCACGTGGTTCCAAGTTCATGC
CAGATAAGAAAGATTTTGGTTATAGTTTCCCTTGCGATGGTCCAGGACGAGGCGGTACT
TGTGATATTTCGGCTTGGGATGCATTTTATTTGGCGGTTTTCTGGATGTTAAATACCATTG
GATGGGTTACTTTTTATTGGCATTGGAAGCATATCACTTTATGGCAGGGTAATCCTTCAC
AATTTAATGAATCTTCCACTTATTTGATGGGATGGTTAAGAGATTATCTATGGTTAAACTC
TTCACAACTTATCAATGGATATAACCCTTTCGGTACAAATAGTTTATCTGTCTGGGCGTG
GATGTTCCTATTTGGGCATCTGGTTTGGGCTACTGGATTCATGTTTTTAATTTCCTGGCG
AGGATATTGGCAGGAATTGATTGAAACTCTAGCATGGGCTCATGAACGCACACCTTTGG
CTAATTTGATTCGATGGAGGGATAAACCTGTGGCTCTTTCCATTGTTCAAGCAAGACTG
GTTGGCTTAGCCCACTTTTCTGTAGGTTATATATTTACTTATGCAGCTTTCTTAATTGCCT
CTACATCGGGCAAATTTGGTATGTCACATTCAGTAAAGATTTATGATACATGTATAGGGT
GTACACAATGCGTACGAGCCTGCCCCACGGATGTATTGGAAATGATACCTTGGGACGGA
TGTAAAGCTAAACAAATAGCTTCTGCTCCAAGAACAGAGGACTGTGTAGGTTGTAAGA
GATGTGAATCCGCCTGTCCAACGGATTTCTTGAGTGTACGTGTTTATTTATGGCATGAGA
C C A C T C G T A G C A T G G G T C T A G C T T A

T???
?????????????????????ATGCGAGATATAAAAACATATCTCTCTGTGGCGCCCGTGTTAACT
ACTCTATGGTTCGGGGCTTTAGCAGGTCTATTGATAGAGATTAATCGTTTCTTCCCAGAT
GCGCTGGCATTCCCCTTTTTT---------------CTGCTAGAGAGACGCGAAAGCACAAGCTTA
TGGGGGCGCTTCTGCAACTGGATAACCAGCACCGAAAACCGTCTTTACATTGGGTGGT
TCGGTGTTTTGATGATCCCTACCTTATTGACCGCAACTTCTGTATTTATTATCGCCTTCAT
TGCGGCTCCTCCAGTAGATATTGATGGGATCCGTGAACCTGTTTCTGGTTCTCTACTTTA
TGGAAACAATATTCTTTCTGGTGCCATTATTCCAACCTCTGCAGCTATAGGTTTGCATTT
TTACCCAATATGGGAAGCGGCATCCGTTGATGAATGGTTATACAATGGTGGTCCTTATGA
GTTAATTGTCCTACACTTCTTACTTAGTGTAGCTTGTTACATGGGTCGTGAGTGGGAACT
TAGTTTCCGTCTGGGTATGCGCCCTTGGATTGCTGTTGCATATTCAGCTCCTGTTGCAGC
TGCTACTGCTGTTTTCTTGATCTACCCTATTGGTCAAGGAAGTTTCTCAGATGGTATGCC
TCTAGGAATATCTGGTATTTTCAACTTGATGATTGTATTCCAGGCGGAGCACAACATCCT
TATGCACCCATTTCACATGTTAGGCGTAGCTGGTGTATTCGGCGGCTCCCTATTCAGTGC
TATGCATGGTTCCTTGGTAACCTCTAGTTTGATCAGGGAAACCACTGAAAATGAGTCTG
CTAATGCAGGTTACAGATTCGGTCAAGAGGAAGAAACCTATAATATCGTAGCTGCTCAT
GGTTATTTTGGTCGATTGATCTTCCAATATGCTAGTTTCAACAATTCTCGTTCCTTACATT
TCTTCCTAGCTGCTTGGCCCGTAGTAGGTATTTGGTTCACTGCTTTGGGTATTAGCACTA
TGGCTTTCAACCTAAATGGTTTCAATTTCAACCAATCCGTAGTTGACAGTCAAGGTCGT
GTCATCAACACTTGGGCTGATATAATCAACCGTGCTAACCTTGGTATGGAAGTTATGCAT
GAACGTAATGCTCACAATTTCCCTCTAGACTTAGCTGCTGTTGAAGCTCCATCTACAAA
TGGAATGGGTTTGCCTTGGTATCGTGTTCATACCGTTGTCTTGAATGATCCCGGTCGGTT
GCTTTCTGTCCATATAATGCATACAGCTCTAGTTTCTGGTTGGGCCGGTTCGATGGCTCT
CTATGAATTAGCTGTTTTTGATCCCTCTGATCCCATTCTTGATCCAATGTGGAGACAAGG
TATGTTCGTTATACCCTTCATGACCCGTTTAGGAATAACAAATTCATGGGGCGGGTGGA
GTATCACA---GGAGGAACTGTAACGAATCCGGGTATTTGGAGTTACGAAGGTGTGGCC
GGGGCACATATTGTGTTTTCTGGTTTGTGCTTTTTGGCATCTATCTGGCATTGGGTGTAT
TGGGATCTAGAAATATTCTGTGATGAACGCACAGGAAAACCCTCTTTGGATTTGCCCAA
AATCTTTGGAATTCATTTATTTCTCTCAGGGGTGGCTTGCTTTGGGTTTGGCGCATTTCA
TGTCACAGGTTTGTATGGTCCTGGAATATGGGTGTCTGATCCTTATGGACTAACGGGAA
AAGTGCAATCTGTAAATCCGGCATGGGGCGCTGAAGGTTTTGATCCTTTTGTTCCGGGG
GGAATAGCTTCTCATCATATCGCAGCCGGGACATTGGGTATACTAGCAGGTCTATTCCAT
CTTAGTGTCCGCCCACCCCAACGTCTCTACAAAGGATTACGTATGGGTAATATTGAAAC
TGTTCTTTCCAGTAGTATCGCTGCTGTATTTTTTGCAGCTTTCATTGTTGCTGGAACTAT
GTGGTACGGTTCAGCAACTACTCCGATTGAATTATTTGGTCCCACTCGTTATCAGTGGG
ATCAAGGATACTTTCAGCAAGAAATATATCGAAGAGTTGGTACCGGGCTGGCCGAAAA
TCTAAGTTTATCGGAAGCTTGGTCCAAAATTCCTGATAAACTAGCTTTTTATGATTACAT
CGGTAATAATCCGGCGAAAGGAGGATTATTCAGAGCAGGCTCAATGGACAACGGAGAT
GGAATAGCTGTTGGGTGGTTAGGACACCCGATCTTTAGAGATAAGGAAGGGCATGAAC
TCTTTGTACGTCGTATGCCTACTTTTTTTGAAACATTTCCAGTAGTTTTGGTAGATGGAG
ACGGAATTGTGAGAGCTGATGTTCCTTTTAGAAGGGCAGAATCAAAGTACAGTGTCGA
ACAAGTAGGTGTAACTGTTGAGTTCTATGGTGGTGAACTCAATAGAGTAAGTTATAGCG
ATCCTGCTACTGTGAAAAAATATGCTAGACGTGCCCAATTGGGTGAGATTTTTGAATTA
GATCGAGCTACTTTGAAATCCGATGGTGTTTTTCGTAGCAGTCCAAGGGGTTGGTTCAC

TTTTGGACATGCTTCGTTTGCTTTGCTCTTCTTTTTCGGACACATTTGGCATGGTGCTAG
AACCTTGTTTAGAGATGTTTTTGCTGGTATCGACCCGGATTTGGATGCCCAAGTAGAAT
TTGGAGCATTCCAAAAAATAGGAGATCCAACTACAAGGAGACAAATAGTCATGAAAAC
CTTATATTCCCTGAGGAGGTTCTACCCCGTGGAAACGCTCTTTAATGGAACTTTAGCTTT
AGCTGGTCGTGACCAAGAAACCACAGGTTTTGCTTGGTGGGCCGGGAATGCCCGACTT
ATCAATTTGTCTGGTAAATTACTCGGGGCTCACGTGGCCCATGCCGGATTAATCGTATTC
TGGGCGGGAGCAATGAATCTATTTGAAGTGGCTCATTTTGTACCAGAGAAACCTATGTA
TGAACAAGGATTAATTTACTTCCCCATCTAGCTACTCTAGGTTGGGGGGTAGGTCCTG
GGGGGGAAGTTATAGACACCTTTCCATACTTTGTATCTGGAGTACTTCATTTAATTTCCT
CTGCAGTCCTAGGCTTTGGTGGTATTTATCATGCACTTCTTGGACCTGAGACTCTTGAA
GAATCCTTTCCATTCTTCGGTTATGTATGGAAAGATAGAAATAAAATGACCACTATTTTG
GGTATTCACTTAATTTTATTAGGTGTAGGCGCTTTTCTCCTAGTGTTCAAGGCTCTTTATT
TTGGAGGCGTATATGATACCTGGGCCCCCGGTGGCGGAGATGTAAGAAAAATAACCAA
CCTGACACTTAGCCCAAGTGTTATATTTGGTTATTTGCTAAAATCTCCTTTTGGGGGAGA
AGGATGGATTGTTAGTGTGGATGATTTAGAAGATATAATTGGAGGACACGTATGGTTAG
GTTTCATTTGTATACTTGGTGGAATATGGCATATCTTAACCAAACCTTTTGCGTGGGCTC
GCCGCGCATTTGTATGGTCTGGAGAAGCTTACTTGTCTTATAGTTTGGGGGCTTTATCTA
TCTTTGGTTTCATTGCTTGCTGTTTCGTCTGGTTCAATAATACGGCTTATCCTAGTGAGT
TTTACGGTCCTACCGGCCCAGAAGCTTCTCAAGCTCAAGCGTTTACTTTTCTAGTTAGA
GACCAACGTCTTGGGGCTAACGTAGGATCTGCTCAAGGACCCACCGGTTTAGGTAAAT
ATCTAATGCGTTCTCCAACTGGGGAGGTCATTTTTGGAGGAGAAACTATGCGCTTCTGG
GATCTCCGTGCTCCCTGGTTGGAACCACTAAGGGGTCCCAACGGTTTGGACTTGAGTA
GGCTGAAAAAAGACATACAGCCCTGGCAAGAACGACGTTCGGCGGAATATATGACTCA
TGCTCCTTTAGGTTCTTTAAATTCCGTGGGCGGCGTAGCTACAGAGATCAATGCAGTCA
ATTATGTCTCTCCCAGAAGTTGGTTAGCTACCTCTCATTTTGTTCTAGGATTTTTCCTATT
CGTGGGTCATTTGTGGCATGCAGGAAGGGCCCGTGCAGCTGCAGCAGGATTTGAAAA
AGGAATCGATCGTGATTTTGAACCTGTTCTTTCCATGACTCCTCTTAACATGACTATAGC
CCTTGGTAGGTTTTCCAAAGAGGAA---GATTTATTTGATATTATGGATGACTGGCTACGG
AGGGACCGCTTCGTTTTTGTAGGTTGGTCTGGTCTATTGCTTTTTCCTTGTGCCTATTTT
GCTTTAGGAGGGTGGTTCACAGGTACAACCTTTGTAACTTCATGGTACACCCATGGATT
AGCTAGCTCCTATTTGGAAGGATGCAATTTTTTAACCGCTGCAGTTTCTACTCCTGCTAA
TAGTTTAGCACATTCTTTGTTGTTACTGTGGGGTCCTGAAGCACAAGGAGATTTTACTC
GTTGGTGTCAATTGGGGGGTCTGTGGACTTTTGTTGCTCTCCATGGTGCTTTCGGACTA
ATAGGTTTCATGTTGCGTCAATTCGAACTTGCTCGATCTGTTCAATTGCGACCTTATAAC
GCAATCGCATTCTCCGCTCCAATTGCTGTTTTTGTTTCTGTATTCCTGATTTATCCACTAG
GTCAGTCTGGTTGGTTCTTTGCACCGAGTTTCGGTGTAGCAGCTATATTTCGATTCATTC
TCTTCTTCCAAGGATTTCATAATTGGACGTTGAATCCATTTCATATGATGGGAGTTGCTG
GAGTATTGGGTGCTGCTCTGCTATGCGCTATTCATGGTGCTACCGTAGAAAATACTTTAT
TTGAAGATGGTGATGGTGCAAATACATTCCGTGCTTTTAACCCAACTCAAGCTGAAGA
GACTTATTCGATGGTCACTGCTAACCGCTTTTGGTCCCAAATCTTTGGGGTTGCTTTTTC
CAATAAACGTTGGTTACATTTCTTTATGTTATTTGTACCAGTAACCGGTTTATGGATGAG
TGCTCTTGGAGTAGTCGGTCTGGCTTTGAACCTACGTGCCTATGACTTCGTTTCCCAAG
AAATCCGTGCAGCGGAAGATCCTGAATTTGAGACTTTCTATACCAAAAATATTCTCTTA
AACGAAGGTATTCGTGCTTGGATGGCGGCTCAGGATCAGCCCCATGAAAACCTTATATT

CCCTGAGGAGGTTCTACCCCGTGGAAACGCTCTTATGTCTGGAAGTACGGGAGAACGT
TCTTTTGCTGATATTATTACCAGTATTCGATACTGGGTCATTCATAGCATTACTATACCTTC
CCTATTCATTGCAGGTTGGTTATTCGTCAGCACAGGCTTAGCTTACGATGTGTTTGGAA
GCCCTCGACCGAACGAGTATTTCACAGAGAGCCGACAAGGGATTCCATTAATAACTGG
CCGTTTCGATTCATTGGAACAACTTGATGAATTTAGTAGATCCTTTATGACCATAGATAG
AACCTATCCGATTTTTACAGTGAGATGGTTGGCTGTTCACGGACTAGCTGTACCTACAG
TTTTTTTCTTGGGGTCAATATCAGCAATGCAGTTCATCCAACGAATGGTTACACAAAGC
GTGGAGGATAGTTCCAGATCTGGT---CCAAGACGGACTATTGTAGGGGATTTATTGAAA
CCGTTGAATTCAGAATATGGTAAAGTAGCTCCTGGATGGGGAACTACCCCCTTTATGGG
GGTCGCAATGGCTCTATTTGCAATATTTCTATCAATTATTTTGGAGATTTATAATTCTTCC
GTTTTACTGGATGGAATCTCAGTGAGTATGCTTACTCTCAAACTGTTCGTTTACACAGTA
GTGATATTCTTTGTTTCTCTATTCATTTTTGGATTCCTATCTAATGACCCAGGACGTAATC
CTGGGCGTGACGAAATGGCCGATACTACCGGAAGGATTCCTCTTTGGCTGATAGGTACT
GTAACTGGTATTCCTGTGATCGGTTCAATGGGCATTTTCTTCTATGGATCATATTCCGGAT
TGGGCTCATCCCTGATGCCTAATATCTTCAGTTTGATCTGTATCTGTCTTCATTCTGCCCT
TCATTCGAGTGGTTTTTTCTTCGCGAAACTGCCCGAGGCCTATGCTGTTTTCAATCCAAT
CGTAGATGTTATGCCGGTCATACCTTTGTTCTTTTTTCTCTTAGCCTTTGTTTGGCAAGC
TGCTGCAAGTTTTCGAACGACACAATCAAACCCAAACGAACAAAATGTTGAATTGAAT
CGTACCAGTCTCTACTGGGGGTTATTACTCATTTTTGTACTTGCTGTTTCATTTTCCAATT
A T T T C T T C A A
T???
???????????????ATGGAAACTGCAACCCTAGTTGCCATCTCCATATCTGGTTCACTTGTAA
GCTTTACTGGGTATGCCTTATATACCGCCTTTGGGCAACCCTCTCAACAACTAAGAGAT
CCATTCGAGGAACATGGGGACATGGAAGCACTGGTTTATACATTCCTGTTGGTATCGAC
TCTAGGGATAATTTTTTTCGCTATCTTTTTTCGAGAACCGCCTAAGGTTCCGAATAAAAA
AATAAAAATGACTATTGCTTTCCAATTGGCTGTTTTTGCATTAATTGCTACTTCATCAATC
TTACTGATTAGTGTACCCGTTGTATTTGCTTCTTCTGATGGTTGGTCAAGTAACAAAAAT
ATTGTATTTTCGGGTACATCATTATGGATTGGATTAGTCTTTTTGGTAGCTATCCTTAATTC
TCTCATCTCT--------------------ATGTCACCAAAAACAGAGACTAAAGCAAGCGCTGGATT
CAAAGCCGGTGTTAAAGATTACAGATTGACTTATTATACTCCTGACTATGAAACCCTAG
CTACTGATATCTTGGCAGCATTCCGAGTAACTCCTCAACCTGGAGTTCCACCTGAGGAA
GCAGGAGCTGCAGTAGCTGCCGAATCTTCCACTGGTACATGGACAACTGTGTGGACCG
ATGGACTTACCAGCCTTGATCGTTACAAAGGTCGATGCTACCACATCGAACCCGTTGCT
GGGGAAGAAAATCAATATATTGCTTATGTAGCTTATCCTTTGGACCTTTTTGAGGAAGGT
TCTGTTACTAACATGTTTACTTCCATCGTAGGTAATGTATTTGGGTTCAAAGCCCTACGA
GCTCTACGTCTAGAGGATCTGCGAATTCCTCCTGCTTATTCCAAAAGTTTCCAAGGCCC
ACCTCATGGCATCCAAGTCGAGAGAGATAAATTGAACAAGTATGGTCGTCCCCTATTAG
GATGTACTATTAAACCAAAATTGGGGTTATCTGCCAAAAACTACGGTAGAGCGGTTTAT
GAATGTCTCCGTGGTGGGCTTGATTTTACCAAGGATGATGAGAATGTGAACTCCCAACC
ATTTATGCGTTGGAGGGACCGTTTCTTATTTTGTGCCGAAGCTCTTTATAAAGCACAGG
CAGAAACAGGTGAAATCAAAGGACATTACTTGAATGCTACTGCAGGTACATGCGAAGA
AATGATGAAAAGGGCCGTATTTGCCAGAGAATTGGGAGTTCCTATCGTAATGCATGACT
ACTTAACAGGAGGATTCACTGCAAATACTAGTTTGGCCCATTATTGCCGAGACAATGGC
CTACTTCTTCACATTCATCGCGCAATGCATGCAGTTATTGATAGACAAAGGAATCATGGT

ATACACTTTCGTGTACTAGCTAAAGCATTGCGTATGTCTGGTGGGGATCATATTCACGCT
GGTACCGTAGTAGGTAAACTGGAAGGGGAACGGGATGTCACTTTGGGTTTTGTTGATC
TACTACGTGATGATTTTATTGAAAAAGACCGAAGTCGCGGTATTTATTTCACTCAAGATT
GGGTATCTATGCCAGGTGTTCTGCCCGTGGCTTCAGGTGGTATTCACGTTTGGCATATGC
CTGCCCTGACCGAGATCTTTGGGGATGATTCCGTATTACAGTTCGGCGGAGGAACTCTG
GGACACCCTTGGGGAAATGCACCCGGTGCAGTAGCTAATCGGGTGGCTTTAGAAGCGT
GCGTACAAGCTCGTAATGAGGGACGTGATCTTGCTCGTGAAGGTAATGAAGTTATCCGT
GAAGCTAGCAGGTGGAGCCCAGAACTAGCTGCTGCTTGTGAGGTATGGAAGGAGATC
AAATTCGAATTCGAAGCAATGGATGTCTTG------ACGGCGATACATTTATACAAAACTTC
TACCCCGAGCACACGCAATGGAGCCGTAGACAGTCAAGTGAAATCCAATCCACGAAA
GAATTTGATCTATGGACAGCATCGTTGTGGTAAAGGTCGTAATGCCAGAGGAATCATTA
CCGCAGGGCATAGAGGGGGGGGTCATAAGCGTCTATACCGTAAAATCGATTTTCGACG
GAATGAAAAAGACATATCTGGTAGAATCGTAACCATAGAATACGACCCTAATCGAAATG
CATACATTTGTCTCATACACTATGGGGATGGTGAGAAGAGATATATTTTACATCCCAGAG
GGGCTATAATTGGAGATACCATTGTTTCTGGTACAGAAGTTCCTATATCAATGGGAAATG
CCCTACCTTTGACCGATATGCCCTTAGGCACGGCCATACATAATATAGAAATAACACTTG
GAAAGGGTGGACAATTAGCTAGAGCAGCAGGTGCTGTAGCGAAACTGATTGCAAAAG
AGGGTAAATCGGCCACATTAAGATTACCATCTGGGGAGGTTCGTTTGATATCCAAAAAC
TGCTCAGCAACAGTCGGACAAGTGGGTAATGTTGGGGTGAACCAGAAAATTTTGGGTA
GAGCCGGATCTAAATGTTGGCTAGGTAAGCGTCCTGTAGTAAGAGGAGTAGTTATGAA
CCCTGTAGACCATCCCCATGGGGGTGGTGAAGGGAGGGCCCCAATTGGTAGAAAAAA
ACCCACAACCCCTTGGGGTTATCCCGCGCTTGGAAGAAGAAGTAGGAAAAAGAAGAA
ATATAGTGATAGTTTCATTCTTCGTCGTCGTAAG---ATGATTCAAGCTCAAACCTATTTGA
ATGTAGCGGACAACAGCGGGGCTCGAGAATTGATGTGTATTCGAATCGTAGGAGCTAG
T---AACCCCCGATATGCTCATATTGGTGACGTTATTGTTGCTGTAATCAAAGAAGCAGTA
CCCAATATGCCCCTGGAAAGATCAGAAGTGATCAGAGCTGTAATTGTACGTACATGTAA
AGAACTCAAACGTGACAACGGTATAATAATACGATATGATGACAATGCAGCAGTTGTTA
TTGATCCAGAGGGAAATCCAAAAGGAACTCGAGTCTTTGGTTCGATCGCTGGGGAATT
GAGACATTTGAATTTCACTAAGATAGTATCATTAGCTCCCGAGGTATTCATGCTTAGTCC
TAAAAGAACCAGATTCCGTAAACAACATAGAGGAAGAATGAAGGGAGTATCTTCTCGG
GGCAATCATATTTGTTTCGGTAGATACGCTCTTCAGGCACTTGAACCTGCTTGGATCACA
TCTAGACAAATAGAAGCGGGTAGACGAGCAATGACACGATACGCTCGTCGCGGCGGA
AAAATATGGGTACGCGTATTCCCCGACAAACCCATTACAGTAAGACCCGCGGAAACAC
GTATGGGTTCGGGAAAGGGATCTCCTGAATATTGGGTTTCTGTAATTAAACCGGGTCGA
ATACTTCATGAAATGGGCGGAGTGCCAGAAACTGTCGCCAGAGCAGCTATGGAAATAG
CCGCGTGCAAAATGCCTATACGAACTCAATTCATTAGTGCGAAAATGACCAGAGTTAG
ACGCGGATATATAGCTCGCAGACGTCGAACAAAAATTCGTTTATTTGCATCGACCTTTC
GAGGGGCCCATTCAAGACTTACTCGAACTGCTACTCAACAGAAAATGAGGGCTTTGGT
TTCCATTTATCGGGATAGAGGTAGGCAAAAGAGAGAATTTCGTCGCTTGTGGATCGCTC
GGATAAATGCAGCAACTCACGAAAAT------GTA------TCCTATAGTCGATTAATAAACGAT
CTGTACAAGAGGCAGTTGCTTCTTAATCGTAAAATACCTGCACAAATAGCTAGATACAA
TCTAAATTCCCTTTACATGATTTCCAATGAAATCATAAAAGAAAAAAGGGAT---------------
------ATGATAAATGAAATACGAGCATTAGCTAAACATATATGTATGTCCCCTCACAAAGCG
CGAAGAGTTATTGATCAGATTCGCGGTTGCTCTTATGAACAAACACTTATGATACTAGA

ACTCATGCCGTATCGAGTATGTTATCCCGTTTTCAAATTGGTTTACTCTGCAGCGGCAAA
TGCTAGTCACAATATGGGTTTGAACGAAGCTGATTCATTCATTAGTAAAGCGGAAGTCC
ATGGAGGTGCCATGATGAAAAAGTTCAAACCTAAAGCTAGGGGACGCAGTTATCCGAT
AAAAAGACCCACTTGTCATATAACTATTGTTTTAAAG------GAAAGAAAA------------------
--ATGGATGGAATCAAATATGCAGTATTTACAGAAAAAAGTATTCGGTTATTGGGGAACA
ATCAA---TATACTTCTAATGTCGAATCAGGATCAACTAGGGCAGAAATAAAGCATTGGAT
CGAACTCTTCTTTGGTGTCAAGGTAATAGCTATGAATAGTCATCGACTCCCGGTCAAGG
GTAGAAGAATGGGACCTCTTATGGGACATAGAATGCATTACAGACGTATGATCATTACG
CTTCAACCGGGTTATTCTATTCCACCTCTTATAGAGAAAAGGACTATGGCAGTCCCAAA
GAAACGTACTTCTATGTCAAAAAAGCATATTCGTAGAAATTATTGGAAGAGAAAGGGAT
ATAAAGCCGCGGTAAAAGCTTTTTCTTTAGGTAAATCTGTTTCTACCGGGCATTCAAAA
AGTTTTTTT------CAAACAACAACAATGGCCAAAGGTAAAGATGCTAGAGTAAGAGTTA
TTTTGGAATGTACCAGTTGTACTCGAAAT------GGTGCTAAGAAGGAATCACCGGGCATT
TCCAGATATATTACTGAAAAGAATCGACACAATACACCTAGTCGATTGGAATTGAAGAA
ATTCTGTCCTTATTGTTCGAAGCATATGATTCACGCGGAGATAAAGAAAATGAAAATAA
GAGCCTCTGTTCGTAAGATTTGTGAAAAATGTCGACTGATTCGTAGGCGAGGACGAATT
ATAGTAATTTGTTCCAATCCGAGACATAAACAGAGACAGGGGATGGTTCGAAAGGAAG
TACCAGTATCCAATCGGACACTACAGTGGAAGTGTGTTGAATCAAAAGCAGACAGTAA
GCGTCTTTATTATGGTCGTTTCGTTCTATCTCCGCTTATGAAAGGTCAAGCTGATACGAT
AGGTACCGCGATGCGAAGGTCTTTACTTGGAGAAATAGAAGGAACATGTATCACATGT
GCAAAATCTGAG------AGGGTACCACATGAATATTCTACAATAGTAGGTATTGAGGAATC
AGTACATGAAATTTTAATGAATTTGAAAGAAATTATAGTTAGAAGCGATCTGTACGGGA
CTCGTGATGCATTTATCTGTGTTAGGGGTCCTAAATATGTAACTGCTCAAGATATAATCTC
ACCACCTTCTGTGAAAATAGTTGATACTACACAGCATATAGCTAGTATCACCGAACCAA
TTAATTTTTGTATTGGATTAGAAATCGAGAGAAATCGCGGATATCGTACGAGAACCACC
AAC------CAAGATGCAAGTTAT------CCTATAGATGCTGTATCTATGCCTGTTCGAAATGCG
AATTATAGTATTCAATGTTATGGAAATGAGAATGAAAAACAAGAGATACTTTTTCTAGA
GATATGGACAAATGGAAGTTTAACTCCTAAAGAGGCACTTTACGAAGCTTCCCGTAATT
TGATCGATTTATTTATTCCTTTTCTACATGCGGAGGAGCGGGACCTCCAT------TTAGATG
ACGATCAA---AACATATTTACTGCGCCTTTTTTCCCCTTTCATGAGGGGTTGGCT---TATA
TAGGGATAGGGGAAAGC---AGAATTGCATTGAAACGTATTTTTATTGACCAATTAGAATT
ATCTCCCAGGACCTATAACTGCCTCAAAAGGTCCAATATCCATACATTATTGGACCTTTT
GAGTAAGAGTCAAGAAGATCTTAGGAAAATTCAACATTTTCGTGTAGAGGATGTCAAA
CGGGTATTGGACATTCTCAAGAAACGT------TTTGCAATGAATTTATTTACC------ATGCTA
CGGGATGGA---AATGAGGGAATGTCTACAATACCTGGATTTAGTCAGATACAATTTGAA
GGATTTTGTAGGTTCGTTGATCAGGGCTTGGCAGAAGAACTTCATAAGTTTCCAAAAAT
TGAAGATACAGATCAAGAAATTGAATTTCAATTACTTGTGGAAACATATCAATTGGCGG
AACCCTTGATAAAAGAAAGAGATGCTGTGTATGAATCACTCACACATTCCTCTGAATTA
TATGTACCCGCGGGGTTAATTTGGAAAGTTGGTAGAGATATGCAAGAACAAACCGTGTT
TATTGGAAACATTCCTCTAATGAATTCTCTGGGAACCTTTATAGTAAATGGAATATACAG
AATTGTGATCAATCAAATATTGCAAAGCCCCGGTATTTATTACAGTTCAGAATTGGACCA
TAACGGGATTTCTGTTTATACCGGCACCATAATATCAGATCGGGGAGGAAGATCAGAAT
TAGAGATTGATAGAAAAGCAAGGATATGGGCCCGTGTGAGCAGGAAACAAAAAATTTC
TATTCTAGTTCTACCATCAGCTATGGGTTCGAATCTAAGAGAAATTCTAGACAATGTTTG

CTACCCCGAAATTTTATTGTATTTCCCGAATGAGAAAGAGAAAAAGAAAATTGGGTCG
AAGGAAAATGCTATTTTGGAGTTTTATCAACAATTTTCTTGTGTAGGCGGGGATCCGGT
ATTTTCTGAGTCCCTCTGTAAGGAATTACAAAAGAGATTTTTTCAACAAAGATGTGAAT
TAGGAAGGATTGGTCGACAAAATATGAACCAGAGATTGAATATTGATATACCCCAGAAC
AATACATTTTTATTACCACGAGACGTATTGGCTGCTACAGATCATTTGATCGGAATGAAA
TTTGGAATGGGTACACTTGACGAT------ATGAATCACTTGAAAAATAAACGTATTCGTTC
TGTAGCAGATCTGTTACAGGATCAATTCGGATTGGCTCTGGTTCGTTTGGAAAATGTGG
TTCGAGGAACTATATGTGGAGCAATCAGG---CATAAATTTATA---CCGACTCCTCAAAAC
TTGGTAACTTCAACTCCA------------TTAACAACCACTTATGAATCGTTTTTCGGTCTACA
CCCTTTATCTCAAGTTTTGGATCGAACTAATCCATTGACACAGATAGTTCATGGGCGAA
AATCAAGCTATTTGGGTCCCGGAGGATTAACAGGGCGAACTGCTAGTTTTCGCATACGA
GATATCCATCCTAGTCACTATGGACGTATTTGCCCAATTGACACGTCCGAAGGAATCAAT
GTTGGACTTATCGGATCCTTAGCTATTCATGCAAGGATCGGCGATTGGGGA---TCTATAC
GAAGTCCGTTTTAT---------GAAATATCTGAGAGATCAAAAGAG------GAACAGATGGTTT
ATTTATCACCAAGGAGAGATGAATACTATATGGTAGCAGCAGGAAATTCTTTGGCCTTG
AATCAGGATATTCAAGACGAACAGGTCGTTCCAGCCCGATACCGTCAAGAATTCGTGA
CTATTGCATGGGAACATATTGATCTTCGAAGCATTTATCCCCTCCAATATTTTTCTATTGG
AGCTTCCCTCATTCCGTTTATCGAACACAATGATGCGAATCGAGCTTTAATGAGTTCTAA
TATGCAGCGTCAAGCAGTTCCGCTTTCTCGGTCCGAGAAATGCATTGTTGGAACTGGG
TTGGAGCGCCAAGCGGCTTTAGATTCGGGGGGTTCAGCTATAGCCCAACACGAGGGAA
AGGTCATTTATACCGATACTGAAAAGATCCTTTTATCAGGGAATGGAGACACTATAAGC
ATTCCATTGCTTATGTATCAGCGTTCCAACAAAAATACTTGTATGCATCAAAAACCTCAG
GTTCATCGGGATAAATATGTTAAAAAGGGACAAGTCTTGGCGGATGGTGCGGCTACAG
TTGGTGGGGAACTCGCTTTGGGAAAAAACGTATTAGTAGCTCATATGCCATGGGAAGG
CTACAATTTTGAAGATGCAGTACTAATTAGCGAACGTCTGGTATATGGAGATATTTATAC
TTCGTTTCACATCCGGAAATATGAGATTCAGACTCATGTGACAAGCCACGGC---CCTGA
AAAGATCACTAACGAAATACCTCATTTAGAAGCTCATTTGCTCCGTAATTTAGACAGAA
ATGGAATTGTGATGCTCGGATCTTGGGTGGAAACGGGTGATGTTTTAGTAGGTAAATTG
ACGCCTCAGACAGCGAAAGAA---TCATCGTATGCCCCGGAAGATAGATTATTACGAGTC
ATACTTGGAATTCAAGTATCCACTGCAAAGGAAACTTGTCTAAAACTACCTATAGGTGG
AAGAGGTCGAGTTATTGATGTGAGATGGATCCAGAAAAAAGGG---GCTTCCAGTTATAA
TCCAGAAAAAATTCGTGTATATATTTCACAGAAACGTGAAATCAAAGTGGGTGATAAA
GTCGCCGGAAGACATGGCAATAAGGGTATCATTTCCAAAATTTTGCCTAGACAAGATAT
GCCTTATTTGCAAGATGGAACACCAGTTGATATGGTCTTCAACCCATTAGGAGTACCTT
CACGAATGAATGTGGGACAGATCTTTGAATGCTCGCTGGGGTTAGCGGGAGATCTGCT
GGACAGACATTATAGAATAACACCCTTTGATGAGAGATATGAGCAAGAGGCTTCGAGA
AAACTAGTGTTTCCTGAATTATATGAAGCCAGTAAGCGAACAGCAAATCCATGGGTATT
TGAACCCGAGTATCCTGGAAAAAGCAGAATATTTGATGGAAGAACAGGAGATCCTTTT
GAACAACCTGTTATAATAGGAAAGTCCTATATGCTGAAATTAATTCATCAAGTAGATGAT
AAAATCCATGGACGTTCCAGTGGACATTATGCACTTGTTACACAACAACCCCTTAGAGG
AAGGGCCAAGCAAGGGGGACAACGAGTAGGAGAAATGGAAGTCTGGGCTCTAGAGG
GATTTGGTGTTGCTCATATTTTACAAGAGATGCTTACTTATAAATCCGACCATATTAGAG
CTCGTCAGGAACTACTTGGTACCACGATCGTTGGAGGAACAATACCTAAGCCTGAGGG
TGCTCCAGAATCTTTTCGATTGCTCGTTCGAGAACTACGATCTTTGGCTCTGGAACTGA

AGCATTTCCTTGTATCTGAGAAGAACTTCCAGATTAATAGGAAGGAAGCT-----------------
---ATGATCGACCGATATAAACATCAACAACTTCGAATTGGATTAGTTTCTCCTCAACAAA
TAACTGCTTGGGCCAACAAAATCCTACCTAATGGAGAGATGGTTGGAGAAGTGACAAA
ACCCTATACTTTTCATTACAAAAGCAATAAACCGGAAAAAGATGGATTGTTTTGTGAAA
GAATCTTTGGGCCTATCAAAAGTGGAATTTGTGCTTGTGGAAATTACCGAGTCATCGGG
GATGAAAAAGAAGACCCGAAATTTTGTGAACAATGCGGAGTCGAATCTGTTGATTCTC
GGATACGAAGATATCAAATGGGATACATCAAACTGGCATGTCCAGTGACTCATGTGTGG
TATTTGAAACGTCTTCCCAGTTATATCGCGAATCTTTCAGATAGACCTCTTAAAGAATTA
GAAGGCCTGGTATACTGCGAT------TTCTCTTTTGCTAGGCCC---ATAGCTAAAAAACCCA
CTTTCTTACGATTACGAGGTTCATTCGAATATGAAATCCAATCCCGGAAATACAGCATCC
CACTTTTTTTTACTACCCAATGCTTCAATCTATTTAGAAATAGAGAAATATCTACTGGAG
CAGGTGCTATCAGAGAACAATTAGCCGACCCGGATTTGCGAATTATTACAGATCGTTCA
TTGGTAGAATGGAAGGAATTGGGGGAAGAAAGATCCGCT---GAGAATGAATGGGAAGA
TAAAAAAATCGTAAGAAGAAAGGATTTTTTGGTTAGACGCATGGAATTGGCTAAGCAT
CTTCTTAGAACAAATGTAGAACCAGAACGGATGGTTTTGTGCCTATTACCAGTTCTTCC
TCCCGAGTTGAGGCCAATCATTCAGATAGATGGGGGTAAACCAATGAGTTCGGATATTA
ATGAACTCTATAGAAGAGTTATCTATCGGAATAATACTCTTATTGATCCATTAACAACAA
GTAGATCTACGCCAGGGGAATCAGTGATGTGTCAGGAAAAATTAGTACAAGAAGCCGT
AGATACACTTCTTGATAATGGGATCCGCGGACAACCAATGAGGGACGGTCACAATAAA
GTTTACAAGTCTTTTTCAGATGTAATTGAAGGCAAAGAGGGAAGATTTCGCGAGACTC
TACTTGGTAAACGGGTCGATTATTCGGGTCGTTCTGTCATTGTGGTGGGCCCTTCGCTTT
CATTACATCGATGTGGATTACCCCGAGAAATAGCAATAGAGCTCTTTCAGACATTTGTAA
TTCGTGGTCTAATCAGACAACATGTTGCTTCCAACATAGGGATTGCTAAAAGTAAAATT
CGGGAAAAAGAACCAATTGTATGGGAAATACTTCAAAAGGTTATGGAAGGACATCCTG
TATTGTTGAATAGAGCACCTACTTTGCATAGATTAGGCATACAGGCGTTCCAACCTATTC
TAGTGGAGGGACGTGCTATTTGTTTACATCCATTAGTTCGTAAGGGATTCAATGCAGAC
TTCGATGGGGATCAGATGGCTGTTCATGTACCTTTGTCTTTGGAAGCTCAAGCGGAAGC
CCGTTTACTTATGTTTTCTCATATGAATCTCTTGTCTCCGGCTATTGGAGATCCTATTTCT
GTACCAACTCAAGATATGCTTATTGGCCTCTATGTACTAACGATTGGGAATCGCCGAGGT
ATTTGTACAAATAGGTATAATCCATGTAACTACAGA------AAC---TATCAAAATGAAATAG
TTGACGATAAT---TATAAGTATACAAAAGAGAAAGAACCTTATTTTTGTAGTTCCTACGA
TGCGCTTGGAGCTTATCGTCAGAAACGAATCGACTTATACAGTCCTTTGTGGCTCCGGT
GGCGGCTAGATCAATGC---GTGATTGCCTCAATAAATAGAGAAGTTCCCATTGAAGTTC
AATATGAATCTTTGGGTATCTACCATGAGATTCACGAGCACTATCGAATAGTAAAAAGC
GTAAAAAAAGAAATCGTTTGTATATACATTCGAACCACTGTTGGTCATATTTCTTTTTAT
CGAGAAATAGAAGAAGCTATACAAGGGTTTTGTCGGACCTAT---------------------------------
-------------ATGGCAGAACGGGCCGATCTGGTCTTTCACAATAAAGCGATAGATGGAACT
GCCATGAAACGACTTATTAGTAGATTAATAGATCATTTTGGAATGGCATATACATCACAC
ATTCTGGATCAAGTAAAGACTCTGGGTTTCCGGCAAGCCACTCTGACATCAATTTCATT
AGGAATTGATGATCTTTTAACAACGCCTTCTAAGGGATGGTTAGTACAAGATGCGGAAC
AACAAAGTTTGATTTTGGAAAAGCACCATCATTATGGGAATGTACACGCAGTAGAAAA
ATTACGCCAATCAATTGAGATATGGTATGCTACAAGTGAATATTTGAGACAAGAAATGA
ATCCAAATTTCAGGATGACTGATCCTTTTAATCCAGTCTATATAATGTCCTTTTCGGGAG
CTAGAGGAAATGCATCTCAGGTCCACCAATTAGTAGGTATGAGAGGATTAATGTCGGAT

CCCCAAGGACAAATGATTGATTTGCCCATTCAAAGCAATTTACGCGAAGGGCTTTCTTT
AACGGAATATACCATTTCCTGTTACGGAGCCCGCAAAGGAGTTGTGGATACTGCTGTAC
GAACATCAGATGCTGGATACCTCACGCGTAGACTTGTTGAAGTAGTTCAACACATTGTT
GTACGTAGAACGGACTGTGGCACTATCCGAGGTATTTCCGTAAGTCCTCGAAATGGGGT
TGGGGTGACGGAAAGGATTTTTATCCAAACACTAATTGGTCGCGTATTAGCGAACGATG
TATATATGGGTCTGCGATGCATTGCGACTCGAAATCAAGATATTGGGATTGGACTTGTCA
ATCGATTCATAACC------------------TCTCGGGCACAACCAATATATATTCGAAGCCCCTTCA
CTTGCAGGAGTACATCTTGGATCTGTCAATTATGTTATGGTCGGAGTACCACTCACGGT
AACCTGGTCGAATTAGGAGAAGCTGTAGGTATTATTGCGGGTCAATCAATTGGAGAAC
CGGGAACTCAACTAACATTAAGAACTTTTCATACCGGTGGAGTATTCACAGGCGGTATC
GCAGAACATGTACGAGCTCCTTCTAATGGAAAAATTAAATTCAATGAGGATTTAGTTCA
TCCCACACGTACACGTCATGGACATCCCGCTTTTCTATGCCATATAGACCTGCATGTAAC
TATTGAGAGTCAGGATATTATACATAAAGTGAATATTCCACCAAAAAGTTTTCTTTTGGT
TCAAAACGATCAATATGTAGAATCAGAACAAGTGATTGCTGAGATTCGCGCTGGAACA
TCCACT---------TTAAAGGAGAGGGTTCAAAAACATATTTATTCTGACTCAGAGGGAGAG
---ATGCACTGGAGTACCGATGTGTACCATACACCTGAATATACGCATGGTAATGTTCATC
TCTTACCCAAAACAAGTCATTTATGGGTATTATCAGGAAGTCCATGCAGA------TCCAGT
ATAGTGCCTTTTCCACTCCACAAGGATCAAGATCAAATGAATGTTCAA------TCTCTTTAT
GTCGAAGAGAGATATATTTCTGACCTCTCG------ATGAATAATGATCGA---GTGAGACAC
AAATTGTTTGGGTGGGATCAA------------AGGGGGAGGGTTTCTTATTATTCA---GGACCT
GATCGAATCATATCCAACAGGAATTGGGATTCCATATATCCTCTT---ATTCTCCACGAGAA
TTCTGAT------TTATTGGCGAAGAGGCGAAGAAATAGATTCATCATCCCATTCCAATATGA
TCAAGAACAGGAGAAGGAACTAAGGCCTCCTGGT---ATCGTGATTAAAATACCCATAAA
A------GGTATTTTACGTAGAAATAGTATTCTTGCCTATTTCGACGATCCCCGATACAGAAG
AAGTAGTTCAGGAATTGCCAAATATGGGACCATAGAAGTCGATTCGATAATAAAAAAA
GAAGAT------TTAATTGAATATCGAAAG---ACAAGAGAGTTTGGACCAAAATACATCAAA
GTAAATCGATTTTTTTTCATTCCTGAAGAAGTGCATATCTTACCTAGATCTTCGTCCATAA
TGGTACGGAATAATAGTATCATTGGAGTAGATACACGAATCACTTTGAATATAAGAAGTC
AAGTAGGCGGCTTGGTCCGAGTGGAGAAAAAAAAAAAAAAGGATTGAATTGAAAATAT
CTTCTGGAGATATCCATTTTCCTGGAGAGACGGATAATATAGCCCGGTACAGCGGCATC
TTGATACCACCTGGAAGGGTAAAAAAC------------ACGGAATCAAAATTC---------AAAAA
TTGGATCTATGTCCAACGG------ATCACACCTATCAAGAAAAAG------TATTTTGTTTCAG
TCCGACCCGTAGTGACATATGAAATCGCCGATGGGATAAATCTAGCAACGTTTTTTCCC
CAGGATCTGTTGCAGGAAAAGAATAATTTGCAACTACGAGTTGTCAATTATATTCTTTAT
GGAGATGGCAAACCCATTCGAGGAATTTTTCACACAAGCATTCAATTAGTTCGGACTTG
CTTAGTATTGAATTGGGATCAAGACCGGGCTGGTTCTATAGAAGAA------GAAGAGGCTT
ATACTTCCCTCGCTGAAGTAAGGGTAAACGATCTGATTCGAAATTTTATAAGAATTGATT
TGGTGAAGTCTCCTATTTCGTCTACTGGAAAAGAGAATGATATGGCGGGTTCAGGATTG
ATTCCTAAT---AATGGATCAGATCGCATCAATACCAATCCTTTTTTTTCCAAGGCGAAGA
CT--------------------------CAATCACTTAGCCAACATCAAGGAACTATTCGTACATTGCTGAA
TAGAAATAAG---GAAGGCCAGGGCGAATCTCTTATGGTTTTGTCATCATCCAATTGTTCT
CGAATTGGTCCA------TTGAATGGTTCGAAATATCACAAT---------GTGACAAAAGAATCA
GATCCCATGATTTCAATTAGGAATTCGTTGGGTCCGTTAGGGACT------GTACCTAACATT
TTGAATTTTTCCTCATCT------TATCATTCAATAACT------CATAATGAGATCTTGTTCAAT---

AAATATTTACTACCTGACAAT---------TCAAGAGAGACTTTCCTAGTACCTAAA---------TCT
TATTTCATGGATGAAAATAGAAGG------ATTTATAATCTTGATCCATGCAGTAACATCATTT
TGACTCCATTCAATTTGAATTGGTGCTTTCTCCATCACGATTATTGGGAAGATACATCAA
CAATAATTAGC---CTTGGACAGTTTCTTTGTGAAAATATATGTATATCCAAAGACGGACC
A---------TGCGTAAAATCTGGTCAAATTATAATTGTTCAT---GTTGACTCTTTAGTAATAAG
ATTAGCTAAGTACCATTTGGCTACCCGCGGAGCAACCGTTCATGGTCATTATGGAGAAA
TCCTTTACGAGGGAGATACATTAGTTACTTTTATATATGAAAAATCAAGATCTGGTGATAT
AACTCAAGGTCTTCCAAAAGTTGAACAAGTGTTAGAAGTTCGTTCGATTGATTCAATAT
CGATGAACCTAGAAAAGAGGGTTGAAGGCTGGAACGAACGCATAACAGGATTTCTTG
GAATTCCCTGGGAATTCTTTATTAGTGCTCAGCTAACCATAGTGCAAAGTCGTATCTCTT
TGGTTAATAAGATCCAAAAGGTTTATCGATCCCAGGGGGTGCAGATCCATAATAGGCAT
ATAGAAACTATTGTACGACAAATAACATCAAAAGTCTTGGTTTCAGAA---------GATGGA
ATGTCTAATGTTTTTTCACCCAGAGAACTAATCGGATTGTTGCGAGCAGAAAGAATAGG
TCGTGCTTTGGAAGACGATATATGTTACCGAGCTATTCTATTGGGAATAACGAGGGCATC
TCTGAATACTCAAAGTTTCATATCAGAAGCGAGTTTTCAAGAAACCACTCGGGTTTTAG
CGAAAGCCGCTCTCCGAAGTCGTATTGATTGGTTGAAAGGCCTGAAAGAGAATGTTGT
TCTGGGGGGGATGATACCTGTTGGTACCGGATTCAAAGGATTCGTTCACCACTCCAGG-
-----GAACACAACAACATTTCT------TTGGAAATAAAAAAGAAGAATCTATTCGATGGGAA
GATGAGA------GATATTTTGTTCTACCACAGAGAATTTTGTGGTTCGTGCATCCCAAAGA
ATTTTCATGATACATCAGAACAA---ATGGCAAGAAGATAT
TGGGACATCAATTTGGAAGAGATGATGGGAGCAGGGGTTCATTTTGGTCATGGTACTA
GGAAATGGAATCCTAGAATGGCACCTTATATCTCTGGAAAGCGTAAAGGCATTCACATT
ACAAATCTTACTAGAACGGCTCGTCCTTTATCAGAAGCCTGCGATTTAGTTTTTGATGC
AGCAAGTAGAGGAAAGCACTTCTTAATAGTAGGTACGAAAGATAAAGCAGCCGATTCA
GTAGCATCAGCTGCAATACGGGCTCGGTGTCATTATGTCAATAAAAAATGGCTTGGTGG
TATGTCAACGAATTGGTCCACTACAGAAACAAGACTTCATAAGTTCAGGGACTTGAGA
GCAGAACAAAAGATGGGTCGATTTAAGCGTCTCCCGAAAAGAGATGCGGCAATGTTG
AAGAGACAATTATCTCACTTGCAAACATATCTGGGCGGGATCAAATATATGACGGGGTT
ACCCGATATTGTAATCATCGTGGATCACCAAGAAGAATATACGGCTATTAGAGAATGCCT
CACTTTGGGTATTCCGACGATTTGTTTAATCGATACAAATTGTGATCCGGATCTCGCAGA
TATTCCGATTCCAGCCAACGATGACGCTATAGCTTCAATCCAATTGATTCTTAACAAATT
AGTATTGGCAATTCGTGAGGGTCATTATAGC---TATCTAGGAAGTCATATGGGACAGAAA
ATAAATCCACTTGGTTTCAGACTTGGCACAACCCAAAGTCATCGTTCCCTTTGGTTCGC
ACAACCAAAGGATTATTCGAGGAATCTACAAGAAGATGAAAAAATACGGGATTGTATC
AAGAATTATGTACAAAAACACATGAGAATATCCTCAGGG---TTCCAAGGAATTGCACGT
CTAGGGATTCAAAAAAGAATCGATCTCATCCAGGTCACAATCTATATTGGGTCCTCCAA
TTTGTTAATTGAGGGC------CCAACCCGA------GGAATCGAAGAATTACGAACAGATGTA
CAAAAAAAGTTAAATTCTATGAACCGGAGACTCAACATTACTATCACAAGAATTGCAA
GACCCTATGAACAACCTAATATTCTTGCAGAATATATTGCTCTGCAATTAAAAAATAGAG
TTTCATTTCGAAAAGCAATGAAAAAAGCTATTGAATTAGCTGAACAAGCAAATACAAA
GGGGATTCGGGTACAAATTGCGGGGCGTCTGAACGGAAAAGAGATTGCCCGTGTCGA
ATGGATCAGAGAAGGCAGGGTTCCCCTACACACTATTCGAGCTAAAATTGATTATTGTT
CTTATATGGTTCAAACTATCTACGGGGTATTAGGGATCAAAATTTGGATATTTGTAGATG
AAGAAATGTCTCGTTACCGAGGACCTCGTTTCAAAAAAATACGCCGTCTGGGGGCTTT

ACCGGGACTAACTAGTAAAAGACCTAGATCTGGAAGTGATCTTAGAAACCAATCCCGT
TCTGGAAAA---AGATCTCAATATCGTATTCGTTTAGAAGAAAAACAGAAATTGCGTTTT
CATTATGGTCTGACGGAGAGACAATTACTTAGATATGTTCGTATCGCCGGAAAAGCCAA
AGGGTCAACAGGACAGGTTTTACTGCAACTACTCGAGATGCGTTTGGATAACATTCTTT
TTCGATTGGGTATGGCTTCGACCATTCCTGGAGCCAGACAATTAGTTAACCATAGACATA
TTTTAGTTAATGGTCGTCTAGTAGATATACCAAGTTATCGCTGCAAACCCGGAGATTTTG
TTACTACGAGGGAGAAAGAAAGATCCAGAGCTCTGATTCAAAATCATATGAATTCATCC
CCCAAC---GAGGAGTTGCCAAAACATTTGACTCTTTACTCGTTGCAGTAT------AAAGGA
TTAGTAAATCAAATAATAGATAGTAAATGGATCGGTTTGAAAATCAATGAATTGTTAGTC
GTAGAATATTATTCCCGTCAGACTATGTCACGTCGAGGTACTGCAGAAGAAAAAACTGC
AAAATCCGATCCAATTTATCGTAATCGATTAGTTAACATGTTGGTTAACCGTATTCTGAA
ACACGGAAAAAAATCATTGGCTTATCAAATTATCTATCGAGCCTTGAAAAAGATTCAAC
AAAAGACAGAAACAAATCCACTATCTGTTTTACGTCAAGCAATACGTGGAGTAACTCC
CGATATAGCAGTAAAAGCAAGACGTGTAGGCGGATCGACTCATCAAGTTCCCATTGAA
ATAGGATCTACACAAGGAAAAGCACTTGCCATTCGTTGGTTATTAGGGGCATCCCGAAA
ACGTCCGGGTCGAAATATGGCTTTCAAATTAAGTTCCGAATTAGTGGATGCTGCCAAAG
GGAACGGCGATGCCATACGCAAAAAGGAAGAGACTCATAGAATGGCAGAGGCCAATA
GAGCTTTTGCACATTTTCGTATGGGTAGGGACACTATTGCCAATATAATAACTTGTATAA
GAAATGCCGACATGGATAAAAAAAGAATGGTTCGAATAGCATCTACCAATATAACCGAA
AACATTGTTAAAATAATTCTACAAGAAGGTTTTATTGAAAACGTTAGGAAACATCGGGA
AAACAACAAAAATTTCCTGGTTTTAACCTTGCGACATAAAAGG------AATAGGAAAGGG
ACATATAGA---------AAGACTTTA------AAACGTATAAGCAGATCCGGTCTACGAATCTATT
CCAACTATCAACGAATTCCTAGGATTTCAGGTGGAATGGGGGTTGTAATTCTTTCTACTT
CTCGAGGTATAATGACAGATCGAGAGGCTCGACGAGAAGGAATTGGAGGAGAGATTTT
ATGTTATATATGGATGAAAAAACCTATACCAAGAATTGGTTCGCGTAGAAATGGACGTAT
TGGTTCACGTAAGAATGGACGTAGAATACCCAAAGGAGTTATTCATGTTCAAGCGAGT
TTCAATAATACCATTGTGACTGTTACAGATGTACGAGGGCGGGTGGTTTCTTGGTGCTC
CGCGGGTACCTGTGGATTCAGAGGCACAAGAAGAGGAACACCGTTTGCTGCTCAAAC
CGCAGCAACAAATGCTATTCGTACGGTAGTGGATCAGGGTATGCAACGAGCAGAAGTT
ATGATAAAGGGACCTGGTCTCGGAAGAGATGCAGCATTACGAGCCATTCGCAGAAGTG
GTGTACTATTAAGTTTCGTACGTGACGTAACTCCCATGCCACATAATGGATGTAGACCCC
CTAAAAAAAGACGCGTGATGCCAACTATTAAACAACTTATTAGAAACACAAGACAGCC
AATCAGAAATGTTACGAAATCTCCCGCTCTTCGAGGATGCCCTCAGCGTAGAGGAACA
TGTACTAGGGTGTATACTATCACCCCCAAAAAACCAAACTCTGCCTTACGTAAAGTTGC
CAGAGTACGATTAACCTCTGGATTTGAAATCACTGCTTATATACCTGGTATTGGCCATAA
TTCACAAGAACATTCTGTAGTATTAGTAAGAGGAGGGAGGGTTAAGGATTTACCCGGT
GTGAGATATCACATTGTTCGAGGAACCCTAGATGCTGTCGGAGTAAAGGATCGTCAAC
AAGGGCGTTCTAAATATGGGGTCAAAAAGCCAAAAATGGCAAGGAAAAGTCTGATTC
AGAGGGAGAGGAAGAGGCAGAAATTGGAACAGAAATATCATTTGATTCGTCGATCCTT
AAAAAAAGAAATAAGC---------AAAACTCCATCGTTGAGTGACAAATGGAAAATTCATG
GAAAATTGCAATCCCCACCACGTAATAGTGCACCTATACGTCTTCATCGACGTTGTTTTT
CGACCGGAAGACCTAGAGCTAACTATCGAGACTTTGGGCTATCCGGACACGTACTCCG
CGAGATGGTTCATGCATGTTTGTTGCCGGGGGCAACAAGATCGAGCTGGATGGTCATA
AATTCGTTTATATCATCTATTTCAAAAGAAGAAAAA---------------AAGGGGTCTGTTGAAT

TTCAAGTATTCTGTTTCACCGATAAGATACGGAAACTTACTTCACATTTGGAACTGCAC
AAAAAGGACTTTTTATCCCAAAGAGGTATGCGTAAAATTCTGGGAAAACGTCAACGGA
TGCTGGCTTATTTGTCAAATAAAAATAAAGTACGTTATAAGAAATTAATCGGTCAGTTGA
ATATTCGGGAGCCAAAGACTCGT------ATGGTAAAACTTCGTTTGAAACGATGTGGTAG
AAAGCAACGAGTCATTTATCGAATCGTTGCAATTGATGTTAGATCCCGAAGAGACGGA
AGAGCTCTTCGGAAAGTGGGTTTTTACGATCCAATAAAGAATCAAACTTATTCAAACGT
TCCTGCTATTCTATATTTCCTTGAAAAAGGAGCTCAGCCTACAGTAACTGTTCATGATAT
TTCAAAGAAGGCGGAGGTATTTAAA--------------------------------ATGGATAAATCCAAGCA
ACCTTTTCGTAAATCCAAGCGATCTTTTCGTAGGCGTTTGCCCCCAATTGGATCGGGGG
ATCGAATTGATTATAGAAACATGAGTTTAATTGGTCGATTTATTAGCGAACAAGGAAAA
ATATTATCCAGACGAGTGAATAGATTGACCTTAAAACAACAACGATTAATTACTATTGCT
ATAAAACAAGCTCGTATTTTATCTCCGTTACCTTTTCTTAATAATGAGAAA------CAATTT
GAGAGAACCGAATCCCTCCCTAGAATTACTGGTACTAGAACCATAAAGAAAATGTCAC
GTTCACTTAAAAAAAATCCTTTTGTAGCTAATAATTTATTATTAAAAATTCATAAGCTTAA
CACGAGGGAGGAAAAAGAAATTATAGTAACTTGGTCCCGGGCATCTACCATTATACCCA
CTATGATTGGTCATACAATTGCTATTCATAATGGAAAAGAGCATTTACCTGTTTATGTAAC
AGATCGTATGGTAGGTCACAAATTGGGAGAATTTGCACCTACTCTTACTTTCCAGGGAC
ATGCGAGAAACGATAGTAAATTTCGTCGTATGATTTTGAAATCTTTTCTACTAGGTAATC
TATTATCCTTATGCATGAAGATAAGAAATTCGGTCGTTGTGGTCGGACTCTATTATGGAT
TTATTACCACATTCTCTATAGGGCCCTCTTATCTCTTCCTTCTCCGAGCTCGGGTTATGGA
AGAAGGA---------ACCGAGAAGGAGGTATCAGCAACAACTGGTTTTATTATGGGACAGT
TCATGATGCTCATATCGATCTATTATACGCCTCTGCATCTAGCATTGGGTAGACCTCATAC
AATAACTGTCCTAGTTCTACCCTATCTTTTGTTTCATTTCTTCTGGAACAATCACAAACA
CTTTTTTTCTTATAGATCTACTACCAGAAATTCAATGCGTAATCTCAGCATTCAATGTGTA
TTCCTGAATAATCTCATTTTTCAATTATTCAACCATTTCATTTTACCAAGTTCAACGTTAG
CCAGATTAGTCAACATTTATATGTTTCGATGCAACAACAAGATGTTATTTGTAACAAGTA
GTTTTGTTGGTTGGTTAATCGGTCACATTTTATTCATGAAATGGGTTGGATTGGTATTATT
CTGG------------------ATACGGCAA---ATTAGATCGAATAAGTACCTTGTGTCAGAATTGAG
AAAGTCTATGGCTCGAATCTTGAGTATTCTCTTGTTTATCACCTGTGTCTACTATTTAGG
CAGAATACCATCGCCTATTTTCACTAAGAAACTGAAAGAAACCGAAGAAGACAAACCC
TTTATTACTTTTCTTTTCGACTATCAACGATGGAATCGACCATTTCGATATATAGAAAATG
ATCAATTTGCAAATACTGTAAGAAATGAAATGTCCCAATATTTTTTTAACACATGTCCAA
GTGATGGAAAACGAGTAATCTCTTTCACATATCCTCCCAGTTTGTCGGTTTTTGGGGAA
ATGATACAAAAAAAAATGTCTTTATGCGGTATGAAAGAACTATATAAAGACAAAAATCT
GTATAATTGGGTTTCTACAAATGAACAAAAAAGGGATAAGGTAAGCAAGGAGTTCATA
AATCGAATAGAAGCTCTATAC------GGGATTGTTCTC---------CAAAAAAGGGTCAGATTA
TGCAATGATCGGGATGAAAAAGAATGCTTACCCAAAATGTCTGATCCCTTTTTAAACGG
ATCATACCGTGGAACAATAAAAGAATTGTATTCAAATGGAGAAAAATTTTGGATAAATA
GAATCCACAGTCTCCTT---------------------GAT---CTAAATGATTCTAGTGAAATTCAAGAA
ATAAAGAAAAAGATACCTCAACGGTTATACAGATTAACTGATTTGGAACAAGACGAGG
GAGAAGAAATTCGTTCAAGAAAAACTAAACATATGGTAATTTATGCTGATGATGTGATA
CGTTACACACAGAAACCTGATTTTCGTCGGGATCTAATCAAAGGATCGATGCGCGCTCA
ACGACGGAAAACAGTTACTTGGGAAATGCTTCAAATAAATTTGCATTCCCCCCTATTTT
TGGATAGAATAGACAAAAACCCTTTTTTTTATTTTGATATC------TCCAGAATCATAAACC

TAATTTTAGGGATAAAAAAGAGGATGAG---------ATAGAAGAAACTTGGGAGACTATTG
CATTTGGTCAAGTAATAAGAGGTTTTCTGTTAGTAACCCAATCTATTCTTAGAAAATACA
TCGTATTACCTTCATTGATAATAGCTAAGAATCTCGTGCGTATGTTATTATTTCAACTCCC
CGAGTGGTATGAGGATTTCAAGGAATGGAGTAGAGAAATGCATATTAAATGCACCTATC
ATGGTGTTCAATTATCAGAAAGAAAATTTCCGAAGAATTGGTTAAAAGCCGGTATTCAG
ATAAAGATTCTATTTCCTTTCTATTTGAAACCTTGGCGCAAATCTAAAAAAAGAAAAAA
ACAAAATTCTTGTTTTTTAACAGTCTGGGGAATGGAAACTGACCTACCCTTTGGTCCTG
CCCGAAAGGGACCTTCCTTTTTTGAGCCTATTCATAAAGAACTTGAAAAAAAGTTTAA
AAAAGGAAAA---AAGAAATGGTTTTTATTTGTAAGAATTTTCAAAGAA--------------------
-----------AAAAAAATATGGGTTATCAAAAGAGTTCTA------TTTATAAAAGGAGTAATGAA
AGAACTTACAAAAGCAAATAATAAGACAATTTCTGAATCACCTATTAGAAAATCACCTA
TTAGAACTGGATCGATGGATTGGACAAATTCTTCACTGACAGAAAGAAAAAGAAAGGA
TCTGTCTGATAAGACAACCACAATCAGAGATCAAATAGAAAGGATCAGGATCACAAGA
GACAAGAGTCCTAATGAGACAAGTTATAGTGATAAAAGATCGGAATCACAGAAACCTA
TTTGGCAAGTATCCAAAAGAAGAAGTACTCGATTACTCTATAAATGGCGCTATTTTATGA
AAAGTTTCATTGAAAGAATACACAGAAATGTCTTTCTATGTATGATTAACCTCCCTAGGA
TCAATACACAACTTTTTATCCACTTTATCTTTTGTTCGTTGTCT---------CAAGCATATGTAT
TTTACAAATTATCGCAAAAACCAGGTATTAAGAAGTATCGCTTGGGATCTTTATTTCAAT
ACGCGTAT--------------TCTATTAAAGATAGAATAATAGAAAGAATATTTCATTCCGAATCA
AAACATAAGAAACCGGGTAATTTCGGAATGAACGAATGGAAAAACTGGTTAATAGGTA
ATTATCAATACAATTTTTCTTATACTAGATGGTCTAGATTAGACCCGCAAAAATGGCGAA
ATAAAGTAAATGAACAGTGTATGATAAAAAATAAGAAAAAAACCGATTCATATTCATAT
GAGAAAGACCCATTAATTAATCACGAGAGACATAAAAAACTTAGCAAACGTTACAGAT
ATGATCTTTTATCATATAGATATATCAATAGGTTTATCGTTGATACAGATAGAAAATATTTT
GATCTGAGAATCATCAAATTTTGGTCTAGATTTTTTGATTGGATGGGAATGAATGAAGA
ACTACTATATCGTTGGTTTTTCCCAGAACTTGTACTACTTAATGACGCATATAAGACTAA
ACCTTGGACCATACCCATAAGATTGCTTCTTTTTAATTCCAAGAGCAACAAGGAAGCAG
AACTGGATTTACTCCTGAAAAGATATTTACTTTTTCAATTCAGATGGGATGATTCTTTGA
AGAAAAAAATGATCAATAACATCAAATTATATAGTCTCCTGCTTAGACTCATGGATCCAA
AAAAAATTGTTATATCCTCTATTCAAAGAGGAGAAATGTGGTTGGATATAATGCTAATTC
ATAAGGATCTACCTCTTACAAAATTGAAAAAAGGGGGAGTATTTACTATGGAACCGCTT
CGTCTATCTAGAAAATGGAATGGACAATTTCTTATGTATCAAACCCTAAGTATTTCATTG
GTCCATAAGATTAAGCAGAAGAATTATGATTTGTTTGTTCCTGAAAATATTTCAGCTCTG
AGACGGCGTAGAGAATTGAGAATTCTAAGTCGTCTCATATTTCTTTGGCCCAACCATCG
ATTAGAAGATTTAGCTTGCATGAATCGCTATTGGTTTGATACCAATAATGGTACTCGTTT
CAGTATGTCAAGGATACGTATGTATCCAATGAAAGGACATCAACTCAAATCCTGGATCT
TCGAATTGAGAGAGATATTGAGAGAGATCAAGAATTCTCACTATTTCTTAGATTCATGG
ACAAAATTGAATTTAGTGGGATCTTTCACTCACATTTTTTTCCACCAAGAACGTTTTATG
AAACTCTTTGACCCCCGAATTTGGAGTATCCTACTTTCA---TGCGATTCG------CAGAGTT
CAACAAGCAATCGATATTTC---------ACGATCAAAGGTGTAGTACTGCTTGTAGTAGCG---
GTCCTTATA------------TATCGTATTAACAATCGAAATATGGTCGAAAGAAAAAATCTCTAT
TTGATGAGGCTTCTT--------------CCTATACCTATGAATTCCATTGGGCCCAGAAATGATAC
ATTGGAAGAATCTTTT---TTGTCTTCCAATATCAATAGGTTGATTGTTTCG---CTCCTGTAT
CTTCCAAAAGGGAAAAAGATCTCTGAGAGTTGTTTCATGGATCCGAAAGAGAGTACTT

GGCTTCTCCCAATAACT------------AAAAAGTGTATCATGCCTGAATCTAACTGGGGTTCG
CGGTGGTGGAGGAACCGGATCGGAAAAAAGAGG------GATTCTAGTTGTAAGATATCT---
------AATGAAACAGTAGCTGGAATTGAGATCTCATTCAAAGAGAAAGATATCAAA---TAT
CTGGAGTTTCTTTTTGTATCCTATACGGATACGGATGATCCGATCCGCAAGGACCATGAT
TTGGAATTGTTTGATCGTCTTTCTCCGGGGAAGAAGCAAAACATAATCAACTTG------AA
TTCGGGACAGCTATTCGAAATCTTAGTGAAACACTTGATTTGTTATCTCATGTCTGCTTT
TCGTGAAAAAAGACCAATTGAAGTGGAGGGT---TTCTTCAAACAACAAGGA---GCTGA
GGCAACTATTCAATCAAATGATATTGAGCATGTTCCCCGT------CTCTTCTCGAAAAACA
AGTGGGGTATTTCTTTGCAAAATTGCGCTCAATTTCATATGTGGCAATTCCACCAGGATC
TCTTCGTT---------AGTTGGGGGAAG---AATCAGCACGAATCGGATTTTTTGAGGAAC---G
TATCGAGAGAGAATTTGATTTGGTTAGGCAGTATGTGGTTGGTAAACAAGGATAGG------
---TTTTTTAGC------AAGGTACGGAATGTATCGTCAAATATTCAATACGATTCC---------ACA
AGA------TCTATTTTCGTTAAAGTAACGGATTCTAGCCAATTGAAAGGATCTTCT---GATC
AATCCAGAGATCATTTCAATTCCATTAGT------------AATGAGGATTCGGAA------------------
------------------TATCACACATTGATACATCAAACAGAAATTGAGCAACTAAAAGAAAGA
TCGATTCTT---TGGGAT------CCTTCCTTTCTTCAAACGGAACGAACAGAGATAGAATCA
GAT------CGATTCCCGAAA------TGCCTTTCTGGATATTTC------TCAATGTCCCGGCTATTC
ACG---GAACGTGAGAAGCAGATGAATAATCATCTGTTTCCGGAAGAAATCGAAGAATTT
ATT------GGGAATCCTACAAGATCAATTCGTTCTTTTTTCTCTGACAGATGG------TCAGA
ACTTCAT---CTGGGGTCGAATGCTACTGAGAGGTCCACTAGAGATCAG------------AAATT
GTTGAAGAAACAACAAGATGTTTCT---TTTGTCCCTTCCAGGCGATCGGAA------------AA
TAAAGAAATGGTTGATATATTCAAGATAATTACGTATTTACAAAAT------ACCGTCTCAAT
TCATCCTATTTCATCAGATCCGGGATGTGATATGGTTCCG---AAGGATGAACCGGAT---AT
GGACAGTTCCAATAAGATTTCATTCTTGAACAAAAATCCATTTTGTGAT------TTATTCCA
CCTATTCCATGGCCGGAACAAAGGTGGATACACGTTA------CACCACGATTTTGAATCAG
AA------GAGAGATTTCAAGAAAGGGCAGATCTATTCACTCTATCAATAACCGAGCCC---G
ATCTGATGTATCATAAGGGATTTGCCTTTTCTATTGATTCCTACGGATTGGATCAAAAAA
AA------TTATTGAATGAGGTATTCAACTCCAGGGATGAATCGAAAAAGAAATCTTTA------
---TTGGTTCTACCTCATATTTTTTAT------GAAGAGAATGAATCTTTTTATCGAAGGATCAG
AACAAAATCGGTCCGG---ATCTCCTGCAGGAATGGTTTGGAAGATCCA------AAAATAGT
GGTATTTGCTAGCAACAACATAATGGAGGCAGTCAATCAATATAGATTGATCCGAAATC
TGATTCAAATCCAATATAGCACCTATGGG------TACATAAGAAATGTATCGAAGAGATTCT
TTTTAATG---------AATAGATCCGATCGCAACTTCGAATATGGAATTCAA---------AGGGAT
CAAATAGGAAATGATACTCTGAATCATATAACTATAATGAAATATACGATCAACCAACAT
TTATCGAATTTGAAAAAG---AGTCAGAAGAAATGGTTCGAT------------CCTCTTATTTCTC
GAACCGAGAGATCCATGAATCGGGATCCTAATGCATATAGATACAAATGGTCCAATGGG
AGCAAGAATTTCCAAGAACATTTGGAACATTTCGTTTCTGAACAGAAGAAC---------CGT
TTTCAAGTAGTGTTCGATCGATTACGTATTAATCAACATTCGATTGATTGGTCCGAGGTT
ATCGACAAACAAGATTTGTCTAAGTCACTTCGTTTCTTTTTGTCCAAGTCA----------------
----------CTTCTCTTTTTGTCT---------------------AAGTCACTT------------------------CCTTTTTTC
TTTGTGAGTATCGGGAATATCCCCATTCATAGGTCCGAG---ATCCACATCTAT---GAATTG
AAAGGTCCGAATGATCAACTCTGCAATCAGTTGTTAGAATCAATAGGTGTTCAAATCGT
TCATTTGAACAAATTGAAACCT------TTCTTATTAGAT---------GATCATGATACTTCC---CA
AAGATCGAAATTATTGATCAATGGAGGAACAATAGCACGATTTTGGTTCAATAAGATAC

CA---AAGTGG---ATGATTGACTCATTCCATACT------AGGAATAATCGCGGGAAATCCTTT
GAGAACACGGATTCCTATTTCTCAATGATATCCCACGATCGAGACAATTGGCTGAATCC
CGTGAAACCCTTTCATAGGAGTTCATTGATATCTTCTTTTTATAAAGCAAATCAACTTCG
ATTCTTGAATAATCCACATCACTTCTGGTTCTAT------TGTAACAAAAGATTCCCTTTTTAT
------------GCGGAAAAGACCCGTATCAATAATTATGAT------CTTACATAT---GGACAATTCCT
CAATATCTCGTTCATTCGCAACAAG---ATATTTTCTTTGTGCGTCGGTAAA------AAAAAA
CATGTTTTTTTGGAG---AGAGATACTATTTCACCTATTGAGTCACAGGTATCTGACATATT
CATACCTAACGATTTTCCACAAAGTGGTGACGAAACG---TATAACTTGTACAAATCT------
---TTCCATTTTCCAACTCGATCCGATCCATTCGTTCGT------AGAGCTATTTACTCGATCGC
AGACATTTCTGGAACACCTCTGACAGAGGAACAAAGAGTCAATTTTGAAGGAACTTAT
TGTCAGCCTCTTTCAGATCTGAATCTATCTGATTCAGAAGGGAAGAACTTGCATCAGTA
TCTCAGT------TTCAATTCAAACATGGGTTTGATTCACACTCCATGTTCTGAGAAATATTT
ACCA------TCCGGAAAGAGGAAAAAA------------------------CGGAATCTTTGTCTAAAT------
AAATGCGTTGAGAAACGGCAGATGTATAGAACCTTTCAACGAGATAGTGCTTTTTCAAA
TATCTCAAAATGGAATCTGTTCCAAACATATATGCCATGGTTCCTTACTTCGACAGGGTG
CAAATATATAAATTTCATCCTTTTAGATACTTTTTCAGACCCATTG------CCGATACTAAGT
AGCAGT---CACAAATTTGTATCCATTTTTCATGATATTATGCATGGATCG------GCATGGTC
AATTCCTCAGAAAAAAATTGCGG------------GCGATTCTTCCACAATGGAATCTG---ATAAG
TGAGATTTCGAGTAAATGTTTACAGAAT------CTTCTTCTGTCCGAAGAAATGATTCATCG
AAATAATGAGTCACCCGTTCCATTGATATGGACACATCTGAGATCACCAAATCCTCGAG
AGTTC---TTCTATTCAAGCCTTTTCCTTCTTCTTGTTGCTGGATAT---CTCGTTCGTACACA
TCTTCTCTTTGTTTCCCGA------GTCTCTAGTGAGTTACATACAGAGTTAGAAAAGATCA
AATCTTTGATGATTCCATCATACATGATGGAGTTGCGAAAACTTCTGGATAGTTATCCTC
CACCTGAACTGAATTCTTTCTGGTTAAAGAATCTCTTTCTAGTTGCTCTGGAACAATTA
GGAGATTCTCCGGAAGAAATACGGGGTTCTGATTCTGGCGGCAACATGCTATTGGGTG
GCGGTCCC---------------GCTTATGGG---GTCAAATCAATACGTTCTAAGAAGAAAGATTTG
AATATCAATCTCATCGATATCATCGATCTCATA---AGTATCATACCAAATCCCGTCAATCC
AATCACTTTTTCGATAAATACGAGACGTTTAAGTCGTACGAGTAAAGAGATCTATTCATT
GATAAGAAAAAGAAAAAACGTGAACAGCGATTGGATTGATGGGAAAATAGAATCCTG
GGTCGCGAACAGTGATTTGATTGATGATGAAGAAAGAGAATTCTTGGTTCAGTTCTCC--
----GCCTTAACGACA------GAAAAAAGGATTGATCAAATTCTATGGAGTCTGACT---CATA
GTGATCCTTTCTCA---------------AAGAATGACTCTGGTTATCAAATGATTGAACAACCGG
GATCAATTTACTTACGATACTTAGTTGACATTCAT------AAAAAGTATCTAATGAATTCTG
AGTTCAATAGATCCTGTTTAGCAGAAAGACGAATATTCCTTGCTCATTATCAGACAATC
ACTTAT------TCACAAACCTCGTGTGGGGCTAATAGTTCTCATTTCCCATCTCATGGA------
---AAACCCTTTTCGCTCCGCTTAGACCTATCCCCTTCT---AGGGGTATTTTAGTGATAGGT
TCTATAGGAACTGGACGATCCTATTTGGTCAAATACCTAGCGACAAACTCCTACCTTCC
TTCCATTACGGTATCTCCGAACAAGTTCCTGGATGACAAGCCTAAAGGTTATCTTATTGA
T---------------GATATTGATAGTATTGATGATAGTGACGATGTCGAT------------ATTGGTGATA
GTGATGATATCGATATT--GATGATGACCTTGATGCGG
AGCTGCTAACTATGACGAATGTGCTA---------ACTATGTATATGACG---------CCGAAAATG
GACCGGTTTGATATCACCCCTCAATTAGAATTAGCAAAAGCAATGTCTCCTTGCATAATA
TGGATTCCAAACATTCATGATCTGTATGTGAATGAGTCGAATTACTTATCCCTCGGTCTA
TTAGTGAACCATCTCTCC---------AGAGATTGTGAAAGATGTTCC---ACTAGAAATATTCT

TGTTATTGCTTCGACTCATATTCCCCAAAAAGTGGATCCCGCTCTAATAGCTCCGAATAA
ATCAAATACATGCATTAAGATACGAAGGCTTCTTATTCCACAACAACGAAAGCACTTTT
TCATTCTTTCATATACTAGGGGATTCCACTTGGAAAAGAAGATGTTCCATACT------AACG
GATTCGGGTCCATAACCATGGGTTCCAATGCACGAGATCTTGTAGCACTTACCAATGAG
GCCCTATCAATTAGTATTACACAGAAGAAATCAATTATAGACACTAATACAATTAGATCA
GCTCTTCATAGACAAACTTGGGATTTGCGA---------TCCCAGGTAAGATCCGTTCAGGAT
CATGGGATCCTTTTCTATCAGATAGGAAGGTCCTTTGCACAAAATGTACTTCTAAGTAAT
TGCTCCATAGATCCTATATCTACCTATATGAAGAAGAAA------TCATGTAAGGAAGGGGAT
TCTTATTTGTACAAATGGTACTTCGAACTTGGAACGAGCATAAAGAAATTAACGACACT
TCTTTATCTTTTGAGTTGTTCTGCCGGATCGGTCGCTCAAGATCTTTGGTCTCCACCC----
--GGACCCGATGAAAAA---AATTGGATCACTTCTTATGGATTCGTTGAGAATGATTCT---G
ATCTAGTTCATGGC---------TTAGAAGGCGCTCTGGTGGGA------TCCTCACGGACAGAAA
AAGATTGCAGTCAGTTTGATAATGAT------CGAGTGACATTGCTTCTTCGGTCCGAACCA
AGGAAT---CCGTTAGATATGATGCAAAATGGATCTTGTTCTATCGTTGATCAGAGATTTCT
ATATGAAAAA------TACGAATCGGAGTTTGAAGAA---GGGGAAGGAGAAGGAACCCTC
GACCCGCAA------------CAGATAGAGGAGGAT------------TTATTCAATCAC------ATAGTTTG
GGCTCCT------AGAATATGGCGCCCT---------TGTGGCAATCTATTTGATTGTATCGAAAGG
CCCAATGAATCGAGATTTCCC---TATCGGTCCAGGTCATTTCGG---------GGCAAGCAGAT
CATTTCTCATAAAGAGGATGAGCTTCAAGAGAATGATTTGGAGTTCTTGCAGAGTGGA
ACCATGCAGTACCAGACACGAGATAGATCCTCCAAAGAACAAGGGTTTTTTCAAATAA
GCCAATTCATTTGGGACCCCGCAGATCCATTCTTTTTCCTATTCAAA---GATCAGCCCTT
TGTCTCTGTGTTTGCACGTCGAGAATTCTTTGCAGATGAAGAGATGTCAAAGGGGCTTA
TTACTTCCCAAACACATTCTCCTACATCTATATATGAACGCTGGTTGATC------AAGAATA
CGCAAGAAAAGCACTTCGAATTGTTGATTCATCGCCAGAGATGGCTTAGCACC---------A
ATAGTTCATTATCT------AATGGATCTTTCCGTTCTAAT------ACGCCATCCGAGAGTTATCA
GTATTTATCAAATCTGTTCCTATCTAAC---GGAACGCTATTGGATCAAATGGCAAAGACA
TTGTTGAGAAAGAGATGGCTTTTCCCGGATGAAATGAAACATTTGATT------CATGTAAC
AGGAGAAAGATTTCCCATTCCTATGCCTAGATCTCGG---ATAAATGGCAATTTTATCGAT
AAGACCTCTTCAATTGTAGCCAATATCTTATTACGGATCATTCCGACGACTTCAGGAGA
AAAAGAGGCATTTACCTATTACAGAGATGGG------ATGTCAGCTCAATCTGAAGGAAATT
ATGCGGAAGCTTTACAGAATTATTATGAAGCTACGCGACCAGAAATTGATCCCTATGAT
CGAAGTTATATACTCTATAATATAGGACTTATCCACACAAGCAACGGAGAACATACGAA
GGCTTTGGAATATTATTTTCGGGCACTAGAACGAAATCCATTCTTACCACAAGCTTCTAA
TAATATGGCCGTGATCTGTCATTAC---CGGGGAGAACAGGCCATTCGACAGGGAGATTC
TGAAATCGCAGAGACTTGGTCCGATCAAGCAGCTGAGTATTGGAAACAAGCTATAGCG
CTTACTCCAGGTAATTATATTGAAGCACAGAATTGGTTGAAGATCACGAGGCGCTTCGA
A------ATGAACTGGCGATCAGAACGTATATGGATAGAACTTATAACAGGTTCTCGAAAAA
CAAGTAATTTATGTTGGGCCTGTATACTTTTTTTAGGTTCACTAGGGTTCTTATTAGTTGG
AACTTCCAGTTATCTTGGCAGGAATCTGATATCCTTATTTCCGTCTCAGCAAATCCTTTT
TTTTCCACAAGGGATTGTGATGTCTTTCTATGGTATCGCAGGTCTGTTCATTAGCTCCTA
TTTGTGGTGCACAATTCTGTGGAATGTAGGTAGTGGTTATGATAGATTTGATAGAAAAG
AAGGAATAGTGTGTATTTTTCGTTGGGGATTTCCTGGAAGAAATCGTCGTATCTTCTTTC
GATTCCTTATGAGAGATATTCGGTCAATCAGAATGGAAGTGAAAGAAGGTATTTATCCT
CGTCGTGTCCTTTCTATTGAAATAAGAAGCCAGGGATCCATTCCCTTGACTCGTACTGA

TGAAAAT---TTCACTCCACGAGAAATTGAGCAAAAAGCTGCTGAATTGGCCTATTTCTT
GCGCGTACCAATTGAAGTATTT
>Nymphaea
ATGGAAAAAATGGTGGTTAAATTCAATGTTGTCTCTAAGT---------GCGAGTCTTGGCCCTA
TTGGAAATACCAGCGGGAGTGAAGAACCCATTATAAATGATAGTGAGAAAAACGTTAA
T---TGCAGTAATGTTGATAACTTGGGCGGTGTCAAGGACGTTTGGAGTCTCATCTCTGG
TGAGACTTTTTGGGTTAGGGATAGTAATGGTGACAGTTATTCCATATATTTCGATATTGA
AAATAAGATTTTTGAGATTGACACTGAT------AGTTACTTTCTGGGTGAACTAGAAAGTT
TATTTTCTAGTTATCTG---------AATCTAAATAATGGGTCTAAGAGTTACAATCGCTATTAT
GATCATTACGTGTATGATACTAGGCATAGTTGGAAGAGTCACATAAATAGTTGCATTGAC
AGTTATATTCGTTCTGAAACCAAT------ATGGATAGTTGCATTCCAAATGGTAGCAACAAT
TCAAGTGACAATTACATTTATAGTTACATTTGTAGCGACCGTGGGAGTTCCCTAAAAAC
TAGTGGTAGTGAT---------------TTCGGAAGACAT---GATTTAGATAGAAATAAAAGATACA
GACATTTATGGGTTCAATGTGAAAATTGTTATGGATTAAATTATAAGAAATTTTTTAGTTC
AAAAATGAATATTTGTGAACAATGTGGATACCACTTGAAAATGAGTAGTTCAGATAGAA
TCGAACTTTTGATTGATCCAGGCACTTGGGACCCTATGGATGAAAACATGGTCTCTATG
GACCCTATTGAATTTCATTCTGAGGAGGACCCTTATAGGGATCGTATCAATTCTTATCAA
ATAGAGACGGGTTTAGCTGAGGCTGTTCAAACAGGCATAGGCAAACTAAATGGTATTC
CTATAGCAATTGGTGTTATGGATTTTAAGTTCATGGGGGGTAGTATGGGATCCGTAGTAG
GCGAGAAAATCACACGTTTGATCGAGTATGCTACTGATAGATCTCTCCCAGTTATTATGG
TGTGTGCTTCTGGAGGAGCGCGCATGCAAGAAGGAAGTTTGAGCTTGATGCAAATGGC
TAAAATATCTTCCGCTTTATACAATTATCAATCAAATAAAAAGTTATTCTATGTATCAATC
CTTACATCCCCTACAACTGGTGGAGTGACAGCCAGTTTTGGTATGTTGGGAGATATCAT
TATTGCCGAACCCAATGCTTACATTGCATTCGCGGGTAAAAGAGTAATTGAACAAACAT
TGAAGAAGACAGTACCTGAGGGTTCGCAAGTAGCGGAGTATTTATTCAATAAGGGTTTA
TTTGATCCAATCGTACCGCGTAATCTTTTAAAAGGTGTTCCGAGTGAGTTATTTCAGTTC
CATGGTTTCTTTCCCCGCCCT---------------ATGGTAACCATTCGAGCCGAAGAAATTAGTA
ATATTATCCGCGAACGGATTGAGCAATATAATAGAGAAGTAAAGATTGTGAACACTGGT
ACCGTACTTCAAGTGGGCGACGGCATTGCTCGTATTCATGGTCTTGATGAAGTAATGGC
AGGTGAATTAGTAGAGTTTGCAGAGGGTACGATAGGTATTGCTCTTAATTTGGAATCAA
ATAATGTTGGCGTTGTATTAATGGGTGACGGTTTGATGATACAAGAGGGTAGTTCCGTG
AAAGCAACAGGACGAATTGCTCAGATACCGGTGAGTGAGGCTTATTTGGGTCGTGTTA
TAAATGCCCTAGCTAAACCTATTGATGGGAGGGGTGAAATTTTAGCTTCCGAATATCGG
TTAATTGAGTCTCCCGCTCCAGGTATTATTTCGAGACGTTCCGTATATGAGCCTCTTCAA
ACGGGGCTTATTGCTATTGATTCGATGATCCCTATAGGACGCGGTCAGCGAGAATTAATT
ATCGGGGACAGACAGACCGGTAAAACAGCAGTAGCTACAGATACTATTCTCAATCAAA
AAGGTCAAAATGTAATATGCGTTTATGTAGCTATTGGTCAAAAAGCATCTTCCGTGGCC
CAGGTAGTGACTACTTTCCAGGAACGGGGAGCAATGGAATACACCATTGTGGTAGCGG
AAACAGCGGATTCGCCTGCTACATTACAATACCTCGCTCCTTATACAGGAGCCGCTCTG
GCTGAATATTTTATGTACCGTCAACGACATACCTTAATAATTTATGATGATCTCTCCAAAC
AAGCACAAGCTTATCGCCAAATGTCCCTTCTATTAAGAAGACCACCCGGTCGCGAAGC
TTATCCAGGAGACGTTTTTTATTTGCATTCCCGCCTTTTGGAAAGAGCCGCTAAACCAA
GTTCTCGTTTAGGCGAAGGAAGTATGACTGCTTTACCAATAGTTGAGACTCAATCTGGG
GACGTTTCGGCTTATATTCCCACTAATGTAATTTCCATTACAGATGGGCAAATCTTCTTAT

CCGCGGATTTATTCAATGCTGGAATCCGTCCAGCTATTAATGTGGGTATTTCCGTCTCCA
GAGTAGGATCCGCAGCTCAAATTAAAGCCATGAAACAAGTAGCCGGCAAATTAAAATT
AGAATTGGCTCAATTTGCAGAGTTAGAAGCCTTTGCACAATTCGCTTCCGATCTAGATA
AAGCTACTCAGAATCAATTGGCAAGAGGTCAACGATTACGGGAGTTGCTCAAACAATC
CCAATCATCCCCTCTCGCGGTGGATGAACAGATAGTTACTATTTATACCGGAACGAATG
GATATCTTGATCAATTAGAAATTGGACAGGTAAAGAAATTTATTGTTCAGTTACGTACCC
ATTTAAGAACGAATAAGCCTCAGTTCCAAGAAATCATATCTTCTACCAAGGTATTCACC
GAGCAAGCGGAAGCCCTTTTGAAAGAGGCTATTCAGGAGCAGATGGAACTATTTCTAC
TTCAGGAACAAACAATGAGAATAAATCCTACTACTTCTGGTCTCGGAGTTTCAACACTT
GTGGAAAAAAATCAGGGACGTATCGCTCAAATCATCGGTCCAGTACTGGATGTAGCTTT
TCCCCCGGGGAAGATGCCTAATATTTACAACTCTTTGGTAGTTAAGGGTCGAGATACCG
CAGGTCAGGAAATTAATGTGACTTGTGAGGTACAGCAATTATTAGGAAATAATCGAGTT
AGAGCTGTAGCTATGAGTGCTACAGATGGGCTGACGAGAGGAATGGAAGTGATTGACA
CGGGAGCTCCTCTAAGTGTTCCAGTCGGTGGAGCCACTCTAGGACGAATTTTCAATGT
TCTTGGAGAACCTGTTGATAATTTAGGTCCTGTAGATACTCGCACAACATCTCCTATTCA
TAGAGCCGCACCCGCTTTTACACAATTAGATACCAAATTATCAATCTTTGAAACGGGCA
TTAAAGTGGTGGATCTTTTAGCGCCTTATCGGCGTGGGGGAAAAATCGGACTATTCGGG
GGAGCCGGAGTGGGGAAAACAGTACTCATCATGGAATTAATCAACAACATTGCCAAAG
CTCATGGGGGTGTATCCGTATTCGGCGGAGTAGGAGAACGCACTCGTGAAGGAAATGA
TCTTTACATGGAAATGAAAGAATCCGGGGTGATTAATGAAGAAAATATTGCAGAATCTA
AAGTAGCTCTAGTATATGGACAGATGAATGAACCGCCGGGAGCTCGTATGAGAGTCGG
TTTGACTGCCCTAACCATGGCGGAATATTTCCGTGATGTTAATGAACAAGACGTACTTCT
ATTTATTGACAATATCTTTCGCTTCGTTCAAGCGGGGTCAGAAGTATCTGCCTTATTAGG
TAGAATGCCTTCCGCCGTGGGTTATCAGCCTACCCTTAGTACAGAAATGGGTTCTTTGC
AAGAAAGAATCACTTCTACTAAAGAGGGATCTATAACTTCCATTCAAGCAGTTTATGTA
CCTGCGGACGATTTGACTGATCCTGCTCCTGCCACGACATTTGCACATTTAGATGCTACT
ACCGTACTATCAAGAGGATTAGCTGCCAAAGGTATCTATCCAGCAGTAGATCCTTTAGA
TTCAACGTCAACTATGCTCCAACCTCGGATTGTTGGCGAAGAACATTACGAAACTGCG
CAAAGAGTTAAGCAAACTTTACAACGTTACAAAGAACTTCAGGACATTATAGCTATTCT
TGGACTGGACGAATTATCCGAAGAGGATCGTTTAACCGTAGCAAGAGCGCGAAAAATT
GAACGTTTCTTATCACAACCCTTCTTCGTAGCAGAAGTATTTACTGGTTCTCCAGGGAA
ATATGTTGGTCTCGCAGAAACAATTAGGGGGTTTCAACTGATCCTTTCCGGAGAATTAG
ATAGTTTTCCCGAGCAGGCCTTTTATTTGGTAGGTAATATCGATGAAGCTACCGCGAAG
GCTATGAACTTAGAAGTAGAGAGCAAATTGAAGAAAATGACCCTAAATCTTTGTGTACT
GACTCCTAATAGAATTATTTGGGATTCAGAAGTGAAAGAAATCATTTTATCTACTAATAG
TGGCCAAATCGGCGTATTACCAAACCACGCCCCTATTGCCACGGCCATAGATATTGGTAT
TTTAAGAATACGCCTCGACGACCAATGGTTAACGATGGCTCTGATGGGGGGGTTTGCC
AGAATAGGCAATAATGAAATCACCATATTAGTAAATGATGCGGAGAAGGGTAGTGACAT
TGATCCGCAAGAAGCTCAGCGAACTCTTGAAATAGCTGAGGCTAACTTGAGTAAAGCA
GAAGGCAAGAGACAAGTAATTGAGGCGAATTTAGCTCTCAGACGAGCTAGGGCACGA
GTAGAGGCTATCAATGCTATTTCGATGAAAAATCTAACCGATTCTTTCGTTTTTTTGGGT
CACTGGCCATCCGCCGGGAGTTTCGGGTTTAATACCGATATTTTAGCAACAAATCTAATA
AATCTAAGTGTAGTGCTTGGTGTATTGATCTTTTTTGGAAAGGGAGTGTTAAGTGATTTA
TTAGATAATCGAAAGCAGAGGATTTTGAGTACTATTCGCAATTCAGAAGAACTATGCGG

AGGAGCCGTTGAGCAGCTTGAAAAAGCACGGGCTCGCTTACGGAAAGTGGAAAGGG
AAGCGGAAGAGTTTCGAGTGAATGGATACTCTGAGATAGAACAAGAAAAGATGAATTT
GATTAATGCCGCTTATCAGAATTTGGAACAATTAGAAAATTACAAAAACGAAACCATTC
ATTTCGAACAACAAAGAGCGATTAATCAGGTCCAACAACGAGTTTTCCAACAAGCCTT
ACAAGGGGCTCTAGGAACTCTGAATAATTGTTTGAACGGCGAGTTACATTTACGCACCA
TCGGTGCTAATATTGGCATACTTGGGGCCATGAAAGAAATAACCGATATGAATCCCCTAA
TTTCTGCTGCTTCCGTTATTGCTGCTGGATTGGCCGTAGGACTTGCTTCTATTGGACCCG
GAGTCGGTCAAGGTACTGCTGCGGGCCAAGCCGTAGAAGGTATTGCTAGACAACCAG
AAGCAGAGGGTAAAATACGAGGTACTTTATTGCTTAGTCTGGCTTTTATGGAAGCTTTA
ACAATTTACGGACTGGTCGTGGCATTAGCACTTTTATTTGCTAATCCCTTTGTGATGAAT
GTTCTACCATGTTCCATCAACACACTAAAGGGGTTATACGAGATATCCGGCGTGGAAGT
AGGCCAACATTTCTATTGGCAAATAGGGGGTTTCCAAGTGCATGCCCAAGTACTTATAA
CTTCCTGGGTCGTAATTGCTATCTTATTAGGTTCAGCCGCTATAGCCGTTCGGAATCCAC
AAACTATCCCGACCGACGGTCAGAATTTTTTTGAATATGTTCTTGAATTCATTCGAGAC
GTGAGCAAAACTCAAATTGGAGAAGAATATGGTCCTTGGGTTCCTTTTATTGGAACTCT
GTTCCTATTTATTTTTGTTTCTAACTGGTCAGGTGCTCTTTTACCTTGGAGAATCATACA
GTTACCTCATGGGGAGTTAGCTGCACCCACGAATGATATAAATACTACTGTTGCTTTAGC
TTTACCCACGTCAGTGGCATATTTCTATGCAGGCCTTACTAAAAAGGGATTGGGTTATTT
CGGTAAATATATTCAACCAACTCCAATACTTTTACCAATTAATGTCTTAGAAGATTTCAC
AAAACCTTTATCACTTAGTTTTCGACTTTTCGGGAATATATTGGCCGATGAATTAGTAGT
TGTTGTTCTTGTTTCTTTAGTACCTTTAGTGATTCCTATACCTGTGATGTTCCTCGGATTA
TTCACAAGCGGTATTCAAGCTCTCATTTTTGCAACCTTAGCCGCGGCTTATATAGGTGAA
TCCATGGAAGGTCATCATATGATATTTGCAACTTTAGAACATATATTAACTCACATATCTT
TTTCGATCATTTCAATTGTGATTCCTACTCATCTGATGACC---TTGGTCTATGAAATTGTA
GGACTATGTGATTCGTCAGAAAAAGGCATGATAACTACTTTTTTCTGTATAACAGGATTA
TTAGTTACTCGTTGGATTTATTCGGGACATGTACCATTAAGTGACCTATATGAATCATTAA
TGTTCCTTTCATGGAGTTTCTCTCTTATTCATATAGTTCCATATTTTAGAAACTATAAGAA
TTTTTTCAGTAAAATAACCGCCCCAAGCGCTATTTTAACCCAAGGCTTTGCCACTTCGG
GTCTTTTAACTAAAATGCATCAATCCGCAATATTAGTACCTGCTCTCCAATCCCGTTGGT
TAATGATGCACGTAAGTATGATGTTGTTGAGTTATGCAGCTCTTTTATGTGGATCATTATT
ATCCATAACTCTCCTGGTCATTACGTTTAGAAGAAAGATAGATATTTTCGGTAAGACCAA
TGAGACCCAATACGTGAAT--------------------------------TTTTCGTTTAGAAATTATCACAGA
TATCAATTGACTCAGCGATTGGATTATTGGAGTTATCGTGTCATTGGCTTGGGTTTTACC
CTTTTAACTATAGGTATTCTTTCAGGAGCAGTATGGGCTAATGAGGCATGGGGATCTTAT
TGGAATTGGGACCCCAAAGAAACTTGGGCATTTATTACTTGGACCGTATTCGCGATTTA
TTTACATACTAGAACAAATAAAAGTTTGCAAGGTGCAAATTCAGCAATTGTGGCTTCTA
TGGGATTTCTTATAATTTGGATCTGCTATTTTGGGGTCAATCTATTAGGAAGAGGGCTAC
ATAGTTATGGTTCATTCACATTAAACGTGACGAAAAAA---GCATTGAATCCCCTACCATA
TCTTGCATCTATCGTATTTCTGCCTTGGGGAATTTCTCTCTCATTTAATAAAAGTATGGAA
CCTTGGGTGACTAATTGGTGGAATACCAGCCAATCCGAAACTTTTTTGAATGATATTCA
AGAAAAGAACATTCTAGAAGGATTCATAAAATTAGAAGAACTGTTCTTGTTGGACGAA
ATGATAAAGGAGTATCCGGAGACACATATACAAAAGCTTCGTATAGGAATCCACAAAG
AAACGATACAATTGGTAAGAATGCACAATCAAGATCATATACATATTATTTTGCATTTCT
CGACAAATATAACCTGTTTCGCTATTCTAAGTGCTTATTCTATTCTGGGTAATGAAGAAC

TTATCACTCTTAATTCTTGGGTTCAGGAATTCCTCTATAACTTAAGCGACACAATAAAAG
CTTTTTCCATTCTCTTATTAACCGATTTATGTATTGGATTCCATTCGCCCCACGCTTGGGA
ACTAATGATTGGTTCGTTCTACAAAGATTTTGGATTTGTTCAGAATGAGAAAATTATATC
CGGTCTCGTTTCTACCTTTCCGGTCATTCTAGATACAATTTTGAAATATTGGATCTTTCAT
TATTTAAACCGTGTATCTCCTTCACTTGTAGTGATTTATCATTCACTGAATGAGATGCCCA
TTGGTGTTCCAAAAGTCCCTTTTCGGAGTCCTGGAGAGGAAGATGCAGTTTGGGTTGA
CATATACAACCGACTTCATCGAGAAAGATTACTTTTTCTAGGCCAAGGCGTTGATAGCG
AGATCTCGAATCAACTTGTTGGTCTCATGGTATATCTCAGTATGGAAGATAATACCAGGG
ATCTCTATTTGTTTATAAATTCTCCCGGCGGATGGGTAATACCGGGAATAGCTATTTATGA
TGCTATGCAAATTGTTCCACCAGACGTACATACAATATGCATGGGATTAGCCGCTTCGAT
GGGATCTTTCATCCTGGTCGGAGGAGAATTTACCAAACGTCTAGCATTCCCTCACGCTA
GGGTTATGATCCACCAACCTGCTAGTTCTTTTTATGAGGCACAAGCAGGAGAATTTATC
CTGGAAGCGGAAGAACTGTTGAAACTTCGCGAAATACTAACAAGAGTTTATGTACAAA
GAACGGGCAAACCTTTATGGGTTGTATCTGAAGACATGGAAAGGGATGTTTTTATGTCA
GCAACAGAAGCCCAAGCTCATGGCATTGTTGATCTTGTAGCAGTCGAAATGAAAGAAC
AAAAATTGATCCATGAAGGTTTAATTACTGAATCACTTCCCAATGGCATGTTCTGGGTT
CGCTTAGATAACGAAGACCTGGTTCTAGGTTATGTTTCAGGGCGGATACGACGTAGTTT
TATACGAATACTACCAGGAGATAGAGTCAAAATAGAAGTCAGCCGTTATGATTCAACAA
GGGGACGTATAATTTATAGACTTCGCAATAAAGATTCAAACGATCAATACGAATTGCAA
GGCTATTTAGAAATAGATAGATACCGGAAACAGCGCTTCTTATATCCACTTCTTTTTCGG
GAGTATATCTATGCACTTGCTCATGATCATGGTTTATTAGGTTATGACAAT---TCTAGTTCA
CTAATTGTGAAACGCTTAATTACTCGACTGCATCAACAGAATCATTTGACCATTTCGGTT
AATGATTCT---------AGGTTCGTTGGGAGGAGTTTTTATTCTCAAACGATACCAGAGGGTT
TTGCAGGAATTATGGAAATTCCATTCTCGGTGCGATTAGTATCTTCCTTAATAGCAAAAT
ATCAT---TTACGATCTATTCATTCAATATTTCCCTTTTTAGAGGACAAATTATCACATTTAT
ATTATGTTTCAGATATACTAATACCCTACCCAATCCATCTGGAAATCTTGCTTCAAACTCT
TCGCACTCGGATACGAGATGCTCCTTCTTTGCATTTATTGAGATGTTTTCTACACGAGCA
TCATAATTGGAATAGCCTTATTACTAAATCCATTTCCATTTTTTCAAAGCAAAGATTATTC
TTGTTCTTGTATAATTCTCATGTATATGAATGCGAATCCGTATTAGTTTTCCTTCGTAAAC
AATCCTCTCATTTACGGTCAATATCTTCTCTAGCCTTTCTTGAGAGAACACATTTTTATG
GAAAAATAAAACAT---GTAGTGACGCCTCGTAATCAAAGGCCCCTCTGGTTCTTCAAGG
AACCTTTGATGCATTATGTTAGGTATCAAGGAAAATCAATTATGGCTTCAAGGTGTACTA
ATTTACTGATGAAGAAATGGAAATATTACCTTGTCAATTTCTGGCAATGTCATTTTCACT
TATGGTCTCAACCGGGTAGGATCCATATAAATGAATTATCCAATCATTCTTTCTATTTTCT
GGGCTATCTTTCAGGTGTACGACTAACGCCTTGGGTGATAAGGAGTCAAATGCTAGAG
AATTCATTTATGATCGATACTGCTATT------AAGAGATTCGATACAATAGTCCCAATTTTTC
CTCTGATTGGATCGTTGGTTAAAGCTAAATTCTGTAACGTATCAGGGTATCCTATTAGTA
AGTCAGTCTGGGCCGATTCGTCGGATTCTGATATTATTGCTCGATTCGGGTGGATATGCA
GAAATCTCTCTCATTATCACAGCGGATCCTCAAAAAAACACAGTTTGTGTCGAATAAAG
TATATACTTCGACTTTCGTGTGCTAGAACTCTAGCTCGTAAACATAAAAGTACGGTACG
CGCAATCTGTAAGAGATTAGGTTCAAAACTATTGGAAGAGTTCCTTACAGAGGAACAC
GAAATTGTTTCTTTCATCTTCCGAAGACGGATTTGGTATTTGGATATTATCCGTATCCATG
GTCTGGTCCCCCATTCAATGATAATTGAAGAAGCACAA------------GCTATCAATTCTTTTT
TCAGATCGGAATCC---TCAAAAGAGGTCTATGGGCTCATATGGTTACTTGTACCTATTTT

GACTCTTGTATTGGGAATCACAATAGGGGTATTAGTAATTGTGTGGCTAGAAAGAAAAA
TATCCGCAGGGATACAACGACGAATTGGTCCTGAGTATGCCGGGCCCTTGGGCATTCTT
CAAGCTCTAGCGGATGGGGTCAAACTACTTTTCAAAGAAGATCTTCTTCCATCTAGAGG
AGATATTCGTTTATTTAGTGTCGGCCCATCCGTAGCGGTCGTATCAATTCTACTGAGTTAT
TCAGTAATTCCCTTTGGCTATCACCTTGTACTAACCGATCTCAGTATAGGTGTTTTTCTAT
GGATCGCTATTTCAAGTATTGCCCCTATCGGACTTCTTATGTCCGGATATGGATCAAATAA
TAAATATTCCTTTTCAGGTGGTTTACGAGCTGCTGCTCAATCTATAAGTTATGAAATACC
GTTAACTCCATGTGTGTTATCAATATCTCTACTATCTAACAGTTCAAGTACGGTTGATATA
GTTGAGGCGCAGTCAAAATATGGTTTTTGGGGGTGGAATCTTTGGCGTCAACCTATAGG
GTTTCTAGTTTTTATAATTTCTTCACTAGCAGAATGTGAGAGATTGCCCTTTGATTTACC
GGAAGCCGAGGAGGAATTAGTAGCAGGTTATCAAACTGAATATTCAGGTATCAAATTTG
GTTTATTTTATGTTGCTTCTTACCTAAATTTACTAGTTTCTTCATTATTCGTAACAGTTCTG
TACTTGGGAGGGTGGAATCTTCCTATTCCATATATACCAATTACTGAACTTTTCGAAATA
AATAAAACTAGTGAAGTCTTTGGAACAACAATTAGTCTCCTCATTACATTAGCTAAAGC
TTATTTGTTCCTGTTCATTCCTATCTCAACAAGATGGACTTTACCTAGGCTGAGAATGGA
TCAACTATTAAATCTTGGGTGGAAATCTCTTTTACCTATTGCTCTCGGTAATCTATTATTG
ACAACTTCTTCCCAACTTGTTTCGCTGATGATCTGGCATGTCCAGAATGAAAACTTCAT
TCTCGATTCTACGAGAATTTTTATGAAAGCATTTCATTTGCTTCTCTTCAATGGAAGTTT
TATTTTCCCAGAATGTATCCTAATTTTTGGCCTAATTCTTCTTCTGATGATCGATTTAACC
TCTGATCAAAAAGATACACCTTGGTTATATTTCATCTCTTCCACAAGTTTAGTAATGAGC
ATAACCGCCCTATTGTTCCGATGGAGAGAAGAACCTATGATTAGCTTTTCGGGAAATTT
CCAAACGAACAATTTCAACGAAATCTTTCAATTCCTTATTTTACTATGTTCAACTCTATG
TATTCCTCTATCCGTAGAGTACATTGAATGTACAGAAATGGCTATAACAGAGTTTCTGTT
ATTTGTATTAACAGCTACTCTAGGAGGAATGTTTTTATGTGGTGCTAACGATTTAATAAC
TATCTTTGTAGCTCCAGAATGTTTCAGTTTATGCTCCTACCTATTATCTGGATATACCAAG
AGAGATGTACGGTCTAATGAGGCTACTATGAAATATTTACTCATGGGTGGGGCAAGCTC
TTCTATTCTGGTTTATGGTTTCTCTTGGCTATATGGTTCATCCGGGGGAGAGATCGAGCT
TCAAGAAATAGTGAACGGTCTTATCAATACACAAATGTATAACTCCCCAGGAATTTCGA
TTGCGCTTATATCCATCACTGTAGGAATTGGGTTCAAGCTTTCCCCAGCCCCTTTTCATC
AATGGACTCCTGACGTATACGAAGGATCTCCCACTCCAGTCGTTGCTTTTCTTTCTGTTA
CTTCGAAAGTAGCTGCTTCAGCTTCAGCCACTCGAATTTTCGATATTCCTTTCTATTTCT
CATCGAACGAATGGCATCTTCTTCTGGAAATCCTAGCTATTCTTAGCATGATATTGGGGA
ATCTCATTGCTATTACTCAAACAAGCATGAAACGTATGCTTGCATATTCGTCCATAGGTC
AAATCGGATATGTAATTATTGGAATAATTGTTGGAGACTCAAATGATGGATATGCAAGCA
TGATAACTTATATGCTGTTCTATATCGCCATGAATCTAGGAACTTTTGCTTGCATTGTATT
ATTTGGTCTACGTACTGGAACTGATAACATTCGGGATTATGCAGGATTATACACGAAAG
ATCCTTTTTTGGCTCTCTCTTCAGCCCTATGTCTCCTATCCCTAGGGGGTATTCCTCCATT
AGCAGGTTTTTTTGGAAAACTCTATCTATTCTGGTGTGGATGGCAGGCAGGCCTATATTT
CTTGGTTTCAATAGGACTCCTTACGAGCGTTGTTTCTATCTACTATTATCTAAAAATAATC
AAGTTATTAATGACTGGACGAAACAAAGAAATAACCCCTCACGTGCGAAATTATAGAA
GATCTCCTTTAAGATCAAACAATTCCATCGAATTGAGTATGATTGTATGTGTAATAGCAT
CTACTATACCAGGAATATCAATGAACCCAATTATTGCAATTGCTCAGGATACCCTCTTTAT
GTTTCTGCTTTACGAATATGATATTTTCTGGGCATTTCTAATGATATCAAGTGTTATTCCTA
TTTTGGCATTTCTAATTTCCGGGGTTTTAGCCCCAATTAGTGAAGGACCAGAGAAGCTC

TCCAGTTATGAATCGGGTATAGAACCAATAGGGGATGCTTGGATACAATTTCGAATCCG
CTATTACATGTTTGCTCTCGTTTTTGTTGTTTTTGATGTCGAAACGGTCTTTCTTTATCCA
TGGGCCGTGAGTTTTGATATATTGGGCGTATACGTATTTATAGAAGCTTTGATTTTCGTGC
TTATCCCAGTTGTTGGTTCAGTTTATGCATGGCGAAAAGGAGCATTGGAATGGTCTATG
AGTGATTTTTATTGGTTAACAATCATTGTTGTTTTGCCCATATCCGCGGGTTCCTTAATTG
CTCTTCTCCCTCATAGAGGAAATAAGGTAGTTCGATGGTATACTATCTGTATCTGCTTATT
TGAACTCCTTCTAACGACCTACGTATTCTGTTATCATTTTAAATTGGACGATCCATTAATC
CAATTGGAAGAAGACTTTAACTGGATAAATCTTTTTGATTTTCACTGGAGACTCGGAAT
CGACGGACTTTCTATAGGACCCATTTTATTGACAGGATTTATCACTACTTTAGCTACTTC
AGCGGCTTGGCCAGTTACTCGAAATTCGCGATTGTTCCATTTCCTGATGTTAGCAATGT
ACAGTGGTCAAATAGGATCATTTTCTTCTCGAGACCTCTTACTTTTTTTCATCATGTGGG
AATTAGAATTAATTCCTGTTTACCTACTTTTATCCATGTGGGGAGGAAAGAAACGTCTGT
ACTCAGCTACAAAGTTCATTTTGTACACTGCAGGAGGTTCCATTTTTCTCTTAATGGGA
GTTCTAGGTATGGGTTTATATGGTTCCAATGAACCAACATTAAATTTTGAAACATTAGCT
AATCAATCGTATCCCGTGGCATTGGAAATAATATTTTATTTTGGTTTCCTTATTGCTTATG
CTGTCAAATCACCGATCATACCTTTACATACATGGTTGCCAGATACCCATGGAGAGGCG
CATTACAGTACATGTATGCTTCTAGCTGGAATCTTATTAAAAATGGGAGCATACGGGTTG
GTTCGGGTCAATATGGAATTATTACCCCACGCCCATTCCATATTTTCTCCTTGGTTGATGA
TAGTAGGGACTATTCAAATAATCTATGCAGCTTTAACTTCTCCTGGTCAACGCAATTTAA
AAAAGAGAATAGCCTATTCTTCCGTATCTCATATGGGTTTCATAATAATAGGAATTAGTT
CTATAACCGATGCGGGACTCAATGGAGCTATTTTACAAATAATCTCTCATGGATTTATTG
GTGCTGCACTTTTTTTCCTGGCAGGAACAAGTTATGATAGAATACGTCTTCGTTATCTTA
ACGAAATGGGGGGAATAGCTATACTAATGCCAAGAATCTTTACGATGTTCAGTAGCTTC
TCGATGGCTTCTCTCGCATTACCAGGAATGAGTGGTTTTGTTGCAGAATTTGTGATATTT
TTGGGAATAATTACTAGCCCAAAATACCTTGTAATGTCAAAAATACTAATTACTTTTGTA
ATGGCAATTGGAATGATATTAACTCCTATTTATTCATTATCTATGTCACGCCAGATGTTCT
ATGGATACAGGTTATTCAATGTTCCAAAGTCTCACTTTGTGGATTCTGGACCACGAGAA
ATCTTTATTTTTATGTGTATCCTTTTACCAATAATAGGTATTGGTATTTATCCAGATTTCGT
TCTTTCACTATCAGTTGACAAGGTAGAAACTATTCTATCTAATTACTTTCATGGGATGAT
GCTCGAGCATGTACTTGTTTTAAGTGCCTATTTATTTTCTATTGGTATCTATGGATTGATC
ACAAGTCGAAACATGGTTAGAGCACTTATGTGTCTTGAACTTATACTGAATGCTGTTAA
TATGAATCTCGTAACATTTTCTGATTTATTTGATAGTCGCCAATTAAAGGGAGACATTTT
CTCGATCTTTGTTATCGCTATTGCAGCCGCCGAAGCAGCTATTGGACCAGCTATTGTTTC
TTCAATCTATCGTAACAGAAAATCAACTCGTATCAATCAATCAAATTTATTGAATAAAAT
GGAACGCACATATCAATATGCATGGATTATACCTTTCGTTCCGCTTCTAGTTACTATGTTA
ATAGGATTGGAACTCCTGCTTAATCCGACAGCAACAAAAAATATTCGTCGTATATGGGC
TTTTCCCGCTGTTTTATTGTTAAGTATAGTTATGGTCTTTTCCACCAAGCTGGCGATCCA
GCAAATAAATGGTAGCTCAATTTATGAATATCTATGGTCTTGGAGCATCACTAGTGATTT
TTCCTTAGAGTTCGGCTACTTGATTGATCCACTTACTTCTATTATGTCGATATTAATCACT
ACTGTTGGAATCATGGTTCTTATCTATAGTGATAATTATATGTCTCATGACCGAGGATATT
TGAGATTTTTTGCTTATATGAGTTTTTTCAATACAGCGATGCTGGGATTAGTTACTAGTC
CCAATTTGATACAAATCCATATTTTTTGGGAACTAGTGGGAATGTGTTCCTATCTGTTAAT
AGGTTTTTGGTTTACCCGACCAATTGCAGCAAATGCCTGCCAAAAAGCGTTTGTGACC
AATCGTGTGGGGGATTTTGGTTTATTATTAGGAATCCTAGGTTTTTATTTAATAACAGGTA

GTTTCGAATTTCAGGATTTATTCGAAATATTCAATGATAATGAGATTAATTCCTCATTTGC
TACGCTTTGTGCCTTCTTATTATTCCTTGGTGCAGTTGCTAAATCTGCGCAATTCCCACT
TCATGTATGGTTACCTGATGCTATGGAGGGACCCACTCCTATTTCGGCTCTTATACATGC
GGCTACTATGGTAGCCGCAGGAATTTTTCTTGTAGCTCGGCTTCTTCCTCTTTTCATAGC
CATACCTTACATAATGAATATCATATCTTTGATAGGTGTAATAACGGTACTATTAGGAGCT
ACCTTAGCTCTTGCTCAAAGAGATATTAAGAGAAGTTTAGCTTATTCCACGATGTCTCA
ATTGGGCTACATGATGTTAGCTTTGGGTATAGGTTCTTATCGAGCCGCTTTATTCCATTTG
ATCACTCATGCTTATTCTAAAGCATTATTGTTTTTAGGGTCTGGATCCATCATTCATTCCA
TGGAACCCATTGTTGGGTATTCTCCGGCTAAGAGTCAGAACATGGTTCTTATGGGCGGT
TTAACCAAATATATGCCAATTACAAAAACAACTTTTTTTTTAGGTACACTTTCTCTTTGT
GGTATTCCACCCCTCGCTTGTTTTTGGTCCAAAGACGAAATTCTTAATGATAGTTGGTTG
TATTCACCGATTTTTGCAATAATAGCTTTCTACACAGCAGGATTAACTGCATTTTATATGT
TTCGTATGTATTTACTTACTTTCGAAGGGCATTTACGTAGTCATTTTCAAAATTACAGCG
GACACACAAATAGTTCCTTCTATTCAATATCCATATGGGGGCAAGAAGGTTCCAAACCT
GTTAGCAGT---AATTTGCTTTTAACAACAAGGAATTTTTCC---AATAAAGACTCTCATACT
TTACTATACCCGCATGAATCGGACAATACCATGTTATTTCCCCTGCTTATATTGGCCATAT
TTACTTTGTTCGTTGGATGCATAGGAATTCATTTCGAGGTTGACATATTATCGAAATGGT
TAACCCCATCGATGAAACTTTTCCATCAGAATTCAACTGAT---GAAGATTGGTATAAATT
TTTGACGAATGCATTTTATTCAGTTAGTATAGCCTACTTCGGAATATTTCTAGCATCTGTT
CTATATGGGTCTGTCTATTCAGATCGACAAAATTTGTACTTAATCAATTCATTTGCTATTC
GCTTAGAACAAATAATAAATGTCATATACAACTGGTCATACAATCGCGGATACATAGACG
TTTATTACGAAAAAGTATTCATTAAGGGTGTACGAGAATTAGCTCAACTAGCTCATTCTT
TTGATCGACGAGTAATTGATGGAGTTACTAATGGGATTGGCGTTGCAAGTTTCTTTCTA
GGAGAAGGTATTAAATAT---ATAGGGGGAGGGCGAATTTCTTCTTATCTATTCTTATATTT
AGCTTGTGTATCAATAGTCCTACTAATTTACTATATGGATTTACCTGCGCCAATACATGAT
ATTCTTTTAGTATCTCTGGGATCAGGTCTTATAGTAGGAGGTCTAGGAGTGGTATTACTT
ACCAATCCAATTTATTCTGCCTTTTCGTTGGGATTGGTTCTTGTTTGTATATCTTTATTTTA
TATTCCATCGAACTCTTATTTTGTAGCTGCTGCGCAGCTACTTATTTACGTGGGAGCCAT
AAATGTCTTAATCTTATTTGCTGTGATGTTTATGAATGGTTCAGAATATTACAATTATTTT
CATTTTTGGACTGTCGGAGATGGATTCACTTCGCTAGTTTGTACAAGTATTTTTTTTTCA
CTAATTGCTACTATCCCAAACACGTCATGGTACGGGATTATTTGGACTACAAGATCAAA
CCAGATCATAGAACAGGACCTGACAAGTAATGTTCAACAGATTGGGATTCATTTATCAA
CAGATTTTTATCTTCCATTTGAACTCATTTCTATAATCCTTTTAGTTTCCTTAGTGGGCGC
AATTGCTATGGCTCGCCGGATGACTGTACCAGACGCAAGAAAAGACCTCCTCGTGGTC
AATATGGGTCCTCACCACCCGTCAATGCATGGTGTTCTTCGACTCATTGTTACTCTAGAT
GGCGAAGATGTTATCGACTGTGAACCCATATTGGGTTATTTACACAGAGGAATGGAAAA
AATTGCGGAAAACCGAACAATTATACAATATCTACCTTATGTGACACGTTGGGATTATTT
AGCTACTATGTTCACGGAGGCAATAACCGTTAATGCACCTGAGGAATTGGGAAATATTC
AAGTACCTAAAAGAGCCAGCTATATTAGGGTAATTATGCTGGAGTTGAGTCGTATAGCT
TCGCATTTGTTATGGCTTGGACCTTTTATGGCCGATATCGGTGCACAGACTCCTTTCTTC
TATATTTTCAGAGAGAGGGAATTGTTATATGATCTATTCGAAGCTGCCACAGGTATGCGA
ATGATGCATAATTATTTCCGTATCGGGGGGGTAGCTGCTGATTTACCTCATGGCTGGATA
GATAAATGTTTAGATTTTTGCGATTATTTTTAACGGGAGTTGTTGAATATCAAAAGCTT
ATTACGCGCAATCCCATCTTTTTGGAACGAGTTGAAGGGGTGGGTTTTATTGGGGGAG

AAGAAGCCATAAATTGGGGTTTATCAGGACCAATGCTACGAGCTTCCGGAATCCAATG
GGACCTTCGTAAAGTCGATCGGTATGAGTGTTATGATGAATTCGATTGGGAAGTCCAAT
GGCAAAAAGAAGGAGACTCATTAGCTCGTTATTTAGTAAGAATTGGCGAAATGACGGA
ATCCATAAAAATTATTCAACAGGCTCTAGAAGGAATTCCGGGGGGACCTTATGAAAATT
TAGAATTCCGACGCTTTGCTGGAACAAAAGACTCAGAATTGAACGACTTTGAATATCG
ATTCATTAGTAAAAAGCCTTCTCCCAGTTTTGAATTGTCAAAACAAGAACTTTATGTAA
GAGTGGAAGCCCCAAAGGGAGAATTAGGTATTTTTCTGATAGGAGATAATAGTGTTTTT
CCTTGGAGATGGAAAATCCGTCCACCCGGTTTCATCAATTTGCAAATTCTTCCTCAGCT
AGTTAAAAGAATGAAATTGGCTGATATTATGACAATACTAGGTAGTATAGATATCATTAT
GGGAGAAGTTGATCGTATGTTTCCTATGGTGACTGGGTTCATGAATTATGGTCAACAGA
CAGTACGAGCTGCAAGATACATTGGTCAAAGTTTCATGATTACCTTATCTCACGCAAAT
CGTTTACCTGTAACTATTCAATATCCTTATGAAAAATCGATCACATCAGAGCGTTTCCGT
GGGCGAATCCACTTTGAATTTGATAAATGCATTGCTTGTGAAGTATGTGTTCGCGTATGT
CCGATAGATCTACCTGTTGTTGATTGGCGATTAGAAACAGATATTAGAAAGAAACGATT
GCTTAATTATAGTATTGATTTTGGAATCTGTATATTTTGTGGTAATTGCGTGGAGTATTGC
CCCACAAACTGTTTATCAATGACTGAAGAATATGAACTTTCCACCTACGATCGTCACGA
ATTAAATTATAATCAAATTGCTTTGGGTCGGTTACCAATGTCTGTAATTGGAGATTACAC
AGTTCGAACAATTATGAACTCAAAAATATCTATGGATAAACCCCTTGATTCAAGAACGA
TTACCAAT---ATGCAGGGTCGTTCATCCGCTTGGCTAGTTAAACACGAGCTGGTTCATAG
ATCCTTGGGCTTTGATTACCAAGGAGTAGAGACTTTACAAATAAAGCCCGAGGATTGGT
ACTCCATTGCTGTCATTTCATACGTATATGGTTACAATTATCTACGTTCCCAATGTGCCTA
CGATGTAGCACCGGGTGGATTGCTAGCTAGTGTGTATCATCTGACGAGAATACAGTATG
GTGTGGACCAACCAGAAGAGGTATGCATAAAAGTATTTGTCCCAAGGAGTAATCCTAG
AATCCCGTCTGTTTTCTGGATTTGGAAAAGCGCCGATTTTCAAGAACGGGAGTCTTATG
ATATGTTGGGAATCTCTTATGATAATCATCCACGCATGAAACGGATTTTGATGCCCGAAA
GTTGGATTGGTTGGCCCCTACGCAAGGATTATATTGCCCCTAATTTCTATGAACTACAGG
ATGCTTAT---
--ATGAATTCTATGGAGTTTCCG
CTCTTTGACCGAACAACCCCCAATTCAGTTATTTCAACTACATTGAATGATCTTTCGAAT
TGGTCAAGACTTTCCAGTTTATGGCCGCTTCTCTACGGTACCAGTTGTTGCTTCATTGAA
TTTGCCTCATTAATAGGCTCCCGATTCGATTTTGATCGTTATGGACTGGTGCCAAGATCC
AGTCCTAGGCAAGCCGACCTTATTTTAACAGCCGGTACAGTAACAATGAAAATGGCTC
CTTCTTTAGTAAGACTATATGAACAAATGCCTGAACCAAAATATGTAATTGCTATGGGAG
CCTGTACTATTACAGGAGGAATGTTCAGTACCGATTCTTATAGTACCGTTCGGGGAGTC
GATAAGTTAATCCCCGTCGATGTCTATTTGCCAGGATGCCCACCTAAACCAGAGGCAAT
TATAGACGCTATAACGAAACTTCGTAAGAAGGTATCTCGAGAAATCTATGAAGATAGAA
CGGGGTCCCAACAGGAAAATCGATGTTTTACTACTAATCACAAGTTTCATCTTGGACGC
AGTACTCGTGCCGTAAATTACGATCAAGGA---------CTCTATCAATCGACTTCAGAGATAC
CTTCTGAAGCATTTTTCAAATACAAGAGTTCAGTATCTTCTCACGAATTAGTGAATATGC
AAAAAAATAGAAATTTTTCTTGGGTAAAGGAACAGATGACTCGATGCATTTCCGTATCG
ATGATGATATATGTAATAACTCGGGCATCTATTTCAAATGCATATCCCATTTTTGCGCAGC
AGAGTTATGAAAATCCGCGAGAAGCAACTGGGCGTATTGTATGCGCTAATTGCCACCTG
GCTAACAAGCCCGTGGATATTGAGGTTCCACAAGCTGTGCTTCCTGATACTGTATTTGA
GGCAGTTGTTAGAATCCCCTACGATATGCAACTGAAACAGGTTCTTGCTAATGGTAAAA

AGGGAGGTTTGAATGTGGGGGCTGTTCTCATTTTACCCGAAGGATTTGAATTAGCTCCC
CCCGATCGTATTTCTCCCGAGATGAAAGAAAAGATGGGTAATCTGTCTTTTCAGAGTTA
TCGTCCTAATAAAAAAAACATTCTTGTGGTGGGCCCTGTTCCTGGTCAGAAATATAGTG
AAATCGTCTTTCCCATCCTTTCTCCAGATCCCGCTACTAAGAAAGAGGTTCACTTCTTA
AAATATCCTATATATGTAGGTGGGAACAGGGGAAGGGGTCAGATTTATCCTGATGGGAG
CAAGAGTAACAATACAGTCTATAATGCTTCAGCAGCAGGGATAGTAAGTAAAATAGTAC
GTAAAGAAAAGGGTGGATATGAAATAACCATATCTGATGCATCGAATGGACACGAAAC
GGTTGATATTATACCTCCAGGACCAGAACTTCTTGTTTCTGAAGGTGAATACATCAAGC
TTGATCAGCCATTAACAAGTAATCCCAATGTAGGTGGTTTTGGTCAAGGCGATGCAGAA
ATAGTACTTCAAGATCCATTGCGTATCCAAGGCCTTTTGTTCTTCTTAGCATCTGTTATTC
TGGCGCAAATCTTTTTGGTTCTTAAAAAGAAACAGTTTGAGAAGGTTCAATTAGCCGA
AATGAATTTCATGAGTAAAGTATATGATTGGTTCGAGGAGCGTCTCGAGATTCAAGCGA
TTGCAGATGATATAACTAGTAAATATGTTCCTCCTCATGTCAATATATTTCATTGTCTAGG
AGGGATCACACTTACTTGCTTTTTAGTACAAGTAGCTACGGGTTTTGCTATGACTTTTTA
CTATCGCCCGACCGTCACAGAAGCTTTTGCCTCTGTTCAATACATAATGACTGAAGCCA
ACTTCGGTTGGTTAATCCGATCAGTTCATCGATGGTCAGCAAGTATGATGGTTCTAATGA
TGATCCTGCACGTATTTCGTGTTTATCTCACAGGTGGTTTTAAGAAACCACGTGAATTG
ACTTGGGTTACGGGCGTAGTTCTGGCTGTGTTGACTGCATCCTTTGGGGTAACTGGTTA
TTCTTTACCTTGGGACCAAATTGGGTATTGGGCAGTCAAAATTGTAACAGGTGTACCTG
AGGCTATCCCTATAGTGGGATCGCCCTTGGTAGAGTTATTACGCGGAAGTGCTAGTGTC
GGTCAATCCACTTTGACCCGCTTTTATAGTTTACACACTTTTGTATTACCTCTTCTTACTG
CCGTATTTATGTTAATGCACTTTTCAATGATACGTAAGCAAGGTATTTCAGGTCCTTTAAT
GGGAGTAACAAAGAAACCCGACTTGAATGATCCTGTATTAAGAGCAAAATTGGCTAAG
GGGATGGGACATAATTATTATGGGGAACCGGCATGGCCTAATGATCTTTTATATATTTTTC
CCGTAGTAATTCTAGGTACTATTGCATGTAACGTAGGTCTAGCGGTTCTAGAGCCATCAA
TGATTGGTGAACCGGCAGATCCATTTGCAACTCCTTTGGAAATATTACCCGAATGGTAC
TTCTTTCCCGTATTTCAAATACTCCGTACAGTACCCAATAAGTTATTGGGTGTTCTTTTAA
TGGTTTCGGTACCAGCGGGATTATTGACAGTACCCTTTTTGGAAAATGTTAATAAATTCC
AAAATCCATTCCGTCGCCCAGTAGCTACAACAGTCTTTTTGATTGGTACGGCAGTAGCC
CTTTGGTTAGGTATTGGAGCAACATTACCTATTGATAAGTCTCTAACTTTAGGTCTTTTTA
TGATTGAAGTTTTTTTATTTGGAATCGTGTTAGGTCTAATTCCTATTACTTTAGCCGGATT
GTTTGTAACTGCATATTTACAATACAGACGCGGCGATCAGTTGGACTTTATGCCTACTAT
AGTTAGTTATTTCGGTTTTCTACTGACTGCTTCAACTATAACCCCAGCTCTATTTATTGGT
CTGAGCAAGATACGACTTATTATGGATATAGTAAGTCTCGCTTGGGCTGCTTTAATGGTA
GTTTTTACGTTTTCCCTTTCACTCGTAGTATGGGGAAGAAGTGGACTCATGATTATTCGT
TCGCCGGAACCAGAAGTGAAGATTTTGGTGGATAGGGATCCCGTAAAAACTTCATTTG
AGGAATGGGCCAGACCCGGCCATTTCTCAAGGACAATAGCTAAGGGCCCTGATACTAC
CACTTGGATCTGGAACCTACATGCTGATGCTCATGATTTCGATAGCCATACCAATGATTT
GGAGGAGATCTCCCGAAAAGTATTTAGTGCTCATTTCGGTCAACTATCCATCATCTTTCT
TTGGTTGAGTGGCATGTATTTCCATGGAGCCCGTTTTTCCAATTATGAAGCGTGGCTGA
GCGACCCTACTCACATCGGACCCAGTGCCCAGGTGGTTTGGCCGATAGTGGGTCAAGA
AATATTGAATGGCGATGTAGGCGGAGGTTTCCGAGGAATACAAATAACCTCTGGATTTT
TTCAGATTTGGCGAGCGTCGGGAATAACTAGTGAATTACAACTTTATTGTACTGCAATT
GGAGCATTGGTCTTTGCAGCGTTAATGCTTTTTGCCGGTTGGTTCCATTATCACAAAGC

TGCTCCAAAATTGGCTTGGTTCCAAGATGTAGAATCCATGTTGAACCACCACTTAGCAG
GATTACTAGGACTTGGGTCTCTTTCTTGGGCAGGGCATCAAGTACATGTATCTTTACCTA
TTAACCAATTTCTGAACGCTGGGGTGGATCCTAAGGAAATACCACTTCCTCATGAATTT
ATCTTAAATCGGGATCTTTTGGCTCAACTTTATCCCAGTTTTGCAGAGGGAGCAACCCC
GTTTTTCACCCTGAATTGGTCAAAATACGCGGAATTTCTTACTTTTCGTGGAGGATTAGA
TCCAGTAACAGGAGGTCTATGGCTAACCGATATTGCACACCATCATTTGGCTATTGCAAT
TCTTTTCCTGATAGCTGGTCACATGTATAAGACCAACTGGGGCATTGGTCATGACCTGA
AAGATATTTTGGAGGCTCATAAGGGTCCGTTTACAGGTGAGGGTCATAAGGGCCTCTAT
GAGATCCTAACAACGTCATGGCATGCTCAATTAGCTCTGAATCTAGCTATGTTGGGTTCT
TTAACCATTGTAGTAGCCCACCATATGTACTCTATGCCCCCTTATCCATACCTAGCTACTG
ATTATGGTACACAACTTTCGTTGTTCACACATCACATGTGGATCGGTGGATTTCTCATAG
TTGGTGCTGCTGCACATGCAGCAATTTTTATGGTAAGAGACTATGATCCAACTACTCGAT
ACAACGATCTATTAGATCGTGTCCTTAGGCACCGAGATGCAATCATATCACATCTTAACT
GGGTGTGTATATTTCTAGGCTTTCACAGTTTTGGCTTGTATATTCATAATGATACCATGAG
CGCTTTGGGGCGTCCCCAAGATATGTTTTCAGATACCGCTATACAATTACAACCTATCTT
TGCTCAATGGATACAAAACACTCATGCTTTAGCACCTAGCGCAACAGCTCCTGGTGCA
ACAGCGGGCACCAGCTTGACTTGGGGGGGTGGTGATTTAGTAGCAGTAGGCGGCAAA
GTAGCTTTGTTACCTATTCCACTGGGAACCGCAGATTTTTTGGTACATCACATTCATGCA
TTTACGATCCATGTAACTGTATTGATACTACTGAAAGGTGTTCTATTTGCCCGCAGTTCT
CGTTTGATACCTGATAAAGCAAATCTGGGTTTTCGTTTCCCTTGTGACGGACCCGGAAG
AGGGGGAACATGCCAAGTATCCGCCTGGGATCATGTCTTCCTAGGTCTATTCTGGATGT
ACAATGCGATTTCGGTAGTAATTTTCCATTTCAGCTGGAAAATGCAGTCAGATGTTTGG
GGTAGTATAAGTGATCAAGGGGTGGTAACTCATATCACGGGGGGAAACTTTGCACAGA
GTTCCATTACAATTAATGGGTGGCTACGAGATTTTTTATGGGCACAGTCCTCCCAAGTAA
TCCAGTCTTATGGCTCTTCATTATCCGCCTATGGTCTTTTCTTCTTAGGTGCTCATTTTGT
CTGGGCCTTCAGTTTAATGTTTCTATTTAGCGGTCGTGGTTATTGGCAAGAACTCATTGA
ATCCATCGTCTGGGCTCATAACAAATTAAAAGTTGCTCCTGCTACTCAGCCTAGAGCCT
TGAGCATTATACAAGGACGTGCTGTAGGAGTAACCCATTACCTTCTGGGTGGAATTGCC
ACAACATGGGCATTCTTCTTAGCAAGAATTATTTCAGTAGGAATGGCATTAAGATTTCC
AAGGTTTAGCCAAGGCTTAGCTCAGGACCCCACTACTCGTCGTATTTGGTTTGGTATTG
CTACCGCACATGACTTCGAGAGTCATGATGATATCACCGAGGAACGTCTTTATCAGAAT
ATTTTTGCTTCTCACTTCGGGCAGTTAGCAATAATCTTTCTGTGGACTTCCGGCAATCTG
TTTCATGTGGCTTGGCAAGGAAATTTTGAGTCATGGGTACAGGACCCCTTACATGTAAG
ACCCATTGCTCATACTATTTGGGATCCTCATTTTGGTCAACCGGCTGTAGAAGCTTTTAC
TCGAGGGGGTGCTCTTGGCCCAGTGAATATTGCTTATTCCGGTGTTTATCAATGGTGGTA
TACAATCGGCTTGCGCACCAATGAAGATCTTTATACTGGAGCTCTTTTTCTATTATTTATT
TCTGCCATATCTTTAGTAGGGGGTTGGTTACATCTACAACCCAAATGGAAGCCAAGCGT
TTCGTGGTTCAAAAATGCCGAATCTCGTCTCAATCATCATTTGTCAGGGCTCTTCGGAG
TAAGCTCCTTGGCTTGGGCAGGACATTTAGTTCATGTTGCTATTCCCGGATCAAGGGGT
GAATATGTCAGATGGAATAATTTCTTAGATGTATTACCATATCCCCAAGGATTGGGGCCA
CTTTTTACAGGTCAGTGGAATCTTTATGCCCAAAACCCCGATTCCAGTAGTCATTTATTC
GGTACCTCCCAAGGAGCGGGAACTGCCATTCTAACCCTTCTCGGGGGATTCCATCCGC
AAACTCAAAGTTTATGGCTGACCGATATTGCCCATCATCATTTAGCTATTGCATTTATTTT
TCTCGTTGCTGGTCATATGTATAGAACTAACTTCGGGATTGGGCACAGTATAAAAGATCT

TCTAGAGGCGCATATTCCTCCGGGGGGTCGATTGGGGCGCGGGCATAAAGGCCTTTATG
ACACAATCAATAATTCGATTCATTTTCAATTAGGTCTTGCTCTAGCCTCTTTGGGGGTTA
TTACTTCCTTGGTAGCTCAACACATGTACTCTTTACCTGCTTATGCATTCATAGCACAAG
ACTTTACTACTCAAGCTGCGTTATATACTCATCACCAATACATCGCAGGGTTCATCATGA
CAGGAGCCTTTGCTCATGGAGCTATATTCTTCATTAGGGATTACAACCCGCAGCAGAAT
GAGGATAATGTATTGGCAAGAATGTTAGACCATAAGGAAGCTATCATATCTCATTTAAGT
TGGGCTAGCTTGTTCCTGGGGTTCCATACCTTGGGACTCTATGTTCATAATGACGTCATG
CTTGCTTTTGGTACTCCAGAAAAACAAATCTTGATCGAACCCATATTTGCCCAATGGAT
ACAGTCCGCTCATGGTAAGACTTCATATGGGTTTGATGTACTCTTATCTTCAACGAATGG
CCCAGCATTTAATGCAGGTCAAAGCTTATGGTTACCTGGCTGGTTGAATGCTATTAATGA
A------AATCGTAATTCACTATTCTTAACAATAGGGCCGGGGGACTTCTTGGTACATCATGC
TATTGCTCTAGGGTTGCATACAACTACATTGATCTTAGTAAAAGGTGCTTTAGATGCACG
TGGTTCCAAGTTAATGCCAGATAAAAAAGATTTCGGGTATAGTTTCCCCTGCGACGGTC
CAGGACGAGGTGGTACTTGTGATATTTCGGCTTGGGATGCATTTTATTTGGCAGTTTTCT
GGATGTTAAATACCATTGGATGGGTTACCTTCTATTGGCATTGGAAGCATATCACATTAT
GGCAGGGTAATGTTTCGCAATTTAATGAATCTTCCACTTATTTGATGGGATGGTTAAGAG
ATTATCTATGGTTAAACTCTTCACAACTTATCAACGGATATAATCCTTTTGGTATGAATAG
TTTATCCGTCTGGGCGTGGATGTTCTTATTTGGGCATCTTGTTTGGGCTACTGGATTCAT
GTTCTTAATTTCCTGGCGTGGATATTGGCAGGAATTGATTGAAACTTTAGCATGGGCTCA
TGAACGCACACCTTTGGCTAATTTGATTCGCTGGAGGGATAAACCGGTGGCTCTTTCCA
TTGTACAAGCAAGACTGGTTGGCTTAGCTCACTTTTCTGTAGGTTATATATTTACCTACG
CAGCTTTCTTGATTGCTTCTACATCAGGCAAATTTGGTATGTCACACTCAGTAAAGATTT
ATGATACATGTATAGGGTGTACCCAATGCGTACGAGCGTGTCCTACAGATGTCCTGGAA
ATGATACCCTGGGACGGATGTAAAGCTAAGCAAATTGCTTCCGCACCAAGAACGGAGG
ACTGTGTAGGTTGTAAGAGATGTGAATCCGCTTGTCCAACGGATTTCTTAAGTGTACGG
GTTTATTTATGGCATGAGACAACCCGTAGCATGGGTCTAGCTTATATGACAGATCTCAAC
TTACCCTCTATTTTTGTGCCTTTAGTAGGCCTATTATTTCCGGCAATTGCAATGGTTTCTT
TATTTTTCCATGTTCAAAAAAACAAGATTGTCATGCGAGATATCAAAACATACCTTTCCG
TAGCGCCTGTGCTAACTACTCTATGGTTCGGTTCTTTAGCAGGTCTATTGATAGAGATCA
ATCGTTTATTCCCAGATGCGTTGACATTCCCTTTTTTTTCATTCATGACTGCAATTTTAGA
GAGACGCGAAAGCACAAGCCTATGGGGTCGCTTCTGCAACTGGGTAACCAGCACTGA
AAATCGTCTTTACATTGGATGGTTCGGTGTTTTGATGATCCCTACCCTATTGACCGCTAC
TTCTGTATTTATTATCGCCTTCATTGCGGCTCCTCCAGTAGATATTGATGGTATTCGTGAA
CCTGTTTCTGGTTCTCTACTTTATGGAAATAATATTATTTCTGGTGCCATTGTTCCTACTT
CTGCGGCTATCGGGTTGCATTTTTACCCGATATGGGAAGCATCATCCGTTGATGAATGGT
TATACAATGGCGGTCCTTATGAACTAATTGTTCTACACTTCTTACTTGGTGTAGCTTGTT
ACATGGGTCGTGAGTGGGAGCTTAGCTTCCGTCTGGGTATGCGCCCTTGGATTGCTGTT
GCGTATTCAGCTCCTGTTGCAGCTGCTACTGCTGTTTTCTTGATCTACCCTATTGGTCAA
GGAAGCTTCTCTGATGGTATGCCTCTAGGAATATCTGGTACTTTCAACTTCATGATTGTA
TTCCAGGCTGAACACAACATCCTTATGCATCCATTCCACATGTTAGGTGTGGCTGGTGT
ATTCGGCGGCTCTCTATTCAGTGCTATGCATGGTTCCTTGGTAACCTCTAGTTTGATCAG
GGAAACTACTGAAAATGAGTCTGCTAATGAAGGTTACAGATTCGGTCAAGAGGAAGA
AACCTATAATATCGTAGCTGCTCATGGTTATTTTGGCCGATTGATCTTCCAATATGCTAGT
TTCAACAACTCTCGTTCCTTACACTTCTTCCTAGCTGCTTGGCCTGTTGTAGGTATCTGG

TTCACCGCTTTAGGTATCAGCACTATGGCATTCAACCTAAATGGATTCAATTTCAATCAA
TCCGTAGTTGACAGTCAAGGTCGTGTTATTAACACTTGGGCTGATATCATTAATCGTGCT
AACCTTGGTATGGAAGTTATGCATGAACGTAATGCTCACAACTTCCCTCTAGATCTAGC
TGCTGTCGAAGCTCCATCCACAAATGGAATGGGTTTGCCTTGGTATCGTGTTCATACTG
TCGTATTGAATGATCCTGGTCGCTTGCTTTCTGTCCATATAATGCATACAGCTCTAGTTTC
TGGTTGGGCCGGTTCTATGGCTCTATACGAATTAGCTGTTTTTGATCCCTCTGATCCCGT
TCTTGATCCAATGTGGCGACAAGGTATGTTCGTTATACCCTTCATGACTCGTTTAGGAAT
AACCAATTCATGGGGCGGTTGGAGTATTACA---GGAGGAACTGTAACCAATCCGGGTAT
TTGGAGTTACGAAGGTGTTGCCGGGGCACACATTGTGTTTTCTGGCTTGTGCTTCTTGG
CAGCTATCTGGCATTGGGTGTATTGGGATCTAGAAATATTCTGTGATGAACGTACGGGA
AAACCCTCTTTGGATTTGCCCAAGATCTTTGGAATTCATTTATTTTTATCTGGGGTGGCT
TGCTTTGGGTTTGGTGCATTTCATGTAACAGGTTTGTATGGCCCTGGAATATGGGTGTCT
GATCCTTATGGGCTAACCGGAAAAGTGCAACCTGTAAATCCTTCATGGGGCGCGGAGG
GTTTTGATCCTTTTGTTCCGGGGGGGATAGCTTCTCATCATATTGCAGCGGGCACCTTG
GGAATATTAGCGGGTCTATTCCATCTTAGTGTCCGCCCGCCCCAACGTCTATACAAAGCA
TTACGTATGGGCAATATTGAAACTGTGCTTTCCAGTAGTATCGCTGCTGTGTTTTTTGCA
GCTTTCGTTGTTGCTGGAACTATGTGGTATGGTTCAGCGACTACTCCGATCGAATTATTT
GGTCCTACTCGTTATCAGTGGGATCAGGGATATTTCCAGCAAGAAATATATCGAAGAGT
TAACGCCGGGCTAGCTGAAAATCTGAGTTTATCGGAATCTTGGTCTAAAATTCCCGATA
AACTAGCTTTTTATGATTATATCGGTAATAATCCGGCGAAAGGAGGATTGTTCAGAGCC
GGCTCAATGGACAACGGGGATGGAATAGCTGTTGGGTGGTTAGGACACCCTGTCTTTA
GAGATAAAGAGGGGCATGAACTTTTTGTACGTCGTATGCCTACCTTCTTTGAAACATTT
CCGGTAGTTTTGGTAGATGGAGACGGAATTGTGAGAGCCGATGTTCCTTTTAGAAGGG
CGGAATCAAAGTATAGTGTCGAACAAGTGGGTGTAACTGTTGAGTTCTATGGTGGTGA
ACTCGATGGAGTCAGTTATAATGATCCTGCTACTGTGAAAAAATATGCTAGACGTGCCC
AATTGGGTGAAATTTTTGAATTGGATCGCGCTACTTTGAAATCCGATGGTGTTTTTCGTA
GTAGTCCAAGGGGTTGGTTCACTTTTGGACATGCTTCGTTTGCTTTGCTTTTCTTTTTCG
GCCACATCTGGCATGGTGCTAGAACCTTGTTCAGAGATGTTTTTGCTGGTATCGACCCA
GATTTAGATGCTCAAGTGGAATTTGGAGCCTTCCAAAAACTGGGGGATCCAACTACAA
GGAGACAAGTAGTCATGAAAACCTTATATTCCCTGAGGAGGTTCTACCCCGTGGAAAC
GCTCTTTAATGGAACTTTAGCTTTAGCTGGTCGTGACCAAGAAACCACCGGTTTCGCTT
GGTGGGCGGGGAATGCCCGACTTATCAATCTGTCCGGTAAATTACTCGGAGCTCACGT
GGCCCATGCCGGATTAATCGTATTCTGGGCTGGAGCAATGAACCTATTTGAAGTGGCTC
ATTTCGTACCAGAGAAGCCTATGTATGAACAAGGATTGATTTTACTTCCCCATCTAGCTA
CTCTAGGTTGGGGGGTAGGTCCTGGTGGGGAAGTTGTAGACACCTTTCCATACTTTGTA
TCTGGAGTACTTCACTTAATTTCCTCCGCAGTCCTAGGCTTTGGCGGTATTTATCATGCG
CTTCTTGGACCCGAGACTCTTGAGGAATCCTTTCCATTCTTCGGTTATGTATGGAAAGAT
AGAAATAAAATGACCACAATTTTGGGTATTCACTTAATCTTGTTAGGTCTAGGCGCTTTT
CTTCTAGTGTTCAAGGCTCTTTATTTTGGGGGCGTATATGATACCTGGGCCCCTGGCGGT
GGAGATGTAAGAAGAATTACCAACTTGACCCTTAGCCCAAGTATTATATTTGGTTATTTA
CTAAAGTCTCCCTTTGGGGGAGAAGGCTGGATTGTTAGTGTGGACGATTTAGAAGATAT
AATTGGAGGACATGTATGGTTAGGTTCCATTTGTATACTTGGTGGAATCTGGCATATCTT
AACCAAACCCTTTGCGTGGGCTCGCCGTGCATTTGTATGGTCTGGAGAAGCTTACTTGT
CTTATAGTTTAGGGGCTTTATCCGTCTTTGGTTTCACTGCTTGTTGTTTCGTCTGGTTCA

ATAATACCGCTTATCCTAGTGAGTTTTACGGGCCTACTGGGCCGGAAGCTTCTCAAGCT
CAAGCATTTACTTTTCTAGTTAGAGACCAACGTCTTGGGGCTAACGTGGGATCCGCTCA
AGGACCTACTGGTTTAGGTAAATATCTAATGCGTTCCCCGACCGGAGAGATTATTTTTG
GGGGGGAAACTATGCGTTTTTGGGATCTCCGTGCTCCCTGGTTGGAACCCCTAAGGGG
ACCTAACGGTTTGGACTTGAGTAGGTTGAAAAAAGACATACAACCTTGGCAAGAACG
ACGTTCGGCGGAATATATGACTCATGCTCCTTTAGGTTCTTTAAATTCCGTGGGTGGTGT
AGCTACAGAGATCAATGCAGTCAATTATGTCTCTCCTAGAAGTTGGTTAGCTACCTCTC
ATTTTGTTCTAGGATTCTTCTTTTTTGTGGGTCATTTGTGGCATGCGGGAAGGGCCCGTG
CAGCTGCAGCAGGTTTTGAAAAAGGGATCGATCGCGATTTTGAACCTGTTCTTTTCATG
ACTCCTCTTAATATGACTATAGCCCTTGGTAGATTTACCAAAGAAGAA---GATTTATTTGA
TATTATGGATGACTGGCTACGGAGAGACCGTTTCGTTTTTGTAGGTTGGTCCGGTCTATT
GCTCTTCCCTTGTGCCTATTTCGCTTTAGGAGGGTGGTTCACAGGTACAACCTTTGTAA
CTTCATGGTATACCCATGGATTGGCTAGTTCCTATTTGGAAGGCTGCAATTTCCTAACCG
CTGCAGTTTCTACCCCTGCTAATAGTTTAGCACATTCTTTATTGCTACTATGGGGCCCGG
AAGCACAAGGAGATTTTACTCGTTGGTGCCAATTAGGCGGTCTGTGGACTTTTGTCGCT
CTCCACGGCGCTTTCGGACTAATAGGCTTCATGTTACGTCAATTCGAACTTGCTCGATC
TGTTCAATTGAGACCTTATAACGCAATCGCATTCTCTGGTCCAATTGCTGTTTTTGTTTC
CGTATTCCTGATTTATCCACTAGGTCAGTCTGGCTGGTTCTTTGCGCCTAGTTTTGGTGT
AGCAGCTATATTTCGGTTCATCCTTTTCTTCCAAGGGTTTCATAATTGGACATTGAACCC
ATTTCATATGATGGGAGTTGCCGGAGTATTGGGTGCTGCTCTGCTATGCGCTATTCATGG
TGCTACCGTAGAAAATACTTTATTCGAGGATGGTGACGGTGCAAATACATTCCGTGCCT
TTAACCCAACTCAAGCTGAAGAGACCTATTCTATGGTCACTGCTAACCGCTTTTGGTCA
CAAATCTTTGGGGTTGCTTTTTCCAATAAACGTTGGTTACATTTCTTTATGTTATTTGTAC
CAGTCACCGGTTTATGGATGAGTGCTCTTGGGGTGGTCGGTCTGGCTTTGAACCTACGT
GCCTATGACTTCGTTTCCCAGGAAATCCGTGCAGCAGAAGATCCTGAATTTGAGACTTT
CTACACCAAAAATATTCTTTTAAACGAAGGTATTCGTGCTTGGATGGCGGCTCAGGATC
AGCCTCATGAAAACCTTATATTCCCTGAGGAGGTTCTACCCCGTGGAAACGCTCTTATG
TCTGGAAGTACGGGAGAACGCGCTTTTGCTGATATTATTACCAGTATTCGATACTGGGT
CATTCATAGCATTACTATACCTTCCCTATTCATTGCAGGTTGGTCATTCGTCAGCACGGG
TTTAGCTTACGACGTGTTTGGAAGCCCTCGACCCAACGAATATTTCACAGAAAGCAGA
CAAGGGATTCCATTAATAACTGGCCGTTTCGATTCATTGGAACAACTCGATGAATTTAG
TCGATCCTTTATGACCATAGATAGAACCTATCCAATTTTCACAGTGAGATGGTTGGCTGT
TCACGGACTAGCTGTACCTACCGTTTTTTTCTTGGGGTCAATATCAGCAATGCAGTTCAT
CCAACGAATGGCTACACAAACCATTGAGGGTAGTTCC---AGATCTGGGCCAAGACGGA
CTATTGTAGGCGATTTATTAAAACCATTGAATTCGGAATATGGTAAAGTAGCCCCCGGAT
GGGGAACTACACCCCTTATGGGTGTCGCAATGGCTCTATTTGCAGTATTCCTATCTATTA
TTTTGGAGATTTATAATTCTTCCGTTTTACTAGATGGTATTTCATTAAGTATGCTTACTCTC
AAACTGTTCGTTTATACGGTGGTGATATTCTTTGTTTCGCTCTTCATCTTCGGATTCCTGT
CTAATGACCCAGGACGTAATCCTGGACGCGAAGAAATGGCCGATACTACTGGAAGGAT
TCCTCTTTGGCTGATAGGTACTGTAACTGGTATTCTTGTGATCGGTTTAATCGGCGTTTT
CTTCTACGGCTCATATTCCGGATTGGGATCATCCCTGATGCCTAATATCTTTAGTTTAATC
TGTATCTGTCTTAATTCTGCCCTTCAG---AGTGGTTTTTTCTTCGCAAAATTGCCCGAGG
CTTATGCTTTTTTGAACCCAATCGTGGATTTTATGCCGGTCATACCTGTGCTCTTTTTTCT
CTTAGCCTTTGTGTGGCAAGCTGCTGTAAGTTTTCGAACGACACAATCAAATCCGAAC

GAACAAAACGTTGAATTGAATCGTACCAGTCTCTACTGGGGGTTATTACTCATTTTTGT
ACTTGCTGTTTTATTTTCCAATTATTTCTTCAATATGGAAGTAAATATACTCGCATTTATTG
CTACTGCGCTGTTCATTCTAGTTCCTACTGCTTTTTTACTTATCATCTACGTAAAAACAGT
CAGTCAAAATGATATGGAAACTGCAACTCTAGTCGCCATCTTCATATCTGGTTTACTTGT
AAGCTTTACGGGGTATGCCTTATATACCGCCTTTGGGCAACCCTCTCAACAACTAAGAG
ATCCATTTGAGGAACACGGGGACATGGAAGCATTGGTTTATACATTCCTGTTGGTCTCG
ACTCTGGGGATAATATTTTTTGCTATCTTTTTTCGAGAACCGCCTAAGGTTCCGAATAAA
AAGATGAAAATGACTATTGCTTTCCAATTGGCTGTTTTTGCATTAATTGCTACTTCATCA
ATCTTATTGATTAGTGTACCCGTTGTATTTGCTTCTCCCGATGGTTGGTCAAGTAAGAAA
AATGTTGTATTTTCCGGTACGTCATTATGGATTGGATTAGTATTTCTGGTAGGTATTCTTA
ATTCGCTCATCTAT---------------------ATGTCACCAAAAACAGAAACTAAAGCAAGTGTTG
GATTCAAAGCTGGTGTTAAAGATTACAGATTGACTTATTACACTCCTGAGTATGAAACC
CTTGCTACTGATATCTTGGCAGCATTCCGAGTAACTCCTCAACCTGGAGTTCCGCCTGA
GGAAGCAGGAGCTGCGGTGGCTGCCGAATCTTCCACTGGTACATGGACAACTGTGTGG
ACCGATGGACTTACCAGCCTTGATCGTTACAAAGGACGATGCTACCACATCGAGCCTGT
TGCTGGGGAGGAAAATCAATATATTGCTTATGTAGCTTACCCTTTGGACCTTTTTGAAGA
AGGTTCTGTTACTAACATGTTTACTTCCATTGTGGGTAATGTATTTGGGTTCAAAGCCCT
ACGAGCTCTACGTCTGGAGGATCTGAGAATTCCTCCTGCTTATTCTAAAACTTTTCAGG
GCCCACCTCATGGAATCCAAGTTGAGAGAGATAAATTGAACAAGTATGGTCGTCCCCTA
TTGGGATGTACTATTAAACCAAAATTGGGGTTATCTGCAAAGAACTATGGGAGAGCGGT
TTATGAGTGTCTCCGTGGTGGACTTGATTTTACCAAGGATGATGAAAACGTGAACTCCC
AACCGTTTATGCGTTGGAGAGACCGTTTCTTATTTTGCGCCGAAGCTATTTATAAAGCG
CAGGCCGAAACAGGTGAAATTAAAGGACATTACTTGAATGCTACTGCGGGTACATCCG
AAGAAATGATGAAAAGGGCGGTCTGTGCCCGAGAGTTGGGAGTTCCTATCGTAATGCA
TGACTACTTAACAGGGGGATTCACCGCAAATACTAGCTTGGCTCATTATTGCCGAGACA
ATGGCCTACTTCTTCACATCCACCGCGCAATGCATGCAGTTATTGATAGACAGAGGAAT
CATGGTATTCACTTCCGTGTACTAGCTAAAGCGTTGCGTATGTCTGGGGGGGATCATATT
CACGCTGGTACCGTAGTAGGTAAACTGGAAGGGGAACGAGATGTCACTTTGGGCTTTG
TTGATTTACTACGTGATGATTTTATTGAAAAAGACCGGAGTCGCGGTATTTATTTCACTC
AAGATTGGGTATCTATGCCAGGTGTTCTGCCCGTGGCTTCAGGGGGTATTCACGTTTGG
CATATGCCTGCCCTGACCGAGATATTTGGGGATGATTCCGTGCTACAGTTCGGTGGAGG
AACTTTGGGACACCCTTGGGGGAATGCACCTGGTGCAGTAGCTAATAGGGTAGCTTTA
GAAGCGTGTGTACAAGCTCGTAATGAGGGACGTGATCTTGCTCGTGAAGGTAATGAAG
TTATTCGTGAAGCTAGCAAATGGAGTCCTGAACTGGCTGCTGCTTGTGAGGTATGGAA
AGAGATCAAATTCGAATTCGAAGCAATGGATGTCTTG------ATGGCCATACATTTATACA
AAACTTCTACCCCTAGCACACGCAAAGGAGCCGTAGACAGTCAAGCGAAATCCAATCC
ACGAACAAAATTGATCTATGGACAGCATCGTTGTGGTAAAGGTCGTAATGCCAGAGGA
ATAATTACCGCAAGGCATAGAGGGGGAGGTCATAAGCGTCTATACCGTAAAATCGATTT
TCGACGGAATGAAAAAGACATATCTGGTAGAATCGTAACCATAGAATACGACCCTAATC
GAAATGCATACATTTGTCTCATACACTATGGGGATGGTGAGAAGAGATATATTTTACATC
CCAGAGGGGCTATAATTGGAGATACCATTGTTTCTGGTACAGAAGTTCCTATATCAATGG
GAAATGCCCTACCTTTGACCGATATGCCCTTAGGCACGGCCATACATAACATAGAAATA
ACACTTGGAAAGGGTGGACAATTAGCTAGAGCAGCAGGTGCTGTAGCGAAACTGATT
GCAAAAGAGGGTAAATCGGCCACATTAAGATTACCATCTGGAGAGGTTCGTTTGATATC

CAAAAACTGCTCAGCAACAGTCGGACAAGTGGGTAATGTCGGGGCGAACCAGAAAAG
TTTGGGTAGAGCCGGATCTAAATGTTGGCTAGGTAAGCGTCCTGTAGTAAGAGGAGTA
GTTATGAACCCTGTAGACCATCCCCATGGAGGTGGTGAAGGGAGGGCCCCGATTGGTA
GAAAAAAACCCACAACTCCTTGGGGTTATCCCGCGCTTGGAAGAAGGAGTAGGAAAA
GGAATAAATATAGTGATATTTTTATTCTTCGTCGCCGTAAG---ATGATTCAACCTCAGACC
CTTTTGAATGTAGCGGACAACAGCGGAGCCCGAAAACTTATGTGTATTCGAATCATAGG
AGCTAGT---AATTACCGATATGCTCATATCGGTGACGTTATTGTTGCTGTAATCAAAGAA
GCAGTGCCAAATATGCCCCTGGAAAGATCAGAAGTAATCAGAGCTGTAATTGTACGTAC
ATGTAAAGAACTCAAGCGCGACAACGGTATGATAATCCGGTATGATGACAATGCAGCA
GTTGTCATTGATCAAGAAGGAAATCCAAAAGGCACCCGAGTTTTTGGTTCGATTGCTC
GTGAATTGAGACAGTTAAATTTCACTAAGATAGTCTCATTAGCTCCCGAAGTATTAATGC
TTAGTCCCAAAAGAACCAGGTTTCGTAAACAACATAGAGGAAGAATGAAAGGAGTATC
TTATCGGGGCAATCGTATTTGTTTCGGTAGATATGCGCTTCAGGCACTTGAACCCGCTTG
GATCACATCTAGACAAATAGAAGCAGGGCGCCGGGCAATCTCACGATATGCCCGTCGT
GGCGGAAAAATATGGGTACGTATATTCCCCGACAAACCCGTTACACTAAGACCCGCGG
AAACACGTATGGGTTCGGGGAAGGGATCTCCCGAATATTGGGTATCCGTCGTTAAACCG
GATCGAATACTTTATGAAATGGGCGGAGTATCAGAAACCGTAGCCAGAGCCGCTATGG
AAATAGCGGCGCGCAAAATGCCTATACGAACAAAATTCGTTATTGCGGAAATGACAAG
AGTTAGACGTGGATATATTGCGCGGAGGCGTCGAAGAAAAATTCGTTTATTTGCATCAA
GCTTTCGAGGAGCTCATTCAAGACTTACTCGAACTGCTACTCAACAGAAAATAAGGGC
TTTGGTTTCCAGCCATAGGGGTAGAGGAAGGCAAAAGCGAGATTTTCGTCGTTTGTGG
ATCACTCGGATAAATGCAGTAACCCGGGAGAAT------GTACCC------TATAGTCGATTAATA
CACGATCTGCGCAAGAAGCAGCTCCTTCTGAATCGTAAAATACTTGCACAAATAGCTAT
ATCAAATAGGAATTGCCTTTACATGATTTCCAATGATATAATCAAA------------------------------
----ATGATAAAGGAAATACGAGCTTTAGCTCGACATATAGGTATGTCTGCTCAAAAAGCA
CGAAGGGTAATTGATCAAATTCGCGGTTGCTCCTATGAGCAAACACTTATGATACTGGA
ACTCATGCCTTATCGAGCATGTTACCCCATTTTCAAATTAGTTTATTCCGCAGCAGCAAA
TGCTAGTCACAATAGGGGTTTGAAAGAAGCTGATTTATTTATTAGTAAAGCCGAAGTCA
ACGAAGGTGTTATCGTCAAAAGGTTAAAACCGCGAGCTCGGGGACGTAGTTACCCAAT
AAAAAGACCCACCTGTCATATAACTATTGTATTGAGC------GAAAGACCT---------------------
-ATGGATGGAATCAAATATGCAGTATTTACAGAAAAAAGTATTCGGTTATTGGGGAACAA
TCAA---TATACTTCTAATGTCGAATCAGGATCAACTAGGACAGAAATAAAGCATTGGATC
GAACTCTTCTTTGGTGTCAAGGTAGTAGCTATGAATAGCCATCGACTCCCGGGAAAGG
GTAGAAGAATGGGACCTATTATGCGACATACAATGCATTACAGACGTATGATCATTACGC
TTCAACCGGGTTATTCTATTCCACCTCTTATAGAGAAAAGAACTATGGCGGTTCCAAAA
AAACGTACTTCTATATCAAAAAAGCATATTCGTAGAAATTTTTGGAAGAGAAAGGGATA
TTGGGCAGCGGTAAAAGCTTTTTCTTTAGCTAAATCCATTTCTACCGGTTATTCAAAAG
GTTTTTTT------CAAAAAAAA---ATGCCCAAGGGTAAAAATCCCAGAGTTACGGTTATTT
TGGAATGTACCAGTTGTGTCCGAAATGGT------AAGAAGAAGGAATCACCGGGCATTTC
CAGATATATTACTCAAAAGAATCGACACAATACGCCTGGTCGATTGGAATTGAAGAAAT
TCTGTCCCTATTGTTACAAACATACGATTCACGGGGAGATAAAGAAAATGAAAATAAGA
GCTTCCGTTCGTAAGATTTGTGAAAAATGTCGACTGATCCGTAGGCGAGGACGAATTAT
AGTAATTTGTTCTAATCCGAAACATAAACAGAGACAGGGAATGGTTCGAGAGGAAGTA
CCAGTATCCACTCGGACGCTACAGTGGAAGTGTGTTGAATCAAGAGCAGACAGCAAA

CGCCTTTATTATGGTCGTTTCGTTTTATCCCCACTTATGAAGGGTCAAGCCGATACAATC
GGTATCGCGATGCGAAGGGCTTTACTTGGAGAACTAGAAGGAACATGTATTACGCGCG
CAAAATCTGAT------AAGGTACCACATGAATATTCTACGGTAGTAGGTATTGAAGAATCA
GTCCATGAAATTTTAATGAATTTGAAAAAAATGGTATTTAGAAGTGATCTGTACGGAAC
TCTCGACGCATCTATCTGTGTCAGGGGCCCTAGACACGTAACTGCTCAAGACATCATCT
CACCACCTTCTGTAGAAATAGTTGATACTACACAGCATATAGCTGTCCTAACAGAACCG
GTTGATTTGTGCATTGAATTAAAAATCGAGAGGGGTCGTGGATATTGTACGAGAACCCA
AAATAACTATCAAGACGGAAGCTAT------CCTATAGATGCTGTATCCATGCCTGTTCGAAA
TGCGAATCATAGTATTCATTCTTATGGGAATGGGAATGAGAAACAAGAGATACTTTTTCT
TGAAATATGGACGAATGGAAGTTTAACTCCTAAGGAAGCGCTTTATGAAGCTTCTCGTA
ATTTGATCGATTTATTTATTCCTTTTCTACATGCGGAGGAACAGGACATAAATAGAAATA
TGGAGGACAATCTA---AAAAGGGCTAGTGTGCCCTTTTTTGCCTTTGATGATGGATTGG
AT---AATATAAAAAGA------------GAAATCATATTGAAACGTATTTTTATTGATCAATTAGAA
TTGCCTCCTAGGACTTATAACTGTCTCAAAAGGTCTAATATACATACATTACTGGACCTT
TTGAGTAAAAGTCAAGAAGATCTTATGAGAATTGAGCATTTTCGCGTAGAAGACGTAA
AACAAATATTCGACATTCTACAGAAGGGT------TTCACAATCGACTTGCTGAAAAATTCA
ATGCTCCGGGATGGTGGAGATGAGGGAATGTTTACAATACCTGGATTTAGTCAGATCCA
ATTTGAGGGATTTTGTAGGTTCATTGATCAGGGCTTGATGGAAGAACTTCATCAATTTC
CAAAAATTGAAGATACAGATCAAGAAATTGAATTTCAATTATTTGGGGAATCTTATCAAT
TGGTGGAACCTTTGATAAAAGAAAGAGATGCTGTGTATGAATCAATTACATATTCCTCT
GAGTTATATGTACCCGCGGGGTTAATTTGGAGAACCGGTAGAAATATGCAAGAACAAA
CCGTATTGCTTGGAAACATTCCTCTAATGAATTCCTTGGGAACCTCTATAGTAAATGGAA
TTTACAGAATTGTCATCAATCAAATATTGCAAAGTCCTGGTATTTATTACAGTACAGGAT
TGGATCATAACGGAATTTCTGTCTATACTGGCACCATAATATCAGATTGGGGAGGAAGAT
CAGAATTAGAGATTGATAGAAAAGAAAGGATATGGGCCCGTGTAAGCAGGAAACAAA
AAATTTCTATTCTAGTTCTATCATCAGCTATGGGTTCAAGTCTAAGAGAAATTCTAGACA
ATGTTTGCTACCCTGAAATTTTCTTGTCTTTCCCAAACGAGAAGGAGAAAAAAAAGAT
TGGTTCAAAGGAAAATGCCATTTTAGAGTTTTATCAAAAATTTGCTTGTGTAGGCGGTG
ATCCGGTGTTTTCTGAGTCCTTATGTAAAGAATTACAAAAGAAATTTTTTCAACAAAGA
TGTGAATTAGGAAGGATTGGCCGACGAAATATGAACCAGAGACTGAATCTTGATATACC
CCCGAACAATACGTTTTTGTTACCACGAGATGTATTGGCTGCTGCCGATCATTTGATCGG
AATGAAATTTGGAATGGGTACACTCGACGAT------ATGAATCACTTGAAAAATAAACGTA
TTCGTTCTGTAGCGGATCTGTTACAGGATCAATTCGGATTGGCTTTGGTTCGCTTGGAA
AATGTGGTTCGGGGAACTATATGTGGAGCAATTCGG---CATAAATTGATA---CCGACTCC
TCGTAATTTGGTAACTTCAACTCCA------------TTAACAACAACTTATGAATCATTTTTCGG
CCTACACCCTTTATCTCAAGTCTTGGATCGAACTAATCCATTGACACAAATAGTTCATGG
GCGAAAATCGAGTTATTTGGGTCCTGGGGGATTGACAGGACGAACTGCTAGTTTTCGTA
TACGAGATATCCATCCTAGTCACTATGGGCGTATTTGCCCAATTGATACGTCCGAAGGAA
TCAATGTTGGACTTATTGGGTCCTTAGCTATTCATGCAAGGGTTGGTGATTGGGGG---TC
TATAGAGACCCCATTTTAT---------GAAATATCTGAGAGATCAAAAGAA------GAACAGAT
GGTTTATTTATCGCCGAGTAGAGATGAATACTATATGGTGGCGGCGGGAAATTCTTTGGC
GTTGACTCGGGGTATTCAGGAAGAAGAAGTTGGTCCAGCTCGATACCGTCAAGAATTT
TTGACTATTGCATGGGAACAGATTCATCTTCGAAACATTTATCCCTTCCAATATTTTTCTA
TTGGAGCTTCTCTCATTCCTTTTATCGAGCATAATGATGCGAATCGGGCTTTAATGAGTT

CTAATATGCAGCGTCAAGCAGTTCCGCTTTCTCAGTCCGAAAAGTGTATTGTTGGAACC
GGCTTGGAACGCCAAGCGGCTCTCGATTCAGGGGGTTCGGCTATAGCCGAACGCGAG
GGAAAGATCATTTATACCGATGCTGAAAAGATCGTTTTATCAGGTAATGGGGACACTAT
AAGCATTCCGTTGGTTATGTATCAGCGTTCCAACAAAAATACTTGGATGCATCAAAAAC
CTCAGGTTCATCGGGGTAAATACCTTAAAAAGGGGCAAATTTTGGCGGATGGTGCGGC
TACGGTTGGTGGCGAACTCGCTTTGGGGAAAAATGTATCAGTAGCTTATATGCCATGGG
AAGGTTACAATTCTGAAGATGCCGTACTCATTAGCGAACGTCTAGTCTATGACGATATTT
ATACTTCTTTTCATATCCGGAAATATGAAATTCAGACTCATGTGACAAGCCAAGGA---CC
TGAAAGAATCACTAATGAAATACCTCATTTAGAGCCTTATTTACTCCGCAATTTAGACAG
AAATGGAATTGTGATGCTGGGATCTTGGGTAGAAACTGGTGATGTTTTAGTAGGTAAAT
TAACGCCTCAGACAGCGAAAGAA---TCATCCTATGCTCCAGAAGATAGATTATTACGAG
CCATACTTGGCATTCAGGTATCCACTGCAAAAGAAACTTGTCTAAAGTTGCCTATAGGC
GGAAGAGGTCGAGTTATTGATGTGAGGTGGGGCCAGAAAAAGGGG---GGTTCCATTTA
TAATCCAGAAATGATTCGTGTATATATCTCACAGAAACGTAAAATCAAAGTGGGCGATA
AAGTAGCTGGAAGACATGGGAATAAAGGTATCATTTCAAAAATTTTGCCTAGACAGGAT
ATGCCTTATTTGCAAGATGGAACACCTGTTGATATGGTATTCAATCCATTAGGAGTACCT
TCGCGAATGAATGTGGGACAGATGTTTGAATGCTCGCTCGGGTTAGCGGGAGACCTTC
TGGGCAGACATTATAGAATAACACCCTTTGATGAGAGATACGAGCAAGAGGCTTCGAG
AAAACTAGTGTTTTCTGAATTATATGAAGCCAGTAAGCAAACAGCAAATCCATGGGTAT
TTGAACCCGAGTATCCTGGAAAGAGCAGAATATTTGATGGAAGAACAGGAGATCCTTT
TGAACAACCTGTTATAATAGGAAAGTCCTATATGTTAAAATTAATTCATCAAGTTGATGA
TAAAATCCACGGACGTTCCAGTGGTCATTATGCACTTGTTACACAACAACCCCTTAGGG
GAAGGGCTAAGCAAGGTGGACAACGAGTAGGAGAAATGGAAGTTTGGGCTCTAGAGG
GATTTGGTGTTGCTCATATTTTACAAGAGATGCTCACTTATAAATCCGATCATATTAGAG
CTCGTCAGGAAGTACTTGGTACCACGATCGTTGGGGGAACAATACCTAATCCTGAGGG
TGCGCCAGAATCCTTTCGATTGCTCGTTCGCGAACTACGATCTTTATCTCTGGAACTGA
ATCATTTCCTTGTATCTGAGAAAAACTTCCAGATTAATAGGAAGGAAGTTATGAATCAG
AATTTTTCTTCTATGATCGATCAGTATAAACATCAACAACTTCGAATTGGATTAGTTTCT
CCTAAACAAATACGTGCTTGGGCCAACAAAATCCTACCGAATGGAGAGATAGTTGGAG
AGGTGACAAAACCCTATACTTTTCATTACAAAACCAATAAACCGGAAAAAGATGGGTT
GTTTTGCGAAAGAATTTTTGGACCTATAAAAAGTGGAATTTGTGCTTGTGGAAATTATC
GAGTGATCGGGGGTGAAAAAGAAGAACCGAAATTTTGCGAACAATGCGGAGTCGAAT
CTGTTGATTCTCGGATCCGAAGATATCAAATGGGATACATCAAACTAGCATGCCCGGTG
ACTCATGTGTGGTATTTGAAACGTCTTCCTAGTTATATCGCGAATCTTTTAGATAAACCT
CTTAAGGAATTAGAAGGCCTGGTATACTGCGAT------TTTTCTTTTGCTAGGCCC---ATAG
CTAAAAAACCTACTTTCTTACGATTACGAGGTTCATTCGAATATGAAATCCAATCCCGG
AAATACAGCATCCCACTTTTTTTTACTACCCAAGGCTTCGATACATTTAGAAATCGAGA
AATATCTACCGGAGCTACTGCTATCAGAGAACAATTAGCCGATCTGGATTTGCGAATTAT
TATAGATCGTTCATTGGTGGAATGGAAGGAATTGGGAGAAGAA---GGATCAACCGGGA
ATGACTGGGAAGATAGAAAAATTGGGAGGAGAAAAGATTTTTTGGTTAGACGCATGGA
ATTGGCTAAGCATTTTCTTCGAACAAATGTAGAACCAGAATGGATGGTTTTGTCCCTATT
ACCAGTTCTTCCTCCCGAGTTGAGGCCAATCATTCAGATTGATGGGGGTAAATCAATGA
GTTCAGATATTAATGAACTTTATAGAAGAGTTATCTATCGCAACAATACTCTTACCGATCT
ATTAACAACAAGTAGATCTACGCCGGGGGAATTAGTAATGTGTCAGGAAAAATTGGTA

CAAGAAGCTGTAGATACACTTCTTGATAATGGGATCCGCGGACAACCAATGAGGGACG
GTCACAATAAAGTTTACAAGTCGTTTTCAGATGTAATTGAAGGCAAAGAGGGAAGATT
TCGCGAGACTCTACTTGGTAAACGGGTCGATTATTCGGGTCGTTCCGTCATTGTGGTAG
GCCCTTCGCTTTCACTGCATCAATGTGGATTACCTCGAGAAATAGCAATAGAGCTTTTT
CAGACATTTGTAATTCGTGGTCTAATCAGACAACATCTTGCTTCCAACATAGGACTTGC
TAAAAGTAAAATTCGGGAAAAAGAACCCATTGTATGGGAAATACTTCAAGAAGTTATG
CAGGGGCATCCTGTATTGCTAAATAGAGCACCCACTTTGCATAGATTAGGCATACAGGC
GTTCCAACCTATTTTAGTGGAGGGACGAGCTATTTGTTTACATCCATTGGTTTGTAAGGG
ATTCAATGCGGACTTCGATGGAGATCAAATGGCTGTTCATGTACCTTTATCTTTGGAAG
CTCAAGCAGAAGCTCGCTTACTTATGTTTTCTCATACGAATCTCTTGTCTCCAGCCATTG
GGGATCCCATTTCCGTACCCACTCAAGATATGCTTATGGGGCTCTATGTATTAACGATGG
GGAATCGTAGAGGTATTTGTGCAAATAGGTATAATCCATGTAACCCCAGAAACCAC------
---CAGAATGAAAGAATGGATCATAGTAATTATGAGTATAGGAAAGGGAAAGAACCCTAT
TTTTGTAGTTCCTACGATGCACTTGGGGCTTATCGGCAGAAAGGGATCGATTTATATAGT
ACTTTATGGCTACGGTGGCGACTAGATCAACGT---GTCATTGCCTCAATAAATAGAGAA
GTTCCCATCGAAGTTCAATATGAATCTTTGGGCACTTATCATGAGATTTATGATCACTATC
GAGTAGTAAGAAGTGTAAAAAAAGGAATGCTTTGTATATATATTCGAACTACTGTTGGT
CATATTTCCTTTTATCGAGAAATAGAAGAAGCCATACAGGGGTTTTGTCGGTCCTATTCA
TATGGTACC------------------------------------ATGGCAGAACGGGAT---CTGGTCTTTCACAATA
AAGTGATAGATGGAACTGCCATGAAACGGCTTATTAGCAGATTAATAGATCATTTCGGA
ATAGCCTATACATCACACATTCTGGATCAAGTAAAAACTCTGGGTTTTCAGCAAGCCAC
CGCTACATCAATTTCATTAGGAATTGATGATCTTTTAACAACACCTTCTAAGAGATGGTT
AGTCCAAGATGCTGAACAACAAAGTTTGATTTTGGAAAAGCACCATCATTATGGGAAT
GTACACGCGGTAGAAAAATTACGTCAATCAATCGAGATATGGTATGCTACAAGTGAATA
TTTGAGACAAGAAATGAATCTTAATTTTAAGATGACTGATCCTTCAAATCCAGTCCATAT
AATGTCTTATTCTGGAGCTAGAGGAAATGCATCTCAGGTCCACCAATTAGTAGGTATGA
GAGGATTAATGTCAGATCCCCAAGGACAAATGATTGATTTACCCATTCAAAGCAATTTA
CGCGAAGGACTTTCTTTAACGGAATATACAATTTCCTGCTACGGAGCCCGCAAAGGAG
TTGTGGATACTGCTGTACGAACGTCAGACGCTGGATACCTCACGCGTAGACTTGTTGA
AGTAGTTCAGCATATTGTTGTACGTAGAACAGATTGTGGAAGTACCCGAGGTATTTCCG
TGAGTTTTCGAAAGGGGATGACGGAA------AGAATTTTTATCCAAACGCTAATAGGTCG
CGTATTAGCGAACGATGTATATCTAGGTCTACGATGCATTGCTACCAGAAATCAAGATAT
TGGGATTGGGCTTGTCAATCGATTCATGACC------------------TCTCGGGCGCAGCCAATAT
ATATTCGAACTCCCTTCACTTGCCGAAGTGCATCTTGGATCTGTCGATTATGTTATGGTC
GGAGTCCCACTCATGGTGACCTGGTTGAATTGGGAGAAGCAGTAGGTATTATTGCGGG
TCAATCCATTGGAGAACCTGGGACTCAACTAACATTAAGAACTTTTCATACCGGTGGAG
TATTCACAGGTGGTACTGCAGAACATGTACGAGCTCCTTCTAATGGAAAAATAAAATTC
AATGAGGATTTGGTTCATCCCACACGGACACGCCATGGACATCCTGCCTTTCTCTGCTA
TGTAGACCTATATGTGACTATTGAGAGTCAGGATATTATACATAGTGTGAATATTCCACC
AAAAAGTTTTCTTTTGGTTCAAAATGATCAATATGTGGAATCAGAACAAGTGATTGCTG
AGATTCGTGCTGGAACATCCACTTTC------TTTAAAGAGAGGGTTCGAAAACATATTTAT
TCTGACTCAGAGGGAGAA---ATGCATTGGAGTACCGGTGTGTACCATGCACCTGAATAT
ACACACGGTAATGTTCATTTCTTACCCAAAACAAGTCATTTATGGATCTTATCGGGGGG
TCCGTGCAAA------TCCAGTCTAGTGCCTTTTTCACTCCACAAGGATCAAGATCAAATGA

ATGTTCAA------TCTCTTTCTGTCCAAGAGAGATCCATTTCTGACTTCTCG------GTGAAT
AATAATCGA---GTGAAACACAAATTGTTTGGCTCGGATCCCCTGGCGAGA---GGGAGAA
GGATTTCTGATTATGCA------TCTGAGCGAGTCATATCCAATGGTGATGGGGATTTCATAT
ACCCCGCC---ATTCTCCGAGAAAATTCTTAT------TTATTGGCGAAGAGGCGAAGAAATA
GATTCATCATACCATTCCAATATGATCCGGAATGGGAGAAGGAATTAACGCCTCATTCTA
GTATCACGGTTGAAATACCCGCAAAT------GGTATTTTACGTAGAAATAGTATTCTTGCTT
ATTTCGACGATCCACGATACAGAAGAAGCAGTTCGGGAATTACCAAATATGGC---------G
TCGATTCAATCGTCAAAAAAGAGGGT------CTGGTTGAGTATCGAAGACCAAAA---GAA
TCTAGACCGAAATAC------GTAGATCGATTTTTTGTCATTCCCGAAGAAGTCCATATCTTG
CCCGGGTCTTCATCCATAATGGTACGGAACAATAGTATCATTGGAGTAGATACACGAATT
ACGTTTAATACAAGAAGCCAAATAGGTGGATTGGTGCGAATAGAAAAAAAAAAAAAAG-
--ATTGAACTGAAAATCTTTTCTGGAGGTATCCATTTTCCTGGAGAAACGGATAAGATAT
CCCGACACATTGGCATCTTGATACCACCAGGAGCAAGAAAAAAAAATGGATAAGGGATC
AAAGGGGAAAAATTGGGAAGGGAATAATTGGGTCTATGTCCAACGG------ATCACACCT
ATCAAGAAAAAA------TATTTTGTTTCAGTACGACCCGTAGTGACATATGAAATAGCTGA
TGGGATAAATCTAGTAACGCTTTTCCCTGGGGATATGTTACAGGAAAAGGATAATTTGC
GACTCCAAGTTGTCAATTATATCCTTTATGGGGATGGCAAACCAATTCGAGGAATTTCC-
-----CATATTCAATTGGTTCGTACTTGTTTAGTATTGAATTGGGACCAAGAC------------------
AAATCTATAGAAAAGGTTCAGGCTTCTTCTGCTGAAGTAAGGGCAAATGATCTGATTCG
ATATTTCATAAGAATTGATTTGGTGAAGTCTCCTATTTTGTATACCGGAAAGAGGAATGA
TAGGTCAGGTTCAGTGATTCCTGATACT------GGGTCATATTGCGCCAATACCAATCTTTT
TTCTTCCAAGGTGAAAATTAAATCA---------------------------CTTAGTCAACATCAAGGAAC
CGTTCGTACATTTCTTAATAGA---------AATAAAGAAGGCCAATCTCTTATAGTTTTTTCAT
CATCTAATTGTTCTCGCATCAAT---------------GTTTCGAAATATCACAAT---------GTGACCA
AAGAATCAATTCCAATTCTTAATTTGTTG---------GGTCCCTTAGGTACT------GTACCTAA
AATTCATAATTTTTCCCCATCT------TATCATTCAATAACC------CATAATGAGATCTTGTTA
AAT---AAATATTTAATACTGGATAAT---AAAAATCCAAAACAGACTTTCCAACTACTTAAA
---------TATTATTTAGTGGATGAAAACGGGAGA------ATCTCTAATGCAAATCCATGCAGTG
ACATCATTTTTAATCTATTCGGT------TCGTGCTTTCTCCCTCACGATTATTGTGAGGGGA
CATCCACAACCAGAATAATTCTCGGACAGTTTATTTGTGAAAATGTATGTCTATCCAAAC
ACGGAACA---------CGCATAAAATCTGGTCAAGTTATAATGGTTTAT---CTTGACTCTTTCA
TAATAAGATCAGCTAAACCCTATTTGGCGACCCGAGGAGCAACCGTTCATGGTGATTAT
GGAGAAATCTTTTACGAAGGAGATACATTAGTTACATTTATATATGAAAAATCGAGATCT
GGTGATATAACTCACGGCCTTCCAAAGGTTGAACAAGTGTTAGAAGTTCGTTCGATTGA
TTCAATATCGATGAACCTAGAAAAAAGGGTTGAAGGTTGGAACGAACATATAACAGGC
ATTCTTGGAATTCCTTGGGGATTCCTGATTGGTGCTGAACTCACCATAGCGCAAAGTCG
TGTTTCTTTGGTTAATAAGATCCAAAAGGTTTATCGATCCCAGGGGGTACAGATTCATAA
TAAGCATATAGAGATTATTGTACGACAAATAACATCAAAAGTGTTGGTTTCAGAA---------
GATGGAATGTCTAATGTTTTTTCACCCGGGGAACTAATCGGATTGTTGCGAGCAGAACG
AGCAGGTCGTGCTTTGGAAGAGGCTATTTGTTACCGAGCCGTCTTATTGGGAATAACGA
GAGCATCTCTGAATACTCAAAGTTTCATATCAGAAGCTAGTTTTCAGGAAACCGCTCGA
GTTTTAGCAAAAGCCGCTCTCCGGGGTCGTATTGATTGGTTGAAAGGTCTGAAAGAGA
ATGTTGTTCTGGGGGGGATGATACCCGTTGGTACCGGATTCAAACGATTCGTTCACCGT
TCAAGG------GAATACAACAACATTCCT------TTGGAAATACAAAAGAAGAATTTTTTCG

GGGGGGAAATGAGA------GATATTCTGTTCCACCACAGAGAATTATTTGTTCTTGCATC
CCAAAGCCAAAGAGTTTCCATTCAGAACAACCATTCTATGCTATGGGATCTAATCCAAT
CGTTCATAAGAGCATGACAAGAAGATATTGGAACATCAATTTGGAAGAGATGATGGAA
GCGGGGGTTCATTTTGGTCATGGTACTAGGAAATGGGATCCTAGAATGGCACCTTACAT
CTCTGCAAAGCGTAAAGGTATTCATATTACAAATCTTACGAGAACTGCTCGTTTTTTATC
AGAAGCCTGTGATTTAGTTTTTGACGCAGCAAGTAGAGGAAAACACTTCTTAATAGTT
GGTACGAAAGATAAGGCAGCTGATTCGGTAGCATCAGCTGCAATAAGGGCTCGGTGTC
ATTATGTCAATAAAAAAATGGCTCGGTGGTATGTCAACGAATTGGTCCACTACGGAAACG
AGGCTTCAGAAGTTCAGGGACTTGAGAGTGAGAGCCGAGATGGGGCAATTCAGTCGT
CTCCCGAAACGGGATGCAGCAATATTGAAGAGACAATTATCTCACTTTCAAACATATCT
GGGCGGTATCAAATATATGACGGGGTTACCCGATATTGTAATCATCATTGATCAGCAAGA
AGAATATACGGCCCTTCGAGAATGCGTCACTTTGGGTATTCCAACTATTTGTTTAATCGA
TACAAATTGTGATCCAGATCTCGCAGATATTCCGATTCCAGCCAACGATGACGCTATAG
CTTCCATCCGATTGATTCTTAACAAATTAGTATCCGCAATTTGTCAGGGACGTTCT---AG
CTATATAAGAAGTCGTATGGGACAGAAAATAAATCCACTTGGTTTCAGACTTGGTACAA
CCCAAAGTCATCGTTCCTTTTGGTTCGCACAACCAAAAAATTATTCCAAAGGTCTCCAG
GAAGATGAAAAAATACGGGATTGTATCAAGAATTATGTACAAAAACATATGAGAATATC
CTCAGGT---TTTGAAGGAATTGCACGTATAGATATTAAAAAAAGAATCGATTTGATCCAA
GTCATAATTCATATTGGATTCGCCAATATGTTAATGGAGGGT------AGAGCCCGA------GG
AATCGAAGAATTACAGACTAATGTACAAAAAAGCTTTCATTCTGTGAACCGAAGACTC
AACATTGCTATTGCAAGAGTTGCAAGACCTTACGGGCAACCTAATATTCTTGCAGAATA
TATCGCTCTGCAATTAAAGAATAGAGTTTCCTTTCGAAAGGCAATGAAAAAAGCTATTG
AATTAGCTGAACAAGCGGATGCAAAGGGAATTCAGGTACAAATTGCAGGACGTCTCAA
TGGAAACGAAATTGCCCGCGTTGAATGGATTAGAGAAGGTAGGGTTCCCCTGCAGACC
ATTCGAGTTAAAATTGATCATTGTTCCTATCCAGTTCGAACTATCTATGGAGTATTAGGG
ATAAAAATTTGGATATTTTTGGATGAAGAGATGTCTCGTTACCGAGGACCTCGTTTTAA
AAAAATACGCCGTCTGGGGGCTTTGCCGGGACTAACTAGTAAAAAACCTAGGTCCGCA
AGTGATCTTAGAAACCAATCCCGCTCTGGGAAA---AGATCTCAATATCGTATTCGTTTAG
AAGAAAAACAGAAATTGCGTTTTCATTATGGTCTGACAGAGCGACAACTACTTCGATAT
GTTCGTATCGCTGGAAAAGCAAACGGTTCGACGGGTCAAGTTTTACTGCAACTACTTG
AGATGCGTTTGGATAACATTCTTTTTCGATTGGGTATGGCTTCAACTATTCCTGGAGCCA
GGCAATTAGTTAACCATGGACATATTTTAGTTAATGGTCGTCTAGTAGATATACCAAGTT
ATCGCTGCAAACCCCGAGATATTATTACTACGAAGGATAAACAAGGATCCAGAGCTTTG
ATTCAAAATCATATGGATTCATCCTCCAACGAA------TTGCCAAAACATTTGACTCTGCA
CTCA------TTCCAATATAAAGGGGTAGTCAATCAAATAATAGATAGTAAATGGGTCGGTTT
GAAAGTCAATGAATTGCTAATCGTAGAATATTATTCCCGACAAACTATGTCACGCCGAG
GTACTGCAGAAGAAAAAACTGCAAAATCTGATCCAATTTATCGTAATCGATTAGTTAAC
ATGTTGGTTAACCGTATTCTGAAACACGGAAAAAAAATCATTGGCTTATCAAATTATCTAT
CGAGCCGTGAAAAAGATTCAACAAAAGACAGAAACAAATCCACTATCTGTTTTACGTC
AAGCAATACGTGGAGTAACTCCCAATATAGCAGTAAAAGCAAGACGTGTAGGCGGATC
GACTCATCAAGTTCCCATTGAAATAGGATCTACACAAGGAAAAGCACTTGCCATTCGTT
GGTTATTAGGGGCATCCCGAAAACGTCCGGGTCGAAATATGGCTTTCAAATTAAGTTCC
GAATTAGTCGATGCTGCCAGGGGGAGTGGCGATGCCATACGCAAAAAGGAAGAGACT
CATAGAATGGCAGAGGCCAATAGAGCTTTTGCACATTTTCGTATGGGTAGAGACACTAT

TGCCGATATAATAACTTATATAAGAAATGCTGACATGGATAAAAAAGGAACAGTTCGAA
TACCATCCACTAATATTACCGAAAACATTGTTAAAATACTTCTGCGAGAGGGTTTTATCG
AAAATGTTAGGAAGCACCGGGAAAACAACAAGGATTTCTTGGTTTTAACCCTACGACA
TAGAAGA------AATAGGAAAAGAGCATATAGA---------AATATTTTA------AAGCGTATCAG
CAGACCAGGTCTGCGAATCTATTCCAACTCTCAACGAATTCCTAGGATTTCAGGCGGGA
TAGGGGTTGTAATTCTTTCTACTTCTAGAGGTATAATGACAGATCGAGAAGCTCGACTA
GAAAGAATTGGAGGAGAAATTTTGTTTTATATATGGATGACAAAACCTATACCAAAAAT
TGGTTCACGTAGAAATAGCCGTATTAGTTCACGTAAGAGTGGGTGTAGAACACCAATAG
GAGTTATTCATGTTCAAGCGAGTTTCAACAATACTATTGTTACTGTTACAGACCCACAA
GGTCGGGTCGTTTCTTGGTCTTCCGCAGGTACCTGTGGGTTCAAGGGCACAAGAAGGG
GTACCCCATTTGCTGCTCAAACCGCAGCAGGAAATGCTATTCGTGCGGTAGTGGACCA
GGGTATGCAACGAGCAGAAGTCATGATAAAGGGGCCCGGTCTCGGGAGAGATGCAGC
ATTACGAGCGATTCGTAGAAGTGGTATACTATTAAATTTTGTACGTGACGTAACCCCTAT
GCCGCATAATGGATGTAGACCGCCAAAAAAAAAGGCGCGTTATGCCAACTATTAAACAA
CTTATTAGAAACACAAGACAGCCAATGAGAAATGTCACGAAATCTCCTGCTCTTCGAG
GATGTCCTCAGCGTAGAGGAACATGTACTAGGGTGTATACTATCACCCCCAAAAAACCA
AACTCTGCCTTACGTAAAGTTGCCAGAGTACGATTAACCTCTGGATTTGAAATCACTGC
TTATATACCTGGTATTGGCCATAATTTACAAGAACATTCTGTAGTATTAGTAAGAGGAGG
AAGGGTTAAGGATTTACCCGGTGTGAGATATCACATTGTTCGAGGAACCCTAGATGCTG
TCGGAGTAAAGGATCGTCAACAAGGGCGTTCTAAATATGGGGTCAAAAAGCCAAAAAT
GGCAAGGAAAAGTTTGATTCAGAGGGAGAAGAAGAGGCAGAAATTAGAAGAGAAATA
TCATTTGATTCGTCGATCCTCAAAAAAAGAAATAAGC---------AAAGTTTCATCGTTGGA
CGAGAAATGGGAAATTCATGTAAAATTGCAATCCCCACCACGTAATAGTGCACCTATAC
GTCTTCATCGACGCTGTTTTTTGACCGGAAGACCTAGAGCTAACTATCGAGACTTTGGG
CTATCCGGACACGTACTCCGCGAAATGGTTCATGCATGTTTGTTGCCGGGGGCAACAA
GATCAAGTTGGATGGTAAAGAATTCGCCCATATCCGTTATTCCACAAGAAGAAAAA------
---------AGGGGTTCCGTTGAATTCCAAGTATTTAATTTTACCAATAAGATAGAGAGACTTA
CTTCACATTTGGAACTGCACAGAAGAGACTATTTATCTCAGAGGGGTCTGCGGAAAAT
TCTGGGGAAACGCCAACGACTGTTGGTTTATTTATCAAAGAAAAATCGAGTGCGTTATA
GGGAATTAATTGGTCAATTGGGTATTCGAGAACCAAAAACTGGT------ATGGTAAAACTT
CGTTTGAAACGATGTGGTAGAAGGCAACGAGCCACCTATCGAATCATTGCAATTGATGT
CAGATCCCGAAGAGAGGGAAGAGATCTTCGGAAAGTAGGCTTTTACGACCCGATAAA
GAATCAAACTTATTTAAATGTTCCTGCTATTCTATATTTCCTTGAAAAAGGAGCTCAACC
CACGGGAACTGTTCATGATATTTCAAAAAAGGCAGAGGTATTTAAGGAACTTCGAGTT
AATCAAACAAAA---------ATGGAGAAATTCAAGCGACCCTTTCGTAAATCCAAGCGATCT
TTTCGTAGGCGTTTGCCCCCAATTGGGTTGGGGGATCGAATTGATTATAGAAACATGAG
TTTAATTAGTCGATTTATTAGTGAACAAGGAAAAATATTACCTAGGCGAGTGAATAGATT
GACCTTGAAACAACAACGATTAATTACTATTGCTATAAAACAAGCTCGTATTTTATCTTT
GTTACCTTTTCTTAATAATGAGAAA------CAATTTGAGAGAGCCGAGTCGATTTCTAGAA
CTACCGGCACTAGAACCAGAAAAAAAAGTGGCCCGT---TCTAAAAAAAAATCCTTTTGTA
GCTAATCACTTATTAAGTAAAATTGAGAAGCTCAACAAGGCGGAGGAAAGAAGTATAA
TAGTAACTTGGTCCCGGGCATCCACCATTATATTTGCAATGGTCGGTCATACAATTGCTG
TTCATAACGGGAAGGAGCATTTACCTATTTATATAACGGAGCGTATGGTGGGTCACAAA
TTGGGAGAATTCGCGCCTACTCTGACTTTCCGGGGACACGCGAGAAACGATAATAGAT

CTCGGCGAATGATTTTGAAATATAGTCTACTCGGTAATCTATTATTATTATGGATTAATATA
GTTAATTCGGTCGTTATGGTCGGACTTTATTATGGATTTCTAACCACATTTTCTATAGGTC
CCTCTTATCTCTTACTTCTAAGAACTCGGGTTATGAAAGAAGGG---------AGCGAAAAAG
AAGTATCAGCAACAACCGCTTTTATTATGGGACAGTTCATAGTATTCATATCGACCTATTA
TCCGCCCTTGCATCTAGCATTAAGTAGACCCCATACACTAACTGTCCTAGTTATACCGTA
TCTTTTGTCTCATTTCTTCTGGAACAATAAGAAATCCCTTTTTGATTATAGATCTACTCAC
GGGAATTTCATTCCTAACCTTAGCATTCAATATGTATTCCTGAATAATCTAATTTTTCAAT
TATTCAACCATTTTGTTTTACCAAGTTCAACATTAACCAGATTAGTCGACATTTCTATGTT
TCGATACAACAATAAGATTTTATTTGTAACAAGTAGTTTTTTTGGCTGGTTAATTGGGCA
CATGTTATTGATGAAATGTATTGGCTTAGTATTATCTTGGCCATGGGAAAAA------ATGAG
ATCT---TTTAGATCTAATAAGTATCTTATGTCAAAATGGAGAAATTCTGTATCTCAAATCT
TTAGTATTCTCTTATTTATCATCTGTATCTGCTATCTAGGAAGAATGCCATCACCCATTATA
ACTAAAAAACTGAAAGAAGTCGAAGAAGACAAATCCCTTCTGTCTCTTCTTTTCGATT
ATCAACGATGGAATCGACCCCTGCGATATATAGAAAATGATCGATTTTCAAATGCTGTAA
GAAATGAAATGTCTCAATCTTTTTTTTATACATGCGCAAGTGATGGAAAGCAAAGAATA
TCTTTCACATATCCACCTAGTTTGTCTACTTTTTTTGAAATGATAAAAAAAAAGATGTCT
TTTCGCACAGCAGAAAAACTACCTGAGGAC------CTCTCTAATTGGGTTTCGACCACTA
AAAAACAAAGAGATAACTTAAGGAACGAATTCATAAATCGAATCGAAGCTATAGAC-----
-AAACTTATTCTCAATGTGGTCGAAAAAAGGGCCAGATTGTGCAATGATGAAGAGGAA
CAAGAATGCTTACCTAAGTTTTATGATCCTCTTTTGAACGGACCCTATCGTGGAACAAG
AAAAAAAGGGTATTTATATACCAAAACGGCTTGGATAAATAAGATTCATGCTATCCTTGA
ACGAGAATTTGAACACGAAGAT---CCGAATGAT---CTTCCAATTGAAGAAATTAAGAAA
AAGGTTCCTCGATGGTTATACAAACTGACTGATTTGGATTTTGAAGAAGAGGAGGGGA
TTCGTTCAAGAAAAGGCAAACGTGTAGTAATTTATACTGATGCCTTGATACGTTACTCG
CAACAATCAGATTTTCGCCGTGATCTAATCAAAGGGTCGATGCGGGCTCAAAGACGTA
AAACAGTGACTTGGGAAATGTTTCAAACAGATGCACACTCCCCCCTTTTTTTTGACAG
AATAGACAAAACACCTTTGTTTTCTTTTGATATC------TCAAGGATGATGAACCTAATTTT
TAGGAATAGACAGGAGGAGGAACGGATAGCAATAGCAGAAACTTGGGATAATATTATAT
TTGCTCAAGCAATAAGGGGTTCCATATTAGTAACTCACTCGATTCTACGAAAATACATC
GTGTTACCTTCATTGATAATAGTTAAAAATATTGGTCGTATGTTATTATTTCAATTCCCTG
AGTGGTATGAAGATTTTAACGAATGGAGTAGGGAAATGCATGTCAAATGCACATATAAT
GGTGTTCAATTATCGGAAACAGAATTTCCAAAAGATTGGTTAACAGACGGTATTCAGAT
AAAAATCCTATTTCCTTTCTCTCTGAAACCTTGGCATAGATCTAAGAGAAAAAAACAAA
AAAATTTCTGTTTTTTAACGATCTGGGGGATGGAAACAGAACTGCCCTTTGGCTCTCCC
CGAAAAAAACCTTTCTTTTTTGAACCCATTCATAAAGCATTCGAAAAAAAAATTATAAA
AGTAAAA---AAGAAAGGTTTTTTCTTTCTAATAATTATAAAAGACAACATAAAAGGTTTT
ATAAAGATTCTCAACGAAAAAATAAGATGGATTATCAAAATAGTTCTA------TTTATAAAA
ATCAAAGTG---------------AAGGAAGATTCCAAAATAAGTAATAGGATCATCCATGAATCA
CCCATTAGAATTGTATCCAGAGATTGGACAAATGATTCACTGATAGAAATAAAAATTAAT
GATCTGGCTGATAAGACAACCAAAATCTGGGATCAAGTAGAAAAGATTAGAAAAGACA
AGAAAAAAAGTACTAACAAGGGAAATTGTAGTAATAAAAGAACCGAATCACGGAAAC
ATATTTGGCAAATATCTAAAAAAAGAAGTGCCCGATTAATCTCTAAATGGGACTCTTTTA
TGAAATCTTTCATTGAAAGAATATACATGAGTATCCTTCTATGTATCACTAACATTTACAG
GATCTATGTACAATTTTTTATTCCC------TTCTGCGACTTATCCTCTTTGTCCCAAGCCTAT

GTGTTTTACAAATTATTGCAAACCCAAGTTAGTAACAATATAAGTCAGAATCCATATTT
CATTACACATATCCTTTTATTAAGGATGGAATAAAAGACTATTTCACACGAGGAATATTT
GATTCTGAATCAAAACACAAGAAACTGCGGAATTCTGGAATGAGTGAATGGAAAAAC
TGGTTAAAGAGCCATTATCAATACAATTTATCCCATGACAAATGGTCTAGATTAGCACCT
CTAAAATGGAGAAAGAAAGTCAATCAGCATCGTACGATTCAAAATAACCCAAAATCGG
GTTCATATGAA---GAAAAAGACCAATTAATTCATTCAGGGAAAAAGAAAAAAATAAAA
AAACACTACAGATATGATCTTTTATCATATAAATATCTTAATAAATATACAATTGATCTAGA
TAGAAAATATTTTGAATTGAGAATCACCAATTTTGACCCTAGATTTTTTGATTGGATGGG
AATGAATGAACAAATGTTAGATCGTTGGTTCTTACCAGAATTTGTGCCGCTTTACGATC
GATATACGACTAATCCGTGGATCATACCAATAAATTTGATTCTATTTGATTTCAAGAGCA
AGAAGGAAGCGGAACTAGACTTATTTTTGAAAAAATATTTTATTTTTCAACTCCGATGG
GATGATCCTTTCAATAAAGGAATGAACAATAATATCAAGGTATATTGTCTACTGCTTAGA
CTTATGAATCCGAATGAAATTGCTATATCCTCTATTCAAAGTGGAGAAATGGGTCTAGAT
GTAATGCTGATTAATAGGGGTTTCTCTCTTCCAGAATTGATAAAAAAGGGAATATTTCTT
ATTGAACCGGTTCGTTTGTCTATCAAACGGGATGGAATTTCGATTATGTATCAAACCATA
GCTATTTCATTGACCCATAAGATTCAGCACAATAATTATGATTTGCTTGTTCCTGAACATA
TTTTATCTCCTAGCCATCGTAGAGAATTGAGAATTCTAAGCCGTTTCAAGTTTCTTTGGC
CCACTCATCGATTAGAAGATTTAGCCTGTATGAATCGCTATTGGTTTGATACCAATAATG
GGAGTCGTTTCAGTATGTCAAGGATCCATATGTATCAGATGAAACGGCATCAATTCAAA
TCCTGTATTTTTGAATTGAGAGAGATATTGAGAGAGATCAAGAATTCTCGCTATTTCTTA
GATTCATGGACCAAATTCGATTCAGTGGTGTCTTTCACTCACATTTTTTTCCACCAAGA
ACGTTTTGTGAAACTCCTTGGCCCCCAAACTTGGAGTGTCCTGCTTTCA---CGTGATTC
A------CAGGGTTCAACAAGCAATCGATATTTC---------ACGATCAAAGGTGTAGTACTGCT
TGTAGTAGCG---GTCCTTATA------------TATCGTATTAACAATCGAAATATGGTCGAAAGA
AAAAATCTCTATTTGATGGGGCTTCTT---------------CCTATACCTATGAATTCCATTGGACC
CAGAAATGATACATTGGAAGAATCTTTT---TGGTCTCCCAATATCAATAGGTTGATTGTTT
CG---CTCCTGTATCTTCCAAAAGGGAAAAAGATCTCTGAGAGTTGTTTCATGGATCCGA
AAGAGAGTACTTGGGTCCTCCCAATAACT------------CAAAAGTGTATCATGCCTGAATCT
AACTGGGGTTCGCGGTGGTGGAGGAACTGGATCGGAAAAAAGAGG------GATTCTAGT
TGTAAGATATCT---------AATGAAACAGTAGCTGGAATTGAGATCTCATTCAAAGAGAAA
GATATCAAA---TATCTGGAGTTTCTTTTTGTATCCTATATGGAT------GATCCGATCCGCAA
GGACCATGGTTGGGAATTGTTTGATCGTGTTTCTCCGAGGAAGAAGCGAAACATAATC
AATTTG------AATTCGGGACAGCTATTCGAAATCTTAGCGAAACACTTGATTTGTTATCTC
ATGTCTGCTTTTCGTGAAAAAAGACCAATTGAAGTGGAGGGT---TTCCTCAAACAACA
AGGA---GCTGAGGCAACTATTCCATCAAATGATATTGAGCATGTTTCCCAT------CTCTTC
TCGAGAAACAAGTGGGATATTTCTTTGCAAAATTGTGCTCAATTTCATATGTGGCAATT
CCACCAAGATCTCTTCGTT---------AGTTGGGGGAAG---AATCAGCACGAATCGGATTTTT
TGAGGAAC---GTATCGAGAGAGAATTTGATTTGGTTAGACAATATGTGGTTGGTAAACA
AGGATCGG---------TTTTTTAGC------AAGGTACGGAATGTATCGTCAAATATTCAATATGA
TTCC---------ACAAGA------TCTATTTTCGTTCAAGTAACGGATTCTAGCCAATTGAAAGGA
TCTTCT---GATCAATCCAGAGAGAATTTCGATTCCATTAGT------------AATGAGGATTCGG
AA--TATCACACATTGATCAATCAAACAGAGATTCAGCAAC
TAAAAGAAAGATTGATTCTT---TGGGAT------CCTTCCTCTCTTCAAACGGAACGAACAG
AGATAGAATCAGAT------CGATTCCCGAAA------TACCCTTCTGGATATTCC------TCAATGT

CCCGGCTATTCACG---GAACGTGAAAAGCAGATGAATAATCATCTGTTTCCGGAAAAAA
TCGAAGAATTTCTT------GGGAATCCTACAAGATCAATTCGTTCTTTTTCTCCGACAGAT
GG------TCAGAACTTCAT---CTGGTTTCGAATGCTACTGAGAGGTTCACTAGAGATCAG---
---------AAATTGTTGAAGAAACAACAAGATGTTTTTTCTTTTGTCCCTTCCAGGCGATCG
GAA------------AAGAAAGAAATGGTTGATATATTCAAGATAATTACGTATTTACAAAAT------
ACCGTCTCAATTCATCCTATTTCATCAGATCGGGGATGTGATATGGTTCCG---AAGGATG
AACCGGAT---ATGGACAGTTCTAATAAGATCTCATTCTTGAACAAAAACCCATTTTGTGA
T------TTATTCCATCTATTCCATGACCGGAACAAAGGGAGATACACGTTG------CACCACG
ATTTTGAATCAGAA------GAGAGATTTCAAGAAATGGCAGATCTATTCACTCTATCAATA
ACCGAGCCT---GATCTGGTGTATCATAAGGGATTTGCCTTTTCTATTGATTCCTACGGGTT
GGATCAAAAAAAA------TTCTTGAATGAGGTATTCAACTCCAGGGATGAATCGAAAAAG
AACTCTTTA---------TTGGTTCTACCTCATATTTTTTAT------GAAGAGAATGAATCTTTTTA
TCGAAGGATCAGAAAAAAATGGGTCCGG---ATCTCCTGCAGGAATGGTTTGGAAGATC
CAAAACCCAAAATAGTGGTATTTGCTAGCAACAACATAATGGAGGCAGTCAATCAATAT
AGATTGATCCGAAATCTGATTCAAATCCAATATAGCACCTATGGG------TACATAAGAAAT
CTATCGAATAGATTCTTTTTAATG---------AATAGATCCGATCGCAACTTCGAATATGGAAT
TCAA---------AGGGATCAAATAGGAAATGATACTCTGAATCATATAACTATAATGAAATATA
TGATCAACCAACATTTATTGAATGTGAAAAAG---AGTCAGAAGAAATGGTTCGAT---------
---CCTCTTATTTCTCGAACCGAGAGATCCATGAATCGGGATCCTAATGCATATAGATACA
AATGGTCCAATGGGAGCAAGAATTTCCAGGAGCATTTGGAGCATTTCGTTTCTGAACA
GAAGAAC---------CGTTTTCAAGTGGTGTTCGATCGATTACGTATTAATCAACATTCGATT
GATTGGTCCAAGGTTATCGACAAACAAGATTTGTCTAAGTCACTTCATTTCTTTTTGTCC
AAGTCA---------------------------CTTCTCTTTTTGTCT---------------------AAGTCACTT------------
------------ACTTTTTTCTTTGTGAGTATCGGGAATATCCCCATTCATAGGTCCGAG---ATCC
ACATCTAT---GAATTGAAAGGTCCGAATGATCAACTCTGCAATCAGTTGTTAGAATCAAT
AGGTGTTCAAATCGTTCATTTGAATAAATTGAAACCC------TTCTTATTGGAT---------GAT
CATGATACTTCC---CAAAGATCGAAATTCTTGATCAATGGGGGAACAATATCAGCATTTT
TGTTCAATAAGATACCA---AAGTGG---ATGATTGACTCACTCCATACT------AGGAATAAT
CGCAGGAAATTCTTTGATAACACCGATTCCTATTTCTCAATGATATCCCACGATCGGGAC
AATTGGCTGAATCCCGTGAAACCATTTCATAGAAGTTCATTGATATCTTCTTTTTATAAA
GCAAATCTACTTCGATTCTTGAATAATCCACATCACTTCTGCTTCTAT------TCTAACAAA
AGATTCCCTTTTTAT------------GCGGAAAAGACCCGTATCGATAATTATGAT------CTTACAT
AT---GGACAATTCCTCCATATCTCGTTCATCCGCAACAAG---ATATTTTCTTTGTGCGTCG
GTAAA------AAAAAACATGTTTTTTTGGAG---AGAGATACTATTTCACCAATCGAGTCAC
AGGTATCTGACATATTCATACCTAACGATTTTCCACAAAGTGGTGACGAAACG---TATAA
CTTGTACAAA--TCTCAGC
CTCTTTCA---------------------------ACTTATTGTCAGCCTCTTTCAGATATGAATCTATCTGAT
TCAGAAGGGAAGAACTTGCATCAGTATCTCAGT------TTCAATTCAAACATGGGTTTGAT
TCACACTCCATGTTCTGAGAAAGATTTACCA------TCCGCAAAA------AAA-------------------
-----CGGAATCTTTGTCTAAAG------AAATGCGTTGAGAAACGGCAGATGTATAGAGCCTT
TCAACGAGATAGTGCTTTTTCAAATCTCTCAAAATGGAATCTGTTCCAAACGTATATGC
CATGGTTCCTTACTTCGGCAGGGTGCAAATATATAAATTTCATCCTTTTAGATACTTTTTC
AGACCCATTG------CCGATACTAAGTAGCAGT---CACCAATTTGTATCCTTTTTTTATGATA
TTATGCATGGATCAGATATATCATGGTCAATTCTTCAGATTCCATTGTGG------------GCGAT

TCTTCCACGATGGAATCTG---ATAAGTGAGATTTCGAGTAAGTGTTTACAGAAT------ATT
CTTCTGCCCGAAGAAATGATTCATCGAAATAATGAGTCACCCGTTCCATTGAAGTGGAC
ACATCTGAGATCACCAAATGCTCGGGAGTTC---CTCTATTCAATCCTTTTCCTTCTTCTT
GTTGCTGGATAT---CTCGTTCGTACGCATCTTCTCTTTATTTCCCGA------GTCTCTAGTGA
GTTACAGACAGAGTTAGAAAAGATCAAATCTTTGATGATTCCATCATACATGATTGAGT
TGCGAAAACTTCTGGATAGGTATCCTCCACCTGAACTGAATTCTTTCTGGTTAAAGAAT
CTCTTTCTAGTTGCTCTGGAACAATTAGGAGATTCTCTGGAAGAAATACGGAGTTCTGC
TTCTGGTGGCAACATGCTATTGAGTGGTGGTCCC---------------ACTTATGGG---GTCAAAT
CAATACGTTCTAAGAAGAAAGATTTGAATATCAATCTCATCGATATCATCTATCTCATA---
AGTATCATACCAAATCCCATCAATTCAATCACTTTTTCGAGAAATACGAGACATCTAAGT
CGTACAAGTAAAGAGATCTATTCATTGATAAGA------AAAAACGTGAACAGTGATTGGA
TTGATGATCAAATAGAATCCTGGGTCGCGAACAGTGATTTGATTGATGATGAAGAAAGA
GAATTCTTAGTTCAGTTCTCC------ACCTTAACGACA------GAAAAAAGGATTGATCAAAT
TCTATTGAGTCTGACT---CATAGTGATCATTTATCA---------------AAGAATGACTCTGGTTA
TCAAATGATTGAACAACCGGGATCCATTTACTTACGATACTTAGTTGACATTCAA------A
AAAGGTATCTAATGAATTATGAGTTCAATAGATCCTGTTTAGCAGAAAGACGGATATTCC
TTGCTCATTATCAGACAATTACTTAT------TCACAAACCCCGTGTGGGGCTAATAGTTCTC
ATTTCCCATCTCATGGA---------AAACCCTTTTCGCTCCGCTTAGCCCTACCCCCTTCT---A
GGGGTATTTTAGTGATAGGTTTTATAGGAACTGGACGATCATATTTTGTCAAATACCGAG
TGACAAACTCCTATGTTCCCTTCATTACGGTATTTCCGAACAAGTTCCTGGATGACAAT--
-AAAGGTTATCTTATTGAT---------------GATATCGAT---ATTGATGATCGTGACGATATCGAT--
----------ATTGATGATCGTGAC--GATGATGAC
CTTGATACGGAGCTGCTAACTATGCCGAATGTGCTA---------ACTATGTATATGACG---------
CCGAAAATAGACCAATTTGAGATCACCCTTCCATTAGAATTAGCAAAAGCAATGTCTCC
TTGCATAATATGGATTCCAAACATTCATGATCTGTATGTGAATGAGTCGAATTACTTATCC
CTCGGTCTATTAGTGAACCATCTCCCC---------AGGGATTGTGAAAGATGTTCC---ACTAG
AAATATTCTTGTTATTGCTTCGACTCATATTCCCCAAAAAGTGGATCCCGCTCTAATAGC
TCCGAATAAATCAAATACATGCATTAAGATACGAAGGCTTCTTATTCCACAACAACGAA
AGCACTTTTTCATTCTTTCATATACTAGGGGATTCCGCTTGGAAAAGAAAATGTCCCATA
CT------AATGGATTCGGATCCATAACCATGGGTTCCAATGCACGAGATCTTGTAGCACTT
ACCAATGAGGTCCTATCAATTAGTATTACACAAAAGAAATCAATTATAGACACTAATACA
ATTAGATCAGCTCTTCATAGACAAACTTGGGATTTGCGA---------TCCCAGGTAAGATCC
GTTCAGTATCATGAGATCCTTTTCTATCAGATAGGAAGGGCTGTTGCACAAAATGTACT
TCTAAGTAATTGCTCCATAGATCCTATATCTATCTATATGAAGAAGAAA------TCATGTAA
GGAAGGGGATTCTTATTTGTCCAAATGGTACTTCGAACTTGGAACGAGCATGAAGAAA
TTAACGATACTTCTTTATCTTTTGAGTTGTTCTGCCGGATCGGTCGCTCAAGATCTTTGG
TCTCCACCC------GGACCCGATGAACAA---AATTGGATCACTTCTTATGGATTCGTTGAG
AATGATTCT---GATCTAGTTCATGGC---------TTAGAAAGCGCTCTGGTGGGA------TACTC
ACGGACAGAA------TGCAGTCAGTTTGATAATGAT------CGAGTGACATTGCTTCTTCGGT
CCGAACCAAGGAAT---CAGTTAGATATGATGCAAAATGGATCTTGTTCTATCGTTGATCA
GAGATTTCTATATGAAAAA------TACGAATCGGGGTTTGAAGAA---GGGGAAGGAGAAG
GAACCCTCGACCTGCAA------------CAGATAGAGGAGGATGAG------GATTTATTCAATCA
C------ATAGTTTGGGCTCCT------AGACTATGGCGCCCT---------CATGGCAATCTATTTGATT
GTATCGAAAGGCCCAAGAAATTGGGATTTCCC---TATTGGGTCAGGTCATTTCGG---------

GACAAGCGGATCATTTATCATAAAGAGGATAAGCTTCAAGAGAATGATTCGGAGTTCTT
GCGGAGTGGAACCGTGCAGTACCAGACACGAGATAGATCTTCCAAAGAACAAGGGTT
TTTTCGAATAAGCCAATTCATTTGGGACCCTGCAGATCAATTCTTTTTCCTATTCAAA---
GATCAGCCCTTTGTCTCTGTGTTTTCACGTCGAGAATCTTTTGCAGATGAAGAGATGTC
AAAGGGACTTATTACTTCCCAAACAAATTCTCCTACATCTATATATAAACGCTGGTTGAT
C------AAGAATACGCAAGAAAAGCACTTCGAATTGTTGATTCATCGCCAGAGATGGCTT
AGAACTAAT---ACTAATAGTTCATTATCT------AATGGATCTTTCCGTTCTAAT------ACTCTA
TCCGAGAGTTATCAGTATTTATCAAATCTGTTCCTATCTAATAACGGAACGCTATTGGAT
CAAATGACAAAGACATTGTTGAGAAAGAGATGGCTTTTCCCGGATGAAATGAAACATT
TGATT------CATGCACCAGGAGAAAGATTTCCCATTCCTATGCCTAGATCTCGG---ATAAA
TGGAAATTTTATTGATAAAACCTCTTCAATTGTAGCCAATATCTTATTACGAATAATTCCG
ACAACTTCGGGAGAAAAAGAGGCATTTACCTATTACAGAGATGGG------ATGTCAGCTC
AATCTGAAGGAAATTATGCGGAAGCTTTACAGAATTATTATGAAGCTATGCGACCAGAA
ATTGATCCCTACGATCGAAGTTATATACTCTATAATATAGGCCTTATACACACAAGTAACG
GAGAACATACGAAGGCTTTGGAATATTATTTCCGAGCACTAGAACGAAATCCATTCTTA
CCACAAGCTTCTAATAATATGGCCGTGATCTGCCATTAC---CGGGGAGAAGAGGCCATT
CGACAGGGAGATTCAGAAATTGCGGAGGCTTGGTTCGATCAAGCTGCTGAGTATTGGA
AACAAGCCATAGCGCTTACTCCAGGTAATTATATTGAAGCACAGAATTGGTTGAAGATC
ACAGGGCGGTTCGAA------ATGAATTGGCGATCAGAACATATATGGATAGAACTTATAAC
GGGTTCTAGAAAAATAAGTAACTTCTGCTGGGCCTGTATCCTTTTTTTAGGTTCACTAG
GATTTTTATTGGTTGGAACTTCCAGTTATTTTGGTAGGAATCTGATATCCTTATTCCCATC
TCAGCAAATAGTTTTTTTTCCACAAGGGATCGTAATGTGTTTCTACGGTATCGCGGGTCT
ATTCATTAGCTCCTATTTGTGGTGCACAATTTCGTGGAGTGTAGGTAGCGGTTATGACAA
ATTCGATAGAAAAGAAGGAATAGTGTGTATTTTTCGTTGGGGATTTCCTGGAAGAAATC
GTCGCATCTTCTTTCGATTCCGTATAAGAGATATCCGTTCGATCAGAATAGAAGTTAAAG
AGGGTCTTTTTCCTCGCCGTGTCCTTTATATGGAAATTGGGGGCCGGGGAGACATTCCA
TTGACGCGTACCGATGAGAAT---TTAACTCCACGAGAAATTGAACAAAAAGCTGCCGA
ATCGGCCTATTTTTTTGCGCGTACCAATTGAAGTATTT
>Saccharum
??
??
??
??
??
??
??
??
??
??
??
??
??
??
??

??
????ATGGCAACCCTTCGAGTCGACGAAATTAATAAAATTCTCCGCGAACGTATTGAACA
ATATAATAGGAAAGTAGGGATTGAGAATATCGGTCGCGTAGTGCAAGTGGGGGATGGG
ATTGCTCGTATTATAGGTCTTGGTGAAATAATGTCAGGTGAATTAGTCGAATTTGCAGAA
GGGACGAGAGGTATTGCTCTGAATTTGGAATCCAAAAATGTTGGGATTGTATTAATGGG
CGATGGGTTGATGATACAAGAGGGAAGTTTTGTAAAAGCAACAGGAAGAATTGCTCAG
ATACCCGTGAGCGAGGCTTACTTGGGTCGTGTTATAAATGCTCTCGCTAAACCTATTGAT
GGGAGAGGCGAAATTGTCGCTTCAGAATCTCGCTTAATTGAATCTCCTGCTCCGGGTAT
AATTTCTAGGCGTTCCGTATATGAACCCCTTCAAACAGGGCTTATTGCTATCGATTCGAT
GATCCCCATAGGGCGCGGTCAGCGAGAGTTAATTATTGGGGACAGACAGACTGGCAAA
ACAGCAGTAGCCACAGATACAATTCTCAATCAAAAAGGGCAAGATGTAATATGTGTTTA
TGTAGCTATCGGTCAAAGAGCATCCTCCGTGGCTCAAGTAGTAACTACTTTCCACGAAG
AGGGGGCCATGGAATACACTATTGTAGTAGCTGAAATGGCGGATTCACCCGCTACATTA
CAATATCTCGCTCCTTATACGGGAGCAGCCCTGGCTGAGTATTTTATGTACCGCGAACG
GCATACTTTAATAATTTATGATGATCTCTCCAAACAGGCACAAGCTTATCGCCAAATGTC
CCTTCTATTAAGAAGACCTCCCGGCCGCGAAGCTTATCCAGGGGATGTTTTTTATTTGCA
TTCACGCCTTTTAGAAAGAGCCGCTAAATTAAATTCTCTTTTAGGCGAAGGGAGTATGA
CCGCTTTACCAATAGTGGAGACTCAATCTGGAGACGTTTCCGCCTATATTCCTACTAATG
TAATCTCAATTACAGATGGACAAATATTCTTATCCGCGGATCTATTCAATGCCGGAATTC
GACCTGCTATTAATGTGGGTATTTCAGTTTCCAGAGTGGGATCCGCAGCTCAAATTAAA
GCCATGAAACAAGTAGCTGGCAAATCAAAATTGGAACTAGCTCAATTCGCAGAATTAC
AAGCCTTTGCACAATTCGCCTCTGCTCTGGATAAAACAAGTCAGAATCAATTGGCAAG
GGGTCGACGATTACGGGAATTGCTTAAACAATCCCAATCAAACCCTCTCCCAGTGGAA
GAGCAGGTAGCTACTATTTATACCGGAACGAGAGGATATCTTGATTCGTTAGAAATTGA
ACAGGTAAAGAAATTTCTGGATGAGTTACGTAAACACCTAAAAGATACTAAACCTCAA
TTCCAAGAAATTATATCTTCTAGTAAGACATTCACCGAGCAAGCAGAAACCCTTTTGAA
GGAAGCTATTCAGGAACAGCTTGAACGGTTTTCCCTTCAGGAACAAACAATGAGAACC
AATCCTACTACTTCGCGTCCCGGGGTTTCCACAATTGAAGAAAAAAGCGTAGGGCGTA
TTGATCAAATTATTGGACCCGTGCTGGATATCACTTTTCCCCCGGGCAAGTTGCCTAATA
TTTATAATGCTTTGATAGTCAAGAGTCGAGACACTGCCGATAAGCAAATTAATGTGACT
TGTGAGGTACAACAATTATTAGGAAATAATCGAGTTAGAGCTGTGGCTATGAGTGCTAC
AGACGGGTTGATGAGAGGAATGGAAGTGATTGACACGGGAACTCCTCTCAGTGTTCC
AGTCGGTGGAGCTACTCTCGGACGAATTTTTAACGTTCTTGGGGAGCCTATTGACAATT
TGGGTCCTGTGGATACTAGTGCAACATTCCCTATTCATAGATCTGCGCCTGCCTTTATCG
AGTTAGATACGAAATTATCTATCTTTGAAACAGGTATTAAGGTGGTCGATCTTTTAGCTC
CCTATCGACGTGGAGGAAAAATCGGACTGTTTGGGGGGGCAGGAGTAGGTAAAACAG
TACTCATCATGGAATTAATCAATAACATTGCTAAAGCTCATGGAGGCGTATCCGTATTTG
GCGGAGTAGGGGAACGGACTCGTGAAGGAAATGATCTTTATATGGAAATGAAGGAATC
CGGAGTAATTAATGAAAAAAATATTGAGGAATCAAAGGTAGCTCTAGTCTATGGCCAAA
TGAATGAACCGCCGGGAGCTCGTATGAGAGTTGGTTTAACTGCCCTAACTATGGCAGA
ATATTTCCGAGATGTTAATAAGCAAGACGTGCTTTTATTCATCGATAATATCTTTCGTTTT
GTTCAAGCAGGATCGGAAGTATCCGCCTTATTAGGCAGAATGCCCTCCGCAGTGGGTTA
TCAACCTACCCTTAGTACAGAAATGGGTTCTTTGCAAGAAAGAATTACCTCTACCAAAA
AGGGATCTATAACTTCGATCCAAGCAGTTTATGTACCTGCAGACGATTTGACCGACCCT

GCTCCTGCCACAACATTTGCACATTTGGACGCTACTACCGTACTTTCCAGAGGATTAGC
TTCCAAGGGTATTTATCCCGCAGTAGATCCTTTAGATTCAACCTCAACTATGTTACAGCC
TCGGATCGTTGGCAACGAACATTATGAAACTGCGCAAAGAGTTAAGGAAACTTTACAA
CGTTACAAAGAACTTCAGGACATTATCGCAATTCTTGGATTGGATGAATTATCGGAGGA
GGATCGTTTAACTGTAGCAAGAGCACGAAAAATCGAGCGGTTCTTATCACAACCGTTC
TTTGTGGCAGAAGTTTTTACTGGTTCTCCAGGAAAGTATGTTGGTCTTGCAGAAACAAT
TAGGGGATTTCAACTAATCCTTTCCGGAGAATTAGACGGCCTACCCGAACAGGCTTTTT
ATTTAGTGGGGAACATCGATGAAGCTAGCACGAAAGCTATAAACTTAGAAGAGGAGAG
CAAATTGAAGAAAATGAAATTAAATCTTTATGTACTGACTCCTAAACGAATTATTTGGGA
TTGTGAAGTGAAAGAAATCATTTTATCTACTAATAGTGGCCAAATTGGTGTATTACCAA
ACCACGCCCCCATTAACACAGCTGTAGATATGGGTCCTTTGAGAATACGCCTCAACGAC
CAATGGTTAACGGCGGTTCTGTGGAGTGGTTTTGCGAGAATAGTTAATAATGAGATCAT
CATTTTAGGAAATGATGCAGAACTGGGTAGTGACATTGATCCAGAAGAAGCTCAACAG
GCACTTGAAATAGCCGAAGCTAACTTGAGTAAAGCTGAGGGTACGAAAGAATTGGTTG
AAGCGAAGCTAGCTCTCAGACGAGCTAGGATACGAGTCGAGGCTGTCAATTGGATTCC
CATGAAAAATGTAACCCATTCTTTCGTTTTTTTAGCTCACTGGCCATTCGCTGGGAGTTT
CGGGCTTAATACCGATATTTTAGCAACAAATCTAATAAATCTAACTGTAGTGGTTGGTGT
ATTGATTTTTTTTGGAAAGGGAGTGTTAAAAGATTTATTAGATAATCGAAAACAGAGGA
TCTTAAGTACTATTCGAAATTCGGAAGAATTGCGTAGAGGGACCCTTGAACAACTCGA
AAAAGCTCGTATTCGATTACAGAAAGTCGAACTAGAAGCAGATGAGTATCGAATGAAT
GGATACTCTGAAATAGAACGAGAAAAAGAAAATTTGATTAATGCTACTTCTATTAGTTT
GGAACAATTAGAAAAGTCTAAAAACGAAACCCTTTATTTTGAAAAACAAAGGGCGATG
AATCAGGTCCGACAACGGGTTTTCCAACAAGCTGTACAAGGAGCTCTAGGAACTCTGA
ATAGTTGTTTGAATACCGAGTTACATTTCCGTACGATTCGTGCTAATATTGGCATTCTCG
GGGCCATAGAATGGAAGAGA---ATGAATCCACTAATTGCTGCTGCTTCCGTTATTGCTGC
TGGATTGGCCGTAGGGCTTGCTTCTATTGGGCCCGGAGTTGGTCAAGGTACTGCTGCA
GGACAAGCTGTAGAAGGTATTGCGAGACAGCCAGAAGCAGAAGGTAAAATAAGAGGT
ACTTTATTGCTTAGTCTAGCTTTTATGGAAGCTTTAACAATTTATGGACTGGTTGTGGCA
CTAGCGCTTTTATTTGCGAACCCTTTTGTTATGAATATTACACCATGTTCCATTAAAACA
CTCAAGGGGTTATATGATATATCGGGTGTAGAAGTAGGCCAACACTTCTATTGGCAAATA
GGAGGTTTCCAAATTCATGCCCAAGTACTCATCACTTCTTGGGTCGTAATTACTATTTTG
CTAGGTTCAGTTATCATAGCTGTTCGGAATCCACAAACCATCCCGACCGATGGTCAGAA
TTTCTTCGAATATGTTCTTGAGTTTATTCGAGACTTGAGCAAAACTCAGATTGGAGAAG
AATATGGTCCCTGGGTTCCCTTTATTGGAACTATGTTCCTTTTTATTTTTGTTTCGAATTG
GTCAGGTGCTCTTTTACCTTGGAAAATTATAGAGTTACCCCATGGGGAATTAGCAGCGC
CCACGAATGATATAAATACTACTGTTGCTTTAGCTTTACTAACATCAGCGGCATATTTTTA
TGCAGGTCTTAGCAAAAAAGGATTGAGTTATTTCGAAAAATATATTAAACCAACCCCAA
TCCTTTTACCTATTAACATCCTAGAAGATTTCACAAAACCATTATCACTTAGTTTTCGAC
TTTTCGGAAATATATTGGCGGATGAATTAGTCGTTGTTGTTCTTGTTTCTTTAGTCCCCTT
AGTAGTCCCTATACCGGTCATGTTTCTTGGATTATTTACAAGCGGTATTCAAGCTCTTATT
TTTGCAACGTTAGCCGCAGCCTATATAGGTGAATCCATGGAAGGTCATCATATGCTATTT
GCAACTTTAGAACATATACTAACTCATATCTCTTTCTCAACCATTTCAATTGTGATTACGA
TTCATTTGATAACCTTATTAGTTCGTGAACTTAGGGGATTACGTGATTCGTCAGAAAAA
GGAATGATAGCTACTTTTTTCTCTATAACAGGATTTTTAGTCTCTCGTTGGGTTTCTTCG

GGACATTTTCCATTAAGTAATTTATATGAGTCATTGATCTTCCTTTCATGGACTCTGTATA
TTCTTCATACTATTCCTAAGATACAGAACTCGAAAAATGATTTAAGCACAATAACTACGC
CAAGTACTATTTTAACGCAAGGCTTTGCCACGTCGGGTCTTTTAACTGAAATGCATCAA
TCCACAATACTAGTACCTGCTCTACAATCTCAGTGGTTAATGATGCATGTCAGTATGATG
TTACTAAGCTATGCAACTCTTTTGTGCGGATCCTTATTATCCGCCGCTCTTCTAATCATTA
GATTTCGAAATTCTTTCGATTTCTTTTCA---CTAAAGGAGATTGAATATTTATATGCAAAA
AGAAGTGCTTTAAAAAACACCTCTTTTCCCGTATTTCCAAATTATTACAAATATCAATTA
ACTGAGCGTTTGGATTCTTGGAGTTATCGTGTCATTAGTCTAGGGTTTACTCTTTTAACC
GTGGGTATTCTTTGTGGAGCAGTATGGGCTAATGAGGCATGGGGATCCTATTGGAATTG
GGATCCTAAGGAAACTTGGGCATTTATTACCTGGACCATATTTGCAATATATTTACATAGT
AGAACAAATCCAAATTGGAAGGGTACGAATTCCGCACTTGTAGCTTCGATAGGATTTCT
TATAATTTGGATCTGCTATTTTGGTATCAATCTGTTAGGAATAGGTTTACATAGTTACGGT
TCATTTACATTACCAATGAAAAAAAAAGAAAGCATTGCCTTCTTTCTTATATCTTGTATTTA
TCGTACTTTTGCCCTGGGGAGTCTCTTTCTCTTTTAACAAATGTCTGGAACTTTGGATTA
AGAATTGGTGGAATACCAGGCAATCCGAAACTTTCTTAACTGATATTCAAGAGAAAAG
GATTCTAGAAGGATTCATAGAATTAGAAGAACTTTTTCTCTTGGACGAAATGATAAAAG
AGAAACCGAAGACACATGTACAAAAACTTCCTATAGGAATACACAAGGAAATAATACA
ATTGGCCAAAATAGATAATGAGGATCATCTCCATATCATTTTGCATTTCTCAACAAATATA
ATCTGTTTGGCTATTCTAAGTGGTTCTTTTTTTCTGGGTAAAGAAGAACTTGTCATTTTG
AATTCTTGGGTTCAGGAATTTTTCTATAACTTAAATGATTCAATAAAAGCTTTTTTTATTC
TTTTAGTTACTGATTTTTTTGTTGGATTTCACTCCACCCGCGGTTGGGAACTACTAATTC
GTTGGGTCTACAACAATCTTGGATGGGCTCCTAACGAGCTAATTTTCACTATTTTTGTTT
GTAGTTTTCCTGTGATTCTAGACACGTGTTTGAAATTTTGGGTCTTTTTTTGTTTAAACC
GTCTATCTCCTTCACTTGTAGTCATTTATCATTCAATTAGTGAAATGCCCATTGGTGTTCC
AAAAGTACCTTACCGGATTCCCGGAGATGAAGAAGCGACTTGGGTTGACTTATACAAT
GTTATGTATCGAGAAAGGACACTTTTTTTAGGTCAAGAGATTCGTTGCGAGATCACGAA
TCATATTACAGGTCTGATGGTATATCTCAGTATAGAAGATGGAAATAGCGATATTTTTTTG
TTTATAAACTCCCCTGGCGGGTGGCTAATCTCAGGAATGGCGATTTTTGATACGATGCA
AACGGTGACACCTGATATATATACAATATGCCTCGGAATAGCCGCGTCCATGGCCTCCTT
CATTCTGCTTGGAGGAGAACCCACCAAGCGTATAGCATTCCCTCACGCTAGGATTATGC
TTCACCAACCTGCTAGTGCTTATTATCGGGCAAGGACACCAGAATTTTTACTAGAAGTG
GAAGAGTTACACAAAGTTCGCGAAATGATCACAAGGGTTTATGCACTAAGAACAGGCA
AGCCTTTTTGGGTTGTATCCGAAGACATGGAAAGGGATGTTTTTATGTCAGCAGACGA
AGCCAAAGCTTATGGACTTGTCGATATTGTAGGGGATGAACCGAGAGAAGCAAAAGTA
ACTTTCGAAGGTTTAGTTACGGAAGCCCTACCCAACGGAATGTTCCGCGTTCGCCTAG
AGAATGACACGATCATCCTGGGCTATATTTCAGGAAAGATCCGGTCTAGCTCTATACGA
ATACTGATGGGGGATAGGGTCAAAATTGAAGTAAGTCGTTATGATTCCAGCAAAGGTC
GTATAATTTATAGACTTCCCCATAAGGATTCGAAGGATATGCAAAAATTCGAAGGGTATT
CAGAAAAACAGAAATCTCGTCAACACTACTTCGTCTACCCACTTCTCTTTCAGGAATAT
ATTTATGCATTTGCTCATGATTATGGATTATTTGGTTGTAATAACAAATTTAGTTCAATACT
TGTGAAACGTTTAATTATTCGAATGTATCAGCAGAATTTTTTGATTAATTCGGTTAATTAT
CCTAACCAAGATCGATTGTTTGATTCTGAGTTTTATTCTCAGATTCTATCTGAGGGGTTT
GGGATTGTTGTAGAAATCCCACTCTCGCTAGGGCAACTATCTTGTCCAATACCAAAGTT
TCAAAATTTACAATCTATTCATTCAATATTTCCCTTTTTAGAAGACAAATTTTTGCATTTA

CATTATCTATCACATATAGAAATACCCTATCCTATCCATTTAGAAATCCTGGTTCAACTCC
TTGAATACCGGATTCAAGATGTTCCCTCTTTGCATTTATTGCGATTCTTTCTCCACTATTA
TTCGAATTGGAATAGTCTTATTACTAAATCCATTTTTCTTTTGAAAAAACAAAGACTATT
TCGATTCCTATATAACTCTTATGTATCAGAATATGAATTTTTCTTGTTGTTTCTTCGTAAAC
AATCTTCTTGCTTACGATTAACATCTTCTGGAACCTTTCTGGAACGAATCATCTTTTCTG
GGAAGATGGAACAT------GTAATGTACCCGGGGCGGAAAACCATATGGTTCTTTATGGAT
CCTCTTATGCATTATGTTCGATATCAAGGAAAGGCAATTCTTGCATCAAAAGGCACTCTT
CTTTTGAAGAAGAAATGGAAATCTTACCTTGTCAATTTCTCGCAATATTTTTTCTATTTTT
GGACTCAACCACAAAGGATTCGTCTAAACCAATTAACAAACTCTTGCTTCGATTTTCTG
GGGTACCTTTCAAGTGTACCAATAAATACTTTGTTAGTAAGGAATCAAATGCTGGAGAA
TTCTTTTCTAATAGATACTCGAATG------AAAAAATTCAATACCACAGTCCCTGCAACTC
CCCTCGTTGGATCCTTATCAAAAGCTCAATTTTGTACTGGATCGGGGCATCCTATTAGTA
AACCCGTTTGGACTGATTTATCAGATTGGGATATTCTTGATCGTTTTGGTCGGATATGTA
GAAATCTTTTTCATTATCATAGTGGATCTTCGAAAAAACAGACTTTGTATCGACTAAAAT
ATATACTTCGACTTTCATGTGCTAGAACTTTAGCTCGTAAACATAAAAGTACGGTACGA
ACTTTTATGCAACGATTGGGTTCGGTATTTTTAGAAGAATTTTTTACGGAAGAAGAGCA
AGTTTTTTCTTTGATGTTCACCAAACGTATTTGGTATTTGGATATTATCCGTATCAATGAC
CTGGTGAATCCTCTTATGATAATAGATAGGGTA---GAGGTAGAA---ACTATCAATTCTTTT
TCGAAATCGGAA---TTATTAAAAGAAATCTACGGACTTATATGGATTCTA---CCCATTTTG
GCCCTCCTACTGGGAATCACAATAGAAGTACTCGTAATTGTGTGGTTAGAAAGAGAAAT
ATCCGCATCGATACAACAACGTATTGGTCCTGAATATGCTGGCCCCCTGGGACTGCTTC
AAGCTATAGCAGATGGAACTAAACTACTTTTAAAAGAGGATATCCTCCCATCCCGAGGA
GAGATTCCTTTATTTAGCATTGGTCCCTCTATAGCAGTCATATCCATTTTATTAAGTTTTTT
AGTTATCCCTTTGGGATATCATTTTGTTTTAGCTGATCTTAGTATTGGTGTTTTTTTATGGA
TTGCGATTTCAAGTATTGCTCCTATTGGTCTTCTCATGGCAGGATATAGCTCAAATAATA
AATATTCTTTTTCAGGCGGTCTACGAGCGGCTGCTCAATCTATTAGTTATGAAATACCAT
TAACTTTTTGTGTACTAGCAATATCTCTACTATCTAATAGTTCAAGTACAGTTGATATAGT
TGAAGCACAGTCCAAATATGGTTTTTTTGGATGGAATCTTTGGCGTCAGCCTATAGGTT
TTCTAGTTTTTCTAATTTCTTCTTTAGCAGAATGTGAAAGATTACCCTTTGATTTACCAG
AAGCAGAAGAAGAATTAGTAGCAGGTTATCAAACCGAATATTCTGGTATTAAATATGGT
TTATTTTATCTTGTTTCTTACCTAAATTTATTAGTTTCCTCTTTATTTGTAACTGTTCTATAC
TTAGGCGGGTGGAATTTCTCTATTCCCTATATATCCTTTTTTGGATTTTTCCAAATGAATA
AAATAATTGGAATTTTGGAAATGGTAATAGGTATCTTTATTACATTAACTAAAGCTTATTT
ATTTCTCTTCATTTCTATCACAATAAGATGGACTTTACCCAGGATGAGAATGGATCAGTT
ATTAAATCTTGGATGGAAATTTCTTTTACCTATTTCTCTGGGCAATCTCTTATTAACAACT
TCTTCCCAACTAGTTTCACTAATGATCTGGCATGTACAGAATGAAAACTTCATTCTCGAT
TCTACGAGAATTTTTATGAAAGCGTTTCATTTGCTTCTCTTCAATGGAAGTTTCATTTTC
CCAGAATGTATCCTAATTTTTGGCCTAATTCTTCTTCTGATGATCGATTTAACCTCTGATC
AAAAAGATAGACCTTGGTTCTATTTCATCTCTTCAACAAGTTTAGTAATAAGCATAACG
GCCCTATTGTTCCGATGGAGAGAAGAACCTATAATTAGCTTTTCGGGAAATTTCCAAAC
GAACAATTTCAACGAAATCTTTCAATTTCTTATTTTATTATGTTCAACTTTATGTATTCCT
CTATCCGTAGAGTACATTGAATGTACAGAAATGGCTATAACAGAGTTTCTGTTATTCGTA
TTAACAGCTACTCTAGGGGGAATGTTTTTATGTGGTGCTAACGATTTAATAACTATCTTT
GTAGCTCCAGAATGTTTCAGTTTATGTTCCTACCTATTGTCTGGATATACCAAGAGAGAT

CTACGGTCTAATGAGGCTACTATGAAATATTTACTCATGGGTGGGGCAAGCTCTTCTATT
CTGGTTCATGGTTTCTCTTGGCTATATGGTTCATCTGGGGGGGAGATCGAGCTTCAAGA
AATTGTGAATGGTCTTATCAATACACAAATGTATAACTCCCCAGGAATTTCAATTGCGCT
TATATTCATCACTGTAGGACTTGGGTTCAAGCTTTCCCCAGCCCCTTTTCATCAATGGAC
TCCTGACGTCTACGAAGGATCCCCCACTCCAGTCGTTGCTTTTCTTTCTGTTACTTCGA
AAGTAGCTGCTTCAGCTTTAGCCACGCGAATTCTCGATATTCCTTTTTATTTCTCATCAA
ACGAATGGCATCTTCTTCTGGAAATCCTAGCTATTCTTAGCATGATATTGGGGAATCTCC
TTGCTATTACTCAAACAAGCATGAAACGTATGCTTGCATATTCGTCCATAGGGCAAATCG
GATATGTAATTATTGGAATAATTGTTGGAGACTCAAATGATGGATATGCAAGCATGATAA
CTTATATGCTGTTCTATATCTCCATGAATCTAGGAACTTTTGCTTGCATTGTATTATTTGGT
CTACGTACCGGAACTGATAACATTCGAGATTATGCAGGATTATACACGAAAGATCCTTTT
TTGGCTCTCTCTTTAGCCCTATGTCTCTTATCCCTAGGAGGCCTTCCTCCACTAGCAGGT
TTCTTCGGAAAACTCTATCTATTCTGGTGTGGATGGCAAGCAGGCCTATATTTCTTGGTT
TCAATAGGACTCCTTACGAGCGTTCTTTCTATCTACTATTATCTAAAAATAATCAAGTTAT
TAATGACTGGACGAAACCAAGAAATAACCCCTTATGTGCGAAATTATAGAAGATCCCCT
TTAAGATCAAACAATTCCATCGAATTGAGTATGACTGTATGTGTGATAGCATCTACTATA
CCAGGAATATCAATGAACCCCATTCTTGCAATTGCTCAGGATACCCTCTTTATGTTTCTG
CTTCACGAATATGATATTTTTTGGACATTTCTAATAATAGCAAGCCTTATTCCTATTTTGG
TATTTTGGATTTCAGGACTTTTAGCCCCGGTTAGTGAAGGACCAGAGAAGCTTTCTAGT
TATGAATCGGGTATAGAACCCATGGGGGGGGCTTGGTTACAATTCCGAATACGCTATTA
CATGTTTGCGCTAGTTTTTGTTGTTTTTGATGTGGAAACAGTCTTTCTCTACCCTTGGGC
AATGAGTTTCGACGTATTGGGTGTATCCGTTTTTATCGAAGCTTTCATTTTTGTGCTTATC
CTAGTTGTTGGTTTAGTTTATGCATGGCGAAAAGGAGCCTTGGAATGGTCTATGAGTTA
TTTTCCTTGGTTAACAATACTTGTTGTTTTGCCGATATTTGCAGGTTCATTAATTTTCTTT
TTACCTCATAAAGGAAATAAAATCGTTAGGTGGTATACTATAGCTATTTGTTTATTAGAAT
TCCTTATAATGACTTATGCATTCTGTTATCATTTCCAATTGGAGGATCCCTTAATCCAATT
AAAGGAGGATTCTAAATGGATAGATGTTTTCGATTTCCACTGGAGATTGGGAATCGATG
GACTTTCATTAGGATCTATTTTATTGACAGGATTTATCACTACTTTAGCTACTTTAGCGGC
TTGGCCGGTTACTCGGAATTCGCAATTATTCTATTTCCTGATGCTAGCAATGTATAGCGG
TCAAATAGGATTATTTTCTTCACGAGACCTTTTACTTTTTTTTATCATGTGGGAGTTAGAA
TTAATTCCTGTTTACTTACTTTTATCCATGTGGGGGGGAAAGAGGCGTCTGTATTCAGCT
ACCAAGTTTATTTTGTATACTGCAGGCGGTTCCATTTTTTTCTTAATTGGAGTTCTGGGT
ATGGGATTATATGGTTCCAATGAACCCGGATTAGATTTAGAAAGATTGATTAATCAATCA
TACCCTACAACATTAGAAATACTACTGTATTTTGGCTTCCTTATTGCTTATGCTGTCAAAT
TGCCGATTATACCTTTACATACGTGGTTACCAGATACCCATGGGGAAGCGCATTACAGTA
CATGTATGCTTTTAGCCGGAATTCTATTAAAGATGGGAGCATACGGATTGATTCGGGTCA
ATATGGAATTGTTACCGCATGCTCATTATCTATTTTCCCCCTGGTTGGTAATAATAGGAGC
GGTGCAAATAATCTATGCAGCTTCAACTTCTCTTGGTCAACGAAATTTCAAAAAAAGAA
TAGCCTACTCCTCCGTATCTCACATGGGTTTCATAATTATAGGAATTGGTTCCATAACCA
ACATTGGACTAAATGGAGCTATTTTACAAATATTATCTCATGGATTTATTGGTGCTACACT
TTTTTTCTTGGCGGGAACGGCTTGTGATAGAATGCGTCTTGTTTATCTCGAAGAACTAG
GGGGAATATCTATCCCAATGCCAAAAATTTTTACCATGTTTAGTAGCTTTTCAATGGCTT
CTCTTGCCTTGCCGGGAATGAGCGGTTTTGTTGCAGAATTAGTAGTATTTTTTGGACTAA
TTACTAGTCCTAAATTTATGTTAATGCCAAAAATGCTAATTACTTTTGTAATGGCAATAG

GAATGATATTAACTCCTATTTATTTATTATCTATGTTACGCCAGATGTTCTATGGATACAAG
CTATTTCATGTTCCAAACAAAAATTTTGTAGATTCTGGACCACGGGAACTCTTTCTTTTA
ATCTGTATCTTTTTACCAGTAATAGGAATTGGTATTTATCCAGATTTTGTTCTCTCTCTATC
CGTTGATAGGGTAGAGGTTCTATTATCCAATTATTATACTAAAATGATGTTTGAGCATGTA
CTTTTTTTGAGTGTCTATTTATTTTCGATTGGTATCTATGGATTGATCACAAGCCGAAACA
TGGTTAGAGCTCTAATATGTCTTGAACTTATACTGAATTCAATTAATCTAAATCTCGTAAC
ATTTTCTGATCTATTTGATAGTCGCCAATTAAAAGGAGACATTTTCGCAATTTTTGTTATA
GCCCTTGCGGCTGCTGAAGCAGCTATTGGACTATCCATTCTTTCTTCCATCCATCGTAAC
AGGAAATCAACTCGTATCAATCAATCCAATTTTTTGAATAATATGGAACATACATATCAA
TATGCCTGGGTAATTCCTCTTCTCCCACTTCCAGTTATTATGTCAATGGGATTTGGACTTT
TTCTTATTCCCACAGCAACAAAAAATCTTCGTCGCATATGGGCTTTTCCTAGTATTTTAC
TCTTAAGTATAGCTATGGTATTCTCACTTCACCTGTCTATTCAACAAATAAATGGAAGTT
CTATCTATCAATATCTATGGTCTTGGACCATCAATAATGATTTTTCCTTAGAATTTGGATA
CTTGGTCGACCCCCTTACGTCTATTATGTTAATACTAATTACTACTGTAGGAATCTTAGTT
CTTATTTATAGTGACGATTATATGTCTCACGATGAAGGATATTTGAGATTTTTTGTTTATAT
AAGTTTTTTTAATACTTCCATGTTGGGATTGGTTACTAGTTCCAATTTGATACAAATTTAT
TTTTTTTGGGAACTTGTCGGAATGTGTTCCTATTTATTGATAGGCTTTTGGTTTACGCGG
CCAATTGCAGCGAGTGCTTGTCAAAAAGCTTTTGTAACTAATCGTGTAGGGGATTTTGG
TCTGTTATTAGGAATTTTAGGTTTTTTTTGGATAACGGGTAGTTTGGAGTTTCGGGATTT
GTTCAAAATAGCTAATAACAATGGGATTAATTCCTTACTTACTACTTTGTGTGCTTTTTTA
TTATTCCTTGGTGCAGTTGCAAAATCTGCACAATTTCCTCTTCACGTATGGTTACCTGAT
GCTATGGAAGGACCCACTCCCATTTCGGCTCTTATACACGCAGCAACTATGGTTGCTGC
GGGGATTTTTCTTCTAGCTAGACTTCTTCCTCTTTTCATATCCCTACCCTTGCTAATGAGT
TTCATTTCTTTAGTAGGTACAATAACACTCTTCTTAGGAGCCACTTTAGCTCTTGCTCAG
AGAGATATTAAAAGAAGCTTAGCCTATTCTACAATGTCTCAATTGGGTTATATGATGTTA
GCTCTAGGTATAGGTTCTTATCAAGCTGCTTTATTCCATTTGATCACTCATGCTTATTCGA
AAGCTTTATTGTTCTTAGGATCCGGATCCGTTATTCATTCAATGGAACCTCTTGTTGGAT
ATTCACCAGATAAAAGTCAGAATATGGTTCTTATGGGTGGTTTAAGAAAATACGTTCCA
ATTACAAGAACTACTTTTTTATGTGGTACACTTTCTCTTTGTGGTATTCCACCTCTTGCTT
GCTTCTGGTCCAAAGATGAAATACTTAGTAATAGTTGGTTGTATTCACCCTTTTTTGGAA
TAATAGCCTCTTTTACTGCAGGATTAACTGCATTTTATATGTTTCGGATATATTTACTTACT
TTTGATGGGTATTTGCGTGTTCATTTTCAAAATTACAGTAGTACTAAAGAAGGTTCGTTG
TATTCAATATCCTTATGGGGAAAAAGTATATCCAAAGGAGTCAATAGG---GATTTTGTTTT
ATCAACAATGAAGTTTTTTTCAGCTAAAAAA------ACTTTTGTCTATCCTCATGAAACGG
GAAATACTATGCTATTTCCTCTTCTTATATTACTACTTTTTACTTTGTTCATTGGATCCATA
GGAATCCCTTTTGAGTTAACCATATTATCAAAGTGGCTAACTCCTTCAATAAACTTGTTC
CAGGAAAATTCAAATTCTTCCATAAATTCATATGAATTTCTCACTAATGCAATTTCTTCT
GTAAGTTTAGCAATTTTTGGTCTATTCATAGCATATATCTTTTATGGATCTGCTTATTCTTT
TTTTCAGAATTTGAATTTTCAAAATTCCCTTGTAAGCTTTTTGGATGAAGTAAAAAAAA
AGATATACAGCTGGTCATATAATCGTGGTTATATAGATTTTTTCTATACTAGGGTTTTTATC
CTAGGTATAAGAAGATTAGCTGAACTAACGCATTTTTTTGATAAAGGTGTCATTGATGG
AATTATCAATGGAGTAGGTCTTGCTGGTTTTTGTATAGGAGAAGAAATCAAATAT---GTA
GGGGGAGGGCGAATATCGTCTTATCTATTCTTTTTTTTATGTTATGTATCCTTGTTCTTATT
CTTTATTCCAATGGATTTACCTGGACCAATACATGAGATTCTTGTGCTATTTGGGGGATT

TGTTCTTCTACTAGGAGGTCTAGGAGTAGTATTACTTACCAACCCAATTTATTCTGCCTT
TTCGCTGGGATTAGTTCTTGTTTGTATATCCTTATTCTATTTTTTATTAAATTCCTACTTTG
TAGCTGTCGCACAACTTCTTATTTATGTGGGAGCCATAAATGTCTTGATCATATTTGCTGT
AATGTTTGTAAACGGCTCAGAGTGGTCTAAAGATAAGAATTATTGGACGATTGGAGATG
GGTTTACTTTACTCCTTTGTATAACTATTCCTTTTTCACTAATGACTACTATCCCAGATAC
GTCGTGGTATGGAATTCTTTGGACTACAAGATCAAACCAAATAGTAGAACAGGGTCTCA
TAAATAACGTTCAACAAATTGGGATTCATTTAGCAACCGATTTTTATCTTCCGTTTGAAC
TCATTTCCCTAATTCTTCTAGTTTCTTTAATAGGTGCAATTACTATGGCTCGACAAATGA
GTCTATCGCTTAAAAGAAAAGATCTCATGATAGTGAATATGGGTCCTCAACACCCATCA
ATGCATGGTGTTCTTCGACTGATTGTTACTCTCGATGGTGAAGATGTTATTGATTGCGAA
CCCATATTAGGGTATTTACACAGAGGAATGGAAAAAATTGCAGAAAACAGAAGTATTAT
ACAATACTTGCCTTATGTAACACGATGGGATTATTTAGCTACTATGTTTACAGAAGCAAT
AACGGTAAATGCACCAGAATTCTTGGAGAATATTCAAATACCCCAAAGAGCCAGCTATA
TTAGAGTAATTATGTTAGAATTGAGCCGTATAGCTTCTCATTTGTTATGGCTTGGACCTTT
TATGGCGGATCTCGGGGCACAGACTCCTTTTTTCTACATTTTTAGAGAGAGAGAATTGA
TATATGATCTATTTGAAGCTGCTACAGGTATGCGAATGATGCATAATTACTTTCGCATCGG
AGGGGTAGCTGCCGATCTCCCTTATGGATGGATGGATAAATGTTTAGATTTCTGTGATTA
TTTTTTACAAGGAGTTGTTGAATATCAACAACTTATTACACAGAATCCTATTTTTTTAGA
ACGAGTTGAAGGAGTCGGTTTTATTAGCGGAGAAGAAGCTGTAAATTGGGGCTTATCG
GGACCAATGTTACGAGCTTCTGGAATACAATGGGATCTTCGTAAAATCGATCCTTATGA
GTCTTACAATCAATTCGATTGGAAAGTCCAATGGCAAAAAGAAGGAGATTCGTTAGCT
CGCTATTTAGTACGAGTCGGTGAAATGAGGGAATCCATAAAAATTATTCAACAGGCTGT
AGAGAAAATTCCTGGAGGACCTTATGAGAATTTAGAAGCCCGACGC---AAGAAAGCAA
AGAATCCCGAATGGAATGATTTTGAATATCGATTTCTTGGTAAAAAACCTTCGCCCAAT
TTTGAATTATCAAAGCAAGAGCTTTATGTAAGAGTAGAAGCTCCAAAAGGCGAATTAG
GAATTTATCTGGTAGGAGATGATAGTCTTTTCCCCTGGAGATGGAAAATTCGTCCACCG
GGTTTTATTAATTTGCAAATTCTTCCTCAGCTAGTTAAAAAAATGAAATTGGCTGATATC
ATGACAATATTAGGTAGTATAGATATCATTATGGGGGAAGTTGATCGTATGTTCCCTATGC
TAACTGGGTTCATTAGTTATGGTCAACAAACAATACGCGCCGCAAGATACATTGGTCAA
GGTTTAATAATTACCTTATCCCACACAAATCGTTTACCTATAACGATTCACTACCCTTATG
AAAAATCAATTACATCGGAGCGTTTCCGGGGGCGAATACACTTTGAATTTGATAAATGC
ATTGCTTGTGAAGTATGTGTTCGCGTATGCCCGATAGATCTACCCCTTGTGGATTGGAAA
TTTGAAAAGGATATTAAAAGAAAACAATTGCTTAATTATAGTATTGATTTCGGAGTTTGT
ATATTTTGTGGTAACTGTGTTGAATACTGTCCCACAAATTGTTTATCAATGACTGAAGAA
TATGAACTTTCTACTTATGATCGTCATGAATTGAATTACAATCAAATTGCTTTGAGTCGG
TTACCAATCTCCATAATGGGAGATTACACAATTCAAACAATTAGGAATTCGAAAATAGA
CGAAGAAAAATCTTGGAATTCAAGAACGATTACGGATTACCAGCAGGGTTGGTTATCT
AATTGGCTAGTTAAACATGACGTGGTTCATAGGTCTTTGGGCTTCGATCATCGAGGAGT
CGAGACTTTACAAATAAAAGCGGGGGATTGGGATTCCATTGCTGTCATTTTATATGTATA
TGGTTACAATTATTTACGTTCCCAATGTGCTTATGACGTAGCACCCGGTGGATCTTTAGC
TAGCGTGTATCATCTTACGAGAATACAGTATGGTATAGATAACCCAGAAGAGGTATGCAT
AAAAGTCTTTGCCCAAAAGGATAATCCTAGAATCCCGTCTGTTTTCTGGGTTTGGAGAA
GTGCCGATTTTCAAGAACGCGAATCTTATGATATGGTGGGAATTTCTTATGATAATCATC
CACGTCTTAAACGTATCTTAATGCCTGAAAGTTGGATAGGCTGGCCCTTACGTAAGGAC

TATATAACCCCCAATTTCTATGAAATACAAGATGCTCAT---

-------------------ATGAGTTTGATTGAGTTTCCGTTACTCGACCAAACAAGTTCTAATTCCGT
TATTTCAACTACACCAAACGATCTTTCGAATTGGTCAAGACTCTCAAGTTTATGGCCCC
TTTTATATGGTACCAGTTGTTGTTTCATTGAATTTGCTTCATTAATAGGCTCACGATTCGA
CTTTGATCGTTATGGATTGGTACCAAGATCAAGTCCTAGACAAGCGGACCTAATTTTAA
CAGCTGGTACGGTAACAATGAAAATGGCTCCATCTTTAGTGCGATTATATGAGCAAATG
CCTGAACCGAAATACGTCATTGCTATGGGAGCCTGTACTATTACAGGGGGCATGTTCAG
TACAGATTCCTATAGTACTGTTCGAGGAGTTGATAAGTTAATTCCTGTGGACGTCTACCT
GCCGGGTTGCCCGCCTAAACCAGAGGCTGTTATAGATGCCCTAACAAAACTTCGTAAG
AAGATAGCGCGAGAAATAATTGAGGATCGAACTCTATGTCAAAGTAAAAATAGATCTTT
TACTACCCGTCACAAACTTTATGTTCGGCGCAGTACTCACACTGGAACTTACGAACAA
GAATTGCTCTATCAATCACCATCTACTTTAGATATATCTACT------TTTTTCAAATCCAAAA
GTTCAGTATCTTCCTACAAATTAGTGAATATGGAAAATAGAAAAACTTTTTCTTGGTTAA
AGGAACAGATGATTCGATCGATTTCTGTATCGATCATGATATACGTAATAACTCGCACAT
CTATTTCAAACGCATATCCCATTTTTGCGCAGCAGGGTTATGAAAACCCGCGGGAAGCA
ACTGGACGAATTGTATGTGCCAATTGCCATTTAGCTAATAAGCCTGTAGATATTGAAGTT
CCCCAAGCAGTGCTTCCTGATACTGTATTTGAAGCAGTTCTTCGAATTCCTTATGATATG
CAGCTGAAACAAGTTCTTGCTAATGGTAAAAAGGGAGGGTTGAATGTGGGTGCTGTTC
TTATTTTGCCCGAGGGATTCGAATTAGCGCCGCCCGACCGTATTTCTCCTGAGTTGAAA
GAAAAGATAGGAAATCTCTCTTTTCAGAGTTATCGTCCCAATAAAAAAAATATTCTTGT
GATAGGCCCAGTTCCCGGTAAGAAATATAGTGAAATTGTCTTTCCCATTCTTTCCCCCGA
TCCCGCTACAAAAAAAGACGTTCATTTCTTAAAATATCCCATATATGTAGGGGGAAACC
GAGGAAGGGGACAGATCTATCCTGATGGTACCAAGAGTAACAATACGGTCTATAATGCT
ACGTCAACAGGTATAGTAAAAAAAATACTACGTAAAGAAAAGGGGGGATATGAAATAT
CCATAGTGGATGCGTCGGATGGACGCCAAGTGATTGATATTATACCTCCCGGGCCAGAA
CTTCTTGTTTCAGAGGGGGAATCAATCAAGCTTGATCAACCATTAACAAGCAATCCTAA
TGTAGGAGGGTTTGGTCAGGGGGATGCAGAAATAGTGCTTCAGGATCCATTGCGCGTC
CAAGGCCTTTTGTTCTTCTTCGCATCTGTTATTTTGGCACAAGTTTTTTTGGTTCTCAAA
AAGAAACAGTTTGAAAAGGTTCAATTGTACGAAATGAATTTCATGAGTAAAGTATATGA
TTGGTTTGAAGAACGTCTTGAGATTCAGGCAATTGCGGATGATATAACTAGTAAATATG
TTCCTCCTCATGTCAACATATTTTATTGTTTAGGGGGAATTACACTTACTTGTTTTCTAGT
ACAAGTCGCTACCGGTTTTGCTATGACTTTTTACTACCGACCCACCGTTACAGAGGCTT
TTTCCTCCGTTCAATATATAATGACGGAGGCCAACTTTGGTTGGTTAATCCGATCGGTTC
ATCGATGGTCAGCAAGTATGATGGTTCTAATGATGATCCTGCACGTATTTCGTGTGTATC
TCACAGGTGGATTTAAAAAACCCCGTGAATTAACTTGGGTCACAGGCGTGGTTTTGGC
TGTATTGACTGCATCATTTGGTGTAACTGGTTATTCTTTGCCTTGGGATCAAATTGGTTAT
TGGGCAGTCAAAATTGTTACAGGTGTACCCGAAGCTATTCCGGTAATAGGGTCCCCTTT
AGTGGAATTATTACGTGGAAGTGCTAGTGTGGGCCAATCCACTTTGACTCGTTTTTATA
GTTTACATACCTTTGTACTACCTCTGCTTACTGCCGTATTTATGTTAATGCACTTTCCAAT
GATACGTAAGCAAGGTATTTCGGGTCCTTTAATGGGAGTAACAAAGAAACCTGACTTA
AATGATCCTGTATTAAGAGCAAAATTAGCTAAAGGGATGGGACATAATTATTATGGGGA
ACCCGCGTGGCCCAACGATCTTTTATACATTTTTCCAGTAGTAATTCTAGGTACTATTGC
GTGTAATGTGGGTTTAGCGGTTCTAGAGCCGTCAATGATTGGTGAACCGGCGGATCCGT

TTGCAACTCCTTTGGAAATATTACCCGAGTGGTACTTCTTTCCCGTATTTCAAATACTCC
GTACAGTACCCAATAAGTTATTGGGCGTTCTCTTAATGGTTTCTGTGCCAACGGGCTTAT
TGACAGTACCCTTTCTAGAGAATGTCAATAAATTCCAAAATCCATTTCGTCGCCCAGTA
GCTACGACCGTTTTTTTAATCGGTACTGCAGTAGCTCTTTGGTTAGGTATTGGAGCAAC
ATTACCCATTGATAAATCCTTAACTTTAGGTCTTTTTATGATTGAAGTTTTTCTATTTGGA
ATCGTCTTAGGCCTAATTCCTATTACTTTAGCGGGATTATTCGTGACTGCGTATTTGCAAT
ACAGGCGTGGGGATCAGTTGGATCTTATGCTTACTATAACTAGTTATTTCGGTTTTCTAC
TGGCTGCTTTAACTATAACCCCAGCTCTATTTATTGGCTTGAACAAGATACGTCTTATTAT
GGATATAGTAAGTCTCGCTTGGGCTGCTTTAATGGTAGTCTTTACATTTTCTCTTTCGCTA
GTAGTATGGGGGAGGAGTGGACTCATGATTATTCGTCCGTCGGAACCAGAAGTTAAAA
TTGCTGTGGATAGGGATCCTGTAAAAACATCTTTTGAGGAATGGGCCAGACCCGGGCA
TTTCTCAAGAACAATAGCTAAGGGCCCCGACACTACCACTTGGATCTGGAACCTACAT
GCTGATGCTCACGATTTCGATAGTCATACCGGTGATTTGGAGGAGATCTCCCGAAAAGT
CTTTAGTGCTCATTTCGGCCAACTCTCCATTATCTTTCTTTGGTTGAGTGGCATGTACTT
CCACGGTGCCCGTTTTTCCAATTATGAAGCATGGCTAAGCGATCCTACTCACATTGGAC
CCAGTGCTCAGGTAGTTTGGCCAATAGTAGGACAAGAAATTTTGAATGGTGATGTAGG
CGGGGGTTTCCGAGGAATCCAAATAACCTCTGGTTTTTTTCAGATTTGGCGAGCATCCG
GAATAACTAGTGAATTACAACTCTATTGTACCGCAATTGGTGCATTGATTTTTGCATCGT
TAATGCTTTTTGCCGGTTGGTTCCATTATCACAAAGCCGCTCCCAAATTGGCCTGGTTC
CAAGATGTAGAATCCATGTTGAATCACCACTTAGCGGGGTTATTAGGACTTGGGTCTCT
TTCTTGGGCGGGACACCAAATCCATGTATCTTTACCGATTAACCAATTTCTCGACGCTG
GGGTTGATCCTAAAGAGATACCACTTCCTCATGAATTTATCTTGAATCGCGACCTTTTGG
CTCAACTTTATCCTAGTTTTGCCGAAGGAGCAACCCCCTTTTTCACCTTGAATTGGTCC
AAATATGCGGAATTTCTTAGTTTTCGCGGAGGACTAGATCCAATAACCGGTGGTCTATG
GTTGAGCGATATTGCACACCATCATTTAGCTATTGCTATTCTTTTCCTGATCGCTGGTCAT
ATGTATAGGACCAATTGGGGTATTGGTCATGGACTTAAAGATATTTTGGAGGCTCATAAG
GGCCCATTTACAGGACAAGGCCATAAAGGTCTCTATGAAATCCTAACAACGTCATGGCA
TGCTCAATTATCTCTTAACCTAGCTATGCTAGGCTCTACAACTATTGTTGTAGCTCATCAT
ATGTACTCTATGCCCCCCTATCCATACCTAGCTACTGACTATGGTACACAACTTTCCTTGT
TCACACACCACATGTGGATTGGCGGATTTCTAATAGTTGGTGCTGCTGCACATGCAGCC
ATTTTTATGGTAAGAGACTATGATCCAACTACTCGATACAACGATCTATTAGATCGCGTC
CTTAGACACCGCGATGCAATCATATCCCACCTTAACTGGGTATGTATATTTCTGGGTTTT
CACAGTTTTGGCTTGTACATTCATAATGATACCATGAGTGCTTTAGGCCGTCCCCAAGAT
ATGTTTTCGGATACCGCCATACAATTACAACCCATCTTTGCTCAATGGATACAAAATATC
CATGCTGGCGCGCCTGGCGTAACAGCTCCTGGTGCAACAACAAGTACCAGCTTAACGT
GGGGAGGCGGCGAGTTAGTAGCTGTAGGCGGCAAAGTAGCTTTGTTACCTATTCCATTA
GGAACCGCAGATTTTTTAGTCCATCACATTCACGCATTTACCATCCATGTGACTGTATTA
ATACTTTTGAAAGGTGTTTTATTTGCTCGCAGTTCCCGTCTGATACCTGATAAAGCAAAT
CTTGGCTTTCGCTTCCCTTGCGACGGACCTGGGCGAGGGGGAACATGTCAAGTATCCG
CCTGGGATCATGTTTTCTTAGGTCTATTCTGGATGTACAATTCTATTTCGGTAGTCATTTT
CCATTTCAGTTGGAAAATGCAGTCGGATGTTTGGGGTACTATAAGTGATCAAGGGATAG
TAACTCATATCACAGGGGGAAACTTTGCGCAGAGTTCCATTACGATTAATGGTTGGCTT
CGAGATTTCTTGTGGGCACAGGCATCCCAAGTAATTCAGTCTTATGGTTCTTCATTATCT
GCATATGGTCTTTTTTTCTTAGGCGCTCATTTTGTCTGGGCTTTCAGTTTAATGTTTTTAT

TCAGCGGCCGTGGTTATTGGCAAGAACTCATTGAATCTATCGTTTGGGCTCATAACAAA
TTAAAAGTTGCTCCTGCTACTCAGCCTAGAGCCTTGAGCATTATACAAGGACGTGCTGT
AGGAGTAACCCATTACCTTCTGGGTGGAATTGCCACAACATGGGCATTCTTCTTAGCGA
GAATTATTGCAGTAGGAATGGAATTAAGATTTCCCAGGTTTAGCCAAGGCTTAGCTCAG
GACCCCACTACTCGTCGTATTTGGTTTGGTATTGCTACCGCACATGATTTCGAAAGTCAT
GATGATATTACTGAGGAACGTCTTTATCAGAACATTTTTGCTTCTCACTTTGGGCAGTTA
GCAATAATCTTTCTATGGACGTCCGGAAATCTGTTTCATGTAGCTTGGCAAGGAAATTTT
GAATCATGGATACAGGATCCTTTACACGTAAGACCTATTGCTCATGCCATTTGGGATCCT
CATTTTGGGCAACCCGCCGTGGAAGCCTTTACTCGAGGAGGTGCTGCCGGTCCAGTGA
ATATCGCTTATTCTGGGGTTTATCAGTGGTGGTATACAATTGGATTGCGCACCAATGAAG
ATCTTTATACTGGAGCTCTTTTTCTATTATTTCTTTCTACGCTATCCTTAATAGGGGGTTG
GTTACATCTACAACCCAAATGGAAGCCAAGCCTTTCGTGGTTCAAAAACGCCGAATCT
CGTCTGAATCATCATTTGTCAGGACTTTTCGGAGTAAGTTCTTTGGCTTGGACAGGACA
TTTAGTTCATGTTGCTATTCCCGGATCCAGGGGGGAGTACGTTCGATGGAATAATTTCTT
AGATGTATTACCCTATCCCCAGGGGTTGGGTCCCCTTCTGACGGGTCAGTGGAATCTTT
ATGCCCAAAATCCTGATTCGAGTAATCATTTATTTGGTACCACTCAAGGAGCGGGAACT
GCCATTCTGACCCTTCTTGGGGGATTCCATCCACAAACACAAAGTTTGTGGCTGACCG
ATATTGCTCATCATCATTTAGCTATTGCATTTATTTTTCTCATTGCCGGTCATATGTATCGA
ACTAACTTCGGAATTGGGCACAGTATCAAAGATCTTTTAGAAGCACATACTCCTCCGGG
GGGTCGATTAGGACGTGGGCATAAAGGCCTTTATGATACAATCAATAATTCGATTCATTT
TCAATTAGGCCTTGCTCTAGCTTCCTTAGGGGTTATTACTTCCTTAGTAGCTCAACATAT
GTACTCTTTACCTGCTTATGCATTCATAGCACAAGACTTTACTACTCAAGCTGCTTTATAT
ACTCATCACCAATACATTGCAGGGTTCATCATGACAGGGGCTTTTGCTCATGGAGCTAT
TTTTTTCATTAGGGATTACAATCCGGAACAGAATGAAGATAATGTATTGGCAAGAATGTT
AGACCATAAGGAAGCTATCATATCTCATTTAAGTTGGGCTAGCCTCTTCCTAGGATTCCA
TACCTTGGGCCTTTATGTTCATAACGACGTTATGCTTGCTTTTGGTACTCCAGAAAAGCA
AATCTTGATCGAACCTATATTTGCCCAATGGATACAATCTGCTCATGGTAAGACGACATA
TGGGTTCGATATACTCTTATCTTCAACGAATGGCCCCGCTTTCAATGCAGGTCGAAACAT
ATGGTTGCCCGGATGGTTGAATGCTGTTAATGAG------AATAGTAATTCGCTTTTCTTAAC
AATAGGACCTGGGGATTTCTTGGTTCATCATGCTATTGCTCTAGGTTTGCATACAACTAC
ATTGATTTTAGTAAAGGGTGCTTTAGATGCACGCGGTTCCAAATTAATGCCGGATAAAA
AGGATTTCGGGTATAGTTTTCCTTGCGACGGCCCAGGGCGCGGCGGTACTTGTGATATT
TCTGCTTGGGACGCGTTTTATTTGGCAGTTTTCTGGATGTTAAATACCATTGGATGGGTT
ACTTTTTATTGGCATTGGAAACACATTACATTATGGCAGGGCAACGTTTCACAATTTAAT
GAATCCTCCACTTATTTGATGGGATGGTTAAGAGATTACCTATGGTTAAACTCTTCACAA
CTTATTAATGGATATAATCCTTTTGGGATGAATAGTTTATCAGTATGGGCTTGGATGTTCT
TATTTGGACATCTTGTTTGGGCTACAGGATTTATGTTCTTAATTTCCTGGCGTGGATATTG
GCAGGAATTAATTGAGACTTTAGCATGGGCTCATGAACGCACACCTTTGGCTAATTTAA
TTCGCTGGAGAGATAAGCCCGTGGCTCTTTCCATTGTGCAAGCAAGATTGGTCGGATTA
GCCCACTTTTCCGTGGGTTATATATTCACTTATGCAGCTTTCTTGATTGCCTCAACATCA
GGCAAGTTTGGTATGTCACATTCTGTAAAAATTTATGATACATGTATAGGATGCACTCAA
TGTGTACGAGCTTGCCCAACAGATGTATTAGAAATGATACCTTGGGATGGATGTAAAGC
CAAGCAAATTGCTTCCGCGCCGAGAACCGAAGATTGTGTGGGTTGTAAGAGATGCGAA
TCCGCCTGCCCAACGGATTTTTTAAGTGTCCGCGTTTATTTAGGGCCTGAAACAACCCG

CAGCATGGCTCTATCTTATATGACAGATCTTAACTTACCCTCTATTTTCGTACCTTTAGTA
GGCTTAGTATTTCCGGCAATTGCAATGACTTCCTTATTTCTTTATGTGCAGAAAAATAAG
ATTGTCATGCGGGATATAAAAACATATCTCTCTGTAGCACCCGTGCTAAGTACTCTATGG
TTTGGGGCTTTAGCAGGTTTATTGATAGAAATCAATCGTTTATTCCCAGATGCTTTGTCA
T T C C C T T T T T T T - - - - -
-???
??
??
??
??
??
??
??
??
??
??
??ATGGG
TTTGCCTTGGTATCGTGTTCATACTGTCGTATTGAATGATCCGGGTCGATTGCTTTCGGT
GCATATAATGCACACAGCTCTAGTTTCTGGTTGGGCTGGCTCGATGGCTTTATACGAATT
AGCAGTTTTTGATCCCTCTGATCCTGTTCTGGATCCAATGTGGAGACAAGGTATGTTCG
TCATCCCCTTCATGACTCGTTTAGGAATAACGAATTCGTGGGGTGGTTGGAGTATTTCA-
--GGAGGAACTGTAACGAATCCGGGTATTTGGAGTTATGAAGGTGTGGCAGGTGCGCAT
ATTGTGTTTTCTGGCTTGTGTTTCTTGGCAGCTATCTGGCATTGGGTATATTGGGACCTA
GAAATATTCTGTGATGAGCGGACGGGAAAACCTTCTTTGGATTTGCCCAAGATCTTTGG
AATTCATTTATTTCTTGCAGGGGTGGCTTGTTTTGGCTTTGGTGCATTTCATGTAACGGG
TTTATATGGCCCTGGGATATGGGTGTCCGATCCTTACGGACTAACTGGAAAAGTACAAG
CTGTAAATCCTGCGTGGGGTGCAGAAGGTTTTGATCCTTTCGTTCCGGGAGGAATAGCT
TCGCATCATATTGCTGCGGGTACTTTGGGCATATTAGCGGGCCTATTCCATCTAAGTGTC
CGTCCGCCTCAACGTCTATACAAAGGGTTACGTATGGGCAATATTGAAACTGTACTTTC
CAGTAGTATCGCTGCTGTTTTTTTTGCTGCTTTCGTAGTTGCCGGAACTATGTGGTATGG
ATCAGCAACTACCCCAATCGAATTATTTGGGCCTACTCGTTATCAGTGGGATCAGGGAT
ACTTTCAGCAAGAAATATATCGAAGAGTTAGCGATGGGTTAGCCGAAAATCTTAGTTTA
TCAGAAGCTTGGTCTAAAATTCCCGAAAAATTAGCCTTTTATGATTATATTGGTAATAAT
CCGGCAAAGGGGGGATTATTCAGAGCAGGCTCAATGGACAATGGGGATGGCATAGCTG
TTGGATGGTTAGGACATCCCGTCTTTAGAGATAAAGAAGGACGCGAGCTTTTTGTACGT
CGTATGCCTACTTTTTTTGAAACATTTCCGGTAGTTTTGGTAGATGAAGAGGGAATTGT
GAGAGCGGACGTTCCTTTTAGAAGAGCAGAATCCAAATATAGTGTTGAACAAGTAGGC
GTAACGGTGGAGTTCTATGGTGGCGAACTTAATGGAGTAAGTTATTCTGATCCTGCTAC
TGTAAAAAAATATGCGCGGCGTGCTCAATTAGGGGAAATTTTTGAATTAGATCGAGCTA
CTTTGAAATCAGATGGTGTTTTTCGCAGCAGTCCAAGGGGTTGGTTCACTTTTGGTCAT
GCTACCTTTGCTTTGCTCTTCTTTTTCGGACACATTTGGCATGGCGCTCGAACCTTGTTC
CGAGATGTTTTTGCTGGTATTGATCCAGACTTGGATGCTCAAGTGGAATTTGGAACATT
CCAAAAAGTTGGAGATCCAACTACAAGGAGACAGGCAGCCATGAAAATCTTATATTCC
CTGAGGAGGTTCTACCACGTGGAAACGCTCTTTAATGGAACTTTCGTTTTAGCTGGTCG

TGACCAAGAAACCACCGGCTTTGCTTGGTGGGCTGGGAATGCCAGACTTATCAATTTG
TCCGGTAAACTACTTGGAGCTCATGTAGCCCATGCCGGATTAATCGTATTCTGGGCCGG
AGCAATGAACCTATTTGAAGTGGCCCATTTCGTACCAGAAAAGCCCATGTATGAACAA
GGGTTGATTTTACTTCCGCACTTAGCTACTCTAGGTTGGGGAGTAGGGCCGGGGGGAG
AAGTTCTAGATACTTTTCCTTACTTTGTATCCGGAGTACTTCACTTAATTTCCTCCGCAG
TCTTAGGCTTCGGTGGCATTTATCACGCGCTTCTGGGACCCGAGACTCTTGAAGAATCT
TTTCCATTCTTTGGTTATGTATGGAAAGATAGAAATAAAATGACTACAATTTTGGGTATT
CACTTAATTTTGTTAGGTCTAGGTGCTTTTCTTCTAGTACTCAAGGCTCTTTATTTTGGC
GGCGTATATGATACCTGGGCCCCCGGGGGGGGGGATGTAAGAAAAATTACCAATTTGA
CCCTTAGCCCCGGTGTTATATTTGGTTATTTACTAAAATCCCCTTTTGGGGGAGAAGGAT
GGATTGTTAGTGTGGATGATTTAGAAGATATAATTGGGGGACATGTATGGTTGGGTTCCA
TTTGTGTACTTGGCGGAATTTGGCATATCTTAACCAAACCCTTTGCATGGGCTCGCCGT
GCATTTGTATGGTCTGGCGAAGCTTACTTGTCTTATAGTTTAGCTGCTTTATCTGTTTTTG
GTTTTATCGCTTGTTGTTTTGTCTGGTTCAATAATACGGCTTATCCAAGTGAGTTTTACG
GGCCCACTGGACCAGAAGCTTCTCAAGCTCAAGCATTTACTTTTCTAGTTAGAGACCA
GCGTCTTGGAGCTAATGTAGGGTCTGCCCAAGGACCGACGGGTTTAGGTAAATATCTAA
TGCGTTCCCCAACGGGAGAGGTTATTTTTGGAGGGGAAACTATGCGTTTTTGGGATCTT
CGTGCTCCGTGGTTAGAACCTCTAAGGGGCCCCAACGGTTTGGACTTGAGTAGGTTGA
AAAAAGACATCCAACCTTGGCAAGAACGACGTTCAGCAGAATATATGACCCATGCTCC
TTTAGGCTCTTTAAATTCCGTGGGTGGCGTAGCTACCGAGATCAATGCAGTTAATTATGT
CTCTCCTAGAAGTTGGTTAGCGACCTCCCATTTTGTTCTAGGATTCTTCTTTTTTGTGGG
CCATTTGTGGCATGCAGGAAGAGCCCGAGCTGCTGCAGCAGGCTTTGAAAAGGGAAT
CGATCGTGATTTGGAACCTGTTCTTTACATGACCCCTCTTAACATGACTATAGCCGTTGG
TAGAGTTACTAAAGAAGAAAACGATCTATTTGATATTATGGACGACTGGTTACGAAGGG
ACCGTTTCGTTTTTGTAGGATGGTCCGGCTTATTGCTCTTTCCTTGTGCTTATTTCGCTTT
AGGGGGTTGGTTTACAGGGACTACTTTTGTAACTTCTTGGTATACCCATGGATTGGCTA
GTTCCTATTTGGAAGGTTGCAATTTCTTAACCGCGGCGGTTTCCACACCTGCCAATAGT
TTAGCACACTCTTTGTTGCTACTATGGGGACCGGAAGCACAGGGGGATTTTACTCGTTG
GTGTCAATTAGGTGGTCTTTGGACTTTTGTCGCTCTCCATGGAGCTTTTGCACTAATAG
GTTTCATGCTACGTCAATTTGAACTTGCTCGGTCTGTTCAATTGCGGCCTTATAATGCAA
TTTCATTCTCTGGTCCAATCGCTGTTTTTGTTTCCGTATTCCTTATTTATCCACTGGGACA
ATCTGGTTGGTTCTTTGCGCCGAGTTTTGGCGTAGCAGCTATATTTCGGTTCATCCTTTT
CTTCCAAGGATTTCATAATTGGACGTTGAACCCATTTCATATGATGGGAGTTGCCGGAG
TATTAGGCGCAGCTCTGCTATGCGCTATCCACGGGGCTACCGTAGAAAACACTCTATTC
GAAGATGGCGATGGTGCAAATACCTTCCGTGCTTTTAACCCAACTCAAGCTGAAGAAA
CTTATTCAATGGTCACTGCTAACCGCTTTTGGTCCCAAATCTTTGGTGTTGCTTTTTCCA
ATAAACGTTGGTTACATTTCTTTATGCTATTTGTACCCGTCACCGGTTTATGGATGAGTG
CTATTGGCGTAGTTGGTCTGGCTCTGAACCTACGTGCCTATGACTTCGTTTCCCAGGAA
ATCCGTGCAGCGGAAGATCCTGAATTTGAGACTTTCTACACCAAAAATATTCTTTTAAA
TGAGGGTATTCGTGCGTGGATGGCAGCTCAGGATCAGCCTCATGAAAATCTTATATTCC
CTGAGGAGGTTCTACCACGTGGAAACGCTCTTATGTCTGGAAGCACGGGAGAACGTTC
TTTTGCTGATATTATTACCAGTATTCGATATTGGGTTATTCATAGCATTACTATACCTTCCC
TATTCATTGCGGGTTGGTTATTTGTCAGTACGGGTTTAGCTTATGACGTGTTTGGAAGTC
CTCGACCAAACGAGTATTTCACGGAAAGCCGACAAGGAATTCCATTAATAACCGACCG

TTTTGATTCTTTAGAACAACTAGATGAATTTAGTAGATCCTTTATGACCATAGATCGAAC
CTATCCTATTTTTACAGTGCGATGGCTGGCTGTTCACGGATTAGCTGTACCCACGGTTTT
TTTCTTGGGATCAATATCAGCAATGCAGTTCATCCAACGAATGGCTACACAAACCGTTG
AAGATAGTTCTAGACCTAAG---CCAAAACGGACTGGCGCAGGTAGTTTATTGAAACCCT
TGAATTCGGAATATGGGAAAGTTGCTCCGGGTTGGGGGACTACTCCTTTTATGGGGGTC
GCAATGGCTTTATTCGCGATATTCCTATCTATAATTTTAGAAATTTATAATTCTTCTGTTTT
ACTGGACGGAATTTTAACGAATATGCTTACTCTCAAACTTTTTGTTTATACAGTAGTGAT
ATTCTTTGTTTCCCTCTTTATCTTTGGATTCTTATCTAATGACCCAGGACGTAATCCTGGG
CGTGAGGAGATGGCCGATACTACTGGAAGAATTCCTCTTTGGCTGATAGGTACTGTAAC
TGGTATTCTTGTGATTGGTTTAATAGGTGTTTTCTTTTACGGTTCGTATTCTGGATTGGGT
TCATCTCTAATGCCTAATATACTTAGTTTAACCTGTATCTGTTTTAATTCTGTTCTTTGTCC
GACTAGCTTTTTCTTCGCCAAATTACCCGAAGCTTATGCCATTTTCAACCCAATCGTGGA
TGTTATGCCTGTCATACCTGTACTCTTTTTTCTATTAGCGTTTGTTTGGCAAGCTGCTGTA
AGTTTTCGAATGACACAATCAAACCCGAATGAACAAAATGTTGAATTGAATCGTACCA
GTCTATACTGGGGTTTATTACTCATTTTTGTACTTGCTGTTTTATTTTCCAATTACTTCTTC
AATATGGAAGTCAATATTCTCGCATTTATTGCTACAGCATTGTTCATTCTAGTTCCTACTG
CCTTTTTACTTATTATTTATGTAAAAACAGCCAGTCAAAATGATATGGAAACAGCAACTT
TAGTCGCCATCTCCATATCTGGTTTACTTGTAAGCTTTACTGGGTATGCCTTATATACCGC
GTTTGGGCAACCCTCTCAACAATTAAGAGATCCATTCGAAGAACACGGGGACATGGAA
GCATTGGTTTATACGTTCCTTTTAGTTTCGACTTTAGGGATAATTTTTTTCGCTATCTTCT
TCCGAGAACCACCTAAGGTTCCGACTAAGAAA------ATGACTATTGCTTTCCAATTAGCT
GTTTTTGCATTAATTGCGACTTCCTCAGTTTTAGTAATTAGTGTACCCCTTGTATTTGCTT
CTCCTGATGGTTGGTCAAATAATAAAAACGTTGTATTTTCCGGTACATCATTATGGATTG
GATTAGTCTTTCTGGTAGCTATTCTGAATTCTCTTATTTCT---------------------ATGTCACCA
CAAACAGAAACTAAAGCAAGTGTTGGATTTAAAGCTGGTGTTAAGGATTATAAATTGA
CTTACTACACCCCGGAGTACGAAACCAAGGATACTGATATCTTGGCAGCATTCCGAGTA
ACTCCTCAGCTCGGGGTTCCGCCTGAAGAAGCAGGAGCTGCAGTAGCTGCGGAATCTT
CTACTGGTACATGGACAACTGTTTGGACTGATGGACTTACCAGTCTTGATCGTTACAAA
GGACGATGCTATCACATCGAGCCCGTTCCTGGGGACCCAGATCAATATATCTGTTATGTA
GCTTATCCATTAGACCTATTTGAAGAGGGTTCTGTTACTAACATGTTTACTTCCATTGTG
GGTAACGTATTTGGTTTCAAAGCCTTACGCGCTCTACGTTTGGAGGATCTACGAATTCC
CCCTGCTTATGTAAAAACTTTCCAAGGTCCGCCTCACGGTATCCAAGTTGAAAGGGATA
AGTTGAACAAGTACGGTCGTCCTTTATTGGGATGTACTATTAAACCAAAATTGGGATTAT
CCGCAAAAAATTACGGTAGAGCGTGTTATGAGTGTCTACGCGGTGGACTTGATTTTACC
AAAGATGATGAAAACGTAAACTCACAACCATTTATGCGCTGGAGAGACCGTTTTGTCT
TTTGTGCCGAAGCAATTTATAAAGCACAAGCCGAAACCGGTGAAATCAAGGGGCATTA
CTTGAATGCGACTGCAGGTACATGCGAAGAAATGATTAAGAGAGCTGTATTTGCAAAG
GAATTAGGGGTTCCTATTGTAATGCATGACTACTTAACAGGAGGATTCACCGCAAATAC
TACTTTGTCTCATTATTGCCGCGACAACGGCCTACTTCTTCACATTCACCGAGCAATGCA
TGCAGTTATTGATAGACAGAAAAATCATGGTATGCATTTCCGTGTATTAGCTAAAGCATT
GCGTATGTCGGGGGGAGATCATATCCACTCCGGTACAGTAGTAGGTAAGTTAGAAGGG
GAACGCGAAATAACTTTAGGTTTTGTTGATTTATTGCGCGATGATTTTATTGAAAAAGAT
CGTTCTCGCGGTATCTTTTTCACTCAGGACTGGGTATCCATGCCAGGTGTTATACCGGTG
GCTTCAGGGGGTATTCATGTTTGGCATATGCCAGCTCTGACCGAAATCTTTGGAGATGA

TTCCGTATTACAATTTGGTGGAGGAACTTTAGGACATCCTTGGGGAAATGCACCTGGTG
CAGCAGCTAATCGTGTGGCTTTAGAAGCCTGTGTACAAGCTCGTAACGAAGGGCGCGA
TCTTGCTCGTGAAGGTAATGAAATTATCAAAGCAGCTTGCAAATGGAGTGCTGAACTA
GCCGCAGCTTGTGAAATATGGAAGGAGATCAAATTTGATTTCAAAGCGATGGATACCTT
A------ACGGCGAAACATTTATACAAAACACCTATCCCGAGCACACGCAAGGGAACCGTA
GACAGGCAAGTGAAATCCAATCCACGAAATAAATTGATCCATGGACGGCACCGTTGTG
GTAAAGGTCGTAATGCCAGAGGAATCATTACCGCAAGGCATAGAGGGGGAGGTCATAA
GCGCCTATACCGTAAAATCGATTTTCGACGGAATCAAAAAGACATATCTGGTAGAATCG
TAACCATAGAATACGACCCTAATCGAAATGCATACATTTGTCTCATACACTATGGGGATG
GTGAGAAGAGATATATTTTACATCCCAGAGGGGCTATAATTGGAGATACTATTGTTTCTG
GTACAAAAGTTCCTATATCAATGGGAAATGCCCTACCTTTGACCGATATGCCCTTAGGC
ACGGCCATACATAACATAGAAATCACACGTGGAAGGGGTGGGCAATTAGCTAGAGCAG
CAGGTGCTGTAGCGAAACTGATTGCAAAAGAGGGTAAATTGGCCACTTTAAGATTACC
ATCTGGGGAGGTCCGTTTGGTATCCCAAAACTGCTTAGCAACAGTCGGACAAGTGGGT
AATGTTGGGGTGAACCAAAAAAGTTTGGGTAGAGCCGGATCTAAGTGTTGGCTAGGTA
AACGCCCCGTAGTAAGAGGGGTAGTTATGAACCCTGTGGACCACCCCCATGGGGGTGG
TGAAGGGAAAGCCCCCATTGGTAGAAAAAAACCCACAACCCCTTGGGGTTATCCTGCG
CTTGGAAGAAGAACTAGGAAAAGGAAAAAATATAGTGATAGTTTTATTCTTCGTCGCC
GTAAG---ATGATTCAACCTCAGACCCTTTTAAATGTAGCAGATAACAGTGGAGCTCGAA
AATTGATGTGTATTCGAGTCATAGGAGCCGCTGGTAATCAGCGATATGCTCGTATTGGTG
ATGTTATTATTGCTGTAATCAAAGACGCAGTGCCCCAAATGCCTCTAGAAAGATCCGAA
GTAATTCGAGCTGTAATTGTACGTACACGTAAAGAATTCAAAGGCGACGACGGTATAAT
AATACGCTATGATGACAATGCAGCAGTTATCATTGATCAAAAAGGAAATCCAAAAGGA
ACTCGAGTTTTTGGCGCGGTTGCCGAAGAATTAAGAGAATTGAATTATACTAAAATAGT
TTCATTAGCTCCTGAAGTATTAATGCTTAGTCCCAAAAGAACTAGATTTCGTAAACAAC
ATAGAGGAAGAATGAATGGAAAATCCTGCCGAGGCAATCATATTTGTTTTGGTAGATAT
GCTCTTCAAGTACTTGAACCCGCTTGGATCACCGCGAGACAGATAGAAGCAGGACGA
AGAGCAATGACACGATATGCACGTCGTGGTGGAAAAATATGGGTGCGTATATTTCCCGA
CAAACCGGTTACAATAAGACCCACAGAAACACGTATGGGCTCGGGAAAGGGATCCCC
CGAATATTGGGTAGCCGTTGTTAAACCAGGTCGAATACTTTATGAAATGAGCGGAGTAT
CCGAAACTGTAGCTAGAGCAGCTATCTCTATAGCTGCCAGTAAAATGCCCATACGAAGT
CAATTTCTTCGATTAGAGATGACCAGAGTTCCGCGAGGATATATAGCTCGGAGACGACG
AACAAAAATGCGTTCATTTGCCTCAAACTTTAGAGGGGCTCATTTAAGACTTAATCGAA
TGATTACTCAACAAGTAAGAAGAGCTTTTGTTTCTTCTCATCGAGATAGAGGCAGGCA
AAAGAGGGATTTTCGTCGTTTGTGGATCACTCGGATAAACGCAGCAACACGGGTATAT--
----GTATTCAATAGTTATAGTAAATTAATACACAATCTGTCCAAGAAGGAATTGATTCTTA
ATCGTAAAATGCTTGCACAAGTAGCTGTATCAAATCCAAATAATCTTTACACGATTTCCA
ATAAAATAAGGACTATCAAT---------------------------AGGATAAAGCGGGTTTTCAAAGCAC
AAAAACGCATACATATGTCTGTTTTCAAAGCACAAAGAGTTCTAGATGAGATTCGCTGG
CGTTACTACGAGGAAACCGTTATGATACTGAATCTCATGCCTTATCGAGCATCGTATCCC
ATCTTAAAGTTGGTTTATTCGGCAGCAGCAAATGCTACTCATTATAGGGATTTCGACAA
AACAAATTTATTCATCACTAAAGCCGAAGTCAGTAGGAGTACTATTATGAAAAAATTCA
GACCTCGAGCTCGAGGACGTAGTTATTCTATAAAAAAAACGATGTGTAATATAACAATT
GTACTAAAT---GTAAAGAAATCT---------------------ATGGATGGAATCAAATACGCAGTATTT

ACAGAAAAGAGTCTTCGTTTATTGGGAAAGAAT---CAATATACTTTTAATGTCGAATCGG
GATTCACTAAGACAGAAATAAAGCATTGGGTCGAACTCTTCTTTGGTGTTAAGGTAGTA
GCTGTGAATAGCCATCGACTACCCGGAAAGGGTAGAAGAATGGGACCTATTCTGGGAC
ATACAATGCATTACAGACGTATGATCATTACCCTTCAACCGGGTTATTCTATTCCACTTCT
AGATAGAGAAACGAACATGGCAGTTCCAAAAAAACGTACTTCGATGTCAAAAAAGCG
TATTCGTAAAAATCTTTGGAAAAAAAAGACTTATTTTTCCATAGTACAATCTTATTCTTT
AGCAAAATCATTTTCTAGCGGCAAACCAAAAGGTTTTTCTGGGCAACAAGCAAACAA
AATGGCCAAGGGCAAAGATGTTAGAATCCGAGTTATTTTGGAATGTATTAGTTGTGTTC
GAAAG------GGT------AAGGAATCGACGGGGATTTCTAGATATAGTACTCAAAAGAATCG
CCACAATACGCCCGGACAATTAGAATTAAGAAAATTTTGTCGTTATTGTCGAAAGCATA
CCACTCATAATGAAATAAAGAAAATGAAAATAAGAGCTTCCGTTCGTAAAATTTGTACA
AAATGTCGACTGATTCGCAGGCGTGGGCGAATTAGAGTCATTTGTTCCAATCCGAAGCA
TAAACAAAGACAGGGGATGGTTCGAGAAGAGATAACAGGATCCACTCAAACACTAGA
GTGGAAGTGTGTTGAATCAAGAGTAGATAGTAAACGTCTTTATTATGGTCGTTTCATTCT
GTCTCCGCTTAGAAAAGGGCAAGCGGATACTGTCGGTATTGCCTTGCGAAGAGCTTTA
CTTGGAGAAATAGAAGGAACATGTATCACACGCGCCAAATTTTGG------AACGTGCCAC
ATGAATATTCCACAATAGTAGGTATTGAAGAATCCATACAAGAAATTTTACTAAATTTGA
AAGAAATTGTATTGAGAAGTAATCTCTATGGAGTTAGAGACGCATCAATTTGCGTCAAA
GGTCCTAGATACATAACCGCTCAAGATATAATCTTACCGCCTTCCGTAGAAATCGTTGAT
ACGACACAACCTATAGCTAACTTGAGAGAGCCCGTTGATTTCTGTATTGAGTTACAGAT
CAAGAGAGATCGCGGATATCATACGGAACTCAGAAAGAACTCTCAAGATGGAAGTTAT-
-----CCTATAGATGCTGTATTCATGCCTGTTCGAAATGTGAATTATAGTATTTTTTCTTGTG
GGAATGGAAATGAAAAACACGAGATACTTTTTTTAGAAATATGGACGAATGGAAGTTT
AACTCCTAAAGAAGCGCTTTATGAAGCTTCTCGTAATTTGATTGATTTATTTCTTCCTTTT
ATACACACAGAGGAAGAG------GGCACTAGTTTCGAAGAAAATAAA---AACAGGTTAAC
TCCACCCCTTTTAACCTTTCAAAAAAGATTCACT---AATCTAAAGAAAAACAAAAAA---
GGAATTCCATTGAATTGTATTTTTATTGATCAATTAGAATTGCCTTCTAGAACGTATAATT
GTCTCAAAAGGGCCAATATACATACACTATTGGACCTTTTGAGTAAGACTGAAGAAGAT
CTTATGCGAATTAATAGTTTTCGTATGGAAGATGGAAAACTGATATGGGACACTCTAGA
GAAGCAT------CTCCCAATTGATTTACCTAAGAACAAGATGCTCCGGAATGGAAAT---GA
GGGAATGTCCACAATACCCGGATTTAGTCAGATCCAATTCGAGGGATTTTGTAGATTCA
TTAATCAAGGCTTGGCGGAAGAACTTGAGAAGTTTCCCACAATTAAAGATCCAGATCA
CGAAATTGCATTTCAATTATTTGCGAAAGGATATCAATTGCTAGAACCCTCGATAAAAG
AAAGGAATGCTGTGTATGAATCACTCACCTATTCTTCCGAATTATATGTATCCGCGAGAT
TAATTTTTGGTTTC------GATGTGCAAAAGCAAACCATTTCTATTGGAAACATTCCTATAA
TGAATTCCTTAGGAACCTTTATAATAAATGGAATATACCGAATTGTGATCAATCAAATATT
GCTAAGTCCTGGTATTTACTACCGCTCGGAATTAGACCATAAGGGAATTTCTATATGCAC
CGGTACTATAATATCAGATTGGGGAGGAAGGTCGGAATTAGCAATTGATAAAAAAGAA
AGGATATGGGCTCGCGTGAGTAGAAAACAAAAGATATCTATTTTAGTTCTATCATCAGCT
ATGGGTTCGAATCTAAGAGAAATTTTAGATAATGTTTCCTACCCCGAAATTTTCTTGTCT
TTCCCGAATGCTAAGGAGAAGAAGAGGATTGAGTCAAAAGAAAAAGCTATTTTGGAG
TTTTATCAACAATTTGCTTGTGTAGGTGGGGACCTGGTATTTTCGGAGTCCTTATGCGAG
GAATTACAAAAGAAATTTTTTCAACAAAAATGTGAATTAGGAAGAGTTGGTCGACGAA
ATATGAATCGGAGACTGAATCTTGATATCCCTCAGAACAATACATTCTTGTTACCACGAG

ATGTATTGGCCGCTACGGATCATTTGATTGGAATGAAATTTGGAACGGGTATACTTGAC
GATGACGATATGAATCACTTGAAAAATAAACGTATTCGTTCGGTTGCAGATCTGTTACA
AGATCAATTCGGACTGGCTCTTGGCCGTTTACAACATGCGGTTCAAAAAACTATCCGTA
GAGTATTCATACGTCAATCGAAA------CCGACTCCACAAACTTTGGTAACTCCAACTTCA
ACTTCGATTTTATTAATCACTACTTATGAGACCTTTTTTGGCACATACCCCTTAGCTCAA
GTTTTTGATCAAACCAATCCATTGACACAAACGGTTCATGGGCGAAAAGTGAGTTGTT
TGGGTCCTGGAGGATTGACGGGGAGAACTGCAAGTTTTCGTAGCCGAGATATTCATCC
GAGTCACTATGGGCGTATTTGTCCAATTGACACGTCCGAAGGAATCAATGTTGGACTTA
CCGGATCGTTAGCTATTCATGCGAGAATTGATCACTGGTGGGGATCTATAGAGAGTCCG
TTTTATGAAATATCTGAGAAAGCAAAAGAAAAAAAAGAG------AGACAGGTAGTTTATT
TATCACCAAATAGAGATGAATATTATATGATAGCAGCAGGAAATTCTTTGTCCTTGAATC
AGGGTATTCAGGAAGAACAGGTTGTTCCAGCTAGATACCGTCAAGAATTCCTGACTATT
GCATGGGAACAGATTCATGTTAGAAGTATTTTTCCTTTCCAATATTTTTCTATTGGAGGT
TCTCTCATTCCTTTTATTGAGCATAATGATGCGAATCGGGCTTTAATGAGTTCTAATATGC
AGCGCCAAGCAGTTCCACTTTCTCGGTCCGAGAAGTGCATTGTTGGAACTGGATTGGA
ACGACAAACAGCTCTAGATTCGAGGGTTTCCGTTATAGCCGAACGCGAGGGAAAGATC
ATTTCTAGTGATAGTCACAAGATCCTTTTATCAAGTAGTGGGAAGACCATAAGTATTCCT
TTAGTTGCCCATCGACGCTCTAACAAAAATACTTGTATGCACCAAAAACCTCGGGTTCC
GCGGGGTAAATCCATTAAAAAAGGGCAAATTTTAGCGGAGGGAGCAGCTACAGTTGGC
GGGGAACTTGCTTTAGGAAAAAACGTTTTAGTAGCTTATATGCCCTGGGAGGGTTACAA
TTTTGAAGACGCAGTACTAATTAGTGAACGTTTGGTATATGAGGATATTTATACTTCTTTT
CACATCCGAAAATATGAAATTCAGACGGATACGACAAGCCAAGGCTCCGCTGAAAAAA
TCACTAAACAAATACCACATCTAGAAGAACATTTACTCCGCAATTTGGACAGAAATGG
AGTTGTGAGGTTGGGATCCTGGGTAGAAACTGGCGATATTTTAGTAGGTAAATTAACGC
CTCAGATAGCGAGCGAA---TCGTCGTATATCGCGGAAGCTGGATTATTACGGGCTATTTT
TGGTCTTGAGGTATCCACTTCAAAAGAAACTTCTCTCAAACTACCTATAGGTGGAAGA
GGGCGCGTTATCGATGTGAAATGGATCCAGAGG---------------------GACCCCATAATGGTT
CGTGTATATATTTTACAGAAACGTGAAATCAAAGTTGGGGATAAAGTAGCCGGAAGAC
ACGGGAATAAGGGGATCATTTCCAAAATTTTGCCTAGGCAAGATATGCCCTATTTGCAA
GATGGAACACCTGTTGATATGGTCTTCAATCCCTTAGGAGTACCCTCACGAATGAATGT
GGGACAAATATTTGAAAGCTCGCTCGGATTAGCAGGGGATCTGCTAAAGAAACATTATA
GAATAGCACCCTTTGATGAGAGATATGAGCAAGAGGCTTCAAGAAAACTTGTGTTTTC
GGAATTATATGAAGCCAGTAAACAAACAAAAAATCCATGGGTATTTGAACCCGAGTAC
CCGGGAAAAAGCAGAATATTTGATGGAAGAACAGGAGATCCCTTCGAACAGCCTGTTC
TAATAGGGAAGTCCTATATCTTAAAATTAATTCATCAAGTTGATGAGAAAATTCATGGAC
GTTCTACTGGGCCCTACTCACTTGTTACCCAACAACCCGTTAGAGGAAGAGCCAAGCA
AGGGGGACAACGAATAGGAGAAATGGAAGTTTGGGCTTTAGAAGGATTTGGTGTTGCT
CATATTTTACAAGAGATACTTACTTATAAATCTGATCATCTTATAGCTCGCCAAGAAATAC
TTAATGCTACGATCTGGGGAAAAAGAGTGCCTAATCACGAGGATCCGCCAGAATCTTTT
CGAGTGCTCGTTCGAGAACTACGATCTTTGGCTCTAGAACTGAACCATTTCCTTGTATC
TGAGAAGAACTTCCGGGTTAATAGGGAGGATGTT---------------------ATGATTGACCAATAT
AAACATAAACAACTTCAAATTGGACTCGTTTCCCCTCAACAAATAAAGGCTTGGGCTA
AAAAAATCCTACCTAATGGGGAAGTCGTTGGCGAAGTCACAAGGCCCTCCACTTTTCA
TTATAAAACCGATAAACCAGAAAAAGATGGATTGTTTTGCGAAAGAATCTTTGGACCCA

TAAAAAGCGGAATTTGTGCTTGTGGAAACTCTCGAGCGAGCGTAGCTGAAAACGAAG
ACGAAAGATTTTGCCAAAAATGCGGGGTAGAATTTGTTGATTCTCGGATACGAAGATAT
CAAATGGGATACATCAAACTAGCATGTCCAGTGACTCATGTGTGGTATTTGAAAGGTCT
TCCTAGTTATATTGCGAATCTTTTAGATAAACCCCTTAAGAAATTGGAGGGCCTAGTATA
TGGTGAT------TTCTCTTTTGCTAGGCCC---AGCGCTAAAAAACCTACTTTCTTACGATTA
CGGGGTTTATTCGAAGATGAAATTTCATCCTGTAACCACAGTATTTCTCCCTTTTTTTCT
ACCCCAGGCTTTGCAACATTTCGAAATCGGGAAATTGCAACAGGAGCAGGTGCTATTA
GAGAACAATTAGCGGATTTAGATTTGCGAATTATTATAGAGAATTCTTTGGTTGAATGGA
AGGAATTAGAAGACGAG---GGGTATAGTGGAGATGAATGGGAAGATAGAAAAAGACG
AATAAGAAAAGTTTTTTTGATTAGACGCATGCAATTGGCGAAACATTTTATTCAAACAA
ATGTAGAACCAGAATGGATGGTTTTGTGCTTATTACCGGTTCTTCCTCCCGAATTGAGA
CCCATTGTTTATAGATCTGGGGATAAAGTAGTGACTTCAGATATTAATGAACTTTATAAG
AGAGTTATCCGTCGGAACAACAACCTTGCCTATCTATTAAAAAGAAGTGAATTAGCACC
AGCAGATTTAGTAATGTGCCAGGAAAAATTGGTACAAGAAGCCGTGGATACACTTCTT
GATAGTGGGTCCCGCGGGCAACCGATGAGGGATGGTCACAATAAAGTATACAAATCAC
TTTCAGATGTAATTGAGGGTAAAGAGGGGAGGTTTCGCGAGACTCTGCTTGGGAAACG
GGTCGATTACTCGGGGCGTTCTGTCATTGTTGTGGGTCCTTCGCTTTCATTACATCAATG
TGGATTACCTCTAGAGATAGCAATAAAGCTTTTTCAGCTATTTGTAATTCGCGATTTAAT
CACGAAACGTGCTACTTCTAATGTCAGGATTGCTAAAAGGAAAATTTGGGAAAAGGAA
CCCATTGTATGGGAAATACTTCAAGAAGTTATGCGGGGACATCCTGTACTGTTGAACAG
AGCACCTACCCTGCATAGATTAGGCATACAGGCTTTCCAACCCACTTTAGTAGAGGGAC
GTACTATTTGTTTACATCCATTAGTGTGTAAGGGTTTCAATGCGGACTTTGATGGGGATC
AAATGGCTGTTCACCTACCTTTATCCTTGGAAGCTCAGGCGGAAGCCCGTTTACTTATG
TTTTCTCATATGAATCTCCTGTCTCCCGCTATTGGAGATCCTATTTGCGTGCCAACCCAA
GACATGCTTATCGGACTTTATGTATTAACGATTGGAAACCGTCGAGGTATTTGTGCAAAT
AGATATAATAGTTGCGGAAAC---------------TCTCCAAATAAAAAAATAAACTACAATAATT
ATAAGTATACGAAAGATAAAGAACCCCATTTTTCTAGTTCCTATGATGCACTGGGAGCTT
ATAGACAGAAACGAATCGGTTTAAACAGTCCCTTGTGGCTCCGATGGAAACTAGATCA
ACGC---ATCGTTGGGTCA------AGAGAAGTTCCGATTGAAGTTCAATATGAATCTTTTGG
GACTTATCATGAGATTTATGCCCACTATCTAGTAGTGGGAAATAGAAAAAAAGAAATCC
GTTCTATATACATTCGAACCACTCTTGGTCATATTTCTTTTTATAGAGAAATAGAGGAAG
CCGTACAAGGATTTAGTCGGGCCTATTCATACACTATC------------------------------------ATGG
CAGAACGGGCCAACCTGGTCTTTCATAATAAAGAGATAGACGGAACTGCTATCAAACG
GCTTATTAGCAGATTAATAGATCATTTCGGAATGGGATATACATCCCATATACTGGATCAA
ATAAAGACTCTGGGCTTCCATCAAGCCACTACTACATCGATTTCTTTAGGAATCGAGGA
TCTTTTAACAATACCCTCTAAAGGATGGTTAGTCCAAGACGCGGAACAACAGAGTTTT
CTTTTGGAGAAACACTATTATTATGGAGCTGTACACGCGGTAGAAAAATTACGCCAATC
CGTTGAGATATGGTATGCTACAAGTGAATATTTGAAACAAGAAATGAATTCGAATTTTC
GAATAACGGATCCTTCTAATCCAGTCTATCTAATGTCTTTTTCAGGAGCCAGAGGAAAT
GCATCTCAGGTACACCAATTAGTAGGTATGAGAGGGTTAATGGCGGATCCTCAAGGAC
AAATGATTGATTTACCTATTCAAAGCAATTTACGCGAGGGACTTTCTTTGACAGAATATA
TAATTTCCTGCTACGGAGCCCGAAAAGGGGTTGTAGATACTGCTGTACGAACAGCGGA
TGCTGGCTATCTTACACGTAGACTTGTTGAAGTAGTTCAACATATTATTGTGCGTAGAAG
AGATTGTGGTACTATCCAAGGTATTTCTGTGAGTCCTCAAAATGGGATGACGGAA------A

AACTTTTTGTCCAAACACTAATTGGTCGTGTATTAGCAGACGATATATATATTGGTTCAC
GATGCATTGCTTCTCGAAATCAAGATATTGGAATTGGATTAGTCAATCGATTCATAACTG
CCTTTCGAGCACAACCGTTTCGAGCACAACCAATATATATTAGAACCCCCTTTACTTGC
CGGAGCACATCTTGGATCTGTCAATTATGTTATGGGCGGAGTCCCACTCATGGGGATCT
GGTCGAATTGGGGGAAGCTGTAGGTATTATTGCGGGTCAATCAATTGGGGAGCCGGGG
ACTCAACTAACATTAAGAACTTTTCATACTGGTGGAGTATTCACAGGGGGTACTGCCGA
CCTTATACGATCCCCCTCGAATGGAAAAATCCAATTCAATGAGGATTTGGTTCACCCCA
CACGTACCCGTCATGGGCAGCCTGCTTTTCTATGCTATATAGACTTGCATGTAACTATTC
AGAGTCAGGATATTCTACATAGTGTGAATATTCCGTTAAAAAGCTTGATTCTAGTGCAA
AATGATCAATATGTAGAATCTGAACAAGTAATTGCGGAGATTCGTGCCGGAATGTCCAC
TTTG------TTTAAAGAAAAGGTACAAAAGCATATTTATTCCGAATCAGACGGGGAA---AT
GCACTGGAGTACTGATGTTTACCATGCGCCCGAATATCAATATGGTAATCTTCGTCGATT
ACCAAAAACAAGCCATTTATGGATATTGTCAGTAAGTATGTGCAGA------TCCAGTATAG
CATCTTTTTCACTGCACAAGGATCAAGATCAAATGAATACTTATTCTTTTTCTGTTGATG
GAAGA------TATATCTTTGACTTCTCA------ATGGCTAAT---GATCAAGTAAGCCATAGACT
GTTGGATACTTTTGGT------------GATAGGGAAATTCTTGATTATTTA---ACGCCAGATCGA
ATCGTGTCCAACGGTCATTGGAATTGTTTCTATCCTTCT---ATTCTTCAAGATAAT------TC
GGATTTGTTGGCGAAAAAGCGAAGAAATAGGTTCGTCGTTCCATTACAATATCATCAAG
AACAAGAAAAAGAGCGAATATCCTGTTTGGGTATTTCAATGGAAATACCCTTTATG------
GGTGTTTTACGTAGAAATACTATTTTTGCTTATTTTGACGATCCACGATACAGAAAAGAT-
-----GGAATTGTTAAATTTAGAAGCCCGGAGGAAAATTCTATCTTAAAA------AAAGAGGG
TTTTATTGAGCATCGAGGA---ACAAAAGAATTTAGTCTAAAATAC---GAAGTAGATCGGT
TTTTTTTCATTCTTCAAGAACTGCATATCTTGCCGAGATCCTCATCCCTAAAGGTACTTG
ACAATAGTATTATTGGAGTCGATACACAACTCACAAAAAATACAAGAAGTCGACTAGG
TGGATTGGTCCGAGTGAAGAGAAAAAAAAGCCATACAGAACTCAAAATCTTTTCCGG
AGATATTCATTTTCCTGAAGAGGCGGATAAGATATTAGGT------GGCAGTTTGATACCACC
AGAAAGAGAAAAAAAA------GATTCTAAGGAA---TCAAAA---AAAAGGAAAAATTGGG
TTTATGTTCAACGGAAAAAAATTCTCAAAAGCAAGGAAAAG------TATTTTGTTTCGGTT
CGACCTGCAGTCGCATATGAAATGGACGAAGGGAGAAATTTAGCAACACTTTTCCCGC
AGGATCTCCTGCAAGAAGAGGATAATCTCCAACTTCGACTTGTCAATTTTATTTCTCAT
GAAAATAGCAAGTTAACTCAAAGAATTTATCACACGAAT---CAATTCGTTCGAACTTGC
TTAGTAGTGAATTGGGAACAAGAAGAAAAAGAGGGGGCTCGT--------------------GCTTC
TCTTGTTGAGGTAAGAACAAACGATCTAATTCGCGATTTCCTAAGAATTGAGTTAGTCA
AGTCTACTATTTCGTATACACGAAGAAGGTATGATAGGACAAGTGTAGGACTGATTCCC
AAT---------TTAGATCGCAACAATACCAATTCCTTTTATTCCAAGGCGAAGATTCAATCA---
------------CTTAGCCAACATCAAGAAGTTATTGGT------------ACCTTGTTGAATCGAAATAA
A---------GAATATCCATCTTTGATGATTTTGTTGGCATCCAACTGTTCTCGAATTGGT------T
TATTCAAGAATTCGAAATATCCC---------AATGCGGTAAAAGAATCGAATCCTAGAATTCC
TATT---GATATTTTTGGGCTCTTAGGCGTTATTGTACCT---AGTATATCGAATTTTTCTTCAT
CT---TACTATTTATTAACG---------CATAATCAGATCTTGTTAAAA---AAATACTTGTTCCTT
GAC---------AATTTGAAACAAACCTTCCAAGTACTTCAAGGACTTAAATACTCTTTAATA
GACGAAAATAAAAGG------ATTTCGAATTTTGAC------AGTAACATCATGTTGGAGCCATT
CCATTTGAATTGGCACTTTCTCCATCATGACTCATGGGAAGAGACATTGGCAATAATTC
AC---CTTGGACAATTTATTTGTGAAAATTTATGTCTATTTAAATCACACATA------------AAA

AAATCTGGTCAAATTTTCATTGTTAAT---ATGGACTCCTTTGTTCTAAGAGCAGCTAAGC
CTTATTTGGCCACTATAGGAGCAACTGTTCATGGTCATTATGGAAAAATCCTTTACAAAG
GAGATAGGTTAGTTACGTTTATATATGAAAAATCGAGATCCAGTGATATAACGCAAGGTC
TTCCAAAAGTAGAACAAATCTTCGAAGCGCGTTCAATTGATTCACTATCGCCGAATCTC
GAAAGGAGAATTGAGGATTGGAATGAACGTATACCAAGAATTCTTGGGGTTCCCTGGG
GATTCTTGATTGGAGCTGAGCTAACCATAGCCCAAAGTCGTATCTCTTTGGTTAATAAGA
TCCAAAAGGTTTATCGATCCCAAGGGGTACAGATCCATAATAGACATATAGAGATTATTA
TACGCCAAGTAACATCAAAAGTAAGGGTTTCCGAA---------GATGGAATGTCTAATGTTT
TTTTACCTGGGGAATTAATAGGACTATTGCGAGCGGAACGAGCAGGGCGGGCTTTAGA
TGAATCGATCTATTATCGGGCAATCTTATTGGGAATAACAAGGGCTTCCCTGAATACCCA
AAGTTTCATATCTGAAGCAAGTTTTCAAGAAACTGCTCGAGTTTTAGCAAAAGCTGCC
CTACGAGGTCGTATTGATTGGTTGAAAGGCCTGAAAGAAAACGTAGTTCTGGGGGGGA
TTATACCTGTTGGTACCGGATTCCAAAAATTTGTGCATCGTTCCCCA------CAAGACAAG
AACCTTTAT------TTCGAAATTCAAAAAAAAAATCTATTCGCGTCGGAAATG------AGAGA
TATTTTGTTTCTCCATACAGAATTAGTTTCTTCTGATTCTGACGTAACAAAC---------TCAG
AGACCCCGTTTACCCCTATTTATACGATT------------------ATGACAAGAAGATATTGGAACA
TCAATTTGAAAGAGATGATAGAAGCGGGAGTTCATTTTGGTCATGGTATTAAGAAATGG
AATCCCAAAATGGCCCCTTACATCTCGGCAAAGCGTAAAGGTACTCATATTACAAATCT
TGCTAGAACAGCTCGTTTTTTATCAGAAGCTTGTGATTTAGTTTTTGATGCAGCAAGTC
AGGGAAAAAGCTTCTTAATTGTTGGTACCAAAAAAAGAGCAGCGGATTTAGTAGCATC
AGCTGCAATAAGGTCTCGTTGTCATTATGTTAATAAAAAGTGGTTCAGCGGTATGTTAA
CGAATTGGTCGATTACCAAAACTAGACTTTCTCAATTTAGAGACTTAAGAGCAGAAGA
AAAAATGGGAAAATTCCACCATCTCCCAAAAAGAGATGCGGCAATCTTAAAGAGAAAA
TTATCTACCTTGCAAAGATATCTCGGCGGGATCAAATATATGACGAGGTTGCCTGACATT
GTGATCGTCCTTGATCAGCAAAAAGAGTATATAGCTCTTCGGGAATGTGCCATTTTGGG
GATTCCTACTATTTCTTTAGTCGATACAAATTGTGACCCAGATCTTGCGAATATATCGATT
CCTGCCAACGATGACACTATGACTTCAATTCGATTAATTCTTAACAAATTAGTATTTGCA
ATTTCTGAGGGCCGTTCTCTC---TATATAAGAAATCGTATGGGACAAAAAATAAATCCAC
TCGGTTTCAGACTTGGTACAACCCAAAATCACCATTCCTTTTGGTTCGCACAACCAAA
AAATTATTCTGAAGGTCTACAGGAAGATAAAAAAATACGGAATTGTATCAAGAACTATA
TACAAAAGAATAGGAAAAAAGGCTCGAATAAAATGGAATCAATTACACATATAGAAAT
TCAAAAAGAAATCGATACAATCCACGTCATAATCCATATTGGATTCCCAAAT---TTATTAA
AGAAAAAGGGA---------------GCAATCGAAGAATTAGAGAAAGATCTCCAAAAGGAAGT
TAATTCTGTAAACCAGAGACTTAATATTGCTATCGAAAAGGTTAAAGAACCTTATAGAC
AACCTAATATTCTTGCGGAATATATAGCTTTCCAATTAAAAAATAGAGTTTCATTCCGAA
AAGCAATGAAAAAAGCTATTGAATTAACTAAAAAAGCGGATATAAAGGGAATAAAAAT
CCAAATTGCGGGTCGTCTAGCAGGAAAAGAAATTGCACGTGCCGAATGCATCAAAAA
GGGTAGACTACCCCTCCAAACAATTCGCGCTAAAATTGATTATTGCTGCTATCCAATTCG
AACTATCTATGGAGTATTAGGTGTCAAAATTTGGATATTCGTAGAAGAAGAAATGTCCC
GTTATCGAGGGCCTCGTTTAAAAAAAAATACGCCGTCTGGGAGCTTTACCAGGACTCACT
AGAAAAACGCCTAAATCCGGAAGTAATCAGAAAAAGAAATTCCATTCTGGGAAA---AA
AGAGCAATATCGTATTCGTCTTCAAGAAAAACAGAAATTGCGTTTTCATTATGGTCTGA
CAGAACGACAATTACTTAGATATGTACATATCGCTGGAAAAGCAAAAAGATCCACAGG
TCAGGTTTTACTACAATTACTTGAAATGCGTTTGGATAATATCCTTTTTCGATTGGGTATG

GCTTCAACCATTCCTGGGGCCCGACAATTAGTTAACCATAGACATATTTTAGTTAATGGT
CGTATAGTTGATATACCAAGCTTTCGTTGCAAACCCCGAGATATTATTACTACGAAGGAT
AACCAACGATCAAAACGTTTGGTTCAAAATTATATTGCTTCATCCGATCCGAAA------TT
ACCAAAGCATTTGACGGTTGACACA------TTGCAATATAAAGGACTAGTAAAAAAAATT
CTAGATAGGAAGTGGGTCGGTCTCAAAATAAATGAGTTGTTAGTTGTAGAATATTATTCT
CGCCAGACTATGTCACGTCGAGGTACTGCAGAAAAAAGAACCGCAAAATCCGATCCAA
TTTTTCGTAATCGATTAGTTAACATGGTGGTTAACCGTATTATGAAAGACGGAAAAAAA
TCATTGGCTTATCAAATTCTCTATCGAGCCGTGAAAAAGATTCAACAAAAGACAGAAA
CAAATCCACTATTGGTTTTACGTCAAGCAATACGTAGAGTAACTCCCAATATAGGAGTA
AAAACAAGACGTAAAAAAGGATCGACGCGGAAAGTTCCGATTGAAATAGGATCTAAA
CAAGGAAGAGCACTTGCCATTCGTTGGTTATTAGAAGCATCCCAAAAGCGTCCGGGTC
GAAATATGGCTTTCAAATTAAGTTCCGAATTAGTAGATGCTGCCAAAGGGAGTGGGGG
TGCCATACGCAAAAAGGAAGCGACTCATAGAATGGCAGAGGCAAATAGAGCTCTTGCA
CATTTTCGTATGGGCAAGGACACTATTGCTGATTTACTAACCTCTATAAGAAACGCAGA
CATGAATAAAAAAGGAACTGTTCGAGTAGTATCCACAAATATTACCGAAAACATTGTTA
AAATACTTCTACGAGAGGGTTTTATTGAAAGTGTTCGGAAACATCAGGAAAGTAACAG
ATATTTCTTGGTTTCAACTTTGCGACATCAAAGGAGAAAGACTAGAAAGGGAATATATA
GAACT---------AGAACCTTTTTAAAGCGTATCAGCCGACCTGGCTTACGAATTTATGCCA
ACTATCAAGGAATTCCTAAGGTTTTGGGCGGAATGGGAATTGCTATTCTTTCTACTTCTC
GAGGTATAATGACAGATCGAGAAGCTCGACTAAACAGAATTGGGGGAGAAGTTTTATG
TTATATATGGATGACAAAAGCTATACCAAAAATAGGTTCACGTAAGAAAGTGCGTATTG
GTTTGCGTAAGAGTGCACGTAGAATAACAAAAGGGGTTATTCATGTTCAAGCCAGTTT
CAACAATACCATTATAACCGTTACAGACCCACAAGGTCGGGTCGTTTTCTGGTCCTCCG
CAGGTACTTGTGGATTCAAAAGCTCAAGAAAAGCATCACCCTATGCTGGTCAAAGAAC
AGCAGTAGATGCTATTCGTACAGTGGGT---------TTGCAACGAGCAGAAGTTATGGTAAA
AGGTGCTGGTAGCGGAAGAGATGCCGCATTACGAGCCATTGCTAAAAGTGGTGTACGG
TTAAGTTGTATACGCGATGTAACACCTATGCCGCATAATGGATGTCGACCTCCTAAAAA
AAGACGTCTGATGCCAACGGTTAAACAACTTATTAGAAATGCAAGACAGCCAATACGA
AATGCTAGAAAATCGGCCGCGCTTAAGGGATGTCCTCAGCGTCGAGGAACATGTGCTA
GGGTGTATACTATCAACCCCAAAAAACCCAACTCTGCCTTACGTAAAGTTGCCAGAGT
ACGATTAACCTCTGGATTTGAAATCACTGCTTATATACCTGGTATTGGCCATAATTTACA
AGAACATTCTGTAGTATTAGTAAGAGGAGGAAGGGTTAAGGATTTACCCGGTGTGAGAT
ATCGCATTATTCGAGGAACCCTAGATGCTGTCGCAGTAAAGAATCGTCAACAAGGGCG
TTCTAAATATGGGGCCAAAAAGCCAAAAATGGCAAAAAAAAAGTTTGATTCAGAGGGA
GAAAAAGCGGCATAAATTAGAACAAAAATATCATTTGATTCGTCGATCCTCAAAAAAA
AAGATAAGAAGCAAAGTTTCCCCTTTGAGTTTGAGCGAAAAAACAAAAATGCAAGAA
AAATTGCAATCCCTACCACGTAATAGTGCACCTACACGCCTTCATCGACGTTGTTTTTA
ACCGGAAGACCTAGAGCTAACTATCGAGACTTTGGGCTATCCGGACACATACTTCGAG
AAATGGTTTATGCATGTTTGTTACCGGGTGCAACAAGATCCAGTTGG-----------------------
---ATGGTTAAAGAAGAAAAACAAGAAAAC------AGGGGTTCTGTTGAATTTCAAGTATT
CAGTTTCACCAATAAGATACGGAGACTTGCTTCACATTTAGAATTACACAAAAAAGATT
TTTCATCGGAAAGAGGTCTACGAAGACTTTTGGGAAAACGTCAACGTTTGCTGGCTTA
TTTGGCAAAGAAAAATAGAGTACGTTATAAGAAATTAATCAGTCAGTTGGATATTCGGG
AGAAG---------------ATGGTAAAACTTCGTTTAAAACGATGTGGTAGAAAGCAACAAGCT

ATCTATCGAATCGTTGCAATTGATGTTCGATCTCGAAGAGAAGGAAGAGATCTTCGAAA
AGTAGGTTTTTATGATCCGATAAAGAATCAAACTTGTTTAAATGTTCCAGCTATTCTCTA
TTTCCTTGAAAAGGGTGCTCAACCTACAAGAACAGTTTATGATATTTTAAGGAAGGCA
GAATTCTTTAAAGATAAAGAAAGAACTTTGAGT------------TTTCATAAATCTAAGCAACC
TTTTCGTAAATTCAAACAACCTTTTCGTAAAAGGCGTTCTCGAATAGGCCCGGGGGATC
GAATTGATTATAGAAACATGAGTTTAATTAATCGATTTATTAGTGAACAAGGAAAAATAT
TATCCAGACGAATAAATAGATTAACCTTGAAACAACAACGATTAATTACTCTTGCTATAA
AACAAGCTCGTATTTTATCTTTCTTACCATTTCGTAACTATGAGAATGAGAAGCAATTTC
AA---GCCCAGGCAATTTCAATAATTACTGGTCCTAGACACAGAAAAAATGTGACACGA
AAA---AAAACGAATCCTTTTGTAGCTCGTCATTTATTGGCAAAAATCGAAAAGGTCAAT
ATGAAGGAGGAGAAAGAAATAATAGTAACGTGGTCCCGGGCATCTAGCATTCTACCCG
CAATGGTTGGCCATACAATCGCGATTCATAATGGAAAAGAACATATACCTATTTACATAA
CAAATCCTATGGTAGGTCGCAAATTGGGGGAATTCGTGCCTACTCGGCATTTCACGAGT
T A T A C A A G A A A G G A T A C T A A A T C T C G T C G
T???
??
??
??
??
??
??
??
??
??
??
??
??
??
??
??
??
??
??
??
??
??
??
??
??
??
??
??
??

??
??
??
??
??
??
??
??
??
??
??
??
??
??
??
??
??
??
??
??
??
??
??
??
??
??
??
??
??
??
??
??
??
??
??
??
??
??
??
??
??
??
??
??

???
???
???
???
???
???
???
???
???
???
???
???
???
???
???
???
???
???
???
???
???
???
???
???
???
???
???
???
???
???
???
???
???
???
???
???
???
???
???
???
???
???
???
???

???
???
???
???
???
???
???
???
???
???
???
???
??????????????????????????????????????ATGCCTAGATCCCGT---ATAAATGGAAATTTCATTG
ATAAGACCTCCTCGATTGTAGCCAATATTTTATTGCAAATAATTCCGACAACCTCCGGGG
AAAAAAGGGCATTTACTTATTATAGAGATGGG------ATGTTGGCTCAATCCGAAGGAAAT
TATGCGGAAGCTTTGCAGAATTATTATGAAGCTACGCGACTAGAAATTGATCCCTATGAT
CGAAGTTATATACTATATAACATAGGCCTTATACACACAAGCAATGGAGAGCATACAAA
GGCTTTGGAATATTATTTCCGGGCGCTAGAACGAAACCCCTTCTTACCGCAAGCTTTTA
ATAATATGGCCGTGATCTGTCATTAC---CGAGGAGAACAGGCCATTCTACAGGGCGATTC
GGAAATTGCGGAAGCTTGGTTTGATCAAGCTGCTGAGTATTGGAAACAAGCTATAGCG
CTTACTCCAGGAAATTATATTGAAGCACAGAACTGGTTAAAGATTACGAAGCGCTTTGA
ATTTGAAATGAATTGGCGATCAGAACACATATGGATAGAACTTCTAAAAGGTTCTCGAA
AAAGAGGTAATTTTTTCTGGGCCTGTATTCTTTTTCTAGGTTCATTAGGATTCTTAGCGG
TTGGGGCTTCCAGTTATCTTGGTAAGAATATGATATCTGTACTTCCATCTCAACAAATTC
TTTTTTTTCCACAGGGGGTCGTGATGTCTTTCTACGGAATCGCGGGCCTATTCATTAGCT
CCTATTTGTGGTGCACTATTTTATGGAATGTAGGCAGTGGTTATGACCGATTCGATAGAA
AAGAGGGAATAGTGTGCATTTTTCGTTGGGGATTCCCTGGAATAAAACGTCGCATCTTC
CTTCAATTCCTTGTGCGGGATATCCAATCAATTAGAATTCAGGTTAAAGAGGGTCTTTAT
CCTCGTCGTATCCTTTATATGGAAATCCGGGGCCAGGGGGTCATTCCCTTGACTCGTACT
GATGAGAAGTTTTTTACTCCACGAGAAATTGAACAAAAAGCTGCCGAATTGGCCTATT
TCTTGCGTGTACCAATTGAAGTATTT
>Panax
ATGGAAAGATGGTGGTTCAATTCGATACTGTTTCTAAGTAAATCAACGGGTAGTCTTGG
TCCTATTGAAAATACCAGTGAAAGTGAAGATCCGAATATTAATGATATGAAGAAAAATA
TTCATAGTTACAGTAATGTTGATCATTTATTCGGCGTCAAGGACATTCGGAATTTCATCT
CTGATGAAACTTTTTTAGTTAGGGATAGTAATGGGAACAGTTATTCCATATATTTTGATAT
TGAAAATCATATTTTCGAGATTGACAATGGTCAT------TCTTTTCAGAGTGAACTAGAAA
GTTCTTTTTATAGTTATCAGAATTCTAGTTATCGGAATAATGGATCTAACAGTGACGATCC
CCACTATGATCATTACATGTATGATACTCAATATAGTTGGAATAATAACATTAATAGTTGC
ATTGACAATTATCTTCAG------TCTCAAATCTTTATTGAGACTGACATTGTAAGTGGTAGT
GACAATTACAGTAACAGTTACATTTATAGTTCCATTTGTGGT------GGTTCCGGTATAAGA
ACCAGAACGGATGGTAGTGATTTAACTATAAGAGAAAGTTCTAATGATCTGGATGTAAC
TCAAAAATACAGGCATTTGTGGGTTCAATGTGAAAATTGTTATGGATTAAATTATAAGA
AATCTTTTAAATCAAAAATGAATCTTTGTGAACAATGTGGATATCATTTGAAAATGAGTA

GTTCAGATAGAATCGAACTTTTGATCGATCCGGGTACTTGGGATCCTATGGATGAAGAC
ATGGTTTCTCTGGATCCCATTGAATTTCATTCGGAGGAGGAGCCTTATAAAGATCGTGT
CGATTCTTATCAAAGAAAGACAGGATTAACTGAGGCCGTTCAAACAGGCATAGGCCAA
CTAAACAGTATTCCCGTAGCAATCGGGGTTATGGATTTTCAGTTTATGGGGGGTAGTATG
GGATCCGTGGTCGGGGAGAAAATCACCCGTTTGATTGAGTACGCTACTAAAAAATTTCT
ACCTCTTATTATAGTGTGTGCTTCCGGGGGGGCACGCATGCAAGAAGGAAGTTTGAGC
TTGATGCAAATGGCTAAAATATCTTCTGCTTTATATGATTATCAATCAAATAAAAAGTTAT
TCTATGTACCAATCCTTACATCTCCTACTACAGGTGGGGTGACTGCTAGTTTTGGTATGT
TGGGGGATATCATTATTGCCGAACCCAATGCCTACATTGCATTTGCGGGTAAAAGAGTA
ATTGAACAAACATTGAATAAAACAGTACCTGAAGGTTCACAAGCGGCTGAATATTTATT
CCAGAAGGGCTTATTTGATCTAATCGTACCACGTAATCCTTTAAAAAGCGTTCTGAGTG
AGTTATTTCAGCTCCACGCTTTCTTTCCTTTGAATCAAAAT---------ATGGTAACCATTCG
AGCCGACGAAATTAGTAATATTATCCGTGAACGTATTGAGCAATATAATAGAGAAGTAA
AGATTGTAAATACCGGTACCGTACTGCAAGTAGGCGACGGCATTGCTCGTATTCATGGT
CTTGATGAAGTAATGGCGGGTGAATTAGTAGAATTTGAAGAGGGTACGGTAGGGATTG
CTCTGAATTTGGAATCAAATAATGTCGGTGTTGTATTAATGGGCGATGGTTTGCTGATAC
AAGAAGGAAGTTCTGTAAAAGCAACAGGAAGAATTGCTCAGATACCCGTGAGTGAGG
CCTATTTGGGTCGTGTTGTAAATGCCCTGGCTAAACCTATTGATGGTAGAGGTGAAATTT
CAGCTTCTGAATATCGGTTAATTGAATCTCCCGCTCCAGGTATTATTTCACGACGTTCAG
TATACGAGCCTCTTCAAACGGGGCTTATTGCTATTGATTCTATGATCCCTATAGGACGTG
GTCAGCGAGAATTAATTATTGGGGACAGACAGACCGGTAAAACAGCAGTAGCAACAG
ATACGATTCTCAATCAACAAGGCCAAAATGTAATATGTGTTTATGTAGCTATTGGTCAAA
AAGCATCTTCTGTGGCTCAGGTAGTGACTACTTTCCAGGAAAGGGGGGCGATGGAATA
CACTATTGTGGTAGCTGAAATGGCGGATTCCCCTGCTACATTACAATACCTCGCTCCTTA
TACAGGAGCCGCTCTGGCGGAATATTTTATGTACCGTGAACGACACACTTCAATCATTT
ATGATGATCCCTCCAAACAAGCACAAGCTTATCGACAAATGTCTCTTCTATTACGAAGA
CCGCCCGGTCGCGAAGCTTATCCAGGGGATGTTTTTTATTTGCATTCACGCCTTTTGGA
AAGAGCTGCTAAATTAAGTTCTCGTTTGGGTGAAGGAAGTATGACTGCTTTGCCAATAG
TTGAAACTCAGTCGGGAGATGTTTCGGCTTATATTCCTACTAATGTAATTTCGATTACTG
ATGGACAAATATTCTTATCTGCCGATCTATTCAATGCTGGAATCAGACCTGCTATTAATGT
GGGTATCTCCGTTTCCAGAGTGGGATCTGCAGCTCAAATTAAAGCTATGAAACAAGTC
GCTGGCAAATTAAAATTGGAACTGGCGCAATTCGCAGAATTAGAAGCCTTTGCACAAT
TTGCTTCTGATCTCGATAAAGCTACTCAGAATCAATTGGCAAGAGGTCAACGATTACGT
GAATTGCTCAAACAATCCCAATCAGCCCCTCTCACGGTGGAAGAACAGATAATGACTA
TTTATACCGGAACAAATGGTTATCTTGATTTATTAGAAATTGGACAGGTAAGGAAATTTC
TTGTTGAGTTACGTACTTACCTAAAAACTAATAAACCGAAGTTTCAAGAAATCATATCTT
CTACTAAGACATTCACCGAGGAAGCAGAAGCCCTTTTGAAAGAAGCTATTCAGGAACA
GACGGACCGTTTTATACTTCAGGAACAAGCAATGAGAATAAATCCTACTACTTCAGGTT
CTGGGGTTTCCACGCTTGAAAAAAAAAACCTGGGGAATATCACCCAAATCATTGGTCC
GGTACTGGATATAGCTTTTCCACCGGGCAAGATGCCTAATATTTATAATGCTCTGGTAGT
TAAGGATCGAGATACTGTCGGTCAACAAATTAATGTTACTTGTGAGGTACAGCAATTATT
AGGAAACAATCGAGTTAGGGCTGTAGCTATGAGTGCTACAGACGGTCTAACGAGAGGA
ATGGAAGTGATTGATACGGGAGCTCCTCTAAGTGTTCCGGTCGGTGGAACGACTCTTG
GACGAATTTTCAACGTGCTTGGAGAGCCTGTTGATAATTTAGGGCCTGTAGATACTCGC

ACAACATCTCCTATTCATAGATCTGCGCCCGCCTTTATACAGTTAGATACAAAATTATCTA
TTTTTGAAACCGGAATTAAAGTAGTAGATCTTTTAGCCCCCTACCGCCGTGGAGGAAAA
ATCGGACTATTCGGGGGGGCTGGAGTGGGTAAAACAGTACTCATTATGGAATTGATTAA
CAATATTGCCAAAGCTCATGGGGGTGTATCCGTATTTGGCGGAGTTGGTGAACGTACTC
GTGGAGGAAATGATCTTTACATGGAAATGAAAGAATCTGGGGTGATTAATGAACAAAA
TATTGCAGAATCGAAAGTGGCTCTAGTTTACGGTCAGATGAATGAACCACCGGGAGCT
CGTATGAGAGTTGGTTTGACTGCCCTAACTATGGCTGAATATTTCCGAGATGTTAATGAA
CAAGACGTACTTCTATTTATCGACAATATCTTCCGTTTCGTCCAAGCAGGATCTGAAGTA
TCTGCCTTATTAGGTAGAATGCCTTCCGCTGTGGGTTATCAACCCACCCTTAGTACCGA
AATGGGTACTTTACAGGAAAGAATTACTTCGACCAAAGAAGGGTCCATAACTTCTATTC
AAGCGGTTTATGTACCTGCAGACGATTTGACCGACCCTGCTCCTGCTACGACATTTGCA
CATTTAGATGCTACTACTGTACTATCAAGAGGATTAGCCGCCAAAGGTATCTATCCCGCA
GTAGATCCTTTAGATTCAACATCAACTATGCTCCAACCTCGGATTGTTGGTGAGGAACA
TTATGAAACTGCGCAAAGGGTTAAGCAAACCTTACAACGTTACAAAGAACTTCAGGAC
ATTATAGCTATCCTTGGGTTGGACGAATTATCCGAAGAGGATCGTTTAACCGTAGCAAG
AGCGCGAAAAATTGAGCGTTTCTTATCACAACCCTTTTTCGTAGCAGAAGTTTTTACTG
GTTCTCCAGGGAAATATGTTGGTTTAGCAGAAACAATTAGGGGGTTTCAATTGATCCTT
TCCGGAGAATTAGATGGTCTTCCTGAACAGGCCTTTTATTTGGTAGGTAACATCGACGA
AGCTACCGCGAAGGCTATGAACTTAGAAATGGAGAGCAATTTGAAGAAAATGACCTTA
AATCTTTGTGTACTGACCCCTAATCGAACTGTTTGGGATTCAAAAGTGAACGAAATCAT
TTTATCTACTAATAATGGCCAAATTGGCGTATTACCGGATCACGCCTCTATTGCCACAGC
TGTAGATATAGGTATTTTGAGAATACGCCTTAACGACCAATGGTTAACGATGGCTCTGAT
GGGTGGTTTTGCTAGAATAGGTAATAATGAGATCACTGTTTTAGTAAATGATGCGGAGA
AGAGTAGTGACATCGATTCACAAGAAGCCCAGCAAACGCTTGAAATAGCGGAAGCTAA
TTTAAGAAAAGCTGAAGGAAAGAGACAAAAAATTGAGGCAAATTTAGCTCTCCGACG
AGCTAGGACACGAGTAGAGACGATCAATGCGATTTCGATGAAAAATGTAACCGATTCT
TTCGTTTCTTTGGGCCACTGGCCGTCCGCCGGGAGTTTCGGGTTTAATACCGATATTTTA
GCAACAAATCTAATAAATCTAAGTGTAGTGCTTGGGGTATTGATCTTTTTTGGAAAGGG
AGTGTTAAGTGATTTATTAGATAATCGAAAACAGAGGATTTTGAATACTATTCGAAATTC
AGAAGAACTCCGTGAAGGGGCCATTGAACAGCTGGAAAAAGCCCGGGCGCGCTTACG
AAAAGTGGAAATGGAAGCAGATCAGTTTCGGGTGAATGGATACTCTGAGATAGAGCGA
GAAAAGTGGAATTTTATTAATTCAACTTCTAAGACTTTGGACCAACTAGAAAATTACAA
AAACGAAACTATTCATTTTGAACAACAAAGGGCGATTAATCAAGTCCGACAACGGGTT
TTCCAACAAGCCTTACAAGGGGCTCTAGGAACTCTGAGTGGTTGTTTGAACAACGAGT
TACATTTACGTACCATCAGTGCCAATATTGGCATCTTGGGGGCGATGAAAGAAATAACT
GATATGAATCCACTGATTTCTGCCGCTTCCGTTATTGCTGCTGGATTGGCTGTAGGGCTT
GCTTCTATTGGACCTGGAGTTGGTCAAGGTACTGCTGCGGGCCAAGCTGTAGAAGGTA
TCGCGAGACAGCCCGAGGCGGAGGGAAAAATACGAGGTACTTTATTGCTTAGTCTAGC
TTTTATGGAAGCTTTAACAATTTATGGCCTGGTTGTAGCATTAGCACTTTTATTTGCGAA
TCCTTTTGTTATGAATGTTCTATCCTGTTCCATCAACACACTAAAAGGGTTATATGATATA
TCCGGTGTGGAAGTAGGCCAACATTTCTATTGGAAAATAGGAGGTTTCCAAGTCCACG
GCCAAGTACTTATTACTTCTTGGGTTGTAATTGCTATCTTATTAGGCTCAGCCACTATAGC
TGTTCGGAACCCACAAACCATCCCGACCGGCGGTCAGAATTTCTTCGAATATGTACTTG
AATTCATTCGAGATGTGAGTAAAACTCAAATTGGAGAAGAATATGGTCCTTGGGTTCCT

TTTATTGGAACTATGTTTCTATTTATTTTTGTTTCTAATTGGTCAGGAGCGCTTTTACCTT
GGAAAATCATACAATTACCTCATGGGGAGTTAGCCGCACCCACGAATGATATAAATACT
ACTGTTGCTTTGGCTTTACTCACGTCAGTGGCATATTTCTATGCGGGTCTTACCAAAAA
GGGATTAGGTTATTTCGGGAAATATATTCAACCAACTCCAATCCTTTTACCCATTAACAT
CTTAGAAGATTTCACAAAGCCCTTATCACTTAGTTTTCGACTTTTCGGAGATATATTAGC
TGATGAATTAGTAGTTGTTGTTCTTGTTTCTTTAGTACCTTCAGTGGTTCCTATACCTGTC
ATGTTCCTTGGATTATTTACAAGTGGTATTCAAGCTCTTATTTTTGCAACTTTAGCCGCG
GCTTATATAGGTGAATCCATGGAGGGCCATCATATGATATTTTCAACTTTAGAGCATATAT
TAACTCATATTTCCTTTTCAATCGTTTCAATTGTAATTACAATTCATTTGATAACCTTATTA
GTCGATGAAATCATAAAACTATATGATTCGTCAGAAAAGGGAATGATAGCTATTTTTTTA
TGTATAACAGGATTATTAGTCACTCGTTGGATTTATTCGAGACATTTCCCACTAAGTGAT
TTATATGAATCATTAATCTTTCTTTCATGGAGTTTATCAGTTATTCATATAGTTCCGTAT-----
-AAAAAGAAAAAAAAATTTAAGCACAATAACTGCGTCAAGTGTTATTTTTACCCAGGGCT
TTGCTACTTCAGGTCTTTTAACTGAAATACATCAATCCGCAATATTAGTACCCGCTCTCC
AATCCGAGTGGTTAATAATGCACGTAAGTATGATGATATTGAGCTATGCAGCTCTTCTAT
GTGGATCATTATTATCAGTAGCACTTCTAGTCATTACATTTCGAAAAAACAGAAATATTT
TTTGT------AAAGAAATCCAATACATGAATGAAAGAAACAATGTTTTAAGAACTACT------
------GCTAAGAATTATTACAGGTCCCAGTTGATTCAACAGTTGGATTATTGGAGTTATCGT
GTCATTAGTCTAGGGTTTATCTTTTTAACCATAGGTATTCTTTCGGGAGCAGTATGGGCT
AATGAAGCATGGGGATCATATTGGAATTGGGACCCAAAGGAAACTTGGGCATTTATTAC
TTGGATCGTATTTGCGATTTATTTACACACTCGAACCAATATAAAATTACGGGGTGCAAA
TTCCGCAATTGTGGCGTCTATAGGCTTTCTTATAATTTGGATATGCTATTTTGGAGTCAAT
CTATTAGGAATAGGACTACACAGTTATGGTTCATTTACGTTAACGATGGCAAAAAAGAA
AGCATTCACTCCTCTTTTATATCTTGCATCTCTAGTATTTTTGCCCTGGTGGATTTCTCTT
TCATTTAATAAAAGTCTGGAATCTTGGGTTACTAATTGGTGGAATACTAGGCAATCCGA
AATTTTTTTGAATGGTATTCAAGAAAAGAATATTCTAGAAAAATTCATAGAATTAGAGG
AAATCCTTCTCTTGGAGGAAATGATCAAGGAATACTCGGAGACACATCTACAAAACCT
TCGTATA---ATCCACAAAGAAACGATCCAATTCATCAAGATACACAATGAGGATCGTATC
CATACGATTTTGCACTTCTCGACAAATATAATCTGTTTCGTTATTCTAAGTGGTTATTCTA
TTTGGGGTAATGAAGAACTTGTTATTCTTAACTCTTGGGCTCAGGAATTCCTATATAACT
TAAGTGACACAATAAAAGCTTTTTCTATTCTTTTATTAACTGATTTATGTATCGGATTCCA
TTCGCCCCATGGTTGGGAACTAATTATTGGCTCTGTCTACAAAGATTTTGGATTTGTTCA
TAATGATCAAATTTTATCTGGTCTTGTTTCTACTTTTCCAGTCATTCTAGATACACTTTTT
AAATTTTGGATTTTCCGTTATTTAAATCGTGTTTCTCCGTCACTTGTAGTGATTTATCATT
CAATGAATGATATGCCTATTGGTGTTCCAAAAGTACCCTTTCGAAGTCCTGGAGAGGAA
GATGCATATTGGGTTGACATATACAACCGACTTTATCGAGAACGATTACTTTTTTTAGGC
CAACCGGTTGCTAGCGAGATCTCGAATCAACTTATTGGTCTTATGGTATATCTCAGTATC
GAGGATGATACCAAAGATCTGTATTTGTTTATGAACTCTCCTGGGGGATGGGTACTCCC
TGGAATAGCTATTTATGATACTATGCAATTTGTGCGACCAGATGTACATACAATATGCATG
GGATTAGCTGCTTCAATGGGATCTTTTATCTTGGCCGGAGGAGAAATTACCAAACGTCT
AGCATTCCCTCACGCT---GTAATGATCCATCAACCTGCTAGTTCTTTTTATGAGGCACAA
ACGGGAGAATTTGTCCTGGAAGCGGAAGAACTGCTGAAACTGCGTGAAACCATCACA
AGGGTTTATGTACAAAAAACGGGCAAACCCTTTTGGGTTGTCTCGGAAGACCTGGAAA
GAGATGTTTTTATGTCAGCAACAGAAGCCCAAGCTTATGGAATTGTTGATCTTGTAGCG

GTTGAGATGAAAGAACAAAAATGGATTCATGAAGGTTTAATTACTGAATCGCTTCCCAA
TGGCATGTTCCGGATTCGCTTAGATAATGAAGATATGATTCTAGGTTATGTTTCAGGAAA
GATCCGACGTAGTTTTATACGGATACTGCCAGGCGATAGAGTAAAAATTGAAGTAAGTC
GTTATGATTCAACCAGAGGACGTATAATTTATCGACTTCGCAATAAGAATTCGAAAGATA
TGGAGGAATTCCAAAGATATTTAGAG---GATAGATCTCAACAACACTACTTCTTATATCC
ACTTATCTTTCAGGAGTATATTTATGCACTTGCTCATGATCATGGTTTAGCGGGTTATGAC
AATAAATTCAGTTTACTAATTGTGAAACGTTTAATTGCTCGAATGTATCAACAGAATCAT
TTGATTCTTTCTACTAATGATTCTAACCAAAATAGATTTTTGAGGAAGAATTTGTATTCTC
AAATGATATCAGAGGGGTTTGCAGTCATTGTGGAAATTCCATTTTATCTACAATTAAAAT
CTTCCCTAATAGTCAAATCTCATAATTTACGATCAATTCATTCAATATTTCCTTTTTTAGA
GGACAAAATTTCACATTTAATTTATGGTTTAGAGATACTAATACCCTACCCAGTCCATCT
GGAAATCTTGGTTCAAACTCTTCGCTACTGGGTAAAAGATGCTTCTTCTTTGCATTTATT
ACGATTCTTTCTCCACGAGTATTGTAATTGGAAT---------ACTAAAGCCGGTTCTTCTTTTT
CAAAACAAAGACTATTCTTCTTACTATATAATTCTCATCTATGTGAATACGAATCCATCTT
CATCTTTCTCCGTAACCAATCTTCTCATTTACGCTCAACATCTTCTGGAACCCTTCTTGA
ACGAATCTATTTCTATGGAAAAATAAAATATCTTGTAAAAGTCTTTGTTAAGGCTTTTAA
TCTATTGTTGTTGAAGGATCCTTTCATGCATTATGTTAGGTATCAAGGAAAATCAATTCT
CGCTTCAAAAGGGACGCCCTTTTTGATGAAAAAATGGACATATTACTTTGTTAATTTATG
GCAATGTCATTTTTACCTGTGGTCTCAACCGGGAAGGATCTGTATAAACCAATTATACAA
TCATTCCCTCGACATTCTGGGCTATCTATCAAGTGCGCGGCTAAACCCTTCAATGGTAC
GCGGTCAAATGCTAGAAAATTCATTTCTAATTGATAATGCTATT------AATAAGTTCGATG
CTATTGTTCCAATTATTCCTCTGATTGGATCATTGGCTAAAGCGAAATTTTGTAACGTATT
GGGGCATCCTATTAGTAAGGCGGTTTGGACCGATTTATCAGATTCTGATATTATTGACCA
ATTTGGGCGTATATGCAGAAATCTTTCTCATTATCATAGTGGATCCTCACAAAAAAAGA
GTTTGTATCGAATAAAGTATATACTTCGACTTTCTTGTGCTAGAACTTTGGCTCGTAAAC
ACAAAAGTACTGTACGTGCTTTTTTGAAAAGATTGGGCTCGGGATTATTGGAAGAATTC
TTTACGGCGGAAGAACAAGTTCTTTACTTGACCTTTCCAAGACGGATTTGGTATTTGGA
TATTATTTGTATCAATGATTTGGCCAATCATGAAATGATAATAGAT---ACAACAGAAGTAC
AA---GCTATCAATTCTTTTTTTAGATTGGAATCC---TTAAAAGAGGTATATGGGATCATAT
GGATGCTTATCCCTATTTTTACTCCTGTATTAGGAATCACAATAGGTGTACTAGTAATTGT
TTGGTTAGAAAGAGAAATATCTGCAGGGATACAACAACGTATTGGACCTGAATACGCC
GGGCCTTTGGGAATTCTTCAAGCTTTAGCAGATGGTACAAAATTACTTTTCAAAGAGAA
TCTTCTTCCATCGAGAGGAGATACTCGTTTATTCAGTATCGGACCATCCGTAGCAGTTAC
ATCAATTCTACTAAGTTATTTAGTAATTCCTTTTGGCTATCACCTTGTTCTAGCCGATCTC
AGTATCGGTGTTTTTTTATGGATTGCCATTTCAAGTATTGCTCCCGTTGGCCTTCTTATGT
CAGGCTATGGATCAAATAATAAATATTCCTTTTTAGGTGGTTTACGAGCTGCTGCTCAAT
CAATTAGTTATGAAATACCATTAACTCTATGTGTGTTATCAATATCTCTACTATCTAACAG
TTCAAGTACAGTTGATATAGTTGAAGCGCAGTCAAAATATGGTTTTTGGGGGTGGAATT
TGTGGCGTCAACCTATAGGGTTTATCATTTTTCTAATTTCGTCCCTAGCCGAGTGTGAGA
GATTACCTTTTGATTTACCAGAAGCAGAAGAAGAATTGGTAGCAGGTTATCAAACCGA
ATATTCAGGTATAAAATTTGGTTTATTTTACGTTGCTTCGTATCTGAATCTACTAGTTTCT
TCATTATTTGTAACGGTTCTTTACTTGGGGGGTTGGAATCTTTCTATTCCGTACATACCC
GTTCCTGATATTTTTGAAATAAATAAAGCGAGTAGAGTATTTGGAACAATAACTGGTATC
TTTATTACATTAGCTAAAACTTATTTGTTCTTATTCATTCCTATCACAACAAGATGGACTT

TACCAAGGCTAAGAATGGACCAACTATTAAATCTTGGATGGAAATTTCTTTTACCCATTT
CTCTCGGCAATCTATTATTAACAACCTCTTCCCAACTTCTTTCACTGATGATCTGGCATG
TACAGAATGAAAACTTCATTCTCGATTCTACGAGAATTTTTATGAAAGCCTTTCATTTGC
TTCTCTTCGATGGAAGTTTGATTTTCCCAGAATGTATCCTAATTTTTGGCCTAATTCTTCT
TCTGATGATCGATTCAACCTCTGATCAAAAAGATATACCTTGGTTATATTTCATCTCTTCA
ACAAGTTTAGTAATGAGCATAACGGCCCTCTTGTTCCGATGGAGAGAAGAACCTATGAT
TAGCTTTTCGGGAAATTTCCAAACGAACAATTTCAACGAAATCTTTCAATTTCTTATTTT
ACTATGTTCAACTCTATGTATTCCTCTATCCGTAGAGTACATTGAATGTACAGAAATGGC
TATAACAGAGTTTCTCTTATTCGTATTAACAGCTACTCTAGGAGGAATGTTTTTATGCGG
TGCTAACGATTTAATAACTATCTTTGTAGCTCCAGAATGTTTCAGTTTATGCTCCTACCTA
TTATCTGGATATACCAAGAAAGATGTACGGTCTAATGAGGCTACTATGAAATATTTACTC
ATGGGTGGGGCAAGCTCTTCTATTCTGGTTCATGGTTTCTCTTGGCTATATGGTTCATCC
GGGGGAGAGATCGAGCTTCAAGAAATAGTGAATGGTCTTATCAATACACAAATGTATAA
CTCCCCAGGAATTTCAATTGCGCTCATATTCATCACTGTAGGAATTGGGTTCAAGCTTTC
CCCAGCCCCTTCTCATCAATGGACTCCTGACGTATACGAAGGATCTCCCACTCCAGTCG
TTGCTTTTCTTTCTGTTACTTCGAAAGTAGCTGCTTCAGCTTCAGCCACTCGAATTTTCG
ATATTCCTTTTTATTTCTCATCAAACGAATGGCATCTTCTTCTGGAAATCCTAGCTATTCT
TAGCATGATATTGGGGAATCTCATTGCTATTACTCAAACAAGCATGAAACGTATGCTTGC
ATATTCGTCCATAGGTCAAATCGGATATGTAATTATTGGAATAATTGTTGGAGACTCAAA
TGATGGATATGCAAGCATGATAACTTATATGCTGTTCTATATCTCCATGAATCTAGGAACT
TTTGCTTGCATTGTATTATTTGGTCTACGTACCGGAACTGATAACATTCGAGATTATGCA
GGATTATACACGAAAGATCCTTTTTTGGCTCTCTCTTTAGCCCTATGTCTCTTATCCCTAG
GAGGTCTTCCTCCACTAGCAGGTTTTTTCGGAAAACTCTATTTATTCTGGTGTGGATGG
CAGGCAGGCCTATATTTCTTGGTTTTAATAGGACTCCTTACAAGCGTTGTTTCTATCTAC
TATTATCTAAAAATAATCAAGTTATTAATGATTGGACGAAACCAAGAAATAACCCCTCAC
GTGCGAAATTATAGAAGATCTCCTTTCAGATCAAACAATTCCATCGAATTGAGTATGATT
GTATGTGTGATAGCATCTACTATACCAGGAATATCAATGAACCCGATTATTGCAATTGCT
CAGGATACCCTTTTTATGTTTCTGCTTTACGAATATGATATTTTTTGGGCATTTCTAATAAT
ATCAAGTCTTATTCCTATTTTGGCATTTTTCATTTCCGGGGTTTTAGCCCCGATTAGCAA
AGGGCCAGAGAAACTCTCTAGTTATGAATCGGGTATAGAACCAATGGGCAATGCTTGG
TTACAATTTCGAATCCGTTATTATATGTTTGCTCTAGTTTTTGTTGTTTTTGATGTTGAAA
CAGTTTTTCTTTATCCATGGGCAATGAGTTTCGATGTATTAGGTGTATCTGTATTTATAGA
AGCTTTAATTTTCGTGCTTATCTTAATTGTTGGTTTAGTTTATGCATGGCGAAAGGGGGC
ATTGGAATGGTCTACGAATTTTTTTCCTTGGTTAACAATAATTGTAGTTTTGCCAATATTC
GCGGGCTCCGTAATTTTCTTTCTTCCCCATAGAGGAAATAGAGTAATTCGGTGGTATACC
ATATGTATATGTATTTTAGAACTCCTTCTAACGACCTATGCATTCTGTTATCATTTTCAATT
CGACGATCCATTAATCCAACTGGTGGAGGACTATAAATGGATCAATTTTTTTGCTTTCCA
TTGGAGATTAGGAATAGATGGACTTTCTATAGGACCCATTTTACTAACGGGATTCATCAC
CACTTTAGCTACTTTAGCGGCTCGGCCAGTTACTCGAGATTCTCGATTATTCCATTTCCT
GATGTTAGCAATGTACAGCGGGCAAATAGGATCATTTTCTTCTCGGGACCTTTTACTTTT
TTTCATCATGTGGGAGTTCGAATTAATTCCGGTTTATCTACTTTTATCCATATGGGGGGG
AAAGAGACGACTTTACTCGGCTACAAATTTTATTTTATACACGGCGGGAGGTTCCATTT
TTCTATTAATGGGAGTTCTGGGTGTTGGTTTATATGGTTCTAATGAACCAACATTAAATT
TTGAAACATCAGTTAATCAGTCATATCCTGTGGCCTTGGAAATAATATTCTATATTGGATT

TTTTATTGCTTTTGCTGTCAAATCGCCGATTATACCCCTACATACATGGTTACCAGATACC
CACGGAGAAGCACATTACAGTACTTGTATGCTTCTAGCCGGAATTTTACTAAAAATGGG
AGCGTATGGATTGATTCGGATTAATATGGAATTATTACCCCACGCCCATTCTATATTTTCT
CCGTGGTTGGTGATAGTAGGCACAATACAAATAATCTATGCAGCTTCAACATCTCCCGG
CCAACGTAATTTAAAAAAAAGAATAGCCTATTCCTCTGTATCTCATATGGGTTTCATACT
TATAGGAATTGGTTCTATAACCGATACGGGACTCAATGGAGCTATTTTACAAATAATCTC
TCATGGATTTATCGGTGCTGCGCTTTTTTTCTTGGCAGGAACGAGTTATGATAGAATACG
TCTTGTTTATCTCGACGAAATGGGCGGAATAGCTATCCCAATGCCAAAAATATTCACGAT
GTTCACTAGCTTTTCGATGGCTTCCCTTGCATTACCGGGTATGAGTGGTTTTGTTGCAGA
ATTGATAGTCTTTTTTGGAATAATTACCAGCCAAAAATATCTTTTAATGCCAAAAATCTT
AATTACTTTTGTAATGGCAATTGGGATGATATTAACTCCTATTTATTCATTATCTATGTTAC
GCCAGATGTTCTATGGATACAAGCTATTTAATGCTCCAAACTCTTATGTTTTTGATTCTG
GACCGAGAGAGTTATTTGTTTCGATCTCTATCTTTCTACCCGTAATAGGTATTGGTATGTA
CCCGGATTTTGTTCTTTCACTATCAGTTGACAAGGTCGAAGTTATTCTATCTAATTTTTTT
TATAGAATGATGCTCGAACATGTACTTGTTTTGAGTGCCTATTTATTTTCTGTTGGTCTTT
ATGGATTGATCACGAGTCGAAATATGGTTAGGGCCCTTATGTGTCTTGAACTTATTTTGA
ATGCAGTTAATATCAATTTTGTAACATTTTCTGATTTTTTTGATAGTCGTCAATTAAAAGG
AGCCATTTTCTCCATTTTTGTTATAGCTATTGCAGCCGCTGAAGCAGCTATTGGACTGGC
TATTGTTTCATCAATTTATCGTAACAGAAAATCAATTCGTATCAATCAATCGAATTTGTTG
AATAAGATGGAACAGACATATCAATATGCGTGGATAATACCTTTCCTTCCACTTCCAGTT
CCTATGTTAATAGGAGTGGGACTTCTTCTTTTTCCGACGGCAACAAAAAATCTTCGTCG
TATGTGGGCTTTTCAGAGTGTTTTATTGTTAAGTATAGTCATGATTTTTTCGATCAATCTG
TCTATTCAGCAAATAAATAGCAGTTCTATCTATCAATATGTGTGGTCTTGGATCATCAATA
ATGATTTTTCTTTAGAATTCGGCTACTTGATCGACCCACTTACTTCTATTATGTCAATATT
AATTACTACTGTTGGGATTGTGGTTCTTGTTTATAGTGATAATTATATGTCTCATGATCAA
GGATATTTGAGATTTTTCGCTTATATGAGTTTTTTCAGTACTTCTATGTTAGGATTAGTTA
CTAGTTCTAATTTGATACAAATTTATATTTTTTGGGAATTAGTCGGGATGTGTTCGTATCT
ATTAATAGGGTTTTGGTTCACACGACCTGTTGCGGCAAATGCTTGCCAAAAGGCGTTTG
TAACTAATCGTGTCGGGGATTTTGGTTTATTATTAGGAATTTTAGGTTTTTATTGGATAAC
AGGTAGTTTCGAATTTCGAGATTTATTCAAAATATTCAGTAACAATGAAGTGAATTTTTT
ATTTGTTACTTTGTGTGCCGTTCTATTATTTGCCGGTGCGGTTGCTAAATCTGCACAATT
CCCCCTTCATGTATGGTTACCTGATGCCATGGAGGGGCCTACTCCTATTTCAGCTCTTAT
ACATGCTGCTACTATGGTAGCAGCGGGAATTTTTCTTGTAGCTCGGCTTATTCCTCTTTT
CATAGTCATCCCTCACATAATGAATTTAATCTCTTTGGTAGGGATAATAACAGTATTATTA
GGAGCTACTTTAGCCCTTGCTCAAAAAGACATTAAGAGAGGTTTGGCCTATTCCACAAT
GTCTCAATTGGGTTATATGATGTTAGCTTTAGGTATGGGGTCTTATCGAAGTGCTTTATTT
CATTTGATTACTCATGCTTATTCGAAAGCATTATTGTTTTTAGGATCCGGATCCGTTATTC
ATTCAATGGAAACTCTTGTTGGATATTCTCCAAAGAAAAGCCAGAATATGGTTTTTATG
GGGGGTTTAACAAAGCATGTGCCAATTACCAAAACGTCTTTTTTATTCGGTACACTTTC
TCTTTGTGGTATTCCGCCTCTTGCTTGTTTTTGGTCCAAGGATGAAATTATTAATGATACT
TGGTTGTATTCACCAATTTTTGCAATAATAGCTTGGTCTACAGCGGGATTAACCGCATTT
TATATGTTTCGAATCTATTTACTTACTTTTGAAGGACATTTAAACGTTCATTTTCAAAATT
AC---------AAAAATACCCCCTTCTATTCAATATCTCTATGGGGTAAAGAAGGTTCGAAAA
GGATTAATAAA---TTATTAACTTTATTAACAATGAATTTTTTTTCATTTGGCAATAAGAATA

TTTTTTCATATCCTTATGAATCAGATAATACTATGTTATTTCCTATACTTGTATTGGTTCTAT
TCACTTTGTTCATTGGATTCCTAGGAATTCCTTTCAATTTGGACATATTATCCAAATGGTT
AACCCCGTCTATAAACCTTTTACATCAAAATTTAAAT---TTAATAGATTGGGATGAATTTG
CGAAAGATGCCATCTTTTCAGTTAGTATAGCTTTTTTTGGAATCTTTATAGCCTCCTTTTT
ATATAAACCCGTTTATTCCTCTTTGCAAAATTTGGACTTAATTAACTCGTTTGTTATTTTT
TTGGACAAAATAATAAATGGTATATATGGTTGGTCGTGTAATCGTGGTTACATAGATGCT
TTTTATGCAACATTCTTTACTGGGGGGATAAGAGGATTGGCCGAATTGACTTCTTTTTTT
GATAGACAAGTAATTGATGGAATTACAAATGGAGTTGGTGTTTTGAGTTTTTTTGTAGG
CGAAAGTATCAAATCTGTAGGTGGGGGG---CGCATCTCTTCTTATCTTTTCTTGTATTTGT
CTTATGTATCAATCTTTTTATTAATTTACTACATGGATTTACCTGGACCAATACATGATTTT
CTTTTAGTCTTTCTGGGATCGGGTCTTATATTAGGAGGTCTGGGAGTGGTATTACTTCCC
AATCCAATTTATTCCGCCTTTTCATTGGGATTGGTTCTTGTTTGTACATCTTTATTCTATAT
TCTATCGAACTCCCATTTTGTAGCTGCTGCACAGCTCCTTATTTATGTAGGGGCCATAAA
TGTTTTAATTCTTTTTGCTGTGATGTTCATGAATGGTTCCGAATATTACAAAGATTTTCAT
CTTTGGACCATTGGAGATGGGGTTACTTCGATGGTTTGTACAAGTATTTTTGTTTCACTA
ATTACTACTATTCCAGATACGTCATGGTACGGGATTATTTGGACTACAAAATCAAACCAG
ATTATAGAGCAAGATTTGATAAGCAATAGTCAACAAATTGGGATTCATCTATCAACAGAT
TTTTTTCTTCCATTTGAACTCATTTCAATAATTCTATTAGTTGCGTTAATAGGTGCAATTG
CCGTAGCTCGTCAAATGACTGCATCAGCTACACGAAATGACCTTATGATAGTCAATCTG
GGTCCTCAGCACCCATCAATGCACGGTGTTCTTCGACTCATTGTTACTCTAGATGGTGA
AGATGTTATTGACTGTGAACCAATATTGGGTTATTTACATAGAGGGATGGAAAAAATTG
CGGAAAACCGAACAATTATACAGTATTTACCTTATGTAACACGTTGGGATTATTTAGCTA
CTATGTTCACAGAAGCAATAACTGTAAATGCACCAGAGCAGTTAGGCAATATTCAAGTA
CCTAAAAGGGCCAGCTATATCAGAGTCATTATGTTGGAGTTAAGTCGTATAGCTTCGCAT
TTGTTATGGCTTGGCCCCTTTCTGGCAGATATTGGCGCACAGACCCCTTTCTTCTATATT
TTTCGAGAAAGAGAATTGATATATGACCTATTCGAAGCTGCCACCGGTATGCGAATGAT
GCATAATTATTTTCGGATCGGAGGAGTAGCTGCTGATTTACCTCATGGATGGATAGATAA
ATGTTTGGATTTTTGTGATTTTTTTTTAACAAGGGTTACTGAATATCAAAAACTTATTAC
ACGGAACCCTATTTTTTTAGAACGGGTTGAAGGAGTAGGCATTATTGGCGGAGCGGAG
GCAATAAATTGGGGTTTATCAGGACCAATGCTACGAGCTTCCGGAATACAATGGGATCT
TCGTAAAGTTGATCATTATGAGTCTTACGACGAATTTGATTGGGGGGTCCAATGGCAAA
AAGAGGGGGATTCCTTAGCTCGTTATTTAGTACGAATCAGTGAAATGACAGAATCCATA
AAAATAATTCAACAGGCTTTAGAAGGAATTCCGGGGGGGCCCTATGAGAATTTAGAAA
TCCGACGTTTTGATAAAGTACGGGATCCTGAATGGAATGATTTTGACTATCGATTCATCA
GTAAAAAACCTTCTCCGACTTTTGAATTATCAAAGCAAGAACTTTATGTGAGAGTCGA
AGCCCCCAAGGGAGAATTGGGGATTTTTCTGATAGGGGATAGGGGTGTTTTTCCTTGG
AGATGGAAAATCCGACCACCGGGTTTTATCAATTTGCAAATCCTTCCTCAGTTAGTTAA
AAGAATGAAATTGGCTGATATTATGACGATACTAGGTAGCATAGATATAATTATGGGAGA
AGTTGATCGTATGTTCTCTATGGTAACTGAGTTCATGAACTATGGTCAACAAACAGTAC
GAGCTGCAAGGTATATTGGTCAAGGTTTCATGATTACCTTATCACACGCGAATCGTTTAC
CTGTAACTATTCAATACCCCTACGAAAAATTGATTACATCGGAGCGTTTCCGCGGCCGA
ATTCACTTTGAGTTTGATAAATGCATTGCTTGTGAAGTATGTGTTCGTGTATGTCCTATA
GATCTACCCGTTGTTGATTGGAAATTGGAAATGGATATTCGAAAGAAACGATTGCTTAA
TTACAGTATTGATTTCGGAATCTGTATATTTTGTGGTAATTGCGTTGAGTATTGTCCAACA

AATTGTTTATCAATGACTGAAGAATATGAACTTTCTACGTATGATCGTCACGAATTGAAT
TATAATCAAATTGCTTTGGGACGGTTACCGATGGCAGTAATTGACGATTACACAACTCG
AACTATTTTGAATTTG-------------------------ATGCAGGGTCGTTTGTCTG
CTTGGTTAGTCAAGCATGGGCTAATTCATAGATCTTTGGGGTTTGATTACCAAGGGATA
GAGACTTTACAAATAAAACCCGAGGATTGGCATTCCATTGCTGTCATTTTATATGTATAT
GGTTACAATTATCTACGCTCCCAATGCGCCTATGATGTAGCACCAGGTGGACTGTTAGCT
AGTGTGTATCATCTTACGAGAATAGAGTATGGCGTGGATCAACCAGAAGAGGTATGCAT
AAAAGTATTTGCCCCGAGGAGGAATCCTCGAATTCCGTCTGTTTTCTGGGTTTGGAAA
AGTGTGGATTTTCAAGAACGGGAATCTTATGATATGTTGGGAATCTTTTATGCTACTCAT
CCACGTTTGAAACGCATCTTAATGCCTGAAAGTTGGGTAGGATGGCCTTTACGTAAGGA
TTATATTGCCCCCAATTTTTATGAAATACAAGATGCTCAT--------------------

-----------ATGAATTCCATCGAGTTTCCCTTACTTGATCGAACAACCCAAAATTCAG
TTATTTCAACTACATCAAATGATCTTTCAAATTGGTCAAGACTCTCTAGTTTATGGCCGC
TTCTCTATGGTACTAGTTGTTGCTTCATTGAATTTGCTTCACTAATAGGCTCACGATTCG
ACTTTGATCGTTATGGACTAGTCCCAAGATCGAGTCCTAGACAAGCGGACCTAATTTTA
ACAGCCGGAACAGTAACAATGAAAATGGCTCCTTCTTTAGTGAGATTATATGAGCAAAT
GCCTGAACCAAAATATGTTATTGCTATGGGAGCATGTACAATTTCAGGGGGGATGTTCA
GTACTGATTCTTATAGTACTGTTCGGGGAGTCGATAAGCTAATTCCTGTCGATGTCTATT
TGCCAGGCTGTCCACCTAAACCCGAAGCAGTTATAGATGCTATAACAAAACTTCGTAAA
AAAATATCTCGAGAAATCTATGAAGATAGAATTAAGTCTCAACGGGAGAATCGGTGTTT
TACAACCAATCACAAGTTTCAGGTTGGGCGTAGTATTCATACTGGAAATTATGATCGAG
GGTTCCTTTATCAACCGCCATTTACTTCAGAGATCCCTTCCGAAACATTTTTCAAATACA
AAAGTTCAGTCTCGTCCCACGAATTAGTGAATATGCAAACTAGAAAGACTTTTTCTTGG
ATAAAGGAACAGATTACTCGATCCATTTCCGCATCGCTCATGATATATATCATAACTCGG
ACATCCATTTCAAGTGCATATCCCATTTTTGCACAGCAGGGTTATGAAAATCCACGAGA
AGCGACTGGGCGTATTGTATGTGCCAATTGCCATTTAGCTAATAAGCCCGTGGATATTGA
AGTTCCACAAGCCGTACTTCCTGATACTGTATTTGAAGCAGTTGTTCGAATCCCTTATGA
TATGCAACTAAAACAAGTTCTTGCTAATGGTAAAAAGGGGGCTTTGAATGTAGGGGCT
GTTCTTATTTTACCGGAGGGTTTTGAATTAGCTCCTGTTGATCGGATTTCTCCCGAGATG
AAAGAAAGGATAGGCAATCTTTCTTTTCAGAGCTATCGCCCCACTAAAAAAAATATTCT
TGTGATAGGCCCTGTTCCTGGTCAAAAATATAGTGAAATCACCTTTCCTATTCTTTCCCC
GGACCCCGCTACTAAGAAAGATGTTCACTTCTTAAAATATCCTATATACGTAGGCGGAA
ACAGGGGAAGGGGTCAGATTTATCCCGACGGGAGCAAGAGTAACAATACGGTTTATAA
TGCTACAGCAGCAGGTATAGTAGGCAAAATAATACGAAAAGAAAAGGGGGGTTACGA
AATAACCATAACGGATGCATCGGATGGACGTCAAGTGGTTGATATTATCCCTCCAGGGC
CAGAACTTCTTGTTTCAGAAGGCGAATCTATCAAATTTGATCAACCATTGACGAGTAAT
CCTAATGTGGGCGGATTTGGTCAGGGAGATGCAGAAATAGTACTTCAAGATCCATTACG
TGTCCAAGGCCTTTTGTTCTTCTTGGCATCTGTTATTTTGGCACAAATCTTTTTGGTTCT
TAAAAAGAAACAGTTCGAGAAGGTTCAATTGTCCGAAATGAATTTCATGAGTAAAGTC
TATGATTGGTTTGAAGAACGTCTCGAGATTCAGGCGATTGCGGATGATATAACTAGTAA
ATACGTTCCTCCTCATGTTAACATATTTTATTGTCTAGGAGGTATTACGCTTACTTGTTTT
TTAGTACAAGTGGCTACGGGGTTTGCTATGACTTTTTACTATCGTCCGACCGTTACTGAC
GCTTTTGCTTCGGTTCAATATATAATGACTGAAGCTAATTTTGGTTGGTTAATCCGATCA

GTTCATCGATGGTCGGCAAGTATGATGGTTCTAATGATGATCCTGCATGTATTTCGTGTG
TATCTCACCGGTGGATTTAAAAAACCTCGCGAATTAACTTGGGTTACAGGTGTGGTTCT
GGCTGTATTGACTGCATCTTTTGGTGTAACTGGTTATTCCTTACCTCGGGACCAAATTGG
TTATTGGGCAGTGAAAATTGTAACAGGTGTACCCGAAGCTATTCCTGTAATAGGATCCC
CTTTGGTAGAATTATTGCGTGGAAGTGCTAGTGTGGGACAATCCACTTTGACTCGTTTT
TATAGTTTACACACTTTTGTATTGCCGCTTCTTACTGCCGTATTTATGTTAATGCACTTTC
CAATGATACGTAAACAAGGTATTTCTGGTCCTTTAATGGGAGTAACAAAAAAACCTGAC
TTGAATGATCCTGTATTAAGAGCTAAATTGGCTAAAGGTATGGGTCATAATTATTACGGA
GAGCCCGCCTGGCCCAATGATCTTTTATATATTTTTCCAGTAGTAATTCTAGGGACTATTG
CATGTAACGTAGGCTTAGCGGTTCTAGAACCATCAATGATTGGTGAACCAGCAGATCCA
TTTGCAACCCCTTTGGAAATATTACCCGAATGGTATTTCTTTCCCGTATTTCAAATACTTC
GTACAGTACCCAATAAATTATTGGGTGTTCTTTTAATGGTTTCAGTACCCGCGGGATTAT
TAACAGTACCCTTTTTAGAGAATGTTAATAAATTCCAAAATCCATTTCGTCGTCCAGTAG
CGACAACCGTCTTTTTGATTGGTACCGCAGTGGCCCTTTGGTTAGGTATTGGTGCAACA
TTACCTATTGATAAATCCCTAACTTTAGGTCTTTTTATGATTGAAGTTTTTCTATTTGGAA
TCGTGTTAGGTCTAATTCCTATTACTTTGGCTGGATTATTCGTAACTGCATATTTACAATA
CAGGCGTGGTGATCAGTTGGACCTTATGCCTACTATAACTAGTTATTTCGGTTTTCTACT
AGTGGCTTTAACTATAACCTCAGCTCTATTTATTGGTCTTAGCAAGATACGACTTATCAT
GGATATAGTAAGTCTTGCTTGGGCTGCTTTAATGGTAGTCTTTACATTTTCCCTTTCACT
CGTAGTATGGGGAAGAAGTGGACTCATGATTATTCGTTCGCCGGAACCAGAAGTAAAA
ATTTTGGTAGATAGGGATCACATAAAAACTTCTTTCGAGGAATGGGCCAGACCGGGTCA
TTTCTCAAGAACACTAGCTAAAGGCCCTGACACTACCACTTGGATCTGGAACCTACAT
GCTGATGCTCACGATTTCGATAGCCATACCAGTGATTTGGAGGAGATCTCTCGAAAAGT
CTTTAGTGCCCATTTCGGCCAACTCTCCATCATCTTTCTTTGGCTGAGTGGCATGTATTT
CCACGGTGCTCGTTTTTCTAATTATGAAGCATGGCTAAGCGACCCGACTCACATTGGGC
CGAGTGCCCAGGTGGTTTGGCCAGTAGTGGGCCAAGAAATATTGAATGGTGATGTGGG
TGGGGGCTTCCGAGGAATACAAATAACCTCCGGTTTTTTTCAGATTTGGCGAGCATCTG
GAATAACTAGTGAATTACAACTCTATTGTACTGCAATTGGTGCATTGATCTTTGCAGCAT
TAATGCTTTTTGCTGGTTGGTTTCATTATCATAAAGCTGCTCCAAAATTGGCTTGGTTTC
AAGATGTAGAATCTATGTTGAATCACCATTTAGCGGGGCTACTAGGACTGGGGTCTCTC
TCTTGGGCGGGACATCAAGTACATGTATCTTTACCGATTAACCAGTTTCTAAACGCTGG
AGTAGATCCTAAAGAGATACCACTTCCTCATGAATTTATCTTGAATCGGGATCTTTTGGC
TCAACTTTATCCCAGTTTTGCTGAGGGAGCAACCCCCTTTTTCACCTTGAATTGGTCAA
AATATGCGGACTTTCTTACTTTTCGCGGAGGATTAGATCCAGTAACTGGGGGTCTATGG
CTGACCGATATTGCACACCATCATTTAGCTATTGCAATTCTTTTCCTGATAGCAGGTCATA
TGTATAAGACCAACTGGGGCATTGGTCATGGCCTAAAAGATATTTTAGAAGCTCATAAA
GGTCCATTTACAGGTCAGGGCCATAAAGGCCTATATGAGATCCTAACAACGTCATGGCA
TGCTCAATTATCTCTTAACTTAGCTATGTTAGGCTCTTTAACCATTGTTGTAGCTCACCAT
ATGTATTCCATGCCCCCTTATCCGTATCTAGCTACTGACTATGGTACACAATTGTCGTTGT
TCACACATCACATGTGGATTGGTGGATTTCTCATAGTTGGCGCTGCTGCGCATGCAGCC
ATTTTTATGGTAAGAGACTATGATCCAACTACTCGATACAACGATCTATTAGATCGTGTC
CTTAGGCATCGCGATGCAATCATATCACATCTCAACTGGGCATGTATATTTCTAGGCTTT
CACAGTTTTGGTTTGTATATTCATAATGATACCATGAGCGCTTTAGGGCGTCCTCAAGAT
ATGTTTTCAGATACCGCTATCCAATTACAACCCGTCTTTGCTCAATGGATACAAAACACC

CATGCTTTAGCACCTGGTGCAACGGCTCCTGGTGCAACAACAAGCACCAGTTTAACTT
GGGGGGGCGGTGATTTAGTAGCAGTGGGTGGCAAAGTAGCTTTGTTACCTATTCCATTA
GGAACTGCAGATTTTTTGGTACATCATATTCATGCATTTACGATTCATGTGACGGTATTG
ATACTTCTGAAGGGTGTTCTATTTGCTCGTAGCTCCCGTTTGATACCGGATAAAGCAAAT
CTTGGTTTTCGTTTTCCTTGTGATGGACCTGGAAGAGGGGGAACGTGTCAAGTATCGG
CTTGGGATCATGTCTTCTTAGGACTATTCTGGATGTACAATGCAATTTCGGTAGTTATATT
CCATTTCAGTTGGAAAATGCAGTCAGATGTTTGGGGCAGTATAAGTGATCAAGGGGTA
GTAACTCATATCACGGGAGGAAACTTTGCGCAGAGTTCCATTACTATTAATGGGTGGCT
CCGCGATTTCTTATGGGCACAAGCATCCCAGGTAATTCAGTCTTATGGTTCTTCATTATC
TGCATATGGACTTTTCTTCCTAGGTGCTCATTTTGTATGGGCTTTTAGTTTAATGTTTCTA
TTCAGTGGACGTGGTTATTGGCAAGAACTTATTGAATCCATCGTTTGGGCTCATAATAAA
TTAAAAGTTGCTCCTGCTACTCAGCCGAGAGCCTTAAGCATTGTACAAGGACGTGCTGT
AGGAGTAACCCATTACCTTCTGGGTGGAATTGCCACAACATGGGCGTTCTTCTTAGCAA
GAATTATTGCAGTAGGAATGGCATTAAGATTTCCAAGGTTTAGCCAAGGCTTAGCTCAG
GACCCCACTACTCGTCGTATTTGGTTTGGTATTGCTACCGCACATGACTTCGAGAGTCA
TGATGATATTACTGAGGAACGTCTTTATCAGAATATTTTTGCTTCTCACTTCGGGCAATT
AGCAATAATTTTTCTGTGGACTTCCGGAAATCTGTTTCATGTAGCTTGGCAAGGAAATT
TTGAGTCATGGGTACAGGACCCTTTACACGTAAGACCTATTGCTCATGCAATTTGGGAT
CCTCATTTTGGTCAACCGGCTGTAGAAGCTTTTACTCGAGGGGGTGCTCTTGGCCCAGT
GAATATCGCTTATTCTGGTGTTTATCAATGGTGGTATACAATCGGTTTACGCACTAATGA
AGATCTTTATACTGGAGCTCTTTTTCTATTATTTCTTTCTACCATATCCTTAATAGCAGGTT
GGTTACACCTACAACCGAAATGGAAACCGAGTGTTTCGTGGTTCAAAAATGCCGAATC
TCGTCTGAATCATCATTTGTCAGGACTCTTCGGCGTAAGTTCCTTGGCTTGGACAGGGC
ATTTAGTACATGTGGCTATTCCTGCATCCAGAGGGGAGTACGTTCGATGGAATAATTTCT
TAGATGTATTACCACACCCCCAAGGGTTAGGCCCACTTTTTACAGGTCAATGGAATCTT
TATGCTCAAAACCCCGACTCAAGTAGTCATTTATTTGGTACCTCCCAAGGAGCAGGAAC
TGCCATTCTAACCCTTCTCGGGGGATTCCACCCACAAACGCAAAGTTTATGGTTGACTG
ATATGGCTCATCATCATTTAGCTATTGCATTTATTTTTCTCGTTGCTGGTCATATGTATAGA
ACTAACTTCGGTATTGGGCACAGTATGAAAGATCTTTTAGATGCACATATTCCTCCGGG
GGGGCGATTGGGACGTGGGCATAAGGGTCTTTATGACACAATCAATAATTCCCTTCATT
TTCAATTAGGCCTTGCTCTAGCTTCTTTAGGGGTTATTACTTCCTTGGTAGCTCAACACA
TGTACTCTTTACCTGCTTATGCATTCATAGCACAAGACTTTACTACTCAAGCTGCGTTAT
ATACTCATCACCAATACATCGCAGGATTCATCATGACAGGAGCTTTTGCTCATGGAGCTA
TATTTTTCATTAGAGATTACAGTCCGGAACAGAATGAAGATAATGTATTGGCAAGAATG
TTAGACCATAAAGAAGCTATCATATCTCATTTAAGTTGGGCCAGCCTCTTTCTGGGGTTC
CATACCTTGGGACTTTATGTTCATAATGATGTCATGCTTGCCTTTGGTACTCCGGAGAAA
CAAATCTTGATCGAACCTATATTTGCTCAATGGATACAATCCGCACATGGTAAAACTTCA
TATGGTTTCGATGTCCTTTTATCTTCAACGAATGGCCCGGCATTTAATGCGGGTCGAAGC
ATATGGTTGCCCGGTTGGTTAAATGCTGTTAATGAG------AATAGTAATTCACTATTCTTA
ACAATAGGTCCGGGAGACTTTTTAGTTCATCATGCTATTGCTCTGGGTTTACATACGACT
ACATTGATCTTAGTAAAGGGTGCTTTAGATGCACGTGGTTCCAAGTTAATGCCAGATAA
AAAGGATTTCGGTTATAGTTTTCCGTGCGACGGCCCGGGACGGGGCGGTACTTGTGATA
TTTCGGCTTGGGACGCATTTTATTTGGCAGTTTTTTGGATGTTAAATACTATCGGATGGG
TTACTTTTTATTGGCATTGGAAGCATATCACATTATGGCAGGGTAACGTTTCGCAGTTTA

ATGAATCTTCCACTTATTTGATGGGATGGTTAAGAGATTATTTATGGTTAAACTCTTCAC
AACTTATCAATGGATATAATCCTTTTGGTATGAATAGTTTATCGGTCTGGGCGTGGATGTT
CTTATTTGGACATCTTGTTTGGGCTACTGGATTTATGTTCTTAATTTCCTGGCGTGGCTAT
TGGCAAGAATTGATTGAAACTTTAGCATGGGCTCATGAACGCACACCTTTGGCCAATTT
GATTCGTTGGAGAGATAAACCGGTGGCCCTTTCCATTGTGCAAGCACGATTGGTTGGAT
TAGCCCACTTTTCTGTAGGTTATATATTCACTTATGCGGCTTTCTTGATTGCCTCTACATC
AGGCAAATTTGGTATGTCACATTCAGTAAAGATTTATGATACATGTATAGGATGTACTCA
ATGTGTCCGAGCCTGCCCCACCGATGTATTAGAAATGATACCTTGGGACGGATGTAAAG
CTAAACAAATTGCTTCTGCTCCACGAACAGAGGACTGTGTCGGTTGTAAGAGATGTGA
ATCCGCCTGTCCAACCGATTTCTTGAGTGTTCGAGTTTCTTTATGGCATGAAACAACTC
GCAGCATGGGTCTAGGTTATATGACAACTTTTGACTTTCCCTCTGTGTTGGTGCCTTTAG
TAGGCCTAGTATTTCCGGCAATGGCAATGGCTTCTTTATCTCTTCATGTTCAAAAGAATA
AGACTGTTATGCGAGATCTAAAAACATATCTCTCCGTGGCACCGGTATTAAGTACTCTAT
GGTTCGGGTCTTTAGCAGGTCTATTGATAGAGATTAATCGTTTTTTCCCAGATGCGTTGA
CATTCCCCTTTTTT------ATGACTGCAATTTTAGAGAGACGCGAAAGCGAAAGCCTATGG
GGTCGCTTCTGTAACTGGATAACCAGCACCGAAAACCGTCTTTACATTGGATGGTTTGG
TGTTTTGATGATCCCTACCTTATTGACCGCAACTTCTGTATTTATTATCGCCTTCATTGCT
GCTCCTCCAGTAGATATTGATGGTATTCGTGAACCTGTTTCCGGATCTCTACTTTATGGC
AACAATATTATCTCTGGTGCCATTATTCCTACTTCTGCAGCTATAGGTTTGCACTTTTACC
CAATCTGGGAAGCGGCATCCGTTGACGAATGGTTATACAACGGTGGTCCTTATGAACTA
ATCGTTCTACACTTTTTACTTGGTGTAGCTTGTTACATGGGTCGTGAGTGGGAGCTTAGT
TTCCGTCTGGGTATGCGACCTTGGATTGCTGTTGCATATTCAGCTCCTGTTGCAGCTGCT
GCTGCTGTTTTCTTGATCTACCCAATCGGTCAAGGAAGTTTTTCTGATGGTATGCCTCTA
GGAATCTCTGGTACTTTCAATTTCATGATTGTATTCCAGGCTGAACACAACATCCTTATG
CACCCATTTCACATGTTAGGCGTAGCTGGTGTATTCGGCGGCTCCCTATTCAGTGCTATG
CATGGTTCCTTGGTAACTTCTAGTTTGATCAGGGAAACCACAGAAAATGAATCTGCTAA
TGAAGGTTACAGATTCGGGCAAGAGGAAGAAACTTATAATATCGTAGCCGCTCATGGTT
ATTTTGGTCGATTGATCTTCCAATATGCTAGTTTCAACAACTCTCGTTCTTTACATTTCTT
CCTAGCTGCTTGGCCTGTAGTAGGTATCTGGTTCACTGCTTTAGGTATTAGCACTATGGC
TTTCAACCTAAATGGTTTCAATTTCAACCAATCTGTAGTTGATAGTCAAGGCCGTGTAAT
TAATACTTGGGCTGATATCATTAACCGTGCTAACCTTGGTATGGAAGTTATGCATGAACG
TAATGCTCATAACTTCCCTCTAGACCTAGCTGCTGTCGAAGCTCCATCTACAAATGGAAT
GGGTTTGCCTTGGTATCGTGTTCATACCGTTGTATTGAATGATCCCGGCCGGTTGCTTTC
TGTCCATATAATGCATACAGCTCTAGTTGCTGGTTGGGCGGGTTCGATGGCTCTATATGA
ATTAGCAGTTTTTGATCCCTCTGACCCTGTTCTTGATCCAATGTGGAGACAGGGTATGTT
CGTTATACCCTTCATGACTCGTTTAGGAATAACCAATTCATGGGGCGGTTGGAGTGTCA
CA---GGAGGGGCTATACCGAATCCGGGTATTTGGAGTTACGAAGGTGTGGCCGGGGCA
CATATTGTGTTTTCTGGCTTGTGCTTCTTGGCAGCTATCTGGCATTGGGTTTATTGGGAT
CTAGAAATTTTTTCTGATGAACGTACAGGAAAACCTTCTTTGGATTTGCCCAAGATCTT
TGGAATTCATTTATTTCTCGCAGGGGTGGCTTGCTTTGGTTTTGGTGCATTTCATGTAAC
AGGCTTGTATGGTCCTGGAATATGGGTGTCTGATCCTTATGGACTAACGGGAAAAGTAC
AATCTGTAAATCCAGCGTGGGGTGTGGAAGGTTTTGATCCTTTTGTTCCGGGAGGAATA
GCCTCTCATCATATTGCAGCAGGGACATTGGGTATATTGGCCGGTCTATTCCATCTTAGT
GTCCGCCCGCCCCAACGTCTATACAAAGGATTGCGTATGGGCAATATTGAAACCGTCCT

TTCCAGTAGTATCGCTGCTGTCTTTTTTGCAGCTTTTGTTGTTGCCGGAACTATGTGGTA
TGGTTCAGCAACTACCCCGATCGAATTATTTGGGCCTACTCGTTATCAATGGGATCAGG
GGTACTTCCAGCAAGAGATATATCGAAGAGTTAGTGCTGGGCTAGCCGAAAATCAAAG
TTTATCAGAAGCTTGGTCTAAAATTCCTGAAAAATTAGCTTTTTATGATTACATCGGCAA
TAATCCGGCAAAAGGGGGATTATTCAGAGCGGGTTCAATGGATAACGGGGATGGAATA
GCGGTTGGATGGTTAGGACATCCTATCTTTAGAGATAAAGAAGGGCGTGAACTTTTTGT
ACGTCGTATGCCTACCTTTTTTGAAACCTTTCCGGTCGTTTTGGTAGATGGCGACGGAA
TTGTTAGAGCCGATGTTCCTTTTCGAAGGGCAGAATCGAAGTATAGTGTCGAACAAGT
AGGTGTAACTGTTGAGTTCTACGGCGGCGAACTCAACGGAGTCAGTTATAGTGATCCT
GCTACTGTGAAAAAATATGCTAGACGTGCTCAATTGGGTGAAATTTTTGAATTAGATCG
TGCTACTTTGAAATCCGATGGTGTTTTTCGTAGCAGTCCGAGGGGTTGGTTTACTTTTG
GACATGCTTCTTTTGCTTTGCTCTTCTTTTTTGGACATATTTGGCATGGTGCTAGAACCT
TGTTCAGAGATGTTTTTGCTGGTATTGATCCAGATTTGGATGCTCAAGTAGAATTTGGA
GCATTCCAAAAACTGGGAGATCCAACTACAAGAAGACAAGTAGTCATGAAAACCTTAT
ATTCCCTGAGGAGGTTCTACCCCGTGGAAACGCTCTTTAATGGAACTTTAGCTTTAGCT
GGTCGTGACCAAGAAACCACTGGTTTCGCTTGGTGGCCGGGAATGCCCGGCTTATCA
ATTTATCCGGTAAACTACTCGGGGCTCATGTAGCCCATGCCGGATTAATTGTATTCTGGG
CCGGAGGAATGAACCTATTTGAAGTGGCTCATTTCGTACCAGAGAAGCCTATGTATGAA
CAAGGATTAATTTTACTTCCCCACCTAGCTACTCTAGGTTGGGGGGTAGGTCCTGGTGG
GGAAGTTATAGACACTTTTCCATACTTTGTATCTGGAGTACTTCATTTAATTTCCTCTGC
AGTATTGGGCTTTGGTGGTATTTATCATGCACTTCTAGGGCCTGAGACGCTTGAAGAAT
CTTTTCCATTCTTCGGTTATGTATGGAAAGATAGAAATAAAATGACTACAATTTTAGGTA
TTCACTTAATCTTATTAGGTATAGGTGCTTTTCTTCTAGTATTCAAGGCTCTTTATTTTGG
TGGCGTATATGATACCTGGGCTCCGGGGGGGGGAGATGTAAGAAAAATTACCAACTTG
ACCCTTAGCCCAAGTATTATATTTGGTTATTTACTAAAATCGCCCTTTGGAGGGGAAGGA
TGGATTGTTAGTGTAGATGATTTGGAAGATATAATCGGAGGACATGTATGGTTAGGTTCC
ATTTGTATACTTGGTGGAATCTGGCATATCTTAACCAAACCCTTCGCATGGGCTCGACGC
GCACTTGTGTGGTCTGGGGAGGCTTACTTATCTTATAGTTTAGGGGCTTTATCCGTCTTT
GGTTTCATTGCTTGTTGTTTTGTCTGGTTCAATAATACCGCCTATCCTAGCGAGTTTTAC
GGACCCACTGGACCAGAAGCTTCCCAAGCTCAAGCATTTACTTTTCTAGTTAGAGACC
AACGCCTTGGGGCTAACGTGGGATCCGCTCAAGGACCTACTGGTTTAGGTAAATATTTA
ATGCGTTCCCCGACCGGAGAAGTCATTTTTGGAGGAGAAACTATGCGTTTTTGGGATCT
GCGTGCCCCTTGGTTAGAACCTCTAAGGGGTCCAAATGGGTTGGACTTGAGTAGGCTG
AAAAAAGACATACAACCTTGGCAAGAACGGCGTTCTGCAGAATATATGACCCATGCTC
CTTTAGGTTCTTTAAATTCTGTGGGTGGCGTAGCTACCGAGATCAATGCAGTCAATTATG
TCTCTCCTAGAAGTTGGTTAGCTACTTCTCATTTTGTTCTAGGATTCTTCTTGTTCGTAG
GTCATTTGTGGCACGCGGGAAGGGCTCGTGCAGCTGCAGCAGGGTTTGAAAAAGGAA
TTGATCGTGATTTTGAACCTGTTCTTTCCATGACCCCTCTTAATATGACTATAGCCCTTG
GTAAATTTACCGAAGACGAAAAGGATTTATTTGATATTATGGATGACTGGTTACGGAGG
GACCGTTTCGTTTTTGTAGGTTGGTCCGGTTTATTGCTCTTTCCTTGTGCCTATTTCGCTT
TAGGGGGTTGGTTCACAGGTACAACCTTTGTAACTTCATGGTATACCCATGGATTGGCC
AGTTCCTATTTGGAAGGCTGCAATTTCTTAACTGCCGCAGTTTCTACTCCTGCTAATAGT
TTAGCACATTCTTTATTGTTACTATGGGGTCCTGAAGCACAAGGGGATTTTACTCGTTGG
TGTCAATTAGGCGGTCTGTGGACTTTTGTTGCTCTCCATGGCGCTTTCGGACTAATAGG

TTTCATGTTACGTCAATTCGAACTTGCTCGATCTGTTCAATTGCGACCTTATAATGCAAT
CGCATTCTCTGGTCCAATTGCTGTTTTTGTTTCTGTATTCTTGATTTATCCACTAGGTCAG
TCTGGTTGGTTCTTTGCCCCTAGTTTTGGTGTAGCAGCTATATTTCGATTCATCCTCTTTT
TTCAAGGATTTCATAATTGGACGTTAAACCCATTTCATATGATGGGAGTTGCCGGGGTAT
TGGGCGCTGCTTTGCTATGCGCTATTCATGGTGCTACTGTAGAAAATCTTTATTTTGAAG
ATGGTGATGGTGCAAATACATTCCGTGCTTTTAACCCAACTCAAGCTGAAGAAACTTAT
TCAATGGTCACCGCTAACCGCTTTTGGTCCCAAATCTTTGGGGTTGCTTTTTCCAATAA
ACGTTGGTTACATTTCTTTATGTTATTTGTACCAGTAACCGGTTTATGGATGAGTGCTCTT
GGAGTAGTCGGTCTGGCCCTGAACCTACGTGCCTATGACTTCGTTTCTCAGGAAATTCG
TGCGGCGGAAGATCCTGAATTTGAGACTTTCTACACCAAAAATATTCTCTTAAACGAAG
GTATTCGTGCTTGGATGGCAGCTCAAGATCAGCCTCATGAAAACCTTATATTCCCTGAG
GAGGTTCTACCCCGTGGAAACGCTCTTATGTCTGGAAACACAGGAGAACGTTCTTTTG
CTGATATTATTACCAGTATTCGTTACTGGGTCATTCATAGCATTACTATACCTTCCCTATTC
ATTGCGGGTTGGTTATTCGTCAGCACCGGTTTAGCTTACGATGTGTTTGGAAGCCCTCG
GCCAAACGAGTATTTCACAGAGAACCGACAAGGAATTCCATTAATAACTGGCCGTTTT
GATCCTTTGGAACAACTCGATGAATTTAGTAGATCTTTTATGACCATAGATCGAACCTAT
CCAATTTTTACAGTGCGATGGTTGGCTGTTCACGGCCTAGCTGTACCTACCGTTTCTTTT
TTGGGGTCAATATCAGCAATGCAGTTCATCCAACGAATGGCTACACAAACTGTTGAGA
ACGGTTCTAGA---TCTCGTCCAAAACCAACTACTGTAGGGAATTTATTGAAACCATTGA
ATTCGGAATATGGTAAAGTAGCTCCTGGATGGGGAACGACTCCTTTGATGGGTGTCGCA
ATGGCCCTATTTGCGATATTTCTATCTATTATTTTGGAGATTTATAATTCTTCCGTTTTATT
GGATGAAATTTCAATGAATATGCTTACTCTCAAACTTTTCGTTTACACAGTAGTGATATT
CTTTGTTTCTCTCTTTATCTTTGGATTCCTATCTAATGATCCGGGGCGTAATCCTGGACGT
GAAGAAATGGCTGATACTACTGGAAGGATTCCTCTTTGGATAATAGGTACTGTAGCTGG
TATTCTTGTGATTGGTTTAATAGGTATTTTTTTTTATGGTTCATATTCCGGATTGGGTTCAT
CCCTGATGCTTAATATCTTTAGTTTGATCTGTATCTGTCTTAATTCTGCCCTTTATTCGAGT
AGTCTTTTCTTCGCCAAATTGCCCGAGGCCTATGCTTTTTTGAATCCAATCGTAGATCTT
ATGCCAGTCATACCTTTGTTCTTTTTTCTCTTAGCCTTTGTTTGGCAAGCTGCTGTAAGT
TTTCGAATGACACAATCAAACCCGAACGAACAAAATGTTGAATTGAATCGTACCAGCC
TCTACTGGGGGTTATTACTCATTTTTGTACTTGCTGTTTTATTTTCCAATTATTTCTTCAAT
ATGGAAGTAAATATTCTTGCATTTATTGCTACTGCACTGTTCATTCTAGTTCCTACTGCTT
TTCTGCTTATCATTTACGTAAAAACAGAAAGTCAAAATAAAATGGAAACAGCAACCCT
AGTCGCCATCTTTATATCTGGTTTACTTGTAAGTTTTACCGGGTACGCCTTATATACCGCT
TTTGGGCAACCTTCTCAACAACTAAGAGATCCATTCGAGGAACATGGGGACATGGAAG
CATTGGTTTATACATTCCTCTTAGTCTCGACTCTAGGTATAATTTTTTTCGCTATCTTTTTT
AGAGAACCGCCTAAAGTTCCAACTAAAAAG------ATGACTCTTGCTTTCCAATTAGCTG
TTTTTGCATTAATTGCTACGTCATCAATCTTATTAATTGGCGTACCCGTTGTATTTGCTTC
TCCTGATGGTTGGTCAAGTAACAAAAATGTTGTATTTTCCGGTACATCATTATGGATTGG
ATTAGTCTTTCTGGTGGGTATCCTTAATTCTCTCATCTCT--------------------ATGTCACCAC
AAACAGAGACTAAAGCAAGTGTTGGATTCAAAGCTGGTGTTAAAGATTACAAATTGAC
TTATTATACTCCTGACTATGATCCCAAAGATACTGATATCTTGGCAGCATTCCGAGTAAC
TCCTCAACCTGGAGTTCCAGCTGAAGAAGCAGGGGCCGCGGTAGCTGCCGAATCTTCT
ACTGGTACATGGACAACTGTGTGGACCGATGGACTTACTAGCCTTGATCGTTACAAAG
GGCGATGCTACAAAATAGAGCCCGTTGCTGGAGAAGAAACTCAATTTATTGCTTATGTA

GCTTACCCATTAGACCTTTTTGAAGAAGGTTCTGTTACTAACATGTTTACTTCCATTGTA
GGTAATGTATTTGGGTTCAAAGCCCTGCGTGCTCTACGTCTGGAAGATCTGCGAATCCC
TGTTGCTTATGTTAAAACTTTCCAAGGCCCGCCTCATGGCATCCAAGTTGAGAGAGATA
AATTGAACAAGTATGGTCGTCCCCTGTTGGGATGTACTATTAAACCTAAATTGGGGTTAT
CTGCTAAAAACTACGGTAGAGCGGTTTATGAATGTCTCCGGGGTGGACTTGATTTTACC
AAAGACGATGAGAACGTGAACTCACAACCATTTATGCGTTGGAGAGATCGTTTCTTATT
TTGTGCCGAAGCACTTTATAAAGCACAGGCTGAAACAGGTGAAATCAAAGGGCATTAC
TTGAATGCTACTGCGGGTACATGCGAAGACATGATGAAAAGGGCTGTATTTGCCAGAG
AATTGGGAGTTCCTATCGTAATGCATGATTACATAACAGGGGGATTCACCGCAAATACT
ACCTTGGCTCATTATTGCCGAGATAATGGCCTACTTCTTCACATCCACCGCGCAATGCAT
GCAGTTATTGATAGACAGAAGAATCATGGTATGCACTTTCGTGTACTAGCTAAAGCGTT
ACGTATGTCTGGTGGAGATCATATTCACTCCGGTACCGTAGTAGGTAAACTTGAAGGGG
AAAGAGACATCACTTTGGGCTTTGTTGATTTACTGCGTGATGATTTCATTGAAAAAGAT
CGAAGTCGCGGTATTTATTTCACCCAAGATTGGGTCTCTCTACCAGGTGTTCTGCCCGT
GGCTTCGGGGGGTATTCACGTTTGGCATATGCCTGCTCTGACCGAGATCTTTGGGGATG
ATTCCGTACTACAGTTCGGTGGAGGAACTTTAGGACACCCTTGGGGAAATGCACCCGG
TGCCGTAGCTAATCGAGTAGCTCTAGAAGCATGTGTACAAGCTCGTAATGAGGGACGT
GATCTTGCTCGTGAAGGTAATGAAATTATCCGCGAGGCTGCTAAATGGAGCCCTGAACT
AGCTGCTGCTTGTGAGGTATGGAAGGAGATCAAATTTGAATTTGAAGCAGTGGATATTT
TGGATGTTATGGCGATACATTTATACAAAACTTCTACCCCGAGCACACGCAATCGAGCC
GTAGACAGTCAAGTGAAATCCAATCCACGAAATAATTTGATCTATGGACAGCATCATTG
TGGTAAAGGTCGTAATGCCAGAGGAATCATTACCGCAGGGCATAGAGGGGGAGGTCAT
AAGCGTCTATACCGTAAAATCGATTTTCGACGGAATGAAAAAGACATATATGGTAGAAT
CGTAACCATAGAATACGACCCTAATCGAAATGCATACATTTGTCTCATACACTATGGGGA
TGGTGAGAAGAGATATATTTTACATCCCAGAGGGGCTATAATTGGAGATACCATTGTTTC
TGGTACAGAAGTTCCTATAAAAATGGGAAATGCCCTACCTTTGACCGATATGCCCTTAG
GCACGGCCATACATAACATAGAAATCACACGTGGAAAGGGTGGACAATTAGCTAGAGC
AGCGGGTGCTGTAGCGAAACTGATTGCAAAAGAGGGGAAATCGGCCACATTAAAATTA
CCTTCTGGGGAGGTCCGTTTGATATCCAAAAACTGCTCAGCAACGGTCGGACAAGTGG
GGAATGTTGGGGTGAACCAGAAAAGTTTGGGTAGAGCCGGATCTAAGCGTTGGCTAG
GTAAGCGTCCTGTAGTAAGAGGAGTAGTTATGAACCCTGTAGACCATCCCCATGGGGG
TGGTGAAGGGAGGGCCCCAATTGGGAGAAAAAAACCCACAACCCCTTGGGGTTATCC
TGCACTTGGGAGAAGAAGTAGAAAAAGGAATAAATATAGTGATAATTTGATTCTTCGTC
GCCGTAGTAAAATGATTCAACCTCAGACCCTTTTGAATGTAGCGGATAACAGCGGGGC
TCGAGAATTGATGTGTATTCGAATCATAGGAGCTAGC---AATCGTCGATATGCTCATATCG
GTGACGTTATTGTTGCTGTGATCAAAGAAGCAGTGCCAAACATGCCCCTAGCAAGATC
AGAAGTGGTCAGAGCTGTAATTGTACGTACTTGTAAAGAACTCAAACGTGATAACGGT
ATGATAATACGATATGATGACAATGCTGCGGTTGTCATCGATCAAGAAGGAAACCCAAA
GGGAACTCGGGTTTTTGGTGCAATTGCCCGGGAATTGAGACAGTTAAATTTTACTAAAA
TAGTTTCATTAGCTCCGGAGGTATTAATGCTTAGTCCCAAAAGAACCAGATTCCGTAAA
CAACATAGAGGAAGAATGAAGGGAATATCTTATCGAGGTAATCATATTTCTTTCGGTAA
ATATGCTCTTCAGGCACTTGAACCCGCCTGGATCACATCTAGACAAATAGAAGCAGGTC
GACGAGCAATGACACGAAATGCACGACGTGGTGGAAAAATATGGGTACGTATATTTCC
AGACAAACCGGTTACAGTAAGACCCGCAGAAACACGTATGGGTTCGGGGAAAGGATC

CCCTGAATATTGGGTAGCTGTTGTTAAACCAGGTCGAATCCTTTATGAAATGGGTGGAG
TAACAGAAAATATAGCCAGAAAGGCTATTTCAATAGCATCGTCTAAAATGCCTATACGA
ACTCAATTCATTATTTCGGCAATGACCAGAATTAGACGGGGATATATAGCTCGGAGACG
TAGAACAAAAATTCGTTTATTTGCATCAAGCTTTCGAGGGGCTCATTCAAGGCTTACTC
GAACAATTACTCAACAGAAAATAAGAGCTTTGGTTTCGTCTCATCGGGATAGGGATCG
GCAAAAGAGAAATTTTCGTCGTTTGTGGATCACTCGAATAAACGCAGTAATTCGTGAA
GGG---GGGGTATCCTATAGTTATAGTAGATTAATACATGATCTGTATAAGAAACAGTTACT
TCTTAATCGTAAAATACTTGCACAAATAGCTATATCAAATAAAAATTGTCTTTATATGATT
TCCAATGAGATCATAAAAGAAGTAGATTGGAAAGAATCCACCGGAATAATTATGTTAAA
GACAGTATATGCTTTAGGTCAATATTTATCTATGTCTGCTCATAAAGCGCGAAGAGTAAT
AGATCAAATTCGTGGACGTTCTTATGAAGAAACACTTATGATACTAGAACTCATGCCTTA
TCGAGCATGTTATCCCATTTTTAAATTGGTTTATTCTGCAGCAGCAAATGCTAGTTACAA
TATGGATTCCAACGAATCAAATTTAGTTATTAGTAAAGCTGAAGTCAGCGAGGGTACTA
TCGTGAAGAAATTAAAACCTCGAGCTCGAGGACGTAGTTTTCCGATAAAAAGACCTAC
CTGCCATATAAGTATTGTAGTGAAAGATATATCCTTAGATGGGGGAGTATGGGACAAAA
AAATGGATGGAATCAAATATGCAGTATTTACAGACAAAAGTATTCGGTTATTGGGGAAA
AAT---CAATATACTTCTAATGTCGAATCAGGATCAACTAGGACAGAAATAAAGCATTGGG
TCGAACTCTTCTTTGGTGTCAAGGTAATAGCTATGAATAGTCATCGACTTCCGGGAAGG
GGTAGAAGAATGGGACCTATTATGGGACAGACAATGCATTACAGACGTATGATCATTAC
GCTTCAACCGGGTTATTCTATTCCACCTCTTAGAAAGAAAAGAACTATGGCAGTTCCAA
AAAAACGTACTTCTATATCAAAAAAGCGTATTCGTAAAAATATTTGGAAAAGAAAGGG
ATATTGGGCAGCCTTAAAAGCGTTGTCATTAGGGAAATCTCTTTCTACTGGAAATTCAA
AAGGTTTTTTTGTGCGACAAACAAATAAGATGGCCAAGGGGAAGGATGTCCGAATAAC
CGTTATTTTGGAATGTACCAGTTGTGTTCGAAACGGTGTTAAT------AAGGTATCAACGG
GTATTTCCAGATATATTACTCAAAAGAACCGGCACAATACGCCTAATCGATTGGAATTGA
GAAAATTCTGTCCATATTGTTACAAACATACGATTCATGGGGAGATAAAGAAAATGAAA
ATAAGAGCTTCTGTTCGTAAAATTTGTGAAAAATGTCGACTAATTCGTAGGCGGGGACG
AATTATAGTAATTTGTTCCAACCCAAGACATAAACAAAGACAGGGGATGGTTCAAGAG
AAAGTAAGAGTATCTACTCGGACACTACAGTGGAAGTGTGTTGAAGCAAGAGCGGAC
AGTAAGCGTCTTTATTATGGACGCTTTATTCTGTCTCCACTTATGAAAGGTCAAGCTGAT
ACAATAGGCATTGCAATGCGAAGAGCTTTGCTTGGAGAAATAGAAGGAACATGTATCA
CACGTGCAAAATCTGAG------AAAATACCACACGAATATTCTACCCTAGTGGGTATTCAA
GAATCAGTACATGAAATTTTAATGAATTTGAAAGAAATTATATTGAAAAGTAATCTATAT
GGAACTTGCGACGCGTCTATTTGTATTAGGGGTCCTGGATATGTAACTGCTCAAGATATC
ATCTCACCGCCTTATGTAGAAATCGTTGACAATACACAGCATATAGCTAGCTTGGCGGA
ACCAATTGATTTGTGTATTGGATTACAAATAGAGAGGAATCGCGGATATCTTATAAAAAC
GCCAAATAACAACTTTGATGGAAGTTAT------CCTATAGATGCTGTATTCATGCCTGTTCG
AAACGCGAATCATAGTATTCATTCTTATGGGAATGGGAATGAGAAACAAGAGATACTCT
TTCTTGAAATATGGACAAATGGAAGTTTAACTCCTAAAGAAGCACTTCATCAAGCCTCC
CGGAATTTGATTGATTTATTTATTCCCTTTTTACATACAGAAGAAGAA------AACTTACAT
TTAGCGGACAATCAA---CACACGGTTCCTTTACCCCCTTTTACTTTTCATGAGAAATTGA
CT---AAACTAAGAAAAAACAAAAAA---AAAATAGCATTGAAATCGATTTTTATTGACCA
ATCAGAACTGCCTCCCAGGATCTATAATTGCCTTATAAGGTCCAATATATATACATTATTG
GACCTTTTGAATAACAGTCAAGAAGACCTTATGAAAATTGAACATTTTCGCATAGAAGA

TGTAAAAGCGATATTGGGCATTCTAGAAAAGCAT------TTCGCAATTGATTTACCGAAAA
AGCTGATGCTCCGAGATGGAAAAAATGAGGGAATGTCTACAATACCTGGGTTTAATCA
GATACAATTTGAAGGATTTTGTAGGTTCATTGATCAGGGCTTGACGGAAGAACTTTATA
AGTTTCCAAAAATTGAAGATACCGACCAAGAAATTGAATTTCAATTATTTGTGGAAACA
TATCAATTAGTAGAACCGTTGATAAAAGAAAGAGATGCTGTGTATGAATCACTCACATA
TTCTTCTGAATTATATGTCTCCGCGGGACTCATTTGGAAAACTGGTAGGGATATGCAAG
AACAAACTATTTTTTTTGGAAACATTCCTCTAATGAATTCTCTGGGAACTTCTATAGTAA
ATGGAATATATAGAATTGTGATCAATCAAGTATTGCAAAGCCCCGGTATTTATTACCGGT
CAGAATTGGACCATAACGGAATTTCGGTGTATACCGGCACCATAATATCAGATTGGGGA
GGAAGATCAGAATTAGAGATTGATAGAAAAGCAAGGATATGGGCTCGTGTGAGTAGGA
AACAAAAAATATCTATTCTAGTTTTATCATCAGCTATGGGTTCGAATCTAAGAGAAATTC
TAGAGAATGTTTGCTATCCTGAAATATTTTTGTCTTTTCTGACTGATAAGGAGAAAAAA
AAAATCGGGTCAAAAGAAAATGCCATTTTGGAGTTTTACCAACAATTTGCTTGTGTAG
GCGGGGATCCGGTATTTTCTGAATCCTTATGTAAGGAATTACAAAAGAAATTCTTTCAA
CAAAGATGTGAATTAGGAAGGATTGGTCGACGAAATATGAATCGGAGACTGAACCTTG
ATATATCCCAGAACAATACATTTTTGTTACCGCGAGATATATTGGCAGCCGCGGATCATT
TGATTGGAATGAAATTTGGAATGGGTACACTTGACGAT------ATGAATCATTTGAAAAAT
AAACGTATTCGTTCTGTAGCAGATCTTTTACAAGATCAATTCGGATTGGCTCTGGTTCGT
TTAGAAAATGTGGTTCGAGGAACTATATGTGGAGCACTTAGG---CATAAATTG---ATACC
GACTCCTCAGAATTTGGTAACTTCAACTCCA------------TTAACAACTACTTATGAATCTTT
TTTCGGTTTACACCCATTATCTCAAGTTTTGGATCGAACTAATCCATTGACACAAATAGT
TCATGGGAGAAAATTGAGTTATTTGGGCCCTGGAGGATTGACAGGGCGAACTGCTAGT
TTTCGAATACGAGATATCCATCCTAGTCACTATGGGCGTATTTGCCCAATTGACACGTCT
GAAGGAATAAATGTCGGACTTATTGGATCTTTAGCAATTCATGCGAGGATTGGTCGTTG
GGGA---TCTCTAGAAAGCCCGTTTTAT---------GAAATTTCTGAGAGATCAAAAGGG------
GCACGGATGCTTTATTTATCACCAGGTAAAGATGAATACTATATGGTAGCGGCGGGAAA
TTCTTTGGCCTTGAATCAGGGTATTCAGGAAGAACAGGTTGTTCCAGCTCGATACCGTC
AAGAATTCCTGACTATTGCGTGGGAACAGGTTCATCTTCGAAGTATTTTTTCCTTCCAAT
ATTTTTCTATTGGAGCTTCCCTCATTCCTTTTATCGAGCATAATGATGCGAATCGGGCTTT
AATGAGTTCGAATATGCAACGTCAAGCAGTTCCTCTTTCTCGGTCCGAGAAGTGCATTG
TTGGAACTGGGTTAGAACGCCAAGCGGCTATAGATTCAGGGGCTCTTGCTATAGCGGA
ACACGAGGGAAAGATCATTTATACCGATACTGACAAGATCCTTTTATCAGGTAATGGAA
ATACTCTAAGCATTCCATTAGTTATATATCAACGCTCCAACAAAAATACTTGTATGCATCA
AAAACCCCAGGTTCAGCGGGGTAAATGCATAAAAAAGGGACAAATTTTAGCCCATGGC
GCCGCTACAGTTGGTGGCGAACTCGCTTTGGGGAAAAACGTATTAGTAGCTTATATGCC
ATGGGAAGGTTACAATTTTGAAGATGCAGTACTCATTAGCGAGCGCTTGGTATATGAAG
ATATTTATACTTCTTTTCACATACGAAAATATGAAATTAAGACTCATGTGACAAGCCAAG
GC---CCCGAAAGGGTCACTAATGAAATACCGCATTTAGAAGCCCATTTACTCCGCAATTT
AGACAAAAACGGAATTGTAATGCTGGGATCTTGGGTAGAGACGGGTGATATTTTAGTA
GGTAAATTAACGCCCCAAATGGTGAAAGAA---TCGTCGTATGCCCCGGAAGATAGATTG
TTACGAGCCATACTTGGCATTCAGGTATCTACTTCAAAAGAAACTTGTCTAAAACTACC
TATAGGTGGTAGGGGTCGGGTTATTGATGTGAGATGGATCCAGAAAAGGGGA---GGTTC
TAGTTATAACCCAGAAATGATTCGTGTATATATTTCACAGAAACGCGAAATTAAGGTAG
GCGATAAAGTAGCTGGAAGGCATGGAAATAAGGGTATCATTTCAAAAATTTTGCCTAGA

CAAGATATGCCTTATTTGCAAGATGGAAGACCTGTTGATATGGTCTTCAACCCATTAGG
AGTACCTCCACGAATGAATGTAGGGCAGATATTTGAATGTTCACTCGGGTTAGCGGGGG
GTCTGCTAGACAGACATTATCGAATAGCGCCTTTTGATGAGAGATATGAACAAGAAGCT
TCGAGAAAACTAGTGTTTTCTGAATTATATGAAGCCGGTAAGCAAACAGCGAATCCATG
GGTATTTGAACCCGAGTATCCGGGAAAAAGTAGAATATTTGATGGAAGAACGGGGGAT
CCTTTTGAACAACCTGTTATAATAGGAAAGCCTTATATCTTGAAATTAATTCATCAAGTT
GATGATAAAATCCATGGACGTTCCAGCGGACATTATGCACTTGTTACACAACAACCCCT
TAGAGGAAGGGCCAAGCAAGGTGGACAACGGGTAGGAGAGATGGAGGTTTGGGCTCT
AGAGGGATTTGGTGTTGCTCATATTTTGCAAGAGATGCTTACTTATAAATCGGATCATAT
TAGAGCTCGCCAGGAAGTACTTGGTACTACGATCATTGGAGGAACAATACCTAATCCTC
AGGATGCTCCAGAATCTTTTCGATTGCTCGTTCGAGAACTACGATCTTTGGCTCTGGAA
CTGAATCATTTCTTTGTATCTGAGAAGAATTTCCAGATTAATAGGAAGGAAGCTATGAAT
CAGAATTTTTCTTCTATGATCGATCGGTATAAACATCAACAACTCCGAATTGGATCAGTT
TCTCCTCAACAAATAAGTACTTGGGCCAATAAAATCCTGCCCAATGGAGAGATAGTTGG
AGAGGTGACAAAACCTTATACTTTTCATTACAAAACCAATAAACCGGAAAAAGATGGA
TTATTTTGTGAAAGAATTTTTGGGCCTATCAAAAGCGGAATTTGTGCTTGTGGAAATTAT
CGAGTAATCGGGAATGAAAAAGAAGACCCGAAATTTTGTGAACAATGCGGAGTCGAA
TTTGTTGATTCTCGGATACGAAGATATCAAATGGGCTACATCAAACTCGCATGCCCAGT
AACCCATGTGTGGTATTTGAAACGTCTTCCTAGTTATATTGCGAATCTTTTAGATAAACC
TCTTAAAGAATTAGAAGGCCTAGTATACTGCGAT------TTTTCTTTTGCGAGGCCC---ATA
GCTAAAAAACCTACTTTCTTACGATTACGAGGTTCGTTCGAATATGAAATCCAATCCTG
GAAATACAGCATCCCGCTTTTTTTTACTACCCAAGGCTTCGATACATTTCGAAATCGAG
AAATTTCTACTGGAGCAGGTGCTATCCGAGAACAATTAGCCGATCTCGATTTACGGATT
ATTATAGATTCTTCGTTGGTAGAATGGAAAGAGTTGGGGGAAGAC---GGGCCTACAGGG
AATGAATGGGAAGATCGAAAGGTTGGAAGACGAAAGGATTTTTTGGTTAGACGTATGG
AATTAGCTAAGCATTTTATTCGAACAAATATAGAACCAGAATGGATGGTTTTGTGTCTAT
TACCTGTTCTTCCTCCTGAGTTGAGACCGATCATTCAGATAGATGGGGGTAAACTAATG
AGCTCAGATATTAATGAACTCTATAGAAGAGTTATCTATCGAAACAATACTCTTACCGAC
CTATTAACAACAAGTAGATCAACGCCAGGAGAATTAGTAATGTGTCAGGAGAAATTGG
TACAAGAAGCCGTGGATACACTTCTTGATAATGGAATCCGCGGACAACCGATGAGGGA
CGGTCATAATAAAGTTTACAAGTCATTTTCAGATGTAATTGAAGGCAAAGAGGGAAGAT
TTCGTGAGACTCTGCTTGGCAAACGGGTCGATTATTCAGGGCGTTCCGTCATTGTCGTG
GGTCCGTCACTTTCATTACATCGATGTGGATTGCCCCGCGAAATAGCAATAGAGCTTTT
CCAGACATTTGTAATTCGTGGTCTAATTAGACAACATCTTGCTTCGAACATAGGAGTTG
CTAAGAGTAAAATTCGGGAAAAAGAACCGATTGTATGGGAAATACTTCAGGAAGTTAT
GCAGGGGCATCCCGTATTGCTAAATAGAGCGCCGACTCTGCATAGATTAGGCATACAGG
CATTCCAGCCTGTTTTAGTGGAAGGTCGTGCTATTTGTTTACATCCATTAGTTCGTAAAG
GATTCAATGCAGACTTTGACGGGGATCAAATGGCTGTTCATGTGCCTTTATCTTTGGAG
GCTCAAGCAGAAGCACGTTTACTTATGTTTTCTCATATGAACCTTTTGTCTCCAGCTATT
GGGGATCCTATTTCCGTACCAACTCAAGATATGCTTATTGGACTCTATGTCTTAACGAGC
GGGAATCGTCGGGGTATTTGTGTAAATAGGTATAATCCATGTAATCGC---------AGAAACT
ATCAAAATGAAAGAATTTACGATAATAACTATAAGTATACGAAA------GAACCCTTTTTTT
GTAATTCCTATGATGCAATTGGAGCTTATCGGCAGAAACGAATCAATTTAGATAGTCCTT
TGTGGCTTCGGTGGCGACTAGATCAACGC---GTTATTTCTGCA------AAAGAAGCCCCCC

TCGAAGTTCACTATGAATCTTTGGGTACCTATTATGAAATTTATGGGCAATATCTAATAGT
AAGAAGTATAAAAAAAGAAATTCTATATATCTATATTCGAACTACTGTTGGTCATATTTCT
CTTTATCGAGAAATCGAAGAAGCTATACAGGGGTTTTGTCAGGCCTGTTCATATGGTCC
C----------------------------------ATGGCAGAACGGGCCAATCTGGTCTTTCACAATAAAGTG
ATAGACGGAACTGCCATGAAACGACTTATTAGTAGATTAATAGAACACTTCGGAATGGC
ATATACATCACACATCCTGGATCAAGTAAAGACTTTGGGTTTTCAACAAGCTACTGCTA
CCTCCATTTCATTAGGAATTGACGATCTTTTAACAATCCCTTCTAAGAGATGGCTAGTTC
AAGATGCTGAACAACAAAGTTTAATTTTGGAAAAGCAACACCATTATGGGAATGTACA
CGCGGTAGAAAAATTACGTCAATCCATTGAGATATGGTATGCTACAAGTGAATTTTTGC
GACAAGAAATGAAACCTAATTTTAGGATGACTGACCCCTTTAATCCAGTTCATATAATGT
CTTTTTCGGGAGCTAGAGGAAATGCATCTCAGGTACATCAATTAGTAGGTATGAGAGGA
TTAATGGCGGATCCCCAAGGACAAATGATTGATTTACCCATTCAAAGCAATTTACGCGA
AGGACTCTCTTTAACAGAATATATTATTTCTTGCTACGGAGCCCGTAAAGGGGTTGTGG
ATACTGCTGTACGAACCTCAGATGCTGGATATCTCACGCGCAGACTTGTTGAAGTAGTT
CAACACATTGTTGTACGTCGAAAAGATTGTGGCACCGTCCGAGGTATTTCTGTGAGTCC
CCGAAATGGGATGATGCCG---GAAAGGGTTTTTATCCAAACATTAATTGGTCGTGTATTA
GCGGATGATATATATCTGGGTCCACGATGTATTGCCACCAGAAATCAAGATATTGGTAGT
GGACTTGTCAATCGATTCATAACC------------------TTTCGAGCACAACCAATATATATTCGA
ACTCCCTTTACTTGTAGGAGTACATCTTGGATCTGTCGATTATGTTATGGCCGGAGTCCT
ACTCACGGCGATCTGGCCGAATTGGGAGAAGCTGTGGGTATTATTGCAGGTCAATCTAT
TGGAGAACCTGGTACTCAATTAACATTAAGAACCTTTCATACCGGCGGAGTATTCACAG
GGGGTACTGCAGAACATGTGCGAGCCCCTTCTAATGGAAAAATAAAATTCAATGAGGA
TTTGGTTCATCCGACACGTACACGTCATGGACATCCTGCTTTTCTATGTTCTATAGACTT
GTATGTAACTATTGAGAGTGAAGATATTATACATAATGTGAATATYCCACCTAAAAGTTT
TCTTTTAGTTCAAAATGATCAATATGTAGAATCAGAACAAGTGATTGCTGAGATTCGCG
CAGGAACATCCACT---TTGAATTTTAAAGAGAAGGTTCGAAAACATATTTATTCTGACTC
AGAGGGAGAA---ATGCACTGGAATACCGATGTGTATCATGCACCTGCATTTACTTATGG
GAATGTTCATCTCTTACCAAAAACAAGTCATTTATGGATATTATTAGGAGAGCCATGCAG
A------TCCGATCTAGTCGCCCTCTCGATCCACAAGGATCAAGATCAAATGAACGCCCAT--
----TCTCTTTCTGTCAAGAGAAGATATATTTCTAACCTCTCA------GTAACTAATGATCAA--
-GCGAGACACAAATTCTTTAGTTCGGATTTTTCTGGTAAA---GAAGATAGGATTACTGAT
TAT---TCAGAACTTAATCGA---------ATTGGTCATTGTAATCTCCCATATCTG---GCCATTCT
CCACGCAAATTCT------GATTTATTGGCAAAGAGACGAAGAAATAGATTCATCATTCCAC
TCCAATCGATTCAAGAACACGAGAACGAACTAATGCCCTCTTCGGGTATCTCGATTGAA
ATACCC------ATACATGGTATTTTCCGTAAAAAGAGTATTATTGCTTATTTCGATGATCCTC
GATACAGAAGAAAGAGTTCGGGAATTACTAAATATGGTACTATAGAAGTGCATTCAATC
GTCAAAAAAGAGGAT------TTGATTGAGTATCGAGGA---GTCAAGGAATTTAGGCCAAA
ATACATGAAAGTGGATCGATTCTTTTTCATTCCCGAGGAAGTGCATATCTTACCCGGATC
TTCTTCCATAATGGTACGGAACAATAGTATTATTGGGGTAGATACACAAATCACTTTAAC
TACAAGAAGCCGAGTGGGCGGATTGGTCCGAGTGGAGAGAAAAAAAAAAAGAATTG
AACTTAAAATATTTTCTGGAGATATCCATTTTCCTGGAGAGACGGATAAGATATCCCGAC
ATAGTGGCGTCTTGATACCACCAGGAACC------GGAAAAACAAATTCCAAGGAATCAAA
AAAA------TGGAAAAATTGGATCTATGTCCAACGG------ATCACACCTACCAAG------AAA
AAGTATTTTGTTTTGGTTCGACCTGTAGTCACATATGAAATAACGGACGGTATAAATTTA

GCA---CTTTTCTTCCCGGATCTGTTGCAGGAAAGGGATAATGTGCAACTTCGAGTTGTC
AATTATATCCTTTATGGAACTGGCAAACCAATTCGGGGTTTTTATGACACAAGTATTCAA
TTAGTTAGGACTTGTTTAGTATTAAATTGGGTCCAAGACAAAAAA---------AGTTCTTCTA
TCGAAGAGGCCCGTACTTCCTTTGTTGAAATAAGGATAAATAGTTTGATTCGAGATTTT
CTAAAAATCGACTTAGCGAAATCCCCTATTTTGTATACCGGAAAAAGGAAAGATCCATC
GGGTTCAGGATTGATCTCTGAG---AATGGATCAGATCGCACCAATATCAATCCGTTTTCT
TCCAGTTTTTTCTAT---------TCCAAGGCAAGGATTAAAGAATCCCTTAATCAAAACCAAG
GAACTATTCATACATTGTTGAATAGA---------AATAAGGAATGCCAATCTTTGATAATTTTG
TCATCATCCAATTGTTTTCGAATGGGTCCA------TTCAACGATGTAAAATATCACAATGTG
---------ATAAAAGAATCA---CCCCTAATTCAAATTAGGAATTCGTTGGGCCCTTTAGGAAC
G------GCCCTTCAAATTGCGAATTTTTATTCA------TTTTCCCATTTA------ATAACTTACAAT
CAGATCCTG---GTAACTAACTATTTGCAACTTGACAATTTAAAA---------CAGAGTTTTCA
AGTACTTAAA---------TATTATTTAATGGATGAAAAT------GGGAAAATTTATAATCCCGATC
GATGCTGTAACATTATTTTGAATCCATTCAATTTGAATTGGTTTTTTCTCCATCACAATTA
TTGTGAAGAGACGTCT---ACAACAATGAGTCTTGGGCAGTTTATTTGTGAAAATGTATGT
ATAGCCAAAAATGGACCACAC---CTAAAA------CCGGGTCAAGTTCTAATTGTTCAA---G
TTGACTCTGTAGTAATACGATCCGCTAAGCCTTATTTGGCCACCCCAGGAGCAACTGTT
CATGGCCATTATGGGGAAATCCTTTACGAAGGAGATACATTAGTTACATTTATATATGAA
AAATCGCGATCTGGTGATATAACGCAGGGTCTTCCAAAAGTCGAACAGGTGTTAGAAG
TGCGTTCGATTGATTCAATATCGATGAATCTCGAAAAGAGGGTTGAGGGCTGGAACGA
ATGTATAACAAGAAATCTTGGAATTCCTTGGGGATTCTTGATTGGGGCTGAGCTAACTAT
AGTGCAAAGTCGTATTTCTTTGGTTAATAAGATCCAAAAGGTTTATCGATCCCAGGGGG
TGCAGATCCATAATAGGCATATAGAAATTATTGTACGTCAAATAACATCAAAAGTGTTGG
TTTCAGAA---------GATGGGATGTCTAATGTTTTTTTACCCGGAGAATTTATTGGATTGTT
GCGAGCGGAACGAATGGGTCGCGCTTTGGAAGAAGCGATCTGTTACCAAGCCGTCTTA
TTGGGAATAACAAAAGCATCTCTGAATACTCAAAGTTTCATATCCGAAGCGAGTTTTCA
AGAAACTGCTCGAGTTTTAGCAAAAGCAGCTCTCCGGGGTCGTATCGATTGGTTGAAA
GGCCTGAAAGAGAACGTTGTTCTGGGGGGAATGATACCCGTTGGTACCGGATTCAAAG
GATTG------GTGCCCCCTTCGAGGCAACATAAGAATATTCCC------TTGGAAACCAAAAAT
AAGAATCTATTCGAGGGGGAAATGAGA------GATATTTTGTTCCACCACAGAAAATTTTT
TGATTCTTGCCTTTCAAAGAATTTCCATGATACACCAGAACAATCATTTATAGGA----------
--------TTTAATGATTCCATGACAAAAAGATATTGGAACATCAATTTGGAAGAGATGATGA
AGGCCGGAGTTCATTTTGGACATGGTACTAAGAAATGGAATCCTAAAATGGCCCCTTAT
ATCTCTGCAAAGCGTAAAGGTATTCATATTACAAATCTTACTAGAACTGCTCGTTTTTTA
TTAGAAGCTTGTGATTTGGTTTTTGATGCAGCAAGTAGGGGAAAACAATTCTTAATCGT
TGGCACTAAAAATAAAGCAGCTAACTTAGTAGCATGGGCTGCAATAAAGGCTCGGTGT
CATTATGTTAATAAAAAATGGCTCGGCGGTATGTTAACGAATTGGTCCACTACAGAAAC
AAGACTTCATAAGTTTAGAGACTTGAGAACAGAACAAAAAACGGGGAGACTCAACCG
TCTTCCGAAAAGAGATGCGGCTATGTTGAAAAGACAATTATCCCACTTGCAAACATATC
TGGGCGGGATTAAATATATGACAGGGTTACCAGATATTGTAATCATCGTTGATCAGCACG
AAGAATATACGGCCCTTCGAGAATGTATCACTTTGGGAATTCCAACAATTTGTTTAATCG
ATACAAATTGTGACCCCGATCTTGCAGATATTTCGATTCCAGCGAACGACGATGCTATAT
CYTCAATCCGATTAATTCTTAACAAATTAGTATTCGCAATTTGTGAGGGGCGTTCT---AG
CTATATAAGAAATCCGATGGGACAAAAAATAAATCCACTCGGTTTCAGACTTGGTACAA

CCCAAGGTCATCATTCCCTTTGGTTTGCACAACCAAAAAATTATTCCGAAGGTTTACAA
GAAGATCAAAAAATAAGAGATTTTATCAAGAATTATGTACAAAAAAATATGAAAATTTC
CTCTAGC---GTTGAGGGAATTGCACGTATAGAGATTCAAAAACGAATCGATCTGATCCA
GGTCATAATCTATATGGGATTCCCAAAGTTATTAATAGAAAGTCGACCGCGA-----------G
GAATCGAAGAATTGCAGATGAATCTACAAAAAGAATTTAAGTCTGTGAACCAAAAACT
TAACATTGCTATCACAAGAATTGCAAAACCTTACGGAAGCCCTAATATTCTTGCGGAAT
TTATAGCCGGCCAATTAAAGAATAGAGTTTCATTTCGAAAAGCAATGAAAAAGGCTATT
GAATTAACCGAACAAGCAGATACAAAAGGAATTCAAGTACAAATTGCAGGGCGTATCG
ACGGAAAAGAAATTGCGCGTGTGGAATGGATCAGAGAAGGTAGGGTTCCCCTGCAAA
CCATTCGAGCTAAAATCGATTATTGTTCCTATACTGTTCGAACTATTTATGGAGTATTAGG
CATCAAAATTTGGATATTTATAGACGGGGAAATGTCGCGTTACCGAGGACCTCGTTTCA
AAAAAATACGCCGCCTGGGAGCTTTACCGGGACTAACTAATAAAAGGCCTAGAGCCGG
AAGTGATCTTAGAAATCAATCGCGTTCCGGGAAA---AAATCTCAATATCGTATTCGTCTA
GAAGAAAAACAAAAATTGCGTTTTCATTATGGTCTTACAGAACGACAATTACTTAAATA
CGTTCGTATCGCCGGAAAAGCCAAGGGGTCAACAGGTCAGGTTTTACTCCAATTACTY
GAAATGCGCTTGGATAACATCCTTTTTCGATTGGGTATGGCTCCGACTATTCCCGGAGC
CCGTCAATTAGTTAACCACAGACATATTTTAGTCAATGGTCGTATAGTAGATATAGCAAG
TTATCGCTGCAAACCCCGAGATATTATTACGGCGAGGGATGAAGAAAACTCTAGAACTC
TGATTCAAAATTCTCTCGATTCGTCCTCTCAT---GACGAATTGCCAAAACATTTGACTCT
TCACCCATTCCAA------TATAAAGGATTAGTCAATCAAATTATAGATAGTAAATGGGTCGG
TTTGAAAATAAATGAATTGCTAGTCGTAGAATATTATTCGCGCCAGACTATGTCACGTCG
AGGTACTGCAGAAGAAAAAACTGCAAAATCCGATCCAATTTATCGTAATCGATTAGTTA
ACATGTTGGTTAACCGTATTCTGAAACACGGAAAAAAATCATTGGCTTATCAAATTATCT
ATCGAGCCGTGAAAAAGATTCAACAAAAGACAGAAACAAATCCACTATCTGTTTTACG
TCAAGCAATACGTGGAGTAACTCCCGATATAGCAGTAAAAGCAAGACGTGTAGGTGGA
TCGACTCATCAAGTTCCCATTGAAATAGGATCCACACAAGGAAAAGCACTTGCCATTC
GTTGGTTATTAGCGGCATCCCGAAAACGTCCGGGTCGAAATATGGCTTTCAAATTAAGT
TCCGAATTAGTGGATGCTGCCAAAGGGAGTGGCGATGCCATACGCAAAAAGGAAGAG
ACTCATAGAATGGCAGAGGCAAATAGAGCTTTTGCACATTTTCGTATGGGTAGGGATAC
TATTGCTGACATAATAACCTCTATACGAAATGCTGATATGGATAAAAAAAGAGTGGTTCG
AATAGCATCTACTAATATCACTGAAAATGTTGTTAAAATACTTTTACGAGAAGGTTTTAT
CGAAAACGTGAGAAAACATCAAGAAAACAAAAAGGATTTTTTGGTTTTAACCCTACG
ACATAGAAGG------AATAGGAAAAGGCCTTAT---------AGAAATATTTTAAATTTAAAACG
GATCAGTCGGCCTGGTCTACGAATCTATTCTAACTATCAACGAATTCCTAGAATTTTGGG
CGGGATCGGAATTGTAATTCTTTCTACTTCTCGAGGTATAATGACAGACCGAGAGGCTC
GACTAGAAAGAATTGGCGGAGAAATTTTGTGTTATATATGGATGGCAAAAGCTATACCG
AGAAGTGGTTCACGTAGGAGTGGGCGTATTGGTTCACGTAAGAGTACACGTAGAATAC
CAAAGGGGGTTATTCATGTTCAAGCAAGTTTCAATAATACTATTGTGACTGTTACAGAT
GTACGGGGTCGGGTGGTCTCTTGGTCCTCTGCCGGTACTTGTGGATTCAAGGGTACAA
GAAGGGGGACGCCGTTTGCTGCTCAAACCGCAGCAGGAAACGCTATTCGTACAGTAGT
GGATCAAGGTATGCAACGAGCAGAAGTCATGATAAAAGGACCCGGTCTCGGAAGAGA
CGCAGCATTACGAGCTATTCGCAGAAGTGGTATACTATTAACTTTCGTGCGGGATGTAA
CCCCCATGCCACATAATGGCTGTAGACCTCCGAAAAAACGACGTGTGATGCCAACCATT
AAACAACTTATTAGAAATACAAGACAGCCAATCAGAAATGTCACGAAATCCCCCGCGC

TTCGGGGATGCCCCCAGCGTCGAGGAACATGTACTAGGGTGTATACTATCACCCCCAAA
AAACCAAACTCTGCCTTACGTAAAGTTGCCAGAGTACGATTAACCTCTGGATTTGAAAT
CACTGCTTATATACCCGGTATTGGCCATAATTCACAAGAACATTCTGTAGTCTTAGTAAG
AGGGGGAAGGGTTAAGGATTTACCCGGTGTGAGATATCACATTGTTCGAGGAACCCTA
GATGCTGTCGGAGTAAAGGATCGTCAACAAGGGCGTTCTAAATATGGAGTAAAAAAGC
CAAAAATGGCAAGGAAAAGTTTGATTCAGAGGGAGAAGAAGAGGCAAAAATTGGAA
CAGAAATATCATTCGATTCGCCGATCCTCAAAAAAAGAAATAAGC---------AAAGTTCTA
TCGTTAAGCGACAAATGGGAAATTTATGGAAAGTTACAATCCCCACCGCGGAATAGTG
CACCTACACGTCTTCATCGACGTTGTTTTCGACCGGAAGGCCGAGAGCTAACTATCGA
GACTTTGGACTATCCGGACACATACTTCGTGAAATGGTTCATGCATGTTTGTTGCCAGG
GGCGACAAGATCAAGTTGGATGATAAAAAATCCATTCAGTGGAATTATTTTGAAA------G
AGGAAAACAAAGACAACAGGGGGTCTGCTGAATTTCAAGTAGTCAGTTTCACCAATA
GGATACGAAGACTTACTTCACATTTGGAATTGCACAAAAAAGACTATTTATCTCAGAGA
GGTCTGCGTAAAATTCTAGGAAAGCGTCAACGGCTACTGGCTTATTTGTCAAAAAAAA
ATAGAGTACGTTATAAAGAATTAATTGGTCGGTTGGATATTCGAGAAACAAAAATTCGT-
-----ATGGTAAAACTTCGTTTGAAACGATGTGGTAGAAAGCAACGAGCCGTCTATCGAAT
CGTTGCAATTGATGTTCGATCCCGAAGAGAAGGAAGAGATCTTCGGAAAGTGGGTTTT
TATGATCCGATAAAGAATCAAAGTTGTTTAAATGTTCCCGCTATTCTATATTTCCTTGAA
AAAGGCGCTCAGCCGACAGGAACTGTTCGGGATATTTTAAAGAAGGCGGAGGTTTTTA
AG--------------------------------ATGGATAAATCCAAGCGACCTTTTCTTAAATCCAAGCGATC
TTTTCGTAGGCGTTTGCCCCCGATCCAATCGGGGGATCGAATTGATTATAGAAACATGA
GTTTAATTAGTCGATTTATTAGTGAACAAGGAAAAATATTATCTAGACGGGTGAATAGAT
TGACCTTGAAACAACAACGATTAATTACTATTGCTATAAAACAAGCTCGTATTTTATCTT
TGTTACCTTTTCTTAATAATGATAAA------CAATTTGAAAGAACCGAGTCGACCGCTAGA
ACTGCTGGTCTTAGAGCCAGAAAAAAAGTGACACGTTCACTAAAAAAAAATCCTTTTG
TAGCAAATCGTTTATTAAGAAAAATTGATAAGCTTAACACAAAAGCAGAAAAAGAAAT
AATAATAACTTGGTCCCGGGCATCTACCATTATACCCACAATGATAGGCCATACTATTGC
TATCCATAATGGAAAGGAGCATTTGCCTATTTATATAAACGATCGTATGGTAGGCCACAA
ATTGGGAGAATTTGCACCTACTTTAAATTTCCGAGGACATGCGAAAAGCGATAATAGAT
CTCGTCGTATGATTTTGAAATCTTTTCTACTAGGTAATCTAGTATCCTTATGCATGAAGAT
AATCAATTCGGTCGTTGTGGTCGGACTCTATTATGGATTTCTGACCACATTCTCCATAGG
GCCCTCTTATCTCTTCCTTCTCCGAGCTCGGGTTATGGAAGAAGGAGAAGAAGGAACC
GAGAAGAAGGTATCAGCAACAACAGGTTTTATTACGGGACAGCTCATGATGTTCATATC
GATCTATTATGCGCCTCTGCATCTAGCATTGGGTAGACCTCATACAATAACTGTACTAGC
TCTACCGTATCTTTTGTTTCATTTCTTCTGGAATAATCACAAAAACTTTTTTGATTATGGA
TCTACTACCAGAAATTCAATGCGTAATCTTAGCATTCAATGCGTATTCCTGAATAATCTC
ATTTTTCAATTATTCAACCATTTCATTTTACCAAGTTCAATGTTAGCCAGATTAGTCAACA
TTTATATGTTTCGATGCAACAACAAGATGTTATTTGTAACAAGCAGTTTTGTTGGTTGGT
TAATTGGTCACATTTTATTCATGAAATGGGTTGGATTGGTATTAGTCTGGATACGGCAAA
ATCATTCTATTAGATCG---ATTCGATCTAATAAGTACCTTGTGTCAGAATTGATAAAT------
TCTCGAATCTTTAGTATTCTCTTATTTATTACCTGTGTCTACTATTTAGGCAGAATACCGT
CACCCATTATTACTAAGAAACTGAAAGAAACCGAAGAAGATAAACCTCTTGTGACCCT
TCTTTTCGATTATAAACGATGGAATCGCCCATTACGATACATAAAA---AATAGATTTGAA
AGGACTGCAAGAAATGAAATGTCACAATATTTTTTTATACATGCCGAAGTGATGGAAA

AGAAAGAATATCTTTTACGTATCCGCCTAGCTTGTCAACTTTTGGAGAAATGATACAAA
GAAGGATGTCCTTGCCCACACTAGAAAAACTCTCTTCGGATGAATTGTACAATCATTGG
ATTTATACTAACAAACAAAAAAAGAACAACTTAAACAACGAGTTTTAAATAGAATTAA
GGCTCTAGATATGGGATTTCTTTCTCTAGATATACTCGAAAAAAGTACTAGATTGTATAAT
------ACTAAAAAAGATTACTTGCCTAAAATATATGATCCCATTTTGAAT---TCATATCGTGG
AACAATCAAAAAAAAATTTTCACCTATAGAGACAATGGAAATAAATAAGATTCATAATC
TCCTTTACCAAGAGTTTAAACAGAAAATCGCTAATGGTCAC---------ATAAAAGAAATAA
GTAAAAAAGTCCCTCGATGGTCAGACAAATTTATTGAGTTGGAACAACAGACGGGCGT
ACAGATTCGTTCAAGAAAAATTGACGATATAGTAGTTTTTACTGAT------ATAGAATATTT
GCAAGAATCGGATTTTCGTCGAAACATAATCAAAGGTTCTATGCGTACTCAAAGGCGTA
AAACTATTATTTGGAAACTGTTTGAAGCAAATGCGCACTCCCCGCTTTTTTTGAACAGA
---AGAAAA---AAATCCCCCCTTTTTTCTTTTGATATCTCCGAACTGATGAAACTTATCTTT
AGAAATATAAAGGAAAAAGTGCGGATAGAA------------TGGGATCTCGTTCCATTTTCGA
AAACAACAAGAAGTTTGCTATTAATAATTCAATCGATGCTTAGAAAATATATTATATTAC
CTTCATTGATAATAGTTAAAAATATTGGGCGTATCTTATTATTCCAACCCCCTGAGTGGTC
TGAGGATTTCCATGAATGGAAGAGAGAAAAGCATATTAAATGTACCTATAACGGTGTTC
AATTATCAGAAACAGAACTTCCCGCAAATTGGTTAACAGAAGGTATTCAGATAAAAATA
CTATTTCCCTTCTGTTTGAAACCTTGGTACAGATCTAAGGAGCAAAAAAAAAGATGATTC
TTGTTTTTTAACGGCTAGTGGAATGGAAACTGACCTTCCTTTTGGTTCTCCCCGAAAAA
GAACTTCCTTTTTTGAGCCCATTTTTAAGGATTTTCTA--
-GCTCTAAGAGTTTTAATAAGAAGACATTTTCTACAAGGTTCAAAAGAAACAAAAAGG
GGGTTTATCAAAAACGTTTTATTTCTGTTTCTAAAAAGAATAAAAAAAGAGCTCTCAAA
AGTAAATGGTTCTATAATCAGCAATCAGACAATTCATAACTCGTTTAATAAAATTCGATC
TACAGAT------AATTATTCACCGAGAGAAAAAAAAAATTAAAAATTTAACTGATAGAACAA
GCCCAATCAGAAAGAAAATAAAGAGTATTACAAAAGAAAAAAAAAAA---------AATAA
AAGAAGTTATAAT------GTAGAATCGCCAAAAGATATTTGGCAAATATTA------AAAAATA
CTCGATTAATCTGGAAATTACAGGTTTTTCTAAAAATTTTTATTGAAAAAATATACATAG
ATATCTTTCTATATATCATTAATATTGCCAGAATGTATACACGACTTGTT---------ATTCACT
TTGACTTATCCTCCTTGTCGCAAGCATATGTATTTTACAAATTATCCCGAACCCGAGTTAT
TAATTTATATAAGTTAAGATCTGTTCTTGAATATACTTCTTTTTTTCTTAAGACTGCAATA
AAGGATTCTTTTACACAAGGAATATTTCATTCCGAATTAGAGCATACGAAATTTCCAAG
TTATGGAACGAATCAATGGAAAAACTGGTTAAGAGGT---TATCAATACAATTTATCTCAG
ATTAGATGGTCTGGATTAATACCACAAAAATGGCGAAATACAATCAATCAAGGCCGTAT
AACTCAAAAAAAAACAAAATGGGATTCA------AAAGACCGA------TTACTTCATTACAAA
AAACAA---AATTCTCAAAAATACTATAGATATGACCTTTTATCATATAAATCTATTAATGG
TGATATTATGTATCCAGATAGAAAATATTTTGATTGGAAAATTATCAACTTTTTTTTAAGA
CTTTTTGGTTGGATGGAAATGAATCAAGAAATACTAAATCGCTGGTTCTTCCCAGAATT
TGTGCTACTTTATAATGTATACAAAAAAAAACCGTGGATTATACCAAGCAAATTACTTCT
TTTAAATAACAAAAGTAACGTGAAAGTAGAACTAAATGCCTTACTGAAACAGCATTTG
CTTTTTCAATTGAGATACGACGAGGGTTTA---CAAAGAATGATCAATAATCTCAAGGTA
AGTTGTCTCTTGATTGGACTGAAAAATCCAAGAAATATTACTGTATCGTCTATTCAAAA
CAGAGAAATAAGTCTGGATATAATGCTGATTCGGAAGAGC---TTAACTGTTAGAGAGTT
GATGAAAGGGGTATTGATTATCGAACCCATTCGTCTGTCTGTAAAAAAGGATGGACAAT
TTATTATGTATCAAACCATAGGTATTTCATTAATTCATAAGAGTAAGTACAACCATTATGA

TTTTCTTGTTCCTGAAAAGATTTTATCGTCTAGACGTCGTAGAGAATTGAGAATTTTAAT
TCATTTCGTGTTTCTTGGGCCTAATTATCGATTAGAAGACTTGGCTTGTATAAATCGGTAT
TGGTTTGATACCAATAATGGAAGCAATTTCAGTATGTTAAGGATATATCCACAATTAATG
AAAGGACATCAATTCAAATCCTGGATTTTCGAATTGAGAGAGATATTGAGAGAGATCA
AGAATTCTCACTATTTCTTAGATTCACGGACCCAATTCAATTCAGTGGGATCTTTCAGTC
ACATTTTTTTCCACCAAGAACGTTTTATAAAACTTTTTGACCCCCGAATTTGGAGTATCC
TACTTTCA---CGCAATTCA------CAGGGTTCAACAAGCAATCGATATTTCACG---------ATC
AAGGGT---GTAATACTCTTTGTAGTAGCGGTCCTTATATATCGT------------ATTAACAGTCG
AAATATGGTCGAAAGAAAAAAATCTCTATTTGATAGGGCTTCTTCCT------------ATACCT
ATGAATTCCATTGGACCCAGAAATGATACATTGGAAGAATCCGTT---GGGTCTTCCAATA
TCAATAGGTTGATTGTTTCG---CTCCTGTATCTTCCAAAAGGAAAAAAGATCTCTGAGA
GTTGTTTCCTGAATCCGAAAGAGAGTACTTGGGTTCTCCCAATAACT------------AAAAA
GTGTAGCATGCCTGAATCTAACTGGGGTTCGCGGTGGTGGAGGAACTGGATCGGAAAA
AAGAGG------GATTCT--

AGCCAATTGAAAGGATCTTCT---GATCAATCCAGAGATCCTTTAGATTCCATTAGT---------
---AATGAGGATTCGGAA---------------------------------------TATCACACATTGATCAATCAAAG
AGAGATTCAACAACTAAAAGAAAGATCGATTCTT---TGGGAT------CCTTCCTTTCTTCAA
ACGGAACGAACAGAGATAGAATCAGAC------CGATTCCCGAAA------TGCCTTTCTGTAT
ATTCC------TCAATGTCCCGGCTATTCACG---GAACGTGAGAAGCAGATGATTAATCATCT
GCTTCCGGAAGAAATCGAAGAATTTCTT------GGGAATCCTACAAGATCCGTTCGTTCTT
TTTTCTCTGACAGATGG------TCAGAACTTCAT---CTGGGTTCGAATCCTACTGAGAGGTC
CACTAGAGATCAG------------AAATTGTTGAAGAAACAACAAGATCTTTCT---TTTGTCCC
TTCGAGGCGATCGGAA------------AAGAAAGAAATGGTTAATATATTCAAGATAATTACGT
ATTTACAAAAT------ACCGTCTCAATTCATCCTATTTCATCATATCCGGGATGTGATATGGT
TCCG---AAGGATGAACCGGAT---ATGGATAGTTCCAATAAGATTTCATTCTTGAACAAAA
ATCCATTTTTTGATTTA------TTTCATCTATTCCATGACCGGAACAGGGGAGGATACACGT
TA------CACCACGATTTTGAATCAGAA------GAGAGATTTCAAGAAATGGCAGATTTATTC
ACTCTATCAATAACCGAGCCG---GATCTGGTGTATCATAAGGGATTTGCCTTTTCTATTGA
TTCCTACGGATTGGATCAAAAACAA------TTCTTGAATGAGGTATTCGACTCCAGGGATG
AATCGAAAAAAAAATAT---------TTATTGGCTCTACCTCCTTTTTTTTAT------GAAGAGAAT
GAATCTTTTTATCGAAGGATCAGAAAAAAAATGGGTCCGG---ATCTCCTGCGGGAATGAT
TTGGAAGATCCAAAACCAAAAATAGTGGTATTTGCTAGCAACAACATAATGGAGGCAG
TCAATCAATATAGATTGATCCGAAATCTGATTCAAATCCAATATAGCACCTATGGG------T
ACATAAGAAATGTATTGAATCGATTCTTTTTAATGAAT---------AGATCCGATCGCAACTTC
GAATATGGA---------ATTCAAAGGGATCAAATAGGAAAGGATACTCTGAATCATAGAACT
ATAATGAAATATACGATCAACCAACATTTCTCGAATTTGAAAAAG---AGTCAGAAGAGA
TGGTTCGATCCTCTT------ATTTTGATTTCTCGAACCGAGAGATCCATGAATCGGGATCCT
GATGCATATAGATACAAATGGTCCAATGGGAGCAAGAATTTCCAGGAACATTTGGAACA
TTTCGTTTCTGAGCAGAAGAGCCGT---------TTTCAAGTAGTGTTCGATCGATTACGTATT

AATCAATATTCGATTGATTGGTCTGAGGTTATCGACAAAAAAGGTTTGTCTAAGCCATTT
CGTTTCTTTTTGTCCAAG--TTGCTTT
TCTTTTTGTCTAAC---TCACTTCCTTTTTTCTTTGTGAGTTTCGGGAATATCCCCATTCAT
AGGTCCGAG---ATCTACATCTAT---GAATTGAAAGGTCCGAATGATCAACTCTGCAATCA
GTTGTTAGAATCAATAGGTCTTCAAATCGTTCATTTGAAAAAATGGAAACCCTTC------T
TATTGGATTTATTGGATGATCATGATACTTCC---CAAAAATCGAAATTCTTGATCAATGGA
GGAACAATATCACCATTTTTGTTCAATAAGATACCA---AAGTGG---ATGATTGACTCATTC
CATACTAGA------AATAATCGCAGGAAATCCTTTGATAACACGGATTCCTATTTCTCAACG
ATATTCCACGATCAAGACAATTGGCTGAATCCCGTGAAACCATTTCATAGAAGTTCATT
GATATCTTCTTTTTATAAAGCAAATCGACTTCGATTCTTGAATAATCCACATCACTTCTGC
TTCTAT------TGTAATAAAAGATTCCCTTTTTATGTG------------GAAAAGGCCCGTATCAAT-
-----AATTCTGATTTTACGTATGGA---CAATTCCTCAATATCTTGTTCATTCACAACAAA---A
TTTTTTCTTTGTGCGTCGGTAAA------AAAAAACATGCTTTTTGGGGG---AGAGATACTAT
TTCACCAATCGAGTCACAGGTATCTAACATATTCATACCTAACGATTTTCCACAAAGTGG
TGGTGACGAAACGTATAACTTGTACAAATCT---------TTCCATTTTCCAAGTCGATCCGAC
CCATTCGTTCGT------AGAGCTATTTACTCGATCGCAGACATTTCTGGAACACCTTTAAC
AGAGGGACAAATAGTCAATTTTGAAAGAACTTATTGTCAACCTCTTTCAGATATGAATC
TATCTGATTCAGAAGGGAAGAACTCGCATCAGTATCTCAAT------TTCAATTCAAACATG
GGTTTGATTCACACTCCATGTTCTGAGAAATATTTACCATCCGAAAAG------AGGAAAAA
A------------------------CGGAGTCTTTGTCTAAAG------AAATGCGTTGAGAAAGGGCAGATG
TATAGAACCTTTCAACGAGATAGTGCTTTTTCAACTCTCTCAAAATGGAATCTATTCCAA
ACATATATGCCATGGTTCCTTACTTCGACAGGGTACAAATATCTAAATTTTATATTTTTAG
ATACTTTTTCAGACCTATTG------CCGATACTAAGTCTA---AGTCAAAAATTTGTATCCATT
TTTCATGATATTATGCATGGATCAGATATATCATGGCGAATTCTTCAGAAAAAATTG-------
-----------TGTCTTCTAAAATGGAATCTG---ATAAGTGAGATTTCGAGTAAGTGTTTCCATAA
T------CTTCTTCTGTCCGAAGAAAGGATTCATCGAAAT------AATGAGTCGCCATTGATATC
GACACATCTGAGATCGCCAAATGTTCGGGAGTTC---CTCTATTCAATCCTTTTCCTTCTT
CTTGTTGCTGGATAT---CTCGTTTGTACACATCTTCTCTTTGTTTCCTGG------GCCTCTAG
TGAGTTACAGACAGAGTTTGAAAAGGTCAAATCTTTGATGATTCCATCATCTATGATTG
AGTTGCGAAAACTTCTGGATAGGTATCCTACATCTGAACCGAATTCTTTCTGGTTAAAG
AATCTCTTTCTAGTTGCTCTGGAACAATTAGGAGATTCTCTAGAAGAAATACGGGGTTC
TGCTTCTGGCGGCAACATGCTATTGGGTGGTGATCCCGCT---------------TATGGG---GTCA
AATCAATACGTTCTAAGAAGAAATATTTGAATATCAATCTCATCGATATCATCGATCTCAT
A---AGTATCATACCAAATCCCATCAATCGAATCACTTTTTCGAGAAATACGAGACATCTA
AGTCATACAAGTAAAGAGATCTATTCATTGATAAGAAAAAGAAAAAATGTGAACGGGG
ATTGGATTGATGATAAAATAGAATCCTGGGTCGCAAACAGTGATTCGATTGATGATGAA
GAAAGAGAATTCTTGGTTCAGTTCTCC------ACCTTAACGACA------GAAAAAAGGATTG
ATCAAATTCTATTGAGTCTGACT---CATAGTGATCATTTA---------------TCAAAGAATGACT
CTGGCTATCAAATGATTGAACAACCGGGAGCAATTTACTTACGATACTTAGTTGACATT
CAT------AAAAAGTATCTAATGAATTATGAGTTCAATACATCCTGTTTAGCAGAAAGACG
GATATTCCTTGCTCATTATCAGACAATCACTTAT------TCACAAACCTCGTGTGGGGCTAA
TAGTTTGAATTTCCCATCTCATGGA---------AAACCCTTTTCGCTCCGCTTAGCCTTATCCC
CCTCT---AGGGGTATTTTAGTGATAGGTTCTATAGGAACTGGACGATCCTATTTGGTCAA
ATACCTAGCGACAAACTCCTATGTTCCTTTCATTACGGTATTTCTGAACAAGTTCCTGGA

TAACAAGCCTAAAGGTTTTCTTATTGAT---------------GATATCGAT---ATTGATGCTAGTGA
CGATATTGATGCTAGTGACGATATTGATGCTAGTGACGATATTGATGCT----------------------
-------------AGTGACGATATCGATCGTGACCTTCATACGGAGCTG---GAACTGCTA------------
------ACTATGGATATGATGCCGGAA---------ATAGACCGATTTTATATCACCCTTCAATTCGA
ATTAGCAAAAGCAATGTCTCCTTGCATAATATGGATTCCAAACATTCATGATCTGGATGT
GAATGAGTCGAATTACTTCTCCCTCGGTCTATTAGTGAACCATCTCTCCAGG---------GAT
TGTGAAAGATGTTCC---ACTAGAAATATTCTTGTTATTGCTTCGACTCATATTCCCCAAA
AAGTGGATCCCGCTCTAATAGCTCCGAATAAATTAAATACGTGCATTAAGATACGAAGG
CTTCTTATTCCACAACAACGAAAGCACTTTTTCACCCTTTCATATACTAGGGGATTTCGC
TTGGAAAATAAAATGTTCCATACT------AATGGATTCGGGTCCATAACCATGGGTTCCAAT
GCACGAGATCTTGTAGCACTTACCAATGAGGCCCTATCGATTAGTATTACACAGAAGAA
ATCAATTATAGACACTAATACAATTAGATCCGCTCTTCATAGACAAACTTGGGATTTGCG
A---------TCCCAGGTAAGATCGGTTCAGGATCATGGGATCCTTTTCTATCAGATAGGAAG
GGCTGTAGCACAAAATGTACTTCTAAGTAATTGCCCCATAGATCCTATATCTATCTATATG
AAGAAGAAA------TCATGTAACGAAGGGGATTCTTATTTGTACAAATGGTACTTCGAACT
TGGAACGAGCATGAAGAAATTAACGATACTTCTTTATCTTTTGAGTTGTTCTGCCGGAT
CGGTCGCTCAAGATCTTTGGTCTCTACCC------CGACCCGATGAAAAA---AATGGGATCA
CTTCTTATGGACTCGTTGAGAATGATTCT---GATCTAGTTCATGGCCTATTAGAAGTAGA
AGGCGCTCTGGTGGGA------TCCTCGCGGACAGAAAAAGATTGCAGTCAGTTTGATAAT
GAT------CGAGTGACATTGCTTCTTCGGCCCGAACCAAGGAAT---CCCTTAGATATGATGC
AAAATGGATCTTGTTCTATCCTTGATCAGAGATTTCTCTATGAAAAAAAC------GAATCG
GAGTTTGAAGAA---GGGGAAGGAGAAGGAGCCCTCGACCCGCAACAG------------ATAG
AGGAGGAT------------TTATTTAATCAC------ATAGTTTGGGCTCCT------AGAATATGGCGCC
CT---------TGGGGCTTTCTATTTGATTGTATCGAAAGGCCCAATGAATTGGGATTTCCC---T
ATTGGTCCAGGTCATTTCGGGGCAAG---------CGGATCCTTTATGATGAAGAGGATGAGC
TTCAAGAGAATGATTCGGAGTTCTTGCAGAGTGGAACCATGCAGTACCAGACACGAGA
TAGATCTTCCAAAGAACAAGGCCTTTTTCGAATAAGCCAATTCATTTGGGACCCTGCAG
ATCCACTCTTTTTCCTATTCAAA---GATCAGCCCTTTGTCTCTGTGTTTTCACATCGAGAA
TTATTTGCAGATGAAGAGATGTCAAAGGGGCTTCTTACTTCCCCAACAGATCCTCCTAC
ATCTATATATAAACGCTGGTTTATC------AAGAAGACGCAAGAAAAGCACTTCGAATTGT
TGATTAATCGCCAGAGATGGTTTAGA---------ACCAATAGTTCATTATCT------AATGGATCT
TTCCGTTCTAAT------ACTCTATCCGAGAGTTATCAGTATTTATCAAATCTATTCCTATCTA
AC---GGAACGCTATTGGATCAAATGACAAAGACATTGTTGAGAAAAAGATGGCTTTTCC
CGGATGAAATGAAAATTGGATTC------ATGGAACAGGAGAAAGATTTCCCATTCCTTATG
CCTAGATCGAGG---ATAAATGGAAATTTTATTGATAAGACCTTTTCAATTGTAGCCAATAT
CTTATTACGAATAATTCCCACAACTTCAGGAGAAAAAGAGGCATTTACCTATTACAGAG
ATGGG------ATGTCAGCTCAATCCGAAGGAAATTATGCGGAAGCTTTACAGAATTATTAT
GAAGCTATGCGACTAGAAATTGATCCCTACGATCGAAGTTATATACTCTATAACATAGGA
CTTATCCACACAAGTAACGGAGAACATACAAAAGCTCTGGAATATTATTTTCGAGCGCT
AGAACGAAATCCGTTCTTACCACAAGCTTTTAATAATATGGCTGTGATCTGTCATTAC---
CGGGGAGAACAGGCCATTCGACAGGGAGATTCTGAAATAGCGGAGGCTTGGTTCGATC
AAGCCGCTGAGTATTGGAAACAAGCTATGGCGCTTACTCCTGGTAATTATATTGAAGCG
CATAATTGGTTGAAGATCACGAGGCGTTTCGAA------ATGAGTTGTCGATCAGAACATAT
ATGGATAGAACCTATAACGGGGTCTCGAAAAACAAGTAATTTCTGCTGGGCCGTTATCC

TTTTTTTAGGGTCATTAGGATTCTTATTGGTTGGAACTTCCAGTTATCTTGGTAGAAACT
TGATATCTTTATTTCCGTCTCAGCAAATCCTTTTTTTTCCACAGGGGATCGTGATGTCTTT
CTATGGAATCGCGGGTCTCTTTATTAGCTCCTATTTGTGGTGCACACTTTCGTTGAATGT
AGGGGGTGGTTATGATCGATTTGATAGAAAAGAAGGAATGGTGTGTATTTTTCGTTGGG
GATTTCCTGGAAAAAATCGTCGCATCTTCCTCCGATTCCTTATAAAAGATATTCAGTCCG
TCAGAATAGAAGCTAAAGAGGGTATTTATGCTCGTCGTGTCCTTTATATGGACATCAGA
GGTCAGGGGGCCATCCCCTTGACTCGTACTGATGAGAAT---GTTACTCCACGGGAAATT
GAACAAAAAGCTGCCGAATTGGCCTATTTCTTGCGTGTACCGATTGAAGTCTTT
>Cucumis
ATGGAAAAAAGATGGTTAAATTCGATGTTGTCTCTAAGTAAATCAATCAATAGTCTTGG
TCCTATTGAA------------AGTGAAGGATCAATTATAAATAATATGAATAAAAACATTCCTAG
TTACAGTACTGTTGATGATTTAGTCGGC---------ATTCGAAATTTCGTATCTGATGACACTT
TTTTAGTTAGGGATAGTAATAGCAGCAGTTATTCCATATATTTGGATATTGAAAATCAAAT
TTTTGAGATTGACAATGATCCT------TCTTTTGTAAGTGAACTAGAAAGTTCTTTTTATAG
TTTTCGGAACTCTACTTATCAAAATAATATATCTAAGAATGATGATTCCCACTATGATCGT
TACATGTATGATACTAAATATAGTTGGAATAATCACATTAATAGTTGCATTGACAGTTATC
TTCGG------ACTCAAATCTGTATTGATAGTTACATTTTAAGTGGTAGTCACAATTACAGTG
ACAGTTACATTTATAGTTATATTTGTGGCAGTGAGAGTTTCAGTATAAGAACTAGCACGC
ATGGAAATAATTTAACTATAACAGAA---AGTTCT------GATCGAACTACAAACTACAGCGA
TTTTTGGGTTATATGCGAAAATTGTCATAAATTCAATTATAAGAGACTTTTCAAATCAAA
AATGAATATTTGTGAAGAATGTGGATACCATTTGAAAATGAATAGTTCAGATAGAATCG
AACTTTTGATTGATCCAGGTACTTGGGACGCTCTATGGGAATGGGATTCGGAGGTGGAT
CCCATTCAATGGAAGTCGCAGGTGGATCCCTATAAAGATCGTATTTCTTCTTCTCAAATA
GAGACAGGATTACCTGAGGCTATTCAAACAGGCACAGGTAAATTAAATGGTATTCCCGT
AGCAATTGGGGTTATGGAGTTTGAATTTATGGGGGGTAGTATGGGATCTGTAGTAGGCG
AGAAAATCACCCGTTTGATCGACTATGCTAGCAATCAATTTTTACCTCTTATTTTAGTGT
GTGCTTCGGGAGGAGCACGCATGCAAGAAGGAAGTTTGAGCTTGATGCAAATGGCAA
AAATATCTTCTGCTTTATATGATTATCAATCAAATAAAAAATTATTCTATGTAGCAATCCTT
ACATCTCCTACTACTGGTGGGGTGACAGCTAGTTTTGCTATGTTGGGGGATATCATTATT
GCCGAACCGAATGCCTACATCGCATTTGCGGGTAAAAGAATAATTGAAGAAACATTGA
AGATGGAAGTCCCTGAGGGTTCACAGAAGACTGAACCTTTATTCGAAAAGGGCTTATT
AGATCTAATCGTACCACGTAATCCTTTAAAAGATGTTGTGAGTGAGTTATTTCAGCTCCA
CGCTTTCGTTCCCTCGAATCAAAATTCAATCAAGATGGAAACCATTCGAGCAGATGAAA
TTAGTAATATTATCCGTGAACGTATTGAGCAATATACTAGAGAAGTCAAAATTGTAAATA
CCGGTACTGTACTTCAAGTAGGCGACGGCATTGCTCGTATTTATGGTCTTGATGAAGTA
ATGGCAGGTGAATTAGTAGAATTTGAAGAGGGTACTATAGGCATAGCTCTCAATTTAGA
ATCAAATAATGTTGGTGTTGTATTAATGGGTGATGGTTTGCTGATACAGGAGGGAAGTT
CTGTAAAAGCAACGGGAAGAATTGTTCAGATACCAGTAAGCGAGGCTTATTTAGGTCG
TGTTATAAATGCCCTAGCTAAACCTATTGATGGCCGAGGTGAAATTTCATCTTCTGACTC
TCGGTTAATCGAATCCCCCGCTCCTGGTATTATTTTGAGACGTTCCGTATACGAGCCTCT
TCAAACAGGACTTATTGCTATTGATTCGATGATTCCTATCGGACGTGGTCAACGAGAATT
AATTATTGGGGACAGGCAGACCGGGAAAACTGCAGTAGCCACGGATACGATTCTCAAT
CAACAAGGGCAAAATGTAATATGTGTTTATGTAGCTATTGGTCAAAAAGCATCTTCTGT
GGCTCAAGTAGTGAGTACTTTACAGGAAAGGGGCGCAATGGAATACACTATTATAGTAG

CCGAAACGGCAGATTCTCCGGCTACATTACAATACCTTGCCCCTTATACAGGAGCAGCT
TGGCTGAAT---ATTTTATGTACAGAAAACAGCACACTTCATATTTATGATGATCCCTCCAA
GCAAGCACAAGCTTATCGACAAATGTCTCTTCTTTTACGAAGACCGCCGGGCCGCGAA
GCTTATCCGGGAGATGTTTTTTATTTGCATTCACGCCTTTTGGAAAGAGCCGCTAAATTA
AGTTCTGCTTTAGGTGAAGGAAGTATGACTGCTTTACCAATAGTTGAGACCCAATCGGG
AGATGTTTCAGCTTATATTCCTACTAATGTAATTTCCATTACCGATGGACAGATATTCTTA
TCTGCCGATCTATTCAATGCTGGAATCAGACCTGCTATTAATGTGGGTATTTCCGTCTCC
AGAGTAGGATCTGCAGCTCAAATTAAAGCCATGAAACAAGTAGCCGGAAAATTAAAAT
TGGAATTGGCTCAATTTGCCGAATTAGAAGCCTTTGCGCAATTCGCTTCTGATCTCGAT
AAAGCTACTCAGAATCAATTGGCAAGAGGTCAACGATTACGTGAGTTGCTCAAACAAT
CCCAATCAGCACCTCTCACGGTAGATGAACAGATAATGACTGTTTATACCGGAACTAAT
GGTTATCTTGATTCATTAGAAATTGCACAAGTAAGGAAATTTCTTGTTGAGTTACGTACT
TACGTAAAAACGAATAAACCTCAGTTTCAAGAAATAATCTCTTCCACGAAGACATTCAC
CCCTGAAGCGGAAGTCCTTTTGAAAGAAGCTATTCAGGAACAGATGGAACGTTTTCTA
CTTCAAGATCAAGTAATGAGAATAAATCCTACGACTTCTAGTCCTGAAGTTTCCACGCT
TGAAAAAAACAACTTGGGACGTATCGCTCAAATAATTGGTCCGGTACTAGATGTAGCTT
TTCCCCCGGGCAAAATGCCTAATATTTACAACGCTTTGATAGTTAAAGGTCAAGATATTT
CCGGTCAAGAAATTAATGTGACTTGTGAAGTACAGCAATTATTAGGAAATAATCGAGTT
AGAGCTGTAGCTATGAGTGCTACAGATGGTCTAAGGAGAGGAATGGAAGTGGTTGACA
CGAGAGCTCCTCTAAGTGTTCCAGTCGGCGGAGCGACTCTCGGCCGAATTTTCAACGT
GCTTGGAGAGCCTATTGATAATTTGGGTCCTGTAGATACTCGCACAACATCTCCTATTCA
TAGATCCGCCCCTGCTTTTATCCAGTTAGATACAAAATTATCTATTTTTGAAACAGGAAT
TAAAGTAGTGGATCTGTTAGCCCCTTATCGACGTGGAGGAAAAATAGGACTATTCGGAG
GGGCTGGAGTGGGTAAAACAGTACTCATTATGGAATTGATCAACAACATTGCCAAAGC
TCATGGAGGTGTATCTGTATTTGGAGGAGTAGGTGAACGTACTCGTGAAGGAAATGATC
TTTACATGGAAATGAAAGAATCCGGAGTAATTAATGAAGAAAATTTTGCAGAATCAAA
AGTGGCTCTAGTCTACGGTCAGATGAATGAACCGCCGGGAGCTCGTATGAGAGTTGGT
TTAACTGCCCTAACTATGGCGGAATATTTCCGAGATGTTAATGAACAAGACGTGCTTCTA
TTTATCGACAATATCTTTCGTTTCGTTCAAGCGGGATCCGAAGTATCCGCGTTACTGGGT
AGAATGCCTTCGGCTGTGGGTTATCAACCCACTCTTAGTACCGAAATGGGTTCCTTACA
AGAAAGAATTACTTCTACCAAGGAAGGGTCCATAACTTCTATTCAAGCTGTTTATGTAC
CTGCGGACGATTTGACCGATCCTGCTCCTGCCACCACATTTGCACATTTGGATGCTACT
ACCGTACTATCAAGAGGATTAGCTGCCAAAGGTATCTATCCAGCAGTAGATCCTTTAGA
TTCAACCTCAACTATGCTACAACCGAGAATCGTTGGTGAAGAACATTATGAAACTGCG
CAAAGAGTTAAACAAACCTTACAACGTTACAAAGAACTGCAAGACATTATAGCTATCC
TTGGGTTGGACGAATTATCCGAAGAGGATCGCTTAACCGTAGCACGAGCACGAAAAAT
TGAACGTTTCTTATCACAACCTTTTTTCGTAGCAGAAGTATTTACCGGCTCCCCGGGGA
AATACGTTGGCCTAGCAGAAACAATTAGAGGGTTTAAATTAATCCTTTCCGGAGAATTA
GATGGTCTTCCCGAACAAGCCTTTTATTTGGTAGGTAACATCGATGAAGCTACTGCGAA
GGCTACGAACTTAGAAATGGAGAGCAATTTGAAGAAAATGACCTTAAATCTTAGTGTC
CTGACTCCGAATCGAATTATTTGGGATTCAGAAGTGAAAGAAATCATTTTAGTTACGAA
TAGTGGCCAAATTGGTGTATTACCAGATCACGCACCTATTGCCACAGCTGTAGATATAG
GTATTTTGAAAATACGCCTTACTGATGGATGGTTAACGATGGCTCTGATGGGTGGTTTTG
CTAGAATAGGCAATAATGAGGTCACTATTTTAGTAAATGATGCGGAGAAGGCTAGTGAC

ATTGATCCACAAGAAGCTCAGCAAACTCTTGAAATAGCAGAAGCAAACTTGAGGAAA
GCTCAGGGCAAGAGACAAACAATCGAGGCAAATTTAGCTCTCAGACGAGCTAGGACA
CGAGTAGAGGCTATCAATGGCGTACCTATGAAAAATGTAACCGATTCTTTCATTTCTTTG
GGTTATTGGCCATCCGCCGAGAGTTTCGGATTTAATACCGATATTTTAGCAACAAATCCA
ATAAATCTAAGCGTAGTGCTTGGTGTATTGATTTTTTTTGGAAAGGGAGTGTTAAGTGAT
TTATTAGATAATCGAAAACAGAGGATCTTGAAAACAATTCAAAATTCAGAAGAACTCC
GTGGGGGGGCCATTGAACAGCTGGAAAAAGCCCGGGCTCGCTTACGAAAAGTCGAAA
TGGAGGCGGATCAGTTTCGAGTGAATGGATATTCTGAGATAGAACGAGAAAAATTGAA
TTTGATTAATTCAACTTCTAAAAGTTTGGAACAATTAGAAAATTATAAAAATGAAACTAT
TCGTTTTGAACAACAAAAAGCGATTAATCAAGTACGACAACAGGTTTTCCAACAAGCC
TTACAAGGAGCTCTAGGAACTCTGAATAGTTGTTTGGACAACGAGTTACATTTACGTAC
CATCAGTGCTAATATTGGCATGTTTGGGACGATGAAAGAAATAACTAACATGAATCCAC
TGATTTCTGCCGCTTCCGTTATTGCTGCTGGATTGGCCGTGGGGCTTGCTTCTATTGGAC
CTGGGATTGGTCAAGGTACTGCTGCGGGCCAAGCTGTAGAAGGGATCGCGAGACAAC
CCGAGGCGGAGGGAAAAATCCGAGGTACTTTATTGCTTAGTCTGGCTTTTATGGAAGCT
TTAACAATTTATGGACTGGTTGTAGCATTAGCACTTTTATTTGCGAATCCTTTTGTTATGA
ATATTCTATTATGTTCTATCAACACACAAAAAGGGTTATACGATATATCTGGTGTGGAAG
TCGGCCAACATTTGTATTGGCAAATAGGAGGTTTCCAAGTACATGCCCAAGTACTTATT
ACTTCTTGGGTTGTAATTGCTATCTTATTAGGTTCAGCCATTATAGCTGTTCGTAATCCAC
AAACCATTCCTACTGACGGTCAGAATTTCTTCGAATATGTCCTTGAATTCATTCGAGAC
GTGAGCAAAACTCAAATTGGCGAAGAATATGGTCCATGGGTTCCCTTTATTGGAACTAT
GTTTCTATTTATTTTTGTTTCGAATTGGTCAGGGGCTCTTTTACCTTGGAAAATCATACA
GTTACCTCACGGAGAGTTAGCCGCACCCACAAATGATATAAATACTACTGTTGCTTTAG
CTTTACTCACATCAGTAGCATATTTTTATGCGGGTCTTAGCAAAAAAGGATTAAGTTATT
TCGGTAAATACATTCAACCAACTCCAATCCTTTTACCCATTAACATCTTAGAAGATTTCA
CAAAACCCCTATCACTTAGTTTTCGACTTTTCGGAAATATATTAGCTGATGAATTAGTAG
TTGTTGTTCTTGTTTCTTTAGTACCTTTAGTAGTTCCTATACCTGTTATGTTCCTTGGATTA
TTTACAAGTGGTATTCAAGCTCTTATTTTTGCAACTTTAGCTGCAGCTTATATAGGCGAA
TCAATGGAGGGTCATCATATGATATTTTCAACTTTTGAGCATATATTAACTCATATATCTTT
TTCGGTCATTTCAATTGTCATTACAATTCAGTTAATAACCTTATTAATC---GAAACCGTAG
GACTCTATGTTTCGTCAGAAAAGGGCATGATAGCTACTTTTTTCTGTATAACAGGATTAT
TAGTTACTCGTTGGATTTATTTGAGGCATTTACCATTAAGTGATTTATATGAATCATTACT
ATTTCTTTCATGGGCTTTCTCCATTATTCATCTATTTACGTATTTTAAAAATATAAAA---AC
CATTAAGTGTAATAACCGCGCCAGTACTATTTTTACACAAGGTTTTGCTACTTCAGGTTT
TTTAACTGAAATGCATCCATCCCCACTATTAGTTCCCGCTCTCCAATCTCATTGGTTAAT
GATGCACGTAAGTATGATGGTATTAGGTTATGCCGCTCTTTTATGTGGATCATTATTTTCA
GTAGCTCTTATAGTGATTACCTTTCAAAAAGCTATAAGAATTTTTTGTAAAAACAATGAG
ATCCAATCCATCAACGAAGGGAACTATTTTGTAAGA------TCCTTTTTTTCTTCGAAGAA
TTATTATAAGTCTCAACTGATTCAACAATTAGATCATTGGAGTTGTCGTATTATTAGTCTA
GGGTTTATCTTTTTAAGCATAGGTATTCTTTCAGGGGCAGTATGGGCTAATGAGACCTGG
GGATCGTATTGGAATTGGGACCCAAAGGAAACTTGGGCATTTATTACTTGGACGATATT
CGCAATTTATTTACATACGCGAACAAATAAAAATTTTGAAGGTGTAAATTCCGCAATTGT
GGCCTCTATGGGTTTTCTTATAATTTGGATATGCTATTTTGGGGTCAATCTATTAGGGATA
GGGCTACATAGTTATGGTCCGTTTACATTAACAATGGAAAAAAAGAAAGCATTCATTCC

CCTTCTATATCTTGCATCTATAGTATTTTTGCCCTGGTGGATCTCTCTTTCATTTAATAAAA
GCCTAGAATCTTGGGTTACTAATTGGTGGAATACTGGTCAATCCGAAAATTTTTTGAAT
GATATTCAAGAAAAGAGTATTATAAAAAAAGTTCTAGAATTAGAGGAACTCTTTCTCTT
GGACGAAATGCTAAAGGAATACCCAGAAACACATCTACAAAAGCTTCGTATCGCA---TC
CAAAGAAACGATCCAATTGATCAAGATGCACAATGAGGATCGTATCCATACGATTTTGC
ACTTCTCGACAAATATAATCTGTTTCGTTATTCTAAGTGGTTATTCTATTCTTGGTAATGA
AGAACTTGTTATTCTTAACTCTTGGGTTCAAGAGTTCCTATATAACTTAAGCGACACAAT
AAAAGCTTTTTCTATTCTTTTATTAACTGATTTATGTATAGGATTCCATTCACCCCATGGT
TGGGAACTAATGATTGGTTCTGTCTACAAAGATTTTGGATTTTCTCATAACGATCAAATT
ATATCCAGTCTTGTTTCCACTTTTCCAGTCATTCTTGACACAATTTTTAAATATTGGATCT
TCCGTTATTTAAATCGTGTATCTCCGTCACTTGGAGTGATTTATCATTCACTGAATGACAT
GCCTGTTGGTGTTCCAAAAGTACCCTTTCGACTTCCCGGAGAAGAAGATGCATCGTGG
GTTGACCTATACAACCGACTTTATCGACAAAGATTACTTTTTTTAGGCCAAGATGTTAAT
AATGAGATCTCGAATCAAATTATGGGTCTTATGGTATATCTCAGTATAGAGGATGGTACC
AAAGATCAGTATTTATTTATAAACTCTCCCGGGGGATCGGTAATACCCGGAGTAGGTCTT
TTTGATACTATGCAATTTGTGTCACCAGATGTCCATACAATATGTATGGGATTAGCCGCTT
CAATGGGATCTTTTATTCTAGTCGGAGGAGAAATTACCAAACGTCTAGCATTCCCTCAC
GCTAGGGTAATGATCCATCAACCTGCTAGTTCTTTTTCTAAGGGAAAAACGGGAGAATT
TGTCCTGGAATCGACCGAACTACTGAACCTGCGCGAAACCATCACAAAGGTTTATGTC
CAAAGAACAGGCAAACCCTTATGGGTTATACCCGAAGATATGGAAAGAGATGTTTTTAT
GTCAGCACCAGAAGCCCAAGCTCATGGAATTGTTGATCTTGTAGCGGTT--
-???
??
??ATGGAGGAATTTCAAGGATATTT
AGAACTTGAGAAATCTCGGCAACGTGACTTCCTATACCCACTTATCTTTCGGGAGTATA
TTTACGCATTTTCTCATGATCATTTTTTATCTGGTTATGACAAAAAATCCAGTTTACTAAT
GGTAAAACATTTAATTACTCGAATGTATCACCAGAATCATTTTTTTTTTTTCACTAATTAT
TATAACAAAAACCCTTTTTGGGGGAACAATTTGTATTCTCAAATGTTATCAGAAGGGTT
TGCAGTCATTGCGGAAATTCCACTTTCCCTACGATTAGTATCTTCCTTAATCGCAAAATC
TTATAATTTACGATCAAGTCATTCAATATTTCCTTTTTTAGAGGATAAATTCCCACATTTA
AATTATGTGTCAGATGTATTAATACCCTATCCGCTCCATCTGGAAATTTTAGTTCAAATCC
TTCGCTCCTGGGTGAAAGATGCCTCTTCTTTTCATTTATTACGGTTCTTTTTTCACGAGT
ATTGTAATTTGAATAGTCTTAGTACTAAATTGATTTCTTTTTTTTCAAAACGAAGATTAGT
CTTGTTGCTATACAATTCTTATGTATGTGGATACGAATCCATTTTCCTTTTTCTACGTAAC
CAATCTTCTCATATACGATTAACTTCTTATAAGGGCCTTTTTGAGCGAATATATTTCTATG
GAAAAATCGAACATCTTGTCAAAGTGTTTGCTAATTCGGCTATCTTACGGGTCTTCAAG
GATCCTTTAATACATTATGTTAGATATCAAGCAAAATCTATTCTGGTTTCAAAGGATACG
CCACTTCTGATTAATAAGTGGAAATATTACCTTGTCAATTTATGGCAATGTCATTTTTATG
TGTGGTCACAACCAGAAAGGATCTATATAAACCAATTATCCAAGCGTTCTCTTGACTTT
TTGGGCTATATTTCAAGTGTGCGATTAAATCCTTCAGTGGTATGGACTCAGATGTTAGAA
AATTCATTTCTAATAGATAAT------GCTACGCAGAAACTCGATACACTAGTTCCTATTATTA
CTCTGCTTGCATCATTGGCTAAAGCGAAATTTTGTAACGTCTTAGACATCCCATTAGTAA
GCCGACCTGGATCGATTCGTCGGATTTTGATATTATTGATCGATTTTTGCGTATATCCAGA
AATCTTTCTCATTATTACAGAGGATCCTCAAAAAAAAAGAATTTGTATCGAATACAATAT

ATACTTCGCCTTTCTTGTGTTAAAACTTTGGCTCGTAAACACAAAAGTACTGTACCGTG
CTCTTTTTAAAAGGTTAAATTCGGAATTATTGGAAGAATTCTTTACGGAGCAAGAACAG
GTTCTTTCTTTGATCTTCCCAAGAAAAATTTGGTATTTGGATATTATTTGCATGAATGATT
TGGCCAATCATGAAATGATAATTGATACATCA---------CAGTCCCAGATATCCATTCTTTTC
GAGATGGATTCT------------AAAGATTCTATGAATCTATGGTACTGTCCCTATGTGGACTAC
TGTATTGGAATCACCATAGGCGTACTAGTAATTGTATGGTTAGAAAGAGAAATATCCGC
AGGAATACAACAACGGATTGGACCTGAATATGCTGGTCCTTTGGGAGTTCTTCAAGCTT
TAGCAGATGGTACAAAACTACTTTTTAAAGAAAATTTGCTTCCATCTAGAGGTGATACT
CGTTTATTCAGTATCGGACCATCCATAGCAGTCATATCAATTTTACTAAGCTATTCAGTAA
TTCCTTTTGGTTATCGCCTTGTTTTAGCCGATCTCCCTATCGGTGTTTTTTTATGGATTGC
CATTTCAAGTGTTGCTCCCATCGGACTTCTTATGTCAGGATATGGATCCAATAATAAATAT
TCCTTCTTAGGTGGTCTACGAGCTGCTGCTCAATCAATTAGTTATGAAATACCATTAACT
CTATGTGTATTATCAATATCTCTACTATCTAACAGTTCGAGTACCGTTGATATAGTTGAGG
CTCAATCAAAATATGGTTTTTGGGGTTGGAATTTGTGGCGTCAACCTATAGGGTTTGTTA
TTTTTCTAATTTCTTCCTTAGCAGAATGCGAGAGATTACCTTTTGATTTACCAGAAGCAG
AAGAAGAATTAGTAGCGGGTTATCAAACTGAGTATTCGGGTATAAAATTTGGTTTATTTT
ATGTTGCTTCCTATCTAAATCTATTAGTTTCTTCGTTATTTGTAACTGTTCTTTACTTGGGT
GGTTGGGATATCTCTATTCCATACATATTAGGTTATGAACTTTTTGAAATAAATAAAGTGT
ATGAAGTCTTTGGAATGACAATTAGTATCTTTATTACATTAGCGAAAACTTATTTGTTCTT
GTTCATTTCTATAGCAACAAGATGGACTTTACCCCGACTAAGAATAGATCAACTATTAAA
TCTCGGATGGAAATTTCTTTTACCTATATCTCTCGGTAATCTATTATTAACAACTTCTTTT
CAACTTTTTTCACTGATGATCTGGCATGTACAGAATGAAAACTTCATTCTCGATTCTACG
AGAATTTTTATGAAAGCCTTTCATTTGCTTCTCTTCGATGGAAGTTTTATTTTCCCAGAA
TGTATCCTAATTTTTGGCCTAATTCTTCTTCTGATGATCGATTCAACCTCTGATCAAAAA
GATATACCTTGGTTATATTTCATCTCTTCAACAAGTTTAGTAATGAGCATAACGGCCCTAT
TGTTCCGATGGAGAGAAGAACCTATGATTAGCTTTTCGGGAAATTTCCAAACGAACAA
TTTCAACGAAATCTTTCAATTTCTTATTTTACTATGTTCAACTCTATGTATTCCTCTATCCG
TAGAGTACATTGAATGTACAGAAATGGCTATAACAGAGTTTCTATTATTCGTATTAACAG
CTACTCTAGGAGGAATGTTTTTATGCGGTGCTAATGATTTAATAACTATCTTTGTAGCTCC
AGAATGTTTCAGTTTATGCTCCTACCTATTATCTGGATATACCAAGAAAGATGTACGGTC
TAATGAGGCTACTACGAAATATTTACTCATGGGTGGGGCAAGTTCTTCTATTCTGGTTCA
TGGTTTCTCTTGGCTATATGGTTCATCCGGGGGAGAGATCGAGCTTCAAGAAATAGTAA
ATGGTCTTCTCAATACACAAATGTATAACTCCCCAGGAATTTCAATTGCGCTTATATTCAT
CACTGTAGGAATTGGGTTCAAGCTTTCCCCAGCCCCTTCTCATCAATGGACTCCTGACG
TATACGAAGGATCTCCCACTCCAGTCGTTGCTTTTCTTTCTGTTACTTCGAAAGTAGCTG
CTTCAGCTTCAGCCACTCGAATTTTCGATATTCCTTTTTATTTCTCATCAAACGAATGGC
ATCTTCTTCTGGAAATCCTAGCTATTCTTAGCATGATATTGGGGAATCTCATTGCTATTAC
TCAAACAAGCATGAAACGTATGCTTGCGTATTCGTCCATCGGTCAAATCGGATATGTAA
TTATTGGAATAATTGTTGGAGACTCAAATGGTGGATATGCAAGCATGATAACTTATATGC
TCTTCTATATCGCCATGAATCTAGGAACTTTTGCTCGCATTGTATCATTTGGTCTACGTAC
CGGAACTGATAACATTCGAGATTATGCAGGATTATACACAAAAGATCCTCTTTTGGCTC
TCTCTTTAGCCCTATGTCTCTTATCCTTAGGAGGTCTTCCTCCACTAGCAGGTTTTTTCG
GAAAACTTCATTTATTCTGGTGTGGATGGCAGGCAGGCCTATATTTCTTGGTTTCAATAG
GACTACTTACGAGCGTTGTTTCTATCTACTATTATCTAAAAATAATCAAGTTATTAATGAC

TGGACGAAACCAAGAAATAACCCCTCACGTGCGAAATTATAGAAGATCTCCTTTAAGA
TCAAACAATTCCATCGAATTGAGTATGATTGTATGTGTGATAGCATCTACTATACCAGGA
ATATCAATGAACCCGATTATTGAAATTGCTCAGGATACCCTTTTTATGTTTCTGCTTTACG
AATATGATATTTTCTGGGTATTTCTAATAATATCAAGTGTTATTCCTATTTTGGCATTTCTA
ATTTCTGGAGTTTTAGCCCCTCTTAGCAAAGAGCCAGAGAAACTTTCGAGTTATGAATC
GGGAATAGAACCAATGGGCGATGCTTGGGTACAATTTAGAATCCGTTATTATATGTTTGC
TCTAGTTTTTGTTGTTTTTGATGTTGAAACAGTTTTTCTTTATCCGTGGGCAATGAGTTT
TGATGTATTAGGGGTATCCGTCTTTATAGAAGCTTTAATTTTCGTGCTTATCCTAATTGTC
GGTTTAGTTTATGCATGGCGAAAAGGAGCATTGGAATGGTCTACGAATTATTTTCCTTG
GTTAACAACAGTTGTAGTTTTTCCAATATTTGCGGGTTTATTACTTTTCTTTTTCCCTCAT
AGAGGAAATAAAGTAATGAGATGGTATACTATATGTATCTGTGTACTCGAGCTGCTTCTA
ACAACCTATGCGTTTTGTTATCATTTCGAATTAGACGATCCATTAATCCAATTGATGGAG
GATTATAAATGGATCCCTTTTTTGGATTTTTACTGGGAGATTGGAATCGATGGACTTTCC
ATAGGACCCATTTTACTGACGGGGTTTATCACCACTTTAGCTACTTTAGCGGCTTGGCC
AGTTACTCGAGATTCCCGATTATTCTATTTTCTAATGTTAGCAATGTATAGCGGTCAAATA
GGATCATTTTCTGCTCGAGACCTCTTACTATTTTTTATCATGTGGGAGTTCGAATTAATTC
CCGTTTATCTACTTCTATCGATGTGGGGCGGAAAGAAACGGTTGTATTCAGCTACAAAG
TTTATTTTGTACACTGCGGGAGGTTCCATTTTTTTGTTAATGGGAGTTCTGGGTATCGGT
TTATATGGTTCTAATGAACCAACTTTAAATTTTGAAACATCAGCTAATCAATCGTATCCTA
TAGCACTGGAAATACTATTCTATATCGGATTTCTTATTGCTTTTGCTGTCAAATCACCGAT
TATACCCTTACATACATGGTTACCAGATACCCACGGAGAGGCACATTACAGTACTTGTAT
GCTTCTATCCGGAATCTTATTAGAAATGGGAGCATATGGATTGGTTCGGATCAATATGGA
ATTATTACCCCACGCCCATTCTATCTTTTCACCCTGGTTGATCATAGTAGGCATAATTCAA
ATAATCTATGCAGCTTCAACATCTTCGGGTCAACGCAATTTAAAAAAAAGAATAGCTTA
TTCCTCCGTCTCTCATATGGGTTTCATAATTATAGGAATTGGTTCTATAAGCGATACGGGA
CTGAATGGAGCTATTTTACAAATAATCTCTCATGGATTTATTGGCGCTGCGCTTTTTTTCT
TAGCGGGAACAAGTTATGATAGAATACGTCTGGTTTATCTTGATGAAATGGGAGGACTG
GCTATCCCAATTCCAAAAATATTTACGACTTTCAATATCTTATCGATGGCTTCCCTTGCAT
TGCCCGGTATGAGCGGTTTTGTTGCAGAATTAATAGTCTTTTTTGGAATAATTACCAGCC
AAAAATATCTTTTAATGACAAAAATGGTAATTACTTTGGTAATGGCAATTGGAATTATATT
AACTCCCATTTATTTATTATCTATGTTACGCCAGATGTTCTATGGATATAAGCTTTTTAATG
CTAAAAATTCTTATTTTTTTGATTCGGGACCACGAGAATTGTTTGTTGCGATCTCTATCC
TTATACCTGTCATAGGTATTGGTATTTATCCAGATTTTGTTTTCTCATTATCAGTTGACAA
GGTCGAAGCTATTTTATCTAATTATTTTGCTAGAATCATGCTCGAACATGTACTTATTATA
AGTGCCTATTTATTTTCTATCGGTATCTATGGATTGATCACGAGCCGAAATATGGTTAGGG
CCCTTATGTGTCTTGAGCTTATACTGAATGCAGTTAATATGAATTTCGTAACATTTTCTGA
TTTTTTTGATAGTCGCCAATTAAAAGGGAATATTTTCTCAATTTTTGTTATAGCTATTGCA
GCCGCTGAAGCAGCTATTGGCCCAGCTATTCTTTCGTCAACTTATCGTAACAGAAAATC
AACTCGTATCAATCAATCGAATTTGTTGAATAAGATGGAATATACATATCAATATTCATGG
ATCATACCGTTCATCTCACTTCCAATTCCTATCTTAATAGGAGTGGGCCTTCTACTTTTTC
CGACGGCAACCAAAAACCTTCGACGTCTGTGGTCGTTTCCGAGTATTTTATTGTTAAGT
ATAGTTATGATTTTTGCCGTTTATCTGTCTATTGATCAAATAAATACTAGTTATATCTATCA
ATATGTATGGTCTTGGACTATCAATAATGATTTTTCTTTAGAGTTGGGCTACTTCATCGAT
CCACTTACTTGTATTATGGCAATATTAATCACGACTGTTGGAATTATGGTTCTTATTTATA

GTGACAATTATATGTCTCATGATCAAGGATATTTGAGATTTTTTGCGTATATGAGTTTGTT
CAATACGTCAATGTTGGGATTAGTTACTAGTTCGAATTTGATCCAAATTTATATTTTTTGG
GAATTGGGTGGAATGTGTTCTTATCTATTAATAGGTTTTTGGTTCACTCGACCTAGTGCG
GCGAATGCTGGTCAAAAGGCATTTGTAACTAATCGCGTAGGGGATTTTGGTTTATTATTA
GGAATTTTAGGTCTTTATTGGACAACGGCGAGTTTTGAATTTCGGGATTTATTTCAAATA
TTCAATAAC---GAGGTAAATTTCTTATTTCTTACTTTGTGCGCCGTCCTATTATTTGCAGG
TGCTGTTGCTAAATCTGCTCAATTTCCTCTTCATGTATGGTTACCCGATGCTATGGAGGG
TCCTACCCCCATTTCGGCTCTTATACATGCTGCTACCATGGTAGCAGCGGGAATTTTTCT
TGTCGCCCGGCTTCTTCCGCTTTTTATAGCAATACCTTACATCATGAATCTAATAGCTTTG
ATAGGTATAATAACAGTACTATTTGGAGCTACTTTAGCTCTTGCTCAAAAAGATATTAAG
AGAAGTTTAGCCTATTCTACAATGTCTCAATTGGGCTATATGATGTTAGCTTTGGGTATG
GGGTCATACCAGGCTGCTTTATTTCATTTGATTACTCATGCTTATTCAAAAGCATTGTTG
TTTTTAGGATCGGGGTCCATTATTCACTCAATGGAATCGGTTGTTGGATATTCTCCAGAT
AAAAGTCAGAATATGGTTCTTATGGGTGGTTTAACAAAGCATGTGCCAATTACAAAAAA
TGCTTTTTTATTAGGTACCCTTTCTCTTTGTGGTGTTCCACCCCTTGCCTGCTTTTGGTCC
AAAGATGAAATTCTTAATGATAGTTGGTTGTATTCACCGATTTTCGCAATAATCGCCTGT
TCCACAGCAGGATTAACAGCATTTTATATGTTTCGCATTTACTTACTTACTTTTGATGGA
CATTTAAACCTTTATTGTAAAAACTACAGTGGAAAAACAAATAACTCCCTTTATTCAATA
TCATTATGGGGTAAAGAA---GGATCAAAAATCATTAAAAAATTCTTCTCATTATTAACAA
TGAATTTTTGGATGAGTACTAAAAAT---ACTTTTTACTATCCCTATGAATCAGAGAATACT
ATGCTATTTCCGATGTTTGTGTTATCCCTATTTACTTTGTTTGTTGGAGTCATAGGAACTC
AATTCGATTTGAATATATTAGACAAATGGTTAACTCCGTCTATAAACTTGTTACACGAAA
GTTCAAAA---TCTGAAAATTGG---GAATTTGTTAAAAATGCAACTTTTTCTGTGACTATA
GCTTTTTTCGGAATATTTATAGCGTCTTTCTTCTATAAGCCCACTTATTCATCTTTACAAA
ATTTGAATTTTCGTAATTCATTTGTTTTTTTTTTTGAAAAAATTATAAATATAATATATAAT
TGGTCATATAATCGTGGTTACATAGATACTTTTTATCGAATATCCTTAATTGGGGGTATAA
GAATATTAGCTGAATTAACTTATTTTTTTGATCGACGGATAATTGATGGAATTACCAATGC
GGTGGGTATTCTTAATTTTTTTGTAGCAGAGAGTATTAAATATGTAGGAGGTGGT---CGA
ATCTCGTCTTATATCTTGTTGTATTTAGTTTATTTAGTTATTTTTATATTAGTAATTTACATG
GATTTGCCTGGACCGATACATGATTTTCTTGTAGTCTTGTGGGGATCAGGTCTTATATTA
GGAAGTATGGGAGTACTATTATTTAACAACTCCATTTATTCTGCTTTTTCGTTGGGACTG
GTTCTTGTTTCTATATCCTTATTCTATATTCTAGCAAACGCCCAGTTTGTAGCTGCTGCGC
AACTCCTTATTTACGTGGGAGCTATAAATGTTTTAATCATATTTGCTGTGATGTTCATGAA
GGGTTCAGAATATTCCAAAGATTTTAATCTTTGGACCGTTGGGAATGGAGTTACTTTTCT
GGTTTGTACAAGTATTTTTGTTTCACTAATGACTACTATTGTGGATACGTCATGGTATGGT
ATTATTTGGACTACACGATCAAATCAGATTCTAGAGCAAGATTTAATAAGTAATAGTCAA
CAAATTGGAATTTATTTATCAACATATTTTTTTCTTCCATTTGAACTCATTTCGATCATTCT
TTTAGCTGCTTTGATCGGCGCAATTGCCGTGGCTCGCCAGATGAATGGACCAGCTACAA
GAAAAGACCTCATGATAGTCAATATGGGACCTCACCACCCCTCAATGCACGGTGTTCTT
CGACTCATCCTTACTTTGGATGGTGAAGATGTTATTGACTGTGAACCAATATTGGGTTAT
TTACACAGGGGGATGGAAAAAATTGCAGAAAACCGAACAATTATACAATATCTACCTTA
TGTAACACGTTGGGATTATTTAGCTACTATGTTTACAGAAGCAATAACCGTAAATGGTCC
AGAACAGTTGGGAAATATTCAAGTCCCTAAAAGAGCCAGCTATATCAGAGTAATTATGT
TGGAGTTGAGTCGTATAGCTTCTCATCTGTTATGGCTTGGCCCTTTTATGGCGGATATTG

GCGCACAGACTCCCTTCTTCTATATTTTCAGAGAACGAGAATTAGTATATGATCTATTCG
AAGCAGCTACCGGTATGAGAATGATGCATAATTTTTTTCGTATCGGGGGAGTCGCGGCT
GATTTACCTCATGGATGGATAGATAAATGTTTGGATTTCTGCGATTATTTTTTGACAGCG
GTTGCTGAATATCAAAAACTTATTACACAAAATCCTATTTTTTTAGAACGAGTTGAGGG
AGTAGGTATTATTTCTGGAGAAGAGGTCATAAATTGGGGGTTATCAGGACCAATGCTGC
GAGCTTCCGGAATACCATGGGATCTTCGTAAAGTTGATCGTTATGAGTGTTATGACGAA
TTTGATTGGGAAGTTCAGTGGCAAAAAGAAGGCGATTCATTAGCTCGTTATTTAGTCAG
ACTTGCTGAGATGACGGAATCCGTAAAAATTATTCAACAAGCTTTGGAAGGAATTCCA
GGGGGACCTTATGAAAATTTAGAAATACGATCTTTTGATCGAGCAAGGTCTCCGGAATG
GAATGACTTTGACTATCGATTCATTAGTAAAAAACCTTCGCCAACTTTTGAATTGGCGA
AACAAGAACTTTATGTGAGAGTCGAAGCCCCCAAAGGGGAATTGGGCATTTTTTTGAT
AGGGGATCAGGGTGGTTTTCCTTGGAGATGGAAAATTCGCCCGCCGGGTTTTATCAATT
TGCAAATTCTTCCTCAGTTAGTTAAAAGAATGAAATTGGCTGATATTATGACAATACTAG
GTAGCATAGATATCATTATGGGAGAAGTTGATCGTATGTTCCCTATGGTAACTGGGTTCA
TGAATTATGGTCAACAAACAGTACGAGCTGCAAGGTACATTGGTCAGGGTTTCGTGATT
ACTTTATCCCATGCAAATCGTTTGCCTGTAACTATTCAATATCCTTACGAAAAATTAATAG
CATCGGAGCGTTTCCGCGGTCGAATCCATTTTGAATTCGATAAATGCATTGCTTGTGAA
GCATGTGTTCGTGTATGTCCTATAGATCTCCCCGTTGTTGATTGGAAATTGGAAACGGAT
ATTCGAAAGAAACGATTGCTTAATTACAGTATTGATTTCGGGATCTGTATATTTTGTGGT
AACTGCGTTGAATATTGTCCAACAAATTGTTTATCAATGACTGAAGAATATGAACTTTCT
ACTTATGATCGTCACGAATTGAATTATAATCAAATTTCTTTGGGTCGTTTACCGATGTCA
GTAGTTGATGATTATACAATTAGAACAATTTTGAAT---
---ATGCAGGGTAATTTGTCTTCTTGGCTAGTCAAACATGGACTGGTTCATAGATCTTTGG
GTTTCGATTACCAAGGAATAGAGACTTTACAAATAAAGCCCGAGGAATGGCATTCCATT
GCAGTCATTTTATATGTATATGGTTACAATTATCTACGTTCCCAATGTGCTTATGATGTAG
CACCAGGCGGACTGTTAGCTAGTGTGTATCATCTTACGAGAATCGAGTATGGTATAGAT
CAACCGGAAGAGGTATGCATAAAAGTATTTGCCGCAAGGATAAATCCTAGAATTCCGTC
TGTTTTCTGGGTTTGGAAAAGTGCGGATTTTCCAGAAAGGGAATCTTATGATATGTTGG
GAATTTATTATGATAATCATCCACGTCTAAAGCGTATCTTAATGCCTGAAAGTTGGGTAG
GATGGCCCTTACGTAAGGATTATATTGCCCCCAATTTTTATGAAATACAAGATGCTCAT---
----
---ATGAATTCCATCGAGTTTCCCTTACTTGAT
CGAACAACTCAAACTTCAGTTATTTCAACTACATCAAATGATCTTTCAAATTGGTCAAG
ACTTTCCAGTTTATGGCCGCTTCTCTATGGTACCAGTTGTTGCTTCATTGAATTCGCTTC
ATTAATAGGTTCACGATTCGACTTTGATCGTTATGGACTGGTACCAAGATCTAGTCCTAG
ACAGGCAGACTTAATTTTAACAGCGGGTACGGTAACAATGAAAATGGCTCCTTCTTTAG
TAAGATTATATGAGCAAATGCCTGAACCAAAATATGTTATTGCTATGGGAGCCTGTACAA
TTACAGGAGGGATGTTCAGTACCGATTCTTATAGTACTGTTCGAGGAGTCGATAAGTTA
ATTCCTGTCGATGTGTATTTGCCGGGTTGTCCACCTAAACCCGAGGCTGTTATAGATGCT
ATAACAAAACTTCGTAAGAAAATATCTCGAGAAATCTATGAAGATCGAATTAAGTCT-----
-GAAAATCGGTGTTTTACTACCAATCACAAATTTCATGTTGGCCCAAGTACTCATACTGG
AAATTACGATCAAGAATTACTCTATCAACCATCATCTTCAGAGATCATCCCTCCTGAAAC
TTTTTTCAAATACAAAAGTGCAGTCTCTTCCTACGAATTAGTAAATATGCAAACTAGAA
ATACCTTTTCTTCGATAAAGGAAGAGATTACTCGATCTATTTCCGTATCACTCATGATATA

TATAATAACTTGGGCACCCGTTTCAAATGCATATCCCATTTTTGCACAACAGGGGTATGA
AAATCCACGAGAAGCAACCGGCCGTATTGTCTGCGCCAACTGCCATTTAGCTAATAAA
CCCGTGGATATCGAGGGTCCACAAGCGGTACTTCCTGATACTGTATTTGAAGCAGGTGG
TCGAATCCCTTATGATATGCAACTGAAACAAGTTCTTGCGAATGGGAAAAAGGGCTGG
TTGAATGTGGGGGCCGGTCTTATTTTACCCGAAGGGTTTGAATTAGCCCCCCCCGATCG
GATTTCTCCCGAGATTAAAGAAAAAAAGGGCTCTGTGCTTTTCAGAGCCTATCGTCCC
ACGAAAAAAAATATTCTTGTGATAGGTCCTGTTCCTGGTCAGAAATATAGTGAAATCAC
CTTTCCTATTCTTTCCCCGGACCCTGCGACTAAGAAAGATGTTCACTTCTTAAAATATCC
CATATACGTAGGCGGGAACAGGGGAAGGGGTCAGATTTATCCCGACGGGAGTAAGAGT
AATAATAATGTTTATAATGCTACAGCAGCAGGGATAGTAAGTAAAATAATACGAAAAGA
AAAAGGAGGATATGAAATAACCATAGTGGATGCCGCGAATGGGCGTCAAGTGGTTGAT
ATTATCCCTCCAGGACCAGAACTTCTTGTTTCAGAGGGCGAATCCTTCAAACTTGATCA
CCCATTAACGAGTAATCCTAATGTGGGAGGATTTGGTCAGGGAGATGCGGAAATCGTCC
TTCAAGATCCATTACGTGTCCAAGGCCTTTTGTTCTTTTTTGCATCTGTTATTTTGGCAC
AAATCTTTTTAGTTCTTAAAAAGAAACAGTTTGAGAAGGTCAATTGTCCGAAA---------A
TGAGTAAAGTGTATGATTGGTTCGAAGAACGTCTTGAAATTCAGGCGATTGCGGATGAT
ATAACTAGTAAATACGTTCCTCCTCATGTCAACATATTTTATTGTCTAGGAGGAATTACG
CTTACTTGTTTTTTAGTACAAGTAGCTACCGGGTTTGCTATGACTTTTTACTACCGTCCG
ACCGTTACTGAGGCTTTTGCTTCTGTTCAATATATAATGACGGAAGCAAATTTTGGTTGG
TTAATCCGATCAGTTCATCGATGGTCGGCAAGTATGATGGTCTTAATGATGATCCTGCAC
GTATTTCGCGTGTATCTTACTGGTGGATTTAAAAAACCTCGTGAATTGACTTGGGTTACA
GGTGTGGTTCTGGCTGTATTGACCGCATCCTTTGGTGTAACTGGTTATTCCTTACCTCGG
GACCAAATTGGTTATTGGGCAGTCAAAATTGTAACAGGTGTACCGGAAGCTATTCCAGT
AATAGGGTCGCCTTTGGTAGAGTTATTACGCGGAAGTGCTAGTGTGGGACAATCTACCT
TGACTCGTTTTTATAGTTTACACACTTTTGTATTACCTCTTCTTACTGCCGTATTTATGTTA
ATGCACTTCCCAATGATACGTAAGCAAGGCATTTCTGGTCCTTTAATGGGAGTAACAAA
AAAACCAGATTTGAATGATCCTGTATTAAGAGCTAAATTGGCTAAGGGTATGGGTCATA
ATTATTACGGAGAACCCGCATGGCCCAATGATCTTTTATATATTTTTCCAGTAGTCATTCT
AGGTACTATTGCATGTAACGTGGGCTTAGCGGTTCTAGAACCATCAATGATTGGTGAAC
CCGCAGATCCATTTGCAACTCCTTTGGAAATATTGCCGGAATGGTATTTCTTTCCCGTAT
TTCAAATACTTCGTACAGTGCCAAATAAGTTATTGGGTGTTCTTTTAATGGTTTCCGTAC
CGGCGGGATTATTAACTGTACCTTTTTTGGAGAATGTCAATAAATTCCAAAATCCATTTC
GTCGTCCAGTAGCGACAACCGTCTTTTTGATTGGTACCGCAGTAGCCCTTTGGTTGGGT
ATTGGAGCAACATTACCGATTGATAAATCCCTAACTTTAGGTCTTTTTATGATTGAAGTT
TTTTTATTTGGAATCGTATTAGGTCTAATTCCTATTACTTTGGCTGGATTATCTGTGACCG
CATATTTACAATACAGACGGGGGGATCAGTTGGACCTTATGCTTACTATCACTAGTTATT
TCGGTTTTCTACTAGCAGTTTTAACTATAACCTCAGCTCTATTTATCGGTCTGAACAAGA
TAAGACTTATTATGGATATGGTAAGTCTCGCTTGGGCTGCTTTAATGGTAGTCTTTACATT
TTCTCTTTCCCTCGTAGTATGGGGAAGAAGTGGACTCATGATTATTCGTTCGCCGGAAC
CAGAAGTCAAAATTTTGGTAGATAGGGATCCCATAAAAACTTCTTTCGAGGACTGGGC
CAGACCGGGTCATTTCTCGAGAACAATAGCTAAGGGTCCTGATACTACCACTTGGATCT
GGAACCTTCATGCTGATGCTCACGATTTCGATAGCCATACTAGTGATTTGGAGGAGATC
TCTCGAAAAGTCTTTAGTGCCCATTTCGGCCAACTCTCCATCATCTTTCTTTGGCTGAGT
GGGATGTATTTCCACGGTGCTCGTTTTTCCAATTATGAAGCATGGCTAGGTGATCCTACT

CACATTGGCCCTAGTGCCCAGGTGGTTTGGCCAATAGTGGGCCAAGAAATATTGAATG
GTGATGTAGGCGGGGGTTTTCGAGGAATACAAATAACATCTGGGTTTTTTCAGATGTGG
CGAGCATCTGGAATAACTAATGAATTACAACTCTATTGTACCGCAATTGGTGCATTAGTC
TTTGCAGCCTTAATGCTTTTTGCTGGTTGGTTTCATTATCACAAGGCTGCTCCAAAATTG
GCTTGGTTCCAAGATGTAGAATCCATGTTGAATCACCATTTAGCGGGTCTGCTAGGACT
TGGGTCTCTTTCTTGGGCGGGACATCAAGTCCACGTATCTTTACCGATTAACCAATTTCT
AAATGCTGGAGTAGATCCTAAAGAGATTCCACTTCCTCATGAATTTATCTTGAATCGGG
ATCTTTTGGCGCAACTTTATCCTAGTTTTGCTGAGGGAGCAACCCCATTTTTCACTTTGA
ATTGGTCAAAATATGCGGAATTTCTTACTTTTCGCGGAGGGTTAGATCCAGTAACCGGG
GGTCTATGGTTAACCGATATTGCACACCATCATTTAGCTATTGCAATTCTTTTCCTGATAG
CGGGTCACATGTATAGGACCAACTGGGGCATTGGTCATGGTATAAAAGATATTTTAGAG
GCTCATAAAGGTCCATTTACAGGCCAGGGCCATAAAGGACTATATGAGATCCTAACAAC
GTCATGGCATGCTCAATTATCTATTAACCTAGCTATGTTAGGCTCTTTAACCATTATTGTA
GCTCACCATATGTATGCAATGCCCCCGTATCCATATCTAGCTACTGACTATGGTACACAA
CTTTCATTGTTCACACATCACATGTGGATTGGTGGATTTCTCATAGTTGGTGCTGCTGCG
CATGCAGCCATTTTTATGGTAAGAGACTATGATCCAACTACTCGCTACAACGATCTATTA
GATCGTGTACTTAGGCATCGTGATGCAATCATATCACATCTGAACTGGGTGTGTATATTT
TTAGGCTTTCACAGTTTTGGTTTGTATATTCATAACGATACCATGAGTGCTTTAGGGCGT
CCACAAGATATGTTTTCAGATACCGCTATACAATTACAACCCCGTCTTTGCTCAATGGAT
ACAAACACCCATGCTTTAGCACCTAGGATAACGGCCCCCGGTGCAACAACAGGCACCA
GTTTGACTTGGGGGGGTGGTGATTTAGTGGCAGTGGGCGGCAAAGTTGCTTTGTTACC
TATTCCGTTAGGAACCGCAGATTTTTTGGTACACCATATTCATGCATTTACGATTCATGTA
ACGGTATTGATACTCCTGAAAGGTGTTCTATTTTCTCGTAGCTCGCGTTTGATACCGGAT
AAAGCAAATCTTGGTTTTCGTTTCCCTTGTGATGGACCGGGAAGAGGGGGTACATGTC
AAGTATCCGCTTGGGATCATGTCTTCTTAGGGCTATTTTGGATGTACAATTCCATTTCTGT
AGTAATATTCCATTTCAGTCGGAAAATGCAGTCAGATGTCTGGGGTAGTGTAAGTGATC
AAGGGGTACTAACTCATATTACGGGAGGAAACTTTGCGCAGAGTTCTATTACTATTAAT
GGGTGGCTTCGCGATTTCTTATGGGCACAGGCATCCCAGGTAATTCAGTCTTATGGTTC
TTCATTATCTGCATATGGCCTGTTTTTCCTAGGTGCTCATTTTGTATGGGCTTTTAGTTTA
ATGTTTCTATTCAGCGGGCGTGGTTATTGGCAAGAACTTATTGAATCCATCGTTTGGGCT
CATAATAAATTAAAAGTTGCTCCTGCTACTCAGCCTAGAGCCTTGAGCATTGTCCAAGG
ACGTGCTGTAGGAGTAACCCATTACCTTCTGGGTGGAATTGCCACAACATGGGCGTTCT
TCTTAGCAAGAATTATTGCAGTCGGAATGGCACTAAGATTTCCAAGGTTTAGCCAAGGC
TTAGCTCAGGACCCCACTACTCGTCGTATTTGGTTTGGTATTGCTACCGCGCATGACTTC
GAAAGTCATGATGATATTACTGAAGAACGTCTTTATCAGAATATTTTTGCTTCTCATTTC
GGGCAATTAGCAATAATTTTTCTGTGGACTTCCGGAAATCTGTTTCATGTAGCTTGGCA
AGGAAATTTTGAAGCATGGGTACAGGACCCTTTACATGTAAGACCTATTGCTCATGCAA
TTTGGGATCCTCATTTTGGTCAACCGGCCGTCGAAGCTTTTACTCGAGGTGGTGCTCTT
GGCCCAGTGAATATCGCTTATTCTGGTGTTTATCAGTGGTGGTATACAATCGGTTTACGC
ACTAATGGGGATCTTTATACTGGAGCTCTTTTTCTATTATTTCTTTCTGCGATATCTTTAAT
AGCGGGTTGGTTACATCTACAACCGAAATGGAAACCGAGCGTTTCCTGGTTTAAAAAT
GCCGAATCTCGTCTGAATCATCATTTGTCAGGACTATTCGGAGTAAGTTCCTTGGCTTG
GACAGGACATTTAGTCCATGTCGCTATTCCTGCATCCAGGGGAGAATCTGTTCGATGGA
ATAATTTCTTAGATGTATTGCCACATCCCCAAGGATTAGGACCACTTTTTACAGGTCAGT

GGAATCTTTATGCTCAAAACCCCGATTCAAATAATCATTTATTTGGTACTTCCGAAGGAG
CAGGAACTGCCATTCTAACCCTTCTCGGGGGATTCCATCCGCAAACCCAAAGTTTATGG
CTGACTGATATGGCTCATCATCATTTAGCTATTGCATTTATTTTTCTTGTTGCTGGTCATAT
GTATAGAACTAACTTCGGGATTGGACACAGTATAAAAGATCTTTTAGAAGCACATATTC
CACCGGGGGGACGATTGGGGCGTGGACATAAAGGTCTTTATGACACAATTAATAATTCG
CTTCATTTTCAATTAGGTCTTGCTTTAGCCTCTTTAGGGGTTATTACTTCCTTGGTAGCTC
AACACATGTACGCTTTACCTGCTTATGCGTTCATAGCACAAGACTTTACTACTCAAGCT
GCGTTATATACCCATCACCAATACATAGCAGGATTCATTATGACAGGAGCTTTTGCTCAT
GGAGCTATATTTTTTATTAGAGATTACAATCCAGAACAGAATGAGGATAATGTATTGGCA
AGAATGTTAGACCATAAAGAAGCTATCATATCCCATTTAAGTTGGGCCAGCCTTTTTCTG
GGGTTCCATACTTTGGGGCTTTATGTTCATAATGATGTCATGCTTGCTTTTGGTACTCCG
GAAAAACAAATCTTAATCGAACCCATATTTGCCCAATGGATTCAATCCGCTCATGGTAA
AACTTCATATGGCTTCGATGTACTTTTATCTTCAACAAGCGGCCCGGCGTTCAATGCGG
GTCGAAGCATATGGTTGCCAGGTTGGTTAAATGCTGTTAATGAG------AATAGTAATTCA
CTATTCTTAACAATAGGGCCCGGAGACTTCTTGGTTCATCATGCTATTGCTTTAGGTTTA
CATACAACTACATTGATCTTAGTAAAAGGTGCTTTAGATGCACGTGGTTCCAAATTAATG
CCGGATAAAAAGGATTTTGGTTATAGTTTTCCTTGCGACGGTCCGGGACGAGGTGGTAC
TTGTGATATTTCGGCTTGGGACGCCTTTTATTTGGCAGTTTTCTGGATGTTAAATACCAT
TGGATGGGTTACTTTTTATTGGCACTGGAAGCACATCACATTATGGCAGGGTAACGTTT
CACAGTTTAATGAATCTTCCACTTATTTGATGGGATGGTTAAGAGATTATCTATGGTTAA
ACTCTTCACAACTTATCAATGGATATAATCCTTTTGGTATGAATAGTTTATCAGTCTGGGC
GTGGATGTTCTTATTTGGACATCTTGTTTGGGCTACTGGCTTTATGTTCTTAATTTCCTGG
CGCGGATATTGGCAAGAATTGATTGAAACTTTAGCATGGGCTCACGAACGTACACCTTT
GGCTAATTTGATTCGATGGAGAGATAAACCAGTGGCTCTTTCCATTGTGCAAGCAAGAT
TAGTTGGATTAGCCCACTTTTCTGTAGGTTATATATTTACTTATGCGGCTTTCTTGATTGC
CTCTACATCAGGTAAATTTGGTATGTCACATTCAGTAAAGATTTATGATACATGTATAGG
GTGTACTCAATGTGTCCGAGCTTGCCCCACAGATGTATTAGAAATGATACCTTGGGGCG
GATGTAAAGCTAAGCAAATAGCTTCGGCTCCACGAACGGAAGACTGTGTTGGTTGTAA
GAGATGTGAATCTGCCTGTCCGACGGATTTCTTGAGTGTTCGCGTTTATTTATGGCATGA
AACAACTCGAAGCATGGGTCTAGCTTATATGACAACTCTCACCTTACCCTCTATTTTTGT
GCCTTTAGTGGGCCTAGTATTTCCGGCAATTGCAATGGCTTCTTTATTTCTTCATGTTCA
AAAAAACAAGATTTTTATGCGAGATCTAAAAACATTCTCTCCCGTGGCACCGGTAGTAA
GTACTATATGGTCGGGC---TTTAGCGGGTCTATTAATCGAGATCAATCGTTTATTCCCGGA
TGCGTGGGAATCCCCATTTTTTCCATCATGACTGCAATTTTAGAGAGACGCGAAAGCGA
AAGCCTATGGGGTCGTTTCTGTAACTGGATAACCAGCACTGAAAACCGTCTTTACATTG
GATGGTTTGGTGTTTTGATGATCCCTACCTTATTGACCGCAACTTCTGTATTTATTATTGC
CTTCATTGCTGCTCCTCCAGTGGATATTGATGGTATTCGTGAACCTGTTTCTGGATCTCT
ACTTTACGGAAACAATATTATTTCTGGTGCTATTATCCCTACCTCTGCAGCTATCGGTTTG
CACTTTTACCCAATTTGGGAAGCTGCTTCCGTTGATGAATGGTTATACAACGGTGGTCC
TTATGAGCTAATTGTTCTACACTTCTTACTTGGTGTAGCATGTTACATGGGTCGAGAGTG
GGAACTTAGTTTCCGTCTGGGTATGCGTCCTTGGATTGCTGTTGCATATTCAGCTCCTGT
TGCAGCAGCTACTGCTGTTTTCTTGATTTACCCAATTGGTCAAGGAAGCTTTTCTGACG
GTATGCCTCTAGGAATCTCTGGTACTTTCAACTTCATGATTGTATTCCAGGCTGAGCACA
ACATCCTTATGCACCCATTCCACATGTTAGGTGTAGCTGGTGTATTCGGCGGCTCCCTAT

TCAGTGCTATGCATGGTTCCTTGGTAACTTCTAAGTTGATCAGGGAAACCACAGAAAAT
GAATCTGCTAATGAAGGTTACAGATTCGGTCAAGAGGAAGAAACTTATAATATCGTAGC
TGCTCATGGTTATTTTGGCCGATTGATCTTCCAATATGCTAGTTTCAACAACTCTCGTTC
TTTACATTTCTTCCTAGCTGCTTGGCCTGTAGTAGGTATCTGGTCCACCGCTTTAGGTATT
AGCACTATGGCTTTCAACTTAAATGGTTTCAATTTCAACCAATCTGTAGTTGATAGTCAA
GGTCGTGTAATTAATACCTGGGCTGATATTATTAACCGTGCTAATCTTGGTATGGAAGTT
ATGCATGAACGTAATGCTCACAACTTCCCTCTAGACCTAGCTGCTGTTGAAGTTCCATC
TATCAATGGAATGGGTTTGCCTTGGTATCGTGTTCATACCGTTGTATTGAATGATCCCGG
TCGTTTGCTTTCTGTTCATATAATGCAAACAGCTCTAGTTGCTGGTTGGGCCGGTTCAAT
GGCCCTATACGAATTAGCAGTTTTTGATCCCTCTGATCCTGTTCTTGATCCAATGTGGAG
ACAAGGTATGTTCGTTATACCCTCCATGACTCGTTTAGGAATAACCAATTCATGGGGAG
GTTGGAGTATCACA---GGAGGGATTATAACGAATCCGGGTATTTGGAGTTACGAAGGTG
TGGCCGGAGCACATATTTTGTTTTCTGGATTGTGCTTCTTAGCAGCTATCTGGCATTGGG
TGTATTGGGATCTAGAAATATTTTCTGATGAACGTACAGGAAAACCTTCTTTGGATTTGC
CGAAGATTTTTGGAATTCATTTATTTCTCTCAGGGTTGGGTTGTTTTGGTTTTGGTGCAT
TTCATGTAACCGGATTGTACGGTCCGGGAATATGGGTATCCGATCCTTATGGATTAACCG
GAAGGGTACAAGCCGTAAATCCTGCGTGGGGTGTCGAAGGGTTTGATCCTTTTGTTCC
GGGCGGAATAGCCTCGCATCATATTGCAGCAGGTACATTGGGTATATTAGCGGGCCTATT
CCATCTTAGTGTTCGTCCGCCCCAACGTCTATACAAAGGATTACGTATGGGAAATATTGA
AACCGTCCTTTCGAGTAGTATCGCTGCTGTATTTTTTGCAGCTTTTGTTGTTGCTGGAAC
TATGTGGTATGGTTCAGCAACTACCCCGATTGAATTATTTGGGCCCACTCGTTATCAATG
GGATCAGGGATACTTCCAGCAAGAAATATATCGAAGAGTTAGTACCGGGTTAGCCGAA
AATCAAAGTTTATCAGAAGCTTGGTCTAAAATTCCTGAAAAATTAGCTTTTTATGATTAT
ATCGGGAATAATCCCGCAAAAGGTGGATTATTCAGAGCGGGTTCCATGGACAACGGGG
ATGGAATAGCTGTTGGTTGGTTAGGACACCCTGTTTTTAGAGATAAAGAAGGGCGCGA
ACTTTTTGTACGCCGTATGCCGACCTTTTTTGAAACATTTCCGGTTGTTTTGGTAGACGG
AGATGGAATTGTTAGAGCCGATGTTCCTTTTCGAAGAGCAGAATCGAAGTATAGTGTTG
AACAGGTCGGTGTAACTGTTGAGTTCTATGGCGGTGAACTCAATGGAGTCAGTTATAGT
GATCCTGCTACTGTGAAAAAATATGCTAGACGTGCTCAATTGGGTGAAATTTTTGAATT
AGATCGTGCTACTTTGAAATCCGATGGGGTTTTTCGTAGCAGTCCAAGGGGTTGGTTTA
CTTTTGGGCATGCTTCCTTTGCTTTGCTCTTCTTCTTCGGACACATTTGGCATGGTGCGA
GAACGTTGTTCAGAGATGTCTTTGCTGGGATTGACCCAGATTTGGACACTCAAGTAGA
ATTTGGGGCATTCCAGAAAACTTGGAGATCCAACTACAAAAAGAGTCTAGTC-------------
----------------------GTGGAAACGCTCTTTAATGGAACTTTAGCTTTAGCTGGTCGTGACCA
AGAAACCACCGGTTTCGCTTGGTGGGCCGGGAATGCCCGACTTATCAATTTATCCGGTA
AACTACTGGGGGCTCATGTAGCCCATGCCGGATTAATCGTATTCTGGGCCGGAGCAATG
AACCTATTCGAAGTGGCTCATTTCGTACCGGAGAAGCACATGTACGAACAAGGATTAAT
TTTACTTCCCCACCTAGCTACTTTAGGTTGGGGGGTAGGTCCTGGTGGGGAAGTTATAG
ACACCTTTCCTTACTTTGTGTCTGGAGTACTTCACTTAATTTCCTCCGCAGTATTGGGTT
TTGGCGGTATTTATCATGCACTTCTGGGACCCGAGACCCTTGAAGAATCTTTTCCATTCT
TCGGTTATGTGTGGAAAGATAGAAATAAAATGACTACCATTTTGGGTATTCACTTAATTT
TGTTAGGTATAGGTGCTTTTCTTCTAGTATTCAAAGCTCTTTATTTTGGGGGCGTATATGA
TACCTGGGCTCCCGGGGGGGGAGATGTAAGAAAAATTACCAACTTGACCCTTAGCCCA
AGTGTTATATTTGGTTATTTACTAAAATCTCCTTTTGGCGGAGAAGGATGGATTGTTAGT

GTGGACGATTTGGAAGATATAGTTGGGGGACATGTATGGTTAGGTTCCATTTGTATACTT
GGTGGAATTTGGCATATCTTAACTAAACCCTTTGCATGGGCTCGCCGCGCACTTGTATG
GTCTGGAGAAGCTTACTTGTCTTATAGTTTAGGTGCTTTATCTGTTTTTGGTTTTATTGCT
TGTTGTTTTGTCTGGTTCAATAATACTGCTTATCCGAGTGAGTTTTACGGGCCGACTGG
ACCAGAAGCTTCTCAAGCTCAAGCATTTACTTTTCTAGTTAGAGACCAACGTCTTGGA
GCTAACATTGGATCCGCTCAAGGACCTACTGGTTTAGGTAAATATCTAATGCGTTCTCCG
ACCGGAGAAGTCATTTTTGGGGGAGAGACTATGCGTTTTTGGGATCTGCGTGCTCCGT
GGTTAGAACCACTAAGGGGTCCTAATGGTTTGGACTTGAGTAGACTGAAAAAAGACAT
CCAAACCTTGGCAAGAACGACGTTCCGCGGAATATATGACCCTGCTCCTTTAGGTTCTT
TAAATTCCGTGGGGGGCGTAGCTACCGAAATTAATGCAGTCAATTATGTCTCTCCTAGA
AGTTGGTTAGCTACCTCTCATTTTGTTCTAGGATTCTTCTTATTCGTAGGTCATTTATGGC
ATGCAGGAAGGGCTCGTGCAGCTGCCGCAGGATTTGAAAAAGGAATTGATCGTGATTT
TGAACCTGTTCTGTTCATGACTCCTCTTAACATGACTATAGCCGTTGGTAAATTTACCAA
AGACGAAAATGATTTATTCGATATTATGGATGACTGGTTACGGAGGGACCGTTTCGTTTT
TGTAGGTTGGTCCGGTCTATTGCTCTTTCCTTGTGCCTATTTTGCCGTCGGAGGTTGGTT
TACAGGTACAACCTTTGTAACTTCATGGTATACCCATGGATTGGCAAGTTCCTATTTGGA
AGGATGCAACTTCTTAACCGCCGCAGTTTCGACTCCTGCTAATAGTTTAGCCCACTCTT
TGTTGTTACTATGGGGCCCTGAAGCACAAGGAGATTTTACTCGTTGGTGTCAATTAGGT
GGTCTGTGGACTTTTGTTGCTCTCCACGGTGCTTTCGGACTAATAGGTTTCATGTTACGT
CAATTTGAACTTGCTCGATCTGTTCAATTGCGACCTTATAATGCAATCGCATTCTCGGGC
CCAATTGCTGTTTTTGTTTCTGTATTCCTGATTTATCCACTAGGTCAGTCTGGTTGGTTCT
TTGCGCCTAGTTTTGGTGTAGCAGCTATATTTCGATTCATCCTATTTTTCCAAGGGTTTCA
TAATTGGACATTGAACCCATTTCATATGATGGGAGTTGCGGGGGTATTGGGCGCTGCTC
TGCTATGCGCTATTCATGGTGCTACCGTAGAAAATACCTTATTTGAGGATGGGGATGGTG
CAAATACATTCCGTGCTTTTAACCCAACTCAAGCTGAAGAAACTTATTCAATGGTCACT
GCTAACCGCTTTTGGTCCCAAATCTTTGGGGTTGCTTTTTCCAATAAACGTTGGTTACAT
TTCTTTATGTTATTTGTACCAGTAACTGGTTTATGGATGAGTGCTCTTGGAGTAGTTGGT
CTGGCCCTGAACCTACGTGCCTATGACTTCGTTTCTCAGGAAATCCGTGCAGCGGAAG
ATCCGGAATTTGAGACTTTCTATACCAAAAATATTCTCTTAAACGAAGGTATTCGTGCTT
GGATGGCGGCTCAAGATCAGCCTCATGAAAACCTTATATTCCCTGAGGAGGTTCTACCC
CGTGGAAACGCTCTTATGTCTGGAAGCACAGGAGAACGTTCTTTTGCTGATATTATCAC
CAGTATTCGATACTGGGTCATTCATAGCATTACTATACCTTCTCTATTCATTGCAGGTTGG
TTATTCGTCAGCACGGGTTTAGCTTACGATGTTTTTGGAAGCCCTCGTCCAAATGAGTA
TTTTACAGAGAGCCGACAAGGAATTCCATTAATAACTGGCCGTTTTGATTCTTTGGAAC
AACTCGATGAATTTAGTAGATCTTTTATGACTATCGATAAAACTTATCCTATTTTTACAGT
GCGATGGTTAACTGTTCACGGACTAGCTGTACCTACCGTTTCTTTTTTAGGGTCAATATC
AGCAATGCAATTCATCCAACGAATGGCTACACAAACCGTTGACGATAGTTCTAAATCTG
GG---CCAAGACGAACTGTTGTAGGGGATTTATTGAAACCATTGAATTCGGAATATGGTA
AAGTGGCTCCTGGGTGGGGAACTACACCTTTAATGGGTGTCGCGATGAGTTTATTTGCG
ATATTCCTATGTATTATTTTGGAGATTTATAATTCTTCCATTTTACTGGACGGAATTTCAAG
CAATATGCTTACTCTCAAACTATTTGTTTATACAGTAGTAATATTCTTTGTTTCTCTCTTCA
TTTTCGGATTCCTATCTAATGACCCGGGACGTAATCCGGGGCGTGAAGAAATGGCCGAG
ACTACTGGAAGGATTCCTCTTTGGCTAATCGGTACTGTAACTGGTATTCCTGTGATCGGT
TTAGTCGGTGTTTTCTTTTATGGTTCATATTCCGGCTTAGGTTCATCTCTGATGCTTAATA

TCTTAAGTTTCATCGGTATCTGTCTTAATTCTTTCCTTTATTCAAGTAGTTTTTTCGTCGC
CAAATTGCCCGAGGCCTACGCTTTTTTGAATCCAATAGTAGATGTTATGCCAGTAATACC
TCTATTCTTTTTTCTATTAGCTTTTGTTTGGCAAGCTGCTGTAAGCTTTCGAATGACACA
ATCAAATCCGAACGAACAAAATGTTGAATTGAATCGTACCAGTCTTTACTGGGGGTTAT
TACTCATTTTTGTACTTGCTGTTTTATTTTCCAATTATTTCTTCAATATGGAAGTAAATATT
CTCGCATTTATTGCTACTGCACTGTTCATTCTAGTTCCGACCGCCTTTTTACTTATAATAT
ACGTAAAAACAATCAGTCAAAGTGATATGGAAACAGCAACCCTAGTTGCCATCTCTATA
TCTGGTTTACTTGTAAGTTTTACTGGGTATGCCTTATATACTGCTTTTGGGCAACCCTCTC
AACAACTAAGAGATCCATTCGAGGAACACGGGGACATGGAAGCATTGGTTTATACATT
CCTCTTAGTCTCGACTCTAGGAATAATTTTTTTCGCTATCTTTTTTCGCGAACCGCCTAA
AGTTCCAACTAAAAAGGCGAAAATGACTATTGCTTTCCAATTGGCTGTTTTTGCATTAA
TTGTTACTTCATCAATCTTACTGATTAGTGTGCCCGTTGTATTTGCTTCTCCTGATGGTTG
GTCGGGTAACAAAAATGTTGTATTTTCCGGTACATCGTTATGGATTGGATTAGTCTTTCT
GGTGGGTATCCTTAATTCTCTTATCTCT---------------------ATGTCACCACAAACAGAGACT
AAAGCAAGTGTTGGATTCAAAGCTGGTGTTAAAGATTATAAATTGACTTATTATACTCCT
GAATATGAAACCAAAGATACTGATATCTTGGCAGCATTCCGAGTAACTCCTCAACCGGG
AGTTCCACCTGAGGAAGCAGGGGCCGCTGTAGCTGCTGAATCTTCTACTGGTACATGG
ACAACTGTGTGGACCGATGGGCTTACCAGTCTTGATCGTTACAAAGGACGATGCTATG
GAATCGAGCCTGTTGCTGGAGAAGAAAATCAATATATTGCTTATGTAGCTTATCCTCTAG
ACCTTTTTGAAGAAGGTTCTGTTACTAACATGTTTACTTCCATTGTGGGTAATGTATTTG
GATTCAAGGCTCTACGTGCTCTACGTCTGGAGGATTTGCGAATCCCTACTGCTTATATTA
AAACTTTCCAAGGCCCGCCTCATGGTATCCAGGTTGAAAGAGATAAATTGAACAAGTA
TGGTCGCCCTCTATTGGGATGTACTATTAAACCAAAATTGGGATTATCCGCTAAGAATTA
TGGTAGAGCAGTTTATGAATGTCTACGCGGTGGACTTGATTTTACCAAAGATGATGAAA
ACGTGAATTCCCAACCATTTATGCGTTGGAGAGACCGTTTCCTATTTTGTGCGGAAGCT
ATTTTTAAATCACAGGCTGAAACAGGTGAAATCAAA---GACATACTGAATGCTACTGCG
GGTACATGCGAAGAAATGATGAAAAGGGCTATATTTGCCCGAGAATTGGGAGCTCCTAT
CGTAATGCATGACTACTTAACAGGTGGATTCACTGCAAATACTAGCTTGGCTCATTATTG
CCGAGATAATGGTCTACTTCTTCACATTCACCGTGCAATGCATGCTGTTATTGATAGACA
GAAGAATCATGGTATGCACTTCCGTGTACTAGCTAAAGCGTTACGTATGTCTGGTGGAG
ACCATATTCACGCTGGTACCGTAGTAGGTAAACTTGAAGGGGAAAGAGAAATCACTTT
AGGCTTTGTTGATTTACTACGTGATGATTTTGTTGAAAAAGACCGAAGCCGCGGTATTT
ATTTCACTCAAGATTGGGTCTCTTTACCAGGTGTTCTGCCAGTGGCTTCCGGTGGTATT
CACGTTTGGCATATGCCTGCTCTAACCGAGATTTTTGGAGATGATTCTGTACTACAATTC
GGCGGAGGAACTTTGGGGCACCCTTGGGGTAATGCACCTGGTGCCGTAGCTAACCGAG
TAGCTCTAGAAGCATGTGTACAAGCTCGTAATGAGGGACGTGATCTTGCTCGTGAGGGT
AATGAAATTATCCGTGAGGCTAGTAAATGGAGTCCTGAACTAGTCTGCGTGAGGAAGTA
TGGTAAGGAGATCAAATTTGAATTTGAGGCAATGGATACTTTG------ATGGCGATACATT
TATACAAAACTTCTACCCCGAGCACACGCAATGGAGCCGTAGACAGCCAAGTAAAATC
CAATCCACGAAATAATTTGATCTATGGACAGCATCGTTGTGGTAAAGGTCGTAATGCCA
GAGGAATCATTACCGCAGGGCATAGAGGGGGAGGTCATAAGCGTCTATACCGTAAAAT
CGATTTTCGACGGAATGAAAAAGACATATATGGTAGAATCGTAACCATAGAATACGACC
CTAATCGAAATGCATACATTTGTCTCATACACTATGGGGATGGTGAGAAAAGATATATTT
TACATCCCAGAGGGGCTATAATTGGAGATACCATTGTTTCTGGTACAGAAGTTCCTATAA

AAATGGGAAATGCCCTACCTTTGACCGATATGCCCTTAGGCACGGCCATACATAACATA
GAAATCACACTTGGGAAGGGGGACAATTAGCTAGAGCAGCAGGTGCTGTAGCGAAA
CTGATTGCAAAAGAGGGGAAATCGGCCACATTAAAATTACCTTCTGGGGAGGTCCGTT
TGATATCCAAAAATTGCTCAGCAACAGTCGGACAAGTGGGGAATGCTGGGGTAAACCA
GAAAAGTTTGGGTAGAGCCGGATCTAAATGTTGGCTAGGTAAGCGTCCTGTAGTAAGA
GGAGTAGTTATGAACCCTGTAGACCATCCCCATGGGGGTGGTGAAGGGAGGGCCCCAA
TTGGTAGAAAAAAACCCGCAACCCCTTGGGGTTATCCTGCACTTGGAAGAAGAAGTAG
AAAAAGGAATAAATATAGTGATAATTTGATTCTTCGTCGCCGTAGTAAAATGATTCAACC
TCAAACCCTTTTGAATGTAGCAGATAACAGCGGAGCCCGAGAATTGATGTGTATTCGAA
TCATAGGGGCTAGT---AATCGACGATATGCTCATATTGGTGACGTTATTGTTGCTGTAATC
AAGAAAGCCGTCCCAAATACACCTTTAGAAAGATCAGAAGTGATCAGAGCTGTAATTG
TACGTACTTGTAAAGAACTCAAACGAGAAAATGGTATGATAATACGATATGATGACAAT
GCTGCGGTTGTTATTGATCAAGAAGGAAATCCAAAAGGAACTCGAATTTTTGGTGCGA
TTGCCCGAGAATTGAGACAGTTAAATTTCACAAAAATAATTTCATTAGCACCTGAAGTA
TTAATGCTTAGTCCTAAAAGAACCAGATTCCGTAAACACCATAGAGGAAGAATGAAAG
GAATATCTTATCGAGGCAATAGTATTTGCTTCGGGAGATACGCTATTCAGGCACTTGAAC
CCGCTTGGATCACATCTAGACAAATCGAAGCCGGACGAAGGGCAATGACACGAAATGC
ACGACGCGGTGGAAAAATATGGGTACGTATATTTCCAGACAAACCCGTTACACTAAGA
CCCACAGAAACACGTATGGGTTCAGGGAAAGGATCCCCCGAATATTGGGTAGCTGTTG
TTAAACCAGGTAGAATACTTTATGAAATGAGTGGAGTAGCCGAAAATATAGCCAGAAA
AGCTATTTCAATAGCGGCATCCAAAATGCCTATACGAACCCAATTCATTACTTCGGGAAT
GACTAGAATTAAACGAGGATATATAGCTCGGAGACGTAGAACAAAAATTCGTTTATTTG
CATCAAGCTTTCGAGGGGCTCATTCAAGACTTACTCGAACTATTACTCAACAGAAAATA
AGAGCTTTGGCTTCAGCTTATCGGGATAGAGGTAGGCAAAAAAGAAATTTTCGACAGT
TGTGGATCGCTCGAATAAATGCAGTAATTCGATCTAAT---AATGTAGACTACAGTTATAGT
AGATTCATACACAATCTGTACAAGAGGCAGTTGCTTCTTAATCGTAAAATACTTGCCCA
AATCGCGATATTAAATAGAAATTGTCTTTATATGATTTCCAATGAGATCATAAAA-----------
----------------------ATGATAAAGGAAGTATACGCTTTAGGTCAACATATATGTATGTCCCCTC
ACAAAGCGCGAAGAGTAATTGATCAGATTCGTGGCCGTTCCTACGAAGAAACAATGAT
GATACTCGAACTCATGCCTTATCGAGCATGTTATCCAATTTTAAAATTGGTTTATTCGGC
AGCAGCAAATGCTAGTCACAATATGGGTTTTAACGAAAGAGATTTAGTCATTAGTAAAG
CAGAAGTTAATCAAGGTACTACTGTGAAAAAATTAAAACCTCGAGCTCAAGGGCGAA
GTTATCCCATAAAACGACCCACTTGTTCTATAACTATTGTATTGAAAAATACATCTGTGA
AT---GGATTATGGGACAAAAAAATGGATGGAATCAAATATGCAGTATTTACAGACAAAA
GTATTCGGTTATTGGGGAAAAAT---CAATATACTTCTAATGTCGAATCAGGATCAACTAG
GACAGAAATAAAGCATTGGGTCGAACTCTTCTTTGGTGTCAAGGTAATAGCTATGAATA
GTCATCGACTCCCGGGAAAGGGTAGAAGAATGGGACCTATTATGGGACATACAATGCA
TTACAGACGTATGATCATTACGCTTCAACCGGGTTATTCTATTCCACCTCTTAGAAAGAA
AAGAACTATGGCACTTCCAAAAAAGCGCACTTCTATATCAAAAAAACGGATTCGGAAA
AATATTTGGAAAAGCAAAGGGCGTCGGGCAGCGCTGAAAGCTTTTTCCCCAGCAAAAT
CTCTTTCAACGGGTAATTCAAAAAGTTTT---------------AAATCAATGGCCAAGGGTAAAG
ATGCCCGAGTAACAGTTATTTTGGAAGGACTCGTGTGT------CGAAAC------GTTAATAAG
CAATCAATAGGGATTTCCAGATATATTACTCAAAAGAATCGACACAATACGCCTAGTCG
ATTGGAATTGAGAAAATTCTGTCCCCGGTGTTACAAACATACGATTCATGGGGAGATAA

AGAAAATGAAAATAAGGGCCTCCATTCGTAAAATTTGTGAAAAATGTCGCTTGATCCGT
AGGCGGAGACGAATTATGGTAATTTGTTCCAATCCGAGACATAAACAAAGACAAGGAA
TGGTTCGAGAGAAAATCAGAGTATCGACTCGGACACTAAAGTGGAAGTGTGTTGAATC
AAGAGCAGACAGTAAGCGTCTTTATTATGGACGCTTTATTCTGTCTCCACTTATGAAGG
GGCAAGGGGATACTATAGGCATTGCGATGCGAAAAGCTTTGCTTGGAGAAATAGAGGG
AACATGTATCACACGTGCAAAATCTGAA------AAAATCCCACATGAATACTCTACCATAG
TCGGTATTCAAGAATCAGTACATGAAATTTTAATGAATTTGAAAGAAATTGTATTGAGA
AGTAATCTGTATGGAACTCGTGATGCGTCTATTTGTGTCAAGGGTCCTGGATGCGTAAC
TGCTCAAGACATCATCTTACCACCTTCGGTGGAAATCGTTGATAATACACAGCATATAGC
TAACCTAATGGAACCAATTAATTTGTGTATTGAATTAAAGATCGAGAGGAATCGCGGAT
ATCATATACAAACGCCAAATAATTTTCAAGACGCAAGTTAT------CCTATGGATGCTATATT
CATGCCTGTTCGAAATGTTAATCATAGTATACATTCTTATGTAAATGGGAATGAAAAACA
AGAGATACTTTTTCTCGAAATATGGACCAATGGAAGTTTAACTCCTAAAGAAGCACTTC
ATGAAGCCTCTCGGAATTTGATTGATTTATTTATTCCTTTTTTACATGCGGAAGAAGAAA
AAGAAAACTTCCATTTAAAAAATAATAAA---AAAAAAGTTACTTTACCCCTTTTTACTTT
TCATGAGAAATTGGCGAAA---CTAAGGAAAAAGAAAAAA---GAAATAGCATTAAAATAT
ATTTTTATTGACCAATCAGAATTGCCTCCTAGGATCTATAATTGCCTCAAAAGGTGCAAT
ATACATACATTATTTGACCTTTTGAATAATAGTCCAGACGAACTTATGAAAATCAAACAT
TTTCGCATAGAAGACGTAAAACATATATTAGACATTCTCGAAATGGAAAAGAATTTTGC
A---------------------ATGCTTGGGGGTGGAAAT---GAGAGAATGTCTACAATACCTGCGTTTA
ATCAGATACAATTTGAAGGGTTTTGTAGGTTCATTGATCATGGCTTAACAGAAGAACTT
TCTAAGTTTCCAAAAATTGAAGATACAGATCAAGAAATTGAATTTCAATTATTTGTGGA
AACATATAAATTGGTAGAACCATTGATAAAAGAACGAGATGCTGTATATGAATCACTCA
CATATTCTTCTGAATTATACGTATCCGCAGGATTAATTTGGAAAACCCGTAGGGATATGC
AAGAACAAACAATTTTTATTGGAAACATTCCTCTAATGAATTCCCTGGGAACTTCTATA
GTAAATGGACTATACCGAATTGTGATCAGTCAAATATTGCAAAGCCCCGGTATCTATTAC
CGGTCAGAATTGGACCATAACGGAATTTCGGTCTATACCGGCACCATAATATCTGATTGG
GGAGGAAGATTAGAATTAGAGATTGATCGAAAAGCAAGGATATGGGCTCGTGTGAGTA
GGAAACAGAAAATATCTATTCTAGTTCTATCATCAGCTATGGGTTCGAATCTAAGAGAA
ATTCTCGAGAATGTTTGCTACCCTGAAATTTTTTTGTCTTTCCTGAATGATAAGGAGAA
AAAAAAAAATTGGATCAAAAGAAAATGCCATTTTGGAGTTTTATCAACAATTTTCTTGTG
TAGGCGGAGATCCGGTATTTTCTGAATCCTTATGTAAGGAATTACAAAAGAAATTCTTTC
AACAAAGATGTGAATTAGGAAGGATTGGTCGACGAAATCTGAACCAGAGACTTAATCT
TGATATACCTGAGAACAATACATTTTTGTTACAGCGAGATATATTGGCAGCTGCGGATCA
TTTGATTGGACTTAAATTTGGAATGGGTACACTTGACGAT------ATGAATCATTTGAAAAA
TAAACGGATTCGTTCTGTCGCGGATCTCTTACAAGATCAATTCGGATTGGCTCTAGTTC
GTTTAGAAAATATGGTTAGAGGAACTATATGTGGAGCAATTAGG---CATAAATTGATA---C
CGACTCCTCAGAATTTGGTAACTTCAACTCCA------------TTAACAACCACATTTGAATCT
TTTTTCGGATTACACCCATTATCTCAAGTTTTAGATCGAACTAATCCATTGACACAAATA
GTTCATGGGCGAAAATTGAGTTATTTGGGCCCAGGAGGATTGACAGGGCGAACTGCTA
GTTTTCGGATACGAGATATCCATCCTAGTCATTATGGACGCATTTGCCCAATTGACACGT
CTGAAGGAATTAATGTTGGACTTATTGGATCATTAGCAATTCATGCAAGGATTGGTCATT
GGGGA---TCTCTAGAAACTCCATTTTAT---------GAAATTTCAGAGAGATCAAAAAAA-----
-GTGCGGATGCTTTATTTATCACCAAGTCGAGATGAATACTATATGGTAGCGACAGGAAA

TTCTTTGGCACTGAATCCAGGTATTCAGGAAGAACAGATTGTTCCAGCTCGATACCGTC
AAGAATTCTTGACTATTGAATGGGAACAAGTTCATCTTCGAAGTATTTTTCCCTTCCAAT
ATTTTTCTATTGGAGCTTCCCTCATTCCTTTTATCGAACATAATGATGCGAATCGTGCTTT
AATGAGTTCTAATATGCAACGTCAAGCAGTCCCGCTTTCTCGGTCCGAAAAGTGCATTG
TTGGAACTGGGTTGGAACGCCAAGTGGCTCGAGATTCAGGGGTTGCCGCTATAGCCGA
ACACGGGGGAAAGATCATTTATACCGATACTGACAAGATCATTTTTTCGGGCAATGGAT
ATACTCGAAGAATTCCATTAGTTATGTATCAACGTTCTAACAAAAATACTTGTATGCAGC
AAAAATCCCAGGTTCACCAGGGTAAATGCATAAAAAAAGGGCAAATTTTAGCGGACGG
TGCTGCTACAGTTGGTGGCGAACTTGCTTTGGGAAAAAACGTATTAGTAGCTTATATGC
CATGGGAAGGTTACAATTTTGAAGATGCGGTACTAATTAGCGAGCGTTTGATATATGAA
GATATTTATACTTCTTTCCACATACGGAAATATGAAATTCAAACTCATGTGACAAGCCAC
GGA---CCTGAAAGAATCACTAACGAAATACCACATCTAGAAGCCCGCTTACTCTGTAAT
TTAGACAAAAATGGAATTGTTATGCTGGGATCGTGGGTGGAGACGGGCGATATTTTAGT
AGGTAAATTAACGCCTCAGATGGCGAAAGAA---TCATCGTATGCCCCGGAAGATAGATT
ATTACGAGCCATACTTGGCATTCAGGTATCCACTTCAAAAGAAACCTGTCTAAAACTAC
CTATCGGCGGTAGGGGTCGAGTTATTGATGTGAGATGGATCCAGAAAAAAGGG---GGTT
CGAGTTATAATCCAGAAATTATTCGTGTATATATTTCACAGAAACGTGAAATCAAAGTAG
GCGATAAGGTAGCGGGAAGACATGGAAATAAAGGTATTGTTTCAAAAATTTTGCCCAG
AGAAGATATGCCTTATTTGCAAAATGGAAGACCTGTTGATATGGTTTTCAATCCATTAGG
AGTCCCCTCACGAATGAATGTAGGACAGATATTTGAGTGCTCACTCGGGTTAGCGGGG
AGTTTGCTAGATAGACATTATCGAATAGTACCTTTTGATGAGAGATATGAACAAGAGGC
TTCTAGAAAACTAGTCTTTTCTGAATTATATGAAGCCAGTAAGCAAACAGCGAAGCCAT
GGGTATTTGAACCCGAGTATCCGGGAAAAAGCAGAATATTTGATGGAAGAACGGGAAA
TCCTTTTGAACAACCTGTTATAATAGGAAAGCCTTATATCTTGAAATTAATTCATCAAGT
TGATGATAAAATACACGGACGTTCCAGTGGACATTATGCACTTGTTACACAACAACCCC
TTAGAGGAAGGGCCAAGCAAGGTGGACAACGGGTGGGAGAAATGGAGGTTTGGGCT
CTAGAAGGATTTGGGGTTGCTCATATTTTACAAGAGATGCTTACTTATAAATCTGATCAT
ATTAGAGCTCGGCAAGAAGTACTTGGTACTACGATCATTGGAGGAACAATACCTAAAC
CCGAGGATACTCCAGAATCTTTTCGATTGCTCGTTCGAGAATTACGATCTTTGGCTTTG
GAACTGAATCATTTCCTTGTATCTGAGAAGAACTTCCAGATTAATAGGAAGGAAGCT----
----------------ATGATCGATCGGTATAAACATCAACAACTCCGAATTGGATTAGTTTCTCCT
CAACAAATAAGTGCTTGGGCCAATAAAACTCTACCTAATGGCGAGATAGTTGGAGAGG
TAACAAAACCCTATACTTTTCATTACAAAACCAATAAACCTGAAAAAGATGGATTATTT
TGTGAAAGAATTTTTGGGCCTATAAAAAGCGGGATTTGTGCTTGTGGAAATTATCGAGT
AATCGGAGATAAAAAAGAAGATTCGAAATTTTGTGAACAATGCGGAGTCGAATTTGTT
GATTCTCGGATACGAAGATATCAAATGGGCTATATTAAACTGGCATGCCCAGTAACCCAT
GTGTGGTATTTAAAACGTCTTCCTAGTTATATCGCGAATCTTTTAGATAAACCTCTTAAA
GAATTAGAAGGCTTAGTATATTGCGAT------TTTTCTTTTGCTAGGCCC---ATAGCTAAAA
AACCTACTTTCTTACGATTACGGGGTTCATTCGAATATGAAATCCAATCCTGGAAATACA
GCATCCCACTTTTTTTTACTACCCAAGGCTTCGATACATTTCGAAATCGAGAAATTTCTA
CCGGAGCAGGTGCTATACGAGAACAATTAGCCGATCTGGATTTGCGACTTATTATAGATT
ATTCGTTGGTAGAATGGAAAGAATTAGGCGAAGAA---GGACCCGCAGGTAATGAATGG
GAAGATCGCAAAGTTGGAAGAAGAAAGGATTTTTTGGTTAGACGTATGGAATTAGCTA
AGCATTTTATTCGAACAAATATAGAACCCGAATGGATGGTTTTATGTCTATTACCTGTTC

TTCCTCCCGAGCTGAGACCGATCATTCAGATAGATGGGGGTAAACTAATGAGCTCGGAT
ATTAATGAACTCTATAGAAGAGTTATATATCGGAACAATACTCTTATTGATCTATTAACAA
CAAGTAGATCTACGCCAGGAGAATTAGTAATGTGTCAGGAGAAATTGGTACAAGAAGC
CGTGGATACACTTCTTGATAATGGAATCCGCGGACAACCAATGAGGGATGGCCATAATA
AGGTTTACAAGTCGTTTTCTGATGTAATTGAAGGCAAAGAGGGAAGATTTCGGGAGAC
TCTGCTTGGCAAACGGGTCGATTATTCAGGACGTTCTGTGATTGTTGTAGGGCCCTCAC
TTTCATTACATCGATGTGGATTGCCTCGCGAAATCGCAATAGAGCTTTTCCAGACATTTT
TAATTCGTGGTCTAATTCGACAACATTTTGCTTCGAACATAGGAGTTGCTAAGAGTAAA
ATTCGGGAAAAAGAACCCATTGTATGGGAAATACTTCAGGAAGTTATGCAGGGGCATC
CCGTATTGCTAAATAGAGCGCCTACTCTGCATAGATTAGGCATCCAGGCCTTCCAACCTA
TTTTAGTGGAAGGACGTGCTATTTGTTTACATCCATTAGTTTGTAAGGGATTCAATGCAG
ACTTTGATGGGGATCAAATGGCTGTTCATGTACCTTTATCTTGGGAGGCTCAAGCAGAG
GCGCGTTTACTTATGTTTTCTCATATGAATCTCCTGTCTTCAGCTATTGGGGATCCCATTT
CTGTACCAACTCAAGATATGCTTATTGGACTCTATGTATTAACGAGCGGGAATCGCCGA
GGTATTTGTGCAAATCGGTATAATCCATCTAATCGCAAAAAT---------CATAAAAATGAAA
AAATTTACAATAATAACTATAAATATACG---------GAACCCTTTTTTTGTAATTCCTATGATG
CAATTGGAGCTTATCGCCAGAAAAGGATCAATTTAGATAGTCCTTTGTGGCTCCGATGG
CGACTAGATCAACGC---GTTATTGCTTCA------AGAGAAGCCCCTATCGAAGTTCACTAT
GAATCTTTGGGTACCCATCATGAGATTTATGGGTACTATCTAATAGTCAAAAGTATAAAA
AAAGAAATTCTTTGTATATACATTCGAACCACTGTTGGTCATATTTCTCTTTATCGAGAA
ATCGAAGAGGCTATACAAGGATTTTGCCGGGCCTGCTCATATGGTACC----------------------
-------------ATGGCGGAACGGGCCGATCTGGTCTTTCACAATAAAGTGATAGATGGAACTG
CCATTAAACGACTTATTAGCAGATTAATAGATCACTTCGGAATGGCATATACATCACACA
TCCTGGATCAACTAAAGACTCTGGGGTTCCAGCAGGCCACTGCTACATCCATTTCATTA
GGAATTGATGATCTTTTAACAATCCCTTCTAAGGGATGGCTAGTCCAAGATGCTGAACA
ACAAAGTTTGATTTTGGAAAAACACCATCATTATGGAAACGTACACGCAGTAGAAAAA
TTACGCCAGTCCATTGAGATATGGTATGCTACAAGTGAATATTTGCGACAAGAAATGAA
TCCTAATTTTAGGATGACTGACCCTTTTAATCCAGTCCATATAATGTCTTTTTCGGGAGC
TCGAGGAAATGCATCTCAAGTACACCAATTAGTAGGTATGAGAGGATTAATGTCAGATC
CACAAGGACAAATGATTGATTTACCCATTCAAAGCAATTTACGTGAGGGGCTGTCCTTA
ACGGAATATATAATTTCTTGCTACGGAGCGCGGAAAGGAGTTGTGGATACTGCTGTACG
AACATCGGATGCTGGATATCTTACACGTAGACTTGTTGAAGTAGTTCAACACATTGTTG
TACGTAGAACCGATTGTGGAACCATCCGAGGGATTTTAGTGAGTCCTGGAAATAGGAT
GATTCCG---GAAAGAATTTTTATCCAAACATTAATTGGTCGTGTATTAGCGGACGATATAT
ATATGGGCCCGCGATGCATTGGCGTTCGAAATCAAGATATTGGTATTGGACTTATTAATC
GATTCATAACC------------------TTTCAAACACAACCAATATCTATTCGGACTCCCTTTACTT
GTAGGAGTACATCTTGGATCTGCCGATTATGTTATGGACGAAGTCCTACTCACGGGGAC
CTGGTCGAATTGGGAGAAGCCGTAGGTATTATTGCGGGTCAATCTATTGGAGAACCGG
GTACTCAACTAACATTAAGAACTTTTCATACCGGCGGAGTATTCACAGGGGGGACTGC
AGAACATATACGAGCTCCTTCTAATGGCAAAATAAAATTCAATGAGGATTTGGTTCATC
CCACACGTACACGCCATGGGCATCCTGCTTTTTTATGTTATATAGACTTGTATGTAACTAT
TGAGAGTGAGGATATTATACATAACGTCACTATTCCACCAAAAAGTTTACTTTTAGTTCA
AAACGATCAATATGTAGAATCAGAACAAGTGATTGCTGAGATTCGCGCGGGAACATAC
ACTCTT---AATTTAAAAGAGAGAGTTCGAAAACATATTTATTCTGACTCAGAGGGAGAA

---ATGCATTGGAGTACCGACGTGTACCATGCACCCGAATTTACATATAGTAATGTCCATC
TCTTACCAAAAACAAGTCATTTATGGATATTATCAGGAGGTTCGTGCGGA------TGCAGT
CTAGTCCCTTTTTCACTCTACAAGGATCAAGATCAAATTAACGTTCAT------TCTCTTTGT
GTCGAAAGA---AGATATATTTCTAGCCTT---TCAGTAAATAATGATAAA---GTGGGACAAA
AATTTTATGGTCCGGATCTCTCTGGTAAA---GAAAACGGAATTCCTGATTAT---TCAGAA
CTTAATCCAATCCTATGTACGGGTCAATCTAATCTCACATATCCTGCT---ATTTTCCATGGT
AATTCTGAT------TTATTGGCAAAGAGGCGAAGAAATGGATTCATCATTCAATTTGAATC
ACTTCAAGAACGAGAAAAAGAACTACGACCCCCTTCCGGTATTTCGATTGAAATACCC
ATAAAT------GGTATTTTTCGTAGAAATAGTATTCTTGCTTTTTTCGATGATCCTCAATACA
GAAGAAATAGTTCAGGAATTACTAAATATGGGACTATAGGGGTGCATTCAATCCTCAAA
AAAGAGGAT------TTGATTGAGTATCGA---GGAGTCAAAGACTTTAAGCCAAAATACATG
AAAGTGGATCGATTTTTTTTCATTCCCGAGGAAGTGCATATTTTACCTGAATCTTCTTCC
ATAATGGTACGGAACAATAGTATCATTGGAGTAGCTACACGACTAACTTTAAGTATAAG
AAGCCGAGTAGGCGGATTGGTCCGAGTGGAGAAAAAAAAAAAAAAGGATTGAACTCA
AAATATTTTCTGGAGATATACATTTTCCTGGAGAGATGGATAAGATATCCCGACACAATG
GGATCTTGATACCACCAGAAAGAGTAAAAAAA------AATTCTAAGAAATCAAAAAAA----
--TCGAAAAATTGGATTTATGTCCAGTGG------ATCACACCTACCAAGAAAAAG------TATT
TTGTTTTTGTTCGACCTGTAATCATATATGAACTAGCCGACGGTATAAATTTAGTCAAAC
TTTTCCCACAGGATCTATTGCAAGAAAGGGATAATCTGGAGCTTAGAGTTGCCAATTAT
ATACTTTATGGAAATGGCAAACCAATTCGGGGAATTTCTGGCACAAGCATTCAATTAGT
TCGGACTTGTTTATTGTTGAATTGGGACCGAGACAAA---------AAAAGCTCTTCTATCGA
AGACGCGCGCGCTTCCTTTGTTGAAGTAAGTACAAATGGTCTGGTTCGAAATTTCCTAC
GAATAGACTTAGGGAAATCCGATACTGCGTATATCAGAAAAAGGAAAGATCCGTCAGG
TTCAGGATTGATCTTT---AATAATGAGTCAGATCGTACCAATATCAATCCATTTTTTTCTAT
TTATTCCAAGACAAGG---------------GTTCCACAATCTCCGAGTCAAAATCAAGGAACTAT
TCGTACGTTGTTGAATAGA---------AATAAAGAACGCCAATCTTTGATAATTTTGTCAGCA
TCGAATTGTTTGCAAATAGATCTA------TTCAACGATGTAAAAGATTATAAT---------GTCAT
AAAAGAATCA---CCTCTAATTTCAATTAGGAATTCGTTAGGACCTTTAGGGGCG------GC
CCCTCAAATTGTGAATTTT------TATTCATTTTATTACTTAATAACT------CATAATCCGATCT
CC---CTAACTAAATATTTGCAACTTGAC------AAT---TTAAAACAGATTTTTCAAGTACTT--
----AAA---TATTATTTAATGGATGAAAACGGG---GGGATTTTAAATTCC---GATCCATGCAT
TAACATCGTTTTCAATACATTTAATTTGAATTGGCATTTTCTGCATGATAATTATTGTGAA
GAAACACCTACAAGAATTAGT---CTTGGACAGTTTTTTTTTGAAAATGTATGTATAGCCA
AAAATAGACCA---------CACTTAAAATCGGGTCAAATTATAATTGTTCAA---GTTGATTCT
CTAGTAATAAGATCAGCTAAGCCTTATTTGGCCACTTCAGGAGCAACTGTTCATCGCCA
TTATGGCGAAATCCTTTACGAAGGAGATACTTTAGTTACATTTATATATGAAAAATCACG
ATCTGGTGATATAACGCAGGGTCTTCCAAAAGTCGAACAAGTATTAGAAGTGCGTTCGA
TTGATTCAATATCGATGAGCCTAGAAAAGAGAATTGAGGGTTGGAACGAGCGTATAAC
AAGAATTCTTGGAATTCCTTGGGGATTTTTGATTGGTGCTGAACTAACGATAGTGCAAA
GTCGTATCTCTTTGGTTAATAAGATCCAAAAGGTTTATCGATCCCAGGGGGTGGAGATC
CATAATAGACATATCGAAATTATTGTACGTCAAATAACATCAAAAGTGTTGGTTTCAGAA
---------GATGGAATGTCTAATGTTTTTTCACCTGGAGAACTCATTGGGTTGTTACGAGCG
GAACGAACAGGGCGTGCTTTGGAAGAAGCGATCTGTTACCGAGCCGTATTATTGGGAA
TAACGAAAGCATCTCTAAATACTCAAAGTTTCATATCGGAAGCAAGTTTTCAAGAAACT

GCTCGAGTTTTAGCAAAAGCCGCCCTCCGAGGTCGTATCGATTGGTTGAGAGGTTTGA
AAGAGAACGTTGTTCTAGGAGGCATGATACCCGTTGGTACGGGATTCAGAGAATTAGC
GCACCGTTCG------AGGCAACATAACAATATTCCTTTAGAAACCCCCCCAAAAAAA------
ATTTTCGAGGGGGAAATGAGA------GATATTTTGTTCCACCACAAAGAATTATTTGATTT
TTTCATTTCAACGAATTTACATGATACATCAGAACAAGAATTTCTAGGATTTAATGATTC
A------------------ATGACAAGAAGATATTGGAACATCCATTTGGAAGAGATGATGGAAGCA
GGAGTTCATTTTGGTCATGGTACTAGGAAATGGAATCCTAGAATGGCACCTTATATCTCT
GCAAAACGTAAAGGTATTCATATTATAAATCTTACTAGAACTGCTCGTTTTTTATCAGAA
GCTTGTGATTTAGTTTTTGATGCAGCAACTAGGGGCAAACAATTCTTAATTGTTGGTAC
CAAAAATAAAGCAGCGGATTCAGTAGCCCGGGCTGCAACAAGGACTCGGTGTCATTAT
GTTAATAAAAAATGGCTCGGGGGGATGTTAACAAATTGGTCTACTACAGAAACGAGAC
TTCATAAGTTCAGGGACTTGAGAACGGAACAAAAGACGGGGGGACTCAACCGTCTTC
CGAAAAGGGATGCCGCTATGTTCAAGAGACAATTATCTCACTTGCAAACATATCTGGGC
GGGATTAAATATATGACGGGGTTACCCGATATTGTAATAATCGTTGATCAACAAGAAGAA
TATCGGGCTCTTCAAGAATGTATCACGTTGGGAATTCCAACTATTTGTTTAATTGATACA
AATTGTGACCCCGATCTCGCCGATATTTCGATTCCAGCGAATGATGATGCTATAGCTTCA
ATCCGATTAATTCTTAACAAATTAGTATTTGCTATTTCGGAGGGTCGTTCT---AGCTCTAT
ACGAAATTCTATGGGACAAAAAATAAATCCACTCGGTTTTCGACTTGGTGCAACCCAA
GGGCATCATTCTCTGTGGTTTGCACACCCCAAAAAATATTCTGAAGGTTTACAAGAAGA
TCAAAAAATACGAGATTGTATCCAGAATTATGTACAAAAAAACATGAGAATTTTTTCTG
GT---GTTGAAGGAATTGCACGTATCGAGATTCAAAAAAGAATTGATCTTATTCAAGTGAT
AATCTTTATGGGATTCCCAAAGTTAGTAATAGACGAT------GGG------TCTCGAAAAATCG
AAGAATTACAGATTAATGTACAAAAAGAATTCAATTCTGGGAACCGAAAACTCAACAT
TGCTATTACAAGAATAGGAAACCCTTATGGACACCCTAATATTCTCGCAGAATTTATAGC
CGGACAATTAAAGAATAGAGTTTCATTTCGCAAAGCAATGAAAAAAGCTATTGAATTA
ACTGAACAGGCGGGTACAAAGGGAATTCAAGTACAAATTGCCGGACGTATAGACGGA
AAAGAAATTGCACGTGTCGAATGGATCAGAGAAGGACGGGTTCCTCTACAAACCATTC
GAGCTAAAATTGATTATTGTTGCTATCCAGTTCGAACTATCTATGGGATATTAGGCATAA
AAATTTGGATATTTGTAGACGAGCAAATGTCGCGTTACAGAAGGCCTCGTTTCAAAAA
AATACGCCGTCTGGGGGCTTTACCGGGACTAACTAGTAAAAGGCCCAAAACCGGAAAC
GATCTTAAAAACCAATCGCGTTCCGGTAAA---AAATCTCAATATCGTATTCGTTTAGAAG
AAAAACAAAAATTGCGTTTTCATTATGGTCTTACAGAACGACAATTACTTAAATACGTT
CGTATCGCCGGAAAAGCCAAAGGATCAACGGGTCAAGTTTTACTACAATTACTTGAAA
TGCGTTTGGATAACATCCTTTTTCGATTGGGTATGGCTTCGACTATTCCTCAAGCCCGAC
AATTAGTTAATCATAGACATATTTTAGTTAATGGTTCTATAGTGGATATACCAAGTTATCG
GTGCAAACCCCGAGATATTATTACAGCAAAAGATGAAAAAAAATCGAGAACACTGATT
CAAAATTATCTTGATTCATCCCCCCCT---CAGGAATTGCCAAAACATTTGACTCTTCAGC
CATTGCAA------TATAAAGGATTAGTCAATCAAATAATAGATAGTAAATGGGTCAGTTTAA
AAATAAATGAATTGCTCGTTGTAGAATATTATTCTCGTCAGACTATGTCACGTCGAGGTA
CTGCAGAAGAAAAAATTGAAAAATCCGATCCAATTTATCGTAATCGATTAGTTAACATG
TTGGTTAACCGTATTCTGAAACATGGAAAAAAATCATTGGCTTATCAAATTATCTATCGA
GCCATGAAAAAGATTCAACAAAAGACAGAAACAAATCCACTATCTGTTTTACGTCAAG
CAATACGTGGAGTAACTCCCGATATAGCAGTAAAGGCAAGACGTGTAGGCGGATCAAC
TCATCAAGTTCCCATTGAAATAGGATCCACACAAGGAAAAGCACTTGCCATTCGTTGGT

TATTAGGGGCATCCCGAAAACGTCCCGGTCGAAATATGGCTTTCAAATTCAGTTCCGAA
TTAGTGGATGCTGCCAAAGGGAGTGGCGATGCCATACGTAAAAAGGAAGAGACTCATA
GAATGGCAGAGGCAAATAGAGCTTTTGCACATTTTCGTATGGGTAAGGACACTATTGCT
AACATAATAACCTCTATTCGCAATGCTGACATGAATAGAAAAGGAATGGTTCGAATACC
AGCTACTAACATCACCGAAAACATTGTTAAAATACTTTTACGAGAAGGTTTTATTGAAA
ACGTAAGGAAACATCGGGAAAGCAACAAGGATTTTTGGGTTTTAACCCTACGACATAG
AAGA------AATAGGAAAGGACCATATAAAACG---ATT------TTAAATTTAAAACGGATCAG
TCGACCCGGTCTCCGAATATATTCTAACTATCAAAAAATTCCTAGAATTTTAGGAGGAAT
GGGCATTGTAATTCTTTCTACTTCTCGGGGTATAATGACAGACCGAGAAGCTCGACTAC
AGGGAATCGGCGGCGAAATTTTGTGTTATATATGGATGGCAAAACCCATACCAAGAATC
GGTTCACGTAGAAATGGACGTATTAGCTCACGT---AGTACGCGTAGAATACCAAAGGGC
GTTATTCATGTTCAAGCAAGTTTCAACAATACAATTGTGACTGTTACAGATGTACGGGG
TCGGGTAATTTCTTGGTCCTCCGCCGGTACGTGTGGATTCAAAGGTACAAGAAGAGGG
ACACCATTTGCCGCTCAAACCGCAGCAGGAAATGCTATTCGGGGAGTAGTGGATCAAG
GGATGCAACGAGCCGAAGTTATGATAAAGGGCCCCGGTCTCGGAAGAGATGCAGCATT
AAGAGCTATTCGGAGAAGCGGTATATTATTAAGTTTCATACGGGATGTAACCCCTATGCC
ACATAATGGCTGTAGGCCCCCTAAAAAACGACGTGTGATGCCAACTATTAAACAACTTA
TTAGAAACACAAGACAGCCAATCAGAAATGTCACCAAATCGCCCGCCCTTCGGGGATG
CCCTCAGCGTCGAGGAACATGTACTAGGGTGTATACTATCACCCCCAAAAAACCAAAC
TCTGCCTTACGTAAAGTTGCCAGAGTACGCTTAACCTCGGGATTTGAAATCACTGCTTA
TATACCTGGTATTGGCCATAATTTACAAGAACATTCTGTAGTCTTAGTAAGAGGGGGAA
GGGTTAAGGATTTACCCGGTGTGAGATATCACATTGTTCGAGGAACCCTAGATGCTGTC
GGAGTAAAGGATCGTCAACAAGGGCGTTCTAAATATGGGGTCAAAAAGCCAAAAATG
GCAAGGAAAAGTTTAATTCAGAGGGAGAAGAAGAGGCAAAAATTGGAACAAAAATAT
CATTTGATTCGTCAATCCTTAAAAAACGAAATAAGC---------AAAGTTCCATCGTTGAGT
GAGAAATGGGAAATTCATGGAAAGTTACAATCCCCACCGCGTAATAGTGCACCCACAC
GTCTTCATCGACGTTGTTTTTAACCGGAAGACCGAGAGCTAACTATCGAGACTTTGGG
CTCTCTGGACACATACTTCGTGAAATGGTTCATGCATGCTTGTTGCCTGGGGCAACAAG
ATCAAGTTGGATGGTAAAAAATTCATTCAGCTCAGTTATTTCGCAAGAA---GAA---AAA
AAAGAAAATGGAGGATCCGTTGAATTTCAAGTAGTCAGTTTCACCAATAAGATACGAA
GACTTACTTCACATTTGGAATTGCACAAAAAAGACTATTTATCTCAAAGAGGTCTACGT
AAAATTCTCGGAAAACGCCAACGATTACTTTCTTATTTATCAAAGAAAAATAAAATGCG
TTATAAAGAATTAATTAATCAATTGGATATTCGGGAGTCAAAAACTCAG------ATGGTAA
AACTTCGTTTAAAACGATGTGGTAGAAAGCAACGAGCTGTTTATCGAATCATTGCAATT
GATGTTCGATCCCGAAGAGAAGGAAGAGATCTTCGTAAAGTGGGTTTTTATGATCCGAT
AAAGAATCAAACCTATTTAAATGTTCCTGCGATTCTATATTTCCTTGAAAAGGGTGCTCA
ACCTACAGGAACTGTTCATGATATTTTAAAGAAGGCAGGTGTTTTTACGGAACTTCACC
TTAATCAA---------------ATGGATAAATCCAAGCGACTCTTTCTTAAATCCAAACGATCTTT
TCGTAGGCGTTTGCCCCCGATTCCATCGGGGGATCGAATTGATTATAGAAACATGAGTT
TAATTAGTCGATTTATTAGTGAACAAGGAAAAATATTATCTAGACGGGTAAATAGATTGA
CCTTAAAACAGCAACGCTTAATTACTATTGCTATAAAACAAGCTCGTATTTTATCTTTGT
TACCTTTTCTTAATAATGATAAAAATAAACAATTTGAAAGAAGCGAGTCGACTCCTAGA
ACTATTGGTCTTAGAACCAGGAATAAAATGGCGCGA---TTAAAAAAAAATCCTTTTGTG
GGAAACCATTTATTAAAAAAAATAAATAAGCTTAACACAAAGGGAGAAAAAGAAATAA

TAGTAACTTGGTCCCGAGCATCTACCATTATACCAACAATGATTGGGCATACTATCGCTG
TTCATAATGGAAAGGACCATTTGCCTGTTTATATAACGGATCGCATGGTAGGTCATAAAT
TGGGAGAATTTGCACCCACTCGCAATTTCCGGGGGCATGTGAAAAATGATAATAGATCT
CGTCGTATGATTTTAAAATCTTTTATACTAGGTAATCTAGTATCCCTATGCATGAAGATAA
TCAATTCGGTCGTTGTGGTCGGACTCTATTATGGATTTCTGACCACATTCTCCATAGGGC
CCTCTTATCTCTTCCTTCTCCGAGCTTGGCTTATGGAAGAAGGA---------ACCGAGAAGA
AAGTATCAGCAACAACAGGTTTTATTACGGGACAGCTCATGATGTTCATATCGATCTATT
ATGCGCCTCTGCATCTAGCATTGGGTAGACCTTATACAATAACTGTCCTAGCTCTACCGT
ATCTTTTGTTTCATTTCTTCTGGACCAATCCCAAACACTTTTTTTATTATGGATCTACTAC
CAGAAACTCAATGCGTAATCTTAGCATTCAATGTGTATTCCTGAATAATCTCATTTTTCA
ATTATTCAACCATTTCATTTTACCAAGTTCAATGTTAGTCAGATTAGTCAACATTTCTATG
TTTCGATGCAACAACAAGATGTTATTTGTAACAAGTAGTTTTGTTGGTTGGTTAATTGGT
CACATTTTATTCATGAAATCGGTTGGATTGATATTAGTCTGGATACAGCAAAATAATTCTA
TTATATCT---ATTAGATCTAATAAGTACCTTGTGTCAGAATTGAGAAAT------TCTCCAATC
TTTAGTATTATCTTATTTATTACCTGGGTCTACTATTTAGGCAGAATACCGTCACCCATTC
CACTGAAAGAAACCTCAAAAACCGAAGAAGATAAACCTCTTGGGATTATCCTTTTCGA
CTATAAACGATGGAATCGTCCATTTCGATATATAAAG---GATAAATTTGAC---GCTGTTAG
AAAAGAAATGTCACAATATTTTTTTTATACATGTCAAAGTGACGGAAAAGACCGAATAG
TTTTTACATATCCACCTAGTGTAGCAACTTTTTTCGAACTGATAGAAAAAAAGATATCTT
TGTTTTCAACAGAAAAACTTTTTTATGATGAATTCGATAATCCTTGGAAGTCTTTGAATG
AACAAAAAAAAAAGAATTTTAAACAAAATAGTTATAACTAGAATTCTACTTCTCGATAAG
GGATCCCTAGTTGGAGATGTACTGCAAAAAAGGACTCAATTATGTATAGATGAAACTAA
AAAAGAATATTTACCAAAAATATATGATCCGTTCTTGAGT---CCTTACCGGAGACGAATC
AATTTACTTTTAGGATTCAGTGAGAACATTTGGACAAATAAGATTCATGCTATCATTAAC
AACGGTTTCTTCGATGGAAAATGCAAACGCGTA---------ATTAAGAAGATTTCTACTTCT
ACTCCTACTTCTACTCCTACTTCTACTACTTCTACTTCTACTCCTACTTCTCCTACTTCTA
CTTCTACTTCTACTCCTGCTTCTATTCCTGCTTCGGCTATTATACGCTATGCCAAGCAATG
GGATTTTCGTCGAGAGATAATCAAAGGCTCTATGCGTGCTCAACGACGTAAAATGGGTA
TGTATCAACTGTTTCAAGGT---GCAAATTCTTATCTTTTCTTGGGCAGA---CACAATTTTT
TTTTTTCTGATTTTTGTATTTATATTCCTAGACTAATGGAAAAACTTGATAGACTTAACGA
AAAAAATCAAGAATCGATAAATCTGAAGGAATGGGAGAGCATACGATTTGGTCAACTA
GTAAGAGGTTCTCTGTTAATAACTCAAGCATTTTTGAGAAAATATATTATCTTACCCTCA
GTGATAATAGCTGCCAACCTTACCCGTATACTATTATTGTCACCTCCTCACTTTTTTGAG
GATTTTAAAAATTGGAAAAAAGAAATATATGTAAAGTGCACATATAATGCTATTCCATTA
GCAGAAAACGAATTTCCCCAAAATTGGTTAACAGACGGTATTCAAATAAAGATCTTATT
TCCTTTTCGTCTGCAATCGTGGCACAGATCAAAATTTGGAACGGAAACTGAACAGCCT
TTTGGGTCTGCCGAAAACTTCATAAAAAAAAAAAAAAACAACAATTAAAAAAATGGAGA
ACGAAGTATTTTCGA---ATATTTTTACGTTTAAGA------------------------
AAAAAGGTTTTTAGA---------AAAATTAATATTGCAAAAGAAAAATGGGTCATCAAACCC
ATTTTATTTATAAAAAAAAAGACTAAGAGAA---TTCTGGCAAAGAAACCCAATTAATTCA
ATAATAAGTAATGGAATGATCCAAAGCTCGTCTGGGTCTATAGATTCGATAATTTTGTCA
TTGAGAGAACGAAAGATGAAAGAGTTGACTGATAGAATAAACACCATGCTAAAAATAA
TTGAAAAAATGAAAAAAGACAGGCAAAAAGAGTTGCTA---------AAAATGAGTTATTAT
GATAAAATATTTGAATCTCAGAAAAATATTTTAAAGATATTAAAA------AATGTACGATTT

ATTCGTAAATCGTATAAGTTTCTAAAATTTTTTGTTCAAAACATATACATAGATATCTTTC
AAGGTATCATTAAT------------ATGTCGAGGATTAAT---------------TGGGACGTATCATACTTG
TCGCAAGCATATTTATTTTACAAATTATCACAAACAAAAATTATTAACTTATCTAAGTTAA
GACCTGTCTTTCAATATACATCTCGTTTTCTTAAGAATGAAACACAGGATTATTTTGCAC
ACGAAATATTACATTCCGATCTAAAACAAAAGAAACAAAAGAATCTTGAAAATTCTCCA
TGGAAAAACTGGTTACGGGGT---TATAACTTCGATTTATATCCGATTAGATGGTCAAAAT
TACTCTCCCAAAAATGGCGAAATAGAGTCAAGCAAGATCGT------AAAAATAAAAACCA
ATGTGATTCATATGAAAAAAAAAGGA------TTAATTGATTACAAAAAACAA---AAAAAAA
AAAAAAAATATATATATGATTCTTTATCATATAAATCTATTAATGCTAATATTAGAGGTATG
GATAGAAAATATTTTGATTGGAGAATGCTGCATTTTTCTATTAAATTTTTTGATTGGATGA
GAATGAGTGAAAAAAAAAATAAGTCGTTTCTTTCCA---AAATTTTTGATACTTTATAATGC
ATATAAGATTAAGCCATGGATGATACCAATCAAATCACTTCTTTTCGATTCCAAGAAAAA
GATCGAAGGGAAGCTTGGTGTACTCCGAACAGGCCATTTTCGTTTTCAATTGAACTGG
CCT---------AGTAAAAGCCTTTTCATGAACCTCAACGGATTTTCTTTCATGCTTAGACGGC
TAGATCTCAAAAAACTTAGTCTAGCCGCTATTCGCAATAACGAATTCAGTCTGGAGTTC
CTGCTACCTCCGTCGGACGATATAACTTTTCACGAGTTACTTGAAGGAATATTTATTATC
GAACCCGTTCGTCTGTCTCTAAAAAATGATGGAAAATTTATTATGTCTCAAACCATAGGT
ATTTCATTAGTTCAGAAGAATAAGTACAATCATTATGATTTGCTTGTTCCGGAAAATATC
TTATCCCCTAGAGGTCGGAGAGAGTTTAGA------ATTCTAATTATCTTTCTTTGGCCCAAT
TATCGATTAGAAAATCTAGCTTGTATGAATCGTTATTGGTTTGATACCAATAATAGCAGTC
GTTTTAGTATGCTAAGGATCCATATGTATCCAGTGAAAGGGCAGCAATTCAAATTATGGA
TTTTAGAATTC------------AGAGAGATCAAGAATTCTCATTATTTCTTAGATTCATGGACCC
AATTCAATTCATTGGGATCTTTCATTCACATTTTTTTCCATCAAGAACGTTTTATAAAACT
CTTAGACTCCCGAATTTGGAGTATCCTACTTTCA---CGCAATTCA------CAGGGTTCAACA
AGCAATCGATATTTC---------ACGATCAAGGGTGTAGTACTATTTGTAGTAGCGGTCGTCC
TTATATATCGT------------ATTAACAATCGAAAGATGGTCGAAAGAAAAAATCTCTATTTGA
CAGGGTTTCTTCCT---------------ATACCTATGAATTTCATTGGACCCAGAAATGATACATTG
GAAGAATCTTTT---GGCTCTTCCAATATCAATAGGTTGATTGTTTTG---CTCCTGTATCTTC
CA---

---ACTA
GTTTCGTTCAAGTAACGGATTCTAGCCAATTGAAAGGATCTTCT---GATCAATCCAGAGA
TCATTTCGATTCCATTAGT------------AATGAGGATTCGGAA--
TATCACACATTGATCAATCAAAAAGAGATTCAACAACCG--

--------------------CTTCCGGAAGAAATCGAAGAATTT------CTCGGGAATCCTACAAGATCCA
TTCGTTCTTTTTTCTCTGACAGATGG------TCAGAACTTCAT---ATGGGTTCGAATCCTATT
GAGAGGTCCACTAGAGATCAG------------AAATTGTTGAAGAAAGAACAAGATGTTTCT--
-TTTGTCCCTTCCAGGCGATCGGAA------------AATAAAGAAATAGTTAATATATTCAAGAT
AATTACGTATTTACAAAAT------ACCGTCTCAATTCATCCTATTTCATCAGATCCGGGATGT

GATATGGTTCCG---AAGGATGAACTGGAT---ATGGACAGTTCCAATAAGATTTCATTCTT
GAAGAAAAATCCATTTATTTATTTA------TTTCATCTATTCCATGACCGGAACAGGAGGG
GATACACGTTA------CACCACGATTTTGAATCAGAA------GAGAGATTTCAAGAAATGGC
AGATCTATTCACTCTATCAATAACCGAGCCG---GATCTGGTGTATCATAAGGGATTTGCC
TTTTCTATTGATTCCTACGGATTGGATCAAAAACAA------TTCTTGAATGAGGTATTCAAC
TCCAGGGATGAATCGAAAAAGAAATATTTA---------TTGGTTCTACCTCCTATTTTTTAT----
--GAAGAGAATGACTCTTTTTATCGAAGGATCAGAAAAAAAATGGGTCCGG---ACCTCCC
CCGGGAATGATTTGGAAGATCCAAAACAAAAAAATAGTGGTATTTGCTAGCAACAACAT
AATGGAGGCAGTCAATCAATATAGATTGATCCGAAATCTGATTCAAATCCAATAT------TA
TAGG------TACATAAGAAATGTATTGAATCGATTCTTTTTAATGAAT---------AGATCCGATC
GCGACTTGGAATATGGAATTCAA---------AGGGATCAAATAGGAAATGATACTCTGAATC
ATAGAACTATAATGAAATATACGATCAACCAACATTTATCAAATTTCAAAAAG---GGTCA
GAAGAAATGGTTCGATCCT------CTTATTTTGATTTCTCGAACCGAGAGATCCATGAATC
GGGATCCTAATGCATATAGATACAAATGGTCCAATGGGAGCAAGAATTTCCAGGAACAT-
--------TTCATTTCTGAGCAGAAGAGCCATTTT---CATTTTCAAGTAGTGTTCGATCGATTA
CGTATTAATCAATATTCGATTGATTGGTCTGAGGTTATCGATAAAAAAGATTGGTCTAAG
TCACTTCGTTTCTTTTTGTCCAAGTTACTTGTT---------------------------TTTTTGTCC----------
-----------AAGTTTCTTCTCTTTTTGTCTAACTCACTTCCT---TTTTTCTTTGTGAGTTTCGG
GAATATCCCCATTCATAGGTCCGAG---ATCCACATCTAT---GAATTGAAAGGTCCGAATGA
TCGACTCTGCAATCAGTTGTTAGAATCAATAGGTCTTCAAATCGTTCATTTGAAAAAAT
TGAAACCCTTC------TTATTGGAT---------GATCATTATACTTCC---CAAAAATCGAAATTCT
TGATCAATGGAGGAACAATATCACCATTTTTGTTCAATAAGATACCA---AAGTGG---ATG
ATTGACTCATTCGATACTAGA------AATAATCGCAGGAAATCCTTTGATAACACGGATTCC
TATTTCTCAATGATATCCCACGATCAAGACAATTGGCTAAATCCCGTGAAACCATTTCAT
AGAAGTTCATTGATATCTTCTTTTTATAAAGCAACTCGACTTCGATTCTTGAATAATCCA
CATCACTTCTGCTTCTAT------TGTAACAAAAGATTCCCTTTTTATGTGGATTAT---GTGGA
AAAGGCCCGTATCAATAATTATGAT------TTTACGTATGGA---CAATTCCTCAATATCTTGT
TCATTCGCAACAAA---ATATTTTCTTTGTGCGGCGGTAAA------AAAAAACATGCTTTTTT
GGAG---AGAGATACTATTTCACCAATCGAGTCACAGGTATCTAACATATTCATACCTAAC
GATTTTCCACAAAGTGGTGACGAAAGG---TATAACTTGTACAAATCT---------TTCCATTT
TCCAATTCGATCCAATCCATTCGTTCGT------CGAGCTATTTACTCGATCGCAGACATTTC
TGTAACACCTCTAACAGAGGGACAAATAGTCAATTTTGAAAGAACTTATTGTCAACCTC
TTTCAGATATGAATCTATCTGATTCAGAGGGGAAGAACTTGCATCAGTATCTCAAT------T
TCAATTCAAACATGGGTTTGATTCACACTCCATGTTCTGAGAAATATTTACCATCCGAA
AAG------AGGAAAAAA------------------------CAGAGTCTTTATCGAAAG------AAATGCCTTG
AGAAAGGGCAGATGTATAGAACCTTTCAACGAGATAGTGCTTTTTCAACTCTCTCAAAA
TGGAATCTATTCCAAACATATATGCCATGGTTCTTTACTTCGACAGGGTACAAATATCTA
AATTTGCTATTTTTAGATACTTTTTCAGAC---CTATTGCCGATACTAAGTAGCAGC------CC
AAAATTTGTATCCATTTTTGATGATATTATGCATCGATCAGATAGATCATGGCGAATTCTT
CGGAAAAAATTG------------------TGTCTTCCACAATGGAAT---CTGATAAGTGAGATTTCG
ATTAAGTGTTTACCTAAT------CTTCTTCTGTCCGAAGAAATGATTCATCGAAATAATGAG
TCA------CCATCGATATCGACACATCTGAGATCGCCAAATGTTCGGGAGTTC---CTCTATT
CAATCCTTTTCCTTCTTCTTATTGCTGTATAT---CTCGTTCGTACACATCTTCTCTTTGTTT
CTCGA------GCCTATAGTGAGTTACAGACAGAGTTCGAAAAGGTCAAATCTTTGATGATT

CCATCATACATGATTGAGTTGCGAAAACTTCTGGATAGGTATCCCACATATGAACGGAA
TTCTTTCTGGTTAAAGAATCTCTTTCTAGTTGCTCTGGAACAATTAGGAGATTCTCTAGA
AGAAATATGGGGTTCTGCTTCTGGTGGC------------------GGTCCC---------------GCTTATGG
G---GTCAAATCAATACGTTCGAAGAAGAAAGATTGGAATATCAATCTCATCAAT---------C
TCATA---AGTATCATACCAAATCCCATCAATCGAATCGCTTTTTCGAGAAATACGAGACA
TCTAAGTCATACAAGTAAAGAGATATATTCATTGATAAGA------AAAAATGTGAACGGTG
ATTGGATTGATGATAAAATCCAATCCTGGGTCTGGAACAGTGATTCGATTGATGATAAA
GAAAGAGAATTCTTGGTTCAGTTCTCC------ACCTTAACGACA------GAAAAAAGGATTG
ATCAAATTCTATTGAGTCTGACT---CATAGTGATCATTTATCA---------------AAGAATGACT
CTGGTTATCAAATGATTGAACAACCGGGAGTAATTTACTTACGATACTTAGTTGACATTC
AG------AAAAAGTATCTAATGAATTATAAGTTCAATACATCCTGTTTAGCAGAAAGACGG
ACATTCCTTGCTCATTATCAGACAATCACTCAT------TCACAAACCTCGTGTGGGGCTAAT
AGTTTTCATTTCCCATCTCATGGA---------AAACTCTTTTCGCTCCGCTTAGCCCTCTCCC
CG---------GGTATTTTAGTGATAGGTTCTATAGGAACTGGACGATCCTATTTGGTCAAATA
CCTAGCGACAAACTCCTATCTTCCTTTCATTACAGTATTTCTGAACAAGTTCCGGGATAA
CAAGCCTAAG------TTTATTGAT---------------GATAGTGACGATATTGATGATAGTGGCGATA
TTGATGATAGTGACGATATCGACCGT--GAC
CTTGATATTGATACGGAGCTGGAGCTTCTAACTATGATGAATGCGCTA---------ACTATGG
AGATGAAGCTG---------GAAATAGACCAATTTTATATCACCCTTCAATTCGAATTAATAAA
AGCAATGTCTCCTTGCATAATATGGATTCCAAACATTCATGATCTGTATGTGAATAAGTC
GAGTCACTTATACTTTGGTCTATTAGTGAACTATCTCTAC---------AGGGATTTTGAAAGA
TGTTCC---ACTACAAATATTCTTGTTATTGCTTCGACCCATATTCCCCAAAAAGTGGATCC
CGCTCTAATAGCTCCGAATAAATTGAATACATGCATTAAAATACGAAGGCTTCTTATTCC
ACAACAACGAAAGCACTTTTTCACTCTTTCATATACTAGGGGATTTCACTTGGAAAAGA
AAATGTTCCATACT------AATGGATTCGGTTCCATAACCATGGGTTCCAATGTACGAGATC
TTGTAGCACTTACCAATGAGGCCCTATCGATTAGTATTACGCAGAGGAAATCAATTATAG
ACACTAATATAATTAGATCTGCTCTTCATAGACAAACTTGGGATTTGCGA---------TCCCA
GGTAAGATCAGTTCAGGATCATGGGATCCTTTTCTATCAGATAGGAAGGGCTCTTGCAC
AAAATGTACTTCTAAGTAATTGCTCCATAGATCCTATATCTATCTATATGAAGAAGAAA---
---TCATGTAACGAAGGGGGTTCTTATTTGTACAACTGGTACTTCGAACTTGAAACGAGC
ATGAAGAAATTAACGATACTTCTTTATCTTTTGAATTGTTCTGCCGGATCGGTCGTTCAA
GACCTTTGGTCTCTATCC------GGACCCGATGAAAAA---AATGGGATCACTTCTTATGTAC
TCGTTGAGAATGATTCT---CATCTAGTTCATGGCCTATTAGAAGTAGAAGGCGCTCTGTT
TGGA------TCCTCATGGACAGAAAAAGATTGCAGTCGATTTGATAATGAT------CGAGTGA
CATTGCTTCTTCGGCCCGAACCCAGGAAT---CCCTTAGATATGATACAAAATGGATCTTC
TTCTATCGTTGATCAGATATTTCTCTATCAAAAA------TACGAATCGAAGTTTGAAGAA----
-----GGGGAAGGAGTCCTCGACCCGCAA------------CAGATAGAGGAGGAT------------TTATT
CAATCAC------ATAGTTTGGGCTCCT------AGAATATGGAGCCCT---------TGGGGCTTTCTA
TTTGATTGTATCGAAAGGCCCAATGAATTGGGAGTTCCC---TATTGGGCTAGGTCATTTC
GGGGCAAG---------CGGATCATTTATGATGAAGAGGATGAGCTTCAAGAGAATGATTCGG
AGTTCTTGCAGAGTGGAACCGTGCAGTACCAGACACGAGATAGCTCTTCCAAAGAAC
AAGGCTTTTTTCTAATAAACCAATTCATTTGGGATCCTGCAGATCCACTCTTTTTCCTAT
TCCAA---GATCATCCTTTTGTCTCTGTGTTTTCACATCGAGAATTCTTTGCAGATGAAGA
GATGGCAAAGGGGCTGCTTACTTCCCAACCAGCTTTTCCTACATCTCTAGAAAAACGCT

GGTTTATCAATATCAAGAATACGCAAGAAAAGTACGTCGAATTGTTGATTCATCGCCAG
AGATGGCTTAGAACTAGA---ACCAATAGTTCATTATCTAAATCTAATGGATTTTTCCGTTC
TAAT------ACTCTATCCGAGAGTTATCAGTATTTATCAAATCTGTTCCTATCTAAC---GGAA
CACTATTGGATCAAATGACAAAGACATTGTTGAGAAAAAGATGGCTTTTCCCGGATGA
AATGAAA--ATGCCTAGATCTGGTGGGATAAATGGAAA
TTTTATCGATAAGACCTTTTCAATTGTAGCCAATATCTTATTACGAATAATTCCGACAACT
TCCGGGGAAAAAGAGGCATTCACCTATTACAGAGATGGG------ATGTCGGCTCAATCCG
AAGGAAATTATGCGGAAGCTCTACAGAATTATTATGAAGCTATGCGACTAGAAATCGAT
CCCTATGATCGAAGTTATATACTCTATAACATAGGTCTTATCCACACAAGGAACGGAGAA
CATACGAAAGCTTTGGAATATTATTTTCGGGCACTAGAACGAAACCCGTTCTTACCACA
AGCTTTTAATAATATGGCTGTGATCTGTCATTAC---CGGGGAGAACAGGCCATTCGACAG
GGAGATTCTGAAATTGCGGAGGCTTGGTTCAATCAAGCCGCTGAGTATTGGAAACAAG
CTATAGCGCTTACTCCCGGTAATTATATTGAAGCCCAAAATTGGTTGAAGATCACAAGG
CGTTTCAAA------ATGACATTGGCGATCAGAACGTATATGATAGGATTTCTAAGAGGATC
TCGAAAAATCAGCAATTTCTGCTGGGCCTTTATCCTTTTTTTAGGTTCATTAGGGTTTTT
ATTGGTTGGAACTTCCAGTTATCTTGGTAGGGATTTGATACCTTTATTTCCGTCTCAGCA
AATAATTTTTTTTCCACAGGGGATCGTAATGTCTTTCTATGGGATTGCCGGTCTCTTTATT
AGCTCCTATTTGTGGGGCACAATTTTGTGGAATGTAGGTAGCGGTTATGATCGATTCGAT
AGAAAAGAAGAAATAGTGTCTATTTTTCGTTGGGGGTTTCCTGGAAAAAATCGTCGAA
TCTTCCTACGATTCCTTATGAAAGACATTCAGTCCATCAGAATAGAAGTTAAAGAGGGT
ATTTATGCACGTCGTGTCCTTTATATGGAAATAAGAGGCCAAGGGGCCATTCCCTTGAC
TCGTACAGATCAGAAT---CTGACCCCACGAGAAATTGAGCAAAAGGCTGCCGAATTGG
CCTATTTCTTGCGTGTACCAATTGAAGTTTTT
>Acorus
??
??
??
??
??
??
??
??
??
??
??
??
??
??
??
??
????ATGGCAACCCTTCGAGCCGATGAAATTAGTAATATTATCCGTGAACGTATTGAACAA
TATACTAGAGAAGTGAAGGTTGTGAATACCGGTACAGTCCTTCAAGTAGGCGACGGCA
TTGCTCGTATTCATGGTCTTGATGAAGTAATGGCAGGTGAATTAGTCGAATTTGAAGAG
GGTACGATAGGTATTGCTCTTAATTTGGAATCCAATAATGTTGGCGTTGTACTAATGGGT

GACGGTTTGACAATACAAGAGGGAAGTTCTGTAAAAGCAACAGGAAGAATTGCTCAG
ATACCGGTGAGTGAGGCTTATTTGGGTCGTGTTGTAAATGCTCTGGCTAAACCTATTGAT
GGGAGAGGTGAAATTGCAGCTTCTGAATTCCGGTTAATCGAATCTCCTGCCCCAGGTAT
TATTTCGAGACGTTCCGTATATGAGCCCCTTCAAACAGGTCTTATCGCTATTGATTCGAT
GATCCCTGTAGGGCGCGGTCAGCGAGAATTAATTATTGGGGACAGACAGACCGGTAAA
ACAGCGGTAGCCACAGATACTATTCTCAACCAAAAAGGACAAAATGTAATATGCGTTTA
TGTAGCTATTGGCCAAAAAGCATCTTCCGTCGCGCAGGTAGTGACTAATTTCCAGGAGC
GAGGGGCGATGGAATACACTATTGTGGTAGCCGAAACGGCGGATTCCCCCGCTACATTA
CAATACCTGGCTCCTTATACAGGAGCGGCTTTGGCTGAATATTTTATGTACCGTGAACG
ACATACTTCAATAATTTATGATGATCTCTCCAAACAAGCGCAAGCTTATCGCCAAATGTC
TCTTCTATTAAGAAGACCGCCCGGACGTGAAGCTTACCCAGGAGATGTTTTTTATTTGC
ATTCCCGCCTTTTGGAAAGAGCCGCTAAATCAAGTTCTCGTTTAGGTGAAGGAAGTATG
ACTGCTTTACCAATAGTTGAAACTCAATCTGGGGATGTTTCGGCTTATATTCCTACTAAT
GTAATTTCCATTACAGATGGGCAAATATTCTTATCCGCCGATTTATTCAATGCCGGAATAC
GACCTGCTATTAATGTGGGTATTTCTGTCTCCAGAGTAGGATCCGCAGCTCAAATTAAA
GCCATGAAACAAGTAGCTGGCAAATCAAAATTGGAACTAGCTCAATTCGCGGAGTTAG
AAGCTTTTGCACAATTCGCTTCTGATCTCGATAAAGCTACTCAGAATCAATTGGCAAGA
GGTAAACGATTGCGCGAGTTACTTAAACAATCCCAATCAGAACCTCTCGCGGTGGACG
AACAAGTGGTTACTATTTATACCGGAACGAATGGATATCTTGATACGTTAGAAATTGGGC
AGGTAAAGGAATTTCTCGTTAAGTTACGTACCTACTTAAAAAAGAATAAACCTCAGTTT
CAAGAAATCATATCTTCTACCAAGACCTTTACCGAGGAAGCAGAAGCTCTTTTGAAGG
AAGCTATTCAGGAGCAGCTCGAACTCTTTCTACTTCAGGAACAAACAATGAGAATAAC
TCCTACTACTTCT---TCTGCGGTTCCCTCACTTGACGAGAAAAACTTAGGGCGTATCGTC
CAAATCATTGGCCCAGTACTGGATGTCGCCTTTCCCCCGGGAAAGATGCCTAATATTTA
CAACGCTTTGGTGGTTAAGGGTCGAGATACTGCCGGTCAGCCAATTAATGTGACTTGTG
AGGTACAGCAATTATTAGGAAATAATCGGGTTCGAGCTGTGGCTATGAGTGCTACAGAT
GGTTTGACGAGAGGAATGGAAGTGATTGACACGGGAGCTGCTCTAAGTGTTCCAGTCG
GTGGAGCTACTCTCGGAAGAATTTTCAACGTTCTTGGGGAGCCCGTTGATAATTTAGGC
CCTGTAGATACTCGCACAACATCTCCTATTCATAGATCTGCGCCTGCATTTATACAGTTA
GATACGAAATTATCAATCTTTGAAACAGGCATTAAAGTGGTAGATCTTTTAGCTCCTTAT
CGCCGGGGAGGAAAAATCGGACTATTTGGGGGAGCTGGAGTGGGTAAAACAGTACTC
ATCATGGAATTGATCAACAACATTGCCAAAGCGCATGGAGGTGTATCCGTATTTGGCGG
AGTAGGCGAGCGTACTCGTGAAGGAAATGATCTTTACATGGAAATGAAAGAATCCGGA
GTAATTAATGAAGAAAAGATTGAAGAATCTAAAGTAGCTCTAGTGTATGGTCAGATGAA
TGAACCGCCGGGCGCTCGTATGAGAGTTGGTTTGACTGCCCTAACCATGGCGGAATATT
TCCGGGATGTTAATGAACAGGATGTACTTCTATTTATCGACAATATCTTCCGGTTCGTAC
AAGCGGGATCAGAAGTATCTGCCTTATTAGGGCGGATGCCTTCCGCCGTGGGTTATCAA
CCTACCCTTAGTACAGAAATGGGTTCTTTGCAAGAAAGAATTACTTCTACAAAAGAGG
GATCTATAACGTCTATTCAAGCGGTTTATGTACCTGCGGATGATTTGACTGACCCTGCTC
CTGCCACCACATTTGCACATTTGGATGCTACTACTGTACTATCAAGAGGATTAGCTGCC
AAAGGTATTTATCCGGCAGTAGATCCTTTAGATTCAACGTCAACTATGCTCCAACCTAG
GATCGTTGGCGAGGAACATTATGAAACTGCGCAAAGAGTTAAGCAAACTTTACAACGT
TACAAAGAACTTCAGGACATTATAGCTATCCTTGGGTTGGACGAATTATCCGAAGAGGA
TCGTTTAACCGTAGCAAGAGCACGAAAAATTGAGCGTTTCTTATCACAACCCTTCTTCG

TAGCAGAAGTATTTACTGGTTCTCCAGGTAAATATGTTGGTCTCGCGGAAACGATTAGG
GGGTTTCAACTGATCCTTTCAGGAGAATTAGATGGTCTTCCCGAGCAGGCCTTTTATTT
GGTGGGTAACATCGATGAAGCTACCGCGAAGGCTATGAATTTAGAAGTGGAGAGCAAA
TTGAAGAAAATGAACTTAAAGCTTTGTTTACTGACTCCTAATCGAATTATTTGGGATTC
AGAAGTGCAAGAAATCATTCTATCTACTAATAGTGGACAAATTGGCATATTACCAAATC
ACGCCTCTATTGCCACAGCTGTAGATATCGGTCTTTTGAGAATACGCCGCGACGACCGA
TGGTTAGCGGTGGCTCTGATGGGGGGGTTTGCTAGAATAGGAAATAATGAGATCACCAT
TTTAGGAAATGATGCCGAGATGGGTACTGACATTGATCCGCAAGAAGCTCAACGAGCT
CTTGAAATAGCTGAAGCTAACTTGAGTAAAGCTGAAGGTAAGAGACAAGCAATTGAG
GCAAATTTAGCGCTAAGACGAGCTAGGACACGGGCGGAGGCTATTAATGTTATTTCCAT
GAAAAATATAACCGATTCTTTCGTTTCCTTGGGCCACTGGCCATTCGCCGGGAGTTTCG
GGTTTAATACCGATATTTTAGCAACAAATCTAATAAATCTAAGTGTAGTGCTTGGTGTAT
TGATCTTTTTTGGAAAGGGAGTGTTAAGTGATTTATTAGATAATCGAAGACAGAGGATC
TTGAGTACTATTCGAAATTCAGAAGAACTGCGTAGAGGAGCCCTTGAGCAGCTCGAAA
AAGCCCGGGCTCGCTTACGGAAAGTAGAAATGGAAGCAGATGAGTATCGAGTGAACG
GATACTCCGAGATAGAACGAGAAAAGATGAATTTGATTAATGCCACTTATGAAAATTTG
GAACGATTAGAAAATTACAAAAATGAAACCCTTCATTTTGAACAACAAAGAGCAATTA
ATCAGGTCCGGCAACGAGTTTTCCAACAAGCGTTACAAGGAGCTCTAGGAACTCTGAA
TAATTGTTTGAATAGTGAATTACATTTCCGTACCATCAGTGCTAATATTGGCATGCTTGG
GGCCATGAAAGAAATAACTGATATGAATCCACTGATTTCTGCCGCTTCCGTTATTGCTGC
TGGATTAGCCGTAGGACTTGCTTCTATTGGACCTGGGGTTGGTCAAGGTACTGCTGCAG
GCCAAGCCGTAGAAGGTATTGCGAGACAACCAGAAGCAGAAGGTAAAATACGAGGTA
CTTTATTGCTTAGTCTGGCTTTTATGGAAGCTTTAACAATTTACGGACTGGTCGTAGCAT
TAGCACTTTTATTTGCGAATCCTTTTGTCATGAATGTTATACTATGTTCCAGCAACATGCT
CAAAGGGTTATATGATATATCTGGTGTAGAAGTGGGCCAACATTTATATTGGCAAATAGG
AGGGTTCCAAGTCCATGCCCAAGTACTAATCACTTCTTGGGTCGTAATTGCTATCTTATT
AGGTTCAGTCACTGTCGCTGTTCGGAATCCACAAACCATTCCGACCAACGGTCAGAAT
TTCTTTGAGTATGTTCTTGAATTCATTCGAGACTTGAGCAAAACACAGATTGGAGAAGA
ATATGGTCCTTGGGTTCCCTTTATTGGAACAATGTTCCTATTTATTTTTGTTTCGAATTGG
TCAGGCGCTCTTTTACCTTGGAAACTCATAGAGTTACCTCACGGGGAGTTAGCCGCAC
CCACGAATGATATAAATACTACTGTTGCTTTAGCTTTACCCACATCTGTAGCATATTTCTA
TGCGGGTCTTACCAAGAAAGGATTGGGTTATTTCGGGAAATACATTCAACCAACTCCGA
TCCTTTTACCAATTAATATCCTAGAAGATTTCACAAAACCTTTATCACTGAGTTTTCGAC
TTTTCGGAAATATCTTGGCTGATGAATTAGTAGTTGTTGTTCTTGTTTCTTTAGTACCTTT
GGTAGTTCCTATACCTGTCATGTTTCTTGGATTATTCACAAGCGGCATTCAAGCTCTTAT
TTTTGCAACTTTAGCCGCGGCCTATATAGGCGAATCCATGGAAGGTCATCATATGATATT
TTCAACTTTAGAACATATATTAACTCACATATCTTTTTCGATTATTTCAGTTGTGATTACA
ATTCAGTTGATGAACTTATTAGTCCACGAACTGGTACAACTAGGTGATGCGTCAGAAAA
AGGCATGATAGCTACCTTTTTCTCTATCACGGGATTATTGGTTACTCGTTGGATCTATTCG
GGTCATTTCCCACTAAGTGATTTATATGAATCATTAATCTTCCTTTCATGGAGTTTCTCCA
TTATTCATATGGTTCCATATTTTAGGAACCACAGAAATCATTTCAGTGCAATAACTGCGC
CAAGTGCCATTTTTACCCAAGGCTTTGCTACTTCGGGTCTTTTAACGGAAATGCATCAA
TCCATAATATTAGTACCCGCTCTACAATCCCAGTGGTTAATGATGCATGTAAGTATGATGT
TATTGAGCTATGCAGCTCTTTTATGCGGGTCATTATTATCAGTTGCTCTTCTAGTTATTAC

GTTTCGAAAAAACAGCGATATTTTTGATAAAAGAAATGAGATTGAATACTTGAATGAAA
AACGCAGTGTTTTACAAAACACTTCGTTTGATTCATTTACAAATTATCACAAATATCAAT
TGACTCAACGATTAGATTTTTGGAGTTATCGGGTCATTAGTCTAGGGTTTATCTTTTTAA
CCATAGGTATTCTATCGGGGGCAGTGTGGGCTAATGAGGCATGGGGATCTTATTGGAAT
TGGGACCCAAAGGAAATTTGGGCATTTATTACCTGGGCTATATTCGCAATTTATTTACAT
ACTAGAACGAATCATAGTTTGCAAGGTGCGAATTCGGCAATTGTGGCTTCTATCGGATT
TCTTATAATTTGGATATGTTATTTTGGAGTCAATCTATTAGGAATAGGTTTACATAGTTATG
GTTCATTCACATTAATAGTGCCAAAAAAAGGATTTACT---CCCCTCCCATATCTTGCATCT
ATAGTATTTTTGCCCTGGTGGGTCTCTCTCTCATTTAATAAGAGTCTGGAACCTTGGGTT
ACCAATTGGTGGAATACCAGACAATCTGAAACCTTTTTGAATGATATTCAAGAGAGGA
ACGTTCTAGAAAGATTCATCGAATTAGAAGAACTATTCCTGTTGGACGAAATGCTAAAG
GAGAACCCGGAGACACGTATGAAAAATCTTCGTATAGGAATCCACAACGAAACGATCC
AATTGGTCAAAACAGACAATGAGTATCATCTCCATACCATTTTGCATTTCTCGACAAATA
TAATCTGTTTCGCTATTCTAAGTGTTTATTCTATTCTGGGTAATGAAGAACTTGTCATTCT
TAATTCTTGGGTTCAGGAATTTCTTTATAACTTAAGCGACACAATAAAAGCTTTTTCGAT
TCTTTTAGTTACTGATTTATGGATTGGATTCCATTCACCCCACGGTTGGGAACTAATGAT
TGGTTCGGTCTACAATGATTTTGGATTAGCTCATAACGAACAAATTATATCTGGGCTTGT
TTCCACTTTTCCAGTTATTCTAGATACGATTGTGAAATATTGGATCTTCCATTATTTAAAT
CGTGTCTCTCCTTCACTTGTAGTGATTTATCATTCAATGAATGACATGCCCATTGGTGTT
CCAAAAGTACCTTTTCGGAGTCCTGGAGAGGAAGATGCAGCTTGGGTTGACATATACA
ACCGACTTCATCGAGAAAGATTACTTTTTTTAGGCCAAGAGCTTGATAGCGAAATCTCG
AATCAACTTGTTGGTCTCATGGTATATCTCAGTATAGAGGATAAGACCAGGGATTTTTTT
TTGTTTATAAATTCTCCCGGCGGATGGGTAATTCCGGGAATAGGTCTTTATGACACTATG
CAATTTGTGCCACCCGATGTACATACAATATGCATGGGATTAGCTGCTTCAATGGGATCT
TTCATTCTTGTCGGAGGAGAAATTACCAAACGTCTAGCATTCCCTCACGCTAGGGTTAT
GATCCACCAACCTGCCAGTTCTTTTTATGAGGCACAAGCAGGGGAATTTGTCCTGGAA
GCGGAAGAACTATTGAAACTTCGCGAAACCCTCACAAGAGTTTACGTACAAAGAACT
GGTAAACCATTATGGGTTGTATCCGAAGATATGGAAAGGGATGTTTTTCTGTCAGCAAC
AGAAGCCCAAGCTCATGGTATTGTTGATCTTGTAGGGGACGAAATGAAAGAACAAAAA
TTGATTCATGAAGGTTTAATTACTGAATCACTTCCTAATGGTATGTTTTGGGTGCGTTTA
GATAATGAAGATCTGGTTCTAGGTTATATTTCTGGAAGGATCCGACGCAGTTCTATACGG
ATACTACCGGGAGATAGAGTAAAAATCGAAGTAAGTCGTTATGATTCAACCAGAGGAC
GCATAATTTATAGACTTCGCAATAAAGATTCGAACGAGATGGAAGAATTCAAAGTCTAT
TTCGAAAAAGATGGGTCTCGTCAACAATACTTCCTATATCCACTTCTCTTTCAGGAGTA
CATCTACGCACTTGCTCATAATCATGTTTTATTCGTTTATGATAAT---TTTAGTTTAATAATT
GTAAAACGTTTAATTACTCAAATGTATCAACAGAATTCTTTGAGTAATTTGGTTAATGAT
TCTAACCGAAATCGATTAGTTCGGAAGAATTTTTATTATCAAACGATATTAGAGGGTTTT
TCAGTCATTATGGAAATTCCATTCTCCATAATATTTTTATCTTCCGTTATACCAAAAATTC
AGAATTTACGATCTATTCATGCAACATTTCCCTTTTTAGAAGACAAATTATCACATTTAA
ATCATGTATCAGATATATTAATACCCTATCCCATCCATCTCGAAATCCTGGTTCAAGTTCT
GCAAGGCTGGATACAAGATGTTCCGTCTTTACATTTATTGCGGTTCTTTCTCCATGAGTT
TCATAATTGGAATAGTCTCATTACTAAATCCATTTCTTTTTTTTCAAAGCAAAGACTCTT
CTTGTTCTTATATAATTCTTATGTATTTGAGTGTGAATCCGCATTAGTCTTTCTCCGTAAA
CAATCCTTTTATTTACGATCAACATCTTTTGGAACCTTTGTTGAGCGAACACATTTCTAT

GGAAAAATAGAGCAT------GTAGTGCGTAGGAAGCAAAAGGCCTTATCTTTGTTCAAAG
ATCCTTTCATCCATTATATCCGATATAAAGGAAAATCAATTCTGGCTTCAAAGGGGACTC
ATTTTCTGATGAAGAAATGGAAATATCACCTTCTAAATTTCTGGCAATGTCATTTTGACT
TTTGGTCTCAACCGCATAGGATTCATATAAACCAATTATCAAATCATTCTTTCTATTTTAT
GGGCTATCTGTCAAGTGTACGAATCAATTTTTCGACGGTAAGGAGTCAAATGCTAGAG
AGTTCATTTCTAATGGATACTCCTACT------AAAAAATTCGATACTATAGTCCCAATTATTC
CTCTAATCGGATCATTGGCTAAAGCTAAGTTTTGTAACGTATCGGGGCATCCTGTTAGTA
AGCCGGTCTGGGCTGATTTGTCAGATGCTGATATTATTGATCGATTTGGACGAATATGTA
GAAATCTTTCTCATTATCACAGTGGATCCTCAAAAAAACAGAGTTTGTATCGAGTAAAG
TATATACTTCGCCTTTCGTGTGCGAGAACTTTGGCCCGTAAACATAAAAGTACAGTGCG
CGCTTTTTTCAAACGATTAGGTTCAGAATTCTTAGAAGAATTCTTTACGGATAAAGAAC
AAGTTCTTTCTTTGATCTTCCCTTCCCGTATTTGGTATTTGGATATTATCCGTATCAATGA
CCTGGTGAATAATTCAATGATAATTGCTACGACA---GAAATACAA---GCTATCAATTCTTT
TTCTAGATCGGAATCCTCCTTAAAAGAGGTCTACGGACTCATATGGCTGCTTGTACCTAT
TTTTACTCTTATATTGGTAATTATAATAGGGGTACTAGTGATTGTTTGGTTGGAAAGAGA
AATATCTGCGGGGATACAACAACGTATTGGGCCTGAATATGCCGGCCCATTGGGAATTC
TTCAAGCTCTAGCGGATGGAACCAAACTGCTTTTCAAAGAAGATCTTCTCCCATCTAGA
GGAGATATTAGTTTATTCAGCCTCGGGCCGTCTATAGCGGTCATATCAACTCTACTAAGT
TATTTAGTAATTCCTTTTGGCTATCACCTTGTTTTAGCTGATCTCAGTATCGGCGTTTTTT
TATGGATTGCCATTTCAAGTATTGCTCCTATTGGACTTCTTATGTCAGGATATGGCTCGAA
TAATAAATATTCCTTTTCAGGTGGTCTACGAGCTGCCGCTCAATCTATTAGTTATGAAAT
ACCATTAACTCTATGTGTATTATCAATATCTCTACTATCTAATAGTTCAAGTACAGTTGATA
TAGTTGAGGCACAGTCAAAATATGGTTTTTGGGGATGGAATCTGTGGCGTCAACCTATA
GGATTTTTAGTTTTCCTAGTTTCTTCTCTAGCCGAATGTGAAAGATTACCCTTTGATTTA
CCAGAAGCAGAGGAAGAATTAGTAGCAGGTTATCAAACTGAATATTCAGGCATCAAAT
TTGGTTTATTTTACGTTGCTTCTTACCTAAATCTACTAGTTTCTTCATTATTCGTAACAGT
TCTTTACTTAGGCGGGTGGAATCTCTCTATTCCATACATATTTATTCCTGAACTTTTCGGA
AAAAACAAAACAGGTGGAATCTTTGGAATGACAATTGGTATCCTAATTACATTAGCTAA
AGCTTATTTGTTCCTGTTCATTTCTATCGCAACAAGATGGACTTTACCTAGGTTAAGAAT
AGACCAACTATTAAATCTTGGATGGAAATTTCTCTTACCTATTTCCCTCGGTAATCTATTA
TTGACAACCTCTTCCCAACTTGTTTCACTAATGATCTGGCATGTACAGAATGAAAACTT
CATTCTCGATTCTACGAGAATTTTTATGAAAGCGTTTCATTTGCTTCTCTTCGATGGAAG
TTTCATTTTCCCAGAATGTATCCTAATTTTTGGCCTAATTCTTCTTCTGATGATCGATTCA
ACCTCTGATCAAAAAGATAGACCTTGGTTCTATTTCATATCTTCAACAAGTTTAGTAATG
AGCATAGCGGCCCTATTGTTCCGATGGAGAGAAGAACCTATGATTAGCTTTTCGGGAAA
TTTCCAAACGAACAATTTCAACGAAATCTTTCAATTTCTTATTTTACTATGTTCAACTCT
ATGTATTCCTCTATCCGTAGAGTACATTGAATGTACAGAAATGGCTATAACAGAATTTCT
GTTATTCGTATTAACAGCTACTCTAGGAGGAATGTTTTTATGTGGTGCTAACGATTCAAT
AACTATCTTTGTAGCTCCAGAATGTTTTAGTTTATGCTCCTACCTCTTATCTGGATATACC
AAGAGAGATGTACGGTCTAATGAGGCTACTATGAAATATTTACTCATGGGTGGGGCAAG
CTCTTCTATTCTGGTTCATGGTTTCTCTTGGCTATATGGTTTATCCGGGGGGGAGATCGA
GCTTCAAGAAATAGTGAATGGTCTTATCAATACACAAATGTATAACTCCCCGGGAATTT
CAATTGCGCTTATATTCATCACTGTAGGAATTGGGTTCAAGCTTTCCCTAGCCCCTTTTC
ATCAATGGACTCCTGACGTATACGAAGGATCTCCCACTCCAGTCGTTGCTTTTCTTTCT

GTTACTTCGAAAGTAGCTGCTTCAGCTTCAGCCACGCGAATTTTCGATATTCCTTTTTAT
TTCTCATCAAACGAATGGCATCTTCTTCTGGAAATCCTAGCTATTCTTAGCATGATATTG
GGGAATCTCATTGCTATTACTCAAACAAGCATGAAACGTATGCTTGCATATTCGTCTATC
GGTCAAATCGGATATGTAATTATTGGAATAATTGTTGGAGACTCAAATGATGGATATGCG
AGCATGATAACTTATATGCTGTTCTATATCTCCATGAATCTAGGAACTTTTGCTCGCATTG
TATTATTTGGTCTACGTACCGGAACTGATAACATTCGAGATTATGCAGGATTATACACGA
AAGATCCTTTTTTGGCTCTCTCTTTAGCCCTATGTCTCTTATCCCTAGGAGGTCTTCCTC
CACTAGCAGGTTTTTTCGGAAAACTCCATCTCTTCTGGTGTGGATGGCAGGCAGGCCTA
TATTTCTTGGTTTCAATAGGACTCCTTACGAGCGTTGTTTCTATCTACTATTATCTAAAAA
TAATCAAGTTATTAATGACTGGACGAAACCAAGAAATAACCCCTCACGTGCGAAATTAT
AGAAGATCTCCTTTAAGATCAAACAATTCCATCGAATTGAGTATGATTGTATGTGTGATA
GCATCTACTATACCAGGAATATCAATGAACCCCATTCTTGCAATTGCTCAGGATACCCTC
TTTATGTTTCTGCTTCACGAATATGATATTTTCTGGGCATTTCTACTAATATCAAGTGTTAT
TCCTATTTTAGCATTTTTAATTTCCGGAGTTTTAGCCCCAACGAGGGAAGGACCCGAGA
AGCTTTCTAGTTATGAATCGGGTATAGAACCAATAGGGGATGCTTGGGTACAATTCCGA
ATCCGATATTATATGTTTGCTTTAGTTTTTGTTGTTTTTGATGTTGAAACGGTCTTTCTTTA
TCCATGGGCAATGAGTTTCGATGTATTGGGTGTATCCGTATTTCTAGAAGCTTTGATTTT
CGTGCTTATCCTAATTGTTGGTTCAGTTTATGCATGGCGAAAGGGGGCATTGGAATGGT
CTACGAGTTATTTTCCTTGGTTAACCATAATTGTTGTTTTTCCGATATCCGCAGGTCTTTC
AATTTTCTTTCTCCCGCATAGAGGGAATAAGGTGGTTCGGTGGTATACTATATGTATTTG
TTTGCTAGAACTCCTTTTAATGACCTATGTGTTCTGTTATCACTTTCAATTGAACGATCC
ATTAATCCAATTGGATGAAGATTATGAATGGATAAATATTTTTGATTTTCACTGGAGACC
AGGAATCGATGGACTTTCCATAGGACCCATTCTACTGACGGGATTTATCACTACTTTAGC
TACTTTAGCGGCTTGGCCGGTTACTCGAGATTCGCGATTGTTCCATTTCCTGATGTTAGC
AATGTACAGTGGTCAAATAGGATTATTTTCTTCTCGAGACCTTTTACTTTTTTTTCTCATG
TGGGAGTTAGAATTAATTCCCGTTTACCTACTTTTATCTATGTGGGGTGGGAAGAAACG
GCTGTACTCGGCTACCAAGTTTATTTTGTACACAGCGGGGGGTTCTGTTTTTCTTTTAAT
GGGAGTTCCGGGTATGGGTTTATATGGTTCCAATGAACCAACATTAAATTTTGAAACAT
CGGCTAATCAATCGTATCCTGTGTCATTGGAAATACTATTTTATTTTGGATTCCTTATTGC
TTATGCTGTCAAATTACCGATTATACCCCTCCATACATGGTTACCAGATACCCATGGAGA
AGCCCATTACAGTACATGTATGCTTCTAGCTGGAATCTTATTAAAAATGGGAGCATATGG
ATTAGTTCGGATCAATATGGAATTATTGCCCCACGCTCATTCTATATTTTCCCCCTGGTTG
GTACTAGCGGGAACGTTGCAAATAATCTATGCAGCTTCAACTTCTCTTGGTCAAGTTAA
TTTAAAAAAGAGAATCGCCTATTCCTCCGTATCTCATATGGGTTTCACAATTATAGGAAT
TGGTTCTATAACCGATACAGGACTCAATGGAGCCATTTTACAGTTACTCTCTCATGGATT
TCTTGGTGCTGCACTTTTTTTCTTAGCGGGAACAAGTTGTGATAGAATACGTCTTATTTA
TCTCGACGAAATGGGGGGAATATCCATCCCGATGCCCAAAATATTTACCATGTTCAGTA
GCTTCTCAATGGCTTCTCTTGCATTGCCAGGAATGAGTGGTTTTGTTGCGGAAGCAGTA
GTATTTTTTGGAATAATTACTAGTCAAAAATTTCTTTTTTTGCCAAAAATACTAATTACTT
TTGTAATGGCAATTGGAATGATATTAACTCCTATTTATTTATTATCCATGTTACGCCAGAT
CTTCTATGGATACAAGCTATTTAATATGACAAACTCTTATTTTATGGATTCTGGACCACGA
GAACTATTTGTTTCGATCTGTATCTTTTTGCCCGTAATAGGTATTGGTATTTATCCCGATTT
CGTTCTATCGTTATCAGTTGACAAGGTGGAAGCTATTTTATCTAATTACTTTTATAGAATG
ATGCTGGAGTATGTACTTTTTTTGAGTGCTTATTTATTTTCCATCGGTATCTATGGATTGA

TTACAAGTCGAAACATGGTTAGAGCACTTATGTGTCTTGAACTTATACTGAATGCGGTT
AATATCAATCTCGTAACATTTTCTGATTTATTTGATAGTCGCCAATTAAAAGGAGACATT
TTTTCGATCTTTGTTATAGCTATTGCAGCCGCTGAAGCAGCTATTGGACCAGCTATTGTT
TCATCGATCTATCGTAACAGAAAATCCATTCGTATTAATCAATCAAATTTGTTGAATAAA
ATGGAACATACATATCAATATGCATGGATAATACCTTTCCTTCCATTTCCAGTTACTATAT
CAATAGGGTTGGGACTTCTGCTCGTTCCAACGGCAACGAAAAATCTTCGCCGTATTTG
GGCTTTTTTTAGTGTTTTATTGCTAAGTATAGCTATGGTGTTTTCGGCCGATCTGGCTATT
CAGCAAATAAACGGGAGTTTTATCTATCAATATTCATGGTCTTGGACGATTAATAACACT
TTTTCATTAGAGTTTGGATACTTAATTGATCCACTTACCTCTATTATGTTAATACTAATCA
CTACTGTTGGAATCATGGTTCTTATTTATAGTGATAATTATATGTCTCATGATCAAGGATAT
TTAAGATTTTTTGCTTATATGAGTTTTTTCAATACTTCTATGTTAGGATTAGTTACTAGTTC
TAATTTGATACAAATTTATATTTTTTGGGAACTCGTAGGAATGTGCTCCTATTTATTAATA
GGGTTTTGGTTCACACGACCAACTGCAGCAAATGCTTGTCAAAAAGCGTTTGTAACTA
ATCGTGTAGGGGATTTTGGTTTGTTATTAGGAATCTTAGGTCTTTATTGGATAACAGGTA
GTTTCGAATTTCGGGATTTGTTCGAAATTTTAAAAAACAATGAAGTCAATTCTTTATTTG
CCGCCCTGTGCGCCTCACTATTATTTGTTGGTGCGGTTGCGAAATCCGCACAATTTCCA
CTTCATGTATGGTTACCTGATGCTATGGAGGGCCCTACCCCCATTTCGGCTCTTATACAC
GCTGCTACTATGGTAGCGGCGGGCATTTTTCTTGTAGCTAGGCTTCTTCCTCTTTTCACA
GTCATACCTTACATAATGAATTTCATTTCTTTGATAGGTATAATAACAGTCCTTTTAGGGG
CTACTTTAGCTCTTGCTCAAAGAGACATTAAAAGAAGTTTAGCCTACTCTACAATGTCT
CAGTTAGGTTATATTATGTTAGCTCCAGGTATAGGCTCTTATCGAGCTGCTTTATTCCATT
TGATCACTCACGCCTATTCTAAAGCATTATTGTTTTTAGGATCTGGATCAATTATTCATTC
AATGGAACCTATTGTTGGATATTCACCAGAGAAAAGTCAGAACATGATTCTTATGGGTG
GTTTAAGAAGATATGTTCCAATTACAAAAACCACTTTTTTCTTAGGTACACTTTCTCTTT
GTGGTATGCCCCCTCTTGCTTGTTTTTGGTCCAAAGATGAAATTCTTAATGATACTTGGT
TATATTCACCAATTTTCGCAATAATAGCTTGGTCCACAGCAGGATTAACTGCATTTTATAT
GTTTCGGGTATATCTCCTTACTTTTGATGGTCATTTACAAGTTCATTTTCAAAATTTCAGT
AGTACTAAAAATAGCTCCTTCTATTCAATATCTATATGGGGAAAAGAAGTACCGAAACC
ACTTAACGTA---AATTTGTTTTTATCAACAATGAACAAAATGTCTAATAAGTAC------ACT
TCCATGTATCCCCATGAATCGGACAATACTATGCTTTTTCCGATGCTTGTATTGGTCTTAT
TTACTTTGTTCATTGGATTTATAGGAATTCCTTTCGATTTGGATATATTATCAAAATGGTTA
ACTCCATCGATAAACCTTTTACATTCCAATTCCGGCGATTCCTTTGATTGGTATGAATTT
GTGACAAATGCAATTTATTCAGTCACTATATCCTTTTTGGGGATATTTCTAGCATACATAT
TTTATGGGTCTGTTTATTCATCTTTTCAAAATTTGGACTTAATCAATTCGTTTGTTATTTTA
TCGGATCGAATAATAAATGGGATATACAATTGGTCATATAATCGTGGTTACATCGACGTT
TTTTATGGAAAAGTTTTAAGTAATACTATAAGAGGACTTGCAGAATTAATTCATTTTTTT
GATAGACGAGTTATTGATGGGATTACAAATGGGGTTGGTGTTGTAAGTTTCTTTGTAGG
AGAAGGGATAAAATCT---GTAGGGGGTGGACGAATCTCATCTTATATCTTCTTGTATGCA
TTTTCTGTATCAATTTGTTTAATAATTTATTATATGGATTTACCTGGACCAATACATGATGT
TCTTTTAGTATTTCTGGGATCAGGTCTTATATTAGGAGGTTTAGGAGTGGTATTACTTACC
AATCCAATTTATTCTGCCTTTTCATTGGGATTGGTTCTTGTTTGTATATCCTTATTCTATAT
TCTATCGAACTCCTATTTTGTAGCTGCTGCACAGCTCCTTATTTACGTGGGAGCCGTAAA
TGTCTTAATCATATTTGCTGTGATGTTCATGAATGGTTCAGACTATTCCAACGATTTCTAT
CTTTGGACCGTTGGGGATGGAGTCACTTCACTGGTTTGTACAAGTATTCTTTTTTCACT

AATTACTACTATCCTAGATACGTCATGGTACGGAATTATTTGGAATACAGGATCGAACCA
GATTGTAGAGCAGGACTTAACAAGTAACGTTCAACAAATTGGAATTCATTTATCAACAG
ATTTTTATCTTCCATTTGAACTCGTTTCAATAATTCTTTTAGTTGCCTTGATAGGTGCAAT
TACTATGGCGCGTCAGATGACTGTACCAGCTACAAGACAAGACCTTATGATAGTTAATA
TGGGTCCTCACCACCCATCAATGCACGGTGTTCTTCGCCTGATCGTTACTCTCGACGGT
GAAGATGTTATTGACTGTGAACCCATATTGGGTTATTTACATAGAGGGATGGAAAAAAT
TGCAGAAAACCGAACAATTATACAATATCTGCCTTATGTAACACGTTGGGATTATTTAGC
TACTATGTTCACAGAAGCAATAACAGTAAATGGGCCCGAACAATTGGGAAATATTCAAA
TACCTAAAAGAGCCAGCTACATCAGAGTAATTATGTTAGAGCTGAGTCGGATAGCTTCT
CATTTGTTATGGCTTGGTCCATTTATGGCGGATATCGGTGCACAAACACCCTTTTTTTATA
TTTTCAGAGAGAGGGAATTGATATATGATCTATTCGAAGCTGCTACAGGTATGCGAATGA
TGCATAATTATTTCCGTATAGGAGGAGTTGCTGCTGATCTACCTTATGGCTGGATAGATA
AATGTTTAGATTTCTGCGATTATTTTTTAACAGGAATTGTTGAATATGAAAAGCTTATTAC
ACAGAATCCTATTTTTTTGGAACGAGTTGAAAGAGTGGGCATTATTAGTGGGGAGGAA
GCCATAAATTGGGGTTTATCAGGACCCATGTTACGAGCTTCCGGAATAGAATGGGATCT
TCGTAAAGTTGATAATTATGAGTGTTATAATGAATTTGATTGGGAAGTCCAATGGCAAA
AAGAAGGAGATTCATTAGCTCGTTATTTAGTCCGAATTAGTGAAATGAAAGAATCCATA
AAAATTATTCAACAGGCTCTAGAAGGAATCCCTGGGGGGCCCTATGAAAATTTAGAGG
TTCGGCGC---GATAAAGTCAAGGATTCGGAATGGAATGATTTTGAATATAGGTTTATTAG
TAAAAAGCCTTCGCCTACTTTTGAATTGGCGAAACAAGAACTTTATGTGAGAGTAGAA
GCACCAAAGGGAGAATTAGGAATTTTTCTGATAGGAGATAATAGTGTTTTCCCCTGGAG
ATGGAAAATTCGTCCACCCGGTTTCATCAATTTGCAAATTCTTCCTCAACTAGTTAAAA
GAATGAAATTGGCCGATATCATGACGATACTAGGTAGTATAGATATCATTATGGGAGAAG
TTGATCGTATGTTCCCTATGGTGACTGGGTTCATGAATTATGGTCAACAAACAGTACGA
GCTGCAAGGTACATTGGTCAAAGTTTCATGATCACCTTATCGCACGCGAATCGTTTACC
TGTAACTATTCAATATCCTTATGAAAAATTGATCACATCAGAGCGTTTTCGTGGTCGAAT
CCACTTTGAATTTGATAAATGTATCGCTTGTGAAGTATGTGTTCGTGTATGCCCTATAGAT
CTACCCGTTGTTGATTGGAGATTGGACACAGATGTTAGAAAAAAACAATTGCTTAATTA
TAGTATTGATTTTGGAGTCTGTATATTTTGTGGCAACTGCGTCGAGTATTGTCCAACAAA
CTGTTTATCCATGACTGAAGAATATGAACTTTCTACTTATGATCGTCACGAATTGAATTAT
AATCAAATTGCTTTGGGGCGGTTACCGATGTCAGTAATTGAGGATTACACAATTCGAAC
AACTACAAATTTGAAAATAGCTAAAAATAAACCCCTGGGTTCACGAACGATTACTAAT--
-ATGCAGGGTCGTTTATCTGCTTGGCTAGTCAAGCATGAGCTAGTTCATAGATCTTTGGG
CTTCGATTACCAAGGAATAGAGATTTTACAAATAAAACCCGAGGATTGGGATTCCATTG
CTGTCATTTCATACGTATATGGTTACAATTATTTACGTTCCCAGTGTGCCTATGATGTAGC
ACCCGGCGGATTTTTAGCCAGTGTCTATCATCTTACGAGAATACAGTATGGTGTGGATC
AACCGGAAGAGGTATGCATAAAAGTATTTGCTCCAAGGAGGAATCCTAAAATCCCGTC
TGTTTTCTGGATTTGGAGAAGTGCTGATTTTCAAGAACGGGAATCTTATGATATGTTGG
GAATTTCTTATGAAAATCATCCGCGCCTTAAACGTATTTTGATGCCTGAAAGTTGGATAG
GCTGGCCCTTACGTAAGGATTATATTGCCCCCAATTTCTATGAAATTCAAGATGCTCAT---
----
------------------------------ATGAATTCTATTGAGTTTCCGTTACTTGAC
CGAACAACCCCAAATTCAGTTATTTCAACTACATTGAATGATCTTTCGAACTGGTCAAG
ACTATCCAGTTTATGGCCGCTTCTCTACGGTACCAGTTGTTGCTTCATCGAATTTGCTTC

ATTAATAGGCTCGCGATTCGACTTTGATCGTTATGGATTGGTACCAAGATCGAGTCCTAG
GCAAGCGGACCTAATTTTAACAGCTGGCACAGTAACAATGAAAATGGCTCCCTCTTTA
GTGAGATTGTATGAGCAAATGCCTGAACCAAAATATGTCATTGCTATGGGAGCTTGCAC
TATTACCGGAGGGATGTTCAGTACCGATTCTTATAGTACCGTTCGGGGAGTCGATAAGCT
AATTCCTGTCGATGTCTATTTGCCGGGCTGCCCCCCTAAGCCAGAGGCAGTTATAGATG
CTATAACAAAACTTCGTAAGAAGTTATCTCGAGAAATCTCTGAAGATAGAATGGGGTCT
CAA---GAAAATCGATGTTTTACGACCAATCACAAATTTTATGTTCGACGCAGTACTCATA
CTGGAAATTACGATCAAGGATTACTCTATCAATCGCCACCTACTGCAGAAATACCTACA
GAACCCTTTTTCAAATACAAAAGTTCAGTATCTTCCCGCGAATTAGGGAATATGCAAAA
TCGAAATACCTTTTCTTGGGTAAAGGAACAGATGACTCGATTCATTTCAGTATCGATCAT
GATATATGTAATAACTCGGACATCTATTGCAAATGCATATCCCATTTTTGCACAACAGGG
TTATGAAAATCCACGAGAAGCAACTGGACGCATTGTATGTGCTAATTGCCATTTGGCGA
ATAAGCCCGTGGATATTGAAGTTCCACAAGCCGTGCTTCCTGATACTGTATTTGAAGCA
GTTGTTAGAATCCCTTATGATAAGCAACTGAAACAAGTTCTTGCTAATGGTAAAAAGGG
GTCTTTGAATGTAGGGGCTGTTCTTATTTTACCCGAGGGATTTGAATTAGCCCCCCCCGA
TCGTATTTCTCCAGAAATGAAAGAAAAGATGGGGAATCTGGCTTTTCAGAGTTATCGCC
CTACTAAAAAAAATATTATTGTGATAGGTCCTGTTCCGGGTCAGAAATATAGTGAAATCG
TCTTTCCTATTCTTTCCCCCGACCCTGCTACAAAGAAAGATGTTCACTTCTTAAAATATC
CCATATATGTGGGCGGGAACAGGGGAAGGGGTCAGATTTATCCCGATGGGAGCAAGAG
TAACAATACAGTCTATAATGCTACAGCCGCTGGTGTGGTAAGCAGAATCCTACGTAAGG
AAAAGGGGGGTTATGAAATAACCATAGCTGATGCATCGGATGGACATCAAGTGGTTGAT
ATTATACCTCCAGGACCAGAACTTCTTGTTTCGGAGGGTGAATCCATTAAGCTTGATCA
ACCATTAACGAGTAATCCCAATGTGGGCGGATTCGGGCAGGGCGATGCAGAAATAGTA
CTTCAAGATCCATTACGCGTCCAAGGTCTTTTGTTCTTCTTGGCATCTGTTATTTTGGCA
CAAATCTTTTTGGTTCTTAAAAAGAAACAGTTTGAAAAGGTTCAATTGTACGAAATGA
ATTTTATGAGTAAAGTATATGATTGGTTCGAGGAACGTCTCGAGATTCAGGCGATTGCA
GATGATATAACTAGTAAATATGTTCCTCCTCATGTCAACATATTTTATTGTCTAGGAGGGA
TCACACTTACTTGTTTTTTAGTACAAGTAGCTACGGGCTTTGCTATGACTTTTTACTATC
GTCCAACTGTTACAGATGCTTTTGCATCTGTTCAATATATAATGACTGAGGTCAACTTTG
GTTGGTTAATACGATCAGTTCATCGATGGTCAGCAAGTATGATGGTACTAATGATGATCC
TGCACGTATTTCGTGTGTATCTTACAGGTGGATTTAAAAAACCCCGTGAATTGACTTGG
GTTACAGGTGTGGTTCTGGGTGTATTGACTGCATCTTTTGGTGTAACTGGTTATTCTTTA
CCTTGGGACCAAATCGGTTATTGGGCAGTAAAAATTGTTACAGGCGTACCTGACGCTAT
TCCTGTAATAGGATCTCCTTTGGTAGAGTTATTACGTGGAAGTGCTAGTGTGGGTCAAT
CCACTTTGACTCGTTTTTATAGTTTACACACTTTTGTCTTACCTCTTCTTACTGCCGTATT
TATGTTAATGCACTTCCCAATGATACGTAAGCAAGGGATTTCAGGTCCTTTAATGGGAGT
AACAAAGAAACCTGACTTGAATGATCCTGTATTAAGAGCTAAGTTGGCTAAAGGGATG
GGACATAATTATTATGGAGAACCCGCGTGGCCCAACGACCTTTTATATATCTTTCCAGTA
GTAATTTTAGGTACTATTGCGTGTAATGTGGGCCTGGCGATTCTAGAACCATCAATGATT
GGTGAACCCGCGGATCCATTTGCAACTCCTTTGGAAATATTACCCGAATGGTACTTTTTT
CCCGTATTTCAAATACTCCGTACAGTTCCCAATAAGTTACTGGGTGTTCTTTTAATGGCT
TCAGTACCGCTGGGATTATTTACAGTACCCTTTTTGGAGAATGTTAATAAATTCCAAAAT
CCCTTTCGTCGTCCAGTAGCTACAACAGTCTTTTTGATCGGTACTGCAGTAGCCCTTTG
GTTAGGTATTGGAGCCACATTACCTATTGATAAATCCCTAACTTTAGGTCTTTTTATGATT

GAAGTTTTTCTATTTGGAATCGTATTAGGTCTAATTCCTATTACTTTGGCAGGATTGTTCG
TAACTGCATATTTACAATACAGACGCGGTGATCAGTTGGACCTTATGCCTACTATAACTA
GTTATTTCGGTTTTCTACTGGCTGCTTCAACTATAACCACAGCTCTATTCATTGGTCTGA
GCAAGATACGGCTAATTATGGATATTGTAAGTCTTGCTTGGGCTGCTTTAATGGTAGTCT
TTACATTTTCCCTTTCACTCGTAGTATGGGGAAGAAGTGGACTCATGATTATTCGTTCGC
CGGAACCAGAAGTGAAAATTGTGGTAGATAGGGATCCCATAAAAACTTCTTTCGAGGC
ATGGGCCAGACCCGGCCATTTTTCAAGAACAATAGCTAAAGGCCCTGATACTACCACTT
GGATCTGGAACCTACATGCTGATGCTCACGATTTCGATAGCCATACCAGTGATTTGGAG
GAGATTTCTCGAAAAGTATTTAGTGCTCATTTCGGTCAACTCTCCATCATCTTTCTTTGG
CTGAGTGGCATGTACTTCCATGGCGCCCGTTTTTCTAATTATGAAGCATGGCTGAGTGAT
CCTACTCACATTAGACCCAGTGCCCAGGTAGTTTGGCCAATAGTGGGTCAAGAAATTTT
GAATGGTGATGTGGGCGGGGGTTTCCGCGGAATACAAATAACCTCTGGTTTTTTTCAAA
TTTGGCGAGCATCTGGAATAACTAGTGAATTACAACTTTATTGTACCGCAATTGGCGCG
TTGGTCTTTGCAGCGTTAATGCTTTTTGCAGGTTGGTTCCATTATCACAAAGCTGCTCC
AAAATTGGCTTGGTTCCAAGATGTCGAATCTATGTTGAATCACCACTTAGCGGGGTTAC
TAGGACTGGGGTCTCTTTCTTGGGCGGGACACCAAATACATGTATCTTTGCCGATTAAC
CAATTTCTCGACGCGGGAGTGGATCCTAAAGAGATACCACTTCCTCATGAATTTATATTG
AATCGGGATCTTTTGGCTCAACTTTATCCAAGTTTTGCCGAAGGAGCAACTCCATTTTT
CACCTTGAATTGGTCAAAATATGCAGAATTTCTTACTTTTCGGGGAGGATTAGATCCTAT
AACAGGCGGCCTATGGTTGAGCGATATTGCGCACCATCATTTAGCTATTGCAATTCTTTT
CCTGATAGCTGGTCATATGTATAGAACCAACTGGGGCATTGGTCATGGACTAAAAGATA
TTTTGGAGGCTCATAAGGGTCCATTTACGGGCCAAGGTCATAAAGGCCTTTATGAAATC
CTAACCACGTCATGGCATGCTCAATTAGCTCTTAACCTGGCTATGTTAGGCTCTTTAACC
ATTGTTGTAGCTCACCATATGTATTCCATGCCCCCCTATCCATACCTAGCTACTGACTATG
GTACACAACTTTCGTTGTTCACACATCACATGTGGATCGGTGGATTTCTCATAGTTGGT
GCTGCTGCACATGCAGCCATTTTTATGGTAAGAGACTACGATCCAACTACTCGATACAA
CGATCTATTAGATCGTGTCCTTAGGCACCGTGATGCAATCATATCACATCTAAACTGGGC
ATGTATATTTCTAGGCTTTCACAGTTTTGGTTTGTATATTCATAATGATACCATGAGTGCC
TTAGGGCGCCCCCAGGATATGTTTTCAGATACCGCTATACAATTACAACCCATCTTTGCT
CAATGGGTACAAAACACCCATGCTTTAGCACCTGTTGCGACAGCTCCTGGTGCAACAA
CAGGCACCAGCTTAGCTTGGGGGGGTGGTGAGTTAGTAGCAGTAGGCGGCAAAGTAG
CTTTGTTACCTATTCCATTAGGAACTGCGGATTTCTTGGTACATCACATTCATGCATTTAC
GATCCATGTGACTGTATTGATACTACTGAAAGGTGTTCTGTTTGCTCGGAGCTCCCGTTT
AATACCTGATAAAGCGAATCTTGGCTTTCGTTTCCCTTGTGATGGTCCTGGAAGAGGAG
GAACATGTCAAGTATCCGCCTGGGATCATGTCTTCTTAGGTCTATTCTGGATGTACAATT
CAATTTCGGTAGTCATATTCCATTTCAGTTGGAAAATGCAATCGGATGTTTGGGGTAGTA
TAAGCGATCAAGGGGTGGTAACTCATATCACAGGAGGAAACTTTGCACAGAGTTCCAT
TACTATTAATGGGTGGCTTCGGGATTTCTTATGGGCACAGGCATCTCAGGTAATTCAGTC
TTATGGTTCTTCATTATCTGCATATGGTCTTTTTTTCCTAGGTGCTCATTTTGTATGGGCTT
TCAGTTTAATGTTTCTATTCAGCGGCCGTGGTTATTGGCAAGAACTTATTGAATCCATCG
TTTGGGCTCATAACAAATTAAAAGTTGCTCCTGCTACTCAGCCTAGAGCCTTGAGCATT
GTACAAGGACGTGCTGTAGGAGTAACTCATTACCTTCTGGGTGGAATTGCCACAACAT
GGGCATTCTTCTTAGCAAGAATTATTGCAGTAGGAATGGCATTAAGATTTCCAAGGTTT
AGCCAAGGCTTAGCTCAGGACCCCACTACTCGTCGTATTTGGTTTGGTATTGCTACCGC

ACATGACTTCGAGAGTCATGATGATATTACTGAGGAACGTCTTTATCAGAACATTTTTGC
TTCTCACTTTGGTCAGTTAGCAATAATCTTTCTGTGGACTTCCGGAAATCTGTTTCATGT
AGCTTGGCAAGGAAATTTTGAGTCATGGGTACAGGACCCTTTACACGTAAGACCTATT
GCTCACGCAATTTGGGATCCTCATTTTGGTCAACCGGCTGTCGAAGCCTTTACTCGAGG
AGGTGCTCCTGGACCAGTGAATATCGCTTATTCCGGCGTTTATCAGTGGTGGTATACAAT
CGGATTACGCACCAATGAAGATCTTTATACTGGAGCTCTTTTTCTATTATTTCTTTCTGCT
ATATCCTTAATAGGGGGTTGGTTACACCTACAACCCAAATGGAAACCAAGCGTTTCTTG
GTTCAAAAATGCGGAATCCCGTCTGAATCATCATTTGTCAGGACTTTTCGGAGTAAGTT
CCTTGGCTTGGACAGGGCATTTAGTTCATGTCGCTATTCCCGCATCAAGGGGCGAGTAT
GTCAGATGGAATAATTTCTTAGATGTATTACCGCACCCCCAAGGTTTGGGACCACTTTTT
AGCGGTCAGTGGAATCTTTATGCCCAAAACCCCGATTCCAGTAGTCATTTATTCGGTAC
CTCCCAAGGAGCGGGAACTGCCATTCTAACTCTTCTCGGGGGATTCCATCCGCAAACG
CAAAGTTTATGGCTGACCGATATGGCTCATCATCATTTAGCTATTGCATTTATTTTCCTGA
TAGCTGGTCATATGTATAGAACCAACTTCGGGATTGGGCACAGTATAAAAGATCTTTTA
GAAGCACATACTCCTCCGGGGGGTCGATTGGGGCGTGGGCATAAGGGTCTTTATGATAC
AATCAATAATTCGCTTCATTTTCAATTAGGTCTTGCTCTGGCCTGTTTAGGAGTTATTACT
TCCTTGGTAGCTCAACACATGTACTCTTTACCTGCTTACGCATTCATAGCGCAAGACTTT
ACTACTCAAGCTGCGTCGTATACTCATCACCAATACATCGCAGGTTTCATCATGACGGG
AGCCTTTGCTCACGGAGCTATATTCTTCATTAGAGATTACAACCCGGAACAGAATGAAG
ATAATGTATTGGCGAGAATGTTAGACCATAAAGAAGCTATCATATCTCATTTAAGTTGGG
CCAGTCTGTTTTTGGGGTTCCATACCTTGGGACTTTATGTTCATAACGACGTCATGCTCG
CTTTTGGGACTCCGGAAAAACAAATCTTGATTGAACCTATATTTGCCCAGTGGATACAA
TCCGCTCATGGTAAGACTTTATATGGGTTTGATGTACTCTTATCTTCAACGAGTGGCCCA
GCATTCAATGCTGGTCGAAGCATTTGGTTACCCGGTTGGTTGAGTGCTGTTAATGAG----
--AATAGTAATTCACTATTCTTAACAATAGGTCCTGGAGACTTCTTGGTTCATCATGCTAT
TGCTCTAGGTTTGCATACAACTACATTGATCTTAGTAAAGGGTGCTTTAGATGCGCGTG
GTTCGAAGTTAATGCCAGATAAAAAAGATTTCGGTTATAGTTTTCCTTGCGACGGCCCG
GGACGGGGGGTACTTGTGATATTTCTGCTTGGGATGCATTTTATTTGGCGGTTTTCTGG
ATGTTAAATACCATTGGATGGGTTACTTTTTATTGGCATTGGAAGCACATCACATTATGG
CAAGGTAATGTTTCACAATTTAACGAATCCTCTACTTATTTGATGGGATGGTTAAGAGAT
TATCTATGGTTAAACTCTTCCCAACTTATCAACGGATACAATCCGTTTGGTATGAATAGT
TTATCGGTCTGGGCATGGATGTTCTTATTTGGACATCTTGTTTGGGCTACTGGATTTATGT
TCTTAATTTCCTGGCGTGGATATTGGCAGGGATTAATTGAAACTTTAGCATGGGCTCATG
AACGCACACCTTTGGCTAATTTGATTCGATGGAGAGATAAGCCAGTGGCTCTTTCCATC
GTGCAAGCAAGATTGGTTGGATTAGCCCACTTTTCCGTAGGTTATATATTCACTTATGCA
GCTTTCTTGATTGCCTCTACATCAGGTAAATTTGGTATGTCACATTCAGTAAAGATTTAT
GATACATGTATAGGGTGTACTCAATGTGTACGAGCCTGTCCTACAGATGTATTGGAAATG
ATACCCTGGGACGGATGTAAAGCTAAGCAAATTGCTTCTGCGCCAAGAACAGAGGACT
GTGTAGGTTGTAAGAGATGCGAATCCGCCTGTCCAACAGACTTCTTGAGTGTCCGGGT
TTATTTAGGGAATGAGACAACTCGCAGCATGGGTCTACCTTATATGACAGATCTCAACTT
ACCCTCTATTTTTGTGCCTTTAGTAGGCCTAGTCTTTCCGGCAATTGCAATGGCTTCTTT
ATTTCTTCATGTTCAAAAAAACAAGATTGTCATGCAAGATATAAAAACATATCTCTCTGT
GGCACCTGTGCTAACTACTCTATGGTTTGGGTCTTTAGCAGGTTTATTGATAGAGATCAA
TCGTTTATTCCCGGATGCCTTGTCATTCCCATTTTTT------ATGACTGCAATTTTAGAGAG

ACGCGAAAGTACAAGCCTATGGGGTCGCTTCTGTAACTGGATAACCAGCACCGAAAAC
CGCCTTTACATTGGATGGTTCGGTGTTTTAATGATCCCTACCTTATTGACCGCAACTTCT
GTATTTATTATCGCCTTCATCGCTGCTCCTCCAGTAGATATTGATGGTATCCGTGAACCTG
TTTCCGGGTCTTTACTTTATGGAAACAACATTATTTCTGGTGCCATTATTCCTACATCTGC
AGCTATAGGTTTGCACTTTTACCCAATTTGGGAAGCAGCATCCGTTGATGAGTGGTTAT
ACAATGGTGGTCCTTATGAACTAATTGTTCTACACTTCTTACTTGGTGTAGCTTGTTATAT
GGGTCGTGAGTGGGAACTTAGTTTCCGTCTGGGTATGCGTCCTTGGATTGCTGTCGCAT
ATTCAGCTCCTGTTGCAGCTGCTACTGCTGTTTTCTTGATCTACCCTATTGGTCAAGGAA
GCTTCTCTGATGGTATGCCTCTAGGAATATCTGGTACTTTCAACTTTATGATTGTATTCCA
GGCTGAACACAACATCCTTATGCATCCATTCCACATGTTAGGCGTAGCTGGTGTATTCG
GCGGCTCCCTATTTAGTGCTATGCATGGTTCCTTGGTAACCTCTAGTTTAATCAGGGAAA
CTACTGAAAATGAATCTGCTAATGAAGGTTACAGATTCGGTCAAGAGGAAGAAACTTAT
AATATCGTCGCTGCTCACGGTTATTTTGGCCGATTAATCTTCCAATATGCTAGTTTCAAC
AATTCCCGTTCCCTACATTTCTTCTTGGCTGCTTGGCCTGTAGTAGGTATCTGGTTCACT
GCTTTAGGTATTAGTACTATGGCTTTCAACCTAAACGGTTTCAATTTCAACCAATCCGTA
GTTGACAGTCAAGGTCGTGTTATTAACACTTGGGCTGATATCATCAACCGTGCTAACCT
TGGTATGGAAGTAATGCATGAACGTAATGCTCACAACTTCCCTCTAGACCTAGCTTCTG
TTGAAGCACCTTCTACAAATGGCATGGGTTTGCCTTGGTATCGTGTTCATACCGTCGTAT
TGAATGATCCCGGTCGGTTGCTTTCTGTCCACATAATGCATACAGCTCTAGTTGCTGGTT
GGGCCGGGTCAATGGCTCTATACGAATTAGCGGTTTTTGATCCCTCTGACCCCGTTCTT
GATCCAATGTGGAGACAAGGTATGTTCGTTATACCCTTCATGACTCGTTTAGGAATAAC
CAATTCGTGGGGTGGTTGGAGTATCACA---GGAGGAACTATAACGAATCCGGGTATTTG
GAGTTACGAAGGTGTGGCAGGGGCACATATTGTGTTTTCTGGCTTGTGCTTTTTAGCAG
CTATTTGGCATTGGGTATATTGGGACTTAGAAATATTCTGTGATGAACGTACGGAAAA
CCCTCTTTGGATTTGCCCAAGATCTTTGGAATTCATTTATTTCTCTCAGGAGTGGCTTGC
TTTGGCTTTGGCGCATTTCATGTAACAGGCTTGTATGGTCCTGGAATATGGGTGTCTGAT
CCTTATGGACTAACCGGAAAAGTACAACCCGTAAATCCAGCTTGGGGCGCGGAAGGTT
TTGATCCTTTTGTTCCGGGCGGAATAGCCTCTCATCATATTGCAGCGGGGACTTTGGGC
ATACTGGCAGGCCTATTCCACCTTAGTGTCCGTCCACCCCAGCGTCTATACAAAGGATT
ACGTATGGGCAATATTGAAACTGTACTTTCGAGTAGTATCGCTGCTGTGTTTTTTGCAGC
TTTCGTAGTTGCGGGAACTATGTGGTATGGTTCAGCAACTACCCCCATCGAATTATTTGG
TCCGACGCGTTACCAGTGGGATCAAGGATACTTCCAGCAAGAAATATATCGAAGAGTT
GGTGCTGGACTAGCCGAAAATCTGAGTTTATCGGAAGCTTGGTCTAAAATTCCTGAAA
AATTAGCTTTTTATGATTACATCGGTAATAATCCGGCAAAAGGTGGATTATTTAGAGCAG
GCTCAATGGACAACGGAGATGGAATAGCCGTTGGATGGTTAGGACACCCCGTCTTTCG
AGATAAGGAAGGGCGTGAACTTTTTGTACGCCGTATGCCTACTTTTTTTGAAACATTTC
CGGTAGTTTTGGTAGATGGAGACGGAATTGTGCGAGCCGATGTTCCTTTTCGAAGGGC
AGAATCCAAATATAGTGTCGAACAAGTAGGCGTAACTGTTGAGTTCTATGGTGGCGAA
CTCAATGGAGTCAGTTATAGCGATCCTGCTACTGTTAAAAAATATGCTAGACGTGCCCA
ATTAGGTGAAATTTTTGAATTAGATCGCGCTACTTTAAAATCCGATGGTGTTTTTCGTAG
CAGTCCGAGGGGTTGGTTCACTTTTGGACATGCTTCATTTGCTTTACTCTTCTTTTTCGG
GCACATTTGGCATGGAGCTAGAACCTTGTTCAGAGATGTTTTTGCTGGTATTGATCCAG
ATTTGGATGCTCAAGTGGAATTTGGGGCATTCCAAAAAATTGGAGATCCAACTACAAG
GCGACAAGCAGTTATGAAAACCTTATATTCCCTGAGGAGGTTCTACCCCGTGGAAACG

CTCTTTAATGGAACTTTAGCTTTAGCTGGTCGTGACCAAGAAACCACTGGTTTCGCTTG
GTGGGCCGGGAATGCCAGACTTATCAATTTGTCCGGTAAACTACTCGGGGCTCACGTA
GCCCATGCCGGATTAATCGTATTCTGGGCAGGAGCAATGAACCTCTTTGAAGTGGCTCA
TTTCGTACCAGAGAAGCCCATGTATGAACAAGGATTAATTTTACTTCCCCATCTAGCTAC
TCTAGGTTGGGGGGTAGGTCCGGGTGGGGAAGTTATAGACACCTTTCCATACTTTGTAT
CTGGAGTACTTCATCTAATTTCCTCTGCAGTCTTAGGCTTTGGCGGTATTTATCATGCGC
TTCTTGGACCTGAGACTCTAGAAGAATCCTTTCCGTTCTTCGGTTATGTATGGAAAGAT
AGAAATAAAATGACCACAATTTTAGGTATTCATCTAATTTTGTTAGGGATAGGTGCTTTT
CTTCTAGTATTCAAGGCTCTTTATTTTGGGGGTGTATATGACACCTGGGCTCCTGGGGGG
GGAGATGTAAGAAAAATAACCAACTTAACACTTAGCCCAAGTGTTATATTTGGTTATTT
ACTAAAATCTCCCTTTGGAGGAGAAGGATGGATTGTTAGTGTGGACGATTTAGAAGATA
TAATTGGAGGACATGTATGGTTAGGTTCCATTTGTATACTTGGTGGAATCTGGCATATCT
TGACCAAACCCTTTGCATGGGCTCGCCGCGCATTTGTATGGTCTGGAGAGGCTTACTTG
TCTTATAGTTTAGGTGCTTTAGCTGTCTTTGGGTTCATCGCTTGTTGTTTCGTCTGGTTC
AATAATACCGCCTATCCTAGTGAGTTTTATGGACCTACTGGGCCGGAGGCTTCTCAAGC
TCAAGCATTTACTTTTCTAGTTAGAGACCAACGTCTTGGGGCTAACGTGGGATCCGCTC
AGGGACCTACTGGTTTAGGTAAATATCTAATGCGGTCCCCAACGGGAGAGGTCATTTTT
GGAGGAGAAACCATGCGCTTTTGGGATCTCCGTGCTCCTTGGTTGGAACCTCTAAGGG
GTCCCAATGGTTTGGACTTGAGTAGGCTGAAAAAAGACATACAACCTTGGCAAGAAC
GACGTTCGGCGGAATATATGACTCATGCTCCTTTAGGGTCTTTAAATTCCGTGGGTGGT
GTAGCTACCGAGATCAATGCAGTCAATTATGTCTCTCCTAGAAGTTGGTTAGCTACTTCT
CATTTTGTTCTAGGATTCTTCTTTTTTGTGGGTCATTTGTGGCACGCAGGAAGAGCCCG
CGCAGCGGCAGCAGGATTTGAAAAAGGAATCGATCGTGATTTAGAACCTGTTCTTTCC
ATGACTCCTCTTAATATGACTATAGCCCTTGGTAGATTTACCAAAGAGGAA---GATTTATT
CGATATTATGGACGACTGGTTACGGAGAGACCGTTTCGTTTTTGTAGGTTGGTCCGGTC
TATTGCTCTTCCCTTGTGCTTATTTCGCTTTAGGAGGTTGGTTCACAGGTACAACTTTTG
TAACTTCGTGGTATACCCACGGATTGGCTAGTTCATATTTAGAAGGCTGCAATTTCTTAA
CCGCTGCAGTTTCTACTCCTGCTAATAGTTTAGCACATTCTTTGTTGCTACTATGGGGTC
CTGAAGCACAAGGCGATTTTACTCGTTGGTGTCAATTAGGCGGTCTGTGGACTTTTGTT
GCTCTACACGGTGCTTTCGGACTAATCGGTTTCATGTTACGTCAATTCGAACTTGCTCG
ATCTGTTCAATTGCGACCTTATAATGCAATCGCATTTTCTGGTCCAATTGCTGTTTTCGTT
TCTGTATTCCTAATTTATCCACTGGGTCAGTCTGGTTGGTTCTTTGCACCCAGTTTTGGT
GTGGCAGCTATATTTCGATTCATCCTCTTCTTCCAGGGGTTTCATAATTGGACCCTAAAC
CCATTTCATATGATGGGAGTTGCCGGAGTATTGGGTGCTGCTCTGCTATGCGCTATTCAT
GGTGCTACCGTAGAAAATACTTTATTTGAGGACGGTGACGGCGCAAATACATTCCGTGC
TTTTAACCCAACGCAAGCCGAAGAGACTTATTCGATGGTCACTGCTAACCGATTTTGGT
CCCAAATCTTTGGGGTTGCTTTTTCTAATAAACGTTGGTTACATTTCTTTATGTTATTTGT
ACCAGTAACCGGTTTATGGATGAGTGCCCTTGGAGTAGTTGGTCTGGCTCTGAACCTAC
GTGCCTATGACTTCGTTTCCCAGGAAATCCGTGCCGCGGAAGATCCTGAATTTGAGACT
TTCTACACCAAAAATATTCTCTTAAATGAGGGTATTCGTGCTTGGATGGCAGCTCAGGA
TCAGCCTCATGAAAACCTTATATTCCCTGAGGAGGTTCTACCCCGTGGAAACGCTCTTA
TGTCTGGAAGCACGGGAGAACGCTCTTTTGCTGATATTATTACCAGTATTCGATACTGG
GTTATTCATAGCATTACTATACCTTCCCTATTCATTGCGGGTTGGTTATTCGTCAGTACAG
GTTTAGCTTACGATGTGTTTGGAAGTCCTCGTCCAAACGAGTATTTTACAGAAAGCCGG

CAAGGGATTCCATTAATAACTGGCCGTTTTGATCCTTTGGACCAACTTGATGAATTTAGT
AGATCCTTTATGACCATAGATCGAACCTATCCAATTTTTACAGTAAGATGGTTGGCTGTT
CATGGACTAGCTGTACCTACCGTTTCTTTTTTGGGGTCAATATCAGCAATGCAGTTCATC
CAACGAATGGCTACACAAACCGTTGAGGGTAGTTCTAGATCTGGG---CCAAGACGAAC
AATTGTAGGGGATTTATTGAAACCATTGAATTCAGAATATGGTAAAGTAGCCCCGGGAT
GGGGAACTACTCCTTTTATGGGAGTCGCAATGGCCCTATTTGCGATATTCCTATGTATTAT
TTTGGAGATTTATAATTCTTCCGTTTTACTGGATGGAGTTTCACCGAATATGCTTACTCTC
AAACTCTTCGTTTACACAGTAGTGATATTTTTTGTTTCTCTATTTATCTTCGGATTCCTAT
CTAATGATCCAGGACGTAATCCTGGACGTGAGGAAATGGCTGATACTACTGGAAGGATT
CCTCTTTGGCTGATAGGTACTGTAGCTGGTATTCCTGTGATCGGTTCAGTGGGTGTTTTC
TTTTACGGTTCATATTCCGGATTGGGCTCATCTCTGATGCTTAATATACTTAATTTGATCT
GTATCTGTCTTAATTCTGCCCCTTATTCGAGTAGTTTTTTCTGCGCTAAATTGCCCGAGG
CTTATGCTGTTTTCAATCCAATTGTAGATTTTATGCCAGTCATACCTGTACTCTTTTTTCT
CTTAGCCTTTGTTTGGCAAGCCGCTGTAAGTTTTCGCATGACACAATCAAACCCAAAC
GAACAAAATGTTGAATTGAATCGTACCAGTCTATACTGGGGGTTATTACTCATTTTTGTA
CTTGCTGTTTTATTTTCCAATTATTTCTTCAATATGGAAGTAAATATTCTCGCATTTATTGC
TACTGCACTGTTCATTCTAGTTCCGACTGCCTTTTTACTTATCATCTACGTAAAAACAGT
CAGTCAAAATGATATGGAAACAGCAACCTTAGTCGCCATCTTCATATCTGGTTTACTTGT
AAGCTTTACTGGGTATGCCTTATATACCGCTTTTGGGCAACCCTCTCAACAACTAAGAG
ATCCATTCGAGGAACACGGAGACATGGAAGCATTGGTTTATACATTCCTGTTAGTCTCG
ACTCTAGGGATAATTTTTTTCGCTATCTTCTTTCGAGAACCGCCAAAGGTTCCTACTAAA
AAGATGAAAATGACTATTGCTTTTCAATTGGCTATTTTTGCATTAATTGCGACTTCATCG
ATCTTATTGATTAGTGTACCCGTTGTATTTGCTTCTCCTGATGGTTGGTCAAGTAACAAA
AATGTTGTATTTTCCGGTACCTCATTATGGATTGGGTTAGTCTTTCTGGTAGGTATCCTTA
ATTCGCTCATCTCTATGAGTTGTAGGGAGGGACTTATGTCACCACAAACAGAGACTAAA
GCAGGTGTTGGATTCAAAGCTGGTGTTAAAGATTACAAATTGACTTATTATACTCCTGA
ATATGAAACCAAAGATACTGATATCTTGGCAGCATTTCGAGTAACTCCTCAACCCGGAG
TTCCACCCGAGGAAGCAGGGGCTGCGGTAGCTGCCGAATCCTCTACTGGTACATGGAC
AACTGTGTGGACCGATGGACTTACCAGCCTTGATCGTTACAAAGGCCGATGCTACCAC
ATCGAGCCCGTTGTTGGGGAGCAAAATCAATATATTGCTTATGTAGCTTATCCTTTAGAC
CTTTTTGAAGAAGGTTCTGTTACTAACATGTTTACTTCCATTGTGGGTAATGTATTTGGG
TTCAAAGCCCTACGAGCTCTACGTTTGGAGGATCTTCGAATTCCCCCTGCTTATTCCAA
AACTTTCCAAGGCCCGCCCCATGGAATCCAGGTTGAGAGAGATAAATTGAACAAGTAT
GGTCGTCCCCTATTGGGATGTACTATTAAACCAAAATTGGGGTTATCCGCGAAGAACTA
CGGTAGAGCAGTTTATGAATGTCTCCGTGGTGGACTTGATTTACCAAGGATGATGAGA
ACGTGAACTCACAGCCATTTATGCGTTGGAGAGACCGTTTCTTATTTTGTGCTGAAGCA
ATTTATAAAGCGCAAGCGGAAACAGGTGAAATCAAAGGACATTATTTGAATGCTACTG
CGGGTACATGTGAAGAAATGATGAGAAGGGCCCAATGTGCCAGAGAATTGGGAGTTCC
TATCGTAATGCATGACTACTTAACAGGTGGATTCACTGCAAATACTAGCTTGGCTATTTA
TTGCCGAAACAACGGCCTACTTCTTCACATCCATCGTGCAATGCATGCAGTTATTGATA
GACAGAAGAATCATGGTATGCACTTTCGTGTACTAGCTAAAGCGCTACGTATGTCTGGT
GGAGATCACATTCACGCCGGTACAGTAGTAGGTAAACTGGAAGGGGAACGTGAAATG
ACTTTAGGTTTTGTTGATTTACTACGTGATGATTATATTGAAAAAGACCGAAGTCGTGGT
ATTTTTTTCACTCAAGATTGGGTCTCTATGCCAGGTGTTTTGCCCGTAGCTTCCGGTGGT

ATTCACGTTTGGCATATGCCTGCCTTGACCGAGATCTTTGGGGATGATTCTGTACTACAG
TTCGGTGGAGGAACTTTAGGACACCCTTGGGGAAATGCACCTGGTGCAGTAGCCAATC
GGGTAGCTTTAGAAGCGTGTGTACAAGCTCGTAATGAGGGACGTGATCTTGCTCGCGA
AAGTACTCAAATTATCCGTGAAGCTTGTAAATGGAGCCCTGAGCTAGCCGCTGCTTGTG
AAGTATGGAAGGAAATCAAATTTGAGTTCGAACCAGTAGATAAGCTAGATGTTACGGC
GATACATTTATACAAAACTTCTACCTCGAGCACACGCAATGGAGCCGTAGACAGTCAA
GTGAAATCCAATCCACGAAATAATTTGATCTATGGACAGCATCATTGTGGTAAAGGTCG
TAATGCCAGAGGAATCATTACCGCAGGGCATAGAGGGGGAGGTCATAAGCGTCTATAC
CGTAAAATCGATTTTCGACGGAATAAAAAAGACATATCTGGTAGAATCGTAACCATAGA
ATACGACCCTAATCGAAATGCATACATTTGTCTCATACACTATGGGGATGGTGAGAAGA
GATATATTTTACATCCCAGAGGGGCTATAATTGGAGATACCATTGTTTCTGGTACAGAAG
TTCCTATATCAATGGGAAATGCCCTACCTTTGACCGATATGCCCTTAGGCACGGCCATAC
ATAACATAGAAATCACACTTGGAAAGGGTGGACAATTAGCTAGAGCAGCGGGTGCTGT
AGCGAAACTGATTGCAAAAGAGGGTAAATCGGCCACATTAAGATTACCATCCGGGGAG
GTCCGTTTGATATCCAAAAACTGCTCAGCAACAGTCGGACAAGTAGGTAATGTTGGGG
CGAACCAACAAAGTTTGGGTAGAGCCGGATCTAAGTGTTGGCTAGGTAAGCGTCCTGT
AGTAAGAGGAGTAGTTATGAACCCTGTGGACCATCCCCACGGGGGTGGTGAAGGGAG
AGCCCCAATTGGTAGAAAAAAACCCACAACCCCTTGGGGTTACCCTGCGCTTGGAAG
AAGAAGTAGGAAAAGGAATAAATATAGTGATCGTTTTATTCTTCGTCGCCGTAAA---ATG
ATTCAACCTCAGACCCTTTTAAATGTAGCGGATAACAGCGGGGCTCGAGAATTGATGTG
TATTCGAATCATAGGAGCCAGT---AATCGCCGATATGCTCATATTGGTGACGTTATTGTTG
CTGTAATCAAAGAAGCAGTACCCAATATGTCTCTAGAAAGATCAGAAGTGGTCAGAGC
TGTAATTGTACGTACATGTAAAGAACTCAAACGTGACAACGGTATGATAATACGATATG
ATGACAATGCAGCAGTTGTCATTGATCAAGAAGGGAATCCAAAAGGAACTCGAGTTTT
TGGTGCGATCGCTCGGGAATTGAGACATTTGAATTTCACTAAAATAGTTTCCCTAGCTC
CTGAGGTATTAATGCTTAGTCCCAAAAGAACCAGATTCCGTAAACAACATAGAGGAAG
AATGAAGGGAATGTCTTATCGAGGCAGTCATATTTGTTTCGGAAGATACGCTCTTCAGG
CACTTGAACCATCTTGGATCACATCTAGACAAATAGAAGCGGGGCGAAGGGCAATGAC
ACGATATGCACGTCGTGGTGGAAAAATCTGGGTACGTATATTTCCGGACAAACCCGTTA
CAATAAGACCCGCGGAAACACGTATGGGTTCGGGGAAGGGATCCCCCGAATACTGGGT
ATCCGTTGTTAAACCGGGTCGAATACTTTATGAGATGGGTGGAGTATCAGAAACTGTAG
CCAGAGCCGCTATTGAAATAGCCGCGTCCAAAATGCCTATAAGAACGCAATTTATTATT
GCGGGAATGACCAGAGTTAGGCGAGGATATATAGCTCGGAGACGTCGAACAAAAATTC
GTTTATTTGCAGCAACCTTTCGAGGGGCTCATTCAAGACTTACTCGAGCTGCCACTCAA
CAGAAAATGAGAGCTTTGGTTTCTGCTCATCGAGATAGAGGCAAGCAAAAGAGAGATT
TTCGTCGTTTGTGGATCACTCGGATAAATGCGGTAACTCGTGAGAAT------GTATGCTATA
GTTATAGTCGATTAATGCACAATCTGTATAAGAGGCAGTTGCTTCTTAATCGTAAAATAC
TTGCACAAATAGCTATATTAAATAAGAATTGTCTTCACATAATTTCCAATGAGATCATCA
AA---------------------------------ATGATAAAGCGGGCTTTA---GCTCAAAATATATCT---ATGTC
TGCTTACAAAGCGAGAAGAGTAATTGATCAGATTCGTGGGCGTTCCTATGAGGAAACA
ATTATGATACTAGAACTGATGCCTTATCGAGCATCTTTTCCTATTTTAAAATTGGTTTACT
CTGCAGCAGCAAATGCTATTAACAATATGGGTTTGAGCGAAGCTGATTTATTCATTAGTA
AAGCAGAAGTCAATGGAGGTACTTTCGTGAAAAAGTTACGACCCCGAGCTCGGGGAC
GTAGTTATGCGATAAAAAGGCCCACTTGTCATATAACAATTGTATTGAAG---AAAGATAA

ATCG--------------------ATGGATGGAATCAAATATGCAGTATTTACCGAAAAAAGTATTCGG
TTATTGGGGAACAATCAA---TATACTTCTAATGTCGAATCAGGATCAACTAGGACAGAAA
TAAAGCATTGGGTCGAACTCTTCTTTGGCGTCAAGGTAATAGCTATGAATAGTCATCGA
CTCCCGGGAAAGGGTAGAAGAGTGGGACCTATTAAGGGACATACAATGCATTACAGAC
GTATGATCATTACGCTTCAACCGGGTTATTCTATTCCACCTCTTATAGAGAAAAGAACTA
TGGCGGTTCCAAAGAAACGTACTTCTATATCAAAAAAACGTATTCGTAAAAATTTTTGG
AAGAAAAAGGGATATTTTGCCGCGGTAAAAGCTTTTTCTTTAGCTAAATCAGTTTCCAC
CGGACAGTCAAAAAGTTTTTTTGTGCGACAAACAAGTAATATGGCCAAGGGTAAAGAT
GCCAGATTAACAGTTATTTTGGAATGTACCAGTTGTGTCCGAAATGGT------GTCAATAA
GGAA------GGTATTTCCAGATATATTACTCAAAAAAATCGACACAATACATCGAGTCGAT
TAGAATTGAGAAAATATTGTCGCTATTGTTATAAGCATACGATTCACGGGGAAATAAAG
AAAATGAAAATAAGAGCCTCTGTTCGTAAAATTTGCGAAAAATGTCGACTAATCCGTA
GGCGGGGACGAATTATAGTTATTTGTTATAATCCGAAACATAAACAAAGACAGGGGATG
GTTCGAGAGGAAGTAGTAGGATCCACTCGGGCACTACAGTGGAAGTGTGTTGAATCAA
GAGCAGACAGTAAGCGCCTTTATTATGGTCGTTTCATTCTGTCCCCACTTCTGAAAGGA
CAAGCCGATACTATAGGTATCGCCATGCGAAGGGCTTTACTTGGAGAAATAGAAGGAA
CATGTATCACACGTGCAAAATCTGAG------AAGGTACCACATGAATATTCTACGATAGCG
GGCATTGAAGAATCAGTACATGAAATTTTAATGAATTTGAAAGAAATTGTATTGAGAAG
TAATCTTTATGGAACTCGAGACGCATCTATTTGCGTCAGAGGTCCTAGATGCGTAACTG
CTCAAGACATCATCTCACCACCTTCCGTAGAAGTAGTTGATACTACACAACATATAGCT
AGCCTGACGGAACCGATTGATTTGTGTATTGGATTACAAATCCAGAGGGATCGCGGATA
TCGTATGAAAACCACAAAAAACTCTCAAGATGGAAGTTAT------CCGATAGATGCTGTAT
CCATGCCTGTTCGAAATGCGAATCATAGTATTCATTCTTATGGAAATGGTAATCAGAAAC
AAGAGATACTTTTCCTCGAAATATGGACAAATGGAAGTTTAACTCCTAAAGAAGCACTT
CACGAAGCTTCCCGTAATTTGATTGATTTATTTATTCCTTTTCTACATGCAGAGGAAGAG
------GACATTAATTTCGAGGAAAATCAA---AACAGGTTTACTGTACCCCCTTTTACCTTTC
CTGATAGATTGGCT---AATCTAAAGAAAAACAAAAAA---GAAATTGCATTGAAATGTATT
TTTATTGACCAATCAGAATTGCCTCCCAGGACCTATAATTGTCTCAAAAGGTCCAATATA
CATACATTATTGGACCTTTTGAGTAACAGTCAAGAAGATCTTATGAAAATTGAATATTTT
CGAATAGAAGATGTAAAACAGATATTGGACACTCTACAGAAGCAT------TTCGCAATTGA
TTTACCTAAGAATAAGATGCCCCGGGAT---GGAAATGAGGGAATGTTCACAATACCTGG
ATTTAGTCAGATCCAATTTGAGGGATTTTGTAGGTTCATTGATCAGGGCTTGATGGAAG
AATTTCATAAGTTTCCAAAAATTGAAGATACAGACCAAGAAATTGAATTTCAATTATTT
GTGGAAAGATATCAATTGGTAGAACCCTTGATAAAAGAAAGAGATGCTGTATATGAATC
ACTCACATATTCCTCGGAATTATATGTACCCGCGGGATTAATTTGGAAAACCGGTAGAGA
TATGCAAGAACAAACCATATTTATTGGAAACATTCCTCTAATGAATTCCCTGGGAACCTT
TATAGTAAATGGAATATACAGAATTGTAATCAATCAAATATTGCAAAGCCCCGGTATTTA
CTACCGTTCAGAATTGGACCATAACGGAATTTCTGTTTATACCAGTACCATAATATCAGA
TTGGGGAGGAAGATCAGAATTAGAAATCGATAGAAAATCAAGGATATGGGCCCGTGTG
AGTAGGAAACAAAAAATATCTATTCTAGTTTTATCATCAGCTATGGGTTCGAATCTAAGA
GAAATTCTAGATAATGTTTGCTACCCCGAAATTTTCTTGTCTTTTCTGAATGATAGGGAG
AAAAAAAAGATTGGGTCAAAAGAAAATGCCATTTTGGAATTTTATCAACAATTTGCTTG
TGTAGGTGGAGATCCGGTATTTTCTGAATCCTTATGTAAAGAATTACAAAAGAAATTTTT
TCAACAAAGATGTGAATTAGGAAGGATTGGACGACGAAATATGAACCGGAGATTAAAT

CTTGATATACCTCAGAGCAATACATTTTTGTTACCACGAGATGTATTGGCTGCCGCGGAT
CATTTGATCGGAATGAAATTTGGAATGGGTACACTTGACGAT------ATGAATCACTTGAA
AAATAAACGTATTCGTTCTGTAGCAGATCTGTTACAGGATCAATTCGGCTTGGCTCTTGT
TCGTTTAGAAAATGCAGTTCGAGGAACTATATGTGGAGCAATCAGG---CATAAATTGATA
---CTGACTCCTCAAAATTTGGTCAGTTCAACTTCA-----------TTAACAACCACTTATGAAT
CGTTTTTCGGCCTACACCCTTTATCTCAAGTTTTGGATCGAACTAATCCATTGACGCAAA
TCGTTCATGGGCGAAAATTGAGTTATTTGGGTCCTGGAGGATTGACAGGGCGAACTGC
TAGTTTTCGAATACGAGATATCCATCCTAGTCACTATGGACGGATTTGCCCAATTGACAC
GTCCGAAGGAATCAATGTTGGACTTATTGGATCCTTAGCTATTCATGCCAGGATTGGTCA
TTGGGGG---TCTATAGAGAGTCCGTTTTAT---------GAAGTATATCAGAGATCAAAAGAG--
----ACAAAGATGGTTTTTTTATCACCAAGTAGAGATGAATATTATACGGTAGCGACAGGA
AATTCTTTGGCTTTGAATCGAGGTATTCAGGAAGAACAGATTGTTCCCGCCCGATACCG
TCAAGAATTCCTGACTATTGCATGGGAACAGATTCATCTTCGAAGTATTTTTCCCTTCCA
ATATTTTTCTATTGGAGCTTCCCTGATTCCGTTTATCGAGCATAATGATGCGAATCGGGCT
TTAATGAGTTCGAATATGCAGCGTCAAGCAGTTCCGCTTTCCCGGTCCGAGAAGTGCAT
TGTTGGGACTGGATTGGAACGACAAGCGGCTCTGGATTCGGGGGTTTCAGCTATAGCC
GAATGCGAGGGAAAGATTATTTATACCGATACTCATAAGATCGTTTTATCAGGTCATGGA
GACACTATAAGCATTCCATTGGTTATGTATCAACGTTCGAACAAAAATACTTGTATGCAT
CAAAATCCTCAGGTTCGGCGGGGGAAATGCATTAAAAAAGGACAAATTTTAGCGGATG
GTGCGGCTACAGTTGGTGGCGAACTCGCTTTAGGAAAAAACGTATTAGTAGCTTATATG
CCATGGGAAGGTTACAATTTTGAGGACGCGGTACTCATTAGCGAACGTCTGGTATATGA
AGATATTTATACTTCTTTTCACATCCGTAAATATGAAATTCAGACTCATGTGACAAGCCA
GGGT---CCTGAAAGAATTACTCATGAAATACCGCATTTGGAAGCTCATTTACTCCGCAAT
TTAGACAGAAATGGAATTGTGGCGCTGGGATCTTGGGTAGAAACAGGCGATATTTTAGT
AGGTAAATTAACCCCTCAGACAGCCAACGAA---TCATCGTATGCCCCGGAAGATAGATT
ATTACGAGCCATACTTGGGATTCAGGTATCCACTGCAAAAGAAACTTGTCTAAAACTAC
CTATAGGCGGAAGGGGTCGAGTTATTGATGTGAGATGGATCCAGAAAAAGGGA---GGT
TCCAGCTATAATCCAGAAACGATTCGTGTATATATTTCACAGAAACGTGAAATCAAAGT
GGGTGATAAAGTAGCTGGAAGACACGGGAATAAAGGTATCATTTCCAAAATTTTATCTA
GACAAGATATGCCTTATTTGCAAGATGGAACGCCGGTTGATATGGTTTTCAACCCATTA
GGAGTACCTTCACGAATGAATGTGGGACAAATATTTGAATGCTCGCTCGGGTTAGCGG
GGGACCTGCTAGACAGACATTATAGAATAGCACCTTTTGATGAGAGATATGAGCAAGA
GGCTTCGAGAAAACTAGTGTTTTCTGAATTATATGAAGCCAGTAAGCAAACAGCAAAT
CCATGGGTATTTGAACCCGAATATCCCGGAAAAAGCAGAATATTTGATGGAAGAACGG
GAGATCCTTTTGAACAGCCTGTTCTAATAGGAAAGTCCTATATCCTGAAATTAATTCATC
AAGTTGACGATAAAATCCACGGTCGTTCCAGTGGACATTACGCACTTGTTACACAACA
ACCTCTTAGAGGAAGGGCCAAACAGGGCGGACAGCGCGTAGGAGAAATGGAAGTTTG
GGCTCTCGAAGGGTTTGGTGTTGCTCATATTTTACAAGAGATGCTTACTTATAAATCTGA
TCATATTAGAGCTCGTCAGGAAGTACTTGGTACTACAATCATTGGAGGAACAATACCTA
CCCCCGAGGATGCTCCAGAATCTTTTCGATTGCTCGTTCGCGAATTACGATCTTTGGCTT
TGGAACTGAATCATTTCCTTGTATCTGAGAAGAACTTCCAGATTAATAGGAAGGAAGCT
--------------------ATGATCGACCGGTATAAACATCAACAACTTCAAATTGGATCAGTTTCTC
CTCAACAAATAAGTGCTTGGGCCAACAAAATCCTACCTAATGGAGAGATAGTTGGAGA
GGTCACAAAACCCTATACTTTTCATTACAAAACCAATAAACCGGAAAAAGATGGATTAT

TTTGTGAAAGAATCTTTGGACCTATAAAAAGTGGAATTTGCGCTTGTGGAAATTATCGA
GTAATTGGCGCTGAAAAAGAAGACCCCAAATTTTGTGAACAATGTGGAGTCGAATTTA
TTGATTCTCGGATACGAAGATATCAAATGGGATACATCAAGCTCGCATGTCCAGTGACT
CATGTGTGGTATTTGAAACGTCTTCCTAGTTATATCGCGAATCTTTTAGATAAACCCCTT
AAGGAATTAGAAGGCCTAGTATACTGC------GATTTTTCTTTTGCTAGGCCC---ATAGCTA
AAAAACCGACTTTCTTACGATTACGAGGTTTATTCGAATATGAAATCCAATCCTGGAAAT
ACAGCATCCCCCTTTTTTTTACTACCCAAGGCTTCGATACATTTCGAAATAGAGAAATCT
CTACCGGAGCGGGTGCTATCAGGGAACAATTAGCCGATTTGGATTTGCGAATTGTTATC
GATAATTCATCGGTAGAATGGAAGGACTTAGGAGACGAA---GGGTCCACTGGGAATGA
ATGGGAAGATAGAAAAATTGGACGAAGAAAGGATTTTTTAGTTAGACGCATGGAATTA
GCTAAACATTTTATTCGAACAAATGTAGAACCAGAGCGGATGGTTTTGTGCCTATTACC
GGTTCTTCCTCCCGAGTTGAGACCAATTATTCAGATAGATGGGGGTAAACTAATGAGTT
CGGATATTAATGAACTCTATAGAAGAGTTATCTATCGGAACAATACTCTTACCGATCTATT
AAAAACAAGTAGATCTACGCCAGGGGAATTAGTAATGTGTCAGGAGAAATTGGTGCAA
GAAGCCGTAGATACACTTCTTGATAATGGAATCCGCGGACAACCAATGAGGGATGGCC
ATAATAAAGTGTACAAGTCATTTTCAGATGTAATTGAAGGCAAGGAGGGGAGATTTCGT
GAGACTTTACTTGGTAAACGGGTCGATTATTCGGGTCGTTCCGTCATTGTCGTAGGCCC
TTCACTTTCATTACATCGATGCGGATTACCTCGAGAAATAGCAATAGAGCTTTTCCAGA
CATTTGTAATTCGCGGTCTAATCAGACAACATCTTGCTTCTAACATAGGAATTGCTAAAA
GCAAAATTCGGGAAAAAGAACCGATCGTATGGGAAATACTTCAAGAAGTTATGCAGGG
ACATCCTGTATTGCTAAATAGAGCGCCCACCCTGCATAGATTAGGCATACAGGCGTTCC
AACCCGTTTTAGTGGAGGGACGTGCTATTTGTTTACATCCATTAGTTTGTAAGGGATTCA
ATGCAGATTTTGATGGAGATCAAATGGCTGTTCATGTACCTTTATCTTTGGAAGCTCAA
GCAGAGGCTCGTTTACTTATGTTTTCTCATATGAATCTCTTGTCACCAGCTATTGGAGAT
CCCATTTCTGTACCAACTCAAGATATGCTTATTGGCCTCTATGTATTAACGATGGGGAAT
CGTCGAGGTATTTTTGTAAATAGGTATAATCCATGTAATCGCAGAAATTAT---------CAAAA
TAAAACAGTTGACAATAATAACTATAAACATACGAAAGAGAAAAAACCCTATTTTTTGA
GTTCCTATGATGCACTTGGAGCTTATCAGCAGAAACGAATCAATTTACATAGTCCTTTGT
GGCTCCGGTGGCGACTAGATCAACGT---GTCATTGGTTCA------AGAGAAGTTCCTATCG
AAGTTCAATATGAATCTTTGGGTACCTATCAGGAGATTTATGGGCACTATCTAATAGTAA
GAAGTGTAAAAAAAGAAATACTTTGTATATACATTCGAACCACTGTTGGTCATATTTCTT
TTTATCGAGAAATAGAAGAATCCGTACAAGGGTTTTGTCGAGCCTACTCATATGGTACC-
------------------------------------ATGGCAGAACGGGCCGATCTGGTCTATCACAATAAAGCGAT
AGATGGAACTGCCATGAAACGACTTATTAGCAGATTAATAGATCACTTCGGAATGGCAT
ATACATCACACATCCTGGATCAAGTAAAGACTCTGGGTTTTCGGCAAGCCACTGCTACA
TCTATTTCATTAGGAATTGATGATCTTTTAACAATACCTTCTAAAGGATGGCTAGTCCAA
GATGCTGAACAACAAAGTTTTATTTTGGAGAAACACCATTCTTATGGAAATGTACACGC
GGTAGAAAAATTACGTCAATCCATTGAGATATGGTATGCTACAAGTGAATATTTGAGAC
AAGAAATGAATCCTAATTTTCGGATGACTGATCCTTCTAATCCAGTCCATATAATGTCCT
TTTCGGGAGCTAGAGGAAATGCATCTCAGGTGCACCAATTAGTAGGTATGAGAGGACT
AATGTCGGATCCCCAGGGACAAATGATTGATTTACCCATCCAAAGCAATTTACGTGAAG
GACTTTCTTTAACCGAATATATAATTTCCTGCTACGGAGCCCGCAAAGGAGTTGTAGATA
CTGCTGTACGAACATCAGATGCCGGATACCTCACTCGTAGACTTGTTGAAGTAGTTCAA
CACATTGTTGTACGCAGAACAGATTGTGGCACTATCCGAGGTATTTCTGTGCAACCTCG

AAACGGGATGACGGAA------AGGATGTTTTTCAAACACTAATCGGTCGTGTATTAGCA
GACGATGTATATATAGGTCTACGATGCATTGCCGCTCGAAATCAAGATATTGGGATTGGA
CTTGTCAATCGATTCATAACC------------------TTTCGAGCACAACCAGTATATATTCGAACT
CCCTTTACTTGTAGGAGTACCTCTTGGATCTGTCAATTATGTTATGGCCGGAGTCCTACT
CACGGCGACCTGGTCGAATTGGGGGAAGCGGTAGGTATTATTGCGGGACAATCAATTG
GGGAACCGGGGACTCAACTAACATTAAGAACCTTTCATACTGGTGGAGTATTCACAGG
GGGTACTGCAGAACATGTACGAGCTCCTTCTAATGGAAAAATAAAATTCAATGAGGAG
TTGGTTCATCCCACACGTACACGTCACGGACATCCTGCTTTTCTATGTTCTATAGACCTG
TATGTAACTGTTGAGGGTCGGGATATTATACATAATGTCAATATTCCACCAAAAAGTTTG
ATTTTAGTTCAAAATGATCAATATGTAGAATCAGAACAAGTGATTGCCGAGATTCGCGC
CGGAACATCCACTTTT------TTTAAAGAGAGGGTCCAGAAACATATTTATTCCGAATCAG
CGGGAGAA---ATGCACTGGAGTACCGATGTCTATCATGCACCTGAATATACATATGGTAA
TGTTCATCTATTACCAAAAACAAGTCATTTATGGATATTATCCGGAGGGCCATACAGA----
--TCCAGTATAGTGTCTTCGTCGCTCCACAAGGATCAAGATCAAACGAATGCTCATTCTT
TTTCTGTTGAACGGCGA------TATATTTCTGACCTCTCA------ATGACTAAT---GGTCGAGT
GAGACACAAATTATTTAGTTCGGATCTTTCT---GGT---GGGGATAGGATTCTTGATTATTC
A---AGACCCGATCAAATCATATCCAAGGGTCATTGGAATTTTATATACCCTTCT---ATTCT
CCACGAGAAT------TCAGATTTCTTAGCGAAGAGGCGAAAAAATAGATTCATCATCCCGT
TCCAATATGATCAAGAAGGGGAGAAAGAACTAATACCGCATTCTGGTATTTCGATCGAA
ATACCCATAAAC------GGTATGTTGCGTAGAAATAGTATTCTTGCTTATTTTGACGATACA
CGATACAGAAGAAGC------GGAATTACTAAATAC---ACCGTAGAGATAGATTCAATCGTT
AAA------AAAGAGGATTTCATTGAGTATCGAGGA---ACAAAAGAATTTAGTTCGAAATAC
------GTAGATCGATTTTTTTTCATTCCCGAAGAAGTGCATATCTTACCTGGATCTTCGTCC
ATAATGGTCCGGAACAACAGTCTCATCGGAGTAGACACACGAATCACTTTAAATATAAG
AAGCCGAGTGGGTGGATTGGTTCGAGTGGAGAGAAAAAAAAAAAAATATTGAACTCAA
AATTGTTTCTGGAGATATCCATTTTCCTGGAGAGACAGATAAGATATCCAGGCACAGCG
GTATCTTGATACCGCCAGGAACGGAAAAAAAA------AACTCTAAGGAATCAAAAACA---
AAATTGAAAAATTGGATCTATGTCCAGCGG------ATCACGCCTACTAAGAAAAAG------TA
TTTTGTTTTGGTCCGACCCGTAGCCACATATGAAATAGCAGATGGGATCAATTTAGCAA
CGCTTTTCCCTCAGGATCTATTGCAGGAAAGGGATAATGTTCAACTTCGAGTTGTTAATT
ATATCCTTTATGGAAATGGCAAACCAATTCGAGGAATTTCTCACACAAGT---CAATTAGT
TCGTACTTGCTTAGTATTGAATTGGGACCAAGAACAAAATGGTTCTATAGAA------------G
GTGTTCGTGCTTCCTTTGTTGAAGTAAGGGCAAACGATCTTATTAGAGATTTCATAAGA
ATTGAGTTATGCAAGTCCGCTATTTTGTATACCGGAAAGAGGAAGGATATCGCAGGTTC
AGGATTGATCCACGAT------------GATCGCACAAATATCAATCCTGTTTATTTGAAGGATAA
GATTCCATCA---------------TTTATTCAACATCAAGGGACTGTTGGT------------ACGTTGTTG
AATCGAAATAAG---------GAATGCCAATCTTTGATTCTTTTGTCATCATCTAATTGTTTTCG
AATTGGT------CCATTTAATGGTTCAAAATATCAC---------AATGTGCCAAAAGAATCAGAT
CCTATAATTCCAATT---GATTCGCTGGGTCTTTTAGGTACTACTGTACCA---AAAATTGCG
AATTTTTTTTATTTATCTTACCATGTAATAACT---------CATAATCATATCTTGTTAACT---AA
ATATTTGCTACGTGAC---------CATTTAAAACAGGCTTTCCAAGTACTTAGA---------TATTG
TTTAATGGATGAAAATAGGAGA------ATTTATAATCCCGATCCGTGCAGTAACATCATTTT
TAATGCATTCGATTTGAATTGGCGCTTTCTCCATCACGATTATTCTGAAGAGACATCCAC
AATACTTAGC---CTTGGACAATTTGTTTGTGAAAATGTATGTCTATTTAAACACGGACCA-

--------CAGATAAAATCGGGTCAAGTTATAATTGTTCAT---GTTGACTCCTTTGTAATAAGA
GCAGCCAAGCCGTATTTGGCCACTCCAGGAGCAACTGTTCATGGCCATTATGGAGAAAT
ACTTTACGGAGGAGATACATTAATTACATTTATATATGAAAAATCAAGATCTGGTGATAT
AACGCAGGGTCTTCCAAAAGTAGAACAAGTGTTAGAAGTACGTTCAATTGATTCCATAT
CGACGAACCTCGAAAAAAGGGTTGAAGGTTGGAACGAACATATAACAAAAATTCTTG
GAATTCCCTGGGGATTCTTGATCGGCGCCGAGTTAACCATAGCGCAAAGTCGTATCTCT
TTGGTTAATAAGATCCAAAAAGTTTATCGATCTCAGGGGGTGCAGATTCATAATAAGCAT
ATAGAGATTATTGTACGTCAAATAACATCAAAAGTGTTGGTTTCAGAA---------GATGGA
ATGTCTAATGTTTTTTCACCGGGAGAACTTATCGGATTGTTGCGGGCGGAACGAACAG
GTCGCGCTTTGGAAGAAGCGATCTGTTATCGAGCAATCTTATTGGGAATAACAAGAGC
GTCTTTGAATACTCAAAGTTTCATATCCGAAGCGAGTTTTCAAGAAACTGCTCGAGTTT
TGGCAAAAGCGGCTCTACGGGGTCGTGTTGATTGGTTGAAAGGCCTGAAAGAGAACG
TTGTTCTAGGGGCGATGATACCCGTCGGTACGGGATTCAAAGCATTAGTACACCGGTCA
AGA------AAACCCAACAACATTCAT------TTGGAAATCAAAAAGAATAATCTATTTGAGG
GGCAAGTGGGA---GATGATATTTTGTTCTACCACAGAGAATTATTTGGTTCTTGCGGCCC
AAAT---------------TCAGAACAGTCATTTATGAAATTTCATGATTCC------------------ATGACA
AAAAGATATTGGAACATCAATTTGGAAGAGATGATGGAAGCAGGAGTGCATTTCGGTC
ACGGTACTAGGAAATGGAATCCTAGAATGGCACCTTACATTTCTGCAAAGCGTAAAGGT
ATTCATATTACAAATCTTACTAGAACTGCTCGTTTTTTATCAGAAGCCTGTGATTTACTTT
TTGATGCAGCAAGTGGGGGAAAACACTTCTTAATCGTTGGTACCAAAAAGAAAGCAG
CTGATTCAGTAGCATCGGCTGCAATAAGGGCTCGGTGTCATTATGTTAATAAAAAATGG
CTTGGTGGTATGTCAACGAATTGGTCCACTACAGAAACGAGACTTCAAAATTTTAGGG
ACTTAAGAGCAGAACAAAAGGCTGGGAAAATCGACCGTCTCCCGAAAAGGGATGCAG
CAATGTTGAAGAGACAATTATCTACCTTGCAAACATATCTGGGTGGGATCAAATATATGA
CGGGTTTACCTGATATTGTAATCATCGTTGATCAGCAAGAAGACTATACGGCTCTTCGA
GAATGTGTCATTTTAGGAATTCCGACTATTTGTTTAATCGATACAAATTCTGACCCGGAT
CTCGCAGACATTTCGATTCCCGCCAACGATGACGCTATAGCTTCAATCCGATTGATTCTT
AACAAATTAGTATTCGCAATTTGCGAGGGTCGTTCTAGC---TATATAAGAAATCGTATGG
GACAAAAAATAAATCCACTTGGTTTCAGACTTGGTGCAACCCAAAGTCATCTTTCCCTT
TGGTTCGCACAACCAAAAAGTTATTCTATGGGTTTACAGGAAGATGAAAAAATACGGG
AGTGTATCAAGAATTATGTACAAAAAAACCCGAGACTGTCTTCAGGA---TTCGAAGGAA
TAGCGCGTATAGAAATTAAAAAAAGAATCGATCTGATCCAGGTCATAATCTATATCGGAT
TCCCAAATTTATTCATAGAGGGT------CGAACGCAA------GGAATCAAAGAATTACAGATG
AGTGTACAAAAAAGATTAAATTCTGTGAATCAGAGACTCAACATTGCTATCGCAAGAAT
TTCAAAACCTTATGGGCAACCCAATATTCTTGCTGAATATATAGCTTTACAACTAAAAAA
TAGAGTTTCGTTTCGAAAAGCAATGAAAAAAGCTATTGAATTGACTGAACAAGCAGAT
ACAAAAGGAATTCAAATTCAAATCGCAGGACGTATCGATGGAAAAGAAATTGCACGTG
TCGAATGGATAAGAGAAGGTAGGGTTCCCCTACAAACCATTCGAGCTAAAATTGATCAT
TGTTTCCATAAAGTTCAAACTATCTATGGGGTATTAGGCATAAAAATTTGGATATTTGTA
GACGAGGAAATGTCTCGCTACCGAGGGCCTCGTTTAAAAAAAATACGCCGTCTGGGGG
CTTTACCCGGACTAACTAGTAAAGGAACTAGACCCGGAAGTGATCTTAGAAATCAATTT
CGTTCTGGTAAA---AGATCCCAATATCGTATTCGTCTTGAAGAAAAACAGAAATTGCGT
TTTCATTATGGTCTGACAGAGCGACAATTACTTAGATATGTTCATATCGCCGGAAAAGCC
AAAGGATCAACAGGTCAGGTTTTACTACAACTACTTGAAATGCGTTTGGATAACATCCT

TTTTCGATTGGGTATGGCTCCTACGATTCCTGGAGCCCGGCAATTAGTTAACCATAGACA
TATTTTAGTTAATGGTCGTATAGTAGATATACCAAGTTATCGTTGCAAACCCAGAGACAT
TATTACTACGAAGGATAAGCAAAGATCGAAAGTTCTGATTCAAAATAATATGGATTCATC
GACCCGCGAA------TTGCCAAAACATTTGACTCTTGACTCA------TTCCAACATAAAGGA
TTAGTAAATCAAATCATAGATAGTAAATGGGTCGGTTTGAAAATTAATGAGTTGTTAGTT
GTGGAATATTATTCCCGCCAGACTATGTCACGTCGAGGTACTGCAGAAGAAAAAACTG
CAAAATCCGATCCAATTTATCGTAATCGATTAGTTAACATGTTGGTTAACCGTATTCTGA
AACACGGAAAAAAATCATTGGCTTATCAAATTATCTATCGAGCCTTGAAAAAGATTCAA
CAAAAGACAGAAACAAATCCACTATCTGTTTTACGTCAAGCAATACGTGGAGTAACTC
CCGATATAGCAGTAAAATCAAGACGTGTAGGCGGATCGACTCATCAAGTTCCTATTGAA
ATAGGATCTACACAAGGAAAAGCACTTGCCATTCGTTGGTTATTAGCGGCATCCCGAAA
ACGTCCGGGTCGAAATATGGCTTTCAAATTAAGTTCCGAATTAGTAGATGCTGCCAAAG
GGAGTGGCGATGCCATACGCAAAAAGGAAGAGACTCATAAAATGGCAGAGGCAAATA
GAGCTTTTGCACATTTTCGTATGGGTAAGGACACTATTGCCGATATAATAACTTCTATAA
GAAATGCTCAAATGGCTAAAAAAGGGACAGTTCGAATAGCATCTACGAATCTTACCGA
AAACGTTGTTAAAATACTTCTGCGAGAAGGTTTTATTGAAAACGTTAGAAAACATCGG
GAAAGTAATAAAGATTTCTTGGTTTTAACCCTGCGACATAGAAGG------ACTAGGAAAG
GGATATATAGA---------------ACTATTTTAAAGCGTATCAGCCGACCTGGTTTACGAATCTAT
TCCAACTATCAAGGAATTCCCAAGATTTTAGGCGGAATAGGAATTGTAATTGTTTCTACT
TCTAGAGGTATAATGACAGATCGGGAGGCTCGACTACAGGGAATTGGAGGAGAAATGT
TATGTTATATATGTATGACAAAAACTATACCAAGAATTGGTTCACGTAGGAATGGACGTA
TTGGTTTACGTAAGACTGGACGTAGAATACCAAAAGGAATTATTCATGTTCAGGCAAGT
TTCAACAATACCATTGTGACTGTTACAGATGTTCGGGGTCGAGTAGTTTCTTGGTCCTC
CGCCGGTACTTGTGGATTCAAAGGCACAAGAAGAGGGACGCCGTTTGCGGCTCAAAC
CGCAGCGGGAAATGCTATTCGTACAGTAGTAGATCAGGGTATGCAACGAGCAGAAGTC
ATGATAAAAGGTCCTGGGCTCGGAAGAGACGCAGCATTACGAGCCATTCGTAGAAGCG
GCATATTATTAAATTTCGTACGTGACGTGACCCCTATGCCACATAATGGATGTAGACCTC
CTAAAAAAAGACGTGTGATGCCAACTATTAAACAACTTATTAGAAACACAAGACAGCC
AATCCGAAATGTCACAAAATCCCCCGCTCTTCGAGGATGTCCTCAGCGTCGAGGAACA
TGTACTAGGGTGTATACTATCACCCCCAAAAAACCAAACTCTGCCTTACGTAAAGTTGC
CAGAGTACGATTAACCTCTGGATTTGAAATCACTGCTTATATACCTGGTATTGGCCATAA
TTTACAAGAACATTCTGTAGTCTTAGTAAGAGGTGGGAGGGTTAAGGATTTACCCGGTG
TGAGATATCACATTGTTCGAGGAACCCTAGATGCTGTCGGAGTAAAGGATCGTCAACA
AGGGCGTTCTAAATATGGGGTCAAAAAGCCAAAAATGGCAAGGAAAGGTTTGATTGA
GAGGGAGAAGAAGAGGAAGAAATTGGAACAGAAATATCATTCGATTCGTGGATCCTCA
AAAAAAGAAATAAGA---------AAAGTTCCATCGTTGAGTGACAAATGGGAAATTCATGG
AAAATTGCAATCCCCACCACGTAATAGTGCACCTGTACGTCTTCATCGACGTTGTTTTTT
GACCGGAAGACCCAGAGCTAACTATAGGGATTTTGGGTTATCCGGACACGTACTTCGA
GAAATGGTTCAGGCATGTTTGTTGCCGGGCGCAACAAGATCAAGTTGGATGGTCAAAA
ATTCATTTATCTCGCTTATTCCGCAAGAAGAAAAAGAAGAA------AACCGGGGATCTGTT
GAATTTCAAGTATTCAGTTTCACCAATAAGATACGGAGACTTACTTCACACTTGGAATT
ACACAGAAAAGATTATTTATCCCAGAGAGGTTTACGAAAAATTCTAGGAAAACGTCAA
CGGCTGCTGGCTTATTTGTCAAAGAAAAATAGAGTACGTTATAATAAATTAATTGGTCA
GTTGGATATTCGGGAGCCAAAAACTCGT------ATGGTAAAACTTCGTTTAAAACGATGCG

GTAGAAAGCAACGAGCCATTTATCGAATCGTTGCAATTGATGTTCGATCCCGAAGAGA
GGGAAGAGATCTTTGTAAAGTGGGTTTTTATGATCCGATAAAAAATCAAACTTATTTAA
ATGTTCCTGTTATTCTAAATTTTCTGGAAAAGGGGGCTCAACCTACAGAAACTGTTCAT
GATATTTTAAAGAAGGCAGAGGTATTTAAGGAACTTCAAGGAAATCAAACGAAA--------
-ATGGATAAATCCAAGCAACTTTTTCGTAAATCCAAGCGATCTTTTCGTAGGCGTTTGCC
CCCAATTGGATCGGGGGATCGAATTGATTATAGAAACATGAGTTTAATTAGTCGATTTAT
TAGTGAACAGGGAAAAATATTATCTAGACGAGTGAATAGATTGACCTTGAAACAACAA
CGATTAATTACTATTGCTATAAAACAAGCCCGTATTTTATCTTCGTTACCTTTTCTTAATA
ACGAGAAA------CAATTTGAAAGAACCGAGTCAATACCTAGACCTACCGGTCCTAGAAG
CAGAAATAAAGTGACACGTTCATTAAAAAAAAAATCCTTTTGTGGCCAATCATTTATTGG
AAAAAATTGAGAAGCTCAACATGAAAGAGGAGAAAGAAATAATAGTAACTTGGTCCC
GGGCATCTACCATTATACCCACAATGATCGGTCATACAATTGCTATTCATAATGGAAAGG
AACACTTACCCATTTATATAACAGATCGTATGGTCGGTCACAAATTGGGAGAATTCGCA
CCTACTCTTACTTTCCGGGGACACGCGAGAAACGATAATAAATCTCGTCGTATGATTTTC
AAATCATTTCTACTAGGTAATCTTGGATCCTTATACATAAAGATAATAAATTCAGTCGTTG
TGGTCGGACTATATTATGGATTTTTGACAACATTCTCCATAGGGCCCTCTTATTTCTTCCT
[truncated: 378,507 more chars]
