# Supplementary material for: A precise chloroplast genome of Nelumbo nucifera (Nelumbonaceae) evaluated with Sanger, Illumina MiSeq, and PacBio RS II sequencing platforms: insight into the plastid evolution of basal eudicots
Source: BMC Plant Biol. 2014 Nov 19;14:289. doi: 10.1186/s12870-014-0289-0 (PMC4245832; doi:10.1186/s12870-014-0289-0)
Supplement: Additional file 6 — The amino acid sequences alignment of the proteins from 133 species used in the phylogenetic analyses. [file 12870_2014_289_MOESM6_ESM.pdf]

Additional file 6. The amino acid sequences alignment of the proteins from 133 species used in the phylogenetic analyses.

>Aucuba
MERWWFNSMFFLSKSMDSLGPIENISE--DPTINDTTKKIHSYSNVDNLFGIKDIGNFIADDT
FLVRDSNGDSYSIYFDIENQIFEIDNDH--SFLNELESSFYSYRN-----NGSKSDDPYYDRYMY
DTQYSWNNHINSCIDSYLE--SQIRIDTSIGSGSDNYG--YIYSSICGSEGSGIQTSTNGSDLTIR
ESSNDLDVTQKYRHLWIQCENCYGLNYKKFFKSKMNICEQCGYHLKMSSSDRIEFSIDPG
TWDPMDEDMVSLDPIEFHSEEEPYKDRIDSYQRKTGLTEAVQTGIGQLNGIPIAIGVMDFQ
FMGGSMGSVVGEKITRLIEYATNQFLPLIIVCASGGARMQEGSLSLMQMAKISSALYDYQS
NKKLFYVAILTSPTTGGVTASFGMLGDIIIAEPNAYIAFAGKRVIEQTLNKTVPEGSQAAEY
LFQKGLFDLIVPRNPLKDVLSELLQLHAFFPLT-----MVTIRADEISNIIRERIEQYNREVKIVN
TGTVLQVGDGIARIHGLDEVMAGELVEFEEGTIGIALNLESNNVGVVLMGDGLMIQEGSS
VKATGKIAQIPVSEAYLGRVINALAKPIDGRGEISASESRLIESPAPGIISRRSVYEPLQTGLIA
IDSMIPIGRGQRELIIGDRQTGKTAVATDTILNQQGQNVICVYVAIGQKASSVAQVVTTFQE
RGAMEYTIVVAEAADSPATLQYLAPYTGAALAEYFMYRERHTSIIYDDPSKQAQAYRQMS
LLLRRPPGREAYPGDVFYLHSRLLERAAKSSSSLGEGSMTALPIVETQSGDVSAYIPTNVISI
TDGQIFLSADLFNAGIRPAINVGISVSRVGSAAQIKAMKQVAGKLKLELAQFAELEAFAQF
ASDLDKATQNQLARGQRLRELLKQSQAAPLTVEEQIMTIYTGTNGYLDSLEIGQVRKFLV
ELRTYLKTNKPQFQEIISSTKTFTEEAETLLKEAIQEQMERFILQEQAMRINPTSSGSGISTLE
KKNLGRIAQIIGPVLDVAFPPGKMPNIYNALVVKGRDIAGQPINVTCEVQQLLGNNRVRAV
AMSATDGLTRGMEVIDTGAPLSVPVGGSTLGRIFNVLGEPVDNLGPVDTRITSPIHRSAPAF
IQLDTKFSIFETGIKVVDLLAPYRRGGKIGLFGGAGVGKTVLIMELINNIAKAHGGVSVFG
GVGERTREGNDLYMEMKESGVINEQNIAESKVALVYGQMNEPPGARMRVGLTALTMAEY
FRDVNEQDVLLFIDNIFRFVQAGSEVSALLGRMPSAVGYQPTLSTEMGTLQERITSTKEGSI
TSIQAVYVPADDLTDPAPATTFAHLDATTVLSRGLAAKGIYPAVDPLDSTSTMLQPRIVGEE
HYETAQRVKETLQRYKELQDIIAILGLDELSEEDRLTVARARKIERFLSQPFFVAEVFTGSPG
KYVGLAETIRGFQLILSGELDSLPEQAFYLVGNIDEATAKAMNLEMESNLKKMSLNLCVLT
PNRIVWDSEVKEIILSTNSGQIGVLPNHAPIATAVDIGILRIRLNDQWVTMALMGGFARIGN
NEITILVNDAEKGSDIDPQEARETLEIAEANLRKAEGKRQTIEANLALRRARTRVESINAIS
MKNVTDSFVSLGHWPSAGSFGFNTDILATNPINLSVVLGVLIFFGKGVLSDLLDNRKQRIL
NTIRNSEELREGAIEQLEKARARLRKVEMEADRFRVNGYSEIEREKLNLINSTYKTLEQLE
NYKNETIHFEQQRAINQVRQRVFQQALQGALGTLNSCLNNELHLRTISANIGMLGAMKEI
TDMNPLISAASVIAAGLAVGLASIGPGVGQGTAAGQAVEGIARQPEAEGKIRGTLLLSLAF
MEALTIYGLVVALALLFANPFVMNVLSCSINTLKGLYDISGVEVGQHFYWKIGGFQVHGQ
VLITSWVVIAILLGSATIAVRNPQTIPTGGQNFFEYVLEFIRDVSKTQIGEEYGPWVPFIGTM
FLFIFVSNWSGALLPWKIIQLPHGELAAPTNDINTTVALALLTSVAYFYAGLTKKGLGYFGK
YIQPTPILLPINILEDFTKPLSLSFRLFGNILADELVVVVLVSLVPSVIPIPVMFLGLFTSGIQA
LIFATLAAAYIGESMEGHHMIFLTLEHILTHISFSIVSIVITIHLITLLVDEIVKLYDSSEKGMIA
IFFCITGLLATRWIYSGHFPLSNLYESLIFLSWSFSIIHIVPY--KKKNYLSTITAPSVIFTQGFVT
SGLLIEIHQSRILVPALQSEWLIMHVSMMILGYAALLCGSLLSVALLVITFRKNGKIFC--KEI
EYMNERDNVLRNTS---AKNYYRSQLIQQLDYWSYRVISLGFLFLTIGILSGAIWANEAWGS
YWNWDPKETWAFITWIVFAIYLHTRTNKNLQGANSAIVASIGFFIIWICYFGVNLLGIGLHS
YGSFTLTMAKKKAFTPLLYLASLVFLPWWISLSFNKSLESWVTNWWNTRQSETFLNDIQE

KSILEKFIELEELLFLDEMIKEYSETQLQKLRI-IHKETIQLIKIHNEDRIHTILHFSTNIICFVIL
SGCSILGNEELVILNSWAQEFLYNLSDTVKAFSILLLTDLCIGFHSPHGWELMIGYVYKDFG
FVHNDQIISGLVSTFPVILDTIFKYWIFRYLNRVSPSLVVIYHSMNDMPIGVPKVPFRNPGEE
DASWVDVYNRLYRERLLFLGQEVDSEISNQLIGLMVYLSIEDDTKDLYLFINSPGGWVIPG
VAIYDTMQFVRPDVHTICMGLAASMGSFILVGGEITKRLAFPHARVMIHQPASSFYEAQTG
EFILEAEELLKLRESLTRVYVQRTGKPLWVVSEDMERDVFMSATEAQAYGIVDLVAVEMK
EQKWIHEGLITESLPNGMFRVRLDNEEMILGYVSGKIRRSFIRILPGDRVKIEVSRYDSTRG
RIIYRLRNKDSKDMEEFQRYLQ-DRSQQHDFLYPLIFQEYIYALAHDHGLTGSDNKSSLLIV
KRLIIRMYQQNHLIIPVNDSNQNQLFGKNLYSQMISEVFAVIVEIPFSLRLIASLIVKSHNLRS
IHSIFPFFENNFSHLNYVLDMLIPHPVHLEILVQTLRYWVKDASSLHLLRFFLYEYRNWNSL
ITKPSSSFSKQRLFFFLYNAHVCEYESIFVFLCNQSSYLRSTSSEAFLERIYFYGKIECLVEVF
AKVFNLWLFKDPNMHYVRYQGKSIMASKGTSLLMNKWKYYLINFWQCHFYLWSHPGRI
YINQLSNHSFDFMGYFSSVRLNPSMVRSQMLENSFLIDNAI--KKFDTIVPIIPLIGSLAKAKF
CNVLGYPISKPVRADLSDSDIIDRFGRICRNFSHYHSGSSKKKSLYRIKYILRLSCARTLARK
HKSSVRFFLKRLGSELLEEFFMAEEQVLPLTFPRRVWYLDIFCINDLANHEMIID-ITEVQ-AI
NSFSRLES-LKEIYGIIWMLVPILTLVLGITIGVLVIVWLEREISAGIQQRIGPEYAGPLGILQA
LADGTKLLFKENIFPSRGDTRLFSIGPSIAVISIFLSYSVIPFGYRLVLADLSIGVFLWIAISSIA
PVGLLMSGYGSNNKYSFLGGLRAAAQSISYEIPLTLCVLSISLLSNSSSTVDIVEAQSKYGF
GGWNLWRQPIGLIVFLISSLAECERLPFDLPEAEEELVAGYQTEYSGIKFGLFYIASYLNLLV
SSLFVTILYLGGWNLSIPYIFVPELFDINKTGRVFGTIIGIFITLAKTYLFLFIPITTRWTLPRLR
MDQLLNLGWKFLLPISLGNLLLTTSSQLFSLMIWHVQNENFILDSTRIFMKAFHLLLFDGS
FIFPECILIFGLILLLMIDSTSDQKDIPWLYFISSTSLVMSITALLFRWREEPMISFSGNFQTNN
FNEIFQFLILLCSTLCIPLSVEYIECTEMAITEFLLFVLTATLGGMFLCGANDLITIFVAPECFS
LCSYLLSGYTKKDVRSNEATMKYLLMGGASSSILVHGFSWLYGSSGGEIELQEIVNGLINT
QMYNSPGISIALIFITVGIGFKLSPAPSHQWTPDVYEGSPTPVVAFLSVTSKVAASASATRIF
DIPFYFSSNEWHLLLEILAIFSMILGNLIAITQTSMKRMLAYSSIGQIGYVIIGIIVGDSNDGY
ASMITYMLFYISMNLGTFACIVLFGLRTGTDNIRDYAGLYTKDPFLALSLALCLLSLGGLPP
LAGFFGKLYLFWCGWQAGLYFLVLIGLLTSVVSIYYYLKIIKLLMTGRNQEITPHVRNYRR
SPLRSNNSIELSMIVCVIASTIPGISMNPIIAIAQDTLFMFLLYEYDIFWAFLIISSIVPILAFVIS
GILAPISKGPEKLSSYESGIEPMGDAWLQFRIRYYMFALVFVVFDVETVFLYPWAMSFDVL
GVSVFIEAFIFVLILIVGSVYAWRKGALEWSTNYFPWLTIIVVFPISAGSLIFFLPHRGNRVIR
WYTIFICILELLLMTYVFRYHFQSDDPLIQLVEDYKWINFFDFRWRLGIDGFSIGLILLTGFIT
TLATLAAWPVTRDSRLFHFLMLAMYSGQIGSFSSRDLLLFFIMWELELIPIYLLLAIWGGK
KRLYSATKFILYTAGGSVFLLLGVLGVGLYGSYEPTLNFETSANQSYPVALEIIFYIGFFIAFA
VKSPIIPLHTWLPDTHGEAHYSTCMLLAGILLKMGAYGLVRINMELLPHAHFLFSPWLIIV
GTMQIIYAASTSSGQRNLKKRIAYSSVSHMGFIIIGIGSITETGLNGALLQIISHGFIGAALFFL
AGTSYDRIRLVYLDEMGGIAIPMPKIFTMFSSFSMASLALPGMSGFIAELIVFFGIITSQKFL
LMPKILITFVMAIGMILTPIYSLSMSRQMFYGYKLCNAPNSSFFDSGPRELFLSLSIFLPAIGI
GIYPDFVLSLSVDKVEVILSHFFYRMMLEHVLVLSAYLFSIGIYGLITSRNMVRALMCLELI
LNAVNLNFVTFSDFFDSRQLKGAIFSIFVIAIAAAEAAIGLAIVSSIYRNRKSTRINQSNLLN
KMEQTYQYAWIIPFVPLPVPILIGVGLLLFPTATKNLRRMWAFYSVLLLSIVMIFSINISIHQI
NSSSIYQYVWSWIINNDFSLDFGYLIDPLSSIMSILITTVGIMVLIYSDNYMSHDQGYLRFFA
YMGLFSTSMLGLVTSSNLIQIYIFWELVGICSYLLIGFWFTRPVAANASQKAFVTNRVGDF
GLLLGILGFYWITGSFEFRDLFEIFNNNEVNFLFVILCAVLLFAGAVAKSAQFPLHVWLPDA

MEGPTPISALIHAATMVAAGIFLIARLIPLFIVIPYIMNLISLIGIITVLLGATLALAQKDIKRGL
AYSTMSQLGYMMLALGMGSYRSALFHLITHAYSKALLFLGSGSIIHSMETIVGYSPDKSQ
NMVLMGGLTKHVPITKTAFLLGTLSLCGIPPLACFWSKDEILNDTWLYSPIFAIIAWATVGL
TAFYMFRIYLLTFEGHLNVNFQNYSGKKNTSFYSISLWGKRSSNKINK-LVTLLKMNFFSFG
NKNNSSYPYESDNTMLFPLLVLVLFTLFVGALGIPFDLDILSNWLTPSINLLHQNLN-DIDW
YEFVKDAVFSISMAFFGIFIASFLYKPVYSSLQNLDLLNSFVILWDKIINGIYDWSYNRAYID
SFYTTSLTGGIRRLAELTHFFDRRIIDGITNGVGVMSFFVGEGIKYVGGG-RISSYLFLYLSY
VSIFLFIFSMDLPGPIHDFLLVFLGSGLILGALGVVLLPNPIYSAFSLGLVLVCISLFYILSNSH
FVAAAQLLIYVGAINVLIIFAVMFMNGSEYYKDFHLWTIGDGVTSMVCTSIFVSLMTTIPDT
SWYGIIWTTRSNQIVEQDLISNSQQIGIHLSTDFFLPFELISIILLVALIGAIAVAR-MTAQATRK
DLMIVNMGPQHPSMHGVLRLIVTLDGEDVIDCEPILGYLHRGMEKIAENRTIIQYLPYVTR
WDYLATMFTEAITVNAPEQLGNIQVPKRASYIRVIMLELSRIASHLLWLGPFMADIGAQTP
FFYIFRERELIYDLFEAATGMRMMHNYFRIGGVAADLPHGWIDKCLDFCDYFLTRIAEYQ
KLITRNPIFLERVEGVGFISGEEAINWGLSGPMLRASGIQWDLRKVDHYECYDEFDWEVQ
WQKEGDSLARYLVRIGEMTESIKIIQQALEGIPGGPYENLEIRRFDKVRDADWNDFEYRFIS
KKPSPTFELSKQELYVRVEAPKGELGIFLIGDQSVFPWRWKIRPPGFINLQILPQLVKKMKL
ADIMTILGSIDIIMGEVDRMFSMVTEFMNYGQQTIRAARYIGQGFMITLSHANRLPVTIQY
PYEKLITPERFRGRIHFEFDKCIACEVCVRVCPIDLPVVDWKFETDIRKKRLLNYSIDFGICI
FCGNCVEYCPTNCLSMTEEYELSTYDRHELNYNQIALGRLPMSIIDDYTIRTIVNS-----------
----MQGRLSAWLVKHGLIHRSLGFDYQGIETLQIKPEDWHSIAVILYVYGYNYLRSQCAYD
VAPGGLLASVYHLTRIEYGVDQPEEVCIKVFTPRRNPRIPSVFWVWKSVDFQERESYDML
GISYDNHPRLKRILMPESWIGWPLRKDYIAPNFYEIQDAH----------------------------MAKGG
IGMVLAPEYSDNKKKKGKNKIET-IMNSIEFPLLDRTTPNSVISTTLNDLSNWSRLSSLWPLL
YGTSCCFIEFASLIGSRFDFDRYGLVPRSSPRQADLILTAGTVTMKMAPSLVRLYEQMPEPK
YVIAMGACTITGGMFSTDSYSTVRGVDKLIPVDVYLPGCPPKPEAVIDAITKLRKKISREIY
EDRIRSQRENRCFTTNHKFHVGRSIHTGKYDQGFLYQPPSIAEISPETFFKYKSSVSSHKLV
NMQTRNTFSWIKEQITRSISVSLMIYIITRTSISSAYPIFAQQGYENPREATGRIVCANCHLAN
KPVDIEVPQAVLPDTVFEAVVRIPYDIQMKQVLANGKKGGLNVGAVLILPEGFELAPPDRI
SPEMKEKIGNLSFQGYRPNKKNILVIGPVPGQKYSEITFPILSPDPATKKDVHFLKYPIYVGG
NRGRGQIYPDGSKSNNTVYNATAAGIVSKIIRKEKGGYEITITDASDGRQAVNIIPPGPELLV
SEGESIKFDQPLTSNPNVGGFGQGDAEIVLQDPLRVQGLLFFLASVILAQIFLVLKKKQFEK
VQLSEMNFMSKVYDWFEERLEIQAIADDITSKYVPPHVNIFYCLGGITLTCFLVQVATGFA
MTFYYRPTVTEAFASVQYIMTEANFGWLIRSVHRWSASMMVLMMILHVFRVYLTGGFKK
PRELTWVTGVVLGVLTASFGVTGYSLPRDQIGYWAVKIVTGVPEAIPVIGSPLVELLRGSAS
VGQSTLTRFYSLHTFVLPLLTAVFMLMHFPMIRKQGISGPLMGVTKKPDLNDPVLRAKLA
KGMGHNYYGEPAWPNDLLYIFPVVILGTIACNVGLAVLEPSMIGEPADPFATPLEILPEWYF
FPVFQILRTVPNKLLGVLLMVSVPAGLLTVPFLENVNKFQNPFRRPVATTVFLIGTAVALWL
GIGATLPIDKSLTLGLFMIEVFLFGIVLGLIPITLAGLFVTAYLQYRRGDQLDLMPTITSYFGF
LLVSLTITSALFIGLSKIRLIMDIVSLAWAALMVVFTFSLSLVVWGRSGLMIIRSPEPEVKILV
DRDHIKTSFEEWARPGHFSRTIAKGPDTTTWIWNLHADAHDFDSHTSDLEEISRKVFSAHF
GQLSIIFLWLSGMYFHGARFSNYEAWLSDPTHIGPSAQVVWPIVGQEILNGDVGGGFRGIQ
ITSGFFQIWRASGITSELQLYCTAIGALVFAALMLFAGWFHYHKAAPKLAWFQDVESMLN
HHLAGLLGLGSLAWAGHQVHVSLPINQFLNAGVDPKEIPLPHEFILNRDLLAQLYPSFAEG
ATPFFTLNWSKYAEFLTFRGGLDPVTGGLWLTDIVHHHLAIAILFLIAGHMYRTNWGIGHG

LKDILEAHKGPFTGQGHKGLYEILTTSWHAQLSLNLAMLGSLTIVVAHHMYSMPPYPYLA
TDYGTQLSLFTHHMWIGGFLIVGAAAHAAIFMVRDYDPTTRYNDLLDRVLRHRDAIISHL
NWACIFLGFHSFGLYIHNDTMSALGRPQDMFSDTAIQLQPVFAQWIQNTHALAPGATAPGA
TTSTSLTWGGGDLVAVGGKVALLPIPLGTADFLVHHIHAFTIHVTVLILLKGVLFARSSRLIP
DKANLGFRFPCDGPGRGGTCQVSAWDHVFLGLFWMYNAISVVIFHFSWKMQSDVWGSI
SDQGVVTHITGGNFAQSSITINGWLRDFLWAQASQVIQSYGSSLSAYGLFFLGAHFVWAFS
LMFLFSGRGYWQELIESIVWAHNKLKVAPATQPRALSIVQGRAVGVTHYLLGGIATTWAFF
LARIIAVGMALRFPRFSQGLAQDPTTRRIWFGIATAHDFESHDDITEERLYQNIFASHFGQL
AIIFLWTSGNLFHVAWQGNFEAWVQDPLHVRPIAHAIWDPHFGQPAVEAFTRGGALGPVN
IAYSGVYQWWYTIGLRTNEDLYTGAFFLLFLSAISLIASWLHLQPKWKPSVSWFKNAESRL
NHHLSGLFGVSSLAWTGHLVHVAIPGSRGEYVRWNNFLDVLPHPQGLGPLFTGQWNLYA
QNPDSSSHLFGTSQGAGTAILTLLGGFHPQTQSLWLTDMAHHHLAIAFIFLVAGHMYRTNF
GIGHSMNDLLDAHIPPGGRLGRGHKGLYDTINNSLHFQLGLALASLGVITSLVAQHMYSLP
AYAFIAQDFTTQAALYTHHQYIAGFIMTGAFAHGAIFFIRDYNPEQNEDNVLARMLDHKE
AIISHLSWASLFLGFHTLGLYVHNDVMLAFGTPEKQILIEPIFAQWIQSAHGKTSYGFDVLL
SSTSGPAFNAGRSIWLPGWLNAVNE--NSNSLFLTIGPGDFLVHHAIALGLHTTTLILVKGAL
DARGSKLMPDKKDFGYSFPCDGPGRGGTCDISAWDAFYLAVFWMLNTIGWVTFYWHW
KHITLWQGNVSQFNESSTYLMGWLRDYLWLNSSQLINGYNPFGMNSLSVWAWMFLFGH
LVWATGFMFLISWRGYWQELIETLAWAHERTPLANLIRWRDKPVALSIVQARLVGLAHFS
VGYIFTYAAFLIASTSGKFGMSHSVKIYDTCIGCTQCVRACPTDVLEMIPWDGCKAKQIAS
APRTEDCVGCKRCESACPTDFLSVRVYLGNESTRSMGLGYMTTFNFPSIFVPLVGLVFPAIA
MASLSLHVQKNKIVMRDLKTYLSVAPVLSTLWLGSLAGLLIEINRFFPDALTFPFFSFMTAI
LERRESESLWGRFCNWITSTENRLYIGWFGVLMIPTLLTATSVFIIAFIAAPPVDIDGIREPVS
GSLLYGNNIISGAIIPTSAAIGLHFYPIWEAASVDEWLYNGGPYELIVLHFLLGVACYMGRE
WELSFRLGMRPWIAVAYSAPVAAATAVFLIYPIGQGSFSDGMPLGISGTFNFMIVFQAEHNI
LMHPFHMLGVAGVFGGSLFSAMHGSLVTSSLIRETTENESANEGYRFGQEGETYNIVAAH
GYFGRLIFQYASFNNSRSLHFFLAAWPVVGIWFTALGISTMAFNLNGFNFNQSVVDSQGRV
INTWADIINRANLGMEVMHERNAHNFPLDLAAIEAPSTNGMGLPWYRVHTVVLNDPGRL
LSVHIMHTALVAGWAGSMALYELAVFDPSDPVLDPMWRQGMFVIPFMTRLGITNSWGGW
SIT-GGAITNPGIWSYEGVAGAHIVFSGLCFLAAIWHWVYWDLEIFCDERTGKPSLDLPKIF
GIHLFLSGVACFGFGAFHVTGLYGPGIWVSDPYGLTGKVQPVNPAWGVEGFDPFVPGGIAS
HHIAAGTLGILAGLFHLSVRPPQRLYKGLRMGNIETVLSSSIAAVFFAAFVVAGTMWYGSA
TTPIELFGPTRYQWDQGYFQQEIYRRVSAGLAENRSLSEVWSKIPEKLAFYDYIGNNPAKG
GLFRAGSMDNGDGIAVGWLGHPIFRDKEGRELFVRRMPTFFETFPVVLVDGDGIVRADVP
FRRAESKYSVEQVGVTVEFYGGELNGVSYSDPATVKKYARRAQLGEIFELDRATLKSDGV
FRSSPRGWFTFGHASFALLFFFGHIWHGARTLFRDVFAGIDPDLDAQVEFGAFQKLGDPTT
RRQVV------------VETLFNGTLALAGRDQETTGFAWWAGNARLINLSGKLLGAHVAHAGLI
VFWAGAMNLFEVAHFVPEKPMYEQGLILLPHLATLGWGVGPGGEVIDTFPYFVSGVLHLI
SSAVLGFGGIYHALLGPETLEESFPFFGYVWKDRNKMTTILGIHLILLGIGAFLLVFKALYF
GGIYDTWAPGGGDVRKITNLTLSPSIIFGYLLKSPFGGEGWIVSVDDLEDIIGGHVWLGSICI
LGGIWHILTKPFAWARRALVWSGEAYLSYSLGALAVFGVIACCFVWFNNTAYPSEFYGPTG
PEASQAQAFTFLVRDQRLGANVGSAQGPTGLGKYLMRSPTGEVIFGGETMRFWDLRAPW
LEPLRGPNGLDLSRLKKDIQPWQERRSAEYMTHAPLGSLNSVGGVATEINAVNYVSPRSW
LATSHFVLGFFLFVGHLWHAGRARAAAAGFEKGIDRDFEPVLSMTPLNMTIALGKFTKDE

N-LFDIMDDWLRRDRFVFVGWSGLLLFPCAYFALGGWFTGTTFVTSWYTHGLASSYLEG
CNFLTAAVSTPANSLAHSLLLLWGPEAQGDFTRWCQLGGLWIFVALHGAFGLIGFMLRQF
ELARSVQLRPYNAIAFSAPIAVFVSVFLIYPLGQSGWFFAPSFGVAAIFRFILFFQGFHNWTL
NPFHMMGVAGVLGAALLCAIHGATVENTLFEDGDGANTFRAFNPTQAEETYSMVTANRF
WSQIFGVAFSNKRWLHFFMLFVPVTGLWMSALGVVGLALNLRAYDFVSQEIRAAEDPEFE
TFYTKNILLNEGIRAWMAAQDQPHENLIFPEEVLPRGNALMSGSTGERSFADIITSIRYWVI
HSITIPSLFIAGWLFVSTGLAYDVFGSPRPNEYFTESRQGIPLITGRFDSLEQLDEFSRSFMTI
DRTYPIFTVRWLAVHGLAVPTVSFLGSISAMQFIQRMATQTVENSSRSG-PRRTTVGSLLKP
LNSEYGKVAPGWGTTPLMGVAMALFAIFLSIILEIYNSSVLLDGISMNMLTLKLFVYTVVIF
FVSLFIFGFLSNDPGRNPGREEMADTTGRIPLWIIGTVTGILVIGLIGIFFYGSYSGLGSSLML
NIFSLICICLNSALY-SSFFFTKLPEAYAFLNPIVDVMPVIPLFFFLLAFVWQAAVSFRMTQSN
PNEQNVELNRTSLYWGLLLIFVLAVLFSNYFFNMEVNILAFIATALFILVPTAFLLIIYVKTIS
QNDMETATLVAIFISGLLVSFTGYALYTAFGQPSQQLRDPFEEHGDMEALVYTFLLVSTLGII
FFAIFFREPPKVPTKKMKMTLAFQLAVFALIATSSILLISVPVVFASPDGWSSNKNVVFSGTS
LWIGLVFLVGILNSLIS-------MSPQTETKASVGFKAGVKDYKLTYYTPDYETKDTDILAAFR
VTPQPGVPPEEAGAAVAAESSTGTWTTVWTDGLTSLDRYKGRCYHIEPVAGEESQFIAYVA
YPLDLFEEGSVTNMFTSIVGNVFGFKALRALRLEDLRIPVAYTKTFQGPPHGIQVERDKLN
KYGRPLLGCTIKPKLGLSAKNYGRAVYECLRGGLDFTKDDENVNSQPFMRWRDRFLFCA
EAIYKAQAETGEIKGHYLNATAGTCEDMMKRAVFARELGVPIVMHDYLTGGFTANTSLAF
YCRDNGLLLHIHRAMHAVIDRQKNHGMHFRVLAKALRMSGGDHIHAGTVVGKLEGEREI
TLGFVDLLRDDFIEKDRSRGIYFTQDWVSLPGVLPVASGGIHVWHMPALTEIFGDDSVLQF
GGGTIGHPWGNAPGAVANRVALEACVQARNEGRDLAREGNEIIREASKWSPELAAACEV
WKEIKFEFPAMDTL--MAIHLYKTSTPSTRNGTVDSQVKSNPRNNLISGQHRCGKGRNARG
IITAGHRGGGHKRLYRKIDFRRNEKDIYGRIVTIEYDPNRNAYICLIHYGDGEKRYILHPRG
AIIGDTVVSGTEVPIKMGNALPLTDMPLGTAIHNIEITLGKGGQLARAAGAVAKLIAKEGKS
ATLKLPSGEVRLISKNCSATVGQVGNVGVNQKSLGRAGSKRWLGKRPVVRGVVMNPVD
HPHGGGEGKAPIGRKKPTTPWGYPALGRRSRKRNKYSDNLILRRRSKMIQPQTYLNVADN
SGARELMCIRIIGAS-NRRYAHIGDVIVAVIKEAVPNMSLERSEVVRAVIVRTCKELKRDNG
MIIRYDDNAAVVIDQEGNPKGTRVFGAIARELRQLNFTKIVSLAPEVLMLSPKRTRFRKQH
RGRMKGISYRGNHICFGKYALQALEPAWITSRQIEAGRRAMTRNARRGGKIWVRIFPDKP
VTIRPTETRMGSGKGSPEYWVAVVKPGRILYEMGGVTENIARRAISIAASKMPIRTQFIISG
MTRIKRGYIARRRRTKIRLFASSFRGAHSRLTRTISQQKIRALVSAHRDRNKQKRNFRRLWI
TRINAVIRE-RGVSYSYSRLIHDLYKRQLLLNRKILAQIAISNRNCFYMISNEIIKEVDWKEST
GIIMLKEVYALGQHICMSADKARRVIDQIRGRSYEETLMILELMPYRACYPIFKLVYSAAA
NASYNMGSNEADLIISKAEVNEGTTAKKFKPRARGRSFPIKRPTCHITIVVKDISLDGGVW
DKKMDGIKYAVFTDKSIRLLGKN-QYTSNVESGSTRTEIKHWVELFFGVKVIAMNSQRLPG
KGRRMGPIMGHTMHYRRMIITLQPGYSIPPLRKKRTMAVPKKRTSISKKHIRKNIWKRKG
YWAALKAFSLGKSLSTGNSKSFFVQQTQTMAKGKDVRVTVILECTSCARNGVN--KVSRG
ISRYITQKNRRNTPNRLELRKFCPYCYKHTSHGEIKKMKIRASVRKICEKCRLIRRRGRIRVI
CSNPRHKQRQGMVREKVTVSTRTLQWKCVESRADSKRLYYGRFILSPLMKSQADTIGIA
MRRALLGEIEGTCITRAKSE--KIPHEYSTIVGIQESIHEILMNLKEIVLRSNLYGTCDTSICVR
GPGYITAQDIILPPYVEIVDKTQHIASLTEPIDLCIGLQIERNRGYLIKTPN-NFDGSY--PIDAV
FMPVRNVNYSIHSYGNGNEKQEILFLEIWTNGSLTPKEALHEASRNLIDLFIPFLHMEEE--N
LHLEDNQ-HTVPLSPFTFHDKLA-KLRKNKN-KIALKSIFIDQSELPPRIYNCLKRSNIYTLLD

LLNNSQEDLMKIEQFRIQDVKQILGILEKH--FAIDEKEVLMLRDG-NEGMSTIPGFNQIQFE
GFCRFIDQGLTEELSKFPKIEDTDQEIEFQLFVETYQLVEPLIKERDAVYESLTYSAELYVSA
GLIWKTSRDMQEQTIFIGNIPLMNSLGTSIVNGIYRIVINQILQSPGIYYRSELDHNGISVYT
GTIISDWGGRSELEIDRKARIWARVSRKQKISILVLSSAMGSNLREILENVCYPEIFLSFLND
KERKKIGSKENAILEFYQQFACVGGDPVFSESLCKELQKKFFQQRCELGRIGRRNMNRRL
NLDIPQNNTFLLPRDILAAADHLIGIKFGMGTLDD--MNHLKNKRIRSVADLLQDQFGLAL
VRLENAVRGTICGAIR-HKL-IPTPQNLVTSTP----LTTTYESFFGLYPLSQVLDRTNPLTQIVH
GRKLSYLGPGGLTGRTASFRIRDIHPSHYGRICPIDTSEGINVGLIGSLAIHARIGHWG-SLES
PFY---KISERSTG--VRILYLSPGRDEYYRVAAGNSLALNQGIQEEQVVPARYRQEFLTIAWE
QVHLRSIFPFQYFSIGASLIPFIEHNDANRALMSSNMQRQAVPLSRSEKCIVGTGLERQAAL
DSGALAIAEREGKIIYTDTDKILLSGNGETLSIPLVMYQRSNKNTCMHQKPQVQRGKCIKK
GQILADGAATVGGELALGKNVLVAYMPWEGYNFEDAVLISERLVYEDIYTSFHIRKYEIKT
HVTSQG-PERVTNEIPHLEAHLLRNLDKNGIVMLGSWVETGDILVGKLTPQMVKE-SSYAP
EDRLLRAILGIQVSTSKETCLKLPIGGRGRVIDVRWIQKKG-GSSYNPETIRVYISQKREIKV
GDKVAGRHGNKGIISKILPRQDMPYLQDGRPVDMVFNPLGVPSRMNVGQIFECSLGLAGG
LLDRHYRIAPFDERYEQEASRKLVFSELYEASKQTANPWVFEPEYPGKSRIFDGRTGDLFE
QPVIIGKPYILKLIHQVDDKIHGRSSGHYALVTQQPLRGRAKQGGQRVGEMEVWALEGFG
VAHILQEMLTYKSDHIRARQEVLGTTIIGGTIPNPEDAPESFRLLVRELRSLALELNHFLVSE
KNFQINKKEAMNQNFSSMIDRYKHQQLRIGSVSPQQISAWATKILPNGEIVGEVTKPYTFH
YKTNKPEKDGLFCERIFGPIKSGICACGNYRVIGNEKEDPKFCEQCGVEFIDSRIRRYQMGY
IKLACPVTHVWYLKRLPSYIANLLDKPLKELEGLVYCD--FSFARP-IAKKPTFLRLRGTFEY
EIQSWKYSIPLFFTTQGFDTFRNREISTGAGAIREQLADLDLRIIIDNSLVEWKELGEE-GTL
GNEWEDRKVGRRKDFLVRRMELAKNFIRTNIEPEWMVLCLLPVLPPELRPIIQIDGGKLMS
SDINELYRRVIYRNNTLTDLLTTSRSTPGELVMCQEKLVQEAVDTLLDNGIRGQPMRDGHN
KVYKSFSDVIEGKEGRFRETLLGKRVDYSGRSVIVVGPSLSLHRCGLPREIAIELFQTFVIR
GLIRQHLASNIGVAKSKIREKEPIVWEILQEVIQGHPVLLNRAPTLHRLGIQAFQPILVEGRA
ICLHPLVCKGFNADFDGDQMAVHVPLSLEAQAEARLLMFSHMNLLSPAIGDPISVPTQDM
LIGLYVLTSGNRRGICVNRYNPWNR---RNYQNERSDHNNYMDT---EPFFCNSYDAIGAYRQ
KRINLDSPLWLRWRLDQR-VIAS--REVPIEVHYESLGTYYEIYGHYLIVRSIKKEILFIYIRTT
VGHISLYREIEEAIQGFCQACSYGT--------MEVLMAERANLVFHNKVIDGTAMKRLISRLID
HFGMAYTSHILDQVKTLGFQQATATSISLGIDDLLTIPSKGWLVQDAEQQSLILEKHHHYG
NVHAVEKLRQSIEIWYATSEYLRQEMNPNFRMTDPFNPVHIMSFSGARGNASQVHQLVG
MRGLMSDPQGQMIDLPIQSNLREGLSLTEYIISCYGARKGVVDTAVRTSDAGYLTRRLVEV
VQHIVVRRTDCGTVRGISVSPRNGMMP-ERFFIQTLIGRVLADDIYMGPRCIATRNQDIGIGL
VNRFIT------FRAQPISIRTPFTCRSTSWICRLCYGRSPTHGDLVELGEAVGIIAGQSIGEPGTQ
LTLRTFHTGGVFTGGTAEHVRAPSNGKIKFNEDLVHPTRTRHGHPAFLCSIDLYVTIESEDII
HNVNIPPKSFLLVQNDQYVESEQVIAEIRAGTST-LNLKEKVRKHIYSDSEGE-MHWSTDV
YHVPEFTYGNVHLLPKTSHLWILLGGPCR--SSLVSLPLHKDQDQMNVH--SLSVKRRDISN
LS--VTNDQ-ARHKLFSSDFSGK-EYRIHDD-SEQNRIICTGRCNLIYP-AILHENS--DLLAKRR
RNRFIISLQSSQERENELMPRSGISIEIP--INGIFRRNSILAYFDDPRYRRKSSGITKYGTIEMP
SIVKKDD--LIEYRG-VKEFRPKYMKVDRFFFIPEEVYLLPGSSSIMVRNNSIIGVDTQITLNT
RSRVGGLVRVERKKKRIELKIFSGDIHFPGETDKISRHSGVLIP-PG-TVKTNFKESKK--LKD
WIYVQR--ITPSK--KKFFVFVRPVVTYEIRDGINLATLFPPDLLQERDNVQFRVVNYILYGN
GKPIRGISDTSIQLVRTCLVLNWDQDKK---SASSKEARASFVEIRTNVLIRDFLRIDLVKCPIS

YIGKRNDPSGSGLISD-NGSDRTNITPFSSIYSY---SKARIQQPLNQNQGTIHTLLNR---NKEC
QSLIILSSSNCSRMGP--LNDVKYHNV---IKES-PLILIRNLFGPLGT--ALQIGNFDS--FYHL--IT
HNQIL-VTNYLQLDN---LKQTFQGLK---YYLMDEN--GKIYNPDPCSNIILNPFNLNWYFLH
HNYCEETS-TIISLGQFICENVCIAKNGPH-LK--SGQVLIVQ-VDSVVIRSAKSYLATPGATVH
GHYGEILYEGDTLVTFIYEKSRSGDITQGLPKVEQVLEVRSIDSISMNLEKRVEGWNECITR
ILGIPWGFLIGAELTIAQSRISLVNKIQRVYRSQGVQIHNRHLEIIVRQITSKVLVSE---DGMS
NVFLPGELIGLLRAERMGRALEEGICYRAVLLGITRASLNTQSFISEASFQETARVLAKAAL
RGRIDWLKGLKDNVVLGGMIPVGTGFKGL--VHLSRQHNNIP--LETKKKNLFEREMR--DI
LFHHRKLLDSCLSKNFHDISEQPFIG------FNDFMTRRYWNINLEEMMEAGVHFGHGTRK
WNPKMASYISAKRKGIHITNLTRTARFLSEACDLVFDAASRGKQFLIVGTKNKVADSVARA
AIKARCHYVNKKWLGGMLTNWSTTETRLHKFRDLRTEQKTGRLNRLPKRDAAMLKRQL
SRLQTYLGGIKYMTGLPDIVIIVDQHEEYTALRECITLGIPTICLLDTNCDPDLADISIPANDD
AISSIRLILNKLVFAICEGRS-SYIRNPMGQKINPLGFRLGTTQGHHSLWFAQPKNYSEGLQE
DQKLRDCIKNYVQKNMKIPSG-VEGIARIEIQKRIDLIQVIIFMGFPKLLIESRPR----GIEELQ
MNLQKEFNCVNRKLNIAITRIAKPYGNPNILAEFIAGQLKNRVSFRKAMKKAIELTEQADT
KGIQIQIAGRIDGKEIARVEWIREGRVPLQTIRAKIDYCSYTVRTIYGVLGIKIWIFIDQEMSR
YRGPRFKKIRRLGALPGLTNKRPRAGSDLRNQSRSGK-KSQYRIRLEEKQKLRFHYGLTER
QLLKYVRIARKAKGSTGQVLLQLLEMRLDNILFRLGMASTIPGARQLVNHRHILVNGRIV
DIPSYRCKPRDIITARDEQKSRALIQNSLDSSTR-EELPKHLTLHPFQ--YKGLVNQIIDSKWV
GLKINELLVVEYYSRQTMSRRGTAEEKTAKSDPIYRNRLVNMLVNRILKHGKKSLAYQIIY
RAVKKIQQKTETNPLSVLRQAIRGVTPDIAVKARRVGGSTHQVPIEIGSTQGKALAIRWLL
AASRKRPGRNMAFKLSSELVDAAKGSGDAIRKKEETHRMAEANRAFAHFRMGRDTIAEII
TSIRNADMDRKRVVRIASTNITENIIKILLREGFIENVRKHRENNKDFLVLTLRHRR--NKKG
PY---INFLNLKRISRPGLRIYSNYQRISRILGGMGIVILSTSRGIMTDREARLEGIGGEILCYIW
MAKAIPRIGSRRTGRIGSRKSARRIPKGVIHVQASFNNTIVTITDVRGRVVSWSSAGTCGFK
GTRRGTPFAAQTAAGNAIRTVVDQGMQRAEVMIKGPGLGRDAALRAIRRSGILLTFVRDV
TPMPHNGCRPPKKRRVMPTIKQLIRNTRQPIRNVMKSPALRGCPQRRGTCTRVYTITPKKP
NSALRKVARVRLTSGFEITAYIPGIGHNSQEHSVVLVRGGRVKDLPGVRYHIVRGTLDAVG
VKDRQQGRSKYGVKKPKMARKSLIQREKKRKNLEQKYHLIRRSSKKEIS---KVPSLSDKW
EIYGKLQSPPRNSAPTRLHRRCFSTGRPRANYRDFGLSGHILREMVHACLLPGATRSSWM
VKNSFISVISQ--EENTENRGSVEFQVFSFTNKIRRLTSHLELHKKDYLSQKGLRKILGKRQR
LLTYLSKKNKVRYKELIGQLNIREIKTR--MVKLRLKRCGRKQRAVYRIVAIDVRSRREGRD
LRRVGFYDPIKNQTYLNVPTILYFLEKGAQPTGTVQDILKKAEVFKELRPN------MDKSKRP
FLKSKRSFRRRLPPIQSGDRIDYRNMSLISRFISEQGKILSRRVNRLTLKQQRLITIAIKQARI
LSLLPFLNNEK--QFERAESTARTTGLRTRNKVTRSLKKNPFVANHLLRKIEKLNTKADKEII
VTWSRASTIIPTMIGHTIAIHNGKEHLPIYITDRMVGHKLGEFSPTLNFRGHAKSDNRSRR
MILKSFLLGNLVSLCMKIINSVVVVGLYYGFLTTFSIGPSYLFLLRAQVMEEGEEGTEKKVS
ATTGFITGQLMMFISIYYAPLHLALGRPHTITVLALPYLLFHFFWNNHKHFFDYGSTTRNS
MRNLSIQCVFLNNLIFQLFNHFILPSSMLARLVNIYMFRCNNKMLFVTSSFVGWLIGHILF
MKWVGLVLVWIRQNHSIRSYIRSNKYLGSELRNSTARIFSILLFITCVYYLGRIPSPIFTKKL
KETEEDKPLVTLLFDYNRWNRPFRYIKNNRFEKAVRNEMSQYFFYTCRNDGKQRISFTYP
LSLSTFWEMIAKRISLSTLEKLSSDEPDSDWVYTNKQKSN-LNNEFLNRIEALNKRSLPLDI
LEKRTRLCNDETKKEYLHKMYDPLLNGPYRGTIKKRVSPIEPFGINRIHAILYKEFEQKYPN
GKSIGIKEISKKVPRWSYKLIDLEQQS-GQIRSRKAKRVVIFTDALIRYSQQSDFRQNIIKGS

MRAQRRKTFIWELFQANVHSPLFLDRLNKTSLFSFDI--SGMMKIFFQNRKEKPRIEIAEAW
DTIPFAQVIRGSMLITQSILRKYIILPSLIIAKNLSRMLLLQLPEWSEDLEEWNKEMHVKCTY
NGVQLSETEFPKNWLTDGIQIKILFPFCLKPWHKSKKGQKDDFCFLTVWGMEAELPFGSP
RKRPSFFEPIFKELEKKIGKLKKKYFLV-LRVLKERTKLLLTVSKEIKKWVSKSVL--FLKRIL
KELSKVNDSIISNQIIHESFSQIGSPDWTNYSLTEKKMKDLADRTRTIRNQIERITKEKKKSP
NKTSYNDKRLESPKNIWQIFKRKNDRFICKLHYFIKLFVEKIYIYIFLCIINIPRINTQLF-HFIS
YDLSSLSQAYVFYKLSQIQLINLYKLRSVLQYTSFFLKTEIKDFFTQGIFHSELSHKKPQSYG
MNQWKNWLRGRYQYDLSQIRWARLIPQKWRNRVNWRRTAQNQNKWDSYEKDQ--LIHY
KKQ-NVKKCYKYDFLSYKSINGNIMYSDRKFFDWKILNFCLRFFDWMGMNEEILNRWFF
PEFVLLYNSYKIKSWVIPNKLLLLRYKSNTEAELKSFLKRYLLFQLRWDDALNQRMINNIK
VYCLLLRLINPRKITISSIQRREMSLDIMLVQKNLTLPELMK-GVLIIEPLRLSVKNDGQFFM
YQTIGISLVHKSKYNHYDLFVPENILSAKRRRELRILICFRFIWPNYRLEDLACMNRYWFD
TNNGSRFSMLKIHMYPMKGHQFKSWIFELREILREIKNSHYFLDSWTQFNSVGSFIHIFFH
QERFIKLFDPRIWSILLS-RNS--QGSTSNRYFT---IKG-VILFVVAVLIYR----INNRNMVERKNL
YLIGLLP-----IPMNSIGPRNDTLEESV-GSSNINRLIVS-LLYLPKGKNISESCFLNPKESTWVL
PIT----KKFSMPESNWGSRWWRNWIGKKR--DSSCKISN---ETVAGIEILFKEKDIK-YLEFLF
VYYMD--DPIRKDHDWELFDRLSLRKRRNIINL--NSGPLFEILVKHWICYLMSAFREKIPIEV
EG-FFKQQG-AGSTIQSNEIEHVSH--LFSRNKWPISLQNCAQFHMWQFRQDLFV---SWGK-
NPHESDFLRN-VSRENLIWLDNVWLVNKDR---FFS--KVRNVSSNIQYDS---TR--SSFVQVT
DSSQLKGSS-DQSRDHLDSIS----NEDSE-------------YHTLINQREIQQLKERSIL-WD--PSFLQ
TERTEIESD--RFPK--CLSGYS--SMSRLFA-EREKQMINHLLPEEIEEFL--GNPTRSVRSFFSD
RW--SELH-LGSNPTERSTRDQ----KLLKKQQDLS-FVPSRRSE----NQEMVNIFKIITYLQN--T
VSIHPISSDPGCDMVP-KDEPD-MDSSNKISFLKKNPFFDL--FHLFHDRNREGYTL--HHDFE
SE--ERFQEMADLFTLSITEP-DLVYHKGFAFSIDSYGLDQKQ--FLNEVFNSRDESKKKS---L
LVLPPIFY--EENESFYRRIRKKWVR-ISCGNDLEDPKPKIVVFASNNIMEAVNQYRLIRNLIQI
QYSTYG--CIRNVLNQFFLMN---RSDRNFEYG---IQRDQIGKDTLNHRTIMKYTINQHLSNL
KK-SQKKWFDP----LISRTERSMNRDPDAYRYKWSNGSKNFQEHLEHFVSEQKSR---FQVV
FDRLRINQYSIDWSEVIDKKDLSKPLRFFLSK---------SLLFLS-------KLFFFLSN-SLPFFFVSF
GNIPIHRSE-IYIY-ELKGPNDQLCNQLLESIGLQIVHLKKLKPF--LLD---DHDTS-QKSKFLIN
GGTISPFLFNKIP-KW-MIDSFHTR--NNCRKSFDNTDSYFSMIFHDQDNWLNPVKPFHRSSL
ISSFYKANRLRFLNNPHHFCFY--CNKRFPFYV----ERARIN--NYDFTYG-QFLNILFIRNK-IFS
LCVGK--KKHAFWG-RDTISPIESQVSNIFIPNDFPQSG-DETYNLYKS---FHFPSRSDPFVR--R
AIYSIADISGTPLTEGQIVNFERTYCQPLSDMNLSDSEGKNLHQYLN--FNSNMGLIHTPCSE
KYLPSEK--RKK--------RSLCLK--KCVEKGQMYRTFQRDSAFSTLSKWNLFQTYMPWFLTS
TGYKYLNLIFLDTFSDLL--PILSS-SQKFVSIFHDIMHGSDISWRILQKKL------CLPQWNL-IN
EISSKCLHN--LLLSEERIHRN--NESPLISTHLRSPNVREF-LYSILFLLLVAGY-LVRTHLLFVS
R--ASSELQTEFEKVKSLMIPSSMIELRKLLDRYPTSEPNSFWLKNLFLVALEQLGDSLEEIW
GSASGGNMLLGGGPT-----YG-VS---SKKKYLNINLIDLISLI----PNPINRITFSRNTRHLSHTS
KEIYSLIRKRKNVNGDWIDDKIESWVANSDSIDDEEREFLVQFS--TLTT--EKRIDQILLSLT-H
SDHL-----SKNDSGYQIIEQPGAIYLRYLVDIH--KKYLMNYEFNTSCLAERRIFLAHYQTITY-
-SQTSCGANSFHFPSHG---KPFSLRLALSPS-RGILVIGSIGTGRSYLVKYLATNSYVPFITVFL
NKFLDNKPKGFLID-----DID-IDDSDDIDDSD------------------------DIDRDLDTEL-ELLTRMN
ALTMDMMSE---IDQFYITLQFELAKAMSPCIIWIPNIHDLDVNESNYLSLGLLVNHLSR---D
CERCS-TRNILVIASTHIPQKVDPALIAPNKLNTCIKIRRLLIPQQRKHFFTLSYTRGFHLEKK

MFHT--NGFGSITMGSNARDLVALTNEALSISITQKKSIIDTNTIRCALHRQTWDLR---SQVRS
VQDHGILFYQIGRAVAQNVLLSNCPIDPISIYMKKK--SCNEGDSYLYKWYFELGTSMKKLT
ILLYLLSCSAGSVAQDLWSLP--GPDEK-NGITSYGLVENDS-DLVHGLLEVEGALVG--SSRT
EKDCSQFDND--RVTLLLRPEPRN-PLDMMQNGSCSILDQRFLYEKY--ESEFEE-GEGEGAL
DPQQ----IEED----LFNH--IVWAP--RIWRP---WGFLFDCIERPNELGFP-YGSRSFRGK---RIIY
DEEDELQENDSEFLQSGTVQYQTRDRSSKEQGLFRISQFIWDPADPLFFLFK-DQPLGSVFS
HRELFADEEMSKGLLTSQTDPPTSIYKRWFI--KNTQEKHFELLINRQRWLR---TNSSLS--NG
SFRSN--TLSESYQYLSNLFLSN-GRLLDQMTKTLLRKRWLFPDEMKIGF--M---------MPRSR
-INGNFIDKTFSIVANILLRIIPTTSGEKEAFTYYRDG--MSAQSEGNYAEALQNYYEAMRLEI
DPYDRSYILYNIGLIHTSNGEHTKALEYYFRALERNPFLPQAFNNMAVICHY-RGEQAIRQG
DSEIAEAWFDQAAEYWKQAIALTPGNYIEAHNWLKITRRFE--MSWRSEHIWIELIRGSRKI
SNFCWAFILFLGSLGFLLVGTSSYLGRNLISFFSSQQIIFFPQGIVMSFYGIAGFFISSYLWCTI
LWNVGSGYDRFDRKEGIVCIFRWGFPGKNRRIFLRFLIKDIQSVRIEAKESIYARRVLYMDI
RGQGAIPLTRTDEN-LTPREIEQKAAELAYFLRVPIEVF
>Berberidopsis
MEKRWFNSMLSLSKSMDSLGPIENTSGSEDPSINDTDKNIHSYSNVDHLFVVRDIRNFISD
DTFLVRDSNGDSYSIYFDIENHIFEVDNDH--SFLSELESYFYSYRNSSYLNTGSKSDDPYYN
RYMYDTKYSWNNHINSCIDSYLQ--SQIRIDS---YGSGNYS--YIYSYICGSESFSIKTSMNGG
DLTIRESSNDLDVTQKYRHLWVQCENCYGLNYKKFFKSKMNICEQCGYHLKMSSSDRIEL
SIDPGTWDPMDEDMVSLDPIEFHSEEEPYKDRIDSYQRKTGLTEAVQTGIGQLNGIPIAIGV
MDFQFMGGSMGSVVGEKITRLIEYATNKFLPLIIVCASGGARMQEGSLSLMQMAKISSAL
YDYQSNKKLFYVSILTSPTTGGVTASFGMLGDIIIAEPNAYIAFAGKRVIEQTLNKTVPEGS
QAAEYLFHKGLFDLIVPRNPLKGVLSELFKLHAFFPLN-----MVTIRADEISNIIRERIEQYNR
EVKIVNTGTVLQVGDGIARIHGLDEVMAGELVEFEEGTIGIALNLESNNVGVVLMGDGLM
IQEGSSVKATGRIAQIPVSEAYLGRVINALAKPIDGRGEISASESRLIESPAPGIISRRSVYEPL
QTGLIAIDSMIPIGRGQRELIIGDRQTGKTAVATDTILNQQGQNVICVYVAIGQKASSVAQV
VNTFQERGAMEYTIVVAETADSPATLQYLAPYTGAALAEYFMYRERHTSIIYDDLSKQAQ
AYRQMSLLLRRPPGREAYPGDVFYLHSRLLERAAKSSSRLGEGSMTALPIVETQSGDVSAY
IPTNVISITDGQIFLSADLFNAGIRPAINVGISVSRVGSAAQIKAMKQVAGKLKLELAQFAEL
EAFAQFASDLDKATQNQLARGQRLRELLKQSQSAPLTVEEQIMTIYTGTNGYLDSLEIGQV
RKFLVELRTYLKTNKPQFQEIISSTKTFTEEAEALLKEAIQEQMERFLLQEQAMRINPTTSG
PGVSTLEKKNLGRIAQIIGPVLDVAFPPGKMPNIYNALVVKGRDTVGQPINVTCEVQQLLG
NNRVRAVAMSATDGLMRGMEVIDTGAPLSVPVGGATLGRIFNVLGEPVDNLGPVDTRTTS
PIHRSAPAFIQLDTKLSIFETGIKVVDLLAPYRRGGKIGLFGGAGVGKTVLIMELINNIAKA
HGGVSVFGGVGERTREGNDLYMEMKESGVINEQNIAESKVALVYGQMNEPPGARMRVGL
TALTMAEYFRDVNEQDVLLFIDNIFRFVQAGSEVSALLGRMPSAVGYQPTLSTEMGTLQE
RITSTKEGSITSIQAVYVPADDLTDPAPATTFAHLDATTVLSRGLAAKGIYPAVDPLDSTSTM
LQPRIVGEEHYETAQQVKQTLQRYKELQDIIAILGLDELSEEDRLTVARARKIERFLSQPFFV
AEVFTGSPGKYVGLAETIRGFQLILSGELDGLPEQAFYLVGNIDEATAKAMNLEMENKLK
KMTLNLCVLTPNRIVWDSEVKEIILSTNSGQIGVLPNHAPIATAVDIGILRIRLNDQWLTMA
LMGGFARIGNNEITVLVNDAEKGSDIDPQEAQQTLEIAEANLRKAEGKRQTIEANLALRRA
RTRVEAINVISMKNVTDSFVSLGHWPSAGSFGFNTDILATNPINLSVVLGVLIFFGKGVLSD
LLDNRKQRILNTIRNSEELREGAIEQLEKARARLRKVEMEADEFRVNGYSEIEREKLNLINS
TYKTLEQLENYKNETIHFEEQRAINQVRQRVFQQALQGALGTLNSCLNNELHLRTISANIG

MFGAMKEITDMNPLIAAASVIAAGLAVGLASIGPGVGQGTAAGQAVEGIARQPEAEGKIR
GTLLLSLAFMEALTIYGLVVALALLFANPFVMNVLSCSINTLKGIYDISGVEVGQHFYWQI
GGFQVHAQVLITSWVVIAILLGSATIAVRNPQTIPTDGQNFFEYVLEFIRDVSKTQIGEEYGP
WVPFIGTLFLFIFVSNWSGALLPWKIIQLPHGELAAPTNDINTTVALALLTSVAYFYAGLTK
KGLGYFGKYIQPTPILLPINILEDFTKPLSLSFRLFGNILADELVVVVLVSLVPSVVPIPVMFL
GLFTSGIQALIFATLAAAYIGESMEGHHMIFSTLEHILTHISFSIVSIVITIHLITLLVDEIIGLYD
SSEKGMIATFFCITGLLVTRWVYSGHFPLSDLYESLIFLSWSFAIIHMVPYF-KYKNHLSAITA
PSAIFTQGFATSGLLTEMHQSAILVPALQSQWLMMHVSMMVLGYAALLCGSLLSVALLVIT
FRKNIRIFGKSNEIQYMDERNNVLQNTSFFSVRNYYRSQLIQQLDRWSYRVISLGFIFLTIGI
LSGAVWANEAWGSYWNWDPKETWAFITWTIFAIYLHTRTNTNLQGVNSAIVASLGFLIIWI
CYFGVNLLGIGLHSYGSFTLTMTKKKAFTPLLYLASIVFLPWWISLSFNKSLESWVTNWW
NTRQSETFLNDIQEKNILEKFIELEELLLLDEMIKEYPGTHLQKLRIG-HKETIQLIKMHNED
RIHTILHFSTNIICFVILSGYSILGNEELVILNSWVQEFLYNLSDTIKAFSILLLTDLCIGFHSPH
GWELMIGSVYKDFGFAHNDQIISGLVSTFPVILDTIFKYWIFRYLNRVSPSLVVIYHSMNDM
PIGVPKVPFRSPGEEDASWVDVYNRLYRERLLFLGQEVDSEISNQLIGLMVYLSIEDDTKN
LYLFINSPGGWVIPGVAIYDTMQFVRPNVHTICMGLAASMGSFILAGGEITKRLAFPHARV
MIHQPASSFYEAQTGEFILEAEELLRLRESLTRVYAQRTGKSLWVVSEDMERDVFMSATEA
QTHGIVDLVAVEMKEQKWIHEGLITESLPNGMFRVRLDNEDLILGYVSGRIRRSFIRILPGD
RVKIEVSRYDSTRGRIIYRLRNKDSSDMEEFQGYLE-DKSRQHDFLYPLIFQEYIYALAHDH-
-AGYDNKSSSLIVKRLITRMYQQNHLIISANDSNQNPFFGKNLYSQMISEGFAVIVEIPFSLR
LVSSLIVKSHKLRSIHSIFPFLEDKFSHLNYVLDILIPHPVHLEILVQTLRYWVKDASSLHLL
RLFLHEYRN--WNSLKSISIFSKQRLLLFLYNSHVCEYESVFVFLRNQSSHLLSTSSGALLERI
YFYGKIEHVE-VFAKDFILWLCKDPFMHYVRYQGKSILASKGTPLLMNKWKYYLVNFWQ
CHFYVWAQPGRIYINQLSNHSLNFMGYLSSVRLNLSVVRSQMLENAFLIDKSS--KKFDPIV
PIIPLIGSLAKAKFCNVLGHPISKPVWADLSDSDIIDRFGRICRNLSHYHSGSSKKKSLYRIK
YILRLSCARTLARKHKSTVRAFLKRLGSELLGEFLTEEEQVLSLIFPRRIWYLDIICINDLAN
HEMIID-TTEVQ-AINSFSRLES-LKEVYGIIWMFVPIFTPVLGITIGVLVIVWLEREISAGIQQ
RIGPEYAGPLGILQALADGTKLLFKENLLPSRGDTRLFSIGPSIAVVSILLSYSVIPFGYRLVL
ADLSIGVFLWIAISSIAPIGLLMSGYGSNNKYSFLGGLRAAAQSISYEIPLTLCVLSISLLSNS
SSTVDIVEAQSKYGFWGWNLWRQPIGFIVFLISSLAECERLPFDLPEAEEELVAGYQTEYSG
IKFGLFYVASYLNLLVSSLFVTVLYLGGWNLSIPYIFVPELFEINKVNGVFGTTIGIFITLAKT
YLFLFIPITTRWTLPRLRMDQLLNLGWKFLLPISLGNLLLTTSSQLLSLMIWHVQNENFILD
STRIFMKAFHLLLFDGSFIFPECILIFGLILLLMIDSTSDQKDIPWLYFISSTSLVMSITALLFR
WREEPMISFSGNFQTNNFNEIFQFLILLCSTLCIPLSVEYIECTEMAITEFLLFILTATLGGMFL
CGANDLITIFVAPECFSLCSYLLSGYTKKDVRSNEATTKYLLMGGASSSILVHGFSWLYGSS
GGEIELQEIVNGLINTQMYNSPGISIALIFITVGIGFKLSPAPSHQWTPDVYEGSPTPVVAFLS
VTSKVAASASATRIFDIPFYFSSNEWHLLLEILAILSMILGNLIAITQTSMKRMLAYSSIGQIG
YVIIGIIVGDSNDGYASMITYMLFYISMNLGTFARIVSFGLRTGTDNIRDYAGLYTKDPFLAL
SLALCLLSLGGLPPLAGFFGKLHLFWCGWQAGLYFLVSIGLLTSVVSIYYYLKIIKLLMTGR
NQEITPHVRNYRRSPLRSNNSIELSMIVCVIASTIPGISMNPIIAIAQDTLFMFLLYEYDIFWA
FLIISSVIPILAFLISGVLAPISKGPEKLSSYESGIEPMGDAWLQFRIRYYMFALVFVVFDVET
VFLYPWAMSFDVLGVSVFIEAFIFVLILIVGSVYAWRKGALEWSTNYFPWLTIIVVLPISAG
SLIFFLPHRGNKIIRWYTICICILELLLTTYAFCYHFQFDDPLIQLAEDYKWIDFFDFHWRLGI
DGLSIGPILLTGFITTLATLAAWPVTRDSRLFHFLMLAMYSGQIGSFSSRDLLLFFIMWELEL

IPVYLLLSMWGGKKRLYSATKFILYTAGGSVFLLMGVLGVGLYGSNEPTLNFETSTNQSYP
VPLEIIFYIGFLIAFAVKSPIIPLHTWLPDTHGEAHYSTCMLLAGILLKMGAYGLVRINMELL
PHAHSIFSPWLMIVGTMQIIYAALTSPGQRNLKKRIAYSSVSHMGFIIIGICSITDTGLNGAIL
QIISHGFIGAALFFLAGMSYDRIRLVYLDEMGGIAIPMPKIFTMFSSFSMASLALPGMSGFV
AELIVFFGIITSQKYLLMPKILITFVMAIGMILTPIYSLSMSRQMFYGYKLFNAPNSYFFDSG
PRELFVSISIFLPVIGIGIYPDFVLSLSVDKVEAILSNYFYRMMLEHVLVLSAYLFSIGIYGLIT
SRNMVRALMCLELILNAVNMNFVTFSDFFDSRQLKGDIFSIFVIAIAAAEAAIGPAIVSSIYR
NRKSTRINQSNLLNKMEHTYQYAWIIPFVPLPVPMLIGVGLLLFPTATKNLRRMWAFPSVL
LLLIVMIFSIDLSIQQINSSSIYQYVWSWIINNDFSLEFGYLIDPLTSIMSILITTVGIMVLIYSD
NYMSHDQGYLRFFAYMSFSNTSMLGLVTSSNLIQIYIFWELVGMCSYLLIGFWFTRPIAAN
ACQKAFVTNRVGDFGLLLGILGFYWITGSFEFRDLFEIFNNNEVNFVFVTLCASLLFAGAV
AKSAQFPLHVWLPDAMEGPTPISALIHAATMVAAGIFLVARLLPLFIVIPYIMNLISLIGIITV
LLGATLALAQKDIKRGLAYSTMSQLGYMMLALGMGSYRAALFHLITHAYSKALLFLGSG
SVIHSMEAVVGYSPDKSQNMVLMGGLTKHVPITKTAFLLGTLSLCGIPPLACFWSKDEILN
DSWLYSPIFAIIACSTAGLTAFYMFRIYLLTFEGHLNVHFQNYSGKKNSLFYSISLWGKEGP
KTINKNLLTLLTMNFFSFGNKKTSSYPRESGNTMLFPLLVLGLFTLFVGSIGIPFGLDILSKW
LTPSINLLHQNSN-SVDWYEFVTNAIFSVSIAYFGIFIAFFLYKPVYSSLQNLNLINSFATLWD
KIINVLYDWSYNRGYIDAFYATSLTKGIRGLAELTHFFDRRVIDGITNGFGVTSFFVGEGIKY
VGGG-RISSYLFFYLSYI--------MDLPGPIHDFLLVFLGSGLILGGLGVVLLTNPIYSAFSLGL
VLICISLFYILSNSHFVAAAQLLIYVGAINVLIIFAVMFMNGSEYYKDFHLWTIGDGVTSLVC
TSIFVSLITTIPDTSWYGIIWTTRSNQIIEQDLISNSQQIGIHLSTDFFLPFELISIILLVALIGAIA
VARQMTVPATRKDLMIVNMGPHHPSMHGVLRLIVTLDGEDVIDCEPILGYLHRGMEKIAE
NRTIIQYLPYVTRWDYLATMFTEAITVNAPEQLGNIQVPKRASYIRVIMLELSRIASHLLWL
GPFMADIGAQTPFFYIFRERELVYDLFEAATGMRMMHNYFRIGGVAADLPHGWIDKCLDF
CDYFLTGVAEYQKLITRNPVFLERVEGVGIIGGEEAINWGLSGPMLRASGIQWDLRKVDH
YECYDEFDWEVQWQKEGDSLARYLVRISEMTESIKIIQQALEGIPGGPYENLEIRRFDRVR
DPEWNDFEYRFISKKPSPTFELSKQELYVRVEAPKGELGIFLIGDQSVFPWRWKIRPPGFIN
LQILPQLVKRMKLADIMTILGSIDIIMGEVDRMFPMVTGFMNYGQQTVRAARYIGQSFMV
TLSHANRLPVTIQYPYEKLITSERFRGRIHFEFDKCIACEVCVRVCPIDLPVVDWKLETDIR
KKRLLNYSIDFGICIFCGNCVEYCPTNCLSMTEEYELSTYDRHELNYNQIALGRLPMSVID
DYTIRTILNS-IKNR-----------MQGRLSAWLVKHGLVHRSLGFDYQGIETLQIKPEDWHSIAVI
LYVVYGYNYLRSQCAYDVAPGGLLASVYHLTRIEYGVDQPEEVCIKVFAPRRNPRIPSVFW
VWKSADFQERESYDMLGISYDNHPRLKRILMPESWIGWPLRKDYIAPNFYEIQDAHMGN
EFRCIGCICIYRSFHFRAYPNCWFSLCMAKRSIGMVLAPEYSDNKKKKGKKKNIETVMNSI
EFPLLDRTTQNSAILTTSNDLSNWSRLSSLWPLLYGTSCCFIEFASLIGSRFDFDRYGLVPRSS
PRQADLILTAGTVTMKMAPSLVRLYEQMPEPKYVIAMGACTITGGMFSTDSYSTVRGVDK
LIPVDVYLPGCPPKPEAVIDAITKLRKKISREIYEDRIRSQEENRCFTTNHKFHVGRSTHTGN
YNQGLLYQSPSTSEIPPETFFKYKSSVSSHELVNMQTRNTFSWIKEQITRSISVSLMIYIITRT
SISNAYPIFAQQGYENPREATGRIVCANCHLANKPVDIEVPQAVLPDTVFEAVVRIPYDMQL
KQVLANGKKGALNVGAVLILPEGFELAPPDRISPEMKEKIGNLSFQSYRPTKKNILVIGPVP
GQKYSEITFPILSPDPATKKDVHFLKYPIYVGGNRGRGQIYPDGSKSNNTVYNATAAGIVS
KIIRKEKRGYEITIADASDGRQVVDIIPPGPELLVSEGESIKLDQPLTSNPNVGGFGQGDAEI
VLQDPLRVQGLLFFLASVILAQIFLVLKKKQFEKVQLSEMNFMSKVYDWFEERLEIQAIAD
DITSKYVPPHVNIFYCLGGITLTCFLVQVATGFAMTFYYRPTVTEAFASVQYIMTEANFGW

LIRSVHRWSASMMVLMMILHVFRVYLTGGFKKPRELTWVTGVVLAVLTASFGVTGYSLPR
DQIGYWAVKIVTGVPEAIPVIGSPLVELLRGSASVGQSTLTRFYSLHTFVLPLLTAVFMLMH
FPMIRKQGISGPLMGVTKKPDLNDPVLRAKLAKGMGHNYYGEPAWPNDLLYIFPVVILGT
IACNVGLAVLEPSMIGEPADPFATPLEILPEWYFFPVFQILRTVPNKLLGVLLMVSVPAGLLT
VPFLENVNKFQNPFRRPVATTVFLVGTAVALWLGIGATLPIDKSLTLGLFMIEVFLFGIVLGL
IPITLAGLFVTAYLQYRRGDQLDLMPTITSYFGFLLAALTITSALFIGLSKIRLIMDIVSLAWA
ALMVVFTFSLSLVVWGRSGLMIIRSPEPEVKILVDRDPIKTSFEEWARPGHFSRTIAKGPDT
TTWIWNLHADAHDFDSHTSDLEEISRKVFSAHFGQLSIIFLWLSGMYFHGARFSNYEAWL
SDPTHIGPSAQVVWPIVGQEILNGDVGGGFRGIQITSGFFQLWRASGITSELQLYCTAIGAL
VFAALMLFAGWFHYHKAAPKLAWFQDVESMLNHHLAGLLGLGSLSWAGHQVHVSLPIN
QFLNAGVDPKEIPLPHEFILNRDLLAQLYPSFAEGATPFFTLNWSKYAEFLTFRGGLDPVTG
GLWLTDIAHHHLAIAILFLIAGHMYRTNWGIGHGLKDILEAHKGPFTGQGHKGLYEILTTS
WHAQLSLNLAMLGSLTIVVAHHMYSMPPYPYLATDYGTQLSLFTHHMWIGGFLIVGAAA
HAAIFMVRDYDPTTRYNDLLDRVLRHRDAIISHLNWACIFLGFHSFGLYIHNDTMSALGRP
QDMFSDTAIQLQPVFAQWIQNTHALAPGATAPGATTSTSLTWGGGDLVAVGGKVALLPIPL
GTADFLVHHIHAFTIHVTVLILLKGVLFARSSRLIPDKANLGFRFPCDGPGRGGTCQVSAW
DHVFLGLFWMYNAISVVIFHFSWKMQSDVWGSISDQGVVTHITGGNFAQSSITINGWLRD
FLWAQASQVIQSYGSSLSAYGLFFLGAHFVWAFSLMFLFSGRGYWQELIESIVWAHNKLK
VAPATQPRALSIVQGRAVGVTHYLLGGIATTWAFFLARIIAVGMALRFPRFSQGLAQDPTTR
RIWFGIATAHDFESHDDITEERLYQNIFASHFGQLAIIFLWTSGNLFHVAWQGNFESWVQDP
LHVRPIAHAIWDPHFGQPAVEAFTRGGALGPVNIAYSGVYQWWYTIGLRTNEDLYTGALF
LLFLSAISLIAGWLHLQPKWKPSVSWFKNAESRLNHHLSGLFGVSSLAWTGHLVHVAIPG
ARGEYVRWNNFLDVLPHPQGLGPLFTGQWNLYAQNPDSSSHLFGTSQGAGTAILTLLGGF
HPQTQSLWLTDIAHHHLAIAFIFLIAGHMYRTNFGIGHSMKDLLEAHIPPGGRLGRGHKGL
YDTINNSIHFQLGLALASLGVITSLVAQHMYSLPAYAFIAQDFTTQAALYTHHQYIAGFIMT
GAFAHGAIFFIRDYNPEQNEDNVLARMLDHKEAIISHLSWASLFLGFHTLGLYVHNDVML
AFGTPEKQILIEPIFAQWIQSAHGKTSYGFDVLLSSTNGPAFNAGRSIWLPGWLNAVNE--NS
NSLFLTIGPGDFLVHHAIALGLHTTTLILVKGALDARGSKLMPDKKDFGYSFPCDGPGRGG
TCDISAWDAFYLAVFWMLNTIGWVTFYWHWKHITLWQGNVSQFNESSTYLMGWLRDYL
WLNSSQLINGYNPFGMNSLSVWAWMFLFGHLVWATGFMFLISWRGYWQELIETLAWAHE
RTPLANLIRWRDKPVALSIVQARLVGLAHFSVGYIFTYAAFLIASTSGKFGMSHSVKIYDTC
IGCTQCVRACPTDVLEMIPWDGCKAKQIASAPRTEDCVGCKRCESACPTDFLSVRVYLWH
ETTRSMGLAYMTTINFPSIFVPLVGLVFPAIAMASLSLHVQKNKIVMRDLKTYLSVAPVLST
LWFGSLAGLLIEINRFFPDALTFPFFSFMTVILERRESESLWGRFCNWITSTENRLYIGWFGV
VMIPTLLTATSVFIIAFIAAPPVDIDGIREPVSGSLLYGNNIISGAIIPTSAAIGLHFYPIWEAAS
VDEWLYNGGPYELIVLHFLLGVACYMGREWELSFRLGMRPWIAVAYSAPVAAATAVFLIY
PIGQGSFSDGMPLGISGTFNFMIVFQAEHNILMHPFHMLGVAGVFGGSLFSAMHGSLVTSS
LIRETTENESANEGYRFGQEEETYNIVAAHGYFGRLIFQYASFNNSRSLHFFLAAWPVVGI
WFTALGISTMAFNLNGFNFNQSVVDSQGRVINTWADIINRANLGMEVMHERNAHNFPLD
LAAIEAPSTNG-GLPWYRVHTVVLNDPGRLLSVHIMHTALVAGWAGSMALYELAVFDPSD
PVLDPMWRQGMFVIPFMTRLGITNSWGGWSIT-GGTITNPGIWSYEGVAGAHIVFSGLCFL
AAIWHWVYWDLEIFCDERTGKPSLDLPKIFGIHLFLSGVACFGFGAFHVTGLYGPGIWVSD
PYGLTGKVQSVNPAWGVEGFDPFVPGGIASHHIAAGTLGILAGLFHLSVRPPQRLYKGLR
MGNIETVLSSSIAAVFFAAFVVAGTMWYGSATTPIELFGPTRYQWDQGYFQQEIYRRVGA

GLSENQSLSEAWSKIPEKLAFYDYIGNNPAKGGLFRAGSMDNGDGIAVGWLGHPIFRDKE
GRELFVRRMPTFFETFPVVLVDGDGIVRADVPFRRAESKYSVEQVGVTVEFYGGELNGVS
YSEPATVKKYARRAQLGEIFELDRATLKSDGVFRSSPRGWFTFGHASFALLFFFGHIWHGA
RTLFRDVFAGIDPDLDAQVEFGAFQKLGDPTTKRQVV------------VETLFNGTLALAGRDQE
TTGFAWWAGNARLINLSGKLLGAHVAHAGLIVFWAGAMNLFEVAHFVPEKPMYEQGLIL
LPHLATLGWGVGPGGEVIDTFPYFVSGVLHLISSAVLGFGGIYHALLGPETLEESFPFFGYV
WKDRNKMTTILGIHLILLGIGAFLLVFKALYFGGVYDTWAPGGGDVRKITNLTLSPSVIFG
YLLKSPFGGEGWIVSVDDLEDIIGGHVWLGSICILGGIWHILTKPFAWARRALVWSGEAYL
SYSLGALAVFGFIACCFVWFNNTAYPSEFYGPTGPEASQAQAFTFLVRDQRLGANVGSAQ
GPTGLGKYLMRSPTGEVIFGGETMRFWDLRAPWLEPLRGPNGLDLSRLKKDIQPWQERR
SAEYMTHAPLGSLNSVGGVATEINAVNYVSPRSWLSTSHFVLGFFLFVGHLWHAGRARA
AAAGFEKGIDRDFEPVLSMTPLNMTIALGKFTKDEN-LFDIMDDWLRRDRFVFVGWSGLL
LFPCAYFALGGWFTGTTFVTSWYTHGLASSYLEGCNFLTAAVSTPANSLAHSLLLLWGPEA
QGDFTRWCQLGGLWTFVALHGAFGLIGFMLRQFELARSVQLRPYNAIAFSAPIAVFVSVFL
IYPLGQSGWFFAPSFGVAAIFRFILFFQGFHNWTLNPFHMMGVAGVLGAALLCAIHGATVE
NTLFEDGDGANTFRAFNPTQAEETYSMVTANRFWSQIFGVAFSNKRWLHFFMLFVPVTGL
WMSALGVVGLALNLRAYDFVSQEIRAAEDPEFETFYTKNILLNEGIRAWMAAQDQPHEN
LIFPEEVLPRGNALMSGSTGERSFADIITSIRYWVIHSITIPSLFIAGWLFVSTGLAYDVFGSP
RPNEYFTESRQGIPLITGRFDSLEQLDEFSRSFMTIDRTYPIFTVRWLAVHGLAVPTVSFLGSI
SAMQFIQRMATQTVEGSSRSG-PRRTIIGDLLKPLNSEYGKVAPGWGTTPLMGVAMALFAI
FLSIILEIYNSSVLLDGILMNMLTLKLFVYTVVIFFVSLFIFGFLSNDPGRNPGREEMADTTG
RIPLWLIGTVTGIPVIGLIGIFFYGSYSGLGSSLMLNIFSLICICLNSALY-SNFFFAKLPEAYAF
LNPIVDFMPVIPVLFFLLAFVWQAAVSFRTTQSNPNEQNVELNRTSLYWGLLLIFVLAVLFS
NYFFNMEVNILAFIATALFILVPTAFLLIIYVKTVSQNDMETATLVAISISGLLVSFTGYALYT
AFGQPSQQLRDPFEEHGDMEALVYTFLLVSTLGIIFFAIFFREPPKVPTKKLKMTIAFQLAVF
ALIATSSILLISVPVVFASPDGWSSNKNVVFSGTSLWIGLVFLVGILNSLIS-------MSPQTETK
ASAGFKAGVKDYKLTYYTPEYETKDTDILAAFRVTPQPGVPPEEAGAAVAAESSTGTWTT
VWTDGLTSLDRYKGRCYHIEPVAGAENQFIAYVAYPLDLFEEGSVTNMFTSIVGNVFGFKA
LRALRLEDLRIPPAYSKTFQGPPHGIQVERDKLNKYGRPLLGCTIKPKLGLSAKNYGRAVY
ECLRGGLDFTKDDENVNSQPFMRWRDRFLFCAEALYKAQAETGEIKGHYLNATAGTCED
MMKRAVFARELGVPIVMHDYLTGGFTANTTLAHYCRDNGLLLHIHRAMHAVIDRQKNHG
IHFRVLAKALRMSGGDHIHSGTVVGKLEGERDITLGFVDLLRDDYIEKDRSRGIYFPQDW
VSLPGVLPVASGGIHVWHMPALTEIFGDDSVLQFGGGTLGHPWGNAPGAVANRVALEACV
QARNEGRDLAREGNEIIREASNWSPELAAACAVWKEIKFEFEAMDILDKMAIHLYKTSTPS
TRNGAVDSQVKSNPRNNLIYGQHRCGKGRNARGIITAGHRGGGHKRLYRKIDFRRNEKDI
YGRIVTIEYDPNRNAYICLIHYGDGEKRYILHPRGAIIGDTIVSGTEVPIKMGNALPLTDMPL
GTAIHNIEITLGKGGQLARAAGAVAKLIAKEGKSATLKLPSGEVRLISKNCSATVGQVGNV
GVNQKSLGRAGSKCWLGKRPVVRGVVMNPVDHPHGGGEGRAPIGRKKPTTPWGYPALG
RRSRKRNKYSDNLILRRRSKMIQPQTHLNVADNSGARELMCIRIIGAS-NRRYAHIGDVIVA
VIKEAVPNMPLERSEVIRAVIVRTCKELKRDNGMIIRYDDNAAVVIDQEGNPKGTRVFGAI
ARELRQLNFTKIVSLAPEVLMLSPKRTRFRKQHRGRMKGISYRGNRICFGRYALQALEPA
WITSRQIEAGRRAMTRNARRGGKIWVRIFPDKPVTVRPTETRMGSGKGSPEYWVAVVKP
GRILYEMGGVAENIARKAISIAASKMPIRTQFIISGMTRIRRGYIARRRRTKLRLFASSFRGA
HSRLTRTITQQKIRALVSAHRDRDRQKRDFRRLWITRINAVIRE-NRVSYSYSRLIHDLYKRQ

LLLNRKILAQIAISNRNCLYMISNEIIK--------------EVYALGQHISMSAHKARRVIDQIRGRSY
EETLMILELMPYRACYPIFKLVYSAAANASHNMGFNEADLVISKAEVNEGTTVKKLKPRA
RGRSFPIKRPTCHITIVLKDISLDEGVWDKKMDGIKYAVFTDKSIRLLGKN-QYTSNVESGS
TRTEIKHWIELFFGVKVIAMNSHRLPGKGRRMGPIMGHTMHYRRMIITLQPGYSIPPLRKK
RTMAVPKKRTSLSKKRIRKNIWKRKGYWAALKAFSLAKSLSTGNSKSFFVQQNK-MAKG
KDVRITVILECTSC-ARN--VNKE--GISRYITQKNRHNTPSRLELRKFCPYCYKHTIHGEIKK
MKIRASVRKICEKCRLIRRRGRIIVICSNPRHKQRQGMVREKVTVSTRTLQWKCVESRADS
KRLYYGRFILSPLMKGQADTIGIAMRRALLGEIEGTCITRAKSE--KIPHEYSTIVGIQESVHE
ILMNLKEIVLRSNLYGTCDASICVRGPGYVTAQDIISPPSVEIVDNTQHIASLTEPIDLCIGLQ
IERNRGYRIKTLN--NDGSY--PIDAVFMPVRNANHSIHSYGNGNEKQEILFLEIWTNGSLTPK
EALHEASRNLIDLFIPFLHAEEE--NLHLEDNQ-HKATFPLFTFHDRLA-KLRKNKK--IALKYI
FIDQLELPPRIYNCLKRSNIHTLSDLLNNSQEDLMKIEHFRIEDVKQILGILEKH--FTIDLPK
NKMLRDGN-EGMSTIPGFNQIQFEGFCRFIDQGLTEELFKFPKIEDTDQEIEFQLFVERYQLV
EPLIKERNAVYESLTYSSELYVSAGLIWKTSRDMQEQTIFIGNIPLMNSLGTSIVNGIYRIVIN
QILQSPGIYYRSELDHNGISVYTGTIISDWGGRSELEIDRKARIWARVSRKQKISILVLSSAM
GSNLREILENVCYPEIFLSFLNDKEKKKIGSKENAILEFYQQFACVGGDPVFSESLCKELQK
KFFQQRCELGRIGRRNMNRRLNLDIPQNNTFLLPRDILAAVDHLIGMKFGMGTLDD--MN
HLKNKRIRSVADLLQDQFGLALVRLENVVRGTICGAIR-HKLI-PTPQNLVTSTP----LTTTYE
SFFGLHPLSQVLDRTNPLTQIVHGRKSSYLGPGGLTGRTASFRIRDIHPSHYGRICPIDTSEGI
NVGLIGSLAIHARIGHWG-SLESPFY---EIS--ERSKKVRMLYLSPSRDEYYMVAAGNSLALN
QGIQEEQVVPARYRQEFLTIAWEQVHLRSIFPFQYFSIGASLIPFIEHNDANRALMSSNMQR
QAVPLSRPEKCIVGTGLERQAALDSGVLAIAEHEGKIIYTATDKIILSGNGDTLSIPLLMYQR
SNKNTCMHQKPQVRRGKCIKKGQILADGAATVGGELALGKNVLVAYMPWEGYNSEDAV
LISERLVYGDIYTSFHIRKYEIQTHVTSQG-PERVTKEIPHLEAHLLRNLDKNGIVMLGSWV
EAGDILVGKLTPQMAKE-SSYAPEDRLLRAILGIQVSTSKETCLKLPIGGRGRVIDVRWIPKK
G-GSSYNPEMIRVYISQKREIKVGDKVAGRHGNKGIVSKILPRQDMPYLQDGRPVDMVFN
PLGVPSRMNVGQIFECSLGLTGGLLDRHYRIAPFDERYEQEASRKLVFSELYEASKQTANP
WVFEPEYPGKSRIFDGRTGDPFEQPVIIGKPYILKLIHQVDDKIHGRSSGHYALVTQQPLRG
RAKQGGQRVGEMEVWALEGFGVANILQEMLTYKSDHIRARQEVLGTTIIGGTIPNPEDAPE
SFRLLVRELRSLALELNHFLVSEKNFQINRKEA-------MIDRYKHQQLRIGSVSPQQISAWAK
KILPNGEIVGEVTKPYTFHYKTNKPEKDGLFCERIFGPIKSGICACGNYRVIGDEKEDPKFC
EQCGVEFVDSRIRRYQMGYIKLACPVTHVWYLKRLPSYIANLLDKPLKELEGLVYCD--FS
FARP-IAKKPTFLRLRGSFEYEIQSWKYSIPLFFTTQGFDTFRNREISTGAGAIREQLADLDL
RIIIDDSLVEWKELGEE-GPTGNEWEDRKIGRRKDFLVRRMELAKHFIRTNVKPEWMVLCL
LPVLPPELRPIIQIDGGKLMSSDINELYRRVIYRNNTLTDLLTTSRSTPGELVMCQEKLVQEA
VDTLLDNGIRGQPMRDGHNKVYKSFSDVIEGKEGRFRETLLGKRVDYSGRSVIVVGPSLS
LHRCGLPREIAIELFQTFVIRGLIRQHLASNIGVAKSKIREKEPIVWEILQEVMQGHPVLLNR
APTLHRLGIQAFQPILVEGRAICLHPLVRKGFNADFDGDQMAVHVPLSLEAQAEARLLMFS
HMNLLSPAIGDPISVPTQDMLIGLYVLTSGNRRGICANRYNPCNRRN---YQNERIDDNNYK
YTKEKEPFFCNSYDAIGAYRQKRINLDSPLWLRWRLDQR-VIAS--REAPIEVHYESLGTYH
EIYGHYLIVRSVKKEILFIYIRTTVGHISLYREIEEAIQGFCRACSYGT--------MEVLMAERAN
LVFHNKVIDGTAMKRLISRLIDHFGMAYTSHILDQVKTLGFQQATATSISLGIDDLLTIPSKG
WLAQDAEQQSLILEKHHHYGNVHAVEKLRQSIEIWYATSEYLRQEMNPNFRMTDPFNPV
HIMSFSGARGNASQVHQLVGMRGLMSDPQGQMIDLPIQSNLREGLSLTEYIISCYGARKG

VVDTAVRTSDAGYLTRRLVEVVQHIVVRRTDCGTIRGISVSPRNGMMP-ERIFIQTLIGRVL
ADDIYMGPRCIAARNQDIGIGLVNRFIT------FRAQPISIRTPFTCRSTSWICRLCYGRSPTHG
DLVELGEAVGIIAGQSIGEPGTQLTLRTFHTGGVFTGGTAEHVRAPSNGKIKFNEDLVHPTR
TRHGHPAFLCYIDLYVTIESEDILHNVNIPPKSFLLVQNDQYVESEQVIAEIRAGTST-LNFKE
RVRKHIYSDSEGE-MHWSTDVYHAPEFTYGNVHLLPKTSHLWILSGGPCR--SSVAPFSLHK
DQDQMNVH--SLSVERRYISNLS--VTNDQ-VRHKFFSSYFSGKKEGRIPDY-SELNRIIGTSR
CNLIYP-AILYENS--DLLAKRRKNRFIIPFQSIQEREKELMPRSGISIEIP--INGIFRRNSILAYF
DDPRYRRKSSGITKYGTIEVHSIVKKED--LIEYRG-VKEFRPKYMKVDRFFFIPEEVHILPGS
SSIMVRNNSIIGVNTQITLNTRSRVGGLVRVERKKKRIELKIFSGDIHFPGETDKISRHSGILIP
PGT--GKANSKESKK--LKSWIYVQR--ITPTK--KKYFVLVRPVVTYEIADGINLATLFPQDLL
QERDNVQLRVVNYILYGNSKPIRRISDTSIQLVRTCLVLNWDQDKK---SSSIEEAHASFVEV
KTNGLIRDFLRIDLVKSPISYTGKRNDPSGSGLISD-SGSDRTNINPFYSIYSKAR-IQQSLSQ--
--NQGTIRTLLNR---NKECQSLIILSSSNCSRMGP--FNSVKYHNV---IKESDPLIPIRNLLGPLG
T--ALQIANFYS--FYHL--ITHNQIL-VTNYLQLDN---LKQA-QVLK---YYLMDEN--GRIYNPD
PCSNILLNPFNLNWYFLHHNYCEETS-TKMSLGQFICENVCIAKNGPH-LK--SGQVLIVQ-V
DYVVIRLAKPYLATPGATVHGHYGEILYEGDTLVTFIYEKSRSGDITQGLPKVEQVLEVRSI
DSISMNLEKRVKGWNEHITRILGIPWGFLIGAELTIVQSRISLVNKIQKVYRSQGVQIHNRHI
EIIVRQITSKVLVSE---DGMSNVFSPGELIGLLRAERTGRALEEAICYRAILLGITRASLNTQS
FISEASFQETARVLAKAALRGRIDWLKGLKENVVLGGMIPVGTGFKGL--VHRSRQHNNI--
LLETKKKNLFEGEMR--DILFHHRELFDSCVSKNFHDTSEQSFMG------LNVNMTRRYWNI
NLEEMMEAGVHFGHGTRKWNPRMAPYISAKRKGIHITNLTRTARFLSEACDLVFDAASRG
KQFLIVGTKNKAADSVAWAAIRARCHYVNKKWLGGMLTNWSTTETRLHKFRDLRTEQK
TGRLNRLPKRDAAMLKRQLSHLQTYLGGIKYMTGLPDIVIIVDQQEEYTALRECITLGIPTI
CLIDTNCDPDLADISIPANDDAIASIRLILNKLVFAICEGRS-SYIRNPMGQKINPLGFRLGTT
QGHHSLWFAQPKNYSEGLQEDQKIRDCIKNYVQKNMRISSG-VEGIARIEIQKRIDLIQVIIY
MGFPKLLIEGRPR----GIEELQMNIQKEFNCVNRKLNIAITRIAKPYGHPNILAEFIAGQLKN
RVSFRKAMKKAIELTEQADTKGIQVQIAGRIDGKEIARVEWIREGRVPLQTIRAKIDYCSYT
VGTIYGVLGIKIWIFIDEEMSRYRGPRFKKIRRLGALPGLTNKRPRAGSDLRNQSRSGK-RS
QYRIRLEEKQKLRFHYGLTERQLLKYVRIAGKAKGSTGQVLLQLLEMRLDNILFRLGMAS
TIPGARQLVNHRHILVNGRIVDIPSYRCKPRDIITARDEQKSKALIQNYLDSSPH-EELPNHLT
LHPFQ--YKGLVNQIIDSKWVGLKINELLVVEYYSRQTMSRRGTAEEKPAKSDPIYRNRLVN
MLVNRILKHGKKSLAYQIIYRAVKKIQQKTETNPLSVLRQAIRGVTPDIAVKARRVGGSTH
QVPIEIGSTQGKALAIRWLLGASRKRPGRNMAFKLSSELVDAAKGSGDAIRKKEETHRMA
EANRAFAHFRMGRDTIADIITSIRNADIDRKGTVRIAFTNITENIVKILLREGFIENVRKHRE
SNKYFLVSTLRHRR--NRKRPYR---NILNLKRISRPGLRIYSNYQRIPRILGGMGIVILSTSRGI
MTDREARLEGIGGEILCYIWMAKPIPRIGSRRNGRIGSRKSARRIPKGVIHVQASFNNTIVT
VTDVRGRVVSWSSAGTCGFRGTRRGTPFAAQTAAGNAIRTVVDQGMQRAEVMIKGPGL
GRDAALRAIRRSGILLSFVRDVTPMPHNGCRPPKKRRVMPTIKQLIRNTRQPIRNVTKSPAL
RGCPQRRGTCTRVYTITPKKPNSALRKVARVRLTSGFEITAYIPGIGHNSQEHSVVLVRGGR
VKDLPGVRYHIVRGTLDAVGVKDRQQGRSKYGVKKPKMARKSLIQREKKRQKLEQKYH
LIRRSSKKEIS---KVPSLSDKWEIHGKLQSPPRNSAPTRLHRRCFSTGRPRANYRDFGLSGHI
LREMVHACLLPGATRSSWMVKNSFISVISQEE---NR--GSVEFQVFSFTNKIRKLTSHLELHR
KDYLSQRGLRKILGKRQRLLAYLSKKNRVRYKELIGQLNIREPKTR--MVKLRLKRCGRKQ
RAVYRIVAIDVRSRREGRDLRKVGFYDPIKNQTYLNVPDILYFLEKGAQPTGTVHDILKKA

EVFKKLRLNQPKLN-MDKSKRPFFKSKRSFRRRLPPIQSGDRIDYRNMSLISRFISEQGKILS
RRVNRLTLKQQRLITIAIKQARILSSLPFLNNEK--QFERIESTPRTTGLRTRNKVTRSLKKNP
FVANHLLRKIDKLNTKAEKEIIVTWSRASTIIPTMIGHTIAIHNGKEHLPIYITDRMVGHKLG
EFAPTLNFRGHAKNDNRSRRMIFKSFLLGNLVSLCMKIINSVVVVGLYYGFLTTFSIGPSYL
FLLRAQVMEEGEDGTEKKVSATTGFITGQLMMFISIYYAPLHLALGRPHTITVVALPYLLF
HFFWNNHKHFFDYGSTTRNSMRNLSIQCVFLNNLIFQLFNHFILPSSMLARLVNIYMFRCN
NKMLFVTSSFVGWLIGHILFMKWVGLVLVWIRQNNSIRSLIRSNKYLVSELRNSMARIFSIL
LFITCVYYLGRIPSPILTKKLKETEEDKPLVTLLFDYKRWNRPLRYIKNDRFANAVRNEMSQ
YFFYTCRSDGKQRISFTYPPSLSTFGEMIQRKMSLSTTEKLSSDELYNHWIYTNEQKRN-LS
NEFIKRIAALDKGSLSMDVLEKRIRLCNDETKKEYLPKMYDPFLNGPYRGTIKKMFSSIET
VWLNKIHGIL-REFEQKDLNDQSIGINEMSKKVPRWSYKLIDLEQQEGGEIRSRKAKRVVIF
TDALIRYSQQSDFRRDIIKGSMRAQRRKTVIWELFQANAHSPLFLDRIDKSPFFSFDI--SGL
MKLIFRNRKEKARIEIAEVWDTILFAQVIRGFMLVTQSILRKYVIFPSLIIAKNIGRILLFQFP
EWSEDLKGWNREMHVKCTYNGVQLSETEFPKNWLTDGIQIKILFPFCLKPWHRSKKGQK
NDFCFLTVWGMEAELPFGSPRKRPSFFEPIFKELEKKIIKLKKKCFLV-LRGLKERTNLFLEV
SKEKKKWVLKSVL--FIKRITKELSKANDYIISNRMIHESSIQIRSMDWTNYSLTEKKMKDL
TDRTSTIRNQIERITKDKKKSPNKTSYDAKRLESPKNIWQVVKRRNARLIRKSHYFLKFLIE
RIYIDIFLCIINIPRVNAQLF-HFISCDLSSLSQAYVFYKLAQAQVINLYKLRSVLQYTSFFLK
KKRKDYFTQGIFHSELRHKKLRNSGMNQWKNWLKGHYQYDLSQIRWSRLVPQKWRNRI
NERRTAQN-NKWDSYEKDQ--LIHYKKQDNFKKHYRYDLLSYKYINDDIMYPNRKYFDW
RILHFCLRLFDWMGMNEEILNRWFFPELVLLYNAYKIKPWVIPIKLLLLNSKSNTEAELDFF
LKRYLLFQLRWNDPLNQRLINNIKVYCLLLRLINPREIAISSIQRREMSLDIMLIQKNLTLTE
LMK-GILVIEPVRLSVKSDGQFIMYQTIVISLVHKSKHNHSDLLVPENILSPRRRRELRILICF
RFLWPNYRLEDLACMNRYWFDTNNGSRFSMVRIHMYPMKGHQFKSWIFELREILREIKN
SHYFLDSWTQFNSVGSFIHIFFHQERFIKLFDPRIWSILLS-RNS--QGSTSNRYF---TIKGVVL
FVVA-VLIYR----INNRNMVERKNLYLIGLLP-----IPMNSIGPRNDTLEESF-GSSNINRLIAS-L
LYLPKGKKISESSFLDPKESTWVLPIT----KKCIMPESNWGSRWWRNWIGKKR--NSSCKISN
---ETVAGIEISFKEKDIK-YLEFLFVYYMD--DPIRKDHDWELFDRLSLRKRRNIINL--NSGQL
FEILVKHWICYLMSAFREKIPIEVEG-FFKQQG-AGSTIQSNDIEHLSH--LFSRNKWAISLQN
CAQFHMWQFRQDLFV---SWGK-NPHESDFLRN-VSRENWIWLDNVWLVNKDR---FFS--KV
RNVSSNIQYDS---TR--SSFVQVTDSSQLKGSS-DQSRDHLDSIS----NEDSE------------YHTLI
NQREIQQLKERSIL-WD--PSFLQTERTEIESD--RFPK--CLSGYS--SMSRLFT-EREKQMNNH
LLPEEIEEF--LGNPTRSIRSFFSDRW--SELH-LGSNPTERSTRDQ----KLLKKEQDVS-FVPSR
RSE----NKEMVNIFKIITYLQN--TVSIHPISSDPGCDMVP-KDEPD-MDSSNKISFLNKNPFFD
L--FHLFHDRNRGGYTL--HHDFESE--ERFQEMADLFTLSITEP-DLVYHKGFAFSIDSYGLD
QKQ--FLNEVFNSRDESKKKSL---LVLPPIFY--EENESFYRRIRKKWVR-ISCGNDLEDPKPKI
VVFASNNIMEAVNQYRLIRNLIQIQYSTYG--YIRNVLNRFFLMN---RSDRNFEYGIQ---RDQ
IGNDTLNHRTRMKYTINQHLSNLKK-SQKKWFDP--LIFISRTERSMNRDPDAYRYKWSNG
SKNFQEHLEHFVSEQKSR---FQVVFDRLRINQYSIDWSEVIDKKDLSKSLRFFLSKSLL------
---FLS-------KLLLFLSNSLP-FFFVSFGNIPIHRSE-IHIY-ELKGPNDQLCSQLLESIGLQIVHLK
KWKPF--LLD---DHDTS-QKSKFLINGGTISPFLFNKIP-KW-MIDSFHTR--NNRRKSFDNTDS
YFSMISHDQDNWLNPVKPFHRSSLISSFYKANRLRFLNNPHHFCFY--CNKRFPF---Y-VEKA
RINNYD--FTYG-QFLNILFIRNK-IFSLYVGK--KKHAFWG-RDTISPIESQVSNIFIPNDFPQSG
DET-YNLYKS---FHFPIRSDPFVR--RAIYSIADISGTPLTEGQIVNFERTYCQPLSDMNLSDSE

GKNLHQYLN--FNSNMGLIHTPCSEKYLPSEK--RKK--------RSLCLK--KCVEKGQMYRTFQ
RDSAFSTLSKWNLFQTYMPWFLTSTGYKYLNLIFLDTFSD-LLPILSSS--QKFVSLFHDIMH
GSDISWRILQKKL------CLPQWN-LISEISSKCLHN--LLLSEEMIHRNNES--PLISTHLRSPNV
REF-LYSILFLLLVAGY-LVRTHLLFVSR--ASSELQTEFEKVKSLMIPSYMIELRKLLDKYPTS
EPNSFWLKNLFLVALEQLGDSLEEIWGSVSGGNMLLGGGP-----AYG-VKSIRSKKKYLNIN
LIDIIDLI-SIIPNPINRITFSRNTRHLSHTSKEIYSLIRKRKNVNGDWIDDKIESWVANSDSIDD
EEREFLVQFS--TLTT--EKRIDQILLSLT-HSDHLS-----KNDSGYQMIEQPGAIYLRYLVDIH--K
KYLMNYEFNTSCLAERRIFLAHYQTITY--SQTSCGANSFHFPSHG---KPFSLRLALSPS-RGI
LVIGSIGTGRSYLVKYLATNSYVPFITVFLNKFLDNKPKGFLID-----DIDDIDASDDIDASDDI
DDS----------------DDIDRDLDTELELLTMMNAL---TMDMMP---EIDRFYITLQFELAKAMSP
CIIWIPNIHDLDVNESNYLSLGLLVNYLS---RDCERCS-TRNILVIASTHIPQKVDPALIAPNK
LNTCIKIRRLLIPQQRKHFFTLSYTRGFHLEKKMFHT--NGFGSITMGSNARDLVALTNEALS
ISITQKKSIIDTNIIRSALHRQTWDLR---SQVRSVQDHGILFYQIGRAIAQNVLLSNCPIDPISI
YMKKK--SCNEGDSYLYKWYFELGTSMKKLTILLYLLSCSAGSVAQDLWSLP--GPDEK-NG
ITSYGLVENDS-DLVHGLLEVEGALVG--SSRTEKDCSQFDND--RVTLLLRPEPRN-PLDMM
QNGSCSIVDQRFLYEK--YESEFEE-GEGEGALNPQ----QIEED----LFNH--IVWAP--RIWRP---
WGFLFDCIERPNELGFP-YWARSFRGK---RIIYDEEDELQENDSEFLQSGTVQYQTRDRSSK
EQGFFRISQFIWDPADPLFFLFK-DQPFVSVFSHREFFADEEMSKGLLTSQTDPPTSIYKRWFI
--KNTQEKHFELLINHQRWLR---TNSSLS--NGSFRSN--TPSESYQYLSNLFLSN-GTLLDQMT
KTLLRKRWLFPDEMKIGF--M---------MPRSR-INGNFIDKTFSIVANILLRIIPTTSGEKEAFTY
YRDG--MSAQSEGNYAEALQNYYEAMRLEIDPYDRSYILYNIGLIHTSNGEHTKALEYYFR
ALERNPFLPQAFNNMAVICHY-RGEQAIRQGDSEIAEAWFDQAAEYWKQAIALTPGNYIEA
HNWLKITRRFE--MSWRSERIWIELITGSRKTSNFCWAFILFLGSLGFLLVGTSSYLGRNLISL
FPSQQIIFFPQGIVMSFYGIAGLFISSYLWCTISWNVGSGYDRFDRKEGIVCIFRWGFPGKNR
RIFLRFLMKDIQSIRIEVKEGIYARRVLYMETRGQGAIPLTRTDEN-LTPREIEQKAAELAYFL
RVPIEVF
>Bulnesia
--MKNHEN----------
------YQDSYYN---SDTESNWNNHINRCINSYFN--SQMN---------------YIFNYNENESQSSIKT
SINESE--IKND-----QKPDISQLWVQCENCFELNYKRFLKSKMNICEHCGYHLKMSSSDRIEL
SIDPGTWDPMDEEMVLDEPEEESRLSDPYPDYISSYQRKTGLTEAVQTGTGRLNGIPVAIG
VMDFQFIGGSMGSVVGEKITRLIDYAINQHLPLIIVCASGGARMQEGSFSLMQMAKISSVL
YSYKKKKGLFYVSILTSPTTGGVTASFGMLG--------------------------------------ISLLPSLMP--I
LHLRVKE----------------MVTIRADEIRNIIRERIEQYNREVKIVNTGTVLQVGDGIARIYGLDE
VMAGELVEFEEGTVGIALNLESKNVGVVLMSDGLMIQEGSSVKSTGRIAQIPVSEAYLGR
VLNALAKPIDGRGEISASESRLIESPAPGIVSRRSVYEPLQTGLIAIDSMIPIGRGQRELIIGDR
QTGKTAVATDTILNQQGENVICVYVAIGQKASSVAQVVTTLQEKGAMEYTIVVAEPADSPA
TLQYLAPYTGAALAEYFMYRKRHTLVIYDDLSKQAQAYRQMSLLLRRPPGREAYPGDVF
YLHSRLLERAAKLNSSLGEGSMTALPIVETQSGDVSAYIPTNVISITDGQIFLSADLFNAGIR
PAIDVGISVSRVGSAAQIKAMKQVAGKSKLDLAQFTELESFAQFASDLDKTTQKQLARGR
RLRELLKQSQSDPLPVEEQILTIYTGTNGYLDSLEIDQVKKFLGDLRTYLKTNKPKFKEIISS
TKTFTNEAEALLKEAIQEQMERFLLQEQVMRTNPSTSGSGVSALEKK?LGHIVQIIGPVLDV
AFPPGKMPKIYNALVVKAENTVGQEINVTCEVQQLLGNNRVRAVAMSATDGLMRGMEVI
DTGAPLSVPVGGVTLGRIFNVLGEPVDNLGPVDTSTTSPIHKSAPAFIQLDTKLSIFETGIKV

VDLLAPYRRGGKIGLFGGAGVGKTVLIMELINNIAKAHGGVSVFGGVGERTREGNDLYM
EMKESGVINETNLSESKVALVYGQMNEPPGARMRVGLTALTMAEYFRDVNKQDVLLFID
NIFRFVQAGSEVSALLGRMPSAVGYQPTLSTEMGSLQERITSTKQGSITSIQAVYVPADDLT
DPAPATTFAHLDATTVLSRGLAAKGIYPAVDPLDSTSTMLQPQIVGEEHYETAQQVKQTLQ
RYKELQDIIAILGLDELSEEDRLTVARARKIERFLSQPFFVAEVFTGSPGKYVGLAETIRGFQ
LILSGELDSLPEQAFYLVGNIDEATAKATNLE-------MTFNLCVLTRNRIVWDSEVKEIILSTN
SGQIGILPNHAPIATAVDISILKIRLNEQWITMFLMDGFARIRNNEVTILVKDAEKGSDIDPQ
EAKQTLEMAEADLKKAEGKRQTIEATLALKRAKTRVEAINFVNMNNLTDSFISLGHWPSA
ESFGFNTDILATNPINLGIVIGVLIFFGKGVLSDLLDNRKQRILNTLRNSEELSEGAIEQLEK
ARTRLQKVEMEADQFRVNGYSEIEKQKLNLINSTYKTLEQLENYKNETIRFEEQRAINQVR
QRVFQQALQGALVTLNSCLNNELHFRTINANIDLFGKMKEITDMNPLISAASVIAAGLAVG
LASIGPGIGQGTAAGQAVEGIARQPQAEGKIRGTLLLSLAFMEALTIYGLVVALALLFANPF
VMTVLLCSMNTLKGLYDLSGVEVGQHFYWQLGAFQVHGQVLITSWVVIFILLGSSIIAVR
NPQTIPTNGQNFFEYVLEFIRDVSKTQIGEEYGPWVPFIGTMFLFIFVSNWSGALLPWKIIQ
LPHGELAAPTNDINTTVALALLTSVAYFYAGISKKGLSYFGKYIKPTPILLPINILEDFTKPLS
LSFRLFGNILADELVVVVLVSLVPLLVPIPVMFLGLFTSGIQALIFATLAAAYIGESMEGHHM
IFLTLEHILTHISFSIISIVIIIHLIILFIDEIIELYDSSEKGMIAIFLCITGLLFTRWISLGHFPLSDL
YESLICLSWSLSIVHIVSYWKKHANPFSTITRSSAILTQGFATSGLLTGMHQSVILVPALQSQ
WLMMHVSMMILGYAALLFGSLLSVALLVIQFRKFFNIFDKNKEIQYMKEKGNILL------TL
NYYKFQLIEQLDHWSSRIISLGFIFLTIGILSGAVWANEAWGSYWNWDPKETWAFITWLIFA
IYLHIRTNKNFESINSAIVSSVGFFIIWICYFGVNLLGIGLHSYGSFT--MKKKKTSISFIYLVS
MVFLPWWISFSLNKSLKSWIINWWNNRQSEIFMNSIPERYVIEKFIELEELSLLDEMIKEDP
DPQMQKLHIGIHNETIELINIYNEDRIQMIFDLSTNIIAFIILSGYYILGNEELVILNSWVQEFL
YNLSDTIKAFAILFLTDLCIGFHSPHLWELMISSVYKDFGFDHNDQIISFLVSTLPVILDTILK
YWIFRYLNSVSPSLVVIYHSLNDMPVGVPKVPFLIPGDEDASWVDLYNRLYRQRLLFLGQE
VNDEISNQLIGLMVYLSLEDSTLNQYLFINSPGGLILPGIGIVSTMSTLVPEVQTICMGSAIS
TGCFILVGGEITKRMAFPHARVMLHQPASSFLEAPMRDMILEGEEILNLYEIVTTTYVIRTG
K P L W V I V E D M K K D V F M S A E E A K N Y G L V D L I S E
G???MKKFRG
YLELHRSRQYKFLYPLLFREYIYVLAHDHSFVGYDNQYSLLIVKRLINRMYQQNDWRIPS
KDSKQNSLFGKNSYFQQISEGFAVILEIPFSLRFLYSLIAKSYNLRSIHSIFPFLEDKLSHLNY
VLDVLIPYPIHPEIFIQILRYCLKDASALYLLRLFIHDYWNFHSLITQFISIFSKPRFSFFLYNS
YVCEYESIFLFLGNQFSHLRSSSYGPFIERIHFYQKTEHLLKVFANLPIFWFFKDPFLLYVRY
KEKSIFASKDTPLWIHKWKSYFVNLFQYHFYVWPQPERIYINQLSKHNLYLLGYFSSVQLN
SSLVRSQMLENSFLIEN--AMNKLDTIVPMIPMIRSLAKAQFCNSLGHPISKPNWIDLSDSDII
DQFARICRNLSHYFSGSSKKKNLYRIKYILRLSCVKTLARKHKSTVRALLKKLGSEFLEEF?
IQEEHTLSFLFSRRIWYLDIISRNDLSNYQMIINAT-EVQ-DINSFSIFQL-QKESYEILWALIPIL
TLVLGITIGVLVIVWLEREISAVIQQRIGPEYAGPFGILQSLADGTKLLFKENLFPSRGDTNLF
SIGPSIAVISILLSYSVIPFGYNLILSDLNIGVFLWIAVSSIAPIGLLMSGYGSNNKYSFLGGLR
AAAQSISYEIPLTLCVLSISLLSNSSSTVDIVETQSKYGLWGWNLWRQPIGFIIFLISSLAECE
RLPFDLPEAEEELVAGYQTEYSGIKFGLFYVASYLNLLVSSLFVTVLYLGGWNLSIPYIFVPE
LFEINRTSSIFQMTINIFITLAKTYLFLFISIATRWTLPRLRMDQLLNLGWKFLLPISLGNLLLT
TSFQLLSL------------------MKSFHLLLFDGSLIFPECILIFGLILLLMIDLTSDQKDIPWLYFISST
SLVMSITVLLFRWREEPMISFSGNFQTNNFNELFQFLILLCSTLCIPISVEYIECTEMAITEFL

LFILTATLGGMFLCGANDLITIFVAPECFSLCSYLLSGYTKKDVRSNEATTKYLLMGGASSSI
LVHAFSWLYGSSGGEIELQEIVNGLINTQMYNSPGISIALIFITVGIGFKLSPAPSHQWTPDV
YEGSPTPVVAFLSVTSKVAASASATRIFDIPFYFSSNEWHLLLEILAILSMILGNFIAITQTNM
KRMLAYSSIGQIGYVIIGIIVGDFNGGYASMITYMLFYISMNLGTFACIVSFGLRTGTDNIRD
YAGLYTKDPFLALSLALCLLSLGGLPPLAGFFGKLHLFWCGWQAGLYLLVSIGLFTSVVSI
YYYLKIIKLFMTGRNQEITPHVRNYRRSPFRSKNSIELSMIVCVIASTIPGISMNPIIEIAQDT
LFMFLLYEYDLFWAFLIISSVIPILAFFISGVLAPISKGPEKLSSYESGIEPMGDAWLQFRIRY
YMFALVFVVFDVETVFLYPWAMSFDILGVSVFIEALIFVLILIVGLVYAWRKGALEWSTND
FPWLTLIVVFPILAGSFIFFLPQRGNKTIRWYTICVCILELLLTTYAFCYYFKLDDPLIQLGED
YQWIPFFDFDWRLGIDGLSIGPILLTGFITTLATLAAWPITRDSRFFHFLMLAMYSGQIGLFS
SRDILLFFIMWELELIPVYLLLSMWGGKKRLYSATKFILYTAGGSIFLLIGVLGIGLYGSNQP
ILNFETSANQSYPIALEIIFYIGFFIAFAVKLPILPLHTWLPDTHGEAHYSTCMLLAGILLKMG
AYGLVRINMELLPHAHSLFSPCLVIIGTLQIIYAASTSPGQRNLKKRIAYSSVSHMGFIIIGIGS
INDMGLNGVILQIISHGFIGAALFFLAGTSYDRIRLVYLDEMGGMAIPIPKIFTMFNILSMAS
LALPGMSGFVSELIVFFGIITSQKYLLMAKILITFVMAIGMILTPIYSLSMLRQMFYGYKLFS
TSNSYFFDSGPRELFISISILLPVISIGIYPDFIISLSVDKVEAILSKYFYRMMLEHVLVLSAYL
FSIGIYGLITSRNMVRALMCLELMLNAVNINFVTFSDFFDSRQLKGNIFSIFVIAIAAAEAAI
GLAIVSSIYRNRKSTCINQSNLLNKMEHMYQYSWIIPFLPLPVPILIGVGLILFPTATKNFRRI
WAFPSILLLSIAMIFSTELSIQQINSNSIYQYVGSWTINNDFSFELGYCIDPLTCIMSILITTVAI
LVLFYSDNYMFYDEGYLRFFAYISFFTTSMLGLVTSSNLIQIYIFWELVGLCSYLLIGFWFTR
PSAASACQKAFVTNRVGDFGLLLGILGFYWITGSFEFRDLFEISKN-EINSLFLSLCSFLLFT
GAVAKSAQFPLHVWLPDAMEGPTPISALIHAATMVAAGIFLVARLLPLFIVVPYIMNIIAILG
IITLLFGATLALAQKDVKRGLAYSTMSQLGYMMLALGMGSYRAGLFHLITHAYSKALLFL
GSGSIIHSMESLVGYSPNKSQNLVIMGGLKKHVPITKTAFLLGTLSLCGIPPLACFWSKDEIL
YDSWLYSSIFAIIACSTAGLTAFYMFRIYLLIFEGHFNFHFQNYSGKKSSSFYSISIWGKEGK
KIIKNNNLFTPNINFLKFTKN-IFLYPRESDNTMLFPMIILVLFTLFIGAIG--LDFDLLSKLLTP
SINLLEQTSN-SEGW-QFLTNATFSVSIAFIGIFIASFLYNPFYSSLTNFHLMNLFYILWDKIINF
IYDWSYNRGYIDYFYEILFIKSIRKLSEFTHFFDRRIIDGIPNGFGITSFFVAEGIRYVGSG-RIS
SYLLLYLSYVLIFLLIYYMDLPGPIHDFILVFLGSGLILGSLGVVLFSNSMYSAFSLGLVLFCI
SLLYISSNSHFVAAAQLLIYVGAINVLIIFAVMFMNNSEYSKNFNILTFRNGITSLVCISIFFSL
VTTILDTSWYSIIWTTRSNHIIEQELINNSQQIGIHLSTDFFLPFELISIILLVSLIGAIAMARQM
NIPATRKDLMIINMGPHHPSMHGVLRLIVTLDGEDVIDCEPILGYLHRGMEKIAENRTIIQY
LPYVTRWDYLATMFTEAITVNGPEQLGNIQIPKRASYIRVIMLELSRIASHLLWLGPFMADI
GAQTPFFYIFREREIIYDLFEAATGMRMMHNYFRIGGVAADLPYGWIDKCLDFCDYFSKG
ISEYKKLITRNPIFLERVEGVGIIGEEEAINWGLSGPMLRASGIKWDLRKVDHYESYDEFD
WEVQWQKEGDSLARYLVRISEMAESIKIIQQALEGIPGGPYENLETRCFDKKRGPKWGDF
EYRFISKKPSPTFELPKQELYVRVEAPKGELGIFLIGDQSGFPWRWKIRPPGFINLQILPQLV
KRMKLADIMTILGSIDIIMGEVDRMFPMVTGFMNYGQQTVRAARYIGQGFMITLSHANRL
PVTIQYPYEKLITSERFRGRIHFEFDKCIACEVCVRVCPIDLPVVDWKFENDIRKKRLFNYSI
DFGICIFCGNCVEYCPTNCLSMTEEYELSTYDRHELNYNQIALGRLPIPVFDDYTIRTILNS--
-------------MQGPLSAWLFKHGLVHRSLGFDYQGIETLQVKSKDWYSIAVILYVYGYNYLRS
QCAYDVAPGGLLASVYHLTRITYGIDQPEEVCIKVFAPRVDPKIPSVFWVWKSADFQERES
YDMLGIFYDNHPRLKRILMPESWIGWPLRKDYIAPNFYEIQDAH-------------------------------
---------------------------MNSIEFPLLDRTTKNSVISTTLNDFSNWSRLSSLWPLLYGTSCCFIEF

ASLIGSRFDFDRYGLVPRSSPRQADLILTAGTVTMKMAPSLVRLYEQMPEPKYVIAMGACT
ITGGMFSTDSYSTVRGVDKLIPVDIYLPGCPPKPEAVIDAITKLRKKISREIYEDRIQYQ-ENR
CFTTTHKFHVRRSTHTGNYEQGLLYQSPFTSEIPT--FLKYKSSVSSHEFLNMQTRKIFSWIN
EKITQSISISFLFIIMTDASISNAYPIFAQQGYENPREATGRIVCANCHLANKPVDIEVPQAVL
PDTVFEAVIRIPYDKQLKQVLANGKKGSLNVGAVLILPEGFELAPSDRISPEMREKIGNLYF
QSYRPNQKNILVIGPVSGQKYSEITFPILSPDPSTNKDVYFLKYPIYVGGNRGRGQIYPDGS
KSNNTVYNATAGGIVSKILRKEKGGYEITISDPSNGRQVVDIIPPGPELLVSEGESIKLDQPL
TSNPNVGGFGQGDAEIVLQDPLRVQGLLFFFASVILAQIFLVLKKKQFEKVQLSEMNFMSK
VYDWFEERLEIQAIADDITSKYVPPHVNIFYCLGGITLTCFLVQVATGFAMTFYYRPTVTDA
FASVQYIMTEANFGWLIRSVHRWSASMMVLMMILHVFRVYLTGGFKKPRELTWVTGVIL
AVLTASFGVTGYSLPWDQIGYWAVKIVTGVPEAIPVIGSPLVELLRGSASVGQSTLTRFYSL
HTFVLPLLTAVFMLMHFPMIRKQGISGPLMGVTKKPDLNDPVLRAKLAKGMGHNYYGEP
AWPNDLLYIFPVVILGTIACNVGLAVLEPSMIGEPADPFATPLEILPEWYFFPVFQILRTVPN
KLLGVLLMVSVPAGLLIVPFLENVNKFQNPFRRPVATTVFLIGTVVALWLGIGATLPIDQSL
TLGLFMIEVFLFGIVLGLIPITLAGLFVTAYLQYRRGDQLDFMPTITSYFGFLLAALTITSALF
IGLSKIRLIMDIISLSWAALMVVFTFSLSLVVWGRSGLMIIRSPEQEVKILVDRDPVKTSFEE
WAKPGHFSRTIAKGPDTTTWIWNLHADAHDFDSHTNDLEEISRKVFSAHFGQLSIIFLWLS
GMYFHGARFSNYEAWLSDPTHIGPSAQVVWPIVGQEILNGDVGGGFRGIQITSGFFQMWR
ASGITNELQLYCTAIGALIFAALMLFAGWFHYHKAAPKLSWFQDVESMLNHHLAGLLGL
GSLGWAGHQIHVSLPINQFLNAGVDPKEIPLPHEFILNRDLLAQLYPSFAEGATPFFTLNWS
KYAEFLTFRGGLDPVTGGLWLTDIAHHHLAIAILFLIAGHMYRTNWGIGHDIKDLLEAHKG
PFTGQGHKGLYEILTTSWHAQLSLNLAMLGSLTIIVAHHMYSMPPYPYLATDYGTQLSLFT
HHMWIGGFLIVGAAAHAAIFMVRDYDPTTRYNDLLDRVLRHRDAIISHLNWVCIFLGFHS
FGLYIHNDTMSALGRPQDMFSDTAIQLQPVFAQWIQNTHALAPSTTAPGATTSTSLTWGAS
NLVAVGGKVALLPIPLGTADFLVHHIHAFTIHVTVLILLKGVLFARSSRLIPDKANLGFRFPC
DGPGRGGTCQVSAWDHVFLGLFWMYNAISVVIFHFSWKMQSDVWGSISDQGVVTHITG
GNFAQSSITINGWLRDFLWAQASQVIQSYGSSLSAYGLFFLGAHFVWAFSLMFLFSGRGY
WQELIESIVWAHNKLKVAPAIQPRALSIVQGRAVGVTHYLLGGIATTWAFFLARIIAVGMA
LRFPRFSQGLAQDPTTRRIWFGIATAHDFESHDDITEERLYQNIFASHFGQLAIIFLWTSGNL
FHVAWQGNFEQWVQDPLHVRPIAHAIWDPHFGQPAVEAFTRGGALGPVNIAYSGVYQW
WYTIGLRTNEDLYTGSLFLLFLSALFLIAGWLHLQPKWKPSVSWFKNAESRLNHHLSGLF
GVSSLAWTGHLVHVAIPGSRGEYVRWNNFLDVLPHPQGLGPFSQVQWNLYAQNPDSSSH
LFGTSQGAGTAILTLLGGFHPQTQSLWLTDIAHHHLAIAFIFLVAGHMYRTNFGIGHSIKDL
LETHIPPGGRLGRGHKGLYDTINNSLHFQLGLALASLGVITSLVAQHMYSLPAYAFIAQDFT
TQAALYTHHQYIAGFIMTGAFAHGAIFFIRDYNPEQNKDNVLARMLDHKEAIISHLSWASL
FLGFHTLGLYVHNDVMLAFGTPEKQILIEPIFAQWIQSAHGKTSYGFDVLLSSKTSPAFNA
GQSIWLPGWLNAINE--NSNSLFLTIGPGDFLVHHAIALGLHTTTLILVKGALDARGSKLMP
DKKDFGYSFPCDGPGRGGTCDISAWDAFYLAVFWMLNTIGWVTFYWHWKHITLWQGNV
SQFNESSTYLMGWLRDYLWLNSSQLINGYNPFGMNSLSVWAWMFLFGHLVWATGFMFLI
SWRGYWQELIETLAWAHERTPLANLIRWRDKPVALSIVQARLVGLAHFSVGYIFTYAAFLI
ASTSGKFGMSHSVKIYDTCIGCTQCVRACPTDVLEMIPWDGCKAKQIASAPRTEDCVGCK
RCESACPTDFLSVRVYLWHETTRSMALAYMTFLSFPSIFVPLVGLVFPAIAMASLFLYIQKN
KISMRDLKTYLSTAPVLSTLWFGSLAGLLIEINRFFPDALTFPFFSFMTAILERRESESLWGR
FCNWITSTENRLYIGWFGVLMIPTLLTATSVFIIAFIAAPPVDIDGIREPVSGSLLYGNNIISGA

IIPTSAAIGLHFYPIWEAASVDEWLYNGGPYELIVLHFLLGVACYMGREWELSFRLGMRP
WIAVAYSAPVAAATAVFLIYPIGQGSFSDGMPLGISGTFNFMIVFQAEHNILMHPFHMLGVA
GVFGGSLFSAMHGSLVTSSLIRETTENESANAGYRFGQEEETYNIVAAHGYFGRLIFQYAS
FNNSRSLHFFLAAWPVVGIWFTSLGISTMAFNLNGFNFNQSVVDSQGRIINTWADIINRAN
LGMEVMHERNAHNFPLDLASVEAPSINGMGLPWYRVHTVVLNDPGRLLSVHIMHTALVA
GWAGSMALYELAVFDPSDPILDPMWRQGMFVIPFMTRLGITNSWGGWSIT-GGTVTNPGI
WSYEGVAAAHIVFSGLCFLAAIWHWVYWDLEIFCDERTGKPSLDLPKIFGIHLFLSGLACF
GFGAFHVTGLYGPGIWVSDPYGLTGKVQPVSPAWGVEGFDPFVPGGIASHHIAAGTLGILA
GLFHLSVRPPQRLYKGLRMGNIETVLSSSIAAVFFAAFVVAGTMWYGSATTPIELFGPTRY
QWDQGYFQQEIYRRVNAALSENQSLSEAWSQIPEKLAFYDYIGNNPAKGGLFRAGSMDN
GDGIAVGWLGHPIFRDKEGRELFVRRMPTFFETFPVVLVDGDGIVRADVPFRRAESKYSV
EQVGVTVEFYGGELNGVSYSDPATVKKYARRAQLGEIFELDRATLKSDGVFRSSPRGWFT
FGHASFALLFFFGHIWHGARTLFRDVFAGIDPDLDTQVEFGAFQKIGDPTTKRQVV----------
--VETLFNGTLALAGRDQETTGFAWWAGNARLINLSGKLLGAHVAHAGLIVFWAGAMNLF
EVAHFVPEKPMYEQGLILLPHLATLGWGVGPGGEVIDTFPYFVSGVLHLISSAVLGFGGIY
HALLGPETLEESFPFFGYVWKDRNKMTTILGIHLILLGLGAFLLVFKALYFGGVYDTWAPG
GGDVRKISNLTLNPSVIFGYLLKSPFGGEGWIVSVDDLEDIIGGHVWLGSICILGGIWHILTK
PFAWARRALVWSGEAYLSYSLGALSVFGFIACCFVWFNNTAYPSEFYGPTGPEASQAQAFT
FLVRDQRLGANVGSAQGPTGLGKYLMRSPTGEIIFGGETMRFWDLRAPWLEPLRGPNGL
DLSRIKKDIQPWQERRSAEYMTHAPLGSLNSVGGVATEINAVNYVSPRSWLATSHFVLGFF
LFVGHLWHAGRARAAAAGFEKGIDRDTEPVLFMTPLNMTIALGKFTKDEN-LFDIMDDW
LRRDRFVFVGWSGLLLFPCAYFALGGWFTGTTFVTSWYTHGLASSYLEGCNFLTAAVSTP
ANSLAHSLLLLWGPEAQGDFTRWCQLGGLWTFVALHGAFGLIGFMLRQFELARSVQLRP
YNAISFSAPIAVFVSVFLIYPLGQSGWFFAPSFGVAAIFRFILFFQGFHNWTLNPFHMMGVA
GVLGAALLCAIHGATVENTLFEDGDGANTFRAFNPTQAEETYSMVTANRFWSQIFGVAFS
NKRWLHFFMLFVPVTGLWMSALGVVGLALNLRAYDFVSQEIRAAEDPEFETFYTKNILLN
EGIRAWMAAQDQPHENLIFPEEVLPRGNALMSGSTGERSFADIITSIRYWVIHSITIPSLFIA
GWLFVSTGLAYDVFGSPRPNEYFTESRQGIPLITERFDSLEQLNEFSRSFMTIDRTYPIFTVR
WLAVHGLAVPTVSFLGSISAMQFIQRMATQTAESNSRSS-PRRTPVGSLLKPLNSEYGKVAP
GWGTTPLMGVAMALFAVFLSIILEIYNSSVLLDGISMNMLTLKLFVYTVVIFFVSLFIFGFLS
NDPGRNPGREEMADTTGRIPLWIIGTLTGILLIGLIGIFFYGSYSGLGSSLMLNIFSLICIWFN
SDLYSNHFLSAKLPEAYAFLSPIVDFMPVIPLLFFLLAFVWQASVSFRMTRSNPNEQNVELN
RTSLYWGLLLIFVLAVLFSNYFFNMEVNIAAFIATALFVLVPTAFLIIIYVKTVSQN-METATL
VAISISGLLVSFTGYALYTAFGQPSQQLRDPFEEHGDMEALVYTFLLVSTLGIIFFAIFFREPP
KVPTKKMKMTIAFQLAVFALIITSSILLISVPVVFASPEGWSSNKKIVFSGTSLWIGLVFLVGI
LNSLIS-------MSPQTETKASVGFKAGVKDYKLTYYTPEYETKDTDILAAFRVTPQPGVPPE
EAGAAVAAESSTGTWTTVWTDGLTSLDRYKGRCYNIEPVAGEENQYIAYVAYPLDLFEEG
SVTNMFTSIVGNVFGFKALRALRLEDLRIPKSYIKTFEGPPHGIQVERDKLNKYGRPLLGC
TIKPKLGLSAKNYGRAVYECLRGGLDFTKDDENVNSQPFMRWRDRFLFCAEALFKAQAE
TGEIKGHYLNATAGTCEEMIKRAVCARELGAPIVMHDYLTGGFTANTSLAHYCRDNGLLL
HIHRAMHAVIDRQKNHGMHFRVLAKALRLSGGDHIHAGTVVGKLEGEREITLGFVDLLR
DDFVEKDRSRGIYFTQDWVSLPGVIPVASGGIHVWHMPALTEIFGDDSVLQFGGGTLGHP
WGNAPGAVANRVALEACVQARNEGRDLAREGNTIIREASKWSPELAAACEVWKEIKFEFP
AMDTL--MAIHLYKTSTPRTRNGALDSQVKSNPRKNLIYGQHRC-KGRNARGIITVRHRGG

GHKRLYRKIDFRRKEKDIYGRIVTIEYDPNRNAYICLIHYGDGEKRYILHPRGAIIGDTIVSG
TEVPIQMGNALPLTEMPLGTAIHNIEITFGKGGQLVRAAGAVAKLIAKEGKSATLKLPSGEV
RLISKNCSATVGQVGNVGVNQKSLDRAGSKCWLGKRPVVRGVVMNPVDHPHGGGEGR
APIGRKKPATPWGYPALGRRSRKRNKYSENLILRRRS-MIQPQTHLNVADNSGAQELMCIRI
IGAS-NRRYAHIGDIIVAVIKEAIPNTPIEKSEVIRAVIVRTSKELKRDNGMIIRYDDNAAVIID
QEGNPKGTRIFGAIPRELRQFNFTKIVSLAPEVLMLSPKRTRFRKQHRGRLKGRSYRGNRI
CFARYALQALEPAWITSRQIEAGRRAMTRNIRRGGKIWVRIFPDKPVTMRPAETRMGSGK
GSPEYWVAVVKPGRILYEMGGVSENIARRAISIAASKMPIRTQFIIAEMTRIKRGYIAHRRR
AKIRLFVSGFRGAHSRITRSITQQRIRALVSSDRDRDRKKRDFRRLWIIRINAVIREN-ILSYS
YSKFIHNIYKKQLLLNRKILSQIAIFNRNCLYMIANEI------------IINQAYAFGDNIRMSANK
ARRIIDQIRGRSYEETLMILELMPYRGCYPIFQLVYSAAANASHNQGLEKKNLVITQAEVN
ESTTAKKFKPRARGRSYSIKRRTCNITIVLTDVSL--------IDGIKYSVVTDKGIRLLLKN-QYT
FNVESGSTRTEIKHWVELFFGVKVIAVNSHRLPGKSRRMGPIMGHTMHYKRMIVTLPPGY
YIPPLRRKELMAVPKKRTSISKKRIRKNIWKKKGYWTALKSFSLAKSLFSGNSKSFFVQQIK
IMAKGKDIRITIILECTEC-ARK--IDKK--GISRYITQKNRHNTPNRLELKKFCSYCFKYTIHK
EIQKMKIRASVRKICEKCRLIRRRGRIIVICSNPKHKQRQGMVREKIEVSTRTLQWKCVESR
ADSKRLYYGRFLLSPLKKGQADTIGIAMRRVLLGEIEGTSITSAKSE--NIPHEYSSIVGVEES
VHEILMNLKEIVLRSHLYGICDASICVKGPGCVTAQDIILPSYVEIVDNTQHIANLTEPIDFCI
RLQIERNRGYRIKTPNQFQEGIY--PIDAVFMPVRNANHSIHSYGNGSETQEILFLEIWTNGSL
TPKEALHEASRNLIDLFIPFLHAEEE--NLHLENNQ-NKYTLPLFSFHDQFN-QIKKNQK-EIE
FKSIFIDQLELTPKIYNCLKRSNIHTLFDLLNNRKEDLMRIEHFRIEDINHIFHILEKNFATTIN
LPKKKMLMDEN-EVMSTIPGLNQIQFEGFCKFIDRGLIEELYKFQKIEDADQEIEFQLFVET
YKLVEPSITERDAIYQSLTYSSELHVSAGLIWKTVRGMQEQTIFIGNIPLMNSLGTFIVNGIY
RIVINQILQSPGIYYRSELDHNGIPVYIGTIISDWGGRLELEIDRKARIWARVSRKQKISILVL
SSAMGLNLREILENVCYPEMFLSFLNDKEKKKIGSKENAILEFYQQFASVGGDPVFSESLC
KELQKKFFQQRCELGKIGRRNMNLRFNLDIPQNNTFLLPRDILAATDHLIGIKFGIGTLDD--
MNHLKNKRIRSVSDLLQDQLGLALVRLENVIKGTISGAIR-HKLI-PTPQNLVTSTP----LTTT
YESFFGLHPLSQVFDRTNPLTQIVHGRKLSYLGPGGLTARTANFRIRDIHPSHYGRICPIDTS
EGINVGLIGSLAIHARIGHWG-SLESPFY---EISEQSK--KVRILSLSPNRDEYYMIAAGNSLA
LNRDIQEEQVVPARYRQEFLTIAWERIHLRSIFPFQYFSIGASLIPFIEHNDANRALMSSNMQ
RQAVPLSQSEKCIVGTGLERQAALDSGVPVISEQEGKIIYSDTKKVVLSTNGNTLNIPLIMY
QRSNKNTCIHQKARVQYGKWIKKGQLLADGASTVGGELALGKNILVAYMPWEGYNFED
AVLISERLVYENIYTSFHIRKYEIQTHVTSQG-PEKITKEIPHLEAHLLRNLDKNGIVMLGSW
VETGDVLVGKLTPQMTKE-SSYAPEDRLLRAILGIQVSTSKETCLKLPIGGRGRVIDVRWIQ
KKG-GSSYNPEIIRVYILQKREIKVGDKVAGRHGNKGIISKILTRQDMPYLQDGRCVDMVF
NPLGVPSRMNVGQIFECSLGLAGSLLDRHYRIAPFDERYEKEASRKLVFSELYEASKQTAN
PWVFEPEYPGKSKIFDGRTGDPFEQPVLIGKPYILKLIHQVDDKIHGRSSGHYALVTQQPLR
GRAKQGGQRVGEMEVWALEGFGVAHILQEMLTYKSDHIKARQEVLGTTIIGGTIPKPQDA
PESFRLLVRELRSLALELNHFLVSEKNLKMTRKEAMNNYFSSMIDQYKHQQLRIGSVSPQ
QISAWATKILPNGEIVGEVTKPYTFHYKTNKPEKDGLFCERIFGPIKSGICACGNYRVIGDE
KEK-KFCEQCGVEFVDSRVRRYQMGYIQLACPVTHVWYLKRLPSYIANILDKPLKELEGL
VYCDF--SFARP-VIKKPTFLRLRGLFEYEIQSWKYSIPLFFTTQGFDTFRNREISSGAGAIRE
QLSDLDLRSIINSSLVEWKELVEE-GPTGNEWEDRKIGRRKDFLVRRMELAKHFLRTNIEPE
WMVLCLLPVLPPELRPIIQIDGGKLMSSDINELYRRVIYRNNTLKDLLITSRSTPGELVMCQ

EKLVQEAVDTLLDNGIRGQPMRDSHNKVYKSFSDVIEGKEGRFRETLLGKRVDYSGRSVI
VVGPSLSLHQCGLPGEIAIELFQTFVIRGLIRKNLASNIGVAKSQIREQEPIIWEILQEVMRG
HPVLLNRAPTLHRLGIQAFQPILVEGRAICLHPLVCKGFNADFDGDQMAVHVPLSLEAQAE
ARLLMFSHMNLLSPAIGDPISVPTQDMLMGLYVLTSGNRRGICANRYNPCNR---KNYKNE
KILNKNCEYTKE-EPFFVNSYDAIGAYQQKRIYLDSPLWLRWKLDQR-VIAS--RETPIEVQY
ESLGIYHEIYGNYIIVRGIKKEILWIYIRTTVGHIFLYREIEEAIQGFSRAGLYGT--------MEVL
MAERANLIYHNKAIDGTAIKRLISRFIDHFGMAYTSHILDQIKTLGFQQATSASISLGIDDLL
TIPSKGWLVQDAEQQSFIFEKHHNYGNVHAVEKLRQSIEIWYATSEYLRHEMNPNFRITDP
LNPVHIMSFSGARGNVSQVHQLVGMRGLMSDPQGQMIDLPIQSNLREGLSLTEYIISCYGA
RKGVVDTAVRTSDAGYLTRRLVEVVQHIVVRRTDCGTIRGISVSPNKGMMP-ERIFIQTLIG
RVLADDIYIGSRCIAIRNQDIGIGLVNRFIT------FRKQSILIRTPFTCRSTSWICRLCYGRSPTH
GDLVELGEAVGIIAGQSIGEPGTQLTLRTFHTGGVFTGGTAEHVRAPSNGKIQFNEDLVHPT
RTRHGHPAFICDIDLYVTIESEDILHNVTIPSKSFLLVKNNQYVQSEQVIAEIRARTDNL-IVK
ERVRKHIYSDSEGE-MHWSTDVYHAPEWTYSNVHLLPKTSHLWILSGGSCK--SSVLPLSL
HKDQDQLLSS--GKKKTILPYSSVF--NPSRY-MG--------------------------------HYDFPA-ILHEN
YD--LLGKRRRNRFLIPFQLMAEQYNEEMLMPRLWIQIPP--KGIFYRNSILAYFDDPQYRRK
SSGIIKYGTIGTHSIAKKED--LIEHK-GVKEFQPKYMKVDQFFFIPEEVHILPESSSIMVRDNS
LIGVDTQITLNTRSRVGGLVRVEKKKKRIELKIFSGDIHFPSDTDKISRHSGILIPP-GMIKTIS
K-ESTK--LKNWIYVQR--IILTKTK--YFVLVRPVMPYEITDGVNLGALFLQDLFQEKDNLKL
KVVNYILYENGK---PISDTSIQLVRTCLMLSVGQDK---KNSSIEEAYTSFIGVTINGLIRDFLK
VDLMK-HISYIRKRKDTSGSRFISHNNASDSININPFSIQSNYKD--KEQIQNLLIQNNNTIRC
TLSNQTQNKECRSLIILSPSNCFRFQIKPFNNLKYQN---VIKESEKDSLNFNSLGPFGT--SIPI
ANFYS--FDYFIT--YNPIS-IIKYLQVDN---LKWTFQIL--Q-SYLIDEREQ--IYNPDSCSNIVLN
AFHLNWYFLYYQ----KSTIIS-LGEFICENVSIAKNGP---PLKSGQVLIVQ-VDSVIIRSAKPYL
ATPGATVHGHYGKIIDEGDTLVTFLYEKSRSGDITQGLPKVEQVLEVRSIDSISMNLEKRIES
WNKGIARVLGIPWRFLIGAELTIAQSRLSLVNKIQKVYRSQGVQIHNRHIEIIVRQITSKVLV
SE---DGISNVFLPGELIGLLRAERTGRALEETICYRAILLGITKASLNTQSFLSEASFQETARV
LAKSALRGRIDWLKGLKENVVLGGMIPVGTGFKGLVYCS--RKHNKMI--WETKKNHLFE
QE-V-RDIFFHHRKLLNS-------------------------MTKRYWNINLEEMMEAGVHFGHGTRKWN
PKMGPYISAKRKGIHILNITRTARFLSEACDLVFDAASKGKQFLIVGTKTKAADLVARAAIK
SRSHYVNKKWPGGMLTNWSTTETRLHKFRDLRMKQKMGRLNRLPKRDAAMLKRKLSH
LQTYLGGIQYMKGLPDIVIIVDQQEEYTALRECITLGIPTICLIDTNCDPDLADISIPANDDAI
ASIRFILNKLVFAICEGRS-SYIRNLMGQKINPLGFRLGITQSHHSIWFAQPKNYSEGLQEDK
KIRDCIQNYIQKNMKISS-GVEGIACIEIQKRIDLIQIIIYMGFPKLLIKGRSQ----KIEELQTNIK
KEIHCVNRKINIAIKRISNPYGHPKILAEFIAGQLKNRVSFRKAMKKAIELSEQTDTKGIKV
QIAGRLDGKEIARVEWIREGRVPLQTIRAPIDYCSYTVRTIYGVLGIKIWIFI---MSRYRGPRF
KKIRRLGALPGLTNKRPKAKTITRNQSRSGK-KSQYRIRLEEKQKLRFHYGLTERQLLKYV
RIARKTKGSTGQVLLQLIEMRLDNILFRLGMASTIPQARQFVNHRHILVNDHIVNIPSYRCK
PRDIITVRDNQKSRTLVQNYLDS-PN-QELAKHLTLQPFQ--YKGLVNQIIDSKWVGLRINEL
LVVEYYSRQTMSRRGTAEKKTAKSDPIYRNRLVNMLVNRILKHGKKSLAYQILYRAMKNI
QQKTEKNPLSVLRQAIRGVTPDIAVKARRVSGSTHQVPIEIGSTQGKALAIRWLLAASRKR
PGRNMAFKLSSELVDAAKGSGDAIRKKEETHRMAEANRAFAHFGMSKDTIADIITSIRNA
DMNRKGTVRITYTNITENFVKILLREGFIKNVRKHQERGKYFLVLTLRYKR--NTKGSHKT-
M--LNLKRISRPGLRIYSNYQKIPKILGGMGILILSTSRGIMTDREARLEKIGGEILCCIWMA

KPIKRIGSRRNVRITSRKNTRKIPKGIIHVQASFNNTIVTVTDVRGRVISWSSAGTCGFKGTR
RGTPFAAQTAAGNAIRTVVDQGMQRAEVMIKGPGLGRDAALRAIRRSGLLLSFVRDVTP
MPHNGCRPPKKRRVMPTIKQLIRNARQPIRNVTKSPALRGCPQRRGTCTRVYTITPKKPNS
ALRKVARVRLTSGFEITAYIPGIGHNLQEHSVVLVRGGRVKDLPGVRYHIVRGTLDAVGVK
DRQQGRSKYGVKKPKMAKKSLIQREKKRQKLEQKYHLTRRFLKNERNK--VSLSDKEKW
EIKGKLQSLPRNSAPTRLHRRCFLTGRPRGNYRDFGLSRHILREMFHACLLPGATRSSWMV
KNSFISAISQQEKEENK--GSVEFQILSFTNRIQKLTSHLELHKKDYSSQRGLRKILGKRQRLL
Y Y L S R K N P I S Y K E L I N Q L G I R E L K K N G
-???
MDKSKRLFLKSKRSFRRRLPPIQSGDRIDYRNLSLLNRFLSEQGKILSRRVNRLTLKQQRLV
TIAIKQARILSLLPFMNNET--HFERRKSTARTPGFRTKKKMTR-KKKNPFVAHPLLKKIKRL
NTKSEKEIIVTWSRASTIIPIMIGHTIAIHNGKEHIPIYIEDRMVGHKLGEFVSTLNFRGHAK
N D S R S R
R???
??
??
??
??
??
??
??
??
??
??
??
??
??
??
??
??
??
??
??
??
??
??
??
??
??
??
??
??
??
??

??
??
??
??
??
??
??
??
??
??
??
??
??MARSR-INGNFIDKTFSIVANILLRIIPTTSGEK
EAFTYYRDG--MSAQSEGNYAEALQNYYEAMRLEIDPYDRSYILYNIGLIHTSNGEHTKAL
EYYFRALERNPFLPQAFNNMAVICHY-RGEQAIRQGDSEIAEAWFDQAAEYWKQAIALTP
GNYIEAQNWLKITRRFE--MSLRSELIWIELIGGSRKTSNFFWAFILFFGSLGFLLVGISSYLG
RNLISSFPSQQILFFPQGIVMSFYGIAGLFISSYLWFTIWWNVGSGYDRFDKKQGLVCIFRW
GFPGKNRRIFLRFLIKDIQSIRIEVKEGVYARRVLYMEIRGQGAIPLTHSDDN-WTPREIEQK
AAELAYFLRVPMEVF
>Cornus
MEKWWFNSMLFLSKSTGSLGPIENTSESEDPDRN---KNIHSNSNVDHLFSVKDIRNFISDD
TFLVRDNNGDSYSIYFDIENQIFEIDNDH--SFLSELESSFYSYRN-----KGSKSNDPYYDRYM
YDTQYSWNNHINSCIDSYLH--SQICIDTYILSDSDNYSDSYIYSSICGSDSSGIKTSMNGSDL
TIRESSNDLDVTQKYKHLWVQCENCYGLNYKKFLKSKMNICEQCGYHLKMSSSDRIELSI
DPGTWDPLDEDMVSLDPIEFHSDEEPYKDRIDSYQIKTGLTEAVQTGIGQLNGIPIAIGVMD
FQFMGGSMGSVVGEKITRLVEYATNKFLPLIIVCASGGARMQEGSLSLMQMAKISSALYD
YQSNKKLFYVSILTSPTTGGVTASFGMLGDIIIAEPNAYIAFAGKRVIEQTLHKTVPEGSQA
AEYLFQKGLFDLIVPRNPLKGVLSELFQLHAFFPLNQNSSKMVTIRADEISNIIRERIEQYNR
EVKIVNTGTVLQVGDGIARIHGLDEVMAGELVEFEEGTIGIALNLESNNVGVVLMGDGLM
IQEGSSVKATGRIAQIPVSEAYLGRVINALAKPIDGRGEISASESRLIESPAPGIISRRSVYEPL
QTGLIAIDSMIPIGRGQRELIIGDRQTGKTAVATDTILNQQGQNVICVYVAIGQKASSVAQV
VTNFQERGAMEYTIVVAETADSPATLQYLAPYTGAALAEYFMYRERHTSIIYDDLSKQAQ
AYRQMSLLLRRPPGREAYPGDVFYLHSRLLERAAKLSSRLGEGSMTALPIVETQSGDVSA
YIPTNVISITDGQIFLSSDLFNAGIRPAINVGISVSRVGSAAQIKAMKQVAGKLKLELAQFAE
LEAFAQFASDLDKATQNQLARGQRLRELLKQSQSAPLAVEEQIMTIYTGTNGYLDSLEIGQ
VRKFLVELRTYLKTNKPQFQEIISSTKTFTEEAEALLKEAIREQMERFILQEQAMRINPTTSG
PGISTLEKKNLGRIAQIIGPVLDVAFPPGKMPNIYNALVVKGRDTAGQPINVTCEVQQLLG
NNRVRAVAMSATEGLTRGMEVVDTGAPLSVPVGGATLGRIFNVLGEPVDNLGPVDTRITS
PIHRSAPAFIQLDTKLSIFETGIKVVDLLAPYRRGGKIGLFGGAGVGKTVLIMELINNIAKA
HGGVSVFGGVGERTREGNDLYMEMKESGVINEQNIAESKVALVYGQMNEPPGARMRVGL
TALTMAEYFRDVNEQDVLLFIDNIFRFVQAGSEVSALLGRMPSAVGYQPTLSTEMGSLQE
RITSTKEGSITSIQAVYVPADDLTDPAPATTFAHLDATTVLSRGLAAKGIYPAVDPLDSTSTM
LQPRIVGEEHYETAQRVKQTLQRYKELQDIIAILGLDELSEEDRLTVARARKIERFLSQPFFV
AEVFTGSPGKYVGLAETIRGFQLILSGELDGLPEQAFYLVGNIDEATAKAMNLEMESNLKK

MTLNLCVLTPNRIVWDSEVKEIILSTNSGQIGVLPNHAPIATAVDIGILRIRLNDQWLTMAL
MGGFARIGNNEITVLVNDAERGSDIDPQEAQQTLEIAEANLRKAEGKRQTIEANLALRRAR
TRVEAINVISMKNVTDSFVSLGHWPFAGSFGFNTDILATNPINLSVVLGVLIFFGKGVLSDL
LDNRKQRILNTIRNSEELRGGAIEQLEKARARLRKVEMEADQFRVNGYSEIEREKLNLINS
TYKTLEQLENYKNETINFEQQRAINQVRQRVFQQALQGALGTLNSCLNNELHLRTISANIG
IFGAMKEITDMNPLISAASVIAAGLAVGLASIGPGVGQGTAAGQAVEGIARQPEAEGKIRG
TLLLSLAFMEALTIYGLVVALALLFANPFVMNVLSCSINTLKGLYDISGVEVGQHFYWKIG
GFQVHGQVLITSWVVMAILLGSATIAVRNPQTIPTGGQNFFEYVLEFIRDVSKTQIGEEYGP
WVPFIGTMFLFIFVSNWSGALLPWKIIQLPHGELAAPTNDINTTVALALLTSVAYFYAGLTK
KGLGYFGKYIQPTPILLPINILEDFTKPLSLSFRLFGNILADELVVVVLVSLVPSVVPIPVMFL
GLFTSGIQALIFATLAAAYIGESMEGHHMIFSTLEHILTHISFSIVSIVITIHLITLLVDEIVRLY
DSSEKGMIAALFCITGLLVTRWIYSGHFPLSDLYESLIFLSWSFSIIHIVPY--KKKNHLSAITA
PSAIFTQGFATSGLLTEIHQSTILVPALQSEWLIMHVSMMVLGYAALLCGSLLSVALLVITFR
KDIKIFC--KEIQYINKKSNVLGNTS---AGNYYRSQLIQQLDHWSYRVISLGFIFLTIGILSGAV
WANEAWGSYWNWDPKETWAFITWTIFAIYLHTRTNKNLQGANSAIVASIGFLIIWICYFGV
NLLGIGLHSYGSFTLTMKKKIKHS-LLFFVSIVFLPWWISLSFNKSLESWVTNWWNTRQSEI
FLNDIQEKSILEKFIELEDLLLLEEMIKGYSKIHLQKLHIGIHKETIQLIKIHNEDRIHTILHFS
TNIICFIILSGYSILGNEELVILNSWTQEFLYNLSDTIKAFSILLLTDLCIGFHSPHGWELMIGS
VYKDFGFVHNDQIISGLVSTFPVILDTIFKYWIFRYLNRVSPSLVVIYHSMNDMPIGVPKVP
FRSPGEEDASWVDVYNRLYRERLLFLGQEVDSEISNQLIGLMVYLSIEDDTKDLYLFINSP
GGWVIPGIAIFDTMQFVQPDVHTICMGMAASMGSFILVGGEITKRLAFPHARVMIHQPASA
YYEAQTGEFILEAEELLKLRETLTRVYVQRTGKPLWVVSEDMERDVFMSATEAQAYGIVD
LVAVEMKEQKWIHEGLITESLPNGMFRVRLNNEDLSLGYVSGKIRRSFIRILPGDRVKIEVS
RYDSTRGRIIYRLRNKDSKDMEEFQRYLE-AKSRQHDFLYPLIFQEYIYTLAHDR--ADYDN
KSSLLIVKRLITRMYQQNHLLISANDSNQNPFWGKNLYFQMISEGFAVIVEIPFSLRLISSLIV
KSQNLRSIHSIFPFLEDTFLHLNYVLDIRIPYPVHLEILVQILRYWVKDASSLHLLRFFLHEY
RNRNWNNLKLSSSFSKRRLFLFLYNSHVCEYESIFLFFRNQSSHLRSTSFGALLERIYFYGK
IEHVE-VFAKDFILWLFKDPFIHYVRYQGKSILAAKGTPLLMNKWKYYFVNFWKCHFYV
WYQPVTIYINQLSNYSLDFLGYLSNVRPNPAMVRSQMLENSFLIDNAI--KKFDTIVPIIPLIG
SLAKAKFCNVLGHPISKPVWADLSDSDIIDRFGHICRNLSHYYSGSSKKKSLYRIKYILRLS
CARTLARKHKSTVRAFLKRLGSELLEEFLMEEEQVLSLTFPRRIWYLDIICINDLANHEMII
D-TTEVQ-AINSFSRLES-LKEVYEIIWMLVPILTLVLGITIGVLVIVWLEREISAGIQQRIGPEY
AGPLGILQALADGTKLLFKENLLPSRGDPRLFSIGPSIAVISILLSYLVIPFGYRLVLADLSIG
VFLWIAISSIAPVGLLMSGYGSNNKYSFLGGLRAAAQSISYEIPLTLCVLSISLLSNSSSTVDI
VEAQSKYGFWGWNLWRQPIGFIIFLISSLAECERLPFDLPEAEEELVAGYQTEYSGIKFGLF
YVASYLNLLVSSLFVTVLYLGGWNLSIPYILVPELFEINKAGIVFGTIISIFITLAKTYLFLFISI
TTRWTLPRLRMDQLLNLGWKFLLPISLGNLLLTTSSQLMSLMIWHVQNENFILDSTRIFMK
AFHLLLFDGSFIFPECILIFGLILLLMIDSTSDQKDISWLYFISSTSLVMSITALLFRWREEPMI
SFSGNFQTNNFNEIFQFLILLCSTLCIPLSVEYIECTEMAITEFLLFVLTATLGGMFLCGANDL
ITIFVAPECFSLCSYLLSGYTKKDVRSNEATMKYLLMGGASSSILVHGFSWLYGSSGGEIEL
QEIVNGLINTQMYNSPGISIALIFITVGIGFKLSLAPSHQWTPDVYEGSPTPVVAFLSVTSKV
AASASATRIFDIPFYFSSNEWHLLLEILAILSMILGNLIAITQTSMKRMLAYSSIGQIGYVIIGI
IVGDSNDGYASMITYMLFYISMNLGTFACIVLFGLRTGTDNIRDYAGLYTKDPFLALSLAL
CLLSLGGLPPLAGFFGKLYLFWCGWQAGLYFLVSIGLLTSVVSIYYYLKIIKLLMTGRNQEI

TPHVRNYRRSPLRSNNSIELSMIVCVIASTIPGISMNPIIAIAQDTLFMFLLYEYDIFWAFLIIS
SIIPILAFFISGVLAPISKGPEKLSSYESGIEPMGDAWLQFRIRYYMFALVFVVFDVETVFLYP
WAMSFDVLGVSVFIEALIFVLILIVGSIYAWRKGALEWSTNDFPWLTIIVVLPIAAGSLIFFLP
HRGNRVIKWYTICICILELLLTTYAFCYHFQSDDPLIQLVEDYKWIDFFDFHWRLGIDGLSI
GPILLTGFITTLATLAAWPVTRDSRLFHFLMLAMYSGQIGSFSSRDLLLFFIMWELELIPVYL
LLSMWGGKKRLYSATKFILYTAGGSIFLLMGVLGVGLYGSNEPTLNFETSANQSYPVVLEII
FYIGFFIAFAVKSPIIPLHTWLPDTHGEAHYSTCMLLAGILLKMGAYGLVRINMELLPHAHS
IFSPWFIIVGTTQIIYAASTSLGQRNLKKRIAYSSVSHMGFIIIGIGSITDTGLNGAILQIISHGFI
GAALFFLAGTSYDRIRLAYLDEMGGIAILMPKIFTMFSSFSMASLALPGMSGFVAELIVFFG
IITSQKYLLMTKILITFVMAIGMILTPIYSLSMSRQMFYGYKLFNAPNSYFFDSGPRELFVSI
SIFLPVIGIGIYPDFVLSLSVDKVELILSNYFSRMMLEHVLILSAYLFSIGIYGLITSRNMVRA
LMCLELILNAVNINFVTFSDFFDSRQLKGTIFAIFVIAIAAAEAAIGLAIVSSIYRNRKSTRIN
QSNLLNKMEHTYQYAWIIPLIPLPVPMLIGVGLLLFPTATKNLRRIWAFPSVLLLSIVMIFSID
LSIQQMNSSSIYQYVWSWIINNDFSLELGYVIDPLTSIMSILITSVGIMVLIYSDNYMSHDQG
YLRFFAYMSFFSTSMLGLVTSSNLIQIYIFWELVGMCSYLLIGFWFTRPVAANACQKAFVT
NRVGDFGLLLGILGFYWITGSFEFRDLFEIVNNNEVNLSFITVCAVLVFAGAVAKSAQFPLH
VWLPDAMEGPTPISALIHAATMVAAGVFIVARLLPLFTVIPYILNLISLIGIITVLLGATLALA
QKDIKRSLAYSTMSQLGYMMLALGMGSYRTALFHLITHAYSKALLFLGSGSVIHSMETIV
GYSPDKSQNMVLMGGLTKHVPITKISFLLGTLSLCGIPPLACFWSKDEILNDSWLYSPIFAII
ACSTAGLTAFYMFRIYLLTFEGHLNVHFQNYSGKKNTPFYSISLWGKVGSKTINK-LLTLLT
MNFFSFCNKNTYSYPYESDNTMLFPLLVLALFTLFVGSIGIPFDLDILSKWLNPAINLLHQN
SN-LMDWYEFVKDAIFSVSIAYFGIFIASFLYKPVYSSSQNLNLINSFVTLRDKIINGIYDWS
YNRGYIDAFYAAFLTGGIRGLAKLIHFFDRRIIDGITNGVGIMSFFVGEGIKYIGGG-RISSYL
FLYSSYVSIFLVIY-MDLPGPIHDFLLVFLGLGIILGGLGVVLLNNTIFSAFSLGLVLVCISLFYI
LSNSHFVAAAQLLIYVGAINILIIFAVMFMNGSEYHKDFYLWTVGDGVTSMVCTSIFVSLIT
TILDTSWYGIIWTTRSNQILEQDLINNSQQIGIHLSTDFFLPFELISIILLVALIGAIAVARQMTA
TATRKDLMIVNMGPQHPSMHGVLRLIVTLDGEDVIDCEPILGYLHRGMEKIAENRTIIQYL
PYVTRWDYLATMFTEAITVNAPEQLGNIQVPKRASYIRVIMLELSRIASHLLWLGPFMADI
GAQTPFFYIFRERELIYDLFEAATGMRMMHNYFRIGGVAADLPHGWIDKCLDFCDYFLTG
VAEYQKLITRNPIFLERVEGVGIIGGEEAINWGLSGPMLRASGIQWDLRKVDHYECYDEFD
WEVQWQKEGDSLARYLVRIGEMKESIKIIQQALEGIPGGPYENLEIRRFDKVRNPEWNDFE
YRFISKKPSPTFELSNQELYVRVEAPKGELGIFLIGDQSVFPWRWKIRPPGFINLQILPQLVK
KMKLADIMTILGSIDIIMGEVDRMFPMVTGFMNYGQQTIRAARYIGQSFMITLSHANRLPV
TIQYPYEKLITSERFRGRIHFEFDKCIACEVCVRVCPIDLPVVDWKLETDIRKKRLLNYSIDF
GICIFCGNCVEYCPTNCLSMTEEYELSTYDRHELNYNQIALGRLPISVIDDSTVRTIWNS-----
-----------MQGRLSAWLVKHGLIHRSLGFDYRGIETLQIKPEDWHSIAVILYVYGYNYLRSQC
AYDVAPGGLLASVYHLTRIEYGVDQPEEVCIKVFAPRRNPRIPSVFWVWKSVDFQERESY
DMLGISYDNHPRLKRILMPESWIGWPLRKDYISPNFYEIQDAH-----------------------------MA
KGGIGMVLAPEYSDNKKKK---NTIEMVMNSIDFPLLDRTTPNSVISTTSNDLSNWSRLSSL
WPLLYGTSCCFIEFASLIGSRFDFDRYGLVPRSSPRQADLILTAGTVTMKMAPSLVRLYEQM
PEPKYVIAMGACTITGGMFSTDSYSTVRGVDKLIPVDVYLPGCPPKPEAVIDAITKLRKKIS
QEIYEDRIRSHQENRCFTTNHKFHVGRSIHTGNYNQGLLYQPPSTSEILPETFFKYKSSVSS
HELVNMQTRNTFSWIKEQVTRSISVSLMIYIITRASISSAYPIFAQQGYENPREATGRIVCAN
CHLANKPVDIEVPQAVLPDTVFEAVVRIPYDMQLKQVLANGKRGALNVGAVLILPEGFEL

APPDRISPEMKEKIGNLSFQSYRPNKKNILVIGPVPGQKYSEITFPILSPDPATKKDVHFLKY
PIYVGGNRGRGQIYPDGSKSNNTVYNATAAGIVSKIVRKGKGGYEITIADASDGRQVVDSI
PPGPELLVSEGESIKFDQPLTSNPNVGGFGQGDAEIVLQDPLRVQGLLFFLASVILAQIFLVL
KKKQFEKVQLSEMNFMSKVYDWFEERLEIQAIADDITSKYVPPHVNIFYCLGGITLTCFLV
QVATGFAMTFYYRPTVTEAFASVQYIMTEANFGWLIRSVHRWSASMMVLMMILHVFRVY
LTGGFKKPRELTWVTGVVLAVLTASFGVTGYSLPWDQIGYWAVKIVTGVPEAIPVIGSPLV
ELLRGSASVGQSTLTRFYSLHTFVLPLLTAVFMLMHFLMIRKQGISGPLMGVTKKPDLNDP
VLRAKLAKGMGHNYYGEPAWPNDLLYIFPVVILGTIACNVGLAVLEPSMIGEPADPFATPL
EILPEWYFFPVFQILRTVPNKLLGVLLMVSVPAGLLTVPFLENVNKFQNPFRRPVATTVFLI
GTAVALWLGIGATLPIDKSLTLGLFMIEVFLFGIVLGLIPITLAGLFVTAYLQYRRGDQLDLM
PTITSYFGFLLAALTITSALFIGLSKIRLIMDIVSLAWAALMVVFTFSLSLVVWGRSGLMIIRS
PEPEVKILVDRDHIKTSFEEWARPGHFSRTLAKGPDTTTWIWNLHADAHDFDSHTSDLEEI
SRKVFSAHFGQLSIIFLWLSGMYFHGARFSNYEAWLSDPTHIGPSAQVVWPIVGQEILNGD
VGGGFRGIQITSGFFQIWRASGITSELQLYCTAIGALVFAALMLFAGWFHYHKAAPKLAWF
QDVESMLNHHLAGLLGLGSLSWAGHQVHVSLPINQFLNAGVDPKEIPLPHEFILNRDLLA
QLYPSFAEGATPFFTLNWSKYAEFLTFRGGLDPVTGGLWLTDIAHHHLAIAILFLIAGHMYK
TNWGIGHGLKDILEAHKGPFTGQGHKGLYEILTTSWHAQLSLNLAMLGSLTIVVAHHMYS
MPPYPYLATDYGTQLSLFTHHMWIGGFLIVGAAAHAAIFMVRDYDPTTRYNDLLDRVLR
HRDAIISHLNWACIFLGFHSFGLYIHNDTMSALGRPQDMFSDTAIQLQPVFAQWIQNTHAL
APGSTAPGATASTSLTWGGGDLVAVGGKVALLPIPLGTADFLVHHIHAFTIHVTVLILLKGV
LFARSSRLIPDKANLGFRFPCDGPGRGGTCQVSAWDHVFLGLFWMYNAISVVIFHFSWKM
QSDVWGSISDQGVVTHITGGNFAQSSITINGWLRDFLWAQASQVIQSYGSSLSAYGLFFLG
AHFVWAFSLMFLFSGRGYWQELIESIVWAHNKLKVAPATQPRALSIVQGRAVGVTHYLLG
GIATTWAFFLARIIAVGMALRFPRFSQGLAQDPTTRRIWFGIATAHDFESHDDITEERLYQNI
FASHFGQLAIIFLWTSGNLFHVAWQGNFESWVQDPLHVRPIAHAIWDPHFGQPAVEAFTRG
GAPGPVNIAYSGVYQWWYTIGLRTNEDLYTGALFLLFLSAISLIAGWLHLQPKWKPSVSW
FKNAESRLNHHLSGLFGVSSLAWTGHLVHVAIPASRGEYVRWNNFLDVLPHPQGLEPLFT
GQWNLYAQNPDSSSHLFGTSQGAGTAILTLLGGFHPQTQSLWLTDMAHHHLAIAFIFLIAG
HMYRTNFGIGHSMKDLLDAHIPPGGRLGRGHKGLYDTINNSIHFQLGLALASLGVVTSLV
AQHMYSLPPYAFIAQDFTTQAALYTHHQYIAGFIMTGAFAHGAIFFIRDYNPEQNEDNVLA
RMLDHKEAIISHLSWASLFLGFHTLGLYVHNDVMLAFGTPEKQILIEPIFAQWIQSAHGKTS
YGFDVLLSSTNGPAFNAGRSIWLPGWLNAVNE--NSNSLFLTIGPGDFLVHHAIALGLHTTT
LILVKGALDARGSKLMPDKKDFGYSFPCDGPGRGGTCDISAWDAFYLAVFWMLNTIGWV
TFYWHWKHITLWQGNVSQFNESSTYLMGWLRDYLWLNSSQLINGYNPFGMNSLSVWAW
MFLFGHLVWATGFMFLISWRGYWQELIETLAWAHERTPLANLIRWRDKPVALSIVQARLV
GLAHFSVGYIFTYAAFLIASTSGKFGMSHSVKIYDTCIGCTQCVRACPTDVLEMIPWDGCK
AKQIASAPRTEDCVGCKRCESACPTDFLSVRVYLWHETTRSMGLAYMATFDFSSILVPVVG
IVFPAIAMASLSLHVQNNKIVMRDLKTYLSVAPVLSTLWFGALAGLLIEINRFFPDALTFPF
FSFMTAILERRESESLWGRFCNWITSTENRLYIGWFGVVMIPTLLTATSVFIIAFIAAPPVDID
GIREPVSGSLLYGNNIISGAIIPTSAAIGLHFYPIWEAASVDEWLYNGGPYELIVLHFLLGVA
CYMGREWELSFRLGMRPWIAVAYSAPVAAATAVFLIYPIGQGSFSDGMPLGISGTFNFMIVF
QAEHNILMHPFHMLGVAGVFGGSLFSAMHGSLVTSSLIRETTENESANEGYRFGQEEETY
NIVAAHGYFGRLIFQYASFNNSRSLHFFLAAWPVVGIWFTALGISTMAFNLNGFNFNQSVV
DSQGRVINTWADIINRANLGMEVMHERNAHNFPLDLAAIEAPSTNGMGLPWYRVHTVVL

NDPGRLLSVHIMHTALVAGWAGSMALYELAVFDPSDPVLDPMWRQGMFVIPFMTRLGIT
NSWGGWNIT-GGTITNPGIWSYEGVAGAHIVFSGLCFLAAIWHWVYWDLEIFCDERTGKP
SLDLPKIFGIHLFLSGVACFGFGAFHVTGLYGPGIWVSDPYGLTGKVQPVNPAWGVEGFDP
FVPGGIASHHIAAGTLGILAGLFHLSVRPPQRLYKGLRMGNIETVLSSSIAAVFFAAFVVAG
TMWYGSATTPIELFGPTRYQWDQGYFQQEIYRRVSAGLAENQSLSEAWSKIPEKLAFYDY
IGNNPAKGGLFRAGSMDNGDGIAVGWLGHPVFRDKEGRELFVRRMPTFFETFPVVLVDG
DGIVRADVPFRRAESKYSVEQVGVTVEFYGGELNGVSYSDPATVKKYARRAQLGEIFELD
RATLKSDGVFRSSPRGWFTFGHASFALLFFFGHIWHGARTLFRDVFAGIDPDLDAQVEFGA
FQKLGDPTTRRQVV------------VETLFNGTLALSGRDQETTGFAWWAGNARLINLSGKLLG
AHVAHAGLIVFWAGAMNLFEVAHFVPEKPMYEQGLILLPHLATLGWGVGPGGEVIDTFPY
FVSGVLHLISSAVLGFGGIYHALLGPETLEESFPFFGYVWKDRNKMTTILGIHLILLGIGAFL
LVFKALYFGGIYDTWAPGGGDVRKITNLTLSPSIIFGYLLKSPFGGEGWIVSVDDLEDIIGG
HVWLGSICILGGIWHILTKPFAWARRALVWSGEAYLSYSLGALAVFGFIACCFVWFNNTAY
PSEFYGPTGPEASQAQAFTFLVRDQRLGANVGSAQGPTGLGKYLMRSPTGEVIFGGETMR
FWDLRAPWLEPLRGPNGLDLSRLKKDIQPWQERRSAEYMTHAPLGSLNSVGGVATEINAV
NYVSPRSWLATSHFVLGFFFFVGHLWHAGRARAAAAGFEKGIDRDFEPVLSMTPLNMTIA
LGKFTKDEN-LFDIMDDWLRRDRFVFVGWSGLLLFPCAYFALGGWFTGTTFVTSWYTHG
LASSYLEGCNFLTAAVSTPANSLAHSLLLLWGPEAQGDFTRWCQLGGLWTFVALHGAFGL
IGFMLRQFELARSVQLRPYNAIAFSAPIAVFVSVFLIYPLGQSGWFFAPSFGVAAIFRFILFFQ
GFHNWTLNPFHMMGVAGVLGAALLCAIHGATVENTLFEDGDGANTFRAFNPTQAEETYS
MVTANRFWSQIFGVAFSNKRWLHFFMLFVPVTGLWMSALGVVGLALNLRAYDFVSQEIR
AAEDPEFETFYTKNILLNEGIRAWMAAQDQPHENLIFPEEVLPRGNALMSGSTGERSFADII
TSIRYWVIHSITIPSLFIAGWLFVSTGLAYDVFGSPRPNEYFTESRQGIPLITGRFDPLEQLDE
FNRSFMTIDRTYPIFTVRWLAVHGLAVPTVSFLGSISAMQFIQRMATQTVEGSSRSG-PRRTT
VGSLLKPLNSEYGKVAPGWGTTPLMGVAMALFAVFLSIILEIYNSSVLLDGISMNMLTLKL
FVYTVVIFFVSLFIFGFLSNDPGRNPGREEMADTTGRIPLWLIGTVTGILVIGLIGIFFYGSYS
GLGSSLMLNIFSLICICLNSALY-SSFFIAKLPESYAFLNPIVNVMPVIPVLFFLLAFVWQAAV
SFRMTQSNPNEQNVELNRTSLYWGLLLIFVLAVLFSNYFFNMEVNILAFIATALFILVPTAFL
LIIYVKTISQNDMETATLVAIFISGLLVSFTGYALYTAFGQPSQQLRDPFEEHG
D???????????????????????????????????MTIAFQLAVFALIATSSILLIGVPLVFASPDGWSSNK
NVVFSGTSLWIGLVFLVGILNSLIS-------MSPQTETKASVGFKAGVKEYKLTYYTPTYETKD
TDILAAFRVTPQPGVPPEEAGAAVAAESSTGTWTTVWTDGLTSLDRYKGRCYHIEPVAGEE
SQFIAYVAYPLDLFEEGSVTNMFTSIVGNVFGFKALRALRLEDLRIPVAYVKTFQGPPHGIQ
VERDKLNKYGRPLLGCTIKPKLGLSAKNYGRAVYECLRGGLDFTKDDENVNSQPFMRWR
DRFLFCAEALYKAQAETGEIKGHYLNATAGTCEEMMKRAIFARELGVPIVMHDYLTGGFT
ANTSLAHYCRDNGLLLHIHRAMHAVIDRQKNHGIHFRVLAKALRMSGGDHIHSGTVVGK
LEGEREITLGFVDLLRDDFVEKDRSRGIYFTQDWVSLPGVLPVASGGIHVWHMPALTEIFG
DDSVLQFGGGTLGHPWGNAPGAVANRVALEACVQARNEGRDLAREGNEIIREASKWSPE
LAAACEVWKEIKFEFEAMDTL--MAIHLYKTSTPSTRNGAVDSQVKSNPRKNLIYGQHRCG
KGRNARGIITAGHRGGGHKRLYRKIDFRRNEKDIYGRIVTIEYDPNRNAYICLIHYGDGEK
RYILHPRGAIIGDTIVSGTEVPIKMGNALPLTDMPLGTAIHNIEITLGKGGQLARAAGAVAK
LIAKEGKSATLKLPSGEVRLISKNCSATVGQVGNVGVNQKSLGRAGSKRWLGKRPVVRG
VVMNPVDHPHGGGEGRAPIGRKKPTTPWGYPALGRRSRKRNKYSDNLILRRRSKMIQPQ
THLNVADNSGARELMCIQIIGTS-NRRYAHIGDVIVAVIKEAVPNMPLERSEVVRAVIVRTCK

ELKRDNGMIIRYDDNAAVVIDQEGNPKGTRVFGAIARELRQLNFTKIVSLAPEVLMLSPKR
TRFRKQHRGRMKGISYRGNHICFGKYALQALEPAWITSRQIEAGRRAMTRNARRGGKIW
VRIFPDKPVTVRPTETRMGSGKGSPEFWVAVVKPGRILYEMSGVTETIARKAISIAASKMPI
RTQFIISGMTRIKRGYIARRRRTKIRLFASSFRGAHSRLTRTITQQKIKALVSANRDRDRQKR
NFRRLWITRINAGIRE-GGVSYSYSRLIHDLYKRQLLLNRKILAQVAILNRNCLYMISNEIIK-
----------MLRKVYAFSRYISMSAHKARRVIDQIRGRSYEETLMILELMPYRACYPIFKLVYSA
AANASYSLGSDETNLIISKAEVNEGTTIKKLKPRARGRSYPIKRPTCHITIVITDISIE-------M
DGIKYAVVTDKSIQLLGKN-QYTSNVESGSTRTEIKHWVELFFGVKVIAMNSHRLPGKGRR
MGPIMGHTMHYRRMIITLQPGYSIPPLRKKTTMAVPKKRTSISKKRIRKNMWKKKGYWA
ALKAFSLAKSLSTGNSKSFFVRQTNKMAKGKDVRVTVILECTSCVRNGVNVNKASTGISR
YITQKNRHNTPNRLELRKFCPYCHKHTIHGEIKKMKIRASVRKICEKCRLIRRRGRIIVICSN
PRHKQRQGMVREKVTVSTRTLQWKCVESRADSKRLYYGRFILSPLMKGQADTIGIAMRR
ALLGEIEGTCITRAKSE--KIQHEYSTIVGIQESVHEILMNLKEIVLRSTLYGTCDASICVKGP
GYVTAQDIISPPYVEIVDNTQYIASLTEPIDLCIGLQIERNRGYRIKPPN-NFDGNY--PIDAVF
MPVRNANHSIHSYGNGNEKQEILFLEIWTNGSLTPKEALHEASRNLIDLFIPFLHTEEE--NL
HLEDNQ-HKVTLPTFTFHDKLA-KLRKNKK-KIAFKYIFIDQSELSPRIYNCLKRSNIYTLLD
LLNNSQEDLMKMKDFRIEDVKEILDILEKR--FATDLLKNRMLRDG-NEGMSTIPEFNQIQF
EGFCRFIDRGLTEELYKFPKIEDTDQEIEFQLFVETYQLVEPLIKERDAVYESLTYSSELYVS
AGLIWKTGRDMQEQTIFIGNIPLMNSLGTSIVNGIYRVVINQILQSPGIYYRSELDHNGISVY
TGTIISDWGGRSELEIDRKARIWARVSRKQKISILVLSSAMGSNLREILENVCYPEIFLSFLN
DKEKKKIGSKENAILEFYQQFACVGGDPVFSDSLCKELQKKFFQQRCELGRIGRRNMNRR
LNLDIPQNNTFLLPRDILAAADHLIGMKFGMGTLDD--MNHLKNKRIRSVADLLQDQFGLA
LVRLENVVRGTICGAIR-HKL-IPTPQNLVTSTP----LTTTYESFFGLHPLSQVLDRTNPLTQIV
HGRKLSYLGPGGLTGRTASFRIRDIHPSHYGRICPIDTSEGINVGLIGSLAIHARIGHLG-SLE
SPFY---EISERPKK--VRVLYLSPGRDEYYMVAAGNSLALNQGIQEEQVVPARYRQEFLTIAW
EQVHLRSIFPFQYFSIGASLIPFIEHNDANRALMSSNMQRQAVPLSQSEKCIVGTGLERQAA
LDSGALAIAEHEGKIVYTDTDKIVLSGNGNTISIPLVMYQRSNKNTCMHQKPRVQGGKCIK
RGQILADGAATVGGELALGKNILVAYMPWEGYNSEDAVLISERLVYEDVYTSFHIRKYEV
QTHVTSQG-PERVTSEIPHLEAHLLRNLDKNGIVMLGSWVETGDILVGKLTPQMVKE-SSY
APEDRLLRAILGIQVSTSKETCLKLPIGGRGRVIDVRWIQKRG-GSSYNPETIRVYISQKREI
KVGDKVAGRHGNKGIISKILPRQDMPYLQDGRPVDMVFNPLGVPSRMNVGQIFECSLGLA
GGLLDRHYRIAPFDERYEQEASRKLVFSELYEASKQTANPWVFEPEYPGKSRIFDGRTGDP
FEQPVLIGKPYILKLIHQVDDKIHGRSSGHYALVTQQPLRGRAKQGGQRVGEMEVWALEG
FGVAHILQEMLTYKSDHIRARQEVLGTTIVGGTIPNPEDAPESFRLLVRELRSLALELNHFL
VSEKNLQINRKEAMNQNFSSMIDRYKHQQLRIGLVSPQQISAWAKKILPNGEIVGEVTKPY
TFHYKTNKPEKDGLFCERIFGPIKSGICACGNYRVIGDEKEDPKFCEQCGVEFVDSRIRRYQ
MGYIKLACPVTHVWYLKRLPSYIANLLDKPLKELEGLVYCD--FSFARP-IAKKPTFLRLRG
SFEYEIQSWKYSIPLFFTTQGFDTFRNREISTGAGAIREQLSDLDLRIIIDYSLVEWKELGEE-
GLTGNEWEDRKIGRRKDFLVRRMELAKHFIRTNIEPEWMVLCLLPVLPPELRPIIQIDGGKL
MSSDINELYRRVIYRNNTLTDLLTTSRSTPGELVMCQEKLVQEAVDTLLDNGIRGQPMRDG
HNKVYKSFSDVIEGKEGRFRETLLGKRVDYSGRSVIVVGPSLSLHRCGLPREIAIELFQTFV
IRGLIKQHLASNIGVAKSKIREKEPIVWEILQEVMQGHPVLLNRAPTLHRLGIQAFQPILVE
GRAICLHPLVRKGFNADFDGDQMAVHVPLSLEAQAEARLLMFSHMNLLSPAIGDPISVPT
QDMLIGLYVLTSGNRRGICVNRYNPCNR---RDYQNENI----YKYTK--EPFFCNSYDAIGAYR

QKRIHLDSPLWLRWRLDQR-VIAS--REAPIEVHYESLGTYYEIYEHYLIIRSIKKEIILIYIRTT
VGHISLYREIEEAIQGFCRACSYGT--------MEVLMAERANLVYHNKAIDGTAMKRLISRLID
HFGMAYTSHILDQVKTLGFQQATATSISLGIDDLLTIPSKGWLVQDAEQQSLILEKHHHYG
NVHAVEKLRQSIEIWYATSEYLRQEMNPNFRMTDPFNPVHIMSFSGARGNASQVHQLVG
MRGLMSDPQGQMIDLPIQSNLREGLSLTEYIISCYGARKGVVDTAVRTSDAGYLTRRLVEV
VQHIVVRRTDCGTVRGISVSPQNGMIP-ERIFIQTLIGRVLADDIYMGPRCIATRNQDIGIGLI
NRFIT------FRAQPISIRTPFTCRSTSWICRLCYGRSPTHGDLVELGEAVGIIAGQSIGEPGTQL
TLRTFHTGGVFTGGTAEHVRAPSNGKIKFNEDLVHPTRTRHGHPAFLCSIDLYVTIESEDIIH
NVNIPPQSFLLVQNDQYVESEQVIAEIRAGTST-LNFKERVRKHIYSDSEGE-MHWSTDVYH
APEFTYGNVQLLPKTSHLWILSGRPCR--SSLASLSLHKDQDKMNAH--SLSVKRRYISNLS--
VTNDQ-ARHKFFSSDFSGK-QDRIPDY-SERNRIICTGHCNLTYP-AIFHENS--DLLAKRRRN
RFIIPLQSIQERKKKRIPRSGISIEIP--INGIFRRNSILAYFDDPRYRRKSSGITKYGTIEAHSIVK
KED--LIEYRG-VKEFRPKYMKVDRFFFIPEEVHILPGSSSIMVRNNSLIGVDTQIAFNTRSRV
GGLVRVERKKKRIELKIFSGDIHFPGETDKISRHSGILIP-PG-TGKKNSTESKK--LKNWIYV
QR--ITPTK--KKYFVLVRPVVAYEITDGINLATLFPPDLLQERDNVQLRVVNYILYGNGKPIR
GIPDTSIQLVRTCLVLNWGQDKK---SSYIDEAHASFVEIRTNGLIQDFLRIDLVKSPISYTSKR
NDPSGSGLISE-NGSDRTNINPFSSIY-----SKAKIQQSLNQNQGTIHTLLNR---NKECQSLIILS
SSNCSQMGP--FNDVKYHNV---IKES-PLIRIRNLLGPLGT--ALPIYNFDS--FYHLNLITHNQL
L-VTNYLQLDN---LKQAFQVLK---YYLMDEN--GKIYNPDPCSNIFLNPLNLNWYFLHHNY
CEETS-TIISLGQFICENVCIAKNGPH-LK--SGQVLIVQ-IDSLVIRSAKPYLATPGATVHGHY
GETLYEGDTLVTFIYEKSRSGDITQGLPKVEQVLEVRSIDSISMNLEKRVEGWNECITRILGI
PWGFLIGAELTIAQSRISLVNKIQRVYRSQGVQIHNRHIEIIVRQITSKVLVSE---DGMSNVFS
PGELIGLLRAERMGRALEEAICYRAILLGITRASLNTQSFISEASFQETARVLAKAALRGRID
WLKGLKENVVLGGRIPVGTGFKGL--AHPSRQHNNIP--LETKKKSLFEGKMR--DILFHHRK
LFDSCLSKNFHDTSEQSFIG------FNDSMTRRYWNINLEEMMESGVHFGHGTRKWNPRMA
PYISAKRKGIHITNLTRTARFLSEACDLVFDAASKGKQFLIVGTKTKAADSVARAALKARC
HYVNKKWLGGMLTNWATTETRLHKFRDLRTEQKTGKLNRLPKRDAAMLKRQLSHLQTY
LGGIKYMTGVPDIVIIVDQHEEYTALRECITLGIPTICLIDTNCDPDLADISIPANDDAIASIRL
ILNKLVFAICEGRS-SYIRNSMGQKINPLGFRLGITQGHHSLWFAQPKNYSEGLQEDQKIRD
CVKNYVQKNMRISSG-VEGIARIEIQKRIDLIQVIIYMGFPKLLIESRPR----GIEELQVNLQK
EFNCVNRKLNIAITRIAKPYGNPNILAEFIAGQLKNRVSFRKAMKKAIELTEQEDTKGIQVQ
IAGRIDGKEIARVEWVREGRVPLQTIRAKIDYCSYTARTIYGVLGIKIWIFIDEEMSRYRGPR
FKKIRRLGALPGLTNKRPRAGSDLRNQSRSGK-RSQYRIRLEEKQKLRFHYGLTERQLLKY
VRIAGKAKGSTGQVLLQLLEMRLDNILFRLGMASTIPGARQLVNHRHILVNGRIVDIPSYR
CKPQDIITARDEQKSRALIQNSLESSPH-EELPKHLTVHPFQ--YKGLVNQIIDSKWVGLKINE
LLVVEYYSRQTMSRRGTAEEKTAKSDPIYRNRLVNMLVNRILKHGKKSLAYQILYRAVKKI
QQKTETNPLSVLRQAIRGVTPDIAVKARRVGGSTHQVPIEIGSTQGKALAIRWLLAASRKR
PGRNMAFKLSSELVDAAKGSGDAIRKKEETHRMAEANRAFAHFRMGRDTIADVITSIRNA
DMDRKRVVRVPSTTITENIVKILLREGFIENVRKHWESNKYFLVSTLRHRR--NRRRPYR---
NLLNLKRISRPGLRIYSNYQRIPRILGGMGIVILSTSRGIMTDREARQEGIGGEILCYIWMAK
AIPRIGSRRNARIGSRKNVRRIPKGVIHVQASFNNTIVTVTDVRGRVVSWSSAGSCGFRGT
RRGTPFAAQTAAGNAIRTVVDQGMQRAEVMIKGPGLGRDAALRAIRRSGILLTFVRDVTP
MPHNGCRPPKKRRVMPTIKQLIRNTRQPIRNVTKSPALRGCPQRRGTCTRVYTITPKKPNS
ALRKVARVRLTSGFEITAYIPGIGHNSQEHSVVLVRGGRVKDLPGVRYHIVRGTLDAVGVK

DRQQGRSKYGVKKPKMARKSLIQREKKRQKLEQKYHLIRRSSKKEIS---KVPSLSDKWEI
HGKLQSPPRNSAPTRLHRRCFLTGRPRANYRDFGLSGHILREMVHACLLPGATRSSWMVK
NPFISVISQE---KEEKRGSVEFQVFVFTNKIQRLTSHLELHKKDYLSQRGLRKILGKRQRLL
AYLSKKNRVRYKELISQLGIRETKTR--MVKLRLKRCGRKQRAVYRIVAIDVRSRREGRDL
RKVGFYDPIKNQTYLNVPAILYFLEKGAQPTGTVHDILKKAEVFKELRSN------MDKSKRP
FLKSKRSFRRRLPPIQSGDRIDYRNMSLISRFISEQGKILSRRVNRLTLKQQRLITIAIKQARI
LSSLPFLNNEK--QFERTESTARTTGLKTRNKVTRSLKKNPFVANHLLKKIDKLNTKAEKEII
VTWSRASTIIPTMIGHTIAIHNGKEHLPIYITDRMVGHKLGEFAPTLNFRGHAKSDNRSRR
MIFKSFLLGNLVSLCMKIINSVVVVGLYYGFLTTFSIGPSYLFLLRAQAMEEGEEGTEKKVS
ATTGFITGQLMMFISIYYAPLHLALGRPHTITVLALPYLLFHFFWNNHKHFFDYGSTTRNS
MRNLSIQCVFLNNLIFQLFNHFILPSSMLARLVNIYMFRCNNKMLFVTSSFVGWLIGHILF
MKWVGLVLVWIRQNHSIRSLIRSNKYLVSELRNSMARIFSILLFITCVYYLGRIPSPILTKKL
KETEEDKSLVILLFDYNRWNRPLRYIKNQHFEKAVRNEMSQYFFYTCQSDGKQKISFTYPP
SLSTFWEMIQKKMSLYTIENPPSDELYNNWIYTNEEKSK-LSNEFINRIEALDGGSLSLNLLE
KKTRLCNDETRQEYLPKMYDPFLNGSSRGTIKKGFSPIEKIWINKIHGILYREFEQKDPNDQ
SIEIQEIIKKVPRWSYKLIDLEQQQERQIRSRKAKRVVIFTNALIRYSQQSDFRRNIIKGSMR
AQRRKIVIEELFQANVHSPLFLDRIDKSLLFSFDI--SRLMKLILKNRKEKTRIEIAEAWDTIP
LAQVIRGSMLVTQSIFRKYIILPSLIIAKNIGRILLLQFPEWSEDLKEWAGEMHVKCTYNGV
QLSETEFPKNWLLDGIQIKILFPFCLKPWHKPKKVKKADFCFLTVWGMETDLPFGSPRKRP
SFFEPIFKELENQIGQLKNKSFIV-LRVLKERIEFFLRVLNETKKWVIKSIL--FIKRILKELSKV
NDSTINDQKIHESFSQIRSTNWTNYSLTEKKMKDLTDRTSTIRNQIERITKEKKTSPNKTSY
NVKRLESPKTIWQMLKRRNVRLLRKLHYFIKFFIEKISMDIFLYIINIPRINTQLF-HFISCDLP
SLSQAYVFYKLSQTQVINLYKLRSVLQYTSFFLKNTIKDYFTQGIFHFELSHKKLRNSGMN
QWKNWLKGHYQYDFSRIKWSRLIPQKWRNRVNQRRTAQTKNKRDSYEKDQ--LIHYKKQ
-NFQKNYRYDLLSYKSINGDIGYPDRKYFDWKIINFCLRFLDWMGMNEEILNRWFFPEFLL
LYNIYKIKPWVIPSKLLLLNYKNNTEAELHFFLNKYLLFQLRWNETLNQRMINNIKVYCLL
LRLRNPKEIAIFSIQRREISLDIMLIQKNLTLTELMK-GILIIEPVRLFLKNDGQFIMYQTLGISL
VHKSKHNHYDLLIPENILSSRRCRELRILICFRFLWPNYRLEDLACMNRYWFDTNNGSRFS
MLRIHMYPMKGHQFKSWIFELREILREIKNSHYFLDSWTQFNSVGSFIHIFFHQERFIKLFD
PRIWSILLS-RNS--QGSTSNRYFT---IKG-VILFVVAVLLYR----INNQNMVERKNLYLIGLLP---
--IPMNSIGPRNDTLEESF-WSSNINRLIVS-LLYLPKGKKISESCFLDPKESTWVLPST----KKC
IMPESNWGSRWWRSWIGKKR--DSSWKISN---ETVAGIEILFKEKDIK-YLEFCFVYYMD--D
PIRKDHDWELFDRLSLRKKRRNIINL--NSGPLFEILVKHWICYLMSAFREKIPIEVEG-FFKQQ
G-AGSTIQSNDIEHVSH--LFSRNKWAISLQNCAQFHMWQFRQDLLV---SWGK-NPHESDFL
RN-VSRENWIWLDNVWLVNKGR---FFS--KVRNVSSNIQYDS---TR--SSFVQVTDSSQLKGS
S-DQSRDHLDSIS----NEDSE-------------YYTLINQREIQQLKERSIL-WD--PSFLQTERTEIESD-
-RFPK--CFSGYS--SMSRLFT-EREKQMINHLLPEEIEEFL--GNPTRSIRSFFSDRW--SELH-LG
SNPTERSTRDQ----KLLKKQQDLS-FVPSRRSE----NKEMVNIFKIITYLQN--TVSIHPISSDPG
CDMVP-KDEPD-MDSSNKISFLNKNPFFDL--FHLFHDRNRGGYTL--HHDFESE--ERFQEM
ADLFTLSITEP-DLVYHKGFAFSIDSYGLDQKQ--FLNEVFNSRDESKKKS---LLVLPPIFY--EE
NESFYRRIRKKWVR-ISCRNDLEDPKPKIVVFASNNIMEAVKQYRLIRNLIQIQYSIYG--YIR
NVLNRFFLMN---RSDRNFEYG---IQRDQIRKDTLNHRPIMKYTINQHLSNLKK-SQKKWFD
PL--IFISRTERSMNRDPNAYRYKWSNGSKNFQEHLEHFVSEQKSR---FQVVFDRLRINQYSI
DWSEVIDKKDLSKPLRFFLSK---------SLLFLS-------KLLFFLSN-SLPFFFVSFGNIPIHRSE-IY

VY-ELKGPNDQLCNQLLESIGLQIVHLKKWKPF--LLD---DHDTS-QKSKFLINGGRISPFLFN
KIP-KW-MIDSFHTR--NNRRKSFDNMDSYFSMISHDQDNWLNPVKPFHRSLLISSFYKANR
LRFLNNPHHFCFY--CNKRFPFYV----EKARIN--NYDFTYG-QFLNILFIRNK-IFSLCVGK--KK
HAFGG-RDTISPIESPVSNIFIPNDFPQSG-DETYNLYKS---FHLPSRSDPFVR--RAIYSIADISG
TPLTEGQIVNFERTYCQPLSDMNLSDSEGKNLHQYLN--FNSNMGLIHTPCSEKYLPSGK--R
KK--------RSLWLK--KCVEKGQMYRTFQRDSAFSTLSKWNLFQTYMPWFLTSTGYKYLNFI
FLDTFSDLL--PILSS-SQKFVSIFHDIMHGSDISWRILQKKL------CLPQWNW-ISEISSKCLHN
--LLLSEEMIHRN--NESPLISTHLRSPNVREF-LYSILFLLLVAGY-LVRTHLLFVSR--ASSELQT
EFEKVKSLMIPSSMIELRKLLDRYPTSEPNSFWLKNLFLVALEQLGDSLEEIRGSASGGNML
LGGGPA-----YG-VKSIRSKKKYLNINLIDLISII----PNPINRITFSRNTRHLSHTSKEIYSLIRKR
KNVNGDWIDDKIESWVANSDSIDDEEREFLVQFS--TLTT--EKRIDQILLSLT-HSDHLS---ISK
NDSGYQMIEQPGAIYLRYLVDIH--KKYLMNYEFNTSCLAERRIFLAHYQTITY--SQTSCGA
NSFHFPSHG---KPFSLRLALSPS-RGILVIGSIGTGRSYLVKSLATNSYVPFITVFLNKFLDNKP
KGFLID-----DIN-IEDSDDIDDSDDIDASDDID---------------DIDHDLDTEL-ELLTMMNALTM
DMMPE---IDRFYIILQFELAKAMSPCIIWIPNIHDLDVNESNYLSLGLLVNHLSR---DCERCS-
TRNILVIASTHIPQKVDPALIAPNKLNTCIKIRRLLIPQQRKHFFTLSYTRGFHLEKKMFHT--
NGFGSITMGSNALDLVALTNEALSISITQKKSIIDTNTIRSVLHRQTWDLR---SQVRSVQDH
GILFYQIGRAVAQNVFLSNCPIDPISIYMKKK--SCNEGDSYLYKWYFELGTSMKKLTILLYL
LSCSAGSVAQDLWSLP--GPDEK-NGITSYGLVENDS-DLVHGLLEVEGALAG--SSRTEKYC
SQFDND--RVTLLLRPESRN-PLDMMKNGSCSILDQRFLYEKD--ESEFEE-GEGEGALDPQQ-
---IEED----LFNH--IVWAP--RIWRP---WGFLFDCIERPNELGFP-YWSRSFRGK---RIIYDEEDE
LQENDSEFLQSGTMQYQTRDRSSKEQGLFRISQFIWDPADPLFFLFK-DRPFVSVFSHRELF
ADEEMSKGLLTSQTDPPTSIYKRWFI--KNTQEKHFELLINRQRWLR---TNSSLS--NGSFRSN
--TLSESYQYLSNLFLSN-GTLLDQMTKTLLRKRWLFPDEMKIGF--M---------MPRSR-INGN
FIDKTFSIVANILLRIIPTTSGEKEAFTYYRDG--MSAQSEGNYAEALQNYYEAMRLEIDPYD
RSYILYNIGLIHTSNGEHTKALEYYFRALERNPFLPQAFNNMAVICHY-RGEQAIRQGDSEI
AEAWFDQAAEYWKQAIALTPGNYIEAHNWLKITRRFE--MSWRSERIWIELITGSRKTGNF
CWAFILFLGSLGFLLVGISSYLGRNLISLFPSQQIIFFPQGLVMSFYGIAGLFISSYLWSTISWN
VGSGYDRFDIKEGIVCIFRWGFPGKNRRIFLRFLIKDIQSVRIEVKEGIYARRVLYMEIRGQG
AIPLTRTDEN-LTPREIEQKAAELAYFLRVPIEVF
>Dillenia
MEKRWFNSMLSISKSMHSLGPIENTSGSKDSIINDTDKNIHSYSNIDYLFSLRDIRNFISDDIF
VIKDSNGGSYSIYFDIENQIFEIDNDH--SFLNERESSFYSYQNSSYLNNGSKRDDSYYDRYI
YDTQSSWNNHINSCIDSYLH--SQIRIDSYILSGSHNYSDSYIYSYIWS--SSNIRTNTNDSDLTI
KESSNDLDVTQKYKHLWVQCENCYGLNYKKFCKSKMNICEQCGYHLKMSSSDRIELSID
PGTWDPMDEDMVSLDPIEFHSEEEPYKDRIHSYQKKTGLSEAVQTGTGQLNGIPIAIGVMD
FQFMGGSMGSVVGEKITRLIEYATNKFLPLIIVCASGGARMQEGSLSLMQMAKISSALYDY
QSNKKLFYVSILTSPTTGGVTASFGMLGDIIIAEPNAYIAFAGKRVIEQTLNKTVPEGSQAAE
YLFHKGLFDSIVPRNLLKGVLSELFQLHAFFPLNQNSIKMVTIRADEISNIIRERIEQYNREV
KIVNTGTVLQVGDGIARIHGLDEVMAGELVEFEEGTIGIALNLESNNVGVVLMGDGLMIQ
EGSSVKATGKIAQIPVSEAYLGRVINALAKPIDGRGEISASESRLIESPAPGIISRRSVYEPLQT
GLIAIDSMIPIGRGQRELIIGDRQTGKTAVATDTILNQQGQNVICVYVAIGQKASSVAQVVTT
FQERGAMEYTIVVAETADSPATLQYLAPYTGAALAEYFMYREQHTSIIYDDLSKQAQAYR
QMSLLLRRPPGREAYPGDVFYLHSRLLERAAKSSSLLGEGSMTALPIVETQSGDVSAYIPT

NVISITDGQIFLSADLFNAGIRPAINVGISVSRVGSAAQIKAMKQVAGKSKLELAQFAELEAF
AQFASDLDKATQNQLARGQRLRELLKQSQSAPLTVEEQIMTIYTGTNGYLDSLEIGQVRKF
LVELRTYLKTNKPQFQEIISSTKTFTEEAEALLKDAIQEQMKRFLLQEQAMRINPTTSGPGV
STLEKKSLGRIAQIIGPVLDVAFPPGKMPNIYNALLVKGRDTAGQQINVTCEVQQLLGNNR
VRAVAMSATDGLMRGMEVIDTEAPLSVPVGGATLGRIFNVLGEPIDNLGPVDTRTTSPIHR
SAPAFIQLDTKLSIFETGIKVVDLLAPYRRGGKIGLFGGAGVGKTVLIMELINNIAKAHGGV
SVFGGVGERTREGNDLYMEMKESGVINEQNIAESKVALVYGQMNEPPGARMRVGLTALT
MAEYFRDVNEQDVLLFIDNIFRFVQAGSEVSALLGRMPSAVGYQPTLSTEMGSLQERITST
KRGSITSIQAVYVPADDLTDPAPATTFAHLDATTVLSRGLAAKGIYPAVDPLDSTSTMLQPRI
VGEEHYETAQKVKQTLQRYKELQDIIAILGLDELSEEDRLTVARARKIERFLSQPFFVAEVF
TGSPGKYVGLAETIRGFQLILSGELDGLPEQAFYLVGNIDEATAKAMNLEMESNLKKMTL
NLCVLTPNRIVLDSGVKEIILSTNSGQIGILPNHAPIATAVDIGILRIRLNDQWLTMALMGGF
ARISNNEITILVNDAEKGSDIDPQEAQQTLEIAEANLRKAEGKRQTIEANLALRRARTRVEA
INVISMKNVTDSFVS-GHWPYAGSFGFNTDILATNPINLSVVIGVLIFFGKGVLSDLLDNRK
QKILNSIRNSEELRGGAIDQLEKARARLRKVEMEADQFRVNGYSEIEREKLNLINSTYKTL
EQLENYKNETIQFEQQRALNQVRQRVFQQALQGALGTLNSCLNNELHLRTINVNIGMFGA
IKEITDMNPLISAASVIAAGLAVGLASIGPGVGQGTAAGQAVEGIARQPEAEGKIRGTLLLS
LAFMEALTIYGLVVALALLFANPFVMNVLSCSINTLKVLYDISGVEVGQHFYWQIGGFQV
HAQVLITSWVVIAILLGSAIIAVRNPQTIPTDGQNFFEYVLEFIRDVSKTQIGEEYGPWVPFI
GTMFLFIFVSNWSGALLPWKIIQLPHGELAAPTNDINTTVALALLTSVAYFYAGLSKKGLG
YFGKYIQPTPILLPINILEDFTKPLSLSFRLFGNILADELVVVVLVSLVPLVVPIPVMFLGLFTS
GIQALIFATLAAAYIGESMEGHHMIFLTLEHILTHISFSIISILITIHFITLLSDETVGLSNSKKEG
MIVTFSCITGLLITRWIYSQHFPLSDLYESVIFLSWAFSIIHMIPYF-MHKNYLSAITAPSAIFT
QGFATSGFLTEIHQSKILVPALQSQWLMMHVSMMVLGYAALLCGSLLSVALLVITFRKDI---
SKSNQIQRMNERYNILQNPSLISARNYYRSQLIQQLDYWSYRVISLGFLFLTIGILSGAVWA
NEAWGSYWNWDPKEIWAFITWTIFAIYLHTRTNTNLKGANSAIVSFMGFLIIWICYFGINLL
GIGLHSYGSFTLIMEQKKLFTPFLYLLSIVFLPWGISLSFNKSMESWVINWWNTSKSETFLN
DIQEKSILEKFIKLEELFLLDEMIKEYPETHLQKLCIEIYKETIQLIKVYNEDRIHAILHFSTNI
ISFIILSGYSILGNEELVILNSWVQEFLYNLSDTIKVFSILLFTDLCIGFHSPHGWELMIGSVY
KDFGFVHNDQIISGLVSTFPVILDTIFKYWIFRYLNRVSPSLVVIYHSMNDMPIGVPKVPFRS
PGEEDASWVDVYNRLYRERLLFLGQEVDNEISNQLIGLMVYLSIEDDTKDLYLFINSPGG
WVIPGVAIYDTIQFVQPDVHTICMGLAASMGSFILVGGEITKRLAFPHARVMIHQPASSFYE
AQTGEFILEAEELLKLRETITRVYVQRTGKPLWLVSEDMERDVFMSAREAQAYGIIDLVAV
EMKETKWIHEGLITESLPNGMFRVRLDNEDMILGYVSGRIRRSFIRILPGDRVKIEISRYDST
RGRIIYRLRNKDSNDMEEFQVYLERDISRQHDFLYPLIFQEYIYALAHDHG-VGYDNKSSFL
IVKRLITRMYQWNHLNISLNDSNKKKFVVNNLDFQMILEGFSVIAEIPFSLQLLSFLIEKFH
NLRSIHSIFPFLEDKFSHLNYVSDILIPHPVHLEILIQTLRYWVKDASSLHFLRFFLHEYRNW
TSFITKSIFFFSKPRFFFFLYNSHVYEYESIFGFLCNQSSHLQSTSYGALLGRIFFYEKRKHLV
ETLANDFNLWLCKDPFMHYVRHQEKIILASKGTALLMNKWKYYFVNLWQCNFDVWAQP
GRIHIKQLSNNCLHFLGYLSSVRLNPSMVWSQILENAFLIDNTI--KKLDALVPIFPLIESLAK
AGFCNGLGHPISKPVWADSSDSDIIDRFGRICRNLSHYHSGSSKKKSLYQIKYILRISCARTL
ARKHKSTIRAFLKRLGSELLEEFLTEDEQIISLILPGQIWYLDIIYIADLADHSMIINTT-EVQ-
NIHFFSRLEF-LKEIYEIIWMLVLLLTLVLGLTISVLVIVWLEREISAGIQQRIGPEYAGPLGIL
QALADGIKLLFKESLLPSRGDTRLFSIGPSISVISILLSYSVIPFGYGLVLADLSIGVFLWISFS

SIAPIGLLMSGYGSNNKYSFLGGLRAAAQSISYEIPLALCVLSISLLSNSSSTVDIVEAQAKY
GLWGWNFWRQPIGVLVFLISSLAECERLPFDLPEAEEELVAGYQTEYSGIKFALFYVASYL
NLLVSSLFVTVLYFGGWDLSIPYIFVPELFEINKTGGVFQTTIGIFITLIKTYLFLFISITTRWT
LPRLRIDQLLNLGWKFLLPISLGNLLLTTSFQLFSLMIWHVQNENFILDSTRIFMKAFHLLL
FDGSLIFPECILIFGLILLLMIDLTSDQKDIPWLYFISSTSLVMSITALLFRWREEPMISFSGNF
QTNNFNEIFQFLILLCSTLCIPLSVEYIECTEMAITEFLLFVLTATLGGMFLCGANDLITIFVAP
ECFSLCSYLLSGYTKKDVRSNEATTKYLLMGGASSSILVHGFSWLYGSSGGEIELQEIVNG
LINTQMYNSPGISIALIFITVGIGFKLSPAPSHQWTPDVYEGSPTPVVAFLSVTSKVAASASA
TRILDIPFYFSSNEWHLLLEILAILSMILGNLIAITQTSMKRMLAYSSIGQIGYVIIGIIVGDSN
DGYASMITYMLFYISMNLGTFACIVLFGLRTGTDNIRDYAGLYTKDPFLALSLALCLLSLG
GLPPLAGFFGKLHLFWCGWQAGLYFLVSIGLLTSVLSIYYYLKIIKLLMTGRNQEITPHVRN
YRRSPLRSNNSIELSMIVCVIASTIPGISMNPIIAIAQDTLFMFLLYEYDIFWTFLIISSFIPILAF
IISGVLAPIRKGPEKLSSYESGIEPMGDAWLQFRIRYYMFALVFVVFDVETVFLYPWAMSFD
LLGVSVFIEALIFVLILIVGSVYAWRKGALEWSTNFFPWLTIIVVLPIFAGSLIFFLPHRGNKI
FRWYTLCICFLELLLTTYVFCYHFQLNDPLIQLAEDYNWINFINFHWRLGIDGLSIGPILLTG
FITTLATLAAWPVTRDSRLFHFLMLAMYSGQIGSFSSRDLLLFFIMWEFELIPVYLLLSMW
GGKKRLYSATKFILYTAGGSVFLLMGVLGISLYGFNEPIFYFETSANQSYPLALEILFYIGFLI
AFAVKLPIIPLHTWLPDTHGEAHYSTCMLLAGILLKMGAYGLVRINMELLSHAHYIFSPWL
VIVGTIQIIYAASTSPGQRNLKKRIAYSSVSHMGFIIIGIGSITDTGLNGALLQIISHGFIGAAL
FFLAGTSYDRLRLVYLDEMGGIAISMPKIFTMFSSFSMASLALPSMSGFVAELIVFFGIITSQ
KFFLMPKILITFIMAIGMILTPIYSLSMLRQMFYGYKLFNVPNSYFFDSGPRELFVLISIFLPV
IGIGIYPDFVLSLSVDKVEYILSNYFYRMILEHVLVLSSYLFSIGIYGLITSRNMVRALICLEL
ILNAVNINFITFSDFFDSRQLKGDIFSIFVIAIAAAEAAIGPAIVSSIYRNRKSTRINQSNLLNK
MEHTYRYAWIVPFIPLTVPMLIGLGLLFFPTGTKNLRRIWAFPSVLLLSIAMIFSTDLSIQQIN
DSYSYQYVWSWTINNDFSLEFGHLIDPLTSIMSILITTVGITVLIYSDNYMSHDQGYLRFFA
YMSFSNSAMLGLVTSSNLVQIYIFWELVGMCSYLLIGFWFTRPIAANACQKAFITNRVGDF
GLLLGILGLYWVTGSFEFQDLFEIFNNREVHYLFVTLCAFLLFLGAVAKSAQFPLHVWLPD
AMEGPTPISALIHAATMVAAGIFLVARLFPLFIALPYIMNLISLIGIITVLLGATLALAQKDIK
KTLAYSTMSQLGYTMLALGMGSYRAALFHLITHAYSKALLFLGSGSIIHSMEAIVGYSPEK
SQNMALMGGLAKHIPITKTAFLLGTLSLCGIPPLACFWSKDEILNDSWLYSTIFAIMACSTA
GFTAFYMFRIYLLTFEGYFNIHFQNYSGKKNSLVYSISLWGKEGTKPIKKNLLTLLTLNFFSF
ENKNTF-CPHESKNTMIFPILLLVLFTLFVGFIGIPFHFDILSKLINPSINLLHQNLNNLEDWY
EFITNSIRSVSIAYLGIFIAAFFYKPVSSSLQNLDLINLFANLWNKIKNVIYDWSYNRGYIDA
FYVVSLTKGIKGLAELTHFFDKQVIDGIINGVGILNFFVGEVIKYIGGG-RISFYLFFYLSSVFI
LLLIYYMGLPGLIHDFLLVFLGLGLILGGLGVVLLTNPIFSAFSLGLVLVCISLFYISANSHFV
AAAQLLIYVGAINVLIIFAVMFMNGSEYYKDFHLWTIGDGISSLVCTSIFVSLITTIPDTSWY
GIIWTTRSNQIIEQDLISNGQQIGIHLSTDFVLPFELISIILLVALIGAIAAARQMTVSATKKDF
MIVNMGPHHPSMHGVLRLIVTLDGEDVIDCEPILGYLHRGMEKIAENRTIIQYLPYVTRWD
YLATMFTEAITVNAPERLGNIQVPKRASYIRVIMLELSRIASHLLWLGPFLADIGAQTPFFYI
FREREFVYDLFEAATGMRMMHNFFRIGGVAADLPHGWIDKCLDFCDYFLTGVSEYQKLIT
RNPIFLERVEGVGIINEEEAKNWGLSGPMLRASGIQWDLRKVDHYECYDEFDWEAKWQK
EGDSLARYLVRISEMMESIKIIQQALEGIPGGPYENLEIRRFDRVINSEWNDFEYRFISKKPS
PTFELSKQELYVRVEAPKGELGIFLIGDQSVFPWRWKIRPPGFINLQILPQLVKRMKLADIM
TILGSIDIIMGEVDRMFPIVTGFMSYGQQTIRAARYIGQSFMITLSHANRLPVTIQYPYEKSI

TSERFRGRIHFEFDKCIACEVCVRVCPIDLPVVDWKFETDIRKKRLFNYSIDFGICIFCGNCV
EYCPTNCLSMTEEYELSTYDRHELNYNQIALGRLPMSIMNDYTIRTILNS---QIKNR--------
MQGRLSAWLVKHELVHRSLGFDYQGVETLQIKPEDWHSIAVIFYVYGYNYLRSQCGYDV
APGGLLASVYHLTRIEYGVDQPEEVCIKVFTSRGNPRIPSVFWVWKSADFQERESYDMLGI
FYDNHPRLKRILMPESWIGWPLRKDYIAPNFYEIQDAH-------------------------------------MVL
VPDYSVNKKKKGKKKIETIMNSIEFPLLDRTTQNSVISTTSNDLSNWSRLSSLWPLLYGTSC
CFIEFASLIGSRFDFDRYGLVPRSSPRQADLILTAGTVTMKMAPSLVRLYEQMAEPKYVIAM
GACTITGGMFSTDSYSTVRGVDKLIPVDVYLPGCPPKPEAVIDAITKLRKKISREIYEDRIQS
--ENRCFTTNHKFHVGRSIHTGNYNKELLYQSPSISKTPPETFVKYKSSGLSHELVNMQIINT
FSWIKEQITRFISVSLMIYIITRTSISNAYPIFAQQGYENPREATGRIVCANCHLANKPVDIEV
PQAVLPDTVFEAVVRIPYDMQLKQVLANGKKGALNVGAVLILPEGFELAPPDRISPEMKE
KIGNLSFQSYRPTKKNILVIGPVPGQKYSEIIFPILSPDPATKKDVHFLKYPIYVGGNRGRGQI
YPDGSKSNNTVYNATAAGIVSKIIRKEKGGYEITIADASDGHQVVDIIPPGPELLVSEGESIK
LDQPLTSNPNVGGFGQGDAEIVLQDPLRIQGLLFFLASIILAQIFLVLKKKQFEKVQLSEMN
FMSKVYDWFEERLEIQAIADDITSKYVPPHVNIFYCLGGITLTCFLVQVATGFAMTFYYRPT
VTEAFASVQYIMTEANFGWLIRSVHRWSASMMVLMMILHVFRVYLTGGFKKPRELTWVT
GVVLAVLTASFGVTGYSLPWDQIGYWAVKIVTGVPEAIPVIGSPLVELLRGSASVGQSTLTR
FYSLHTFVLPLLTAVFMLMHFPMIRKQGISGPLMGVTKKPDLNDPVLRAKLAKGMGHNY
YGEPAWPNDLLYIFPVVILGTIACNVGLAILEPSMIGEPADPFATPLEILPEWYFFPVFQILRT
VPNKLLGVLLMVSVPAGLLTVPFLENVNKFQNPFRRPVATTIFLIGTVVALWLGIGATLPID
KSLTLGLFMIEVFLFGIVLGLIPITLAGLFVTAYLQYRRGDQLDLMPTITSYFGFLLAALTITS
ALFIGLSKIRLIMDIVSLAWAALMVVFTFSLSLVVWGRSGLMIIRSPEPEVKILVDRDPIKTS
FEEWARPGHFSRTIAKGPDTTTWIWNLHADAHDFDSHTSDLEEISRKVFSAHFGQLSIIFLW
LSGMYFHGARFSNYEAWLSDPTHIGPSAQVVWPIVGQEILNGDVGGGFRGIQITSGFFQIW
RASGITSELQLYCTAIGALIFAALMLFAGWFHYHKAAPKLTWFQDVESMLNHHLAGLLGL
GSLSWAGHQIHVSLPINQFLNAGVDPKEIPLPHEFILNRDLLAQLYPSFAEGATPFFTLNWS
KYAEFLTFRGGLDPVTGGLWLTDIAHHHLAIAILFLIAGHMYRTNWGIGHGLKDILEAHKG
PFTGQGHKGLYEILTTSWHAQLSLNLAMLGSLTIVVAHHMYSMPPYPYLATDYGTQLSLF
THHMWIGGFLIVGAAAHAAIFMVRDYDPTTRYNDLLDRVLRHRDAIISHLNWACIFLGFH
SFGLYIHNDTMSALGRPQDMFSDTAIQLQPVFAQWIQNTHALAPGATAPGAITSTSLTWGG
GDLVAVGGKVALLPIPLGTADFLVHHIHAFTIHVTVLILLKGVLFARSSRLIPDKANLGFRFP
CDGPGRGGTCQVSAWDHVFLGLFWMYNAISVVIFHFSWKMQSDVWGSISDQGVVTHIT
GGNFAQSSITINGWLRDFLWAQASQVIQSYGSSLSAYGLFFLGAHFVWAFSLMFLFSGRGY
WQELIESIVWAHNKLKVAPATQPRALSIVQGRAVGVTHYLLGGIATTWAFFLARIIAVGMA
LRFPRFSQGLAQDPTTRRIWFGIATAHDFESHDDITEERLYQNIFASHFGQLAIIFLWTSGNL
FHVAWQGNFESWVQDPLHVRPIAHAIWDPHFGQPAVEAFTRGGALGPVNIAYSGVYQWW
YTIGLRTNEDLYTGALFLLFLSAISLTAGWLHLQPKWKPSVSWFKNAESRLNHHLSGLFGV
SSLAWTGHLVHVAIPGSRGEYIRWNNFLDVLPHPQGLGPLFTGQWNLYAQNPDSSSHLFG
TSQGAGTAILTLLGGFHPQTQSLWLTDIAHHHLAIAFIFLVAGHMYRTNFGIGHSMKDLLE
AHIPPGGRLGRGHKGLYDTINNSIHFQLGLALASLGVITSLVAQHMYSLPAYAFIAQDFTTQ
AALYTHHQYIAGFIMTGAFAHGAIFFIRDYNPEQNEDNVLARMLDHKEAIISHLSWASLFL
GFHTLGLYVHNDVMLAFGTPEKQILIEPIFAQWIQSAHGKTSYGFDVLLSSTTGPAFNAGR
SIWLPGWLNAVNE--NSNSLFLTIGPGDFLVHHAIALGLHTTTLILVKGSLDARGSKLMPDK
KDFGYSFPCDGPGRGGTCDISAWDAFYLAVFWMLNTIGWVTFYWHWKHITLWQGNVSQ

FNESSTYLMGWLRDYLWLNSSQLINGYNPFGMNSLSVWAWMFLFGHLVWATGFMFLISW
RGYWQELIETLAWAHERTPLANLIRWRDKPVALSIVQARLVGLVHFSVGYIFTYAAFLIAST
SGKFGMSHSVKIYDTCIGCTQCVRACPTDVLEMIPWDGCKAKQIASAPRTEDCVGCKRCE
SACPTDFLSVRVYLWHETTRSMGLAYMTTFNFPSIFVPLVGLVFPTIAMASLFLHVQKNKI
VMRDLKTYLSVAPVLSTLWFGSLAGLLIEINRLFPDALTFPFFSFMTAILERRESESLWGRF
CNWITSTENRLYIGWFGVLMIPTLLTATSVFIIAFIAAPPVDIDGIREPVSGSLLYGNNIISGAII
PTSAAIGLHFYPIWEAASVDEWLYNGGPYELIVLHFLLGVACYMGREWELSFRLGMRPWI
AVAYSAPVAAAAAVFLIYPIGQGSFSDGMPLGISGTFNFMIVFQAEHNILMHPFHMLGVAG
VFGGSLFSAMHGSLVTSSLIRETTENESANAGYRFGQEEETYNIVAAHGYFGRLIFQYASF
NNSRSLHFFLAAWPVVGIWFTALGISTMAFNLNGFNFNQSVVDSQGRVINTWADIINRANL
GMEVMHERNAHNFPLDLAAVEAPSINGMGLPWYRVHTVVLNDPGRLLSVHIMHTALVAG
WAGSMALYELAVFDPSDPVLDPMWRQGMFVIPFMTRLGITNSWGGWSIT-GGTITNPGIW
SYEGVAGAHIVFSGLCFLAAIWHWVYWDLEIFCDERTGKPSLDLPKIFGIHLFLSGVACFG
FGAFHVTGLYGPGIWVSDPYGLTGKVQSVNPAWGVEGFDPFVPGGIASHHIAAGTLGILA
GLFHLSVRPPQRLYKGLRMGNIETVLSSSIAAVFFAAFVVAGTMWYGSATTPIELFGPTRY
QWDQGYFQQEIYRRVGAGLAENQSLSEAWSKIPEKLAFYDYIGNNPAKGGLFRAGSMDN
GDGIAVGWLGHPIFRDKGGRELFVRRMPTFFETFPVVLVDGDGIVRADVPFRRAESKYSV
EQVGVTVEFYGGELNGVSYSDPATVKKYARRAQLGEIFELDRATLKSDGVFRSSPRGWFT
FGHASFALLFFFGHIWHGSRTLFRDVFAGIDPDLDAQVEFGAFQKVGDPTTKRQVV---------
---VETLFNGTLALAGRDQETTGFAWWAGNARLINLSGKLLGAHVAHAGLIVFWAGAMNL
FEVAHFVPEKPMYEQGLILLPHLATLGWGVGPGGEVIDTFPYFVSGVLHLISSAVLGFGGIY
HALLGPETLEESFPFFGYVWKDRNKMTTILGIHLILLGLGAFLLVFKALYFGGVYDTWAPG
GGDVRKITNLTLSPSVIFGYLLKSPFGGEGWIVSVDDLEDIIGGHVWLGSICILGGIWHILTK
PFAWARRALVWSGEAYLSYSLGALSVCGFIACCFVWFNNTAYPSEFYGPTGPEASQAQAF
TFLVRDQRLGANVGSAQGPTGLGKYLMRSPTGEVIFGGETMRFWDLRAPWLEPLRGPNG
LDLSRLKKDIQPWQERRSAEYMTHAPLGSLNSVGGVATEINAVNYVSPRSWLATSHFVLG
FFLFVGHLWHAGRARAAAAGFEKGIDRDFEPVLSMTPLNMTIALGKFTKDENDLFDIMD
DWLRRDRFVFVGWSGLLLFPCAYFALGGWFTGTTFVTSWYTHGLASSYLEGCNFLTAAV
STPANSLAHSLLLLWGPEAQGDFTRWCQLGGLWTFIALHGAFALIGFMLRQFELARSVQL
RPYNAIAFSGPIAVFVSVFLIYPLGQSGWFFAPSFGVAAIFRFILFFQGFHNWTLNPFHMMG
VAGVLGAALLCAIHGATVENTLFEDGDGANTFRAFNPTQAEETYSMVTANRFWSQIFGVA
FSNKRWLHFFMLFVPVTGLWMSALGVVGLALNLRAYDFVSQEIRAAEDPEFETFYTKNIL
LNEGIRAWMAAQDQPHENLIFPEEVLPRGNALMSGSTGERSFADIITSIRYWVIHSITIPSLFI
AGWLFVSTGLAYDVFGSPRPNEYFTESRQGIPLITGRFDPLEQLDEFSRSFMTIDRTYPIFTV
RWLAVHGLAVPTVSFLGSISAMQFIQRMATQSVEDSSRSG-PRRTIIGDLLKPLNSEYGKVA
PGWGTTPLMGVAMALFAVFLSIILEIYNSSVLLDGISMNMLTLKLFVYTVVIFFVSLFIFGFL
SNDPGRNPGREEMADTTGRIPLWLIGTVTGILVIGLIGIFFYGSYSGLGSSLMFNIFSLTCICL
NSVLYSSNFFLSKLPEAYAFLNPIVDVMPVIPLLFFLLAFVWQAAVSFRTTQSNPNEQNVEL
NRTSLYWGLLLIFVLAVLFSNYFFNMEVNILAFIATALFILVPTAFLLIIYVKTVSQNDMETA
TLVAIFISGLLVSFTGYALYTAFGQPSQQLRDPFEEHGDMEALVYTFLLVSTLGIIFFAIFFREP
PKVPTKKIKMTIAFQLAVFALIATSSILLISVPVVFSSPDGWSSNKNVVFSGTSLWIGLVFLV
GILNSLIS-------MSPQTETKASVGFKAGVKDYKLTYYTPDYETKDTDILAAFRVTPQPGVPP
EEAGAAVAAESSTGTWTTVWTDGLTSLDRYKGRCYHIEPVPGEESQFIAYVAYPLDLFEEG
SVTNMFTSIVGNVFGFKALRALRLEDLRIPPAYTKTFWGPPHGIQVERDKLNKYGRPLLGC

TIKPKLGLSAKNYGRAVYECLRGGLDFTKDDENVNSQPFMRWRDRFLFCAEAIFKAQAET
GEIKGHYLNATAGTCEEMMKRAVFARELGVPIIMHDYLTGGFTANTSLAHYCRDNGLLLH
IHRAMHAVIDRQKNHGMHFRVLAKALRMSGGDHIHAGTVVGKLEGERDITLGFVDLLRD
DFVEKDRSRGIFFTQDWVSLAGVLPVASGGIHVWHMPALTEIFGDDSVLQFGGGTLGHPW
GNAPGAVANRVAVEACVQARNEGRDLAREGNEIIEEASKWSPELAAACAIWKKIKFEFEP
VDTI--MAIHLYKTSTPSTRNGAVDSQVKYNPRNNLIYGQHRCGKGRNARGIITAGHRGGG
HKRLYRKIDFRRNEKDIYGRIVTIEYDPNRNAYICLIHYGDGEKRYILHPRGARIGDTIVSGT
EVPIKMGNALPLTDMPLGTAIHNIEITLGKGGQLARAAGAVAKLIAKEGKSATLKLPSGEV
RLISKNCSATVGQVGNVGVNQKSLGRAGSKCWLGKRPVVRGVVMNPVDHPHGGGEGR
APIGRKKPTTPWGYPALGRRSRKRNKYSDKLILRRRSKMIQPQTHLNVADNSGARELMCI
RIIGAS-NRRYAHIGDVIVAVIKEAVPNMPLERSEVIRAVIVRTCKELKRDNGMIIRYDDNAA
VVIDQEGNPKGTRIFGAIARELRQLNFTKIVSLAPEVLMLSPKRTKFRKQHRGRLKGISYR
GNHICFGRYALQTLEPAWITSRQIEAGRRAMARSARRGGKIWVRIFPDKPITLRPTETRMG
SGKGSPEYWVAVVKPGRILYEMGGVPENTARKAISIAASKMPIRTQFIISGMTRIRRGYIAR
RRRTKIRLFTSSFRGAHSRLTRTITQQKIRALVSAHQDRSRQKRDFRRLWITRINAAIRGS--V
SYSYSKFIHDLYKSQLLLNRKILAQIAISNKNCLYMISNEIIK-----------MIKEVYALGHYIRM
SAHKARRVIDQIRGRSYEETLMILELMPYRACYPIFKLIYSAAANASHNMGINEADLIISKA
EVNESTTMKKLKPQARGRSYLIKRPTCHITIVLQDISLY-------MDGIKYAVFTDKSIRLLGK
N-QYTSNVESGSTRTEIKHWVELFFGVKVIAMNSHRLPGKDRRMGPIMGHTMHYRRMIIT
LQPGYSIPPLRKKRNMAVPKKRTSMSKKHIRKNIWKRKGCWSALKAFSLAKSLSTGNSKS
FFVRQNK-MAKGKDARVTVILECTSC-VRN--VNKESTGIFRYITQKNRHNTPGRLELRKFC
PYCYKHTIHGEIKKMKIRASVRKICEKCRLIRRRGRIIVICSNPRHKQRQGMVREQIRVSIRT
LQWKCVESRIDSKRLYYGRFILSPLMKGQADTIGIAMRRALLGEIEGTCITRAKSE--KIPHE
YSTMRGIQESVHEILMNLKEIVLRSNLYGTRNASICARGPGYVTAQDIILPPSVEIIDNTQHI
ASLTEPINLYIELQIERNRGYHIKTPNNFQDESY--PIDAVFMPVRNANHSIHSYGNGNEKQEI
LFLEIWTNGSLTPKEALHEASRNFIDLFIPFIHVEKEN--LYLGDNQ-DKDTFPLFTFHDELA-
KLRKNKK-EIALKYLFIDQLELPPRIYNCLKRSNIHTLLDLLNNSQEDLMKIEYFRREDVKQ
IFGILEKH--LNFFLTKNKMLGDGN-EVMSTIPGFNQIQFEGFCRFIDQGLAEELSKFPKIEDT
DQEIEFQLFVETYQLVEPLIKERDAVYESLTYSSELYVSAGLIWKTNRDMQEQTIFIGNIPL
MNSLGTSIINGIYRIVINQILQSPGIYYRSELDHKGISVYTGAIISDWGGRSELEIDRKARIWA
RVSRKQKISILVLSSAMGANLKAILENVYYPEIFLSFLNDKEKKKIGSKENAILEFYQQFAC
VGGDPVFSESLCKELQKKFFQQRCELGRIGRRNINRRLNLDIPQNNTFLLPRDILAAADHLI
GLKFGMGTLDD--MNHLKNKRIRSVADLLQDQFGLALVRLENLVRGTICGAIR-HKLL-PTP
QNLVTSTP----LTTTYESFFGLHPLSQVLDRTNPLTQIVHGRKSSYLGPGGLTGRTASFRIRDI
HPSHYGRICPIDTSEGINVGLIGSLAIHTRIGHWG-SLESPFYDY-EISEKSKK--VRMLYLSPS
GDEYYMVAAGNSLALNHGIQEEQVAPARYRQEFLTIAWEQVHLRSIFPFQYFSIGASLIPFIE
HNDANRALMSSNMQRQAVPLSRSEKCIVGTGLERQVTLDSGVLTIAEHEGNIIYTDTAKIL
LSGNGDIRSIPLVMYQRSNKNTCMHQKPQVQWGKYIKKGQILADGAATVGGELALGKNV
LVAYMPWEGYNSEDAVLISERMVYGDVYTSFHIRKYEIQTHVTSQG-PERITNEIPHLEAHL
LRNLDKNGIVMLGSWVETGDILVGKLTPQMAKE-SSYAPEDRLLRAILGIQVSTSKETCLK
LPIGGRGRVIDVRWIRKKG-GYSYNPETIRVYISQKREIKVGDKVAGRHGNKGIVSKILPRQ
DMPYFQDGSPVDMVFNPLGVPSRMNVGQIFECSLGLAGGLLNRHYRILPFDERYEQEASR
KLVFSELYKASKQTANPWIFEPEYPGKSRIFDGRTGDPFEQPVIIGKPYILKLIHQVDDKIHG
RSSGHYALVTQQPLRGRAKQGGQRVGEMEVWALEGFGVAHILQEMLTYKSDHIRARQEV

LGTTIIGGTIPNPEDAPESFRLLVRELRSLALELNHFLVSEKNFRINRKEAMNQNFSSMIDRY
KHQQLRIGSVSPQQISAWANKTLPNGEIVGEVTKPYTFHYKTNKPEKDGLFCERIFGPIKSG
ICACGNYRVIGDEKEDPKFCEQCGVEFVDSRIRRYQMGYIKLACPVTHVWYLKRLPSYIA
NLLDKPLKELEGLVYCD--FSFARP-IAKKPTFLRLRGSFEYEIQSWKYSIPLFFTTSGFETFRS
REISTGAGAIREQLSDLDLRIIINYSLVEWKELGEE-GPTGNEWEDRKIGRRKDFLVRRMEL
AKHFIRTNVEPEWMVLCLLPVLPPELRPIIQIDGGKLMSSDINELYRRVIYRNNTLTDLLTTN
RSTPGELVMCQEKLVQEAVDTLLDNGIRGQPMRDSHNKVYKSFSDVIEGKEGRFRETLLG
KRVDYSGRSVIVVGPSLSLHRCGLPREIAIELFQTFVIRDLIRQHLASNIGVAKSKIREKEPIV
WEILQEVMQGHPVLLNRAPTLHRLGIQAFQPILVEGRAICLHPLVCKGFNADFDGDQMAV
HVPLSLEAQAEARLLMFSHMNLLSPAIGDPISIPTQDMLIGLYVLTSGNRRGICATRYNPCN
CKN---YQNERIDDITYKYTK--EPLFSNSYDAIGAFRQKRINLDSPLWLRWRLDQR-IIAS--R
EAPIEVHYESLGTYHEIYGHYLIVRSVKKEILCIYIRTTVGHISLYREIEEAIQGFCRACSYGT
-----------MAECANLVFHNKVIDGTAMKRLIRRLIDHFGMAYTSHILDQIKTLGFQQATATSI
SLGIDDLLTTPSKGWLVQDTEQQSLILEKHHHYGNVHAVEKLRQSIEIWYATSEYLRQEMN
PNFRMTDPFNPVHIMSFSGARGNVSQVHQLVGMRGLMSDPQGQMIDLPIQSNLREGLSLT
EYIISCYGARKGVVDTAVRTSDAGYLTRRLVEVVQHIVVRKTDCATTRGISVSPRNGMMP-
ERIFIQTLIGRVLADDIYMGPRCIATRNQDIGIGLIKRFIN------LRAQSIYIRTPFTCKSISWIC
QLCYGRSPTHGDLVELGEAVGIIAGQSIGEPGTQLTLRTFHTGGVFTGGTAEHVRAPSNGKI
KFNEDLVHPTRTRHGHPAFLCYIDLYVTIESEDIIHNVNIPPKSFILVQNDQYVESEHVIAEIR
TGTST-LNFKERVRKHIYSDSEGE-MHWSTDVYHAPEFIYGNVHRLPKTSHLWILSGGPCR-
-SSVVPFSLHKDQDQMNIH--SLSVEQKYISNSS--LTNDK-VRHKIGNSGFSDKKEGGIPDY-S
EINRIRCTSHSNLIYP-AILYGNS--NLLAKRRRNGFIIPFQSIQKREKELIPPSGISIEIP--INSIFC
INSIYAYFDDPRYRRRSSGITKYGTVEVHSIVKKED--LIEYRG-VKEFRPKYRKGDRFFFIPE
EVHILPGSSFIMVRNNSIIGVDTRITLNTRSRVGGLVRVERKKKSIELQIFSGDIHFPGETDKI
SRHSGILIPPGT--RKTNSKESKK--LRNWIYVQR--IALTK--KKYFVLIRPVVTYEIADGINLAT
LFPQDPSRERDNVKLRVVNYILYGNGKPIRGIFDTSIQLVRTCLILNWDQDKK---SSSIGEAH
ASFVEVTTNWLIRDFLRIDLAKSPISYTGKRNDPSGSGLIFD-NESDR--INPFYSIYSKAR-IQ
KSLKQ----NQGTIRILLNR---NKECQSLIILSSSNCSRMGL--FNNVKYYNG---IKESDPLIPIRN
FFGPLGT--ALQIANFYS--FYHL--ITHNQIL-VIKYYQLDN---LKQAFQLLK---YSLMDEN--G
RILNPDPCSNIILNAFNLNWYFFHHNSCDATS-TIISLGQFICQNGYIAKNRSH-LK--SGQVLI
VQ-VDSLVIRSAKPYLATSGATVHGHYGEIFYEGDTLITFIYEKSRSGDITQGLPKVEQVLE
VRSIDSISMNLEKRIESWNERITRILGIPWGFLIGAELTIAQSRISLVNKIQKVYRSQGVQIHN
RHIEIIVRQITSKVLVSE---DGMSNVFSPGELIGLLRAERTGRALEEAICYRAVLLGITKASLN
TQSFISEASFQETARVLAKAALRGRIDWLKGLKENVVLGGMIPVGTGFKGL--VHPSRQHN
NIP--LETKKKNLFEGEMR--DILFHHKVLH-------FKEFS---------------MTRRYWNINLEEMM
EAGVHFGHGTKKWNPRMAPYISAKRKGIHITNLTKTARFLSEACDLVFNAASRGKQFLIV
GTKNKAADSVAWAAIRARCHYVNKKWLGGMLTNWSTTETRLHKFRDLRTEQKTGRLNR
LPKRDAAMLKRQLSHLQTYLGGIKYMTGLPDIVIIVDQQEEYTALRECITFGIPTICLIDTN
CDPDLADISIPANDDAIASIRLILNKLVFAICEGRS-SYIRNPMGQKINPLGFRLGTTQGHHSI
WFTQPKNYSEDLQEDQKIRNCIKNYVQKNMRISSG-VEGIAHIEIKKKIDLIQIIIYMGFPKL
LIEGRPR----GIEELEMNVQKELNCINKKLNIAVARITKPYGHPNILAEFIAGQLKNRVSFRK
AMKKAIELTKQADIKGIQIQIAGRIDGKEIARVEWIREGRVPLQTIRAKIDYCPYTVRTIYGV
LGIKIWIFVGKDMSRYRGPRFKKIRRLGSLPGLTSKRPKTRSDLRNQSRSGK-RSQYRIRLE
EKQKLRFHYGLTERQLLKYVRIAGKAKGSTGQVLLQLLEMRLDNILFRLGMTSTIPGARQ

LVNHRHILVNDRIVDIPSYRCKPRDILTVKDEHKSRALIQNYLDSSPH-EELPKHLTLHPFQY
--KGLVNQIIDSKCVGLKINELLVVEYYSRQTMSRRGTTEEKTAKSDPIYRNRLVNMLVNRI
LKHGKKSLAYQIIYRAVKKIQQKTETNPLSVLRQAIRGVTPDIAVKARRVGGSTHQVPIEIG
STQGKALAIRWLLGASRKRPGRNMAFKLSSELVDAAKGSGDAIRKKEETHRMAEANRAF
AHFRMGRDTIADIITSLRNADMNRKGTVRIPSTNLTENIVKILLREGFIENVRKHQENKKSF
LVLTLRHKR--NRKGTYRT-IF--NLKRISRPGLRIYSNYKRIPRILGGMGILILSTSRGIMTDRE
ARLEGIGGEILCYIWMAKLIPRIGSRKNERISSRKNARRIPKGVIHVQASFNNTIVTVTDVR
GRVVSWSSAGTCGFKGTRRGTPFAAQTAVGNAIRTVVDQGMQRAEVMIKGPGLGRDAAL
RAIRRSGILLNFVRDVTPMPHNGCRPPKKRRVMPTIKQLIRNTRQPIRNVTKSPALRGCPQR
RGTCTRVYTITPKKPNSALRKVARVRLTSGFEITAYIPGIGHNSQEHSVVLVRGGRVKDLPG
VRYHIVRGTLDAVGVKDRQQGRSKYGVKKPKMARKSLIQREKKRQKLEQKYHLIRRSSK
KEIS---KVPLLSDKWEIHGKLQSSPRNSAPTRLHRRCFSTGRPRANYRDFGLSGHILREKVH
ACLLPGATRSSWMVKNSFISVNSHE--EKEEKKGSVEFQVFSFTNKIRRLTSHLELHRKDYL
SHRGLRKILGKRQRLLAYLSKKNKVRYKKLISQLGIRETKTP--MVKLRLKRCGRKQRAVY
RIVAIDVRSRREGRDLRKVGFYDPIKNKTYLNVPAILYFLDKGAQPTGTVYDISKKVEVFK
ELRLNETKFNSMDKSKRPFLKSKRSFRRRLPPIQSGDQIDYRNMSLISRFISEQGKILSRRVN
RLTLKQQRFITIAIKQARILSSLPFLNNEK--QFERTESTTRTTGLRTRNKVTR-LKKNPFVAN
HLLKKINKLNTKAEKEVIISWSRASTIIPTMIGHTIAIHNGKEHLPIYITDRMVGHKLGEFVP
TLNFRGHAKNDTRSRRMVFKSFILGN----CMKIINSVVVVGLYYGFLTTFSIGPSYLFLLRA
QVMEEGEEGTEKKISATTGFITGQLMMFISIYYAPLHLALGRPHTITILALPYLLFHFFWNN
HKHFFDYGSTTRNSMRNLSIQCVFLNNLIFQLFNHFILPSSMLARLVNIYMFRCNNKMLFV
TSSFVGWLIGHILFMKWVGLVLVWIRQNHSIRSLIRSNKYLVSELRNSMARIFSILLFITCVY
YLGRIPSPILT--LKEKEDPKPIVTILFDYKRWNRPFRYIKNNKFDKAVINKMSQYFFYTCQS
DGKQRISFTYSLSLATFFEMIQKKISLSTTEKGLN-FLYNHWVFINEKTKNNLSKEFLNRIKS
LNKGS--LNGFEKRTRLCNDETKNEYLTQIYDLLINGPYRGTKKNLQ--FDTIWINKIHSIL-R
KFEQKDSNDQ-IGINEISKKVPQHSYKLIDLEQQEGQQIRSRKFKRVVIFTDALIRYSQQSDF
RRGIIKGSMRAQRRKTITWKFFQANVHSPLFLDRIDKSIFLSFEI--SGLIKQIFINREEKARIK
IAEAWDTILFAQVIRGSMLVIQSILRKSIVLPSLIIAKNISRILLFQFPEWSEDLKEWNREMHV
KCTYNGVQLSETEFPKNWLTDGIQIKILFPFCLKPWHKSKREKTN-FCFLTVWGMEAELPF
GSPRKRPSFFEPIFKELKNKIRKLKKECFFV-FTILKERTKQKFSFSNETKKWFIKNAL--FIKQ
IIKELSKINGLIINNQMIQEPSISIQSMDLQNYSLTEKKIKDLTDKTRIIRNQIEQITKDKKKNT
KTKQKNSNIKEPQKNTWQIRKRKNAQLIRKFYYFIQFFIERIYMDTLLCIININ-ITTQLFIHFI
S-DLSSLSQAYVFYKLVQTQLSNLYSLKPILLYLKFKLKNEIKDYFIQGIIHSELKQNKFRNC
EMTQWKNWLTG-YQYNLYQIKLSKLVPQKWRNKIHQSFMFQNKSRCYLYEKDQ--LIDYE
KE--FKKHYLYDLLSYKYIK-----TP-KYFDWGIPHFFFIIFDWMGMNEEILNRWFFPEFVFLY
NTYKIKQWGIPIKLLLFNYKSHTEAELDLFLKKYLLFQLRWDDSLNQRMINNVKVYCFLL
RLRNSKEIAISSIKKEEMSLDIMLIQKNVKFTELMQKGILIIEPIRLSKTTNRQFIMYQTIRIPL
VHKSKYYDFDLFVLENILSSRHRRELRIRIYFIFLWPNYRLEDLACMNRYWFDTNNGSRFS
TLRVHMYPMKGHQFKSWIFELREILREIKNSHYFLDSWTQFNSVGSFIHIFFHQERFIKLFD
PRIWSILLS-RNS--QGSTSNRYF---TIKGVVLFVVA-VLIYR----INNRNMVERKNLYLIGLLP--
---ILMNSIGPRNDTLEESF-GSSNINRLILS-LLYLPKGKKISESCFLDPKESTWVLPRT----KKC
IVPESNWSSRWWRNWIGKKR--DSSCKISN---ETVAGIEISFKEKDIK-YLEFLFVYYMD--DP
IRKDHDWELFDRLSPRKRRNIINLNLNSGQLFEILVKHWICYLMSAFREKIPIEVEG-FFKQQ
G-AGSTIQSNDIEHLSP--LFSRNKWAISLQNCAQFHMWQFRQNPFV---SWGK-NPHESDFLR

K-VSRENWIWLDNVWLVNKDR---VFS--KVRNVSSNIQYDS---TR--SSFVQVTDSSQLKGSS
-DQSRDHSDSIS----NEDSE-------------YHTLLNQREIQQLKERSIL-WD--PSFLQTERTEIESD--
RFPK--RLSGYS--SMSRLFA-EREKQMNHHLLPEEIEEF--LGNPTRSIRSFFSDRW--SELH-LG
SNPTERSTR------KLLKKQQGLY-FVPSRRSE----NKEMVNIFKIITYLQN--TVSIHPISSDPGC
DMVP-KDEPD-MDSSNKISFLNKNPFFDF--FHLFHDQNRGGYTL--HHDFESE--ERFQEMA
DLFTLSITEP-DLVYHKGFAFSIDSYGLDQKQ--FLNEVFNSRDESKKKFL---LVLPPIFY--EEN
ESFYRRIRKKWVR-ISCGNDLEDPKPKKVVFASNNIMEAVNPYRLIRNLIQIQYSTYGYGYI
RNGLNRFFLMN---RSDRNFEYGIQ---KDQIGNDTLNHRTIMKYMINQHLSNLKK-SQKKWF
DP--LIFLSRTERSMNRDPDAYRYKWSNENKNFQEHLEHLVSEQKSR---FQVVFDRLRINQY
SIDWSEVIDKKDLSKSLRFFLSKSLL---------FLS-------KLLLFLSNSLP-FFFVSFENIPIHRSE-
IHIY-ELKGPNDQLCNQLLESIGLQIVHLKKWKAF--LLD---DHDTS-QKSKFLINGGTISPFLF
NKIP-KW-MIDSFHTR--NNRGKSFDNTDSYFSMISHDQDSWLNPVKPFHRSSLISSFYKANR
LRFLNNPHHFFFY--CNKRFPF---Y-VEKGRIENYD--FTYG-QFLNILFIRNK-IFSLCVGK--KK
HAFLE-RDTISPIESQVSNIFIPNDFPQSGDER-YNLYKS---FYFPIRSDPFVR--RAIFSIADIFGT
PLTEGQIVNFERTYCQPLSDMNLSDSEGKNLHQYLN--FNSNMGLIHTPCSEKYLPSEK--RK
K--------RSLCLK--KCVEKGQMYRTFQRDSAFSTLSKWNLFQTYMPWFLTLTGYKYLNFIFL
DTFSD-LLPILNSS--QKFLSIFHDIMHGADISWRILQKKL------CLPQWN-LISEISSKCLHN--L
LLSEEMIHQKNES--PLISTHLRSPNVREF-LYSILFLLLVAGY-LVRTHLLFFSR--ASSELQTEF
EKVKSLMIPSYMIELRKLLDRYPTSELNSFWLKNLFLVALEQLGDSLEEIRGSASGGNMLL
GGGP-----AYG-VKSIRSKKKYLNINLINIIDLI-SIIPNPINRITFSKNTRHISHTSKEIYSLIRKR
KNVNGDWIDDKIESWVANSDSIDDEEREFLIQFS--TLTT--EKRIDQILLSLT-HSDHLS-----K
NDSGYQMIEQPGAIYLRYLVDIH--KKYLMNYEFNTSCLAERRIFLAHYQTITY--SQTLCGA
NSFHFPSHG---KPFSLRLALSPS-SGILVIGSIGTGRSYLVKYLATNSYVPFITVFLNKFLDNK
PKGFLID-----DIDDIDDSDDIDDSDDIDDS----------------DDIDRDLDTELELLTMMNAL---TM
DMMP---EIDRFYITLQFELAKAMSPCIIWIPNIHDLDVNESNYLSLGLLVNHLS---RDCERCS
-TRNILVIASTHIPQKVDPALITPNKLNTCIKIRRLLIPQQRKHFFTLSYTRGFHLEKKMFHT--
NGFGSITMGSNARDLVALTNEALSISITQKKSILDTNTIRSALHRQTWDLR---SQVRSVQDH
GILFYQIGRAVAQNVLLSNCPIDPISIYMKKK--SCNEGDSYLYKWYFELGTSMKKLTILLYL
LSCSAGSVAQDLWSLP--GPDEK-NGITSYGLVENDS-DLVHGLLEVEGALVG--SSRTEKDCS
QFDND--RVTLLLRPEPKN-PLDMMQNGSCSIVDQRFLYEK--YESEFEE-GEGEGALDPQ----
QIEED----LFNH--IVWAP--RIWRP---WGFLFDCIERPNELGFP-YWARSFRGE---RIIYDEEDE
LQENDSEFLQSGSMQYQTRDRSSKEQGFFRISQFIWDPADPLFFLFK-DQPFVSVFSHREFF
ADEEMSKGLLTSQTDPPTSIYKRWFI--KNTQEKHFELLIHRQRWLR---TNSSLS--NGSFRSN
--TLSESYQYLSNLFLSN-GRLLDQMTKTLLRKRWLFPDEMQI-------------MPRSR-INGNFID
KTFSIVANILLRIIPTTSGEKEAFTYYRDG--MLAQSEGNYAEALQNYYEAMRLEIDPYDRS
YILYNIGLIHTSNGEHTKALEYYFRALERNPFLPQALNNMAVICHY-RGEQAIRQGDSEIAE
AWFDQAAEYWKQAIALTPGNYIEAHNWLKITRRFE--MSWRSERIWIELIAGSRKRSNFCW
AFILFLGSLGFLLVGTSSYLGRNLISLFPSQQIVFVPQGIVMSFYGIAGLFISSYLWCTISWNV
GSGYDRFDRKEGIVCIFRWGFPGKNRRIFLRFFMKDIQSIRIEVKEGIYARRVLYMEIRGQG
AVPLTRTDEN-LTPREIEQKAAELAYFLRVPIEIF

>Ehretia

MERWWFNSMLFLNKSMDSPGPIENTSESEDPNLKDRAKNIHSYSNLDSLFGVKDIRNFISD
DTFLVRDSNGDSYSIYFDIENQIFEIDNDH--SFLSELESSFYSYRNSSYLNNRHTSEDPYYNR
YMYDTQYSWNNHINSCIDSYLQ--SQICIETSIISGSDNYS--YISRSIWGSEGSSIRTRTKGSDL

TIRESSNDLDVTQKYRHLWVQCENCYGLNYKKILKSKMNICEQCGYHLKMSSSDRIELSI
DPGTWDPMDEDMVSLDPIEFHSEEEPYKDRIDSYQRKTGLTEAVQTGIGRLNGIPLAIGVM
DFQFMGGSMGSVVGEKITRLIEYATNKFLPLIIVCASGGARMQEGSLSLMQMAKISSALYD
YQSNKKLFYVSILTSPTTGGVTASFGMLGDIIIAEPNAYIAFAGKRVIEQTLNKTVPEGSQA
AEYLFQKGLFDLIVPRNPLKSVLSELLKLHAFFPLDQNSIKMVTIRADEISNIIRERIEQYNR
EVKIVNTGTVLQVGDGIARIHGLDEVMAGELVEFEEGTIGIALNLESNNVGVVLMGDGLM
IQEGSSVKATGRIAQIPVSEAYLGRVINALAKPIDGRGEISASESRLIESPAPGIISRRSVYEPL
QTGLIAIDSMIPIGRGQRELIIGDRQTGKTAVATDTILNQQGQNVICVYVAIGQKASSVAQV
VNTLQERGAMEYTIVVAETADSPATLQYLAPYTGAALAEYFMYRERHTSIIYDDPSKQAQ
AYRQMSLLLRRPPGREAYPGDVFYLHSRLLERAAKSSSSLGEGSMTALPIVETQSGDVSAY
IPTNVISITDGQIFLSADLFNAGIRPAINVGISVSRVGSAAQIKAMKQVAGKLKLELAQFAEL
EAFAQFASDLDKATQNQLARGQRLRELLKQSQAAPLAVEEQIMTIYTGTNGYLDSLEIGQ
VRKFLVELRTYVKTNKPQFQEIISSTKTFTEEAEALLKEAIQEQMDRFLLQEQAMRINPTAS
GSGVSTLEKKNLGRIVQIIGPVLDVAFPPGKMPNIYNALVVKGRDTVGQPINVTCEVQQLL
GNNRVRAVAMSATDGLTRGMEVVDTGAPLSVPVGGATLGRIFNVLGEPVDNLGPVDTRT
TSPIHRPAPAFIQLDTKLSIFETGIKVVDLLAPYRRGGKIGLFGGAGVGKTVLIMELINNIAK
AHGGVSVFGGVGERTREGNDLYMEMKESGVINEENIAESKVALVYGQMNEPPGARMRV
GLTALTMAEYFRDVNEQDVLLFIDNIFRFVQAGSEVSALLGRMPSAVGYQPTLSTEMGSL
QERITSTKAGSITSIQAVYVPADDLTDPAPATTFAHLDATTVLSRGLAAKGIYPAVDPLDSTS
TMLQPRIVGEEHYETAQRVKQTLQRYKELQDIIAILGLDELSEEDRLTVARARKIERFLSQP
FFVAEVFTGSPGKYVGLAETIRGFQLILSGELDGLPEQAFYLVGNIDEATAKAMNLEMESN
LKKMTLNLCVLTPNRIVWDSEVKEIILSTNSGQIGVLPNHAPIATAVDIGILRIRLNDQWVT
MALMGGFARIGNNEITVLVNDAEKASDIDPQEAQKTLEIAEANLRKAEGKRQTIEANLAL
RRARTRVEAINAMIMKNVTDSFVS-GHWPSAGSFGFNTDILATNPINLSVVIGVLIFFGKGV
LSDLLDNRKQRILNTIRNSEELRGGAIEQLEKARSRLRKVEMEAEQFRVNGYSEIEREKLN
LINSTYKTLEQLENYKNETIQFEQQRAINQVRQRVFQQALQGALGTLNSCLNNELHLRTIS
ANIGMLGAMKEITDMNPLISAASVIAAGLAVGLASIGPGVGQGTAAGQAVEGIARQPEAE
GKIRGTLLLSLAFMEALTIYGLVVALALLFANPFVMNVLSCSINTLKGLYDISGVEVGQHF
YWQIGGFQVHGQVLITSWVVIAILLGSAAIAVRNPQTIPTGGQNFFEYVLEFIRDVSKTQIG
EEYGPWVPFIGTMFLFIFVSNWSGALLPWKIIQLPHGELAAPTNDINTTVALALLTSVAYFY
AGLSKKGLGYFGKYIQPTPILLPINILEDFTKPLSLSFRLFGNILADELVVVVLVSLVPSVVPI
PVMFLGLFTSGIQALIFATLAAAYIGESMEGHHMIFSTLEHILTHISFSIVSIVITIHLITFLVDK
TVKLYDSSEKGMIVTFFCITGLLVTRWIYSGHFPLSDLYESLIFLSWSFSLIHIVPY--KNKND
LSTITGPSAIFTQGFATSGLLTEIHQSEILVPALQSEWLIMHVSMMILGYAALLCGSLLSVALL
VITFRKRRKIFC--KEIEYMNERSNVLQNTS---AKNYYRSQLIHQLDYWSYRIISLGFIFLTVG
ILSGAVWANEAWGSYWNWDPKETWAFITWIVFAIYLHTRTNINLQGANSAIVASIGFLIIWI
CYFGVNLLGIGLHSYGSFALTMAKKKVFTPLFYLASIVFLPWWISLSFNKSLESWVTNWW
NTGQSEIFLNDIQEKSILEKFIELEELLFLDEMIKEYSETHLQKLRI-IHKETIQLIKIHNEDRIH
TILHFSTNLICFVILSGYSILGNEELVILNSWTQEFLYNLSDTVKAFSILLLTDLCIGFHSPHG
WELMIGSVYKDFGFVHNDQIISGLVSTFPVILDTIFKYWIFRYLNRLSPSLVVIYHSMNDMP
IGVPKVPFRSPGEEDASWVDVYNRLYRERLLFLGQEVDSEISNQLIGLMVYLSIEDDTKDL
YLFINSPGGWVIPGVAIYDTMQFVRPDVHTICMGLAASMGSFILVGGEITKRLAFPHARVM
IHQPASSFYEAQTGEFILEAEELLKLRETLTRVYVQRTGKPLWVVSEDMERDVFMSATEAQ
AYGIVDLVAVEMKEQKWIHEGLITESLPNGMFRVRLDNEDLILGYVSGKIRRSFIRILPGDR

VKIEVSRYDSTRGRIIYRLRNKDSKDMEEIQRYLQ-DRSQQHDCLYPLIFQEYIYALAHDHG
LPGYDNKSSLLIVKRLITRMYQQNHFFISVNDSNQNQFFGKNLYSQMISEGFAFIVEMPFSL
RLISSLIFKSHNLRSIHSIFPFLEDNFSHLNCVLDILIPHPVHPEILVQTLRFWVKDASSLHLL
RFFLHEYWNN--LITPS-SSFSKQRLFFFLYNSHVCEYESIFVFLRNQSSHLRSTSFGALLERIY
FYGKIERLVEVFA-DFTLWLFKDPFIHYVRYQGKLILASKGTPLLMNKWKYYLVNFWQCY
FYLWSRPGRIYINQLSNHSLDFMGYLSSVRLNPSMVRSQMLENAFLVNNVI--KKFDTLLPI
IPLIGSLAKAKFCNILGNPISKPVRADLSDSDIIDRFGRICRNLSHYHSGSSKKKSLYRIKYIL
RLSCAKTLARKHKSSVRAFLKRFGSELLEEFLTSEEQVLSLTFPRRIWYLDIICINDLANHQ
MIID-TTEIE-AINSFSRLES-LKEIYGIIWILVPILILVLGITIGVLVIVWLEREISAGIQQRIGPEY
AGPLGILQALADGTKLLFKENLLPSRGDARLFNIGPSIAVISILLSYSVIPFGYHLVLADLSIG
VFLWIAISSIAPVGLLMSGYGSNNKYSFLGGLRAAAQSISYEIPLTLCVLSISLLSNSSSTVDI
VEAQSKYGFWGWNLWRQPIGFIIFLISSLAECERLPFDLPEAEEELVAGYQTEYSGIKFGLF
YVASYLNLLVSSLFVTVLYLGGWNFSIPYILVPELFDINKAGKVFGTIIGIFITLSKTYLFLFIP
IATRWTLPRLRMDQLLNLGWKFLLPISLGNLLLTTSSQLLSLMIWHVQNENFILDSTRIFMK
AFHLLLFDGSLIFPECILIFGLILLLMIDSTSDQKDIPWLYFISSTSLVMSITALLFRWREEPMI
SFSGNFQTNNFNEIFQFLILLCSTLCIPLSVEYIECTEMAITEFLLFVLTATLGGMFLCGANDL
ITIFVAPECFSLCSYLLSGYTKKDVRSNEATMKYLLMGGASSSILVHGFSWLYGSSGGEIEL
QEIVNGLINTQMYNSPGISIALIFITVGIGFKLSPAPSHQWTPDVYEGSPTPVVAFLSVTSKV
AASASATRIFDIPFYFSSNEWHLLLEILAILSMILGNLIAITQTSMKRMLAYSSIGQIGYVIIGI
IVGDSNDGYASMITYMLFYISMNLGTFACIVLFGLRTGTDNIRDYAGLYTKDPFLALSLAL
CLLSLGGLPPLAGFFGKLYLFWCGWQAGLYFLVLIGLLTSVVSIYYYLKIIKLLMTGRNQEI
TPHVRNYRRSPLRSNNSIELSMIVCVIASTIPGISMNPIIAIAQDTLFMFLLYEYDIFWAFLIIS
SLIPILAFLISGVLAPTSKGPEKLSSYESGIEPMGDAWLQFRIRYYMFALVFVVFDVETVFLY
PWAMSFDVLGVSVFIEALIFVLILIVGLVYAWRKGALEWSTNDFPWLTTIVVLPIFAGSLIFF
LPHRGNRVIRWYTICICILELLLTTYAFCYHFQSDDPLIQLVEDFKWIKFFDFHWRLGIDGLS
IGPILLTGFITTLATLAARPITRDSRLFHFLMLAMYSGQIGSFSSRDLLLFFIMWELELIPVYL
LLSMWGGKKRLYSATKFILYTAGGSVFLLMGVLGISLYGSNEPTLNFETSVNQSYPVALEII
FYIGFFIAFAVKSPIIPLHTWLPDTHGEAHYSTCMLLAGILLKMGAYGLVRINMELLPHAHS
IFSPWLVIVGTMQIIYAASTSLGQRNLKKRIAYSSVSHMGFIIIGIGSITDTGLNGALLQIISHG
FIGAALFFLAGTTYDRIRLVYLDEMGGIAIPLPKIFTMFSSFSMASLALPGMSGFVAELIVFF
GIITSQKYLLMPKILITFVMAIGMILTPIYSLSMLRQMFYGYKLFNAPNSYFFDSGPRELFLS
ICIFLPVIAIGMYPDFVLSLSVDKVEVILSNSFYRMILEHVLVLSAYLFSIGIYGLITSRNMVR
ALMCLELILNAVNINFVTFSDFFDNRQLKGDIFSIFVIAIAAAEAAIGPAIVSSIYRNRKSTRI
NQSNLLNKMEQTYQYAWIIPLLPLTVPMLIGVGLLLFPTATKNLRRMWAFQSILLLSIVMIF
SINLSIQQINSSSIYQNVWSWIINNDFSLEFGYLIDPLTSIMSILITTVGIMVLIYSDNYMSHD
QGYLRFFAYMSFFSTSMLGLVTSSNLIQIYIFWELVGMCSYLLIGFWFARPVAANACQKAF
VTNRVGDFGLLLGILGFYWITGSFEFRDLFEVFNNNEVNLFFATLCAILLFAGAVAKSAQFP
LHVWLPDAMEGPTPISALIHAATMVAAGIFLVARLLPLFIVIPYIMNFIALIGIITVFLGATLA
LAQKDIKRGLAYSTMSQLGYMMLALGMGSYRNALFHLITHAYSKALLFLGSGSVIHSME
TVVGYSPDKSQNMVLMGGLTKHVPITKTAFLLGTLSLCGIPPLACFWSKDEILNDSWLYSP
IVAIIAWATAGFTAFYMFRIYLLTFEGHLNVHFQNYSGKQNTPFYSLSLWGKRGSKGINK-L
VT---INFFSFKNKKPYSYPSESDNTMLFPLLILGLFTLFVGSLGIPF-VDILSKWLAPSINLLH
QKSN-SMDWYEFLKDAVFSVSIAYFGILIASFLYKPIYSSLQNFDLINSFVIVWDKILNGIYD
WSYNRAYIDAFYRTSLTGGIRGLAELTHLFDRRVIDGITNGVGVMSFFVGEGIKYVGGG-RI

SSYLFLYLFSVSIFLLIYYMDLPGPIHDFLLVFLGSGLILGGLGVVLLPNPIYSAFSLGLVLVC
ISLFYILSNSYFVAAAQLLIYVGAINVLIIFAVMFMNGSEYYKDFHLWTVGDGVTSMVCTS
LFISLITTILDTSWYGIIWTTKSNQIIEQDLISNSQQIGIHLSTDFFLPFEFISIILLVALIGAIAVA
RQMTAPVTRKDLMIVNMGPHHPSMHGVLRLIVTLDGEDVIDCEPILGYLHRGMEKIAENR
TIIQYLPYVTRWDYLATMFTEAITVNGPEQLGNIQVPKRASYIRVIMLELSRIASHLLWLGP
FMADIGAQTPFFYIFRERELIYDLFEAATGMRMMHNYFRIGGVAADLPYGWIDKCLDFCD
YFLTGVAEYQKLITRNPIFLERVEGVGIIGGEEAKNWGLSGPMLRASGIQWDLRKVDHYE
CYDEFDWEVQWQKEGDSLARYLVRISEMTESIKIIQQALEGIPGGPYENLEIRRFDKVKDP
EWNDFEYRFISKKPSPTFELSKQELYVRVEAPKGELGIFLIGDQSVFPWRWKIRPPGFINLQI
LPHLVKRMKLADIMTILGSIDIIMGEVDRMFPMVTEFMNYGQQTIRAARYIGQGFMITLSH
ANRLPVTIQYPYEKLIASERFRGRIHFEFDKCIACEVCVRVCPIDLPVVDWKLETDIRKKQL
LNYSIDFGVCIFCGNCVEYCPTNCLSMTEEYELSTYDRHELNYNQIALGRLPMSVIDDYTI
RTISNS----------------MQGRLSAWLVKHGLIHRSLGFDYQGIETLQIKPEDWHSIAVILYVYG
YNYLRSQCAYDVAPGGLLASVYHLTRIEYGVDQPEEVCIKVFASRRNPRIPSVFWVWKSV
DFQERESYDMLGISYDNHPRLKRILMPESWIGWPLRKDYIAPNFYEIQDAHMGNEFRCIG
CICIYRSFNFRAYLNCWFSLCMAKGGIGMVLAPECSDNKKKKGKKKIET-VMNSIEFPLLD
RTTPNSVISTTLNDLSNWSRLSSLWPLLYGTSCCFIEFASLIGSRFDFDRYGLVPRSSPRQAD
LILTAGTVTMKMAPSLVRLYEQMPEPKYVIAMGACTITGGMFSTDSYSTVRGVDKLIPVD
VYLPGCPPKPEAVIDAITKLRKKISREIYEDRIRSQRANRCFTTTHKFHVGRSIHTGNYDQR
FLYQPSSTSEIPTETFFKYKSSASSHELVNMQTRNTFSWIKEQITRSISVSLMIYIITRTYISNA
YPIFAQQGYENPREATGRIVCANCHLANKPVEIEVPQAVLPDTVFEAVVRIPYDMQLKQVL
ANGKKGGLNVGAVLILPEGFELAPPDRISPELKEKIGNLSFQSYRPNKKNILVIGPVPGQKY
SEITFPILSPDPATKKDVHFLKYPIYVGGNRGRGQIYPDGSKSNNTVYNATATGIVSKIIRKE
KGGYEITITDASDGRQVVDIIPPGPELLVSEGESIKFDQPLTSNPNVGGFGQGDAEIVLQDPL
RVQGLLFFLASVILAQIFLVLKKKQFEKVQLAEMNFMSKVYDWFEERLEIQAIADDITSKY
VPPHVNIFYCLGGITLTCFLVQVATGFAMTFYYRPTVTEAFASVQYIMTEANFGWLIRSVH
RWSASMMVLMMILHVFRVYLTGGFKKPRELTWVTGVVLAVLTASFGVTGYSLPRDQIGY
WAVKIVTGVPEAIPVIGSPLVELLRGSASVGQSTLTRFYSLHTFVLPLLTAVFMLMHFLMIR
KQGISGPLMGVTKKPDLNDPVLRAKLAKGMGHNYYGEPAWPNDLLYIFPVVILGTIACNV
GLAVLEPSMIGEPADPFATPLEILPEWYFFPVFQILRTVPNKLLGVLLMVSVPAGLLTVPFLE
NVNKFQNPFRRPVATTVFLIGTAVALWLGIGATLPIDKSLTLGLFMIEVFLFGIVLGLIPITLA
GLFVTAYLQYRRGDQLDLMLTITSYFGFLLAALTITSALFIGLSKIRLIMDIVSLAWAALMV
VFTFSLSLVVWGRSGLMIIRSPEPEVKILVDRDPVKTSFEEWARPGHFSRTIAKGPDTTTWI
WNLHADAHDFDSHTSDLEEISRKVFSAHFGQLSIIFLWLSGMYFHGARFSNYEAWLSDPT
HIGPSAQVVWPIVGQEILNGDVGGGFRGIQITSGFFQIWRASGITSELQLYCTAIGALVFAAL
MLFAGWFHYHKAAPKLAWFQDVESMLNHHLAGLLGLGSLSWAGHQVHVSLPINQFLNA
GVDPKEIPLPHEFILNRDLLAQLYPSFAEGATPLFTLNWSKYAEFLTFRGGLDPVTGGLWLT
DIAHHHLAIAILFLIAGHMYRTNWGIGHGIKDILEAHKGPFTGQGHKGLYEILTTSWHAQL
SLNLAMLGSLTIVVAHHMYSMPPYPYLATDYGTQLSLFTHHMWIGGFLIVGAAAHAAIFM
VRDYDPTTRYNDLLDRVLRHRDAIISHLNWACIFLGFHSFGLYIHNDTMSALGRPQDMFSD
TAIQLQPVFAQWIQNTHALAPGATAPGATASTSLTWGGGDLVAVGGKVALLPIPLGTADFL
VHHIHAFTIHVTVLILLKGVLFARSSRLIPDKANLGFRFPCDGPGRGGTCQVSAWDHVFLG
LFWMYNAISVVIFHFSWKMQSDVWGSISDQGVVTHITGGNFAQSSITINGWLRDFLWAQA
SQVIQSYGSSLSAYGLFFLGAHFVWAFSLMFLFSGRGYWQELIESIVWAHNKLKVAPATQP

RALSIVQGRAVGVTHYLLGGIATTWAFFLARIIAVGMALRFPRFSQGLAQDPTTRRIWFGIA
TAHDFESHDDITEERLYQNIFASHFGQLAIIFLWTSGNLFHVAWQGNFESWVQDPLHVRPIA
HAIWDPHFGQPAVEAYTRGGALGPVNIAYSGVYQWWYTIGLRTNEDLYTGALFLLFLSAL
FLIAGWLHLQPKWKPSVSWFKNAESRLNHHLSGLFGVSSLAWTGHLVHVAIPGSRGEYVR
WNNFLDVLPHPQGLGPLFTGQWNLYAQNPDSSSHLFGTSQGAGTAILTLLGGFHPQTQSL
WLTDIAHHHLAIAFIFLVAGHMYRTNFGIGHSIKDLLDAHIPPGGRLGRGHKGLYDTINNSL
HFQLGLALASLGVITSLVAQHMYSLPAYAFIAQDFTTQAALYTHHQYIAGFIMTGAFAHGA
IFFIRDYNPEQNEDNVLARMLEHKEAIISHLSWASLFLGFHTLGLYVHNDVMLAFGTPEKQ
ILIEPIFAQWIQSAHGKTSYGFDVLLSSTSGPAFNAGRSIWLPGWLNAVNE--NSNSLFLTIGP
GDFLVHHAIALGLHTTTLILVKGALDARGSKLMPDKKDFGYSFPCDGPGRGGTCDISAWD
AFYLAVFWMLNTIGWVTFYWHWKHITLWQGNVSQFNESSTYLMGWLRDYLWLNSSQLI
NGYNPFGMNSLSVWAWMFLFGHLVWATGFMFLISWRGYWQELIETLAWAHERTPLANLI
RWRDKPVALSIVQARLVGLAHFSVGYIFTYAAFLIASTSGKFGMSHSVKIYDTCIGCTQCV
RACPTDVLEMIPWDGCKAKQIASAPRTEDCVGCKRCESACPTDFLSVRVYLWHETTRSM
GLAYMTTFNFPSIFVPLVGLVFPAIAMASLFLYVQKNKIVMRDLKTYLSVAPVLSTLWFGA
LAGLLIEINRFFPDALTFPFFSFMTAILERRESESLWGRFCNWITSTENRLYIGWFGVLMIPT
LLTATSVFIIAFIAAPPVDIDGIREPVSGSLLYGNNIISGAIIPTSAAIGLHFYPIWEAASVDEW
LYNGGPYELIVLHFLLGVACYMGREWELSFRLGMRPWIAVAYSAPVAAATAVFLIYPIGQG
SFSDGMPLGISGTFNFMIVFQAEHNILMHPFHMLGVAGVFGGSLFSAMHGSLVTSSLIRET
TENESANAGYRFGQEEETYNIVAAHGYFGRLIFQYASFNNSRSLHFFLAAWPVVGIWFTAL
GISTMAFNLNGFNFNQSVVDSQGRVINTWADIINRANLGMEVMHERNAHNFPLDLAAIEA
PSTNGMGLPWYRVHTVVLNDPGRLLSVHIMHTALVAGWAGSMALYELAVFDPSDPVLDP
MWRQGMFVIPFMTRLGITNSWGGWSIT-GGTVTNPGIWSYEGVAGAHIVFSGLCFLAAIW
HWVYWDLEIFCDERTGKPSLDLPKIFGIHLFLSGVACFGFGAFHVTGLYGPGIWVSDPYGL
TGKVQPVNPAWGVEGFDPFVPGGIASHHIAAGTLGILAGLFHLSVRPPQRLYKGLRMGNIE
TVLSSSIAAVFFAAFVVAGTMWYGSATTPIELFGPTRYQWDQGYFQQEIYRRVSAGLAENQ
SLSEAWSKIPEKLAFYDYIGNNPAKGGLFRAGSMDNGDGIAVGWLGHPIFRDKEGRELFV
RRMPTFFETFPVVLVDGDGIVRADVPFRRAESKYSVEQVGVTVEFYGGELNGVSYSDPAT
VKKYARRAQLGEIFELDRATLKSDGVFRSSPRGWFTFGHASFALLFFFGHIWHGARTLFRD
VFAGIDPDLDAQVEFGAFQKLGDPTTRRQVV------------VETLFNGTLALAGRDQETTGFA
WWAGNARLINLSGKLLGAHVAHAGLIVFWAGAMNLFEVAHFVPEKPMYEQGLILLPHLA
TLGWGVGPGGEVIDTFPYFVSGVLHLISSAVLGFGGIYHALLGPETLEESFPFFGYVWKDR
NKMTTILGIHLILLGLGAFLLVFKALYFGGVYDTWAPGGGDVRKITNLTLSPNIIFGYLLKS
PFGGEGWIVSVDDLEDIIGGHVWLGSICILGGIWHILTKPFAWARRAFVWSGEAYLSYSLG
ALAVFGFIACCFVWFNNTAYPSEFYGPTGPEASQAQAFTFLVRDQRLGANVGSAQGPTGL
GKYLMRSPTGEVIFGGETMRFWDLRAPWLEPLRGPNGLDLSRLKKDIQPWQERRSAEYM
THAPLGSLNSVGGVATEINAVNYVSPRSWLATSHFVLGFFFFVGHLWHAGRARAAAAGFE
KGIDRDFEPVLSMTPLNMTIALGKFTKDEN-LFDIMDDWLRRDRFVFVGWSGLLLFPCAY
FALGGWFTGTTFVTSWYTHGLASSYLEGCNFLTAAVSTPANSLAHSLLLLWGPEAQGDFT
RWCQLGGLWTFVALHGAFGLIGFMLRQFELARSVQLRPYNAIAFSAPIAVFVSVFLIYPLG
QSGWFFAPSFGVAAIFRFILFFQGFHNWTLNPFHMMGVAGVLGAALLCAIHGATVENTLF
EDGDGANTFRAFNPTQAEETYSMVTANRFWSQIFGVAFSNKRWLHFFMLFVPVTGLWMS
ALGVVGLALNLRAYDFVSQEIRAAEDPEFETFYTKNILLNEGIRAWMAAQDQPHENLIFPE
EVLPRGNALMSGSTGERSFADIITSIRYWVIHSITIPSLFIAGWLFVSTGLAYDVFGSPRPNE

YFTESRQGIPLITGRFDPLEQLDEFSRSFMTIDRTYPIFTVRWLAVHGLAVPTVFFLGSISAM
QFIQRMATQTVEKSSRSG-PRRTTVGSLLKPLNSEYGKVAPGWGTTPLMGVAMALFAIFLSI
ILEIYNSSVLLDGISMNMLTLKLFVYTVVIFFVSLFIFGFLSNDPGRNPGREEMADTTGRIPL
WIIGTVTGILVIGLIGIFFYGSYSGLGSSLMLNIFSL--ICLNSALH-SSFFFGKLPEAYAFLNPIV
DVMPVIPVLFFLLAFVWQAAVSFRMTQSNPNEQSVELNRTSLYWGLLLIFVLAVLFSNYFF
NMEVNILAFIATALFILVPTAFLLIIYVKTVSQNDMETATLVAIFISGLLVSFTGYALYTAFGQ
PSQQLRDPFEEHGDMEALVYTFLLVSTLGIIFFAIFFREPPKVPTKKMKMTLAFQLAVFALIA
TSSILLISVPVVFASPDGWSSNKNVVFSGTSLWIGLVFLVGILNSLIS-------MSPQTETKASV
GFKAGVKEYKLTYYTPQYQTKDTDILAAFRVTPQPGVPPEEAGAAVAAESSTGTWTTVW
TDGLTSLDRYKGRCYYIEPVPGEKEQFIAYVAYPLDLFEEGSVTNMFTSIVGNVFGFKALR
ALRLEDLRIPPAYIKTFQGPPHGIQVERDKLNKYGRPLLGCTIKPKLGLSAKNYGRAVYEC
LRGGLDFTKDDENVNSQPFMRWRDRFLFCAEALYKAQAETGEIKGHYLNATAGNCEEM
MKRAVFARELAVPIVMHDYLTGGFTANTTLASYCRDNGLLLHIHRAMHAVIDRQKNHGIH
FRVLAKALRMSGGDHIHSGTVVGKLEGERNITLGFVDLLRDDYIEKDRSRGIFFTQDWVS
LPGVLPVASGGIHVWHMPALVEIFGDDSVLQFGGGTLGHPWGNAPGAVANRVSLEACVQ
ARNEGRNLATEGNEIVREASKWSPELAAACEVWKEIKFEFEDVDTLDKMAIHLYKTSTPS
TRNGTVDSQVKSNPRNNLIYGQHRCGKGRNARGIITARHRGGGHKRLYRKIDFRRNEKDI
YGRIVTIEYDPNRNAYICLIHYGDGEKRYILHPRGAIIGDTIVSGTEVPIKMGNALPLTDMPL
GTAIHNIEITLGKGGQLARAAGAVAKLIAKEGKSATLKLPSGEVRLISKNCSATVGQVGNV
GVNQKSLGRAGSKRWLGKRPVVRGVVMNPVDHPHGGGEGRAPIGRKKPTTPWGYPALG
RRSRKRKKYSDNLILRRRSKMIQPQTHLNVADNSGARELMCIRIIGAS-NRRYAHIGDVIVA
VIKEAVPNMPLERSEVVRAVIVRTCKELKRDNGMIIRYDDNAAVVIDQEGNPKGTRIFGAI
ARELRQLNFTKIVSLAPEVLMLSPKRTRFRKQHRGRMKGISYRGNHICFGKYALQALEPA
WITSRQIEAGRRAMTRNARRGGKIWVRIFPDKPVTLRPAETRMGSGKGSPEYWVAVVKPG
RILYEMSGVTENIARRAISIAASKMPIQTQFIISGMTRIKRGYVARRRRTKIRLFASSFRGAHS
RLTRTITQQKIRALVSAHRDRDRQKRNFRRLWITRINAVIRE-RGVSYSYSRLIHDLYKKQL
LLNRKILAQIAISNRNCLYMISNEIRKEVDWKESTGIIMLKEVYALGQHISMSADKARRVID
QIRGRSYEETLMILELMPYRACYPIFKLVYSAAANASYNMGSNEANLVISQAEVNEGSTV
KKFKPRAKGRSYPIKRPTCHITIVMKDISLDGGIWDKKMDGIKYAVFTDKSIRLLGKN-QY
TSNVESGSTRTEIKHWVELFFGVKVIAMNSHRLPGKGRRMGPIMGHTMHYRRMIITLQPG
YSIPPLRKKRTMAVPKKRTSISKKRIRKNIWKRKGYWTALKAFSLGKSLSTGKSKSFVVRQ
TNKMAKRKDVRVTVILECTSCVRNGA--NKISTGISRYITQKNRQNTPNRLELRKFCRYCY
KHTIHGEIKKMKIRASVRKICEKCRLIRRRGRIIVICSNPRHKQRQGMVREKVIVSTRTLQW
KCVESRTDSKRLYYGRFILSPLMKGQADTIGIAMRRALLGEIEGTSITRVKSESEKVPHEYS
TISGIQESVHEILMNLKEIVLRSNLYGTCDASICVRGPGYVTAQDIILPPYVEIVDNTQHIASL
TEPIDLCIGLQIERNRGYLIKTPH-NFDGSY--PIDAVFMPVRNANHSIHSYGNGNEKQEILFL
EIWTNGSLTPKEALHEASRNLIDLFIPFLHMEEE--NVHLEDNQ-HTVPLSPFIFHDKLA-KLR
KKKK-KIALKSIFIDQSELPPRIYNCLKRSNIYTLLDLLNNSQEDLMKIEHFRIEDVKQILGIL
EKH--FAIDLSKSKMLGDG-NEGMSTIPGFNQIQFEGFCRFIDQGLTEELYKFPKIEDTDQEIE
FQLFVETYQLVEPLIKERDAVYESLTYSSELYVSAGLIWKTSRDMQEQTIFIGNIPLMNSLG
TSIVNGIYRIVINQILQSPGIYYRSELDHNGISVYTGTIISDWGGRPELEIDRKARIWARVSRK
QKISILVLSSAMGSNLREILENVCYPEIFLSFLNDKERKKIGSKENAILEFYQQFACVGGDPV
FSESLCKELQKKFFQQRCELGRIGRRNMNRRLNLDIPQNNTFLLPRDILTAADHLIGLKFG
MGTLDD--MNHLKNKRIRSVADLLQDQFGLALVRLENVVRGTICGAIR-HKL-IPTPQNLVT

STP----LTTTYESFFGLHPLSQVLDRTNPLTQIVHGRKLSYLGPGGLTGRTASFRIRDIHPSHY
GRICPIDTSEGINVGLIGSLAIHARIGHWG-SLESPFY---EISERSTG--VRMLYLSPGRDEYYM
VAAGNSLALNQDIQEEQVVPARYRQEFLTIAWEQVHLRSIFPFQYFSIGASLIPFIEHNDANR
ALMSSNMQRQAVPLSRSEKCIVGTGLERQAALDSGALAIAEGEGKIISTDTDKILLSGNGN
TLSIPLVMYQRSNKNTCMHQKPQVRRGKCIKKGQILADGAATVGGELALGKNVLVAYMP
WEGYNFEDAVLISERLVYEDIYTSFHIRKYEIQTHVTSQG-PERVTNEIPHLEAHLLRNLDK
NGIVMLGSWVETGDILVGKLTPQMVKE-SSYAPEDRLLRAILGIQVSTSKETCLKLPIGGRG
RVIDVRWIQKKG-GSSYNPETIRVYISQKREIKVGDKVAGRHGNKGIISKILPRQDMPYLQD
GRPVDMVFNPLGVPSRMNVGQIFECSLGLAGSLLDRHYRIAPFDERYEQEASRKLVFSELY
EASKQTANPWVFEPEYPGKSRIFDGRTGNPFEQPVIIGKPYILKLIHQVDDKIHGRSSGHYA
LVTQQPLRGRAKQGGQRVGEMEVWALEGFGVAHILQEMLTYKSDHIRARQEVLGTTIIGG
TIPNPEDAPESFRLLVRELRSLALELNHFLVSEKNFQINRKEAMNQNFSSMIDRYKHQQLRI
GLVSPQQISAWATKILPNGEIVGEVTKPYTFHYKTNKPEKDGLFCERIFGPIKSGICACGNY
RVIGDEKEDPKFCEQCGVEFVDSRIRRYQMGYIKLACPVTHVWYLKRLPSYIANLLDKPL
KELEGLVYCD--FSFARP-ITKKPTFLRLRGLFEYEIQSWKYSIPLFFTTQGFDTFRNREISTG
AGAIREQLADLDLQIIIDKSLVEWKELGEG-GPTGNEWEDRKVGRRKDFLVRRMELAKHFI
RTNIEPEWMVLCLLPVLPPELRPIIQIDGGKLMSSDINELYRRVIYRNNTLTDLLTTSRSTPG
ELVMCQEKLVQEAVDTLLDNGIRGQPMRDGHNKVYKSFSDVIEGKEGRFRETLLGKRVD
YSGRSVIVVGPSLSLHRCGLPREIAIELFQTFVIRGLIRQHLASNIGVAKSKIREKEPIVWEIL
QEVMQGHPVLLNRAPTLHRLGIQAFQPVLVEGRAICLHPLVCKGFNADFDGDQMAVHVP
LSLEAQAEARLLMFSHMNLLSPAIGDPISIPTQDMLIGLYVLTSGNRRGICVNRYNPCNR---
RNYQNQRSDNNNYKYT---EPFFCNSYDAIGAYRQKRINLDSPLWLRWRLDQR-VIAL--RET
PIEVHYESLGTYYEIYGHYLIVRSIKKEILFIYIRTTVGHISLYREIEEAIQGFSQACSYGT------
--MEVLMAERANLVFHNKVIDGTAMKRLISRLIDHFGMAYTSHILDQVKTLGFQQATATSIS
LGIDDLLTIPSKGWLVQDAEQQSLILEKHHHYGNVHAVEKLRQSIEIWYATSEYLRQEMNP
NFRMTDPFNPVHMMSFSGARGNASQVHQLVGMRGLMSDPQGQMIDLPIQSNLREGLSLT
EYIISCYGARKGVVDTAVRTSDAGYLTRRLVEVVQHIVVRRTDCGSTRGISVSPRNGMMP-
ERIFIQTLIGRVLADDIYMGPQCIATRNQDIGIGLVNRFLT------FRAQPISIRTPFTCRSTSWIC
RLCYGRSPTHGDLVELGEAVGIIAGQSIGEPGTQLTLRTFHTGGVFTGGTAEHVRAPSNGKI
KFNEDLVHPTRTRHGHPAFLCSIDLYVTIESEDIIHNVNIPPKSFLLVQNDQYVESEQVIAEIR
AGTST-LNFKEKVRKHIYSDSDGE-IHWSTDVYHAPEFTYGNVHLLPKTSHLWILLGGPYR-
-SSLVFLSLHKDQDQMNAH--SLSVKGGDTSNRS--VTNDQ-VRHKFFSSDFSGK-EDKIPDY-
SDLNRIICTGSCNLIYP-AILHDNS--DLLSKRRRNRFIIPLQWIQECENELMSCLGISIEIP--TN
GIFRRNSILAYFDDPRYRRKSSGITKYGTIEMHSILKKED--LIEYRG-VKEFRPKYMKVDRF
FFIPEEVHILPGSSSIMVRNNSLIGVDTPITLNIRSRVGGLVRVEKKKKRIELKIFSGDIHFPGE
TDKISRHSGVLIP--PGTGKTNSKESKK--LKNWIYVQR--ITPSK--KKYFVLVRPVVTYEITD
GINLSTLFPPDLLQERDNVQLRVVNYILYGNGKPIRGISDTSIQLVRTCLVLSWDQETK---SS
SIEEARASFVEIRTNGLIRHFLRIDLVKSPISYIGKRNDPLDSRLLSD-NGSDCTNINPFSSIYS
Y---SKGRIQQSLNQNQGTIHTLLNR---NKECQSLIILSSSNCFRMGP--FNDVKYHSV---IKES
DSRIPIRNSLGPLGT--ALQIANFYS--FYHL--ITHNQIL-VTHYLQLD--N-LKHTFQVIK---YYL
MDEN--GKIYNPDPGSNIILNPFHLNWYFLHHNCCEETF-TIISLGQFICENVCIAKNRPH-LK-
-SGQVILVQ-VDSIVIRSAKPYLATPGATVHGHYGEILYEGDTLVTFIYEKSRSGDITQGLPKV
EQVLEVRSIDSISMNLEKRVEVWNECITRILGIPWGFLIGAELTIVQSRISLVNKIQKVYRSQ
GVQIHNRHIEIIVRQITSKVLVSE---DGMSNVFSPGELIGLLRAERMGRALEEAICYRAILLG

ITRASLNTQSFISEASFQETARVLAKAALRGRIDWLKGLKENVVLGGMIPVGTGFKGL--V
HLSKQHNKIP--LETKKKNLFEGEMR--DILFHHRKLFDSSLSKNFHDTSE----------------MTRR
YWNINLEEMMEAGVHFGHGTRKWNPKMAPYISAKRKGIHIINLTRTARFLSEACDLVFDA
ARRGKQFLIVGTKNKAADSVAWAAIRARCHYVNKKWLGGMLTNWSTTETRLHKFRDLR
MEQKTGRLNRLPKRDAAMLKRQLFRLQTYLGGIQYMTGLPDIVIIVDQHEEYTALRECIT
LGIPTICLIDTNCDPDLADISIPANDDAISSIRLILNKLVFAICEGRS-SYIRNPMGQKINPLGFR
LGTTQGHHSLWFAQPKNYSEGLQEDQKIRDCIKNYVQKNMRISSG-VEGIARIEIQKRIDLI
QVIIFMGFPKLLIESRPR----GIEELQTNLQKEFNCVSRKLNIAITRIAKPYGNPNILAEFIAGQ
LKNRVSFRKAMKKAIELTEQADTKGIQVQIAGRIDGKEIARVEWIREGRVPLQTIRAKIDY
CSYTVRTIYGVLGIKIWIFIDEEMSRYRGPRFKKIRRLGALPGLTNKRPRAGSDLRNQSRSG
K-KSQYRIRLEEKQKLRFHYGLTERQLLKYVRIARKAKGSTGQVLLQLLEMRLDNILFRLG
MALTIPAARQLVNHRHILVNGRIVDIPSYRCKPRDIITAKDEQKSRTLIQNSLDSSPH-EGLPN
HLTLHPFQ--YKGLVNQIIDSKWVGLKINELLVVEYYSRQTMSRRGTAEKKTAKSDPIYRNR
LVNMLVNRIMKHGKKSLAYQIIYRAVKKIQQKTETNPLSVLRQAIRGVTPDITVKARRVGG
STHQVPIEIGSTQGKALAIRWLLAASRKRPGRNMAFKLSSELVDAAKGSGDAIRKKEETH
RMAEANRAFAHFRMSRDTIAEIITSIRNADMDRKRVVRIASTNITESIVKILLREGFIENVRK
HQENNKSFLVLTLRHRR--NRKRPY---RNIFNLKRISRPGLRIYSNYQRIPRILGGMGIVILSTS
RGIMTDREAREQRIGGEILCYIWMAKAMPRIGSRRNGRIGSRKSTRRIPKGVIHVQASFNN
TIVTVTDVRGRVVSWASAGTCGFKGTRRGTPFAAQTAAGNAIRTVVDQGMQRAEVMIKG
PGLGRDAALRAIRRSGILLTFVRDVTPMPHNGCRPPKKRRVMPTIKQLIRNTRQPMRNVT
KSPALRGCPQRRGTCTRVYTITPKKPNSALRKVARVRLTSGFEITAYIPGIGHNLQEHSVVL
VRGGRVKDLPGVRYHIVRGTLDAVGVKDRQQGRSKYGVKKPKMARKSLIQREKKRQKL
EQKYHLIRRSSKKEIS---KVPSLSDKWEIYGKLQSPPRNSAPTRLHRRCFSTGRPRANYRDF
GLSGHILREMVHACLLPGATRSSWMVKNSFISVISQ--EENKENRGSVEFQVFSFTNKIRRL
TSHLELHKKDYLSQRGLRKILGKRQRLLAYLSKKNRVRYKELIGQLDIRETKTR--MVKLR
LKRCGRKQRAVYRIVAIDVRSRREGKDLRKVGFYDPIKNQTYLNVPAILYFLEKGAQPTGT
VQDILKKAEFFKERRPNQTEFN-MDKSKRPFLKSKRSFRKRLPPIQSGDRIDYRNMSLISRFI
SEQGKILSRRVNRLTLKQQRLITIAIKQARILSLLPFLNNEK--QFERTESAARTTGIRTSNNV
TRSLKKNPFVANHLLKKIDKLNTKAEKEIIVTWSRASTIIPTMIGHTIAIHNGKEHLPVYITD
RMVGHKLGEFAPTLNFRGHAKTDNRSRRMIFQSFLLGNLVSLCMKIINSVVVVGLYYGFL
TTFSIGPSYLFLLRAQVMEEG---TEKKVSATTGFITGQLMMFISIYYAPLHLALGRPHTITVL
ALPYLLFHFFWNNHKHF---GSTTRNSMRNLSIQCVFLNNLIFQLFNHFILPSSMLARLVNIY
MFRCNNKMLFVTSSFLGWLIGHILFMKWLGLVLVWIRQNHSIRSLIRSNKYLVSELRNSM
ARIFSILLFITCVYYLGRIPSPILTKKLKQTEEDKPLGTLLFDYNRWNRPFRYIKNNRFEKAV
RNEMSQYFFDTCRSDGKQRISFTYPPSLSTFLKMIKKRIFLSTLEKTPSDELDNRWVYTNK
EKSN-LTNEFLNRIEVLDREPLSLDIIETRTRLCNDNTKKEYLPKMYDPFLNGPYRGTIKKW
FSPKKRVGINRIHSILYQELVNSDPNGQSIGIKEISKKAPRRSYKLIDLEQQS-GQIRSRKAKR
VVIFTIALIRYSQQSDFRRGIIKGSMRAQRRKTVIWELFQANAHSPLFLDRIKKSPLFSFDI--
SRLINPIFRNRKEKARIQVAEAWDTIPFAQGIRGSMLITQSILRKYILLPSLIIAKNIGRMLLFQ
LPEWSEDLQEWNREMHVKCTYNGVQLSETEFPKNWFTDGIQIKILFPFRLKPWHRPKKK
QKDDFCFLTVSGMEAELPFGSPRKRPSFFEPIIRKLKKKIAKLKKKYFLA-LRVLKGKTKLL
LKVSKETKKWVMKNIL--FRKIIRNELSKVNDSIISNQIIHESFNQIQSPDWTNSSLTEKKMK
DLTDRISIIRNQIERITKEKKKSLTKRSYNAKRLELPKNIWQIVKRRNPRLTYKLHYFLKFFI
ERMYIDIFLSIINIPRINTQLF-RFISSDLSYLSQAYVFYKLSQTQVRNLYKLRSVLQYTSFFL

KTEIKDSFTLRIFQPELRHKKLKNYGMNHWKNWLRGHYQYDLSQIRWSRLIPQKWRNRI
NQCRMTQKKNKWDSYEKDQ--LIHYKKQ-NFSKYYKYDLLSYKSLNDDFLYFDRKYLDW
KIINFYLRLFDWMEMNEEILNRWFFPEFVRLYNAYKMKPWVIPSKLLLLNYKSNTEAELDI
FLKRYLLFQLRWDDSLNQRMINNIKVYCLLLRLINPRKITISSIQRREISLDIMLIEKNLTLTE
LMK-GVLIIEPLRLSVKNDGQFIMYQTIGISLVHKSKHNHFNLLVPENIFSSRHRRELRILICF
RFLWPNYRLEDLACMNRYWFDTNNGSCFSMLRIHMYPMKGHQFKSWIFELREILREIKNS
HYFLDSWTQFNSVGSFIHIFFHQERFIKLFDPRIWSILLS-RNS--QGSTSNRYFT---IKG-LILF
VVAVLIYR----INNRNMVERKNLYLIGLLP-----IPMNSIGPRNDTLEESV-GSSNINRLIVS-LL
YLPKGKKISESCFLNPKESTWENPST----KKCSMPESNWGSRWWRNWIGKRR--DSNCKISN
---ETVAGIEILFKEKDIK-YLEFLFVYYMD--DPIRKDHDWELFDRLSLRKRRNIINL--NSGPL
FEILVKHWISYLMSAFREKIPIEVEG-FFKQQG-AGSTIQSNDIEHVSH--LFSRNKWAFSLQN
CAQFHMWQFRQDLFV---SWGK-NPHESDFLKN-VSRENLIWLDNVWLVNKDR---FFS--KV
RNVSSNIQYDS---TR--SSFVQVTDSSQLKGSS-DQSRDHLDSIS----NEDSE-------------YHTLI
NQREIQQLKERSIL-WD--PSFLQTERTEIESD--RFPK--CLSGYS--SMSRLFT-EREKQMINHL
LPEEIEEFL--GNPTRSVRSFFSDRW--SELH-LGSNPTERSTRDQ----KLLKKQQDLS-FVPSRR
SE----NKEMVNIFKIITYLQN--TVSIHPISSDPGCDMVP-KDEPD-MDSSNKISFLNKNPFLDL-
-FHLFHDRNRGGYTL--HHDFESE--ERFQEMADLFTLSITEP-DLVYHKGFAFSIDSYGLDQK
Q--FLNEVFNSRDESKKKS---LLVLPPSFY--EENESFSRRIRKKWVR-ISCGNDLEDPKPEIVV
FASNNIMEAVNQYRLIRNLIQIQYSTYG--YIRNVLNRFFLMN---RSDRNFEYG---IQRDQIG
KDTLNHRTIMKYTINQHFSNLKK-SQKKWFDPL--ILISRTERSMNWDPDAYRYKWSNGSK
NFQEHLEHFVSEQKSR---FQVVFDRLRINQYSIDWSEVIDKKDLSKPLRFFLSK---------SLL
FLS-------KLLFFLSN-SLPFFCVSFGNIPIHRSE-IYIY-ELKGPNDQLSNQLLESIGLQIVHLKK
LKPF--LLD---DHDTS-QKSKFLINGGTISPFLFNKIP-KW-MIDSFHTR--NNRRKSFDNTDSYF
SMIFHDQDNWLNPLKPFHRSSLISSFYKANRLRFLNNPHYFCFY--CNTRFPFSV----EKARIN
--NYDFTYG-QFLNILFIRNK-IFSLCVGK--KKHAFGG-RDTISPIESQVSNIFIPNDFPQSG-DE
AYNLYKS---FHFPSRSDPFVR--RAIYSIADISGTPLTEGQIVNFERTYCQPLSDMNLSDSEGK
NLHQYLN--FNSNMGLIQTPYSEKYLPSEK--RKK--------RSLCLK--KCVEKGQMYRTFQRD
SAFSTLSKWNLFQTYMPWFLTSTGYKYLNLLFLDTFSDLL--PILSS-SQKFVSIFHDIMHGS
GISWRILQKKL------CLPQWNL-ISEISSKCLHN--LLLSEEMIHRN--NESPLISTHLRSPKVRE
F-LYSILFLLLVAGY-LVRTHLLFVSR--ASSELQTEFEKVKSLMIPSSMIELRKLLDRYPTSEP
NSFWLKNLFLVALEQLGYSLEEIRDSASGGNMLG---PA-----YG-VKSIRSKKKYLNIN---LID
LI----PNPINRITFSRNTRHLSHTSKEIYSLIRKRKNVNGDWIDDKIESWVANSDSIDDEEREF
LVQFS--TLTT--EKRIDQILLSLT-HSDHL-----SKNDSGYQMIEQPGAIYLRYLVDIH--KTYLM
NYEFNTSCLAERRIFLAHYQTITY--SQTSCGANSFHFPSHG---KPFSLRLALSPS-RGILVIGSI
GTGRSYLVKYLATNSYVPFITVFLNKFLDNKPKGFLID-----DID-IDDSDDIDASDDIDASD--
----------------DIDRDLDTELLELLTRMNALTMDMMPE---IDRFYITLQFELAKATSPCIIWIPNI
HDLDVNESNYLSLGLVVNHLSR---DCERCS-TRNILVIASTHIPQKVDPALIAPNKLNTCIKI
RRLLIPQQRKHFFTLSYTRGFHLEKKMFHT--NGFGSITMGSNARDLVALTNEALSISITQKK
SIIDTNTIRSAIHRQTWDLR---SQVRSVQDHGILFYQIGRAVAQNVLLSNCPIDPISIYMKKK-
-SCNEGDSYLYKWYFELGTSMKKLTILLYLLSCSAGSVAQDLWSLP--GPDEK-NGITSYGLV
ENDS-DLVHGLLEVEGALVG--SSRTEKDCSQFDND--RVTLLLRPEPRN-PLDMMQNGSCSI
LDQRFLYEKY--ESEFEE-GEGEGALDPQQ----IEEY----LFNH--IVWAP--RIWRP---WGFLFDC
IERPNELGFP-YWSRSFRGK---RIIYDEEDELQENDSEFLQSGTVQYQTRDRSSKEQGFFRIS
QFIWDPADPLFFLFK-DQPPGSVFSHRELFADEEMSKGLLTSQTDPPTSIYKRWFI--KNTQE

KHFELLINRQRWLR---TNSSLS--NGSFRSN--TLSESYQYLSNLFLSN-GTLLDQMTKTLLRK
RWLFPDEMKIGF--M---------MPRSR-INGNFIDKTFSIVANILLRIIPTTSGEKEAFTYYRDG--
MSAQSEGNYAEALQNYYEAMRLEIDPYDRSYILYNIGLIHTSNGEHTKALEYYFRALERN
PFLPQAFNNMAVICHY-RGEQAIRQGDSEIAEAWFDQAAEYWKQAIALTPGNYIEAHNWL
KITRRFE--MSWRSEHIWIELIRGSRKISNFCWAFILFLGSLGFLLVGISSYLGRNLISFFPSQQI
VFFPQGIVMSFYGIAGLFISSYLWCAISWNVGSGYDRFDRKEGIVCIFRWGFPGKNRRIFLR
FLIKDIQSVRIEVKEGISARRVLYMDIRGRGAIPLTRTDEN-LTPREIEQKAAELAYFLRVPIE
VF
>Euonymus
MEKRWFNSMLSLSKSMDSLGPIENVSGREDPIENDRDKKMHPYSNLDHLAGGRGFRNFIS
DGTFLLKDNNRNAYSVHFDIENQTFEIDNEH--SFISELESSFYSYRISSYLNNESDSEDSDPP
SVVYNTKHSWNNHINSCIDTYLRFRSPIGSDSYILRGGGNYSESYIYNSICRNESSSLRPITN
DIDLTLRESSNDLDVTQKYRHLWVQCENCYGLNYKKFFKSKMNICEQCGSHLKMSSSDRI
ELSIDPGTWNPMDEDMVSVDPIEFHSEEEPYKDRIDSYQRKTGLTEAVQTGTGQLNGIPVA
IGVMDFMFMGGSMGSVVGEKITRLIEYATNQFLPLILVCASGGARMQEGSLSLMQMAKIS
SALFDYQSNKKLFYVSILTSPTTGGVTASFGMLGDIIIAEPNAYIAFAGKRVIEQTLNKTVPE
GSQAAEYLFHKGLFDQIIPRNLLKGVLSELFQFHAFFPLNQNSIKMVTIRADEISNIIRERIE
QYNREIKIVNIGTVLQVGDGIARIYGLDEVMAGELVEFEEGTIGIALNLESNNVGVVLMGG
GLMIEEGSSVKATGRIAQIPVSEAYLGRVINALAKPIDGRGEISASESRLIESPAPGIISRRSVY
ESLQTGLIAIDSMIPIGRGQRELIIGDRQTGKTAVATDTILNQQGENVICVYVAIGQKASSVA
QVVTTLQERGAMEYTIVVAEMADSPATLQYLAPYTGAALAEYFMYREQHTLIIYDDLSKQ
AQAYRQMSLLLRRPPGREAYPGDVFYLHSRLLERAAKSSSSLGEGSMTALPIVETQSGDVS
AYIPTNVISITDGQIFLSADLFNAGIRPAINVGISVSRVGSAAQIKAMKQVAGKLKLELAQFA
ELEAFAQFASDLDKVTQNQLARGQRLRELLKQSQSAPLAVEDQIMTIYTGTNGYLDSLEIG
QVRKFLVELRTYLKTNKPQFQEIISFTKVFTEEAEALLKEAIQEQMKRFILPEQLMKKNPTT
SGMEVSALEKKNLGRIAQIIGPVLDVAFSPGKMPNIYNALVVKGRDTSGHTINVICEVQQL
LGNNQVRAVAMSATDGLMRGMDVIDTGAPLSVPVGVATLGRIFNVLGEPVDNLGPVDTR
TTSLIHKSAPAFIQLETKLSIFETGIKVVDLLAPYRRGGKIGLFGGAGVGKTVLIMELINNIA
KAHGGVSVFGGVGERTREGNDLYMEMKESGVINEQNIAESKVALVYGQMNEPPGARMR
VGLTALTMAEYFRDVNEQDVLLFIDNIFRFVQAGSEVSALLGRMPSAVGYQPTLSTEMGS
LQERITSTKKGSITSIQAVYVPADDLTDPAPATTFAHLDATTVLSRGLAAKGIYPAVDPLDST
STMLQPRIVGEEHYETAQRVKQTLQRYKELQDIIAILGLDELSEEDRLNVARARKIERFLSQ
PFFVAEVFTGSPGKYVGLAETIRGFKLILSGELDGFPEQAFYLVGNIDEATTKATNLEMENK
LKKMTLNLCVLTPNRIIWDSEVKEIILSTNSGQIGVLPNHAPIATAIDIGILRIRLNDQWLTM
ALMGGFARIGNNEITVLVNDAEKSIDIDPQEARKTLEMAEDNLKKAEGKRQTIEANLALR
RARTRVEAINGMAMKNVTDSFLFVGHWPYAGSFGFNTDILATNPINLSVVLGVLIFFGKG
VLSDLLDNRKQRILNTIRNSEELREGAIEQLEKAWDRLRKAELEADQFRADGYSEIEREKF
NLINSTSKILEQLENYKNETIHFEQERAINQVRQRVFQQALQEALGTLNSSLTDELHLRTIN
ANIGIFGTMKEITNMNPLISAASVIAAGLAVGLASIGPGVGQGTAAGQAVEGIARQPEAEG
KIRGTLLLSLAFMEALTIYGLVVALALLFANPFVMNVLLCSISTLKGLYDLSGVEVGQHFY
WEIGGFKIHGQVLITSWVVIAILVGSAIVAVRNPQTIPTASQNFFEYVLEFIRDVSKTQIGEE
YGPWVPFIGTMFLFIFVSNWSGALLPWKLIQLPQGELSAPTNDINTTVALALLTSVAYFYAG
LTKKGLGYFSKYIQPTPILLPINVLEDFTKPLSLSFRLFGNILADELVVVVLVSLVPLVVPIPV
MFLGLFTSGIQALIFATLAAAYIGESMEGHHMIFSTLEHILTHISVSVVSIVITIHLITILLDEIV

GLYDSSEKGTIATFFCITGLLVTRCIYLGHLPLSNLYESLVFLSWSLFIIHIIPYFKKHQKNFSL
ITAPSALFTQGFATSGFLTEMHRSALLVPALQSQWLVMHVSMMVLAYAALLCGSLLSVALL
VITFRKIIRIPFLFKEIQYMNGSGSVLR-TSFLSSKNYYRSQLIHQLDCWSYHIISIGFILLTIGI
LSGAVWANEAWGSYWSWDPKETWAFITWAIFAIYLHTRTHKKWEGVNSAIVAFIGFLLIWI
CYLGVNLLGIGLHSYGSFTLIMAKKKAFTPLLYLASIVFLPWCVSLSFQKSLESWITNWWN
TRESETFLNDIKEKTILEKFIELDELFLLEEMIKEYPETHLQKLRIGIHKEMIKLIKMYNEDGI
HTIFQFSTNIIYFLILSGYSILGNQELRFLNSWVQEFLYNLSDTIKAFSILLLTDLCIGFHSPHG
WELMIGSIYKDFGFTHNDQIISGLVSTFPVILDTIFKYWIFRYLNRLSPSLVVIYHSMNDMPI
GVPRIPFRNPGENASSWVDIYNRLYRERLLFLGQIINTHLANQLIALILYLTMESDTKDLYLF
INSPGGWVIPGIALYDTMQFVRPDVQTICLGLAASMGSFILVGGKITKRLAFPHARVMIHQ
PFSAFFMAQVGEFVLEAEELLKLRETITRVYVQRTGKPLWMVSEDMERDAFMSATEAQA
HGIVDRVAVEMKEQKWIHEGLITESLPNGMFRVRLDNEDFILGYISGRIRRSFIRILPGDRVK
IEVSRYDSTRGRIIYRLRNKD---KEKFQRYLELDPSRQHDFLYPLIFREYSYVLSHDYGLVS
YENKSSLLIVKRLIIRMDQQNHLIISTHYSNQNIFFKKNLYYQMISEGFSVIVEIPFSLRFAPS
LIVKSHNLRSIHSIFPFLEDKFSHLTYVLDVLIPYPIHLEILVQILRCWVKDVSSLHLLRFVLH
EYWNWNSLLTKSIYIFSQPRFFLFLYNSYVSEYESIFLFLRKQDSHLWSTSSRVLLERIHFYG
KIEHLAEVFPSQVILWLLKDPCTHYVRYQGKCILASKDMPFLMNKWKFYLVNLFQCHFFA
GSQPERIYINQLSKHSLDFLGYLSSVQLNSSVVRSQMVDNAFLIDN--AMKKLDTRVPLISLI
RLLAKARFCNVLGHPISKTTRADSSDSDIIDRFVRICINLSHYYSGSSKKKSLYRIKYVLRLS
CVKTLARKHKSTVRAFLKRLGSELLEEFFTEDEEILSLSFPRRIWYFDIICINDLAPVLMILD
TP-EIQDIH-SFSGLES-LKEIYNIIWMLAPILTLVLGITIGVLVIVWLEREIAAGVQQRIGPEYA
GPLGVLQALADGIKLLFKENLLPSRGDTRLFSVGPSIAVISILLSYSVIPFGYHLVLADLKIG
VFLWIAISSIAPIGLLMSGYGSNNKYSFLGGLRAAAQSISYEIPLTLCVLSISLLSNSLSTVDI
VEAQSKYGLWGWNLWRQPIGFIVFLISSLAECERLPFDLPEAEEELVAGYQTEYSGIKFGLF
YVASYLNLLVSSLFVTVLYLGGWNISIPHFFVPKLFELNKVSGVFGTTTGIFIILAKTYLFLFI
SITTRWTLPRLRMDQLLNLGWKFLLPISLGNLLLTTSSQLLSLMIWHVQNENFILDSTRIFM
KAFHLLLFDGSLIFPECILIFGLILLLMIDSTSDQKDIPWLYFISSTSLVMSITALLFRWREEPM
ISFSGNFQTNNFNEIFQFLILLCSTLCIPLSVEYIECTEMAITEFLLFVLTATLGGMFLCGAND
LITIFVAPECFSLCSYLLSGYTKKDVRSNEATMKYLLMGGASSSILVHGFSWLYGSSGGEIE
LQEIVNGLINTQMYNSPGISIALIFITVGIGFKLSLVPSHQWTPDVYEGSPTPVVAFLSVTSK
VAASASATRIFDIPFYFSSNEWHLLLEILAILSMILGNLIAITQTSMKRMLAYSSIGQIGYVIIG
IIVGDSNGGYASMITYMLFYISMNLGTFACIVSFGLRTGTDNIRDYAGLYTKDPFLALSLAL
CLLSLGGLPPLAGFFGKLHLFWCGWQAGLYFLVSIGLLTSVVSIYYYLKIIKLLMTGRNQEI
TPHVRNYRRSPLRSNNSIELSMIVCVIASTIPGISMNPIMEIAQDTLFMVLLYEYDLFWAFLII
SSVIPVLAFLISGVLAPLNKRPEKLSSYESGIEPMGDAWLQFRIRYYMFALVFVIFDVETVFL
YPWAMSFDVVGVSVFIEAFIFVLILIAGLVYAWRKGALEWSTNFFPWLTIFVVFPISAGSLIF
FLPQGGNKVIRWYAICICVLELLLMTYVFCYRFQSDDLLIQLAENYKWVDFFGFYWRLGI
DGLSIGPILLTGFITTLATLAAWPVTRDSRLFHFLMLAMYSGQIGLFSSRDLLLFFIMWELEL
IPVYLLIFMWGGKQRLYSATKFILYTAGGSLFLLIGILGICLYGSNEPTLNFETSANQPYPLA
LEIIFYIGFLIAFAVKLPIIPLHTWLPDTHGEAHYSTCMLLAGILLKMGAYGLVRINMELLPH
AHSLVSPWLIIIGTIQIIYAASTSPSQRNLKKRIAYSSVSHMGFIIIGICSLSDTGLNGALLQIIS
HGFIGAALFFVAGTSYDRIRLLYLDKMGGMAISVPKIFTMFSISSMASLALPGMSGFFAELII
FFGIITSQKFLLLAKILIIFVMAIGTILTPVYLLSMLHQMFYGYKLFNASNSSFLDFGPRELFI
SISILLPVIGIGIYPDFVFSLSGDKVEAILYNY---MMLEHILVLSAYLFCIGIYGLITSQNMVRA

LMCLELLMNAININFVTFSDFFDSRQLKGTICSIFVIAIAAAEAAIGPAIVSSIYRNRKSTRIN
QSNLLNKMEHIYQYSGIIPFITLPVPMVIMGGLLLFPTVTKKIRRMLSFSSVVLLSIVMLFSL
NLLLQQIYSSSIYKSVWSWTINNDFSLEFGYLIDPLTSIMLILITTVGIMVLIYSDNYMSHDQ
GYLRFFASMSFFNTSMLGLVSSCNLIQIYIFWELVGMCSYLLIGFWFTRPLVGTACQKAFIT
NRVGDFGLLLGILGFYWITGSFEFRDLFEIFNN-EIDLLFVTLCSLLLFSGAVAKSAQFPLHV
WLPDAMEGPTPISALIHAATMVAAGIFLVARLLPLFLVIPYVMNLIALIGILTVLLGATLALA
QKDIKRGLAYSTMSQLGYIMLALGLGSSRAALFHLITHAYSKALLFLGSGSIIHSMEAIVGY
SPDKSQNMVLMGGLTKHVPITKISFLLGTLSLCGIPPFACFWSKDEILNDSWLYSPIFSIIACS
AAGLTAFYMFRIYLLTFEGHLNPHFQDYSGKKGSSFYSISLWVKRGQVR?KK-YYLY?KINL
FCNTLN---PYPHESNNTMLAPILILALFTLFSGVIGN--GLDILSKLLTPPINLLDQNSK-SKDW
-QFLTNATFPVSIAFGGILFASFLYKPVYSSLQNLNFLNLVVILRDKLIYLIYRLSYNRGYIDA
FYAKFITRGLRRLAELTHFFDRQIIDRITNVMGLTSFFLGEVIKYVGGG-RISSYLLLYLFAVF
IFLVIKLMDLPGSIHDFLLVFLGLGLILLGLGVVLLTNPIYSAFSLGLVLICIALFYILSNSYFV
AAAQLLIYVGAINVLIIFAVMFMNGSEYHNAFYLWNAGGGVTSLVCTSIFLLLITTIPDTSW
YGIIWTTRSNQIIEKDLITNSQQIGIRLSTDFFLPFELISIILLVALIGAIAAARQMNVSSPRQD
LMIVNMGPHHPSMHGVLRLIVTLDGEDVIDCEPILGYLHRGMEKIAENRTIIQYLPYVTRW
DYLATMFTEAITVNGPERLGNIQIPKRASHIRVIMLELSRIASHLLWLGPFMADIGAQTPFF
YIFREREL VYDLFEAATGMRMMHNFFRIGGVVADLPHGWIDKCLDFCDYFLTRAAEYQN
LITRNPIFLERVEGVGIISAEEAINWGLSGPMLRASGIQWDLRKVDHYECYNEFDWEAPW
QKEGDSLARYLVRISEMKESIKIIQQALERIPGGPYENLETRCFDRERDQNWNDFEYRFISK
KTSPTFELPKQELYVRVEAPKGELGIFLIGDQSGFPWRWKIRPPGFINLQILPQLVKRMKLA
DIMTILGSIDIIMGEVDRMLPMVTGFRNYGQQTIRAVRYIGQSFMITLSHANRLPVTIQYPY
EKLITSERFRGRIHFEFDKCIACEVCVRVCPIDLPVVDWQFETNIRKKRLLNYSIDFGICIFC
GNCVEYCPTNCLSMTEEYELSTYDRHELNYNQIALGRLPISVIDDYTIRTISTLD--------------
-MQGSLSARLVKHGLVHRSLGFDYQGIETLQIKPADWHSIAVILYVYGYNYLRSQCAYDVA
PGGLLASVYHLTRVAYSIDQPEEVCIKVFAPRRNPRILSVFWVWKSADFQERESYDMFGIS
YENHPRLKRILMPESWIGWPLRKDYIAPDFYEIQDAH--
--------------MNSIAFPLLDRTTQ-SVISTTSNDLSNWSRLSSLWPLLYGTSCCFIEFASLIGSRFD
FDRYGLVPRSSPRQADLILTAGTVTMKMAPSLVRLYEQMPEPKYVIAMGACTITGGMFSTE
SYSTVRGVDKLIPVDVYLPGCPPKPEAVIDAITKLRKKISREISEDRIRAQQENRCFTTNHQF
RLGRSTHTGNYDQGLLYQPLSTSEIPTETFFKYTAGVSSHDLVNMQTRNTFSWIKEEITRSF
SVSLIISIMTWASISNAYPIFAQQGYENPREATGRIVCANCHLANKPVDIEVPQAVLPDTVFE
AVIRIPYDKQLKQVLANGKKGALNVGAVLILPEGFELAPPDRISPEMKEKMGNLSFQSYRP
TKKNILVIGPVPGQKYSEITFPILSPDPTTKKDVHFLKYPIYVGGNRGRGQIYPDGSKSNNT
VYNATAAGIVSKIIRKEKGGYEITITDASDGRQVIDIIPPGPELLVSEGESIKLDQPFTSNPNV
GGFGQGDAEIVLQDPARVQSLLFFFASVILAQIFLVLKKKQFEKVQLSEMNFMSKVYDWF
EERLEIQAIADDITSKYVPPHVNIFYCLGGITLTCFLVQVATGFAMTFYYRPTVTEAFASVQ
YIMTEANFGWLIRSVHRWSASMMVLMMILHVFRVYLTGGFKKPRELTWITGVVLAVLTAS
FGVTGYSLPWDQIGYWAVKIVTGVPEAIPVIGSPLVELLRGSASVGQSTLTRFYSLHTFVLP
LLTAVFMLMHFLMIRKQGISGPLMGVTKKPDLNDPVLRAKLAKGMGHNYYGEPAWPND
LLYIFPVVILGTIACNVGLAVLEPSMVGEPAEPFATPLEILPEWYFFPVFQILRTVPNKLLGVL
LMVSVPAGLLTVPFLESVNKFQNPFRRPVATTVFLIGTVVALWLGIGATLPIDKSLTLGLFMI
EVFLFGIVLGLIPITLAGLFVTAYLQYRRGDQLGLMPTITSYFGFLLAALTITSVLFIGLSKIR
LIMDIVSLAWAALMVVFTFSLSLVVWGRSGLMIIRSPEPEVKILVDRDPIKTSFEEWARPGH

FSRTIAKGPDTTTWIWNLHADAHDFDSHTSDLEEISRKVFSAHFGQLSIIFLWLSGMYFHG
ARFSNYEAWLSDPTHIGPSAQVVWPIVGQEILNGDVGGGFRGIQITSGFFQIWRASGITSEL
QLYCTAIGALVFAALMLFAGWFHYHKAAPKLAWFQDVESMLNHHLAGLLGLGSLSWAG
HQVHVSLPINQFLNAGVDPKEIPLPHEFILNRDLLAQLYPSFAEGATPFFTLNWSKYAEFLT
FRGGLDPITGGLWLTDIAHHHLAIAILFLIAGHMYRTNWGIGHDIKDILEAHKGPFTGQGH
KGLYEILTTSWHAQLSLNLAMLGSLTIVVAHHMYSMPPYPYLATDYGTQLSLFTHHMWIG
GFLIVGAAAHAAIFMVRDYDPTTRYNDLLDRVLRHRDAIISHLNWVCIFLGFHSFGLYIHN
DTMSALGRPQDMFSDTAIQLQPVFAQWIQNTHALAPGATAPGATTSTSLTWGGSDLVEVG
GKVALLPIPLGTADFLVHHIHAFTIHVTVLILLKGVLFARSSRLIPDKANLGFRFPCDGPGRG
GTCQVSAWDHVFLGLFWMYNAISVVIFHFSWKMQSDVWGSISNQGVVTHITGGNFAQSS
ITINGWLRDFLWAQASQVIQSYGSALSAYGLFFLGAHFVWAFSLMFLFSGRGYWQELIESI
VWAHNKLKVAPATQPRALSIVQGRAVGVTHYLLGGIATTWAFFLARIIAVGMALRFPRFSQ
GLAQDPTTRRIWFGIATAHDFESHDDITEERLYQNIFVSHFGQLAIIFLWTSGNLFHVAWQG
NFEAWVQDPLHVRPIAHAIWDPHFGQPAVEAFTRGGALGPVNIAYSGVYQWWYTTGIRT
NGDLYIGALFLLFLSALSLIAGWFHLQPKWKPSVSWFKNAESRLNHHLSGLFGVSSLAWT
GHLVHVAIPGSRGQYVRWNNFLDVAPHPQGLGPLFTGQWNLYAQNPDSSSHLFGTSQGA
GTAILTLLGGFHPQTQSLWLTDIAHHHLAIAILFLIAGHMYRTNFGIGHSIKDLLEAHVPPG
GRLGRGHKGLYDTINNSLHFQLGLALASLGVITSLVAQHMYSLPAYAFIAQDFTTQAALYT
HHQYIAGFIMTGAFAHGAIFFIRDYNPEQNEDNVLARMLDHKEAIISHLSWASLFLGFHTL
GLYVHNDVMLAFGTPEKQILIEPIFAQWIQSAHGKTSYGFDVLLSSTNSPAFNAGRNIWLP
GWLNAINE--NSNSLFLTIGPGDFLVHHAIALGLHTTTLILVKGALDARGSKLMPDKKDFGY
SFPCDGPGRGGTCDISAWDAFYLAVFWMLNTIGWVTFYWHWKHITLWQGNVSQFNESST
YLMGWLRDYLWLNSSQLINGYNPFGMNSLSVWAWMFLFGHLVWATGFMFLISWRGYW
QELIETLAWAHERTPLANLIRWRDKPVALSIVQARLVGLAHFSVGYIFTYAAFLIASTSGKF
GMSHSVKIYDTCIGCTQCVRACPTDVLEMIPWDGCKAKQIASAPRTEDCVGCKRCESACP
TDFLSVRVYLWHETTRSMGLAYMTTFNVPSIFVPLVGLVVPAIAMASLSLHVQRNKIVMR
DLKTYLSAAPVLSTLWFGSLAGLLIEINRFFPDALTFPFFSFMTAILERRESESLWGRFCNWI
TSTENRLYIGWFGVLMIPTLLTATSVFIIAFIAAPPVDIDGIREPVSGSLLYGNNIISGAIIPTSA
AIGLHFYPIWEAASVDEWLYNGGPYELIVLHFLLGVACYMGREWELSFRLGMRPWIAVAY
SAPVAAATAVFLIYPIGQGSFSDGMPLGISGTFNFMIVFQAEHNILMHPFHMLGVAGVFGGS
LFSAMHGSLVTSSLIRETTENESANAGYRFGQEEETYNIVAAHGYFGRLIFQYASFNNSRSL
HFFLAAWPVVGIWFTALGISTMAFNLNGFNFNQSVVDSQGRVINTWADIINRANLGMEVM
HERNAHNFPLDLATVEAPSTNVMGLPWYRVHTVVLNDPGRLLAVHIMHTALVAGWAGS
MALYELAVFDPSDPVLDPMWRQGMFVIPFMTRLGITDSWGGWTIT-GGTITNPGIWSYEGV
AGAHIVFSGLCFLAAIWHWVYWDLEIFTDERTGKPSLDLPKIFGIHLFLSGVACFGFGAFH
VTGLYGPGIWVSDPYGLTGKVQSVNPAWGVEGFDPFAPGGIASHHIAAGTLGILAGLFHLS
VRPPQRLYKGLRMGNIETVLSSSIAAVFFAAFVVAGTMWYGSATTPIELFGPTRYQWDQG
YFQQEIYRRVVAGLAENQSLSEAWSKIPEKLAFYDYIGNNPAKGGLFRAGSMDNGDGIAV
GWLGHPVFRDKEGRELFVRRMPTFFETFPVVLVDGDGIVRADVPFRRAESKYSVEQVGV
TVEFYGGELNGVSYSDPATVKKYARRAQLGEIFELDRATLKSDGVFRSSPRGWFTFGHASF
ALLFFFGHIWHGARTLFRDVFAGIDPDLDAQVEFGAFQKLGDPTTKRQVV------------VETL
FNGTLALAGRDQESTGFAWWAGNARLINLSGKLLGAHVAHAGLIVFWAGAMNLFEVAHF
VPEKPMYEQGLILLPHLATLGWGVGPGGEVIDTFPYFVSGVLHLISSAVLGFGGIYHALLG
PETLEESFPFFGYVWKDRNKMTTILGIHLILLGLGAFLLVFKALYFGGVYDTWAPGGGDV

RKITNLTLSPSVIFGYLLKSPFGGEGWIVSVDDFEDIIGGHVWIGSICILGGIWHILTKPFAWA
RRALVWSGEAYLSYSLAALSVFGFIACCFVWFNNTAYPSEFYGPTGPEASQAQAFTFLVRD
QRLGANVGSAQGPTGLGKYLMRSPTGEVIFGGETMRFWDLRAPWLEPLRGPNGLDLSRL
KKDIQPWQERRSAEYMTHAPLGSLNSVGGVATEINAVNYVSPRSWLATSHFVLGFFLFVG
HLWHAGRARAAAAGFEKGIDRDFEPVLSMTPLNMTIALGKFTKEEKDLFDIMDDWLRRD
RFVFVGWSGLLLFPCAYFALGGWFTGTTFVTSWYTHGLASSYLEGCNFLTAAVSTPANSL
AHSLLLLWGPEAQGDFTRWCQLGGLWTFVALHGAFGLIGFMLRQFELARSVQLRPYNAIS
FSAPIAVFVSVFLIYPLGQSGWFFAPSFGVAAIFRFILFFQGFHNWTLNPFHMMGVAGVLGA
ALLCAIHGATVENTLFEDGDGANTFRAFNPTQAEETYSMVTANRFWSQIFGVAFSNKRWL
HFFMLFVPVTGLWMSALGVVGLALNLRAYDFVSQEIRAAEDPEFETFYTKNILLNEGIRA
WMAAQDQPHENLIFPEEVLPRGNALMSGSTGERSFADIITSIRYWVIHSITIPSLFIAGWLFV
STGLAYDVFGSPRPNEYFTESRQGIPLITGRFDPLEQLDKLSRSFMTIDRTYPIFTVRWLAVH
GLAVPTVFFLGSISAMQFIQRMVTQSVEGSSR-SRPKRTGVGGLLKPLNSEYGKVAPGWGT
TPLMGVTMALFAIFLSIILEIYNSSVLLDGISMNMLTLKLFVYTVVIFFVSLFIFGFLSNDPGR
NPGREEMADTTGRIPLWIIGTVTGILVIGLIGIFFYGSYSGLGSSLMLNIFSLIWICFNSLLYSS
SFLFAKLPEAYAFLKPIIDVMPVIPVLFLLLAFVWQAAVSFRTTQSNPNEQNVELNRTSLYW
GLLLIFVLAVLFSNYFFNMEVNILAFIATALFILVPTAFLLIIYVKTISQSDMEIATLVAIFISGL
LVSFTGYALYTAFGQPSQQLRDPFEEHGDMEALVYTFLLVSTLGIIFFAIFFREPPKVPTKKM
KMTIAFQLAVFALIATSLILLISVPVVFASPEGWSSNKNVVFSGTSLWIGLVFLVGILNSLIS---
----MSPQTETKASVGFKAGVKDYKLTYYTPDYETKDTDILAAFRVTPQPGVPPEEAGAAVA
AESSTGTWTTVWTDGLTNLDRYKGRCYHIEPVAGEESQFIAYVAYPLDLFEEGSVTNMFTS
IVGNVFGFKALRALRLEDLRIPTAYSKTFQGPPHGIQVERDKLNKYGRPLLGCTIKPKLGLS
AKNYGRAVYECLRGGLDFTKDDENVNSQPFMRWRDRFLFCAEALYKAQAETGEIKGHY
LNATAGTCEEMMKRAVCARELGVPIVMHDYLTGGFTANTTLAHYCRDNGLLLHIHRAMH
AVIDRQKNHGIHFRVLAKALRMSGGDHIHAGTVVGKLEGERDITLGFVDLLRDDFIEKDR
SRGIYFTQDWVSLPGVLPVASGGIHVWHMPALTEIFGDDSVLQFGGGTLGHPWGNAPGAV
ANRVALEACVQARNEGRDLAREGNEIIREASKWSPELAAACEVWKEIKFEFQAMDTL--M
AIHLYKTSTPSTRNGAVDSQVKSNKRNNLIYGQHRCGKGRNARGIITAGHRGGGHKRLYR
QIDFRRKEKDIYGRIVTIEYDPNRNAYICLIHYGDGEKRYILHPRGAIIGDTIVSGTEVPIKM
GNDLPLTDMPLGTAIHNIEITLGKGGQLARAAGAVAKLIAKEGKSATLKLPSGEVRLISKNC
SATVGQVGNVGVNQKSLGKAGSKCWLGKRPIVRGVVMNPVDHPHGGGEGRAPIGRKKP
VTPWGYPALGRRSRKRNKYSDNLILRRRSKMIQPQTHLNVADNSGARELMCIRIIGTS-NR
RYAEIGDVIVAVIKEALPNTSLEKSEVIRAVIVRTCKEFKRDNGMIIRYDDNAAVVIDQEGNP
KGTRIFGAITRELRQLNFTKIVSLAPEVLMLSPKRTRFRKQHRGRMKGISYRGNHISFGRYA
LQALEPAWITSRQIEAGRRAMTRNVRRGGKIWVRLFPDKPVTVRPTETRMGSGKGSPEY
WVVVVKPGRILYEMDGVGENIAQKAISIAASKMPIRTQFIISVMTRIRRGYIARNRRTKMR
LFASSFRGARSRLTRTITQQKIRALVSAHRDRDRQKRDFRRLWITRINAVIRER-RVSYSYSIL
MHSLYKRQLLLNRKILAQMAILNKKSLYMISNEIIK-----------MIKKVYAIGQHMSMSAHKA
KIVIDQIRGRFYEETLRILELMPFERCYPVFKLVYSAAANASHNIESNKSNLIISKAEVNEGG
TLKKFKPRARGRSYPIKRRTCHITIVLKDISLG-------MDGIKYAVFTDKSIRLLGKN-QYTSN
VESGSTRTEIKHWVELFFGVKVIAMNSHRPPGKGRRMGPIVGHTMHYRRMIITLQPGYSIP
PLRKKRTMAVPKKRTSISKKRIRKNSWKRKGYWAALKAFSLAKSLATGNSKSFFVR--QIM
ARGKDTRVKVILECTGC-VRTGVVNKK--GISRYITQKNRHNTPGRLELRKFCPYCYKHRIH
GEIKKMKIRASVRKICEKCRLIRRRGRIMVICSNPRHKQRQGMVREKVKVSTRTLQWKCV

ESRTDSKRLFYGRFILSPLMKGQADTIGIAMRRALLGEIEGTSITRAKSE--KIPHEYSTVVGI
QETVHELLMNLKEIVLRGNLYGIQNGSICIKGPGYVTAQDLVLPPSVEIVDNTQHIAILTEPI
YFCIELQIERHRGYHIKTPNNFQEGSF--PIDAVFMPVRNTNYSIQSYRNGSEKEEILFLEIWT
NGSLTPKEALHEASRNLIDLFIPFLHAEEE--NLHLENDW-YKVTLPLFTFHDRLT-KLKKNQ
K-EMALKYIFIDQLELTSRTYNCLKRSNIHTLFDLLNKSHEYLMKMEHFHIEDVKHILNILE
KKK-FATDLPKNKMLGDGN-EGTSTIPGFNQIQFEGFCRFIDHGLTEELYKFPKIEDTEQEIEF
QLFAETYQLVEPLIKERDAVYEAITYSSELYVSARLIWKTSRDIQKQTLFFGNIPLMNSLGTS
IVNGIYRTAINQILQSPGIYYRSEADHNGISVYTGSIISDWGGRFELEIDRKARIWARVSRKQ
KISILVLSSAMGLNLREILENVCYPEIFLSFLNEKEKKKIGSKETAILEFYQQFTCVGGDPVF
SESLCKELQKKFFQQRCELGRIGRRNMNQRLNLDLPPTNTFLLPRDILAAADYLIGMKFG
MGTLDD--MNHLKNKRIRSVADLLQDQFGLALVRLENMVRGTICGAIR-HKLI-PTPQSLVP
STP----LTTTYESFFGLHPLSQVLDRTNPLTQTVHGRKLSYLGPGGLTGRTANFRIRDIHPSH
YGRICPIDTSEGINVGLIGSLAIHAKIGHCG-SLESPFY---EIFEESKSKKNRMFYLSPNRDEY
CMIAAGNSLALSRGIQEEQVVPARYRQEFLTIAWEQVHLRSIFPFQYFSIGASLIPFIEHNDA
NRALMSSNMQRQAVPLSQSEKCIVGTGAERQVALDSGVSVIAEHEGRIIYTDTDKIFLLGN
EDIFNIPLVVYQRSNKNTCMHQKGRVQRDKCIKKGQILGDGAATVGGELTLGKNILVAYM
PWEGYNSEDAVLISERMIYGDIYTSFHIRKYEIQAHVTSQG-SERITNEIPHLEAHLLRNLDK
NGIVALGSWVETGDILVGKLTPQMAKE-SSYAPEDRLLRAILGIQVSSSKETCLKLPTGGRG
RVIDVRWVQKKG-GSDSNPETIRVYILQKREIKVGDKVAGRHGNKGIVSKILPRQDMPYLQ
DGGTVDMVFNPLGVPSRMNVGQIFECSLGLAGDLLDRHYRIAPFDERYEQEASRKLVFSE
LYEASKQTANPWVFEPEYPGKSRIFDGRTGNPFEQPVIIGKPYILKLIHQVDDKIHGRSSGH
YALVTQQPLRGRAKQGGQRIGEMEVWALEGFGVSHILQEMLTYKSDHIKARQEVLGTTIV
GGTIPKPEDAPESFRLLVRELRSLALELNHFFVSEKNFQINRKEA-------MIDRYKHQQLRIG
SVSPQQISAWANKILPNGELVGEVTKPYTFHYKTNKPEKEGLFCERIFGPIKSGICACGNYR
GIGDEKEDLNFCEQCGVEFVNSRIRRYQMGYIKLACPVTHVWYLKRLPSFIANLLDKSIKE
LEGLVYCD--FSFARP-IAKKPTFLRLRGSFEYEIQSWKYSIPLFFTTQGFDTFRNREISTGAG
VIRDQLADLDLRIIIEYSLVEWNELGEE-RPMDNEWEDRKVVRRKDFLVRRMELAKHFLR
TNIEPEWMVLCLLPVLPPELRPIIQIDGGKLMSSDINELYRRVIYRNNTLADLLTTSRSTPGE
LVMCQEKLVQEAVDTLLDNGIRGQPMRDGHNKVYKSFSDVIEGKEGRFRETLLGKRVDY
SGRSVIVVGPSLPLHRCGLPREIAIELFQTFVIRGLIRQHLASNIGVAKSKIREKEPIVWEILQ
EVMQGHPVLLNRAPTLHRLGVQAFQPILVEGRAICLHPLVCKGFNADFDGDQMAVHVPL
SLEAQAEARLLMFSHTNLLSPAIVDPIALPTQDMLIGLYVLTNGDRRGICANRYNPCNRRN-
--FQNERIPYNNYRDTKKKVIFFCNSYHAIGAYRQKRIDLDSSLWLRWQLDQR-VIAS--REA
PIEVHYESLGTYHEIYGHYLIVRSIKREILCIYLRTTVGHISLYREIEEAIQGFCRAFSYGI------
--TEVLMAEGANLVYHNKAIDGTVIKRLISRLIDHFGMAYTSHILDQVKTLGFHQATATSISL
GVDDLLTIPSKGWLVQDAEQQSFLLEKQHHYGNVHAVEKLRQSIEIWYATSEYLRQEMNP
NFRMTEPGNPVHIMSFSGARGNASQVHQLVGMRGLMSDPQGQMIDLPIQSNLREGLSLTE
YIISCYGARKGVVDTAVRTSDAGYLTRRLVEVVQHIVVRRTDCGTSRGISVSPQNEIMS-ER
LFIQTLIGRVLADDIYMGSRCIAVRNQDIGVWLVNRFIT------FQTQFISLRTPFTCRSMSWIC
RLCYGRSPTHGDLVELGEAVGIIAGQSIGEPGTQLTLRTFHTGGVFTGGTAEHVRAPSNGKI
KFNEDLVHPTRTRHGHPAFLCYMDLYVTIESEDILYNVTIPQKSFLLVQNDQYVESEQVIAE
IRAGTYTL-NFKERVRKHIYSDSEGE-IHWSTDVYHTPEFAYSNVHLLPRTSHLWILKGSSCR
--YRVVSFSIHKDQDQVHSL--SVSAEG-RYISSF-SGNNDQ-VRSRFFSSDLSGK-QSKITEY-S
EHNRIICTGHCNLIYPP-ILHESYD--LLAKRRRNRFIIPFQSIPEREKELMSPADISIEIPIN--GIF

RRKSIFAYFDDLQYRRNSSGITKYGTIGVHSILKKED--LIEYR-GVKEFKPKYMKIDRFFFIP
EEVHILPESSSIMVRNNSIIRVDTRIALNTRSRVGGLVRVEKKKKEIELKIFSGDIHFPGETDK
ISRHSGILIPPGTIKK--NSKESKN--LQNWIYVQR--ITPTKKK--YFIFVRPVIIYEIADGLNLST
LFPQDLLQERENLQLRVVNYILYGYGKPVLGISDPSIQLIRTCLSLDWDQEK---KSSSIEEAH
ASFIEVSTRGLIRYFLRIDLVKSHSLYSRKRKDFSSSGFLY-DNASERANINPFY---LKAR-----I
QQSLRQNQGTISTLLNR---NKEYQSLIILSSPNCFRMGP--FNNVKYHN---VIKER-PIILIRNY
LGPLG--TALQIANYSSYSFYHFI--THNQIS-VTKYLQLELDN-LKKNVQVIQIK-YYLMNEN
GR--VSKPDPCSNIILNQFNLNWHFLYHDYCEERSPIIS-LGQLICENLCIAKNGP---HLKSGQ
VIIVQ-VDSVVIRSAKPYLTTPGATLHGHYGEILSEGDTLVTFIYEKSRSGDITQGLPKVEQV
LEVRSIDSISSNLEKRVEGWNACITRILGIPWTFLIGGELTIVQSRISLVNKIQKVYRSQGVQI
HNRHIEIIVRQITSKVLVSE---EGMSNVFLPGELIGLLRAERTGRALEEALCYRAILLGITRAS
LNTQSFISEASFQETARVLAKAALKSRIDWLKGLKENVVLGGMIPVGTGFKGFVRFS--QQ
HNNISLKTKKTN--LFKGEVR--DILFYHREFFDSCISKNFHDPAEQPFIGFNHS------MTRRY
WNINLKEMVEAGVHFGHGTRKWNPRMAPYISEKRKGIHITNLTKTARFLSEACDLVFDAA
SKGKQFLIVGTKTKAADSVARAAIRARCHYVNKKWLGGILTNWSTTETRLHKFRDFRME
QKTGGFNRLPKRDAAVLKRQLSHLQTYLGGIKYMMGLPDIVIIVDQQKEYTALRECIILGI
PTICLIDTNCDPDLADISIPTNDDAIASIRLILNKLVFAICEGRS-SYIRNPMGQKINPLGFRLG
TNQSHHSLWFVQPTNYSEGLQEDQKIRNFIKNYVQQNIKISSG-VEGIARIEIQKRIDLIQVLI
YIGFPKLLLESR----TRRIEELQRKLQKELNCMNRKLNIAITRIANPYGHPTILAEFLAGQLK
SRVSCRKAMKKAIELAEQANTKGIQVQIAGRINGKEIARVEWIREGRVPLQTIRAKIDFCSY
TAQTLYGVLGIKIWIFGDEEMSRYRGPRFKKIRRLGVLPGLTSKRPRVGNDLRNQSRSGK-
KSQYRIRLEEKQKLRFHYGLTERQLLKYVRIAGKAKGSTGQILLQLLEMRLDNILFRLGM
ASTVPQARQLVNHRHILVNGRIVDIPSYRCKPRDIITAKDEQKSRALIQNYLDSFPH-EEVPK
HLTLHPIQ--YKGLVNQIIDSKSIGLKINELLIVEYYSRQAMSRRGTAEEKTAKSDPIYRNRLV
NMLVNRILKHGKKSLAYQILYRAMKKIQQKTETNPLSVLRQAIRGVTPDIAVKARRVGGS
THQVPIEIGSTQGKALAIRWLLGASRKRPGRNMAFKLSSELVDAAKGSGDAIRKKEETHR
MAEANRAFAHFRMGRDTLADIINSLRNAAMNRKGTIRIPFTNITKNIIKILLREGFIENARK
HRESDKYFLVLTLRHR-----KGRTS-----LNLKRISRPGLRIYSNYQQIPRILGGMGIVILSTSRG
IMTDREARLEGIGGEILCYIWMAKTITKTSSRRNVRIGSRKNTRRIPKGVIHVQASFNNTIV
TVTDVRGRVISWSSAGTCGFKGTRRGTPFAAQTAAGTAIRAVVDQGMQRAEVMIKGPGL
GRDAALRAIRRSGILLSFVRDVTPMPHNGCRPPKKRRVMPTLKQLIRNTRQPIKNVTKSPA
LRGCPQRRGTCIRVYTITPKKPNSALRKVARVRLTSGFEITAYIPGIGHNSQEHSVVLVRGGR
VKDLPGVRYHIVRGTLDAVGVKDRQQGRSKYGVKKPKMARKGLIQREKTRQKLEQKYH
LIRRSSRKEIS---KVLSLSDQWEIHGKLQSPPRNSAATRLHRRCFSTGRPRANYRDFGLSGH
ILREMVHAGLLPGATRSSWMVKNAFISVISQNEKEKNKNKGSVELQVFVFTNKIRRITSHL
ELHRKDYLSQRGLRKIIGKRQRLLSYLSKKNEVRYKQLISQLDIREPKTR--MLKLRLRRCG
RKQRTIYRIVAIDVRSRREGKPLRKVGFYDPINNHTYLNFPDILYFLEKGAQPTETVQDISK
KSGVFTQFCLNQ-SKFNMHKFKRLFHKSKRSLRRRLPPIKSGDRIAYRNMSLISRFISEQGKI
LSKRVNRLTLKQQRLITIAIKQARILSLLPFLNNEK--QFEKGESTARTAGLRTRNQVAR-LKK
NPFVANHLLGKINKLNTKAEKEIIVTWSRASTIIPTMIGHTIAIHNGKEHLPLYITDRMVGH
KLGEFAPTLNFRGHAKNDNRSRRMIFKSFILGNLVSLSLKIINSVVVVGLYYGFLTTFSIGPS
YLFLLRARVMEEGEEGTEKKVSATTGFITGQLMMFISIYYAPLYLAFGRPHTITVLALPYLL
FHFFWNNHKHFFDYGSTTTNSMRNLSIQCVFLNNLIFQLFNHFILPSSMLVRLVNIYMFRC
NNKMLFVTSSFVGWLIGHILFMKWVGFVLVWIQQKNSIRS-IRSNKYLVSELRNSMARILSI

LLFITCVYYLGRIPSPLFTKKLKETEEDKTLVTRLFDYKRWNRPFRYIKNNGREKAVRNEM
SQFFFYTCLSDGKEKIPFTYLPSLSIFFDLIERKMPIFTTEKMTSPKLYNRWSYMNDQKKNK
LNNEFLNRVETLDLHFLAFTLLEKRTRLCK--NTKEYLPKIYDPLLNGPSRRKIKKLFLFPPW
ITINRIHGLL---FDQKRGEDRKIGINEISKKVPRWSYKLIELEQQEREEIRSRKSKRVIIFTEAL
IRYSQQADFRRDIIKGSMRPQRRKTITWELFQPNIHSPLFLDRLDKPLLFSFDI--SQMMKIM
FINREEKARVEIAEAWDNILFAHLIRGSLLVIQSILRKYIFLPLLIIAKNTIRTLLFQFPEWSED
LKDWNKEMHVKCTYNGVQLSEKEFPKNWLTEGIQIKILFPFRLKPWHRVKKGKKKNFCF
LTVFGMETEFPFGSTKRRLFDFKPICKKLEEKVRKFTKKSFLF-IRILKKRPKLNFTKSKKSK
KRVIKKSPFFFFKEQIKELI-----ITNKKTIHESSIQIIPMDWTNSSLAEARMQDLTNKKRTIIN--
-QIKKKKEKSPNKITYGTKRFGSLKK---ILKRRNARVVRKFHFLKKKNIEKIYIDIFLSIIQILR
INAELF---IN-DLSSLSQAYVFYKLSQTKKLNLYKLRSALQYTTIFLKNKIKGYFVQGIFHSQ
LRHKKPI-STMNQWKNWLKAGYQYDLSQIRWSRLVPQKWRTIVNQGCIAENKSDSYEKS
YEKDRLIPYEKK-NFKKHYRYDLLAYKFINDEIVDPARKYFDWRILRFCIRLFDWMEMNER
ILSHWFFPEILILYNAYKKTPVSIPIKLLLFDYKSSTEAELDFLLKRYFCFQLRWNASVNQRV
LNNIKVFCLLLRLINPNEIAISSIQRRELSLDILLIQNNLSLTEFLKKGILIIEPVRLAVKNVGQ
FLMYQTINISLGHKSKHNLDDLFVPENIFLSQRRREFGILISLQVFWTNYRLEDLACMNRC
WFDTNNGSRFNMVRIHMYPMKGHQFQSWIFELREILREIKNSHYFLDSWTQFNSVGSFIHI
FFHQERFIKLLDSRILSILLS-RNS--QGSTGNRYF---TIKGVVLFVVA-VFIYR----INNRKMVE
RKNLYLTGLLP-----IPMNSIGPRNEKLEESF-GSSNINRLILS-LLYLPKGKKISESGFLDPKES
TWVLPIT----KKCIMPESNWSSRWWRNWIGKKR--DSSCKISN---ETVAGIEISFKEKDLK-YL
EFLFVYYMD--DPIRKDQDWELFDRLSPRKRRNIINL--NSGRLFEILVKDWICYLMFAFREK
IPIEVEG-FFKQQG-AGSTIQSNEIEHVSH--LFSRKKWAISLKNCAQFHMWQFRQDLFV---S
WGK-NPHESDFLRN-ISRENWIWLYNVWLVNKDR---FFS--KVRNVSSNIQYDS---TR--SSFL
QVRDSSQLKGSS-DQSRDHFDSIS----NEDSE------------YHTLINQREIQQLKERSIL-WD--PS
FLQTELTEIESD--RFPK--CLSGYS--SMSRLFA-EREKEMNNHLLPEEIEEF--LGNPTRSIRSFF
SDRW--SELH-LGSNPTERSTRDR----KLLKKEQDVS-FVPSRRSE----NKEIVNIFKIITYLQN--
TVSIHPILSDPGCDRVP-KDELD-MDSSNKISFLNKNPFLDL--FHLFHDRNRGGYTL--HHDF
ESE--ERFQEMADLFTLSITEP-DLVYHKGFTFSIDSYGLDQKQ--FLNEVLD---ESKKKSL---L
VLPPIFY--EENESFYRRIRKKWVR-ISCGNDLEDPKAKRVVFASNNIMEAVNQYRLIRNLIQI
QYSTYG--YIRNVLNRFFLMN---RPDRNFEYVIQ---RDQIGNDTLNHRTIMKYTINQHLSNL
KK-SQKKWFDP--LIWISRTERSMNRDPNAYRYKWSNGNKNFQEHLEHFISEPKSHF-HFQV
VFDRLRINQYSIDWSEVIDKKDLSKSLRFFLSKLLL---------FLS-------KFLLFLSNSLP-FFFV
SFGNIPIHRSE-IHIY-ELKGPNNQLCNQLLESIGLQIVHLKKWKPF--LLD---DHDTS-QKSKF
LINGGTISPFLFNKIP-KW-MIDSFHTR--NNRGKSFDKTDSYFSMISHDQDNWPNPVKPFHIS
SLISSFYKANRLRFVKNPHHFYFY--CNKRFPF---Y-VEKARINNYD--FTYG-QFLNILFIRNK-
IFSLYAGK--KKHAFLE-RDTISPIESQVSNIFIPNDFSESGDER-YNLYKS---FHFPIRSDPFVR--
RAIYSIADISGTPLTEEQIVNFEKTYCQPLSDMNLSDSERKNLHQYLN--FNSNMGLIHTPCS
EKYLPSEK--RKK--------RSLCLK--KCVEKGQMYRTFQRDSAFSTLSKWNLFKTYMPWFLT
STGYKYLNWRFLDTFSD-LLPILSSSS-QKFVSIFRDIMHGSDISWRILQKKW------GLPQWN
-LISDISSKCLHN--LLLSEEMVHRNNES--PLISTHLRSPNVREL-LYSILFLLLVAGY-LVRTHL
LVVSR--AYSELQTEFEKVKSLMIPSYMIELRKLLDRY---ELNSFWLKNLFLVALEQLGDSLE
EIRGSASGGNMLWGGGP-----AYG-VKSIRSKKKYLNIN---LIDLI-SIIPNPINRITFSRNTRHL
SHTSKEIYSLIRKRKNVNGDWIDDKIESLVANSDSIDDKEREFLVQFS--TLTT--EKRIDQILL
SLT-HSDHLS-----KNDSGYQMIEQPGAIYLRYLVDIH--KKYLMHYEFNTSCLAERRLFLAH

YQTITY--SQISCGANSFHFPSHG---KPFSLRLALSPP-RAILVIGSIGTGRSYLVKYLAENSYV
PFITVFLNKFLDNKPKGFLID-----DSDDIDLSDDIDR---------------------------DLDTELELLTR
MNAL---TMDMML---EIDRFYITLQFELAKAMSPCIIWIPNIHDLNVNESNYLSLGLLVNYLS
---RDCERCS-TRDILVIASTHIPKKVDPALIAPNKLNTCIKIRRLLIPQQRKHFFTLSYTRGFHL
EKKMFHT--NGFGSITMGSKVRDLVALTNEALSISITQKKSIIDTNIIRSALHRQTWDLR---SQ
VRSVQDHGILFYQIGRAVAQNVLLSNCTMDPISIYMKKK--SCNEGDSYLYKWYFELGTSM
KKLTILLYLLSCSAGSVAQDLWSLP--GPDEK-NGITYYGLVENDS-DLVHGLLEVEGALVG--
SSRTEKDCSQFDND--RVTLLLRPEPRN-PLDMMQNGSCSIVDQRFLYEK--YESEFEE-VEGE
GVLDPQ----QREED----LFNH--IVWAP--RIWRY---WGFLFACIERPNELGFP-YWARSFRGK---
RIIYDEEDELQENDSEFLQSGTMQYQIRDRSSKEQGFFRISQFIWDPADPLFFLFK-DQPFVS
VFSHREFFADEEMSKGLLTSQGDPPTSIYKRWFI--KNTQEKHFELLIHRQRWLR---TNSSLS-
-NGFFRSN--TLSESYQYLSNLFLSN-GTLLDQMTKTVLRKRWLFPDEMKIGF--M---------MP
RSR-INGNFIDKTFSIVANILLRIIPTTSGEKEAFTYYRDG--MSAQSEGNYAEALQNYYEAM
RLEIDPYDRSYILYNIGLIHTSNGEHTKALEYYFRALERNPFLPQAFNNMAVICHY-RGEQAI
RQGDSEIAEAWFDQAAEYWKQAIALTPGNYIEAQNWLKITRRFE--MSCRSERIWIEFIAGS
RKTSNLCWAFVLFLGSLGFLLVGTSSYLGQNLISLFPSQQIIFFPQGIVMSFYGIAGLFIGSYL
WCTISWNVGSGYDLFDKKEGIVCIFRWGFPGKNRRIFLRFFMKDIQSLRIEVKEGIYARRVL
YMEIRGQGAIPLTRTDEN-LTPREIEQKAGELAYFLRVPIEVF
>Gunnera
MEKCWFNSMLSLSKSMDSLGPIENTSGSEDPTINDTDKNIHNSSNVDLLFSVRDIKNLISD
DTFLVRDSNGDSYSIYFDMENHIFEVDNDH--SFLSELESSSYSYRNSSYLTNGSQSDDPYY
DRYMYDTKYSWNNHIISCIDVYLR--SQIRIDSSTLSGSDNYSYIYIYSYICGSESSSIRTSTNG
SDLIIRESSNDLDVTQKYRHLWIQCENCYGLNYKKFFKSKMNICEQCGYHLKMSSSERIEL
SIDPGTWDPMDEDMVSLDPIEFHSEEEPYKDRIDSYQITTGLTEAVQTGIGQLNGIPIAIGV
MDFQFMGGSMGSVVGEKITRLIEYATKRFLPLIIVCASGGARMQEGSLSLMQMAKISSALY
DYQLNKKLFYIAILTSPTTGGVTASFGMLGDIIIAEPNAYIAFAGKRVIEQTLNKTVPEGSQA
AEYLFHKGLFDPIVPRNPLKGVLSELFQLHAFFPLNQNSIKMVTLQADEISNIIRERIEQYTI
EVKIVNTGTVLQVGDGIARIHGLDEVMAGELVEFEEGTIGIALNLESNNVGVVLMGDGLM
IQEGSSVKATGRIAQIPVSEAYLGRVINALAKPIDGRGEISASESRLIESPAPGIISRRSVYEPL
QTGLIAIDSMIPIGRGQRELIIGDRQTGKTAVATDTILNQQGQNVICVYVAIGQKASSVAQV
VTTFQERGAMDYTIVVAEMADSPATLQYLAPYTGAALAEYFMYRERHTSIIYDDPSKQAQ
AYRQMSLLLRRPPGREAYPGDVFYLHSRLLERAAKSSSRLGEGSMTALPIVETQSGDVSAY
IPTNVISITDGQIFLSGDLFNAGIRPAINVGISVSRVGSAAQIKAMKKVAGKLKLELAQFAEL
EAFAQFASDLDKATQNQLARGQRLRELLKQSQSSPLTVEEQIMTIYTGTNGYLDSLEIGQV
SKFLVELRTYLKTNKPQFQEIISSTKILTEEAEDLLKEAIQEQMERFLLQ---MRINPTTSGPG
VSTLEKKNLGRIAQIIGPVLDVAFPPGKMPNIYNALVVKGRDTVGQQINVTCEVQQLLGN
NRVRAVAMSATDGLTRGMEVIDTGAPLSVPVGGATLGRIFNVLGEPVDNLGPVETRTTSPI
HRSAPAFIQLDTKLSIFETGIKVVDLLAPYRRGGKIGLFGGAGVGKTVLIMELINNIAKAHG
GVSVFGGVGERTREGNDLYMEMKESGVINEQNIAESKVALVYGQMNEPPGARMRVGLTA
LTMAEYFRDVNEQDVLLFIDNIFRFVQAGSEVSALLGRMPSAVGYQPTLSTEMGSLQERIT
STKEGSITSIQAVYVPADDLTDPAPATTFAHLDATTVLSRGLAAKGIYPAVDPLDSTSTMLQP
RIVGEEHYEIAQRVKQTLQRYKELQDIIAILGLDELSEEDRLTVARARKIERFLSQPFFVAEV
FTGSPGKYVGLTETIRGFQLILSGELDSLPEQAFYLVGNIDEATAKAINLELEMEESKMTLN
LCVLTPNKIVWDSEVKEIILSTNSGQIGVLPNHAPIATAVDIGILRIRLNDQWLTMALMGGF

ARIGNNEITVLVNDAEKGSDIDPQEAQQTLEIAEANLRKAEGKRQTIEANLALRRARTRVE
ALNVISMKNVTDSFVSLGHWPSAGSFGFNTDILATNPINLSVVLGVLIFFGKGVLSDLLDN
RKQRILNTIRNSEELRGGAIEQLEKAQARLRKVEIEADQFRVNGYSEIEREKLNLINSTYKT
LEQLENYKNETIHFEQQRAINQVRQRVFQQALQGALGTLNSRLNNELHLRTISANIGVFGA
MKEITDMNPLISAASVIAAGLAVGLASIGPGVGQGTAAGQAVEGIARQPEAEGKIRGTLLL
SLAFMEALTIYGLVVALALLFANPFVMNVLSCSINTLNVLYDISGVEVGQHFYWQIGGFQV
HAQVLITSWVVIAILLGSATIAVRNPQTIPTDGQNFFEYVLEFIRDVSKTQIGEEYGPWVPFI
GTMFLFIFVSNWSGALLPWKIIQLPHGELAAPTNDINTTVALALLTSVAYFYAGLTKKGLG
YFGKYIQPTPILLPINILEDFTKPLSLSFRLFGNILADELVVIVLVSLVPSVVPIPVMFLGLFTS
GIQALIFATLAAAYIGESMEGHHMIFLTLEHILTHISFSIVSIVITIYLITLLVNGIVELYDSSEK
GMIATFFCITGLLVTRWIFSGHFPLSDLYESLIFLSWSFSIINMVPY--KYQKTLSTITAPSAIFT
QGFATSGLLTEMHQSTILVPALQSQWLMMHVSMMVLSYAALLCGSLLSVALLVITFRKDI
MIFC--KETQYMNEGNNILRNTS---ARNYYRSQLIQQLDHWSYRVISIGFIFLTIGILSGAVWA
NEAWGSYWNWDPKETWAFITWTIFAIYLHTRTNKKFQGPNSAIVASMGFLIIWICYFGVNL
LGIGLHSYGSFILTMAKKKAFTPLLYLASIVFLPWWISLSFSKSLESWVTNWWNTSKSEFFL
NDIQEKSILEKFIEIEELFLLDEMIKEYPETHLQKLHI-IHKETIQLIKMHNEDRIHTILHFSTNI
ICFVILSGYSILVNDELVILNSWVQEFLYNLSDTIKAFSILFLTDFCIGFHSPPGWELIIGSVSK
DFGFYHNDQIISGLVCTFPVILDTIFKYLIFDYLSRVSPSLVVIYHSMNDMPIGVPRVPHRIPG
EEDAYWVDIYNRLYRNRIMFLGQEVSSEISNQLISLLVYLTIEEDRKDVFLFINSPGGWVVP
GVALYDTMRFVQPEIHTICMGMAASMGSFLLAGGEITKRIAFPHARVMIHQPASSFYETRT
GEFILESEELLKLREIITKVYVQRTGKPFWVISEDMERDVFMSATEAQAYGLIDIILIQMKEQ
KWIHEGLITESLPNGMFRVRLDNEDLILGYVSGRIRRSFIRILPGDRVKIEVSRYDSTRGRIIY
RLRNKDSGDMEEFKIYLE-DRFRQHYFLYPLIFQEYIYALAHDHGLFGYDNKYSSLIVKRLI
TRMYHQKYLISSANDSNQNPFFGNNLYSQLISEGLAVIVEIPFSLRLISYRIAKYHNLRSIHSI
FPFLEDKFSHLNYVSDILIPHPIHLEILVQTLRYWVKDAPSLHLVRFFLHEFRNWNSLITNKS
IYFFSQRLFLLLYNSHVCEYEYMFIFLRNQSYHLRSTSSEVLLERIYFYGKIEQLVGVFINDF
ILCMFKDPFMHYVRYQGKSILASKGTILMMNKWKYYFINFWQCHFYVWSQPGRIYINQL
YNHPLDLLGYLSSVRLSPSVLRSQMLENSFIIDTAI--KKFDTIIPIITLIGSLAKAKFCNVLGH
PISKPAWADSSDSDIIDRFGRICRNLSHYHSGSSKKNNLYRIKYILRLSCAKTLARKHKSTV
RAFLKRLGSELLEEFFTEEEHVLSLILPRRIWYLDIICINDLVIHEMIIDTT-EVQ-AINSFSRLE
N-LIELYGIIWMLVPIFTPVLGITIGVLVIVWLEREISAGIQQRIGPEYAGPLGILQAIADGTKL
LFKEDLLPSRGDTRLFSIGPSIAVISILLSYSVIPFSYHLVLADLSIGVFLWIAISSIAPIGLLISG
YGSNNKYSFLGGLRAAAQSISYEIPLTLCVLSISLLSNSSSTVDIVEAQSKYGFWGWNLWR
QPIGFIVFIISSLAECERLPFDLPEAEEELVAGYQTEYSGIKFGLFYVASYLNLLVSSLFVTVL
YLGGWNLSIPYIWISEFFEINKVGGVFITTIGIFITLAKTYLFLFISITTRWTLPRLRMDQLLN
LGWKFLLPISLGNLLLTTSFQLLSLMIWHVQNENFILDSTRIFMKAFHLLLFDGSFIFPECILI
FGLILLLMIDSTSDQKDIPWLYFISSTSLVMSITALLFRWREEPMISFSGNFQTNNFNEIFQFL
ILLCSTLCIPLSVEYIECTEMAITEFLLFVLTATLGGMFLCGANDLITIFVAPECFSLCSYLLSG
YTKKDVRSNEATTKYLLMGGASSSILVHGFSWLYGSSGGEIELQEIVNGLINTQMYNSPGI
SIALIFITVGIGFKLSPAPSHQWTPDVYEGSPTPVVAFLSVTSKVAASASATRIFDIPFYFSSNE
WHLLLEILAILSMILGNLIAITQTSMKRMLAYSSIGQIGYVIIGIIVGDSNDGYASMITYMLF
YISMNLGTFACIVSFGLRTGTDNIRDYAGLYTKDPFLALSLALCLLSLGGLPPLAGFFGKLH
LFWCGWQAGLYFLVSIGLLTSVVSIYYYLKIIKLLMTGRNQEITPHVRNYRRSPLRSNNSIE
LSMIVCVIASTIPGISMNPIIAIAQDTLFMFLLYEYDIFWAFLIISSIIPILAFLISGVLAPISKGP

EKLSSYESGIEPMGDAWLQFRIRYYMFALVFVVFDVETVFLYPWAMSLDVLGVSVFIEAFI
FVLILIVGSVYAWRKGALEWSTNHFPWLTIIVVFPISAGSLILFLPQRGNKVIRWYTLCICIL
ELLLTTYAFCYHFQLDDPLMQLSEDYKWINFFDFHWRLGIDGLSIGPILLTGFITTLATLSA
WPVTRDPRLFYFLMLAMYSGQIGSFSSQDLLLFFIMWELELIPVYLLLSMWGGKKRLYSA
TKFILYTAGGSIFLLMGVLGIGLYGSNKPTLNFETSANQSYPVALEIIFYIGFLIAFAVKLPIIP
LHTWLPDTHGEAHYSTCMLLAGILLKMGAYGLVRINMELLPHAHSIFSPWLMIVGTVQIIY
AASTSPGQRNLKKRIAYSSVSHMGFLIIGIGSLTDTGLNGAILQIISHGFIGAALFFLSGTSYD
RIRLVYLDEMGGIAIEMPKIFTMFSSFSMASLALPGMSGFVAELIVFFGIITSQKYLLIPKILIT
FVMAIGMILTPIYSLSMSRQMFYGYKLFNAPNSYFFDFGPRELFVSIFIFLPVIGIGIYPDFVL
SLSVDKVEAILSNYFYRMMLEHVLVLSAYLFSIGIYGLITSRNMVRALMCLELILNAVNINL
VTFSDFFDSRQLKGDIFSIFVIAIAAAEAAIGPAIVSSIYRNRKSIRINQSNLLNKMEHTYQYA
WIIPFIPLPVPMLIGVGLLLFPTSTKNLRRMWAFTSVLLLSIVLIFSIDLSIQQINSSSINQYVW
SWTINNDFSLEFGHFIDPLTSIMLLLITTVGIMVLIYSDNYMSHDQGYLRFFAYMSFSNTSM
LGLVTSSNLIQIYIFWELVGMCSYLLIGFWFTRPIAANACQKAFVTNRVGDFGLLLGILGIY
CITGSFEFRDFFKIFNNNEVNFLFITFCASLLFAGAVAKSAQFPLHVWLPDAMEGPTPISALI
HAATMVAAGIFLVARLLPLFILIPYIMNLISLIGIITLLLGATLALAQKDIKRGLAYSTMSQLG
YTMLALGMGSYQAALFHLITHAYSKALLFLGSGSIIHSMEAIVGYSPDKSQNMVLMGGLT
KHVPITKIAFLLGTLSLCGIPPLACFWSKDEILNDSWLYSPSFALIAYSTAGLTAFYMFRIYLL
TFEGHFNVNLKNYSGKKNNSLYSISIWGKEGSKTIQKKLLTLLTRNFFSFGNRGTYLYPHES
DNTMLFPLLLLVLFTLFIGFIGIPFYNNILLKWLTPSISLLHKNLE-SSDWYEFVTNSIFSVSIA
YLGIFIASLLYRPVYSSLKNLNLINSFAIFWDKIINGVYNWSYNRGYIDAFYTTYLIGGIRGL
AELTNFFDRRIIDGITNGVGITSFFVGEGIKYIGGG-RISSYLFLYLSYVSIFLLI-YMDLPGPIH
DFLLVFLGSGLILGGLGVVLFTNPIYSAFSLGLVLVCISLLYIPSNSHFVAAAQLLIYVGAINV
LIIFAVMFMNGSEYYKDFHFWTVGDGVTSLVCTSIFVSLITTISDTSWYGIIWTTRSNQIIEQ
DLISNIQQIGIHLLTDFFLPFELISIILLVALIGAISVARQMTVPATRKDLMIINMGPQHPSMHG
VLRLIVTLDGEDVIDCEPILGYLHRGMEKIAENRTIIQYLPYVTRWDYLATMFTEAITVNAP
EQLGNIQVPKRASYIRVIMLELSRIASHLLWLGPFMADIGAQTPFFYIFRERELIYDLFEAAT
GMRMMHNYFRIGGVAADLPHGWIDKCLDFCDYFLTGVAEYQKLITRNPIFLERVEGVGII
GGEEAINWGLSGPMLRASGIQWDLRKVDHYESYDEFDWEVQWQKEGDSLARYLVRIGE
MKESIKIIQQALEGIPGGPYENLEIRRFDRARDSEWNEFEYRFISKKPSPTFELSKQELYVRV
EAPKGELGIFMIGDQSVFPWRWKIRPPGFINLQILPQLVKRMKLADIMTILGSIDIIMGEVD
RMFPMVTGFMNYGQQTVRAARYIGQSFMITLSHANRLPVTIQYPYEKLITSERFRGRIHFE
FDKCIACEVCVRVCPIDLPVVDWKLETNIRKKQLLNYSIDFGICIFCGNCVEYCPTNCLSM
TEEYELSTYDRHELNYNQIALGRLPISVIDDYTIRTILNS-IKNG-----------MQGRLSAWLVKH
GLVHRSLGFDYQGIETLQIKPEDWHSVAVILYVYGYNYLRSQCAYDVAPGGLLASVYHITR
IQYGADQPEEVCIKVFAPRRNPRIPSVFWIWKSADFQERESYDMLGISYDNHPRLKRILMP
ESWIGWPLRKDYIAPNFYEIQDAHMGNEFRCIGCISIYRSLHFCAYPNCWFS--LCMAKRSV
GMVLAPEYSDNKKVKKNNIETVMNSIEFPLLDRTTQNSVISTTSNDLSNWSRLSSLWPLLY
GTSCCFIEFASLIGSRFDFDRYGLVPRSSPRQADLILTAGTVTMKMAPSLVRLYEQMPEPKY
VIAMGACTITGGMFSTDSYSTVRGVDKLIPVDVYLPGCPPKPEAVIDAITKLRKKISREIYE
DRIRSQQDNRCFTTNHKFHVERSTHTGNYDQGLLYQSPSTSEITSETFLKYKSSVSSHELVN
MQTKNTFSWIKEQITRSISVLFMIYIITRTSISNAYPIFAQQGYENPREATGRIVCANCHLAN
KPVDIEVPQAVLPDTVFEAVVRIPYDMQLKQVLANGKRGALNVGAVLILPEGFELAPPDRI
SPEMKEKIGKLSFQSYRPTKKNILVIGPVPGQKYSEITFPILSPDPATKKDVNFLKYPIYVGG

NRGRGQIYPDGSKSNNTVYNATAAGIVSKITRKEKGGYEITIADASDGRQVVDIIPPGPELL
VSEGESIKLDQPLTSNPNVGGFGQGDAEIVLQDPLRVQGLLFFLASVILAQIFLVLKKKQFE
KVQLSEMNFMSKVYDWFEERLEIQAIADDITSKYVPPHVNIFYCLGGITLTCFLVQVATGF
AMTFYYRPTVTEAFASVQYIMTEANFGWLIRSVHRWSASMMVLMMILHVFRVYLTGGFK
KPRELTWVTGVVLAVLTASFGVTGYSLPWDQIGYWAVKIVTGVPEAIPVIGSPVVELLRGS
ASVGQSTLTRFYSLHTFVLPLLTAVFMLMHFSMIRKQGISGPLMGVTKKPDLNDPVLRAK
LAKGMGHNYYGEPAWPNDLLYIFPVVILGTIACNVGLAVLEPSMIGEPADPFATPLEILPEW
YFFPVFQILRTVPNKLLGVLLMVSVPAGLLTVPFLENVNKFQNPFRRPVATTVFLIGTAAAL
WLGIGATLPIDKSLTLGLFMIEVFLFGIVLGLIPITLAGLFVTAYLQYRRGDQLDLMPTITSY
FGFLLAASTITSALFIGLSKIRLIMDIVSLAWAALMVVFTFSLSLVVWGRSGLMIIRSPEPEV
KILVDRDPVKTSFEEWARPGHFSRTIAKGPDTTTWIWNLHADAHDFDSHTSDLEEISRKVF
SAHFGQLSIIFLWLSGMYFHGARFSNYEAWLSDPTHIGPSAQVVWPIVGQEILNGDVGGGF
RGIQITSGFFQIWRASGITSELQLYCTAIGALVFAALMLFAGWFHYHKAAPKLAWFQDVES
MLNHHLAGLLGLGSLSWAGHQVHVSLPINQFLNAGVDPKEIPLPHEFILNRDLLAQLYPSF
AEGATPFFTLNWSKYAEFLTFRGGLDPVTGGLWLTDIAHHHLAIAILFLIAGHMYRTNWGI
GHGLKDILEAHKGPFTGQGHKGLYEILTTSWHAQLSLNLAMLGSLTIVVAHHMYSMPPYP
YLATDYGTQLSLFTHHMWIGGFLIVGAAAHAAIFMVRDYDPTTRYNDLLDRVLRHRDAII
SHLNWACIFLGFHSFGLYIHNDTMSALGRPQDMFSDTAIQLQPVFAQWIQNTHALAPGATA
PGATTSTSLTWGGGDLIAVGGKVALLPIPLGTADFLVHHIHAFTIHVTVLILLKGVLFARSSR
LIPDKANLGFRFPCDGPGRGGTCQVSAWDHVFLGLFWMYNAISVVIFHFSWKMQSDVW
GSISDQGIVTHITGGNFAQSSITINGWLRDFLWAQASQVIQSYGSSLSAYGLFFLGAHFVWA
FSLMFLFSGRGYWQELIESIVWAHNKLKVAPATQPRALSIVQGRAVGVTHYLLGGIATTWA
FFLARIIAVGMALRFPRFSQGLAQDPTTRRIWFGIATAHDFESHDDITEERLYQNIFASHFGQ
LAIIFLWTSGNLFHVAWQGNFESWVQDPLHVRPIAHAIWDPHFGQPAVEAFTRGGALGPV
NIAYSGVYQWWYTIGLRNNEDLYTGALFLLFLSAISLIGGWLHLQPKWKPSVSWFKNAES
RLNHHLSGLFGVSSLAWTGHLVHVAIPASRGEYVRWNNFLNVLPHPQGLGPLFTGQWNLY
AQNPDSGSHLFSTSQGAGTAILTLLGGFHPQTQSLWLTDIAHHHLAIAFIFLVAGHMYRTNF
GIGHSMKDLLEAHIPPGGRLGRGHKGLYDTINNSIHFQLGLALASLGVITSLVAQHMYSLP
AYAFIAQDFTTQAALYTHHQYIAGFIMTGAFAHGAIFFIRDYNPEQNEDNVLARMLDHKE
AIKSHLSWASLFLGFHTLGLYVHNDVMLAFGTPEKQILIEPIFAQWIQSAHGKTSYGFDVL
LSSTNGPAFNAGRSIWLPGWLNAINE--NSNSLFLTIGPGDFLVHHAIALGLHTTTLILVKGA
LDARGSKLMPDKKDFGYSFPCDGPGRGGTCDISAWDAFYLAVFWMLNTIGWVTFYWHW
KHITLWQGNVSQFNESSTYLMGWLRDYLWLNSSQLINGYNPFGMNSLSVWAWMFLFGH
LVWATGFMFLISWRGYWQELIETLAWAHERTPLANLIRWRDKPVALSIVQARLVGLAHFS
VGYIFTYAAFLIASTSGKFGMSHSVKIYDTCIGCTQCVRACPTDVLEMIPWDGCKAKQIAS
APRTEDCVGCKRCESACPTDFLSVRVYLWHETTRSMGLAYMIFLNLPSIFVPLVGLVFPAIA
MASLSIHIQKNKIVMQDLKTYLSVAPVLSTLWFGSLAGLLIEINRLFPDALTFPFFSFMTAIL
ERRESESLWGRFCNWITSTENRLYIGWFGVLMIPTLLTATSVFIIAFIAAPPVDIDGIREPVSG
SLLYGNNIISGAIIPTSAAIGLHFYPIWEAASVDEWLYNGGPYELIVLHFLLGVACYMGREW
ELSFRLGMRPWIAVAYSAPVAAATAVFLIYPIGQGSFSDGMPLGISGTFNFMIVFQAEHNIL
MHPFHMLGVAGVFGGSLFSAMHGSLVTSSLIRETTENESANEGYRFGQEEETYNIVAAHG
YFGRLIFQYASFNNSRSLHFFLAAWPVVGIWFTALGISTMAFNLNGFNFNQSVVDSQGRVI
NTWADIINRANLGMEVMHERNAHNFPLDLAAVEAPSTNG-GLPWYRVHTVVLNDPGRLL
SVHIMHTALVAGWAGSMTLYELAVFDPSDPVLDPMWRQGMFVIPFMTRLGITNSWGGWS

IT-GGAITNPGIWSYEGVAGAHIMFSGLCFLAAIWHWVYWDLEIFSDERTGKPSLDLPKIFG
IHLFLSGVACFGFGAFHVTGLYGPGIWVSDPYGLTGKVQPVNPAWGVEGFDPFVPGGIASH
HIAAGTLGILAGLFHLSVRPPQRLYKGLRMGNIETVLSSSIAAVFFAAFVVAGTMWYGSAT
TPIELFGPTRYQWDQGYFQQEIYRRIGAGLAENQSLSEVWSKIPEKLAFYDYIGNNPAKGG
LFRAGSMDNGDGIAIGWLGHPIFRDKEGRELFVRRMPTFFETFPVVLVDGDGIVRADVPF
RRAESKYSVEQVGVTVEFYGGELNGVSYSDPATVKKYARRAQLGEIFELDRATLKSDGVF
RSSPRGWFTFGHASFALLFFFGHIWHGARTLFRDVFAGIDPDLDTQVEFGAFQKLGDPTTK
RQVV------------VETLFNGTLALAGRDQETTGFAWWAGNARLINLSGKLLGAHVAHAGLIV
FWAGAMNLFEVAHFVPEKPMYEQGLILLPHLATLGWGVGPGGEVIDTFPYFVSGVLHLIS
SAVLGFGGIYHALLGPETLEESFPFFGYVWKDRNKMTTILGIHLILLGIGAFLLVFKALFFG
GVYDTWAPGGGDVRKITNLTLSPSVIFGYLLKSPFGGEGWIVSVDDLEDIIGGHVWLGSICI
LGGIWHILTKPFAWARRALVWSGEAYLSYSLGALSVFGFIACCFVWFNNTAYPSEFYGPTG
PEASQAQAFTFLVRDQRLGANVGSAQGPTGLGKYLMRSPTGEVIFGGETMRFWDLRAPW
LEPLRGPNGLDLSRLKKDIQPWQERRSAEYMTHAPLGSLNSVGGVATEINAVNYVSPRSW
LATSHFVLGFFLFVGHLWHAGRARAAAAGFEKGIDRDFEPVLSMTPLNMTIALGRFTKDE
KDLFDTMDDWLRRDRFVFVGWSGLLLFPCAYFALGGWFTGTTFVTSWYTHGLASSYLE
GCNFLTAAVSTPANSLAHSLLLLWGPEAQGDFTRWCQLGGLWTFVALHGAFGLIGFMLRQ
FELARSVQLRPYNAIAFSGPIAVFVSVFLIYPLGQSGWFFAPSFGVAAIFRFILFFQGFHNWT
LNPFHMMGVAGVLGAALLCAIHGATVENTLFEDGDGANTFRAFNPTQAEETYSMVTANR
FWSQIFGVAFSNKRWLHFFMLFVPVTGLWMSALGVVGLALNLRAYDFVSQEIRAAEDPEF
ETFYTKNILLNEGIRAWMAAQDQPHENLIFPEEVLPRGNALMSGNTGERSFADIITSIRYW
VIHSITIPSLFIAGWLFVSTGLAYDVFGSPRPNEYFTENRQGIPLITGRFDPLEQLDEFSRSFM
T I D R T F P I F T V R W L A V H G L A V P T V S F L G S I S A M Q F I Q
R??MLTLKLFVY
TVVIFFVSLFIFGFLSNDPGRNPGREEMADTTGRIPLWLIGTVTGIPVIGLIGIFFYGSYSGLG
SSLMLNILSLICICLNSTLYSSSFFLGKLPEAYAFLNPIVDFMPVIPLLFFLLAFVWQAAVSFR
TTQSNPNEQSVELNRTSLYWGLLLIFVLAVLFSNYFFNMEVNILAFIATALFILVPTAFLLIIY
VKTVSQNDMETATLVAISISGLLVSFTGYALYTAFGQPSQQLRDPFEEHGDMEALVYTFLLV
STLGIIFFAIFFREPPKVPTKKIKMTIAFQLAVFALIATSLILLISVPVVFASPDGWSSNKNVVF
SGTSLWIGLVFLVGILNSLIS-------MSPQTETKAGVGFKAGVKDYKLTYYTPDYETKDTDIL
AAFRMTPQPGVPPEEAGAAVAAESSTGTWTTVWTDGLTSLDRYKGRCYGIEPVAGEENQ
YIAYVAYPLDLFEEGSVTNMFTSIVGNVFGFKALRALRLEDLRIPPAYVKTFQGPPHGIQVE
RDKLNKYGRPLLGCTIKPKLGLSAKNYGRAVYECLRGGLDFTKDDENVNSQPFMRWRDR
FLFCAEAIYKAQAETGEIKGHYLNATAGTCEEMIKRAVFARELGVPIVMHDYLTGGFTANT
SLAFYCRDNGLLLHIHRAMHAVIDRQKNHGIHFRVLAKALRMSGGDHIHSGTVVGKLEG
EREITLGFVDLLRDDFIEKDRSRGIYFTQDWVSLPGVLPVASGGIHVWHMPALTEIFGDDS
VLQFGGGTLGHPWGNAPGAVANRVALEACVQARNEGRDLAREGNEIIREASKWSPELAA
ACEVWKEIKFEFEAMDTL--MAIHLYKTSTPSTRNGAVDSQVKSNPRNNLIYGQNRCGKGR
NARGIITARHRGGGHKRLYRKIDFRRNAKDIYGRIVTIEYDPNRNAYICLIHYGDGEKRYIL
HPRGAIIGDTIVSGTEVPIKMGNALPLTDMPLGTAIHNIEITLGKGGQLARAAGAVAKLIAK
EGKSAKLKLPSGEVRLISKNCSATVGQVGNVGVNQKNLGRAGSKCWLGKRPVVRGVVM
NPVDHPHGGGEGRAPIGRKKPTTPWGYPALGRRSRKRNKYSDNLIVHRRSKMIQPQTHL
NVADNSGARELMCIRIIGAS-NRRYAHIGDVIVAVIKEAIPNMPLQRSEVIRAVIVRTCKELK
RDNGMIIRYDDNAAVVIDQEGNPKGTRVFGAIARELRQLNFTKIVSLAPEVLMLSPKRTRF

RKQHRGRMKGISYRGNRICFGRYALQALEPTWITSRQIEAGRRAITRHARRGGKIWVRIFP
DKPITVRPAETRMGSGKGSPEYWVAVVKPGRILYEMGGVTENAAREAISLAASKMPIRTQF
IISGMTRIRRGYIARRRRTKIRLFVSSFRGAHSRLTRTITQQKIRALVSAHRDRDKQKRNFRR
LWITRINAVIRD-NRVSYSYSRLINDLYKRRLLLNRKILAQIAISNRNCLHMISNEIIKIK-------
---MINVYALGQHICISANKARRVIDQIRGRSYEETLMILELMPYRASYPIFKLIYSAVANATH
NRGFNEANLFISKMEVNEGTTVKKLKPRARGRSYPIKRSTCHIKIVLTDISLYGGVWDKK
MDGIKYSVFTDKSIRLLVKN-QYTSNVESGSTRTEIKHWVELFFGVKVIAMNSHRLPGKGR
RMGPIMGHTMHYRRMIITLQPGYSIPPVRKKRTMAVPKKRTSLSKKRIRKNIWKKKGYWA
ALKAFSLAKSLSSGNSKSFF--LRKKMS--KDVRVTVMLECISCVQNG--GDKESKGVSRYIT
EKNRHNTPGRLELKKFCPFCSKQTIHVEIKKMKIRASVRKICEKCRLIRRRGRIIVICSNPRH
KQRQGMVREKITVSTRTLQWKCVESRTDSKRLYYGRFIMSPLMKGQADTIGIAMRRALL
GEIEGTCITRAKSE--KIPHEYSTIVGIQESVHEILMNLKEIILKSNIYGTRDASICVRGPGYVT
AQDIISPPSVEIVDNTQHIASLTEPIDLCIQLQIEKNRGYRIKTPNTYQDGSY--PIDAVFMPVR
NANHSIHSYGNGKEKQEILFLEIWTNGSLTPKEALHEASRNLIDLFIPFLHAEEE--NLHLED
NQ-YKVTLPFFTFHDRLA-KLRKNKK-EIALKSIFIDQSELPSRTYNCLKSSNIHTLLDLLNNS
QEDLMKIKHFSIEDVKQILDILEKQ-------FHN-MLRDGN-EEMSTIPGFSQIQFEGFCRFIDQ
GLTEELYKFPKIEDTDQEIEFQLFVETYQLVEPLIKERDAVYESITYSSELYVSAGLIWKTSR
DMQEQTIFLGNIPIMNSLGTSIVNGIYRIVINQILQSPGIYYRSELDHNGISVYTGTIISDWGG
RLELEIDRKARIWARVSRKQKISILVLSSAMGSNLREILDNACYPEIFLSFLNDKEKKKIGSK
ENAILEFYQQFSCVGGDPVFSESLCKELQKKFFQQRCELGRIGRRNMNQRLNLDIPQNNTF
LLPRDILAAADHLIGMRFGMGTLDD--MNHLKNKRIRSVADLLQDQLGLALARLENVVRG
TICGAIR-HKLI-PTPQNLVTSTP----LTTTYESFFGLHPLSQVLDRTNPLTQIVHGRKLSYLGP
GGLTGRTASFRIRDIHPSHYGRICPIDTSEGINVGLIGSLAIHARIGHWG-SLESPFY---EISERS
KK--VRMLYLSPSRDEYYMVAAGNSLALNQGIQEEQVVPARYRQEFLTIAWEQVHLRSIFP
FQYFSIGASLIPFIEHNDANRALMSSNMQRQAVPLSRSEKCIVGTGLERQAALDSGVSAIAE
HEGKIIYTDTDKIILSGNGDTLSIPLVMYERSNKNTCMHQKPQVQRGNCIKKGQILADGAA
TVGGELTLGKNLLVAYMPWEGYNSEDAVLISERLVYGDIYTSFHIRKYEIQTHVTSQG-PER
ITNEIPHLEAYLLRNLDKNGIVMLGSWVETGDILVGKLTPQMAKE-SSYAPEDRLLRAILGI
QVSTSKETCLKLPIGGRGRVIDVRWIQKKR-GSSYNPEMIRVYISQKREIKVGDKVAGRHG
NKGIISKILPRQDMPYLQDGRPVDMVFNPLGVPSRMNVGQIFECSLGLAGGLLDRHYRIA
PFDERYEQEASRKLVFSELYEASKQTANPWVFEPEYPGKSRIFDGRTGDPFEQPVIIGKSYIL
KLIHQVDDKIHGRSSGHYALVTQQPLRGRAKQGGQRVGEMEVWALEGFGVAHILQEMLT
YKSDHIRARQEVLGTTIIGGTIPNPEDAPESFRLLVRELRSLALELNHFLVSEKNFQINRKEA
-------MIDQYKHQQLRIGSVSPQQISAWANKILPNGEIVGEVTKPYTFHYKTNKPEKDGLFC
ERIFGPIKSGICACGNYRVIGDKKENPKFCEQCGVEFVDSRIRRYQMGYIKLACPVTHVWY
LKRLPSYIANLLDKPLKELEGLVYCD--FSFARP-IAKKPTFLRLRGSFEYEIQSWKYSIPLFF
TTQGFDTFRNREISTGAGAIREQLSDLDLRIIIDYSLVEWKELGEE-GTTGNEWEDRKIGRR
KDFLVRRMELAKHFIRTNVEPERMVLCLLPVLPPELRPIIQIDGGKLMSSDINELYRRVIYR
NNTLTDLLTTSRSTPGELVMCQEKLVQEAVDTLLDNGIRGQPMRDGHNKVYKSFSDVIEG
KEGRFRETLLGKRVDYSGRSVIVVGPSLSLHRCGLPREIAIELFQTFVIRGLIRQHVASNIGV
AKSKIREKEPIVWEILQEVMQGHPVLLNRAPTLHRLGIQAFQPILVEGRAICLHPLVRKGFN
ADFDGDQMAVHVPLSLEAQAEARLLMFSHMNLLSPAIGDPISVPTQDMLIGLYVLTSGNR
RGICANRYNPCNRRN---YKNKRIADNK--YTKEKEPFFCNSYDAIGAYRQKIINLDSPLWLR
WRLDQR-VIAS--REVPIEVHYESLGTYHEIYGHYLIVRSVKKEILCIYIRTTVGHISFYREIEE

AIQGFCRACSYGI--------MEVLMAERANLVFHNKVIDGTAMKRLISRLIDHFGMAYTSHIL
DQVKTLGFQQATATSISLGIDDLLTIPSKGWLVQDAEQQSFILEKNHQYGNVHAVEKLRQSI
EIWYATSEYLRQEMNPNFRMTDPFNPVHIMSFSGARGNASQVHQLVGMRGLMSDPQGQ
MIDLPIQSNLREGLSLTEYIISCYGARKGVVDTAVRTSDAGYLTRRLVEVVQHIVVRRTDCG
TVRGISVSPRNGMMP-ERIFIQTLIGRVLADDIYIGPRCIATRNQDIGIGLVNRFIT------FRTQP
ISIRTPFTCRSTSWICRLCYGRSPTHGDLVELGEAVGIIAGQSIGEPGTQLTLRTFHTGGVFTG
GTAEHVRAPYNGKIKFNEDLVHPTRTRHGHPAFLCYIDLYVTIESEDIIHNVNIPPKSFLLVQ
NDQYVESEQVIAEIRAGTST-LNFKERVRKHIYSDSEGE-MHWSTDVYHVPEYTYGNVHLL
PKTSHLWILSGGPCR--SSVVSFSLHKDQDQMNVH--SLSVEQRYISNFS--AINNQ-VRHKLF
SSDLYGKKEGSIPDY-SELNRIICTGHCNFIYP-AILHENF--YLLAKRRRNRFIIPFQSIQEREK
ELMPRSGISIEIP--INGIFRRNSILAYFDDFRYRRTGSGITKYGTIEMHSIIKKED--LIEYRG-V
KEFRPKYIKVDRFFFIPEEVHILPGSSYVMVRNNSIIGVDTQITFNTRSRVGGLVRVEKKKK
SIELKIFSGNIHFPGETDKISRHSGILIPPGT--GKTNSKESKE--LKNWIYVQR--ITPTK--KKYF
VLVRPVITYEITDGINLATLFPQDLLQERDNVQFRVVNYILYGNGNQIRGISDTSIQLVRTCL
VLNWDQDKK---NSSIEEAPASFVEVRTNGLIRDFLRIDLVKSPISYTGKRNDPAGSGLISD-N
GSDRTNINPFYSIYSKGR-IQQSLSQ--NKNQGTIRTLLNR---NKECQFFLILSSSNCSRMGP--F
NGVKYKNV---IKESDPLIPIRDSLGPLGT--ALKIANFYS--FYHL--ITHNHIL-VTKYLQLDN---
LKQTFQVLT---YYLMDEN--GRIYNPDLCSNIILNTFNLNWYFLHHNFREETS-TIISLGQFIC
ENVCIAKNGPH-LK--SGQVIIVQ-VGSVVIRSAKPYLATPGTTVHGHYGEILYEGDTLVTFIY
EKSRSGDITQGLPKVEQVLEVRSIDSISLNLEKRIEGWNNRITRILGIPWGFLIGAELTIVQSR
ISLVNKIQKVYRSQGVQIHNRHIEIIVRQITSKVLVSE---DGMSNVFSPGELIGLLRAERTGR
ALEEAICYRAILLGITRASLNTQSFISEASFQETARVLAKAALRGRVDWLKGLKENVVLGG
MIPVGTGFKGL--VY----YNNIP--LEPKKKNLFEGEMR--DILFHHRELFDSFILKNLHDTSEQ
SFMG------FNDCMTKRYWNINLEEMMEAGVHFGHGTRKWNPRMAPYISAKRKGIHITNL
TRTARFLSEACDLVFDAASRGKQFLIVGTKNKAADSVAWAAIRARCHYVNKKWLGGMST
NWSTTETRLHKFRDLRAEQKTGRLNRLQKRDAAVLKRQLSHLETYLGGIKYMTWLPDIV
IIVDQQEEYTALRECVTLGIPTICLIDTNCDPDIADISIPANDDAIASIRLILNKLVFAICEGRSS
-YIRNPMGQKINPLGFRLGTTQGHHSLWFAQPKNYSEGLQEDQKIRNCIKNYIQKNIKISSG
-VEGIARIEIKKRIDLIQVIIYMGFPKLLIEGRPQ----GVEELQINVQKEFNSVNKKLNIAITRIE
KPYGNPNILAEFIAGQLKNRVSFRKAIKKAIELTEQADTKGIQVQIAGRIDGKEIARVEWIR
EGRVPLQTIRAKIDYCSYTVRTIYGVLGIKIWIFVDEEMSRYRGPRFKKIRRLGALPGLTSK
RPRAGSDLRNQSRSVK-RSQYRIRLEEKQKLRFHYGLTERQLLKYVRIAGKAKGSTGQVL
LQLLEMRLDNILFRLGMASTIPGARQLVNHRHILVNGRIVDIPSYRCKPRDIITARDEEKSR
ALIQNFLDSSTH-AELPKHLTRHPFQ--YKGLVNQIIDSKWVSLKINELLVVEYYSRQIMSRR
GTAEEKTAKSDPIYRNRLVNMLVNRILKHGKKSLAYQIIYRAMKKIQQKTETNPLSVLRQA
IRGVTPDIAVKARRVGGSTHQVPIEIGSTQGKALAIRWLLGASRKRPGRNMAFKLSSELVD
AAKGSGDAIRKKEETHRMAEANRAFAHFRMGKDTIANIITSIRNADMDRKGTVRIAFTNIT
ENFVKILLQEGFIENVRKHQERNKYFLVLTLRHRR--NRKGPYRT-ILN--LKRISRPGLRIYSN
YQRIPRILGGMGIVILSTSRGIMTDREARLERIGGEILCYIWMAKPIPRIGSRRNGRIGSRKN
ARRIPKGVIHVQASFNNTIVTVTDIRGRVVSWSSAGTCGFSGTRRGTPFAAQTAAGNAIRT
VVDQGMQRAEVMIKGPGLGRDAALRAIRRSGILLSFVRDVTPMPHNGCRPPKKRRVMPTI
KQLIRNTRQPIRNVTKSPALRGCPQRRGTCTRVYTITPKKPNSALRKVARVRLTSGFEITAYI
PGIGHNSQEHSVVLVRGGRVKDLPGVRYHIVRGTLDAVGVKDRQQGRSKYGVKKPKMA
RKSLIQREKKRQKLEQKYHLIRRSSKKEIS---KVQSLNDKWEIHGKLQSPPRNSAPTRLHR

RCFSTGRPRANYRDFELSGHILREMVDACLLPGATRSSWMAKNSFISVISQKEKEK--NTGS
IEFQVLSFTNKIRRLTSHLKLHRKDYLSQRGLRKILGKRQRMLTYLSKKNRVRYKELIDQL
DIRDTKTR--MVKLRLKRCGRKQRAVYRIVAIDVRSRREGRDLQKVGFYDPIKNQTYLNVP
AILYFLEKGAQPTGTVYDISKKAEVFKELRVNQTKCN-MDKSKRPFLKSKRSFRRRLPPIQS
GDRIDYRNMSLISRFISEQGKILSRRVNRLTLKQQRLITIAIKQARILSSLPFLNNEK--QFERI
ESTARTTGLRTRNKVTRSLKKNPFVANQLLRKIDKLNTKAEKEIIVTWSRTSTIIPTMIGHTI
AIHNGKEHLPIYITDRMVGHKLGEFAPTLNFRGHAKNDNKSRRMILKSFLLSNLVSLCMKI
INSVVVVGLYYGFLTTFSIGPSYLLLLRAQVMEEG---TENKVSATTGFITGQLMMFISIYYAP
LHLALGRPHTITVLALPYLLFHFFSNNHKNFFYYGSTSRNSMRNFSIQCVFLNNLIIQLFNL
FLLPSSMLARLVNIYMFRCNNKMLFVISSFVGWLIGHILFMKWVGLVLVWIRQNRS----IRS
NKYLVSELRN--SRIVSILLFITCVYYLGRIPSPIFTKKLKETEEDKPLVTLLFDYKRWNRPLR
YIR-YEFENTLKNEMSQYFFYTCRSNGKERISFMYPPSLSNFFEMIQKKMFLYTKETFLSDE
RYNHWVYTNEKKRNNLSNEFINRIKALDKRPIFLDIFEKITR--LNNNKKKYLPKTYDPFLA
-PYRGTINKLSSPIEIASINKIYSILYQEFEQKDISNI-----EISKKVPLWTHKFIEFEQIEREEIRS
RKVKHAAYVSDNVIDFETESDFRRDIIKGSMRAQRRKTVIFKLFQSNAHSPLFLDR-TDPLF
YFDSFGLTKLIS---RNKESNEQKIKIEEWDNIIFGHVIRSCILLIQSILRKYMIFPLLIIIKNIGRI
LLFQVAEWGEDFADLKKEIHVISNKSGVELSEKEVRITSFLNGIQIKILFPFCLKPWHKYKD
ENTTKKKKNKVKKMTAERPFGFPQLEPSFFGPIFNELK-----------------KKIFQYILMVSNKIK
KWVINGILFIKIIIMKGLSQVNPIDSIISNQMINYP----YIQILSTDQKNYLLTEKQIKDSAEIIR
TIKN----------KIERKDKKIESPKNIWQILK--NVRVIHKSHYFLKLFIEKIYIDILISIINSIIDIPRI
N-----HDLSYLSQAYVFYKLSQNQVSNLS---FVLQYTSFFLKNEIKDYLTHGIFHSELQHNKI
HNYEMNQWKNWLRVHYQYDFSLIRWSRLVIQKRQNKVNQCRTVQNKNKWDSYEKDR--
LIHYEKQ-NLKKHYIYNLLSYKYLNINLDRPDRKYFGFGILNFCLR--------------FLP-EFRQL
YNAYKIKPWVLPIKVLFFDKHINIETEPDFLLKKYLLFQLAWY------IFNNVKAYYILFRAIE
AREISIAYIKNGQVSLDLMR-IHDHLTLNELVKRLLLIDPIRLSINNDRQIIMYQTIGIALVHK
NKHNNYDFLVPENILSSRRRRELR--ILSVFLWPNYRLEDLACMNRYWFNTNNGSRFSMLR
IHMYPMKGHQFKFWIFELREILREIKNSHSFLNSWTQFNSVGSFIHIFFHQERFIKLFDPRIL
SILLS-RNS--QGSTSNRYFT---IKGVLLFVVA-VLIYR----INNRNMVERKNLYLIGLLP-----IS
MNSIGPRNDTLEESF-GSSNINRLIVS-LLYLPKVKNISESCFLDPKESTLVLPIT----KKCIMPE
SNWGSRWWRNWIGKKR--DSSCKISN---ETVAGIEISFKEKDIK-YLEFIFVYYMD--DPIRKD
HDWELFDRLSPRKRRNIINL--NSGELFEILVKHWICYLMSAFREKIPIEVEG-FFKQQG-AGS
TIQSNDIEHVSH--LFSRNKWAISLQNCAQFHMWQFRQDLFV---SWGK-NPHESDFLRN-VS
RENWIWLDNAWLVNKDR---FFS--KVRNVSSNIQYDS---TR--SSFVQVTDSSQLKGSS-DQS
RDHFDSIS----NEDSE------------YHTLINQRE---LKERSIL-WD--LSFLQTERTEIESD--RFPK--
CLSGYS--SRSRLFA-EREKQMNNHLLPEEIEEFL--GNPTRSIRSFFSDRW--SELH-LGSNPTE
RSTRDQ----KLLKKQQDVS-FVPSRRSE----NKEMVNIFKIITYLQN--TASIHPISSDPGSGMV
P-KDEPD-MGSSNKISFLNKNPFFYL--FHLFHDRNRGGYTL--DQDFESE--ERFQEMADLFT
LSITEP-DLLYHKGFAFSIDSYGLDQKQ--FLNEVFNSRDESKNKSL---LVLPPIFY--EENESFY
RRIRKEWVR-ISCRNDLEDPKQKIVVFASNNIMEAVNQYRLIRNLIQIQYSTYG--YIRNVLN
RFFLMN---RSDRNFEYGIQ---RDQIGNDTLNHRTIMKYTINQHLSNLKK-SQKKWFDP--LIFI
SRTERSMNRDPDAYRYKWSNGSKNLQEHLEHFVSEQKSR---FQVVFDRLRINQYSIDWSE
VIDKKDLSKSLRFFLSKL----------------------LFFLSN-SLPFFFVSFGNIPIHRSE-IHIY-ELKGP
NDQLCNQLLESISLQIVHLKKLKPF--LLD---DHDTS-QKSKFLINGGTISPFLFNKIP-KW-MI
DSFHTR--NNRRKSFDNTDSYLSMISHDQDNWLNPVKPFHRSSLISSFYKANRLRFLNNPH

HFCFY--CNKRFPFYV----EKARINNYD--FTYG-QFLNILFIRNK-IFSLCVGK--KKHAFLE-RD
TISPIESQVSSIFIPNDFPQSGDET-YNLYKS---FHFPIRSDPFVR--RAIYSIADISGTPLTEGQIV
NFERTYCQPLSDMNLSDSERKNLHQHFN--FNSNMGLIHTPCSEKYLP--SEKREK--------RS
LCLN--KCVEKGQMYRTFQRDSAFSTLSKWNLFQTYMPWFLTSTGYKYLNLIFLDTFSDLL
P-ILSRSI-KKFVSIFHDIMHGSDILWRILQEKL------CLPEWNP-ISKILSKCLHKLKLLLLEEM
IHRNNES--PLISTHLRPPNVREF-LYSILFLLLVAGY-LVWTHLIFVSISAASSELQTEFEKVKS
LMIPSYMIELRKLLDRYPTSELNSFWLKNLFLVALEQLGDSLEEIWGSAFGGNMLLGGGP--
---TYG-VKSIRSKKKYLNINLIDIIDLI-SIIPNPINRITFSRNTRHLSHTSKELYSLIRKRKKVK
GAWIYDKIESWVANSDSIDDEEREFLVQFS--TLTT--EKRIDQILLSMT-QSDHLS-----KNDSG
YQMIEQPGAIYLRYLVDIH--KKYLMNYEFNTSCLAERRIFLAHYQTITY--SQTSCGANSFN
FPSHG---KPFSLRLALSPS-RGILVIGSIGTGRSYLVKYLATNSYVPFITVFLNKFLNNKLKGFI
FDDSDSDDIDDIDDSDDIDASDDIDASDDID-----------DNDDIDRDLDPELELLMDALTI-TM
DMMP---EIDLFYITLQFELAKAMSPCIIWIPNIHDLDVNESNYLSLGLLVNYLS---RDCERCS
-TRNILVIASTHIPQKVDPALIAPNKLNTCIKIRRLLIPQQRKHFFILSYTRGFHLEKKMFHT--
NGFGSITMGSNARYLAVLTNEALSISITQKKSIIDTNTIRSALHRQTWDLR---SQVRSAQDH
GILFYQIGRAVAQNVLLSNCLIDPISIYIKKK--SCNEGDSYLYKWYFELGTSMKKLTILLYLL
SCSAGSVAQDLWSLP--GPDEK-NGITSYGLVENDS-DLVHGLLEVEGALVG--SSRTEKDCS
QFDND--RVTLVLRPEPRN-PLDMMQNGSCSIVDQRFIYEK--YESEFEE-GEGEGALDPQ----
QIEED----LFNH--IVWAP--RIWRP---WGFLFDCIERPNELGFP-YWARSFRGK---RIIYDEEDE
LQENDSEFLQSGTMQYQTLDRSSKEQGFFRISQFIWDPADSLFFLFK-DQPFVSVFSHREFF
ADEEMSKGFLTSQTDPPTSIYKRWFI--KNTQEKHFELLIYRQRWLRT---NSSLS--NGAFRSN
--TPSESYQYLSNLFLSN-GTLLDQMTKTLLRKRWLFPDEMKIGF--M---------MPRSR-INGN
FIDKTFSIVANILLRIIPTTSGEKEAFTYYRDG--MSAQSEGNYAEALQNYYEAMRLEIDPYD
RSYILYNIGLIHTSNGEHTKALEYYFRALERNPFLPQAFNNMAVICHY-RGEQAIRQGDSEI
AEAWFDQAAEYWKQAIALTPGNYIEAHNWLKITRRFE--MNWRSERIWIELITGSRKISNFC
WAFILFLGSLGFLLVGTSSYLGRNLIYLFPSQQILFFPQGIVMSFYGIAGLFISSYLWCTISWN
IGSGYDRFDRKEGIVCIFRWGFPGKNRRIFLRFLMKDIQSIRIEVKEGIYARRVLYMEIRGQG
AIPLTPTDEN-FTPREIEKKAAELAYFLRVPIEVF
>Heuchera
MEKRRFNSMLSLSKSMDSLGPIENTCGSEDPIITDTYKKIPRYNNIDHLFGVRDIRNFISDDT
FLVKDSNGNSYSIYFDIENQIFEIDNDH--SFLSEVESSFYSYRNSSYLNHVSKGDDPHYDRY
MYDTKYSWNNHINSCIDSYLR--SQIHIDSYILSNSDNYG--YIYSYICGNKNSSIRTSTNGSD
LTK--SSNDLEVTQKYRHLWVQCENCYGLNYKKFFKSKMHICEQCGYHLKMSSSDRIELSI
DPGTWDPLDEDMVSLDPIEFHSEEEPYKERIDSYQRKTGLTEAVQTGTGQLNGIPIAIGVM
DFQFMGGSMGSVVGEKITRLVEYATNKFLPLILVCASGGARMQEGSLSLMQMAKISSALY
DYQSNKKLFYVSILTSPTTGGVTASFGMLGDIIIAEPNAYIAFAGKRVIEQTLNKTVPEGSQ
AAEYLFHKGLFDPIVPRNLLKGVLNELFQLHAFFPLKKNAIKMVTIRADEISNIIRERIEQY
NREVKIVNTGTVLQVGDGIARIHGLDEVMAGELVEFEEGTIGIALNLESNNVGVVLMGDG
LMIQEGSSVKATGRIAQIPVSEAYLGRVINALAKPIDGRGEIPASESRLIESPAPGIISRRSVYE
PLQTGLIAIDAMIPIGRGQRELIIGDRQTGKTAVATDTILNQQGQNVICVYVAIGQKASSVAQ
VVTNFQERGAMEYTIVVAETADSPATLQYLAPYTGAALAEYFMYRERHTSIIYDDLSKQA
QAYRQMSLLLRRPPGREAYPGDVFYLHSRLLERAAKSSSRLGEGSMTALPIVETQSGDVSA
YIPTNVISITDGQIFLSADLFNSGIRPAINVGISVSRVGSAAQIKAMKQVAGKSKLELAQFAE
LEAFAQFASDLDKATQNQLARGQRLRELLKQSQSAPLTVEEQIMTIYTGTNGYLDSLEIGQ

VRKFLVELRTYLKTNKPQFQEIISSTKTFTEEAEALLKEAIQEQMERFLLQEQAMRINPTTS
DPGVSTLEKKNLGRIVQIIGPVLDVAFPPGKMPNIYNALVVKGRDTVGQEINVTCEVQQLL
GNNRVRAVAMSATDGLRRGMDVIDTGAALSVPVGGATLGRIFNVLGEPVDNLGPVDTRT
TSPIHRSAPAFIQLDTKLSIFETGIKVVDLLAPYRRGGKIGLFGGAGVGKTVLIMELINNIAK
AHGGVSVFGGVGERTREGNDLYMEMKESGVINEQNIAESKVALVYGQMNEPPGARMRV
GLTALTMAEYFRDVNEQDVLLFIDNIFRFVQAGSEVSALLGRMPSAVGYQPTLSTEMGTL
QERITSTKEGSITSIQAVYVPADDLTDPAPATTFAHLDATTVLSRGLAAKGIYPAVDPLDSTS
TMLQPRIVGEEHYEIAQRVKQTLQRYKELQDIIAILGLDELSEEDRLTVARARKIERFLSQPF
FVAEVFTGSPGKYVGLAETIRGFQLILSGELDSLPEQAFYLVGNIDEVTAKAMNLEMESNL
KKMTLNLCVLTPNRIVWDSEVKEIILSTNSGQIGVLPNHAPIATAVDIGILRIRLNDQWLTM
ALMGGFARIGNNEITILVNDAEKGSDIDPQEAQQTLEIAETNLKKAEGKRQTIEANLALRR
ARTRVEAINVISMKNVTDSFVSLGHWPSAGSFGFNTDILATNLINLSVVLGVLIFFGKGVLS
DLLDNRKQRILNTIRNSEELRGGAIEQLEKARARLRKVEMEADQFRVNGYSEIEREKLNLI
NSTYKTLEQLENYKNETINFEQQRAINQVRQRVFQQALQGALGTLNSCLNNELHLRTISA
NIGMFGAMKEITDMNPLISAASVIAAGLAVGLASIGPGVGQGTAAGQAVEGIARQPEAEG
KIRGTLLLSLAFMEALTIYGLVVALALLFANPFVMNVLSCSITLN-GLYDISGVEVGQHFYW
QIGGLQVHAQVLITSWVVIAILLGSATIAVRNPQTIPTDGQNFFEYVLEFIRDVSRTQIGEEY
GTWVPFIGTMFLFIFVSNWSGALLPWKIIQLPHGELAAPTNDINTTVALALLTSVAYFYAGL
SKKGLGYFGKYIQPTPILLPINILEDFTKPLSLSFRLFGNILADELVVVVLVSLVPLVVPIPVM
FLGLFTSGIQALIFATLAAAYIGESMEGHHMIFSTFEHILTHISFSIVSILITIYFITLLVDEIVGL
DDSSEKGMIATFFCITGLLIARWIYSRHFPLSDLYESLIFLSWSFSIIHMVPYF-KHKNHLSAIT
APSAIFTQGFATSGLLTEMHQSAILVPALQSQWLMMHVSMMVLGYAALLCGSLLSVALLVI
TFRKDIRIFGKSNEIQYINKRSNVLRTTSFLSDRNYYKYQLIQQLDHWGYRVISLGFIFLTIGI
LSGAVWANEAWGSYWNWDPKETWAFITWTIFAIYLHTRTNTNLQGANSAIVASMGFLIIW
ICYFGVNLLGIGLHSYGSFNI-MAKKKAFTPLLYLASIVFLPWWISLLFNKSLESWVTNWW
NTRQSETFLNDIQEKSILEKFIELEELFLLNEMIKEYPETHLQKLSLG-HKETIQLIKMHNED
RIHTILHFSTNLICFVILSGYSILGNEELVILNSWVQEFLYNLSDTIKAFSILLLTDLCIGFHSP
HGWELMLGSVYKDFGFAHNDQIISGLVSTFPVILDTILKYWIFRYLNRVSPSLVVIYHSMN
DMPIGVPKVPFRSPGEEDASWVDVYNRLYRERLLFLGQEVDSEISNQLIGLMVYLSIEDDT
KDLYLFINSPGGWVIPGVAIYDTMQFVRPDVHTICMGLAASMGSFILVGGEITKRLAFPHA
RVMIHQPASSFYEAQTGEFILEAEELLKLRETITRVYVQRTGKPLWVVSEDMERDVFMSAT
EAQAHGIVDLVAVEMKEQKWVHEGLITESLPNGMFRVRLDNEDLILGYVSGRIRRSFIRILP
GDRVKIEVSRYDSTRGRIIYRLRNKDSNDMGEFQGYLE-DKFRQHDFLYPLIFQEYIYALAH
DHVLFGYDNKSSSLIVKRLITRMCQQNHFIISANDSNQNQFFGKNLYSQMISEGFAVIVEIPF
SLRFVSSLLVKSHNLRSIHSIFPFLEDNFLHLNYVSDILIPHPIHLEILVQTLRYWVKDASSLH
LLRFFLYEYQNWNSLITKKSISIVSQRLFLFLYNSYVCEYESIFIFLCNQSSHLRSTSSGTLFE
RIHFYGKIKHVVEVFSNDFILWLFKDPFMHYVRYQGKSILASKGTPLLLNKWKYYLVNLW
QCNFYVWSQPGRIHINQLSNHSLDFLGYLSSVQLNPSVVRSQMLENSLIMDNAI--KKLDII
VPIIPLIRSLAKAKFCNVVGDPISKPAWADSSDSDIIDRFVRICRNISHYHSGSSKKKSLYRIK
YILRLSCARTLARKHKSTVRAFLKRLGSELLEEFLTEDEQVLSLIFPRRVWYLDIICINDLVN
HEMIID-TTEVQ-DINSFSRLES-LKEVYGIIWMLIPILTLVLGITIGVLVIVWLEREISAGIQQRI
GPEYAGPLGILQALADGTKLLFKENILPSRGDTGLFSIGPSIAVISILLSYSVIPFGYHLVLAD
LNIGVFLWIAISSIAPIGLLMSGYGSNNKYSFLGGLRAAAQSISYEIPLTLCVLSISLLSNSLS
TVDIVEAQSKYGFWGWNLWRQPIGFIVFLISSLAECERLPFDLPEAEEELVAGYQTEYSGIK

FGLFYVASYLNLLVSSFFVTVLYLGGWNLSIPYLFVPELFEINKASGVFGTTLGLFITLAKT
YLFLFIPITTRWTLPRLRMDQLLNLGWKFLLPISLGNLLLTTSSQLLSLMIWHVQNENFILD
STRIFMKAFHLLLFDGSFIFPECILIFGLILLLMIDSTSDQKDIPWLYFISSTSLVMSITALLFR
WREEPMISFSGNFQTNNFNEIFQFLILLCSTLCIPLSVEYIECTEMAITEFLLFVLTATLGGMF
LCGANDLITIFVAPECFSLCSYLLSGYTKKDVRSNEATTKYLLMGGASSSILVHGFSWLYGS
SGGEMELQEIVNGLINTQMYNSPGISIALIFITVGIGFKLSPAPSHQWTPDVYEGSPTPVVAF
LSVTSKVAASASATRIFDIPFYFSSNEWHLLLEILAILSMILGNLIAITQTSMKRMLAYSSIGQ
IGYVIIGIIVGDSNGGYASMITYMLFYISMNLGTFACIVSFGLRTGTDNIRDYAGLYTKDPFL
ALSLALCLLSLGGLPPLAGFFGKLHLFWCGWQAGLYFLVSIGLLTSVVSIYYYLKIIKLLMT
GRNQEITPHVRNYRRSPLRSNNSIELSMIVCVIASTIPGISMNPIIAIAQDTLFMFLLYEYDIF
WAFLIISSLIPILAFLISGVLAPISKGPEKLSSYESGIEPMGDAWVQFRIRYYMFALVFVVFDV
ETVFLYPWAMSFDVLGVSAFIEALIFVLILIVGSVYAWRKGALEWSTNYFPWLTIIVVLPIFA
GSLIFFLPHRGNKIIRWYTICICILELLLTTYAFGYHFQLDDPLIQFSEDYKWINFFDFYWRL
GIDGLSIGPILLTGFITTLATLAGRPVTRDSRLFHFLMLAMYSGQIGSFSSRDLLLFFIMWEL
ELIPVYLLLSMWGGKKRLYSATKFILYTAGGSIFLLMGVLGIGLYGSNEPTLNFETLANQSY
PIALEILFYLVFLIAFAVKSPIIPLHTWLPDTHGEAHYSTCMLLAGILLKMGAYGLIRINMEL
LPRAHSIFSPWLLIVGTIQIIYAASTSSGQRNLKKRIAYSSVSHMGFIIIGISSLTDTGLNGAIL
QIISHGFIGAALFFLAGTSYDRIRLVYLDEMGGIAIPMPKIFTMFSILSMASLALPGMSGFVA
ELLVFFGIITSQKYLLMPKIIITCVMAIGMILTPIYSLSMSRQMFYGYKLFNAPNSYFFDSGP
RELFVSISIFLPVIGIGIYPDFVLSLSVDKVETILSNFFYRMMLEHVLILSAYLFSIGIYGLITSR
NMVRALMCLELILNAVNINFVTFSDFFDNRQLRGDIFSIFVIAIAAAEAAIGLAIVSSIYRNR
NSIRINQSNLLNKMEHTYPYAWIIPFVPLPVPVLIGVGLLLFPTATKNLRRMWAFPSVLLLSI
VMIFSADLSIQQINSNSIYQYVWSWTINNDFSLEFGHLIDPLTSIMLILITTVGIMVLIYSDNY
MSHDQGYLRFFAYMSFFNTSMLGLVTSSNLIQIYIFWELVGMCSYLLIGFWFTRPIAANAC
QKAFVTNRIGDFGLFLGILGLYWITGSFEFRDLFEIFNNNELNLLFVTLCAFLLFSGAVAKS
AQFPLHVWLPDAMEGPTPISALIHAATMVAAGIFLVARLLPLFIVIPYIMNLISLISIITVLLGA
TLALAQKDIKRSLAYSTMSQLGYTMLALGMGSYRAALFHLITHAYSKALLFLGSGSIIHSM
EAIVGYSPDKSQNMVLMGGLTKHVPITKTAFFLGTLSLCGIPPLACFWSKDEILNDTWLYS
PIFAIIACSTAGLTAFYMFRIYLLTFEGHLNVHFQNYSGKKTSSFYSISLWGKEGPKTTKTKL
FTLLTMNSFSTWNKKTFSYPHESDNTMLFSLLVLVLFTLFVGAIGIPFDLDILSKWLTPSINL
LHKNLT-SVDWYAFVTNAIFSVSIAYFGILIASLLYKPVYSSLQNLNLINSFVIFWDKIIYVIY
GWSYNRGYIDAFYATSFTKGIRGLAELTHFFDRRVIDGITNGVGITSFFVAEGIKYVGGG-RI
SSYLFLYLSYVSIFLLSYYMDLPGPIHDFLLVFLGSALILGGLGVVLLTNPIYSAFSLGLVLV
CISLFYISSNSHFVAAAQLLIYVGAINVLIIFAVMFMNGSGSYKDFTLWTVGDGVTSVVCTS
IFVSLITIIPDTSWYGIIWTTRSNQIIEQDLISNSQQIGIHLSTDFFLPFELISIILLVALIGAITVA
RQMNVPATRKDLMIVNMGPHHPSMHGVLRLIITLDGEDVIDCEPILGYLHRGMEKIAENR
TIIQYLPYVTRWDYLATMFTEAITVNAPEQLGNIQVPKRASYIRVIMLELSRIASHLLWLGP
FMADIGAQTPFFYIFKERELVYDLFEAATGMRMMHNYFRIGGVAVDLPHGWIDKCLDFCD
YFLTGIAEYQKLITRNPIFLERVEGVGIISGEEAINWGLSGPMLRASGIQWDLRKVDHYECY
DEFDWEVQWQKEGDSLARYLVRISEMTESIKIIQQALEGIPGGPYENLEIRSFDRVRDPEW
NDFEYRFISKKPSPTFELSKQELYVRVEAPKGELGIFLIGDQSVFPWRWKIRPPGFINLQILP
QLVKRMKLADIMTILGSIDIIMGEVDRMFPMVTGFMNYGQQTVRAARYIGQSFMITLSHA
NRLPVTIQYPYEKLITSERFRGRIHFEFDKCIACEVCVRVCPIDLPVVDWKLETDIRKKRLL
NYSIDFGICIFCGNCVEYCPTNCLSMTEEYELSTYDRHELNYNQIALGRLPMPVIDDYTIRT

ILNS-IKNG-----------MQGRLSAWLVKHGLVHRSLGFDYQGIETLQIKPEDWHSIAVILYVYG
YNYLRSQCAYDVAPGGLLASVYHLTRIEYAVDQPEEVCIKVFAPRRNPRIPSVFWVWKSA
DFQERESYDMLGISYDNHPRLKRILMPESWIGWPLRKDYIAPNFYEIQDAH-------------------
-----------MAKGSIGMVLAPEYSDNKKKKGTKKKIETVMNSIEFPLLDRTTQNSVISTTSNDL
SNWSRLSSLWPLLYGTSCCFIEFASLIGSRFDFDRYGLVPRSSPRQADLILTAGTVTMKMAP
SLVRLYEQMPEPKYVIAMGACTITGGMFSTDSYSTVRGVDKLIPVDVYLPGCPPKPEAVID
AITKLRKKISREIYEDRIRSQRGNRCFTTNHKFHVGRSTHTGNYDQGLLYQSPSTPEIPPETF
FKYKSSVSSHELVNMQTRNTFSWTKEQITRSISVSLMIYIITRTYISNAYPIFAQQGYENPRE
ATGRIVCANCHLASKPVDIEVPQTVLPDTVFEAVVRIPYDMQLKQVLANGKKGGLNVGA
VLILPEGFELAPPDRISPEMKEKIGNLSFQSYRPTKKNILVIGPVPGQKYSEITFPILSPDPASK
KDVHFLKYPIYVGGNRGRGQIYPDGSKSNNTVYNATAAGIVNKIIRKEKGGYEITIADPSD
GRQVVDIIPPGPELLVSEGESIKLDQPLTSNPNVGGFGQGDAEIVLQDPLRVQGLLFFLASVI
LAQIFLVLKKKQFEKVQLSEMNFMSKVYDWFEERLEIQAIADDITSKYVPPHVNIFYCLGG
ITLTCFLVQVATGFAMTFYYRPTVTEAFASVQYIMTEANFGWLIRSVHRWSASMMVLMMI
LHVFRVYLTGGFKKPRELTWVTGVVLGVLTASFGVTGYSLPWDQIGYWAVKIVTGVPDAI
PVIGSPLVELLRGSASVGQSTLTRFYSLHTFVLPLLTAVFMLMHFPMIRKQGISGPLMGVTK
KPDLNDPVLRAKLAKGMGHNYYGEPAWPNDLLYIFPVVILGTIACNVGLAVLEPSMIGEP
ADPFATPLEILPEWYFFPVFQILRTVPNKLLGVLLMVSVPAGLLTVPFLENVNKFQNPFRRP
VATTVFLIGTAVALWLGIGATLPIDKSLTLGLFMIEVFLFGIVLGLIPITLAGLFVTAYLQYRR
GDQLDLMPTITSYFGFLLAASTITSALFIGLSKIRLIMDIVSLAWAALMVVFTFSLSLVVWG
RSGLMIIRSPEPEVKILVDRDPIKTSFEEWARPGHFSRTIAKGPETTTWIWNLHADAHDFDS
HTSDLEEISRKVFSAHFGQLSIIFLWLSGMYFHGARFSNYEAWLSDPTHIGPSAQVVWPIV
GQEILNGDVGGGFRGIQITSGFFQIWRASGITSELQLYCTAIGALVFAALMLFAGWFHYHK
AAPKLAWFQDVESMLNHHLAGLLGLGSLSWAGHQVHVSLPINQFLNAGVDPKEIPLPHEF
ILNRDLLAQLYPSFAEGATPFFTLNWSKYAEFLTFRGGLDPVTGGLWLTDIAHHHLAIAILF
LIAGHMYRTNWGIGHGLKDILEAHKGPFTGQGHKGLYEILTTSWHAQLSLNLAMLGSLTI
VVAHHMYSMPPYPYLATDYGTVLSLFTHHMWIGGFLIVGAAAHAAIFMVRDYDPTTRYN
DLLDRVLRHRDAIISHLNWACIFLGFHSFGLYIHNDTMSALGRPQDMFSDTAIQLQPVFAQ
WIQNTHALAPGATAPGATTSTSLTWGGGDLVAVGGKVALLPIPLGTADFLVHHIHAFTIHVT
VLILLKGVLFARSSRLIPDKANLGFRFPCDGPGRGGTCQVSAWDHVFLGLFWMYNAISVV
IFHFSWKMQSDVWGSISDQGVVTHITGGNFAQSSITINGWLRDFLWAQASQVIQSYGSSLS
AYGLFFLGAHFVWAFSLMFLFSGRGYWQELIESIVWAHNKLKVAPATQPRALSIVQGRAV
GVTHYLLGGIATTWAFFLARIIAVGMALRFPRFSQGLAQDPTTRRIWFGIATAHDFESHDDI
TEERLYQNIFASHFGQLAIIFLWTSGNLFHVAWQGNFESWVQDPLHVRPIAHAIWDPHFGQ
PAVEAFTRGGALGPVNIAYSGVYQWWYTIGLRTNEDLYTGALFLLFLSAVSLIAGWLHLQ
PKWKPSVSWFKNAESRLNHHLSGLFGVSSLAWTGHLVHVAIPGSRGEYVRWNNFLDVLP
HPQGLGPLFTGQWNLYAQNPDSSSHLFGTSQGAGTAILTLLGGFHPQTQSLWLTDMAHHH
LAIAFIFLVAGHMYRTNFGIGHSMKDLLEAHVPPGGRLGRGHKGLYDTINNSIHFQLGLAL
ASLGVVTSLVAQHMYSLPAYAFIAQDFTTQAALYTHHQYIAGFIMTGAFAHGAIFFIRDYN
PEQNEDNVLARMLDHKEAIISHLSWASLFLGFHTLGLYVHNDVMLAFGTPEKQILIEPIFA
QWIQSAHGKTSYGFDVLLSSTNGPAFNAGRSIWLPGWLNAINE--NSNSLFLTIGPGDFLVH
HAIALGLHTTTLILVKGALDARGSKLMPDKKDFGYSFPCDGPGRGGTCDISAWDAFYLAV
FWMLNTIGWVTFYWHWKHITLWQGNVSQFNESSTYLMGWLRDYLWLNSSQLINGYNPF
GMNSLSVWAWMFLFGHLVWATGFMFLISWRGYWQELIETLAWAHERTPLANLIRWRDKP

VALSIVQARLVGLAHFSVGYIFTYAAFLIASTSGKFGMSHSVKIYDTCIGCTQCVRACPTDV
LEMIPWDGCKAKQIASAPRTEDCVGCKRCESACPTDFLSVRVYLWHETTRSMGLAYMTT
FNLPSIFVPLVGLVFPAIAMASLSLHVQRNRIFMRDLKTYLSVAPVLSTLWFGALAGLLIEIN
RLFPDALTFPFFSFMTAILERRESESLWGRFCNWITSTENRLYIGWFGVLMIPTLLTATSVFII
AFIAAPPVDIDGIREPVSGSLLYGNNIISGAIIPTSAAIGLHFYPIWEAASVDEWLYNGGPYE
LIVLHFLLGVACYMGREWELSFRLGMRPWIAVAYSAPVAAATAVFLIYPIGQGSFSDGMPL
GISGTFNFMIVFQAEHNILMHPFHMLGVAGVFGGSLFSAMHGSLVTSSLIRETTENESANE
GYRFGQEEETYNIVAAHGYFGRLIFQYASFNNSRSLHFFLAAWPVVGIWFTALGISTMAFN
LNGFNFNQSVVDSQGRVINTWADIINRANLGMEVMHERNAHNFPLDLAAVEAPSTNGMG
LPWYRVHTVVLNDPGRLLAVHIMHTALVAGWAGSMALYELAVFDPSDPVLDPMWRQGM
FVIPFMTRLGITNSWGGWSIT-GGTITNPGIWSYEGVAGAHIVFSGLCFLAAIWHWVYWDL
EIFSDERTGKPSLDLPKIFGIHLFLSGVACFGFGAFHVTGLYGPGIWVSDPYGLTGKVQSVN
PAWGVEGFDPFVPGGIASHHIAAGTLGILAGLFHLSVRPPQRLYKGLRMGNIETVLSSSIAA
VFFAAFVVAGTMWYGSATTPIELFGPTRYQWDQGYFQQEIYRRISAGLAENQSLSEAWSKI
PEKLAFYDYIGNNPAKGGLFRAGSMDNGDGIAVGWLGHPVFRDKEGRELFVRRMPTFFE
TFPVVLVDGDGIVRADVPFRRAESKYSVEQVGVTVEFYGGELNGVSYSDPATVKKYARR
AQLGEIFELDRATLKSDGVFRSSPRGWFTFGHASFALLFFFGHIWHGARTLFRDVFAGIDP
DLDAQVEFGAFQKLGDPTTRRQVV------------VETLFNGTLALAGRDQETTGFAWWAGNA
RLINLSGKLLGAHVAHAGLIVFWAGAMNLFEVAHFVPEKPMYEQGLILLPHLATLGWGV
GPGGEVIDTFPYFVSGVLHLISSAVLGFGGIYHALLGPETLEESFPFFGYVWKDRNKMTTIL
GIHLILLGLGAFLLVFKALYFGGIYDTWAPGGGDVRKITNLTLSPSVIFGYLLKSPFGGEGW
IVSVDDLEDIIGGHVWLGSICILGGIWHILTKPFAWARRALVWSGEAYLSYSLAALSIFGFIA
CCFVWFNNTAYPSEFYGPTGPEASQAQAFTFLVRDQRLGANVGSAQGPTGLGKYLMRSP
TGEVIFGGETMRFWDLRAPWLEPLRGPNGLDLSRLKKDIQPWQERRSAEYMTHAPLGSL
NSVGGVATEINAVNYVSPRSWLSTSHFVLGFFLFVGHLWHAGRARAAAAGFEKGIDRDFE
PVLSMTPLNMTIALGKYTKDENDLFDIMDDWLRRDRFVFVGWSGLLLFPCAYFALGGWF
TGTTFVTSWYTHGLASSYLEGCNFLTAAVSTPANSLAHSLLLLWGPEAQGDFTRWCQLGG
LWTFVALHGAFGLIGFMLRQFELARSVQLRPYNAIAFSGPIAVFVSVFLIYPLGQSGWFFAP
SFGVAAIFRFILFFQGFHNWTLNPFHMMGVAGVLGAALLCAIHGATVENTLFEDGDGANT
FRAFNPTQAEETYSMVTANRFWSQIFGVAFSNKRWLHFFMLFVPVTGLWMSALGVVGLA
LNLRAYDFVSQEIRAAEDPEFETFYTKNILLNEGIRAWMAAQDQPHENLIFPEEVLPRGNA
LMSGSTGERSFADIITSIRYWVIHSITIPSLFIAGWLFVSTGLAYDVFGSPRPNEYFTESRQGI
PLITGRFDSLEQLDEFSRSFMTIDRTYPIFTVRWLAIHGLAVPTVSFLGSISAMQFIQRMATQ
TVESSSRSG-PRQTNVGNLLKPLNSEYGKVAPGWGTTPLMGVAMALFAIFLSIILEIYNSSV
LLDGISMNMLTLKLFVYTVVIFFVSLFIFGFLSNDPGRNPGREEMADTTGRIPLWLIGTVAG
ILVIGLIGIFFYGSYSGLGSSLMLNIFSLIGICLNSALY-SSFFFAKLPEAYAFLNPIVDFMPVIPL
LFFLLAFVWQAAVSFRTTQSNPNEQSVELNRTSLYWGLLLIFVLAVLFSNYFFNMEVNILA
FIATALFILVPTAFLLIIYVKTVSQNNMETATLVAISISGLLVSFTGYALYTAFGQPSQQLRDPF
EEHGDMEALVYTFLLVSTLGIIFFAIFFREPPKVPTKK--MTIAFQLAVFALIATSSILLISVPVV
FASPDGWSSKKNVVFSGTSLWIGLVFLVGILNSLIS-------MSPQTETKASVGFKAGVKDYK
LTYYTPDYETKDTDILAAFRVTPQPGVPPEEAGAAVAAESSTGTWTTVWTDGLTNLDRYK
GRCYHIEPVAGEESQFIAYVAYPLDLFEEGSVTNMFTSIVGNVFGFKALRALRLEDLRIPPA
YVKTFQGPPHGIQVERDKLNKYGRPLLGCTIKPKLGLSAKNYGRAVYECLRGGLDFTKD
DENVNSQPFMRWRDRFLFCAEALYKAQAETGEIKGHYLNATAGTCEEMIKRAVFARELG

VPIVMHDYLTGGFTANTSLSFYCRDNGLLLHIHRAMHAVIDRQKNHGIHFRVLAKALRMS
GGDHIHSGTVVGKLEGEREITLGFVDLLRDDFIEKDRSRGIYFTQDWVSLPGVLPVASGGI
HVWHMPALTEIFGDDSVLQFGGGTLGHPWGNAPGAVANRVALEACVQARNEGRDLARE
GNEIIREACKWSPELAAACEVWKEIKFEFEAMDTL--MAIHLYKTSTPSTRNGAVDSQVKS
NPRNNLIYGQHHCGKGRNARGIITAGHRGGGHKRLYRKIDFRRNKKDIYGRIVTIEYDPNR
NAYICLIHYGDGEKRYILHPRGAIIGDTIVSGTEVPIKMGNALPLTDMPLGTAIHNIEITLGK
GGQLARAAGAVAKLIAKEGKSATLKLPSGEVRLISKNCSATVGQVGNVGVNQKSLGRAG
SKRWLGKRPVVRGVVMNPVDHPHGGGEGRAPIGRKRPTTPWGYPALGRRSRKKNKYSD
NLILRRRSKMIQPQTYLNVADNSGARELMCIRIIGAS-NRRYAHIGDVIVAVIKEAVPNMPLK
RSEVIRAVIVRTCKELKRDNGMIIRYDDNAAVVIDQEGNPKGTRVFGAIARELRRLNFTKIV
SLAPEVLMLSPKRTRFRKQHRGRMKGISYRGNHICFGRYALQALEPAWITSRQIEAGRRA
MTRNARRGGKIWVRIFPDKPVTVRPAETRMGSGKGSPEYWVAVVKPGRILYEMGGVTENI
ARKAISIAASKMPIRTQFIIS-MTRIRRGYVARKRRTKIRLFASSFRGAHSRLTRTITQQKIRAL
VSAHRDRDRQKRDFRRLWITRINAVIRGS-KVSYSYSRLIHDLYKRKLLLNRKILAQIAILN
RNCLYMISNEIIREGDCKESTGKIMLKEVYALGQHICMSAHKARRVVDQIRGRSYEETLMI
LELMPYRACYPIFKLVYSAAANASHNMGFNEADLIISKAEVNEGTTVKKLKPRARGRGYP
IKRSTCHITIVLKNTSL--------MDGIKYAVFTDKSIRLLRKN-QYTSNVESGSTRTEIKHWVEL
FFGVKVIAMNSHRLPGKGRRMGPIMGHTMHYRRMIITLQPGYSIPPLRKKRTMAVPKKRT
SMSKKRIRKNYWKKKGYWAALKAFSLSKSLSTGNSKSFF----PTMAKSKDVRVTVILECTS
CVRNGVN--KESMGISRYITQKNRHNTPSRLELRKFCPYCYKHTIHGEIKKMKIRASVRKIC
EKCRLIRRRGRIIVICSNPRHKQRQGMVREKVTVSTRTLEWKCVESRADSKRLYYGRFILS
PLMKGQADTIGIAMRRALLGEIEGTCITRAKSE--KIPHEYSTIVGIQESVHEILMNLKEVVL
RSNLYGTRNASICVSGPGYVTAQDIISPPSVEIVDNTQHIASLTEPIDLCIGLQIEKNRGYRIK
TPN--NDGSY--PIDAVFMPVRNANHSIHSYGNGNEKQEILFLEIWTNGSLTPKEALHEASRN
LIDLFIPFLHAEEE--NLHLEDNQ-HKVTLPLFTFHDRLA-KLRKNKK--IALKYIFIDQSELPP
RIYTCLKRSNIHTLLDLLNNSQEDLMKIEHFRIEDVKEILGFLEKH--FEIDLPKNKMLRNGN
-EGMSTIPGFNQIQFEGFCRFIDQGLTEELYKFPKIEDTDQEIEFQLFVETYQLVEPLIKERDA
VYESLTYSSELYVSAGLIWKTSRDMQEQTIFIGNIPLMNSLGTSIVNGIYRIVINQILQSPGIY
YRSELDHNGISVYTGTIISDWGGRLELEIDRKARIWARVSRKQKISILVLSSAMGSNLREILE
NVCYPEIFLSFLNDKEKKKIGSKENAILEFYQQFACVGGDPVFSESVCKELQKKFFQQRCE
LGRVGRRNMNRRLNLDIPQNNTFLLPRDILAAADHLIGMKFGMGTLDD--MNHLKNKRIR
SVADLLQDQFGLALVRLENVVRGTIGGAIR-HKLI-PTPQNLVTSTP----LTTTYESFFGLHPL
SQVLDRTNPLTQIVHARKLSYLGPGGLTGRTASFRIRDIHSSHYGRICPIDTSEGINVGLIGSL
AIHARIGHWG-SLESPFY---EISESERSKKVRMLYLSPSRDEYYMIAAGNSLALNQGIQEEQ
VVPARYRQEFLSIAWEQVHLRSIFPFQYFSIGASLIPFIEHNDANRALMSSNMQRQAVPLSR
SEKCIVGTGLERQAALDSGVPAIAEHEGKIIYTDTDKIILSGNGNTLSIPLIMYERSNKNTC
MHQKPQVHRGKCIKKGQILADGAAIVGGELALGKNVLVAYMPWEGYNSEDAVLISERLV
YGDIYTSFHIRKYEIQTHVTSQG-PERITNEIPHLEAHLLRNLDKNGIVRLGSWVETGDILVG
KLTPQMAKE-SSYAPEDRLLRAILGIQISTSKETCLKLPIGGKGRVIDVRWIQKKG-GSSYNP
ETIRVYISQKREIKVGDKVAGRHGNKGIISKILPRQDMPYLQDGRPVDMVFNPLGVPSRMN
VGQIFECSLGLAGSLLDRHYRIAPFDERYEQEASRKLVFSELYEASKQTANPWVFEPEYPG
KSRIFDGRTGDPFEQPVIIGKPYILKLIHQVDDKIHGRSSGHYALVTQQPLRGRAKQGGQRV
GEMEVWALEGFGVAHILQEMLTYKSDHIRARQEVLGTTIIGGTIPNPEDAPESFRLLVRELR
SLALELNHFLVSEKNFQINRKEAMNQNFSSMIDRYKHQQLRIGLVSPQQISAWANKILPNG

EIVGEVTKPYTFHYKTNKPEKDGLFCERIFGPIKSGICACGNYRVIGDEKEDPKFCEQCGVE
FVDSRVRRYQMGYIKLACPVTHVWYLKRLPSYIANLLDKPLKELEGLVYCDF--SFARP-VA
KKPTFLRLRGLFEYEIQSWKYSIPLFFTTQGFDTFRNREISTGAGAIREQLADLDLRLIIDSSL
VEWKELGEE-GPTGNEWEDRKIGRRKDFLVRRMELAKHFIRTNIEPEWMVLCLLPVLPPE
LRPIIQIDGGKLMSSDINELYRRVIYRNNTLNDLLTTSRSTPGELVMCQEKLVQEAVDTLLD
NGIRGQPMRDGHNKVYKSFSDVIEGKEGRFRETLLGKRVDYSGRSVIVVGPSLSLHRCGL
PREIAIELFQTFVIRGLIRQHLASNIGVAKSKIREKEPIVWEILQEVMQGHPVLLNRAPTLHR
LGIQAFQPVLVEGRAICLHPLVRKGFNADFDGDQMAVHVPLSLEAQAEARLLMFSHMNL
LSPAIGDPISVPTQDMLIGLYVLTRGNRRGICANRYNPRNC---INYQNERIRTNNYKYTKE-E
PFFCNSYDAIGAYRQKRINLDSPLWLRWRLDQR-VIAS--REAPVEVHYESLGTYHEIYGHY
LIVRSVKKEIICIYIRTTVGHISLYREIEEAIQGFCRACSYRT--------MEVLMAERANLVFHNK
VIDGTAMKRLISRLIDHFGMAYTSHILDQVKTLGFQQATATSISLGIDDLLTIPSKGWLVQD
AEQQSLILEKHHHYGNVHAVEKLRQSIEIWYATSEYLRQEMNPNFRMTDPFNPVHMMSFS
GARGNASQVHQLVGMRGLMSDPQGQMIDLPIQSNLREGLSLTEYIISCYGARKGVVDTAV
RTSDAGYLTRRLVEVVQHIVVRRTDCGTSRGISVSPQNGMMP-ERIFIQTLIGRVLADDIYM
GSRCIAIRNQDIGIGLVNRFIT------FRAQPISIRTPFTCRSTSWICRLCYGRSPTHGDLVELGE
AVGIIAGQSIGEPGTQLTLRTFHTGGVFTGGTAEHVRAPSNGKIKFNEDLVHPTRTRHGHPA
FLCSIDLYVTIESEDIIHNVNIPPKSFILVKNDQYVESEQVIAEIRAGTST-FNFKERVRKHIYS
DSQGE-MHWSTDVYHAPEFTYGNVHLLPKTSHLWILSGSSCR--SNVVPFSLHKDQDQLNV
H--SLSVERKYISNFS--VTNDQ-VRHKLFSSDRSGK---RIPDY-SELNRIICTDHCNFIYP-AILH
ENY--DLLSKRRKNRLIIPFQSIQEREKEQMPHSGISIEIP--INGIFRKNSIMAYFDDPRYRRNS
SGITKYGTIEVHSIVKKED--LIEYRG-GKEFKPRYMQVDRFFFIPEEVHILPGSSSIMVRNNSI
IGVSTRITLNKRSRVGGLVRMERKKRGIELKIFSGDIYFAGETDKIARHSGILIPPGT--GKTN
SKESKK--LKNWLYVQR--ITPTK--KKYFVLVRPVVTYEIADGINLATLFPEDLLQERDNMK
LRVVNYILYGNGKPIRGISDTSIQLVRTCLVLNWDQDKK---SSSVGEAHASFVEVSTTGLIR
DFLRIDVVKSPISYTGKRNSPSSSGLVYD-NGSDRTNINPFY---SKAK-IQQSLSQ----NQGTIR
TLFNR---NKEFQSLIILSSSNCSRMGP--FN---DHNV---IKESDFLIPIRNSLGPLGT--ALQIENF
YS--FYDL--ITRNQIL-VTKYLQLEN---LKQIFQVLK---YFLMDEN--GRIYNPDPCSTIILNPFN
LNWYFLHHNYCEETT-TKVSLGQFICENVCIAKNGPH-LK--SGQVLIVQ-VDSIVIRSAKTY
LATPGATVRGHYGEILYEGDTLVTFIYEKSRSGDITQGLPKVEQVLEVRSIDSISINLEKRVE
GWNERITRILGIPWGFLIGAELTIVQSRISLVNKIQKVYRSQGVQIHNRHIEIIVRQITSKVLV
SE---DGMSNVFSPGELIGLLRAERTGRALEEVICYRAILLGITRASLNTQSFISEASFQETAR
VLAKAALRGRIDWLKGLKENVVLGGMIPVGTGFKRL--VHHSRQHKNI--SLETKKKNLFE
GEMR--DILFHHRELFNFCISKNFHDTSEQYGI-----------MTRRYWNINLEEMMEAGVHFGH
GTRKWNPRMAPYISAKRKGIHITNLTRTARFLSEACDLVFDAASRGKQFLIVGTKNKAAD
SVARAAIKARCHYVNKKWLGGMLTNWSTTETRLHKFRDLRTEQKTGGLNRLPKRDAAM
LKRQLSHLQTYLGGIKYMTGVPDIVIIVDQQEEYTALRECITLGIPTICLIDTNCDPDLADISI
PANDDAIASIRLILNKLVFAICEGRS-SYIRNPMGQKINPLGFRLGTTQGHHSLWFAQPKNYS
EGLQEDQKIRDCIKNYVQKNMRISSG-VEGIARIEIQKRIDLIQVIIYMGFPKLLIEGRPR----R
IEDLQMNVQKELNYVNRKLNIAITRIAKPYGNPNILAEFIAGQLKNRVSFRKAMKKAIELT
EQADTKGIQVQIAGRIDGKEIARVEWIREGRVPLQTIRAKIDYCSYTVRTIYGVLGIKIWIFV
DE-MSRYRGPRFKKIRRLGALPGLTSKRPRAGSDLRNQSRSGK-RSQYRIRLEEKQKLRFH
YGLTERQLLKYVRIAGKAKGSTGQVLLQLLEMRLDNILFRLGMASTIPGARQLVNHRHIL
VNGRIVDIPSYRCKPRDIITAKDEQKSRTLIQNYLDSSPH-EELPKYLTLHPLQY--KGLVNQII

DNKWVGLKINELLVVEYYSRQTMSRRGTAEEKTAKSDPIYRNRLVNMLVNRILKHGKKS
LAYQIIYRAVKKIQQKTETNPLSVLRQAIRGVTPDIAVKARRVGGSTHQVPIEIGSTQGKAL
AIRWLLWASRKRPGRNMAFKLSSELVDAAKGSGDAIRKKEETHKMAEANRAFAHFRMG
RDTIAEIITSIRNADMHRKGTVRIASTNITENIVKILLREGFIENVRKHRENNKDFLVLTLQH
RR--NRKGPYKT-I--LNLKRISRPGLRIYSNYQRIPRILGGVGIVILSTSRGIMTDREARLEGIG
GEILCYIWMAKPIPRIGSRRNGRIGSRKSARRLPKGVIHVQASFNNTIVTVTDVRGRVVSW
SSAGTCGFRGTRRGTPFAAQTAAGNAIRTVVDQGMQRAEVMIKGPGLGRDAALRAIRRS
GILLSFVRDVTPMPHNGCRPPKKRRVMPTIKQLIRNTRQPIRNVTKSPALRGCPQRRGTCT
RVYTITPKKPNSALRKVARVRLTSGFEITAYIPGIGHNSQEHSVVLVRGGRVKDLPGVRYHI
VRGTLDAVGVKDRQQGRSKYGVKKPKMARKSLIQREKKRQKLEQKYHLIRRSSKKEIS---
KVTSLSDKWEIHGKLQSPPRNSAPTRLHRRCFSTGRPRANYRDFGLSGHILREMVHACLL
PGATRSSWMVKNSFISVFLQE-E-KEENRGSVEFQVFSFTNKIRRLTSHLELHRKDYLSQRG
LRKILGKRQRLLAYLSKKNRVRYKELIGRLDIRELKTH--MVKLRLKRCGRKQRAVYRIVAI
DVRSRREGRDLQKVGFYDPIKNQTYSNVPAILYFLAKGAQPTGTVHDILKRAEVFNEFRF
NQTKFN-MDKSKRPFLKSKRSFRRRLPPIQSGDRIDYRNMSLISRFISEQGKILSRRVNRLTL
KQQRLITIAIKQARILSLLPFLNNEK--QFERTESTARTTGFRTRNKVAR-LKKNPFVANNLLK
KITKLNTKSEKEIIVTWSRASTIIPTMIGHTIAIHNGKEHLPIYITDRMVGHKLGEFAPTLNFR
GHAKNDNRSRRMILKSFLLGNLVSLCMKIINSVVVVGLYYGFLTTFSIGPSYLFLLRAQVM
EEGEEGTQKKVSATTGFITGQLMMFISIYYAPLHLALGRPHTITVLALPYLLFHFFWNNHK
HFFDYGSTTRNSMRNLSIQCVFLNNLIFQLFNYFILPSSMLARLVNIYMFRCNNKMLFVTS
SFVGWLIGHILFMKWVGLVLVWIRQNNSIRSLIRSNKYLVSEVRNSMARIFSILLFITCVYY
LGRIPSPTVTKKLKETEEDKPLVTRLFDYKRWTRPLRYIKNDRFENAVRNEMSQYFFYTCR
SDGKQRISFTSPPSLSTFLEMIQRKMSLYTTENPSSYELYNQWVYTNEQNEN-LSNEFLSRIE
ALDKGSLPLDVLEKKTRLCNDETKKEYLPKKYDPFLNGPYRGTIKFIFPPIGTLWLNKIHGI
LDREFQQKDTNDQGIGINEITKKIPRWSYKLIDLEQQER-QIRSRKAKRVVIFTDALIRYSQQ
SDFRRDIIKGSMRAQRRKLAIWELFQANVHSPLFLDRIDNPPLFSFDI--SELMKLIFITIEEK
VRIEIAEAWDTIIFAQVIRGLVLVTQSILRKSIILPSLIIAKNIGRMLLFQFPEWSEDLKDWKR
EMHVKCTYNGVQLSETEFPKNWLTDGIQIKILFPFCLKPWHRSKKGKKDDFCFLTVWGM
EAELPFGSPRKRPSFFEPVFQELGKKILKWKRNCFLA-LRVFKERTNFFLIVPKEIKKWIIKKI
L--LIQRIIKELSKLNDSIISNSMIYESSSQIQSIDWTNYSLTEKKIKDLTDRTSLIRTQIGKITKE
KKTSANKKSHDAKRLESAPIFWQMLKRINIRLIRKSYFFIKFFVERIYIDIFLCIINIPRINTQL
F-HFI--NLSSLSQAYVFYKLSQTQVISLDKLRSILQYTSLFLKNEIKDYFAQGILHSELKHKK
LRNSGMNQWKSWLRGHYQYNFSKIRWSRLVPQKWRNRVNQGWRAQN-NKWDSYEKD
Q--VIHYEK-ENFKKHYRYDLLSSKSINDGIMDPDRKYFDWKILHFCLK-FDWMGMNEEIL
NRWFFPEFALLYNSYKIQPWAIPIKLLLFNYKSNTEAELDLFLKKYLLFQLRWDDSLNQRM
INNIKVYCLLLRLINPREIAISSIERGEMSLDIMLIQKDLTLTELMK-GIFIIEPVRLSVKNDGQ
FIMYQTISISLVHKSKDNHYALLVPENILSPRRRREFRILICFRFLWPTYRLEDLACMNRYWF
DTTNGSRFGMVRIHMYPMKGHQFKSWIFELREILREIKNSHYFLDSWTQFNSVGSFIHIFF
HQERFIKLFDPRIWSILLS-RNS--QGSTSNRYF---TIKGVVLFVVA-VLIYR----INNRNMVESK
NLYLTGLLP-----IPMNSIGPRNDTLEESF-GSSNINRLIVS-LLYLPKGKKISESCFLDPKESTW
VLPIT----KKCIMSESNWGWRWWRNWIGKKR--DSSCKISN---ETVAGIEISFKEKDIK-YLEF
LFVYYMD--DPIRKDHDWELFDRLSPRKRRNIINL--NSGQLFEILVKHWICYLMSAFREKIPI
EVEG-FFKQQG-AGSTIQSNDIEHVSH--LFSRNKWAISLQNCAQFHMWQFRQDLFV---SWG
K-NPHESDFLRN-VSRENWIWLDNVWLVNKDR---FFS--KVRNVSSNIQYDS---TR--SSFVQ

VTNSSQLKGSS-DQSRDHFDSIS----NEDSE-------------YHTLINQREIQQLKERSIL-WD--PSF
LQTERTEIESD--RFPK--CLSGYS--SMSRLFT-EREKQMNNHLLPEEIEEF--IGNPTRSIRSFFA
DRW--SELH-LGSNPTEGSTRDQ----KLLKKEQYLS-FVPSRRSE----NKEMVNIFKIITYLQN--
TVSIHPISSDPGCDMVP-KDEPD-MDSSNKISVLNKNPFFDL--FHLFHDRNRGGYTL--HHDF
ESE--ERFQEMADLFTLSITEP-DLVYHKGFAFSIDSYGLDQKQ--FLNEVFNSRDESKKKSL--
-LVLPPIFY--EENESFYRRIRKKWVR-ISCGNDLEDPKPKIVVFASNNIMEAVNQYRLIQNLIQ
IQY--YG--YIRNVLNRFFLMN---RSDHNFEYGIQ---RDQIGNDTLNHRTIMKYRINQHLSNLK
K-SQKKWFDP--LIFISRTERSTNRDLNAYRYKWSNGSKNFQEHLEHFVSEQKSR---FQVVF
DRLRINQYSIDWSEVIDKKDLSKSLRFFLSKSLL---------FLS-------KLLLFLSNSLP-FFFVSF
GNIPIHRSE-IHIY-ELKGPNDQLCNQLLESIGLQIVHLKKLKPF--LLD---DHDTS-QKSKFLIN
GGTISPFLFNKIP-KW-MIDSFHTKTKNNRRKSFDNTDSYFSMISHDQDNWLNPVKPFHGSS
LISSFYKANRLRFLNNPHHFCFY--CNKRFPF---Y-VEKARINNYD--FTYG-QFLNILFIRNK-I
FSLCGSQ--KKYAFWE-RDTISPIESQVSNIFIPNDFPQSGDET-YNLSKS---FYFPIRSDPFVR--
RAIYSIADISGTPLTEGQIVNFERTYCQPLSDMNLSDSEGKNLHQYLN--FNSNMGLIHTPCS
EKDLPSEK--RKK--------RSLCLK--KCVEKGQMYRTFQRDSAFSTLSKWNLFQTYMPWFLT
STGYKYLNLIFLDTFSD-LLPILSSS--QKFVSIFHDIMYGSDISWRVLQKKL------CLPQRN-LI
SDISRKCLHN--LLLSEEMIHRNNES--PLISTHLRSPNVREF-LYSILFLLLVAGY-LVCTHLLF
VSR--ASSELQTEFEKVKSLMIPSYMIELRKLLDRYPTSELNSFWLKNLFLVALEQLGDSLEE
IRGSASGGNMLWGGGP-----AYG-VKSIRSKKKYLNIN---LIDLI-SIIPNPINRITFSRNTRHLS
HTSKEIYSLIRKRKNVNGDWIDDKIESWVANTDSIDDEEREFLVQFS--TLTT--EKRIDQILLS
MT-HSDHLSSDHLSKNDSGYQMIEQPGAIYLRYLVDIH--KKCLMNYEFNTSCLAERRIFLA
HYQTITY--SP--CGANSFHFPSHG---KPFSLRLALSPS-RGILVIGSIGTGRSYLVKYLATNSYV
PFITVFLNKFLDNKPKGFLID-----DID------DIDASDDIDDS---------------DDIDRDLDTELELL
TMMNAL---TMDMMP---EIDRFYITLQFELAKAMSPCIIWIPNIHDLDVNESNYLSLGLLVN
YLS---RDCERCS-TRNILVIASTHIPQKVDPALIAPNKLNTCIKIRRLLIPQQRKHFFTLSYTRG
FHLEKKMFHT--NRFGSISMGSNARDLVALTNEALSISITQKKSIIDTNTIRSALHRQTWDLR-
--SQVRSVQDHGILFYQIGRAVAQNVLLSNCPIDPISIYMKKK--SCNEGDSYLYKWYFELGT
SMKKLTILLYLLSCSAGSVAQDLWSLP--RPDEK-NGITSYGLVENDS-DLVHGLLEVEGALV
G--SSRTEKDCSQFDND--RVTLFLRPEPRN-PLDMIQNGSCSIVDQRFLYEK--YESEFEE---GE
GALDPQ----QIEED----LFNH--IVWAP--RIWRP---WGFLFDCIERPNELGFP-YWARSFRGK---
RIIYDEEDELQENDSEFLQSGTVQYQTRERSSKEQGFFRISQFIWDPADPLFFLFK-DQPPVS
VFSHREFFADEEMSKGLLTSQTDPPTSIYKRWFI--KNTQEKHFELLIHRQRWLR---TNSSLS-
-NGSFRSN--TLSESYQYLSNLFLSN-GTLLDQMTKTLLRKRWLFPDEIKIGF--M---------MPR
SR-INGNFIDKTFSVVANILLQIIPTTSGEKEAFTYYRDG--MSAQSEGNYAEALQNYYEAMR
LEIDPYDRSYILYNIGLIHTSNGEHTKALEYYFRALERNPFLPQAFNNMAVICHY-RGEQAIR
QGDSEIAEAWFDQAAEYWKQAIALTPGNYIEAHNWLKITRRFE--MSWRSEHIWIELITGSR
KISNFCWAFILFLGSLGFLLVGTSSYLGRNLISLFPSQQIIFFPQGIVMSFYGIAGLFISSYLWC
TISWNVGSGYDRFDRKEGVVCIFRWGFPGKNRRIFLRFLMKDIQSIRIEVKEGISARRVLY
MDIRGQGAIPLTRTDEN-LTPREIEQKAAELAYFLRVPIEVF
>Ilex
MERCRFNSMLFLSKSMDSLGPIENTSESEEPNINDPDKNIYSSSNVDHLFSIKDIRNLISDDT
FLVRDSNGDSYSIYFDIENQIFEINNDH--SFLSELESSLYSYRNSSYLNNGSKSDDPYYDRY
MYDTQYSWNNHINSCIDSYLQ--SQICIDTYIVSGSDNYS--YIYSSICGNEGS--STSTNGSDL
TIRESSNDLDVTQKYRHLWVQCENCYGLNYKKFFKSKMNICEQCGYHLKMSSSDRIELSI

DPGTWDPMDEDMVSLDPIEFHSEEEPYKDRIDSYQRKTGLTEAVQTGIGQLNGIPVALGV
MDFQFMGGSMGSVVGEKITRLIEYATKKFLPLIIVCASGGARMQEGSLSLMQMAKISSAL
YDYQSNKKLFYVSILTSPTTGGVTASFGMLGDIIIAEPNAYIAFAGKRVIEQTLNKTVPEGS
QAAEYLFQKGLFDLIVPRNLLKSVLSELFQLHAFFPLNQNSIKMVTIRADEISNIIRERIEQY
NREVKIVNTGTVLQVGDGIARIHGLDEVMAGELVEFEEGTIGIALNLESNNVGVVLMGDG
LMIQEGSSVKATGKIAQIPVSEAYLGRVINALAKPIDGRGEISASESRLIESPAPGIISRRSVY
EPLQTGLIAIDSMIPIGRGQRELIIGDRQTGKTAVATDTILNQQGQNVICVYVAIGQKASSVA
QVVTTFQERGAMDYTIVVAETADSPATLQYLAPYTGASLAEYFMYRKEHTLIIYDDPSKQ
AQAYRQMSLLLRRPPGREAYPGDVFYLHSRLLERAAKSSSHLGEGSMTALPIVETQSGDV
SAYIPTNVISITDGQIFLSADLFNSGIRPAINVGISVSRVGSAAQIKAMKQVAGKLKLELAQF
AELEAFAQFSSDLDKATQNQLARGQRLRELLKQSQAAPLTVEEQIMTIYTGTNGYLDSLEI
GQVRKFLVELRTYLKSNKPQFQEIISSTKTFTEEAEALLKEAIQEQLERFMLQEQAMRNNP
TTSGSGVSTLEKKNLGRIAQIIGPVLDVAFAPGKMPNIYNALVVKGRDTAGQPINVTCEVQ
QLLGNNRVRAVAMSATDGLTRGMEVIDTGAPLSVPVGGATLGRIFNVLGEPVDNLGPVDT
RTTSPIHRSAPAFIQLDTKLSIFETGIKVVDLLAPYRRGGKIGLFGGAGVGKTVLIMELINNI
AKAHGGVSVFGGVGERTREGNDLYMEMKESGVINEQNIAESKVALVYGQMNEPPGARM
RVGLTALTMAEYFRDVNEQDVLLFIDNIFRFVQAGSEVSALLGRMPSAVGYQPTLSTEMG
TLQERITSTKEGSITSIQAVYVPADDLTDPAPATTFAHLDATTVLSRGLAAKGIYPAVDPLDS
TSTMLQPRIVGEEHYETAQEVKQTLQRYKELQDIIAILGLDELSEEDRLTVARARKIERFLS
QPFFVAEVFTGSPGKYVGLAETIRGFQLILSGELDGLPEQAFYLVGNIDEATAKAMNLEME
SNLKKMILNLCVLTPNRIVWDSEVKEIILSTNSGQIGVLPNHAPIATAVDIGILRIRLNDQWL
TMALMGGFARIGNNEITVLVNDAEKSSDIDPQEAQQTLEIAEANLRKAEGKRQTIEANLAL
RRARTRVEAINAISMKNVTDSFVSLGHWPSAGSFGFNTDILSTNPINLSVVLGVLIFFGKGV
LSDLLDNRKQRILNTIRNSEELRGRAIEQLEKARARLRKVEMEAEQFRVNGYSEIEREKLN
LINSTYKTLEQLENYKNETIHFEQQRVINEVRQRVFQQALQGALGTLNSCLNNELHLRTIS
ANISMLGAMKEITDMNPLISAASVIAAGLAVGLASIGPGVGQGTAAGQAVEGIARQPEAEG
KIRGTLLLSLAFMEALTIYGLVVALALLFANPFVMNVLACSINTLKGLYDISGVEVGQHFY
WKIGGFQVHGQVLITSWVVIAILLGSATIAVRNPQTIPTGGQNFFEYVLEFIRDVSKTQIGEE
YGPWVPFIGTMFLFIFVSNWSGALLPWKIIQLPHGELAAPTNDINTTVALALLTSVAYFYAG
LSKKGLGYFGKYIQPTPILLPINILEDFTKPLSLSFRLFGNILADELVVVVLVSLVPLVIPIPV
MFLGLFTSGIQALIFATLAAAYIGESMEGHHMIFLTLEHILTHISFSMVSIVITIHLITLFFDEIV
KLYDSSEKGMITTFFCITGLLVTRWIYSGHFPLSNLYESLIFLSWSFSIIHIVPY--KKKNHLSTI
TAPSAIFTQGFATSGILTEIHQSEILVPALQSEWLIMHVSMMILGYAALLCGSLLSVALLIITFR
KNRKILC--KKIQYMNERNDVLQNTS---DKNYYRSQLIQQLGYWSYRVISLGFIFLTIGILSG
AVWANEAWGSYWNWDPKETWAFVTWIVFAIYLHTRTNKNLQDTNSAIVASIGFLIIWICY
FGVNLLGIGLHSYGSFMLTMAKKKAFTPLLYLASIVCLPWWISLSFNKSLESWVTNWWNT
GQSETFLNDIQEKSILEKFIELEELLLLDEMIKEYSETHLQKLRI-IHKETIQLIKIHNEDRIHTI
LHFSTNIICFVILSGYSIWGNEELVILNSWAQEFLYNLSDTLKAFSILLLTDLCIGFHSPHGWE
LMIGSVYKDFGFVHNDQIISGLVSTFPVILDTILKYWIFRDLNRVSPSLVVIYHSMNDMPIG
VPKVPFRSPGEEDASWVDVYNRLYRERLLFLGQEVDSEISNQLIGLMVYLSIEDDTKDLYL
FINSPGGWVIPGVAIYDTMQFVRPDVHTICMGLAASMGSFILVGGEITKRLAFPHARVMIH
QPASSFYEAQTGEFILEAEELLKLRETLTRVYVQRTGKPLWVVSEDMERDVFMSATEAQA
YGIVDLVAVAMKEQKWIHEGLITESLPNGMFRVRLDNEDLILGYVSGKIRRSFIRILPGDRV
KIEVSRYDSTRGRIIYRLRNKDSKDMEEFQRYFE-DRSQQHDFLYPLIFQEYIYALAHDHGL

AGYDNKSSLLIVKRLITRMYQQNHLILSTNDSNQNKIFGKNLYSQMISEGFAVIVEIPFSVR
LISSLIIKSHNLRSIHSIFPFLEDKFLHLNYVLDILIPHPVHLEILVQTLRYWVKDTSSLHLLRF
FLYEDRNWNSLITKPGSSFSKQRFFFFLYNSHVCEYESIFVFLRNQSSHLRSTSSGVLLERIY
FYGKIEYLVEVFAKAFNLWLFKDPFIHYVRYQEKSILASKGTSLLMNKWKHYLVNFWQC
YFYLWSQPGRIHINQFSNYYLDFMGYFSSVRLNPLMVRNQMLENAFLIDNAI--KKFDISVP
IIPLIGSLAKAKFCNGLGHPISKPVRADLSDSDIIDRFGRIYRNLFHYHSGSSKKKSLYRIKYI
LRFSCIRTLARKHKSTVRTFLKRLGSELLEEFFKAEDQVLSLTFPKRIWYLDIICINDLVNHE
MIID-TTEVQ-AINSFSRLES-LQEVYGIIWMLVYILTTVLGITIGVLVIVWLEREISAGIQQRIG
PEYAGPLGILQALADGTKLLFKENLLPSRGDNRLFSIGPSIAVISILLSYSVIPFGYHLVLADL
SIGVFLWIAISSIAPVGLLMSGYGSNNKYSFLGGLRAAAQSISYEIPLTLCVLSISLLSNSSST
VDIVEAQSKYGFWGWNLWRQPIGFIIFLISSLAECERLPFDLPEAEEELVAGYQTEYSGIKF
GLFYVASYLNLLLSSLFVTVLYLGGWNLSIPYIFVPELFEINKAGRVFGTIIGIFITLAKTYLF
LFISITTRWTLPRLRMDQLLNLGWKFLLPISLGNLLLTISSQLLSLMIWHVQNENFILDSTRI
FMKAFHLLLFDGSFIFPECILIFGLILLLMIDSTSDQKDIPWLYFISSTSLVMSITALLFRWREE
PMISFSGNFQTNNFNEIFQFLILLCSTLCIPLSVEYIECTEMAITEFLLFVLTATLGGMFLCGA
NDLITIFVALECFSLCSYLLSGYTKKDVRSNEATMKYLLMGGASSSILVHGFSWLYGSSGG
EIELQEIVNGLINTQMYNSPGISIALIFITVGIGFKLSPAPSHQWTPDVYEGSPTPVVAFLSVT
SKVAASASATRIFDIPFYFSSNEWHLLLEILAILSMILGNLIAITQTSMKRMLAYSSIGQIGYV
IIGIIVGDSNDGYASMITYMLFYISMNLGTFACIVLFGLRTGTDNIRDYAGLYTKDPFLALSL
ALCLLSLGGLPPLAGFFGKLYLFWCGWQAGLYFLVLIGLLTSVVSIYYYLKIIKLLMTGRN
QEITPHVRNYRRSPLRSNNSIELSMIVCVIASTIPGISMNPIIAIAQDTLFMFLLYEYDIFWTF
LIISSLIPILAFFISGVLAPISKGPEKLSSYESGIEPMGDAWLQFRIRYYMFALVFVVFDVETV
FLYPWAMSFDVLGVSVFIEALIFVLILIVGSVYAWRKGALEWSTNYFPWLTIIVVLPISAGSL
IFFLPHKGNRVIRWYTIFISILELLLTTYAFCYHFQLDDPLIQLVDDYKWINFFDFHWRLGID
GLSIGPILLTGFITTLATLAARPVTRDSRLFHFLMLAMYSGQIGPFSSRDLLLFFIMWELELIP
VYLLLSRWGGKKRLYSATKFILYTAGGSIFLLMGVLGIGLYGSNEPTLNFETSANQSYPVAL
EIIFYIGFFIAFAVKSPIIPLHTWLPDTHGEAHYSTCMLLAGILLKMGAYGLIRINMELLPHA
HSIFSPWLIIVGTIQIIYAALTSPGQRNLKKRIAYSSVSHMGFILIGIGSITATGLNGAILQIISH
GFIGAALFFLAGTSYDRIRLVYLDAMGGIAIPMPKIFTMFSSFSMASLALPGMSGFVAELIV
FFGIITSQKYLLMPKILITLVMAIGMILTPIYSLSMSRQMFYGYKLFNAPNSYFFDSGPRELF
VSISIFLPVIGIGMYPDFVLSLSVDKVAVILSNYFYRMILEHVLVLSAYLFSVGLYGLITSRN
MVRALMCLELILNAINMNFVTFSYFFDSRQLKGDIFSIFVIAIAAAEAAIGLAIVSSIYRNRK
STCINQSNLLNKMEQTYQYAWIIPFVPLPVPMLIGVGLLLFPTATKNLRRMWSFHSILLLSI
VMIFSIDLSIQQINSSSIYQYVWSWIITNDFSLEFGYLIDPLTSIMSILITTVGIMVLIYSDNYM
SHDQGYVRFFAYMSFFSTSMLGLVTSSNLIQIYIFWELVGICSYLLIGFWFTRPVAANACQK
AFVTNRVGDFGLLLGILGFYWITGSFEFRDLFEIFNNNEVNFLFVTLCGVLLFAGAVAKSA
QFPLHVWLADAMEGPTPISALIHAATMVAAGIFLVARLFPLFIVIPSLMNLISLVGIITVFLGA
TLALAQKDIKRGLAYSTMSQLGYMMLALGMGSYQSALFHLITHAYSKALLFLGSGSVIHS
METLVGYSPDKSQNMVLMGGLTKHVPITKNAFLLGTLSLCGIPPLACFWSKDEILHDSWL
YSPLFAIIAWSTAGLTAFYMFRIYLLTFEGHLNVHFQNYSGKKNTPFYSISLWGKGDSRGIN
T-LLSLLRMNLKTFGNKKTYLYPYESDNTMLFPLLLLVLFTLFVGFLGIPFDLDILSKWLTPS
INLLHENSN-STSWYEFVKDVGFSVSIAYLGIFIASFLYKPLYSSLQNLDLINSFVTLWDKIIN
VIYSWSYNRGYIDAFYARSLTGGIRGLAELTHFFDRRVVDGITNGVGVMSFFVGEGIKSVG
GG-RISSYLFLYLSYVSIFLFIYSMDLPGPIHDFLLVFLGSGLILGGLGVVLLPNPIYSAFSLGL

VLVCISLFYILSNSHFVAAAQLLIYVGAINVLIIFAVMFMNGSEYYKDFHLWTVGDGVTSIV
CTSLFVSLMTTIPDTSWHGIIWTTKSNQIIEQDLISNSQQIGIHLSTDFFLPFELISIILLVALIG
AIAVARQMTAPATRKDLMIVNMGPHHPSMHGVLRLIVTLDGEDVIDCEPILGYLHRGMEK
IAENRTIIQYLPYVTRWDYLATMFTEAITVNAPEQLGNIQVPKRASYIRVIMLELSRIASHLL
WLGPFMADIGAQTPFFYIFRERELIYDLFEAATGMRMMHNYFRLGGVAADLPHGWIDKC
LDFCDYFLTGVAEYQKLITQNPIFLERVEGVGIIGGEEAINWGLSGPMLRASGIQWDLRKV
DHYECYDEFDWEVQWQKEGDSLARYLVRISEMTESIKIIQQALEGIPGGPYENLEIRRFDR
VRDPEWNDFDYRFISKKPSPTFELSQQELYVRVETPKGELGIFLIGDQSVFPWRWKIRPPGF
INLQILPQLVKKMKLADIMTILGSIDIIMGEVDRMFPMVTEFINYGQQTVRAARYIGQGFTI
TLSHANRLPVTIQYPYEKLIISERFRGRIHFEFDKCIACEVCVRVCPIDLPVVDWKLETDIRK
KRLLNYSIDFGICIFCGNCVEYCPTNCLSMTEEYELSTYDRHELNYNQIALGRLPMSVIDD
YTIRTVLNS----------------MQGRLSAWLVKYGLIHRFLGFDYQGIETLQIKPEDWPSIAVILYV
YGYNYLRSQCAYDVAPGGLLASVYHLTRIGYGVDQPEEVCIKVFAPRRNPRIPSVFWVWK
SVDFQERESYDMLGISYDNHPRLKRILMPESWIGWPLRKDYIAPNFYEIQDAH---------------
---MNSIQFPLLERTTQNSVISTTSNDLSNWSRLSSLWPLLY
GTSCCFIEFASLIGSRFDFDRYGLVPRSSPRQADLILTAGTVTMKMAPSLVRLYEQMPEPKY
VIAMGACTITGGMFSTDSYSTVRGVDKLIPVDVYLPGCPPKPEAVIDAITKLRKKISREISE
DRIRSQRENRCFTTNHKFHVGRSIHTGNYDRGFLYQPPSTSEIPPETFFKYKSSVSSQELVN
MQARNTFSWIKEQITRSISVSLMIYIITRTSISSAYPIFAQQGYENPREATGRIVCANCHLAN
KPVDIEVPQAVLPDTVFEAVVRIPYDMQLKQILANGKKGALNVGAVLILPEGFELAPPDRIS
PEMKEKIGNLSFQSYRPNKKNILVIGPVPGQKYSEIIFPILSPDPATKKDVHFLKYPIYVGGN
RGRGQIYPDGSKSNNTVYNATAAGIVSRIIRKEKGGYEITITDASDGRQVVDIIPPGPELLVS
EGESIKFDQPLTSNPNVGGFGQGDAEIVLQDPLRVQGLLFFLASVILAQIFLVLKKKQFEKV
QLSEMNFMSKVYDWFEERLEIQAIADDITSKYVPPHVNIFYCLGGITLTCFLVQVATGFAM
TFYYRPTVTEAFASVQYIMTEANFGWLIRSVHRWSASMMVLMMILHVFRVYLTGGFKKP
RELTWVTGVVLGVLTASFGVTGYSLPRDQIGYWAVKIVTGVPEAIPVIGSPLVELLRGSAS
VGQSTLTRFYSLHTFVLPLLTAVFMLMHFPMIRKQGISGPLMGVTKKPDLNDPVLRAKLA
KGMGHNYYGEPAWPNDLLYIFPVVILGTIACNVGLAVLEPSMIGEPADPFATPLEILPEWYF
FPVFQILRTVPNKLLGVLLMVSVPTGLLTVPFLENVNKFQNPFRRPVATTVFLVGTVVALW
LGIGATLPIDKSLTLGLFMIEVFLFGIVLGLIPITLAGLFVTAYLQYRRGDQLDLMPTITSYFG
FLLAALTITSALFIGLSRIRLIMDIVSLAWAALMVVFTFSLSLVVWGRSGLMIIRSPEPEVKIL
VDRDHIKTSFEEWARPGHFSRTIAKGPDTTTWIWNLHADAHDFDSHTSDLEEISRKVFSAH
FGQLSIIFLWLSGMYFHGARFSNYEAWLSDPTHIGPSAQVVWPIVGQEILNGDVGGGFRGI
QITSGFFQLWRASGITSELQLYCTAIGALVFAALMLFAGWFHYHKAAPKLAWFQDVESML
NHHLAGLLGLGSLSWAGHQVHVSLPINQFLNAGVDPKEIPLPHEFILNRDLLAQLYPSFAE
GATPFFTLNWSKYADFLTFRGGLDPVTGGLWLTDIAHHHLAIAILFLIAGHMYRTNWGIGH
GLKDILEAHKGPFTGQGHKGLYEILTTSWHAQLSLNLAMLGSLTIVVAHHMYSMPPYPYL
ATDYGTQLSLFTHHMWIGGFLIVGAAAHAAIFMVRDYDPTTRYNDLLDRVLRHRDAIISH
LNWACIFLGFHSFGLYIHNDTMSALGRPQDMFSDTAIQLQPVFAQWIQNTHALAPSATAPG
ATTSTSLTWGGGDLVAVGGKVALLPIPLGTADFLVHHIHAFTIHVTVLILLKGVLFARSSRLI
PDKANLGFRFPCDGPGRGGTCQVSAWDHVFLGLFWMYNAISVVIFHFSWKMQSDVWGS
ISDQGIVTHITGGNFAQSSITINGWLRDFLWAQASQVIQSYGSSLSAYGLFFLGAHFVWAFS
LMFLFSGRGYWQELIESIVWAHNKLKVAPATQPRALSIIQGRAVGVTHYLLGGIATTWAFF
LARIIAVGMALRFPRFSQGLAQDPTTRRIWFGIATAHDFESHDDITEERLYQNIFASHFGQL

AIIFLWTSGNLFHVAWQGNFESWVQDPLHVRPIAHAIWDPHFGQPAVEAFTRGGALGPVNI
AYSGVYQWWYTIGLRTNEDLYTGALFLLFLSAVSLIAGWLHLQPKWKPSVSWFKNAESR
LNHHLSGLFGVSSLAWTGHLVHVAIPGSRGESVRWNNFLDVLPHPQGLGPLFTGQWNLYA
QNPDSSSHLFGTSQGAGTAILTLLGGFHPQTQSLWLTDIAHHHLAIAFIFLIAGHMYRTNFGI
GHSMKDLLDAHIPPGGGLGRGHKGLYDTINNSIHFQLGLALASLGVITSLVAQHMYSLPAY
AFIAQDFTTQAALYTHHQYIAGFIMTGAFAHGAIFFIRDYNPEQNEDNVLARMLDHKEAII
SHLSWASLFLGFHTLGLYVHNDVMLAFGTPEKQILIEPIFAQWIQSAHGKTSYGFDVLLSST
NGPAFNAGRSIWLPGWLNAVNE--NSNSLFLTIGPGDFLVHHAIALGLHTTTLILVKGALDA
RGSKLMPDKKDFGYSFPCDGPGRGGTCDISAWDAFYLAVFWMLNTIGWVTFYWHWKHI
TLWQGNVSQFNESSTYLMGWLRDYLWLNSSQLINGYNPFGMNSLSVWAWMFLFGHLVW
ATGFMFLISWRGYWQELIETLAWAHERTPLANLIRWRDKPVALSIVQARLVGLAHFSVGYI
FTYAAFLIASTSGKFGMSHSVKIYDTCIGCTQCVRACPTDVLEMIPWDGCKAKQIASAPRT
EDCVGCKRCESACPTDFLSVRVYLGNETTRSMGLAYMITLNLPSIFVPLIGLVFPAIAMASL
FLHVQKNKIVMRDLKTYLSVAPVISTLWFGSLAGLLIEINRFFPDALTFPFFSFMTAILERRE
SESLWGRFCNWITSTENRLYIGWFGVLMIPTLLTATSVFIIAFIAAPPVDIDGIREPVSGSLLY
GNNIISGAIIPTSAAIGLHFYPIWEAASVDEWLYNGGPYELIVLHFLLGVACYMGREWELSF
RLGMRPWIAVAYSAPVAAAAAVFLIYPIGQGSFSDGMPLGISGTFNFMIVFQAEHNILMHPF
HMLGVAGVFGGSLFSAMHGSLVTSSLIRETTENESANAGYRFGQEEETYNIVAAHGYFGR
LIFQYASFNNSRSLHFFLAAWPVVGIWFTALGISTMAFNLNGFNFNQSVVDSQGRVINTWA
DIINRANLGMEVMHERNAHNFPLDLAAIEAPSTNGMGLPWYRVHTVVLNDPGRLLSVHI
MHTALVAGWAGSMALYELAVFDPSDPVLDPMWRQGMFVIPFMTRLGITNSWGGWSIT-G
GTVTNPGIWSYEGVAGAHIVFSGLCFLAAIWHWVYWDLEIFCDERTGKPSLDLPKIFGIHL
FLAGVACFGFGAFHVTGLYGPGIWVSDPYGLTGKVQSVNPAWGVEGFDPFVPGGIASHHI
AAGTLGILAGLFHLSVRPPQRLYKGLRMGNIETVLSSSIAAVFFAAFVVAGTMWYGSATTP
IELFGPTRYQWDQGYFQQEIYRRVSAGLAENQSLSEAWSKIPEKLAFYDYIGNNPAKGGLF
RAGSMDNGDGIAVGWLGHPIFRDKEGRELFVRRMPTFFETFPVVLIDGDGIVRADVPFRR
AESKYSVEQVGVTVEFYGGELNGVSYSDPATVKKYARRAQLGEIFELDRATLKSDGVFRS
SPRGWFTFGHASFALLFFFGHIWHGARTLFRDVFAGIDPDLDAQVEFGAFQKLGDPTTRR
QVV------------VETLFNGTLALAGRDQETTGFAWWAGNARLINLSGKLLGAHVAHAGLIVF
WAGAMNLFEVAHFVPEKPMYEQGLILLPHLATLGWGVGPGGEVIDTFPYFVSGVLHLISS
AVLGFGGIYHALLGPETLEESFPFFGYVWKDRNKMTTILGIHLILLGIGAFLLVFKALYFGG
VYDTWAPGGGDVRKITNLTLSPSIIFGYLLKSPFGGEGWIVSVDDLEDIIGGHVWLGSICIL
GGIWHILTKPFAWARRALVWSGEAYLSYSLGAISVFGFIACCFVWFNNTAYPSEFYGPTGP
EASQAQAFTFLVRDQRLGANVGSAQGPTGLGKYLMRSPTGEVIFGGETMRFWDLRAPWL
EPLRGPNGLDLSRLKKDIQPWQERRSAEYMTHAPLGSLNSVGGVATEINAVNYVSPRSWL
ATSHFVLGFFFFVGHLWHAGRARAAAAGFEKGIDRDFEPVLSMTPLNMTIALGKFTKDEN
-LFDIMDDWLRRDRFVFVGWSGLLLFPCAYFALGGWFTGTTFVTSWYTHGLASSYLEGC
NFLTAAVSTPANSLAHSLLLLWGPEAQGDFTRWCQLGGLWTFVALHGAFGLIGFMLRQFE
LARSVQLRPYNAIAFSAPIAVFVSVFLIYPLGQSGWFFAPSFGVAAIFRFILFFQGFHNWTLN
PFHMMGVAGVLGAALLCAIHGATVENTLFEDGDGANTFRAFNPTQAEETYSMVTANRF
WSQIFGVAFSNKRWLHFFMLFVPVTGLWMSALGVVGLALNLRAYDFVSQEIRAAEDPEFE
TFYTKNILLNEGIRAWMAAQDQPHENLIFPEEVLPRGNALMSGSTGERSFADIITSIRYWVI
HSITIPSLFIAGWLFVSTGLAYDVFGSPRPNEYFTESRQGIPLITGRFDSLEQLDEFSRSFMTI
DRTYPIFTVRWLAVHGLAVPTVSFLGSISAMQFIQRMATQTVENASRSG-PRRTTVGSLLKP

LNSEYGKVAPGWGTTPLMGVAMALFAIFLSIILEIYNSSVLLDGISMQMLTLKLFVYTVVIF
FVSLFIFGFLSNDPGRNPGREEMADTTGRIPLWIIGTVTGILVIGLLGIFFYGSYSGLGSSLM
LNIFSLICICLNSTLYSSSPFLAKLPEAYAFLNPIVDIMPVIPLLFFLLAFVWQAAVSFRMTQS
NPNEQSVELNRTSLYWGLLLIFVLAVLFSNYFFNMEVNILAFIATALFILVPTAFLLIIYVKTV
SQNDMETATLVTIFISGLLVSFTGYALYTAFGQPSQQLRDPFEEHGDMEALVYTFLLVSTLGI
IFFAIFFREPPKVPTKKMKMTLAFQLAVFALIATSSILLISVPVVFASPDGWSSNKNVVFSGT
SLWIGLVFLVGILNSLIS-------MSPQTETKASVGFKAGVKDYKLNYYTPDYVTKDTDILAA
FRVSPQPGVPPEEAGAAVAAESSTGTWTTVWTDGLTSLDRYKGRCYQIEPVAGEENQFIAY
VAYPLDLFEEGSVTNMFTSIVGNVFGFKALRALRLEDLRIPPAYTKTFQGPPHGIQVERDKL
NKYGRPLLGCTIKPKLGLSAKNYGRAVYECLRGGLDFTKDDENVNSQPFMRWRDRFLFC
TEALFKAQAETGEIKGHYLNATAGTCEEMMKRAVFARELGVPIVMHDYLTGGFTANTTLA
HYCRDNGLLLHIHRAMHAVIDRQKNHGMHFRVLAKGLRMSGGDHIHAGTVVGKLEGER
EITLGFVDLLRDDFIEKDRSRGIYFTQDWVSLPGVLPVASGGIHVWHMPALTEIFGDDSVL
QFGGGTLGHPWGNAPGAVANRVALEACVKARNEGRDLAREGNEIIREASKWSPELAAAC
EIWKEIKFEFEAMDTL--MAIHLYKTSTPSTRNGVVDSQVKSNPRNHLIYGQHRCGKGRNA
RGIITAGHRGGGHKRLYRKIDFRRNEKDIYGRIVTIEYDPNRNAYICLIHYGDGEKRYILHP
RGAIIGDTIVSGTEVPIKMGNALPLTDMPLGTAIHNIEITLGKGGQLARAAGAVAKLIAKEG
KSATLKLPSGEVRLISKNCSATVGQVGNVGVNQKSLGRAGSKRWLGKRPVVRGVVMNP
VDHPHGGGEGRAPIGRKKPTTPWGYPALGRRSRKRNKYSDNLILRRRSKMIQPQTHLNVA
DNSGARELMCIRIIGAS-NRRYAHIGDIIVAVIKEAVPNMPLEKSEVVRAVIVRTCKELKRDN
GMIIRYDDNAAVVIDQEGNPKGTRVFGAIARELRQLNFTKIVSLAPEVLMLSPKRTRFRKQ
HRGRMKGISYRGNHICFGKYALQALEPAWITSRQIEAGRRAMTRNARRGGKIWVRIFPDK
PVTVRPTETRMGSGKGSPEYWVAVVKPGRILYEMGGVTENIARKAISIAASKMPIRTQFIIS
GMTRIRRGYIARRRRTKIRLFASSFRGAHSRLTRTITQQKIRALVSAHRDRNRQKRNFRRL
WITRINAVIRE-RGVSYSYSRLIHDLYKRQLLLNRKILAQIAISNRNCIYMISNEIIKEVDWKE
STGRIMLKEVYALGQHISMSAHKARRVIDQIRGRSYEETLMILELMPYRACYPIFKLVYSA
AANASYNMDSNEANLFISKAEVNEGTTVKKLKPRARGHSYPIKRPTCRITIVVKDISLDGG
VWDKKMDGIKYAVFTDKSIRLLGKN-QYTSNVESGSTRTEIKHWVELFFGVKVIAMNSHR
LPGKGRRMGPIMGHTMHYRRMIITLQPGYSIPPLRKKRTMAVPKKRTSISKKRIRKNIWKR
KGYWAALKAFSLGKSLLTGNSKSFFVRQTNKMAKGKDVRVTVILECTSCVRNSVN--KGS
TGISRYITQKNRHNTPNRLELRKFCPYCYKHTIHGEIKKMKIRASVRKICEKCRLIRRRGRII
VICSNSRHKQRQGMVREKVTVSTRTLQWKCVESRADSKRLYYGRFILSPLMKGQADTIGI
AMRRALLGEIEGTCITRAKFE--KIPHEYSTIVGIQESVHEILMNLKEIVLRSNLYGTCDASIC
VRGPGYVTTQDIILPPYVEIVDNTQHIASLTEPIDLCIGLQIERNRGYLIKTPN-NFDGSY--PI
DAVFMPVRNANHSIHSYRNGNEKQEILFLEIWTNGSLTPKEALHEASRNLIDLFIPFLHMEE
E--NLHLEDNQ-HTIPLPPFNFHDKLA-KLRKNKK-KIALKSIFIDQLELPPRIYNCLKRSNIYT
LLDLLNNVQEDLMKIEHFRIEDVKQILGVLEKH--FAIDLPKNKMLRDG-NEGMSTIPGFNQ
IQFEGFCRFIDQGLTEELYKFPKIEDTDQEIEFQLFVETYQLVEPLIKERDAVYESLTYSSELY
VSAGLIWKTSRDMQDQTIFIGNIPLMNSLGTSIINGIYRIVINQILQSPGIYYKSELDHNGISV
YTGTIISDWGGKSELEIDKKARIWARVSRKQKISILVLSSAMGSNLREILENVCYPEIFLSFL
NDKEKKKIGSKENAILEFYQQFACVGGDPVFSESLCKELQKKFFQQRCELGRIGRRNMNR
RLNLDIPQNNTFLLPRDILAAADHLIGMKFGMGTLDD--MNHLKNKRIRSVADLLQDQFGL
ALVRLENVVRGTICGAIR-HKF-IPTPQNLVTSTP----LTTTYESFFGLHPLSQVLDRTNPLTQI
VHGRKLSYLGPGGLTGRTASFRIRDIHPSHYGRICPIDTSEGINVGLIGSLAIHARIGHWG-SL

ESPFY---EISERSKG--VRVLYLSPHRDEYYMVAAGNSLALNQGIPEEQVVPARYRQEFLTIA
WEQVHLRSIFPFQYFSIGASLIPFIEHNDANRALMSSNMQRQAVPLSRSEKCIVGTGLERQA
ALDSGALAISEHEGKIIYTDTDKIFLSGNGDTLSIPLVMYQRSNKNTCMHQKTQVRRGKCI
KKGQILADGTATVGGELALGKNVLVAYMPWEGYNSEDAVLISERLVYEDIYTSFHIRKYEI
QTHVTSQG-PEKVTNEIPHLEAHLLRNLDKNGIVMLGSWVETGDILVGKLTPQMVKE-SSY
APEHRLLRAILGIQVSTSKETCLKLPIGGRGRVIDVRWIQKKG-GSSYNPETICVYISQKREI
KVGDKVAGRHGNKGIISKILPRQDMPYLQDGRPVDMIFNPLGVPSRMNVGQIFECSLGLA
GGLLDRHYRIAPFDERYEQEASRKLVFSELYEASTQTANPWVFEPEYPGKSRIFDGRTGDP
FEQPVIIGKPYILKLIHQVDDKIHGRSSGHYALVTQQPLRGRAKQGGQRVGEMEVWALEG
FGVAHILQEMLTYKSDHIRARQEVLGTTIIGGTIPNPEDAPESFRLLVRELRSLALELNHFLV
SEKNFQITRKEAMNQNFSSMIDRYKHQQLRIGSVSPQQISAWATKILPNGEIVGEVTKPYTF
HYKTNKPEKDGLFCERIFGPIKSGICACGNYRVIGDEKEDSKFCEQCGVEFVDSRIRRYQM
GYIKLACPVTHVWYLKRLPSYIANLLDKPLKELEGLVYCDPNFSFARP-VAKKPTFLRLRG
LFEYEIQSWKYSIPLFFTTQGFDTFRNREISTGAGAIREQLADLDLRIIIDYSLVEWKELGEE-
GFTGNEWEDRKVGRRKDFLVRRMELAKHFIRTNIEPERMVLCLLPVLPPELRPIIQIDGGK
LMSSDINELYRRVIYRNNTLTDLLTTSRSTPGELVMCQEKLVQEAVDTLLDNGIRGQPMRD
GHNKIYKSFSDVIEGKEGRFRETLLGKRVDYSGRSVIVVGPSLSLHRCGLPREIAIELFQTF
VIRGLIRQHLASNIGVAKSKIREKEPLVWEILQDVMQGHPVLLNRAPTLHRLGIQAFQPVL
VEGRAICLHPLVCKGFNADFDGDQMAVHVPLSLEAQAEARLLMFSHMNLVSPAIGDPISV
PTQDMLIGLYVLTSGNRRGICVNRYNPCNL---RNYQNERIYDNNYKYTK--EPFFCNSYDAI
GAYRQKQINLDSPLWLRWRLDQR-VIAS--REVPIEVHYESLGTYYEIYGHYLIVRSIKKEIL
FIYIRTTVGHISLYREIEEAIQGFCQACSYGP--------MEVLMAERANLVFHNKVIDGTAMKR
LISRLIDHFGMAYTSHILDQVKTLGFQQATATSISLGIDDLLTIPSKGWLVQDAEQQSLILEK
HHHYGNVHAVEKLRQSIEIWYATSEYLRQEMNPNFRMTDPFNPVHIMSFSGARGNASQV
HQLVGMRGLMSDPQGQMIDLPIQSNLREGLSLTEYIISCYGARKGVVDTAVRTSDAGYLTR
RLVEVVQHIVVRRTDCGTVRGISVSPRNGMMP-ERIFIQTLIGRVLADDIYMGARCVATRN
QDIGIGLVNRFIT------FRAQPIPIRTPFTCRSTSWICRLCYGRSPTHADLVELGEAVGIIAGQSI
GEPGTQLTLRTFHTGGVFTGGTAEQVRAPSNGKIKFNEDLVQPTRTRHGHPAFLCSIDLYV
TIESEDILHNVNIPPKSFLLVQNDQYVESEQVIAEIRAGTST-LNFKERVRKHIYSDSEGE-MH
WSTDVYHAPEFTYGNVHLLPKTSHLWILLGQPCR--SSLVSPSLHKDQDQMNAY--SLSVKR
RYISNLS--VTNNQ-VRHKFFRSDFSGK-EDRIPDY-SELNRIICTGRCNLIYP-AILPENS--DLL
AKSRRNRFIIPLQSIQERENELMPRSDISIEIP--INGIFRRKSILAYFDDPRYRRKSLGITKYGTI
EVHSIVKKED--LIEYRG-VKEVRPKYMKVDRFFFIPEEVHILPGSSSIMVRNNSIIGVDTQIT
LNTRSRVGGLVRVERKKKRIELKIFSGDIHFPGETDKISRHSGVLIP-PG-TGKTNSKESKK--
LKNWIYVQR--ITPIK--KKYFVLVRPVVTYEITDGINLATLFPLDLLQERDNVQLRVINYILY
GNGKPIRGISDTSIQLVRTCLVLNWDQDKK---SSSIEEARASFVEIKTKGLIRDFLRIDLVKSS
ISYTRKRNDPSGAGLISD-NGSDRTNINPFSFIYTY---SKARIQESLNQNQGTIHTVLNK---NK
ECQSLIILSSSNCSRMGP--FNDVKYHNM---IKES-PPIPIRNSLGSLGT--AFQIANFYS--FYHL-
-ITYNPIL-VNNYFQLDN---LKQTFQVLK---YYLMDEN--GKFYNPDPRSNIILNPFNLNWYF
LHHNYCEETS-TIISLGQFICENVCIAKNGPH-LK--SGQVLIVQ-GDSVVIRSAKPYLATPGAT
VNGHYGEILYEGDILVTFIYEKSRSGDITQGLPKVEQVLEVRSINSIAMNLEKRVEGWNECI
TGILGIPWGFLIGAELTIVQSRISLVNKIQKVYRSQGVQIHNRHIEIIVRQITSKVLVSE---DG
MSNVFSPGELIGLLRAERMGRALEEAICYRAVLLGITRASLNTQSFISEASFQETARVLAKA
ALGGRIDWLKGLKENVVLGGMIPVGTGFKGL--VHPSRQHNNIP--LETKKKNLFEGEMR--

DILFHHRKLLDSCLSKNFHDISNNHL------------MTRRYWNINLEEMMEAGVHFGHGTRKW
NPKMAPYISAKRKGIHITNLTRTARFLSEACDLVFDAASMGKKFLIVGTKNKAADSVAWA
AIRARCHYVNKKWLGGMLTNWSTTETRLHKFRDLRTEQKTGRLNRLPKRDAAMLKRQL
AHLQTYLGGIKYMTGLPDIVIIVDQHEEYTALRECITLGIPTICLIDTNCDPDLADISIPANDD
AISSIRLILNKLVFAICEGRS-SYIRNTMGQKINPLGFRLGTTQGHHSLWFAPPKNYSEGLQE
DQKIRDCIKNYVQKNSKISSG-VEGIARIEIQKRIDLIQVIIYMGFPKFLIESRPR----GIEELQ
MNLQKEFNGVNRKLNIAITRIAKPYGNPNILAEFIAGQLKNRVSFRKAMKKAIELTEQADT
KGIQVQIAGRIDGKEIARVEWIREGRVPLQTIRAKIDYCSYTVRTIYGVLGIKIWIFIDEEMS
RYRGPRFKKIRRLGALPGLTNKRPRAGNDLRNQSRSGK-KSQYRIRLEEKQKLRFHYGLTE
RQLLKYVRIAGKAKGSTGQVLLQLLEMRLDNILFRLGMAPTIPGARQLVNHRHILVNGRI
VDIPSYRCKPRDIITARDEQKSIVLVQNSLDSSPH-EELPKHLTLHPFQ--YKGLVNQIIDSKW
VGLKINELLVVEYYSRQTMSRRGTAEEKTAKSDPIYRNRLVNMLVNRILKHGKKSLAYQI
VYRAVKKIQQKTETNPLSVLRQAIRGVTPDIAVKARRVGGSTHQVPIEIGSTQGKALAIRW
LLAASRKRPGRNMAFKLSSELVDAAKGSGDAIRKKEETHRMAEANRAFAHFRMGRDTIA
DIITSIRNADMDRKRVVRIASTNITENLVKILLREGFIENVRKPREKNKYFLVLTLRHRR--NR
KGPY---RNILNLKRISRPGLRIYSNYQRIPRILGGIGIVILSTSRGIMTDREARLEKIGGEILCY
IWMAKAIPRVGSRRNGRIGSRKSARRIPKGVIHVQASFNNTIVTVTDVRGRVVSWSSAGTC
GFRGTRRGTPFAAQTAAGNAIRAVVDQGMQRAEVMIKGPGLGRDAALRAIRRSGILLTFV
RDVTPMPHNGCRPPKKRRVMPTIKQLIRNTRQPIRNVTKSPALGGCPQRRGTCTRVYTITP
KKPNSALRKVARVRLTSGFEITAYIPGIGHNSQEHSVVLVRGGRVKDLPGVRYHIVRGTLD
AVGVKDRQQGRSKYGVKKPKMARKSLIQREKKRQKLEQKYHLIRRSSKKEIS---KVPLLS
DKWKVYGKLQSPPRNSAPTRLHRRCFSTGRPRANYRDFGLSGHILREMVHACLLPGATRS
SWMVKNSFISVTSQ--EEKKENRGSVEFQVVSFTNKIRRLTSHLELHKKDYLSQRGLRKILG
KRQRLLAYLSKKNRVRYKEIIGQLDIREIKTR--MVKLRLKRCGRKQRAVYRIVAIDVRSRR
EGRDLRKVGFYDPIKNQTYLNVPAILYFLEKGAQPTGTVQDILKKAEVFKELRSNQTKFN-
MDKSKRPFFKSKRSFRRRLPPIQSGDRIDYRNMSLISRFISEQGKILSRRVNRLTLKQQRLIT
TAIKQARILSSLPFLNNEK--QFERTESTARTTGLRTRNKVTRSLKKNPFVANHLLRKIEKLN
TKAEKEIIVTWSRASTIIPTMIGHTIAIYNGKEHLPIYITDRMVGHKLGEFAPTLHFRGHAKS
DNRSRRMIFKSFLLGNLVPLCMKIINSVVVVGLYYGFLTTFSIGPSYLFLLRAQIMEEG---TE
KKVSATTGFITGQLMMFISIYYAPLHLALGRPHTITVLALPYLLFHFFWNNHKHFFDYGST
TRNSMRNLSVQCVFLNNLIFQLFNHFILPSSMLARLVNIYMFRCNNKMLFVTSSFVGWLIG
HILFMKWVGLVLVWIRQNHSIRSLIRSNKYLVSELRNSMARIFSILLFITCVYYLGRIPSPILT
KKLKETEEDKPLVTLLFDYNRWNRPFRYIKNKRFEKNVRNETSQYFFYTCQNDGKQRISF
TYPLSLSTFWEMIQRRMSLSTLGKIASDELYTHWIYTNKQQNN-INNEFINRIETLDMGSLF
LDILEKRTRLCNDETKKDYLPKMYDPFLNGPYRGTIKKWFSPIETIGINKIHGILCREFEQK
DPNDQSIGINEISKKIPRWSYKLIDLEQQS-GQIRSRKAKRVVIFTSALIRYSQQSDFRRNIIK
GSMRSQMRKIVIWELFQANVHSPLFLDRINKSPLFSFDI--SGLMKLIFRNRKEKARIEIAEA
WDTIPFAQVIRGSMLITQSILRKYIILPLLIVAKNMGRMLLLQLPEWSKDLQEWNREMHVK
CTYNGVQLSETEFPKNWLTDGIQIKILFPLCLKPWHRSKIKKKDDFCFLTVWGMEAELPFG
SPRKRPSFFEPIFKELKKKLKKLKKKYFLV-LRVLKGRTNFFLTVSKETKNWVIKSVL--FIKR
IRKVLSKVNDSIINNQIIHESFNQSRSTDWTNYSLTEKKRKDLTDRTRTIRNQIEKITNDKKK
NSNKTTYNAKRLESLKNIWQILKRRNARLISKLHYFIKIFIERIYIDVFRCIINIPQINIQLF-HF
ISCDLSSLSQAYVFYNLSQTHLD---KLRSVLQYTSFFLKTGIKDSFTQGIIHSELRYKKFRSY
GANQWKNWLRGHYQYDLSQIRWSRLIPQKWRNRVNQCRTAQKKKKWNSYEKNQ--LLY

YKKQ-NFQKYFKYDLLSYQSINGDIMYPDRKYFDWKILKFCLRFFDWMGMNEEILNCWF
FPEFVLLYNAYKMKPWIIPSKLLLLNYKSNTEAELNFFLKRYLLFQLRWDDALNQRMINNI
KVYCLLLRLRNPRKITISSIQRREMSLNIMLIQKNLPLTELMK-GILIIESVRLSVKNDGQFIM
YQTIGISLVHKSKRNHSNLLVPENILSSRRRRELRILICFRFLWPNYRLEDLACMNRYWFDT
NNGSRINMLRLRMYPMKGHQFKSWIFELREILREIKNSHYFLDSWTQFNSVGSFIHIFFHQ
ERFIKLFDPRIWSILLS-RNS--QGSTSNRYFT---IKG-VILFVLAVLIYR----INNRKMVERKNL
YLIGLLP-----IPMNSIGPRNDTLEESV-GSSNINRLIVS-LLYLPKGKKISESCFLNPKESTWVL
PIT----KKCSMPESNWGSRWWRNWIGKKR--DSSCKISN---ETVAGIEILFKEKDIK-YLEFLF
VYYMD--DPIRKNHDWELFDRLSLRKRRNIINL--NSGPLFEILVKHWICYLMSAFREKIPIEV
EG-FFKQQG-AGSTIQSNDIEHVSH--LFSRNKWAISLQNCAQFHMWQFRQDLFV---SWGK-
NPPQSDFLRN-VSRENLIWLDNVWLVNKDR---FFS--KVQNVSSNIQYDS---TR--SSFVQVT
DSSQLKGSS-DQSRDHLDSIS----NEDSD------------YHTLINQREIQQLKERSIL-WD--PSFLQ
TERTEIESD--RFPK--CLSGYS--SMSRLFT-EREKQMINHLFPEEIEEFL--GNPTRSVRSFFSDR
W--SELH-LGSNPTERSTRDQ----KLLKKQQDLS-FVPSRRSE----NKEMVNIFKIITYLQN--TV
SIHPISSDPGCDMVP-KDEPD-MDSSNKISFLNKNPFFDL--FHLFHDRNRGGYTL--HHDFES
E--ERFQEMADLFTLSITEP-DLVYHKGFAFSIDSYGLDQKQ--FLNEVFNSRDESKKKS---LL
VLPPIFY--EENESFYRRIRKKWVR-ISCGNDLEDSKPKIVVFASNNIMEAVNQYRLIRNLIQI
QYSTYG--YIRNVLNRFFLMN---RSDRNFEYG---IQRDQIGKDTMNHRTIMKYTINQHLSNL
KK-NQKKWFDPL--IFISRTERSMNRDPNAYRYKWSNGSKNFQEHLEHFVSEQKSR---FQV
VFDRLRINQYSIDWSEVIDKKDLSKPLRFFLSK---------SLLFLS-------KLLFFLSN-SLPFFFV
SFGNIPIHRSE-IYIY-ELKGPNDQLCNQLLESIGLQIVHLKKWKPF--LLD---DHDTS-QKSKF
LINGGTISPFLFNKIP-KW-MIDSFHTR--NNRRKSFDNTDSYFSMIFHDQDNWLNPVKPFHR
SSLISSFYKANRLRFLNNPHHFCFY--CNKRFPFYV----EKARIN--NYDFTYG-QFLNILFIRNK
-IFSLCVGK--KKHAFWG-RDTISPIESQVSNIFIPNDFPQSG-DETYNLYKS---FHFPSRSDPFV
R--RAIYSIADISGTPLTEGQIVNFERTYCQPLSDMNLSDSEGKNLHQYLN--FNSTMGLIHTP
CSEKYLPSEK--RKK--------RSLCLK--KCVEKGQMYRTFQRDSAFSTLSKWNLFQTYMPWF
LTSTGYKYLNLIFLDTFSDLL--PILSS-SQKFVSIFHDIMHGSDTSWRILQKKW------CLPQW
NL-ISEISSKCLHN--LLLSEEMIHRN--NESPLISTHLRSPNVREF-LYSILFLLLVAGY-LVRTHL
LFVSR--ASSELQTEFEKVKSLMIPSSMIDLRKLLDRYPTSEPNSFWLKNLFLVALEQLGDSL
EEIRGSASGGNMLLGGGPA-----YG-VKSIRSKKKYLNINLIDLISII----PNPINRITFSRNTRHL
SHTSKEIYSLIRKRKNVNGDWIDDKIESWVANSDSIDDEEREFLVQFS--TLTT--EKRIDQILL
SLT-HSDHL-----SKNDSGYQMIEQPGAIYLRYLVDIH--KKYLMNYEFNTSCLAERRIFLAHY
QTITY--SQTSRGANSFHFPSHG---KPFSLRLALSPS-RGILVIGSIGTGRSYLVKYLATNSYVP
FITVFLNKFLDNKPKGFLID-----DID-IDDSDDIDDSDDIDASDNIDA-----------SDDIDRDLD
TEL-ELLTMMNALTMDMMPE---IDRFYITLQFELAKAMSPCIIWIPNIHDLDVNESNYLSLG
LLVNHLSR---DCERYS-TRNILVIASTHIPQKVDPALIAPNKLNTCIKIRRLLIPQQRKHFFTLS
YTRGFHLEKKMFHT--NGFGSITMGSNARDLVALTNEALSISITQKKSIIDTNTIRSALHRQT
WDLR---SQVRSVQDHGILFYQIGRAVAQNVLLSNCPIDPISIYMKKK--SCNEGDSYLYKWY
FELGTSMKKLTRLLYLLSCSAGSVAQDLWSLP--GPDEK-NGITSYGLVENDS-DLVHGLLEV
EGALVG--SSRTEKDCSQFYND--RVTLLLRPEPRN-PLDMMQNGSCSILDQRFLYEKY--ESE
FEE-GEGEGALDPHQ----IEED----LFNH--IVWAP--RIWRP---WGFLFDCIERPNELGFP-YWS
RSFRGK---RIIYDEEDELQENDSEFLQSGTMQYQTRDRSSKEQGLFRISQFIWDPADPLFFLF
K-DQPFVSVFSHRELFADEEMSKGLLTSQTDPPTSIYKRWFI--KNTQEKHFKLLINRQRWLR
---TNSSLS--NGSFRSN--TLSESYQYLSNLFLSN-GTLLDQMTKTLLRKRWLFPDEMKIGF--

M---------MPRSR-INGNFIDKTFSIVANILLRIIPTTSGEKEAFTYYRDG--MSAQSEGNYAEAL
QNYYEAMRLEIDPYDRSYILYNIGLIHTSNGEHTKALEYYFRALERNPFLPQAFNNMAVIC
HY-RGEQAIRQGDSEIAEAWFDQAAEYWKQAIALTPGNYIEAHNWLKITGRFE--MSWRSE
HIWIEPIMGSRKASNFWWAIILFLGSLGFLLVGTSSYLGRNLISLFPSQQILFFPQGIVMSFY
GIAGLFISSYLWCTISWNVGSGYDRFDRKEGIVCIFRWGFPGKNRRIFLRFLIKDIQSVRIEV
KEGIYARRVLYMDIRGQGAIPLTRTDEN-FTPREIEQKAAELAYFLRVPIEVF
>Lonicera
LKKLWFHLMLLESDPEEGNDPFHDEEGNDNPHDSESDPRLPAYSGAPDLLEKRLLIYFPKD
DPNATRKKNPKGLCYYFRHWGSYLDRDRDRGKNREKEKHIAMVRARLYLYKDKGHTTD
DPSYERFIVDPKYFPKDNDREKIDNFFNEASHIVKSFSIVERYPSYGGDYGGDSDSGDYGS
DESDPDYGSDPSLQKKRNRKNYTHKYKHLWLMCDECYTLNYRRFLKERLYICEGCGFHL
KMNSSERLKLLIDPDTWDPMNEKMASLDPIEFHSEEDPYIDRVNSYRKKTGLTEAIQTGLC
QLNGIPTAMGVMEFGFMGGSMGSVVGEKITRLVEYATNQALPLIIVCASGGARMQEGSLS
LMQMAKISSSLYDFQTKKKLFYVSILTSPTTGGVTASFGMLGDVIVAEPDAYIAFAGKRVIE
LTLNKEVPEGSQETEYLFEKGLFDLVIPRKNLKSILNKLFGSHGFFPFTLNQNHMVTIRADE
ISNIIRQRIEQYNREVKIVNTGTVLQVGDGIARIHGLDEVMAGELVEFGEGTIGIALNLESN
NVGVVLMGGGLMIQEGSSVKATGRIAQIPVSEAYLGRVINALAKPIDGRGEIASSEYRLIES
PAPGIISRRSVYEPLQTGLIAIDSMIPIGRGQRELIIGDRQTGKTAVATDTILNQQGQNVICVY
VAIGQKASSVAQVVTTFREKRAMEYTIVVAETADSPATLQYLAPYTGASLAEYFMYRERH
TSIIYDDLSKQAQAYRQMSLLLRRPPGREAYPGDVFYLHSRLLERAAKLSSQLGEGSMTAL
PIVETQSGDVSAYIPTNVISITDGQIFLSADLFNAGIRPAINVGLSVSRVGSAAQIKAMKQVA
GKLKLDLAQFTELEVFAQFSSDLEKTTQNQLARGRRLRELLKQSQANPLAVEEQIMTIYTG
TNGYLDSFEVGQVRGFLDKLREYLKNNKPQFQEIISSTKTFTEEAEALLKEAIQEQTERFIL
QEEVMRIDPTTSGSGVSTLEKKNLGRIVQIIGPVLDVVFPPGKMPNIYNALVVKGRDTAGQ
PINVTCEVQQLLGNNRVRAVAMSATDGLTRGMEVIDTGAPLSVPVGGATLGRIFNVLGEP
VDNLGPVDTRTTSPIHRSAPAFIELDTKLSIFETGIKVVDLLAPYRRGGKIGLFGGAGVGKT
VLIMELINNIAKAHGGVSVFGGVGERTREGNDLYKEMKESGVINEQNIAESKVALVYGQM
NEPPGARMRVGLTALTMAEYFRDVNKQNVLLFIDNIFRFVQAGSEVSALLGRMPSAVGYQ
PTLSTEMGTLQERITSTKKGSITSIQAVYVPADDLTDPAPATTFAHLDATTVLSRGLAAKGIY
PAVDPLDSTSTMLQPGIVGKNHYETAQRVKQTLQRYKELQDIIAILGLDELSEEDRLTVARA
RKIERFLSQPFFVAEVFTGSPGKYVGLTETIRGFQLILSGELDVLPEQAFYLVGTIDEATAKA
MNLEMESNLKKMTLNLCVLTPNRIVWDSEVKEIILSTNSGQIGVLPNHAPIATAVDIGILRIR
LNDQWVTMALMGGFARIGNNEITVLVNNAEKSSDIDPHEAQQTLEIAEAGLKKAEGKRQ
TIEANLALRRARTRVETIRAISMKNVTDSFVSLGHWPSAGSFGFNTDILATNPINLSVVLGV
LIFFGKGVLSDLLDNRKQRILNAIRNSEELRGGAIERLEKARAHLRKVEMEADQYRVNGY
SEIERERLILINSTYKTLEQLENDNNETIHFEQQRAINQVRQRVFQQALQGALGTLNSCLNN
ELHLRIISANIDILGAMNEITDMNPLISAASVIAAGLAVGLASIGPGVGQGTAAGQAVEGIA
RQPEAEGKIRGTLLLSLAFMEALTIYGLVVALALLFANPFVMNVLSCSINTLKGLYDISGVE
VGQHFYWKIGGFQVHGQVLITSWVVIAILLGSATIAVRNPQTIPTGSQNFFEYVLEFIRDVS
KTQIGEEYGPWVPFIGTMFLFIFVSNWSGALLPWKIIQLPHGELAAPTNDINTTVALALLTS
VAYFYAGLSKRGLGYFGKYIQPTPILLPINILEDFTKPLSLSFRLFGNILADELVVVVLVSLVP
SVVPIPVMFLGLFTSGIQALIFATLAAAYIGESMEGHHMRFLTLEHILTHISFSTVSIVITIHLI
TLLADKIRKLYDSSAKGMIAAFLCITGLLVTRWVYSGHFPLSDLYESLIFLSWSFSVFHIVPY
--KKKQNLSTITGSSVIFTQGFATSGILDEIHQSTILVPALQSEWLIMHVSMMILGYAALLCGS

LLSVALLVITFRKNRKIFC--KEIQYMNERGNVLRKTS---AKNYYRCQLIQQLDYWSYRIISL
GFLFLTIGILSGAVWANEAWGSYWNWDPKETWAFITWTVFAIYLHTRTNKKLRGTNSAIV
ASIGFLIIWICYFGVNLLGIGLHSYGSFNLGMAKKKTFTPLLYLASLVFLPWWISLSVNKSL
ESWVTNWWNTRQSEIFLNDSQEKSILEKFIELEELALLDEMIKEYSETHLQNLRI-IHKETIQ
LIKIHNEDHIRMILHFSTNIICFIILSGYSILGTEELVILNSWAQEFLYNLSDTIKAFSILLVVDF
WVGFHSPHVWELMIGSVYKDFGFVHNDQILSVLVSTIPVILDTLFKYWLFFYLNRVSPSLV
VIYHSMNDMPVGVPKIPFQISGDEEASWVDVYNGLYRRGFLFIVQEIDTELSNQIVGLMVF
LSLEGEVNDLYFFISSLGGSMVDGFAIFDMLVYLPPTMHTLSIGECASMASLIVAGGTFTQR
IAFPHSRIMIHQPNTFPFETPTGETFLEIKEFCRLRALVGEAYAKITGQPLWVVEIDLERDIFM
SAQEAKDYGIVDLVASGQKDPKRIHEGLITESLPNGMFRVHLDNGDLIIGYISGKIRRSFIRI
LPGDKVQVEVSRYDSTRGRIIYRLRNKDSKDMEEFKRDFE-AGSQQHYFLYPLIFQEYIYAL
AYDHGLVGYDNKSSLLFVKRLITRMYQKNHLIFSVKDSNQNVFLGKNLYSQMISEGFAVI
VEIPFSLRFLSSLRVKSQNLRSIHSVFPFLEDKFSHLNYVLDILIPYSAHLEILVQTLRYWVK
DAPSLHLLRFFLHEYPNLTSLIIKTGSSFSKQRLFFFLYNSHVCEYEFIFVFLRNQSSHLRSTS
SGALLERIYFYEKIEHLVEVFAKDFNLWLFKDPFMHYVRYQGKSIFASKGTFLLMNKWKY
YFVRFWQCHFYQWFQPERISINQLSKHSLDLLGYLSSVRLNPLMARSQMLENAFLIDNAG
--KKFDTIVPISPLIGSLAKAKFCNVLGHPVSKVLWADLSDSEIIDRFGRICRNLSHYHSGSSQ
KKSLDRIKYILRLSCARTLARKHKSTVRAFLKRSGSEFLEDFFTAEEQVLFLTLPRTIWYLDI
ICINDLANHEMIID-TTEVQ-AINSFSRLES-LKEVDGIIWMLVPIFSLVLGITIGVLVIVWLERE
ISAGIQQRIGPEYAGPFGILQALADGTKLLFKENLLPSRGDIRLFSIGPSIAVISILLSYLVIPFG
YHLVLADLSIGVFLWIAISSIAPIGLLMSGYGSNNKYSFLGGLRAAAQSISYEIPLTLCVLSIS
LLSNSSSTVDVVEAQSKYGFWGWNLWRQPIGFIVFLISSLAECERLPFDLPEAEEELVAGY
QTEYSGIKFGLFYVASYLNLLVSSLFVTVLYLGGWNLSIPYILVPPLFEINKESRVFGTIMGIF
ITLAKTYLFLFIPIITRWTLPRLRMDQLLNLGWKFLLPISLGNLLLTTSSQLLSLMIWHIQNE
NFILDSTRIFMKAFHLLLFDGSLIFPECILIFGLILLLMIDSTSDQKDIPWLYFISSTSLVMSITA
LLFRWREEPMISFSGNFQTNNFNEIFQFLILLCSTLCIPLSVEYIECTEMAITEFLLFVLTATLG
GMFLCGANDLITIFVAPECFSLCSYLLSGYTKKDVRSNEATMKYLLMGGASSSILVHGFSW
LYGLSGGEIELQEIVNGLINTQMYNSPGISIALIFITVGIGFKLSPAPSHQWTPDVYEGSPTPV
VAFLSVTSKVAASASATRIFDIPFYFSSNEWHLLLEILAILSMILGNLIAITQTSMKRMLAYSS
IGQIGYVIIGIIVGDSNDGYASMITYMLFYISMNLGTFACIVLFGLRTGTDNIRDYAGLYTKD
PFLALSLALCLLSLGGLPPLAGFFGKLYLFWCGWQAGLYFLVLIGLLTSVVSIYYYLKIIKL
LMTGRNQEITPHVRNYRRSPFRSKNSIELSMIVCVIASTIPGISMNPIIAIAQDTLFMFLLYEY
DIFWTFLIISSLIPILAFFISGVLAPISKGPEKLSSYESGIEPMGDAWVQFRIRYYMFALVFVVF
DVETVFLYPWAMSFDVLGVSVFIEAFIFVLILIVGSVYAWRKGALEWSTNDFPWLTIIVVLP
ISAGSLICFLPHRGNKVIRWYTICICVLELLLTTYAFCYHFQSDDPLIQLVEDYKWIHFFDFH
WRLGIDGLSIGLILLTGFITTLATLAARPVTRDSRFFHFLMLAMYSGQIGSFSARDLLLFFIM
WELELIPVYLLLSMWGGKKRLYSATKFILYTAGGSVFLLMGVLGVGLYGSNEPTLNFETS
VNQRYPVALEIIFYIGFFIAFAVKSPIIPLHTWLPDTHGEAHYSTCMLLAGILLKMGAYGLVR
INMELLHHAHSIFSPWLVIVGTIQIIYAASTSPGQRNLKKRIAYSSVSHMGFILIGIGSITDTG
LNGAILQIISHGFIGAALFFLAGTGYDRIRLVYLDEMGGIAIQMPKIFTMFSTFSMASLALPG
MSGFAGEFIVFFGIITSQKYLLMPKILITFVMGIGMILTPIYSLAMSRQMFYGYKLFNAPNS
YVFDSGPRELFVSISIFLPILGIGMYPDFVLSLSVDKVEVILSNFFYRMMLEHVLVLSAYLFS
VGLYGLITSRNMVRALMCLELILNAVNINFVIFSDFFDSRQLKGAIFSIFVIAIAAAEAAIGL
AIVSSIYRNRKSTRINQSNLLNKMEQTSQYAWIIPFLPLPVPMLIGLGLLLFPTATKNFRRM

WAFQSVLLLSIVMIFSMNLSIQQLNSSSIYQYVWSWIINNDFSFELGYLIDPLTTIMSILITTV
GIMVLVYSDNYMSHDQGYLRFFAYMSFFSSSMLGLVTSSNLIQIYIFWELVGMCSYLLIGF
WFTRPVAANACQKAFVTNRVGDFGLLLGILGFYWLTGSFEFRDLFEIFNNNEVHFLFVILC
AVLFFAGAVAKSAQFPLHVWLPDAMEGPTPISALIHAATMVAAGVFLVARLLPLFIIIPYIMN
LISLLGIITVLLGATLALAQKDIKRGLAYSTMSQLGYMMLALGMGSYRSALFHLITHAFSK
ALLFLGSGSIIHAMETIVGYSPNKSQNMALMGGLTKHVPITKTAFLLGTLSLCGIPPLACFW
SKDEILNDSWLYSPIFAIIAWSTAALTAFYMFRIYLLTFEGHLNANFQNYSGKKNKPFDSISL
WGKEGSKRIKK-LLTLLTINFFSLGNKKSYLYPYESDNTMLFPILVLGLFTLFVGFLGIPFNL
DILSKWLTPYINLLHPNLN-SMDWYEFAKDAVFSVSIAYFGIVIASLLYKPVYSSFQNLYLIN
SFVILSDKIINGLYDWSYNRGYIDAFYATSLTGGIRGLAEFTHFFDRRVIDGITNGVGIMSFF
VGEGIKSVGGG-RISSYLFLYLSYVSIFLLIYYMDLPGPIHDFLLVFLGLGFILGGLGVVLLP
NPIYSAFSLGLVLVCISLFYILSNSYFVAAAQLLIYVGAINVLIIFAVMFMKGSEYYKDFHLL
AVGDGLTAMVCTSLFVSLITTIPDTSWYGILWTTKSSQIIEQDLISNSQQIGIHLSTDFFLPFE
LISIILLVSLIGAIAVARQMTTPVTRKDLMIVNMGPHHPSMHGVLRLIVTLDGEDVIDCEPV
LGYLHRGMEKIAENRTIIQYLPYVTRWDYLATMFTEAITVNAPEQLGNIQVPKRASYIRVI
MLELSRIASHLLWLGPFMADIGAQTPFFYIFRERELIYDLFEAATGMRMMHNFFRIGGVAA
DLPHGWIDKCLDFCDYFLTEVAEYQNLITRNPIFLKRVEGVGIIGGKEAINWGLSGPMLRA
SGIQWDLRKVDQYECYDEFDWEVQWQNEGDSLARYLVRINEMAESIKMIQQALEGIPGG
PYENLEIRRFDPLRDPKWNDFDYRFISKKPSPTFELSKQELYVRVEAPKGELGIFLIGDKNV
FPWRWKIRPPGFINLQILPQLVKRMKLADIMTILGSIDIIMGEVDRMFSMITELVNYGQQTV
RAARYIGQSFTITLSHANRLPVTIQYPYEKLITSERFRGRIHFEFDKCIACEVCVRVCPIDLPI
VDWKLETDIRKKRLLNYSIDFGICIFCGNCIEYCPTNCLSMTEEYELSVYDRHELNYNQIA
LGRLPMSVIDDYTIRTILNL----------------MQGHLSTWLVKHGLIHRSLGFDYQGTETLQIKP
EDWHSIAVILYVYGYNYLRSQCAYDVAPGGLLASVYHLTRIEYGVDQPEEVCIKVFAPRRN
PRIPSVFWVWKSVDFQERESYDMLGISYDNHPRLKRILMPENWIGWPLRKDYIAPNFYEI
QDAH--------------------------------------MVLAPEYSDNKQKNEKNKIDTVMNSIEFPLLDQTTQ
NSVISTTLNDLSNWSRLSSLWPLLYGTSCCFIEFASLIGSRFDFDRYGLVPRSSPRQADLILTA
GTVTMKMAPSLVRLYEQMPEPKYVIAMGACTITGGMFSTDSYSTVRGVDKLIPVDVYLP
GCPPKPEAVIDAITKLRKKISREIYEDRIRYQQENRCFTTNHKFQVWRSSHTGNYDQGFLY
QVPPTSEIP-ETFFKYKGSVSSHELVNMQTRNIFSWIKEQSTRSISVLLMIYIITRTSISSAYPIF
AQQGYENPREATGRIVCANCHLANKPVDIEVPQAILPDTVFEAVVRIPYDIQLKQVLANGK
KGALNVGAVLILPEGFELAPPDRISPEMKEKIGNLSFQSYRPNKKNILVIGPVPGQKYSEITF
PILSPDPVTKKDVHFLKYPIYVGGNRGRGQIYPDGSKSNNTVYNATAAGIVNKIIRKEKGG
YEITIADTSDGHQVVDIIPPGPELLVSEGESIKFDQPLTSNPNVGGFGQGDAEIVLQDPLRVQ
GLLFFLASVILAQIFLVLKKKQFEKVQLSEMNFMSKVYDWFEERLEIQAIADDITSKYVPP
HVNIFYCLGGITLTCFLVQVATGFAMTFYYRPTVTDAFASVQYIMTEANFGWLIRSVHRWS
ASMMVLMMILHVFRVYLTGGFKKPRELTWVTGVVLAVLTASFGVTGYSLPWDQIGYWAV
KIVTGVPEAIPVIGPPLVELLRGSASVGQSTLTRFYSLHTFVLPLLTAVFMLMHFPMIRKQGI
SGPLMGVTKKPDLNDPVLRAKLAKGMGHNYYGEPAWPNDLLYIFPVVILGTIACNVGLA
VLEPSMIGEPADPFATPLEILPEWYFFPVFQILRTVPNKLLGVLLMVSVPAGLLTVPFLENVN
KFQNPFRRPVATTVFLIGTAVALWLGIGATLPIDKSLTLGLFMIEVFLFGIVLGLIPITLAGLFV
TAYLQYRRGDQLDLMPTITSYFGFLLAALTIASVLFIGLSKIRLIMDIVSLAWAALMVVFTF
SLSLVVWGRSGLMIIRSPEPEVKILVDRDHIKTSFEEWAKPGHFSRTIAKGPDTTTWIWNLH
ADAHDFDSHTGDLEEISRKVFSAHFGQLSIIFLWLSGMYFHGARFSNYEAWLSDPTHIGPS

AQVVWPIVGQEILNGDVGGGFRGIQITSGFFQLWRASGITSELQLYCTAIGALIFAALMLFA
GWFHYHKAAPKLAWFQDVESMLNHHLAGLLGLGSLSWAGHQVHVSLPINQFLNAGVDP
KEIPLPHEFILNRDLLAQLYPSFAEGATPFFTLNWSKYADFLTFRGGLDPVTGGLWLTDIAH
HHLAIAILFLIAGHMYRTNWGIGHGLKDILEAHKGPFTGQGHKGLYEILTTSWHAQLSLNL
AMLGSLTIVVAHHMYSMPPYPYLATDYGTQLSLFTHHMWIGGFLIVGAAAHAAIFMVRD
YDPTTRYNDLLDRVLRHRDAIISHLNWACIFLGFHSFGLYIHNDTMSALGRPQDMFSDTAI
QLQPVFAQWIQNTHALAPGATAPGATTSTSLTWGGGDLVAVGGKVALLPIPLGTADFLVHH
IHAFTIHVTVLILLKGVLFARSSRLIPDKANLGFRFPCDGPGRGGTCQVSAWDHVFLGLFW
MYNAISVVIFHFSWKMQSDVWGSISDQGVVTHITGGNFAQSSITINGWLRDFLWAQASQV
IQSYGSSLSAYGLFFLGAHFVWAFSLMFLFSGRGYWQELIESIVWAHNKLKVAPATQPRAL
SIVQGRAVGVTHYLLGGIATTWAFFLARIIAVGMALRFPRFSQGLAQDPTTRRIWFGIATAH
DFESHDDITEERLYQNIFASHFGQLAIIFLWTSGNLFHVAWQGNFESWVQDPLHVRPIAHAI
WDPHFGQPAVEAFTRGGALGPVNIAYSGVYQWWYTIGLRTNEDLYTGALFLLFLSAISLIA
GWLHLQPKWKPSVSWFKNAESRLNHHLSGLFGVSSLAWTGHLVHVAIPGSRGQYVRWN
NFLDVVPHPQGLGPLFTGQWNLYAQNPDSTSHLFGTSQGAGTAILTLLGGFHPQTQSLWLT
DMAHHHLAIAFIFLVAGHMYRTNFGIGHSMKDLLDVHLPPGGRLGRGHRGLYDTINNSIH
FQLGLALASLGVITSLVAQHMYSLPAYAFIAQDFTTQAALYTHHQYIAGFIMTGAFAHGAIF
FIRDYNPEQNEDNVLARMLDHKEAIISHLSWASLFLGFHTLGLYVHNDVMLAFGTPEKQI
LIEPIFAQWIQSAHGKTSYGFDVLLSSTNGPAFNAGRSIWLPGWLNAINE--NSNSLFLTIGP
GDFLVHHAIALGLHTTTLILVKGALDARGSKLMPDKKDFGYSFPCDGPGRGGTCDISAWD
AFYLAVFWMLNTIGWVTFYWHWKHITLWQGNVSQFNESSTYLMGWLRDYLWLNSSQLI
NGYNPFGMNSLSVWAWMFLFGHLVWATGFMFLISWRGYWQELIETLAWAHERTPLANLI
RWRDKPVALSIVQARLVGLAHFSVGYIFTYAAFLIASTSGKFGMSHSVKIYDTCIGCTQCV
RACPTDVLEMIPWDGCKAKQIASAPRTEDCVGCKRCESACPTDFLSVRVYLWHETTRSM
GLAYMTTFNFPSVFVPLVGLVFPAIAMASLSLHVQKNKIVMRDLKTYLSAAPVLSTLWFG
SLAGLLIEINRFFPDALTFPFFSFMTAILERRESESLWGRFCNWITSTENRLYIGWFGVLMIPT
LLTATSVFIIAFIAAPPVDIDGIREPVSGSLLYGNNIISGAIIPTSAAIGLHFYPIWEAASVDEW
LYNGGPYELIVLHFLLGVACYMGREWELSFRLGMRPWIAVAYSAPVAAATAVFLIYPIGQG
SFSDGMPLGISGTFNFMIVFQAEHNILMHPFHMLGVAGVFGGSLFSAMHGSLVTSSLIRET
TENESANEGYRFGQEEETYNIVAAHGYFGRLIFQYASFNNSRSLHFFLAAWPVVGIWFTAL
GISTMAFNLNGFNFNQSVVDSQGRVINTWADIINRANLGMEVMHERNAHNFPLDLAALE
APSTNGMGLPWYRVHTVVLNDPGRLLSVHIMHTALVAGWAGSMALYELAVFDPSDPVLD
PMWRQGMFVIPFMTRLGITNSWGGWGIT-GGTITNPGIWSYEGVAGAHIVFSGLCFLAAIW
HWVYWDLEIFCDERTGKPSLDLPKIFGIHLFLAGVACFGFGAFHVTGLYGPGIWVSDPYGL
TGKVQSVNPAWGVEGFDPFVPGGIASHHIAAGALGILAGLFHLSVRPPQRLYKGLRMGNI
ETVLSSSIAAVFFAAFVVAGTMWYGSATTPIELFGPTRYQWDQGYFQQEIYRRVSAGLAEN
QSLSEAWSKIPEKLAFYDYIGNNPAKGGLFRAGSMDNGDGIAVGWLGHPIFRDKEGRELF
VRRMPTFFETFPVVLVDGDGIVRADVPFRRAESKYSVEQVGVTVEFYGGELNGVSYSDPA
TVKKYARRAQLGEIFELDRATLKSDGVFRSSPRGWFTFGHASFALLFFFGHIWHGARTLFR
DVFAGIDPDLDAQVEFGAFQKLGDPTTRRQVV------------VETLFNGTLALAGRDQETTGFA
WWAGNARLINLSGKLLGAHVAHAGLIVFWAGAMNLFEVAHFVPEKPMYEQGLILLPHLA
TLGWGVGPGGEVIDTFPYFVSGVLHLISSAVLGFGGIYHALLGPETLEESFPFFGYVWKDR
NKMTTILGIHLILLGIGAFLLVFKALYFGGVYDTWAPGGGDVRKITNLTLSPSIIFSYLLKSP
FGGEGWIVSVDDLEDIIGGHVWVGSICILGGIWHILTKPFAWARRALVWSGEAYLSYSLAA

LSVFGFIACCFVWFNNTAYPSEFYGPTGPEASQAQAFTFLVRDQRLGANVGSAQGPTGLG
KYLMRSPTGEVIFGGETMRFWDLRAPWLEPLRGPNGLDLSRLKKDIQPWQERRSAEYMT
HAPLGSLNSVGGVATEINAVNYVSPRSWLATSHFVLGFFLFVGHLWHAGRARAAAAGFE
KGIDRDFEPVLSMTPLNMTIALGKFTKDENDLFDIMDDWLRRDRFVFVGWSGLLLFPCAY
FAVGGWFTGTTFVTSWYTHGLASSYLEGCNFLTAAVSTPANSLAHSLLLLWGPEAQGDFT
RWCQLGGLWTFVALHGAFGLIGFMLRQFELARSVQLRPYNAIAFSGPIAVFVSVFLIYPLG
QSGWFFAPSFGVAAIFRFILFFQGFHNWTLNPFHMMGVAGVLGAALLCAIHGATVENTLF
EDGDGANTFRAFNPTQAEETYSMVTANRFWSQIFGVAFSNKRWLHFFMLFVPVTGLWMS
ALGVVGLALNLRAYDFVSQEIRAAEDPEFETFYTKNILLNEGIRAWMAAQDQPHENLIFPE
EVLPRGNALMSGSTGERSFADIITSIRYWVIHSITIPSLFIAGWLFVSTGLAYDVFGSPRPNE
YFAENRQGIPLITGRFDPLEQLDEFSRSFMTIDRTYPIFTVRWLAVHGLAVPTVSFLGSISAM
QFIQRMATQTVESGSRSG-PRRTNVGSLLKPLNSEYGKVAPGWGTTPLMGVAMALFAIFLS
IILEIYNSSVLLDGISMNMLTLKLFVYTVVIFFVSLFIFGFLSNDPGRNPGREEMANTTGRIP
LWIIGTVAGILVIGLIGIFFYGSYSGLGSSLMLNIFSLIYICLNSTLYSTSLFFAKLPEAYAFLNP
IVDVMPVIPLFFFLLAFVWQAAVSFRTTQSNPNEQNVELNRTSLYWGLLLIFVLAVLFSNYF
FNMEVNILAFIATALFILVPTAFLLIIYVKTISQNDMEAATLLAIFISGLLVSFTGYALYTAFGQ
PSQHLRDPFEEHGDMEALVYTFLLVSTLGIIFFAIFFREPPKVPTKK--MTLAFQLAVFALIAT
SSILLISVPVVFASPDGWSSNKNVVFSGTSLWIGLVFLVGILNSLIS-------MSPQTETKASVG
FKAGVKDYKLTYYTPDYETKDTDILAAFRVTPQPGVPPEEAGAAVAAESSTGTWTTVWT
DGLTSLDRYKGRCYHIEPVAGEENQFIAYVAYPLDLFEEGSVTNMFTSIVGNVFGFKALRA
LRLEDLRIPVSYVKTFQGPPHGIQVERDKLNKYGRPLLGCTIKPKLGLSAKNYGRAVYECL
RGGLDFTKDDENVNSQPFMRWRDRFLFCAEALFKAQVETGEIKGHYLNATAGTCEEMIK
RAVFARELGVPIVMHDYLTGGFTANTSLAHYCRDNGLLLHIHRAMHAVIDRQKNHGIHFR
VLAKALRMSGGDHIHSGTVVGKLEGEREITLGFVDLLRDDFIEKDRSRGIYFTQDWVSLP
GVLPVASGGIHVWHMPALTEIFGDDSVLQFGGGTLGHPWGNAPGAVANRVALEACVQAR
NEGRDLARDGNEIIRRASKWSPELAAACEVWKEIKFEFQAMDTL--MAIHLYKTSTPSTRN
GTVNGQVKPNPRNNLIYGQHRCGKGRNARGIITAGHRGGGHKRLYRKIDFRRNEKNIYGR
IVTIEYDPNRNASICLIHYGDGEKRYILHPRGAIIGDTIVSGTEVPIKMGNALPLTNMPLGTA
IHNIEMTLGKGGQLARAAGAVAKLISKEGKSATLKLPSGEVRLISKNCSATVGQVGNVRVN
QKSLGRAGSKRWLGKRPVVRGVVMNPVDHPHGGGEGKAPIGRKKPTTPWGYPALGKRS
RKRNKYSDNLILRRRNKMIQPQTYLNVADNSGARELMCIRIIGAS-NRRYAHIGDVIVAVIK
EAVPKMPLERSEVVRAVIVRTCKELKRDSGMIIRYDDNAAVLIDQEGNPKGTRVFGAIAGE
LRQLNFTKIVSLAPEVLMLSPKRTRFRKQHRGRMKGISYRGNHICFGKYALQALEPTWITS
RQIEAGRRAMTRNARRGGKIWVRIFPDKPVTVRSAETRMGSGKGSPEYWVAVVKPGRILY
EMGGVTENIARKAISIAASKMPIRAQFIIAGMTRTKRGSIARRRRTKVRSFVSSFQGAHSRL
TRTMSQQKIRALDSAHRDRDRQKRNFRRLWITRINAVMRE-RGVSYSYSRLIHDLYKRQLL
LNRKILAQIAISNRNSISIISNEIVKEVDWKESAGIIMFEDVYALGQHIAMSAHKARRVIDQI
RGRSYEETLMILELMPYRACYPIFKLVYSAAANASYILGSNEANLIISKAEVNEGTAVKKL
KPRARGRSYPIKRPTCHITIIVKDMSLPGGVWDKKMDGIKYAVFTDKSIRLLSKN-QYTSN
VESGLTRTEIKHWGELFFGVKIIAMNSHRLQGKGRRMGPSMGQTMHYRRMIITLQPGYSI
PPLRKNLNMAVPKKRTSISKKRIHKNIWKGKGYWFALKALSLGKSLSTGNSKSFFVRQTN
KMAKSKDVRITVILECTSCVRNGVNVDKVSTGISRYMTQKNQHNTPNRLELRKFCPYCY
KHTIHGEIKKMKIRASVRKICDKCRLIRRRGRIIVICSNPRHKQRQGMVQEKVTVSTRTLE
WKCVESRADSKRLYYGRFILSPLMKGQADTIGIAMRRALLGEIEGTCITRAKSE--KIPHEY

STIVGIQESVHEILMNLKEIVLRSNLYGTCDASICVRGPGNVTAQDIISPPYVEIIDNTQHIVS
LTEPIDFCIGLQIERNRGYLIKTPN-HFDGSY--PIDAVFMPVRNANHSIHSYGNENEKQEILFL
EIWTNGSLTPKEALHEASRNLIDLFIPFLHTEEE--NLHLEDNQ-HTVPLTPFTFHEKLA-KLR
KKK---MLQDGN-EGMYTIPGLNQI
QFEGFCRFIDQGLMEELSKFPKIEDTDQKAEFQLFMETYQFVEPLIKERDAVYKLLTYSSEL
YVSAGLIWKTRRDMQEQTIFIGNIPLMNSLGTSIVKGIYRIVINQILQSPGIYYRSELDHNGI
EAYTGTIISDWGGRSGLEIDRKARIWARVSRKQKISILVLSSAMGSNLREILENVCYPEIFLS
FLNAKEKKKIGSKENAILEFYQQFACVGGDPVFSESLSKELQKKFFQQKCELGRIGRRNM
NRRLNLDIPQNNTFLLPRDILAAADHLIGMKFGMGPLDD--MNHLKNKRIRSVADLLQDQF
GLALSRLENVIRGTISGALR-HKLI-PTPQNLVTSTP----LTTTYESFFSLHPLSQVLDRTNPLT
QIVHGRKLSYLGPGGLTGRTASFRIRDIHPSYYGRICPIDTSEGINVGLIGSLAIHARIGRWG-
SLESPFY---EISEKPKG--ARMLYLSPGRDEYSMVAAGNSLALNQSIQEEQVVPARYRQEFLT
VAWEQVHLRSIFSFQYFSIGASLIPFIEHNDANRALMSSNMQRQAVPLSRSEKCIVGTGLER
QAALDSGALAIAKHEGKISYIDTDKILLSSNRDTLSI---MGRGSNKKTCMHQKPQVRRVKC
IKKGQILGYGAATVGGELALGKNVLVAYMPWEGYNSEDAVLISERLVYEDIYTSFHIRKYE
TKICVTSQG-PERVTNEIPHLEAHLLRNLDKRGIVMLGSWVEAGDILVGKLTPKIVKE-SSS
APEARLLRAILGIQAATSKETCLKLPIGGRGRVIDVRWIQQNEVSGSN-PERICVSILQKREI
KVGDKVAGRHGNKGIISKILPRQDMPYLQDGRPVDMVFNPLGVPSRMNVGQIFECSLGLA
GDLLNRHYRIAPFDERYEQEASRKLVFSELYEASKQTANPWVFEPEYPGKSRIFDGRTGDP
FEQPVIIGKPYILKLIHQVDDKIHGRSSGQYAVVTQQAVRGRSRGGGQRVGEMEVWALEG
FGVAHILQEMLTYKSDHMRARQGILGTTLIGGTIPNPEGAPESFRLLVRELRSLALELNHFL
VSEKNFQIHREEA-------MIDRYKHQQLRIGLVSPQQISTWANKILPNGEIVGEVTKSSTFHY
KTKKPQKGGLFCERIFGPIKSGICACGNYRVIGDAKDDPKFCEQCGVEFVDSRIRRYQMGY
IKLACPVTHVWYLKRLPSYIANLLDKPLKELEGLVYCD--FSFARP-ITKKPTFLRLRGSFEY
EIQSWKSIIPPFFTSQGFDTFRNREISTGAGAIREQLADLDLRIIVDYSLVEWKELGEE-GLTG
NEWEDRKVGRRKHFLARRMELAKHFIRTNIEPEWMVLCLLPVLPPELRPLFRTDSSRLVRS
DINELYRRVIYRNNTLTDLLTTSRFAPGELVMCQEKLVQEAVDSLLDNGIGGQPTKDGSNQ
VYKSFSDRIAGKEGRFRETLLGKRVDYSGRSVIVVGPSLSLHRCGLPREIAIELFQTFVIRGL
IRQHLASNIRVAKSQIREKEPIVWEILQEVMQGHPVLLNRAPTLHRLGIQAFQPVLVEGRAI
CLHPLVCKGFNADFDGDQMAVHVPLSLEAQAEARLLMFSHLNLLSPAIGNPISVPTQDMLI
GLYVLTRGNRRGVCVNRYNPWNC---RNYQNERIFDNNYKYTK--EPLFSNSYDAIGAYRQ
KRINLDSPLWLRWRLDQR-VIAT--REAPIEVHYESLGTYYEIYGHYLIVRSIKKEILYIYIRTT
VGHIFLYREIEEAIQGFCQACS-----------MEVLMAERANLVFHNKVIDGTAIKRLISRLIDHF
GMAYTSHILDQVKTLGFQQATATSISLGIDDLLTIPSKRWLVQDAEQQSLILEKHHHYGNV
HAVEKLRQSIEIWYATSEYLRQEMNPNFRMTDPFNPVHIMSFSGARGNASQVHQLVGMRG
LMSDPQGQMIDLPIQSNLREGLSLTEYIISCYGARKGVVDTAVRTSDAGYLTRRLVEVVQH
IVVRRTDCGTAQGISVSPRNGTMA-ESILIQTLIGRVLADDIYMGPRCIAARNQDIGIGLVNR
FIT------FRAQPISIRTPFTCRSTSWICRLCYGRSPTHGDLVELGEAVGIIAGQSIGEPGTQLTL
RTFHTGGVFTGGTAEYVRAPSNGKIKFNEDLIHPTRTRHGYPAFLCSIDLYVTIESEDIIHNV
NIPPKSFLLVQNEQYVESEQVIAEIRAGTST-LNFKEKVRKHIYSDSGGE-MHWNTDVYHAP
EFTYGNVHLLPKTSHLWVLLGQPCR--SSLVSLSSHKDQDQMNAH--SLSVKRKYISNPS--V
TNDQ-ARHKFFSSDFYGQ-EDRIPDY-SELTRIICIGRCNLIYP-AILHENS--DLLAKRRRNRFII
PLQSIQEGENELMLSSGITIEMP--INGIFRRNSILAYFDDPQYRRKGSGITKYGILEVDSILKK
ED--LDEYRE-VEEFSPKNMKVDRFFFIPEEVHILSESSSLMVQNNSIIGVDTQMTLTTRSRA

AGLVRVERKKNRIELKIFSGDIHFPGETDKISRHSGVLIPQGM--GKKNSK--KS--MKDWIYV
QR--ITPTP--KKYFVLVRPVVTYEITDGINLA-LFPPDLLQERDNVQLRIVNYILYGNGKSIRG
ISDTSIQLVRICLVLNWDQDKK---SSLVEEGRPSFVEIRTNGLIRDFLRIDLAKSPILYTGKRN
DTSGSGLISE-NGSDRTNINPFSSIFSY---SGAKIQESVNQNQGTIHTLLNR---NKECQSLIIFSS
SNCSRIGS--CNDVKYDNV---IKEL-PLI---NSLGPLGT--ALPIENFYL--FSHL--ITHNQIL-VTN
YLQLDNLKQIKQTFQVLN---YYFIDEN--AKIYNSDPCRNIILNPFKLNWYLIPHNYFEKTS-T
IMSLGQFICENGCIAKNRPH-LK--SGQVLIVQ-VDSVVIRSAKPYLAIPGATVHGHYGRILYA
GDTLVTFIYEKSRSGDITQGLPKVEQVFEVRSIDSISMNLEQRVEGWNECITRILGIPWGFLI
GAELTIVQSRISLVNKIQKVYRSQGVQIHNRHIEIIVRQITSKVLVSE---DGMSNVFLPGELIG
LLRAERTGRALEEAICYRAILLGITRASLNTQSFISEASFQETARVLAKAALRGRIDWLKGL
KANVVLGGMIPVGTGFKGL--VHPSRKHNNIP--LETKKKNLFEGEMR--DILFHHKNVFDS
CFSQNFYDTPEQSFIT------FNDSMTRRYWNINLEEMMEAGVHFGHGIRKWNPKMAPYISA
KRKGNHITNLTRTARFLSEACNLVFDAASRRKQFLIVGTKKKAADSVARAAIRARCHYVN
KKWPGGMLTNWSTTEMRLHKFRDLRTQQKTGRLNRLPKRDAAMLKRRLSHLQTYLGGI
KYMTGLPDIVIVVDQHEEYTALRECITLGIPTICLIDTNCDPDLADLPIPANDDAISSIRLILN
KLVFAICEGRS-SYLRNPMGQKINPLGFRLGTTQSHHSLWFAQPKNYSEGLQEDQKIRNSIK
NYIQKNMRIASG-VEGIGRIEIQKSIDLIQVIIYMGFSKLLIKSRMREIRNLQEELQMNLQKE
FHWVNRKLNIAITEIEKPYGNPNILAEFIANQLKNRVSVRKAMKKVIELTEKEGTKGIQVQI
AGRINGKDIARVVWIREGRVPLQTIRAKIDYCSYTVRTIHGVLGIKIWIFIDGEMSRYRGPR
FKKIRRLGALPGLTKKGPIG------QSRSVK-KSQYRIRLEEKQKLRFHYGLTERQLLKYVRIA
GKAKGSTGQVLLQLLEMRLDNILFRLGMAPTIPGARQLVNHRHILVNGRIVDIPSYRCKPR
DIITARDEQNSRALIQNSLNSSPHKNKLPKHLTLQPFQ--YKGLVNQIIDSKWVGLKINELLV
VEYYSRQTMSRRGTAEKKTAKYDPIYRNRLVNMLVNRILKHGKKALAYKILYRAIKKIHQ
ETKRNPLSVLREAIRKVTPKIAVKARRVSGSTRQVPIEIGSKKGKVLAIRWLLAASRKRPG
QDMAFKLSSEFMDASKGSGDAIRKKEETRRMAEANRTFAHFRMGRDTIADIITSIRNADM
DRKRVVRIASTNITENIVKILLREGFIENARKHRENSKSFLVLTLQHRR--NRQKPYR-KRNG
LNLKRISRPGLRIYSNYQRIPRILGGMGVVILSTSRGIMTDREARLERIGGEILCYIWMAKAI
PRIGSHRKGRFGSRKSTRRIPKGVIHVQASFHNTIVTVTDVRGQVVSWSSAGTCGFRGRRR
GTPFAAQTIAGNAIRTVVDQGMKRANVMIKGPGLGRDAALRAIRRSGILLTFIRDVTPLPH
NGCRPPKRRRVMPTIKQLIRNTRQPIRNVTKAPALRGCPQRRGTCTRVYTITPKKPNSALR
KVARVRLTSGFEITAYIPGIGHNSKEHSVVLVRGGRVKDLPGVRYHIVRGTLDADKVEGRQ
QGRSKYGVKKPKMARKSLIQREKKRQKLEQKYHLIRRSSKKEIR---KVPSLSDKREIYGKL
QSPPRNSAPTRLHRRCFLTGRPRANYRDFGLSGHILREMVHACLLPGTTRSSWMLKDAFV
SIILQ--EENKENRGSAEFQVVNFTNKIRKLTLHLKLHKKDYSSQRGLLKILGKRQRLLAYL
SNKNRIR-----------------MVKLRLKRCGRKQRAVYRIVAIDVRCRREGRALRKVGFYDPIKN
QTYLNVPAILYFLEKGAQPTGTVGDILRKAEAFKELRPKQTQLRKMDKSKRPFLKSKRPFR
KRLPPIQPGDQIEYRNMSLISRFISDQGKILPRRVKRLTLKQQRLITLAIKQARILSSLPFLNN
RKL-KTKNKKNKRKKRLKNKFHPVTHSFKKNPFVANNLLRKMDKLNTKEEKEIIITWSRA
STIIPTMVGHTIAIHNGKEHLPIYITDRMVGHKLGEFAPTLNFRGHAKSDNRSRRMTLPFFQ
LANLVSLCMKIINSAVVVGLYYGFLTTFCVGSSYLFLLRAHVIEEAR---EKKISATTGFLMG
QLIMFISIYYVPLHLALGRPHTITVLVCPYLWFHVRWNNDKKKKFFASTTRDSMRNLSTQG
LFLNHLVFQSLNPYFLPSAMLARLVNVYMFRCNNKILFVTSSFVGWLIGFILLMK----LLV
WIQHNRFIRLYVRFNKYLMSEFTYRILRTFSIILFITCVYYLGRMPSPLLTKKLEKFEFVKLP
VILLFDYKDWNRPLRYIKNRDFKGAVKSETAQYFFDTCRSDGKQRISFTYPPSLSTFLEMIQ

RRIDEFTLPP--YDELYNHWVYTKTKKKNKIKNELLNRIKALDMGFLSRDILEKRTRLVKD
RPNQKLLSQEYDPFLNGPYRGTPQQRFSP-KTVKMNKIHGILQKEFEQKNLRVPGIGIKEID
KKVPRWSSKLIEMEAEFLDHLCSRALKEIVLSKEIYLDILKEPDFERDLIMSSTRAQRRKVII
LKLLHPKPQSPLFWGRIKSVLGYFFTH----PIKFIFINKEELMRIQVESAWENLPYGPGIRSSL
LLMQSFLRKNISLPLLIIAKNIGRLLFLQVPEWAEDFQELNRERHFPCTHLGVGLNDPEVLA
HWAEEGFDIKILYPFRLKPWHRSKKEVEDDFGFLTILGMETEYPFGSPRKRTSFFKPIFKKL
GKQ-------------------------IKKKKAFEDFKVLG-VFKKVISEFSKRN--TINNQIIEESFTQIRSID
RANKNLTEQEMKNLTDRTSTIKNKIQRLTKDKKK-------TNSNVESQPLIWQILKRRNARLIS
QFHYFLKIFIEKIYIDIFPRIINITRINTQPL------DLSSLSQRDVFFKLSQPQAINLSKLRSALQ
YTSFFLKTAIKDSFIQGIFHSELKHKKPR--SYDQWKNWLRGH-QYNLSPIRWSRLIPQKWR
NRVNQRCTAENKQKWSSDENEKDLLFHYTNP-NFQKYYRYGLLSYKSLNANIIDPDRKYF
DWKMQNFALNFFDWIKIDKERLRNWFFPEHVLLYTAYKVKPWIIPNPLFLGNGEAFRGRIR
TPDLVQYWLFQLKWHNYFGETIPGLMKQFSAQLEWLNYQKWVITYLNNKLFSINQHLIH
YETTLEMGELGEVVILESNRLYLGTYSQIIMYQTIRISLVDQSKQNHYDFLVPETILSSRRRR
ELRILISFKFLWPNYRLEDLACMNRYWFDTNNGSRFSMLRIYMYPMIRYSFQPLIFELREIL
REIKNSHHLLDSWTQFNSVASFIHIFFHQERFIKLFDPRIWSILLS-RNS--QGSTSSRYFR---IK
GVILFGVA-VLLYR----INSRNMVERKNLYLIGLLP-----IPMNSIGPRNDTLEESV-GSSNINRLI
VS-LLYLPKGKKISESCFLNPKESTWVLPIT----KKCSMPESNWGSRWWRNWIGKKR---------
----
--DS----------------SQLKGSS
-DQSRDPLDSVS----NEDSE------------YHPLINQREIQQLKERSIL-WD--PSFLQTERTEIESD--
RFPK--CLSGYS--SMSRLFT-EREKQMINHLLPEEIEEFL--GNPTRSVRSFFSDRW--SELH-LG
SNPTERSTRDQ----KLLKKQEDLS-FVPSRRSE----NKEMVNIFKIITYLQN--TVSIHPISSDPG
CDMVP-KDEPD-MDSSNKISFLNKNPFLDL--FHLFHDRNRGGYTL--HHDFESE--ERFPEMA
DLFTLSITEP-DLVYHRGFAFSIDSYGLDQKQ--FLNEVFNSRDESKKKSL---LVLPPIFY--EEN
ESFYRRIRKKWVM-ISCGNDLEDPKPKRVVFASNNIMEAVTQSRLIGNLIQIQYSTYG--YIR
NVWNRFFLMN---RSDRNFEYGIQ---GDQIGKDTLNHRTIMKYTINQHLSNLKK-SQKRWFD
P--LILISRTERSMNRDPDAYRYKWSNGSKNFQEH---FVSEQKSR---FQVVFDRLRINQYSID
WSEVIDKKDLSKPLRFFLSKPLRFFWSKSLRFFLSKSPLFLSKLLFFLSN-SLPFFFVSFGNIPI
HRSE-IYIY-ELKGPNDQLCNQLLESIGLQIVHLKKWKPF--LLD---DHDTS-QKSKFLINGGT
TSPFLFNKIP-KW-MIDSFHTR--KNRRKSFDNPDSYFSMIFHDQDNWLNPVKPFHRSSLISA
FYKANRLRFLNNPHHFCFY--CNKRFPFYV----EKARINNYD--FTYG-QFLNILFIRNK-IFSLC
VGK--KKHAFWG-RGTISPIESQVSNIFIPNDFPQSGDET-YNLYKS---FHFPSRPDPFVR--RAI
YSIADISGTPLTEGQMVHFERTYCQPLSDMNLSDSEGKNLHQY----FNSNMGLIHTPCSEKY
LPSEK--RKK--------RSLCLK--KCVKKGQMYRTFQRDSSFSTLSKWNLFQTYMPWFFTSTG
YKYLNLILLDIFSDLLPILSRSQ---KFVSNIHIMRRLG-ISWRILQRKWG------LQ-WNW-ISEIS
SKCLH--QLLLSEEMIHRNNES--PLISTHLRSPNVREF-LYSILFLLLVAGY-LVPTHLLFVSR--
ASSELQTEFEKVKSLMIPSSMIELQELLESDPPSEPNDPIFKHPLQARLEQLADFLVWKVFF
SVGDMLFNLS------------------IDLVDIDIIDLISNI----PNPNDQITFSRNTRHLSHTSKEIYSLIR
KVKVGNGDWADDQVESWVANSDYVDDEEREFLVQFST-LTTEK-RIEIDQILWSLT-HSDHF
S-----KNDSGYQMIEQPGEIYLRYLVDIH--KKYLMNYEFNTSCLAERRIFLAHYQTITY--SQT
PCGANSFDFPSPG---KLFSLRLALSPA-RGILVIGSIGTGRSYLVKYLATNSYVPFITVLYEEEL
DNDPSNFAAYKDVEMFFEVIDDIDDSKIDESDIDESQIDE------------SPIDDRTIDRQMDHRL
DGITAWDALGMEVLSLRNHYYINLQFELAKAMSPCIIWMPNIHDLRVKESDCLSLGLLVN

HLSG---DCERCS-TRNILVIASTHIPQKVDPAVIAPNKLTTCIKIRKLLIPHQRKHFLTLSYTRG
FRLENQMFHT--NRFGYITMGSNPQDLAALTNEALSISIIQKKSIIDTNTIRFALHRQTWDLR-
--SQVRSAQDRGILFYQIGRAVAQNVLLSNCLIDPISIYLKKK--SCNEGDSYLYKWYFELGTS
MKKLTILLYLLSCSAGSVAQDLWSLP--GPDEK-TGIAYYRPAENDS-DLVHGLLEVEGALVG
--DSRTEKDCSQFDND--RVTLLLRPEPRN-PLDMMQIGYFSILDVGFLYQR--SELEFED---GE
GALDLE-------QEF--FTN----IVWAP--RIWSP---WDFLLDCIDIPTELGFPFYGSDEKYAG-DKE
EYYDEEEENDEEEDELQENDSEFLQTRDISYKKESLFRLSRFIWNPGDPLFGLFV-DESSVS
VFLHRELFADEAQSLLTSVTSEEPSRSMMKGWFID-NKAQEKHFEFLLNWHRWFRT---PSS
LS--NGSFRSN--TLFESYQYLSNLFLSN-ETLLDQMRNTLLRKRWLFPDENWIHG--TGERFK
DM--MPRSR-INGNFIDKTFSIVANILLRIIPTTSGEKEAFTYYRDG--MSAQSEGNFAEALQN
YYEAMRLEIDPYDRSYILYNIGLIHTSNGEHTKALEYYFRALERNPFLPQAFNNMAVICHY-
RGEQAIRQGDSEIAEAWFDQAAEYWKRAIALTPGNYIEAHNWLKITRRFE--MSCRSEHIWI
EPITGSRKTSNFCWAIILFLGSLGFLLVGTSSYFGRNFTSLFPSQQIVFFPQGLVMSFYGIAGL
FISSYLWCTISWNVGSGYDRFDRKEGIVCIFRWGFPGKNRRICLRFFIKDIQSIRIEVKEGLY
ACRVLYMDIRGQGAIPLTRTDEN-LTPREIEQKAAELAYFLRVPIEVF
>Meliosma
MEKWWFNSILSLSKSMGSLGPIRKTNGRENPFINDTEKNIPSCSNVDHLFGVRNIRSFIFDD
TLLVRDSNGYSYSIYFDIENQLFEIDND--HSFLSELEKELSSYLNSNYLNTGSKSDNHYYDR
YMYDTKYSWNNHINSCIDSYLCSEIS--IDSYISNGSDNYSDSYIYSYICSDSRSSIR---NDSD-
NKRGRY-DLEINKRYRTLWVQCENCYGLNYKKLFRSKMNICEQCGYHLKMSSSDRIELSID
PGTWDPMDENMVSTDPIEFHSEADPYKDRIDFYQRKTGLTEAVQTGIGQLNGIPVAMGVM
DFKFMGGSMGSVVGEKITRLIEYATHRSLPLIIVCASGGARMQEGSLSLMQMAKISSASYN
YQSNKKLFYVSILTSPTTGGVTASFGMLGDIIIAEPNAYIAFAGKRVIEQTLNKTVPEGSQA
AEYSFHKGLFDSIVPRNLLKGVLSELFYLHGFFPLNQNSIKMVTLRADEISNIIRERIEQYNR
EVKIVNTGTVLQVGDGIARIHGLDEVMAGELVEFEEGTIGIALNLESNNVGVVLMGDGLM
IQEGSSVKATGRIAQIPVGDAYLGRVINALAKPIDGRGEISASESRLIESPAPGIISRRSVYEPL
QTGLIAIDSMIPIGRGQRELIIGDRQTGKTAVATDTILNQKGQNVICVYVAIGQKASSVAQV
VTTFQERGAMEYTIVVAETADSPATLQYLAPYTGAALAEYFMYRERHTSIIYDDLSKQAQ
AYRQMSLLLRRPPGREAYPGDVFYLHSRLLERAAKSSSRLGEGSMTALPIVETQSGDVSAY
IPTNVISITDGQIFLSADLFNAGIRPAINVGISVSRVGSAAQIKAMKQVAGKSKLELAQFAEL
EAFAQFASDLDKATQNQLARGQRLRELLKQSQSAPLTVEEQILTIYTGTNGYLDSLEIGQV
KKFLVQLRNYLKTNKPQFQEIISSTKTFTEEAETLLKEAIPEQIERFLLQEQTMRINPTTSGP
GVPALEEKNLGRIAQIIGPVLDVAFPPGKMPNIYNALVVKGRDTVGQQINVTCEVQQLLG
NNRVRAVAMSATDGLMRGMDVIDTGAPLSVPVGGATLGRIFNVLGEPVDNLGPVDTRTTS
PIHRSAPAFIQLDTKLSIFETGIKVVDLLAPYRRGGKIGLFGGAGVGKTVLIMELINNIAKA
HGGVSVFGGVGERTREGNDLYMEMKESGVINEQNIAESKVALVYGQMNEPPGARMRVGL
TALTMAEYFRDVNEQDVLLFIDNIFRFVQAGSEVSALLGRMPSAVGYQPTLSTEMGSLQE
RITSTKEGSITSIQAVYVPADDLTDPAPATTFAHLDATTVLSRGLAAKGIYPAVDPLDSTSTM
LQPRIVGEEHYETAQRVKQTLQRYKELQDIIAILGLDELSEEDRLTVARARKIERFLSQPFFV
AEVFTGSPGKYVGLAETIRGFQLILSGELDGLPEQAFYLVGNIDEATAKAMNLEVESNLKK
MTLNLCVLTPNRIVWDSEVKEIILSTNSGQIGVLPNHAPIATAVDIGILRIRLNDQWLTMAL
MGGFARIGNNEITILVNDAEKGSDIDPQEAQRTLEIAEANLSRAEGKRQTIEANLALRRART
RVEAINVISMKNVTDSFVSLGYWPSAGSFGFNTDILATNPINLSVVLGVLIFFGKGVLSDLL
DNRKQRILSTIRSSEELREGAIQQLEKARARLRKVEMEADEFRVNGYSEIEREKLNLINSTY

KNLEQLENYKNETIHFEQQRAINQVRQRVFQQALQGALGTLNSCLNSELHLRTINANIGM
FGAMKEITDMNPLISAASVIAAGLAVGLASIGPGVGQGTAAGQAVEGIARQPEAEGKIRGT
LLLSLAFMEALTIYGLVVALALLFANPFVMNVLPCSINTLKEFYDISGVEVGQHFYWQIGG
FQVHAQVLITSWFVIAILLGSATIAVRNPQTIPTDGQNFFEYVLEFIRDLSKTQIGEEYGPWV
PFIGTMFLFIFVSNWSGALLPWKIIQLPHGELAAPTNDINTTVALALPTSVAYFYAGLTKKG
LGYFGKYIQPTPILLPINILEDFTKPLSLSFRLFGNILADELVVVVLVSLVPLVVPIPVMFLGL
FTSGIQALIFATLAAAYIGESMEGHHMIFLTLEHILTYISFSIVSIVITIHLMTLLVDEIIGLYDS
SEKGMIVTFFFITGLLVTRWIYSGHFPLSDLYESLMFLSWSFSIIHMVTKIRNHQNHLSAITA
PSAIFTQGFVISGLLTGMHQSAILVPALQSQWLMMHVTMMLLSYAALLCGSLLSVALLVIT
FRKNIEIVGKSNKIQSMNERNTLLQNTSFLSFRNYHRYQLIQQLDHWSYRVISLGFIFLTIGI
LSGAVWANEAWGSYWNWDPKETWAFITWTIFAIYFHIRTNNNLQGANSAIVASMGFLIIWI
CYFGVNLLGIGLHSYGSFILTMPKKKAFTPPPYLASILFLPWWISLSFNKSLESWVTNWWN
TRQSETFLNDIQEKCVLEKFIELEELFLLDEMIKEYPETHIQKLRIGIHKETIQLVKMYNEDH
IHAILDFSTNIICFAILSGYSILGNEELVSLNSWVQEFLYNLSDTIKAFSILLLTDLCIGFHSPH
GWELMIGSVYKDFGFSHNDQIISGLVSTFPVILDTIFKYWIFRYLNRVSPSLVVIYHSMNDM
PIGVPKVPFRSPGEEDAIWVDVYNRLHRERLLFLGQGVESEISNQLIGLMVYLSIEDDTRDL
YLFINSPGGWVIPGIAIYDTMQFVPPDVHTICMGLAASMGSFILVGGEITKRLAFPHARVMI
HQPASSFYEAQTGEFILEAEELLKLRETLTRVYVQRTGNPLWVVSEDMERDVFMSATEAQ
AHGIVDLVAVEMKEQKWIHEGLITESLPNGMFRVRLDNEDLILGYVSGRIRRSFIRILPGDR
VKIEVSRYDSTRGRIIYRLRNKEANDMEELQEYLEIDGSWQQDFLYPLLFQEYIYALAHDH
GLENFG-KSSLLIVKRLITQMYQQNHLSIPANDSNQNQLVVKNLDSQMISEGFSVIVEIPFLL
RLVSSLIEKSHNLRSIHSIFPFLEDKFSHLNYVSHILIPHPIHWEVMVQNLRCWVQDAPSLH
LLRFFLHEYHNSNSLITKSISILKKQRLFCFLYNSYIYECESIVVFIRKKSSHLRSTSSGALLE
RAHFYGKMEHLVVVFRNDFNLWFFKEPFIHYARYQGKAILASKGTPLLMKKWKYHLLNL
WQCHFYLWSQPSRIHINRLSNHSLQFLGYLSSVRLNPSVVRSQLLETSFIIDIAI--KKFDTIIP
IIPLIGSLAKAKICNVSGHPISKPVWADSSDSDIIDRFGWICRNLSHYHSGSSKKNGLYRIKYI
LRLSCAKTLARKHKSTVRAFLKRLGSELLEEFLTKEEQVLSLIFPRRIWYLDIIRINDLANY
EMIIDTT-EVQ-AINPFSRSE-SLKEVYELIWLFVPIFTPVLGITIGVLVIVWLERQISAGIQQRI
GPEYAGPLGILQALADGTKLLFKEDLLPSRGDSRLFSIGPSIAVISILLSYSVIPFGYRLVLAD
LSIGVFLWIAISSIAPIGLLMSGYGSNNKYSFSGGLRAAAQSISYEIPLTLCVLSISLLSNSSST
VDIVEAQSKYGFWGWNLWRQPIGFIVFLISSLAECERLPFDLPEAEEELVAGYQTEYSSIKF
GLFYVASYLNLLVSSLFVTVLYLGGWNFSIPYIFIPELLRIN---GVFGTTIGIFITLAKAYMFL
FISITTRWTLPRMRMDQLLNLGWKFLLPISLGNLLLTTSFQLFSLMIWHVQNENFILDSTRI
FMKAFHLFLFHGSFIFPECILIFGLILLLMIDSTSDQKDIPWLYFISSTSLVMSITALLFRWREE
PMISFSGNFQTNSFNEIFQFLILLCSTLCIPLSVEYIECTEMAITEFLLFVLTATLGGMFLCGA
NDLITIFVAPECFSLCSYLLSGYTKRDVRSNEATTKYLLMGGASSSILVHGFSWLYGSSGGE
IELQEIVNGLINTQMYNSPGISIALIFITVGIGFKLSPAPSHQWTPDVYEGSPTPVVAFLSVTS
KVAASASATRIFDIPFYFSSNEWHLLLEILAILSMILGNLIAITQTSMKRMLAYSSIGQIGYVII
GIIVGDSNDGYASMITYMLFYISMNLGTFARIVSFGLRTGTDNIRDYAGLYTKDPFLALSLA
LCLLSLGGLPPLAGFFGKLHLFWCGWQAGLYFLVSIGLLTSVVSIYYYLKIIKLLMTGRNQ
EITPHVRNYRRSPLRSNNSIEFSMIVCVIASTIPGISMNPIIAIAQDTLFMFLLHEYDIFWAFLI
ISSIIPILAFLISGVLAPISAGPEKLSSYESGIEPMGDAWLQFRIRYYLFALVFVVFDVETVFLY
PWAMSFDVLGVSVFIEALIFVLIPIVGSVYAWRKGALEWSTNYFPWLTIIVVLPISAGSLIFF
LPYRGNKVIRWYTICICLLELLLTTYAFCYHFQLDDPLIQLEEEYKWINFFDFHWRLGIDGL

SIGPILLTGFITTLATLAAWPVTRDSRLFHFLMLAMYSGQIGSFSSRDLLLFFIMWELELIPV
YLLLSMWGGKKRLYSATKFILYTAGGSIFLLMGVSGMGLYGSNEPTLNFETSASQSYPVAL
EIIFYFGFLIAYAVKSPIIPLHTWLPDTHGEAHYSTCMLLAGILLKMGAYGLVRINMELLPH
AHSIFSPWLMIIGTIQIIYAASTSPGQRNLKKRIAYSSVSHMGFTIIGIGSITDTGLNGAILQIIS
HGFIGAALFFLAGTSYDRIRLVYLDEMGGIAIPMPKIFMMFSSFSMASLALPGMSGFVAELI
VFFGIITSQKYLVLPKIVIIFVMAVGMILTPIYSLSMSRQMFYGYKLFNAPNYSFFDSGPREL
FVSISIFLPVIGIGIYPDFVLSLSVDKVEAILSNYFYRMMLEHVLVLSAYLFSIGIYGLITSRH
MVRALMCLELILNAVNINFVTFSDFFDSRQLKGDIFSIFVIAIAAAEAAIGPAIVSSIYRNRKS
TRINQSNLLNKMEQTYQYAWIIPFVPLPVPMLIGVGLLLLPKATKNLRRIWAFPSVLLLSVV
MILAADLSIQQINGSYIYQYVWSWTINNDFSLEFGYLIDPLTSIMSILITTVGILVLIYSDNY
MSHDQGYLRFFAYMSFSNTSMLGLVTSSNLIQIYIFWELVGMCSYLLIGFWFTRPIAANAC
QKAFVTNRVGDFGLLLGILGLYWITGSFEFRDLFEIFNNNEVNSLFVTLCASLLFVGAVAKS
AQFPLHVWLPDAMEGPTPISALIHAATMVAAGIFLVARLLPLFTAIPYIMYLISLIGIITILLGA
TLALAQKDIKRGLAYSTMSQLGYIMLALGMGSYQAALFHLITHAYSKALLFLGSGSIIHSM
ETIVGYSPDKSQNIVLMGGLTKYVPITKTSFLLGTLSLCGIPPLACFWSKDEILNDSWLYSPI
FAIIAWVTAGLTGFYMFRMYLLTFEGQLNVYFQNYSGKKNISGYSISIWGKEGPKPIQKI----
---FLFAVGNKETSSYPHESDNTMLLPLLVLALFTLVVGSIGIPFDFDILSKWLTPSINLLHPNS
N-SFDWYEFVTNGIFSVSIAYFGIVIASLLYTPVYSSFQNFDVINSFVILWDKIINVIYNWSYN
RGYIDVFYATTLTRGIRGLAELTYFFDRRVIDGITNGVGITSFFVGEGIKYFGGG-RISSYLFL
YLSYVSIFLLIYYMDLPGPIHDFLLVFLGSGLILGGLGVVLLTNPIFSAFSLGLVLVCISLFHIP
SNSHFVAAAQLLIYVGAINVLILFAVMFMSGSEYYNDFQLWTVGDGVTSLVCTSIFVSLITT
IPDTSWYGIIWTTRSNQIIEQDLINNSQQIGIHLSTDFFLPFELISIILLVALIGAISMARQMTVP
AIKKDLMIVNMGPQHPSMHGVLRLVVTLDGEDVIDCEPILGYLHRGMEKIAENRTIIQYLP
YVTRWDYLATMFTEAITVNAPEQLGNIQVPKRASYIRVIMLELSRIASHLLWLGPFMADIG
AQTPFFYIFRERELIYDLFEAATGMRMMHNYFRIGGVAADLPHGWIDKCLDFCDYFLTGV
TEYQNLIRRNPIFLERVEGVGIIGREEAINWGLSGPMLRASGIQWDLRKVDHYECYDEFD
WEVQWQKEGDSLARYLVRISEMTESIKIIQQALEGIPGGPYENLEVRRFDRAKDSEWNDF
EYRFISKKPSPTFELSKQELYVRVEAPKGELGIFLIGDQSVFPWRCKIRPPGFINLQILPQLVK
RMKLADIMTILGSIDIIMGEVDRMFPMLTGLMNYGQQTVRAARYIGQSFMITLSHTNRLPI
TIQYPYEKLITSERFRGRIHFEFDKCIACEVCVRVCPIDLPVVDWRLETDIRKKRLLNYSIDF
GICIFCGNCVEYCPTNCLSMTEEYELSTYDRHELNYNQIALGRLPMSIIGDYTIRTIMNSKIT
TGNPLDSKTITNYMQGRLSAWLVKHGLVHRSLGFDYQGIETLQIKPEDWHSIAVISYVVYGY
NYLRSLCAYDVAPGGLLASVYHLTRIQSGVEQPEEVCIKIFAPRRNPRIPSVFWIWKSADFQ
ERESYDMFGISYDNHPRLKRILMPESWIGWPLRKDYIAPNFYEIQDAHMGDEFRCIGCICI
YRGFNFCAYPNCWFSLCMAKRSIGMVLAPKYSDNKKQKEEKNDIETVMNSIEFPLLERTT
QNSVISTTSNDLSNWSRLSSLWPLLYGTSCCFIEFASLIGSRFDFDRYGLVPRSSPRQADLILT
AGTVTMKMAPSLVRLYEQMPEPKYVIAMGACTITGGMFSTDSYSTVRGVDKLIPVDVYL
PGCPPKPEAVIDAITKLRKKVSREIYEDRIGSQQENRCFTTNHKFHVGRSTHTGNYDQELL
YQSPSTSEIPSETFFKYKSGTSSHELVNMQNRNTFSWIKEQMIRSISVSIMINVITQTSISNAY
PIFAQQGYENPREATGRIVCANCHLANKPVDIEVPQAVLPDTVFEAVVRIPYDMQLKQVLA
NGKKGALNVGAVLILPEGFELAPPDRISPKMKEKLGNLSFQSYRPTKKNILVIGPVPGQKY
SEITFPILSPDPATKKDVHFLKYPIYVGGNRGRGQIYPDGSKSNNTVYNATAAGIVSKVVRK
EKGGYEINIADASDGHQVVDIIPPGPELLVSEGESIKLDQPLTSNPNVGGFGQGDAEIVLQD
PLRVQGLLFFLASVILAQIFLVLKKKQFEKVQLSEMNFMSKVYDWFEERLEIQAIADDITS

KYVPPHVNIFYCLGGITLTCFLVQVATGFAMTFYYRPTVTEAFASVQYIMTEANFGWLIRS
VHRWSASMMVLMMILHVFRVYLTGGFKKPRELTWVTGVVLAVLTASFGVTGYSLPRDQI
GYWAVKIVTGVPEAIPVIGSPLVELLRGSASVGQSTLTRFYSLHTFVLPLLTAVFMLMHFPM
IRKQGISGPLMGVTKKPDLNDPVLRAKLAKGMGHNYYGEPAWPNDLLYIFPVVILGTIAC
NVGLAVLEPSMIGEPADPFATPLEILPEWYFFPVFQILRTVPNKLLGVLLMVSVPAGLLTVPF
LENVNKFQNPFRRPVATTVFLIGTVVALWLGIGATLPIEKSLTLGLFMIEVFLFGIVLGLIPIT
LAGLFVTAYLQYRRGDQLDLMPTITSYFGFLLAASTITPALFIGLSKIRLIMDIVNLAWAAL
MVVFTFSLSLVVWGRSGLMIIRSPESEMKILVDRDPVKTSFEEWARPGHFSRTIAKGPDTTT
WIWNLHADAHDFDSHTSDLEEISRKVFSAHFGQLSIIFIWLSGMYFHGARFSNYEAWLSDP
THIGPSAQVVWPIVGQEILNGDVGGGFRGIQITSGFFQLWRASGITNELQLYCTAIGALVFA
ALMLFAGWFHYHKAAPKLAWFQDVESMLNHHLAGLLGLGSLGWAGHQVHVSLPINQFL
DAGVDPKEIPLPHEFILNRDLLAQLYPSFAEGATPFFTLNWSKYAEFLTFRGGLDPVTGGLW
LTDIAHHHLAIAVLFLVAGHMYRTNWGIGHGLKDILEAHKGPFTGQGHKGLYEILTTSWH
AQLSLNLAMLGSLTIVVAHHMYSMPPYPYLATDYGTQLSLFTHHMWIGGFLIVGAAAHA
AIFMVRDYDPTTRYNDLLDRVLRHRDAIISHLNWACIFLGFHSFGLYIHNDTMSALGRPQD
MFSDTAIQLQPVFAQWVQNTHALAPGATAPGATASTSLTWGGGDLVAVGGKVALLPIPLGT
ADFLVHHIHAFTIHVTVLILLKGVLFARSSRLIPDKANLGFRFPCDGPGRGGTCQVSAWDH
VFLGLFWMYNSISVVIFHFSWKMQSDVWGSISDQGVVTHITGGNFAQSSITINGWLRDFL
WAQASQVIQSYGSSLSAYGLFFLGAHFVWAFSLMFLFSGRGYWQELIESIVWAHNKLKVA
PATQPRALSIVQGRAVGVTHYLLGGIATTWAFFLARIIAVGMALRFPRFSQGLAQDPTTRRI
WFGIATAHDFESHDDITEERLYQNIFASHFGQLAVIFLWTSGNLFHVAWQGNFESWVQDPL
HVRPIAHAIWDPHFGQPAVEAFTRGGALGPVNIAYSGVYQWWYTIGLRTNEDLYTGALFL
LFLSAISLIAGWLHLQPKWKPSVSWFKNAESRLNHHLSGLFGVSSLAWTGHLVHVAIPGSR
GQSVRWNNFLDVLPHPQGLGPLFTGQWNIYAQNPDSGSHLFGTSQGAGTAILTLLGGFHP
QTQSLWLTDMAHHHLAIAVLFLVAGHMYRTNFGIGHSMKDLLEAHIPPGGRLGRGHKGL
YDTINNSIHFQLGLALASLGVITSLVAQHMYSLPAYAFIAQDFTTQAALYTHHQYIAGFIMT
GAFAHGAIFFIRDYNPEQNEDNVLARMLDHKEAIKSHLSWASLFLGFHTLGLYVHNDVML
AFGTPEKQILIEPIFAQWIQSAHGKTSYGFDVLLSSTTGPAFNAGRSIWLPGWLNAVNE--NS
NSLFLTIGPGDFLVHHAIALGLHTTTLILVKGALDARGSKLMPDKKDFGYSFPCDGPGRGG
TCDISAWDAFYLAVFWMLNTIGWVTFYWHWKHITLWQGNVSQFNESSTYLMGWLRDYL
WLNSSQLINGYNPFGMNSLSVWAWMFLFGHLVWATGFMFLISWRGYWQELIETLAWAHE
RTPLANLIRWRDKPVALSIVQARLVGLAHFSVGYIFTYAAFLIASTSGKFGMSHSVKIYDTC
IGCTQCVRACPTDVLEMIPWDGCKAKQIASAPRTEDCVGCKRCESACPTDFLSVRVYLWH
ETTRSMGLAYMITLNLPSIFVPLVGLVFPAIAMVSLFLHVQKNKIVMRDIKTYLSVAPVLST
LWFGALAGLLIEINRLFPDALAFPFF--MTAILERRESESLWGRFCNWITSTENRLYIGWFGV
LMIPTLLTATSVFIIAFIAAPPVDIDGIREPVSGSLLYGNNIISGAIIPTSAAIGLHFYPIWEAAS
VDEWLYNGGPYELIVLHFLLGVACYMGREWELSFRLGMRPWIAVAYSAPVAAAAAVFLIY
PIGQGSFSDGMPLGISGTFNFMIVFQAEHNILMHPFHMLGVAGVFGGSLFSAMHGSLVTSS
LIRETTENESANEGYRFGQEEETYNIVAAHGYFGRLIFQYASFNNSRSLHFFLAAWPVVGI
WFTALGISTMAFNLNGFNFNQSVVDSQGRVINTWADIINRANLGMEVMHERNAHNFPLD
LAAVEAPSTNGMGLPWYRVHTVVLNDPGRLLSVHIMHTALVSGWAGSMALYELAVFDPS
DPVLDPMWRQGMFVIPFMTRLGITNSWGGWSIT-GGTITNPGIWSYEGVAGAHIVFSGLCF
LAAIWHWVYWDLEIFCDERTGKPSLDLPKIFGIHLFLSGVACFGFGAFHVTGLYGPGIWVS
DPYGLTGKVQSVNPAWGVEGFDPFVPGGIASHHIAAGTLGILAGLFHLSVRPPQRLYKGLR

MGNIETVLSSSIAAVFFAAFVVAGTMWYGSATTPIELFGPTRYQWDQGYFQQEIYRRVGTG
LAENQSLSEAWSKIPEKLAFYDYIGNNPAKGGLFRAGSMDNGDGIAVGWLGHPIFRDKEG
RELFVRRMPTFFETFPVVLVDGDGIVRADVPFRRAESKYSVEQVGVTVEFYGGELNGVSY
SDPATVKKYARRAQLGEIFELDRATLKSDGVFRSSPRGWFTFGHASFALLFFFGHIWHGAR
TLFRDVFAGIDPDLDAQVEFGAFQKLGDPTTKRQVV------------VETLFNGTLALAGRDQET
TGFAWWAGNARLINLSGKLLGAHVAHAGLIVFWAGAMNLFEVAHFVPEKPMYEQGLILL
PHLATLGWGVGPGGEVIDTFPYFVSGVLHLISSAVLGFGGIYHALLGPETLEESFPFFGYV
WKDRNKMTTILGIHLILLGIGAFLLVLKALYFGGVYDTWAPGGGDVRKITNLTLSPSVIFG
YLLKSPFGGEGWIVSVDDLEDIIGGHVWLGSICILGGIWHILTKPFAWARRALVWSGEAYL
SYSLGALAVFGFIACCFVWFNNTAYPSEFYGPTGPEASQAQAFTFLVRDQRLGANVGSAQ
GPTGLGKYLMRSPTGEVIFGGETMRFWDLRAPWLEPLRGPNGLDLSRLKKDIQPWQERR
SAEYMTHAPLGSLNSVGGVATEINAVNYVSPRSWLATSHFVLGFFLFVGHLWHAGRARA
AAAGFEKGIDRDFEPVLSMTPLNMTIALGRFTKDENDLFDIMDDWLRRDRFVFVGWSGL
LLFPCAYFALGGWFTGTTFVTSWYTHGLASSYLEGCNFLTAAVSTPANSLAHSLLLLWGPE
AQGDFTRWCQLGGLWTFVALHGAFGLIGFMLRQFELARSVQLRPYNAIAFSAPIAVFVSV
FLIYPLGQSGWFFAPSFGVAAIFRFILFFQGFHNWTLNPFHMMGVAGVLGAALLCAIHGAT
VENTLFEDGDGANTFRAFNPTQAEETYSMVTANRFWSQIFGVAFSNKRWLHFFMLFVPVT
GLWMSALGVVGLALNLRAYDFVSQEIRAAEDPEFETFYTKNILLNEGIRAWMAAQDQPH
ENLIFPEEVLPRGNALMSGSTGERSFADIITSIRYWVIHSITIPSLFIAGWLFVSTGLAYDVFG
SPRPNEYFTESRQGIPLITGRFDPLAQLDEFSRSFMTIDRTYPIFTVRWLAVHGLAVPTVSFL
GSISAMQFIQRMATQTVEGSSR-SGPRQTGVGSLLKPLNSEYGKVAPGWGTTPLMGVAMA
LFAIFLSIILEIYNSSVLLDGISMSMLTLKLVVYTVVIFFVSLFIFGFLSNDPGRNPGREEMAD
TTGRIPLWLIGTVTGIPVIGLIGIFFYGSYSGLGSSLMLNILSLIWICLNSALHSSSFLFAKLPE
AYAFFNPIVDVMPVIPVLFFLLAFVWQAAVSFRTTQSNPNEQNVELNRTSLYWGLLLIFVLA
VLFSNYFFNMEVNILAFIATALFILVPTAFLLIIYVKTVSQNDMETATLVAIFISGLLVSFTGYA
LYTAFGQPSQQLRDPFEEHGDMEALVYTFLLVSTLGIIFFAIFFREPPKVPTKKMKMTIAFQ
LAVFALIATSSILLISVPVVFASPDGWSSNKNVVFSGTSLWIGLVFLVGILNSLIS-------MSPQ
TETKASVGFKAGVKDYKLTYYTPEYETKDTDILAAFRVTPQPGVPPEEAGAAVAAESSTG
TWTTVWTDGLTSLDRYKGRCYHIEPVAGEESQFIAYVAYPLDLFEEGSVTNMFTSIVGNVF
GFKALRALRLEDLRIPVAYVKTFQGPPHGIQVERDKLNKYGRPLLGCTIKPKLGLSAKNY
GRAVYECLRGGLDFTKDDENVNSQPFMRWRDRFLFCAEALFKAQTETGEIKGHYLNATA
GTCEEMMKRAVFARELGVPIVMHDYLTGGFTANTTLAHYCRDNGLLLHIHRAMHAVIDR
QKNHGMHFRVLAKALRMSGGDHIHAGTVVGKLEGEREITLGFVDLLRDDFIEKDRSRGIY
FTQDWVSLPGVLPVASGGIHVWHMPALTEIFGDDSVLQFGGGTLGHPWGNAPGAVANRV
ALEACVQARNEGRDLAREGNEIIREASKWSPELAAACEVWKEIKFEFEAMDTL--TAIHLY
KTSTPSTRNGAVDSQAKSNPRNNLIYGQHRCGQGRNARGIITAGHRGGGHKRLYRKIDFR
RNEKDIPGRIVTIEYDPNRNAYICLIHYGDGEKRYILHPRGAIIGDTIVSGTEVPIKIGNALPL
TDMPLGTAIHNIEITLGKGGQLARAAGAVAKLIAKEGKSATLKLPSGEVRLISKNCSATVG
QVGNVGVNQKSLGRAGSKCWLGKRPVVRGVVMNPVDHPHGGGEGRAPIGRKKPTTPW
GYPALGRRSRKRNKYSDSLILRRRSKMIQPQTHLNVADNSGARELMCIRIIGAS-NRRYAHI
GDVIVAVIKEAVPNMPLERSEVIRAVIVRTCKELKRDNGMIIRYDDNAAVVIDQEGNPKGTR
VFGAVARELRQLNFTKIVSLAPEVLMLSPKRTRFRKQHRGRMKGKSYRGSRICFGRYALQ
ALEPAWITSRQIEAGRRAMTRYARRGGKIWIRIFPDKPVTLRPTETRMGSGKGSPEYWVSV
VKPGRILYEMGGVSEIIAREAISIAASKMPIRTQFVIAGMTRIRRGYIARRRRTKIRLFSSTFR

GAHSRLTRTITQQKMRALVSAHRDRGRQKRDFRRLWITRINAVIREN--VFYSYSRLIHDLY
KGQLFLNRKILAQIAISNRNCLYMIFNEIIK-----------MIKEVYALGQHICMSAHKARRVIDQI
RGRSYEETLMILELMPYRACYSIFKLIYSAAANASHNMGFNEADSIISKVEVNEGTTMKRL
KPRARGRSFPIKRPTCHITIVLKDISLDGVIWDKKMDGIKYAVFTDKSIRLLVANNQYTSNV
ESGSTRTEIKHWVELFFGVKVISMNSHRLPGKGRRMGPIMGHTMHYRRMIITLQPGYSIPP
LRKKRTMAVPKKRTSISKKRIRKNIWKRKGYWAAVKAFSLAKSLSTGNSNSFFVR----MAK
GKDVRVTVILECTSCVRNGVN--KESPGISRYITQKNRHNTPSRLELRKFCPYCYKHTIHGEI
KKMKIRASVRKICEKCRLIRRRGRILLICSNPRHKQRQGMVREEVAVSTRTLQWKCVESRA
DSKRLYYGRFIMSPLMKGQADTIGIAMRRALLGEIEGTCITRAKSE--TIPHEYSTIVGIEESV
HEILMNLKEIVLRSNLYGTRDASICVRGPGYVTAQDIISPSSVEIVDTTQHIASLTEPINLCIG
LQIERNHGYRMKTPKNSQDGSY--PIDAVFMPVRNANHSIHSYGNGNEKQEILFLEIWTNGS
LTPKEALHEASRNLIDLFIPFLHTEEE--DIIIEDNQ-NRFTVPFFTFHDRLA-KLRKNKK--IAL
KFIFIDQSELPPRTYNCLKRSNIHTLLDLLNKSQEDLMRIEHFRIEDVKHILGILQKH--FEINL
PKNKMFWDGN-EGMSTIPGFSQIQFEGFCKFIDQGLTEELYKFPKIEDPDQEIEFQLFVETY
QLVEPFIKERDAVYESLTYSSELYVPAGLIWKTGRDMQEQTIFIGNIPLMNSLGTSIVNGIYR
ILINQILQSPGIYYRSELDHNGISVYTGTIISDWGGRSELEIDRKARIWARVSRKQKISILVLS
SAMGSNLKEILDNVCYPEIFLSFLNDKEKKKIGSKENAILEFYQQFACVGGDPVFSESLCKE
LQKKFFQQRCELGRIGRRNMNRRLNLDISQNNTFLLPRDVLAAGDQLIGMKFGMGTLDD-
-MNHLKNKRIRSVADLLQDQFGLALVRLENVVRGTICGAIR-HKWI-PTPHNLVTSTP----LT
TTYESFFGLHPLSQVLDQTNPLTQIVHGRKLSYLGPGGLTGRTASFRIRDIHPSHYGRICPID
TSEGINVGLIGSLAIHARIGHWG-SLESPFY---EISERS--KKVRMVYLSPSRDEYYIVAAGNS
LALNQNVQEEEVVPARYRQEFLTIAWEQVHLRSIFPFQYFSIGASLIPFIEHNDANRALMSS
NMQRQAVPLSRSEKCIVGTGLERQAALDSGVSAIAEHEGKIIYTDIDKIILSGNGDTLKIPLV
MYQRSNKNTCMHQKPQVPRGKCMKKGQILADGAATVGGELALGKNVLVAYMPWEGYN
SEDAVLISERLVYGDIYTSFHIRKYEIQTHVTNQG-PERITNEIPHLEVHLLRNLDRNGIVML
GSWVETGDILVGKLTPQMAKE-SSYAPEDRLLRAILGIQVSTSKETCLKLPIGGRGRVIDVR
WIQKKG-GSSYNPETIRVYISQKREIKVGDKVAGRHGNKGIISKILPRQDMPYLQDGTPVD
MVFNPLGVPSRMNVGQIFECSLGLAGDLLDRHYRIAPFDERYEQEASRKLVFSELYEASK
QTANPWVFEPEYPGKGIIFDGRTGDPFEQPVIIGKSYILKLIHQVDDKIHGRSSGHYALVTQ
QPLRGRAKQGGQRVGEMEVWALEGFGVAHISQEMLTYKSDHIRARQEVLGTTIIGGTIPNP
ADAPESFRLLVRELRSLALELNHFLVSEKNFQINRKEA-------MIDRYKHQQLRIGLVSPKQI
SAWASKTLPNGEIVGEVTKPYTFHYKTNKPEKDGLFCERIFGPIKSGICACGNYRVIGDEKE
DPKFCEQCGVEFVDSRIRRYQMGYIKLACLVTHVWYLKRLPSYIANLLDKPLKELEGLVY
CD--FAFARP-VAKKPTFLRLRGSFEYEIQSRKYSIPLFFTTQGFNTFRNREISTGAGAIKEQL
ADLDLRIITDYSLVEWKELGEE-GPTGNEWEDRKIGRRKDFLVRRMELAKHFIRTNVEPER
MVLCLLPVLPPELRPIIQIDGGKLMSSDINELYRRVIYRNNTLTDLLTTSRSTPGELVMCQEK
LVQEAVDTLLDNGIRGQPMRDGYNKVYKSFSDVIEGKEGRFRETLLGKRVDYSGRSVIVV
GPSLSLHRCGLPREIAIELFQTFVIRGLIRQHVAPNIGVAKSKIREKEPIVWEILQEVMQGHP
VLLNRAPTLHRLGIQAFQPILVEGRAICLHPLVCKGFNADFDGDQMAVHVPLSLEAQAEA
RLLMFSHMNLLSPAIGDPISVPTQDMLIGLYVLTIGNRRGICANRYNPCNHRN---YQNKRID
DNNEKYTKEKEPYFCSSYDVLGAYQQKRITLDSPLWFRWRLDQR-VIAS--REVPVEVQYE
SLGTYHEIYGHYLIVRSVKKEILCIYIRTTVGHISFYREIEEAIQGFWRACSYTVPKLSNYMI
MEALMAERADLVFHNKVIDGTAMKRLISRLIDHFGMAYTSHILDQIKTLGFQQATATSISL
GIDDLLTIPSKGWLVQDAEQQSLILEKHHHYGNVHAVEKLRQSIEIWYATSEYLRQEMNPN

FRMTDPFNPVHIMSFSGARGNASQVHQLVGMRGLMSDPQGQMIDLPIQSNLREGLSLTEY
IISCYGARKGVVDTAVRTSDAGYLTRRLVEVVQHIVVRRTDCGTIRGISVSPRNGMMT-ERI
FIQTLIGRVLADDIYMGPRCIAIRNQDIGIGLVNRFIT------FRAQPISIRTPFTCRSTSWICRLC
YGRSPTHGDLVELGEAVGIIAGQSIGEPGTQLTLRTFHTGGVFTGGTAEHVRAPSNGKIKFN
EDLVHPTRTRHGHPAFLCYIDLYVTIESQDIIHNVNIPPKSFLLVQNDQYVESEQVIAEIRAG
TSTL-NFKERVRKHIYSDSEGE-MHWSTDVYHAPEYTYGNVHLLPKTSHLWILSGGPCR--S
SIAPFSLHKDQDQINLH--SLSVERRSISN--LSVTNDQ-VRHKLFSSDLSG--RGRILDY-SGPD
RIICNDHCNFRYPT-TLHENSD--LLAKRRRNRFIIPFQSNQEREKELMLCSGISIEIPIN--GLFR
RNSILAYFDDPRYRRNSSGITKYGTIEVHSILKKED--LIEYRR--KEFRPKYMKVDRFFFIPEE
VHILPGSSSIMVQNNSIIGVDTRITLNTRSRVGGLVRVERKKKKIELKIFSGDIHFPGETDKIS
RHSGILIPPGTKKK--NSKESK--KWKNWIYVQR--ITPTKKK--YFVLVRPVVTYEIADGINLA
TLFPQDLLQERDNVQLRVVNYILYGNGKPIRGISHTSIQLVRTCLVLNWDQDKK---DSSIEE
VHASCVEVRAIDLIRDFIRMDLVKPPISYTVKRNDPAGSGLIPD-NGSDRTNINPFY---SKAR-
----IQQSLSQHQGTIRTLLNR---NKECHSLIILSSSNCFR--MGLFNGLKYHN---VTKESDSLIPI
RNSLGPLGT--VPKIANFDL--SYHLIT--HNQIL-RNKYLLLDN---FKKIFQVLK---YYLMDEN
GG--INNPDPSSNIILNPVNLNWYFLHHDYCEETPIIIS-LGQFICENVCIYKKRP---HLKSGQV
LIVH-VDSLVIRSAKPYLATTGATVHGHYGEILYEGDTLVTFIYEKSRSGDITQGLPKVEQVL
EVRSIDSISMNLEKRVEGWNEHITRILGIPWGFLIGAELTIAQSRISLVNKIQTVYRSQGVQI
HNRHIEIIVRQITSKVLVSE---DGMSNVFSPGELIGLLRAERTGRTLEEAICYRAVLLGITRAS
LNTQSFISEASFQETARVLARAALRGRIDWLKGLKENVVLGGMIPVGTGFKGLVHCS--RQ
HKNIP--LEIKTKNLFGGEMR--DILFHHRELFDSCIQKNFHDTSEQ-------------MTRRYWNIN
LEEMMEAGVHFGHGTRKWNPRMAPYISAKRKGIHITNLTRTARFLSEACDLVFDAASREK
QFLIVGTKNKAADSVASAAIRARCHYVNKKWLGGMSTNWSTTETRLHKFRDLRVEQKT
GRLNRLPKRDAAMLKRQLSHLQTYLGGIKYMTGLPDIVIIVDQQEEYTALRECVTLGIPTI
CLIDTNCDPDLADISIPANDDAIASIRLILNKLVSAICEGRSS-YRRNRMGQKINPLGFRLGTT
QGHHSVWFAQPKKYSEGLEEDQKIRDCIKNYVQKNMRISSG-VEGIARIEIQKRIDLIQVIIY
IGFPKLLIEGRPR----GVEELQINVQKKFHSVNRKLNIAITRIAKPYGQPNILAEFIAGQLKNR
VSFRKAMKKAIELTEQADTKGIQVQIAGRIDGKEIARVEWIREGRVPLQTIRAKIDYCSYTV
RTIYGVLGIKIWIFVDEEMSRYRGPRFKKIRRLGALPGLTSKRPRAGSDLRNQSRSGK-RSQ
YRIRLEEKQKLRFHYGLTERQLLKYVRIAGKAKGSTGKVLLQLLEMRLDNILFRLGMSSTI
PGARQLVNHRHILVNGRIVDIPSYRCNPRDLITAKDEQKSRALIQNYLDSSPH-EELPKHLTL
HSFQY--KGLVNQIIDSKWVGLKINELLVVEYYSRQTMSRRGTAEEKTAKSDPIYRNRLVN
MLVNRILKHGKKSLAYQILYRAVKKIQQKTETNPLSVLRQAIRGVTPDIAVKARRVGGSTH
QVPIEIGSTQGKALAIRWLLGASRKRPGRNMAFKLSSELVDAAKGSGDAIRKKEETHRMA
EANRAFAHFRMGRDTISDIITSIRNADMEKKGTVRIAFTNITENIVKILLREGFIENVRKHRE
SNKYFLVSTLRHRR--NRKGPYR---TIL--KRISRPGLRIFSNYQRIPRILGGMGIVILSTSRGIM
TDREARLEGIGGEILCYIWMAKPILRIGSRRTGRIGSRKNGRRIPKGVIHVQASFNNTIVTVT
DVRGRVISWSSAGTCGFRGTRRGTPFAAQTAAGNAIRTVMEQGMQRAEVMIKGPGLGRD
AALRAIRRSGILLSFVRDVTPMPHNGCRPPKKRRVMPTIKQLIRNTRQPIRNVTKSPALRGC
PQRRGTCTRVYTITPKKPNSALRKVARVRLTSGFEITAYIPGIGHNSQEHSVVLVRGGRVKD
LPGVRYHIVRGTLDAVGVKDRQQGRSKYGVKKPKMARKSLIQREKKRQKLEEKYHLIRR
SSKKEIS---KVPSLRDKWEIHGKLQSPPRNSALTRLHRRCFLTGRPRANYRDFGLSGHLLRE
MVHACLLPGATRSSWMVKNTFISVISQD--EKVASSGSVEFQVFRFTNKIRRLTSHLKLHRK
DYLSQRGLRKILGKRQRLLAYFSKKNRVRYKELIFQLDIREPKTR--MVKLRLKRCGRKQR

VIYRIVAIDVRSRREGRDLRKVGFYDPIKNQTYLNVPAILYFLEKGAQPTGTVHDISKRAEV
FKELRLNQTKSNYMDKFKRPFLKSKRSFRRRLPPIGSGDRIDYRNMSLISRFISEQGKILSRR
VNRLTLKQQRLITIAIKQARILSSLPFLNNEK--QFERTESTPRTAGPRTRNKVTRSLKKNPFV
ANHLLGKIEKLNMRAEKEIILTWSRASTIIPTMIGHTIAIHNGKEHLPVYITDRMVGHKLGE
FAPTLNFRGHARNDNSSRRMILKSFLLGNLLSLCMKIINSVVVVGLYYGFLTTFFIGPSYLF
LLRARVMEEG---TEKEVSATTGFITGQLMMFISIYYAPLHLALGRPHTITVLVLPYLLFHFF
WNNHKHFFDYGSTTRNSMRNLSIQCVFLNNLIFQLFNHFILPSSTLARLVNIYMFRCNNKM
LFVTSSFVGWLIGHILFMKWVGLVLFWIRQNHSIRS--VLIKYLVSALRNSTARIFSILLFITC
VYYLGRIPSPIVTKKLKETEEDKPLVTLLFDYKRWNRPLRYIKNNRFENAVRNEMSQYFFD
TCRSDGKQRISFTYPPSLSTFWEMIQRKMSLCTTEKLS-EELYNHWIYTNEQKRNSLKNEF
LNRMKALNKGF--RDVLEKRTRLCNDETEQKCFPKMYDPFLNG--RYRVTTNYSHDYKVW
INKIHGMLYDTFEQKNVNDQ-IEIKEIRKKVPRWSYKLIDLEIQKRKGIRSRKAKRVVIFTD
ALIRYSQQSDFRRHFIKGSIRAQRCKTVPWKLFQRNVHSPLFLDRIDKPFFFSFDI--SGTMK
LIFSNKDKNERITIAETWDTFIFAQVIRGSMLVTQSILRKYIILPSLIIAKNIGRMLLFQFPEW
YEDLTEWSREMHVKCTYNGVQLSEIEFPKNWLTDGIQIKILFPFCLKPWHKSKEKKEDNF
CFLTVWGMEAELPFGSPRKRPSFFEPIYKELEKKNFKVK-KKCFVVLRVLKERTKWFLKVS
KEKKRWVSKKVL--FIKTKTNELVKGN-SLVSNDIFHESSIGIRSIDWTNYSLTETKMKDLAD
RTRTIRNQIERITKDNKKSPNETSWDDKRSKSPKNIWQILKRRNARLMRKWHYFLKFFFER
ISMYILLCIINIPRINTTFFIHFISCDLAALSQAYVFYKLSQTKILNKYHLRSVLKYTSLFLKD
RIKYFFRQGIFHSESEHKKLRNSGMNEWKTWLRGHYQYDLSQTRWSRLVPKKWRTRINQ
CHTVQNKNRFASYEKDQ--LIHYEKQEKFKKQYRYGLLSHKYINDSIIDPDRKYFDWRILN
FFISNFNWMGMNEEILNRLFLPEFLLFYDAYKMKPWVIPTKLLFFNFKNNTKAELNFFLKR
YFLFQLRWGDPLNQRIINNIKVYCFLLRLINPKEIAISSIQRGEMDLDVMLSQKDLSLKELL
KKGIFLIEPIRLSIKWDGQFIMYKTIGISLVHKSKHNHYDLLVPENILSSRCRRELRILICFRFL
WPNYRLEDLACMNRYWFDTTNGSRFSMSRIHMYPMKRHQFQSWIFELREILREIKNSHYF
LDSWTRFDSLGSFTPIFFHQERFMKLFDPRIWSLLLS-RDS--RGSKSNRYFT---IKGVGLLVV
A-VLIY----RINNRNRV--KNLYLTGLL-----PIPMNSIGPRNETLEESF-GSSNINRLIVS-LLYLPK
GKKISESCFMDPKESTWVLPIT----KKCIMPESNWGSRWWRNRIRKKR--DSSCKIS---NETV
AGIEISFKEKDSK-YLEFLFVSYTD--DPIRKDHDWELFDRLSPRKKRNIINL--NSGQLFEILV
KHWICYLMSAFREKRPIEVEG-FFKQQG-AESTIQSNDIEHVSH--LFSRNKWGISLQNCAQF
HMWQFRQDLFV---SWGK-NPHESDFLRN-VSRENWIWLDNVWLVNKDR---FFS--KVRNVS
SNIQYDS---TR--SIFVQVTDSSQLKGSS-DQSRDHFDSIS----NEDSE------------YHTLINQREI
QQLKERSIL-GD--PSFLQTERTEIESD--RFPK--CLSGYS--SMSRLFT-EREKQMNNHLLPEEI
EEFI--GNPTRSIRSFFSDRW--SELH-LGSNPTERSTRDQ----KLLKKQQDVS-FVPSRRSE----K
KEMVDIFKIITYLQN--TVSIHPISSDPGCDMVP-KDEPD-MDTSNKISFLNKNPFFD--LFHLF
HDRNRGGYTL--HHDFESE--ERFQEMADLFTLSITEP-DLVYHKGFAFSIDSYGLDQKK--FL
NEVFNSRDESKKKSL---LVLPPIFY--EENESFYRRIRKKSVR-ISCGNDLEDPKPKIVVFASN
NIMEAVNQSRLIRNLIQIQYSTYG--YIRNVSNRFF---LMNRSDRNFEYGIQ---RDQIGNDTL
NHRTIMKYMINQHFSNLKK-SQKKWFDP----LISRTERSMNRDPDAYRYKWSNGSKNFQE
HLEHFVSEQKS---RFQVVFDRLRINQYSIDWFEVIDKQDLSKSLRFFLSKS---------LLFLS----
---KSLLFLSKKSLPFFFVSFGNIPIHRSE-IHIY-ELKGPNDQLCNQLLESIGVQIVHLNKLKPF
LLLLD---DHDTS-QKSKFWINGGTISPFLFNKIP-KW-MIDSFHT--RKNRRKSFDNTDSYFSM
ISHDRDNWLNPVKPFHRSSLISSFYKANRLRFLNNPHHFWFY--CNKRFPFYV----EKARIKN
SD--LTY-GQFLNILFIRN----KMCVGK--KKHAFLE-RDTISPIESQVSNISIPNDFPQSGDET-Y

NLYKS---FHFPIRSDPFVR--R---AIADISGTPLTEGQIVNFERTYCQPLSDMNLSDSEGKNLH
QYLN--FNSNMGLIHTPCSEKYLP--SEKRKK--------RSLCLK--KCVEKRQMYRTFQRDSAFS
NLSKWNLFQTYMPWFLTSTGCKYLNFTLLDTFSGLL--PILNSS-QKFVSIFHDIMHGSDISW
PIPQKKR------GLPQWNL-ISEISNKCLHN--LLLSEEMIHRNNESPVPLIWTHLRSPNARKF-L
YSILFLLLVAGY-LVRPHLLFVSR--ASSELQTEFEKIKSLMIPSYMIELRKLLDRYPTSELNSF
WLKNLFLVALEQLGDSLEEIRGSASGGNMLLGGGP-----AYG-VKSIRSKKKYLNINLIDLID
LI-SIIPNPINRITFSRNTRHLSRTSKEIYSLIRKRKNVNGDWIDDKIESWVANSDSIDDEEREF
LVQFS--TLTT--EKRIDQILLSLT-QSDHLS-----KNDSGYQMIEQPGSIYLRYLVDIH--KKYLM
NYEFNRSCLAERRIFLAHYQTITY--SQTSCGTNSFHFPSHG---KPFSLRLALSPS-RGILVIGSI
GTGRSYLVKYLATNSYVPFITVFPNKFLDDKPKGYPIDD---IDDSDIDDRDDID---------------
--------DSDDIDDDLDTELLTMTNAL---TMYMT---PKIDRFDITLQFELAKAMSPCIIWIPNIHD
LYVNESNYLSLGLLVNYLS---GGCERCS-TRNILVIASTHIPQKVDPALIAPNKLNTCIKIRRL
LIPQQRKHFFTLSYTRGFRLEKKMFHT--NGFGSITMGSNARDLVALTNEALSISITQKKSIID
TNTIRSALHRQTWDLR---SQVRSVQDHGILFYQIGRAVAQNVLLSNCPIDPISIYMKKK--SC
KEGDSYLYKWYFELGTSMKKLTRLLYLLSCSAGSVAQDLWSLP--GPDEK-NWITSYGFVE
NDS-DLVHGLLEVEGALVG--SSRTEKDCSQFDND--RVTLLLRSEPRN-PLDMIQNGSCSIVD
QRFLYEK--YKSEFEE-GE--GALDPQ----QIEED----LFNH--IVWAP--RIWRP---CGNLFDCIER
PNELGFP-YWARSFR---GKRIIYHKEDELQENDSKFLQSGTMQYQTRDRSSKEQGFFRISQF
IWDPADPFFFLFK-DQPFVSVFSRREFFADEEMSKGLLTSQTNPPTSIYKRWFI--KNTQEKHF
ELLIHRQRWLRT---NSSLS--NGSFRSN--TPSESYQYLSNLFLSN-GTLLDQMIKTLLRKRWL
FPDEMKHLI--HVTGERFPIPMPRSR-INGNFIDKTSSIVANILLRIIPTSSGEKEAFTYYRDG--
MSAQSEGNYAEALQNYYEATRLEIDPYDRSYILYNIGLIHTSNGEHTKALEYYFRALERNP
FLPQAFNNMAVICHY-RGEQAIRQGDSEIAEAWSDQAAEYWKQAIALTPGNYIEAHNWLK
ITRRFE--MNWRSERIWIELITGSRKISNFCWAFILFLGSLGFLLVGISSYLGRNLISLFPSQQII
FFPQGLVMSFYGIAGLFISSYLWCTISWNVGSGYDRFDRKGGIVCIFRWGFPGINRRIFLRF
LMRDIQSIRIEVKEGLYPRRVLYMEIRGQGAIPLTRTDEN-LTPREIEQKAAELAYFLRVPIEV
F

>Arabidopsis
MEKSWFNFMFSLSKAMDS-FPGEKTTISQDRFIYDMDKNFYGSNNVDLLVSSKDIRNFISD
DTFFVRDSNKNSYSIFFDKKKKIFEIDND-----FSDLEKFFYSYCSSSYLNNRSKGDN-HYDP
YIKDTKYNCTNHINSCIDSYFR--SYICIDNNFLIDSNNFNESYIYNFICSSKNYKIRTNRNRS
NLIS---SK-FDITQNYNQLWIQCDNCYGLMYK---KVKMNVCEQCGHYLKMSSSERIELSID
PGTWNPMDEDMVSADPIKFHSKEEPYKNRIDSAQKTTGLTDAVQTGTGQLNGIPVALGV
MDFRFMGGSMGSVVGEKITRLIEYATNQCLPLILVCSSGGARMQEGSLSLMQMAKISSVL
CDYQSSKKLFYISILTSPTTGGVTASFGMLGDIIIAEPYAYIAFAGKRVIEQTLKKAVPEGSQ
AAESLLRKGLLDAIVPRNLLKGVLSELFQLHAFFPLNTN---MVTIRADEISNIIRERIEQYNR
EVTIVNTGTVLQVGDGIARIYGLDEVMAGELVEFEEGTIGIALNLESNNVGVVLMGDGLM
IQEGSSVKATGKIAQIPVSEAYLGRVINALANPIDGRGKISASESRLIESPAPGIISRRSVYEPL
QTGLIAIDSMIPIGRGQRELIIGDRQTGKTAVATDTILNQQGQNVICVYVAIGQKASSVAQV
VTSLQERGAMEYTIVVAETADSPATLQYLAPYTGAALAEYFMYREQHTLIIYDDLSKQAQ
AYRQMSLLLRRPPGREAYPGDVFYLHSRLLERAAKLSSQLGEGSMTALPIVETQSGDVSA
YIPTNVISITDGQIFLSADLFNAGIRPAINVGISVSRVGSAAQIKAMKQVAGKLKLELAQFAE
LEAFSQFSSDLDKATQNQLARGQRLRELLKQSQSAPLTVEEQIMTIYTGTNGYLDGLEIGQ
VRKFLVQLRTYLKTNKPQFQEIIASTKTLTAEAESFLKEGIQEQLERFLLQEKVMRTNPTTS

NPEVSIREKKNLGRIAQIIGPVLDVAFPPGKMPNIYNALVVKGRDTLGQEINVTCEVQQLL
GNNRVRAVAMSATEGLKRGMDVVDMGNPLSVPVGGATLGRIFNVLGEPVDNLGPVDTRT
TSPIHKSAPAFIELDTKLSIFETGIKVVDLLAPYRRGGKIGLFGGAGVGKTVLIMELINNIAK
AHGGVSVFGGVGERTREGNDLYMEMKESGVINEQNLAESKVALVYGQMNEPPGARMRV
GLTALTMAEYFRDVNEQDVLLFIDNIFRFVQAGSEVSALLGRMPSAVGYQPTLSTEMGTL
QERITSTKKGSITSIQAVYVPADDLTDPAPATTFAHLDATTVLSRGLAAKGIYPAVDPLDSTS
TMLQPRIVGEEHYETAQQVKQTLQRYKELQDIIAILGLDELSEEDRLTVARARKIERFLSQP
FFVAEVFTGSPGKYVGLAETIRGFNLILSGEFDSLPEQAFYLVGNIDEATAKATNLEMESKL
KKMTLNLCVLTPNRIVWDSEVKEIILSTNSGQIGVLANHAPIATAVDIGILKIRLANQWLTM
ALMGGFARIGNNEITILVNDAEKNSDIDPQEAQQTLEIAEANLRKAEGKRQTIEANLALRR
ARTRVEALNTI-MKNLTDSFVYLGHWPSAGSFGFNTDILATNPINLSVVFGVLIFFGKGVLN
DLLDNRKQRILNTIRNSEELREGAIQQLENARARLRNVETEADKFRVNGYSEIEREKLNLI
NSTYKTLKQLENYKNETILFEQQRTINQVRERVFQQALQGAIGTLNSCLSNELHLRTINANI
GMFGTMKEITDMNPLVSAASVIAAGLAVGLASIGPGVGQGTAAGQAVEGIARQPEAEGKI
RGTLLLSLAFMEALTIYGLVVALALLFANPFVMNVLSCSINTLIKLYEISGVEVGQHFYWQI
GGFQVHAQVLITSWVVIAILLGSAVLAIRNPQTIPTDGQNFFEFVLEFIRDVSKTQIGEEYGP
WVPFIGTLFLFIFVSNWSGALLPWKIIQLPQGELAAPTNDINTTVALALLTSVAYFYAGLSK
KGLGYFSKYIQPTPILLPINILEDFTKPLSLSFRLFGNILADELVVVVLVSLVPLVVPIPVMFL
GLFTSGIQALIFATLAAAYIGESMEGHHMIFSILEHILTHISFSVVSIVLTIYFLTLLVNEIIGFF
DSSDKGIIITFFGITGLLLTRWIYSGHFPLSNLYESLIFLSWAFSIIHMVSYFNKQQNKLNTITA
PSVIFIQGFATSGLLNKMPQSAILVPALQSQWLMMHVSMMILGYGALLCGSLLSIALLVITF
RKVGPTFWKKNVLYYINERNSILLNINFSFSRNYYRYQLIQQLDFWSFRIISLGFIFLTVGILS
GAVWANETWGSYWNWDPKETWAFITWTIFAIYLHIKTNRNVRGINSAIVALIGFILIWICYF
GVNLLGIGLHSYGSFTSNMAKKKAFIPFFYFLSIVFLPWLISLCCNKSLKTWITNWWNTRQ
CETFLNDIQEKSFLEKFIQLEELFQLDEMIKEYPETNLQQFRLG-HKETIQFIKIHNEYNIHTI
LHFSTNLISFVILSGYSFWGKEKLFILNSWVQEFLYNLSDTIKAFSILLLTDLCIGFHSPHGW
ELMIGYIYKDFGFAHYEQILSGLVSTFPVILDTIFKYWIFRYLNRVSPSLVVIYHAINDMPIG
VPKVPFRSPGEGDTSWVDIYNRLYRERLFFLGQEVDTEISNQLISLMIYLSIEKDTKDLYLFI
NSPGGWVISGMAIYDTMQFVRPDVQTICMGLAASIASFILVGGAITKRIAFPHA-VMIHQPA
SSFYEAQTGEFILEAEELLKLRETITRVYVQRTGKPIWVISEDMERDVFMSATEAQAHGIV
DLVAVQ??M
DKFQGYLEFDGARQQSFLYPLFFREYIYVLAYDHGLADYDKKYSSLITKRLILRMYEQNR
LIIPTKDVNQNSFLGSLFYYQMISVLFAVIVEIPFSLRLGSSFLKKSYNLQSIHSIFPFLEDKLG
HFNYVLDVLIPYPIHLEILVQTLRYRVKDASSLHFFRFCLYEYC--NWKNFK-----SIPRFFLFL
YNSHVCEYESIFFFLRKRSSHLRSTSYEVLFERIVFYGKIHHFFKVFVNPAILGLLKDPFIHY
VRYHGRCILATKDTPLLMNKWKYYFVNLWQCYFSVWFQSQKVNINQLSKDNLEFLGYLS
SLRLNPLVVRSQMLENSFLIDN--VRIKLDSKIPISSIIGSLAKDKFCNVLGHPISKATWTDSS
DSDILNRFVRICRNISHYYSGSSKKKNLYRIKYILRLCCVKTLARKHKSTVRTFLKRLGSGL
LEEFLTGEDQVLSLIFPRRIWYLDILYLNDLVNHEMIIYAT-AVQTIN-SFVKLES-LKEVYGLI
WIFVPIFSLVLGIITGVLVIVWLEREISAGIQQRIGPEYAGPLGILQALADGTKLLFKENLRPS
RGNTPLFSIGPSIAVISILLSYSVIPFSNHLVLADLNIGIFLWIAISSIAPIGLLMSGYGSNNKYS
FLGGLRAAAQSISYEIPLTLCVLSISLLSNSLSTVDIVEAQSKYGFWGWNLWRQPIGFIIFLIS
SLAECERLPFDLPEAEEELIAGYQTEYSGIKFGLFYVASYLNLLISSLFVTVLYLGGWNISIP
YISILELFQR---DQIFGTTIGIFITLAKTYLFLFVSIATRWTLPRLRMDQLLNLGWKFLLPISL

GNLLLTTSFQLFSLMIWHVQNENFILDSTRIFMKAFHLLLFDGSFIFPECILIFGLILLLMIDS
TSDQKDIPWLYFISSTSFVMSITALLFRWREEPMISFSGNFQTNNFNEIFQFLILLCSTLCIPLS
VEYIECTEMAITEFLLFILTATLGGMFLCGANDLITIFVAPECFSLCSYLLSGYTKKDIRSNEA
TMKYLLMGGASSSILVHGFSWLYGSSGGEIELQEIVNGLINTQMYNSPGISIALIFITVGIGF
KLSLAPSHQWTPDVYEGSPTPVVAFLSVTSKVAASASATRIFDIPFYFSSNEWHLLLEILAIL
SMIFGNLIAITQTSMKRMLAYSSIGQIGYVIIGIIVGDSNGGYASMITYMLFYIAMNLGTFAC
IILFGLRTGTDNIRDYAGLYTKDPFLALSLALCLLSLGGLPPLAGFFGKLHLFWCGWQAGL
YFLVSIGLLTSVLSIYYYLKIIKLLMTGRNQEITPHMRNYRISPLRSNNSIELSMIVCVIASTIP
GISMNPIIAIAQDTLFMFLLYEYDIFWAFLLISSAIPVLAFLISGVLSPIRKGPEKLSSYESGIEP
IGDAWLQFRIRYYMFALVFVVFDVETVFLYPWAMSFDVLGVSAFIEAFIFVLILILGLVYAW
RKGALEWSTNDFPWLTIIVVFPISAGSLMLFLPHRGNKVNKWYTICICILELLLTTYAFCYN
FKMDDPLIQLSEDYKWIDFFDFYWRMGIDGLSIGTILLTGFITTLATLAAFPVTRDSRFFHF
LMLAMYSGQIGSFSSRDLLLFFIMWELELIPVYLLLSMWGGKKRLYSATKFILYTAGSSIFL
LIGVLGISLYGSNEPTLNLELLANKSYPVTLEILFYIGFLIAFAVKSPIIPLHTWLPDTHGEAH
YSTCMLLAGILLKMGAYGLVRINMELLPHAHSMFSPWLLVVGTIQIIYAASTSPGQRNLKK
RIAYSSVSHMGFIIIGISSITDPGLNGAILQIISHGFIGAALFFLAGTSYDRIRLVYLDEMGGM
AISIPKIFTMFTILSMASLALPGMSGFIAEFIVFFGIITSQKYFLISKIFIIFVMAIGMILTPIYLLS
MLRQMFYGYKLINIKNFSFFDSGPRELFLSISILLPIIGIGIYPDFVLSLASDKVESILSNYFYG
MILEHVLVLSAYLFLIGLYGLITSRNMVRALMCLELILNAVNMNFVTFSDFFDNSQLKGEIF
CIFVIAIAAAEAAIGLAIVSSIYRNRKSIRINQSTLLNKMEHTYQYSWIIPFIPLPVPILLGVGL
LLFPTATKNLRRMWTFLSIFLLSIVMIFSIYLSIQQIFLSCIHQNVWSWTINNEFSFEFGYFIDP
LTSIMSILITTVGILVLIYSDNYMSHDQGYLRFFAYMGFFNTSMLGLVTSSNLIQVYFFWEL
VGMCSYLLIGFWFTRPIAANACQKAFVTNRVGDFGLLLGILGLYWITGSFEFQDLFEIFNN-
RVNLLFLTLCAFLLFVGPIAKSAQFPLHVWLPDAMEGPTPISALIHAATMVAAGIFLVARLL
PLFIVIPSIMYIISLIGIITVLLGATLALAQKDIKRGLAYSTMSQLGYMMLALGMGSYRSALF
HLITHAYSKALLFLGSGSIIHSMEAIVGYSPDKSQNMILMGGLTKHVPITKTAFLIGTLSLCG
IPPLACFWSKDEILNDSLLFSPIFAIIACSTAGLTAFYMFRIYLLTFEGHLNTYFLNYSGKKSG
SFYSLSLWGKEEEKKLNK-LVPLLTMNFFCGLNTRTFYYPHESDNTILFPMLILVLFTLFIGA
IG--FDFDILSKFFTPSINLLHKNSQ-FVDW-EFLRNATFSVSIAFFGIFIAYCLYKPFYSSLLNLT
LLNSFQIHWEKLINFVYNWSYNRGYIDSFFKTSLIESIRRLAKQTTFFDKRIIDGITNGVGIT
SFFVGEVTKYIGGS-RISSYLFLYLSYVLIFLMILFMDLPGPIHDFLLVFLGSGLLVGGLGVV
LLPNPIFSAFSLGFVLVCISLLYILSNSHFVAAAQLLIYVGAINVLIIFAVMFMNDSEYSTDFN
LWTIGNGITSLVCTTILFLLMSTILDTSWYGVIWTTKLNQILEQDLISNSQQIGIHLSTDFFLP
FELISIILLVALIGAISVARQMKRPVTGKDLMIVNMGPHHPSMHGVLRLIVTLDGEDVVDCE
PILGYLHRGMEKIAENRAIIQYLPYVTRWDYLATMFTEAITVNGPEQLGNIQVPKRASYIR
VIMLELSRIASHLLWLGPFMADIGAQTPFFYIFREREFVYDLFEAATGMRMMHNFFRIGGI
AADLPYGWIDKCLDFCDYFLTEVVEYQKLITRNPIFLERVEGVGIIGGEEAINWGLSGPML
RASGIPWDLRKIDRYESYDEFEWEIQWQKQGDSLARYLVRLSEMTESIKIIQQALEGLPGG
PYENLESRGFDRKRNPEWNDFEYRFISKKPSPTFELSKQELYVRVEAPKGELGIFLIGDQSG
FPWRWKIRPPGFINLQILPELVKRMKLADIMTILGSIDIIMGEVDRMLPMITGFMNYGQQTL
RAARYIGQGFMITLSHTNRLPVTIQYPYEKLITSERFRGRIHFEFDKCIACEVCVRVCPIDLP
VVDWKLETNIRKKRLLNYSIDFGICIFCGNCVEYCPTNCLSMTEEYEFSTYDRHELNYNQI
ALGRLPMSVIDDYTIRTIWNS--GVNPLI--------MQGTLSVWLAKRGLVHRSLGFDYQGIET
LQIKPEDWHSIAVILYVYGYNYLRSQCAYDVAPGGLLASVYHLTRIEYGVNQAEEVCIKVF

THRSNPRIPSVFWVWKSTDFQERESYDMLGITYDSHPRLKRILMPESWIGWPLRKDYIAP
NFYEIQDAY---MNSIKFPILDRTTKNSVISTTL
NDLSNWSRLSSLWPLLYGTSCCFIEFASLIGSRFDFDRYGLVPRSSPRQADLILTAGTVTMK
MAPSLVRLYEQMPEPKYVIAMGACTITGGMFSTDSYSTVRGVDKLIPVDVYLPGCPPKPE
AVIDAITKLRKKIAREIYKDRIRPQQGNRCFTTNHKFFVVRSPHIGNYDQELLYPPSSTSEIS-
ETFFKYKSPVSSHELVNMQTRNTFSWIREEITRSISVSLIIYIITWASISSAYPIFAQQNYENPR
EATGRIVCANCHLANKPVDIEVPQTVLPDTVFEAVVKIPYDMQLKQVLANGKKGALNVG
AVLILPEGFELAPPDRISPEMKEKIGNLSFQNYRPNKKNILVIGPVPGQKYSEITFPILAPDPA
TNKDVHFLKYPIYVGGNRGRGQIYPDGSKSNNTVYNATAGGIISKILRKEKGGYEITIVDA
SNGREVIDIIPRGLELLVSEGESIKLDQPLTSNPNVGGFGQGDAEIVLQDPLRVQGLLFFLGS
VVLAQIFLVLKKKQFEKVQLSEMNFMSKVYDWFEERLEIQAIADDITSKYVPPHVNIFYCL
GGITLTCFLVQVATGFAMTFYYRPTVTEAFASVQYIMTEANFGWLIRSVHRWSASMMVLM
MILHVFRVYLTGGFKKPRELTWVTGVVLGVLTASFGVTGYSLPWDQIGYWAVKIVTGVPD
AIPVIGSPLVELLRGSASVGQSTLTRFYSLHTFVLPLLTAVFMLMHFLMIRKQGISGPLMGV
TKKPDLNDPVLRAKLAKGMGHNYYGEPAWPNDLLYIFPVVILGTIACNVGLAVLEPSMIG
EPADPFATPLEILPEWYFFPVFQILRTVPNKLLGVLLMVSVPAGLLTVPFLENVNKFQNPFR
RPVATTVFLIGTAAALWLGIGATLPIDKSLTLGLFMIEVFLFGIVLGLIPITLAGLFVTAYLQY
RRGDQLDFMPTITSYFGFLLAALTITSVLFIGLSKIRLIMDIVSLAWAALMVVFTFSLSLVV
WGRSGLMIIRSPEPEVKILVDRDPIKTSFEEWAKPGHFSRTIAKGPDTTTWIWNLHADAHD
FDSHTSDLEEISRKVFSAHFGQLSIIFLWLSGMYFHGARFSNYEAWLSDPTHIGPSAQVVW
PIVGQEILNGDVGGGFRGIQITSGFFQIWRASGITSELQLYCTAIGALVFAALMLFAGWFHY
HKAAPKLAWFQDVESMLNHHLAGLLGLGSLSWAGHQVHVSLPINQFLNAGVDPKEIPLP
HEFILNRDLLAQLYPSFAEGATPFFTLNWSKYSEFLTFRGGLDPVTGGLWLTDIAHHHLAIA
ILFLIAGHMYRTNWGIGHGIKDILEAHKGPFTGQGHKGLYEILTTSWHAQLSLNLAMLGSL
TIIVAHHMYSMPPYPYLATDYATQLSLFTHHMWIGGFLIVGAAAHAAIFMVRDYDPTNRY
NDLLDRVLRHRDAIISHLNWVCIFLGFHSFGLYIHNDTMSALGRPQDMFSDTAIQLQPVFA
QWIQNTHALAPGVTAPGETASTSLTWGGGELVAVGGKVALLPIPLGTADFLVHHIHAFTIH
VTVLILLKGVLFARSSRLIPDKANLGFRFPCDGPGRGGTCQVSAWDHVFLGLFWMYNAIS
VVIFHFSWKMQSDVWGSISDQGVVTHITGGNFAQSSITINGWLRDFLWAQASQVIQSYGS
SLSAYGLFFLGAHFVWAFSLMFLFSGRGYWQELIESIVWAHNKLKVAPATQPRALSIIQGR
AVGVTHYLLGGIATTWAFFLARIIAVGMALRFPRFSQGLAQDPTTRRIWFGIATAHDFESHD
DITEERLYQNIFASHFGQLAIIFLWTSGNLFHVAWQGNFETWVQDPLHVRPIAHAIWDPHF
GQPAVEAFTRGGALGPVNIAYSGVYQWWYTIGLRTNEDLYTGALFLLFLSALSLIGGWLH
LQPKWKPRVSWFKNAESRLNHHLSGLFGVSSLAWTGHLVHVAIPASRGEYVRWNNFLNV
LPHPQGLGPLFTGQWNLYAQNPDSSSHLFGTSQGSGTAILTLLGGFHPQTQSLWLTDMAH
HHLAIAILFLIAGHMYRTNFGIGHSIKDLLEAHIPPGGRLGRGHKGLYDTINNSIHFQLGLA
LASLGVITSLVAQHMYSLPAYAFIAQDFTTQAALYTHHQYIAGFIMTGAFAHGAIFFIRDYN
PEQNEDNVLARMLDHKEAIISHLSWASLFLGFHTLGLYVHNDVMLAFGTPEKQILIEPIFA
QWIQSAHGKTSYGFDVLLSSTSGPAFNAGRSIWLPGWLNAINE--NSNSLFLTIGPGDFLVH
HAIALGLHTTTLILVKGALDARGSKLMPDKKDFGYSFPCDGPGRGGTCDISAWDAFYLAV
FWMLNTIGWVTFYWHWKHITLWQGNVSQFNESSTYLMGWLRDYLWLNSSQLINGYNPF
GMNSLSVWAWMFLFGHLVWATGFMFLISWRGYWQELIETLAWAHERTPLANLIRWKDKP
VALSIVQARLVGLAHFSVGYIFTYAAFLIASTSGKFGMSHSVKIYDTCIGCTQCVRACPTDV
LEMIPWDGCKAKQIASAPRTEDCVGCKRCESACPTDFLSVRVYLWHETTRSMGLAYMTT

FNLPSIFVPLVGLVFPAIAMASLFLHIQKNKIFMRDLKTYLSVAPVLSTLWFGSLAGLLIEIN
RLFPDALTFPFFSFMTAILERRESESLWGRFCNWITSTENRLYIGWFGVLMIPTLLTATSVFII
AFIAAPPVDIDGIREPVSGSLLYGNNIISGAIIPTSAAIGLHFYPIWEAASVDEWLYNGGPYE
LIVLHFLLGVACYMGREWELSFRLGMRPWIAVAYSAPVAAATAVFLIYPIGQGSFSDGMPL
GISGTFNFMIVFQAEHNILMHPFHMLGVAGVFGGSLFSAMHGSLVTSSLIRETTENESANE
GYRFGQEEETYNIVAAHGYFGRLIFQYASFNNSRSLHFFLAAWPVVGIWFTALGISTMAFN
LNGFNFNQSVVDSQGRVINTWADIINRANLGMEVMHERNAHNFPLDLAAVEAPSTNGMG
LPWYRVHTVVLNDPGRLLAVHIMHTALVAGWAGSMALYELAVFDPSDPVLDPMWRQGM
FVIPFMTRLGITNSWGGWNIT-GGTITNPGLWSYEGVAGAHIVFSGLCFLAAIWHWVYWD
LEIFCDERTGKPSLDLPKIFGIHLFLSGVACFGFGAFHVTGLYGPGIWVSDPYGLTGKVQPV
NPAWGVEGFDPFVPGGIASHHIAAGTLGILAGLFHLSVRPPQRLYKGLRMGNIETVLSSSIA
AVFFAAFVVAGTMWYGSATTPIELFGPTRYQWDQGYFQQEIYRRVSAGLAENQSLSEAWA
KIPEKLAFYDYIGNNPAKGGLFRAGSMDNGDGIAVGWLGHPVFRNKEGRELFVRRMPTFF
ETFPVVLVDGDGIVRADVPFRRAESKYSVEQVGVTVEFYGGELNGVSYSDPATVKKYARR
AQLGEIFELDRATLKSDGVFRSSPRGWFTFGHASFALLFFFGHIWHGARTLFRDVFAGIDP
DLDAQVEFGAFQKLGDPTTKRQAVMKTLYSLRRFYHVETLFNGTLALAGRDQETTGFAW
WAGNARLINLSGKLLGAHVAHAGLIVFWAGAMNLFEVAHFVPEKPMYEQGLILLPHLATL
GWGVGPGGEVIDTFPYFVSGVLHLISSAVLGFGGIYHALLGPETLEESFPFFGYVWKDRNK
MTTILGIHLILLGVGAFLLVFKALYFGGVYDTWAPGGGDVRKITNLTLSPSVIFGYLLKSPF
GGEGWIVSVDDLEDIIGGHVWLGSICIFGGIWHILTKPFAWARRALVWSGEAYLSYSLAAL
SVCGFIACCFVWFNNTAYPSEFYGPTGPEASQAQAFTFLVRDQRLGANVGSAQGPTGLGK
YLMRSPTGEVIFGGETMRFWDLRAPWLEPLRGPNGLDLSRLKKDIQPWQERRSAEYMTH
APLGSLNSVGGVATEINAVNYVSPRSWLSTSHFVLGFFLFVGHLWHAGRARAAAAGFEKG
IDRDFEPVLSMTPLNMTIALGKFTKDEKDLFDIMDDWLRRDRFVFVGWSGLLLFPCAYFA
LGGWFTGTTFVTSWYTHGLASSYLEGCNFLTAAVSTPANSLAHSLLLLWGPEAQGDFTRW
CQLGGLWAFVALHGAFALIGFMLRQFELARSVQLRPYNAIAFSGPIAVFVSVFLIYPLGQSG
WFFAPSFGVAAIFRFILFFQGFHNWTLNPFHMMGVAGVLGAALLCAIHGATVENTLFEDG
DGANTFRAFNPTQAEETYSMVTANRFWSQIFGVAFSNKRWLHFFMLFVPVTGLWMSALG
VVGLALNLRAYDFVSQEIRAAEDPEFETFYTKNILLNEGIRAWMAAQDQPHENLIFPEEVL
PRGNALMSGSTGERSFADIITSIRYWVIHSITIPSLFIAGWLFVSTGLAYDVFGSPRPNEYFTE
SRQGIPLITGRFDPLEQLDEFSRSFMTIDRTYPIFTVRWLAVHGLAVPTVSFLGSISAMQFIQ
RMATQTVEDSSRSG-PRSTTVGKLLKPLNSEYGKVAPGWGTTPLMGVAMALFAVFLSIILEI
YNSSVLLDGISVNMLTLKLFVYTVVIFFVSLFIFGFLSNDPGRNPGREEMADTTGRIPLWVI
GTVAGILVIGLIGIFFYGSYSGLGSSLMLNIFNLICIFFNSTLFSSTFLVAKLPEAYAFLNPIVD
VMPVIPLFFLLLAFVWQAAVSFRMTQSNPNEQSVELNRTSLYWGLLLIFVLAVLFSNYFFN
MEVNILAFIATALFILVPTAFLLIIYVKTVSQNDMETATLVAIFISGLLVSFTGYALYTAFGQPS
QQLRDPFEEHGDMEALVYTFLLVSTLGIIFFAIFFREPPKISTKK--MTIAFQLAVFALIITSSIL
LISVPVVFASPDGWSSNKNVVFSGTSLWIGLVFLVGILNSLIS-------MSPQTETKASVGFKA
GVKEYKLTYYTPEYETKDTDILAAFRVTPQPGVPPEEAGAAVAAESSTGTWTTVWTDGLT
SLDRYKGRCYHIEPVPGEETQFIAYVAYPLDLFEEGSVTNMFTSIVGNVFGFKALAALRLE
DLRIPPAYTKTFQGPPHGIQVERDKLNKYGRPLLGCTIKPKLGLSAKNYGRAVYECLRGGL
DFTKDDENVNSQPFMRWRDRFLFCAEAIYKSQAETGEIKGHYLNATAGTCEEMIKRAVFA
RELGVPIVMHDYLTGGFTANTSLSHYCRDNGLLLHIHRAMHAVIDRQKNHGMHFRVLAK
ALRLSGGDHIHAGTVVGKLEGDRESTLGFVDLLRDDYVEKDRSRGIFFTQDWVSLPGVLP

VASGGIHVWHMPALTEIFGDDSVLQFGGGTLGHPWGNAPGAVANRVALEACVQARNEGR
DLAVEGNEIIREACKWSPELAAACEVWKEITFNFPTIDKLDGMAIHLYKTSTPSTRNGAVD
SQVKSNPRNNLICGQHHCGKGRNARGIITARHRGGGHKRLYRKIDFRRNAKDIYGRIVTIE
YDPNRNAYICLIHYGDGEKRYILHPRGAIIGDTIVSGTEVPIKMGNALPLTDMPLGTAIHNIE
ITLGRGGQLARAAGAVAKLIAKEGKSATLKLPSGEVRLISKNCSATVGQVGNVGVNQKSL
GRAGSKCWLGKRPVVRGVVMNPVDHPHGGGEGRAPIGRKKPVTPWGYPALGRRTRKRK
KYSETLILRRRSKMIQPQTYLNVADNSGARELMCIRIIGAS-NRRYAHIGDVIVAVIKEAIPN
TPLERSEVIRAVIVRTCKELKRNNGTIIRYDDNAAVVIDQEGNPKGTRVFGAIPRELRQLNF
TKIVSLAPEVLMLSPKRTRFRKQHRGRLKGISSRGNRICFGRYALQTLEPAWITSRQIEAGR
RAMTRNVRRGGKIWVRIFPDKPVTVRPAETRMGSGKGSPEYWVAVVKPGKILYEMGGVP
ENIARKAISIAASKMPIKTQFIISEMTRIKRGYIARRRRTKLRLFASSFRGAHSRLTRTMTQQ
RIRALVSAHRDRGKRKRDFRRLWITRINAVIHEM-GVFYSYNEFIHNLYKKQLLLNRKILAQ
IALLNRSCLYTISNDIKK-----------MIKEVYALGQYISMSAHKARRVIDQIRGRSYEEALMILE
LMPYRGCYPIFKLVYSAAANASHNKGFKETNLVISKAEVNQGNTVKKLKPRARGRSYPIK
RSTCHITIVLEDISFYGGLWDKKMDGIKYAVFTDKSIRLLGKN-QYTFNVESGSTRTEIKHW
VELFFGVKVIAMNSHRLPGKVKRMGPILGHTMHYRRMIITLQPGYSIPPLRKKRTMAVPK
KRTSISKKRIRKKIWKRKGYWTSLKAFSLGKSLSTGNSKSFFVQQNK-MAKGKDVRVTIIL
ECTSCVRNDI--KKEAAGISRYITQKNRHNTPSRLELRKFCPYCYKHTIHGEIKKMKIRASV
RKICEKCRLIRRRGRIIVICSNPRHKQRQGMVREKVKVSTRTLQWKCVESKRDSKRLYYG
RFILSPLMKGQADTIGIAMRRALLGEIEGTCITRAKSE--NIPHDYSNIAGIQESVHEILMNLN
EIVLRSNLYGTRNALICVQGPGYITARDIILPPAVEIIDNTQHIATLTEPIDLCIELKIERNRGYS
LKMSNNFE-DR-SYPIDAVFMPVENANHSIHSYGNGNEKQEILFLEIWTNGSLTPKEALHQA
SRNLINLFIPFLHVEEE--TFYLENNQ-HQVTLPFFPFHNRLV-NLRKKKKKELAFQYIFIDQL
ELPPRIYNCLKKSNIHTLLDLLNNSQEDLIKIEHFHVEDVKKILDILEKK-----------MLGDEK-
EGTSAIPGFNQIQFEGFYRFIDQGLIEELAKFPKIEDIDHEIEFQLFVETYQLVEPLIKERDAV
YESLTYSSELYVSAGLIWKTSRNMQEQRIFIGNIPLMNSLGTSIVNGIYRIVINQILQSPGIYY
QSELDHNGISVYTGTIISDWGGRLELEIDKKARIWARVSRKQKISILVLSSAMGLNLREILE
NVCYPEIFLSFLTDKEKKKIGSKENAILEFYQQFSCVGGDPIFSESLCKELQKKFFHQRCEL
GRIGRRNINWRLNLNIPQNNIFLLPRDVLAAADHLIGMKFGMGTLDD--MNHLKNKRIRSV
ADLLQDQLGLALARLENVVKGTISGAIR-HKLI-PTPQNLVTSTP----LTTTYESFFGLHPLSQ
VLDRTNPLTQIVHGRKLSYLGPGGLTGRTANFRIRDIHPSHYGRICPIDTSEGINVGLIGSLSI
HARIGDWG-SLESPFY---ELFEKSKKARIRMLFLSPSQDEYYMIAAGNSLALNRGIQEEQAV
PARYRQEFLTIAWEEVHLRSIFPFQYFSIGASLIPFIEHNDANRALMSSNMQRQAVPLSRSEK
CIVGTGLERQVALDSGVPAIAEHEGKILYTDTEKIVFSGNGDTLSIPLIMYQRSNKNTCMHQ
KPQVRRGKCIKKGQILADGAATVGGELALGKNILVAYMPWEGYNFEDAVLISECLVYGDI
YTSFHIRKYEIQTHVTTQG-PERITKEIPHLEGRLLRNLDKNGIVMLGSWVETGDILVGKLT
PQVAKE-SSYAPEDRLLRAILGIQVSTSKETCLKLPIGGRGRVIDVRWVQKKG-GSSYNPEII
RVYISQKREIKVGDKVAGRHGNKGIISKILPRQDMPYLQDGRPVDMVFNPLGVPSRMNVG
QIFECSLGLAGSLLDRHYRIAPFDERYEQEASRKLVFSELYEASKQTANPWVFEPEYPGKS
RIFDGRTGDPFEQPVIIGKPYILKLIHQVDDKIHGRSSGHYALVTQQPLRGRSKQGGQRVGE
MEVWALEGFGVAHILQEMLTYKSDHIRARQEVLGTTIIGGTIPKPEDAPESFRLLVRELRSL
ALELNHFLVSEKNFQINRKEV-------MIDRYKHQQLRIGLVSPQQISAWATKIIPNGEIVGEVT
KPYTFHYKTNKPEKDGLFCERIFGPIKSGICACGNYRVIGDEKEDPKFCEQCGVEFVDSRIR
RYQMGYIKLTCPVTHVWYLKRLPSYIANLLDKPLKELEGLVYCD--FSFARP-ITKKPTFLRL

RGSFEYEIQSWKYSIPLFFTTQGFDIFRNREISTGAGAIREQLADLDLRIIIENSLVEWKQLG
EE-GPTGNEWEDRKIVRRKDFLVRRMELAKHFIRTNIEPEWMVLCLLPVLPPELRPIIQIEG
GKLMSSDINELYRRVIYRNNTLTDLLTTSRSTPGELVMCQEKLVQEAVDTLLDNGIRGQPM
RDGHNKVYKSFSDVIEGKEGRFRETLLGKRVDYSGRSVIVVGPSLSLHRCGLPREIAIELFQ
TFVIRGLIRQHLASNIGVAKSQIREKKPIVWEILQEVMQGHPVLLNRAPTLHRLGIQSFQPIL
VEGRTICLHPLVCKGFNADFDGDQMAVHVPLSLEAQAEARLLMFSHMNLLSPAIGDPISVP
TQDMLIGLYVLTSGTRRGICANRYNPCNRKN---YQNERIYETNYKYT--KEPFFCNSYDAIG
AYRQKKINLDSPLWLRWQLDQR-VIAS--REVPIEVHYESFGNYHEIYAHYLIVRSVKKENF
CIYIRTTVGHISFYREIEEAIQGFSQACSYDT-----------MAERANLVFHNKVIDGTAIKRLISR
LIDHFGMAYTSHILDQVKTLGFQQATATSISLGIDDLLTIPSKGWLVQDAEQQSWILEKHHH
YGNVHAVEKLRQSIEIWYATSEYLRQEMNPNFRMTDPFNPVHMMSFSGARGNASQVHQL
VGMRGLMSDPQGQMIDLPIQSNLREGLSLTEYIISCYGARKGVVDTAVRTSDAGYLTRRLV
EVVQHIVVRRTDCGTIRGISVSPKNRMMS-ERIFIQTLIGRVLADDIYIGSRCVAFRNQDLGI
GLVNRLIT------FGTQSISIRTPFTCRSTSWICRLCYGRSPTHGDLVELGEAVGIIAGQSIGEPG
TQLTLRTFHTGGVFTGGTAEHVRAPYNGKIKFNEDLVHPTRTRHGHPAFLCYIDLSVIIESE
DIIHSVTIPPKSFLLVQNDQYVESEQVIAEIREGTYTF-HFKERVRKYIYSDSEGE-MHWSTD
VSHAPEFTYSNVHLLPKTSHLWILSGGSCG--SSLIRFSIHKDQDQMNIP--FLSAER-KSIS--L
SVNNDQ-VSQKFFSSDFADPK-LGIYDY-SELNGNLGTSHYNLIYSA-IFHENSD--LLAKRRR
NRFLIPFQSIQEQEKEFIPQ-GISVEIPIN--GIFRRNSIFAFFDDPRYRRKSSGILKYGTLKADSII
QKED--MIEYR-GVQKIKTKYMKVDRFFFIPEEVHILPESSAIMVQNYSIIGVDTRLTLNIRSQ
VGGLIRVEKKKKRIELKIFSGDIHFPDKTDKISRHSGILIPPGRGKK--NSKESKK--FKNWIY
VQR--ITPTKKK--FFVLVRPVATYEIADSINLATLFPQDLFREKDNIQLRVFNYILYGNGKPTR
GISDTSIQLVRTCLVLNWD------KNSSLEEVRAFFVEVSTKGLIQDFIRIGLVKSHISYIRKRN
NSPDSGLIS----ADHMN--PFYSISPKSGI----LQQSLRQNHGTIRMFLNR---NKESQSLLILSSS
NCFRMGP--FNHVKHHN---VINQSNTLITIKNSSGPLGT--ATPISNFYS--FLPLLT--YNQIS-LI
KYFQLDN---LKYIFQKI--N-SYLIDENGI--ILNLDPYSNVVLNPFKLNWYFLHQNYCEETSTI
IS-LGQFFCENVCIAKKEP---HLKSGQVLIVQ-RDSAVIRSAKPYLATPGAKVHGHYSEILYE
GDTLVTFIYEKSRSGDITQGLPKVEQVLEVRSIDSISLNLEKRIKGWNKCITRILGIPWGFLI
GAELTIVQSRISLVNKIQKVYRSQGVQIHNRHIEIIVRQITSKVLVSE---EGMSNVFLPGELIG
LLRAERTGRALEEAICYRAVLLGITRASLNTQSFISEASFQETARVLAKAALRGRIDWLKGL
KENVVLGGVIPAGTGFKGLVHCS--RQHTNIILEKKTKNLALFEGDMR--DILFYHREFCDSS
ISKSDFSRI----------------MTKRYWNIDLEEMMRAGVHFGHGTRKWNPRMAPYISAKRKGI
HIINLTRTARFLSEACDLVFDAASRGKQFLIVGTKNKAADLVSRAAIRARCHYVNKKWLG
GMLTNWSTTEKRLHKFRDLRTEQKTEGFNRLPKRDAAVLKRQLSRLETYLGGIKYMTGL
PDIVIILDQQEEYTALRECITLGIPTISLIDTNCNPDLADISIPANDDAIASIRFILNKLVFAICEG
RSS-YIQNSMGQKINPLGFRLGTTQSHHSLWFAQPKKYSEGLEEDKKIRDCIKNYVQKNIRI
SSG-MEGIARIEIQKRIDLIQIIIYMGFPKLLIEDK----PRRVEELQMNVQKELNCVNRKLNIAI
TRISNPYGDPNILAEFIAGQLKNRVSFRKAMKKAIELTEQANTKGIQVQIAGRIDGKEIARV
EWIREGRVPLQTIEAKIDYCSYTVRTIYGVLGIKIWIFVDEEMSRYRGPRFKKIRRLGALPG
LTSKRPKAGSDLRNQSRSVK-KSQYRIRLEEKQKLRFHYGLTEHQLLKYVRIAGKAKGSTG
QVLLQLLEMRLDNILFRLGMALTIPQARQLVNHGHILVNGRIVDIPSYRCKPRDIITVKDEQ
NSRTLVQNLLDSSAP-EELPNHLTLHTFQ--YEGLVNQIIDRKCVGLKINELLVVEYYSRQTM
SRRGTAEEKTAKSDPIYRNRLVNMLVNRILKHGKKSLAYQIIYRALKKIQQKTETNPLSVL
RQAIRGVTPDIAVKARRVGGSTHQVPIEIGSTQGKALAIRWLLGASRKRPGRNMAFKLSSE

LVDAAKGSGDAIRKKEETHRMAEANRAFAHFRMGKDTIADIITSIRNADMNRKGTVRIGS
TNITESIVKILLREGFIENVRKHRENNQYFLILTLRHRR--NKKESYK---TILNLKRISRPGLRI
YSNSQRIPRILGGIGIVILSTSQGIMTDREARLKRIGGEILCYIWMAKPILRIGSRKNTRSGSR
KNVRRIPKGVIHVQASFNNTIVTVTDVRGRVISWSSAGTCGFRGTRRGTPFAAQTAAGNAI
RAVVDQGMQRAEVRIKGPGLGRDAALRAIRRSGILLSFVRDVTPMPHNGCRPPKKRRVM
PTIKQLIRNTRQPIRNVTKSPALRGCPQRRGTCTRVYTITPKKPNSALRKVARVRLTSGFEIT
AYIPGIGHNLQEHSVVLVRGGRVKDLPGVRYHIVRGTLDAVGVKDRQQGRSKYGVKKPK
MAKKSLIYREKKRQKLEKKYHLIRRSSKKEIS---KIPSLSEKWKIHGKLQSPPRNSAPTRLH
RRCFSTGRPRANYRDFGLSGHILREMVQACLLPGATRSSWMIKNIVIS--FEE-Q-KEESRGS
VEFQVFSFTNKIRRLTSHLELHRKDYLSQRGLRKILGKRQRLLAYLSKKNRVRYKELINQL
NIRELKTR--MVKLRLKRCGRKQRAVYRILAIDVRYRREGRDLSKVGFYDPITNQTFLNLS
AILDFLKKGAQPTRTAHDISKKAGIFTE----------MNKSKRLFTKSKRSFRRRLPPIQSGDRID
YRNMSLISRFISEQGKILSRRVNRVTLKQQRLITIAIKQARILSLLPFLNNQ--KQFERSESTPR
TTSLRTRKKVTRSLKKNPFVAKHLLRKIEKLNTKAEKEIIITWSRASTIIPTMIGHTIAIHNGR
EHLPVYIIDLMVGHKLGEFSPTINFRGHAKNDNRSRRMVFQSFILGNLVSLCMKIINSVVV
VGLYYGFLTTFSIGPSYLFLLRARVMDEGEEGTEKKVSATTGFIAGQLMMFISIYYAPLHLA
LGRPHTITVLALPYLLFHFFWNNHKHFFDYGSTTRNEMRNLRIQCVFLNNLIFQLFNHFILP
SSMLARLVNIYMFRCNNKMLFVTSSFVGWLIGHILFMKWVGLVLVWIQQNNSIRS-IRSNK
FLVSELRNSMARIFSILLFITCVYYLGRIPSPIFTKKLKGTEEAKPFVTLVFDYKRWNRPNRY
IKNDKIENIVRNEMSQYFFYTCQSDGKERISFTYPPNLSTFFEMIQKRIPSFTKEKKTFDQVS
TYWSLIHEEKRENLKKEFLNRIEALD---SVENILEKTTRFCYNEAKKEYLPKIYDPFLHGIS
RGRIKKLPPFLGGSWINKIHGLLYKKFEQTDSNDQD--FPEINKKVPRWSYKLIELEELEGE
GIRSRKAKRVVVFTDALIRYSQQSDFRREIIKGSMRSQRRKTVIWEFFQAKVHSPLFFDRID
K--LFFFSFDIWGLKKKIIKNREETRRIEIAETWDSFLFAQIIRGSLLVTQSILRKYIILPLLIIIK
NSVRMLLFQFPEWSQDLKDWKREMHVKCTYNGVQLSETEFPRNWLTDGIQIKILFPFYLK
PWHKSKKGEKNDFCFLTVWGMETELPFGSAQRKPSFFEPISKELKKRIKKLKKKSFVV-LK
IFKERAPIFLKVAKETKNWILKNFI--FIKG----ISKRNDSIISNQMIHEL-VQNKSLEWTN-SLS
EKKIKNLIDRKKTIRNQIEEISKEKQNSCTKLRYDSKIIESSKKIWQTFK--NTRLIRKSIFFFK
FCIEQMSIAIFLGIINIPRITTQL------YDLCSLSQAYVFYKLSQI-VSNFCKLKAVLEYTSFFVK
NKIKVFFEHGIFH-ELKNKTFL-SEVNQWKNWLRSQYQYNLPQISWARLVTQNWKNKINK
DSLVLNPTKEDSYEKKK--FDNYKKQ-NVKKDSIYNLFCYKSIHYNIRGRHRKYLDWRILN
FWFTFFDWMGMNEEILNQFFFPEFFLFSSTYKMKPWVIPIKLLLLNSN--TEAELDLFLTRYS
RFQLRWN---NQKILNNVKVYCLLVRLNNPNEIAVSSIERGEMSLDILMIEKNFTFAKLMKK
GILIIEPVRLSVQNDGQLIIYRTIGISLVHKNKHNYD-FFVPEKILSPKRRREFRILICFTFLWP
NFKLEDLACMNRYWFNTTNGNHFSMIRIRMYTMKGHQFKSWIFELREIVREIKNAHYFL
DSWTQFNSVGSFIHIFFHQERFRKLLDPRIFSILLL-RNS--QGSTSNRYF---TIKGVVLFVVA-
ALLYR----INNRNMVESKNLYLKGLLP-----IPMNSIGPRNDTSEESF-GSCNINRLIVS-LLYLT
KGKKISESCFRDPKESTWVLPIT----QKCIMPESNWSSRWWRNWIGKKR--GFCCKISN---ET
VAGIDISFKEKDIK-YLEFLFVYYMD--DPIRKGHDWELFDRLSPSKRRNIINL--NSGQLFEIL
VKDWICYLMFAFREKIPIEVEG-FCKQQG-AGSTIQSNDIEHVSH--LFSRNKWAISLQNCAQ
FHMWQFHQDLFV---SWGK-NPHESDFFRK-ISRENWIWLDNVWLVNKDR---FFS--KVRNV
SSNIQYDS---TR--SSFVQVTDSSQLNGSS-DQFIDPFDSIS----NEDSEYH----------YHTLINQR
EIQQLKERSIL-LD--PSFIQTEGREIESD--RFPK--YLSGYS--SMPRLFT-EREKRMNNHLLPEE
SEEF--LGNPTRAIRSFFSDRW--SELH-LGSNPTERSTRDQ----KLLKKEQDVS-FVPSRRSE----

NKEIVNIFKIITYLQN--TVSIHPISSDLGCDMVP-KDELD-MDSSNKISFLNKNPFFDL--FHLF
HERKRGGYTL--RH--ESE--ERFQEMADLFTLSITEP-DLVYHKGFAFSIDSYGLDQRQ--FLK
EVFNFRDESKKKSL---LVLPPIFY--EENESFYRRLRKIWVR-ISCGNYLEDQK--RVVFASNNI
MEAVNQYRLIRNMIQIQFQYSPYGYIRNVLNRFFLMK---RPDRNFEYGIQ---RDLIGNDTLN
HRTIMKDTINQHLSNLKK-SQKKWFDP--LIFLSQTERSINRDPNAYRYKWSNGSKNFQEHL
EHFVSERKSR---FQVVFDQLCINQYSIDWSEVIDKKDLSKSLRFFLSKLLRFFLSK-LLLFLS-
------KLLLFLSNSLP-FFFVSFENIPIHRSE-IHIY-ELKGPNDQLCNQLLESIGLQIVHLKKLKPF
--LLD---DHNTS-QKSKFLINGGTISPFLFNKIP-KW-MIDSFHTR--KNRRKSFDNTDSYFSIVS
HDQDNWLNPVKPFQRSSLISSFSKANRLRFLNNPHHFCFY--CNKRFPF---Y-VEKARLNNS
DFTFTYG-QFLTILFIHNK-TFSSCGGK--KKHAFLE-RDTISPIESQVSNIFISNDFPQSGDER-Y
NLYKS---FHFPIRSDPLVR--RAIYSIADISGTPLIEGQRVNFERTYCQTLSDMNLSDSEEKSLH
QYLN--FNSNMGLIHTPCSEKYLQ-----RKK--------RSLCLK--KCVDKGQMDRTFQRDSAFST
LSKWNLFQTYMPWFFTSTGYKYLNLIFLDTFSD-LLRILSSS--QKFVSIFHDIMHGLDISWRI
LQKKL------CLPQRN-LISEISSKSLHN--LLLSEEMIHRNNES--SLISTHLRSPNVREV-LYSILF
LLLVAGY-IVRTHLLFVSR--AYSELQTEFEKIKSLMIPSYMIELRKLLDRYPTSELNSFWLKN
LFLVALEQLGDCLEEIRGS--GGNMLWGGDP-----AYG-VKSIRSKKKDLKINFIDIIDLI-SIIPN
PINRITFSRNTRHLSHTSKEIYSLIRKRKNVSGDWIDDKIESWVANSDSIDDKEREFLVQFS--
TLRA--EKRIDQILLSLT-HSDHLS-----KNDSGYQMIEQPGTIYLRYLVDIH--KKYLMNYEFN
TSCLAERRIFLAHYQTITY--SQTSCGANSFHFPSHG---KPFSLRLALSPS-RSILVIGSIGTGRS
YLVKYLATNSYVPFITVFLNKFLDNKPKGFFID-----DID--DDSDDIDASN---------------------D
IDRELDTELELLTMMNAL---TMDMML---EIDRFYITLQFELAKAMSPCIIWIPNIHDLDVNE
SSYLALGLLVNSLS---RDCERCS-TRNILVIASTHIPQKVDPALIAPNKLNTCIKIRRLLIPQQR
KHFFTLSYTRGFHLEKKMFHT--NGFESITMGSSARDLVALTNEALSISITQKKSIIDTNTIRS
ALHRQTWDLR---SQVRSVQDHGILFYQIGRAVAQNVLISNCPIDPISIYMKKK--SCNEGDS
YLYKWYFELGTSMKKFTILLYLLSCSAGSVAQDLWSLP--VPDEK-NRITSYGFVENDS-DLV
HGLLEVQGALVG--SSRTEKDCSQFDND--RVTLLFRSEPRD-PLYMMQDGSCSIVDQRFLYE
K--YESEFEE-GEGEGVLDPQ----QIEED----LFNH--IVWAP--RIWRP---RGFLFDCIERPNELGF
P-YSAGSFRGK---RIIYDEKYELQENDSEFLQSGTMQYQRRDRSSKEQGFFRISQFIWDPAD
PLFFLFK-DQPFVSVFSHREFFADEEMSKGLLTSQTDPPTSIYKRWFI--KNTQEKHFELLIQR
QRWLR---TNSSLS--NGFFRSN--TRSESYQYLSNLFISN-GTLLDRMTKTLLKKRWLFSDEM
KIGF--M---------MPRSR-INGNFIDKTFTIVADILLRVIPTTSGEKEAFTYYRDG--MSAQSEGN
YAEALQNYYEAMRLEIDPYDRSYILYNIGLIHTSNGEHTKALEYYFRALERNPFLPQAFNN
MAVICHYR-GEQAIQQGDSEMAEAWFAQAAEYWKQAITLTPGNYIEAQNWLTITRRFE--M
SWRSESIWIEFITGSRKTSNFCWAFILFLGSLGFLLVGTSSYLGRNVISLFPSQQIIFFPQGIV
MSFYGIAGLFISCYLWCTILWNVGSGYDLFDRKEGIVRIFRWGFPGKSRRIFLRFFMKDIQS
IRIEVKEGVSARRVLYMEIRGQGAIPLIRTDEN-FTTREIEQKAAELAYFLRVPIEVF
>Oryza
??
??
??
??
??
???????????????????????????????MATLRVDEIHKILRERIEQYNRKVGIENIGRVVQVGDGIA
RIIGLGEIMSGELVEFAEGTRGIALNLESKNVGIVLMGDGLMIQEGSFVKATGRIAQIPVSEA

YLGRVINALAKPIDGRGEIVASESRLIESPAPGIISRRSVYEPLQTGLIAIDSMIPIGRGQRELII
GDRQTGKTAVATDTILNQKGQDVICVYVAIGQRASSVAQVVTTFHEEGAMEYTIVVAEMA
DSPATLQYLAPYTGAALAEYFMYRERHTLIIYDDLSKQAQAYRQMSLLLRRPPGREAYPG
DVFYLHSRLLERAAKLNSLLGEGSMTALPIVETQSGDVSAYIPTNVISITDGQIFLSADLFN
AGIRPAINVGISVSRVGSAAQIKAMKQVAGKSKLELAQFAELQAFAQFASALDKTSQNQLA
RGRRLRELLKQSQANPLPVEEQIATIYIGTRGYLDSLEIGQVKKFLDELRKHLKDTKPQFQ
EIISSSKTFTEEAEILLKEAIQEQLERFSLQEQTMRTNPTTSRPGVSTIEEKSTGRIDQIIGPVL
DVTFPRGKLPYIYNALVVKSRDTDGKQINVTCEVQQLLGNNRVRAVAMSATDGLMRGME
VIDTGAPLSVPVGGATLGRIFNVLGEPVDNLGPVDTSATFPIHRSAPAFIELDTKLSIFETGIK
VVDLLAPYRRGGKIGLFGGAGVGKTVLIMELINNIAKAHGGVSVFGGVGERTREGNDLY
MEMKESGVINEKNLEESKVALVYGQMNEPPGARMRVGLTALTMAEYFRDVNKQDVLLFI
DNIFRFVQAGSEVSALLGRMPSAVGYQPTLSTEMGSLQERITSTKKGSITSIQAVYVPADDL
TDPAPATTFAHLDATTVLSRGLASKGIYPAVDPLDSTSTMLQPRIVGNEHYETAQRVKQTL
QRYKELQDIIAILGLDELSEEDRLTVARARKIERFLSQPFFVAEVFTGSPGKYVGLAETIRGF
QLILSGELDGLPEQAFYLVGNIDEASTKAINLEEENKLKKMKLNLYVLTPKRIIWDCEVKEI
ILSTNSGQIGVLPNHAPINTAVDMGPLRIRLNDQWLTAVLWSGFARIVNNEIIILGNDAELGS
DIDPEEAQQALEIAEANVSRAEGTKELVEAKVALRRARIRVEAVNWIPMKNVTHSFVFLA
HWPSAGSFGLNTDILATNLINLTVVVGVLIYFGKGVLKDLLDNRKQRILSTIRNSEELRRGT
IEQLEKARIRLQKVELEADEYRMNGYSEIEREKANLINATSISLEQLEKSKNETLYFEKQRA
MNQVRQRVFQQAVQGALGTLNSCLNTELHFRTIRANISILGAME----MNPLIAAASVIAAG
LAVGLASIGPGVGQGTAAGQAVEGIARQPEAEGKIRGTLLLSLAFMEALTIYGLVVALALLF
ANPFVMNIIPCSIKTLKGLYDISGVEVGQHFYWQIGGFQIHAQVLITSWVVITILLGSVIIAV
RNPQTIPTDGQNFFEYVLEFIRDLSKTQIGEEYGPWVPFIGTMFLFIFVSNWSGALLPWKII
QLPHGELAAPTNDINTTVALALLTSAAYFYAGLSKKGLSYFEKYIKPTPILLPINILEDFTKP
LSLSFRLFGNILADELVVVVLVSLVPLVVPIPVMFLGLFTSGIQALIFATLAAAYIGESMEGH
HMLFATLEHILTHISFSTISIVITIHLITLLVRELGGLRDSSEKGMIATFFCITGFLVSRWASSGH
FPLSNLYESLIFLSWALYILHMIPKIQNSKNDLSTITTPSTILTQGFATSGLLTEMHQSTILVPA
LQSQWLMMHVSMMLLSYATLLCGSLLSAALLMIRFRKNLDFFSKKKEIEYFYAKRSALKS
TFFPLFPNYYKYQLIERLDSWSYRVISLGFTLLTIGILCGAVWANEAWGSYWNWDPKETW
AFITWTIFAIYLHSRTNPNWKGTKSAFVASIGFLIIWICYFGINLLGIGLHSYGSFTLPMKKK
KALPSFLYLVFIVLLPWGVSFSFNKCLELWIKNWWNTRQSQTLLTAIQEKRVLERFMELED
LFILDEMIKEKPNTHVQNPPIGIRKEIIQLAKIDNEGHLHIILHFSTNIICLAILSGSFFLGKEEL
VILNSWVQEFFYNLNDSVKAFFILLVTDFFVGFHSTRGWELLIRWVYNDLGWVPNELIFTI
FVCSFPVILDTCLKFWVFFCLNRLSPSLVVIYHSISEMPIGVPKVPYRIPGDEEATWVDLYN
VMYRERTLFLGQEIRCEVTNHITGLMVYLSIEDGSSDIFLFINSPGGWLISGMAIFDTMQTV
TPNIYTTCLGIAASMPSFILLGGEPTKRIAFPHARIMLHQPASAYYRARTPEFLLEVEELHKV
REMITRVYALRTGKPFWVVSEDMERDVFMSADEAKAYGLVDIVGDEPREAKITFEGLVME
ALPNGMFRVRLENDTIILGYISGKIRSSSIRILMGDRVKIEVSRYDSSKGRIIYRLPHKDSKD
MEKFEGYSEKLKFPRQYFVYPLLFQEYIYVFAHDYGLIGSNNKFSSLLVKRLMIRMYQQN
FWINLVNHPNQDRLLDSEFYSQILSEGFAIVVEIPFSLREQSCPIPKFQNLRSIHSIFPFLEDKF
LHLHYLAHIEIPYPIHLDILLQLLQYRIQDVPSLHLLRFFLNYYSNWNSFITKSIFILKKKRLF
RFLYNSYVSEYEFFLLFLRKQSSCLRLTSSGTFLERIIFSRKMEH--LMYPARKTIWFVMDPL
MHYVRYQGKAILASKGTLLLKKKWKCYLVRLWQYSFSFWTQPQRIHLNQLENSCFDFLG
YFSSVPINSLLVRNQMLENSFLIDTQM--KKFDTKVPVTPLIGSLAKAQFCTGSGHPISKPIW

TDLSDWDILDRFGRICRNLFHYHSGSSKKKTLYRLKYILRLSCARTLARKHKSTVRAFMQ
WLGSVFLEEFFTEEEQVFSLMFAKRIWYLDILRINDLVNPLMIIDRV-QVE-AINSFSNLE-LL
KEVYGLIWIL-PILTLLLGITIEVLVIVWLEREISASIQQRIGPEYAGPLGLLQAIADGTKLLFK
EDILPSRGDIPLFSIGPSIAVISILLSFLVIPLGYRFVLADLSIGVFLWIAISSIAPIGLLMAGYSS
NNKYSFSGGLRAAAQSISYEIPLTFCVLAISLLSNSSSTVDIVEAQSKYGFFGWNLWRQPIG
FLVFLISSLAECERLPFDLPEAEEELVAGYQTEYSGIKYGLFYLVSYLNLLVSSLFVTVLYLG
GWNLSIPYISFFGFFQMNKMVGILEMTMSIFITLTKAYLFLFISITIRWTLPRMRMDQLLNL
GWKFLLPISLGNLLLTTSSQLVSLMIWHVQNENFILDSTRIFMKAFHLLLFQGSFIFPECILIF
GLILLLMIDLTSDQKDRPWFYFISSTSLVISITALLFRWREEPIISFSGNFQTNNFNEIFQFLILL
CSTLCIPLSVEYIECTEMAITEFLLFVLTATLGGMFLCGANDLITIFVAPECFSLCSYLLSGYT
KRDLRSNEATMKYLLMGGASSSILVHGFSWLYGSSGGEIELQEIVNGLINTQMYNSPGISIA
LISITVGLGFKLSPAPFHQWTPDVYEGSPTPVVAFLSVTSKVAASASATRILDIPFYFSSNEW
HLLLEILAILSMILGNLLAITQTSMKRMLAYSSIGQIGYVIIGIIVGDSNDGYASMITYMLFYI
SMNLGTFACIVLFGLRTGTDNIRDYAGLYTKDPFLALSLALCLLSLGGLPPLAGFFGKLYLF
WCGWQAGLYFLVSIGLLTSVLSIYYYLKIVKLLMTGRNQEITPYVRNYRRSPLRSNNSIELS
MTVCVIASTIPGISMNPILAIAQDTLFMFLLHEYDIFWAFLIIASLIPILAFWISALLAPVREGP
EKLSSYESGIEPMGGAWLQFRIRYYMFALVFVVFDVETVFLYPWAMSFDVLGISVFIEAFIF
VLILVVGLVYAWRKGALEWSMSSFPWLTILVVLPIFAGSLIFFLPHRGNKIVRWYTMSICLL
EFLLMTYAFCYHFQLEDPLIQLKEDSKWIDVFNFHWRLGIDGLSLGSILLTGFMTTLATLA
AWPVTRNSRLFYFLMLAMYSGQIGLFSSRDLLLFFIMWELELIPVYLLLSMWGGKRRLYS
ATKFILYTAGGSIFFLIGVLGMGLYGSNEPRLDLERLINQSYPATLEILFYFGFLIAYAVKLPII
PLHTWLPDTHGEAHYSTCMLLAGILLKMGAYGLIRINMELLPHAHYLFSPWLVIIGAMQII
YAASTSLGQRNFKKRIAYSSVSHMGFIIIGIGSITNIGLNGAILQILSHGFIGATLFFLAGTACD
RMRLVYLEELGGASIPMPKIFTMFSSFSMASLALPGMSGFVAELVVFFGLITSPKFLLMPK
MLITFVMAIGMILTPIYLLSMLRQMFYGYKLFHVPNENFEDSGPRELFLLICIFLPVIGIGIYP
DFVLSLSVDRVEALLSNYYPKMMFEHVLFLSVYLFSIGIYGLITSRNMVRALICLELILNSI
NLNLVTFSDLFDSRQLKGDIFAIFVIALAAAEAAIGLSILSSIHRNRKSTRINQSNFLNNMEH
TYQYAWVIPLLPLPVIMSMGFGLFLVPTATKNLRRIWAFPSVLLLSIAMVFSVHLSIQQINGS
SIYQYLWSWTVNNDFSLEFGYLIDPLTSIMLILITTVGILVLIYSDDYMSHDEGYLRFFVYIS
FFNTSMLGLVTSSNLIQIYFFWELVGMCSYLLIGFWFTRPIAASACQKAFVTNRVGDFGLL
LGILGFFWITGSLEFRDLFKIANNNEINSLLTILCAFLLFLGAVAKSAQFPLHVWLPDAMEG
PTPISALIHATTMVAAGIFLIARLLPLFISLPLIMSFISLIGTLTLFLGATLALAQRDIKRSLAYS
TMSQLGYMMLALGIGSYQAALFHLITHAYSKALLFLGSGSVIHSMEPLVGYSPDKSQNMV
LMGGLRKYIPITRTCFLWGTLSLCGIPPLACFWSKDEILSNSWLYSPFFGIIASFTAGLTAFY
MFRIYLLTFDGYLRVHFQNYSSTKEDSLYSISLWGKRISKGVNR-DFVLSTAKFFSTKN--TF
VYPHEPGNTMLFPLLILLLCTLFIGSIGIHFELTILSKWLTPSINFFQESSNSSINSYEFITNAIS
SVSLAIFGLFIGIYVLWICLLFFSEFDLINSFVYFFHQLKKKIYSWSYNRGYIDIFYTRTFTLG
IRGLTELTQFFDKGVIDGITNGVGLASFCIGEEIKY-VGGGRISSYLFFFLCYVSVFLFFFLMD
LPGPIHEILVLFGGFVLLLGGLGVVLLTNPTFSAFSLGLVLVCISLFYILLNSYFVAVAQLLIY
VGAINVLIIFAVMFVNGSEWSKDKNFWTIGDGFTSLVCITIPFSLMTTIPDTSWYGILWTTRS
NQIVEQGLINNVQQIGIHLATDFYLPFELISIILLVSLIGAITMARQMSLPLTRKDLMIVNMG
PQHPSMHGVLRLIVTLDGEDVIDCEPILGYLHRGMEKIAENRTIIQYLPYVTRWDYLATMF
TEAITVNAPEFLENIQIPQRASYIRVIMLELSRIASHLLWLGPFMADLGAQTPFFYIFRERELI
YDLFEAATGMRMMHNYFRIGGVAADLPYGWIDKCLDFCDYFLRGVIEYQQLITQNPIFLE

RVEGVGFISGEEAVNWGLSGPMLRASGIQWDLRKVDLYESYNQFDWKVQWQKEGDSLA
RYLVRIGEMRESIKIIQQAVEKIPGGPYENLEVRR-KKAKNSEWNDFEYRFLGKKPSPNFEL
SKQELYARVEAPKGELGIYLVGDDSLFPWRWKIRPPGFINLQILPQLVKKMKLADIMTILGS
IDIIMGEVDRMFPMVTGFM--GQQTIRAARYIGQSFIITLSHTNRLPITIHYPYEKSITSERFRG
RIHFEFDKCIACEVCVRVCPIDLPLVDWRFEKDIKRKQLLNYSIDFGVCIFCGNCVEYCPTN
CLSMTEEYELSTYDRHELNYNQIALSRLPISIMGDYTIQTIRNSKIDEEKSWNSRTITDYQQ
GWLSNWLVKHEVVHRSLGFDHRGIETLQIKAEDWDSIAVILYVYGYNYLRSQCAYDVAP
GGSLASVYHLTRIQYGIDNPEEVCIKVFAQKDNPRIPSVFWIWRSSDFQERESFDMVGISYD
NHPRLKRILMPESWIGWPLRKDYITPNFYEIQDAH--
---------MSLIEFPLLDQRSSNSVISTTLKDLSNWSRLSSLWPLLYGTSCCFIEFASLIGSRFDFD
RYGLVPRSSPRQADLILTAGTVTMKMAPSLVRLYEQMPEPKYVIAMGACTITGGMFSTDS
YSTVRGVDKLIPVDVYLPGCPPKPEAVIDALTKLRKKISREIVEDRTLSQ-KNRCFTTSHKL
YVRRSTNTGTYEQELLYQSPSTLDIST--FFKSKSPVSSYKLVNMENRNTFSWVKEQMTRSI
SVSIMIYVITRTSISNAYPIFAQQGYENPREATGRIVCANCHLANKPVDIEVPQAVLPDTVFE
AVLRIPYDMQLKQVLANGKKGGLNVGAVLILPEGFELAPPDRISPELKEKIGNLSFQSYRP
NKKNILVIGPVPGKKYSEIVFPILSPDPAMKKDVHFLKYPIYVGGNRGRGQIYPDGSKSNN
TVYNATSTGVVRKILRKEKGGYEISIVDASDGRQVIDLIPPGPELLVSEGESIKLDQPLTSNP
NVGGFGQGDAEIVLQDPLRVQGLLFFFASVILAQVFLVLKKKQFEKVQLYEMNFMSKVY
DWFEERLEIQAIADDITSKYVPPHVNIFYCLGGITLTCFLVQVATGFAMTFYYRPTVTEAFS
SVQYIMTEANFGWLIRSVHRWSASMMVLMMILHVFRVYLTGGFKKPRELTWVTGVVLA
VLTASFGVTGYSLPWDQIGYWAVKIVTGVPDAIPVIGSPLVELLRGSASVGQSTLTRFYSLH
TFVLPLLTAVFMLMHFLMIRKQGISGPLMGVTKKPDLNDPVLRAKLAKGMGHNYYGEPA
WPNDLLYIFPVVILGTIACNVGLAVLEPSMIGEPADPFATPLEILPEWYFFPVFQILRTVPNKL
LGVLLMVSVPTGLLTVPFLENVNKFQNPFRRPVATTVFLIGTAVALWLGIGATLPIEKSLTLG
LFMIEVFLFGIVLGLIPITLAGLFVTAYLQYRRGDQLDLMLTITSYFGFLLAALTLTLALFIGL
NKIRLIMDIVSLAWAALMVVFTFSLSLVVWGRSGLMMIRSPEPEVKIVVDRDPVKTSFEEW
ARPGHFSRTLAKGPDTTTWIWNLHADAHDFDSHTGDLEEISRKVFSAHFGQLSIIFLWLSG
MYFHGARFSNYEAWLSDPTHIGPSAQVVWPIVGQEILNGDVGGGFRGIQISSGFFQIWRAS
GITSELQLYCTAIGALIFASLMLFAGWFHYHKAAPKLAWSHDVESMLNHHLAGLLGLGSL
SWAGHQIHVSLPINQFLDAGVDPKEIPLPHEFILNRDLLAQLYPSFAERATPFFTLNWSKYA
EFLSFRGGLDPITGGLWLSDIAHHHLAIAILFLIAGHMYRTNWGIGHGLKDILEAHKGPFTG
QRHKGLYEILTTSWHAQLSLNLAMLGSTTIVVAHHMYSMPPYPYLATDYGTQLSLFTHH
MWIGGFLIVGAAAHAAIFMVRDYDPTTRYNDLLDRVLRHRDAIISHLNWVCIFLGFHSFG
LYIHNDTMSALGRPQDMFSDTAIQLQPIFAQWVQNLHAGAPRLTAPGATTSTSLTWGGGEL
VAVGGKVALLPIPLGTADFLVHHIHAFTIHVTVLILLKGVLFARSSRLIPDKANLGFRFPCDG
PGRGGTCQVSAWDHVFLGLFWMYNSISVVIFHFSWKMQSDVWGTISDQGVVTHITGGNF
AQSSITINGWLRDFLWAQASQVIQSYGSSLSAYGLFFLGAHFVWAFSLMFLFSGRGYWQE
LIESIVWAHNKLKVAPATQPRALSIIQGRAVGVTHYLLGGIATTWAFFLARIIAVGMELRFPR
FSQGLAQDPTTRRIWFGIATAHDFESHDDITEERLYQNIFASHFGQLAIIFLWTSGNLFHVA
WQGNFESWIQDPLHVRPIAHAIWDPHFGQPAVEAFTRGGAAGPVNIAYSGVYQWWYTIG
LRTNEDLYTGALFLLFLSTLSLIGGWLHLQPKWKPSLSWFKNAESRLNHHLSGLFGVSSLA
WTGHLVHVAIPASRGEYVRWNNFLDVLPYPQGLGPLLTGQWNLYAQNPDSSNHLFGTTQ
GAGTAILTLLGGFHPQTQSLWLTDIAHHHLAIAFIFLIAGHMYRTNFGIGHSIKDLLEAHTPP
GGRLGRGHKGLYDTINNSIHFQLGLALASLGVITSLVAQHMYSLPSYAFIAQDFTTQAALY

THHQYIAGFIMTGAFAHGAIFFIRDYNPEQNEDNVLARMLDHKEAIISHLSWASLFLGFHT
LGLYVHNDVMLAFGTPEKQILIEPIFAQWIQSAHGKTTYGFDILLSSTSGPAFNAGRTLWLP
GWLNAVNE--NSNSLFLTIGPGDFLVHHAIALGLHTTTLILVKGALDARGSKLMPDKKDFG
YSFPCDGPGRGGTCDISAWDAFYLAVFWMLNTIGWVTFYWHWKHITLWQGNVSQFNES
STYLMGWLRDYLWLNSSQLINGYNPFGMNSLSVWAWMFLFGHLVWATGFMFLISWRGY
WQELIETLAWAHERTPLANLIRWRDKPVALSIVQARLVGLAHFSVGYIFTYAAFLIASTSGK
FGMSHSVKIYDTCIGCTQCVRACPTDVLEMIPWDGCKAKQIASAPRTEDCVGCKRCESAC
PTDFLSVRVYLGPETTRSMALSYMMDFNLPSIFVPLVGLVFPAIAMASLFLYVQKNKI
V??MTAILERRESTSLWGRFCNWITSTENRLYI
GWFGVLMIPTLLTATSVFIIAFIAAPPVDIDGIREPVSGSLLYGNNIISGAIIPTSAAIGLHFYPI
WEAASVDEWLYNGGPYELIVLHFLLGVACYMGREWELSFRLGMRPWIAVAYSAPVAAAT
AVFLIYPIGQGSFSDGMPLGISGTFNFMIVFQAEHNILMHPFHMLGVAGVFGGSLFSAMHG
SLVTSSLIRETTENESANEGYRFGQEEETYNIVAAHGYFGRLIFQYASFNNSRSLHFFLAAW
PVVGIWFTALGISTMAFNLNGFNFNQSVVDSQGRVINTWADIINRANLGMEVMHERNAH
NFPLDLAALEVPSLNGMGLPWYRVHTVVLNDPGRLLSVHIMHTALVSGWAGSMALYELA
VFDPSDPVLDPMWRQGMFVIPFMTRLGITNSWGGWSIS-GGTVTNPGIWSYEGVAGAHIV
FSGLCFLAAIWHWVYWDLEIFCDERTGKPSLDLPKIFGIHLFLAGVACFGFGAFHVTGLYG
PGIWVSDPYGLTGKVQAVNPAWGAEGFDPFVPGGIASHHIAAGTLGILAGLFHLSVRPPQR
LYKGLRMGNIETVLSSSIAAVFFAAFVVAGTMWYGSATTPIELFGPTRYQWDQGYFQQEIY
RRVSDGLAENLSLSEAWSKIPEKLAFYDYIGNNPAKGGLFRAGSMDNGDGIAVGWLGHPI
FRDKEGRELFVRRMPTFFETFPVVLVDEEGIVRADVPFRRAESKYSVEQVGVTVEFYGGE
LNGVSYSDPATVKKYARRSQLGEIFELDRATLKSDGVFRSSPRGWFTFGHATFALLFFFGHI
WHGARTLFADVFAGIDPDLDAQVEFGAIQKRGDPTTGRQPVMKILYSLRRFYHVETLFNG
TFVLAGRDQETTGFAWWAGNARLINLSGKLLGAHVAHAGLIVFWAGAMNLFEVAHFVPE
KPMYEQGLILLPHLATLGWGVGPGGEVLDTFPYFVSGVLHLISSAVLGFGGIYHALLGPET
LEESFPFFGYVWKDRNKMTTILGIHLILLGIGAFLLVLKALYFGGIYDTWAPGGGDVRKIT
NLTLSPGVIFGYLLKSPFGGEGWIVSVDDLEDIIGGHVWLGFICVFGGIWHILTKPFAWARR
AFVWSGEAYLSYSLGALSVFGFIACCFVWFNNTAYPSEFYGPTGPEASQAQAFTFLVRDQ
RLGANVGSAQGPTGLGKYLMRSPTGEVIFGGETMRFWDLRAPWLEPLRGPNGLDLSRLK
KDIQPWQERRSAEYMTHAPLGSLNSVGGVATEINAVNYVSPRSWLATSHFVLGFFFFVGH
LWHAGRARAAAAGFEKGIDRDLEPVLYMTPLNMTIALGRVTKEENDLFDIMDDWLRRDR
FVFVGWSGLLLFPCAYFALGGWFTGTTFVTSWYTHGLASSYLEGCNFLTAAVSTPANSLA
HSLLLLWGPEAQGDFTRWCQLGGLWTFVALHGAFALIGFMLRQFELARSVQLRPYNAISF
SGPIAVFVSVFLIYPLGQSGWFFAPSFGVAAIFRFILFFQGFHNWTLNPFHMMGVAGVLGA
ALLCAIHGATVENTLFEDGDGANTFRAFNPTQAEETYSMVTANRFWSQIFGVAFSNKRWL
HFFMLFVPVTGLWMSAIGVVGLALNLRAYDFVSQEIRAAEDPEFETFYTKNILLNEGIRAW
MAAQDQPHENLIFPEEVLPRGNALMSGSTGERSFADIITSIRYWVIHSITIPSLFIAGWLFVS
TGLAYDVFGSPRPNEYFTESRQGIPLITDRFDSLEQLDEFSRSFMTIDRTYPIFTVRWLAVHG
LAVPTVFFLGSISAMQFIQRMATQTVEDSSRPG-PRQTRVGNLLKPLNSEYGKVAPGWGTT
PFMGVAMALFAVFLSIILEIYNSSVLLDGILMNMLTLKLFVYTVVIFFVSLFIFGFLSNDPGR
NPGRDEMADTTGRIPLWLIGTVTGIAVIGLIGVFFYGSYSGLGSSLMPNILSLTCICFNSVIYP
TSFFFAKLPEAYAIFNPIVDFMPVIPVLFFLLAFVWQAAVSFRMTQSNPNEQNVELNRTSLY
WGLLLIFVLAVLFSNYFFNMEVNILAFIATALFILVPTAFLLIIYVKTVSQNDMETATLVAISIS
GLLVSFTGYALYTAFGQPSQQLRDPFEEHGDMEALVYTFLLVSTLGIIFFAIFFREPPKVPTK

KVKMTIAFQLAVFALIVTSSVLVISVPLVFASPDGWSNNKNVVFSGTSLWIGLVFLVAILNSL
IS-------MSPQTETKASVGFKAGVKDYKLTYYTPEYETKDTDILAAFRVTPQPGVPPEEAGA
AVAAESSTGTWTTVWTDGLTSLDRYKGRCYHIEPVVGEDNQYIAYVAYPLDLFEEGSVTN
MFTSIVGNVFGFKALRALRLEDLRIPPTYSKTFQGPPHGIQVERDKLNKYGRPLLGCTIKP
KLGLSAKNYGRACYECLRGGLDFTKDDENVNSQPFMRWRDRFVFCAEAIYKSQAETGEI
KGHYLNATAGTCEEMIKRAVFARELGVPIVMHDYLTGGFTANTSLAHYCRDNGLLLHIHR
AMHAVIDRQKNHGMHFRVLAKALRMSGGDHIHAGTVVGKLEGEREMTLGFVDLLRDDF
IEKDRARGIFFTQDWVSMPGVIPVASGGIHVWHMPALTEIFGDDSVLQFGGGTLGHPWGN
APGAAANRVALEACVQARNEGRDLAREGNEIIRSACKWSPELAAACEIWKAIKFEFEPVD
KLDSTAKHLYKTPIPSTRKGTIDRQVKSNPRNNLIHGRHRCGKGRNSRGIITARHRGGGHK
RLYRKIDFRRNQKDISGRIVTIEYDPNRNAYICLIHYGDGEKGYILHPRGAIIGDTIVSGTKV
PISMGNALPLTDMPLGTAIHNIEITRGRGGQLARAAGAVAKLIAKEGKSATLRLPSGEVRLV
SQNCLATVGQVGNVGVNQKSLGRAGSKCWLGKRPVVRGVVMNPVDHPHGGGEGKAPI
GRKKPTTPWGYPALGRRTRKRKKYSDSFILRRRK-MIQPQTLLNVADNSGARKLMCIRVIG
AASNQRYARIGDVIVAVIKDAVPQMPLERSEVIRAVIVRTCKEFKCEDGIIIRYDDNAAVIID
QKGNPKGTRVFGAIAEELRELNFTKIVSLAPEVLMLSPKRTRFRKQHRGRMKGKSYRGNC
ICFGRYALQALEPTWITARQIEAGRRAMTRYARRGGKIWVRIFPDKPVTIRPTETRMGSGK
GSPEYWVAVVKPGRILYEMGGVSETVARRAISIAASKMPIRSQFLRLEMTRVPRGYIARRR
RAKMRSFASNFRGAHLRLNRMITQQVRRAFVSSHRDRVRQKRDFRRLWISRINAATRIH--
VFDNYSKLIHNLYKKELILNRKILAQVAVLNSNNLYTISNKIKIIN---------RIKRVFKAQKRIH
MSVFKVQRVLDEIRWRYYEETVMILNLMPYRASYPILKLVYSAAANATHYRDFDKANLFI
TKAEVSRSTIMNKFRPRARGRSSPIKKTMCHITIVLN-VKKS-------MDGIKYAVFTEKSLRL
LGKN-QYTFNVESGFTKTEIKHWVELFFGVKVVAVNSHRLPGKGRRMGPILGHTMHYRR
MIITLQPGYSIPLLDREKNMAVPKKRTSMSKKRIRKNLWKKKTYFSIVQSYSLAKSFSGVK
PKGFSRQQTNNMAKGKDVRIRVILQCVSCVRK--G--EESAGISRYSTQKNRHNTPGQLELR
KFCRYCRKHTIHAEIKKMKIRASVRKICTKCRLIRRRGRIRVICSNPKHKQRQGMVREEVA
GSTQTLQWKCVESRVDSKRLYYGRFILSPLRKGQADTVGIALRRALLGETEGTCITHAKFG
--SVPHEYSTIAGIEESVQEILLNLKEIVLRSNLYGVRTASICVKGPRYITAQDIILPPSVEIVDT
AQPIANLTEPTDFRIELRIKRDRGYHTEVRKNTQDGSY--PIDAVSMPVRNVNYSIFACGNG
NAKYEILFLEIWTNGSLTPKEALYEASRNLIDLFLPFLHTEEE--GTRFQENK-NRFTSPLLSF
QKRLT-NLKKNKK-RIPLNCIFIDQLELPSRTYNCLKRANIHTLLDLLSKTEEDLMRIDSFRM
QDGKQIWDTLEKH--LPMDLPKNKMLRNGN-EGMSTIPGFSQIQFEGFCRFINQGLAEELE
KFPTIKDPDHEISFQLFAKGYQLLEPSIKERDAVYESLTYSSELYVSARLIFGF--DVQKQTISI
GNIPIMNSLGTFIINGIYRIVINQILLSPGIYYRSELDHKGISIYTGTIISDWGGRSELAIDKKE
RIWARVSRKQKISILVLSSAMGSNLKEILDNVSYPEIFLSFPNAKEKKRIESKEKAILEFYQQ
FACVGGDLVFSESLCEELQKKFFQQKCELGRIGRRNMNRRLNLDIPQNSTFLLPRDVLAAT
DHLIGMKFETGILDDDDMNHLKNKRIRSVADLLQDQFGLALGRLQHAVQKTIRRVFIRQS
K--PTPQTLVTPTSTSILLITTYETFFGTYPLSQVFDQTNPLTQTVHGRKVSCLGPGGLTGRTA
SFRSRDIHPSHYGRICPIDTSEGINVGLTGSLAIHARIDHWWGSVESPFYEISEKAKKKKE--
RQVVYLSPNRDEYYMIAAGNSLSLNRGIQEEQVVPARYRQEFLTIAWEQIHVRSIFPFQYFS
IGGSLIPFIEHNDANRALMSSNMQRQAVPLSRSEKCIVGTGLERQTALDSRVSVIAEREGKII
STNSHKILLSSSGKTISIPLVTHRRSNKNTCMHQKPRVPRGKSIKKGQILAEGAATVGGELA
LGKNVLVAYMPWEGYNFEDAVLISERLVYEDIYTSFHIRKYEIQTDTTSQGSAEKITKEIPH
LEEHLLRNLDRNGVVKLGSWVETGDILVGKLTPQIASE-SSYIAEAGLLRAIFGLEVSTSKE

TSLKLPIGGRGRVIDVKWIQR-------DPIMVRVYILQKREIKVGDKVAGRHGNKGIISKILPR
QDMPYLQDGTPVDMVFNPLGVPSRMNVGQIFESSLGLAGDLLKKHYRIAPFDERYEQEA
SRKLVFSELYEASKQTKNPWVFEPEYPGKSRIFDGRTGDPFEQPVLIGKSYILKLIHQVDEK
IHGRSTGPYSLVTQQPVRGRAKQGGQRIGEMEVWALEGFGVAHILQEILTYKSDHLIARQE
ILNATIWGKRVPNHEDPPESFRVLVRELRSLALELNHFLVSQKNFQVNREEV-------MIDQY
KHQQLQIGLVSPQQIKAWANKTLPNGEVVGEVTRPSTFHYKTDKPEKDGLFCERIFGPIKS
RICACGNSRASGAENEDERFCQKCGVEFVDSRIRRYQMGYIKLACPVTHVWYLKGLPSYI
ANLLDKPLKKLEGLVYGD--FSFARP-SAKKPTFLRLRGLFEDEISSCNHSISPFFSTPGFTTF
RNREIATGAGAIREQLADLDLRIILENSSVEWKELEDE-GYSGDEWEDRKRRIRKVFLIRR
MQLAKHFIQTNVEPEWMVLCLLPVLPPELRPIVYRSGDKVVTSDINELYKRVIRRNNNLA
YLLKRSELAPADLVMCQEKLVQEAVDTLLDSGSRGQPTRDGHNKVYKSLSDVIEGKEGRF
RETLLGKRVDYSGRSVIVVGPSLSLHQCGLPLEIAIKLFQLFVIRDLITKRATSNVRIAKRKI
WEKEPIVWEILQEVMRGHPVLLNRAPTLHRLGIQAFQPTLVEGRTICLHPLVCKGFNADFD
GDQMAVHLPLSLEAQAEARLLMFSHMNLLSPAIGDPICVPTQDMLIGLYVLTIGNRRGICA
NRYNSCGN-----YPNQKVNYNN-KYTKDKESLFSSSYDALGAYRQKQICLDSPLWLRWKL
DQR-VIGL--REVPIEVQYESLGTYREIYAHYLVVGNRKKEIRSIYIRTTLGHISFYREIEEAIQ
GFSQAYSYTI------------MAERANLVFQNKEIDGTAMKRLISRLIDHFGMGYTSHILDQIKTL
GFHQATTTSISLGIEDLLTIPSKGWLVQDAEQQSFLLEKHYYYGAVHAVEKLRQSVEIWYA
TSEYLKHEMNSNFRITDPSNPVYLMSFSGARGNASQVHQLVGMRGLMADPQGQMIDLPI
QSNLREGLSLTEYIISCYGARKGVVDTAVRTADAGYLTRRLVEVVQHIIVRRRDCGTIQAIS
VSPQNGMTE--KLFVQTLIGRVLANDIYIGSRCIATRNQDIGIGLVNRFITTFRAQPFRAQPIYI
RTPFTCRSTSWICQLCYGRSSTHGDLVELGEAVGVIAGQSIGEPGTQLTLRTFHTGGVFTGG
TADLVRSPSNGKIQFNGDLVHPTRTRHGQPAFLCYIDLHITIQSQDILHSVTIPSKSLILVQND
QYVESEQVIAEIRAGTSAL--FKEKVQKHIYSESDGE-MHWSTDVYHAPEYQYGNLRRLPK
TSHLWILSVSMCR--SSIASFSLHKDQDQMNTYSFSVDGR--YIFGLS--MAD-DEVRHRLLDT
FG----DREILDYS-TPDRIMSNGHWNFVYPS-ILQNN--FDLLAKKRRNRFAIPLQYHQEQEKE
PISCFGISIEIPFM--GVLRRNTIVAYFDDPRYKKD--GIVKFRNPEEDSVLK--KGVLIEHRG-T
KEFSLKY-EVDRFFFILQELHILPRSSSLKVLDNSIIGVDTQLTKNTRSRLGGLVRVKRKKSH
TELKIFSGDIHFPEEADKILG--GSLIPLEREKK--DSKE-SK-KRENWVYVQWKKILKSKEK--
YFVLVRPAVAYEMNEGRNLATLFPQDLFEEEGNLQLRLVNFISHENSKLTQRIYHTN-QFVR
TCLVLNWEQEEKEEAR-------ASLVEIRANGLIRDFLRIGLIKSTISYTRKRYDSRSAGLILH---
LDRTNTNSFYSKAKIQS-----LSQHQEAIG----TLLNRNK---EYQSLMVLSASNCSRIG--FFKN
SKNP---NGVKESNPRIPIPNFSGLLGTIAP-SISNFSSS-YYLLT---YNQILLK-KHLLLD---NLK
QNFKVLQGLKHSLINENQR--TSNFD--SNIMLDPFQLNWHFLPHDSWEETSAKIH-LGQFIC
ENVCLFKSHI----KKSGQIFIVN-IDSFVIRAAKPYLATTGATVHGHYGEILYKGDRLVTFIYE
KARSSDITQGLPKVEQIFEARSIDSLSPNLERRIEDWNERIPRILGGPWGFLIGAELTIAQSRI
SLVNKIQKVYRSQGVQIHNRHIEIIIRQVTSKVRVSE---DGMSNVFSPGELIGLLRAERAGR
ALDESIYYRAILLGITRVSLNTQSFISEASFQETARVLAKAALRGRIDWLKGLKENVVLGGII
PVGTGFQKFVHRYP--QNKNLY--FEIQKKKLFASEM--RDILFLHTELVSSDSDVTN---SESPF
TPFI--------MTRRYWNINLKEMIEAGVHFGHGIKKWNPKMAPYISAKRKGTHITNLARTTR
FLSEACDLVFDAASQGKSFLIVGTKKRAADLVASAAIRARCHYVNKKWFSGMLTNWSITK
TRLSQFRDLRAEEKMEKFHHLPKRDVAILKRKLSTLQRYLGGIKYMTRLPDIVIVLDQQKE
YIALRECAILGIPTISLADTNCDPDLANISIPANDDTMTSIRLILNKLVFAICEGRSL-YIRNHM
GQKINPLGFRLGTTQNHHSFWFAQPKNYSEGLQEDKKIRNCIKNYIQKNRKKGSNKIEAIT

HIEIQKEIDTIHVIIHIGFPN-LLKKKG-----AIEELEKDLQKEVNSVNQRLNIGIEKVKEPYRQ
PNILAEYIAFQLKNRVSFRKAMKKAIELTKKTDIKGVKVKIAGRLAGKEIARAECIKKGRL
PLQTIRAKIDYCCYPIRTIYGVLGVKIWIFVDEEMSRYRGPRFKKIRRLGALPGLTRKTPKS
GSNLKKKFHSGK-KEQYRIRLQEKQKLRFHYGLTERQLLRYVHIAGKAKSSTGQVLLQLL
EMRLDNILFRLGMASTIPEARQLVNHRHILVNGRIVDIPSFRCKPRDIITTKDNQRSKRLVQ
NSIASSDPK--LPKHLTIDT--LQYKGLVKKILDRKWVGLKINELLVVEYYSRQTMSRRGTAE
KRTAKSDPIFRNRLVNMVVNRIMKDGKKSLAYQILYRAVKKIQQKTETNPLLVLRQAIRRV
TPNIGVKTRRKKGSTRKVPIEIGSKQGRALAIRWLLEASQKRPGRNMAFKLSSELVDAAK
GGGGAIRKKEATHRMAEANRALAHFRMGKDTIADLLTSIRNADMNKKGTVRVVSTNITE
NIVKILLREGFIESVRKHQESNRYFLVSTLRHQKRKTRKGIYRT---RTFLKRISRPGLRIYAN
YQGIPKVLGGMGIAILSTSRGIMTDREARLNRIGGEVLCYIWMTKAIPKIGSRRKVRIGLR
KSARRITKGVIHVQASFNNTIITVTDPQGRVVFWSSAGTCGFKSSRKASPYAGQRTAVDAIR
TVG---LQRAEVMVKGAGSGRDAALRAIAKSGVRLSCIRDVTPMPHNGCRPPKKRRLMPT
VKQLIRNARQPIRNARKSAALKGCPQRRGTCARVYTINPKKPNSALRKVARVRLTSGFEIT
AYIPGIGHNLQEHSVVLVRGGRVKDLPGVRYRIIRGALDAVAVKNRQQGRSKYGVKKPKM
AKKSLIQRERKRQKLEQKYHLIRRSSKKKIRSKVYPLSLSEKTKMREKLQSLPRNSAPTRL
HRRCFLTGRPRANYRDFGLSGHILREMVYACLLPGATRSSWMKKRKIFGFMVKEEKEEN--
WGSVEFQVFSFTNKIRRLASHLELHKKDFSSERGLRRLLGKRQRLLAYLAKKNRVRYKKL
ISQLDIRER-----MLKLRLKRCGRKQ-----------R------------FYDPIKNQTCLNVPAILYFLEKGA
QPTRTVSDILRKAEFFKEKERTLS----FRKSKQTFRKFKQPFRKRRPRIGPGDRIDYRNMSLI
NRFISEQGKILSRRINRLTLKQQRLITLAIKQARILSFLPFRNYENEKQFQ-AQSISIITGPRPR
KNVTRK-KTNPFVAHHLLAKIEKVNMKEEKETIVTWSRASSILPAMVGHTIAIHNGKEHIPI
Y I T N P M V G R K L G E F V P T R H F T S Y A R K D T K S R
R???
???
???
???
???
???
???
???
???
???
???
???
???
???
???
???
???
???
???
???
???

???
???
???
???
???
???
???
???
???
???
???
???
???
???
???
???
???
???
???
???
???
???
???MPRSR-INGNFIDKTFSIVANILLRIIPTTSGEK
RAFTYYRDG--MLAQSEGNYAEALQNYYEATRLESDPYDRSYILYNIGLIHTSNGEHTKAL
EYYFRALERNPFLPQAFNNMAVICHY--GEQAILQGDSEIAEAWFDQAAEYWKQAIGLTPG
NYIEAQNWLKITKRFEFEMNWRSEHIWIELLKGSRKRGNFFWACILFLGSLGFLAVGASSY
LGKNIISVLPSQQILFFPQGVVMSFYGIAGLFISAYLWCTILWNVGSGYDRFDRKEGVVCIF
RWGFPGIKRRVFLRFLMRDIQSIRIQVKEGLFPRRILYMEIRGQGAIPLTRTDEKFFTPREIEQ
KAAELAYFLRIPMEVF
>Epifagus
MERWRFSLM--IKKSLDSVSSIENTIENKDSNIKGRAQNIRSYNNIIYLFGVEYNRNLIPNDT
FLFRDNKGDSFY-SFNIENHIFEIDIN--FSCLSELEIEFYSYHGFDYFNNGS-IEDYLYN--MYD
TPS--NNHITSCIESYLQ--SQICVNSIISDSS-SY----IFGCF-DSRRSGIRNRAQSSYLTIRESFNN
LDSTKKYKHLWVQCENCYGLNYKKFLKSKINLCEQCGYHFKMSSSERIEVLVDPDTWYP
MDEDMSSLDPIEFHSEEE-YKDRIYSYQKRTGLTEAVQTGLGQLNGIPIAIGVMDFQFMGG
SMGSVVGEKITRLIEYATNKILPLIIVCASGGARMQEGSLSLMQMAKISSALFDYQSNKKLL
YVSILTSPTTGGVTASFGMLGDIIIAEPNSYIAFAGKRVIEQTLHKIVPEGSQAAEYLFQKGL
F D L I I P R N L L K S V L G E L F K L H A F F P L N - - - -
-??
???
???
???
???
???

??
??
??
??
??
??
??
??
??
??
??
??
??
??
??
??
??
??
???MPIGVPKVPFQIPGEEDASWVDVY
NRLYRERLLFLGQEVDSDISNQLIGLMVYLSIEDDTKEIYLFINSPGGWVIPGVAIYDTMQF
VRPEVHTICMGLAASMGSFLLVGGEITKRLAFPHARVMIHQPASYFFGAQTGEFILEAEEL
LKLRETLTRVYVQRTGKPLWVISEDMERDVFMSAKDAQAYGIVDLVAVEMKEQKWIHEG
LITESLTNGMFWVRLDNKDLIIGYVSGNIRHSFIRILPGDKVKIEVSRYNSTRGRIIYRLRNK
YYKD---MYKQNLFIISSNDSKYNFL-LKKLYYEIIL
EGLTFILEFFFFIRLISYLIEKLNNLRSILSIFPFLEDNFSNLKFILDILIPRKVHAEILVQTLRYW
IKDATYLHLLRFWLNN--NWNSIINDG--YFYPNKILLLLYNSYVCKYESIFVFLRNQSYHFR
STPFIMLLERIYFYFKIERLVNPFLKDFNLWLIKEPCLHYFRYRRQYILASKGTSLFLNKWK
CYVITFWQWYFSLWFFSRSIYIKKLLNNSFEILTYHSSLNINPSFVLSQILETPFISNNTI--KK
VNILVPIILSIS--DKEKFFNVLGHPTSNLFRVDLSDYNIIGRLECIYRNLYHYLSGSSKKNSLY
LIKYILLLSCVRTLARKHKSTVRTFAKRLCLELLEGFFISEEDILFLKFQKQFWYLNIYSI--N
Y L A
-???
??
??
??
??
??
??
??
??
??
??
??
??

??
??
??
??
??
??
??
??
??
??
??
??
??
??
??
??
??
??
??
??
??
??
??
??
??
??
??
??
??
??
??
??
??
??
??
??
??
??
??
??
??
??
??
??

???
???
???
???
???
???
???
???
???
???
???
???
???
???
???
???
???
???
???
???
???
???
???
???
???
???
???
???
???
???
???
???
???
???
???
???
???
???
???
???
??????MAIHLYKTSTPSTRNGTVYSQVKSNPRKNLIYGQHHCGKGRNVRGIITTRHRGGGH
KRLYRKISFIRNEKYIYGRIITIEYDPNRNAYICLIHYGDGDKRYILHPRGAIIGDTLVSGTEV
PIIIGNALPLTDMPLGTAIHNIEITLGKGGQLVRAAGAVAKLIAKEGKLATLKLPSGEVRLIS
KNCSATVGQVGNVGVNKKSLGRAGSKRWLGKRPVVRGVVMNPIDHPHGGGEGRAPIGR

K K P T T P W G Y P A L G R R S R K I N K Y S D N F I V R R R S
K???
??MLSPQKTRFRKQHRGRMKGISYRGNNICFGKYGLK
ALEPAWITPRQIEAGRRAITRKFRRGGKIWVRVFPDKPVTVRSSETRMGSGKGSHKYFVAV
VKPGLILYEIGGVTENIAKRAILIAASKMPMQTQFIISGMTRIKRGYIARRRIKKFRLFASSFL
NAHSRLTRTITQQKIRALVSSDRDRNNKKRKFRSLWITRINAVIREE-GVSYSYKNFIYAQY
K I Q L L I N R K I L A Q I A I L N R N F F Y M I F N E I R K E A D L K E Y I R I
N???
??
???-
-????MAKSKDARVAVILECTSCIRNS--VNKVLTGISRYITQKNRRNTPNRLELRKFCPYCYK
H M I H G E I K K M K I R A S I R K I C E K C R L I C R R R R I I V I C S N P R H K Q R Q
G???
??
??
??
??
??
??
??
??
??
??
??
??
??
??
??
??
??MLSPQKTRFRKQHRGRMKGISYRGNNICFGKYGLK??????????????
??
??????????????????????????????GGVTENIAKRAILIAASKMPMQTQFIISGMTRIKRGYIARRRIKKF??????????????
??
???????????????????????????????????ILAQIAILNRNFFYMIFNEIRKEADLKEYIRI?????????????????????????
??
??
??
??
??
??
??
??
??
??

??
??
??
??
??
??
??
??
???MTRRYWNIDLEEMIGAGVSFGHG
TRKWNPKMAPYISVKHKGIHFTNLTKTARFLSEACDLVFYAASRGKQFLIVDTKNKAADS
AAWAAIKARCHCVNKKWPGGMLTNWSTTETRLHKLRDLIMEQKAGRLNRLKKKDEAAV
KRQLARLQTYLGGIKYMTRLPDIVIIVDQHEEYKALHECITIGIPTIGLIDTNCDPDLADISIP
ANDDAISSIRLILNKLVFAICEGRS-GYIIKNMGQKINPLGFRLGTTQSHHSFWFAQPKNYY
KGIQEDQKIRDFIKNYVKNNIIISPD-TEGIAYIEIQKRIDFLKIMIFIGFK-FLIENRQLGI--IKE
ALHIDLKKNFHYVNRKLIIDIIRITKPYRNPNILAEFIADQLKNRVSFRKTMKKAIELTESED
TKGIQVQISGRIDGKEIARVEWIREGRVPLQTIQAKINYCSYMVRTTHGVLGIKIWIFIEKE
MSRYRGPSLKKIRRLGALPGLTNKRSKAENDFIKKLRSDK-KSQYRIRLEEKQKLRFNYGL
RERQLRKYFSIAIKTRGSTGKVLMQLLEMRLDNIIFRLGMASTIPAARQLVNHRHVLVNGR
IVDIPSYRCKSRDIIMARDEQQSNTFINNCINYSTHRMEAPNHLTLLHP---FKGLVNQIIDSK
WVGFKINELLVVEYYFRKTMSRRGTAEEKTAKPDPIYWNRLVNMLVNRILKHGKKSLAY
QIIYRALKKIQQKTEKNPLYVLRQAIRGVTPDIAVKARRVGGSTHQVPIEIGSTQGKALAVR
WLLVASKKRPGQNMAFKLSSELVDAAKGSGDAIRKKEETHKMAEASRAFAHLRMGRDTI
LEIINSIRNADRGRKRVVRITSTNITENFVKILFIEGFIENARKHREKNKYYFTLTLRHRR--N
SKRPY---INILNLKRISRPGLRIYSNSQQIPLILGGIGIVILYTSRGIMTDREARLKGIGGELLC
YIWMAKAIPITGSRRNVHVGSRKSSFRIQKGVIHVQTSFNNTIVAVTDIKGRVVSWSSAGT
CGFKGTRRGTSFAAQIAATNAIR--IVQGMQRAEVMIKGPGIGRDAVLRAIRGSGVLLTFVR
DVTPMPHNGCRPPKKRRVMPTIKQLIRKKRQPNLNVTKSPALRGCPQRRGTCTRVYTITPK
KPNSALRKVARVRLTSGIEITAYIPGIGHNSQEHSSVLVRGGRVKDLPGVRYHIIRGTLDAVG
VKDRQQGRSKYGVKIKNMARKSLIQREKGRLKLENKYHFIRRSSKNEIS---KVPSLSDKW
EIYGKLESPPRNSAPTRLRRRCFYTGRPRANYRDFGLCGHILREMVNACLLPGATRSS
W??
??
????????MYKFK-------RSFRRRLSPIGSGNLIYYRNMSLISRFISEQGKILSRRVNRLTLKQQR
LITIAIKQARILSLLPFINNEK--QFERIESITRVKG-FIKK-VIHSLKKNLFVANHLRAKINKLN
NKKKKEIIVTWSRASTIIPIMIGHMISIHNGKEHLPIYITDHMVGHKLGEFVPTLNFRGHAK
S D N R S R
R??
??
??
??
??
??
??
??

???
???
???
???
???
???
?????????MKEHPYKSWILEL----REIKNSHYFLDSWTKFNSVGSYINIFSHQERFIKLFDPRIL
SILLS-RNS--QGSTSNRYFT---IKGVILFVVA-VLIYR----INNRNMVEIKNIYWRGLLP-----IPM
NSIGPRNDTLEELV-GSYNINRFIVS-LLYLTKGKNISESFFLNLKESTLVLPIT----KKCSMPES
NWGSRWWRNWTGKNR--DYSCKISN---ETVAGIEILFKEKDKK-YLEFIFFYYMD--DPIRKD
RDWELFDRLSPSKRLNKINF--YSGPLFEILVKRRIYYLMSAFREKIPIEVVKGFFKQQK-VG
STIQSNDIEHVSH--FFSRNKRAISLKNSAQFNMWQFRQDLLV---SWGE-NPHESDFLRN-VS
RANWIWLNNVWLVNKYR---FCR--KVRNVSSNIKYKY-DSTRSRSSFVQVTDSSQLKGSY-
YKSSGHFYSVIS---NEDSE-------------YHTLINQREIKPLK---SIFFD--PSFLQTEATEIESD--QL
QK--RPSGYS----STLFT-EHEKQMINHMLPEEIEEFIG--NPTRLVHSFLSDRW--SELH-LGSNP
TERSTRDH----KLLKKQQDLS-FVPSRRSE----NKELVNILKIITYLKN--TVSIHPISSDPGCDG
VL-KDEPD-MDSSNKISVFNKNTFIYL--FHLFHDWNRVGYTLNLHHDFELE--ERFQEKADL
FTLSITEP-DLVYHKGFSFSIY---MDQKQ---KM
VVFASNNIMEAVNQSRFIRNMIKIQYSTYG--YIRNVLHRFFLMN---RSDHNLEYEIK---RDQ
IGKDTLNHRTIIKYMINQHLSNFKK-SQNKWFNP--ILFFSRTERSVNRNPDAYRYKRSNGSN
NFLEHLEHFVSEQKSHF-KFKIVFDLIRFNQYSIDWSAFIDTKDLSKPLRFFLSK----------------
-------LLFFLSN-SLPFFCVSFGNIPIHRSE-IYIY-ELKDPNDQLCNQFLEPIDLKIVHLKKRKPF
--LLG---YHGTS-RKLKLLITGGR--PFLFNKIP-RC-MIDSFHTI--NNRSKSFDNTDSYLSMIFH
NKDNWLNLVKPFHRSSLISYFYKANRLQFLNNPHNFCFY--CNTRLPFYV----EKAHIHNYY-
-FTYG-QFLNILFIRNK-IFSLCVDK--KKHAFWGGRDTISPIESQVSKIFIPKNFPQSGDET-YN
LSQP---FHFPSRYDPFVR------LIANIYGTPLTEGQIVNLGRTYCQPLSDMNLSDSEGKNFHQ
YLNF--NSNMGLIHTPCSDKYLPSEK--RKK--------RSLCINKYKCVEKGQMYRTFQRKVAFS
TLSKWNLFQTYMPWFLTSAGYKYINLIFLDTFSELLSILSSS---KKFVSIFNNIMHGSGISWR
IINKKR------CLP----LISEISSKCLHN--LLLSEETIRQNNES--PLISTHLRSPNVREF-LYSILFLL
LVVGY-LVRTHLLFVSR--ASSELQTEFKRVKSLMIPSSMIELRKLLNRYPT-ASNSFWLKNLF
IVAMEQLVYSLEEIRASGGN-----LLGPA-----YGVKSICS-KNKYFNIN------LI-DLIPNPINRIIF
SRNMRHLSHTSKEIYSLIRKRKNVNGDWIDDIIESWVANSDSIDDEEREFLVQFS--ALTT--E
KRIYQILLSLT-HSDHLS-----KNDSGYKMIEQPGAIYLRYLVDIH--KKYLLNYECNTSCLVER
RVFLAHSQTITY--SQTSRGTNTLHFPSQG---KPFSISLALSPS-KGILVIGSIGTGRSFLVKYLA
TNSYVPFITVFLNKFLDNKPKGFLVD-----DND-NDSSDDIYASDDINS---------------------DLD
TELELITMMNAL---TMDMML---ELDRFFTTLQLELAKAMSPWIIWIPNIHDLDVNESNYLS
FGLLVNHLS------ERCS-TNNIIVIASTHIPKKVDPALLAPNKLNTCIKIRRLLIPQQRKHFCTL
SYTRGFHLENKIFHT--NGFGSITMGSSARDLVALTNEALSISITQNKSILDTNTIRSALHRQT
WDLR---SGVRSFQDNGILSYQIGRAITQNVLLSNCPIDPISIYMKKKSCTCNGGDYYFYKW
YFGLGTSMKKLTILLYLLSCSAGSVAQDLWSLP--GPAEK-NGITSYGLVENDS-DLVRGLLE
VEGALVG--SSRTEKDCSPFDNDRVIFTLILRPEPGN-PLDIIKKGSCSIFDHRFIYEK--YESEF
EE-GYGEGALDPQQ----IEED----LFNH--IVWAP--RIWRP---WGFIFYCIERPNELGFP-YWSRS
FR---GKRIVYDKDGELQENDSELLKSGTVQYQTRDRSSKEQGLLKINQFIWDPADPLFFLL
K-DQPPGSVFSHRRFFADEEMSKGLLTSQKD-PTSIYKRWFI--KNTQEQHFELLINRQRWLR

T---KSSLSKSNGSFRSN--TLFESYQYLSTLFLSN-GTLFDKMTKTLLIKRWLFPDEMQM------
- - - - - - -
-??
???
???
???
????
>Zea
???
???
???
???
???
??????????????????????????????????MATLRVDEINKILRERIEQYNRKVGIENIGRVVQVGDGIA
RIIGLGEIMSGELVEFAEGTRGIALNLESKNVGIVLMGDGLMIQEGSFVKATGRIAQIPVSEA
YLGRVINALAKPIDGRGEIVASESRLIESPAPGIISRRSVYEPLQTGLIAIDSMIPIGRGQRELII
GDRQTGKTAVATDTILNQKGQDVICVYVAIGQRASSVAQVVTTFHEEGAMEYTIVVAEMA
DSPATLQYLAPYTGAALAEYFMYRERHTLIIYDDLSKQAQAYRQMSLLLRRPPGREAYLG
DVFYLHSRLLERAAKLNSLLGEGSMTALPIVETQSGDVSAYIPTNVISITDGQIFLSADLFN
AGIRPAINVGISVSRVGSAAQIKAMKQVAGKSKLELAQFAELQAFAQFASALDKTSQNQLA
RGRRLRELLKQSQSNPLPVEEQVATIYTGTRGYLDSLEIEQVKKFLDELRKHLKDTKPQFQ
EIISSSKTFTEQAETLLKEAIQEQLERFSLQEQTMRTNPTTSRPGISTIEEKSVGRIDQIIGPVL
DITFPPGKLPYIYNALIVKSRDTADKQINVTCEVQQLLGNNRVRAVAMSATEGLMRGMEVI
DTGTPLSVPVGGATLGRIFNVLGEPIDNLGPVDTSATFPIHRSAPAFIELDTKLSIFETGIKVV
DLLAPYRRGGKIGLFGGAGVGKTVLIMELINNIAKAHGGVSVFGGVGERTREGNDLYME
MKESGVINEKNIEESKVALVYGQMNEPPGARMRVGLTALTMAEYFRDVNKQDVLLFIDNI
FRFVQAGSEVSALLGRMPSAVGYQPTLSTEMGSLQERITSTKKGSITSIQAVYVPADDLTDP
APATTFAHLDATTVLSRGLASKGIYPAVDPLDSTSTMLQPRIVGNEHYETAQRVKETLQRY
KELQDIIAILGLDELSEEDRLTVARARKIERFLSQPFFVAEVFTGSPGKYVGLAETIRGFQLIL
SGELDGLPEQAFYLVGNIDEASTKAINLEEESKLKKMKLNLYVLTPKRIIWDCEVKEIILST
NSGQIGVLPNHAPINTAVDMGPLRIRLNDQWLTAVLWSGFARIVNNEIIILGNDAELGSDID
PEEAQQALEIAEANLSKAEGTKELVEAKLALRRARIRVEAVNWIPMKNVTHSFVFLAHWP
FAGSFGLNTDILATNLINLTVVVGVLIFFGKGVLKDLLDNRKQRILSTIRNSEELRKGTLEQ
LEKARIRLQKVELEADEYRMNGYSEIEREKENLINATSISLEQLEKSKNETLYFEKQRAMN
QVRQQGFQQAVQGALGTLNSCLNTELHFRTIRANIGILGAIEWKR-MNPLIAAASVIAAGL
AVGLASIGPGVGQGTAAGQAVEGIARQPEAEGKIRGTLLLSLAFMEALTIYGLVVALALLF
ANPFVMNITPCSIKTLKGLYDISGVEVGQHFYWQIGGFQIHAQVLITSWVVITILLGSVIIAV
RNPQTIPTDGQNFFEYVLEFIRDLSKTQIGEEYGPWVPFIGTMFLFIFVSNWSGALLPWKIIE
LPHGELAAPTNDINTTVALALLTSAAYFYAGLSKKGLSYFEKYIKPTPILLPINILEDFTKPLS
LSFRLFGNILADELVVVVLVSLVPLVVPIPVMFLGLFTSGIQALIFATLAAAYIGESMEGHH
MLFATLEHILTHISFSTISIVITIHLITLLVRELRGLRDSSEKGMIATFFSITGFLVSRWVSSGHF
PLSNLYESLIFLSWTLYILHTIPKIQNSKNDLSTITTPSTILTQGFATSGLLTEMHQSTILVPAL
QSQWLMMHVSMMLLSYATLLCGSLLSAALLIIRFRKNFDFFS-LKEIEYLYAKRSALKNTSF
PVFPNYYKYQLTERLDSWSYRVISLGFTLLTVGILCGAVWANEAWGSYWNWDPKETWAFI

TWTIFAIYLHSRKNPNWKGTNSALVASIGFLIIWICYFGINLLGIGLHSYGSFTLPMKKKKA
LPSFLYLVFIVLLPWGVSFSFNKCLELWIKNWWNTRQSETFLTDIQEKRILEGFIELEELFLL
DEMIKEKPKTHVQKLPIGIHKEIIQLAKIDNEDHLHIILHFSTNIICLAILSGSFFLGKEELVIL
NSWVQEFFYNLNDSIKAFFILLVTDFFVGFHSTRGWELLIRWVYNNLGWAPNELIFTIFVCS
FPVILDTCLKFWVFFCLNRLSPSLVVIYHSISEMPIGVPKVPYRIPGDEEATWVDLYNVMYR
ERTLFLGQEIRCEITNHITGLMVYLSIEDGNSDIFLFINSLGGWLISGMAIFDTMQTVTPDIY
TICLGIAASMASFILLGGEPTKRIAFPHARIMLHQPASAYYRARTPEFLLEVEELHKVREMIT
RVYALRTGKPFWVVSEDMERDVFMSADEAKAYGLVDIVGDEPREAKVTFEGLVTEALPN
GMFRVRLENDTIILGYISGKIRSSSIRILMGDRVKIEISRYDSSKGRIIYRLPHKDSKDMEKFE
GYSEKQKSRQHYFVYPLLFQEYIYAFAHDYGLFGCNNKFSSLLVNRLIIRMYQQNFLINSV
NYPNQDRLLDSEFYSQILSEGFAIVVEIPLSLGQLSCPIPNFQNLQSIHSIFPFLEDKFLHLHY
LSHIKIPYPIHLEILVQLLEYRSQDVPSLHLLRFFLYYYSNWNSFITKSIFLLKKKRLFRFLYN
SYVSEYEFFLLFLHKQSSCLRLTSSGTFLERIIFSGKMEH--VMYPGRKTIWFFMDPLMHYV
RYQGKAILASKGTLLLKKKWKSYLVNFSQYFFSFWTQPQRIRLNQLTNSCFDFLGYLSSVP
INTLLVRNQMLENSFLIDTRM--KKFDTTVLATPLVGSLSKAQFCTGSGHPISKPVWTDLSD
WDILDRFGRICRNIFHYHSGSSKKQTLYRLKYILRLSCARTLARKHKSTVRTFMQRLGSVF
LEEFFTEEEQVFSLMFTKCIWYLDIIRINDLVNPLMIIDRV-EVE-TINSFSKSE-LFKEIYGLIW
IL-PIFALLLGITIEVLVIVWLEREISASIQQRIGPEYAGPLGLLQAIADGTKLLLKEDILPSRG
DIPLFSIGPSIAVISILLSFLVIPLGYRFVLADLSIGVFLWIAISSIAPIGLLMAGYSSNNKYSFS
GGLRAAAQSISYEIPLTFCVLAISLLSNSSSTVDIVEAQSKYGFFGWNLWRQPIGFLVFLISS
LAECERLPFDLPEAEEELVAGYQTEYSGIKYGLFYLVSYLNLLVSSLFVTVLYLGGWNFSIP
YISFFGFFQMNKIIGILEMVIGIFITLTKAYLFLFISITIRWTLPRMRMDQLLNLGWKFLLPISL
GNLLLTTSSQLVSLMIWHVQNENFILDSTRIFMKAFHLLLFHGSFIFPECILIFGLILLLMIDL
TSDQKDRPWFYFISSTSLVISITALLFRWREEPIISFSGNFQTNNFNEIFQFLILLCSTLCIPLSV
EYIECTEMAITEFLLFVLTATLGGMFLCGANDLITIFVAPECFSLCSYLLSGYTKRDLRSNEA
TMKYLLMGGASSSILVHGFSWLYGSSGGEIELQEIVNGLINTQMYNSPGISIALIFITVGLGF
KLSPAPFHQWTPDVYEGSPTPVVAFLSVTSKVAASALATRILDIPFYFSSNEWHLLLEILAIL
SMILGNLLAITQTSMKRMLAYSSIGQIGYVIIGIIVGDSNDGYASMITYMLFYISMNLGTFA
CIVLFGLRTGTDNIRDYAGLYTKDPFLALSLALCLLSLGGLPPLAGFFGKLYLFWCGWQAG
LYFLVSIGLLTSVLSIYYYLKIIKLLMTGRNQEITPYVRNYRRSPLRSNNSIELSMTVCVIAST
IPGISMNPILAIAQDTLFMFLLHEYDIFWTFLIIASLIPILVFWISGLLAPVSEGPEKLSSYESGI
EPMGGAWLQFRIRYYMFALVFVVFDVETVFLYPWAMSFDVLGVSVFIEAFIFVLILVVGLV
YAWRKGALEWSMSYFPWLTILVVLPIFAGSLIFFLPHKGNKIVRWYTIAICLLEFLLMTYAF
CYHFQLEDPLIQLKEDSKWIDVFDFHWRLGIDPLSLGSILLTGFITTLATLAAWPVTRNSQL
FYFLMLAMYSGQIGLFSSRDLLLFFIMWELELIPVYLLLSMWGGKRRLYSATKFILYTAGG
SIFFLIGVLGMGLYGSNEPGLDLERLINQSYPTTWEILLYFGFLIAYAVKLPIIPLHTWLPYTH
GEAHYSTCMLLAGILLKMGAYGLIRVNMELLPHAHYLFSPWLVIIGAVQIIYAASTSLGQR
NFKKRIAYSSVSHMGFIIIGIGSITNIGLNGAILQILSHGFIGATLFFLAGTACDRMRLVYLEE
LGGISIPMPKIFTMFSSFSMASLALPGMSGFVAELVVFFGLITSPKFMLMPKMLITFVMAIG
MILTPIYLLSMLRQMFYGYKLFHVPNKNFVDSGPRELFLLICIFLPVIGIGIYPDLVLSLSVD
RVEVLLSNYYTKMMFERVLFLSVYLFSIGIYGLITSRNMVRALICLELILNSINLNLVTFSDL
FDSRQLKGDIFAIFVIALAAAEAAIGLSILSSIHRNRKSTRINQSNFLNNMEHTYQYAWVIPL
LPLPVIMSMGFGLFLIPTATKNLRRIWAFPSILLLSIAMVFSLHLSIQQINGSSIYQYLWSWTI
NNDFSLEFGYLVDPLTSIMLILITTVGILVLIYSDDYMSHDEGYLRFFVYISFFNTSMLGLVT

SSNLIQIYFFWELVGMCSYLLIGFWFTRPIAASACQKAFVTNRVGDFGLLLGILGFFWITGS
LEFRDLFKIANNNGINSLLTTLCAFLLFLGAVAKSAQFPLHVWLPDAMEGPTPISALIHAAT
MVAAGIFLLARLLPLFISLPWIMSFISLIGTITLFLGATLALAQRDIKRSLAYSTMSQLGYMM
LALGIGSYQAALFHLITHAYSKALLFLGSGSVIHSMEPLVGYSPDKSQNMVLMGGLRKYV
PITRTTFLCGTLSLCGIPPLACFWSKDEILSNSWLYSPFFGIIASFTAGLTAFYMFRIYLLTFD
GYLRVHFQNYSSTKEGSLYSISLWGKSISKGVNR-DFVLSTMKFFSAKN--TFVYPHETGNT
MLFPLLILLLFTLFIGSIGIHFELTILSKWLTPSINLFQENSNSSINSYEFLTNAISSVSLAIFGLF
IAYIFYGSAYSFFQNLNFQNSLVSFLDEVKKKIYSWSYNRGYIDFFYTRVFILGIRKLAELTH
FFDKGVIDGITNGVGLAGFCIGEEIKY-VGGGRISSYLFFFLCYVSLFLFFIPMDLPGPIHEILV
LFGGFGLLLGGLGVVLLTNPIYSAFSLGLVLVCISLFYFLLNSYFVAVAQLLIYVGAINVLIIF
AVMFVNGSEWSKDKNYWTIGDGFTLLLCITIPFSLMTTIPDTSWYGILWTTRSNQIVEQGLI
NNVQQIGIHLATDFYLPFELISLILLVSLIGAITMARQMSLSLKRKDLMIVNMGPQHPSMHG
VLRLIVTLDGEDVIDCEPILGYLHRGMEKIAENRSIIQYLPYVTRWDYLATMFTEAITVNAP
EFLENIQIPKRASYIRVIMLELSRIASHLLWLGPFMADLGAQTPFFYIFRERELIYDLFEAVT
GMRMMHNYFRIGGVAADLPYGWMDKCLDFCDYFLQGVVEYQELITQNPIFLERVEGVG
FISGEEAVNWGLSGPMLRASGIQWDLRKIDPYESYNQFDWKVQWQKEGDSLARYLVRVG
EMRESIKIIQQAVEKIPGGPYENLEARR-KKAKNPEWNDFEYRFLGKKPSPNFELSKQELYV
RVEAPKGELGIYLVGDDSLFPWRWKIRPPGFINLQILPQLVKKMKLADIMTILGSIDIIMGE
VDRMFPMLTGFISYGQQTIRAARYIGQSFIITLSHTNRLPITIHYPYEKSITSERFRGRIHFEF
DKCIACEVCVRVCPIDLPLVDWRFEKDIKRKQLLNYSIDFGVCIFCGNCVEYCPTNCLSMT
EEYELSTYDRHELNYNQIALSRLPISIMGDYTIQTIRNSKIDEEKSWNSRTITDYQQGWLSN
WLVKHDVVHRSLGFDHRGVETLQIKAGDWDSIAVILYVVYGYNYLRSQCAYDVAPGGSLA
SVYHLTRIQYGIDNPEEVCIKVFAQKDNPRIPSVFWVWRSADFQERESYDMVGISYDNHP
RLKRILMPESWIGWPLRKDYITPNFYEIQDAH---
---MSLIEFPLLDQTSSNSVISTTPNDLSNWSRLSSLWPLLYGTSCCFIEFASLIGSRFDFDRYG
LVPRSSPRQADLILTAGTVTMKMAPSLVRLYEQMPEPKYVIAMGACTITGGMFSTDSYSTV
RGVDKLIPVDVYLPGCPPKPEAVIDALTKLRKKIAREIIEDRTLCQSKNRSFTTRHKLYVRR
STHTGTYEQELLYQSPSTLDIST--FFKSKSSVSSYKLVNMENRKTFSWLKEQMIRSISVSIMI
YVITRTSISNAYPIFAQQGYENPREATGRIVCANCHLANKPVDIEVPQAVLPDTVFEAVLRIP
YDMQLKQVLANGKKGGLNVGAVLILPEGFELAPPDRISPELKEKIGNLSFQSYRPNKKNIL
VIGPVPGKKYSEIVFPILSPDPATKKDVHFLKYPIYVGGNRGRGQIYPDGSKSNNTVYNATS
TGIVKKILRKEKGGYEISIVDASDGRQVIDIIPPGPELLFSEGESIKLDQPLTSNPNVGGFGQ
GDAEIVLQDPLRVQGLLFFFASVILAQVFLVLKKKQFEKVQLYEMNFLNKVYDWFEERLEI
QAIADDITSKYVPPHVNIFYCLGGITLTCFLVQVATGFAMTFYYRPTVTEAFSSVQYIMTEA
NFGWLIRSVHRWSASMMVLMMILHVFRVYLTGGFKKPRELTWVTGVVLAVLTASFGVTG
YSLPWDQIGYWAVKIVTGVPEAIPVIGSPLVELLRGSASVGQSTLTRFYSLHTFVLPLLTAVF
MLMHFPMIRKQGISGPLMGVTKKPDLNDPVLRAKLAKGMGHNYYGEPAWPNDLLYIFPV
VILGTIACNVGLAVLEPSMIGEPADPFATPLEILPEWYFFPVFQILRTVPNKLLGVLLMVSVP
TGLLTVPFLENVNKFQNPFRRPVATTVFLIGTAVALWLGIGATLPIDKSLTLGLFMIEVFLFGI
VLGLIPITLAGLFVTAYLQYRRGDQLDLMLTITSYFGFLLAALTITPALFISLNKIRLIMDIVS
LTWAALMVVFTFSLSLVVWGRSGLMIIRSSEPEVKIAVDRDPIKTSFEEWARPGHFSRTIAK
GPDTTTWIWNLHADAHDFDSHTGDLEEISRKVFSAHFGQLSIIFLWLSGMYFHGARFSNY
EAWLSDPTHIGPSAQVVWPIVGQEILNGDVGGGFRGIQITSGFFQIWRASGITSELQLYCTA
IGALIFASLMLFAGWFHYHKAAPKLAWFQDVESMLNHHLAGLLGLGSLSWAGHQIHVSL

PINQFLDAGVDPKEIPLPHEFILNRDLLAQLYPSFAEGATPFFTLNWSKYAEFLSFRGGLDPI
TGGLWLSDIAHHHLAIAILFLIAGHMYRTNWGIGHGLKDILEAHKGPFTGQGHKGLYEILT
TSWHAQLSLNLAMLGSTTIVVAHHMYSMPPYPYLATDYGTQLSLFTHHMWIGGFLIVGA
AAHAAIFMVRDYDPTTRYNDLLDRVLRHRDAIISHLNWVCIFLGFHSFGLYIHNDTMSAL
GRPQDMFSDAAIQLQPIFAQWIQNIHAGAPGVTAPGATTSTSLTWGGGELVAIGGKVALLPI
PLGTADFLVHHIHAFTIHVTVLILLKGVLFARSSRLIPDKANLGFRFPCDGPGRGGTCQVSA
WDHVFLGLFWMYNSISVVIFHFSWKMQSDVWGTISDQGIVTHITGGNFAQSSITINGWLR
DFLWAQASQVIQSYGSSLSAYGLFFLGAHFVWAFSLMFLFSGRGYWQELIESIVWAHNKL
KVAPATQPRALSIIQGRAVGVTHYLLGGIATTWAFFLARIIAVGMELRFPRFSQGLAQDPTT
RRIWFGIATAHDFESHDDITEERLYQNIFASHFGQLAIIFLWTSGNLFHVAWQGNFESWIQD
PLHVRPIAHAIWDPHFGQPAVEAFTRGGAAGPVNIAYSGVYQWWYTIGLRTNEDLYTGAL
FLLFLSTLSLIGGWLHLQPKWKPSLSWFKNAESRLNHHLSGLFGVSSLAWTGHLVHVAIPG
SRGEYVRWNNFLDVLPYPQGLGPLLTGQWNLYAQNPDSSNHLFGTTQGAGTAILTLLGGF
HPQTQSLWLTDIAHHHLAIAFIFLIAGHMYRTNFGIGHSIKDLLEAHTPPGGRLGRGHKGL
YDTINNSIHFQLGLALASLGVITSLVAQHMYSLPAYAFIAQDFTTQAALYTHHQYIAGFIMT
GAFAHGAIFFIRDYNPEQNEDNVLARMLDHKEAIISHLSWASLFLGFHTLGPYVHNDVML
AFGTPEKQILIEPIFAQWIQSAHGKTTYGFDILLSSTNGPTFNAGRNIWLPGWLNAVNE--NS
NSLFLTIGPGDFLVHHAIALGLHTTTLILVKGALDARGSKLMPDKKDFGYSFPCDGPGRGG
TCDISAWDAFYLAVFWMLNTIGWVTFYWHWKHITLWQGNVSQFNESSTYLMGWLRDYL
WLNSSQLINGYNPFGMNSLSVWAWMFLFGHLVWATGFMFLISWRGYWQELIETLAWAHE
RTPLANLIRWRDKPVALSVVQARLVGLAHFSVGYIFTYAAFLIASTSGKFGMSHSVKIYDT
CIGCTHCVRACPTDVLEMIPWDGCKAKQIASAPRTEDCVGCKRCESACPTDFLSVRVYLG
PETTRSMALSYMTDFNLPSIFVPLVGLVFPAIAMTSLFLYVQKNKIVMRDIKTYLSVAPVLS
TLWFGALAGLLIEINRLFPDALSFPFF--MTAILERRESTSLWGRFCNWITSTENRLYIGWFG
VLMIPTLLTATSVFIIAFIAAPPVDIDGIREPVSGSLLYGNNIISGAIIPTSAAIGLHFYPIWEAA
SVDEWLYNGGPYELIVLHFLLGVACYMGREWELSFRLGMRPWIAVAYSAPVAAATAVFLI
YPIGQGSFSDGMPLGISGTFNFMIVFQAEHNILMHPFHMLGVAGVFGGSLFSAMHGSLVTS
SLIRETTENESANEGYKFGQEEETYNIVAAHGYFGRLIFQYASFNNSRSLHFFLAAWPVVGI
WFTALGISTMAFNLNGFNFNQSVVDSQGRVINTWADIINRANLGMEVMHERNAHNFPLD
LAALEVPYLNGMGLPWYRVHTVVLNDPGRLLSVHIMHTALVSGWAGSMALYELAVFDPS
DPVLDPMWRQGMFVIPFMTRLGITNSWGGWSIS-GGTVTNPGIWSYEGVAGAHIVFSGLC
FLAAIWHWVYWDLEIFCDERTGKPSLDLPKIFGIHLFLAGVACFGFGAFHVTGLYGPGIWV
SDPYGLTGKVQAVNPAWGAEGFDPFVPGGIASHHIAAGTLGILAGLFHLSVRPPQRLYKGL
RMGNIETVLSSSIAAVFFAAFVVAGTMWYGSATTPIELFGPTRYQWDQGYFQQEIYRRVSD
GLAENLSLSEAWSKIPEKLAFYDYIGNNPAKGGLFRAGSMDNGDGIAVGWLGHPVFRDK
EGRELFVRRMPTFFETFPVVLVDEEGIVRADVPFRRAESKYSVEQVGVTVEFYGGELNGV
SYSDPATVKKYARRAQLGEIFELDRATLKSDGVFRSSPRGWFTFGHATFALLFFFGHIWHG
ARTLFRDVFAGIDPDLDAQVEFGTFQKVGDPTTRRQAAMKILYSLRRFYHVETLFNGTFV
LAGRDQETTGFPWWAGNARLINLSGKLLGAHVAHAGLIVFWAGAMNLFEVAHFVPEKP
MYEQGLILLPHLATLGWGVGSGGEVLDTFPYFVSGVLHLISSAVLGFGGIYHALLGPETLE
ESFPFFGYVWQDRNKMTTLLGIHLILLGLGAFLLVLKALYFGGVYDTWAPGGGDVRKITN
LTLSPGVIFGYLLKSPFGGEGWIVSVDDLEDIIGGHVWLGSICVLGGIWHILTKPFAWARRA
FVWSGEAYLSYSLGALSVFGFIACCFVWFNNTAYPSEFYGPTGPEASQAQAFTFLVRDQRL
GANVGSAQGPTGLGKYLMRSPTGEVIFGGETMRFWDLRAPWLEPLRGPNGLDLSRLKKD

GQPWQERRSGEYMTHAPLGSLNSVGGVATEINAVNYVSPRSWLATSHFVLGFFFFVGHLW
HAGRARAAAAGFEKGIDRDLEPVVYMTPLNMTIAVGRVTKEENDLFPLIDDWLRRDRFV
FVGWSGLLLFPCAYFALGGWFTGTTFVTSWYTHGLASSYLEGCNFLTAAVSTPANSLAHS
LLLLWGPEAQGDFTRWSQLGGLWTFLALHGAFALIGFMLRQFELARPVQLRPYNAISFSG
PIAVFLSVFLIYPLGQSGWFFSPSFGVAAIFRFILFFQGFHNWTLKPFHMMGVAGVLGAVLL
CAIHGATVENTLFEDGDGANTFRAFNPTQAEETYSMVTANRFWSQIFGVAFSNKRWLHFF
MLFVPVTGLWMSAIGVVGLALNLRAYDFVSQEIRAAEDPEFETFYTKNILLNEGIRAWMA
AQDQPHENLIFPEEVLPRGNALMSGSTGERSFADIITSIRYWVIHSITIPSLFIAGWLFVSTGL
AYDVFGSPRPNEYFTESRQGIPLITDRFDSLEQLDEFSRSFMTIDRTYPIFTVRWLAVHGLAV
PTVFFLGSISAMQFIQRMATQTVEDSSRPK-PKRTGAGSLLKPLNSEYGKVAPGWGTTPFM
GVAMALFAIFLSIILEIYNSSVLLDGILTNMLTLKLFVYTVVIFFVSLFIFGFLSNDPGRNPGR
EEMADTTGRIPLWLIGTVTGILVIGLIGFFFYGSYSGLGSSLMPNILSLTCICFNSVLCPTSFFF
AKLPEAYAIFNPIVDVMPVIPVLFFLLAFVWQAAVSFRMTQSNPNEQNVELNRTSLYWGLL
LIFVLAVLFSNYFFNMEVNILAFIATALFILVPTAFLLIIYVKTASQNDMETATLVAISISGLLV
SFTGYALYTAFGQPSQQLRDPFEEHGDMEALVYTFLLVSTLGIIFFAIFFREPPKVPTKK--M
NIAFQLAVFALIATSSVLVIRGHLVFASPDGWSNNKNVVFSGTSLWIGLVFLVAILNSLIS-----
--MSPQTETKASVGFKAGVKDYKLTYYTPEYETKDTDILAAFRVTPQLGVPPEEAGAAVAA
ESSTGTWTTVWTDGLTSLDRYKGRCYHIEPVPGDPDQYICYVAYPLDLFEEGSVTNMFTSI
VGNVFGFKALRALRLEDLRIPPAYSKTFQGPPHGIQVERDKLNKYGRPLLGCTIKPKLGLS
AKNYGRACYECLRGGLDFTKDDENVNSQPFMRWRDRFVFCAEAIYKAQAETGEIKGHY
LNATAGTCEEMIKRAVFARELGVPIVMHDYLTGGFTANTTLSHYCRDNGLLLHIHRAMHA
VIDRQKNHGMHFRVLAKALRMSGGDHIHSGTVVGKLEGEREITLGFVDLLRDDFIEKDRS
RGIFFTQDWVSMPGVIPVASGGIHVWHMPALTEIFGDDSVLQFGGGTLGHPWGNAPGAA
ANRVALEACVQARNEGRDLAREGNEIIKAACKWSAELAAACEIWKEIKFDFKAMDTI--TA
KHLYKTPIPSTRKGTVDRQVKSNPRNKLIHGRHRCGKGRNARGIITARHRGGGHKRLYRK
IDFRRNQKDISGRIITIEYDPNRNAYICLIHYGDGEKRYILHPRGAIIGDTIVSGTKVPISMGN
ALPLTDMPLGTAIHNIEITRGRGGQLARAAGAVAKLIAKEGKLATLRLPSGEVRLVSQNCL
ATVGQVGNVGVNQKSLGRAGSKCWLGKRPVVRGVVMNPVDHPHGGGEGKAPIGRKKP
TTPWGYPALGRRTRKRKKYSDSFILRRRK-MIQPQTLLNVADNSGARKLMCIRVIGAAGN
QRYARIGDVIIAVIKDAVPQMPLERSEVIRAVIVRTRKEFKGDDGIIIRYDDNAAVIIDQKGNP
KGTRVFGAVAEELRELNLTKIVSLAPEVLMLSPKRTRFRKQHRGRMKGKSCRGNHICFGR
YALQVLEPAWITARQIEAGRRAMTRYARRGGKIWVRIFPDKPVTIRPTETRMGSGKGSPEY
WVAVVKPGRILYEMSGVSETVARAAISIAASKMPIRSQFLRLEMTRVPRGYIARRRRTKMR
SFASNFRGAHLRLNRVITQQVRRAFVSSHRDRGRQKRDFRRLWITRINAATRVY--VFNSYS
KLIHNLSKKELILNRKMLAQVAVSNPNNLYTISNKIRIIN---------RIKRVFKAQKRIHMSVFK
AQRVLDEIRWRYYEETVMILNLMPYRASYPILKLVYSAAANATHYRDFDKTNLFITKAEV
SRSTIMKKFRPRARGRSYSIKKTMCNITIVLN-VKKS-------MDGIKYAVFTEKSLRLLGKN-
QYTFNVESGFTKTEIKHWVELFFGVKVVAVNSHRLPGKGRRMGPILGHTMHYRRMIITLQ
PGYSIPLLDRETNMAVPKKRTSMSKKRIRKNLWKKKTYFSIVQSYSLAKSFSRGKPKGFSG
QQANKMAKGKDVRIRVILECISCVRK--G--KESTGISRYSTQKNRHNTPGQLELRKFCRYC
RKHTTHNEIKKMKIRASVRKICTKCRLIRRRGRIRVICSNPKHKQRQGMVREEITGSTQTLE
WKCVESRVDSKRLYYGRFILSPLRKGQADTVGIALRRALLGEIEGTCITRAKFG--NVPHEY
STIVGIEESIQEILLNLKEIVLRSNLYGVRDASICVKGPRYITAQDIILPPSVEIVDTTQPIANL
REPVDFCIELQIKRDRAYHTELRKNSQDGSY--PIDAVFMPVRNVNYSIFSCGNGNEKHEILF

LEIWTNGSLTPKEALYEASRNLIDLFLPFLHTEEE--GTSFEENK-NRLTPPLLTFQKRFT-NLK
KNKK-GIPLNCIFIDQLELPSRTYNCLKRANIHTLLDLLSKTEEDLMQINSFRMEDGKLIWD
TLEKH--LPIDLPKNKMLRNGN-EGMSTIPGFSQIQFEGFCRFINQGLAEELEKFPTIKDPDHE
IAFQLFAKGYQLLEPSIKERNAVYESLTYSSELYVSARLIFGF--DVQKETISIGNIPIMNSLGT
FIINGIYRIVINQILLSPGIYYRSELDHKGISIYTGTIISDWGGRSELAIDKKERIWARVSRKQK
ISILVLSSAMGSNLREILDNVSYPEIFLSFPNAKEKKRIESKEKAILEFYQQFACVGGDLVFS
ESLCEELQKKFFQQKCELGRVGRRNMNRRLNLDIPQNNTFLLPRDVLAATDHLIGMKFGT
GILDDDDMNHLKNKRIRSVADLLQDQFGLALGRLQHAVQKTIRRVFIRQSK--PTPQTLVTP
TSTSILLITTYETFFGTYPLAQVFDQTNPLTQTVHGRKVSCLGPGGLTGRTASFRSRDIHPSH
YGRICPIDTSEGINVGLTGSLAIHARIDHWWGSIESPFYEISEKAKEKKE--RQVVYLSPNRD
EYYMIAAGNSLSLNQGIQEEQVVPARYRQEFLTIAWEQIHVRSIFPFQYFSIGGSLIPFIEHN
DANRALMSSNMQRQAVPLSRSEKCIVGTGLERQTALDSRVSVIAQREGKIISSDSHKILLSS
SGKTISIPLVAHRRSNKNTCMHQKPRVPRGKSIKKGQILAEGAATVGGELALGKNVLVAY
MPWEGYNFEDAVLISERLVYEDIYTSFHIRKYEIQTDTTSQGSAEKITKQIPHLEEHLLRNL
DRNGVVRLGSWVETGDILVGKLTPQIASE-SSYIAEAGLLRAIFGLEVSTSKETSLKLPIGGR
GRVIDVKWIQR-------DPIMVRVYILQKREIKVGDKVAGRHGNKGIISKILPRQDMPYLQDG
APVDMVFNPLGVPSRMNVGQIFESSLGLAGDLLKKHYRIAPFDERYEQEASRKLVFSELYE
ASKQTKNPWVFEPEYPGKSRIFDGRTGDPFEQPVLIGKSYILKLIHQVDEKIHGRSTGPYSL
VTQQPVRGRAKQGGQRIGEMEVWALEGFGVAHILQEILTYKSDHLIARQEILNATIWGKR
MPNHEDPPESFRVLVRELRSLALELNHFLVSEKNFQVNREDV-------MIDQYKHKQLQIGLV
SPQQIKAWAKKILPNGEVVGEVTRPSTFHYKTDKPEKDGLFCERIFGPIKSGICACGNSRAS
VRENEDERFCQKCGVEFVDSRIRRYQMGYIKLACPVTHVWYLKGLPSYIANLLDKPLKK
LEGLVYGD--FSFARP-SAKKPTFLRLRGLFEDEISSCNHSISPFFSTPGFATFRNREIATGAGAI
REQLADLDLRIIIENSLVEWKELEDE-GYSGDEWEDRKRRIRKVFLIRRMQLAKHFIQTNV
EPEWMVLCLLPVLPPELRPIVYRSGDKVVTSDINELYKRVIRRNNNLAYLLKRSELAPADL
VMCQEKLVQEAVDTLLDSGSRGQPTRDGHNKVYKSLSDVIEGKEGRFRETLLGKRVDYS
GRSVIVVGPSLSLHQCGLPLEIAIKLFQLFVIRDLITKRATSNVRIAKRKIWEKEPIVWEILQ
EVMRGHPVLLNRAPTLHRLGIQAFQPTLVEGRTICLHPLVCKGFNADFDGDQMAVHLPLS
LEAQAEARLLMFSHMNLLSPAIGDPICVPTQDMLIGLYVLTIGNRLGICANRYNSCGN-----S
PNKKVNYNNYKYTKDKEPHFSSSYDALGAYRQKRIGLNSPLWLRWKLDQR-IVGS--REVP
IEVQYESFGTYHEIYAHYLVVGNRKKEIRSIYIRTTLGHISFYREIEEAIQGFSRAYSYTI-------
-----MAERANLVFHNKEIDGTAMKRLISRLIDHFGMGYTSHILDQIKTLGFHQATTTSISLGIE
DLLTIPSKGWLVQDAEQQSFLLEKHYYYGAIHAVEKLRQSVEIWYATSEYLKQEMNSNFRI
TDPSNPVYLMSFSGARGNASQVHQLVGMRGLMADPQGQMIHLPIQSNLREGLSLTEYIISC
YGARKGVVDTAVRTADAGYLTRRLVEVVQHIIVRRRDCGTIQGISVSPQNGMTE--KLFVQ
TLIGRVLADDIYIGSRCIASRNQDIGIGLVNRFITAFRAQPFRAQPIYIRTPFTCRSTSWICQLC
YGRSPTHGDLVELGEAVGIIAGQSIGEPGTQLTLRTFHTGGVFTGGTADLIRSPSNGKIQFNE
DLVHPTRTRHGQPAFLCYIDLHVTIQSQDILHSVNIPLKSLILVQNDQYVESEQVIAEIRAGT
STL--FKEKVQKHIYSESDGE-MHWSTDVYHAPEYQYGNLRRLPKTSHLWILSVSMCR--SSI
ASFSLHKDQDQMNTYSFSVDGR--YIFDFS--MAN-DQVSHRLLDTFG----DREILDYL-TPDR
IVFNGHWNCFYPS-ILQDN--SDLLAKKRRNRLVVPLQYHQEQEKERISCLGISMEIPFM--G
VLRRNTIFAYFDDPRYRKD--GIVKFRSPEEDSILK--KEGFIEHRG-TKEFSLKY-EVDRFFFIL
QELHILPRSSSLKVLDNSIIGVDTQLTKNTRSRLGGLVRVKRKKSHTELKIFSGDIHFPEEAD
KILG--GSLIPPEREKK--DSKE-SK-KRKNWVYVQRKKFLKSKEK--YFVSVRPAVAYEMDEG

INLATLFPQDLLQEEDNLQLRLVNFISHENSKLTQRIYHTN-QFVRTCLVVNWEQEEKEGAR
-------ASLVEVKTNDLIRDFLRIELVKSTILYTRRRYDRTSVGLIPN---LDRNNTNSFYSKAKI
QS-----LSQHQEVIG----TLLNRNK---EYPSLMILLASNCSRIG--LFKNSKYP---NAVKESNPRI
PI-DIFGLLGVIVP-SISNFSSS-YYLLT---HNQILLK-KYLFLD---NLKQTLQVLQGLKYSLIDE
NKR--ISNFD--SNIMLEPFHLNWHFLHHDSWEETLAIIH-LGQFICENLCLFKLHI----KKSGQI
FIVN-MDSFVLRAAKPYLATIGATVHGHYGKILYKGDRLVTFIYEKSRSSDITQGLPKVEQIF
EARSIDSLSPNLERRIEDWNERIPRILGVPWGFLIGAELTIAQSRISLVNKIQKVYRSQGVQI
HNRHIEIIIRQVTSKVRVSE---DGMSNVFLPGELIGLLRAERAGRALDESIYYRAILLGITRA
SLNTQSFISEASFQETARVLAKAALRGRIDWLKGLKENVVLGGIIPVGTGFQKFVHRSP--Q
DKNLY--LEIQKKNLFASEM--RDILFLHTELVSSDSDVTN---SETPFTPIYTI------MTRRYWNI
NLKEMIEAGVHFGHGIKKWNPKMAPYISAKRKGTHITNLARTARFLSEACDLVFDAASQG
KSFLIVGTKKRAADLVASAAIRSRCHYVNKKWFSGMLTNWSITKTRLSQFRDLRAEEKMG
KFHHLPKRDAAILKRKLSTLQRYLGGIKYMTRLPDIVIVLDQQKEYIALQECAILGIPTISLV
DTNCDPDLANISIPANDDTMTSIRLILNKLVFAISEGRSL-YIRNRMGQKINPLGFRLGTTQN
HHSFWFAQPKNYSEGLQEDKKIRNCIKNYIQKNRKKGSNKMESITHIEIQKEIDTIHVIIHIG
FPN-LLKKKG-----AIEELEKDLQKEVNSVNQRLNIAIEKVKEPYRQPNILAEYIAFQLKNRV
SFRKAMKKAIELTKKADIKGIKIQIAGRLAGKEIARAECIKKGRLPLQTIRAKIDYCCYPIRT
IYGVLGVKIWIFVEEEMSRYRGPRLKKIRRLGALPGLTRKTPKSGSNQKKKFHSGK-KEQY
RIRLQEKQKLRFHYGLTERQLLRYVHIAGKAKRSTGQVLLQLLEMRLDNILFRLGMASTIP
GARQLVNHRHILVNGRIVDIPSFRCKPRDIITTKDNQRSKRLVQNYIASSDPK--LPKHLTVD
T--LQYKGLVKKILDRKWVGLKINELLVVEYYSRQTMSRRGTAEKRTAKSDPIFRNRLVNM
VVNRIMKDGKKSLAYQILYRAVKKIQQKTETNPLLVLRQAIRRVTPNIGVKTRRKKGSTRK
VPIEIGSKQGRALAIRWLLEASQKRPGRNMAFKLSSELVDAAKGSGGAIRKKEATHRMAE
ANRALAHFRMGKDTIADLLTSIRNADMNKKGTVRVVSTNITENIVKILLREGFIESVRKHQ
ESNRYFLVSTLRHQRRKTRKGIYRT---RTFLKRISRPGLRIYANYQGIPKVLGGMGIAILSTS
RGIMTDREARLNRIGGEVLCYIWMTKAIPKIGSRKKVRIGLRKSARRITKGIIHVQASFNNT
IITVTDPQGRVVFWSSAGTCGFKSSRKASPYAGQRTAVDAIRTVG---LQRAEVMVKGAGSG
RDAALRAIAKSGVRLSCIRDVTPMPHNGCRPPKKRRLMPTVKQLIRNARQPIRNARKSAA
LKGCPQRRGTCARVYTINPKKPNSALRKVARVRLTSGFEITAYIPGIGHNLQEHSVVLVRGG
RVKDLPGVRYRIIRGTLDAVAVKNRQQGRSKYGAKKPKMAKKSLIQREKKRQKLEQKYH
LIRRSSKKKIRSKVSPLSLSEKTKMQEKLQSLPRNSAPTRLHRRCFLTGRPRANYRDFGLSG
HILREMVYACLLPGATRSSW---------MVKEEKQEN--RGSVEFQVFSFTNKIRRLASHLELHK
KDFSSERGLRRLLGKRQRLLAYLAKKNRVRYKKLISQLDIREK-----MVKLRLKRCGRKQQ
AIYRIVAIDVRSRREGRDLRKVGFYDPIKNQTCLNVPAILYFLEKGAQPTRTVYDILRKAEF
FKDKERTLS----FHKSKQPFRKFKQPFRKRRSRIGPGDRIDYRNMSLINRFISEQGKILSRRIN
RLTLKQQRLITLAIKQARILSFLPFRNYENEKQFQ-AQAISIITGPRHRKNVTRK-KTNPFVAR
HLLAKIEKVNMKEEKEIIVTWSRASSILPAMVGHTIAIHNGKEHIPIYITNPMVGRKLGEFV
P T R H F T S Y T R K D T K S R
R???
???
???
???
???
???

??
??
??
??
??
??
??
??
??
??
??
??
??
??
??
??
??
??
??
??
??
??
??
??
??
??
??
??
??
??
??
??
??
??
??
??
??
??MPRSR-INGNFIDKTSSIVANILLQIIPTTSGEK
RAFTYYRDG--MLAQSEGNYAEALQNYYEATRLEIDPYDRSYILYNIGLIHTSNGEHTKALE
YYFRALERNPFLPQAFNNMAVICHY-RGEQAILQGDSEIAEAWFDQAAEYWKQAIALTPG
NYIEAQNWLKITKRFEFEMNWRSEHIWIELLKGSRKRGNFFWACILFLGSLGFLAVGASSY
LGKNMISVLPSQQILFFPQGVVMSFYGIAGLFISSYLWCTILWNVGSGYDRFDRKEGIVCIF
RWGFPGIKRRIFLQFLVRDIQSIRIQVKEGLYPRRILYMEIRGQGVIPLTRTDEKFFTPREIEQ
KAAELAYFLRVPIEVF

>Nicotiana

MERWWFNSMLFLNKSMGSLGPIENTNE--DPNRK--VKNIHSCSNVDYLFGVKDIRNFISDD
TFLVSDRNGDSYSIYFDIENHIFEIDNDH--SFLSELESSFYSYRNSNYRNNGFRGEDPYYNS
YMYDTQYSWNNHINSCIDSYLQ--SQICIDTSIISGSENYG--YIYRAVCGNEGSSRRTRTKGS
DLTIRESSNDLEVTQKYRHLWVQCENCYGLNYKKFLKSKMNICEQCGYHLKMSSSDRIEL
LIDPGTWDPMDEDMVSLDPIEFHSEEEPYKDRIDSYQRKTGLTEAVQTGIGQLNGIPVAIGV
MDFQFMGGSMGSVVGEKITRLIEYAANQILPLIIVCASGGARMQEGSLSLMQMAKISSAL
YDYQLNKKLFYVSILTSPTTGGVTASFGMLGDIIIAEPNAYIAFAGKRVIEQTLNKTVPEGS
QAAEYLFQKGLFDLIVPRNLLKSVLSELFKLHAFFPLNQKSSKMVTIRADEISNIIRERIEQY
NREVKIVNTGTVLQVGDGIARIHGLDEVMAGELVEFEEGTIGIALNLESNNVGVVLMGDG
LLIQEGSSVKATGRIAQIPVSEAYLGRVINALAKPIDGRGEISASEFRLIESAAPGIISRRSVYE
PLQTGLIAIDSMIPIGRGQRELIIGDRQTGKTAVATDTILNQQGQNVICVYVAIGQKASSVAQ
VVTTLQERGAMEYTIVVAETADSPATLQYLAPYTGAALAEYFMYRERHTLIIYDDPSKQA
QAYRQMSLLLRRPPGREAYPGDVFYLHSRLLERAAKLSSSLGEGSMTALPIVETQSGDVSA
YIPTNVISITDGQIFLSADLFNSGIRPAINVGISVSRVGSAAQIKAMKQVAGKLKLELAQFAE
LEAFAQFASDLDKATQNQLARGQRLRELLKQSQSAPLTVEEQIMTIYTGTNGYLDSLEVG
QVRKFLVELRTYLKTNKPQFQEIISSTKTFTEEAEALLKEAIQEQMDRFILQEQAMRINPTT
SGSGVSTLEKKNPGRVVQIIGPVLDVAFPPGKMPNIYNALVVQGRDSVGQPINVACEVQQL
LGNNRVRAVAMSATEGLTRGMEVIDTGAPISVPVGGATLGRIFNVLGEPVDNLGPVDTSTT
SPIHRSAPAFIQLDTKLSIFETGIKVVDLLAPYRRGGKIGLFGGAGVGKTVLIMELINNIAKA
HGGVSVFGGVGERTREGNDLYMEMKESGVINEENIAESKVALVYGQMNEPPGARMRVGL
TALTMAEYFRDVNEQDVLLFIDNIFRFVQAGSEVSALLGRMPSAVGYQPTLSTEMGSLQE
RITSTKEGSITSIQAVYVPADDLTDPAPATTFAHLDATTVLSRGLAAKGIYPAVDPLDSTSTM
LQPRIVGEEHYETAQRVKQTLQRYKELQDIIAILGLDELSEEDRLLVARARKIERFLSQPFFV
AEVFTGSPGKYVGLAETIRGFQLILSGELDGLPEQAFYLVGNIDEATAKAMNLEMESNLKK
MTLNLSVLTPNRIVWDSEVEEIVLSTNSGQIGILPNHAPIATAVDIGILRIRLNDQWLTMAL
MGGFARIGNNEITVLVNDAEKGSDIDPQEAQQTLELAEANVKKAEGRRQKIEANLALRRA
RTRVEAINPISMKNVTDSFVS-GHWPSAGSFGFNTDILATNPINLSVVLGVLIFFGKGVLSDL
LDNRKQRILNTIRNSEELRGGAIEQLEKARSRLRKVESEAEQFRVNGYSEIEREKLNLINST
YKTLEQLENYKNETIQFEQQRAINQVRQRVFQQALRGALGTLNSCLNNELHLRTISANIG
MLGTMKEITDMNPLISAASVIAAGLAVGLASIGPGVGQGTAAGQAVEGIARQPEAEGKIRG
TLLLSLAFMEALTIYGLVVALALLFANPFVMNVLSCSINTLKGLYDISGVEVGQHFYWQIG
GFQVHGQVLITSWVVIAILLGSATIAVRNPQTIPTGGQNFFEYVLEFIRDVSKTQIGEEYGP
WVPFIGTMFLFIFVSNWSGALLPWKIIQLPHGELAAPTNDINTTVALALLTSVAYFYAGLTK
KGLGYFGKYIQPTPILLPINILEDFTKPLSLSFRLFGNILADELVVVVLVSLVPLVVPIPVMLL
GLFTSGIQALIFATLAAAYIGESMEGHHMIFSTLEHILTHISFSIVSIVITIHLITFLVDEIVKLY
DSSEKGIIVTFFCITGLLVTRWISSGHFPLSDLYESLIFLSWSFSLIHIIPY--KNVLILSKITGPS
AIFTQGFATSGILTEIHQSVILVPALQSEWLIMHVSMMILGYAALLCGSLLSVALLVITFRKN
RQLFY--KENVLQNTSF----------AKNYYRSQLIQQLDYWSYRVISLGFTFLTIGILSGAVWAN
EAWGSYWNWDPKETWAFITWIVFAIYLHTRTNRNLRGANSAIVASIGFLIIWICYFGVNLL
GIGLHSYGSFPSTMAKKKAFTPLFYLASIVFLPWWISFSVNKCLESWVTNWWNTGQSEIF
LNNIQEKSLLEKFIELEELLFLDEMIKEYSETHLEEFGI-IHKETIQLIKIQNENRIHTILHFSTN
IICFIILSGYSILGNEKLVILNSWAQEFLYNLSDTVKAFSILLLTDLCIGFHSPHGWELMIGSIY
KDFGFVHNDQIISGLVSTFPVILDTIFKYWIFRYLNRLSPSLVVIYHSMNDMPIGVPKVPFRS

PGEEDASWVDVYNRLYRERLLFLGQEVDSEISNQLIGLMVYLSIEDETKDLYLFINSPGGW
VIPGVAIYDTMQFVRPDVHTICMGLAASMGSFILVGGEITKRLAFPHARVMIHQPASSFYE
AQTGEFVLEAEELLKLRETLTRVYVQRTGKPLWVVSEDMERDVFMSATEAQAYGIVDLVA
VE??MEEIQR
YLQ-DRSQQHNFLYPLIFQEYIYALAHDHGLPGYNNKLSFLIVKRLITRMYQQNHFLISTND
SNKNSFLGKSLYSQMISEGFAFIVEIPFSLRLISSLIFKSYNLRSIHSTFPFLEDNFSHLNYVLD
ILIPYPVHLEILVQTLRYWVKDASSLHLLRFFLHEFWNLNSLITPG-YSFSKQRFFFFLYNSY
VYECESTFVFLRNQSSHLRSTSFGALLERIYFYGKIERLVEVFA-DFTLWLFKDPFMHYVRY
QGKSILASKGTFLLMNKWKFYLVNFWQCHCSLCFHTGRIHINQLSNHSRDFMGYLSSVRL
NPSMVRSQMLENSFLINNAI--KKFDTLVPIIPLIGSLAKANFCTVLGHPISKPVWSDLSDSDI
IDRFGRICRNLFHYYSGSSKKKTLYRIKYILRLSCARTLARKHKSTVRTFLKRSGSELLEEFL
TSEEQVLSLTFPRRIWYLDIFCINDLANYQMIID-TTEIE-TINSFSKLES-LKEVYGIIWMLFPI
LTLVLGITIGVLVIVWLEREISAGIQQRIGPEYAGPLGILQALADGTKLLLKENLIPSTGDTRL
FSIGPSIAVISIFLSYSVIPFGDHLVLADLSIGVFFWIAISSIAPVGLLMSGYGSNNKYSFLGGL
RAAAQSISYEIPLALCVLSISLLSNSLSTVDIVEAQSKYGFWGWNLWRQPIGFIVFLISSLAE
CERLPFDLPEAEEELVAGYQTEYSGIKFGLFYIASYLNLLVSSLFVTVLYLGGWNLSIPYIFV
PELFGINKRGKVFGTLIGIFITLAKTYLFLFIPIATRWTLPRLRMDQLLNLGWKFLLPISLGN
LLLTTSSQLLSLMIWHVQNENFILDSTRIFMKAFHLLLFDGSLIFPECILIFGLILLLMIDSTS
DQKDIPWLYFISSTSLVMSITALLFRWREEPMISFSGNFQTNNFNEIFQFLILLCSTLCIPLSV
EYIECTEMAITEFLLFVLTATLGGMFLCGANDLITIFVAPECFSLCSYLLSGYTKKDVRSNEA
TMKYLLMGGASSSILVHGFSWLYGSSGGEIELQEIVNGLINTQMYNSPGISIALIFITVGIGF
KLSPAPSHQWTPDVYEGSPTPVVAFLSVTSKVAASASATRIFDIPFYFSSNEWHLLLEILAIL
SMILGNLIAITQTSMKRMLAYSSIGQIGYVIIGIIVGDSNDGYASMITYMLFYISMNLGTFAC
IVLFGLRTGTDNIRDYAGLYTKDPFLALSLALCLLSLGGLPPLAGFFGKLYLFWCGWQAGL
YFLVLIGLLTSVVSIYYYLKIIKLLMTGRNQEITPHVRNYRRSPLRSNNSIELSMIVCVIASTI
PGISMNPIIAIAQDSLFMFLLYEYDFFWAFLIISILVPILAFLISGVLAPISKGPEKLSTYESGIE
PMGDAWLQFRIRYYMFALVFVVFDVETVFLYPWAMSFDVLGVSVFIEAFIFVLILIIGLVYA
WRKGALEWSTNYFPWLTIIVVFPIFAGSLIFFLPHKGNRVIRWYTICICILELLLTTYAFCYHF
QSDDPLIQLVEDYKWINFFDFHWRLGIDGLSIGPILLTGFITTLATLAAWPVTRDSRLFHFL
MLAMYSGQIGSFSSRDLLLFFIMWELELIPVYLLLCMWGGKKRLYSATKFILYTAGGSVFL
LMGVLGLALYGSNEPTLNFETSVNQSYPVVLEIIFYIGFFIAFAVKSPIIPLHTWLPDTHGEA
HYSTCMLLAGILLKMGAYGLIRINMELLPHAHSIFSPWLMIIGTIQIIYAALTSLGQRNLKKR
IAYSSVSHMGFIIIGISSLTDTGLNGALLQIISHGFIGAALFFLAGTTYDRIRLVYLDEMGGIAI
PMPKMFTMFSSFSMASLALPGMSGFVAELIVFFGIITGQKYLLIPKILITFVMAIGMILTPIYS
LSMSRQMFYGYKLFNAPKDSFFDSGPRELFLSISIFLPVIGIGIYPDFVLSLAVDKVEVILSNF
FYRMILEHVLVLSAYLFSIGIYGLITSRNMVRALMCLELILNAVNINFVTFSDFFDNRQLKG
DIFSIFVIAIAAAEAAIGLAIVSSIYRNRKSTRINQSNLLNNMEQTYEYAWIIPFIPLPVPMLIG
AGLFLFPTATKSFRRMWAFQSVLLLSIVMVFSIYLSIQQINSSSFYQYVWSWIINNDFSLDF
GYLIDPLTSIMSILITTVGIMVLIYSDNYMAHDQGYLRFFAYMSFFSTSMLGLVTSSNLIQIYI
FWELVGLCSYLLIGFWFTRPVAANACQKAFVTNRVGDFGLLLGILGFYWITGSFEFRDLFE
IFNNNEVDFLFVTLCAVLLFAGAVAKSAQFPLHVWLPDAMEGPTPISALIHAATMVAAGIFL
VARLLPLFRVIPYIMYLISVIGIITVLLGATLALAQKDIKRGLAYSTMSQLGYMMLALGMGS
YRSALFHLITHAYSKALLFLGSGSIIHSMETIVGYSPAKSQNMGLMGGLRKHVPISKITFLL
GTLSLCGIPPLACFWSKDEILNDSWLYSPIFAIIAWATAGLTAFYMFRIYLLTFEGHLNAHFP

NYGGKQKTPFYSISLWGK---NGVKK-LLT---MNFFAFEHKAVYSYPYESDNTMLFPIFVLGL
FTLFVGSIGIPFNLDILSKWLAPSINLLHQKSN-SMDWNEFLKDAVLSVSIAYFGIFIASFLYK
PIYSSLKNFELINSFVILWDKIINGIYDWSYNRAYIDAFYTRFLVGGIRGLAEFTHFFDRRVID
GMTNGVGVISFIVGEGIKYIGGG-RISSYLFLYLAYVSIFLLVYYMDLSEPIHDFLLVFLGSG
LILGGLGVVLLPNPIYSAFSLGLVLVCTSLFYILSNSYFVAAAQLLIYVGAINVLIIFAVMFM
NGSEYYKDFHLWTVGDGITSMVCISLFISLITTISDTSWYGIIWTTRSNQIIEQDFISNSQQIG
IHLSTDFFLPFELISIILLVALIGAIAVARQMTAPTTRKDLMIVNMGPQHPSMHGVLRLIVTL
DGEDVVDCEPILGYLHRGMEKIAENRTIIQYLPYVTRWDYLATMFTEAITINGPEQLGNIQ
VPKRASYIRVIMLELSRIASHLLWLGPFMADIGAQTPFFYIFRERELIYDLFEAATGMRMM
HNYFRIGGVAADLPYGWIDKCLDFCDYFLTGVAEYQKLITRNPIFLERVEGVGIIGGDEAL
NWGLSGPMLRASGIEWDLRKVDHYESYDEFDWQVQWQREGDSLARYLVRIGEMTESIKI
IQQALEGIPGGPYENLEIRRFDRLKDPEWNDFEYRFISKKPSPTFELSKQELYVRVEAPKGE
LGIFLIGDQSVFPWRWKIRPPGFINLQILPQLVKRMKLADIMTILGSIDIIMGEVDRMLPMIT
EFINYGQQTIRAARYIGQGFMITLSHANRLPVTIQYPYEKLITSERFRGRIHFEFDKCIACEV
CVRVCPIDLPVVDWKLETDIRKKRLLNYSIDFGICIFCGNCVEYCPTNCLSMTEEYELSTY
DRHELNYNQIALGRLPMSVIDDYTIRTISNL----------------MQGRLSAWLVKHGLIHRSLGFD
YQGIETLQIKPEDWHSIAVIFYVYGYNYLRSQCAYDVAPGGLLASVYHLTRIEDGVDQPEE
VCIKVFASRRNPRIPSVFWVWKSVDFQERESYDMLGISYDNHPRLKRILMPESWIGWPLR
KDYIAPNFYEIQDAH-------------------------------------MVLAPEYSDNKKKNGKNKIET-VMNS
IQFPLLDRTTQNSVISTTLNDLSNWSRLSSLWPLLYGTSCCFIEFASLIGSRFDFDRYGLVPRS
SPRQADLILTAGTVTMKMAPSLVRLYEQMPEPKYVIAMGACTITGGMFSTDSYSTVRGVD
KLIPVDVYLPGCPPKPEAVIDAITKLRKKISRELYEDRIRSQRANRCFTTNHKFHVQHSIHT
GNYDQRVLYQPPSTSEIPTEIFFKYKNSVSSPELVNMQTRNAFSWLKKQITRSISVSLMIYIL
TRTSISSAYPIFAQQGYENPREATGRIVCANCHLANKPVEIEVPQAVLPDTVFEAVVRIPYD
MQLKQVLANGKRGGLNVGAVLILPEGFELAPPDRISPEMKEKIGNLSFQSYRPNKKNILVI
GPVPGQKYSEITFPILSPDPATKKDVHFLKYPIYVGGNRGRGQIYPDGSKSNNTVYNATAA
GIVSKIIRKEKGGYEITITDASDGRQVVDIIPPGPELLVSEGESIKFDQPLTSNPNVGGFGQG
DAEIVLQDPLRVQGLLFFLASVILAQIFLVLKKKQFEKVQLAEMNFMSKVYDWFEERLEIQ
AIADDITSKYVPPHVNIFYCLGGITLTCFLVQVATGFAMTFYYRPTVTEAFASVQYIMTEAN
FGWLIRSVHRWSASMMVLMMILHVFRVYLTGGFKKPRELTWVTGVVLAVLTASFGVTGY
SLPWDQVGYWAVKIVTGVPDAIPVIGSPLVELLRGSASVGQSTLTRFYSLHTFVLPLLTAVF
MLMHFPMIRKQGISGPLMGVTKKPDLNDPVLRAKLAKGMGHNYYGEPAWPNDLLYIFPV
VILGTIACNVGLAVLEPSMIGEPADPFATPLEILPEWYFFPVFQILRTVPNKLLGVLLMVSVP
AGLLTVPFLENVNKFQNPFRRPVATTVFLIGTAVALWLGIGATLPIDKSLTLGLFMIEVFLFGI
VLGLIPITLAGLFVTAYLQYRRGDQLDLMLTITSYFGFLLAALTITSALFIGLSKIRLIMDIVS
LAWAALMVVFTFSLSLVVWGRSGLMIIRSPEPEVKILVDRDPVKTSFEEWARPGHFSRTIA
KGPDTTTWIWNLHADAHDFDSHTSDLEEISRKVFSAHFGQLSIIFLWLSGMYFHGARFSN
YEAWLSDPTHIGPSAQVVWPIVGQEILNGDVGGGFRGIQITSGFFQIWRASGITSELQLYCT
AIGALVFAALMLFAGWFHYHKAAPKLAWFQDVESMLNHHLAGLLGLGSLSWAGHQVHV
SLPINQFLNAGVDPKEIPLPHEFILNRDLLAQLYPSFAEGATPFFTLNWSKYADFLTFRGGLD
PVTGGLWLTDIAHHHLAIAILFLIAGHMYRTNWGIGHGLKDILEAHKGPFTGQGHKGLYEI
LTTSWHAQLSLNLAMLGSLTIVVAHHMYSMPPYPYLATDYGTQLSLFTHHMWIGGFLIVG
AAAHAAIFMVRDYDPTTRYNDLLDRVLRHRDAIISHLNWACIFLGFHSFGLYIHNDTMSAL
GRPQDMFSDTAIQLQPVFAQWIQNTHALAPGATAPGATASTSLTWGGGDLVAVGGKVALL

PIPLGTADFLVHHIHAFTIHVTVLILLKGVLFARSSRLIPDKANLGFRFPCDGPGRGGTCQVS
AWDHVFLGLFWMYNAISVVIFHFSWKMQSDVWGSVSDQGVVTHITGGNFAQSSITINGW
LRDFLWAQASQVIQSYGSSLSAYGLFFLGAHFVWAFSLMFLFSGRGYWQELIESIVWAHN
KLKVAPATQPRALSIIQGRAVGVTHYLLGGIATTWAFFLARIIAVGMALRFPRFSQGLAQDP
TTRRIWFGIATAHDFESHDDITEERLYQNIFASHFGQLAIIFLWTSGNLFHVAWQGNFESWV
QDPLHVRPIAHAIWDPHFGQPAVEAFTRGGALGPVNIAYSGVYQWWYTIGLRTNEDLYTG
ALFLLFLSAISLIAGWLHLQPKWKPSVSWFKNAESRLNHHLSGLFGVSSLAWTGHLVHVAI
PASRGEYVRWNNFLDVLPHPQGLGPLFTGQWNLYAQNPDSSSHLFGTAQGAGTAILTLLG
GFHPQTQSLWLTDIAHHHLAIAFIFLVAGHMYRTNFGIGHSMKDLLDAHIPPGGRLGRGHK
GLYDTINNSLHFQLGLALASLGVITSLVAQHMYSLPAYAFIAQDFTTQAALYTHHQYIAGFI
MTGAFAHGAIFFIRDYNPEQNEDNVLARMLEHKEAIISHLSWASLFLGFHTLGLYVHNDV
MLAFGTPEKQILIEPIFAQWIQSAHGKTSYGFDVLLSSTSGPAFNAGRSIWLPGWLNAVNE-
-NSNSLFLTIGPGDFLVHHAIALGLHTTTLILVKGALDARGSKLMPDKKDFGYSFPCDGPGR
GGTCDISAWDAFYLAVFWMLNTIGWVTFYWHWKHITLWQGNVSQFNESSTYLMGWLR
DYLWLNSSQLINGYNPFGMNSLSVWAWMFLFGHLVWATGFMFLISWRGYWQELIETLAW
AHERTPLANLIRWRDKPVALSIVQARLVGLAHFSVGYIFTYAAFLIASTSGKFGMSHSVKIY
DTCIGCTQCVRACPTDVLEMIPWDGCKAKQIASAPRTEDCVGCKRCESACPTDFLSVRVY
LWHETTRSMGLAYMTNLNLPSIFVPLVGLVFPAIAMASLFLHVQKNKIVMRDLKTYLSVA
PVLSTLWFGALAGLLIEINRFFPDALTFPFFSFMTAILERRESESLWGRFCNWITSTENRLYI
GWFGVLMIPTLLTATSVFIIAFIAAPPVDIDGIREPVSGSLLYGNNIISGAIIPTSAAIGLHFYPI
WEAASVDEWLYNGGPYELIVLHFLLGVACYMGREWELSFRLGMRPWIAVAYSAPVAAAT
AVFLIYPIGQGSFSDGMPLGISGTFNFMIVFQAEHNILMHPFHMLGVAGVFGGSLFSAMHG
SLVTSSLIRETTENESANEGYRFGQEEETYNIVAAHGYFGRLIFQYASFNNSRSLHFFLAAW
PVVGIWFTALGISTMAFNLNGFNFNQSVVDSQGRVINTWADIINRANLGMEVMHERNAH
NFPLDLAAIEAPSTNGMGLPWYRVHTVVLNDPGRLLSVHIMHTALVAGWAGSMALYELA
VFDPSDPVLDPMWRQGMFVIPFMTRLGITNSWGGWSIT-GGTVTNPGIWSYEGVAGAHIV
FSGLCFLAAIWHWVYWDLEIFCDERTGKPSLDLPKIFGIHLFLSGVACFGFGAFHVTGLYG
PGIWVSDPYGLTGKVQPVNPAWGVEGFDPFVPGGIASHHIAAGTLGILAGLFHLSVRPPQR
LYKGLRMGNIETVLSSSIAAVFFAAFVVAGTMWYGSATTPIELFGPTRYQWDQGYFQQEIY
RRVSAGLAENQSLSEAWSKIPEKLAFYDYIGNNPAKGGLFRAGSMDNGDGIAVGWLGHPI
FRDKEGRELFVRRMPTFFETFPVVLVDGDGIVRADVPFRRAESKYSVEQVGVTVEFYGGE
LNGVSYSDPATVKKYARRAQLGEIFELDRATLKSDGVFRSSPRGWFTFGHASFALLFFFGH
IWHGARTLFRDVFAGIDPDLDAQVEFGAFQKLGDPTTKRQAA-----------VETLFNGTLALA
GRDQETTGFAWWAGNARLINLSGKLLGAHVAHAGLIVFWAGAMNLFEVAHFVPEKPMY
EQGLILLPHLATLGWGVGPGGEVIDTFPYFVSGVLHLISSAVLGFGGIYHALLGPETLEESF
PFFGYVWKDRNKMTTILGIHLILLGLGAFLLVFKALYFGGVYDTWAPGGGDVRKITNLTL
SPSIIFGYLLKSPFGGEGWIVSVDDLEDIIGGHVWLGSICILGGIWHILTKPFAWARRALVWS
GEAYLSYSLGALSVFGFIACCFVWFNNTAYPSEFYGPTGPEASQAQAFTFLVRDQRLGAN
VGSAQGPTGLGKYLMRSPTGEVIFGGETMRFWDLRAPWLEPLRGPNGLDLSRLKKDIQP
WQERRSAEYMTHAPLGSLNSVGGVATEINAVNYVSPRSWLATSHFVLGFFFFVGHLWHA
GRARAAAAGFEKGIDRDFEPVLSMTPLNMTIALGKFTKDENDLFDIMDDWLRRDRFVFV
GWSGLLLFPCAYFAVGGWFTGTTFVTSWYTHGLASSYLEGCNFLTAAVSTPANSLAHSLL
LLWGPEAQGDFTRWCQLGGLWTFVALHGAFGLIGFMLRQFELARSVQLRPYNAIAFSGPI
AVFVSVFLIYPLGQSGWFFAPSFGVAAIFRFILFFQGFHNWTLNPFHMMGVAGVLGAALLC

AIHGATVENTLFEDGDGANTFRAFNPTQAEETYSMVTANRFWSQIFGVAFSNKRWLHFFM
LFVPVTGLWMSALGVVGLALNLRAYDFVSQEIRAAEDPEFETFYTKNILLNEGIRAWMAA
QDQPHENLIFPEEVLPRGNALMSGSTGERSFADIITSIRYWVIHSITIPSLFIAGWLFVSTGLA
YDVFGSPRPNEYFTESRQGIPLITGRFDPLEQLDEFSRSFMTIDRTYPIFTVRWLAVHGLAVP
TVFFLGSISAMQFIQRMATQTVENSS-RSGPRRTAVGDLLKPLNSEYGKVAPGWGTTPLMG
VAMALFAVFLSIILEIYNSSVLLDGISMNMLTLKLFVYTVVIFFVSLFIFGFLSNDPGRNPGR
EEMADTTGRIPLWIIGTVAGILVIGLIGIFFYGSYSGLGSSLMLNTFSLIGICLNSTLFSSSFFF
GKLPEAYAFLNPIVDIMPVIPLFFFLLAFVWQAAVSFRTTQSNPNEQNVELNRTSLYWGLLL
IFVLAVLFSNYFFNMEVNILAFIATALFILVPTAFLLIIYVKTVSQNDMETATLVAIFISGLLVS
FTGYALYTAFGQPSQQLRDPFEEHGDMEALVYTFLLVSTLGIIFFAIFFREPPKVPTKKN-MT
LAFQLAVFALIATSLILLISVPVVFASPDGWSSNKNVVFSGTSLWIGLVFLVGILNSLIS-------
MSPQTETKASVGFKAGVKEYKLTYYTPEYQTKDTDILAAFRVTPQPGVPPEEAGAAVAAE
SSTGTWTTVWTDGLTSLDRYKGRCYRIERVVGEKDQYIAYVAYPLDLFEEGSVTNMFTSI
VGNVFGFKALRALRLEDLRIPPAYVKTFQGPPHGIQVERDKLNKYGRPLLGCTIKPKLGLS
AKNYGRAVYECLRGGLDFTKDDENVNSQPFMRWRDRFLFCAEALYKAQAETGEIKGHY
LNATAGTCEEMIKRAVFARELGVPIVMHDYLTGGFTANTSLAHYCRDNGLLLHIHRAMHA
VIDRQKNHGIHFRVLAKALRMSGGDHIHSGTVVGKLEGERDITLGFVDLLRDDFVEQDRS
RGIYFTQDWVSLPGVLPVASGGIHVWHMPALTEIFGDDSVLQFGGGTLGHPWGNAPGAVA
NRVALEACVKARNEGRDLAQEGNEIIREACKWSPELAAACEVWKEIVFNFAAVDVLDKM
AIHLYKTSTPSTRNGTVDSQVKSNPRNNLIYGQHHCGKGRNARGIITARHRGGGHKRLYR
KIDFRRNEKDIYGRIVTIEYDPNRNAYICLIHYGDGEKRYILHPRGAIIGDTIVSGTEVPIKM
GNALPLTDMPLGTAIHNIEITLGKGGQLARAAGAVAKLIAKEGKSATLKLPSGEVRLISKNC
SATVGQVGNVGVNQKSLGRAGSKRWLGKRPVVRGVVMNPVDHPHGGGEGRAPIGRKK
PTTPWGYPALGRRSRKRNKYSDNLILRRRSKMIQPQTHLNVADNSGARELMCIRIIGAS-N
RRYAHIGDVIVAVIKEAVPNMPLERSEVVRAVIVRTCKELKRDNGMIIRYDDNAAVVIDQE
GNPKGTRIFGAIARELRELNFTKIVSLAPEVLMLSPKRTRFRKQHRGRMKGISHRGNHISF
GKYALQALEPAWITSRQIEAGRRAMTRNARRGGKIWVRIFPDKPVTLRPAETRMGSGKGS
PEYWVAVVKPGRILYEMGGVTENIARRAISLAASKMPIRTQFIIS-MTRIKRGYIARRRRTKI
RLFASSFRGAHSRLTRTITQQKIRALVSAHRDRDRKKRDFRRLWITRINAVIRE-RGVSYSYS
RLIHDLYKRQLLLNRKILAQIAISNRNCLYMISNEIIKEVDWKESTRIIMLKEVYALGEHISM
SADKARRVINQIRGRSYEETLMILELMPYRACYPILKLIYSAAANASYNMGSSEANLVISK
AEVNGGTTVKKLKPRARGRSFPIKRSTCHITIVMKDISLDGGLWDKKMDGIKYAVFTDKSI
RLLGKN-QYTSNVESGSTRTEIKHWVELFFGVKVIAMNSHRLPGKSRRMGPIMGHTMHY
RRMIITLQPGYSIPPLRKKRTMAVPKKRTSTSKKRIRKNIWKRKGYSIALKAFSLAKSLSTG
NSKSFFVRQTKIMAKGKDVRVTVILECTSCVRNSVD--KVSRGISRYITQKNRHNTPNRLEL
KKFCPYCYKHTIHGEIKKMKIRASVRKICEKCRLIRRRGRIIVICSNPRHKQRQGMVREKV
TVSTRTLQWKCVESRTDSKRLYYGRFILSPLMKGQADTIGIAMRRALLGEIEGTCITRVKSE
--KVPHEYSTITGIQESVHEILMNLKEIILRSNLYGTSDASICVKGPGSVTAQDIILPPYVEIVD
NTQHIASLTEPIDFCIGLQIERNRGYLIKTPH-NFDGSY--PIDAVFMPVRNANHSIHSYGNGN
EKQEILFLEIWTNGSLTPKEALHEASRNLIDLFIPFLHMEED--NLYLQDNQ-HTVPLSPFTFH
DKLA-KLIKNKK-KIALKSIFIDQSELPSRIYNCLKMSNIYTLLDLLNNSQEDLMKIEHFRSE
DVKRILGILEKY--FVIDLAKNKMLGDG-NEGISTIPGFNQIQFEGFCRFIDQGLTEELYKFPKI
EDTDQEIEFQLFVETYQLVEPLIKERDAVYESLTYSSELYVSAGLIWKNSRDMQEQTIFIGNI
PLMNSLGTSIVNGIYRIVINQILQSPGIYYRSELDHNGISVYTGTIISDWGGRSELEIDRKARI

WARVSRKQKISILVLSSAMGLNLREILENVCYPEIFLSFLSDKERKKIGSKENAILEFYQQFA
CVGGDPVFSESLCKELQKKFFQQRCELGRIGRRNMNRRLNLDIPQNNTFLLPRDILAAAD
HLIGLKFGMGALDD--MNHLKNKRIRSVADLLQDQFGLALVRLENVVRGTICGAIR-HKL-I
PTPQNLVTSTP----LTTTYESFFGLHPLSQVLDRTNPLTQIVHGRKLSYLGPGGLTGRTASFRI
RDIHPSHYGRICPIDTSEGINVGLIGSLAIHARIGHWG-SLESPFY---EISERSTG--VRMLYLSP
GRDEYYMVAAGNSLALNQDIQEEQVVPARYRQEFLTIAWEQVHLRSIFPFQYFSIGASLIPF
IEHNDANRALMSSNMQRQAVPLSRSEKCIVGTGLERQAALDSGALAIAEREGRVVYTNTD
KILLAGNGDILSIPLVIYQRSNKNTCMHQKLQVPRGKCIKKGQILADGAATVGGELALGK
NVLVAYMPWEGYNSEDAVLISERLVYEDIYTSFHIRKYEIQTHVTSQG-PEKVTNEIPHLEA
HLLRNLDKNGIVMLGSWVETGDILVGKLTPQVVKE-SSYAPEDRLLRAILGIQVSTSKETCL
KLPIGGRGRVIDVRWIQKRG-GSSYNPETIRVYILQKREIKVGDKVAGRHGNKGIISKILPRQ
DMPYLQDGRSVDMVFNPLGVPSRMNVGQIFECSLGLAGSLLDRHYRIAPFDERYEQEASR
KLVFSELYEASKQTANPWVFEPEYPGKSRIFDGRTGNPFEQPVIIGKPYILKLIHQVDDKIH
GRSSGHYALVTQQPLRGRAKQGGQRVGEMEVWALEGFGVAHILQEMLTYKSDHIRARQE
VLGTTIIGGTIPNPEDAPESFRLLVRELRSLALELNHFLVSEKNFQINRKEAMNNNFSSMIDR
YKHQQLRIGSVSPQQISAWATKILPNGEIVGEVTKPYTFHYKTNKPEKDGLFCERIFGPIKS
GICACGNYRVIGDEKEDPKFCEQCGVEFVDSRIRRYQMGYIKLACPVTHVWYLKRLPSYI
ANLLDKPLKELEGLVYCD--FSFARP-ITKKPTFLRLRGLFEYEIQSWKYSIPLFFTTQGFDTF
RNREISTGAGAIREQLADLDLRIIIENSLVEWEELGEE-GHTGNEWEDRKVGRRKDFLVRR
VELAKHFIRTNIEPEWMVLCLLPVLPPELRPIIQIDGGKLMSSDINELYRRVIYRNNTLTDLL
TTSRSTPGELVMCQEKLVQEAVDTLLDNGIRGQPMRDGHNKVYKSFSDVIEGKEGRFRET
LLGKRVDYSGRSVIVVGPSLSLHRCGLPREIAIELFQTFVIRGLIRQHLASNIGVAKSKIREK
EPIVWEILQEVMQGHPVLLNRAPTLHRLGIQAFQPVLVEGRAICLHPLVCKGFNADFDGD
QMAVHVPLSLEAQVEARLLMFSHMNLLSPAIGDPISVPTQDMLIGLYVLTSGNHRGICVNR
YNPCNR---RNYQNQKRSDNSYKYT---EPFFSNSYDAIGAYRQKRINLDSPLWLRWRLDQR-
VIAS--RETPIEVHYESLGTFYEIYGHYLIVRSLKKQILFIYIRTTVGHIALYREIEEAIQGFSRA
YSSGT------------MAERANLVFHNKAINGTAMKRLISRLIDHFGMAYTSHILDQVKTLGFQQ
ATATSISLGIDDLLTIPSKGWLVQDAEQQSLILEKHHHYGNVHAVEKLRQSIEIWYATSEYL
RQEMNPNFRMTDPFNPVHIMSFSGARGNASQVHQLVGMRGLMSDPQGQMIDLPIQSNLR
EGLSLTEYIISCYGARKGVVDTAVRTSDAGYLTRRLVEVVQHIVVRRTDCGTARGISVSPRN
GMMP-ERIFIQTLIGRVLADDIYMGPRCIATRNQDIGIGLVNRFIT------FRAQPISIRTPFTCRS
TSWICRLCYGRSPTHGDLVELGEAVGIIAGQSIGEPGTQLTLRTFHTGGVFTGGTAEHVRAP
SNGKIKFNEDLVHPTRTRHGHPAFLCSIDLYVTIESEDILHNVNIPPKSLLLVQNDQYVESEQ
VIAEIRAGIST-LNFKEKVRKHIYSDSDGE-MHWSTDVYHAPEFTYGNVHLLPKTSHLWILL
GRPCR--SSLVYLSIHKDQDQMNAH--FLSGKRRYTSNLS--VTNDQ-ARQKLFSSDFSGK-ED
RIPDY-SDLNRIICAGQYNLVYS-PILHENS--DLLSKRRRNKFIIPLHSIQELENELMPCSGISIE
IP--VNGIFRRNSILAYFDDPRYRRKSSGIIKYGTVETHSVIKKED--LLEYRG-VKEFRPKYM
KVDRFFFIPEEVHILPGSSSIMVRNNSIVGVDTQITLNLRSRVGGLVRVERKKKRIELKIFSG
DIHFPGETDKISRHTGVLIP-PG-TGKRNSKESKK--VKNWIYVQR--ITPSK--KKFFVLVRPV
VTYEITDGINLATLFPPDPLQERDNVQLRIVNYILYGNGKPIRGISDTSIQLVRTCLVLNWNQ
DKK---SSSCEEARASFVEIRTNGLIRHFLRINLVKSPISYIGKRNDPSGSGLLSD-NGSDCTNI
NPFSSIYSY---SKAKIQQSINQPQGTIHTLLNR---NKECQSLIILSAANCSRMGP--FKDVKYH
SV---IKKS-PLIPIRNSLGPLGT--SLPIENFYS--SYHL--ITHNQIL-VTNYLQLDN---LKQTFQVI
KFK-YYLMDEN--GKIFNPDPCRNIILNPFNLNWYFLHHNYCEETS-KIISLGQFICENVCIAK

NGPP-LK--SGQVILVQ-VDSIVIRSAKPYLATPGATVHGHYGETLYEGDTLVTFIYEKSRSGD
ITQGLPKVEQVLEVRSVDSISMNLEKRIEGWNKCITRILGIPWGFLIGAELTIAQSRISLVNKI
QQVYRSQGVQIHNRHLEIIVRQITSKVLVSE---DGMSNVFSPGELIGLLRAERMGRALEEAI
CYRVVLLGITRASLNTQSFISEASFQETARVLAKAALRGRIDWLKGLKENVVLGGVIPVGT
GFKGL--VHPSKQHNNIP--LETKKKNLFEGEMR--DILFHHKKLFDSCLSKNFHDIPEQSFIG-
-----FNDSMTRRYWNINLEEMMEAGVHFGHGTRKWNPKMAPYISAKRKGIHITNLTRTARF
LSEACDLVFDAASRGKQFLIVGTKNKAADSVEWAAIRARCHYVNKKWLGGMLTNWSTT
ETRLHKFRDLRMEQKTGRLNRLPKRDAAMLKRQLSRLQTYLGGIKYMTGVPDIVIIVDQ
HEEYTALRECITLGIPTICLTDTNCDPDLADISIPANDDAISSIRLILNKLVFAICEGRS-SYIRN
PMGQKINPLGFRLGTTQGHHSLWFSQPKNYSEGLQEDQKIRDCIKNYVQKNMRTSSG-VE
GIARIEIQKRIDLIQVIIFMGFPKLLIESRPR----GIEELQTTLQKEFHCVNRKLNIAVTRIAKPY
GNPNILAEFIAGQLKNRVSFRKAMKKAIELTEQADTKGIQIQIAGRIDGKEIARVEWIREGR
VPLQTIRAKIDYCSYTVRTIYGVLGIKIWIFLDEEMSRYRGPRFKKIRRLGALPGLTNKKPR
NGSDLRNQSRSGK-KSQYRIRLEEKQKLRFHYGLTERQLLKYVRIARKAKGSTGQVLLQL
LEMRLDNILFRLGMASTIPAARQLVNHRHILVNGRIVDIPSYRCKPRDIITAKDEQKSRALIQ
ISLDSSPH-EELPNHLTLHPFQ--YKGLVNQIIDSKWVGLKINELLVVEYYSRQTMSRRGTAE
KKTAKSDPIYRNRLVNMLVNRILKHGKKSLAYQIIYRAVKKIQQKTETNPLSVLRQAIRGV
TPDITVKARRVGGSTHQVPIEIGSTQGKALAIRWLLAASRKRPGRNMAFKLSSELVDAAK
GSGDAIRKKEETHRMAEANRAFAHFRMGRDTIAEIITSIRNADMDRKRVVRIASTNITENI
VQILLREGFIENVRKHREKNKYFLVLTLRHRR--NRKRPY---RNILNLKRISRPGLRIYSNYQ
RIPRILGGMGIVILSTSRGIMTDREARLEGIGGEILCYIWMAKAIPKISSRRNGRIGSRKGAR
RIPKGVIHVQASFNNTIVTVTDVRGRVVSWSSAGTSGFKGTRRGTPFAAQTAAANAIRTVV
DQGMQRAEVMIKGPGLGRDAALRAIRRSGILLTFVRDVTPMPHNGCRPPKKRRVMPTIKQ
LIRNTRQPIRNVTKSPALRGCPQRRGTCTRVYTITPKKPNSALRKVARVRLTSGFEITAYIPGI
GHNLQEHSVVLVRGGRVKDLPGVRYHIVRGTLDAVGVKDRQQGRSKYGVKKPKMARKS
LIQREKKRQKLEQKYHSIRRSSKKEIS---KVPSLSDKWEIYGKLQSPPRNSAPTRLHRRCFL
TGRPRANYRDFGLSGHILREMVHACLLPGATRSSWMVKNSVISVISQE--EK---RGSVEFQV
FNFTNKIRRLTSHLELHKKDYLSQRGLKKILGKRQRLLAYLSKKNRVRYKELINQLDIRET
KTR--MVKLRLKRCGRKQRAVYRIVAIDVRSRREGKDLRKVGFYDPIKNQTYLNVPAILYF
LEKGAQPTGTVQDILKKAEVFKELRPNQS----MDKSKRPFLKFKRSFRRRLPPIQSGDRIDY
RNMSLISRFISEQGKILSRRVNRLTLKQQRLITLAIKQARILSLLPFLNNEK--QFERTESTART
TGFKARNKVTRSLKKNPFVANHLLKKIDKLNTKAEKEIIVTWSRASTIIPTMIGHTIAIHNG
KEHLPIYITDSMVGHKLGEFAPTLNFRGHAKSDNRSRRMIFQSFLLGNLVSLCMKIINSVV
VVGLYYGFLTTFSIGPSYLFLLRALVMEEG---TEKKVSATTGFITGQLMMFISIYYAPLHLAL
GRPHTITVLALPYLLFHFFWNNHKHFFDYGSTTRNSMRNLSIQCVFLNNLIFQLFNHFILPS
SMLARLVNIYLFRCNSKILFVTSGFVGWLIGHILFMKWLGLVLVWIRQNHSIRSYIRSNKYL
VLELRNSMARIFSILLFITCVYYLGRIPSPILTKKLKETEEDQPLVSLLFDYNRWNRPFRYIK
NNRFEQAIRTEMSQYFFNTCKSDGKQRISFTYPPSLSTFWKMIKRRIPLLSLQKTLPNELDN
QWISTNKEKSN-LNKEFLNRLEVLDKESFSLDILETRTRLCNDDTKKEYVPKMYDPLLNGP
YRGTIKKKFSPKERVRINRIHTIFYQELEQKAPNGTSIGIKEISKKIPRWSHKLIELEQQS-GQI
RSRKAKRVVIFTAALIRYSQQPDFRRGIIKGSMRAQRRKTVIWKLFQANVHSPLFLDRITPP
FLFSFDI--SGLIKPIFRNRKEKARIEIAEAWDTIPFAQIIRGYMLITQSILRKYIVLPSLIIAKNL
GRMLVLQLPEWSEDLQEWNREMHIKCTYNGVQLSETEFPKNWLKDGIQIKILFPFCLKPW
HISKKKQKDDFCFLTVWGMEAELPFGSPRKRPSFFEPIFKELEKKIGKFKKKYFIT-LKVFK

GKIKLFRRISKETKKWLIKSSL--FIKKMKKELSKVNDSLMSNQIINESFSQIESGNWPNSSLI
ESKMKDLTDRTSTIKNQIERITKEKKKSPNK-TNNIKKFESPKKIFQILKRRNTRLIWKFHYF
LKLFIQRLYIDLFLSIINIPRINTQLF--FISFDLSYLSQAYVFYKLSQPQVINLSKLRSVLQYTS
FFLKTKIKDYFTLGIFHSELKHKKLQSYRINQWKNWLRRHYQYDLSQIRWSRLMPQKWR
NRVNQGCMAQNRNKWNSYEKDQ--LIHYKKE-NFQKCYRYDLLAYKSINGNILYLDRKYF
DWKIIHFSLRFFDWMGMNEKILNRWFFPEFVPLYNVYKIKPWIIPSKLLLLNYKSNTEAEL
DLFLKRYLLFQLRWNDALNQRMIENIKVYCLLLRLINPSKIAISSIQRREMSLDIMLIQKNL
TLTELMK-GILIIEPIRLSVKNNGQFIMYQTIGISLVHKSKHNHFDLLVPENILWSRRRRELRI
RSLFRFLWPNYRLEDLACMNRYWFDTNNGSRFSILRIHMYPMRGHQFKSWIFELREILREI
KNSHHFLDSWTQFNSAGSFIHIFFHQERFLKLFDPRIWSILLS-RNS--QGSTSNRYFT---IKG-
VILFVVAVLIYR----INNRNMVERKNLYLIGLLP-----IPMNSIGPRNDTLEESV-GSSNINRLIV
S-LLYLPKGKKISESCFLNPKESTWVLPIT----KKCSMPESNWGSRWWRNWIGKKR--DSSC
KISN---ETVAGIEILFKEKDLK-YLEFLFVYYMD--DPIRKDHDWELFDRLSLRKSRNRINL--
NSGPLFEILVKHWISYLMSAFREKIPIEVEG-FFKQQG-AGSTIQSNDIEHVSH--LFSRNKWAI
SLQNCAQFHMWQFRQDLFV---SWGK-NPPESDFLRN-VSRENWIWLDNVWLVNKDR---FF
S--KVQNVSSNIQYDS---TR--SSFVQVTDSSQLKGSS-DQSRDHLDSIS----NEDSE------------
YHTLINQREIQQRKERSIL-WD--PSFLQTERKEIESG--RFPK--CLSGYS--SMSRLFT-EREKQ
MINHLFPEEIEEFL--GNPTRSVRSFFSDRW--SELH-LGSNPTERSTRDQ----KLLKKQQDLS-F
VPSKRSE----NKEMVNIFKIITYLQN--TVSIHPISSDPGCDMVP-KDEPD-MDSSNKISFLNKN
PFFDL--FHLFHDRNRGGYTL--HYDFESE--ERFQEMADLFTLSITEP-DLVYHKGFAFSIDSC
GLDQKQ--FLNEARDE---SKKKS---LLVLPPIFY--EENESFSRRIRKKWVR-ISCGNDLEDPKP
KIVVFASNNIMEAVTQYRLIRNLIQIQYSTYG--YIRNVLNRFFLMN---RSDRNFEYG---IQRD
QIGKDTLNHRTIMKYTINQYLSNLKK-SQKKWFEPL--ILISRTERSMNRDPDAYRYKWSNG
SKNFQEHLEQSVSEQKSR---FQVVFDRLRINQYSIDWSEVIDKKDLSKPLRFFLSK---------S
LLFLS-------KLLFFLSN-SLPFFCVSFGNIPIHRSE-IYIY-ELKGPNDQLCNQLLESIGLQIVHL
KKWKPF--LLD---DHDTS-QKSKFLINGGTISPFLFNKIP-KW-MIDSFHTR--NNRRKSFDNPD
SYFSMIFHDQDNWLNPVKPFHRSSLISSFYKANRLRFLNNPHHFCFY--WNTRFPFSV----EK
ARIN--NSDFTYG-QFLNILFIRNK-IFSLCVGK--KKHAFWG-RDTISPIESQVSNIFIPNDFPQS
G-DETYNLYKS---FHFPSRSDPFVR--RAIYSIADISGTPLTEGQIVNFERTYCQPLSDMNLSDS
EGKNLHQYLN--FNSNMGLIHTPCSEKDLSSEK--RKK--------RSLCLK--KCVEKGQMYRTF
QRDSAFSTLSKWNLFQTYMPWFLTSTGYKYLNLIFLDTFSDLL--PILSS-SQKFVPIFHDIM
HGSGISWRILQKKL------CLPQWNL-ISEISSKCLHN--LLLSEEMIHRN--NESPLISTHLRSPN
AREF-LYSILFLLLVAGY-LVRTHLLFVSR--ASSELQTEFEKVKSLMIPSSMIELRKLLDRYPT
SEPNSFWLKNLFLVALEQLGDSLEEIRGSASGGNMLG---PA-----YG-VKSIRSKKKDWNINL
IEIIDLI----PNPINRITFSRNTRHLSHTSKEIYSLIRKRKNVNGDWIDDKIESWVANSDSIDDE
EREFLVQFS--TLTT--ENRIDQILLSLT-HSDRL-----SKNDSGYQMIEQPGAIYLRYLVDIH--KK
HLMNYEFNPSCLAERRIFLAHYQTITY--SQTSCGENSFHFPSHG---KPFSLRLALSPS-RGILV
IGSIGTGRSYLVKYLATNSYVPFITVFLNKFLDNKPKGFLLD-----EID-IDDSDDIDDSDNLD
ASD-----------------DIDRDLDTEL-KLLTRMNGLTMDMMPE---IDRFYITLQFELAKAMSPCII
WIPNIHDLDVNESNDLALGLLVNHLSR---DCERCS-TRNILVIASTHIPQKVDPALIAPNKLN
TCIKIRRLLLPQQRKHFFTLSYTRGFHLEKKMFHT--NGFGSITMGSNARDLVALTNEVLSISI
TQKKSIIDTNTIRSALHRQTWDLR---SQVRSVQDHGILFYQIGRAVAQNVLLSNCPIDPISIY
MKKK--SCNEGDSYLYKWYFELGTSMKRLTILLYLLSCSAGSVAQDLWSLS--GPDEK-NGI
TSYGLVENDS-DLVHGLLEVEGALVG--SSRTEKDCSQFDND--RVTLLLRPEPRN-PLDMMQ

NGSCSILDQRFLYEKY--ESEFEE-GEGEGALDPQ-------ED----LFNH--IVWAP--RIWRP---WG
FLFDCIERPNELGFP-YWSRSFRGK---RIIYDEEDELQENDSEFLQSGTMQYQTRDRSSKEQ
GLFRISQFIWDPADPLFFLFK-DQPPGSVFSHRELFADEEMSKGLLTSQTDPPTSIYKRWFI--
KNTQEKHFELLINRQRWLR---TNSSLS--NGSFRSN--TLSESYQYLSNLFLSN-GTLLDQMT
KTLLRKRWLFPDEMKIGF--M---------MPRSR-INGNFIDKTFSIVANILLRVIPTTSGEKEAFT
YYRDG--MSAQSEGNYAEALQNYYEAMRLEIDPYDRSYILYNIGLIHTSNGEHTKALEYYF
RALERNPFLPQAFNNMAVICHY-RGEQAIQQGDSEIAEAWFDQAAEYWKQAIALTPGNYIE
AHNWLKITRRFE--MTWRSEHIWIELITGSRKISNFCWAFILFLGSLGFLLVGTSSYLGRNLIS
FFPPQQIIFFPQGLVMSFYGIAGLFISSYLWCTISWNVGSGYDRFDRKEGIVCIFRWGFPGK
NRRIFLRFLIKDIQSVRIEVKEGISARRVLYMDIRGQGSIPLTRTDEN-LTPREIEQKAAELAY
FLRVPIEVF
>Spinacia
MKKWWFNSMLSLSKSMDSLGPIENTSTKEDPSLNDPEKQIHSYSNVNHLVRGIDIQNFISD
DTFLLIDTKGDSYSIYFDIENQIFEIDNDHS--FLSKLQSSFSNYWNSSYLNSRSKNGDTYNG
HSLYYTNDSWNNHINNCIDSYLH--SQIRSDSSILSGNDSYILSYIFN----NRSESFSKRSNGSN
LTRRESS-NLDVTQKYRHLWVQCESCYALNYKKLLKSKMGICEQCGYHLKISSSDRIELLI
DPGTWNPMDDDMVSMDPIGFHSEEEAYKDRIDSYQIKTGLTEAVQTGIGQLNGIPVAIGV
MDFQFMGGSMGSVVGEKITRLIEYASNKFIPLIIVCASGGARMQEGSLSLMQMAKISSVLY
DYQSNKKLFYVSILTSPTTGGVTASFGMLGDIIIAEPNAYIAFAGKRVIEQTLNKTVPEGSQ
AAEFLFHKGLFDPIVPRNLLKGVLSELFELHAFFPLNQNKGEMATIRADEISKIIRERIEGYN
REVKVVNTGTVLQVGDGIARIHGLDEVMAGELVEFEEGTIGIALNLESNNVGVVLMGDG
LMIQEGSSVKATGRIAQIPVSEAYLGRVINALAKPIDGRGEITASESRLIESPAPGIMSRRSVY
EPLQTGLIAIDAMIPVGRGQRELIIGDRQTGKTAVATDTILNQQGQNVICVYVAIGQKASSV
AQVVTNFQERGAMEYTIVVAETADSPATLQYLAPYTGAALAEYFMYRERHTLIIYDDLSK
QAQAYRQMSLLLRRPPGREAYPGDVFYLHSRLLERAAKLSSLLGEGSMTALPIVETQAGD
VSAYIPTNVISITDGQIFLSADLFNAGIRPAINVGISVSRVGSAAQIKAMKKVAGKLKLELAQ
FAELEAFAQFASDLDKATQNQLARGQRLRELLKQPQSAPLTVEEQVMTIYTGTNGYLDSL
ELDQVRKYLVELRTYVKTNKPEFQEIISSTKTFTEEAEALLKEAIQEQMERFLLQEQAMRI
NPTTSDPGVSTLEKKNLGRIAQIIGPVLDVAFPPGKMPNIYNALIVKGRDTAGQPMNVTCE
VQQLLGNNRVRAVAMSATDGLTRGMEVIDTGAPLSVPVGGATLGRIFNVLGEPVDNLGPV
DTRTTSPIHRSAPAFTQLDTKLSIFETGIKVVDLLAPYRRGGKIGLFGGAGVGKTVLIMELI
NNIAKAHGGVSVFGGVGERTREGNDLYMEMKESGVINEQNIAESKVALVYGQMNEPPGA
RMRVGLTALTMAEYFRDVNEQDVLLFIDNIFRFVQAGSEVSALLGRMPSAVGYQPTLSTE
MGSLQERITSTKEGSITSIQAVYVPADDLTDPAPATTFAHLDATTVLSRGLAAKGIYPAVDPL
DSTSTMLQPRIVGEEHYEIAQRVKETLQRYKELQDIIAILGLDELSEEDRLTVARARKIERFL
SQPFFVAEVFTGSPGKYVGLAETIRGFQLILSGELDSLPEQAFYLVGNIDEATAKAMNLEM
ESKLKKMTLNLCVLTPNRSIWNSEVKEIILSTNSGQIGVLPNHAPTATAVDIGILRIRLNDQ
WLTLALMGGFARIGNNEITILVNDAERGSDIDPQEAQQTLEIAEANLRKAEGKRQKIEANL
ALRRARTRVEASNTISMKNVTDSFVFLGHWPSAGSFGFNTDILATNLINLSVVLGVLIFFGK
GVLSDLLDNRKQRILNTIRNSEELRGKAIEQLEKARARLKKVEMDADQFRVNGYSEIERE
KMNLINSTYKTLEQFENYKNETIQFEQQKAINQVRQRVFQQALQGALGTLNSCLNNELHL
RTINANIGMFGAMNEITDMNPLIAAASVIAAGLAVGLASIGPGVGQGTAAGQAVEGIARQP
EAEGKIRGTLLLSLAFMEALTIYGLVVALALLFANPFVMNVLSYSINPLK-LYAISGVEVGQ
HFYWQIGGFQIHGQVLITSWVVIAILLGSAAIAVRSPQTIPTGGQNFFEYVLEFIRDVSKTQI

GEEYRPWVPFIGTMFLFIFVSNWSGALLPWKIIQLPHGELAAPTNDINTTVALALLTSVAYF
YAGLTKKGLGYFGKYIQPTPILLPINILEDFTKPLSLSFRLFGNILADELVVVVLVSLVPLVVP
IPVMFLGLFTSGIQALIFATLAAAYIGESLEGHHMIFSTLEHILTHISFSTVSVVITLHLITLLV
NEIVGLYNSLEKGMLVTFFCITGLLVTRWVYWKHFPLSDLYESLIFLSWSFYLIHMIPS--FL
KKKNSLITAPSAIFTQGFATSGLLTEMHQSGILVPALQSQWLMMHVSMMVLGYAALLGGS
LLSVTLLIIIFQKDIQVFDKRKEIQYINEKSNIVQ-ASPSYVRNYYRSQLIEQLDHWSYRVISL
GFIFLTIGILSGAVWANEAWGSYWNWDPKETWAFITWTIFAIYLHIRTNKNLRGANSAIVAF
IGFLIIWICYFGVNLLGIGLHSYGSFTLTMEKKKVFIPFLYLISIVFLPWWIYLSFQKSLESWV
TTWWNTKQSETFLNDIQEKKLLEKFIELEELRLLDEMIKEYPETQLQKLGIGIHNETIQLIK
MHNEDCIHMILHFSTNLICFLILGGYSILGNKELILLNSWVQEFLYNLSDTIKAFSILLVTDL
CIGFHSPQGWELLIESIYKDFGFADNDQIISSLVSTFPVILDTILKYWIFRSLNRVSPSLVVIYH
SMNDMPIGVPKVPFRSPGEEDASWVDVYNRLYRERLLFLGQEVDSEISNQLIGLMVYLSIE
DDTKDLYLFINSPGGWVIPGVAIYDTMQFVRPDVHTICMGLAASMGSFILVGGEITKRLAF
PHARVMIHQPASSFYEAQTGEFLLEAEELLKLRETLTKVYGQRTGKPLWVVSEDMERDVF
MSATEAQAHGIIDLVAVEMKEQKWIHEGLITESLPNGMFWVRLDNEDPILGYVSGRIRRSSI
RILPGDRVKIEVSRYDSTRGRIIYRLRNKDSNDMEEFQRHIE-DRSWQHNFLYPLIFQEYIYA
FAYDH--SGNSK-YSLLLVKRLITRMYQQNHLILSANDSNQNVILGKNLYSQMITEGFAVIVE
VPFSLLLISSLIVKSHNLRSIHSIFPFLEDKLLHLNYVLDILITYPAHLEILVQTLRYWVKDAS
SLHLLRFFLYEYRN--LNSLELISFLKKQRLFLFLYNLHVCEYESLFVFLCNQSSYLRPTSFG
ALIERIYFCGKLKYVK-VFTKDFILWIFREPFPHYVRYQGKSILASKGTSLLMHKWKYYLIY
FGQCHFSVWSQPKRLYINRLSNHSLDFMGFLSRVRLNSSVIRSQMLKNSFLIENIS--KKFDT
VVPIIPLVGSLAKAKFCNVLGHPISKSVWTDLSDSDILDRFGRICRNISHYYSGSSRKKSLYR
IKYILRLSCARTLSRKHKSTVRAFLKRLGSEFLEEFFTEEEKALSLILPRRIWYLDIICIHNLA
NDEMIIDTTTTKVQAINSFSRLE-FLKEVYETIWMLFPILILVLGITIGVLVIVWLEREISASIQ
QRIGPEYAGPLGILQALADGTKLLFKENLLPSRGDTYLFSIGPSIAVISILLGYLIIPFGSRLVL
ADLSIGVFLWIAVSSIAPIGLLMSGYGSNNKYSFLGGLRAAAQSISYEIPLTLCVLSISLLSNS
SSTVDIVEAQSKYGFWGWNLWRQPIGFIVFIISSLAECERLPFDLPEAEEELVAGYQTEYSGI
KFGLFYVASYLNLLISSLFVTVLYLGGWNLSIPYIFISEFFEINKIDGVFGTTIGIFITLAKTFL
FLFIPITTRWTLPRLRMDQLLNLGWKFLLPISLGNLLLTTSSQLFSLMIWHVQNENFILDST
RIFMKAFHLLLFDGSLIFPECILIFGLILLLMIDSTSDQKDIPWLYFISSTSLVMSITALLFRWR
EEPMISFSGNFQTNNFNEIFQFLILLCSTLCIPLSVEYIECTEMALTEFLLFILTATLGGMFLCG
ANDLITIFVAPECFSLCSYLLSGYTKKDVRSNEATTKYLLMGGASSSILVHGFSWLYGSSGG
EIELQEIVNGLINTQMYNSPGISIALIFITVGIGFKLSPAPSHQWTPDVYEGSPTPVVAFLSVT
SKVAASASATRIFDIPFYFSSNEWHLLLEILAILSMILGNLIAITQTSMKRMLAYSSIGQIGYV
IIGIIVGDSNDGYASMITYMLFYISMNLGTFACIVLFGLRTGTDNIRDYAGLYTKDPFLALSL
ALCLLSLGGLPPLAGFFGKIYLFWCGWQAGLYFLVLIGLLTSVLSIYYYLKIIKLLMTGRNQ
EITPHVRNYRRSPLRSKNSIEFSMIVCVIASTIPGISMNPIITIAQDTLFMFLLYEYDIFWAFLII
SSVIPILAFLFSGILAPISKGPEKLSSYESGIEPMGDAWLQFRIRYYMFALVFVVFDVETVFL
YPWAMSFDILGVSVFIEALIFVLILIVGLVYAWRKGALEWSTNSFPWLTTIVVLPIFAGSLIFL
FPHRGNKVIRWYTICISMIELLLMTYVFFYHFQPDDPLIQLVEDYKWINFFDFHWRLGIDG
LSIGPILLTGFITTLATLAAWPVTRNSQLFHFLMLAMYSAQIGLFSSRDLLLFFIMWELELIP
VYLLLSMWGGKKRLYSATKFILYTAGGSIFLLMGVLGVGLYGSNEPTLNLETLVNQSYPV
ALEIIFYIGFFIAFAVKLPIIPLHTWLPDTHGEAHYSTCMLLAGILLKMGAYGLVRINMELLP
HAHSIFSPWLMIIGTMQIIYAASTSPGQRNLKKRIAYSSVSHMGFIIIGISSITDTGLNGAILQI

ISHGFIGAALFFLAGTSYDRIRLVYLDEMGGIAIPMPKIFTLFSSFSMASLALPGMSGFIAELI
VFFGLITSQKYLLIPKLLITFGMAIGMILTPIYLLSMSRQMFYGYKLFNISNSSFFDSGPRELF
VSTSIFLPVIGIGVYPDLVLSLSVEKVEAILSNYFYRMILEHVLVLSAFLFSIGIYGLVTSRNL
VRALMCLELILNAVNLNFVTFSDFFDSRQLKGNIFSIFVIAIAAAEAAIGPAIVSSIYRNRKSI
RINQSNLLNKMEHIYQYAWIIPFLPLPVPLLIGAGLLFFPTATKNLRRIWAFSSISLLSIVMIFS
MKLAIQQINSNSIYQYLWSWTINNDFSLEFGYLMDPLTSIMSMLITTVAILVLIYSDNYMSH
DQGYLRFFAYMSFFNTSMLGLVTSSNLIQIYIFWELVGMCSYLLIGFWFTRPIAANACQKA
FVTNRVGDFGLLLGILGLYWITGSFEFRDLFEIFNN-EVNSLFCILCAFLLFAGAVAKSAQFP
LHVWLPDAMEGPTPISALIHAATMVAAGIFLVARLLPLFVVIPYIMYVISFIGIITVLLGATLA
LAQKDIKRSLAYYTMSQLGYMMLALGMGSYRTALFHLITHAYSKALLFLASGSLIHSMGT
IVGYSPDKSQNMVLMGGLTKHVPITKTSFLIGTLSLCGIPPLACFWSKDEILNDSWVYSPIF
AIIAYFTAGLTAFYMFRIYLLTFEGHLNFFCKNYSGKKSSSFYSISLWGKKELKTINQKLLNL
LTMNFFSFENKNISLYPYESDNTMLFPLIILIMFTLFVGFIGIPFDLDILTKWLTPSINLLHSNS
ENFVDWYEFVINAIFSISIAFFGIFIAFFFYKPIYSSLKNFDLINSFDILGDNIITIIYNWSANRG
YIDAFYSTFLIKGIRSLSELVSFFDRRIIDGIPNGFGVTSFFVGEGIKYVGGG-RISSYLFWYLL
YVSIFLFIFTMDLPGPIHDFLLVFLGSGLILGALGVVLFTNPIFSAFSLGLVLVCISLFYILANS
HFVASAQLLIYVGAINVLIIFSVMFMSGPEYDKKFQLWTVGDGVTSLVCISLFVSLISTILNT
SWYGIIWTTKSNQILEQDLINASQQIGIHLSTDFFLPFELISIILLVSLIGAIAVARQMAVPTTR
KDLMIVNMGPHHPSMHGVLRLIVTLDGEDVIDCEPIVGYLHRGMEKIAENRTIIQYLPYVT
RWDYLATMFTEAITVNGPEQLGNIQVPKRASYIRVIMLELSRIASHLLWLGPFMADIGAQT
PFFYILRERELIYDLFEAATGMRMMHNYFRIGGVAADLPYGWIDKCLDFCDYFLIGLTEYQ
KLITRNPIFLERVENVGIIGGEEAINWGLSGPMLRASGIQWDLRKVDHYECYDEFDWEVQ
WQKEGDSLARYLIRIGEMAESVKIIQQALEGIPGGPYENLEIRRFNRIKYPEWNDFEYRFIS
KKPSPAFELSKQELYVRVEAPKGELGIFLIGDQSVFPWRWKIRPPGFINLQILPQLVKKMKL
ADIMTILGSIDIIMGEVDRMFPMVTGFINYGQQTIRAARYIGQSFMITLSHANRLPVTIQYP
YEKLITSERFRGRIHFEFDKCIACEVCVRACPIDLPVVDWKLETDIRKKRLLNYSIDFGICIF
CGNCVEYCPTNCLSMTEEYELSTYDRHELNYNQIALGRLPISITDDYTIRTILNS--------------
--MQGRLSAWLVKHGLVHRSLGFDYQGIETLQIKPEDWHSIAVILYVVYGYNYLRSQCAYDV
APGGLLASVYHLTRIEYGVDQPEEVCIKVFAPRRNPRIPSVFWVWKSADFQERESYDMFGI
SYDNHPRLKRILMPESWIGWPLRKDYIVPNFYEIQDAY--
--------------MNSIEFPLLDQIAQNSVISTTSNDLSNWSRLSSLWPLLYGTSCCFIEFASLIGSRF
DFDRYGLVPRASPRQADLILTAGTVTMKMAPSLLRLYEQMPEPKYVIAMGACTITGGMFS
TDSYSTVRGVDKLIPVDVYLPGCPPKPEAVIDAITKLRKKISREIYEDRIKS--KNRCFTINHK
FRVGRSIHTGNYDQALLYKSPSTSEIPPETFFKYKNAASSRELVNMQTINTFSWIKEQITRSI
SISLILYIITRSSIANAYPIFAQQGYENPREATGRIVCANCHLANKPVDIEVPQAVLPDTVFEA
VVRIPYDMQLKQVLANGKKGGLNVGAVLILPEGFELAPPDRISPEMKEKMGNLSFQSYRP
NKQNILVIGPVPGQKYSEITFPILAPDPATKKDVHFLKYPIYVGGNRGRGQIYPDGSKSNNT
VYNSTATGIVKKIVRKEKGGYEINIADASDGREVVDIIPRGPELLVSEGESIKLDQPLTSNPN
VGGFGQGDAEVVLQDPLRIQGLLFFFASVILAQIFLVLKKKQFEKVQLSEMNFMSKVYDW
FEERLEIQAIADDITSKYVPPHVNIFYCLGGITLTCFLVQVATGFAMTFYYRPTVTDAFASVQ
YIMTEVNFGWLIRSVHRWSASMMVLMMILHVFRVYLTGGFKKPRELTWVTGVVLGVLTA
SFGVTGYSLPWDQIGYWAVKIVTGVPDAIPVIGSPLVELLRGSASVGQSTLTRFYSLHTFVL
PLLTAVFMLMHFLMIRKQGISGPLMGVTKKPDLNDPVLRAKLAKGMGHNYYGEPAWPN
DLLYIFPVVILGTIACNVGLAVLEPSMIGEPADPFATPLEILPEWYFFPVFQILRTVPNKLLGV

LLMASVPAGLLTVPFLENVNKFQNPFRRPVATTVFLVGTVVALWLGIGATLPIDKSLTLGLF
MIEVFLFGIVLGLIPITLAGLFVTAYLQYRRGDQLDLMSTLTSYFGFLLAALTITSALFIGLN
KIRLIMDIVSLAWAALMVVFTFSLSLVVWGRSGLMIIRSPEPEVKILVDRDPVKTSFEAWAK
PGHFSRTIAKGPETTTWIWNLHADAHDFDSHTSDLEEISRKIFSAHFGQLSIIFLWLSGMYF
HGARFSNYEAWLSDPTHIGPSAQVVWPIVGQEILNGDVGGGFRGIQITSGFFQIWRASGITS
ELQLYCTAIGALVFAALMLFAGWFHYHKAAPKLAWFQDVESMLNHHLAGLLGLGSLSW
AGHQIHVSLPINQFLNAGVDPKEIPLPHELILNRDLLAQLYPSFAEGATPFFTLNWSKYADF
LTFRGGLDPVTGGLWLTDTAHHHLAIAILFLIAGHMYRTNWGIGHGLKDILEAHKGPFTG
QGHKGLYEILTTSWHAQLALNLAMLGSLTIVVAHHMYAMPPYPYLATDYGTQLSLFTHH
MWIGGFLIVGAAAHAAIFMVRDYDPTTRYNDLLDRVLRHRDAIISHLNWACIFLGFHSFGL
YIHNDTMSALGRPQDMFSDTAIQLQPVFAQWIQNTHALAPSATAPGATASTSLTWGGSDLV
AVGGKVALLPIPLGTADFLVHHIHAFTIHVTVLILLKGVLFARSSRLIPDKANLGFRFPCDGP
GRGGTCQVSAWDHVFLGLFWMYNSISVVIFHFSWKMQSDVWGSISDQGVVTHITGGNFA
QSSITINGWLRDFLWAQASQVIQSYGSSLSAYGLFFLGAHFVWAFSLMFLFSGRGYWQELI
ESIVWAHNKLKVAPATQPRALSIVQGRAVGVTHYLLGGIATTWAFFLARIIAVGMALRFPRF
SQGLAQDPTTRRIWFGIATAHDFESHDDITEERLYQNIFASHFGQLAIIFLWTSGNLFHVAW
QGNFESWVQDPLHVRPIAHAIWDPHFGQPAVEAFTRGGALGPVNIAYSGVYQWWYTIGL
RTNEDLYTGALFLLFLSVISLLGGWLHLQPKWKPSVSWFKNAESRLNHHLSGLFGVSSLA
WTGHLVHVAIPGSRGEYVRWNNFLDVLPHPQGLGPLFTGQWNLYAQNPDSSSHLFGTSQ
GAGTAILTLLGGFHPQTQSLWLTDMAHHHLAIAFVFLVAGHMYRTNFGIGHSMKDLLEAH
IPPGGRLGRGHKGLYDTINNSLHFQLGLALASLGVITSLVAQHMYSLPAYAFIAQDFTTQAA
LYTHHQYIAGFIMTGAFAHGAIFFIRDYNPEQNEDNVLARMLDHKEAIISHLSWASLFLGF
HTLGLYVHNDVMLAFGTPEKQILIEPIFAQWIQSAHGKTSYGFDVLLSSTSGPAFNAGRSI
WLPGWLNAVNE--NSNSLFLTIGPGDFLVHHAIALGLHTTTLILVKGALDARGSKLMPDKK
DFGYSFPCDGPGRGGTCDISAWDAFYLAVFWMLNTIGWVTFYWHWKHITLWQGNVSQF
NESSTYLMGWLRDYLWLNSSQLINGYNPFGMNSLSVWAWMFLFGHLVWATGFMFLISW
RGYWQELIETLAWAHERTPLANLIRWRDKPVALSIVQARLVGLAHFSVGYIFTYAAFLIAS
TSGKFGMSHSVKIYDTCIGCTQCVRACPTDVLEMIPWDGCKAKQIASAPRTEDCVGCKRC
ESACPTDFLSVRVYLWHETTRSMGLGY---MNFPSIFVPLVGLVFPAIAMASLFLYVQKNKIV
MRDFKTYLSVAPVLSTLWFGSLAGLLIEINRFFPDALTFPFFSFMTAILERRESESLWGRFCN
WITSTENRLYIGWFGVLMIPTLLTATSVFIIAFIAAPPVDIDGIREPVSGSLLYGNNIISGAIIPT
SAAIGLHFYPIWEAASVDEWLYNGGPYELIVLHFLLGVACYMGREWELSFRLGMRPWIAV
AYSAPVAAATAVFLIYPIGQGSFSDGMPLGISGTFNFMIVFQAEHNILMHPFHMLGVAGVF
GGSLFSAMHGSLVTSSLIRETTENESANEGYRFGQEEETYNIVAAHGYFGRLIFQYASFNNS
RSLHFFLAAWPVVGIWFTALGISTMAFNLNGFNFNQSVVDSQGRVINTWADIINRANLGM
EVMHERNAHNFPLDLAAIEAPSTNGMGLPWYRVHTVVLNDPGRLISVHIMHTALVAGWA
GSMALYELAVFDPSDPVLDPMWRQGMFVIPFMTRLGITNSWGGWSIT-GGTITDPGIWSYE
GVAGAHIMFSGLCFLAAIWHWVYWDLEIFSDERTGKPSLDLPKIFGIHLFLSGVACFGFGA
FHVTGLYGPGIWVSDPYGLTGKVQPVSPAWGVEGFDPFVPGGIASHHIAAGTLGILAGLFH
LSVRPPQRLYKGLRMGNIETVLSSSIAAVFFAAFVVAGTMWYGSATTPIELFGPTRYQWDQ
GYFQQEIYRRVSAGLAENQSFSEAWSKIPEKLAFYDYIGNNPAKGGLFRAGSMDNGDGIA
VGWLGHPIFRDKEGRELFVRRMPTFFETFPVVLIDGDGIVRADVPFRRAESKYSVEQVGV
TVEFYGGELNGVSYSDPATVKKYARRAQLGEIFELDRATLKSDGVFRSSPRGWFTFGHASF
ALLFFFGHIWHGSRTLFRDVFAGIDPDLDVQVEFGAFQKIGDPTTRRQGVMKTLYSLRRFY

PVETLFNGTLTLAGRDQETTGFAWWAGNARLINLSGKLLGAHVAHAGLIVFWAGAMNLF
EVAHFVPEKPMYEQGLILLPHLATLGWGVGPGGEVIDTFPYFVSGVLHLISSAVLGFGGIY
HALLGPETLEESFPFFGYVWKDRNKMTTILGIHLILLGIGAFLLVFKALYFGGVYDTWAPG
GGDVRKITNVTLSPSIIFGCLLKSPFGGEGWIVSVDDLEDIIGGHVWIGVICILGGIWHILTK
PFAWARRALVWSGEAYLSYSLAALSVFGFIACCFVWFNNTAYPSEFYGPTGPEASQAQAFT
FLVRDQRLGANVGSAQGPTGLGKYLMRSPTGEVIFGGETMRFWDLRAPWLEPLRGPNGL
DLSRLKKDIQPWQERRSAEYMTHAPLGSLNSVGGVATEINAVNYVSPRSWLSTSHFVLGF
FLFVGHLWHAGRARAAAAGFEKGIDRDFEPVLSMTPLNMTIAVGKFTKDEKDLFDSMDD
WLRRDRFVFVGWSGLLLFPCAYFALGGWFTGTTFVTSWYTHGLASSYLEGCNFLTAAVST
PANSLAHSLLLLWGPEAQGDFTRWCQLGGLWAFVALHGAFALIGFMLRQFELARSVQLRP
YNAIAFSGPIAVFVSVFLIYPLGQSGWFFAPSFGVAAIFRFILFFQGFHNWTLNPFHMMGVA
GVLGAALLCAIHGATVENTLFEDGDGANTFRAFNPTQAEETYSMVTANRFWSQIFGVAFS
NKRWLHFFMLFVPVTGLWMSALGVVGLALNLRAYDFVSQEIRAAEDPEFETFYTKNILLN
EGIRAWMAAQDQPHENLIFPEEVLPRGNALMSGSTGERSFADIITSIRYWVIHSITIPSLFIA
GWLFVSTGLAYDVFGSPRPNEYFTESRQGIPLITGRFDSLEQLDEFSRSFMTIDRTYPIFTVR
WLAIHGLAVPTVSFLGSISAMQFIQRMATQTVESSSR-SRPKPTTVGALLKPLNSEYGKVAP
GWGTTPLMGVAMALFAVFLSIILEIYNSSVLLDGISMNMLTLKLFVYTVVIFFVSLFIFGFLS
NDPGRNPGREEMADTTGRIPLWIIGTVAGILVIGLVGIFFYGSYSGLGSSLMLNIFSLIC--LN
SALYSSSFFFGKLPEAYAFLSPIVDFMPVIPLFFFLLAFVWQAAVSFRTTQSNPNEQNVELNR
TSLYWGLLLIFVLAVLFSNYFFNMEVNILAFIATALFILVPTAFLLIIYVKTVSQNDMETATLV
AIFISGLLVSFTGYALYTAFGQPSQQLRDPFEEHGDMEALVYTFLLVSTLGIIFFAIFFREPPKI
STKK--MTIAFQLAVFALIATSSILLISVPVVFASPDGWSSNKNIVFSGTSLWLGLVFLVGILNS
LIS-------MSPQTETKASVGFKAGVKDYKLTYYTPEYETLDTDILAAFRVSPQPGVPPEEAG
AAVAAESSTGTWTTVWTDGLTNLDRYKGRCYHIEPVAGEENQYICYVAYPLDLFEEGSVT
NMFTSIVGNVFGFKALRALRLEDLRIPVAYVKTFQGPPHGIQVERDKLNKYGRPLLGCTIK
PKLGLSAKNYGRAVYECLRGGLDFTKDDENVNSQPFMRWRDRFLFCAEALYKAQAETGE
IKGHYLNATAGTCEDMMKRAVFARELGVPIVMHDYLTGGFTANTTLSHYCRDNGLLLHIH
RAMHAVIDRQKNHGMHFRVLAKALRLSGGDHIHSGTVVGKLEGERDITLGFVDLLRDDY
TEKDRSRGIYFTQSWVSTPGVLPVASGGIHVWHMPALTEIFGDDSVLQFGGGTLGHPWGN
APGAVANRVALEACVQARNEGRDLAREGNTIIREATKWSPELAAACEVWKEIKFEFPAMD
TV--MAIHLYKTSTSSTRNGAV--QVKSNPRNNLISGQRRCGKGRNARGIITARHRGGGHKRL
YRKIDFRRNEKDIYGKIVTIEYDPNRNAYICLIHYGDGEKRYILHPRGAIIGDTIVSGTEVPI
KMGNALPLTDMPLGTAIHNIEITLGRGGQLARAAGAVAKLIAKEGKSATLKLPSGEVRLIS
KNCSATVGQVGNVGVNQKRLGRAGSKRWLGKRPVVRGVVMNPVDHPHGGGEGRAPIG
RKSPTTPWGYPALGRRSRKRNKYSDNFIIRRRSKMIQPQTHLNVADNSGARELMCIRIIGA
S-NRRYARIGDVIVAVIKEAIPNTPLERSEVIRAVVVRTCKELKRDNGMIIRYDDNAAVIIDQE
GNPKGTRIFGAIARELRQK-FAKIVSLAPEVLMLSPKRTRFRKQHRGRMKGISYRGNRICFG
RYALQALEPAWITSRQIEAGRRAMTRNARRGGKIWVRIFPDKPVTVRPAETRMGSGKGSP
EYWVAVVKPGRILYEISGVAENIARRAVAIAASKMPIRTQFIISGMTRVKRGYIARRRRKKIR
FFASSFRGAHSRLTRTIAQQKIRALVSAHRDRDRQKRDFRRLWITRINAAIRE-RGVYYNYS
KFIHDLYKRQLLLNRKILAQIAILNPNCIYMIYNEIIKKEDCKKYLEIIFFKEITTRGYSISMS
VDKARRVIDQIRGRSYAETLMILELMPYRACYPIFKLIYSAAANASHNKQFNKANLIISKAE
V N K G I T L K K V K P R A R G R S Y M I K R P T C H I T I V L R D I T H F Y E V M - -
-???

??????MAVPKKRTSIYKKRIRKNIWKKKGYWAALKAFSLAKSLSTGNSKSFFVRKISNMAK
GKDVRVKVILECTGC-VRK--VNKG--GVSRYITQKNRHNTPSRLELRKFCPYCYKHTIHGEI
KKMKIRASVRPICEKCRLIRRRGRIIVICSNPKHKQRQGMVREKIRVSTQTLQWKCVESRT
DSKCLHYGRFILSPLMKGQADTIGIAMRRALLGEIEGTCITRAKSE--KIPHEYSTILGIQESV
HEILMNLKEIVLRSNLYGTCEASICVRGPRGVTAQDIILPPYVEIVDNTQHIASLTEPIDLCIG
LQLERNRGYHIKAPNNFQDGSF--PIDALFMPVRNVNHSIHSYGNGNEKQEILFLEIWTNGS
LTPKEALYEASRNLIDLLIPFLHAEEN---VNLEDNQ-HKVSLPLFTFHNRLA-EIRKNKK-KIA
LKFIFIDQLELPPRIYNCLKKSNIHTLLDLLNNSQEDLIKMKHFRIEDVKQIFGTLEKH--FVI
DLKNKRMLRDGN-EGMSTIPGFNQIQFEGFWRFIDQGLTEELSKFPKMEDTDQEIEFQLFV
ETYQLAEPLIKEKDAVYESLTYSSELYVSAGLIWKTRREMQEQTILIGNIPLMNSLGTFIVN
GIYRIVINQILQSPGIYYRSELDHNGISVYTGTIISDWGGRSELEIDRKARIWARVSRKQKISI
LVLSSAMGSNLREILDNVCYPEIFLSFLNDKEKKKIGSKENAILEFYQQFACVGGDPVFSES
LCKDLQKKFFQQRCELGRIGRRNMNRRLNLDIPENNTFLLPRDILAAADHLIGMKFGMGT
LDD--MNHLKHKRIRSVADLLQDQFGLALVRLENVVRGTISGAIR-HKLI-PTPQNLVTSTP---
-LTTTFESFFGLHPLSQVLDRTNPLTQIVHGRKLSYLGPGGLTGRTASFRIRDIHPSHYGRICP
IDTSEGINVGLIGSLAIHARIGPWG-SLESPYY---EISERSK--RVQMLYLSPSRDEYYMLASG
NSLALNQGIQEEQVVPARYRQEFLTIAWEQVHFRSIFSFQYFSIGASLIPFIEHNDANRALMS
SNMQRQAVPLSQSEKCIVGTGLERQVALDSGVLAIAEHEGKIIYTNTDKIVLLGNGNTVSIP
LVMYQRSNKNTCMHQKPQIPRGKCVKKGQILADGAATVGGELALGKNVLVAYMPWEGY
NFEDAVLISERLVYEDIYTSFHIRKYEIQTYVTSQG-PEKVTSEIPHLEAHLLRNLDKNGIVR
LGSWVETGDILVGKLTPQMAKE-SSYAPEDRLLRAILGIQVSTSKETCLKLPIGGRGRVIDV
RWIQKKG-GSSYNPETIHVYISQKREIKVGDKVAGRHGNKGIISRILLRQDMPYLQDGRPV
DMIFNPLGVPSRMNVGQIFECSLGLAGSLLDRHYRIAPFDERYEQEASRKLVFSELYEASK
QTANPWVFEPEYPGKSRIFDGRTGDPFEQPVIIGNPYILKLIHQVDDKIHGRSSGHYALVTQ
QPLRGRAKQGGQRVGEMEVWALEGFGVAHILQEMLTYKSDHIKARQEVLGTTIIGGTIPN
PEDAPESFRLLVRELRSLALELNHFLVSERNFQINRMEA-------MIDQYKHQQLRIGSVSPQQ
ISAWATKILPNGEIVGEVTKPYTFHYKTNKPEKDGLFCERIFGPIKSGICACGNYRVIGDEKE
DPKFCEQCGVEFVDSRIRRYQMGYIKLACPVTHVWYLKRLPSYIANFLDKPLKELEGLVY
CDF--SFARP-IAKKPTFLRLRGLFEYEIQSWKYSIPLFFTTQGFDTFRNREISTGAGAIREQLA
DLDLRTIIDYSFAEWKELGEE-GSTGNEWEDRKVGRRKDFLVRRMELVKHFIRTNIEPEWM
VLCLLPVLPPELRPIIQIDGGKLMSSDINELYRRVIYRNNTLTDLLSTSRSTPGELVMCQEKL
VQEAVDTLLDNGIRGQPMRDGHNKVYKSFSDVIEGKEGRFRETLLGKRVDYSGRSVIVVG
PSLSLHRCGLPREIAIELFQTFVIRGLIRQHLASNIGVAKRKIREKEPIVWKILQEVMQGHPV
LLNRAPTLHRLGIQAFQPILVEGRAICLHPLVCKGFNADFDGDQMAVHVPLSLEAQAEARL
LMFSHMNLLSPAIGDPISVPTQDMLIGLYILTSGNRRGICANRYNPWNH---KTYQNERIDDT
NYKSMK--EPFFCNFYDAIGAYRQKRIHLDSPLWLRWQLDQR-IIAS--KEAPIEVHYESLGT
YHEIYAHYLIIRSVKKEIIDIYIRTTVGHISLYREIEEAIQGFYQACS--------------MAERANLV
FHNKAIDGTAMKRLISRLIDHFGMAYTSHILDQLKTLGFQQATATSISLGIDDLLTIPSKGWL
VQDAEQQSLILEKHHHYGNVHAVEKLRQSIEIWYSTSEYLRQEMNPNFRMTDPYNPVHIM
SFSGARGNVSQVHQLVGMRGLMSDPQGQMIDLPIQSNLREGLSLTEYIISCYGARKGVVD
TAVRTSDAGYLTRRLVEVVQHIVVRRRDCGTIRGISVSPQNSTMP-ERILIQTLIGRVLADDI
YMGSRCIATRNQDIGVGLVNRFIT------LRTQLISIRTPFTCRSASWICRLCYGRSPTHGGLVE
LGEAVGIIAGQSIGEPGTQLTLRTFHTGGVFTGGTAEHVRAPSNGKIQFNEDLVHPTRTRHG
HPAFLCYIDLYVTIESDDILHNVNIPPKSFLLVQNDQYVESEQVIAEIRAGTSTL-NFKERVR

KHIYSDSEGE-MHWSTDVYHAPEFTYGNVHLLPKTSHLWVLSGKPYR--SSVVPFSLSKDQ
DQMNTH--SLSFEQIYISNPS--VTNDQ-VKDKLSDS-FSKK-EDRITDY-SELNRIG---HCNLIY
P----AKNLD--LLAKKRRNRFIIPFQGSQERKKELMSLSGISIEIPIN--GIFRKNSIFAYFDDPRY
RRKSSGITKYGTIEMHSIVKKED--LIEYR-GVKEFRPKYMKVDRFFFIPEEVHILAGSSSIMV
RNNSIIGVDTWITLNTRSRIGGVVRVERKKKKIELTIFSGDIHFPGETDKISRHSGILIPPSRK-
---NSKDSKN--LKKWIYVQR--ITPTKKK--YFVLVRPVVPYEITDGINLATLFPQDLLQERDN
VQLRVVNYILYGNGKVTRGISDTSIQLVRTCLVLNWNQDK---KGSSIEEARGSFVEVRTNG
MIQDFLKVNLVKPAISYISKRNDPS-SEKKEG---SDHTNMNPFYSIYIYPKT---KLQKSFNQN
QGTVRTLLGI---NKECQFFLILSSSNCFRIGP-----FKG------VKYPDPLIPIRNSFGPLGT--ALQI
ANFFS--FYYLIT--HNQIL-VTNYLQLDN---LKQTFQPFKFQ-YYLMDENGR--IYNPDPCSNII
FNPFKLNWYFLHYHFCEETSTKID-LGQFVCENVCITKKGT---HLKSGQVLIVQ-FDSVVIRS
AKPYLATPGATLHGHYGEIIYEGDTLVTFIYEKSRSGDITQGLPKVEQVLEVRSIDSISINLE
KRIDSWNERITRILGSPWGFLIGAELTIAQSRISLVNKIQKVYRSQGVQIHNRHIEIIVRQITS
KVLVSE---DGMSNVFLPGELIGLFRAERTGRALEEAICYRATLLGITRASLNTQSFISEASFQ
ETARVLAKAALRGRIDWLKGLKENVVLGGMIPVGTGFKGFVHHS--SQHK--DIPLKTKKQ
NLFEGEMG--DILFYHRELFESCLSKN--------------------MTRRYWNINLEEMMEAGVHFGH
GTRKWNPRMSPYISAKCKGIHIINLTRTARFLSEACDLVFDASSRGKQFLIVGTKNKAADSV
ARAAIRARCHYVNKKWLGGMLTNWSTTETRLHKFRDLRMEQTAGRLARLPKRDAAVVK
RQLSHLQTYLGGIKYMTGLPDIVIIVDQQEEYTALRECITLGIPTICLIDTNCNPDLADISIPA
NDDAIASIRLILTKLVFAICEGRS-SYIRNPMGQKINPLGFRLGTTQSHYSLWFSQPKNYAEG
LQEDQKIRDCIKNYVQKNTKTSSG-VEGIARIEIQKRIDLIQVIIHMGFPKLLIENRPQ----GV
EDLKINVQKELNCVNRKLNIAITRIAKPYGDPNILAEFIAGQLKSRVSFRKAMKKAIELTEQ
ADTKGIQIQIAGRIDGKEIARIEWIREGRVPLQTIRAKIDYCAYTVRTIYGVLGIKIWIFMGE
EMSRYRGPRFKKIRRLGALPGLTNKRPRAGSDLRNQSRSGK-RSQYRIRLEEKQKLRFHYG
ITERQLLKYVRIARKAKGSTGQVLLQLLEMRLDNILFRLGMAPTIPGARQLVNHRHILVNG
RIVDIPSYRCKPQDTIMARDEQKSIALIQNSLDLSPR-EELPKHLTLNPFP--YKGLVNQIIDSK
WVGLKINELLVVEYYSRQTMSRRGTVEEKTAKSDPIYRNRLVNMLVNRILKHGKKSLAYQ
ILYRAVKKIQQKTETNPLSVLRQAIRGVTPDIAVKARRVGGSTHQVPIEIGSTQGKALAIRW
LLGAARKRPGRNMAFKLSSELVDAAKGSGDAVRKKEETHRMAEANRAFAHFRMGKDTI
ADIITCIRNADMNRKGTVRIVSTNITENIVKILLREGFIENARKHQERNKYFLVLTLRHRR--
NKKGPYLN---TFHLKRVSRPGLRIYSNYQRIPRILGGMGIAILSTSRGIMTDREARLEGIGGE
ILCYIWMAKPIPKIGSRRNGRISSRKSARKIPKGVIHVQASFNNTIVTVTDVRGRVVSWASA
GTCGFRGTKRGTPFAAQTAAGNAIRTVVEQGMQRAEVMIKGPGLGRDAALRAIRRSGILL
SFVRDVTPMPHNGCRPPKKRRVMPTIKQLIRNTRQPIRNVTKSPALRGCPQRRGTCTRVYT
ITPKKPNSALRKVARVRLTSGFEITAYIPGIGHNLQEHSVVLVRGGRVKDLPGVRYHIVRGT
LDAVGVKDRQQGRSKYGVKKPKMARKSLIQREKKRRNLEQKYHLIRRSSKQEIR---KVTS
LSDKWEIHGKLQSPPRNSAPARLHRRCFLTGRPRANIRDFGLSGHILREMVHTCLLPGATR
SSWMKKNSFISVISDE--KKEENKGSVEFQVFCFTNKIRRLTLHLELHKKDYSSQRGLRKTL
GKRQRLLAYLLKINGVRYKELISKLNIRELKTR--MVKLRLKRCGRKQRAVYRIVAIDVRSR
REGRDLQKVGFYDPIKSQTYLNVPAILDFLEKGAQPTETVYDILKRAEVFKEFRLNQTKFN
-MDKSKRPFIKSKRSFRRRLPPIQSGDRIDYRNMSLISRFISEQGKILSRRVNRLTLKQQRLIT
SAIKQARILSLLPFLNNEK--QFERTESTTRTANFRTKNKVTRSLKKNPFVANHLLRKIEKLN
KKAEKEIIVTWSRASTIIPTMIGHTIAIHNGREHLPIYITDRMVGHKLGEFAPTLNFRGHAK
NDNKSRRMIFQSFLLGNLVSLCMKIINSVVVVGLYYGFLTTFSIGPSYLFLLRAQVMEEGEE

GTEKKVSGTTGFIMGQLMMFISIYYTPLHLALGRPHTITVLALPYLLFHFFWNNHKHFFDY
GSTSRNSMRNLSIQCVFLNNLIFQLFNYFILPSSMLARLVNIYMFRCNNKMLFVTSSFVGW
LIGHILFMKWVGLVLVWIQQNNSIRSYLVS------ELRNSMARIFSILFFITCVYYLGRMPSPIF
T--LKQEEDPKPLIFLLFDYKRWNRPMRYIKNNRFENAVRNEMSQYFFYTCQNDGKQRISF
TYPPSLSIFWEMIQRKISLATTEKFLDDELYNYWIYTNEQKKN-LSNEFANRITVLDKGL--ID
VLDKKTRLC--KSKNEYLQKDHDPLLNGSYRGIKKTLLPFIDEIFINKIHSVLYQEFEYKDSF
TQ-IGIKEISKKVPHWSYQLIESEQEEMEQIRSRSGKEVVFFTDVLTRYPQESDFRRDIIKGS
MRSQRRKIVIWELFQANIHSPLFLDRTNKSSFFSITF--SRLIKRIFKNKQEQIRLDIAETWDTI
PGAQIIRSLILLTQSILRKYILLPLLITGKNIGRILLFQLPEWSDDFKEWTSEMHIKCTYNGVQ
LSEKEFPKNWLTDGMQIKILSPFCLKPWHKSMQNQIDAFCFLTVVGLETDIPFGPPRKRPSF
FQPIFKQLDKKIEKLIKGNFQV-RKRLKE--KFFLKLQNETNNWIIEIFP--FFKKIIRKMSTVN
DSRIKNHMIHESSVQIRFLNQTNSSVTEKKMKDLANRTRIIKNKIEKISNDKLK---------KTR
YGTKNLGQILKRRNARLIRNSNYILKFFRERIYGDIFLYIINIPKINTQLFIQFIS-DFSFLSQAY
VFYKLSQAKILNLYKLRLVLQYISLFLKNEIKDFFTQGITNSELKTKKLPNSGMNQWKNW
LKLKYQYNLSQLKWSRLVPQKWRNRVTEHCEVEN---TNLYQNEE--LINSKKH--FQKNYR
YDVLSYKFFN-----DKNKYLDFKILDFSLRLFDWMGMNEKILSRWFFSEFFSFYNAYKMKP
WVIPINLLFSNPKSVIEAEFDLFLKRYLLFQLKWADSLNEKLMDNIQVYCLVLRLINPIEILI
SSIERKELSMDIMLDRKDFNCPNWKQKRVLIIEPIRLSIRGDGQFLLYQTIGISLVHKSKHNN
FDLLAPENLLSPRRRRELRILLCL-FLWPNYRLEDLACMNRFWFDTNNGSRFSILRIHMYP
MKGHQFKSWIFELREILREIKNSHYFLDSWTQFNSVGSFIHIFFYQERFLKLFDPRIWSILLS
-PNS--QGSTSNRYF---TIKGVVLFVVV-VLIYR----ITNRNMVERKNLYLIGLFP-----IPMNSIG
PRNDTLEKSF-GSSNINRLIVS-LLYLPKGKKISESYFLDPKESTWFLPIT----KKCIMPESNRG
SRWWRNWIGKRR--DSSCKISN---ETVAGIEISFKEKDIQ-YLEFPFVYYMD--DPIRKDHDW
ELFDCLSL--
------FLRN-VSRENWIWLDNVRLVNKDR---FFS--KVRNVSSNIQYDF---TR--SSFVQVTDSS
QLKESS-DQSRDRSNSIS----NADSE------------YHTLINKREIQQLKERSIL-RD--PSFLQTEGT
EIESD--RFPK--CLSGYS--SMPRLFT-AREKQMIIHLLPEEIEQL--LENPTRSIRSFFSGRW--SE
LH-LGSNPTERSTRDP----QLLKKQQDVS-FAPSRQSE----NKEMVNIFKIIKYLQN--TVSIHPI
SSDPGCDMVP-KDELD-MDSSDKISFLNKNSFFDL--FHLFHDRNRGGYAL--HHDFESE--EK
FQEMADLFTLSITDP-DLVYHRGFSFSIDSCGLDQKQ--FLNEVFNSRDESKKKSL---LVLSPI
FY--EENESFYRRIRKKGVR-IS---RNVLNRFFLIN---RSDR
SFEYGIQ---RDQIGNDTLNHRTIRKYMINQDFSNLKK-SQKKWFDP--LIFLSRTERFMNRDP
DAYRYKWFNGSKNFQEHLEHFVSEQKSR---FQVVFDQLRINQYSIDWSEVIDKKDLSKSL
RFFLSKSLR--------FFLS-------KLLLFLSNSLP-FFFVSFGNIPINRSE-IRIY-ELKGPNDQLYNP
LVESIGLQIVHLKKWKAF--LLD---DHDTF-QKSKFLINGGTISPFLFNKIP-KW-MIDSFHTR--
NNSGKSFDNTDSYFSMISHDQNNWLNPVKPFHRSSLISSFYKANQLRFLNNPHHFCFY--C
NKRFPF---Y-MEKARINNSD--FTYR-QFLNILFIHNK-LFSLCVGK--KKHAFLE-RDTISPIESQ
VSNIFLPNDFP--------------------IRSDLLVR--RTIYSIADISGTPLTEGQLVHFERTYCQPLSDM
NLSDSEKKNLHQYLN--FNSNMGFIYTPCSEKYLLSEK--RKK--------RSLCLK--KCVEKGQ
MYRTFQRDSAFSTLSKWNLFQTYMPWFLTSTGYKYLNFLFLDTFSD-LLPILSSS--QKFLSI
LHDIMHGSGISWRILQKKL------CLPPWN-LISEISSKCLHN--LLLPEEMIHRNNES--PLIWTH
LASPNVREF-FYSILFLLFVAGY-LVRTHLLFVFR--ASSELQTEFERVKSLMIPSYMIELRKLL
DRYPTSEPNSFWLKNLFLVALEQLGDSLEEIRGSASGDNMLLGGGPGP---AYG-FKSIRSKK
KYLNINLIDILDLI-SIIPNPINRITFSRNTRHLSHTSKEIYSLIRKRKRVNGDWIDDKIESWVA

SSDSIDDEEREFLVQFS--TLTT--EKRIDQILLSLT-HSDHLS-----KNDSGYQLIEQPGAIYLRYL
VDIH--KKYLMNYEFNTSCLAERRVFLAHYQTITY--SQTSCGANSFHFPSHG---KPFSLRLA
LSPS-RGILVIGSIGTGRSYLVKYLATNSYVPFITVFLNKFLDNKPKGSLID-----AS------DDID
R---------------------------DLDTELELLTMMNAL---TMDMMP---EIDQFSITLQFELAKAMSPCI
IWIPNIHDLDVNESNYLSLGLFVNYLS---RDCERGS-TRNILVIASTHIPQKVDPALIAPNQLN
TCIKIRRLRIPQQRKHFFTLSYTRGFHLEKKMFHT--NGFGSITMGSNVRDLVAFINEALSISI
TQKKSIIDTNTIRSALHRQTWDLR---SQVRSVQDHGILFYQIGRAVAQNVLLSNCPIDPISTY
MKKK--SCNEGDSYLYKWYFELGTSMKKLTILLYLLSCSAGSVAQDLWSLP--GPDEK-NGI
TSYGLVENDS-YLVHGLLEVEGALVG--SSR--IEKACSQND--RVTLFLRPELRN-PLDMMQN
GSCSILDHRFLYEK--YESELEE---GEGALDPQ----QIEED----LFNH--IVWAP--RIWNP---WGF
LFDCIERPNELGFP-YWARSFRGK---RSIYDKEDELQENDSEFLQSGTMQYQTRDRSSKEQ
GFFRISQFIWDPADPLFFLFK-DQPFVSVFSHREFFADEEISKGLLTSQMNPPISIFQRWFI--K
NTQEKHFELLINRQRWLR---TNSSLS--NGSFRSN--TLSESYQYLSNLFLSN-GTLLDQMTKT
LLRKRWLFPDEMKIGF--MQEEKDFPFLMSR----NGNFIDKTFSVVANILLQIIPTTSGEKEAF
TYYRDG--MSAQSEGNYAEALQNYYEAMRLEIDPYDRSYILYNIGLIHTSNGEHTKALEYY
FRALERNPFLPQAFNNMAVICHY-RGEQAIRQGDSEIAEAWFDQAAEYWKQALTLTPGNYI
EAHNWLKITGRFE--MNWRSERIWVEFITGSRKISNFCWAFILFLGSSGFLLVGISSYLGKNF
ISLFPPQQILFFPQGLVMSFYGIAGLFISAYLWCAISWNVGSGYDRFDRKEGIVCIFRWGFPG
KNRRIFFRYLIKDIQSIRIELKEGIYTRRVLYLEIRGQGAIPLTRTDEN-LTPREMEQKAAELA
YFLRVPIEVF
>Oenothera
MKPTKGPKKPNNKEDLEGPKPNKSNEEAEEL--GDNKEDLEGKSNEE-AEELGDKQKDKK
DGIFVLNYDDEYEEDLYDDEYEEDLEYDDEYEEDLEYDEEYDYEEDLEG-DNKPHKEGD
NK---PHKEDLEGDNKPHKEDLEGDKQKEEEEKQKEEEKLKDCPHWFHQ-IYPKHWG-DR
KSVPAKEVPAQSTKRDTDPEASLKSNYAHLWVHCKLCSGFNYKKILKSKNNVCEQCGSH
LKMHSSDRIDLMLDPKTWAPMHEGLLSLDPIEFHSEKDPYKDRVASYKRKTGLSEAIQTG
RGYLKRIHLGIGLMDFQFMGGSMGSVVGERITRLVEYATNRVLPLILVCASGGARMQEGS
LSLMQMAKISSALSDYQFNRTVFYVALLTSPTTGGVTASFGMLGDIILAEPNAYIAFAGKR
VIEQTLNIEVPEGSQTAEYLFDKGLFDQIVPRNPLKGSLSELFNFHGFVTLNSQVINMATIR
ADEISNIIRERIEQYNREVKIVNTGTVLQVGDGIARIYGLDEVMAGELVEFEEGTIGIALNLE
SKNVGVVLMGDGLLIQEGSSVKATGRIAQIPVSEAYLGRVINALAKPIDGRGEISSSESRLIE
SPAPGIISRRSVYEPLQTGLIAIDAMIPIGRGQRELIIGDRQTGKTAVATDTILNQQGNNVICV
YVAIGQKASSVAQVVNALQERGAMEYTIVVAEAADSPATLQYLAPYTGAALAEYFMYRE
RHTLIIYDDPSKQAQAYRQMSLLLRRPPGREAYPGDVFYLHSRLLERAAKLSSRLGEGSMT
ALPIVETQSGDVSAYIPTNVISITDGQIFLSADLFNAGIRPAINVGISVSRVGSAAQIKAMKQ
VAGKLKLELAQFAELEAFAQFSSDLDKATQNQLARGQRLRELLKQSQAKPLTVAEQILTIY
TGTNGYLDSFEIAQVRKFLDELRDYVKTRKPQFEEIISSTKIFTEEAQALLKDAIQEQKELF
LVQE--MRINPTTSGPGVSTLEKKKSGRIAQIIGPVLDVTFPPGKMPNIYNALVVKGRDTGG
QEINVTCEVQQLLGNNRVRAVAMSATDGLTRGMEVIDTGAPLSVPVGGATLGRIFNVLGE
PVDELGPVDTRTTSPIHRSAPAFIQLDTKLSIFETGIKVVDLLAPYRRGGKIGLFGGAGVGK
TVLIMELINNIAKAHGGVSVFGGVGERTREGNDLYMEMKESGVINEQNIAESKVALVYGQ
MNEPPGARMRVGLTALTMAEYFRDVNKQNVLLFIDNIFRFVQAGSEVSALLGRMPSAVG
YQPTLSTEMGSLQERITSTKAGSITSIQAVYVPADDLTDPAPATTFAHLDATTVLSRGLAAK
GIYPAVDPLDSTSTMLQPRIVGDEHYETAQRVKETLQRYKELQDIISILGLDELSEEDRLTVA

RARKIERFLSQPFFVAEVFTGSPGKYVGLAETIRGFKLILSGELDGLPEQAFYLVGTIDEATA
KAANLEMESDLKKMTLNLCVLTPNRIVWDSEVKEIILSTNSGQIGVLPNHAPIATAVDIGIL
RIRQNGQWLTMALMGGFARIGNNEITILVNDAEKGSDIDPQEAQETLGLAEANFRKAEGK
RQTIEANLALRRARTRVEAINVISMKNVTDSFVSLVHWPSAGSFGFNTDILATNPINLSVVL
GVLIFFGKGVLSDLLDNRKQRILNTIRNSEELREGAIEQLEKARARLQDVQIEAEGYRAYG
YFGIDEQRHESINSTYKTLEQLENNKNESIHFEQQRAINQVRQQIFQQALQGALGTLNSCL
NNELHLRTISANIGLFGSMKELTDMNPLISAASVIAAGLAVGLASIGPGIGQGTAAGQAVEG
IARQPEAEGKIRGTLLLSLAFMEALTIYGLVVALALLFANPFVMDVLSCSNNTLKGLYDISG
VEVGQHFYWQIGGFQVHGQVLITSWVVIAILLGSASIAVRNPQTIPNDSQNFFEYILEFIRD
VSKTQIGEEYGPWVPFIGTMFLFIFVSNWSGALLPWKLVELPHGELAAPTNDINTTVALAL
LTSVAYFYAGLSKKGLGYFSKYIQPTPILLPINILEDFTKPLSLSFRLFGNILADELVVVVLVS
LVPSVVPIPVMFLGLFTSGIQALIFATLAAAYIGESMEGHHMIFYTLEHILTHISFSLVSIGITIF
LITLSV-EIIGLYDSSEKGVIGTFLCITGLLVTRWAYSGHFPLSNLYESLLFLSWSFAIIHMFPY
FKKQKSYVRTITSSSTIFTQGLVTSGLLSEMQQSEILVPALQSQWLMMHVSMMVLGYAAL
LCGSLLSVALLVITFRKALRIFSKKKEIQYRNEPSNVLL--SFISSKNYYRAQLIQQLDRWSSR
IISLGFIFLTIGILSGAVWANEAWGSYWNWDPKETWAFITWTMFAIYLHTRTNPNFQSVNSA
IVAFLGFIIIWICYFGVNLLGIGLHSYGSFNLH---------------IVFFPWWISLLFNKGLESWVTN
WWNTTHSETFLTDMQEKSILDKFIELEELLLLDEMINEYPETHLQTLRIGIHKEMVRLIKM
RNEDHIHTILHLSTNIICFIIFRGYSILGNKELLILNSWMQEFLYNLSDTIKAFSILLLTDFCIGF
HSPHGWELMIAYVYKDFGFAQNDQIISGLVSTFPVILDTIFKYWIFRYLNRVSPSLVVIYDS
MNDMPIGMPKIPFLLDGEDDATWVDLYNVLYRTRSIFLGDAIHFEVANHIAGLMIFLTIQDA
TQNLYFFINSPGGLAVAGLLIYDTMQYVTPPVYTLGLGVLASMASFLLVGGETSKRLMGP
NGRVMIHQPEDYTHKDQSLEVQLDSGEVEDIRKMVIRVYLERTRLPREVLNDHLERNYF
M T A T E A K Y Y G I V D D I G I
Q???MEEFPG
YFELDRSRQHDFLYPLIFRESIYALAHDHGLVDYDKKYSLIIVKRLITRMYQRNHLIISANG
SVQNPFWGQNLYSKILSEGFAVIVEIPFSLRVLSSFIAKSPTLRSIHSIFPFLEDQFSHLDYLSH
VLIPYPIHLEIAVQTLRYWVKDASSLHLLRIFLHEYWN--SFSTKHITLFLKSRFFLFLYNSYV
CEYESIFLFIRNQSSHFQSTSSGVFFERILFYVKIDHLVEVFVGFLIRSFFKDPNMHYVRYQG
KSILASKDTPLLMNKWKYYLVNLWQYHFSVWSQPGRININQLGKYSLDFLGYFSNVQLK
SSVVRNQTLENSFLINN--AMKKLETTVPILPLIGSLSRAKFCNALGHPISKPTRNDSSDSDII
DRFVRICRNLSHYHSGSSKKKSLYRIKYILRLSCVKTLARKHKSSVRAFLKRLGSELGDEFL
TEEGVVLAVIFP-RIWYLDIPCINDRVGDAMIIDTT-AVQ-DMNSFSRLQS-LKEVSGIIWMLV
PILSLVLGITLGVLVIVWLEREISAGIQQRIGPEYAGPMGILQALADGIKLIFKENLLPSRGDT
RLFSIGPSIAVISILLSYSVIPFSSHLVLSDLNIGVFLWIAVSSIAPIGLLMSGYGSNNKYSFLG
GLRAAAQSISYEIPLTLCLLSISLLSNSSSTVDIVEAQSKYGLWGWNLWRQPIGFLVFLISSL
AECERLPFDLPEAEEELVAGYQTEYSGIKFGLFYVASYLNLLVSSLFVTVLYLGGWNISISYI
FVPGLF--EKVGRVFGTTIGIFTTLAKTYLFLFISITTRWTLPRLRMDQLLNLGWKFLLPISLG
NLLLTTSSQLLSLMIWHVQNENLILDSTRIFMKAFHLPLFDGSFIFPEGILIFGLILLLMIDST
SDQTDIPWFYFISSISLVMSITALLFRWREEPRILFSGNFQTNNFNEIFQFLILLCSTLCIPLSV
EYIECTEMAITEFLLFVLTATLGGMFLCGANDLITIFVAPECFSLCSYLLSGYTKKDVRSNEA
TMKYLLMGGASSSILVHGFSWLYGSSGGEIELQEIVNGLINTQMYNSPGISIALIFITVGIGF
KLSPAPSHQWTPDVYEGSPTPVVAFLSVTSKVAASASATRIFDIPFYFSSNEWHPLLEILAIL
SMILGNLIAITQTSMKRMLAYSSIGQIGYVIIGIIVGDANGGYASMITYMLFYISMNLGTFAC

IVLFGLRTGTDNIRDYAGLYTKDPFLALSLALCLLSLGGLPPLAGFFGKLHLFWCGWQAGL
YFLVSIGLFTSVVSIYYYLKIIKLLMTGRKQEITPHVRNYRRSPLRSNNSIELSMIVCVIASTI
PGISMNPIIAIAQDTLFMFLLYEYDIFWAFLIISSVIPILAFRISGLLAPTSIGPEKLSSYESGIEP
MGDAWLQFRIRYYMFALVFVVFDVETIFLYPWALSFDILGVSVFIEALIFVLILVLGLVYAW
RKGALEWSTNSFPWLTIIVVFPILTGSLIFLLPHRGNKVMKWYTLCICILELLLTTYTFCYHF
QLDDPLTQLTENYKWIHFFDFYWRLGIDGLSIGPILLTGFITTLATLAAWPVTRDAQLFHFL
MLAMYSGQIGSFSSRDLLLFFLMWEFELIPVYLLLSMWGGKKRLYSATKFILYTAGGSIFL
LIGVLGIGLYGSNEPTLNFETLANQSYPVALEVIFYVGFLIAFAVKLPIIPFHTWLPDTHGEA
HYSTCMLLAGILLKMGAYGLVRINMELLPHAHCLFSPGLIIVGAIQIIYAASTSPGQLNLKK
RIAYSSISHMGFIIIGIGSLSDTGLNGAILQIISHGFIGAALFFLAGTSYDRIRLLYLDEMGGM
AIPLPKLFTMLSILSMASLALPGLSGFVAELLVFFGIITSQKYLLMPKILIAFLMAIGMILTPIY
SLSMLRQMFYGYKLFNVPNYYFFDSGPRELFVSISLLLPIIGIGIYPDFVLSLSVEKVEAIISH
FFFSMILEHVLVLSAYLFSIGIYGLITSRNMVRALMCLELILNSVNLNFVTFSDFFDSRQLKG
DIFSIFIIAIAAAEAAIGLAIVSSIYRNRKSIRINQSNLLNKMEYTYQYSWIIPFIPLPVPILIGM
GLLLFPTATKNHRRVWSFPSILLLSMVMLLSVYLSIQQINRSFIYQYVWSWTINNDFSLEFG
HLIDPLASIMLILITTVGILVLFYSDNYMSHDQGYLRFFAYLSFFNTSMLGLVTSSNLIQIYIF
WELVGMCSYLLIGFWFTRPIAATACQKAFVTNRVGDFGLLLGILGLYWITGSFEFRDLFEIV
NN-QVHFLFVTLCSFLLFAGAVAKSAQFPLHVWLPDAMEGPTPISALIHAATMVAAGIFLVA
RLLPLFVITPYIMNLISLIGIITVLLGATLALAQKDIKRSLAYSTMSQLGYMMLALGMGSYR
AALFHLITHAYSKALLFLGSGSIIHSMESIVGYSPDKSQNMVLMGGLKKHVPITKTAFLVG
TLSLCGIPPLACFWSKDEILNDSWLYSPIFAIIACSTAGFTAFYMFRVYLLTFDGHLNVHFQN
YSGQKSSSVYSISLWGKQVPKRIQN-LLNLLTMNFFWPNRK-TFSYPHESDNTMLFSLFVLV
LFTLFVAAIG--FDCDILSKLLNPSINLLHQNSN-FTDW-EFVTNASFSVSIALLGIFIATFLYKPI
YSSLQNFNLLNSFYVMWDKIQNWIYDWSYNRGYIDSFYTISLTGGIRGLAELSHFFDRRVI
DGILNGFGLTSFFLGESLKYFGGG-RISSYLLLYSIFIFIFLLMDSMDLPGPIHDFLLVFLGSGL
IVGGLGVVLLTNPIFSAFSLGLVLVCISLFFSLSNSYFVAAAQLLIYVGAINVLILFAVMFMN
GSEYSKDLTLWTVGDGITSLVCTSIFISLITTILDTSWYGIIWTTKSNQIIEQDLIGNSQQIGIH
LSTDFFLPFELISIILLVSLIGAIAVARQMNVTTTRKDLMIVNMGPHHPSMHGVLRLILTLDG
EDVIDCEPILGYLHRGMEKIAENRTVIQYLPYVTRWDYLATMFTEAITINGPEQLGNIQVPK
RASYIRIIMLELSRIASHLLWLGPFMADIGAQTPFFYIFRERELVYDLFEAATGMRMMHNY
FRIGGVAADLPYGWIDKCLDFCDYFLTAVSEYQKLITRNPIFLERVEGVGIIGGEEAINWGL
SGPMLRASGIEWDLRKVDRYECYGELDWEIRWQKEGDSLARYLVRMSEMTESIKIIQQAL
EGIPGGPYENLEIRCFDREKDPEWDGFEYRFISKKPSPTFELPKQELYVRVEAPKGELGIFLI
GDQSGFPWRWKIRPPGFINLQILPQLVKRMKLADIMTILGSIDIIMGEVDRMFPMVTGFMN
YGQQTVRAARYIGQGFMITLSHANRLPVTIQYPYEKLITSERFRGRIHFEFDKCIACEVCVR
VCPIDLPVVDWKLETGVRKKRLLNYSIDFGVCIFCGNCVEYCPTNCLSMTEEYELSTYDR
HELNYNQIALGRLPMSVIDDYTIRTILNS---------------MQGRLSAWLVKHGLVHRSLGFDY
QGIETLQIKPEEWHSIAVILYVYGYNYLRSQCAYDVAPGGLLASVYHLTRIEYGVDQAEEV
CIKVFAPRNNPRIPSVFWVWKSADFQERESYDMLGIRYDNHPRLKRILMPESWIGWPLRK
DYIAPNFYEIQDAH--MNSIEFTLLARRTQNS
VISTTSNDLSNWSRLSSLWPLLYGTSCCFIEFASLIGSRFDFDRYGLVPRSSPRQADLILTAGT
VTMKMAPSLVRLYEQMPEPKYVIAMGACTITGGMFSTDSYSTVRGVDKLIPVDVYLPGCP
PKPEAIIDAITKLRKKISREIYEDRIRSQEENRCFTTNHKFHVGPSMHTGNYDPGLLYQLPST
SEIASETFFKYKSSVSAHELVN--MKNTFSWIKKEITRSISLSLMIYIITRTSISNAYPIFAQQGY

ENPREATGRIVCANCHLANKPVDIEVPQAVLPDTVFEAVVRIPYDRQVKQVLANGKKGGL
NVGAVLILPEGFELAPPARISPEMKERIGNPSFQSYRPTKKNILVIGPVPGQKYSEITFPILSP
DPATNKDVHFLKYPIYVGGNRGRGQIYPDGSKSNNTVYNATAAGIVSKIIRKEKGGYEITIT
DASDGRQVVDIIPSGPELLVSEGESIKLDQPLTSNPNVGGFGQGDAEVVLQDPLRVQGLLF
FLASVILAQIFLVLKKKQFEKVQLSEMNFMSKVYDWFEERLEIQAIADDITSKYVPPHVNIF
YCLGGITLTCFLVQVATGFAMTFYYRPTVTEAFASVQYIMTEANFGWLIRSVHRWSASMM
VLMMILHVFRVYLTGGFKKPRELTWVTGVVLAVLTASFGVTGYSLPWDQIGYWAVKIVTG
VPDAIPVIGSPLVELLRGSASVGQSTLTRFYSLHTFVLPLLTAVFMLMHFLMIRKQGISGPL
MGVTKKPDLNDPVLRAKLAKGMGHNYYGEPAWPNDLLYIFPVVILGTIACNVGLAILEPS
MLGEPADPFATPLEILPEWYFFPVFQILRTVPNKLLGVLLMVSVPSGLLTVPFLENVNKFQN
PFRRPVATTVFLIGTVVALWLGIGATLPIDKSLTLGLFMIEVFLFGIVLGLIPITLAGLFVTAYL
QYRRGDQLDLMLTITSFFGFLLAALTITSVLFIGLTKIRLIMDIVSLAWAALMVVFTFSLSLV
VWGRSGLMIIRSPEPEVKILVDRDPIKTSFEEWAKPGHFSRTIAKGPETTTWIWNLHADAH
DFDSHTSDLEEISRKVFSAHFGQLSIIFLWLSGMYFHGARFSNYEAWLSDPTHIGPSAQVV
WPIVGQEILNGDVGGGFRGIQITSGFFQLWRASGITSELQLYCTAIGALVFAALMLFAGWF
HYHKAAPKLAWFQDVESMLNHHLAGLLGLGSLSWAGHQVHVSLPINQFLNAGVDPKEIP
LPHEFILNRDLLAQLYPSFAEGATPFFTLNWSKYAEFLTFRGGLDPVTGGLWLTDIAHHHL
AIAILFLIAGHMYRTNWGIGHGLKDILEAHKGPFTGQGHKGLYEILTTSWHAQLSLNLAM
LGSLTIVVAHHMYSMPPYPYLATDYGTQLSLFTHHMWIGGFLIVGAAAHAAIFMVRDYDP
TTRYNDLLDRVLRHRDAIISHLNWVCIFLGFHSFGLYIHNDTMSALGRPQDMFSDTAIQLQ
PVFAQWIQNTHALAPGATAPGATTSTSLAWGGGDLVAVGGKVALLPIPLGTADFLVHHIHA
FTIHVTVLILLKGVLFARSSRLIPDKANLGFRFPCDGPGRGGTCQVSAWDHVFLGLFWMY
NAISVVIFHFSWKMQSDVWGSISDQGVVTHITGGNFAQSSITINGWLRDFLWAQASQVIQS
YGSSLSAYGLFFLGAHFVWAFSLMFLFSGRGYWQELIESIVWAHNKLKVAPATQPRALSIV
QGRTVGVTHYLLGGIATTWAFFLARIIAVGMALRFPRFSQGLAQDPTTRRIWFGIATAHDF
ESHDDITEERLYQNIFASHFGQLAIIFLWTSGNLFHVAWQGNFEAWVQDPLHVRPIAHAIW
DPHFGQPAVEAFTRGGALGPVNIAYSGVYQWWYTIGLRTNEDLYTGALFLLFLSAISLIAG
WLHLQPKWKPSVSWFKNAESRLNHHLSGLFGVSSLAWTGHLVHVAIPGSRGESVRWNNF
LDVLPHPEGLGPLFTGQWNLYAQNPDSSSHLFGTSQGSGTAILTLLGGFHPQTQSLWLTDM
AHHHLAIAFLFLIAGHMYRTNFGIGHSIKDLLEAHTPPGGRLGRGHKGLYDTINNSIHFQL
GLALASLGVITSLVAQHMYSLPAYAFIAQDFTTQAALYTHHQYIAGFIMTGAFAHGAIFFIR
DYNPEQNEDNVLARMLDHKEAIISHLSWASLFLGFHTLGLYVHNDVMLAFGTPEKQILIEP
IFAQWIQSAHGKTSYGFDVLLSSTNSPAFNAGRSIWLPGWLNAINE--NSNSLFLTIGPGDFL
VHHAIALGLHTTTLILVKGALDARGSKLMPDKKDFGYSFPCDGPGRGGTCDISAWDAFYL
AVFWMLNTIGWVTFYWHWKHITLWQGNVSQFNESSTYLMGWLRDYLWLNSSQLINGY
NPFGMNSLSVWAWMFLFGHLVWATGFMFLISWRGYWQELIETLAWAHERTPLANLIRWR
DKPVALSIVQARLVGLAHFSVGYIFTYAAFLIASTSGKFGMSHSVKIYDTCIGCTQCVRACP
TDVLEMIPWDGCKAKQIASAPRTEDCVGCKRCESACPTDFLSVRVYLWHETTRSMGLAY
MT--DFPSIFVPLVGLVFPAIAMASLSLHVENNKIVMRDLKTYLSVAPVLSALWFGALAGLLI
EINRFFPDALTFPFFS-MTAILERRESESLWGRFCNWITSTENRLYIGWFGVLMIPTLLTATSV
FIIAFIAAPPVDIDGIREPVSGSLLYGNNIISGAIIPTSAAIGLHFYPIWEAASVDEWLYNGGP
YELIVLHFLLGVACYMGREWELSFRLGMRPWIAVAYSAPVAAATAVFLIYPIGQGSFSDGM
PLGISGTFNFMIVFQAEHNILMHPFHMLGVAGVFGGSLFSAMHGSLVTSSLIRETTENESA
NEGYRFGQEEETYNIVAAHGYFGRLIFQYASFNNSRSLHFFLAAWPVVGIWFTALGISTMA

FNLNGFNFNQSVVDSQGRVINTWADIINRANLGMEVMHERNAHNFPLDLAAVEAPSTNG
MGLPWYRVHTVVLNDPGRLLAVHIMHTALVAGWAGSMALYELAVFDPSDPVLDPMWRQ
GMFVIPFMTRLGITNSWGGWSIT-GGTVTNPGIWSYEGVAGSHILFSGLCFLAAIWHWVY
WDLAIFSDERTGKPSLDLPKIFGIHLFLSGLACFGFGAFHVTGLYGPGIWVSDPYGLTGEVQ
PVNPAWGVEGFDPFVPGGIASHHIAAGTLGILAGLFHLSVRPPQRLYKGLRMGNIETVLSS
SIAAVFFAAFVVAGTMWYGSATTPIELFGPTRYQWDQGYFQQEIYRRVGAGLAKNQSLSE
AWSKIPEKLAFYDYIGNNPAKGGLFRAGSMDSGDGIAVGWLGHPIFRDKEGRELFVRRMP
TFFETFPVVLVDGDGIVRADVPFRRAESKYSVEQVGVTIEFYGGELNGVSYSDPATVKKYA
RRAQLGEIFELDRATLKSDGVFRSSPRGWFTFGHASFALLFFFGHIWHGARTLFRDVFAGI
DPDLDTQVEFGAFQKLGDPTTRRQAVMKTLYSLRRFYPVETLFNGTLALAGRDQETTGFA
WWAGNARLINLSGKLLGAHVAHAGLIVFWAGAMNLFEVAHFVPEKPMYEQGLILLPHLA
TLGWGVGPGGEVIDTFPYFVSGVLHLISSAVLGFGGIYHALLGPETLEESFPFFGYVWKDR
NKMTTILGIHLILLGLGAFLLVFKALYFGGVYDTWAPGGGDVRKITNLTLSPSILFGYLLKS
PFGGEGWIVSVDDLEDIIGGHVWLGSICILGGIWHILTKPFAWARRALVWSGEAYLSYSLA
ALSVFGFIACCFVWFNNTAYPSEFYGPTGPEASQAQAFTFLVRDQRLGANVGSAQGPTGL
GKYLMRSPTGEVIFGGETMRFWDLRAPWLEPLRGPNGLDLSRLKKDIQPWQERRSAEYM
THAPLGSLNSVGGVATEINAVNYVSPRSWLATSHFVLGFFLFVGHLWHAGRARAAAAGFE
KGIDRDFEPALSMTPLNMTIALGKFTKDEKDLFDIMDDWLRRDRFVFVGWSGLLLFPCAY
FALGGWFTGTTFVTSWYTHGLASSYLEGCNFLTAAVSTPANSLAHSLLLLWGPEAQGDFT
RWCQLGGLWTFVALHGAFALIGFMLRQFEIARSVQLRPYNAIAFSGPIAVFVSVFLIYPLGQ
SGWFFAPSFGVAAIFRFILFFQGFHNWTLNPFHMMGVAGVLGAALLCAIHGATVENTLFE
DGDGANTFRAFNPTQAEETYSMVTANRFWSQIFGVAFSNKRWLHFFMLFVPVTGLWMSA
LGVVGLALNLRAYDFVSQEIRAAEDPEFETFYTKNILLNEGIRAWMAAQDQPHENLIFPEE
VLPRGNALMSGSTGERSFADIITSIRYWVIHSITIPSLFIAGWLFVSTGLAYDVFGSPRPNEY
FTESRQGIPLITGRFDSLEQLDEFSRSFMTIDRTYPIFTVRWLAVHGLAVPTVSFLGSISAMQ
FIQRMATQTAEESSR-ARPKKTGLGGLLKPLNSEYGKVAPGWGTTPLMGLAMALFAVFLSI
ILEIYNSSVLLDGISMNMLTLKLFVYTVVIFFVSLFIFGFLSNDPGRNPGREEMADTTGRIPL
WIIGTVAGILVIGLIGIFFYGAYSGLGSSLMLNIFSL--ICLNSDLYSSRFFLAKLPEAYAFLNPI
VDVMPVIPLFFLLLAFVWQAAVSFRMTQSNPNEQDVELNRTSLYWGLLLIFVLAVLFSNYF
FNMEVNILAFIATALFILVPTAFLLIIYVKTVSQSDMETATLVAISISGLLVSFTGYALYTAFGQ
PSQQLRDPFEEHGDMEALVYTFLLVSTLGIIFFAIFFREPPKIQTKKTKMTIAFQLAVFALIAT
SSLLLISVPVVFASPEGWSSNKNVVFSGTSLWIGLVFLVGILNSLIS-------MSPQTETKASVG
FKAGVKDYKLTYYTPEYETKDTDILAAFRVTPQPGVPPEEAGAAVAAESSTGTWTTVWTD
GLTSLDRYKGRCYHIEPVAGEENQYICYVAYPLDLFEEGSVTNMFTSIVGNVFGFKALRAL
RLEDLRIPTAYVKTFQGPPHGIQVERDKLNKYGRPLLGCTIKPKLGLSAKNYGRAVYECLR
GGLDFTKDDENVNSQPFMRWRDRFLFCAEAIYKSQAETGEIKGHYLNATAGTCEEMMKR
AIFARELGVPIVMHDYLTGGFTANTSLAHYCRDNGLLLHIHRAMHAVIDRQKNHGIHFRV
LAKALRMSGGDHIHSGTVVGKLEGERDITLGFVDLLRDDFIEKDRSRGIYFTQDWVSLPG
VLPVASGGIHVWHMPALTEIFGDDSVLQFGGGTLGHPWGNAPGAVANRVALEACVQARN
EGRDLAREGNEIIREACKWSPELAAACEVWKEIKFEFQAMDTL--MAIHLYKTSTPSTRNG
AVDSQVKSNTRKNLIYGQPRCSKGRNARGIITAGHRGGGHKRLYRKIDFRRNERDIYGRIV
SIEYDPNRNASICLIHYGDGEKRYILHPRGAIIGDTIVSGTEVPIKMGNALPLTKMPLGTALH
NIEITLGKGGQLARAAGAVAKLIAKEGKSATLKLPSGEVRLISNNCSATVGQVGNVGVNQ
KSLGRAGSKRWLGKRPVVRGVVMNPVDHPHGGGEGRAPIGRKKPATPWGYPALGRRSR

KRNKYSENLILRRRSKMIQPQTRLNVADNSGARELMCIRIIGAS-NRRYAHIGDIIVAVIKEA
LPSTSLERSEVVRAVIVRTCKELKCDDGIIIRYDDNAAVVIDQEGNPKGTRVFGAIAHELRE
LSFTKIVSLAPEVLMLSPKRTRFRKQHRGRMRGISYRGNRICFGKYALQALEPAWITSRQIE
AGRRAMTRNVRRGGKTWVRIFPDKPVTLRAAETRMGSGKGNPEYWVAVVKPGRILYEM
GGVAENIARKAISIAASKMPIRTQFIISGMTRIRRGYIARRRRTKTRFFASSWRGARGNLTRA
IIQQRIRAWFSSHRDRTRQKRDFRRLWITRINAAIREN--RSS--SKLIHNLYKRQLFLNRKML
AQLAILNRNCLYMISNQILK-EVDWQESATIMKKEVYALGQYISMSAPKARRVIDQIRGRS
YEETLMLLALMPYRACDPILKLVNSAAANARHNMSFNEATLVISKAEVNEGTTVKKLKPR
ARGRSYPIRRPTCHIRIVLQDTSFD-------MDGIKYAVFTDKGVRLLGKN-QYTSNVESGSTR
TEIKHWVELFFGVKVQAMNSHRLPGKGRRMGPIMGHTMHYRRMIITLQPGYSIPPLRKKR
TMAVPKKRTSISKKRIRKTIWKKKAYWASLKAFSLAKSLSTGNSKSFFYSKF--MARGKDA
RVTVILECTNCVRGG--VTKESTGISRYITEKNRHNTPGQLELKKFCPYCYKQTIHGEIKKM
KIRASVRKICTKCRLIRRRGRIIVICSNPRHKQRQGMVREKVRVSTRTLQWKCVESRVDSK
RLYYGRFILSPLMKGQADTIGIAMRRALLGEIEGTCITRAKFE--KIPHEYSTIVGIQESVHEI
LMNLKEIVLRSNLYGTCNASICVKGPGCVTAQDILLPPSVEIIDNTQHIASLMEPIHLCIGLQ
IERNRGYHIKSPNNFQDGSY--PIDAVFMPVRNANHSIHSYGNQNEKQEILFLEIWTNGSLTP
KEALHEASRNLIDLFLPFLHAEEE--NLHLGKNK-YNVTFPLFTFHKKLAK-LRKPKN-EIAL
KSIFIDQLELPPRIYNSLKGSKIHTLSDLLNKSQEDLMKMKHFRIEDVKQILDILEIEKAFHN
RFNEENMLGDGN-GGMCTIPGFNQIQFEGFCRFIDQGLTEELSKFPKIEDTDQEIEFQLFVET
YHLVEPLIKERDAVYELLTYSSELYISAGLIWKSSRDMQKQTIFIGNIPLMNSLGTSIVNGIY
RIVINQILQSPGIYYRSELDHNGISVYTGTIISDWGGRLELEIDRKARIWARVSRKQKISILVL
SSAMGSNLREILENVCYPEMFLSFLNEKEKKTIGSKENAILEFYQQFSCVGGDPVFSESLC
KELHTKFFQQRCELGRIGRQNMNRRLNLDIPQHNTFLLPRDILAAADHLIGMKFGMGTLD
D--TNHLKNKRIRSVADLLQDQFGLALVRLESVVRRTICGAIR-HKLI-PTPHNLVTSTP----LT
TTYESFFGLHPLSQVLDRTNPLTQIVHGRKLSYLGPGGLTARTASFRMRDIHPSYYGRICPID
TSEGINVGLIGSLAIHARIGRGG-SLESPFY---EISERS--NKGRMLYFSPSRDEYYMVAAGNS
LALNRGIQEEQVVPARYRQEFLTLAWDQVHLRSIFPFQYFSIGASLIPFIEHNDANRALMSS
NMQRQAVPLSQSERCIVGTGLERQASLDSGVTAIAEREGKIIYTNSDKIILSGSGDTLSIPLV
MYQRSNKNTWMHQKPQVPRGKCIKKGQILADGAATVGGELALGKNVLVAYMPWEGYN
FEDAVLISERLVYEDIYTSFHIRKYEMQTHVTSQG-PERITNEIPHLEAHLLRNLDKNGIVM
MGSWVEAGEILVGKLTPQMAEEESSYAPEERLLRAILGIQISTSKETCLKLPIGGRGRVIDV
RWIQKRG-GSSYNPETIRVYILQKREIKVGDKVAGRHGNKGIISKILPRQDMPYLQDGRPV
DMVFNPLGVPSRMNVGQIFECSLGFAGDLLERHYRIAPFDERYEQEASRKLVFSELYEASK
QTANPWVFEPEYPGKSRLFDGRTGDPFEQPVIIGKPYILKLIHQVDDKIHGRSSGHYARVTQ
QPLRGRAKQGGQRVGEMEVWALEGFGVAHLLQEMLTYKSDHIRARQGVLGTTILGGTIP
KPEDAPESFRLLVRELRSLALELNHFLVSEKNFQINRKEA-------MIDRYKHQQLRIGSVSPQ
QISTWANKILPNGEIVGEVTKPYTFHYKTNKPERDGLFCERIFGPIKSGICACGTYRVIGDK
KEDPNFCEQCGVEFVDSRIRRYQMGYIKLACPATHVWYLKRLPSYIANLLDKPLKELEGL
VYCD--FSFARP-VAKKPTFLRLRGLFEYEIQSWKYSIPLFFTTQGFDTFRNREISTGAGAIRE
QLAGLDLRVIIDYSLVEWKELGEE-RSTGNEWEDRKIGRRKQFLVRRVELAKHFIRTNIEPE
WMVLCLLPVLPPELRPIIQMDGGKLMSSDINELYRRVIYRNNILADLLTTSRSTPGDLVMG
QEKLVQEAVDTLLDNGIRSRPVRDGQNKVYKSFSDVIEGKEGRFRETLLGKRVDYSGRSVI
VVGPTLPLHRCGLPREIAIELFQTFLIRGLIRQHLASDIVGAKSQIREKEPIVWEILQQVMQG
HPVLLNRAPTLHRLGIQAFQPILVEGRAICLHPLVCKGFNADFDGDQMAVHVPLSLEAQTE

ARLLMFSHMNLLSPAMGDPISVPTQDMLIGLYILTSGNPRGICTNRYNPWNRSN---YQNERI
SDNN--WKKK-EPFFCNSYDAIGAYRQKRIHLDSPLWLRWRLDQR-VIAS--REVPIEVQYES
LGTYHEIYGHYIIVRSVKTEILWMYIRTTVGHISLFREMEEAIQGFCRARWYLS-----------M
DEGVNLVFHKKVIDGTAIKRLISRLIDHFGMAHTSHILDQVKTLGFQQATATSISLGIDDLLT
IPSKGWLVQDAEQQSLSLEKHHHYGNVHAVEKLRQSIEVWYATSEYLRQEMNPNFRMTD
PFNPVHIMSFSGARGNASQVHQLVGMRGLMSDPQGQMIDLPIQSNLREGLSLTEYIISCYG
ARKGVVDTAVRTSDAGYLTRRLVEVVQHIVVRRTDCGTLRGISVSPRR--MP-ERIFIQTLIG
RVLADDIYIGSRCIAIRNQDIGIGLVNRFIT------FRIQPISIRTPFTCRSTSWICRLCYGRSPTH
GDLVELGEAVGIIAGQSIGEPGTQLTLRTFHTGGVFTGGTAEHVRAPSNGKIKFNEALVHPA
RTRHGHPALLCSMDLDVTIESEDILHNLTIPPKSFLLVQNNQYVESEQVIAEICAGTSTF-HF
KERVRKHIYSDSEGE-MHWSTDVYHAPEFTYSNVHLLPKTSHLWILSGGSCR--SRGAPFSL
HKDQDQMNPR--STERER-RYLSSL-SANNDQ-IRYKFFSSSFSGKKDDRSPGY-SEMNRIICT
LHCNLIYPS-ILRENSD--LLAKRRRNRLVIPVQSSQEREKELIPHSGISIELPIN--GIFRKKSIL
AFFDDPRYRTKSSGITQYETMGMHSIVKKEG--LVDYR-GINEFKPKYMTIDRFFFIPEEVHI
LPESSSIMVRNNSLIGVDTRIALNTRSRAGGLVRVERKKRGIALQIFSGTIHFPGETDKISWD
SGILIPPGTGKR--NSKESKK--WKNGIYVQR--ITPTKKK--HFVLFRPVVTYEIADGLNLARL
FPPDLCQEKDNMQLQIVNYIVYGNGKPIREISDTSIQLVRTWFILNWDQDK---KSASAEAA
HASFVEVRAKGLIRDFLRIDLVKSPILDPRKRNDPSGSGLIS-DNVSDHTNINPFY---SKPK---
--MKQSPRQNHGTIRTLLNQ---NKECPSLMILSASNCFRMGP--FNDVKSQN---VIKES-AIIQI
RNSIGPLGT--ALQVVNF--DSFYYFIT--HNQVL-LTKYLQVEN---LKQTFQVL--Q-YYLMDES
GR--IYNPDPRSNIVLNSFNLSWYFLPHNSCEEISTIVS-LGQFICENGCIAKNGP---YLRSGQ
VLIVQ-LDSVVIRSAKPYLATPGATVHGHYGEILYDGDTVVTFLYEKSRSGDITQGLPKVEQ
VLEVRSVDSISVNLEKRVENWNEHITRILGFPWGFLIGAELTIVQSRISLVNKIQKVYRSQG
VQIHNRHIEIIVRQITSKVLVSE---DGMSNVFLPRELIGLLRAERTGRALEESICYKAFLLGIT
RTSLNTQSFISEASFQETARVLAKAALRGRIDWLKGLKENVVIGGMIPVGTGFKGLVHCS--
KQHKSIP---KNKH--FFEGEIR--DILFHHRELFDSCISKNFHDTPEQSFRVFNDS------MTRRY
WNINLEEMMEAGVHFGHGIKKWNPRMAPYIYANRKGIHITNLTKTARFLAEACDLVFDA
ASRGGQFLIVGTKKQAAALVARAAIKARCHYVNKKWLGGMLTNWSTTETRLHQFRDLR
TEQKTGRLNRLPKRDAAILKRQLSHLQTYLGGIKYMTGLPDILIILDQQEEYTALRECITLG
IPTICLIDTDCDPDLADLPIPANDDAMASIRLILNKLVFAICEGRS-SSIRNPMAQKINPLGFRL
GTTQSHHSIWFAQPKDYSDGLQEDQKIRDCIQNYIQKNMQISSDGVEGIARIEIRKRIDLIQ
VKIYMGFPKLLIEDGTR----RIEELQRIVQKEINSVNRKINITITKITKPYGDPNILAEFIAGQL
NNRVSFRKAIQKAIELAEQADTKGIRVQIGGRINGGEIARVLWMKEGRVPLQTIRAKIDYC
VYTVRTIHGLLGIKIWIFRED-MSRYRGPRFKKIRRLGALPGLTSKRPRAGSDPRNQELSGN-
KSQYRIRLEEKQKLRFHYGLTERQLLKYVRIAGKAKGPTGQVLLQLLEMRLDNILFRLGM
ASTIPQARQLVNHRHILVNGRIVDIPSYRCKPRDIITAKDDQKSKAMIQNSLESPPH-EEVPK
HLTFHPFQ--YKGLVNQIIDSEWVGLKINELLVVEYYSRQTMSRRGTAEEKTAKSDPIYRNR
LVNMLINRILKHGKKSLAYQILYRAMKKIQQKTETNPLSVLRQAIRRVTPDIAVKARRASG
STHPVPIEIGSTQGRALAIRWLLGASRKRPGRNMAFKLSSELVDATKGRGGAIRKREETHR
MAEANRAFAHFRMGRDTIADIITSIRNVDMNRKGTVRIESTNMAEKIVKILLREGFIENVR
KHQENKKSFLVLTLRHRR--NRKGPSRT-FLNLNLKRISRPGLRVYSNYQKIPRILGGMGIVIL
STSRGIMTDREARLEGIGGEILCYIWMAKSIPSAGLR--LRLRLRKSTRKIPKGVIHVQASFH
NTIVTVTDVRGRVISWSSAGTCGFKSTRKGTPFAAQTAAGDAIRPVVDQGMQRAEVRIKG
PGLGRDAALRAIRRSGIRLSCIRDVTPLPHNGCRPPKKRRVMPTIKQLIRNTRQPIRNVTKS

PALRGCPQRRGTCTRVYTINPKKPNSALRKVARVRLTSGFEITAYIPGIGHNSQEHSVVLVR
GGRVKDLPGVRYHIVRGTLDAVGVKDRQQGRSKYGVKRPKMARKGLIQREKKREKLEQ
KYRLIRRSSKKEIS---TAPSLSEKWKIHGKLQSSPRNSAPTRLHRRCFSTGRPRANYRDFRL
SGHILREMVHACLLPGATRSSWMVKKAFISVISQ-----EENRGSVEFQVVSFTNKIRRLTSHL
EFHRKDFLSQRGLRKILGKRQRLLSYLSKKDKVRYTELISQLDIRELTTR--MVKLRLKRCG
KKQRAVYRIVAIDVRSRREGRDLWKVGFYDPINNKTYLDIPGILYFLEKGAQPTGTVYDIL
KKAGVFTEFSLNQMEVN-MDKSKRLFLKSKRSFRRRLPPIQSGDRIDYRNISLISRFISQQG
KILSRRVNRLTLKQQRLITIAIKQARILSLLPFRPKAQ--RFKRSQSTARTVGLRTRNKMKRS
LKNNPFVANPLLRKMEKLNRREDKILIRTWSRASTIILTMIGHTIAIHNGKEHLPIYITDYMV
GHKLGEFAPTINFHEHAKNDNKSRR-------MVNLVYVCMKINNSVVMVGLYYGFISAFSIG
SSYLFLLRPRFLNDDPDAIEKKASETAGFFTGQLLIFISILYGPLHLALGRPHTILLLLAPYFF
FHYLFSNSGQWPRFA-LLTKSMRNRRFQLVFLNNLLFQLFSLSLLGRPMLTRLSYIYIFRCN
NKMLFVLSSFVGWLIGHILVLKWAGLVFVWLLQVIRSKTKQVESSSLISGLKINFYHLFSIIL
FAIFLLYLDQRKLSNETKKLEEAQKDIPIITSLFDPQKPLRPLRYIKTAGVEKAVKNEMSQYF
FYACRSDGKQRICFTYPPSLATFWEMIQRKMASRFPYAKAAPGSYRQWISRNKKKKNSLS
TEFQNRIKTLDKKKSLQNVLETRKRLCNNKTKKEYLPEIADPFLTGALRGKLKNNITMRM
KNPVNKLHLLFVKTLVNREKYSFIELEEEINKKVPRWSYQLIELEQLEGAEIRILPFKRVAVF
TEAIRFLGHMSDFRRGLIKGSARQDRRKAYVCRTTQVNARSPLFALGLVNLAVQVKFFYE
TRIKGEIVDDVEDDIRENVTEMWENIDYGQVIRTFILLLHIFLRKKVVFPAFIIGKNIARMLL
LQATEWKIDFARLKRERYAICTYNGMKVSEKIFPPDWADDGIQILVTNPFYLKPWYRSKPK
KEPWYRFKTRFIKDPKKEKGPKKEKPWYRRFFFQKDPKKDRGVRFFGILTGDLITPDWGV
NPIRNELKKKIRQFEKKHSIILSKRFRNVLKKWIDNLKNEFRMSRNLQGPVRALKD-SLPEE
KVADPSDLDNELRAEIDKVTKERKK------SPDKLVQAKKNILKKLERIHKFYFLRIRYVLLF
FIKRISKRIYLNAISIRKIHPQRFIFKESSDLASMSQAYVFYKLYQTQTQLLDKLRYVLQYTS
RFLKKELKDYFAQELFHSKLKHKNSL-SGKNQWKNWLKAQHQYSVSPIIWNSLSPQKWR
TKVNQERMDENTNKRYSNEKRKQFFEANSLD-DIKNSIKYDLFSYQSINTATNSTAATSIKP
GVFWQNSQENDPIEDGRQNVIQQNLIDFFLEKKKDTNQELHAQAKARIWDQVQENKIPYT
AFGSDIRNFQVILPEDDEEDRLDELKLNAYELSRQKITDNLLSSIKRERLKMEFSTRNNSLA
TIMLTHG-IFSIEPLRISRQNQDASLIYQLIKISLVEQLDPSDSDLFVPETILSTKRRRELRILISF
RFLWPNYRLEDLACMNRYWFNAQNGSRFSMLRIRMYPVNLNPFRFWVFELREILREIKNS
--PFN------SVGSFIHIFVHQERFLKLLDPRIWSVLRSQGS----------------TGVVLFLVA-VLIYR
----INNRNMIERKNIYLTGLLP-----IPTNFAGPRNETLEESF-LSSNINRLIVS-LLHLPKGKRLS
ESCFLDPKESTRVLPITK----------------WRNWIGKRR--

--DSSQLKGSS-DQSRDHFDSIG----TEDSE-----
--------YHTLINQREIQQRKERSSLLD---PSFLQTERTEIESD--RFSK--GLSGSS--SKSRLFT-EG
EKEMNNHLPPEEIEEFL--GNPTRSILSFFSDEW--SELH-LGSNPTERSTVDQ----KLLKKEQE
VS-FAPFRRSE----TKEIVNLFKTMAYLQK--TVSIHPISSDPGCDMVP-KDELD-S---------------
--------------------------EERFQEMADLFTLSITEP-DLVYHKGFAFSIDSSVLDQKQ--FLAEA---
RDESKKKSL---LVLPPVFY--QENESFYRRIRKRG-VQISCGNDLEDPKPKIVVFASNNIVEAV
NQYR-IRNLIQIQYSTHG--YIRNVLNRFFLMN---RSDRNFEYGIQR---DQIGNDTLNHRTFM
KYTINQHLSNLKK-SQKKGSDP--LILIS--ERSVNRDPNAYRYKWSKGSKNFQEHLEHFVSE
QKSR---FQVVFDRYRINQYSSDRSEVSDKKDLAKFRSFVFSK----------------------LLLFLSN-
SLPFFFVSFGNPPIQRSE-IRVS-ELKGPNDRLCNQFLESIGLQLVYLKKLKPF--LLD---DHET

S-QKSKLLFN----------KKP-EG-MIDSFHTR--NNRGKSLD---SYFSMISHDQDNWLNPVKPFH
RSSLISSFYKANRLRFLNNPHDFGFF--CNKRFPFYVD-------IKNLD--FTYG-QFLNILFIRNT-
KFSLCGDK--KKHAFLE-RDTISSIESQVSNLFK--DFPQSGDER-YNFYKY---FHLAMRSDPL
VR--RAIYSIADISGTPLTEGQRVNFERTYCQPLSDMNLSDSEGKNLYQYLN--FNSNMGLIY-
--SEKCFS--SEKRKKKK-----PEKRKEK--KPEKRKEKKPE---KRKE------KKPEKRKEKKPEK
RKEKKPE-KRKEKKPEKRKESLTSTGSPKFVSICYAISDPCGISWRILQKKLCLLQ---------WI
SAISNKCFHK--LLLSEESIHRNNESPSMTDLRWP---NLGAF-LYSILFLLFVAGH-LVFSHLLF
LSQ--DFSELQRDFARAQSLMIPSYIVELRELLDMYPAPRS----FKKLFLAAREKLVN-------YL
RWGGGRK---------------------SFLIH---LFELLNITPNPIDR-IAFLKNTRHLSHTSKELYSLITEL
GNGPCCLIDDKIESWISNCDAIEDKEREFLVPFCN--FTR--ETRIDQILLSLT-HSDHLS-----NN
DSASQMSEEPGAFYLRHLVDIH--KKGLMNYECNTSCLAERRIFLAHYQTIT----YSPCGDN
RSHFPSHG---KTFSLRLPLHPS-RATLVIGSIGSGRSYLVKSLATNSYVPLITVVLNKFLKNW
TPQGFDIH-ESGVYD-YEDRDSDDYDEPG--------------------ASDDYED-RDMEDFVDSEMTE
WLTKTNVPLVYQLFYITLQFELAKAMSPCILWIPNIHDLDAKESDYLSLGLLVNHLSR---DC
GRRSTKNEILVIASTHIPQKVDPSLIGPDGLSTCIKTRRLLVPQQQQCLFTLSYTRGFHLENK
MFHTHTNEFESTILGPSVPDLVALTNEALSISITQKKSIIDTTTIRYALHRKTWDLEAD-RNLS
PAKEHGTLFYQVGRAFAHTVLLRNCPIDPISIYIKKN--LCEAGDSSLYKWYFELGTSMKKL
TILLYLLTCSAGSIAQDLLSPPG---PDEQNLITSYGLVENDS-DLVHGLLELEGALVGSSGEG
TEKDSSQFDND--RVTLLLRPKPRN-PLDIQRLIYQHQKY-ESELEE---DDDDDE--DVFAPQK
ML-------ED----LFSE--LVWSP--RIWHP---WDFILDCDEEDERLEAQ-QEDELLEEEHEEEEEE
EEEDELQENDSEFFRSETQQPQARDGFSEEEGCFRISQFMWVPGDPLSFLYK-DTPFVEVLS
YPEEAT--EISKELLR-LLNAPKRARQRWWT--KKKQDKHYELVLDRQRWLIT---KSSLSKSN
GFFRSN--TPSESYQYLSNLFLSNR-RLLDQITKTFFRKKWLFPDEMKIGF--MEQ-------MPRS
R-INGNFIDKTFSIVANILLRIIPTTSGEKEAFTYYRDG--MSAQSEGNYAEALQNYYEAMRL
EIDPYDRSYILYNIGLIHTSNGEHTKALEYYFRALERNPFLPQAFNNMAVICHY-RGEQAIR
QGDSEIAEAWFDQAAEYWKQAIALTPGNYIEAQNWLKITRRFE--MIWRSEHIWIELIVGSR
KISNFCWAFLLFLGSLGFVLVGTSSYLGKNLISLVSSQQIHFFPQGLVMSFYGIAGLFISSYL
WCTISWNVGSGYDRFDRKEGIVCIFRWGFPGKNRRIFLRFRMKDIQSIRIEVKEGIYARRVL
YMEIKGQGAIPLTRSDDN-LTLREIEQKAAELAYFLRVPIEVF
>Lotus
MEKWWLNSMLRLSKSTDRFGPIENTSVSEDPILTDMDKNIHSYNNVDYLVGVENIRNFLS
NKSFLVRDSKRNSYSIYFAIENQILEIDNDH--LFLSELESFFSRYTNSIYLNNSSKGDGD---H
YMYDTESSWNNNINSFIENYIR--SQICIDNYILGDGDKYND-YFYSYICSRERTSIITSTNDS
DFTKIEGSNDLDETQKYKHLWIECENCYGLNYKKILKSKMNICEHCGYHLKMSSSDRIEL
SIDPGTWNPMDEDMISVDPIEFHSEEEPYKDRIDSYQKTTGLTEAVQTGTGHLNGIPVAIGI
MDFEFMGGSMGSVVGEKITRLVEYATNQLLPLIVVCASGGARMQEGSLSLMQMAKISSAL
YDYQLNKKLFYVSILTSPTTGGVTASFGMLGDIIIAEPNAYIAFAGKRVIEQTLNKAVPEGS
QAAEYLFHKGLFDSIVPRNPLKGILSELFQLHAFFPLIQNEKEMVTIRADEISKIIRERIEQY
NTEIKIVNTGTVLQVGDGIARIYGLDEVMAGELVEFEEGTIGIALNLESKNVGVVLMGDG
LMIQEGSSVKATGRIAQIPVSEGYLGRVINALAKPIDGRGEISSSESRLIESPAPGIISRRSVYE
PLQTGLIAIDSMIPIGRGQRELIIGDRQTGKTAVATDTILNQQGQNVICVYVAVGQKASSVAQ
VVNTLQERGAMEYTIVVAETADSPATLQYLAPYTGAALAEFFMYRERHTLIIYDDLSKQA
QAYRQMSLLLRRPPGREAYPGDVFYLHSRLLERAAKLSSQLGEGSMTALPIVETQSGDVS
AYIPTNVISITDGQIFLSADLFNAGIRPAINVGISVSRVGSAAQIKAMKQVAGKLKLELAQFA

ELEAFAQFASDLDKATQNQLARGQRLRELLKQSQSAPLTVEEQVITIYTGTNGYLDSLEIR
QVRKFLVELRAYLKTNKPQFNEIISSTKTFTGEAEALLKEAIQEQMELFLLQEQVMIINPTT
SDPEVSALEKKNTGRIAQIIGPVLDVTFPPGKMPNIYNALIVKGRDTVGQQINVTCEVQQL
LGNNRVRAVAMSATDGLTRGMEVIDTGAALSVPVGGVTLGRIFNVLGEPIDNLGPVDTRT
TSPIHRSAPAFIQLDTKLSIFETGIKVVDLLAPYRRGGKIGLFGGAGVGKTVLIMELINNIAK
AHGGVSVFGGVGERTREGNDLYMEMKESGVINEKNIAESKVALVYGQMNEPPGARMRV
GLTALTMAEYFRDVNEQDVLLFIDNIFRFVQAGSEVSALLGRMPSAVGYQPTLSTEMGSL
QERITSTKEGSITSIQAVYVPADDLTDPAPATTFAHLDATTVLSRGLAAKGIYPAVDPLDSTS
TMLQPRIVGEEHYETAQRVKQTLQRYKELQDIIAILGLDELSEEDRLTVARARKIERFLSQP
FFVAEVFTGSPGKYVGLAETIRGFKLILSGELDSLPEQAFYLVGNIDEATVKATNLEKESKL
KKMTLNLCVLTPNRIVWDSDVKEIILSTNSGQVGILPNHAPLAMALDIGILRIRLNDQWLT
MALMGGFARIGNNEITVLVNDAEKGSDIDPQEAQQTLEIAEANLKEAKGKRQTIEANLAL
RRARTRVESINMISMKNITDPFPCLGYWPSAGSFGFNTDILATNPINLSIVIGVLIFFGKGVLS
DLLDNRKQRILRTIRNSEELREGAIEQLEKAQARLKKVETEADRFRVNGYSEIEREKLNLIN
SIYTTLEQLENYKNETIHFEQQRAINQVRQRVLQQALQGALGTLNSCLNNELHFRTIAANI
GMFGAMKEKNNMNPIISAASVIAAGLAVGLASIGPGVGQGTAAGQAVEGIARQPEAEGKI
RGTLLLSLAFMEALTIYGLVVALALLFANPFVMNVLLCSINTFKRLYDISAVEVGQHFYWQ
MGDFQVHAQVLITSWVVIAILLGSTILVVRNPQTIPNFGQNFFEYVLEFIRDVSKTQIGEEY
GPWVPFIGTLFLFIFVSNWSGALLPWKIIQLPHGELAAPTNDINTTVALALLTSVAYFYAGIS
KKGLAYFGKYIQPTPILLPINILEDFTKPLSLSFRLFGNILADELVVVVLVSLVPLVVPIPVMF
LGLFTSGIQALIFATLAAAYIGESMEGHHMIFSTLEHILTHISFSVVSIVISIHLITLFVNQIVGF
YDSSKKGMIITFLCITGLLITRWFFSGHLPFSDLYESLIFLSWGFSIFYMVPRFKKQKNDLSTI
IAPSVIFTQGFATSGLLTEMHQSVILVPALQSHWLMMHVSMMILGYAALLCGSLLSVAILVI
TFQELIPILGKSKEIKYINMNENVLRKTSFSSYRNYYRYQFIQQLDRWGYRIISLGFIFLTIGI
LSGAVWANEAWGSYWNWDPKETWAFITWTIFAIYLHSRKNKKLEGLNSSIVASIGFLIIWI
CYFGVNLLGIGLHNYGSFTSNMAKKKASIPFLSLTSIVFLPWCISFTCKKGMEYWVTNWW
NTKQSEIFLNIIQEKSILKKFMELEELFFLDELLKEYSETRLQILRTG-HKETIQLIKTHNEDRI
YTILHFSTNIIYFIILSGYSILGNQELIILNSWVQEFLYNLSDTIKAFSILLVTDLCIGFHSTHG
WELLIGSVYKDFGFIQNDQIISGLVSTFPVILDTILKYWIFRYLNRVSPSLVVIYHSMNDMPI
GVPKVPFRSPGEEDASWVDIY-RLYRERLLFLGQEVNSEISNQLIGLMVYLSIEDDKKDLYL
FINSPGGWVIPGIAIYDTMQFVQPDVQTVCMGLAASMGSFVLAGGKITKRLAFPHA-VMI
HQPASSFYEAQTGEFILEAEELLKLRETITRVYVQRTGKPLWLVSEDMERDVFMSAAEAQA
Y G I V D L V A V
E??MEEYQL
YLELDRSRQQDFLYPLVFHEYIYGLAYSHDLFGYDNKYSLLIVKRLITRMYQQNHLIISAN
DSNKNRFLRKNFYSQIISEGFAIIVEILFSLQLSSSLIIKSYNLRSIHSIFPFFEDKVTYLNYISDI
RVPYPIHLEILVQILRYWVKDAPLFHLLRLFLYDYCNWNSIFIKSIYTFSKPRFFFFLYNFYV
CEYESIFFFLRTKSFHLRLKSFRVFFERIFFYAKKGHLVEVFVKFSTLTFFKDPFIHYVRYQG
KSILASKNLPILMNKWKYYFIHLWQCYFDVWSQPRTIRINQLSEYSFHFLGYFLKVGLKHS
VVRGQMLQNGFLIKI--IIKKLDIIVPIIPIIRLLAKSKFCNVLGNPLSKPSWADLSDFEIIARFL
RICRNLSHYYNGSSKKKSLYRIKYILRLSCIKTLACKHKSTVRAFSKRLGSELLEEFFTEEEE
ILSLIFPTRIWYLDILFSNDLINHDMIIDTT-EVQDIN-SFSRLES-FKEVYGVLWVLAPILIIVL
GITISVLAIVWLEREISAGMQQRIGPEYAGPFGVLQALADGTKLLFKENLIPSRGDIRLFSIG
PSISVISILITYLVIPFSYNFVLSDLNIGVFLWIAISSIAPIGLLMSGYGSNNKYSFLGGLRAAA

QSISYEIPLTLCVLSISLLSNSLSTVDIVDAQSKYGFWGWNLWRQPMGFVVFLISSLAECER
LPFDLPEAEEELVAGYQTEYSGIKFGLFYVASYLNLLISSLFVTVLYLGGSNISIPYISLFEFFE
INKEYGVFGTTIGIFITLAKTYLFLFVSIITRWTLPRLRMDQLLNLGWKFLLPISLGNLLLTT
SSQLFSLMI*HVQNENLILDSTRIFMKAFHLLLFDGSFIFPECILIFGLILLLMIDSTSDQKDIS
WFYFISSTSLVMSIVVLLFRWREEPMISFSGNFQTNNFNEIFQFLILLSSTLCIPLSVEYIECTE
MAITEFLLFVLTATLGGMFLCSANDLITIFVAPECFSLCSYLLSGYTKKDVRSNEATMKYLL
MGGASSSILVHGFSWLYGSSGGEIELQEIVNGLINTQMYNSPGISIALIFITVGIGFKLSLAPS
HQWTPDVYEGSPTPVVAFLSVTSKVAASALATRIFDIPFYFSSNEWHLLLEILAILSMILGNL
IAITQTSMKRMLAYSSIGQIGYVIIGIIVGDSNGGYASMITYMLFYISMNLGTFACIVSFGLR
TGTDNIRDYAGLYTKDPFLALSLALCLLSLGGLPPLAGFFGKLYLFWCGWQAGLYFLVSIG
LLMSVVSIYYYLKIIKLLMTGRNQEITPHVRNYKRSPLRSNNSIELSMIVCVIASTISGISMN
PIIEIAQDTLFMFLLYEYDIFWAFLIISIFIPILAFTISGFLAPINKGPEKLSSYESGIEPMGDAW
LQFQIRYYMFALVFVVFDVETVFLYPWAMSFDVLGISVFIEALIFVLILIVGSVYAWRKGAL
EWSTNYFPWLTLVVILPIAGGSLIFLFPHRGNKVIRWYTVCICLIDLLLTTYAFCYHFQLDDP
LIQLTESYKWINFFDFYWRFGIDGLSIGPILLTGFITTLATLAAQPITRECKLFYFLMLAMYS
GQIGPFSSRDILLFFIMWELELIPVYLLLAMWGGKKRLYSATKFILYTAGSSVFLLMATLGI
GLYGSNEPTLNFETLNNQSYPLALEIIVYIGFLIAFAVKSPIIPLHTWLPDTHGEAHYSTCML
LAGILLKMGAYGLVRINMELFSHAHSIFCPWLMILGSIQIIYAASASLGQRNLKKRIAYSSV
SHMGFLIIGIGSISDTGLNGAILQIISHGFIGAALFFLSGTSYDRLRLLYLDEMGGMALPMPK
IFTVFTILSMASLALPGMSGFFAELIVFWGIITSQKYFLIMKILITFVTAIGMILTPIYSLSILRQ
MFYGYKFFNTPNSYFFDSGPRELFISISILIPIIGIGIYPDFIFSFSVDKVEAVLSHF---MMLEH
VLVLSAYLFSIGIYGLITSRNMVRALMCLELILNAVNMNLVTFSDFFDNRQLKGNIFSIFVIA
IAAAEAAIGPAIVSSISRNRKSIRINQSNLLNKMEYTHQSSWIIPFTPLPVPILIGVGLLLFPTA
TKNLRRMWAFPSIFLLIIVMIFSVDLSIHQIKNSSIYQYVWSWTINTDLSLEFGYLIDSLTSIM
SILITTVGILVLIYSDNYMSHDQGYLRFFAYLSFFNTSMLGLVTSSNLIQVYIFWELVGMCS
YLLIGFWFTRPIAANACQKAFVTNRVGDFGLLLGILGLYWITGSLEFRDLFQIINN-EVNIFFI
TLCALLLFCGSVAKSAQFPLHVWLPDAMEGPTPISALIHAATMVAAGIFLVARLLPFFIAIPS
IMNGIAFIGIITVVLGATLAIAQKDIKKNLAYSTMSQLGYMMLALGMGSYRIALFHLITHA
YSKALLFLGSGSIIHSMEAIVGYSPNKSQNMLLMGGLTKHVPITKTAFLIGTLSLCGIPPFAC
FWSKDEILNDSWLYSPIFAIIACSTAGLTAFYMFRIYLLVFEGYFNVHFQNFNGKKNSSFYSI
SLWGK-EGKKKLKNLLSLLTMNLFRGTKKFPCFYPHESDNTMLFSMLVLVLFTFFVGAIG--
FDLDILSKLLSPSIDLLHQNSN-SVDW-EFFTNATFSVSIAFFGIFIASFFYKPVYSTLQNLNLL
NLFEILADTIINGIYDWSYNRGYIDGFYEISLIASVRKLAKLNSFFDRQVIDGIPNAVGITSFL
IGEAFKYVGSG-RISSYILFFVFFVLLFLIIFYMDLPKTLHDFLLVFLGSGLILGSLGVVLLTN
PIFSAFSLGLTLVCISLLYILSNSHFVAASQLLIYVGAINVLIIFGVMFMNGSEYYQDFRLWT
VGDGMTLMVCTSIFISLITTIPDTSWYGIIWTTRSNQIIEQDLISTSQQIGIHLSTDFFLPFELIS
IILLAALIGAIVVARQMNVPATRKDLMIVNMGPHHPSMHGVLRLIVTLDGEDVIDCEPILG
YLHRGMEKIAENRTIIQYLPYVTRWDYLATMFTEAITVNGPEQLGNIQVPKRASYIRVIML
ELSRIASHLLWLGPFMADIGAQTPFFYIFREREFIYDLFEAATGMRMMHNFFRIGGVAADL
PHGWIDKCFDFCNYFFTRVVEYQKLITRNPIFLERVEGVGVVGGEEVINWGLSGPMLRAS
GIQWDLRQVDNYECYEEFDWEVQWQKEGDSLARYLVRIGEMMESIKIIQQALEGIPGGPY
ENLEIRCFGREKEPEWNDFEYRFIGKKPSPTFELPKQELYVRVEAPKGELGIFLIGDQNGFP
WRWKIRPPGFINLQILPQLVKRMKLADIMTILGSIDIIMGEVDRMFLMVTGFMNYSQQTVR
AARYIGQSFMITLSHANRLPVTIQYPYEKLITSERFRGRIHFEFDKCIACEVCVRVCPIDLPV

VDWKLETNIRKKRLLNYSIDFGICIFCGNCIEYCPTNCLSMTEEYELSAYDRHELNYNQIAL
GRLPMSVIDDYTIRTIQIK----------------MQGRLSAWLVKHGLVHRSLGFDYQGIETLQIKPE
DWHSIAVILYVYGYNYLRSQCAYDVSPGGLLASVYHLTRIECGIYQPEEVCIKIFVPRNNPR
IPSIFWVWKSADFQERESYDMLGISYDNHPRLKRILMPESWIGWPLRKDYITPNFYEIQDA
H---MNSIEFPLIDRTTQNSVISTTLNDLSNWSR
LSSLWPLLYGTSCCFIEFASLIGSRFDFDRYGLVPRSSPRQADLILTAGTVTMKMAPSLVRLY
EQMPEPKYVIAMGACTITGGMFSTDSYSTVRGVDKLIPVDVYLPGCPPKPESILDAITKLR
KKISREIHEDQLSSQ-ENRCFTTNHKFYVERSTHTGNYDQVLFHQPPSTSEIST--FFRYQKV
QYPNEIVNMQTRNAFSYIKEEITRSISVLLVIYIIIRAPISNAYPIFAQQGYENPREATGRIVCA
NCHLANKPVDIEVPQTVLPDTVFEAVVRIPYDMQVKQVLANGKRGALNVGAVLILPEGFE
LAPTDRISPEIKEKMGNLSFQSYRPTKKNILVVGPVPGQKYSEITFPILSPDPATNRDVNFLK
YPIYVGGNRGRGQIYPDGSKSNNNVYNATTSGIINKIIRKDKGGYEITIVDASDGREVIDIIP
PGPELLVSEGESIKLDQPLTSNPNVGGFGQGDAEIVLQDPLRVQGLLFFLASIIFAQIFLVLK
KKQFEKVQLSEMNFMSKVYDWFEERLEIQAIADDITSKYVPPHVNIFYCLGGITLTCFLVQ
VATGFAMTFYYRPTVTEAFASVQYIMTEANFGWLIRSVHRWSASMMVLMMILHVFRVYL
TGGFKKPRELTWVTGVVLGVLTASFGVTGYSLPWDQIGYWAVKIVTGVPEAIPVIGSSVVE
LLRGSASVGQSTLTRFYSLHTFVLPLLTAVFMLMHFSMIRKQGISGPLMGVTKKPDLNDPV
LRAKLAKGMGHNYYGEPAWPNDLLYIFPVVILGTIACNVGLAVLEPSMIGEPADPFATPLEI
LPEWYFFPVFQILRTVPNKLLGVLLMVSVPAGLVTVPFLENVNKFQNPFRRPVATTVFLIGT
AVSLWLGIGATLPIEKSLTLGLFMIEVFLFGIVLGLIPITLAGLFVTAYLQYRRGDQLDLMPTI
TSYFGFLLAVLTITSGLFISLRKLRLIMDIVSLAWAALMVVFTFSLSLVVWGRSGLMIIRSPE
PEVKILVDRDPIKTSFEEWAKPGHFSRTIAKGPDTTTWIWNLHADAHDFDSHTSDLEDISR
KIFSAHFGQLSIIFLWLSGMYFHGARFSNYEAWLSDPTHIRPSAQVVWPIVGQEILNGDVG
GGFRGIQITSGFFQIWRASGITSELQLYCTAIGGLVFAALMLFAGWFHYHKAAPKLAWFQD
VESMLNHHLAGLLGLGSLSWAGHQVHVSLPINQFLNAGVDPKEIPLPHEFILNRDLLAQL
YPSFAEGATPFFTLNWAKYADFLTFRGGLDPVTGGLWLTDIAHHHLAIAILFLIAGHMYRT
NWGIGHGIKDILEAHKGPFTGQGHKGLYEILTTSWHAQLSINLAMLGSLTIIVAHHMYSMP
PYPYLATDYGTQLSLFTHHMWIGGFLIVGAAAHAAIFMVRDYDPTTRYNDLLDRVLRHR
DAIISHLNWVCIFLGFHSFGLYIHNDTMSALGRPQDMFSDTAIQLQPIFAQWIQNTHALAPG
ITAPGATTGTSLTWGGGDLVAVGNKVALLPIPLGTADFLVHHIHAFTIHVTVLILLKGVLFAR
SSRLIPDKANLGFRFPCDGPGRGGTCQVSAWDHVFLGLFWMYNAISVVIFHFSWKMQSD
VWGSISDQGVVTHITGGNFAQSSITINGWLRDFLWAQASQVIQSYGSSLSAYGLFFLGAHF
VWAFSLMFLFSGRGYWQELIESIVWAHNKLKVAPATQPRALSIVQGRAVGVTHYLLGGIAT
TWAFFLARIIAVGMALRFPRFSQGLAQDPTTRRIWFGIATAHDFESHDDITEERLYQNIFASH
FGQLAIIFLWTSGNLFHVAWQGNFEAWVQDPLHVRPIAHAIWDPHFGQPAVEAFTRGGAL
GPVNIAYSGVYQWWYTIGLRTNGDLYSGALFLLFLSAISLLAGWLHLQPKWKPSVSWFK
NAESRLNHHLSGLFGVSSLAWTGHLVHVAIPGSRGEYVRWNNFLSILPHPQGLGPLFTGQ
WNLYAQNPDSSSHLFGTSQGAGTAILTLLGGFHPQTQSLWLTDMAHHHLAIAILFLLAGH
MYRTNFGIGHSIKDLLEAHIPPGGRLGRGHKGLYDTINNSIHFQLGLALASLGVITSLVAQH
MYSLPAYAFIAQDYTTQAALYTHHQYIAGFIMTGAFAHGAIFFIRDYNPEQNEDNVLARM
LDHKEAIISHLSWASLFLGFHTLGLYVHNDVMLAFGTPEKQILIEPIFAQWIQSAHGKTSYG
FDVLLSSTNSPAFNAGRSIWLPGWLNAINE--NSNSLFLTIGPGDFLVHHAIALGLHTTTLILV
KGALDARGSKLMPDKKDFGYSFPCDGPGRGGTCDISAWDAFYLAVFWMLNTIGWVTFY
WHWKHITLWQGNVSQFNESSTYLMGWLRDYLWLNSSQLINGYNPFGMNSLSVWAWMF

LFGHLVWATGFMFLISWRGYWQELIETLAWAHERTPLANLIRWRDKPVALSIVQARLVGL
AHFSVGYIFTYAAFLIASTSGKFGMSHSVKIYDTCIGCTQCVRACPTDVLEMIPWDGCKAK
QIASAPRTEDCVGCKRCESACPTDFLSVRVYLWHETTRSMGLAYMT--NLPSIFVPLVGLVF
PAMAMASLFLYLQKNKIFMRDLKTYLSVAPVVSTLWFAALAGLLIEINRLFPDALIFPFFSF
MTAILERRESENLWGRFCNWITSTENRLYIGWFGVLMIPTLLTATSVFIIAFIAAPPVDIDGIR
EPVSGSLLYGNNIISGAIIPTSAAIGLHFYPIWEAASVDEWLYNGGPYELIVLHFLLGVACY
MGREWELSFRLGMRPWIAVAYSAPVAAATAVFLIYPIGQGSFSDGMPLGISGTFNFMIVFQ
AEHNILMHPFHMLGVAGVFGGSLFSAMHGSLVTSSLIRETTENESANEGYRFGQEEETYNI
VAAHGYFGRLIFQYASFNNSRSLHFFLAAWPVVGIWFTALGISTMAFNLNGFNFNQSVVDS
QGRVINTWADIINRANLGMEVMHERNAHNFPLDLAAVEAPSINGMGLPWYRVHTVVLND
PGRLLAVHIMHTALVAGWAGSMALYELAVFDPSDPVLDPMWRQGMFVIPFMTRLGITNS
WGGWNIT-GGTITNPGIWSYEGVAGAHIVFSGLCFLAAIWHWVYWDLEIFSDERTGKPSLD
LPKIFGIHLFLAGLACFGFGAFHVTGLYGPGIWVSDPYGLTGRVQPVNPAWGVEGFDPFVP
GGVASHHIAAGTLGILAGLFHLSVRPPQRLYKGLRMGNIETVLSSSIAAVFFAAFVVAGTM
WYGSATTPIELFGPTRYQWDQGYFQQEIYRRVGAGLAENQSFSEAWSKIPEKLAFYDYIG
NNPAKGGLFRAGSMDNGDGIAVGWLGHPIFRDKEGRELFVRRMPTFFETFPVVLVDGDGI
VRADVPFRRAESKYSVEQVGVTVEFYGGELNGVSYSDPATVKKYARRAQLGEIFELDRAT
LKSDGVFRSSPRGWFTFGHASFALLFFFGHIWHGSRTLFRDVFAGIDPDLDSQVEFGAFQK
LGDPTTRRQVVMKTLYSLRRFYHVETLFNGTLALTGRDQETTGFAWWAGNARLINLSGK
LLGAHVAHAGLIVFWAGAMNLFEVAHFVPEKPMYEQGLILLPHLATLGWGVGPGGEVID
TFPYFVSGVLHLISSAVLGFGGIYHALLGPETLEESFPFFGYVWKDRNKMTTILGIHLILLGI
GAFLLVFKASYFGGIYDTWAPGGGDVRKITNLTLSPSIIFGYLLKSPFGGEGWIVSVDDLED
IIGGHVWLGSICILGGIWHILTKPFAWARRALVWSGEAYLSYSLAALSVFGFIACCFVWFNN
TAYPSEFYGPTGPEASQAQAFTFLVRDQRLGANVGSAQGPTGLGKYLMRSPTGEVIFGGE
TMRFWDLRAPWLEPLRGPNGLDLSRLKKDIQPWQERRSAEYMTHAPLGSLNSVGGVATE
INAVNYVSPRSWLATSHFVLGFFLFVGHLWHAGRARAAAAGFEKGIDRDFEPVLSMTPLN
MTIALGKFTKDENDLFDIMDDWLRRDRFVFVGWSGLLLFPCAYFALGGWFTGTTFVTSW
YTHGLASSYLEGCNFLTAAVSTPANSLAHSLLLLWGPEAQGDFTRWCQLGGLWTFVALHG
AFGLIGFMLRQFELARSVQLRPYNAIAFSGPIAVFVSVFLIYPLGQSGWFFAPSFGVAAIFRF
ILFFQGFHNWTLNPFHMMGVAGVLGAALLCAIHGATVENTLFEDGDGANTFRAFNPTQA
EETYSMVTANRFWSQIFGVAFSNKRWLHFFMLFVPVTGLWMSALGVVGLALNLRAYDFV
SQEIRAAEDPEFETFYTKNILLNEGIRAWMAAQDQPHENLIFPEEVLPRGNALMSGSTGER
SFADIITSIRYWIIHSITIPSLFIAGWLFVSTGLAYDVFGSPRPNEYFTESRQGIPLITGRFDPLE
QLDEFSRSFMTIDRTFPIFTVRWLAVHGLAVPTVSFLGSISAMQFIQRMATQTVEDSSR-ARP
RQTSVGSLLKPLNSEYGKVAPGWGTTPLMGIAMALFAIFLSIILEIYNSSILLDGISMNMLTL
KLFVYTVVIFFVSLFIFGFLSNDPGRNPGREEMADTTGRIPLWIIGTVTGITVIGLIGIFFYGS
YSGLGSSLMINIVSLVCIYINSVPYSSIFFLDKLPEAYAFLNPIVDIMPVIPLFFFLLAFVWQA
AVSFRTTQSNPNEQSVELNRTSLYWGLLLIFVLAVLFSNYFFNMEVNILAFIATALFILVPTA
FLLIIYVKTVSQSDMETATLVAISISGLLVSFTGYALYTAFGQPSQQLRDPFEEHGDMEALVY
TFLLVSTLGIIFFAIFFREPPKVPTKK--MTIAFQLAVFALIATSSILLISVPVVFASPDGWSSNK
NVVFSGTSLWIALVFLVGILNSLIS-------MSPQTETKASVGFKAGVKDYKLTYYTPDYETK
DTDILAAFRVTPQPGVPPEEAGAAVAAESSTGTWTTVWTDGLTSLDRYKGRCYHIEPVPG
EESQFIAYVAYPLDLFEEGSVTNMFTSIVGNVFGFKALRALRLEDLRIPNAYVKTFQGPPHG
IQVERDKLNKYGRPLLGCTIKPKLGLSAKNYGRAVYECLRGGLDFTKDDENVNSQPFMR

WRDRFLFCAEALFKAQEETGEIKGHYLNATAGTCEEMMKRAVFARELGVPIVMHDYLTG
GFTANTTLAHYCRDNGLLLHIHRAMHAVIDRQKNHGMHFRVLAKALRMSGGDHIHAGT
VVGKLEGEREITLGFVDLLRDDFVEKDRSRGIYFTQDWVSLPGVLPVASGGIHVWHMPAL
TEIFGDDSVLQFGGGTLGHPWGNAPGAVANRVALEACVQARNEGRDLAREGNEIIREASK
WSPELAAACEIWKEIKFEFQAMDTL--MAIHLYKTSTPSTRNRAVDSQVKSNPRNRLIYGL
HCCSKGRNARGIITAGHRGGGHKRLYRKIDFRRNEKNIYGKIVTIEYDPNRNAYICLIHYG
DGEKRYILHPRGAIIGDTIVSGTEVPIKMGNALPLTDMPLGTAIHNIEITFGKGGKLARAAG
AVAKLIAKEGKSATLKLPSGEVRLISKNCSATVGQVGNVGVNQKSLGRAGSKCWLGKRP
VVRGVVMNPVDHPHGGGEGRAPIGRKKPVTPWGYPALGRRSRKRKKYSDNLILRRRTK
MIQPQTHLNVADNSGARKLMCIRIIGAS-NRRYAYIGDIVVAVIKEAVPNTPLERSEVIRAVIV
RTCKELKRSNGIIIQYDDNAAVVIDQEGNPKGTRIFCAIARELRQFNFTKIVSLAPEILMLSP
KRTRFRKQHRGRMKGISHRGNQICFGRYALQALEPAWITSRQIEAGRRAMSRNVRRGGQI
WVRIFPDKPVTVRPTETRMGSGKGSPEYWVAVVKPGKIVYEMGGVAENIARKAISIAASK
MPIRTQFIISGMTRIKRGYIARKRRTKIRLFTSSFRGAHSRLTRTISQQKIKALVSAHRDRNR
KKREFRGLWISRINAGIGDNKNIYYSYSNFMYNLYKKQLLLNRKIVAQIAIFKGNCLFMIA
N E I I T - - - - - - - - - -
-???
??????????????????????????????????MNGIKYAVFTDKSIRLLGKN-QYTSNVESGSTRTEIKH
WVELFFGVKVKAMNSHRLPAKGRKVRLIMGHTMHYRRMIITLQPGYSIPPLRKKRTMAV
PKKRTSISKKLIRKNFWKKRGYWTALKAFSLAQSIFTGNSKSFF--K---MAKGKDIRIIVILE
CTGC-DQK--VNKE--GISRYITQKNRHNTPSRLELIKFCPCCRKHMIHAEIKKMKIGASVRKI
CEKCRLIRRRGRIIVICSNPRHKQRQGMVREKVRVSTRTLQWKCVESRIDSKRLYYGRFILS
PLMKGQADTIGIAMRRILLGEMEGTCITRAKSE--KISHEYSTIVGIQEPVHEILMNLKEIVL
KSNLYGIRDASICFKGPGYVTAQNIILPPSVVIVDNTQHIANVTEPIQLCIGLQIERDRGYRIN
TLKNFQDGSY--NIDAIFMPVRNANHSIHSYVNGNEKQEILFLEIWTNGSLTPKEALYEASR
NLIDLFIPFLHAEEE--KLNFENNE-HKVTLPLFTCHDLLAKKLKKKKK-EIAFKSIFIDQLELP
PKIYNCLKKSNIHTLLELLTKSKEDLMKIEHFRVEDVKHILDILEIEKHFP-------MLGVGN-E
GMSTLPGLNQIQFEGFCRFIDRGLTEELFKFPKIEDTDQEIEFQLFVETYQLVEPSIKEKDAV
YESLTYSSELYVSAGLIWKNSKNIQEQTIFIGNIPLMNSLGTSIVNGIYRIVINQILQSPGIYY
QSELDHKGISVYTGTIISDWGGRLELEIDRKARIWARVSRKQKISILVLSSAMGSNLNEILEN
VCYPEIFLSFLNDKEEKKIGSKESAILEFYRQFACVGGDPVFPESLCRELQKKFFQQRCELG
EIGRRNMNRRLNLDIPQNNTFLLPRDILTAADHLIGMKFRMGTLDD--MNHLKNKRIRSVA
DLLQDQFGLALVRLENMVRGTICGAIR-YKLI-PTPQNLVTSTP----LTTTYESFFGLHPLSQV
LDRTNPLTQIVHGRKLSYLGPGGLTGRTASFRIRDIHSSHYGRICPIDTSEGINVGLIGSLAIH
ARIGRWG-SIESPFF---EISERSKR--IHMLYLSPSRDEYYMIATGNYLALNQGNQEEQIVPAR
YRQEFLTIAWEQVHLRSIFSFQYFSIGASLIPFIEHNDANRALMSSNMQRQAVPLSQSEKCIV
GTGLERQVALDSGTLAIAEHEGKIIYKDTNKIVLFGSGETLSVPLVIYRRSNKNTCMHQKS
QVQRGKCIKRGQILADGAATVGGELSLGKNVLVAYMPWEGYNSEDAVLISDRLVYEDIYT
SFHIRKYEIQTHVTSNG-PERITNKIPHLEVHLLRNLDKNGLVILGSWVEAGDILVGKLTPQ
MAKE-SSYAPEDRLLRAILGIQVSTSKETCLKLPIGGRGRVIDVRWLHKKG-GSGYNPETIHI
YILQKREIKVGDKVAGRHGNKGIVSKILARQDMPYLQDGRPVDMVFNPLGVPSRMNVGQ
IFECSLGLAGDVLDRHYRIAPFDERYEQEASRKLVFSELYQASKQTSNPWIFEPEYPGKSRI
FDGRTGTPFEQPVIIGNPYILKLIHQVDDKIHGRSSGHYALVTQQPLKGRAKQGGQRVGEM
EVWALEGFGVAHILQEMLTYKSDHIKARQEVLGTTIIGGTISKPVDAPESFRLLVRELRSLA

LELNHFLVSEKNFRIHRKEV-------MIDQYKHQQLRIGSVSPQQISAWATKILPNGEIVGEVT
KPYTFHYKTNKPEKDGLFCERIFGPIKSGICTCGNYRVIGDKKDEPKFCEQCGVEFVDSRIR
RYQMGYIKLACPVTHVWYLKRLPSYIANLLDKPLKELEGLVYCDF--SFARP-VVKKPTFL
RLRGLFEYEIQSWKYSIPLFFATHGFDTFRNREISSGAGAIREQLVDLDLRIVIDSSLVEWKE
LGEE-RSTHNEWEDRKVGRRKNFLVRRMELAKHFIRTNIEPEWMVLCLLPVLPPELRPIIQI
DGGKLMSSDINELYRRVIYRNNTLIDLLTTSRSTPGELVMCQEKLVQEAVDTLLDNGIRGQ
PMKDGHNKVYKSFSDIIEGKEGRFRETLLGKRVDYSGRSVIVVGPSLSLHRCGLPREIAIEL
FQTFLIRSLIRKHFASNIGVAKSKIREKEPIVWEILQEVMRGHPILLNRAPTLHRLGIQAFQPI
LVEGRAICLHPLVCKGFNADFDGDQMAIHVPLSLEAQAEARLLMFSHTNLLSPAIGDPIAV
PTQDMLIGLYILTSGNRRGICANRYNRSNW---KNSKNEKIRDK--KYMKK-EPFFSNSYDAI
GAFRQKKINFDSPFWLRWRLDKC-IMSS--REAPIEVHYESLGTYHDIYEHYLVIRSIKKQIC
CIYIRTTIGHISFYREIEEAIQGFCRGYSYGI-----------MAERANLVFHNKVIGGTAIKRIISRLI
DHFGMAYTSHILDQVKTLGFRQATTTSISLGIDDLLTIPSKGWLVQDAEQQSLILEKHHHY
GNVHAVEKLRQSIEIWYATSEYLRQEMNPNFSMTDPFNPVHIMSFSGARGNASQVHQLVG
MRGLMSDPQGQMIDLPIQSNLREGLSLTEYIISCYGARKGVVDTAVRTSDAGYLTRRLVEV
VQHIVVRRTDCGTVRGISVNTRNRMMS-ERILIQTLIGRVLADDIYIGSRCIVVRNQDIGIGL
INRFIN------FQTQPIFIRTPFTCRNTSWICRLCYGRSPIHGNLVELGEAVGIIAGQSIGEPGTQL
TLRTFHTGGVFTGGTAEYVRSPSNGKIKFNENSAYPTRTRHGHPAFLCYIDLYVTIESNDIM
HNVIIPPKSFLLVQNDQYVKSEQIIAEIRAGTYTL-NLKEKVRKHIFSDSEGE-MHWSTNIYH
VSEFAYSNVHILPKTSHLWILSGNSHK--SDTVSLSLLKDQDQMSTH--SLPTAKRNTSNFL--
VSNNQ-VR-------------------------LCPDHCHFMHPT-ISPDTSN--LLAKKRRNRFIIPFLFIRERN
NELMP--DISVEIPI--DGIIHKNSILAYFDDPQYRTQSSGIAKYKTIGIHSIFQKED--LIEYR-GI
REFKPKYIKVDRFFFIPQEVHILSESSSIMVRNNSIIGVNTPITLNKKSRVGGLVRVEKNKKK
IELKIFSGDIHFPGEIDKISQHSAILIPP-EMVKKKNSKESKK--KTNWRYIQW--ITTTKKK--Y
FVLVRPVILYDIADSINLVKLFPQDLFKEWDNLELKVLNFILYGNGKSIRGILDTSIQLVRTC
LVLNWNEDE---KSSSIEEALASFVEVSTNGLIRYFLRIDLVK-HISYIRKRNDPSSSGLISY-NE
SDRININPF---FSIY---KENIQQSLSQKH-TIRMLLN---RNKENRSFIILSSSNCF--QMGPFNNV
KYHN---GIKEENHKIPIKISLGPLGV--APQIANFFS--FYHLIT--HNKISSIKKNLQLNK---FKE
TFQVI--K-YYLMDENER--IYKPDLYNNIILNPFHLNWDFIHPN----TFPIIS-LGQFICENVCIV
QNGP---NLKSGQVITVQ-MDFVGIRLANPYLATPGATIHGHYGEMLYEGDILVTFIYEKSRS
GDITQGLPKVEQVLEVRSIDSISMNLEKRIDAWNERITGILGIPWRFLIGAELTIAQSRISLVN
KIQRVYRSQGVHIHNRHIEIIVRQITSKVLVSE---DGMSNVFSPGELIGLLRAQRTGRALEESI
CYRTLLLGITKTSLNTQSFISEASFQETARVLAKAALRGRIDWLKGLKENLVLGGIIPVGTG
FKKIGDRSSARQDTKIT--LETIK-------------IIRGRN-----------------------------MTKRYWNITFE
EMMEAGVHFGHGTRKWNPKMAPYISMKRKGIHIINLTRTARFLSEACDLVFDAASRGKRF
LIVGTKKKAADLVARAAIRARCHYVNKKWLGGMLTNWYTTETRLRKFRELRTEQKTGK
LNCLPKRDAAILKRQLSHLETYLGGIKYMTGLPDIVIIVDQQEEYTALRECITLGIPTICLID
TNCDPDLADISIPANDDAIASIRLILNKLVFAICEGHSS-YIRNFMGQKINPLGFRLGTTQSHD
SIWFAQPTNYSENLKEDKKIRDCIKTYIQKTIKISS-GVEGIGRIKIQKRIDLIQVIIYMGFPKL
LIDGKPR----RIEELQINVQKKMNYVNRKLNIAITRIANAYRDPNILAEFIAGQLKNRVSFRK
AMKKAIELTEQAGTKGVQVQIAGRIDGKEIARVEWIREGRVPLQTIRAKIDYCSYTVRTIY
GVLGIKVWIFLNKEMSRYRGPRFKKIRRLGALPGLTSKRPTVGSELRNQSRSTK-KSQYRIR
LEEKQKLRFHYGLTERQLLKYVRIAGKAKGSTGQVLLQLLEMRLDNILFRLGMASTIPQA
RQLVNHRHVFVNGHIVDIPSYRCKPQDIITAKDNKKSKTLIQNSLESAPR-EELPTHLTLQPF

Q--YKGLVNQIIDSKWVGLKINELLVVEYYSRQTMSRRGTAEKKTAKSDPIYRNRLVNMLV
NRILKHGKKSLAYQIIYRAMKKIQQKTETNPLSVLRQAIRGVTPDIAVKARRVGGSTHQVP
IEIGSTQGKALAIRWLLGASRKRPGRNMAFKLSSELVDAAKGSGDAIRKKEETHRMAEAN
RAFAHFRMGKDTIADIITSIRNADMNRKRTVQIPFTNITENTVKILLREGFIENVRKHRESD
KSFLVLTLRYRR--NTKGSYKT-F--LNLKRISTPGLRIYYNYQKIPRILGGMGIVILSTSRGIM
TDREARLEKIGGEVLCYIWMAKPIPKIGSRKNARSGS-KHLRKIPKGIIHVQASFNNTIVTV
TDVRGRVISWSSAGTCGFKGTRRGTPFAAQTAAGNAIRTVADQGMQRAEVMIKGPGLGR
DAALRAIRRSGILLNFIRDVTPMPHNGCRSPKKRRVMPTMKQLIRKTRQPIRNVTKSPALR
GCPQRRGTCTRVYTITPKKPNSALRKVARVRLTSGFEITAYIPGIGHNLQEHSVVLVRGGRV
KDLPGVRYHIVRGTLDAVGVKDRQQGRSKYGAKKPKMARKSLIQREKKRQKLEQKYHLI
RRSSKKEIS---KVPSLSEKWKIHGKLESLPRNSAPTRLHRRCFSTGRPRANYRDFGLSGHTL
REMVHECLLPGATRSSWMVKKSFIPVISQE-KKEE-NPGSVEFQVFNFTNKIRRLTSHFELH
RKDYLSQRGLRKILGKRQRLLSYLSKKNKIRYKKLINLLDIRESKIR--MVKLRLKRCGKK
QRAVYRIVAIDVRSRREGRDLRKVGFYDPIKNQTYLNIPVILNFLEQGAQPTGTVQDISKK
AGFFMDL---------MDKSKRLFLKSKRFFRRRLPPIQSGDRIDYKNMSLISRFISEQGKILSRR
VNRLTLKQQRLITIAIKQARILSSLPFINNEKK-QFEKSETATRTTTVFKTKKVTR-LTKNPFV
ANRLLRKINNLNTKSEKEIIITWSRTSTIIPAMIGHTIAIHNGKEHLPIYVTDRMVGHKLGEF
SPTLNFRGHAKNDNRSRRMIFQSFILDNLVSLCLKIINSVIVVGLYYGFMTTFSTGPSYLFLL
RAHVMEEG---TEKKISATTGFITGQLVMFISIYYAPLHIALDRPHTITVITLPYLLLYFLGNNQ
KNFLNYVYKNQNSIRHFSIQRIFFQNLFFQLLNPFFLPSSILMRLANIYIFQSNNKVLFLTSSF
VGWLIGHVFFMKWIGLMLVWIQEKNNSIK-IRSNKGVLAKFRKS--QIFLIFFFITCLYYLGRI
PPIYFFKMSEIEEKLKPLVTILFDYNRWNRPLRYIKNDRFENVVRNEISQFFFFTCQSDGKER
ISFTYPPNLSTFQKMMEMKISLFTR-DIIYEELSNSWRSTNEEKKKKLTNEFLNRVEVLDKE
S--VDIFENRIRLCNDEKKQKYLTKEYDPFLNGPCRGQIQKWFSP--SLFINKIHGILYPKFEQ
KDLNDNCIGINEIVKKLPRWSYNLIELEQLEGKQIRSRKAKRVVLLTKALIRYSQQPDFRRD
IIKGSIRAQRRKTVTCKLFQRSVDSPLFLEKMEKTSFFCFDILDSSKIFFMFKNKEEKRRIEI
GEAWDSIIFAQVIRGCLLITQSILRKYILLPSLIITKNIVRILLFQFPEWSEDFRDWQREMYIK
CTYNGVQLSETEFPKKWLTDGIQIKILFPFRLKPWHRSKKVKKKNFCFLTIFGMEVELPFSG
YPRNFSFFDPILKELKKK------KKLKNILKIVNERTKNFITTLKETSKRIIQSILKKVLFLNKKI
KK--L---ITKNIIYESTILIQAINKTNCSLTEKKIKAINAKTKKIIKKIERMTK-ENKNSKKTSSN
TKGLELEKKILQILQRRNVQLTHKLYSFFKFLLNFMKTDIFLCIVSVPRINVQFF---------ALS
QAYVFFKISQIQ--NKYKFKYVFDYRSFFIKDEIKDYFIQGIIHSKLRHKNSP-SLKNQWTNW
LKVHYQYDLSQNRWSRLVQKNLKNRINKHRLDQNKTKCDSYKKTQ--LIVSKNK-KIKKQ
SRYDLLLYKFINDAIRYRDRKYFDWVVMD--------VKIQNNSISNSFFFKFLRFYDAYRNKP
WIIPIKFLFLHYKNKIEAEVNLLLRKYLNFHLNWKGSLNKRVINNVKVYCLLIRLKNIKQIA
ISSIQRGELSLDIMMIQNEKDSTLTGFRGIFIIEPVRLSRKNNEQFFMYETARLLLIHKSKRK
HYALVVIENILSARRRRELRILICF-ILWPNYRLEDLACINRYWFDTYNGSRFSIVRIHMYPM
KGHQFKSWIFELREILKEIKNSRYFLYSWTQFNSTGSFIHIFFHQESFIKLLDSRIWSILLS-GN
S--QGSTSNRYF---TIKDVVLFVVA-ILLYR----INNRKMVERKNLYLTGLLP-----IPMNSIGPR
NDTLEESF-ESSNINRLIVP-LLYLPKEKKISESSFLDPKESTQVLPIT----KKCIMPESNWGSR
WWRDWIRKKR--DSSCKISN---ETVAGIEISFKEKDIK-YLEFPFVYYMD--DPIRKDHDWEL
FDRLSPRKRRNIINL--NSGQLFEILVKDWISYLMFAFREKKPIEVEG-FFKQQG-AGSTIQSN
DIEHVSH--LFLRNKRAISLQNCAQFHMWQFRQDLFV---SWGKKQPHESDFLRN-ISRENWI
WLDNVWLVNKDR---FFS--KIRNVSSNIQYDS---TR--SSFIQVTDSSQLKGSS-DQSRDHFDSI

R----NEDSK-------------YDTLINQREIQQLKERSILCWD--PSFLQTERTDIESERFRFPK--SLSG
YS--SMCRLFM-QREKQMNNHLLPEEIEEF--LGNPTRADRFFFSDRW--SELH-LGSNPTERS
TRDQ----KLLKKEQDVS-FVLSKRSE----KKEIVNIFKIILYLQN--TVSIHPISSDPVCDMVP-K
DEP---DSSNKMSFLNKN---GL--FHLFHDRNRGVYTL--HHDFESE--EIFQEMADLFNLSITEP
-DLVYHKGFAFSIDSSGLDQKQ--FLNEVFNSRDESKKKSL---LVLPTLFY--EENESFYRRIIK
KWVQ-TSCGNDLEDPKPKIVVFTSNNIMEAVNQYRLIRNLIPIQYITYGYGYIRNVLNRFIQ
KN---RFDRNFEYRIQ---RYQIENDTLNHRTRMKYTINQHFSNLKKKSQKKWFDS--LILISRT
ERSMNRDPNAYRYKWSNGNKNFQEHLDHFISEQNSR---FQVVFDRLRINQYSIDWSEVID
KKDLSKSLCFFLYKFLL---------FFS-------KFLLFLSKSLP-FFFVSFGSIPIHRSE-IHIY-ELKSP
NHPLCNQLFESIGLQIVHLKKWKPF--LLD---DHDTS-QKSRFLINGGTISPFLFNKIP-KW-MI
DSFDTI--KNRRKFFDNTDSYFSMISHDEDNWLNPVKPFHRSSLISSFYKANRLRFLNNRYH
FCFY--CNKRLPF---Y-VEKACINNYD--FTYG-QFLNILFIRNK-RFSLCGGK--KKHAFLE-RD
TISPIESRVFNILILNDFPQSGDEG-YNLYKS---FHFPIRSDPFVH--RAIYSIADISVTPLTEGQI
VNFERTYCQPLSDMNLPDSEGKNLHQYLK--FNSNMGLIHIPCSEKYLPSEN--RKK--------GI
PCLK--KCLEKGQMYRTFQRDSVFSTLSKWNLFQTYIPWFLTSTGYKYLNFIFLDTFSD-LLP
ILSSS--QKFVSIFHDIMHRSDISWRILQKKW------CLSQWN-LISEISSKCFHN--LLLSEEIIHR
NNES--PLISTHLRSLNVREF-LYSILFLLLVAGY-LVRTHLLFVSR--VYSELQTEFEKVKSLMI
PSYMIELRKLLDRYPTSEQNSFWLKNLFLVTLEQLGNSLEEIRSSASGGNMLWGGGS-----A
YG-VKSIRSKKKYLNL-----IDLI-SIIPNPINRIAFSRNMRHLSHTSKAIYSLIRKIKNVNGDWI
DDKIESWVSNSDSIDDKEREFLVQFS--TLTT--EKRIDQILLSLT-HSDHLS-----KNNSGYQMI
EQPGTIYLRYLVDIQ--KKYLMNYEFNTSCLVEKRIFLAHYQTITY--SQTLCGTNSFHFSSHG
---KPFSLRLALSPP-RGLLVIGSIGTGRSYLVKYLAANSYVPFIRVFLNKFLDNKPKGFLID----
-DSDDSHDIDDIDDSNDSDD-------------------IDRDLDTELELLTMMNAL---TMDMMP---EID
RFYITLQFELAKAMSPCIIWIPNIHDLDVNESNSLSLGLLANHLS---RDCERCS-TRNILVIAS
THIPQKVDPALIAPNKLNTCIKIRRLLIPQQRKHFFTLSYTRRFHFEKKMFHT--NGFGSITLG
SNVQDLVALTNEALSISIIQKKSIIDTNLIRSALHKQTWDLR---SQVRSVQDHGILFYQIGRA
VSQNVLLSNCSIDPISIYMKKK--SCNEGDSYLYKWYFELGTSMKNLTILLYLLSCSAGAVA
QDLWSLP--GPDEK-NGITSYGLVENDS-DLVHGLLEVEGALVG--SSRTEKDCSQFDND--RV
TLLLRPEPSN-PLNMIQNGSCSIVDQRFLYEK--YESEFEE---GEGVLDLQ----QIEED----LFNH
--IVWAP--RIWCP---WGFLFDCIERPNELGFP-YWARSFRGK---RIIYDEEDELQENDSEFLQG
GTMQYQTQDRSSKEQGFFRISQFIWDPTDPLFFLFK-DQPFVSVFSHREFFADEEISRGLLTF
QTDLPTSIYKRWFI--KNTQEKHFELLIHRQRWLR---TNNSLS--NGFFRSN--TLSESYQYLSN
MFLSN-GTLLDQMTKTLLRKRWLFPDEMVVAI--CSNNESLV--MPRSR-INGNFIDKTFSVV
ANILLQIIPTTSGEKEAFTYYRDG--MSAQSEGNYAEALQNYYEAMRLEVDPYDRSYILYNI
GLIHTSNGEHTKALEYYFRALERNPFLPQAFNNMAVICHY-RGEQAIRQGDSEIAESWFDQ
AAEYWKQAIALTPGNYIEAQNWLKITRRFE--MTWISEDTWVDFVTGCRNPRDFFWAFITL
LGSVGFLLVAASSYLHKNFLSFISSELIRFLPQGATITVYGTAGLFVSLFLWLTIFWNVGSGY
DLFDKKRGIVCIFRYGFPGKNRRIFIRVLINDIQSLIIQSKEGRYLRSVLYMQTREQGAIPLTA
VDDS-WNPPKIAQKAGDLSKLLRVPIEIF
>Triticum
???
???
???
???

??
????????????????????????????????MATLRVDEIHKILRERIEQYNRKVGIENIGRVVQVGDGIA
RIIGLGEIMSGELVEFAEGTRGIALNLESKNVGIVLMGDGLMIQEGSFVKATGRIAQIPVSEA
YLGRVVNALAKPIDGKGEIIASESRLIESPAPSIISRRSVYEPLQTGLIAIDSMIPIGRGQRELII
GDRQTGKTAVATDTILNQKGQGVICVYVAIGQRASSVAQVVTTFHEEGAMEYTIVVAEMA
DSPATLQYLAPYTGAALAEYFMYRERHTLIIYDDLSKQAQAYRQMSLLLRRPPGREAYPG
DVFYLHSRLLERAAKLNSLLGEGSMTALPIVETQSGDVSAYIPTNVISITDGQIFLSADLFN
AGIRPAINVGISVSRVGSAAQIKAMKQVAGKSKLELAQFAELQAFAQFASALDKTSQNQLA
RGRRLRELLKQSQANPLPVEEQIATIYTGTRGYLDSLEIEQVNKFLDELRKHLKDTKPQFQ
EIISSSKTFTEQAEILLKEAIQEQLERFSLQ---MRTNPTTSPPGASTIEEKSTGRIDQIIGPVLD
VTFPPGKLPYIYNALVVQSRDTDDKQINVTCEVQQLLGNNRVRAVAMSATDGLMRGMEV
IDTGAPLSVPVGGATLGRIFNVLGEPVDNLGPVDSSATFPIHRSAPAFIELDTKLSIFETGIKV
VDLLAPYRRGGKIGLFGGAGVGKTVLIMELINNIAKAHGGVSVFGGVGERTREGNDLYM
EMKESGVINEKNIEESKVALVYGQMNEPPGARMRVGLTALTMAEYFRDVNKQDVLLFIDN
IFRFVQAGSEVSALLGRMPSAVGYQPTLSTEMGSLQERIASTKKGSITSIQAVYVPADDLTD
PAPATTFAHLDATTVLSRGLASKGIYPAVDPLDSTSTMLQPRIVGNEHYETAQRVKETLQRY
KELQDIIAILGLDELSEEDRLTVARARKIERFLSQPFFVAEVFTGSPGKYVALAETIRGFQLIL
SGELDGLPEQAFYLVGNIDEASTKAITLEEENKSQKMKLNLYVLTPKRIIWDCEVKEIILST
NSGQIGVLPNHAPINTAVDMGPLRIRLNDQWLTAVLWSGFARIVNNEIIILGNDAELGSDID
PEEAQKALEIAEANLSKAEGTKDLVEAKLALRRARIRIEAVNWIPMKNVTHSFVFLAHWP
SAGSFGLNTDILATNLINLTVVVGVLIFFGKGVLKDLLDNRKQRILSTIRNSEELRRGTIEQL
EKARIRLQKVELEADEYRMNGYSEIEREKANLINATSISLEQLEKSKNETLYFEKQRAMNQ
VRQRVFQQAVQGALGTLNSCLNTELHFRTIRANIGILGSLEWKR-MNPLIAAASVIAAGLA
VGLASIGPGVGQGTAAGQAVEGIARQPEAEGKIRGTLLLSLAFMEALTIYGLVVALALLFA
NPFVMNIIPCSIKTLKGLYDISGVEVGQHFYWQIGGFQIHAQVLITSWVVITILLGSVVIAVR
NPQTIPTDGQNFFEYVLEFIRDLSKTQIGEEYGPWVPFIGTMFLFIFVSNWSGALLPWKIIEL
PHGELAAPTNDINTTVALALLTSAAYFYAGLSKKGLSYFEKYIKPTPILLPINILEDFTKPLSL
SFRLFGNILADELVVVVLVSLVPLVIPIPVMFLGLFTSGIQALIFATLAAAYIGESMEGHHML
FATLEHILTHISFSTISIVITIHLITLLVRELGGLRDSSEKGMIVTFFSITGFLVSRWASSGHFPLS
NLYESLISLSWALYILHTIPKIQNSKNDLSTITTPSTILTQGFATSGLLTEMHQSTILVPALQSQ
WLMMHVSMMLLSYATLLGGPPLSAAILIIRFRNNFHFFSKKKDIKYFYAKRSALKRTSVPS
FPNYYKYQLTERLDSWSYRVISLGFTLLTGGILGGAVWANEAWGAYWNWDPKETWAFIT
WTIFAIYLHSRTHPNWKGTNSALIASIGFLIIWICYFGINLLGIGLHSYGSFTLTMKKKKALP
SLLYLVFIVLLPWGVSSSFNKCLELWIKNWWNTRQSETLLTDIQEKRILERFIELEELSLLDE
MIKGKLKTHVQKPPTGIHKEIIQWVKINNEDHLHTILHFSTNIICLAILSGSFFLGKEELVILN
SWVQEFFYNLNDSIKAFFILLVTDFFVGFHSTRGWELVIRWVYNDFGWAPNELIFTIFVCSF
PVILDTCLKFWVFFCLNRLSPSLVVIYHSISEMPIGVPKVPYRIPGDEEATWVDLYNVMYRE
RTLFLGQEIRCEITNHITGLMVYLSIEDGISDIFLFINSPGGWLISGMAIFDTMQTVTPDIYTI
CLGIAASMASFILLGGEPTKRIAFPHARIMLHQPASAYYRARTPEFLLEVEELHKVREMITR
VYAVRTGKPFWVVSEDMERDVFMSADEAKAYGLVDIVGDEPREAKVTFEGLVTEALPNG
MFRVRLENDTIILGYISGKIRSSSIRILMGDRVKIEVSRYDSSKGRIIYRLPHKDSKDMEKFE
GYSEKHKSRQQYFVYPLLFQEYIYAFAPDYGLVGCNNKFSSLLVKRLIIRMYQQNFLDNSV
NHPNQDRLLDSEFYSQILSEGFAIVVEIPFSLRELFCPIPKFQNLRSIHSIFPFFEDKFLHLDYL
SHIEIPYPIHLEILVQLLQYRIQDVPSLHLLRFLLNYYSNWNSFITKSIFIFKKKRLFRFLYNS

YVSEYEFFLLFLRKQSSCLPLASSGTFLERIHFSRKMEH--IMYPGRKTLWFFMDPLMHYVR
YQGKAILASKGTFLLKKKWKCYLINLWQYYFCFWTQPRRIHINQLANSCFDFMGYLSSVP
KSSLLVRNQMLENSFLIDTRM--KKFDTIVHATLLIGYLSKAQFCTGSGHPISKPIWTDLSD
WDILDRFGRICRNLFHYHSGSSKKKTLYRLKYILRLSCARTLGPKHKSTVRAFMQWLGSV
FLEEFFREEEQVFSLMFAKRIWYLDILRINDLVNPLMIIDRV-EVE-TINSFWKLE-LLKEVYG
LISIL-PILTLLLGITIEVLVIVWLEREISASIQQRIGPEYAGPLGLLQAIADGTKLLFKEDILPSR
GDISLFSIGPSIAVISVLLSFLVIPLGYHFVLADLSIGVFLWIAISSIAPIGLLMAGYSSNNKYS
FSGGLRAAAQSISYEIPLTFCVLAISLLSNSSSTVDIVEAQSKYGFFGWNIWRQPIGFLVFLIS
SLAECERLPFDLPEAEEELVAGYQTEYSGIKYGLFYLVSYLNLLVSSLFVTVLYLGGWNLSI
PYISFFDFFQMNKAVGILEMTMGIFITLTKAYLFLFISITIRWTLPRMRMDQLLNLGWKFLL
PISLGNLLLTTSSQLVSLMIWHVQNENFILDSTSIFMKAFHLLLFNGSFIFPECILIFGLILLLM
FDSTSVQKDRPWFYFISSTCLVISITALLFRWREEPIISFSGNFQTNNFNEIFQFLILLCSTLCIP
LSVEYIECTEMAITEFLLFVLTATLGGMFLCGANDLITIFVAPECFSLCSYLLSGYTERDLRS
NGASL*YLLMGGAGSSILVHGFSWLYGSSGGEIELQEIVNGLINTQMYNSPGISLALISITVG
LGFKLSPAPFHQWTPDVYEGSLLHFVAFHSITSKVAASASATRILEFSLYFSSNEWHLLLEIL
AILSMILGNLLAITQTSMKRMLAYSSIGQIGYVIIGIIVGDSNDGYASMITYMLFYISMNLGT
FACIVLFGLRTGTDNIRDYAGLYTKDPFLALSLALCLLSLGGLPPLAGFFGKLYLFWCGWQ
AGLYFLVSIGLLTSVLSIYYYLKIIKLLMTGRNQEITPYVRNYRRSPLRSNNSIELSMTVCVI
ASTIPGISMNPILAIAQDTLFMFLLHEYDIFWTFLIIASLIPILAFSISGLLAPVSEGPEKLSSYE
SGIEPMGGAWVQFRIRYYMFALVFVVFDVETVFLYPWAMSFDVLGVSVFIEALIFVLILVV
GLVYAWRKGALEWSMSYFPWLTILVVLPIFAGSLIFFLPHKGNKVVRWYTISICLLEFLLMT
YAFCYHFQLEDPLIQLKEDYKWIDVFDFHWRLGIDGLSLGSILLTGFITTLATLAAWPITRN
SRLFYFLMLAMYSGQIGLFSSRDLLLFFIMWELELIPVYLLLSMWGGKRRLYSATKFILYT
AGGSIFFLIGVLGMGLYGSNEPGLDLERLINQSYPATLEILLYFGFLIAYAVKLPIIPLHTWLP
DTHGEAHYSTCMLLAGILLKMGAYGLIRINMELLPHAHYLFSPWLVIIGAIQIIYAASTSLG
QRNFKKRIAYSSVSHMGFIIIGIGSITNIGLNGAILQILSHGFIGATLFFLAGTASDRMRLVYL
EELGGISIPMPKIFTMFSSFSMASLALPGMSGFVAELVVFFGLITSPKFLLMPKTLITFVMAI
GMILTPIYLLSMLRQMFYGYKLFNVPNANFVDSGPRELFILICIFLPVIGIGIYPDFVLSLSVD
RVEALLSNYYPKMMFEHVLFLSVYLFSIGIYGLITSRNMVRALICLELILNSINLNLVTFSDL
FDSRQLKGDIFAIFVIALAAAEAAIGLSILSSIHRNRKSTRINQSNLLNNMEHTYQYAWVIPL
LPLPVIMSMGFGLILIPTATKNLRRIWAFPSVLLLSIAMVFSVQLSIQQINGSSIYQYLWSWT
VNNDFSLEFGYLIDPLTSIMLILITTVGILVLIYSDGYMSHDEGYLRFFVYISFFNTSMLGLV
TSSNLIQIYFFWELVGMCSYLLIGFWFTRPIAASACQKAFVTNRVGDFGLLLGILGFFWITG
SLEFRDLFKIANNNGVNSLLTTLCAFLLFLGAVAKSAQFPLHVWLPDAMEGPTPISALIQAA
TMVAAGIFLLARLLPLFISLPLIMSFISLVGTITLFLGATLALAQRDIKRSLAYSTMSQLGYM
MLALGIGSYQAALFHLITHAYSKALLFLGSGSVIHSMEPLVGYSPDKSQNMVLMGGLRKY
IPITRTTFLWGTLSLCGIPPLACFWSKDEILSNSWLYSPFFGIIASFTAGLTAFYMFRIYLLTFD
GYLRVHFQNYSSTKESSLYSISLWGKRIPKGVNR-DFVLSTTKFFSAKN--TFAYPHETGNTM
LFPLLILLLFTLFIGFIGISFELTILSKWLTPSINFTQESSNSFVNSYEFITNAISSVTLAIFGLFIA
YIFYGSAYSFFQNLDLINSFYEFLDQVKKNIYSWSYNRGYIDIFYTRVFTLGIRGLTELTEFF
DKGVIYGITNGVGLPSFCIGEEIKY-VGGGRISSYLFFFLCYVSLFLFFFLMDLPGPIHEILML
FGGFVLLLGGLGVVLLTNPIYSAFSLGLVLVCISLFYFLLNSYFVAVAQLLIYVGAINVLIIFA
VMFVNGSEWSKDKNYWTIGDGFTSLVCITIVFSLMTTIPDTSWYGILWTTRSNQIVEQGLI
NNVQQIGIHLATDFYLPFELISIILLVSLIGAITMARQMSLPLTRKDLMIVNMGPQHPSMHG

VLRLIVTLDGEDVIDCEPILGYLHRGMEKIAENRTIIQYLPYVTRWDYLATMFTEAITVNAP
EFLENIQIPQRASYIRVIMLELSRIASHLLWLGPFMADLGAQTPFFYIFRERELIYDLFEAAT
GMRMMHNYFRIGGVAADLPYGWIEKCLDFCDYFLRGVVEYQQLITQNPIFLERVEGVGFI
SGEEAVNWGLSGPMLRASGIQWDLRKVDPYESYNQFDWKVQWQKEGDSLARYLVRVGE
MRESIKIIQQAVEKIPGGPYENLEVRR-KKEKNSEWNDFEYKFLGKKPSPNFELSRQELYVR
VEAPKGELGIYLVGDDSLFPWRWKIRPPGFINLQILPQLVKKMKLADIMTILGSIDIIMGEV
DRMFPMVTGFMSYGQQTIRATRYIGQSFITTLSHTNRLPITIHYPYEKSITPERFRGRIHFEF
DKCIACEVCVRVCPIDLPVVDWRFEKDIKRKQLLNYSIDFGVCIFCGNCVEYCPTSCLSMT
EEYELSTYDRHELNYNQIALSRLPISIMGDYTIQTIRNSKINKEKSSNSRTITDYQQGWLSN
WLVKHEVVHRSLGFDHRGIETLQIKAGDWDSIAVILYVVYGYNYLRSQCAYDVAPGGSLAS
VYHLTRIQYGIDNPEEVCIKVFAQKDNPRIPSVFWIWRSADFQERESYDMVGISYDNHPRL
KRILMPESWIGWPLRKDYITPNFYEIQDAH------------------------------------MVLT-EYLD-KK
K-EGKDSIETVMNLIEFPLLDQTSSNSVISTTPNDLSNWSRLSSLWPLLYGTSCCFIEFASLIG
SRFDFDRYGLVPRSSPRQADLILTAGTVTMKMAPSLVRLYEQMPEPKYVIAMGACTITGG
MFSTDSYSTVRGVDKLIPVDVYLPGCPPKPEAVIDALTKLRKKISREIVEDRTLSQ-KNRCF
TTSHKLYVRRSTHTGTYEQELLYQSPSTLDIST--FFKSKSSVPSYKLVNMENRNTFSWVKE
QITRSISVSIMIYVITRTSISNAYPIFAQQGYENPREATGRIVCANCHLASKPVDIEVPQAVLP
DTVFEAVLRIPYDMQLKQVLANGKKGGLNVGAVLILPEGFELAPPDRISPELKEKIGNLAF
QSYRPDKKNILVIGPVPGKKYSEIVFPILSPDPATKKDAHFLKYPIYVGGNRGRGQIYPDGS
KSNNTVYNATSTGIVRKILRKEKGGYEISIVDASDGRQVIDIIPPGPELLVSEGESIKLDQPLT
SNPNVGGFGQGDAEIVLQDPLRVQGLLNVLESVILAQVFWFSSTKQFEKVQLYEMNFMSK
VYDWFEERLEIQAIADDITSKYVPPHVNIFYCLGGITLTCFLVQVATGFAMTFYYRPTVTEA
FSSVQYIMTEANFGWLIRSVHRWSASMMVLMMILHVFRVYLTGGFKKPRELTWVTGVVL
AVLTASFGVTGYSLPWDQIGYWAVKIVTGVPDAIPVIGSPLVELLRGSASVGQSTLTRFYSL
HTFVLPLLTAVFMLMHFPMIRKQGISGPLMGVTKKPDLNDPVLRAKLAKGMGHNYYGEP
AWPNDLLYIFPVVILGTIACNVGLAVLEPSMIGEPADPFATPLEILPEWYFFPVFQILRTVPN
KLLGVLLMVSVPTGLLTVPFLENVNKFQNPFRRPVATTVFLIGTVVALWLGIGATLPIDKSL
TLGLFMIEVFLFGIVLGLIPITLAGLFVTAYLQYRRGDQLDLMLTLTSYFGFLLAALTITPAL
FIGLNKIRLIMDIVSLAWAALMVVFTFSLSLVVWGRSGLMIIRSPEPEVKIVVDRDPVKTSF
EEWARPGHFSRTLAKGPDTTTWIWNLHADAHDFDSHTGDLEEISRKVFSAHFGQLSIIFLW
LSGMYFHGARFSNYEAWLSDPTHIGPSAQVVWPIVGQEILNGDVGGGFRGIQITSGFFQL
WRASGITSELQLYCTAIGALIFAALMLFAGWFHYHKAAPKLAWFQDVESMLNHHLAGLL
GLGSLSWAGHQIHVSLPINQFLDAGVDPKEIPLPHEFILNRDLLAQLYPSFAEGATPFFTLN
WSKYAEFLTFRGGLDPVTGGLWLTDIAHHHLAIAILFLIAGHMYRTNWGIGHGLKDILEAH
KGPFTGQGHKGLYEILTTSWHAQLSLNLAMLGSTTIVVAHHMYSMPPYPYLATDYGTQLS
LFTHHMWIGGFLIVGAAAHAAIFMVRDYDPTTRYNDLLDRVLRHRDAIISHLNWVCIFLG
FHSFGLYIHNDTMSALGRPQDMFSDTAIQLQPIFAQWVQNIHATAPGVTAPGATTSTSLTW
GGGELVAVGGKVALLPIPLGTADFLVHHIHAFTIHVTVLILLKGVLFARSSRLIPDKANLGFR
FPCDGPGRGGTCQVSAWDHVFLGLFWMYNAISVVIFHFSWKMQSDVWGTISDQGVVTHI
TGGNFAQSSITINGWLRDFLWAQASQVIQSYGSSLSAYGLFFLGAHFVWAFSLMFLFSGRG
YWQELIESIVWAHNKLKVAPATQPRALSIIQGRAVGVTHYLLGGIATTWAFFLARIIAVGME
LRFPRFSQGLAQDPTTRRIWFGIATAHDFESHDDITEERLYQNIFASHFGQLAIIFLWTSGNL
FHVAWQGNFESWIQDPLHVRPIAHAIWDPHFGQPAVEAFTRGGAAGPVNIAYSGVYQWW
YTIGLRTNEDLYTGALFLLFLSTLSLIAGWLHLQPKWKPSLSWFKNAESRLNHHLSGLFGV

SSLAWTGHLVHVAIPASRGEYVRWNNFLDVLPYPQGLGPLLTGQWNLYAQNPDSSNHLFG
TAQGAGTAILTLLGGFHPQTQSLWLTDMAHHHLAIAFIFLIAGHMYRTNFGIGHSIKDLLEA
HTPPGGRLGRGHKGLYDTINNSIHFQLGLALASLGVITSLVAQHMYSLPPYAFIAQDFTTQ
AALYTHHQYIAGFIMTGAFAHGAIFFIRDYNPEQNEDNVLARMLDHKEAIISHLSWASLFL
GFHTLGLYVHNDVMLAFGTPEKQILIEPIFAQWIQSAHGKTTYGFDILLSSTNGPAFNAGRS
LWLPGWLNAVNE--NSNSLFLTIGPGDFLVHHAIALGLHTTTLILVKGALDARGSKLMPDK
KDFGYSFPCDGPGRGGTCDISAWDAFYLAVFWMLNTIGWVTFYWHWKHITLWQGNVSQ
FNESSTYLMGWLRDYLWLNSSQLINGYNPFGMNSLSVWAWMFLFGHLVWATGFMFLISW
RGYWQELIETLAWAHERTPLANLIRWRDKPVALSIVQARLVGLAHFSVGYIFTYAAFLIAS
TSGKFGMSHSVKIYDTCIGCTQCVRACPTDVLEMIPWDGCKAKQIASAPRTEDCVGCKRC
ESACPTDFLSVRVYLGPETTRSMALSYMTDLNLPSIFVPLVGLVFPAIAMTSLFLYVQKNKI
VMRDIKTYLSVAPVLSTLWFGALAGLLIEINRLFPDALSFPFF--MTAILERRESTSLWGRFC
NWITSTENRLYIGWFGVLMIPTLLTATSVFIIAFIAAPPVDIDGIREPVSGSLLYGNNIISGAIIP
TSAAIGLHFYPIWEAASVDEWLYNGGPYELIVLHFLLGVACYMGREWELSFRLGMRPWIA
VAYSAPVAAATAVFLIYPIGQGSFSDGMPLGISGTFNFMIVFQAEHNILMHPFHMLGVAGVF
GGSLFSAMHGSLVTSSLIRETTENESANEGYKFGQEEETYNIVAAHGYFGRLIFQYASFNNS
RSLHFFLAAWPVVGIWFTALGISTMAFNLNGFNFNQSVVDSQGRVINTWADIINRANLGM
EVMHERNAHNFPLDLAAVEVPSINGMGLPWYRVHTVVLNDPGRLLAVHIMHTALVSGWA
GSMALYELAVFDPSDPVLDPMWRQGMFVIPFMTRLGITDSWGGWSIS-GGTVTNPGIWSY
EGVAGTHIVFSGLCFLAAIWHWVYWDLEIFSDERTGKPSLDLPKIFGIHLFLAGVACFGFG
AFHVTGLYGPGIWVSDPYGLTGKVQAVNPAWGAEGFDPFVPGGIASHHIAAGTLGILAGL
FHLSVRPPQRLYKGLRMGNIETVLSSSIAAVFFAAFVVAGTMWYGSATTPIELFGPTRYQW
DQGYFQQEIYRRVSNGLAENLSLSEAWSKIPEKLAFYDYIGNNPAKGGLFRAGSMDNGDG
IAVGWLGHPVFRDKEGRELFVRRMPTFFETFPVVLVDEEGIVRADVPFRRAESKYSVEQV
GVTVEFYGGELNGVSYSDPATVKKYARRSQLGEIFELDRATLKSDGVFRSSPRGWFTFGH
ATFALLFFFGHIWHGARTLFRDVFAGIDPDLDAQVEFGTFQKVGDPTTRKQAAMKILYSLR
RFYHVETLFNGTFVLAGRDQETTGFAWWAGNARLINLSGKLLGAHVAHAGLIVFWAGAM
NLFEVAHFVPEKPMYGQGLILLPHLATLGWGVGPGGEVLDTLPYFVSGVLHLISSAVLGFG
GIYHALLGPETLEESFPFFGYVWKDRNKMTTILGTHLILLGLGASLLVLKALYFAGVYDTW
APGGGDVRKITNLTLSPSVIFGYFLKPPFGGEGWIVSVDDLEDIIGGHVWLGFICVLGGIW
HILTKPFAWARRAFVWCGEAYLSYSLAALSVFGFIACCFVWFNNTAYPSEFYGPTGPEASQ
AQAFTFLVRDQRLGANVGSAQGPTGLGKYLMRSPTGEVIFGGETMRFWDLRAPWLEPLR
GPNGLDLSRLKKDIQPWQERRSAEYMTHAPLGSLNSVGGVATEINAVNYVSPRSWLASSH
FVLGFFFFVGHFWHAGRARAAAAGFEKGIDRDLEPVLYMNPLNMTIALGRIPKEENDLFD
TMDDWLRRDRFVFVGWSGLLLFPCAYFALGGVFTGTTFVTSWYTHGVASSYLEGCNFLT
AAVSTPANSLAHSLLLLWGPEAQGDFTRWCQLGGLWTFVALHGAFALIGFMLRQFELARS
VQLRPYNAISFSGPIAVFVSVFLIYPLGQSGWFFAPSFGVAAIFRFILFFQGFHNWTLNPFHM
MGVAGVLGAALLCAIHGATVENTLFEDGDGANTFRAFNPTQAEETYSMVTANRFWSQIF
GVAFSNKRWLHFFMLFVPVTGLWMSAIGVVGLALNLRAYDFVSQEIRAAEDPEFETFYTK
NILLNEGIRAWMAAQDQPHENLIFPEEVLPRGNALMSGSTGERSFADIITSIRYWVIHSITIP
SLFIAGWLFVSTGLAYDVFGSLRPNEYFTESRQGIPLITDRFDSLEQLDEFSRSFMTIDRTYP
IFTVRWLAIHGLAVPTVSFLGSISAMQFIQRMATQTVEDSSKPR-PKQTGAGSLLKPLNSEY
GKVAPGWGTTPFMGVAMALFAIFLSIILEIYNSSVLLDGILTNMLTLKLFVYTVVIFFVSLFI
FGFLSNDPGRNPGREEMSDTTGRIPLCLIGTVAGIAVIGLVGVFFYGSYSGLGSSLMPNILSL

TCICFNSVIYPTSFFFAKLPEAYAIFNPIVDFMPVIPLFFFLLAFVWQAAVSFRMTQSNPNEQ
NVELNRTSLYWGLLLIFVPAVLFANYFFNMEVNILAFIATALFILVPTSFLLIIYVKTVSQNN
METATLVAISISGLLVSFTGYALYTAFGQPSQQLRDPFEEHGDMEALVYTFLLVSTLGIIFFAI
FFREPPKVPTKRIKMTIAFQLAVFALIATSSVLVISVPLVFASPDGWSNNKNVVFSGTSLWIG
LVFLVAILNSLIS-------MSPQTETKAGVGFKAGVKDYKLTYYTPEYETKDTDILAAFRVSPQ
PGVPPEEAGAAVAAESSTGTWTTVWTDGLTSLDRYKGRCYHIEPVAGEDSQWICYVAYPL
DLFEEGSVTNMFTSIVGNVFGFKALRALRLEDLRIPPTYSKTFQGPPHGIQVERDKLNKYG
RPLLGCTIKPKLGLSAKNYGRACYECLRGGLDFTKDDENVNSQPFMRWRDRFVFCAEAI
YKSQAETGEIKGHYLNATAGTCEEMIKRAVFARELGVPIVMHDYLTGGFTANTTLAHYCR
DNGLLLHIHRAMHAVIDRQKNHGMHFRVLAKALRMSGGDHIHSGTVVGKLEGEREMTL
GFVDLLRDDFIEKDRARGIFFTQDWVSMPGVIPVASGGIHVWHMPALTEIFGDDSVLQFGG
GTLGHPWGNAPGAAANRVALEACVQARNEGRDLAREGNEIIRAACKWSPELAAACEVW
KAIKFEFEPVDTIDKTAKHLYKTPIPSTRKGTLDRQVKSNPRNNLIHGRHRCGKGRNSRGII
TASIEGEVIKRLYRKIDFRRNQKDISGRIVTIESDPNRNAYICLIHYGDGEKRYILHPRGAIIG
DTIVSGTKVPISMGNALPLTDMPLGTVMHNKENTRGRGGQLARAAGAVAKHKAKEGKM
ATLRLPSGEGRIVTQNSLATVGQEGKVGVNQKSLGRAGSKCWLGKRPVVRGVVMNPVD
HPHGGGEGKAPIGRKKPTTPWGYPALGRRTRKRKKYSDSFILRRRK-MIQPQTLLNVADNS
GARKLMCIRVIGAAGNQRYARIGDVIVAVIKDALPQMPLERSEVIRAVIVRTCKEFKCEDGII
IRYDDNAAVIIDQKGNPKGTRVFGAIAEELRGLNFTKIVSLAPEVLMLSPKRTRFRKQHRG
RMKGKSCRGNRICFGRYALQALEPAWITARQIEAGRRAITRYARRGGKIWVRIFPDKPVTL
RPTETRMGSGKGSPEYWVSVVKPGRILYEMGGVSETVARAAISIAASKMPIRSQFIRLEMT
RVPRGYIARRRRTKMRSFASNFRGAHLRLNRMITQQVKRAFVSSHRDRGRQKRDFRRLWI
TRINAATRVY--VFDSYSKLIHNLYKKKLILNRKMLAQVAVSNPNNLYTISNKIKIIN---------R
IKRVFKAQKRIPMSVFKAQRVLDEIRWRYYEETVMILNLMPYRASYPILKLVYSAAANAT
HYRDFDKANLFITKAEVSRSTIMKKFRPRARGRSFPIKKSMCHITIVLN-VKKS-------MDGI
KYAVFTEKSLRLLGKN-QYTFNVESGFTKTEIKHWVELFFGVKVVAVNSHRLPGKGRRMG
PILGHTMHYRRMIITLQPGYSIPLLDREKNMAVPKKRTSMSKKRIRKNIWKKKTYFSIVQS
YSLVKSFSSGKPKGFSGQQTNNMAKGKDVRIRVILECISCVRK--G--EESTGISRYSTQKNR
HNTPGQLEFKKFCRYCRKHTTHHEIKKMKIRASVRKICTKCRLIRRRGRIRVICSNPKHKQ
RQGMVREEVAGSTQTLQWKCVESRVDSKRLYYGRFILSPLRKGQADTVGIALRRALLGEI
EGTCITRAKFG--SVPHEYSTIAGIEESVQEILLNLKEIVLRSNLYGVRDASICVKGPRYITAQ
DIILPPSVEIVDTAQPIANLTEPIDFCIDLQIKRDRGYQTELRKNYQDGSY--PIDAVSMPVRN
VNYSIFSCGNGNEKHEILFLEIWTNGSLTPKEALYEASRNLIDLFLPFLHAEEE--GASFEEN
K-NRFTPPLFTFQKRLT-NLKKNKK-GIPLNCIFIDQLELTSRTYNCLKRANIHTLLDLLSKTE
EDLLRIDSFRMEDRKHIWDTLEKH--LPIDLLKNKMLRNGN-EGMSTIPGFSQIQFEGFCRFI
NQALAEELDKFPTIKDPDHEIAFQLFAKGYQLLEPSIKERDAVYESLTYSSELYVSARLIFGF
--DVQKQTISIGNIPIMNSLGTFIINGIYRIVINQILLSPGIYYRSELDHKGISIYTGTIISDWGGR
SELAIDKKERIWARVSRKQKISILVLSSAMGSNLREILDNVSYPEIFLSFPNAKEKKRIESKE
KAILEFYQQFACVGGDLVFSESLCEELQKKFFQQKCELGRIGRRNMNRRLNLDIPQNNTFL
LPRDVLAATDHLIGMKFGTGILDDDDMNHLKNKRIRSVADLLQDQFGLALGRLQHAVQK
TIRRVFIRQSK--PTPQTLVTPTSTSILLITTYETFFGTYPLSQVFDQTNPLTQTVHGRKVSCLG
PGGLTGRTASFRSRDIHPSHYGRICPIDTSEGINVGLTGSLAIHARIDHLWGSIESPFYEISEK
AKEKKE--RQVVYLSPNRDEYYMIAAGNSLSLNQGIQEEQVVPARYRQEFLTIAWEQIHVR
SIFPFQYFSIGGSLIPFIEHNDANRALMSSNMQRQAVPLSRSEKCIVGTGLERQTALDSRVSII

AEREGKIISTDSHKILLSSSGKTISIPLVNHRRSNKNTCMHQKPRVPRGKSIKKGQILAEGAA
TVGGELALGKNVLVAYMPWEGYNFEDAVLISERLVYEDIYTSFHIRKYEIQTDTTSQGSAE
KITKEIPHLEEHLLRNLDRNGVVRLGSWVETGDILVGKLTPQIASE-SSYIAEAGLLRAIFGL
EVSTSKETSLKLPIGGRGRVIDVKWIQR-------DPIMVRVYILQKREIKVGDKVAGRHGNKGI
ISKILPRQDMPYLQDGTPVDMVFNPLGVPSRMNVGQIFESSLGLAGDLLKKHYRIAPFDER
YEQEASRKLVFSELYEASKETKNPWVFEPEYPGKSRIFDGRTGDPFEQPVLIGKSYILKLIH
QVDEKIHGRSTGPYSLVTQQPVRGRAKQGGQRVGEMEVWALEGFGVAHILQEILTYKSDH
LIARQEILNATIWGKRIPNHEDPPESFRVLVRELRSLALELNHFLVSEKNFQVNREEV-------
MIDQYKHQQLQIGLVSPQQIKAWANKNLPNGEVVGEVTRPSTFHYKTDKPEKDGLFCERI
FGPIKSGICACGNSRASGAENEDERFCQKCGVEFVDSRIRRYQMGYIKLACPVTHVWYLK
GLPSYIANLLDKPLKKLEGLVYGD--FSFARP-STKKPTFLRLRGLFEEEIASCNHSISPFFSTP
GFATFRNREIATGAGAIREQLADLDLRIIIENSLVEWKELEDE-GYSGDEWEDRKRRIRKVF
LIRRMQLAKHFIQTNVEPEWMVLCLLPVLPPELRPIVYRSGDKVVTSDINELYKRVIRRNN
NLAYLLKRSELAPADLVMCQEKLVQEAVDTLLDSGSRGQPTRDGHNKVYKSLSDVIEGKE
GRFRETLLGKRVDYSGRSVIVVGPSLSLHQCGLPLEIAIKLFQLFVIRDLITKRATSNVRIAK
RKIWEKEPIVWEILQEVMRGHPVLLNRAPTLHRLGIQAFQPTLVEGRTISLHPLVCKGFNA
DFDGDQMAVHLPLSLEAQAEARLLMFSHMNLLSPAIGDPICVPTQDMLIGLYVLTIGKRRG
ICANRYNSCRN-----YPNLKVNYNNSKYRKDKEPHFSSSYDALGAYRQKLISLDSPLWLRW
NLDQR-VIGS--REVPIEVQYESLGTYHEIYAHYLIMGNRKKEIRSIYIRTTLGHISFYREIEEA
IQGFSQAYSYTT------------MAERANLVFHNKEIDGTGMKRLISRLIDHFGMGYTSHILDQLK
TLGFHQATTTSISLGIEDLLTIPSKGWLVQDAEQQSFLLEKHYYYGAVHAVEKLRQSVEIW
YATSEYLKQEMNSNFRITDPSNPVYLMSFSGARGNASQVHQLVGMRGLMSDPQGQMIDL
PIQSNLREGLSLTEYIISCYGARKGVVDTAVRTADAGYLTRRLVEVVQHIIVRRRDCGTIRGI
SVSPQNGMTE--KLFVQTLIGRVLADDIYIGSRCIAARNQDIGIGLVNRFITAFRAQPFRAQPI
YIRTPFTCRSTSWICQLCYGRSPTHSDLVELGEAVGIIAGQSIGEPGTQLTLRTFHTGGVFTG
GTADLVRSPSNGKIKFNENLVHPTRTRHGQPAFLCYIDLHVTIQSQDILYSVNIPSKSLILVQ
NDQYVKSEQVIAEIRAGTSTL--FKERVQKHIYSESDGE-MHWSTDVYHAPEYQYGNLRRL
PKTSHLWILSVSMCR--SSIASFSLHKDQDQMNTY-------------------------------G----DREILDY
S-TSDRIMSNGHWNFIYPS-IFQDN--SDLLAKKRRNRFVIPLQYHQEQEKELISCFGISIEIPL
M--GVLRRNTIFAYFDDPRYRKD--GIVKFRSPEEDSILK--KEGLIEHRG-TKEFSLKY-EVDRF
FFILQELHILPRSSSLKILDNSIIGVDTQLTKNTRSRLGGLVRVKRKKSHTELKIFSGDIHFPE
EADKILG--GCLIPPERQKK--DSKE-SK-KRKNWVYVQRKKILKSKEK--YFVSVRPTVAYEM
DEGRNLATLFPQDLLQEENNLQIRLVNFISHENSKLTQRIYHTN-QFVRTCLVVNWEQEEKE
KAG-------ASLVEVRANDLIRDFLRIELVKSTISYTRKRYDRTSAGPIPH---LDRANINSFYSK
AKIES-----LSQHPEAIG----TLLNRNK---EYHSLMILSASNCSRIG--LFKNSKHP---NAIKEWN
PRIPI-EIFGPLGAIVA-SISHFSSS-YYLLT---HNKILLK-KYLFVD---NLKQTFQVLQELKYSLI
DENKR--ISNFD--SNIMLDPFLLNCHFVHHDSWEETLAIIH-LGQFICENVCLFKSHI----KKS
GQIFIVN-MNSFVIRAAKPYLATTGATVNGHYGEILYKGDRLVTFIYEKSRSSDITQGLPKVE
QIFEARSIDSLSPNLERRIEDWNERIPRILGVPWGFLIGAELTIAQSRISLVNKIQKVYRSQGV
QIHNRHIEIIIRQVTSKVRVSE---DGMSNVFSPGELIGLLRAERAGRALDESIYYRAILLGITR
ASLNTQSFISEASFQETARVLAKAALRGRIDWLKGLKENVVLGGIIPVGTGFQKFVHRSP--
QDKNLY--FEIKKKNLFASEM--RDFLFLHTELVSSDSDVTN-------------------MTRRYWNINL
KEMIEAGVHFGHGIKKWNPKMAPYISAKRKGTHIINLARTARFLSEACDLVFDAASQGKS
FLIVGTKKRATDLVASAAIRARCHYVNKKWFSGMLTNWSITKTRLSQFRDLRAEEKMGKF

HHLPKRDVAILKRKLSTLQRYLGGIKYMTRLPDIVIVLDQQKEYIALRECAILGIPTISLVDT
NCDPDLANISIPANDDTMTSIRLILNKLVFSICEGRSL-YIRNRMGQKINPLGFRLGTTQKHH
SFWFAQPKNYSEGLQEDKKIRDCIKNYIQKNRKKGSNKIESITHIEIQKEIDTIHVIIHIGFPN-
LLKKKG-----AIEELEKDLQKEINSVNQRFNISIEKVKEPYRQPNILAEYIAFQLKNRVSFRK
AMKKAIELTKKADIRGVKVKIAGRLGGKEIARAESIKKGRLPLQTIRAKIDYCCYPIRTIYG
VLGVKIWIFVDEEMSRYRGPRLKKIRRLGALPGLTRKTPKSGSNLKKKFNSGK-KEQYRIR
LQEKQKLRFHYGLTERQLLRYVHIAGKAKRSTGQVLLQLLEMRLDNILFRLGMASTIPGA
RQLVNHRHILVNGRIVNIPSFRCKPRDIITTKDNQRSKGLVQNYIASSDPK--LPKHLAIDT--L
EYKGLVNKILDRKWVGLKINELLVVEYYSRQTMSRRGTAEKRTAKSDPIFRNRLVNMVV
NRIMKDGKKSLAYQILYRAVKKIQQKTETNPLLVLRQAIRRVTPNIGVKTRRKKGSTRKVP
IEIGSKQGRALAIRWLLEASQKRPGRNMAFKLSSELVDAAKGGGGAIRKKEATHRMAEAN
RALAHFRMGKDTIADLLTSIRNADMNKKGTVRVVSTNITENIVKILLREGFIESVRKHQER
NRYFLVSTLRHQKRKTRKGIYRT---RTFLKRISRPGLRIYTNYQGIPKVLGGMGIAILSTSRG
IMTDREARLNRIGGEVLCYIWMAKAIPKIGSRKKVRIGLRKSARRITKGVIHVQASFNNTII
TVTDPQGRVVFWSSAGTCGFKSSRKASPYAGQRTAVDAIRTVG---LQRAEVMVKGAGSGR
DAALRAIAKSGVRLSCIRDVTPMPHNGCRPPKKRRLMPTVKQLIRNARQPIRNARKTAAL
KGCPQRRGTCARVY--NPKKPNSALRKVARVRLTSGFEITAYIPGIGHNLQEHSVVLVRGGR
VKDLPGVRYRIIRGTLDAVAVKNRQQGRLQYGVKKPKMAKKSLIQREKKRQKLEQKYHL
IRQSLKKKIRSKVSPLSLSEKTKMREKLQSLPRNSAPTRLHRRCFLTGRPRANYRHFGLSG
HVLREMVYECLLPGATRSSWMKKRKIFGFMVKEEKEEN--RGSVEFQVFSFTNKIRRLASH
LELHKKDFSSERGLRRLLGKRRRLLAYLAKKNRVRYKKLIGQLNIREQ-----MLKLRLKRC
GRKQQAVYRIVAIDVRSRREGRDLRKVGFYDPIKNQTCLNVPAILYFLEKGAQPTRTVYDI
LRKAEFFKEKESTLS----FNKSKQPFRKSKQTFRKRRPRIGPGDRIDYRNMSLINRFISEQGK
ILSRRINRLTLKQQRLITLAIKQARILSFLPFRNYENEKQFQ-AQSISIITGSRPRKNVTRK-KT
NPFVAHHLLAKIEKVNMKEEKETIVTWSRASSILPTMVGHTIAIHNGKEHIPIYITNPMVGR
K L G E F V P T R H F T S Y A R K D T K S R
R???
???
???
???
???
???
???
???
???
???
???
???
???
???
???
???
???
???

??
??
??
??
??
??
??
??
??
??
??
??
??
??
??
??
??
??
??
??
??
??
??
??
??
???MPRSR-VNGNFIDKTSSIVANILLRIIPTTSGEK
KAFTYYRDG--MLAQSEGNYAEALQNYYEATRLEIDPYDRSYILYNIGLIHTSNGEHTKALE
YYFRALERNPFLPQAFNNMAVICHY-RGEQAILQGDSEIAEAWFDQAAEYWKQAIALTPG
NYIEAQNWLKITKRFEFEMNWRSEHIWVELLKGSRKRGNFFWACILFLGSLGFLSVGISSY
LGKNIISILPSQEILFFPQGVVMSFYGIAGLFISSYLWCTILWNVGSGYDRFDRKEGIVCIFR
WGFPGIKRRVFLRFLMRDIQSIRIQVKEGLYPRRILYMEIRGQGIIPLTRTDDKFFTPREIEQK
AAELAYFLRVPIEVF
>Atropa
MERWWFNSMLFLNKSMGSLGPIENTSE--DPNRK--VKNIH-CSNVDYLFGVKDIRNFISDD
TFLVSDRNGDSYSIYFDIENQIFEIDNDH--SFLSELESSFYSYRNSSYLNNGFRGEDPYYNSY
MYDTQYSWNNHINSCIDNYLQ--SQICIDTSIISGSENYG--YIYRTICGNEGSSKRTRTKGSD
LTIRESSNDLEVTQKYRHLWVQCENCYGLNYKKFLKSKMNICEQCGYHLKMSSSDRIELL
IDAGTWDPMDEDMVSLDPIEFHSEEEPYKDRIDSYQRKTGLTEAVQTGIGQLNGIPVAIGV
MDFQFMGGSMGSVVGEKITRLIEHAANQILPLIIVCASGGARMQEGSLSLMQMAKISSAL
YDYQLNKKLFYVSILTSPTTGGVTASFGMLGDIIIAEPNAYIAFAGKRVIEQTLNKTVPEGS
QAAEYLFQKGLFDQIVPRNLLKSVLSELFKLHAFFPLNQKSSKMVTIRADEISNIIRERIEQ
YNREVKIVNTGTVLQVGDGIARIHGLDEVMAGELVEFEEGTIGIALNLESNNVGVVLMGD
GLLIQEGSSVKATGRIAQIPVSEAYLGRVVNALAKPIDGRGEISASEFRLIESAAPGIISRRSV
YEPLQTGLIAIDSMIPIGRGQRELIIGDRQTGKTAVATDTILNQQGQNVICVYVAIGQKASSV

AQVVTTLQERGAMEYTIVVAETADSPATLQYLAPYTGASLAEYFMYRERHTLIIYDDLSK
QAQAYRQMSLLLRRPPGREAYPGDVFYLHSRLLERAAKLSSSLGEGSMTALPIVETQSGD
VSAYIPTNVISITDGQIFLSADLFNSGIRPAINVGISVSRVGSAAQIKAMKQVAGKLKLELAQ
FAELEAFAQFASDLDKATQNQLARGQRLRELLKQSQSAPLTVEEQIMTIYTGTNGYLDSLE
VGQVRKFLVELRTYLKTNKPQFQEIISSTKTFTEEAEALLKEAIQEQMKRFILQEQAMRINP
TTSGSGVSTLEKKNLGRVVQIIGPVLDVAFPPGKMPNIYNALVVQGRDSVGQPINVACEVQ
QLLGNNRVRAVAMSATDGLTRGMEVIDKGAPISVPVGGATLGRIFNVLGEPVDNLGPVDT
STMSPIHRSAPAFIQLDTKLSIFETGIKVVDLLAPYRRGGKIGLFGGAGVGKTVLIMELINNI
AKAHGGVSVFGGVGERTREGNDLYMEMKESGVINEENIAESKVALVYGQMNEPPGARM
RVGLTALTMAEYFRDVNEQDVLLFIDNIFRFVQAGSEVSALLGRMPSAVGYQPTLSTEMG
SLQERITSTKEGSITSIQAVYVPADDLTDPAPATTFAHLDATTVLSRGLAAKGIYPAVDPLDS
TSTMLQPRIVGEEHYETAQRVKQTLQRYKELQDIIAILGLDELSEEDRLLVARARKIERFLS
QPFFVAEVFTGSPGKYVGLAETIRGFQLILSGELDGLPEQAFYLVGNIDEATAKAMNLEME
SNLKKMTLNLSVLTPNRIVWDSEVEEIVLSTNSGQIGILSNHAPIATAVDIGILRIRLNDQWL
TMALMGGFARIGNNEITVLVNDAEKGSDIDPQEAQQTLEIAEANVKKAEGRRQKIEANLA
LRRARTRVEAINPISMKNVTDSFVS-GHWPSAGSFGFNTDILATNPINLSVVLGVLIFFGKG
VLSDLLDNRKQRILNTIRNSEELREGAIEQLEKARSRLRKVETEAEQFRVNGYSEIEREKLN
LINSTYKTLEQLENYKNETIQFEQQRAINQVRQRVFQQALRGALGTLNSCLNNELHLRTIS
ANIGMLGTMKEITDMNPLVFAASVIAAGLAVGLASIGPGVGQGTAAGQAVEGIARQPEAE
GKIRGTLLLSLAFMEALTIYGLVVALALLFANPFVMNVLSCSINTLKGLYDISGVEVGQHF
YWQIGGFQVHGQVLITSWVVIAILLGSATIAVRNPQTIPTGGQNFFEYVLEFIRDVSKTQIG
EEYGPWVPFIGTMFLFIFVSNWSGALLPWKIIQLPHGELAAPTNDINTTVALALLTSVAYFY
AGLTKKGLGYFGKYIQPTPILLPINILEDFTKPLSLSFRLFGNILADELVVVVLVSLVPLVVPI
PVMLLGLFTSGIQALIFATLAAAYIGESMEGHHMIFSTLEHILTHISFSIVSIVITIHLITFLVDE
IVKLYDSSEKGIIVTFFCITGLLVTRWISSGHFPLSDLYESLIFLSWSFSLIHIIPY--NNVLILSKI
TGPSAIFTQGFATSGILTEIHQSGILVPALQSEWLIMHVSMMILGYAALLCGSLLSVALLVITF
RKNRKLFY--KENVLENTS-----------AKNYYRSQLIQQLDYWSYRVISLGFTFLTIGILSGAVW
ANEAWGSYWNWDPKETWAFITWIVFAIYLHTRTNINLRGANSAIIATIGFLIIWICYFGVNL
LGIGLHSYGSFTSTMAKKKAFTPLFYLASIVFLPWWISFSVNKCLESWVTNWWNTGQSEI
FLNNIQEKSLLEKFIELEELLLLDEMIKEYSETHLEEFGI-IHKETIQLIKIQNENRIHTILHFST
NIICFIILSGYSILGNEKLVILNSWAQEFLYNLSDTVKAFSILLLTDLCIGFHSPHGWELMIGSI
YKDFGFVHNDQIISGLVSTFPVILDTIFKYWIFRYLNRLSPSLVVIYHSMNDMPIGVPKVPFR
SPGEEDASWVDVYNRLYRERLLFLGQGINSEISNQLIGLMVYLSIEDETKELYLFINSPGGW
VIPGIAIYDTMQFVRPDVHTVCMGLAASMGSFILVGGEITKRLAFPHARVMMHQPASGYY
EAQTGEFVLEAEELLKLRETLTRVYVQRTGKPLWVVSEDMEKDVFMSATEAQAYGIVDLV
AVE???MEEIQ
RYLQ-DSSQQHNFLYPLIFQEYIYALAHDHGLPGYNNQFSLLIVKRLITRMYQQNHFLISTN
DSNKNTFLGKSLYSQMISEGFAFIVEIPFSLRLISSLILKSHNLRSIHSTFPFLEDNFSHLNYVL
DILIPYPVHLEILVQTLRYWVKDASSLHLLRFFLHEYWNLNSLITPG-YSFSKKRFFFFLYNS
YVYECESTFVFLRNQSSHLRPTSFGALLERIYFYGKIERLVEVFA-DLTLWLFKDPFMHYVR
YQGKSILASKGTFLLINKWKFYLVNFWQCHFSLCFHTGRIHINQLSNHSRDFMGYLSSVRL
NPSMVRSQMLENSFIINNAI--KKFDTLVPIIPLIGSLAKENFCTVLGHPISKPVWSDLSDSDII
DRFGRICRNLFHYYSGSSKKKTLYRIKYILRLSCARTLARKHKSTVRTFLKRSGSELLEEFL
TSEEQVLSLTFPRRIWYLDIFCINDLANYQMIID-TTEIE-TINSFSKLES-LKEVYGIIWMLVPI

LTLVLGITIGVLVIVWLEREISAGIQQRIGPEYAGPLGILQALADGTKLLFKENLIPSTGDPRL
FSIGPSIAVISIFLSYSVIPFGDHLVLADLSIGVFLWIAISSVAPVGLLMSGYGSNNKYSFLGG
LRAAAQSISYEIPLALCVLSISLLSNSSSTVDIVEAQSKYGFLGWNLWRQPIGFIVFLISSLAE
CERLPFDLPEAEEELVAGYQTEYSGIKFGLFYIASYLNLLVSSLFVTVLYLGGWNLSIPYIFV
PELFGINKRGKVFGTLIGIFITLAKTYLFLFIPIATRWTLPRLRMDQLLNLGWKFLLPISLGN
LLLTTSSQLLSLMIWHVQNENFILDSTRIFMKAFHLLLFDGSLIFPECILIFGLILLLMIDSTS
DQKDIPWLYFISSTSLVMSITALLFRWREEPMISFSGNFQTNNFNEIFQFLILLCSTLCIPLSV
EYIECTEMAITEFLLFVLTATLGGMFLCGANDLITIFVAPECFSLCSYLLSGYTKKDVRSNEA
TMKYLLMGGASSSILVHGFSWLYGSSGGEIELQEIVNGLINTQMYNSPGISIALIFITVGIGF
KLSPAPSHQWTPDVYEGSPTPVVAFLSVTSKVAASASATRIFDIPFYFSSNEWHLLLEILAIL
SMILGNLIAITQTSMKRMLAYSSIGQIGYVIIGIIVGDSNDGYASMITYMLFYISMNLGTFAC
IVLFGLRTGTDNIRDYAGLYTKDPFLALSLALCLLSLGGLPPLAGFFGKLYLFWCGWQAGL
YFLVLIGLLTSVVSIYYYLKIIKLLMTGRNQEITPHVRNYRRSPLRSNNSIELSMIVCVIASTI
PGISMNPIIAIAQDSLFMFLLYEYDFFWAFLIISILVPILAFFISGVLAPISKGPEKLSTYESGIE
PMGDAWLQFRIRYYMFALVFVVFDVETVFLYPWAMSFDVLGVSVFIEAFIFVLILIIGLVYA
WRKGALEWSTNYFPWLTIIVVFPIFAGSLIFFLPHKGNRVIRWYTICICILELLLTTYAFCYHF
QLDDPLIQLVEDYKWIDFFDFHWRLGIDGLSIGPILLTGFITTLATLAAWPITRDSRLFHFIM
LAMYSGQIGSFSSRDLLLFFIMWELELIPVYLLLAMWGGKKRLYSATKFILYTAGGSVFLL
MGVLGVALYGSNEPTLNFETLVNQSYPVVLEIIFYIGFFIAFAVKLPIIPLHTWLPDTHGEAH
YSTCMLLAGILLKMGAYGLIRINMELLPHAHSIFSPWLMIIGTIQIIYAASTSLGQRNLKKRI
AYSSVSHMGFIIIGISSLTDTGLNGALLQIISHGFIGAALFFLAGTTYDRIRLVYLDEMGGIAI
PMPKMFTMFSSFSMASLALPGMSGFVAELIVFFGIITGQKYVLIPKILITFVMAIGMILTPIYS
LSMSRQMFYGYKLFNAPKDSFFDSGPRELFLSISIFLPVIGIGIYPDFVLSLAVDKVEVILSNF
FYRMILEHVLVLSAYLFSIGIYGLITSRNMVRALMCLELILNAVNINFVTFSDFFDNRQLKG
DIFSIFVIAIAAAEAAIGLAIVSSIYRNRKSTRINQSNLLNNMEQTYEYAWIIPFIPLPVPMLIG
AGLILFPTATKSFRRMWAFQSVLLLSIVMIFSIYLSIQQINSSSFYQYVWSWIINNDFSLDFG
YLIDPLTSIMSILITTVGIMVLIYSDNYMAHDQGYLRFFAYMSFFSTSMLGLVTSSNLIQIYIF
WELVGLCSYLLIGFWFTRPVAANACQKAFVTNRVGDFGLLLGILGFYWITGSFEFRDLFEI
FNNNEVNFLFVTLCAVLLFAGAVAKSAQFPLHVWLPDAMEGPTPISALIHAATMVAAGIFL
VARLLPLFRVIPYIMYLISVIGIITVLLGATLALAQKDIKRGLAYSTMSQLGYMMLALGMGS
YRSALFHLITHAYSKALLFLGSGSIIHSMETIVGYSPAKSQNMGLMGGLRKHVPITQITFLL
GTLSLCGIPPLACFWSKDEILNDSWLYSPIFAIIAWATAGLTAFYMFRIYLLTFEGHLNVHFQ
NYGGKQKTPFYSISLWGK---KGVKK-LLT---MNFFSFEHKTVYSYPYESDNTMLFPIFVLGL
FTLFVGSIGIPFNLDILSKWLAPSINLLHQKSN-SMDWNEFLKDAVLSVSIAYFGIFLASFLY
KPIYSSLKNFELINSFVILWDKIINGIYDWSYNRAYIDAFYTRFFVGGIRGLAEFIHFFDRRVI
DGMTNGVGVISFIVGEGIKYIGGG-RISSYLFLYLAYVSVFLLVVYYMDLSEPIHDFLLVFLGS
GLILGGLGVVLLPNPIYSAFSLGLVLVCTSLFYILSNSYFVAAAQLLIYVGAINVLIIFAVMF
MNGSEYYKDFHLWTVGDGITSMVCISLFISLITTISDTSWYGIIWTTRSNQIIEQDFLSNSQQ
IGIHLSTDFFLPFELISIILLDALIGAIAVARQMTTPTTRKDLMIVNMGPQHPSMHGVLRLIVT
LDGEDVVDCEPILGYLHRGMEKIAENRTIIQYLPYVTRWDYLATMFTEAITINGPEQLGNI
QVPKRASYIRVIMLELSRIASHLLWLGPFMADIGAQTPFFYIFRERELIYDLFEAATGMRM
MHNYFRIGGVAADLPYGWIDKCLDFCDYFLTGIAEYQKLITRNPIFLERVEGVGIIGGDEAL
NWGLSGPMLRASGIEWDLRKVDHYESYDEFDWQVQWQREGDSLARYLVRIGEMTESIKI
IQQALEGIPGGPYENLEIRRFDRLKDPEWNDFEYRFISKKPSPTFELSKQELYVRVEAPKGE

LGIFLIGDQSVFPWRWKIRPPGFINLQILPQLVKRMKLADIMTILGSIDIIMGEVDRMLPMIT
EFINYGQQTIRAARYIGQGFMITLSHANRLPVTIQYPYEKLITSERFRGRIHFEFDKCIACEV
CVRVCPIDLPVVDWKLETDIRKKRLLNYSIDFGICIFCGNCVEYCPTNCLSMTEEYELSTY
DRHELNYNQIALGRLPMSVIDDYTIRTISNL----------------MQGRLSAWLVKHGLIHRSLGFD
YQGIETLQIKPEDWHSIAVIFYVYGYNYLRSQCAYDVAPGGLLASVYHLTRIEDGVDQPEE
VCIKVFASRRNPRIPSVFWVWKSVDFQERESYDMLGISYDNHPRLKRILMPESWIGWPLR
KDYIAPNFYEIQDAH------------------------------------MVLAPEYSDNKKKNGKNKIET-VMNS
IQFPLLDRTAQNSVISTTLNDLSNWSRLSSLWPLLYGTSCCFIEFASLIGSRFDFDRYGLVPRS
SPRQADLILTAGTVTMKMAPSLVRLYEQMPEPKYVIAMGACTITGGMFSTDSYSTVRGVD
KLIPVDVYLPGCPPKPEAVIDAITKLRKKISRELYEDRIRSQRVNRCFTTNHKFHVRRSIHTG
NYDQRVLYQPPSTSEIPTEIFFKYKNSVSSPELVNMQTRNAFSWLKKQITRSISVSLMIYILT
RTSISSAYPIFAQQGYENPREATGRIVCANCHLANKPVEIEVPQAVLPDTVFEAVVRIPYDM
QLKQVLANGKKGGLNVGAVLILPEGFELAPSDRISPEMKEKIGNLSFQSYRPNKKNILVIGP
VPGKKYSEITFPILSPDPATKKDVHFLKYPIYVGGNRGRGQIYPDGSKSNNTVYNATAAGI
VSKIIRKEKGGYEITITDASEGRQVVDIIPPGPELLVSEGESIKFDQPLTSNPNVGGFGQGDA
EIVLQDPLRVQGLLFFLASVILAQIFLVLKKKQFEKVQLAEMNFMSKVYDWFEERLEIQAI
ADDITSKYVPPHVNIFYCLGGITLTCFLVQVATGFAMTFYYRPTVTEAFASVQYIMTEANFG
WLIRSVHRWSASMMVLMMILHVFRVYLTGGFKKPRELTWVTGVVLAVLTASFGVTGYSL
PWDQIGYWAVKIVTGVPDAIPVIGSPLVELLRGSASVGQSTLTRFYSLHTFVLPLLTAVFML
MHFPMIRKQGISGPLMGVTKKPDLNDPVLRAKLAKGMGHNYYGEPAWPNDLLYIFPVVI
LGTIACNVGLAVLEPSMIGEPADPFATPLEILPEWYFFPVFQILRTVPNKLLGVLLMVSVPA
GLLTVPFLENVNKFQNPFRRPVATTVFLIGTAVALWLGIGATLPIDKSLTLGLFMIEVFLFGIV
LGLIPITLAGLFVTAYLQYRRGDQLDLMLTITSYFGFLLAALTITSALFIGLSKIRLIMDIVSL
AWAALMVVFTFSLSLVVWGRSGLMIIRSPEPEVKILVDRDPVKTSFEEWARPGHFSRTIAK
GPDTTTWIWNLHADAHDFDSHTSDLEEISRKVFSAHFGQLSIIFLWLSGMYFHGARFSNYE
AWLSDPTHIGPSAQVVWPIVGQEILNGDVRGGFRGIQITSGFFQIWRASGITSELQLYCTAI
GALVFAALMLFAGWFHYHKAAPKLAWFQDVESMLNHHLAGLLGLGSLSWAGHQVHVS
LPINQFLNAGVDPKEIPLPHEYILNRDLLAQLYPSFAEGATPFFTLNWSKYADFLTFRGGLD
PVTGGLWLTDIAHHHLAIAILFLIAGHMYRTNWGIGHGLKDILEAHKGPFTGQGHKGLYEI
LTTSWHAQLSLNLAMLGSLTIVVAHHMYSMPPYPYLATDYGTQLSLFTHHMWIGGFLIVG
AAAHAAIFMVRDYDPTTRYNDLLDRVLRHRDAIISHLNWACIFLGFHSFGLYIHNDTMSAL
GRPQDMFSDTAIQLQPVFAQWIQNTHALAPGATAPGATASTSLTWGGGDLVAVGGKVALL
PIPLGTADFLVHHIHAFTIHVTVLILLKGVLFARSSRLIPDKANLGFRFPCDGPERGGTCQVS
AWDHVFLGLFWMYNAISVVIFHFSWKMQSDVWGSVSDQGVVTHITGGNFAQSSITINGW
LRDFLWAQASQVIQSYGSSLSAYGLFFLGAHFVWAFSLMFLFSGRGYWQELIESIVWAHN
KLKVAPATQPRALSIIQGRAVGVTHYLLGGIATTWAFFLARIITVGMALRFPRFSQGLAQDP
TTRRIWFGIATAHDFESHDDITEERLYQNIFASHFGQLAIIFLWTSGNLFHVAWQGNFESWV
QDPLHVRPIAHAIWDPHFGQPAVEAFTRGGALGPVNIAYSGVYQWWYTIGLRTNEDLYTG
ALFLLFLSAISLIAGWLHLQPKWKPSVSWFKNAESRLNHHLSGLFGVSSLAWTGHLVHVAI
PASRGESVRWNNFLDVLPHPQGLGPLFTGQWNLYAQNPDSSSHLFGTAEGAGTAILTLLG
GFHPQTQSLWLTDIAHHHLAIAFIFLVAGHMYRTNFGIGHSMKDLLDAHIPPGGRLGRGHK
GLYDTINNSLHFQLGLALASLGVITSLVAQHMYSLPAYAFIAQDFTTQAALYTHHQYIAGFI
MTGAFAHGAILVIRDYNPEQNEDNVLARMLDHKEAIISHLSWASLFLGFHTLGLYVHNDV
MLAFGTPEKQILIEPIFAQWIQSAHGKTSYGFDVLLSSTSGPAFNAGRSIWLPGWLNAVNE-

-NSNSLFLTIGPGDFLVHHAIALGLHTTTLILVKGALDARGSKLMPDKKDFGYSFPCDGPGR
GGTCDISAWDAFYLAVFWMLNTIGWVTFYWHWKHITLWQGNVSQFNESSTYLMGWLR
DYLWLNSSQLINGYNPFGMNSLSVWAWMFLFGHLVWATGFMFLISWRGYWQELIETLAW
AHERTPLANLIRWRDKPVALSIVQARLVGLAHFSVGYIFTYAAFLIASTSGKFGMSHSVKIY
DTCIGCTQCVRACPTDVLEMIPWDGCKAKQIASAPRTEDCVGCKRCESACPTDFLSVRVY
LWHETTRSMGLAYMTNLNLPSIFVPLVGLVFPAIAMASLFLHVQKNKIVMRDLKTYLSVA
PVLSTLWFVALAGLLIEINRFFPDALTFPFF--MTAILERRESESLWGRFCNWITSTENRLYIG
WFGVLMIPTLLTATSVFIIAFIAAPPVDIDGIREPVSGSLLYGNNIISGAIIPTSAAIGLHFYPIW
EAASVDEWLYNGGPYELIVLHFLLGVACYMGREWELSFRLGMRPWIAVAYSAPVAAATAV
FLIYPIGQGSFSDGMPLGISGTFNFMIVFQAEHNILMHPFHMLGVAGVFGGSLFSAMHGSL
VTSSLIRETTENESANEGYRFGQEEETYNIVAAHGYFGRLIFQYASFNNSRSLHFFLAAWPV
VGIWFTALGISTMAFNLNGFNFNQSVVDSQGRVINTWADIINRANLGMEVMHERNAHNFP
LDLAAIEAPSTNGMGLPWYRVHTVVLNDPGRLLSVHIMHTALVAGWAGSMALYELAVFD
PSDPVLDPMWRQGMFVIPFMTRLGITNSWGGWSIT-GGTVTNPGIWSYEGVAGAHIVFSG
LCFLAAIWHWVYWDLEIFCDERTGKPSLDLPKIFGIHLFLSGVACFGFGAFHVTGLYGPGI
WVSDPYGLTGKVQPVNPAWGVEGFDPFVPGGIASHHIAAGTLGILAGLFHLSVRPPQRLY
KGLRMGNIETVLSSSIAAVFFAAFVVAGTMWYGSATTPIELFGPTRYQWDQGYFQQEIYRR
VSAGLAENQSLSEAWSKIPEKLAFYDYIGNNPAKGGLFRAGSMDNGDGIAVGWLGHPIFR
DKEGRELFVRRMPTFFETFPVVLVDGDGIVRADVPFRRAESKYSVEQVGVTVEFYGGELN
GVSYSDPATVKKYARRAQLGEIFELDRATLKSDGVFRSSPRGWFTFGHASFALLFFFGHIW
HGARTLFRDVFAGIDPDLDAQVEFGAFQKLGDPTTKRQAAMKTLYSLRRFYHVETLFNGT
LALAGRDQETTGFAWWAGNARLINLSGKLLGAHVAHAGLIVFWAGAMNLFEVAHFVPEK
PMYEQGLILLPHLATLGWGVGPGGEVIDTFPYFVSGVLHLISSAVLGFGGIYHALLGPETLE
ESFPFFGYVWKDRNKMTTILGIHLILLGIGAFLLVFKALYFGGVYDTWAPGGGDVRKITNL
TLSPSIIFGYLLKSPFGGEGWIVSVDDLEDIIGGHVWLGSICILGGIWHILTKPFAWARRALV
WSGEAYLSYSLGALAVFGFIACCFVWFNNTAYPSEFYGPTGPEASQAQAFTFLVRDQRLG
ANVGSAQGPTGLGKYLMRSPTGEVIFGGETMRFWDLRAPWLEPLRGPNGLDLSRLKKDI
QPWQERRSAEYMTHAPLGSLNSVGGVATEINAVNYVSPRSWLATSHFVLGFFFFVGHLWH
AGRARAAAAGFEKGIDRDFEPVLSMTPLNMTIALDKFTKDENDLFDIMDDWLRRDRFVF
VGWSGLLLFPCAYFAVGGWFTGTTFVTSWYTHGLASSYLEGCNFLTAAVSTPANSLAHSL
LLLWGPEAQGDFTRWCQLGGLWTFVALHGAFGLIGFMLRQFELARSVQLRPYNAIAFSGP
IAVFVSVFLIYPLGQSGWFFAPSFGVAAIFRFILFFQGFHNWTLNPFHMMGVAGVLGAALL
CAIHGATVENTLFEDGDGANTFRAFNPTQAEETYSMVTANRFWSQIFGVAFSNKRWLHFF
MLFVPVTGLWMSALGVVGLALNLRAYDFVSQEIRAAEDPEFETFYTKNILLNEGIRAWM
AAQDQPHENLIFPEEVLPRGNALMSGSTGERSFADIITSIRYWVIHSITIPSLFIAGWLFVSTG
LAYDVFGSPRPNEYFTESRQGIPLITGRFDSLEQLDEFSRSFMTIDRTYPIFTVRWLAVHGLA
VPTVFFLGSISAMQFIQRMATQTVENSS-RSGPRRTAVGDLLKPLNSEYGKVAPGWGTTPL
MGVAMALFAVFLSIILEIYNSSVLLDGISMNMLTLKLFVYTVVIFFVSLFIFGFLSNDPGRNP
GREEMADTTGRIPLWIIGTVAGILVIGLIGIFFYGSYSGLGSSLMLNTFSLIGICLNSTLYSSSF
FFGKLPEAYAFLNPIVDIMPVIPLFFFLLAFVWQAAVSFRTTQSNPNEQSVELNRTSLYWGL
LLIFVLAVLFSNYFFNMEVNILAFIATALFILVPTAFLLIIYVKTVSQNDMETATLVAIFISGLL
VSFTGYALYTAFGQPSQQLRDPFEEHGDMEALVYTFLLVSTLGIIFFAIFFREPPKLPTKKN-
MTLAFQLAVFALIATSLILLISVPVVFASPDGWSSNKNVVFSGTSLWIGLVFLVGILNSLIS----
---MSPQTETKASVGFKAGVKEYKLTYYTPEYQTKDTDILAAFRVTPQPGVPPEEAGAAVA

AESSTGTWTTVWTDGLTSLDRYKGRCYRIERVVGEKDQYIAYVAYPLDLFEEGSVTNMFT
SIVGNVFGFKALRALRLEDLRIPPAYVKTFQGPPHGIQVERDKLNKYGRPLLGCTIKPKLG
LSAKNYGRAVYECLRGGLDFTKDDENVNSQPFMRWRDRFLFCAEALYKAQAETGEIKGH
YLNATAGTCEEMIKRAVFARELGVPIVMHDYLTGGFTANTSLAHYCRDNGLLLHIHRAMH
AVIDRQKNHGIHFRVLAKALRMSGGDHIHSGTVVGKLEGERDITLGFVDLLRDDFVEQDR
SRGIYFTQDWVSLPGVLPVASGGIHVWHMPALTEIFGDDSVLQFGGGTLGHPWGNPPGAV
ANRVALEACVKARNEGRDLAQEGNEIIREACKWSPELAAACEVWKEIVFNFAAVDVLDK
MAIHLYKTSTPSTRNGTVDSQVKSNPRNNLIYGQRRCGKGRNARGIITARHRGGGHKRLY
RKIDFRRNEKDIYGRIVTIEYDPNRNAYICLIHYGDGEKRYILHPRGAIIGDTIVSGTEVPIK
MGNALPLTDMPLGTAIHNIEITLGKGGQLARAAGAVPKLIAKEGKSATLKLPSGEVRLISK
NCSATVGQVGNVGVNQKSLGRAGSKRWLGKRPVVRGVVMNPVNHPHGGGEGRAPIGR
KKPTTPWGYPALGRRSRKRNKYSDNLILRRRSKMIQPQTHLNVADNSGARELMCIRIIGAS
-NRRYAHIGDVIVAVIKEAVPNMPLERSEIVRAVIVRTCKELKRDNGMIIRYDDNAAVVIDQ
EGNPKGTRIFGAIARELRELNFTKIVSLAPEVLMLSPKRTRFRKQHRGRMKGISYRGNHISF
GKYALQALEPAWITSRQIEAGRRAMTRNARRGGKIWVRIFPDKPVTLRPAETRMGSGKGS
PEYWVAVVKPGRILYEMGGVTENIARRAISLAASKMPIRTQFIIS-MTRIKRGYIARRRRTKI
RLFASSFRGAHSRLTRTITQQKIRALVSAHRDRDRKKRDFRRLWITRINAVIRE-RGVSYSYS
RLIHDLYKRQLLLNRKILAQIAISNRNCLYMISNEIIKEVDWKESTRIIMLKEVYALGEHISM
SADKARRVIDQIRGRSYEETLMILELMPYRACYPILKLVYSAAANASYNMGSSEANLVISK
AEVNGGTTVKKLKPRARGRSFPIKRSTCHITIVMKDISLNGGLWDKKMDGIKYAVFTDKSI
RLLGKN-QYTSNVESGSTRTEIKHWVELFFGVKVIAMNSHRLPGKSRRMGPIMGHTMHY
RRMIITLQPGYSIPPLRKKRTMAVPKKRTSTSKKRIRKNIWKRKGYWVALKAFSLAKSLST
GNSKSFFVRQTKIMAKGKDVRVTVILECTSCVRNSVD--KVSRGISRYITQKNRHNTPNRLE
LKKFCPYCYKHTIHGEIKKMKIRASVRKICEKCRLIRRRGRIIVICSNPRHKQRQGMVREK
VTVSTRTLQWKCVESRTDSKRLYYGRFILSPLMKGQADTIGIAMRRALLGEIEGTCITRVK
FE--KVPHEYSTITGIQESVHEIIMNLKEIVLRSNLYGTSEASVCVKGPGYVTAQDIILPPYVE
IVDNTQHIASLTEPIDFCIGLQIERNRGYLIKTPQ-NFDGSY--PIDAVFMPVRNANHSIHSYG
NGNEKQEILFLEIWTNGSLTPKEALHEASRNLIDLFIPFLHMEED--NLYLQDNQ-HTVPLSP
FTFHDKLA-KLIKNNK-KIALKSIFIDQSELPSRIYNCLKMSNIYTLLDLLNNSQEDLMKIEH
FRSEDIKQILGILEKY--FVIDLAKNKMLGDG-NEGISTIPGFNQIQFEGFCRFIDQGLTEELYK
FPKIEDTDQEIEFQLFVETYQLVEPLIKERDAVYESLTYSSELYVSAGLIWKNSRDMQEQTIF
IGNIPLMNSLGTSIVNGIYRIVINQILQSPGIYYRSELDHNGISVYTGTIISDWGGRSELEIDR
KARIWARVSRKQKISILVLSSAMGLNLREILENVCYPEIFLSFLSDKERKKIGSKENAILEFY
QQFACVSGDPVFSESLCKELQKKFFQQRCELGRIGRRNMNRRLNLDIPQNNTFLLPRDILA
AADHLIGLKFGMGALDD--MNHLKNKRIRSVADLLQDQFGLALVRLENVVRGTICGAIR-H
KL-IPTPQNLVTSTP----LTTTYESFFGLHPLSQVLDRTNPLTQIVHGRKLSYLGPGGLTGRTA
SFRIRDIHPSHYGRICPIDTSEGINVGLIGSLAIHARIGHWG-SLESPFY---EISERSTG--VRML
YLSPGRDEYYMVAAGNSLALNRDIQEEQVVPARYRQEFLTIAWEQVHLRSIFPFQYFSIGA
SLIPFIEHNDANRALMSSNMQRQAVPLSRSEKCIVGTGLERQAALDSGALAIAEREGRVVY
TNTDKILLAGNGDILSIPLVIYQRSNKNTCMHQKLQVPRGKCIKKGQILADGAATVGGELA
LGKNVLVAYMPWEGYNSEDAVLISERLVYEDIYTSFHIRKYEIQTHVTSQG-PEKVTNEIPH
LEAHLLRNLDKNGIVMLGSWVETGDILVGKLTPQVVKE-SSYAPEDRLLRAILGIQVSTSK
ETCLKLPIGGRGRVIDVRWIQKRG-GSSYNPETIRVYISQKREIKVGDKVAGRHGNKGIISKI
LPRQDMPYLQDGRSVDMVFNPLGVPSRMNVGQIFECSLGLAGSLLDRHYRIAPFDERYEQ

EASRKLVFSELYEASKQTANPWVFEPEYPGKSRIFDGRTGNPFEQPVIIGKPYILKLIHQVD
DKIHGRSSGHYALVTQQPLRGRAKQGGQRVGEMEVWALEGFGVAHILQEMLTYKSDHIR
ARQEVLGTTIIGGTIPNPEDAPESFRLLVRELRSLALELNHFLVSEKNFQINRKEA-------MID
RYKHQQLRIGSVSPQQISAWATKILPNGEIVGEVTKPYTFHYKTNKPEKDGLFCERIFGPIK
SGICACGNYRVIGDEKEDPKFCEQCGVEFVDSRIRRYQMGYIKLACPVTHVWYLKRLPSY
IANLLDKPLKELEGLVYCD--FSFARP-ITKKPTFLRLRGLFEYEIQSWKYSIPLFFTTQGFDT
FRNREISTGAGAIREQLADLDLRIIIENSLVEWEELGEE-GHTGNEWEDRKVGRRKDFLVRR
VELAKHFIRTNIEPEWMVLCLLPVLPPELRPIIQIDGGKLMSSDINELYRRVIYRNNTLTDLL
TTSRSTPGELVMCQEKLVQEAVDTLLDNGIRGQPMRDGHNKVYKSFSDVIEGKEGRFRET
LLGKRVDYSGRSVIVVGPSLSLHRCGLPREIAIELFQTFVIRGLIRQHLASNIGVAKSKIREK
EPIVWEILQEVMQGHPVLLNRAPTLHRLGIQAFQPVLVEGRAICLHPLVCKGFNADFDGD
QMAVHVPLSLEAQVEARLLMFSHMNLLSPAIGDPISVPTQDMLIGLYVLTSGNRRGICVNR
YNPCNR---RNYQNQKRSDNSYKYT---EPFFSNSYDAIGAYRQKRINLDSPLWLRWRLDQR-
VIAS--RETPIEVHYESLGTFYEIYGHYLIVRSLKKQILFIYIRTTVGHIALYREIEEAIQGFSRA
YSYGT------------MAERANLVFHNKAIDGTAMKRLISRLIEHFGMAYTSHILDQVKTLGFQQ
ATATSISLGIDDLLTIPSKGWLVQDAEQQSLILEKHHHYGNVHAVEKLRQSIEIWYATSEYL
RQEMNPNFRMTDPFNPVHIMSFSGARGNASQVHQLVGMRGLMSDPQGQMIDLPIQSNLR
EGLSLTEYIISCYGARKGVVDTAVRTSDAGYLTRRLVEVVQHIVVRRTDCGTARGISVSPRN
GMMP-ERIFSQTLIGRVLADDIYMGPRCIATRNQDIGIGLVNRFIT------FRAQPISIRTPFTCR
STSWICRLCYGRSPTHGDLVELGEAVGIIAGQSIGEPGTQLTLRTFHTGGVFTGGTAEHVRA
PSNGKIKFNEDLVHPTRTRHGHPAFLCSIDLYVTIESEDILHNVNIPPKSLLLVQNDQYVESE
QVIAEIRAGIST-LNFKEKVRKHIYSDSDGE-MHWSTDVYHAPEFTYGNVHLLPKTSHLWIL
LGGPCK--SSLVYLSIHKDQDQMNAH--SLSGKQRYTSNLS--VTNDQ-ARQKLFSSGFSGK-E
DRIPDY-SDLNRIICTGQYNLVYS-PILHENS--DLLSKRRRNKFIIPLHSIQELENELMPCSGISI
EIP--VNGIFRRNSILAYFDDPRYRRKSSGIIKYGTIETHSVIKKED--LIEYRG-VKEFRPKYM
KVDRFFFIPEEVHILPGSSSIMVRNNSIVGVDTQITLNLRSRVGGLVRVERKKKRIELKIFSG
DIHFPGETDKISRHTGLLIP-PG-TGKINSKESKK--VKKWIYVQR--ITPSK--KKFFVLVRPVV
TYEITDGINFETLFPPDPLQERDNVQLRIVNYILYGNGKPIRGISDTSIQLVRTCLVLNWNQD
KK---SSSCEEARASFVEIRTNGLIRHFLRINLVKSPISYIGKRNDPSGSGLLSD-NGSDCTNIN
PFSSIYSY---SKAKIQQSLNQPQGTIHTLLNR---NKECQSLIILSSANCSRMG-----TLKYHSV--
-IKDS-PLIPIRNSLGPLGT--CLPIENFYS--SYRL--ITHNQIL-VTNYLQLDN---LKQTFQVIKFK
-YYLMDEN--GKIFNPDPCRNIILNPFNLNWYFLHHNYCEETS-KIISLGQFICENVCIAKNGP
P-LK--SGQVILVQ-VDSIVIRSAKPYLATPGATVHGHYGETLYEGDTLVTFIYEKSRSGDITQ
GLPKVEQVLEVRSIDSISMNLEKRVEGWNKCITRILGIPWGFLIGAELTIAQSRISLVNKIQQ
VYRSQGVQIHNRHIEIIVRQITSKVLVSE---DGMSNVFSPGELIGLLRAERMGRALEEAICYR
VVLLGITRASLNTQSFISEASFQETARVLAKAALRGRIDWLKGLKENVVLGGVIPVGTGFK
GL--VHPSKQHNNIP--LETKKKNLFEGEMR--DILFHHRKLFDSCLSKKFHDIPEQSFIG------F
NDSMTRRYWNINLEEMMEAGVHFGHGTRKWNPKMAPYISAKRKGIHITNLTRTARFLSE
ACDLVFDAASRGKQFLIVGTKNKAADSVEWAAIRARCHYVNKKWLGGMLTNWSTTETR
LHKFRDLRMEQKTGRLNRLPKRDAAMLKRQLSRLQTYLGGIKYMTGVPDIVIIVDQHEE
YTALRECITLGIPTICLTDTNCDPDLADISIPANDDAISSIRLILNKLVFAICEGRS-SYIRNPMG
QKINPLGFRLGTTQGHHSLWFSQPKNYSEGLQEDQKIRDCIKNYVQKNMRTSSG-VEGIAR
IEIQKRIDLIQVIIFMGFPKLLIESRPR----GIEELQMTLQKELNCVNRKLNIAVTRIAKPYGNP
NILAEFIAGQLKNRVSFRKAMKKAIELTEQADTKGIQIQIAGRIDGKEIARVEWIREGRVPL

QTIRAKIDYCAYTVRTIYGVLGIKIWIFLDEEMSRYRGPRFKKIRRLGALPGLTNKKPRTGS
DLRNQSRSGK-KSQYRIRLEEKQKLRFHYGLTERQLLKYVRIARKAKGSTGQVLLQLLEM
RLDNILFRLGMASTIPAARQLVNHRHILVNGRIVDIPSYRCKPRDIITAKDEQKSRALIQISL
DSSPH-EELPNHLTLHPFQ--YKGLVNQIIDSKWVGLKINELLVVEYYSRQTMSRRGTAEKK
TAKSDPIYRNRLVNMLVNRILKHGKKSLAYQIIYRAVKKIQQKTETNPLSVLRQAIRGVTP
DITVKARRVGGSTHQVPIEIGSTQGKALAIRWLLAASRKRPGRNMAFKLSSELVDAAKGS
GDAIRKKEETHRMAEANRAFAHFRMGRDTIAEIITSIRNADMDRKRVVRIASTNITENIVQI
LLREGFIENVRKHQENNKYFLVLTLRHRR--NKKRPY---RNILNLKRISRPGLRIYSNYQRIP
RILGGMGIVILSTSRGIMTDREARLEGIGGEILCYIWMAKAIPKISSRRNGRIGSRKGARRIP
KGVIHVQASFNNTIVTVTDVRGRVVSWSSAGTSGFKGTRRGTPFAAQTAAANAIRTVVDQ
GMQRAEVMIKGPGLGRDAALRAIRRSGILLTFVRDVTPMPHNGCRPPKKRRVMPTINQLI
RNTRQPIRNVTKSPALRGCPQRRGTCTRVYTITPKKPNSALRKVARVRLTSGFEITAYIPGIG
HNLQEHSVVLVRGGRVKDLPGVRYHIVRGTLDAVGVKDRQQGRSKYGVKKPKMAKKSL
IQREKKRQKLEQKYHSIRRSSKKEIS---KVPSLSDKWEIYGKLQSLPRNSAPTRLHRRCFLT
GRPRANYRDFGLSGHILREMVHACLLPGATRSSWMVKNSVISIIFQE--EK---RGSVEFQVF
NFTNKIRRLTSHLELHKKDYLSQRGLKKILGKRQRLLAYLAKKNRVRYKELINQLDIRETK
TR--MVKLRLKRCGRKQRAVYRIVAIDVRSRREGKDLRKVGFYDPIKNQTYLNVPAILYFL
EKGAQPTGTVQDILKKAEVFKELRPNQPKFN-MDKSKRPFLKFKRSFRRRLPPIQSGDRID
YRNMSLISRFISEQGKILSRRVNRLTLKQQRLITLAIKQARILSLLPFLNNEK--QFERTESTAR
TTGFKARNKVTRSLKKNPFVANHLLKKIDKLNTKAEKEIIVTWSRASTIIPTMIGHTIAIHN
GKEHLPIYITDSMVGHKLGEFAPTLNFRGHAKSDNRSRRMIFQSFLLGNLVSLCMKIINSV
VVVGLYYGFLTTFSIGPSYLFLLRALVMEEG---TEKKVSATAGFITGQLMMFISIYYAPLHL
ALGRPHTITVLALPYLLFHFFWNNHKHFFDYGSTTRNSMRNLSIQCVFLNNLIFQLFNHFIL
PSSMLARLVNIYLFRCNNKILFVTSGFVGWLIGHILFMKWLGLVLVWIRQNHSIRPYIRSNK
YLVLELRNSMARIFSILLFITCVYYLGRIPSPILTKKLKETEEDKPLVSLLFNYNRWNRPFRYI
KNNRFEQAVRTEMSQYFFDTCKSDGKQRISFTYPPSLSTFWKMIKGRLPLLSLQKTLPNEL
DNQWVSTNKEKSN-LNKEFLNRLEVLDKESLSLDILETRTRLSNDDTKKEYVPKMYDPLL
NGPYRGTIKKGFSPKERVILNRIHTIFYQEFEQKDPNGTSIGIKEISKKIPRWSHKLIELEQQL
-GQIRSRKARRVVIFTTALISYSQQSDFRRGIIKGSMRAQRRKTVIWKLFQANVHSPFFLDRI
TPPRLFSFDI--SELIKPIFRNKKEKARIAIAEAWDSILFAQIIRGSMLITQSILRKYILLPSLIIAK
NLGRMLFLQLPEWSEDLEEWNREMHIKCTYNGVQLSETEFPKNWLRDGIQIKILFPFCLK
PWHISKKKQKDDFCFLTVWGMEAELPFGSPRSRPSFFEPIFKELEKKNGKLKKKYFIT-LKV
LKGKTKLFRRISKETKKWLIKSIL--FLKKIRKELSKIKDSLISNQIINESFSQIESVNWPNSSLI
ETKMKDLTDRTSTIKNQIERITKEKKKSRNK-TNNIKKFESTKNIFQILQRRNTRLIWKFHYF
IKLFIQRLYINLFLSIINIPRINTQLF--FISFDLSYLSQAYVFYKLSQTQVINLSKFRSVLQYTSF
FLKTKIKDYFTLGIFHSELKHKKLQSYRINQWKNWLRRHYQYDLSQIRWSRLMPQKWRN
RVNQSCMAQNKNKWNSYENDQ--LIHYKKE-NFQKCYRYDLLSYKSINGNILYLDRKYFD
WKIIHFSLRFFDWMGMNEKIFNRWFFPEFVLLYNVYKIKPWIIPSKLLLLNYKSNTEAELD
LFLKRYLLFQLKWNDDLNQRMIENIKVYCLLLRLINPTKISISSVQRGEMSLDIMLIQGNLT
LTELMK-GILIIEPIRLSVKNNGQFIMYQTIGISLVHKSKHNHFDLLVPENILWSRRRRELRIR
SFFRFLWPNYRLEDLACMNRYWFDNNNGSRFSILRIHMYPMRGHQFKSWILELREILREIK
NSHHFLDSWTQFTSVGSFIHIFFHQERFLKLFDPRIWSILLS-RNS--QGSTSNRYFT---IKG-VI
LFVVAVLIYR----INNRNMVERKNLYLIGLLP-----IPMNSIGPRNDTLEESV-GSSNINRLIVS-L
LYLPKGKKISESCFLNPKESTWVLPIT----KKCSMPESNWGSRWWRNWIGKKR--DSSCKIS

N---ETVAGIEILFKEKDLK-YLEFLFVYYMD--DPIRKDHDWELFDRLSLRKSRNRINL--NSG
PLFEILVKHWISYLMSAFREKIPIEVEG-FFKQQG-AGSTIQSNDIEHVSH--LFSRNKWAISLQ
NCAQFHMWQFRQDLFV---SWGK-NPPESDFLRN-VSRENWIWLDNVWLVNKDR---FFS--K
VQNVSSNIQYDS---TR--SSFVQVTDSSQLKGSS-DQSRDHLDSIS----NEDSE------------YHT
LINQREIQQRKERSIL-WD--PSFLQMERKEIESG--RFPK--CLSGYS--SMSRLFT-EREKQMIN
HLFPEEIEEFL--GNPTRSVRSFFSDRW--SELH-LGSNPTERSTRDQ----KLLKKQQDLS-FVPS
RRSE----NKEMVNIFKIITYLQN--TVSIHPISSDPGCDMVP-KDEPD-MDSSNKISFLNKNPFF
DL--FHLFHDRNRGGYTL--HYDFESE--ERFQEMADLFTLSITEP-DLVYHKGFAFSIDSCGL
DQKQ--FLNEARDE---SKKKS---LLVLPPIFY--EENESFSRRIRKKWVR-ISCGNDLEDPKPKI
VVFASNNIMEAVTQYRLIRNLIQIQYSTYG--YIRNVLNRFFLMN---RSDRNFEYG---IQRDQI
GKDTLNHRTIMKYTINQYLSNLKK-SQKKWFEPL--ILISRTERSMNRDPDAYRYKWSNGS
KNFQEHLEQSVSEQKSR---FQVVFDRLRINQYSIDWSEVIDKKDLSKPLRFFLSKPLRFFLS
K-SLLFLS-------KLLFFLSN-SLPFFCVSFGNIPIHRSE-IYIY-ELKGPNDQLCNQLLESIGLQIV
HLKKLKPF--LLD---DHDTS-QKSKFLINGGTISPFLFNKIP-KW-MIDSFHTR--NNRRKSFDN
PDSNFSMIFHDQDNWLNPVKPFHRSSLISSFYKANRLRFLNNPHHFCFY--WNTRFPFSV----
EKARIN--NSYFTYG-QFLNILFIRNK-IFSLCVGN--KKHAFWG-RDTISPIESQVSNIFIPNDFP
QSG-DETYNLYKS---FHFPSRSDPFVR--RAIYSIADISGTPLTEGQIVNFERTYCQPLSDMNLS
DSEGKNLHQYLN--FNSNMGLIHTPCSEKDLSSEK--RKK--------RSLCLK--KCVEKGQMYR
TFQRDSAFSTLSKWNLFQTYMPWFLTSTGYKYLNLIFLDTFSDLL--PILSS-SQKFVSIFPDI
MHGSGISWRILQKKL------CLPQWNL-ISEISSKCLHN--LLLSEEMIHRN--NESPLISTHLRSP
NAREF-LYSILFLLLVAGY-LVRTHLLFVSR--ASSELQTEFEKVKSLMIPSSMIELRKLLDRYP
TSEPNSFWLKNLFLVALEQLGDSLEEIRGSASGGNMLG---PA-----YG-VKSIRSKKKDWNIN
LIEIIDLI----PNPINRITFSRNTRHLSHTSKEIYSLIRKRKNVNGDWIDDKIESWVANSDSIAD
EEREFLVQFS--TLTT--ENRIDQILLSLT-HSDHL-----SKNDSGYQMIEQPGAIYLRYLVDIH--K
KHLMNYEFNPSCLAERRIFLAHYQTITY--SQTSCGENSFHFPSHG---KPFSLRLALSPS-RGIL
VIGSIGTGRSYLVKYLATNSYVPFITVFLNKFLDNKPKGFLLD-----EID-IDDSDDIDDSDNL
DASD-----------------DIGRDLDTEL-ELLTRMNGLTMDMMPE---IDRFYITLQFELAKAMSPC
IIWIPNIHDLDVNESNDLSLGLLVNHLSR---DCERCS-TRNILVIASTHIPQKVDPALIAPNKL
NTCIKIRRLLIPQQRKHFFTLSYTRGFHLEKKMFHT--NGFGSITMGSNARDLVALTNEVLSI
SITQKKSIIDTNTIRSALHRQTWDLR---SQVRSVQDHGILFYQIGRAVAQNVLLSNCPIDPISI
YMKKK--SCNEGDSYLYKWYFELGTSMKRLTILLYLLSCSAGSVAQDLWSLS--GPDEK-NG
ITSYGLVENDS-DLVHGLLEVEGALVG--SSRTEKDCSQFDND--RVTLLLRPEPRN-PLDMM
QKGSCSILDQRFLYEKY--ESEFEE-GEGEGALDPQ-------ED----LFNH--IVWAP--RIWRP---W
GFLFDCIERPNELGFP-YWSRSFRGK---RIIYDEEDELQENDSGFLQSGTMQYQTRDRSSKE
QGLFRISQFIWDPADPLFFLFK-DQPPGSVFSHRELFADEEMSKGLLTSQTDPPTSIYKRWFI-
-KNTQEKHFELLINRQRWLR---TNSSLS--NGSFRSN--TLSESYQYLSNLFLSN-GTLLDQMT
KTLLRKRWLFPDEMKIGF--M---------MPRSR-INGNFIDQTFSIVANILLRVIPTTSGEKEAFT
YYRDG--MSAQSEGNYAEALQNYYEAMRLEIDPYDRSYILYNIGLIHTSNGEHTKALEYYF
RALERNPFLPQAFNNMAVICHY-RGEQAIQQGDSEIAEAWFDQAAEYWKQAIALTPGNYIE
AHNWLKITRRFE--MTWRSEHIWIELITGSRKISNFCWAFILFLGSLGFLLVGTSSYLGRNLIS
FFPTQQIVFFPQGIVMSFYGIAGLFISSYLWCTISWNVGSGYDRFDRKEGIVCIFRWGFPGK
NRRIFLRFLIKDIQSVRIEVKEGISARRVLYMDIRGQGSIPLTRTDEN-LTPREIEQKAAELAY
FLRVPIEVF
>Calycanthus

MGKWWFN---SLSKSMDSLGLIGNTSGSEDPTLNDTNKNIP-CNNVDHFFSVRDIWSFISDG
TFLVRDNNGNCYSVYLDIENQVFEIDND--SSFLSELESSFSSYMN-SYLNSGSKSDNRYYDR
YTYDIKYSWNNHINSCIDSYLRSEIN--IDSYISSGSDNYRDSYIYSYICSKSGSSIRTGGNSSD
FNRRGRS-DFDLNKKYRHLWVQCENCYGLNYKKFVSFKMHICEQCGYHLKMSSSERIELS
IDSGTWDPMDEDMVSMDPIQFHSEEEPYRDRIDSYQRKTGLTEAVQTGIGQLNGIPVAIGV
MDFQFMGGSMGSVVGEKITRLIEYATNRSLPVIMVCASGGARMQEGSLSLMQMAKISSAS
YDYQLNKKLFYVSILTSPTTGGVTASFGMLGDIIIAEPNAYIAFAGKRVIEQTLNKTVPDGS
QAAEYSFHKGLFDPIVPRNPLKGVLNELFRLHGFFPLNQNSSRMVTIRADEISNIIRERIEQY
NREVKIVNTGTVLQVGDGIARIHGLDEVMAGELVEFEEGTIGIALNLESNNVGVVLMGDG
LTIQEGSSVKATGRIAQIPVSEAYLGRVINALAKPIDGRGEISASESRLIESPAPGIISRRSVYE
PLQTGLIAIDSMIPIGRGQRELIIGDRQTGKAAVATDTILNQKGQNVVCVYVAIGQKASSVA
QVVTTFQERGAMEYTIVVAETADSPATLQYLAPYTGAALAEYFMYRERHTSIIYDDLSKQ
AQAYRQMSLLLRRPPGREAYPGDVFYLHSRLLERAAKLSSPLGEGSMTALPIVETQSGDV
SAYIPTNVISITDGQIFLSADLFNAGIRPAINVGISVSRVGSAAQIKAMKQVAGKSKLELAQF
AELEAFAQFASDLDKATQNQLARGQRLRELLKQSQAAPLTVEEQIVTIYTGANGYLDPLEI
GQVKKFLVQLRTYLKTNKPQLQEIISSTKTFTEEVEALLKEAIPEQIELFLLQEQTMRINPTT
SGPGVSTLEEKNLGRIAQIIGPVLDVAFPPGKMPNIYNALVVKGRDTVGQQINVTCEVQQL
LGNNRVRAVAMSATDGLMRGMEVIDTGAPLSVPVGGATLGRIFNVLGEPVDNLGPVDTRT
TSPIHRSAPAFIQLDTKLSIFETGIKVVDLLAPYRRGGKIGLFGGAGVGKTVLIMELINNIAK
AHGGVSVFGGVGERTREGNDLYMEMKESGVINEQNIAESKVALVHGQMNEPPGARMRV
GLTALTMAEYFRDVNEQDVLLFIDNIFRFVQAGSEVSALLGRMPSAVGYQPTLSTEMGSL
QERITSTKEGSITSIQAVYVPADDLTDPAPATTFAHLDATTVLSRGLAAKGIYPAVDPLDSTS
TMLQPRIVGEEHYEIAQRVKQTSQRYKELQDIIAILGLDELSEEDRLTVARARKIERFLSQPF
FVAEVFTGSPGKYVGLAETIRGFQLILSGELDGLPEQAFYLVGNIDEATAKAMNLEVESKL
KKMTLNLCVLTPNRIIWDSEVKEIILSTNSGQIGVLPNHAPIATAVDIGILRIRLNGQWLMM
AVMGGFARIGNNEITILVNDAEKGSDIDPQEAQRTLEIAEANLSKAEGKRQVIEANLALRR
ARTRVEAINVI-MKNVTDSFVSLGHWPFAGSFGFNTDILATNLINLSVVLGVLIFFGKGVLS
DLLDNRKQRIWSTIRNSDELREVAIEQLEKARARLRKVEREADEFRVNGYSEIEREKWNLI
NATYENLERLENYKNETIHFEQQRAINQVRQRVFQQALQGALGTLNSRSNSELHLRTISAN
IGMLGAMKEITDMNPLISAASVIAAGLAVGLASIGPGVGQGTAAGQAVEGIARQPEAEGKI
RGTLLLSLAFMEALTIYGLVVALALLFANPFVMNVLPCSINTLKGLYDISEVEVGQHFYWQ
IGGFQVHAQVLITSWVVIAILLGSAAIAVRNPQTIPTDGQNFFEYVLEFIRDLTKTQIGEEYG
PWVPFIGTMFLFIFVSNWSGALLPRKIIQLPHGELAAPTNDINTTVALALLTSVAYFYAGLTK
KGLGYFGKYIQPTPILLPINILEDFTKPLSLSFRLFGNILADELVVVVLVSLVPLVVPIPVMFL
GLFTSGIQALIFATLAAAYIGESMEGHHMIFSTLEHILTHISFSIISIVITIHLLNLLVHDTVGLC
DSSEKGMMATFFCITGLLVTRWIYSRHFPLSDLYESLMFLSWSFSIIHMVPYFRNHKNSLSA
ITAPSAIFTQGFATSGLLTEMHQSAILVPALQSQWLMMHVSMMLLGYASLLCGSLLSVALL
VITFRKTLGIPGKNNEIRYFNEKRSVLKKTSFFSFRNYHRYQLTQRLDHCSYRIISLGFTFSTI
GILSGAVWANEAWGSYWNWDPKETWAFITWTIFAIYLHTRTNQRFQDVGPAIVASMGFLII
WICYFGVNLLGIGLHSYGSFALTVPKK-ALTPFPYLASIVFLPWWISLSFTKSLEPWVTNW
WNTGQSKTFLNDIQEKNVLERFIELEQLFLLDEMIKEYPETQIQKFHIGIHKETLQLVKMH
NEDHIHIVLHFSTNIICFAILSGYYFLGNEELVILNSWVQEFLYNLSDTIKAFSILLVTDLCIGF
HSPRGWELMIGSVYKDFGFAHNDQIISGLVSTFPVILDTILKYWIFHYLNRVSPSLVVIYHS
MNEMPIGVPKVPFRSPGEEDAVWVDVYNRLHRERLLFLGQEVDSEISNQLVGLMVYLTIE

DDTKDLYLFINSPGGWVIPGIAIYDTMQFVSPDVHTICMGLAASMGSFILVGGEITKRLAFP
HARVMIHQPASSFYEAPTGEFILEAEELLKLRETLTRVYVQRTGNPLWVVSEDMERDVFM
SATEAQAHGIVDLVAIEMKEQKLIHEGLITESLPNGMFRVRLDNEDLILGYVSGRIRRSFIRI
LPGDRVKIEVSSYDSTRGRIIYRLRNKDSNDMEELQGYLEIDGFRQHHFLYPLLLQEYIYAL
AHDHGLLSHDN-SSSLIVKRLITRMHQQNHFIISVNDSNQKGFVGKNFHSQKISEGFAVIVEI
PFSLQLVSSLIAKFH-SRSIHSIFPFFEDKLSHLNHVSDILIPYPIHLEILVQTLRCWIQDAPSLH
LLRFFLHEYWNSNSLITKSISFFSKQRLFLFLYNSHVYECESVFIFLRKQSSHLRSTSFGSFLE
RTHFYGKIEH-VVVLGNPK-LWLFKDPFVHYVRYQGKSILASRGTQFLIKKWKYHLVNFW
QCHFYLWSQPDRIHLNQLCNHSFYFLGYLSSVQLNSSVVRSQMLENAFLMDTAI--KKFETI
VPIIPLIGSLAKAKFCNGSGHPISKPFRTDLSDSEIINRFGRICKNLSHYHSGSSKKQSLYRIK
FILRLSCARTLSRKHKSTVRAFLKRLGSELLEEFLTEEEQVLSLLFPRRIWYLDIICINDLAN
HEMIIDTT-EVQ-AINSFSRSE-SLKEVYDLLWLLVPIFTPVLGITIGVLVIVWLEREISAGIQQ
RIGPEYAGPLGILQALADGTKLLFKEDLLPSRGDIRLFSIGPSIAVISILLSYSVIPFGYRLVLA
DLGIGVFLWIAISSIAPIGLLMSGYGSNNKYSFSGGLRAAAQSISYEIPLTPCVLSISLLSNSS
STVDIVEAQSKYGFWGWNLWRQPIGFIVFLISSLAECERLPFDLPEAEEELVAGYQTEYSGI
KFGLFYVASYLNLLVSSLFVTVLYLGGWNLPIPYIFIPEPFGINKTDGVFGTTIDILITLAKSY
LFLFVPIITRWTLPRMRMDQLLNLGWKFLLPISLGNLLLITSFQLVSLMIWHVQNENFILDS
TRIFMKAFHLLLFHGSFIFPECILIFGLILLLMIDSTSDQKDIPWLYFISSTSLVMSITALLFRW
REEPMISFSGNFQTNNFNEIFQFLILLCSTLCIPLSVEYIECTEMAITEFLLFVLTATLGGMFL
CGANDLITIFVAPESFSLCSYLLSGYTKRDVRSNEATTKYLLMGGASSSILVHGFSWLYGSS
GGEIELQEIVNGLINTQMYNSPGISIALISITVGIGFKLSPAPSHQWTPDVYEGSPTPVVAFLS
VTSKVAASASATRIFDIPFYFSSNEWHLLLEILAILSMILGNLIAITQTSMKRMLAYSSIGQIG
YVIIGIIVGDSNDGYASMITYMLFYISMNLGTFARIVSFGLRTGTDNIRDYAGLYTKDPFLAL
SSALCLLSLGGLPPLAGFFGKLHLFWCGWQAGLYFLVSIGLLTSVVSIYYYLKIIKLLMTGR
NQEITPHVRNYRRSLLRSNNSIELSMIVCVIASTIPGISMNPIIAIAQDTLFMFLLHEYDIFWA
FLIISSVIPILAFLISGVLAPIREGPEKLSSYESGIEPMGDAWLQFRIRYYMFALVFVVFDVET
VFLYPWAMSFDVLGVSVFIEALIFVLIPIVGSVYAWRKGALEWSTSYFPWLTVIVVLPISAG
SSIFFVPYRGNKVIRWYTICICLLEILLTTYAFCYHFQSDDPLIQLEEAYKWIHIFDFHWRPGI
DGLSIGPILLTGFITTLATLAARPVTRDSRLFHFLMLAMYSGQVGSFSSRDLLLFFLMWELE
LIPVYLLLSMWGGKKRMYSATKFILYTAGGSIFLLMGVSGMGLYGSNEPTLNFETLANQS
YPLGLEIIFYFGFLIAYAVKSPIIPLHTWLPDTHGEAHYSTCMLLAGILLKMGAYGLVRINM
ELLPHAHSIFSPWLVIVGAIQIIYAASTSFGQRNFKKRIAYSSVSHMGFTLIGIGSITDTGLNG
AILQIISHGFIGAALFFLAGTSYDRIRLVYLDEMGGIAIPMPKIFTMFSSFSMASLALPGMSG
FVAELVVFFGIITSSKYLLMPKIVISFVMAIGMILTPIYSLSMLRRMFYGYKLFNVPDFYFLD
SGPRELFVSICIFLPVVGIGIYPDFVLSLSVDRVEAILSNYFHKMMTEYVLILSAYLFSIGIYG
LITSRNMVRALMCLELILNAVNMNLVTFSDLFDSRQLKGDIFSIFVIAIAAAEAAIGPAIVSSI
HRNRKSTRINQSNLLNKMEHTYQHAWIIPFAPLPFTMSIGLGLLLVPTATKNLRRMWTFPS
VSLLSIAMVFSVNLSIQQINGSSIYQHLWSWTINNDFSFEFGHLIDPLTSIMSILITTVGIMVLI
YSDNYMSHDQGYLRFFAYMSFSNTSMLGLVTSSNLIQIYIFWELVGMCSYLLIGFWFTRPT
AANACQKAFVTNRVGDFGLLLGILGFYWITGSFEFRDLFEIFTNNGVNSLFATLCAFLLFV
GAVAKSAQFPLHVWLPDAMEGPTPISALIHAATMVAAGIFLVARLLPLFTVIPYIMNLISLIG
VITLLLGATLALAQRDIKRSLAYSTMSQLGYIMLAPGIGSYRAALFHLITHAYSKALLFLGS
GSIIHSMEPIVGYSPDKSQNVVLMGGLTKYLPITKTTFLLGTLSLCGIPPLACFWSKDEILN
DSWLYSPIFAIIACSTAGLTAFYMVRVYLLTFDGHLHVHFQNYSGTRNSSFYSISIWGKEGT

KFVNG-NLPLSTLKKISNKE--TFVYPHESDNTMLLPLLGLVLFTLFVGSVGIPFEFDILSKW
LNPSINLLHPNSNVSVDWYEFGTNAIYSVSIACFGIFIASLFYGSIYSSFQNLDFINSFVIFLD
RIINVIYNWSYNRGYIDVFYTTCFTKGIRGLAELTYLLDRRLIDGIANGIGMASFFVGEGIK
Y-VGGGRISSYLFVYLSYVSILLLIYYMDLPGPIHDILLVFLGLGLILGGLGVVLLTNPIYSAF
SLGLVLVCISLFHIPSNSYFVAAAQLLIYVGAVNVLIVFAVMFMNGSEYSNDFHLWTVGDG
VTSLVCTSILFSLITTIWNTSWYGIIWTTRSNQIIEQDLTSNVQQIGIRLATDFYLPFELISIILL
AALIGAIAMARQMNVPATRKDLMIVNMGPHHPSMHGVLRLIVTLDGEDVIDCEPVLGYL
HRGMEKIAENRTIIQYLPYVTRWDYLATMFTEAITVNAPEQLGNIQVPKRASYIRVIMLELS
RIASHLLWLGPFMADIGAQTPFFYIFRERELLYDLFEAATGMRMMHNYFRIGGVAADLPH
GWIDKCLDFCDYFLTGVVEYQKLITRNPIFLERVEGVGIIGGEEAINWGLSGPMLRASGIQ
WDLRKVDHYECYDEFDWQVQWQKEGDSLARYLVRISEMTESIKILQQALEGIPGGPYEN
LEGRRFDRESDSEWNDFDYRFISKKPSPTFELSKQELYVRVEAPKGELGIFLIGDNSVFPWR
WKIRPPGFINLQILPHLVKRMKLADIMTILGSIDIIMGEVDRMFPMVTGFMNYGQQTVRAA
RYIGQSFMITLSHANRLPVTIQYPYEKSITSERFRGRIHFEFDKCIACEVCVRVCPIDLPVVH
WRLETDIRKKRLLNYSIDFGICIFCGNCVEYCPTNCLSMTEEYELSAYNRHELNYNQIALG
RLPMSVIEDYTIRTTRNSKIAMDKPLNARTVTNFMQGRLSAWLVKHGLVHRSLGFDYQGI
ETLQIKPEDWYSIAVISYVYGYNYLRFQCAYDVAPGGLLASVYYLTRIQYGVDQPEEVCIK
VFAPRRNPKIPSVFWIWKSADFQERESYDMLGISYENHPRLKRILMPESWIGWPLRKDYIA
PNFYEIQDAH---MNSTEFPLLDRTTQNSVISTT
LNDLSNWSRLSSLWPLLYGTSCCFIEFASLIGSRFDFDRYGLVPRSSPRQADLILTAGTVTM
KMAPSLVRLYEQMPEPKYVIAMGACTITGGMFSTDSYSTVRGVDKLIPVDVYLPGCPPKP
EAVIDAITKLRKKVSREIYQDRIGSQQENRCFTTNHKFHIERSTHTGNYGQGLLYQSP-TSEI
-SETFFKYKSSVSSHELMNMQNRNTFSWVKEEMTRFISVSIMIYVITRTSISNAYPIFAQQGY
ENPREATGRIVCANCHLANKPVDIEVPQAVLPDTVFEAVVRIPYDMQLKQVLANGKKGAL
NVGAVLILPEGFELAPPDRISPELKEKMGNLSFQSYRPNKRNILVVGPVPGQKYSEIVFPILS
PDPATKKDVHFLKYPIYVGGNRGRGQIYPDGSKSNNTVYNATAAGIVSRIVRKEKGGYEIS
IADASDGRQVVDIIPPGPELLVSEGESIKLDQPLTSNPNVGGFGQGDAEIVLQDPLRVQGLL
FFLASVILAQIFLVLKKKQFEKVQLSEMNFMSKVYDWFEERLEIQAIADDITSKYVPPHVNI
FHCLGGITLTCFLVQVATGFAMTFYYRPTVTEAFASVQYIMTEANFGWLIRSVHRWSASM
MVLMMILHVFRVYLTGGFKKPRELTWVTGVVLAVLTASFGVTGYSLPWDQIGYWAVKIV
TGVPEAIPVIGSPLVELLRGSASVGQSTLTRFYSLHTFVLPLLTAVFMLMHFPMIRKQGISGP
LMGVTKKPDLNDPVLRAKLAKGMGHNYYGEPAWPNDLLYISPVVILGTIACNVGLAVLE
PSMIGEPADPFATPLEILPEWYFFPVFQILRTVPNKLLGVLLMVLVPTGLLTVPFLENVNKFQ
NPFRRPVATTVFLIGTAVALWLGIGATLPIDKSLTLGLFMIEVFLFGIVLGLIPITLAGLFVTAY
LQYRRGDQLDLMPTITSYFGFLLAASTITPALLIGLNKIRLIMDIVSLAWAALMVVFTFSLSL
VVWGRSGLMIIRSPESEVKIMVDRDPIKTSFEEWARPGHFSRTIAKGPDTTTWIWNLHADA
HDFDSHTSDLEEISRKVFSAHFGQLSIIFLWLSGMYFHGARFSNYEAWLSDPTHIGPSAQV
VWPIVGQEILNGDVGGGFRGIQITSGFFQLWRASGITNELQLYCTAIGALVFAALMLFAGW
FHYHKAAPKLAWFQDVESMLNHHLAGLLGLGSLSWAGHQVHVSLPINQFLDAGVDPKEI
PLPHEFILNRDLLAQLYPSFAEGATPFFTLNWSKYADFLSFRGGLDPVTGGLWLTDIAHHHL
AIAILFLVAGHMYKTNWGIGHSLKDILEAHKGPFTGQGHKGLYEILTTSWHAQLSLNLAM
LGSSTIVVAHHMYSMPPYPYLAIDYGTQLSLFTHHMWIGGFLIVGAAAHAAIFMVRDYDP
TTRYNDLLDRVLRHRDAIISHLNWACIFLGFHSFGLYIHNDTMSALGRPQDMFSDTAIQLQ
PIFAQWVQNTHALAPSATAPGATASTSLTWGGGDLVAVGGKVALLPIPLGTADFLVHHIHA

FTIHVTVLILLKGVLFARSSRLIPDKANLGFRFPCDGPGRGGTCQVSAWDHVFLGLFWMY
NAISVVIFHFSWKMQSDVWGSISDQGVVTHITGGNFAQSSITINGWLRDFLWAQASQVIQS
YGSSLSAYGLFFLGAHFVWAFSLMFLFSGRGYWQELIESIVWAHNKLKVAPATQPRALSIV
QGRAVGVTHYLLGGIATTWAFFLARIIAVGMALRFPRFSQGLAQDPTTRRIWFGIATAHDF
ESHDDITEERLYQNIFASHFGQLAIIFLWTSGNLFHVAWQGNFESWVQDPLHVRPIAHAIW
DPHFGQPAVEAFTRGGALGPVNIAYSGVYQWWYTIGLRTNEDLYTGALFLLFLSAISLIAG
WLHLQPKWKPSVSWFKNAESRLNHHLSGLFGVSSLAWTGHLVHVAIPGARGEYVRWNN
FLDVLPHPQGLAPLFTGQWNLYAQNPDSSSHLFGTSQGAGTAILTLLGGFHPQTQSLWLTD
IAHHHLAIAFIFLVAGHMYRTNFGIGHSMKDLLEAHIPPGGRLGRGHKGLYDTINNSIHFQL
GLALASLGVITSLVAQHMYSLPAYAFIAQDFTTQAALYTHHQYIAGFIMTGAFAHGAIFFIR
DYNPEQNEDNVLARMLDHKEAIISHLSWASLFLGFHTLGLYVHNDVMLAFGTPEKQILIEP
IFAQWIQSAHGKTSYGFDVLLSSTNGPAFNAGRSIWLPGWLNAVNE--NSNSLFLTIGPGDF
LVHHAIALGLHTTTLILVKGALDARGSKLMPDKKDFGYSFPCDGPGRGGTCDISAWDAFY
LAVFWMLNTIGWVTFYWHWKHITLWQGNVSQFNESSTYLMGWLRDYLWLNSSQLINGY
NPFGTNSLSVWAWMFLFGHLVWATGFMFLISWRGYWQELIETLAWAHERTPLANLIRWR
DKPVALSIVQARLVGLAHFSVGYIFTYAAFLIASTSGKFGMSHSVKIYDTCIGCTQCVRACP
TDVLEMIPWDGCKAKQIASAPRTEDCVGCKRCESACPTDFLSVRVYLWHETTRSMGLAY
MTDLNLPSIFVPLVGLMFPAIAMASLSLHIQKNKIIMRDIKTYLSTAPVLATLWFGSLAGLLI
EINRLFPDALTFPFFSFMTAILERRESTSLWGRFCNWITSTENRLYIGWFGVLMIPTLLTATS
VFIIAFIAAPPVDIDGIREPVSGSLLYGNNIISGAIIPTSAAIGLHFYPIWEAASVDEWLYNGG
PYELIVLHFLLGVACYMGREWELSFRLGMRPWIAVAYSAPVAAATAVFLIYPIGQGSFSDG
MPLGISGTFNFMIVFQAEHNILMHPFHMLGVAGVFGGSLFSAMHGSLVTSSLIRETTENES
ANAGYRFGQEEETYNIVAAHGYFGRLIFQYASFNNSRSLHFFLAAWPVVGIWFTALGISTM
AFNLNGFNFNQSVVDSQGRVINTWADIINRANLGMEVMHERNAHNFPLDLAAVEVPSTN
GMGLPWYRVHTVVLNDPGRLLSVHIMHTALVSGWAGSMALYELAVFDPSDPVLDPMWR
QGMFVIPFMTRLGITNSWGGWSIT-GGTITNPGIWSYEGVAGAHIVFSGLCFLAAIWHWVY
WDLEIFCDERTGKPSLDLPKIFGIHLFLSGVACFGFGAFHVTGLYGPGIWVSDPYGLTGKV
QPVNPAWGAEGFDPFVPGGIASHHIAAGTLGILAGLFHLSVRPPQRLYKGLRMGNIETVLS
SSIAAVFFAAFVVAGTMWYGSATTPIELFGPTRYQWDQGYFQQEIYRRVGAGLAENLSLSE
AWSKIPEKLAFYDYIGNNPAKGGLFRAGSMDNGDGIAVGWLGHPVFRDKEGHELFVRRM
PTFFETFPVVLVDGDGIVRADVPFRRAESKYSVEQVGVTVEFYGGELNGVSYSDPATVKK
YARRAQLGEIFELDRATLKSDGVFRSSPRGWFTFGHATFALLFFFGHIWHGARTLFRDVFA
GIDPDLDAQVEFGAFQKLGDPTTRRQVVMKTLYSLRRFYPVETLFNGTLALAGRDQETTG
FAWWAGNARLINLSGKLLGAHVAHAGLIVFWAGAMNLFEVAHFVPEKPMYEQGLILLPH
LATLGWGVGPGGEVIDTFPYFVSGVLHLISSAVLGFGGIYHALLGPETLEESFPFFGYVWK
DRNKMTTILGIHLILLGIGAFLLVLKALYFGGVYDTWAPGGGDVRKITNLTLSPGVIFGYLL
KSPFGGEGWIVSVDDLEDIIGGHVWLGSICILGGIWHILTKPFAWARRAFVWSGEAYLSYS
LGALSVFGFIACCFVWFNNTAYPSEFYGPTGPEASQAQAFTFLVRDQRLGANVGSAQGPT
GLGKYLMRSPTGEVIFGGETMRFWDLRAPWLEPLRGPNGLDLSRLKKDIQPWQERRSAE
YMTHAPLGSLNSVGGVATEINAVNYVSPRSWLATSHFVLGFFLFVGHLWHAGRARAAAA
GFEKGIDRDFEPVLSMTPLNMTIALGRFTKEENDLFDIMDDWLRRDRFVFVGWSGLLLFP
CAYFALGGWFTGTTFVTSWYTHGLASSYLEGCNFLTAAVSTPANSLAHSLLLLWGPEAQG
DFTRWCQLGGLWTFVALHGAFGLIGFMLRQFELARSVQLRPYNAIAFSAPIAVFVSVFLIY
PLGQSGWFFAPSFGVAAIFRFILFFQGFHNWTLNPFHMMGVAGVLGAALLCAIHGATVEN

TLFEDGDGANTFRAFNPTQAEETYSMVTANRFWSQIFGVAFSNKRWLHFFMLFVPVTGL
WMSAIGVVGLALNLRAYDFVSQEIRAAEDPEFETFYTKNILLNEGIRAWMAAQDQPHENL
IFPEEVLPRGNALMSGSTGERSFADIITSIRYWVIHSITIPSLFIAGWLFVSTGLAYDVFGSPR
PNEYFTESRQGIPLITGRFDPLAQLDEFSRSFMTIDRTYPIFTVRWLAVHGLAVPTVSFLGSIS
AMQFIQRMATQTVEGSSRSG-PRRTLTGDLLKPLNSEYGKVAPGWGTTPFMGVAMALFAIF
LSIILEIYNSSVLLDGISTSMLTLKLFVYTVVIFFVSLFIFGFLSNDPGRNPGREEMADTTGRI
PLWLIGTVTGIPVIGSIGIFFYGSYSGLGSSLMLNIFSLIC--LNSALHSSSFFFAKLPEAYAFFN
PIVDVMPVIPVLFFLLALVWQAAVSFRTTQSNPNEQSVELNRTSLYWGLLLIFVLAVLFSNY
FFNMEVNILAFIATALFILVPTAFLLIIYVKTVSQSDMETATLVAISISGSLVSFTGYALYTAFG
QPSQQLRDPFEEHGDMEALVYTFLLVSTLGIIFFAIFFREPPKVPTKKMKMIIAFQLAVFALI
ATSSILLISVPVVFASPDGWSNNKNVVFSGTSLWIGLVFLVAILNSLIS-------MSPKTETKAG
VGFKAGVKDYKLTYYTPDYETKDTDILAAFRVTPQPGVPAEEAGAAVAAESSTGTWTTV
WTDGLTSLDRYKGRCYHIEPVAGEESQFIAYVAYPLDLFEEGSVTNMFTSIVGNVFGFKAL
RALRLEDLRIPPAYTKTFLGPPHGIQVERDKLNKYGRPLLGCTIKPKLGLSAKNYGRAVYE
CLRGGLDFTKDDENVNSQPFMRWRDRFLFCAEAIFKSQAETGEIKGHYLNATAGTCEEMI
KRAVFARELGVPIVMHDYLTGGFTANTSLAHYCRDNGLLLHIHRAMHAVIDRQKNHGMH
FRVLAKALRMSGGDHIHAGTVVGKLEGEREITLGFVDLLRDDFIEKDRSRGIYFTQDWVS
MPGVLPVASGGIHVWHMPALTEIFGDDSVLQFGGGTLGHPWGNAPGAVANRVALEACVQ
ARNEGRNLAREGNEIIREASKWSPELAAACEVWKEIKFEFEAMDTL--MAIHLYKTSTPST
RNGAVDGQVKSNPRNNLIYGQHHCSKGRNARGIITARHRGGGHKRLYRKIDFRRNEKDIS
GRIVTIEYDPNRNAYICLIHYGDGEKRYILHPRGAIIGDTIVSGTEVPISMGNALPLTDMPLG
TAIHNIEITLGKGGQLARAAGAVAKLIAKEGKSATLRLPSGEVRLISKNCSATVGQVGNVG
VNQKSLGRAGSKRWLGKRPVVRGVVMNPVDHPHGGGEGRAPIGRKKPTTPWGYPALGR
RSRKRNKYSDSFILRRRK-MIQPQTHLNVADNSGARELMCIRIIGAS-NRRYAHIGDVIVAVI
KEAVPNMPLERSEVIRAVIVRTCKELKRDNGMIIRYDDNAAVVIDQEGNPKGTRVFGAIAR
ELRKLNFTKIVSLAPEVLMLSPKRTRFRKQHRGRMKGISYRGNHICFGRYALQALEPSWIT
SRQIEAGRRAMTRYARRGGKIWVRIFPDKPVTVRPTETRMGSGKGSPEYWVSVVKPGRIL
YEMGGVSETVARAAISIAACKMPIRTQFIIARMTRIRRGYIARRRRTKIRLFASTFRGAHSRL
TRTITQQKMRALVSTQRDRGRRKRDFRRLWITRINAVTREN--VSYSYSRLIHDLYKKRLLL
NRKILAQIAISNRNCLDTISNEILK-----------KMKQVQALAQHISMSAHKARRVVDQIRGRS
YAETLMLLELMPYRASYPIFKLVYSAAANASHNKSFNEADSFISKAEVNGGTIVKKFKPRA
KGRSYPIKRPTCHITIVLKDRSKK-------MDGIKYAVFTEKSIRLLGNNQ-YTSNVESGSTRTE
IKHWVELFFGVKVIAMNSHRLPGKGRRMG----HTMHYRRMIITLQPGYSIPPLIEKRTMAV
PKKRTSVSKKHIRRNIWKRKGYRAASKALSLAKSISTGHSKSFFVRQTSNMAKGKDVRVII
ILECTGCARN--G--KESRGISRYITQKNRHNTPSRLELKKFCSYCYKHTIHAEIKKMKIRASV
RKICEKCRLIRRRGRIIVICSNPRHKQRQGMVREEVAVSTRTLQWKCVESRTDSNRLYYGR
FVLSPLMKGQADTIGIAMRKALLGEIEGTCITRAKSE--KVPHEYSTIVGIEESVHEILMNLK
EIVLKSNLYGTRDASICVRGPKYVTAQDIISPPSVEIVDTTQHIASLTEPINLCIELQIERDRG
YRMKAPNNYQDGSY--PIDAVSMPVRNANHSIHSYGSGNEKQEILFLEIWTNGSLTPKEAL
HEASRNLIDLFIPFLHAEEQDIHLNLEDNQ-NRFTVSPFTLHDRLGN-IRKNKK-EIALKCIFI
DQSELPTRTYNCLKRSNIHTLLDLLNNSQEDLMRIEHFRIEDIKQILDILQKH--FGIDLPKNK
TFRDG-NEGMPTIPGFSQIQFEGFCRFIDQGLTEELHKFPKIEDTDQEIEFQLFVETYQLVEPL
IKERDAVYESLTYSSELYVSAGLIWKTGRDMQEQTIFIGNIPLMNSLGNSIVSGIYRIVINQIL
QSPGIYYRSELDHNGISVYTGTIISDWGGRPELEIDRKARIWARVSRKQKISILVPSSAMGSN

LREILDNVCYPEIFLSFPNDKEKKKIGSRENAILEFYKQFACVGGDPVFSESLCKELQKKFF
QQRCELGRIGRRNMNRRLNIDIPQNNTFLLPRDVLAVVDHLIGMKFGMGTLDD--MNHLK
NKRIRSVADLLQDQFGLALVRLENAVRGTICGAIR-HKLI-PTPHNLVTSTP----LTTTYESFFG
LHPLSQVLDRTNPLTQIVHGRKSSYLGPGGLTGRTASFRIRDIHPSHYGRICPIDTSEGINVG
LIGSLAIHARIGHWG-SIESPFY---EISEKSK----EIVYLPPSRDEYYMVAAGNSLALNRGIQEE
QVVPARYRQEFLTIAWEQIHLRSIFPFQYFSIGASLIPFIEHNDANRALMSSNMQRQAVPLSR
SEKCIVGTGLEGQAALDSGVSAIAEHEGKIIYTDTDKIVLSGNRDTISIPLVMYQRSNKNTC
MHQKPRVPRGKCIKKGQILADGAATVGGELALGKNVLVAHMPWEGYNSEDAVLISERLIY
GDIYTSFHIRKYEIQTHVTSQG-PERITNEIPHLEAHLLRNLDKNGIVMLGSWIERGDILVGK
LTPQAAKE-SSYAPEDRLLRAILGIQVSTAKETCLKLPIGGRGRVIDVRWIQKKG-GSSYNPE
TIRVSILQKREIKVGDKVAGRHGNKGIVSKILPRQDMPYLQDGTPVDMVFNPLGVPSRMN
VGQMFECSLGLAGYLLDKHYRIAPFDERYEQEASRKLVFPELYSASKQTVNPWVFEPEYP
GKSRIFDGRTGDPFEQPVIIGKSYILKLIHQVDDKIHGRSSGHYALVTQQPLRGRAKQGGQR
VGEMEVWALEGFGVAHISQEMLTYKSDHIRARQEVLGATIIGGTIPKPEDAPESFRLLVREL
RSLALELNHFLVSEKNFQINRKEA-------MIDRYKHQQLRIGSVSPQQIRAWANKTLPNGEII
GEVTKPYTFHYKTNKPEKDGLFCERIFGPIKSGICACGNYRVIGDEKEDPKFCEQCGVEFV
DSRIRRYQMGYIKLACPVTHVWYLKRLPSYIANLLDKPLKEFEGLVYCD--FSFARP-IAKK
PTFLRLRGSFESEIQSRKYSIPLFFTTQGFDTFRNREISTGASAIREQLADPDLRIITDHSLVE
WKELGEEGST-GNEWEDRKIGRRKDFLVRRIELAKHFIRTNVEPERMVLCLLPVLPPELRPI
IQIDGGKLMSSDINELYRRVIYRNNTLTDPLTTSTSTPGESVMCQEKLVQEAVDTLLDNGIR
GQPMRDGHNKVYKSFSDVIEGKEGRFRETLLGKRVDYSGRSVIVVGPSLSLHRCGLPREI
AIELFQTFVIRGLIRQHVASNIGIAKSKIREKEPIVWEILQEVMQGHPVLLNRAPTLHRLGIQ
AFQPILVEGRAICLHPLVRKGFNADFDGDQMAVHVPLSLEAQAEARLLMFSHMNLLSPTI
GDPISVPTQDMLIGLYVLTIGNRRGICANRYNPWNR--GN-SQNERVNDT-YMYTK-KEPYF
CSSSDALGAYRQKRINLDSPLWLRWRLDQR-VIAS--REVPIEVQYESLGTYHEIYGHYLIVR
SIKKEILCIYIRTTVGHISFYREIEEAIQGFCRAYS---------------MAERADLVFHNKVIDGAAM
KRLISRLIDHFGMAYTSHILDQVKTLGFQQATTTSISLGIDDLLTIPSKGWLVQDAEQQSLIL
EKHHHYGNVHAVEKLRQSIEIWYATSEYLRQEMHPNFRMTDPSNPVHIMSFSGARGNASQ
VHQLVGMRGLMSDPQGQMIDLPIQSNLREGLSLTEYIISCYGARKGVVDTAVRTSDAGYL
TRRLVEVVQHIVVRRTDCGTIRGISVSPRN--GMTERIWIQTLIGRVLAYDIYMGPRCIAARN
QDIGIGLVNRFIT------FRAQPIYIRTPFLCRSTSWICRLCYGRSPAHGDLVELGEAVGIIAGQS
IGEPGTQLTLRTFHTGGVFTGGTAEHVRAPFNGKIQFNEDLVHPTRTRHGHPAFLCYTDLY
VTIESHDILHNVNIPQKSFLLVQNDQYVESEQVIAEIRAGTSTF-KVKEKVRKHIYSDSEGE-
MHWSTDVYHAPEYTYGNVHLLPKTSHLWILSGGL--CRSSIVPFSLHKDQDQMNVH--SLS
VEQRYISDLS--VTNVR-VRHKLFSSDSSGK-GGRAPDYS-GPDRIISNGHWNFIYPA-ILHENS
D--LLAKRRRNRFIIPFQSDQEREKELMPRSGISIEIPIN--GILRRNSILAYFDDPRYRRSSSGIT
KYGTIGVDSIVKKED--LIEYRR-AKEFRPKYMKVDRFFFIPEEVHILPASSPVMVRNNSIIGV
NTRIALNTRSRVGGLVRVERKKKRIELKIFSGDIHFPGETDKISRHSGILIPPGTGKK--NVKE
SK--KWKNWIYVQR--ITPTKKK--YFVLVRPVVTYEIADGINLETLFPQDPLQERDNVQLRV
VNYILYGNGKPIRGISHTSLQLVRTCLVLNWDQDRNG----SIEEVHASFVEVRANGLIRDFL
KIDLVKSPILYSGKRNDTTSSGFIPN-NGSDRTNINPFYFKAR---------IQSLTQHQGTIRTLLN
R---NKECQSFLILSSSDCSRIGS--FNGSKSHKVT---KESDPTIPIRNSLGPLGT--VPKIANFDSS
--YYLIT--HNQILLN-KYLSLDN---LKQTFQVLKYY---LMDENGR--IYNPDPRSNIIFNPFDLN
WCFLPHDYCEETSTIIN-PGQFICENVCISKYGP---HIKSGQILIVR-VDSLVIRSAKPHLATPG

ATVHGHCGEILYEGDTLVTFIYEKSRSGDITQGLPKVEQVLEARSIDSISMNLEKRVEGWN
ERITKILGIPWGFLIGAELTIAQSRISLVNKIQKVYRSQGVQIHNRHIEIIVRQITSRVLVSE---
DGMSNVFSPGELIGLLRAERTGRALEEAICYRAILLGITRASLNTQSFISEASFQETARVLAK
AALRGRIDWLKGLKENVVLGGMMPVGTGFKGLVRRSR--QHNNIP--LEIKKKNLFEGEMR
--DILFHHRELFDSCIPNNFPDT----------------MTRRYWHINLEEMMEAGVHFGHGTRKWNP
RMAPYISAKRKGIHITNLTRTARFLSEACDLVFDAASIGKHFLIVGTKKKAADSVASAAIRA
RCHYVNKKWLGGMSTNWSTTETRLHKFRDLRAEQKAGKLNRLPKRDAAMLKRQLSHL
QTYLGGIKYMTGLPDIVIIIDQQEEYTALRECLTLGIPTICLIDTNCDPDLADISIPANDDAIA
SIRLILNKLVSAICEGRSS-YIRNCMGQKINPLGFRLG-EQSHHSLWFAQPKSYCRGLQEDEK
IRDCIKIYVQKNMRISSG-FEGIAHIEIQKKMDLIQVIISIGFPNFLIEG--GTR--GIEELQMNVQ
KRFHSANRRLNIAITRVAKPYGQPNILAEYIARQLKNRVSFRKAMKKVIELTEQADTKGIQ
VQIAGRIDGKEIARVEWIREGRVPLQTIRAKIDHCSYTVRTIYGVLGIKIWIFVDEQMSRYR
GPRFKKIRRLGALPGLTSKRPRSGSDLRNQSRSGK-KSQYRIRLEEKQKLRFHYGLTERQLL
RYVRIAGKAKGSTGQVLLQLLEMRLDNILFRLGMASTIPGARQLVNHRHILVNGRIVDIPS
YRCKPRDIITTRDEQRSRALIQNYLDSPSH-EELPKHLTFHPSQY--KGLVNQIIDSKWVGLKI
NELLVVEYYSRQTMSRRGTAEEKTAKSDPIYRNRLVNMLVNRILKHGKKSLAYQIVYQAV
KKIQQKTETNPLSVLRQAICRVTPDIAVKARRVGGSTHQVPIEIGSTQGKALAIRWLLGASR
KRPGRNMAFKLSSELVDAAKGSGDAIRKKEETHRMAEANRAFAHFRMGRDTIADILTSIR
NANMDKKGTVRIASTNITENIVKILLREGFIENVRKHQENNKYFLVSTLQHRR--NRKGRY
R---TIL--KRISRPGLRIYSNYQRIPRILGGMGIVILSTSQGIMTDREARLEGIGGEILCYIWMT
KSIPRIGSRRGGRIASRKNARRIPKGVIHVQASFNNTIVTVTDVTGRVVSWSSAGTCGFRGT
RRGTPFAAQTAAANAIRTVIDQGMQRAEVMIKGPGLGRDAALRAIRRSGILLSFVRDVTP
MPHNGCRPPKKRRVMPTIKQLIRNTRQPIRNVTKSPALRGCPQRRGTCTRVYTITPKKPNS
ALRKVARVRLTSGFEITAYIPGIGHNSQEHSVVLVRGGRVKDLPGVRYHIVRGTLDAVGVK
DRQQGRSKYGVKKPKMARKGLIQRERKRQKLEQKYHLIRRSPKKEIG---KVSSLSDKWKI
HGKLQSPPRNSAPTRLHRRCFLTGRPRANYRDFGLSGHILREKVHACLLPGATRSSWMVK
NSFISVIPQEEKNK----GSVEFQVFSFTNKIRKLTSHLELHRKDYSSQIGLRRILGKRQRLLAY
LSKKNRVRYKELIDQLGIREPKTR--MVKLRLKRCGRKQRAIYRIVAIDVRSRREGRDLRKV
GFYDPINNQTYLNVPLILYFLEKGAQPTGTVYDILKKAEVFKELQINQKKSN-MDKSKRPF
RKSKRSFRRRLPPIGSGDRIDYRNMSLISQFISEQGKILSRRVNRLTLKQQRLITIAIKQARIL
SSLPFLNNEK--QFERTESIPRTTGPRTRK-VTRSLKKNPFVANHLLAKIEKLNMREEKETIVT
WSRASTIIPTMIGHTIAIHNGKEHLPIFITDRMVGHKLGEFAPTLTFRGHARTDTRSRRMILK
SFLLGNLLSLCMKIINSVVVVGLYYGFLTTFSIGPSYLFLLRARVMEEG---TEKEVSATTGFI
TGQLMIFISIYYAPLHLAMGRPYTITVLGLPYLLFHFFWKNHKHFFDYGSTNLNSMRNFSI
QSIFLNNFIFQLLNHFVLPSSTLIRLVNISMFRCNNKTLFVISSFVGWLIGHILFMKWVGLILF
WIQRNRSIRS-IRSNKYLVSELRNFLSRMVNIFLFITCVYYLGRIPSPILTKKLKEAEEDKPIV
TLLFDYKRWDRPLRYIKNDRFANAVRNEMSQYFFHKCPVDGKQKISFTYPPSLSIFFEMIE
KHLSLCTTEKLSPEDLYNHWVYTNEKKRCNLNNEFINRIEALN--KLSMHVLEKRTRLYND
KNEQECLPRMYDPFLNGPYRGTIKKVYSHLRVVWINKIDDILYQEFEEKDTPNYSIGIEEIG
KKVPRWSYKLTDLQQQEREAIRSRKSKRVVIYTDALIRYSQQSDFRRDLIKGSRRAQRRKT
VTWEIFQANVHSPLFLDRIDKTFFFSFDI--SGTINLAFRNKEENERIAIAETWDTIIFAQAIRG
IMLVTQSILRKYIVLPSLIIAKNLVRMLLFQFPEWYEDLKEWNREMHVKCTYNGVQLSETE
FPKDWLTDGIQIKILFPFCLKPWHRSKKGKKDNFCFLTVWGMETELPFGSSRKRPSFFEPIL
KELEKKIRKAK-KKYFLVLSVL---KEWFRKVSKEKTRWIRKIVL--FIKKIIKEFAKVN-TIISN

KILSEPTIRIRSMDWTNYSLTEKKMKDLADRTTTIRNQIERITKEKRTSPNETSCDDKRSES
QKHIWQISKRRSAWFIRKWRYFIKIFSERIYIDIFLCAINIPRVNAQLF-KLISWDLSSLSQAYL
FYKLSQTQVINKYRLKSVLQY-DLFLRDRIRDYLTRGIFDSK--FKKLPNSGMGEWKNWLR
GHYQYNLSQTRWSRLVSKKWRDRVNQHRTIQNKK-RDSYEK--DQFIHYEKQEKFQKHYR
YDLFSYKYINKYIIDRDKKYLEWKILNFCLRFFGRMGMNQGMLYRWFFSEFVPLYDAYKI
KPWIIPIKLLLFNSKSNKGAELQFFLKRYLLFQLRWDDPLNQRMMNNVKVYCLLLRLINP
REIAISSIQRGEMRLDVMLIQKDLTLTELIKRGILIIEPVRLSIKWDGQFIMYQTIGISLVHKN
KHNDYDLLVPENILSPRRRRELRILISFRFLWPNYRLEDLACINRYWFDTNNGSRFSMLRIH
MYPMKRHQFKSWIFEL----REIKNSHYFLDSWTKFDSVGSFTHIFFHQERFMKLFDPRIWSI
LLS-RDS--QGSTSNRYF---TIKGVVLLVVV-VLI----YRINNRNMVERKNLYLMGLL-----PIPM
NSIGPRNDTLEESF-WSSNLNRLIVS-LLYLPKGKKISESCFMDPKESTWVLPIT----KKCIMP
ESNWGSRWWRNRIGKKR--DSSCKIS---NETVAGIEISFKEKDIK-YLEFLFVSYTD--DPIRKD
HDWEFFDRLSPRKKRNIINL--NSGQLFEILVKHLICYLMSAFREKRPIEVEG-FFKQQG-AEA
TIQSNDIEHVSH--LFSRNKRGISLQNCAQFHMWQFRQDLFV---SWGK-NRHELDFLRN-VS
RENLIWLDNVWLVNKDR---FFS--KVRNVSSNIQYDS---TR--SIFVQVTDSSQLKGSS-DQSR
DPFDSIS----NEDSE-------------YHTLINQTEIQQLKERSIL-WG--PSFLQTERTEIESD--RLLK--
CLSGYS--SMSRLFT-EREKQMNNHLLPEEIEEFL--GNPTRSIRSFFSDRW--SELH-LGSNPTE
RSTRDH----KLLKKQQDVS-FVPSRRSE----NQEIVDIFKIITYLQN--TVSIHPISSDPGCDMVP
-KDEPD-MDSSNKISFLNKNPFLD--LFHLFHDRNKGGYRL--HHDFESE--ERFQEMADLFTL
SITEP-GLVDHKGFAFSIDSYGLDQKK--FLNEVFNSRDESKKKSL---LVLPPIFY--EENESFYR
RIRKKSVR-ISCGNDLEDPKPKIVVFANNNIMEAVNQYRLIRNLMQIQYSTYG--YIRNVSNR
FFLM---NRSDRNFEYGIQ---RDQIGNDTLNHLTIIKYTINQHLSNLKK-SQKKWFD----PLISR
TERSMNRDPDAYRYKWSNGSKNFQEHLEHFVSEQKH---RFQVVFDRLRINQYSIDWSEVI
DKQDLSKSLRFFLSKS---------LLFLS-------KSL-LFLSKSLPFFFVSIGNIPIHRSE-IHIY-ELKG
PNDQLCNQLLESIGVQIVHLNKLKP--FLLD---DHDTS-QRSKFLINGGTISP---NKIP-KW-MI
DSFHT--RNNRRKSFENTDSYFSMISHDRDNWLNPVKPFHRSSLISSFYKANRLRFLNHPHH
FWFY--CNKGFPFYV----EKTRINNYD--LTY-GQFLNILFIRNK-IFSLCVGK--KKHVFLE-RDT
ISPIESQVSDIFIPNDFPQSGDET-YNLYKS---FHFPIRSDPFVR--RAIYSIADISGTPLTEEQIVN
FERTYCQPLSDMNLSDSEGKNLHQYLS--FNSNMGLIHTPCSEKYLP--SGKRKK--------RSL
CLK--KCVEKRQMDRTFQRDSAFSNLSKWNLFQTYMPWFLTSTGCKYLNLTLLDTFSDPL-
-PILSSS-QKFVSIFHDIMHGSDISWPIPQKKLW----AILPQWNL-ISESSSKCLQN--LLLSEEMI
HRNNESPVPLIWTHLRSPNAWEF-LYSILFLFLVAGY-LVRTHLLFVSR--ASSELQTGLERIKS
LMIPSYMIELRKLLDRYPTSEPNSFWLKNLFLVALEQLGDSLEKIRGSASGGNMLLGGGP--
---AYG-VKSIRSKKKYLNIN---LIDLI-SIIPNPINRITFSRNTRHLSRTSKEIYSLIRKRKNVNG
DWIDDKIESWVANSDSIDDEEREFLVQFS--TLTT--EKGIDQILLSLT-HSDHLS-----KNDSGY
QMIEQPGSIYLRYLVDIH--KKYLMNYEFNRSCLAERRIFLAHYQTITY--SQTSCGANSSHFP
SHG---KPFSLRLALSPS-RGILVIGSIGTGRSYLVKYLATNSYVPFITVFPNKFLDDKPKGYRI
D-----DID-IDDSDDID----IDDSDDI-----------------DDDLDTELLTMTNVL---TMYMT---PKIDR
FDITLQFELAKAMSPCIIWIPNIHDLYVNESNYLSLGLLVNYLS---RDCERCS-TRNILVIAST
HIPQKVDPALIAPNKSNTCIKIRRLLIPQQRKHFFILSYTRGFHLEKKMFHT--NGFGSITMGS
NARDLVALTNEALSISITQKKSIIDTNTIRSALHRQTWDLR---SQVRSVQDHGILFYQIGRAV
AQNVLLSNCPIDPISIYMKKK--SCKEGDSYLYKWYFELGTSMKKLTILLYLLSCSAGSVAQ
DLWSPP--GPDEK-NWITSYGFVENDS-DLVHG-LEVEGALLG--SSRTEKDCSQFDND--RVT
LFLRSEPRN-PLDMMQNGSCSIVDQRFLYEK--YESEFEE-GEREGALDPQ----QIEED----LFN

H--IVWAP--RIWRP---CGNLFDCIERTNELGFP-YWARSFR---GKRIIYHKEDELQENDSEFLQ
SGTMQYQTRDRSSKEQGFFRISQFIWDPADPFFFLFK-DQPFVSVFSRREFFADEEMSKGLI
TSQTNPPTSIYKRWFI--KNTQEKHFELLIHRQRWLRT---NSSLS--NGSFRSN--TPSESYQYLS
NLFLSN-GTLLDQIAKALLRKRWLFPDEMKHLI--HVTGERFPIPMPRSR-INGNFIDKTSSIV
ANILLRIIPTTSGEKEAFTYYRDG--MSAQSEGNYAEALQNYYEATRLEIDPYDRSYILYNIG
LIHTSNGEHTKALEYYFRAIERNPFLPQAFNNMAVICHY-RGEQAIRQGDSEIAEAWSDQA
AEYWKQAIALTPGNYIEAHNWLKIARRFE--MNWRSERIWIELITGSRKTSNFCWACILFLG
SLGFLLVGTSSYLGRNLISLFPSQQIIFFPQGIVMSFYGIAGLFISSYLWCTISWNVGSGYDRF
DRKEGIVCIFRWGFPGINRRIFLRFLMRDIQSIRMEVKGGLYSRRVLYMEIRGQGAIPLTRTD
EN-FTPREIEQKAAELAYFLRVPIELK
>Amborella
MEKCGFNSMLSLSKSMDSLGLIGNTGAGGGPLIYDDTFKLK-YFDIDEIDDDSDSLSDIEN
QNQIFFDIYNDSDSLELDSENKNFEIHNE--SDFMSKLESSFFSYLTYSNLNSGSKSYNPYYD
PYMHDTRYSWNNNINSCIDSYIRCEID--S---ISSGSDNCSDSYIYSYICSDNGSSIRTRTSGSS
YHIRGRS-NFEKNKKLKKLWVQCENCYALNYNKLFRSKMNVCEQCGYHLKMSSSDRIEL
SIDPGTWHPMDEDMVSTDPIEFHSEEEPYRDRIDSYQRQAGLTDAVQTGIGQLEGIPIAIGV
MDFQFMGGSMGSVVGEKITRLIEYAIDRSLPVVIVCASGGARMQEGGLSLMQMAKISSAS
YNYQSNKKLFYVSILTSPTTGGVTASFGMLGDIIIAEPNAYVAFAGKRVIEETLNKKIPDGL
QVAEYSFHKGLFDSIVPRNPLKGVPSELFQLHGFFSSTFHPLKMVTIRADEISNIIRERIEQY
NREVKIVNTGTVLQVGDGIARIHGLDEVMAGELVEFEESTIGIAPNLESNNVGVVLMGDG
LMIQEGSSVKATGRIAQIPVSEAYLGRVINALAKPIDGRGEISSSESRLIESPAPGIISRRSVYE
PLQTGLIAIDSMIPIGRGQRELIIGDRQTGKTAVATDTILNQKGQNVICVYVAIGQKASSVAQ
VVTTFQEQGAMEYTIVVAETADSPATLQYLAPYTGAALAEYFMYRERHTLIIHDDPSKQA
QAYRQMSLLLRRPPGREAYPGDVFYLHSRLLERAAKSSSRLGEGSMTALPIVETQSGDVSA
YIPTNVISITDGQIFLSADLFNAGIRPAINVGISVSRVGSAAQIKAMKQVAGKSKLELAQFAE
LEAFAQFASDLDKATQNQLARGQRLRELLKQSQAAPLAVEEQVVTIYTGANGYLDPLEIG
QVKKFLVQLRTYLRTNKPQFQEIISSTRTFTEEAEAILKEAIQEQIELFLFQEQTMRINPTTSG
SGASTLAEKSVGRIAQIIGPVLDVAFPPGKMPNIYNSLVVKGRDTAGQQINVTCEVQQLLG
NNRVRTVAMSATDGLMRGMEVIDTGAPLSVPVGEATLGRIFNVLGEPVDNLGPVDTRTTS
PIHRSAPAFIQLDTKLSIFETGIKVVDLLAPYRRGGKIGLFGGAGVGKTVLIMELINNIAKA
HGGVSVFGGVGERTREGNDLYMEMKESGVINEQNIAESKVALVYGQMNEPPGARMRVGL
TALTMAEYFRDVNEQDVLLFIDNISRFVQAGSEVSALLGRMPSAVGYQPTLSTEMGSLQE
RITSTKEGSITSIQAVYVPADDLTDPAPATTFAHLDATTVLSRGLAAKGIYPAVDPLDSTSTM
LQPRIVGEEHYETAQRVKQTLQRYKELQDIIAILGPDELSEEDRLTVARARKIERFLSQPFFV
AEVFTGSPGKYVGLAETIRGFQLILSGELDGLPEQAFYLVGNIDEATAKAINLEVESKLKK
MTLNLCVLTPNRIIWDSEVKEIILSTNSGQIGVLPNHAPIATAVDIGILKIRLQEQWLTMALM
GGFARIGNNEITILVNDAEKGSDIDPQEAQQTLEIAEANLSRAEGKRQTIEANLALRRARTR
VEAINAISMKNVTDSFVSLGHWPSAGSFGFNTDIFATNPINLSVVLGVLIFFGKGVLSDLLD
NRKQRILSTIRNSEELRGGAIEQLEKARARLRKVEIEADEFRVNGYSEIEREKSNLINAAYE
NLERLENYKNESIHFEQQRAMNQVRQRVFQQALQGALETLNSYLNSELHLRTISANIGML
GTMKNITDMNPLISAASVIAAGLAVGLASIGPGVGQGTAAGQAVEGIARQPEAEGKIRGTL
LLSLAFMEALTIYGLVVALALLFANPFVMSVLPCSINTLKGIYDLSGVEVGQHFYWQIGGF
QVHAQVLITSWVVIAILLGSATIVVRNPQTIPTDGQNFFEYVLEFIRDLTKTQIGEEYGPWV
PFIGTMFLFIFVSNWSGALLPRKIIELPQGELAAPTNDINTTVALALPTSVAYFYAGISKKGL

GYFGKYIQPTPILLPINILEDFTKPLSLSFRLFGNILADELVVVVLVSLVPSLVPIPVMFLGLFT
SGIQALIFATLAAAYIGESMEGHHMIFATLEHILTHISFSIISIVITTHLMTFAR-EIAGLSDLSE
KGMIAVFLRITGLLVTRWIYSGHLPLSNLYESLIFLSWGFSLIHMISKIQNHKNFLSSITAPSA
ILTQGFVTSGLLTEMHQSAILVPALQSHWLIMHVSMMLLSYAALLCGSLLSIALLVITFRKK
LDIIF---EIQYLNEKRSILQNTS-FSFRNYHKYQLTQQLDHWSYRVIGIGFTLLTLGILSGAVW
ANEAWGSYWNWDPKETWAFITWTISAIYLHTRTNKNLQSANSAIVASIGFLIIWICYFGVN
LLGIGLHSYGSFALRVSKK-ALTSLPYLASIVFLPWGISLSFNKSLEPWVTNWWNTSQYETL
LNEIQENNVLERFIELEELFLLDEMIRDYPETHIQKLRIGIHKETIELVKMYNQDYIHIISHFS
TNIMSFAILSAYSILGNEELVTLNSWVQEFFYNLSDTIKAFSILLLTDSCIGFHSPHGWELMI
GSVYKDFGFSHNDQIISGLVSTFPVILDTILKYWIFHHLNRISPSLVVIYHSMNEMPVGVPK
VPFRSPGEEDATWVDIYNRLHRERLLFLGQEVEGEISNQIVGLMVYLSIEDDTRDLYLFINS
PGGWVIPGIAIYDAMQFVPPDVHTICMGLAASMGSFVLAGGEITKRLAFPHARVMIHQPA
SSFYEAPAGEFILEAGELLKLRETITSVYVQRTGNPLWVVSEDMERDVFMSATEAQAHGIV
DLIAVEMKEQKLIHEGLITESLPNGMFQVRLDNGDLVLGYVSGRIRRSFIRILPGDRVKIEV
SRYDSTRGRIIYRLRNKDSNDMEELRGYLEIDRPHQQRFLYPLLFQESIYALAHNHGLLGH
DK-SSSLNVKRLIIQMYQQNHFIISFNDSNQNRFLGRNSYFQMVSEGFAVIVEIPFSMRLVSS
LGSESH-FQSIHSIFPFLEDKLSYLNYVLDILIPHPIHLEILVQSLRQWIRDLPSLHLLRFFLHE
HQNWNSFITKCSSFFSRQRLFLFLYNFHVYQFESLFVFLRKQFFHLRSISFGSFLERTHFYG
KIEN-VVSMRNQNILWLFKDLFMHYVRYKGKSIMASRGTYLLMNKWKSHLVNFWQFRF
YFWSQPGRIHINELSNHSFYFPGYLSGLRLNPSMVRSEMLENSFMIDAVI--KRFDTVVPTIF
LIGSLAKVKLCNVSGHPISKSVWADSSDSDILDQFGRICRNLSHYHSGSYKKHSLCRIKYIL
RLSCARTLARKHKSTVRAILKRLGSEFLDEFLTEEQEILSLIFPKRIWYLDIIRIHSLANH-MII
DTT-EVQ-AINSFSRLE-SSKEVYGLIWLFIPIFTPVSGITIGVLVIVWLEREISAGIQQRIGPEY
AGPLGILQAIADGTKLLFKEDLLPSRGDIRLFSIGPSVVVISILLSHLVIPFGYRLVLADLSIG
VSLWIAISSIAPIGLLMSGYASNNKYSFSGGLRAAAQSISYEIPLTLCVLSISLLSNSSSTVDIV
EAQYKYGFWGWNLWRQPIGFLAFLISSLAECERLPFDLPEAEEELVAGYQTEYSGIKFGLF
YLASYLNLLVSSLFVTVLYLGGWNLSIPYIAIPELFRINRIGGVFGTTISIFFTLAKAYLFLFIP
ITTRWTLPRMRMDQLLNLGWKFLLPIALGNLLLTTSSQLFSLMIWHVQNENFILDSTRIFM
KAFHLLLFHGSFIFPECILIFGLILLLMIDSTSDQKDRPWFYFISSTSLVMSITALLFRWREEP
MISFSGNFQTNNFNEIFQFLILLCSTLCIPLSVEYIECTEMAITEFLLFVLTATLGGMFLCGAN
DLITIFVAPECFSLCSYLLSGYTKRDVRSNEATTKYLLMGGASSSILVHGFSWLYGSSGGEI
ELQEIVNGLINTQMYNSPGISIALLSITVGIGFKLSPAPFHQWTPDVYEGSPTPVVAFLSVTS
KVAASALATRIFDIPFYFSSNEWHLLLEILAILSMILGNLIAITQTSMKRMLAYSSIGQIGYVI
IGIIVGDSNDGYASMITYMLFYISMNLGTFARIVSFGPRTGTDNIRDYAGLYTKDPFLALSSA
LCLLSLGGIPPLAGFFGKLHLFWCGWQAGLYFLVSIGLLTSVVSIYYYLKIIKLLMTGRNKE
ITPHVRNYRRSPLRSNNSIELSMIVCVIASTIPGISMNPIIAIAQDTFFMFLLHEYDIFWAFLM
ISSVIPILAFLISGVLAPISQGPEKVSSYESGIEPMGDAWIQFRIRYYMFALVFVVFDVETVFL
YPWAMSFDVLGVSVFIEALIFVLIPIVGSVYAWRKGALEWSTNNFPWLTAILLLPISAGSSIL
FIPQRGNKAVRWYTICICLLELLLMTYVFYYNFQLDDPLIQLEEDCNWINLFDFHWRLGID
GLSIGPILLTGFITTLATSAAWPVTRNSRLFHFLMLAMYSGQIGSFSSRDLLLFFIMWELELI
PVYLLLSMWGGKKRLYSATKFILYTAGGSIFLLIGVLGMGLYSSNQPMLNFETSANQSYPV
GLEILFYFGFLIAYAAKPPIIPLHTWLPDTHGEAHYSTCMLLAGILLKMGAYGLVRINMELL
PHAHSLFSPWLVIVGTLQIIYAASTSLGQRNLKKRIAYSSVSHMGSTIIGIGSMTDTALNGAI
LQIISHGFIGAALFFLAGTSYDRIRFLYLDEMGGIAIPMPKIFTMFSIFSMASLASPGMSGFV

AEFLVFLGIITSPKYLFIPKILITVVMAIGMILTPIYLLSMSRQMFYGYKLFNVPNSYFVDSGP
REIFILISILLPVMGIGIYPDFVLSLSVDKVEAILANYFNGMMFEYALVLSSYLFSMGIYGLIT
SRNMVRALMCLELILNAVNMNLVTFSDLFDSRQLKGDIFSIFVIAIAAAEAAIGPAIVSSIHR
NRKSTRINQSNLLKKMERTYQYAWIIPFLTLAVPILIGLGLLLVPPATKSIRRIWAFPSVLLLSI
VMVFSTNLSIQQINGNSIYQYLWSWTINSDSSLEFGYLIDSLTSIMSILIATVGMMVLIYSDN
YMSHDKGYLRFFAYMSFFNTSMLGLVISPNLIQIYIFWELVGMCSYLSIGFWFTRPSAANA
CQKAFVTNRVGDFGLLLGILGLYWIAGSFEFRDLFDIFNDNVLNSSFAILCASLLFLGAVAK
SAQFPLHVWLPDAMEGPTPISALIHAATMVAAGIFLVARLLPLLTVIPYIMNLISLIGVITVLL
GATLALAQKDIKRSLAYSTMSQLGYIMLASGIGSYRAALFHSITHAYSKALLFLGSGSIIHS
MEPILGYSPDKSQNMVFMGGLSKYVPITKATFLLGTLSLCGIPPLACFWSKDEILNNSWLY
SPIFAIIASSTTGLTAFYMFRMYLLTFEGHLRVHFK---GDTNSSLYSISIWGKEGPEVFSR-NLI
LLSMNVSSKKE--TFPYPHESDNTMLFPLLLLAILTLFVGSVGIRFDFDVLSKWLIPSMDLFH
EYLNPSADWYEFAQNAIYSVSIAFFGILIANLLYGSVHSSFQNLDLINSFAIFSDQALNVIYN
WSYNRGYIDLYYATILTRGIRGLAESTHFFDQRIIDGVTNAVGITNFFVGEAIKY-MAGGRIS
SYLFFSLSSLSIALILVYMDLLGPIHDILLVSLGSGIILGSLGVVLLTNPIYSAFSSGLVLVRISL
FHILSNSYFVAAAQLLIYVGAINVLIIFAVMFMNGSEYYNNFHLWTVGDGISSVVCTSILFS
LIATILDTSWYGIIWTTRSNQIIEQDLTSNVQQIGIHLSTDFFLPFELISIILLVALVGAIAMAR
AMTVPATRNDLMIVNMGPHHPSMHGVLRLIVTLDGEDVIDCEPILGYLHRGMEKIAENRT
IIQYLPYVTRWDYLATMFTEAITVNAPEELGNIQVPRRASYIRVIMLELSRIASHLLWLGPF
MADIGAQTPFFYIFRERELLYDLFETATGMRMMHNFFRIGGVAADLPHGWIDKCLDFCDY
FLPEVAEYQKLITRNPIFLERVEGVGFIAEEEAINWGLSGPMLRASGIPWDLRKVDHYECY
DEFDWEVQWQKEGDSLARYLVRISEMIESVKIIQQALEGIPGGPYENLEARRFDRVRNIEW
NDFEYRFISKKPSPTFELSKQELYVRVEAPKGELGIFLIGDNSVFPWRWKIRPPGLINLQILP
QLVKRMKLADIMTILGSIDIIMGEVDRMFPMVTGFMNYGQQAVRAARYIGQSFMITLSHA
NRLPVTIQYPYEKSITSERFRGRIHFEFDKCIACEVCVRVCPIDLPVVDWRFETDIRKKRLL
NYSIDFGICIFCGNCVEYCPTNCLSMTEEYELSTYDRHELNYNQIALGRLPIPVVGDYTIQT
IMNSKIAMNKPLDSRTITNYMQGRLSAWLVKHELVHRSLGFDYQGIETLQIKSEDWYSIAV
ISYVVYGYNYLRSQCAYDVAPGGLLASVYHLTRIQYGVDQPEEVCIKVFAQRRNPRIPSVFW
IWKSSDFQERESYDMLGIYYDNHPRLKRILMPESWIGWPLRKDYIAPNFYEIQDAH---------
--MNSMEFPLLDRTTPNSVISTTPNDLSNWSRLSSLW
PLLYGTSCCFIEFASLIGSRFDFDRYGLVPRSSPRQADLILTAGTVTMKMAPSLVRLYEQMP
EPKYVIAMGACTITGGMFSTDSYSTVRGVDKLIPVDVYLPGCPPKPEAIIDAITKLRKKVS
REIYEDRIASQQEDRCFTTNHKFRVGRSIHTGNYDQE---LYQSTSEILSETLFKYKSSLSSHE
LVNMQNRNTFSWVKEQMTRSIFVSMMIYIITRASISNAYPIFAQQGYENPREATGRIVCAN
CHLANKPVDIEVPQAVLPDTVFEAVVRIPYDMQLKQVLANGKKGGLNVGAVLILPDGFEL
APPDRISPAMKEKIGNLSFQSYRPTKKNILVIGPVPGQKYNEILFPILSPDPATKKEVHFLKY
PIYVGGNRGRGQIYPDGSKSNNTVYNASASGIISRIIRKEKGGYEITIANALDGRQVVDIIPP
GPELLVSEGESIKLDQPLTSNPNVGGFGQGDAEIVLQDPLRIQGLFLFLASVILAQIFLVLKK
KQFEKVQLAEMNFMSKVYDWFEERLEIQAIADDITSKYVPPHVNIFHCLGGITLTCFLVQV
ATGFAMTFYYRPTVTEAFASVQYIMTEVNFGWLIRSVHRWSASMMVLMMILHVFRVYLT
GGFKKPRELTWVTGVVLAVLTASFGVTGYSLPRDQIGYWAVKIVTGVPEAIPIIGSPLVELL
RGSASVGQSTLTRFYSLHTFVLPLLTAVFMLMHFPMIRKQGISGPLMGVTKKPDLNDPVLR
AKLAKGMGHNYYGEPAWPNDLLYIFPVVILGTIACNVGLAVLEPSMIGEPADPFATPLEILP
EWYFFPVFQILRTVPNKLLGVLLMVSVPTGLLTVPFLENVNKFQNPFRRPVATTVFLFGTA

LSLWLGIGATLPIDKSLTLGLFMIEVFPFGIVLGLIPITLAGLFVTAYLQYRRGDQLD
L???????????????????????????????MDIVSLAWAALMVVFTFSLSLVVWGRSGLMIIRSPESEV
KIMVDRDPIKTSFEEWARPGHFSRTIAKGPNTTTWIWNLHADAHDFDSHTSDLEEISRKVF
SAHFGQLSIIFLWLSGMYFHGARFSNYEAWLSDPTHIAPSAQVVWPIVGQEILNGDVGGGF
RGIQITSGFFQIWRASGITSELQLYCTAIGALVFAALMLFAGWFHYHKAAPKLAWFQDVES
MLNHHLAGLLGLGSLSWAGHQVHVSLPINQFLDAGVDPKEIPLPHEFILNRDLLAQLYPSF
AEGSTPFFTLNWSKYAEFLTFRGGLDPVTGGLWLTDIAHHHLAIAIIFLIAGHMYRTNWGI
GHGLKEILEAHKGPFTGEGHKGLYEILTTSWHAQLALNLAMLGSLTIVVAHHMYSMPPYP
YLAIDYGTQLSLFTHHMWIGGFLIVGAAAHAAIFMVRDYDPTTRYNDLLDRVLRHRDAII
SHLNWACIFLGFHSFGLYIHNDTMSALGRPQDMFSDTAIQLQPIFAQWIQNTHALAPGATA
PGATTSTSLTWGGGDLVAVGGKVALLPIPLGTADFLVHHIHAFTIHVTVLILLKGVLFARSS
RLIPDKANLGFRFPCDGPGRGGTCQVSAWDHVFLGLFWMYNAISVVIFHFSWKMQSDV
WGSISNQGVVTHITGGNFAQSSITINGWLRDFLWAQASQVIQSYGSSLSAYGLFFLGAHFV
WAFSLMFLFSGRGYWQELIESIVWAHNKLKVAPATQPRALSIVQGRAVGVTHYLLGGIATT
WAFFLARIIAVGMALRFPRFSQGLAQDPTTRRIWFGIATAHDFESHDDITEEGLYQNIFASHF
GQLAIIFLWTSGNLFHVAWQGNFESWVQDPLHVRPIAHAIWDPHFGQPAVEAFTRGGALG
PVNIAYSGLYQWWYTIGLRTNEDLYTGALFLLFLSAISLIAGWLHLQPKWKPSVSWFKNA
ESRLNHHLSGLFGVSSLAWTGHLVHVAIPGSRGEYVRWNNFLDVLPSPQGLGPLFTGQWN
LYAQNPDSSGHLFGTSQGAGTAILTLLGGFHPQTQSLWLTDIAHHHLAIAFVFLVAGHMYR
TNFGIGHSIKDLLEAHTPPGGRLGRAHKGLYDTINNSIHFQLGLALASLGVITSLVAQHMYS
LPPYAFIAQDFTTQAALYTHHQYIAGFIMTGAFAHGAIFFIRDYNPEQNEDNVLARMLDHK
EAIKSHLSWASLFLGFHTLGLYVHNDVMLAFGTPEKQILIEPIFAQWIQSAHGKTSYGFDV
LLSSTNGPAFNAGRSLWLPGWLNAINE--NGNSLFLTIGPGDFLVHHAIALGLHTTTLILVKG
ALDARGSKFMPDKKDFGYSFPCDGPGRGGTCDISAWDAFYLAVFWMLNTIGWVTFYWH
WKHITLWQGNPSQFNESSTYLMGWLRDYLWLNSSQLINGYNPFGTNSLSVWAWMFLFG
HLVWATGFMFLISWRGYWQELIETLAWAHERTPLANLIRWRDKPVALSIVQARLVGLAHF
SVGYIFTYAAFLIASTSGKFGMSHSVKIYDTCIGCTQCVRACPTDVLEMIPWDGCKAKQIA
S A P R T E D C V G C K R C E S A C P T D F L S V R V Y L W H E T T R S M G L A
Y?????????????????????????????????????MRDIKTYLSVAPVLTTLWFGALAGLLIEINRFFPDA
LAFPFF-----LLERRESTSLWGRFCNWITSTENRLYIGWFGVLMIPTLLTATSVFIIAFIAAPPV
DIDGIREPVSGSLLYGNNILSGAIIPTSAAIGLHFYPIWEAASVDEWLYNGGPYELIVLHFLL
SVACYMGREWELSFRLGMRPWIAVAYSAPVAAATAVFLIYPIGQGSFSDGMPLGISGIFNL
MIVFQAEHNILMHPFHMLGVAGVFGGSLFSAMHGSLVTSSLIRETTENESANAGYRFGQE
EETYNIVAAHGYFGRLIFQYASFNNSRSLHFFLAAWPVVGIWFTALGISTMAFNLNGFNFN
QSVVDSQGRVINTWADIINRANLGMEVMHERNAHNFPLDLAAVEAPSTNGMGLPWYRV
HTVVLNDPGRLLSVHIMHTALVSGWAGSMALYELAVFDPSDPILDPMWRQGMFVIPFMTR
LGITNSWGGWSIT-GGTVTNPGIWSYEGVAGAHIVFSGLCFLASIWHWVYWDLEIFCDERT
GKPSLDLPKIFGIHLFLSGVACFGFGAFHVTGLYGPGIWVSDPYGLTGKVQSVNPAWGAEG
FDPFVPGGIASHHIAAGTLGILAGLFHLSVRPPQRLYKGLRMGNIETVLSSSIAAVFFAAFIV
AGTMWYGSATTPIELFGPTRYQWDQGYFQQEIYRRVGTGLAENLSLSEAWSKIPDKLAFY
DYIGNNPAKGGLFRAGSMDNGDGIAVGWLGHPIFRDKEGHELFVRRMPTFFETFPVVLVD
GDGIVRADVPFRRAESKYSVEQVGVTVEFYGGELNRVSYSDPATVKKYARRAQLGEIFEL
DRATLKSDGVFRSSPRGWFTFGHASFALLFFFGHIWHGARTLFRDVFAGIDPDLDAQVEFG
AFQKIGDPTTRRQIVMKTLYSLRRFYPVETLFNGTLALAGRDQETTGFAWWAGNARLINL

SGKLLGAHVAHAGLIVFWAGAMNLFEVAHFVPEKPMYEQGLILLPHLATLGWGVGPGGE
VIDTFPYFVSGVLHLISSAVLGFGGIYHALLGPETLEESFPFFGYVWKDRNKMTTILGIHLIL
LGVGAFLLVFKALYFGGVYDTWAPGGGDVRKITNLTLSPSVIFGYLLKSPFGGEGWIVSVD
DLEDIIGGHVWLGFICILGGIWHILTKPFAWARRAFVWSGEAYLSYSLGALSIFGFIACCFV
WFNNTAYPSEFYGPTGPEASQAQAFTFLVRDQRLGANVGSAQGPTGLGKYLMRSPTGEVI
FGGETMRFWDLRAPWLEPLRGPNGLDLSRLKKDIQPWQERRSAEYMTHAPLGSLNSVGG
VATEINAVNYVSPRSWLATSHFVLGFFLFVGHLWHAGRARAAAAGFEKGIDRDFEPVLSM
TPLNMTIALGRFSKEE-DLFDIMDDWLRRDRFVFVGWSGLLLFPCAYFALGGWFTGTTFV
TSWYTHGLASSYLEGCNFLTAAVSTPANSLAHSLLLLWGPEAQGDFTRWCQLGGLWTFVA
LHGAFGLIGFMLRQFELARSVQLRPYNAIAFSAPIAVFVSVFLIYPLGQSGWFFAPSFGVAA
IFRFILFFQGFHNWTLNPFHMMGVAGVLGAALLCAIHGATVENTLFEDGDGANTFRAFNP
TQAEETYSMVTANRFWSQIFGVAFSNKRWLHFFMLFVPVTGLWMSALGVVGLALNLRAY
DFVSQEIRAAEDPEFETFYTKNILLNEGIRAWMAAQDQPHENLIFPEEVLPRGNALMSGST
GERSFADIITSIRYWVIHSITIPSLFIAGWLFVSTGLAYDVFGSPRPNEYFTESRQGIPLITGRF
DSLEQLDEFSRSFMTIDRTYPIFTVRWLAVHGLAVPTVFFLGSISAMQFIQRMVTQSVEDSS
RSG-PRRTIVGDLLKPLNSEYGKVAPGWGTTPFMGVAMALFAIFLSIILEIYNSSVLLDGISV
SMLTLKLFVYTVVIFFVSLFIFGFLSNDPGRNPGRDEMADTTGRIPLWLIGTVTGIPVIGSM
GIFFYGSYSGLGSSLMPNIFSLICICLHSALHSSGFFFAKLPEAYAVFNPIVDVMPVIPLFFFLL
A F V W Q A A A S F R T T Q S N P N E Q N V E L N R T S L Y W G L L L I F V L A V S F S N Y F F
N??????????????????????????????????METATLVAISISGSLVSFTGYALYTAFGQPSQQLRDPF
EEHGDMEALVYTFLLVSTLGIIFFAIFFREPPKVPNKKIKMTIAFQLAVFALIATSSILLISVPV
VFASSDGWSSNKNIVFSGTSLWIGLVFLVAILNSLIS-------MSPKTETKASAGFKAGVKDYR
LTYYTPDYETLATDILAAFRVTPQPGVPPEEAGAAVAAESSTGTWTTVWTDGLTSLDRYK
GRCYHIEPVAGEENQYIAYVAYPLDLFEEGSVTNMFTSIVGNVFGFKALRALRLEDLRIPPA
YSKSFQGPPHGIQVERDKLNKYGRPLLGCTIKPKLGLSAKNYGRAVYECLRGGLDFTKDD
ENVNSQPFMRWRDRFLFCAEALYKAQAETGEIKGHYLNATAGTCEEMMKRAVFARELGV
PIVMHDYLTGGFTANTSLAHYCRDNGLLLHIHRAMHAVIDRQRNHGIHFRVLAKALRMS
GGDHIHAGTVVGKLEGERDVTLGFVDLLRDDFIEKDRSRGIYFTQDWVSMPGVLPVASG
GIHVWHMPALTEIFGDDSVLQFGGGTLGHPWGNAPGAVANRVALEACVQARNEGRDLAR
EGNEVIREASRWSPELAAACEVWKEIKFEFEAMDVL--TAIHLYKTSTPSTRNGAVDSQVKS
NPRKNLIYGQHRCGKGRNARGIITAGHRGGGHKRLYRKIDFRRNEKDISGRIVTIEYDPNR
NAYICLIHYGDGEKRYILHPRGAIIGDTIVSGTEVPISMGNALPLTDMPLGTAIHNIEITLGK
GGQLARAAGAVAKLIAKEGKSATLRLPSGEVRLISKNCSATVGQVGNVGVNQKILGRAGS
KCWLGKRPVVRGVVMNPVDHPHGGGEGRAPIGRKKPTTPWGYPALGRRSRKKKKYSDS
FILRRRK-MIQAQTYLNVADNSGARELMCIRIVGAS-NPRYAHIGDVIVAVIKEAVPNMPLER
SEVIRAVIVRTCKELKRDNGIIIRYDDNAAVVIDPEGNPKGTRVFGSIAGELRHLNFTKIVSL
APEVFMLSPKRTRFRKQHRGRMKGVSSRGNHICFGRYALQALEPAWITSRQIEAGRRAMT
RYARRGGKIWVRVFPDKPITVRPAETRMGSGKGSPEYWVSVIKPGRILHEMGGVPETVAR
AAMEIAACKMPIRTQFISAKMTRVRRGYIARRRRTKIRLFASTFRGAHSRLTRTATQQKMR
ALVSIYRDRGRQKREFRRLWIARINAATHEN--V--SYSRLINDLYKRQLLLNRKIPAQIARYN
LNSLYMISNEIIKEKRD-------MINEIRALAKHICMSPHKARRVIDQIRGCSYEQTLMILELMP
YRVCYPVFKLVYSAAANASHNMGLNEADSFISKAEVHGGAMMKKFKPKARGRSYPIKRP
TCHITIVLK--ERK-------MDGIKYAVFTEKSIRLLGNNQ-YTSNVESGSTRAEIKHWIELFFGV
KVIAMNSHRLPVKGRRMGPLMGHRMHYRRMIITLQPGYSIPPLIEKRTMAVPKKRTSMSK

KHIRRNYWKRKGYKAAVKAFSLGKSVSTGHSKSFF--QTTTMAKGKDARVRVILECTSCT
RN--GAKKESPGISRYITEKNRHNTPSRLELKKFCPYCSKHMIHAEIKKMKIRASVRKICEK
CRLIRRRGRIIVICSNPRHKQRQGMVRKEVPVSNRTLQWKCVESKADSKRLYYGRFVLSP
LMKGQADTIGTAMRRSLLGEIEGTCITCAKSE--RVPHEYSTIVGIEESVHEILMNLKEIIVRS
DLYGTRDAFICVRGPKYVTAQDIISPPSVKIVDTTQHIASITEPINFCIGLEIERNRGYRTRTT
N--QDASY--PIDAVSMPVRNANYSIQCYGNENEKQEILFLEIWTNGSLTPKEALYEASRNLI
DLFIPFLHAEERDLH--LDDDQ-NIFTAPFFPFHEGLA-YIGIGES-RIALKRIFIDQLELSPRTYN
CLKRSNIHTLLDLLSKSQEDLRKIQHFRVEDVKRVLDILKKR--FAMNLFT--MLRDG-NEGM
STIPGFSQIQFEGFCRFVDQGLAEELHKFPKIEDTDQEIEFQLLVETYQLAEPLIKERDAVYE
SLTHSSELYVPAGLIWKVGRDMQEQTVFIGNIPLMNSLGTFIVNGIYRIVINQILQSPGIYYS
SELDHNGISVYTGTIISDRGGRSELEIDRKARIWARVSRKQKISILVLPSAMGSNLREILDNV
CYPEILLYFPNEKEKKKIGSKENAILEFYQQFSCVGGDPVFSESLCKELQKRFFQQRCELGR
IGRQNMNQRLNIDIPQNNTFLLPRDVLAATDHLIGMKFGMGTLDD--MNHLKNKRIRSVAD
LLQDQFGLALVRLENVVRGTICGAIR-HKFI-PTPQNLVTSTP----LTTTYESFFGLHPLSQVL
DRTNPLTQIVHGRKSSYLGPGGLTGRTASFRIRDIHPSHYGRICPIDTSEGINVGLIGSLAIHA
RIGDWG-SIRSPFY---EISERSKE--EQMVYLSPRRDEYYMVAAGNSLALNQDIQDEQVVPAR
YRQEFVTIAWEHIDLRSIYPLQYFSIGASLIPFIEHNDANRALMSSNMQRQAVPLSRSEKCIV
GTGLERQAALDSGGSAIAQHEGKVIYTDTEKILLSGNGDTISIPLLMYQRSNKNTCMHQK
PQVHRDKYVKKGQVLADGAATVGGELALGKNVLVAHMPWEGYNFEDAVLISERLVYGD
IYTSFHIRKYEIQTHVTSHG-PEKITNEIPHLEAHLLRNLDRNGIVMLGSWVETGDVLVGKL
TPQTAKE-SSYAPEDRLLRVILGIQVSTAKETCLKLPIGGRGRVIDVRWIQKKG-ASSYNPEK
IRVYISQKREIKVGDKVAGRHGNKGIISKILPRQDMPYLQDGTPVDMVFNPLGVPSRMNV
GQIFECSLGLAGDLLDRHYRITPFDERYEQEASRKLVFPELYEASKRTANPWVFEPEYPGK
SRIFDGRTGDPFEQPVIIGKSYMLKLIHQVDDKIHGRSSGHYALVTQQPLRGRAKQGGQRV
GEMEVWALEGFGVAHILQEMLTYKSDHIRARQELLGTTIVGGTIPKPEGAPESFRLLVREL
RSLALELKHFLVSEKNFQINRKEA-------MIDRYKHQQLRIGLVSPQQITAWANKILPNGEM
VGEVTKPYTFHYKSNKPEKDGLFCERIFGPIKSGICACGNYRVIGDEKEDPKFCEQCGVES
VDSRIRRYQMGYIKLACPVTHVWYLKRLPSYIANLSDRPLKELEGLVYCD--FSFARP-IAK
KPTFLRLRGSFEYEIQSRKYSIPLFFTTQCFNLFRNREISTGAGAIREQLADPDLRIITDRSLV
EWKELGEERSA-ENEWEDKKIVRRKDFLVRRMELAKHLLRTNVEPERMVLCLLPVLPPEL
RPIIQIDGGKPMSSDINELYRRVIYRNNTLIDPLTTSRSTPGESVMCQEKLVQEAVDTLLDNG
IRGQPMRDGHNKVYKSFSDVIEGKEGRFRETLLGKRVDYSGRSVIVVGPSLSLHRCGLPRE
IAIELFQTFVIRGLIRQHVASNIGIAKSKIREKEPIVWEILQKVMEGHPVLLNRAPTLHRLGI
QAFQPILVEGRAICLHPLVRKGFNADFDGDQMAVHVPLSLEAQAEARLLMFSHMNLLSPA
IGDPISVPTQDMLIGLYVLTIGNRRGICTNRYNPCNYR--N-YQNEIVDDN-YKYTKEKEPYF
CSSYDALGAYRQKRIDLYSPLWLRWRLDQC-VIASINREVPIEVQYESLGIYHEIHEHYRIV
KSVKKEIVCIYIRTTVGHISFYREIEEAIQGFCRTY---------------MAERADLVFHNKAIDGTA
MKRLISRLIDHFGMAYTSHILDQVKTLGFRQATLTSISLGIDDLLTTPSKGWLVQDAEQQSL
ILEKHHHYGNVHAVEKLRQSIEIWYATSEYLRQEMNPNFRMTDPFNPVYIMSFSGARGNA
SQVHQLVGMRGLMSDPQGQMIDLPIQSNLREGLSLTEYTISCYGARKGVVDTAVRTSDAG
YLTRRLVEVVQHIVVRRTDCGTIRGISVSPRNGVGVTERIFIQTLIGRVLANDVYMGLRCIA
TRNQDIGIGLVNRFIT------SRAQPIYIRSPFTCRSTSWICQLCYGRSTTHGNLVELGEAVGIIA
GQSIGEPGTQLTLRTFHTGGVFTGGIAEHVRAPSNGKIKFNEDLVHPTRTRHGHPAFLCHID
LHVTIESQDIIHKVNIPPKSFLLVQNDQYVESEQVIAEIRAGTST---LKERVQKHIYSDSEGE-

MHWSTDVYHTPEYTHGNVHLLPKTSHLWVLSGSPCR--SSIVPFPLHKDQDQMNVQ--SLY
VEERYISDLS--MNNDR-VRHKLFGWDQ----RGRVSYYS-GPDRIISNRNWDSIYPL-ILHENS
D--LLAKRRRNRFIIPFQYDQEQEKELRPPG-IVIKIPIK--GILRRNSILAYFDDPRYRRSSSGIA
KYGTIEVDSIIKKED--LIEYRK-TREFGPKYIKVNRFFFIPEEVHILPRSSSIMVRNNSIIGVDT
RITLNIRSQVGGLVRVEKKKKRIELKISSGDIHFPGETDNIARYSGILIPPGRVKN----TESKF--
-KNWIYVQR--ITPIKKK--YFVSVRPVVTYEIADGINLATFFPQDLLQEKNNLQLRVVNYILY
GDGKPIRGIFHTSIQLVRTCLVLNWDQDRAGSIEE--EEAYTSLAEVRVNDLIRNFIRIDLVKS
PISSTGKENDMAGSGLIPN-NGSDRINTNPFFSKAKT---------QSLSQHQGTIRTLLNRNK-EG
QGESLMVLSSSNCSRIGP--LNGSKYHN---VTKESDPMISIRNSLGPLGT--VPNILNFSSS--YH
SIT--HNEILFN-KYLLPDN---SRETFLVPK---SYFMDENRR--IYNLDPCSNIILTPFNLNWCFL
HHDYWEDTSTIIS-LGQFLCENICISKDGP---CVKSGQIIIVH-VDSLVIRLAKYHLATRGATV
HGHYGEILYEGDTLVTFIYEKSRSGDITQGLPKVEQVLEVRSIDSISMNLEKRVEGWNERIT
GFLGIPWEFFISAQLTIVQSRISLVNKIQKVYRSQGVQIHNRHIETIVRQITSKVLVSE---DGM
SNVFSPRELIGLLRAERIGRALEDDICYRAILLGITRASLNTQSFISEASFQETTRVLAKAAL
RSRIDWLKGLKENVVLGGMIPVGTGFKGFVHHSR--EHNNIS--LEIKKKNLFDGKMR--DIL
FYHREFCGSCIPKNFHDTSEQ-------------MARRYWDINLEEMMGAGVHFGHGTRKWNPR
MAPYISGKRKGIHITNLTRTARPLSEACDLVFDAASRGKHFLIVGTKDKAADSVASAAIRA
RCHYVNKKWLGGMSTNWSTTETRLHKFRDLRAEQKMGRFKRLPKRDAAMLKRQLSHL
QTYLGGIKYMTGLPDIVIIVDHQEEYTAIRECLTLGIPTICLIDTNCDPDLADIPIPANDDAIA
SIQLILNKLVLAIREGHYS-YLGSHMGQKINPLGFRLGTTQSHRSLWFAQPKDYSRNLQEDE
KIRDCIKNYVQKHMRISSG-FQGIARLGIQKRIDLIQVTIYIGSSNLLIEG--PTR--GIEELRTD
VQKKLNSMNRRLNITITRIARPYEQPNILAEYIALQLKNRVSFRKAMKKAIELAEQANTKG
IRVQIAGRLNGKEIARVEWIREGRVPLHTIRAKIDYCSYMVQTIYGVLGIKIWIFVDEEMSR
YRGPRFKKIRRLGALPGLTSKRPRSGSDLRNQSRSGK-RSQYRIRLEEKQKLRFHYGLTERQ
LLRYVRIAGKAKGSTGQVLLQLLEMRLDNILFRLGMASTIPGARQLVNHRHILVNGRLVDI
PSYRCKPGDFVTTREKERSRALIQNHMNSSPN-EELPKHLTLYSLQY--KGLVNQIIDSKWIG
LKINELLVVEYYSRQTMSRRGTAEEKTAKSDPIYRNRLVNMLVNRILKHGKKSLAYQIIYR
ALKKIQQKTETNPLSVLRQAIRGVTPDIAVKARRVGGSTHQVPIEIGSTQGKALAIRWLLG
ASRKRPGRNMAFKLSSELVDAAKGNGDAIRKKEETHRMAEANRAFAHFRMGRDTIANIIT
CIRNADMDKKRMVRIASTNITENIVKIILQEGFIENVRKHRENNKNFLVLTLRHKR--NRKG
TYR---KTL--KRISRSGLRIYSNYQRIPRISGGMGVVILSTSRGIMTDREARREGIGGEILCYI
WMKKPIPRIGSRRNGRIGSRKNGRRIPKGVIHVQASFNNTIVTVTDVRGRVVSWCSAGTC
GFRGTRRGTPFAAQTAATNAIRTVVDQGMQRAEVMIKGPGLGRDAALRAIRRSGVLLSFV
RDVTPMPHNGCRPPKKRRVMPTIKQLIRNTRQPIRNVTKSPALRGCPQRRGTCTRVYTITP
KKPNSALRKVARVRLTSGFEITAYIPGIGHNSQEHSVVLVRGGRVKDLPGVRYHIVRGTLD
AVGVKDRQQGRSKYGVKKPKMARKSLIQRERKRQKLEQKYHLIRRSLKKEIS---KTPSLS
DKWKIHGKLQSPPRNSAPIRLHRRCFSTGRPRANYRDFGLSGHVLREMVHACLLPGATRS
SWMVINSFISSISKEEK-----KGSVEFQVFCFTDKIRKLTSHLELHKKDFLSQRGMRKILGKR
QRMLAYLSNKNKVRYKKLIGQLNIREPKTR--MVKLRLKRCGRKQRVIYRIVAIDVRSRRD
GRALRKVGFYDPIKNQTYSNVPAILYFLEKGAQPTVTVHDISKKAEVFK----------MDKSK
QPFRKSKRSFRRRLPPIGSGDRIDYRNMSLIGRFISEQGKILSRRVNRLTLKQQRLITIAIKQA
RILSPLPFLNNEK--QFERTESLPRITGTRTIKKMSRSLKKNPFVANNLLLKIHKLNTREEKEII
VTWSRASTIIPTMIGHTIAIHNGKEHLPVYVTDRMVGHKLGEFAPTLTFQGHARNDSKFRR
MILKSFLLGNLLSLCMKIRNSVVVVGLYYGFITTFSIGPSYLFLLRARVMEEG---TEKEVSA

TTGFIMGQFMMLISIYYTPLHLALGRPHTITVLVLPYLLFHFFWNNHKHFFSYRSTTRNSM
RNLSIQCVFLNNLIFQLFNHFILPSSTLARLVNIYMFRCNNKMLFVTSSFVGWLIGHILFMK
WVGLVLFW------IRQ-IRSNKYLVSELRKSMARILSILLFITCVYYLGRIPSPIFTKKLKETEED
KPFITFLFDYQRWNRPFRYIENDQFANTVRNEMSQYFFNTCPSDGKRVISFTYPPSLSVFGE
MIQKKMSLCGMKELYKDKNLYNWVSTNEQKRDKVSKEFINRIEALY--GIVL---QKRVRLC
NDRDEKECLPKMSDPFLNGSYRGTIKELYSNGEKFWINRIHSLL-------D-LNDSSEIQEIKK
KIPQRLYRLTDLEQDEGEEIRSRKTKHMVIYADDVIRYTQKPDFRRDLIKGSMRAQRRKTV
TWEMLQINLHSPLFLDRIDKNPFFYFDI--SRIINLIFRDKKEDE---IEETWETIAFGQVIRGFL
LVTQSILRKYIVLPSLIIAKNLVRMLLFQLPEWYEDFKEWSREMHIKCTYHGVQLSERKFP
KNWLKAGIQIKILFPFYLKPWRKSKKRKKQNSCFLTVWGMETDLPFGPARKGPSFFEPIHK
ELEKKFKKGK-KKWFLFVRIFKE-----------KKIWVIKRVL--FIKGVMKELTKANNKTISESPI
RKSPIRTGSMDWTNSSLTERKRKDLSDKTTTIRDQIERIRITRDKSPNETSYSDKRSESQKPI
WQVSKRRSTRLLYKWRYFMKSFIERIHRNVFLCMINLPRINTQLFIHFIFCSLS---QAYVFY
KLSQKPGIKKYRLGSLFQYAY-----SIKDRIIERIFHSESKHKKPGNFGMNEWKNWLIGNYQ
YNFSYTRWSRLDPQKWRNKVNEQCMIKNKKKTDSYSYEKDPLINHERHKKLSKRYRYD
LLSYRYINRFIVDTDRKYFDLRIIKFWSRFFDWMGMNEELLYRWFFPELVLLNDAYKTKP
WTIPIRLLLFNSKSNKEAELDLLLKRYLLFQFRWDDSLKKKMINNIKLYSLLLRLMDPKKI
VISSIQRGEMWLDIMLIHKDLPLTKLKKGGVFTMEPLRLSRKWNGQFLMYQTLSISLVHKI
KQKNYDLFVPENISALRRRRELRILSRLIFLWPNHRLEDLACMNRYWFDTNNGTRFSMSRI
RMYPMKGHQLKSWIFELREILREIKNSHYFLDSWTKLNLVGSFTHIFFHQERFMKLFDPRI
WSILLS-CDS--QSSTSNRYF---TIKGVVLLVVA-VLI----YRINNRNMVERKNLYLMRLL-----P
IPMNSIGPRNDTLEESF-LSSNINRLIVS-LLYLPKGKKISESCFMDPKESTWLLPIT----KKCIM
PESNWGSRWWRNRIGKKR--DSSCKIS---NETVAGIEISFKEKDIK-YLEFLFVSYTDTDDPIR
KDHDLELFDRLSPGKKQNIINL--NSGQLFEILVKHLICYLMSAFREKRPIEVEG-FFKQQG-A
EATIQSNDIEHVPR--LFSKNKWGISLQNCAQFHMWQFHQDLFV---SWGK-NQHESDFLRN-
VSRENLIWLGSMWLVNKDR---FFS--KVRNVSSNIQYDS---TR--SIFVKVTDSSQLKGSS-DQ
SRDHFNSIS----NEDSE-------------YHTLIHQTEIEQLKERSIL-WD--PSFLQTERTEIESD--RFP
K--CLSGYF--SMSRLFT-EREKQMNNHLFPEEIEEFI--GNPTRSIRSFFSDRW--SELH-LGSNA
TERSTRDQ----KLLKKQQDVS-FVPSRRSE----NKEMVDIFKIITYLQN--TVSIHPISSDPGCD
MVP-KDEPD-MDSSNKISFLNKNPFCD--LFHLFHGRNKGGYTL--HHDFESE--ERFQERADL
FTLSITEP-DLMYHKGFAFSIDSYGLDQKK--LLNEVFNSRDESKKKSL---LVLPHIFY--EENE
SFYRRIRTKSVR-ISCRNGLEDP--KIVVFASNNIMEAVNQYRLIRNLIQIQYSTYG--YIRNVS
KRFFLM---NRSDRNFEYGIQ---RDQIGNDTLNHITIMKYTINQHLSNLKK-SQKKWFD----PL
ISRTERSMNRDPNAYRYKWSNGSKNFQEHLEHFVSEQKN---RFQVVFDRLRINQHSIDWS
EVIDKQDLSKSLRFFLSKS---------LLFLS-------KSL--------PFFFVSIGNIPIHRSE-IHIY-ELKG
PNDQLCNQLLESIGVQIVHLNKLKP--FLLD---DHDTS-QRSKLLINGGTIARFWFNKIP-KW-
MIDSFHT--RNNRGKSFENTDSYFSMISHDRDNWLNPVKPFHRSSLISSFYKANQLRFLNNP
HHFWFY--CNKRFPFY----AEKTRINNYD--LTY-GQFLNISFIRNK-IFSLCVGK--KKHVFLE-R
DTISPIESQVSDIFIPNDFPQSGDET-YNLYKS---FHFPTRSDPFVR--RAIYSIADISGTPLTEEQ
RVNFEGTYCQPLSDLNLSDSEGKNLHQYLS--FNSNMGLIHTPCSEKYLP--SGKRKK--------
RNLCLN--KCVEKRQMYRTFQRDSAFSNISKWNLFQTYMPWFLTSTGCKYINFILLDTFSD
PL--PILSSS-HKFVSIFHDIMHGS--AWSIPQKKLR----AILPQWNL-ISEISSKCLQN--LLLSEE
MIHRNNESPVPLIWTHLRSPNPREF-FYSSLFLLLVAGY-LVRTHLLFVSR--VSSELHTELEKI
KSLMIPSYMMELRKLLDSYPPPELNSFWLKNLFLVALEQLGDSPEEIRGSDSGGNMLLGG

GP-----AYG-VKSIRSKKKDLNINLIDIIDLI-SIIPNPVNPITFSINTRRLSRTSKEIYSLIRKRKN
VNSDWIDGKIESWVANSDLIDDEEREFLVQFS--ALTT--EKRIDQILWSLT-HSDPFS-----KND
SGYQMIEQPGSIYLRYLVDIH--KKYLMNSEFNRSCLAERRIFLAHYQTITY--SQTSCGANSS
HFPSHG---KPFSLRLDLSPS-RGILVIGSIGTGRSYLVKYLATNSYLPSITVSPNKFLDDKPKG
YLID-----DIDSIDDSDDVD----IGDSDDIDI---------------DDDLDAELLTMTNVL---TMYMT---
PKMDRFDITPQLELAKAMSPCIIWIPNIHDLYVNESNYLSLGLLVNHLS---RDCERCS-TRNI
LVIASTHIPQKVDPALIAPNKSNTCIKIRRLLIPQQRKHFFILSYTRGFHLEKKMFHT--NGFG
SITMGSNARDLVALTNEALSISITQKKSIIDTNTIRSALHRQTWDLR---SQVRSVQDHGILFY
QIGRSFAQNVLLSNCSIDPISTYMKKK--SCKEGDSYLYKWYFELGTSIKKLTTLLYLLSCSA
GSVAQDLWSPP--GPDEK-NWITSYGFVENDS-DLVHG---LEGALVG--SSRTEKDCSQFDND-
-RVTLLLRSEPRN-PLDMMQNGSCSIVDQRFLYEK--YESEFEE-GEGEGTLDPQ----QIEED---
-LFNH--IVWAP--RIWRP---CGNLFDCIERPNESRFP-YRSRSFR---GKQIISHKEDELQENDLE
FLQSGTMQYQTRDRSSKEQGFFQISQFIWDPADPFFFLFK-DQPFVSVFARREFFADEEMSK
GLITSQTHSPTSIYERWLI--KNTQEKHFELLIHRQRWLST---NSSLS--NGSFRSN--TPSESYQ
YLSNLFLSN-GTLLDQMAKTLLRKRWLFPDEMKHLI--HVTGERFPIPMPRSR-INGNFIDKT
SSIVANILLRIIPTTSGEKEAFTYYRDG--MSAQSEGNYAEALQNYYEATRPEIDPYDRSYILY
NIGLIHTSNGEHTKALEYYFRALERNPFLPQASNNMAVICHY-RGEQAIRQGDSEIAETWSD
QAAEYWKQAIALTPGNYIEAQNWLKITRRFE--MNWRSERIWIELITGSRKTSNLCWACILF
LGSLGFLLVGTSSYLGRNLISLFPSQQILFFPQGIVMSFYGIAGLFISSYLWCTILWNVGSGY
DRFDRKEGIVCIFRWGFPGRNRRIFFRFLMRDIRSIRMEVKEGIYPRRVLSIEIRSQGSIPLTR
TDEN-FTPREIEQKAAELAYFLRVPIEVF
>Nymphaea
MEKWWLNSMLSLS---ASLGPIGNTSGSEEPIINDSEKNVN-CSNVDNLGGVKDVWSLISGE
TFWVRDSNGDSYSIYFDIENKIFEIDTD--SYFLGELESLFSSYL---NLNNGSKSYNRYYDHY
VYDTRHSWKSHINSCIDSYIRSETN--MDSCIPNGSNNSSDNYIYSYICSDRGSSLKTSGSD---
--FGRH-DLDRNKRYRHLWVQCENCYGLNYKKFFSSKMNICEQCGYHLKMSSSDRIELLID
PGTWDPMDENMVSMDPIEFHSEEDPYRDRINSYQIETGLAEAVQTGIGKLNGIPIAIGVMD
FKFMGGSMGSVVGEKITRLIEYATDRSLPVIMVCASGGARMQEGSLSLMQMAKISSALYN
YQSNKKLFYVSILTSPTTGGVTASFGMLGDIIIAEPNAYIAFAGKRVIEQTLKKTVPEGSQVA
EYLFNKGLFDPIVPRNLLKGVPSELFQFHGFFPRP-----MVTIRAEEISNIIRERIEQYNREVKI
VNTGTVLQVGDGIARIHGLDEVMAGELVEFAEGTIGIALNLESNNVGVVLMGDGLMIQEG
SSVKATGRIAQIPVSEAYLGRVINALAKPIDGRGEILASEYRLIESPAPGIISRRSVYEPLQTG
LIAIDSMIPIGRGQRELIIGDRQTGKTAVATDTILNQKGQNVICVYVAIGQKASSVAQVVTTF
QERGAMEYTIVVAETADSPATLQYLAPYTGAALAEYFMYRQRHTLIIYDDLSKQAQAYRQ
MSLLLRRPPGREAYPGDVFYLHSRLLERAAKPSSRLGEGSMTALPIVETQSGDVSAYIPTN
VISITDGQIFLSADLFNAGIRPAINVGISVSRVGSAAQIKAMKQVAGKLKLELAQFAELEAF
AQFASDLDKATQNQLARGQRLRELLKQSQSSPLAVDEQIVTIYTGTNGYLDQLEIGQVKK
FIVQLRTHLRTNKPQFQEIISSTKVFTEQAEALLKEAIQEQMELFLLQEQTMRINPTTSGLG
VSTLVEKNQGRIAQIIGPVLDVAFPPGKMPNIYNSLVVKGRDTAGQEINVTCEVQQLLGNN
RVRAVAMSATDGLTRGMEVIDTGAPLSVPVGGATLGRIFNVLGEPVDNLGPVDTRTTSPIH
RAAPAFTQLDTKLSIFETGIKVVDLLAPYRRGGKIGLFGGAGVGKTVLIMELINNIAKAHG
GVSVFGGVGERTREGNDLYMEMKESGVINEENIAESKVALVYGQMNEPPGARMRVGLTA
LTMAEYFRDVNEQDVLLFIDNIFRFVQAGSEVSALLGRMPSAVGYQPTLSTEMGSLQERIT
STKEGSITSIQAVYVPADDLTDPAPATTFAHLDATTVLSRGLAAKGIYPAVDPLDSTSTMLQP

RIVGEEHYETAQRVKQTLQRYKELQDIIAILGLDELSEEDRLTVARARKIERFLSQPFFVAEV
FTGSPGKYVGLAETIRGFQLILSGELDSFPEQAFYLVGNIDEATAKAMNLEVESKLKKMTL
NLCVLTPNRIIWDSEVKEIILSTNSGQIGVLPNHAPIATAIDIGILRIRLDDQWLTMALMGGF
ARIGNNEITILVNDAEKGSDIDPQEAQRTLEIAEANLSKAEGKRQVIEANLALRRARARVE
AINAISMKNLTDSFVFLGHWPSAGSFGFNTDILATNLINLSVVLGVLIFFGKGVLSDLLDNR
KQRILSTIRNSEELCGGAVEQLEKARARLRKVEREAEEFRVNGYSEIEQEKMNLINAAYQN
LEQLENYKNETIHFEQQRAINQVQQRVFQQALQGALGTLNNCLNGELHLRTIGANIGILGA
MKEITDMNPLISAASVIAAGLAVGLASIGPGVGQGTAAGQAVEGIARQPEAEGKIRGTLLL
SLAFMEALTIYGLVVALALLFANPFVMNVLPCSINTLKGLYEISGVEVGQHFYWQIGGFQV
HAQVLITSWVVIAILLGSAAIAVRNPQTIPTDGQNFFEYVLEFIRDVSKTQIGEEYGPWVPFI
GTLFLFIFVSNWSGALLPWRIIQLPHGELAAPTNDINTTVALALPTSVAYFYAGLTKKGLGY
FGKYIQPTPILLPINVLEDFTKPLSLSFRLFGNILADELVVVVLVSLVPLVIPIPVMFLGLFTSG
IQALIFATLAAAYIGESMEGHHMIFATLEHILTHISFSIISIVIPTHLMT-LVYEIVGLCDSSEKG
MITTFFCITGLLVTRWIYSGHVPLSDLYESLMFLSWSFSLIHIVPYFRNYKNFFSKITAPSAIL
TQGFATSGLLTKMHQSAILVPALQSRWLMMHVSMMLLSYAALLCGSLLSITLLVITFRRKI
DIFGKTNETQYVN-----------FSFRNYHRYQLTQRLDYWSYRVIGLGFTLLTIGILSGAVWAN
EAWGSYWNWDPKETWAFITWTVFAIYLHTRTNKSLQGANSAIVASMGFLIIWICYFGVNL
LGRGLHSYGSFTLNVTKK-ALNPLPYLASIVFLPWGISLSFNKSMEPWVTNWWNTSQSET
FLNDIQEKNILEGFIKLEELFLLDEMIKEYPETHIQKLRIGIHKETIQLVRMHNQDHIHIILHF
STNITCFAILSAYSILGNEELITLNSWVQEFLYNLSDTIKAFSILLLTDLCIGFHSPHAWELMI
GSFYKDFGFVQNEKIISGLVSTFPVILDTILKYWIFHYLNRVSPSLVVIYHSLNEMPIGVPKV
PFRSPGEEDAVWVDIYNRLHRERLLFLGQGVDSEISNQLVGLMVYLSMEDNTRDLYLFINS
PGGWVIPGIAIYDAMQIVPPDVHTICMGLAASMGSFILVGGEFTKRLAFPHARVMIHQPAS
SFYEAQAGEFILEAEELLKLREILTRVYVQRTGKPLWVVSEDMERDVFMSATEAQAHGIV
DLVAVEMKEQKLIHEGLITESLPNGMFWVRLDNEDLVLGYVSGRIRRSFIRILPGDRVKIEV
SRYDSTRGRIIYRLRNKDSNDQYELQGYLEIDRYRKQRFLYPLLFREYIYALAHDHGLLGY
DN-SSSLIVKRLITRLHQQNHLTISVNDS---RFVGRSFYSQTIPEGFAGIMEIPFSVRLVSSLIA
KYH-LRSIHSIFPFLEDKLSHLYYVSDILIPYPIHLEILLQTLRTRIRDAPSLHLLRCFLHEHHN
WNSLITKSISIFSKQRLFLFLYNSHVYECESVLVFLRKQSSHLRSISSLAFLERTHFYGKIKH-
VVTPRNQRPLWFFKEPLMHYVRYQGKSIMASRCTNLLMKKWKYYLVNFWQCHFHLWS
QPGRIHINELSNHSFYFLGYLSGVRLTPWVIRSQMLENSFMIDTAI--KRFDTIVPIFPLIGSLV
KAKFCNVSGYPISKSVWADSSDSDIIARFGWICRNLSHYHSGSSKKHSLCRIKYILRLSCAR
TLARKHKSTVRAICKRLGSKLLEEFLTEEHEIVSFIFRRRIWYLDIIRIHGLVPHSMIIEEAQ---
-AINSFFRSES-SKEVYGLIWLLVPILTLVLGITIGVLVIVWLERKISAGIQRRIGPEYAGPLGIL
QALADGVKLLFKEDLLPSRGDIRLFSVGPSVAVVSILLSYSVIPFGYHLVLTDLSIGVFLWIA
ISSIAPIGLLMSGYGSNNKYSFSGGLRAAAQSISYEIPLTPCVLSISLLSNSSSTVDIVEAQSK
YGFWGWNLWRQPIGFLVFIISSLAECERLPFDLPEAEEELVAGYQTEYSGIKFGLFYVASYL
NLLVSSLFVTVLYLGGWNLPIPYIPITELFEINKTSEVFGTTISLLITLAKAYLFLFIPISTRWT
LPRLRMDQLLNLGWKSLLPIALGNLLLTTSSQLVSLMIWHVQNENFILDSTRIFMKAFHLL
LFNGSFIFPECILIFGLILLLMIDLTSDQKDTPWLYFISSTSLVMSITALLFRWREEPMISFSGN
FQTNNFNEIFQFLILLCSTLCIPLSVEYIECTEMAITEFLLFVLTATLGGMFLCGANDLITIFVA
PECFSLCSYLLSGYTKRDVRSNEATMKYLLMGGASSSILVYGFSWLYGSSGGEIELQEIVN
GLINTQMYNSPGISIALISITVGIGFKLSPAPFHQWTPDVYEGSPTPVVAFLSVTSKVAASAS
ATRIFDIPFYFSSNEWHLLLEILAILSMILGNLIAITQTSMKRMLAYSSIGQIGYVIIGIIVGDS

NDGYASMITYMLFYIAMNLGTFACIVLFGLRTGTDNIRDYAGLYTKDPFLALSSALCLLSL
GGIPPLAGFFGKLYLFWCGWQAGLYFLVSIGLLTSVVSIYYYLKIIKLLMTGRNKEITPHVR
NYRRSPLRSNNSIELSMIVCVIASTIPGISMNPIIAIAQDTLFMFLLYEYDIFWAFLMISSVIPI
LAFLISGVLAPISEGPEKLSSYESGIEPIGDAWIQFRIRYYMFALVFVVFDVETVFLYPWAVS
FDILGVYVFIEALIFVLIPVVGSVYAWRKGALEWSMSDFYWLTIIVVLPISAGSLIALLPHRG
NKVVRWYTICICLFELLLTTYVFCYHFKLDDPLIQLEEDFNWINLFDFHWRLGIDGLSIGPI
LLTGFITTLATSAAWPVTRNSRLFHFLMLAMYSGQIGSFSSRDLLLFFIMWELELIPVYLLL
SMWGGKKRLYSATKFILYTAGGSIFLLMGVLGMGLYGSNEPTLNFETLANQSYPVALEIIF
YFGFLIAYAVKSPIIPLHTWLPDTHGEAHYSTCMLLAGILLKMGAYGLVRVNMELLPHAHS
IFSPWLMIVGTIQIIYAALTSPGQRNLKKRIAYSSVSHMGFIIIGISSITDAGLNGAILQIISHGF
IGAALFFLAGTSYDRIRLRYLNEMGGIAILMPRIFTMFSSFSMASLALPGMSGFVAEFVIFL
GIITSPKYLVMSKILITFVMAIGMILTPIYSLSMSRQMFYGYRLFNVPKSHFVDSGPREIFIFM
CILLPIIGIGIYPDFVLSLSVDKVETILSNYFHGMMLEHVLVLSAYLFSIGIYGLITSRNMVRA
LMCLELILNAVNMNLVTFSDLFDSRQLKGDIFSIFVIAIAAAEAAIGPAIVSSIYRNRKSTRIN
QSNLLNKMERTYQYAWIIPFVPLLVTMLIGLELLLNPTATKNIRRIWAFPAVLLLSIVMVFST
KLAIQQINGSSIYEYLWSWSITSDFSLEFGYLIDPLTSIMSILITTVGIMVLIYSDNYMSHDRG
YLRFFAYMSFFNTAMLGLVTSPNLIQIHIFWELVGMCSYLLIGFWFTRPIAANACQKAFVT
NRVGDFGLLLGILGFYLITGSFEFQDLFEIFNDNEINSSFATLCAFLLFLGAVAKSAQFPLHV
WLPDAMEGPTPISALIHAATMVAAGIFLVARLLPLFIAIPYIMNIISLIGVITVLLGATLALAQ
RDIKRSLAYSTMSQLGYMMLALGIGSYRAALFHLITHAYSKALLFLGSGSIIHSMEPIVGYS
PAKSQNMVLMGGLTKYMPITKTTFFLGTLSLCGIPPLACFWSKDEILNDSWLYSPIFAIIAF
YTAGLTAFYMFRMYLLTFEGHLRSHFQNYSGHTNSSFYSISIWGQEGSKPVSS-NLLLTTRN
FS-NKDSHTLLYPHESDNTMLFPLLILAIFTLFVGCIGIHFEVDILSKWLTPSMKLFHQNSTD-
EDWYKFLTNAFYSVSIAYFGIFLASVLYGSVYSDRQNLYLINSFAIRLEQIINVIYNWSYNR
GYIDVYYEKVFIKGVRELAQLAHSFDRRVIDGVTNGIGVASFFLGEGIKY-IGGGRISSYLFL
YLACVSIVLLIYYMDLPAPIHDILLVSLGSGLIVGGLGVVLLTNPIYSAFSLGLVLVCISLFYI
PSNSYFVAAAQLLIYVGAINVLILFAVMFMNGSEYYNYFHFWTVGDGFTSLVCTSIFFSLIA
TIPNTSWYGIIWTTRSNQIIEQDLTSNVQQIGIHLSTDFYLPFELISIILLVSLVGAIAMARRM
TVPDARKDLLVVNMGPHHPSMHGVLRLIVTLDGEDVIDCEPILGYLHRGMEKIAENRTIIQ
YLPYVTRWDYLATMFTEAITVNAPEELGNIQVPKRASYIRVIMLELSRIASHLLWLGPFMA
DIGAQTPFFYIFRERELLYDLFEAATGMRMMHNYFRIGGVAADLPHGWIDKCLDFCDYFL
TGVVEYQKLITRNPIFLERVEGVGFIGGEEAINWGLSGPMLRASGIQWDLRKVDRYECYD
EFDWEVQWQKEGDSLARYLVRIGEMTESIKIIQQALEGIPGGPYENLEFRRFAGTKDSELN
DFEYRFISKKPSPSFELSKQELYVRVEAPKGELGIFLIGDNSVFPWRWKIRPPGFINLQILPQL
VKRMKLADIMTILGSIDIIMGEVDRMFPMVTGFMNYGQQTVRAARYIGQSFMITLSHANR
LPVTIQYPYEKSITSERFRGRIHFEFDKCIACEVCVRVCPIDLPVVDWRLETDIRKKRLLNY
SIDFGICIFCGNCVEYCPTNCLSMTEEYELSTYDRHELNYNQIALGRLPMSVIGDYTVRTIM
NSKISMDKPLDSRTITN-MQGRSSAWLVKHELVHRSLGFDYQGVETLQIKPEDWYSIAVISY
VYGYNYLRSQCAYDVAPGGLLASVYHLTRIQYGVDQPEEVCIKVFVPRSNPRIPSVFWIW
KSADFQERESYDMLGISYDNHPRMKRILMPESWIGWPLRKDYIAPNFYELQDAY-----------
---MNSMEFPLFDRTTPNSVISTTLNDLSNWSRLSSLWP
LLYGTSCCFIEFASLIGSRFDFDRYGLVPRSSPRQADLILTAGTVTMKMAPSLVRLYEQMPE
PKYVIAMGACTITGGMFSTDSYSTVRGVDKLIPVDVYLPGCPPKPEAIIDAITKLRKKVSR
EIYEDRTGSQQENRCFTTNHKFHLGRSTRAVNYDQG---LYQSTSEIPSEAFFKYKSSVSSHE

LVNMQKNRNFSWVKEQMTRCISVSMMIYVITRASISNAYPIFAQQSYENPREATGRIVCAN
CHLANKPVDIEVPQAVLPDTVFEAVVRIPYDMQLKQVLANGKKGGLNVGAVLILPEGFEL
APPDRISPEMKEKMGNLSFQSYRPNKKNILVVGPVPGQKYSEIVFPILSPDPATKKEVHFLK
YPIYVGGNRGRGQIYPDGSKSNNTVYNASAAGIVSKIVRKEKGGYEITISDASNGHETVDII
PPGPELLVSEGEYIKLDQPLTSNPNVGGFGQGDAEIVLQDPLRIQGLLFFLASVILAQIFLVL
KKKQFEKVQLAEMNFMSKVYDWFEERLEIQAIADDITSKYVPPHVNIFHCLGGITLTCFLV
QVATGFAMTFYYRPTVTEAFASVQYIMTEANFGWLIRSVHRWSASMMVLMMILHVFRVY
LTGGFKKPRELTWVTGVVLAVLTASFGVTGYSLPWDQIGYWAVKIVTGVPEAIPIVGSPLV
ELLRGSASVGQSTLTRFYSLHTFVLPLLTAVFMLMHFSMIRKQGISGPLMGVTKKPDLNDP
VLRAKLAKGMGHNYYGEPAWPNDLLYIFPVVILGTIACNVGLAVLEPSMIGEPADPFATPL
EILPEWYFFPVFQILRTVPNKLLGVLLMVSVPAGLLTVPFLENVNKFQNPFRRPVATTVFLI
GTAVALWLGIGATLPIDKSLTLGLFMIEVFLFGIVLGLIPITLAGLFVTAYLQYRRGDQLDFM
PTIVSYFGFLLTASTITPALFIGLSKIRLIMDIVSLAWAALMVVFTFSLSLVVWGRSGLMIIRS
PEPEVKILVDRDPVKTSFEEWARPGHFSRTIAKGPDTTTWIWNLHADAHDFDSHTNDLEEI
SRKVFSAHFGQLSIIFLWLSGMYFHGARFSNYEAWLSDPTHIGPSAQVVWPIVGQEILNGD
VGGGFRGIQITSGFFQIWRASGITSELQLYCTAIGALVFAALMLFAGWFHYHKAAPKLAWF
QDVESMLNHHLAGLLGLGSLSWAGHQVHVSLPINQFLNAGVDPKEIPLPHEFILNRDLLA
QLYPSFAEGATPFFTLNWSKYAEFLTFRGGLDPVTGGLWLTDIAHHHLAIAILFLIAGHMYK
TNWGIGHDLKDILEAHKGPFTGEGHKGLYEILTTSWHAQLALNLAMLGSLTIVVAHHMYS
MPPYPYLATDYGTQLSLFTHHMWIGGFLIVGAAAHAAIFMVRDYDPTTRYNDLLDRVLR
HRDAIISHLNWVCIFLGFHSFGLYIHNDTMSALGRPQDMFSDTAIQLQPIFAQWIQNTHAL
APSATAPGATAGTSLTWGGGDLVAVGGKVALLPIPLGTADFLVHHIHAFTIHVTVLILLKGV
LFARSSRLIPDKANLGFRFPCDGPGRGGTCQVSAWDHVFLGLFWMYNAISVVIFHFSWKM
QSDVWGSISDQGVVTHITGGNFAQSSITINGWLRDFLWAQSSQVIQSYGSSLSAYGLFFLG
AHFVWAFSLMFLFSGRGYWQELIESIVWAHNKLKVAPATQPRALSIIQGRAVGVTHYLLG
GIATTWAFFLARIISVGMALRFPRFSQGLAQDPTTRRIWFGIATAHDFESHDDITEERLYQNI
FASHFGQLAIIFLWTSGNLFHVAWQGNFESWVQDPLHVRPIAHTIWDPHFGQPAVEAFTRG
GALGPVNIAYSGVYQWWYTIGLRTNEDLYTGALFLLFISAISLVGGWLHLQPKWKPSVSW
FKNAESRLNHHLSGLFGVSSLAWAGHLVHVAIPGSRGEYVRWNNFLDVLPYPQGLGPLFT
GQWNLYAQNPDSSSHLFGTSQGAGTAILTLLGGFHPQTQSLWLTDIAHHHLAIAFIFLVAGH
MYRTNFGIGHSIKDLLEAHIPPGGRLGRGHKGLYDTINNSIHFQLGLALASLGVITSLVAQH
MYSLPAYAFIAQDFTTQAALYTHHQYIAGFIMTGAFAHGAIFFIRDYNPQQNEDNVLARML
DHKEAIISHLSWASLFLGFHTLGLYVHNDVMLAFGTPEKQILIEPIFAQWIQSAHGKTSYGF
DVLLSSTNGPAFNAGQSLWLPGWLNAINE--NRNSLFLTIGPGDFLVHHAIALGLHTTTLILV
KGALDARGSKLMPDKKDFGYSFPCDGPGRGGTCDISAWDAFYLAVFWMLNTIGWVTFY
WHWKHITLWQGNVSQFNESSTYLMGWLRDYLWLNSSQLINGYNPFGMNSLSVWAWMF
LFGHLVWATGFMFLISWRGYWQELIETLAWAHERTPLANLIRWRDKPVALSIVQARLVGL
AHFSVGYIFTYAAFLIASTSGKFGMSHSVKIYDTCIGCTQCVRACPTDVLEMIPWDGCKAK
QIASAPRTEDCVGCKRCESACPTDFLSVRVYLWHETTRSMGLAYMTDLNLPSIFVPLVGLL
FPAIAMVSLFFHVQKNKIVMRDIKTYLSVAPVLTTLWFGSLAGLLIEINRLFPDALTFPFFSF
MTAILERRESTSLWGRFCNWVTSTENRLYIGWFGVLMIPTLLTATSVFIIAFIAAPPVDIDGI
REPVSGSLLYGNNIISGAIVPTSAAIGLHFYPIWEASSVDEWLYNGGPYELIVLHFLLGVAC
YMGREWELSFRLGMRPWIAVAYSAPVAAATAVFLIYPIGQGSFSDGMPLGISGTFNFMIVF
QAEHNILMHPFHMLGVAGVFGGSLFSAMHGSLVTSSLIRETTENESANEGYRFGQEEETY

NIVAAHGYFGRLIFQYASFNNSRSLHFFLAAWPVVGIWFTALGISTMAFNLNGFNFNQSVV
DSQGRVINTWADIINRANLGMEVMHERNAHNFPLDLAAVEAPSTNGMGLPWYRVHTVVL
NDPGRLLSVHIMHTALVSGWAGSMALYELAVFDPSDPVLDPMWRQGMFVIPFMTRLGITN
SWGGWSIT-GGTVTNPGIWSYEGVAGAHIVFSGLCFLAAIWHWVYWDLEIFCDERTGKPS
LDLPKIFGIHLFLSGVACFGFGAFHVTGLYGPGIWVSDPYGLTGKVQPVNPSWGAEGFDPF
VPGGIASHHIAAGTLGILAGLFHLSVRPPQRLYKALRMGNIETVLSSSIAAVFFAAFVVAGT
MWYGSATTPIELFGPTRYQWDQGYFQQEIYRRVNAGLAENLSLSESWSKIPDKLAFYDYI
GNNPAKGGLFRAGSMDNGDGIAVGWLGHPVFRDKEGHELFVRRMPTFFETFPVVLVDGD
GIVRADVPFRRAESKYSVEQVGVTVEFYGGELDGVSYNDPATVKKYARRAQLGEIFELDR
ATLKSDGVFRSSPRGWFTFGHASFALLFFFGHIWHGARTLFRDVFAGIDPDLDAQVEFGAF
QKLGDPTTRRQVVMKTLYSLRRFYPVETLFNGTLALAGRDQETTGFAWWAGNARLINLS
GKLLGAHVAHAGLIVFWAGAMNLFEVAHFVPEKPMYEQGLILLPHLATLGWGVGPGGEV
VDTFPYFVSGVLHLISSAVLGFGGIYHALLGPETLEESFPFFGYVWKDRNKMTTILGIHLIL
LGLGAFLLVFKALYFGGVYDTWAPGGGDVRRITNLTLSPSIIFGYLLKSPFGGEGWIVSVD
DLEDIIGGHVWLGSICILGGIWHILTKPFAWARRAFVWSGEAYLSYSLGALSVFGFTACCFV
WFNNTAYPSEFYGPTGPEASQAQAFTFLVRDQRLGANVGSAQGPTGLGKYLMRSPTGEII
FGGETMRFWDLRAPWLEPLRGPNGLDLSRLKKDIQPWQERRSAEYMTHAPLGSLNSVGG
VATEINAVNYVSPRSWLATSHFVLGFFFFVGHLWHAGRARAAAAGFEKGIDRDFEPVLFM
TPLNMTIALGRFTKEE-DLFDIMDDWLRRDRFVFVGWSGLLLFPCAYFALGGWFTGTTFV
TSWYTHGLASSYLEGCNFLTAAVSTPANSLAHSLLLLWGPEAQGDFTRWCQLGGLWTFVA
LHGAFGLIGFMLRQFELARSVQLRPYNAIAFSGPIAVFVSVFLIYPLGQSGWFFAPSFGVAA
IFRFILFFQGFHNWTLNPFHMMGVAGVLGAALLCAIHGATVENTLFEDGDGANTFRAFNP
TQAEETYSMVTANRFWSQIFGVAFSNKRWLHFFMLFVPVTGLWMSALGVVGLALNLRAY
DFVSQEIRAAEDPEFETFYTKNILLNEGIRAWMAAQDQPHENLIFPEEVLPRGNALMSGST
GERAFADIITSIRYWVIHSITIPSLFIAGWSFVSTGLAYDVFGSPRPNEYFTESRQGIPLITGRF
DSLEQLDEFSRSFMTIDRTYPIFTVRWLAVHGLAVPTVFFLGSISAMQFIQRMATQTIEGSS-
RSGPRRTIVGDLLKPLNSEYGKVAPGWGTTPLMGVAMALFAVFLSIILEIYNSSVLLDGISL
SMLTLKLFVYTVVIFFVSLFIFGFLSNDPGRNPGREEMADTTGRIPLWLIGTVTGILVIGLIG
VFFYGSYSGLGSSLMPNIFSLICICLNSALQ-SGFFFAKLPEAYAFLNPIVDFMPVIPVLFFLL
AFVWQAAVSFRTTQSNPNEQNVELNRTSLYWGLLLIFVLAVLFSNYFFNMEVNILAFIATA
LFILVPTAFLLIIYVKTVSQNDMETATLVAIFISGLLVSFTGYALYTAFGQPSQQLRDPFEEHG
DMEALVYTFLLVSTLGIIFFAIFFREPPKVPNKKMKMTIAFQLAVFALIATSSILLISVPVVFA
SPDGWSSKKNVVFSGTSLWIGLVFLVGILNSLIY-------MSPKTETKASVGFKAGVKDYRLT
YYTPEYETLATDILAAFRVTPQPGVPPEEAGAAVAAESSTGTWTTVWTDGLTSLDRYKGR
CYHIEPVAGEENQYIAYVAYPLDLFEEGSVTNMFTSIVGNVFGFKALRALRLEDLRIPPAYS
KTFQGPPHGIQVERDKLNKYGRPLLGCTIKPKLGLSAKNYGRAVYECLRGGLDFTKDDEN
VNSQPFMRWRDRFLFCAEAIYKAQAETGEIKGHYLNATAGTSEEMMKRAVCARELGVPI
VMHDYLTGGFTANTSLAHYCRDNGLLLHIHRAMHAVIDRQRNHGIHFRVLAKALRMSGG
DHIHAGTVVGKLEGERDVTLGFVDLLRDDFIEKDRSRGIYFTQDWVSMPGVLPVASGGIH
VWHMPALTEIFGDDSVLQFGGGTLGHPWGNAPGAVANRVALEACVQARNEGRDLAREG
NEVIREASKWSPELAAACEVWKEIKFEFEAMDVL--MAIHLYKTSTPSTRKGAVDSQAKSN
PRTKLIYGQHRCGKGRNARGIITARHRGGGHKRLYRKIDFRRNEKDISGRIVTIEYDPNRN
AYICLIHYGDGEKRYILHPRGAIIGDTIVSGTEVPISMGNALPLTDMPLGTAIHNIEITLGKG
GQLARAAGAVAKLIAKEGKSATLRLPSGEVRLISKNCSATVGQVGNVGANQKSLGRAGSK

CWLGKRPVVRGVVMNPVDHPHGGGEGRAPIGRKKPTTPWGYPALGRRSRKRNKYSDIFI
LRRRK-MIQPQTLLNVADNSGARKLMCIRIIGAS-NYRYAHIGDVIVAVIKEAVPNMPLERSE
VIRAVIVRTCKELKRDNGMIIRYDDNAAVVIDQEGNPKGTRVFGSIARELRQLNFTKIVSLA
PEVLMLSPKRTRFRKQHRGRMKGVSYRGNRICFGRYALQALEPAWITSRQIEAGRRAISRY
ARRGGKIWVRIFPDKPVTLRPAETRMGSGKGSPEYWVSVVKPDRILYEMGGVSETVARA
AMEIAARKMPIRTKFVIAEMTRVRRGYIARRRRRKIRLFASSFRGAHSRLTRTATQQKIRAL
VSSHRGRGRQKRDFRRLWITRINAVTREN--VP--YSRLIHDLRKKQLLLNRKILAQIAISNR
NCLYMISNDIIK-----------MIKEIRALARHIGMSAQKARRVIDQIRGCSYEQTLMILELMPYR
ACYPIFKLVYSAAANASHNRGLKEADLFISKAEVNEGVIVKRLKPRARGRSYPIKRPTCHI
TIVLS--ERP-------MDGIKYAVFTEKSIRLLGNNQ-YTSNVESGSTRTEIKHWIELFFGVKVVA
MNSHRLPGKGRRMGPIMRHTMHYRRMIITLQPGYSIPPLIEKRTMAVPKKRTSISKKHIRR
NFWKRKGYWAAVKAFSLAKSISTGYSKGFF--QKK-MPKGKNPRVTVILECTSCVRNG--K
KKESPGISRYITQKNRHNTPGRLELKKFCPYCYKHTIHGEIKKMKIRASVRKICEKCRLIRR
RGRIIVICSNPKHKQRQGMVREEVPVSTRTLQWKCVESRADSKRLYYGRFVLSPLMKGQA
DTIGIAMRRALLGELEGTCITRAKSD--KVPHEYSTVVGIEESVHEILMNLKKMVFRSDLYG
TLDASICVRGPRHVTAQDIISPPSVEIVDTTQHIAVLTEPVDLCIELKIERGRGYCTRTQNNY
QDGSY--PIDAVSMPVRNANHSIHSYGNGNEKQEILFLEIWTNGSLTPKEALYEASRNLIDLF
IPFLHAEEQDINRNMEDNL-KRASVPFFAFDDGLD-NIKR----EIILKRIFIDQLELPPRTYNCL
KRSNIHTLLDLLSKSQEDLMRIEHFRVEDVKQIFDILQKG--FTIDLLKNSMLRDGGDEGMF
TIPGFSQIQFEGFCRFIDQGLMEELHQFPKIEDTDQEIEFQLFGESYQLVEPLIKERDAVYESI
TYSSELYVPAGLIWRTGRNMQEQTVLLGNIPLMNSLGTSIVNGIYRIVINQILQSPGIYYSTG
LDHNGISVYTGTIISDWGGRSELEIDRKERIWARVSRKQKISILVLSSAMGSSLREILDNVCY
PEIFLSFPNEKEKKKIGSKENAILEFYQKFACVGGDPVFSESLCKELQKKFFQQRCELGRIG
RRNMNQRLNLDIPPNNTFLLPRDVLAAADHLIGMKFGMGTLDD--MNHLKNKRIRSVADL
LQDQFGLALVRLENVVRGTICGAIR-HKLI-PTPRNLVTSTP----LTTTYESFFGLHPLSQVLD
RTNPLTQIVHGRKSSYLGPGGLTGRTASFRIRDIHPSHYGRICPIDTSEGINVGLIGSLAIHAR
VGDWG-SIETPFY---EISERSKE--EQMVYLSPSRDEYYMVAAGNSLALTRGIQEEEVGPARY
RQEFLTIAWEQIHLRNIYPFQYFSIGASLIPFIEHNDANRALMSSNMQRQAVPLSQSEKCIVG
TGLERQAALDSGGSAIAEREGKIIYTDAEKIVLSGNGDTISIPLVMYQRSNKNTWMHQKPQ
VHRGKYLKKGQILADGAATVGGELALGKNVSVAYMPWEGYNSEDAVLISERLVYDDIYT
SFHIRKYEIQTHVTSQG-PERITNEIPHLEPYLLRNLDRNGIVMLGSWVETGDVLVGKLTPQ
TAKE-SSYAPEDRLLRAILGIQVSTAKETCLKLPIGGRGRVIDVRWGQKKG-GSIYNPEMIRV
YISQKRKIKVGDKVAGRHGNKGIISKILPRQDMPYLQDGTPVDMVFNPLGVPSRMNVGQ
MFECSLGLAGDLLGRHYRITPFDERYEQEASRKLVFSELYEASKQTANPWVFEPEYPGKSR
IFDGRTGDPFEQPVIIGKSYMLKLIHQVDDKIHGRSSGHYALVTQQPLRGRAKQGGQRVGE
MEVWALEGFGVAHILQEMLTYKSDHIRARQEVLGTTIVGGTIPNPEGAPESFRLLVRELRS
LSLELNHFLVSEKNFQINRKEVMNQNFSSMIDQYKHQQLRIGLVSPKQIRAWANKILPNGE
IVGEVTKPYTFHYKTNKPEKDGLFCERIFGPIKSGICACGNYRVIGGEKEEPKFCEQCGVES
VDSRIRRYQMGYIKLACPVTHVWYLKRLPSYIANLLDKPLKELEGLVYCD--FSFARP-IAK
KPTFLRLRGSFEYEIQSRKYSIPLFFTTQGFDTFRNREISTGATAIREQLADLDLRIIIDRSLVE
WKELGEE-GSTGNDWEDRKIGRRKDFLVRRMELAKHFLRTNVEPEWMVLSLLPVLPPEL
RPIIQIDGGKSMSSDINELYRRVIYRNNTLTDLLTTSRSTPGELVMCQEKLVQEAVDTLLDN
GIRGQPMRDGHNKVYKSFSDVIEGKEGRFRETLLGKRVDYSGRSVIVVGPSLSLHQCGLP
REIAIELFQTFVIRGLIRQHLASNIGLAKSKIREKEPIVWEILQEVMQGHPVLLNRAPTLHRL

GIQAFQPILVEGRAICLHPLVCKGFNADFDGDQMAVHVPLSLEAQAEARLLMFSHTNLLSP
AIGDPISVPTQDMLMGLYVLTMGNRRGICANRYNPCNPRNH---QNERMDHSNYEYRKGK
EPYFCSSYDALGAYRQKGIDLYSTLWLRWRLDQR-VIASINREVPIEVQYESLGTYHEIYDH
YRVVRSVKKGMLCIYIRTTVGHISFYREIEEAIQGFCRSYSYGT-----------MAERD-LVFHNK
VIDGTAMKRLISRLIDHFGIAYTSHILDQVKTLGFQQATATSISLGIDDLLTTPSKRWLVQDA
EQQSLILEKHHHYGNVHAVEKLRQSIEIWYATSEYLRQEMNLNFKMTDPSNPVHIMSYSG
ARGNASQVHQLVGMRGLMSDPQGQMIDLPIQSNLREGLSLTEYTISCYGARKGVVDTAVR
TSDAGYLTRRLVEVVQHIVVRRTDCGSTRGISVSFRKGMTE--RIFIQTLIGRVLANDVYLG
LRCIATRNQDIGIGLVNRFMT------SRAQPIYIRTPFTCRSASWICRLCYGRSPTHGDLVELGE
AVGIIAGQSIGEPGTQLTLRTFHTGGVFTGGTAEHVRAPSNGKIKFNEDLVHPTRTRHGHPA
FLCYVDLYVTIESQDIIHSVNIPPKSFLLVQNDQYVESEQVIAEIRAGTSTF--FKERVRKHIY
SDSEGE-MHWSTGVYHAPEYTHGNVHFLPKTSHLWILSGGPCK--SSLVPFSLHKDQDQMN
VQ--SLSVQERSISDFS--VNNNR-VKHKLFGSDPLAR-GRRISDYA--SERVISNGDGDFIYPA-I
LRENSY--LLAKRRRNRFIIPFQYDPEWEKELTPHSSITVEIPAN--GILRRNSILAYFDDPRYR
RSSSGITKYG---VDSIVKKEG--LVEYRRPK-ESRPKY--VDRFFVIPEEVHILPGSSSIMVRNN
SIIGVDTRITFNTRSQIGGLVRIEKKKK-IELKIFSGGIHFPGETDKISRHIGILIPPGARKKMD
KGSKGKNWEGNNWVYVQR--ITPIKKK--YFVSVRPVVTYEIADGINLVTLFPGDMLQEKD
NLRLQVVNYILYGDGKPIRGIS--HIQLVRTCLVLNWDQD------KSIEKVQASSAEVRANDLI
RYFIRIDLVKSPILYTGKRNDRSGSVIPDT--GSYCANTNLFSSKVKIKS---------LSQHQGTVR
TFLNR---NKEGQSLIVFSSSNCSRIN-----VSKYHN---VTKESIPILNLL---GPLGT--VPKIHNFS
PS--YHSIT--HNEILLN-KYLILDN-KNPKQTFQLLK---YYLVDENGR--ISNANPCSDIIFNLFG-
-SCFLPHDYCEGTSTTRIILGQFICENVCLSKHGT---RIKSGQVIMVY-LDSFIIRSAKPYLATR
GATVHGDYGEIFYEGDTLVTFIYEKSRSGDITHGLPKVEQVLEVRSIDSISMNLEKRVEGW
NEHITGILGIPWGFLIGAELTIAQSRVSLVNKIQKVYRSQGVQIHNKHIEIIVRQITSKVLVSE-
--DGMSNVFSPGELIGLLRAERAGRALEEAICYRAVLLGITRASLNTQSFISEASFQETARVL
AKAALRGRIDWLKGLKENVVLGGMIPVGTGFKRFVHRSR--EYNNIP--LEIQKKNFFGGE
MR--DILFHHRELFCSCIPKPKSFHSEQPFYAMGSNPIVHKSMTRRYWNINLEEMMEAGVH
FGHGTRKWDPRMAPYISAKRKGIHITNLTRTARFLSEACDLVFDAASRGKHFLIVGTKDK
AADSVASAAIRARCHYVNKKWLGGMSTNWSTTETRLQKFRDLRVRAEMGQFSRLPKRD
AAILKRQLSHFQTYLGGIKYMTGLPDIVIIIDQQEEYTALRECVTLGIPTICLIDTNCDPDLA
DIPIPANDDAIASIRLILNKLVSAICQGRS-SYIRSRMGQKINPLGFRLGTTQSHRSFWFAQPK
NYSKGLQEDEKIRDCIKNYVQKHMRISSG-FEGIARIDIKKRIDLIQVIIHIGFANMLMEG--R
AR--GIEELQTNVQKSFHSVNRRLNIAIARVARPYGQPNILAEYIALQLKNRVSFRKAMKKA
IELAEQADAKGIQVQIAGRLNGNEIARVEWIREGRVPLQTIRVKIDHCSYPVRTIYGVLGIKI
WIFLDEEMSRYRGPRFKKIRRLGALPGLTSKKPRSASDLRNQSRSGK-RSQYRIRLEEKQKL
RFHYGLTERQLLRYVRIAGKANGSTGQVLLQLLEMRLDNILFRLGMASTIPGARQLVNHG
HILVNGRLVDIPSYRCKPRDIITTKDKQGSRALIQNHMDSSSNE--LPKHLTLHS--FQYKGV
VNQIIDSKWVGLKVNELLIVEYYSRQTMSRRGTAEEKTAKSDPIYRNRLVNMLVNRILKH
GKKSLAYQIIYRAVKKIQQKTETNPLSVLRQAIRGVTPNIAVKARRVGGSTHQVPIEIGSTQ
GKALAIRWLLGASRKRPGRNMAFKLSSELVDAARGSGDAIRKKEETHRMAEANRAFAHF
RMGRDTIADIITYIRNADMDKKGTVRIPSTNITENIVKILLREGFIENVRKHRENNKDFLVLT
LRHRR--NRKRAYR---NIL--KRISRPGLRIYSNSQRIPRISGGIGVVILSTSRGIMTDREARLER
IGGEILFYIWMTKPIPKIGSRRNSRISSRKSGCRTPIGVIHVQASFNNTIVTVTDPQGRVVSW
SSAGTCGFKGTRRGTPFAAQTAAGNAIRAVVDQGMQRAEVMIKGPGLGRDAALRAIRRS

GILLNFVRDVTPMPHNGCRPPKKRRVMPTIKQLIRNTRQPMRNVTKSPALRGCPQRRGTC
TRVYTITPKKPNSALRKVARVRLTSGFEITAYIPGIGHNLQEHSVVLVRGGRVKDLPGVRYH
IVRGTLDAVGVKDRQQGRSKYGVKKPKMARKSLIQREKKRQKLEEKYHLIRRSSKKEIS--
-KVSSLDEKWEIHVKLQSPPRNSAPIRLHRRCFLTGRPRANYRDFGLSGHVLREMVHACLL
PGATRSSWMVKNSPISVIPQEEK-----RGSVEFQVFNFTNKIERLTSHLELHRRDYLSQRGLR
KILGKRQRLLVYLSKKNRVRYRELIGQLGIREPKTG--MVKLRLKRCGRRQRATYRIIAIDV
RSRREGRDLRKVGFYDPIKNQTYLNVPAILYFLEKGAQPTGTVHDISKKAEVFKELRVNQT
K---MEKFKRPFRKSKRSFRRRLPPIGLGDRIDYRNMSLISRFISEQGKILPRRVNRLTLKQQR
LITIAIKQARILSLLPFLNNEK--QFERAESISRTTGTRTRKKVAR-SKKNPFVANHLLSKIEKL
NKAEERSIIVTWSRASTIIFAMVGHTIAVHNGKEHLPIYITERMVGHKLGEFAPTLTFRGHA
RNDNRSRRMILKYSLLGNLLLLWINIVNSVVMVGLYYGFLTTFSIGPSYLLLLRTRVMKEG
---SEKEVSATTAFIMGQFIVFISTYYPPLHLALSRPHTLTVLVIPYLLSHFFWNNKKSLFDYR
STHGNFIPNLSIQYVFLNNLIFQLFNHFVLPSSTLTRLVDISMFRYNNKILFVTSSFFGWLIGH
MLLMKCIGLVLSWPWEK--MRS-FRSNKYLMSKWRNSVSQIFSILLFIICICYLGRMPSPIITK
KLKEVEEDKSLLSLLFDYQRWNRPLRYIENDRFSNAVRNEMSQSFFYTCASDGKQRISFTY
PPSLSTFFEMIKKKMSFRTAEKLPED--LSNWVSTTKKQRDNLRNEFINRIEAID--KLILNVV
EKRARLCNDEEEQECLPKFYDPLLNGPYRGTRKKGYLYTKTAWINKIHAILEREFEHED-P
ND-LPIEEIKKKVPRWLYKLTDLDFEEEEGIRSRKGKRVVIYTDALIRYSQQSDFRRDLIKGS
MRAQRRKTVTWEMFQTDAHSPLFFDRIDKTPLFSFDI--SRMMNLIFRNRQEEERIAIAETW
DNIIFAQAIRGSILVTHSILRKYIVLPSLIIVKNIGRMLLFQFPEWYEDFNEWSREMHVKCTY
NGVQLSETEFPKDWLTDGIQIKILFPFSLKPWHRSKRKKQKNFCFLTIWGMETELPFGSPR
KKPFFFEPIHKAFEKKIIKVK-KKGFFFLIIIKDNIKGFIKILNEKIRWIIKIVL--FIKIKV-----KE
DSKISNRIIHESPIRIVSRDWTNDSLIEIKINDLADKTTKIWDQVEKIRKDKKKSTNKGNCSN
KRTESRKHIWQISKKRSARLISKWDSFMKSFIERIYMSILLCITNIYRIYVQFFIP--FCDLSSL
SQAYVFYKLLQTQVSNKYKSESIFHYTYPFIKDGIKDYFTRGIFDSESKHKKLRNSGMSEW
KNWLKSHYQYNLSHDKWSRLAPLKWRKKVNQHRTIQNNPKSGSYE-EKDQLIHSGKKK
KIKKHYRYDLLSYKYLNKYTIDLDRKYFELRITNFDPRFFDWMGMNEQMLDRWFLPEFV
PLYDRYTTNPWIIPINLILFDFKSKKEAELDLFLKKYFIFQLRWDDPFNKGMNNNIKVYCLL
LRLMNPNEIAISSIQSGEMGLDVMLINRGFSLPELIKKGIFLIEPVRLSIKRDGISIMYQTIAIS
LTHKIQHNNYDLLVPEHILSPSHRRELRILSRFKFLWPTHRLEDLACMNRYWFDTNNGSRF
SMSRIHMYQMKRHQFKSCIFELREILREIKNSRYFLDSWTKFDSVVSFTHIFFHQERFVKLL
GPQTWSVLLS-RDS--QGSTSNRYF---TIKGVVLLVVA-VLI----YRINNRNMVERKNLYLMG
LL-----PIPMNSIGPRNDTLEESF-WSPNINRLIVS-LLYLPKGKKISESCFMDPKESTWVLPIT--
--QKCIMPESNWGSRWWRNWIGKKR--DSSCKIS---NETVAGIEISFKEKDIK-YLEFLFVSYM
D--DPIRKDHGWELFDRVSPRKKRNIINL--NSGQLFEILAKHLICYLMSAFREKRPIEVEG-F
LKQQG-AEATIPSNDIEHVSH--LFSRNKWDISLQNCAQFHMWQFHQDLFV---SWGK-NQH
ESDFLRN-VSRENLIWLDNMWLVNKDR---FFS--KVRNVSSNIQYDS---TR--SIFVQVTDSSQ
LKGSS-DQSRENFDSIS----NEDSE------------YHTLINQTEIQQLKERLIL-WD--PSSLQTERTE
IESD--RFPK--YPSGYS--SMSRLFT-EREKQMNNHLFPEKIEEFL--GNPTRSIRSFFSDRW--SE
LH-LVSNATERFTRDQ----KLLKKQQDVFSFVPSRRSE----KKEMVDIFKIITYLQN--TVSIHPI
SSDRGCDMVP-KDEPD-MDSSNKISFLNKNPFCD--LFHLFHDRNKGRYTL--HHDFESE--ER
FQEMADLFTLSITEP-DLVYHKGFAFSIDSYGLDQKK--FLNEVFNSRDESKKNSL---LVLPHI
FY--EENESFYRRIRKKWVR-ISCRNGLEDPKPKIVVFASNNIMEAVNQYRLIRNLIQIQYSTY
G--YIRNLSNRFFLM---NRSDRNFEYGIQ---RDQIGNDTLNHITIMKYMINQHLLNVKK-SQK

KWFD----PLISRTERSMNRDPNAYRYKWSNGSKNFQEHLEHFVSEQKN---RFQVVFDRLRI
NQHSIDWSKVIDKQDLSKSLHFFLSKS---------LLFLS-------KSL--------TFFFVSIGNIPIHRSE-
IHIY-ELKGPNDQLCNQLLESIGVQIVHLNKLKP--FLLD---DHDTS-QRSKFLINGGTISAFLF
NKIP-KW-MIDSLHT--RNNRRKFFDNTDSYFSMISHDRDNWLNPVKPFHRSSLISSFYKANL
LRFLNNPHHFCFY--SNKRFPFY----AEKTRIDNYD--LTY-GQFLHISFIRNK-IFSLCVGK--KK
HVFLE-RDTISPIESQVSDIFIPNDFPQSGDET-YNLYK---------------------------SQPLS---------T
YCQPLSDMNLSDSEGKNLHQYLS--FNSNMGLIHTPCSEKDLP--SAK--K--------RNLCLK--
KCVEKRQMYRAFQRDSAFSNLSKWNLFQTYMPWFLTSAGCKYINFILLDTFSDPL--PILSS
S-HQFVSFFYDIMHGSDISWSILQIPLW----AILPRWNL-ISEISSKCLQN--ILLPEEMIHRNNES
PVPLKWTHLRSPNAREF-LYSILFLLLVAGY-LVRTHLLFISR--VSSELQTELEKIKSLMIPSY
MIELRKLLDRYPPPELNSFWLKNLFLVALEQLGDSLEEIRSSASGGNMLLSGGP-----TYG-V
KSIRSKKKDLNINLIDIIYLI-SIIPNPINSITFSRNTRHLSRTSKEIYSLIR--KNVNSDWIDDQIE
SWVANSDLIDDEEREFLVQFS--TLTT--EKRIDQILLSLT-HSDHLS-----KNDSGYQMIEQPGSI
YLRYLVDIQ--KRYLMNYEFNRSCLAERRIFLAHYQTITY--SQTPCGANSSHFPSHG---KPFS
LRLALPPS-RGILVIGFIGTGRSYFVKYRVTNSYVPFITVFPNKFLDDN-KGYLID-----DID-ID
DRDDID----IDDRD-------------------DDDLDTELLTMPNVL---TMYMT---PKIDQFEITLPLELA
KAMSPCIIWIPNIHDLYVNESNYLSLGLLVNHLP---RDCERCS-TRNILVIASTHIPQKVDPAL
IAPNKSNTCIKIRRLLIPQQRKHFFILSYTRGFRLEKKMSHT--NGFGSITMGSNARDLVALTN
EVLSISITQKKSIIDTNTIRSALHRQTWDLR---SQVRSVQYHEILFYQIGRAVAQNVLLSNCSI
DPISIYMKKK--SCKEGDSYLSKWYFELGTSMKKLTILLYLLSCSAGSVAQDLWSPP--GPDE
Q-NWITSYGFVENDS-DLVHG---LESALVG--YSRTE--CSQFDND--RVTLLLRSEPRN-QLDM
MQNGSCSIVDQRFLYEK--YESGFEE-GEGEGTLDLQ----QIEEDE--DLFNH--IVWAP--RLWR
P---HGNLFDCIERPKKLGFP-YWVRSFR---DKRIIYHKEDKLQENDSEFLRSGTVQYQTRDR
SSKEQGFFRISQFIWDPADQFFFLFK-DQPFVSVFSRRESFADEEMSKGLITSQTNSPTSIYKR
WLI--KNTQEKHFELLIHRQRWLRTN-TNSSLS--NGSFRSN--TLSESYQYLSNLFLSNNGTLL
DQMTKTLLRKRWLFPDEMKHLI--HAPGERFPIPMPRSR-INGNFIDKTSSIVANILLRIIPTTS
GEKEAFTYYRDG--MSAQSEGNYAEALQNYYEAMRPEIDPYDRSYILYNIGLIHTSNGEHT
KALEYYFRALERNPFLPQASNNMAVICHY-RGEEAIRQGDSEIAEAWFDQAAEYWKQAIA
LTPGNYIEAQNWLKITGRFE--MNWRSEHIWIELITGSRKISNFCWACILFLGSLGFLLVGTS
SYFGRNLISLFPSQQIVFFPQGIVMCFYGIAGLFISSYLWCTISWSVGSGYDKFDRKEGIVCI
FRWGFPGRNRRIFFRFRIRDIRSIRIEVKEGLFPRRVLYMEIGGRGDIPLTRTDEN-LTPREIEQ
KAAESAYFLRVPIEVF
>Saccharum
???
???
???
???
???
???????????????????????????????MATLRVDEINKILRERIEQYNRKVGIENIGRVVQVGDGIA
RIIGLGEIMSGELVEFAEGTRGIALNLESKNVGIVLMGDGLMIQEGSFVKATGRIAQIPVSEA
YLGRVINALAKPIDGRGEIVASESRLIESPAPGIISRRSVYEPLQTGLIAIDSMIPIGRGQRELII
GDRQTGKTAVATDTILNQKGQDVICVYVAIGQRASSVAQVVTTFHEEGAMEYTIVVAEMA
DSPATLQYLAPYTGAALAEYFMYRERHTLIIYDDLSKQAQAYRQMSLLLRRPPGREAYPG
DVFYLHSRLLERAAKLNSLLGEGSMTALPIVETQSGDVSAYIPTNVISITDGQIFLSADLFN

AGIRPAINVGISVSRVGSAAQIKAMKQVAGKSKLELAQFAELQAFAQFASALDKTSQNQLA
RGRRLRELLKQSQSNPLPVEEQVATIYTGTRGYLDSLEIEQVKKFLDELRKHLKDTKPQFQ
EIISSSKTFTEQAETLLKEAIQEQLERFSLQEQTMRTNPTTSRPGVSTIEEKSVGRIDQIIGPV
LDITFPPGKLPNIYNALIVKSRDTADKQINVTCEVQQLLGNNRVRAVAMSATDGLMRGME
VIDTGTPLSVPVGGATLGRIFNVLGEPIDNLGPVDTSATFPIHRSAPAFIELDTKLSIFETGIK
VVDLLAPYRRGGKIGLFGGAGVGKTVLIMELINNIAKAHGGVSVFGGVGERTREGNDLY
MEMKESGVINEKNIEESKVALVYGQMNEPPGARMRVGLTALTMAEYFRDVNKQDVLLFI
DNIFRFVQAGSEVSALLGRMPSAVGYQPTLSTEMGSLQERITSTKKGSITSIQAVYVPADDL
TDPAPATTFAHLDATTVLSRGLASKGIYPAVDPLDSTSTMLQPRIVGNEHYETAQRVKETLQ
RYKELQDIIAILGLDELSEEDRLTVARARKIERFLSQPFFVAEVFTGSPGKYVGLAETIRGFQ
LILSGELDGLPEQAFYLVGNIDEASTKAINLEEESKLKKMKLNLYVLTPKRIIWDCEVKEIIL
STNSGQIGVLPNHAPINTAVDMGPLRIRLNDQWLTAVLWSGFARIVNNEIIILGNDAELGSDI
DPEEAQQALEIAEANLSKAEGTKELVEAKLALRRARIRVEAVNWIPMKNVTHSFVFLAHW
PFAGSFGLNTDILATNLINLTVVVGVLIFFGKGVLKDLLDNRKQRILSTIRNSEELRRGTLE
QLEKARIRLQKVELEADEYRMNGYSEIEREKENLINATSISLEQLEKSKNETLYFEKQRAM
NQVRQRVFQQAVQGALGTLNSCLNTELHFRTIRANIGILGAIEWKR-MNPLIAAASVIAAG
LAVGLASIGPGVGQGTAAGQAVEGIARQPEAEGKIRGTLLLSLAFMEALTIYGLVVALALLF
ANPFVMNITPCSIKTLKGLYDISGVEVGQHFYWQIGGFQIHAQVLITSWVVITILLGSVIIAV
RNPQTIPTDGQNFFEYVLEFIRDLSKTQIGEEYGPWVPFIGTMFLFIFVSNWSGALLPWKIIE
LPHGELAAPTNDINTTVALALLTSAAYFYAGLSKKGLSYFEKYIKPTPILLPINILEDFTKPLS
LSFRLFGNILADELVVVVLVSLVPLVVPIPVMFLGLFTSGIQALIFATLAAAYIGESMEGHH
MLFATLEHILTHISFSTISIVITIHLITLLVRELRGLRDSSEKGMIATFFSITGFLVSRWVSSGHF
PLSNLYESLIFLSWTLYILHTIPKIQNSKNDLSTITTPSTILTQGFATSGLLTEMHQSTILVPAL
QSQWLMMHVSMMLLSYATLLCGSLLSAALLIIRFRNSFDFFS-LKEIEYLYAKRSALKNTSF
PVFPNYYKYQLTERLDSWSYRVISLGFTLLTVGILCGAVWANEAWGSYWNWDPKETWAFI
TWTIFAIYLHSRTNPNWKGTNSALVASIGFLIIWICYFGINLLGIGLHSYGSFTLPMKKKKAL
PSFLYLVFIVLLPWGVSFSFNKCLELWIKNWWNTRQSETFLTDIQEKRILEGFIELEELFLLD
EMIKEKPKTHVQKLPIGIHKEIIQLAKIDNEDHLHIILHFSTNIICLAILSGSFFLGKEELVILN
SWVQEFFYNLNDSIKAFFILLVTDFFVGFHSTRGWELLIRWVYNNLGWAPNELIFTIFVCSF
PVILDTCLKFWVFFCLNRLSPSLVVIYHSISEMPIGVPKVPYRIPGDEEATWVDLYNVMYRE
RTLFLGQEIRCEITNHITGLMVYLSIEDGNSDIFLFINSPGGWLISGMAIFDTMQTVTPDIYTI
CLGIAASMASFILLGGEPTKRIAFPHARIMLHQPASAYYRARTPEFLLEVEELHKVREMITR
VYALRTGKPFWVVSEDMERDVFMSADEAKAYGLVDIVGDEPREAKVTFEGLVTEALPNG
MFRVRLENDTIILGYISGKIRSSSIRILMGDRVKIEVSRYDSSKGRIIYRLPHKDSKDMQKFE
GYSEKQKSRQHYFVYPLLFQEYIYAFAHDYGLFGCNNKFSSILVKRLIIRMYQQNFLINSV
NYPNQDRLFDSEFYSQILSEGFGIVVEIPLSLGQLSCPIPKFQNLQSIHSIFPFLEDKFLHLHY
LSHIEIPYPIHLEILVQLLEYRIQDVPSLHLLRFFLHYYSNWNSLITKSIFLLKKQRLFRFLYN
SYVSEYEFFLLFLRKQSSCLRLTSSGTFLERIIFSGKMEH--VMYPGRKTIWFFMDPLMHYV
RYQGKAILASKGTLLLKKKWKSYLVNFSQYFFYFWTQPQRIRLNQLTNSCFDFLGYLSSV
PINTLLVRNQMLENSFLIDTRM--KKFNTTVPATPLVGSLSKAQFCTGSGHPISKPVWTDLS
DWDILDRFGRICRNLFHYHSGSSKKQTLYRLKYILRLSCARTLARKHKSTVRTFMQRLGS
VFLEEFFTEEEQVFSLMFTKRIWYLDIIRINDLVNPLMIIDRV-EVE-TINSFSKSE-LLKEIYGL
IWIL-PILALLLGITIEVLVIVWLEREISASIQQRIGPEYAGPLGLLQAIADGTKLLLKEDILPS
RGEIPLFSIGPSIAVISILLSFLVIPLGYHFVLADLSIGVFLWIAISSIAPIGLLMAGYSSNNKYS

FSGGLRAAAQSISYEIPLTFCVLAISLLSNSSSTVDIVEAQSKYGFFGWNLWRQPIGFLVFLI
SSLAECERLPFDLPEAEEELVAGYQTEYSGIKYGLFYLVSYLNLLVSSLFVTVLYLGGWNFS
IPYISFFGFFQMNKIIGILEMVIGIFITLTKAYLFLFISITIRWTLPRMRMDQLLNLGWKFLLPI
SLGNLLLTTSSQLVSLMIWHVQNENFILDSTRIFMKAFHLLLFNGSFIFPECILIFGLILLLMI
DLTSDQKDRPWFYFISSTSLVISITALLFRWREEPIISFSGNFQTNNFNEIFQFLILLCSTLCIPL
SVEYIECTEMAITEFLLFVLTATLGGMFLCGANDLITIFVAPECFSLCSYLLSGYTKRDLRSN
EATMKYLLMGGASSSILVHGFSWLYGSSGGEIELQEIVNGLINTQMYNSPGISIALIFITVGL
GFKLSPAPFHQWTPDVYEGSPTPVVAFLSVTSKVAASALATRILDIPFYFSSNEWHLLLEIL
AILSMILGNLLAITQTSMKRMLAYSSIGQIGYVIIGIIVGDSNDGYASMITYMLFYISMNLGT
FACIVLFGLRTGTDNIRDYAGLYTKDPFLALSLALCLLSLGGLPPLAGFFGKLYLFWCGWQ
AGLYFLVSIGLLTSVLSIYYYLKIIKLLMTGRNQEITPYVRNYRRSPLRSNNSIELSMTVCVI
ASTIPGISMNPILAIAQDTLFMFLLHEYDIFWTFLIIASLIPILVFWISGLLAPVSEGPEKLSSY
ESGIEPMGGAWLQFRIRYYMFALVFVVFDVETVFLYPWAMSFDVLGVSVFIEAFIFVLILVV
GLVYAWRKGALEWSMSYFPWLTILVVLPIFAGSLIFFLPHKGNKIVRWYTIAICLLEFLIMT
YAFCYHFQLEDPLIQLKEDSKWIDVFDFHWRLGIDGLSLGSILLTGFITTLATLAAWPVTRN
SQLFYFLMLAMYSGQIGLFSSRDLLLFFIMWELELIPVYLLLSMWGGKRRLYSATKFILYT
AGGSIFFLIGVLGMGLYGSNEPGLDLERLINQSYPTTLEILLYFGFLIAYAVKLPIIPLHTWLP
DTHGEAHYSTCMLLAGILLKMGAYGLIRVNMELLPHAHYLFSPWLVIIGAVQIIYAASTSL
GQRNFKKRIAYSSVSHMGFIIIGIGSITNIGLNGAILQILSHGFIGATLFFLAGTACDRMRLVY
LEELGGISIPMPKIFTMFSSFSMASLALPGMSGFVAELVVFFGLITSPKFMLMPKMLITFVM
AIGMILTPIYLLSMLRQMFYGYKLFHVPNKNFVDSGPRELFLLICIFLPVIGIGIYPDFVLSLS
VDRVEVLLSNYYTKMMFEHVLFLSVYLFSIGIYGLITSRNMVRALICLELILNSINLNLVTF
SDLFDSRQLKGDIFAIFVIALAAAEAAIGLSILSSIHRNRKSTRINQSNFLNNMEHTYQYAW
VIPLLPLPVIMSMGFGLFLIPTATKNLRRIWAFPSILLLSIAMVFSLHLSIQQINGSSIYQYLWS
WTINNDFSLEFGYLVDPLTSIMLILITTVGILVLIYSDDYMSHDEGYLRFFVYISFFNTSMLG
LVTSSNLIQIYFFWELVGMCSYLLIGFWFTRPIAASACQKAFVTNRVGDFGLLLGILGFFWI
TGSLEFRDLFKIANNNGINSLLTTLCAFLLFLGAVAKSAQFPLHVWLPDAMEGPTPISALIH
AATMVAAGIFLLARLLPLFISLPLLMSFISLVGTITLFLGATLALAQRDIKRSLAYSTMSQLG
YMMLALGIGSYQAALFHLITHAYSKALLFLGSGSVIHSMEPLVGYSPDKSQNMVLMGGL
RKYVPITRTTFLCGTLSLCGIPPLACFWSKDEILSNSWLYSPFFGIIASFTAGLTAFYMFRIYL
LTFDGYLRVHFQNYSSTKEGSLYSISLWGKSISKGVNR-DFVLSTMKFFSAKK--TFVYPHET
GNTMLFPLLILLLFTLFIGSIGIPFELTILSKWLTPSINLFQENSNSSINSYEFLTNAISSVSLAIF
GLFIAYIFYGSAYSFFQNLNFQNSLVSFLDEVKKKIYSWSYNRGYIDFFYTRVFILGIRRLAE
LTHFFDKGVIDGIINGVGLAGFCIGEEIKY-VGGGRISSYLFFFLCYVSLFLFFIPMDLPGPIHE
ILVLFGGFVLLLGGLGVVLLTNPIYSAFSLGLVLVCISLFYFLLNSYFVAVAQLLIYVGAINV
LIIFAVMFVNGSEWSKDKNYWTIGDGFTLLLCITIPFSLMTTIPDTSWYGILWTTRSNQIVE
QGLINNVQQIGIHLATDFYLPFELISLILLVSLIGAITMARQMSLSLKRKDLMIVNMGPQHPS
MHGVLRLIVTLDGEDVIDCEPILGYLHRGMEKIAENRSIIQYLPYVTRWDYLATMFTEAIT
VNAPEFLENIQIPQRASYIRVIMLELSRIASHLLWLGPFMADLGAQTPFFYIFRERELIYDLF
EAATGMRMMHNYFRIGGVAADLPYGWMDKCLDFCDYFLQGVVEYQQLITQNPIFLERVE
GVGFISGEEAVNWGLSGPMLRASGIQWDLRKIDPYESYNQFDWKVQWQKEGDSLARYL
VRVGEMRESIKIIQQAVEKIPGGPYENLEARR-KKAKNPEWNDFEYRFLGKKPSPNFELSK
QELYVRVEAPKGELGIYLVGDDSLFPWRWKIRPPGFINLQILPQLVKKMKLADIMTILGSID
IIMGEVDRMFPMLTGFISYGQQTIRAARYIGQGLIITLSHTNRLPITIHYPYEKSITSERFRGRI

HFEFDKCIACEVCVRVCPIDLPLVDWKFEKDIKRKQLLNYSIDFGVCIFCGNCVEYCPTNC
LSMTEEYELSTYDRHELNYNQIALSRLPISIMGDYTIQTIRNSKIDEEKSWNSRTITDYQQG
WLSNWLVKHDVVHRSLGFDHRGVETLQIKAGDWDSIAVILYVYGYNYLRSQCAYDVAPG
GSLASVYHLTRIQYGIDNPEEVCIKVFAQKDNPRIPSVFWVWRSADFQERESYDMVGISYD
NHPRLKRILMPESWIGWPLRKDYITPNFYEIQDAH--
---------MSLIEFPLLDQTSSNSVISTTPNDLSNWSRLSSLWPLLYGTSCCFIEFASLIGSRFDFD
RYGLVPRSSPRQADLILTAGTVTMKMAPSLVRLYEQMPEPKYVIAMGACTITGGMFSTDS
YSTVRGVDKLIPVDVYLPGCPPKPEAVIDALTKLRKKIAREIIEDRTLCQSKNRSFTTRHKL
YVRRSTHTGTYEQELLYQSPSTLDIST--FFKSKSSVSSYKLVNMENRKTFSWLKEQMIRSIS
VSIMIYVITRTSISNAYPIFAQQGYENPREATGRIVCANCHLANKPVDIEVPQAVLPDTVFEA
VLRIPYDMQLKQVLANGKKGGLNVGAVLILPEGFELAPPDRISPELKEKIGNLSFQSYRPN
KKNILVIGPVPGKKYSEIVFPILSPDPATKKDVHFLKYPIYVGGNRGRGQIYPDGTKSNNTV
YNATSTGIVKKILRKEKGGYEISIVDASDGRQVIDIIPPGPELLVSEGESIKLDQPLTSNPNVG
GFGQGDAEIVLQDPLRVQGLLFFFASVILAQVFLVLKKKQFEKVQLYEMNFMSKVYDWFE
ERLEIQAIADDITSKYVPPHVNIFYCLGGITLTCFLVQVATGFAMTFYYRPTVTEAFSSVQYI
MTEANFGWLIRSVHRWSASMMVLMMILHVFRVYLTGGFKKPRELTWVTGVVLAVLTASF
GVTGYSLPWDQIGYWAVKIVTGVPEAIPVIGSPLVELLRGSASVGQSTLTRFYSLHTFVLPL
LTAVFMLMHFPMIRKQGISGPLMGVTKKPDLNDPVLRAKLAKGMGHNYYGEPAWPNDLL
YIFPVVILGTIACNVGLAVLEPSMIGEPADPFATPLEILPEWYFFPVFQILRTVPNKLLGVLL
MVSVPTGLLTVPFLENVNKFQNPFRRPVATTVFLIGTAVALWLGIGATLPIDKSLTLGLFMIE
VFLFGIVLGLIPITLAGLFVTAYLQYRRGDQLDLMLTITSYFGFLLAALTITPALFIGLNKIRLI
MDIVSLAWAALMVVFTFSLSLVVWGRSGLMIIRPSEPEVKIAVDRDPVKTSFEEWARPGHF
SRTIAKGPDTTTWIWNLHADAHDFDSHTGDLEEISRKVFSAHFGQLSIIFLWLSGMYFHGA
RFSNYEAWLSDPTHIGPSAQVVWPIVGQEILNGDVGGGFRGIQITSGFFQIWRASGITSELQ
LYCTAIGALIFASLMLFAGWFHYHKAAPKLAWFQDVESMLNHHLAGLLGLGSLSWAGHQ
IHVSLPINQFLDAGVDPKEIPLPHEFILNRDLLAQLYPSFAEGATPFFTLNWSKYAEFLSFRG
GLDPITGGLWLSDIAHHHLAIAILFLIAGHMYRTNWGIGHGLKDILEAHKGPFTGQGHKGL
YEILTTSWHAQLSLNLAMLGSTTIVVAHHMYSMPPYPYLATDYGTQLSLFTHHMWIGGFL
IVGAAAHAAIFMVRDYDPTTRYNDLLDRVLRHRDAIISHLNWVCIFLGFHSFGLYIHNDTM
SALGRPQDMFSDTAIQLQPIFAQWIQNIHAGAPGVTAPGATTSTSLTWGGGELVAVGGKVA
LLPIPLGTADFLVHHIHAFTIHVTVLILLKGVLFARSSRLIPDKANLGFRFPCDGPGRGGTCQ
VSAWDHVFLGLFWMYNSISVVIFHFSWKMQSDVWGTISDQGIVTHITGGNFAQSSITING
WLRDFLWAQASQVIQSYGSSLSAYGLFFLGAHFVWAFSLMFLFSGRGYWQELIESIVWAH
NKLKVAPATQPRALSIIQGRAVGVTHYLLGGIATTWAFFLARIIAVGMELRFPRFSQGLAQD
PTTRRIWFGIATAHDFESHDDITEERLYQNIFASHFGQLAIIFLWTSGNLFHVAWQGNFESWI
QDPLHVRPIAHAIWDPHFGQPAVEAFTRGGAAGPVNIAYSGVYQWWYTIGLRTNEDLYTG
ALFLLFLSTLSLIGGWLHLQPKWKPSLSWFKNAESRLNHHLSGLFGVSSLAWTGHLVHVA
IPGSRGEYVRWNNFLDVLPYPQGLGPLLTGQWNLYAQNPDSSNHLFGTTQGAGTAILTLL
GGFHPQTQSLWLTDIAHHHLAIAFIFLIAGHMYRTNFGIGHSIKDLLEAHTPPGGRLGRGH
KGLYDTINNSIHFQLGLALASLGVITSLVAQHMYSLPAYAFIAQDFTTQAALYTHHQYIAGF
IMTGAFAHGAIFFIRDYNPEQNEDNVLARMLDHKEAIISHLSWASLFLGFHTLGLYVHNDV
MLAFGTPEKQILIEPIFAQWIQSAHGKTTYGFDILLSSTNGPAFNAGRNIWLPGWLNAVNE-
-NSNSLFLTIGPGDFLVHHAIALGLHTTTLILVKGALDARGSKLMPDKKDFGYSFPCDGPGR
GGTCDISAWDAFYLAVFWMLNTIGWVTFYWHWKHITLWQGNVSQFNESSTYLMGWLR

DYLWLNSSQLINGYNPFGMNSLSVWAWMFLFGHLVWATGFMFLISWRGYWQELIETLAW
AHERTPLANLIRWRDKPVALSIVQARLVGLAHFSVGYIFTYAAFLIASTSGKFGMSHSVKIY
DTCIGCTQCVRACPTDVLEMIPWDGCKAKQIASAPRTEDCVGCKRCESACPTDFLSVRVY
LGPETTRSMALSYMTDLNLPSIFVPLVGLVFPAIAMTSLFLYVQKNKIVMRDIKTYLSVAPV
L S T L W F G A L A G L L I E I N R L F P D A L S F P F F -
-???
??
??
???M
GLPWYRVHTVVLNDPGRLLSVHIMHTALVSGWAGSMALYELAVFDPSDPVLDPMWRQG
MFVIPFMTRLGITNSWGGWSIS-GGTVTNPGIWSYEGVAGAHIVFSGLCFLAAIWHWVYW
DLEIFCDERTGKPSLDLPKIFGIHLFLAGVACFGFGAFHVTGLYGPGIWVSDPYGLTGKVQA
VNPAWGAEGFDPFVPGGIASHHIAAGTLGILAGLFHLSVRPPQRLYKGLRMGNIETVLSSSI
AAVFFAAFVVAGTMWYGSATTPIELFGPTRYQWDQGYFQQEIYRRVSDGLAENLSLSEAW
SKIPEKLAFYDYIGNNPAKGGLFRAGSMDNGDGIAVGWLGHPVFRDKEGRELFVRRMPTF
FETFPVVLVDEEGIVRADVPFRRAESKYSVEQVGVTVEFYGGELNGVSYSDPATVKKYAR
RAQLGEIFELDRATLKSDGVFRSSPRGWFTFGHATFALLFFFGHIWHGARTLFRDVFAGIDP
DLDAQVEFGTFQKVGDPTTRRQAAMKILYSLRRFYHVETLFNGTFVLAGRDQETTGFAW
WAGNARLINLSGKLLGAHVAHAGLIVFWAGAMNLFEVAHFVPEKPMYEQGLILLPHLATL
GWGVGPGGEVLDTFPYFVSGVLHLISSAVLGFGGIYHALLGPETLEESFPFFGYVWKDRN
KMTTILGIHLILLGLGAFLLVLKALYFGGVYDTWAPGGGDVRKITNLTLSPGVIFGYLLKSP
FGGEGWIVSVDDLEDIIGGHVWLGSICVLGGIWHILTKPFAWARRAFVWSGEAYLSYSLA
ALSVFGFIACCFVWFNNTAYPSEFYGPTGPEASQAQAFTFLVRDQRLGANVGSAQGPTGL
GKYLMRSPTGEVIFGGETMRFWDLRAPWLEPLRGPNGLDLSRLKKDIQPWQERRSAEYM
THAPLGSLNSVGGVATEINAVNYVSPRSWLATSHFVLGFFFFVGHLWHAGRARAAAAGFE
KGIDRDLEPVLYMTPLNMTIAVGRVTKEENDLFDIMDDWLRRDRFVFVGWSGLLLFPCAY
FALGGWFTGTTFVTSWYTHGLASSYLEGCNFLTAAVSTPANSLAHSLLLLWGPEAQGDFT
RWCQLGGLWTFVALHGAFALIGFMLRQFELARSVQLRPYNAISFSGPIAVFVSVFLIYPLGQ
SGWFFAPSFGVAAIFRFILFFQGFHNWTLNPFHMMGVAGVLGAALLCAIHGATVENTLFE
DGDGANTFRAFNPTQAEETYSMVTANRFWSQIFGVAFSNKRWLHFFMLFVPVTGLWMSA
IGVVGLALNLRAYDFVSQEIRAAEDPEFETFYTKNILLNEGIRAWMAAQDQPHENLIFPEE
VLPRGNALMSGSTGERSFADIITSIRYWVIHSITIPSLFIAGWLFVSTGLAYDVFGSPRPNEY
FTESRQGIPLITDRFDSLEQLDEFSRSFMTIDRTYPIFTVRWLAVHGLAVPTVFFLGSISAMQ
FIQRMATQTVEDSSRPK-PKRTGAGSLLKPLNSEYGKVAPGWGTTPFMGVAMALFAIFLSII
LEIYNSSVLLDGILTNMLTLKLFVYTVVIFFVSLFIFGFLSNDPGRNPGREEMADTTGRIPLW
LIGTVTGILVIGLIGVFFYGSYSGLGSSLMPNILSLTCICFNSVLCPTSFFFAKLPEAYAIFNPIV
DVMPVIPVLFFLLAFVWQAAVSFRMTQSNPNEQNVELNRTSLYWGLLLIFVLAVLFSNYFF
NMEVNILAFIATALFILVPTAFLLIIYVKTASQNDMETATLVAISISGLLVSFTGYALYTAFGQP
SQQLRDPFEEHGDMEALVYTFLLVSTLGIIFFAIFFREPPKVPTKK--MTIAFQLAVFALIATSS
VLVISVPLVFASPDGWSNNKNVVFSGTSLWIGLVFLVAILNSLIS-------MSPQTETKASVGFK
AGVKDYKLTYYTPEYETKDTDILAAFRVTPQLGVPPEEAGAAVAAESSTGTWTTVWTDGL
TSLDRYKGRCYHIEPVPGDPDQYICYVAYPLDLFEEGSVTNMFTSIVGNVFGFKALRALRL
EDLRIPPAYVKTFQGPPHGIQVERDKLNKYGRPLLGCTIKPKLGLSAKNYGRACYECLRG
GLDFTKDDENVNSQPFMRWRDRFVFCAEAIYKAQAETGEIKGHYLNATAGTCEEMIKRA

VFAKELGVPIVMHDYLTGGFTANTTLSHYCRDNGLLLHIHRAMHAVIDRQKNHGMHFRV
LAKALRMSGGDHIHSGTVVGKLEGEREITLGFVDLLRDDFIEKDRSRGIFFTQDWVSMPG
VIPVASGGIHVWHMPALTEIFGDDSVLQFGGGTLGHPWGNAPGAAANRVALEACVQARN
EGRDLAREGNEIIKAACKWSAELAAACEIWKEIKFDFKAMDTL--TAKHLYKTPIPSTRKGT
VDRQVKSNPRNKLIHGRHRCGKGRNARGIITARHRGGGHKRLYRKIDFRRNQKDISGRIV
TIEYDPNRNAYICLIHYGDGEKRYILHPRGAIIGDTIVSGTKVPISMGNALPLTDMPLGTAIH
NIEITRGRGGQLARAAGAVAKLIAKEGKLATLRLPSGEVRLVSQNCLATVGQVGNVGVNQ
KSLGRAGSKCWLGKRPVVRGVVMNPVDHPHGGGEGKAPIGRKKPTTPWGYPALGRRTR
KRKKYSDSFILRRRK-MIQPQTLLNVADNSGARKLMCIRVIGAAGNQRYARIGDVIIAVIKD
AVPQMPLERSEVIRAVIVRTRKEFKGDDGIIIRYDDNAAVIIDQKGNPKGTRVFGAVAEELR
ELNYTKIVSLAPEVLMLSPKRTRFRKQHRGRMNGKSCRGNHICFGRYALQVLEPAWITAR
QIEAGRRAMTRYARRGGKIWVRIFPDKPVTIRPTETRMGSGKGSPEYWVAVVKPGRILYE
MSGVSETVARAAISIAASKMPIRSQFLRLEMTRVPRGYIARRRRTKMRSFASNFRGAHLRL
NRMITQQVRRAFVSSHRDRGRQKRDFRRLWITRINAATRVY--VFNSYSKLIHNLSKKELIL
NRKMLAQVAVSNPNNLYTISNKIRTIN---------RIKRVFKAQKRIHMSVFKAQRVLDEIRWR
YYEETVMILNLMPYRASYPILKLVYSAAANATHYRDFDKTNLFITKAEVSRSTIMKKFRPR
ARGRSYSIKKTMCNITIVLN-VKKS-------MDGIKYAVFTEKSLRLLGKN-QYTFNVESGFTK
TEIKHWVELFFGVKVVAVNSHRLPGKGRRMGPILGHTMHYRRMIITLQPGYSIPLLDRETN
MAVPKKRTSMSKKRIRKNLWKKKTYFSIVQSYSLAKSFSSGKPKGFSGQQANKMAKGKD
VRIRVILECISCVRK--G--KESTGISRYSTQKNRHNTPGQLELRKFCRYCRKHTTHNEIKKM
KIRASVRKICTKCRLIRRRGRIRVICSNPKHKQRQGMVREEITGSTQTLEWKCVESRVDSK
RLYYGRFILSPLRKGQADTVGIALRRALLGEIEGTCITRAKFW--NVPHEYSTIVGIEESIQEI
LLNLKEIVLRSNLYGVRDASICVKGPRYITAQDIILPPSVEIVDTTQPIANLREPVDFCIELQI
KRDRGYHTELRKNSQDGSY--PIDAVFMPVRNVNYSIFSCGNGNEKHEILFLEIWTNGSLTP
KEALYEASRNLIDLFLPFIHTEEE--GTSFEENK-NRLTPPLLTFQKRFT-NLKKNKK-GIPLNCI
FIDQLELPSRTYNCLKRANIHTLLDLLSKTEEDLMRINSFRMEDGKLIWDTLEKH--LPIDLP
KNKMLRNGN-EGMSTIPGFSQIQFEGFCRFINQGLAEELEKFPTIKDPDHEIAFQLFAKGYQ
LLEPSIKERNAVYESLTYSSELYVSARLIFGF--DVQKQTISIGNIPIMNSLGTFIINGIYRIVIN
QILLSPGIYYRSELDHKGISICTGTIISDWGGRSELAIDKKERIWARVSRKQKISILVLSSAMG
SNLREILDNVSYPEIFLSFPNAKEKKRIESKEKAILEFYQQFACVGGDLVFSESLCEELQKKF
FQQKCELGRVGRRNMNRRLNLDIPQNNTFLLPRDVLAATDHLIGMKFGTGILDDDDMNH
LKNKRIRSVADLLQDQFGLALGRLQHAVQKTIRRVFIRQSK--PTPQTLVTPTSTSILLITTYE
TFFGTYPLAQVFDQTNPLTQTVHGRKVSCLGPGGLTGRTASFRSRDIHPSHYGRICPIDTSE
GINVGLTGSLAIHARIDHWWGSIESPFYEISEKAKEKKE--RQVVVYLSPNRDEYYMIAAGNS
LSLNQGIQEEQVVPARYRQEFLTIAWEQIHVRSIFPFQYFSIGGSLIPFIEHNDANRALMSSN
MQRQAVPLSRSEKCIVGTGLERQTALDSRVSVIAEREGKIISSDSHKILLSSSGKTISIPLVAH
RRSNKNTCMHQKPRVPRGKSIKKGQILAEGAATVGGELALGKNVLVAYMPWEGYNFEDA
VLISERLVYEDIYTSFHIRKYEIQTDTTSQGSAEKITKQIPHLEEHLLRNLDRNGVVRLGSW
VETGDILVGKLTPQIASE-SSYIAEAGLLRAIFGLEVSTSKETSLKLPIGGRGRVIDVKWIQR--
-----DPIMVRVYILQKREIKVGDKVAGRHGNKGIISKILPRQDMPYLQDGTPVDMVFNPLGV
PSRMNVGQIFESSLGLAGDLLKKHYRIAPFDERYEQEASRKLVFSELYEASKQTKNPWVFE
PEYPGKSRIFDGRTGDPFEQPVLIGKSYILKLIHQVDEKIHGRSTGPYSLVTQQPVRGRAKQ
GGQRIGEMEVWALEGFGVAHILQEILTYKSDHLIARQEILNATIWGKRVPNHEDPPESFRVL
VRELRSLALELNHFLVSEKNFRVNREDV-------MIDQYKHKQLQIGLVSPQQIKAWAKKILP

NGEVVGEVTRPSTFHYKTDKPEKDGLFCERIFGPIKSGICACGNSRASVAENEDERFCQKC
GVEFVDSRIRRYQMGYIKLACPVTHVWYLKGLPSYIANLLDKPLKKLEGLVYGD--FSFAR
P-SAKKPTFLRLRGLFEDEISSCNHSISPFFSTPGFATFRNREIATGAGAIREQLADLDLRIIIEN
SLVEWKELEDE-GYSGDEWEDRKRRIRKVFLIRRMQLAKHFIQTNVEPEWMVLCLLPVLP
PELRPIVYRSGDKVVTSDINELYKRVIRRNNNLAYLLKRSELAPADLVMCQEKLVQEAVDT
LLDSGSRGQPMRDGHNKVYKSLSDVIEGKEGRFRETLLGKRVDYSGRSVIVVGPSLSLHQ
CGLPLEIAIKLFQLFVIRDLITKRATSNVRIAKRKIWEKEPIVWEILQEVMRGHPVLLNRAP
TLHRLGIQAFQPTLVEGRTICLHPLVCKGFNADFDGDQMAVHLPLSLEAQAEARLLMFSH
MNLLSPAIGDPICVPTQDMLIGLYVLTIGNRRGICANRYNSCGN-----SPNKKINYNNYKYTK
DKEPHFSSSYDALGAYRQKRIGLNSPLWLRWKLDQR-IVGS--REVPIEVQYESFGTYHEIY
AHYLVVGNRKKEIRSIYIRTTLGHISFYREIEEAVQGFSRAYSYTI-----------MAERANLVFH
NKEIDGTAIKRLISRLIDHFGMGYTSHILDQIKTLGFHQATTTSISLGIEDLLTIPSKGWLVQD
AEQQSFLLEKHYYYGAVHAVEKLRQSVEIWYATSEYLKQEMNSNFRITDPSNPVYLMSFS
GARGNASQVHQLVGMRGLMADPQGQMIDLPIQSNLREGLSLTEYIISCYGARKGVVDTAV
RTADAGYLTRRLVEVVQHIIVRRRDCGTIQGISVSPQNGMTE--KLFVQTLIGRVLADDIYIG
SRCIASRNQDIGIGLVNRFITAFRAQPFRAQPIYIRTPFTCRSTSWICQLCYGRSPTHGDLVEL
GEAVGIIAGQSIGEPGTQLTLRTFHTGGVFTGGTADLIRSPSNGKIQFNEDLVHPTRTRHGQP
AFLCYIDLHVTIQSQDILHSVNIPLKSLILVQNDQYVESEQVIAEIRAGMSTL--FKEKVQKHI
YSESDGE-MHWSTDVYHAPEYQYGNLRRLPKTSHLWILSVSMCR--SSIASFSLHKDQDQM
NTYSFSVDGR--YIFDFS--MAN-DQVSHRLLDTFG----DREILDYL-TPDRIVSNGHWNCFYP
S-ILQDN--SDLLAKKRRNRFVVPLQYHQEQEKERISCLGISMEIPFM--GVLRRNTIFAYFDD
PRYRKD--GIVKFRSPEENSILK--KEGFIEHRG-TKEFSLKY-EVDRFFFILQELHILPRSSSLK
VLDNSIIGVDTQLTKNTRSRLGGLVRVKRKKSHTELKIFSGDIHFPEEADKILG--GSLIPPER
EKK--DSKE-SK-KRKNWVYVQRKKILKSKEK--YFVSVRPAVAYEMDEGRNLATLFPQDLL
QEEDNLQLRLVNFISHENSKLTQRIYHTN-QFVRTCLVVNWEQEEKEGAR-------ASLVEVR
TNDLIRDFLRIELVKSTISYTRRRYDRTSVGLIPN---LDRNNTNSFYSKAKIQS-----LSQHQE
VIG----TLLNRNK---EYPSLMILLASNCSRIG--LFKNSKYP---NAVKESNPRIPI-DIFGLLGVIV
P-SISNFSSS-YYLLT---HNQILLK-KYLFLD---NLKQTFQVLQGLKYSLIDENKR--ISNFD--SN
IMLEPFHLNWHFLHHDSWEETLAIIH-LGQFICENLCLFKSHI----KKSGQIFIVN-MDSFVLR
AAKPYLATIGATVHGHYGKILYKGDRLVTFIYEKSRSSDITQGLPKVEQIFEARSIDSLSPNL
ERRIEDWNERIPRILGVPWGFLIGAELTIAQSRISLVNKIQKVYRSQGVQIHNRHIEIIIRQVT
SKVRVSE---DGMSNVFLPGELIGLLRAERAGRALDESIYYRAILLGITRASLNTQSFISEASF
QETARVLAKAALRGRIDWLKGLKENVVLGGIIPVGTGFQKFVHRSP--QDKNLY--FEIQKK
NLFASEM--RDILFLHTELVSSDSDVTN---SETPFTPIYTI------MTRRYWNINLKEMIEAGVH
FGHGIKKWNPKMAPYISAKRKGTHITNLARTARFLSEACDLVFDAASQGKSFLIVGTKKR
AADLVASAAIRSRCHYVNKKWFSGMLTNWSITKTRLSQFRDLRAEEKMGKFHHLPKRDA
AILKRKLSTLQRYLGGIKYMTRLPDIVIVLDQQKEYIALRECAILGIPTISLVDTNCDPDLAN
ISIPANDDTMTSIRLILNKLVFAISEGRSL-YIRNRMGQKINPLGFRLGTTQNHHSFWFAQPK
NYSEGLQEDKKIRNCIKNYIQKNRKKGSNKMESITHIEIQKEIDTIHVIIHIGFPN-LLKKKG--
---AIEELEKDLQKEVNSVNQRLNIAIEKVKEPYRQPNILAEYIAFQLKNRVSFRKAMKKAIE
LTKKADIKGIKIQIAGRLAGKEIARAECIKKGRLPLQTIRAKIDYCCYPIRTIYGVLGVKIWI
FVEEEMSRYRGPRLKKIRRLGALPGLTRKTPKSGSNQKKKFHSGK-KEQYRIRLQEKQKLR
FHYGLTERQLLRYVHIAGKAKRSTGQVLLQLLEMRLDNILFRLGMASTIPGARQLVNHRH
ILVNGRIVDIPSFRCKPRDIITTKDNQRSKRLVQNYIASSDPK--LPKHLTVDT--LQYKGLVK

KILDRKWVGLKINELLVVEYYSRQTMSRRGTAEKRTAKSDPIFRNRLVNMVVNRIMKDG
KKSLAYQILYRAVKKIQQKTETNPLLVLRQAIRRVTPNIGVKTRRKKGSTRKVPIEIGSKQG
RALAIRWLLEASQKRPGRNMAFKLSSELVDAAKGSGGAIRKKEATHRMAEANRALAHFR
MGKDTIADLLTSIRNADMNKKGTVRVVSTNITENIVKILLREGFIESVRKHQESNRYFLVST
LRHQRRKTRKGIYRT---RTFLKRISRPGLRIYANYQGIPKVLGGMGIAILSTSRGIMTDREAR
LNRIGGEVLCYIWMTKAIPKIGSRKKVRIGLRKSARRITKGVIHVQASFNNTIITVTDPQGR
VVFWSSAGTCGFKSSRKASPYAGQRTAVDAIRTVG---LQRAEVMVKGAGSGRDAALRAIA
KSGVRLSCIRDVTPMPHNGCRPPKKRRLMPTVKQLIRNARQPIRNARKSAALKGCPQRRG
TCARVYTINPKKPNSALRKVARVRLTSGFEITAYIPGIGHNLQEHSVVLVRGGRVKDLPGVR
YRIIRGTLDAVAVKNRQQGRSKYGAKKPKMAKKSLIQREKKRHKLEQKYHLIRRSSKKKI
RSKVSPLSLSEKTKMQEKLQSLPRNSAPTRLHRRCFLTGRPRANYRDFGLSGHILREMVYA
CLLPGATRSSW---------MVKEEKQEN--RGSVEFQVFSFTNKIRRLASHLELHKKDFSSERGL
RRLLGKRQRLLAYLAKKNRVRYKKLISQLDIREK-----MVKLRLKRCGRKQQAIYRIVAIDV
RSRREGRDLRKVGFYDPIKNQTCLNVPAILYFLEKGAQPTRTVYDILRKAEFFKDKERTLS-
---FHKSKQPFRKFKQPFRKRRSRIGPGDRIDYRNMSLINRFISEQGKILSRRINRLTLKQQRLI
TLAIKQARILSFLPFRNYENEKQFQ-AQAISIITGPRHRKNVTRK-KTNPFVARHLLAKIEKV
NMKEEKEIIVTWSRASSILPAMVGHTIAIHNGKEHIPIYITNPMVGRKLGEFVPTRHFTSYT
R K D T K S R
R??
???
???
???
???
???
???
???
???
???
???
???
???
???
???
???
???
???
???
???
???
???
???
???
???

??
??
??
??
??
??
??
??
??
??
??
??
??
??
??
??
??
???MPRSR-INGNFIDKTSSIVANILLQIIPTTSGEK
RAFTYYRDG--MLAQSEGNYAEALQNYYEATRLEIDPYDRSYILYNIGLIHTSNGEHTKALE
YYFRALERNPFLPQAFNNMAVICHY-RGEQAILQGDSEIAEAWFDQAAEYWKQAIALTPG
NYIEAQNWLKITKRFEFEMNWRSEHIWIELLKGSRKRGNFFWACILFLGSLGFLAVGASSY
LGKNMISVLPSQQILFFPQGVVMSFYGIAGLFISSYLWCTILWNVGSGYDRFDRKEGIVCIF
RWGFPGIKRRIFLQFLVRDIQSIRIQVKEGLYPRRILYMEIRGQGVIPLTRTDEKFFTPREIEQ
KAAELAYFLRVPIEVF
>Panax
MERWWFNSILFLSKSTGSLGPIENTSESEDPNINDMKKNIHSYSNVDHLFGVKDIRNFISDE
TFLVRDSNGNSYSIYFDIENHIFEIDNGH--SFQSELESSFYSYQNSSYRNNGSNSDDPHYDH
YMYDTQYSWNNNINSCIDNYLQ--SQIFIETDIVSGSDNYSNSYIYSSICG--GSGIRTRTDGS
DLTIRESSNDLDVTQKYRHLWVQCENCYGLNYKKSFKSKMNLCEQCGYHLKMSSSDRIE
LLIDPGTWDPMDEDMVSLDPIEFHSEEEPYKDRVDSYQRKTGLTEAVQTGIGQLNSIPVAIG
VMDFQFMGGSMGSVVGEKITRLIEYATKKFLPLIIVCASGGARMQEGSLSLMQMAKISSA
LYDYQSNKKLFYVPILTSPTTGGVTASFGMLGDIIIAEPNAYIAFAGKRVIEQTLNKTVPEGS
QAAEYLFQKGLFDLIVPRNPLKSVLSELFQLHAFFPLNQN---MVTIRADEISNIIRERIEQYN
REVKIVNTGTVLQVGDGIARIHGLDEVMAGELVEFEEGTVGIALNLESNNVGVVLMGDG
LLIQEGSSVKATGRIAQIPVSEAYLGRVVNALAKPIDGRGEISASEYRLIESPAPGIISRRSVY
EPLQTGLIAIDSMIPIGRGQRELIIGDRQTGKTAVATDTILNQQGQNVICVYVAIGQKASSVA
QVVTTFQERGAMEYTIVVAEMADSPATLQYLAPYTGAALAEYFMYRERHTSIIYDDPSKQ
AQAYRQMSLLLRRPPGREAYPGDVFYLHSRLLERAAKLSSRLGEGSMTALPIVETQSGDV
SAYIPTNVISITDGQIFLSADLFNAGIRPAINVGISVSRVGSAAQIKAMKQVAGKLKLELAQF
AELEAFAQFASDLDKATQNQLARGQRLRELLKQSQSAPLTVEEQIMTIYTGTNGYLDLLEI
GQVRKFLVELRTYLKTNKPKFQEIISSTKTFTEEAEALLKEAIQEQTDRFILQEQAMRINPT
TSGSGVSTLEKKNLGNITQIIGPVLDIAFPPGKMPNIYNALVVKDRDTVGQQINVTCEVQQ
LLGNNRVRAVAMSATDGLTRGMEVIDTGAPLSVPVGGTTLGRIFNVLGEPVDNLGPVDTR
TTSPIHRSAPAFIQLDTKLSIFETGIKVVDLLAPYRRGGKIGLFGGAGVGKTVLIMELINNIA

KAHGGVSVFGGVGERTRGGNDLYMEMKESGVINEQNIAESKVALVYGQMNEPPGARMR
VGLTALTMAEYFRDVNEQDVLLFIDNIFRFVQAGSEVSALLGRMPSAVGYQPTLSTEMGT
LQERITSTKEGSITSIQAVYVPADDLTDPAPATTFAHLDATTVLSRGLAAKGIYPAVDPLDST
STMLQPRIVGEEHYETAQRVKQTLQRYKELQDIIAILGLDELSEEDRLTVARARKIERFLSQ
PFFVAEVFTGSPGKYVGLAETIRGFQLILSGELDGLPEQAFYLVGNIDEATAKAMNLEMES
NLKKMTLNLCVLTPNRTVWDSKVNEIILSTNNGQIGVLPDHASIATAVDIGILRIRLNDQWL
TMALMGGFARIGNNEITVLVNDAEKSSDIDSQEAQQTLEIAEANLRKAEGKRQKIEANLA
LRRARTRVETINAISMKNVTDSFVSLGHWPSAGSFGFNTDILATNLINLSVVLGVLIFFGKG
VLSDLLDNRKQRILNTIRNSEELREGAIEQLEKARARLRKVEMEADQFRVNGYSEIEREK
WNFINSTSKTLDQLENYKNETIHFEQQRAINQVRQRVFQQALQGALGTLSGCLNNELHLR
TISANIGILGAMKEITDMNPLISAASVIAAGLAVGLASIGPGVGQGTAAGQAVEGIARQPEA
EGKIRGTLLLSLAFMEALTIYGLVVALALLFANPFVMNVLSCSINTLKGLYDISGVEVGQHF
YWKIGGFQVHGQVLITSWVVIAILLGSATIAVRNPQTIPTGGQNFFEYVLEFIRDVSKTQIG
EEYGPWVPFIGTMFLFIFVSNWSGALLPWKIIQLPHGELAAPTNDINTTVALALLTSVAYFY
AGLTKKGLGYFGKYIQPTPILLPINILEDFTKPLSLSFRLFGDILADELVVVVLVSLVPSVVPI
PVMFLGLFTSGIQALIFATLAAAYIGESMEGHHMIFSTLEHILTHISFSIVSIVITIHLITLLVDE
IIKLYDSSEKGMIAIFLCITGLLVTRWIYSRHFPLSDLYESLIFLSWSLSVIHIVPY--KKKKNLS
TITASSVIFTQGFATSGLLTEIHQSAILVPALQSEWLIMHVSMMILSYAALLCGSLLSVALLVI
TFRKNRNIFC--KEIQYMNERNNVLRTT----AKNYYRSQLIQQLDYWSYRVISLGFIFLTIGIL
SGAVWANEAWGSYWNWDPKETWAFITWIVFAIYLHTRTNIKLRGANSAIVASIGFLIIWIC
YFGVNLLGIGLHSYGSFTLTMAKKKAFTPLLYLASLVFLPWWISLSFNKSLESWVTNWWN
TRQSEIFLNGIQEKNILEKFIELEEILLLEEMIKEYSETHLQNLRI-IHKETIQFIKIHNEDRIHTI
LHFSTNIICFVILSGYSIWGNEELVILNSWAQEFLYNLSDTIKAFSILLLTDLCIGFHSPHGWE
LIIGSVYKDFGFVHNDQILSGLVSTFPVILDTLFKFWIFRYLNRVSPSLVVIYHSMNDMPIGV
PKVPFRSPGEEDAYWVDIYNRLYRERLLFLGQPVASEISNQLIGLMVYLSIEDDTKDLYLF
MNSPGGWVLPGIAIYDTMQFVRPDVHTICMGLAASMGSFILAGGEITKRLAFPHA-VMIH
QPASSFYEAQTGEFVLEAEELLKLRETITRVYVQKTGKPFWVVSEDLERDVFMSATEAQA
YGIVDLVAVEMKEQKWIHEGLITESLPNGMFRIRLDNEDMILGYVSGKIRRSFIRILPGDRV
KIEVSRYDSTRGRIIYRLRNKNSKDMEEFQRYLE-DRSQQHYFLYPLIFQEYIYALAHDHGL
AGYDNKFSLLIVKRLIARMYQQNHLILSTNDSNQNRFLRKNLYSQMISEGFAVIVEIPFYLQ
LKSSLIVKSHNLRSIHSIFPFLEDKISHLIYGLEILIPYPVHLEILVQTLRYWVKDASSLHLLRF
FLHEYCNWN---TKAGSSFSKQRLFFLLYNSHLCEYESIFIFLRNQSSHLRSTSSGTLLERIYF
YGKIKYLVKVFVKAFNLLLLKDPFMHYVRYQGKSILASKGTPFLMKKWTYYFVNLWQC
HFYLWSQPGRICINQLYNHSLDILGYLSSARLNPSMVRGQMLENSFLIDNAI--NKFDAIVPII
PLIGSLAKAKFCNVLGHPISKAVWTDLSDSDIIDQFGRICRNLSHYHSGSSQKKSLYRIKYIL
RLSCARTLARKHKSTVRAFLKRLGSGLLEEFFTAEEQVLYLTFPRRIWYLDIICINDLANHE
MIID-TTEVQ-AINSFFRLES-LKEVYGIIWMLIPIFTPVLGITIGVLVIVWLEREISAGIQQRIGP
EYAGPLGILQALADGTKLLFKENLLPSRGDTRLFSIGPSVAVTSILLSYLVIPFGYHLVLADL
SIGVFLWIAISSIAPVGLLMSGYGSNNKYSFLGGLRAAAQSISYEIPLTLCVLSISLLSNSSST
VDIVEAQSKYGFWGWNLWRQPIGFIIFLISSLAECERLPFDLPEAEEELVAGYQTEYSGIKF
GLFYVASYLNLLVSSLFVTVLYLGGWNLSIPYIPVPDIFEINKASRVFGTITGIFITLAKTYLF
LFIPITTRWTLPRLRMDQLLNLGWKFLLPISLGNLLLTTSSQLLSLMIWHVQNENFILDSTRI
FMKAFHLLLFDGSLIFPECILIFGLILLLMIDSTSDQKDIPWLYFISSTSLVMSITALLFRWREE
PMISFSGNFQTNNFNEIFQFLILLCSTLCIPLSVEYIECTEMAITEFLLFVLTATLGGMFLCGA

NDLITIFVAPECFSLCSYLLSGYTKKDVRSNEATMKYLLMGGASSSILVHGFSWLYGSSGG
EIELQEIVNGLINTQMYNSPGISIALIFITVGIGFKLSPAPSHQWTPDVYEGSPTPVVAFLSVT
SKVAASASATRIFDIPFYFSSNEWHLLLEILAILSMILGNLIAITQTSMKRMLAYSSIGQIGYV
IIGIIVGDSNDGYASMITYMLFYISMNLGTFACIVLFGLRTGTDNIRDYAGLYTKDPFLALSL
ALCLLSLGGLPPLAGFFGKLYLFWCGWQAGLYFLVLIGLLTSVVSIYYYLKIIKLLMIGRNQ
EITPHVRNYRRSPFRSNNSIELSMIVCVIASTIPGISMNPIIAIAQDTLFMFLLYEYDIFWAFLII
SSLIPILAFFISGVLAPISKGPEKLSSYESGIEPMGNAWLQFRIRYYMFALVFVVFDVETVFL
YPWAMSFDVLGVSVFIEALIFVLILIVGLVYAWRKGALEWSTNFFPWLTIIVVLPIFAGSVIF
FLPHRGNRVIRWYTICICILELLLTTYAFCYHFQFDDPLIQLVEDYKWINFFAFHWRLGIDGL
SIGPILLTGFITTLATLAARPVTRDSRLFHFLMLAMYSGQIGSFSSRDLLLFFIMWEFELIPVY
LLLSIWGGKRRLYSATNFILYTAGGSIFLLMGVLGVGLYGSNEPTLNFETSVNQSYPVALEII
FYIGFFIAFAVKSPIIPLHTWLPDTHGEAHYSTCMLLAGILLKMGAYGLIRINMELLPHAHSI
FSPWLVIVGTIQIIYAASTSPGQRNLKKRIAYSSVSHMGFILIGIGSITDTGLNGAILQIISHGFI
GAALFFLAGTSYDRIRLVYLDEMGGIAIPMPKIFTMFTSFSMASLALPGMSGFVAELIVFFG
IITSQKYLLMPKILITFVMAIGMILTPIYSLSMLRQMFYGYKLFNAPNSYVFDSGPRELFVSI
SIFLPVIGIGMYPDFVLSLSVDKVEVILSNFFYRMMLEHVLVLSAYLFSVGLYGLITSRNMV
RALMCLELILNAVNINFVTFSDFFDSRQLKGAIFSIFVIAIAAAEAAIGLAIVSSIYRNRKSIRI
NQSNLLNKMEQTYQYAWIIPFLPLPVPMLIGVGLLLFPTATKNLRRMWAFQSVLLLSIVMI
FSINLSIQQINSSSIYQYVWSWIINNDFSLEFGYLIDPLTSIMSILITTVGIVVLVYSDNYMSH
DQGYLRFFAYMSFFSTSMLGLVTSSNLIQIYIFWELVGMCSYLLIGFWFTRPVAANACQKA
FVTNRVGDFGLLLGILGFYWITGSFEFRDLFKIFSNNEVNFLFVTLCAVLLFAGAVAKSAQF
PLHVWLPDAMEGPTPISALIHAATMVAAGIFLVARLIPLFIVIPHIMNLISLVGIITVLLGATL
ALAQKDIKRGLAYSTMSQLGYMMLALGMGSYRSALFHLITHAYSKALLFLGSGSVIHSM
ETLVGYSPKKSQNMVFMGGLTKHVPITKTSFLFGTLSLCGIPPLACFWSKDEIINDTWLYSP
IFAIIAWSTAGLTAFYMFRIYLLTFEGHLNVHFQNY---KNTPFYSISLWGKEGSKRINK-LLTL
LTMNFFSFGNKNIFSYPYESDNTMLFPILVLVLFTLFIGFLGIPFNLDILSKWLTPSINLLHQN
LN-LIDWDEFAKDAIFSVSIAFFGIFIASFLYKPVYSSLQNLDLINSFVIFLDKIINGIYGWSCN
RGYIDAFYATFFTGGIRGLAELTSFFDRQVIDGITNGVGVLSFFVGESIKSVGGG-RISSYLFL
YLSYVSIFLLIYYMDLPGPIHDFLLVFLGSGLILGGLGVVLLPNPIYSAFSLGLVLVCTSLFYI
LSNSHFVAAAQLLIYVGAINVLILFAVMFMNGSEYYKDFHLWTIGDGVTSMVCTSIFVSLIT
TIPDTSWYGIIWTTKSNQIIEQDLISNSQQIGIHLSTDFFLPFELISIILLVALIGAIAVARQMTA
SATRNDLMIVNLGPQHPSMHGVLRLIVTLDGEDVIDCEPILGYLHRGMEKIAENRTIIQYLP
YVTRWDYLATMFTEAITVNAPEQLGNIQVPKRASYIRVIMLELSRIASHLLWLGPFLADIG
AQTPFFYIFRERELIYDLFEAATGMRMMHNYFRIGGVAADLPHGWIDKCLDFCDFFLTRVT
EYQKLITRNPIFLERVEGVGIIGGAEAINWGLSGPMLRASGIQWDLRKVDHYESYDEFDW
GVQWQKEGDSLARYLVRISEMTESIKIIQQALEGIPGGPYENLEIRRFDKVRDPEWNDFDY
RFISKKPSPTFELSKQELYVRVEAPKGELGIFLIGDRGVFPWRWKIRPPGFINLQILPQLVKR
MKLADIMTILGSIDIIMGEVDRMFSMVTEFMNYGQQTVRAARYIGQGFMITLSHANRLPV
TIQYPYEKLITSERFRGRIHFEFDKCIACEVCVRVCPIDLPVVDWKLEMDIRKKRLLNYSID
FGICIFCGNCVEYCPTNCLSMTEEYELSTYDRHELNYNQIALGRLPMAVIDDYTTRTILNL--
--------------MQGRLSAWLVKHGLIHRSLGFDYQGIETLQIKPEDWHSIAVILYVYGYNYLRS
QCAYDVAPGGLLASVYHLTRIEYGVDQPEEVCIKVFAPRRNPRIPSVFWVWKSVDFQERE
SYDMLGIFYATHPRLKRILMPESWVGWPLRKDYIAPNFYEIQDAH-----------------------------
---------------------------MNSIEFPLLDRTTQNSVISTTSNDLSNWSRLSSLWPLLYGTSCCFIEF

ASLIGSRFDFDRYGLVPRSSPRQADLILTAGTVTMKMAPSLVRLYEQMPEPKYVIAMGACT
ISGGMFSTDSYSTVRGVDKLIPVDVYLPGCPPKPEAVIDAITKLRKKISREIYEDRIKSQREN
RCFTTNHKFQVGRSIHTGNYDRGFLYQPPFTSEIPSETFFKYKSSVSSHELVNMQTRKTFS
WIKEQITRSISASLMIYIITRTSISSAYPIFAQQGYENPREATGRIVCANCHLANKPVDIEVPQ
AVLPDTVFEAVVRIPYDMQLKQVLANGKKGALNVGAVLILPEGFELAPVDRISPEMKERIG
NLSFQSYRPTKKNILVIGPVPGQKYSEITFPILSPDPATKKDVHFLKYPIYVGGNRGRGQIYP
DGSKSNNTVYNATAAGIVGKIIRKEKGGYEITITDASDGRQVVDIIPPGPELLVSEGESIKFD
QPLTSNPNVGGFGQGDAEIVLQDPLRVQGLLFFLASVILAQIFLVLKKKQFEKVQLSEMNF
MSKVYDWFEERLEIQAIADDITSKYVPPHVNIFYCLGGITLTCFLVQVATGFAMTFYYRPT
VTDAFASVQYIMTEANFGWLIRSVHRWSASMMVLMMILHVFRVYLTGGFKKPRELTWVT
GVVLAVLTASFGVTGYSLPRDQIGYWAVKIVTGVPEAIPVIGSPLVELLRGSASVGQSTLTR
FYSLHTFVLPLLTAVFMLMHFPMIRKQGISGPLMGVTKKPDLNDPVLRAKLAKGMGHNY
YGEPAWPNDLLYIFPVVILGTIACNVGLAVLEPSMIGEPADPFATPLEILPEWYFFPVFQILRT
VPNKLLGVLLMVSVPAGLLTVPFLENVNKFQNPFRRPVATTVFLIGTAVALWLGIGATLPID
KSLTLGLFMIEVFLFGIVLGLIPITLAGLFVTAYLQYRRGDQLDLMPTITSYFGFLLVALTITS
ALFIGLSKIRLIMDIVSLAWAALMVVFTFSLSLVVWGRSGLMIIRSPEPEVKILVDRDHIKTS
FEEWARPGHFSRTLAKGPDTTTWIWNLHADAHDFDSHTSDLEEISRKVFSAHFGQLSIIFL
WLSGMYFHGARFSNYEAWLSDPTHIGPSAQVVWPVVGQEILNGDVGGGFRGIQITSGFFQ
IWRASGITSELQLYCTAIGALIFAALMLFAGWFHYHKAAPKLAWFQDVESMLNHHLAGLL
GLGSLSWAGHQVHVSLPINQFLNAGVDPKEIPLPHEFILNRDLLAQLYPSFAEGATPFFTLN
WSKYADFLTFRGGLDPVTGGLWLTDIAHHHLAIAILFLIAGHMYKTNWGIGHGLKDILEA
HKGPFTGQGHKGLYEILTTSWHAQLSLNLAMLGSLTIVVAHHMYSMPPYPYLATDYGTQL
SLFTHHMWIGGFLIVGAAAHAAIFMVRDYDPTTRYNDLLDRVLRHRDAIISHLNWACIFL
GFHSFGLYIHNDTMSALGRPQDMFSDTAIQLQPVFAQWIQNTHALAPGATAPGATTSTSLT
WGGGDLVAVGGKVALLPIPLGTADFLVHHIHAFTIHVTVLILLKGVLFARSSRLIPDKANLG
FRFPCDGPGRGGTCQVSAWDHVFLGLFWMYNAISVVIFHFSWKMQSDVWGSISDQGVV
THITGGNFAQSSITINGWLRDFLWAQASQVIQSYGSSLSAYGLFFLGAHFVWAFSLMFLFS
GRGYWQELIESIVWAHNKLKVAPATQPRALSIVQGRAVGVTHYLLGGIATTWAFFLARIIA
VGMALRFPRFSQGLAQDPTTRRIWFGIATAHDFESHDDITEERLYQNIFASHFGQLAIIFLW
TSGNLFHVAWQGNFESWVQDPLHVRPIAHAIWDPHFGQPAVEAFTRGGALGPVNIAYSGV
YQWWYTIGLRTNEDLYTGALFLLFLSTISLIAGWLHLQPKWKPSVSWFKNAESRLNHHLS
GLFGVSSLAWTGHLVHVAIPASRGEYVRWNNFLDVLPHPQGLGPLFTGQWNLYAQNPDSS
SHLFGTSQGAGTAILTLLGGFHPQTQSLWLTDMAHHHLAIAFIFLVAGHMYRTNFGIGHSM
KDLLDAHIPPGGRLGRGHKGLYDTINNSLHFQLGLALASLGVITSLVAQHMYSLPAYAFIA
QDFTTQAALYTHHQYIAGFIMTGAFAHGAIFFIRDYSPEQNEDNVLARMLDHKEAIISHLS
WASLFLGFHTLGLYVHNDVMLAFGTPEKQILIEPIFAQWIQSAHGKTSYGFDVLLSSTNGP
AFNAGRSIWLPGWLNAVNE--NSNSLFLTIGPGDFLVHHAIALGLHTTTLILVKGALDARGS
KLMPDKKDFGYSFPCDGPGRGGTCDISAWDAFYLAVFWMLNTIGWVTFYWHWKHITLW
QGNVSQFNESSTYLMGWLRDYLWLNSSQLINGYNPFGMNSLSVWAWMFLFGHLVWATG
FMFLISWRGYWQELIETLAWAHERTPLANLIRWRDKPVALSIVQARLVGLAHFSVGYIFTY
AAFLIASTSGKFGMSHSVKIYDTCIGCTQCVRACPTDVLEMIPWDGCKAKQIASAPRTEDC
VGCKRCESACPTDFLSVRVSLWHETTRSMGLGYMTTFDFPSVLVPLVGLVFPAMAMASLS
LHVQKNKTVMRDLKTYLSVAPVLSTLWFGSLAGLLIEINRFFPDALTFPFF--MTAILERRES
ESLWGRFCNWITSTENRLYIGWFGVLMIPTLLTATSVFIIAFIAAPPVDIDGIREPVSGSLLYG

NNIISGAIIPTSAAIGLHFYPIWEAASVDEWLYNGGPYELIVLHFLLGVACYMGREWELSFR
LGMRPWIAVAYSAPVAAAAAVFLIYPIGQGSFSDGMPLGISGTFNFMIVFQAEHNILMHPFH
MLGVAGVFGGSLFSAMHGSLVTSSLIRETTENESANEGYRFGQEEETYNIVAAHGYFGRLI
FQYASFNNSRSLHFFLAAWPVVGIWFTALGISTMAFNLNGFNFNQSVVDSQGRVINTWADI
INRANLGMEVMHERNAHNFPLDLAAVEAPSTNGMGLPWYRVHTVVLNDPGRLLSVHIM
HTALVAGWAGSMALYELAVFDPSDPVLDPMWRQGMFVIPFMTRLGITNSWGGWSVT-GG
AIPNPGIWSYEGVAGAHIVFSGLCFLAAIWHWVYWDLEIFSDERTGKPSLDLPKIFGIHLFL
AGVACFGFGAFHVTGLYGPGIWVSDPYGLTGKVQSVNPAWGVEGFDPFVPGGIASHHIAA
GTLGILAGLFHLSVRPPQRLYKGLRMGNIETVLSSSIAAVFFAAFVVAGTMWYGSATTPIEL
FGPTRYQWDQGYFQQEIYRRVSAGLAENQSLSEAWSKIPEKLAFYDYIGNNPAKGGLFRA
GSMDNGDGIAVGWLGHPIFRDKEGRELFVRRMPTFFETFPVVLVDGDGIVRADVPFRRAE
SKYSVEQVGVTVEFYGGELNGVSYSDPATVKKYARRAQLGEIFELDRATLKSDGVFRSSP
RGWFTFGHASFALLFFFGHIWHGARTLFRDVFAGIDPDLDAQVEFGAFQKLGDPTTRRQV
VMKTLYSLRRFYPVETLFNGTLALAGRDQETTGFAWWAGNARLINLSGKLLGAHVAHAG
LIVFWAGGMNLFEVAHFVPEKPMYEQGLILLPHLATLGWGVGPGGEVIDTFPYFVSGVLH
LISSAVLGFGGIYHALLGPETLEESFPFFGYVWKDRNKMTTILGIHLILLGIGAFLLVFKALY
FGGVYDTWAPGGGDVRKITNLTLSPSIIFGYLLKSPFGGEGWIVSVDDLEDIIGGHVWLGSI
CILGGIWHILTKPFAWARRALVWSGEAYLSYSLGALSVFGFIACCFVWFNNTAYPSEFYGP
TGPEASQAQAFTFLVRDQRLGANVGSAQGPTGLGKYLMRSPTGEVIFGGETMRFWDLRA
PWLEPLRGPNGLDLSRLKKDIQPWQERRSAEYMTHAPLGSLNSVGGVATEINAVNYVSPR
SWLATSHFVLGFFLFVGHLWHAGRARAAAAGFEKGIDRDFEPVLSMTPLNMTIALGKFTE
DEKDLFDIMDDWLRRDRFVFVGWSGLLLFPCAYFALGGWFTGTTFVTSWYTHGLASSYL
EGCNFLTAAVSTPANSLAHSLLLLWGPEAQGDFTRWCQLGGLWTFVALHGAFGLIGFMLR
QFELARSVQLRPYNAIAFSGPIAVFVSVFLIYPLGQSGWFFAPSFGVAAIFRFILFFQGFHNW
TLNPFHMMGVAGVLGAALLCAIHGATVENLYFEDGDGANTFRAFNPTQAEETYSMVTAN
RFWSQIFGVAFSNKRWLHFFMLFVPVTGLWMSALGVVGLALNLRAYDFVSQEIRAAEDP
EFETFYTKNILLNEGIRAWMAAQDQPHENLIFPEEVLPRGNALMSGNTGERSFADIITSIRY
WVIHSITIPSLFIAGWLFVSTGLAYDVFGSPRPNEYFTENRQGIPLITGRFDPLEQLDEFSRSF
MTIDRTYPIFTVRWLAVHGLAVPTVSFLGSISAMQFIQRMATQTVENGSR-SRPKPTTVGNL
LKPLNSEYGKVAPGWGTTPLMGVAMALFAIFLSIILEIYNSSVLLDEISMNMLTLKLFVYTV
VIFFVSLFIFGFLSNDPGRNPGREEMADTTGRIPLWIIGTVAGILVIGLIGIFFYGSYSGLGSSL
MLNIFSLICICLNSALYSSSLFFAKLPEAYAFLNPIVDLMPVIPLFFFLLAFVWQAAVSFRMT
QSNPNEQNVELNRTSLYWGLLLIFVLAVLFSNYFFNMEVNILAFIATALFILVPTAFLLIIYV
KTESQNKMETATLVAIFISGLLVSFTGYALYTAFGQPSQQLRDPFEEHGDMEALVYTFLLVS
TLGIIFFAIFFREPPKVPTKK--MTLAFQLAVFALIATSSILLIGVPVVFASPDGWSSNKNVVFS
GTSLWIGLVFLVGILNSLIS-------MSPQTETKASVGFKAGVKDYKLTYYTPDYDPKDTDILA
AFRVTPQPGVPAEEAGAAVAAESSTGTWTTVWTDGLTSLDRYKGRCYKIEPVAGEETQFIA
YVAYPLDLFEEGSVTNMFTSIVGNVFGFKALRALRLEDLRIPVAYVKTFQGPPHGIQVERD
KLNKYGRPLLGCTIKPKLGLSAKNYGRAVYECLRGGLDFTKDDENVNSQPFMRWRDRFL
FCAEALYKAQAETGEIKGHYLNATAGTCEDMMKRAVFARELGVPIVMHDYITGGFTANTT
LAHYCRDNGLLLHIHRAMHAVIDRQKNHGMHFRVLAKALRMSGGDHIHSGTVVGKLEG
ERDITLGFVDLLRDDFIEKDRSRGIYFTQDWVSLPGVLPVASGGIHVWHMPALTEIFGDDS
VLQFGGGTLGHPWGNAPGAVANRVALEACVQARNEGRDLAREGNEIIREAAKWSPELAA
ACEVWKEIKFEFEAVDILDVMAIHLYKTSTPSTRNRAVDSQVKSNPRNNLIYGQHHCGKG

RNARGIITAGHRGGGHKRLYRKIDFRRNEKDIYGRIVTIEYDPNRNAYICLIHYGDGEKRYI
LHPRGAIIGDTIVSGTEVPIKMGNALPLTDMPLGTAIHNIEITRGKGGQLARAAGAVAKLIA
KEGKSATLKLPSGEVRLISKNCSATVGQVGNVGVNQKSLGRAGSKRWLGKRPVVRGVV
MNPVDHPHGGGEGRAPIGRKKPTTPWGYPALGRRSRKRNKYSDNLILRRRSKMIQPQTLL
NVADNSGARELMCIRIIGAS-NRRYAHIGDVIVAVIKEAVPNMPLARSEVVRAVIVRTCKEL
KRDNGMIIRYDDNAAVVIDQEGNPKGTRVFGAIARELRQLNFTKIVSLAPEVLMLSPKRTR
FRKQHRGRMKGISYRGNHISFGKYALQALEPAWITSRQIEAGRRAMTRNARRGGKIWVRI
FPDKPVTVRPAETRMGSGKGSPEYWVAVVKPGRILYEMGGVTENIARKAISIASSKMPIRT
QFIISAMTRIRRGYIARRRRTKIRLFASSFRGAHSRLTRTITQQKIRALVSSHRDRDRQKRNF
RRLWITRINAVIREG-GVSYSYSRLIHDLYKKQLLLNRKILAQIAISNKNCLYMISNEIIKEVD
WKESTGIIMLKTVYALGQYLSMSAHKARRVIDQIRGRSYEETLMILELMPYRACYPIFKLV
YSAAANASYNMDSNESNLVISKAEVSEGTIVKKLKPRARGRSFPIKRPTCHISIVVKDISLD
GGVWDKKMDGIKYAVFTDKSIRLLGKN-QYTSNVESGSTRTEIKHWVELFFGVKVIAMNS
HRLPGRGRRMGPIMGQTMHYRRMIITLQPGYSIPPLRKKRTMAVPKKRTSISKKRIRKNIW
KRKGYWAALKALSLGKSLSTGNSKGFFVRQTNKMAKGKDVRITVILECTSCVRNGVN--K
VSTGISRYITQKNRHNTPNRLELRKFCPYCYKHTIHGEIKKMKIRASVRKICEKCRLIRRRG
RIIVICSNPRHKQRQGMVQEKVRVSTRTLQWKCVEARADSKRLYYGRFILSPLMKGQADT
IGIAMRRALLGEIEGTCITRAKSE--KIPHEYSTLVGIQESVHEILMNLKEIILKSNLYGTCDAS
ICIRGPGYVTAQDIISPPYVEIVDNTQHIASLAEPIDLCIGLQIERNRGYLIKTPNNNFDGSY--
PIDAVFMPVRNANHSIHSYGNGNEKQEILFLEIWTNGSLTPKEALHQASRNLIDLFIPFLHT
EEE--NLHLADNQ-HTVPLPPFTFHEKLT-KLRKNKK-KIALKSIFIDQSELPPRIYNCLIRSNIY
TLLDLLNNSQEDLMKIEHFRIEDVKAILGILEKH--FAIDLPKKLMLRDGKNEGMSTIPGFN
QIQFEGFCRFIDQGLTEELYKFPKIEDTDQEIEFQLFVETYQLVEPLIKERDAVYESLTYSSEL
YVSAGLIWKTGRDMQEQTIFFGNIPLMNSLGTSIVNGIYRIVINQVLQSPGIYYRSELDHNG
ISVYTGTIISDWGGRSELEIDRKARIWARVSRKQKISILVLSSAMGSNLREILENVCYPEIFLS
FLTDKEKKKIGSKENAILEFYQQFACVGGDPVFSESLCKELQKKFFQQRCELGRIGRRNMN
RRLNLDISQNNTFLLPRDILAAADHLIGMKFGMGTLDD--MNHLKNKRIRSVADLLQDQFG
LALVRLENVVRGTICGALR-HKL-IPTPQNLVTSTP----LTTTYESFFGLHPLSQVLDRTNPLT
QIVHGRKLSYLGPGGLTGRTASFRIRDIHPSHYGRICPIDTSEGINVGLIGSLAIHARIGRWG-
SLESPFY---EISERSKG--ARMLYLSPGKDEYYMVAAGNSLALNQGIQEEQVVPARYRQEFL
TIAWEQVHLRSIFSFQYFSIGASLIPFIEHNDANRALMSSNMQRQAVPLSRSEKCIVGTGLE
RQAAIDSGALAIAEHEGKIIYTDTDKILLSGNGNTLSIPLVIYQRSNKNTCMHQKPQVQRG
KCIKKGQILAHGAATVGGELALGKNVLVAYMPWEGYNFEDAVLISERLVYEDIYTSFHIRK
YEIKTHVTSQG-PERVTNEIPHLEAHLLRNLDKNGIVMLGSWVETGDILVGKLTPQMVKE-
SSYAPEDRLLRAILGIQVSTSKETCLKLPIGGRGRVIDVRWIQKRG-GSSYNPEMIRVYISQK
REIKVGDKVAGRHGNKGIISKILPRQDMPYLQDGRPVDMVFNPLGVPPRMNVGQIFECSL
GLAGGLLDRHYRIAPFDERYEQEASRKLVFSELYEAGKQTANPWVFEPEYPGKSRIFDGRT
GDPFEQPVIIGKPYILKLIHQVDDKIHGRSSGHYALVTQQPLRGRAKQGGQRVGEMEVWA
LEGFGVAHILQEMLTYKSDHIRARQEVLGTTIIGGTIPNPQDAPESFRLLVRELRSLALELN
HFFVSEKNFQINRKEAMNQNFSSMIDRYKHQQLRIGSVSPQQISTWANKILPNGEIVGEVT
KPYTFHYKTNKPEKDGLFCERIFGPIKSGICACGNYRVIGNEKEDPKFCEQCGVEFVDSRIR
RYQMGYIKLACPVTHVWYLKRLPSYIANLLDKPLKELEGLVYCD--FSFARP-IAKKPTFLR
LRGSFEYEIQSWKYSIPLFFTTQGFDTFRNREISTGAGAIREQLADLDLRIIIDSSLVEWKEL
GED-GPTGNEWEDRKVGRRKDFLVRRMELAKHFIRTNIEPEWMVLCLLPVLPPELRPIIQID

GGKLMSSDINELYRRVIYRNNTLTDLLTTSRSTPGELVMCQEKLVQEAVDTLLDNGIRGQP
MRDGHNKVYKSFSDVIEGKEGRFRETLLGKRVDYSGRSVIVVGPSLSLHRCGLPREIAIEL
FQTFVIRGLIRQHLASNIGVAKSKIREKEPIVWEILQEVMQGHPVLLNRAPTLHRLGIQAFQ
PVLVEGRAICLHPLVRKGFNADFDGDQMAVHVPLSLEAQAEARLLMFSHMNLLSPAIGDP
ISVPTQDMLIGLYVLTSGNRRGICVNRYNPCNR---RNYQNERIYDNNYKYTK--EPFFCNSY
DAIGAYRQKRINLDSPLWLRWRLDQR-VISA--KEAPLEVHYESLGTYYEIYGQYLIVRSIK
KEILYIYIRTTVGHISLYREIEEAIQGFCQACSYGP-----------MAERANLVFHNKVIDGTAMK
RLISRLIEHFGMAYTSHILDQVKTLGFQQATATSISLGIDDLLTIPSKRWLVQDAEQQSLILE
KQHHYGNVHAVEKLRQSIEIWYATSEFLRQEMKPNFRMTDPFNPVHIMSFSGARGNASQV
HQLVGMRGLMADPQGQMIDLPIQSNLREGLSLTEYIISCYGARKGVVDTAVRTSDAGYLT
RRLVEVVQHIVVRRKDCGTVRGISVSPRNGMMP-ERVFIQTLIGRVLADDIYLGPRCIATRN
QDIGSGLVNRFIT------FRAQPIYIRTPFTCRSTSWICRLCYGRSPTHGDLAELGEAVGIIAGQ
SIGEPGTQLTLRTFHTGGVFTGGTAEHVRAPSNGKIKFNEDLVHPTRTRHGHPAFLCSIDLY
VTIESEDIIHNVN?PPKSFLLVQNDQYVESEQVIAEIRAGTST-LNFKEKVRKHIYSDSEGE-M
HWNTDVYHAPAFTYGNVHLLPKTSHLWILLGEPCR--SDLVALSIHKDQDQMNAH--SLSV
KRRYISNLS--VTNDQ-ARHKFFSSDFSGK-EDRITDY-SELNR---IGHCNLPYL-AILHANS--D
LLAKRRRNRFIIPLQSIQEHENELMPSSGISIEIP--IHGIFRKKSIIAYFDDPRYRRKSSGITKY
GTIEVHSIVKKED--LIEYRG-VKEFRPKYMKVDRFFFIPEEVHILPGSSSIMVRNNSIIGVDT
QITLTTRSRVGGLVRVERKKKRIELKIFSGDIHFPGETDKISRHSGVLIPPGT--GKTNSKESK
K--WKNWIYVQR--ITPTK--KKYFVLVRPVVTYEITDGINLA-LFFPDLLQERDNVQLRVVN
YILYGTGKPIRGFYDTSIQLVRTCLVLNWVQDKK---SSSIEEARTSFVEIRINSLIRDFLKIDL
AKSPILYTGKRKDPSGSGLISE-NGSDRTNINPFSSSFFY---SKARIKESLNQNQGTIHTLLNR-
--NKECQSLIILSSSNCFRMGP--FNDVKYHNV---IKES-PLIQIRNSLGPLGT--ALQIANFYS--F
SHL--ITYNQIL-VTNYLQLDNLK---QSFQVLK---YYLMDEN--GKIYNPDRCCNIILNPFNLN
WFFLHHNYCEETS-TTMSLGQFICENVCIAKNGPH-LK--PGQVLIVQ-VDSVVIRSAKPYLA
TPGATVHGHYGEILYEGDTLVTFIYEKSRSGDITQGLPKVEQVLEVRSIDSISMNLEKRVEG
WNECITRNLGIPWGFLIGAELTIVQSRISLVNKIQKVYRSQGVQIHNRHIEIIVRQITSKVLVS
E---DGMSNVFLPGEFIGLLRAERMGRALEEAICYQAVLLGITKASLNTQSFISEASFQETAR
VLAKAALRGRIDWLKGLKENVVLGGMIPVGTGFKGL--VPPSRQHKNIP--LETKNKNLFE
GEMR--DILFHHRKFFDSCLSKNFHDTPEQSFIG------FNDSMTKRYWNINLEEMMKAGVH
FGHGTKKWNPKMAPYISAKRKGIHITNLTRTARFLLEACDLVFDAASRGKQFLIVGTKNK
AANLVAWAAIKARCHYVNKKWLGGMLTNWSTTETRLHKFRDLRTEQKTGRLNRLPKRD
AAMLKRQLSHLQTYLGGIKYMTGLPDIVIIVDQHEEYTALRECITLGIPTICLIDTNCDPDL
ADISIPANDDAI?SIRLILNKLVFAICEGRS-SYIRNPMGQKINPLGFRLGTTQGHHSLWFAQP
KNYSEGLQEDQKIRDFIKNYVQKNMKISSS-VEGIARIEIQKRIDLIQVIIYMGFPKLLIESRP
R----GIEELQMNLQKEFKSVNQKLNIAITRIAKPYGSPNILAEFIAGQLKNRVSFRKAMKKAI
ELTEQADTKGIQVQIAGRIDGKEIARVEWIREGRVPLQTIRAKIDYCSYTVRTIYGVLGIKI
WIFIDGEMSRYRGPRFKKIRRLGALPGLTNKRPRAGSDLRNQSRSGK-KSQYRIRLEEKQK
LRFHYGLTERQLLKYVRIAGKAKGSTGQVLLQL?EMRLDNILFRLGMAPTIPGARQLVNH
RHILVNGRIVDIASYRCKPRDIITARDEENSRTLIQNSLDSSSH-DELPKHLTLHPFQ--YKGLV
NQIIDSKWVGLKINELLVVEYYSRQTMSRRGTAEEKTAKSDPIYRNRLVNMLVNRILKHG
KKSLAYQIIYRAVKKIQQKTETNPLSVLRQAIRGVTPDIAVKARRVGGSTHQVPIEIGSTQG
KALAIRWLLAASRKRPGRNMAFKLSSELVDAAKGSGDAIRKKEETHRMAEANRAFAHFR
MGRDTIADIITSIRNADMDKKRVVRIASTNITENVVKILLREGFIENVRKHQENKKDFLVLT

LRHRR--NRKRPY---RNILNLKRISRPGLRIYSNYQRIPRILGGIGIVILSTSRGIMTDREARLE
RIGGEILCYIWMAKAIPRSGSRRSGRIGSRKSTRRIPKGVIHVQASFNNTIVTVTDVRGRVV
SWSSAGTCGFKGTRRGTPFAAQTAAGNAIRTVVDQGMQRAEVMIKGPGLGRDAALRAIR
RSGILLTFVRDVTPMPHNGCRPPKKRRVMPTIKQLIRNTRQPIRNVTKSPALRGCPQRRGT
CTRVYTITPKKPNSALRKVARVRLTSGFEITAYIPGIGHNSQEHSVVLVRGGRVKDLPGVRY
HIVRGTLDAVGVKDRQQGRSKYGVKKPKMARKSLIQREKKRQKLEQKYHSIRRSSKKEIS
---KVLSLSDKWEIYGKLQSPPRNSAPTRLHRRCFSTGRPRANYRDFGLSGHILREMVHACL
LPGATRSSWMIKNPFSGIILK--EENKDNRGSAEFQVVSFTNRIRRLTSHLELHKKDYLSQR
GLRKILGKRQRLLAYLSKKNRVRYKELIGRLDIRETKIR--MVKLRLKRCGRKQRAVYRIVA
IDVRSRREGRDLRKVGFYDPIKNQSCLNVPAILYFLEKGAQPTGTVRDILKKAEVFK--------
---MDKSKRPFLKSKRSFRRRLPPIQSGDRIDYRNMSLISRFISEQGKILSRRVNRLTLKQQRL
ITIAIKQARILSLLPFLNNDK--QFERTESTARTAGLRARKKVTRSLKKNPFVANRLLRKIDKL
NTKAEKEIIITWSRASTIIPTMIGHTIAIHNGKEHLPIYINDRMVGHKLGEFAPTLNFRGHAK
SDNRSRRMILKSFLLGNLVSLCMKIINSVVVVGLYYGFLTTFSIGPSYLFLLRARVMEEGEE
GTEKKVSATTGFITGQLMMFISIYYAPLHLALGRPHTITVLALPYLLFHFFWNNHKNFFDY
GSTTRNSMRNLSIQCVFLNNLIFQLFNHFILPSSMLARLVNIYMFRCNNKMLFVTSSFVGW
LIGHILFMKWVGLVLVWIRQNHSIRS-IRSNKYLVSELIN--SRIFSILLFITCVYYLGRIPSPIIT
KKLKETEEDKPLVTLLFDYKRWNRPLRYIK-NRFERTARNEMSQYFFYTCRSDGKERISFT
YPPSLSTFGEMIQRRMSLPTLEKLSSDELYNHWIYTNKQKKNNLNNEFLNRIKALDMGFL
SLDILEKSTRLYN--TKKDYLPKIYDPILN-SYRGTIKKKFSPIETMEINKIHNLLYQEFKQKIA
NGH---IKEISKKVPRWSDKFIELEQQTGVQIRSRKIDDIVVFTD--IEYLQESDFRRNIIKGSM
RTQRRKTIIWKLFEANAHSPLFLNR-RK-KSPLFSFDISELMKLIFRNIKEKVRIE----WDLVPF
SKTTRSLLLIIQSMLRKYIILPSLIIVKNIGRILLFQPPEWSEDFHEWKREKHIKCTYNGVQLS
ETELPANWLTEGIQIKILFPFCLKPWYRSKEQKKDDSCFLTASGMETDLPFGSPRKRTSFFEP
IFKDFL--------------ALRVLIRRHFLQGSKETKRGFIKNVLFLFLKRIKKELSKVNGSIISNQTI
HNSFNKIRSTD--NYSPREKKIKNLTDRTSPIRKKIKSITKEKKK---NKRSYN--VESPKDIWQI
L--KNTRLIWKLQVFLKIFIEKIYIDIFLYIINIARMYTRLV---IHFDLSSLSQAYVFYKLSRTRVI
NLYKLRSVLEYTSFFLKTAIKDSFTQGIFHSELEHTKFPSYGTNQWKNWLRG-YQYNLSQI
RWSGLIPQKWRNTINQGRITQKKTKWDS--KDR--LLHYKKQ-NSQKYYRYDLLSYKSING
DIMYPDRKYFDWKIINFFLRLFGWMEMNQEILNRWFFPEFVLLYNVYKKKPWIIPSKLLLL
NNKSNVKVELNALLKQHLLFQLRYDEGL-QRMINNLKVSCLLIGLKNPRNITVSSIQNREIS
LDIMLIRKS-LTVRELMKGVLIIEPIRLSVKKDGQFIMYQTIGISLIHKSKYNHYDFLVPEKIL
SSRRRRELRILIHFVFLGPNYRLEDLACINRYWFDTNNGSNFSMLRIYPQLMKGHQFKSWI
FELREILREIKNSHYFLDSRTQFNSVGSFSHIFFHQERFIKLFDPRIWSILLS-RNS--QGSTSNR
YFT---IKG-VILFVVAVLIYR----INSRNMVERKNLYLIGLLP-----IPMNSIGPRNDTLEESV-GS
SNINRLIVS-LLYLPKGKKISESCFLNPKESTWVLPIT----KKCSMPESNWGSRWWRNWIGK
KR--DS--

SQLKGSS-DQSRDPLDSIS----NEDSE------------YHTLINQREIQQLKERSIL-WD--PSFLQTER
TEIESD--RFPK--CLSVYS--SMSRLFT-EREKQMINHLLPEEIEEFL--GNPTRSVRSFFSDRW--
SELH-LGSNPTERSTRDQ----KLLKKQQDLS-FVPSRRSE----KKEMVNIFKIITYLQN--TVSIH
PISSYPGCDMVP-KDEPD-MDSSNKISFLNKNPFFDL--FHLFHDRNRGGYTL--HHDFESE--E
RFQEMADLFTLSITEP-DLVYHKGFAFSIDSYGLDQKQ--FLNEVFDSRDESKKKY---LLALP
PFFY--EENESFYRRIRKKWVR-ISCGNDLEDPKPKIVVFASNNIMEAVNQYRLIRNLIQIQYS

TYG--YIRNVLNRFFLMN---RSDRNFEYG---IQRDQIGKDTLNHRTIMKYTINQHFSNLKK-S
QKRWFDPL--ILISRTERSMNRDPDAYRYKWSNGSKNFQEHLEHFVSEQKSR---FQVVFDR
LRINQYSIDWSEVIDKKGLSKPFRFFLSK----------------------LLFFLSN-SLPFFFVSFGNIPIH
RSE-IYIY-ELKGPNDQLCNQLLESIGLQIVHLKKWKPF--LLDLLDDHDTS-QKSKFLINGGTI
SPFLFNKIP-KW-MIDSFHTR--NNRRKSFDNTDSYFSTIFHDQDNWLNPVKPFHRSSLISSFY
KANRLRFLNNPHHFCFY--CNKRFPFYV----EKARIN--NSDFTYG-QFLNILFIHNK-IFSLCVG
K--KKHAFWG-RDTISPIESQVSNIFIPNDFPQSGGDETYNLYKS---FHFPSRSDPFVR--RAIYS
IADISGTPLTEGQIVNFERTYCQPLSDMNLSDSEGKNSHQYLN--FNSNMGLIHTPCSEKYLP
SEK--RKK--------RSLCLK--KCVEKGQMYRTFQRDSAFSTLSKWNLFQTYMPWFLTSTGYK
YLNFIFLDTFSDLL--PILSL-SQKFVSIFHDIMHGSDISWRILQKKL------CLLKWNL-ISEISSK
CFHN--LLLSEERIHRN--NESPLISTHLRSPNVREF-LYSILFLLLVAGY-LVCTHLLFVSW--AS
SELQTEFEKVKSLMIPSSMIELRKLLDRYPTSEPNSFWLKNLFLVALEQLGDSLEEIRGSAS
GGNMLLGGDPA-----YG-VKSIRSKKKYLNINLIDIIDLI-SIIPNPINRITFSRNTRHLSHTSKEI
YSLIRKRKNVNGDWIDDKIESWVANSDSIDDEEREFLVQFS--TLTT--EKRIDQILLSLT-HSD
HL-----SKNDSGYQMIEQPGAIYLRYLVDIH--KKYLMNYEFNTSCLAERRIFLAHYQTITY--
SQTSCGANSLNFPSHG---KPFSLRLALSPS-RGILVIGSIGTGRSYLVKYLATNSYVPFITVFL
NKFLDNKPKGFLID-----DID-IDASDDIDASDDIDASDDIDA------------SDDIDRDLHTEL-EL
L------TMDMMPE---IDRFYITLQFELAKAMSPCIIWIPNIHDLDVNESNYFSLGLLVNHLSR--
-DCERCS-TRNILVIASTHIPQKVDPALIAPNKLNTCIKIRRLLIPQQRKHFFTLSYTRGFRLEN
KMFHT--NGFGSITMGSNARDLVALTNEALSISITQKKSIIDTNTIRSALHRQTWDLR---SQV
RSVQDHGILFYQIGRAVAQNVLLSNCPIDPISIYMKKK--SCNEGDSYLYKWYFELGTSMKK
LTILLYLLSCSAGSVAQDLWSLP--RPDEK-NGITSYGLVENDS-DLVHGLLEVEGALVG--SSR
TEKDCSQFDND--RVTLLLRPEPRN-PLDMMQNGSCSILDQRFLYEKN--ESEFEE-GEGEGA
LDPQQ----IEED----LFNH--IVWAP--RIWRP---WGFLFDCIERPNELGFP-YWSRSFRGK---RIL
YDEEDELQENDSEFLQSGTMQYQTRDRSSKEQGLFRISQFIWDPADPLFFLFK-DQPFVSVF
SHRELFADEEMSKGLLTSPTDPPTSIYKRWFI--KKTQEKHFELLINRQRWFR---TNSSLS--N
GSFRSN--TLSESYQYLSNLFLSN-GTLLDQMTKTLLRKRWLFPDEMKIGF--MEQEKDFPFL
MPRSR-INGNFIDKTFSIVANILLRIIPTTSGEKEAFTYYRDG--MSAQSEGNYAEALQNYYE
AMRLEIDPYDRSYILYNIGLIHTSNGEHTKALEYYFRALERNPFLPQAFNNMAVICHY-RGE
QAIRQGDSEIAEAWFDQAAEYWKQAMALTPGNYIEAHNWLKITRRFE--MSCRSEHIWIEP
ITGSRKTSNFCWAVILFLGSLGFLLVGTSSYLGRNLISLFPSQQILFFPQGIVMSFYGIAGLFIS
SYLWCTLSLNVGGGYDRFDRKEGMVCIFRWGFPGKNRRIFLRFLIKDIQSVRIEAKEGIYA
RRVLYMDIRGQGAIPLTRTDEN-VTPREIEQKAAELAYFLRVPIEVF
>Cucumis
MEKRWLNSMLSLSKSINSLGPIE----SEGSIINNMNKNIPSYSTVDDLVG---IRNFVSDDTFLV
RDSNSSSYSIYLDIENQIFEIDNDP--SFVSELESSFYSFRNSTYQNNISKNDDSHYDRYMYD
TKYSWNNHINSCIDSYLR--TQICIDSYILSGSHNYSDSYIYSYICGSESFSIRTSTHGNNLTIT
E-SS--DRTTNYSDFWVICENCHKFNYKRLFKSKMNICEECGYHLKMNSSDRIELLIDPGTW
DALWEWDSEVDPIQWKSQVDPYKDRISSSQIETGLPEAIQTGTGKLNGIPVAIGVMEFEFM
GGSMGSVVGEKITRLIDYASNQFLPLILVCASGGARMQEGSLSLMQMAKISSALYDYQSN
KKLFYVAILTSPTTGGVTASFAMLGDIIIAEPNAYIAFAGKRIIEETLKMEVPEGSQKTEPLFE
KGLLDLIVPRNPLKDVVSELFQLHAFVPSNQNSIKMETIRADEISNIIRERIEQYTREVKIVN
TGTVLQVGDGIARIYGLDEVMAGELVEFEEGTIGIALNLESNNVGVVLMGDGLLIQEGSS
VKATGRIVQIPVSEAYLGRVINALAKPIDGRGEISSSDSRLIESPAPGIILRRSVYEPLQTGLIA

IDSMIPIGRGQRELIIGDRQTGKTAVATDTILNQQGQNVICVYVAIGQKASSVAQVVSTLQE
RGAMEYTIIVAETADSPATLQYLAPYTGAAWLN-ILCTENSTLHIYDDPSKQAQAYRQMSL
LLRRPPGREAYPGDVFYLHSRLLERAAKLSSALGEGSMTALPIVETQSGDVSAYIPTNVISI
TDGQIFLSADLFNAGIRPAINVGISVSRVGSAAQIKAMKQVAGKLKLELAQFAELEAFAQF
ASDLDKATQNQLARGQRLRELLKQSQSAPLTVDEQIMTVYTGTNGYLDSLEIAQVRKFLV
ELRTYVKTNKPQFQEIISSTKTFTPEAEVLLKEAIQEQMERFLLQDQVMRINPTTSSPEVST
LEKNNLGRIAQIIGPVLDVAFPPGKMPNIYNALIVKGQDISGQEINVTCEVQQLLGNNRVRA
VAMSATDGLRRGMEVVDTRAPLSVPVGGATLGRIFNVLGEPIDNLGPVDTRTTSPIHRSAP
AFIQLDTKLSIFETGIKVVDLLAPYRRGGKIGLFGGAGVGKTVLIMELINNIAKAHGGVSV
FGGVGERTREGNDLYMEMKESGVINEENFAESKVALVYGQMNEPPGARMRVGLTALTMA
EYFRDVNEQDVLLFIDNIFRFVQAGSEVSALLGRMPSAVGYQPTLSTEMGSLQERITSTKE
GSITSIQAVYVPADDLTDPAPATTFAHLDATTVLSRGLAAKGIYPAVDPLDSTSTMLQPRIVG
EEHYETAQRVKQTLQRYKELQDIIAILGLDELSEEDRLTVARARKIERFLSQPFFVAEVFTGS
PGKYVGLAETIRGFKLILSGELDGLPEQAFYLVGNIDEATAKATNLEMESNLKKMTLNLSV
LTPNRIIWDSEVKEIILVTNSGQIGVLPDHAPIATAVDIGILKIRLTDGWLTMALMGGFARIG
NNEVTILVNDAEKASDIDPQEAQQTLEIAEANLRKAQGKRQTIEANLALRRARTRVEAING
VPMKNVTDSFISLGYWPSAESFGFNTDILATNPINLSVVLGVLIFFGKGVLSDLLDNRKQRI
LKTIQNSEELRGGAIEQLEKARARLRKVEMEADQFRVNGYSEIEREKLNLINSTSKSLEQL
ENYKNETIRFEQQKAINQVRQQVFQQALQGALGTLNSCLDNELHLRTISANIGMFGTMKE
ITNMNPLISAASVIAAGLAVGLASIGPGIGQGTAAGQAVEGIARQPEAEGKIRGTLLLSLAF
MEALTIYGLVVALALLFANPFVMNILLCSINTQKGLYDISGVEVGQHLYWQIGGFQVHAQ
VLITSWVVIAILLGSAIIAVRNPQTIPTDGQNFFEYVLEFIRDVSKTQIGEEYGPWVPFIGTM
FLFIFVSNWSGALLPWKIIQLPHGELAAPTNDINTTVALALLTSVAYFYAGLSKKGLSYFGK
YIQPTPILLPINILEDFTKPLSLSFRLFGNILADELVVVVLVSLVPLVVPIPVMFLGLFTSGIQA
LIFATLAAAYIGESMEGHHMIFSTFEHILTHISFSVISIVITIQLITLLI-ETVGLYVSSEKGMIAT
FFCITGLLVTRWIYLRHLPLSDLYESLLFLSWAFSIIHLFTYFKNIK-TIKCNNRASTIFTQGFA
TSGFLTEMHPSPLLVPALQSHWLMMHVSMMVLGYAALLCGSLFSVALIVITFQKAIRIFCK
NNEIQSINEGNYFVR--SFFSSKNYYKSQLIQQLDHWSCRIISLGFIFLSIGILSGAVWANETW
GSYWNWDPKETWAFITWTIFAIYLHTRTNKNFEGVNSAIVASMGFLIIWICYFGVNLLGIG
LHSYGPFTLTMEKKKAFIPLLYLASIVFLPWWISLSFNKSLESWVTNWWNTGQSENFLNDI
QEKSIIKKVLELEELFLLDEMLKEYPETHLQKLRIA-SKETIQLIKMHNEDRIHTILHFSTNII
CFVILSGYSILGNEELVILNSWVQEFLYNLSDTIKAFSILLLTDLCIGFHSPHGWELMIGSVY
KDFGFSHNDQIISSLVSTFPVILDTIFKYWIFRYLNRVSPSLGVIYHSLNDMPVGVPKVPFRL
PGEEDASWVDLYNRLYRQRLLFLGQDVNNEISNQIMGLMVYLSIEDGTKDQYLFINSPGG
SVIPGVGLFDTMQFVSPDVHTICMGLAASMGSFILVGGEITKRLAFPHARVMIHQPASSFSK
GKTGEFVLESTELLNLRETITKVYVQRTGKPLWVIPEDMERDVFMSAPEAQAHGIVDLVAV
-??MEEFQGY
LELEKSRQRDFLYPLIFREYIYAFSHDHFLSGYDKKSSLLMVKHLITRMYHQNHFFFFTNY
YNKNPFWGNNLYSQMLSEGFAVIAEIPLSLRLVSSLIAKSYNLRSSHSIFPFLEDKFPHLNYV
SDVLIPYPLHLEILVQILRSWVKDASSFHLLRFFFHEYCNLNSLSTKLISFFSKRRLVLLLYN
SYVCGYESIFLFLRNQSSHIRLTSYKGLFERIYFYGKIEHLVKVFANSAILRVFKDPLIHYVR
YQAKSILVSKDTPLLINKWKYYLVNLWQCHFYVWSQPERIYINQLSKRSLDFLGYISSVRL
NPSVVWTQMLENSFLIDN--ATQKLDTLVPIITLLASLAKAKFCNVLDIPLVSRPGSIRRILIL
LIDFCVYPEIFLIITEDPQKKRICIEYNIYFAFLVLKLWLVNTKVLYRALFKRLNSELLEEFFT

EQEQVLSLIFPRKIWYLDIICMNDLANHEMIIDTS---QSQISILFEMDS----KDSMNLWYCPY
VDYCIGITIGVLVIVWLEREISAGIQQRIGPEYAGPLGVLQALADGTKLLFKENLLPSRGDT
RLFSIGPSIAVISILLSYSVIPFGYRLVLADLPIGVFLWIAISSVAPIGLLMSGYGSNNKYSFLG
GLRAAAQSISYEIPLTLCVLSISLLSNSSSTVDIVEAQSKYGFWGWNLWRQPIGFVIFLISSL
AECERLPFDLPEAEEELVAGYQTEYSGIKFGLFYVASYLNLLVSSLFVTVLYLGGWDISIPYI
LGYELFEINKVYEVFGMTISIFITLAKTYLFLFISIATRWTLPRLRIDQLLNLGWKFLLPISLG
NLLLTTSFQLFSLMIWHVQNENFILDSTRIFMKAFHLLLFDGSFIFPECILIFGLILLLMIDSTS
DQKDIPWLYFISSTSLVMSITALLFRWREEPMISFSGNFQTNNFNEIFQFLILLCSTLCIPLSV
EYIECTEMAITEFLLFVLTATLGGMFLCGANDLITIFVAPECFSLCSYLLSGYTKKDVRSNEA
TTKYLLMGGASSSILVHGFSWLYGSSGGEIELQEIVNGLLNTQMYNSPGISIALIFITVGIGF
KLSPAPSHQWTPDVYEGSPTPVVAFLSVTSKVAASASATRIFDIPFYFSSNEWHLLLEILAIL
SMILGNLIAITQTSMKRMLAYSSIGQIGYVIIGIIVGDSNGGYASMITYMLFYIAMNLGTFAR
IVSFGLRTGTDNIRDYAGLYTKDPLLALSLALCLLSLGGLPPLAGFFGKLHLFWCGWQAGL
YFLVSIGLLTSVVSIYYYLKIIKLLMTGRNQEITPHVRNYRRSPLRSNNSIELSMIVCVIASTI
PGISMNPIIEIAQDTLFMFLLYEYDIFWVFLIISSVIPILAFLISGVLAPLSKEPEKLSSYESGIE
PMGDAWVQFRIRYYMFALVFVVFDVETVFLYPWAMSFDVLGVSVFIEALIFVLILIVGLVY
AWRKGALEWSTNYFPWLTTVVVFPIFAGLLLFFFPHRGNKVMRWYTICICVLELLLTTYAF
CYHFELDDPLIQLMEDYKWIPFLDFYWEIGIDGLSIGPILLTGFITTLATLAAWPVTRDSRLF
YFLMLAMYSGQIGSFSARDLLLFFIMWEFELIPVYLLLSMWGGKKRLYSATKFILYTAGGS
IFLLMGVLGIGLYGSNEPTLNFETSANQSYPIALEILFYIGFLIAFAVKSPIIPLHTWLPDTHG
EAHYSTCMLLSGILLEMGAYGLVRINMELLPHAHSIFSPWLIIVGIIQIIYAASTSSGQRNLK
KRIAYSSVSHMGFIIIGIGSISDTGLNGAILQIISHGFIGAALFFLAGTSYDRIRLVYLDEMGG
LAIPIPKIFTTFNILSMASLALPGMSGFVAELIVFFGIITSQKYLLMTKMVITLVMAIGIILTPI
YLLSMLRQMFYGYKLFNAKNSYFFDSGPRELFVAISILIPVIGIGIYPDFVFSLSVDKVEAIL
SNYFARIMLEHVLIISAYLFSIGIYGLITSRNMVRALMCLELILNAVNMNFVTFSDFFDSRQL
KGNIFSIFVIAIAAAEAAIGPAILSSTYRNRKSTRINQSNLLNKMEYTYQYSWIIPFISLPIPILI
GVGLLLFPTATKNLRRLWSFPSILLLSIVMIFAVYLSIDQINTSYIYQYVWSWTINNDFSLEL
GYFIDPLTCIMAILITTVGIMVLIYSDNYMSHDQGYLRFFAYMSLFNTSMLGLVTSSNLIQIY
IFWELGGMCSYLLIGFWFTRPSAANAGQKAFVTNRVGDFGLLLGILGLYWTTASFEFRDL
FQIFNN-EVNFLFLTLCAVLLFAGAVAKSAQFPLHVWLPDAMEGPTPISALIHAATMVAAGI
FLVARLLPLFIAIPYIMNLIALIGIITVLFGATLALAQKDIKRSLAYSTMSQLGYMMLALGM
GSYQAALFHLITHAYSKALLFLGSGSIIHSMESVVGYSPDKSQNMVLMGGLTKHVPITKN
AFLLGTLSLCGVPPLACFWSKDEILNDSWLYSPIFAIIACSTAGLTAFYMFRIYLLTFDGHLN
LYCKNYSGKTNNSLYSISLWGKE-GSKIIKKFFSLLTMNFWMSTKN-TFYYPYESENTMLFP
MFVLSLFTLFVGVIGTQFDLNILDKWLTPSINLLHESSK-SENW-EFVKNATFSVTIAFFGIFI
ASFFYKPTYSSLQNLNFRNSFVFFFEKIINIIYNWSYNRGYIDTFYRISLIGGIRILAELTYFFD
RRIIDGITNAVGILNFFVAESIKYVGGG-RISSYILLYLVYLVIFILVIYMDLPGPIHDFLVVLW
GSGLILGSMGVLLFNNSIYSAFSLGLVLVSISLFYILANAQFVAAAQLLIYVGAINVLIIFAV
MFMKGSEYSKDFNLWTVGNGVTFLVCTSIFVSLMTTIVDTSWYGIIWTTRSNQILEQDLIS
NSQQIGIYLSTYFFLPFELISIILLAALIGAIAVARQMNGPATRKDLMIVNMGPHHPSMHGVL
RLILTLDGEDVIDCEPILGYLHRGMEKIAENRTIIQYLPYVTRWDYLATMFTEAITVNGPEQ
LGNIQVPKRASYIRVIMLELSRIASHLLWLGPFMADIGAQTPFFYIFRERELVYDLFEAATG
MRMMHNFFRIGGVAADLPHGWIDKCLDFCDYFLTAVAEYQKLITQNPIFLERVEGVGIISG
EEVINWGLSGPMLRASGIPWDLRKVDRYECYDEFDWEVQWQKEGDSLARYLVRLAEMT

ESVKIIQQALEGIPGGPYENLEIRSFDRARSPEWNDFDYRFISKKPSPTFELAKQELYVRVEA
PKGELGIFLIGDQGGFPWRWKIRPPGFINLQILPQLVKRMKLADIMTILGSIDIIMGEVDRM
FPMVTGFMNYGQQTVRAARYIGQGFVITLSHANRLPVTIQYPYEKLIASERFRGRIHFEFD
KCIACEACVRVCPIDLPVVDWKLETDIRKKRLLNYSIDFGICIFCGNCVEYCPTNCLSMTEE
YELSTYDRHELNYNQISLGRLPMSVVDDYTIRTILN-----------------MQGNLSSWLVKHGLV
HRSLGFDYQGIETLQIKPEEWHSIAVILYVYGYNYLRSQCAYDVAPGGLLASVYHLTRIEY
GIDQPEEVCIKVFAARINPRIPSVFWVWKSADFPERESYDMLGIYYDNHPRLKRILMPESW
VGWPLRKDYIAPNFYEIQDAH---MNSIEFPLL
DRTTQTSVISTTSNDLSNWSRLSSLWPLLYGTSCCFIEFASLIGSRFDFDRYGLVPRSSPRQA
DLILTAGTVTMKMAPSLVRLYEQMPEPKYVIAMGACTITGGMFSTDSYSTVRGVDKLIPV
DVYLPGCPPKPEAVIDAITKLRKKISREIYEDRIKS--ENRCFTTNHKFHVGPSTHTGNYDQE
LLYQPSSSEIIPPETFFKYKSAVSSYELVNMQTRNTFSSIKEEITRSISVSLMIYIITWAPVSNA
YPIFAQQGYENPREATGRIVCANCHLANKPVDIEGPQAVLPDTVFEAGGRIPYDMQLKQV
LANGKKGWLNVGAGLILPEGFELAPPDRISPEIKEKKGSVLFRAYRPTKKNILVIGPVPGQK
YSEITFPILSPDPATKKDVHFLKYPIYVGGNRGRGQIYPDGSKSNNNVYNATAAGIVSKIIR
KEKGGYEITIVDAANGRQVVDIIPPGPELLVSEGESFKLDHPLTSNPNVGGFGQGDAEIVLQ
DPLRVQGLLFFFASVILAQIFLVLKKKQFEKVNCPK---MSKVYDWFEERLEIQAIADDITSK
YVPPHVNIFYCLGGITLTCFLVQVATGFAMTFYYRPTVTEAFASVQYIMTEANFGWLIRSV
HRWSASMMVLMMILHVFRVYLTGGFKKPRELTWVTGVVLAVLTASFGVTGYSLPRDQIG
YWAVKIVTGVPEAIPVIGSPLVELLRGSASVGQSTLTRFYSLHTFVLPLLTAVFMLMHFPMI
RKQGISGPLMGVTKKPDLNDPVLRAKLAKGMGHNYYGEPAWPNDLLYIFPVVILGTIACN
VGLAVLEPSMIGEPADPFATPLEILPEWYFFPVFQILRTVPNKLLGVLLMVSVPAGLLTVPFL
ENVNKFQNPFRRPVATTVFLIGTAVALWLGIGATLPIDKSLTLGLFMIEVFLFGIVLGLIPITL
AGLSVTAYLQYRRGDQLDLMLTITSYFGFLLAVLTITSALFIGLNKIRLIMDMVSLAWAAL
MVVFTFSLSLVVWGRSGLMIIRSPEPEVKILVDRDPIKTSFEDWARPGHFSRTIAKGPDTTT
WIWNLHADAHDFDSHTSDLEEISRKVFSAHFGQLSIIFLWLSGMYFHGARFSNYEAWLGD
PTHIGPSAQVVWPIVGQEILNGDVGGGFRGIQITSGFFQMWRASGITNELQLYCTAIGALVF
AALMLFAGWFHYHKAAPKLAWFQDVESMLNHHLAGLLGLGSLSWAGHQVHVSLPINQF
LNAGVDPKEIPLPHEFILNRDLLAQLYPSFAEGATPFFTLNWSKYAEFLTFRGGLDPVTGGL
WLTDIAHHHLAIAILFLIAGHMYRTNWGIGHGIKDILEAHKGPFTGQGHKGLYEILTTSWH
AQLSINLAMLGSLTIIVAHHMYAMPPYPYLATDYGTQLSLFTHHMWIGGFLIVGAAAHAAI
FMVRDYDPTTRYNDLLDRVLRHRDAIISHLNWVCIFLGFHSFGLYIHNDTMSALGRPQDM
FSDTAIQLQPRLCSMDTNTHALAPRITAPGATTGTSLTWGGGDLVAVGGKVALLPIPLGTAD
FLVHHIHAFTIHVTVLILLKGVLFSRSSRLIPDKANLGFRFPCDGPGRGGTCQVSAWDHVF
LGLFWMYNSISVVIFHFSRKMQSDVWGSVSDQGVLTHITGGNFAQSSITINGWLRDFLWA
QASQVIQSYGSSLSAYGLFFLGAHFVWAFSLMFLFSGRGYWQELIESIVWAHNKLKVAPAT
QPRALSIVQGRAVGVTHYLLGGIATTWAFFLARIIAVGMALRFPRFSQGLAQDPTTRRIWF
GIATAHDFESHDDITEERLYQNIFASHFGQLAIIFLWTSGNLFHVAWQGNFEAWVQDPLHV
RPIAHAIWDPHFGQPAVEAFTRGGALGPVNIAYSGVYQWWYTIGLRTNGDLYTGALFLLF
LSAISLIAGWLHLQPKWKPSVSWFKNAESRLNHHLSGLFGVSSLAWTGHLVHVAIPASRG
ESVRWNNFLDVLPHPQGLGPLFTGQWNLYAQNPDSNNHLFGTSEGAGTAILTLLGGFHPQ
TQSLWLTDMAHHHLAIAFIFLVAGHMYRTNFGIGHSIKDLLEAHIPPGGRLGRGHKGLYDT
INNSLHFQLGLALASLGVITSLVAQHMYALPAYAFIAQDFTTQAALYTHHQYIAGFIMTGAF
AHGAIFFIRDYNPEQNEDNVLARMLDHKEAIISHLSWASLFLGFHTLGLYVHNDVMLAFG

TPEKQILIEPIFAQWIQSAHGKTSYGFDVLLSSTSGPAFNAGRSIWLPGWLNAVNE--NSNSL
FLTIGPGDFLVHHAIALGLHTTTLILVKGALDARGSKLMPDKKDFGYSFPCDGPGRGGTCD
ISAWDAFYLAVFWMLNTIGWVTFYWHWKHITLWQGNVSQFNESSTYLMGWLRDYLWL
NSSQLINGYNPFGMNSLSVWAWMFLFGHLVWATGFMFLISWRGYWQELIETLAWAHERT
PLANLIRWRDKPVALSIVQARLVGLAHFSVGYIFTYAAFLIASTSGKFGMSHSVKIYDTCIG
CTQCVRACPTDVLEMIPWGGCKAKQIASAPRTEDCVGCKRCESACPTDFLSVRVYLWHE
TTRSMGLAYMTTLTLPSIFVPLVGLVFPAIAMASLFLHVQKNKIFMRDLKTFSPVAPVVSTI
WSG-FSGSINRDQSFIPGCVGIPIFSIMTAILERRESESLWGRFCNWITSTENRLYIGWFGVL
MIPTLLTATSVFIIAFIAAPPVDIDGIREPVSGSLLYGNNIISGAIIPTSAAIGLHFYPIWEAASV
DEWLYNGGPYELIVLHFLLGVACYMGREWELSFRLGMRPWIAVAYSAPVAAATAVFLIYPI
GQGSFSDGMPLGISGTFNFMIVFQAEHNILMHPFHMLGVAGVFGGSLFSAMHGSLVTSKLI
RETTENESANEGYRFGQEEETYNIVAAHGYFGRLIFQYASFNNSRSLHFFLAAWPVVGIWS
TALGISTMAFNLNGFNFNQSVVDSQGRVINTWADIINRANLGMEVMHERNAHNFPLDLAA
VEVPSINGMGLPWYRVHTVVLNDPGRLLSVHIMQTALVAGWAGSMALYELAVFDPSDPV
LDPMWRQGMFVIPSMTRLGITNSWGGWSIT-GGIITNPGIWSYEGVAGAHILFSGLCFLAAI
WHWVYWDLEIFSDERTGKPSLDLPKIFGIHLFLSGLGCFGFGAFHVTGLYGPGIWVSDPY
GLTGRVQAVNPAWGVEGFDPFVPGGIASHHIAAGTLGILAGLFHLSVRPPQRLYKGLRMG
NIETVLSSSIAAVFFAAFVVAGTMWYGSATTPIELFGPTRYQWDQGYFQQEIYRRVSTGLA
ENQSLSEAWSKIPEKLAFYDYIGNNPAKGGLFRAGSMDNGDGIAVGWLGHPVFRDKEGR
ELFVRRMPTFFETFPVVLVDGDGIVRADVPFRRAESKYSVEQVGVTVEFYGGELNGVSYS
DPATVKKYARRAQLGEIFELDRATLKSDGVFRSSPRGWFTFGHASFALLFFFGHIWHGART
LFRDVFAGIDPDLDTQVEFGAFQKTWRSNYKKSLV------------VETLFNGTLALAGRDQETT
GFAWWAGNARLINLSGKLLGAHVAHAGLIVFWAGAMNLFEVAHFVPEKHMYEQGLILLP
HLATLGWGVGPGGEVIDTFPYFVSGVLHLISSAVLGFGGIYHALLGPETLEESFPFFGYVW
KDRNKMTTILGIHLILLGIGAFLLVFKALYFGGVYDTWAPGGGDVRKITNLTLSPSVIFGYL
LKSPFGGEGWIVSVDDLEDIVGGHVWLGSICILGGIWHILTKPFAWARRALVWSGEAYLSY
SLGALSVFGFIACCFVWFNNTAYPSEFYGPTGPEASQAQAFTFLVRDQRLGANIGSAQGPT
GLGKYLMRSPTGEVIFGGETMRFWDLRAPWLEPLRGPNGLDLSRLKKDIQTLARTTFRGI
YDPAPLGSLNSVGGVATEINAVNYVSPRSWLATSHFVLGFFLFVGHLWHAGRARAAAAGF
EKGIDRDFEPVLFMTPLNMTIAVGKFTKDENDLFDIMDDWLRRDRFVFVGWSGLLLFPCA
YFAVGGWFTGTTFVTSWYTHGLASSYLEGCNFLTAAVSTPANSLAHSLLLLWGPEAQGDF
TRWCQLGGLWTFVALHGAFGLIGFMLRQFELARSVQLRPYNAIAFSGPIAVFVSVFLIYPL
GQSGWFFAPSFGVAAIFRFILFFQGFHNWTLNPFHMMGVAGVLGAALLCAIHGATVENTL
FEDGDGANTFRAFNPTQAEETYSMVTANRFWSQIFGVAFSNKRWLHFFMLFVPVTGLWM
SALGVVGLALNLRAYDFVSQEIRAAEDPEFETFYTKNILLNEGIRAWMAAQDQPHENLIFP
EEVLPRGNALMSGSTGERSFADIITSIRYWVIHSITIPSLFIAGWLFVSTGLAYDVFGSPRPN
EYFTESRQGIPLITGRFDSLEQLDEFSRSFMTIDKTYPIFTVRWLTVHGLAVPTVSFLGSISA
MQFIQRMATQTVDDSSKSG-PRRTVVGDLLKPLNSEYGKVAPGWGTTPLMGVAMSLFAIF
LCIILEIYNSSILLDGISSNMLTLKLFVYTVVIFFVSLFIFGFLSNDPGRNPGREEMAETTGRIP
LWLIGTVTGIPVIGLVGVFFYGSYSGLGSSLMLNILSFIGICLNSFLYSSSFFVAKLPEAYAFL
NPIVDVMPVIPLFFFLLAFVWQAAVSFRMTQSNPNEQNVELNRTSLYWGLLLIFVLAVLFS
NYFFNMEVNILAFIATALFILVPTAFLLIIYVKTISQSDMETATLVAISISGLLVSFTGYALYTA
FGQPSQQLRDPFEEHGDMEALVYTFLLVSTLGIIFFAIFFREPPKVPTKKAKMTIAFQLAVFA
LIVTSSILLISVPVVFASPDGWSGNKNVVFSGTSLWIGLVFLVGILNSLIS-------MSPQTETKA

SVGFKAGVKDYKLTYYTPEYETKDTDILAAFRVTPQPGVPPEEAGAAVAAESSTGTWTTV
WTDGLTSLDRYKGRCYGIEPVAGEENQYIAYVAYPLDLFEEGSVTNMFTSIVGNVFGFKAL
RALRLEDLRIPTAYIKTFQGPPHGIQVERDKLNKYGRPLLGCTIKPKLGLSAKNYGRAVYE
CLRGGLDFTKDDENVNSQPFMRWRDRFLFCAEAIFKSQAETGEIK-DILNATAGTCEEMM
KRAIFARELGAPIVMHDYLTGGFTANTSLAHYCRDNGLLLHIHRAMHAVIDRQKNHGMH
FRVLAKALRMSGGDHIHAGTVVGKLEGEREITLGFVDLLRDDFVEKDRSRGIYFTQDWVS
LPGVLPVASGGIHVWHMPALTEIFGDDSVLQFGGGTLGHPWGNAPGAVANRVALEACVQ
ARNEGRDLAREGNEIIREASKWSPELVCVRKYGKEIKFEFEAMDTL--MAIHLYKTSTPSTR
NGAVDSQVKSNPRNNLIYGQHRCGKGRNARGIITAGHRGGGHKRLYRKIDFRRNEKDIYG
RIVTIEYDPNRNAYICLIHYGDGEKRYILHPRGAIIGDTIVSGTEVPIKMGNALPLTDMPLGT
AIHNIEITLGKGGQLARAAGAVAKLIAKEGKSATLKLPSGEVRLISKNCSATVGQVGNAGV
NQKSLGRAGSKCWLGKRPVVRGVVMNPVDHPHGGGEGRAPIGRKKPATPWGYPALGRR
SRKRNKYSDNLILRRRSKMIQPQTLLNVADNSGARELMCIRIIGAS-NRRYAHIGDVIVAVIK
KAVPNTPLERSEVIRAVIVRTCKELKRENGMIIRYDDNAAVVIDQEGNPKGTRIFGAIAREL
RQLNFTKIISLAPEVLMLSPKRTRFRKHHRGRMKGISYRGNSICFGRYAIQALEPAWITSRQI
EAGRRAMTRNARRGGKIWVRIFPDKPVTLRPTETRMGSGKGSPEYWVAVVKPGRILYEM
SGVAENIARKAISIAASKMPIRTQFITSGMTRIKRGYIARRRRTKIRLFASSFRGAHSRLTRTI
TQQKIRALASAYRDRGRQKRNFRQLWIARINAVIRSN-NVDYSYSRFIHNLYKRQLLLNRKI
LAQIAILNRNCLYMISNEIIK-----------MIKEVYALGQHICMSPHKARRVIDQIRGRSYEETM
MILELMPYRACYPILKLVYSAAANASHNMGFNERDLVISKAEVNQGTTVKKLKPRAQGR
SYPIKRPTCSITIVLKNTSVN-GLWDKKMDGIKYAVFTDKSIRLLGKN-QYTSNVESGSTRTE
IKHWVELFFGVKVIAMNSHRLPGKGRRMGPIMGHTMHYRRMIITLQPGYSIPPLRKKRTM
ALPKKRTSISKKRIRKNIWKSKGRRAALKAFSPAKSLSTGNSKSF-----KSMAKGKDARVTV
ILEGLVC--RN--VNKQSIGISRYITQKNRHNTPSRLELRKFCPRCYKHTIHGEIKKMKIRASIR
KICEKCRLIRRRRRIMVICSNPRHKQRQGMVREKIRVSTRTLKWKCVESRADSKRLYYGRF
ILSPLMKGQGDTIGIAMRKALLGEIEGTCITRAKSE--KIPHEYSTIVGIQESVHEILMNLKEI
VLRSNLYGTRDASICVKGPGCVTAQDIILPPSVEIVDNTQHIANLMEPINLCIELKIERNRGY
HIQTPNNFQDASY--PMDAIFMPVRNVNHSIHSYVVNGNEKQEILFLEIWTNGSLTPKEALHE
ASRNLIDLFIPFLHAEEEKENFHLKNNK-KKVTLPLFTFHEKLAK-LRKKKK-EIALKYIFID
QSELPPRIYNCLKRCNIHTLFDLLNNSPDELMKIKHFRIEDVKHILDILEMEKNFA-------ML
GGGN-ERMSTIPAFNQIQFEGFCRFIDHGLTEELSKFPKIEDTDQEIEFQLFVETYKLVEPLIK
ERDAVYESLTYSSELYVSAGLIWKTRRDMQEQTIFIGNIPLMNSLGTSIVNGLYRIVISQILQ
SPGIYYRSELDHNGISVYTGTIISDWGGRLELEIDRKARIWARVSRKQKISILVLSSAMGSNL
REILENVCYPEIFLSFLNDKEKKKIGSKENAILEFYQQFSCVGGDPVFSESLCKELQKKFFQ
QRCELGRIGRRNLNQRLNLDIPENNTFLLQRDILAAADHLIGLKFGMGTLDD--MNHLKNK
RIRSVADLLQDQFGLALVRLENMVRGTICGAIR-HKLI-PTPQNLVTSTP----LTTTFESFFGLH
PLSQVLDRTNPLTQIVHGRKLSYLGPGGLTGRTASFRIRDIHPSHYGRICPIDTSEGINVGLIG
SLAIHARIGHWG-SLETPFY---EISERSKK--VRMLYLSPSRDEYYMVATGNSLALNPGIQEEQ
IVPARYRQEFLTIEWEQVHLRSIFPFQYFSIGASLIPFIEHNDANRALMSSNMQRQAVPLSRS
EKCIVGTGLERQVARDSGVAAIAEHGGKIIYTDTDKIIFSGNGYTRRIPLVMYQRSNKNTC
MQQKSQVHQGKCIKKGQILADGAATVGGELALGKNVLVAYMPWEGYNFEDAVLISERLI
YEDIYTSFHIRKYEIQTHVTSHG-PERITNEIPHLEARLLCNLDKNGIVMLGSWVETGDILV
GKLTPQMAKE-SSYAPEDRLLRAILGIQVSTSKETCLKLPIGGRGRVIDVRWIQKKG-GSSY
NPEIIRVYISQKREIKVGDKVAGRHGNKGIVSKILPREDMPYLQNGRPVDMVFNPLGVPSR

MNVGQIFECSLGLAGSLLDRHYRIVPFDERYEQEASRKLVFSELYEASKQTAKPWVFEPEY
PGKSRIFDGRTGNPFEQPVIIGKPYILKLIHQVDDKIHGRSSGHYALVTQQPLRGRAKQGGQ
RVGEMEVWALEGFGVAHILQEMLTYKSDHIRARQEVLGTTIIGGTIPKPEDTPESFRLLVRE
LRSLALELNHFLVSEKNFQINRKEA-------MIDRYKHQQLRIGLVSPQQISAWANKTLPNGEI
VGEVTKPYTFHYKTNKPEKDGLFCERIFGPIKSGICACGNYRVIGDKKEDSKFCEQCGVEF
VDSRIRRYQMGYIKLACPVTHVWYLKRLPSYIANLLDKPLKELEGLVYCD--FSFARP-IAK
KPTFLRLRGSFEYEIQSWKYSIPLFFTTQGFDTFRNREISTGAGAIREQLADLDLRLIIDYSL
VEWKELGEE-GPAGNEWEDRKVGRRKDFLVRRMELAKHFIRTNIEPEWMVLCLLPVLPPE
LRPIIQIDGGKLMSSDINELYRRVIYRNNTLIDLLTTSRSTPGELVMCQEKLVQEAVDTLLDN
GIRGQPMRDGHNKVYKSFSDVIEGKEGRFRETLLGKRVDYSGRSVIVVGPSLSLHRCGLP
REIAIELFQTFLIRGLIRQHFASNIGVAKSKIREKEPIVWEILQEVMQGHPVLLNRAPTLHRL
GIQAFQPILVEGRAICLHPLVCKGFNADFDGDQMAVHVPLSWEAQAEARLLMFSHMNLLS
SAIGDPISVPTQDMLIGLYVLTSGNRRGICANRYNPSNRKN---HKNEKIYNNNYKYT---EPF
FCNSYDAIGAYRQKRINLDSPLWLRWRLDQR-VIAS--REAPIEVHYESLGTHHEIYGYYLIV
KSIKKEILCIYIRTTVGHISLYREIEEAIQGFCRACSYGT-----------MAERADLVFHNKVIDGT
AIKRLISRLIDHFGMAYTSHILDQLKTLGFQQATATSISLGIDDLLTIPSKGWLVQDAEQQSLI
LEKHHHYGNVHAVEKLRQSIEIWYATSEYLRQEMNPNFRMTDPFNPVHIMSFSGARGNAS
QVHQLVGMRGLMSDPQGQMIDLPIQSNLREGLSLTEYIISCYGARKGVVDTAVRTSDAGY
LTRRLVEVVQHIVVRRTDCGTIRGILVSPGNRMIP-ERIFIQTLIGRVLADDIYMGPRCIGVRN
QDIGIGLINRFIT------FQTQPISIRTPFTCRSTSWICRLCYGRSPTHGDLVELGEAVGIIAGQSI
GEPGTQLTLRTFHTGGVFTGGTAEHIRAPSNGKIKFNEDLVHPTRTRHGHPAFLCYIDLYVT
IESEDIIHNVTIPPKSLLLVQNDQYVESEQVIAEIRAGTYTL-NLKERVRKHIYSDSEGE-MH
WSTDVYHAPEFTYSNVHLLPKTSHLWILSGGSCG--CSLVPFSLYKDQDQINVH--SLCVER-
RYISSL-SVNNDK-VGQKFYGPDLSGK-ENGIPDY-SELNPILCTGQSNLTYPA-IFHGNSD--LL
AKRRRNGFIIQFESLQEREKELRPPSGISIEIPIN--GIFRRNSILAFFDDPQYRRNSSGITKYGT
IGVHSILKKED--LIEYR-GVKDFKPKYMKVDRFFFIPEEVHILPESSSIMVRNNSIIGVATRLT
LSIRSRVGGLVRVEKKKKRIELKIFSGDIHFPGEMDKISRHNGILIPPERVKK--NSKKSKK--S
KNWIYVQW--ITPTKKK--YFVFVRPVIIYELADGINLVKLFPQDLLQERDNLELRVANYILY
GNGKPIRGISGTSIQLVRTCLLLNWDRDK---KSSSIEDARASFVEVSTNGLVRNFLRIDLGKS
DTAYIRKRKDPSGSGLIF-NNESDRTNINPFFSIYSKTR-----VPQSPSQNQGTIRTLLNR---NK
ERQSLIILSASNCLQIDL--FNDVKDYN---VIKES-PLISIRNSLGPLGA--APQIVNF--YSFYYLI
T--HNPIS-LTKYLQLD--N-LKQIFQVL--K-YYLMDENG-GILNS-DPCINIVFNTFNLNWHFLH
DNYCEETPTRIS-LGQFFFENVCIAKNRP---HLKSGQIIIVQ-VDSLVIRSAKPYLATSGATVH
RHYGEILYEGDTLVTFIYEKSRSGDITQGLPKVEQVLEVRSIDSISMSLEKRIEGWNERITRI
LGIPWGFLIGAELTIVQSRISLVNKIQKVYRSQGVEIHNRHIEIIVRQITSKVLVSE---DGMSN
VFSPGELIGLLRAERTGRALEEAICYRAVLLGITKASLNTQSFISEASFQETARVLAKAALR
GRIDWLRGLKENVVLGGMIPVGTGFRELAHRS--RQHNNIPLETPPKK--IFEGEMR--DILFH
HKELFDFFISTNLHDTSEQEFLGFNDS------MTRRYWNIHLEEMMEAGVHFGHGTRKWNP
RMAPYISAKRKGIHIINLTRTARFLSEACDLVFDAATRGKQFLIVGTKNKAADSVARAATRT
RCHYVNKKWLGGMLTNWSTTETRLHKFRDLRTEQKTGGLNRLPKRDAAMFKRQLSHLQ
TYLGGIKYMTGLPDIVIIVDQQEEYRALQECITLGIPTICLIDTNCDPDLADISIPANDDAIAS
IRLILNKLVFAISEGRS-SSIRNSMGQKINPLGFRLGATQGHHSLWFAHPKKYSEGLQEDQKI
RDCIQNYVQKNMRIFSG-VEGIARIEIQKRIDLIQVIIFMGFPKLVIDD--G--SRKIEELQINVQ
KEFNSGNRKLNIAITRIGNPYGHPNILAEFIAGQLKNRVSFRKAMKKAIELTEQAGTKGIQV

QIAGRIDGKEIARVEWIREGRVPLQTIRAKIDYCCYPVRTIYGILGIKIWIFVDEQMSRYRRP
RFKKIRRLGALPGLTSKRPKTGNDLKNQSRSGK-KSQYRIRLEEKQKLRFHYGLTERQLLK
YVRIAGKAKGSTGQVLLQLLEMRLDNILFRLGMASTIPQARQLVNHRHILVNGSIVDIPSY
RCKPRDIITAKDEKKSRTLIQNYLDSSPP-QELPKHLTLQPLQ--YKGLVNQIIDSKWVSLKIN
ELLVVEYYSRQTMSRRGTAEEKIEKSDPIYRNRLVNMLVNRILKHGKKSLAYQIIYRAMKK
IQQKTETNPLSVLRQAIRGVTPDIAVKARRVGGSTHQVPIEIGSTQGKALAIRWLLGASRKR
PGRNMAFKFSSELVDAAKGSGDAIRKKEETHRMAEANRAFAHFRMGKDTIANIITSIRNA
DMNRKGMVRIPATNITENIVKILLREGFIENVRKHRESNKDFWVLTLRHRR--NRKGPYKT-I
--LNLKRISRPGLRIYSNYQKIPRILGGMGIVILSTSRGIMTDREARLQGIGGEILCYIWMAKP
IPRIGSRRNGRISSR-STRRIPKGVIHVQASFNNTIVTVTDVRGRVISWSSAGTCGFKGTRRG
TPFAAQTAAGNAIRGVVDQGMQRAEVMIKGPGLGRDAALRAIRRSGILLSFIRDVTPMPH
NGCRPPKKRRVMPTIKQLIRNTRQPIRNVTKSPALRGCPQRRGTCTRVYTITPKKPNSALR
KVARVRLTSGFEITAYIPGIGHNLQEHSVVLVRGGRVKDLPGVRYHIVRGTLDAVGVKDRQ
QGRSKYGVKKPKMARKSLIQREKKRQKLEQKYHLIRQSLKNEIS---KVPSLSEKWEIHGK
LQSPPRNSAPTRLHRRCFLTGRPRANYRDFGLSGHILREMVHACLLPGATRSSWMVKNSF
SSVISQE-E-KKENGGSVEFQVVSFTNKIRRLTSHLELHKKDYLSQRGLRKILGKRQRLLSY
LSKKNKMRYKELINQLDIRESKTQ--MVKLRLKRCGRKQRAVYRIIAIDVRSRREGRDLRK
VGFYDPIKNQTYLNVPAILYFLEKGAQPTGTVHDILKKAGVFTELHLNQ-----MDKSKRLFL
KSKRSFRRRLPPIPSGDRIDYRNMSLISRFISEQGKILSRRVNRLTLKQQRLITIAIKQARILSL
LPFLNNDKNKQFERSESTPRTIGLRTRNKMAR-LKKNPFVGNHLLKKINKLNTKGEKEIIVT
WSRASTIIPTMIGHTIAVHNGKDHLPVYITDRMVGHKLGEFAPTRNFRGHVKNDNRSRRM
ILKSFILGNLVSLCMKIINSVVVVGLYYGFLTTFSIGPSYLFLLRAWLMEEG---TEKKVSATT
GFITGQLMMFISIYYAPLHLALGRPYTITVLALPYLLFHFFWTNPKHFFYYGSTTRNSMRN
LSIQCVFLNNLIFQLFNHFILPSSMLVRLVNISMFRCNNKMLFVTSSFVGWLIGHILFMKSV
GLILVWIQQNNSIIS-IRSNKYLVSELRN--SPIFSIILFITWVYYLGRIPSPIPLKETSKTEEDKP
LGIILFDYKRWNRPFRYIK-DKFD-AVRKEMSQYFFYTCQSDGKDRIVFTYPPSVATFFELIE
KKISLFSTEKLFYDEFDNPWKSLNEQKKRILNKIVITRILLLDKGSLVGDVLQKRTQLCIDE
TKKEYLPKIYDPFLS-PYRRRINLLLGFSENIWTNKIHAIINNGFFDGKCKRV---IKKISTSTPT
STPTSTTSTSTPTSPTSTSTSTPASIPASAIIRYAKQWDFRREIIKGSMRAQRRKMGMYQLFQ
G-ANSYLFLGR-HNFFFSDFCIYIPRLMEKLDRLNEKNQESINLKEWESIRFGQLVRGSLLIT
QAFLRKYIILPSVIIAANLTRILLLSPPHFFEDFKNWKKEIYVKCTYNAIPLAENEFPQNWLT
DGIQIKILFPFRLQSWHRSKFGTETEQPFGSAENFIKKKKQQLKKWRTKYFR-IFLRLR-------
----------KKVFR---KINIAKEKWVIKPILFIKKRLRE-FWQRNPINSIISNGMIQSSSGSIDSIILSL
RERKMKELTDRINTMLKIIEKMKKDRQKELL---KMSYYDKIFESQKNILKILK--NVRFIRKS
YKFLKFFVQNIYIDIFQGIIN----MSRIN-----WDVSYLSQAYLFYKLSQTKIINLSKLRPVFQY
TSRFLKNETQDYFAHEILHSDLKQKKQKNLENSPWKNWLRG-YNFDLYPIRWSKLLSQKW
RNRVKQDR--KNKNQCDSYEKKG--LIDYKKQ-KKKKKYIYDSLSYKSINANIRGMDRKYF
DWRMLHFSIKFFDWMRMSEKKISRFFP-KFLILYNAYKIKPWMIPIKSLLFDSKKKIEGKLG
VLRTGHFRFQLNWP---SKSLFMNLNGFSFMLRRLDLKKLSLAAIRNNEFSLEFLLPPSDDIT
FHELLEGIFIIEPVRLSLKNDGKFIMSQTIGISLVQKNKYNHYDLLVPENILSPRGRREFR--IL
IIFLWPNYRLENLACMNRYWFDTNNSSRFSMLRIHMYPVKGQQFKLWILEF----REIKNSH
YFLDSWTQFNSLGSFIHIFFHQERFIKLLDSRIWSILLS-RNS--QGSTSNRYF---TIKGVVLFV
VAVVLIYR----INNRKMVERKNLYLTGFLP-----IPMNFIGPRNDTLEESF-GSSNINRLIVL-LL
YLP---

---------------------------------------TSFVQVTDSSQLKGSS-DQSRDHFDSIS----NEDSE-----------
-YHTLINQKEIQQP---LPEEIEEF--LGNPTRSIRS
FFSDRW--SELH-MGSNPIERSTRDQ----KLLKKEQDVS-FVPSRRSE----NKEIVNIFKIITYLQ
N--TVSIHPISSDPGCDMVP-KDELD-MDSSNKISFLKKNPFIYL--FHLFHDRNRRGYTL--HH
DFESE--ERFQEMADLFTLSITEP-DLVYHKGFAFSIDSYGLDQKQ--FLNEVFNSRDESKKKY
L---LVLPPIFY--EENDSFYRRIRKKWVR-TSPGNDLEDPKQKIVVFASNNIMEAVNQYRLIRN
LIQIQY--YR--YIRNVLNRFFLMN---RSDRDLEYGIQ---RDQIGNDTLNHRTIMKYTINQHLS
NFKK-GQKKWFDP--LILISRTERSMNRDPNAYRYKWSNGSKNFQEH---FISEQKSHF-HFQV
VFDRLRINQYSIDWSEVIDKKDWSKSLRFFLSKLLV---------FLS-------KFLLFLSNSLP-FFFV
SFGNIPIHRSE-IHIY-ELKGPNDRLCNQLLESIGLQIVHLKKLKPF--LLD---DHYTS-QKSKFL
INGGTISPFLFNKIP-KW-MIDSFDTR--NNRRKSFDNTDSYFSMISHDQDNWLNPVKPFHRS
SLISSFYKATRLRFLNNPHHFCFY--CNKRFPFYVDY-VEKARINNYD--FTYG-QFLNILFIRN
K-IFSLCGGK--KKHAFLE-RDTISPIESQVSNIFIPNDFPQSGDER-YNLYKS---FHFPIRSNPFV
R--RAIYSIADISVTPLTEGQIVNFERTYCQPLSDMNLSDSEGKNLHQYLN--FNSNMGLIHTP
CSEKYLPSEK--RKK--------QSLYRK--KCLEKGQMYRTFQRDSAFSTLSKWNLFQTYMPWF
FTSTGYKYLNLLFLDTFSD-LLPILSSS--PKFVSIFDDIMHRSDRSWRILRKKL------CLPQW
N-LISEISIKCLPN--LLLSEEMIHRNNES--PSISTHLRSPNVREF-LYSILFLLLIAVY-LVRTHLL
FVSR--AYSELQTEFEKVKSLMIPSYMIELRKLLDRYPTYERNSFWLKNLFLVALEQLGDSL
EEIWGSASGG------GP-----AYG-VKSIRSKKKDWNINLIN---LI-SIIPNPINRIAFSRNTRHLSH
TSKEIYSLIR--KNVNGDWIDDKIQSWVWNSDSIDDKEREFLVQFS--TLTT--EKRIDQILLSLT
-HSDHLS-----KNDSGYQMIEQPGVIYLRYLVDIQ--KKYLMNYKFNTSCLAERRTFLAHYQT
ITH--SQTSCGANSFHFPSHG---KLFSLRLALSP---GILVIGSIGTGRSYLVKYLATNSYLPFITV
FLNKFRDNKPK--FID-----DSDDIDDSGDIDDSDDIDR--------------------DLDIDTELELLTMM
NAL---TMEMKL---EIDQFYITLQFELIKAMSPCIIWIPNIHDLYVNKSSHLYFGLLVNYLY---R
DFERCS-TTNILVIASTHIPQKVDPALIAPNKLNTCIKIRRLLIPQQRKHFFTLSYTRGFHLEK
KMFHT--NGFGSITMGSNVRDLVALTNEALSISITQRKSIIDTNIIRSALHRQTWDLR---SQVR
SVQDHGILFYQIGRALAQNVLLSNCSIDPISIYMKKK--SCNEGGSYLYNWYFELETSMKKL
TILLYLLNCSAGSVVQDLWSLS--GPDEK-NGITSYVLVENDS-HLVHGLLEVEGALFG--SS
WTEKDCSRFDND--RVTLLLRPEPRN-PLDMIQNGSSSIVDQIFLYQK--YESKFEE---GEGVL
DPQ----QIEED----LFNH--IVWAP--RIWSP---WGFLFDCIERPNELGVP-YWARSFRGK---RIIY
DEEDELQENDSEFLQSGTVQYQTRDSSSKEQGFFLINQFIWDPADPLFFLFQ-DHPFVSVFS
HREFFADEEMAKGLLTSQPAFPTSLEKRWFINIKNTQEKYVELLIHRQRWLRTR-TNSSLSK
SNGFFRSN--TLSESYQYLSNLFLSN-GTLLDQMTKTLLRKRWLFPDEMK--------------MPRS
GGINGNFIDKTFSIVANILLRIIPTTSGEKEAFTYYRDG--MSAQSEGNYAEALQNYYEAMRL
EIDPYDRSYILYNIGLIHTRNGEHTKALEYYFRALERNPFLPQAFNNMAVICHY-RGEQAIR
QGDSEIAEAWFNQAAEYWKQAIALTPGNYIEAQNWLKITRRFK--MTLAIRTYMIGFLRGS
RKISNFCWAFILFLGSLGFLLVGTSSYLGRDLIPLFPSQQIIFFPQGIVMSFYGIAGLFISSYLW
GTILWNVGSGYDRFDRKEEIVSIFRWGFPGKNRRIFLRFLMKDIQSIRIEVKEGIYARRVLY
MEIRGQGAIPLTRTDQN-LTPREIEQKAAELAYFLRVPIEVF
>Acorus
??
??
??

??
??
???????????????????????????????MATLRADEISNIIRERIEQYTREVKVVNTGTVLQVGDGIA
RIHGLDEVMAGELVEFEEGTIGIALNLESNNVGVVLMGDGLTIQEGSSVKATGRIAQIPVSE
AYLGRVVNALAKPIDGRGEIAASEFRLIESPAPGIISRRSVYEPLQTGLIAIDSMIPVGRGQR
ELIIGDRQTGKTAVATDTILNQKGQNVICVYVAIGQKASSVAQVVTNFQERGAMEYTIVVA
ETADSPATLQYLAPYTGAALAEYFMYRERHTSIIYDDLSKQAQAYRQMSLLLRRPPGREA
YPGDVFYLHSRLLERAAKSSSRLGEGSMTALPIVETQSGDVSAYIPTNVISITDGQIFLSADL
FNAGIRPAINVGISVSRVGSAAQIKAMKQVAGKSKLELAQFAELEAFAQFASDLDKATQNQ
LARGKRLRELLKQSQSEPLAVDEQVVTIYTGTNGYLDTLEIGQVKEFLVKLRTYLKKNKP
QFQEIISSTKTFTEEAEALLKEAIQEQLELFLLQEQTMRITPTTS-SAVPSLDEKNLGRIVQIIG
PVLDVAFPPGKMPNIYNALVVKGRDTAGQPINVTCEVQQLLGNNRVRAVAMSATDGLTRG
MEVIDTGAALSVPVGGATLGRIFNVLGEPVDNLGPVDTRTTSPIHRSAPAFIQLDTKLSIFET
GIKVVDLLAPYRRGGKIGLFGGAGVGKTVLIMELINNIAKAHGGVSVFGGVGERTREGND
LYMEMKESGVINEEKIEESKVALVYGQMNEPPGARMRVGLTALTMAEYFRDVNEQDVLL
FIDNIFRFVQAGSEVSALLGRMPSAVGYQPTLSTEMGSLQERITSTKEGSITSIQAVYVPAD
DLTDPAPATTFAHLDATTVLSRGLAAKGIYPAVDPLDSTSTMLQPRIVGEEHYETAQRVKQT
LQRYKELQDIIAILGLDELSEEDRLTVARARKIERFLSQPFFVAEVFTGSPGKYVGLAETIRG
FQLILSGELDGLPEQAFYLVGNIDEATAKAMNLEVESKLKKMNLKLCLLTPNRIIWDSEVQ
EIILSTNSGQIGILPNHASIATAVDIGLLRIRRDDRWLAVALMGGFARIGNNEITILGNDAEM
GTDIDPQEAQRALEIAEANLSKAEGKRQAIEANLALRRARTRAEAINVISMKNITDSFVSL
GHWPFAGSFGFNTDILATNLINLSVVLGVLIFFGKGVLSDLLDNRRQRILSTIRNSEELRRG
ALEQLEKARARLRKVEMEADEYRVNGYSEIEREKMNLINATYENLERLENYKNETLHFE
QQRAINQVRQRVFQQALQGALGTLNNCLNSELHFRTISANIGMLGAMKEITDMNPLISAA
SVIAAGLAVGLASIGPGVGQGTAAGQAVEGIARQPEAEGKIRGTLLLSLAFMEALTIYGLV
VALALLFANPFVMNVILCSSNMLKGLYDISGVEVGQHLYWQIGGFQVHAQVLITSWVVIA
ILLGSVTVAVRNPQTIPTNGQNFFEYVLEFIRDLSKTQIGEEYGPWVPFIGTMFLFIFVSNWS
GALLPWKLIELPHGELAAPTNDINTTVALALPTSVAYFYAGLTKKGLGYFGKYIQPTPILLPI
NILEDFTKPLSLSFRLFGNILADELVVVVLVSLVPLVVPIPVMFLGLFTSGIQALIFATLAAAY
IGESMEGHHMIFSTLEHILTHISFSIISVVITIQLMNLLVHELVQLGDASEKGMIATFFSITGLL
VTRWIYSGHFPLSDLYESLIFLSWSFSIIHMVPYFRNHRNHFSAITAPSAIFTQGFATSGLLTE
MHQSIILVPALQSQWLMMHVSMMLLSYAALLCGSLLSVALLVITFRKNSDIFDKRNEIEYL
NEKRSVLQNTSFDSFTNYHKYQLTQRLDFWSYRVISLGFIFLTIGILSGAVWANEAWGSYW
NWDPKEIWAFITWAIFAIYLHTRTNHSLQGANSAIVASIGFLIIWICYFGVNLLGIGLHSYGS
FTLIVPKKGFT-PLPYLASIVFLPWWVSLSFNKSLEPWVTNWWNTRQSETFLNDIQERNVL
ERFIELEELFLLDEMLKENPETRMKNLRIGIHNETIQLVKTDNEYHLHTILHFSTNIICFAILS
VYSILGNEELVILNSWVQEFLYNLSDTIKAFSILLVTDLWIGFHSPHGWELMIGSVYNDFGL
AHNEQIISGLVSTFPVILDTIVKYWIFHYLNRVSPSLVVIYHSMNDMPIGVPKVPFRSPGEED
AAWVDIYNRLHRERLLFLGQELDSEISNQLVGLMVYLSIEDKTRDFFLFINSPGGWVIPGIG
LYDTMQFVPPDVHTICMGLAASMGSFILVGGEITKRLAFPHARVMIHQPASSFYEAQAGEF
VLEAEELLKLRETLTRVYVQRTGKPLWVVSEDMERDVFLSATEAQAHGIVDLVGDEMKE
QKLIHEGLITESLPNGMFWVRLDNEDLVLGYISGRIRRSSIRILPGDRVKIEVSRYDSTRGRII
YRLRNKDSNEMEEFKVYFEKDGSRQQYFLYPLLFQEYIYALAHNHVLFVYDN-FSLIIVKR
LITQMYQQNSLSNLVNDSNRNRLVRKNFYYQTILEGFSVIMEIPFSIIFLSSVIPKIQNLRSIH

ATFPFLEDKLSHLNHVSDILIPYPIHLEILVQVLQGWIQDVPSLHLLRFFLHEFHNWNSLITK
SISFFSKQRLFLFLYNSYVFECESALVFLRKQSFYLRSTSFGTFVERTHFYGKIEH--VVRRK
QKALSLFKDPFIHYIRYKGKSILASKGTHFLMKKWKYHLLNFWQCHFDFWSQPHRIHINQ
LSNHSFYFMGYLSSVRINFSTVRSQMLESSFLMDTPT--KKFDTIVPIIPLIGSLAKAKFCNVS
GHPVSKPVWADLSDADIIDRFGRICRNLSHYHSGSSKKQSLYRVKYILRLSCARTLARKHK
STVRAFFKRLGSEFLEEFFTDKEQVLSLIFPSRIWYLDIIRINDLVNNSMIIATT-EIQ-AINSFS
RSESSLKEVYGLIWLLVPIFTLILVIIIGVLVIVWLEREISAGIQQRIGPEYAGPLGILQALADG
TKLLFKEDLLPSRGDISLFSLGPSIAVISTLLSYLVIPFGYHLVLADLSIGVFLWIAISSIAPIGL
LMSGYGSNNKYSFSGGLRAAAQSISYEIPLTLCVLSISLLSNSSSTVDIVEAQSKYGFWGW
NLWRQPIGFLVFLVSSLAECERLPFDLPEAEEELVAGYQTEYSGIKFGLFYVASYLNLLVSSL
FVTVLYLGGWNLSIPYIFIPELFGKNKTGGIFGMTIGILITLAKAYLFLFISIATRWTLPRLRID
QLLNLGWKFLLPISLGNLLLTTSSQLVSLMIWHVQNENFILDSTRIFMKAFHLLLFDGSFIFP
ECILIFGLILLLMIDSTSDQKDRPWFYFISSTSLVMSIAALLFRWREEPMISFSGNFQTNNFN
EIFQFLILLCSTLCIPLSVEYIECTEMAITEFLLFVLTATLGGMFLCGANDSITIFVAPECFSLC
SYLLSGYTKRDVRSNEATMKYLLMGGASSSILVHGFSWLYGLSGGEIELQEIVNGLINTQM
YNSPGISIALIFITVGIGFKLSLAPFHQWTPDVYEGSPTPVVAFLSVTSKVAASASATRIFDIP
FYFSSNEWHLLLEILAILSMILGNLIAITQTSMKRMLAYSSIGQIGYVIIGIIVGDSNDGYAS
MITYMLFYISMNLGTFARIVLFGLRTGTDNIRDYAGLYTKDPFLALSLALCLLSLGGLPPLA
GFFGKLHLFWCGWQAGLYFLVSIGLLTSVVSIYYYLKIIKLLMTGRNQEITPHVRNYRRSP
LRSNNSIELSMIVCVIASTIPGISMNPILAIAQDTLFMFLLHEYDIFWAFLLISSVIPILAFLISG
VLAPTREGPEKLSSYESGIEPIGDAWVQFRIRYYMFALVFVVFDVETVFLYPWAMSFDVLG
VSVFLEALIFVLILIVGSVYAWRKGALEWSTSYFPWLTIIVVFPISAGLSIFFLPHRGNKVVR
WYTICICLLELLLMTYVFCYHFQLNDPLIQLDEDYEWINIFDFHWRPGIDGLSIGPILLTGFI
TTLATLAAWPVTRDSRLFHFLMLAMYSGQIGLFSSRDLLLFFLMWELELIPVYLLLSMWG
GKKRLYSATKFILYTAGGSVFLLMGVPGMGLYGSNEPTLNFETSANQSYPVSLEILFYFGFL
IAYAVKLPIIPLHTWLPDTHGEAHYSTCMLLAGILLKMGAYGLVRINMELLPHAHSIFSPWL
VLAGTLQIIYAASTSLGQVNLKKRIAYSSVSHMGFTIIGIGSITDTGLNGAILQLLSHGFLGA
ALFFLAGTSCDRIRLIYLDEMGGISIPMPKIFTMFSSFSMASLALPGMSGFVAEAVVFFGIITS
QKFLFLPKILITFVMAIGMILTPIYLLSMLRQIFYGYKLFNMTNSYFMDSGPRELFVSICIFLP
VIGIGIYPDFVLSLSVDKVEAILSNYFYRMMLEYVLFLSAYLFSIGIYGLITSRNMVRALMC
LELILNAVNINLVTFSDLFDSRQLKGDIFSIFVIAIAAAEAAIGPAIVSSIYRNRKSIRINQSNL
LNKMEHTYQYAWIIPFLPFPVTISIGLGLLLVPTATKNLRRIWAFFSVLLLSIAMVFSADLAI
QQINGSFIYQYSWSWTINNTFSLEFGYLIDPLTSIMLILITTVGIMVLIYSDNYMSHDQGYLR
FFAYMSFFNTSMLGLVTSSNLIQIYIFWELVGMCSYLLIGFWFTRPTAANACQKAFVTNRV
GDFGLLLGILGLYWITGSFEFRDLFEILKNNEVNSLFAALCASLLFVGAVAKSAQFPLHVW
LPDAMEGPTPISALIHAATMVAAGIFLVARLLPLFTVIPYIMNFISLIGIITVLLGATLALAQR
DIKRSLAYSTMSQLGYIMLAPGIGSYRAALFHLITHAYSKALLFLGSGSIIHSMEPIVGYSPE
KSQNMILMGGLRRYVPITKTTFFLGTLSLCGMPPLACFWSKDEILNDTWLYSPIFAIIAWST
AGLTAFYMFRVYLLTFDGHLQVHFQNFSSTKNSSFYSISIWGKEVPKPLNV-NLFLSTMNK
MSNKY--TSMYPHESDNTMLFPMLVLVLFTLFIGFIGIPFDLDILSKWLTPSINLLHSNSGDSF
DWYEFVTNAIYSVTISFLGIFLAYIFYGSVYSSFQNLDLINSFVILSDRIINGIYNWSYNRGYI
DVFYGKVLSNTIRGLAELIHFFDRRVIDGITNGVGVVSFFVGEGIKS-VGGGRISSYIFLYAFS
VSICLIIYYMDLPGPIHDVLLVFLGSGLILGGLGVVLLTNPIYSAFSLGLVLVCISLFYILSNS
YFVAAAQLLIYVGAVNVLIIFAVMFMNGSDYSNDFYLWTVGDGVTSLVCTSILFSLITTILD

TSWYGIIWNTGSNQIVEQDLTSNVQQIGIHLSTDFYLPFELVSIILLVALIGAITMARQMTVP
ATRQDLMIVNMGPHHPSMHGVLRLIVTLDGEDVIDCEPILGYLHRGMEKIAENRTIIQYLP
YVTRWDYLATMFTEAITVNGPEQLGNIQIPKRASYIRVIMLELSRIASHLLWLGPFMADIG
AQTPFFYIFRERELIYDLFEAATGMRMMHNYFRIGGVAADLPYGWIDKCLDFCDYFLTGIV
EYEKLITQNPIFLERVERVGIISGEEAINWGLSGPMLRASGIEWDLRKVDNYECYNEFDWE
VQWQKEGDSLARYLVRISEMKESIKIIQQALEGIPGGPYENLEVRR-DKVKDSEWNDFEYR
FISKKPSPTFELAKQELYVRVEAPKGELGIFLIGDNSVFPWRWKIRPPGFINLQILPQLVKRM
KLADIMTILGSIDIIMGEVDRMFPMVTGFMNYGQQTVRAARYIGQSFMITLSHANRLPVTI
QYPYEKLITSERFRGRIHFEFDKCIACEVCVRVCPIDLPVVDWRLDTDVRKKQLLNYSIDF
GVCIFCGNCVEYCPTNCLSMTEEYELSTYDRHELNYNQIALGRLPMSVIEDYTIRTTTNLKI
AKNKPLGSRTITN-MQGRLSAWLVKHELVHRSLGFDYQGIEILQIKPEDWDSIAVISYVYGY
NYLRSQCAYDVAPGGFLASVYHLTRIQYGVDQPEEVCIKVFAPRRNPKIPSVFWIWRSADF
QERESYDMLGISYENHPRLKRILMPESWIGWPLRKDYIAPNFYEIQDAH----------------------
-------------------------------------MNSIEFPLLDRTTPNSVISTTLNDLSNWSRLSSLWPLLYGTSC
CFIEFASLIGSRFDFDRYGLVPRSSPRQADLILTAGTVTMKMAPSLVRLYEQMPEPKYVIAM
GACTITGGMFSTDSYSTVRGVDKLIPVDVYLPGCPPKPEAVIDAITKLRKKLSREISEDRMG
SQ-ENRCFTTNHKFYVRRSTHTGNYDQGLLYQSPPTAEIPTEPFFKYKSSVSSRELGNMQN
RNTFSWVKEQMTRFISVSIMIYVITRTSIANAYPIFAQQGYENPREATGRIVCANCHLANKP
VDIEVPQAVLPDTVFEAVVRIPYDKQLKQVLANGKKGSLNVGAVLILPEGFELAPPDRISPE
MKEKMGNLAFQSYRPTKKNIIVIGPVPGQKYSEIVFPILSPDPATKKDVHFLKYPIYVGGNR
GRGQIYPDGSKSNNTVYNATAAGVVSRILRKEKGGYEITIADASDGHQVVDIIPPGPELLVS
EGESIKLDQPLTSNPNVGGFGQGDAEIVLQDPLRVQGLLFFLASVILAQIFLVLKKKQFEKV
QLYEMNFMSKVYDWFEERLEIQAIADDITSKYVPPHVNIFYCLGGITLTCFLVQVATGFAM
TFYYRPTVTDAFASVQYIMTEVNFGWLIRSVHRWSASMMVLMMILHVFRVYLTGGFKKP
RELTWVTGVVLGVLTASFGVTGYSLPWDQIGYWAVKIVTGVPDAIPVIGSPLVELLRGSAS
VGQSTLTRFYSLHTFVLPLLTAVFMLMHFPMIRKQGISGPLMGVTKKPDLNDPVLRAKLA
KGMGHNYYGEPAWPNDLLYIFPVVILGTIACNVGLAILEPSMIGEPADPFATPLEILPEWYF
FPVFQILRTVPNKLLGVLLMASVPLGLFTVPFLENVNKFQNPFRRPVATTVFLIGTAVALWL
GIGATLPIDKSLTLGLFMIEVFLFGIVLGLIPITLAGLFVTAYLQYRRGDQLDLMPTITSYFGF
LLAASTITTALFIGLSKIRLIMDIVSLAWAALMVVFTFSLSLVVWGRSGLMIIRSPEPEVKIV
VDRDPIKTSFEAWARPGHFSRTIAKGPDTTTWIWNLHADAHDFDSHTSDLEEISRKVFSAH
FGQLSIIFLWLSGMYFHGARFSNYEAWLSDPTHIRPSAQVVWPIVGQEILNGDVGGGFRGI
QITSGFFQIWRASGITSELQLYCTAIGALVFAALMLFAGWFHYHKAAPKLAWFQDVESML
NHHLAGLLGLGSLSWAGHQIHVSLPINQFLDAGVDPKEIPLPHEFILNRDLLAQLYPSFAEG
ATPFFTLNWSKYAEFLTFRGGLDPITGGLWLSDIAHHHLAIAILFLIAGHMYRTNWGIGHGL
KDILEAHKGPFTGQGHKGLYEILTTSWHAQLALNLAMLGSLTIVVAHHMYSMPPYPYLAT
DYGTQLSLFTHHMWIGGFLIVGAAAHAAIFMVRDYDPTTRYNDLLDRVLRHRDAIISHLN
WACIFLGFHSFGLYIHNDTMSALGRPQDMFSDTAIQLQPIFAQWVQNTHALAPVATAPGAT
TGTSLAWGGGELVAVGGKVALLPIPLGTADFLVHHIHAFTIHVTVLILLKGVLFARSSRLIPD
KANLGFRFPCDGPGRGGTCQVSAWDHVFLGLFWMYNSISVVIFHFSWKMQSDVWGSISD
QGVVTHITGGNFAQSSITINGWLRDFLWAQASQVIQSYGSSLSAYGLFFLGAHFVWAFSLM
FLFSGRGYWQELIESIVWAHNKLKVAPATQPRALSIVQGRAVGVTHYLLGGIATTWAFFLA
RIIAVGMALRFPRFSQGLAQDPTTRRIWFGIATAHDFESHDDITEERLYQNIFASHFGQLAIIF
LWTSGNLFHVAWQGNFESWVQDPLHVRPIAHAIWDPHFGQPAVEAFTRGGAPGPVNIAYS

GVYQWWYTIGLRTNEDLYTGALFLLFLSAISLIGGWLHLQPKWKPSVSWFKNAESRLNH
HLSGLFGVSSLAWTGHLVHVAIPASRGEYVRWNNFLDVLPHPQGLGPLFSGQWNLYAQNP
DSSSHLFGTSQGAGTAILTLLGGFHPQTQSLWLTDMAHHHLAIAFIFLIAGHMYRTNFGIGH
SIKDLLEAHTPPGGRLGRGHKGLYDTINNSLHFQLGLALACLGVITSLVAQHMYSLPAYAFI
AQDFTTQAASYTHHQYIAGFIMTGAFAHGAIFFIRDYNPEQNEDNVLARMLDHKEAIISHL
SWASLFLGFHTLGLYVHNDVMLAFGTPEKQILIEPIFAQWIQSAHGKTLYGFDVLLSSTSGP
AFNAGRSIWLPGWLSAVNE--NSNSLFLTIGPGDFLVHHAIALGLHTTTLILVKGALDARGS
KLMPDKKDFGYSFPCDGPGRGGTCDISAWDAFYLAVFWMLNTIGWVTFYWHWKHITLW
QGNVSQFNESSTYLMGWLRDYLWLNSSQLINGYNPFGMNSLSVWAWMFLFGHLVWATG
FMFLISWRGYWQGLIETLAWAHERTPLANLIRWRDKPVALSIVQARLVGLAHFSVGYIFTY
AAFLIASTSGKFGMSHSVKIYDTCIGCTQCVRACPTDVLEMIPWDGCKAKQIASAPRTEDC
VGCKRCESACPTDFLSVRVYLGNETTRSMGLPYMTDLNLPSIFVPLVGLVFPAIAMASLFL
HVQKNKIVMQDIKTYLSVAPVLTTLWFGSLAGLLIEINRLFPDALSFPFF--MTAILERRESTS
LWGRFCNWITSTENRLYIGWFGVLMIPTLLTATSVFIIAFIAAPPVDIDGIREPVSGSLLYGN
NIISGAIIPTSAAIGLHFYPIWEAASVDEWLYNGGPYELIVLHFLLGVACYMGREWELSFRL
GMRPWIAVAYSAPVAAATAVFLIYPIGQGSFSDGMPLGISGTFNFMIVFQAEHNILMHPFHM
LGVAGVFGGSLFSAMHGSLVTSSLIRETTENESANEGYRFGQEEETYNIVAAHGYFGRLIF
QYASFNNSRSLHFFLAAWPVVGIWFTALGISTMAFNLNGFNFNQSVVDSQGRVINTWADII
NRANLGMEVMHERNAHNFPLDLASVEAPSTNGMGLPWYRVHTVVLNDPGRLLSVHIMH
TALVAGWAGSMALYELAVFDPSDPVLDPMWRQGMFVIPFMTRLGITNSWGGWSIT-GGTI
TNPGIWSYEGVAGAHIVFSGLCFLAAIWHWVYWDLEIFCDERTGKPSLDLPKIFGIHLFLS
GVACFGFGAFHVTGLYGPGIWVSDPYGLTGKVQPVNPAWGAEGFDPFVPGGIASHHIAAG
TLGILAGLFHLSVRPPQRLYKGLRMGNIETVLSSSIAAVFFAAFVVAGTMWYGSATTPIELF
GPTRYQWDQGYFQQEIYRRVGAGLAENLSLSEAWSKIPEKLAFYDYIGNNPAKGGLFRAG
SMDNGDGIAVGWLGHPVFRDKEGRELFVRRMPTFFETFPVVLVDGDGIVRADVPFRRAES
KYSVEQVGVTVEFYGGELNGVSYSDPATVKKYARRAQLGEIFELDRATLKSDGVFRSSPR
GWFTFGHASFALLFFFGHIWHGARTLFRDVFAGIDPDLDAQVEFGAFQKIGDPTTRRQAV
MKTLYSLRRFYPVETLFNGTLALAGRDQETTGFAWWAGNARLINLSGKLLGAHVAHAGL
IVFWAGAMNLFEVAHFVPEKPMYEQGLILLPHLATLGWGVGPGGEVIDTFPYFVSGVLHLI
SSAVLGFGGIYHALLGPETLEESFPFFGYVWKDRNKMTTILGIHLILLGIGAFLLVFKALYF
GGVYDTWAPGGGDVRKITNLTLSPSVIFGYLLKSPFGGEGWIVSVDDLEDIIGGHVWLGSI
CILGGIWHILTKPFAWARRAFVWSGEAYLSYSLGALAVFGFIACCFVWFNNTAYPSEFYGP
TGPEASQAQAFTFLVRDQRLGANVGSAQGPTGLGKYLMRSPTGEVIFGGETMRFWDLRA
PWLEPLRGPNGLDLSRLKKDIQPWQERRSAEYMTHAPLGSLNSVGGVATEINAVNYVSPR
SWLATSHFVLGFFFFVGHLWHAGRARAAAAGFEKGIDRDLEPVLSMTPLNMTIALGRFTK
EE-DLFDIMDDWLRRDRFVFVGWSGLLLFPCAYFALGGWFTGTTFVTSWYTHGLASSYLE
GCNFLTAAVSTPANSLAHSLLLLWGPEAQGDFTRWCQLGGLWTFVALHGAFGLIGFMLRQ
FELARSVQLRPYNAIAFSGPIAVFVSVFLIYPLGQSGWFFAPSFGVAAIFRFILFFQGFHNWT
LNPFHMMGVAGVLGAALLCAIHGATVENTLFEDGDGANTFRAFNPTQAEETYSMVTANR
FWSQIFGVAFSNKRWLHFFMLFVPVTGLWMSALGVVGLALNLRAYDFVSQEIRAAEDPEF
ETFYTKNILLNEGIRAWMAAQDQPHENLIFPEEVLPRGNALMSGSTGERSFADIITSIRYWV
IHSITIPSLFIAGWLFVSTGLAYDVFGSPRPNEYFTESRQGIPLITGRFDPLDQLDEFSRSFMT
IDRTYPIFTVRWLAVHGLAVPTVSFLGSISAMQFIQRMATQTVEGSSRSG-PRRTIVGDLLKP
LNSEYGKVAPGWGTTPFMGVAMALFAIFLCIILEIYNSSVLLDGVSPNMLTLKLFVYTVVIF

FVSLFIFGFLSNDPGRNPGREEMADTTGRIPLWLIGTVAGIPVIGSVGVFFYGSYSGLGSSL
MLNILNLICICLNSAPYSSSFFCAKLPEAYAVFNPIVDFMPVIPVLFFLLAFVWQAAVSFRMT
QSNPNEQNVELNRTSLYWGLLLIFVLAVLFSNYFFNMEVNILAFIATALFILVPTAFLLIIYV
KTVSQNDMETATLVAIFISGLLVSFTGYALYTAFGQPSQQLRDPFEEHGDMEALVYTFLLVS
TLGIIFFAIFFREPPKVPTKKMKMTIAFQLAIFALIATSSILLISVPVVFASPDGWSSNKNVVF
SGTSLWIGLVFLVGILNSLISMSCREGLMSPQTETKAGVGFKAGVKDYKLTYYTPEYETKD
TDILAAFRVTPQPGVPPEEAGAAVAAESSTGTWTTVWTDGLTSLDRYKGRCYHIEPVVGE
QNQYIAYVAYPLDLFEEGSVTNMFTSIVGNVFGFKALRALRLEDLRIPPAYSKTFQGPPHGI
QVERDKLNKYGRPLLGCTIKPKLGLSAKNYGRAVYECLRGGLDFTKDDENVNSQPFMRW
RDRFLFCAEAIYKAQAETGEIKGHYLNATAGTCEEMMRRAQCARELGVPIVMHDYLTGG
FTANTSLAIYCRNNGLLLHIHRAMHAVIDRQKNHGMHFRVLAKALRMSGGDHIHAGTVV
GKLEGEREMTLGFVDLLRDDYIEKDRSRGIFFTQDWVSMPGVLPVASGGIHVWHMPALTE
IFGDDSVLQFGGGTLGHPWGNAPGAVANRVALEACVQARNEGRDLARESTQIIREACKWS
PELAAACEVWKEIKFEFEPVDKLDVTAIHLYKTSTSSTRNGAVDSQVKSNPRNNLIYGQHH
CGKGRNARGIITAGHRGGGHKRLYRKIDFRRNKKDISGRIVTIEYDPNRNAYICLIHYGDG
EKRYILHPRGAIIGDTIVSGTEVPISMGNALPLTDMPLGTAIHNIEITLGKGGQLARAAGAVA
KLIAKEGKSATLRLPSGEVRLISKNCSATVGQVGNVGANQQSLGRAGSKCWLGKRPVVR
GVVMNPVDHPHGGGEGRAPIGRKKPTTPWGYPALGRRSRKRNKYSDRFILRRRK-MIQPQ
TLLNVADNSGARELMCIRIIGAS-NRRYAHIGDVIVAVIKEAVPNMSLERSEVVRAVIVRTCK
ELKRDNGMIIRYDDNAAVVIDQEGNPKGTRVFGAIARELRHLNFTKIVSLAPEVLMLSPKR
TRFRKQHRGRMKGMSYRGSHICFGRYALQALEPSWITSRQIEAGRRAMTRYARRGGKIW
VRIFPDKPVTIRPAETRMGSGKGSPEYWVSVVKPGRILYEMGGVSETVARAAIEIAASKMP
IRTQFIIAGMTRVRRGYIARRRRTKIRLFAATFRGAHSRLTRAATQQKMRALVSAHRDRGK
QKRDFRRLWITRINAVTREN--VCYSYSRLMHNLYKRQLLLNRKILAQIAILNKNCLHIISNE
IIK-----------MIKRAL-AQNIS-MSAYKARRVIDQIRGRSYEETIMILELMPYRASFPILKLVYS
AAANAINNMGLSEADLFISKAEVNGGTFVKKLRPRARGRSYAIKRPTCHITIVLK-KDKS---
----MDGIKYAVFTEKSIRLLGNNQ-YTSNVESGSTRTEIKHWVELFFGVKVIAMNSHRLPGK
GRRVGPIKGHTMHYRRMIITLQPGYSIPPLIEKRTMAVPKKRTSISKKRIRKNFWKKKGYFA
AVKAFSLAKSVSTGQSKSFFVRQTSNMAKGKDARLTVILECTSCVRNG--VNKE--GISRYIT
QKNRHNTSSRLELRKYCRYCYKHTIHGEIKKMKIRASVRKICEKCRLIRRRGRIIVICYNPK
HKQRQGMVREEVVGSTRALQWKCVESRADSKRLYYGRFILSPLLKGQADTIGIAMRRAL
LGEIEGTCITRAKSE--KVPHEYSTIAGIEESVHEILMNLKEIVLRSNLYGTRDASICVRGPRC
VTAQDIISPPSVEVVDTTQHIASLTEPIDLCIGLQIQRDRGYRMKTTKNSQDGSY--PIDAVSM
PVRNANHSIHSYGNGNQKQEILFLEIWTNGSLTPKEALHEASRNLIDLFIPFLHAEEE--DINF
EENQ-NRFTVPPFTFPDRLA-NLKKNKK-EIALKCIFIDQSELPPRTYNCLKRSNIHTLLDLLS
NSQEDLMKIEYFRIEDVKQILDTLQKH--FAIDLPKNKMPRD-GNEGMFTIPGFSQIQFEGFC
RFIDQGLMEEFHKFPKIEDTDQEIEFQLFVERYQLVEPLIKERDAVYESLTYSSELYVPAGLI
WKTGRDMQEQTIFIGNIPLMNSLGTFIVNGIYRIVINQILQSPGIYYRSELDHNGISVYTSTII
SDWGGRSELEIDRKSRIWARVSRKQKISILVLSSAMGSNLREILDNVCYPEIFLSFLNDREK
KKIGSKENAILEFYQQFACVGGDPVFSESLCKELQKKFFQQRCELGRIGRRNMNRRLNLDI
PQSNTFLLPRDVLAAADHLIGMKFGMGTLDD--MNHLKNKRIRSVADLLQDQFGLALVRL
ENAVRGTICGAIR-HKLI-LTPQNLVSSTS----LTTTYESFFGLHPLSQVLDRTNPLTQIVHGRK
LSYLGPGGLTGRTASFRIRDIHPSHYGRICPIDTSEGINVGLIGSLAIHARIGHWG-SIESPFY--
-EVVYQRSKE--TKMVFLSPSRDEYYTVATGNSLALNRGIQEEQIVPARYRQEFLTIAWEQIHL

RSIFPFQYFSIGASLIPFIEHNDANRALMSSNMQRQAVPLSRSEKCIVGTGLERQAALDSGV
SAIAECEGKIIYTDTHKIVLSGHGDTISIPLVMYQRSNKNTCMHQNPQVRRGKCIKKGQIL
ADGAATVGGELALGKNVLVAYMPWEGYNFEDAVLISERLVYEDIYTSFHIRKYEIQTHVTS
QG-PERITHEIPHLEAHLLRNLDRNGIVALGSWVETGDILVGKLTPQTANE-SSYAPEDRLLR
AILGIQVSTAKETCLKLPIGGRGRVIDVRWIQKKG-GSSYNPETIRVYISQKREIKVGDKVAG
RHGNKGIISKILSRQDMPYLQDGTPVDMVFNPLGVPSRMNVGQIFECSLGLAGDLLDRHY
RIAPFDERYEQEASRKLVFSELYEASKQTANPWVFEPEYPGKSRIFDGRTGDPFEQPVLIGK
SYILKLIHQVDDKIHGRSSGHYALVTQQPLRGRAKQGGQRVGEMEVWALEGFGVAHILQE
MLTYKSDHIRARQEVLGTTIIGGTIPTPEDAPESFRLLVRELRSLALELNHFLVSEKNFQINR
KEA-------MIDRYKHQQLQIGSVSPQQISAWANKILPNGEIVGEVTKPYTFHYKTNKPEKDG
LFCERIFGPIKSGICACGNYRVIGAEKEDPKFCEQCGVEFIDSRIRRYQMGYIKLACPVTHV
WYLKRLPSYIANLLDKPLKELEGLVYC--DFSFARP-IAKKPTFLRLRGLFEYEIQSWKYSIP
LFFTTQGFDTFRNREISTGAGAIREQLADLDLRIVIDNSSVEWKDLGDE-GSTGNEWEDRKI
GRRKDFLVRRMELAKHFIRTNVEPERMVLCLLPVLPPELRPIIQIDGGKLMSSDINELYRRV
IYRNNTLTDLLKTSRSTPGELVMCQEKLVQEAVDTLLDNGIRGQPMRDGHNKVYKSFSDV
IEGKEGRFRETLLGKRVDYSGRSVIVVGPSLSLHRCGLPREIAIELFQTFVIRGLIRQHLASN
IGIAKSKIREKEPIVWEILQEVMQGHPVLLNRAPTLHRLGIQAFQPVLVEGRAICLHPLVCK
GFNADFDGDQMAVHVPLSLEAQAEARLLMFSHMNLLSPAIGDPISVPTQDMLIGLYVLTM
GNRRGIFVNRYNPCNRRNY---QNKTVDNNNYKHTKEKKPYFLSSYDALGAYQQKRINLH
SPLWLRWRLDQR-VIGS--REVPIEVQYESLGTYQEIYGHYLIVRSVKKEILCIYIRTTVGHIS
FYREIEESVQGFCRAYSYGT-----------MAERADLVYHNKAIDGTAMKRLISRLIDHFGMAY
TSHILDQVKTLGFRQATATSISLGIDDLLTIPSKGWLVQDAEQQSFILEKHHSYGNVHAVEK
LRQSIEIWYATSEYLRQEMNPNFRMTDPSNPVHIMSFSGARGNASQVHQLVGMRGLMSDP
QGQMIDLPIQSNLREGLSLTEYIISCYGARKGVVDTAVRTSDAGYLTRRLVEVVQHIVVRR
TDCGTIRGISVQPRNGMTE--RMFFQTLIGRVLADDVYIGLRCIAARNQDIGIGLVNRFIT-----
-FRAQPVYIRTPFTCRSTSWICQLCYGRSPTHGDLVELGEAVGIIAGQSIGEPGTQLTLRTFH
TGGVFTGGTAEHVRAPSNGKIKFNEELVHPTRTRHGHPAFLCSIDLYVTVEGRDIIHNVNIP
PKSLILVQNDQYVESEQVIAEIRAGTSTF--FKERVQKHIYSESAGE-MHWSTDVYHAPEYT
YGNVHLLPKTSHLWILSGGPYR--SSIVSSSLHKDQDQTNAHSFSVERR--YISDLS--MTN-G
RVRHKLFSSDLS-G-GDRILDYS-RPDQIISKGHWNFIYPS-ILHEN--SDFLAKRRKNRFIIPFQ
YDQEGEKELIPHSGISIEIPIN--GMLRRNSILAYFDDTRYRRS--GITKY-TVEIDSIVK--KEDFI
EYRG-TKEFSSKY--VDRFFFIPEEVHILPGSSSIMVRNNSLIGVDTRITLNIRSRVGGLVRVER
KKKNIELKIVSGDIHFPGETDKISRHSGILIPPGTEKK--NSKESKT-KLKNWIYVQR--ITPTK
KK--YFVLVRPVATYEIADGINLATLFPQDLLQERDNVQLRVVNYILYGNGKPIRGISHTS-Q
LVRTCLVLNWDQEQNGSIE----GVRASFVEVRANDLIRDFIRIELCKSAILYTGKRKDIAGSG
LIHD----DRTNINPVYLKDKIPS-----FIQHQGTVG----TLLNRNK---ECQSLILLSSSNCFRIG--P
FNGSKYH---NVPKESDPIIPI-DSLGLLGTTVP-KIANFFYLSYHVIT---HNHILLT-KYLLRD---
HLKQAFQVLR---YCLMDENRR--IYNPDPCSNIIFNAFDLNWRFLHHDYSEETSTILS-LGQF
VCENVCLFKHGP---QIKSGQVIIVH-VDSFVIRAAKPYLATPGATVHGHYGEILYGGDTLITF
IYEKSRSGDITQGLPKVEQVLEVRSIDSISTNLEKRVEGWNEHITKILGIPWGFLIGAELTIA
QSRISLVNKIQKVYRSQGVQIHNKHIEIIVRQITSKVLVSE---DGMSNVFSPGELIGLLRAER
TGRALEEAICYRAILLGITRASLNTQSFISEASFQETARVLAKAALRGRVDWLKGLKENVV
LGAMIPVGTGFKALVHRSR--KPNNIH--LEIKKNNLFEGQVG-DDILFYHRELFGSCGPN-----
SEQSFMKFHDS------MTKRYWNINLEEMMEAGVHFGHGTRKWNPRMAPYISAKRKGIHIT

NLTRTARFLSEACDLLFDAASGGKHFLIVGTKKKAADSVASAAIRARCHYVNKKWLGGM
STNWSTTETRLQNFRDLRAEQKAGKIDRLPKRDAAMLKRQLSTLQTYLGGIKYMTGLPDI
VIIVDQQEDYTALRECVILGIPTICLIDTNSDPDLADISIPANDDAIASIRLILNKLVFAICEGRS
S-YIRNRMGQKINPLGFRLGATQSHLSLWFAQPKSYSMGLQEDEKIRECIKNYVQKNPRLS
SG-FEGIARIEIKKRIDLIQVIIYIGFPNLFIEG--RTQ--GIKELQMSVQKRLNSVNQRLNIAIAR
ISKPYGQPNILAEYIALQLKNRVSFRKAMKKAIELTEQADTKGIQIQIAGRIDGKEIARVEWI
REGRVPLQTIRAKIDHCFHKVQTIYGVLGIKIWIFVDEEMSRYRGPRLKKIRRLGALPGLTS
KGTRPGSDLRNQFRSGK-RSQYRIRLEEKQKLRFHYGLTERQLLRYVHIAGKAKGSTGQV
LLQLLEMRLDNILFRLGMAPTIPGARQLVNHRHILVNGRIVDIPSYRCKPRDIITTKDKQRS
KVLIQNNMDSSTRE--LPKHLTLDS--FQHKGLVNQIIDSKWVGLKINELLVVEYYSRQTMS
RRGTAEEKTAKSDPIYRNRLVNMLVNRILKHGKKSLAYQIIYRALKKIQQKTETNPLSVLR
QAIRGVTPDIAVKSRRVGGSTHQVPIEIGSTQGKALAIRWLLAASRKRPGRNMAFKLSSEL
VDAAKGSGDAIRKKEETHKMAEANRAFAHFRMGKDTIADIITSIRNAQMAKKGTVRIAST
NLTENVVKILLREGFIENVRKHRESNKDFLVLTLRHRR--TRKGIYR-----TILKRISRPGLRIY
SNYQGIPKILGGIGIVIVSTSRGIMTDREARLQGIGGEMLCYICMTKTIPRIGSRRNGRIGLR
KTGRRIPKGIIHVQASFNNTIVTVTDVRGRVVSWSSAGTCGFKGTRRGTPFAAQTAAGNAI
RTVVDQGMQRAEVMIKGPGLGRDAALRAIRRSGILLNFVRDVTPMPHNGCRPPKKRRVM
PTIKQLIRNTRQPIRNVTKSPALRGCPQRRGTCTRVYTITPKKPNSALRKVARVRLTSGFEIT
AYIPGIGHNLQEHSVVLVRGGRVKDLPGVRYHIVRGTLDAVGVKDRQQGRSKYGVKKPK
MARKGLIEREKKRKKLEQKYHSIRGSSKKEIR---KVPSLSDKWEIHGKLQSPPRNSAPVRL
HRRCFLTGRPRANYRDFGLSGHVLREMVQACLLPGATRSSWMVKNSFISLIPQEEKEE--N
RGSVEFQVFSFTNKIRRLTSHLELHRKDYLSQRGLRKILGKRQRLLAYLSKKNRVRYNKLI
GQLDIREPKTR--MVKLRLKRCGRKQRAIYRIVAIDVRSRREGRDLCKVGFYDPIKNQTYL
NVPVILNFLEKGAQPTETVHDILKKAEVFKELQGNQTK---MDKSKQLFRKSKRSFRRRLPP
IGSGDRIDYRNMSLISRFISEQGKILSRRVNRLTLKQQRLITIAIKQARILSSLPFLNNEK--QF
ERTESIPRPTGPRSRNKVTRSLKKNPFVANHLLEKIEKLNMKEEKEIIVTWSRASTIIPTMIG
HTIAIHNGKEHLPIYITDRMVGHKLGEFAPTLTFRGHARNDNKSRRMIFKSFLLGNLGSLYI
KIINSVVVVGLYYGFLTTFSIGPSYFFLLRARIMEEG---TEREVSATTGFITGQLMMFISIYYA
PLHLALGRPHTITVLVIPYLLFHFFSNNQKQFFDYGSTTRNSMRNLSIQCVFLTNLIFQLFNH
LMLPSSTLARLVNIYMFRCNNKMLFVTSSFVGWLIGHILFMKWVGLVVSWIRQNHSIRS-I
RSNKYLGLKLKSAIAQILSIIFFIACVNYLARLPDPSVTKKLNETEQDKPLVTCLFDYKRWN
RPFRYIKNKTFENAIRDEMSQYFFYTCLNNGKKKISFTYPPSLATFSEIIERKMSLYTTKKLT-
-DDDLYWVSNNEEKKNNLSNELINRIKVLDKGS---DVLEKRTRLCDDENEQECLPKKYDP
LLNGSYRGRIKKLESELEKIWKNKIHSLI-REFEHQDANNQFIEIEKIDKKIPRWVYKLIQQE
QQEEEGIRSRKGRRVVIYTDALIRYAQQSDFSRDLINGSIRAKRRKIVLWEMLQANAHSPL
FLDQVAKMVSFDLFDDLRETMNLIFRNREEDERIIISETWDNVMCAQAIRGCMLVTHSFIR
KYIILPLLIIAKNIGRILLFQLSEWDEDFKDWNREMHVKCTYNGVQLSETEFPQNWLKDGI
QIKILFPFSLKPWRESKKRKNDDFCFLTVWGMEAELPFGPPRNRPSFFKPIFEELDTNIRKV
E--NQSFVFKEKTKD------FLKKKTGWVTKIVL--LKNKIRNFFTKVNSIIS-NKITHESTVQIPS
SNWTNYSPIEKKMKDLSDRTITTRSQIERITKDKQKRSNETSCNAKRTELQKDILRIAKKRS
IRFIRKLHSFVKSFIERIYLDIFLCTINIPRINLQLFIHFISWDFSLLSQAYVLYKLSQTQVIKRY
QLKSVLQYAYPFLKDRIKDLFTPRIVHAKSRPKKLPISRINAWKNWLRGHYQYNLSQTRW
SELVPKKWREKFNQR-TIKNKRKLNSSEKEKNQQAHYAKEGKLKKDYRYDLLSHKYINE
YFIGSDRKYFDRRILRFDLRFFDWMGMREEILYRWFFPEFVLRYDAYKSKPRIISIKSLLLDF

KSKKEAELDFFLKKYLLFQLRWDELLNKKMMTNIKVYCLLLRLKNLKEIAISSIERGEMC
LDLMLMQKDLSLRELIKKGIFIIEPIRLSRKWDGKLIMYQTIGISLVAENKDNDYDLLVPENI
LSPRRRRELRILICFGFLWPNYRLEDLACMNRYWFDTTNGSRFSMLRIHMYPMKRHQFRF
WIFELREILREIKNSPHFLDSWTKFDSVRSLIHIFFHQERLMKLFDPRIWSILLS-RDS--QGST
SNRYFI---IKGLVLLVVA-VLIS----RINNRNMVERKNLYLRGLLP-----IPMNSIGPGNETLEES
F-WSSDINRLIVL-LLYLPKGKKTSESCFMDPKESLWLFPINKIN-QKCIMPESNRGSRWWRN
RIGKKR--DSSCKIS---TKTVAGIEISSKEKDLK-YLEFLFLSYT--DDPIRK------------------------
----
DR---FFS--KVQNVSSNIQYDS---TR--SIFVQVTDFSQSKGSS-DQSRDHFDSIS----KEDSE-----
--------YHTLIDQTEIPQLKGRSIP-GD--PSFLQTERTEIESD--RFPK--CLSGSS--PMSPLFP-EHE
KQMIIHRLPEEIDEFL--GNPTRSIRSFFSDRW--SELH-MGSNPTDRSTRDQ----KWLKKQQD
VS-FVSSRRSE----NKEMVDIFKIITYLQN--TVSIHPISSDPGCDMVP-KDEPD-MDSSNKISFL
NKNPFFD--LFHLFHDRNKGGYTL--HHDFESE--ERFQEMADLFTLSITEP-DLVYHRGFAFSI
DSCGLDQKK--LVNGKEKTKDESKKKSL---LVLPPLFY--EENAFFYRSIRKKPVW-IHCGND
L---------FASNNIMEAVNQYRLIRNLIQIQYSAYG--YIRNVLNRFFLM---NRSDRNFEYGIQ---
RDQIGNDTLNHITIMKYTINQHLSNLKK-SQKKWFDP----LISRTERSMNRDPNAYRYKWS
NGSKNFQEHLEHFVSEQKN---RFQVVFDQLRINQYSINWSEAIDKQDLSKSLRFFLSKS-----
----LLFLS-------KPLRFFLSKSLPLFFVSIGNSPIHRSE-IHIY-ELKGPNEPLCNQLLKSIGVQII
HLHKLKP---------DHDTS-QRSKFLINGGTISPFLFNKIP-KW-MIDSFHT--RKNRSKSFANTDS
YFSMISHDRDNWLNPVKPFHRSSLISSFYKANQLRFLNNPHHFWFY--CNKRFPFYV----EK
TRINNYD--RTY-GQFLNILFIRNK-IFSLCVGK----------KKHLLPIYSQVSDIFIPNDFPQSGDE
T-YNLYKS---FRFPIRPDPFVR--RALYSIADISGTPLTEEQIVNFERTYCQPLSDMNLSDSEGK
NLHQYLS--FNSNIGLIHTPCSEKNLP--YVKRKKQSLYLRKKRSLYLK--KCVEKGQMYRTS
QQRD--SAFSKWNLFQTYMPWFLTSTGCKYLTSVLLDIFSDRL--SILSSS-PKLVSILNSIMHR
SDISRLIK--KWW----TILPQWNL-ISEISSKCLQN--LLLSEEMIHRNNESPVSMILTHLRSTNA
REF-LYSILFLLLVAGY-LVRTHLLFVSR--VSNELQTELEKIKSLMIPSYMIELQKLLYRYPT---
-----------STSKSESESESEYEYELNSFLLNNLF-----ALE----------QLDWN----IDLI-SIISN---TIT
FSRNTRHLSRTSKEIYSLIRERKNVNSDWIEDRVESWVTINDLIDEDERDLAVQFS--TLTT--E
KRIDQILWSLT-HSHPVS-----KNDLDYQIIEQPGLISLRYLVDIH--KKDLMNYEFNGSCLAEK
RIFLANYRTITY--SQT---------PSRG---KPFSLRLDLSPS-RGILVIGSIGTGRSHLVKYLATNSY
VPFITVFPDRGLSDRPIYCFMDED--TDPDYIDDDIDID----------------------------SDNFDTELE
TIADVVT--MDNML---LKVIRFDILGQFELAKTMSPCIIWIPNIHDLYLNESNYLSLGLLTNFL
S---SLYGDCETTRNILVFASTHLPQLVDPVLIDPKKFNTCIKIRRLLSPQQRKHFFNLLYTRGF
HSENKMFHA--NGFGSITMGSNARDLVALTNEVLSISITQKKSILETNTIRLALHRQTWDLR-
--SHVRSVPDHGILFYQIGRAVAQNVLLSNCCTDPISIYMKKK--SCNEGFSYLYKWYYELGT
SMKKLTILLYLLSCSAGSVAQDLWSPP--GPDEN-SWITSDRFVENDS-DLVDSLLEIEGALVG
------------------SWLLRSEPRN-PLDMMQKRSCS------IFKK--DESEFAE-GEGALALDPQ------
--EM--LFYNQ--IVWAP--RLWRPP--CGKLFDCIEYSQE--------------------------DDDDWEFWQ
SGTKYYWMRD-SEGGQGFWVSQKFLWNPADPFLFLFK-DQPFVSDSDFSSYHQPFSNFLVE
LMPPFKSSVSLYKGWVN--KKLQEMCLEYLSDRERWLRT---NSSLS--HGSFRSN--TLSESY
QYLANLFLSN-ATLLDQMTKTLLRKRWLFPDEMKDLI--HGTGGGTELTMPRSR-INANFID
KTSTIVANILLRIIPTTSGEKEAFTYYRDG--MSAQSEGNYAEALQNYYEATRPEIDPYDRSYI
LYNIGLIHTSNGEHTKALEYYFRALERNPFLPQAFNNMAVICHY-RGEQAIRQGDSEIAEAW
SDQAAEYWKQAIALTPGNYIEAQNWLKITRRFE--MNWRSEHIWIEFITGSRKTSNFGWACI

LFLGSLGFLVVGASSYLGRNLISVFPSQQIVFFPQGIVMSFYGIAGLFISSYLWCTISWNVGS
GYDRFDRKEGIVCIFRWGFPGINRRIFLRFFIRDIQSIRIEVKEGLYSRRVLYMEIRGQGAIPL
TRTDEN-LTPREIEQKAADSAYFLRVPIEVF
>Phalaenopsis
MKKYWFNSMLSLSKSMDGLDALGHTSGSEETK--DADKKIPSFSNIHSLFDRRNLWSLISD
HTFLVRNSNGDTYSVYFDIENQIFDIDNAS--SFLSEL---------SYLKSGSNNSNYSYY-SMYD
TQSNWNNHINSCIDSYLR--FEISLNSDIYSDID-----YISSFICTNGNSSIKTSS--SYFNRIEKSN
DFDLNTKYKQLWVQCENCYGLNYKKFFSSKMNICEYCGYHLKMSSSDRIELFIDPGTWEP
MDEDMVSIDPIEFHSEEEPYIDRISFYQTKTGLTEAVQTGVGQLNSIPIAIGVMDFQFMGGS
MGSVVGEKITRLIEYATNRSLPVIIVCASGGARMQEGALSLMQMAKISSASYDYQSNKKL
FFVSILTSPTTGGVTASFGMLGDIIIAEPNAYIAFAGKRVIEQTLKKTVPDGLQVAEYLFHKG
LFDPIVPRNPLKGVLNELFQLHGFLPLNQD---MGTLRADEISNIIRERIEQYNREVKIVNTGT
VLQVGDGIARIHGLDEVMAGELIEFEEGTIGIALNLESNNVGVVLMGDGLMIKEGSSVKA
TGRIAQIPVSEAFLGRVINALAKPIDGRGEISSSESRLIESPAPGIISRRSVYEPLQTGLIAIDS
MIPIGRGQRELIIGDRQTGKTAVATDTILNQKGQNVICVYVAIGQKASSVAQVVTTFQEGGA
MEYTIVVAETADSPATLQYLAPYTGAALAEYFMYRERHTSIIYDDLSKQAQAYRQMSLLL
RRPPGREAYPGDVFYLHSRLLERAAKLSSRLGEGSMTALPIVETQSGDVSAYIPTNVISITD
GQIFLSADLFNAGIRPAINVGISVSRVGSAAQIKAMKQVAGKSKLELAQFAELEAFAQFASD
LDKATQNQLARGQRLRELLKQSQSDPLAVEDQIATIYTGTNGYLDALEIGQVKKFLVGLRT
YLTKKKPKFQEILSSTKIFTEEAETLLREAIQEQIELFLLQEQTMRNNPITSDPVVSTLKEQN
QGRIAQIIGPVLDVIFPSGKMPNIYNALIIKGRDTVGHQINVTCEVQQLLGNNRVRAVAMSA
TDGLMRGMKVIDTGAPLSVPVGGATLGRIFNVLGEPVDNLGPVDTRTTFPIHRSAPAFIQL
DTKLSIFETGIKVVDLLAPYRRGGKIGLFGGAGVGKTVLIMELINNIAKAHGGVSVFGGV
GERTREGNDLYMEMKESGVINEKNIAESKVALVYGQMNEPPGARMRVGLTALTMAEYFR
DVNEQDVLLFIDNIFRFVQAGSEVSALLGRMPSAVGYQPTLSTEMGSLQERITSTKEGSITS
IQAVYVPADDLTDPAPATTFAHLDATTVLSRGLAAKGIYPAVDPLDSTSTMLQPRIVGEEHY
ETAQRVKQTSQRYKELQDIIAILGLDELSEEDRLTVARARKIERFLSQPFFVAEVFTGSPGKY
VGLAETIRGFQFILSGELDGLPEQAFYLVGNIDEATAKAMNLEEESKLKKMTLNLCVLTPN
RMIWDSEVKEIILSTNSGQIGVLPNHAPIATAVDIGLLRIRLK-RWLTVALMGGFARISNNEIT
ILGNGAEISTDIDPQEAQQTLEIAEANLSKAEGKRQAIEANLALRRARTRVEAIKVISMKNI
TDSFVSVGHWPSAGSFEFNTDILATNPINLSVVLGVLIFFGKGVLNDLLDKRKQRILSTIRN
SEELRRGAIEQLERARVRLRKVEIEADEYRTNGYYEIEREKGNLINATCNSLERLENYKNE
TLFFEKQRAINKVRQEVLQQALQRALGTLNSCLNIEVHFRTISANIDILGSMEEITDMNPLIS
AASVIAAGLAVGLASIGPGVGQGTAAGQAVEGIARQPEAEGKIRGTLLLSLAFMEALTIYG
LVVALALLFANPFVMNVIPCSIKTLKGLYDISGVEVGQHLYWQIGGLQIHAQVLITSWVVI
AILLGSVIIAVRNPQTIPTNGQNFFEYVLEFIQDLSKTQIGEEYGPWVPFIGTMFLFIFVSNW
SGALLPWKIIKLPHGELAAPTNDINTTVALALPTSVAYFYAGLRKKGLGYFGKYIQPTPILL
PINILEDFTKPLSLSFRLFGNILADELVVVVLVSLVPSVVPIPVMFLGLFTSGIQALIFATLAA
AYIGESMEGHHMIFATLEHILTHISFSIISIVIIIHLMNLLVYEIEELRNSLEKGMIATFSSITGFL
VTRWISSGHFPLSNLYESLIFLSWSLSIIHIILYIRNYKNDLNAITAPSAIFTQGFATSGLSTEM
HQPAILVPALQSQWLMMHVSMMLLSYAALLCGSLLSVALIVITFQKKTDFFLKNKNMEYL
NEK-SILKKTSLLSFKNFYKYQLIQHLDSWSYRVISLGFTFLTIGILSGAVWANEAWGSYWN
WDPKETWAFITWTIFAIYLHTRKNSKLQDTNSAFVASIGFLLIWICYFGINLLGIGFHSYGSF
Q--MKKKALA-SLPYLVSIIFLPWWVSLSFNKCLETWVINWWNTRQSEIPLNDIQDKNVLE

KFMELEELFLLDEMIKEYSETHMQRLHIGMHKETIQLVQRQNESHFHIILHFSTNLICFAILS
GYFFLGNKELFIFNSWIQEFLYNLSDTIKAFSILLVTDLWIGFHSTHGWELMIGSIYNDFGLA
QNDQIISGLVSTFPVILDTIVKYWIFHFLNRVSPSLVVIYHSMNEMPIGVPKVPFRNPGEEDA
VWVDVYNRLHRERLLFLGQEVDSEISNQIVGLMVYLSIEDDTRDLYLFINSPGGWVIPGIA
IYDTMQFVLPDVHTICMGLAASMGSFILVGGEITKRLAFPHARVMIHQPASSFYEAQARDF
ILEAEELLKLRETLTKVYVQRTGNPLWVISEDMERDVFMSATEAQAHGIVDLVAIEMKEQ
KLIHEGLITESLPNGMFRVRLDNEDLILGYISGRIRRSFIRILPGDRVKIEMSRYDSTRGRIIY
RLRKKDSND---IIFRLKKERFWQQNFLYPLLLQEYIYSLAHYHSFIGYDN-SSLVLVKRLITR
MYQQKYLISSVSDSNQNGFSLYHFYSQMVSEGFGVILEISFSSRLVSSLIPKSQNLRSIHSIFP
FLEDKLSHLNYVSDLLIPYPIHMEILVQILQCWIKDVPSLHLLRFIFHEYHNLNSLITKSIYVF
SKKRFLWFLHNFYVYECEYIFLFLRKQSSYLRSISSGVFLERTHFYGKIEY--VMSCNQRILW
FLKDIFIHYVRYQGKAILVSKGTLILMNKWKFHFVNFWQFYFHFWFQPYRIRIKQLPNYSF
SFMGYFSSVLKNPLVVRNQMLENSFLINTLT--NKLDTIAPVIFLIRSLSKAQFCTVLGHPISK
SIWTNLSDSDILDRFCRICRNLCRYHSGSSKKQVLYRIKYILRLSCARTLARKHKSTVRTFM
R R L G S A F L E E F F F E E E Q S L S L V F L Q R I W Y L D I I R M N D L V D H
S??
???
???
???
???
???
???
???
???
???
???
???
???
???
???
???
???
???
???
???
???
???
???
???
???
???
???
???
???

??
??
??
??
??
??
??
??
??
??
?????????????MQNRKTFSWVKEQMTRSIYVSIMIYVITRASISNAYPIFAQQVYENPREATG
RIVCANCHLANKPVDIEVPQAVLPDTVFEAVVRIPYDMQLKQVLANGKKGALNVGAVIIL
PEGFELAPPDRISPEIKEKMGNLSFQFYRPNKRNILVIGPVPGQKYSEIVFPILSPDPATKKDV
HFLKYPIYVGGNRGRGQIYPDGNKSNNTVYNATSAGIVSRIARKEKGGYEITIVDASEGRQ
VVDIIPPGPELLVSEGESIKLDQPLTSNPNVGGFGQGDTEIVLQDPLRVQGLLFFLASVILAQ
IFLVLKKKQFEKVQLYEMNFMSKVYDWFEERLEIQAIADDITSKYVPPHVNIFYCLGGITLT
CFLVQVATGFAMTFYYRPTVTEAFSSVQYIMTEANFGWLIRSVHRWSASMMVLMMILHV
FRVYLTGGFKKPRELTWVTGVVLAVLTASFGVTGYSLPWDQIGYWAVKIVTGVPEAIPIIGS
PLVELLRGSASVGQSTLTRFYSLHTFVLPLLTAVFMLMHFPMIRKQGISGPLMGVTKKPDL
NDPVLRAKLAKGMGHNYYGEPAWPNDLLYIFPVVILGTIACNVGLAVLEPSMIGEPADPFA
TPLEILPEWYFFPVFQILRTVPNKLLGVLLMVSVPLGLLTVPFLENVNKFQNPFRRPVATTV
FLIGTAVALWLGIGATLPIEKSLTLGLFMIEVFLFGIVLGLIPITLTGLFVTAYLQYRRGDQLD
LMPILTSYFGFLLAASTITPALFIGLNKIRLIMDIVSFAWAALMVVFTFSLSLVVWGRSGLMI
IRSPEPEVKIVVDRNPIKTSFEEWARPGHFSRTIAKGPDTTTWIWNLHADAHDFDSHTSDLE
EISRKVFSAHFGQLSIIFLWLSGMYFHGARFSNYEAWLSDPTHIAPSAQVVWPIVGQEILNG
DVGGGFRGIQITSGFFQIWRASGITSELQLYCTAIGALVFASLMLFAGWFHYHKAAPKLAW
FQDVESMLNHHLAGLLGLGSLSWAGHQIHVSLPINQFLDAGVDPKEIPLPHEFILNRDLLA
QLYPSFAEGATPFFTLNWAKYAEFLSFRGGLDPITGGLWLSDIAHHHLAIAILFLIAGHMYR
TNWAIGHGLKDILEAHKGPFTGQGHKGLYEILTTSWHAQLSLNLAMLGSLTIVVAHHMYS
MPPYPYLAIDYGTQLSLFTHHMWIGGFLIVGAAAHAAIFMVRDYDPTTRYNDLLDRVLR
HRDAIISHLNWACIFLGFHSFGLYIHNDTMSALGRPQDMFSDTAIQLQPIFAQWVQNTHAL
APNVTAPGATTSTSLIWGGGELVAVGGKVALLPIPLGTADFLVHHIHAFTIHVTVLILLKGV
LFARSSRLIPDKANLGFRFPCDGPGRGGTCQVSAWDHVFLGLFWMYNAISVVIFHFSWKM
QSDVWGTISDQGVVTHITGGNFAQSSITINGWLRDFLWAQASQVIQSYGSSLSAYGLFFLG
AHFVWAFSLMFLFSGRGYWQELIESIVWAHNKLKVAPATQPRALSIVQGRAVGVTHYLLG
GIATTWAFFLARIIAVGMTLRFPRFSQGLAQDPTTRRIWFGIATAHDFESHDDITEERLYQNI
FASHFGQLAIIFLWTSGNLFHVAWQGNFESWIQDPLHVRPIAHAIWDPHFGQPAVEAFTRG
GAIGPVNIAYSGVYQWWYTIGLRTNGDLYTGALFLLLLSAISLIASWLHLQPKWKPSVSW
FKNAESRLNHHLSGLFGVSSLAWTGHLVHVAIPGSRGEYVRWNNFLEVLPYPQGLGPLFM
GQWNLYAQNPDSSNHLFGTSQGAGTAILTLLGGFHPQTQSLWLTDIAHHHLAIAFLFLVAG
HMYRTNFGIGHSIKDLLEAHIPPGGRLGRGHKGLYDTINNSLHFQLGLALASLGVITSLVA
QHMYSLPAYAFIAQDFTTQAALYTHHQYIAGFIMTGAFAHGAIFFIRDYNPEQNEDNVLAR
MLDHKEAITSHLSWASLFLGFHTLGLYVHNDVMLAFGTPEKQILIEPIFAQWIQSAHGKTS
YGFDVLLSSTNSPAFNAGRSIWLPGWLNAINE--NSNSLFLTIGPGDFLVHHAIALGLHTTTL

ILVKGALDARGSKLMPDKKDFGYSFPCDGPGRGGTCDISAWDAFYLAVFWMLNTIGWVT
FYWHWKHITLWQGNVSQFNESSTYLMGWLRDYLWLNSSQLINGYNPFGMNSLSVWAW
MFLFGHLVWATGFMFLISWRGYWQELIETLAWAHERTPLANLIRWKDKPVALSIVQARLV
GLVHFSVGYIFTYAAFLIASTSGKFGMSHSVKIYDTCIGCTQCVRACPTDVLEMIPWDGCK
AKQIASAPRTEDCVGCKRCESACPTDFLSVRVYLWHETTRSMGLSYMIDLNFPSIFVPLVG
LVFPAIAMASLSLYVQRNKIVMQDIKTYLSTAPVLTTLWFGSLAGLLIEINRLFPDALSFPFF
SSMTAILERRESTSLWGRFCNWITSTENRLYIGWFGVLMIPTLLTATSVFIIAFIAAPPVDIDG
IREPVSGSLLYGNNIISGAIIPTSAAIGLHFYPIWEAASVDEWLYNGGPYELIVLHFLLGVAC
YMGREWELSFRLGMRPWIAVAYSAPVAAATAVFLIYPIGQGSFSDGMPLGISGTFNFMIVF
QAEHNILMHPFHMLGVAGVFGGSLFSAMHGSLVTSSLIRETTENESANEGYRFGQEEETY
NIVAAHGYFGRLIFQYASFNNSRSLHFFLAAWPVVGIWFTALGISTMAFNLNGFNFNQSVV
DSQGRVINTWADIINRANLGMEVMHERNAHNFPLDLASVEAPSINGMGLPWYRVHTVVL
NDPGRLLSVHIMHTALVSGWAGSMALYELAVFDPSDPVLDPMWRQGMFVIPFMTRLGITN
SWGGWSIS-GGTITNPGIWSYEGVAGAHILFSGLCFLAAIWHWVYWDLEIFCDERTGKPSL
DLPKIFGIHLFLSGIACFGFGAFHVTGLYGPGIWVSDPYGLTGKVQSVNPAWGAEGFDPFV
PGGIASHHIAAGTLGILAGLFHLSVRPPQRLYKGLRMGNIETVLSSSIAAVFFAAFVVAGTM
WYGSATTPIELFGPTRYQWDQGYFQQEIYRRVGAGLAENLSLSEAWSKIPEKLAFYDYIG
NNPAKGGLFRAGSMDNGDGIAVGWLGHPIFRDKEGHELFVRRMPTFFETFPVVLVDADGI
VRADVPFRRAESKYSVEQVGVTVEFYGGELNGVIYSDPATVKKYARRAQLGEIFELDRAT
LKSDGVFRSSPRGWFTFGHATFALLFFFGHIWHGARTLFRDVFAGIDPDLDAQVEFGAFQK
LGDPTTRRQVVMKTLYSLRRFYPVETLFNGTLALVGRDQETTGFAWWAGNARLINLSGK
LLGAHVAHAGLIVFWAGAMNLFEVAHFVPEKPMYEQGLILLPHLATLGWGVGPGGEVID
TFPYFVSGVLHLISSAVLGFGGIYHALLGPETLEESFPFFGYVWKDRNKMTTILGIHLILLGI
GAFLLVLKALYFGGVYDTWAPGGGDVRKITNLTLSPSVIFGYLLKSPFGGEGWIVSVDDLE
DIIGGHVWLGSICILGGIWHILTKPFAWARRAFVWSGEAYLSYSLGALSVFGFIACCFVWFN
NTAYPSEFYGPTGPEASQAQAFTFLVRDQRLGANVGSAQGPTGLGKYLMRSPTGEVIFGG
ETMRFWDLRAPWLEPLRGPNGLDLSRLKKDIQPWQERRSAEYMTHAPLGSLNSVGGVAT
EINAVNYVSPRSWLATSHFVLGFFLFVGHLWHAGRARAAAAGFEKGIDRDLEPVLSMTPL
SMTIAFGRFIKEENDLFDIMDDWLRRDRFVFVGWSGLLLFPCAYFALGGWFTGTTFVTSW
YTHGLASSYLEGCNFLTAAVSTPANSLAHSLLLLWGPEAQGDFTRWCQLGGLWTFVALHG
AFGLIGFMLRQFELARSVQLRPYNAIAFSAPIAVFVSVFLIYPLGQSGWFFAPSFGVAAIFRF
ILFFQGFHNWTLNPFHMMGVAGVLGAALLCAIHGATVENTLFEDGDGANTFRAFNPTQA
EETYSMVTANRFWSQIFGVAFSNKRWLHFFMLFVPVTGLWMSAIGVVGLALNLRAYDFV
SQEIRAAEDPEFETFYTKNILLNEGIRAWMAAQDQPHENLIFPEEVLPRGNALMSGSTGER
SFADIITSIRYWVIHSITIPSLFIAGWLFVSTGLAYDVFGSPRPNEYFTESRQGIPLITGRFDSL
EQLDKFSNPFMTIDRTYPIFTVRWLAVHGLAVPTVSFLGSISAMQFIQRMATKTIESSSRSG-
PRRTGVGSLLKPLNSEYGKVAPGWGTTPLMGVAMALFAIFLSIILEIYNSSVLLDGISINMLT
LKLFVYTVVIFFVSLFIFGFLSNDPGRNPGREEMTDTTGRIPLWLIGTLTGILVIGLIGIFFYG
SYSGLGSSLMLNIFCLICICLNSTLYSSSFFFAKLPEAYAFFNPIIDVMPIIPVLFFLLAFVWQA
AVSFRMTQSNPNEQNVELNRTSLYWGLLLIFVLAVLFSNYFFNMEVNILAFIATALFILVPT
AFLLIIYVKTVSQNDMETATLVAISISGLLVSFTGYALYTAFGQPSQKLRDPFEEHGDMEAL
VYTFLLVSTLGIIFFAIFFREPPKVPTKKTKMTIAFQLAVFALIATSLILLISVPVVFASSDGWS
SNKNIVFSGTSLWIGLVFLVAILNSLISMSCREGLMSPQTETKASVGFKAGVKDYKLTYYTP
DYETKDTDILAAFRVTPQPGVPPEEAGAAVAAESSTGTWTTVWTDGLTSLDRYKGRCYHI

EVVVGEENQYIAYVAYPLDLFEEGSVTNMFTSIVGNVFGFKALRALRLEDLRIPPSYSKTF
QGPPHGIQVERDKLNKYGRPLLGCTIKPKLGLSAKNYGRAVYECLRGGLDFTKDDENVN
SQPFMRWRDRFLFCAEAIYKAQAETGEIKGHYLNATAGTCEEMIKRAVFARELGVPIVMH
DYLTGGFTANTSLAHYCRDNGLLLHIHRAMHAVIDRQKNHGMHFRVLAKALRMSGGDHI
HAGTVVGKLEGEREMTLGFVDLLRDDYIEKDRSRGIFFTQDWVSMPGVIPVASGGIHVW
HMPALTEIFGDDSVLQFGGGTLGHPWGNAPGAVANRVALEACVQARNEGRDLAREGNDII
REASKWSPELAAACEVWKEITFDFDPVDKPDITAIHLYKTSTSSTRNGAVDSQVKSNPRNN
LIYGQHRCGKGRNARGIITAGHRGGGHKRLYRKIDFRRNEKEISGRIVTIEYDPNRNAYICL
IHYGDGEKRYILHPRGAIIGDTICSGTEVPISMGNALPLTDMPLGTAIHNIEITLGKGGQLAR
AAGAVAKLIAKEGKSATLRLPSGEVRLISKNCLATVGQVGNVGVNQKSLGRAGSKCWLG
KRPVVRGVVMNPVDHPHGGGEGRAPIGRKKPTTPWGYPALGRRSRKRNKYSDSLILRRR
K-MIQPQTLLNVADNSGAQKLMCIRIIGTG-NHRYAHLGDVIVAVIKEAVPNMPLERSEIIRA
VIVRTCKELKRDNGMIIRYDDNAAVIIDQEGNPKGTRIFGAIARELRQLNFTKIVSLAPEVL
MLSPKRTRFRKQHRGRMKGISCRGNHICFGRYALQALEPAWITARQIEAGRRAMTRYARR
GGKIWVRIFPDKPVTVRPTETRMGSGKGSPEYWVSVVKPGRIIYEMSGVSEAVAKAAMEI
AACKMPIRTQFVISGMTRVKRGYTARRRRKKIRLFASTFIGAHSRLTRTTTQQRMRALISSH
RDRSRKKRDFRRLWITRINAVTRED--LFYSYSLFIHNLYKRQLLLNRKILAQIALSNKNCFD
IISNKIIN-----------MIKKVL-AQNIC-MSVFKARRVIDQIRGRSYEEALMILELMPYRASYPIL
KLVYSAAANASHNMGLNEVDLFISKAEVNRGTIMKKLKPRARGRSYLIKKTTCHIKIFLK-
KYKV-------MDGIKYAVFTEKSIRLLGNNQ-YTSNVESGSTRTEIKHWVELFFGVKVIAMNS
HRLPGKGRRMGPIMGHTMHYRRMIITLQPGYSIPPLIEKRTMAVPKKRTSMSKKHIRRNL
WKKKGSLAAVKAFSLAQSISTGQSKSFF--QKKVMAKGKDIRIIVILECTCC-VRK--VNKE--
GISRYITQKNRHNTPNRLEFRKFCRYCQKYTIHGEIKKMKIRASVRKICEKCRLIRRQGRIIV
ICSNPRHKQRQGMVREDVVGSTRTLQWKCVESRVDSKRLYYGRFVLSPLMKGQADTIGI
AIRRALLGEIEGTCITRATSE--NVLHEYSTIAGIEESVHEILLNLKDIVLRSNLYGVRNASICV
RGPKYITAQDIISPPSVEIVDTTQHIANLTEPINLCIELQIKRDRGYRMKSTNDSHDGSY--PID
IVSMPVRNANHSIHSYGNGNEKQEILFIEIWTNGSLTPKEALYEASRNLIDLLIPFLHAEEE--
DMNFKENE-NRWNLPPFSFQDKFT-NFKKNKK-GIPLTCIFIDQSELSSRTYNCLKRSNIHTLL
DLLSHSQEDLMKMEYFRTEDVKQILSTVQKH--FAINLLNKKMLQN-GNEGMSTIPGFSQIQ
FEGFCRFINQGLTEEFHKFQKIEDRDQEIEFQLFVETYQLVEPLITERDAVYESLTYSSELYVP
AVLIWKTGRNMQEQTVFIGNIPIMNSFGTSIVNGIYRIVINQIVQSPGIYYRSELEHNGVSVY
TSTIISDWGGRSELEIDRKARIWARVSRKQKISILVLSSAMGSNIREILDNVCYPEIFLSFPND
KEKKKIGSKENAILEFYQQFACVGGDPVFSESLCKELQKKFFQQRCELGKIGRQNLNRRL
NLDIPQNNTFLLPRDLLAAVDHLIGMKLGMGTLDD--MSHLKNKRIRSVADLLQDQFGLAL
VRLENTVRGTICGAIR-HKLI-PTPQNLVTSTS----LTTTYESFFGLHPLSQVLDRTNPLTQIVH
GRKWSYLGPGGLTGRTASFRIRDIHPSHYGRICPIDTSEGINVGLMGSLAIHVKVGYWG-SI
ESPFY---GLSERSKE--AQMVYLSPNRDEYYMVAAGNSLALNRDIQEQQVVPARYRQEFLTI
AWEEIHLRSIFPFQYFSIGASLIPFIEHNDANRALMSSNMQRQAVPLSRSEKCIVGTGLEGQT
ALDSGVSVIAECKGKIIYTDTQKIFLSSNGDTLSIPLVMYQRSNKNTCMNQKTQVQRGKYI
KKGQILAGGAATAGGELALGKNVLVAYMPWEGYNFEDAVLISERLVYKDIYTSFHIRKYEI
QTHVTSQG-PERITKEIPHLEARLLRNLDRNGIVMLGSWIETGDILVGKLTPQTASE-SSYAP
EDRLLRAILGIQVSTSKETSLRLPIGGRGRVIDVRWIHRRG-VSNSNPERIRVYISQKREIKV
GDKVAGRHGNKGIISKILSRQDMPYLQDGTPVDMVFNPLGVPSRMNVGQIFECSLGLAGD
LLKKHYRIGPFDERYEQEASRKLVFSELYEASKKTKNPWVFEPEYPGKSRIFDGRTGDLFE

QPVLIGKSYILKLIHQVDDKIHGRSSGHYALVTQQPLRGRAKQGGQRVGEMEVWALEGFG
VAHILQEMLTYKSDHIRARQEVLGAMIIGATVPNPESAPESFRLLVRELRSLSLELNHFLVSE
KNFQMDRKEA-------MIDQYKHQQLRIGSVSPQQIRAWAKKILPNGEIVGEVTKPYTFHYK
TNKPEKDGLFCERISGPIKSGICACGNYRGIGTEKEDPKFCEECGVEFVDSRIRRYQMGYIK
LTCPVTHVWYLKRLPSYIANLLDKPLRELEGLVYCD--FSFARS-IAKKPTFLRLRGSFEYEI
QSWQYSIPLFFTTQGFETFRNREISTGAGAIREQLADSDLRIITDNSLLEWKELGDE-ESAG
NEWEEKKIRRRKDFLVRRIELAKHFLRTNVDPEWMVLCLLPVLPPELRPIIQIDGGKLMSS
DINELYRRVIYRNNTLTDLLATSRSTPGELVMCQEKLVQEAVDTLFDNGIRGQPMRDGHNK
VYKSFSDVIEGKEGRFRETLLGKRVDYSGRSVIVVGPLLSLHQCGLPREIAIELFQAFVIRG
LIRQDVASNTGIAKSKIREKEPIVWEILQEVMQGHPVLLNRAPTLHRLGIQAFQPILVEGRA
ICLHPLVCKGFNADFDGDQMAVHVPLSLEAQAEARLLMFSHMNLLSPAIGDPVSVPTQDM
LIGLYVLTIGNPRGICANRYNQSNSNCRN-YKKEKVYKNDFKYTK--ELYFSSSYDALGAYR
QKRIHLDSPLWLRWRLDQR-VVGS--REVPIEIQYESFGNYNEIYKHYQIIGSVKIEICCIYIRT
TAGHISFYREIEEAIQGFWRAYS---------------MAERTNLVFHNKVIDGTAIKRLISRLIDHFG
MAYTSHILDQVKALGLRRATATSISLGIDDLLTIPSKRWLVQDAEQQSFLLEKNHHYGNVH
VVEKLRQSIEVWYATSEYLRQEMNPNFQMTDPSNPVHLMSFSGARGNASQVHQLVGMR
GLMSDPQGQMIDLPIQSNLREGLSVTEYIISCYGARKGVVDTAVRTSDAGYLTRRLVEVVQ
HILVRRTDCGTIRGISVSPQNGMTE--KIFVQTLIGRVLADDIYIGLRCIGTRNKDIGIGLVNR
FIT------FQAHPIYIRTPFTCRSTSWICQLCYGRSPTHGDLVELGEAVGIIAGQSIGEPGTQLTL
RTFHTGGVFTGGTAEHVRAPSNGKIKFDEGLVHPTRTRHGHPAFLCFIDFYVTIESRDIRHN
VNIPTKSLILVQNDQYVESEQVIAEIRAETSTF--FKERVQKHIYSESEGE-MHWSTDGYHTP
KYPYSNVHLLPKTSHLWILAGGLCRSNSNIVSFSLHKDQDQMNANSFSLKD----LFDLS--L
TN-DQVRHKLLDTFG----DRESLDYS-KPYRIISKGHWNLIEPS-TYIRQ--ENSLAKKRRNRF
VIPLQYDQEQENKLIPCFGISIKIPIN--GILRRNSIIAYFDDPRYRRS--GITKY-IVEVDSIVK--K
EDLIEYRG-AKDFSPKY-KVDQFFFIPEEIHILPGSSPIMVRNNSIIGVDTRLVLNTRSRVGGL
VRVERKKKSLELKIFSGDIHFYGEVDKISRHSGILIPPGIGKK--DSKG-SK-KLKNWIYVQR--
ITPTKKK--YFVLVRPVVTYEIADGINLATLFSQDLLQEKNNVQLRIVNYILYENGKSIRGISH
TS-QLVRACLVLTWDQEKKSSIEE---KVHASFVEIRTNDLLCYFIRIELVKSTLSYTEKRYDT
AGSGLISN---LDRIHRNPFDSKAKIQI-----FPQHQGTLG----TLLNRNK---ECQSFLILSSSNCS
RIG--PFNTSRYN---NATKELDPMTPI-YLLGPLGTIVP-RIVNSRSS-YYLRT---YNQVFLK-KS
FLLE---NFQQTFQVLQVPQYCFLDENSR--IYNPDPCSNIIWNSFHLNWCFLHHDSCEEAWPI
IS-LGQFLCENVYLLKYGS--RIKKSGQIFIVH-VDSLVIRSAKPYLVTPGATVHGHYGETFYE
GDTLITFIYEKSRSGDITQGLPKVEQILEVRSIDSISMNLERRVEGWDERIRRILGIPWGFLIG
AELTIAQSRISLVNKIQKVYRSQGVQIHNRHLEIIVRQVTSRVLVSE---DGMSNVFLPGEFIG
LLRAERAGRVLAESICYRAILLGITKSSLNTQSFISEASFQETARVLAKAALRGRIDWLKGL
KENVVLGGIIPVGTGFNKLVHRSK--QDKNIH--LKIKRKNLFELEM--SDILLHHRELFCSCA
QE-----QTNLLRNVS--------MGRRYWNINLEEMMEAGIHFGHVTNKWNPRMAPYISSKRKG
IHITNLTITARFLSEACDLVFDAASRGKRFLIVGTKKKAADLVASAAIKARCHYVNKKWLG
GMLTNWSTTETRLQKFRDLRAEQKMGKLNRLPKGDAAMLKRKLSTLQAYLGGIKYMTG
LPDIVIIVDQQEEYTVLRECVILGIPTICLIDTNCDPDLADISIPANDDAIASIRLILTKLVSAIC
EGHSS-YIKKGMGQKINPLGFRLGTTQSHHSFWFAKPKNFSIGLQEDERIRNCIKDYVKKN
KKISSG-FEGIAYIGIQKRIDFIQVIIYIGFPNLLIEG--RTR--GVEELQMNVQKEFHSVNRRLNI
AITRIEKPYGQPNILAEYIALQLKNRVSFRKAMKKAIELTEQTGTKGIQVQIAGRIDGKEIAR
VEWIREGRVPLQTIRAKIDHCSHTVRTIYGILGIKIWIFVNQEMSRYRGPRFKKIRRLGVLP

GLTSKRPRSRSDLQTQLRFGK-RSQYRIRLEEKQKLRFHYGLTERQLLKYVHIAGKAKGST
GQVLLQLLEMRLDNILFRLGMASTIPGARQLVNHRHILVNGRIVDIPSYRCKPLDIITTKDK
ERSKALIQNYLVSSPRE--LPNHLTIDS--LQYKGFVNQIIDSKWIGLKINELLVVEYYSRQTM
SRRGTAEEKTAKSDPIYRNRLVNMLVNRILKNGKKSLAYQIIYRAVKKIQQKTETNPLSVL
RQAIRGVTPDIAVKARRVGGSTHQVPIEIGSTQGKALAIRWLLGASRKRPGRNMDFRLSSE
LVDAAKGSGDAIRKKEETHRMAEANRAFAHFRMGRDIIANIITSIRNADIDQKGRVKIVST
NITKNIVKILLREGFIENVRKHQESQKNFLVSTLRHRK--TRKG------NKINLKRISRPGLRIYS
NYQQIPKILGGMGIVILSTSRGIITDREARLEKIGGEILCYIWMTKPIAKISLRKNARIGSNK
NERRIPKGVIHVQASFNNTIVTVTDVRGRVVSWASAGTSGFRGTRKGTPYAAQAAAFNAI
RTLVDQGMQRAEVMIKGPGLGRDAALRAIRRNGILLSFVRDVTPMPHNGCRPPKKRRVM
PTIKQLIRNARQPIRNVTKSPALRGCPQRRGTCTRVYTITPKKPNSALRKVARVRLTSGFEIT
AYIPGIGHNSQEHSVVLVRGGRVKDLPGVRYHIVRGTLDAVGVKDRQQGRSKYGVKKPK
MARKSLIQREKKRQKLEQKYYWIRQSLKKEIS---KVPSLREQWKIHGKLQSSPRNSAPIRL
HRRCFLTGRPRANYRDFGLSGHILREMVHACLLPGATRSSWMVKNSFISVITQEEKEEN--S
GSVEFQVFHFTSKIRRLTSHLELHKRDFLSQKGLRIILGKRQRLLTYLSKKNKVRYKKLTD
QLNIREPKIS--MVKLRLKRCGRKQRAIYRIVAIDVRSRREGRDLQKVGFYDPIKNQTYSNV
PAILYFLEKGAQPTETVYDILRKTEFFKEFRISFDKKRKMDKSKQPFRKSKRSFRRRLPPIGS
GDQIDYRNMSLISRFLSEQGKILSRRTNRLTLKQQRLITIAIKQARILSLLPFRNNEK--QLER
VESIPRTTGPRTKNKVTRSLKKNPFVANHLLGRIEKLNRREEKEIIVTWSRASTIIPTMIGHTI
A I H N G K E H L P I Y I T D R M V G H K L G E F A P T L T F V R H A R N D K K S R
R??
???
???
???
???
???
???
???
???
???
???
???
???
???
?????????MKRHQFKSWIFELREILREIKNSHYFLDSWIKFDSVGSFTHIFFHQERFMKLFDPR
IWSILLS-RDS--QGATSNRYFT---IKGLVLLVVV-VLLS----RINNRKMVERKNLYLMGLLP----
-IPMNSIGPRKETLEESF-WSSNRNRLIVS-LLYLPKGKKISESCFMDPQESTWVIPIK----KKRI
MPESNRGSRWWRNRIGKKR--DSSCQIS---NETIAGIEISFKEKDSK-YLEFLFLSYT--DDPIR
KDHDWELFDRLSPRKKRNIINL--NSGQLFEILGKDLICYLMSAFREKRPIQGES-FFKQQG-
AGATMQSNDIEHVSH--LFSRNKWGLSLQNCAQFHMWQFRQDLFV---SWGK-NQHESDFL
RN-VSRENWIWLDNVWLVNKDR---FFS--KVRNVLSNIQYDS---TR--SIFVQVTDSSQWKGS
SSDQSRDHFDSVR----NENSE-------------YHTLIDQTEIQQLKERSIL-WD--PSFLQTERTEIES
D--RFPK--CLFGSS--SMSWLFT-EPEKRMNNHLLPEEIEEFL--GNPTRSIRSFFSDRW--SELH-
LGSNPTERSTRDH----KLLKKKQDVS-FVPSRRAE----KKEMVDIFKIITYLQD--TVSIHPSEP

YHIPDLVP-KDEPD-MDSSNKISFLNKNPFFD--FFHLFHDRNKGGYAL--RHDFFES-EERFPE
MADLFTLSITEP-DLVFHRGFAFYIDSYGLDQKR--FLNEVFNSRDESKKK-----LVLPPLFY--E
ENESFSRRIRKKSVR-IYCGNELEDPKLKTAVFASNNIMEAVN-----QYRLIRNLQYYG--YIR
NVSNRFFLM---NRSDRNFEYGIQ---RDQIGNDTLNHITIMKYTINQHLSNLKK-SQKKWFDP
----LISRTERSMNQDPDAYRYKGSDGSKNFQEHLEHFVSEQKNPFQVVQVVFDQLRIHQYS
IDWSEVIDKEDLSKSLRFFLSKS---------LLFLS-------KSLRFFLSKSLPFFFVSIGNIPIHRSE-I
RIY-ELKGPNDQLCNQLLESIGVQIVHLKKLKP--FLLD---DHDTS-QRPKFLINGGTILPFLFK
KIP-KR-VIDSFHT--RKNRRKSFDNTDSYFSMISHDRDNWLNPVKAFHRSSLISSFYKANRL
RFLNDPHHFWFY--CNKRFPFDV----EKTRINNYD--LTY-GQFLNILSIRNK-IFSLCVGK--KGS
LFLE-RETLSPIESQVSDIFIPNDFPQSGDET-YNLYKLYKSFHFPIRSDPFVR--GAIYSIADISA
TPLTEEQIVNLEKTYCQPLSDMNLSDSEGNNLHQYLS--FNSNMGLIHTPCSEKYLP--SGKR
KK--------RSLCLK--KCVEKGQMYRTFQRDSAFSNLSKWNLFQTYMPWFLTSTGCKYLNFT
LLDTLSNPL--PILSSS-QKFVSIFHDMMHGSDISWPIPQK--------ILPQWTL-ISEISSQCLQN--
LLLSEEMIHRNNESPVPLIWAHLRSTNAREF-LYSIFFLLLVAGY-LVRIHLLFVSR--ASSELQ
TELEKIKSLMIPSYMMEFRKLLDRYPTSELNPFWLKNLFLVVLEQLGDSLGEIRGSASGGN
MLLGGGP-----AYG-VKSIRSKKKYWKIN---LIDLV-SIIPNPINRIIFSRNTRHLSRTSKEIYSLI
RKRKNVNGDWIDDKIEFWVANSDSIDDEEREFLVQFS--ALTT--EKRIDQILLSLT-HSDHLS-
----KNDSGYQMIEQPGSISLRYLVDIH--QKDLMNYEFNRSCLAERRIFLAHYHTITY--SQTL
CGANIFHSPSP--HGKPFSLRLALSPS-RGILVIGSIGTGRSCLVKYLATNSYVPFITVFPNKFL
DDKPKGYPIDD-----ID-IDDSDDID----DS--------------------NDDLDIDKELLTMTNVL---TMY
MT---TKIDRFDITLQFELAKAMSPCIIWIPNIHDLHVNESNYLSLDLLENYLS---RDCERCS-T
GKILVIASTHIPQKVDPALIAPNKLNTCIKIRRLLLPQQLKHFFILSYTRGFHLEKKMFHT--N
GFGSITMGSNARDLVALINEALSISITQKKSIIETNTIRSALHRKTWDFR---SQIRSVQDHGIL
FYQIGRAVTQNVLLSNCPIDPISIYMKKK--SCKGGDSYLYKWYFELGTSMKKLTILLYLLS
CSAGSVAQDLWSSS--RHDEK-NWITSYGFVENDS-DLVHGLLEVEGALALAGSSRTEKDCS
QFDNN--RVTLLLRSEPRN-QLDMMQNGSCSIVDQRFLYEK--YESEFEE-GEG--ALDPQ----Q
IEED----LFNH--IVWAP--RIWR--------FDCIERPTELGFP-YWTGSFR---GKWIIYHKEGELQE
NDSEFLQSGTMQYQTRDRSSKEQGFFRTSQFIWDPADPSFSLFK-DQPSVSVFSRREFFADE
EMSKGLIASQTNPPTSIYKRWFI--KNTQEKHFELLIHRQRWFRT---NSSLS--NGSFRSN--TLS
ESYQYLSNLFLSN-GTLLDQMTKTLLRKRWLFPDEMKHLI--HVTGERFPIPMPRSR-INGNF
IDKTSSIVANILLRIIPTTSGEKKAFTYYRDG--MSAQSEGNYAEALQNYYEATRPEIDPYDR
SYILYNIGLIHTSNGEHTKALEYYFRALERNPFLPQAFNNMAVICHY-RGEQAILQGDSEIAE
AWFDQAAEYWKQAIALTPGNYIEAHNWLKITRRFE--MNWRSEHIWIELITGSRKTSNFCW
ACILFLGSLGFLVVGTSSYLGKNLISVFPSQQILFFPQGIVMSFYGIAGLFISSYLWCTISWN
VGSGYDRFDRKEGIMSLFRWGFPGRDRRIFLRFFLKDIQSIRMEVREGLFPRRVLYMEIKG
QGAIPLTRTDED-LTPREIEQKAAELAYFLRVPIEVF
>Lactuca
MKRWWFNSMLFLSKSMGSLGPIENASESKDPNRNDTDKNIQGYSNVDLFFGVKDIRNFFS
DDTFLVKDSNGDSYSIYFDIENHIFEIANDH--PFCSELESSFY--RNSSDLNNGSKSKNPDHD
RYMDDTQYTWNNHINSCIDSYLQ--YQICIDNYIVSGNDNSSNNYISSSISS--NSSQNEDITT
SDQTIPESSTHMGVTQQYRHLWVQCENCYGLNYKKFFKSKMHLCEQCGYHLKMSSSDRI
ELLIDPGTWEPMDEDMVSLDPIEFHSEEEPYKNRIDSYQRNTGLTEAVQTGRGQLNGITVAI
GVMDFQFMGGSMGSVVGEKITRLIEYATKEFLPLIIVCASGGARMQEGSVSLMQMAKISS
ALYDYQSNKKLFYVPILTSPTTGGVTASFGMLGDIIIAEPNAYIAFAGKRVIEQTLNKTVPEG

SQAAEYLFQKGLFDLIVPRNPLKSVLSELFQLHTFFPLNQN---MVTIQADEISNIIRERIEQY
NREVKIVNTGTVLQVGDGIARIHGLDEVMAGELVEFEEGTIGIALNLESTNVGVVLMGDG
LLIQEGSSVKATGRIAQIPVSEAYLGRVINALAKPIDGRGEISSSEYRLIESPAPGIISRRSVYE
PLQTGLIAIDSMIPIGRGQRELIIGDRQTGKTAVATDTILNQQGKNVICVYVAIGQKASSVAQ
VVTNFQERGAMEYTIVVAETADSPATLQYLAPYTGAALAEYFMYRERHTSIIYDDPSKQA
QAYRQMSLLLRRPPGREAYPGDVFYLHSRLLERAAKLSSLLGEGSMTALPIVETQSGDVSA
YIPTNVISITDGQIFLSADLFNAGIRPAINVGISVSRVGSAAQIKAMKQVAGKLKLELAQFAE
LEAFAQFASDLDKATQNQLARGQRLRELLKQSQSAPLGVEEQVLTIYTGTNGYLDSLEIGQ
VRKFLVELRTYLKTNKPQFQEIISSTKTFTEEAEAILKEAIKEQRERFILQEQAMRMNPTTS
GSGVTTLDKKTLGRIAQIIGPVLDVAFPPGKMPNIYNALVVKGRDTAGQPINVTCEVQQLL
GNNRVRAVAMSATDGLTRGMDVIDTGAPLSVPVGGATLGRIFNVLGEPVDNLGPVDTSTT
FPIHRSAPAFIQLDTKLSIFETGIKVVDLLAPYRRGGKIGLFGGAGVGKTVLIMELINNIAKA
HGGVSVFGGVGERTREGNDLYMEMKESGVINEKNIPESKVALVYGQMNEPPGARMRVGL
TALTMAEYFRDVNEQDVLLFIDNIFRFVQAGSEVSALLGRMPSAVGYQPTLSTEMGSLQE
RITSTKEGSITSIQAVYVPADDLTDPAPATTFAHLDATTVLSRGLAAKGIYPAVDPLDSTSTM
LQPRIVGEEHYDTAQEVKQTLQRYKELQDIIAILGLDELSEEDRLTVARARKIERFLSQPFFV
AEVFTGSPGKYVGLAETIRGFQLILSGELDGLPEQAFYLVGNIDEATAKAMNLEMESNLKK
MTLNLCVLTPNRIVWDSEVKEIILSTNSGQIGVLPNHAPIATSVDIGILRIRLNDQWLTMAL
MGGFARIGNNEITVLVNDAEKSGDIDPQEAQQTLEIAEAALRKAEGKRQTIEANLALRRAR
TRVEAINAISMKNVTDSFVSLGHWPSAGSFGFNTDILATNLINLSVVLGVLIFFGKGVLSDL
LDNRKQRILNTIRNSEELREGAIEQLEKARARLRKVEIEADQFRVNGYSEIEREKLNLIDST
YKTLEQLENYKNETINFEQQKASNQVRQRVFQQALQGALGTLNSCLNSELHLRTISANIGI
LGAMKEITDMNPLISAASVIAAGLAVGLASIGPGVGQGTAAGQAVEGIARQPEAEGKIRGT
LLLSLAFMEALTIYGLVVALALLFANPFVMNVLSCSINTLNGLYDISGVEVGQHFYWKIGG
FQVHGQVLITSWVVIAILLASATLAVRNPQTIPTSGQNFFEYVLEFIRDVSKTQIGEEYGPW
VPFIGTMFLFIFVSNWSGALLPWKIIQLPHGELAAPTNDINTTVALALLTSVAYFYAGLSKK
GLGYFGKYIQPTPILLPINILEDFTKPLSLSFRLFGNILADELVVVVLVSLVPSVVPIPVMFLG
LFTSGIQALIFATLAAAYIGESMEGHHMIFSTLEHIFTHISFSIVSIVIIIHLITLLGNEIIKPYDS
SEKGMIVTFLCLTGLLITRWIYSGHFPLSDLYESLIFLSWSFSLIHIVPY--IRKNYLTEITASSTI
FTQGFATSGLLTEIRKPTILVPALQSEWLIMHVSMMILSYAALLCGSLLSVALLVITFRK---IF
YSYKEIQYKNERNNILKKNY---AKNYYKAQLIQQLDYWSYRVISLGFIFLTIGILSGAVWAN
EAWGSYWSWDPKETWAFITWIVFAIYLHIRTNKNFQGANSAIVATLGFLIIWICYFGVNLL
GIGLHSYGSFTLTMEKKKAFTPLLYLASIIFLPWWISLSFQKSMESWVTNWWNTRQSEPFL
NDIEEKSILEKFIELEELLFLEEMIKEYSETHLQNLRI-IHKETIQLIKIHNEGRIHTILHFSTNII
CFIILSGYSLLGNKELVILNSWVQEFLYNLSDTIKAFSLLLLTDLCIGFHSPHGWELMIGFVY
KDFGFVHNEQIISGLVSTFPVILDTIFKYWIFRYLNRVSPSLVVIYHSMNDMPIGVPKVPFRS
PGEEDASWVDIYNRLYRERLLFLGQEVDSEISNQLIGLMIYLSIEDDTKDLYLFINSPGGWV
IPGVALYDTMQFVQPDVHTICMGSAASMGSFILVGGEITKRLAFPHARVMIHQPAGSFSEVA
TGEFILEVGELLKLRETLTRVYVQRTGKPLWVVSEDMERDVFMSATEAQAYGIVDLVAVE
MKEQKWIHEGLITESLPNGMFRVRLDNEDMILGYVSGKIRRSFIRILPGDRVKIEVSRYDST
RGRIIYRLRNKDSKDMEKFQSYLG-DRSQQHHFLYPLIFQEYIYVLAHDHGLAGYDNKSSL
LIVKRLINRMYQQNHLILSVNNSKQTPFLGKNFYSQVMSEVSSIIMEIPLSLRLISYLVVKSD
NLRSIHSIFSFLEDNFSHLNYVLDILIPYPAHLEILVQALRYWIKDASSLHLLRFFLHECHNW
DSLITKASSSFSKHRLFFFLYTSHLCEYESGFLFLRNQSSHLRSTSSGALIERIYFYGKIDHLA

EVFARAFNLWLFKDPFMHYVRYQGKSILGSKGTFLLMNKWKYYFVNFWKSYFYLWSQP
GRIYINQLSNHSLDFLGYRSSVRLKPSMVRSQMLENAFIIENAI--KKFETIVPIMPLIGSLAK
SKFCNALGHPIGKAIWADFSDSDIIDRFGRIYRNLSHYHSGSSKKKSLYRVKYILRLSCART
LARKHKSTVRAFLKRFGSELLEEFFTEEEQVFSLTFPRRIWYLDIICINDLANHEMIID-TTE
VQ-AINSFSILES-LKEVYGIIWMLIPIFTLVLGITIGVLVIVWLEREISAGIQQRIGPEYAGPLG
ILQALADGTKLLFKENLLPSRGDTRLFSIGPSIAVISILLSYLVIPFSYHLVLADLSIGVFLWIA
ISSIAPVGLLMSGYGSNNKYSFLGGLRAAAQSISYEIPLTLCVLSISLLSNSSSTVDIVEAQS
KYGFWGWNLWRQPIGFLVFLISSLAECERLPFDLPEAEEELVAGYQTEYSGIKFGLFYVAS
YLNLLVSSLFVTVLYLGGWNLSIPYIFVPEVFEITKRGRVFGTIIGIFITLAKTYLFLFIPIATR
WTLPRLRMDQLLNLGWKFLLPISLGNLLLTTCSQLISLMIWHVQNENFILDSTRIFMKAFH
LLLFDGSLIFPECILIFGLILLLMIDSTSDQKDIPWLYFISSTSLVMSITSLLFRWREEPMISFSG
NFQTNNFNEIFQFLILLCSTLCIPLSVEYIECTEMAITEFLLFVLTATIGGMFLCGANDLITIFV
APECFSLCSYLLSGYTKKDVRSNEATMKYLLMGGASSSILVHGFSWLYGSSGGEIELQEIV
NGLINTQMYNSPGISIALIFITVGIGFKLSPAPSHQWTPDVYEGSPTPVVAFLSVTSKVAASA
SATRIFDIPFYFSSNEWHLLLEILAILSMILGNLIAITQTSMKRMLAYSSIGQIGYVIIGIIVGD
SNDGYASMITYMLFYISMNLGTFACIVLFGLRTGTENIRDYAGLYTKDPFLALSLALCLLSL
GGLPPLAGFFGKLYLFWCGWQAGLYFLVLIGLLTSVVSIYYYLKIIKLLMTGRNQEITPHV
RNYRRSPLRSNNSIELSMIVCVIASTIPGISMNPIIAIAQDTLFMFLLYEYDIFWAFLIISSLIPIL
VFFISGFLAPISKGPEKLSSYESGIEPIGDAWLQFRIRYYMFALVFVVFDVETVFLYPWAMSF
DVLGVSVFVEALIFVLILIVGLVYAWRKGALEWSTNKFPWLTIIVVLPIFAGSFILFLPHKGN
RVIRWYTICICMLELLLTTYAFCYHFQLDDPLIQLVEDYKWINFFDFRWKLGIDGLSIGPVL
LTGFITTLATLAAWPVTRDSRLFHFLMLAMYSGQIGSFSSRDLLLFFIMWELELIPVYLLLS
MWGGKKRLYSATKFILYTAGGSIFLLMGVLGVGLSGSNEPTLNFETSVNQSYPVALEIIFYI
GFFIAFAVKLPILPLHTWLPDTHGEAHYSTCMLLAGILLKMGAYGLIRINMELLPHAHSIFS
PWLMIVGTIQIIYAASTSPGQRNLKKRIAYSSVSHMGFILIGIASITDTGLNGAILQIISHGFIG
AALFFLAGTSYDRIRLVYLDQMGGVAIPMPKIFTMFSSFSMASLALPGMSGFVAEIVIVFLGI
ITSQKYLLMPKIAITFVMAIGMILTPIYSLSMLRQMFYGYKLFNTPNAYVFDSGPRELFVSIS
IFLPVIGIGMYPDFVLSLSVDKVEGILSNYFYRMMLEHVLVLSAYLFSVGLYGLITSRNMV
RALMCLELILNAVNLNFVTFSDFFDSRQLKGAIFSIFVIAIAAAEAAIGLAIVSSIYRNRKST
RINQSNLLNKMEQTYQYAWIIPFLPLPVPMLIGLGLLLFPTATKSLRRMWSFQSVLLLSIVM
IFSMNLSIQQINSSYVYQYVWSWIINNDFSLEFGYLIDPLTSIMLILITTVGIMVLIYSDNYM
SHDHGYLRFFAYMSFFSTSMLGLVTSSNLIQIYIFWELVGICSYLLIGFWFTRPVAAKACQK
AFVTNRVGDFGLLLGILGFYWITGSFEFRDLFQIFNNNEVNFLFVTLCAILLFAGAIAKSAQ
FPLHVWLPDAMEGPTPISALIHAATMVAAGIFLVARLMPLFIVIPHIMNFISLIGIITVFLGAT
LALAQKDIKRGLAYSTMSQLGYMMLALGMGSYRSALFHLITHAYSKALLFLGSGSVIHS
METLVGYCPKKSQNMVLMGGLTKHLPITKNSFLLGTLSLCGIPPLACFWSKDEILNDSWL
YSPIFGIIAWSTAGLTAFYMCRIYLLTFEGHLNVHFQNYSGKRNTPFYSISLWGKEGSKISN
K-LVTLLKMKFFSFGNTKTYSYPYESDNTMLFPILILILFTLFVGFLGIPF-VDILSKWLNPSI
NLLHQNSN-SIDWYEFSKDAFFSVSIASFGIFIAFFLYKPVYSSFQNLEFLNTFVIFYDKIKNS
IYDWSYNRGYIDAFYGRFLTAGIRKLANFAHFFDRRIIDAIPNGVGLMSFFGAEVIKSVGGG
-RISSYLFFYFSYVAIFLLIYYMDLPGLIHDFLLVFLGLGLILGGLGVVLLPNPIYSAFSLGLV
FVCISLFYILSNSHFVAAAQLLIYVGAINVLIIFAVMFINGSEYSKDFHLWTVGDGITSVVCT
SLFVSLITTIPDTSWYGIIWTTKANQIIEQDLISNSQQIGIHLSTDFFLPFEFISIILLVALIGAIAV
ARQMTGPATRKDLMIVNMGPHHPSMHGVLRLIVTLDGEDVIDCEPILGYLHRGMEKIAEN

RTIIQYLPYVTRWDYLATMFTEAITVNAPEQLGNIQVPKRASYIRVIMLELSRIASHLLWLG
PFMADIGAQTPFFYIFRERELIYDLFEAATGMRMMHNFFRIGGIAADLPHGWIDKCLDFCD
YFLTGIAEYQKLITRNPIFLERVEGVGIIGGEEAINWGLSGPMLRASGIQWDLRKVDHYEC
YDEFDWEVQWQNEGDSLARYLVRISEMTESIKIIQQALEGIPGGPYENLEIRRFDRVKDTV
WNEFDYRFISKKPSPTFELSKQELYARVEAPKGELGIFLIGDKGVFPWRYKIRPPGFINLQIL
PQLVKRMKLADIMTILGSIDIIMGEVDRMFPMVTEFMNYGQQTVRAARYIGQGFMITLSH
ANRLPVTIQYPYEKLITSERFRGRIHFEFDKCIACEVCVRVCPIDLPVVDWKLETDIRKKRL
LNYSIDFGICIFCGNCVEYCPTNCLSMTEEYELSTYDRHELNYNQIALGRLPMSVIDDYTIR
TIFNL----------------MQGHLSAWLVKHGLIHRSLGFDYQGIETLQIKPGDWHSIAVILYVYGY
NYLRSQCAYDVAPGGLLASVYHLTRIEYGADQPEEVCIKVFAPRRDPRIPSVFWVWKSVD
FQERESYDMLGISYDNHPRLKRILMPESWIGWPLRKDYIAPNFYEIQDAH---------------------
--------------------------------------MNSIEFPLFDRTTQNSVISTTLNDLSNWSRLSSLWPLLYGTS
CCFIEFASLIGSRFDFDRYGLVPRSSPRQADLILTAGTVTMKMAPSLVRLYEQMPEPKYVIA
MGACTITGGMFSTDSYSTVRGVDKLIPVDVYLPGCPPKPEAIIDAITKLRKKISREIYPDRT
MSQRENRCFTTNHKFQVGHSIHTGNYDQGFLYQPTSTSEIPPETFFKYKSSVSSPELVNMQ
TRNNFSWIKEQITRSISVSLMIYIITRASISNAYPIFAQKGYENPREATGRIVCANCHLANKP
VDIEVPQTVLPDTVFEAVVRIPYDMQLKQVLANGKKGALNVGAVLILPEGFELAPPDRISP
EIKEKMGNLSFQSYRPNQKNILVIGPVPGQKYSEITFPILSPDPATKKDIHFLKYPIYVGGNR
GRGQIYPDGSKSNNTVYNATASGIVSKILRKEKGGYEITIADASDGRQVVDIIPPGPELLVSE
GESIKFEQPLTSNPNVGGFGQGDAEIVLQDPLRVQGLLFFLASVILAQIFLVLKKKQFEKVQ
LSEMNFMSKVYDWFEERLEIQAIADDITSKYVPPHVNIFYCLGGITLTCFLVQVATGFAMTF
YYRPTVTDAFASVQYIMTEANFGWLIRSVHRWSASMMVLMMILHVFRVYLTGGFKKPRE
LTWVTGVVLGVLTASFGVTGYSLPRDQIGYWAVKIVTGVPEAIPVIGSPLVELLRGSASVG
QSTLTRFYSLHTFVLPLLTAVFMLMHFPMIRKQGISGPLIPITKKPDLNDPVLRAKLAKGMG
HNYYGEPAWPNDLLYIFPVVILGTIACNVGLAVLEPSMIGEPADPFATPLEILPEWYFFPVFQ
ILRTVPNKLLGVLLMVSVPAGLLTVPFLENVNKFQNPFRRPVATTVFLIGTAVALWLGIGAT
LPIDKSLTLGLFMIEVFLFGIVLGLIPITLAGLFVTAYLQYRRGDQLDLMLTITSYFGFLLTAL
TITSALFIGLSKIRLIMDIVSLAWAALMVVFTFSLSLVVWGRSGLMIIRSPEPEVKILVDRDHI
KTSFEEWARPGHFSRTIAKGPETTTWIWNLHADAHDFDSHTSDLEEISRKVFSAHFGQLSII
FLWLSGMYFHGARFSNYEAWLSDPTHIRPSAQVVWPIVGQEILNGDVGGGFRGIQITSGFF
QIWRASGITSELQLYCTAIGGLVFAALMLFAGWFHYHKAAPKLAWFQDVESMLNHHLAG
LLGLGSLSWAGHQVHVSLPINQFLNAGVDPKEIPLPHEFILNRDLLAQLYPSFAEGATPFFT
LNWSKYADFLTFRGGLDPVTGGLWLTDTAHHHLAIAILFLIAGHMYRTNWGIGHGLKDIL
EAHKGPFTGQGHKGLYEILTTSWHAQLSLNLAMLGSLTIVVAHHMYAMPPYPYLATDYGT
QLSLFTHHMWIGGFLIVGAAAHAAIFMVRDYDPTTRYNDLLDRVLRHRDAIISHLNWACI
FLGFHSFGLYIHNDTMSALGRPQDMFSDTAIQLQPVFAQWIQNTHALAPGATAPGATASTS
LTWGGGDLVAVGGKVALLPIPLGTADFLVHHIHAFTIHVTVLILLKGVLFARSSRLIPDKAN
LGFRFPCDGPGRGGTCQVSAWDHVFLGLFWMYNSISVVIFHFSWKMQSDVWGSISDQGV
VTHITGGNFAQSSITINGWLRDFLWAQASQVIQSYGSSLSAYGLFFLGAHFVWAFSLMFLF
SGRGYWQELIESIVWAHNKLKVAPATQPRALSIVQGRAVGVTHYLLGGIATTWAFFLARIIA
VGMALRFPRFSQGLAQDPTTRRIWFGIATAHDFESHDDITEERLYQNIFASHFGQLAIIFLW
TSGNLFHVAWQGNFESWVQDPLHVRPIAHAIWDPHFGQPAVEAFTRGGALGPVNIAYSGV
YQWWYTIGLRTNEDLYTGALFLLLISAISLIAGWLHLQPKWKPSVSWFKNAESRLNHHLS
GLFGVSSLAWTGHLVHVAIPASRGEYVRWNNFLDVLPHPQGLGPLFTGQWNLYAQNPDS

GSHLFGTSQGAGTAILTLLGGFHPQTQSLWLTDMAHHHLAIAFLFLIAGHMYRTNFGIGHS
MKDLLDAHIPPGGRLGRGHKGLYDTINNSLHFQLGLALASLGVITSLVAQHMYSLPAYAFI
AQDFTTQAALYTHHQYIAGFIMTGAFAHGAIFFIRDYNPEQNEDNVLARMLEHKEAIISHL
SWASLFLGFHTLGLYVHNDVMLAFGTPEKQILIEPIFAQWIQSAHGKTSYGFDILLSSTNGP
AFNAGRSIWLPGWLNAINE--NSNSLFLTIGPGDFLVHHAIALGLHTTTLILVKGALDARGS
KLMPDKKDFGYSFPCDGPGRGGTCDISAWDAFYLAVFWMLNTIGWVTFYWHWKHITLW
QGNVSQFNESSTYLMGWLRDYLWLNSSQLINGYNPFGMNSLSVWAWMFLFGHLVWATG
FMFLISWRGYWQELIETLAWAHERTPLANLIRWRDKPVALSIVQARLVGLAHFSVGYIFTY
AAFLIASTSGKFGMSHSVKIYDTCIGCTQCVRACPTDVLEMIPWDGCKAKQIASAPRTEDC
VGCKRCESACPTDFLSVRVYLWHETTRSMGIAYMTTLNFPSVLVPLVGLVFPAIAMASLFL
HVQKNKIVMRDLKTYLSVAPVLSTLWFGSLAGLLIEINRFFPDALTFPFFSFMTAILERRESE
SLWGRFCNWITSTENRLYIGWFGVLMIPTLLTATSVFIIAFIAAPPVDIDGIREPVSGSLLYGN
NIISGAIIPTSAAIGLHFYPIWEAASVDEWLYNGGPYELIVLHFLLGVACYMGREWELSFRL
GMRPWIAVAYSAPVAAATAVFLIYPIGQGSFSDGMPLGISGTFNFMIVFQAEHNILMHPFHM
LGVAGVFGGSLFSAMHGSLVTSSLIRETTENESANEGYRFGQEEETYNIVAAHGYFGRLIF
QYASFNNSRSLHFFLAAWPVVGIWFTALGISTMAFNLNGFNFNQSVVDSQGRVINTWADII
NRANLGMEVMHERNAHNFPLDLAAIEAPSTNGMGLPWYRVHTVVLNDPGRLLSVHIMH
TALVAGWAGSMALYELAVFDPSDPVLDPMWRQGMFVIPFMTRLGITNSWGGWSIT-GGTI
TNPGIWSYEGVAGAHIVFSGLCFLAAIWHWVYWDLEIFSDERTGKPSLDLPKIFGIHLFLA
GVACFGFGAFHVTGLYGPGIWVSDPYGLTGKVQAVNPSWGVEGFDPFVPGGIASHHIAAG
TLGILAGLFHLSVRPPQRLYKGLRMGNIETVLSSSIAAVFFAAFVVAGTMWYGSATTPIELF
GPTRYQWDQGYFQQEIYRRVSAGLAENQSLSEAWSKIPEKLAFYDYIGNNPAKGGLFRAG
SMDNGDGIAVGWLGHPIFRDKEGRELFVRRMPTFFETFPVVLVDGDGIVRADVPFRRAES
KYSVEQVGVTVEFYGGELNGVSYSDPVTVKKYARRAQLGEIFELDRATLKSDGVFRSSPR
GWFTFGHASFALLFFFGHIWHGARTLFRDVFAGIDPDLDAQVEFGAFQKLGDPTTRRQIG
MKTLYSLRRFYPVETLFNGTLALAGRDQETTGFAWWAGNARLINLSGKLLGAHVAHAGL
IVFWAGAMNLFEVAHFVPEKPMYEQGLILLPHLATLGWGVGPGGEVIDTFPYFVSGVLHLI
SSAVLGFGGIYHALLGPETLEESFPFFGYVWKDRNKMTTILGIHLILLGIGAFLLVFKALYF
GGVYDTWAPGGGDVRKITNLTLSPSIIFGYLLKSPFGGEGWIVSVDDLEDIIGGHVWLGSIC
ILGGIWHILTKPFAWARRALVWSGEAYLSYSLAAISVFGFIACCFVWFNNTAYPSEFYGPTG
PEASQAQAFTFLVRDQRLGANVGSAQGPTGLGKYLMRSPTGEVIFGGETMRFWDLRAPW
LEPLRGPNGLDLSRLKKDIQPWQERRSAEYMTHAPLGSLNSVGGVATEINAVNYVSPRSW
LATSHFVLGFFFFVGHLWHAGRARAAAAGFEKGIDRDFEPVLSMTPLNMTIALGKVTKDE
NDLFDIMDDWLRRDRFVFVGWSGLLLFPCAYFAVGGWFTGTTFVTSWYTHGLASSYLEG
CNFLTAAVSTPANSLAHSLLLLWGPEAQGDFTRWCQLGGLWTFVALHGAFGLIGFMLRQF
ELARSVQLRPYNAIAFSGPIAVFVSVFLIYPLGQSGWFFAPSFGVAAIFRFILFFQGFHNWTL
NPFHMMGVAGVLGAALLCAIHGATVENTLFEDGDGANTFRAFNPTQAEETYSMVTANRF
WSQIFGVAFSNKRWLHFFMLFVPVTGLWMSALGVVGLALNLRAYDFVSQEIRAAEDPEFE
TFYTKNILLNEGIRAWMAAQDQPHENLIFPEEVLPRGNALMSGSTGERSFADIITSIRYWVI
HSITIPSLFIAGWLFVSTGLAYDVFGSPRPNEYFTENRQGIPLITGRFDSLEQLDEFSRSFMTI
DRTYPIFTVRWLTVHGLAVPTVSFLGSISAMQFIQRMATQTVENGARSG-PRRTTVGDLLKP
LNSEYGKVAPGWGTTPLMGVAMALFAVFLSIILEIYNSSVLLDGISMNMLTLKLFVYTVVI
FFVSLFIFGFLSNDPGRNPGREEMADTTGRIPLWIIGTVAGILVIGLVGVFFYGSYSGLGSSL
MLNIFSLIC--LNSALYPSSLFFAKLPEAYAFLNPIVDVMPVIPLFFFLLAFVWQAAVSFRTTQ

SNPNEQNVELNRTSLYWGLLLIFVLAVLFSNYFFNMEVNILAFIATALFILVPTAFLLIIYVKT
VSQNNMETATLVAIFISGLLVSFTGYALYTAFGQPSQQLRDPFEEHGDMEALVYTFLLVSTL
GIIFFAIFFREPPKVPTKK--MTLAFQLAVFALIATSSILLISVPVVFASPDGWSSNKNVVFSGT
SLWIGLVFLVGILNSLIS-------MSPQTETKASVGFKAGVKDYKLTYYTPEYETKDTDILAAF
RVTPQPGVPPEEAGAAVAAESSTGTWTTVWTDGLTSLDRYKGRCYGIEPVPGEENQYIAY
VAYPLDLFEEGSVTNMFTSIVGNVFGFKALRALRLEDLRIPTAYVKTFQGPPHGIQVERDK
LNKYGRPLLGCTIKPKLGLSAKNYGRAVYECLRGGLDFTKDDENVNSQPFMRWRDRFLF
CAEAIFKSQAETGEIKGHYLNATAGTCEEMMKRAIFARELGVPIVMHDYLTGGFTANTSL
AHYCRDNGLLLHIHRAMHAVIDRQKNHGIHFRVLAKALRMSGGDHIHSGTVVGKLEGER
EITLGFVDLLRDDFIEKDRSRGIYFTQDWVSLPGVLPVASGGIHVWHMPALTEIFGDDSVL
QFGGGTLGHPWGNAPGAVANRVALEACVQARNEGRDLATEGNEIIREATKWSPELAAAC
EVWKEIKFEFQAMDTLDQMAIHLYKTSTPSTRNGAVDSKVKSNPRNNLIYGQHHCGKGR
NARGIITAGHRGGGHKRLYRKIDFRRNEKDIYGRIVTIEYDPNRNAYICLIHYRDGEKRYIL
HPRGAIIGDTIVSGTEVPIKMGNALPLTDMPLGTAIHNIEITLGKGGQLARAAGAVAKLIAK
EGKSATLKLPSGEVRLISKNCSATVGQVGNVGVNQKSLGRAGSKRWLGKRPVVRGVVM
NPVDHPHGGGEGRAPIGRKQPTTPWGYPALGKRSRKRNKYSDNLILRRRSKMIQPQTHLN
VADNSGARELMCIRIIGAS-NRRYAHIGDVIVAVIKDAVPNMPLERSEVVRAVIVRTCKELK
RDNGMIIRYDDNAAVVIDQEGNPKGTRVFGAIARELRQFNFTKIVSLAPEVLMLSPKRTRF
RKQHRGRMKGISYRGNAICFGKYALQALEPAWITSRQIEAGRRAMTRNARRGGKIWVRIF
PDKPVTVRPAETRMGSGKGSPEYWVAVVKPGRILYEMGGVTENIARRAISIAASKMPIRAQ
FIISGMTRIRRGYIARRRRTKIRLFASSFRGAHSRLTRTITQQKIRALVSAHRDRDKQKINFR
RLWITRINAAIRE-RGVCYSYSRLINGLYKRQLLLNRKILAQIAISNRNCLYMISNEIIKEVG
WKESTG--MLNEVYALGQHISMSAHKARRVIDQIRGRSYEETLMILELMPYRACYPIFKLV
YSAAANASFNMGSNEVNLVISKAEVNEGTIVKRLKPRARGRSFAIQKPTCHITIVMKDISL
DGGTWDKKMDGIRYAVFTDKSIQLLGKN-QYTSNVESGSTRTEIKHWVELFFGVKVIAMN
SHRLRGKARRMGPIMGQTMHYRRMIITLQPGYSIPPLRKKRTMAVPKKRTSISKKRIRKNI
WKRKGYWAALKALSLGKSLSTGNSKSFFVRQTNKMAKGKDVRIPVLLECTACVRNGVN
VNKASTGISRYITQKNRHNTPNRLELRKFCPYCYKHTIHGEVKKMKIRASVRKICEKCRLI
RRRGRIRVICSNPRHKQRQGMVREKITVSTRTLQWKCVESAADSKRLLYGRFILSPLMKG
QADTIGIAMRRALLGEIEGTCITRAKSE--KISHEYSTIMGIQESVHEILMNLKEIVLRSNLYG
TCEASICVRGPGYVTAQDIILPPYVEILDNTQHIASLTEPIELVIGLQIEKNRGYLIKAPN-TFD
GSY--PIDPVFMPVRNANHSIHSYENGN--KEILFLEIWTNGSLTPKEALYEASRNLIDLLIPFL
HTKEE--NLNLEGNQ-HMVPLPPFTFYDKLA-KLTKNKK-KMALKSIFIDQSELPPRIYNCLK
RSNIYTLLDLLNNSQEDLMKMEHFRIEDVKQILGILEKN--FVIDLPKNK---------MSTIPGFN
QIQFEGFCRFIDQGLTEELSKFPKIEDTNQEIDFELFLERYQLVEPSIKERDAVYESLTYSSEL
YVSARLIWKNRRYIQEQTILIGKIPLMTSLGAFIVNGIYRIVINQILQSPGIYYQSELNDNGIS
VYTGTIISDWGGRLELEIDRKTRIWVRVSRQQKLSILVLLSAMGLNIREILENVCYPELFLSF
LNDK--KQIGSKENAILEFYQQFACVEGDPVFSESLSKDLQKKFFQQRCELGGIGRRNMNR
RLNLDIPQNNTFLLPRDILAAADRLIRIKFGMGTLDD--MNHLQNKRIRSVADLLQEQFGLA
LVRLENMARGNIYAALK-HNWT-PTPQNLVNSTP----LTDTYKVFFRLHPLSQVLDRTNPLT
QIVHGRKLSYLGPGGLTARTATFPIRDIHPSHYGRICPIDTSEGINVGLIGSLAIHARIGRWG-
SLESPFY---KISERSKG--ARMLYLSPGRDEYYMVAAGNSLALNQGIQEEQVVPARYRQEFL
TIAWEQVHLRSIFSFQYFSIGASLIPFIEHNDANRALMSSNMQRQAVPLSQSEKCIVGTGLE
GQAALDSGALAIAEHEGEIIYTDTDKILLSGNGDTLRIPLVMYQRSNKNTCMHQKPQVQR

GKCIKKGQILAYGAATVGGELALGKNVLVAYMPWEGYNFEDAVLISERLVYEDIYTSFHIR
KYEIQIN---QG-SERVTNEIPHLEVHLLRNLDKNGIVMLGSWVETGDILVGKLTPQMVKE-S
SYAPEDRLLRTILGMRVYTSKETCLKLPIGGRGRVIDVRWVQSSKTDETEKTESIRVYILQK
REIKVGDKVAGRHGNKGIISKILPRQDMPYLQDGRPVDMVFNPLGVPSRMNVGQIFESSL
GLAGGLLDRHYRIAPFDERYEQEASRKLVFSELYEASKQTVNPWIFEPESPGKSRIFDGRTG
DPFEQPVIIGKPYILKLIHQVDDKIHGRSSGRYSRLTQQPLKGRAKKGGQRVGEMEVWALE
GFGVAYILQEMLTYKSDHIRARQEVLGTIIFGGRIPTPEDAPESFRLFVRELRSLALELNHFL
VSEKTFQLNRKEA-------MIDRYTHQQLRIGLVSPQQISTWSKKILPNGEIVGEVTKPYTFHY
KTNKPEKDGLFCERIFGPIKSGICACGNYRVIGDEKEDPQFCEQCGVEFVDSRIRRYQMGYI
KLAYPVMHVWYLKRLPSYIVTLLDKPLNELEDLVYCN--FYFARP-IDKKPTFLRLRGLLEY
EIQPWKYRIPIFFTTRSFDTFRNREMSTGGGSIRQQLANLDLRIIIDYSLVEWKELEEE-EPTG
NEWEDRKVGRRKDFLLRRMELAKHFIRTNIEPKWMVLRLLPVLPPELRPIYHIDEDKLVTS
DINEIYRRIIYRNNTLTDLLTTSIATPEELIISQEKLLQEAVDALLDNGICGQPMRDDHNRVY
KSLSDVIEGKEGRVRETLLGKRVDYSGRSVIVVGPSLSLHRCGLPREIAIELFQAFVIRDLIR
KHLASNIGVAKSQIRKKKPIVWEILQEILDDHPVLLNRAPTLHRLGIQAFLPVLVEGRAICL
HPLVCKGFNADFDGDQMAVHVPLSLEAQAEARLLMFSHMNLLSPTIGDPISAPTQDMLSG
LYVLTSGNRRGICVNRYNPCNRRN---YQNED---NNYKYTKKKEPFFCNPYDAIGAYRQKRI
NLGSPLWLRWRLDQR-VIAA--REVPIEIHYESVGTYYEIYGHYLIVRSIKKEILYIYIRTTLG
HISLYREIEEAIQGFWQCNSMLPTGIRVSPGMEVLMAERPTQVFHNKVIDGTAMKRLISRFI
DHYGIGYTSHILDQVKTLGFRQATAASISLGIDDLLTIPSKRWLVQDAEQQSFILEKHHHYG
NVHAVEKLRQSIEIWYATSEYLRQEMNPNFRMTDPFNPVHIMSFSGARGNASQVHQLVG
MRGLMSDPQGQMIDLPIQSNLREGLSLTEYIISCYGARKGVVDTAIRTSDAGYLTRRLVEV
VQHIVVRRTDCGTVRGISVSPRNGMMT-DRIFIQTLIGRVLADDIYIGSRCIATRNQDIGVGL
VSRFIT------FRAQPISIRTPFTCRSTSWICQLCYGRSPAHDDLVELGEAVGIIAGQSIGEPGTQ
LTLRTFHTGGVFTGGTAEHVRAPSNGKIKFNEDLVHPTRTRHGHPAFLCSRDLYVTIESEDII
HNVCIPPKSFLLVQNDQYVESEQVIAEIRARTST-LNLKEKVRKHIYSDSEGE-MHWNTDV
YHAPEFTYGNIHLLPKTSHLWILLGEPWR--YSLGPCSIHKDQDQMNAY--SLSVKPRYIANP
S--VTNNQ-VRHKFFSSYFSGKNGDRIPDC-SELNRMTCTDHSNLRYP-AILDGNS--DLLAKR
RRNRFIIPLESIQEGENQLIPSSGISMEIP--RNGILRRNSILAYFDDPRYIRKSSGLTKYETREL
NSIVNEEN--LIEYRG-VKVFWPKYKEVNPFFFIPVEVHILSESSSIMVRHNSIIGADTQITFNR
RSRVGGLVRVKKKAEKMKLIIFSGDIHFPGKTNKAFR----LIPPGG--GKPNSKEYKK--LKN
WLYIQR--MKLSRYEKKYFVLVQPVVPYKKTDGINLG-LFPPDLLQESDNLQLRVVNYILYY
DP--ILEIWDTSIQLVRTSLVLNWDQDKK------IEKACASFVEIRTNGLLRYFLRIDLAKSPISY
TGKRNDLSGSGLISE-NGSDRANVNPFSSIYSYS---KSRIKESLNPNQGTIHTLLNR---NKES
QSLIILSSSNCFRIGP--FNDVKSPNV---IKES-PLIPIRNSLGPLGT--GFPIYNFDL--FSHL--ITH
NQIL-VTNYLQLDNFK---QIFQILK---YYLLDEN--GQIYNPYSCSNIILNPFHLNWYFLHYNY
CEETS-PIVSLGQFLCENVCIAKKGPH-LK--SGQVLIVQ-VDSVVIRSAKPYLATPGATVHGH
YGEILYEGDTLVTFIYEKSRSGDITQGLPKVEQVLEVRSIDSISMNLEKRIEGWNKSITRILGI
PWAFLIGAELTIVQSRISLVNKVQKVYRSQGVQIHNRHIEIIVRQITSKVLVSE---DEMSNVF
SPGELIGLLRAERMGRALEEAICYQAVLLGITRASMNTQSFISEASFQETARVLAKAALLGR
IDWLKGLKENVVLGGMIPVGSGFKTP-SSEPNNIPNNIA--FELQKKNLLEGEMK--DILFYH
RKLFDSCLSNNFHDTQEQSFF-----------MTRRYWNINLEEMMEAGVHFGHGTRKWNPKM
APYISAKRKGIHITNLTRTARFLSEACDLVFDAASRGKQFLIVGTKNKEADSVAWAAIRARC
HYVNKKWLGGMLTNWSTTETRLHKFRDLRTEQKTGGLDRLPKRDAAMLKRQLSHLQTY

LGGIKYMTGLPDIVIIVDQHEEYTALQECITLGIPTICLIDTNCDPDLADISIPANDDAISSIRLI
LNKLVFAICEGRS-GYIRNPMGQKINPIGFRLGTTQGHHSLWFAQPKNYSEGLQEDKKIRTY
IQNYVQKNMKTSSG-VEGIARIEIQKRIDLIQIIIYMGFPKILIESRPRGI----EELQMNLQKEF
HSVNRKLNIAITRIEKPYGNPNILAEFIAGQLKNRVSFRKAMKKAIELTEQADTKGIQVQIA
GRIDGKEIARVEWIREGRVPLQTIRAKIDYCCYTVRTIYGVLGIKIWIFIDGEMSRYRGPRFK
KIRRLGALPGLTNKRPRAGSDLRNQSRSGK-KSQYRIRLEEKQKLRFHYGLTERQLLKYVR
IAGKAKGSTGQVLLQLLEMRLDNILFRLGMAPTIPGARQLVNHRHILVNGRIVDIPSYRCK
PRDTIAARDEQKSKVLIQNSLDSSPH-EELPNHLTLQPFQ--YKGLVNQIIDSKWVGLKINEL
LVVEYYSRQTMSRRGTAEEKTAKSDPIYRNRLVNMLVNRILKHGKKSLAYQIIYRAVKKIQ
QKTETNPLSVLRQAIHGVTPGIAVKARRVGGSTQQVPIEIGSTQGKALAIRWLLAASRKRP
GRNMAFKLSSELVDAAKGSGDAIRKREETHKMAESNRAFAHFRMGSDTIADIITSIRNAD
MYRKSVVRVASTNISQSIVKILLREGFIENVRKHRENNKSFLVLTLRHRR--NRKRTY---RNL
LNLKRISRPGLRIYSNYQRIPRILGGMGIVILSTSQGIMTDREARLERIGGEILCYIWMAKAI
PKKGSR--GRIGSRKSTRKIPKGVIHIQASFNNTIVTVTDVRGRVVSWSSAGTSGFRGTKRG
TPFAAQTAAGHAIRAVVDQGMQRAEVMIKGPGLGRDAALRAIRRSGILLTFVRDVTPMPH
NGCRPPKKRRVMPTIKQLIRNTRQPIRNVTKSPALRGCPQRRGTCTRVY-ITPKKPNSALRK
VARVRLTSGFEITAYIPGIGHNSQEHSVVLVRGGRVKDLPGVRYHIVRGTLDAVGVKDRQQ
GRS-SAL----MAKKSLIQREKKRQKLEQKYHLIRRSSKKEIS---KVRSLSDKWEIYGKLQSPP
RNSAPTRLHRRCFSTGRPRANYRDFGLSGHILREMVHACLLPGATRSSWMVKNSFISIIFQ
EQEENKENRGSVEFQVVSFTNKIRKLTSHLELHKKDYLSQRGLRKILGKRQRLLAYLSKK
NRARYKELIGQLNIRERKTR--MVKLRLKRCGRKQRAVYRIVAIDVRSRREGRDLRKVGFY
DPIKNQTYLNVPAILYFLEKGAQPTGTVQDILKKAEVFKELCPNQTKFN-MDKSKRTFLKS
KRSFRRRLPPIQSGDRIDYKNMSLISRFISEQGKILSRRVNRLTLKQQRLITIAIKQARILSLLP
FLNNEK--QFERTESTTRTPSLRARKRVTRSLKKNPFVANNLLKKINKLNTKAEKEIIITWSR
ASTIIPIMVGHTIAIHNGKEHLPIYITDRMVGHKLGEFAPTLNFRGHAKSDNRSR
R???
??
??
??
??
??
??
??
??
??
??
??
??
??
?????????MTGHEFKSWILELREILREIKNSHYFLDSWTQFNSVGSFIHIFFHQERFIKLFDSRI
WSILLS-HNS--QGSTSNRYFT---IKG-VILFGVAVLIYR----INNRNMVERKNLYLIGLLP-----IP
MNSIGPRNDTLEESV-GSSNINRLIVS-LLYLPKGKKIYESSFLNPKESTWVLPIT----KKCSMP
ESNWGSRWWRDWIGKKR--DSSCKISN---ETVAGIEILFKEKDLK-YLEFFFVYYRD--DPIR
KDHDWELFDRLSLRKRQNRINL--NSGPLFEILVKHWICYLMSAFREKIPIEVEG-FFKQQG-

AGSTIKSNDIEHVSH--LFSRNKSAISLQNCAQFHMWQFRQDLFV---SWGK-NPPESDLLRN-
VSRENLIWLDNVWLVNKDR---FFR--KVRNVSSNIQYDS---TR--SSFVQVRDSSQLKGSS-D
QSRDHFDSIS----NEDSE-------------YHTLINQREIQQLKERSIL-WD--PSFLQTEGTEIESN--RF
PK--CLSGYS--SMSRLFT-EREKQMINHLLPEEIEEFL--GNPTRSVRSFFSDRW--SEFH-LGSN
PTERSTRDQ----KLLKKQQDLS-FL--RRSE----NKEMVNLFKIITYLQN--TVSIHPISSDSGCD
MVP-KDEPD-MDSSNKISFLNKNPFFDL--FHLFHDRNRGGYTL--HHDFESE--ERFQELADL
FTLSITEP-DLVYHKRFAFSIDSYGLDPKQ--FLNGVFNSRYEWKTTSLLVLLVLLPIFY--EEN
ESFYRRIRKKRVR-ISCGNDLEEPKPKIVVFASNNIMEAANQYRLIRNLIQIQHSTHR--YIRN
VLNRFFLMN---RSDRNFKYG---IQRDQIGKDTLNHRTLMKYMINQHLSNLKK-SQKRWFD
PL--IFFSRTKRSMNRDPDAYRYKWSTGSKNFQEH---FVSEQKSR---FQVVFDRLRINQYSID
WSEVIDKKDLSKPLRFFLSK-----------------------LLLFLSN-SLPFLFVSFGNIPIHRSE-IYIY-EL
KGPND---PQFLESIGLQIVHLKKLKPF--LLD---DHETC-QKSKFLINGGTISPFLFNKIP-KW-
MIDSFHTR--NNRRKSFDNTDSYFSMIFHDQYNWLNPVKSFHRSSLRSSFYKANQLRFLNN
PHHFCFY--CNKRFPFYV----EKARIN--NYDFTYG-QFLNILFIRNK-IFSLCVGK--KKHAFWG
-RDTISAIESQVSNIFIPKAFPQSG-DETYNLYKS---FHFPSRSNPFVR--RAIYSIADISGTPLTE
GQIVNFERTYCQPLSDMNLSDSEGKNLYQYLN--FNSNMGLIHTPCYEKYLPSEK--RKK----
----RSLCLK--KCVEKGQMYRTFQRDSAYSTLSKWNLFQTYMPWFLTSTGYRYLKFLFLDT
FSDLL--PILSS-SQKFVSIFHDIMHGSNISWRILQKKF------CLPQRNL-ISEISSKCLHN--LLLS
EEMIHRN--NESPLISTHLT--NVREF-LYAILFLLLVAAY-LACTRLLFVFG--ASSELQTEFEKV
KSLMIPSSMIELRKLLDRYPTSEPNSFWF-------LKQLGDSL--------GGNMLLGGGPA-----YR-
VKSIRSKKKYLNINLIDIIDLI-SIIPNPINRITFSRNTRHLSHTSKEIYSLIRKRKNVNGDWIDD
KIESWVANSDSIDDEKREFLVQFS--TLTT--EKRIDQILLSLT-HSDHF-----SKNDSGYQMIEQP
GAIYLRYLVDIH--KKYLMNYEFNTSSLAERRIFLAHYQTITY--SQTSCGANSLHFPSHG---K
PFSLRLALSLS-RGTLVIGSIGTGRSYLVKYLAKNSYLPFITVFLNKSLDNKSQGFDNI-----D
VD-SDDSD---------ASDDIDA------------SDDI---LDMEL-ELLTSMNALTMDMMPED--EDLLYI
TLQFELAKAMSPCIIWIPNIHDLDVNESNYLSPGLLVNLLSR---DYE----TRNILVIASTHIPQ
KVDPALIAPNKLNTCIKIRRLLIPQQRKHFFTLSYTRGFHLEKKMFHT--NGFGSITMGSNAR
DLVALTNEALSISITQNKSIIDTNTIRSALHRQIWDLR---SQVRSVQDHGILFYKIGRAVAQN
VLLSNCPIDPISIYMKKK--SCNEVDYYLYNWYFELGTSMKKLTILLYLLSCSAGSVTQDLW
SLP--GPDEK-NGITPYGLVENDS-GLVRGLLEVEGALVG--SSR---TCSQFDKD--RVTLLLRPE
PRN-PLDMMQNGSCSILDQRFLYEKD--ESEFEE-GDE-----RQQ----IEED----LFNH--IVWAP--
RIWRP---WGFLFDCIERPNELGFP-YWSRSFRGK---RIVYDEEDELQENDSEFLQNGTVQYQ
TRDISSKEQGLFRISQFIWDPADPLFFLFK-AQPFVSVFSHRELFADEEMSKGLLTPQKNRPT
SLYKRWFI--KKTQEKHFELLINRQRWLR---TNRSLS--NGSFRSN--TLSESYQYLSNLFLSN-
GTLLDQMTKALLRKRWLFPDEMQIGF--MEQDKDFPFL---
-MSAQSEGNYAEALQNYYEAMRLEIDPYDRSYILYNIGLIHTSNGEHTKALEYYFRALERN
PFLPQAFNNMAVICHY----------------------------VRLSP---------L---------MSCRSEHIWIEPITGA
RKTSNFCWAVILFLGSLGFLLVGTSSYLGRNLISLFPSQEIVFFPQGIVMSFYGIAGLFISSYL
WCTISWNVGSGYDRFDRKDGIVCIFRWGFPGKNRRVFLQFLIKDIQSVRIEVKEGIYARRV
LYMDIRGQGAIPLTRTDEN-FTPREMEQKAAELAYFLRVPIEVF
>Glycine
MEKWWFNSMLFLSKSMDSLGPIENTSLREDPILTDIEKKIHR--DLDYL-------------------------
----------------------EMEGFFS-----SDLNTVSKNDDD---HYMYETQFSFNNNITSFIDS--------CIE
SFNLGDIDKYND-YFYSYIFLNSSTSIITSTNDSDSTIGESSNNLDESQKYKHLWLECENCYG

LNYKKFFKSKMNICEYCGYHLKMGSSDRIELSIDSGTWNPMDEDMVSLDPIEFHSEEEPY
KDRIDSYQRKTGLTEAVQTGTGQLNGIPVAIGIMDFQFMGGSMGSAVGEKITRLVEYATNQ
LLPLILVCASGGARMQEGSLSLIQMAKISSALYDYQKNKKLFYVSILTSPTTGGVTASFGM
LGDIIIAEPNAYIAFAGKRVIEQTLNKAVPEGSQAAEYLFHKGLFDSIVPRNLLKGVLSELFQ
FHNFFSLTKNDKAMVTIRADEISKIIRERIEQYNTEVKIVNTGTVLQVGDGIARIYGLDEVM
AGELVEFEEGTIGIALNLESKNVGVVLMGDGLMIQEGSSVKATGRIAQIPVSEAYLGRVIN
ALAKPIDGRGEISASESRLIESPAPGIISRRSVYEPLQTGLIAIDSMIPIGRGQRELIIGDRQTG
KTAVATDTILNQQGQNVICVYVAIGQKASSVAQVVNTLQERGAMEYTIVVAETADSPATLQ
YLAPYTGAALAEYFMYRERHTLIIYDDLSKQAQAYRQMSLLLRRPPGREAYPGDVFYLHS
RLLERAAKLSSQLGEGSMTALPIVETQSGDVSAYIPTNVISITDGQIFLSADLFNAGIRPAIN
VGISVSRVGSAAQIKAMKQVAGKLKLELAQFAELEAFAQFSSDLDKATQNQLARGQRLRE
LLKQSQSAPLTVEEQIITIYTGTNGYLDSLEIGQVRKFLVELRAYLNTNKPQFKEIISSTKTFT
GEAEVLLKEAIQEQMELFLLQEQVMRINPTTSGPEVSALEKKNLGRIAQIIGPVLDVAFPPG
KMPNIYNALIVKGRDTVGQQINVTCEVQQLLGNNRIRAVAMSATEGLMRGMEVIDTGAA
LSVPVGGATLGRIFNVLGEPIDNLGPVDTRTTSPIHRSAPAFIQLDTKLAIFETGIKVVDLLA
PYRRGGKIGLFGGAGVGKTVLIMELINNIAKAHGGVSVFGGVGERTREGNDLYMEMKES
GVINEQNIAESKVALVYGQMNEPPGARMRVGLTALTMAEYFRDVNEQDVLLFIDNIFRFV
QAGSEVSALLGRMPSAVGYQPTLSTEMGSLQERITSTKEGSITSIQAVYVPADDLTDPAPAT
TFAHLDATTVLSRGLAAKGIYPAVDPLDSTSTMLQPRIVGEEHYETAQRVKQTLQRYKELQ
DIIAILGLDELSEEDRLTVARARKIERFLSQPFFVAEVFTGSPGKYVSLAETIRGFKLILSGEL
DGLPEQAFYLVGNIDEATAKATNLEMESNLKKMTLNLCVLTPNRIVWDSEVKEIILSTNSG
QIGVLPNHAPIATAVDIGILRIRLKDQWLTMALMGGFARINNNEITVLVNDAEKGSDIDPQE
AQQTLEIAEANLNKAEGKRQTIEANLALRRARTRVEAINVISMKNITDSFLCLGYWPSAGS
FGFNTDILATNPINLSVVLGVLVFFGKGVLSDLLDNRKQKILRTIRNSEELREGAIEQLEKA
QARLRKVETEADRFRVNGYSEIEREKLNLINSIYTTLEQLENYKNEAIHFEQQRVINQVRQ
RVLQQALQGALGTLKSCLNNELHLRTVSANIGMFGTMKEKITMNPIISAASVIAAGLAVGL
ASIGPGVGQGTAAGQAVEGIARQPEAEGKIRGTLLLSLAFMEALTIYGLVVALALLFANPF
VMNVLLCSINTLKRLYDISAVEVGQHFYWQIGSFQVHAQVLITSWVVIALLLVSAILIIRNL
QTIPAFGQNFFEYVLEFIRDVSKTQIGEEYGPWVPFIGTLFLFIFVSNWSGALLPWKIIQLPH
GELAAPTNDINTTVALALLTSIAYFYAGLSKKGLAYFNKYIQPTPILLPINILEDFTKPLSLSF
RLFGNILADELVVVVLVSLVPLVVPIPVMFLGLFTSGIQALIFATLAAAYIGESMEGHHMVF
ASLEHILTHISFSVVSILISIHLITLLFVEIIGLSDSSKKGMIITFFCITGLLVTRWVFSGHLPFSD
LYESLIFLSWTFSIFYMVPYFKKSKNYLNTIITPSVIFTQGFATSGLLTKMHESLILVPALQSH
WLMMHVSMMILGYATLLCGSLLSVAILVITFQELIQIIGKSKEIKYMNMTDNVLQKTSFLS
YRNYYRSQFLQQLDRWGYRTISLGFIFLTIGIISGAVWANEAWGSYWNWDPKETWAFITW
TIFAIYLHTRKNKKLEDLNSSIVASIGFLIIWVCYLGINLLGIGLHSYGSFTPNMTKKKTFIPL
LYLTSIVFLPWCISFTFKKSLESWFIDWWNTRQSEIFLNDIKEKSILKKFIEFEELFFLDDML
KECPETHLQNLRTG-YKETIQLIKTHNEDRMNTILHFSTNIICFFILSGYSILGNQELVLINSL
VREFIYNLSDTIKAFSILLLTDLCIGFHSTHGWELVIGFVYKDFGFAQNDQIISGLVSTFPVIL
DTILKYWIFRYLNRVSPSLVVIYHSMNDMPIGVPRVPFRSPGEEDASWVDIY-RLYRERLLF
LGQEVDSEISNQLISLMVYLSIEEENKDLYLFINSPGGWVIPGIAIYDTMQFVQPDVQTVCM
GLAASMGSFLLAGGEITKRLAFPHA-VMIHQPASSFYEAQTGEFILEAEELLKMRETITRVY
V Q R T G K P L W V I S E D M E R D V F M S A A E A Q A H G I V D L V A V
E???MEESRAY

LELHRSRHQDTLYPLFFRESIYGLACGH--VGYNNKFSLLIVKRLITRMYQQTHFIIFTNDSN
KNPFMGNHFYSQIILEGFVVVVEILFSLQFCISLIVKSYNLRSIHSIFPFFEDKLIYLNHESDIR
IPYPIHLEILVQILRYWIKDVSFFHLLRFFFSYYYNWNSIFTKWISTFFSPRFFLFLYNLYVWE
YESIFLFLRNKSSKLRLKYFRVFFERIFFYEKIEHLVEVSVKSYTLSFFKDTFMHYVRYQGK
SILVSKNTPLLINKWKYYFIYLWQCHFDIWSRPGTIHINQLSQHSFHFLGYFLSIRPNLSVVR
NQMLQNSFLIKM--VMKRLDTIVPIIPLIRSLAKAKFCNVFGHPISKPVWANLSDFDIIDRFL
RICRNFSHYYNGSAKKKSLYQIRYILRLSCIKTLARKHKSTARTFLKRLGSKLLEEFFTEEEE
TFSLIFPRRIWYLDILVRNDFVNHFMIIYLT-EIQDIH-FFFRLES-FKEIYGIIWVFAPILILILGIT
ISVLAIVWLEREISAGIQQRIGPEYTGPFGVLQALADGTKLLFKENLIPSRGDIRLFSIGPSIS
VISIIISYSVIPFGYNFVLSDLNIGVFLWIAISSIAPIGLLMSGYGSNNKYSFLGGLRAAAQSIS
YEIPLTLCVLSISLLSNSLSTVDIVDAQSKYGFWGWNLWRQPMGFIVFLISSLAECERLPFD
LPEAEEELVAGYQTEYSGIKFGLFYVASYLNLFISSLFVTVLYFGGSNISIPYIFVSDFFEINQ
TYGVFVTIIGIFITLAKTYLFLFLSIATRWTLPRLRIDQLLNLGWKFLLPISLGNLLLTTSSQL
FSLMIWHVQNENFILDSTRIFMKAFHLPLFDGSFIFPECILIFGLILLLMIDSTSDQKDISWFY
FISSTSLVMSITALLFRWREEPMISFSGNFQTNNFNEIFQFLILLCSTLCIPLSVEYIECTEMAI
TEFLLFILTATLGGMFLCGANDLITIFVALECFSLCSYLLSGYTKKDVRSNEATTKYLLMGG
ASSSILVHGFSWLYGSSGGEIELQEIVNGLINTQMYNSPGILIALLFITVGIGFKLSPAPSHQW
TPDVYEGSPTPVVAFLSVTSKVAASASATRIFDIPFYFSSNEWHLLLEILAILSMILGNLIAIT
QTSMKRMLAYSSIGQIGYVIIGIIVGDSNGGYASMITYMLFYISMNLGTFACIVSFGLRTGT
DNIRDYAGLYTKDPYLALSLALCLLSLGGLPPLSGFFGKLHLFWCGWQAGLYFLVSIGLLT
SVVSIYYYLKIIKLLMTGRNQEITPHVRNYRRPPLRSNNSIELSMIVCVIASTIPGISMNPIIEI
AQDTLFMFLLYEYDIFWAFLIISSFIPILAFLISGILAPISKGPEKLSSYESGIEPIGDAWLQFRI
RYYMFALIFVVFDVETVFLYPWAMSFDVLGVSVFIEAFIFVLILIVGSVYAWRKGALEWST
NYFPWLTTVVILPIVGGSLIFLFPHKGNKVIKWYTICICLIDLLITSYVFCYHFELDDPLIQLT
ENYKWINFFDFYWSFGIDGLSLGPILLTGFITTLATLAAQPVTRESKLFYFLMLAMYSGQIG
TFSSQDILLFFIMWEFELIPVYLLLSMWGGKKRLYSATKFILYTAGSSVFLLLGILGMSFYSS
NEPTLNFESLTNQSYPVALEIIFYIGFLIAFAVKSPIIPLHTWLPDTHGEAHYSTCMLLAGILL
KMGAYGLVRINMEFLSRAHSIFSPWLILLGSIQIIYAASTSLGQRNLKKRIAYSSVSHMGFLL
LGIGSISDTGLNGAILQIISHGFIGAALFFLAGTSYDRLRLLYLDEMGGMAIPMPKIFTVFTIL
SMASLALPGMSGFVAELIVLLGIITSQKYLLITKILITFVTAIGMILTPIYSLSMLRQMFYGYK
LFNTPNSYFFDSGPRELFISISILIPVISIGIYPDFIFSFSADKVEAILSNFL--MIFEHALVLSAYL
FSIGIYGLITSRNMVRALMCLELILNAVNINLVTFSDFFDSRQLKGNIFSIFVIAIAAAEAAIG
PAIVSSIYRNRKSTRINQSNLLNKMEYTHQYSWIIPFIPFPVPMLIGVGLLLFPTATKYLRRM
WAFPSILLLTIVMMFSLDLSIHQINNSSFYQYVWSWTINNDLSLEFGYLIDSLTSIMSILITTV
GILVLIYSDNYMSHDQGYLRFFAYLTLFNISMLGLVTSSNLIQIYVFWELVGMCSYLLIGFW
FTRPIAANACQKAFVTNRVGDFGLLLGILGLYWITGSLEFRDLFQIINN-EVNIFFLTLVALLL
FCGSVAKSAQFPLHVWLPDAMEGPTPISALIHAATMVAAGIFLVARLLPLFIVLPAIMNGIAF
IGIITVVLGATLAIAQQDIKKNLAYSTMSQLGYMMLALGMGSYRAALFHLITHAYSKALLF
LGSGSIIHSMEALVGYSPAKSQNMVLMGGLTKHVPITKTSFLVGTLSLCGIPPFACFWSKDE
ILNDSWLYSPIFAIIACCTAGLTAFYMFRIYLLVFEGYLNVHFLNFNGKKNSSFYSISLWGKK
EVKQKLKNFLALLRMKFFIGTKKTACLYPNESDNTMRFSILVLVLFTLFVGTIG--FDFDILS
KWLIPFIDLLHKNSK-FVDW-EFLTNAAFSVILTFLGIFIASFFYKPVYSDLQNLNLLNLFEKV
ADYFQNVIYDWSYNRGYIDVFYDISLITSVRKLVQFNYFFDKKIIDAIPNGIGITSFFMGEAI
KYVGGG-RISSYILLYIFYIVIFILIWYMDLPEPLHDFLLVFLGSGLILGSLGVVLFTNPIFSAF

SLGLVLVCISLFYILSNSHFVAASQLLIYVGAINVLIIFAVMFMNGSEYYQDFRLWTVGDGIT
LMVCTSIFVSLITTILDTSWHGIIWTTRPNQIIEQDLISTSQQIGIHLSTDFFLPFELISIILLVAL
IGAIVVARQMNISTTRKDFMIVNMGPHHPSMHGVLRLIVTLDGEDVIDCEPILGYLHRGME
KIAENRTIIQYLPYVTRWDYLATMFTEAITVNGPEQLGNIQVPKRASYIRVIMLELSRIASHL
LWLGPFMADIGAQTPFFYIFRERELIYDLFEAATGMRMMHNFFRIGGVATDLPYGWVDKC
YDFCDYFLTRIVEYQKLITRNPIFLERVEGVGVVDIKEVINWGLSGPMLRASGIQWDLRKV
DNYECYEEFDWEVQWQKEGDSLARYLVRIGEMMESIKIIQQALEGIPGGPYENLEIRCFDR
EKEPEWNEFEYRFISKKPSPTFELPKQELYVRIEAPKGELGIFLIGDQNGFPWRWKIRPPGFI
NLQILPQLVKRMKLADIMTILGSIDIIMGEVDRMFLMVSGFINYSQQTVRAARYIGQGFTIT
LSHANRLPVTIQYPYEKIISSERFRGRIHFEFDKCIACEVCVRVCPIDLPVVDWKLETDIRKK
QLLNYSIDFGICIFCGNCIEYCPTNCLSMTEEYELSTYDRHELNYNQIALGRLPVSVIDDYTI
RTIQIK----------------MQGRLSSWLVKHGLIHRSLGFDYQGIETLQIKPEDWHSIAVILYVYG
YNYLRSQCAYDVAPGGLLASVYHLTRLEYGIDQPEEVCIKIFVARKNPRIPSIFWVWKSAD
FQEKESYDMLGISYDNHPRLRRILMPESWIGWPLRKDYIAPNFYEIQDAH--------------------
--------------------------------------MNSIEFPLLDQTTKNSVISTTLNDLSNWSRLSSLWPLLYGTS
CCFIEFASLIGSRFDFDRYGLVPRSSPRQADLILTAGTVTMKMAPSLVRLYEQMPEPKYVIA
MGACTITGGMFSTDSYSTVRGVDKLIPVDVYLPGCPPKPEAIIDAITKLRKKISREIYENQM
SSQ-ENRCFTTNHKFHIGYSTHTGNYGQELFYQLPSTSEIPT--FF------------MQTRNAFSCIK
EGITRSISISIMIYIIIRAPISNAYPIFAQQGYENPREATGRIVCANCHLANKPVDIEVPQAVLP
DTVFEAVVRIPYDMQVKQVLANGKKGALNVGAVLILPEGFELAPPDRISPEIKEKIGNLSF
QNYRPTKKNILVVGPVPGQKYKEITFPILSPDPTTKRDVHFLKYPIYVGGNRGRGQIYLDG
SKSNNNVYNATAAGMVKKIIRKEKGGYEITIVDALDGREVIDIIPPGPELLVSEGESIKLDQP
LTSNPNVGGFGQGDAEIVLQDPLRVQGLLFFFASIILAQIFLVLKKKQFEKVQLSEMNFMS
KVYDWFEERLEIQAIADDITSKYVPPHVNIFYCLGGITLTCFLVQVATGFAMTFYYRPTVTE
AFASVQYIMTEANFGWLIRSVHRWSASMMVLMMILHVFRVYLTGGFKKPRELTWVTGV
VLAVLTASFGVTGYSLPWDQIGYWAVKIVTGVPDAIPVIGSSLVELLRGSSSVGQSTLTRFY
SLHTFVLPLLTAVFMLMHFSMIRKQGISGPLMGVTKKPDLNDPVLRAKLAKGMGHNYYG
EPAWPNDLLYIFPVVILGTIACNVGLAVLEPSMIGEPADPFATPLEILPEWYFFPVFQILRTVP
NKLLGVLLMVSVPTGLLTVPFLENVNKFQNPFRRPVATTIFLIGTVVALWLGIGATLPIEKSL
TLGLFMIEVFLFGIVLGLIPITLAGLFVTAYLQYRRGDQLDLMLTITSYFGFLLVVLIITSSLFI
GLSKIRLIMDIVSLAWAALMVVFSFSLSLVVWGRSGLMIIRSPEPEVKILVDRDPIKTSFEEW
AKPGHFSRTIAKGPDTTTWIWNLHADAHDFDSHTNDLEEISRKVFSAHFGQLSIIFLWLSG
MYFHGARFSNYEAWLSDPTHIRPSAQVVWPIVGQEILNGDVGGGFRGIQITSGFFQIWRAS
GITSELQLYCTAIGALVFAALMLFAGWFHYHKAAPKLAWFQDVESMLNHHLTGLLGLGSL
SWAGHQIHVSLPINQFLNAAVDPKEIPLPHEFILNRDLLAQLYPSFAEGATPFFTLNWSKYA
EFLTFRGGLDPVTGGLWLTDIIHHHLAIAILFLIAGHMYRTNWGIGHSIKDILEAHKGPFTG
QGHKGLYEILTTSWHAQLSINLAMLGSLTIVVAHHMYSMPPYPYLATDYGTQLSLFTHHM
WIGGFLIVGAAAHAAIFMVRDYDPTIRYNDLLDRVLRHRDAIISHLNWVCIFLGFHSFGLYI
HNDTMSALGRPQDMFSDTAIQLQPVFAQWIQNTHALAPGTTAPGATTSTSLTWGGDNLVA
VGGKVALLPIPLGTADFLVHHIHAFTIHVTVLILLKGVLFARSSRLIPDKANLGFRFPCDGPG
RGGTCQVSAWDHVFLGLFWMYNSISVVIFHFSWKMQSDVWGSISDQGIVNHITGGNFAQ
SSITINGWLRDFLWAQASQVIQSYGSSLSAYGLFFLGAHFVWAFSLMFLFSGRGYWQELIE
SIVWAHNKLKVAPATQPRALSIVQGRAVGVTHYLLGGIATTWAFFLARIIAVGMALRFPRFS
QGLAQDPTTRRIWFGIATAHDFESHDDITEERLYQNIFASHFGQLAIIFLWTSGNLFHVAWQ

GNFEAWVQDPLHVRPIAHAIWDPHFGQPAVEAFTRGGALGPVNIAYSGVYQWWYTIGLR
TNGDLYTGALFLLFLSTISLIAGWLHLQPKWKPSVSWFKNAESRLNHHLSGLFGVSSLAW
TGHLVHVAIPGSRGEYVRWNNLLSILPHPEGLGPFFTGQWNLYAQNPDSNSHIFGTPQGAG
TAILTLLGGFHPQTQSLWLTDIAHHHLAIAFLFLVAGHMYRTNFGIGHSIKDLLEAHTPPGG
RLGRGHKGLYDTINNSIHFQLGLALASLGVITSLVAQHMYSLPAYAFIAQDFTTQAALYTH
HQYIAGFIMTGAFAHGAIFFIRDYNPEQNEDNVLARMLDHKEAIISHLSWASLFLGFHTLG
LYVHNDVMLAFGTPEKQILIEPIFAQWIQSAHGKTSYGFDILLSSTNSPAFNAGRSIWLPGW
LNAVNE--NSNSLFLTIGPGDFLVHHAIALGLHTTTLILVKGALDARGSKLMPDKKDFGYSF
PCDGPGRGGTCDISAWDAFYLAVFWMLNTIGWVTFYWHWKHITLWQGNVSQFNESSTY
LMGWLRDYLWLNSSQLINGYNPFGMNSLSVWAWMFLFGHLVWATGFMFLISWRGYWQ
ELIETLAWAHERTPLANLIRWRDKPVALSIVQARLVGLAHFSVGYIFTYAAFLIASTSGKFG
MSHSVKIYDTCIGCTQCVRACPTDVLEMIPWDGCKAKQIASAPRTEDCVGCKRCESACPT
DFLSVRVYLWHETTRSMGLAYMI--NFPSIFVPLVGLVFPAIAMASLFLHVQKNKIFMRDLK
TYLSVAPVVSTLWFGALAGLLIEINRFFPDALIFPFFSFMTAILERRESESLWGRFCNWITST
ENRLYIGWFGVLMIPTLLTATSVFIIAFIAAPPVDIDGIREPVSGSLLYGNNIISGAIIPTSAAIG
LHFYPIWEAASVDEWLYNGGPYELIVLHFLLGVACYMGREWELSFRLGMRPWIAVAYSAP
VAAATAVFLIYPIGQGSFSDGMPLGISGTFNFMIVFQAEHNILMHPFHMLGVAGVFGGSLFS
AMHGSLVTSSLIRETTENESANEGYRFGQEEETYNIVAAHGYFGRLIFQYASFNNSRSLHFF
LAAWPVVGIWFTALGISTMAFNLNGFNFNQSVVDSQGRVINTWADIINRANLGMEVMHE
RNAHNFPLDLAAIDAPSINGMGLPWYRVHTVVLNDPGRLLSVHIMHTALVAGWAGSMAL
YELAVFDPSDPVLDPMWRQGMFVIPFMTRLGITNSWGGWNIT-GGTITNPGIWSYEGVAG
AHIVFSGLCFLAAIWHWVYWDLEIFCDERTGKPSLDLPKIFGIHLFLAGVACFGFGAFHVT
GLYGPGIWVSDPYGLTGRIQSVNPAWGVEGFDPFVPGGVASHHIAAGTLGILAGLFHLSVR
PPQRLYKGLRMGNIETVLSSSIAAVFFAAFVVAGTMWYGSATTPIELFGPTRYQWDQGYFQ
QEIYRRVGAGLAENQSLSEAWSKIPEKLAFYDYIGNNPAKGGLFRAGSMDNGDGIAVGWL
GHPVFRDKEGHELFVRRMPTFFETFPVVLVDGDGIVRADVPFRRAESKYSVEQVGVIVEF
YGGELNGVSYSDPATVKKYARRAQLGEIFELDRATLKSDGVFRSSPRGWFTFGHASFALLF
FFGHIWHGARTLFRDVFAGIDPDLDAQVEFGAFQKLGDPTTKKQVVMKTLYSLRRFYHV
ETLFNGTLALTGRDQETTGFAWWAGNARLINLSGKLLGAHVAHAGLIVFWAGAMNLFEV
AHFVPEKPMYEQGLILLPHLATLGWGVGPGGEVIDTFPYFVSGVLHLISSAVLGFGGIYHA
LLGPETLEESFPFFGYVWKDRNKMTTILGIHLILLGIGAFLLVFKALYFGGIYDTWAPGGGD
VRKITNLTLSPSIIFGYLLKSPFGGEGWIVSVDDLEDIIGGHVWLGSICILGGIWHILTKPFAW
ARRALVWSGEAYLSYSLGALSVFGFIACCFVWFNNTAYPSEFYGPTGPEASQAQAFTFLVR
DQRLGANVGSAQGPTGLGKYLMRSPTGEVIFGGETMRFWDLRAPWLEPLRGPNGLDLSR
LKKDIQPWQERRSAEYMTHAPLGSLNSVGGVATEINAVNYVSPRSWLATSHFVLGFFLFV
GHLWHAGRARAAAAGFEKGIDRDFEPVLSMTPLNMTIALGKFTKDENDLFDIMDDWLRR
DRFVFVGWSGLLLFPCAYFALGGWFTGTTFVTSWYTHGLASSYLEGCNFLTAAVSTPANS
LAHSLLLLWGPEAQGDFTRWCQLGGLWTFVALHGAFALIGFMLRQFELARSVQLRPYNAI
AFSGPIAVFVSVFLIYPLGQSGWFFAPSFGVAAIFRFILFFQGFHNWTLNPFHMMGVAGVLG
AALLCAIHGATVENTLFEDGDGANTFRAFNPTQAEETYSMVTANRFWSQIFGVAFSNKRW
LHFFMLFVPVTGLWMSALGVVGLALNLRAYDFVSQEIRAAEDPEFETFYTKNILLNEGIRA
WMAAQDQPHENLIFPEEVLPRGNALMSGSTGERSFADIITSIRYWIIHSITIPSLFIAGWLFV
STGLAYDVFGSPRPNEYFTESRQGIPLITGRFDPLEQLDEFSRSFMTIDRTYPIFTVRWLAVH
GLAVPTVSFLGSISAMQFIQRMATQTVEDNSRSG-PRRTVVGDLLKPLNSEYGKVAPGWGT

TPLMGVAMALFAIFLSIILEIYNSSILLDGISMNMLTLKLFVYTVVIFFVSLFIFGFLSNDPGR
NPGREEMADTTGRIPLWIIGTVTGITVIGLIGIFFYGSYSGLGSSLMLNIFNLVCICIHSVLYS
SSFFSAKLPEAYAFLNPIVDIMPVIPLLFFLLAFVWQAAVSFRTTQSNPNEQNVELNRTSLY
WGLLLIFVLAVLFSNYFFNMEVNILAFIATALFILVPTAFLLIIYVKTVSQSDMETATLIAISIS
GLLVSFTGYALYIAFGQPSQQLRDPFEEHGDMEALVYTFLLVSTLGIIFFAIFFREPPKVPTK
KG-MTIAFQLAVFALIAISFILLISVPVVFASPEGWSNNKNVVFSGTSLWIGLVFLVGILNSLIS
-------MSPQTETKASVGFKAGVKDYKLTYYTPDYETKDTDILAAFRVTPQPGVPPEEAGAA
VAAESSTGTWTTVWTDGLTSLDRYKGRCYGLEPVAGEENQYIAYVAYPLDLFEEGSVTNM
FTSIVGNVFGFKALRALRLEDLRIPTAYIKTFQGPPHGIQVERDKLNKYGRPLLGCTIKPKL
GLSAKNYGRAVYECLRGGLDFTKDDENVNSQPFMRWRDRFLFCAEAIFKSQAETGEIKG
HYLNATAGTCEEMMKRAVFARELGVPIVMHDYLTGGFTANTSLAHYCRDNGLLLHIHRA
MHAVIDRQKNHGMHFRVLAKALRLSGGDHVHAGTVVGKLEGEREITLGFVDLLRDDFV
EKDRSRGIYFTQDWVSLPGVLPVASGGIHVWHMPALTEIFGDDSVLQFGGGTLGHPWGN
APGAVANRVALEACVQARNEGRDLAREGNEIIREASKWSPELAAACEVWKEIKFEFEAMD
TL--MAIHLYKTSTPSTRNGAVDSQVKSNPRNHLIYGQHRCGKGRNARGIITAGHRGGGHK
RLYRKIDFRRNEKNIYGRIVTIEYDPNRNAYICLIHYGDGEKKYILHPRGAIIGDTIVSGTEV
PIKMGNALPLSDMPLGTAIHNIEITLGKGGQLARAAGAVAKLIAKEGKSATLKLPSGEVRLI
SKNCSATVGQVGNVGVNQKNLGRAGSKCWLGKRPVVRGVVMNPVDHPHGGGEGRAPI
GRKKPATPWGFPALGRRSRKRKKYSDNLILRRRTKMIQPQTHLNVADNSGARELMCIRILG
AS-NRRYAYIGDIVVAVIKQAVPNTNLERSEVIRAVIVRTCKELKRSNGIIIQYDDNAAVVIDQ
EGNPKGTRIFCAIARELRQLNFTKIVSLAPEVLMLSPQRTRFRKQHRGRMKGISYRGNHIC
FGRYALQALEPAWITSRQIEAGRRAMSRNVRRGGQIWVRIFPDKPVTVRPTETRMGSGKG
SPEYWVAVVKPGKILYEMGGVPENIARKAISIASSKMPIRTQFIISGMTRIKRGYIARKRRTK
IRLFTSSFRGAHSRLTRTITQQKIKALVSAHRDRDRKKRDFRGLWISRINAVIRGNIKVYYIY
S N L V Y S L Y T G Q L L L N R K I V A Q I A I L K Q N C L F M I A N D I I K T K N P L R L Y S G
K??
???????????????????????????????????MNGIKYAVFTDKSIRLLGKN-QYTFNVESGSTRTEIKH
WVELFFDVKVIAMNSHRLPVKGRRVRPIMGHTMHYRRMIITLQPGYSIPPLRKKKTMAVP
KKRTSISKKRIRNTLWKKKGYFTTLKAFSLAQSIFTGNSKSFF--KYKRMAKGKDIRVIVILE
CTGC-DKK--VNKE--GISRYITKKNRQNTPSRLELRKFCPRCCKHTIHAEIKKMKIRASVRKI
CEKCRLIRRRGRIIVICSNPRHKQRQGMVREKIRVSTRTLQWKCVESRIDSKRLYYGRFILS
PLMKGQADTIGIAMRRVLLGEIEGTCITRVKSE--KIPHEYSTIIGIEESVHEILMNLKEIVLRS
NLYGTRDASICFKGPGYVTAQDIILPPSVEIVDNTQHIANVTEPVNLCIELKIERNRGYRIKT
LKNFQDGSY--PIDATFMPVRNVNHSIHSYVNGNEKQEILFLEIWTNGSLTPKEALYEASRN
LIDLFIPFLHAEED--NFNLENNQ-HKVTLPLFTFHDILAKKLRKKKK-EIALKSIFIDQLELPP
RIYNCLKRSNIHTLLELLNNSQEDLLKIEHFRVEDVKYILDFLEIEKHFA-------MLGDGN-E
GMSTLPGLNQIQFEGFCRFIDRGLPEGLFKFPKIEDTDQEIEFQLFVETYQLLEPLINEKDAV
YESLTYSAELYVSAGLIWKSSRDIQEQTIFVGNIPLMNSLGTSIVNGIYRIVINQILQSPGIYY
RSELDPSGISVYTGTIISDWGGRLELEIDRKARIWARVSRKQKISILVLSSAMGSNLSEILEN
VCYPEIFVSFLNDKDKKKIGSKENAILEFYRQFACVGGDPVFSESLCKELQKKFFQQRCEL
GRIGRRNMNQKLNLDIPQNNTFLLPRDILTAADHLIGMKFGMGILDD--INHLKNKRIRSVA
DLLQDQFGLALVRLENMVRGTICGAIR-HKLI-PTPQNLVTSTP----LTTTYESFFGLHPLSQV
LDQTNPLTQIVHGRKLSYLGPGGLTGRTASFRIRDIHPSHYGRICPIDTSEGINVGLIGSLAIH
ARIGSWG-SIESPFY---EISERSKR--IRMLYLSPSRDEYYMVATGNSLALNRDIQEEQTVPAR

YRQEFLTIAWEQVHLRSIFPFQYFSIGASLIPFIEHNDANRALMSSNMQRQAVPLSQSEKCIV
GTGLERQVALDSGVSAIAEHEGNIIYTNTDRIFLFGNGDTLSIPLTIYQRSNKNTCMHQKPQ
VRRGKCIKKGQILADGAATVDGELALGKNVLVAYMPWEGYNSEDAVLINERLVYEDIYTS
FHIRKYEIQTHMTSYG-SERITNKIPHLEAHLLRNLDKNGIVILGSWVETGDILVGKLTPQM
AKE-SSYSPEDRLLRAILGIQVSTSKETCLKLPTGGRGRVIDVRWIQKKG-GSSYNPETIRIYI
LQKREIKVGDKVAGRHGNKGIVSKILSRQDMPYLQDGRPVDMVFNPLGVPSRMNVGQIF
ECSLGLAGGMLERHYRITPFDERYEQEASRKLVFSELYEASKQTSNPWIFEPEYPGKSKIFD
GRTGNSFKQPAIMGKPYILKLIHQVDDKIHGRSSGHYALVTQQPLRGRAKQGGQRVGEME
VWALEGFGVAHILQEMLTYKSDHIKTRQEVLGTTIIGGTIPKPTDAPESFRLLVRELRSLAM
ELNHFLVSEKNFRIHRKEA-------MIDQYKHQQLRIGSVSPQQISAWAKKILPNGEIVGEVTK
PYTFHYKTNKPEKDGLFCERIFGPIKSGICACGNYRVIRDKKDDPKFCEQCGVEFIDSRIRR
YQMGYIKLACLVTHVWYLKRLPSYIANLLDKSLKELESLVYCDF--SFARP-VVKKPTFLRL
RGSFEYEIQSWKHSIPLFFTTQGFDIFRNREISSGAGAIREQLADLDLRILMDSSLIEWKELG
EE-GSPDNEWEDRKVGRRKNFLVRRIELAKHFLRTNIEPEWMVLCLLPVLPPELRPIIQIDG
GKLMSSDINELYRRVIYRNNTLIDLLTTSRSTPGELVMCQEKLVQEAVDTLLDNGIRGQPM
RDGHNKVYKSFSDIIEGKEGRFRETLLGKRVDYSGRSVIVVGPSLSLHRCGLPREIAIELFQ
TFLIRGLIRKHFASNIGIAKSKIREKEPIVWEILQEVMQGHPVLLNRAPTLHRLGIQAFQPIL
VEGRAICLHPLVCKGFNADFDGDQMAVHVPLSLEAQAEARLLMFSHTNLLSPAIGDPISVP
TQDMLIGLYILTSGNRRGIYSNRYNPRNCGNFRNLKNERIRDNNYKYTKK-EPFFCNSYDAI
GAYQQKRINFDSPLWLRWRLDQR-IISS--REVPIEVHYESLGTYHEIYEHYLVVRSTKKEIRS
IYIRTNVGHISFYREIEEAIQGFCRAYSYDI------------MAERANLVFHNKVIGGTAIKRLISRLI
DHFGMAYTSHILDQVKTLGFRQATDTSISLGIDDLLTIPSKGWLVQDAEQQSLILEQHHHY
GNVHAVEKLRQSIEIWYATSEYFRQEMNPNFRMTDPFNPVHIMSFSGARGNASQVHQLVG
MRGLMSDPQGQMIDLPIQSNLREGLSLTEYIISCYGARKGVVDTAVRTSDAGYLTRRLVEV
VQHIVVRRTDCGTIQGISVNTQNGMMP-ERIWIQTLIGRVLADDIYRGSRCIAVRNQDIGIG
LINRFKT------LQTQPISIRTPFTCRNTSWICRLCYGQSPTHGHLVELGEAVGIIAGQSIGEPGT
QLTLRTFHTGGVFTGGTAEQVRAPYNGKIQFNEDLVHPTRTRHGHPAFLCYIDLYVTIESG
DIIHNVTIPPKSFLLVQNNQYVKSEQVIAEIRAGTYTF-NLKEKVRKHVYSDLEGE-MHWST
DVYHASEFRYSNVHILPKTSHLWILSGKSYR--SGSVSFSIRKDQDQMNIH--YLSTGERDFC
NLL--ASKKK-VRHKLFRFNPSDKKERRISDY-SIFNQIISIDHCHFTHPT-IFHDTTD--LLAKR
RRNRLIIPFQFIQERDKELMLASSISIEIPI--NGIFRRNSILAYFDDPQYRTQSSGITKYRTIGIN
SIFKKEDF-LIEYQ-GVKEFKTKYIKVDQFFFIPEEVHILPESSSIMVRNNSIVEVDTLITLNIR
SRVRGLVRLEKKKKKIELKIFSGDIYFPGEMDKISRHSAVLIPP-RTVKKNSK--EKK--MKN
WIYVQW--ITITKKK--YFVLVRPVILYEIADRINLVKLFPQDMFQERDNLELKVINYILSGNG
KSIRGISDTSIQLVRTCLVLNWDQDK---KFSSIENAHASFVEISIKGLVRYFLRIDLVK-HISYI
RKRNNPPGSRFILD-NESDRTTINPF---FSIDS--REKIQQSLSKNH-TIRMLLN---RNEKCRSLII
LSSSNCF--QIRSFNHGKYYN---GIKEGDAMIPIKNSLGPLGI--APQVAHFD---FYRLIT--HNQ
IS-IIKNGQLDK---LKETFQVF--Q-YYFLDENER--IYKPDLCSNIILNPFYLNWHFLHHN----TF
TIMS-LGQFICENVCIVQNAP---HLKSGQILTVQ-MDSVGIRSANPYLATPGATVHGHYGEIL
SEGDILVTFIYEKSRSGDITQGLPKVEQVLEVRSIDSISMNLEKRVDTWNGRITKILGIPWGF
LIGAELTIAQSRISLVNKIQKVYRSQGVHIHNRHIEIIVRQITSKVLVSE---DGMSNVFLPGEL
IGLLRAERAGRSLEEAICYRVLLLGITKTSLNTQSFISEASFQETARVLSKAALRGRIDWLK
GLKENVVLGGMIPVGTGFKRIMNRSKQRQYNKMT--SETKKKNLFEGE-M-RDILFHHREF
FFFVSKNLCDTSQQSSI------------MTKRYWNIILEEMMEAGVHFGHGTRKWNPKMSPYISA

KRKGIHITNLIRTARFLSEACDLVFDAASEGKQFLIVGTKKKAADSVARAAIRARCHYVNK
KWLGGMLTNWYTTETRLQKFRDLRMQQKTGRLNSFPKRDAAILKRHLAHLETYLGGIK
YMTGLPDIVIIVDQQEEYTALRECITLEIPTICLIDTNCDPDLADISIPANDDAIASIRLILNKL
VFAICEGRSS-YIRNSMGQKINPLGFRLGTTQSHDSIWFAQPTNYSENIQEDKKIRDCIKNYI
QKNIRISS-GVEGIGQIKIQKRIDLIQVIIYMGFPKLLIEGKSQ----KIEELQTNMQKKLNCVN
RKLNIAIVKVANAYKHPNIIAEFIAGQLKNRVSFRKAMKKAIELTEQAGTKGVQVQIAGRI
DGKEIARVEWIREGRVPLQTIRAKIDYCCYTVRTIYGVLGIKVWIFSK--MSRYRGPRFKKIR
RLGSLPGLTSKRPTVKSELRNQSRSSK-KSQYRIRLEEKQKLRFHYGLTERQLLKYVRIAGK
AKGSTGQVLLQLLEMRLDNILFRLGMAATIPQARQLINHRHVLVNGHIVDIPSYRCKPQDII
TAKDEQKSKTLIQNYLDSAPR-EKLPNHLTLHPFQ--YKGLINQIIDNKWVGLKINELLVVEY
YSRQTMSRRGTAEEKTAKSDPIYRNRLVNMLVNRILKHGKKSLAYQIIYRAMKKIQQKTE
TNPLSVLRQAIRGVTPDIAVKARRVGGSTHQVPVEIGSTQGKALAIRWLLGASRKRPGRN
MAFKLSSELVDAAKGSGDAIRKKEETHRMAEANRAFAHFRMGKDTIADIITYIRNADMN
KKGMVQIPFTNITENIVKILLREGFVENVRKHRENDKYFLVLTLRYRR--NRKGSYKS-F--L
NLKRISTPGLRIYSNYQRIPRILGGMGIVILSTSRGIMTDREARLERIGGEVLCYIWMAKPIS
KKGSRKNVRIGS-KHTRKIPKGVIHVQASFNNTIVTVTDVRGRVISWSSAGTCGFKGTRRG
TPFAAQTAAGNAIRTVSDQGMQRAEVMIKGPGLGRDAALRAIRRSGILLNFIRDVTPMPH
NGCRSPKKRRVMPTMKQLIRNTRQPIRNVTKSPALRGCPQRRGTCTRVYTITPKKPNSALR
KVARVRLTSGFEITAYIPGIGHNLQEHSVVLVRGGRVKDLPGVRYHIVRGTLDAVGVKDRQ
QGRSKYGAKKPKMARKSVIQREKKRQKLEQKYHLIRRSSKKEIS---KVPSLSDKWKIHGK
LESLPRNSAPIRLHRRCFSTGRPRANYRDFGLSGHILREMVHACLLPGATRSSWMVKNSIIP
VISQE-KKEK-NPGSVEFQIFKFTDRIRRLTSHFELHRKDYLSQRGLRKILGKRQRLLSYLSK
KDRIRYKKLINQFDIRESQIR--MVKLRLKRCGKKQRAVYRIVAIDVRSRREGRDLRKVGFY
DPIKNQTYLNIPVILYFLERGAQPTGTVQDISKRAGVFMELSSNQQTKFHMEKSKRLFMKS
KRSFRRRLPPIQSGDRIDYKNMGLISRFISEQGKILSRRVNRLTLKQQRLITIAIKQARILSSL
PFLNNEK--QFEKSESTARTTTLRKKKYVTR-LKKNPFVANHLLRKINKLNTKAEKDIIITWS
RASTIIPTMIGHTIAIHNGKEHLPIYITDRMVGHKLGEFSPTLNFRGHAKNDNRSRRMIFQS
FILDNLVFLCMKIINSIVVVGLYYGFLTTFSIGPSYLFLLRARLVEEG---TEKKISATTGFITGQ
LIMFISIYYAPLHLALGRPHIITVIALPYLLFQFFGNNHKNFLNYGYKNPNSIRNFSIQRIFFH
NLIFQLLNPFFLPSSILIRLVNIYLFR--------------------------FKY--

--MKGHQFKSWIFELREILREIK
NSRYFLDSWTQFNSAGFFIHIFFHQESFIKLLDSRIWSILLS-RNS--QGSTSNRYF---TIKYVV
LFVVA-VLIYR----INNRKMVERKNPYLTRLLT-----IPMNSIGPKNDTLEESS-ESSNINRLIVP-
LLYLPKGKKISESYFLDPKESTRVLPIT----KKYIMPESNWGSRWWRNWIGKKS--DSSCKIS
N---ETIAGIEISFKEKDIK-YLEFLFVYYMD--DPIRKDHDWEFFDRLSPRKRRNIINL--NSGQ
LFEILVKDWIYYLMFAFREKIPKEVEG-FFKQQG-TGSIIQSNDIEHVSH--LFLRNKRAISLQ

NCAQFHMWQFRQDLFV---SWGK-SPHESDFLRNNMSRENWIWLDNVWLVNKDR---FFS--KVRNVSSNIQYDS---TR--SSFIQVTGSSQLKGSS-DQSKDYFDSIR----NEDSK-------------YHTLINQREIQQLKERSILCWD--PSFLQTERTEIESE--RFPK--ILSGYS--SMCRLFM-EREKQMNNHLLPQEIEEF--LGNPARATRSFFSDRW--SELH-LSSNPTERSTRDQ----KLLKKEQKKH-LVLSRRSE----NKEIVNLFKIIMYLQN--TVSIHPISSYPGCDMVP-KGEL---DSSNKISFLNKNPFWGL--FHLFHDRNSGRYTL--HHDFESE--EIFQEMADLFTLSITEP-DLVYHKGFAFSIDSSGLGQKH--FLNELFNSRDESKNHSL---LVLPPLFY--EENESFYRRIIKKWVQ-TSCGNNLEDPKPKIVVFTSNNIMEAVNQYRLIRNLIQIQYSTH--GYIRNVLN---------RFNCNFEYGIQ---RYQIGNDTLNHRTIMKYTINQHLSNLKK-SQKKWFDP--LIFISRTERSMNRDPNAYRYKWSNGSKNFQEHLDYFISEQNSR---FQVVFDRFHINQYSIDWSEVIDKKDLSKSLCFFLSKLLL---------FLP-------KFLLFLSNSLPSFFFVSFGGIPIHRSE-IHIY-ELKGPNNPLCNQLLESIGLQIFHLKKWKPL--LLD---DQDTS-QKSKFLINGGTISPFLFNKIP-KW-MIDSFHTR--KNRRKSFDNTDSYFSMISHDPDNWLNPVKPFHRSSLIYSFYKANRLRFLNNQYHFCFY--CNKRFPF---Y-VEKACINNYD--FTYG-QFLNILFIRNK-IFSFCDGQ--KKHAFLK-RDTISPIESQVSNILIPNDFPQSGDEG-YNLYKS---FHFPIRYDLFVR--GAIYSIADISGTPLTEGQIVHFEKTYCQPLSDMNIPDSEGKNLHQYLN--FNSNMGLIHTPCSEKYLPSEK--RKK--------RSPCLK--KCLEKGQMYRTFQQDSVFSTLSKWNLFQTYIPWFLTSTGYKYLNFIFLDTFSD-LLPILSSS--QKFVSIFHDIMHGSDILWRIRQIPL------CLPQWNLLISEIPGNCLHN--LLLSEEIIHRNNEL--LLISTHLRSLNVQEF-FYSILFLLLVAGY-LVRTHLLFVSR--VYSELQTEFEKVKSLMIPSYMIELRKLLDRYPTSELNSFWLKNLFLVALEQLGDSLEEIRSFAFGGNMLWGGGP-----TYG-VKSIRSKKKYLNL-----IDLI-SIIPNPINRIAFSRNTRHLSHPSKTIYSLIRKIKNVNGDWIDDKIESWVLNSNSIDDKEREFLVQFS--TLTT--EKRIDQILLSLT-HSHHLS-----KNNSGYQMIEQPGAIYLRYLVDIH--KKYLMIYEFNTSCLAERRIFLANYQTITY--SQTLRGANSFHFPSHG---KPFSLRLALPPPSRGILVIGSIGTGRSYLVKYLATNSYVPFITVFLNKFLDNKPKGFLID-----DSD------DIDDSYDSDD------------------IDRDLDMELELLTMMNTL---TMDMMP---EIDRFYITFQFELAKAMSPCIIWIPNIHDLDVNESNYLSLGLLVNYLS---RDCERCS-TRNILVIASTHIPQKVDPALIAPNKLNTCIKIRRLLISQQRKHFFTLSYTRGFHLEKKMSHT--NGFGSTTMGSNVRDLVALTNEALSISIIQKKSIIDTNIIRSVLHRQTWDFR---SQVRSVQDHGILFYQIGRAVSQNVLLSNCSIDPISIYMKKK--SCDGGDSYLYKWYFELGTSMKKLTILLYLLSCSAGSVAQDLWSLP--GPDEK-NGITSYGLVENNS-DLVHGLLEVEGALVG--SSRTEKDCSQFDKD--RVTLLLRSEPRN-PLNMIQNGSYSIVDQRFLYEK--YESEFEE---GGGVLDPQ----QIEED----FFNH--IVWAP--RIWSP---WGFLFYCIERPNELGFP-YWARSFRDK---RIIYDEEDELQENDSEFLQGGTMQYQTRDRSSKEQGFFRISQFIWDPADPLFFLFK-DQPFVSVFSHRQFFTDEEMSRELLTSQTDLPTSIYKHWFI--KNTQEKHFELLIHCQRWLR---INSSLS--NGFFRSN--TLSESYQYLSNLFLSN-EALLDQMTKTLLRKRWLFPDEMVVAI--CSNNESLV--MPRSR-INENFIDKTFSIVANILLRIIPTTSGEKRAFTYYRDG--MSAQSEGNYAEALQNYYEAMRLEIDPYDRSYILYNIGLIHTSNGEHTKALEYYFRALERNPFLPQAFNNMAVICHY-RGEQAIRQGDSEVAESWFNQAAEYWKQAIALTPGNYIEAQNWLKITG R F E --??

>Solanum

MERWGFNSMLFLNKSMGSLGPIENTSE--DPNLK--VKNIH-CSNVDYLFGVKDIWNFISDDTFLVSDRNGDSYSIYFDIENQIFEVDNDH--SFLSELESSFSSYRNSSYLNNGFRGEDPYYNS

YMYDTQYSWNNHINSCIDNYLQ--SQICIDTSIISGGESYG--YIYRAICSNEGSSRRTRTKGS
DLTIRESSNDLEVTQKYKHLWVQCENCYGLNYKKFLKSKMNICEQCGYHLKMSSSDRIEL
LIDPGTWDPMDEDMVSLDPIEFHSEEEPYKDRIDSYQRKTGLTEAVQTGIGQLNGIPVAIGV
MDFQFMGGSMGSVVGEKITRLIEHAANQNLPLIIVCASGGARMQEGSLSLMQMAKISSAL
YDYQLNKKLFYVSILTSPTTGGVTASFGMLGDIIIAEPNAYIAFAGKRVIEQTLNKTVPEGS
QAAEYLFQKGLFDLIVPRNLLKSVLSELFKLHAFFPLNQKSSKMVTIRADEISNIIRERIEQY
NREVKIVNTGTVLQVGDGIARIHGLDEVMAGELVEFEEGTIGIALNLESNNVGVVLMGDG
LLIQEGSSVKATGRIAQIPVSEAYLGRVVNALAKPIDGRGEISASEFRLIESAAPGIISRRSVY
EPLQTGLIAIDSMIPIGRGQRELIIGDRQTGKTAVATDTILNQQGQNVICVYVAIGQKASSVA
QVVTTLQERGAMEYTIVVAETADSPATLQYLAPYTGAALAEYFMYRERHTLIIYDDLSKQ
AQAYRQMSLLLRRPPGREAYPGDVFYLHSRLLERAAKLSSSLGEGSMTALPIVETQSGDV
SAYIPTNVISITDGQIFLSADLFNSGIRPAINVGISVSRVGSAAQIKAMKQVAGKLKLELAQF
AELEAFAQFASDLDKATQNQLARGQRLRELLKQSQSAPLTVEEQIMTIYTGTNGYLDSLEV
GQVRKFLVELRTYLKTTKPQFQEIISSTKTFTEEAEALLKEAIQEQMDRFILQEQAMRINPT
TSGSGVSTLEKKNPGRVVQIIGPVLDVAFPPGKMPNIYNALVVQGRDSVGQPINVACEVQQ
LLGNNRVRAVAMSATDGLTRGMAVIDTGAPISVPVGGATLGRIFNVLGEPVDNLGPVDTST
TSPIHRSAPAFIQLDTKLSIFETGIKVVDLLAPYRRGGKIGLFGGAGVGKTVLIMELINNIAK
AHGGVSVFGGVGERTREGNDLYMEMKESGVINKENIAESKVALVYGQMNEPPGARMRV
GLTALTMAEYFRDVNEQDVLLFIDNIFRFVQAGSEVSALLGRMPSAVGYQPTLSTEMGSL
QERITSTKDGSITSIQAVYVPADDLTDPAPATTFAHLDATTVLSRGLAAKGIYPAVDPLDSTS
TMLQPRIVGEEHYETAQRVKQTLQRYKELQDIIAILGLDELSEEDRLLVARARKIERFLSQP
FFVAEVFTGSPGKYVGLAETIRGFQLILSGELDGLPEQAFYLVGTIDEATAKAMNLEMESN
LKKMTLNLSVLTPNRIVWDSEVEEIVLSTNSGQIGILPNHAPIATAVDIGILRIRLNDQWLTM
ALMGGFARIGNNEITVLVNDAEKGSDINPQEAQQTLEIAEANVKKAEGRRQKIEANLALR
RARTRVEASNPISMKNVTDSFVS-GHWPSAGSFGFNTDILATNPINLSVVLGVLIFFGKGVL
SDLLDNRKQRILNTIRNSEELRGGAIEQLEKARSRLRKVETEAEQFRVNGYSEIEREKLNLI
NSTYKTLEQLENYKNETIQFEQQRAINQVRQRVFQQALRGALGTLNSCLNNELHLRTISA
NIGMLGTMKEITDMNPLIAAASVIAAGLAVGLASIGPGVGQGTAAGQAVEGIARQPEAEG
KIRGTLLLSLAFMEALTIYGLVVALALLFANPFVMNVLSCSINTLKGLYDISGVEVGQHFY
WQIGGFQVHGQVLITSWVVIAILLGSATIAVRNPQTIPTGGQNFFEYVLEFIRDVSKTQIGEE
YGPWVPFIGTMFLFIFVSNWSGALLPWKIIQLPHGELAAPTNDINTTVALALLTSVAYFYAG
LTKKGLGYFGKYIQPTPILLPINILEDFTKPLSLSFRLFGNILADELVVVVLVSLVPLVVPIPV
MLLGLFTSGIQALIFATLAAAYIGESMEGHHMIFSTLEHILTHISFSIVSIVITIHLITFLVDEIV
KLYDSSEKGIIVTFFCITGLLVTRWISSGHFPLSDLYESLIFLSWSFSLIHIIPY--KNVLILSKIT
GPSAILTQGFATSGILTEIHQSGILVPALQSEWLIMHVSMMILGYAALLCGSLLSVALLVITFR
KNRKLFY--KENVVENTSF----------AKNYYRSQLIQQLDYWSYRVISLGFTFLTIGILSGAVW
ANEAWGSYWNWDPKETWAFITWIVFAIYLHTRTNRNLRGPNSAIVASIGFLIIWICYFGVN
LLGIGLHSYGSFPSTMAKKKAFTPLFYLASIVFLPWWISFSVNKCLESWVTNWWNTGQSQ
IVLNNIQEKSLLEKFRELEELLFLDEMIKEYSETHLEEFGI-IHKETIQLITIQNENRMDTILHF
STNIIWFGILSGYSILGKEKLVILNSWAQEFLYNLSDTAKALCLLLVTEFFLGYHSPPGWEFA
IRSIYNEVGVVANEQTITILVCILPVIFDTCFKYWLFRYLTSLSPSILLIYDSITEMPIGVPKVL
FRNPGDPISSWVDVYNRLYRERLLFLGQGISTDLSNQLIGLMMYLSMEDENKELYLFVNSP
GGWVIPGIAIYDTMQFVRPDIHTICIGLAASMGSFILAGGQLTKRIAFPHARVMIHEPYSAF
YMAQVGEFVMEAVELMKLRETLTRVYAERTGKPFWVVHEDMERDIFMSATEAQAYGIVD

FVAVQ---IQILPGDRVKIEVSPYDSTKGHIIYRLHNKD-KDM
EEIHRYLQ-DSSQQHNFLYPLIFQEYIYALAQDHGLSGYNNKFSFLIVKRLITRMYQQNHFII
STNDSNKNPFLGKSLYSQMISEGFACIVEIPFSIRLISSLIFKSHNLRSIHSTFPFLEDNFSHLN
YVLDILIPYPVHLEILVQTLRYWVKDASSLHLLRFFLHEYCNLNSLITPG-YSFSKQRFFFFL
YNSYVYECESTFVFLRNQSSHLRSTSFGALLERIYFYGKIERLVEVFA-DFTLWLFKDPFMH
YVRYEGKSILASKGTFPLMNKWKFYLVNFWQCHFSMYFHTGRIHINQLSNHSRDFMGYL
SSVRLNHSMVRSQMLENSFLINNPI--KKFETLVPIIPLIGSLAKAHFCTVLGHPISKPVWSDL
SDSDIIDRFGRICRNLFHYYSGSSKKKTLYRIKYILRLSCARTLARKHKSTVRTFLKRSGSEL
LEEFLTSEEQVLSLTFPRRIWYLDIFCINDLANYQMIID-TTEIE-TINSFSKLES-LKEVYGIIW
MLVPIVTLVLGITIGVLVIVWLEREISAGIQQRIGPEYAGPLGILQALADGTKLLLKENLIPST
GDTRLFSIGPSIAVISIFLSYSVIPFGDHLVLADLSIGVFFWIAISSIAPVGLLMSGYGSNNKYS
FLGGLRAAAQSISYEIPLALCVLSISLLSNSSSTVDIVEAQSKYGFWGWNLWRQPIGFIVFLI
SSLAECERLPFDLPEAEEELVAGYQTEYSGIKFGLFYIASYLNLLVSSLFVTVLYLGGWNLSI
PYIFVPELFGINKGGKVFGTLIGIFITLAKTYLFLFIPIATRWTLPRLRMDQLLNLGWKFLLPI
SLGNLLLTTSSQLLSLMIWHVQNENFILDSTRIFMKAFHLLLFDGSLIFPECILIFGLILLLMI
DSTSDQKDIPWLYFISSTSLVMSITALLFRWREEPMISFSGNFQTNNFNEIFQFLILLCSTLCIP
LSVEYIECTEMAITEFLLFVLTATLGGMFLCGANDLITIFVAPECFSLCSYLLSGYTKKDVRS
NEATMKYLLMGGASSSILVHGFSWLYGSSGGEIELQEIVNGLINTQMYNSPGISIALIFITVG
IGFKLSPAPSHQWTPDVYEGSPTPVVAFLSVTSKVAASASATRIFDIPFYFSSNEWHLLLEIL
AILSMILGNLIAITQTSMKRMLAYSSIGQIGYVIIGIIVGDSNDGYASMITYMLFYISMNLGT
FACIVLFGLRTGTDNIRDYAGLYTKDPFLALSLALCLLSLGGLPPLAGFFGKLYLFWCGWQ
AGLYFLVLIGLLTSVVSIYYYLKIIKLLMTGRNQEITPHVRNYRRSPLRSNNSIELSMIVCVI
ASTIPGISMNPIIAIAQDSLFMFLLYEYDFFWAFLIISILVPILAFFISGVLAPISKGPEKLSTYE
SGIEPMGDAWLQFRIRYYMFALVFVVFDVETVFLYPWAMSFDVLGVSVFIEAFIFVLILIIGL
VYAWRKGALEWSTNYFPWLTIIVVFPIFAGSLIFFLPHKGNRVIRWYTICICILELLLTTYAFC
YHFQSDDPLIQLVEDYKWIDFFDFHWRLGIDGLSIGPILLTGFITTLATLAAWPVTRDSRLF
HFLMLAMYSGQIGSFSSRDLLLFFIMWELELIPVYLLLAMWGGKKRLYSATKFILYTAGGS
VFLLMGVLGVALYGSNEPTLNFETSVNQSYPVVLEIIFYIGFFIAFAVKSPIIPLHTWLPDTH
GEAHYSTCMLLAGILLKMGAYGLIRINMELLPHAHSIFSPWLMIIGTIQIIYAASTSLGQRNL
KKRIAYSSVSHMGFIIIGISSLTDTGLNGALLQIISHGFIGAALFFLAGTTYDRIRLVYLDEMG
GIAIPMPKMFTMFSSFSMASLALPGMSGFVAELIVFFGIITGQKYLLIPKLLITFVMAIGIILT
PIYSLSMPRQMFYGYKLFNAPKDSFFDSGPRELFLSISIFLPVIGIGIYPDFVLSLAVDKVEVI
LSNFFYRMILEHVLVLSAYLFSIGIYGLITSRNMVRALMCLELILNAVNINFVTFSDFFDNRQ
LKGDIFSIFVIAIAAAEAAIGLAIVSSIYRNRKSTRINQSNLLNNMEQTYEYAWIIPFIPLPVP
MLIGAGLILFPTATKSFRRMWAFQSVLLLSIVMIFSIYLSIQQINSSSVYQYVWSWIINNDFS
LDFGYLIDPLTSIMSILITTVGIMVLIYSDNYMAHDQGYLRFFAYMSFFSTSMLGLVTSSNLI
QIYIFWELVGLCSYLLIGFWFTRPVAANACQKAFVTNRVGDFGLLLGILGFYWITGSFEFR
DLFEIFNNNEVNFLFVTLCAVLLFAGAVSKSAQFPLHVWLPDAMEGPTPISALIHAATMVA
AGIFLVARLLPLFRVIPYIMYLISVIGIITVLLGATLALAQKDIKRGLAYSTMSQLGYMMLAL
GMGSYRSALFHLITHAYSKALLFLGSGSIIHSMETIVGYSPAKSQNMGLMGGLRKHVPITK
ITFLLGTLSLCGIPPLACFWSKDEILNDSWLYSPIFAIIAWATAGLTAFYMFRIYLLTFEGHLN
VHFQNYGGKHKTPFYSISLWGK---NGVKK-LLT---MNFLSFEHKAVSSYPYESDNTMLFPIF
VLGLFTLFVGAIGIPFNLDILSKWLAPSINLLHPKSN-SQDWNEFLKDAVVSVSIAYFGIFIAS
FLYKPVYSSLKNLEFINSFVILWDKIINGIYDWSYNRAYIDAFYTRFFVGGIRGLAEFTHFFD

RRVIDGMTNGVGVISFIVGEGIKYIGGG-RISSYLFLYLAYVSVFLLVYYMDLSEPIHDFLLV
FLGSGLILGGLGVVLLPNPIYSAFSLGLVLVCTSLFYILSNSYFVAAAQLLIYVGAINVLIIFA
VMFMNGSEYYKDFHLWTVGNGITSMVCISLFISLITTISDTSWYGIIWTTRSNQIIEQDFLSN
SQQIGIHLSTDFFLPFELISIILLVALIGAIAVARQMTAPTTRKDLMIVNMGPQHPSMHGVLR
LIVTLDGEDVVDCEPILGYLHRGMEKIAENRTIIQYLPYVTRWDYLATMFTEAITINGPEQL
GNIQVPKRASYIRVIMLELSRIASHLLWLGPFMADIGAQTPFFYIFRERELIYDLFEAATGM
RMMHNFFRIGGVAADLPYGWIDKCLDFCDYFLTGVAEYQKLITRNPIFLERVEGVGIIGRD
EALNWGLSGPMLRASGIEWDLRKVDHYESYDEFDWQVQWQREGDSLARYLVRIGEMTE
SIKIIQQALEGIPGGPYENLEMRRFDRLKDPEWNDFEYRFISKKPSPTFELSKQELYVRVEA
PKGELGIFMIGDQSVFPWRWKIRPPGFINLQILPQLVKRMKLADIMTILGSIDIIMGEVDRM
LPMITEFINYGQQTIRAARYIGQGFMITLSHANRLPVTIQYPYEKLITSERFRGRIHFEFDKCI
ACEVCVRVCPIDLPVVDWKLETDIRKKRLLNYSIDFGICIFCGNCVEYCPTNCLSMTEEYE
LSTYDRHELNYNQIALGRLPMSVIDDYTIRTISNL---------------MQGRLSAWLVKHGLIHRS
LGFDYQGIETLQIKPEDWHSIAVIFYVYGYNYLRSQCAYDVAPGGLLASVYHLTRIEDGVD
QPEELCIKVFASRRNPRIPSVFWVWKSVDFQERESYDMLGISYDNHPRLKRILMPESWIGW
PLRKDYIAPNFYEIQEAHMGNEFRRIGCICIYRSFHFRAYLNYWFSLCMAKGGIGMVLAPE
YSDNKKKNGKNKIET-VMNSIQFPLLDRTAPNSVISTTLNDLSNWSRLSSLWPLLYGTSCCF
IEFASLIGSRFDFDRYGLVPRSSPRQSDLILTAGTVTMKMAPSLVRLYEQMPEPKYVIAMGA
CTITGGMFSTDSYSTVRGVDKLIPVDVYLPGCPPKPEAVIDAITKLRKKISRELYEDRIRSQR
ANRCFTTNHKFHVRRSIHTGNYDQRVLYQPPSTSEIPTEIFFKYKNSVSSAELVNMQTRNA
FSWLKKQITRSISVSLMIYILTRTSISSAYPIFAQQGYENPREATGRIVCANCHLANKPVEIEV
PQAVLPDTVFEAVVRIPYDMQLKQVLANGKKGGLNVGAVLILPEGFELAPPDRISPEMKE
KIGNLSFQSYRPNKTNILVVGPVPGKKYSEITFPILSPDPATKKDVHFLKYPIYVGGNRGRG
QIYPDGNKSNNTVYNATAAGIVSKIIRKEKGGYEITITDASEGRQVVDIIPPGPELLVSEGESI
KFDQPLTSNPNVGGFGQGDAEIVLQDPLRVQGLLFFLASVILAQIFLVLKKKQFEKVQLAE
MNFMSKVYDWFEERLEIQAIADDITSKYVPPHVNIFYCLGGITLTCFLVQVATGFAMTFYY
RPTVTEAFASVQYIMTEANFGWLIRSVHRWSASMMVLMMILHVFRVYLTGGFKKPRELT
WVTGVVLAVLTASFGVTGYSLPWDQIGYWAVKIVTGVPDAIPVIGSPLVELLRGSASVGQS
TLTRFYSLHTFVLPLLTAVFMLMHFPMIRKQGISGPLMGITKKPDLNDPVLRAKLAKGMG
HNYYGEPAWPNDLLYIFPVVILGTIACNVGLAVLEPSMIGEPADPFATPLEILPEWYFFPVFQ
ILRTVPNKLLGVLLMVSVPAGLLTVPFLENVNKFQNPFRRPVATTVFLIGTAVALWLGIGAT
LPIDKSLTLGLFMIEVFLFGIVLGLIPITLAGLFVTAYLQYRRGDQLDLMLTITSYFGFLLAAL
TITSALFIGLSKIRLIMDIVSLAWAALMVVFTFSLSLVVWGRSGLMIIRSPEPEVKILVDRDP
VKTSFEEWARPGHFSRTIAKGPDTTTWIWNLHADAHDFDSHTSDLEEISRKVFSAHFGQLS
IIFLWLSGMYFHGARFSNYEAWLSDPTHIGPSAQVVWPIVGQEILNGDVGGGFRGIQITSG
FFQLWRASGITSELQLYCTAIGALVFAALMLFAGWFHYHKAAPKLAWFQDVESMLNHHL
AGLLGLGSLSWAGHQVHVSLPINQFLNAGVDPKEIPLPHEFILNRDLLAQLYPSFAEGATPF
FTLNWSKYADFLTFRGGLDPVTGGLWLTDIAHHHLAIAILFLIAGHMYRTNWGIGHGLKDI
LEAHKGPFTGQGHKGLYEILTTSWHAQLSLNLAMLGSLTIVVAHHMYSMPPYPYLATDYG
TQLSLFTHHMWIGGFLIVGAAAHAAIFMVRDYDPTTRYNDLLDRVLRHRDAIISHLNWAC
IFLGFHSFGLYIHNDTMSALGRPQDMFSDTAIQLQPVFAQWIQNTHALAPGATAPGATASTS
LTWGGGDLVAVGGKVALLPIPLGTADFLVHHIHAFTIHVTVLILLKGVLFARSSRLIPDKAN
LGFRFPCDGPGRGGTCQVSAWDHVFLGLFWMYNSISVVIFHFSWKMQSDVWGSVSDQG
VVTHITGGNFAQSSITINGWLRDFLWAQASQVIQSYGSSLSAYGLFFLGAHFVWAFSLMFL

FSGRGYWQELIESIVWAHNKLKVAPATQPRALSIIQGRAVGVTHYLLGGIATTWAFFLARII
AVGMALRFPRFSQGLAQDPTTRRIWFGIATAHDFESHDDITEERLYQNIFASHFGQLAIIFL
WTSGNLFHVAWQGNFESWVQDPLHVRPIAHAIWDPHFGQPAVEAFTRGGALGPVNIAYS
GVYQWWYTIGLRTNEDLYTGALFLLFLSAISLIAGWLHLQPKWKPSVSWFKNAESRLNH
HLSGLFGVSSLAWTGHLVHVAIPASRGEYVRWNNFLDVLPHPQGLGPLFTGQWNLYAQNP
DSSSHLFGTAEGAGTAILTLLGGFHPQTQSLWLTDIAHHHLAIAFIFLVAGHMYRTNFGIGH
SMKDLLDAHIPPGGRLGRGHKGLYDTINNSLHFQLGLALASLGVITSLVAQHMYSLPAYAF
IAQDFTTQAALYTHHQYIAGFIMTGAFAHGAIFFIRDYNPEQNEDNVLARMLDHKEAIISH
LSWASLFLGFHTLGLYVHNDVMLAFGTPEKQILIEPIFAQWIQSAHGKTSYGFDVLLSSTT
GPAFNAGRSIWLPGWLNAVNE--NSNSLFLTIGPGDFLVHHAIALGLHTTTLILVKGALDAR
GSKLMPDKKDFGYSFPCDGPGRGGTCDISAWDAFYLAVFWMLNTIGWVTFYWHWKHIT
LWQGNVSQFNESSTYLMGWLRDYLWLNSSQLINGYNPFGMNSLSVWAWMFLFGHLVWA
TGFMFLISWRGYWQELIETLAWAHERTPLANLIRWRDKPVALSIVQARLVGLAHFSVGYIF
TYAAFLIASTSGKFGMSHSVKIYDTCIGCTQCVRACPTDVLEMIPWDGCKAKQIASAPRTE
DCVGCKRCESACPTDFLSVRVYLWHETTRSMGLAYMTNLNLPSIFVPLVGLVFPAIAMASL
FLHVQKNKIVMRDLKTYLSVAPVLSTLWFGALAGLLIEINRFFPDALTFPFFSFMTAILERR
ESESLWGRFCNWITSTENRLYIGWFGVLMIPTLLTATSVFIIAFIAAPPVDIDGIREPVSGSLL
YGNNIISGAIIPTSAAIGLHFYPIWEAASVDEWLYNGGPYELIVLHFLLGVACYMGREWEL
SFRLGMRPWIAVAYSAPVAAATAVFLIYPIGQGSFSDGMPLGISGTFNFMIVFQAEHNILMH
PFHMLGVAGVFGGSLFSAMHGSLVTSSLIRETTENESANEGYRFGQEEETYNIVAAHGYFG
RLIFQYASFNNSRSLHFFLAAWPVVGIWFTALGISTMAFNLNGFNFNQSVVDSQGRVINTW
ADIINRANLGMEVMHERNAHNFPLDLAAIEAPSTNGMGLPWYRVHTVVLNDPGRLLSVH
IMHTALVAGWAGSMALYELAVFDPSDPVLDPMWRQGMFVIPFMTRLGITNSWGGWSIT-G
GTVTNPGIWSYEGVAGAHIVFSGLCFLAAIWHWVYWDLEIFCDERTGKPSLDLPKIFGIHL
FLSGVACFGFGAFHVTGLYGPGIWVSDPYGLTGKVQPVNPAWGVEGFDPFVPGGIASHHI
AAGTLGILAGLFHLSVRPPQRLYKGLRMGNIETVLSSSIAAVFFAAFVVAGTMWYGSATTP
IELFGPTRYQWDQGYFQQEIYRRVSAGLAENQSLSEAWSKIPEKLAFYDYIGNNPAKGGLF
RAGSMDNGDGIAVGWLGHPIFRDKEGRELFVRRMPTFFETFPVVLVDGDGIVRADVPFRR
AESKYSVEQVGVTVEFYGGELNGVSYSDPATVKKYARRAQLGEIFELDRATLKSDGVFRS
SPRGWFTFGHASFALLFFFGHIWHGARTLFRDVFAGIDPDLDAQVEFGAFQKLGDPTTKR
QAAMKTLYSRRRFYHVETLFNGTLALAGRDQETTGFAWWAGNARLINLSGKLLGAHVA
HAGLIVFWAGAMNLFEVAHFVPEKPMYEQGLILLPHLATLGWGVGPGGEVIDTFPYFVSG
VLHLISSAVLGFGGIYHALLGPETLEESFPFFGYVWKDRNKMTTILGIHLILLGIGAFLLVFK
ALYFGGVYDTWAPGGGDVRKITNLTLSPSIIFGYLLKSPFGGEGWIVSVDDLEDIIGGHVW
LGSICILGGIWHILTKPFAWARRALVWSGEAYLSYSLGALAVFGFIACCFVWFNNTAYPSEF
YGPTGPEASQAQAFTFLVRDQRLGANVGSAQGPTGLGKYLMRSPTGEVIFGGETMRFWD
LRAPWLEPLRGPNGLDLSRLKKDIQPWQERRSAEYMTHAPLGSLNSVGGVATEINAVNYV
SPRSWLATSHFVLGFFFFVGHLWHAGRARAAAAGFEKGIDRDFEPVLSMTPLNMTIAIGK
FTKDENDLFDIMDDWLRRDRFVFVGWSGLLLFPCAYFAVGGWFTGTTFVTSWYTHGLAS
SYLEGCNFLTAAVSTPANSLAHSLLLLWGPEAQGDFTRWCQLGGLWTFVALHGAFGLIGF
MLRQFELARSVQLRPYNAIAFSGPIAVFVSVFLIYPLGQSGWFFAPSFGVAAIFRFILFFQGF
HNWTLNPFHMMGVAGVLGAALLCAIHGATVENTLFEDGDGANTFRAFNPTQAEETYSM
VTANRFWSQIFGVAFSNKRWLHFFMLFVPVTGLWMSALGVVGLALNLRAYDFVSQEIRA
AEDPEFETFYTKNILLNEGIRAWMAAQDQPHENLIFPEEVLPRGNALMSGSTGERSFADIIT

SIRYWVIHSITIPSLFIAGWLFVSTGLAYDVFGSPRPNEYFTESRQGIPLITGRFDPLEQLDEF
SRSFMTIDRTYPIFTVRWLAVHGLAVPTVFFLGSISAMQFIQRMATQTVENSS-RSGPRRTAV
GDLLKPLNSEYGKVAPGWGTTPLMGVAMALFAVFLSIILEIYNSSVLLDGISMNMLTLKLF
VYTVVIFFVSLFIFGFLSNDPGRNPGREEMADTTGRIPLWIIGTVAGILVIGLIGIFFYGSYSG
LGSSLMLNTFSLIGICLNSTLYSSSFFFGKLPEAYAFLNPIVDIMPVIPLFFFLLAFVWQAAVS
FRTTQSNPNEQNVELNRTSLYWGLLLIFVLAVLFSNYFFNMEVNILAFIATALFILVPTAFLLI
IYVKTVSQNDMETATLVAIFISGLLVSFTGYALYTAFGQPSQQLRDPFEEHGDMEALVYTFL
LVSTLGIIFFAIFFREPPKVPTKKN-MTLAFQLAVFALIATSLILLISVPVVFASPDGWSSNKN
VVFSGTSLWIGLVFLVGILNSLIS-------MSPQTETKASVGFKAGVKEYKLTYYTPEYQTKDT
DILAAFRVTPQPGVPPEEAGAAVAAESSTGTWTTVWTDGLTSLDRYKGRCYRIERVVGEK
DQYIAYVAYPLDLFEEGSVTNMFTSIVGNVFGFKALRALRLEDLRIPTAYVKTFQGPPHGIQ
VERDKLNKYGRPLLGCTIKPKLGLSAKNYGRAVYECLRGGLDFTKDDENVNSQPFMRWR
DRFLFCAEALFKAQTETGEIKGHYLNATAGTCEEMMKRAVFARELGVPIVMHDYLTGGFT
ANTTLAHYCRDNGLLLHIHRAMHAVIDRQKNHGIHFRVLAKALRMSGGDHIHSGTVVGK
LEGERDITLGFVDLLRDDFIEQDRSRGIYFTQDWVSLPGVLPVASGGIHVWHMPALTEIFG
DDSVLQFGGGTLGHPWGNAPGAVANRVALEACVKARNEGRDLAREGNEIIREASKWSPE
LAAACEVWKEIVFNFAAVDVLDKMAIHLYKTSTPSTRNGTVDSQVKSNPRNNLIYGQRRC
GKGRNARGIITARHRGGGHKRLYRKIDFRRNEKDIYGRIVTIEYDPNRNAYICLIHYGDGE
KRYILHPRGAIIGDTIVSGTEVPIKMGNALPLTDMPLGTAIHNIEITLGKGGQLARAAGAVA
KLIAKEGKSATLKLPSGEVRLISKNCSATVGQVGNVGVNQKSLGRAGSKRWLGKRPVVR
GVVMNPVDHPHGGGEGRAPIGRKKPTTPWGYPALGRRSRKRNKYSDNLILRRRSKMIQP
QTHLNVADNSGARELMCIRIIGAS-NRRYAHIGDVIVAVIKEAVPNMPLERSEVVRAVIVRTC
KELKRDNGMIIRYDDNAAVVIDQEGNPKGTRIFGAIARELRELNFTKIVSLAPEVLMLSPK
RTRFRKQHRGRMKGISSRGNRISFGKYALQALEPAWITSRQIEAGRRAMTRNARRGGKIW
VRIFPDKPVTLRPAETRMGSGKGSPEYWVAVVKPGRILYEMGGVTENIARRAISLAASKMP
IRTQFIIS-MTRIKRGYIARRRRTKIRLFASSFRGAHSRLTRTITQQKIRALVSAHRDRDRKKR
DFRRLWITRINAVIRE-RGVSYSYSRLIHDLYKRQLLLNRKILAQIAISNRNCLYMISNEIIKE
VDWKESTRIIMLKEVYALGEHISMSADKARRVIDQIRGRSYEETLMILELMPYRACYPILK
LVYSAAANASYNMGSSETNLVISKAEVNEGTTVKKLKPRARGRSFPIKRSTCHITIVMKDI
SLDGGLWDKKMDGIKYAVFTDKSIRLLGKN-QYTSNVESGSTRTEIKHWVELFFGVKVIA
MNSHRLPGKSRRMGPIMGHTMHYRRMIITLQPGYSIPPLRKKRTMAVPKKRTSTSKKRIR
KNIWKRKGYWVALKAFSLAKSLSTGNSKSFFVRQTKIMAKGKDVRVTVILECTSCVRNS
VD--KVSRGISRYITQKNRHNTPNRFELKKFCPYCYKHTIHGEIKKMKIRASVRKICEKCRLI
RRRGRIIVICSNPRHKQRQGMVREKVTVSTRTLQWKCVESRTDSKRLYYGRFILSPLMKG
QADTIGIAMRRALLGEIEGTCITRVKSE--KVPHEYSTITGIQESVHEILMNLKEIVLRSNLYG
TSDASICVKGPGYVTAQDIILPPYVEIVDNTQHIASLTEPIDFCIGLQIERNRGYLIKTPH-NF
DGSY--PIDAVFMPVRNANHSIHSYGNGNEKQEILFIEIWTNGSLTPKEALHDASRNLIDLFIP
FLHMEED--NLYLQDNQ-HTVPLSPFTFHDKLA-KLIKNKK-KIALKSIFIDQSELSSRIYNCL
KMSNIYTLLDLLNNSQEDLMKIEHFRSEDIKQILGILEKY--FVIDLAKNKMLGDG-NEGISTI
PGFNQIQFEGFCRFIDQGLTEELYKFPKIEDTDQEIEFQLFVETYQLVEPLIKERDAVYESLTY
SSELYVSAGLIWKNSRDMQEQTIFIGNIPLMNSLGTSIVNGIYRIVINQILQSPGIYYRSELD
HNGISVYTGTIISDWGGRSELEIDRKARIWARVSRKQKISILVLSSAMGLNLREILENVCYPE
IFLSFLNDKERKKIGSKENSILEFYQQFACVGGDPVFSESLCKELQKKFFQQRCELGRIGRR
NMNRKLNLDIPQNNTFLLPRDILAAADHLIGLKFGMGALDD--MNHLKNKRIRSVADLLQ

DQFGLALVRLENVVRGTICGAIR-HKL-IPTPQNLVTSPP----LTTTYESFFGLHPLSQVLDRT
NPLTQIVHGRKLSYLGPGGLTGRTASFRIRDIHPSHYGRICPIDTSEGINVGLIGSLSIHARIG
HWG-SLESPFY---EISERSTG--VRMLYLSPGSDEYYMVAAGNSLALNRDIQEEQVVPARYR
QEFLTIAWEQVHLRSIFPFQYFSIGASLIPFIEHNDANRALMSSNMQRQAVPLSRSEKCIVGT
GLERQAALDSGALAIAEREGRIVYTNTDKILLAGNGDILSIPLVIYQRSNKNTCMHQKLRV
PRGKCIKKGQILADGAATVGGELALGKNVLVAYMPWEGYNSEDAVLISERLVYEDIYTSF
HIRKYEIHTHVTSQG-PEKVTNEIPHLEAHLLRNLDKKGIVMLGSWVETGDILVGKLTPQV
VKE-SSYAPEDRLLRAILGIQVSTSKETCLKLPIGGRGRVIDVRWIQKRG-GSSYNPETIRVYI
SQKREIKVGDKVAGRHGNKGIISKILPRQDMPYLQDGRSVDMVFNPLGVPSRMNVGQIFE
CSLGLAGSLLDRHYRIAPFDERYEQEASRKLVFSELYEASKQTANPWVFEPEYPGKSRIFD
GRTGNPFEQPVIIGKPYILKLIHQVDDKIHGRSSGHYALVTQQPLRGRAKQGGQRVGEMEV
WALEGFGVAHILQEMLTYKSDHIRARQEVLGTTIIGGTIPNPEDAPESFRLLVRELRSLALE
LNHFLVSEKNFQINRKEAMNNNFSSMIDRYKHQQLRIGSVSPQQISAWATKILPNGEIVGE
VTKPYTFHYKTNKPEKDGLFCERIFGPIKSGICACGNYRVIGDEKEDPKFCEQCGVEFVDS
RIRRYQMGYIKLACPVTHVWYLKRLPSYIANLLDKPLKELEGLVYCD--FSFARP-ITKKPTF
LRLRGLFEYEIQSWKYSIPLFFTTQGFDTFRNREISTGAGAIREQLADLDLRIIIENSLVEWE
ELGEE-GHTGNEWEDRKVGRRKDFLVRRVELAKHFIRTNIEPEWMVLCLLPVLPPELRPIIQ
IDGGKLMSSDINELYRRVIYRNNTLTDLLTTSRSTPGELVMCQEKLVQEAVDTLLDNGIRG
QPMRDGHNKVYKSFSDVIEGKEGRFRETLLGKRVDYSGRSVIVVGPSLSLHRCGLPREIAI
ELFQTFVIRGLIRQHLASNIGVAKSKIREKEPIVWEILQEVMQGHPVLLNRAPTLHRLGIQA
FQPVLVEGRAICLHPLVCKGFNADFDGDQMAVHVPLSLEAQVEARLLMFSHMNLLSPAIG
DPISVPTQDMLIGLYVLTSGNHRGICVNRYNPCNR---RNYQNQKRSDNSYKYT---EPFFSNS
YDAIGAYRQKRINLDSPLWLRWRLDQR-VIAS--RETPIEVHYESLGTFYEIYGHYLIVRSLK
KKILFIYIRTTVGHIALYREIEEAIQGFSRAYSYAT--------MEVLMAERANLVFHNKAIDGTA
MKRLISRLIEHFGMAYTSHILDQVKTLGFQQATATSISLGIDDLLTIPSKGWLVQDAEQQSLI
LEKHHHYGNVHAVEKLRQSIEIWYATSEYLRQEMNPNFRMTDPFNPVHIMSFSGARGNAS
QVHQLVGMRGLMSDPQGQMIDLPIQSNLREGLSLTEYIISCYGARKGVVDTAVRTSDAGY
LTRRLVEVVQHIVVRRTDCGTARGISVSPRNGIMP-ERIFSQTLIGRVLADDIYMGSRCIATR
NQAIGIGLVNRFIT------FRAQPISIRTPFTCRSTSWICRLCYGRSPTHGDLVELGEAVGIIAGQ
SIGEPGTQLTLRTFHTGGVFTGGTAEHVRAPSNGKIKFNEDLVHPTRTRHGHPAFLCSIDLY
VTIESEDILHNVNIPPKSLLLVQNDQYVESEQVIAEIRAGIST-LNFKEKVRKHIYSDSDGE-M
HWSTDVYHAPEFTYGNVHLLPKTSHLWILLGGPCR--SSLVYLSIHKDQDQMNAH--SLSG
KPRYTSNLS--VTNDQ-ARQKLFSSDFYGQ-EDRIPDY-SDLNRIICTGQYNLVYS-PILHGNS--
DLLSKRRRNKFIIPLHSIQELENELMPCSGISIEIP--VNGIFRRNSILAYFDDPRYRRKSSGIIK
YGTIETHSVIKKED--LIEYRG-VKEFRPKYMKVDRFFFIPEEVHILPGSSSIMVRNNSIVGVD
TQITLNLRSRVGGLVRVERKKKRIELKIFSGDIHFPGETDKISRHTGVLIP-PG-TGKRNSKEY
KK--VKNWIYVQR--ITPSK--KRFFVLVRPVVTYEITDGINLGTLFPPDPLQERDNVQLRIVN
YILYGNGKPIRGISDTSIQLVRTCLVLNWNQDKK---SSSCEEARASFVEIRTNGLIRHFLRINL
VKSPISYIGKRNDPSGSGLLSD-NGSDCTNINPFSAIYSY---SKAKIQQSLNQPQGTIHTLLNR
---NKECQSLIILSAANCSRMEP--FKDVKYHSV---IKES-PLIPIRNSLGPLGT--CLPIENFYS--S
YHL--ITHNQIL-VTKYLQLDN---LKQTFQVIKLK-YYLMDEN--GKIFNPDPCRNIILNPFNLN
WSFLHHNYCAETS-KIISLGQFICENVCIAKNGPP-LK--SGQVILVQ-VDSIVIRSAKPYLATP
GATVHGHYGETLYEGDTLVTFIYEKSRSGDITQGLPKVEQVLEVRSIDSISMNLEKRVESW
NKCIPRILGIPWGFLIGAELTIAQSRISLVNKIQQVYRSQGVQIHNRHIEIIVRQITSKVLISE---

DGMSNVFSPGELIGLLRAERMGRALEEAICYRVVLLGITRASLNTQSFISEASFQETARVLA
KAALRGRIDWLKGLKENVVLGGVIPVGTGFKGL--VHPSKQHNNIP--LETKKTNLFEGEM
R--DILFHHRKLFDSCLSKKFHDIPEQSFIG------FNDSMTRRYWNINLEEMMEAGVHFGHG
TRKWNPKMAPYISAKRKGIHITNLTRTARFLSEACDLVFDAASRGKQFLIVGTKNKAADS
VEWAAIRARCHYVNKKWLGGMLTNWSTTETRLHKFRDLRMEQKTGRLNRLPKRDAAM
LKRQLSRLQTYLGGIKYMTGVPDIVIIVDQHEEYTALRECITLGIPTICLTDTNCDPDLADISI
PANDDAISSIRLILNKLVFAICEGRS-SYIRNPMGQKINPLGFRLGTTQGHHSLWFSQPKNYS
EGLQEDKKIRDCIKNYVQKNMRTSSG-IEGIARIEIQKRIDLIQVIIFMGFPKLLIESRPR----GI
EELQMTLQKEFNCVNRKLNIAVTRIAKPYGNPNILAEFIAGQLKNRVSFRKAMKKAIELTE
QADTKGIQIQIAGRIDGKEIARVEWIREGRVPLQTIRAKIDYCSYTVRTIYGILGIKIWIFLDE
EMSRYRGPRFKKIRRLGALPGLTNKKPRTGSDLRNQSRSGK-KSQYRIRLEEKQKLRFHYG
LTERQLLKYVRIARKAKGSTGQVLLQLLEMRLDNILFRLGMASTIPAARQLVNHRHILVNG
HIVDIPSYRCKPRDIITAKDEQKSRALIQISLDSSPH-EELPNHLTLQPFQ--YKGLVNQIIDSK
WVGLKINELLVVEYYSRQTMSRRGTAEKKTAKSDPIYRNRLVNMLVNRILKHGKKSLAY
QIIYRAVKKIQQKTETNPLSVLRQAIRGVTPDITVKARRVGGSTHQVPIEIGSTQGKALAIR
WLLAASRKRPGRNMAFKLSSELVDAAKGSGDAIRKKEETHRMAEANRAFAHFRMGRDTI
AEIITSIRNADMDRKRVVRIASTNITENIIQILLREGFIENVRKHRENNKYFLVLTLRHRR--N
RKRPY---RNILNLKRISRPGLRIYSNYQRIPRILGGMGIVILSTSRGIMTDREARLEGIGGEIL
CYIWMAKAIPKISSRRNGRISSRKGARRIPKGVIHVQASFNNTIVTVTDVRGRVVSWSSAG
TSGFKGTRRGTPFAAQTAAANAIRTVVDQGMQRAEVMIKGPGLGRDAALRAIRRSGILLT
FVRDVTPMPHNGCRPPKKRRVMPTIKQLIRNTRQPIRNVTKSPALRGCPQRRGTCTRVYTI
TPKKPNSALRKVARVRLTSGFEITAYIPGIGHNSQEHSVVLVRGGRVKDLPGVRYHIVRGTL
DAVGVKDRQQGRSKYGVKKPKMARKSLIQREKKRQKLEQKYHSIRRSSKKEIS---KVPSL
SDKWEIYGKLQSLPRNSAPTRLHRRCFLTGRPRANYRDFGLSGHILREMVHACLLPGATR
SSWMVKNSVISVIFQE--EK---KGSVEFQVFNFTNKIRRLTSHLELHKKDYLSQRGLKKILG
KRQRLLAYLAKKNRVRYKELINQLDIRETKTH--MVKLRLKRCGRKQRAVYRIVAIDVRSR
REGKDLQKVGFYDPIKNQTYLNVPAILYFLEKGAQPTETVQDILKKAEVFKELRLNQPKF
N-MDKSKRPFLKFKRSFRRRLPPIQSGDRIDYRNMSLISRFISEQGKILSRRVNRLTLKQQRL
ITLAIKQARILSLLPFLNNEK--QFERTESTARTTGFKARNKVTRSLKKNPFVANHLLKKIDK
LNTKAEKEIIVTWSRASTIIPTMIGHTIAIHNGKEHLPIYITDSMVGHKLGEFAPTLNFRGHA
KSDNRSRRMIFQSFLLGNLVSLCMKIINSVVVVGLYYGFLTTFSIGPSYLFLLRALVMEEG--
-TEKKVSATTGFITGQLMMFISIYYAPLHLALGRPHTITVLALPYLLFHFFWNNHKHFFDYG
STTRNSMRNLSIQCVFLNNLIFQLFNHFILPSSMLARLVNIYLFRCNNKILFVTSGFVGWLIG
HILFMKWLGLVLVWIRQNHSIRSYIRSNKYLVLELRNSMARIFSILLFITCVYYLGRIPSPILT
KKLKETEEDKPLVSLLFDYNRWNRPFRYIKNNRFEQAVRTEMSQYFFDTCKSDGKQRISFT
YPPSLSTFWKMIKRKIPLLSLQKTLPNKLDTQWVSTNKEKSN-LNKEFLNRLEILDKESLS
MDILETRTRLCNDDTKKEYVPKMYDPLLNGPYRGTIKKGVSSEKRVRLNRIHTIFYQEFEQ
KDPNEPSIGIKEISKKVPRWSHKLIELDQQM-GQLRSRKAKRVVIFTDALISYSQQSDFRRGI
IKGSMRAQRRKTFISKLFQANVHSPLFVDRITPLRLFSFDI--SELIKPIFRNRKEQARIAIEEA
WDNIPFAQIIRGYMLITQSILRKYILLPSLIIAKNLGRMLFLQLPEWSEDLQEWNREMQIKC
TYNGVQLSETEFPKDWLRDGIQIKILFPFCLKPWHISKKKQKDDFCFLTVWGMEAELPFGS
PRKRPSFFEPIFKELEKKIGKFKKKYFLT-LKILKGKTKLFRSVSKETKKWFIQSIG--FLKKIK
KELSKVNDYLISNQIINESFSQIESGNWPNSSLIEKKMKDLTDRTSTIKNQIERITKEKKKNP
NK-TNNIKKLESPKKFFQILKSRNTRVIWKFHYFLKLFIQRLYIDLFLSIINIPRITTQLF-IFISC

DLSYLSQAYVFYKLSQTQVINLSKFRSVLQYTSCFLKTKIKDYFKLGIFHSELKHKKLQSY
RINQWKNWLRWHYQYDLSQIRWSRLMPKKWRTRVNQSCMAQNKNKWNSYEKDQ--LIH
YKKE-NFKKCYRYGLLAYKSINVNILDLDRKYFDWKIIHFSLRFFDWMGMNEKIFNRWFF
PEFVLLYNVYKIKPWIIPSKFLLFNYKSNTEAELDLFLKRYLLFQLRWNDALNQRMLENIK
VYCLLLRLINPTKITISSIQRREMSLDIMLIQANLPLTDLMK-GVLIIEPIRLSVKHNGQFIMY
QTIGISLVHKSKHTHFDLLVPENILWSRRRRELRIRSFFRFLWPNYRLEDLACMNRYWFDT
NNGSRFSILRIHMYPMRGHQFKSWIFELREILREIKNSHHFLDSWTQFNSVGSFIHIFFHQE
RFLKLFDPRIWSILLS-RNS--QGSTSNRYFT---IKG-VILFVVAVLIYR----INNRNMVERKNLY
LIGLLP-----IPMNSIGPRNDTLEESV-GSSNINRLIVS-LLYLPKGKKISESCFLNPKESTWVLPI
T----KKCSMPESNWGSRWWRNWIGKKR--DSSCKISN---ETVAGIEILFKEKDLK-YLEFLFV
YYMD--DPIRKDHDWELFDRLSLRKSRNRINL--NSGLLFEILVKHWISYLMSAFREKIPIEV
EG-FFKQQG-AGSTIQSNDIEHVSH--LFSRNKWAISLQNCAQFHMWQFRQDLFV---SWGK-
NPPESDFLRN-VSRENWIWLDNVWLVNKDR---FFS--KVQNVSSNIQYDS---TR--SSFVQVT
DSSQLKGSS-DQSRDHLDSIS----NEDSE-------------YHTLINQREIQQRKERSIL-WD--PSFLQ
TERKEIESG--RFPK--CLSGYS--SMSRLFT-EREKQMINHLFPEEIEEFL--GNPTRSVRSFFSD
RW--SELH-LGSNPTERSTRDQ----KLLKKQQDLS-FVPSRRSE----KKEMVNIFKIITYLQN--T
VSIHPISSDPGCDMVP-KDEPD-MDSSNKISFLNKNPFFDL--FHLFHDRNRGGYTL--HYDFA
SE--ERFQEMADLFTLSITEP-DLVYHKGFAFSIDSCGLDQKQ--FLNEARDE---SKKKS---LLV
LPPIFY--EENESFSRRIRKKWVR-ISCGNDLEDPKPKIVVFASNNIMEAVTQYRLIRNLIQIQY
STYG--YIRNVLNRFFLMN---RSDRNFEYG---IQRDQIGKDTLNHRTIMKYTINQYLSNLKK-
SQKKWFEPL--ILISRTERSMNRDPDAYRYKWSNGSKSFQEHLEQSVSEQKSR---FQVVFDR
LRINQYSIDWSEVIDKKDLSKSLRFFLSK---------SLLFLS-------KLLLFLSN-SLPFFCVSFGNI
PIHRSE-IYIYEELKGPNDQLCNQLLESIGLQIVHLKKLKPF--LLD---DHDTS-QKSKFLINGG
TISPFLFNKIP-KW-MIDSFHTR--NNRRKSFDNPDSYFSMIFHDQDNWLNPVKPFHRSSLISS
FYKANRLRFLNNPHHFCFY--WNTRFPFSV----EKARIN--NSDFTYG-QFLNILFIRNK-IFSLC
VGK--KKHAFWG-RDTISPIESQVSNIFIPNDFPQSG-DETYNLYKS---FHFPSRSDPFVR--RAI
YSIADISGTPLTEGQIVNFERTYCQPLSDMNLSDSEGKNLHQYLN--FNSNMGLIHTPCSEK
DLSSEK--RKK--------WSLCLK--KCVEKGQMYRTFQRDSAFSTLSKWNLFQTYMPWFLTST
GYKYLNLIFLDTFSDLL--PILSS-SQKFVSIFPDIMHGSGISWRILQKKL------CLPQWNL-ISEI
SSKCLHN--LLLSEEMIHRN--NESPLISTHLRSPNAREF-LYSILFLLLVAGY-LVRTHLLFVSR-
-ASSELQTEFEKVKSLMIPSSMIELRKLLDRYPTSEPNSFWLKNLFLVALEQLGDSLEEIRGS
ASGGNMLG---PA-----YG-VKSIRSKKKDWNINLIEIIDLI----PNPINRITFSRNTRHLSHTSKEI
YSLIRKRKNVNGDWIDEKIESWVANSDSIDDEEREFLVQFS--TLTT--ENRIDQILLSLT-HSD
HL-----SKNDSGYQMIEQPGAIYLRYLVDIH--KKHLMNYEFNPSCLAERRIFLAHYQTITY--S
QTSCGENSFHFPSPG---KPFSLRLALSPS-RGILVIGSIGTGRSYLVKYLATNSYVPFITVFLNK
FLDNKSKGFLLD-----EID-IDDSDDIDDSDNLDASD------------------DIDRDLDTEL-ELLTRM
NGLTVDMMPE---IDRFYITLQFELAKAMSPCIIWIPNIHDLDVNESNDLSLGLLVNHLSR---
DCERCS-TRNILVIASTHIPQKVDPALIAPNKLNTCIKIRRLLIPQQRKHFFTLSYTRGFHLEK
KMFHT--NGFGSITMGSNARDLVALTNEVLSISITQKKSIIDTNTIRSALHRQTWDLR---SQV
RSVQDHGILFYQIGRAVAQNVLLSNCPIDPISIYMKKK--SCNEGDSYLYKWYFELGTSMKR
LTILLYLLSCSAGSVAQDLWSLS--VPDEK-NGITSYGLVENDS-DLVHGLLEVEGALVG--SSR
TEKDCSQFDND--RVTLLLRPEPRN-PLDMMQKGSWSILDQRFLYEKY--ESEFEE-GEGEGA
LDPQ-------ED----LFNH--IVWAP--RIWRP---WGFLFDCIERPNELGFP-YWSRSFRGK---RIIY
DEEDELQENDSGFLQSGTMQYQTRDRS---QGLFRISQFIWDPADPLFFLFK-DQPPGSVFSH

RELFADEEMSKGLLTSQTDPPTSIYKRWFI--KNTQEKHFELLINRQRWLR---TNSSLS--NGS
FRSN--TLSESYQYLSNLFLSN-GTLLDQMPKTLLRKRWLFPDEMKIGF--M---------MPRSR-
TNGNFIDKTFSIVANILLRVIPTTSGEKEAFTYYRDG--MSAQSEGNYAEALQNYYEAMRLE
IDPYDRSYILYNIGLIHTSNGEHTKALEYYFRALERNPFLPQAFNNMAVICHY-RGEQAIQQ
GDSEIAEAWFDQAAEYWKQAIALTPGNYIEAHNWLKITRRFE--MTWRSDDIWIELITGSR
KISNFCWALILFLGSLGFLLVGTSSYLGRNLLSFFPPQQIIFFPQGIVMSFYGIAGLFISSYLW
CTISWNVGSGYDRFDRKEGIVCIFRWGFPGKNRRIFLRFLIKDIQSVRIEVKEGIYARRVLY
MDIRGQGSIPLTRTDEN-LTPREIEQKAAELAYFLRVPIEVF
>Gossypium
MEKSWFNLILSLSKSMDSRLPVENTTVNEDPTRNDTDKNIHDYSKVDHLVDVKDIRNFIS
DDTFLIRDSNQDRYSIYFDSENQIFELNNDH--SFLSELESFFYSYHNSSYMNNGSKNDEPHF
NLYDNDTNCGWNNHINSCIDSYLR--SQICIDSSILSGSDNSNDNYISNYICGGKNFDIITREN
GNDLTLKESSN-LDL---YKDLWVQCENCYGVNYKKSLNSKMNICEQCGYHLKMRSSDRIE
LSIDPGTWGPMDEDMISLDPIEFQSEEELYKDRIDFYQRKTGLTEAIQTGTGQLNGIPIAIGV
MDFQFMGGSMGSVVGEKITRLIEYATNNFLPLILVCASGGARMQEGSLSLMQMAKISSAL
YDYQSNKKLFYVSILTSPTTGGVTASFGMLGDIIIAEPNAYIAFAGKRVIEQTLNKTIPEGSQ
AAEYLFHKGLFDPIVPRNPLKGVLSELVQLHGFFPLNQNSIKMVTIRADEISNIIRERIEQYN
REVKIVNTGTVLQVGDGIARIHGLDEVMAGELVEFEEGTIGIALNLESNNVGVVLMGDGL
MIQEGSSVKATGKIAQIPVSEAYLGRVINALAKPIDGRGEISASESRLIESPAPGIISRRSVYEP
LQTGLIAIDSMIPIGRGQRELIIGDRQTGKTAVATDTILNQQGQNVICVYVAIGQKASSVAQV
VTTFQERGAMEYTIVVAETADSPATLQYLAPYTGAALAEYFMYRERHTLIIYDDLSKQAQ
AYRQMSLLLRRPPGREAYPGDVFYLHSRLLERAAKSSSQLGEGSMTALPIVETQSGDVSA
YIPTNVISITDGQIFLSADLFNAGIRPAINVGISVSRVGSAAQIKAMKQVAGKSKLELAQFAE
LEAFAQFASDLDKATQNQLARGQRLRELLKQSQSAPLTVAEQISTIYTGTNGYLDSLEIGQ
VRKFLVELRTYLKTNKPQFQEIISSTKTFTEEAETLLKDAIQDQMERFRLQEQLMKINPTTS
VPGVSTLEKENLGRISQIIGPVLDVAFPPGKMPNIYNALVVKGQDTAGQQINVTCEVQQLL
GNNRVRAVAMSATDGLTRGMEVIDTGAALSVPVGGATLGRIFNVLGEPVDNLGPVDTRTT
SPIHKSAPAFIQLDTKLSIFETGIKVVDLLAPYRRGGKIGLFGGAGVGKTVLIMELINNIAKA
HGGVSVFGGVGERTREGNDLYMEMKESGVINEQNLAESKVALVYGQMNEPPGARMRVG
LTALTMAEYFRDVNEQDVLLFIDNIFRFVQAGSEVSALLGRMPSAVGYQPTLSTEMGTLQ
ERITSTKEGSITSIQAVYVPADDLTDPAPATTFAHLDATTVLSRGLAAKGIYPAVDPLDSTST
MLQPRIVGEEHYETAQRVKQTLQRYKELQDIIAILGLDELSEEDRLTVARARKIERFLSQPF
FVAEVFTGSPGKYVGLAETIRGFKLILSGELDGLPEQAFYLVGNIDEATAKATNLEMESKL
KKMTLNLCVLTPNRIVWDSEVKEIILSTNSGQIGVLPNHAPIATAVDIGILRIRLNDQWLTM
ALMGGFARIGNNEITILVNDAEKGSDIDPQEAQQALEIAEANLRKAEGKRQTIEANLALRR
ARTRVEAINAISMKNVTDSFVSLGHWPSAGSFGVNTDILATNPINLSVVLGVLIFFGKGVLS
DLLDNRKERILNTIRNSEELRGGAIERLEKARARLRKVEMEADQFRVNGYSEIEREKLNLI
NSTYKILEQLENYKNETIYFEQQRAINQVRQRVFQQALQGALGTLNSSLNNELHLRTISAN
IGLFGVMKEITDMNPLISAASVIAAGLAVGLASIGPGVGQGTAAGQAVEGIARQPEAEGKI
RGTLLLSLAFMEALTIYGLVVALALLFANPFV---MNGISNALN-LYDISGVEVGQHFYWQIA
GFQVHAQVLITSWVVIAILLGSAVIAVRNPQTIPTAGQNFFEYVLEFIRDVSKTQIGEEYGP
WVPFIGTMFLFIFVSNWSGALLPWKIIQLPHGELAAPTNDINTTVALALLTSVAYFYAGLSK
KGLGYFSKYIQPTPILLPINILEDFTKPLSLSFRLFGNILADELVVVVLVSLVPSVVPIPVMFL
GLFTSGIQALIFATLAAAYIGESMEGHHMIFSTLEHILTHISFSVVSIVITIHFLTLFLLEVVGL

YDSSEKGMIVTFFCITGLLVTRWIYSGHFPLSDLYESLIFLSWGFSLIHMVSYLKKRKNNLS
AITAPSAIFTQGFATSGLLTKMHQSAILAPALQSQWLMMHVSMMVLGYAALLCGSLLSVA
LLVITFRKAIKIIGENNKIQYMNERSNVLL--YFLSSKNYYRYQLTQQLDRWSYRIISLGFIFL
TIGILSGAVWANEAWGSYWNWDPKETWAFITWTVFAIYFHTRTNTNLEGVNSALVASMGF
LIIWICYFGVNLLGIGLHSYGSFTLNMAKKKAFTPLLYLASIVFLPWWISLSFNKSLKSWIT
NWWDTRQSETFLNDIQEKSILEQFIEVEELFLLDEMIKEYPETHLQKLRIG-QKETIQLIKLH
NEDHIHTILHFSTNLICFVILSGYSILGNEELLILNSWVQEFLYNLSDTIKAFSILLLTDLCIGF
HSPHGWELMIGSIYKDFGFAHNDQIISGLVSTFPVILDTIFKYWIFRYLNRVSPSLVVIYHSM
NDMPIGVPKVPFRNPGEEDAVWVDVYNRLYRERLLFLGQEVDSEISNQLIGLMVYLSIEN
DTKDLYLFINSPGGWVIPGVAIYDTMQFVQPDVHTICMGLAASMGSFLLAGGEITKRLAFP
HARVMIHQPASSFYEAQTGEFILEAEELLKLRESLTRVYVQRTGKPLWVVSEDMERDVFM
S A T E A Q A H G I V D L V A V
K???MEEFQV
YLELNRSRRHDFLYPLIFREYIYALAHEHGLQGYGNKFSSLIVKRLILRMDQQNRLISSAND
SNQNPVFGNNLYSQMIAVGFAVIVEIPFSLRLISYSVAKSHNLQSIHSIFPFLEDKFSHLNYVL
EALIPHPIHLEILVQALRYWVKDASSLHLLRFSLYEYC--NLKSFKS---ISIPRLFLFLYNSHTC
EYESIFLFLRNQSSHLRSTSSGVFLERIFFYGKIKYLGEVFYNQNNLWLFKDPFIHFIRYQGK
SILASKDTSLLINKWKYYFVDLWQYYFYLWSQSGRVRINQLSKYSLDFLGYLSSVRLNPS
VVRSQMLENSFLIDN--AVKTLDTRIPIISLIGSLSKAKFCNTLGHPISKPTWADSPDSDIIDRF
VRISRNLSHYHSGSSKKKSLYRIKYILRFSCVKTLARKHKSTVRAFLKKLGSEFLEEFFTEE
EHVFSLIFPRRIWYLDIICINALVNHSMIIYTT-EVEDIN-SFYTLES-LKEVYGIIWMLVPILTL
VLGITIGVLVIVWLEREISAGIQQRIGPEYAGPLGILQALADGTKLLFKENILPSRGNTRLFSI
GPAIAVISILLSFSVIPFSSRLILADLNIGIFLWIAISSIAPVGLLMSGYGSNNKYSFLGGLRAA
AQSISYEIPLTLCVLSISLLSNSSSTVDIVEAQSKYGFWGWNLWRQPIGFIVFLISSLAECERL
PFDLPEAEEELVAGYQTEYSGIKFGLFYVASYLNLLLSSLFVTVLYLGGWNISIPYVFAPELF
EINKINGIIGTTIGIFITLAKTYLFLFISIATRWTLPRLRMDQLLNLGWKFLLPISLGNLLLTTS
FQLLSLMIWHVQNENFILDSTRIFMKAFHLLLFDGSFIFPECILIFGLILLLMIDSTSDQKDIP
WLYFISSTSLVMSITALLFRWREEPMISFSGNFQTNNFNEIFQFLILLCSTLCIPLSVEYIECTE
MAIAEFLLFVLTATLGGMFLCGANDLITIFVAPECFSLCSYLLSGYTKKDVRSNEATTKYLL
MGGASSSILVHGFSWLYGSSGGEIELQEIVNGLINTQMYNSPGISIALIFITVGIGFKLSPAPS
HQWTPDVYEGSPTPVVAFLSVTSKVAASASATRIFDIPFYFSSNEWHLLLEILAILSMILGNL
IAITQTSMKRMLAYSSIGQIGYVIIGIIVGDSNGGYASMITYMLFYISMNLGTFACIVLFGLR
TGTDNIRDYAGLYTKDPFLALSLALCLLSLGGLPPLAGFFGKLHLFWCGWQAGLYFLVSIG
LLTSVVSIYYYLKIIKLLMTGRNQEITPHVRNYRRSPLRSNNSIELSMIVCVIASTIPGISMNP
IIAIAQDTLFMFLLYEYDIFWAFLIISSAIPILAFLISGVLAPIRKGPEKLSSYESGIEPMGDAW
LQFRIRYYMFALVFVVFDVETVFLYPWAMSFDVLGVPVFIEAFIFVLILIVGSVYAWRKGAL
EWSTNYFPWLTIIVFLPISAGSLLFFLPHKGNKLIKWYTICICILELLLTTYAFCYHFRLDDPL
IQLAEDYKWINFFDFYWRLGIDGLSIGPILLTGFITTLATLAAWPVTRDSRLFHFLMLAMYS
GQIGSFSSRDLLLFFIMWEFELIPVYLLLSMWGGKKRLYSATKFILYTAGGSVFLLIGVLGL
GLYGSNEPTLNFETLANQSYPVALEIIFYIGFLIAFAVKSPIIPLHTWLPDTHGEAHYSTCML
LAGILLKMGAYGLVRINMELLPHAHSIFSPWLIIVGTMQIIYAASTSLGQRNLKKRIAYSSV
SHMGFIIIGIGSITDTGLNGAILQIISHGFIGAALFFLAGTSYDRMRLVYLDEMGGMAVSIPKI
FTMFSILSMASLALPGMSGFVAELIVFFGIITSQKYFLMPKILITFVMAIGMILTPIYSLSMSR
QMFYGYKLFNAPSSYFFDSGPRELFVSISIFLPVIGIGIYPDFVLSLSGEKVETILYNYFYRM

MLEHILVLSAYLFSIGIYGLITSRNMVRALMCLELILNAVNINFVTFSDFFDSRQLKGNIFSIF
VIAIAAAEAAIGSAIVSSIYRNRKSTRINQSTLLNKMEHADQYSWIIPFVPLPIPILIGMGLLL
FPTATKNLRRMWAFPNILLLSIVMIFSLDLSIQQINGSSIYQYVWSWTINNDFSFEFGYFIDS
LTSIMSILITTVGIFVLIYSDNYMSHDEGYLRFFAYMSLFNTSMLGLVTSCNLIQIYIFWELV
GMCSYLLIGFWFTRPAAANACQKAFVTNRIGDFGLLLGILGFYWITGSFEFQDLFEIFNN-E
VHFLFVTLCASLLFAGAVAKSAQFPLHVWLPDAMEGPTPISALIHAATMVAAGIFLVARLLP
LFIVIPYIMNLISLIGIITVLLGATLALAQNDIKRGLAYSTMSQLGYMMLALGMGSYRAALF
HLITHAYSKALLFLASGSIIHSMEAIVGYSPEKSQNMVFMGGLRKHVPVTQIAFLVGTLSL
CGIPPLACFWSKDEILSDSWLYSPIFAIIAWSTAGLTAFYMFRIYLLTFEGHLNVHFQKYSGK
KSSSFYSIKLWGKEEQKIINR-LFPLLTLT---GYKK-AFSYPHESDNTMLFPMLILLLFTLFVG
AIA--FHLDILSKLLTPSINLLHQNSN-FEDS-QFFKNATFSVSIACFGIFTAFLLYKPFYSSVQN
LNLLNSFVILLDKMIYLIYDWSYNRGYIDMFYSISLTKGIRGLAELTHFFDRRVIDGITNGV
GITSFFVGESVKYLGGS-RISFYLLLYLVYVFIFLVISYMDLPGPIHDFLLVFLGSGLILGGLG
VVLLTNPIYSAFSLGLVLVCISLFYILSNSHFVAAAQLLIYVGAINVLILFAVMFMNGSEYYK
DFNLWTIGNGLTSLVCTSILVSLITTILDTSWYGIIWTTRSNQIIEQDLISNSQQIGIHLATDFF
LPFEFISIILLVALIGAIAVARQMNLSATEKDLMIVNMGPHHPSMHGVLRLIVTLDGEDVVD
CEPILGYLHRGMEKIAENRTIIQYLPYVTRWDYLATMFTEAITVNGPEQLGNIQVPKRASYI
RVIMLELSRIASHLLWLGPFMADIGAQTPFFYIFRERELVYDLFEAATGMRMMHNYFRIGG
VAADLPYGWLDKCLDFCDYFLTGIVEYQKLITRNPIFLERVEGIGVIGGEEAINWGLSGPM
LRASGIKWDLRKVDHYECYDEFDWEIQWQKEGDSLARYLVRISEMTESIKIIQQALEGIPG
GPYENLEIRCFDRERDPEWNDFEYRFISKKPSPTFELPRQELYARMEAPKGELGIFLIGDQS
GFPWRWKIRPPGFINLQILPQLVKRMKLADIMTILGSIDIIMGEVDRMFPMVTGFMNYGHQ
TVRAARYIGQGFMITLSHANRLPVTIQYPYEKLITSERFRGRIHFEFDKCIACEVCVRVCPID
LPVVDWKFETDIRKKRLLNYSIDFGICIFCGNCVEYCPTNCLSMTEEYELSTYDRHELNYN
QIALGRLPMSVIDDYTIRTVLNS----------------MQGRLSVWLVKHGLVHRSLGFDYQGIETL
QIKPEDWHSIAVILYVYGYNYLRSQCAYDVAPGGLLASVYHLTRIEYGVDQPEEVCIKVFA
PRSNPRIPSVFWVWKSSDFQERESYDMLGISYENHPRLKRILMPESWIGWPLRKDYIAPNF
YEIQDAH---MNSIEFPLLDRTTQNSVISTTLND
LSNWSRLSSLWPLLYGTSCCFIEFASLIGSRFDFDRYGLVPRSSPRQADLILTAGTVTMKMA
PSLVRLYEQMPEPKYVIAMGACTITGGMFSTDSYSTVRGVDKLIPVDVYLPGCPPKPEAVI
DAITKLRKKISREIYEDRIRSQQGDRCFTTNHKFCLVRSTRTGNYNQGLLYQPPSTSEIP-ET
FFNYKGSLSSHELVNMQTRNTFSWIKEEITRSISVSLMIYIITGASISNAYPIFAQQGYENPRE
ATGRIVCANCHLANKPVDIEVPQAVLPDTVFEAVVRIPYDMQLKQVLANGKKGALNVGA
VLILPEGFELAPPDRISPEMKEKIGNLSFQNYRPTKKNILVIGPVPGKKYSEITFPILSPDPAS
NKDAHFLKYPIYVGGNRGRGQIYPDGNKSNNTVYNATATGIVSKIIRKEKGGYEITITDAL
DGHQVVDIIPPGPELLVSEGESIKLDQPLTINPNVGGFGQGDAEIVLQDPLRVQGLLFFLASI
VFAQIFLVLKKKQFEKVQVSEMNFLNKVYDWFEERLEIQAIADDITSKYVPPHVNIFYCLG
GITLTCFLVQVATGFAMTFYYRPTVTEAFASVQYIMTEANFGWLIRSVHRWSASMMVLM
MILHVFRVYLTGGFKKPRELTWVTGVVLGVLTASFGVTGYSLPRDQIGYWAVKIVTGVPE
AIPVIGSPLVELLRGSASVGQSTLTRFYSLHTFVLPLLTAVFMLMHFLMIRKQGISGPLMGV
TKKPDLNDPVLRAKLAKGMGHNYYGEPAWPNDLLYIFPVVILGTIACNVGLAVLEPSMIG
EPADPFATPLEILPEWYFFPVFQILRTVPNKLLGVLLMVSVPAGLLTVPFLENVNKFQNPFR
RPVATTVFLIGTAVALWLGIGATLPIDKSLTLGLFMIEVFLFGIVLGLIPITLAGLFVTAYLQY
RRGDQLDLMPTITSYFGFLLAALTITSALFIGLSKIRLIMDIVSLAWAALMVVFTFSLSLVV

WGRSGLMIIRSPEPEVKILVDRDPVKTSFEEWARPGHFSRTIAKGPDTTTWIWNLHADAHD
FDSHTSDLEEISRKIFSAHFGQLSIIFLWLSGMYFHGARFSNYEAWLSDPTHIGPSAQVVWP
IVGQEILNGDVGGGFRGIQITSGFFQIWRASGITSELQLYCTAIGALVFAALMLFAGWFHYH
KAAPKLAWFQDVESMLNHHLAGLLGLGSLSWAGHQVHVSLPINQFLNAGVDPKEIPLPH
EFILNRDLLAQLYPSFAEGATPFFTLNWSKYAEFLTFRGGLDPVTGGLWLTDIAHHHLAIAI
LFLIAGHMYKTNWGIGHSLKDILEAHKGPFTGQGHKGLYEILTTSWHAQLSLNLAMLGSL
TIVVAHHMYSMPPYPYLATDYGTQLSLFTHHMWIGGFLIVGAAAHAAIFMVRDYDPTTR
YNDLLDRVLRHRDAIISHLNWVCIFLGFHSFGLYIHNDTMSALGRPQDMFSDTAIQLQPVF
AQWIQNTHTLAPGATAPGATASTSLTWGGGDLVAVGGKVALLPIPLGTADFLVHHIHAFTIH
VTVLILLKGVLFARSSRLIPDKANLGFRFPCDGPGRGGTCQVSAWDHVFLGLFWMYNSIS
VVIFHFSWKMQSDVWGTISDQGVVTHITGGNFAQSSITINGWLRDFLWAQASQVIQSYGS
SLSAYGLLFLGAHFVWAFSLMFLFSGRGYWQELIESIVWAHNKLKVAPATQPRALSIVQGR
AVGVTHYLLGGIATTWAFFLARIIAVGMALRFPRFSQGLAQDPTTRRIWFGIATAHDFESHD
DITEERLYQNIFASHFGQLAIIFLWTSGNLFHVAWQGNFEAWVQDPLHVRPIAHAIWDPHF
GQPAVEAFTRGGAPGPVNIAYSGVYQWWYTIGLRTNEDLYTGALFLLFLSALSLIAGWLH
LQPKWKPSVSWFKNAESRLNHHLSGLFGVSSLAWTGHLVHVAIPGSRGEYVRWNNFLDV
LPHPQGLGPFFSGQWNLYAQNPDSSSHLFGTSQGSGTAILTLLGGFHPQTQSLWLTDIAHH
HLAIAILFLIAGHMYRTNFGIGHSIKDLLEAHIPPGGRLGRGHKGLYDTINNSIHFQLGLAL
ASLGVITSLVAQHMYSLPAYAFIAQDFTTQAALYTHHQYIAGFIMTGAFAHGAIFFIRDYNP
EQNEDNVLARMLDHKEAIISHLSWASLFLGFHTLGLYVHNDVMLAFGTPEKQILIEPIFAQ
WIQSAHGKTSYGFDVLLSSTNGPAFNAGRSIWLPGWLNAVNE--NSNSLFLTIGPGDFLVHH
AIALGLHTTTLILVKGALDARGSKLMPDKKDFGYSFPCDGPGRGGTCDISAWDAFYLAVF
WMLNTIGWVTFYWHWKHITLWQGNVSQFNESSTYLMGWLRDYLWLNSSQLINGYNPF
GMNSLSVWAWMFLFGHLVWATGFMFLISWRGYWQELIETLAWAHERTPLANLIRWRDKP
VALSIVQARLVGLAHFSVGYIFTYAAFLIASTSGKFGMSHSVKIYDTCIGCTQCVRACPTDV
LEMIPWDGCKAKQIASAPRTEDCVGCKRCESACPTDFLSVRVYLWHETTRSMGLAYMTT
FSFPSIFVPLVGLVFPAIAMASLSLYVQKTKIFMRDLKTYLSVAPVLSTLWFGSLAGLLIEIN
RFFPDALTFPFF--MTAILERRESESLWGRFCNWITSTENRLYIGWFGVLMIPTLLTATSVFIIA
FIAAPPVDIDGIREPVSGSLLYGNNIISGAIIPTSAAIGLHFYPIWEAASVDEWLYNGGPYELI
VLHFLLGVACYMGREWELSFRLGMRPWIAVAYSAPVAAATAVFLIYPIGQGSFSDGMPLGI
SGTFNFMIVFQAEHNILMHPFHMLGVAGVFGGSLFSAMHGSLVTSSLIRETTENESANEGY
RFGQEEETYNIVAAHGYFGRLIFQYASFNNSRSLHFFLAAWPVVGIWFTALGISTMAFNLN
GFNFNQSVVDSQGRVINTWADIINRANLGMEVMHERNAHNFPLDLAAIEAPSTNGMGLP
WYRVHTVVLNDPGRLLSVHIMHTALVAGWAGSMALYELAVFDPSDPVLDPMWRQGMFV
IPFMTRLGITNSWGGWSIT-GGTITNPGIWSYEGVAGAHIVFSGLCFLAAIWHWVYWDLEI
FCDERTGKPSLDLPKIFGIHLFLSGVACFGFGAFHVTGLYGPGIWVSDPYGLTGKVQPVNPA
WGVEGFDPFVPGGIASHHIAAGTLGILAGLFHLSVRPPQRLYKGLRMGNIETVLSSSIAAVF
FAAFVVAGTMWYGSATTPIELFGPTRYQWDQGYFQQEIYRRVSAGLAENQSLSEAWSKIP
EKLAFYDYIGNNPAKGGLFRAGSMDNGDGIAVGWLGHPIFRDKDGRELFVRRMPTFFETF
PVVLVDGDGIVRADVPFRRAESKYSVEQVGVTVEFYGGELNGVSYSDPATVKKYARRAQ
LGEIFELDRATLKSDGVFRSSPRGWFTFGHASFALLFFFGHIWHGARTLFRDVFAGIDPDLD
AQVEFGAFQKLGDPTTRRQVVMKTLYSLRRFYPVETLFNGTLALAGRDQETTGFAWWAG
NARLINLSGKLLGAHVAHAGLIVFWAGAMNLFEVAHFVPEKPMYEQGLILLPHLATLGW
GVGPGGEVIDTFPYFVSGVLHLISSAVLGFGGIYHALLGPETLEESFPFFGYVWKDRNKMT

TILGIHLILLGIGAFLLVFKALYFGGVYDTWAPGGGDVRKITNLTLSPSVIFGYLLKSPFGGE
GWIVSVDDLEDIIGGHVWLGSICIFGGIWHILTKPFAWARRALVWSGEAYLSYSLGALSVF
GFIACCFVWFNNTAYPSEFYGPTGPEASQAQAFTFLVRDQRLGANVGSAQGPTGLGKYL
MRSPTGEVIFGGETMRFWDLRAPWLEPLRGPNGLDLSRLKKDIQPWQERRSAEYMTHAP
LGSLNSVGGVATEINAVNYVSPRSWLATSHFVLGFFLFVGHLWHAGRARAAAAGFEKGID
RDFEPVLSMTPLNMTIALGKFTKDENDLFDIMDDWLRRDRFVFVGWSGLLLFPCAYFALG
GWFTGTTFVTSWYTHGLASSYLEGCNFLTAAVSTPANSLAHSLLLLWGPEAQGDFTRWCQ
LGGLWTFVALHGAFGLIGFMLRQFELARSVQLRPYNAIAFSGPIAVFVSVFLIYPLGQSGW
FFAPSFGVAAIFRFILFFQGFHNWTLNPFHMMGVAGVLGAALLCAIHGATVENTLFEDGDG
ANTFRAFNPTQAEETYSMVTANRFWSQIFGVAFSNKRWLHFFMLFVPVTGLWMSALGVV
GLALNLRAYDFVSQEIRAAEDPEFETFYTKNILLNEGIRAWMAAQDQPHENLIFPEEVLPR
GNALMSGSTGERSFADIITSIRYWVIHSITIPSLFIAGWLFVSTGLAYDVFGSPRPNEYFTESR
QGIPLITGRFDSLEQLDEFSRSFMTIDRTYPIFTVRWLAVHGLAVPTVSFLGSISAMQFIQRM
ATQTVEGSSRSG-PRRTVVGDFLKPLNSEYGKVAPGWGTTPLMGVAMALFAIFLSIILEIYN
SSVLLDGISMNMLTLKLFVYTVVIFFVSLFIFGFLSNDPGRNPGREEMADTTGRIPLWIIGT
VTGIPVIGLIGIFFYGSYSGLGSSLMLNIFNLICICFNSALFSSSFLFAKLPEAYAFLNPIVDFM
PVIPVLFFLLAFVWQAAVSFRMTQSNPNEQNVELNRTSLYWGLLLIFVLAVLFSNYFFNME
VNILAFIATALFILVPTAFLLIIYVKTVSQSDMETATLVAISISGLLVSFTGYALYTAFGQPSQQ
LRDPFEEHGDMEALVYTFLLVSTLGIIFFAIFFREPPKILTKKMKMTIAFQLAVFALIATSSILL
ISVPVVFASPDGWLSNKNIVFSGTSLWIGLVFLVGILNSLIS-------MSPQTETKASVGFKAGV
KEYKLTYYTPEYEVKDTDILAAFRVTPQPGVPPEEAGAAVAAESSTGTWTTVWTDGLTSL
DRYKGRCYDIEPVPGEEDQYICYVAYPLDLFEEGSVTNMFTSIVGNVFGFKALRALRLEDL
RVPTAYIKTFQGPPHGIQVERDKLNKYGRPLLGCTIKPKLGLSAKNYGRAVYECLRGGLDF
TKDDENVNSQPFMRWRDRFLFCAEAIFKSQAETGEIKGHYLNATAGTCEEMIKRAMCAR
ELGVPIVMHDYLTGGFTANTSLAHYCRDNGLLLHIHRAMHAVIDRQKNHGMHFRVLAKA
LRMSGGDHIHAGTVVGKLEGERDITLGFVDLLRDDFIEKDRSRGIYFTQDWVSMPGVLPV
ASGGIHVWHMPALTEIFGDDSVLQFGGGTLGHPWGNAPGAVANRVALEACVQARNEGRD
LAREGNEIIREASKWSPELAAACEVWKAIKFEFDAVDKLDKMAIHLYKTSTPGTRNGAVD
SQVKSNPRNNLIYGQHRCGKGRNARGIITARHRGGGHKRLYRKIDFRRNEKDIYGRIVTIE
YDPNRNAYICLIHYGDGEKRYILHPRGAIIGDTIVSGTEVPIKMGNALPLTDMPLGTAIHNIE
ITLGRGGQLARAAGAVAKLIAKEGKSATLKLPSGEVRLISKNCSATVGQVGNVGVNQKSL
GRAGSKCWLGKRPVVRGVVMNPVDHPHGGGEGRAPIGRKKPATPWGYPALGRRSRKRN
KYSDNLILRRRSKMIQPQTHLNVADNSGARELMCIRVIGAS-NRRYAHIGDVIVAVIKEAVP
NTPLERSEVIRAVIVRTRKELKRDNGMIIRYDDNAAVVIDQEGNPKGTRIFGAIARELRQLN
FTKIVSLAPEVLMLSPKRNRFRKQHRGRMKGISYRGNRICFGRYALQALEPAWITSRQIEA
GRRAMTRNVRRGGKIWVRIFPDKPVTVRPTETRMGSGKGSPEYWVAVVKPGRILYEMSG
VAENIARKAISIAASKMPIKTQFIISGMTRIKRGYIARRRRKKISLFASSFRGAHSRLTRTITQ
QRIRALVSAHRDRDRKKRDFRRLWITRINAVIRGV-GVSYSYSRLIHNLYKKQLLLNRKILA
Q I A I S N R N C L Y M I S N E I R K - - - - - - - - - -
-??
?????????????????????????????????MDGIKYVVVTDKSIRLLVKN-QYTSNVESGSTRTEIKH
WVELFFGVKVIAMNSHRLPGKGRRMGPIMGHTMHYRRMIITLQPGYSIPPLRTKRTMAVP
KKRTSTSKKRIRKNVWKKKGYWAALKAFSLAKSLSTGNSKSFFVRQIN-MAKSKDVRVTI
ILECTSCVRNGVN--KESTGISRYITQKNRHNTPSRLELKKFCPYCYKHTIHGEIKKMKIRAS

VRKICEKCRLIRRRGRIIVICFNPRHKQRQGMVREKVKVSTRTRQWKCVESRTDSKRLYY
GRFILSPLMKGQADTIGIAMRRALLGELEGTCITRAKSE--KIPHEYSTIVGIQESVHEILMNL
KEIVLRGNLYGTRNAFICAKGPGYVTAQDIILPPSVEIVDNTQHVASLTEPIDLCIGLQIERN
RGYGIKTPKNFH-DG-SYPIDAVFMPVRNANHSIHCYGNDNEKQEILFLEIWTNGSLTPKEA
LHEASRNLIDLFIPFLHTEEE--NLHLENNQ-HDVTLPFFPFHDRLV-KLTKKK-KEIALKYIFI
DQSELPPRIYNCLKKSNIHTLLDLLNNSREDLMKIEHFRIEDVKQILGILEKK-----------MLG
DEN-GEMSTIPGLNQIQFEGFCGFMDRGLTEELYKFPKIEDTEQEIEFQLFVETYQLVEPLIK
ERDAVYESLTYSSELYVSAGLIWKTSKDMQEQTIFIGNIPLMNSLGTSIVNGIYRIVINQILQ
SPGIYYRSELDHNGISVYTGTIISDWGGRLELEIDRKARIWARVSRKQKISILVLSSAMGSNL
REILENVCYPEIFLSFLTDKEKKKIGSKENAILEFYQQFSCVGGDPVFSESLCKELQKKFFQ
QRCELGRIGRRNMNQRLNLNIPQNNTFLLPRDILAAADRLIGMKFGMGPLDD--MNHLKN
KRIRSVADLLQDQFGLALVRLENVVRGTICGAIR-HKLI-PTPQNLVTSTP----LTTTYESFFGL
HPLSQVLDRTNPLTQIVHGRKLSYLGPGGLTGRTANFRIRDIHPSHYGRICPIDTSEGINVGL
IGSLAIHARIGHWG-SLESPFY---KIFERSKKA--QMLYLSPSRDEYYMVAAGNSLALNQGIQ
EEQVVPARYRQEFLTIAWEQVHLRSIFPFQYFSIGASLIPFIEHNDANRALMSSNMQRQAVP
LSRSEKCIVGTGLERQVALDSGVPAIADHEGKIISTDTDKIILSGNGDALGIPLVMYQRSNK
NTCMHQTARVRRGKCIKKGQILADGAATVGGELALGKNVLVAYMPWEGYNFEDAVLISE
RLVYEDIYTSFHIRKYEIQTHVTSQG-PERITNEIPHLEAHLLRNLDKNGIVMLGSWVETGD
ILVGKLTPQVAKE-SSYAPEDRLLRAILGIQVSTSKETCLKLPIGGRGRVIDVRWVQKKG-GS
SYNPETIRVYISQKREIKVGDKVAGRHGNKGIISKILPRQDMPYLQDGRPVDMVFNPLGVP
SRMNVGQLFECSLGLAGSLLDRHYRIAPFDERYEQEASRKLVFSELYQASKQTANPWVFE
PEYPGKSRIFDGRTGGPFEQPVIIGKPYILKLIHQVDDKIHGRSSGHYALVTQQPLRGRSKQ
GGQRVGEMEVWALEGFGVAHILQEMLTYKSDHIRARQEVLGTTIIGGTIPKPEDAPESFRL
LVRELRSLALELNHFLVSEKNFQINRKEA-------MIDRYKHQQLRIGSVSPQQISAWAKKILP
NGETVGEVTKPYTFHYKTNKPEKDGLFCERIFGPIKSGICACGNYRVIGNQKEGPKFCEQC
GVEFVDSRIRRYQMGYIRLACPVTHVWYLKRLPSYIANLLDKPLKELEGLVYCD--FSFAR
P-IAKKPTFLRLRGSFEYEIQSWKYSIPLFFTTQGFDTFRSREISTGAGAIREQLADLDLRILI
DYSVVEWKELGEE-GLTGNEWEDRKIGRRKDFLVRRMELAKHFIRTNIEPEWMVLCLLPV
LPPELRPIIQIDGGKLMSSDINELYRRVIYRNNTLTDLLTTSRSTPGELVMCQEKLVQEAVDT
LLDNGIRGQPMRDGHNKVYKSFSDVIEGKEGRFRETLLGKRVDYSGRSVIVVGPSLSLHR
CGLPREIAIELFQTFVIRGLIRQHLAPNIGVAKSKIREKGPIVWEILQEVMRGHPVLLNRAPT
LHRLGIQAFQPILVEGRAICLHPLVCKGFNADFDGDQMAVHVPLSLEAQAEARLLMFSHM
NLLSPAIGDPISVPTQDMLIGLYVLTSGNRRGICANRYNPWNRKS---YQNERIDDNNYKST--
REPFFCNSYDAIGAYRQKRINLDSPLWLRWQLEQC-VIAS--REAPIEVHYESSGTYHEIYGH
YLIVRSLKKKILCIYIRTTVGHISLYREIEEAIQGFFRAYSYDTQSYGI-------MAERANLVFHN
KVIDGTAIKRLISRLIDHFGMAYTSHILDQVKALGFQQATATSISLGIDDLLTIPSKGWLVQD
AEQQSLILEKHHHFGNVHAVEKLRQSIEIWYATSEYLRQEMNPNFRMTDPFNPVHIMSFSG
ARGNASQVHQLVGMRGLMSDPQGQMIDLPIQSNLREGLSLTEYIISCYGARKGVVDTAVR
TSDAGYLTRRLVEVVQHIVVRRTDCGTTRGISVSPQKRTLP-ERIFIQTLIGRVLADDIYMGP
RCIAIRNQDIGLGLVDRFRA------FRTQPISIRTPFTCRSTSWICRLCYGRSPTHGDLVELGEA
VGIIAGQSIGEPGTQLTLRTFHTGGVFTGGTAEHVRAPFNGKIKFNEDLVHPTRTRHGHPAF
LCYRDLYVIIESEDIIHKVAIPPKSFLLVQNDQYVESEQVIAEIRAGTYTL-NLKERVRKHIYS
DSEGE-MHWSTDVYHSPEFTYSNVHLLPKTSHLWILSGGSYK--FSVVPFSLHKDQDQISIH-
-YLSAER-RYIS--FSVNNDQ-VRHNLFSSDFSDKK-ERIYDY-SELNRIIGTGHCDFIYSA-ILHE

NAD--LLAKRRRNRFIIPFQLIQDQEKELMLHSGISMEIPIN--GIFRRKSILAFFDDPRYRRKS
SGITKYGTLGAHSIVKRED--VIEYR-GVKKVKPKYMKVDRFFFIPEEVHILSESSSIMVRNN
SIIGVDTPITLNTRSQVGGLVRVERKKKRIELKIFSGNIYFPGERDKISRHSGILIPPGTGKT--
NSKESKK--LKNWIYVQR--ITPTKKK--YFVLVRPVTPYEIPDGLNLATLFPQDPFQEKDNM
QLRAVNYILYGNGKPTRRISDTSIQLVRTCLVLSWDQDN---KSSFAEEVCASFVEVRTNGLI
RDFLRIDLVKSHIFYIRKRNDPSGSELISD-NRSDRTNKNPFYSIYSNAR-----IQQSFSQNHGT
IHTLLNR---NKESQSLIILSASNCFRMGP--FNDVKYHN---VIKQSDPLIPIKNLLGPLGT--AP
KIANFYSS-FYPLIT--HNQTS-VAKYFELDN---LKQAFQVL--N-YYLIAENGR--IYNFDPCRNI
FLNAVNLNWYFPHHHYCEETSTIIS-LGQFICENVCIAKSGP---RLKSGQVFIVQ-ADSIVIRS
AKPYLATPGATVHGHYGETLYEGDTLVTFIYEKSRSGDITQGLPKVEQVLEVRSIDSISMNL
EKRIEGWNECITRILGIPWGFVIGAELTIVQSRLSLVNKIQKVYRSQGVQIHNRHIEIIVRQIT
SKVLVSE---DGMSNVFLPGELIGLLRAERTGRALEEAICYRAVLLGITRASLNTQSFISEASF
QETARVLAKAALRGRIDWLKGLKENVVLGGMIPAGTGFKGLVHRS--RQHNNILLETKKK
N--FFGGEMR--DIFFHHRELFDSCISNNLHDTSGRSFIGIEFNDS----MARRYWNINLEEMLE
AGVHFGHGTRKWNPRMAPYISAKRKGIHITNLTRTARFLSEACDLVFDAASKGKQFLIVG
TKNKAADSVARAAIRARCHYVNKKWLGGMLTNWPTTETRLHKFRDLRTEQKTGGLNRL
PKRDAAMLKRQLSRLQTYLGGIKYMTRLPDIVIIVDQQEEYTALRECITLGIPTICLIDTNSD
PDLADISIPANDDAIASIRLILNKLVVAICEGRS-SYIRNPMGQKINPLGFRLGTTQSHHSLWF
AQPKKYSEGLQEDKKIRDCIKNYVQKNTRLSSG-VEGIARIEIQKRLDLIQVIIYMGFPKLLI
EDK----PRKLEELQMNVQKELNCMNRKLNIAITRIGNPYGHPNILAEFIAGQLKNRVSFRK
AMKKAIELTEQADTKGIQIQIAGRIDGKEIARVEWIREGRVPLQTIGAKIEYCSYRVRTIYGV
LGIKIWIFIDEEMSRYRGPRFKKIRRLGALPGLTSKRPRAGSDLRNQSRPGK-KSQYRIRLEE
KQKLRFHYGLTERQLLKYVRIARKAKGSTGQVLLQLLEMRLDNILFRLGMASTIPQARQL
VNHRHILVNGRTVDIPSYRCKPRDIISARDEQKSRTLIQNYLDSSTN-EELPKHLTFHTLQ--Y
KGLVNQIIDRKWVGLKINELLVVEYYSRQTMSRRGTAEEKTAKSDPIYRNRLVNMLVNRIL
KHGKKSLAYQIIYRALKKIQQKTETNPLSVLRQAIRGVTPDIAVKARRVGGSTHQVPIEIGS
TQGKALAIRWLLGASRKRPGRNMAFKLSSELVDAAKGSGDAIRKKEETHRMAEANRAFA
HFRMGKDTIADIITSIRNADMNRKGTIQIGSTNITENIVKILLREGFIDNVRKHRERNKYFLV
LTLRHRR--NRKGPHRT-I--LNLRRISRPGLRIYSNYQQIPRILGGMGIVILSTSRGIMTDREAR
LEGIGGEILCSIWMAKPIPKVGSRRNGRSSARKSARRIPKGVIHVQASFNNTIVTVTDVRGR
VISWSSAGTCGFKGTRRGTPFAAQTAAGNAIRAVVDQGMQRAEVMIKGPGLGRDAALRA
IRRSGILLSFVRDVTPMPHNGCRPPKKRRVMPTIKQLIRNARQPIRNVTKSPALGGCPQRRG
TCTRVYTITPKKPNSALRKVARVRLTSGFEITAYIPGIGHNSQEHSVVLVRGGRVKDLPGVR
YHIVRGTLDAVGVKDRQQGRSKYGVKKPKMAKKSLIHREKKRQKLEQKYHLIRRSSKKE
IS---KVPSLSEKWKIHGKLQSSPRNSAPTRLHRRCFSTGRPRANYRDFGLSGHILREMVHA
CLLPGATRSSWMVKNSFISVIFQE-KKEE-NRGSAEFQIVSFTNKIRRLTSHLELHKKDYLSQ
RGLRKILGKRQRLLSYLSKTNKIRYKELIGELDIRESKNR--MVKLRLKRCGRKQRAVYRIV
AIDVRSRREGRDLRKVGFYDPINNQTYLNVPAILYFLEKGAQPTATVHDILKKAGVFTELT
LNQ-TKFTMDKSKRLFLKSKRSFRRRLPPIQSGDRIDYRNMSLISRFISEQGKILSRRVNRLT
LKQQRLITIAIKQARILSSLPFLNNEK--QFERSESTTRTTALRTRNKMAR-LKKNPFVANHLL
KKIERLNTKAEKEIIITWSRASTIIPTMIGHTIAIHNGKEHLPIYITDRMVGHKLGEFAPTINF
RGHAKNDNKSRRMILKSFIPGNLISLYMTIINSVVMVGLYYGFLTTLSIGPSYIFLLRARFME
EGEEGTEKRVSATTGFIAGQLMMFISIYYVPLHLALGKPHTITVLALPYLLFHFFWNNHKD
FFDHRRPTRNSMRNLSIQCVFLNNLIIQLFNHFILPSSMLARLVNIYMFRCNNNMLFVTSSF

VGWLIGHILLMKWVGLVLVWIQQNNLIRS-IRSNKYLVSEFRNSMARIFSILLFITCVYYLG
RIPSPILTKKLKGTEENKPLVTLLFDSKRWNRPLRYIKNDRVENAIKNEMSQYFFYTCQSDG
KEKISFTYPPSLATFGEMIQKKIYFFTPEKWFSDELYTDWSFINELKRRNLSKEFIKRVESLD
---FDLGILEKRTRFSNNETKREYLPKLYDPFLHGPYRGRIKKWGSPTDPFWINKIHALLYHD
LEQTNPNNQG--IKEISKKVPRWSYKLIDLEQQEGEEIRSRKSKRVVIFTNALIRYSQQSDFR
RDIIKGSMRAQRRKTITWELFQANVHSPLFLDRVDKP--LFFSFDISGPLKRISRNREEKMRI
EIAEAWDSLLLAQVIRGSVLITQSILRKYIILPSLIIVKNIARMLLFQFPEWSEDLTDWNREM
HVKCTYNGVQLSETEFPKNWLTDGIQIKILFPFCLKPWHRFK-GQKNDFCFLTVWGMETE
LPFGSPRKRRSFFEPIFKELKKKIKKWKTKCFIA-LTILKEKTKLFRKASKETKKWITQSIL--F
LKGIIKELSKINDSIISNKMIHKS-LQIQSMAWTN-SLTEKKIKDLTKRTNTIKNQIQKILIIKD
KSSNKISYQDKILESSKKFWQIVK--NIRLIRKFYFFINFFIEKIYMDILLYIINIPRTNTQL------
CDLSSLSQAYVFYKLSQT-VISFYKFKPILQYTSLFLKNEIKDYFERGVSHSGLRQKIVW-SG
MNEWKNWLRSRYQYDLVQNRWLKLGPGQWRNRVNQHYLAQNKTKWDSDEKER--LIH
YEKKSNFKKHYGYDFLSYKSINDILDASHRKFFNWRILDFCLRHFDWMGLNEEILSHWFF
PEFVLLYNAYKVKPWIIPIKLLLFNSN--TEAELDFFLKRYLRFQLRWD---NQRIINNIKVYCL
LLRLINPNEIVISSIQRGEMNLDILMIQKDLTLRELMKKGILIIDPVRLSVKNDGQFILYQTISI
LLVHKNKHHYD-LFVPENILSPNHRRELRIRISFRFLWPNYRLEDLACMNRYWFNTNNGSR
FSMIRIRMYPMKGHQFKSWIFELREILREIKNSHYFLDSWTQFNSVGSFIHIFFHQERFIKLL
DPRIWSILLS-RNS--QGSTSNRYF---TIKGVVLFVVA-VLIYR----INNRNMVERKNLYLTGLL
P-----IPMNSIGPRNDTLEESF-GSSNINRLIVS-LLYLPKGKKISESCFLDPKESTWVLPIT----K
KCIMPESNRGSRWWRNWIGKKR--DSSCKISN---ETVAGIEISFKEKDIK-YLEFLFVYYMD--
DSIRKDHDWELFDRLSPSKRRNIINL--NSGQLFEILVKDWICYLMFAFREKIPIEVEG-FFKQ
QG-AGSTIQSNDIEHVSH--LFSRGKWAISLQNCAQFHMWQFRQDLFV---SWGK-NPHESDF
LRN-ISRENWIWLDNVWLVNRDR---FFS--KVRNVSSNIQYDS---TR--SSFVQVTDSRQLKG
SS-DQSRDRFDSIS----NEDSE-------------YHTLINQREIQQLKERSIL-WD--PSFLQTERTEIES
D--RFPK--CLSGYS--SMSRLFT-EREKRMNNHLLPEEIEEF--LGNPTRSIRSFFSDRW--SELH-
LGSNPTERSTRDQ----KLLKKEQDVS-FVPSRRSE----NKEIVNIFKIITYLQN--TVSIHPISSDP
GCDMVP-KD--E-LGSSNKISFLNKNPFFDL--FHLFHDQNRRGYTL--HHDFESE--ERFQEMA
DLFTLSITEP-DLVYYKGFAFSIDSYGLDQKQ--FLNEVFNSRDESKKKSL---SALPPIFY--EE
NESFYRRIRKKWVR-ISCGNDLEDPKPKIVVFASNNIMEAVNQDRLIRNLIQIQ--YSTYGYI
RNVLNRFIKKN---RSDRNFEYGIQ---RDQIGNDTLNHRTIMKYTINQHLSNLKK-SQKKWF
DP--LILISRTERSMNRDPNAYRYKWSNGSKNFQEHLEHFVSEQKSR---FQVVFDRLRINQY
SIDWSEVIDKKDLSKSLRFFLSKLLL---------FLS-------KLLLFLSNSLP-FFFVSFGNIPIHRSE-
IHIY-ELKGPNDQLCNQLLESIGLQIVHLKKLKPF--LLD---DHNTS-QKPKFLINGGTISPFLV
NKIP-KW-MIDSFHTR--NNRRKSFDNMDF--SMISHDQDNWLNPVKPFHRSSLISSFYKANR
LRFLNNPHHFCFY--CNKRFPF---Y-VEKARINNYD--FTYG-QFLNILFIRNK-IFSLCGGK--KK
HAFLG-RDTISPIESQVSNIFISNDFPQSGDER-YNLYKS---FQFAIRSDPLVR--RAIYSIADISG
TPLTEGQIVNFERTYCQPLSDMNPSDSEEKNLHQYLN--FNSNMGLIHTPCSEKYLPSEK--R
KK--------RSLCLK--KCVEKGWMYRTFQRDSAFSTLSKRNLFQTYMPWFLTSTGYKYLNLI
FLDTFSD-LLPILSSS--QKFVSIFHDIMHGSDISWRILQKKL------CLPQWN-LISEISSKCLHN-
-FLLSEEMIHRNNES--PLISTHLRSPNVQEF-LYSILFLLLVAGY-LVRTHLLFVSR--AYSELQT
EFEKVKSLMIPSYMIELRKLLDRYPTSELNSFWLKNLFLVALEQLGDSLEEIRGSAFGGNM
LWGGGP-----AYG-VKSIRSKKKYLNINLIDIIDLI-SIIPNPINRIIFSRNTRHLSHTSKEIYSLIR
KRKNVSGDWIDEKIESWVANSDSIDDKEREFLVQFS--TLTT--EKRIDQILLSLT-HSDHLS----

-KNDSGYQMIEQPGPIYLRYLVDIH--KKYLMNYEFNTSCLAERRIFLAHYQTITY--SQTSCG
ANSFHFPSRG---KPFSLRLALSPS-RGILVIGSIGTGRSYLFKYLATNSYVPFITVFLNKFLDN
KLKGFLID-----DID--DDSDDIDASDDIDASDAID----------DSDAIDRDLDTELELLTMMNAL-
--TMDMMS---EIDRFYITLQFELAKAMSPCIIWIPNIHDLDVNEANYLSLGLLVNYLS---KDC
ERCS-TRNILVIASTHIPQKVDPTLIAPNKLNTCIKIRRLLIPQQRKHFFTLSYTRGFHLEKKM
FHT--NGFGSITVGSNARDLVALTNEALSISITQKKSIIDTNTIRSALHRQTWDLR---SQVRSV
QDHGILFYQIGRAVVQNVLLSNCPLDPISIYMKKK--SCNEGDSYLYKWYFELGTSMKKLTI
LLYLLSCSAGSVAQDLWSLP--GPDEK-NGITSYGFVENDS-DLVHGLLEVEGALVG--SSRTE
KDCGQFDND--RVTLLLRSEPGN-PLYMMQNGSCSIVDQRNLYEK--YESEFEE-GEGEGVLD
PQ----QIEED----LFNH--IVWAP--RIWRP---WGFLFDCIERPNELGFP-YWAGSFRGK---RIIYD
EKDELQENDSAFLQSGTMQYQARDRSSKEQGFFRISQFIWDPADPLFFLFK-DQPFVSVFS
HREFFADEEMSKGLLTSQTDPPTSIYKRWFI--KNTQEKHFELLIHRQRWLR---TNSSLSNAN
GFFRSN--TPSESYQYLSNLFLSN-GRLLDQMTKTLLRKRWLFPDEMKIGF--M---------MPRS
Q-INGNFIDKTFSIVANILLRIIPTTSGEKEAFTYYRDG--MSAQSEGNYAEALQNYYEAMRL
EIDPYDRSYILYNIGLIHTSNGEHTKALEYYFRALERNPFLPQAFNNMAVICHY-RGEQAIR
QGDSEIAEAWFDQAAEYWKQAIALTPGNYIEAQNWLKITRRFE--MSWRSESIWIEFIVGSR
KTSNFCWAFILFFGSLGFLLVGTSSYLGRNLISLFPSQQIVFFPQGIVMSFYGIAGLFISSYLW
CTIFWNVGSGYDRFDRKEGIVCIFRWGFPGKNRRIFLRFLMKDIQSIRIEVKEGIYARRVLY
MEIRGQGAVPLTRTDEN-LTPREIEQKAAELAYFLRVPIEVF
>Vitis
MEKWWFNSMLSLSKSTDSPDPIENTSGSEDRVINNTDKNINNYSNVDHLFGIRDIRNFISD
DTFLVRDRNGVSYFIYFDIENQIFEIDNDQ--SFLSELESFFYSYRNSSYLNNVSKSNHPHYD
RYMYDTKYSWNKHINSCIN------------NYILSGSNNYS--YIYSYICGHGSSSKQTSTNGSDLK
---SSNVLYVAQKYRHLWIQCENCYGLNYKKNLKSKINICEQCGYHLKMSSSDRIELSIDPG
TWDPVDEDMVSLDPIEFHSGEEPYKERIDFYQRKTGLTEAVQTGTGQLNGIPVAIGVMDFQ
FMGGSMGSVVGEKITRLIEYATNEFLPLILVCSSGGARMQEGSLSLMQMAKISSALYDYQS
NKKLFYVSILTSPTTGGVTASFGMLGDIIIAEPNSYIAFAGKRVIEQTLKKTVPEGSQAAEYL
FHKGLFDPIVPRNTLKGVLSELFQLHAFFPLNPKKIKMVTIRADEISNIIRERIEQYNREVKI
VNTGTVLQVGDGIARIHGLDEVMAGELVEFEEGTIGIALNLESNNVGVVLMGDGLMIQEG
SSVKATGRIAQIPVSEAYLGRVINALAKPIDGRGEISASESRLIESPAPGIISRRSVYEPLQTGL
IAIDSMIPIGRGQRELIIGDRQTGKTAVATDTILNQQGQNVICVYVAIGQKASSVAQVVTTFQ
ERGAMEYTIVVAETADSPATLQYLAPYTGAALAEYFMYRKRHTLIIYDDPSKQAQAYRQM
SLLLRRPPGREAYPGDVFYLHSRLLERAAKSSSSLGEGSMTALPIVETQSGDVSAYIPTNVI
SITDGQIFLSADLFNAGIRPAINVGISVSRVGSAAQIKAMKQVAGKSKLELAQFAELEAFAQ
FSSDLDKATQNQLARGQRLRELLKQSQAAPLTVEEQIMTIYTGTNGYLDSLEIGQVRKFLA
ELRNYLKTNKPQFQEIISSTKTFTEEAETLLKETIQEQMERFLLQEQAMRINPTTSGPGVST
LEKKNLGRIAQIIGPVLDVAFPPGKMPNIYNALVVKGRDTVGQQINVTCEVQQLLGNNRV
RAVAMSATDGLMRGMEVIDTGAPLSVPVGGATLGRIFNVLGEPVDNLGPVDTRTTSPIHRS
APAFIQLDTKLSIFETGIKVVDLLAPYRRGGKIGLFGGAGVGKTVLIMELINNIAKAHGGVS
VFGGVGERTREGNDLYMEMKESGVINEKNISESKVALVYGQMNEPPGARMRVGLTALTM
AEYFRDVNEQDVLLFIDNIFRFVQAGSEVSALLGRMPSAVGYQPTLSTEMGTLQERITSTK
EGSITSIQAVYVPADDLTDPAPATTFAHLDATTVLSRGLAAKGIYPAVDPLDSTSTMLQPRIV
GEEHYETAQRVKQTLQRYKELQDIIAILGLDELSEEDRLTVARARKIERFLSQPFFVAEVFT
GSPGKYVGLAETIRGFQLILSGELDGLPEQAFYLVGNIDEATAKAMNLEMESKLKKMTLN

LCVLTPNRIVWDSKVKEIILSTNSGQIGILPNHAPIATAVDIGILRIRLNDQWLTMALMGGFA
RIGNNEITVLVNDAEKGSDIEPQEAQQTLEIAEANLRKAEGKRQIIEANLALRRARTRVEAI
NVTSMKNVTDSFVSLGHWPSAGGFGFNTDILATNPINLSVVLGVLIFFGKGVLSDLLDNR
KQRILNTIRNSEELREGAIEQLEKARARLRKVEMEADQYRVNGYSEIEREKLNLINSTYNT
LEQLENYKNETIHFEQQRAINQVRQRVLQQALQGALGTINSCLNKELHLRTISANIGMFGS
MKEIRNMNPLISAASVIAAGLAVGLASIGPGVGQGTAAGQAVEGIARQPEAEGKIRGTLLL
SLAFMEALTIYGLVVALALLFANPFVMNVLSCSINTLKGLYDISGVEVGQHFYWQIGGFQV
HAQVLITSWVVIAILLGSATLAVRNPQTIPTDGQNFFEYVLEFIRDVSKTQIGEEYGSWVPFI
GTMFLFIFVSNWSGALLPWKIIQLPHGELAAPTNDINTTVALALLTSVAYFYAGLTKKGLG
YFGKYIQPTPILLPINILEDFTKPLSLSFRLFGNILADELVVVVLVSLVPSIVPIPVMFLGLFTS
GIQALIFATLAAAYIGESMEGHHMIFSTLEHILTHISFSVVSIVITIHLITLLVDEIVGLSDSSEK
GMIATFFCITGLLVTRWIYLGHFPLSDLYESLIFLSWSFSIIHIVPYF-KHKNHLSALTAPSAIF
TQGFATSGLLTEMHQSEILVPALQSQWLMMHVSMMVLGYAALLCGSLLSVALLVITFRQDI
RILCKNNEIQYMNKGNNVLQNTSFLSVRNYYRAQFIQQLDHWSYRVISLGFLFLTIGILSGA
VWANEAWGSYWNWDPKETWAFITWTIFAIYLHTRTNKNLQVANSAIVASMGFLIIWICYF
GVNLLGIGLHSYGSFTFSMTKKKAFTPLLYLASIVFLPWWISLSLNKSLESWVTNWWNTR
QSETFLNDIQEKSILEKFIELEELFLLDEMIKECPETHLQKLRMG-HKETIQLIKMYNEDPIH
TILHFSTNIICFVILSGYSILGNEELLILNSWIQQFLYNLSDTIKAFSILLLTDLCIGFHSPHGW
ELMIGSVYKDFGFAHNDQIISGLVSTFPVILDTILKYWIFRYLNRVSPSLVVIYHSMNDMPIG
VPKVPFRNPGEDDISWIDVYNRLYRERLLFLGQEVESEISNQLIGLMIYLSIEDENKDLYFFI
NSPGGWVLPGIAIYDTMQFVPPEVHTICLGLAASMGSFILVGGTITKRLAFPHARVMIHQP
AAAFYEAQAGEFVMEAEELLKLREIITKVYVQRTGKPLWVVSEDLERDVFMSATEAQTH
GIVDLVAVQMKEQKWIHEGLITESLPNGMFRVRLDNENMILGYVSGRIRRSFIRILPGDRVK
IEVSRYDSTKGRIIYRIRNKDSNNMEGVQRYFK-DRSRQDDFLYPLIFQEYIYALSYDHGLV
GYDNKSSLLIVKRLITRMYQQNHLIFS--DSNQNPFFGKNFYSQMISEGFGVIVEIPFSLRLVS
SLIAKSHNLRSIHSIFPFLEDKFPHLKYVSDILIPHPIHLEILVQALRYWVKDASSLHLLRFFL
HEYHNWNSMITKKSISIFSQRFFLFLYNFHVCEYDSIFIFIRNQSYHLRSTSYGALLGRIFFY
GKIEHFVEVFANDFILWLFKNPFMHYVRYQGKSILASKGAPLLMNKWKYYLVNFWQCHF
YVWSQPVRIHINQLSKHSLDFLGYLSSVRLNPSVVRSQMLENAFIIDNAI--KKFDTIVPIIPII
GSLAKARFCNALGHPISKPSWADSSDYDIIDRFVRICRNISHYHSGSSKKKNLYRIKYILRLS
CARTLARKHKSTVRAFLKRLGSEFLEEFLTEEEQALSLILPRRFWYFDIICINDLANHEMIID
-TPEVQ-DINSFSRLEY-LKEVYGIIWMLVPIFTPVLGITIGVLAIVWLEREISAGIQQRIGPEY
AGPLGILQALADGTKLLFKENLLPSRGDTRLFSIGPSIAVISILLSYLIIPFGSNLVLSDLNIGV
FLWIAISSIAPIGLLMSGYGSNNKYSFLGGLRAAAQSISYEIPLTLCVLSISLLSNSSSTVDIV
EAQSKYGFWGWNLWRQPIGFIVFLISSLAECERLPFDLPEAEEELVAGYQTEYSGIKFGLFY
VASYLNLLVSSLFVTVLYLGGWDLSIPYIFVPEFFEINKAGEVFGTTIGIFITLAKTYLFLFIPI
TTRWTLPRLRMDQLLNLGWKFLLPISLGNLLLTTSSQLLSLMIWHIQNENFILDSTRIFMKA
FHLLLFDGSFIFPECILIFGLILLLMIDSTSDQKDIPWLYFISSTSLVMSITALLFRWREEPMISF
SGNFQTNNFNEIFQFLILLCSTLCIPLSVEYIECTEMAITEFLLFVLTATLGGMFLCGANDLIT
IFVAPECFSLCSYLLSGYTKKDVRSNEATTKYLLMGGASSSILVHGFSWLYGSSGGEIELQE
IVNGLINTQMYNSPGISIALIFITVGIGFKLSPAPSHQWTPDVYEGSPTPVVAFLSVTSKVAA
SASATRIFDIPFYFSSNEWHLLLEILAILSMILGNLIAITQTSMKRMLAYSSIGQIGYVIIGIIV
GDSNGGYASMITYMLFYISMNLGTFACIVSFGLRTGTDNIRDYAGLYTKDPFLALSLALCL
LSLGGLPPLAGFFGKLHLFWCGWQAGLYFLVSIGLLTSVVSIYYYLKIIKLLMTGRNQEITP

HVRNYIRSPLRSNNSIELSMIVCVIASTIPGISMNPIIAIAQDTLFMFLLYEYDIFWAFLIISSVI
PILAFFISGVLAPISKGPEKLSSYESGIEPMGDAWLQFRIRYYMFALVFVVFDVETVFLYPW
AMSFDVLGVSVFIEALIFVLILIVGSVYAWRKGALEWSTNYFPWLTLIVVLPISAGSLIFFLP
HRGNKVIRWYTIFICMLELLLTTYVFCYHFQLDDPLIQLVEDYKWINFFDFDWRLGIDGLSI
GPILLTGFITTLATLAAWPVTRDSRLFHFLMLAMYSGQIGSFSSRDLLLFFIMWELELIPVYL
LLSMWGGKKRLYSATKFILYTAGGSIFLLLGVLGIGLYGSNEPTLNFETSANQSYPVALEIIF
YIGFFIAFAVKSPIIPLHTWLPDTHGEAHYSTCMLLAGILLKMGAYGLVRINMELLPHAHSI
FSPWLVVVGTMQIIYAASTSSGQRNLKKRIAYSSVSHMGFIIIGIGSITDTGLNGAILQIISHG
FLGAALFFLAGTSYDRIRLIYLDEMGGIAIPMPKIFTMFSILSMASLALPGMSGFVAELIVFF
GIITSQKFLLMPKILITFVMAIGMILTPIYSLSMSRQMFYGYKLFNAPNSYFFDSGPRELFVL
ISIFLPVIGIGIYPDFVLSLSVDKVEAILSNFFYRMMLEHVLVLSAYLFSIGIYGLITSRNMVR
ALMCLELILNAVNINFVTFSDFFDNRQLKGDIFSIFVIAIAAAEAAIGPAIVSSIYRNRKSTRI
NQSNLLNKMEHTYQYAWIIPFLPLPIPMLLGVGLLLFPTATKNLRRMWSFTSVLLLSIVMT
FSVNLSIHQINSSSIYQYVWSWTINNDFSLEFGYLIDPLTSIMSILITTVGIMVLIYSDNYMSH
DQGYLRFFAYMSFFNASMLGLVTSSNLIQIYIFWELVGMCSYLLIGFWFTRPIAANACQKA
FVTNRVGDFGLLLGILGLYWITGSFEFRDLFEIFNNNGTNFLFVSLCALFLFVGAVAKSAQF
PLHVWLPDAMEGPTPISALIHAATMVAAGIFLVARLFPLFIVIPYIMNLISLIGIITILLGATLA
LAQKDIKKSLAYSTMSQLGYTMLALGMGSYRAALFHLITHAYSKALLFLGSGSIIHSMEAI
VGYSPDKSQNMVLMGGLTKHVPITKNTFLLGTLSLCGIPPLACFWSKDEILNDSWLYSPIF
AIIACLTAGLTAFYMFRIYLLTFEGYFNVHCKNYSGKKSSLFYSISLWGKEGQKTIKKKLLT
LLTTNFFSFDNKNTFLYPQESDNTMLFPLFALALFTLFVGAIGIPFDFDILSKWLNPATNLLH
KNLN-YGDWYEFVINAIFSVSISYFGIFLASLLYNPVYSSLQNLDLINSVAIFWDKIINKVYN
WSYNRGYIDAFYETSLTKGIRGLAELTHFFDGRVIDGITNGVGVTSFFVGESIKYVGGG-RI
SSYLFLYLSSVSILLLISYMDLPGPIHDFLLVFLGSGLILGGLGVVLLTNPIYSAFSLGLVFVCI
SLFYIPSNSHFVAAAQLLIYVGAINVLIIFAVMFMNGSEYYKDFNLWTVGDGVTSVVCTSIF
ASLITTILDTSWYGIIWTTRSNQIIEQDLISNSQQIGIHLSTDFFLPFELISIILLVALIGAIAVAR
QMNVPATREDLMIVNMGPHHPSMHGVLRLIVTLDGEDVIDCEPILGYLHRGMEKIAENRT
IIQYLPYVTRWDYLATMFTEAITVNAPEQLGNIQVPKRASYIRVIMLELSRIASHLLWLGPF
MADIGAQTPFFYIFREREL VYDLFEAATGMRMMHNYFRIGGVAADLPHGWIDKCLDFCD
YFLTGVAEYQKLITRNPIFLERVEGVGIIGVEEAINWGLSGPMLRASGIQWDLRKVDHYEC
YDEFDWEVQWQKEGDSLSRYLVRIGEMVASIKIIQQALEGIPGGPYENLEIRCFDRARDPE
LNDFEYRFISKKPSPTFELSKQELYVRVEAPKGELGIFLIGDQNVFPWRWKIRPPGFINLQIL
PQLVKRMKLADIMTILGSIDIIMGEVDRMFPMVTGFMNYGQQTVRAARYIGQSFMITLSH
ANRLPVTIQYPYEKLITSERFRGRIHFEFDKCIACEVCVRVCPIDLPVVDWKLETDIRKKQL
LNYSIDFGICIFCGNCVEYCPTNCLSMTEEYELSTYDRHELNYNQIALGRLPMSVIDDYTIR
TILNS-ITSE-----------MQGRLSAWLVKHGLVHRSLGFDYQGIETLQIKPEDWHSIAVILYVYG
YNYLRSQCAYDVAPGGLLASVYHLTRIEYGVDQPEEVCIKVFASRRNPRIPSVFWVWKSA
DFQERESYDMLGISYDNHPRLKRILMPESWIGWPLRKDYIAPNFYEIQDAH------------------
--MNSIEFPLLDRTTQNSVISTTSNDLSNWSRLSSLWPLLYGT
SCCFIEFASLIGSRFDFDRYGLVPRSSPRQADLILTAGTVTMKMAPSLVRLYEQMPEPKYVI
AMGACTITGGMFSTDSYSTVRGVDKLIPVDVYLPGCPPKPEAVIDAITKLRKKISREIYEDR
IRS--ENRCFTTNHKFHVGRSTHTGNYDQRLLYQSP-TSEIPLETFFKYKSSVSSPEFVNMQT
RNTFSWIKEQITRSISVSLMIYIITRTSISNAYPIFAQQGYENPREATGRIVCANCHLANKPVD
IEVPQAVLPDTVFEAVVRIPYDMQMKQVLANGKRGALNVGAVLILPEGFELAPPDRISPEM

KEKIGNLSFQNYRPTKKNILVIGPVPGQKYSEITFPILSPDPATKKDVHFLKYPIYVGGNRGR
GQIYPDGSKSNNTVYNATAAGIVSKIIRKEKGGYEITIADASDGRQVVDIIPPGPELLVSEGE
SIKLDQPLTSNPNVGGFGQGDAEIVLQDPLRIQGLLFFLSSVILAQIFLVLKKKQFEKVQLSE
MNFMSKVYDWFEERLEIQAIADDITSKYVPPHVNIFYCLGGITLTCFLVQVATGFAMTFYY
RPTVTEAFASVQYIMTEANFGWLIRSVHRWSASMMVLMMILHVFRVYLTGGFKKPRELT
WVTGVVLGVLTASFGVTGYSLPRDQIGYWAVKIVTGVPEAIPVIGSPLVELLRGSASVGQS
TLTRFYSLHTFVLPLLTAVFMLMHFPMIRKQGISGPLMGVTKKPDLNDPVLRAKLAKGMG
HNYYGEPAWPNDLLYIFPVVILGTIACNVGLAVLEPSMIGEPADPFATPLEILPEWYFFPVFQ
ILRTVPNKLLGVLLMVSVPAGLLTVPFLENVNKFQNPFRRPVATTVFLIGTAVALWLGIGAT
LPIDKSLTLGLFMIEVFLFGIVLGLIPITLAGLFVTAYLQYRRGDQLDLMPTITSYFGFLLAA
STITSVLFIGLSKIRLIMDIVSLAWAALMVVFTFSLSLVVWGRSGLMIIRSPEPEVKILVDRD
PIKTSFEEWARPGHFSRTIAKGPDTTTWIWNLHADAHDFDSHTSDLEEISRKVFSAHFGQL
SIIFLWLSGMYFHGARFSNYEAWLSDPTHIGPSAQVVWPIVGQEILNGDVGGGFRGIQITS
GFFQMWRASGITNELQLYCTAIGALVFAALMLFAGWFHYHKAAPKLAWFQDVESMLNH
HLAGLLGLGSLSWAGHQVHVSLPINQFLNAGVDPKEIPLPHEFILNRDLLAQLYPSFAEGA
TPFFTLNWSKYAEFLTFRGGLDPVTGGLWLTDIAHHHLAIAILFLIAGHMYRTNWGIGHGL
KDILEAHKGPFTGQGHKGLYEILTTSWHAQLSLNLAMLGSLTIVVAHHMYSMPPYPYLAT
DYGTQLSLFTHHMWIGGFLIVGAAAHAAIFMVRDYDPTTRYNDLLDRVLRHRDAIISHLN
WACIFLGFHSFGLYIHNDTMSALGRPQDMFSDTAIQLQPVFAQWIQNTHALAPSATAPGAT
TSTSLTWGGGDLVAVGGKVALLPIPLGTADFLVHHIHAFTIHVTVLILLKGVLFARSSRLIPD
KANLGFRFPCDGPGRGGTCQVSAWDHVFLGLFWMYNSISVVIFHFSWKMQSDVWGSISD
QGVLTHITGGNFAQSSITINGWLRDFLWAQASQVIQSYGSSLSAYGLFFLGAHFVWAFSLM
FLFSGRGYWQELIESIVWAHNKLKVAPATQPRALSIVQGRAVGVTHYLLGGIATTWAFFLA
RIIAVGMALRFPRFSQGLAQDPTTRRIWFGIATAHDFESHDDITEERLYQNIFASHFGQLAIIF
LWTSGNLFHVAWQGNFESWVQDPLHVRPIAHAIWDPHFGQPAVEAFTRGGAPGPVNIAYS
GVYQWWYTIGLRTNEDLYTGALFLLFLSAISLIAGWLHLQPKWKPSVSWFKNAESRLNH
HLSGLFGVSSLAWTGHLVHVAIPGSRGEYVRWNNFLDVLPHPQGLGPLFTGQWNLYAQN
PDSSSHLFGTSQGAGTAILTLLGGFHPQTQSLWLTDIAHHHLAIAFIFLVAGHMYRTNFGIG
HSMKDLLEAHIPPGGRLGRGHKGLYDTINNSIHFQLGLALASLGVITSLVAQHMYSLPAYA
FIAQDFTTQAALYTHHQYIAGFIMTGAFAHGAIFFIRDYNPEQNEDNVLARMLDHKEAIIS
HLSWASLFLGFHTLGLYVHNDVMLAFGTPEKQILIEPIFAQWIQSAHGKTSYGFDVLLSST
TGPAFNAGRSIWLPGWLNAVNE--NSNSLFLTIGPGDFLVHHAIALGLHTTTLILVKGALDA
RGSKLMPDKKDFGYSFPCDGPGRGGTCDISAWDAFYLAVFWMLNTIGWVTFYWHWKHI
TLWQGNVSQFNESSTYLMGWLRDYLWLNSSQLINGYNPFGMNSLSVWAWMFLFGHLVW
ATGFMFLISWRGYWQELIETLAWAHERTPLANLIRWRDKPVALSIVQARLVGLAHFSVGYI
FTYAAFLIASTSGKFGMSHSVKIYDTCIGCTQCVRACPTDVLEMIPWDGCKAKQIASAPRT
EDCVGCKRCESACPTDFLSVRVYLWHETTRSMGLAYMITLNLPSILVPLVGLVFPAIAMAS
LFLHVQKNKIFMRDLKTYLSTAPVLSTIWFGSLAGLLIEINRLFPDALTFPFFN-MTVILERR
ESESLWGRFCNWITSTENRLYIGWFGVLMIPTLLTATSVFIIAFIAAPPVDIDGIREPVSGSLL
YGNNIISGAIIPTSAAIGLHFYPIWEAASVDEWLYNGGPYELIVLHFLLGVACYMGREWEL
SFRLGMRPWIAVAYSAPVAAAAAVFLIYPIGQGSFSDGMPLGISGTFNFMIVFQAEHNILMH
PFHMLGVAGVFGGSLFSAMHGSLVTSSLIRETTENESANAGYRFGQEEETYNIVAAHGYF
GRLIFQYASFNNSRSLHFFLAAWPVVGIWFTALGISTMAFNLNGFNFNQSVVDSQGRVINT
WADIINRANLGMEVMHERNAHNFPLDLAAVEAPSINGMGLPWYRVHTVVLNDPGRLISV

HIMHTALVAGWAGSMALYELAVFDPSDPVLDPMWRQGMFVIPFMTRLGITNSWGGWSIT-GGTITNPGIWSYEGVAGAHIVFSGLCFLAAIWHWVYWDLEIFCDERTGKPSLDLPKIFGIH LFLSGLACFGFGAFHVTGLYGPGIWVSDPYGLTGKVQSVNPAWGVEGFDPFVPGGIASHHI AAGTLGILAGLFHLSVRPPQRLYKGLRMGNIETVLSSSIAAVFFAAFVVAGTMWYGSATTP IELFGPTRYQWDQGYFQQEIYRRVGAGLAENQSLSEAWSKIPEKLAFYDYIGNNPAKGGL FRAGSMDNGDGIAVGWLGHPIFRDKEGRELFVRRMPTFFETFPVVLVDGDGIVRADVPFR RAESKYSVEQVGVTVEFYGGELNGVSYSDPATVKKYARRAQLGEIFELDRATLKSDGVFR SSPRGWFTFGHASFALLFFFGHIWHGARTLFRDVFAGIDPDLDAQVEFGTFQKLGDPTTRR QVVMKTLYSLRRFYPVETLFNGTLALAGRDQETTGFAWWAGNARLINLSGKLLGAHVAH AGLIVFWAGAMNLFEVAHFVPEKPMYEQGLILLPHLATLGWGVGPGGEVIDTFPYFVSGV LHLISSAVLGFGGIYHALLGPETLEESFPFFGYVWKDRNKMTTILGIHLILLGIGAFLLVFKA LYFGGVYDTWAPGGGDVRKITNLTLSPSVIFGYLLKSPFGGEGWIVSVDDLEDIIGGHVWL GSICIFGGIWHILTKPFAWARRALVWSGEAYLSYSLGALAVFGFIACCFVWFNNTAYPSEFY GPTGPEASQAQAFTFLVRDQRLGANVGSAQGPTGLGKYLMRSPTGEVIFGGETMRFWDL RAPWLEPLRGPNGLDLSRLKKDIQPWQERRSAEYMTHAPLGSLNSVGGVATEINAVNYVS PRSWLATSHFVLGFFLFVGHLWHAGRARAAAAGFEKGIDRDFEPVLSMTPLNMTIALGKF TKDES-LFDIMDDWLRRDRFVFVGWSGLLLFPCAYFALGGWFTGTTFVTSWYTHGLASSY LEGCNFLTAAVSTPANSLAHSLLLLWGPEAQGDFTRWCQLGGLWTFVALHGAFGLIGFML RQFELARSVQLRPYNAIAFSAPIAVFVSVFLIYPLGQSGWFFAPSFGVAAIFRFILFFQGFHN WTLNPFHMMGVAGVLGAALLCAIHGATVENTLFEDGDGANTFRAFNPTQAEETYSMVT ANRFWSQIFGVAFSNKRWLHFFMLFVPVTGLWMSALGVVGLALNLRAYDFVSQEIRAAE DPEFETFYTKNILLNEGIRAWMAAQDQPHENLIFPEEVLPRGNALMSGSTGERSFADIITSIR YWVIHSITIPSLFIAGWLFVSTGLAYDVFGSPRPNEYFTESRQGIPLITGRFDPLEQLDEFSRS FMTIDRTYPIFTVRWLAVHGLAVPTVSFLGSISAMQFIQRMATKTVEGSSR-SRPKPTTVGE LLKPLNSEYGKVAPGWGTTPLMGVAMALFAVFLSIILEIYNSSVLLDGISLNMLTLKLFVYT VVIFFVSLFIFGFLSNDPGRNPGREEMADTTGRIPLWLIGTVTGIPVIGLIGIFFYGSYSGLGS SLMLNIFSLICICLNSALYSSNFFFAKLPEAYAFLNPIVDVMPVIPLFFFLLAFVWQAAVSFRT TQSNPNEQNVELNRTSLYWGLLLIFVLAVLFSNYFFNMEVNILAFIATALFILVPTAFLLIIYV KTVSQNNMETATLVAISISGLLVSFTGYALYTAFGQPSQQLRDPFEEHGDMEALVYTFLLVS TLGIIFFAIFFREPPKVPTKKVKMTIAFQLAVFALIATSSILLISVPVVFASPDGWSSNKNIVFS GTSLWIGLVFLVGILNSLIS-------MSPQTETKASVGFKAGVKDYKLTYYTPEYETKPTDILA AFRVTPQPGVPPEEAGAAVAAESSTGTWTTVWTDGLTSLDRYKGRCYHIEPVAGEESQFIA YVAYPLDLFEEGSVTNMFTSIVGNVFGFKALRALRLEDLRIPPAYSKTFQGPPHGIQVERDK LNKYGRPLLGCTIKPKLGLSAKNYGRAVYECLRGGLDFTKDDENVNSQPFMRWRDRFLF CAEAIFKSQAETGEIKGHYLNATAGTCEEMIKRAVFARELGVPIVMHDYLTGGFTANTSLA HYCRDNGLLLHIHRAMHAVIDRQKNHGMHFRVLAKALRLSGGDHIHAGTVVGKLEGER EITLGFVDLLRDDFVEKDRSRGIYFTQDWVSLPGVLPVASGGIHVWHMPALTEIFGDDSVL QFGGGTLGHPWGNAPGAVANRVALEACVQARNEGRDLAREGNEIIRAASKWSPELAAAC EVWKEIKFEFPAMDTL--MAIHLYKTSTPSTRNRAVDSQVKSNPRNNLIYGQHRCGKGRNA RGIITAGHRGGGHKRLYRKIDFRRNEKDIYGRIVTIEYDPNRNAYICLIHYGDGEKRYILHP RGAIIGDTIVSGTEVPIKMGNALPLTDMPLGTAIHNIEITLGKGGQLARAAGAVAKLIAKEG KSATLKLPSGEVRLISKNCSATVGQVGNVGVNQKSLGRAGSKCWLGKRPVVRGVVMNP VDHPHGGGEGRAPIGRKKPTTPWGYPALGRRSRKRNKYSDNLILRRRSKMIQPQTHLNVA DNSGARELMCIRIIGTS-NRRYAHIGDVIVAVIKEVVPNMPLERSEVIRAVIVRTCKELKRDN

GMIIRYDDNAAVIIDQEGNPKGTRIFGAIVRELRQLNFTKIVSLAPEVLMLSPKRTRFRKQH
RGRMKGISYRGNRICFGRYALQALEPAWITSRQIEAGRRAMTRNVRRGGKIWVRIFPDKP
VTVRPTETRMGSGKGSPEYWVAVVKPGRILYEMGGVAENIARKAISIAASKMPIRTQFIISG
MTRTRRGYIARRRRTKIRLFTSSFRGAHSRLTRTITQQKIRALVSTHRDRDRQKRDFRRLWI
TRINAVIHEN--ASYSYSRFIHDLYKRQLLLNRKILAQIAISNRNCLYMISNEIIK------------EE
VYALSQSISMSAHKARRVLDQILGRSYRETLTILELMPYRACYPIFKLIYSAAANAKHNMG
LNEEYLVITKATVNEGTTVKKLKPRARGRSYAIKRPTCHITIVLKDTALYGGVWDKKMDG
IKYAVFTDKSIRLLGKN-QYTSNVESGSTRTEIKHWVELFFGVKVIAMNSHRLPGKGRRMG
PIMGHTMHYRRMIITLQPGYSIPPLRKKRTMAVPKKRTSMSKKRIHKNIWKRKGWRAALK
AFALAKSLSTGNSKSFFVRQLNNMAKSKDARIRVLLECTSCVRGG--VNKESTGISRYITEK
NRHNTPGRLELQKFCPYCYKHTIHGEIKKMKIRASVRKICEKCRLIRRRGRIIVICPNPRHK
QRQGMVREKVTVSTRTLQWKCVESRVDNKRLCYGRFILSPLIKGQADTIGIAMRRALLGE
IEGTCITRAKSE--KIPHEYSTIVGIQESVHEILMNLKEIVLRSNLYGTRGAFICARGPGYVTA
QDIISPPSVEIVDNTQHIARLMEPIDLCIGLQIERNRGYSIKRPN--NDGSY--PIDAVFMPVRN
ANHSIHSYGNGNERQEILFLEIWTNGSLTPKEALHEASRNLIDLFIPFLHAEEE--TLHLEYN
Q-HKSTLPPLTFHDRLA-KLRKNKK--IELKYIFIDQLELPPRIYNCLKRSNIHTLLDLLNKSQ
KDLMKIEDFRIEDVKQLLGILEKH--FTID-----MFRGGN-EGMSTIPGFNQIQFEGFCRFIDQG
LTEELYKFPKIEDTDQEIEFQLFVETYQLVEPLIKERDAVYESLTYSSELYVSAGLIWKTSRD
MQEQTIFIGNIPLMNSLGTSIVNGLYRIVINQILQSPGIYYRSELDHNGISVYTGTIVSDWGG
RLELEIDRKARIWARVSRKQKISILVLSSAMGSNLREILENVCYPEIFLSFLNDKEKKKIGSK
ESAILEFYQQFACVGGDPVFSESLCKELQKKFFQQRCELGRIGRRNINRRLNLDIPQNNTFL
LPQDILAAADHLIGMKFGMGILDD--MNHLKNKRIRSVADLLQDQFGLALIRLENVVRGTI
CGAIR-HKLI-PTPQNLVTSTP----VTTTYESFFGLHPLSQVLDRTNPLTQIVHGRKSSYLGPG
GLTGRTASFRIRDIHPSHYGRICPIDTSEGINVGLIGSLAIHARIGHWG-SLESPFY---EIS--ERS
KKVRMLYLSPSRDEYYMVAAGNSLALNQGIQEEQVVPARYRQEFLTIAWEQVHLRSIFPF
QYFSIGASLIPFIEHNDANRALMSSNMQRQAVPLSRSEKCIVGTGLERQAALDSGVPVIAE
HEGKIVYTDTDKIILSGNGDTLSIPLVMYQRSNKNTCMHQKAQVRRGKCIKKGQILADGA
ATVGGELALGKNVLVAYMPWEGYNSEDAVLISERLVYGDIYTSFHIRKYEIQTHVTSQG-P
ERITNKIPHLEAHLLRNLDKNGIVILGSWVETGDILVGKLTPQMAKE-SSYAPEDRLLRAIL
GIQVSTSKETCLKLPIGGRGRVIDVRWIQKKG-GSSYNPETIRVYISQKREIKVGDKVAGRH
GNKGIISKILPRQDMPYLQDGRPVDMVFNPLGVPSRMNVGQIFECSLGLAGGLLDRHYRI
APFDEKYEQEASRKLVFSELYEASKQTANPWVFEPEYPGKSRIFDGRTGDPFEQPVIIGKPY
ILKLIHQVDDKIHGRSSGHYALVTQQPLRGRAKQGGQRVGEMEVWALEGFGVAHILQEML
TYKSDHIRARQEVLGTTIIGGTIPNPEDAPESFRLLVRELRSLALELNHFLVSEKNFQINRKE
A-------MIDRYKHQQLRIGSVSPQQISAWANKILPNGEIIGEVTKPYTFHYKTNKPEKDGLFC
ERIFGPIKSGICACGNYRVIGDEKDDPKFCEQCGVEFVDSRIRRYQMGYIKLACPVTHVWY
LKRLPSYIANLLDKPLKELEGLVYCD--FSFARP-IEKKPTFLRLRGSFEYEIQSWKYSIPLFFT
TQGFDTFRNREISTGAGAIREQLDDLDLRIIIDYSLVEWKELGEE-GPTGNEWEDRKIGRRK
DFLVRRMELAKHFIRTNIEPEWMVLCLLPVLPPELRPIIQIDGGKLMSSDINELYRRVIYRN
NTLTDLLTTSRSTPGELVMCQEKLVQEAVDTLLDNGIRGQPMRDGHNKVYKSFSDVIEGK
EGRFRETLLGKRVDYSGRSVIVVGPSLSLHQCGLPREIAIELFQTFLIRGLIRQHLASNIGVA
KSQIREKEPIVWEILQEVMRGHPVLLNRAPTLHRLGIQAFQPILVEGRAICLHPLVRKGFNA
DFDGDQMAVHVPLSLEAQSEARLLMFSHMNLLSPAIGDPISVPTQDMLIGLYVLTSGNRRG
ICANRYNPCNRRN---YQNERIDDNNYRYTKEKEPFFCNSYDAIGAYRHKRINLYSPLWLRW

QLDQR-LIAS--KEAPIEVHYESLGTYHEIYGHYLIVRSVKKEIPCIYIRTTVGHISLYREIEEAI
QGFCRACSYET--------MGVLMTERANLVFHNKVIDGTAMKRLISRLIDHFGMAYTSHILDQ
VKTLGFQQATATSISLGIDDLLTIPSKGWLVQDAEQQSLILEKHHHYGNVHAVEKLRQSIEI
WYATSEYLRQEMNTNFRMTDPFNPVHIMSFSGARGNASQVHQLVGMRGLMSDPQGQMI
DLPIQSNLREGLSLTEYIISCYGARKGVVDTAVRTSDAGYLTRRLVEVVQHIVVRRTDCGTI
RGISVSPRNGMIP-ERIFIQTLIGRVLADDIYMGPRCIAIRNQDIGIGLVNRFIT------FQAQTISI
RTPFTCKSTSWICRLCYGRSPTHGDLVELGEAVGIIAGQSIGEPGTQLTLRTFHTGGVFTGG
TAEHVRAPSNGKIKFNEDWVHPTRTRHGHPAFLCYIDLYVTIESEGIIHNVNIPPKSFLLVQ
NDQYVESEQVIAEIRAGTYT-FNFKERVRKHIYSDSEGE-MHWSTDVYHAPEFTYGNVHLL
PKTSHLWILSGGSCR--FSIVPFSIHKDQDQMSVR--SLSVERRYISNPS--VTNDQ-VIHEFFSS
DLSGKKEGRIPAY-SELNRIIYTGRYNLIDP-AILHENS--DLLAKRRRNRFIIPFQSIQEGEKEL
MPPSGISIEIP--INGIFRRNSILAYFDDPRYRRNSSGITKYGTIEAHSIVKKED--LIEYRG-VKE
FKPKYMKVDRFFFIPEEVHILPGSASIMVRNNSIIGVDTRITLNTRSRVSGLVRVERKKKRIE
LKIFSGDIQFPGETNKISRHIGILIPPGT--RKKNSKESKK--LKNWIYVQG--ITPTK--KKYFVL
VRPVVTYEIADGINLATLFPQDLLQERDNMHLQVVNYILYGNGKPIRGISDTSIQLVRTCLV
LNWYQEKK---SSPIEKTHASFMEVRTNGLIRDFLTINLVKSYISYTGKRNDPSSSGLIAD-NG
SDHTNINPFYSIYPKER-IQQLLRQRQKKNQGTIRTLLNR---NKECQSLIILSSSNCSRMGP--F
NDVKYHNV---IKESDTPIPIRNSLGPLGT--ALQITNFYS--FYHL--ITHNHIL-VTKYFQLDN---
FKQTFQTLK---YYLMDEN--GITYNPDPCSNIILNPFNLNWYFLHHNYCEETS-TIISLGQFIC
ENVCIAKNGPH-LK--SGQVLIVQ-VDSVVIRSAKPYLATLGATVHGHYGEILYEGDTLVTFI
YEKSRSGDITQGLPKVEQVLEVRSIDSISMNLEKRVEGWNEHITRILGIPWGFFIGAELTIAQ
SRISLVNKIQKVYRSQGVQIHNRHIEIIVRQITSKVLVSE---DGMSNVFSPGELIGLLRAERTG
RAFEEAICYRTILLGITRASLNTQSFISEASFQETARVLAKAALWGRIDWLKGLKENVVLG
GMIPVGTGFKGL--VHRSRQHNNIPLEMETKKNNLFEREMR--DILFHHRELFDSCSSKNFH
DTSEQSFMG------FNDSMTRRYWNINLEEMMEAGVHFGHGTRKWNPRMAPYISAKRKGI
HITNLTRTARFLSEACDLVFDAASRGKQFLIVGTKNKAADSVARAAIKARCHYVNKKWLG
GMSTNWSTTETRLHKFRDLRTEQKTGRLNRLPKRDAAMLKRQLSHLQTYLGGIKYMTGL
PDIVIIVDQQEEYTALRECITLGIPTICLIDTNCDPDLADISIPANDDAIASIRLILNKLVFAICE
GRS-SYIRNPMGQKINPLGFRLGTTQGHHSLWFAQPKNYSKGLQEDQKIRDCIKNYVQKNI
RISSG-VEGIARIEIQKRIDLIQVIIYMGFPKLLVEGKPR----RIEELQMNVQKELNYVNRKLN
IAITRITKPYGHPNILAEFIAGQLRNRVSFRKAIKKAIELTEQADTKGIQVQIAGRIDGKEIAR
VEWIREGRVPLQTIRAKIDYCSYRVRTIYGVLGIKIWIFVDEEMSRYRGPRFKKIRRLGALP
GLTSKRPRAGSDLRNQSRSGK-RSQYRIRLEEKQKLRFHYGLTERQLLNYVRIAGKAKGAT
GRVLLQLLEMRLDNILFRLGMASTIPGARQLVNHRHILVNGRIVDIPSYRCKPRDIITAGNE
QKSRALIQNVFDSSSH-EELPKHLTLHPFQ--FKGLVNQIIDSNWVGLKINELLVVEYYSRQT
MSRRGTAEEKTAKSDPIYRNRLVNMLVNRILKHGKKSLAYQIIYRSVKKIQQKTETNPLSV
LRQAIRGVTPDIAVKARRVGGSTHQVPIEIGSTQGKALAIRWLLGASRKRPGRNMAFKLSS
ELVDAAKGSGDAIRKKEETHRMAEANRAFAHFRMGRDTIADIITSIRNVDMDRKVTVRIA
STNITENIVKILLREGFIENVRKHQESNKYFLVLTLRHRR--NRKGTYRT-ILN--LKRISRPGL
RIYSNYQRIPRILGGMGIVILSTSRGIMTDREARLERIGGEILCYIWMAKPIPRIGSRRNGRIG
SRKSARRIPKGVIHVQASFNNTIVTVTDVRGRVVSWSSAGTCGFRGTRRGTPFAAQTAAG
NAIRTVVDQGMQRAEVMIKGPGLGRDAALRAIRRSGILLSFVRDVTPMPHNGCRPPKKRR
VHPTCSSALRTPPKSGGFRDISDWLS-CVSNK-LFNSWHTITPKKPNSALRKVARVRLTSGF
EITAYIPGIGHNSQEHSVVLVRGGRVKDLPGVRYHIVRGTLDAVGVKDRQQGRSKYGVKK

PKMARKSLIQRERKRQKLEQKYHLIRRSSKKEIS---KVPSLSDKWEIHGKLQSSPRNSAPTR
LHRRCFLTGRPGANYRYFGLSGHILREMVHACLLPGATRSSWMVKNLFISVISQ----KEAN
RGSVEFQVFSFTNKIRRLTSHLELHRKDYLSQRGLRKILGKRQRLLSYLAKKNRVRYKELI
GQLGIRETKIR--MVKLRLKRCGRKQRAVYRIVAIDARSRREGRDLRKVGFYDPIKNQTYS
NVPAILYFLEKGAQPTGTVHDISKKAEVFTELRLNQTKFKFMDKSKRPFLKSKRSFRRRLP
PIQSGDRIDYRNMSLISRFISEQGKILSRRVNRLTLKQQRLITIAIKQARILSSLPFLNNEK--Q
FERTESTARTTGLRTRNKVTR-LKKNPFIAKHLLRKIAMLNTTWEKEIIVTWSRTSTIIPTMI
GHTIAVHNGKEHFPIYITDRMVGHKLGEFSSTLNFRGHAKNDNRSRRMILKSFLLGNLVSL
CMKIINSVVVVGLYYGFLTTFSIGPSYLFLLRAQVMEEGEEGTEKKVSAITGFITGQLMMFI
SIYYAPLHLALGRPHTITVLALPYLLFHFFWNNHKHFFDYGSTTRNSMRNLSIQCVFLNNL
IFQLLNHFILPSSMLARLVNIYMFRCNNKMLFVTSSFVGWLIGHILFMKWVGLVLVWIRQN
PSIRSLIRSNKYLVSELRNSMARIFSILLFITCVYYLGRIPSPILTKKMKETEEDKPLVTLLFD
YKRWNRPLRYIKAFQIKKAVRNEMSQYFFYTCLSDGKQRISFTYPPSLSIFLEMIQRKMSLS
TTERLSYDELYNHWIYTNEQKEN-LRKEFITRIEALDNGSLTLDVLEKRTRLCNDATKKEY
LPKIYDPFLNGPYRGTIKKMFSPIEKFRINKIYGIL-REFEHTDPKDQSIGIKEISKKVPRWSY
KLMNEE-----EIRSRKSKHVVIFTDALIRYSQQSDFRRDIIKGSMRAQRRKIVIRELFQANVH
SPLFLDRIYKPIFFSFDI--SGLIKLIFENREEKARIKIAEVWDTVLFAQVIRGCLLLTQSIFRKY
IILPSLIIAKNIGRMLLFQSPEWSEDLKDWNREMHVKCTYNGVQLSETEFPQNWLRDGIQI
KILFPFCLKPWHKPQKVKK--FCFLTVWGMETDLPFGSPRKRPSFFKPILKELEKRIRKFKN
KCFLV-LTVLKERTKIFLRVSKETKKWVIKSVLFINIKKIIKELLKINDSIINNQMIHKSSIQIRS
MNWTNYSLTEKKVKDLTDRTSTIRKKIEKIIKDKKKSPNKASYNAKRLESQKSIWQILKRR
NSRLIRKLHYFIKFFIERIYIDILLCLINIPRTNAQFF-HFISCDLSSLSQAYVFYKLSQTQVINL
YKLRSVLQYTSLFLKNERKDYFAHEIFHYELRHKKLRNSGMNQWKNWLRGHYQYKLSPI
IWSRLVPQKWRNRVNQHCVAQNQSKWDLSQKDQ--LIHYNKQENFKKHYRYDLLSYKYI
NDDIMYPDRKYFDWKILHFCFRLFDWMGMNEEILNRWFFPEFVLLYNTYKMKPWVIPIK
LLLLNFKNHTEAELDLFLKRYLLFQLRWDDSLNQKMINNIKVYCLLLRLINPREICISAIQR
GEMSLDLIPVHKDLTLTELMKMGIFIIEPIRLSVKNDGQFIMYQTIGISLVHKSKYNHYDLL
VPENILSPRRRRKFRILICFRFLWPNYRLEDLACMNRYWFDTNNGSCFSMIRIHMYPMKGH
QFKSWIFELREILREIKNSHYFLDSWTQFTSVGSFIHIFFHQERFIKLFDPRIWSILLS-RNS--Q
GSTSNRYF---TIKGVVLFVVA-VLIYR----INNRNMVERKNLYLTGLLP-----IPMNSIGPRNDT
LEESF-GSSNINRLIVS-LLYLPKGKKISESCFLDPKESTWVLPIT----KKCIMPESNWGSRWW
RNWIGKKR--DSSCKISN---ETVAGIEISFKEKDIK-YLEFLFVYYMD--DPIHKDHDWELFDR
LSPRKRRNIINL--NSGQLFEILVKHWICYLMSAFREKIPIEVEG-FFKQQG-AGSTIQSNDIEH
VSH--LFSRNKWAISLQNCAQFHMWQFRQDLFV---SWGK-NPHESDFLRN-VSRENWIWLD
NVWLVNKDR---FFS--KVRNVSSNIQYDS---TR--SSFVQVTDSSQLKGSS-DQSRDHFDSIS---
-NEDSK-------------YHTLINQREIQQLKERSIL-WD--PSFLQTERTEIESD--RFPK--CLSGYSS
MSMSRLFT-EREKQMIIHLLPEEIEEF--LGNPTRSIRSFFSDRW--SELH-LGSNPIERSTRDQ---
-KLLKKQQDVS-FVPSRRSE----NKEMVNIFKIITYLQN--TVSIHPISSDPGCDMVP-KDEPD-
MDSSNKISFLNKNPFFDL--FHLFHDRNRGGYTL--HHDFESE--ERFQEMADRFTLSITEP-DL
VYHKGFAFSIDSYGLDQKQ--FLNEVFNSRDESNKKSL---LVLPPSFY--EENESFYRRIRKK
WVR-ISCGNDLEDPKPKIVVFASNNIMEAVNQYILIRNLIQIQYSTYG--YIRNVLNRFFLMN-
--RSDRNFEYGIQ---RDQIGNDTLNHRTIMKYTINQHLSNLKK-SQKKWFDP--LIFISRTERS
MNRDPHAYRYKWSNGSKNFQEHLEHFVSEQKSR---FQVVFDRLRINQYSIDWSEVIDKKD
LSKSLRFFLSKSLL---------FLS-------KLLLFLSNSLP-FFFVSFGNIPIHRSE-IHIY-ELKGPNDQ

LCNQLLESIGLQIIHLKKWKLF--LLD---DHDTS-QKSKFLINGGTISPFLFNKIP-KW-MIDSF
HSR--NNRRKSFDNTDSYFSMISHDQDNWLNPVKPFHRSSLISSFYKANRLRFLNNPHRFCF
Y--CNKRFPF---Y-VEKARINNSD--FTYG-QFLNILFIRKK-IFSLCGGK--KKHAFLE-RDTISPI
ESQVSNIFIPNDFPQSGDET-YNLYKS---FHFPIRSNPFVR--RAIYSIADISGTPLTEGQIVNFER
TYCQPLSDMNLSDSEGKNLHQYLN--FNSNMGLIHTPCSEKYLPSEK--RKK--------RSLCIK-
-KCVEKGQMYRTFQRDSAFSTLSKWNLFQTYMPWFLTSTGYKYLNLIFLDTFSD-LLPILSS
S--PKFVSIFHDIMYGSDISWRILQKKL------CLPQRN-LISEISSKCLHN--LLLSEEMIHRNNE
S--PLISTHLRSPNVREF-LYSILFLLLVAGY-LVRTHLLFVSR--ASSELQTEFEKVKSLMIPSY
MIELRKLLDRYPTSELNSFWLKNLFLVALEQLGDSLEEIRGSASGGNMLLGGGP-----AYG-
VKSIRSKKKYLNINLIDIIDLI-SIIPNPINRITFSRNTRCLSHTSKEIYSLIRKRKNVNGDWIDD
KIESWVANSDSIDDEEREFLVQFS--TLTT--EKRIDQILLSLT-HSDHLS-----KNDSGYQMIEQP
GAIYLRYLVDIH--KKYLMNYEFNTSCLAERRIFLAHYQTITY--SQTSCGANSFHFPSHG---K
PFSLRLALSPS-RGILVIGSIGTGRSYLVKYLATNSYVPFITVFLNKFLDNKPKGFLFD-----DI
DDIDASDDIDASDDIDAS----------------DDIDRDLDTELELLTMMNAL---TMDMMP---EIDRF
YITLQFELAKAMSPCIIWIPNIHDLDVNESNYLSLGLLVNYLS---RDCERCS-TRNILVIASTH
IPQKVDPALIAPNKLNTCIKIRRLLIPQQRKHFFTLSYTRGFHLEKKMFHT--NGFGSITMGS
NARDLVALTNEALSISITQKKSIIDTNAIRSALHRQTWDLR---SQVRSVQDHGILFYQIGRAV
AQNVLLSNCPIDPISIYMKKK--SCNEGDSYLYKWYFELGTSMKKLTILLYLLSCSAGSVAQ
DLWSLP--GPDEK-NGITSYGLVENDS-DLVHGLLEVEGALVG--SSRTEKDCSQFDND--RVT
LLLRPEPRN-PLDMMQNGSCSIVDQRFLYEK--YESEFEE-GAGEGALDPQ----QIEED----LFN
H--IVWAP--RIWRP---WGFLFDCIERTNELGFP-YWARSFRGK---RIIYDEEDELQENDSEFLQ
SGTMQYQTRDRSSKEQGFFRISQFIWDPADPLFFLFK-DQPFVSVFSHREFFADEEMSKGLL
TSQTDPPTSIYKRWFI--KNTQEKHFELLIHRQRWLR---TNSSLS--NGSFRSN--TPSESYQYL
SNLFLSN-ARLLDQMTKTLLRKRWLFPDEMKIGF--M---------MPRSR-INGNFIDKTFSIVANI
LLRIIPTTSGEKEAFTYYRDG--MSAQSEGNYAEALQNYYEAMRLEIDPYDRSYILYNIGLIH
TSNGEHTKALEYYFRALERNPFLPQAFNNMAVICHY-RGEQAIRQGDSEIAEAWFDQAAE
YWKQAIALTPGNYIEAHNWLKITRRFE--MSWRSERIWIELITGSRKTSNFCWAFILFLGSLG
FLLVGTSSYLGRNLISLFPSQQIIFFPQGIVMSFYGIAGLFISSYLWCTISWNVGSGYDRFDR
KEGIVCIFRWGFPGKNRRIFLRLLMKDIQSIRIAVKEDIYARRVLYMEIRGQGAIPLTRTDEN-
LTPREMEQKAAELAYFLRVPIEVF
>Helianthus
MKRWWLNSMLFLSKSTSSLGPIENASESKDPNINDTDKNIQSYSNVDLFFGVKDLRNLISD
DTFLVKDSNGDIYSLYFDIENHIFEIDNDH-----PELESSFY--RNSSYLNNGSKSKNPEHDPY
MNDTQYTWNNHINSCIDSYLQ--SQICIDSYIVSGSDNSSNNYISSSICG--NSSKNEDARTSD
PIIRESSTDLDVTQKYRHLWVQCENCYGLNYKKFFKSKMNLCEQCGYHLKMSSSDRIELS
IDPGTWEPMDEDMVSLDPIEFHSEEEPYKNRIDSYQRKTGLTEAVQTGIGQLDGINVAIAV
MDFQFMGGSMGSVVGEKITRLIEYATKEFLPLIIVCASGGARMQEGSLSLMQMAKISSALY
DYQSNKKLFYVPILTSPTTGGVTASFGMLGDIIIAEPNAYIAFAGKRVIEQTLNKTVPDGSQ
AAEYLFQKGLFDLIVPRNPLKSVLSELFQLHTFFPLNQN---MVTIQADEISNIIRERIEQYNR
EVKIVNTGTVLQVGDGIARIHGLDEVMAGELVEFQEGTIGIALNLESTNVGVVLMGDGLL
IQEGSSVKATGKIAQIPVSEAYLGRVINALAKPIDGRGEISSSEYRLIESPAPGIISRRSVYEPL
QTGLIAIDSMIPIGRGQRELIIGDRQTGKTAVATDTILNQQGQNVICVYVAIGQKASSVAQV
VTTFQEKGAMEYTIVVAETADSPATLQYLAPYTGAALAEYFMYRERHTLIIYDDLSKQAQ
AYRQMSLLLRRPPGREAYPGDVFYLHSRLLERAAKLSSLLGEGSMTALPIVETQSGDVSAY

IPTNVISITDGQIFLSADLFNAGIRPAINVGISVSRVGSAAQIKAMKQVAGKLKLELAQFAEL
EAFAQFASDLDKATQNQLARGQRLRELLKQSQSAPLAVEEQILTIYTGTNGYLDSLEVGQV
RKFLVELRTYLKTNKPQFQEIISSTKIFTEEAEAILKEAIQEQRERFILQEQAMRMNPTTSSS
GVATLDKKNLGRIAQIIGPVLDVAFPPGKMPNIYNALVVKGRDTVGQPINVTCEVQQLLGN
NRVRAVAMSATDGLTRGMDVIDTGAPLSVPVGGATLGRIFNVLGEPIDNLGPVDNSTTFPI
HRSAPAFIQLDTKLSIFETGIKVVDLLAPYRRGGKIGLFGGAGVGKTVLIMELINNIAKAHG
GVSVFGGVGERTREGNDLYMEMKESGVINEQNIAESKVALVYGQMNEPPGARMRVGLTA
LTMAEYFRDVNEQDVLLFVDNIFRFVQAGSEVSALLGRMPSAVGYQPTLSTEMGSLQERI
TSTKEGSITSIQAVYVPADDLTDPAPATTFAHLDATTVLSRGLAAKGIYPAVDPLDSTSTMLQ
PRIVGDEHYETAQQVKQTLQRYKELQDIIAILGLDELSEEDRLTVARARKIERFLSQPFFVA
EVFTGSPGKYVGLAETIRGFQLILSGELDGLPEQAFYLVGNIDEATAKAMNLEMESNSKK
MTLNLCVLTPNRIVWDSEVKEIILSTNSGQIGVLPNHAPIATAVDIGILRIRLNDQWLTMAL
MGGFARIGNNEITVLVNDAEKSSDIDPQEAQQTLEIAEAALRKAEGKRQTIEANLALRRAR
TRVEAINAISMKNVTDSFVSLGHWPSAGSFGFNTDILATNLINLSVVLGVLIFFGKGVLSDL
LDNRKQRILNTIRNSEELREGAIEQLEKARARLRKIEIEADEFRVNGYSEIEREKLNLIDSTY
KTLEQLENYKNETINFEQQKASNQVRQRVFQQALQGALGTLNSCLNNELHLRTISANIGIL
AAMKQITDMNPLISAASVIAAGLAVGLASIGPGVGQGTAAGQAVEGIARQPEAEGKIRGTL
LLSLAFMEALTIYGLVVALALLFANPFVMNVLSCSINTLNGLYDLSGVEVGQHFYWKIGGF
QVHGQVLITSWVVIAILLGSAALAVRNPQTIPTGGQNFFEYVLEFIRDVSKTQIGEEYGPW
VPFIGTMFLFIFVSNWSGALLPWKIIQLPHGELAAPTNDINTTVALALLTSVAYFYAGLTKK
GLSYFGKYIQPTPILLPINILEDFTKPLSLSFRLFGNILADELVVVVLVSLVPSVVPIPVMFLG
LFTSGIQALIFATLAAAYIGESMEGHHMIFSTLEHILTHISFSIVSIVITLHLITLLGNEIIKPYD
SSEKGMIATFLCLTGLLTTRWIYSGHFPLSDLYESLIFLSWSFSLIHIVPY--IRKNDLTTITASIT
IFTQGFATSGLLNEIHKPTILVPALQSEWLIMHVSMMILSYAALLCGSLLSVALLVITFRN---I
FYSSKEIQYKNERNNIFQKTS---DKNYYKAQFIQQLDYWSYRVISLGFIFLTIGILSGAVWAN
EAWGSYWSWDPKETWAFITWIVFAIYLHTRTNKNLQGANSAIVATLGFLIIWICYFGVNLL
GIGLHSYGSFTLTMAKKKAFTPLLYLVSIVFLPWWISLLFQKSLESWVTNWWNTRQSETFL
NDIEEKSILEKFIELEELLFLEEMIKEYSETHLQNLRI-IHKETIQLIKIQNEGRIHTILHFSTNII
CFIILSGYSILGNKELVILNSWAQEFLYNLSDTIKAFSLLLLTDLCIGFHSPHGWELMIGFVY
KDFGFVHNDQIISGLVSTFPVILDTILKYWIFRYLNRVSPSLVVIYHSMNDMPIGVPKVPFRS
PGEEDASWVDIYNRLYRERLLFLGQEVDSEISNQLIGLMIYLSIEDDTQDLYLFINSPGGWV
IPGVALYDTMQFVQPDVHTICMGSAASMGSFILVGGEITKRLAFPHARVMIHQPAGSFSEVA
TGEFILEVGELLKLRETLTRVYVQRTGKPVWVVSEDMERDVFMSATEAQAYGIVDLVAVE
MKEQKWIHEGLITESLPNGMFRVRLDNEDMILGYVSGKIRRSFIRILPGDRVKIEVSRYDST
RGRIIYRLRNKDSKDMEKFQR----DRS--HYFLYPLIFQEYIYVLAHDHGLAGYDNKSSLLIV
KRLIIRMYQQNHLILSVNDSKQTAFLGKNFYSQVMSEVSSTIMEIPLSLRLISSLVVKSDNL
RSIHSIFSFLEDNFSHLNYVLDILIPYPAHLEILVQALRYWIKDASSLHLLRFFLHECHNWDS
LITKASSFFSKHRLFFFLYTSYVCEYESGFLFLRNQSSHLRSTSSGALIERIYFYGKIEHLAEV
FARTFNLWFFKDSFMHYVRYQGKSILASKGTFLLMNKRKDYFFNFWKSYFYLWSYPGRIS
INQLSNHSLDFLGYRSSVRLKPSMVRGQMLENTFLINNAI--KKFDSIVPIMPLVGSLAKSKF
CNALGHPIGKAIWADLSDSDIIERFGRIYRNLSHYHSGSSKKKSLYRVKYILRLSCARTLAR
KHKSTVRAFLKRFGSQLLEEFFTEEEQVFSLTFPRRIWYLDIVCINDLANH-MIID-TTEVQ-
AINSFSRLES-LKEVYGIIWVLIPIFTPVLGITIGVLVIVWLEREISAGIQQRIGPEYAGPLGILQ
ALADGTKLLFKENLLPSRGDTRLFSIGPSIAVISILLSYLVIPFGYRLVLADLSIGVFLWIAISS

IAPVGLLMSGYGSNNKYSFLGGLRAAAQSISYEIPLTLCVLSISLLSNSSSTVDIVEAQSKY
GFWGWNLWRQPIGFLVFLISSLAECERLPFDLPEAEEELVAGYQTEYSGIKFGLFYVASYLN
LLVSSLFVTVLYLGGWNLSIPYIPVPELFDITKRGRVFGTIIGIFITLAKTYLFLFIPIATRWTL
PRLRMDQLLNLGWKFLLPISLGNLLLTTSSQLLSLMIWHVQNENFILDSTRIFMKAFHLLL
FDGSLIFPECILIFGLILLLMIDSTSDQKDIPWLYFISSTSLVMSITALLFRWREEPMISFSGNF
QTNNFNEIFQFLILLCSTLCIPLSVEYIECTEMAITEFLLFILTATIGGMFLCGANDLITIFVAPE
CFSLCSYLLSGYTKKDVRSNEATMKYLLMGGASSSILVHGFSWLYGSSGGEIELQEIVNGL
INTQMYNSPGISIALIFITVGIGFKLSPAPSHQWTPDVYEGSPTPVVAFLSVTSKVAASASAT
RIFDIPFYFSSNEWHLLLEILAILSMILGNLIAITQTSMKRMLAYSSIGQIGYVIIGIIVGDSND
GYASMITYMLFYISMNLGTFACIVLFGLRTGTENIRDYAGLYTKDPFLALSLALCLLSLGGL
PPLAGFFGKLYLFWCGWQAGLYLLVLIGLLTSVVSIYYYLKIIKLLMTGRNQEITPHVRNY
RRSPLRSNNSIELSMIVCVIASTIPGISMNPIIAIAQDTLFMFLLYEYDIFWAFLIISSLIPILVFFI
SGFLAPSSKGPEKLSSYESGIEPIGDAWLQFRIRYYMFALVFVVFDVETVFLYPWAMSFDVL
GVSVFVEALIFVLILIVGLVYAWRKGALEWSTNKFPWLTIIVVLPIFAGSLIFFLPHKGNRVI
RWYTICICMLELLLTTYAFCYHFQLDDPLIQLVEDYKWINFFDFRWKLGIDGLSLGPVLLT
GFITTLATLAAWPVTRDSRLFHFLMLAMYSGQIGSFSSRDLLLFFIMWELELIPVYLLLSM
WGGKKRLYSATKFILYTAGGSIFLLMGVLGVGLYGSNEPTLNFETSVNQSYPVALEIIFYIG
FLIAFAVKLPILPLHTWLPDTHGEAHYSTCMLLAGILLKMGAYGLIRINMELLPHAHSIFSP
WLMVVGTIQIIYAASTSLGQRNLKKRIAYSSVSHMGFILIGIASITDTGLNGAILQIISHGFIG
AALFFLAGTSYDRIRLVYLDEMGGVAIPMPKIFTMFSSFSMASLALPGMSGFVAEVLVFLGI
ITSQKYLLMPKIAITFVMAIGMILTPIYLLSMSRQIFYGYKLFNTPNSYVFDSGPRELFVSISI
FIPVIGIGMYPDFVLSLSVDKVEGILSNYFYRMMLEHVLVLSAYLFSVGLYGLITSRNMVR
ALMCLELILNAVNLNFVTFSDFFDSRQLKGAIFSIFVIAIAAAEAAIGLAIVSSIYRNRKSTRI
NQSNLLNKMEQTYQYAWIIPFLPLPVPMLIGLGLLLFPTATKSLRRMWAFQSVLLLSIVMIF
SLNLSIQQINSSSVYQYVWSWIINNDFSLEFGYLIDPLTSIMSILITTVGIMVLIYSDNYMSH
DHGYLRFFAYMSFFSTSMLGLVTSSNLIQIYIFWELVGMCSYLLIGFWFTRPVAAKACQKA
FVTNRVGDFGLLLGILGFYWITGSFEFRDLFQIFNNNEVNSVFVTLCAVLLFAGAIAKSAQF
PLHVWLPDAMEGPTPISALIHAATMVAAGIFLVARLMPLFIVIPHIMNFISFIGIITVFFGATL
ALAQKDIKRGLAYSTMSQLGYMMLALGMGSYRSALFHLITHAYSKALLFLGSGSVIHSM
ETLVGYCPKKSQNMVLMGGLKKHVPITKNSFLLGTLSLCGIPPLACFWSKDEILNDSWLY
SPIFAIIAWSTAGFTAFYMCRIYLLTFEGHLNVHFQNYSGKRNTPLYLISLWGKEGSKISNKN
LVTLLKMNFFSFSNTKTSLYPYESDNTMLFPILILILFTLFVGFLGIPF-VDILSKWLTPSINLL
HQSSN-SIDWYEFCKDAVFSVSIACFGIYIAFFLYKPVYSSFQNLDLINSVVIFSDKIKNAIYD
WSYNRGYIDAFYGTFFTAGVRKLAEFTHFFDRRIIDGIPNGVGFMSFFVAEVIKSVGGG-RI
SSYLFFYFSYVSIFLLIYYMDLPGLIHDFLLVFLGLGLILGGLGVVLLANPIYSAFSLGLVFV
CISLFYILSNSHFVAAAQLLIYVGAINVLIIFAVMFINGSEYSKDFHLWTVGDGVTSVVCTSL
FVSLITTIPDTSWYGIIWTTKANQIIEQDLISNSQQIGIHLSTDFFIPFEFISIILLVALIGAIAVAR
QMTVPSTRKDLMIVNMGPHHPSMHGVLRLIVTLDGEDVIDCEPILGYLHRGMEKIAENRT
IIQYLPYVTRWDYLATMFTEAITVNAPEQLGNIQVPKRASYIRVIMLELSRIASHLLWLGPF
MADIGAQTPFFYIFRERELIYDLFEAATGMRMMHNFFRIGGVAADLPHGWIDKCLDFCDY
FLTGIAEYQKLITRNPIFLERVEGVGIIGGEEAINWGLSGPMLRASGIEWDLRKVDHYECYD
EFDWEVQWQKEGDSLARYLVRISEMTESIKIIQQALEGIPGGPYENLEIRRFDRVKDTVWN
EFDYRFISKKPSPTFELSKQELYARVEAPKGELGIFLIGDKGVFPWRYKIRPPGFINLQILPQL
VKRMKLADIMTILGSIDIIMGEVDRMFPMVTEFMNYGQQTVRAARYIGQGFMITLSHANR

LPVTIQYPYEKLITSERFRGRIHFEFDKCIACEVCVRVCPIDLPVVDWKLETDIRKKRLLNY
SIDFGICIFCGNCVEYCPTNCLSMTEEYELSTYDRHELNYNQIALGRLPMSIIDDYTIRTILN
L----------------MQGNLSAWLVKHALIHRSLGFDYQGIETLQIKPGDWHSIAVILYVYGYNYL
RSQCAYDVAPGGLLASVYHLTRIEYGVDQPEEVCIKVFAPRKDPRIPSVFWVWKSVDFQE
RESYDMLGISYDNHPRLKRILMPESWIGWPLRKDYIAPNFYEIQDAH--------------------------
--------------------------------MNSIEFPLFHRTTQNSVISTTLNDLSNWSRLSSLWPLLYGTSCCF
IEFASLIGSRFDFDRYGLVPRSSPRQADLILTAGTVTMKMAPSLVRLYEQMPEPKYVIAMGA
CTITGGMFSTDSYSTVRGVDKLIPVDVYLPGCPPKPEAIIDAITKLRKKISREIYPDRIMSQR
ENRCFTTNHKFQVGHSIHTGNYDQGFLYQPPSTSEIPPETFFKYKSSVSSHELVNMQTRNTF
SWIKEQITRSISASLMIYIITRTSISNAYPIFAQQGYENPREATGRIVCANCHLANKPVDIEVP
QAVLPDTVFEAVVRIPYDTQLKQVLANGKKGGLNVGAVLILPEGFELAPPDRISPEIKEKM
GNLSFQSYRPNQKNILVIGPVPGQKYSEITFPILSPDPATKKDIHFLKYPIYVGGNRGRGQIY
PDGSKSNNTVYNATASGIVSKILRKEKGGYEITIADASDGRQVVDIIPPGPELLVSEGESIKL
DQPLTSNPNVGGFGQGDAEIVLQDPLRVQGLLFFFAAVILAQIFLVLKKKQFEKVQLSEMN
FMSKVYDWFEERLEIQAIADDITSKYVPPHVNIFYCLGGITLTCFLVQVATGFAMTFYYRPT
VTDAFASVQYIMTEANFGWLIRSVHRWSASMMVLMMILHVFRVYLTGGFKKPRELTWVT
GVVLAVLTASFGVTGYSLPWDQIGYWAVKIVTGVPDAIPVIGSPLVELLRGSASVGQSTLT
RFYSLHTFVLPLLTAVFMLMHFPMIRKQGISGPLMGVTKKPDLNDPVLRAKLAKGMGHN
YYGEPAWPNDLLYIFPVVILGTIACNVGLAVLEPSMIGEPADPFATPLEILPEWYFFPVFQILR
TVPNKLLGVLLMVSVPAGLLTVPFLENVNKFQNPFRRPVATTVFLIGTAVALWLGIGATLPI
DKSLTLGLFMIEVFLFGIVLGLIPITLAGLFVTAYLQYRRGDQLDLMPIITSYFGFLLTALTIA
SALFIGLSKIRLIMDIVSLAWAALMVVFTFSLSLVVWGRSGLMIIRSPEPEVKILVDRDHIKT
SFEEWARPGHFSRTIAKGPETTTWIWNLHADAHDFDSHTSDLEEISRKVFSAHFGQLSIIFL
WLSGMYFHGARFSNYEAWLSDPTHIGPSAQVVWPIVGQEILNGDVGGGFRGIQITSGFFQ
LWRASGITSELQLYCTAIGALIFAALMLFAGWFHYHKAAPKLAWFQDVESMLNHHLAGL
LGLGSLSWAGHQVHVSLPINQFLNAGVDPKEIPLPHEFILNRDLLAQLYPSFAEGATPFFTL
NWSKYADFLTFRGGLDPVTGGLWLTDTAHHHLAIAILFLIAGHMYRTNWGIGHGLKDILE
AHKGPFTGQGHKGLYEILTTSWHAQLSLNLAMLGSLTIVVAHHMYAMPPYPYLATDYGT
QLSLFTHHMWIGGFLIVGAAAHAAIFMVRDYDPTTRYNDLLDRVLRHRDAIISHLNWACI
FLGFHSFGLYIHNDTMSALGRPQDMFSDTAIQLQPVFAQWIQNTHALAPGATAPGATASTS
LTWGGGDLVAVGGKVALLPIPLGTADFLVHHIHAFTIHVTVLILLKGVLFARSSRLIPDKAN
LGFRFPCDGPGRGGTCQVSAWDHVFLGLFWMYNSISVVIFHFSWKMQSDVWGSISDQGV
VTHITGGNFAQSSITINGWLRDFLWAQASQVIQSYGSSLSAYGLFFLGAHFVWAFSLMFLF
SGRGYWQELIESIVWAHNKLKVAPATQPRALSIVQGRAVGVTHYLLGGIATTWAFFLARIIA
VGMALRFPRFSQGLAQDPTTRRIWFGIATAHDFESHDDITEERLYQNIFASHFGQLAIIFLW
TSGNLFHVAWQGNFESWVQDPLHVRPIAHAIWDPHFGQPAVEAFTRGGAPGPVNIAYSGV
YQWWYTIGLRTNEDLYTGALFLLFISAISLIAGWLHLQPKWKPSVSWFKNAESRLNHHLS
GLFGVSSLAWTGHLVHVAIPASRGESVRWNNFLDVLPHPQGLGPLFTGQWNLYAQNPDSS
SHLFGTSQGAGTAILTLLGGFHPQTQSLWLTDMAHHHLAIAFIFLIAGHMYRTNFGIGHSM
KDLLDAHIPPGGRLGRGHKGLYDTINNSIHFQLGLALASLGVITSLVAQHMYSLPAYAFIAQ
DFTTQAALYTHHQYIAGFIMTGAFAHGAIFFIRDYNPEQNEDNVLARMLEHKEAIISHLSW
ASLFLGFHTLGLYVHNDVMLAFGTPEKQILIEPIFAQWIQSAHGKTSYGFDILLSSTSGPAF
NAGRSIWLPGWLNAVNE--NSNSLFLTIGPGDFLVHHAIALGLHTTTLILVKGALDARGSKL
MPDKKDFGYSFPCDGPGRGGTCDISAWDAFYLAVFWMLNTIGWVTFYWHWKHITLWQG

NVSQFNESSTYLMGWLRDYLWLNSSQLINGYNPFGMNSLSVWAWMFLFGHLVWATGFM
FLISWRGYWQELIETLAWAHERTPLANLIRWRDKPVALSIVQARLVGLAHFSVGYIFTYAA
FLIASTSGKFGMSHSVKIYDTCIGCTQCVRACPTDVLEMIPWDGCKAKQIASAPRTEDCVG
CKRCESACPTDFLSVRVYLWHETTRSMGIAYMTTLNFPSVLVPLVGLVFPAIAMASLFLHV
QKNKIVMRDLKTYLSVAPVLSTLWFGALAGLLIEINRFFPDALTFPFF--MTAILERRESESL
WGRFCNWITSTENRLYIGWFGVLMIPTLLTATSVFIIAFIAAPPVDIDGIREPVSGSLLYGNNI
ISGAIIPTSAAIGLHFYPIWEAASVDEWLYNGGPYELIVLHFLLGVACYMGREWELSFRLG
MRPWIAVAYSAPVAAATAVFLIYPIGQGSFSDGMPLGISGTFNFMIVFQAEHNILMHPFHML
GVAGVFGGSLFSAMHGSLVTSSLIRETTENESANEGYRFGQEEETYNIVAAHGYFGRLIFQ
YASFNNSRSLHFFLAAWPVVGIWFTALGISTMAFNLNGFNFNQSVVDSQGRVINTWADIIN
RANLGMEVMHERNAHNFPLDLAAIEAPSTNGMGLPWYRVHTVVLNDPGRLLSVHIMHT
ALVAGWAGSMALYELAVFDPSDPVLDPMWRQGMFVIPFMTRLGITNSWGGWSIT-GGTIT
NPGIWSYEGVAGAHIVFSGLCFLAAIWHWVYWDLEIFCDERTGKPSLDLPKIFGIHLFLAG
LACFGFGAFHVTGLYGPGIWVSDPYGLTGKVQSVNPSWGVEGFDPFVPGGIASHHIAAGT
LGILAGLFHLSVRPPQRLYKGLRMGNIETVLSSSIAAVFFAAFVVAGTMWYGSATTPIELFG
PTRYQWDQGYFQQEIYRRVSAGLAENQSLSEVWSKIPEKLAFYDYIGNNPAKGGLFRAGS
MDNGDGIAVGWLGHPIFRDKEGRELFVRRMPTFFETFPVVLVDGDGIVRADVPFRRAESK
YSVEQVGVTVEFYGGELNGVSYSDPVTVKKYARRAQLGEIFELDRATLKSDGVFRSSPRG
WFTFGHASFALLFFFGHIWHGSRTLFRDVFAGIDPDLDAQVEFGAFQKLGDPTTRRQAIM
KTLYSLRRFYPVETLFNGTLALAGRDQETTGFAWWAGNARLINLSGKLLGAHVAHAGLIV
FWAGAMNLFEVAHFVPEKPMYEQGLILLPHLATLGWGVGPGGEVIDTFPYFVSGVLHLIS
SAVLGFGGIYHALLGPETLEESFPFFGYVWKDRNKMTTILGIHLILLGLGAFLLVFKALYFG
GVYDTWAPGGGDVRKITNLTLSPSIIFGYLLKSPFGGEGWIVSVDDLEDIIGGHVWLGSICI
LGGIWHILTKPFAWARRALVWSGEAYLSYSLAAISVFGFIACCFVWFNNTAYPSEFYGPTG
PEASQAQAFTFLVRDQRLGANVGSAQGPTGLGKYLMRSPTGEVIFGGETMRFWDLRAPW
LEPLRGPNGLDLSRLKKDIQPWQERRSAEYMTHAPLGSLNSVGGVATEINAVNYVSPRSW
LATSHFVLGFFFFVGHLWHAGRARAAAAGFEKGIDRDFEPVLSMTPLNMTIALGKFTKDE
NDLFDIMDDWLRRDRFVFVGWSGLLLFPCAYFAVGGWFTGTTFVTSWYTHGLASSYLEG
CNFLTAAVSTPANSLAHSLLLLWGPEAQGDFTRWCQLGGLWTFVALHGAFGLIGFMLRQF
ELARSVQLRPYNAIAFSGPIAVFVSVFLIYPLGQSGWFFAPSFGVAAIFRFILFFQGFHNWTL
NPFHMMGVAGVLGAALLCAIHGATVENTLFEDGDGANTFRAFNPTQAEETYSMVTANRF
WSQIFGVAFSNKRWLHFFMLFVPVTGLWMSALGVVGLALNLRAYDFVSQEIRAAEDPEFE
TFYTKNILLNEGIRAWMAAQDQPHENLIFPEEVLPRGNALMSGSTGERSFADIITSIRYWVI
HSITIPSLFIAGWLFVSTGLAYDVFGSPRPNEYFTENRQGIPLITGRFDSLEQLDEFSRSFMTI
DRTYPIFTVRWLAVHGLAVPTVSFLGSISAMQFIQRMATQTVENGSKSG-PRRTTVGNLLKP
LNSEYGKVAPGWGTTPLMGVAMALFAVFLSIILEIYNSSVLLDGISMNMLTLKLFVYTVVI
FFVSLFIFGFLSNDPGRNPGREEMADTTGRIPLWIIGTVAGILVIGLVGVFFYGSYSGLGSSL
MLNIFSLIC--LNSALYPSSLFFAKLPEAYAFLNPIVDVMPVIPLFFFLLAFVWQAAVSFRTTQ
SNPNEQNVELNRTSLYWGLLLIFVLAVLFSNYFFNMEVNILAFIATALFILVPTAFLLIIYVKT
VSQNDMETATLVAIFISGLLVSFTGYALYTAFGQPSQQLRDPFEEHGDMEALVYTFLLVSTL
GIIFFAIFFREPPTISTKK--MTLAFQLAVFALIATSSILLIGVPVVFASPDGWSSNKNVVFSGTS
LWIGLVFLVGILNSLIS-------MSPQTETKASVGFKAGVKDYKLTYYTPEYETKDTDILAAFR
VTPQPGVPPEEAGAAVAAESSTGTWTTVWTDGLTSLDRYKGRCYGLEPVPGEDNQFIAYV
AYPLDLFEEGSVTNMFTSIVGNVFGFKALRALRLEDLRIPTAYVKTFDGPPHGIQVERDKL

NKYGRPLLGCTIKPKLGLSAKNYGRACYECLRGGLDFTKDDENVNSQPFMRWRDRFLFC
AEAIYKAQAETGEIKGHYLNATAGNCEDMMKRAVFARELGVPIVMHDYLTGGFTANTSLS
QYCRDNGLLLHIHRAMHAVIDRQKNHGMHFRVLAKALRMSGGDHIHSGTVVGKLEGER
EITLGFVDLLRDDFIEKDRSRGIYFTQDWVSLPGVLPVASGGIHVWHMPALTEIFGDDSVL
QFGGGTLGHPWGNAPGAVANRVALEACVQARNEGRDLATEGNEIIREATKWSPELAAAC
EVWKEIKFEFQAMDTLDTMAINLYKTSTPSTRNGTVDSQVKSNPRNNLIYGQHHCGKGR
NARGIITAGHRGGGHKRLYRKIDFRRNEKDIYGRIVTIEYDPNRNAYICLIHYRDGEKRYIL
HPRGAIIGDTIVSGTEVPIKMGNALPLTDMPLGTAIHNIEITLGKGGQLARAAGAVAKLIAK
EGKSATLKLPSGEVRLISKNCSATVGQVGNVGVNQKNLGRAGSKRWLGKRPVVRGVVM
NPVDHPHGGGEGRAPIGRKKPTTPWGYPALGKRSRKRNKYSDNLILRRRSKMIQPQTHLN
VADNSGARELMCIRIIGAS-NRRYAHIGDVIVAVIKDAVPNMPLERSEVVRAVIVRTCKELK
RDNGMIIRYDDNAAVVIDQEGNPKGTRVFGAIARELRQFNFTKIVSLAPEVLNYNPKRTRF
RKQHRGRMKGISYRGNTICFGKYALQALEPAWITSRQIEAGRRAMTRNARRGGKIWVRIF
PDKPVTVRPAETRMGSGKGSPEYWVAVVKPGRILYEMGGVTENIARRAISIAASKMPIRAQ
FIISGMTRIRRGYIARRRRTKIRLFASSFRGAHSRLTRTITQQKIRALVSAHRDRDRQKINFR
RLWITRINAAIRE-RGVCYSYSRFINDLYKRQLLLNRKILAQIAISNRNCLYMISNEIIKEVSW
KESTG--MLNEVYALGQHISMSAHKARRVIDQIRGRSYEETLMILELMPYRACYPIFKLVYS
AAANASSNMGSNEANLVISKAEVNKGTIMKRLKPRARGRSFAIQKPTCHITIVMKDISLDG
GTWDKKMDGIRYAVFTDKSIRLLGKN-QYTSNVESGSTRTEIKHWVELFFGVKVIAMNSH
RLRGKARRMGPIMGQTMHYRRMIITLQPGYSIPPLRKKRTMAVPKKRTSISKKRIRKNIWK
RKGYGAALKALSLGKSLSTGSSKSFFVRQTNKMAKGKDARIPVLLECTACVRNG--VNKE
STGISRYITQKNRHNTPNRLELLKFCPYCYKHTIHGEIKKMKIRASVRKICEKCRLIRRRGRI
IVICSNPRHKQRQGMVREKITVSTRTLQWKCVESAADSKRLLYGRFILSPLMKGQADTIGI
AMRRALLGEIEGTCITRAKSE--KISHEYATIMGIQESVHEILMNLKEIVLRSNLYGTCEASIC
VRGPGYVTAQDIILPPYVEIVDNTQHIASLTEPIELVIGLQIEKNRGYLIKAPN-TFDGSY--PI
DPVFMPVRNANHSIHSYENGN--KEILFLEIWTNGSLTPKEALHEASRNLIDLLIPFLHTKEE-
-NLSLEDNQ-HIVPLPPFTFYDKLA-KLTKNKK-KMALKSIFIDQSELPPRIYNCLKRSNIYTL
LDLLNNSQEDLMKMEHFRIEDVKQILGILEKN--FVIDLPKNK---------MSTIPGFNQIQFEGF
CRFIDQGLAEELSKFPKIEDTNQEIDFEFFLERYQLVEPLIKERDAVYESLTYSSELYVSARLI
WKNRRYIQEQTILIGKIPIMTSLGAFIVNGIYRIVINQILQSPGIYYQSELNDNGISVYTGTIIS
DWGGRLELEIDRKARIWVRVSRQQKLSILVLLSAMGLNIREILENVCYPELFLSFLNDK--K
KIGSKENAILEFYQQFACVEGDPVFSESLSKDLQKKFFQQRCELGGIGRRNMNRRLNLDIP
QNNTFLLPRDILAAADRLIRIKFGMGTLDD--MNHLQNKRIRSVADLLQEQFGLALVRLEN
MARGNIYAALK-HNWT-PTPQNLVNSTP----LTDTYKVFFRLHPLSQVLDRTNPLTQIVHGR
KLSYLGPGGLTARTATFPIRDIHPSHYGRICPIDTSEGINVGLIGSLAIHARIGRWG-SLESPFY
---QISERSKG--AQMLYLSPGRDEYYMVAAGNSLALNQGIQEEQVVPARYRQEFLTIAWEQ
VQLRSIFAFQYFSIGASLIPFIEHNDANRALMSSNMQRQAVPLSQSEKCIVGTGLEGQAALD
SGALAIAEHEGKIFYTDTDKILLSGNGDTLRIPLVMYQRSNKNTCMHQKPQVRRGKCIKK
GQILAYGAATVGGELALGKNVLVAYMPWEGYNFEDAVLISERLVYEDIYTSFHIRKYEIQI
N---QG-PERVTNEIPHLEVHLLRNLDKNGIVMLGSWVETGDILVGKLTPQMVKE-SSYAPED
RLLRTILGMRVYTSKETCLKLPIGGRGRVIDVRWVQSSKTDETEKTESIRVYILQKREIKVG
DKVAGRHGNKGIISKILPRQDMPYLQDGRPVDMVFNPLGVPSRMNVGQIFESSLGLAGDL
LDRHYRIAPFDERYEQEASRKLVFSELYEASKQTANPWIFEPESPGKSRIFDGRTGDPFEQP
VIIGKPYILKLIHQVDDKIHGRSSGRYSRLTQQPLKGRAKKGGQRVGEMEVWALEGFGVA

YILQEMLTYKSDHIRARQEVLGTIIFGGRIPTPEDAPESFRLFVRELRSLALELNHFLVSEKT
FQLNRKEA-------MIDRYTHQQLRIGLVSPQQISTWSKKILPNGEIVGEVTKPYTFHYKTNKP
EKDGLFCERIFGPIKSGICACGNYRVIGDEKEDPQFCEQCGVEFVDSRIRRYQMGYIKLAYP
VMHVWYLKRLPSYIVNLLDKPLNELEDLVYCG--FYFARP-IDKKPTFLRLRGLLEYEIQPW
KYRIPIFFTTRSFDTFRNREMSTGGGSIRQQLANLDLRMIIDYSLVEWKELEEE-ESTGNEW
EDRKVGRRKDFLLRRMELAKHFIRTNIEPKWMVLRLLPVLPPELRPIYHIDEDKLVTSDIN
EIYRRIIYRNNTLTDLLTTSIATPEELIISQEKLLQEAVDALLDNGICGQPMRDDHNRIYKSLS
DVIEGKEGRVRETLLGKRVDYSGRSVIVVGPSLSLHRCGLPREIAIELFQAFVIRDLIRKHL
ASNIGVAKSQIRKKKPIVWEILQEILDDHPVLLNRAPTLHRLGIQAFLPVLVEGRAICLHPLV
CKGFNADFDGDQMAVHVPLSLEAQAEARLLMFSHMNLLSPTIGDPISAPTQDMLSGLYVL
TSGNRRGICVNRYNPCNRRN---YQNED---NNYKYTKKKEPFFCNAYDAIGAYRQKRINLG
SPLWLRWRLDQR-VIAA--REAPIEIHYESLGTYYEIYGHYLIVRSIKKEILYIYIRTTLGHISLY
REIEEAIQGFWQYNSMLPARIRVSPGMEVLMAERPTQVFHNKVIDGTAMKRLISRFIDHYG
IGYTSHILDQVKTLGFRQATAASISLGIDDLLTIPSKRWLVQDAEQQSVLLEKHHHYGNVH
AVEKLRQSIEIWYATSEYLRQEMTPNFRMTDPFNPVHIMSFSGARGNASQVHQLVGMRGL
MSDPQGQMIDLPIQSNLREGLSLTEYIISCYGARKGVVDTAIRTSDAGYLTRRLVEVVQHIV
VRRTDCGTVRGISVSPRNGMMA-DRIFIQTLIGRVLADDIYIGSRCIATRNQDIGVGLVNRFI
T------FRAQPISIRTPFTCRSTSWICQLCYGRSPAHDDLVELGEAVGIIAGQSIGEPGTQLTLRT
FHTGGVFTGGTAEHVRAPFNGKIKFNEDLVHPTRTRHGHPAFLCSRDLYVTIESEDIIHNVC
IPPKSFLLVQNDQYVESEQVIAEIRARTST-LNLKEKVRKHIYSDSEGE-MHWNTDVYHAPE
FTYGNIHLLPKTSHLWILLGEPWR--SSLGPCSIHKDQDQMNAY--SLSVKRRYISNPS--VTN
NQ-VRHKFFSSYFSGKNGDRIPDY-SELNRMTCTGRCNLRYP-GILDGNS--DLLAKRRRNRV
IIPLDSIQEGENQLIPSSGISIEIP--RNGILRRNSILAYFDDPRYIRKSSGLTKYETRELNSIVNE
EN--LVEYRG-VKVFWPKYKEVNPFFFIPVEVHILAESSSIMVRHNSIIGVDTQITLNRRSRVG
GLVRVKKKAEKIKLIIFSGDIHFPGKTNKAFR----LIPPGG--GKQNSKEYKK--LKNWLYIQR-
-MKLSRYEKKYFVLVQPVVPYKKTDGINLG-LFPADLLQESDNLQLRVVNYILYYDP--ILEI
WDTSIQLVRTCLVLNWDQDKK------IEKACASFVEIRTNGLIRDFLRIDLAKSPISYTGKRND
LPGSGLISE-NGSDRANGNPFSSIYSYS---KATIQESLNPNQGTIHTLLNR---NKESQSLIILSS
SNCSRIGP--FNDVKYPNV---IKDS-PLIPIRNSLGPLGT--GFPIYHFDL--FSHL--ITHNQIL-VT
NYLQLDNLK---QIFQILK---YYLLDEN--GKIYNPYSCSNIILNPFKLNWYFLHYNYCEETS-T
IVSLGQFLCENVCIAKKGPH-LK--SGQVLILQ-VDSVVIRSAKPYLATPGATVHGHYGEILY
EGDTLVTFIYEKSRSGDITQGLPKVEQVLEVRSIDSISMNLEKRIEGWNKCITRILGIPWAFFI
GAKLTIVQSRISLVNKVQKVYRSQGVQIHNRHIEIIVRQITSKVLVSE---DEMSNVFSPGELI
GLLRAERMGRALEEAICYQAVLLGITRASMNTQSFISEASFQETARVLAKAALLGRIDWLK
GLKENVVLGGMIPVGSGFKTP-SSEPNNIPNNIA--FELKKKNLLEGEMK--DILFYHRK-------
-----------------------MTRRYWNINLEEMMEAGVHFGHGTRKWNPKMAPYISAKRKGIHITNL
TRTARFLSEACDLVFDAASRGKQFLIVGTKNKEADSVAWAAIRARCHYVNKKWLGGMLT
NWSTTETRLHKFRDLRTEQKTGGLSRLPKRDAAMLKRQLSHLQTYLGGIKYMTGLPDIVI
IVDQHEEYTALQECITLGIPTICLIDTNCDPDLADISIPANDDAISSIRLILNKLVFAICEGRS-G
YIRNPMGQKINPIGFRLGTTQGHHSLWFAQPKNYSEGLQEDQKIRNYIKNYVQKNMKTSS
G-VEGIARIEIQKRIDLIQIIIYMGFPKILIESRPRGI----EELQMNLQKEFNSVNRKLNIAITRIE
KPYGNPNILAEFIAGQLKNRVSFRKAMKKAIELTEQADTKGIQVQIAGRIDGKEIARVEWI
REGRVPLQTIRAKIDYCSYTVRTIYGVLGIKIWIFIEGEMSRYRGPRFKKIRRLGALPGLTNK
RPRAGSDLRNQSRSGK-KSQYRIRLEEKQKLRFHYGLTERQLLKYVRIAGKAKGSTGQVL

LQLLEMRLDNILFRLGMAPTIPGARQLVNHRHILVNGRIVDIPSYRCKPRDTIAARDEQKSR
ALIQNSLDSSPP-EELPNHLTLQPFQ--YKGLVNQIIDSKWVGLKINELLVVEYYSRQTMSRR
GTAEEKTAKSDPIYRNRLVNMLVNRILKHGKKSLAYQIIYRAVKKIQQKTETNPLSVLRQAI
HGVTPGIAVKARRVGGSTHQVPIEIGSTQGKALAIRWLLAASRKRPGRNMAFKLSSELVD
AAKGSGDAIRKREETHRMAEANRAFAHFRMSSDTIADIITSIRNADMYRKSVVRVASTNIS
QSIVKILLREGFIENVRKHRENNKDFLVLTLRHRR--NRKRPY---RNLLNLKRISRPGLRIYSN
YQRIPRILGGMGVVILSTSRGIMTDREARLERIGGEILCYIWMAKAIPKKGSR--GRISSRKSI
RKIPKGVIHIQASFNNTIVTVTDVRGRVVSWSSAGTCGFQGTRRGTPFAAQTAAANAIRAV
VDQGMQRAEVMIKGPGLGRDAALRAIRRSGILLTFVRDVTPMPHNGCRPPKKRRVHPTCS
STLRASPKRGGFCDISNWLS-CISNK-LFNRWHTITPKKPNSALRKVARVRLTSGFEITAYIPG
IGHNSQEHSVVLVRGGRVKDLPGVRYHIVRGTLDAVGVKDRQQGRS--SAL---MAKKSLIQ
REKKRQKLEQKYHLIRRSSKEEIS---KVRSLSDKWEIYGKLQSPPRNSAPTRLHRRCFSTGR
PRANYRDFGLSGHILREMVHACLLPGATRSSWMVKNSFISIILKEQEENKENRGSVEFQVV
SFTNKIRRLTSHLELHKKDYLSQRGLRKILGKRQRLLAYLSKKNRVRYKELIGQLDIRERK
TR--MVKLRLKRCGRKQ----RIVAIDVRSRREGRDLRKVGFYDPIKNQTYLNVPAILYFLEK
GAQPTGTVQDILKKAEVFKELGPNQTKFN-MDKSKRTFLKSKRSFRKRLPPIQSGDRIDYK
NMSLISRFISEQGKILSRRVNRLTLKQQRLITIAIKQARILSLLPFLNNEK--QFERTESTTRTPS
LRARKRVTRSLKKNPFVANHLLKKINKLNTKEEKEIIITWSRASTIIPIMVGHTIAIHNGKEH
LPIYITDRMVGHKLGEFAPTLNFRGHAKSDNRSRRMILKSFLLGNLVSLCMKIINSVVVVG
LYYGFLTTFSIGPSYLFLLRAHVMEEGEEGAEKKVSATTGFITGQLIMFISIYYAPLHLALGR
PHTITVLALPYLLFHFFCNSKKNFLDYGSTTRNSMRNLSIQCVFLNNLIFQLLNHFILPSSM
LARLVNIFMFRCNSKMLFVTSSFVGWIIGHILFMKWVGLLLVWIRQNRSIR--IQANKYLVL
ELKNSMSGIFSIFLLVTCVHYLGRIPSPIFSTKLNMNEEEKPILTFFFDYNRWNRPLRYIRKK
KLKGSVRKEASQYFFYTCQSDGKQRISFTYPPSLSTFGEMIARRISLSTLEKISPDELYTEW
VFTNKEKKNNLNNEFINRIEALETVFLSLNILEAKTRLSNTKNKNNCLVKMYDPFLN-MYR
GRMKKLFSSTETAELNKIHDILSPENEQKNPKRE---INEIGKKPPRWSYKLIELEQYFKKQL
RTRKFKHIAFLTRGFISYLEEPDFRRAVIKDSMRVQRRKMVIWGPSQGNPHSPLFLEK-ME-
DFPFP---ISDLMKLFFN-IKKNNQED----WENIPYAQKSRSVLLLAQSIFRRYIKLPLLIIAKNI
GRILLRQSPEWYEDFQDWNREIYLKCNYNGLQFSKTEFPKNWLIEGFQIKILYPFHLKPWH
RSKQQDDFDSCFLTVLGMETEYPFGPPRKTPSFFEPIFKDID-----------------YKVEIRNFRIRR
ALKKIKKKEAKVFFFVKQILKELLKGKDSIISNKIIHESLSQNRPKGLTNYSQTEEEMKHRI
DRRNTIRNQIEIMKKNKK----KKINKKNNGEALPTIWKILK------RNNIELIVKIFIEKIYIDIFL
CIINMRRIALQLF-----FELSYLSQKYVFLKLSQTQVIHFNKLRSILQYPSFFLKNEIKDFFRQ
GIFDYEVRHKKLPNSGMNPWKNWLRG-YQYDLSQITWSRLVPQKWRNGKNQCQPSQNK
NKWYSYEKDQ--LFDYKKK-NFQKNYRYDRLSYKYIHSDIIYTDRKYLGWKLN-----PFDW
MGMNEKFLILFLFPEFLILYHKYKEKPWFIPSNLLLFN--------LQILIKGDFPLQLRWTGRLN
QKMMNNIQIYGLLVSLINVRKISISYIQRKEMNLDIMRRR---LNLTQLTKGILIMEPARLSVK
NDGQFFMYQIIGISLVHKSKHKNSDLLVPEKILSSRRRRELRILISFVFFWPNYRLEDLACM
NRYWFDTNNGSRFSMLRIYMYPMTGHEFKSWILELREILREIKNSHYFLDSWTQFNSVGS
FIHIFFHQERFIKLFDSRIWSILLS-HNS--QGSTSNRYFT---IKG-VILFGVAVLIYR----INNRNM
VERKNLYLIGLLP-----IPMNSIGPRNDTLEESV-GSSNINRLIVS-LLYLPKGKKIYESSFLNPK
ESTWVLPIT----KKCSMPESNWGSRWWRDWIGKKS--DSSCKISN---ETVAGIEILFKEKDLK
-YLEFVFVYYRD--DPIRKDHGWEFFDRLSLRKRQNRINL--NSGPLFEILVKHWICYLMSAF
REKIPIEVEG-FFKQQG-AGSTIQSNDIEHVSH--LFSRNK---

------------------------------SRLFT-EREKQMINHMLPEEIEEFL--GNPTRSVRSFFSDRW--SELH-
LGSNPTERSTRDQ----KLLKKQQDLS-FL--RRSE----KKEMVNLFKIITYLQN--TVSIHPISLD
SGCDMVP-KDEPD-MDSSNKISFLNKNPFFDL--FHLFHDRNRGGYTL--HHDFESE--DRFQE
MADLFTLSITEP-DLVYHKRFAFSIDSYGLDPKQ--FLNGVFNSRYEWKTTS---LLVLFPIFY--
EENESFYRRIRKNRVR-ISCGNDLEEPKPKIVVFASNNIMEAVNQYRLIRNLIQIQHSTHR--Y
IRNVLNRFFLMN---RSDRNFEYG---IQRDQIRKDTLNHRTLMKYTINQHLSNLKK-SQKRW
FDPL--IFFYRTERSMNRDPDAYRYKWSTGSNNFQEHLEHFVSEQKSR---LQVVFDRLRINP
YSIDWSEVIDKKDLSKPLRFFLSK-----------------------LLLFLSN-SLPFLFVSFGNIPIHRSE-IY
IY-QLKGPND---PQFLESIGLQIVHLKKLKPF--LLD---DHETC-QKSKFLINGGTISPFLFNKIP-
KW-MIDSFHTR--NNRRKSFDNTDSYFSMIFHDQYNWLNPVKSFHRSSLRSSFYKGNQLRF
LNNPHHFCFY--CNKRFPFYV----EKARIN--NYDFTYG-QFLNILFIRNK-IFSLCVGK--KKHA
FWG-RDTISAIESQVSNIFIPKAFPQSG-DETYNLYKS---FHFPSRSNPFVR--RAIYSIADISGT
PLTEGQIVNFERTYCQPLSDMNLSDSEGKNLYQYLN--FNSNMGLIHTPCSEKYLPSEK--RK
K--------RSLCLK--KCVEKGQMYRTFQRDSAYSTLSKWNLFQTYMPWFLTSTGYRYLKFLF
LDTFSDLL--PILSS-SQKFVSIFHDIMHGSDISWRILQKKF------CLPQWNL-ISEISSKCFHN--
LLLSEEMIHRN--NESPLISTHLT--NVREF-LYAILFLLLVAAY-LVCTHLLFVFG--ASSELQAEF
EKVKSLMIPSSMIELRKILDRYPTSEPNSFWLKNLFLVALKQLGDSL--------GGNMLLGGGP
A-----YG-VKSIRSKKKYLNINLIDIIDLI-SIIPNPINRITFSRNTRHLSHTSKEIYSLIRKRKNVN
GDWIDDKIESWVANSDSIDDEKREFLVQFS--TLTT--EKRIDQILLSLT-HSDHL-----SKNDSG
YQMIEQPGAIYLRDLVDIH--KKYLMNYEFNTSSLAERRIFLAHYQTITY--SQTSCGANSFH
FPSHG---KPFSLRLALSLS-RGILVIGSIGTGRSYLVKYLAKNSYLPFITVFLNKFLDNKSQGF
DDI-----DID-LDASDDLDASDDLDASDDLDA------------SDDI---LDMEL-ELLTSMNALTMN
MMPED--EDQLYITLQFELAKAMSPCIIWIPNIHDLDVNESNYFSLGLLVNLLSR---DYE----T
RNILVIASTHIPQKVDPALIAPNKLNTCIKIRRLLIPQQRKHFFTLSYTRGFHLEKKMFHT--N
GFGSITMGSNARDLVALTNEALSISITQKKSIIDTNTIRSALHRQIWDLR---SQVRSVQDHGI
LFYQIGRAVAQNVLLSNCPIDPISIYMKKK--SCNEVDFYLYNWYFELGTSMKKLTILLYLLS
CSAGSVTQDLWSLP--GPDEK-NGITPYGLVENDS-GLVRGLLEVEGALVG--SSR---TCSQFD
KD--RVTLLLRPEPRN-PLDMMQKGSCSILDQRFLYEKD--ESEFEE-GEG--ALDRQQ----IEED
----LFNH--IVWAP--RIWRP---WGFLFDCIERPNELGFP-YWSRSFRGK---RIIYDKEDELQEN
DSEFLQSGTAQYQTRDRSSKEQGLFRISQFIWDPADPLFFLFK-AQPFVSVFSHRELFADEE
MSKGLLTPQTNPPTSLYKRWFI--KKTQEKHFELLINRQRWLR---TNRSLS--NGSFRSN--TLS
ESYQYLSNLFLSN-GTLLDQMTKALLRKRWLFPDEMQIGF--MEQEKDFPFLMSRSR-INGN
FIDKTFSIVANILLRIIPTTSGEKEAFTYYRDG--MSAQSEGNYAEALQNYYEAMRLEIDPYD
RSYILYNIGLIHTSNGEHTKALEYYFRALERNPFLPQAFNNMAVICHY-RGEQAIRQGDSEI
AEAWFDQAAEYWKQAIALTPGNYIEAHNWLKITRRFE--MSCRSEHIWIEPIKGARKTSNF
CWAIILFLGSLGFLLVGTSSYLGRNLISLFPSQEIVFFPQGIVMSFYGIAGLFISSYLWCTISW
NVGSGYDRFDIKDGIVCIFRWGFPGKNRRVFLRFLIKDIQSVRIEVKEGIYARRVLYMDIRG
QGAIPLTRTDEN-FTPREMEQKAAELAYFLRVPIEVF
>Eucalyptus
MEKWWFNSMLYLSKSIDRFGPIEKTSVSEDPVRNDTDKKIHSYSNATHFVGVRDVRNFIS
DDTFLVRDSNRNIYSIYFDITNQIFEIDNDP--SLLSERESSFFSYRTYAYLKNGSKNDDPPCD
RSMYDTKYSWNNHITSCINSYLG--SQICIESSILSSSDNSSDSYISSYIYGRGSSSIRTSRNGI
DFTRRENST-FDLTQKYRHLWVQCENCYGLNYKKLLKSKMNICDQCGYHLKMSSSDRIEL

SIDPGTWDPMDEDMVSLDPIEFHSEEEPYKDRIDSYQRKTGLTDAVQTGTGRLNGIPIAIGV
MDFQFMGGSMGSVVGEKITRLIEYATNQFLPLILVCASGGARMQEGSLSLMQMAKISAAL
YDYQSHKKLFYVSILTSPTTGGVTASFGMLGDIIIAEPNAYIAFAGKRVIEQTLNKIVPEGSQ
AAEYLFHKGLFDPIVPRNPLKGVLSELFKLHAFFPLN-----MVTIRADEISNIIRERIEQYNRE
VKIVNTGTVLQVGDGIARIYGLDEVMAGELVEFEEGTIGIALNLESNNVGVVLMGDGLMI
QEGSSVKATGRIAQIPVSEAYLGRVINALAKPIDGRGEIYASESRLIESPAPGIISRRSVYEPL
QTGLIAIDSMIPIGRGQRELIIGDRQTGKTAVATDTILNQQGQNVICVYVAIGQKASSVAQV
VNTLQERGAMEYTIVVAETADSPATLQYLAPYTGAALAEYFMFRERHTLIIYDDLSKQAQ
AYRQMSLLLRRPPGREAYPGDVFYLHSRLLERAAKLSSLLGEGSMTALPIVETQSGDVSAY
IPTNVISITDGQIFLSADLFNAGIRPAINVGISVSRVGSAAQIKAMKQVAGKLKLELAQFAEL
EAFAQFASDLDKATQNQLARGQRLRELLKQSQAAPLTVEEQIMTIYTGTNGYLDSLEIGQV
RKFLVELRTYVKTNKPQFQEIISSTKTFTEEAEALLKEAIQEQKERFLLQEQGMRINPTTSG
PGVSTLEKKNLGRISQIIGPVLDVAFPPGKMPNIYNALVVKGRDTIGQEINVTCEVQQLLG
NNRVRAVAMSATDGLMRGMEVIDTGAPLSVPVGGATLGRIFNVLGEPVDDLGPVDTRTTS
PIHRSAPAFIQLDTKLSIFETGIKVVDLLAPYRRGGKIGLFGGAGVGKTVLIMELINNIAKA
HGGVSVFGGVGERTREGNDLYMEMKESGVINEQNIAESKVALVYGQMNEPPGARMRVGL
TALTMAEYFRDVNEQDVLLFIDNIFRFVQAGSEVSALLGRMPSAVGYQPTLSTEMGTLQE
RITSTKEGSITSIQAVYVPADDLTDPAPATTFAHLDATTVLSRGLAAKGIYPAVDPLDSTSTM
LQPRIVGEEHYETAQRVKQTLQRYKELQDIIAILGLDELSEEDRLTVARARKIERFLSQPFFV
AEVFTGSPGKYVGLAETIRGFKLILSGELDGLPEQAFYLVGNIDEATAKATNLEMESKLKK
MTLNLCVLTPNRIVWDSEVKEIILSTNTGQIGVLPNHAPIATAVDIGILRIRLNDQWLTMAL
MGGFARIGNNEITILVNDAEKGSDIDPQEAQETLELAEANLRKAEGKRQTIEANLALRRAR
TRVEAINAISMKNVTDSFVSLGHWPSAGSFGFNTDILATNPINLSVVLGVLIFFGKGVLSDL
LDNRKQRILNTIRNSEELRGGAIEQLEKARARLRKVEMEAEQFRVNGYSEIEQEKLNLINS
TYKTLEQLENYKNETIHFEQQRAINQVRQRVFQQALQGALGTLNSCLNNELHLRTISANIG
MFGAMKEITDMNPLISAASVIAAGLAVGLASIGPGVGQGTAAGQAVEGIARQPEAEGKIR
GTLLLSLAFMEALTIYGLVVALALLFANPFVMNVLSCSTNTLKGLYDISGVEVGQHFYWQI
GGFQVHGQVLITSWVVIAILLGSASIAVRNPQTIPNDSQNFFEYVLEFIRDVSKTQIGEEYGP
WVPFIGTMFLFIFVSNWSGALLPWKIIELPHGELAAPTNDINTTVALALLTSVAYFYAGLTK
KGLSYFSKYIQPTPILLPINILEDFTKPLSLSFRLFGNILADELVVVVLVSLVPLVVPIPVMFL
GLFTSGIQALIFATLAAAYIGESMEGHHMIFSTLEHILTHISFSIISIVITIFLISLSV-EIVGLYDS
SEKGMTATFFCITGLLVTRWIYSGHFPLSDLYESLIFLSWSFSVIHMVPYFKKHKNYLSTITA
PSTIFTQGFATSGLLTEMHQSEIVVPALQSQWLMMHVSMMILGYAALLCGSLLSVALLVIT
LRKAIRVFSKKNEIQYMNERSNVLL--SFCSSRNYYRAQLIQQLDQWSYRIISLGFLFLTIGIL
SGAVWANEAWGSYWNWDPKETWAFITWTLFAIYLHTRTNKNWESFNCAIVASIGFLIIWIC
YFGVNLLGIGLHSYGSFNLHMVKKKAFLPLLYLTSIVFLPWWISLSFNKSLEFWITNWWN
TRQSETFLTDIQEKSILEKFIELEELLLLDEMIKEYPETHLQTLRIGIHKETIQLIKMHNEDHI
HMILHLSTNITSFIILSGYSILGNEELAILNSWVQEFLYNLSDTIKAFSILLLTDLCIGFHSPHG
WELMIGYVYKDFGFAHNDQIISGLVSTFPVILDTIFKYWIFRYLNRISPSLVVIYHSMNDMPI
GVPKVPFRSPGEEDASWVDVYNRLYRERLLFLGQEVDSEISNQLIGLMVYLSIENDNKDL
YLFINSPGGWVIPGVAIYDTMQFVQPDVHTICMGLAASMGSFVLVGGEITKRLAFPHARV
MIHQPASSFYEAQTGEFILEAEELLKLRETITRVYVQRTGKPLWVVSEDMERDVFMSATEA
Q A H G I V D L V A I
E???MEEFQG

YFELDRSRQHDFLYPLLFREYIYALAHDHGLAGYDNKSSSIIVKRLITRMYQQNPLIFSAND
SIQNPFFGKNFYSQIISEGFAVIVEIPFSLRLVSSLIAKSHKLRAIHSIFPFLEDKFSHLDYVSD
VLIPYHIHLEILVQTLRYWVNDASSLHLLRFFLHEYWN--SLITNHITIFSKPRLFLFLYNSHIC
EYEYFFLFLRNQSSHLRSTSSGIFFERIYFYVKIEHFVKVFYDFQILWFFKDPFMHYVRYQG
KSILASKDTPLLMKKWKYYLVNLWQYHFYAWFQPGRIDINQLCKYSLYFLGYRSSVRLNP
SVVRSQMLENSFLINN--AMKKFETIVPIIPLIGSLSKANFCDTLGHPISKPTRADSSDSDIIDR
FLRISRNLSHYHSGSSKKKSLYRVKYILRLSCVKTLARKHKKTVRTFLKRLGSEFLEEFLTE
EEVVLSLIFPRRIWYLDITSINDLVNYEMIIDTT-EVQ-DLNSFSRLES-LKEVYGIIGMFLPILT
LVLGITIGVLVIVWLEREISAGIQQRIGPEYAGPLGILQALADGTKLIFKENLFPSRGDTRLFS
IGPSIAVISILLSYSVIPFSYHLVLSDLNIGVFLWIAISSIAPIGLLMSGYGSNNKYSFLSGLRA
AAQSISYEIPLTLCVINIS-LSNSSSTVDIVEAQSKYGFWGWNLWRQPIGFFIFLISSLAECER
LPFDLPEAEEELVAGYQTEYSGIKFGLFYVASYLNLLVSSLFVTVLYLGGWNISIPYIFVPEL
F--EKVGRVFGTTIGIFITLAKTYFFLFISITTRWTLPRLRIDQLLNLGWKFLLPISLGNLLLTT
SFQLLSLMIWHVQNENLILDSTRIFMKAFHLLLFDGSFIFPECILIFGLILLLMIDSTSDQKDI
PWFYFISSTSLVMSITALLFRWREEPMIIFSGNFQTNNFNEIFQFLILLCSTLCIPLSVEYIECT
EMAITEFLLFVLTATLGGMFLCGANDLITIFVAPECFSLCSYLLSGYTKKDVRSNEATMKYL
LMGGASSSILVHGFSWLYGSSGGEIELQEIVNGLINTQMYNSPGILIALLFITVGIGFKLSLA
PSHQWTPDVYEGSPTPVVAFLSVTSKVAASASATRIFDIPFYFSSNEWHLLLEILAILSMILG
NLIAITQTSMKRMLAYSSIGQIGYVIIGIIVGDSNGGYASMITYMLFYISMNLGTFACIVLFG
LRTGTDNIRDYAGLYTKDPFLALSLALCLLSLGGLPPLAGFFGKLHLFWCGWQAGLYFLV
SIGLLTSVISIYYYLKIIKLLMTGRNQEITPHVRNYRRSPLRSNNSIELSMIVCVIASTIPGISM
NPIIAIAQDTLFMFLLYEYDIFWAFLIISSVIPILAF?ISGILAPISKGPEKLSSYESGIEPMGDA
WLQFRIRYYMFALVFVVFDVETVFLYPWAMSFDVLGVSVFIEALIFVLILIVGLVYAWRKG
ALEWSTNYFPWLTIIVLLPIFAGSLIFFLPHRGNKVIRWYTICICSLELLLTTYTFCYHFQLND
PLTQLTEDYKWINFFDFYWRLGIDGLSIGPILLTGFITTLATLAARPVTRDSRLFHFLMLAM
YSGQIGSFSSRDLLLFFIMWELELIPVYLLLAMWGGKKRLYSATKFILYTAGGSIFLLIGVL
GIGLYGSNEPTLNLETLSNQSYPVALEIILYIGFLIAFSVKLPIIPLHTWLPDTHGEAHYSTCM
LLAGILLKMGAYGLVRINMELLPHAHSIFSPWLIIVGAIQIIYAALTSPGQRNLKKRIAYSSV
SHMGFIIIGIGSITDTGLNGAILQIISHGFIGAALFFLAGTSYDRIRLVYLDEMGGMAIHLPKI
FTIFSILSMASLALPGMSGFLAELIVFFGIITSHQYLLMPKILITFVMAIGMILTPIYLLSMLRQ
MFYGYKLFNVPNSYFLDFGPRELFVSISILLPIIAIGIYPDFVLSLSVDKVEAIISNYFYQMIL
EHVLVLSAYLFSIGIYGLITSRNMVRALMCLELILNAVNINFVTFSDFFDSRQLKGDIFSIFVI
AIAAAEAAIGLAIVSSIYRNRKSTRINQSNLLNKMEYTYQYSWIIPFIPLPVPMLIGVGLLLF
PTATKNLRRMWAFPSIFLLSIVMILSVYLSIQQINRSFIYQYVWSWTINNDFSFEFGHLIDPL
TSIMLILITTVGILVLFYSDNYMSHDQGYLRFFAYMSFFNTSMLGLVTSSNLIQIYIFWELVG
MCSYLLIGFWFTRPSAATACQKAFVTNRVGDFGLLLGILGLYWITGSFEFRDLFQILNN-EV
PFLFLTLCYFLLFAGAVAKSAQFPLHVWLPDAMEGPTPISALIHAATMVAAGIFLVARLLPL
FIIIPYIMNFISLIGIITVLLGATLALAQKDIKRGLAYSTMSQLGYMMLALGMGSYRAALFH
LITHAYSKALLFLGSGSIIHSMEAIVGYSPDKSQNMVLMGGLKKHVPITKTAFLVGTLSLC
GIPPLACFWSKDEILNDSWLYSPIFAIIACSTAGLTAFYMFRIYLLTFEGHFNVHFQNYNGQK
SSSCYSISLWGKEVPKTIKN-LLSLLTMNFFSVTKN-TFSYPHESDNTMLFSMVILVLFTLFV
GVVGA-FHLDILSKLLNPSINLLHPNSN-SVDW-EFVTNASFSVSIAFFGIFIASFLYKPIYSSL
QNLNLLNSFSILGDRIRNGIYDWSYNRGYIDAFYTISLTQGIRGLAELIHFLDRRLIDGITNG
FGLTSFFFGEGIKYVGGG-RISSYLLLY----------YSMDLPGPIHDFLLVFLGSGLILGSLGVVL

LTNPIYSAFSLGLVLVCISLFYILSNSHFVAAAQLLIYVGAINILILFAVMFMNGSEYYKDLNL
WTVGDGITSLVCTSILVSLMTTILDTSWYGIIWTTKSNQIIEQDLIGNSQQIGIHLSTDFFLPF
ELISIILLVALIGAIAVARQMNVTTTRKDLMIVNMGPHHPSMHGVLRLILTLDGEDVIDCEPI
LGYLHRGMEKIAENRTVIQYLPYVTRWDYLATMFTEAITVNGPEQLGNIQVPKRASYIRVI
MLELSRIASHLLWLGPFMADIGAQTPFFYIFRERELVYDLFEAATGMRMMHNYFRIGGVA
ADLPYGWIDKCLDFCDYFLTAVSEYQKLITRNPIFLERVEGVGIIGPEEAINWGLSGPMLRA
SGIQWDLRKVDHYECYDELDWEIFWQKEGDSLARYLVRIGEMRESIKIIQQALEGIPGGPY
ENLETRCFARERDPEWNDFEYRFISKKPSPTFELPKQELYVRVEAPKGELGIFLIGDQSGFP
WRWKIRPPGFINLQILPQLVKRMKLADIMTILGSIDIIMGEVDRMFPMVTGFMNYGQQTV
RAARYIGQGFMITLSHANRLPVTIQYPYEKLITSERFRGRIHFEFDKCIACEVCVRVCPIDLP
VVDWKLETGIRKKRLLNYSIDFGICIFCGNCVEYCPTNCLSMTEEYELSTYDRHELNYNQI
ALGRLPMSVINDYTIRTISNS---IIK----------MQGRLSAWLVKHGLVHRSLGFDYQGIETLQI
KPEDWHSIAVILYVYGYNYLRSQCAYDVAPGGLLASVYHLTRIEYGVDQPEEVCIKVFAPR
RNPRIPSVFWIWKSADFQERESYDMLGICYDTHPRLKRILMPETWIGWPLRKDYIAPNFYE
IQDAHMGNEFRCIGGIRIYRSFNFRAYPNCWFSLCMAKRSIGMVLVPEYSDNKKSK-KKNN
IETVMNSIEFPLLARTTQNSVISTTLNDLSNWSRLSSLWPLLYGTSCCFIEFASLIGSRFDFDR
YGLVPRSSPRQADLILTAGTVTMKMAPSLVRLYEQMPEPKYVIAMGACTITGGMFSTDSY
STVRGVDKLIPVDVYLPGCPPKPEAVIDAITKLRKKIAREIYEERIRSQEENRCFTTNHKFH
VGRSIHTGNYDQGLLYQSPSTSEIPPETFFKYKSSVSSHELVNMQTKNTFSWIKKEIIRSISV
SLMIYIIARTSISNAYPIFAQQGYENPREATGRIVCANCHLANKPVDIEVPQAVLPDTVFEAV
VRIPYDMQVKQVLANGKRGGLNVGAVLILPEGFELAPPDRISPEMKEKIGNLSFQSYRPNK
KNILVIGPVSGQKYSEVTFPILSPDPATNKDVHFLKYPIYVGGNRGRGQIYPDGSKSNNTVY
NATAAGVVSKIIRKEKGGYEITISDASDERQVVDIIPPGPELLVSEGESIKLDQPLTSNPNVG
GFGQGDAEIVLQDPFRVQGLLFFLASVILAQIFLVLKKKQFEKVQLSEMNFMSKVYDWFE
ERLEIQAIADDITSKYVPPHVNIFYCLGGITLTCFLVQVATGFAMTFYYRPTVTEAFASVQYI
MTEANFGWLIRSVHRWSASMMVLMMILHVFRVYLTGGFKKPRELTWVTGVVLAVLTASF
GVTGYSLPWDQIGYWAVKIVTGVPEAIPVIGSPLVELLRGSASVGQSTLTRFYSLHTFVLPL
LTAVFMLMHFLMIRKQGISGPLMGVTKKPDLNDPVLRAKLAKGMGHNYYGEPAWPNDLL
YIFPVVILGTIACNVGLAVLEPSMIGEPADPFATPLEILPEWYFFPVFQILRTVPNKLLGVLL
MVSVPAGLLTVPFLENVNKFQNPFRRPVATTVFLIGTVVALWLGIGATLPIDKSLTLGLFMI
EVFLFGIVLGLIPITLAGLFVTAYLQYRRGDQLDLMLTITSYFGFLLAALTITSALFIGLTKIR
LIMDIVSLAWAALMVVFTFSLSLVVWGRSGLMIIRSPEPEVKILVDRDPIKTSFEEWAKPGH
FSRTIAKGPDTTTWIWNLHADAHDFDSHTNDLEEISRKVFSAHFGQLSIIFLWLSGMYFHG
ARFSNYEAWLSDPTHIGPSAQVVWPIVGQEILNGDVGGGFRGIQITSGFFQIWRASGITSEL
QLYCTAIGALVFAALMLFAGWFHYHKAAPKLAWFQDVESMLNHHLAGLLGLGSLSWAG
HQVHVSLPINQFLNAGVDPKEIPLPHEFILNRDLLAQLYPSFAEGATPFFTLNWSKYAEFLT
FRGGLDPVTGGLWLTDIAHHHLAIAILFLIAGHMYRTNWGIGHGIKDILEAHKGPFTGQGH
KGLYEILTTSWHAQLSLNLAMLGSLTIVVAHHMYSMPPYPYLATDYGTQLSLFTHHMWIG
GFLIVGAAAHAAIFMVRDYDPTTRYNDLLDRVLRHRDAIISHLNWVCIFLGFHSFGLYIHN
DTMSALGRPQDMFSDTAIQLQPVFAQWIQNTHALAPAATAPGATASTSLTWGGGDLVAVG
GKVALLPIPLGTADFLVHHIHAFTIHVTVLILLKGVLFARSSRLIPDKANLGFRFPCDGPGRG
GTCQVSAWDHVFLGLFWMYNAISVVIFHFSWKMQSDVWGSISDQGVVTHITGGNFAQSS
ITINGWLRDFLWAQASQVIQSYGSSLSAYGLFFLGAHFVWAFSLMFLFSGRGYWQELIESI
VWAHNKLKVAPATQPRALSIVQGRAVGVTHYLLGGIATTWAFFLARIIAVGMALRFPRFSQ

GLAQDPTTRRIWFGIATAHDFESHDDITEERLYQNIFASHFGQLAIIFLWTSGNLFHVAWQG
NFEAWVQDPLHVRPIAHAIWDPHFGQPAVEAFTRGGALGPVNIAYSGVYQWWYTIGLRT
NEDLYTGALFLLFLSAISLIAGWLHLQPKWKPSVSWFKNAESRLNHHLSGLFGVSSLAWT
GHLVHVAIPGSRGEYVRWNNFLDVLPHPEGLGPLFTGQWNLYAQNPDSNSHLFGTSQGSG
TAILTLLGGFHPQTQSLWLTDIAHHHLAIAFIFLVAGHMYRTNFGIGHSIKDLLEAHTPPGGR
LGRGHKGLYDTINNSIHFQLGLALASLGVITSLVAQHMYSLPAYAFIAQDFTTQAALYTHH
QYIAGFIMTGAFAHGAIFFIRDYNPEQNEDNVLARMLDHKEAIISHLSWASLFLGFHTLGL
YVHNDVMLAFGTPEKQILIEPIFAQWIQSAHGKTSYGFDVLLSSTNSPAFNAGQSIWLPGW
LNAINE--NSNSLFLTIGPGDFLVHHAIALGLHTTTLILVKGALDARGSKLMPDKKDFGYSFP
CDGPGRGGTCDISAWDAFYLAVFWMLNTIGWVTFYWHWKHITLWQGNVSQFNESSTYL
MGWLRDYLWVNSSQLINGYNPFGMNSLSVWAWMFLFGHLVWATGFMFLISWRGYWQE
LIETLAWAHERTPLANLIRWRDKPVALSIVQARLVGLAHFSVGYIFTYAAFLIASTSGKFGM
SHSVKIYDTCIGCTQCVRACPTDVLEMIPWDGCKAKQIASAPRTEDCVGCKRCESACPTD
FLSVRVYLWHETTRSMGLAYMIIFNLPSFFVPLVGLVFPAIAMASLSLHVQKNKIFMRDLK
TYLSVAPVLSTLWFGALAGLLIEINRLFPDALTFPFFSFMT?ILERRESESLWGRFCNWITST
ENRLYIGWFGVLMIPTLLTATSVFIIAFIAAPPVDIDGIREPVSGSLLYGNNIISGAIIPTSAAIG
LHFYPIWEAASVDEWLYNGGPYELIVLHFLLGVACYMGREWELSFRLGMRPWIAVAYSAP
VAAATAVFLIYPIGQGSFSDGMPLGISGTFNFMIVFQAEHNILMHPFHMLGVAGVFGGSLFS
AMHGSLVTSSLIRETTENESANEGYRFGQEEETYNIVAAHGYFGRLIFQYASFNNSRSLHFF
LAAWPVVGIWFTALGISTMAFNLNGFNFNQSVVDSQGRVINTWADIINRANLGMEVMHE
RNAHNFPLDLAAVEVPSTNGMGLPWYRVHTVVLNDPGRLLSVHIMHTALVAGWAGSMA
LYELAVFDPSDPVLDPMWRQGMFVIPFMTRLGITNSWGGWSIT-GGTITNPGIWSYEGVAG
AHIVFSGLCFLAAIWHWVYWDLEIFCDERTGKPSLDLPKIFGIHLFLSGVACFGFGAFHVT
GLYGPGIWVSDPYGLTGKVQPVNPAWGVEGFDPFVPGGIASHHIAAGTLGILAGLFHLSVR
PPQRLYKGLRMGNIETVLSSSIAAVFFAAFVVAGTMWYGSATTPIELFGPTRYQWDQGYFQ
QEIYRRVGAGLAKNQSLSEAWSKIPEKLAFYDYIGNNPAKGGLFRAGSMDNGDGIAVGW
LGHPIFRDKEGRELFVRRMPTFFETFPVVLVDGDGIVRADVPFRRAESKYSVEQVGVTVEF
YGGELNGVSYSDPATVKKYARRAQLGEIFELDRATLKSDGVFRSSPRGWFTFGHASFALLF
FFGHIWHGARTLFRDVFAGIDPDLDAQVEFGAFQKLGDPTTRRQVVMKTLYSLRRFYPVE
TLFNGTLA?AGRDQETTGFAWWAGNARLINLSGKLLGAHVAHAGLIVFWAGAMNLFEVA
HFVPEKPMYEQGLILLPHLATLGWGVGPGGEVIDTFPYFVSGVLHLISSAVLGFGGIYHAL
LGPETLEESFPFFGYVWKDRNKMTTILGIHLILLGIGAFLLVFKALYFGGVYDTWAPGGGD
VRKITNLTLSPSVIFGYLLKSPFGGEGWIVSVDDLEDIIGGHVWLGSICILGGIWHILTKPFA
WARRALVWSGEAYLSYSLAALSVFGFIACCFVWFNNTAYPSEFYGPTGPEASQAQAFTFL
VRDQRLGANVGSAQGPTGLGKYLMRSPTGEVIFGGETMRFWDLRAPWLEPLRGPNGLD
LSRLKKDIQPWQERRSAEYMTHAPLGSLNSVGGVATEINAVNYVSPRSWLATSHFVLGFF
LFVGHLWHAGRARAAAAGFEKGIDRDFEPVLSMTPLNMTIALGKFTKDENDLFDIMDDW
LRRDRFVFVGWSGLLLFPCAYFALGGWFTGTTFVTSWYTHGLASSYLEGCNFLTAAVSTP
ANSLAHSLLLLWGPEAQGDFTRWCQLGGLWTFVALHGAFGLIGFMLRQFELARSVQLRP
YNAIAFSGPIAVFVSVFLIYPLGQSGWFFAPSFGVAAIFRFILFFQGFHNWTLNPFHMMGVA
GVLGAALLCAIHGATVENTLFEDGDGANTFRAFNPTQAEETYSMVTANRFWSQIFGVAFS
NKRWLHFFMLFVPVTGLWMSALGVVGLALNLRAYDFVSQEIRAAEDPEFETFYTKNILLN
EGIRAWMAAQDQPHENLIFPEEVLPRGNALMSGSTGERSFADIITSIRYWVIHSITIPSLFIA
GWLFVSTGLAYDVFGSPRPNEYFTESRQGIPLITGRFDPLEQLDEFSRSFMTIDRTYPIFTVR

WLAVHGLAVPTVSFLGSISAMQFIQRMATQTVEGSSRSG-PRRTIVGDLLKPLNSEYGKVAP
GWGTTPLMGVAMALFAVFLSIILEIYNSSVLLDGISMNMLTLKLFVYTVVIFFVSLFIFGFLS
NDPGRNPGREEMADTTGRIPLWIIGTVTGILVIGLIGIFFYGSYSGLGSSLMLNIFSLICICLH
S T L Y S S S F F L A K L P E A Y A F L N P I V D F M P V I P L L F F L L A F V W Q A A V S F
R???????????????????????????????????????MEVNILAFIATALFILVPTAFLLIIYVKTVSQSDME
TATLVAISISGLLVSFTGYALYTAFGQPSQQLRDPFEEHGDMEALVYTFLLVSTLGIIFFAIFFR
EPPKVPTKKVKMTIAFQLAVFALIATSLILLISVPVVFASPDGWSSNKNVVFSGTSLWIGLVF
LVGILNSLIS-------MSPQTETKASVGFKAGVKDYKLTYYTPDYETKDTDILAAFRVTPQPG
VPPEEAGAAVAAESSTGTWTTVWTDGLTSLDRYKGRCYHIEPVAGDENQYICYVAYPLDL
FEEGSVTNMFTSIVGNVFGFKALRALRLEDLRIPPSYTKTFQGPPHGIQVERDKLNKYGRP
LLGCTIKPKLGLSAKNYGRAVYECLRGGLDFTKDDENVNSQPFMRWRDRFLFCAEAIFKS
QAETGEIKGHYLNATAGTCEEMMKRAVFARELGVPIVMHDYLTGGFTANTSLAHYCRDN
GLLLHIHRAMHAVIDRQKNHGIHFRVLAKALRMSGGDHIHAGTVVGKLEGERDITLGFVD
LLRDDFIEKDRSRGIYFTQDWVSLPGVLPVASGGIHVWHMPALTEIFGDDSVLQFGGGTLG
HPWGNAPGAVANRVALEACVQARNEGRDLAREGNEIIREASKWSPELAAACEVWKEIKF
EFEAMDTL--MAIHLYKTSTPSTRNGAVDSQVKSNTRNNLIYGQHRCSKGRNARGIITAGH
RGGGHKRLYRKIDFRRNEKDIYGRIVSIEYDPNRNASICLIHYGDGEKRYILHPRGAIIGDTI
VSGTEVPIKMGNALPLTDMPLGTAIHNIEITLGKGGQLARAAGAVAKLIAKEGKSATLKLP
SGEVRLISKNCSATVGQVGNVEVNQKKLGRAGSKCWLGKRPVVRGVVMNPVDHPHGG
GEGRAPIGRKKPATPWGYPALGRRSRKRKKYSDNLILRRRSKMIQPQSHLNVADNSGARE
LMCIRIIGAS-NRRYAHIGDIIVAVIKEALPNTPLERSEVIRAVIVRTCKELKRDNGMIIRYDD
NAAVVIDQEGNPKGTRVFGAIARELRQLNFTKIVSLAPEVLMLSPKRTRFRKQHRGRMKG
ISYRGNRICFGKYALQALEPAWITSRQIEAGRRAMTRNVRRGGKIWVRIFPDKPVTVRPTE
TRMGSGKGSPEYWVAVVKPGRILYEMGGVAENIARKAISIAASKMPIRTQFIISGMTRIRRG
YIARRRRTKIRLFASSFRGARSRLTRTIIQQKIRSLVSSHRDRDRKKIDFRRLWITRINAVIRG
N-RVSYSYSRLIHNLYKRQLLLNRKILAQIAILNRNCLYMISNEIRS-----------MRKEVYALGQ
HISMSAHKARRVIDQIRGRSYEETLMILELMPYRACYPIFKLVYSAAANASHNMGFNEASL
VISKAEVNEGTTMKKLKPRARGRSYAIKRPTCHIRIVLKDKSFY-GLWDKKMDGIKYAVFT
DKSIRLLGKN-QYTFNVESGSTRTEIKHWVELFFGVKVKAMNSHRLPGKGRRMGPILGHT
MHYRRMIITLQPGYSIPPLRKKRTMAVPKKSTSISKKRIRKNIWKKKVYRAALKAFSLAKS
LSTGNSKSFFCTTIK-MAKGKDVRVTVILECTNC-VRT--LNKESRGVSRYITQKNRHNTPSR
LELRKFCPYCYKHTIHGEIKKMKIRASVRKICEKCRLIRRRGRIIVICSNPRHKQRQGMVRE
KVRVSTRTLQWKCVESRADSKRLYYGRFILSPLMKGQADTIGIAMRRALLGEIEGTCITRA
KFE--KIPHEYSTIVGIQESVHEILMNLKEIVLRSNLYGTRNASICVKGPGCVTAQDIILPTSV
EIVDNTQYIANLTEPINLCIGLQIERNRGYHIKTPKNFQDGSY--PIDAVFMPVRNANHSIHSY
GNGNEKQEILFLEIWTNGSLTPKEALHEASRNLIDLFIPFLHAEEE--NLHLENNQ-YKVTLP
FFTFHDRLAK-LRKTKK-EIALKSIFIDQFELPPRTYNCLKRYNIHTLFDLLNKSQEDLMKME
YFRIEDVKQILDSLEIEKAFHNPFTEE-MLGDGN-EGMSTIPGFNQIQFEGFCRFIDQGLTEEL
IKFPKIEDTDQEIEFQLFVETYQLVEPLIKERDAVYESLTYSSELYVSAGLIWKTSRDMQEQT
LFIGNIPLMNSLGTSIVNGIYRIVINQILQSPGIYYRSELDHNGISVYTGTIISDWGGRLELEI
DRKARIWARVSRKQKISILVLSSAMGSNLREILENVCYPEIFLSFLNDKEKKKIGSKENAILE
FYQQFACVGGDPVFSESLCKELQKKFFQQRCELGRIGRRNMNRRLNLDIPQNNTFLLPRDI
LAAADHLIGMKFGMGTLDD--MNHLKNKRIRSVADLLQDQFGLALVRLENVVKGTICGAI
R-HKLI-PTPQNLVTSTP----LTTTYESFFGLHPLSQVLDRTNPLTQIVHGRKLSYLGPGGLTG

RTASFRIRDIHPSHYGRICPIDTSEGINVGLIGSLAIHARIGQGG-SLESPFY---EISERSKSKKV
RMLYLSPSRDEYYMIAAGNSLALNQRIQEEQVVPARYRQEFLTIAWEQVNLRSIFPFQYFSI
GASLIPFIEHNDANRALMSSNMQRQAVPLSRSEKCIVGTGLERQAALDSGVTAIAEHEGKII
YTNTDKIILLGNGDTLSIPLVMYQRSNKNTCMHQKPQVPRGKCIKKGQILADGAATVGGE
LALGKNVLVAYLPWEGYNFEDAVLISERLVYEDIYTSFHIRKYEIQTHVTSQG-PERITNEIP
HLEAHLLRNLDKNGIVMLGSWVEAGDVLVGKLTPQTAKE-SSYAPEDRLLRAILGIQVSTS
KETCLKLPIGGRGRVIDVRWIQKKG-GSNYNPETICVYILQKREIKVGDKVAGRHGNKGIIS
KILPRQDMPYLQDGRPVDMVFNPLGVPSRMNVGQIFECSLGLAGDLLDRHYRIAPFDERY
EQEASRKLVFSELYEASQQTANPWVFEPEYPGKSRIFDGRTGDPFEQPVIIGKPYILKLIHQV
DDKIHGRSSGHYALVTQQPLRGRAKQGGQRVGEMEVWALEGFGVAHILQEMLTYKSDHI
RARQEVLGTTIIGGTIPKPKDAPESFRLLVRELRSLSLELNHFLVSEKNFQINRKEAMNHNF
SSMIDRYKHQQLRIGSVSPQQISAWAKKILPNGEIVGEVTKPYTFHYKTNKPEKDGLFCERI
FGPIKSGICACGNYRVIGDEKEDPKFCEQCGVEFVDSRIRRYQMGYIKLACPVTHVWYLK
RLPSYIANILDKPLKELEGLVYCDF--SFARP-IAKKPTFLRLRGLFEYEIQSWKYSIPLFFTTQ
GFDTFRNREISTGAGAIREQLADLDLRIIIDYSLVEWKELGEE-RPTGNEWEDRKIGRRKDF
LVRRMELAKHFLRTNIEPEWMVLCLLPVLPPELRPIIQIDGGKLMSSDINELYRRVIYRNNT
LTDLLTTSRSTPGELVMCQEKLVQEAVDTLLDNGIRGQPMRDGHNKVYKSFSDVIEGKEG
RFRETLLGKRVDYSGRSVIVVGPSLPLHRCGLPREIAIELFQTFVIRGLIRQHLASNIGVAKS
KIREKEPIVWEILQEVMQGHPVLLNRAPTLHRLGIQAFQPILVEGRAICLHPLVRKGFNADF
DGDQMAVHVPLSLEAQAEARLLMFSHMNLLSPAIGDPISVPTQDMLIGLYVLTSGNRRGIC
TNRYNPWNR---RNYQNERTYDNNYKYRKK-EPFFCNSYDAIGAYRQKRINLDSPLWLRW
RLDQR-IIAS--REVPIEIHYESLGTYHEIYGHYLIVRSVKKEIICIYIRTTVGHISLFREIEEAIQ
GFCRACSFGT--------MEVLMAERVNLVFHNKVIDGTAIKRLISRLIDHFGMAYTSHILDQVK
TLGFQQATATSISLGIDDLLTIPSKGWLVQDAEQQSLILEKHHHYGNVHAVEKLRQSIEVWY
ATSEYLRQEMNPNFRMTDPFNPVHIMSFSGARGNASQVHQLVGMRGLMSDPQGQMIDLP
IQSNLREGLSLTEYIISCYGARKGVVDTAVRTSDAGYLTRRLVEVVQHIVVRRTDCGTIRGV
SVSPRNGMMP-ERIFIQTLIGRVLADDIYMGPRCIAIRNQDIGIGLVNRFIT------FRTQPISIRT
PFTCRSTSWICRLCYGRSPTHGDLVELGEAVGIIAGQSIGEPGTQLTLRTFHTGGVFTGGTA
EHVRAPSNGKIKFNDDLVHPTRTRHGHPAFLCSIDLYVTIESEDILHNVTIPPKSFLLVQNNQ
YVESEQVIAEIRAGTYTF-NLKERVRKHIYSESEGE-MHWSTDVYHAPEFTYSNVHLLPKTS
HLWILSGGSCR--SSRVPFSLYKDQDQMNLR--STERER-RYISSL-SVNNDQ-MRYELCSSDFS
GKIEDRIPDY-SELNRIISIVHCNLKYPT-TFDENSD--LLAKRRRNRFLIPLQSIQERKKELMP
HSGISIELPIN--GILRRNSILAYFDDPRYRRKSSGIIKYGTLGVHSVVKKED--LIEYR-GIKEF
KQKCMKLDPFFFIPEEVHIFPESSSIMVRNNSLIGVDTRIALNTRSRVGGLVRVERKKKRIEL
KIFSGDIHFPGETDKISRHSGILIPPGTGKT--NFKESKK--WKNWIYVQR--ITPTKKK--YFVL
VRPVVTYEVLDGINLATLFPPDLLQERDNMQLRVVNYIVYRNGKPIRGISDTSIQLVRTCLI
LNWDQDK---KSSSIEEAHTSFVEVSTNGLIRDFLRIDLVKFPISYLRKRNDPSGSGLISDDNV
SDHTNSNPFY---SKTK-----IQQLLSQNQGTIRTLLNK---NKECPSLIILSSSNCFRMGP--FNAG
KYHN---VIKES-PIIKIRNSIGPLGT--VLQFVNF--YSFYYLIT--HNPIL-VTKYLQLEN---LKQT
FQVI--N-YYLMDENGR--ILNPDSCSNIVLNSFNLNWYFLHHNYFEERSTIIS-LGQFICENVC
ISKNGP---HLKSGQVLIVQ-VDSVVIRSAKPYLATPGATVHGHYGEILYEGDTLVTFIYEKSR
SGDITQGLPKVEQVLEVRSIDSISMNLEKRIEGWNEHIKKILGIPWGFLIGAELTIVQSRISLV
NKIQKVYRSQGVQIHNRHIEIIVRQITSKVLVSE---DGMSNVFLPGELIGLLRAARTGRALE
ESICYRAILLGITRASLNTQSFISEASFQETARVLAKAALRGRIDWLKGLKENVVIGGMIPV

GTGFKGLVHRS--RQHKNIPLKTKKKN--FFEGEIG--DILFHHRELFDSSISKKFHDTSEQSFR
GFNDS------MTRRYWNINLEEMMEAGVHFGHGTRKWNPRMAPYISAKRKGIHITNLTKTA
RFLSEACDLVFDAASRGKQFLIVGTKNKAADSVARAAIRARCHYVNKKWLGGMLTNWS
TTETRLHKFRDLRTEQKAGRLNRLPKRDAAMLKRQLSHLQTYLGGIKYMTGLPDIVIIVD
QQEEYTALRECITLGIPTICLIDTNCDPDLADISIPANDDAIASIRLILNKLVFAICEGRS-SYIR
NPMGQKINPLGFRLGTTQGHHSIWFAQAKNYSDGLQEDQKIRNCIKNYLQKNMRISS-GV
EGIARIEIRKRIDLIQVIIYMGFPKLLLEGKTR----RIEELQMNVQKELNCVNRKLNIAITRIT
NPYGHPNILAEFIAGQLKNRVSFRKAIKKAIELTEQADTKGIQVQIAGRLDGKEIARVEWM
REGRVPLQTIRAKIDYCSYGVRTVYGVLGIKIWIFWE--MSRYRGPRFKKIRRLGALPGLTS
KRPRAGSDLRNQSRSGK-KSQYRIRLEEKQKLRFHYGLTERQLLKYVRIAGKAKGSTGQV
LLQLLEMRLDNILFRLGMASTIPQARQLVNHRHILVNGCIVDIPSYRCKPRDIITARDDQKS
RAMIQNYFDSSPH-EEVPKHLTLHPFQ--YKGLVNQIIDSKWIGLKINELLVVEYYSRQTMSR
RGTAEEKTAKSDPIYRNRLVNMLVNRILKHGKKSLAYQIIYRAMKKIQQKTETNPLSVLRQ
AIRGVTPDIAVKARRVGGSTHQVPIEIGSTQGKALAIRWLLGASRKRPGRNMAFKLSSELV
DAAKGSGDAIRKKEETHRMAEANRAFAHFRMGRDTIADIITSIRNADMNRKGTIQIASTNI
TENIVKILLREGFIKNVRKHQENNKYFLVLTLRHRR--NRKGPYRT-I--LNLKRISRPGLRIYS
NYQRIPRILGGMGIVILSTSRGIMTDREARLEGIGGEILCYIWMAKSIPRTGSRRNVRSGSR
KSTRRIPKGVIHVQASFNNTIVTVTDVRGRVISWSSAGTCGFNGTRRGTPFAAQTAAGNAI
RTVVDQGMQRAEVMIKGPGLGRDAALRAIRRSGILLSFVRDVTPMPHNGCRPPKKRRVH
PTCSSTLRTSPKSGGFRDISDWLS-CVSNK-LFNSWHTITPKKPNSALRKVARVRLTSGFEITA
YIPGIGHNSQEHSVVLVRGGRVKDLPGVRYHIVRGTLDAVGVKDRQQGRSQYGVKKPKM
ARKSLIQREKKRQKLEQKYHLIRRSSKKEIS---KVPSLSEKWKIHGKLQSSPRNSAPTRLHR
RCFSTGRPRANYRDFGLSGHILREMVHACLLPGATRSSWMVKNSFISVISQE-EEKEENRG
SIEFQVFRFTNKIRRLTSHLELHRKDYLSQRGLRKILGKRQRLLSYLSKKNRIRYKELISQL
DIRESKTR--MVKLRLKRCGRKQ-PVYRIVAIDVRSRREGRDLRKVGFYDPINNQTYLNIPAI
LFFLEKGAQPTGTVYDILKKAGVS--F---------MEKSKRLFLKSKRSFRRRLPPIQSGDRIDYR
NMSLISRFISEQGKILSRRVNRLTLKQQRLITIAIKQARILSLLPFLNNEK--QFERSESTAGAT
GLRTINKVTRSLKKNPFVANHLLRKIEKLNTKAEKEIIITWSRASTIIPTMIGHTIAIHNGREH
LPIYITDRMVGHKLGEFSPTINFRGHAKNDNRSRRMILKSFLLGNPVSLCMNIINSVVMVG
LYYGFLTTFSIGPSYLFLLRARVLEEGEEGTEKKVSATTGFIAGQLMMFISIYYAPLHLALG
RPHTITVLALPYLLFHFFWNNHKHFFDYGSTTRNSMRNLSIQCVFLNNLIFQLFNHFILPSS
MLARLVNIYMFRCNNKMLFVTSSFVGWLIGHIFFMKWVELVLVWIQQNNSIRS-IRSNKYL
VSELRNSMARIFSILLFITCVYYLGRIPSPIVTKKFKETEEDKPLVTLLFDYKRWNRPLRYIK
NNHFENAVRNEVSQYFFYICRNNGKERISFTYPPSLSTFLKMIERKISLLTKEKLPSDELYDH
WIYTNDQKKNNLSKKFRNQMETVN---ITLDVFEKRTRLCNDENKKKYLPKIYDPFLNGPG
RGRVKFFFSSIETGWINKIHSILQEKLEEKEPKTKG--IKEISTKVPSWSYKLIDLEQQEGEEIR
SRKTKHVLIFTDTFIRYSQQSDFRRDIIKGSMRAQRRKTVICELFQANVHSPLFLDRIGKP---
LFSFDISGMIKFILKKRQEKARIEIAQTWDRVLFAQAIRGSLLVTQSFLRKYILFPSVIIAKNI
ARILLFQFPEWSEDLKDWNREMHVKCTYNGVQLSETEFPKNWLTDGIQIKILFPFYIKPWH
RSK--QKNDFCFLTVWGMETEVPFGSPRKQRSFFEPIFKELRKKIIKLQKNPFLV-IQVLKERI
NLFLNLSKERKKSVMKTIL--FLKGIIKELSKRNNGVISNGMINES-IQIRFMDLTD-SLTEKK
MKDLDDRTSTIRNQIEKITKDNKKSSNKISYPTKLLASIKNIWQKLKKFNVRLIRKSYSFFK
FLIERIYINILICIINSPRITTQL--------LSSFSQAYVFYQLYQT-FCNLYKLRYVLQYTSFFLKN
EIKDYFTQGIFYPELKHKNIL-SEMIQWKNWLRGHYQYDLSPIRWYRLVPHKWRNRFNQT

RLVQNKNKRHSDEKDRLILINYKKL-NLQKYYGYDLFSYKSINDDILNTDRKYLDWRIFDF
CLRLFDWMAMNEEILSRWFFPEFVILYNTYIMKPWIIPIQLLLLNSN--TEVELYFFLKRYLRI
QLRWD---NQRMINNIKIYCLLLRLINPREIAISSIQRGEMSLDILITQKDLTLTELMKKGILIIE
PIRLSIKNDGQLIMYQSLNISLFHKSKPDYDLLFVPEKVLSTKRRRELRIRICFKFLWPNYRL
EDLSCMNRYWFDTNNGGRFTMIRIHMYPMKGHQFKSWIFELREILREIKNSHHFLDSWTQ
FNSVGSFIHIFFHQERFIKLLDPRIWSILLS-RNS--QGSTSNRYF---TIKGVVLFVVA-VLLYR--
--INNRNMVERKNLYLTGLLP-----IPMNSIGPRNDTLEESF-GSSNINRLIVS-LLYLPKGKKIS
ESCFLDPKESTWVLPIT----KKCIMPESNWGSRWWRNWIGKKR--DSSCKISN---ETVAGIEIS
FKEKDIK-YLEFLFVYYMD--DPICKDHDWEFLDRLSPSKRRNIINL--NSRQLFEILVKDWIC
YLMFAFREKIPIEVEG-FFKQQG-AGSTIQSNDIEPISH--LFSRKKWAISLQNCAQFHMWQFR
QDLFV---SWGK-NPPESDFLRN-ISRENWIWLDNVWLVNKDR---FFS--KVRNVSSNIQYDS--
-TR--SSFVQVTDSSQLKGSS-DQSRDHFDSIS----NEDSE------------YHTLINQREIQQLKERSI
L-WD--PSFLQTERTELESD--RFSK--CLSGYS-----RLFT-EREKEMKNHLLPEEIEEF--LGNPT
RSILSFFSDRW--SELH-LGSNPTERSTRDQ----KLLKKEQDVS-FVPSRRSE----NKEIVNIFKII
TYLQN--TVSIHPISSDPGCDMVP-KDELD-MHSSHKISFLNKNTFFDL--FHLFHDRNRGGYT
L--HHDFESE--ERFQEMADLFTLSISEP-DLVYHKGFAFSMDSYVLDQKQ--FLNEVFNSRDE
SKKKSL---LVLPPLFY--EENESFYRRIRKKWVR-ISCGNDLEDPKPKIVVFASNNIMEAVNQ
YRWIRNLIQIQ--YSTYGYIRNVWNRFFLMN---RSDRNFEYGIQ---RDQIGNDTLNHRTIMK
YTINQHLSNLKK-SQKKWFNP--LIFISRTERSVNRDPNAYRYKWSNGSKNFQEHLEHFVSK
QKSR---FQVVFDRLRINQYSIDWSEVIDKKDLSKSLRFVLSKLLL---------FLS-------KFLLFL
SNSLP-FFFVSFGNTPIHRSE-IHVY-ELKGPNDQLCNQLLESIGLQIVHLKKWKPL--LLD---D
HDTS-QKSKLLINGGTISPFFFNKIP-KW-MIDSFHTR--NNRRKSFDNTDSYFSMISHDQDN
WLNPVKPFHRSSLISSFYKANRLRFLNNPHHFCFY--CNKRFPF---Y-VERARINNSD--FTYG-
QFLNILFIRNK-IFSLCGGK--KKYAFLE-RDTISPIESQVSNIFIPNDFPQSGDER-SNLYKS---F
HFAIRSDPLVR--RAIYSIADISGTPLTEGQIVNFERTYCQPLSDMNLSDSEGKNLYQYLN--F
NSNMGLIHTPCSEKYLPSEK--RKK--------RSLCLK--KCVEKGQMYRTFQRDSAFSTLSKW
NLFQTYMPWFLTSTGYKYLNWIFLDTFSD-LLPILSSS--QKFVSIFHDIMHGSDISWQILQK
KF------CLPQWN-LISEISSKCLHN--LLLSEEMIHRNNES--PLISTHLRSPNVREF-LYSILFLLL
VATY-IVRTHLLFVSR--AYSELQTEFEKVKSWMIPSYMMELRKLLDRYPTSELNSFWLKNL
FLVALEKLGDSLEEIRGSASGGNMLWGGGP-----AYG-FKSIRSKKKYWNIN---LIDLI-SIIPN
PIHRITFSKKTRHLSHTSKEIYSLIRKRKNVNGDWIDDKIESWVANSDSIDDKEREFLVQFS-
-TLTT--EKGIDQILLSLT-HSDHLS-----KNDSGYQMIEQPGAIYLRYLVDIH--KKYLMNYEFN
TFCLAERRIFLAHYQTITY--SQTSCGTNSFHFTSHG---KPFSLRLALSPS-RGILVIGSIGTGRS
YLVKYLATNSYVPFITVFLNKFLDNKPKGFLID-----DID--EDSDDIEDSD---------------------N
IDRDLDTELELLTRMNAL---TMDMMP---EIDRFYITLQFELAKAMSPCIIWIPNIHDLDVNE
SNYLSLGLLVNYLS---RDCERCS-TQNILVIASTHIPQKVDPALIAPNKLNTCIKIRRLLIPQQ
RKHFFTLSYTRGFHLEKKMFHT--NGFGSITMGSNVRDLVALTNEALSISITQKKSILDTNTI
RSALHRQTWDLR---SQVRSVQDHGILFYQIGRAVAQNVLLSNCPIDPISIYMKKK--SCNEG
DSYLYKWYFELGTSMKKLTILLYLLSCSAGSIAQDLWSLP--GPDEK-NEITSYGLVENDS-D
LVHGLLEVEGALVG--SSRTEKDYSQFDND--RVTLLLRPKPRN-PLDMMQNGSCSIVDQRFL
YEK--YESEFEE-GEGEGVLDPQ----QIEED----LFNH--IVWAP--RIWRP---WGFLFDCIERPNS
LGFP-YWARSFRGK---RIIYDEKEELQENDSEFLQSGTMQYQTRDRSSKEQGFFRISQFIWD
PADPLFFLFK-DQPFVSVFSHREFFADEEMSKGLLTSQTDSPTSIYKRWFI--KNTQEKHFELL
IHRQRWLR---TNSSLS--NGFFRSN--TPSESYQYLSNLFLSN-GRLLDQMTKTLLRKRWLFP

DEMKIGF--M---------MPRSR-INGNFIDKTFSIVANILLRIIPTTSGEKEAFTYYRDG--MSAQS
EGNYAEALQNYYEAMRLEIDPYDRSYILYNIGLIHTSNGEHTKALEYYFRALERNPFLPQA
FNNMAVICHY-RGEQAIRQGDSEIAEAWFDQAAEYWKQAIALTPGNYIEAQNWLKITRRFE
--MSCRSEHIWIELIVGSRKISNFCWAFILFLGSLGFVLVGSSSYLGKNLISLVPSQQILFFPQG
IVMSFYGIAGLFISSYLWCTISWNVGSGYDRFDRKEGIVCIFRWGFPGKNRRIFLRFRMKDI
QSIRIEVKEGISARRVLYMEIKGQGAVPLTRTDEN-LTPREIEQKAAELAYFLRVPIEVF
>Daucus
MERWWFDSILFLSKSMGGLGPIENTSESEDPNRNDMKKNSHSYSNVDYLFGVKDIWNFIS
DETFLVVDRNGNSYSIYLDIEKHIFEIDSGH--FFQSGLESSFYSYWNLSYLNNGSKTDDPHD
DHYMDDTQYSWNNHINSYIDIYLE--SQIFIDTYIVSGSDNYSNSYISRSVCGSKGSNISTST
NGS--TIIESSNDLDITQKYRHLWVQCENCYGLNYKKILKSKMNLCEQCGYHLKMSSSDRI
ELSIDPDTWDAMDEDMVSLDPIEFHSEEEPYKDRIDSYQRKTGLTEAVQTGIGQLNGIPIAL
GVMDFQFMGGSMGSVVGEKITRLIEYATNQLLPLIIVCASGGARMQEGSLSLMQMAKISS
ALYDYQSNKKLFYVPILTSPTTGGVTASFGMLGDIIIAEPNAYIAFAGKRVIEQTLNKTVPEG
SQAAEYLFQKGLFDLIVPRNLLKSVLSELFQLHAFFPLNKNSIEMVTIRADEISKIIRERIEE
YNREVKIVNTGTVLQVGDGIARIHGLDEVMAGELVEFQEGTVGIALNLESTNVGVVLMG
DGLLIQEGSSVKATGRIAQIPVSEAYLGRVVNALAKPIDGRGEISASEYRLIESPAPGIISRRS
VYEPLQTGLIAIDSMIPIGRGQRELIIGDRQTGKTAVATDTILNQQGQNVICVYVAIGQKASS
VAQVVTNFQERGAMEYTIVVAETADSPATLQYLAPYTGAALAEFFMYRKRHTLIIYDDPS
KQAQAYRQMSLLLRRPPGREAYPGDVFYLHSRLLERAAKLGSLLGEGSMTALPIVETQSG
DVSAYIPTNVISITDGQIFLSADLFNAGIRPAINVGISVSRVGSAAQIKAMKQVAGKLKLELA
QFAELEAFAQFASDLDKATQNQLARGQRLRELLKQSQSAPLAVEEQIMTIYTGTNGYLDSL
EIGQVRKFLVELRTYLKTNKPQFQEIISSTKIFTEEAEALLKEAIQEQMERFILQEQVMKINP
TNSSSLTSTLEKKNLGNITQIIGPVLDVAFPPGKMPNIYNALVVKGRDTAGQQINVTCEVQQ
LLGNNRVRAVAMSATDGLTRGMEVMDTGAPLSVPVGGTTLGRIFNVLGEPVDNLGPVDT
RTTSPIHRSAPAFIQLDTKLSIFETGIKVVDLLAPYRRGGKIGLFGGAGVGKTVLIMELINNI
AKAHGGVSVFGGVGERTREGNDLYMEMKESGVINEENIAESKVALVYGQMNEPPGARM
RVGLTALTMAEYFRDVNEQDVLLFIDNIFRFVQAGSEVSALLGRMPSAVGYQPTLSTEMG
TLQERITSTKEGSITSIQAVYVPADDLTDPAPATTFAHLDATTVLSRGLAAKGIYPAVDPLDS
TSTMLQPRIVGEDHYETAQKVKQTLQRYKELQDIIAILGLDELSEEDRLTVARARKIERFLS
QPFFVAEVFTGSPGKYVGLSETIRGFRLILSGELDSLPEQAFYLVGNIDEATAKAMNLEMES
NSNKMTLNLCVLTPNRTVWNSKVNEIILSTNSGQIGVLPNHASVATAVDIGILKIRLDGQWL
TMALMGGFAIIGNNEITVLVNDAEKGSDIDSQEAQQTLEIAEANFRKAEGKRQKIEANLAL
RRARTRVETINAISMKNVTDSFVSLGHWPSAGSFGFNTDILATNLINLSVVLGVLVFFGKG
VLSDLLDNRKQRILNTIRNSEELRGGAIEQLEKARTRLRKVEMEADQFRVNGYSEIERERL
NFINSTSKTLKQLENYKNETINFEQQRAINQVRQLVFQQALQGALGTLSSCLNNELHLRTI
RANIGMLGAITD---MNPLISAASVIAAGLAVGLASIGPGVGQGTAAGQAVEGIARQPEAEG
KIRGTLLLSLAFMEALTIYGLVVALALLFANPFVMNVLSCSINKLNGLYDISGVEVGQHFY
WKIGGFQVHGQVLITSWVVIAILLGSATLAVRNPQTIPTSGQNFFEYVLEFIRDVSKTQIGE
EYGPWVPFIGTMFLFIFVSNWSGALLPWKIIQLPHGELAAPTNDINTTVALALLTSVAYFYA
GISKKGLGYFGKYIQPTPILLPINILEDFTKPLSLSFRLFGNILADELVVVVLVSLVPLVVPIPV
MFLGLFTSGIQALIFATLAAAYIGESMEGHHMIFSTLEHILTHISFSIVCIVITIHLITLLIDEIIK
LNNSSEKGMIATFLCITVLLVTRCIYSGHLPLSDLYESLIFLSWSLSFIHIVPY--KNKSHLSTIT
ASSVIFTQGFATSGLLTEIHQSAILVPALQSEWLIMHVSMMILGYAALLCGSLLSVALLVLTF

RKNRNLLC--KEIQYINEINNIFGNAS---DNNYYRSQLIQQLDYWSYRVISLGFIFLTIGILSGA
VWANEAWGSYWNWDPKETWAFITWIVFAVYLHTRTNTNLQVENSAIVASMGFLIIWICYF
GVNLLGIGLHTYGSFTLTMSKKKAFTSLLYLASIVFLPWWISLSFNKSLESWVTDWWNTR
QSEIFWNDIQEKNIIKNFIELEEILLLEEMIKEDSETHLQNLRI-IHKETLQLIKIHNEDHIHTIL
HFSTNIICFVILSGYSILGNEELVILNSWAQEFIYNLSDTIKAFWILLLTDFFIGFHSTRGWEL
MIGFVYKDFGFAHNDQILSSLVCIFPVILDTLFKFWVFRYLNCVSPSLVVIYHSMNEMPIGV
PKVPFRSPGEEDASWVDVYNRLYRERLLFLGQEVDSEISNQLIGLMVYLSIENDTKDLYLF
INSPGGWVIPGVAIYDTMQFVRPDVQTICMGLAASMGSFILAGGEITKRLAFPHARVMIHQ
PASSFYEAQTGEFILEAEELLKLRETLTRVYVQRTDKPLWVVSEDMERDVFMSATEAQAY
GIVDLVAVVMKEQKWIHEGLITESLPNGMFRIRLDNQDMILGYVSGKIRRSFIRILPGDRVK
IEVSRYDSTRGRIIYRLRNKDSKDMEEFQRYLK-NRSQQHYFLYPLIFQEYIYALAHDHGLV
GYN-KFSLLIVKRAIERMYQQKHLILFDNDFNQNRFFGNNFSFQMISEGFAVIVEIPFYLRLL
SSLIVKSNNLRSIHSIFPFLEDNFSHLIYVLEILIPYPAHLEILIQTLRYWVKDASSLHLLRFFL
HEYRSWN---TKASSFFSKQRFFFVLYNSHICEYESIFVFLRNQSSHLCSTSSGTLLERIYFYG
KLEHLVEAFAKAFNLWLFKDPFMHYVRYQGKSILASKGTPPLMKKWTYYFVNLWECRFY
LWSQPGRICINQLYNNSLSLLGYISSAGLNPSMVRSQMLENAFIIDNPI--KKFDTLVPIVPLI
GSLAKARFCNVLGHPISKAVWTDLSDSDIIVRFGRICRNLSHFFSGSSQKKSLYRIKYILRLS
CARTLARKHKSTVRAFLKRSGSGLLEEFFTAEEQVLYLTFPRRIWYLDIICINDLASHEMIID
-TTEVQ-AINSFFRLES-LKEVYDIIWMLVPILTLVLGITIGVLVIVWLEREISAGIQQRIGPEYA
GPLGILQALADGTKLLFKENLLPSRGDTRLFSIGPSIAVTSILLSYLVIPFGYRLVLADVSIGV
FLWIAISSIAPVGLLMSGYGSNNKYSFLGGLRAAAQSISYEIPLTLCVLSISLLSNSSSTVDIV
EAQSKYGFWGWNLWRQPIGFIVFLISSLAECERLPFDLPEAEEELVAGYQTEYSGIKFGLFY
VASYLNLLVSSLFVTVLYLGGWNLSLPHIALPFFFEINKAGRVFGTIIGIFITLAKTYLFLFIAI
TTRWTLPRLRMDQLLNLGWKFLLPISLGNLLLTTSSQLLSLMIWHVQNENFILDSTRIFMK
AFHLLLFDGSLIVPECILIFGLILLLMIDSTSDQKDIPWLYFISSTSLVMSITALLFRWREEPVI
SFSGNFQTNNFNEIFQFLILLCSTLCIPLSVEYIECTEMAITEFLLFVLTATLGGMFLCGANDL
ITIFVAPECFSLCSYLLSGYTKKDVRSNEATMKYLLMGGASSSILVHGFSWLYGSSGGEIEL
QEIVNGLINTQMYNSPGISIALIFITVGIGFKLSPAPSHQWTPDVYEGSPTPVVAFLSVTSKV
AASASATRIFDIPFYFSSNEWHLLLETLAILSMILGNLIAITQTSMKRMLAYSSIGQIGYVIIGI
IVGDSNDGYASMITYMLFYISMNLGTFACIVLFGLRTGTDNIRDYAGLYTKDPFLALSLAL
CLLSLGGLPPLAGFFGKLYLFWCGWQAGLYFLVLIGLLTSVVSIYYYLKIIKLLMTGRTQEI
TPHVRNYRRSPFRSNNSIELSMIVCVIASTIPGISMNPIIAIAQDTLFMFLLYEYDIFWAFLIIS
SLIPILAFFVSGVLAPINKGPEKLSSYESGIEPMGNAWLQFRIRYYMFALVFVVFDVETVFL
YPWAMSFDVLGISVFVEALIFVLILIVGLVYAWRKGALEWSTNFFPWLTIIVILPIFAGSIILF
LPHRGNRVIRWYTICICILELLLTTYAFCYHFQLDDPLIQLVEDFKWIDFFDFHWRLGIDGLS
IGPILLTGFITTLATLAAWPVTRDSRLFHFLMLAMYSGQIGLFASRDLLLFFIMWELELIPVY
LLLSMWGGKKRLYSATKFILYTAGGSVFLLMGVLGVGLYGSTEPTLNFATLVNQSYPVALE
IILYIGFFIAFAVKSPIIPLHTWLPDTHGEAHYSTCMLLAGILLKMGAYGLIRINMELLSHAH
SIFSPWLVIVGTIQIIYAASTSLGQRNLKKRIAYSSVSHMGFILIGIGSINDTGLNGAILQIVSH
GFIGAALFFLAGTSYDRIRLVYLDEMGGIAIPMPKIFTMFSSFSMASLALPGMSGFVAEFIV
FFGIITSQKYLLISKLGITFVMAIGIILTPIYSLSMLRQMFYGYKLFNAPNSYVFDSGPRELFV
SIAIFIPVIGIGMYPDFVLSLSVDKVEVLLSNFVYRMMLEHVLVLSAYLFSVGLYGLITSRN
MVRALMCLELILNAVNINFVTFSDFFDSRQLKGSIFSIFVIAIAAAEAAIGLAIVSSIYRNRKS
TRINQSNLLNKMEQTYQYAWIIPFLPLPVPMLIGAGLLLFPSATKSLRRMWAFQSVFLLSIV

MLFSINLSIQQINGSSIYQYVWSWIINNDFSLEFGYLIDPLTSIMSILITTIGIMVLIYSDNYMS
YDQGYLRFFAYMSFFSTSMLGLVTSSNLIQIYIFWELVGMCSYLLIGFWFTRPVAANACQK
AFVTNRVGDFGLLLGILGFYWITGSFEFRDLFKILNNNEVNFLFVTFCAVLLFAGAVAKSA
QFPLHVWLPDAMEGPTPISALIHAATMVAAGIFLVARLIPLFIVIPHIMNFISLVGVITILFGAT
FALAQKDIKRGLAYSTMSQLGYMMLALGMGSYRSALFHLITHAYSKALLFLGSGSVIHSM
ETLVGYSPNKSQNMVFMGGLTKHVPITKTSFLLGTLSLCGIPPLACFWSKDEILNDTWLYS
PIFAIIAWFTAGLTAFYMFRIYLLTFEGHLNVHFQNYSGKKNTPFYSISLWGKENSKRINK---
SLLTMKFFSFGNKKFFLYPYESDNTMLFPILVLVLFTLFVGSLGIPFRLDILSKWLTPSINLLH
KNLN-SIGWYEFLKDALFSVSIAIFGIFIAFFLYKPVYSSLQNWDLINSFVILLDKIINGIYDW
SYNRGYIDAFYARFFIGGIRGLAKLTSFFDRRVIDGITNGVGVLSFFLGEGIKSIGGG-RISSY
LFLYFFFVAILLLIINMDLPGPIHDFLLVFLGSGLILGGLGVVLLPNPIYSAFSLGLVLVCTSLF
YILSNSHFVAAAQLLIYVGAINVLIIFAVMFMNGSEYYKDFHLWTVGDGVTSMVCTSIFVS
LITTIPDTSWYGIIWTTKSNQIVEQDLISNSQQIGIHLSTDFFLPFELISIILLVSLIGAITVARQ
MTAPATRNDLMIVNMGPQHPSMHGVLRLIVTLDGEDVIDCEPILGYLHRGMEKIAENRTII
QYLPYVTRWDYLATMFTEAITVNAPEQLGNIQVPKRASYIRVIMLELSRIASHLLWLGPFM
ADIGAQTPFFYIFRERELIYDLFEAATGMRMMHNFFRIGGVAADLPHGWIDKCLDFCDYFL
TRVTEYQRLITRNPIFLERVEGVGIIGGEEAINWGLSGPMLRASGIQWDLRKVDHYESYDE
FDWGVQWQKEGDSLARYLVRISEMTESVKIIQQALEGITGGPYENLEIRRFDKVWDPEWN
DFDYRFISKKPSPTFELSKQELYVRVEAPKGELGIFLIGDKGIFPWRWKIRPPGFINLQILPQL
VKRMKLADIMTILGSIDIIMGEVDRMFSMVTEFMNYSQQTVRAARYIGQGFMITLSHASR
LPVTIQYPYEKLITSERFRGRIHFEFDKCIACEVCVRVCPIDLPVVDWKLEKDIRKKRLLNY
SIDFGICIFCGNCVEYCPTNCLSMTEEYELSTYDRHELNYNQIALGRLPMSIINDYTTRTILN
L---------------MQGRLSAWLVKHGLIHRSLGFDYQGIETLQIKPEDWHSIAVILYIYGYNYLR
SQCAYDVAPGGLLASVYHLTRIEYGVDQPEEVCIKVFSPRKNPRIPSVFWVWKGVDFQER
ESFDMLGISYDNHPRLKRILMPESWIGWPLRKDYIAPNFYEIQDAH----------------------------
------------------------------MNSIEFPLLDRTTQNSVISTTSNDLSNWSRLSSLWPLLYGTSCCFIE
FASLIGSRFDFDRYGLVPRSSPRQADLILTAGTVTMKMAPSLVRLYEQMPEPKYVIAMGAC
TISGGMFSTDSYSTVRGVDKLIPVDVYLPGCPPKPEAVIDAITKLRKKISREIYEDRIKSQRE
NRCFTTNHKLKVGRSIHTGNYDREFLYQPTSTSEIPPETFFKYKSSVSSHELVNMQTRKTFS
WIKEQINRSISVSLMIYIITRPSISIAYPIFAQQGYENPREATGRIVCANCHLANKPVDIEVPQ
TVLPDTVFEAVVRIPYDMQLKQVLANGKRGTLNVGAVLILPEGFELAPADRISPEMKEKIG
NLSFQSYRPNKKNILVIGPVPGQKYSEITFPILSPDPATTKEVHFLKYPIYVGGNRGRGQIYP
DGSKSNNTVYNATSAGIVGKIIRKEKGGYEITIADASDGRQVVDIIPPGPELLVSEGESIKLD
QPLTSNPNVGGFGQGDAEIVLQDPLRVQGLLFFLASVILAQIFLVLKKKQFEKVQLSEMNF
MSKVYDWFEERLEIQAIADDITSKYVPPHVNIFYCLGGITLTCFLVQVATGFAMTFYYRPT
VTDAFASVQYIMTEANFGWLIRSVHRWSASMMVLMMILHVFRVYLTGGFKKPRELTWVT
GVVLAVLTASFGVTGYSLPRDQIGYWAVKIVTGVPEAIPVIGSPLVELLRGSASVGQSTLTR
FYSLHTFVLPLLTAVFMLMHFPMIRKQGISGPLMGVTKKPDLNDPVLRAKLAKGMGHNY
YGEPAWPNDLLYIFPVVILGTIACNVGLAVLEPSMIGEPADPFATPLEILPEWYFFPVFQILRT
VPNKLLGVLLMVSVPVGLLTVPFLENVNKFQNPFRRPVATTVFLIGTAVALWLGIGATLPID
KSLTLGLFMIEVFLFGIVLGLIPVTLAGLFVTAYLQYRRGDQSDLMPTITSYFGFLLAALTV
TSALFIGLSKIRLIMDIVSLAWAALMVVFTFSLSLVVWGRSGLMIIRSPEPEVKILVDRDHIK
TSFEEWARPGHFSRTLAKGPDTTTWIWNLHADAHDFDSHTSDLEEISRKVFSAHFGQLSIIF
LWLSGMYFHGARFSNYEAWLSDPTHIGPSAQVVWPIVGQEILNGDVGGGFRGIQITSGFF

QLWRASGITSELQLYCTAIGALIFAALMLFAGWFHYHKAAPKLAWFQDVESMLNHHLAG
LLGLGSLSWAGHQVHVSLPINQFLNAGVDPKEIPLPHEFILNRDLLAQLYPSFAEGATPFFT
LDWSKYSDFLTFRGGLDPVTGGLWLTDIAHHHLAIAILFLIAGHMYKTNWGIGHGLKDIL
EAHKGPFTGQGHKGLYEILTTSWHAQLSLNLAMLGSLTIIVAHHMYSMPPYPYLATDYGT
QLSLFTHHMWIGGFLIVGAAAHAAIFMVRDYDPTTRYNDLLDRVLRHRDAIISHLNWACI
FLGFHSFGLYIHNDTMSALGRPQDMFSDTAIQLQPVFAQWIQNTHALAPSATAPGATTSTSL
TWGGGDLVAVGGKVALLPIPLGTADFLVHHIHAFTIHVTVLILLKGVLFARSSRLIPDKANL
GFRFPCDGPGRGGTCQVSAWDHVFLGLFWMYNAISVVIFHFSWKMQSDVWGSISDQGV
VTHITGGNFAQSSITINGWLRDFLWAQASQVIQSYGSSLSAYGLFFLGAHFVWAFSLMFLF
SGRGYWQELIESIVWAHNKLKVAPATQPRALSIVQGRAVGVTHYLLGGIATTWAFFLARIIA
VGMALRFPRFSQGLAQDPTTRRIWFGIATAHDFESHDDITEERLYQNIFASHFGQLAIIFLW
TSGNLFHVAWQGNFESWVQDPLHVRPIAHAIWDPHFGQPAVEAFTRGGALGPVNIAYSGV
YQWWYTIGLRTNEDLYTGALFLLFLSAISLIAGWLHLQPKWKPSVSWFKNAESRLNHHLS
GLFGVSSLAWTGHLVHVAIPASRGEYVRWNNLLDVLPHPQGLGPLFTGQWNLYAQNPDSS
SHLFGTSQGAGTAILTLLGGFHPQTQSLWLTDMAHHHLAIAFIFLVAGHMYRTNFGIGHSM
KDLLDAHIPPGGRLGRGHKGLYDTINNSLHFQLGLALASLGVITSLVAQHMYSLPAYAFIA
QDFTTQAALYTHHQYIAGFIMTGAFAHGAIFFIRDYSPEQNEDNVLARMLDHKEAIISHLS
WASLFLGFHTLGLYVHNDVMLAFGTPEKQILIEPIFAQWIQSAHGKTSYGFDVLLSSTNGP
AFNAGRSIWLPGWLNAVNE--TSNSLFLTIGPGDFLVHHAIALGLHTTTLILVKGALDARGS
KLMPDKKDFGYSFPCDGPGRGGTCDISAWDAFYLAVFWMLNTIGWVTFYWHWKHITLW
QGNVSQFNESSTYLMGWLRDYLWLNSSQLINGYNPFGMNSLSVWAWMFLFGHLVWATG
FMFLISWRGYWQELIETLAWAHERTPLANLIRWRDKPVALSIVQARLVGLAHFSVGYIFTY
AAFLIASTSGKFGMSHSVKIYDTCIGCTQCVRACPTDVLEMIPWDGCKAKQIASAPRTEDC
VGCKRCESACPTDFLSVRVSLWNETTRSMGLAYMTTFDFPSVLVPLVGLIFPAMAMASLFL
HVQNNKTVMRDLKTYLSVAPVLSTLWFGSLAGLLIEINRFFPDALTFPFF--MTAILERRESE
SLWGRFCNWITSTENRLYIGWFGVLMIPTLLTATSVFIIAFIAAPPVDIDGIREPVSGSLLYGN
NIISGAIIPTSAAIGLHFYPIWEAASVDEWLYNGGPYELIVLHFLLGVACYMGREWELSFRL
GMRPWIAVAYSAPVAAATAVFLIYPIGQGSFSDGMPLGISGTFNFMIVFQAEHNILMHPFHM
LGVAGVFGGSLFSAMHGSLVTSSLIRETTENESANEGYRFGQEEETYNIVAAHGYFGRLIF
QYASFNNSRSLHFFLAAWPVVGIWFTALGISTMAFNLNGFNFNQSVVDSQGRVINTWADII
NRANLGMEVMHERNAHNFPLDLAAVEAPSTNGMGLPWYRVHTVVLNDPGRLLAVHIMH
TALVAGWAGSMALYELAVFDPSDPVLDPMWRQGMFVIPFMTRLGITSSWGGWSIT-GGAT
PNPGIWSYEGVAGAHIVFSGLCFLAAIWHWTYWDLAIFCDERTGKPSLDLPKIFGIHLFLA
GLACFGFGAFHVTGLYGPGIWVSDPYGLTGKVQSVNPAWGVEGFDPFVPGGIASHHIAAG
TLGILAGLFHLSVRPPQRLYKGLRMGNIETVLSSSIAAVFFAAFVVAGTMWYGSATTPIELF
GPTRYQWDQGYFQQEIYRRVSAGLAENQSLSEAWSKIPEKLAFYDYIGNNPAKGGLFRAG
SMDNGDGIAVGWLGHPIFRDKEGRELFVRRMPTFFETFPVVLVDGDGIVRADVPFRRAES
KYSVEQVGVTVEFYGGELNGVSYSDPATVKKYARRAQLGEIFELDRATLKSDGVFRSSPR
GWFTFGHASFALLFFFGHIWHGARTLFRDVFAGIDPDLDAQVEFGAFQKLGDPTTRRQTV
MKTLYSLRRFYPVETLFNGTLALAGRDQETTGFAWWAGNARLINLSGKLLGAHVAHAGL
IVFWAGGMNLFEVAHFVPEKPMYEQGLILLPHLATLGWGVGPGGEVIDTFPYFVSGVLHLI
SSAVLGFGGIYHALLGPETLEESFPFFGYVWKDRNKMTTILGIHLILLGIGAFLLVFKALYF
GGVYDTWAPGGGDVRKITNLTLNPSIIFGYLLKSPFGGEGWIVSVDDLEDIIGGHVWLGSI
CIFGGIWHILTKPFAWARRALVWSGEAYLSYSLAALSVFGFIACCFVWFNNTAYPSEFYGPT

GPEASQAQAFTFLVRDQRLGANVGSAQGPTGLGKYLMRSPTGEVIFGGETMRFWDLRAP
WLEPLRGPNGLDLSRLKKDIQPWQERRSAEYMTHAPLGSLNSVGGVATEINAVNYVSPRS
WLATSHFVLGFFLFVGHLWHAGRARAAAAGFEKGIDRDFEPVLSMTPLNMTIALGKFTK
DEKDLFDIMDDWLRRDRFVFVGWSGLLLFPCAYFAVGGWFTGTTFVTSWYTHGLASSYL
EGCNFLTAAVSTPANSLAHSLLLLWGPEAQGDFTRWCQLGGLWTFVALHGAFGLIGFMLR
QFELARSVQLRPYNAIAFSGPIAVFVSVFLIYPLGQSGWFFAPSFGVAAIFRFILFFQGFHNW
TLNPFHMMGVAGVLGAALLCAIHGATVENTLFEDGDGANTFRAFNPTQAEETYSMVTAN
RFWSQIFGVAFSNKRWLHFFMLFVPVTGLWMSALGVVGLALNLRAYDFVSQEIRAAEDP
EFETFYTKNILLNEGIRAWMAAQDQPHENLIFPEEVLPRGNALMSGSTGERSFADIITSIRY
WVIHSITIPSLFIAGWLFVSTGLAYDVFGSPRPNEYFTENRQGIPLITGRFDPLEQLDEFSRSF
MTIDRTYPIFTVRWLAVHGLAVPTVSFLGSISAMQFIQRMATQTVENVSR-SRPKPTTVGGL
LKPLNSEYGKVAPGWGTAPLMGVAMALFAIFLSIILEIYNSSVLLDGISMNMLTLKLFVYT
VVIFFVSLFIFGFLSNDPGRNPGREEMADTTGRIPLWIIGTVAGILVIGLIGIFFYGSYSGLGSS
LMLNIFSLICICLNSAFYSSSLFFAKLPEAYAFLNPIVDLMPVIPLFFFLLAFVWQAAVSFRM
TQSNPNEQDVELNRTSLYWGLLLIFVLAVLFSNYFFNMEVNILAFIATALFILVPTAFLLIIYV
KTETQNKMETATLVAIFISGLLVSFTGYALYTAFGQPSQQLRDPFEEHGDMEALVYTFLLVS
TLGIIFFAIFFREPPKVPTKK--MTLAFQLAVFALIATSSILLIGVPVVFASPDGWSSNKNVLFS
GTSLWIGLVFLVGILNSLIS-------MSPQTETKAGVGFKAGVKDYKLTYYTPDYETKDTDIL
AAFRVTPQPGVPPEEAGAAVAAESSTGTWTTVWTDGLTSLDRYKGRCYGIEPVAGEENQF
IAYVAYPLDLFEEGSVTNMFTSIVGNVFGFKALRALRLEDLRIPVAYVKTFQGPPHGIQVER
DKLNKYGRPLLGCTIKPKLGLSAKNYGRAVYECLRGGLDFTKDDENVNSQPFMRWRDRF
LFCAEAIYKAQAETGEIKGHYLNATAGTCEEMMKRAIFARELGVPIVMHDYLTGGFTANT
SLAHYCRDNGLLLHIHRAMHAVIDRQKNHGIHFRVLAKALRMSGGDHIHSGTVVGKLEG
ERDITLGFVDLLRDDFIEKDRSRGIYFTQDWVSLPGVLPVASGGIHVWHMPALTEIFGDDS
VLQFGGGTLGHPWGNAPGAVANRVALEACVQARNEGRDLAREGNEIIREATKWSPELAA
ACEVWKEIKFEFEAMDTL--MAIHLYKTSTPSTRNRTVDSRVKSNPRNNLIYGQHRCGKGR
NARGIITAGHRGGGHKRLYRKIDFRRNEKDIYGRIVTIEYDPNRNAYICLIHYGDGEKRYIL
HPRGAIIGDTIVSGTEVPIKMGNALPLTDMPLGTAIHNIEITRGRGGQLARAAGAVAKLIAK
EGKSATLKLPSGEVRLISKNCSATVGQVGNVGVNQKSLGRAGSKRWLGKRPVVRGVVM
NPVDHPHGGGEGRAPIGRKKPTTPWGYPALGRRSRKRNKYSDNLILRRRSKMIQPQTLLN
VADNSGARELMCIRIIGAS-NRRYAHIGDVIVAVIKEAVPNMPLARSEVVRAVIVRTCKELK
RDNGMIIRYDDNAAVVIDQEGNPKGTRIFGAIARELRQLNFTKIVSLAPEVLMLSPKRTRFR
KQHRGRMKGISYRGNHISFGKYALQALEPAWITSRQIEAGRRAMTRNARRGGKIWVRIFP
DKPVTVRPAETRMGSGKGSPEYWVAVVKPGRILYEMGGVTENIARRAISIASSKMPIRTQFI
ISGMTRIRRGNIARRRRTKIRLFASSFRGAHSRLTRTITQQKIRALVSSHRDRDKQKRNFRRL
WITRINAVIREI-GVSYSYSRLIHALYKKQVLLNRKILAQIAISNKNCLYMISNEIIKEVDWKE
STGIIMLKEVYALGQHISMSAHKARRVVDQIRGRSYEETLMILELMPYRACYPILKLVYSA
AANANYNMDSNESNLVISKAEVSEGTATKKLKPRARGRSFTIKRPTCHIAIVVKDISLDGGI
WDKKMDGIKYAVFTDKSIRLLGKN-QYTSNVESGSTRTEIKHWVELFFGVKVIAMNSHRL
PGKGRRMGPIMGQTMHYRRMIITLQPGYSIPPLRKKRTMAVPKKRTSISKKRIRKNIWKGK
GSWAALKALSLGKSLSTGNSKSFF---QKKMAKGKDVRITVILECTGCVRNGVN--KVSTGI
SRYITEKNRHNTPNRLELRKFCPFCYKHTMHGEIKKMKIRASVRKICEKCRLIRRRGRIIVI
CSNPRHKQRQGMVREKVTVSTRTLQWKCVESKADNKRLYYGRFILSPLMKGQADTIGIS
MRRALLGEIEGTCITRAKSE--KIPHEYSTLVGIQESVHDILMNLKEIVLRSNLYGTCDASIC

VRGPGYVTAQDIILPPYVEVVDNTQHIASLTEPIELCIGLQIERNRGYLIKTPNNNSDGSY--P
IDAVFMPVRNANHSIHSYGNGNDKQEILFLEIWTNGSLTPKEALHEASRNLIDLFIPFLHTE
EE--NLHLANNQ-HMVPLPPFTFHDKLD-KLRKNKK-KRALKSIFIDQSELPPRIYNCLKRSNI
YTLLDLLNNSQEDLMKIEHFRIKDVKQILGILEKN--FSINLGKKPMLRDG-NEGMSTIPGFN
QIQFEGFCRFMDQGLTEELYKFPKIEDTDQEIEFQLFVETYQLVEPLIKERDAVYESLTYSSE
FYISAGLIWKTSRDMQEQTIFLGNIPLMNSLGTSIVNGIYRIVINQILQSPGIYYRSELDHNGI
SVYTGTIISDWGGRLELEIDKKARIWARVSRKQKISILVLSSAMGSNLREILENVCYPEIFLS
FLTDKEKKKIGSKENAILEFYQQFACVGGDPVFSESLCKELQKKFFQQKCELGKIGRRNM
NRRLRLDISQNNTFLLPRDILAAADHLIGMKFGMGTLDD--MNHLKNKRIRSVADLIQDQL
GLALVRLENMIRGTIGGALR-HKL-IPSPQNLVTSTP----LTSTYESFFGLHPLSQVLDRTNPLT
QIVHGRKLSYLGPGGLTGRTASFRIRDIHPSHYGRICPIDTSEGINVGLIGSLAIHAKIGRGG-
SLESPFY---EISQRSKG--ARMLYLSPGKDEYYMVAAGNPLALNQGLQEEQVVPARYRQEFL
TIAWEQVHLRSIFSFQYFSIGASLIPFIEHNDANRALMSSNMQRQAVPLSRSEKCIVGTGLE
RQAALDSGVLAIAEHEGKVIYTDTDKILLSGNGDTLNIPLVMYQRSNKNTCMHQKPQVQ
RGKYIKKGQILAYGAATIGGELALGKNVLVAYMPWEGYNFEDAVLISERLVYEDIYTSFHI
RKYEIQTHVTSQG-PERVTREIPHLEAHLLRNLDKNGIVMLGSWVETGEILVGKLTPQMVK
E-SSYAPEDRLLRAILGIQVSTSKETCLKLPIGGRGRVIDVRWIQKRV-GSSYNPETIRVYILQ
KREIKVGDKVAGRHGNKGIISKILPRQDMPYLQDGRPVDMVFNPLGVPSRMNVGQIFECS
LGLAGGLLDRHYRIAPFDERYEQEASRKLVFSELYQASKQTATPWVFEPEYPGKSRIFDGR
TGDPFEQPVIIGKPYILKLIHQVDDKIHGRSSGHYALVTQQPLRGRAKQGGQRVGEMEVW
ALEGFGVAYILQEMLTYKSDHIRARQEVLGTTIIGGAIPNPEDAPESFRLLVRELRSLALELN
HFFVSEKTFKIKRKEAMNPNFSSMIDQYKHQQLRIGSVSPQQISTWANKILPNGERVGEVT
KPYTFHYKTNKPEKDGLFCERIFGPIKSGICACGNYRVIGDKKKDRKSCEQCGVEFVDSRI
RRYQMGYIKLACPVTHVWYLKRLPSYIANLLDKPLKELEGLVYCD--FSFARP-IAKKPTFL
RLRGLFEYEIQSWKYSIPLFFTTQGFDTFRNREISTGAGAIREQLADLDLRIIIDSSLVEWKE
LGED-GPTGNEWEDRKVGRRKDFLVRRMELAKHFIRTNIDPKWMVLCLLPVLPPELRPIV
QIDGGKLMSSDINELYRRVIYRNNTLTDLLTTSRSTPGELIMCQEKLVQEAVDTLLDNGIRG
QPMRDGHNKVYKSFSDVIEGKEGRFRETLLGKRVDYSGRSVIVVGPSLSLYQCGLPREIAI
ELFQTFVIRSLIRQQLASNIGVAKSKIREKKPIVWEILREVMRGHPVLLNRAPTLHRLGIQA
FQPVLVEGRAICLHPLVRKGFNADFDGDQMAVHVPLSFEAQAEARLLMFSHINLLSPAIGD
PISVPTQDMLIGLYVLTSGNRRGICVNRYNPSNH---RN-QNKRIYENNYKYTK--EPFFCNSY
DAIGAYRQKRINLDSPLWLRWRLDQR-VIAA--REAPLEVHYESLGTYYDIYGQYLIVRSIK
KEILSIYIRTTVGHISIYREIEEAIQGFCQAC-----------MEVLMAERANLVFHNKVIDGTAMK
RLISRLIDHFGMAYTSHILDQVKTLGFQQATATSISLGIDDLLTIPSKRWLVQDAEEQSFILEK
HHHSGNVHAVEKLRQSIEIWYATSEFLRQEMNPNFRMTDPFNPVHIMSFSGARGNASQVH
QLVGMRGLMSDPQGQMIDLPIQSNLREGLSLTEYIISCYGARKGVVDTAVRTSDAGYLTRR
LVEVVQHIVVRRKDCGTARGISVSLGNGMMS-ENIFIQTLIGRVLADDIYMGTRCIATRNQ
DIGSGLVNQFIT------FRAQPIYIRTPFTCRSTSWICQLCYGRSPTHGDLVELGEAVGIIAGQSI
GEPGTQLTLRTFHTGGVFTGGIAEHVRAPSNGKIKFNEDLVHPTRTRHGHPAFVCSIDLYVT
IESEDILHNVNIPPKSFLLVQNDQYVESEQVIAEIRAGTST-LNFKEKVRKHIYSDSEGE-MH
WNTDVYHAPEFTYGNVHLLPKTSHLWILLGEPRR--SDLISLSIHKDQDQMNAR--SFSVKK
RSISNLS--VTNDQ-VRHKFFSSDFFGK-EEEHPDY-SELNR---IVRCNLRYP-TIPYADY--DLL
AKRRRKRFIIPLQSIQERENELMPPSGISIEIP--INGIFRINSILAFFDDPRYRRKSSGITKYGTI
EVDPIAKKED--LIEYRG-VKEFKPKYIKVDRFFFIPEEMHILPGSSSIMVRNNSIIGIDTQIALT

TRSRVGGLVRVEIKKKRIELKIFSGDIHFPGETDKISRHSGVLIPPGT--GKTNSKESKK--GKN
WIYVQR--ITPTN--KKYFVLVRPVVTYEITDGINLV-LFPPDLLQEMDNVQLRVVNYILYGN
GKPIREIYNTSIQLVRTCLVLNCTQDKK---SSYIEETRTSFVEIGINGLSRDFIKIDLVKSPISYT
AKRNDPSRSGLIYE-NGSDCTNINPFSSRFFYY--SNARIKESLNQNQGTIHTLFNR---NKEYQ
SLIILSSSNCFRMGP--FNNVKYHNV---IKES-PPIPIRNLLGPLGT--ALKTANFYP--FSHL--ITY
NQIL-VINYLQLDNLK---QTFQVIK---YFLMDEI--GKIYNYDLCSNIILNPFNLNWYFLQHNY
CEGMS-TIMSLGQFICENVCIAKNGPH-LK--SGQVLIVK-MDSVVIRSAKPYLATPGATVHG
HYGEILYEGDTLVTFIYEKSRSGDITQGLPKVEQVLEVRSIDSISMNLEKRVEGWHECITRT
LGIPWGFLIGAELTIVQSRIALVNKIQKVYRSQGVQIHNRHIEIIVRQITSKVLVSE---DGMSN
VFLPGELIGLLRAERMGRALEEAICYRAVLLGITKASLNTQSFISEASFQETARVLAKAALR
GRIDWLKGLKENVVLGGMIPVGTGFEGL--VHSSRQHTNLS--LETKNNNIFEGEMR--DILF
HHRKFFDSCFSKNFHDTSEQSFIGI----GFNDSMTKRYWNIDLEEMMKAGVHFGHGTRKW
NPKMAPYISAKRKGIHIINLTRTARFLSEACDLVFDAASRGKQFLIVGTKNKAADLVAWAAI
RARCHYVNKKWLGGMLTNWSTTETRLHKFRDLRTEQKTGRLRRLPKRDAAVLKRQLSH
LQTYLGGIKYMTGLPDIVIIDQHEEYTALQECITLGIPTISLIDTNCDPDLADISIPANDDAIS
SIRLILNKLVFAIREGRS-SYIRNPMGQKINPLGFRLGTTQSHHSLWFTQPKNYSEGLQEDQ
KIRDFIKNYVQKNLKISSG-VEGIARIEIHKRIDLIQVIIYMGFPKLLIENRPG----GIEKLQMN
LQKEFNCVNRKLNITITRIAKPYGSPNILAEFIAGQLKNRVSFRKAMKKAIELTEQADTKGI
QVQISGRIDGKEIARVEWIREGRVPLQTIRAKIDYCSYTVRTIYGVLGIKIWIFLDGEMSRYR
GPRFKKIRRLGALPGLTNKRPRAGSDLRNQSRSGK-KSQYRIRLEEKQKLRFHYGLTERQL
LKYVRIAGKAKGSTGQVLLQLLEMRLDNILFRLGMATTIPGARQLVNHRHILVNGRIVDIP
SYRCKPRDIITARDEQNSRALIQNSFNSPSQ-DEMPKHLTLQPFQ--YKGLVNQIIDSKWVGL
KINELLVVEYYSRQTMSRRGTAEEKTAKSDPIYRNRLVNMLVNRILKHGKKSLAYQIIYRA
VKKIQQKTETNPLSVLRQAIRGVTPDIAVKARRVGGSTHQVPIEIGSTQGKALAIRWLLAA
SRKRPGRNMAFKLSSELVDAAKGSGDAIRKKEETHRMAEANRAFAHFRMGRDIIADIITSI
RNADMDRKRAVRIASTNITENIVKILLREGFIENVRKHKETKKDFLVLTLRHRR--NRKRSY-
--RNFLNLKRISRPGLRIYSNYQRIPRILGGMGIVIISTSRGIMTDREARLERIGGEVLCYIWM
AKTIPRIGSRKNGRIGSRKNTRRIPKGVIHVQASFNNTIVTVTDVRGRVVSWSSAGTCGFK
GTRRGTPFAAQTAAGNAIRTVVDQGMQRAEVMIKGPGLGRDAALRAIRRSGILLTFVRDV
TPMPHNGCRPPKKRRVMPTIKQLIRNTRQPIRNVTKSPALRGCPQRRGTCTRVYTITPKKP
NSALRKVARVRLTSGFEITAYIPGIGHNSQEHSVVLVRGGRVKDLPGVRYHIVRGTLDAVG
VKDRQQGRSKYGVKKPKMARKGLIQRENKRQKLEQKYHSIRRSSKKEIR---QVLSLSDK
WEIYGKLQSPPRNSAPTRLHRRCFSTGRPRANYRDFGLSGQILREMVHACLLPGATRSSW
MIKNSFSAIFFK--EENEDNKGSVEFQVVSFTNRIRKLTSHLELHKKDYLSQRGLRKILGKR
QRLLAYLSKKNRVRYKELIGQLEIRETKKN--MVKLRLKRCGRKQRAVYRIVAIDVRSRRE
GRDLRNVGFYDPIKNQSYLNVPAILYFLEKGAQPTGTVRDLLKKAEVFK-----------MDKSK
RPFLKSKRSFRRRLPPIQSGDRIDYRNMSLISRFISEQGKILSRRVNRLTLKQQRLITIAIKQA
RILSLLPFLNNEK--QFERTESTTRTAGLRARNKVTRSLKKNPFVANRLLRKIDNLNTKAEK
EIIITWSRASTIIPTMIGHTIAIHNGKEHLPIYITDRMVGHKLGEFAPTLNFRGHAKSDNRSR
RMILKSFLLGNLVSLCMKIINSVVVVGLYYGFLTTFSIGPSYLFLLRAQVMEEGEEGTEKK
VSATTGFITGQLMMFISIYYAPLHLALGRPHTITVLALPYLLFHFFWKNHKNFFDYGSTTR
NSMRNLSIQCVFLNNLIFQLFNHFILPSSMLARLVNIYMFRCNNKMLFVTSSFVGWLIGHIL
FMKWVGLVLVWIRQNNSIRS-IRSNK---SELIN--YRIFSILLFITCVYYLGRIPSPIFTKKLN-T
EEDKPLLTFLFDYKRWNRPLRYIK-NRFERTTRKEMSQYFFYTCRSDGKERISFTYPPSLSTF

GEMIQRRMSLPTLEKLSSAELYNHWVYTNQHKNNNLNNEFLNRIEALDTGFFSLDILEKS
TRLCN--TRKYYLPKMYDPILN-LYRGTIQKKISPIETLEINKIHSLLYQEFEQKSPNDQ---IKE
ISKKVPRWSYKLIELEFLSGTQIRSRKRDGIIIYRP--RDYSEESDFRREFIKGSMRVQRRKTV
IWKLFQANAHSPLFLDR-IK-KYPLFSFNIPERMKSFFRNIKEKASIK----WDFIPYPQATRSLI
LIIQSIFRKYILLPSLIIVKNIGRILLSQPPEWTEDFDEWNREKHIICTYNGVQVSEFELPKNW
FKDGIQIKIVFPFYLKPWHRSKKEEQPKFYFLTAWGAQTTLNFGSIPPKPSFFKPILKELKKK
-------MQKTLKVLRVLKERDFLQDSKEPKEVIIKNVLFLFIKRIKKELFKIHDYIIRNQQIHDS
FIKIKSTDNKNDSLRDKKIKNLTDRTSTIRTKTKGLMKEKNS---NKTSFNRKKKSPNILLKIL
KIKSTRLIYKFYLFITIFIKRIYIDIFVCIIHIARISTQLL-KEINFDLSSLSQAYVFYKLSQTPIIH
FYKLRDIFEYTPFFLKTAMKNYFTQGIFHSELRHTKLSSYETNQWKSWLRG-YQYNLSQIR
WSGLIPKKWRTTINEGGMTPNKTKCNSYEKDR--LLYHKKQ-NFLKYYIYDLFSYKSINGDI
MYPDRKYFDWTILNFFIRFFDWMEMNEEILNPTFFPEFVPLYNVYKTKPWIIPSKLLLLNIR
NKMRREIIFLRKHYLLFQWIIDDGLTERMINNIKIYCYLLGLRNPRDTTISSIQRKELNLDIM
VIRQK-LTPIELNKGIFFLDPTRLSVKNDGYFIIYQTVGISLVHKSKYKHYDFVVPENILSSRR
RRELRILLSFVFLWPNYRLEDLACMNRYWFNTNNGSRFSILRIYPQFMKGHQFKFWIFELR
EILREIKNSHYFLDSWTQFNSVGSFIHIFFHQEHFIKLFDPRIWSILLS-RNS--QGSTSNRYFT-
--IKGVVILFVVAVLIYR----INSRNMVERKNLYLIGLLP-----IPMNSIGPRNDTLEESV-GSSNI
NRLIVS-LLYLPKGKKISESCFLNPKESTWVLPIT----KKCSMPESNWGSRWWRNWIGKKR--
DS--
--SQL
KGSS-DQSRDPLDSIS----NEDSE------------YHTLINQRKIQQLKERSIL-WD--PSFLQTERTEI
ESD--RFPK--SLSGYS--SMSRLFT-EREKQVINHLLPEEIEEFL--GNPTRSVRSFFSDRW--SEL
H-LGSNPTERSTRDH----KLLKKQQDLS-FVPSRRSE----KKEMVNIFKIITYLQN--TVSIHPIS
SYPGCDMVP-KDEPD-MDSSNKISFLNKNPFLDL--FHLFHDRNMGGYTL--HHDFESE--ERF
QEMADLFTLSITEP-DLVYHKGFAFSIDSYGLDQKQ--FLNEVFNSRDESKKKS---LLALPPF
FY--EENESFYRRIIKKWVR-ISCGNDLEDPKPKKMVFASNN---------------------------LNVLNR
FFLMN---RSDRNFEYG---IQRDQIGKDTLNHRTRMKYMINQHLSNLKK-SQKRWFDPL--ILI
SRTERSTNRDPDAYRYKWSNGSNNFQEHLDHFVSERKSR---FRVVFDRLRINQYSIDWSEV
IDKKGLSKPFRFFLSKSLLFLSK--SLLFLS-------KFLFFLSN-SLPFFFVSFGNIPIHRSE-IYIY-
ELKGPNDQLCNQLLESIGLQIVHLKKLKPF--LLD---DHDTS-QKSKFLINGGTISPFLFNKIP-
KW-MIYSFHTR--NNRRKSFANTDSYFSTIFHDQDYWLNPVKPFHRSSLISSFYKANQLRFL
NNPHHFCFY--CNKRFPFYV----EKARIN--NSDFTYG-QFLNILFIHNK-IFSLCVGK--KKHAF
WG-RDTISPIESRVSNIFIPNDFPQGGGDETYNLYKS---SHFPSRSDPFVR--RAIYSIADISGTP
LTEGQIVNFERTYCQPLSDLNLSDSEGKNSHQYLN--FNSNMGLIHTPCSEKYLPSEK--RKN
--------RSLFLK--KYVEKGQMYRTFQRDSAFSTLSKWNLFQTYIPWFLTSTGHKYLNWIFLD
TFSGLL--PIHLLPSQKFVSIFHDIMHVLDISWRILQKKL------GLPQRNP-IRKISSKCLHN--LL
LSEEMIHRN--NESPLISTHLRSPNVREF-LYSILFLLLVAGY-LVRTHLIFVSR--ASSELQIEFE
KVKSLMISSYMIELRKLLDRYPTSEPNSFWLKNLFLVALEQLGDSLEEIWGFASGGNMLLG
GDSA-----YG-VKSIRSKKKYLNIN---LIDLI-SIIPNPISRITFSKNTRHLSHTSKEIYSLIRKRKN
VNGDWIDDKIESWVANSDSIDDEEREFLVQLS--TLTT--EKRIDQILLSLT-HSDHL-----SKND
SGYQMIEQRGAIYLRYLVDIH--KKYLMNYEFNTSCLAERRIFLAHYQTITY--SQTSCGANS
FHLPSHG---KPFSLRLALSPS-RGILVIGSIGTGRSYLVKYLATNSYVPFITVFLNKFLDN-------
------LSE-IDASEDIDASEDIDASEDIDA------------SDDIDRDLHTEL-ELL------TMDMMSE---K
DRFYITLQFELAKAMSPCIIWIPNIHDLDVNESNYFSLGLLVNHLSR---DCERCS-TRNILVIA

STHIPQKVDPALIAPNKLNTCIKIRRLLIPQQRKHFFTLSYTRGFRLEKKMFHT--NGFGSITM
GSNARDLVALTNEALSISITQKKSIIDTNTIRSAFHRQTWDLR---SQVRSVQDHGILFYQIGR
AVAQNVLLSNCPIDPISIYIKKK--SCNEGDSYLYKWYFELGTSMKKLTILLYLLSCSAGSVA
QDLWSLP--GPDER-NGITSYGLVENDS-DLVHGLLQVEGALVG--SSRTEKDCSQFDND--RV
TLLLRPEPRN-PLDMMQNGSCSILDQRFLYEKN--ESEFEE-GEG--ALDPQQ----IEED----LFN
H--IVWAP--RIWHP---WGILFDCIERPNELGFP-YWSRSFRGK---RILYDEEDELQENDSEFLQ
SGTMQYQTRDRSSKEQGFFRISQFIWDPADPLFVLFK-DQSSVSVFSHRELFADEEMSKGLL
TSQTDPPTSIYKRWFI--KKTQEKHFELLINRQRWFR---TTSSLS--NGSFRSN--TLSESYQYLS
NLFLSN-GTLLDQMTKTLLRKRWLFPDEMKIGF--MEQEKDFPFLMPRSR-INGNFIDKTFSII
ANILLRIIPTTSGEKEAFTYYRDG--MSAQSEGNYAEALQNYYEAMRLEIDPYDRSYILYNI
GLIHTSNGEHTKALEYYFRALERNPFLPQAFNNMAVICHY-RGEQAIRQGDSEIAEAWFDQ
AAEYWKQAIALTPGNYIEAHNWLKITRRFE--MSCRSEHIWIQPITGSRKTSNLCWAIILFLG
SLGFLLVGTSSYLGRNLISLFPSQQILFFPQGIVMSFYGIAGLFISSYLWCTISWNVGGGYDR
FDRKEGMVCLFRWGFPGKNRRIFLRFLIKDIQSVRIEVKEGIYARRVLYMDIRGQGAIPLTR
TDEN-VTPREIEQKAAELAYFLRVPIEVF
>Liriodendron
MEKWWFNSMLSLSKSMDSLGPIGNTSGSEDPIINDTDKNIH-CSNVDHFLGVRDIWSFISD
ETFLVRDSNGNSYSVYFDIENHIFEIDND--SSFLSELESSFS-----SYLNNGSKSDNRYYDCD
MYDTKYSWNNHINSCIDSYLRSEIS--IDSYISSGSDNCSDSYIYSYICSDSGSSIRTDGNGS--
DIRGRS-DFDINQKYRHLWVQCENCYGLNYKKFFKSKMNICEQCGYHLKMSSSDRIELSID
PGTWDPMDEDMVSIDPIEFHSEEEPYRDRIDSYQRKTGLTEAVQTGIGQLNGIPIAIGVMDF
EFMGGSMGSVVGEKITRLIEYATNRSLPVIMVCASGGARMQEGSLSLMQMAKISSALYDY
QSNKKLFYVSILTSPTTGGVTASFGMLGDIIIAEPNAYIAFAGKRVIEQTLNKTVPDGSQAA
EYSFHKGLFDPIVPRNLLKGVLSELFQLHGFFPLTQN---MVTIRADEISNIIRERIEQYNREV
TIVNTGTVLQVGDGIARIHGLDEVMAGELVEFEEGTIGIALNLESNNVGVVLMGDGLLIQE
GSSVKATGRIAQIPVSEAYLGRVINALAKPIDGRGEISASESRLIESPAPGIISRRSVYEPLQT
GLIAIDSMIPIGRGQRELIIGDRQTGKTAVATDTILNQKGQNVICVYVAIGQKASSVAQVVTT
FQEQGAMEYTIVVAETADSPATLQYLAPYTGAALAEYFMYRERHTSIIYDDPSKQAQAYR
QMSLLLRRPPGREAYPGDVFYLHSRLLERAAKSSSRLGEGSMTALPIVETQSGDVSAYIPT
NVISITDGQIFLSADLFNAGIRPAINVGISVSRVGSAAQIKAMKQVAGKSKLELAQFAELEAF
AQFASDLDKATQNQLARGQRLRELLKQSQSTPLTVEDQIVTIYTGANGYLDPLEIGQVKKF
LVQLRTYLKTNKPQLQEIISSTKTFTEQAEALLKEAIPEQIELFLLQEQTMRINPTTSSPGVS
TLEEKNLGRIVQIIGPVLDVAFPPGKMPNIYNALVVKGRDTVGQQINVTCEVQQLLGNNR
VRAVAMSATDGLMRGMEVIDTGAPLSVPVGGATLGRIFNVLGEPVDNLGPVDTRTTSPIH
RSAPAFIQLDTKLSIFETGIKVVDLLAPYRRGGKIGLFGGAGVGKTVLIMELINNIAKAHGG
VSVFGGVGERTREGNDLYMEMKESGVINEQNIAESKVALVYGQMNEPPGARMRVGLTAL
TMAEYFRDVNEQDVLLFIDNIFRFVQAGSEVSALLGRMPSAVGYQPTLSTEMGSLQERITS
TKEGSITSIQAVYVPADDLTDPAPATTFAHLDATTVLSRGLAAKGIYPAVDPLDSTSTMLQP
RIVGEEHYETAQRVKQTSQRYKELQDIIAILGLDELSEEDRLTVARARKIERFLSQPFFVAEV
FTGSPGKYVGLAETIRGFQLILSGELDGLPEQAFYLVGNIDEATAKAMNLEVESKLKKMTL
NLCVLTPNRIIWDSEVKEIILSTNSGQIGVLPNHAPIATAVDIGILRVRLNDQWLTMALMGG
FARIGNNEITILVNDAEKGSDIDPQEAQRTLEIAEANLSRAEGKRQAIEANLALRRARTRVE
AINVISMKNVTDSFVSLGYWPSAGSFGFNTDILATNLINLSVVLGVLIFFGKGVLSDLLDN
RKQRILSTIRNSEELREGAIEQLEKARARLRKVEMEADEFRVNGYSEIEREKQNLINATYEN

LERLENYKNETIHFEEQRAINQVRQRVFQQALQGALGTLNSCSNSELHLRTISANIGVLGA
MKEITDMNPLISAASVIAAGLAVGLASIGPGVGQGTAAGQAVEGIARQPEAEGKIRGTLLL
SLAFMEALTIYGLVVALALLFANPFVMNVLPCSINTLKGLYDISGVEVGQHFYWQIGGFQV
HAQVLITSWVVIAILLGSATIAVRNPQTIPTDGQNFFEYVLEFIRDLSKTQIGEEYGPWVPFI
GTMFLFIFVSNWSGALLPRKIIQLPHGELAAPTNDINTTVALALPTSMAYFYAGFTKKGLS
YFGKYIQPTPILLPINILEDFTKPLSLSFRLFGNILADELVVVVLVSLVPSVVPIPVMFLGLFT
SGIQALIFATLAAAYIGESMEGHHMIFATLEHILTHISFSIISIVITIHLVTLLVHEIVGLCDPSE
KGMIAAFFCITGLLVTRWIYSRHFPLSDLYESLMFLSWSFSIIHMVPKIWNHKNDLSAITAP
SAIFTQGFATSGLSTEMHQSAILVPALQSQWLMMHVSMMLLSYAALLCGSLLSVALLVITF
RKNIGIVGKSNEIHYLNEKRNVLQNTSFLSFRNYHRYQLTQRLDYWSYRVISLGFTFLTIGI
LSGAVWANEAWGSYWNWDPKETWAFITWTIFAIYLHTRTNRSLQGVNSAIVASMGFLIIWI
CYFGVNLLGIGLHSYGSFTLTVPKK-ALTPLPYLASIVFLPWWISISFNKSLEPWVTNWWNT
RQSETFLNDIQEKNVLERFIELEQLFLLDEMIKEYPGTQIQKLRIGIHKETIQLVKMHNEDHI
HIILHFSTNIICFAILSGYSILGNEELVILNSWVQEFLYNLSDTIKAFSILLLTDLCIGFHSPHG
WELMIGSVYKDFGFAHNDQIISGLVSTFPVILDTILKYWIFHYLNRVSPSLVVVYHSMNEM
PIGVPKVPFRSPGEEDAIWVDVFNRLYRERLLFLGQEVDSEISNQLVGLMVYLSIEDDTRDL
YLFINSPGGWVIPGISIYDTMQFVPPDVHTICMGLAASMGSFILVGGEITKRLAFPHARVMI
HQPASSFYEAPTGEFILEAEELLKLRETLTRVYVQRTGNPLWVVSEDMERDVFMSAAEAQ
AHGIVDLVAVEMKEQKLIHEGLITESLPNGMFRVRLDNEDLILGYVSGRIRRSFIRILPGDRV
KIEVSRYDSTRGRIIYRLRNKDSNDMEELQGYLEIDRSRQQHFLYPLLFQEYIYALAHDHG
LLGYDN-SSSLIVKRLITRMHQQNHLIISVNDSNENGFVGKSFYSQMVSEGFAVIMEIPFSLR
LVSSLIAKSH-LRSIHSIFPFFEDKLSHLNHVSDILIPHPIHLEILVQTLHCWIQDAPSLHLLRFF
LHEYRNSNSLITKSISLFSKQRFFLFLYNSHVYECESVLVFLRKQSSHLRSTSSGTFLERTHF
YGKIEH-VVVLRNQK-LWLFKDPFMHYVRYQGKYILASKGTHLLMKKWKSHLVNFWQC
HFYLWSRPDRIHINQLYNHSFYFLGYLSSVRLNTSAVRIQMLENSFLIDTSI--NKFETLVPIIS
LIGSVAKAKFCNVSGHPISKSVRADSSDSDIINRFGRIYRNLSHYHSGSSKKQTLYRIKYILR
LSCARTLARKHKSTVRAFLKRLGSEFLEEFLTEEEQVLSLIFQRRIWYLDIIRINDLANHSMI
IDTT-EVQ-AINSFSRSE-SLKEVYGLVWLLVPIFTPVLGITIGVLVIVWLEREISAGIQQRIGPE
YAGPLGILQALADGTKLLFKEDLLPSRGDIRLFSIGPSIAVISILLSYSVIPFGYRLVLADLSIG
VFLWIAISSIAPIGLLMSGYGSNNKYSFSGGLRAAAQSISYEIPLTPCVLSISLLSNSSSTVDI
VEAQSKYGFGGWNLWRQPIGFIVFLISSLAECERLPFDLPEAEEELVAGYQTEYSGIKSGLF
YVASYLNLLVSSLFVTVLYLGGWNLSIPYIFIPELFGINKTGGVFGTTIGIFITLAKAYLFLFIP
ITTRWTLPRMRMDQLLNLGWKFLLPISLGNLLLTTSSQLLSLMIWHVQNENFILDSTRIFM
KAFHLLLFHGSFIFPECILIFGLILLLMIDSTSDQKDIPWLYFISSTSLVMSITALLFRWREEPM
ISFSGNFQTNNFNEIFQFLILLCSTLCIPLSVEYIECTEMAITEFLLFVLTATLGGMFLCGAND
SITIFVAPECFSLCSYLLSGYTKRDVRSNEATTKYLLMGGASSSILVHGFSWLYGSSGGEIEL
QEIVNGLIKTQMYNSPGISIALIFITVGIGFKLSPAPSHQWTPDVYEGSPTPVVAFLSVTSKV
AASASATRIFDIPFYFSSNEWHLLLEILAILSMILGNLIAITQTSMKRMLAYSSIGQIGYVIIGI
IVGDSNDGYASMITYMLFYISMNLGTFARIVSFGLRTGTDNIRDYAGLYTKDPFLALSSALC
LLSLGGLPPLAGFFGKLHLFWCGWQAGLYFLVSIGLLTSVVSIYYYLKIIKLLMTGRNQEIT
PHVRNYRRSPLRSNNSIELSMIVCVIASTIPGISMNPIIAIAQDTLFMFLLHEYDIFWAFLIISS
VIPILAFLISGVLAPISEGPEKLSSYESGIEPMGDAWLQFRIRYYMFALVFVVFDVETVFLYP
WAMSFDVLGVSVFIEALIFVLIPIVGSVYAWRKGALEWSTSYFPWLTIIVVLPIFAGSSIFFFP
HRGNKVVRWYTICICLLELLLTTYAFCYHFQLDDPLIQLEEDYKWINIFDFHWRLGIDGLSI

GPILLTGFITTLATLAARPVTRDSRLFHFLMLAMYSGQIGSFSSRDLLLFFIMWELELIPVYL
LLSMWGGKKRLYSATKFILYTAGGSIFLLMGVPGMGLYGSNEPILNFETSANQSYPLALEII
FYFGFLIAYAVKSPIIPLHTWLPDTHGEAHYSTCMLLAGILLKMGAYGLVRINMELLPHAH
SIFSPWLMIVGTIQIIYAASTSSGQSNLKKRIAYSSVSHMGFTIIGIGSITDTGLNGAILQIISH
GFIGAALFFLAGTSYDRIRLVYLDEMGGIAIPMPKIFTMFSSFSMASLALPGMSGFVAESVV
FWGIITSPKYLLMPKILITFVMAIGMILTPIYSLSMSRQMFYGYKLFNVPNSYFLDSGPRELF
VSICIFLPVIGIGIYPDFVLSLSVDKVEAILANYFYKMMTEHVLILSAYLFSIGIYGLITSRNM
VRALMCLELILNAVNLNLVTFSDLFDSRQLKGDIFSIFVIAIAAAEAAIGPAIVSSIYRNRKST
RINQSNLLNKMEHTYQYAWIIPFVPLPVTMSIGLGLLLVPTATKNLRRMWAFPSVSLLSIVM
VFSADLSIQQINGSSIYQYLCSWTINNDFSLEFGHLIDPLTSIMSILITTVGIMVLIYSDNYMS
HDQGYLRFFAYMSFSNTSMLGLVTSSNLIQIHIFWELVGMCSYLLIGFWFTRPIAANACQK
AFVTNRVGDFGLLLGILGFYWITGSLEFRDLFEIFNNNGVNSLFATLCAFLLFVGAVAKSAQ
FPLHVWLPDAMEGPTPISALIHAATMVAAGIFLVARLLPIFTVIPYIMNLISLLGIITVLLGAT
LALAQKDIKRSLAYSTMSQLGYTMLAPGIGSYRAALFHLITHAYSKALLFLGSGSIIHSMEP
IVGYSPDKSQNMVLMGGLTKYVPITKTTFLLGTLSLCGIPPLACFWSKDEILNDSWLYSPIF
AIIACSTAGLTAFYMFRMYLLTFEGHLHIRFQNYSGTKNSSFCSISIWGKEGPEPVKK-KLLL
SVMNKVSNKD--TSMYPCESDNTMLFPLLVLVLFTLFVGSIGIPFDFDILSKWLTPSINLLHQ
NSNYSVDWYEFVTNAIYSVSISYFGIFIASLLYGSVYSSFQNLDLINSFVLFLDRIINVIYNW
SYNRGYIDVFYATFLTKGIRGLAELTNFFDRRVIDGITNGVGIASFFVGEGIKY-VGGGRISS
YLFVYLSYVSTFLLIYYMDLPGPIHDILLVFLGSGLILGGLGVVLLTNPIYSAFSLGLVLVCIS
LFHIPSNSYFVAAAQLLIYVGAVNVLIVFAVMFMNGSEYSKDLYLWTVGDGVTSLVCTSIL
FSLITTISDTSWYGIIWTTRSNQIIEQDLTSNVQQIGIHLSTDFYLPFELISIILLVALIGAISMA
RQMTVPDIRKDFMIVNMGPHHPSMHGVLRLIVTLDGEDVIDCEPVLGYLHRGMEKIAEN
RTIIQYLPYVTRWDYLATMFTEAITVNAPEQLGNIQVPKRASYIRVIMLELSRIASHLLWLG
PFMADIGAQTPFFYIFRERELLYDLFEAATGMRMMHNYFRIGGVAADLPHGWIDKCLDFC
DYSLTGVVEYQKLITRNPIFLERVEGVGIIGGEEAINWGLSGPMLRASGIQWDLRKIDHYE
CYDEFDWEVQWQKEGDSLARYLVRISEMAESIKIIQQVLEGIPGGPYENLEVRRFDKARD
SEWNDFEYRFISKKPSPTFELSKQELYVRVEAPKGELGIFLIGDNSVFPWRWKIRPPGFINL
QILPQLVKRMKLADIMTILGSIDIIMGEVDRMFPMMTGFMNYGQQTVRAARYIGQSFMIT
LSHANRLPVTIQYPYEKSITSERFRGRIHFEFDKCIACEVCVRVCPIDLPVVDWRLETDIRK
KRLLNYSIDFGICIFCGNCVEYCPTNCLSMTEEYELSTYDRHELNYNQIALGRLPMSVIGD
YTIRTIMNSKIATDNPLDSRTITNYMQGRSSAWLVKHELVHRSLGFDYQGIETLQIKPEDW
YSIAVISYVYGYNYLRSQCAYDVAPGGLLASVYHLTKIEYGVDQPEEVCIKVFAPRRNPRIP
SVFWIWKSADFQERESYDMLGISYENHPRLKRILMPESWIGWPLRKDYIAPNFYEIQDAY--
---MNSIEFPLLDRTTQNSVISTTSNDLSNWSRLS
SLWPLLYGTSCCFIEFASLIGSRFDFDRYGLVPRSSPRQADLILTAGTVTMKMAPSLVRLYE
QMPEPKYVIAMGACTITGGMFSTDSYSTVRGVDKLIPVDVYLPGCPPKPEAVIDAITKLRK
KVSREISEDRIGSQQENRCFTTNHKFHVGRSTHTGNYDQGLLYQSPSTSEISSETFFKYKSS
VSSHELVNMQNRNTFSWVKEQMTRFISVSIMIYVITRTSISNAYPIFAQQGYENPREATGRI
VCANCHLANKPVDIEVPQAVLPDTVFEAVVRIPYDMQLKQVLANGKKGALNVGAVLILP
EGFELAPPDRISPEMKEKIGNLSFQSYRPTKRNILVVGPVPGQKYSEIVFPILSPDPATKKDV
HFLKYPIYVGGNRGRGQIYPDGNKSNNTVYNATAAGIVNRIVRKEKGGYEITIADASDGH
QVVDIIPPGPELLVSEGESIKLDQPLTSNPNVGGFGQGDAEIVLQDPLRVQGLLFFLASVILA
QIFLVLKKKQFEKVQLSEMNFMSKVYDWFEERLEIQAIADDITSKYVPPHVNIFYCLGGIT

LTCFLVQVATGFAMTFYYRPTVTEAFASVQYIMTEANFGWLIRSVHRWSASMMVLMMIL
HVFRVYLTGGFKKPRELTWVTGVILAVLTASFGVTGYSLPRDQIGYWAVKIVTGVPEAIPVI
GSPLVELLRGSASVGQSTLTRFYSLHTFVLPLLTAVFMLMHFPMIRKQGISGPL--ITKKPDL
NDPVLRAKLAKGMGHNYYGEPAWPNDLLYISPVVILGTIACNVGLAVLEPSMIGEPADPFA
TPLEILPEWYFFPVFQILRTVPNKLLGVLLMVSVPTGLLTVPFLENVNKFQNPFRRPVATTV
FLIGTVIALWLGIGATLPIDKSLTLGLFMIEVFLFGIVLGLIPITLAGLFVTAYLQYRRGDQLD
LMSTITSYFGFLLAASTITPALLIGLSKIRLIMDIVSLAWAALMVVFTFSLSLVVWGRSGLMI
IRSPEPEVKIVVDRDPIKTSFEEWARPGHFSRTIAKGPDTTTWIWNLHADAHDFDSHTSDLE
EISRKVFSAHFGQLSIIFLWLSGMYFHGARFSNYEAWLSDPTHIGPSAQVVWPIVGQEILNG
DVGGGFRGIQITSGFFQIWRASGITSELQLYCTAIGALVFAALMLFAGWFHYHKAAPKLAW
FQDVESMLNHHLAGLLGLGSLSWAGHQVHVSLPINQFLDAGVDPKEIPLPHEFILNRDLL
AQLYPSFAEGATPFFTLNWSKYAEFLSFRGGLNPVTGGLWLTDIAHHHLAIAILFLIAGHMY
RTNWGIGHGLKDILEAHKGPFTGQGHKGLYEILTTSWHAQLSLNLAMLGSSTIVVAHHMY
SMPPYPYLAIDYGTQLSLFTHHMWIGGFLIVGAAAHAAIFMVRDYDPTTRYNDLLDRVLR
HRDAIISHLNWACIFLGFHSFGLYIHNDTMSALGRPQDMFSDTAIQLQPIFAQWVQNTHAL
APGATAPGATTSTSLTWGGSDLVAVGGKVALLPIPLGTADFLVHHIHAFTIHVTVLILLKGV
LFARSSRLIPDKANLGFRFPCDGPGRGGTCQVSAWDHVFLGLFWMYNAISVVIFHFSWKM
QSDVWGSISDQGVVTHITGGNFAQSSITINGWLRDFLWAQASQVIQSYGSSLSAYGLFFLG
AHFVWAFSLMFLFSGRGYWQELIESIVWAHNKLKVAPATQPRALSIVQGRAVGVTHYLLG
GIATTWAFFLARIIAVGMALRFPRFSQGLAQDPTTRRIWFGIATAHDFESHDDITEERLYQNI
FASHFGQLAIIFLWTSGNLFHVAWQGNFESWVQDPLHVRPIAHAIWDPHFGQPAVEAFTRG
GALGPVNIAYSGVYQWWYTIGLRTNEDLYTGALFLLFLSAISLIAGWLHLQPKWKPSVSW
FKNAESRLNHHLSGLFGVSSLAWTGHLVHVAIPGSRGEYVRWNNFLDVLPYPQGLGPLFT
GQWNLYAQNPDSSSHLFGTSQGAGTAILTLLGGFHPQTQSLWLTDIAHHHLAIAFIFLVAGH
MYRTNFGIGHSMKDLLEAHTPPGGRLGRGHKGLYDTINNSIHFQLGLALASLGVITSLVAQ
HMYSLPAYAFIAQDFTTQAALYTHHQYIAGFIMTGAFAHGAIFFIRDYNPEQNEDNVLARM
LDHKEAIKSHLSWVSLFLGFHTLGLYVHNDVMLAFGTPEKQILIEPIFAQWIQSAHGKTSY
GFDVLLSSTSGPAFNAGRSIWLPGWLNAVNE--NSNSLFLTIGPGDFLVHHAIALGLHTTTLI
LVKGALDARGSKLMPDKKDFGYSFPCDGPGRGGTCDISAWDAFYLAVFWMLNTIGWVT
FYWHWKHITLWQGNVSQFNESSTYLMGWLRDYLWLNSSQLINGYNPFGTNSLSVWAWM
FLFGHLVWATGFMFLISWRGYWQELIETLAWAHERTPLANLIRWRDKPVALSIVQARLVGL
AHFSVGYIFTYAAFLIASTSGKFGMSHSVKIYDTCIGCTQCVRACPTDVLEMIPWDGCKAK
QIAPAPRTEDCVGCKRCESACPTDFLSVRVYLWHETTRSMGLAYMTDFNLPSIFVPLVGLV
FPAIAMASLSLHVQKNKIVMRDIKTYLSVAPVLTTLWFGSLAGLLIEINRLFPDALVFPFFSF
MTAILERRESTSLWGRFCNWITSTENRLYIGWFGVLMIPTLLTATSVFIIAFIAAPPVDIDGIR
EPVSGSLLYGNNIISGAIIPTSAAIGLHFYPIWEAASVDEWLYNGGPYELIVLHFLLGVACY
MGREWELSFRLGMRPWIAVAYSAPVAAATAVFLIYPIGQGSFSDGMPLGISGTFNFMIVFQ
AEHNILMHPFHMLGVAGVFGGSLFSAMHGSLVTSSLIRETTENESANEGYRFGQEEETYNI
VAAHGYFGRLIFQYASFNNSRSLHFFLAAWPVVGIWFTALGISTMAFNLNGFNFNQSVVDS
QGRVINTWADIINRANLGMEVMHERNAHNFPLDLAAVEAPSTNGMGLPWYRVHTVVLN
DPGRLLSVHIMHTALVSGWAGSMALYELAVFDPSDPVLDPMWRQGMFVIPFMTRLGINNS
WGGWSIT-GGTITNPGIWSYEGVAGAHIVFSGLCFLAAIWHWVYWDLEIFCDERTGKPSLD
LPKIFGIHLFLSGVACFGFGAFHVTGLYGPGIWVSDPYGLTGKVQSVNPAWGAEGFDPFVP
GGIASHHIAAGTLGILAGLFHLSVRPPQRLYKGLRMGNIETVLSSSIAAVFFAAFVVAGTM

WYGSATTPIELFGPTRYQWDQGYFQQEIYRRVGAGLAENLSLSEAWSKIPEKLAFYDYIG
NNPAKGGLFRAGSMDNGDGIAVGWLGHPIFRDKEGHELFVRRMPTFFETFPVVLVDGDGI
VRADVPFRRAESKYSVEQVGVTVEFYGGELNGVSYSDPATVKKYARRAQLGEIFELDRAT
LKSDGVFRSSPRGWFTFGHATFALLFFFGHIWHGARTLFRDVFAGIDPDLDAQVEFGAFQK
LGDPTTRRQVVMKTLYSLRRFYPVETLFNGTLSLAGRDQETTGFAWWAGNARLINLSGKL
LGAHVAHAGLIVFWAGAMNLFEVAHFVPEKPMYEQGLILLPHLATLGWGVGPGGEVVDT
FPYFVSGVLHLISSAVLGFGGIYHALLGPETLEESFPFFGYVWKDRNKMTTILGIHLILLGIG
AFLLVLKALYFGGVYDTWAPGGGDVRKITNLTLSPSVIFGYLLKSPFGGEGWIVSVDDLED
IIGGHVWLGSICILGGIWHILTKPFAWARRAFVWSGEAYLSYSLGALSVFGFIACCFVWFNN
TAYPSEFYGPTGPEASQAQAFTFLVRDQRLGANVGSAQGPTGLGKYLMRSPTGEVIFGGE
TMRFWDLRAPWLEPLRGPNGLDLSRLKKDIQPWQERRSAEYMTHAPLGSLNSVGGVATE
INAVNYVSPRSWLATSHFVLGFFLFVGHLWHAGRARAAAAGFEKGIDRDFEPVLSMTPLN
MTIALGRFTKEENDLFDIMDDWLRRDRFVFVGWSGLLLFPCAYFALGGWFTGTTFVTSW
YTHGLASSYLEGCNFLTAAVSTPANSLAHSLLLLWGPEAQGDFTRWCQLGGLWTFVALHG
AFGLIGFMLRQFELARSVQLRPYNAIAFSAPIAVFVSVFLIYPLGQSGWFFAPSFGVAAIFRF
ILFFQGFHNWTLNPFHMMGVAGVLGAALLCAIHGATVENTLFEDGDGANTFRAFNPTQA
EETYSMVTANRFWSQIFGVAFSNKRWLHFFMLFVPVTGLWMSAIGVVGLALNLRAYDFV
SQEIRAAEDPEFETFYTKNILLNEGIRAWMAAQDQPHENLIFPEEVLPRGNALMSGSTGER
SFADIITSIRYWVIHSITIPSLFIAGWLFVSTGLAYDVFGSPRPNEYFTESRQGIPLITGRFDPL
AQLDEFSRSFMTIDRTYPIFTVRWLAVHGLAVPTVSFLGSISAMQFIQRMATQTVEGSSRSG
-PRRTITGDLLKPLNSEYGKVAPGWGTTPFMGVAMALFAIFLSIILEIYNSSVLLDGISMSML
TLKLFVYTVVIFFVSLFIFGFLSNDPGRNPGREEMADTTGRIPLWLIGTVTGIPVIGSMGIFF
YGSYSGLGSSLMLNIFSLICICINSALHSSSFFFAKLPEAYAFFNPIVDVMPVIPVLFFLLALV
WQAAVSFRTTQSNPNEQNVELNRTSLYWGLLLIFVLAVLFSNYFFNMEVNILAFIATALFIL
VPTAFLLIIYVKTVSQNDMETATLVAISISGSLVSFTGYALYTAFGQPSQQLRDPFEEHGDME
ALVYTFLLVSTLGIIFFAIFFREPPKVPTKKMKMTIAFQLAVFALIATSSILLISVPVVFASSDG
WSSNKNVVFSGTSLWIGLVFLVAILNSLIS-------MSPQTETKASVGFKAGVKEYKLTYYTPE
YETKDTDILAAFRVTPQLGVPPEEAGAAVAAESSTGTWTTVWTDGLTSLDRYKGRCYHIE
PVAGETDQYIVYVAYPLDLFEEGSVTNMFTSIVGNVFGFKALRALRLEDLRIPPAYIKTFQG
PPHGIQVERDKLNKYGRPLLGCTIKPKLGLSAKNYGRAVYECLRGGLDFTKDDENVNSQP
FMRWRDRFLFCAEALYKAQSETGEIKGHYLNATAGTCEEMMKRAVFARELGVPIVMHDY
LTGGFTANTSLAHYCRDNGLLLHIHRAMHAVIDRQKNHGIHFRVLAKALRMSGGDHIHSG
TVVGKLEGERDITLGFVDLLRDDFIEKDRSRGIYFSQDWVSLPGVLPVASGGIHVWHMPA
LTEIFGDDSVLQFGGGTLGHPWGNAPGAVANRVALEACVQARNEGRDLASQGNEIIREAS
KWSPELAAACEVWKEIKFDFKAMDTL---AIHLYKTSTPSTRNGAVDSQVKSNPRNNLIHG
QHHCGKGRNARGIITAGHRGGGHKRLYRKIDFRRNEKDTSGRIVTIEYDPNRNAYICLIHY
GDGEKRYILHPRGAIIGDTIVSGTEVPISMGNALPLTDMPLGTAIHNIEITLGKGGQLARAA
GAVAKLIAKEGKSATLRLPSGEVRLISKNCSATVGQVGNVGVNQKSLGRAGSKCWLGKRP
VVRGVVMNPVDHPHGGGEGRAPIGRKKPTTPWGYPALGRRSRKRNKYSDSLILRRRK-MI
QPQTHLNVADNSGARELMCIRIIGAS-NHRYAHIGDVIVAVIKEAVPNMPLERSEVIRAVIVR
TCKELRRDNGMIIRYDDNAAVVIDQEGNPKGTRVFGAIARELRQLNFTKIVSLAPEVL---P
KRTRFRKQHRGRMKGISYRGNHICFGRYALQALEPAWITSRQIEAGRRAMTRYARRGGKI
WVRIFPDKPVTVRPTETRMGSGKGSPEYWVSVVKPGRILYEMGGVSETVARAAIKIAACK
MPIRTQFIISGMTRVRRGYIARRRRTKIRLFASTFRGAHSRLTRTTTQQKMRALVSTHRDRG

RRKRDFRHLWITRINAVTREN--VSYSYSRLIHDLYKRQLLLNRKIPAQIAISNRNCLYTISNE
I I K - - - - - - - - - -
-??
?????????????????????????????????MDGIKYEVFTEKSIRLLENNQ-YTSNVESGSTRTEIKH
WVELFFGVKVIAMNSHRLPGKGIRMGPIMGHTMHYRRMIITLQPGYSILTLIQK-RMAVPK
KRTSMSKKRIRRNIWKKKGYWAAVKALSLAKSISTGHSKSFFVRQTSNMAKGKDVRGIVI
LQCTSCVRNDFN--KESRGTSRYITQKNRHNTPSRLELRKFCPYCYKHTIHGEIKKMKIRAS
VRKICEKCRLIRRRGRIIVICFNPRHKQRQGMVREEVAVSTRTLQWKCVESRTDSKRLYYG
RFILAPLMKGQADTIGIAMRRALLGEIEGTCITRVKSE--KVPNEYYTIVGIEESVHEILMNL
KEIVLRSHLYGTRDASICVRGPRYVTAQDIIPPPSVEIVDTTQHIASLTEPIDLCIELQIERDRG
YRMKTPNNYQDGSY--PIDAVSMPVRNANHSIHSYGNRNEKQEILFLEIWTNGSLTPKEAL
HEASRNLIDLFIPFLHAEEQDIVL--EDNP-NRFTVPLFTFHDRLAN-IRKNKK-GIALKCIFID
QSELPPRTYNCLKRSNIHTLLDLLSNSQEDLMRIEHFRIEDVKQILDILQKH--FTIDLPKNK
MLRDG-NEGMSTIPGFSQIQFEGFCRFIDQGLTEELHKFPKIEDTDQEIEFQLFVETYQLVEP
LIKERDAVYESLTYSSELYVPAGLIWKTGRDMQEQTVFIGNIPLMNSLGTSIVNGIYRIVINQ
ILQSPGIYYRSELDHNGISVYTGTIISDWGGRSELEIDRKARIWARVSRKQKISILVLSSAMG
SNLREILDNVCYPEIFLSFPNDKEKKKIGSRENAILEFYQQFACVGGDPVFSESLCKELQKK
FFQQRCELGRIGRRNMNRRLNLDIPQNNTFLLPRDVLAAADHLIGMKFGMGTLDD--MNH
LKNKRIRSVADLLQDQFGLALVRLENAVRGTICGAIR-HKLI-PTPQNLVTSTP----LTTTYES
FFGLHPLSQVLDRTNPLTQIVHGRKSSYLGPGGLTGRTASFRIRDIHPSHYGRICPIDTSEGIN
VGLIGSLAIHARIGHWG-SIESPFY---EISERSKE--VQMVYLSPSRDEYYMVAAGNSLALNW
GIQEEQVVPARYRQEFLTIAWEQIHLRSIFPFQYFSIGASLIPFIEHNDANRALMSSNMQRQA
VPLSRSEKCIVGTGLECQAALDSGVSAIAEHEGKIVYTDTDKIVLSGNGDTISIPLVMYQRS
NKNTCMHQKPQVRRGKCIKKGQILADGAATVGGELALGKNVLVAYMPWEGYNSEDAVL
ISERLVYGDIYTSFHIRKYEIQTHVTSQG-PERITNEIPHLEAHLLRNLDKNGIVMLGSWIET
GDILVGKLTPQTAKE-SSYAPEDRLLRAILGIQVSTAKETCLKLPIGGRGRVIDVRWIQKKG-
GSSYNPEMIRVYISQKREIKVGDKVAGRHGNKGIISKILPRQDMPYLQDGTPVDMVFNPLG
VPSRMNVGQIFECSLGLAGDLLDRHYRIAPFDERYEQEASRKLVFSELYSASKQTANPWVF
EPECPGKSRIFDGRTGDPFEQPVIIGKSYILKLIHQVDDKIHGRSSGHYALVTQQPLRGRAK
QGGQRVGEMEVWALEGFGVAHISQEMLTYKSDHIRARQEVLGTTIIGGTIPNPEDAPESFR
LLVRELRSLALELNHFLVSEKNFQINRKEA-------MIDRYKHQQLRIGSVSPQQISAWANKIL
PNGEIVGEVTKPYTFHYKTNKPEKDGLFCERIFGPIKSGICACGNYRVIGNEKEDPKFCEQC
GVEFVDSRIRRYQMGYIKLACPVTHVWYLKRLPSYIASLLDKPLKELEGLVYCD--FSFARP
-IAKKPTFLRLRGSFESEIQSRKYSIPLFFTTQDFDTFRNREISTGAGAIKEQLADPDLRIITDH
SLVEWKELGEEGSADGNEWEDRKIGRRKDFLVRRMELAKHFIRTNVEPERMVLCLLPVLP
PELRPIIQIDGGKPMSSDINELYRRVIYRNNTLTDPLTTSRSTPGESVMCQEKLVQEAVDTLL
DNGIRGQPMRDGHNKVYKSFSDVIEGKEGRFRETLLGKRVDYSGRSVIVVGPSLSLHRCG
LPREIAIELFQTFVIRGLIRQHVASNIGIAKSKIREKEPIVWEILQEVMQGHPVLLNRAPTLH
RLGIQAFQPILVEGRAICLHPLVRKGFNADFDGDQMAVHVPLSLEAQAEARLLMFSHMNL
LSPAIGDPISVPTQDMLIGLYILTIGNRRGICSNRYNPCNRR--N-YQNETVDYN--KYTKEKEP
YFCSSYDALGAYRQKRINLDSPLWLRWRLDQR-VIAS--REVPIEVQYESLGTYHEIYGHYL
IVRSVKKEILCIYIRTTVGHISFYREIEEAIQGFCRAYLYDT--------MEVLMAERADLVFHNK
VIDATAMKRLISRLIDHFGMAYTSHILDQVKTLGFQQATATSISLGIDDLLTIPSKGWLVQD
AEQQSLILEKHHHYGNVHAVEKLRQSIEIWYATSEYLRQEMHPNFRMTDPSNPVHIMSFSG

ARGNASQVHQLVGMRGLMSDPQGQMIDLPIQSNLREGLSLTEYIISCYGARKGVVDTAVR
TSDAGYLTRRLVEVVQHIVVRRTDCGTIRGISVSPRN--GMTEKILIQTLIGRVLADDIYMGL
RCIATRNQDIGIGLVNRFIT------FRAQSIYIRTPFICRSTSWICRLCYGRSPTHGDLVELGEAV
GIIAGQSIGEPGTQLTLRTFHTGGVFTGGTAEHVRAPSNGKIKFNECLVHPTRTRHGHPAFL
CYIDLYVTIESQDIIHNVNIPPKSFLLVQNDQYVESEQVIAEIRAGTSTF--FKERVRKHIYSDS
EGE-MHWSTDVYHAPEYRYGNVHLLPKTSHLWILSGGP--CRSSIVPFSLHKDQDQMNVH-
-SLSVERRYISDLS--VTNDR-VRHKLFSSDPSGK-KERILDYS-GPDRIVSNGHWNFLYPA-IL
HENSD--LLAKRRRNRFIIPFQYDQEREKELMPRSGISIEIPIN--GILRRDTILAYFDDPRYRRS
SSGITKYGTIEVDSIVKKED--LIEYRG-AKEFRPKYMKVDRFFFIPEEVHILPGSSPIMVRNN
SIIGVDTRIALNTRSRVGGLVRVERKKKKIELKIFSGDIHFPGETDKISRHSGILIPPGTGKK--
NSKESK--KWKNWIYVQR--ITPTKKK--YFVSVRPVVTYEIADGINLGTLFPQDLLQERDNV
QLRVVNYILYGNGKPIRGIYHTSIQLVRTCLVLNWDQDRNG----SIEEVHASFVEVGTNDLI
RDFIRIDLVKSPISYIGKRDDTTGSGLIPD-NESDRTNINTFYSKTR---------IQSLTQHQGTIRT
FLNR---NKECQSFLILSSSDCSRIGP--FNGSKSHKVT---KESDPMIPIRNSLGPLGT--VPKIAN
FYSS--YYLIT--HNQILLN-KYLLLDN---LKQTFQVLKYY---LMDENGR--IYNPNLHSNIIFNP
FDLNWCFLRHDYCEETSTIIS-LGQFICENVCISKYGP---HIKSGQVLIVH-VDSLVIRSAKPH
LATPGATVHGHYGEILSEGDTLVTFIYEKSRSGDITQGLPKVEQVLEVRSIDSISMNLEKRIE
GWNEHITRILGIPWGFLIGAELTIAQSRISLVNKIQKVYRSQGVQIHNRHIEIIVRQITSKVLV
SE---DGMSNVFSPGELIGLLRAERTGRALEEGICYRAILLGITRASLNTQSFISEASFQETAR
VLAKAALRGRIDWLKGLKENVVLGGMIPVGTGFKGLVHRSR--QHNNIP--LEIKKKNLFE
GEMR--DILFHHRELLSSCIPKNFHDTSFTGFNDS---------MTRRYWNIHLEEMMEAGVHFG
HGTRKWNPRMAPYISAKRKGIHITNLTRTARFLSEACDLVFDAASRGKNFLLVGTKNKAA
DSVASAAIRARCHYVNKKWLGGMLTNWSTTETRLHKFRDLRAEQKTGKLNRLPKRDAA
MLKRQLSHLQTYLGGIKYMTGLPDIVIIVDQQEEYTALRECLTLGIPTICLIDTNCDPDLADI
SIPANDDAIASIRLILNKLVSAICEGRSS-SIRNRMGQKINPLGFRLGGEQKHRSLWFAQPKS
YSIGLQEDEKIRDCIKNYVQKNMRISSG-FEGIAHIEIKKKIDLIQVIIYIGFPNLLIEG--RTR--
GIEELQINVQKGFHSVNRRLNIAITRVAKPYGQPNILAEYIALQLKNRVSFRKAMKKAIELT
EQADTKGIQVQIAGRIDGKEIARVEWIREGRVPLQTIRAKIDHCSYTVRTIYGVLGIKIWIFV
DEQMSRYRGPRFKKIRRLGALPGLTSKRPRPGSDLRNQSRSGK-RSQYRIRLEEKQKLRFH
YGLTERQLLRYVRIAGKAKGSTGQVLLQLLEMRLDNILFRLGMASTIPGARQLVNHRHIL
VNGRIVDIPSYRCKPRDIITTRGEQRSRALIQNYIDSSPH-EELAKHLTFHSSQY--KGLVNQII
DSKWIGLKINELLVVEYYSRQTMSRRGTAEEKTAKSDPIYRNRLVNMLVNRILKHGKKSL
AYQIIYRAVKKIQQKTETNPLSVLRQAIRGVTPDIAVKARRVGGSTHQVPIEIGSTQGKALA
IRWLLGASRKRPGRNMAFKLSSELVDAAKGSGDAIRKKEETHRMAEANRAFAHFRMGRD
TIADILTSIRNADMDKKGTVRIASTNMAESVVKILLREGFIENVRKHRENNKYFLVSTLRH
RR--NRKGTYR---NIL--KRISRPGLRIYSNYQRIPRILGGMGIVILSTSRGIMTDREARLEGIG
GEVLCYIWMTKPIPRIGSRRNGRIGSRKSGRRIPKGVIHVQASFNNTIVTVTDVLGQVVSW
SSAGTCGFRGTRRGTPFAAQTAAGNAIRTVVDQGMQRAEVMIKGPGLGRDAALRAIRRS
GILLSFVRDVTPMPHNGCRPPKKRRVMPTIKQLIRNTRQPIRNVTKSPALRGCPQRRGTCT
RVY-ITPKKPNSALRKVARVRLTSGFEITAYIPGIGHNSQEHSVVLVRGGRVKDLPGVRYHIV
RGTLDAVGVKDRQQGRS-SAL----MARKSLIQRERKRQKLEQKYHLIRRSSKKEIS---KVSS
LSDKWEIHGKLQSPPRNSAPTRLHRRCFSTGRPRANYRDFGLSGHILRERVHACLLPGATR
SSWMVKNSFISVIPQEEKEK--NKGSVEFQVFSFTNKIRRLTSHLELHRKDYLSQRGLRKIL
GKRQRLLAYLSKKNRVRYKELIGRLDIREPKTR--MVKLRLKRCGRKQRAIYRIVAIDVRSR

REGRDLRKVGFYDPIKNQTYSNVPAILYFLEKGAQPTGTVHDISKKAEVFKELRINQTK---
MDKSKRLFRKSKRSFRRRLPPIGSGDRIDYRNMSLISQFISEQGKILSRRVNRLTLKQQRLIT
IAIKQARILSSLPFLNNEK--QFEKTESIPRTTGPRTRNKVTRSLKKNPFVANNFLGKIEKLNM
REEKEIIITWSRASTIIPTMIGHTIAIHNGKDHLPIYITDRMVGHKLGEFAPTLTFKGHARND
NRSRRMILKSFLLGNLLSLCMKIINSVVGVGLYYGFLTTFSIGPSYLFLLRARVMEEG---TE
KEVSATTGFITGQLMMFISIYYAPLHLALDRPHTITVLVLPYLLFHFFWNNHKHFFDYGSTT
RNSMRNLSIQCVFLNNLIFQLFNHFILPSSTLARLVNIYMFRCNNKMLFVTSSFVGWLIGHI
FFMKWVGLVLFWIRQNHSIRS-IRSNKYLVSELRNSMARIFSILLFITCVYYLGRIPSPIVTKK
LKETEEDKPLVTLLFDYKRWNRPLRYIKNDRFEKTVRNEMSQYFFHTCPSDGKKRISFTYP
PSLSTFGEIIQRKMSLYTTDKLLPEDPYNHWVYTNEQKRHNLSNEFINRIEALD--RLTLDVL
EKSTRLCNNENEQEFFPKIYDPFLNGPYRGTTKKVYSRIEMVWINKIHDIFYREFEQKD-PN
DYIGIEEIGKKVPRWSYKLTHLEEQEKEGIRSRKAKRVVIYTDALIRYSQQSDFRRDLIKGS
MRAQRRKTVTWEMFQANVHSPLFLDRIDKTFFFSFDI--SGMMNLIFRNKEENERIAIAET
WDTIIFAQAIRGSMLVTQSILRKYIVLPSLIIAKNLGRMLLFQFPEWYEDWKEWNREMHV
KCTYNGVQLSETEFPKDWLTDGIQIKILFPFCLKPWHRSKKGKKENFCFLTVWGMETELPF
GSSRKRPSFFEPICKELEKKIRKVK-KKCFLVLRALGERTKWFLRVLKEKTRWILKTVL--FI
KRIIKEFAKVN-SITNNKINHESIIRIRSVDWTNYSLTEKKMKDLADRTTTIRNKIERITKDKR
KSPNETGCDDKRSESQKHIWQISKRRSARLIRKWHYFMKSFIERIYMDIFLCTINIPRRNAH
LFIHFISCDLSSLSQAYVFYNLLQIQVINKYYLRFVLQYAYLFLKDRIKDHFTRGILDAKSRP
KKPPNSGMNEWKNWLRGHYQYNLSQTRWSRLVPQKWRNRVNQRRTIQNKK-KDSYEK--
DQFIHYEKQEKFKKHYRYDLLSHKYIHESIIDPDRKYLEWRILNFFLRFFDWMGMNEEML
YRWFFSEFVLLYDAYKIKPWIIPIKLLIFNSKSNKEAELNFFLKRYLLFQLRWDDPLNQRMI
NNIKVYCLLLRLINPKEIAISSIQRGEMRLDVMLIQKDLTLTELIKRGILIIEPVRLSIKWDGQ
WIMYQTIGISLVHKNKYNNYALLVPENIPSPRRRRELRILICLRFLCPNYRLEDLACMNRY
WFDTNNGSRFSMSRIHMYPMKRHQFKSWIFEL----REIKNSHYFLDSWTKFDSVGSFTHIF
FHQERFMKLFDPRIWSILLS-RDS--QGSTSNRYF---TIKGVVLLVVA-VLI----YRINNRNMVE
RKNLYLMGLLPIPILPIPMNSIGPRNDTLEESF-WSSNINRLIVS-LLYLPKGKKISESCFMDPK
ESTWVLPIT----KKCIMPESNWGSRWWRNRIGKKR--DSSCKIS---NETVAGIEISFKEKDIK-
YLEFLFVSYTD--DPIRKDLDWEFFDRLSPRKKRNIINL--NSGQLFEILVKHLICYLMSAFRE
KRPIEVEG-FFKQQG-AEATIQSNDIEHVSH--LFSRNKWGISLQNCAQFHMWQFRQDLFV---
SWGK-NQHESDFLRN-VSRENWIWLDNVWLVNKDW---FFS--KVRNVSSNIQYDS---TR--SI
FVQVTDSSQLKGSS-DQSRDPFDSIS----NEDSE------------YHTLINQTEIQQLKERSIL-WD--
PSFLQTERTEIESD--RFPK--CLSGYS--SMSRLFT-EREKQMNNHLLPEEIEEFL--GNPTRSIRS
FFSDRW--SELH-LGSNPTERSTRDQ----KLLKKQQDVS-FVPSRRSE----NKEMVDIFKIITYL
QN--TVSIHPISSDPGCDMVP-KDEPD-MGSSNKISFLNKNPFLD--LFHLFHDRNKGGYTL--H
HDFESE--ERFQEMADLFTLSITEP-DLVYHKGFAFSIDSYGLDQKK--FLNEVFNSRDESKKK
SL---WVLPPIFD--EENESFYRRIRKKSVR-ISCGNDLEDPKPKIVVFASNNIMEAVNQYRLIR
NLIQIQYSTYG--YIRNVSNRFFLM---NRSDRNFEYGIQ---RDQIGNDTLNHLTRIKYTINQHL
SNLKK-SQKKWFD----PLISRTERSMNRDPDAYRYKWSNGSKNFQEHLEHFVSEQKH---RF
QVVFDRLRINQYSIDWSEVIDKQDLSKSLRFFLSKS---------LLFLS-------KSL-LFLSKSLPFF
FVSIGNIPIHRSE-IHIY-ELKVPNDQLCNQLLESIGVQIVHLNKLKP--FLLD---DHDTS-QRSK
FLINGGTISPFLFNKIP-KW-MIDSFHT--RNNRRKSFDNTDSYFSMISRDRDNWLNPVKPFHR
SSLISSFYKANRLRFLNNPHHFWFY--CNKRFPFYV----EKTRINNYD--LTY-GQFLNILFIRNK
-IFSLCVGK--KKHVFLE-RDTISPIESQVSDIFIPNDFPQSGGET-YNLYKS---FHFPIRSDPFVR-

-RAIYSIADISGTPLTEEQIVNFERTYCQPLSDMNLSDSEGKNLHQYLS--FNSNMGLIHTPCS
EKDLP--SGKRKK--------RSLCLK--KCVEKRQMYRTFQRDSAFSNLSKWNLFQTYMPWFLT
STGCKYLNFTLLDTFSDPL--PILSSS-QKFVSIFHDIMHGSDISWPIPQKKLW----AILPQWNL-
ISEISSKCLQN--LLLSEEMIHRNNESPVPLIWTHLRSPNAREF-LYSILFLLLVAGY-LVRTHLL
FVSR--ASSELQTELEKIKSLMIPSYMIELRKLLDRYPTSELNSFWLKNLFLVALEQLGDSLE
EIRGSASGGNMLLGGGP-----AYG-VKSIRSKKKYLNIN---LIDLI-SIIPNPINRITFSRNTRHLS
RTSKEIYSLIRKRKNVNGDWIDDKIESWVANSDSIDDEEREFLVQFS--TLTT--EKRIDQILLS
LT-HSDHLS-----KNDSGYQMIEQPGSIYLRYLVDIH--KKYLMNYEFNRSCLAERRIFLAHYQ
TITY--SQTSCGANSSHFPSHG---KPFSLRLALSPS-RGILVIGSIGIGRSYLVKYLATNSYVPFI
TVFPNKFLDDKPKGYLID-----DID-IDDSDDID----IDDSDDI-----------------DDDLDTELLTM
TNVL---TMYMT---PKIDRFDITPQLELAKAMSPCIIWIPNIHDLYVNESNYLSLGLLVNYLS--
-RDCERCS-TRNILVIASTHIPKKVDPALIAPNKLNTCIKIRRLLIPQQRKHFFILSYTRGFHLE
KKMFHT--NGFGSMTMGSNARDLVALTNEALSISITQKKSIIDTNTIRSALHRQTWDLR---SQ
VRSVQDHGILFYQIGRAVAQNVLLSNCPIDPISIYMKKK--SCKEGDSYLYKWYFELGTSMK
KLTILLYLLSCSAGSVAQDLWSPP--GPDEK-NGITSYGFVENDS-DLVHG-LEVEGALVG--SS
RTEKDCSQFDND--RVTLLLRSEPRN-QLDMMQNGSCSIVDQRFLYEK--YESEFEG-GEGEG
ALDPQ----QIEED----LFNH--IVWAP--RIWRP---CGNLFDCIERTNELGFP-YWARSFR---GKRI
IYHKEDELQENDSEFLQSGTMQYQTRDRSSKEQGFFRISQFVWDPADPFFFLFK-DQPFVS
VFSRREFFADEEMSKGLITSQTNPPTSIYKRWFI--KNTQEKHFELLIHRQRWLRT---NSSLS--
NGSFRSN--TPSESYQYLSNLFLSN-GTLLDQMTKALLRKRWLFPDEMKHLI--HVR---FPIP
MPRSR-INGNFIDKTSSIVANILLRIIPTTSGEKEAFTYYRDG--MSAQSEGNYAEALQNYYEA
TRPEIDPYDRSYILYNIGLIHTSNGEHTKALEYYFRALERNPFLPQAFNNMAVICHY-RGEQ
AIRQGDSEIAEAWSDQAAEYWKQAIALTPGNYIEAHNWLKITRRFE--MNWQSERIWIELIT
GSRKTSNFCWACILFLGSLGFLLVGTSSYLGRNLISLFPSQQILFFPQGIVMSFYGIAGLFISS
YLWCTISWNVGSGYDLFDRKEGIVCIFRWGFPGINRRIFLRFLMRDIQSIRMEVKEGLYPRR
VLYMEIRGQGAIPLTRTDEN-FTPREIEQKAAESAYFLRVPIEVF
>Citrus
MKKWWFNSMLSLSKSMESPGPIKNPSVSEELIRNDKNKNIHSYS---KLAGVRDIHNFISDD
TFFVKDSNRDSYSIYFDIENQILELDNAH--SFLSELESSFYSFRNYSSRNNGSKSADPDSDR
YMYDTKSSWNNHIHNCLDSYL--HSQICIDSHVLSSSDNYSASYIYNFIC-SESSNIQSSTNGS
DLTISESSNESDVTQKYRHLWVQCENCYGLNYKKFLKSKMYLCEQCGYHLKMISSDRIDL
LVDPGTWDPMDDDMVSIDPIEFDSEEEPYKNRIDSYQSKTGLTEAVQTGTGQLNGIPIAIGV
MDFQFMGGSMGSVVGEKITRLIEYAANKFLPLLLVCASGGARMQEGSLSLMQMAKISSAL
YDYQSNKKLFYVSILTSPTTGGVTASFGMLGDIIIAEPNAYIAFAGKRVIEQTLNKTVPEGS
QEAEYLFDKGLFDPIVPRNPLKGVLSELFLFHGRFPLNQN---MTTIKADEISNIIRERIEQYN
REVKIVNIGTVLQVGDGIARIYGLDEVMAGELVEFEEGTIGIALNLESTNVGVVLMGDGL
MIQEGSSVKATGKIAQIPVSEAYLGRVINALAKPIDGRGEISASESRLIESPAPGIISRRSVYEP
LQTGLIAIDSMIPIGRGQRELIIGDRQTGKTAVATDTILNQQGQNVICVYVAIGQKASSVAQV
VNTFQERGAMDYTIVVAETADSPATLQYLAPYTGAALAEYFMYRERHTLIIYDDPSKQAQ
AYRQMSLLLRRPPGREAYPGDVFYLHSRLLERAAKLGSQLGEGSMTALPIVETQSGDVSA
YIPTNVISITDGQIFLSADLFNAGIRPAINVGISVSRVGSAAQIKAMKQVAGKLKLELAQFAE
LEAFAQFASDLDKATQNQLARGQRLRELLKQAQAAPLTVEEQIMTIYTGTNGYLDSLEIG
QVRKFLVELRTYLKTNKPQFQEIISSTKIFTEEAEALLQEAIQEQKERFLLQEQFMRINPTTS
GPGVSAFANKNLGHIAQIIGPVLDVAFPPGKMPNIYNALVVKGRDTVDQPINVTCEVQQLL

GNNRVRAVAMSATDGLTRGMEVIDTGAPLSVPVGGVTLGRIFNVLGEPVDNLGPVDTRTT
SPIHKSAPAFIQLDTRLSIFETGIKVVDLLAPYRRGGKIGLFGGAGVGKTVLIMELINNIAKA
HGGVSVFGGVGERTREGNDLYMEMKESGVINDQNLSESKVALVYGQMNEPPGARMRVG
LTALTMAEYFRDVNEQDVLLFIDNIFRFVQAGSEVSALLGRMPSAVGYQPTLSTEMGSLQE
RITSTKEGSITSIQAVYVPADDLTDPAPATTFAHLDATTVLSRGLAAKGIYPAVDPLDSTSTM
LQPRIVGEEHYETAQRVKQTLQRYKELQDIIAILGLDELSEEDRLTVARARKIERFLSQPFFV
AEVFTGSPGKYVGLAETIRGFKLILSGELDGLPEQAFYLVGNIDEVTAKATNLEMESNLKK
MTLNLCVLTPNRIVWDSEVKEIILSTNSGQIGVLPNHAPIATAVDIGILRIRFNDQWLTMAL
MGGFARIGNNEITILVNDAEKSSDIDPQEAQQTLEIAEANLRKAESKRQTIEANLALRRART
RVEVINAISMKNVTDSFVSLVNWPFAGSFGLNTDILATNPINLSVVLGVLIFFGKGVLSDLL
DNRKLRILNSIRNSEELQRGAIERLEKARARLRKIEIEADQFRVNGYSEIEREKFNLINSTYK
TLDQLENYKNETIHFEQQRAINQVRQRVFQQALQGALGTLNSCLNKELHLRTISANIGMF
GAMKEITDMNPLISAASVIAAGLAVGLASIGPGVGQGTAAGQAVEGIARQPEAEGKIRGTL
LLSLAFMEALTIYGLVVALALLFANPFVMNVLSCSMNTLR-LYDISGVEVGQHFYWQIGGF
QVHAQVLITSWVVIAILLGSAFIAVRNPQTVPTATQNFFEYVLEFIRDVSKTQIGEEYGPWV
PFIGTLFLFIFVSNWSGALLPWKIIELPHGELAAPTNDINTTVALALLTSIAYFYAGLSKKGL
GYFSKYIQPTPILLPINILEDFTKPLSLSFRLFGNILADELVVVVLVSLVPLVVPIPVMFLGLFT
SGIQALIFATLAAAYIGESMEGHHMIFSTLERILTHISFSVVSIGITIYLITFLVDEIRGLYASSE
RGMTATAFCLTGLLITRWVYSRHFPLSDLYESLIFLSWSLSIIHKIFDFKNNQNHLSAITAPSA
FFTQGFATSGFLTKMHQSRILVPALQVQWLMMHVSMMVLGYAALLCGSLLSTALLVITFR
KVIRLFLKRKEIQYMNEKKNVFL-S--LSARNYYRYQVIQQLDRWSYRIISLGFIFLTTGILSG
AVWANEAWGSYWNWDPKETWAFITWTIFGIYLHTRTNTKWEGVNSAIVASMGFLIIWICY
FGVNLLGIGLHSYGSFN--MTKKNASIPLRYLSSVVFLPWWIPLSFNKSLESWVTNWWNTS
QSETFLNDIQEKAILEKFIELEELFLLDEMIKEFPERRLEKLRI-LQKETIQLIKMHDEDHIHTI
FHFSTNTICFVILSGYSILCNEELFILNSWVQEFLYNLSDTIKAFSILFVTDLCIGFHSPRGWE
LLIGYVVYNDFGLAHNDIILSVLVSTFPVVLDTFFKYWLFSYLNRVSPSLVVIYHSMTEMPIG
VPKVPYRSPGDKHPSWVDIYNRLYRERLLFLGQMVESDISNQLIGIMVYLSIENETKDLYL
FINSPGGWVIPGIAIYDTMQFVRPDVQTICMGLAASMGSFLLAAGASTKRLAFPHARVMIH
QPIGAFYGAQTGEFILDTEELLRLREILTMVYVQRSGKPLWVVSEDMERDTFMSATEAQA
H G L V D L V A V
G???MEEFQV
YLELDRSQQHDFLYPLLFREYIYVLAHDHGLGFYDNKSSSLSVKRLITRMYQRINLSIAAN
DSNQNPIFGNKLYSQIISEVFAAVVEIPFSLRLVAFLIEKSPNFQSIHSIFPFFEDKLSHLNYVL
DVRIPYPICPEILVQTLREWVKDASSLHLLRFFLHEYFNSNSLITNSISVFLKPRLLLFLYNSH
VYEYESILFFLCNQSSHLQSTSFQVLVERTYFYGKVEHLVEVFAKFQILGLVKDPFMHYVR
YQGKSILASKDTPLLMNKWKYYLVGLWQWHFHASSQPGRVQLNYLGKYAINFLGYLSG
VRLNSLLVRSQMLENSFLIDN--SMKKVDTTVPIIHLIGSLTKARFCNALGHPISKSTWSDFS
DSHLIDRFVRICRNLSHYYSGSSKKKSLYRVKYILRLSCVKSLVRKHKSTVRAFLKRLGSE
LLEEFLMEEEHVLALLFPRRIWYLDIFCINDLVNYQMIIATP-EVQDIN-SFSRLES-LQEVYG
ILWVLAPISIYVLAITIGVLVIVWLEREISAGIQQRIGPEYASPLGILQALADGTKLLFKENLL
PSRGNSSLFSIGPSIAVIAILLSYSVIPFSYNLVLADLNIGIFLWIAISSIAPIGLLMSGYGSNNK
YSFLGGLRAAAQSISYEIPLTLCVLSISLLSNSLSTVDIVGAQSKYGFWGWNLWRQPIGFIV
FLISSLAECERLPFDLPEAEEELVAGYQTEYSGIKFGLFYVASYLNLLVSSLFVTVLYLGGSN
LSIPYIFVPGLVEINKADGIFGTTIGIFITLAKTYLFLFIPIATRWTLPRLRMDQLLNLGWKFL

LPISLGNLLLTTSSQLLSLMIWHVQNENFILDSTRIFMKAFHLLLFDGSFIFPECILIFGLILLL
MIDSTSDQKDIPWLYFISSTSLVMSITALLFRWREEPMISFSGNFQTNNFNEIFQFLILLCSTL
CIPLSVEYIECTEMAITEFLLFVLTATLGGMFLCGANDLITIFVAPECFSLCSYLLSGYTKKD
VRSNEATMKYLLMGGASSSILVHGFSWLYGSSGGEIELQEIVNGLINTQMYNSPGISIALIFI
TVGIGFKLSLAPSHQWTPDVYEGSPTPVVAFLSVTSKVAASASATRIFDIPFYFSSNEWHLL
LEILAILSMILGNLIAITQTSMKRMLAYSSIGQIGYVIIGIIVGDSNGGYASMITYMLFYISMN
LGTFACIVLFGLRTGTDNIRDYAGLYTKDPFLALSLALCLLSLGGLPPLAGFFGKLHLFWC
GWQAGLYFLVSIGLLTSVVSIYYYLKIIKLLMTGRKQEITPHVRNYRGSPLRSNNSIELSMI
VCVIASTILGISMNPIIAIAQDTLFMFLLYKYDIFWAFLIISSVIPILAFLISAVLAPINKGPEKL
SSYESGIEPMGDAWLQFRIRYYMFALVFVVFDVETVFLYPWAMSFDVLGVPVFIEAFIFMLI
LIVGLVYAWRKGALEWSTNYFPWLTLIVVFPIAAGSLIFFLPHRGNKVTRGYTIYICVLELL
LTTYAFCYNFQLDDPLIQLAEDYKWITFFEFDWRLGIDGLSIGPILLTGFITTLATLAARPVT
LNSRLFHFLMLAMYSGQIGSFSSRDLLLFFIMWELELIPVYLLLSVWGGKKRLYSATKFILY
TAGGSVFLLIGVLGIGLYGSNEPTLNLETLVNRSYPVALEIIFYIGFFIAFAVKLPIIPFHTWLP
DTHGEAHYSTCMLLAGILLKMGAYGLIRINMELLPHAHSIFSPWFMIVGTVQIIYAASTSPG
QRNLKKRIAYSSVSHMGFIILGIGSITDTGLNGAVLQIISHGLISAALFFLAGTSYDRIRFAYL
DEMGGLAIPIPKIFTLFSILSMASLALPGMSGFVAELIVFFGIITSQKFFLMPKIVITFVMAIG
MILTPIYSLSMLRQMFYGYKLFNAPNSYFFDSGPRELFVLISILLPVIGIGIYPDFVLSLSADK
AEAIISNFFL-MMLEHVLVLSAYLFSIGIYGLITSRNMVRALMCLELILNAVNINFVTFSDFF
DSRQLKGDIFSIFVIAIAAAEAAIGSAIVSSIYRNRKSTRINQSTLLNKMEHTYQYSWIIPLVP
LPVPMLIGVGLLFFPTATKNLRRMWAFISILLLSIVMIFSIDLSIEQIDRTSIYQSLRTWTITSD
LSFEFGYFIDPLTSIMSILITTVGILVLIYSDNYMSHDQGYLRFFAYMSFFNASMLGLVTSSN
FIQIYIFWELVGMCSYLLIGFWFTRPIAAGACQKAFVTNRVGDFGLLLGILGLYWLTGSFEF
RDLFEILKN-EVNLLFVTLCAFLLFAGPVAKSAQFPLHVWLPDAMEGPTPISALIHAATMVA
AGIFLVARLLPLFIVIPYAMNLISLIGIITVFLGATLALAQQDIKRGLAYSTMSQLGYMMLAL
GMGSYRAALFHLITHAYSKALLFLGSGSIIHSMEAIVGYFPDKSQNMVLMGGLRKHVPIT
KTAFLVGTLSLCGIPPLACFWSKDEILNDTWLYSPIFATIAFFTAGLTAFYMFRIYLLTFEGPF
NFCLQNYSGKKRNSLYSISLWGKEEPKPIKN-LVALLTMNFFAGAAK-TFCYPHESDNTILF
VMLVLVLFPLFVGAIG--LESDILSKLLTPSINLLQQNST-FVDW-EVVKNPTLSVSITYFGILL
AYFLYKPFYSSLHNWNILNFFAILWDKILNFLYDWSYNRAYIDAFYTRSLTEGIRGLAELTH
LFDRRVIDGITNGVGITSFFVGEGIKYLGGS-RISFYLLLYLFSLLFFLIFLFMDLPGPIHDFIL
VFLGSGLILGGLGVVLFTNPIYSAFSLGLVLVCISLFYILSNSHFVAAAQLLIYVGAVNVLIIF
AVMFMNGSDYSKDFNLWTIGDGLTSLVCTSIFFSLITTILDTSWYGIIWTTRANQIIEQDLIS
NSQQIGIHLSTDFFLPFELVSIILLVALIGAIAVARQMSILATEKEFMIVNMGPHHPSMHGVLR
LIVTLDGEDVIDCEPILGYLHRGMEKIAENRTIIQYLPYVTRWDYLATMFTEAITVNGPEQL
GNIRVPKRASYIRVIMLELSRIASHLLWLGPFMADIGAQTPFFYIFRERELVYDLFEAATGM
RMMHNYFRIGGVAADLPYGWIDKCLDFCDYFLTGVAEYQKLITRNPIFLERVEGVGIIGGE
EAINWGLSGPMLRASGIEWDLRKVDHYECYDEFDWEVQWQKEGDSLARYLVRIGEMTE
SVKIIQQALEGIPGGPYENLEIRCFDRESDPERNDFENRFISKKPSPTFELTKQELYARVEAP
KGELGIFLIGDQSGFPWRWKIRPPGFINLQILPQLVKRMKLADIMTILGSIDIIMGEVDRMFP
MVTGFMNYGQQTIRAARYIGQSFMITLSQANRLPVTIQYPYEKLIPSERFRGRIHFEFDKCI
ACEVCVRVCPIDLPVVDWKFETDIRKKRLLNYSIDFGICIFCGNCVEYCPTNCLSMTEEYE
LATYDRHELNYNQIALGRLPMSVIDDFTIRTVLNS----------------MQGRLSAWLVKHGLVHR
SLGFDYQGIETLQIKPEDWHSVAVILYIYGYNYLRSQCAYDVAPGGLLASVYHLTRIEYGV

DQPEEVCIKVFAPRSNPKIPSVFWVWKSANFQERESYDMLGILYDNHPRLKRILMPESWIG
WPLRKDYIAPNFYEIQDAY--MNSIEFSLLDRT
TPNSFISTTSNDLSNWSRLSSLWPLLYGTSCCFIEFASLIGSRFDFDRYGLVPRSSPRQADLIL
TAGTVTMKMAPSLVRLYEQMPEPKYVIAMGACTITGGMFSTDSYSTVRGVDKLIPVDVY
LPGCPPKPEAVIDAITKLRKKISREIYEDRIRL--ENRTFTTNHKFRVVCSTNTGNYDQGLLY
QPP-TSEIPPETFFKYKSSVSSPEFINMQIRNTFSSLKGEITRFISVSLMIYIITRASISNAYPIFA
QQGFENPREATGRIVCANCHLANKPVDIEVPQAVLPDTVFEAVVRIPYDMQLKQVLANGK
KGALNVGAVLILPEGFELAPPDRISPEMKEKIGKLSFQTYRPTKKNILVIGPVPGQKYSEITF
PILSPNPATNKDAYFLKYPIYVGGNRGRGQIYPDGNKSNNTVYNATSAGIVSKIIRKEKGGY
EITITDASNGREVVDIIPPGPELRVSEGQSIKLDQPLTSNPNVGGFGQADAEIVLQDPLRVQG
LLFFLASVVLAQIFLVLKKKQFEKVQLSEMNFLNKVYDWFEERLEIQAIADDITSKYVPPH
VNIFYCLGGITLTCFLVQVATGFAMTFYYRPTVTEAFASVQYIMTEVNFGWLIRSVHRWSA
SMMVLMTILHVFRVYLTGGFKKPRELTWVTGVVLAVLTASFGVTGYSLPWDQIGYWAVKI
VTGVPEAIPVIGSPLVELLRGSASVGQSTLTRFYSLHTFVLPLLTAVFMLMHFLMIRKQGIS
GPLMGVTKKPDLNDPVLRAKLAKGMGHNYYGEPAWPNDLLYIFPVVILGTIACTVGLAV
LEPSMIGEPADPFATPLEILPEWYFFPVFQILRTVPNKLLGVLLMVSVPTGLLTVPFLENVNK
FQNPFRRPVATTVFLIGTAVALCLGIGATLPIEKSLTLGLFMIEVFLFGIVLGLIPITLAGLFVT
AYLQYRRGDQLDLMPTITSYFGFLLAALTITSALFIGLSKIGLIMDIVSLAWAALMVVFTFS
LSLVVWGRSGLMIIRSPEPEVKILVDRDPVKTSFEEWAKPGHFSRTIAKGPETTTWIWNLH
ADAHDFDSHTSDLEEISRKVFSAHFGQLSIIFLWLSGMYFHGARFSNYEAWLSDPTHIGPS
AQVVWPIVGQEILNGDVGGGFRGIQITSGFFQLWRASGITSELQLYCTAIGALIFAALMLFA
GWFHYHKAAPKLAWFQDVESMLNHHLAGLLGLGSLSWAGHQVHVSLPINQFLNAGVDP
KEIPLPHEFILNRDLLAQLYPSFSEGATPFFTLNWSKYAEFLTFRGGLDPVTGGLWLTDIAH
HHLAIAILFLIAGHMYRTNWGIGHGLKDILEAHKGPFTGQGHKGLYEILTTSWHAQLSLNL
AMLGSLTIIVAHHMYSMPPYPYLATDYGTQLSLFTHHMWIGGFLIVGAAAHAAIFMVRDY
DPTTRYNDLLDRVLRHRDAIISHLNWVCIFLGFHSFGLYIHNDTMSALGRPQDMFSDTAIQ
LQPVFAQWIQNTHALAPGGTAPGATASTSLTWGGVDLVAVGGKVALLPIPLGTADFLVHHI
HAFTIHVTVLILLKGVLFARSSRLIPDKANLGFRFPCDGPGRGGTCQVSAWDHVFLGLFW
MYNAISVVIFHFSWKMQSDVWGSISDQGVVTHITGGNFAQSSITINGWLRDFLWAQASQV
IQSYGSSLSAYGLFFLGAHFVWAFSLMFLFSGRGYWQELIESIVWAHNKLKVAPATQPRAL
SIVQGRAVGVTHYLLGGIATTWAFFLARIIAVGMALRFPRFSQGLAQDPTTRRIWFGIATAH
DFESHDDITEERLYQNIFASHFGQLAIIFLWTSGNLFHVAWQGNFEAWVQDPLHVRPIAHAI
WDPHFGQPAVEAFSRGGALGPVNIAYSGVYQWWYTIGLRTNEDLYTGALFLLFLSAISLIA
GWLHLQPKWKPSVSWFKNAESRLNHHLSGLFGVSSLAWTGHLVHVAIPGSRGEYVRWN
NFLDVLPHPQGLGPLFTGQWNLYAQNPDSSSHLFGTSQGSGTAILTLLGGFHPQTQSLWLT
DIAHHHLAIAFIFLVAGHMYRTNFGIGHSIKDLLEAHIPPGGRLGRGHKGLYDTINNSLHFQ
LGLALASLGVITSLVAQHMYSLPAYAFIAQDFTTQAALYTHHQYIAGFIMTGAFAHGAIFFI
RDYNPEQNEDNVLARMLDHKEAIISHLSWASLFLGFHTLGLYVHNDVMLAFGTPEKQILI
EPIFAQWIQSAHGKTSYGFDVLLSSTDGPAFNAGRSIWLPGWLSAVNE--NSNSLFLTIGPGD
FLVHHAIALGLHTTTLILVKGALDARGSKLMPDKKDFGYSFPCDGPGRGGTCDISAWDAF
YLAVFWMLNTIGWVTFYWHWNHITLWQGNVSQFNESSTYLMGWLRDYLWLNSSQLING
YNPFGMNSLSVWAWMFLFGHLVWATGFMFLISWRGYWQELIETLAWAHERTPLANLIRW
RDKPVALSIVQARLVGLAHFSVGYIFTYAAFLIASTSGKFGMSHSVKIYDTCIGCTQCVRA
CPTDVLEMIPWDGCKAKQIASAPRTEDCVGCKRCESACPTDFLSVRVYLWHETTRSMGLA

YMTILSFPSIFVPLVGLVFPAMAMASLFLYLEKNKILMRDLKTYLSVAPVLSTLWFGSLAGL
LIEINRLFPDALTFPFFSFMTAILERRESERLWGRFCNWITSTENRLYIGWFGVLMIPTLLTAT
SVFIIAFIAAPPVDIDGIREPVSGSLLYGNNIISGAIIPTSAAIGLHFYPIWEAASVDEWLYNG
GPYELIVLHFLLGVACYMGREWELSFRLGMRPWIAVAYSAPVAAATAVFLIYPIGQGSFSD
GMPLGISGTFNFMIVFQAEHNILMHPFHMLGVAGVFGGSLFSAMHGSLVTSSLIRETTENE
SANAGYRFGQEEETYNIVAAHGYFGRLIFQYASFNNSRSLHFFLAAWPVVGIWFTALGIST
MAFNLNGFNFNQSVVDSQGRVINTWADIINRANLGMEVMHERNAHNFPLDLAAIEAPST
NGMGLPWYRVHTVVLNDPGRLIAVHIMHTALVAGWAGSMALYELAVFDPSDPVLDPMW
RQGMFVIPFMTRLGITDSWGGWSIT-GGTVTNPGIWSYEGVAGAHIVFSGLCFLAAIWHW
VYWDLAIFVDDRTGKRSLDLPKIFGIHLFLSGVACFGFGTFHVTGLYGPGIWVSDPYGLTG
KVQSVNPAWGVEGFDPFVPGGIASHHIAAGTLGILAGLFHLSVRPPQRLYKGLRMGNIETV
LSSSIAAVFFAAFVVAGTMWYGSATTPIELFGPTRYQWDQGYFQQEIYRRVSAGLAENQSL
SEAWSKIPEKLAFYDYIGNNPAKGGLFRAGSMDNGDGIAVGWLGHPIFRDKEGRELFVRR
MPTFFETFPVVLVDGDGIVRADVPFRRAESKYSVEQVGVTVEFYGGELNGVSYSDPATVK
KYARRAQLGEIFELDRATLKSDGVFRSSPRGWFTFGHASFALLFFFGHIWHGARTLFRDVF
AGIDPDLDAQVEFGVFQKLGDPTTRRQVV------------VETLFNGTLAVAGRDQETTGFAWW
AGNARLINLSGKLLGAHVAHAGLIVFWAGAMNLFEVAHFVPEKPMYEQGLILLPHLATLG
WGVGPGGEVIDTFPYFVSGVLHLISSAVLGFGGIYHALLGPETLEESFPFFGYVWKDRNK
MTTILGIHLILLGIGAFLLVFKALYFGGVYDTWAPGGGELRKITNLTLSPSVIFGYLLKSFFG
GEGWIVSVDDLEDIIGGHVWLGSICIFGGIWHILTKPFAWARRALVWSGEAYLSYSLGALA
VFGFIACCFVWFNNTAYPSEFYGPTGPEASQAQAFTFLVRDQRLGANVGSAQGPTGLGKY
LMRSPTGEVIFGGETMRFWDLRAPWLEPLRGPNGLDLSRLKKDIQPWQERRSAEYMTHA
PLGSLNSVGGVATEINAVNYVSPRTWLSTSHFVLGFFFFVGHLWHAGRARAAAAGFEKGI
DRDFEPVLSMTPINMTIALGKFTKEEKDLFDIMDDWLRRDRFVFVGWSGLLLFPCAYFAL
GGWFTGTTFVTSWYTHGLASSYLEGCNFLTAAVSTPANSLAHSLLLLWGPEAQGDFTRWC
QLGGLWTFVALHGAFGLIGFMLRQFELARSVQLRPYNAIAFSAPIAVFVSVFLIYPLGQSG
WFFAPSFGVAAIFRFILFFQGFHNWTLNPFHMMGVAGVLGAALLCAIHGATVENTLFEDG
DGANTFRAFNPTQAEETYSMVTANRFWSQIFGVAFSNKRWLHFFMLFVPVTGLWMSALG
VVGLALNLRAYDFVSQEIRAAEDPEFETFYTKNILLNEGIRAWMAAQDQPHENLIFPEEVL
PRGNALMSGSTGERSFADIITSIRYWVIHSITIPSLFIAGWLFVSTGLAYDVFGSPRPNEYFTE
SRQGIPLITGRFDSLEQLNEFSRSFMTIDRTYPIFTVRWLAVHGLAVPTVSFLGSISAMQFIQ
RMATQTVEGSSR-ARPKRTSAGGLLKPLNSEYGKVAPGWGTTPLMGVAMALFAVFLSIILE
IYNSSVLLDGISLNMLTLKLFVYTVVIFFVSLFIFGFLSNDPGRNPGREEMADTTGRIPLWII
GTVTGIPVIGLIGIFFYGSYSGLGSSLMLNIFSLMYICLNSALY-SSFLFAKLPEAYAFLNPIVD
VMPVIPVLFFLLAFVWQAAVSFRMTQSNPNEQNVELNRTSLYWGLLLIFVLAVLFSNYFFN
MEVNILAFIATTLFVLVPTAFLLIIYVKTVSQSDMETATLVAISISGLLVSFTGYALYTAFGQP
SQQLRDPFEEHGDMEALVYTFLLVSTLGIIFFAIFFREPPKVPTKKTKMTIAFQLAVFALIATS
SILLISVPVVFASPDGWSSNKNVVFSGTSLWIGLVFLVGILNSLIS-------MSPQTETKASVGF
KAGVKDYKLTYYTPDYVTKDTDILAAFRVTPQPGVPPEEAGAAVAAESSTGTWTAVWTD
GLTSLDRYKGRCYNIEPVAGEENQYICYVAYPLDLFEEGSVTNMFTSIVGNVFGFKALRAL
RLEDLRIPPAYTKTFQGPPHGIQVERDKLNKYGRPLLGCTIKPKLGLSAKNYGRAVYECLR
GGLDFTKDDENVNSQPFMRWRDRFLFCAEALYKAQAETGEIKGHYLNATAGTCEEMLKR
AVFARELGVPIVMHDYLTGGFTANTTLAHYCRDNGLLLHIHRAMHAVIDRQKNHGMHFR
VLAKALRLSGGDHIHAGTVVGKLEGERDITLGFVDLLRDDFVEKDRSRGIYFTQDWVSIP

GVIPVASGGIHVWHMPALTEIFGDDSVLQFGGGTLGHPWGNAPGAVANRVALEACVQAR
NEGRDLAREGNEIIREASKWSPELAAACEVWKSIKFEFAAMDTL--MAIHLYKTSTPSTRN
GAVDSQVKSNPRNNLIYGQHRCGKGRNARGIITAGHRGGGHKRLYRKIDFRRNEKDIYGR
IVTIEYDPNRNAYICLIHYGDGEKRYILHPRGAIIGDTIVSGTEVPIKMGNALPLTDMPLGTA
IHNIEITLGKGGQLARAAGAVAKLIAKEGKSATLKLPSGEVRLISKNCSATVGQVGNVGAN
QKSLGRAGSKCWLGKRPVVRGVVMNPVDHPHGGGEGRAPIGRKRPATPWGYPALGRRS
RKRNKYSDNLILRRRTKMIQPQTHLNVADNSGARELMCIRIIGAS-NRRYAHIGDVIVAVIK
EAVPNTPLERSEVIRAVIVRTCKELRRDNGMIIRYDDNAAVVIDHEGNPKGTRVFGAIAREL
RQLNFTKIVSLAPEVLMLSPKRTRFRKQHRGRMKGISYRGNHICFGRYALQALEPAWITSR
QIEAGRRAMTRNVRRGGKIWVRIFPDKPVTLRPTETRMGSGKGSPEYWVAVVKPGRILYE
MSGVAENIARKAISIAASKMPIRTQFIISGMTRIRRGYIARRRRTKMRLFVSSFRGAHSRLSR
TITQQKIRALVSAHRDRDRKKRDFRRLWITRINAVIRGN-LVSYSYSRLIYNLYKAQLVLNR
KILAQIAISNRNCLYMISNEIIK-----------MIREVSALGQHICMSAHKARRVIDQIRGRSYEET
LMILELMPYRACYPILKLVYSAAANGIHNLGFNEGSLFIIKAE------------------------------------
--------MDGIKYAVFTDKSIRLLGKN-QYTSNVESGSTRTEIKHWVELFFGVKVIAMNSHQLP
RKGRRMGPIMAHTMHYRRMIITLQPGYSIPPLRKKRTMAVPKKRTSILKKRIRKNLWKKG
GYWAALKAFSLAKSLATGNSKSFF----TTMAKGKEVRVRVILECTSCVRNGVN--KESRGIS
RYITQKNRHNTPSRLELRKFCPYCYKHTLHGEIKKMKIRASVRKICEKCRLIRRRGRIIVICS
NPRHKQRQGMVREKVKVSTRTLQWKCVESRADSKRLYYGRFILSPLMKGQADTIGIAMR
RVLLGEIEGTCITRAKSE--KIPHEYSTIVGIQESVHEILMNLKDIVLRSNLYGTCDALICVKG
PGYVTAQDILLPPSVEIVDNTQHIASLTEPIDLSIGLQIERSRGYNIKTPNTFQ-DGNCYPIDA
VFMPVRNANHSIQSYGNGNEKQEILFLEIWTNGSLTPKEALHEASRSLIDLFIPFLQAEDE--
NLPLENNQ-YKVTLPFFTFHDRLA-KLTKKK-KEIALKSIFIDQSEMSPRIYNCLKKSNIHTLF
DLLNTRQEDLMKIEHFRIEDVKQIMSILEKK-----------MLGDGN-AGMSTIPGLNQIQFEGFC
RFIDQGLTEELYKFPKIEDTDQEIEFQLFVETYQLVEPLLKERDAVYESFTYSSELYVSAGLI
WKSRGDMQEQTIFIGNIPLMNSLGTSIVNGIYRIVINQILQSPGIYYRSELGHNGISVYTGTII
SDWGGRFELEIDRKARIWARVSRKQKVSILVLSAAMGSNLREILENICYPEIFLSFLTNKEK
KKIGSKENAILEFYQQFACVGGDPVFSESLCKELQKKFFHQRCELGKIGRRNMNQRLNLNI
PQNNTFLLPRDVLAAVDHLIGLKFGMGTLDD--MNHLKNKRIRSVANLLQDQFGLALVRL
ENVIRGTICGAIR-HKLM-PTPQNLVTSTP----LTTTYDSFFGLHPLSQVLDRTNPLTQIVHGR
KLSYLGPGGLTGRTASFRVRDIHPSHYGRICPIDTSEGINVGLIGSLAIHARIGYWG-SLESPF
Y---EIFEKSKKV--RMLYLSPSRDEYYMVAAGNSLALNQGSPEEQVVPTRYRQEFLTIAWEQ
VHLRSIFPFQYFSIGASLIPFIEHNDANRALMSSNMQRQAVPLVRSEKCIVGTGLEPQVALD
SGVPAIAEHEGKIIYTDIDKIVLSGNGNTYSIPLIMYQRSNKNTCMHQKPQVGRGKCIKKG
QVLADGAATVGGELALGKNILVAYMPWEGYNFEDAVLISERLIYRDIYTSFHIRKYEIQTH
VTSQG-PERITNEIPHLEARLLRNLDKNGIVMLGSWVETGDILVGKLTPQAAKE-SSYAPED
RLLRAILGIQVSTSKETCLKLPIGGRGRVIDVRWVQKKG-GSSYNPETICVYISQKREIKVG
DKVAGRHGNKGIVSKILPRQDMPYLQDGRPVDMVFNPLGVPSRMNVGQIFECSLGLAGG
LLNRHYRIAPFDERYEQEASRKLVFSELYEASKQTANPWVFEPEYPGKSRIFDGRTGDPFEE
PVLIGKPYILKLIHQVDDKVHGRSSGHYALVTQQPLRGRSKQGGQRVGEMEVWALEGFGV
AHILQEMLTYKSDHIRARQEVLGTTIIGETIPNPEDAPESFRLLVRELRSLALELNHFVVSEK
NFQINKKEA-------MIDRYKHQQLRIGSVSPQQIRAWANKILPNGEIIGEVTKPYTFHYKTNK
PEKDGLFCERIFGPIKSGICACGNYRIIGDEKEDPQFCEQCGVEFVDSRIRRYQMGYIKLGC
PVTHVWYLKRLPSYIANLLDKPLKELEGLVYCD--FSFARP-IAKKPTFLRLRGSFEYEIQSW

KYSIPLFFTTQGFDKFRNREISTGAVAIREQLADLDLRIILDNSLLEWKELGEE-GPAGNDWE
DRKIGRRRDFLVRRMELAKHFLRTNIEPEWMVLCLLPVLPPELRPIIQIDGGKLMSSDINEL
YRRVIYRNNTLIDLLTTSRSTPGELVMCQEKLVQEAVDTLLDNGIRGQPMRDGHNKIYKSF
SDVIEGKEGRFRETLLGKRVDYSGRSVIVVGPSLSLHQCGLPREIAIELFQTFVICGLIRQHL
ASNIGVAKSKIREKGPIIWEILQEVMQGHPVLLNRAPTLHRLGVQAFQPILVEGRAICLHPL
VCKGFNADFDGDQMAVHVPLSLEAQAEARLLMFSHMNLLSPTIGDPISIPTQDMLIGLYVL
TSGNRRGICANRYNTWNRRN---YPDERIDD--YTYTK--EPLFCNSYDAIGAYRQKRINLDSP
LWLRWRLDQR-LIAS--REAPIEVHYESLGTSHEIYGHYLIVRSVKKEILSIYIRTTVGHISLYR
EIEEAIQGFCRACSYGT------------MAERVSLVFHNKMIDGTAIKRLISRLIDHFGMAYTSHIL
DQVKTLGFQQATATSISLGIDDLLTIPSKRWLVQDAEQQSFILEKHYHYGNVHAIEKLRQSI
EIWYATSEYLRQEMNPNFRMTEPFNPVHIMSFSGARGNASQVHQLVGMRGLMSDPQGQM
IDLPIQSNLREGLSLTEYIISCYGARKGVVDTAVRTSDAGYLTRRLVEVVQHIIVRRTDCGT
VRGISVSPQNRMMS-ERVFSQTLIGRVLADDIYMGQRSIAIRNQDIGIGLVNRLIT------LRTQ
AISIRTPFTCRSTSWICRLCYGRSPTHGDLVELGEAVGIIAGQSIGEPGTQLTLRTFHTGGVFT
GGTAEHVRAPSNGKIKFNEDLVHPTRTRHGHPAFLCSIDLDVIIEGEDIMHNVTIPPKSFLLV
QNDQYVESEQVIAEIRAGAYTL-NFKERVRKHIYSDSEGE-MHWSTDVYHAPEFTYSNVHL
LPKTSHLWILSGGSRR--SSVVSFSLHKDQDQMNIH--SLSVER-REISSLLSVNNDQ-TGHKF
FSSDFSAKKILEMSDY-SGFNRIISTGHCNLIHPA-ILHANSD--LLAKRQRNGFLIPFHSIQEQE
KELMPH-GISIEIPVR--GIFRRNSILAYFDDPRYRRKSSGITKWGTLGAHSIVKKED--LIEYR-
GLKKIKPKYMKIDRLFFIPEEVHIFPESSYLMVRNNSIIGVDTQITLNIRSRVGGLVRMERKR
GGVELKIFSGDIHFPGEIDKISRHSGILIPPESEKTKLATKESKK--LKKWIYVQR--ITPTKKK-
-SFVLVRPVVTYEIANGINLATLFPQDPLREKDNMQFRVVNYVLYGKGKAAGGISDTSLQL
VRTCLVLNWDQDN---KSSSVEEVCASFVEVRTNGLIRNFLRINLVKSNISYLRKRDHPSGS
GLISD-NGSARTNSNPFYSVFGKAG-----VEQSLRQNQGTIRTLLNK---NKECQVLIILSSSNY
FRMGP--LNDVKYHN---VIKQSDSLTPIKTSLGPLGT--FLQIANFYS--FYYLIT--HNHIS-VTK
YLKLDN---LKQPFQVL--K-YYLMDETGE--IYNPDTDSKILLNPFNLNWYFLQP-YCEEMSPI
IS-LGQFLCENVRITKRGP---YLKSGQVLIVQ-VNSVVIRSAKPYLATPGATVHGHYGAILYE
GDTLVTFIYEKSRSGDITQGLPKVEQVLEVRSLDSISMNLEKRVEGWNASITRILGIPWGFLI
GAELTIVQSRISLVNKIQKVYRSQGVQIHNRHIEIIVRQITSKVLVSE---DGMSNIFLPGELIG
LLRAERMGRALEEVICYRAILLGITRASLNTQSFISEASFQETTRVLAKAALRGRIDWLKGL
KENVVLGGIIPVGTGFKGLVHCS--RQHNNILLERQKGN--LFGGEMR--DIFLHHRELFDSCI
STTFHDTSEHNCLEGLMSPRSEFFMARRYWNIHLEEMMEAGIHFGHGTRKWNPRMAPYI
SAKHKGIHITNLTRTARFLSEACDLVFDAASRGKQFLIVGTKNKAADLVARAAIRARCHYV
NQKWLGGMLTNWSTTETRLHKFRDLRTEQKGGRLDSLPKRDAAILKRQLSRLQTYLGGI
KYMTRVPDIVIVVDQQEEYTALRECITLGIPTICLIDTNCDPDLADISIPANDDAIASIRLILN
KLVFAICEGRS-SYIRNPMGQKINPLGFRLGTTQSHHSLWFAKPKNYCEGLQEDQKIRNFIK
NYIKKNIRISSG-VEGIARIEIQKRIDLIQVIIYIGFPKLLLENR----PRRIEELQMNVQKELNCV
NRKINIAITRITNPYGHPNILAEFIAGQLKNRVSFRKAMKKAIELTEQADTKGIQVQIAGRL
DGKEIARAEWIREGRVPLQTIGAKIDYCSYTVRTIYGVLGIKIWIFVDEKMSRYRGPRFKKI
RRLGALPGLTSKRPRAGSDFRNQLRSVK-KSQYRIRLEEKQKLRFHYGLTERQLLNMFVSP
EKPKGQPVWFYYNYLKCVWITFFF------------DWVWLQLFLKPANWLTIDIF------------------
--MSRRGTTEEKTAKSDPIYRNRLVNMLVNRIL
KHGKKSLAYQIIYRALKKIQQKTEKNPLSVLRQAIRGVTPDIAVKARRVGGSTHQVPIEIGS
AQGKALAVRWLLGASRKRPGRNMAFKLSSELVDAAKGSGDAIRKKEETHRMAEANRAF

AHFRMGKDTIADIITSIRNADMNRKGTVRIASTNITENVVKILLREGFIENARKLVENKKEF
LVLTLRHRR--NRKGPYRP-ILN--LKRISRPGLRIYFNYRRIPRILDGMGIVILSTSRGIMTDRA
ARLERIGGEILCYIWMAKSPPRSGSRRPGRIGSRKSGRRIPKGVIHVQASFNNTIVTVTDVR
GRVISWSSAGTCGFRGTRRGTPFAAQTAAGNAIRAVADQGMQRAEVMIKGPGLGRDAAL
RAIRRSGILLNFVRDVTPMPHNGCRPPKKRRVMPTIKQLIRNPRQPIRNVTKSPALRGCPQR
RGTCTRVYTITPKKPNSALRKVARVRLTSGFEITAYIPGIGHNSQEHSVVLVRGGRVKDLPG
VRYHIVRGTLDAVGVKDRQQGRSKYGVKKPKMARQSLIQREKKRHKLEQKYHLIRRSSK
KEIN---KAPSLSDKWKIHGKLQSSPRNSAPTRLHRRCFLTGRPRANYRDFGLSGHILREMV
HACLLPGATRSSWMVKNAFISVRVQE--KKEKNSGSVEFQVFRFTNTIRRLTSHLELHRKD
YSSQRGLRKILEKRQRLLAYLSKKNRVRYKELISKLNIRESKNR--MVKLRLKRCGRKQRA
VYRIVAIDGRSRREGRDLRKVGFYDPINNQTHLNVPAILYFLAKGAQPTGTVHDISQKAGV
FTEVSLNQ-TKFYMDKTKRLFLKSKRSFRRRLPPIQSGDRIDYRNMTLISRFLSEQGKILSRR
VNRLTLKEQRLITIAIKQARILSSLPFLNNEK--QFERSGLTARPPGLRTRKKVTRSLKKNPFV
ANHLLRKIDKLNTKAEKEIIITWSRASTIIPTMVGHTIAIHNGREHLPIYITDRMVGHKLGEF
APTINFRGHAKNDNRSRRMIFQSFILGNLVSLCMKIINSVVVVGLYYGFMTTFSIGPSYLFL
LRARVMEEGEEGTEKKVSATTGFIAGQLMMFISIYYAPLHLALGRPHTITVLALPYLLFHFF
WNNPKHFFDYGSTTRNSMRNLSIQCVFLNNLIFQLFNHFLLPSSMLARLVNIYMFRCNNK
MLFVTSSFVGWLIGHILFMKWVGLVLVWMQQKNSIRS-IRFNKYLVSELRNSMARIFSILLF
ITCIYYLGRIPSPIFTKKLKVAEEDKPLVTLLFDYKRWTRPLRYKKNNRFENAVRNEMSHYF
FYACRSDGKERISFTYPASLSTFLDMIKKNNYFFATEKLSSDEFSTHWNYTNNQKIKNLSKE
FRNRLEGLD---LIRDILEKRTQLCNDKTKKKYLPKTYDPLLNGPYRGRIKKFFSPIRTPWIN
KIHNLIYHEFEQTDPNDQ----KEISKRVPRWSYKLINVYQELGEAFRSRKAKRLVVAVDSLP
HYLEQSDFRRYIIKGSMRAQRRKTVIWKPVQANAHSPLFLDRIDKT--LYLSFDISQLMKVI
LRNRQEMVRIKQAEGWDKRLLTRILRSSMLVIQSILRKYIVLPLLIIAKNVVRILLFQDPEW
SEDFKDWNREIYVKCTYSGVNLSETEFPKNWLIEGIQIKILFPFHLKPWHGST----TDVCFLT
IFGLETDMPFGSPQKRRSFFEPIFKELKKKIRKLKTKSFIV-LRDFKE---------------------------GK
IKELSKINNSIISKQMIHEP-IEISSMDWTK-SRTEKKMKDLTNRTSRIRNQIYKITKEKKKSS
NKPTYSVKILDPSKRIWRILK--NARLIRKSYLFLKFGIEKIYRNIFISTLTIPRINTKP------CNL
LSFSQAYVFYKLLQV-ILNFYNLRPVLQYTSLFLKNEIKDFFKHGIFN-QLRHKPLW-FGRNT
RKHCLSGHYQYDLSRIKWARLVPQEWRNRANQHCMAQNKIKRDSYEKNG--LTHYEKQ-
NFKKHYRYDLLSYKSLNDDIMDLYRKYFDWRILNFCLRFCDWMGMNEEILSCWFFPEFV
LLFNAYRTKPWIIPIKLLLFNRE--TEVQLDFFLKRYLCLQLRWRGA-REKILNDMQVYCHL
VRLINPSDVAIASIQRGEMSLPILITKKKFALKELTKGGMLIIEPRRLSVKNDGQFFLYQIVGI
ALVHKNKRHYD-LLVPETILLPKRRRELRTLICFKFIWANSRLEDLICMNRYWFNTNNGSRF
SMVRVHMYPMKGHQFKSWIFELREILREIKNSHYFLDSWTQFNSVGSFIHIFFHQERFIKLL
DPRIWSILLS-RNS--QGSTSNRYF---TIKCVVLFVVA-ILIYR----INNRNMVERKNLYLTGLLP
-----IPMNSIGPRTDTLEESF-GSSNINRLIVS-LLYLPKGKKISESFFLDPKESTWVLPIT----KRC
IMPESNWGSRWWRNWLGKKR--DSSCKISN---ETVAGIEISFKEKDLK-YLEFLFVYYMD--
DPIRKDHDWELFDRLSPSKRRNIINL--NSGHLFEILVKDWICYLMFAFREKIPIEVGG-FLKQ
QG-AGSTIQSNDIERFSH--LFLRNKWAISLQNCAQFHMWQFRQDLFV---SWGK-NPHESDF
LRN-IARENWIWLDNVWLVNKDR---FFT--KVRNVSSNIQYDS---TR--SSFVQVTDSSQLKG
SS-DQSRDHFDSIS----NEDSE-------------YHTLINQREIQQLKERSIL-WD--PSFLQAERREIES
D--RFPK--CLSGYS--SMSRLFT-ERERQMNKHLLPEEIEEF--LGNPARSIRSFFSDRW--SELH-
LGSNPTERSTRDQ----KLLKKEEDVS-FVPSRRSE----NKEIVNIFKIITYLQN--TVSIHPISSDP

GCDMVP-KDELD-MDSSNKISFLNKNPFFDL--FHLFHDRNRGGSTL--HHDFESE--ERFQEM
ADLFTLSITEP-DLVYHKGFAFSIFSYGLEQKQ--FLNEVFNSRNESKKKSL---LVLPPIFY--EE
NESFYRRIRKKWVR-ISCGNDLEDPKPKIVVFASNNIMEAVNQSRLIRNRIQIQ--YSTYGYIR
NVLNRFFLMN---RSDRNFEYGIH---RDQIGNDTLNHRTIMKYTINQHLSNLKK-SQKKWFA
P--LILISRTERFMNRDPNAYRYKWSNGSKNFQEHLKHFVSEQKSR---FQVVFDRLRINQYSI
DWSEVIDKKDLSKPLPFFLS----------------------KFLFFLSNSLP-FFFVSFGNIPIHRSE-IHIY-E
LKGPNDQLCNQLLESIGLQIVHLKKLKPF--LLD---DHNTS-QKSKFLINGGRISPFLFNKIP-K
W-MIDSFHTR--KNRRKSFDNTDSYFSMISHDQDNWLNPVKPFHRSSLISSFYKANRLRFLN
NPHRFRFY--CNKRFTF---Y-VEKVRINNYD--FTYG-QFLNILFIRNK-IFSLCGGK--KKHAFL
E-RDTISPIESQVSNIFIPNDFPQSGDER-YNLYKF---FPFPIRSDLLVR--RAIYSIAAISGTPLTE
GQIVNFERTYCQPLSDMNRSDSDEKNLHQYLN--FNSNMGLIHTPCSEKYLPSEKRKRKK--
------RSLCLK--KCVEKGQMSRTFQRDSAFSTLSKWNRFQTYMPWFLTSTGYKYLNLIFLDT
FSD-LLPVLSSS--QKFVSIFHDIMHGSDRAWRILQKKW------CLPQWN-LISEISSKCFHN--LL
LSEEMIHRNNES--PLISTHLRSPNAREF-LYSILFLLLVAGY-LVHTHLLFVSR--AYSELETEFE
RVKSLMIPPYMIELRKLLDRYPTSELNSFWLKNLFLVALEQLGDSLEEIRGSAFGGNMLWG
GGP-----ADG-VKSIRSKTKDLNINLVDITDLI-SIIPNPINRITFSRNTRHLSHTSKEIYSLIRKR
KKGNGDWIDDKIESWVANSDSIDDKEREFLVQFSTLTLTT--EKRIDQILSSLT-HSDHLS-----
KNDSGYQMIEQPGTIYLRYLVDIH--KKHFMNYEFNTYCLAERRIFLAHYQTITY--SQTSCG
ANSFHFPSHG---KPFSLRLALSPS-RGILVIGSIGTGRSYLVKYLAANSYVPFITVFLNKFLDN
KPKGFLID-----DID--DASDDIDASD---------------------DIDRDLDTELELLTMMNAL---TIDM
MS---EIGRFYITLQFELAKAMSPCIIWIPNIHDLDVNESNYLSLGLLVNYLS---RDCERCS-TR
NILVIASTHIPQKVDPALIAPNKLNTCIKIRRLLIPQQRKHFFTLSYTRGFHLEKKMFHT--NG
FGSITMGSNARDLVALTNEALSISITQKKSIIDTNTIRSALHRQTWDLR---SQVRSVQDHGIL
FYQIGRAVAQNVLLSNCPIDPISIYMKKK--SCNEGDSYLYKWYFELGTSMKKLTILLYLLSC
SAGSVAQDLWSLP--GPDEK-NGITSSGLVENDS-DLVHGLLEVEGALVG--SSRTEKDCSRFD
ND--RVTLLLRPEPRN-PLDMMQKGSCSIVDQRFLYEK--YESEFEE-GEGEGVLDPQ----QIEE
D----LFNH--IVWAP--RIWRP---WGFLFDCIERPNELGFP-YRAGSFRGK---RIIYDEKDELQEN
DSEFLQSRTMQYQTRDRSSNEQGFFRISQFIWEPADPLFFLFK-DQPLVSVFSHREFFADEE
MSKGLLTSQPDPPTSIYKRWFI--KNTQEKHFELLIHRQRWLR---TNSSLS--NGFFRSN--TPS
ESYQYLSNLFLSN-GTLLDQMTKTLLRKRWLFPDEMKIGF--M---------MPRSR-INGNFIDK
TFSIVANILLRIIPTTVGEKEAFTYYRDG--MSAQSEGNYAEALQNYYEAMRLEIDPYDRSYI
LYNIGLIHTSNGEHTKALEYYFRALERNPFLPQAFNNMAVICHY-RGEQAVRQGDSEIAEA
WFNQAAEYWKQAIALTPGNYIQAQNWLKITGRFE--MSWRSEYIWVEFIAGSRKPGNFFW
AFILFLGSLGFLVVGISSYLDRNLLSLFPSQQINFFPQGIVMSFYGIAGLFISSYLWCTIIWNV
GSGYDRFDTKEGIVCIFRWGFPGKNRRIFLRFLMKDIQSIRIEVKEGIYARRVLYIESRGLGA
IPLNRTDEN-LTPREIEQKAAELAYFLRVPIEGF
>Platanus
MEKWWFNSMLSLSKSMDSLGAIGNSSGSEDPVINDTDKNIH-YSNVDHLFGVRNIRNFISD
DTFLVRDRTGDSYSIYLDIENEIFEIDND--RSFLSELESYFSSYLN-----NRSKSDTHYYDRYM
YDTKYTWNNHINSCIDSYIRSEIS--IDSYISSGSDNYSDSYISSYICSDSGSYIRTS-NGSD-NI
RGRY-DLDINKKYRHLWIQCENCYGLNYKKFFRSKMNICEQCGYHLKMSSSDRIELSIDPG
TWDPMDEDMVSIDPIEFHSEEEPYKDRIDSYQRKTGLTEAVQTGIGQLNGIPIAIGVMDFQF
MGGSMGSVVGEKITRLIEYATNGSLPLIIVCASGGARMQEGSLSLMQMAKISSASYDYQS
NKKLFYVSILTSPTTGGVTASFGMLGDIIIAEPNAYIAFAGKRVIEQTLNKTVPEGSQAAEYS

FHKGLFDSIVPRNLLKGVLSELFQLHGFFPLNQNSIKMVTLRADEISNIIRERIEQYNREVK
VVNTGTVLQVGDGIARIHGLDEVMAGELVEFEEGTIGIALNLESNNVGVVLMGDGLMIQE
GSSVKATGRIAQIPVSEAYLGRVINALAKPIDGRGEISASESRLIESPAPGIISRRSVYEPLQT
GLIAIDSMIPIGRGQRELIIGDRQTGKTAVATDTILNQKGQNVICVYVAIGQKASSVAQVVTT
FQERGAMEYTIVVAETADSPATLQYLAPYTGAALAEYFMYRERHTSIIYDDPSKQAQAYR
QMSLLLRRPPGREAYPGDVFYLHSRLLERAAKSSSLLGEGSMTALPIVETQSGDVSAYIPT
NVISITDGQIFLSADLFNSGIRPAINVGISVSRVGSAAQIKAMKQVAGKLKLELAQFAELEAF
AQFASDLDKATQNQLARGQRLRELLKQSQSAPLTVEEQIVTIYTGTNGYLDSLEIGQVKKF
LVQLRTYLKTNKPQFQEIISSSKTFTEEAEALLKEAIQEQMERFLLQEQTMRINPTTSGPGV
SALEEKNLGRIAQIIGPVLDVAFPPGKMPNIYNALVVKGRDTVGQQINVTCEVQQLLGNNR
VRAVAMSATDGLTRGMEVIDTGAPLSVPVGGATLGRIFNVLGEPVDNLGPVDTRTTSPIHR
SAPAFIQLDTKLSIFETGIKVVDLLAPYRRGGKIGLFGGAGVGKTVLIMELINNIAKAHGGV
SVFGGVGERTREGNDLYMEMKESGVINEQNIAESKVALVYGQMNEPPGARMRVGLTALT
MAEYFRDVNEQDVLLFIDNIFRFVQAGSEVSALLGRMPSAVGYQPTLSTEMGSLQERITST
KEGSITSIQAVYVPADDLTDPAPATTFAHLDATTVLSRGLAAKGIYPAVDPLDSTSTMLQPRI
VGEEHYETAQRVKQTLQRYKELQDIIAILGLDELSEEDRLTVARARKIERFLSQPFFVAEVF
TGSPGKYVGLAETIRGFQLILSGELDGLPEQAFYLVGNIDEATAKAMNLEVESKLKKMTL
NLCVLTPNRIVWDSEVKEIILSTSSGQIGVLPNHAPIATAVDIGILRIRLNEQWLTMALMGGF
ARIGNNKITILVNDAEKGSDIDPQEAQRTLEIAEANLSRAEGKRQTIEANLALRRARTRVET
INVISMQNLTDSFVSLGHWPSAGSFGFNTDILATNPINLSVVLGVLIFFGKGVLSDLLDNRK
QRILSTIRNSEELRGGAIEQLEKARARLRKVEMEADEFRVNGYSEIEREKLNLINSTYKNLE
RLENYKNETIQFEQQRAINQVRQRVFQQALQRALGTLNSCLNNELHLRTISANIGMFGTM
KEITDMNPLISAASVIAAGLAVGLASIGPGVGQGTAAGQAVEGIARQPEAEGKIRGTLLLSL
AFMEALTIYGLVVALALLFANPFVMNVLPCSINTLKGLYDISGVEVGQHFYWQIGGFQVH
AQVLITSWVVIAILLGSATVAVRNPQTIPTDGQNFFEYVLEFIRDLSKTQIGEEYGPWVPFIG
TMFLFIFVSNWSGALLPWKIIQLPHGELAAPTNDINTTVALALPTSVAYFYAGLTKKGLGYF
GKYIQPTPILLPINILEDFTKPLSLSFRLFGNILADELVVVVLVSLVPSVVPIPVMFLGLFTSGI
QALIFATLAAAYIGESMEGHHMIFLTLEHIFTHISFSIVSIVITIHLMTLLVDGIVGLYDSSEKG
MIATFLCITGFLVTRWIYSRHFPLSDLYESLIFLSWSFSIIHMVPKIRNHKNHLSAITAPSAIFT
QGFATSGLLTEMHQSAILVPALQSQWLMMHVSMMVLSYAALLCGSLLSIALLVIRFRKNIE
IFGKDNESQYVNERSNILRNTSFLSFRNYYRYQLIQQLDHWSYRVISLGFIFLTIGILSGAVW
ANEAWGAYWNWDPKETWAFITWTIFAIYLHTRTNKDLQGANSAIVASIGFLIIWICYFGVN
LLGIGLHSYGSFTLTMPKKKAFTPLPYLASIVFLPWWISLSFNKSLESWITNWWNTRQSEIF
LNDIQEKNVLEKFIELEELFLLDEMIKEYPETHIQKLRIGIHKETIELVKIHNEGHIHTILHFS
TNITCFAILSGYSILGNEELVILNSWLQEFLYNLSDTIKAFSILLLTDLCIGFHSPHGWELMIS
SVYRDFGFAHNDPIISGLVSTFPVILDTIFKYWIFRYLNRVSPSLVVIYHSMNDMPIGVPKVP
FRSPGEEDAIWVDVYNRLHRERLLFLGQEVDSEISNQLIGLMVYLSIEDDTRDLYLFINSPG
GWVIPGIAIYDTMQFVPPDVHTICMGLAASMGSFILVGGEITKRLAFPHARVMIHQPASSF
YEAQTGEFILEAEELLKLRETLTRVYVQRTGNPLWVVSEDMERDVFMSATEAQAHGIVDL
VAVEMKEQKWIHEGLITESLPNGMFRVRLDNEDLILGYVSGKIRRSFIRILPGDRVKIEVSR
YDSTRGRIIYRLRSKDSNDMEELQGYLEIDRSRQQDFLYPLLFQEYIYALAHDHSLENLG-K
SSSLIVKRFITRMYQQNHLIISANDSNQNKFVGKNLDSQTISEGFAIIVEIPFSLRLVSSLIVKS
RNLRSIHSIFSFLEDKLSHLNYVSDILIPHPIHPEILVQVLRSWVQDAPSLHLLRLFLHEYRN
WTSLITKSISIFSKQRFFLFLYNFHICECESIFVFLRKQSSHLRSTSFRALLERTHFYGKMEHF

VVVFRNNFILWLVKDPFMHYVRYQGKSFLASKGTPLLMNKWKYYLVNFWQYYFYLWSQ
PSRIHINQLSNHSLDFLGYLSSVRLNPSVVSSQMLENLYLMDIAI--KKFDTIIPIIPLIGSLAK
AKFCNVSGHPVSKPVRADSSDSDIIDRFGRICKNLSHYHSGSSKKKSLYRIKYILRLSCART
LARKHKSTVRAFLKRLGSELLEEFFTEEEQVLSLIFPRQIWYLDIIRINDLANHEMIIDTT-EV
Q-AINSFSRSE-SLKEAYGLIWLLVPIFTPVSGITIGVLVIVWLEREISAGIQQRIGPEYAGPLGI
LQALADGTKLLFKEDLLPSRGDTRLFSIGPSIAIISILLSYSVIPFGYRLVLADLSIGVFLWIAI
SSIAPIGLLMSGYGSNNKYSFSGGLRAAAQSISYEIPLTLCVLSISLLSNSLSTVDIVEAQSK
YGFWGWNLWRQPIGFAVFLISSLAECERLPFDLPEAEEELVAGYQTEYSGIKFGLFYVASYL
NLLVSSLFVTVLYLGGWNLSIPYIFIPELFEINKVSGVFGTTIGIFITLAKAYLFLFISITTRWT
LPRMRMDQLLNLGWKFLLPISLGNLLLTTSSQLFSLMIWHVQNENFILDSTRIFMKAFHLL
LFHGSFIFPECILIFGLILLLMIDSTSDQKDIPWLYFISSTSLVMSITALLFRWREEPMISFSGNF
QTNNFNEIFQFLILLCSTLCIPLSVEYIECTEMAITEFLLFVLTATLGGMFLCGANDLITIFVAP
ECFSLCSYLLSGYTKRDVRSNEATTKYLLMGGASSSILVHGFSWLYGSSGGEIELQEIVNG
LINTQMYNSPGISIALIFITVGIGFKLSPAPSHQWTPDVYEGSPTPVVAFLSVTSKVAASASA
TRIFDIPFYFSSNEWHLLLEILAILSMILGNLIAITQTSMKRMLAYSSIGQIGYVIIGIIVGDSN
DGYASMITYMLFYISMNLGTFARIVSFGLRTGTDNIRDYAGLYTKDPFLALSSALCLLSLGG
LPPLAGFFGKLHLFWCGWQAGLYFLVSIGLLTSVVSIYYYLKIIKLLMTGRNQEITPHVRN
YRRSPLRSNNSIELSMIVCVIASTIPGISMNPIIAIAQDTLFMFLLHEYDIFWAFLIISSVIPILA
FLISGVLAPISEGPEKLSSYESGIEPMGDAWLQFRIRYYMFALVFVVFDVETVFLYPWAMSF
DVLGVPVFIEALIFVLILIVGSVYAWRKGALEWSTNFFPWLTIIVVLPISAGSLIFFLPHRGN
KAIRWYTICICLLELLLITYVFCYHFQLDDPLIQLEEDYKWINIFDFHWRLGIDGLSIGPILLT
GFITTLATLAAWPITRDSRLFHFLMLAMYSGQIGSFSSRDLLLFFIMWELELIPVYLLLSMW
GGKKRLYSATKFILYTAGGSIFLLMGVLGMGLYGSNEPTLNFETSANQSYPVALEIMFYFG
FLIAYAVKSPIIPLHTWLPDTHGEAHYSTCMLLAGILLKMGAYGLVRINMELLPHAHSIFSP
WLMIVGTIQIIYAASTSPGQRNLKKRIAYSSVSHMGFTIIGIGSITDAGLNGAVLQIISHGFIG
AALFFLAGTSYDRIRLVYLDEMGGIAISMPKIFTMFSSFSMASLALPGMSGFVAELIVFFGII
TSPKYFLMPKILITFVMAIGMILTPIYSLSMSRQMFYGYKLFNAPNSYFFDSGPRELFVSISIF
LPVIGIGIYPDFVLSLSVDKVEAILSNYFYKMILEHVLVLSAYLFSIGIYGLITSRNMVRALM
CLELILNAVNINFVTFSDFFDSRQLRGDIFSIFVIAIAAAEAAIGPAILSSIFRNRKSTRINQSN
LLNKMEHTYQYAWIIPFLPLPVPMLIGVGLLLVPRATKNLRRMWAFPSVLLLSIVMIFSADL
SIQQLNGSSIYQYVWSWTINNDFSLEFGYLIDPLTSIMSILITTVGIMVLVYSDNYMSHDQG
YLRFFAYMSFSNTSMLGLVTSSNLIQIYIFWELVGMCSYLLIGFWFTRPIAANACQKAFVTN
RVGDFGLLLGILGLYWITGSFEFRDLFEIFNNNRVNSLFVTLCASLLFVGAVAKSAQFPLHI
WLPDAMEGPTPISALIHAATMVAAGIFLVARLFPLFTVIPYIMNLISLIGIITVLLGATLALAQ
KDIKRSLAYSTMSQLGYTMLALGMGSYRAALFHLITHAYSKALLFLGSGSIIHSMETVVG
YSPEKSQNMALMGGLTKYAPITKTAFLLGTLSLCGIPPLACFWSKDEILNDSWLYSPIFAIIA
CSTAGLTAFYMFRMYLLTFEGHLNVHFQNYSGKKNSSFYSISIWGKKETKTINIRLSTLST
MNYFLFGNKETFPYPHESDNTMLLPLLVLVLFTLFVGFIGIPFDLDILSKWLAPSINLLHPNL
S-SVDWYEFITNAIFSVSIAYFGIFIASLLYRPVYSSFQNLELINFFVILWDQLLNIIYNWSYN
RGYIDFFYAKTFIRGIRGLAELTHFFDRRVIDGITNGVGVTSFFVGEGIKYVGGG-RISSYLFL
YLSYVLIFLLISYMDLPGPIHDFLLVFLGSGLILGGLGVVLLTNPIYSAFSLGLVLVCISLFYIP
SNSHFVAAAQLLIYVGAINVLIIFAVMFMNGSEYYNDFHLWTVGDGVTSLLCTSIFVSLITT
IPDTSWYGVIWTTRSNQIIEQDLISNGQQIGIHLSTDFFLPFELISIILLVALIGAIAMARQMTV
PATRKDLMIVNMGPHHPSMHGVLRLIVTLDGEDVIDCEPVLGYLHRGMEKIAENRTIIQYL

PYVTRWDYLATMFTEAITVNAPEQLGNIQVPKRASYIRAIMLELSRIASHLLWLGPFMADI
GAQTPFFYIFRERELIYDLFEAATGMRMMHNYFRIGGVAADLPHGWIDKCLDFCDYFLTGI
AEYQKLITRNPIFLERVEGVGIIGGEEAINWGLSGPMLRASGIQWDLRKVDHYGCYDEFD
WEVQWQKEGDSLARYLVRISEMTESVKIIQQALEGIPGGPYENLEVRRFDRARDSEWNDF
EYRFISKKPSPTFELSKQELYVRVEAPKGELGIFLIGDQSVFPWRWKIRPPGFINLQILPQLV
KRMKLADIMTILGSIDIIMGEVDRMLPMVTGFMNYGQQTVRAARYIGQSFMITLSHANRL
PVTIQYPYEKLITSERFRGRIHFEFDKCIACEVCVRVCPIDLPVVNWRLETDIRKKRLLNYSI
DFGICIFCGNCVEYCPTNCLSMTEEYELSTYDRHELNYNQIALGRLPISVIGDYTIRTIMNS
KIATGKPLDSKTITNYMQGRLSAWLVKHGLVHRSLGFDYQGIETLQIKSEDWHSIAVISYV
YGYNYLRSQCAYDVAPGGLLASVYHLTRIQYGVDQPEEVCIKVFAARRNPRIPSVFWIWK
SADFQERESYDMLGISYDNHPRLKRILMPESWIGWPLRKDYIAPNFYEIQDAH--------------
---MNSIEFPLLDRTTQNSVISTTPNDLSNWSRLSSLWPLLY
GTSCCFIEFASLIGSRFDFDRYGLVPRSSPRQADLILTAGTVTMKMAPSLVRLYEQMPEPKY
VIAMGACTITGGMFSTDSYSTVRGVDKLIPVDVYLPGCPPKPEAVIDAITKLRKKVSREIYE
DRIWPQQESRCFTTNHKFHVGRSTHTGNYDQGLLYQSPSTSEIPSEIFFKYKSSVSSHELVN
MQNRNTFSWIKEQMTRSISVSIMIYVITRTAISNAYPIFAQQGYENPREATGRIVCANCHLA
NKPVDIEVPQAVLPDTVFEAVVRIPYDMQVKQVLANGKKGALNVGAVLILPEGFELAPPD
RISPEMKEKIGKLSFQSYRPTKKNILVIGPVPGQKYSEITFPILSPDPATKKDVHFLKYPIYVG
GNRGRGQIYPDGSKSNNTVYNATAAGIISKIVRKEKGGYEISIADASDGHQVVDIIPPGPEL
LVSEGESIKLDQPLTSNPNVGGFGQGDAEIVLQDPLRVQGLLFFLASVILAQIFLVLKKKQF
EKVQLSEMNFMSKVYDWFEERLEIQAIADDITSKYVPPHVNIFYCLGGITLTCFLVQVATGF
AMTFYYRPTVTEAFASVQYIMTEANFGWLIRSVHRWSASMMVLMMILHVFRVYLTGGFK
KPRELTWVTGVVLAVLTASFGVTGYSLPRDQIGYWAVKIVTGVPEAIPVIGSPLVELLRGSA
SVGQSTLTRFYSLHTFVLPLLTAVFMLMHFPMIRKQGISGPLMGVTKKPDLNDPVLRAKL
AKGMGHNYYGEPAWPNDLLYIFPVVILGTIACNVGLAVLEPSTIGEPADPFATPLEILPEWY
FFPVFQILRTVPNKLLGVLLMVSVPAGLLTVPFLENVNKFQNPFRRPVATTVFLIGTAVALW
LGIGATLPIDKSLTLGLFMVEVFLFGIVLGLIPITLAGLFVTAYLQYRRGDQLDLMPTITSYF
GFLLAALTITSALFIGLSKIRLIMDIVSLAWAALMVVFTFSLSLVVWGRSGLMIIRSPESEVK
ILVDRDPVKTSFEEWARPGHFSRTLAKGPETTTWIWNLHADAHDFDSHTSDLEEISRKVFS
AHFGQLSIIFLWLSGMYFHGARFSNYEAWLSDPTHIRPSAQVVWPIVGQEILNGDVGGGF
RGIQITSGFFQIWRASGITSELQLYCTAIGALVFAALMLFAGWFHYHKAAPKLAWFQDVES
MLNHHLAGLLGLGSLSWAGHQVHVSLPINQFLDAGVDPKEIPLPHEFILNRDLLAQLYPSF
AEGATPFFTLNWSKYADFLTFRGGLDPVTGGLWLTDIAHHHLAIAVLFLIAGHMYRTNWAI
GHGLKDILEAHKGPFTGQGHKGLYEILTTSWHAQLSLNLAMLGSLTIVVAHHMYSMPPYP
YLATDYGTQLSLFTHHMWIGGFLIVGAAAHAAIFMVRDYDPTTRYNDLLDRVLRHRDAII
SHLNWACIFLGFHSFGLYIHNDTMSALGRPQDMFSDTAIQLQPVFAQWVQNTHALAPGAT
APGATTSTSLTWGGGDLVAVGGKVALLPIPLGTADFLVHHIHAFTIHVTVLILLKGVLFARS
SRLIPDKANLGFRFPCDGPGRGGTCQVSAWDHVFLGLFWMYNAISVVIFHFSWKMQSDV
WGSISDQGVVTHITGGNFAQSSITINGWLRDFLWAQASQVIQSYGSSLSAYGLFFLGAHFV
WAFSLMFLFSGRGYWQELIESIVWAHNKLKVAPAIQPRALSIVQGRAVGVTHYLLGGIATT
WAFFLARIIAVGMALRFPRFSQGLAQDPTTRRIWFGIATAHDFESHDDITEERLYQNIFASHF
GQLAIIFLWTSGNLFHVAWQGNFESWVRDPLHVRPIAHAIWDPHFGQPAVEAFTRGGALG
PVNIAYSGVYQWWYTIGLRTNEDLYTGALFLLFLSAVSLIAGWLHLQPKWKPSVSWFKN
AESRLNHHLSGLFGVSSLAWTGHLVHVAIPGSRGEYVRWNNFLDVLPHPQGLGPLFTGQ

WNLYAQNPDSSSHLFGTAQGAGTAILTLLGGFHPQTQSLWLTDIAHHHLAIALIFLVAGHM
YRTNFGIGHSMKDLLEAHTPPGGRLGRGHKGLYDTINNSVHFQLGLALASLGVITSLVAQ
HMYSLPAYAFIAQDFTTQAALYTHHQYIAGFIMTGAFAHGAIFFIRDYNPEQNKDNVLAR
MLDHKEAIISHLSWASLFLGFHTLGLYVHNDVMLAFGTPEKQILIEPIFAQWIQSAHGKTSY
GFDVLLSSTNSPAFNAGRSIWLPGWLNAVNE--NSNSLFLTIGPGDFLVHHAIALGLHTTTLI
LVKGALDARGSKLMPDKKDFGYSFPCDGPGRGGTCDISAWDAFYLAVFWMLNTIGWVT
FYWHWKHITLWQGNVSQFNESSTYLMGWLRDYLWLNSSQLINGYNPFGMNSLSVWAW
MFLFGHLVWATGFMFLISWRGYWQELIETLAWAHERTPLANLIRWRDKPVALSIVQARLV
GLAHFSVGYIFTYAAFLIASTSGKFGMSHSVKIYDTCIGCTQCVRACPTDVLEMIPWDGCK
AKQIASAPRTEDCVGCKRCESACPTDFLSVRVYLWHETTRSMGLAYMIDLLFPAFLVPFVG
IVLPAIAMASLFIYIERNEIVMRDIKTYLSVAPVLSTLWFGALAGLLIEINRFFPDALTFPFF--
MTAILERRESESLWGRFCNWITSTENRLYIGWFGVLMIPTLLTATSVFIIAFIAAPPVDIDGIR
EPVSGSLLYGNNIISGAIIPTSAAIGLHFYPIWEAASVDEWLYNGGPYELIVLHFLLGVACY
MGREWELSFRLGMRPWIAVAYSAPVAAATAVFLIYPIGQGSFSDGMPLGISGTFNFMIVFQ
AEHNILMHPFHMLGVAGVFGGSLFSAMHGSLVTSSLIRETTENESANEGYRFGQEEETYNI
VAAHGYFGRLIFQYASFNNSRSLHFFLAAWPVVGIWFTALGISTMAFNLNGFNFNQSVVDS
QGRVINTWADIINRANLGMEVMHERNAHNFPLDLAAVEAPSTNGMGLPWYRVHTVVLN
DPGRLLSVHIMHTALVSGWAGSMALYELAVFDPSDPVLDPMWRQGMFVIPFMTRLGITNS
WGGWSIT-GGTITNPGIWSYEGVAGAHIVFSGLCFLAAIWHWVYWDLEIFCDERTGKPSLD
LPKIFGIHLFLSGVACFGFGAFHVTGLYGPGIWVSDPYGLTGKVQPVNPAWGVEGFDPFVP
GGIASHHIAAGTLGILAGLFHLSVRPPQRLYKGLRMGNIETVLSSSIAAVFFAAFVVAGTM
WYGSATTPIELFGPTRYQWDQGYFQQEIYRRVGTGLAENQSLSEAWSKIPEKLAFYDYIG
NNPAKGGLFRAGSMDNGDGIAVGWLGHPIFRDKEGRELFVRRMPTFFETFPVVLVDGDGI
VRADVPFRRAESKYSVEQVGVTVEFYGGELNGVSYSDPATVKKYARRAQLGEIFELDRAT
LKSDGVFRSSPRGWFTFGHASFALLFFFGHIWHGARTLFRDVFAGIDPDLDAQVEFGAFQ
KLGDPTTRRQVV------------VETLFNGTLALAGRDQETTGFAWWAGNARLINLSGKLLGAH
VAHAGLIVFWAGAMNLFEVAHFVPEKPMYEQGLILLPHLATLGWGVGPGGEVIDTFPYFV
SGVLHLISSAVLGFGGIYHALLGPETLEESFPFFGYVWKDRNKMTTILGIHLILLGIGAFLLV
LKALYFGGVYDTWAPGGGDVRKITNLTLSPSVIFGYLLKSPFGGEGWIVSVDDLEDIIGGH
VWLGSICILGGIWHILTKPFAWARRALVWSGEAYLSYSLGALSVFGFIACCFVWFNNTAYP
SEFYGPTGPEASQAQAFTFLVRDQRLGANVGSAQGPTGLGKYLMRSPTGEVIFGGETMRF
WDLRAPWLEPLRGPNGLDLSRLKKDIQPWQERRSAEYMTHAPLGSLNSVGGVATEINAV
NYVSPRSWLATSHFVLGFFLFVGHLWHAGRARAAAAGFEKGIDRDFEPVLSMTPLNMTIA
LGRFTKDENDLFDIMDDWLRRDRFVFVGWSGLLLFPCAYFALGGWFTGTTFVTSWYTHG
LASSYLEGCNFLTAAVSTPANSLAHSLLLLWGPEAQGDFTRWCQLGGLWTFVALHGAFGL
IGFMLRQFELARSVQLRPYNAIAFSAPIAVFVSVFLIYPLGQSGWFFAPSFGVAAIFRFILFFQ
GFHNWTLNPFHMMGVAGVLGAALLCAIHGATVENTLFEDGDGANTFRAFNPTQAEETYS
MVTANRFWSQIFGVAFSNKRWLHFFMLFVPVTGLWMSALGVVGLALNLRAYDFVSQEIR
AAEDPEFETFYTKNILLNEGIRAWMAAQDQPHENLIFPEEVLPRGNALMSGSTGERSFADII
TSIRYWVIHSITIPSLFIAGWLFVSTGLAYDVFGSPRPNEYFTESRQGIPLITGRFDPLAQLDE
FSRSFMTIDRTYPIFTVRWLAVHGLAVPTVSFLGSISAMQFIQRMATQSVEGSSRSG-PRRTI
TGDLLKPLNSEYGKVAPGWGTTPLMGVAMALFAIFLSIILEIYNSSVLLDGISMSMLTLKLF
VYTVVIFFVSLFIFGFLSNDPGRNPGREEMADTTGRIPLWLIGTVTGIPVIGSIGIFFYGSYSG
LGSSLMLNIFGLIC--LNSALHSSSFFFAKLPEAYAFFNPIVDVMPVIPVLFFLLALVWQAAVS

FRTTQSNPNEQNVELNRTSLYWGLLLIFVLAVLFSNYFFNMEVNILAFIATALFILVPTAFLLI
IYVKTVSQNDMETATLVAISISGSLVSFTGYALYTAFGQPSQQLRDPFEEHGDMEALVYTFL
LVSTLGIIFFAIFFREPPKVPTKKMKMTIAFQLAVFALIATSSILLISVPVVFASPEGWSSNKN
VVFSGTSLWIGLVFLVGILNSLIS-------MSPQTETKASVGFKAGVKDYKLTYYTPDYETKD
TDILAAFRVTPQPGVPPEEAGAAVAAESSTGTWTTVWTDGLTSLDRYKGRCYHIEPVAGEE
NQYIAYVAYPLDLFEEGSVTNMFTSIVGNVFGFKALRALRLEDLRIPPAYSKTFQGPPHGIQ
VERDKLNKYGRPLLGCTIKPKLGLSAKNYGRAVYECLRGGLDFTKDDENVNSQPFMRWR
DRFLFCAEAIYKAQAETGEIKGHYLNATAGTCEEMIKRAVFARELGVPIVMHDYLTGGFTA
NTSLAHYCRDNGLLLHIHRAMHAVIDRQKNHGIHFRVLAKALRMSGGDHIHAGTVVGKL
EGEREITLGFVDLLRDDFIEKDRSRGIYFTQDWVSLPGVLPVASGGIHVWHMPALTEIFGD
DSVLQFGGGTLGHPWGNAPGAVANRVALEACVQARNEGRDLAREGNEIIREACKWSPEL
AAACEVWKEIKFEFEAMDTL--TAIHLYKTSTPSTRNGAVDSQVKSNPRNNLIYGQHRCGK
GRNARGIITAGHRGGGHKRLYRKIDFRRNKKNIEGRIVTIEYDPNRNAYICLIHYGDGEKR
YILHPRGAIIGDSIVSGTEVSIKMGNALPLTDMPLGTAIHNIEITLGKGGQLARAAGAVAKLI
AKEGKSATLKLPSGEVRLISKNCSATFGQVGNVGVNQKSLGRAGSKCWLGKRPVVRGVV
MNPVDHPHGGGEGRAPIGRKNPTTPWGYPALGRRSRKRNKYSDSLILRRRTKMIQPQTHL
NVADNSGARELMCIRIIGAS-NRRYAHIGDVIVAVIKEAVPNMPLERSEVIRAVIVRTCKELK
RDNGMIIRYDDNAAVVIDQEGNPKGTRVFGAIARELRQLSFTKIVSLAPEVLMLSPKRTRF
RKQHRGRMKGISYRGNRISFGRYALRALEPAWITSRQIEAGRRAMTRYARRGGKIWVRIFP
DKPVTVRPTETRMGSGKGSPEYWVSVVKPGRILYEMGGISEIVAREAISIAASKMPIRTQFV
IAGMTRIRRGYIARRRRTKIRLFASTFRGAHSRLTRTTTQQKMRALVSAHRDRGRQKRNFR
RLWITRINAVIREN--VSYSYSRLIHDLYKRQLLLNRKILAQIAISNRNCLYMISNEIIK----------
-MINEVYALGQHICMSAHKARRVIDQIRGRSYEETLMILELMPYRACYPIFKLVYSAAANA
SHNMGFNEADLIISKAEVNEGPTMKRLKPRARGRSYPIKRPTCHITIVLKDISLDGIVG---M
DGIKYAVFTDKGIRLLGNN-QYTSNVESGSTRTEIKHWVELFFGVKVIAMNSHRLPGKGRR
MGPIMGHTMHYRRMIITLQPGYSIPPLRKKRTMAVPKKRTSISKKRIRKNIWKRKADWAS
VKAFSLAKSLSTGNSKSFFVRQI-NMAKGKDVRVRVILECINCVRNGVN--KESPGVSRYIT
QKNRHNTPSRLELRKFCPYCFKHTIHGEIKKMKIRASVRKICEKCRLIRRRGRIIVICSNPRH
KQRQGMVREKVTVSTRTLQWKCVESRADSKRLYYGRFILSPLIKGQADTIGIAMRRALLG
EIEGTCITRAKSE--KIPHEYSTIVGIEESVHEILMNLKEIVLRSNLYGTRNASICIRGPGYVTA
QDIISPPSVETVDNTQHIANLTEPIDFCIELEIERNRGYRMKTPNNSQDGSY--PIDAVFMPVR
NANHSIHSYANGNEKQEILFLEIWTNGSLTPKEALHEASRNLIDLFIPFLHAQEE--NILLEDN
Q-NGVTLPFFTFHDRLA-KIRKNEK-EIALKYIFIDQSELPPRTYNCLKRSNIHTLLDLFNNSQ
EDLMKIEDFRIEDVKQILGILQKH--FTVDLPKNKMRRDGN-EGMSTIPGFNQIQFEGFCRFI
DQGLTEELYKFPKIEDTDQEIEFQLFVETYQLVEPLIKERDAVYESLTYSSEVYVPAGLIWKP
GRDMQEQTIFIGNIPLMNSLGTSIVNGIYRIVINQILQSPGIYYRSESDHNGISVYTGTIISDW
GGRSELEIDRKARIWARVSRKQKISILVPSSAMGSNLREILDNVCYPEIFLSFLNDKEKKKIG
SKENAILEFYQQFACVGGDPVFSESLCKELQKKFFQQRCELGRIGRRNMNRRLNLDIPQN
NTFLLPRDVLAAADHLIGMKFGMGTLDD--MNHLKNKRIRSVADLLQDQFGLALIRLENV
VRGTICGAIK-HKLI-PTPQNLVTSTP----LTTTYESFFGLHPLSQVLDRTNPLTQMVHGRKLS
YLGPGGLTGRTASFRIRDIHSSHYGRICPIDTSEGINVGLIGSLAIYARIGHWG-SLESPFY---EI
SDRS--KEVQMLYLSPSRDEYYMVAAGNSLALNQSIPEEQVVPARYRQEFLTIAWEQVHFR
SIFPFQYFSIGASLIPFIEHNDANRALMSSNMQRQAVPLSQSEKCIVGTGLERQAALDSGVS
TIAEHEGKIIYTDTDKIILLGNGDTLSIPLVMYQRSNKNTCMYQKPQVRRGKFIKKGQIVAD

GAATVGGELALGKNVLVAYMPWEGYNSEDAVLISERLVYGDIYTSFHIRKYEIQTHVTSQ
G-PERITNEIPHLEAHLLRNLDRNGIVMLGSWVETGDILVGKLTPQMAKE-SSYAPEDRLLR
AILGIQVSTSKETCLKLPIGGRGRVIDVRWIQKKG-GSSYNPETICVYISQKREIKVGDKVAG
RHGNKGIISKILPRQDMPYLQDGTPIDMVFNPLGVPSRMNVGQIFECSLGLAGDLLDRHY
RIAPFDERYEQEASRKLVFSELYKASKQTANPWVFEPEYPGKSRIFDGRTGDPFEQPVLIGK
SYILKLIHQVDDKIHGRSSGHYALVTQQPLRGRAKQGGQRVGEMEVWALEGFGVAHILQE
MLTYKSDHIRARQEVLGTTIVGGTIPSPEDAPESFRLLVRELRSLALELNHFLVSEKNFQIN
RKEA-------MIDRYKHQQLRIGSVSPQQISAWAKKILPNGEIVGEVTKPYTFHYKTNKPEKD
GLFCERIFGPIKSGICACGNYRVIGDEKEDPKFCEQCGVEFVDSRVRRYQMGYIKLACPVT
HVWYLKRLPSYIANLLDKPLRELEGLVYCD--FSFARP-IAKKPTFLRLRGSFEYEIQSWKYS
IPLFFTTQGFDTFRNREISTGAGAIREQLADLDLRIITAYSLVEWKELGEE-GPTGNEWEDRK
IGRRKDFLVRRMELAKHFIRTNVEPEWMVLCLLPVLPPELRPIIQIDGGKLMSSDINELYRR
VIYRNNTLTDLLTTSRSTPGESVMCQEKLVQEAVDTLLDNGIRGQPMRDGHNKVYKSFSD
VIEGKEGRFRETLLGKRVDYSGRSVIVVGPSLSLHRCGLPREIAIELFQTFVIRGLIRQHLAS
NIGVAKSKIREREPIVWEILQKVIQGHPVLLNRAPTLHRLGIQAFQPILVEGCAICLHPLVRK
GFNADFDGDQMAVHVPLSLEAQAEARLLMFSHMNLLSPAIGDPISVPTQDMLMGLYVLTI
GNRRGICANRYNPRNRRN---YQNERIDDNNYRYTKEKEPYFCSSYDALGAYRQKRINLDS
PLWLRWRLDQR-VIAS--REVPIEVQYESLGTYHEIYGHYLIVRSVKKEILCIYIRTTVGHISF
YREIEEAIQGFCRACSYGT--------MEVLMAERADLVFHNKAIDGTAMKRLISRLIDHFGMA
YTSHILDQVKTLGFQQATATSISLGIDDLLTIPSKGWLVQDAEQQSLILEKHHHYGNVHAVE
KLRQSIEIWYATSEYLRQEMNPNFRMTNPSNPVHIMSFSGARGNASQVHQLVGMRGLMS
DPQGQMIDLPIQSNLREGLSLTEYIISCYGARKGVVDTAVRTSDAGYLTRRLVEVVQHIVV
RRTDCGTLQGISVSPRNGMIT-ERILIQTLIGRVLADDIYMGPRCIAARNQDIGIGLVNRFIT---
---FGAQPISIRTPFTCRSISWICRLCYGRSPTHGDLVELGEAVGIIAGQSIGEPGTQLTLRTFHT
GGVFTGGTAEHVRAPSNGKIKFNEDLVHPTRTRHGHPAFLCYIDLYVTIESQDIIHNVNIPP
KSFLLVQNDQYVESEQVIAEIRAGTSTL-NFKERVRKHIYSDSEGE-MHWSTNVYHAPEYT
YSHVHLLPKTSHLWILSGGSYR--SSIVPLSLHKDQDQMNVH--SLSVERRYISN--FSVTNDQ
-VRHKLFNSYPSS--GERILDY-SGTDRIICNGHCNFIYPS-ILHENSD--LLAKRRRNRFIIPFQS
NQEREKELMSRSDISIEIPIN--GIFRRNSILAYFDDPRYRRNNSGITKYGTIEVHAIVKKED--L
IEYRR--KEFRPKYMKVDRFFFIPEEVHILPGSSSIMVRNNSIIGVDTRITLNARSRVGGLVRV
ERKKKRIELKIFSGDIHFPGETDKISRHSGILIPPGTGKK--NSKKSKK-KLQNWIYVQR--ITPI
KKK--YFVFVRPVVTYEIADGINLATLFPQDLLQERDNVQLRVVNYILYANGKPIRGISHTTI
QLVRTCLVLNWDQGKN---GSSIKEVHASFVEVRANDLIQDFIRMDLVKSTISHTGKRNDPA
GSGLIPD-NGSDRTNINPFY---SKAR-----IQQSLSRHQGTLRSLLNR---NKECQSLIILSSSNCS-
-QMGPLNTLKYHN---VTKESDPPIPIRNSLGPLGT--VPKITNFYSS-YYQLIT--HNQIL-VSKY
LLLAN---LKQTLQVFK---YYLMDENGK--IYNPDPCSNIIFNPFNLNWYFLHHDYCEETSTII
S-LGQFFCENVCISKKGP---HLKSGQVLIVH-VDSLVIRSAKPYLATPGATVHGHYGEILYKG
DTLVTFIYEKSRSGDITQGLPKVEQVLEVRSIDSISMTLENRVEGWNERITRILGIPWEYLIG
AELTIAQSRISLVNKIQKVYRSQGVQIHNRHIEIIVRQITSKVLVSE---DGMSNVFSPGELIGL
FRAERTGRALEEAICYRAVLLGITRASLNTQSFISEASFQETARVLAKAALRGRIDWLKGLK
ENVVLGGMIPVGTGFKGLVHRS--REHNNIP--LEIKKKNLFEGEMR--DTLFHHRELFDSCIP
KNFHDTSKQSFTGFNDF------MTRRYWNINLEEMMEAGVHFGHGTRKWNPRMAPYISAK
RKGIHIINLTRTARFLSEACDLVFDAARRGKRFLIVGTKNKVVDLVASAAIRARCHYVNKK
WLGGMSTNWSTTETRLHKFRDLRAEQKAGRLNHLPKRDAAMLKRQLSHLETYLGGIKY

MTGLPDIVIIVDQQEEYTALRECVTLGIPTICLIDTNCDPDLADISIPANDDAIASIRLVLNKL
VSAICEGRSS-YIRNHMGQKINPLGFRLGTTQNHHSVWFAQPKSYSEGLQEDQKIRDCIKN
YVQKNKRIP-L-VEGIARIEIQKRIDLIQVIIYMGFPKLLIEDRPR----GIEELQINLQKEFNSMN
RKLNIAITRIAKPYGQPNILAEYIAGQLKSRVSFRKAMKKAIELTEQTDTKGIQVQIAGRID
GKEIARVEWIREGRVPLQTIRAKIDYCSYTVRTIYGVLGIKIWIFVDEEMSRYRGPRFKKIR
RLGALPGLTSKSPKAGSDLRNQLRSGK-RSQYRIRLEEKQKLRFHYGLTERQLLKYVRIAG
KAKGSTGQVLLQLLEMRLDNILFRLGMASTIPGARQLVNHRHILVNGRIVDIPSYRCKPRD
IITTRDEQKSRALIQNYLDSSSH-EELPKHLTLHSFQY--KGLVNQIIDSKWVGLQINELLVVE
YYSRQTMSRRGTAEEKTAKSDPIYRNRLVNMLVNRILKHGKKSLAYQIIYRAVKKIQQKTE
TNPLSVLRQAIRGVTPDIAVKARRVGGSTHQVPIEIGSTQGKALAIRWLLGASRKRPGRNM
AFKLSSELVDAAKGSGDAIRKKEETHRMAEANRAFAHFRMGRDTIADIITSIRNADMDKK
GRVRIGYTNIAENIIKILLREGFIENVRKHRESNKYFLVSTLRHRR--NRKRPYK---TIL--KRIS
RPGLRIYSNYQRIPRILGGIGIVILSTSRGIMTDREARLEGIGGEILCYIWMAKPIQRIGSRRN
GPIGSRKNGRRIPKGVIHVQASFNNTIVTVTDVRGRVVSWSSAGACGFRGTRRGTPFAAQT
AAGNAIRTVVDQGMQRAEVMIKGPGLGRDAALRAIRRSGILLSFVRDVTPMPHNGCRPP
KKRRVMPTIKQLIRNTRQPVRNVTKSPALRGCPQRRGTCTRVYTITPKKPNSALRKVARVR
LTSGFEITAYIPGIGHNLQEHSVVLVRGGRVKDLPGVRYHIVRGTLDAVGVKDRQQGRSKY
GVKKPKMAKKSLIQREKKRQKLEQKYHLIRRSSKKEIS---KIPSLSGKWEIHGKLQSLPRN
SAATRLHRRCFSTGRPRANYRDFGLSGHILREMVHACLLPGATRSSWMVKNAFISVISQEE
KEENK--GSAEFQVFNFTNKIRRLTSHLELHRKDYLSQRGLRKILGKRQRLLAYLSKKNKV
RYKELISQLDIREPKIR--MVKLRLKRCGRKQRAIYRIVAIDVRSRREGRDLRKVGFYDPIKN
QTYLNVPVILYFLEKGAQPTGTVHDISKKAEVFKELRINQTKSN-MDKPKRLFLKSKRSFR
RRLPPIGSGDRIDYRNMSLISRFISEQGKILSRRVNRLTLKQQRLITIAIKQARILSSLPFLNNE
K--QFERAESTPRTPGPRTRNKVTRSLKKNPFVANHLLRKIEKLNTKAEKEIIVTWSRASTIIP
TMIGHTIAIHNGKEHLPIYITDRMVGHKLGEFAPTMNFRGHAKNDNRSRRMILKSFLLGNL
LSLCMKIINSVVVVGLYYGFLTTFSIGPSYLFLLRARVMEEG---TEKEVSATTGFITGQLMM
FISIYYAPLHLALGRPHTITVLVLPYLLFHFFWHNHKYFLNYGSTIRNSIRNLNIQCVFLNNL
IFPLFNHFILPSSTLARLINIYMFQCNNKMLFVTSSFVGWLIGHILFMKWVELVLIWIRQNH
FFRSSNVLIKNLVPELRNSMARIFSILLFITCIYYLGRMPSTLVTKKMKETEEDKPLVTLLFD
YKRWNRPLRYIKNNQLENAVRNEMSQYFFYTCQSDGKQRISFTYPPSLSTFWEMIQRKMS
LYTTEKLSPEELYNHWVYTNEQKRNNVSKEFINRIEALDKGSLTLDVLEKRTRLCNDKTE
QQCLPKVYDPFLNGPYRGTIKKVSSSK--LWINKIHGILYREFEQKDPNNQ--VINEICKKVPR
WSYKLIDLEQQEGEQIRSRKAKRVVIFTDALIRYSQQPDFRRDIIKGSMRAQRRKIVIWELF
QANVHSPLFLDKIDKTFFFSFDI--SEMTKFIFRNKEENERIAVAETWDSILFAQPIRGSMLVT
QSILRKYIVLPLLIIAKNTARILLFQFPEWFEDWEEWNREIHVKCTYNGVPLSETEFPRNWL
KDGIQIKILFPLCLKPWHRFKKGKKYNFCFLTVWGMETELPFGSPRNQPSFFEPILKELEKK
IRKVK-NQFFRILRVVKERTKWFLTVLKEKKRWVIEIIL--FIKRIMKELAKINITIISNQIIHESP
IRIRSTDWTNYSLTEKKMKNLADRTSTIRNQIERITKDKKKSPKKMSCGDKRSESLKSFWE
IVKKRSIRLIRKWHFFLKFVIEKIY---LLCIINIPRINAQLFIHFIS-DLSSLSQAYVFYKLSQTK
VINKYHLRSVLEYRSLFLKDRIKDYFTQGIFHFESRHKKLRNSGMNDWKNWLKGHYQYD
LSQTRWARLVPQKWQNRVNQRRTTQNKKKLGSYEKDQ--LIHYQKQEKLKKHYGYDLLS
HKYINDYIIEPDRKYFDWGILHFCLRPFDWMGMNEEILNRWFVPEFLLLYDTYKMKPWGI
PIKLLLVNFKSKAEVELDLLLKKYLLFQLRWNGSLNQRILNNIKIYCFLLRLINPKEITISSIQ
KGEMSLGIMLIKRDLTFTELIKRGIFIIEPVRLSIKWDGQFIMYQTIGISLVHKSKHNHYDLL

VPENISSPRRRRELRILTCFRFLWPNYRLEDLACMNRYWFDTNNGSRFSMSRIHMYPMKR
HQFKSWIFELREILREIKNSHYFLDSWTKFDSVGSFTHIFFHQERFIKLFDPRIWSILLS-RDS-
-QGSTSNRYFT---IKGVVLLVVA-VLLY----RINNRNMVERKNLYLMGLL-----PIPMNSIGPRN
DTLEESF-GSSNINRLIVS-LLYLPKGKKISESCFMDPKESTWVLPIT----KKCIMPESNWGSR
WWRNRIGKKR--DSSCKIS---NETVARIEVSFKEKDIK-YLEFLFVSYTD--DPIRKDHDWELF
DRLSPRKKRNIINL--NSGQLFEILVKHWICYLMSAFREKRPIEVEG-FFKQQG-AESTIQSND
IEHVSH--LFSRNKWGISLQNCAQFHMWQFRQDLFV---SWGK-NPHESDFLRN-VSRENWI
WLDNVWLVNKDR---FFS--KVRNVSSNIQYDS---TR--SIFVQVTDSSQLKGSS-DQSRDRFD
SISNS--NEDLE------------YHTLINQREIQQLKERSIL-WD--PSFLQTERTEIESE--RFPK--CLS
GYS--SMSRLFT-EREKQMNNHLLPEEIEEFL--GNPTRSIRSFFSDRW--SELH-LGSNPTERST
RDQ----KLLKKQQDVS-FVPSRRSE----NKEMVDIFKIITYLQN--TVSIHPISSDPGCDMVP-K
DEPD-MDSSNKISFLNKNQFFD--LFHLFHDRNRGGYTL--HHDFESE--ERFQEMVDLFTLSI
TEP-DLVYHKGFAFSIDSYGLDQKK--FLNEVFNSRDESKKKSL---LVLPPIFY--EENESFYRR
IRKKSVR-ISCGNDLEDPKPKRVVFASNNIMEAVNQDRLIRNLIQIQYSTYR--YIRNVSNRFF
---LMNRSDRNFEYGIQ---RDQIGTDTLNHRTIMKYTINQHLSNLKK-SQKKWFDP----LISRT
ERSMNRDPDAYRYKWSNGSKNFQEHLEHFVSEQKS---RFQVVFDRLRINQYSIDWSEVID
KQDLSKSLRFFLSKS---------LLFLS-------KSLLFLSK-SLPFFFVSFGNIPIHRSE-IHIY-ELKGP
NDQLCNQLLESIGVQIVHLNKLKPF--LLA---DHDTS-QKSKFLINGGTISPFLFNKIS-KW-MI
DSFHT--RNNRRKSFDNTDSYFSMISHDRDNWLNPVKPFHRSSLISSFYKANRLRFLNNPHH
FWFY--CNKRFPFYV----EKARINNYD--LTY-GQFLNILFIRNK-IFSLCVGK--KKHAFLE-RDT
ISPIESQVSNIFIPNDFPQSGDET-YNLYKS---FHFPIRSDPFVR--RAIYSIADISGTPLTEGQIVN
FERTYCQPLSDMNLSDSEGKNLDQYLN--FNSNMGLIHTLCSEKSLP--SEKRKK--------RSL
CLK--KCVEKRQMYRTFQRDSAFSNLSKWNLFQTYMPWFLTSTGCKYLNFTLLDTFSDLL-
-PILSSS-QKFVSIFHDIMHGSDISWPIPQKKW------CLPQWNL-ISEISSKCLHN--LLLSEEMIH
RNNESPVPLIWTHLRSPNAREF-LYSILFLLLVAGY-LVRTHLLFVSR--ASSELHTEFEKIKSL
MIPSYMIELRKLLDRYPTSELNSFWLKNLFLVALQQLGDSLEEIRGSASGGNMLLGGGP----
-AYG-VKSIRSKKKYLNINLIDIIDLI-SIIPNPINRITFSRNTRHLSRTSKEIYSLIRKRKNVNGD
WIDDKIESWVANSDSIDDEEREFLVQFS--TLTT--EKRMDQILLSLT-HSDNLS-----KNDSGYQ
MIEQPGSIYLRYLVDIH--KKYLMNYEFNRSCLAERRIFLAHYQTITY--SQTSCGANSFYFPS
HG---KPFSLRLALSPS-RGILVIGSIGTGRSYLVKYLATNSYVPFITVFPNKFLDDKPKGYLID
D---IDID------DID-----------------------DSDDIDDDLDTELLTMMNAL---TMDMT---PKIDRFDI
TLQFELAKAMSPCIIWIPNIHDLYVNESNYLSLGLLVNYLS---RDCERCS-TRNILVIASTHIP
QKVDPALIAPNKSNTCIKIRRLLIPQQRKHFFTLSYTRGFHLEKKIFHT--NGFGSITMGSNAR
DLVALTNEALSISITQKKSIIDTNTIRSALHRQTWDLR---SQVRSVQDHGILFYQIGRAVAQN
VLLSNCPIDPISIYMKKK--SCKEGDSYLYKWYFELGTSMKKLTILLYLLSCSAGSVAQDLW
SLP--GPDEK-NGITSYGFVENDS-DLVHGLLEVEGVLVG--SSRTEKDCSQFDND--RVTLLLR
SEPRN-PLDMMQNGSCSIVDQRFLYEK--YESEFEE-GE--GALDPQ----QIEED----LFNH--IVW
AP--RIWRP---CGNLFDCIERPNELGFP-YWARSFR---GKRIIYHKEDELQENDSEFLQSGTM
QYQTRDRSSKEQGFFRISQFIWDPGGPFFFLFK-DQPFVSVFSRREFFADEEMSKGLLTSQT
NPPTSIYKRWFI--KNTQEKHFELLIHRQRWLRT---NSSLS--NGSFRSN--TPSESYQYLSNLFL
SN-GTLLDQMTKTLLRKRWLFPDEMKHLI--HVTGERFPIPMPRSR-INGNFIDKTSSIVANIL
LRIIPTTSGEKEAFTYYRDG--MSAQSEGNYAEALQNYYEAMRLEIDPYDRSYILYNIGLIHT
SNGEHTKALEYYFRALERNPFLPQAFNNMAVICHY-RGEQAIRQGDSEIAEAWFDQAAEY
WKQAIALTPGNYIEAHNWLKITRRFE--MNWRSERIWIELITGSRKTSNFCWAFILFLGSLGF

LLVGTSSYLGRNLISLFPSQQIIFFPQGIVMSFYGIAGLFISSYLWCTISWNVGSGYDRFDRK
EGIVCIFRWGFPGKNRRIFLRFFMKDIQSIRIEVKEGLYPRRVLYMEIRGQGAIPLTRTDEN-L
TPREIEQKAAELAYFLRVPIEVF
>Nandina
KEKWWFNSMLFLSKSTDGIGPIGNTNGSEDPVID---KNIH-CNNVDRLFGVRGIRNFISDDT
FLVRDSNGDNYSIYFDIENHIFEIDND-----RYELESSFSSYLNSSYLNTGSKSNNHYYDRYM
YDTKYSWNNHINSCIDNYLDSEIR--INSYFSSGGDNYSKSYIYSYICSANASSIK---NESDSH
MRGKY-DLDINKKYRHLWVQCENCYGLNYKKFFRSKFNICEQCGYHLKMSSSDRIELSID
PGTWDPMDDDMVSVDPIEFHSEEEPYKDRIDSYQKKTGLTEAVQTGIGQLNGIPIAIGVMD
FQFMGGSMGSVVGEKITRLIEYATNRSLPVIIVCASGGARMQEGSLSLMQMAKISSASYNY
QSNKKLLYVSILTSPTTGGVTASFGMLGDIIIAEPNAYIAFAGKRVIEQTLNKIVPDGSQAAE
YLFHKGLFDPIVPRNLLKGVLSELFQLHGFFPVNSNSIKMATLRADEISNIIRERIEQYTTEV
KIVNTGTVLQVGDGIARIHGLDEVMAGELVEFEEGTIGIALNLESNNVGVVLMGDGLMIQ
EGSSVKATGRIAQIPVSEAYLGRVINALAKPIDGRGEIPASESRLIESPAPGIISRRSVYEPMQ
TGLIAIDSMIPIGRGQRELIIGDRQTGKTAVATDTILNQKGQNVICVYVAIGQKASSVAQVVT
TFQEQGAMEYTIVVAETADAPATLQYLAPYTGAALAEYFMYRERHTSIIYDDPSKQAQAY
RQMSLLLRRPPGREAYPGDVFYLHSRLLERAAKSSSRLGEGSMTALPIVETQAGDVSAYIP
TNVISITDGQIFLSADLFNSGIRPAINVGISVSRVGSAAQIKAMKQVAGKLKLELAQFAELE
AFAQFASDLDKATQNQLARGQRLRELLKQSQAAPLTVGEQILTIYTGTNGYLDSLEIGQVK
KFLVELRTYLKTNKPQFQEIISSTKTLTEEAEVLLKEAIQEQTERFILQEQTMRINPTTSGPG
VSALEEKNQGRIVQIIGPVLDVAFPPGKMPNIYNALVVKGQDTVGQQINVTCEVQQLLGN
NRVRAVAMSATDGLMRGMEVIDTGAPLSVPVGGATLGRIFNVLGEPVDNLGPVDTRTTSPI
HRSAPAFIQLDTKLSIFETGIKVVDLLAPYRRGGKIGLFGGAGVGKTVLIMELINNIAKAHG
GVSVFGGVGERTREGNDLYMEMKESGVINEQNIAESKVALVYGQMNEPPGARMRVGLTA
LTMAEYFRDVNEQDVLLFIDNIFRFVQAGSEVSALLGRMPSAVGYQPTLSTEMGSLQERIT
STKEGSITSIQAVYVPADDLTDPAPATTFAHLDATTVLSRGLAAKGIYPAVDPLDSTSTMLQP
RIVGEEHYETAQRVKQTLQRYKELQDIIAILGLDELSEDDRLTVARARKIERFLSQPFFVAE
VFTGSPGKYVGLTETIRGFQLILSGELDSLPEQAFYLVGNIDEATAKAMNLDVESKLKKMT
LNLCVLTPNRIVWNSEVNEIILSTNSGQIGVLPNHAPIAAAVDIGILRIRLNDQWLTIALMGG
FARIGNNEITVLVNDAERGSDIDPQEARQTLEIAEANLSRAEGKRQTIEANLALRRARTRVE
AINAISMKNVTDSFVSLGHWPSAGSFGFNTDILATNPINLSVVLGVLIFFGKGVLSDLLDNR
KQRILNTIRNSEELREGAIERLEKARARLRKVEKEAEDYIVNGYQEIEREKLNLRNSIGKNL
ERLENYKNETIQFEQQRAINQVRQRVFQQALQGALGTLNSCLNNELHLRTISANIGMFGA
MKEITDMNPLISAASVIAAGLAVGLASIGPGVGQGTAAGQAVEGIARQPEAEGKIRGTLLL
SLAFMEALTIYGLVVALALLFANPFVMNVLPCSINTLKGLYEISGVEVGQHFYWQIGGFQV
HAQVLITSWVVMAILLGSATIVVRNPQTIPTDGQNFFEYVLEFIRDLSKTQIGEEYGPWVPF
IGTMFLFIFVSNWSGALLPWKIIQLPHGELAAPTNDINTTVALALPTSVAYFYAGLTKKGLG
YFGKYIQPTPILLPINILEDFTKPLSLSFRLFGNILADELVVVVLVSLVPSVVPIPVMFLGLFT
SGIQALIFATLAAAYIGESMEGHHMIFLTLEHILTHISFSIVSIVITIYLMTLLVHETVGLYDSS
EKGLIATFFCITGFLVIRWIYSGHFPLSDLYESLMFLSWSFSIIHMVPYFTNHKNLLSTITAPS
AIFTQGFATSGLLTEMHRSTKLVPALQSQWLMMHVSMMILSYAALLCGSLLSIALLVITFR
KNVENFGKSKEIKYVNEIN-VLRNTFLISFRNFRRYQVIQQLDCWSCRVISLGFIFLTIGILSG
AVWANEAWGSYWNWDPKETWAFITWTIFAIYLHTRTNKNLQGINSAIVASIGFLIIWICYF
GVNLLGIGLHSYGSFTSNMTKKKAFTPLPYLASIVFLPWWISFSFNKSLESWVIHWWNTS

QPEAFLNDIQEKNVLEKFIELEELFLLDEMIKEYPKTHIQKFRIGIHKETIQLVKMHNEGHIH
TFLQFSTNIISFAILSGYSILGNEELVVLNSWIREFLYNLSDTIKAFSILLLTDLCIGFHSPHGW
ELMIDFVYKDFGFSHNDQIISGLVSTFPVILDTIFKYWIFRYLNRVSPSLVVIYHSMNDMPIG
VPKVPFRSPGEEDAVWVDVYNRLYRERLLFLGQEVDSEISNQLIGLMVYLSIEDETKDLYL
FINSPGGWVIPGVAIYDTMQFVPPDVHTICMGLAASMGSFILVGGEITKRLAFPHARVMIH
QPASSFYEAQTGEFVLEAEELLKLRETLTRVYVQRTGNPLWVVSEDMERDVFMSATEAQA
HGIVDLVAVEMKEQKWIHEGLITESLPNGMFRVRLDNEDLILGYVSGRIRRSFIRILPGDRV
KIEVSRYDSTRGRIIYRLRNKDSNDMEELKGYLEIDRSRQQDFLYPLLFQEYIYALAHDHG
LENLGYKSSSLVVKRLITRMYQQKHLVFFDNDPNQNKFVDKNFYSQMISEGFAVIVEIPFS
LRLVSSLITKSHNLRSIHSTFPFLEDQFSHLNHVLDILIPYPIHLELLIQLLRCWIQDAPSLHL
LRVLLYEYQNWNSLITKNFSVFSNQRLFLFLYNFYVYECESIFVFLRKQSVHLRSTSSRAFL
ERTHFYRKIENFIVVFRNVFNLWLFKDPFMHYVRYQGKSILASKGTPLLMNKWKYYLVNF
WQCHFYLWSQPDRIHINQLSNHSLDFLGHLSSVRLKTSVVRSQTLDNSFIIDIAI--KKFDTI
VPIIPLIGSLAKAKFCNVSGHPISKPAWTDSSDSDIIDRFGRICRNLSHYYSGSSKKNSLYRV
KYILRLSCARTLSRKHKSTVRSFLKRLGSKLLEEFLTEEEQVLSLIFPRRIWYLDIIRINDLA
NHSMIIDTT-KVQQAINSFSRSE-SLKEVYGLIWLLVPIFTPLLGIIIGVLVIVWLERQISAGVQ
QRIGPEYAGPLGILQALADGTKLLFKEDLLPSRGDILLFSLGPSIAVISILLSYSVIPFGYHLV
LADLSIGVFLWIAISSIAPIGLLMSGYGSNNKYSFLGGLRAAAQSISYEIPLTPCVLSISLLSN
SSSTVDIVEAQSKYGFWGWNLWRQPIGFMVFLISSLAECERLPFDLPEAEEELVAGYQTEY
SGIKFGLFYVASYLNLLVSSLFVTVLYLGGWNLSIPFIFIPELFRINEVCGIFGMTIGIFITLAK
AYLFLFISITTRWTLPRMRMDQLLNLGWKFLLPISLGNLLLTTSFQLLSLMIWHVQNENFIL
DSTRIFMKAFHLLLFHGSFIFPECILIFGLILLLMIDSTSDQKDIPWLYFISSTSLVMSIAALLF
RWREEPMISFSGNFQTNNFNEIFQFLILLCSTLCIPLSVEYIECTEMAITEFLLFVLTATLGGM
FLCGANDLITIFVAPECFSLCSYLLSGYTKRDVRSNEATTKYLLMGGASSSILVHGFSWLYG
SSGGEIELQEIVNGLINTQMYNSPGISIALIFITVGIGFKLSPAPSHQWTPDVYEGSPTPVVAF
LSVTSKVAASALATRIFDIPFYFSSNEWHLLLEILAILSMILGNLIAITQTSMKRMLAYSSIGQ
IGYVIIGIIVGDSNDGYASMITYMLFYISMNLGTFACIVLFGLRTGTDNIRDYAGLYTKDPFL
ALSLALCLLSLGGLPPLAGFFGKLHLFWCGWQAGLYFLVSIGLLTSVVSIYYYLKIIKLLMT
GRNQEITPHVRNYRRSPLRSNNSIELSMIVCVIASTIPGISMNPIIAIAQDTLFMFLLHEYDIF
WAFLIISSVIPILAFLISGVLAPISEGPEKLSSYESGIEPMGDAWLQFRIRYYMFALVFVVFDV
ETVFLYPWAMSFDVLGVSVFIEALIFVLILIVGLVYAWRKGALEWSTNSFPWLTIIVVLPIFA
GSSIFFFPHRGNKVIRWYTICICLLELLLTTYAFCYHFQLDDPLVQLEQDYKWINIFDFHWR
LGIDGLSIGPVLLTGFITTLATLAAWPVTRDSRLFHFLMLAMYSGQIGSFSSRDLLLFFIMW
ELELIPVYLLLSMWGGKKRLYSATKFILYTAGGSIFLLIGVSGMGLYGSNEPTLNFETSANQ
SYPVALEILLYFGFFIAYAVKLPIIPLHTWLPDTHGEAHYSTCMLLAGILLKMGAYGLVRIN
MELLPHAHSIFSPWLMIVGTIQIIYAASTSLGQRNLKKRIAYSSVSHMGFTILGISSITDTGLN
GAILQMISHGFIGAALFFLAGTSYDRIRLVYLDEMGGIAIPMPKIFTLFSSFSMASLALPGMS
GFVAELVVFFGIITSEKFFLMPKILITFVTAIGMILTPIYSLSMLRQIFYGYKQFNVPNSHFFD
SGPRELFVSICIFLPVIGIGIYPDFVFSLSVDKVEAILSNYFYRMMLEHVLVLSAYLLSIGIYG
LITSRNMVRALMCLELILNAVNMNFVTFSDLFDSRQIKGDIFSIFVIAIAAAEAAIGLAIVSSI
YRNRKSTRINQSNLLNKMEHTYQYAWIIPFVPLPVPIAIGVGLLLVPTATKSLRRMWAFLSV
LLLSIIMIFSADLSIQQIKGSLIHQYVWSWTINNDFSLEFGYLIDPLTSIMLILITTVGIMVLIY
SDNYMSHDQGYLRFFAYMSFSNASMLGLVTSCNLIQIYIFWELVGMCSYLLIGFWFTRPIA
ASACQKAFVTNRVGDFGLLLGILGFYFITGSFEFRDLFEITNNGGVNSLFLTLCASLLFVGA

IAKSAQFPLHVWLPDAMEGPTPISALIHAATMVAAGIFLVARLLPLFTVIPYVMNFISLIGIIT
ILLGATLALAQRDIKRSLAYSTMSQLGYTMLALGMGSYRAALFHLITHAYSKALLFLGSGS
IIHSMETVIGYSPEKSQNMVLMGGLTKYVPITKTAFLLGTLSLCGIPPLACFWSKDEILNDS
WLYSPIFAIIACFTAGLTAFYMFRMYLLTFEGHLNAHFQNYSGKKNSSFYSISIWGKEGAKA
IKQN-SVLSTMNLFSFSNKKTSPYPHESDNTMLLPLLILVLFTLFVGFIGIPFDFDILSKLLTPS
INLLHSNSN-SVDWYEFVTNAFFSVSIAYSGIFIASLLYRPVYSSFQNLDLINSFVIFWDKIIN
VIYNWSYNRGYIDVFYTTTFTRGIRGVAELIYFFDRQVIDGITNGVGITSFFVGEGIKYVGG
G-RISSYLFLYLFYVSIFLLIYYMDLPAPIHDFLLVFLGLGLIVGGLGVVLLTNPIYSAFSLGL
VLVCISLFHIPANSHFVAAAQLLIYVGAINVLIVFAVMFMNGSEYYNDFHLWTVGDGVTSL
VCTSIFVSLITTISDTSWYGIIWTTRSNQIIEQDLINKGQQLGIHLSTDFFLPFELISIILLVALIG
AIAMARQMTVPTTRKDLMIVNMGPQHPSMHGVLRLIVTLDGEDVIDCEPILGYLHRGME
KIAENRTIIQYLPYVTRWDYLATMFTEAITVNAPEQLGNIQVPKRASYIRVIMLELSRIASHL
LWLGPFMADIGAQTPFFYIFRERELIYDLFEAATGMRMMHNYFRIGGVAADLPHGWIDKC
LDFCDYFVTGVTEYQKLITRNPIFLERVEGVGFISGEEAINWGLSGPMLRASGIQWDLRKV
DHYECYDEFDWEVQWQKEGDSLARYLVRINEMTESIKIIQQALERIPGGPYENLEVRRFD
RTRDSEWNDFDYRFISKKPSPTFELSKQELYVRVEAPKGELGIFLIGDQSVFPWRWKIRPPG
FINLQILPQLVKRMKLADIMTILGSIDIIMGEVDRMFPMVTGFMNYGQQTVRAARYIGQSF
MITLSHANRLPVTIQYPYEKLITSERFRGRIHFEFDKCIACEVCVRVCPIDLPVVDWKLETDI
RKKRLLNYSIDFGICIFCGNCVEYCPTNCLSMTEEYELSTYDRHELNYNQIALGRLPMSVI
GDYTIRTILNSKKTAGNPLDSKTITNFMQGRLSAWLVKHGLVHRSLGFDYQGIETLQIKPE
DWHSIAVISYVVYGYNYLRSQCAYDVAPGGLLASVYHLTRIQYGVDQPEEVCIKVFAPRRN
PRIPSVFWIWKSADFQERESYDMLGISYDNHPRLKRILMPESWIGWPLRKDYIAPNFYEIQ
DAH--MNSIEFPLLDRITQNSVISTTSNDLSN
WSRLSSLWPLLYGTSCCFIEFASLIGSRFDFDRYGLVPRSSPRQADLILTAGTVTMKMAPSL
VRLYEQMPEPKYVIAMGACTITGGMFSTDSYSTVRGVDKLIPVDVYLPGCPPKPEAVIDAI
TKLRKKVSREIYEDRTGSQQETRCFTTNHKFRVGRSIHTGNYNQGLLYQSPSTSETPSETFF
KYKSSVSPHELVNMQNRNIFSWV-EQTTRSISVSIMILIITWTSISHAYPIFAQQSYENPREAT
GRIVCANCHLANKPVDIEVPQAVLPDTVFEAVVRIPYDMQLKQVLSNGKRGSLNVGAVLI
LPEGFELAPPDRISPEMKEKMGNLSFQNYRPTKKNILVIGPVPGQKYSEITFPILSPDPATKK
DVYFLKYPIYVGGNRGRGQIYPDGSKSNNTVYNATAAGIVSKIVRKEKGGYELTIADASE
GRQVVDIIPPGPELLVSEGESIKLDQPLTSNPNVGGFGQGDAEIVLQDPSRVQGLFFFFASVI
LAQIFLVLKKKQFEKVQLSEMNFMSKVYDWFEERLEIQAIADDITSKYVPPHVNIFYCLGG
ITLTCFLVQVATGFAMTFYYRPTVTEAFTSVQYIMTEANFGWLIRSVHRWSASMMVLMMI
LHVFRVYLTGGFKKPRELTWVTGVVLGVLTASFGVTGYSLPRDQIGYWAVKIVTGVPDAIP
VIGSPLVELLRGSASVGQSTLTRFYSLHTFVLPLLTAVFMLMHFLMIRKQGISGPLMGVTK
KPDLNDPVLRAKLAKGMGHNYYGEPAWPNDLLYIFPVVILGTIACSVGLAVLEPSMIGEPA
DPFATPLEILPEWYFFPVFQILRTVPNKLLGVLLMVSVPAGLLTVPFLENVNKFQNPFRRPV
ATTVFLIGTAVALWLGIGATLPIDKSLTLGLFMIEVLLFGIVLGLIPITLAGLFVTAYLQYRRG
DQLDLMLTITSYFGFLLAALTITSVLFIGLNKIRLIMDIVSLAWAALMVVFTFSLSLVVWGR
SGLMIIRSPEPEVKILVDRDPVKTSFEEWARPGHFSRTLAKGPETTTWIWNLHADAHDFDS
HTSDLEEISRKIFSAHFGQLSIIFLWLSGMYFHGARFSNYEAWLSDPTHIRPSAQVVWPIVG
QEILNGDVGGGFRGIQITSGFFQIWRASGITSELQLYCTAIGALVFAALMLFAGWFHYHKA
APKLAWFQDVESMLNHHLAGLLGLGSLSWAGHQVHVSLPINQFLDAGVDPKEIPLPHEFI
LNRDLLAQLYPSFAEGATPFFTLNWSKYAEFLTFRGGLDPVTGGLWLTDIAHHHLAIAILFL

IAGHMYRTNWGIGHGLKDILEAHKGPFTGQGHKGLYEILTTSWHAQLALNLAMLGSLTIV
VAHHMYSMPPYPYLATDYGTQLSLFTHHMWIGGFLIVGAAAHAAIFMVRDYDPTTRYND
LLDRVLRHRDAIISHLNWACIFLGFHSFGLYIHNDTMSALGRPQDMFSDTAIQLQPVFAQW
IQNTHALAPSATAPGATTSTSLTWGGGGLVAVGGKVALLPIPLGTADFLVHHIHAFTIHVTV
LILLKGVLFARSSRLIPDKANLGFRFPCDGPGRGGTCQVSAWDHVFLGLFWMYNAISVVIF
HFSWKMQSDVWGSINDQGVVTHITGGNFAQSSITINGWLRDFLWAQASQVIQSYGSSLSA
YGLFFLGAHFVWAFSLMFLFSGRGYWQELIESIVWAHNKLKVAPATQPRALSIVQGRAVG
VTHYLLGGIATTWAFFLARIIAVGMALRFPRFSQGLAQDPTTRRIWFGIATAHDFESHDDIT
EERLYQNIFASHFGQLAIIFLWTSGNLFHVAWQGNFESWVQDPLHVRPIAHAIWDPHFGQP
AVEAFTRGGALGPVNIAYSGVYQWWYTIGLRTNEDLYTGALFLLFLSAISLIAGWLHLQP
KWKPSVSWFKNAESRLNHHLSGLFGVSSLAWTGHLVHVAIPGSRGEYVRWNNFLDVLPH
PQGLGPLFTGQWNLYAQNPDSGSHLFGTSQGAGTAILTLLGGFHPQTQSLWLTDMAHHHL
AIAFIFLVAGHMYRTNFGIGHSMKDLLEAHIPPGGRLGRGHKGLYDTINNSIHFQLGLALAS
LGVITSLVAQHMYSLPAYAFIAQDFTTQAALYTHHQYIAGFIMTGAFAHGAIFFIRDYNPEQ
NEDNVLARMLDHKEAIISHLSWASLFLGFHTLGLYVHNDVMLAFGTPEKQILIEPIFAQWI
QSAHGKTSYGFDVLLSSTNGPAFNAGRSIWLPGWLNAVNE--NSNSLFLTIGPGDFLVHHAI
ALGLHTTTLILVKGALDARGSKLMPDKKDFGYSFPCDGPGRGGTCDISAWDAFYLAVFW
MLNTIGWVTFYWHWKHITLWQGNVSQFNESSTYLMGWLRDYLWLNSSQLINGYNPFGM
NSLSVWAWMFLFGHLVWATGFMFLISWRGYWQELIETLAWAHERTPLANLIRWRDKPVA
LSIVQARLVGLAHFSVGYIFTYAAFLIASTSGKFGMSHSVKIYDTCIGCTQCVRACPTDVLE
MIPWDGCKAKQIASAPRTEDCVGCKRCESACPTDFLSVRVYLWHETTRSMGLAYMTNFN
FPSIFVPLVGLVFPAIAMASLSIHVQKNKIVMRDLKTYLSVAPVLSTLWFGSLAGLLIEINRL
FPDALTFPFFSFMTAILERRESESLWGRFCNWITSTENRLYIGWFGVLMIPTLLTATSVFIIAFI
AAPPVDIDGIREPVSGSLLYGNNIISGAIIPTSAAIGLHFYPIWEAASVDEWLYNGGPYELIV
LHFLLGVACYMGREWELSFRLGMRPWIAVAYSAPVAAAAAVFLIYPIGQGSFSDGMPLGIS
GTFNFMIVFQAEHNILMHPFHMLGVAGVFGGSLFSAMHGSLVTSSLIRETTENESANAGY
RFGQEEETYNIVAAHGYFGRLIFQYASFNNSRSLHFFLAAWPVVGIWFTALGISTMAFNLN
GFNFNQSVVDSQGRVINTWADIINRANLGMEVMHERNAHNFPLDLAAVEAPSTNGMGLP
WYRVHTVVLNDPGRLLAVHIMHTALVSGWAGSMALYELAVFDPSDPVLDPMWRQGMFV
IPFMTRLGITNSWGGWSIT-GGTVTNPGIWSYEGVAGAHIVFSGLCFLAAIWHWVYWDLEI
FRDERTGKPSLDLPKIFGIHLFLSGLACFGFGAFHVTGLYGPGIWVSDPYGLTGKVQSVSPA
WGVEGFDPFVPGGIASHHIAAGTLGILAGLFHLSVRPPQRLYKGLRMGNIETVLSSSIAAVF
FAAFVVAGTMWYGSATTPIELFGPTRYQWDQGYFQQEIYRRVGAGLAENQSLAEAWSKIP
EKLAFYDYIGNNPAKGGLFRAGSMDNGDGIAVGWLGHPIFRDKEGRELFVRRMPTFFETF
PVVLVDGDGIVRADVPFRRAESKYSVEQVGVTVEFYGGELNGVSYSDPATVKKYARRAQ
LGEIFELDRATLKSDGVFRSSPRGWFTFGHASFALLFFFGHIWHGARTLFRDVFAGIDPDLD
AQVEFGAFQKLGDPTTKRQVV-----------VETLFNGTLALAGRDQETTGFAWWAGNARLIN
LSGKLLGAHVAHAGLIVFWAGAMNLFEVAHFVPEKPMYEQGLILLPHLATLGWGVGPGG
EVIDTFPYFVSGVLHLISSAVLGFGGIYHSLLGPETLEESFPFFGYVWKDRNKMTTILGIHLI
LLGIGAFLLVLKALYFGGVYDTWAPGGGDVRKITNLTLSPSVIFGYLLKSPFGGEGWIVSV
DDLEDIIGGHVWLGSICIFGGIWHILTKPFAWARRALVWSGEAYLSYSLAALSIFGFTACCF
VWFNNTAYPSEFYGPTGPEASQAQAFTFLVRDQRLGANVGSAQGPTGLGKYLMRSPTGE
VIFGGETMRFWDLRAPWLEPLRGPNGLDLSRLKKDIQPWQERRSAEYMTHAPLGSLNSV
GGVATEINAVNYVSPRSWLATSHFVLGFFLFVGHLWHAGRARAAAAGFEKGIDRDLEPVL

FMTPLNMTIALGRFTKDEKDLFDIMDDWLRRDRFVFVGWSGLLLFPCAYFALGGWFTGT
TFVTSWYTHGLASSYLEGCNFLTAAVSTPANSLAHSLLLLWGPEAQGDFTRWCQLGGLWT
FVALHGAFGLIGFMLRQFELARSVQLRPYNAIAFSGPIAVFVSVFLIYPLGQSGWFFAPSFG
VAAIFRFILFFQGFHNWTLNPFHMMGVAGVLGAALLCAIHGATVENTLFEDGDGANTFRA
FNPTQAEETYSMVTANRFWSQIFGVAFSNKRWLHFFMLFVPVTGLWMSALGVVGLALNL
RAYDFVSQEIRAAEDPEFETFYTKNILLNEGIRAWMAAQDQPHENLIFPEEVLPRGNALMS
GSTGERSFADIITSIRYWVIHSITIPSLFIAGWLFVSTGLAYDVFGSPRPNEYFTESRQGIPLIT
GRFDSLAQLDEFSRSFMTIDRTYPIFTVRWLAVHGLAVPTVSFLGSISAMQFIQRMATQTVE
GNSR-SGPRQTGLGNLLKPLNSEYGKVAPGWGTTPLMGVAMALFAVFLSIILEIYNSSVLLD
GISMNMLTLKLFVYTVVIFFVSLFIFGFLSNDPGRNPGREEMADTTGRIPLWLIGTVAGILII
GLLGVFFYGSYSGLGSSLMLNIFSLICICLNSALHSSSFFFAKLPEAYAFFNPIVDFMPVIPVL
FFLLALVWQAAVSFRMTQSNPNEQNVELNRTSLYWGLLLIFVLAVLFSNYFFNMEVNILAF
IATALFILIPTAFLLIIYVKTVSQNDMETATLVAIFISGLLVSFTGYALYTAFGQPSQQLRDPFE
EHGDMEALVYTFLLVSTLGIIFFAIFFREPPKVPTKRVKMTIAFQLAVFALIATSSILLISVPVV
FASSDGWSSNKNIVFSGTSLWIGLVFLVAILNSLIS-------MSPQTETKASVGFKAGVKDYKL
NYYTPDYVTKDTDILAAFRVTPQPGVPPEEAGAAVAAESSTGTWTTVWTDGLTSLDRYKG
RCYHIEPVAGEDNQYICYVAYPLDLFEEGSVTNMFTSIVGNVFGFKALRALRLEDLRIPTSY
VKTFQGPPHGIQVERDKLNKYGRPLLGCTIKPKLGLSAKNYGRAVYECLRGGLDFTKDDE
NVNSQPFMRWRDRFLFCAEALFKAQAETGEIKGHYLNATAGTCEEMMKRAVFARELGVP
IVMHDYLTGGFTANTTLAHYCRDNGLLLHIHRAMHAVIDRQKNHGIHFRVLAKALRMSG
GDHIHAGTVVGKLEGEREITLGFVDLLRDDFIEKDRSRGIYFTQDWVSLPGVLPVASGGIH
VWHMPALTEIFGDDSVLQFGGGTLGHPWGNAPGAVANRVALEACVQARNEGRDLAREG
NEIIRAACKWSPELAAACEVWKEIKFEFEAMDTL--TAIHLYKTSTPSTRNGTVGSQVKSNP
RNNLIYGQRRCGKGRNARGIITARHRGGGHKRLYRKIDFRRNEKDISGRIVTIEYDPNRNA
YICLIHYGDGEKRYILHPRGAIIGDTIVSGTEVPISMGNALPLTDMPLGTAIHNIEITLGKGG
QLARAAGAVAKLIAKEGKSATLKLPSGEVRLISKNCSATVGQVGNVGVNQKSLGRAGSKR
WLGKRPVVRGVVMNPVDHPHGGGEGRAPIGRKKPTTPWGYPALGRRSRKRNKYSDSLIL
RRRSKMIQPQTHLNVADNSGARELMCIRIIGAS-NRRYAHIGDVIVAVIKEAVPHMPLQRSE
VIRAVIVRTRKELKRDNGMIIQYDDNAAVVIDQEGNPRGTRVFGAIARELRQLNFTKIVSL
APEVLMLSPKRTRFRKQHRGRMKGISYRGNHICFGRYALQALEPAWITSRQIEAGRKSMA
RYARRGGKIWIRIFPDKPVTVRPTETRMGSGKGSPEYWVSVVKPGRILYEMGGVSEIAARE
AISIAASKMPIRTQFVIAGMTRIRRGYIARRRRTKIRLFASTFRGAHSRLTRTIIQQKMRALA
SSHRDRGRQKRNFRRLWITRINAAIREI--VSYSYSRSIKDLYKSQLLLNRKILAQIAISNTNC
LYMISKEIIKFK------------EVYALGQHICMSAHKARRVIDQIRGRPYDEILMILELMPYRACY
PIFKLVYSAAANATNNKGFNKTALIISQAEVNEGTVVKKLKPRARGRSYPIKRPTCHITIVL
QDISKDEVVWDKKMDGIKYAVFTDKSIRLLGNN-QYTSNVESGSTRTEIKHWVELFFGVK
VIAMNSHRLPGKGRRMGPIMGHTMHYRRMIITLQPGYSIPPLRKKRTMAVPKKRTSISKKR
IRKNIWKLKGHWAAKKAISLAKSISTGNSKSFFVQQTSNMAKGKDVRIRVILECNSCVRN
DLN--KESPGISRYITQKNRHNTPSRLELRKFCPYCYKHTIHGEIKKMKIRASVRKICEKCRL
IRRRGRIIVICSNPRHKQRQGMLREEVAVSTRTLQWRCVESRTDSKRLYYGRFVLSPLMKG
QADTIGIAMRRALLGEIEGTCITHAKSE--KIPHEYSTIVGIEESVHEILMNLKEIVLRSNLYG
TCDASICVKGPINITAKDIISPPSVEIVDTTQHIASLTEPFDLCIGLQIERNRGYRMKTPNKAQ
NGSY--PIDAVFMPVRNANHSIHSYGNGNEKQEILFLEIWTNGSLTPKEALHEASRNLIDLFI
PFLHAEEQ--DIILEDNQ-NRVTLPFFTFHDGLA-KLKK-KN--IALKFIFIDQSELPSRTYNCLK

KSNINTLLDLLNKSQEDLLKIEHFRIEDVKRVLGILQKR--FVIDLPKNKMLRDAN-EGMSTI
PGFSQIQFEGFCRFIDRGLMEELNKFPKIEDTDQEIEFQLFVETYQLVEPLIKERDAVHESLT
YCSELYVPAGLIWKTGKDIQEQTIFIGNIPLMNSLGTSIVNGIYRIVINQILQSPGIYYRSELD
HNGISVYTGTIISDWGGRSELEIDRKARIWARVSRKQKISILVPSSAMGSNLKEIIDNVCYPE
IFLSFPNDKEKKKIGSKENAILEFYQQFACVGGDPVFSESLCKELQKKFFQQRCELGRIGRR
NMNRRLNLDIPHNITFLLPRDVLAAADHLIGMKFGMGTLDD--MNHLKNKRIRSVADLLQ
DQFGLAMVRLENAVRGTIGGAIR-QKLI-PTPHNLVTSTP----LTTTYESFFGLHPLSQVLDRT
NPLTQIVHGRKSSYLGPGGLTGRTASFRIRDIHPSHYGRICPIDTSEGINVGLIGSLAIHARIG
HWG-SLERPFY---EISERS--KGVRVIYLSPSIDEYYMVTAGNSLALNHGIQEEQVVPARYRQ
EFLTIAWEQIQLRSIFPFQYFSVGASLIPFIEHNDANRALMSSNMQRQAVPLSRSEKCIVGTG
LERQAALDSGVSAIAEHEGKIIYTDTDKIILSGNGATLSIPLVMYQRSNKNTCMHQKPQVS
RGKCIKKGQILADGAATVGGELALGKNVLVAYMPWEGYNFEDAVLISERLVYGDIYTSFHI
RKYEIQTHVTSQG-PERITNEIPHLEAHLLRNLDRNGIVMLGSWVETGDILVGKLTPQVAKE
-SSYAPEDRLLRAILGIQVSTAKETCLKLPIGGRGRVIDVRWIHKKG-GSSYNPETIRVYISQK
REIKVGDKVAGRHGNKGIISKILPRQDMPYLQDGTPVDMVFNPLGVPSRMNVGQIFECSL
GLAGGLLDRHYRIAPFDERYEQEASRKLVFSELYEASKQTANPWVFEPEYPGKSRIFDGRT
GDPFAQPVIIGKSYILKLIHQVDDKIHGRSSGHYALVTQQPLRGRAKQGGQRVGEMEVWA
LEGFGVAHILQEMLTYKSDHIRARQEVLGTTIIGGTIPNPEDAPESFRLLVRELRSLALELNH
FLVSEKNFQINRKEA-------MIDRYKHQQLRIGSVSPQQISAWAKKILPNGEMVGEVTKPYT
FHYKTNKPEKDGLFCERIFGPIKSGICACGNYRVIGDKKEDPKFCEQCGVEFVDSRVRRYQ
MGYIKLACPVTHVWYLKRLPSYIANLLDKPLKELEGLVYCD--FLFARP-VAKKPTFLRLRG
SFEYEIQSWKYSIPLFFTTQGFDTFRNREISTGAGAIREQLADLDLRLITDSSLVEWKELGEE
-GATGNEWEDRKIGRRKDFLVRRMELAKHFIRTNVEPERMVLCLLPVLPPELRPIIQIDGGK
PMSSDINELYRRVIYRNNTLTDLLTTSRSTPGELVMCQEKLVQEAVDTLLDNGIRGQPMRD
SHNKVYKSFSDVIEGKEGRFRETLLGKRVDYSGRSVIVVGPSLSLHRCGLPREIAIELFQTF
VIRGLIRQHAASNIGVAKSKIREKEPIVWEILQEVMQGHPVLLNRAPTLHRLGIQAFQPILV
EGRAIFLHPLVCKGFNADFDGDQMAVHVPLSLEAQAEARLLMFSHMNLLSPAIGDPISVPT
QDMLMGLYVLTTGNHRGICANRYNPSNHRN---YQNEKIDDNNYKYT--KEPYFCSSYDAL
GAYRQKRIYLDSPLWLRWRLDQR-VIAS--REVPIEVQYESLGTYHEIYGHYLIVRNVKKEI
LCIDIRTTVGHISFYREIEEAIQGFCRACSYGT-----------MAERADLVFHNKAIDGTAMKRLI
SRLIDHFGMAYTSHILDQIKTLGFQQATATSISLGIDDLLTIPSKGWLVQDAEQQSLILEKHH
HYGNVHAVEKLRQSIEIWYATSEYLRQEMNPNFRMTDPSNPVHIMSFSGARGNASQVHQL
VGMRGLMSDPQGQMIDLPIQSNLREGLSLTEYIISCYGARKGVVDTAVRTSDAGYLTRRLV
EVVQHIVVRRTDCGTLRGSSVSPRNGTIT-ERIFIQTLIGRVLADDIYIGSRCIAIRNQDIGIGL
VNRFIT------FRAQPISIRTPFTCKSTSWICRLCYGRSPTHGDLVELGEAVGIIAGQSIGEPGTQ
LTLRTFHTGGVFTGGTAEHVRAPSNGKIKFKEDLVHPTRTRHGHPAFLCYIDLYVTIEGQDI
RHNVTIPPKSFLLVQNDQYVESEQVIAEIRAGTSTL-NFKERVRKHIYSDSEGE-MHWSTAV
YHAPEYTYGNVHLLPKTSHLWILSGGPYR--SSIVSFSLHKDQDQMNVH--SLSAEQKSISN--
LLVTNNQ-VRHKFLSSEPSG--GERILDY-SGPNRIICNGHCNFIYPA-ILHENS--YLLAKRRRN
RFIIPFQYNQEQEKELITRSGISIEIPIN--GIFRRNSILAYFDDPRYRRNSSGITKYGTIEAHSIV
KKED--LIEYRK-----RPKYTKIDRFFFIPEEVHILPGSSSVMVRNNSIIGVNTRITFKTRSRVGG
LVRVEKKKKKIELKIFSGDIHFPGETDKISWHSGILIPPGTGKK--NSKESK--NFKNWIYVQR
--ITPTKKK--YFVLVRPVVTYEIADGINLATLFPQDPLQERDNVQLRVVNYILYGNGKPIRGI
SHTNIQLVRTCLVLNWDQEKN---GSSMEEVHASFVEVRVNNLIRDFIRMDLVKSPISYTRK

RNAPAGSELILA-NGSDYTNLNP-FYS--KAR-----IQQSLTQHQGTIRTLLNR---NKECQSLIFL
SSSNCFR--IGPFKGLKYNN---MTKKSDSLLLIRNSLGPLGI--VPKIANFSS--FYYLIT--HNQIL
-INKYLLLDN---LKQTFQK--LK-YYLMDENGR--IYNPDPCSNMILNPFNWNWYFLHHDYC
EETSTIIS-LGQFFCENVCISKYGP---HLKSGQVIIVH-VDSLVIRSAKPYLATPGATVHGHYG
EILYKGDTLVTFIYEKSRSGDITQGLPKVEQVLEVRSIDSISMNLEKRVEGWNERITRILGIP
WGFLIGAELTIAQSRVSLVNKIQKVYRSQGVQIHNRHIEIIVRQITSKVLVSE---DGMSNVFS
PGELIGLLRAERTGRALEEAICYRAVLLGITRASLNTQSFISEASFQETARVLAKAALRGRID
WLKGLKENVVLGGMIPVGTGFKGLVHRS--SQHSNIS--LEIQNTNLFEGGMR--DILFHHRE
LFYSCIPKNFHDTSEQKFTGFIDS------MTRRYWNINLEEMMEAGVHFGHGTRKWNPRMA
PYISAKRKGIHITNLTRTARFLSEACDLVFDAASRGKQFLIVGTKNKAADSVASAAIRARCH
YVNKKWLGGMSTNWSTTETRLHKFRDLRAEQKTGRFNRLPKRDAAMLKRQLSHLQTYL
GGIKYMTGLPDIVIIVDQQEEYTALRECVTLGIPTICLIDTNCDPDLADISIPANDDAIASIRFI
LNKLVSAICEGRSS-YIRNRMGQKINPLGFRLGTTQSHHSLWFAQPKNYSEGLQEDQKIQD
CIRNYVQKNMRISSG-VEGIARIEIQKRIDLIQVIIYMGFPKLLIEGRSR----GIEELQSSVQKR
FNSVNRKLNIAITRIAKPYGHPNILAEFIAGQLKNRVSFRKAIKKAIELTEQADTKGIQVQIA
GRIDGKEIARVEWIREGRVPLQTIRAKIDYCSYTVRTIYGALGIKIWIFTDEEMSRYRGPRF
KKIRRLGALPGLTSKRPTPGSDLRNQSRSGK-RSQYRIRLEEKQKLRFHYGLTERQLLKYV
RIAGKAKGSTGQVLLQLLEMRLDNILFRLGMASTIPGARQLVNHRHILVNGRIVDIPSYRC
KPRDIITTRDEQKSRALIQNYLDSSPH-EELPKHLTLHSSQY--KGLVNQIIDSKWVGLKINEL
LVVEYYSRQTMSRRGTAEEKTAKSDPIYRNRLVNMLVNRILKHGKKSLAYQIIYRALKKIQ
QKTETNPLCVLRQAIRGVTPDIAVKARRVGGSTHQVPIEIGSTQGKALAIRWLLGASRKRP
GRNMAFKLSSELVDAAKGSGDAIRKKEETHRMAEANRAFAHFRMGRDTIADIITSIRNAD
MDKKGTVRIASTNITETIVKILLREGFIENVRKHRENDQFFLVSTLRHRR--NRKGPYR---TI
L--KRISRPGLRIYSNYQRIPRILGGMGIVILSTSRGIMTDREARLEGIGGEILCYIWMTKPISR
IGSRRNVRIGSRKSGRRIPKGVIHVQASFNNTIVTVTDIRGRVVSWSSAGTCGFRGTRRGTP
FAAQTAASNAIRTVVDQGMQRAEVRIKGPGLGRDAALRAIRRSGILLSFVRDVTPMPHNG
CRPPKKRRVMPTIKQLIRNTRQPIKNVTKSPALRGCPQRKGTCTRVYTITPKKPNSALRKV
ARVRLTSGFEITAYIPGIGHNLQEHSVVLVRGGRVKDLPGVRYHIVRGTLDAVGVKDRQQG
RSKYGVKKPKMARKSLIQREKKRQRLEQKYHLIRRSSKKEIK---KVPSLSEKMEIHGKLQS
PPRNSAPTRLHRRCFSTGRPRATYRDFGLSGHILREMLHACLLPGATRSSWMIKNPFISVIS
QEEKEENK--GSVEYQILSFTNKIRRLTSHLELHRKDFLSQRGLRKILGKRQRLLAYLSKKN
RVRYKELISQLDIREPKTR--MVKLRLKRCGRKQRAIYRIVAIDVRSRREGRDLQKVGFYDP
IKNQTYSNVPAILYFLEKGAQPTGTVHDILKKAEIFKELHVN------MDKSK-LFLKSKRSFRR
RLPPIGSGDRIDYRNMSLISRFISEQGKILSRRVNRLTLKQQRLITIAIKQARILSSLPFLNNEK
--QFERTESTPRTTGPRTRKKVTRSLKKNPFVANHLLGKIEKLNMRAEKEIIVTWSRASTIIPI
MIGHTIAIHNGKEHLPIYITESMVGHKLGEFAPTLTFRGHARNDKKSRRMIFNSFLLGNLLS
LCMKIINSVVVVGLYYGFLTTFSIGPSYLFLLRARVMEEG---TEKEVSATTGFITGQLMMFIS
IYYAPLHLALGRPHTITVLVLPYLLFHFFWNNHKHFFDYGSTTRNSMRNLSIQCVFLNNLIF
QLFNHFILPSPTLARLVNIYMFRCNNKMLFVTSSFVGWLIGHILFMKWVGLVLFWIRQNRS
IRS-----KYLVSELRNSMARIFSILLFITCVYYLGRIPSPIVTKKLKEAEKEKPLVTLLFDYKR
WNRPLRYIKNERFENAVRNEMSQYFFYTCISDGKQRISFTYPPSLSTFFEMIQKKLFLCTTE
KISSEELYNHWAYTNEQKRNNLSKEFINRIETLDEGSLDLDILEKKTRLCN-ENEQKCLPKI
YDPFLNGPHRGTIQNLYSHT--LWINKYQGILCREFEKEYPNDQ-LEINEIKKTVPRWLYKLT
DLEEEEIEGIRSRKAKRVVIFTDALTRYSQQPDFRRDLIKGSMRAQRRKTVTWELFQATVR

SPLFLDRVDKTFFFSFDI--SGTLKYFFRNRQENERIRISETWDTIIFAQAIRGYMLVTQSILRK
YIVLPSLIIAKNIGRMLLFQFPEWHEDLKAWNKEMHVKCTYNGVQLSEKEFPKNWLTDGI
QIKILFPFCLKPWHRSKKGKPENFCFLTVWGMEAELPFGSPRKRPSFFEPIWKEIEKNIIKM
K-KKCFIALRILKERTKWFIRVSKEKTRWVIKIFL--FIKRIMKELAKAK--IKSNQIIHESPIRIR
SLDWINYSLTAKKIKDVVDRTSTIRNQIEKITKDKKKNPNKIGCDDKGLESLKKIWQKLKR
RSNRLIRKCYYFLKFFIERIY-DILFCIINSLRIKVKLFIHFIS-DLDSLSQAYVFYKLSQTQVFN
KYHLRSVLQYTLLFLKDRIKDSFTQGIFHSESGHKKLRNFRMNEWKNWLRGRYQYDFSQ
TRWSRLVPQKWRNRIHQRCMVQTKTYSDLYEKDQ--LIHYEKQFKNYKYDKYDRLSHKYI
HD--SDLDRKYFDWKIFNFCLRSFDWMAMNEEILNCWFFPEFVSFYNAYKIQPWTIPINLLL
LNSTGNIAAELDLFLKRYFLFQLRWDNPLNKKIINNVKVYCLLLRLKNPKEIAIASIQRDE
MSLDVMPISKTLTLAKLINKGMLIIEPARLSIKRDGQFLLYQTITISRVHKNKHN--PLLVLEN
ILSSQRRRELRIRICLRLLWPNYRLEDLACINRYWFDTNNGSRFSMSRIRMYPAKRHPFKS
WIFELREILREIKNSHYFLDSWTKFDSVGSFTHIFFHQERFMKLFDPRIWSILLS-RDS--QGS
TSNRYFT---TKGVVLLVVA-VLIY----RINNRNMVERKNIYLMGLLP-----IPMNSIGPRNDPLE
ESF-GSSNINRLIVS-LLYLPKGKKISESCFMDPKESTWVLPIT----KKCIMPESNWGSRWWR
NRVGKKR--DSSCKIS---NETVAGIEISFKEKDIK-YLESPFVSYTD--DPIRKDHDWELFDRLS
PRKKRNIINL--NSGQLFEILVKHWICYLMSSFREKRLIEVEG-FFKQQG-AGSTIQSNDIEHV
SH--LFSRNKWGISLQNCAQFHMWQFRQDLFV---SWEK-NPHESDFFRN-VSRENWIWLDN
VWLVNKDRFFSFFS--KVRNVSSNMQYDS---TR--SIFVQVTDSSQLKGSS-DQSRDHFDSIS--
--NEDSE-------------YHTLINQREIQQLKERSIL-WD--PSFLQTERTEIESD--RFPK--CLSGYS--
SMSRLFT-EREKQMKNHLLPEEIEEFL--GNPTRSIRSFFSDRW--SELH-LGSNPTERSTRDQ--
--KLLKKQQDVS-FVPSRRSE----NKEMVDIFKIITYLQN--TVSIHPISSDPGCDMVP-KDEPD-
MDSSNKISFLNQN--------DLFHDRNRGGYTL--HHDFESE--ERFQEMADLLTLSITEP-DLVY
HKGFAFSIDSYGLDQKK--FLNEVFNSRDESKKKSL---LVLPPIFY--EENESFYRRIRKKWVR
-ISCGNALEDPKPKRVVFASNNIMKAVNQYRLIQNLIQIQYSIHG--YIRNVSNQFFLM---NRP
DRNFEYGIQ---RDQIGNDTLNHRTIMKYTINQHLSNLKK-SQKKWFDP----LISRTERSMNR
DPDAYRYKWSNGSKNFQEHLEHFVSEQKSR---FQVVFDRLR--QYSIDWSEVIDKQDLSKS
LRFFLS----------KLLLFL-------SKLLLFLSKSLPFFFVSIGNIPIHRSE-IHIY-ELKGPNDQLCNQ
LLESIGVQIVHLNKLKP--FLLD---DHDTS-QKSKLLINGGTISPFLFNKIP-KW-MIDSFHT--R
KNRRKSLDNTDSYFSMISHDRDNWLNPVKPFHRSSLISSFYKANRLRFLNNPHHFWFY--C
NKRFPFYV----ERARINNYD--LTY-GQFLNILFIRNK-IFSLCVGK--KKHVFLE-RDTP--IESQV
SNIFIPNDFPPSGDET-YNLYKS---FHFPIRSAPFVR--RALYSIADISGTPLTEGQIVNFERTYC
QPLSDMNPSDSEGKNLHQYL--NFNSNMGLIHIPCSEKYLP--SEKRKK--------RSLCLK--KC
VEKRQMYRPFQRDSAFSNFSKWNLFQTYMPWFLTSTGCKYLNFTLLDTFSDLL--PILSSS-
QNFVSIFHDIMHGSDISWPIPQKKLW----VILPQWNL-ISEISSKCLHN--LLLSEEMIRRNNES
PIPLIWAHLRSPNAREF-LYSILFLLLVAGY-LVRTHLLFVSR--ASSELQTEFEKIKSLMIPSYM
IELRKLLDGYPTSELNSFWLKNLFLVALKQLGDFLEEIRGSASGGNMLLGGGP-----AYG-V
KSIRSKKKYLNINLIDIIDFL-SIIPNPINRITFSRNTRHLSRTSKEIYSLIRKRKNVNGDWIDDK
IESWVANSDSIDDEEREFLVQFS--TLMK--EKRIDKILLSLT-HSDHLS-----KNDSGYQMIEQP
GSVYLRYLVYIH--KKYLMNYEFNRSCLAERRIFLAHYQTITY--SQTSCGANSFHFPSHG---
KPFSLRLALSPS-RGILVIGSIGTGRSYLVKYLTTNSYVPFITVFPNKFLDDKPKGYLIDDI---N
ID-IDDSDD------------------------------IDDDLDIDTELLTMMNAL---TMD---MTPKIDRFYITLQL
ELAKAMSPCIIWIPNIHDLSVNESNYLSLGLLVNHLS---RDCERSS-TRNILVIASTHIPQKVD
PTLIAPNKSNTCIKIRRLLIPQQRKHFFTLSYTRGFHLEKKMFHT--NGFGSITMGSNARDLV

ALTNEALSISITQKKSIIDTNTIRSALHRQTWDLR---SQVRSVQDHGILFYQIGRAVAQNVLL
SNCPIDPISIYMKKK--SCTEGDSYLYKWYFELGTSMKKLTILLYLLSCSAGSVAQDLWSPP--
GPDEK-NWITSYGFVENDS-DLVHGLLEVEDALVG--SSRTEKDCSQFDND--RVTLLLRSEP
RN-PLDMMQNGSCSIVDQRFLYEK--YESEFEE-GEGEGALDPQ----QLEED----LFNH--IVWA
P--RIWRP---CGNLFDCIESPNELGFP-YWARSFR---GKRIIDHEHDELQENDSEFLQSGTMQY
QTRDRSFKEQGFFRISQFIWDPADPFFFLFK-DQPFVSVFSRREFFADQEMSKGLLTSQTDPS
TSMNKRWFI--NNTQEKHFELLIHRQRWLRT---NSSLS--NGSFRSN--TPSESYQYLSNLFLSN
-GTLLDQMTKTLLRKRWLFPDEMKHLI--HVTGERFPIPMPRSR-INGNFIDKTFSIVANILLRI
IPTTSGEKEAFTYYRDG--MSAQSEGNYAEALQNYYEATRLEIDPYDRSYILYNIGLIHTSNG
EHTKALEYYFRALERNPFLPQAFNNMAVICHY-RGEQAIQQGDSEIAESWFDQAAEYWKQ
AIALTPGNYIEAHNWLKITRRFE--MNWRSERIWIELITGSRKTSNFFWACILFLGSLGFLLV
GTSSYLGRNLISLFPSQQILFFPQGIVMSFYGIAGLFISSYLWCTIWWNVGSGYDQFDRKEG
IVCIFRWGFPGRNRRIFLRFLMRDIQSIRIEVKEGLYPRRVLYMEIRGQGALPLTRIDEN-LTP
REIEQKAAELAYFLRVPIEVF
>Morus
MEKWRFDSMLFLSKSINSLGPIE-TSVNEDPIINDMDKDIPSYSNVDHLVGVRYIRNFLSDD
TFLVRDSNRDSYTIYFDIENQIFEIDNHD--SFISEPENSFYSYQNSSYLNNVSKNDDPRYDRY
VYDTQYSWNNHINSCIGSYLR--YQICIDSDILTSSDNYNDNYIYTYICGSESSNIRTGADDS
DLTTIRESS---VTQKYRHLWVQCENCYGLNYKRFLKSKMNICEHCGCHLKMSSSDRIELLI
DPGTWDPMDEDMVSLDPIEFNSEEEPYKDRIDSYQRKTGLTEAVQTGTGKLNGIPVAIGV
MDFQFMGGSMGSVVGEKITRLIEYATNQSLPLILVCASGGARMQEGSLSLMQMAKISSAL
YDYQSNKKLFYIAILTSPTTGGVTASFGMLGDIIIAEPNAYIAFAGKRVIEQTLNKTVPEGSQ
VAEYLFHKGLFDPIVPRNPLKGVLSELFQLHAFFPLNQNSIKMVTIRADEISDIIRERIEQYN
REVKILNTGTVLQVGDGIARIYGLDEVMAGELVEFEEGTIGIALNLESNNVGVVLMGDGL
MIQEGSSVKATGRIAQIPVSEAFLGRVINALAKPIDGRGEISASESRLIESPAPGIISRRSVYEP
LQTGLIAIDSMIPIGRGQRELIIGDRQTGKTAVATDTILNQQGQNVICVYVAIGQKASSVAQV
VTTLQERGAMEYTIVVAETADSPATLQYLAPYTGATLAEYFMYREQHTSIIYDDPSKQAQA
YRQMSLLLRRPPGREAYPGDVFYLHSRLLERAAKSSSRLGEGSMTALPIVETQSGDVSAYI
PTNVISITDGQIFLSADLFNAGIRPAINVGISVSRVGSAAQIKAMKQVAGKLKLELAQFAEL
EAFAQFASDLDKATQNQLARGQRLRELLKQSQSAPLAVEEQIITIYTGTNGYLDSLEIGQV
RKFLVELRTYLKTNKPQFQEIISSTKTFTKEAEALLKEAIQEQMERFLLQEQVMRINPTTSS
PGVPALEKKNLGRIAQIIGPVLDVAFPPGKMPNIYNALVVKGRDTVGQQINVTCEVQQLLG
NNRVRAVAMSATDGLMRGMEVIDTGAPLSVPVGGATLGRIFNVLGEPIDNLGPVDTRTTSP
IHRSAPAFIQLDTKLSIFETGIKVVDLLAPYRRGGKIGLFGGAGVGKTVLIMELINNIAKAH
GGVSVFGGVGERTREGNDLYMEMKESGVINEQNIAESKVALVYGQMNEPPGARMRVGLT
ALTMAEYFRDVNEQDVLLFIDNIFRFVQAGSEVSALLGRMPSAVGYQPTLSTEMGSLQERI
TSTKEGSITSIQAVYVPADDLTDPAPATTFAHLDATTVLSRGLAAKGIYPAVDPLDSTSTMLQ
PRIVGEEHYETAQRVRQTLQRYKELQDIIAILGLDELSEEDRLTVARARKIERFLSQPFFVAE
VFTGSPGKYVGLAETIRGFKLILSGELDGLPEQAFYLVGNIDEATAKAMNLEMENKLKKM
TLNLCVLTPNRIVWDSEVKEIILSTNSGQIGILPNHAPIATAVDIGILRIRLNDQWLTMALMG
GFARIGNNEITVLVNDAEKGSDIDPQEAQQTLEMAEANLSKAEGKRQTIEANLALRRART
RVEAINMMSMKNVTDPFLSLSYWPSAGSFGFNTDILATNPINLSVVLGVLIFFGKGVLSDL
LDNRKQRILKTIRNSEELREGALEQLEKARSRLRKVEKEADQFRVNGYSEIEREKLNLINS
TSKTLEQLENYKNETIHFEQQRAINEVRQRVFQQALEGALGTLNSCLNNELHLRTISTNIG

MFGTMKEITDMNPLISAASVIAAGLAVGLASIGPGVGQGTAAGQAVEGIARQPEAEGKIRG
TLLLSLAFMEALTIYGLVVALALLFANPFVMNVLLCSINTLKGLYDISGVEVGQHLYWKIG
GFQVHAQVLITSWVVIAILLGSSIIAVRNPQTIPTDGQNFFEYVLEFIRDVSRTQIGEEYGPW
VPFIGTMFLFIFVSNWSGALLPWKIIQLPHGELAAPTNDINTTVALALLTSVAYFYAGLTKK
GLGYFGKYIQPTPILLPINILEDFTKPLSLSFRLFGNILADELVVVVLVSLVPLVVPIPVMFLG
LFTSGIQALIFATLAAAYIGESMEGHHMIFSTLEHILTHISFSVVSIVITIHLITLLV-EIIGLYDS
LEKGMMTTFFCITGLLMTRWIYLRYLPLSNLYESLIFLSWSFSIIHMVPYFKKYKNYLKAIT
APSAIFTQGFATSGLLTEMHQSEILVPALQSHWLMMHVSMMVLGYAALLCGSLLSLALLVI
TFRKFISIFSKSNEIQYVREKNNLLP--SFLSSRNYYRFQLIQQLDLWSYRIISLGFIFLTIGILS
GAVWANEAWGSYWNWDPKETWAFITWTLFSIYLHTRTNKNFESVNSAIVASMGFLIIWIC
YFGVNLLGIGLHSYGSFTLTMAKKKAFISLLYLASIVFLPWWISFTFNKSLESWVNNWWN
TRPSEILLNDIQEKSILKKFIELEELSLFDEMLKEYSERRLQKLHVG-YNETIQWIKMHNEG
RIHTILHFSTNIISFVILSVFSILSNEELIFLNSCLQEFLYNLSDTIKAFSILLLTDLCIGFHSPHG
WELMIGSIYKDFGFAHNDQIISGVVSTFPVILDTIFKYWIFRYLNRVSPSLVVIYHSMNDMPI
GVPKVPFRSPGDEDASWVDVINRLYRERLLFLGQEVDSEISNQLIGLMVFLSIEDDTKDLY
LFINSPGGWVIPGLGIYDTMQFVQPDVQTICMGLAASMGSFILVGGEITKRLAFPHA-VMIH
QPASSFYEAQTGEFILEAEELLKLRETITRVYVQRTGKSLWVISEDMERDVFMSATEAQAH
GLVDLVAVE???
MAEFQGYLELDRSWKHDLLYPLIFREYIYVFAHDLGLVGYDNKSSLLIVKRLISRMYQQN
HFIISANDSNQNQLFGKNLYSQMISEGFAVIVEIPFSLRLVSSLIVKYYNLRSIHSIFPFLEDKF
SQLNYVSDVLIPYPIHLEILVQTLRYWVKDASSLHLLRFFLHDYYNWNTLIIKYISIFSKPRL
FLFLYNSHVCEYESILLFLRNQSSHLRLTSSGGFFERIYFYGKIKHVEEVFANPTSLWFLEDL
FMHYVRYQGKSILASKDTPLFMNKWKYYLVLLWQCHFYVWSQPGRMYINQLCKHSFSF
LGYLSSMQINLSVVRSQMLENSFLMDN--AMKKIDTLVPISLLIGSLDKMKFCNVLGHPVS
KSTWADSSDFDIIDRFVCICRNLFHYYSGSSKKKSLYRIKYILRLSCVKTLARKHKSTVRTF
LKKLGSNLLEEFFTEEEEVLSLIFPRRIWYLDIFCINDLVNHKMISHTPE--VQDINSFSRFEF-
LKETYGIIWAFIPIFTPVLGITIGVLVIVWLEREISAGIQQRIGPEYAGPLGVLQALADGAKLL
FKENLFPSRGETRLFSIGPSIAVISILVSYSVVPFGSHLVLVDLNIGVFLWIAISSIAPIGLLMSG
YGSNNKYSFLGGLRAAAQSISYEIPLTLCVLSISLLSNSSSTVDIVEAQSKYGFWGWNLWR
QPIGFIIFLISSLAECERLPFDLPEAEEELIAGYQTEYSGIKFGLFYVASYLNLLVSSLFVTILY
LGGWNISIPYIFVFDFFEINKTYGVFEPTIGMFITLAKTYLFLFIPITTRWTLPRLRMDQLLNL
GWKFLLPISLGNLLLTTSSQLFSLMIWHVQNENFILDSTRIFMKAFHLLLFDGSFIFPECILIF
GLILLLMIDSTSDQKDIPWLYFISSTSLVMSITALLFRWREEPMISFSGNFQTNNFNEIFQFLI
LLCSTLCIPLSVEYIECTEMAITEFLLFVLTATLGGMFLCGANDLITIFVAPECFSLCSYLLSG
YTKKDVRSNEATTKYLLMGGASSSILVHGFSWLYGSSGGEIELQEIVNGLINTQMYNSPGI
SIALIFITVGIGFKLSPAPSHQWTPDVYEGSPTPVVAFLSVTSKVAASASATRIFDIPFYFSSNE
WHLLLEILAILSMILGNLIAITQTSMKRMLAYSSIGQIGYVIIGIIVGDSNGGYASMITYMLF
YISMNLGTFACIVSFGLRTGTDNIRDYAGLYTKDAFLALSLALCLLSLGGLPPLAGFFGKLH
LFWCGWQAGLYFLVSIGLLTSVVSIYYYLKIIKLLMTGRNQEITPHVQNYRRSPLRSNNSIE
LSMIVCVIASTIPGISMNPIIEIAQDTLFMFLLYEYDIFWAFLIISSVIPIFAFIISGVLAPISKGPE
KLSSYESGIEPMGDAWLQFRICYYMFALVFVVFDVETVFLYPWAMSFDILGVSVFIEALIFV
LILIVGSIYAWRKGALEWSTNYFPWLTIIIVLPIFAGFLILFLPHRGNKVIRWYTLCICIIELLL
TTYAFGYHFQLDDPLIQLAEDYKWIDFLDFSWRLGIDGISIGPILLTGFITTLATLAARPVTR
ESRVFHFLMLAMYSGQIGPFSSQNLLLFFIMWELELIPVYLLLSMWGGKKRLYSATKFILYT

AGSSAFLLVGILGICLSASNEPTLNFETSANQSYPVALEMLFYIGFFIAFAVKSPIIPLHTWLP
DTHGEAHYSTCMLLAGILLKMGAYGLVRINMELLPHAHSIFSPWLMIVGTIQIIYAASTSPG
QRNLKKRIAYSSVSHMGFIIIGLGSINDTGLNGAILQIISHGFIGAALFFLAGTSYDRIRLVYL
DEMGGMAITIPKIFTTFSILSMASLALPGMSGFVAELILFFGILTSQKYLLMTKILITFVMAIG
MILTPIYSLSMLRQMFYGYKLFTATNSYFVDSGPREFFVSISILLPVIAIGIYPDFVFSLSVDK
VEALLSNYFYPMMLEHVLVLSAYLFSIGIYGLITSRNMVRALMCLELILNAVNINFVTFSDF
FDSRQLKGNIFSIFVIAIAAAEAAIGPAIVSSIYRNRKSTRINQSNLLNKMEYIYQYFWIITFIS
LPVPILIGVGLLLFPTATKSLRRMWVFPSVLLLSIIMLFSTYLAIQQVNNPSIYLSIWSWTINN
DFSLEFGYLVDPLTSIMLILITTVGILVLIYSDNYMSHDQGYLRFFAYMSFFNTSMVGLVTSS
NLIQIYFFWELVGMCSYLLIGFWFTRPTAANACQKAFVTNRVGDFGLLLGILGFYWITGSL
EFRDLFEIFNN-EVNLLFATLCAFLLFAGAIAKSAQFPLHVWLPDAMEGPTPISALIHAATM
VAAGIFLGARLLPLFIVMPYIMNLIALLGIITLLLGATLAFAQKDIKRGLAYSTMSQLGYMM
LALGMGSYRAALFHLITHAYSKALLFLGSGSIIHSMESIVGYSPDKSQNLVLMGGLKKHVP
ITKTSFLLGTLSLCGIPPLGCFWSKDEILNDSWLYSPIFAIIAFSTAGLTAFYMFRIYLLTFEGH
LNLYFQTYSGKKRSSVYSISLWGRE-EQKWIKTLLPLLTMNFFFGTKN-SFSYPHESENTML
FSMLVLGLFTLFIGIIG-FFQLDILTKLLTPSINLLYQNKK-LMDWDEFITNATYSVSIASFGILI
ASFLYNPGYSSLQNFNLFNSFVFFEDKIINVTYDWSYNRGYIDFFYATFLIQGIRILSELIHFF
DRQVIDGITNGVGISSFFVGEGIKYVGVG-RISSYLLVYISYVLIFLLIYSMDLPGPIHDFLLV
FLGLGLILGGIGVVLLTNPIYSAFSLGLVLVCISLLYILSNSYFVAAAQLLIYVGAINVLIIFAV
MFMNGSEYYKDFNLWTVGNGFTLLICTSIFGLLITTITDTSWYGIIWTTRSNQIIEHDLISNS
QQIGIHLSTDFFLPFEFISIILLAALIGAITVARQMNIPAPRKDLMIVNMGPHHPSMHGVLRLI
VTLDGEDVIDCEPILGYLHRGMEKIAENRTIIQYLPYVTRWDYLATMFTEAITINGPELLGN
IQVPKRASYIRAIMLELSRIASHLLWLGPFLADIGAQTPFFYIFRERELVYDLFEAATGMRM
MHNFFRIGGVAADLPHGWIDKCLDFCDYFLIGVTEYQKLITRNPIFLERVEGVGVIGREEVI
NWGLSGPMLRASGIQWDLRKVDHYECYDEFDWEVQWQKEGDSLARYLVRIGEMTESIKI
IQQALEGIPGGPYENLEIRYFDRERNPEWNDFDYRFISKKPSPTFELPKQELYVRVEAPKGE
LGIFLIGDQSGFPWRWKIRPPGFINLQILPQLVKRMKLADIMTILGSIDIIMGEVDRMFPMIT
GFMNYGQQTVRAARYIGQSFMITLSHVNRLPITIQYPYEKVIAAERFRGRIHFEFDKCIACE
VCVRVCPIDLPVVDWKLETDIRKKQLLNYSIDFGICIFCGNCVEYCPTNCLSMTEEYELST
YDRHELNYNQIALGRLPMSVIDDYTIRTILNS-IKNK-----------MQGRLSVWLIKHGLVHRSL
GFDYQGIETLQIKPEDWHSIAVILYVYGYNYLRSQCAYDVAPGGLLASVYHLTRIEYGIDQ
PEEVCIKVFAPRRNPRIPSVFWIWKSADFQERESYDMLGISYNNHPRLKRILMPESWIGWP
LRKDYIAPNFYEIQDAHMGNEFRYIGGIRIYRSFNFRTYPNCWFNLCMAKR----SIGMVLAL
EYSSDNKKDNIETVMNIIEFPLLDRTTQ-SVISTTSSDLSNWSRLSSLWPLLYGTSCCFIEFAS
LIGSRFDFDRYGLVPRSSPRQADLILTAGTVTMKMAPSLVRLYEQMPEPKYVIAMGACTIT
GGMFSTDSYSTVRGVDKLIPVDVYLPGCPPKPEAVIDAITKLRKKISREIYEDRIRSQQENR
CFTTSHKFRVGRSTHTGNYNQGLLYQSPSTSEIPTETFFEYKS-VSSHELVNMQMRNTFSWI
KEEIIRFIAVSLIIYIITRAPISNAYPIFAQQSYENPREATGRIVCANCHLANKPVDIEVPQAVL
PDTVFEAVVRIPYDLQLKQVLANGKKGALNVGAVLILPEGFELAPPDRISPEIKEKISNLSF
QSYRPTKKNILVIGPVPGQKYSEITFPILSPDPATKKDVHFLKYPIYVGGNRGRGQIYPDGS
KSNNNVYNATSAGIVSKIIRKEKGGYEITIVDASDGRQVVDIIPPGPELLVSEGESIKLDQPL
TSNPNVGGFGQGDAEIVLQDPLRVQGLLLFLASIILAQIFLVLKKKQFEKVQLSEMNFMSKI
YDWFEERLEIQAIADDITSKYVPPHVNIFYCLGGITLTCFLVQVATGFAMTFYYRPTVTEAF
ASVQYIMTETNFGWLIRSVHRWSASMMVLMMILHVFRVYLTGGFKKPRELTWVTGVVLA

VLTASFGVTGYSLPWDQIGYWAVKIVTGVPEAIPVIGSPLVELLRGSASVGQSTLTRFYSLH
TFVLPLLTAVFMLMHFLMIRKQGISGPLMGVTKKPDLNDPVLRAKLAKGMGHNYYGEPA
WPNDLLYIFPVVILGTIACNVGLAVLEPSMIGEPADPFATPLEILPEWYFFPVFQILRTVPNKL
LGVLLMVSVPAGLLTVPFLENVNKFQNPFRRPVATTVFLIGTAVALWLGIGATLPIDKSLTL
GLFMIEVFLFGIVLGLIPITLAGLFVTAYLQYRRGDQLDLMSTITSYFGFLLAALTITSAIFIG
LNKIRLIMDIVSLAWAALMVVFTFSLSLVVWGRSGLMIIRSPEPDVKILVDRDPIKTSFEEW
ARPGHFSRTIAKGPDTTTWIWNLHADAHDFDSQTSDLEEISRKVFSAHFGQLSIIFLWLSG
MYFHGARFSNYEAWLSDPTHIGPSAQVVWPIVGQEILNGDVGGGFRGIQITSGFFQIWRAS
GITNELQLYCTAIGALVFAALMLFAGWFHYHKAAPKLAWFQDVESMLNHHLAGLLGLGS
LSWAGHQVHVSLPINQFLNAGVDPKEIPLPHEFILNRDLLAQLYPSFAEGATPFFTLNWSKY
AEFLTFRGGLDPVTGGLWLTDIAHHHLAIAILFLVAGHMYRTNWGIGHGIKDILEAHKGPF
TGQGHKGLYEILTTSWHAQLSLNLAMLGSLTIVVAHHMYSMPPYPYLATDYGTQLSLFTH
HMWIGGFLIVGAAAHAAIFMVRDYDPTTRYNDLLDRVLRHRDAIISHLNWVCIFLGFHSF
GLYIHNDTMSALGRPQDMFSDTAIQLQPVFAQWIQNTHALAPGATAPGATTSTSLTWGGG
DLVAVGGKVALLPIPLGTADFLVHHIHAFTIHVTVLILLKGVLFARSSRLIPDKANLGFRFPC
DGPGRGGTCQVSAWDHVFLGLFWMYNSISVVIFHFSWKMQSDVWGSISDQGVVTHITGG
NFAQSSTTINGWLRDFLWAQASQVIQSYGSSLSAYGLFFLGAHFVWAFSLMFLFSGRGYW
QELIESIVWAHNKLKVAPATQPRALSIVQGRAVGVTHYLLGGIATTWAFFLARIIAVGMALR
FPRFSQGLAQDPTTRRIWFGIATAHDFESHDDITEERLYQNIFASHFGQLAIIFLWTSGNLFH
VAWQGNFEAWVQDPLHVRPIAHAIWDPHFGQPAVEAFTRGGALGPVNIAYSGVYQWWY
TIGLRTNGDLYNGALFLLFISAISLIAGWLHLQPKWKPSVSWFKNAESRLNHHLSGLFGVS
SLAWTGHLVHVAIPGSRGEYVRWNNFLDVLPHPQGLGPLFTGQWNLYAQNPDSSSHLFGN
SQGAGTAILTLLGGFHPQTQSLWLTDIAHHHLAIAFIFLIAGHMYRTNFGIGHSIKNLLEAHI
PPGGRLGRGHKGLYDTINNSIHFQLGLALASLGVITSLVAQHMYSLPAYAFIAQDFTTQAAL
YTHHQYIAGFIMTGAFAHGAIFFIRDYNPEQNEDNVLARMLDHKEAIISHLSWASLFLGFH
TLGLYVHNDVMLAFGTPEKQILIEPIFAQWIQSAHGKTSYGFDVLLSSTNGPAFNAGRSIW
LPGWLNAINE--NSNSLFLTIGPGDFLVHHAIALGLHTTTLILVKGALDARGSKLMPDKKDF
GYSFPCDGPGRGGTCDISAWDAFYLAVFWMLNTIGWVTFYWHWKHITLWQGNVSQFNE
SSTYLMGWLRDYLWLNSSQLINGYNPFGMNSLSVWAWMFLFGHLVWATGFMFLISWRG
YWQELIETLAWAHERTPLANLIRWRDKPVALSIVQARLVGLAHFSVGYIFTYAAFLIASTSG
KFGMSHSVKIYDTCIGCTQCVRACPTDVLEMIPWDGCKAKQIASAPRTEDCVGCKRCESA
CPTDFLSVRVYLWHETTRSMGLAYMTTINLPAIFVPLVGLVFPAIAMVSLSLHVQKNKIFM
RNLKTYLSVAPVVSTLWFGSLAGLLIEINRLFPDALIFPFFSLMTAILERRESESLWGRFCN
WITSTENRLYIGWFGVLMIPTLLTATSVFIIAFIAAPPVDIDGIREPVSGSLLYGNNIISGAIIPT
SAAIGLHFYPIWEAASVDEWLYNGGPYELIVLHFLLGVACYMGREWELSFRLGMRPWIAV
AYSAPVAAATAVFLIYPIGQGSFSDGMPLGISGTFNFMIVFQAEHNILMHPFHMLGVAGVF
GGSLFSAMHGSLVTSSLIRETTENESANAGYRFGQEEETYNIVAAHGYFGRLIFQYASFNN
SRSLHFFLAAWPVVGIWFTALGISTMAFNLNGFNFNQSVVDSQGRVINTWADIINRANLG
MEVMHERNAHNFPLDLAGVEVPSTNGMGLPWYRVHTVVLNDPGRLISVHIMHTALVAG
WAGSMALYELAVFDPSDPVLDPMWRQGMFVIPFMTRLGITNSWGGWSIT-GGTITNPGIW
SYEGVAGAHIVFSGLCFLAAIWHWVYWDLEIFCDERTGKPSLDLPKIFGIHLFLSGVACFG
FGAFHVTGLYGPGIWVSDPYGLTGRVQAVNPAWGVEGFDPFVPGGIASHHIAAGTLGILA
GLFHLSVRPPQRLYKGLRMGNIETVLSSSIAAVFFAAFVVAGTMWYGSATTPIELFGPTRY
QWDQGYFQQEIYRRVSAGLAENKSLSEAWSKIPEKLAFYDYIGNNPAKGGLFRAGSMDN

GDGIAVGWLGHPIFRDKEGRELYVRRMPTFFETFPVVLVDGDGIVRADVPFRRAESKYSV
EQVGVTVEFYGGELNGVSYSDPATVKKYARRAQLGEIFELDRATLKSDGVFRSSPRGWFT
FGHASFALLFFFGHIWHGARTLFRDVFAGIDPDLDAQVEFGAFQKLGDPTTRRQVVMKTL
YSLRRFYPVETLFNGTLALAGRDQETTGFAWWAGNARLINLSGKLLGAHVAHAGLIVFW
AGAMNLFEVAHFVPEKPMYEQGLILLPHLATLGWGVGPGGEVLDTFPYFVSGVLHLISSA
VLGFGGIYHALLGPETLEESFPFFGYVWKDRNKMTTILGIHLILLGIGAFLLVFKALYFGGV
YDTWAPGGGDVRKITNLTLSPSIVFGYLLKSPFGGEGWIVSVDDLEDIIGGHVWLGSICILG
GIWHILTKPFAWARRALVWSGEAYLSYSLGALSIFGFVACCFVWFNNTAYPSEFYGPTGPE
ASQAQAFTFLVRDQRLGANVGSAQGPTGLGKYLMRSPTGEVIFGGETMRFWDLRAPWLE
PLRGPNGLDLSRLKKDIQPWQERRSAEYMTHAPLGSLNSVGGVATEINAVNYVSPRSWLA
TSHFVLGFFLFVGHLWHAGRARAAAAGFEKGIDRDFEPVLSMTPLNMTIALGKFTKDEN
DLFDIMDDWLRRDRFVFVGWSGLLLFPCAYFALGGWFTGTTFVTSWYTHGLASSYLEGC
NFLTAAVSTPANSLAHSLLLLWGPEAQGDFTRWCQLGGLWTFVALHGAFALIGFMLRQFE
LARSVQLRPYNAIAFSAPIAVFVSVFLIYPLGQSGWFFAPSFGVAAIFRFILFFQGFHNWTLN
PFHMMGVAGVLGAALLCAIHGATVENTLFEDGDGANTFRAFNPTQAEETYSMVTANRF
WSQIFGVAFSNKRWLHFFMLFVPVTGLWMSALGVVGLALNLRAYDFVSQEIRAAEDPEFE
TFYTKNILLNEGIRAWMAAQDQPHENLIFPEEVLPRGNALMSGSTGERSFADIITSIRYWVI
HSITIPSLFIAGWLFVSTGLAYDVFGSPRPNEYFTESRQGIPLITGRFDPLEQLDEFSRSFMTI
DRTYPIFTVRWLAVHGLAVPTVSFLGSISAMQFIQRMATQTVEGSSRSG-SRRTSVGNLLKP
LNSEYGKVAPGWGTTPLMGIAMALFAIFLSIILEIYNSSILLDGISMNMLTLKLFVYTVVIFF
VSLFIFGFLSNDPGRNPGREEMADTTGRIPLWIIGTVTGIIVIGLIGIFFYGSYSGLGSSLMLN
IFSLICICLNSVLY-SSFFVAKLPEAYAFLNPIVDVMPVIPVLFFLLAFVWQAAVSFRTTQSNP
NEQNVELNRTSLYWGLLLIFVLAVLFSNYFFNMEVNILAFIATALFILIPTAFLLIIYVKTVSQ
SDMETATLVAIFISGLLVSFTGYALYTAFGQPSQQLRDPFEEHGDMEALVYTFLLVSTLGIIFF
AIFFREPPKVPTKKMKMTIAFQLAVFALIATSSILLISVPVVFASPDGWLGNKNVVFSGTSL
WITLVFLVGILNSLIS-------MSPQTETKASVGFKAGVKDYKLTYYTPEYEVKDTDILAAFRV
TPQPGVPPEEAGAAVAAESSTGTWTTVWTDGLTSLDRYKGRCYNIEPVAGEESQFIAYVAY
PLDLFEEGSVTNMFTSIVGNVFGFKALRALRLEDLRIPNAYIKTFQGPPHGIQVERDKLNK
YGRPLLGCTIKPKLGLSAKNYGRAVYECLRGGLDFTKDDENVNSQPFMRWRDRFLFCAE
AIYKSQAETGEIKGHYLNATAGTCEEMMKRAVFARELGVPIVMHDYLTGGFTANTTLAHY
CRDNGLLLHIHRAMHAVIDRQKNHGMHFRVLAKALRMSGGDHIHAGTVVGKLEGEREIT
LGFVDLLRDDFIEKDRSRGIYFTQDWVSLPGVLPVASGGIHVWHMPALTEIFGDDSVLQFG
GGTLGHPWGNAPGAVANRVALEACVKARNEGRDLAVEGNEIIREASKWSPELAAACEVW
KEIKFEFEAMDTL--MAIHLYKTSTPSTRKGAVDRQVKSNPRNNLIYGQHRCGKGRNARGII
TAGHRGGGHKRLYRKIDFRRNEKDIYGKIVTIEYDPNRNAYICLIHYGGGEKRYILHPRGA
LIGDTIVSGTEVPIKMGNALPLTDMPLGTAIHNIEITLGKGGQLARAAGAVAKLIAKEGKSA
TLKLPSGEVRLIPKNCSATVGQVGNVGVNQKSLGRAGSKCWLGKRPVVRGVVMNPVDH
PHGGGEGRAPIGRKKPATPWGYPALGRRSRKRNKYSDNLILRRRSKMIQPQTHLNVADNS
GARKLMCIRIIGAS-NRRYAKIGDVVVAVIKEAIPNTPLERSEVIRAVIVRTCKELKRDNGMII
RYDDNAAVVIDQERNPKGTRIFGAIARELRQLNFTKIVSLAPEVLMLSPKRTRFRKHHRGR
MKGISYRGNHICFGRYALQALEPAWITSRQIEAGRRAMTRNARRGGKIWVRIFPDKPITVR
PTETRMGSGKGSPEYWVAVVKPGRILYEMSGVPENIARKAIAIAASKMPIRTQFIISGMTRI
KRGYIARRRRTKIRLFASSFQGARSRLTRTITQQKIRALVSAHQDRGRQKRDFRRLWITRIN
AVIRAN-KVYYSYSTLIHHLYKRQLLLNRKILAQIAILNRNCLYMISNEILK----------MIKEV

YAIAQYISMSAHKARRVIDQIRGRSYAETLIILELMPYRACYPILKLVYSAAANANHNMRF
NEANLIISKAEVNEGNTVKKLKPRARGRGYPIKRPTCHITIVLKDLDVE-------MDGIKYFV
VTDKSIRLLLKN-QYTSNVESGSTRTEIKHWVELFFGVKVIAMNSHRLPGKGRRMGPIMG
HTMHYRRMIITLQPGYYIPPLIKKSTMAVPKKRTSISKKRIRKNIWKKKGYWAALKAFSLG
KSLSTRNSKSFF----PTMAKGKDARVTIILECTSC-LRN--VNKESRGISRYITQKNRHNTPSR
LELRKFCPSCYKHTIHGELKKMKIRASVRKICEKCRLIRRRGRIIVICSNPRHKQRQGMGQ
EKVTVSTRPLQWKCVESRADSKRLYYGRFLLAPLMRGQADTIGIAMRRALLGEIEGTCIT
RAKSE--KIPHEYSTIVGIQESVHEILMNLKEIVLRSNLYGTRDASICVKGPGYVTAQDIILPP
SVEIVDNTQHIANLTEPITLCIELQIERNRGYRIKTPNNFQNGSY--PIDAVFMPVRNANHSIH
SYVNGNENQEILFLEIWTNGSLTPKEALHEASRNLIDLFIPFFHAEE-----HLENNQ-HKGTLP
LFNFHGRLAK-PRKTKK-EIALKYIYIDQSELPPRVYNCLKRSNINTFLELLNNSQEELMKIQ
DFRIEDVKHILDVLEI------------MLEGGN-EEISTIPGFNQIQFEGFCRFIDQGLTEELYKFPKI
EDTDQEIEFQLFVETYQLVEPLIKERDAVYESLTYSSELYVSAGLIWKTSRDMQEQTIFIGNI
PLMNSLGTSIVNGIYRIVINQILQSPGIYYRSELDHNGISVYTGTIISDWGGRLELEIDRKARI
WARVSRKQKISILVLLSAMGLNLREILENVCYPEIFLSFLKDKEKKKIKSKENAILEFYQQF
ACVGGDPVFSESLCKELQKKFFQQRCELGRIGRRNMNRRLNLDIPQNTTFLLPRDILAAA
DHLIGMKFGMGILDD--MNHLKNKRIRSVADLLQDQFGLALVRLENMVRGTMCGAIR-HK
LI-PTPQNLVTSTT----LTTTYESFFGLHPLSQVLDRTNPLTQIVHGRKASYLGPGGLTGRTAS
FRIRDIHPSHYGRICPIDTSEGINVGLIGSLAIHARIGHWG-SLESPFY---EISERSKK--VRMLY
LSPSIDEYYMVAAGNSLALNRGSQEEQVVPARYRQEFLTIEWEQVHLRSIFPFQYFSIGASL
IPFIEHNDANRALMSSNMQRQAVPLSRSEKCIVGTGLECQVALDSGVPTIAEHQGKIIYTDT
EKIILSGNRDTLSIPLVIYQRSNKNTCMHQKPQVSRGKCIKKGQILADGAATVGGELALGK
NVLVAYMPWEGYNSEDAVLINERLVYEDIYTSFHIRKYEIHTHVTSHG-PERITNEIPHLEAH
LLRNLDKNGIVMLGSWVETGDILVGKLTPQMAKE-SSYAPEDRLLRAILGIQISISKETCLK
LPIGGRGRVIDVRWIQKKG-GSSYNPETIRVYISQKREIKVGDKVAGRHGNKGIVSKILPRQ
DMPYLQDGRPVDMVFNPLGVPSRMNVGQIFECSLGLAGGLLDRHYRIAPFDERYEQEAS
RKLVFSELYEASKQTANPWVFEPEYPGKSRIFDGRTGDPFEQPVIIGKPYILKLIHQVDDKIH
GRCSGHYALVTQQPLRGRAKQGGQRVGEMEVWALEGFGVAHILQEMLTYKSDHIRARQE
VLGTTMVGGPIPKPEDAPESFRLLVRELRSLALELNHFLVSEKNFQINRKDV-------MIDRYK
HQQLRIGSVSPQQISAWAKKILPNGEIIGEVTKPYTFHYKTNKPEKDGLFCERIFGPIKSGIC
ACGNYRVIGNDKEDPKFCEQCGVEFVDSRIRRYQMGYIKLACPVTHVWYLKRLPSYIANL
LDKPLKELEGLVYCD--FSFARP-IAKKPTFLRLRGSFEYEIQSWKYSIPLFFTTQGFDAFRNR
EISTGAGAIREQLADLDLPIIIDYSLVEWKELGEE-RPTVNEWEDRKVGRRKDFLVRRMEL
AKHFIRTNIEPEWMVLCLLPVLPPELRPIIQIDGGKLMSSDINELYRRVIYRNNTLIDLLTTSR
STPGELVMCQEKLVQEAVDTLFDNGIRGQPMRDGHNKVYKSFSDIIEGKEGRFRETLLGK
RVDYSGRSVIVVGPSLSLHRCGLPREIAIELFQTFVIRGLIRQHFASNIGVAKSKIREKEPVV
WEILQDVMQGHPVLLNRAPTLHRLGIQAFQPILVEGHAICLHPLVCKGFNADFDGDQMAV
HVPLSLEAQAEARLLMFSHTNLLSPAIGDPISVPTQDMLIGLYVLTSGNRRGICANRYNPCN
YRN---YQNERIDDNKYKYTKTQEPFFCNSYDAIGAYRQKRIHLDSPLWLRWRLDQR-VITS
--REAPIEVHYQSLGTYHEIYGHLLIVRSIKKEILCIYLRTTVGHISLYREIEEAIQGFFRACSY
GT--------MEVLMAERADLVFRNKVIDGTAIKRLISRLIDHFGMAYTSHILDQVKTLGFRQAT
ATSISLGIDDLLTIPSKGWLVQDAEQQRLILEKHHHYGNVHAVEKLRQSIEIWYATSEYLRQ
EMNPNFRMTDPFNPVHMMSFSGARGNASQVHQLVGMRGLMSDPQGQMIDLPIQSNLRE
GLSLTEYIISCYGARKGVVDTAVRTSDAGYLTRRLVEVVQHIVVRRTDCGTIQGISVSPPNG

MMP-ERIFIQTLIGRVLADYIYIGSRCIAVRNQDIGIGLVNRFIT------FQTQPISIRTPFTCKSTS
WICRLCYGRSPTHGDLVELGEAVGIIAGQSIGEPGTQLTLRTFHTGGVFTGGTAEHVRAPSN
GKIKFNEDLVHPTRTRHGHPAFLCSIDLYVTIESEDIIHNVTIPPKSFILVQNDQYVESEQVIA
EIRAGTYTF-HFKERVRKHIYSDSEGE-MHWSTDVYHAPDFTYSNVHLLPKTSHLWILSGG
SYK--SSVVPFSIHKDQDQTNVY--FLSAAKGKARNSF-SVNNDQQGKHKFFTSGLSGK-ESG
IPDY-SEFNRILDTDHSNLIFPS-ILHKDSD-FLLAKRRRNRFIIPFQWIQEREKELWPRSSISIEI
PIN--GIFRKNSILAYFDDLQYRRKGSGITKYGAIGLHSILKKED--LIEYG-GVKEFKPKYTK
VDQFFFIPEEVHILPESFSIMVRNNSIIGVDTRITLNTRSRVGGLVRVEKKNKRIELKIFSGDI
HFPVEMDKIFRHSGILIPPGRVKK--EFKESKK--WKNWIYVQS--ITPTKKK--YFVLVRPVIIY
EIADGINLETLFPQDPLQEKDNLELRVVNYILYGNGKPILGISGTCIQLVRTCLVLNWDQGN
---KSSSSEETHASFVEVSTRDLIRDFLRINLVKSHISYIRKRNDPLGSVLIS-DNRSDRT--NPF
YSIYSKEK-----IRQLLKENQGTIHTLLNR---SKESQSLIILSSSNCFEMGP--FNDVKHYN---VI
KES-PLIPIRNSLGPLGT--ACQVANF--Y--YILKT--HNQIS-VTKNLQLE--N-LKQTFQVL--K-Y
YLMDENGR--IYNSDPCSNILFNPFNLNWHFLHH-YCEKKSTIIS-LGQFFFENVCITKHGP---
HLKSGQVIIVQ-IDSVVIRSAKSYLATPGATVHGHYGEILSEGDTLVTFIYEKSRSGDITQGL
PKVEQVLEVRSIDSISMNLEKRVEGWNERITRILGIPWGFLIGAELTIVQSRISLVNKIQKVY
RSQGVQIHNRHIEIIVRQITSKVLVSE---DGMSNVFLPGELIGLFRAERTGRALKEAICYQAI
LLGITKASLNTQSFISEASFQETARVLAKAALRGRIDWLKGLKENVVLGGMIPVGTGFKGL
VHRS--KQHNNIPLETKKKN--LFECEMR--DILFHHKELFDFCISTNIHDTSEHLFLGFNDS----
--MTRRYWNINLEEMMDAGIHFGHGTRKWNPKMAPYISAKRKGIHIINLTRTARFLSEACD
LVFDAASRGKQFLIVGTKNKAADSVAQAAIKARCHYVNKKWLGGMLTNWYTTETRLHK
FRDLRTEQKTGRLKRLPKRDAAVLKRQISHLQTYLGGIKYMTGLPDIVIIVDQQEEYTALQ
ECITLRIPTICLIDTNCDPDLADISIPANDDAIASIRLILNKLVFAICEGRS-SYIRNSMGQKINP
LGFRLGATQGHHSLWFAQPKNYSEDLQEDQKIRDCIKNYIKKNMKIFSS-VEGIACIEIQKRI
DLIQVIIYMGFPKLLIEH------QKIEELQMNIQKELNSVNRKLNMAITRIANPYGHPNILAEFI
AGQLKNRVSFRKAMKKAIELTEQAGTKGIQIQIAGRIDGKEIARVEWIREGRVPLQTIKAKI
DYCSYTVRTIYGALGIKIWIFVDE-MSRYRGPRFKKIRRLGALPGLTSKKPRAGSNLRNQLR
PGK-KSQYRIRLEEKQKLRFHYGLTEQQLLKYVRIAGKAKGSTGQVLLQLLEMRLDNILFR
LGMASTIPQARQLVNHRHILVNGRIVDIPSYRCKPRDIIRVKDEQKSKSLVQNYLDSSSR-EE
LPKHLTLHPFQ--YKGSVNQIIDSKCVGLKINELLVVEYYSRQTMSRRGTAEEKTAKSDPIY
RNRLVNMLVNRILKHGKKSLAYQIIYRAMKKIQQKTETNPLSVLRQAIRGVTPDIAVKARR
VGGSTHQVPIEIGSTQGKALAIRWLLGASRKRPGRNMVFKLSSELVDAAKGSGDAIRKKE
ETHRMAEANRAFAHFRMSKDTIANILTSIRNADMNKKRTVRIPYTNITENILKILLREGFIE
NMRKHQESNNLFFVLTLRHRRKRNRKGPDKT-V--LNLKRISRPGLRIYSNYQRIPRILGGM
GIVILSTSRGIMTDREARLEGIGGEILCYIWMAKPLPRIGSRKNRHMGSR-NVRRIPKGVIH
VQASFNNTIVTVTDVRGRVISWSSAGTCGFKGTKRGTPFAAQTAAGNAIRTVVDQGMQR
AEVMIKGPGLGRDAALRAIRRSGILLSFIRDVTPMPHNGCRPPKKRRVMPTIKQLIRNTRQ
PIKNVTKSPALRGCPQRRGTCTRVYTITPKKPNSALRKVARVRLTSGFEITAYIPGIGHNLQE
HSVVLVRGGRVKDLPGVRYHIVRGTLDAVGVKDRQQGRSKYGVKKPKMARKSLIQREK
KRQKLEQKYHLIRRSSKKEIS---KVPSLNEKWKIHGKLQSLPRNSAPIRLHRRCFSTGRPRA
NYRDFGLSGHILREMVQACLLPGATRSSWMIKKSFISVISHEEKEKNR--GSVTFQILSLTNR
IRKLSSHLELHRKDYLSQRGLRKILGKRQRMLSYLSKNNRIRYKELINQLDIRESKTR--MV
KLRLKRCGRRQRAIYRIVAIDVRSRREGRALRKVGFYDPIKNQTYLNVPVILYFLEKGAQP
TGTVHDISKKAGVFMELRLNHQTKFNMDKSKRLFLKSKRSFRRRLPPIQSGDRIDYRNMS

LISRFISEQGKILSRRVNRLTLKQQRLITIAIKQARILCLLPFLNNE--KQFERSESTPRTTGLRI
KNKVAR-LKKNPFVANHLLRKINMLNTKAEKEIIVTWSRASTIIPTMIGHTIAVHNGKEHLP
IYITDRMVGHKLGEFAPTLNFRGHAKNDNRSRRMIFQSFILGNLVSLCMKIINSVVVVGLY
YGFLTTFSIGPSYLFLLRARVMEEGEEGTEKKVSATTGFITGQLMMFISIYYAPLHLALGRP
HTITVLALPYLLFHFFWNNHKHFFDYGSTNRNSMRNLSIQCVFLNNLIFQLFNHFILPSSML
VRLVNIYMFRCNNKMLFVTSSFVGWLIGHILFMKWVGLVLVWIQQNNSIRS----NKYLMS
ELRNFMVRIFSILLFITCVYYLGRIPSPILTKKLKETEEDKPLVTILFDYKRWNRPFRYIKND
KFENAVKNEMSQYFFHTCESDGKKRISFTYPPSLSTFFEMIQRKMSLFTTDKLYSDELYNN
WSYTNEQKRKNLNNKLINIIKDLDKALLILDVLEKRPRLCHDNTKKKYLPKVYDPFLNGA
YRGQIKKRFSLIERGWINKIHGLFYLEFEEKDPNNKAIGIKEIRKKIPQWSYKLIDLEQEEGE
QIRSRKCKRVLIFNHALIRYSQQSDFRRDIIKGSMRAQRRKTVIWKTFQANVHSLPFLDRID
KDV--YFSFDMLRPIKKKFQHRRENARIEIAETWDSIVVAQVIRGFVLVIQSILRKYIILPSLII
VKNIGRILLFQFPEWSEDVKDWKREVHVNCTYNGVQLAEKEFPKNWLIDGIQIKILFPFRL
KPWHKSKKEQQNDFCFLTVWGMETELPFGSPRKQLSFFEPIFKELKKRLIKLKKKCFRI-LII
LKERTKLFLNVSKERKKWVIKNIV--FLKKIIKELSKMNNLIIDNELIHEP-INIQSTNWTT-SL
TKKKIQDLTNRTNTTINQIQKISKDNKKNK--MSYNDKRLELPKNILQLLK--NVRLIRKSNY
FIKFFIERIYRNIFLFISNIPKINAQLF-----CNLSYLSQAYVFYKLSQSRFFNKLRLRSVFQYAF
LFLKNEIKDYFTQKIFHSELRHKNIP-YEINQWKNCLRGYYQNNLSQ---SRLVPQKGINIVN
QHSIAQNNN----------KYDSYEKN-NVKKKYRYDLLSYNFINDNIIDPDRKNFDWIILNFCLR
LFDWMRMNEEILSCWFFPEFLILYTSYKIKPWYIPIKFLLLNTN--MEAEFDFLLKRYLLFQL
RWD---NKKMIDNIKVYCSLLRPINLREITIASIQRGEINLDLLMIQKNFILTELIKKGILLIEPV
RLSRKNEGQFIMYQTIGISLVHKSKHHYD-LFVPETILSPRHRRELRILICFSFLWPNYRLEDF
ACMNRYWFDTNNGSRFSMVRIHMYPMKGHQFKSWIFELREILREIKNSHYFLDSWTQLN
SVVSFIHIFFHQERFIKLLDSRIWSILLS-RNSHLQGSTSNRYL---TIKGVVLFVVS-VLIYR----
INNRKMVERKNLYLTGLLP-----IPMNFIGPRNDTLEESF-GSSNINRLIVS-LLYLPKGKKISES
CFLDPKESTWVLPIT----KKCIMPESNWGSRWWRNWLGKKR--DSSCKISN---ETVTGIEISF
KEKDIK-YLEFLFVYYMD--DPIRKDHDWELFDRLSPRKRRNIINL--NSGQLFEILVKDWIC
YLMFAFREKIPIEAEG-FFKQQG-AGSTIQSNDIEHVSH--LFSRNKWAISLQNCAQFHMWQF
RQDLFF---SWGK-NPHESDFLRN-ISRENWIWLDNVWLENKDR---FFS--KVRNVSSNIQYD
S---TR--SSFVQVTDFSQLKGSS-DRSRDHFDSIR----NEDSE-------------YHTLINQREIQQLKE
RSIL-WD--PSFLQMERTEIESD--RSPK--CLSGYS--SMSRLFT-EREKQMNNHLLPEEIEEFLV
LGNRTRSIRSFFSDRW--SELH-LGSNPTERSTRDH----KLLKKEQDVS-FVPSRRSE----NKEIV
NIFKIITYLQN--TVSIHPISSDPGCDMVP-KDELD-MDSSNKISFLNKNPFFDL--FHLFHDRN
RGGYTL--HHDFESE--ERFQEMADLFTLSITEP-DLVYHKGFALSIDSYRLDQKQ--FLNEVF
NSRDESKKKSL---LVLPPIFY--EENESFYRRIRKKWVR-TSCGNDLEDPKPKIVVFASNNIME
AVNQYRLIRNLIQIQYSTYG--YIRNVLNRFLLMN---RSDRNFEYGIQ---RDQIGNDTLNHRT
IMKYTINQHLSNLKK-SQKKWFDP--LIFISRTERSMNRDPNAYRYKWSNGSKNLQEHLEHF
ISEQKS---RFQVVFDRLRINQYSIDWSEVIDKKDLSKSLPFFLSKLLL---------FLS-------KFLL
FLSNSLP-FFFVSFGNIPIHRSE-IHIY-ELKGPNDQLCNQLLESIGLQIVHLKKLKPF--LLD---D
HDTS-QKSKFLINGGTISPFLFNKIP-KW-MIDSFHTR--KNRRKSFDNTDSYFSMISHDQDN
WLNPVKPFHRSSLISSFYKANRLRFLNNPHHFCFY--CNKRFPF---Y-VEKARINNYD--FTYG
-QFLNILFIRNK-IFSLRGGK--KKYAFLE-RDTISPIELQVSNIFIPNDFPQNGDER-YNFYKS---
FHFPIRSDPFVR--RAIYSIADISGTLLTEGQIVNFERTYCQPLSDMNLSDSEGKNLHQYLN--F
NSNMGLIHTPCSEKYLPSEK--RKK--------RGLCLK--KCLEKGQMYRTFQRDSAFSTLSKW

NIFQTYMPWFLTSTGYNYLNLLFLDTFSD-LLPILSSS--QKFVSIFHDIMHGLDISWRILQKR
L------CLPQWN-LISEISSKCLHN--LLLSKEMIHRNNES--PLISTHLRSPNVREF-LYSILFLLLV
AGY-LVRTHLLFVSR--TYSELQTEFERVKSLMIPSYMIELRKLLDRYPTSELNSFWLKNLFL
VAREQLGDSLEEIRGSASGGNMLWGGGP-----TYG-VKSIRSKKKYLNINLIDIIDFI-SIIPNPI
NRIAFSRNTRHLSHISKETYSLIRKRKKVNGDWIDDKIESWVSNSDSVDDKEREFLVQFS--
TLTT--EKRIDQILLSLT-QSDHLS-----KNDSGYQMIEQPGAIYLRYLVDIH--KKYLMNYEFNT
SCLAERRIFLAHYQTITY--SQTSCGANSFHFPSHG---KPFSLRLALSLS-RGILVIGSIGTGRSY
LVKYLATNSYVPFITVFLNKFLDNKPKGFLID-----DSD------DIDDSDDID---------------------R
DLDTELELLTMMNAL---TMDMMP---EIDRFYITLQFELAKAMSPCIIWIPNIHDLDVNESNY
LSLGLLVNYLS---RDCERCS-TRNILVIASTHIPQKVDPALIAPNKLNTCIKIRRLLIPQQRKH
FFTLSYTRGFHLEKKMFHT--NGFGSITMGSNVRDLVALTNEALSISITQKKSIIDTNIIRSAFH
RQTWDLR---SQVRSVQDHGILFYQIGRAVAQNVLLSNCSIDPISIYMKKK--SCNEGDSYLY
KWYFELGTSMKKLTILLYLLSCSAGSVAQDLWSLP--ELDEK-NGITSYGLVENDS-DLVHGL
LEVEGTLVG--SSRTEKDCSQFDND--RATLLLRPEPRN-PLDMMQNGSCSIVDQRFLYEQ--Y
KSEFEE-GEGEGVLDPQ----RIEED----LFNH--IVWAP--RIWRP---WGFLFDCIESPNELGFP-Y
WARSFRGK---RIIYDEEDGLQENDSEFLQSGTMQYQTRDRSSKEQGFFRISQFIWDPADPLF
FLFK-DQPFVSVFSHREFFADEEMSKGLLTSQTDLPTSIYKRWFI--KNTQEKYFELLIYRQR
WLR---TNSSLS--NGFFRSN--TPSESYQYLSKLFLSN-GTLLDQMTKTLLRKRWLFPDEMVL
AI--CYNNESLV--MSRSR-INGNFIDKTFSIVANILLRIIPTTSGEKEAFAYYRDG--MSAQSEG
NYAEALQNYYEAMRLEIDPYDRSYILYNIGLIHTRNGEHTKALEYYFRALERNPFLPQAFN
NMAVICHY-RGEQAIRQGDSEIAEAWFDQAAEYWKQALALTPGNYIEAQNWLKITGRFE--
MSWRSDHICIELIAGSRKPSNVCWAFILFLGSLGFLLVGTSSYLGRNFIPFFPSQQIIFFPQGI
VMSFYGIAGLFISSYLWCTISWNVGSGYDRFDIKEGIVCIFRWGFPGKNRRIFLRFLMKDIQ
SIRIEVKEGIYARRVLYMEIRGQGSIPLTRTDEN-LTLREIEQKAAELAYFLRVPIEVF
>Jasminum
??
??
??
??
??
???????????????????????????????MVSIRADEISNTLRERIEQYNREVKIVNTGTVLQVGDGIA
RIHGLDEVMAGELVEFEEGTIGIALNLESTNVGVVLMGDGLMIEEGSSVKATGRIAQIPVS
EAYLGRVLNALAKPIDGRGEISASESRLIESPAPGIISRRSVYEPLQTGLVAIDSMIPIGRGQR
ELIIGDRQTGKTAVATDTILNQQGQNVICVYVAIGQKASSVAQVVTTLQEGGAMEYTIVVA
EMADSPATLQYLAPYTGAALAEYFMYRKQHTLIIYDDLSKQAQAYRQMSLLLRRPPGREA
YPGDVFYLHSRLLERAAKSNSSLGEGSMTALPIVETQSGDVSAYIPTNVISITDGQIFLSAD
LFNAGIRPAINVGISVSRVGSAAQTKAMKQVAGKLKLELAQFTELEAFAQFASDLDKATQS
QLARGRRLRELLKQSQSAPLTVGEQIMTVYTGTKGYLDSLEIGQVSKFLDALRTHLKTNK
PQFQEIISSTKTFTEEAEVLLKEAIQEQMERFLLQ---MRMNPTTSGSGVSTLEKKNLGRIVQ
IIGPVLDVSFPSGKMPNIYNALVVQGRDTVGQAINVTCEVQQLLGNNRVRAVAMSATDGL
MRGMEVIDTGAPLSVPVGGATLGRIFNVLGEPVDELGPVDTRTTSPIHRSAPAFIQLDTKLS
IFETGIKVVDLLAPYRRGGKIGLFGGAGVGKTVLIMELINNIAKAHGGVSVFGGVGERTRE
GNDLYMEMKESGVINEENIAESKVALVYGQMNEPPGARMRVGLTALTMAEYFRDVNEQD
VLLFIDNIFRFVQAGSEVSALLGRMPSAVGYQPTLSTEMGTLQERITSTKEGSITSIQAVYVP

ADDLTDPAPATTFAHLDATTVLSRGLAAKGIYPAVDPLDSTSTMLQPRIVGEEHYKIAQRV
KQTLQRYKELQDIIAILGLDELSEEDRLTVARARKIERFLSQPFFVAEVFTGSPGKYVGLAE
TIRGFQLILSGELDGLPEQAFYLVGNIDEATAKAMNLEMESNLKKMTFNLCVLTPNRIIWD
SEVKEIILSTNSGQIGVLPNHAPIATAVDIGILRIRFNDQWLTVALMGGFARIGNNEITVLVN
DAERGSDIDPQEAQQTLEIAEANLRKAEGKRQRIEANLALRRARTRLEAVNVISMKNVTD
SFVSLGHWPSAGSFGWNTDILATNLINLSVVIGVLIFFGKGVLSDLLDNRKQRILNTIRNSE
ELRGGAIEQLEKARARLRKMEMEADQFRVNGYSEIERDKWNLINSTSKTLEQFENFKNET
IQFEQQRAINQVRQRVFQQALQGALGTLNSCLNKELHLRTISANIGILGAMKEITDMNPLIS
AASVIAAGLAVGLASIGPGVGQGTAAGQAVEGIARQPEAEGKIRGTLLLSLAFMEALTIYG
LVVALALLFANPFVMNVLSCSINTLKGLYDISGVEVGQHFYWQIGDFQVHGQVLITSWVVI
AILLGSATLAVRNPQTIPTGGQNFFEYVLEFIRDMSKTQIGEEYGPWVPFIGTMFLFIFVSN
WSGALLPWKIIQLPHGELAAPTNDINTTVALALLTSVAYFYAGLTKRGLSYFGKYIQPTPIL
LPINILEDFTKPLSLSFRLFGNILADELVVVVLVSLVPSVVPIPVMFLGLFTSGIQALIFATLA
AAYIGESMEGHHMIFSTLEHILTHISFSIVSILVTIHLITFLVDEIRKLYDSFEKGMILAFFCITG
LLVIRWIYSGHFPLSNLYESLMFLSWSLSLIHRVPY--KNQKFLSTIIGPSAIFTQAFATSGLLT
EIHESTILVPALQSEWLIMHVSMMILGYAALLCGSLLSVALLVIAVRKNKKSFF--TEIEYMD
ERINVLQNTPNTSTKNYYRSQFIQQLDYWSYRVISLGFLFLTIGILSGAVWANEAWGSYWS
WDPKETWAFITWIVFAIFLHIRTNVKWKGTNSAIVASIGFLIIWICYFGVNLLGIGLHSYGSF
TLTMAKKKAFTPLLYFASIVFLPWWISLSFTKSMESWVTNWWNTGQSEIFLNDIQEKSILE
RFIELEEILLLDEMIKEHSETRLQKLGI-IHKETIQLIKIQNEDRIHTILHLSTNIISFVILSGYSIF
GNEELVILNSLAQEFLYNLSDTVKAFSILLLTDLCIGFHSPRGWELMIGSVYKDFGFVHND
QMISGLVSTFPVILDTLLKYWIFRYLNRVSPSLVVIYHSMNDMPIGLPKVPFFDYEERKQA
WTDLYSKLYHGGILFLGDVIDTELANELIAAMVYLDSHHPEIRCYMYINSPGGGVFDGLAI
YDAMKIMTQEVRTTVYGQAASMASLLLASGEKGHRIALPHSEIMLHEPSMSPTRGKVEFL
LSECDVLASLKERMLNIYVLETGKDRLVFRSPTKRSLFGTRNSPRMWNY-------EKEAKWV
VEGLVTEGHPSGMFRVQLDNGARVLGYISGKIRRKAIRIMQGDRVQIELSHYDLTKGRIIY
RLKPKDSDDMQEIKRYLQ-DRPQQHDFLYPLIFQEYIYALAHDRGFPGYDNKSSLLIVKRLI
TRMYQQNHFWISSNDSNQNPFFGKNLYPQILSEGFAFILELPFSLRLILSLIVKSQNLRSIHSI
FPFLEDNFSHFPFVLDILIPHPIHLEILVQTLRCWVKDASSLHLLRFFLHEYWNWNSLSTAS-
SSFSKQRLFFFLYNSHVCEYESIFVFLRNQSSHLRSTSSGALLERIYFYGKIERLVNVFL-DFN
LCLLKDPFMHYVRYQEKSILASKGTPLFMTKWKYYLVPFWQCYFSLWFHPRRIYLNQLS
KNFLEFGGYLSSVRLNASVIRSQILENSFLINNAI--NKFDTFVPIIPLIGSLAKAQFCNVLGY
PISKLVRTDLSDSDIIDRFGRICRNLSHYHSGSSKRKSLYRIKYILRLSCARTLARKHKSTVR
AFLKRLGSELLEEFLMSEEQVLSLTFPKRIWFLDIICLHDLVNQKMIIYNTTELQ-AIHSFSRL
EF-LKEVYGSIWVLIPILTLVLGITLGVLVIVWLEREISAGIQQRIGPEYAGPLGILQALADGT
KLLFKENLFPSRGDTRLFTIGPSIAVISILLSYSVIPFGYRLILGDLSIGVFLWIAVSSLAPIGLL
MSGYGSNNKYSFLGGLRAAAQSISYEIPLTLCVLSISLLSNSFSTVDIVEAQSKYGFWGWN
LWRQPIGFIVFLISSLAECERLPFDLPEAEEELVAGYQTEYSGIKFGLFYVASYLNLLVSSLF
VTVLYLGGWNLSIPYLFVPELFE---RGKVFETIICIFITLAKTYLFLFISIATRWTLPRLRMDQ
LLNLGWKFLLPISLGNLLLTTSFQLLSLMIWHVQNENFILDSTRIFMKAFHLLLFDGSLIFPE
CILIFGLILLLMIDSTSDQKDLPWFYFISSTSLVMSITALLFRWREEPMISFSGNFQTNNFNEI
FQFLILLCSTLCIPLSVEYIECTEMAITEFLLFVLTATLGGMFLCGANDLITIFVAPECFSFCSY
LLSGYTKKDVRSNEATMKYLLMGGASSSILVHAFSWLYGSSGGEIELQEIVNGLINTQMY
NSPGISIALLFITVGIGFKLSPAPSHQWTPDVYEGSPTPVVAFLSVTSKVAASASATRIFDIPF

YFSSNEWHLLLEILAILSMILGNIIAITQTSMKRMLAYSSIGQIGYVIIGIIVGDSNDGYASMI
TYMLFYISMNLGTFACIVLFGLRTGTDNIRDYAGLYTKDPFLALSLALCLLSLGGLPPLAGF
FGKLYLFRCGWQAGLYSLVLIGLLTSVVSIYYYLKIIKLLMTGRNQEITPHVRNYRRSPLRS
NNSIELSMIVCVIASTIPGISMNPIIAIAQDTLFMFLLYEYDIFWAFLIISSLIPILAFFISGILAPI
RKGPEKLSSYESGIEPMGDAWFQFRIRYYMFALVFVVFDVETVFLYPWAMSFDVLGLSVF
LEAFVFVLILIVGLVYAWRKGALEWSTNHFPWLTIIVVLPIFAGSLIFFLPHRGNRVIFWYTI
CISILELLLTTYAFCYHFQSDDPLIQLVEDYKWIDFFDFHWRLGIDGLSIGPILLTGFITTLAT
LAARPVTRDARLFHFLMLVMYSGQIGLFSCRDLLLFFLMWEFELIPVYLLLSMWGGKKRL
YSATKFILYTAGGSVFLLMGALGIGLYGSNEPTFNLEILANQSYPVALERIFYIGFFIAFAVKL
PIIPLHTWLPDTHGEAHYSTCMLLAGILLKMGAYGLVRINMELLSHAHSLFSPWLMIVGA
MQIIYAASTSLGQRNLKKRIAYSSVSHMGFLIIGIGSITDIGLDGALLQIISHGFIGAALFFLA
GTTYDRIRLVYLDEMGGIAIPMPKIFTMFSIFSMASLALPGMSGFVTEFIVFFGLITSPKYLL
MAKILIPFVMAIGIILTPIYSLSMSRQMFYGYKIFNAPNSYFLDSGPRELFLSISIFLPILGIGLY
PDFVLSLSVDKVEVILSNSFDRMMLEHILVLSAYLFSIGIYGLITSRNMVRALMCLELIFNS
VNINFVTFSGFFDNRQLRGDIFSVFVFAIAAAEAAIGLAIVSSIYRNRKSTRINQSNLLNKME
QTYQYAWIIPFIPLPVPILIGVGLLLFPTTTKNLRRIWAFPSILLLSIVMIFSINLSIQQIKRSPIY
QYVWSWTINNDFSFEFGYLIDPLTSIMSMLITTVGIMVLVYSDNYMAHDQGYLRFFASMS
FFSTSMLGLVTSSNLIQIYIFWELVGMCSYLLIGFWFTRPLAANACQKAFVTNRVGDFCLL
LGILGFYWITGSFEFRDFFEILKNNEVNSSFLTLCASLLFTGAVAKSAQFPLHVWLPDAMEG
PTPISALIHAATMVAAGIFLVARLLPLFIVIPYIMNFISLIGIITILLGATLALAQKDIKRGLAYS
TMSQLGYMMLALGMGSYRSALFHLITHAYSKALLFLGSGSIIHSMETLVGYSPDKSQNMV
LMGGLRKHIPSSQISFLLGTLSLCGIPPLACFWSKDEILNDSGFYSPIFAIIVWTAAGLTLFRH
FCGNLLTFEGHLNVHFQNYSGNKNTPFYSISLWGKEGSKR----NYRLFKMKFFSFENKKSY
SYPYESDNTMLFPLLVLGSFTLFIGSLGIPFELDVLTKWLTPSINLLHQKLN-SIDWYKFFED
SIFSVSIAYFGIFLASFLYKPIYSSFQNFDLTNSFLSISDKIINGLYNWSYNRAYIDTFYTTSLIE
TLRGLSLLARFFDRRAIDGITNGVGVLSFFLAEGIKYVGGG-RISSYLFFYLSCLSIFLLGLY
MDLPGPIHDFLLVFLELGLILGSLGVVFLPNPIYSAFSLGLVLFCISLFYILLNSYFVAAAQLL
IYVGAINVLIIFAVMFMNGSEYYKDFYLWTVGDGVTSMVCTSLFISLITTIPDTSWYGIIWT
TKSNQIIEKDLISNSQQIGIHLVTDFFLPFELISIILLVALIGAIAIARQMIAPTTRKDLMIINMG
PHHPSMHGVLRLIITLDGEDVIDCEPILGYLHRGMEKIAENRTILQYLPYVTRWDYLATMF
TEAISVNGPEQLGNIQVPKRASYIRTIMLELSRIASHLLWLGPFMADIGAQTPFFYIFREREFI
YDLFEAATGMRMMHNFFRIGGIAADLPHGWIDKCLDFCDYFLRGVVEYQKLITRNPIFLE
RVEGVGIIGAEEALNWGLSGPMLRASGIQWDLRKMDHYECYDEFDWEVQWQKEGDSLA
RYLVRISEMTESIKIIQQALERIPGGPFENLEIRRFDRIKDTEWNDFEYRFISKKPSPTFELSK
QELYVRVEAPKGELGIFLIGDQSVFPWRWKIRPPGFINLQILSQLVKRMKLADIMTILGSIDI
IMGEVDRMFPMVTEFMNYGQQTIRAARYIGQGFMITLSHANRLPVTIQYPYEKLITSERFR
GRIHFEFDKCIACEVCVRVCPIDLPVVDWKLETDIRKKRLLNYSIDFGICIFCGNCVEYCPT
NCLSMTEEYELSTYNRHELNYNQIALGRLPVSIIDDYTIRTISSS---------------MQGRLSAWL
VKRGLIHRSLGFDYQGIETLQIKPKDWHSIAVILYVYGYNYLRSQCAYDVAPGGLLASVYH
LTRIEYGGDQPEEVCIKVFASRRNPRIPSVFWVWKSVDFQERESYDMLGISYDNHPRLKRI
LMPESWIGWPLRKDYIAPNFYEIQDAH---MN
SIEFPLLDQTVQNSVFSTTLNDLSNWSRLSSLWPLLYGTSCCFIEFASLIGSRFDFDRYGLVP
RSSPRQADLILTAGTVTMKMAPSLVRLYEQMPEPKYVIAMGACTITGGMFSTDSYSTVRG
VDKLIPVDVYLPGCPPKPEAVIDAITKLRKKISREIYEDRIRSQQANRCFTTNHKFHVGRSM

NTGNYDQGFLYQPPSTSEIPTETFLK------------MQTRKTFSWIKEQIARSISVSLLIYIITRTSI
SSAYPIFAQQGYENPREATGRIVCANCHLANKPVDVEVPQAVLPDTVFEAVVRIPYDKQLK
QVLANGKKGGLNVGAVLILPEGFELAPTDRISPEMKEKIGNLSFQSYRPNKKNILVIGPVPG
QKYSEIIFPILSPDPATNKDVHFLKYPIYVGGNRGRGQIYPDGSKSNNTVYNATAAGIVSKII
RKERGGYEISITDPSDGRQVVDIIPPGPELLVSEGESIQFDQPLTSNPNVGGFGQGDAEIVLQ
DPLRVQGLLFFLASVILAQIFLVLKKKQFEKVQLAEMNFMSKVYDWFEERLEIQAIADDIT
SKYVPPHVNIFYCLGGITLTCFLVQVATGFAMTFYYRPTVTEAFASVQYIMTEANFGWLIRS
VHRWSASMMVLMMILHVFRVYLTGGFKKPRELTWVTGVVLGVLTASFGVTGYSLPRDQI
GYWAVKIVTGVPEAIPVIGAPLVELLRGSASVGQSTLTRFYSLHTFVLPLLTAVFMLMHFP
MIRKQGISGPLMGVTKKPDLNDPVLRAKLAKGMGHNYYGEPAWPNDLLYIFPVVILGTIA
CNVGLAVLEPSMIGEPADPFATPLEILPEWYFFPVFQILRTVPNKLLGVLLMVSVPAGLLTV
PFLENVNKFQNPFRRPVATTVFLIGTAVALWLGIGATLPIDKSLTLGLFMIEVFLFGIVLGLIPI
TLAGLFVTAYLQYRRGDQLDLMLTITSYFGFLLVVLTITSALFIGLSKIRLIMDIVSLAWAAL
MVVFTFSLSLVVWGRSGLMIIRSPEPEVKILVDRDHIKTSFEEWARPGHFSRTIAKGPDTTT
WIWNLHADAHDFDSHTSDLEEISRKVFSAHFGQLSIIFLWLSGMYFHGARFSNYEAWLSD
PTHIGPSAQVVWPIVGQEILNGDVGGGFRGIQITSGFFQIWRASGITSELQLYCTAIGALVFA
ALMLFAGWFHYHKAAPKLAWFQDVESMLNHHLAGLLGLGSLSWAGHQVHVSLPINQFL
NAGVDPKEIPLPHEFILNRELLAQLYPSFAEGATPFFTLNWSKYAEFLSFHGGLDPVTGGLW
LTDIAHHHLAIAILFLIAGHMYRTNWGIGHGLKDILEAHKGPFTGQGHKGLYEILTTSWHA
QLSLNLAMLGSLTIVVAHHMYSMPPYPYLATDYGTQLSLFTHHMWIGGFLIVGAAAHAAI
FMVRDYDPTTRYNDLLDRVLRHRDAIISHLNWACIFLGFHSFGLYIHNDTMSALGRPQDM
FSDTAIQLQPVFAQWIQNTHALAPGATAPGATASTSLTWGGGDLVAVGGKVALLPIPLGTA
DFLVHHIHAFTIHVTVLILLKGVLFARSSRLIPDKANLGFRFPCDGPGRGGTCQVSAWDHV
FLGLFWMYNSISVVIFHFSWKMQSDVWGSLSDQGVVTHITGGNFAQSSITINGWLRDFLW
AQASQVIQSYGSSLSAYGLFFLGAHFVWAFSLMFLFSGRGYWQELIESIVWAHNKLKVAPA
TQPRALSIVQGRAVGVTHYLLGGIATTWAFFLARIIAVGMALRFPRFSQGLAQDPTTRRIW
FGIATAHDFESHDDITEERLYQNIFASHFGQLAIIFLWTSGNLFHVAWQGNFESWVQDPLHV
RPIAHAIWDPHFGQPAVEAFTRGGSLGPVNIAYSGVYQWWYTIGLRTNEDLYTGALFLLFL
SAISLLAGWFHLQPKWKPSVSWFKNAESRLNHHLSGLFGVSSLAWTGHLVHVAIPGSRGE
YVRWNNFLEVLPHPQGLGPLFTGQWNLYAQNPDSSSHLFGTSQGAGTAVLTLLGGFHPQT
QSLWLTDIAHHHLAIAFIFLVAGHMYRTNFGIGHSIKDLLDAHIPPGGRLGRGHKGLYDTIN
NSLHFQLGLALASLGVITSLVAQHMYSLPAYAFIAQDFTTQAALYTHHQYIAGFIMTGAFA
HGAIFFIRDYNPEQNEDNVLARMLDHKEAIISHLSWASLFLGFHTLGLYVHNDVMLAFGT
PEKQILIEPIFAQWIQSAHGKTSYGFDVLLSSTSGPAFNAGRNIWLPGWLNAINE--NSNSLF
LTIGPGDFLVHHAIALGLHTTTLILVKGALDARGSKLMPDKKDFGYSFPCDGPGRGGTCDI
SAWDAFYLAVFWMLNTIGWVTFYWHWKHITLWQGNVSQFNESSTYLMGWLRDYLWLN
SSQLINGYNPFGMNSLSVWAWMFLFGHLVWATGFMFLIFWRGYWQELIETLAWAHERTP
LANLIRWRDKPVALSIVQARLVGLAHFSVGYIFTYAAFLIASTSGKFGMSHSVKIYDTCIGC
TQCVRACPTDVLEMIPWDGCKAKQIASAPRTEDCVGCKRCESACPTDFLSVRVYLWHET
TRSMGLAYMTTFHFPSIFVPLVGLVFPAIAIASLFLHVQKNKIVMRDLKTYLSVAPVLSTLW
FGALAGLLIEINRFFPDALTLSL---MTAILERRESESLWGRFCNWITSTENRLYIGWFGVLMI
PTLLTATSVFIIAFIAAPPVDIDGIREPVSGSLLYGNNIISGAIIPTSAAIGLHFYPIWEAASVDE
WLYNGGPYELIVLHFLLGVACYMGREWELSFRLGMRPWIAVAYSAPVAAATAVFLIYPIGQ
GSFSDGMPLGISGTFNFMIVFQAEHNILMHPFHMLGVAGVFGGSLFSAMHGSLVTSSLIRE

TTENESANEGYRFGQEEETYNIVAAHGYFGRLIFQYASFNNSRSLHFFLAAWPVVGIWFTA
LGISTMAFNLNGFNFNQSVVDSQGRVINTWADIINRANLGMEVMHERNAHNFPLDLAAIE
APSTNGMGLPWYRVHTVVLNDPGRLLSVHIMHTALVAGWAGSMALYELAVFDPSDPVLD
PMWRQGMFVIPFMTRLGITNSWGGWSIT-GGTVTNPGIWSYEGVAGSHIVFSGLCFLAAI
WHWVYWDLEIFCDERTGKPSLDLPKIFGIHLFLSGVACFGFGVFHVTGLYGPGIWVSDPY
GLTGKVQPVNPAWGVEGFDPFVPGGIASHHIAAGTLGILAGLFHLSVRPPQRLYKGLRMG
NIETVLSSSIAAVFFAAFVVAGTMWYGSATTPIELFGPTRYQWDQGYFQQEIYRRVSAGLA
ENKSLSEAWSKIPEKLAFYDYIGNNPAKGGLFRAGSMDNGDGIAVGWLGHPIFRDKEGRE
LFVRRMPTFFETFPVVLVDGDGIVRADVPFRRAESKYSVEQVGVTLEFYGGELNGVSYSD
PAVVKKYARRAQLGEIFELDRATLKSDGVFRSSPRGWFTFGHASFALLFFFGHIWHGARTL
FRDVFAGIDPDLDAQVEFGAFQKLGDPTTRREVVMKTLYSLRRFYHVETLFNGTLAVTGR
DQETTGFAWWAGNARLINLSGKLLGAHVAHAGLIVFWAGAMNLFEVAHFVPEKPMYEQ
GLILLPHLATLGWGVGPGGEVTDTFPYFVSGVLHLISSAVLGFGGIYHALLGPETLEESFPF
FGYVWKDRNKMTTILGIHLILLGLGAFLLVFKALYFGGVYDTWAPGGGDVRKITNLTLSP
SILFGYLLKSPFGGEGWIVSVDDLEDIIGGHVWLGSICILGGIWHILTKPFAWARRALVWSG
EAYLSYSLGALSAFGFIACCFVCFNNTAYPSEFYGPTGPEASQAQAFTFLVRDQRLGANVG
SAQGPTGLGKYLMRSPTGEVIFGGETMRFWDLRAPWLEPLRGPNGLDLSRLKKDIQPWQ
ERRSAEYMTHAPLGSLNSVGGVATEINAVNYVSPRSWLATSHFVLGFFFFVGHLWHAGRA
RAAAAGFEKGIDRDFEPVLSMTPLNMTIALGKFTKDEKDLFDIMDDWLRRDRFVFVGWS
GLLLFPCAYFAVGGWFTGTTFVTSWYTHGLASSYLEGCNFLTAAVSTPANSLAHSLLLLW
GPEAQGDFTRWCQLGGLWTFVALHGAFGLIGFMLRQFELARSVQLRPYNAIAFSGPIAVF
VSVFLIYPLGQSGWFFAPSFGVAAIFRFILFFQGFHNWTLNPFHMMGVAGVLGAALLCAIH
GATVENTLFEDGDGANTFRAFNPTQAEETYSMVTANRFWSQIFGVAFSNKRWLHFFMLF
VPVTGLWMSALGVVGLALNLRAYDFVSQEIRAAEDPEFETFYTKNILLNEGIRAWMAAQ
DQPHENLIFPEEVLPRGNALMSGSTGERSFADIITSIRYWVIHSITIPSLFIAGWLFVSTGLAY
DVFGSPRPNEYFTESRQGIPLITGRFDPLEQLDEFSRSFMTIDRTYPIFTVRWLAVHGLAVPT
VFFLGSISAMQFIQRMATQTVETSSR-SRPKPTIVGSLLKPLNSEYGKVAPGWGTTPLMGVA
MALFAVFLSIILEIYNSSVLLDGISMNMLTLKLFVYTVVIFFVSLFIFGFLSNDPGRNPGQRE
MADTTGRIPLWIIGTVTGILVIGLVGIFFYGSYSGLGSSLMLNIFSLISICLNSALY-SSFFFGKL
PEAYAFLNPIVDVMPVIPVFFFLLAFVWQAAVSFRMTQSNPNEQNVELNRTSLYWGLLLIF
VLAVLFSNYFFNMEVNILAFIATALFILVPTAFLLIIYVKTVSQNDMETATLVAIFLSGLLVSF
TGYALYTAFGQPSQQLRDPFEEHGDMEALVYTFLLVSTLGIIFFAIFFREPPKVPTKKVKMT
LVFQLAVFALIATSSILLISVPVVFASPDGWSSNKNVVFSGTSLWIGLVFLVGILNSLIS-------
MSPQTETKASVGFKAGVKEYKLTYYTPEYETKDTDILAAFRVTPQPGVPPEEAGAAVAAE
SSTGTWTTVWTDGLTSLDRYKGRCYHIEPVPGETDQYICYVAYPLDLFEEGSVTNMFTSIV
GNVFGFKALRALRLEDLRIPPAYIKTFQGPPHGIQVERDKLNKYGRPLLGCTIKPKLGLSA
KNYGRAVYECLRGGLDFTKDDENVNSQPFMRWRDRFLFCAEAIYKSQAETGEIKGHYLN
ATAGTSEEMIKRAVFARELGVPIVMHDYLTGGFTANTSLAHYCRDNGLLLHIHRAMHAVI
DRQKNHGIHFRVLAKALRMSGGDHIHSGTVVGKLEGERDITLGFVDLLRDDFIEKDRSRG
IYFTQDWVSLPGVLPVASGGIHVWHMPALTEIFGDDSVLQFGGGTLGHPWGNAPGAVANR
VALEACVKARNEGRDLASEGNVIIREAAKWSPELSAACEVWKEIRFDFKAVDTLDPMAIH
LYKTSTPSTRNGTVDSQVKSNPRNNLIYGQHHCGKGRNARGVITTRHRGGGHKRLYRQID
FRRNKKDIYGRIVTIEYDPNRNAYICLIHYGDGEKRYILHPRGALIGDTIVSGTKVPIKMGN
ALPLTHMPLGTAIHNIEITLGKGGQLVRAAGAVAKLIAKEGKSATLKLPSGEVRLISQNCSA

TVGQVGNVGAKHKSLGKAGSKRWLGKRPVVRGVVMNPVDHPHGGGEGRAPIGRKKPT
TPWGYPALGSRSRKRNKYSENLILRRRSKMIQPQTHLNVADNSGARKLMCIRIIGAS-NRR
YAHIGDVIVAVIKEAVPHMSLEKSEVVRAVIVRTCKELKRDCGMIIRYDDNAAIVIDQKGNP
KGSRVFGAIPEELRQFGFTKILSIAPEVLMLSPKKTRFRKQHRGRMKGISSRGNYICFGRYA
LQALEPAWITSRQIEAGRRAMTRNVRRGGKIWVRIFPDKPVTARSTEARMGAGKGDPKY
WVAVVKPGRILYEMGGVTKNIARRAISIAASKMPIRTQFIISGMTRIKRGSIARRRRTKAHSF
ASKFREAHARLTRAITQQQIRALFSADRDRDKQKIDFRRLWITRINALIREK---FHNYSKFIN
DLYKSQLLLNRKILAQIAISNRNCLYMIANEIIKKVGFESAVII-MRKEVYALRRHISLSADK
ARRVIDQIRGRSYEETLMILELMPYRACSPISKLVYSAAANANSNMGSNKANLVISNAEVN
EGTTRKKFKPRARGHCYPIKRRTCHITIGVKDISLDGRVWDKKMDGIKYAVFTEKSIRLLG
KN-QYTSNVESGSTRTEIKHWVELFFGVKVIAMNSHRLPGKGRRMGPIMGHTMHYRRMII
TLQPGYSIPPLRKKRTMAVPKKRTSASKKRIRKNSWKGKGYGTALKAFSLGKSLSTGNSK
SFFVPKNSLMAKGKDARVRVILECTGC-VRN--VSR---GVSRYITQKNRHNTSKQLELRKFC
PYCHKHMIHGEIKIMKKRASVRKICQKCRLTRRRGRIRVICSNPRHKKRQGMVREKVRVS
TRTLQWKCVESREDSKRLYYGRFILSPLRKGQADTIAIGIRRALLGEIEGTCITRVKSK--NV
PHEYSTIAGIQESVHEILMNFKEIVLRSNLYGTCDASICVRGPGYVTAQDIILPPYVEIVDNT
QHIASLTEPIDLCIGLQIERNRGYLMKMPH-NSDGSY--PIDAVFMPVRNANHSIHSYGSGNE
KHEILFLEIWTNGSLTPKEALHEACRNLIDLFIPFLHKEEE--TSPLEDNH-HTVPLSPLTFHD
KAD-KLRKRKK-EIKKK---MLGDG-NEG
MSTIPGLNQIQFEGFCRFIDQGLTEELSKFPKIEDTDQEIEFQLFMERYQLVEPLRKERDAVY
ESLTYSSEFYVSAGLIWKTSRDMQEQTILIGNIPLMNSLGTSIVKGIYRIVINQILQSPGIYYR
SELDHNEISVYTGTIISDWGGRSELEIDRKARIWARVSRKQKISILVLSSAMGSNLKEILDNV
FYPEIFLSFLKDKERKKIGSKENAILEFYQQFACVGGDPVFSESLCKELQTKFFQQRCELGR
IGRRNMNRRLNLDIPQNNTFLLPRDILAAADHLIEQKFGMGTLDD--MNHLKNKRIRSVAD
LLQDQLGLALVRLEDEVRETICGPIR-HKWRTPHPQNLVTSTP----LTTTYESFFGSHPLSQV
LDQTNPLTQIVHGRKWSYLDPGGLTGRTASFRIRDIHPSHYGRICPIDTSEGINVGLIGSLAI
HAKIGHWG-SLESPFY---EISERSTG--VQMLYLSPGRDEYYTVAAGNSLALNQDIQEEEVVP
ARYRQEFLTIAWEQVHFRSILPFQYFSIGASLIPFIEHNDANRALMSSNMQRQAVPLSRPEK
CIVGTGLERQVALDSGALAIAEREGKIVSTDTEKILFSGNGDTLSIPLVMYQSSNKNTCMH
QKPQVQRGKCIKKGQILADGAATVGGELALGKNVLVAYMPWEGYNSEDAVLISERLVYE
DIYTSFHIRKYEIQT----AG-TERITNEIPHLEAHLLRNLDKNGIVMLGSWVETGAILVGKLTP
EVVKE-S---PANRLVYDIVGIQVSTSKDTCLKLPIGGRGRVIDVRWIQKRG--DNDNPETIRIY
ISQKREIKVGDKVAGRHGNKGIISKILPRQDMPYLQDGRPVDMVFNPLGVPSRMNVGQIF
ECSLGLAGGLLDRHYRIVPFDERYEQEASRKLVFSELYEASKQTTNPWVFEPEYPGKSRIF
DGRSGNPFEQPILIGKPYILKLIHQVDDKIHGRSVGNYTHITQQPLRGRAKGGGQRVGEME
VWALEGFGVAHILQEMLTYKSDHIRARQKIPGTTMIGGTIPNPEDAPESFRVLVRELRSLAL
ELNHFLVSEKNFQINRKEA-------MIDRYKHQQLRIGLVSPEQISAWATKILPNGEIVGEVTKP
SILHYKTNKAEKGGLFCERIFGPQKSGICACGNYRVIGDEKEDPKFCEQCGVEFVDSRIRR
YQMGYIKLACPVTHVWYLKRLPSYIANLLDKPLKELEGLVYRD--FSFARP-ITKKPTFLRL
RGLFEYEIKSWKYSIPLFFTIQGFDTFRNREISTGAGAIREQLVDLDLRIILDNSLVEWKELG
EE-GPTGNEWEDQKVRRRRGFLVRRMELAKHFIRTNIEPEWMVLCLLPVLPPDLRPVIQIA
EGKVMSSDITKLYQRVIYRNKILTDLLTRSVSTPGDLVTSQEKLVQEAVDALLDNGTRGQP
MRDGQNKVYKSLSDIIEGKEGRFRQTLLGKRVDYSGRSVIVVGPSLSLYRCGLPREIAIELF
QTFVIRGLIRQDPASNIKVAKSQIREKEPIVWEILQEVMQGHPVLLNRAPTLHRLGIQAFQP

VLVEGRAICLHPLVCKGFNADFDGDQMAVHVPLSLEAQAEARLLMFSHMNLLSPAIGDPI
SIPTQDMLVGLYVLTSGNRRGICVNRYNPWNH---RNYENQRSKNNNYRYT---EPLFSNSYD
AIGAYRQKRIKLDSPLWLRWPLDQR-VIAS--RESPIEVHYESLGTYYEIYGHYLIVRSIKKEI
LFLYIRTTVGHISLYREIEEAIEGFSQACSSSYGTYTKLSNMEVLMAERANLVFHNKVIDGT
AMKRLINRLIEHFGMAYTSHILDQVKALGFHQATTTSISLGIDDLLTIPSKRWLVQDAEQQS
LVLEKHHHYGNVHAVEKLRQSVEIWYATSEYLRQEMNPNFRMTDPFNPVHIMSFSGARG
NASQVHQLIGMRGLMSDPQGQMIDLPIQSNLREGLSLTEYIISCYGARKGVVDTAVRTSDA
GYLTRRLVEVVQHIVVRRTDCGTIRGISLSPRNGMMP-ERVFIQTLMGRVLADDIYIGTRCL
ATRNQDIGIGLVNRFIT------FRAQPISIRTPFTCRSASWICRFCYGRSPTHGDLVELGEAVGII
AGQSIGEPGTQLTLRTFHTGGVFTGGTAEHVRAPSNGKIKFNEDLVHPTRTRHGHPAFLCSI
DLYVTIESEDILHNVNIPPKSFLLVQNDQYVESEQVIAEICVGTST-LNFKEKVRKHIFSDSD
GE-MHWSTDVYHAPEFTYGNVHLLPKTSHLWILLGGPCR--SSRVSLSFHKDQDQMNAH--
SPSIKRRYTSNLS--ETNDP-ERQKLFSSYFYGKKKNRISDY-SNLNQILCTGHCNFISP-TTLH
QNS--DLFSKRRKNKFLIPLQSIQEPENKLMPRSSISIEIP--QNGTFRGNSILAYFDDPRYRRK
GSGIPQYGTIEMHSIVKKED--LVEYRG-VKAFRPKYMKVDRLFFIPEEVHILPRSSSIMVRN
NSLIGVDTQLTLNIRSRVGGFVRVERKKKRIELKIFSGDIHFPGETNKISRHSDVLIP-P---GK
PNSKESKK--WKNGIYVQR--ITPRK--KKFFVLVRPVITYEITDGINLATLFPPDLLQERDNVQ
LRVVNYILYENGNPIRGFSHTDSQLVRTCLVLNWDQDKKK--SASSEEARASFVEIRTNGLI
RHFLRIDLVKSPISYIGKRNDPSGSGLPSD-NGSGWTNMNPFSS--IY---SKARIQQSLNQNQG
TIHTLLNR---NTGFQSLRILSSSNCSRMGP--FNDVIKYHK--ESIPIDPFIPIKNSLGPFGT--AFP
IANFDS--FSHL--ITQNRIL-VNNYLELDN---FKQTFQVIQ---YYLMDEN--PKIYNPDLGSNIL
LNPFNWNWYFYHHTYCQETS-PIISLGQFICENVCIAKNAPH-LK--SGQVILVQ-VASVVIRS
AKPYLATPGTTVHGHYGEILYEGDTLITFLYEKSRSSDITQGLPKVEEVFEVRSIDSISMNLE
KRFKGWNKRITRILGMPWGFLIGAELTIVQSRISLVNKIQRVYRSQGVQIHNRHIEIIVRQIT
SKVLVSE---DGMSNVFSPGELIGLLRAERMGRALEEAVYYRAILLGITRASLNTQSFISEAS
FQETARVLAKAALRGRIDWLKGLKENVVLGGMIPLGTGFKGL--VPPSKQDNNSS--LETK
KKNLFEGEMR--DIFFHHRKLFDSCRSKNFHDTSEQSLLG------FHDSMARRYWNINLEEM
MVAGVHFGHPTKQWNPKMAPYIYAKRKGIHIPNLTRTARFLSEACDLVFDAASRGKEFLIV
GTKPKAAGSVVRAAIRARCHYVNKKWLGGMLTNWFTTKTRLHTFRDLRMEQKTGRLN
RLPKRDVAMLKRQLSRLQAYLGGIQYMTGLPDIVILVDQHEEYTALRECITLGIPTICLIDT
NCDPDLADLPIPANDDAKPSIRLILNKLVFAICEGRS-SYIRNPMGQKINPLGFRLGTTQGHH
SLWISQPKNYSKGLKEDQNIRDFIQNYIQKNMRISSRRVEGIARIEIKKTIDLIQIIIFMGFP---I
ENRPRKVKEKIKELQIHLEKEFHYVKKKLKIAITKIAKPYQNPKILAEFLAGQLKDRVSFRK
AMKKAIELAEQADTEGIRVQMAGRINGKDIARVEWIRRGRLPLQTIRTKIDYCCYTIRTIYG
VLGIKIWIFIDEKMPRYLGPRFKKIRRLGALPGLTNKSPKAGSDLRKQPRSRK-KSQYRIRL
EEKQKLCFHYGLREYQLLNYVRIAAKAKGPTGQVLLQLLEMRLDNILFRLGMASTIPAAR
QLVNHRHILVNGRIVDRPSYRCKPRDIIMTKDNQKSRTLIQNSLGSSPQ-KELPNHLTLHPF
Q--YKGLVNQIIDSKWVGLKINELLVVEYYSRQTMSRRGTAEEKTAKSDPFYRNRLVNMLI
NRILKHGKKSLAYQLLYRTLKEVQKKTERNPLSILREAIRRVTPDIAVKARRKGGSTRQVPI
EIGSTQGKALAIRWLLAASRKRPGGNMAFKFSSELVDASKGKGGAIRKKEETHKMAEAN
RTFAHFRMCRDTIAEILTSIRNAYMDRKRVVRIASTNITENIVKILFREGFIENVRKHREKNK
SFLILNLQWNR--KRLHKN---LFSFKLKRISRPSLRIYSNSQRIPRIFGGMGIVILSTSRGIMTD
REARLHGMGGEILCCIWMAKGTRFINFRRYGRFGSRKSVRKIPRGVIHIQTSFHNTIVTVT
DGRGQVVSWSSAGTCRFKGPRKRTPFAAQIVAADAIGPVVDQGLQRAKIKLKGTGRGRD

AALRTISKSGIGFTSVQDVTPVPHNGCRPPNKKRQMPTINQLIRNTRQSIRNVKKSPALQGC
PQRRGICTRVYTINPKKPNSATRKVARVRLTSGFEITAYIPGIGHNSQEHSSVLVRGGRVPDL
PGVKYHIVRGTLDAVGVKDRQQGRSKYGVKKPKMARKSLIQREKKRQQLEQKYHLIRRS
SKKEIS---KIPSLSYKSEFYRKLQSPPRNSAPTRLHRRCFSTGRARAHYRDFGLSGYILREM
FHACLLPGATRSSWMVRNSFISIISQ--KENKENRGSVEFQVLSFTTKIRRLTSHLKLHKTDY
SSERGLRKILGKRQRVLAYLSKKNRVRYQELISQLNIRETKTR--MIKLRLKRCGRKQRAVY
QIVAIDVRSPREGKALRKVGFYDPIKNQTDLNVRAILYFLEKGAQPTGTVQDILKKAEIDK
ELRPNQTKFN-MEKKKRPFRKKKRSFRKRRSPIESGHQIDFTNTSLLNQFISQQGKILPRKV
TRLTLKQQRFMTSAIKQARILSLLPFVFDDKKKQFQKKESTARTTNEKQTNERAR-LKKNP
FVVHHLVRKIDKLNKKKKKGIIKTWSRASTIIPTMIGHTIAVYKGKEHLPIYITDYMTGHKL
GEFAPTLYFRGHTKNDNRSQRMLFHFFLRCNLASLCIKIVNSVVVVGLYYGLLTTFSIGPSY
FFLLRAQVMEEG---TEKKVSATTGFITGQLIMFISIYYAPLHRALARPHTITVLALPYLFVYF
IFYTDKRFMRKNLNTTTSMRNFSIQCVFLNNLIFQLFNHFILPSSMVARLVNIYMFRCNNK
MLFVTSSFVGWLIGHILFWKWLGLVLVWIRQNRSIRI-KYIPKYLVSKWGDR---IFYILVILI
WFYLGGKIPAPLLSMKFDEEKED-HFATLLFDPNRWNRPFRYIKNDRFDNAFRTEMSQYFF
DTCPSAGKERISFTYPPSLSTFWEMIERRISLPTLFSSN--ELSNHWVYTNEQKSNNLKNEFV
NRIEALDKES-----LETTSRLCN--TTKEYLPEPYDPFLNGPYRRTIKKTKSLLETSRDNLETSR
DRDNLETSVFSDE---------DEKGKKYFEYENRIGTNTIAKIRKKVSRWIYNADKIIFPEHGDF
RRDVIWGSIRAQRRKIVIWRTLQRYTHSPLFLDSLKDFRHYFVDFLLDILNIFIIRNPEREPR
LWVSEFWTIIEYAQEVRGCVLLTHSFIRRNIVIPLLIIVKNIGRMLLFQRPELYEDFEDWQRE
SHVKCTYEGIPLSETDFPEDWLVEGIQIKILYPFRLKPWHVPGAEEKGDFCFLTVYGTETNL
PFGYSRLPESFFKPIFQELKKQ----------------------MRN-----SKKNIWKLGKMLFQKVKGTKF
RKVDPITSNQMIPESFS---SPSSTNSEKKRNKMQDLTNRANKARNEIERIRKEKKK------SSN
-------ALQRIKDR----LIRKLPVYLKLFIQRIY-GIFFSIINIRRMS---------YDLSYLSQAYVFYKL
SEIQLSNLDKFKSVLQYIPFFLRSEIKDSFTQGIVHS----KRLP-SEMNQWKNWLRGHSQYD
LSKTRWSILIPQRWRNRVHRMVK--RKNKRNLYEKYE--KDQFIDSDNFQKCCKYDLLSYK
SIHGDIPYPDRKYLDWKMIHF---FLDWMGLNETQTHPNLFPEIVLFYNAYKTQPWLLSSKL
LLLNTSKELQKELDAFLQRYSGYLVKWNRRVVPKSEINWEVYSYLYRLFDDENLVLSSAK
RGELAFEILATRPIQSWKKLLLKELVFFEPIRLFGKNNGQFIMYQTIGISLVHNNKHNHLDL
LVPENIFSFRRRRKLRILNCLRFLWPNYRLEDLACMNRYWFDTNNGSRFSMLRIRLYPVQ
WR----WGERLGEILREIKNSDSFFDSWTQFNSVRSFIPIFFHQERFLKLADPRIWSILLSRTQG
STQGSTSITIKGVILFKGVILFVVA-ALIYR----INNRNMVESKTLYLRGLLP-----IPMNSIGPR
NDTLEESV-GSSNINRLIVS-LLYLPKGKRISESSFLNPKESTWVLPITKK-------SSMPWGSRW
WRNWIGKKR--

-----------DSSQLKGSS-YQSRDHLDSIS----NEDSE-------------YH---NQREIPLPEER--------------
--KIEEFL--ENPTRSVRSFFSDRW--SELH-LGSNPTERSTR
DQ----KLLKKQQDLS-FVPSRRSE----KKEMVHIFKIITYLQN--TVSIHPISSDPGCDRVP-KDE
SD-MDSSNKISFLNKNPFS----------------------------EERFQEMANRFTLSITEP-DLVYPKGSRF-
--CNESIYRRIIK---KWVPRGNVLENLKRQIVVFVSNNIME
AVNQYR-IRNLIQIQYSTYG--YIRNVLNRFFLMN---RSDRNFEYGIQR---DQIGKGTLNHRTL
MKYMINQHLSNFFL-KKSP-FDS--LIVIE---KSKNRDPFAYRYKWSNGSKNFQEHLEHFLSE
QKS--------------------------------RLEIAFDQ-----------------------FQRNYDK-PLPLLLESIA----------
-------------------SIGLQIVHLKKWKPF--LLD---DHDTS-RK-----------------------MIDSFHTR--NN

RRKSFEKTDSYFSMIFDNQDNWLNPVKPFHRSSLISSFFKANRLRFWNNPHHFCFY--SNTR
FPFSVEK----ARISNYD--FTYG-QFLNILFIRNK-IFSLCVGK--KKHAFGG-RDTISPIESQVSNI
FIPNYFPS----------------------------RTIYSIADISGTPLTEEEIVNLERTYCQPLSDRNLSDSEGK
NLNQYLN--FNSSMRLIP---TEKDFPWKSQKRKKQSPCLPCLLCLCLK--KGVEKGEMDRR--
-DSAFSFLSWKDLFQTYMPWFLTSTGYKHLN-IFLEPFSDLLPISLSSS-SLKFVSTFHDSMHE
VGISWQILQIELQLRLFHLSHRLNKWISEISRKCMYS--RLLLEATIHGKNESPLIPTHLRS--V
NVREL-LYSILFLLLLAGY-LVHTHLVFVSR--AFSELQTEFKKLKPFLIPSSRMELEELLDAYP
TSEP------NPFEFELVKSIR------------SKKK----------------------YWNIN---LIDFIPHR-------ITLS
RKMSHLSHTSKELYSLIRKR-NIKNANEDGKIDSWVVNSDLFVEEEFFHLLYDRTFMLTT--
KKSIDPILWSLT-HSDHLSTH-LSKNDSGYQMIEQPGAIYLRYLVDIH--KKSLWNYEFNTSCL
AEKRVFLAHYQTITYS--QTSCGTNTFHFPSHG---KPFSLRL-LSPS-RGVLVIGSIGTGRSYLV
KHLATNSHLPFIRLAVSELLNNNPKFRWIEP--EDSWP-IDASDDIDIDIVAR-------------------G
DIDFDKEEELLTRESDEEKKNPVRVQEKYR---EQFEEDLELAKIISPCIIWIPNIHDLNLREST
SLAAGLFVNKYS------ERCSTR-DILVIASTHIPQKVDPSLISTNRLNRCIKVRRFFIPQKEKH
VFTLLYTRGFHPHLEK-----NAFGSITLGSSAQDLRPLINDALLIGISQRKSIIDTNTIRCALHR
ETWEFQSH-PKWVPNSQ--ILFYQIGRAVVQKVLLSNCLIDPLSIYMKKR--SFAEGDSSLYK
WYFELGTSLKLVTILLYLLSCSAGSVARDLCFLP------GEDRIKLFEN-EMDS-TLFHEVLALE
SPLMGSS--RTEKDCSQFDND--RVPFPFRPEPRK-LLAIQRFLSEKEEWELAEGKE------------
EGLLQPP-------QE----IEGH--IVSAP--KIWRP---WFCLLDGLDRPVEFLEL-GYPYCYGYR-G
KQILFDKEDELEENDSEFLQSFIPRS------SDEHGFFKLNQFIWDPPDPLFLLST-DEFPGSVFS
YRELFADEELR---------KLRTFRTRWLS--KKAQKGRLELLINRQRWLRP---NSDESEDS--LYS
Q--ILSEIFEYLKNLFLSKR-GLLDQTRKVLFRKRWVFPDEMHIFF--M---------MPRSR-INGN
FIDKTFSIVANILLRIIPTTSGEKEAFTYYRDG--ISAQSEGNYAEALQNYYEAMRLEIDPYDR
SYILYNIGLIHTSNGEHTKALEYYFRALERNSFLPQAFNNMAVICHY-RGEQAIRQGDSEIAE
AWFDQAAEYWKQAIALTPGNYIEAHNWLKITRRFE--MSWRSEDIWIELLTGSRKLSNFCW
ALILFLGSLGFLLVGTSSYLGRNWISFVPSQQFIFFPQGIVMSFYGIAGLFISSYLWCTISWNI
GSGYDRFDRKEGIVCIFRWGFPGKNRRIFLRFLIKDIQSVRIEVKEGLYARHVLYMDIRGQG
AIPLTRTDEN-LTPREIEQKAAELAYFLRVPIEVF
>Pelargonium
???
???
???
???
???
??????????????????????????????MVPIRADEIRNLIRERIEQYNREVKIVNTGTVLQVGDGIA
RIYGLNEVMAGELVEFEEGTIGIALNLESNNVGVVLMGAGLMIQEGSSVKATGRIVQIPVS
EAYLGRVINALAKPIDGRGGISASESRLIESPAPGIISRRSVYEPLQTGLIAIDSMIPIGRGQRE
LIIGDRQTGKTAVATDTILNQQGKNVICVYVAIGQKASSVAQVVTTFQEKGAMEYTIVVAE
TAASPATLQYLAPYTGAALAEFFMYRERHTLIIYDDLSKQAQAYRQMSLLLRRPPGREAYP
GDVFYLHSRLLERAAKSSSSLGEGSMTALPIVETQSGDVSAYIPTNVISITDGQIFLSADLFN
SGIRPAINVGISVSRVGSAAQIKAMKQVAGKSKLDLAQFTELEAFAQFATDLDKTTQSQLA
RGRRLRELLKQSQADPLAVEEQIMTVYTGINGYLDSLEIGQVRKFLEDLRKYLKGNKPKF
QEIISSTKTFTEEAEALLKEAIQEQTELFILKEQGMRLNPTASSPGDSTLQKKKLGRIVQIIGP
VLDVAFPRGKMPYIYNALVVQGRDTVGQQIHVTCEVQQLLGNNRVRAVAMSATDGLMR

GMEVIDTGAPLSVPVGGATLGRIFNVLGEPVDNLGPVDNRTTSPIHKSPPAFIELDTKLSIFE
TGIKVVDLLAPYRRGGKIGLFGGAGVGKTVLIMELINNIAKAHGGVSVFGGVGERTREGN
DLYMEMKESGVINEQKVAESKVALVYGQMNEPPGARMRVGLTALTMAEYFRDVNKQDV
LLFIDNIFRFVQAGSEVSALLGRMPSAVGYQPTLSTEMGSLQERITSTKKGSITSIQAVYVPA
DDLTDPAPATTFAHLDATTVLSRGLAAKGIYPAVDPLDSTSTMLQPRIVGNEHYEIAQRVK
QTLQRYKELQDIIAILGLDELSEEDRLTVARARKIERFLSQPFFVAEVFTGSTGKYVGLSETI
RGFHLILSGEFDGLPEQAFYLVGNIDEATAKAMKLEKESNLKKMTLNLCVLTPNRILWDSE
VKEIILPTNSGQIGVLPNHASIVTTIDIGVLRIRFNDQWFTIALMGGFTRIRNNEITILATAAE
KGSDIDPQEAQQTLQIAEDSLRKAEDKRQRIEAKLAIKRASTRVKAINPISMKNVTDSFVSL
GRWPSAGSFGLNTDILATNPINLSVVLGVLIFFGKGVLNDLLDNRKQRILNTIRNSEELRGG
AIEQLEKARARLRKVKTEAARFRVNQYSEAEREKLNLINLTYKSLEDFENYKNDSIRFEQQ
RAIHQVRQRVFQQALRGALETLNSCLNKELHLRTISANIRLFRSMKELTNMNPLISAASVIA
AGLAVGLASIGPGIGQGTAAGQAVEGIARQPEAEGKIRGTLLLSLAFMEALTIYGLVVALAL
LFANPFVMNILSCSINTFKELYDISGVEVGQHFYWQIGGFQVHGQVLITSWVVIAILLGSSIL
AVRNPQTIPTDVQNFFEYVLEFIRDVSKTQIGEEYGPWVPFIGTLFLFIFVSNWSGALFPWK
IIQLPQGELAAPTNDINTTVALALLTSVAYFYAGLTKKGLAYFNKYIQPTPILLPINILEDFTK
PLSLSFRLFGNILADELVVVVLLSLVPLVVPIPVMFLGLFTSGIQALIFATLAAAYIGESMEG
HHMIFLTLEHILTHISFSVVSIVITIHLINLLVNEIVGLYNSSEKGMIVTLFCITGFLIIRWIYSG
HFPLSNLYESLIFLSWNFSIINMLPYLKNLKNHLREITSPSTLFIQGFATSGLLTQIHEQKILVP
ALQSQWLMMHVSMMILSYASLLCGSLLSIALLVITFRKSRSVVGKRNNIQYINERE---IKTS
FTYSINYYRSHLLQQFDFWSYRIISIGFLFLTIGILSGAVWANEAWGSYWSWDPKETWAFIT
WTIFAIYLHIREKKNAEGVKSAIVASIGFLIIWICYFGINILGIGLHSYGSFPISKMKKKRTTPF
LYLASIVFLPWWISLFFKKSLESWVTNWLNISQSEPFLNDIQEKSLIEKFIELEELLLLDEMI
KECPKLDLQKFRIGIHKETIQLMNMYNDDRIHTILHFSTNIISFVILSVCSILGNEELVILNSW
IKEFLYNLSDTLKAFCLLALTDFLFGFHTISAWELLIGSVVHN------DQMRSLLVCLFPSVLH
TIYYFWTFNYLNCVSPSLVVLYDSLVDMPVGVPKVPFLNPNEEKATWVDLYNRLYRQRW
LFLGKDLEEEVANNIVGLMIHLNIEDPFWTQTLYINCLGGLIIPGLALYDTIGFVEPDVKTIC
LGIAASMASVVLVGGTVTERCAFPNARVMIHQPRAKEFDDRFSDLNLEGHLFLQLRNCVT
Q I Y I H R T N K P A W V I I A D L E R D T F M S A T E A R T Y G I I D G V Y A
F??MEEFQG
YLELDRSRQHDFLYPLIFREYIYGLAHGHGLVDYDNKYSFLIVKRLIARMYQQNHFHICEN
DSALNQFIGKNLYSQMISEGFADIVEISLFIQLVSYLTSKSLNLRSIHSIFPFLEDECSHLNYAS
DILIPHPTHLEILVEVLRYSVKDASFLHFLRFLLYEYW--SWKSLKASSIVSKERFFVFLYNSH
VCEYESILLFLRNQSSHLRSTSFGILLERIFFYKKIGGLVAIFGTEHILRFLKDPLIHYVRYQG
KSILISKDRPLLMNKWKYYLVKLWQCHFYVWSQPGRIHINQLSQHSLHLLGYLLSVGTNP
LVVRSQVFDNSFLIDN--AMNKLETRIPLFSLIGSLTKAQFCNPLGQPISKSTWADSSDSDIID
RFVRMCRNLSHYYSGSSKKQSLYRLKYILRLSCVKTLARKHKSTVRAFLKKLGSELLEEFI
KEEEQVLSLLFPRRIWYFDILCINDLANHEMIIDTP-EVQDIN-SFSRFES-LQEIYGIVWMLV
PILTLVVGITIGVLVIVWLEREISAGIQQRIGPEYAGPFGILQALADGTKLLLKENLLPSRGDS
PLFSIGPSMAVISILLSYSVIPFSYRLVLADLNVGVFLWIAISSIAPIGLLMSGYGSNNKYSFL
GGLRAAAQSISYEIPLTLCVLSISLLSNSSSTVDIIEAQSKYGFWGWNLWRQPIGFLIFLISSL
AECERLPFDLPEAEEELVAGYQTEYSGIKFAFFYITSYFNLLVSSLFVTILYLGGWNMSIPYI
DFL-LFEINNVGGIFRTTTELFITLAKTFFFLFISITTRWTLPRLRMDQLLNLGWKFLLPISLG
NLLLTTSSQLLSL----------ILDSTRIFMKAFHLLLFDGSLIFPECILIFGLILLLMIDSTSDQKDI

PWFYFISSTSLVMSITALLFRWREEPMISFSGNFQTNHFNEIFQFLILLCSTLSIPLSVEYIECT
EMAITEFLLFILTATLGGMFLCGANDFITIFVAPECFSLCSYLLSGYTKKDVRSNEATMKYL
LMGGASSSILVHAFSWLYGSSGGEIELQEIVNGLINTQMYNSPGISIALIFITVGIGFKLSPAP
SHQWTPDVYEGSPTPVVAFLSVTSKVAASASATRIFDIPFYFSSNEWHLLLEILAILSMILGN
LIAITQTSMKRMLAYSSIGQIGYVIIGIIVGDSNGGYASMITYMLFYISMNLGTFACIVLFGL
RTGTDNIRDYAGLYTKDPFLALSLALCLLSLGGLPPLAGFFGKLHLFWCGWQAGLSFLVSI
GLLTSVLSIYYYLKIIKLLMTGRNQEITPHVRNYKRSPLRSKNSIELSMIVCVIASTIPGISMN
PIIAIAQDTLFMFLLYEYDLFWVFLIISSLIPILAFLISGVLAPISEGPEKLSSYESGIEPMGDA
WLQFRIRYYIFALVFVVFDVETVFLYPWAMSFDVLGVSVFIEALVFVLILIVGSVYAWRKG
ALEWSTNDFPWLTLIVIFPISAGSFLFFLPHRGNKVTKWYTLCVCSLELLLTTYTFCYHFQL
DDPLIQLVEDSKWIFFFHFDWRLGIDGLSFGPILLTGFITTLATLAAWPVTRDSRLFHFMML
AMYSGQIGSFSSQDLLLFFMMWELELIPVYLLLSMWGGKKRLYSATKFILYTAGSSIFLLIG
VLGIGLYGSNEPTFNFEISAHRSYPAALEIIFYIGFLIAFAVKSPIIPLHTWLPDTHGEAHYSTC
MLLAGILLKMGAYGLVRINMELLPHAHSIFAPYLIIVGAIQIVYAASTSLGQRNLKKRIAYS
SVSHMGFIIIGIGSITDAGLSGALLQIISHGFIGAALFFLAGTSYDRIRLGFFDEMGGMAVAIP
KIFTMFSILSMASLALPGMSGFVAELIVFFGIITSQKFLLMPKILITFVMAIGMILTPIYSLSML
RQMFYGYRLFNAPNPSFFDSGPRELFVSISILLPVIGMGIYPDFLLSLSVDKVELIVSQFFYR
MILEHVLVLSSFLFSIGIYGLITSRNMIRALMSLELMLNGVNLNFVTFSDFFDSRQLKGDIFS
IFVIAIAAAEAAIGPAILSSIYRNKKSIRINQSNLLNKMEHIYQYSWIMPFIPLPVPMLIGVGL
LLFPTTTKHLRRIWAFPTLFLLGLVMLLSAYFFILQLNKSYIYQYVWSWTINNDFSLEFGHL
VDPLTSIMSILITTVGITVLIYSEKYMFHDQGYLRFFSYMTLFHVSMLGLVTSSNLIQIYICW
EFVGMFSYLLIGFWFTRPVAANACQKAFVTNRVGDFSFLLGILGLYWITGSFEFRDLFEIFY
N-EIPFFFLTLCAFLLFGGALAKSAQFPLHVWLPDAMEGPTPISALIHAATMVAAGIFLVARL
LPLFIVMPYILNLISFIGIITVFLGATLALSQKDIKRGLAYSTMSQLGYMMLALGMGSYRAA
LFHLITHAYSKALLFLGAGSIIHSMEAFVGYSPEKSQNMVLMGGLIKHMPITKTSFLLGTLS
LCGIPPLACFWSKDSILNDSWLYSPIFAIIACSTTGFTAFYMFRIYLLTFEGHLNVHFRNYSG
KRSNSFYSISLWGNE-GPKTLQKFKTLLTMNFFCEAKN-TLPYPHESDNTMLFPMVLLVLFT
LFVGAIG--FNLDILSKFLTPSINLLHEKSN-SVDW-EFIPNATLSVSLSYFGIVIASVLYKPNYL
SLTKLNLLNLFVIFGDRIRNRIYDWSYNRGYIDTFYAISLARGLRGFAEFTHFFDARVIDGIT
NGMGILSFFLGEGIKCVGGG-RLSSYLLLYFDCVLLFLFISSMDLPGPIHDFLLVFLGSGIILG
GLGVVLLTNPIYSAFSLGFVFICISLFYILSNSYFVAAAQLLIYVGAINVLILFAVMFMNGSE
YYKDFHLWTLGDGLTSLICTILFLSLITTILDTSWYGILWTTKSNQIIEKDFISNSQQLGILLS
TDFFLPFELMSIILLVALLGAIAAARQMTIPATRKDLMIVNMGPHHPSMHGVLRLILTLDGE
DVIDCEPVLGYLHRGMEKIAENRTIIQYLPYVTRWDYLATMFTEVITVNAPEQLGNIQVPK
RAGYIRTIMLELSRIASHLLWLGPFMADIGAQTPFFYIFRERELIYDLFEAATGMRMMHNY
FRIGGVAADLPHGWIDKCLDFCNYFLTGLAEYEKLITRNPIFLERVEGIGIIGKEEAISWGLS
GPMLRASGMQWDLRKIDNSECYKKFDWEVQWQKEGDSLARYLVRIGEMRESIKMIQQA
LEGIPGGPYENLEIRCFDRERYPEWGGFEYRFISKKPSPTFELPKQELYVRVEAPKGELGVF
LIGDRSSFPWRWKIRPPGFMNLQILPQLVKRMKLADIMTILGSIDIIMGEVDRMFPMVTGF
MNYGQQTVRAARYIGQSLIITLSHSNRFPVTIQYPYEKLMTSERFRGRIHFEFDKCIACEVC
VRVCPIDLPVVDWKFERDIRKKRLLNYSIDFGICIFCGNCVEYCPTNCLSMTEEYELSTYD
RHELNYNQTALGRLPMPVIDDYTIRTIFNL----------------MQGPLSAWLVKRRLVHRSLGFD
YQGIETLEIKSEDWHSVAVILYVYGYNYLRSQCAYDVAPGGLLASVYHFTRIEYGIDQPEE
VCIKVFLARSNPRIPSIFWVWKSADFQERESYDMLGISYDNHPRLKRILMPESWIGWPLRK

DYIAPNFYEIQDAH---MNSVEFPLLDRRTKNS
VISTTSNDLSNWSRLSSLWPLLYGTSCCFIEFASLIGSRFDFDRYGLVPRSSPRQADLVLTAG
TVTMKMAPSLVRLYEQMPEPKYVIAMGACTITGGMFSTDSYSTVRGVDKLIPVDVYLPG
CPPKPEAVIDAITKLRKKISREIYEDRVRS--VNRYFTTNHKFLIGRSIHIGNSDKGFLYQPPAT
SKLPAEKFFKYKSSAPSHELVNMQTRKTLSWIKEEITRSISLSLMLSIITHASLSNAYPIFAQQ
GYENPREATGRIVCANCHLANKPVDIEVPQTVLPDTVFEAVVRIPYDMQIKQVLANGKKG
ALNVGAVLILPEGFELAPPDRISPEMKEKIGNLPFQSYRPTQKNILVIGPVPGQKYSEIAFPIL
SPNPETNKDVHFLKYPIYVGGNRGRGQIYPDGSKSNNTVSNATAAGIVSKIIRKERGGYEIT
IADASNGRQVVDIIPPGPELLVSEGESLKVDQPLTSNPNVGGFGQGDAEIVLQDPLRVQGL
LFFLTSVLLAQIFLVLKKKQFEKVQLSEMNFMSKVYDWFEERLEIQAIADDITSKYVPPHV
NIFYCLGGITLTCFLVQVATGFAMTFYYRPTVTEAFASVQYIMTEANFGWLIRSVHRWSAS
MMVLMMILHVFRVYLTGGFKKPRELTWVTGVVLAVLTASFGVTGYSLPRDQIGYWAVKI
VTGVPDAIPVIGSPLVELLRGSASVGQSTLTRFYSLHTFVLPLVSAVFMLIHFLMIRKQGISG
PLMGVTKKPDLNDPVLRAKLAKGMGHNYYGEPAWPNDLLYIFPVVILGTIACNGGLAVL
EPSMIGEPADPFATPLEILPEWYFFPVFQILRTVPNKLLGVLLMVSVPAGLLTVPFLENVNKF
QNPFRRPVATTVFLVGTLVALWLGIGATLPIEKSLTLGLFMIEVFLFGIVLGLIPITLAGLFVT
AYLQYKRGDRLGLMLTITSYFGFLLVALTITSALFIGLSKIRLIMDIVSLAWAALMVVFTFSL
SLVVWGRSGLMIIRSSEPEVKILVDRDPIKTSFEEWARPGHFSRTIAKGPDTTTWIWNLHAD
AHDFDSHTSDLEEISRKVFSAHFGQLSIIFLWLSGMYFHGARFSNYEAWLSDPTHIGPSAQ
VVWPIVGQEILNGDVGGGFRGIQITSGFFQIWRASGITSELQLYCTAIGALVFAALMLFAG
WFHYHKAAPKLAWFQDVESMLNHHLAGLLGLGSLSWAGHQVHVSLPINQFLNAGVDPK
EIPLPHEFILNRDLLAQLYPSFSEGATPLFTLNWSKYAEFLTFRGGLDPVTGGLWLTDIAHH
HLAIAILFLIAGHMYRTNWGIGHDLKEILEAHKGPFTGQGHKGLYEILTTSWHAQLSLNLA
MLGSLTIVVAHHMYSMPPYPYLATDYGTQLSLFTHHMWIGAFLIVGAAAHAAIFMVRDY
DPTTRYNDLLDRVLRHRDAIISHLNWVCIFLGFHSFGLYIHNDTMSALGRPQDMFSDTAIQ
LQPVFAQWIQNTHALAPGSTAPGATTGTSLTWGGVDLVAVGGKVALLPIPLGTADFLVHHI
HAFTIHVTLLILLKGVLFARSSRLIPDKANLGFRFPCDGPGRGGTCQVSAWDHVFLGLFW
MYNAISVVIFHFSWKMQSDVWGSISEQGVVTHITGGNFAQSSITINGWLRDFLWAQASQV
IQSYGSSLSAYGLFFLGAHFVWAFSLMFLFSGRGYWQELIESIVWAHNKLKVAPATQPRAL
SIVQGRAVGVTHYLLGGIATTWAFFLARIIAVGMALRFPRFSQGLAQDPTTRRIWFGIATAH
DFESHDDITEERLYQNIFASHFGQLAIIFLWTSGNLFHVAWQGNFEAWVQDPLHVRPIAHAI
WDPHFGQPAVEAFTRGGALGPVNIAYSGVYQWWYTIGLRTNEDLYTGALFLLFLSAISLIA
GWLHLQPKWKPSVSWFKNAESRLNHHLSGLFGVSSLAWTGHLVHVAIPASRGEYVRWN
NFLDVLPHPRGLGPLFTGQWNLYAQNPDSNSHFFGTSQGSGTAILTLLGGFHPQTQSLWLT
DIAHHHLAIAFLFLVAGHMYRTNFGIGHSIKDLLEAHIPPGGRLGRGHKGLYDTINNSIHFQ
LGLALASLGVITSLVAQHMYSLPAYAFIAQDFTTQAALYTHHQYIAGFIMTGAFAHGAIFFI
RDYNPEQNEDNVLARMLDHKEAIISHLSWVSLFLGFHTLGLYVHNDVMLAFGTPEKQILI
EPIFAQWIQSAHGKTSYGFDVLLSSTNSPAFIAGRSIWLPGWLNAINE--NSNSLFLKIGPGD
FLVHHAIALGLHTTTLILVKGALDARGSKLMPDKKDFGYSFPCDGPGRGGTCDISAWDAF
YLAVFWMLNTIGWVTFYWHWKHITLWQGNVSQFNESSTYLMGWLRDYLWLNSSQLING
YNPFGMNSLSVWAWMFLFGHLVWATGFMFLISWRGYWQELIETLAWAHERTPLANLIRW
RDKPVALSIVQARLVGLAHFSVGYIFTYAAFLIASTSGKFGMSHSVKIYDTCIGCTQCVRA
CPTDVLEMIPWDGCKAKQIASAPRTEDCVGCKRCESACPTDFLSVRVYLWHETTRSMGLA
YMTTLNLPSILVTLVGLVFPAFAMASLFLHVQKNKILMRDLKTYLSAAPVLSTIWFGALAG

LLIEINRFFPDALTFPFFSFMTAILERRESESLWGRFCNWITSTENRLYIGWFGVLMIPTLLTA
TSVFIIAFIAAPPVDIDGIREPVSGSLLYGNNIISGAIIPTSAAIGLHFYPIWEAASVDEWLYNG
GPYELIVLHFLLGVACYMGREWELSFRLGMRPWIAVAYSAPVAAATAVFLIYPIGQGSFSD
GMPLGISGTFNFMIVFQAEHNILMHPFHMLGVAGVFGGSLFSAMHGSLVTSSLIRETTENE
SANEGYRFGQEEETYNIVAAHGYFGRLIFQYASFNNSRSLHFFLAAWPVVGIWFTALGIST
MAFNLNGFNFNQSVVDSQGRVINTWADIINRANLGMEVMHERNAHNFPLDLAAVEAPST
NGMGLPWYRVHTVVLNDPGRLLSVHIMHTALVAGWAGSMALYELAVFDPSDPVLDPMW
RQGMFVIPFMTRLGITNSWGGWSIT-GGTITNSGIWSYEGVAGAHIVLSGLCFLAAIWHWV
YWDLEIFSDERTGKPSLDLPKIFGIHLFLSGVACFGFGAFHVTGLYGPGIWVSDPYGLTGRV
QSVNPAWGVGGFDPFVPGGIASHHIAAGTLGILAGLFHLSVRPPQRLYKGLRMGNIETVLS
SSIAAVFFAAFVVAGTMWYGSATTPIELFGPTRYQWDQGYFQQEIYRRIGAGLAENQSLSE
AWSKIPEKLAFYDYIGNNPAKGGLFRAGSMDNGDGIAVGWLGHPVFRDKEGRELFVRRM
PTFFETFPVVLVDGDGIVRADVPFRRAESKYSVEQVGVTVEFYGGELNGVSYSDPATVKK
YARRAQLGEIFELDRAALKSDGVFRSSPRGWFTFGHVSFALLFFFGHIWHGARTLFRDVFA
GIDPDLDAQVEFGAFQKLGDPTTKRQAVMKTLYSLRRFYPVETLFNGTLALAGRDQETTG
FAWWAGNARLINLSGKLLGAHVAHAGLIVFWAGAMNLFEVAHFVPEKPMYEQGFILLPH
LATLGWGVGPGGEVIDTFPYFVSGVLHLISSAVLGFGGIYHALLGPETLEESFPFFGYVWK
DRNKMTTILGIHLILLGIGAFLLVFKALSFGGVYDTWAPGGGDVRKITNLTLSPSVLFGYLL
KSPFGGEGWIVSVDDLEDIIGGHVWLGSICILGGIWHILTKPFAWARRALVWSGEAYLSYS
LGALSVFGFIACCFVWFNNTAYPSEFYGPTGPEASQAQAFTFLVRDQRLGANVGSAQGPT
GLGKYLMRSPTGEVIFGGETMRFWDLRAPWLEPLRGPNGLDLSRLKKDIQPWQERRSAE
YMTHAPLGSLNSVGGVATEINAVNYVSPRSWLATSHFVLGFFLFVGHLWHAGRARAAAA
GFEKGIDRDFEPVLSMTPLNMTIALGKFTKEENDLFDIMDDWLRRDRFVFVGWSGLLLFP
CAYFALGGWFTGTTFVTSWYTHGLASSYLEGCNFLTAAVSTPANSLAHSLLLLWGPEAQG
DLTRWCQLGGLWTFVALHGAFGLIGFMLRQFELARSVQLRPYNAIAFSGPIAVFVSVFLIY
PLGQSGWFFAPSFGVAAIFRFILFFQGFHNWTLNPFHMMGVAGVLGAALLCAIHGATVEN
TLFEDGDGANTFRAFNPTQAEETYSMVTANRFWSQIFGVAFSNKRWLHFFMLFVPVTGL
WMSALGVVGLALNLRAYDFVSQEIRAAEDPEFETFYTKNILLNEGIRAWMAAQDQPHEN
LIFPEEVLPRGNALMSGSTGERSFADIITSIRYWVIHSITIPSLFIAGWLFVSTGLAYDVFGSP
RPNEYFTESRQGIPLITGRFDPLEQLDEFSRSFMTIDRTYPIFTVRWLAVHGLAVPTVSFLGSI
SAMQFIQRMATQSVEDSSRSG-PRRTNVGSLLKPLNSEYGKVAPGWGTTPLMGVAMALFA
IFLSIILEIYNSSVLLDGILMNMLTLKLFVYTVVIFFVSLFIFGFLSNDPGRNPGREEMADTT
GRIPLWIIGTVAGIPVIGLIGIFFYGSYSGLGSSLMINIFSFICIYLHSALYSSSFFFGKLPEAYA
FLNPIVDVMPVIPLFFFLLAFVWQAAVSFRTTQSNPNEQNVELNRTSLYWGLLLIFVLAVLF
SNYFFNMEVNILAFIATALFILVPTAFLLIIYVKTVGQSDMETATLVAISISGLLVSFTGYALYT
AFGQPSQQLRDPFEEHGDMEALVYTFLLVSTLGILFFSIFFREPPKVPTQK--MTIAFQLAVF
ALISTSLILLIGVPVVFASPEGWSSNKNIVFSGTSLWIGLVFLVGILNSLIS-------MSPQTETK
ASVGFKAGVKDYKLTYYTPDYETKDTDILAAFRVTPQPGVPPEEAGAAVAAESSTGTWTT
VWTDGLTSLDRYKGRCYHIEPVAGEENQYIAYVAYPLDLFEEGSVTNMFTSIVGNVFGFK
ALRALRLEDLRIPTAYVKTFQGPPHGIQVERDKLNKYGRPLLGCTIKPKLGLSAKNYGRAV
YECLRGGLDFTKDDENVNSQPFMRWRDRFLFCTEALYKAQTETGEIKGHYLNATAGTCE
EMIKRAVFARELGVPIVMHDYLTGGFTANTSLAHYCRDNGLLLHIHRAMHAVIDRQKNHG
IHFRVLAKALRMSGGDHIHSGTVVGKLEGERDITLGFVDLLRDDFIEKDRSRGIYFTQDW
VSLPGVLPVASGGIHVWHMPALTEIFGDDSVLQFGGGTLGHPWGNAPGAVANRVALEACV

QARNEGRDLAREGNEIIRKACKWSRELAAACEVWKEIKFEFEAMDTL--MAIHLYKTSTPS
TRKRAVDSQGKSNPRNHLIYGQHRCGKGRNARGIITAGHRGGGHKRLYRQIDFRRNENNI
YGRIVTIEYDPNRNAYICLIHYGDGEKRYILHPRGARIGDTIVSGTEVPIKMGNALPLTNIPL
GTAIHNIEITLGKGGQLARAAGAVAKLISKEGKSAAVKLPSGEVRLISQNCSATVGQVGNV
GVNRKSLGRAGSKRWLGKRPVVRGVAMNPVDHPHGGGEGKAPIGRKKPATPWGRPALGI
RTRKRKKYNDNLILRRRSKMIQSQTLLNVADNSGARKLMCIRIVGAS-NQRYAHIGDVIVA
VIKEAKPTSALKRSEVIKAVIVRTCKEFKCDDGMIIRYDDNAAVVINKDRNPVGTRIFGAIP
LELHQN-FDKIVSLAREVIMLSPKKTKFRKEHRGRMKGRSSRGSRISFGKYALQALEPAWL
TSRQIEAGRRAMTRNVRRGGKIWVRIFPDKPVTVKPTQTRMGSGKGSPEYWVAVVKPGRI
LYEMGGVAENIARKAIEIAASKMPIRTQFIISKMTRIKRGSIARRRRAKTRLFASGARGAHS
RLTRLIAQQTIRALASAHRDRDKKKRDFRRLWITRINGVIRQN-VLSYSYSQLITNLKKEQL
LLNRKMLAQIAISNRNCLSIIYSAILETKEEGQK----MIKEVSALGKYIPMSTHKARRVIDQI
RGRSYEETLMILELMPYRACYPILKLISSAAANGIHNMNFDETSLIISKAEVNEGPTAKKFK
PRAKGQSYPIKRSTCHITIVLRDISLN-------MDGIKYAVVTDKSLRLVSKN-QYTFNVESGST
RTEIKSWVELFFGVKVIAMNSHRLPGKRRR----MGHTMHYRRMIITLQPGYSIPSLINLIIMT
VPKKRLSSSKKRIRKNIWKGKGHWAALKALSLGKSLSTGNSKSFFTDNKKTMAKGKDNR
GKVTLECTGC-VQN--VNNKKKGIYRYLTQKNPKNTPSPLELRKFCPYCNKHTIHGEIKEM
K I R A S V R K I C T K C Q L I R R K G R I R V I C S N P R H K Q R Q
R???
??
??
??MLGD
RN-EGMATIPALNQIQFEGFCRFLDQGLIEELSQFPFLEYRDEEIDFQLFSKTYHLVKPLIKER
DARYESLTYSSKLYVSAGFLWKD--RPQKKTICIGNLPLMNSVGTFIVNGISRVVINQILQSP
GIYYGSELDYQGFTVYIATIISDWGGRLKLEIDKKERIWARVSRKQKISILVLSSAMGSNLK
EILENACYPEIFLALNLKKECG-FKEKKYAILELYAKYKDFTEDSLDFDFLCETLRKTFFSHK
CELGRVGRQNINRRLNLDISHKNTFLLPQDILAATNHLIGMKFGMGTLDD--MNHLKNKRI
RSVADLLQDQFGLALNRLEDAVCDTIGRAIA-KDKI-PTLRHLATSTP----LTTTYESFFKLHP
LSHVFDETNPLTHIVHGRKWSFLGPGGLTERTARFRVRDIHPSNYGRICPIDTSEGIKVGLIG
YLATHARIGDCG-SLESPFY---EMSEKSKR--VRMLSLSSRKEEYYMIGTGNSLALNQGIQEE
QIIPARYRQEFLTIAWEEVHFRSVFPFQYFSIGVSLIPFLEHNDATRALMSSNMQRQAVPLSR
SEKCIVGTGFEGQVALDSGIPLITEHAGKIISTHTDKILLSVNGETLSSPLVLFQRSNKNTWI
HQTPQVSRGNCTKKGQILADGAATVGGELALGKNVLVGYMPWEGYNFEDAVLISERLVY
EDIYTSFHIRKCEIKIFMTMKG-PEKITNQIPHIDPHLLRNLDKNGVVILGSWVEAGDILVGK
LTPELVELS----PEKRLIEALFDAPIGTTRQSCLKLPIWGRGRVIDVRWIHKGSASRS---ERIRV
YILQKRKIKVGDKVAGRHGNKGIVSKILPIQDMPYLQDGRPLDMVFNPLGVPSRMNVGQI
FECSLGLAGGLLDRHYRIAPFDERYEEEASRKLVFSELYEASKQTGNPWVFEPEYPGKSIIF
DGRTGDPFEQPVIVGQSYILKLIHQVDDKIHGRSTGPYSRVTQQPVKGRARRGGQRVGEM
EVWALQGLGVAHILEEILTYKSDHLTTRNQIFGTLLLGGALPKPEPRPESFRLLVRELRSLSL
ELNYFLVSKNKWKMNRVEV-------MIDKYKHHQLRIGLVSPQQIMAWANKTLPTGEIVGEV
KNYRTFSYDTYKPERDGLFCERIFGPIKSGVCACGESRKIGDEKEKRTFCEKCGVEFVDSRI
RRYQMGYIKLASPMAHVWYLKRIPSYIANLLDEKIKDLENLVYRD--FIFAGP-LTKKPTLL
RLRGSFKTKFSGSRDILSQFLKTKSFQKLRDREFSAGAGAIRKRLANLDLEVIIYSSLAEWK
KRAEIIKPTRTEEEDRKIERRMRVLVRRMELAKHFLETNVKPEWMILRLLPVLPPELRPIFKI

SEVQFISSDLNTLYKEVISNNKILSDSLLGCKFVPEQLLTGQEKIVQVAVDQLLDNRISGQPR
RDRNKQLYKSFSDILAGKEGRFRQTLLGRRVDYSGRSVIVVGPSLALHQCGLPREIALELF
QTFIIRSLIRQRLASSVKTAKKQIREQERIVWDILEEVVQEHPVLLNRAPTLHRLGIQAFQPI
LTKERAIYLHPLVCKGFNADFDGDQMAVHLPLSLEAQAEAYLLMFSTQNLLSPGNGDPIC
VPTQDMLTGLYTLTLGNRRGICATRYNLWNRRN---SQNERIDDKHYGSTKGKELLFCNPD
DAIGAYRQKRINLDSPFWLKVTGQHR--IVS--KELPVEVHYESLGTHHEIYGHCKIVRSVKT
ERHFIYIRTTVGHLFLDREIEDALKDFRAV----------------MKEWPNLAFHNKVIDGAAIKQLI
SRLIQRFGMLYTSHVLDQVKLLGFKQATASSISLGIDDLLTIPSKRWLVQDAQQQSFLLEKY
YQYGNVHAVEKLRQSIEIWDAISAYLRQEMTPNFGMTDPLNPVHIMSFSGARGNASQVN
QLIGMRGLMSDPLGQMIDLPIQSNLREGLSLTEYIISSYGARKGVVDTAIRTADAGYLTRRL
VDVVQHIIVRGTDCGTTRGLSLSPQNGGVP-ES--TQTLIGRVLAKDIYIGPRCIAVRNQDLG
GGLVNRLIT------FKKKPIYVRTPFTCRSTSWICRFCYGRSPTCGDLVELGEAVGIIAGQSIGE
PGTQLTLRTFHTGGVFTGGTAEQVRAPFNGKIKFNEDLVHPTRTRHGQPAFLCYIDLYLTIE
SEDIIHSVPIPPKSFLLVQNDQYVESEQVIAEISAGTSTF-AFKDRVRKHIYSDSSGE-MHSNT
GMYHARKFPFSNVHILPKTSHLWILLGDLCG--SGPVLFSMYKDQDQMSIH--SLSVQGRNT
PSLS--VNNDQ-LKHR--RLSLYDKNSIPILNY-SEMTRSLSTARCNLLYPA-ILHENFS--LLAKK
RRTRFLIPFQSEHRRKNERILCSDISIKIPR--NGLFRGNSILAYFDDPRYKIGVSGITKYGTIEV
DSIVTKES--FINYG----GVQPKDGKVDPLFFIPEEVHIFPETSSIMVRNNSIIGINTKITLNKRS
RVGGLVRVKKIHRGIKLQIFSGDISFPRKRDKRFIPQNKDILIPPQIRKRNFNKSKK--AKNWI
YVQK--MRPTNKN--SFFLLQPVVTYEIADGINLARLFPPDLLQEIDNIQLRVGNYIRYGDGE
LIPGISGKNIQLVRTFLVLNWDQDK---KDSSIEEAHASFVEVSTKGMVRDFLRIHLRKSAYA
YMRKRNDPSGSGFPSD-NELDHSNRNPFSSSYP-----KARVRQSLN----TIRTLLN---RNKECQ
SLLILSSSNCFRLGP--FNNVKYHN---PKKESDPLIPIRNFSGPLGT--VPQIANFDV--FYHLIT--
KNRIS-VFHYLPPE-----KGKLQVI--Q-SFLMDENGR--IYKSDPYSNIFLNPFNFNWYFLQHN
YCEETSTIIN-LGQFLFETVCIAKNGP---RLKSGQVFILQ-VGYVIIRSGKPYLAIPGATVHGH
SGQIVYGGDTLVTFIYEKARSADITQGLPKVDAMFEVRSKNSISMRLKARDEYWNEYITR
NLGIYWGLLIGAKLTIAQSSLSLVNEIQKVYRSQGVQIDNRHIELIVRQITSKVLISE---DGM
SNLFLPGELIGLLQAERTGRALEKGICYRAILVGITKASLNTQSFISEASFQQTARVLAKAAL
RGRIDWLKGLKENVVLGGMSPAGTGFRGLVHPSS-KKYKTLS--LETQRKILFDGK-V-RDL
LFHHKRIV---------------------------MTKRSWNIRLEDMVAARVHLGHDIKKRNPRMAPYISA
KLKDTDITNLKKTARFLSETCDLVFEAASKGKKFLIVGTKKEVANSVAQAARTAGCHYVN
QKWLGGMLTNWSTTKKRLHKFRDLIRQQ--GRLNRLPKRDAAILKRQLFHLQKSLGGVK
YMKELPDIVIILDQHGEFTALRECISLRIPTIGLIDTNCDPDLVDLPIPANDDSIPSIRFILNKLI
LAICKGRSRSHIKKKMGQKINPLGFRLGTTQTHHSIWFEKRKDYSEGLQEDHKIREHIQNY
IEKER-SDS-----ISRIQIDKSIDVVKVIIYMGFSQLLTEDEPPK--TKIKELQVSLKKAIHFIQRK
LNLAVRKMKKPYGDPTILGKYIANQLKNRVSFRKVMKKAVELV--EKKKGIQVQIAGCIGG
KEMARVEWLRKGRVPLQTVTAKIDFCSTQVITIRGVLGIKVWIF----MSRYRGPRVKKIKRL
GSLPGLTTKKPPIVRDPRLSRPKPKKKSQYRIRLEEKQKLRFHYGLTERQLLKYVRIAGKA
KGPTGQVLLQLLEMRLDNTLFRLGMASTIPQARQLVNHRHILVNGRIVDIPSYRCKPRDLI
SGREKEKSKALIQNYLDSTAKPIPLPKHLLLFHSDDPLKGSVKKIIDRKEVGLKIKELLVIEY
YSRQIMSRRGPAEKKTVKRDPFYRNRVVNMLINRILKHGKKSLAYKILYRAMKKIQQKKK
RNPLSILRQAIRRVTPKIAVKRRRVSGSAYQVPIEIKAPQGKVLAIRWLLEAARKRPGRNM
AFKLSSELMDATKGKGNAIRKKEETYRMAEANRTFANFRMGKDTLSEIVTSIRNADMAK
KETVRITFTNIAQNIVTILLREGFIKNAREHRESKKSFLVLTLRHRR--NRKGPSRT-LFN--LK

RVSRPGLRIYSNYQKIPRILGGMGVAILSTSKGIMTDREARLKRIGGEILLYIWMAKTIRRYS
SR-NRRI-SRKSARKIPKGIIHVQASFSNTIVTVTDVGGRVVTSASAGACGFKGRRRGTPFAA
QTTAENAIRTVVTQGMHRAVVLVKGVGRGRDAALRAILRSGVRLHLLRDRTPLPHNGCR
PPKRRRTMPTSNQLLRNSRQPVRKTKKTPALRGCPQRRGRCSKVYIINPKKPNSGNRKVAR
VRLTSGFEITAYIPGVGHNVQEHSVVLVRGGRVKDLPGVRYHIVRGTLDAAGVKDRKQGR
SKYGVKKPKVAKKCWIQREKKRQKLEQKYRLIRRSSKKEIR---EVTSLSEKWEIQRKLQSS
PRNSAPTRLRRRCFSTGRPRATYRDFGLSRHILLKMFRAGLLPGATRSSWMVKTPLISVISQ
E---KEKNRGSVEFQVLCFTKKIDQISSHLKLHRKDYLSQRGLHKILGKRQRLLSYLSKKNR
VRYKELINRLNIRESKTR--MVKLRLKRCGRKKRAVYRIVAIDVRSRREGRDLRKVGLYDPI
NNKTHLNVPAILYFIERGAQPTRTVNDLLKKAGVLKGIPVVGTLVSKMEKWLREFIATKKP
LPPRSSRIKPGDLIDYRNISLIGRFISQQGKILSRRVNRVTLKQQRRLTIAIKQARILSSLPFHA
TDQL-KIKKRRSTARKKRKKGFRKPFPSVKRNPFVANPLLKKIEKLNAKAQKDFLKTWSR
ASTIIPAMIGYTIYIHNGREHVPIPITKYMVNYKLGDFVPTRIFQHNSKSDTKSVR-------MN
HITSKRLDIR-ATLIFGIFYGILITFSKLTSYLFLIRISQDDEG----TGKARALSGFTVGHLLGYL
LIYYLPFYKALSNFYLKIILALGLMLYQFWTNHHLDIF---KSFSTPQR--DQTLAFVYGLSYR
LLNYTFFPNSIFSRMIVGYLVQCQFKILFLYTTFFVWMIGHLICIKWVQFLTLWLQKNYSAF
LKIRSRIAKIFEYKPDMKQILATILFIVALYLLGKSPIPFVPNAVELEEENRGFSHFFFNPRRW
VIPYRYIKTSFYEYVKKGGFSQFCFYKCQSDGKERTSFTYPLSLITFYKMIEGKLSLFRKKR
LPRDRLYTLWVLRNAKKKDSLNKELIKRVQKLKSGSPLRDVLDIRRKLFQSETLFDQKKR
ENDPVWDGPSRGALNQKEQYFEKSLLGKTLRILAPQYIPSNRNTVQLKLRTIMKEVPRWG
YNIDKIDYSDYNYLRTRKAKFRVFRTKYFYRYAKITHFRRNMVKGADRTQRRKVTICGW
YEHRVQSPLFLPKMKKCYRFLRQDSRFQVRVKINRIEAHEIRICIAETWELLQSGHAVRSLI
LLIQSFLRKNVIFPFLIIAKNMARILFFQSPELFQDFADLKKETHTLCDFGGIPLDESQDPIG
WFTDGCQIRIHFPFEWKPWRETEESSVAEDLYLTVFGRITNIPFGTARKPYPFFKTVFEELK
KK-------------KASQELKLTSGIRKLQKKGFLKFKKKGFLRKRLLAFQ----VKVKKR------TN
LKKGPQSK--LTKKKKERKVDKTRAIINEIERVLKEKKKSSTKTSSHSKTSEALRRLSKILKR
RKARFIRKSTIFRKWIIEWIYVKILFDLKNRFDLKKRFL----RFDLSSLSQAYVFYKLSQTEK
SSVSKFQNLRNCWKEWLRGHFDYYLEAQKWRNTAENHNFKSYEIDPQKLKFKKHLRYD
LLAYSFLNAEYKKVIALVSNNQTAISHHKESLVNMLTSNYKHLRDSTALRRRMNYNQILF
HVEDDESFRLEHYIHKICDMHFFSVFFDWMGLNNENELEFLFPEFIQLYNLYAKAEWTTPI
HSFLQNVSTLKPIKYSRFRKTYFRYQVHYSLRYIADFLTNVTRTSQVTNKTNPKLLLVFIKD
LIEMSQFGIMGTTQNQLPTLEDIGYLVLNPIRTFKRLRWEHVTYQILAISLLHKNQFVEKGL
LVPENLLSPRRRRQLRIVNELIFLWPNYRLEDLACMNRYWFDTTNGSRFSMLRIRVYPMD
EQELKFFILELKGILREIKNSHQLLDSWTKLNSLGTLIQIFFNPERAFQLLDPRVWKILLS-R
NS--RGWRKTRHFT----IGRVLLFVVVVLMYR----MSRRNMVENKKSYLTGVLP-----IPLNPI
GHRNDTLEESI-GFSNINRLILP-LLYLPKGKKIPESSFLDPNESTWALPIT----KKSIMPESRWG
SRWWCSWVGKRR--DSSCKRAH---KTVAGIEISFKEKKSK---------------------------------------
----
---YLEFLE------DSE------------YPTLINQREIQQIKEE
SIL-WHPSPSLERT---EEIKEFL--GNSTRSLRSFFSDRW--SEL
Y-LSSNPTERFTRDQ----KLLKQ--DLS-FVPYRRSE----NQEIVNLFKIITYLQK--TVSIYPISSD
PGCDRVP-KDELD-VDSSKKISFFKKNPFFDL--LNLFHDRSRGGYTL--ERDFESE--ERFEER
ADLFTLSITEP-DQVYHN-------SPKMD---
------------------LIHIQEKG----YIRKVLDRFFLMN---RSARNFESGIP---GAQIGNETLSHRTLM

KYTVNRHFSSLKK-SRKKSFDP--LLFIERS---MNRDPAAYRDKWSKGGKTFQEHLEHFVSE
QKNRFKRFKAVFDRFHQSQYSIDWSVFIDKQDLYKSLPFLLSKLP---------LLLS-------KLPL
LVSKKLLPFFFVSCGNIPIHRSE-IRIY-ELKGPTDQPCNPLLESIGLQILHLNKLKPC--LLD---
DHETS-QKSKIVG--------------------------------TIDNTDSYFSILFDDEENWMNPVKAFQRSSLI
SAFYKANRLRFLNNPHRFSFY--CNKIFPFVV----EKARFKNSD--FLYG-QFLKTFFIRKK-PFS
LCGEK----HAFGE-RATLLPLIPQVSKILIPDDFPQSGDER-YNLCKS---FHFPTRSVPLVD--RA
LYSIADISERPLTEGQMVNLERTSFQPLSDIHLSDSERKNLHQDLN--FNSNMGLIHTPYSEK
DLPSE--KRKK--------RNLGRNLKKCVEKGQMFRTLLSKMYRTLLSKWNLFQTYMPWFLT
STGYKYLTLLFLDLFSDLV--PILSLGRQNCVSLFDDIRQGV--PWQILQEKW------VLPQRNR-
IREISRMCLRN--LTLSAERIRRNNES--PLTHTHLRSPNVLEF-LYSTLLLLLVAGY-LVCTYLS
RLSK--DYGELQTELKKVKSLMIPSYTIELRKLMDRYPPSELNSFGLKNLFLVAMEELKESL
SVVG------NMLEGGGR-----AYG----VEAIFSNWNLNLIDISDLI-SLIPNPIDRITFSINTRHLSH
TSKEIYSFIRKRERVYGAWIDDKIESLLSTSVAIDDCDRGNLLQFSTLTLTT--EKGVDQILLSL
T-QSS--------KNASGSQMIEQPGEMYLRHVVDLQ--KKYLMGYEFNTSSLAERRIFLAHYQT
MTY--SKTSCGVNAFHFPSHE---KPFSLRLDLSPP-RGILVIGSIGTGRSYLIKSLAKNTHFPLI
TLHLDEIYGDQLEYVYDDTSLSVEVEDTREDDEIEDQAEMDTRRDLDG---IEYTHAIQDMI
DLGISVDAQLNFLPESILIETELWELWMEEWDRLGISLQFALVRAMTPCILWIPNIHDVDLE
DRTTLAG--LMNSLSG---DSEGRS-TRKTLVIASTHLPKKVDPALIASNRFNTCIKVRRLLSTQ
ERERFFTLSYTRGFHFEKEIFDT--NGFR----SITTQDLAALTNEALSISITQKKSIIDTNTIRFAL
HRQTWAVE---SRSISISDHGILFYQTGRALAQNLFLSQGLIDPISVYIKKK--SCNDGYSYLYR
WYLELGTSMKRLTILLYLLSCFAGSVAQDLWSPP--GPDDK-AEMFSYGLVENDS-HLAQGL
LELEGALVA---SGTEKACSRFDND--QVTLLFRAEPSL---DRMQDGFCSIFEQ-----------EGEE-
------AAPG-----LEKP----LVTH--IVSAP--RIWNP---WLILFDWLQEFQDLEDPQDQEGGLQEL
KEEAYLYQKADELQKYGPGFLESGTIMKKYPTRNRFRQGFFQASQFIWYPADSLFFLLK-E
QALGFVFSQPEFFADEEISKEGLLALTVLPFSTTCHWFI--KKRQERHFEFLIHRERWLRT---N
SSLS--NGFFCSKTQTLFESYQYLSNLFLSN-GRLVDQMTKTLLRKRWIFPDEMKIQIGFMYT
GEGFPITMPRSR-INGNFIDKTFSIVANILLRIIPTTSGEREAFTYYRDG--MSAQSEGNYAEAL
QNYYEAMRLEMDPYDRSYILYNIGLIHTSNGEHTKALEYYFRALERNPFLPQALNNMAVI
CHY-RGEQAIRQGDSEIAEAWFDQAAEYWKQAIALTPGNYIEAQNWLKITRRFE--MSCRS
ENLWLELIAGSRKTSNFLWALILFLGSFAFLLVGTSSYLGRNWISLFPSQQILFFPQGVVMSF
YGIAGLFISSYLWCTISWNVGSGYDRFDRKEGIVCIFRWGFPGRNRRIFLRFLMKDIQSIRIE
AREGFYARRILYMEIRGQGAIPLTRTAEN-WTPREIEQKAAELAYFLRVPMEVF
>Drimys
MEKWWFNSMLSLSKSMNSLGAIGNTSGSEDLIINDTDKNIH-CNNVDDLFGIRDIWSFVSD
DTFLVRDSNGDSYSVYFDIENQIFQIDND--SSFLSELESSFSNYLN-SYLNSGSKSDNRYYDR
YMYDIKYSWNNHINSCIDSYLHSEIS--IESYISSSSDNYGDSYISSFICNDNGSSIRTSGNGSD
FNIRGRS-DFDINQKYRHLWVQCENCYGLNYKKFFRSKMNICEQCGYHLKMSSSDRIELSI
DPGTWDPMDEDMVSIDPIEFHSEEEPYRDRIDSYQRKTGLTEAVQTGIGQLNGIPIAIGVM
DFQFMGGSMGSVVGEKITRLIEYATNGSLPIIMVCASGGARMQEGSLSLMQMAKISSASY
NYQSNKKLFYVSILTSPTTGGVTASFGMLGDIIIAEPNAYIAFAGKRVIEQTLNKTVPDGSQ
AAEYSFHKGLFDSIVPRNPLKGVLSELFQLHGFFPLNQN---MVTLRVDEISNIIRERIEQYNR
EVTIVNTGTVLQVGDGIARIHGLDQVMAGELVEFEEGTVGIALNLESNNVGVVLMGDGL
MIQEGSSVKATGRIAQIPVSEAYLGRVINALAKPIDGRGEISASESRLIESPAPGIISRRSVYEP
LQTGLIAIDSMIPIGRGQRELIIGDRQTGKTAVATDTILNQKGQNVICLYVAIGQKASSVAQV

VTTFQERGAMEYTIVVAETADSPATLQYLAPYTGAALAEYFMYRERHTSIIYDDLSKQAQ
AYRQMSLLLRRPPGREAYPGDVFYLHSRLLERAAKSSSHLGEGSMTALPIVETQSGDVSA
YIPTNVISITDGQIFLSSDLFNAGIRPAINVGISVSRVGSAAQIKAMKQVAGKSKLELAQFAE
LEAFAQFASDLDKATQNQLARGQRLRELLKQSQSAPLTVEEQILTIYTGANGYLDPLDIGQ
VKKFLVQLRTHLKTNKPQFQEIISSTKTFTEQAEALLKEAIPEQMELFILQEQTMRINPTTSG
PGVSTLEEKNLGRIAQIIGPVLDVVFPPGKMPNIYNALVVKGRDTVGQQINVTCEVQQLLG
NNRVRAVAMSATDGLMRGMEVIDTGAPLSVPVGGATLGRIFNVLGEPVDNLGPVDTRTTS
PIHRSAPAFIQLDTRLSIFETGIKVVDLLAPYRRGGKIGLFGGAGVGKTVLIMELINNIAKA
HGGVSVFGGVGERTREGNDLYMEMKESGVINEENIAESKVALVHGQMNEPPGARMRVGL
TALTMAEYFRDVNEQDVLLFIDNIFRFVQAGSEVSALLGRMPSAVGYQPTLSTEMGSLQE
RITSTKEGSITSIQAVYVPADDLTDPAPATTFAHLDATTVLSRGLAAKGIYPAVDPLDSTSTM
LQPRIVGEEHYETAQRVKQTSQRYKELQDIIAILGSDELSEEDRLTVARARKIERFLSQPFFV
AEVFTGSPGKYVGLAETIRGFQLILSGELDGLPEQAFYLVGNIDEATAKAMNLDVESKLKK
MTLNLCVLTPNRIIWDSEVKEIILSTNSGQIGVLPNHAPIATAVDIGILRIRLNDQWLTMALM
GGFARIGNNEITILVNDAERGSDIDPQEAQRTLEIAEASLSRAEGKRQEIEANLALRRARTR
VEAINVISMKNVTDSFVSLGHWSSAGSFGLNTDILATNPINLSVVLGVLIFFGKG-VSDLLD
NRKQRILSTIRNSEELRGRAIEQLEKARARLRKIGMEADEFRVNGYSEIEREKENLINATYN
NLERLENFKNETIRFEEQRAINQVRQRVFQQALQGALGTLNSCLNSELHLRTISANIGMLG
TIKEITDMNPLISAASVIAAGLAVGLASIGPGVGQGTAAGQAVEGIARQPEAEGKIRGTLLL
SLAFMEALTIYGLVVALALLFANPFVMNVLPCSINTLKGLYDLSGVEVGQHFYWQIGGFQ
VHAQVLITSWVVIAILLGSATIAVRNPQTIPTKSQNFFEYVLEFIRDLSKTQIGEDYGPWVPF
IGTMFLFIFVSNWSGALLPRKIVQLPHGELAAPTNDINTTVALALPTSVAYFYAGLTKKGLG
YFGKYIQPTPILLPINILEDFTKPLSLSFRLFGNILADELVVVVLVSLVPLVVPIPVMFLGLFTS
GIQALIFATLAAAYIGESMEGHHMIFATLEHILTHISFSIISIVITIQLIDLLVYQIVGICDSSEKG
IIATFFCITGLLITRWIYSRHFPLSDLYESLMFLSWSFSIIHMVPKAGNHKNDLSAITGPSAIFT
QGFATSDLLTEMHQSAILVPALQSQWLMMHVSMMLLSYAALLCGALLSVAFLVITFRKNT
DIAVKSNEIQYLNEKRSVLQNTSFPSFRNYHRYRLTQRLDHWSYRVISLGFIFSTIGILSGAV
WANEAWGSYWNWDPKETWAFITWTIFAIYLHTRTNQSLQGVNSSIVASIGFLIIWICYFGV
NLLGIGLHSYGSFTLTVPKKKALTPLPYLASIVFLPWWISLSFNKSLEPWVTHWWNTRQSE
TFLNDIQENNVLERFIELEELFLLDEMIKDYPETQIQKFRIGIHKETIQLIKMHNEDHIHIILH
FSANIICFAIFSGYSILGNEELVILNSWVQEFLYNLSDTIKAFSILLLTDLCIGFHSPFGWELMI
GSVYKDFGFAHNDQIISGLVSTFPVILDTILKYWIFHYLNGVSPSLVVIYHSMNEMPIGVPK
VPFRSPGEEDAVWVDVYNRLHRERLLFLGQEVDSEISNQLVGLMVYLSIEDDTRDLYLFIN
SPGGWVIPGIAIYDTMQFVPPDVHTICMGLAASMGSFILVGGEITKRLAFPHARVMIHQPA
SSFYEAPTGEFILEAEELLKLRETLTRVYVQRTGNPLWVISEDMERDVFMSATEAQAHGIV
DVVAVEMKEQKLIHEGLITESLPNGMFRVRLDNEDLILGYVSGRIRRSFIRILPGDRVKIEVS
SYDSTKGRIIYRLRNKDSNDMEELQGYLEIDGSRQQCFLYPLFFQEYIYALAHDHGLFGYD
N-FSSLIGKRLITRMHQQNHLILSVKDSKQNLFVVKYFDSQMMSEVFAVIVEIPFSLRLVSSL
IAKSHSIQSIHSIFLFFEDKFSHLNHVSDILIPHPIHLEILVQTLRCWIQDAPSLHLLRFFLYES
RNSNSLIIKSISFFSKQRFFLFLYNSHIYGCESIFVFLRKQSSHLRSTSSRTLLERTHLYGKIEH-
LVVLRNQR-LWLFKDPFMHYVRYQGKAIMASKGTHFLMKKWKYHLVNLWQCHFYLWS
QPDSIHINQLYNHSFYFLGYLSRVQLNPSVVRSQMLENLFIIDTAI--NKFETIVPMIPLIRSLA
KAKFCNVSGHPISKPARADSSDSDIIDRFGRICRNLFHYHSGSLKKQSLYRIKYILRLSCART
LARKHKSKVRAFLKRLGSGFFQEFLTEEEQVLSLIFPRRIWYLDIIRINDLANHEMIIDTT-E

VQ-AINSFSRSE-SLKEGYGLIGMLVPIFTPVSGITIGVLVIVWLEREISAGIQQRIGPEYAGPL
GILQALADGTKLLFKEDLLPSRGDIRLFSIGPSIAVISILLSYSVIPFGYRLVLADLSIGVFLWI
AISSIAPIGLLMSGYGSNNKYSFSGGLRAAAQSISYEIPLTPCVLSISLLSNSSSTIDIVEAQSK
YGFWGWNLWRQPIGFIVFIISSLAECERLPFDLPEAEEELVAGYQTEYSGIKFGLFYVASYL
NLLVSSLFVTVLYLGGWNLSIPYIFIPELFGINKTGGVFGTTIGILITLAKAYLFLFIPITTRWT
LPRMRMDQLLNLGWKFLLPISLGNLLLTTSSQLLSLMIWHVQNENFILDSTRIFMKAFHLL
LFHGSFIFPECILIFGLILLLMIDSTSDQKDIPWLYFISSTSLVMSITALLFRWREEPMISFSGNF
QTNNFNEIFQFLILLCSTLCIPLSVEYIECTEMAITEFLLFVLTATLGGMFLCGANDSITIFVAP
ECFSLCSYLLSGYTKRDVRSNEATTKYLLMGGASSSILVHGFSWLYGSSGGEIELQEIVNG
LINTQMYNSPGISIALIFITVGIGFKLSPAPFHQWTPDVYEGSPTPVVAFLSVTSKVAASAPAT
RIFDIPFYFSSNEWHLLLEILAILSMILGNLIAITQTSMKRMLAYSSIGQIGYVIIGIIVGDSND
GYASMITYMLFYISMNLGTFARIVLFGLRTGTDNIRDYAGLYTKDPFLALSSALCLLSLGGL
PPLAGFFGKLHLFWCGWQAGLYFLVSIGLLTSVVSIYYYLKIIKLLMTGRKQEITPHVRNY
RRSPLRSNNSIELSMIVCVIASTIPGISMNPIIAIAQDTLFMFLLYEYDIFWAFLIISSLIPILAFL
ISGVLAPISEGPEKLSSYESGIEPMGDAWLQFRIRYYMFALVFVVFDVETVFLYPWAMSFD
VLGVSVFIEALIFVLIPIIGSVYAWRKGALEWSTSYFPWLTIIVVFPISAGSSIFFLPHRGNKV
VRWYTICICLLELLLTTYAFCYHFQLDDPLIQLEEDYKWINILDFHWRLGIDGLSIGPTLLTG
FITTLATLAAWPVTRDSQLFHFLMLAMYSGQIGSFSSRDLLLFFIMWELELIPVYLLLSMW
GGKKRLYSATKFILYTAGGSIFLLMGVPGMGLYGSNEPTLNLETSANRSYPAALEIIFYFGF
FIAYAVKSPIIPLHTWLPDTHGEAHYSTCMLLAGILLKMGAYGLVRINMELLPHAHSIFSPW
LMIVGTIQIIYAASTSPGQPNFKKRIAYSSVSHMGFTIIGIASITDMGLNGAILQIISHGFIGAA
LFFLAGTSYDRIGLVYLDEMGGIAIPMPKIFTMFSSFSMASLALPGMSGFFAELVVFFGIITS
TKYLLMPKILITFVMAIGMILTPIYSLSMLRQMFYGYKLFNVTNSYFVDSGPRELFVSICIFL
PVIGIGIYPDFVLSLSVEKIEAILSNYLHKMMLEHVLILSAYLFSIGIYGLITSRNMVRALMC
LELILNAVNMNLIKFSDLFDSRQLKGDIFSIFVIAIAAAEAAIGPAIVSSIHRNRKSTRINQSN
LLNKMQPIYQYAWIIPFVPLPVTMSIGLGLLLVPTATKNLRRMWAFPSVSLLSIVMVFSADL
SVQQIDGSSIYQYLWSWTINNDFSLEFGHLIDPLTSIMSILITTVGIMVLIYSDKYMSHDQG
YLRFFAYMSFSNTSMLGLVTSSNLIQIYIFWELVGMCSYLLIGFWFTRPIAANACQKALVTN
RVGDFGLLLGILGLYWITGSFEFRDLFEIFNNNGVNSLFATLCASLLFAGAVAKSAQFPLHV
WLPDAMEGPTPISALIHAATMVAAGIFLVARLLPLFTVIPYIMNLISLIGVITVLLGATLALA
QRDIKRSLAYSTMSQLGYIMLAPGIGSYRAALFHLITHAYSKALLFLGSGSIIHSMEPIVGYS
PDKSQNMVLMGGLRKYVPITKMTFLLGTLSLCGIPPLACFWSKDEILNDSWLYSPIFAIIAC
ATAGLTAFYMFRTYLLTFEGYLYAHFQNYSGTQNSSFYSISIWGKEEPKLVNR-NLLLSTINK
VSNKD--TSMYPHESDNTMLLPLLVLVLFTLFVGFIGIPFGLDILSKWLTPSINLLHQNSNYS
VNWYEFATNAFFSVSIAYFGIFIASLLYGSVYLFFQNLELINSFVIFLDQIINVIYNWSYNRG
YIDVFYATSLTKGIRGLAKLTHFFDRRVIDGIMNGVGVSSFFVGEGIKY-LGGGRISSYLFVY
LSYVSIFLLIYIMDLPGPIHDILLVFLGSGLILGGLGVVLFTNPIFSAFSLGLVLVCISLFHILSN
SYFVAAAQLLIYVGAVNVLIIFAVMFMNGSEYYNDFYLWTVGDGVTSLVCTSILFSLITTIS
DTSWYGIIWTTGSNQIIEQDLISNVQQIGIHLSTDFYLPFELISIILLVALIGAIAMARQMTVPA
TKKDLMIINMGPHHPSMHGVLRLIVTLDGEDVIDCEPILGYLHRGMEKIAENRTIIQYLPY
VTRWDYLATMFTEAITVNAPEQLGNIQVPKRASYIRVIMLELSRIASHLLWLGPFMADIGA
QTPFFYIFRERELLYDLFEAATGMRMMHNYFRIGGVAADLPHGWIDKCLDFCDYSLTGVV
EYQKLITRNPIFLERVEEVGIIGGEEAINWGLSGPMLRASGIQWDLRKVDHYECYDEFDWE
VQWQKEGDSLARYLVRISEMTESIKILQQALEGIPGGPYENLEVRRFDRAKDSEWNDFEY

RFISKKPSPTFELLKQELYVRVEAPKGELGIFLIGDNSVFPWRWKIRPPGFINLQILPQLVKR
MKLADIMTILGSIDIIMGEVDRMFPMMTGFMNYGQQTVRAARYIGQSFMITLSHANRLPV
TIQYPYEKSITSERFRGRIHFEFDKCIACEVCVRVCPIDLPVVDWRLETDIRKKRLLNYSIDF
GICIFCGNCVEYCPTNCLSMTEEYELSIYDRHELNYNQIALGRLPMSVIGDYTIRTIMNSKIT
TDKPLDSITITNDMQGRLSAWLVKHELVHRSLGFDYQGIETLQIKPEDWYSIAVISYVYGY
NYLRSQCAYDVAPGGLLASVYHLTRIQYGVDQPEEVCIKVFAPRRNPRIPSVFWIWKSADF
QERESYDMLGISYENHPRLKRILMPESWIGWPLRKDYIAPNFYEIQDAH----------------------
------------------------------------INSIEFPLLDRTTQ-SVISTTSNDLSNWSRLSSLWPLLYGTSCC
FIEFASLIGSRFDFDRYGLVPRSSPRQADLILTAGTVTMKMAPSLVRLYEQMPEPKYVIAMG
ACTITGGMFSTDSYSTVRGVDKLIPVDVYLPGCPPKPEAVIDAITKLRKKVSREIYEDRIGS
QQENRYFTTNHKFHVGRSTHTGNYDQGLLYKSPSTSEIPTETFFKYKS-VSSHELVNMQNR
NTFSWVKEQMTRFISVSIMIYVITRTSISNAYPIFAQQGYENPREATGRIVCANCHLANKPV
DIEVPQAVLPDTVFEAVVRIPYDMQLKQVLANGKKGSLNVGAVLILPDGFELAPPDRISPE
MKEKIGNLSFQSYRPTKKNILVIGPVPGQKYSEIVFPILSPDPATKKDVNFLKYPIYVGGNR
GRGQIYPDGSKSNNTVYNATGAGIVSRIVRKEKGGYEITIADASDGHQVIDIIPPGPELLVSE
GESIKLDQPLTNNPNVGGFGQGDAEIVLQDPLRVQGLLFFLASVILAQIFLVLKKKQFEKV
QLSEMNFMSKVYDWFEERLEIQAIADDITSKYVPPHVNIFHCLGGITLTCFLVQVATGFAM
TFYYRPTVTEAFASVQYIMTEANFGWLIRSVHRWSASMMVLMMILHVFRVYLTGGFKKP
RELTWVTGVVLAVLTASFGVTGYSLPRDQIGYWAVKIVTGVPDAIPVIGSPLVELLRGSAS
VGQSTLTRFYSLHTFVLPLLTAVFMLMHFPMIRKQGISGPLMGITKKPDLNDPVLRAKLAK
GMGHNYYGEPAWPNDLLYIFPVVILGTIACNVGLAVLEPSMIGEPADPFATPLEILPEWYFF
PVFQILRTVPNKLLGVLLMVSVPTGLLTVPFLENVNKFQNPFRRPVATTVFLIGTAVALWLG
IGATLPIDKSLTLGLFMIEVFLFGIVLGLIPITLAGLFVTAYLQYRRGDQLDLMPTITSYFGFL
LAASTITPALLMGLSKIRLIMDIVSLAWAVLMVVFTFSLSLVVWGRSGLMIIRSPEPEVKILV
DRDPVKTSFEEWARPGHFSRTIAKGPETTTWIWNLHADAHDFDSHTSDLEEISRKVFSAHF
GQLSIIFLWLSGMYFHGARFSNYEAWLSDPTHIGPSAQVVWPIVGQEILNGDVGGGFRGIQ
ITSGFFQIWRASGITSELQLYCTAIGALVFAGLMLFAGWFHYHKAAPKLAWFQDVESMLN
HHLAGLLGLGSLSWAGHQVHVSLPINQFLDAGVDPKEIPLPHEFILNRDLLAQLYPSFAEG
ATPFFTLNWSKYAEFLTFRGGLDPVTGGLWLTDIAHHHLAIAILFLIAGHMYRTNWGIGHG
LKDILEAHKGPFTGQGHKGLYEILTTSWHAQLSLNLAMLGSLTIVVAHHMYSMPPYPYLAI
DYGTQLSLFTHHMWIGGFLIVGAAAHAAIFMVRDYDPTTRYNDLLDRVLRHRDAIISHLN
WACIFLGFHSFGLYIHNDTMSALGRPQDMFSDTAIQLQPIFAQWVQNTHALAPGATAPGAT
TSTSLTWGGGDLIAVGGKVALLPIPLGTADFLVHHIHAFTIHVTVLILLKGVLFARSSRLIPD
KANLGFRFPCDGPGRGGTCQVSAWDHVFLGLFWMYNAISVVIFHFSWKMQSDVWGSIS
DQGVVTHITGGNFAQSSITINGWLRDFLWAQASQVIQSYGSSLSAYGLFFLGAHFVWAFSL
MFLFSGRGYWQELIESIVWAHNKLKVAPATQPRALSIVQGRAVGVTHYLLGGIATTWAFFL
ARIIAVGMALRFPRFSQGLAQDPTTRRIWFGIATAHDFESHDDITEERLYQNIFASHFGQLAI
IFLWTSGNLFHVAWQGNFESWVQDPLHVRPIAHAIWDPHFGQPAVEAFTRGGALGPVNIA
YSGVYQWWYTIGLRTNEDLYTGALFLLFLSAISLIAGWLHLQPKWKPSVSWFKNAESRLN
HHLSGLFGVSSLAWTGHLVHVAIPGSRGEYVRWNNFLDVLPYPQGLGPLFTGQWNLYAQ
NPDSSSHLFGTSQGAGTAILTLLGGFHPQTQSLWLTDIAHHHLAIAFLFLVAGHMYRTNFGI
GHSMKDLLEAHTPPGGRLGRGHKGLYDTINNSIHFQLGLALASLGVITSLVAQHMYSLPAY
AFIAQDFTTQAALYTHHQYIAGFIMTGAFAHGAIFFIRDYNPEQNEDNVLARMLDHKEAIK
SHLSWVSLFLGFHTLGLYVHNDVMLAFGTPEKQILIEPIFAQWIQSAHGKTSYGFDVLLSS

TNGPAFNAGRSIWLPGWLNAVNE--NSNSLFLTIGPGDFLVHHAIALGLHTTTLILVKGALD
ARGSKLMPDKKDFGYSFPCDGPGRGGTCDISAWDAFYLAVFWMLNTIGWVTFYWHWK
HITLWQGNVSQFNESSTYLMGWLRDYLWLNSSQLINGYNPFGMNSLSVWAWMFLFGHL
VWATGFMFLISWRGYWQELIETLAWAHERTPLANLIRWRDKPVALSIVQARLVGLAHFSV
GYIFTYAAFLIASTSGKFGMSHSVKIYDTCIGCTQCVRACPTDVLEMIPWDGCKAKQIAPA
PRTEDCVGCKRCESACPTDFLSVRVYLCHETTRSMGLAYMTDLNLPSIFVPLVGLVFPAIA
MASLSLHVQKNKIVMRDIKTYLSVAPVLTTLWFGSLAGLLIEINRLFPDALTFSFFSFMTAIL
ERRESTSLWGRFCNWITSTENRLYIGWFGVLMIPTLLTATSVFIIAFIAAPPVDIDGIREPVSG
SLLYGNNIISGAIIPTSAAIGLHFYPIWEAASVDEWLYNGGPYELIVLHFLLGVACYMGREW
ELSFRLGMRPWIAVAYSAPVAAATAVFLIYPIGQGSFSDGMPLGISGTFNFMIVFQAEHNIL
MHPFHMLGVAGVFGGSLFSAMHGSLVTSSLIRETTENESANEGYRFGQEEETYNIVAAHG
YFGRLIFQYASFNNSRSLHFFLAAWPVVGIWFTALGISTMAFNLNGFNFNQSVVDSQGRVI
NTWADIINRANLGMEVMHERNAHNFPLDLAAVEAPSTNGMGLPWYRVHTVVLNDPGRL
LSVHIMHTALVSGWAGSMALYELAVFDPSDPVLDPMWRQGMFVIPFMTRLGITNSWGGW
SIT-GGTITNPGIWSYEGVAGAHIVFSGLCFLAAIWHWVYWDLEIFCDERTGKPSLDLPKIF
GIHLFLSGVTCFGFGAFHVTGLYGPGIWVSDPYGLTGKVQSVNPAWGTEGFDPFVPGGIAS
HHIAAGTLGILAGLFHLSVRPPQRLYKGLRMGNIETVLSSSIAAVFFAAFVVAGTMWYGSA
TTPIELFGPTRYQWDQGYFQQEIYRRVGAGLAENLSLSEAWSKIPEKLAFYDYIGNNPAKG
GLFRAGSMDNGDGIAVGWLGHPIFRDKEGHELFVRRMPTFFETFPVVLVDGDGIVRADVP
FRRAESKYSVEQVGVTVEFYGGELNGVSYGDPATVKKYARRAQLGEIFELDRATLKSDGV
FRSSPRGWFTFGHATFALLFFFGHIWHGARTLFRDVFAGIDPDLDAQVEFGTFQKLGDPTT
RRQVVMKTLYSLRRFYPVETLFNGTLALAGRDQETTGFAWWAGNARLINLSGKLLGAHV
AHAGLIVFWAGAMNLFEVAHFVPEKPMYEQGLILLPHLATLGWGVGPGGEVIDTFPYFVS
GVLHLISSAVLGFGGIYHALLGPETLEESFPFFGYVWKDRNKMTTILGIHLILLGIGAFLLVL
KALYFGGVYDTWAPGGGDVRKITNLTLSPSVIFGYLLKSPFGGEGWIVSVDDLEDIIGGHI
WLGSICILGGIWHILTKPFAWARRAFVWSGEAYLSYSLAALSVFGFIACCFVWFNNTAYPS
EFYGPTGPEASQAQAFTFLVRDQRLGANVGSAQGPTGLGKYLMRSPTGEVIFGGETMRF
WDLRAPWLEPLRGPNGLDLSRLKKDIQPWQERRSAEYMTHAPLGSLNSVGGVATEINAV
NYVSPRSWLATSHFVLGFFLFVGHLWHAGRARAAAAGFEKGIDRDFEPVLSMTPLNMTIA
LGTFTKEENDLFDIMDDWLRRDRFVFVGWSGLLLFPCAYFALGGWFTGTTFVTSWYTHG
LASSYLEGCNFLTAAVSTPANSLAHSLLLLWGPEAQGDFTRWCQLGGLWTFVALHGAFGL
IGFMLRQFELARSVQLRPYNAIAFSGPIAVFVSVFLIYPLGQSGWFFAPSFGVAAIFRFILFFQ
GFHNWTLNPFHMMGVAGVLGAALLCAIHGATVENTLFEDGDGANTFRAFNPTQAEETYS
MVTANRFWSQIFGVAFSNKRWLHFFMLFVPVTGLWMSAIGVVGLALNLRAYDFVSQEIR
AAEDPEFETFYTKNILLNEGIRAWMAAQDQPHENLIFPEEVLPRGNALMSGSTGERSFADII
TSIRYWVIHSITIPSLFIAGWLFVSTGLAYDVFGSPRPNEYFTESRQGIPLITGRFDPLEQLDE
FSRSFMTIDRTYPIFTVRWLAVHGLAVPTVSFLGSISAMQFIQRMATQTVEGSSR-SRPRRTT
TGNLLKPLNSEYGKVAPGWGTTPLMGVAMALFAIFLSIILEIYNSSVLLDGISMSMLTLKLF
VYTVVIFFVSLFIFGFLSNDPGRNPGREEMADTTGRIPLWLIGTVTGIPVIGSIGIFFYGSYSG
LGSSLMLNIFSLICICLNSALHSSSFFFAKLPEAYAFFNPIVDFMPVIPVLFFLLALVWQAAVS
FRTTQSNPNEQNVELNRTSLYWGLLLIFVLAVLFSNYFFNMEVNILAFIATALFILVPTAFLLI
IYVKTVSQNDMETATLVAISISGSLVSFTGYALYTAFGQPSQQLRDPFEEHGDMEALVYTFL
LVSTLGIIFFAIFFREPPKVPTKRMKMTIAFQLALFALIATSSILLISVPIVFASSDGWSSNKNV
VFSGTSLWIGLVFLVAILNSLIS-------MSPKTETKASVGFKAGVKDYKLTYYTPDYETKDTD

ILAAFRVTPQPGVPPEEAGAAVAAESSTGTWTTVWTDGLTSLDRYKGRCYHIEPVAGEED
QYIAYVAYPLDLFEEGSVTNMFTSIVGNVFGFKALRALRLEDLRIPTAYVKTFQGPPHGIQV
ERDKLNKYGRPLLGCTIKPKLGLSAKNYGRAVYECLRGGLDFTKDDENVNSQPFMRWRD
RFLFCAEALYKAQAETGEIKGHYLNATAGTCEEMMKRAVFARELGVPIVMHDYLTGGFTA
NTTLAHYCRDNGLLLHIHRAMHAVIDRQKNHGIHFRVLAKALRMSGGDHIHAGTVVGKL
EGERDITLGFVDLLRDDFIQKDRSRGIYFTQDWVSMPGVLPVASGGIHVWHMPALTEIFGD
DSVLQFGGGTLGHPWGNAPGAVANRVALEACVQARNEGRDLAREGNEIIREACKWSPEL
AAACEVWKEIKFEFKAVDTLDITAIHLYKTSTPSTRNGAVDSQVKSNPRNNLIYGQHHCGK
GRNARGIITAGHRGGGHKRLYRKIDFRRNEKDISGRIVTIEYDPNRNAYICLIHYGDGEKRY
ILHPRGAIIGDTIVSGTEVPISMGNALPLTDMPLGTAIHNIEITLGKGGQLARAAGAVAKLIA
KEGKSATLRLPSGEVRLIYKNCLATVGQVGNVGVNQKNLGRAGSKCWLGKRPVVRGVA
MNPVDHPHGGGEGRAPIGRKKPTTPWGYPALGRRSRKRNKYSDIYILRRRK-MIQPQTHL
NVADNSGARELMCIRIIGAS-NRRYAHIGDVIVAVIKEAVPNMPLQRSEVIRAVIVRTCKELK
RDNGMIIRYDDNAAVVIDQEGNPKGTRVFGAIARELRQLNFTKIVSLAPEVLMLSPKRTRF
RKQHRGRMKGISYRGNYICFGRYALQALEPAWITSRQIEAGRRAMTRYARRGGKIWVRIF
PDKPVTVRPTETRMGSGKGSPEYWVSVVKPGRILYEMGGVSETVARAAIEIAACKMPIRT
QFVIAGMTRVRRGYIARRRRTKIRLFTSTFRGAHSRLTRTTTQQKMRALVSSHRDRCRQKR
DFRRLWITRINAVTREN--VSYSYSRLMHDLYKRQLLLNRKILAQIAISNRNCIYMISNEIIK--
---------RKKQVQALAQHICMSAHKARRVIDQIRGRSYEKTLMLLELMPYRASYPILKLVYS
AAANASHNKGFNKADLVISQAEVNGGTIIKKLKPRARGHSYPIKRPTCHINIVLKDQSKT---
----MDGIKYAVFTEKSIRLLGNNQ-YTSNVESGSTRTEIKHWVELFFGVKVIAMNSHRLPGK
GRRMGPIMGHTMHYRRMIITLQPGYSIPPLIEKRTMTVPKKRTSMSKKRIRKNIWKRKGY
WAALKALSLAKSISTGHSKSFFVQQTSNMAKGKDVRIRVILECTSCVRNDLN--KESRGISR
YITQKNRHNTPSRLELRKFCPYCYKHTIHGEIKKMKIRASVRKICEKCRLIRRRGRIIVICFN
PRHKQRQGMVREEVAVSTRTLQWKCVESRTDSKRLSYGRFILSPLMKGQADMIGIAMRR
ALLGEIEGTCITRAKSD--KIPHEYSTIVGIEESVHQILMNLKEIVLRSNLYGTCDASIFVRGP
RCVTAQDIISPPSVKMVDTTQHIASLTEPIDLCIGLQIERDRGYRMKTPNNDQDGSYPIPIEA
VSMPVRNANHSIHSYGNGNEKQEILFLEIWTNGSLTPKEALHEASHNLIDLFIPFLHGEEED
INL--EDSL-NRGTLPFFTFQDKLAN-LRKNKK-AIALECIFIDQSELPPRTYNCLKRSNIHTLL
DLLSNSQEDLMRIEHLRIEDVKRILDILQKH--FTIDLPKNKMLRDG-NEGMFTIPGFSQIQFE
GFFRFIDQGLTEELHKFPKIEDTDQEIEFQLFVDTYRLVEPLIKERDAVYESLTYSSELYVPA
GLIWKTGRDMQEQTIFIGNIPLMNSLGTSIVNGIYRIVINQILQSPGIYYRSELDHNGISVYT
GSIISDWGGRSELEIDRKARIWARVSRKQKISILVPSSAMGSNLREILDNVCYPEIFLSFPNE
KEKKKIGSRENAILEFYQQFACVGGDPVFSESLCKELQKKFFQQRCELGRIGRRNMNWRL
NLDIPQNNLFLLPRDVLAAADHLIGMKFGMGTLDD--MNHLKNKRIRSVADLLQDQFGLA
LVRLENVVRGTICGAIR-HKLI-PTPHNLVTSTP----LTTTYESFFGLHPLSQVLDRTNPLTQIV
HGRKSSYLGPGGLTGRTASFRIRDIHPSHYGRICPIDTSEGINVGLIGSLAIHARIGYLG-SIES
PFY---EISQRSKE--VQTVYLSPNRDEYYMVAAGNSLALNQGIQEEQVVPARYRQEFLTIAW
EQIHLRSIFPFQYFSIGASLIPFIEHNDANRALMSSNMQRQAVPLSRSEKCIVGTGLERQAAL
DSGVSAIAEQDGKIIYTDTDKIVLSGNGDTISIPLVIYQRSNKNTCMHQKPQVPRGKCLKK
GQILADGAATVGGELALGKNVLVSYMPWEGYNFEDAVLISERLVYGDIYTSFHIRKYEIQT
HVTSQG-PERITNEIPHLEAHLLRNLDINGIVMLGSWIETGDILVGKLTPQTAKE-SSYAPED
RLLRAILGIQVSTAKETCLKLPIGGRGRVIDVRWIQKGG-GSSYNPEMIRVYISQKREIKVGD
KVAGRHGNKGIISKILPRQDMPYLQDGTPVDMVFNPLGVPSRMNVGQIFECSLGLAGDLL

DRHYRIAPFDERYEQEASRKLVFPELYEASKQTANPWVFEPEYPGKSRIFDGRTGDPFEQP
VIIGKSYILKLIHQVDDKIHGRSSGHYALVTQQPLRGRAKQGGQRVGEMEVWALEGFGVA
HISQEMLTYKSDHIRARQEVLGTTIIGGTIPNPEDAPESFRLLVRELRSLALELNHFLVSEKN
FQIHRKEA-------MIDRYKHQQLRIGSVSPQQISAWANKILPNGEMVGEVTKPYTFHYKTNK
PEKDGLFCERISGPIKSGICACGNYRVIGDEKEDPKFCEQCGVEFVDSRIRRYQMGYIKLAC
PVTHVWYFKRLPSYIANLLDKPLKELESLVYCD--FSFARP-IVKKPTFLRLRGSFEYEIQSR
KYSIPLFFTTQGFDTFRNREISTGASAIREQLADPDLRIITDHSLVEWKELGEEGSA-GNEWE
DRKIGRRKDFLVRRMELAKHFIRTNVEPERMVLCLLPVLPPELRPIIQIDGGKPMSSDINEL
YRRVIYRNNTLTDPLTTSRSTPGESVMCQEKLVQEAVDTLLDNGIRGQPMRNGHNKVYKS
FSDVIEGKEGRFRETLLGKRVDYSGRSVIVVGPSLSLHRCGLPREIAIELFQTFVIRGLIRQHI
ASNIGIAKNKIREKEPIVWEILQEVMQGHPILLNRAPTLHRLGIQAFQPILAEGRAICLHPLV
RKGFNADFDGDQMAVHVPLSLEAQAEARLLMFSHMNLLSPAIGDPISIPTQDMLIGLYVLT
IGNRRGICANRYNPWNRR--N-YQNKTVYEYNYRYTKEKEPYFCSSYDALGAYRQKRINL
DSPLWLRWRLDQR-VIAP--REVPIEVQYESLGTYHEIYGHYLLVRSVKKESLCIYIRTTVGH
ISFYREIEEAIQGFCRAYS-----------MEVLMAERADLVFHNKVIDGTAMKRLISRLIDHFGMA
YTSHILDQVKTMGFQQATATSISLGIDDLLTIPSKGWLVQDAEQQSLILEKHHHYGNVHAV
EKLRQSIEIWYATSEYLRQEMHPNFRMTDPSNPVHIMSFSGARGNASQVHQLVGMRGLMS
DPQGQMIDLPIQSNLREGLSLTEYLISCYGARKGVVDTAVRTSDAGYLTRRLVEVVQHIVV
RRTDCGTIRGISVSPRNGIGMTEKMLIQTLIGRVLADDIYMGLRCIAARNQDIGVGLVNRFI
A------FRAQSIYIRTPFICRSTSWICRLCYGRSPTHGDLVELGEAVGIIAGQSIGEPGTQLTLRT
FHTGGVFTGGTAEHVRAPFNGKIKFNEDLVHPTRTRHGHPAFLCYIDLYVTIESQDILHNV
NIPPKSFLLVQNDQYVESEQVIAEIRARTSTF--FKERVRKHIYSDSEGE-MHWSTDVYHAPE
YTYGNVHLLPKTSHLWILSGGP--RRSSLVPFSLHKDQDQMNIH--SLSVEQRESSDLS--VTN
DR-ARHKLFSSDPSGK-EGKILDYS-GPARIISNGHWNFIYPA-ILHENSY--LLAKRRRNRFIIP
FQYDQEREKELMPRSGISIEIPIN--GILRRNSILAYFDDPRYRRSSSGITKYGTVEVDSIVKKE
D--LIEYRG-AKEFSPKYMKVDRFFFIPEEVHILPGSSSIMVRNNSIIGVDTRITLNTRSRIGGL
VRVERKKKRIELKIFSGDIHFPGEADKISRHSGILIPPGTGKK--NSKESK--KLQNWIYVQR--I
TPTKKK--YFVSVRPVVTYEIADGINLATLFPQDLLQERDNVKFRVVNSILYRNGKPIRGIYY
TSIQLVRTCLVLNWDQDRNG----SIEKVKASIVEVRANDLIRDFIRIDLVKSPISYTGKRNDM
AGSGLIPD-NGSDHTNINPFYSKVRR--------IQSLTQHQGTIRTLLNR---NKECQSFLILSSSNC
SRIGP--FNGSKSHNVT---KESDPMIPIRNSLGPLGT--VPKIANFDSP--YYLIT--HNQILLN-KY
LLLDN---LKQTFQVLKYY---LMDENGR--IYNPYPCRNIIFHPFDLTWCFLHHDYCEKTSTIIV
-LGQFICENVCISKYGP---QIKSGQVLIVH-VDSLVIRSAKPHLATPGATVHGHYGEILYEGDT
LVTFIYEKSRSGDITQGLPKVEQVLEVRSVDSISMNLEKRVEGWNEHIKRILGIPWGFLIGA
ELTIAQSRISLVNKIQKVYRSQGVQIHNRHIEIIVRQITSKVLVSE---DGMSNVFSPGELIGLL
RAERTGRSLEEAICYRAILWGITRASLNTQSFISEASFQETARVLAKAALRGRIDWLKGLKE
NVVLGGMIPVGTGFKGLVHRSR--QDNNIP--LEIKKKNLFEGEIR--DILFHHRELFGSCIPNN
FHNT-----------------MTKRYWNINLEEMMEAGVHFGHGTRKWNPRMAPYISAKRKGIHITN
LTRTARFFSEACDLVFDAASRGKHFLIVGTKNKAADSVASAAIKARCHYVNKKWLGGMS
TNWSTTETRLHKFRDLKAEQKAGKLNRLPKRDAAMLKRQLSHLQTYLGGIKYMTGLPDI
VIIVDQQEEYTALRECLTLGIPTICLIDTNCNPDLADISIPANDDAIASIRLILNKLVSAICEGR
SS-YIRNRMGKKINPLGFRLG-AQSHRSLWFAQPKSYSRGLQEDEKIRDCIKNYVQKNMRS
GS---EGISHIEIKKKIDLIQVIIYIGFPNLLIEG--RTR--GIEELRINVQKGFRSVNRRLNIAITRV
AKPYGQPNILAEYIALQLKNRVSFRKAMKKAIELTEQADTKGIQVQIAGRIDGKEIARVEW

IREGRVPLQTIRAKIDHCSYTVRTIYGVLGIKIWIFVDEQMSRYRGPRFKKIRRLGALPGLTR
KRPRSGSDLRNQSRSGK-RSQYRIRLEEKQKLRFHYGLTERQLLRYVRIAGKAKGSTGQVL
LQLLEMRLDNILFRLGMASTIPGARQLVNHRHILVNGRIVDIPSYRCKPQDIITTRDEQRSR
ALIQNYMDSSPH-EELAKHLSLYSSQY--KGLVNQIIDIKWIGLKINELLVVEYYSRQTMSRR
GTAEEKTAKSDPIYRNRLVNMLVNRILKHGKKSLAYQIIYRAVKKIQQKTETNPLSVLRQAI
RGVTPDIAVKARRVGGSTHQVPIEIGSTQGKALAIRWLLGASRKRPGRNMAFKLSSELVDA
AKGSGDAIRKKEETHRMAEANRAFAHFRMSKDTIADIITSIRNADMDKKGTVRIASTNITE
NIIKILLREGFIENVRKHRENNKYLLILTLRHRR--NRKGTYR---NIL--KCISRPGLRIYSNYK
RIPRILGGIGIVILSTSRGIMTDREARLKGIGGEILCYIWMAKPIPRIGSRRNG--------RRIPKG
VIHVQASFNNTIVTVTDVRGRVVSWSSAGTCGFRGTRRGTPFAAQTAAGNAIRTVVDQG
MQRAEVMIKGPGLGRDAALRAIRRSGILLSFVRDVTPMPHNGCRPPKKRRVMPTIKQLIR
NTRQPIKNVTKSPALRGCPQRRGTCTRVYTITPKKPNSALRKVARVRLTSGFEITAYIPGIGH
NLQEHSVVLVRGGRVKDLPGVRYHIVRGTLDAVGVKDRQQGRSKYGVKKPKMARKSLI
QRERKRQKLEQKYHLIRRSSKKEIS---KVSSLSDKWEIHGKLQSPPRNSAPIRLHRRCFSTG
RSRGNYRDFGLSGHILRERFHACLLPGATRSSWMVKNLFISVIPQEEKEK--NKGSVEFQVF
NFTNKIRRLTAHLEFHRKDYLSQRGLRKILGKRQRLLAYLSKKNRVRYKELIGQLDIREPK
TR--MVKLRLKRCGRKQRAIYRIVAIDVRSQREGRDLRKVGFYDPIKNQTYSNVPAILYFLE
KGAQPTGTVHDISKKAEVFKELRINQTKSNKMDKSKRPFRKSKRSFRRRLPPIGSGDRIDY
RNMSLISRFISEQGKILSRRVNRLTLKQQRLITIAIKQARILSSLPFLNNEK--QFERTESTPRT
AGPKTRNKVTRSLKKNPFVDNHLLGKIEKLNMREEKEIIVTWSRASTIIPTMIGHTIAIHNG
KAHLPIYIIDRMVGHKLGEFAPTLTFRGHARNNNVSRRMILKSFLLGNLLSLCMKIINSVV
VVGLYYGFLTTFSIGPSYLFLLRARVMEEG---TEKEVSATTGFITGQLMMFISIYYAPLHIAL
GRPHTITVLVLPYLLFHFFWNNHKHFFDYGSTTRNSMRNLSIQCVFLNNLIFQLFNHFILPS
STLARLVNIYMFRCNNKMLFVTSSFIGWLIGHILFMKWVGLVLFWIRQKNSIRS-IRSNKYL
VSELRNSMARIFSILLFIICVYYLGRIPSPIVTKKLKETEEDKALSTQLFEKKRWNRPLRYIK
NDRLENSVRTEMSQYFFSTTCTCGKKIISFTFPPSLSTFWKMIQRNISLCTTEKLSPKDLYN
HWVYTNEQKRHNLSNEFRNRMEALD--KLALDVLEKRIRFCNDENEQECLPKVYDPFLNG
PYRGTIKKGYSRTGMVWINRIHGILYQEFEDKD-PNDSIGIEEIGKKVPRWSYKLTDLEEQE
AEGIRSRKAKRVVIYTYALIRYSQQSDFRRNLIKGSMRAQRRKTVTWEMFQANVHSPLFL
DRIDKTFFFSFDI--SGMMNPFLRNKEENERIAISETWDTIIFAQAIRGSMLVTQSIFRKYIILP
SLIIAKNLGRMLLFQFPEWYEDLKEWNKEMHVKCTYNGVQLSETEFPKNWLTDGIQIKIL
FPFCLKPWRGSKKVKKENFCFLTVWGMETELPFGSPRKEPSFFEPIFKELEKKMRKVQ-KK
YFRVLKFLKERTKWCLKVSKEKIRWVIKIVL--FIKRIMKELAKVN-SIISNKITHESTIQIRPM
GWTNYSLTEKKMKDLADSTTTIKNQIERIKKDKKKIPNKTSFDDERSESQKHIWQRSKKR
SARLIRKSQYFIKYFIEIIYMDIFLCTINIPRINAQLFIQFISCDLSFLSQAYVFYKLSQNQVIN
KYHLISVLQYASPFIKHRIKD--TRGIFDSKSRHKKLPNSRMNEWKNWLKRHYQYNLSQNR
WSRLVSKKWRTRVNQRRTIQNKKNLDSYEKEKDQFIHYEKEEKFKKHYRYDLLSYKYIN
ESIMDPDIKYFKWRILHFFLRFFDWMGMNEEMRYRWFFPEFVRLYDTYKMKPWIIPIKLL
LLNYKSNKEAELEFFLKRYLLFQLRWDDSLNQRMINNIKVYCLLLRLINPREIAISSIQRGE
MRLDVMLIHKDLTLTELIKRGIFIIEPARLSIKRDGQFIMYQTIGISLIHKSKHNHYDLLISENI
LSPRRRRNLRILMCFIFLWPNYRLEDLACMNRYWFDTNNGSRFSMSRIYMYPMKRHQFK
SWIFELREILREIKNSHYFLDSWTKFDSVGSFTHIFFHQERFMKLFDPRIWSILLS-RDS--QG
STSNLYF---TIKGVVLLVVA-VLI----YRINNRNMVERKNLYLMGLLPIPILPIPMNSIGPRNDT
LEESF-GSSNINRLIVS-LLYLPKGKKISESCFMDPKESTWVLPIT----KKCIMPESNWGSRWW

RNRIVKKR--DSSCKIS---NETVAEIEISFREKDIK-YLEFLFVSYTD--DPIRKDHDREFFDRLS
PRKKRNIINL--NSGQLFEILVKHLICYLMSAFREKRPIEVEG-FFKQQG-AEATIQSNDIEHVS
H--LFSRNKWGISLQNCAQFHMWQFRQDLFV---SWGK-NQHESDFLRK-VSRENLIWLDNV
WLVNKDR---FFS--KVQNVSSNIQYDS---TR--SIFVQVTDSSQLKGSS-DQSRDHFDSIS----N
EDSE------------YHTLINQTEIQQLKERSIL-WD--PSFLQTERTEIESD--RFPK--CLSGYS--SM
SRLFT-EREKQMNNHLLPEEIDEFL--GNPTRSIRSFFSDRG--SELN-LGSNPTERSTRDQ----K
LLKKQQDVS-FVRSRRSE----NKEMVDIFKIITYLQN--TVSIHPISLDPGCAMVP-KDEPD-M
DSSNKISFLNKNPFFD--LFHLFHDRNKGGYTL--HHDFESE--ERFQEMADLFTLSITEP-DLV
YHKGFAFSIDSYGLDQKK--FLNEVFNSRDESKKKSL---LVLPPIFY--EENESFYRRIRKKSV
R-ISCGNDLEDPKPKIVVFASNNIMEAVNQYRLIRNLIQIQYSTYG--YIRNVSNRFFLM---NR
SDRNFEYGIQ---RDQIGNDTLNHLTIIKYTINQHLSNLKK-SQKKWFD----PLISRTERSMNR
DPDAYRYKWSNGSKNFQEHLEHFVSEQKH---RFQVVFDRLRINQYSIDWSEVIDKQDLSK
PLRFFLSKS---------LLFLS-------KSL-LFLSKSLPFFFASIGNIPIHRSE-IHIY-ELKGPNDQLCN
QLLESIGVQIVHLNKLKP--FLLD---DHDTS-QRSKFLINGGTISPFFFNKIP-KW-MIDSFHT--R
NNRRKSFDNTDSYFSMISHDRDNWLNPVKPFHRSSLISSFYKANRLRFLNNPHHFWFY--C
NKRFPFYV----EKTRINNYD--LTY-RQFLNILFIRNK-IFSLCVGK--KKHVFLE-RDTISPIESQV
SDIFIPNDFPQSGDET-YNLYKS---FHFPIRSDPFVR--RAIYSIADISGTPLTEEEIVNFERTYCQ
PLSDMNLSDSEGKNLHQYLS--FNSNMGLIHTPCSEKSLP--SGKRKK--------RSLCLK--KCV
EKGQMYRTFQRDSAFSNLSKWNLFQTYMPWFLTSTGCKYLNFTLLDTFSDPL--PILSSS-Q
KFVSIFHDIMHGSDISWPIPQKKWW----AILPQWNL-ISEISSKCLQN--LLLSEEMIHRNNESP
VPLIWTHLRSPNAREF-LYSILFLLLVAGY-LVRTHLLFVSR--ASSELQTELEKIKSLMIPSYMI
ELRKLLDRYPTSELNSFWLKNLFLVALEQLGNSLEEIRGSAFGGNMLLGGGP-----AYG-VK
SIRSKKKYLNINLIDIIDLI-GIIPNPINRITFSRNTRHLSRTSKEIYSLIRKRKNVNGDWIDEKI
ESWVANSDSIDDEEREFLVQFS--TLTT--EKRIDQILLSLT-HSDHLS-----KNDSGYQMIEQPG
SIYLRYLVDIH--KKYLMNYEFNRSCLAERRIFLAHYQTITY--SQTSCGANSSPFPSHG---KPF
SLRLDLSPS-RGILVIGSIGTGRSYLVKSLATNSYVPFITVFSNKFLDDKPKGYLID-----DID-I
DDSDDIN----IDDSDDI-----------------DDDLDTELLTMTNVL---TMYMT---PKIDLFDITPQLE
LAKAMSPCIIWIPNIHDLYVNESNYLSLGLLVNYLS---RDCERCS-TRNILVIASTHIPQKVDP
ALIAPNKLNTCIKIRRLLIPQQRKHFFILSYTRGFRLEKKMSHT--NGFGSITMGSNARDLVAL
TNEALSISITQKKSIIDTNTIRSALHRQTWDLR---SEVRSVQDHGILFYQIGRAVAQNVLLSN
CPIDPISIYMKKK--SCKEGDSYLYKWYFELGTSMKKLTILLYLLSCSAGSVAQDLWSPP--G
PDEK-NGITSYGFVENDS-DLVHG-LEVEGALVG--SSRTEKDCSQFDND--RVTLLLRSEPRN-
PLDMMQNGSCSIVDQRFLYEK--YESEFEE-GEGEGALDPQ----QIEED----LFNH--IVWAP--R
IWRP---CGNLFDCIERPNELGFP-YWARSFR---GKRIIYHKEDELQENDSEFLQSGTMQYQT
RDRSSKEQGFFRISQFIWDPADPFFFLFK-DQPFVSVFSRREFFADEEMSKGLITSQTNPPTSI
YKRWFI--KNTQEKHFELLIHRQRWLRT---NSSLS--NGSFRSN--TPSESYQYLSNLFLSN-GT
LLDQMTKALLRKRWLFPDEMKHLI--HVTGERFPIPMPRSR-INGNFIDKTSSIVANILLRIIPT
TSGEKEAFTYYRDG--MSAQSEGNYAEALQNYYEAMRLEIDPYDRSYILYNIGLIHTSNGE
HTKALEYYFRALERNPFLPQAFNNMAVICHY-RGEQAIRQGDSEIAEAWSDQAAEYWKQA
IALTPGNYIEAHNWLKITRRFE--MNWRSERIWIELITGSRKISNFCWACILFLGSLGFLLVGT
SSYLGRNLISLFPSQQIIFFPQGIVMSFYGIAGLFISSYLWCTISWNVGSGYDRFDRKEGVVC
IFRWGFPGINRRIFLRFLMRDIQSIRMEVKEGLYSRRVLYMEIRGQGAIPLTRTDEN-LTPREI
EQKAAESAYFLRVPIEVF
>Piper

MEGRRFDSVLSLSKSMGSFSPIGNISGSGDSTINDRDKTIENCPNVDYLFGVMDILGLVSDD
TFFVRDGSGDTYSIYFDVESQIFEIDND--SSFLSELESSFSSYQN---SRFGPKDDNRDYYCY
MYDTKYSWNTHINSCIDSYLRSEIS--IDSYISSSSDYYNDSYIYTFIFSESGSSIKTITNDHDS
NIREKSNDFDINKKYRHLWVQCENCYGLNYKKFFQSKMNICEQCGYHLKMSSSDRIELSI
DPGTWDPMDEDMVSIDPIEFHSEEEPYKDRLDSYQRKTGLTEAVQTGVGQLNGIPVAIGV
MDFQFMGGSMGSVVGEKITRLIEYATNRSLPVIMVCASGGARMQEGSLSLMQMAKISSVS
YDYQSNQKLFYVSILTSPTTGGVTASFGMLGDIIIAEPNAYIAFAGKRVIEQTLNKTVPEGS
QAAEYLFHKGLFDLIVPRNPLKSVLSEFFQLHGFFPLNSNSKHMETLRADEISNIIRERIEQY
NREVNIVNTGTVLQVGDGIARIHGLDEVMAGELVEFDEGTIGIALNLESNNVGVVLMGDG
LMIQEGSSVKATGRIAQIPVSEAYLGRVINALAKPIDGRGEISASESRLIESPAPGIISRRSVYE
PLQTGLIAIDSMIPIGRGQRELIIGDRQTGKTAVATDTILNQKGQNVICVYVAIGQKASSVAQ
VVTTFQEQGAMEYTIVVAETADSPATLQYLAPYTGAALAEYFMYREQHTSIIYDDPSKQA
QAYRQMSLLLRRPPGREAYPGDVFYLHSRLLERAAKLNSSLGEGSMTALPIVETQSGDVS
AYIPTNVISITDGQIFLSSDLFNAGIRPAINVGISVSRVGSAAQIKAMKQVAGKLKLELAQFA
ELEAFAQFASDLDKATQNQLARGQRLRELLKQSQSAPLAVEEQIVTIYTGANGYLDPLETG
QVKKFLVQLRTYLKTNKPQFQEIISSTKIFTKDAEAILKEVIPEQIELFLLQEQTMRINPTTSG
PGVSTLEEKNQGRIVQIIGPVLDVAFSPGKMPNIYNALIVKGQDTVGQQINVTCEVQQLLG
NNRVRAVAMSATDGLTRGMEVIDTGAPLSVPVGGATLGRIFNVLGEPVDNLGPVDTRTTS
PIHRSAPAFIQLDTKLSIFETGIKVVDLLAPYRRGGKIGLFGGAGVGKTVLIMELINNIAKA
HGGVSVFGGVGERTREGNDLYIEMKESGVINEQNLAESKVALVYGQMNEPPGARMRVGL
TALTMAEYFRDVNEQDVLLFIDNIFRFVQAGSEVSALLGRMPSAVGYQPTLSTEMGSLQE
RITSTKEGSITSIQAVYVPADDLTDPAPATTFAHLDATTVLSRGLAAKGIYPAVDPLDSTSTM
LQPRIVGEEHYETAQRVKQTLQRYKELQDIIAILGLDELSEEDRLTVARARKIERFLSQPFFV
AEVFTGSPGKYVGLPETIRGFRLILSGELDGLPEQAFYLVGNIDEATAKAMNLDEESKLKK
MTLNLCVLTPNRIIWDSEVKEIILPTNSGQIGVLPNHAPIATAVDIGILRIRLDDQWVTMAL
MGGFARIGNNEITILVNDAERGSDIDLQEAQGTLEIAEANLNKAEGKRQVIEANLALRRAR
TRVEAVNTISMKNVTDSFVSLGHWPYAGSFGFNTDILATNPINLSVVLGVLIFFGKGVLSDL
LDNRKQRILSTIRNSEELRGAAIEQLEKARTRLRKVEIEADEFRVNGYSEIEREKSNLINAAS
ENLERLENYKNETIRFEQQKAINQVRQRVFQQALQGALGTLNSCLNKELHLRAISSNISILG
AMKEIMDMNPLISAASVIAAGLAVGLASIGPGIGQGTAAGQAVEGIARQPEAEGKIRGTLL
LSLAFMEALTIYGLVVALALLFANPFVMNVLPCSINTLKGLYDISGVEVGQHLYWQIGGFQ
VHGQVLITSWVVIAILLGSVTIAVRNPQTIPTNSQNFFEYVLEFIRDLSKTQIGEDYGPWVPF
IGTMFLFIFVSNWSGALFPWKIIQLPHGELAAPTNDINTTVALALPTSVAYFYAGLTKKGLG
YFSKYIQPTPILLPINVLEDFTKPLSLSFRLFGNILADELVVVVLVSLVPSVVPIPVMFLGLFT
SGIQALIFATLAAAYIGESMEGHHMISIALEHVLTHISFSIISIVISVHLIKFLVPEIIGLCDSLEK
GIITTSFCITGLLIIRWVYSRHLPLSNLYESLMFLSWGFSTIHIFSKIWNHQNDLSTITATGAIY
CQAFATSGILNEMHQSAILVPALQSQWLMMHVSMMLLSYGALLCGSLLSVSLLVITFRKNI
EILEKMNEVRPLKKKKSFLQNISFLSFKNYHKYQFTQQLDHWSYRIINLGFTLLTIGILSGA
VWANEAWGSYWNWDPKETWAFITWTIFATYLHTRTNQRMPGMHSAIVASIGFLIIWICYF
GVNLLGIGLHSYGAFTSNVPKKALA-PPPYLVSIVFLPWWISLSFQKSLEPWITNWWNSRK
SETFLNDIQEKGVLEKFIELEELLLLDEITKECPETQIQKLYIGIYKETIQLVKIKNEEHIHIIL
QISANIICFATLSGYAILCNEELVIVNSWIQEFLYNLSDTIKAFSILLVTDLCIGFHSPHGWEL
MIGSVYKDFGFAHNEQIISGLVSTFPVILDTILKYWIFHYLNRVSPSLVVIYHSMNEMPIGVP
KVPFRSPGEEDAAWVDVYNRLHRERLLFLGQEVDSEISNQIVGLMVYLSIEDGTRDLYLFI

NSPGGWVIPGIAIYDTMQFVSPDVHTICMGLAASMGSFILVGGEITKRLAFPHAWVMIHQP
ASSFYEAPTGEFILEAEELLKLRETLTRVYVQRTGNPLWVVSEDLERDVFMSATEAQAHGI
VDLVAVEMKEQKLIHEGLITESLPNGMFRVRLDNEDLILGYVSGRIRRSFIRILPGDRVKIEV
SRYDSTRGRIIYRLRNKDSNDMEKFKRYLETFRSEQKYFLYPLLFQEYIYALGHDHGLLGY
GDKSSSLIVKRLIIRMHKQNHFLISCNEFQQNRLLGNNLDSKIISEAFSIIVEIPFSFQLVSCLI
AKSHNLRSIHSIFPFFEDNIFYLYHISDVLIPYPIHPEILVQTLRYWIQDVPSLHLLRIFLYEYC
HSGSFISKKFFSFSKERLSLFIYNSYVYEWESIFLFIRKQSSHLRSISWEALLERVHFYGKIEH
LVVVLCNDFALWVFKDCFMHYVRYQGKSLLISKGTDLLMKKWKYHFIYLWQCNFHLWS
QPHRIHINQLDNRSFHFLGYASSVRINLSVVKSQILENSFLMETSV--KKFETTVPIISLIGSLS
KEKFCNLSGHPTSKAIWADSSDSDIMERFGRVCRNLSHYYSGCSKKQILYRIKYILRLSCAR
TLARKHKSTVRVFLKRLGSGFLKEFLAEEEQVLSFFFPRRIWYLDITHTNDLANHEMIIDTT
-KVQ-AINSFSRSE-SLKEVIGLIWMLVPILTLVLAITIGVLVIVWLERQISAGIQQRIGPEYAGP
LGILQAIADGTKLLFKEDILPSRGDIRLFSIGPSIAVISIILSYSVIPFSYHLVLADLGIGVFLWI
AVSSIAPIGLLMSGYGSNNKYSFSGGLRAAAQSISYEIPLTLCVLSISLLSNSLSTIDIVEVQS
KYGIWGWNLWRQPVGFIVFLISSLAECERLPFDLPEAEEELVAGYQTEYSGIKFGLFYVAS
YLNLLVSSLFVTVLYFGGWNLSIPYTSIFGLFGINQTSGVFGTTMGMFITLAKTYLFLFISVA
TRWTLPRMRMDQLLNLGWKFLLPISLGNLLLTTSFQLLSLMIWHVQNENFILDSTRIFMK
AFHLLLFHGSFIFPECILIFGLILLLMIDSTSDQKDIPWLYFISSTSLVMSITALLFRWREEPMI
SFSGNFQTNNFNEIFQFLILLCSTLCIPLSVEYIECTEMAITEFLLFVLTATLGGMFLCGANDL
ITIFVAPECFSLCSYLLSGYTKRDVRSNEATTKYLLMGGASSSILVHGFSWLYGSSGGEIEL
QEIVNGLINTQMYNSPGISIALIFITVGIGFKLSPAPFHQWTPDVYEGSPTPVVAFLSVTSKV
AASASATRIFDIPFYFSSNEWHLLLEILAILSMILGNLIAITQTSMKRMLAYSSIGQIGYVIIGI
IVGDSNDGYASMITYMLFYISMNLGTFACIVLFGLRTGTDNIRDYAGLYTKDPFLALSSALC
LLSLGGLPPLAGFFGKLYLFWCGWQAGLYFLVSIGLLMSVVSIYYYLKIIKLLMTGRNQEI
TPHMRNYRRYPLRSNNSIELSMILCVIASTIPGISMNPIIAIAQDTLFMFLLHEYDIFWTFLIIS
SVIPILAFLISGVLAPINEGPEKLSSYESGIEPMGDAWVQFRIRYYMFALVFVVFDVETVFLY
PWAMSFDVLGVSVFIEAFIFVLIPIVGSVYAWRKGALEWSTSNFPWLTIIVVFPIFAGLFIFFL
PHRGNKVVRWYTISICLLELLLTTYVFCYHFKLDDPLIQLEENYKWITIFDFHWKLGVDGL
SIGPILLTGFITTLATLAAWPVTRYSRLFHFLMLAMYSGQIGSFSSRDLLLFFLMWELELIPV
YLLLSMWGGEKRLYSATKFILYTAGGSIFLLIGVLGMGLYGSNEPTLNLETLANQSYPLAL
EIIFYIGFLIAYAVKSPIIPLHTWLPDTHGEAHYSTCMLLAGILLKMGAYGLIRINMELLPHA
HSIFSPWLILVGAIQIIYAASTSLGQRNLKRRIAYSSISHMGFTIIGIGSITDTGLNGALLQIISH
GFIGAALFFLAGTSYDRIRLVYLDEMGGIAIPMPKIFTMFSSFSMASLALPGMSGFIAELVIF
FGIIISPQYLLIPKIVITFVVAIGMILTPIYSLSMLRQMFYGYKLFNCKKSHFFDSGPRELFISIC
ILLPVIGIGIYPDFVLSLSVDKVETILSNYF--MMLEHVLFLSAYLFSIGIFGLITSRNMVRAL
MCLELILNAVNLNLVTFSHLFDSRQLKGDIFSIFVITIAAAEAAIGLAIVSSIHRNRKSTRINQ
SNLLNKMERTYPYAWIIPFIPLPVTVLVGLGLLVVPNATKKIRRMWAFISVLLLSIALLFSINI
SIQQIGGSSIYQYLWSWTINNDFSLEFGYLIDSLTSIMLILITTVGIMVLIYSDNYMSHDQGY
LRFFAYLNFFNASMLGLVTSSNLIQIYIFWELVGMCSYLLIGFWFTRPSAANACQKAFVTN
RVGDFGLLLGILGLYWITGSFEFRDLFEIFNINTINLFFAALCASLLFLGAIAKSAQFPLHVW
LPDAMEGPTPISALIHAATMVAAGIFLVARLFPLFINLPYIMYFISLIGIITVLLGATLALAQR
DIKRSLAYSTMSQLGYIMLAMGIGSYRAALFHLITHAYSKALLFLGSGSIIHSMEPIVGYSP
DKSQNMVLMGGLTKYVPITKTTFLLGTLSLCGIPPLACFWSKDEILNDSWLYSPIFAIIAYST
AGLTAFYMFRVYLLTFDGHLRIHFTNLNGAKSRSLYSISIWGKEESNLNNT-NCFLSEINKVF

NEI--VFPYPHESDSTMLLSVCVLGLFTLFVGFIGIPLDFDILSKWLTPSINLLHKNAHYSVD
WYEFVINAIFSVSIALFGIFIASFLYGSVFSSFKSLDLINSFEIFLDRILNVIYNWSDNRGYIDT
LFSLYLTRNIRKLSELTHSFDKRLIDGITNGVGVASFFVGEGFKY-VGGGRISSYLFVYLSYV
SVFLVVIYMGLPGPIHDILLVFLGSGIVLGSLGVILLTNTIYSAFSLGFVLVCISLFYILSNSYF
VAAAQLLIYVGAINVLIIFAVMFMKGSEYSNDFSIWTVGDGVTSLVCTSIFFSLITTILDTSW
YGIIWTTRSNQIMEQDLLSNVQQIGIHLVTDFILPFELISIVLLVALIGAIAMARQMTVPATRK
DLMIVNMGPHHPSMHGVLRLIVTLDGEDVIDCEPILGYLHRGMEKIAENRTIIQYLPYVTR
WDYLATMFTEAITVNAPEQLGNIQVPKRASYIRVIMLELSRIASHLLWLGPFMADIGAQTP
FFYIFRERELLYDLFEAATGMRMMHNYFRIGGVATDLPYGWIDKCFDFCDYFLTGVVEYQ
KLITQNPIFLERVEGIGIIGGEEAINWGLSGPMLRASGIQWDLRKVDRYECYDEFNWEVQW
QKEGDSLSRYLVRIGEMTESIKIIQQALEGIPGGPYENLEVRRFDKTKDSEWNDFEYRFISK
KPSPSFELSKQELYVRVEAPKGELGIFLIGDNSVFPWRWKIRPPGFINLQILPQLVKRMKLA
DIMTILGSIDIIMGEVDRMFPMITGFMNYGQQTVRAARYIGQSFAITLSHTNRLPVTIQYPY
EKSITSERFRGRIHFEFDKCIACEVCVRVCPIDLPVVDWKLETDIRKKRLLNYSIDFGICIFC
GNCVEYCPTNCLSMTEEYELSTYDRHELNYNQIALGRLPISVIEDYTIQTNNYESEKITN----
------MQGRLSAWLVKHELVHRSLGFDYRGIETLQIKSEDWYSIAVILYAYGYNYLRSQCAY
DAAPGGLLASVYHLTRIQYGIDQPEEVCIKVFVPRTNTRIPSVFWIWKSADFQERESYDML
GISYENHPRLKRILMPESWIGWPLRKDYIAPNFYEIQDAH--
-----------------MNSMKFPLLDRTTQSSVISTTLNDLSNWSRLSSLWPLLYGTSCCFIEFASLIG
SRFDFDRYGLVPRSSPRQADLILTAGTVTMKMAPSLVRLYEQMPEPKYVIAMGACTITGG
MFSTDSYSTVRGVDKLIPVDVYLPGCPPKPEAILDAITKLRKKVSREIYEDKIGSQRENRYF
TTNHKFHVPKSTHIENYDQG---LSQSTPEIASENFFKYKSLVPNYDMN-MQNRNTFSWVKE
PINRSISVLIIIYVITQTSISNAYPIFAQQGYENPREATGRIVCANCHLANKPVDIEVPQAVLPD
TVFEAVVRIPYDSQLKQVLANGKKGGLNVGAVLILPEGFELAPPDRISPEMKEKIGNLSFQ
SYRPNKKNILVIGPVPGQKYREILFPILSPDPAAKKDAHFLKYPIYVGGNRGRGQIYPDGSK
SNNTVYNATAAGTITKILRKEKGGYEISIADASDGRQVVDNIPPGPELLVSEGESIKLDQPLT
SNPNVGGFGQGDAEIVLQDPLRVQGLLFFLASVTLAQIFLVLKKKQFEKVQLSEMNFMSK
VYDWFEERLEIQAIADDITSKYVPPHVNIFYCLGGITLTCFLVQVATGFAMTFYYRPTVTEA
FASVQYIMTEANFGWLIRSVHRWSASMMVLMMILHVFRVYLTGGFKKPRELTWVTGVIL
AVLTASFGVTGYSLPWDQIGYWAVKIVTGVPEAIPVIGSPLVELLRGSTSVGQSTLTRFYSL
HTFVLPLLTAVFMLMHFSMIRKQGISGPLMGITKKPDLNDPVLRAKLAKGMGHNYYGEPA
WPNDLLYIFPVVILGTIACNVGLAVLEPSMIGEPADPFATPLEILPEWYFFPVFQILRTVPNKL
LGVLLMVSVPAGLLTVPFLENVNKFQNPFRRPVATTVFLVGTVVALWLGIGATLPIDKSLTL
GLFMIEVFLFGIVLGLIPITLAGLFVTAYLQYRRGDQLDLMPTITSYFGFLLAALTITSALLIG
LSKIRLIMDIVSLAWAALMVVFTFSLSLVVWGRSGLMIIRSPESEVKIVVDRDPVKTSFEEW
AKPGHFSRTIAKGPDTTTWIWNLHADAHDFDSHTSDLEEISRKVFSAHFGQLSIIFLWLSG
MYFHGARFSNYEAWLSDPTHIGPSAQVVWPIVGQEILNGDVGGGFRGIQITSGFFQLWRA
SGITNELQLYCTAIGALVFAGLMLFAGWFHYHKAAPKLSWFQDVESMLNHHLAGLLGLG
SLSWAGHQVHVSLPINQFLDAGVDPKEIPLPHEFILNRDLLAQLYPSFAEGATPLFTLNWSK
YAEFLTFRGGLDPTTGGLWLTDIVHHHLAIAILFLIAGHMYRTNWGIGHSLKDILEAHKGP
FTGQGHKGLYEILTTSWHAQLSLNLAMLGSLTIVVAHHMYSMPPYPYLATDYGTQLSLFT
HHMWIGGFLIVGAAAHAAIFMVRDYDPTTRYNDLLDRVLRHRDAIISHLNWACIFLGFHS
FGLYIHNDTMSALGRPQDMFSDTAIQLQPIFAQWIQNTHALAPSATAPGAIASTSLTWGGA
DLVAVGGKVALLPIPLGTADFLVHHIHAFTIHVTVLILLKGVLFARSSRLIPDKANLGFRFPC

DGPGRGGTCQVSAWDHVFLGLFWMYNAISVVIFHFSWKMQSDVWGSISDQGVVTHITG
GNFAQSSITINGWLRDFLWAQASQVIQSYGSSLSAYGLFFLGAHFVWAFSLMFLFSGRGY
WQELIESIVWAHNKLKVAPATQPRALSIVQGRAVGVTHYLLGGIATTWAFFLARIIAVGMA
LRFPRFSQGLAQDPTTRRIWFGIATAHDFESHDDITEERLYQNIFASHFGQLAIIFLWTSGNL
FHVAWQGNFESWVKDPLHVRPIAHAIWDPHFGQPAVEAFTRGGAFGPVNISYSGVYQWW
YTIGLRTNEDLYTGALFLLFLSAISLVAGWLHLQPKWKPSVSWFKNAESRLNHHLSGLFG
VSSLAWTGHLVHVAIPGSRGEYVRWNNFLDVLPYPQGLTPLFTGQWNLYAQNPDSGSHLF
GTSQGSGTAILTLLGGFHPQTQSLWLTDIAHHHLAIAFIFLVAGHMYRTNFGIGHSMKDLLE
AHTPPGGRLGRGHKGLYDTINNSLHFQLGLALASLGVITSLVAQHMYSLPAYAFIAQDFTS
QAALYTHHQYIAGFIMTGAFAHGAIFFIRDYNPEQNEDNVLARMLDHKEAIISHLSWASLF
LGFHTLGLYVHNDVMLAFGTPEKQILIEPIFAQWIQSAHGKTSYGFDILLSSTNGPAFNAGR
SIWLPGWLSAVND--NSNSLFLTIGPGDFLVHHAIALGLHTTTLILVKGALDARGSKLMPDK
KDFGYSFPCDGPGRGGTCDISAWDAFYLAVFWMLNTIGWVTFYWHWKHITLWQGNVSQ
FNESSTYLMGWLRDYLWLNSSQLINGYNPFGMNSLSVWAWMFLFGHLVWATGFMFLISW
RGYWQELIETLAWAHERTPLANLIRWRDKPVALSIVQARLVGLAHFSVGYIFTYAAFLIAS
TSGKFGMSHSVKIYDTCIGCTQCVRACPTDVLEMIPWDGCKAKQIASAPRTEDCVGCKRC
ESACPTDFLSVRVYLWHETTRSMGLAYMTDFSLPSILVPLVGLVLPAIAMASLSLHVQKNK
IVMRDIKTYLSVAPVLATLWFGSLAGLLIEINRLFPDALVFPFFSFMTAILERRESTSLWGRF
CNWITSTENRLYIGWFGVLMIPTLLTATSVFIIAFIAAPPVDIDGIREPVSGSLLYGNNIISGAII
PTSAAIGLHFYPIWEAASVDEWLYNGGPYELIVLHFLLGVACYMGREWELSFRLGMRPWI
AVAYSAPVAAATAVFLIYPIGQGSFSDGMPLGISGTFNFMIVFQAEHNILMHPFHMLGVAGV
FGGSLFSAMHGSLVTSSLIRETTENESANEGYRFGQEEETYNIVAAHGYFGRLIFQYASFNN
SRSLHFFLAAWPVVGIWFTALGISTMAFNLNGFNFNQSVVDSQGRVINTWADIINRANLG
MEVMHERNAHNFPLDLAAVEAPSTNGMGLPWYRVHTVVLNDPGRLLSVHIMHTALVSG
WAGSMVLYELAVFDPSDPVLDPMWRQGMFVIPFMTRLGITNSWGGWSIT-GGTITNPGIW
SYEGVAGAHIMLSGLCFLAAIWHWVYWDLEIFRDERTGKPSLDLPKIFGIHLFLAGVACFG
FGAFHVTGLYGPGIWVSDPYGLTGKVQPVNPSWGAEGFDPFVPGGIASHHIAAGTLGILA
GLFHLSVRPPQRLYKGLRMGNIETVLSSSIAAVFFAAFVVAGTMWYGSATTPIELFGPTRY
QWDQGYFQQEIYRRVSAGLAENLSLSEAWSKIPEKLAFYDYIGNNPAKGGLFRAGSMDN
GDGIAVGWLGHPVFRDKEGHELFVRRMPTFFETFPVVLVDGDGIVRADVPFRRAESKYSV
EQVGVTVEFYGGELNGVSYSDPATVKKYARRAQLGEIFELDRATLKSDGVFRSSPRGWFT
FGHASFALLFFFGHIWHGARTLFRDVFAGIDPDLDSQVEFGTFQKIGDPTTRRQAVMKTLY
SLRRFYPVETLFNGTLALTGRDQETTGFAWWAGNARLINLSGKLLGAHVAHAGLIVFWAG
AMNLFEVAHFVPEKPMYEQGLILLPHLATLGWGVGPGGEVIDTFPYFVSGVLHLISSAVLG
FGGIYHALLGPETLEESFPFFGYVWKDRNKMTTILGIHLILLGIGAFLLVFKALYFGGVYDT
WAPGGGDVRKITNLTLSPSVIFGYLLKSPFGGEGWIVSVDDLEDIIGGHVWLGSICILGGIW
HILTKPFAWARRAFVWSGEAYLSYSLGALAIFGFVACCFVWFNNTAYPSEFYGPTGPEASQ
AQAFTFLVRDQRLGANVGSAQGPTGLGKYLMRSPTGEVIFGGETMRFWDLRAPWLEPLR
GPNGLDLGRLKKDIQPWQERRSAEYMTHAPLGSLNSVGGVATEINAVNYVSPRSWLATSH
FVLGFFFFVGHLWHAGRARAAAAGFEKGIDRDFEPVLSMTPLNMTIALGRFTKEEEDLFD
IMDDWLRRDRFVFVGWSGLLLFPCAYFALGGWFTGTTFVTSWYTHGLASSYLEGCNFLT
AAVSTPANSLAHSLLLLWGPEAQGDFTRWCQLGGLWTFVALHGAFALIGFMLRQFELARS
VQLRPYNAIAFSGPIAVFVSVFLIYPLGQSGWFFAPSFGVAAIFRFILFFQGFHNWTLNPFH
MMGVAGVLGAALLCAIHGATVENTLFEDGDGANTFRAFNPTQAEETYSMVTANRFWSQI

FGVAFSNKRWLHFFMLFVPVTGLWMSALGVVGLALNLRAYDFVSQEIRAAEDPEFETFYT
KNILLNEGIRAWMAAQDQPHENLVFPEEVLPRGNALMSGSTGERSFADIITSIRYWVIHSITI
PSLFIAGWLFVSTGLAYDVFGSPRPNEYFTESRQGIPLITGRFDPLEQLDEFSRSFMTIDRTY
PIFTVRWLAVHGLAVPTVSFLGSISAMQFIQRMATQTVEDSS-KSGPRRTVIGDLLKPLNSE
YGKVAPGWGTTPLMGVAMALFAIFLSIILEIYNSSVLLDGILVSMLTLKLFVYTVVIFFVSLF
IFGFLSNDPGRNPGREDMADTTGRIPLWLIGTVTGIIVIGLIGIFFYGSYSGLGSSLMLNIFSL
IC--LSSALHSSSFFFAKLPEAYALFNPIVDVMPVIPVLFFLLALVWQAAVSFRTTQSNPNEQS
VELNRTSLYWGLLLIFVLAVLFSNYFFNMEVNILAFIATVLFILVPTAFLLIIYVKTVSQNDM
ETATLVTIFISGSLVSFTGYALYTAFGQPSQQLRDPFEEHGDMEALVYTFLLVSTLGIIFFAIFF
RDPPRISTKK--MTIAFQLAVFALIATSLILVIGVPVVFASPDGWSSNKNIVFSGTSLWIGLVFL
VGILNSLIS-------MSPKTETKAYVGFKAGVKDYKLTYYTPEYETKDTDILAAFRVTPQPGV
PPEEAGAAVAAESSTGTWTSVWTDGLTSLDRYKGRCYDIEPVAGEENQYICYVAYPLDLFE
EGSVTNMFTSIVGNVFGFKALRALRLEDLRIPPAYSKTFQGPPHGIQVERDKLNKYGRPLL
GCTIKPKLGLSAKNYGRAVYECLRGGLDFTKDDENVNSQPFMRWRDRFLFCAEALFKAQ
TETGEIKGHYLNATAGTCEEMIKRAVFARELGVPIVMHDYLTGGFTANTSLAHYCRDNGL
LLHIHRAMHAVIDRQKNHGMHFRVLAKALRMSGGDHVHSGTVVGKLEGERDITLGFVD
LLRDDFIEKDRSRGIYFTQDWVSMPGVLPVASGGIHVWHMPALTEIFGDDSVLQFGGGTL
GHPWGNAPGAVANRVALEACVQARNEGRDLAREGNEIIREAAKWSPELAAACEVWKEIK
FEFAAMDTL--TAINLYKTSIPSTRKGAVDSQVKSNPRNNLIYGQHRCGKGRNARGIITAGH
RGGGHKRLYRKIDFRRNDKDISGRIVTIEYDPNRNAYICLIHYGDGEKRYILHPRGAIIGDTI
VSGTEVPISMGNALPLTDMPLGTAIHNIEITLEKGGQLARAAGAVSKLIAKEGKSATLRLPS
GEVRLISKNCSATVGQVGNVEVNQKSLGRAGSKCWLGKRPVVRGVVMNPVDHPHGGGE
GRAPIGRKKPATPWGYPALGRRSRKRNKYSDRFILRRRK-MIQTQTYLNVADNSGARELM
CIRILGAS-NRRYANIGDIIVAVIKEAVPNMPLERSEVIRAVIVRTCKELKRDNGMIIRYDDNA
AVVIDQEGNPKGTRVFGAIARELRQLNFTKIVSLAPEVLMLSPKRTRFRKQHRGRMKGISY
RGNRICFGRYALQALEPAWITSRQIEAGRRAMARYARRGGKIWVRIFPDKPVTVRPAETRM
GSGKGSPEYWVSVVKPGRILYEIGGISETVARAAISIAASKMPIRTQFVIAGMTRVRRGYIA
RRRRTKIRLFASTFRGAHSRLTRTITQQKMRALVSAHRDRGRKKRDFRRLWITRINAVIRGS
--VSYSYSRLIHDLYKRQLLLNRKILAQIAISNRNCFYMISNQIIKSGECEEFNGIIKAKRVQA
FARDVRISAHKARRVIDQIRGRSYVETLTLLELMPYRASYPIFKLLYSAAASASHNLGFKEA
DSYISKAEVNEGAVLKRLKPRARGRSYLIKKPTCHITIVLKDRTLN-------MDGIKYIVFTEK
SIRLLGNNQ-YTSNVESGSTRTEIKHWVELFFGVKVIAMNSHRLQGKGRRMGPIIGHTMHY
RRMIITLQPGYSIPPLIEKRTMAVPKKRTSISKKRIRRNIWKKRGYWEAKRAFSLAKSISSGR
SKSFF--QKNKMAKGKDARVIVLLECTSC-VRN--FNKQKKGVSRYITQRNRHNTPGRLELR
KFCPYCHKHTIHGEIKKMKIRASARKICEKCRLIRRRGRILVICSNPRHKQRQGMVREEIAV
ATGAPRWKCVESRIDSKRLYYSRFILSPLMKGQADMIGIAMRRALLGEIEGTCITRVKSE--
KVPHEFSTIVGIEESVHEIVMNLKEIVLRSNLYGTLDASICIRGPRYVTAQDIILPPSVEIVDA
TQHIANLTERVDLCIKLQIERDRGYSMKTPHNDEDGSY--PIDAIFMPVRNANHSVHSYGNG
NEKQEILFIEIWTNGSLTPKEALYEASRNLIDLFIPFLHAEER--EIHLEDNA-NGVPLALFTFH
DEFAININRIRKNKK-KMALKSIFIDQSELPSKTYNCLKKSNIHTLLDLLNNSQEDLLKIEHFCM
EDVKRILDILQKH--FGFDLPKNGMLRNR-NEGMSTIPEFSQIQFEGFCRFIDWGLAEELHKF
PKIEDTDQEIEFQLFVETYQLAEPLIKERDAVYESLTYSSELYVPAGLIWKPGKDMQEQTVF
IGNIPLMSSLGIFIVNGIYRIVINQILQSPGIYYRSELDHNGIFIYTGTIISDWGGRSELEIDRK
ARIWARVSRKQKISILVSSSAMGSNLREILENVRYPEIFLSFLNERDKKKIGSRENAILEFYQ

QFACVGGDPVFSESLCRELQKKFFQQRCELGRIGRRNMNRRLNLDIPQNNTFLLPRDVLA
AADHLIGMKFGMGTLDD--MNHLKNKRIRSVADLLQDQFGLALVRLENVVRGTICGAIR-
HKLI-PTPQNLVTSTP----LTTTYESFFGLHPLSQVLDRTNPLTQIVHGRKSSYLGPGGLTGRT
ASFRIRDIHTSHYGRICPIDTSEGINVGLIGSLAIHARVGPWG-SIKTPFY---EISERSKR--LQL
VYLSPSVDEYHMVAAGNSLALNRGIQEEQAVPARYRQEFLTIAWEQIHLRSIFPFQYFSMG
ASLIPFIEHNDANRALMSSNMQRQAVPLSRSEKCIVGTGLERQAALDSGVSAISEREGKVI
YTNTDKIILSGNGDTITIPLVMYQRSNKNTCMHQKTQVSRGKCIKKGQILANGAATVGGE
LALGKNVLVAYMPWEGYNFEDAVLISERLVYEDIYTSFHIRKYEIQTHVTSQG-PERITKEIP
HLETHLLRNLDRNGIVMLGSWVETGDILVGKLTPQTAKE-SSYAPEDRLLRAILGIQVSTSK
ETCLKLPIGGRGRVIDVRWIQKKG-GSSYNPETIRVYISQKREMKVGDKVAGRHGNKGIISK
ILPRQDMPYLQDGTPVDMVFNPLGVPSRMNVGQIFECSLGLAGDFLGRHYRVAPFDERYE
QEASRKLVFSELYEASKQTANPWVFEPEYPGKSRIFDGRTGDPFDQPVLIGKSYILKLIHQV
DDKIHGRSSGHYALVTQQPLRGRAKQGGQRVGEMEVWALEGFGVAHILQEMLTYKSDHI
RSRQEVLGTTIVGGTIANPGDAPESFRLLVRELRSLALELNHFLLSEKDFQIHRKEA-------M
IDRYKHQQLRIGPVSPQQISAWANKILPNGEIVGEVTKPYTFHYKTNKPEKDGLFCERISGP
IKSGICACGNYRVIGDEKEDPNFCEQCGVEFADSRARRYQMGYIKLTCPVTHVWYLKRLP
SYIANLLDKPLKELEGLVYCD--FSFARP-VAKKPTFLRLRGLFEYEIQSRKYSIPLFFTTQGF
DTFRNREISTGASAIREQLADLDLRIIIDRSLVEWKELGEEGST-GNEWEDRKIGRRKDFLV
RRIELAKHFIRTNVEPERMVLSLLPVLPPELRPIIQIDGGKPMSSDINELYRRVIYRNNTLTDL
LTTSRSTPGELVMCQEKLVQEAVDTLLDNGIRGQPMRDGYNKVYKSFSDVIEGKEGRFRE
TLLGKRVDYSGRSVIVVGPSLSLHRCGLPREIAIELFQTFLIRGLIRQRVASNIGIAKSKIREK
EPIVWEILQEIMRGHPVLLNRAPTLHRLGIQAFQPILVEGRAICLHPLVCKGFNADFDGDQ
MAVHVPLSLEAQAEARLLMFSHMNLLSPAIGSPISVPTQDMLIGLYVLTIGNRQGVCANRY
NPWNRR--N-YQNETVDHTKYRYTKEKEPYFCSSYDALGAYRQKRIHLDTPLWLRWRLDQ
R-IISL--REVPIEVQYESLGTYHEIYRPYLIVKSVKKEILCIYIRTTVGHISFYREIEEAIQGFCR
AYSYDGT-------TEVLMAEQADLVFHNKAIDGTAMKRLISRLIDHFGMAYTSHILDQVKTL
GFQQATATSISLGIDDLLTIPSKGWLVRDAEQQGLILEKHHHYGNVHAVEKLRQSIEIWYAT
SEYLRQEMHTNFRMTDPSNPVHIMSFSGARGNASQVHQLVGMRGLMSDPQGQMIDLPIQ
SNLREGLSLTEYIISCYGARKGVVDTAVRTSDAGYLTRRLVEVVQHIVVRRTDCGTIRGISA
SPRNSMEE--KIWIQRLIGHVLAYDIYIGPRCIAARNQDIGIGLVNRFIT------FQAKSIYIRTPFI
CRSTSWICRLCYGRSPTHGDLVELGEAVGVIAGQSIGEPGTQLTLRTFHTGGVFTGGTAEY
VRAPSNGKIKFHEDLVHPTRTRHGHPAFLCYIDLYVTIESRDILHKVNIPPKSFILVQNNQYV
ESEQVIAEIRAGTSAL-SFKEKVRKHIYSDSEGE-MHWSTNVYHTPQYTYGNVHLLPKTSH
LWILSGNPCK--SNGALFSLHKDQDQMNLYSLSLSLEQRSISDFS--VTNDR-VRRKSFSLDPS
REGGGRFFDY-SVPDKSIPNDCWNFIYSA-ILHENSY--FLAKRRRNRFIIPFQY-QEGEKEPMP
CSGISIEIPKT--GILRRNTILAYFDDPRYRRSGSGITKYGTVEVDSLVKKED--LVEYRG-EKE
LRTKYTKVDRFFFIPEEVHILPGSSSIMVRNNSIIGVDTEIALNTRSRVGGLVRVERKKKRTE
LKIFSGNIHFPGETDKISRHSGILLPPGMEKK--NSKESKK--WKNWIYVQR--VTPIKKK--YF
VSVRPVVTYEIADGINLATLFPQDSLQERDNVQLRVVNYILYGNGKPIRGISHTSIQLVRTCL
VLNWDQDNKK----SIEEVFASFVEVRANDLIRDFIRVDLVKPRVLNTGKRNDTAGYGLNPD
-NGSDCPNRNPFYS---------KIKIQSFTQDNGTIHTSLNI---NKQLPPFLILSSSNCFQAGPLKV
NGSKSDNG---KKKSDPAILIRNFFGPLGT--IAKITNLDS--RNYYLT--DNQIL-LKKYLILDN---
FKNLFQDVL---YYLIDEKGR--ILNPDPCSNIILNAFDLNWCFLHHNSCEKTFSIIS-LGQLFY
ENGSIYKYRS---HIKSGQIVTVH-GDSLVIRSAKLHLATPGATVHGHYGEIIYEGDTLVTFIYE

KSRSGDITQGLPKVEQVLEVRSIDSISMNLEKRVEGWNENITKILGIPWGFLIGAELTIVQSR
ISLVNKIQKVYRSQGVQIHNRHIEIIVRQITSKVLVSE---DGMSNAFSPGELIGLLRAERTGR
ALEETICYRAILLGLTRASLNTQSFISEASFQETARVLAKAALRGRIDWLKGLKENVVLGG
MIPVGTGFKGFVRRS--RQHNNIS--LEKKRNNLVK--GEIGNILFHHRELFSSCISKNKKKFS--
-------------MTKRYWNINLEEMMEAGVHFGHGTRKWNPRMAPYISAKRKGIHITNLTRTAR
FLSETCDLVFDAASRGKHFLIVGTKSKAADLVALAAIRARCHYVNKKWLGGMLTNWSTT
ETRLHKFRDLRAEQRAGKLNCLPKRDAAMLKRKLSHLQTYLGGIKYMTGLPDIVIIVDQH
EEYTALRECITLGIPTICLIDTNCDPDLSDISIPANDDAIASIRLILNKLVSALCEGHSS-YIRNR
MGKKINPLGFRLG-AQSHRSIWFAQSKSYSRGLQEDEKIRDCIQHYVEKNTRISSG-FEGISH
IEIQKKIDLIQVIIYMGSPNSLMEG--RTR--GIDELQTNVQKEFLSVNRRLNIAIIRVAKPYAQP
TILAEHIALQLKNRVSFRKAMKSAIELTEQADTKGIQIQIAGRIDGKEIARVEWIREGRVPLQ
TIRAKIDYCSYTVRTIYGILGIKIWIFLDEKMSRYRGPRFKKIRRLGALPGLTSKRPRSESSLR
SQPRSGK-RSQYRIRLEEKQKLRFHYGLTERQLIRYVRIAGKAKGSTGQVLLQLLEMRLDN
ILFRLGMSSTIPGARQLVNHRHVLVNGRIVDIPSYRCKPRDIISTRNEQRSKVLVQNSMDSA
SRE--LPKHLTLDS--SQYKGVVNQIIDSKGVGLKINELLVVEYYSRQTMSRRGTTEEKTAKS
DPIYRNRLVNMLVNRILKHGKKSLAYQIIYRAVKKIQQKTETNPLSVLRQAIHGVTPDIAVK
ARRVGGSTHQVPIEIGSTQGKALAIRWLLWASRKRPGRNMAFKLSSELVDAAKGSGDAIR
KKEETHRMAEANRAFAHFRMGRDTIADIITSIRNAHMDKKGTVRIASTNMTENIVRILLRE
GFIENVRKHRENQKNFLVSTLRHRR--NRKGTSR---TILNLKRISRPGLRIYSNYQKIPRILGG
MGIVILSTSRGVMTDREARLEGIGGEILCYIWMTKPIPRIGSRRSGRIGSRKAGRRIPKGVIH
VQASFNNTIVTVTDVRGRVVSWSSAGTCGFKGTRRGTPFAAQTAAGNAIRTVVDQGMQR
AEVMIKGPGLGRDAALRAIRRSGLLLSFVRDVTPMPHNGCRPPKKRRVMPTIKQLIRNTR
QPIRNVTKSPALRGCPQRRGTCTRVYTITPKKPNSALRKVARVRLTSGFEITAYIPGIGHNLQ
EHSVVLVRGGRVKDLPGVRYHIVRGTLDAVGVKDRQQGRSKYGVKKPKMARKSLIQRER
KRQKLEQKFHSIRRSSKKEIS---KVSSLSGKWEIHGKLQSPPRNSAPIRLHRRCFLTGRPRG
NYRDFGLSGHILRERFHACLLPGATRSSWMVKNSFISAISQEEKEKNK--GSTEFQILNFTKR
IRRLTSHLELHKKDYLSQRGLRKILGKRQRLLTYLSNKNRVRYKELIGQLDIREPKTR--MV
KLRLKRCGRKQRAVYRIVAIDVRSRREGRDLQKVGFYDPIKNQTYLNVPAVLYFLGKGAQ
PTGTVHDISKKAEIFKELRVNQTIRVNMDKTKRPLRKSKRSFRRRLPPIGSGDRIDYRNMSL
ISRFISEQGKILSRRVNRLTLKQQRLITIAIKQARILSSLPFLNNEK--QFERPESIPKTAGPSIRN
KMAR-LKKSPFVANHLLKKIEKLNMKGGKEIIVTWSRASTIIPTMIGHTIAIHNGKEHLPIYI
TDRMVGHKLGEFAPTLTFRGHARNDNRSRRMILKSFLLGNLLSLCMKIINSVVVVGFYYG
FLTTFSIGPSYLFLLRAQVMEEE---TEKEISSTTGFITGQLMMFISIYYAPLHLALGRPHTITV
LVLPHLLFHFFWNNHKHFLDYGSTTRNSMRNLSIQCVFLNNLIFQLFNHFILPSSTLARLVN
IYLFRCNSKILFVTSSFVGWLIGHILFMKWVGLVLSWIRQNHS----IRSNKYLVSELTN--SRIF
SILLFITSVYYLGRMPSPIVTKKLKETEED--GRD-------PSE
------------------------------WK-----------------------------GNI---
----

----MKGHQFQSWIFELREILREVKNSHYFLDSWTQFDSVGSFTHIFFHQERFMKLFDPRIWS
ILLS-RDS--QGSTSNRYF---TVKGVVLLVVA-VLI----YRINNRNIVERKNLYLRGLLPIPILPIP
MNSIGPRNDTLEESF-WSSNINRLIVS-LLYLPKGKKISESCFMDSKESAWVLPITK---KKCIIP
ESNWGSRWWRNRIGKKR--DSSCKISN-ETETIAGIEISFKEKDIQ-YLKFLFVSYTD--DPIRK
DHDWELFDRLSPRKKRNIINL--NSGQLFEILVKHLICYLMSAFREKRPIEMGG-FFKQQGG
AGATIQSNDIEHVSH--LFSRKKWGIFLQNCAQFHMWQFYQDLFV---SWGK-NQHESDFLR
N-VSRDNWIWLDNVWLVNKDR---FFS--KVLNVSSNIQYDS---TR--SIFVQVTDSSQLKGSS-
DQSGDHFDSIR----NENSE-------------YHTLINQTEIQQLKERSIL-WD--PSFLQTERTEIESD--
RFPK--CLSGYS--SISRLFT-EREKQMNNHLLPEEIEEFL--GNPTRSIRSFFSDRW--SELR-LGS
NPTERSTRDQ----KLLKKQQDVS-FVPSRRSE----NKEMVAIFKIITYLQN--TVSIHPISSDPGC
DMVP-KDEPD-IDSSHKISFLNKNPFLD--LFHLFHDRNKGGYTL--HHDFESE--ERFQEMAD
LFTLSITEA-GLVYHKGFTFSIDSYGLDQKK--FLNEVFNSRDESKKKSL---WFLPPIFD--EEN
ESFYGRIRKKSAR-ISCGNDLEDPNPKIVVFSSNNIMGAVNQYRLIRNLIQIQYRTYE--YIRN
VSNRFFLM---NRPDRNFEYGIQ---GDPIGNDTLNHLTIIKHTINQHLSNLKK-SQKKWFD----
PLISRTERCMNRDPDAYRYKWSNGSKNFQEHLEHFVSEQKH---RFQVVFGRLRINQYSID
WSEVIDKQDLSKSLRFFLSKS---------LLFLS-------KSLRFFLSKSLPFFFVSIGNIPIHRFE-IHI
Y-ELKGPNDQLCNPLLESIGVQIVHLNKLKP--FLLD---DHDIS-QRSKFLINGGTISPFLFNKIP
-KW-MIDSFHT--RKNRRKSFDNTDSYFSMISHDRDNWLNPVKPFHRSSSISSFYKANRLRFL
NNPHHLWFY--CNKRFPFDV----EKARINNYD--LTY-GQFLNVLFIRNK-IFSLCVRK--KKHVF
GG-RDAISPIESQVSDIFIPNDFPQSGDET-YNLYKS---FHFPIRSDSFVR--RAIYSIADISGTPL
TEEQIVHFQRTYCQPLSDMNLSDSEGKNLHQYLR--FNSNMGLIHTLCSEKDFP--SGKRKK-
-------RTLCLK--KCVEKWQMYRTFQRDNDFSNLSKWNLFQTYMPWFLTSTGCKYLNFTLL
DTFSDPL--PILSNS-PKFVSIFHDIMHGSDISWPIPQQKGR----AILPQRNW-ISEMSSKCLQN--
LLLYEEIIHRNNESPALLIWTHLRSPNAREL-LYSILFLLLVAGY-LVRTHLLFVSR--ASSELQT
ELEKIKSLMIPSYMMELRKLLDRYPTPELNSFWLKNLFLVALEQLGDSLEEIRGSASGGNIL
LGGGP-----AYG-VKSIRSKKKYLNINLIDIIDFI-SIIPNPINRITFSRNTRHLSHTSKEIYSLIRK
RKNVNGGWIDEKIESWVANSDSIDDEEREFLVQFS--TLTT--EKRIDQILLSLT-HSDHLS-----
KNDSGYQMIEQPGSIYLRYLVDIH--KKYLMNYEFNRSCLAERRIFLAHYQTITY--SQTSCG
TNSSHFPSHG---KPFSLRLALSPS-RGILVIGSIGTGRSYLVKYLATNSYVPFITVFPNKFLDD
KPKGSLFD-----NID-IDDSDDID----IDDSDDIDIDDSDN-----------IDDLDTELLTMTNVL---TM
YMT---PKIDRFDITLQFELAKAMSPCIIWIPNIHDLYVNESNYLSLGLLVNYLS---RDCERCS-
TRNILLIASTHIPQKVDPALIAPNKLNTCIKIRRLLIPQQRKHFFILSYTRGFHLEKKMFHT--N
VFGSITMGSNARDLVALTNEALSISITQKKSIIDTNTIRSAIHRQTWDLR---SQVRWVQDHGI
LFYQIGRAVAQNVLLSDCPIDPISIYMKKK--SCKEGDSYLYKWYFELGTSMKKLTILLYLLS
CSAGSVAQDLWSPP--GPDER-NCITSYGFVENDS-DLVHG-LEVEGALVG--SSRTEKDCSQF
DND--RVILLLLRSEPKN-PFDMIQNGSCSIVDQRFLYEK--YESELEE-GEGEGALDPQ----QIEE
D----LFNH--IVWAP--RIWRP---CGNLFDCIERPNELGFPYWWTRSFR---GKRILYHKEDALQ
ENDSEFLQSGTVRYQRLDRSSKEQGFFRISQFIWDPADPFFFLFK-DQPFV--FSRREFFADEE
MSKGLITSQTNPTTSIYKRWFI--KNTQQNHFELLIHRQRWLRT---NSLLS--NGSFRSN--TLS
ESYQYLSNLFLSN-RTLLDQMTKALLRKRWLFPDEMKHWI--HVTGERFPIPMPRSQ-INGN
FIDKTFSIVANILLRIIPTTSGEKEAFTYYRDG--MSAQSEGNYAEALQNYYEATRLEIDPYDR
SYILYNIGLIHTSNGEHTKALEYYFRALERNPFLPQAFNNMAVICHY-RGEQAIRQGDSEIAE
AWSDQAAEYWKQAISLTPGNYIEAHNWLKITGRFE--MNRRSERIWIEFIMGSRKTSNFCW
ACILLLGSLGFLLVGISSYLGRNLISLFPSQQINFFPQGIVMSFYGIAGLFISSYLWSTILWNIG

SGYDRFDRKEGIVCIFRWGFPGINRRIFLRVFMGDIQSIRIEVKGGVYPRRVLYMDIRGQGS
VPLTRTDEN-FTPREIEQKAAESAYFLRVPIEVF
>Coffea
MERWWFNSMLFLNKSTESLGSIENASEGDEPDIKDRTKNIQSYSKVDHLFVVKDIRNFISD
DTFLVKDSNGDNFSIYFDIENQIFEIDNNH--SFRSELKN---TYWNSSYLNNGSTSEDTYYNH
YMYDTQYSWNNHINSCIDSYLQ--SQIWIETSIVSGGDNYSDSYIYSSICG-EVSDIQTHVKG
SDFTIIESSNDLDVTQKYRHLWVQCENCYGLNYKKFLKSKMNICEQCGYHLKMNSSERIE
LSIDPGTWDPMDEDMASLDPIEFHSEEEPYKDRIDSYQKKTGLTEAVQTGIGQLNGIPVAV
GVMDFQFMGGSMGSVVGEKITRLIEYATKELLPLIIVCASGGARMQEGSLSLMQMAKISS
ALYDYQSNKKLFYVSILTSPTTGGVTASFGMLGDIIIAEPNAYIAFAGKRVIEQTLNTTVPEG
SQAAEYLFQKGLFDLIVPRNLLKSVLSELFKFHAFVPLNQNETEMVTIRADEISNIIRERIEQ
YNREVKIVNTGTVLQVGDGIARIHGLDEVMAGELVEFEEGTIGIALNLESNNVGVVLMGD
GLMIQEGSSVKATGRIAQIPVSEAYLGRVINALAKPIDGRGEISASESRLIESPAPGIISRRSV
YEPLQTGLIAIDSMIPIGRGQRELIIGDRQTGKTAVATDTILNQQGQNVICVYVAIGQKASSV
AQVVTTFQERGAMEYTIVVAETADSPATLQYLAPYTGAALAEYFMYRERHTSIIYDDPSK
QAQAYRQMSLLLRRPPGREAYPGDVFYLHSRLLERAAKSSSNLGEGSMTALPIVETQSGD
VSAYIPTNVISITDGQIFLSADLFNAGIRPAINVGISVSRVGSAAQIKAMKQVAGKLKLELAQ
FAELEAFAQFASDLDKATQNQLARGQRLRELLKQSQAAPLAVEEQIMTIYTGTTGYLDSLE
IGQVRKFLVALRAYVKTNKPQFQEIISSTKTFTEEAESLLKEAIQEQMDRFLLQEQAMKINP
TTSGSGVSTLEKKNMGRIVQIIGPVLDVAFPAGKMPNIYNALVVKGRDTVGQPINVTCEVQ
QLLGNNRVRAVAMSSTDGLTRGMEVIDTGAPLSVPVGGATLGRIFNVLGEPVDNLGAVDT
RTTSPIHRSAPAFIQLDTKLSIFETGIKVVDLLAPYRRGGKIGLFGGAGVGKTVLIMELINNI
AKAHGGVSVFGGVGERTREGNDLYMEMKESGVINKENIAESKVALVYGQMNEPPGARM
RVGLTALTMAEYFRDVNEQDVLLFIDNIFRFVQAGSEVSALLGRMPSAVGYQPTLSTEMG
SLQERITSTKEGSITSIQAVYVPADDLTDPAPATTFAHLDATTVLSRGLAAKGIYPAVDPLDS
TSTMLQPRIVGEEHYETAQRVKQTLQRYKELQDIIAILGLDELSEEDRLTVARARKIERFLS
QPFFVAEVFTGSPGKYVGLAETIRGFQLILSGELDSLPEQAFYLVGNIDEATAKAMNLEME
NNLKKMTLNLCVLTPNRIVWDSEVKEIILPTNSGQIGVLPNHAPIATAVDIGILRIRLNGQW
LTMALMGGFAKIGSNEIIVLVNDAEKGSDIDPQEAQQTLEIAEANLKKAEGKRQTIEANLA
LRRARTRVEAINAV-MKNVTDSFLSLGHWPFAGSFGFNTDILSTNLINLSVVLGVLIFFGKG
VLSDLLDNRKQRILNTIRNSEELRGGAIEQLEKARTRLRKVEMEADQFRVNGYSEIEREKL
NLINSIYKTLEQLENYKNETIQFEQQRTINQVRQRVFQQALQGALGTLNSCLNNELHLRTIS
ANIGILGSIKEKTDMNPLISAASVIAAGLAVGLASIGPGVGQGTAAGQAVEGIARQPEAEGK
IRGTLLLSLAFMEALTIYGLVVALALLFANPFVMNVL---ANTLKGLYDVSGVEVGQHFYW
QIGGFQVHGQVLITSWVVIAILLGSAAIAVRDPQTIPTGGQNFFEYVLEFIRDVSKTQIGEEY
GPWVPFIGTLFLFIFVSNWSGALLPWKIIQLPHGELAAPTNDINTTVALALLTSVAYFYAGIT
KKGLSYFGKYIQPTPILLPINILEDFTKPLSLSFRLFGNILADELVVVVLVSLVPLVVPIPVMF
LGLFTSGIQALIFATLAAAYIGESMEGHHMIFSTVEHILTHISFSIVSIIITMRLISFLVDGIVQL
YDSSEKGMIVTFLCLTGLLVTRWTYSGHFPLSNLYESLIFLSWSFSLIHIVPY--KNKKYLSTI
TGSSVVFTQGFTTSGLLTEIDQSSILVPALQSEWLIMHVTMMILGYASLLCGSLLSIALLVIT
FRKNKKILY--REIEYMNERSNLLQNTS---AKNYYRSQLIQQLDYWSSRGISLGFIFLTIGILS
GAVWANEAWGSYWNWDPKETWAFITWIIFAIYLHTRTTRNPKCANSAIVASIGFLIIWICYF
GVNLLGIGLHSYGSFTLLMTKKKAFTPLLYLVSIVFLPWWLSLSFNKSLESWVNNWWNA
GQSEIFLNDIQEKSILEKFIELEELLVLDELIEEYSETHLQKFRI-IQKEAIQLINIYNEGRIHTIL

HFSTNIICFVILSGFSILGNEELVILNSWTQKFLYNLSDTVKAFSILLLTDLCIGFHSPHGWEL
MVGSVYKDLGFVHNDQIISGLVSTFPVILDTIFKYWIFRYLNRVSPSLVVIYHSMNDMPIGV
PKVPFRSPGEEDASWVDVYNRLYRERLLFLGQAVDSEISNQLIGLMIYLSIEDDTKDLYLFI
NSPGGWVIPGVAIYDTMQFVRPDVHTICMGLAASMGSFILVGGEITKRLAFPHA-VMIHQP
ASSFYEAQAGEFILEAEELLKLRETLTRVYVQRTGKPLWVVSEDMERDVFMSATEAQAYG
IVDLVAVEMKEQKWTHEGLITESLPNGMFRVRLDNEDLILGYVSGKIRRSFIRILPGDRVKI
EVSRYDSTRGRIIYRLRNKDSKDMEEIQGYLQ-DRSQQHGFLYPLIFQEYIYALAHDHSLPV
YDNKSSFLIVKRLITRIYQQNHFIIFANNSNQNLFFGKNLYSQTISEGLAFIVEIPFYIRLISSQI
LKFRNLRSIHSPFPFLENNFSHLNSVLDILIPHPVHLEILVQNLRYWVKDASSLHLLRFFFHE
YWNWNTLIDPS-FDFSPQKLFFLLYNSHVCEYESIFFFLRKQSSHLRSTSFGVFLERIYFYEK
KERLVEVFA-DFSLWLFKDPFIHYVRYQGKSILVSNGTPLLMNKWKSYLVNFWQCHFGIW
FHPGRVYINQLPNYSFNFMGYLSSVRLNHSMVRSQMLENAFLINNAI--KKFDTLVPLIPLIG
SLAKAKFCNLLGHPISKPIWTDLADSDIIDRFWHICRNLSHYYSGSSKKKSLYRIKYILRLS
CAKTLARKHKSTVRAFLKGLGSEFLEKFLTSEEEALSLTFSRRIWYLDIIYINDLTNYQMIID
-TTELQ-AINSFFKLES-LKEVYGIIWILIPIFTLVLGITIGVLVIVWLEREISAGIQQRIGPEYAG
PLGILQALADGTKLLFKENLLPSRGDARLFSIGPSIAVISILLSYSVIPFGYRLVLADLTIGVFL
WIAISSIAPIGLLMSGYGSNNKYSFLGGLRAAAQSISYEIPLTLCVLSISLLSNSSSTVDIVEA
QSKYGFWGWNLWRQPIGFIIFLISSLAECERLPFDLPEAEEELVAGYQTEYSGIKFGLFYVA
SYLNLLVSSLFVTVLYLGGWNLSIPYIFVSEIFDINKAGKVFGPVIGIFITLAKTYLFLFIPIAT
RWTLPRLRMDQLLNLGWKFLLPISLGNLLLTTSSQLLSLMIWHVQNENFILDSTRIFMKAF
HLLLFDGSLIFPECILIFGLILLLMIDSSSDQKDIPWLYFIPSTSLVMSITALLFRWREEPMISFS
GNFQTNNFNEIFQFLILLCSTLCIPLSVEYIECTEMAITEFLLFVLTATLGGMFLCGANDFITI
FVAPECFSLCSYLLSGYTKKDVRSNEATMKYLLMGGASSSILVHGFSWLYGSSGGEIELQE
IVNGLINTQMYNSPGISIALIFITVGIGFKLSPAPSHQWTPDVYEGSPTPVVAFLSVTSKVAA
SASATRIFDIPFYFSSNGWHLLLEILAILSMILGNLIAITQTSMKRMLAYSSIGQIGYVIIGIIV
GDSNDGYASMITYMLFYISMNLGTFACIVLFGLRTGTDNIRDYAGLYTKDPFLALSLALCL
LSLGGLPPLAGFFGKLYLFWCGWQAGLYFLVLIGLLTSVVSIYYYLKIIKLLMTGRNQEITP
HVRNYRRSPLRSNNSIELSMIVCVIASTIPGISMNPIIAIAQDSLFMFLLYEYDIFWTFLIISSLI
PILAFFISGILAPISKGPEKLSSYESGIEPIGDAWLQFRIRYYMFALVFVVFDVETVFLYPWA
MSFDILGVSVFIEALIFVLILIVGLVYAWRKGALEWSTNYFPWLTIIVILPIFAGSLIFFLPHRG
NRVIRWYTIFICIFELLLTAYTFCYHFQTDDPLIQLMEDYKWIQLFDFHWRLGIDGLSIGPILL
TGFITTLATLAAWPVTRDSRLFNFLMLAMYSAQIGLFSSRDLLLFFIMWELELIPVYLLLSM
WGGKKRLYSATKFILYTAGGSIFLLMGVLGIGLYGSNEPTLNFETSANQSYPLPLEIIFYIGF
FIAFAVKLPIIPLHTWLPDTHGEAHYSTCMLLAGILLKMGAYGLVRINMELLSHAHSIFSPW
LLIVGTIQIIYAASTSLGQRNLKKRIAYSSVSHMGFIIIGIGSITDTGLNGALLQIISHGFIGAA
LFFLAGTTYDRIRLVYLDEMGGIAILMPKIFTIFSTFSMASLALPGMSGFVAELIVFFGIITSQ
KYLLMPKILITFVMAIGMILTPIYSLSMSRQMFYGYKFFNIPNSYFFDSGPRELFLSISLFLPV
LGIGMYPDFIFSLSVDKVEVILSNSFSKMMLEHVLVLSAYLFSIGIYGLITSRNMVRALMCL
ELILNAVNINFVTFSDFFDNRQLKGDIFSIFVIAIAAAEAAIGLAIVSSIYRNRKSTRINQSNL
LNNMQQTYQYGWIIPFIPLPIPILIGVGLLLFPTATKNLRRMWSFQSVLLLTIVMIFSINLSIQ
QINSNSIYQYVWSWIINNDFSLEFGYLIDPLTSIMSILITTVGIMVLIYSDNYMAHDQAYLRF
FAYMGFFSTSMLGLVTSSNLIQIYIFWELVGMCSYLLIGFWFTRPLAANACQKAFVTNRIG
DFGLLLGILGFYWITGSFEFRDLFEIFNNNEVNSLFVTLCAGLVFAGAVAKSAQFPLHVWLP
DAMEGPTPISALIHAATMVAAGIFLVARLIPLFIVIPYIMNFISLIGIITVLLGATLALAQKDIK

RGLAFSTMSQLGYMMLALGMGSYRSALFHLITHAYSKALLFLGSGSVIHSMETIVGYSPY
KSQNMVLMGGLTKHIPITKNTFLLGTLSLCGIPPLACFWSKDEILNDSWLYSTIFAIIAWATA
GLTAFYIFRIYLLTFEGHLSVHFQNYIGKEKVFFYSISLWGRGASKKINN-LST---MNFLKFE
NKKFYSYPYEPDNTMLFPLLILGLFTLFVGSIGIPFDL?ILSKWLTPSINLLHQKLS-SMDWY
EFWKDTVSSVSIAYLGIFIASLLYKPPYSSLQNFDLINSILILWDKIINGVYNWSYNRAYIDSF
YTRSFTGGIRGLAKLTHFFDRRIVDGITNGVGVINFFIGEAIKYIGGG-RISSYLFFYLTYVSIF
LLVFFMNLPGPIHDFLLVFLGSGLILGGLGVVLLPNPIYSAFSLGFVLVCISLFYILLNSYFVA
AAQLLIYVGAINVLIIFAVMFMNGSEYYKDFHLWTIGDGVTSVVCTGLFISLITTIPDMSWY
GIIWTTKSNQILEQDLITNSQQIGVHLSTDFFLPFELVSIILLVALIGAIAVARQMTVPATRKD
LMIVNMGPHHPSMHGVLRLIVTLDGEDVIDCEPILGYLHRGMEKIAENRTIVQYLPYVTR
WDYLATMFTEAITVNGPEQLGNIQVPKRASYIRAILLELSRIASHLLWLGPFMADIGAQTPF
FYIFRERELIYDLFEAATGMRMMHNFFRIGGVAADLPHGWIDKCLDFCDYFLTGVAEYQK
FITRNPIFLERVEGVGIIGGEEALNWGLSGPMLRASGIQWDLRKVDHYECYDEFDWEVQW
QNEGDSLARYLVRIREMTESIKIIQQALEGIPGGPYENLEIRRFDRVKDPEWNDFEYRFISK
KPSPTFELSKQELYVRVEAPKGELGIFLVGDRSVFPWRWKIRPPGFINLQILPQLVKRMKLA
DIMTILGSIDIIMGEVDRMFPMLTEFINYGQQTIRAARYIGQGFMITLSHANRLPITIQYPYE
KLITPERFRGRIHFEFDKCIACEVCVRVCPIDLPVVDWKLETDIRKKQLLNYSIDFGVCIFC
GNCVEYCPTNCLSMTEEYELSTYDRHELNYNQISLGRLPTSVIDDYTIRTISNS----------------
MQGRLSAWLVKHGLIHRSLGFDYQGIETLQIKSEDWHSIAVILYVVYGYNYLRSQCAYDVA
PGGLLASVYHLTRIEYGVDQPEEVCIKVFVSRRNPRIPSVFWVWKSVDFQERESYDMLGIS
YDNHPRLKRILMPESWIGWPLRKDYVAPNFYEIQDAH---
---------------MNSIELPLLDRTAQNSVISTTLNDFSNWSRLSSLWPLLYGTSCCFIEFASLIGSRF
DFDRYGLVPRSSPRQADLILTAGTVTMKMAPSLVRLYEQMPEPKYVIAMGACTITGGMFS
TDSYSTVRGVDKLIPVDVYLPGCPPKPEAVIDAITKLRKKISREIYEDRIRPQRSNRCFTTNH
KFHISRSMNTGNYDQGFLYQPASTSEIPTETFFKYKSSVSSHELVNMETRNIFSWIKEQITRS
ISVSLMIYIITRTAVSNAYPIFAQQGYENPREATGRIVCANCHLANKPVDIEVPQAVLPDTVF
EAVVRIPYDKQLKQVLANGKKGGLNVGAVLILPEGFELAPPDRISPEIKEKIGNLSFQSYRP
NKKNILVIGPIPGQKYSEITFPILSPDPATKKDVHFLKYPIYVGGNRGRGQIYPDGSKSNNTV
YNATGAGIVSKIIRKEKGGYEITITDT-DGRQVVDIIPPGPELLVSEGEYIKLDQPLTSNPNVG
GFGQGDAEIVLQDPLRVQGLLFFLASVILAQIFLVLKKKQFEKVQLAEMNFMSKVYDWFE
ERLEIQAIADDITSKYVPPHVNIFYCLGGITLTCFLVQVATGFAMTFYYRPTVTEAFASVQYI
MTEANFGWLIRSVHRWSASMMVLMMILHVFRVYLTGGFKKPRELTWVTGVVLAVLTASF
GVTGYSLPRDQIGYWAVKIVTGVPEAIPVIGSPLVELLRGSASVGQSTLTRFYSLHTFVLPLL
TAVFMLMHFPMIRKQGISGPLMGVTKKPDLNDPVLRAKLAKGMGHNYYGEPAWPNDLL
YIFPVVILGTIACNVGLAVLEPSMVGEPADPFATPLEILPEWYFFPVFQILRTVPNKLLGVLL
MVSVPAGLLTVPFLENVNKFQNPFRRPVATTVFLIGTAVALWLGIGATLPIDKSLTLGLFMIE
VFLFGIVLGLIPITLAGLFVTAYLQYRRGDQLDLMLTITSYFGFLLAALTITSALFIGLNKIRL
IMDIVSLAWASLMVVFTFSLSLVVWGRSGLMIIRSPEPEVKILVDRDPVKTSFEEWAKPGHF
SRTIAKGPDTTTWIWNLHADAHDFDSHTSDLEEISRKVFSAHFGQLSIIFLWLSGMYFHGA
RFSNYEAWLSDPTHIGPSAQVVWPIVGQEILNGDVGGGFRGIQITSGFFQIWRASGITNELQ
LYCTAIGALIFAALMLFAGWFHYHKAAPKLAWFQDVESMLNHHLAGLLGLGSLSWAGHQ
VHVSLPINQFLNAGVDPKEIPLPHEFILNRDLLAQLYPSFAEGATPFFTLNWSKYAEFLTFRG
GLDPVTGGLWLTDIAHHHLAIAILFLIAGHMYRTNWGIGHGLKDILEAHKGPFTGQGHKG
LYEILTTSWHAQLSLNLAMLGSLTIVVAHHMYSMPPYPYLATDYGTQLSLFTHHMWIGGF

LIVGAAAHAAIFMVRDYDPTTRYNDLLDRVLRHRDAIISHLNWACIFLGFHSFGLYIHNDT
MSALGRPQDMFSDTAIQLQPVFAQWIQNTHALAPGATAPGATASTSLTWGGGDLVAVGGK
VALLPIPLGTADFLVHHIHAFTIHVTVLILLKGVLFARSSRLIPDKANLGFRFPCDGPGRGGT
CQVSAWDHVFLGLFWMYNAISVVIFHFSWKMQSDVWGSISDQGVVTHITGGNFAQSSITI
NGWLRDFLWAQASQVIQSYGSSLSAYGLFFLGAHFVWAFSLMFLFSGRGYWQELIESIVW
AHNKLKVAPATQPRALSIVQGRAVGVTHYLLGGIATTWAFFLARIIAVGMALRFPRFSQGL
AQDPTTRRIWFGIATAHDFESHDDITEERLYQNIFASHFGQLAIIFLWTSGNLFHVAWQGNF
ESWVQDPLHVRPIAHAIWDPHFGQPAVEAFTRGGALGPVNIAYSGVYQWWYTIGLRTNE
DLYTGALFLLFISAIFLIAGWLHLQPKWKPSVSWFKNAESRLNHHLSGLFGVSSLAWTGHL
VHVAIPGSRGEYVRWNNFLDVLPHPQGLGPLFTGQWNLYAQNPDSGSHLFGTSQGAGTAI
LTLLGGFHPQTQSLWLTDIAHHHLAIAFIFLVAGHMYRTNFGIGHSMKDLLDAHIPPGGRL
GRGHKGLYDTINNSLHFQLGLALASLGVITSLVAQHMYSLPAYAFIAQDFTTQAALYTHHQ
YIAGFIMTGAFAHGAIFFIRDYNPEQNEDNVLARMLDHKEAIISHLSWASLFLGFHTLGLY
VHNDVMLAFGTPEKQILIEPIFAQWIQSAHGKTSYGFDVLLSSTSGPAFNAGRSIWLPGWL
NAVNE--NSNSLFLTIGPGDFLVHHAIALGLHTTTLILVKGALDARGSKLMPDKKDFGYSFP
CDGPGRGGTCDISAWDAFYLAVFWMLNTIGWVTFYWHWKHITLWQGNVSQFNESSTYL
MGWLRDYLWLNSSQLINGYNPFGMNSLSVWAWMFLFGHLVWAIGFMFLISWRGYWQEL
IETLAWAHERTPLANLIRWRDKPVALSIVQARLVGLAHFSVGYIFTYAAFLIASTSGKFGMS
HSVKIYDTCIGCTQCVRACPTDVLEMIPWDGCKAKQIASAPRTEDCVGCKRCESACPTDF
LSVRVYLWHETTRSMGLAYMITFSFPSIFVPLVGLVFPAIAMASLSLHVQKNKIIMRDFKTY
LSVAPVLSTLWFGALAGLLIEINRFFPDALTFPFFSFMTAILERRESESLWGRFCNWITSTEN
RLYIGWFGVLMIPTLLTATSVFIIAFIAAPPVDIDGIREPVSGSLLYGNNIISGAIIPTSAAIGLH
FYPIWEAASVDEWLYNGGPYELIVLHFLLGVACYMGREWELSFRLGMRPWIAVAYSAPVA
AATAVFLIYPIGQGSFSDGMPLGISGTFNFMIVFQAEHNILMHPFHMLGVAGVFGGSLFSA
MHGSLVTSSLIRETTENESANAGYRFGQEEETYNIVAAHGYFGRLIFQYASFNNSRSLHFFL
AAWPVVGIWFTSLGISTMAFNLNGFNFNQSVVDSQGRVINTWADIINRANLGMEVMHER
NAHNFPLDLAAIDAPSVNGMGLPWYRVHTVVLNDPGRLLSVHIMHTALVAGWAGSMAL
YELAVFDPSDPVLDPMWRQGMFVIPFMTRLGITNSWGGWSIT-GGTVTNAGIWSYEGVAG
AHIVFSGLCFLAAIWHWVYWDLEIFCDERTGKPSLDLPKIFGIHLFLSGVACFGFGAFHVT
GLYGPGIWVSDPYGLTGKVQPVNPAWGVEGFDPFVPGGIASHHIAAGTLGILAGLFHLSVR
PPQRLYKGLRMGNIETVLSSSIAAVFFAAFVVAGTMWYGSATTPIELFGPTRYQWDQGYFQ
QEIYRRVSAGLAENQSLSEAWSKIPEKLAFYDYIGNNPAKGGLFRAGSMDNGDGIAIGWL
GHPIFRDKEGRELFVRRMPTFFETFPVVLVDGDGIVRADVPFRRAESKYSVEQVGVTVEF
YGGELNGVSYSDPATVKKYARRAQLGEIFELDRATLKSDGVFRSSPRGWFTFGHASFALLF
FFGHIWHGARTLFRDVFAGIDPDLDAQVEFGAFQKIGDPTTRRQAVMKTLYSLRRFYHVE
TLFNGTLALAGRDQETTGFAWWAGNARLINLSGKLLGAHVAHAGLIVFWAGAMNLFEVA
HFVPEKPMYEQGLILLPHLATLGWGVGPGGEVIDTFPYFVSGVLHLISSAVLGFGGIYHAL
LGPETLEESFPFFGYVWKDRNKMTTILGIHLILLGIGAFLLVFKALYFGGVYDTWAPGGGD
VRKITNLTLSPGIIFGYLLKSPFGGEGWIVSVDDLEDIIGGHVWLGSICILGGIWHILTKPFAW
ARRALVWSGEAYLSYSLGALSIFGFTACCFVWFNNTVYPSEFYGPTGPEASQAQAFTFLVR
DQRLGANVGSAQGPTGLGKYLMRSPTGEVIFGGETMRFWDLRAPWLEPLRGPNGLDLSR
LKKDIQPWQERRSAEYMTHAPLGSLNSVGGVATEINAVNYVSPRSWLATSHFVLGFFFFV
GHLWHAGRARAAAAGFEKGIDRDFEPVLSMTPLNMTIALGKFTKDEN-LFDIMDDWLRR
DRFVFVGWSGLLLFPCAYFALGGWFTGTTFVTSWYTHGLASSYLEGCNFLTAAVSTPANS

LAHSLLLLWGPEAQGDFTRWCQLGGLWTFVALHGAFGLIGFMLRQFELARSVQLRPYNAI
AFSAPIAVFVSVFLIYPLGQSGWFFAPSFGVAAIFRFILFFQGFHNWTLNPFHMMGVAGVLG
AALLCAIHGATVENTLFEDGDGANTFRAFNPTQAEETYSMVTANRFWSQIFGVAFSNKRW
LHFFMLFVPVTGLWMSALGVVGLALNLRAYDFVSQEIRAAEDPEFETFYTKNILLNEGIRA
WMAAQDQPHENLIFPEEVLPRGNALMSGSTGERSFADIITSIRYWVIHSITIPSLFIAGWLFV
STGLAYDVFGSPRPNEYFTESRQGIPLITGRFDPLEQLDEFSRSFMTIDRTYPIFTVRWLAVH
GLAVPTVFFLGSISAMQFIQRMATQTVDNSSRSG-PRRTTVGSLLKPLNSEYGKVAPGWGT
TPLMGVAMALFAVFLSIILEIYNSSVLLDGISMNMLTLKLFVYTVVIFFVSLFIFGFLSNDPG
RNPGREEMADTTGRIPLWIVGTVTGILVIGLIGVFFYGSYSGLGSSLMLNIFSLIWICLNSAL
Y-SGFFFGKLPEAYAFLNPIIDFMPVIPVFFFLLAFVWQAAVSFRMTQSNPNEQNVELNRTSL
YWGLLLIFVLAVLFSNYFFNMEVNILAFIATALFILVPTAFLLIIYVKTVSQNNMETATLVAIF
ISGLLVSFTGYALYTAFGQPSQQLRDPFEEHGDMEALVYTFLLVSTLGIIFFAIFFREPPKVPT
KKIKMTLAFQLAVFALIATSSILLISVPVVFASPDGWSSNKNVIFSGTSLWIGLVFLVGILNSL
IS-------MSPQTETKASVGFKAGVKEYKLTYYTPEYETKDTDILAAFRVTPQPGVPPEEAGA
AVAAESSTGTWTAVWTDGLTSLDRYKGRCYHIEPVPGEENQYIAYVAYPLDLFEEGSVTN
MFTSIVGNVFGFKALRALRLEDLRVPPAYIKTFQGPPHGIQVERDKLNKYGRPLLGCTIKP
KLGLSAKNYGRAVYECLRGGLDFTKDDENVNSQPFMRWRDRFCFCAEALFKAQAETGEI
KGHYLNATAGTCEEMIKRAVFARELGVPIVMHDYLTGGFTANTSLAHYCRDNGLLLHIHR
AMHAVIDRQKNHGMHFRVLAKGLRMSGGDHIHAGTVVGKLEGERDITLGFVDLLRDDFI
EKDRSRGIYFTQDWVSLPGVIPVASGGIHVWHMPALTEIFGDDSVLQFGGGTLGHPWGNA
PGAVANRVALEACVKARNEGRDLAAEGNEIIREASKWSPELAAACEVWKEIRFNFEAMD
KLDKMAIHLYKTSTPSTRNGTVDSQVKSNPRNNLIYGQHRCGKGRNARGIITAGHRGGGH
KRLYRKIDFRRTEKDIYGRIVTIEYDPNRNAYICLIHYGDGEKRYILHPRGALIGDTIVSGTE
VPIKMGNALPLTDMPLGTAIHNIEITLGKGGQLARAAGAVAKLIAKEGKSATLKLPSGEVR
LISKNCSATVGQVGNVGVNQKSLGRAGSKRWLGKRPVVRGVVMNPVDHPHGGGEGRAP
IGRKKPTTPWGYPALGRRSRKRNKYSDNLILRRRSKMIQPQTHLNVADNSGARELMCIRII
GAS-NRRYAHIGDVIVAVIKEAVPNMPLERSEVVRAVIVRTCKELKRDNGMIIRYDDNAAV
VIDQEGNPKGTRIFGAIARELRQLNFTKIVSLAPEVLMLSPKRTRFRKQHRGRMKGISYRG
NRICFGKFALQALEPAWITSRQIEAGRRAMTRNVRRGGKIWVRIFPDKPITARPAETRMGS
GKGSPEYWVAVVKPGRILYEMGGVTEHIARRAISIAASKMPIRTQFIISRMTRIRRGYIARR
RRTKIRLFASSFRGAHSRLTRTITQQKIKALVSAHRDRDSKKRNFRRLWIIRINAIIRERWALS
YSYSRLIHDLYKRQLLLNRKILAQIAISNRNCLYMISNELYKYKEVEESSGIIMLKEVYALG
QHISMSADKARRVIDQIRGRSYEETLIILELMPYRACYPIFKLVYSAAANASYNMSSNDAN
LLISKAEVNEGTTIKKFKPRARGRSYPIKRPTCHITIVMKDISLDGGVWDKKMDGIKYAVF
TDKSIRLLGKN-QYTFNVESGSTRTEIKHWVELFFGVKVIAMNSHRLPGKGRRMGPIMGH
TMHYRRMIITLQPGYSIPPLRKKRTMAVPKKRTSTSKKRIRKNIWKKKGYWTALKAFSLG
KSLSTGNSKSFFVQQTNKMAKGKDVRVTVILECTDCVRNSVN--KVSTGISRYITQKNRHN
TPNRLELKKFCPYCYKHTVHGEIKKMKIRASVRKICDKCRLIRRRGRIIVICSNPRHKQRQ
GMVREKITVSTRTLEWKCVELRTDSKRLYYGRFILSPLMKGQADTIGIAMRRALLGEIEGT
CITRVKSE--KVAHEYSTIAGIQESVHEILMNLKEIVFRSNLYGTCDASICVRGPGYVTAQDI
VLPPYVEIVDNTQHIARLMEPINLCIRLEIERNRGYLIKTPQ-NFDGSY--PIDAVFMPIRNAN
YSIHSYGNGNEKQEILFLEIWTNGSLTPKEALHQASRNLIDLFIPFLHMEEQ--NSHLENNQ-
HTVPLAPFFFHDKLD-KLRKNKK-EIALKSIFIDQSELPPRIYNCLKRSNIYTLLDLLNNSQE
NLMKIEHFHIEDVKEILGILEKQ--LVIFLPKK-MLGDG-NEGMATIPGFNQIQFEGFCRFIDQ

GLAEELYKFPKIEDTDQEIEFQLFVETYQLVEPLIKERDAVYELLTYSSELYVSAGLIWKTG
RDMQEQTIFIGNIPLMNSLGTFIVNGVYRILINQILQSPGIYYRSELDHNGISVYTGTIISDW
GGRSELEIDRKARIWARVSRKQKISILVLSSAMGSNLREILENVCYPEIFLSFLNDKERKKIG
SKENAILEFYQQFACVGGDPVFSESLCKELQKKFFQQRCELGRIGRRNMNRRLNLDIPQN
NPFLLPRDILAAADHLIGLKFGMGTLDD--MNHLKNKRIRSVADLLQDQFGLALVRLENAV
RGTICGAIR-HKL-IPTPQNLVTSTP----LTTTYESFFGLHPLSQVLDGTNPLTQIVHGRKLSYL
GPGGLTGRTANFRIRDIHPSHYGRICPIDTSEGINVGLIGSLSIHARIGHWG-FLESPFY---EISE
R------RVLFLLPGRDEYYKVAAGNSLALNQDIQEKQVVPARYRQEFLTIAWEQVHLRSIFPF
QYFSIGASLIPFIEHNDANRALMSSNMQRQAVPLSRSEKCIVGTGLEQQAALDSGALSIAER
EGKVLYTDTDKILLSGNGGTLSIPLVMYQRSNKNTCMHQKPRVQRGKCIKKGQILADAAA
TVGGELSLGKNVLVAYMPWEGYNFEDAVLISERLVYEDIYTSFHIRKYEIQTHVTSQG-PER
VTNEIPHLEAHLLRNLDKSGIVMLGSWVETGDILVGKLTPQMVKE-SSYAPEDRLLRAILGI
QVSTSKETCLKLPIGGRGRVIDVRWIQKKG-GSSYNPEMIRVYISQKREIKVGDKVAGRHG
NKGIISKILTRQDMPYLQDGRPVDMVFNPLGVPSRMNVGQIFECSLGLAGSLLDRHYRIAP
FDERYEQEASRKLVFSELYQASKQTANPWVFEAEYPGKSRIFDGRTGNPFEQPVLIGKPYIL
KLIHQVDDKIHGRSSGHYALVTQQPLRGRAKQGGQRVGEMEVWALEGFGVAHILQEMLT
YKSDHIRARQEVLGTTIVGGTIPNPEDAPESFRLLVRELRSLALELKHFLISEKNFRIHRKEA
-------MIDRYKHQQLRIGLVSPQQISAWATKILPNGEIVGEVTKPYTFHYKTNKPEKDGLFCE
RIFGPIKSGICACGNYRVIGDEKEDPKFCEQCGVQFVDSRIRRYQMGYIKLACPVTHVWYL
KRLPSYIANLLDKSLKELEGLVYCD-NFSFARP-ITKKPTFLRLRGSFKYEIQSWKYSIPLFFT
TQGFDTFRNREISTGAVAIREQLADLDLRIILENSLVEWKELGEE-GPTGNEWEDRKVGRR
KDFLVRRMELTKHFIRTNIEPEWMVLFLLPVLPPELRPIIQIDGGKLMSSDINELYRRVIYRN
NTLTDLLTTSRSTPGELVMCQEKLVQEAVDTLLDNGIRGQPMKDGHNKVYKSFSDVIEGK
EGRFRETLLGKRVDYSGRSVIVVGPSLSLHRCGLPREIAIELFQTFVIRGLIRQHLASNIGVA
KNKIREKEPIVWEILQEVMQGHPVLLNRAPTLHRLGIQAFQPVLVEGRAICLHPLVCKGFN
ADFDGDQMAVHVPLSLEAQAEARLLMFSHMNLLSPAIGDPISVPTQDMLMGLYVLTSGN
RRGICVNRYNPWNR---RNYQTKKSDNNNYEYT---EPFFCNSYDAIGAYRQKRINLDSPLWL
RWPLDQR-VIAS--REIPIEVHYESLGIYYEIYGHYLIVKSIKKEILFLYIRTTVGHISLYREIEE
AIQGFSHACSYGT--------MEVLMAERANLVFHNKSIDGTAMKRLISRLIDHFGMAYTSHILD
QVKTLGFKQATATSISLGIDDLLTIPSKGWLVQDAEQQSLILEKHHHYGNVHAVEKLRQSIE
IWYATSEYLRQEMNPNFRMTDPFNPVHIMSFSGARGNVSQVHQLVGMRGLMSDPQGQMI
DLPIQSNLREGLSLTEYIISCYGARKGVVDTAVRTSDAGYLTRRLVEVVQHIVVRRTDCGT
VRGISVSPPNGMMP-EKFFIQTLVGRVLADDIYMGPRCIATRNQDIGIELVNRFIT------FRAQ
PISIRTPFTCRSTSWICRLCYGRSPTHGDLVELGEAVGIIAGQSIGEPGTQLTLRTFHTGGVFA
GGIAEHVRAPSNGKIKFNEDLVHPTRTRHGHPAFLCFIDLYVTIKSENILHNVNIPPKSFLLV
QNDQYVEAEQVIAEIRAGTST-LSFKEKVRKHIYSDSDGE-IHWSTDVYHAPQFTYGNVHL
LPKTSHLWILLGGPCR--SSLVSLSLHKDQDQMNTH--SLSVKRRYTYKLS--VTNDR-VRYK
FFSSDFYGK-KNRIPDY-LDLNRIICTGYCNRIYP-AILHENS--DLLSKRRRNRFIMPLQ---SIQ
ERENESPGISIEIP--INGIFRRNSILSYFDDPRYRRKSSGITKYGTIEMHSLVKKED--LIEYRG-
VKEFRPKYMKVDRFFFIPEEVHILPGSSSIMVQNNSIIGIDTQITLNIRSRVGGLVRVERKKK
GIELKIFSGDIHFPGETDKISRHSGVLIPLPPGTGKINSKESKK--LKNWIYVQR--ITPSK--KG
YFALVRPVITYEKRDGLNLATLFPPDLLQERDNVRLQVVNYILYGNGKPIRGISDTSIQLVR
TCLVLNWDQDKK---SSSSEEAPASFVEIRTNGLIRHFLRIDLVKSPISYIVKRNDPSGSGLLS
D-NGSDCTNINPFSSIYSY---SKARIQQFLNPNQGTIHTLLNK---NREFQSLIILLSSNCSRMGP

--FAGVKYHNV---IKESDPRISIRNSLGPLGS--APPIANFFS--FSHL--LTHNQIL-VTNYLQLD--
N-IKETFQVIK---YYSMDEN--GKIYNPDPCNNIILNPLNLNWYFRHHNYCEETS-TIISLGQFI
CENVCIAKNRPH-LK--PGQVIFVQ-VDSVVIRSAKPYLATPGATVHGHYGETLYEGDILVTFI
YEKSRSGDITQGLPKVEQILEVRSIDSISMNLQKRIEGWNKCITRILGTPWGFLISAELTIVQ
SQISLVNKIQKVYRSQGVQIHNKHIEIIVRQITSKVLISE---DGMSNVFSPGELIGLLRAERM
GRALEEAICYRAVLLGITRASLNTQSFISEASFQETARVLAKAALRGRIDWLKGLKENVVL
GGMIPVGTGFKGL--VKPSKQHNKAL--LETKKKNLFEGEMR--DILFHHRNFFDSFLLKKFH
DPSEQSFIG------FNDSMTRRYWNINLEEMMEAGVHFGHGTRKWNPKMEPYISAKRKGIHI
TNLTRTARFLSEACDLVFDAGSRGKQFLIVGTKNKAADSVAWAAIRARCHYVNKKWLGG
MLTNWSTTETRLHKFRDLRMEQKTGRLNRLPKRDAAMLKRQLSRLQTYLGGIKYMTGL
PDIVIIIDQHEEYTALRECITLGIPTICLIDTNCDPDLADISIPANDDAISSIRLILNKLVFAICEG
RS-SYIRNPMGQKINPLGFRLGTTQGHHSLWFTQAKNYSEGLQEDQQIRNCIKNYVQKNT
KISSG-VEGIARIEIQKRIDLIQVIIFMGFPKPLLENQPR----GIEELQTNLQKKCNSVNRKFNI
AITKIAKPYGNPNILAEYIAGQLKNRVSFRKAMKKAIELTEQANTKGIQVQIAGRIDGKEIA
RVEWIRRGRVPLQTIRAKIDYCSYTVRTIYGVLGIKIWIFIDEEMSRYRGPRFKKIRRLGALP
GLTNKKPRAGNDLRNQLRSGK-KSQYRIRLEEKQKLRFHYGLTERQLLKYVRIAGKAKGS
TGQVLLQLLEMRLDNILFRLGMASTIPAARQLVNHRHILVNGRIVDIPSYRCKPRDIITGKD
EQKSRALIQKSLDSSPQ-EELPSHLTLHPFQ--YKGLVNQIIDSKWVGLKINELLVVEYYSRQ
TMSRRGAAEEKTAKSDPIYRNRLVNMLVNRILKHGKKSLAYQIIYRAVKKIQQKTETNPLS
VLRQAIRGVTPDITVKARRVGGSTHQVPIEIGSTQGKALAIRWLLAASRKRPGRNMAFKLS
SELVDAAKGSGDAIRKKEETHRMAEANRAFAHFRMGRDTVAEIITSIRNADMDKKRVVRI
TSTNITENIVKILLREGFIENARKHRENNKYFLVLTLRHRR--NRKGPH---RNIFNLKRISRPG
LRIYANSQRIPRILGGMGIVIFSTSRGIMTDREARLEGIGGEILCYIWMAKAIPRVGSRKNGR
ISSRKSARRIPKGVIHVQASFHNTIVTVTDVRGRVVSWSSAGTCGFRGTRRGTPFAAQTAA
ANAIRTVVDQGMQRAEVMIKGPGLGRDAALRAIRRSGIVLTFVRDVTPMPHNGCRPPKK
RRVMPTIKQLIRNARQPIRNVTKSPALRGCPQRRGTCTRVYTITPKKPNSALRKVARVRLTS
GFEITAYIPGIGHNLQEHSVVLVRGGRVKDLPGVRYHIVRGTLDAVGVKDRQQGRSKYGV
KKPKMARKGLIQREKKRQKLEQKYHLIRRSSKKEIS---KVQSLNDKWEIYRKLQSPPRNS
APTRLHRRCFATGRPRANYRDFGLSGHILREMVHACLLPGARRSSWMVKNTLISVISEE--E
K---RGSVEFQVFRFTNKIRRLTSHLELHKKDYLSQRGLRKILGKRQRLLAYLAKKNRVRYK
ELIGLLSIRETKTR--MLKLRLKRCGRKQRAVFRIVAIDVRSRREGKDLQKVGFYDPIKNQT
YLNVTAILYFLEQGAQPTGTVQDIFKKAGVFKELRPNQMKFN-MDKSKRSFLKSKRSVRR
RLPPIQSGDLIDYRNMSLISRFISEQGKILSRRVNRLTLKQQRLITVAIKQARILSLLPFLNNE
K--QFERTESTARTTGLRTRNKVTRSLKKNPFVANHLLKKIDKLNTKAEKEIIVTWSRASTII
PTMIGHTIGIHNGKEHLPIYITDRMVGHKLGEFAPTLNFRGHAKSDNRSRRMIFQSFLLGNL
VSLCMKIINSVVVVGLYYGFLTTFSIGPSYLFLLRARVMEEG---TEKKVSATTGFITGQLMM
FISIYYAPLHLALGRPHTITVLALPYLLFHFFWNNHKHFFDYGSTTRNLMRNLSIQCVFLN
NLIFQLFNYFILPSSMLARLVNIYIFRCNNKMLFVTSSFVGWLIGHILFMKWLGLVLVWIRQ
NHSIRSYIRSNKYLVSELRIFMARIFSILLFITCVYYLGRIPSPILTKKLKETEEDKTLVTTLFD
YNRWNHPFRYIKNDRFEKALRKEMSQYFFQIYESNGKQRISFTYLPSLATFGEMIKRKISLY
TLEKSSSNEFDKRWVYTNKQKIK-LNNEFRNRIQALDKEFLFLDILEARTRLCNDDTKNEY
FPKMYDPFLKGAYRGRIKTILSPRDTIGINRIHSILYQEFEQKYPIDQSVGIKEIIKKVPRWSY
KLIDLEQQS-GQIRSRKAKRVVIFTDALIRYSQQSDFRRRIIKGSMRAQRRKIVIWELFQAN
VHSPLFLDRIKKSPRFSFDI--SVLIKLLFRNRKEQARIEIAEAWDTIPFAQVIRGSMLISQSFF

RKYIRLPVLIIGKNIVRMILFQFPEWSEDLEGWNREMHIKCTYNGVQLSETEFPKNWLTDG
IQIKILFPFCLKPWHRSKKKQKDDFCFLTVWGMETELPFGSPRKQSSFFEPAVKELETKIIKF
KRAYFLA-LKVLKRKTKLLLRVSKETKKWIIESFL--FLKRLIKELSKLNDSIISNQIIHESFSKI
NSPNWTNSSLTEKKKKDMSHRTSTIRNQIERIIKEKKKSPNKRNWNTKRLELPKNIWQILK
KRNARLFSKFNSFIKIFVERIYIDIFLFIINITRLNTQLF-YFISCDLSYLSQAYVFYKLSQTKVS
NLYKLRSFFHYTSFFLKTEIKHSFKQGIIQNELSLKKLPSYGMDQWKGWLKGHYQYDLPSI
RWSKLISEKWRNRVNQHHMAQNQFKLNSYEK-----GHYKKQ-NFQKNSRYDLLSYKYING
NIRYTDRKYFDWKIIKFFLRFLDWMGMNEEILNRWFFPEFVLLYNLYKMKPWVIPSKVLL
FNYKSKTEAELDFFLKRYLVFQLRWGDALNQRMINNVKIYCLLLRLINPRKITISSIQRREM
SLDIMLIQKNLSLTELIK-GVLVIEPDRLSVKNDGQFIMYQILGISVVHKNKH-RFDLLVPENI
LSTRCRRELRILICFKFLWPNYRLEDLACMNRYWFDTNNGSRFSMLRIYMYPMKGHQFKS
WIFELREILREIKNSHYFLDSWTQFNSVGSFIHIFFHQERFIKLFDPRIWSILLS-RNS--QGSIS
NRYFT---IKG-VILFVVVVLIYR----INNRNMVESKNLYLIGLLP-----IPMNSIGPKNDTLEESV-
GSSNINRLIVS-LLSLPKGKKISESCFLNPKESTWVLPIT----KKCSIPESNWGSRWWRKWIG
KGG--DSSCKISN---ETAAGIEILFKEKDLK-YLEFLFVYYMD--DPTRKDRDWELFDRLSLR
KRRNTINL--NSGPLFEILVKHWISYLMSAFREKIPIEAEG-FFKQQG-AGSTIQSNDIEHVSH--
LFSRNKWAISLQNCAQFHMWQFRQDLFI---SWGK----ESDFLRN-VSRENLIWLDNVWLVN
KDR---FFS--KVRNVSSNIQYDS---TR--SSFVQVTDSSQLKGSS-DQSRDHLDSIS----NEDSE--
-----------YHTLINQREIQQLKERSIL-LD--PSFLQTERTEIESD--RFPK--CLSGYS--SMSRLFT-E
REKQMINHLLPEEIEEFL--GNPTRSVRSFFSDRW--SELH-LGLNPTERSTRDQ----KLLKKQQ
DLS-FVPSRRSE----NKEMVNIFKIITYLQN--TVSIHPISSDPGCDMVP-KDEPD-MDSSNKISF
LNKNPFFDL--FHLFHARNRGGYTL--HHDFELE--ERFQEMADLFTLSITEP-DLVYHKGFAF
SIDSYGLDQKQ--FLNEVFNSRDESKKKS---LLVLPLIFY--EENESFSRRIRKKWVR-ISCGND
LEDPQPKIVIFASNNIMGAVNQYRLIRNLIQIQSSTYG--YIRNVLNRFFLMN---RSDRNFEYG
IQ-IQRDQIGKDTLNHRTIMKYTINQHLSNLKK-SQKKWLDPL--ILISRTERSMNRDPDAYR
YKGSNGSKNFQEHLEHFVSEQKSH---FQVMFDRLRINQYSIDWSEVIDKKDLSKPLRFFLS
K---------SLLFLS-------KLLFFLSN-SLPFFCVSFGNIPIHRSE-IYIY-EFKGPDDQLCSQLLESIG
LQIVHLKKLKPF--LLD---DHDTS-QKSKFLINGGTISPFLFNKIP-KW-MVDSFHTR--NNRRK
SFDNTDSYFSMIFHDQDNWLNPVKPFHRSSLISSFYKANRLRFLNNPHHFCFY--CNTRFPL
SV----EKARIN--NYDFTYG-QFLNILLRMNK-IFSLCVGK--KKHAFGG-RDTISPIESQVSNIFI
PNDFPQSG-DETYNLYKS---FHFLSRSDPFVR--RAIYSIADISGTPLTEGQIVNFERTYCQPLS
DMNLSDSEGKNLHQYRN--FNSNMGLIHTPYSEKYLPSEK--RKK--------RILCLK--KCVEK
GQMYRTFQRDSAFSTLSKWNLFQTYMPWFLTSTGYKYLNLIFLDTFSDLL--PILSS-SQKLV
SIFHDIMHGSGISWRILQKNL------CLPQWNL-ISEISSKCLHN--LLLSEERIHRNKNNESPLIS
THLRSPNVREF-LYSTLFLLLVAGY-LVRTHLLFVSR--VSSELQTEFEKVKSLIIPSSMIELRKL
LDRYPTSEPNSFWLKNIFLAALEQLGDSLEEIRGSASGGNMLG---PA-----YG-VKSIRSKKK
YLNINLIDIVDLI----PNPINRITFSRNTRHLSHTSKEIYSLIRKRKNVNGDWIDDKIESWVAN
SDSIGDEEREFLVQFS--TLTT--EKGIDQILLSLT-HSDHL-----SKNDSGYQMIEQPGAIYLRYL
VDIH--KKYLMNYEFNTFCLAERRIFLAHYQTITY--SQTSCGANSFHFPSHG---KPFSLRLAL
SPS-RAILVIGSIGSGRSYLVKYLATNSYVPFITVFLNKFLDNKPKGFLID-----DIN-------IDDS
DDIDASD------------------DIDRDLDTEL-ELLTMMNALTMDMMPE---IDRFYITLQFELAKAM
SPCIIWIPNIHDLDVNESNYLSLGLLVNHLSM---DCERCS-TRNILVIASTHIPQKVDPALIAP
NKLNTCIKIRRLLIPQQRKHLFTLSYTRGFHLEKKMFHT--NGFGSITMGSNARDLVALTNE
ALSISITQKKSIIDTNTIRSALYRQTWYLR---SQVRSVQDHGILFYQIGRAVAQNVFLSNCPID

PISIYMKKK--SCNEGDFYLYKWYFELGTSMKKLTILLYLLSCSAGSVAQDLWSLP--GPDEK
-NGITSYGLVENDS-DLVHGLLEVEGALVG--SSRTEKDCSQFDND--RVTLLLRPEPRN-PLD
MMQSGSCSILDQRFLYEKY--ESEFEE-GEGEGALDPQQ----IEED----LFNY--IVWAP--RIWRP
---WAFLFDCIERPNELGFP-YWSRPFWGK---RIIYDEEDELQENDSEFLQNGTVQYQTRDRS
SKEQGPFRISQFIWDPADPLFFLFK-DQPPGSVFSHRELFADEEMSKGLLTSQTDPPTSIYKR
WFI--KNTQEKHFELLINRQRWLR---TNSSLS--NGSFRSN--TLSESYQYLSNLFLSN-GTLLD
QMTKTLLRKRWLFPDEMKIGF--M---------MPRSQ-INGNFIDKTFSIVANILLRIIPTTSGERE
AFTYYRDG--MSAQSEGNYAEALQNYYEAMRLEIDPYDRSYILYNIGLIHTSNGEHTKALE
YYFRALERNPFLPQAFNNMAVICHY-RGEQAIRQGDSEIAEAWFDQAAEYWKQAISLTPGN
YIEAHNWLKITGRFG--MSLRSEHIWIELIKGSRKISNFCWAFILFVGSVGFLLVGISSYLGRN
LISFFPSQQIVFFPQGIVMSFYGIAGLFISSYLWCTISWNVGNGYDRFDRKEGIVCIFRWGFP
GKNRRIFLRFLIKDIQSIRIEVKEGIFARRVLYMEIRGQGTIPLTRTDEN-LTPREIEQKAAELA
YFLRVPIEVF
>Sorghum
??
??
??
??
??
???????????????????????????????MATLRVDEINKILRERIEQYNRKVGIENIGRVVQVGDGIA
RIIGLGEIMSGELVEFAEGTRGIALNLESKNVGIVLMGDGLMIQEGSFVKATGRIAQIPVSEA
YLGRVINALAKPIDGRGEIVASESRLIESPAPGIISRRSVYEPLQTGLIAIDSMIPIGRGQRELII
GDRQTGKTAVATDTILNQKGQDVICVYVAIGQRASSVAQVVTTFHEEGAMEYTIVVAEMA
DSPATLQYLAPYTGAALAEYFMYRERHTLIIYDDLSKQAQAYRQMSLLLRRPPGREAYPG
DVFYLHSRLLERAAKLNSLLGEGSMTALPIVETQSGDVSAYIPTNVISITDGQIFLSADLFN
AGIRPAINVGISVSRVGSAAQIKAMKQVAGKSKLELAQFAELQAFAQFASALDKTSQNQLA
RGRRLRELLKQSQSNPLPVEEQVATIYTGTRGYLDSLEIEQVKKFLDELRKHLKDTKPQFQ
EIISSSKTFTEQAETLLKEAIQEQLERFSLQEQTMRTNPTTSRPGVSTIEEKSVGRIDQIIGPV
LDITFPPGKLPYIYNALIVKSRDTADKQINVTCEVQQLLGNNRVRAVAMSATDGLMRGME
VIDTGTPLSVPVGGATLGRIFNVLGEPIDNLGPVDTSATFPIHRSAPAFIELDTKLSIFETGIK
VVDLLAPYRRGGKIGLFGGAGVGKTVLIMELINNIAKAHGGVSVFGGVGERTREGNDLY
MEMKESGVINEKNIEESKVALVYGQMNEPPGARMRVGLTALTMAEYFRDVNKQDVLLFI
DNIFRFVQAGSEVSALLGRMPSAVGYQPTLSTEMGSLQERITSTKKGSITSIQAVYVPADDL
TDPAPATTFAHLDATTVLSRGLASKGIYPAVDPLDSTSTMLQPRIVGNEHYETAQRVKETLQ
RYKELQDIIAILGLDELSEEDRLTVARARKIERFLSQPFFVAEVFTGSPGKYVGLAETIRGFQ
LILSGELDGLPEQAFYLVGNIDEASTKAINLEEESKLKKMKLNLYVLTPKRIIWDCEVKEIIL
STNSGQIGVLPNHAPINTAVDMGPLRIRLNDQWLTAVLWSGFARIVNNEIIILGNDAELGSDI
DPEEAQQALEIAEANLSKAEGTKELVEAKLALRRARIRVEAVNWIPMKNVTHSFVFLAHW
PFAGSFGLNTDILATNLINLTVVVGVLIFFGKGVLKDLLDNRKQRILSTIRNSEELRRGTLE
QLEKARIRLQKVELEADEYRMNGYSEIEREKENLINATSISLEQLEKSKNETLFYEKQRAM
NQVRQRVFQQAVQGALGTLNSCLNTELHFRTIRANIGILGAIEWKR-MNPLIAAASVIAAG
LAVGLASIGPGVGQGTAAGQAVEGIARQPEAEGKIRGTLLLSLAFMEALTIYGLVVALALLF
ANPFVMNITPCSIKTLKGLYDISGVEVGQHFYWQIGGFQIHAQVLITSWFVITILLGSVIIAV
RNPQTIPTDGQNFFEYVLEFIRDLSKTQIGEEYGPWVPFIGTMFLFIFVSNWSGALLPWKIIE

LPHGELAAPTNDINTTVALALLTSAAYFYAGLSKKGLSYFEKYIKPTPILLPINILEDFTKPLS
LSFRLFGNILADELVVVVLVSLVPLVVPIPVMFLGLFTSGIQALIFATLAAAYIGESMEGHH
MLFATLEHILTHISFSTISIVITIHLITLLVRELRGLRDSSEKGMIATFFSITGFLVSRWVSSGHF
PLSNLYESLIFLSWTLYILHTIPKIQNSKNDLSTITTPSTILTQGFATSGLLTEMHQSTILVPAL
QSQWLMMHVSMMLLSYATLLCGSLLSAALLIIRFRNSFDFFS-LKEIEYLYAKRSALKNTSF
PVFPNYYKYQLTERLDSWSYRVISLGFTLLTVGILCGAVWANEAWGSYWNWDPKETWAFI
TWTIFAIYLHSRTNPNWKGTNSALVASIGFLIIWICYFGINLLGIGLHSYGSFTLPMKKKKAL
PSFLYLVFIVLLPWGVSFSFNKCLELWIKNWWNTRQSETFLTDIQEKRILEGFIELEELFLLD
EMIKEKPKTHVQKLPIGIHKEIIQLAKIDNEDHLHIILHFSTNIICLAILSGSFFLGKEELVILN
SWVQEFFYNLNDSIKAFFILLVTDFFVGFHSTRGWELLIRWVYNNLGWAPNELIFTIFVCSF
PVILDTCLKFWVFFCLNRLSPSLVVIYHSISEMPIGVPKVPYRIPGDEEATWVDLYNVMYRE
RTLFLGQEIRCEITNHITGLMVYLSIEDGNSDIFLFINSPGGWLISGMAIFDTMQTVTPDIYTI
CLGIAASMASFILLGGEPTKRIAFPHARIMLHQPASAYYRARTPEFLLEVEELHKVREMITR
VYALRTGKPFWVVSEDMERDVFMSADEAKAYGLVDIVGDEPREAKVTFEGLVTEALPNG
MFRVRLENDTIILGYISGKIRSSSIRILMGDRVKIEVSRYDSSKGRIIYRLPHKDSKDMEKFE
GYSEKQKSRQHYFVYPLLFQEYIYAFAHDYGLFGCNNKFSSILVKRLIIRMYQQNFWINSV
NYPNQDRLFDSEFYSQILSEGFGIVVEIPLSLGQLSCPIPKFQNLQSIHSIFPFLEDKFLHLHY
LSHIEIPYPIHLEILVQLLEYRIQDVPSLHLLRFFLHYYSNWNSLITKSIFLLKKKRLFRFLYN
SYVSEYEFFLLFLRKQSSCLRLTSSGTFLERIIFSGKMEH--VMYPGRKTIWFFMDPLMHYV
RYQGKAILASKGTLLLKKKWKSYLVNFSQYFFSFWTQPQRIRLNQLTNSCFDFLGYLSSVP
INTLLVRNQMLENSFLIDTRM--KKFDTTVPATPLVGSLSKAQFCTGSGHPISKPVWTDLSD
WDILDRFGRICRNLFHYHSGSSKKQTLYRLKYILRLSCARTLARKHKSTVRTFMQRLGLVF
LEEFFTEEEQVFSLMFTKRIWYLDIIRINDLVNPLMIIDRV-EVE-TINSFSKSE-LLKEIYGLIW
IL-PILALLLGITIEVLVIVWLEREISASIQQRIGPEYAGPLGLLQAIADGTKLLLKEDILPSRG
EIPLFSIGPSIAVISVLLSFLVIPLGYRFVLADLSIGVFLWIAISSIAPIGLLMAGYSSNNKYSFS
GGLRAAAQSISYEIPLTFCVLAISLLSNSSSTVDIVEAQSKYGFFGWNLWRQPIGFLVFLISS
LAECERLPFDLPEAEEELVAGYQTEYSGIKYGLFYLVSYLNLLVSSLFVTVLYLGGWNFSIP
YISFFGFFQMNKIIGILEMVIGIFITLTKAYLFLFISITIRWTLPRMRMDQLLNLGWKFLLPISL
GNLLLTTSSQLVSLMIWHVQNENFILDSTRIFMKAFHLLLFNGSFIFPECILIFGLILLLMIDL
TSDQKDRPWFYFISSTSLVISITALLFRWREEPIISFSGNFQTNNFNEIFQFLILLCSTLCIPLSV
EYIECTEMAITEFLLFVLTATLGGMFLCGANDLITIFVAPECFSLCSYLLSGYTKRDLRSNEA
TMKYLLMGGASSSILVHGFSWLYGSSGGEIELQEIVNGLINTQMYNSPGISIALIFITVGLGF
KLSPAPFHQWTPDVYEGSPTPVVAFLSVTSKVAASALATRILDIPFYFSSNEWHLLLEILAIL
SMILGNLLAITQTSMKRMLAYSSIGQIGYVIIGIIVGDSNDGYASMITYMLFYISMNLGTFA
CIVLFGLRTGTDNIRDYAGLYTKDPFLALSLALCLLSLGGLPPLAGFFGKLYLFWCGWQAG
LYFLVSIGLLTSVLSIYYYLKIIKLLMTGRNQEITPYVRNYRRSPLRSNNSIELSMTVCVIAST
IPGISMNPILAIAQDTLFMFLLHEYDIFWTFLIIASLIPILVFWISGLLAPVSEGPEKLSSYESGI
EPMGGAWLQFRIRYYMFALVFVVFDVETVFLYPWAMSFDVLGVSVFIEAFIFVLILVVGLV
YAWRKGALEWSMSYFPWLTILVVLPIFAGSLIFFLPHKGNKIVRWYTIAICLLEFLIMTYAF
CYHFQLEDPLIQLKEDSKWIDVFDFHWRLGIDGLSLGSILLTGFITTLATLAAWPVTRNSQL
FYFLMLAMYSGQIGLFSSRDLLLFFIMWELELIPVYLLLSMWGGKRRLYSATKFILYTAGG
SIFFLIGVLGMGLYGSNEPGLDLERLINQSYPTTLEILLYFGFLIAYAVKLPIIPLHTWLPDTH
GEAHYSTCMLLAGILLKMGAYGLIRVNMELLPHAHYLFSPWLVIIGAVQIIYAASTSLGQR
NFKKRIAYSSVSHMGFIIIGIGSITNIGLNGAILQILSHGFIGATLFFLAGTACDRMRLVYLEE

LGGISIPMPKIFTMFSSFSMASLALPGMSGFVAELVVFFGLITSPKFMLMPKMLITFVMAIG
MILTPIYLLSMLRQMFYGYKLFHVPNKNFVDSGPRELFLLICIFLPVIGIGIYPDFVLSLSVD
RVEALLSNYYTKMMFEHVLFLSVYLFSIGIYGLITSRNMVRALICLELILNSINLNLVTFSDL
FDSRQLKGDIFAIFVIALAAAEAAIGLSILSSIHRNRKSTRINQSNFLNNMEHTYQYAWVIPL
LPLPVIMSMGFGLFLIPTATKNLRRIWAFPSILLLSIAMVFSLHLSIQQINGSSIYQYLWSWTI
NNDFSLEFGYLVDPLTSIMLILITTVGILVLIYSDDYMSHDEGYLRFFVYISFFNTSMLGLVT
SSNLIQIYFFWELVGMCSYLLIGFWFTRPIAASACQKAFVTNRVGDFGLLLGILGFFWITGS
LEFRDLFKIANNNGINSLLTTLCAFLLFLGAVAKSAQFPLHVWLPDAMEGPTPISALIHAAT
MVAAGIFLLARLLPLFISLPLIMSFISLVGTITLFLGATLALAQRDIKRSLAYSTMSQLGYMM
LALGIGSYQAALFHLITHAYSKALLFLGSGSVIHSMEPLVGYSPDKSQNMVLMGGLRKYV
PITRTTFLCGTLSLCGIPPLACFWSKDEILSNSWLYSPFFGIIASFTAGLTAFYMFRIYLLTFD
GYLRVNFQNYSSTKEGSLYSISLWGKSISKGVNR-DFVLSTMKFFSAKK--TFVYPHETGNT
MLFPLLILLLFTLFIGSIGIPFELTILSKWLTPSINLFQENSNSSINSYEFLTNAISSVSLAIFGLFI
AYIFYGSAYSFFQNLNFQNSLVSFLDEVKKKIYSWSYNRGYIDFFYTRVFILGIRRLAELTHF
FDKGVIDGIINGVGLAGFCIGEEIKY-VGGGRISSYLFFFLCYVSLFLFFIPMDLPGPIHEILVL
FGGFVLLLGGLGVVLLTNPIYSAFSLGLVLVCISLFYFLLNSYFVAVAQLLIYVGAINVLIIFA
VMFVNGSEWSKDKNYWTIGDGFTLLLCITIPFSLMTTIPDTSWYGILWTTRSNQIVEQGLI
NNVQQIGIHLATDFYLPFELISLILLVSLIGAITMARQMSLSLKRKDLMIVNMGPQHPSMHG
VLRLIVTLDGEDVIDCEPILGYLHRGMEKIAENRSIIQYLPYVTRWDYLATMFTEAITVNAP
EFLENIQIPQRASYIRVIMLELSRIASHLLWLGPFMADLGAQTPFFYIFRERELIYDLFEAAT
GMRMMHNYFRIGGVAADLPYGWMDKCLDFCDYFLQGVVEYQQLITRNPIFLERVEGVG
FISGEEAVNWGLSGPMLRASGIQWDLRKIDPYESYNQFDWKVQWQKEGDSLARYLVRVG
EMRESIKIIQQAVEKIPGGPYENLEARR-KKAKNPEWNDFEYRFLGKKPSPNFELSKQELYV
RVEAPKGELGIYLVGDDSLFPWRWKIRPPGFINLQILPQLVKKMKLADIMTILGSIDIIMGE
VDRMFPMLTGFISYGQQTIRAARYIGQGLIITLSHTNRLPITIHYPYEKSITSERFRGRIHFEF
DKCIACEVCVRVCPIDLPLVDWKFEKDIKRKQLLNYSIDFGVCIFCGNCVEYCPTNCLSMT
EEYELSTYDRHELNYNQIALSRLPISIMGDYTIQTIRNSKIDEEKSWNSRTITDYQQGWLSN
WLVKHDVVHRSLGFDHRGVETLQIKAGDWDSIAVILYVYGYNYLRSQCAYDVAPGGSLA
SVYHLTRIQYGIDNPEEVCIKVFAQKDNPRIPSVFWVWRSADFQERESYDMVGISYDNHP
RLKRILMPESWIGWPLRKDYITPNFYEIQDAH--
---MSLIEFPLLDQTSSNSVISTTPNDLSNWSRLSSLWPLLYGTSCCFIEFASLIGSRFDFDRYG
LVPRSSPRQADLILTAGTVTMKMAPSLVRLYEQMPEPKYVIAMGACTITGGMFSTDSYSTV
RGVDKLIPVDVYLPGCPPKPEAVIDALTKLRKKIAREIIEDRTLCQSKNRSFTTRHKLYVRR
STHTGTYEQELLYQSPSTLDIST--FFKSKSSVSSYKLVNMENRKTFSWLKEQMIRSISVSIMI
YVITRTSISNAYPIFAQQGYENPREATGRIVCANCHLANKPVDIEVPQAVLPDTVFEAVLRIP
YDMQLKQVLANGKKGGLNVGAVLILPEGFELAPPDRISPELKEKIGNLSFQSYRPNKKNIL
VIGPVPGKKYSEIVFPILSPDPATKKDVHFLKYPIYVGGNRGRGQIYPDGSKSNNTVYNATS
TGIVKKILRKEKGGYEISIVDASDGRQVIDIIPPGPELLVSEGESIKLDQPLTSNPNVGGFGQ
GDAEIVLQDPLRVQGLLFFFASVILAQVFLVLKKKQFEKVQLYEMNFLNKVYDWFEERLEI
QAIADDITSKYVPPHVNIFYCLGGITLTCFLVQVATGFAMTFYYRPTVTEAFSSVQYIMTEA
NFGWLIRSVHRWSASMMVLMMILHVFRVYLTGGFKKPRELTWVTGVVLAVLTASFGVTG
YSLPWDQIGYWAVKIVTGVPEAIPVIGSPLVELLRGSASVGQSTLTRFYSLHTFVLPLLTAVF
MLMHFPMIRKQGISGPLMGVTKKPDLNDPVLRAKLAKGMGHNYYGEPAWPNDLLYIFPV
VILGTIACNVGLAVLEPSMIGEPADPFATPLEILPEWYFFPVFQILRTVPNKLLGVLLMVSVP

TGLLTVPFLENVNKFQNPFRRPVATTVFLIGTVVALWLGIGATLPIDKSLTLGLFMIEVFLFG
IVLGLIPITLAGLFVTAYLQYRRGDQLDLMLTITSYFGFLLAALTITPALFIGLNKIRLIMDIV
SLAWAALMVVFTFSLSLVVWGRSGLMIIRPSEPEVKIAVDRDPVKTSFEEWARPGHFSRTIA
KGPDTTTWIWNLHADAHDFDSHTGDLEEISRKVFSAHFGQLSIIFLWLSGMYFHGARFSN
YEAWLSDPTHIGPSAQVVWPIVGQEILNGDVGGGFRGIQITSGFFQIWRASGITSELQLYCT
AIGALIFASLMLFAGWFHYHKAAPKLAWFQDVESMLNHHLAGLLGLGSLSWAGHQIHVS
LPINQFLDAGVDPKEIPLPHEFILNRDLLAQLYPSFAEGATPFFTLNWSKYAEFLSFRGGLDP
ITGGLWLSDIAHHHLAIAILFLIAGHMYRTNWGIGHGLKDILEAHKGPFTGQGHKGLYEIL
TTSWHAQLSLNLAMLGSTTIVVAHHMYSMPPYPYLATDYGTQLSLFTHHMWIGGFLIVG
AAAHAAIFMVRDYDPTTRYNDLLDRVLRHRDAIISHLNWVCIFLGFHSFGLYIHNDTMSA
LGRPQDMFSDTAIQLQPIFAQWIQNIHAGAPGVTAPGATTSTSLTWGGGELVAVGGKVALL
PIPLGTADFLVHHIHAFTIHVTVLILLKGVLFARSSRLIPDKANLGFRFPCDGPGRGGTCQVS
AWDHVFLGLFWMYNSISVVIFHFSWKMQSDVWGTISDQGIVTHITGGNFAQSSITINGWL
RDFLWAQASQVIQSYGSSLSAYGLFFLGAHFVWAFSLMFLFSGRGYWQELIESIVWAHNK
LKVAPATQPRALSIIQGRAVGVTHYLLGGIATTWAFFLARIIAVGMELRFPRFSQGLAQDPT
TRRIWFGIATAHDFESHDDITEERLYQNIFASHFGQLAIIFLWTSGNLFHVAWQGNFESWIQ
DPLHVRPIAHAIWDPHFGQPAVEAFTRGGAAGPVNIAYSGVYQWWYTIGLRTNEDLYTGA
LFLLFLSTLSLIGGWLHLQPKWKPSLSWFKNAESRLNHHLSGLFGVSSLAWTGHLVHVAIP
GSRGEYVRWNNFLDVLPYPQGLGPLLTGQWNLYAQNPDSSNHLFGTTQGAGTAILTLLGG
FHPQTQSLWLTDIAHHHLAIAFIFLIAGHMYRTNFGIGHSIKDLLEAHTPPGGRLGRGHKGL
YDTINNSIHFQLGLALASLGVITSLVAQHMYSLPAYAFIAQDFTTQAALYTHHQYIAGFIMT
GAFAHGAIFFIRDYNPEQNEDNVLARMLDHKEAIISHLSWASLFLGFHTLGLYVHNDVML
AFGTPEKQILIEPIFAQWIQSAHGKTTYGFDILLSSTNGPAFNAGRNIWLPGWLNAVNE--NS
NSLFLTIGPGDFLVHHAIALGLHTTTLILVKGALDARGSKLMPDKKDFGYSFPCDGPGRGG
TCDISAWDAFYLAVFWMLNTIGWVTFYWHWKHITLWQGNVSQFNESSTYLMGWLRDYL
WLNSSQLINGYNPFGMNSLSVWAWMFLFGHLVWATGFMFLISWRGYWQELIETLAWAHE
RTPLANLIRWRDKPVALSIVQARLVGLAHFSVGYIFTYAAFLIASTSGKFGMSHSVKIYDTC
IGCTQCVRACPTDVLEMIPWDGCKAKQIASAPRTEDCVGCKRCESACPTDFLSVRVYLRS
ETTRSMALSYMTDLNLPSIFVPLVGLVFPAIAMTSLFLYVQKNKIVMRDIKTYLSVAPVLST
LWFGALAGLLIEINRLFPDALSFPFF--MTAILERRESTSLWGRFCNWITSTENRLYIGWFGV
LMIPTLLTATSVFIIAFIAAPPVDIDGIREPVSGSLLYGNNIISGAIIPTSAAIGLHFYPIWEAAS
VDEWLYNGGPYELIVLHFLLGVACYMGREWELSFRLGMRPWIAVAYSAPVAAATAVFLIY
PIGQGSFSDGMPLGISGTFNFMIVFQAEHNILMHPFHMLGVAGVFGGSLFSAMHGSLVTSS
LIRETTENESANEGYKFGQEEETYNIVAAHGYFGRLIFQYASFNNSRSLHFFLAAWPVVGI
WFTALGISTMAFNLNGFNFNQSVVDSQGRVINTWADIINRANLGMEVMHERNAHNFPLD
LAALEVPSLNGMGLPWYRVHTVVLNDPGRLLSVHIMHTALVSGWAGSMALYELAVFDPS
DPVLDPMWRQGMFVIPFMTRLGITNSWGGWSIS-GGTVTNPGIWSYEGVAGAHIVFSGLC
FLAAIWHWVYWDLEIFCDERTGKPSLDLPKIFGIHLFLAGVACFGFGAFHVTGLYGPGIWV
SDPYGLTGKVQAVNPAWGAEGFDPFVPGGIASHHIAAGTLGILAGLFHLSVRPPQRLYKGL
RMGNIETVLSSSIAAVFFAAFVVAGTMWYGSATTPIELFGPTRYQWDQGYFQQEIYRRVSD
GLAENLSLSEAWSKIPEKLAFYDYIGNNPAKGGLFRAGSMDNGDGIAVGWLGHPVFRDK
EGRELFVRRMPTFFETFPVVLVDEEGIVRADVPFRRAESKYSVEQVGVTVEFYGGELNGV
SYSDPATVKKYARRAQLGEIFELDRATLKSDGVFRSSPRGWFTFGHATFALLFFFGHIWHG
ARTLFRDVFAGIDPDLDAQVEFGTFQKVGDPTTRRQAAMKILYSLRRFYHVETLFNGTFV

LAGRDQETTGFAWWAGNARLINLSGKLLGAHVAHAGLIVFWAGAMNLFEVAHFVPEKP
MYEQGLILLPHLATLGWGVGPGGEVLDTFPYFVSGVLHLISSAVLGFGGIYHALLGPETLE
ESFPFFGYVWKDRNKMTTILGIHLILLGLGAFLLVLKALYFGGVYDTWAPGGGDVRKITN
LTLSPGVIFGYLLKSPFGGEGWIVSVDDLEDIIGGHVWLGSICVLGGIWHILTKPFAWARRA
FVWSGEAYLSYSLGALSVFGFIACCFVWFNNTAYPSEFYGPTGPEASQAQAFTFLVRDQRL
GANVGSAQGPTGLGKYLMRSPTGEVIFGGETMRFWDLRAPWLEPLRGPNGLDLSRLKKD
IQPWQERRSAEYMTHAPLGSLNSVGGVATEINAVNYVSPRSWLATSHFVLGFFFFVGHLW
HAGRARAAAAGFEKGIDRDLEPVLYMTPLNMTIAVGRVTKEENDLFDIMDDWLRRDRFV
FVGWSGLLLFPCAYFALGGWFTGTTFVTSWYTHGLASSYLEGCNFLTAAVSTPANSLAHS
LLLLWGPEAQGDFTRWCQLGGLWTFVALHGAFALIGFMLRQFELARSVQLRPYNAISFSG
PIAVFVSVFLIYPLGQSGWFFAPSFGVAAIFRFILFFQGFHNWTLNPFHMMGVAGVLGAAL
LCAIHGATVENTLFEDGDGANTFRAFNPTQAEETYSMVTANRFWSQIFGVAFSNKRWLHF
FMLFVPVTGLWMSAIGVVGLALNLRAYDFVSQEIRAAEDPEFETFYTKNILLNEGIRAWM
AAQDQPHENLIFPEEVLPRGNALMSGSTGERSFADIITSIRYWVIHSITIPSLFIAGWLFVSTG
LAYDVFGSPRPNEYFTESRQGIPLITDRFDSLEQLDEFSRSFMTIDRTYPIFTVRWLAVHGLA
VPTVFFLGSISAMQFIQRMATQTVEDSSRPK-PKRTGAGSLLKPLNSEYGKVAPGWGTTPF
MGVAMALFAIFLSIILEIYNSSVLLDGILTNMLTLKLFVYTVVIFFVSLFIFGFLSNDPGRNPG
REEMADTTGRIPLWLIGTVTGILVIGLIGVFFYGSYSGLGSSLMPNILSLTCICFNSVLCPTSF
FFAKLPEAYAIFNPIVDVMPVIPVLFFLLAFVWQAAVSFRMTQSNPNEQNVELNRTSLYWG
LLLIFVLAVLFSNYFFNMEVNILAFIATALFILVPTAFLLIIYVKTASQNDMETATLVAISISGL
LVSFTGYALYTAFGQPSQQLRDPFEEHGDMEALVYTFLLVSTLGIIFFAIFFREPPKVPTKK--
MTIAFQLAVFALIATSSVLVISVPLVFASPDGWSNNKNVVFSGTSLWIGLVFLVAILNSLIS----
---MSPQTETKASVGFKAGVKDYKLTYYTPEYETKDTDILAAFRVTPQLGVPPEEAGAAVA
AESSTGTWTTVWTDGLTSLDRYKGRCYHIEPVPGDPDQYICYVAYPLDLFEEGSVTNMFT
SIVGNVFGFKALRALRLEDLRIPPAYVKTFQGPPHGIQVERDKLNKYGRPLLGCTIKPKLG
LSAKNYGRACYECLRGGLDFTKDDENVNSQPFMRWRDRFVFCAEAIYKAQAETGEIKGH
YLNATAGTCEEMIKRAVFAKELGVPIVMHDYLTGGFTANTTLSHYCRDNGLLLHIHRAMH
AVIDRQKNHGMHFRVLAKALRMSGGDHIHSGTVVGKLEGEREITLGFVDLLRDDFIEKDR
SRGIFFTQDWVSMPGVIPVASGGIHVWHMPALTEIFGDDSVLQFGGGTLGHPWGNAPGAA
ANRVALEACVQARNEGRDLAREGNEIIKAACKWSAELAAACEIWKEIKFDFKAMDTL--T
AKHLYKTPIPSTRKGTVDRQVKSNPRNKLIHGRHRCGKGRNARGIITARHRGGGHKRLYR
KIDFRRNQKDISGRIVTIEYDPNRNAYICLIHYGDGEKRYILHPRGAIIGDTIVSGTKVPISM
GNAL--TDMPLGTAIHNIEITRGRGGQLARAAGAVAKLIAKEGKLATLRLPSGEVRLVSQNC
LATVGQVGNVGVNQKSLGRAGSKCWLGKRPVVRGVVMNPVDHPHGGGEGKAPIGRKK
PTTPWGYPALGRRTRKRKKYSDSFILRRRK-MIQPQTLLNVADNSGARKLMCIRVIGAAGN
QRYARIGDVIIAVIKDAVPKMPLERSEVIRAVIVRTRKEFKGDDGIIIRYDDNAAVIIDQKGNP
KGTRVFGAVAEELRELNFTKIVSLAPEVLMLSPKRTRFRKQHRGRMKGKSCRGNHICFGR
YALQVLEPAWITARQIEAGRRAMTRYARRGGKIWVRIFPDKPVTIRPTETRMGSGKGSPEY
WVAVVKPGRILYEMSGVSETVARAAISIAASKMPIRSQFLRLEMTRVPRGYIARRRRTKMR
SFASNFRGAHLRLNRMITQQVRRAFVSSHRDRGRQKRDFRRLWITRINAATRVY--VFNSY
SKLIHNLSKKELILNRKMLAQVAVSNPNNLYTISNKIRTIN---------RIKRVFKAQKRIHMSVF
KAQRVLDEIRWRYYEETVMILNLMPYRASYPILKLVYSAAANATHYRDFDKTNLFITKAE
VSRSTIMKKFRPRARGRSYSIKKTMCNITIVLN-VKKS-------MDGIKYAVFTEKSLRLLGKN
-QYTFNVESGFTKTEIKHWVELFFGVKVVAVNSHRLPGKGRRMGPILGHTMHYRRMIITL

QPGYSIPLLDRETNMAVPKKRTSMSKKRIRKNLWKKKTYFSIVQSYSLAKSFSSGKPKGFS
GQQANKMAKGKDVRIRVILECISCVRK--G--KESTGISRYSTQKNRHNTPGQLELRKFCRY
CRKHTTHNEIKKMKIRASVRKICTKCRLIRRRGRIRVICSNPKHKQRQGMVREEITGSTQK
LEWKCVESRVDSKRLYYGRFILSPLRKGQADTVGIALRRALLGEIEGTCITRAKFG--NVPH
EYSTIVGIEESIQEILLNLKEIVLRSNLYGVRDASICVKGPRYITAQDIILPPSVEIVDTAQPIA
NLREPIDFCIELQIKRDRGYHTELRKNSQDGSY--PIDAVFMPVRNVNYSIFSCGNGNEKHEI
LFLEIWTNGSLTPKEALYEASRNLIDLFLPFIHTEEE--GTSFEESK-NRLTPPLLTFQKRFT-NL
KKNKK-GIPLNCIFIDQLELPSRTYNCLKRANIHTLLDLLSKTEEDLMRINSFRMEDGKLIW
DTLEKH--LPIDLPKNKMLRNGN-EGMSTIPGFSQIQFEGFCRFINQGLAEELEKFPTIKDPD
HEIAFQLFAKGYQLLEPSIKERNAVYESLTYSSELYVSARLIFGF--DVQKQTISIGNIPIMNSL
GTFIINGIYRIVINQILLSPGIYYRSELDHKGISIYTGTIISDWGGRSELAIDKKERIWARVSRK
QKISILVLSSAMGSNLREILDNVSYPEIFLSFPNAKEKKRIESKEKAILEFYQQFACVGGDLV
FSESLCEELQKKFFQQKCELGRVGRRNMNRRLNLDIPQNNTFLLPRDVLAATDHLIGMKF
GTGILDDDDMNHLKNKRIRSVADLLQDQFGLALGRLQHAVQKTIRRVFIRQSK--PTPQTLV
TPTSTSILLITTYETFFGTYPLAQVFDQTNPLTQTVHGRKVSCLGPGGLTGRTASFRSRDIHP
SHYGRICPIDTSEGINVGLTGSLAIHARIDHWWGSIESPFYEISEKAKEKKE--RQVVYLSPN
RDEYYMIAAGNSLSLNQGIQEEQVVPARYRQEFLTIAWEQIHVRSIFPFQYFSIGGSLIPFIE
HNDANRALMSSNMQRQAVPLSRSEKCIVGTGLERQTALDSRVSVIAEREGKIISSDSHKILL
SSSGKTISIPLVAHRRSNKNTCMHQKPRVPRGKSIKKGQILAEGAATVGGELALGKNVLVA
YMPWEGYNFEDAVLISERLVYEDIYTSFHIRKYEIQTDTTSQGSAEKITKQIPHLEEHLLRN
LDRNGVVRLGSWVETGDILVGKLTPQIASE-SSYIAEAGLLRAIFGLEVSTSKETSLKLPIGG
RGRVIDVKWIQR-------DPIMVRVYILQKREIKVGDKVAGRHGNKGIISKILPRQDMPYLQD
GTPVDMVFNPLGVPSRMNVGQIFESSLGLAGDLLKKHYRIAPFDERYEQEASRKLVFSELY
EASKQTKNPWVFEPEYPGKSRIFDGRTGDPFEQPVLIGKSYILKLIHQVDEKIHGRSTGPYS
LVTQQPVRGRAKQGGQRIGEMEVWALEGFGVAHILQEILTYKSDHLIARQEILNATIWGKR
VPNHEDPPESFRVLVRELRSLALELNHFLVSEKNFRVNREDV-------MIDQYKHKQLQIGLV
SPQQIKAWAKKILPNGEVVGEVTRPSTFHYKTDKPEKDGLFCERIFGPIKSGICACGNSRAS
VAENEDERFCQKCGVEFVDSRIRRYQMGYIKLACPVTHVWYLKGLPSYIANLLDKPLKKL
EGLVYGD--FSFARP-SAKKPTFLRLRGLFEDEISSCNHSISPFFSTPGFATFRNREIATGAGAI
REQLADLDLRIIIENSLVEWKELEDE-GYSGDEWEDRKRRIRKVFLIRRMQLAKHFIQTNV
EPEWMVLCLLPVLPPELRPIVYRSGDKVVTSDINELYKRVIRRNNNLAYLLKRSELAPADL
VMCQEKLVQEAVDTLLDSGSRGQPMRDGHNKVYKSLSDVIEGKEGRFRETLLGKRVDYS
GRSVIVVGPSLSLHQCGLPLEIAIKLFQLFVIRDLITKRATSNVRIAKRKIWEKEPIVWEILQ
EVMRGHPVLLNRAPTLHRLGIQAFQPTLVEGRTICLHPLVCKGFNADFDGDQMAVHLPLS
LEAQAEARLLMFSHMNLLSPAIGDPICVPTQDMLIGLYVLTIGNRRGICANRYNSCGN-----S
PNKKINYNNYKYTKDKEPHFSSSYDALGAYRQKRIGLNSPLWLRWKLDQR-IVGS--REVPI
EVQYESFGTYHEIYAHYLVVGNRKKEIRSIYIRTTLGHISFYREIEEAIQGFSRAYSYTI--------
----MAERANLVFHNKEIDGTAMKRLISRLIDHFGMGYTSHILDQIKTLGFHQATTTSISLGIE
DLLTIPSKGWLVQDAEQQSFLLEKHYYYGAVHAVEKLRQSVEIWYATSEYLKQEMNSNFR
ITDPSNPVYLMSFSGARGNASQVHQLVGMRGLMADPQGQMIDLPIQSNLREGLSLTEYIIS
CYGARKGVVDTAVRTADAGYLTRRLVEVVQHIIVRRRDCGTIQGISVSPQNGMTE--KLFV
QTLIGRVLADDIYIGSRCIASRNQDIGIGLVNRFITAFRAQPFRAQPIYIRTPFTCRSTSWICQL
CYGRSPTHGDLVELGEAVGIIAGQSIGEPGTQLTLRTFHTGGVFTGGTADLIRSPSNGKIQFN
EDLVHPTRTRHGQPAFLCYIDLHVTIQSQDILHSVNIPLKSLILVQNDQYVESEQVIAEIRAG

TSTL--FKEKVQKHIYSESDGE-MHWSTDVYHAPEYQYGNLRRLPKTSHLWILSVSMCR--S
SIASFSLHKDQDQMNTYSFSVDGR--YIFDFS--MAN-DQVSHRLLDTFG----DREILDYL-TP
DRIVSNGHWNCFYPS-ILQDN--SDLLAKKRRNRFAVPLQYHQEQEKERISCLGISMEIPFM--
GVLRRNTIFAYFDDPRYRKD--GIVKFRSPEEDSILK--KEGFIEHRG-TKEFSLKY-EVDRFFFI
LQELHILPRSSSLKVLDNSIIGVDTQLTKNTRSRLGGLVRVKRKKSHTELKIFSGDIHFPEEA
DKILG--GSLIPPEREKK--DSKE-SK-KRKNWVYVQRKKILKSKEK--YFVSVRPAVSYEMDE
GRNLATLFPQDLLQEEDNLQLRLVNFISHENSKLTQRIYHTN-QFVRTCLVVNWEQEVKEG
AR-------ASLVEVRTNDLIRDFLRIELVKSTISYTRRRYDRTSVGLIPN---LDRNNTNSFYSKA
KIQS-----LSQHQEVIG----TLLNRNK---EYPSLMILLASNCSRIG--LFKNSKYP---NAVKESNP
RIPI-DVFGLLGVIVP-SISNFSSS-YYLLT---HNQILLK-KYLFLD---NLKQTFQVLQGLKYSLI
DENQR--ISNFG--SNIMLEPFHLNWHFLHHDSWEETLAIIH-LGQFICENLCLFKSHI----KKS
GQIFIVN-MDSFVLRAAKPYLATIGATVHGHYGKILYKGDRLVTFIYEKSRSSDITQGLPKV
EQIFEARSIDSLSPNLERRIEDWNERIPRILGVPWGFLIGAELTIAQSRISLVNKIQKVYRSQG
VQIHNRHIEIIIRQVTSKVRVSE---DGMSNVFLPGELIGLLRAERAGRALDESIYYRAILLGIT
RASLNTQSFISEASFQETARVLAKAALRGRIDWLKGLKENVVLGGIIPVGTGFQKFVHRSP-
-QDKNLY--FEIKKKNLFASEM--RDILFLHTELVSSDSDVTN---SETPFTPIYTI------MTRRYW
NINLKEMIEAGVHFGHGIKKWNPKMAPYISAKRKGTHITNLARTARFLSEACDLVFDAAS
QGKSFLIVGTKKRAADLVASAAIRSRCHYVNKKWFSGMLTNWSITKTRLSQFRDLRAEEK
MGKFHHLPKRDAAILKRKLSTLQRYLGGIKYMTRLPDIVIVLDQQKEYIALRECAILGIPTI
SLVDTNCDPDLANISIPANDDTMTSIRLILNKLVFAISEGRSL-YIRNRMGQKINPLGFRLGTT
QNHHSFWFAQPKNYSEGLQEDKKIRNCIKNYIQKNRKKGSNKMESITHIEIQKEIDTIHVII
HIGFPN-LLKKKG-----AIEELEKDLQKEVNSVNQRLNIAIEKVKEPYRQPNILAEYIAFQLK
NRVSFRKAMKKAIELTKKADIKGIKIQIAGRLAGKEIARAECIKKGRLPLQTIRAKIDYCCY
PIRTIYGVLGVKIWIFVEEEMSRYRGPRLKKIRRLGALPGLTRKTPKSGSNQKKKFHSGK-K
EQYRIRLQEKQKLRFHYGLTERQLLRYVHIAGKAKRSTGQVLLQLLEMRLDNILFRLGMA
STIPGARQLVNHRHILVNGRIVDIPSFRCKPRDIITTKDNQRSKRLVQNYIASSDPK--LPKHL
TVDT--LQYKGLVKKILDRKWVGLKINELLVVEYYSRQTMSRRGTAEKRTAKSDPIFRNRL
VNMVVNRIMKDGKKSLAYQILYRAVKKIQQKTETNPLLVLRQAIRRVTPNIGVKTRRKKG
STRKVPIEIGSKQGRALAIRWLLEASQKRPGRNMAFKLSSELVDAAKGSGGAIRKKEATHR
MAEANRALAHFRMGKDTIADLLTSIRNADMNKKGTVRVVSTNITENIVKILLREGFIESVR
KHQESNRYFLVSTLRHQRRKTRKGIYRT---RTFLKRISRPGLRIYANYQGIPKVLGGMGIAIL
STSRGIMTDREARLNRIGGEVLCYIWMTKAIPKIGSRKKVRIGLRKSARRITKGVIHVQASF
NNTIITVTDPQGRVVFWSSAGTCGFKSSRKASPYAGQRTAVDAIRTVG---LQRAEVMVKG
AGSGRDAALRAIAKSGVRLSCIRDVTPMPHNGCRPPKKRRLHPTCSSTLRTSLKRGRFSSIS
YWLS-CISNK-LFNRWHTINPKKPNSALRKVARVRLTSGFEITAYIPGIGHNLQEHSVVLVRG
GRVKDLPGVRYRIIRGTLDAVAVKNRQQGRSKYGAKKPKMAKKSLIQREKKRHKLEQKY
HLIRRSSKKKIRSKVSPLSLSEKTKMQEKLQSLPRNSAPTRLHRRCFLTGRPRANYRDFGLS
GHILREMVYACLLPGATRSSW---------MVKEEKQEN--RGSVEFQVFSFTNKIRRLASHLELH
KKDFSSERGLRRLLGKRQRLLAYLAKKNRVRYKKLISQLDIREK-----MVKLRLKRCGRKQ
QAIYRIVAIDVRSRREGRDLRKVGFYDPIKNQTCLNVPAILYFLEKGAQPTRTVYDILRKAE
FFKDKERTLS----FHKSKQPFRKFKQPFRKRRSRIGPGDRIDYRNMSLINRFISEQGKILSRRI
NRLTLKQQRLITVAIKQARILSFLPFRNYENEKQFQ-AQAISIITGPRHRKNVTRK-KTNPFVA
RHLLAKIEKVNMKEEKEIIVTWSRASSILPAMVGHTIAIHNGKEHIPIYITNPMVGRKLGEF
V P T R H F T S Y T R K D T K S R

R??
???
???
???
???
???
???
???
???
???
???
???
???
???
???
???
???
???
???
???
???
???
???
???
???
???
???
???
???
???
???
???
???
???
???
???
???
???
???
???
???
???
???
???MPRSR-INGNFIDKTSSIVANILLQIIPTTSGEK

RAFTYYRDG--MLAQSEGNYAEALQNYYEATRLEIDPYDRSYILYNIGLIHTSNGEHTKALE
YYFRALERNPFLPQAFNNMAVICHY-RGEQAILQGDSEIAEAWFDQAAEYWKQAIALTPG
NYIEAQNWLKITKRFEFEMNWRSEHIWIELLKGSRKRGNFFWACILFLGSLGFLAVGASSY
LGKNMISLLPSQQILFFPQGVVMSFYGIAGLFISSYLWCTILWNVGSGYDRFDRKEGIVCIF
RWGFPGIKRRIFLQFLVRDIQSIRIQVKEGLYPRRILYMEIRGQGVIPLTRTDEKFFTPREIEQ
KAAELAYFLGVPIEVF
>Nuphar
MEKWWLNSMLSLS---ASLGPIGNTSGSEEPITNDNEKNIH-YSNVDGLDGVKDIWSLISGD
TFWVRDSNGDSYSVYFDIENKIFEIDTD--SYFLGELESLFSSYL---NLNSGSKDYNRYYDQ
YVYDTRHSWKNHINSCIDSYIRSETN--MDSCISNGSNNSSDNYIYSYICSDRGSSLKTS-SD-
----FGRH-DLDRNLKYGHLWVQCENCYGLNYKKFFSSKMNICEQCGYHLKMSSSDRIELLI
DPGTWDPMDENMVSIDPIEFHSEEEPYRDRINSYQIETGLAEAVQTGIGKLNGIPIAIGVMD
FKFMGGSMGSVVGEKITRLIEYATDRSLPVIMVCASGGARMQEGSLSLMQMAKISSALYN
YQSNKKLFYVSILTSPTTGGVTASFGMLGDIIIAEPNAYIAFAGKRVIEQTLKKTVPEGSQVA
EYSFHKGLFDPIVPRNLLKGVPSELFQFHGFFPRP-----MVTIRAEEISNIIRERIEQYNREVKI
VNTGTVLQVGDGIARIHGLDEVMAGELVEFEEGTIGIAPNLESNNVGVVLMGDGLMIQEG
SSVKATGRIAQIPVSEAYLGRVINALAKPIDGRGEILASEYRLIESPAPGIISRRSVYEPLQTG
LIAIDSMIPIGRGQRELIIGDRQTGKTAVATDTILNQKGQNVICVYVAIGQKASSVAQVVTTF
QERGAMEYTIVVAETADSPATLQYLAPYTGAALAEYFMYRERHTLIIYDDLSKQAQAYRQ
MSLLLRRPPGREAYPGDVFYLHSRLLERAAKPSSRLGEGSMTALPIVETQSGDVSAYIPTN
VISITDGQIFLSADLFNAGIRPAINVGISVSRVGSAAQIKAMKQVAGKSKLELAQFAELEAFA
QFASDLDKATQNQLARGQRLRELLKQSQSSPLAVDEQIVTIYTGTNGYLDQLEIGQVRKFL
VQLRTYLRTNKPQFQEIISSTKVFTEQAEAILKEAIQEQIELFLLQEQTMRINPTTSGPGVST-
--KNLGRIAQIIGPVLDVAFPPGKMPNIYNSLVVKGRDTAGQEINVTCEVQQLLGNNRVRAV
AMSATDGLMRGMEVIDTGAPLSVPVGGATLGRIFNVLGEPVDNLGPVDTRTTSPIHRSAPA
FIQLDTKLSIFETGIKVVDLLAPYRRGGKIGLFGGAGVGKTVLIMELINNIAKAHGGVSVF
GGVGERTREGNDLYMEMKESGVINEENIAESKVALVYGQMNEPPGARMRVGLTALTMAE
YFRDVNEQDVLLFIDNIFRFVQAGSEVSALLGRMPSAVGYQPTLSTEMGSLQERITSTKEG
SITSIQAVYVPADDLTDPAPATTFAHLDATTVLSRGLAAKGIYPAVDPLDSTSTMLQPRIVGE
EHYETAQRVKQTLQRYKELQDIIAILGLDELSEEDRLTVARARKIERFLSQPFFVAEVFTGSP
GKYVGLAETIRGFQLILSGELDSFPEQAFYLVGNIDEATAKAMNLEVESKLKKMTLNLSVL
TPNRIIWDSEVKEIILSTNSGQIGVLPNHAPVATAVDIGILRIRLDDQWLTMALMGGFARIGN
NEITILVNDAEKGSDIDPQEAQQTLEIAEANLRKAEGKRQVIEANLALRRARARVEAINAIS
MKNLTDSFVFLGHWPSAGSFGFNTDILATNLINLSVVLGVLIFFGKGVLSDLLDNRKQRIL
STIRNSEELSGGAVEQLEKARARLRKVEREAEEFRVNGYSEIEQEKVNLINAAYQNLEQLE
NYKNETIHFEQQRAINQVQQRVFQQALQGALGTLNNCLNGELHLRTIGANIGILGAMKEIT
DMNPLISAASVIAAGLAVGLASIGPGVGQGTAAGQAVEGIARQPEAEGKIRGTLLLSLAFM
EALTIYGLVVALALLFANPFVMNVLPCSINTLKGLYEISGVEVGQHFYWQIGGFQVHAQVL
ITSWVVIAILLGSAAIAVRNPQTIPTDGQNFFEYVLEFIRDVSKTQIGEEYGPWVPFIGTLFL
FIFVSNWSGALLPWRIIQLPHGELAAPTNDINTTVALALLTSVAYFYAGLTKKGLGYFGKYI
QPTPILLPINVLEDFTKPLSLSFRLFGNILADELVVVVLVSLVPLVIPIPVMFLGLFTSGIQALI
FATLAAAYIGESMEGHHMIFATLEHILTHISFSIISIVIPTHLMT-SVYEIVGLCDLSEKGMIATF
FCITGLLVTRWIYSGHVPLSDLYESLMFLSWSFSFIHIVPYFRNYKNYFSTITAPSAILTQGFA
TSGLLTKMHQSAILVPALQSRWLMMHVSMMLLSYAALLCGSLLSITLLVITFRRNIDIIGKT

NEIQYLN-----------VSFRNYHRYQLTQRLDHWSYRVIGLGFTLLTIGILSGAVWANEAWGSY
WNWDPKETWAFITWTVFAIYSHTRTNKSLQGANSAIVASIGFLIIWICYFGVNLLGRGLHS
YGSFTLNVTKK-ALNPLPYLASIVFLPWGISLSFNKSMEPWVTNWWNTSQSETFLNDIQEK
NILEGFIKLEELFLLDEMIKEYPETHIQKLRIGIHKETIQLVRMHNQDHIHIILHFSTNITCFAI
LSAYSILGNEELIALNSWVQEFLYNLSDTIKAFSILLLTDLCIGFHSPHAWELMIGSFYKDFG
FVQNDKIISGLVSTFPVILDTILKYWIFHYLNRVSPSLVVIYHSLNEMPIGVPKVPFRSPGEE
DAVWVDIYNRLHRERLLFLGQGVDSEISNQLVGLMVYLSMEDNTRDLYLFINSPGGWVIP
GIAIYDAMQIVPPDVHTICMGLAASMGSFILVGGEFTKRLAFPHARVMIHQPASSFYEAQA
GEFILEAEELLKLREILTRVYVQRTGKPLWVVSEDMERDVFMSATEAQAHGIVDLVAVEM
KEQKLIHEGLITESLPNGMFWVRLDNEDLVLGYVSGRIRRSFIRILPGDRVKIEVSRYDSTR
GRIIYRLRNKDSNDQYELQGYLEIDRYRKQRFLYPLLFREYIYALAHDHGLLGYDN-SSSLI
VKRLITRLHQQNHLIISVNDS---RFVGRSFYSQTISEGFAGIMEIPFSMRLVPSLIAKYQ-LRSI
HSIFPFLEDKLSHLYYVSDILIPHPIHLEILLQTLRTRIRDAPSLHLLRCFLHEHHNWNSLITK
STSIFSKQRLFLFLYNSHVYECESVLVFLRKQSSHLRSISSLAFLERTHFYGKIKH-VVAPRN
QRPLWFFKEPLMHYVRYQGKSIMASRCTNLLMKKWKYYLVNFWQCHFHLWSQPGGIHI
NELSNHSFYFLGYLSGVRLMPWVIRSQMLENSFMIDTAI--KRFDTIVPIFPLIGSLVKAKFC
NVSGYPISKSVWADSSDSDIIARFGWICRNLSHYHSGSSKKHSLCRIKYILRLSCARTLARK
HKSTVRVICKRLGSKLLEEFLTEEQEIVSFIFRGRIWYLDIIRINGLVPHSMIIEEAQ----AINSF
FRSES-SKEVYGLIWLLVPILTLVLGITIGVLVIVWLERKISAGIQRRIGPEYAGPLGILQALA
DGVKLLFKEDLLPSRGDIRLFSVGPSIAVVSILLSYSVIPFGHHLVLTDLSIGVSLWIAISSIAP
IGLLMSGYGSNNKYSFSGGLRAAAQSISYEIPLTPCVLSISLLSNSSSTVDIVEAQSKYGFW
GWNLWRQPIGFIVFIISSLAECERLPFDLPEAEEELVAGYQTEYSGIKFGLFYVASYLNLLVS
SLFVTILYLGGWNLSIPYIPITELFEKNQTSEVFGTTISLLITLAKAYLFLFIPISTRWTLPRMR
MDQLLNLGWKSLLPIALGNLLLTTSSQLVSLMIWHVQNENFILDSTRIFMKAFHLLLFNGS
FIFPECILIFGLILLLMIDSTSDQKDTPWLYFISSTSLVMSITALLFRWREEPMISFSGNFQTNN
FNEIFQFLILLCSTLCIPLSVEYIECTEMAITEFLLFVLTATLGGMFLCGANDLITIFVAPECFS
LCSYLLSGYTKRDVRSNEATTKYLLMGGASSSILVYGFSWLYGSSGGEIELQEIVNGLINT
QMYNSPGISIALISITVGIGFKLSPAPFHQWTPDVYEGSPTPVVAFLSVTSKVAASASATRIF
DIPFYFSSNEWHLLLEILAILSMILGNLIAITQTSMKRMLAYSSIGQIGYVIIGIIVGDSNDGY
ASMITYMLFYISMNLGTFACIVLFGLRTGTDNIRDYAGLYTKDPFSALSSALCLLSLGGIPPL
AGFFGKLYLFWCGWQAGLYFLVSIGLLTSVVSIYYYLKIIKLLMTGRNKEITPHVRNYRRS
PLRSNNSIELSMIVCVIASTIPGISMNPIIAIAQDTLFMFLLHEYDIFWAFLMISSVIPILAFIIS
GVLAPISEGPEKLSSYESGIEPIGDAWIQFRIRYYMFALVFVVFDVETVFLYPWAVSFDVLG
VSVFIEALIFVLIPVVGSVYAWRKGALEWSTSDFYWLTIIVVLPISAGSLIALLPHRGNKVV
RWYTICICLFELLLTTYVFCYHFQLDDPLIQLEEDFNWINLFDFHWRLGIDGLSIGPILLTGFI
TTLATSAARPVTRNSRLFHFLMLAMYSGQIGLFSSRDLLLFFIMWELELIPVYLLLSMWGG
KKRLYSATKFILYTAGGSIFLLMGVLGMGLYGSNEPKLNFETLANQSYPVALEIIFYFGFLIA
YAVKSPIIPLHTWLPDTHGEAHYSTCMLLAGILLKMGAYGLVRINMELLPHAHSIFSPWLM
IVGTIQIIYAASTSLGQRNLKKRIAYSSVSHMGFIIIGISSITDAGLNGAILQIISHGFIGAALFF
LAGTSYDRIRLRYLNEMGGIAILMPRIFTMFSSFSMASLALPGMSGFVAELVIFLGIITSPKY
LVVSKILITFVMAIGMILTPIYSLSMSRQMFYGYRLFNVPNSYFVDSGPREIFILICILLPIIGIG
IYPDFVLSLSVDKVEAILSNYFHGMMLEHVLVLSAYLFSIGIYGLITSRNMVRALMCLELIL
NAVNMNLVTFSDLFDSRQLKGDVFSIFVIAIAAAEAAIGPAIVSSIYRNRKSTRINQSNLLNK
MERTYQYAWIIPFVPLLVTMLIGLGLLLIPTATKNIRRIWAFPAVLLLSIVMVFSTKLAIQQIN

GSSIYEYLWSWSITSDFSLEFGYLIDPLTSIMSILITTVGIMVLIYSDNYMSHDQGYLRFFAY
MSFFNTSMLGLVTSPNLIQIHIFWELVGMCSYLLIGFWFTRPIAANACQKAFVTNRVGDFG
LLLGILGFYLITGSFEFQDLFEIFNDNEINSSFATLCAFLLFLGAVAKSAQFPLHVWLPDAME
GPTPISALIHAATMVAAGIFLVARLLPLFIGIPYIMNIISLIGLITVLLGATLALAQRDIKRSLA
YSTMSQLGYIMLALGIGSYRAALFHLITHAYSKALLFLGSGSIIHSMEPIVGYSPAKSQNMV
LMGGLTKYMPITKTTFFLGTLSLCGIPPLACFWSKDEIINDSWLYSPIFATIACYTAGLTAFY
MFRMYLLTFEGHLRSNFKNYSGHTNSSFYSISIWGQEGSKPVSS-NLLLATRNFS-NKDPYT
LLYPHESDNTMLFPLLILAIFTLFVGCIGIRFEVDILSKWLTPSMKLLHQNST--EDWYKFVT
NAFYSVSIAYFGIFIASVLYGSVYSDRQNLYLINSFAIRLEQIINVIYNWSYNRGYIDVYYEK
VFIRGIRGLAQLAHSFDRRVIDGVTNGVGVASFFLGEGIKY-IGGGRISSYLFLYLACVSIVL
LLVKMDLPAPIHDILLVSLGSGLIVGGLGVVLLTNPIYSAFSSGLVLVCISLFYIPSNSYFVAA
AQLLIYVGAINVLILFAVMFMNGSEYYNSFHFWTIGDGFTSVVCTSIFFSLIATIPNTSWYGI
IWTTRSNQIIEQDLTSNVQQIGIHLSTDFYLPFELISIILLVSLVGAIAMARRMTVPDARKDLL
VVNMGPHHPSMHGVLRLIVTLDGEDVIDCEPILGYLHRGMEKIAENRTIIQYLPYVTRWD
YLATMFTEAITVNAPEELGNIQVPKRASYIRVIMLELSRIASHLLWLGPFMADIGAQTPFFY
IFRERELLYDLFEAATGMRMMHNYFRIGGVAADLPHGWIDKCLDFCDYFLTGVVEYQKLI
TRNPIFLERVEGVGFIGGEEAINWGLSGPMLRASGIQWDLRKVDRYECYDEFDWEVQWQ
KEGDSLARYLVRIGEMTESIKIIQQALEGIPGGPYENLEFRRFAGTKDSELNDFEYRFISKKP
SPSFELSKQELYVRVEAPKGELGIFLIGDNSVFPWRWKIRPPGFINLQILPQLVKRMKLADI
MTILGSIDIIMGEVDRMFPMVTGFMNYGQQTVRAARYIGQSFMITLSHANRLPVTIQYPYE
KSITSERFRGRIHFEFDKCIACEVCVRVCPIDLPVVDWRLETDIRKKRLLNYSIDFGICIFCG
NCVEYCPTNCLSMTEEYELSTYDRHELNYNQIALGRLPMSVIGDYTVRTIMNSKISMDKS
LDSRTITN-MQGRSSAWLVKHELVHRSLGFDYQGIETLQIKPEDWYSIAVISYVYGYNYLRS
QCAYDVAPGGLLASVYHLTRIQYGVDQPEEVCIKVFVPRSNPRIPSVFWIWKSADFQERES
YDMLGISYDNHPRMKRILMPESWVGWPLRKDYIAPNFYELQDAH------------------------------
------------------------------MNSMEFPFLDRTTPNSVISTTLNDLSNWSRLSSLWPLLYGTSCCFI
EFASLIGSRFDFDRYGLVPRSSPRQADLILTAGTVTMKMAPSLVRLYEQMPEPKYVIAMGA
CTITGGMFSTDSYSTVRGVDKLIPVDVYLPGCPPKPEAIIDAITKLRKKVSREIYEDRTESQ
QENRCFTTNHKFHLGRSTRAVNYDQG---LYQSTSEIPSEAFFKYKSSVSSHELVNMQKNRN
FSWVKEQMTRCISVSMMIYVITRASISNAYPIFAQQGYENPREATGRIVCANCHLASKPVDI
EVPQAVLPDTVFEAVVRIPYDMQLKQVLANGKKGGLNVGAVLILPEGFELAPPDRISPEM
KERMGNLSFQSYRPNKKNILVVGPVPGQKYSEIVFPILSPDPATKKEVHFLKYPIYVGGNR
GRGQIYPDGSKSNNTVYNASAAGIVSKIVRKEKGGYEITIADASNGQQTVDIIPRGPELLVS
EGEYIKLDQPLTSNPNVGGFGQGDAEIVLQDPLRIQGLLFFLVSVILAQIFLVLKKKQFEKV
QLAEMNFMSKVYDWFEERLEIQAIADDITSKYVPPHVNIFHCLGGITLTCFLVQVATGFAM
TFYYRPTVTEAFASVQYIMTEANFGWLIRSVHRWSASMMVLMMILHVFRVYLTGGFKKP
RELTWVTGVVLAVLTASFGVTGYSLPWDQIGYWAVKIVTGVPEAIPVVGSPLVELLRGSAS
VGQSTLTRFYSLHTFVLPLLTAVFMLMHFSMIRKQGISGPLMGVTKKPDLNDPVLRAKLA
KGMGHNYYGEPAWPNDLLYIFPVVILGTIACNVGLAVLEPSMIGEPADPFATPLEILPEWYF
FPVFQILRTVPNKLLGVLLMVSVPAGLLTVPFLENVNKFQNPFRRPVATTVFLIGTAVALWL
GIGATLPIDKSLTLGLFMIEVFSFGIVLGLIPITLAGLFVTAYLQYRRGDQLDFMPTIASYFGF
LLTASTITPALFIGLSKIRLIMDIVSLAWAALMVVFTFSLSLVVWGRSGLMIIRSPEPEVKILV
DRDPIKTSFEEWARPGHFSRTIAKGPDTTTWIWNLHADAHDFDSHTSDLEEISRKVFSAHF
GQLSIIFLWLSGMYFHGARFSNYEAWLSDPTHIGPSAQVVWPIVGQEILNGDVGGGFRGIQ

ITSGFFQIWRASGITSELQLYCTAIGALVFAALMLFAGWFHYHKAAPKLAWFQDVESMLN
HHLAGLLGLGSLSWAGHQVHVSLPINQFLNAGVDPKEIPLPHEFILNRDLLAQLYPSFAEG
ATPFFTLNWSKYAEFLTFRGGLDPVTGGLWLTDIAHHHLAIAILFLIAGHMYKTNWGIGHD
LKDILEAHKGPFTGEGHKGLYEILTTSWHAQLALNLAMLGSLTIVVAHHMYSMPPYPYLA
TDYGTQLSLFTHHMWIGGFLIVGAAAHAAIFMVRDYDPTTRYNDLLDRVLRHRDAIISHL
NWACIFLGFHSFGLYIHNDTMSALGRPQDMFSDTAIQLQPIFAQWIQNTHALAPSATAPGA
TTGTSLTWGGGDLVAVGGKVALLPIPLGTADFLVHHIHAFTIHVTVLILLKGVLFARSSRLIP
DKANLGFRFPCDGPGRGGTCQVSAWDHVFLGLFWMYNAISVVIFHFSWKMQSDVWGSI
SDQGVVTHITGGNFAQSSITINGWLRDFLWAQSSQVIQSYGSSLSAYGLFFLGAHFVWAFS
LMFLFSGRGYWQELIESIVWAHNKLKVAPATQPRALSIIQGRAVGVTHYLLGGIATTWAFF
LARIISVGMALRFPRFSQGLAQDPTTRRIWFGIATAHDFESHDDMTEERLYQNIFASHFGQL
AIIFLWTSGNLFHVAWQGNFESWVQDPLHVRPIAHTIWDPHFGQPAVEAFTRGGALGPVNI
AYSGVYQWWYTIGMRTNEDLYTGALFLLFISAISLVAGWLHLQPKWKPSVSWFKNAESR
LNHHLSGLFGVSSLAWAGHLVHVAIPGSRGEYVRWNNFLDVLPYPQGLGPLFTGQWNLY
AQNPDSSSHLFGTSQGAGTAILTLLGGFHPQTQSLWLTDIAHHHLAIAFIFLVAGHMYRTNF
GIGHSIKDLLEAHIPPGGRLGRGHKGLYDTINNSIHFQLGLALASLGVVTSLVAQHMYSLPA
YAFIAQDFTTQAALYTHHQYIAGFIMTGAFAHGAIFFIRDYNPQQNEDNVLARMLDHKEAI
ISHLSWASLFLGFHTLGLYVHNDVMLAFGTPEKQILIEPIFAQWIQSAHGKTSYGFDVLLSS
TNGPAFNAGQSLWLPGWLNAINE--NRNSLFLTIGPGDFLVHHAIALGLHTTTLILVKGALD
ARGSKLMPDKKDFGYSFPCDGPGRGGTCDISAWDAFYLAVFWMLNTIGWVTFYWHWK
HITLWQGNVSQFNESSTYLMGWLRDYLWLNSSQLINGYNPFGMNSLSVWAWMFLFGHL
VWATGFMFLISWRGYWQELIETLAWAHERTPLANLIRWRDKPVALSIVQARLVGLAHFSV
GYIFTYAAFLIASTSGKFGMSHSVKIYDTCIGCTQCVRACPTDVLEMIPWDGCKAKQIASA
PRTEDCVGCKRCESACPTDFLSIRVYLWHETTRSMGLAYMTDLNLPSIFVPLVGLLFPAIA
MASLFLHVQKNKIVMRDIKTYLSVAPVLTTLWFGSLAGLLIEINRLFPDALTFPFFSFMTAIL
ERRESTSLWGRFCNWITSTENRLYIGWFGVLMIPTLLTATSVFIIAFIAAPPVDIDGIREPVSG
SLLYGNNIISGAIIPTSAAIGLHFYPIWEASSVDEWLYNGGPYELIVLHFLLGVACYMGREW
ELSFRLGMRPWIAVAYSAPVAAATAVFLIYPIGQGSFSDGMPLGISGTFNFMIVFQAEHNIL
MHPFHMLGVAGVFGGSLFSAMHGSLVTSSLIRETTENESANAGYRFGQEEETYNIVAAHG
YFGRLIFQYASFNNSRSLHFFLAAWPVVGIWFTALGISTMAFNLNGFNFNQSVVDSQGRVI
NTWADIINRANLGMEVMHERNAHNFPLDLAAVEAPSTNGMGLPWYRVHTVVLNDPGRL
LSVHIMHTALVSGWAGSMALYELAVFDPSDPVLDPMWRQGMFVIPFMTRLGITNSWGGW
SIT-GGTITNPGIWSYEGVAGAHIVFSGLCFLAAIWHWVYWDLEIFCDERTGKPSLDLPKIF
GIHLFLSGVACFGFGAFHVTGLYGPGIWVSDPYGLTGKVQPISPSWGAEGFDPFVPGGIAS
HHIAAGTLGILAGLFHLSVRPPQRLYKALRMGNIETVLSSSIAAVFFAAFVVAGTMWYGSA
TTPIELFGPTRYQWDQGYFQQEIYRRVNAGLAENLSLSESWSKIPDKLAFYDYIGNNPAKG
GLFRAGSMDNGDGIAVGWLGHPIFRDKEGHELFVRRMPTFFETFPVVLVDGDGIVRADVP
FRRAESKYSVEQVGVTVEFYGGELDGVSYNDPATVKKYARRAQLGEIFELDRATLKSDGV
FRSSPRGWFTFGHASFALLFFFGHIWHGARTLFRDVFAGIDPDLDAQVEFGTFQKLGDPTT
RRQVVMKTLYSLRRFYPVETLFNGTLALAGRDQETTGFAWWAGNARLINLSGKLLGAHV
AHAGLIVFWAGAMNLFEVAHFVPEKPMYEQGLILLPHLATLGWGVGPGGEVVDTFPYFV
SGVLHLISSAVLGFGGIYHALLGPETLEESFPFFGYVWKDRNKMTTILGIHLILLGLGAFLL
VFKALYFGGVYDTWAPGGGDVRRITNLTLSPSIIFGYLLKSPFGGEGWIVSVDDLEDIIGGH
VWLGSICILGGIWHILTKPFAWARRAFVWSGEAYLSYSLGALSVFGFIACCFVWFNNTAYP

SEFYGPTGPEASQAQAFTFLVRDQRLGANVGSAQGPTGLGKYLMRSPTGEIIFGGETMRF
WDLRAPWLEPLRGPNGLDLSRLKKDIQPWQERRSAEYMTHAPLGSLNSVGGVATEINAV
NYVSPRSWLATSHFVLGFFFFVGHLWHAGRARAAAAGFEKGIDRDFEPVLFMTPLNMTIA
LGRFTKEE-DLFDIMDDWLRRDRFVFVGWSGLLLFPCAYFALGGWFTGTTFVTSWYTHG
LASSYLEGCNFLTAAVSTPANSLAHSLLLLWGPEAQGDFTRWCQLGGLWTFVALHGAFGL
IGFMLRQFELARSVQLRPYNAIAFSAPIAVFVSVFLIYPLGQSGWFFAPSFGVAAIFRFILFFQ
GFHNWTLNPFHMMGVAGVLGAALLCAIHGATVENTLFEDGDGANTFRAFNPTQAEETYS
MVTANRFWSQIFGVAFSNKRWLHFFMLFVPVTGLWMSALGVVGLALNLRAYDFVSQEIR
AAEDPEFETFYTKNILLNEGIRAWMAAQDQPHENLIFPEEVLPRGNALMSGSTGERAFADI
ITSIRYWVIHSITIPSLFIAGWSFVSTGLAYDVFGSPRPNEYFTESRQGIPLITGRFDSLEQLDE
FSRSFMTIDRTYPIFTVRWLAVHGLAVPTVSFLGSISAMQFIQRMATQTIEGSS-RSGPRRTII
GDLLKPLNSEYGKVAPGWGTTPFMGVAMALFAVFLSIILEIYNSSVLLDGISLSMLTLKLFV
YTVVIFFVSLFIFGFLSNDPGRNPGREEMADTTGRIPLWLIGTVTGIPVIGLVGVFFYGSYSG
LGSSLMPNIFSLICICLNSALQ-SGFFFAKLPEAYAFLNPIVDVMPVIPVLFFLLAFVWQAAVS
FRTTQSNPNEQNVELNRTSLYWGLLLIFVLAVLFSNYFFNMEVNILAFIATALFILVPTAFLLI
IYVKTVSQNDMETATLVAISISGLLVSFTGYALYTAFGQPSQQLRDPFEEHGDMEALVYTFL
LVSTLGIIFFAIFFREPPKVPNKKMKMTIAFQLAVFALIATSSILLISVPVVFASPEGWSSNKN
VLFSGTSLWIGLVFLVGILNSLIY-------MSPKTETKASVGFKAGVKDYRLTYYTPDYETLAT
DILAAFRVTPQPGVPPEEAGAAVAAESSTGTWTTVWTDGLTSLDRYKGRCYHIEPVPGEE
NQYIAYVAYPLDLFEEGSVTNMFTSIVGNVFGFKALRALRLEDLRIPPAYSKTFQGPPHGIQ
VERDKLNKYGRPLLGCTIKPKLGLSAKNYGRAVYECLRGGLDFTKDDENVNSQPFMRWR
DRFLFCAEALYKAQAETGEIKGHYLNATAGTSEEMLKRAACARELGVPIVMHDYLTGGFT
ANTSLAHYCRDNGLLLHIHRAMHAVIDRQRNHGIHFRVLAKALRMSGGDHIHSGTVVGK
LEGERDVTLGFVDLLRDDFIEKDRSRGIYFTQDWVSMPGVLPVASGGIHVWHMPALTEIF
GDDSVLQFGGGTLGHPWGNAPGAVANRVALEACVQARNEGRDLAREGNEVIREASKWS
PELAAACEVWKEIKFEFEAMDVL--MAIHLYKTSTPSTRKGAVDSQVKSNPRTNLIYGQHR
CGKGRNARGIITARHRGGGHKRLYRKIDFRRNEKDISGRIVTIEYDPNRNAYICLIHYGDGE
KRYILHPRGAIIGDTIVSGTEVPISMGNALPLTDMPLGTAIHNIEITLGKGGQLARAAGAVA
KLIAKEGKSATLRLPSGEVRLISKNCSATVGQVGNVGVNQKSLGRAGSKCWLGKRPVVR
GVVMNPVDHPHGGGEGRAPIGRKKPTTPWGYPALGRRSRKRNKYSDIFILRRRK-MIQPQ
THLNVADNSGARKLMCIRIIGAS-NYRYAHIGDVIVAVIKEAVPNMPLERSEVIRAVIVRTCK
ELKRDNGMIIRYDDNAAVVIDQEGNPKGTRVFGSIARELRQLNFTKIVSLAPEVLMLSPKR
TRFRKQHRGRMKGVSYRGNHICFGRYALQALEPAWITSRQIEAGRRAMARYARRGGKIW
VRIFPDKPVTLRPAETRMGSGKGSPEYWVSVVKPDRILYEMGGVSETVARAAMELAARK
MPIRTKFVIEEMTRVRRGYIARRRRRKIRLFASTFRGAHSRLTRTATQQKIRALVSSHRGRG
RQKRDFRRLWITRINAVTREN--VP--YSRLIHDLRKRQLLLNRKILAQIAISNRNCLYMISND
IIK----------MIKEIRALARHIGMSAQKARRVIDQIRGCSYEQTLMILELMPYRACYPIFKLVY
SAAANASHNRGLKEADLFISKAEVNEGVIVKRLKPQARGRSYPIKRPTCHITIVLS--ERP----
---MDGIKYAVFTEKSIRLLGNNQ-YTSNVESGSTRTEIKHWIELFFGVKVVAMNSHRLPGKG
RRMGPIMRHTMHYRRMIITLQPGYSIPPLIEKRTMAVPKKRTSISKKHIRRNFWKRKGYWA
AVKAFSLAKSISTGYSKGFF--QKK-MPKGKNPRVTVILECTSCVRNG--KKKESPGISRYITQ
KNRHNTPGRLELKKFCPYCYKHTIHGEIKKMKIRASVRKICEKCRLIRRRGRIIVICSNPKH
KQRQGMVREEVPVSTRTLQWKCVESRADSKRLYYGRFVLSPLMKGQADTIGIAMRRALL
GELEGTCITRAKSD--KVPHEYSTIVGIEESVHEILMNLKKIVFRSDLYGTLDASICVRGPRH

VTAQDIISPPSVEIVDTTQHIAILTEPVDLCIELKIERGRGYCMRTPNNYQDGSY--PIDAVSM
PVRNANHSIHSYGNGNEKQEILFLEIWTNGSLTPKEALYEASRNLIDLFIPFLHAEEQDIN--I
EDNL-KRATVPFFTFHDGLD-NMKR----EIILKRIFIDQSELPPRTYNCLKKSNIHTLLNLLSKS
QEDLMRIEHFRVEDVKQIFDILQKG--FTIDLLKNKMLRDGGDEGMFTIPGFSQIQFEGFCRF
IDQGLMEELHQFPKIEDTDQEIEFKLFGESYQLVEPLIKERDAVYESITYSSELYVPAGLIWR
TGRNMQEQTVLLGNIPLMNSSGTSIVNGIYRIVINQILQSPGIYYGTGLDHNGISVYTGTIIS
DWGGRSELEIDRKERIWARVSRKQKISILVLPSAMGSSLREILDNVCYPEIFLSFPNEKEKK
KIGSKENAILEFYQKFACVGGDPVFSESLCKELQKKFFQQRCELGRIGRRNMNQRLNLDIP
QNNTFLLPRDVLAAADHLIGIKFGMGTLDD--MNHLKNKRIRSVADLLQDQFGLALVRLE
NVVRGTICGAIR-HKLI-PTPHNLVTSTP----LTTTYESFFGLHPLSQVLDRTNPLTQIVHGRKS
SYLGPGGLTGRTASFRIRDIHPSHYGRICPIDTSEGINVGLIGSLAIHARVGDWG-SIETPFY---
EISERSKE--EQMVYLSPSRDEYYMVAAGNSLALNRGIQEEEVGPARYRQEFLTIAWEQIHL
RNIYPFQYFSIGASLIPFIEHNDANRALMSSNMQRQAVPLSQSEKCIVGTGLERQAALDSG
GSAIAEREGKIIYTDAEKIILSGNGDTISIPLVMYQRSNKNTWMHQKPQVHRGKYLKKGQI
LADGAATVGGELALGKNVSVAYMPWEGYNSEDAVLVSERLVYDDIYTSFHIRKYEIQTHV
TSQG-PERITNEIPHLEPYLLRNLDRNGIVMLGSWVETGDVLVGKLTPQTAKE-SSYAPEDR
LLRAILGIQVSTAKETCLKLPIGGRGRVIDVRWGQKRG-GSIYNPEMIRVYISQKRKIKVGD
KVAGRHGNKGIISKILPRQDMPYLQDGTPVDMVFNPLGVPSRMNVGQMFECSLGLAGDL
LGRHYRITPFDERYEQEASRKLVFSELYEASKQTANPWVFEPEYPGKSRIFDGRTGDPFEQP
VIIGKSYMLKLIHQVDDKIHGRSSGHYALVTQQPLRGRAKQGGQRVGEMEVWALEGFGV
AHILQEMLTYKSDHIRARQEVLGTTIVGGTIPNPEGAPESFRLLVRELRSLSLELNHFLVSE
KNFQINRKEVVNQNFSSMIDQYKHQQLRIGSVSPKQIHAWANKILPNGEIVGEVTKPYTFH
YKTNKPEKDGLFCERIFGPIKSGICACGNYRVIGGEKEEPKFCEQCGVESVDSRIRRYQMG
YIKLACPVTHVWYLKRLPSYIANLSDKPLKELEGLVYCD--FSFARP-IAKKPTFLRLRGSFE
YEIQSRKYSIPLFFTTQGFDTFRNREISTGATAIREQLADPDLRIIIDRSLVEWKELGEE-GST
GNDWEDRKIGRRKDFLVRRMELAKHFLRTNVEPEWMVLCLLPVLPPELRPIIQIDGGKPM
SSDINELYRRVIYRNNTLTDLLTTSRSTPGELVMCQEKLVQEAVDTLLDNGIRGQPMRDGH
NKVYKSFSDVIEGKEGRFRETLLGKRVDYSGRSVIVVGPSLSLHRCGLPREIAIELFQTFVI
RGLIRQNLASNIGLAKSKIREKEPIVWEILQEVMQGHPVLLNRAPTLHRLGIQAFQPILVEG
RAICLHPLVCKGFNADFDGDQMAVHVPLSLEAQAEARLLMFSHTNLLSPAIGDPISVPTQD
MLMGLYVLTMGNRRGICANRYNPCNSRNY---QNERTDHNNYKYRKGKEPYFCSSYDALG
AYRQKGIDLYSTLWLRWRLDQR-VIASINREIPIEVQYESLGTYHEIYDHYRVVRSVKKGM
LCIYIRTTVGHISFYREIEEAVQGFCRSYSYGT--------MEVLMAERD-LVFHNKVIDGTAMKR
LISRLIDHFGIAYTSHILDQVKTLGFHQATATSISLGIDDLLTTPSKGWLVQDAEQQSLILEK
HHHYGNVHAVEKLRQSIEIWYATSEYLRQEMNLNFRMTDPSNPVHIMSYSGARGNASQV
HQLVGMRGLMSDPQGQMIDLPIQSNLREGLSLTEYTISCYGARKGVVDTAVRTSDAGYLT
RRLVEVVQHIVVRRTDCGTTRGISVSLRKGMTE--RIFIQTLIGRVLANDVYLGLRCIATRNQ
DIGIGLVNRFMT------SRAQPIYIRTPFTCRSASWICRLCYGRSPTHGDLVELGEAVGIIAGQS
IGEPGTQLTLRTFHTGGVFTGGTAEHVRAPSNGKIKFNEDLVHPTRTRHGHPAFLCYVDLY
VTIESQDIIHSVNIPPKSFLLVQNDQYVESEQVIAEIRAGTSTF--FKERVRKHIYSDSEGE-MH
WSTDVYHAPEYTHGNVHFLPQTSHLWILSGGPCK--SSLVPFSLHKDQDQMNVQ--SLSVQ
ERYISDFS--VNNNR-VRHKLFGSDPLAK-GQRISDYA--FERVISNGHWDFIYPA-ILRENSY--
LLAKRRRNRFIIPFQYDPEREKELTPRSSIAVEIPAN--GILRRNSILAYFDDPRYRRSSSGITKY
G---VDSIVKKEG--LIEYRGPK-ESRPKY--VDRFFVIPEEVHILPGSSSIMVRNNSIIGVDTRITF

NTRSQIGGLVRVEKKKKKIELKIFSGGIHFPGETDKISRHIGILIPPGARKKMDKGSKGKNW
EGKNWVYVQR--ITPIKKK--YFVSVRPVVTYEIADGINLVTLFPRDMLQEKENLRLRVVNY
ILYGDGKPIRGIS--HIQLVRTCLVLNWDQD------KSIEKVQASSAEVRANDLIRYFIRIDLVKS
PILYTGKRNDRAGSVIPDT--GSYCANTNLFSSKVKMKS---------LSQHQGTVHTFLNR---NK
EGQSLIVFSSSNCSRIN-----VSKYHN---VTKESIPILNSS---GPLGT--VPKIYNFSSS--YHSIT--
HNEILLN-KYLLLDN-NNPKQTFQLLK---YYLVDENGR--ISNPNPCSDIILNLFDSNSCFLPH
DYCEGTSTTTIILGQFICENVCLSKHGT---RIKSGQVIMVY-VDSFIIRSAKPYLATRGATVHG
DYGEIFYEGDTLVTFIYEKSRSGDITHGLPKVEQVLEVRSIDSISMNLEKRVEGWNEHITGI
LGIPWGFLIGAELTIAQSRISLVNKIQKVYRSQGVQIHNRHIEIIVRQITSKVLVSE---DGMSN
VFSPGELIGLLRAERAGRALEEAICYRAVLLGITRASLNTQSFISEASFQETARVLAKAALR
GRIDWLKGLKENVVLGGMIPVGTGFKRFVHRSR--EYNNIP--LEIQKKNLFGGEMR--DILF
HHRELFGSCIPKPKSFHSEQPFYGI---------MTRRYWNISLEEMMEAGVHFGHGTRKWNPR
MAPYISAKRKGIHITNLTRTARFLSEACDLVFDAASRGKHFLIVGTKDKAADSVASAAIRA
RCHYVNKKWLGGMSTNWSTTETRLHKFRDLRVRAEMGQLSRLPKRDAAILKRQLSHFQ
TYLGGIKYMTGLPDIVIIIDQQEEYTALRECVTLGIPTICLIDTNCDPDLADIPIPANDDAIASI
RLILNKLVSAICQGRSSSYIRSRMGQKINPLGFRLGTTQSHRSFWFAQPKNYSKGLQEDEKI
RDCIKNYVQKHMRISSG-FEGIARIDIKKRIDLIQVIIHIGFANMLIEG--RAR--GIEELQTNVQ
KSFHSVNRRLNIAIARVPRPYGQPNILAEYIALQLKNRVSFRKAMKKAIELAEQADAKGIQ
VQIAGRLNGNEIARVEWIREGRVPLQTIRVKIDHCSYPVRTIYGVLGIKIWIFLDEEMSRYR
GPRFKKIRRLGALPGLTSKKPRSASDLRNQSRSGK-RSQYRIRLEEKQKLRFHYGLTERQLL
RYVRIAGKANGSTGQVLLQLLEMRLDNILFRLGMASTIPGARQLVNHGHILVNGRLVDIPS
YRCKPRDIITTKDKQRSRALIQNHMDSSSNE--LPKHLTLHS--LQYKGVVNQIIDSKWVGL
KINELLIVEYYSRQTMSRRGTAEEKTAKSDPIYRNRLVNMLVNRILKHGKKSLAYQIIYRAV
KKIQQKTETNPLSVLRQAIRGVTPNIAVKARRVGGSTHQVPIEIGSTQGKALAIRWLLGAS
RKRPGRNMAFKLSSELIDAAKGSGDAIRKKEETHRMAEANRAFAHFRMGRDTIADIITYIR
NADMDKKGTVRIASTNITENIVKILLREGFIENVRKHRENNKYFLVLTLRHRR--NRKGAYR
---NIL--KRISRPGLRIYSNSQRIPRISGGIGVVVLSTSRGIMTDREARLEGIGGEILFYIWMTK
PIPRIGSRRNSRISSRKSGGRTPIGVIHVQASFNNTIVTVTDPQGRVVSWSSAGTCGFKGTRR
GTPFAAQTAAGNAIRTVVDQGMQRAEVMIKGPGLGRDAALRAIRRSGILLNFVRDVTPMP
HNGCRPPKKRRVMPTIKQLIRNTRQPMRNVTKSPALRGCPQRRGTCTRVYTITPKKPNSAL
RKVARVRLTSGFEITAYIPGIGHNLQEHSVVLVRGGRVKDLPGVRYHIVRGTLDAVGVKDR
QQGRSKYGVKKPKMARKSLIQREKKRQKLEEKYHLIRRSSKKEIS---KVSSLSEKWEIHRK
LQSPPRNSAPIRLHRRCFLTGRPRANYRDFGLSGHVLREMVHACLLPGATRSSWMVKNSS
ISVIPQEEK-----RGSVEFQVFSFTNKIKRLTSHLELHRRDYLSQRGLRKMLGKRQRLLVYLS
KKNRVRYRELIGQLGIREPKTG--MVKLRLKRCGRRQRATYRIIAIDVRSRREGRDLRKVGF
YDPIKNQTYLNVPAILYLLEKGAQPTETVHDISKKAEVFKELLVNQTK---MDKFKRPFRKS
KRSFRRRLPPIGLGDRIDYRNMSLISRFISEQGKILPRRVNRLTLKQQRLITIAIKQARILSLLP
FLNNEK--QFERAESISRTTGTRTRKKVAR-SKKNPFVANHLLSKIEKLNVEEEKRIIVTWSR
ASTIIPAMVGHTIAIHNGKEHLPIYITDRMVGRKLGEFAPTLTFRGHAKNDNRSRRMILKYS
LLGNLLLLWMNIVNSVVLVGLYYGFLTTFSIGPSYLLLLRTRVMKEG---SEKEVSATTAFIM
GQFIIFISTYYPPLHLALSRPHTLTVLVLPYLLLHFFWNNHKSLFDHRSTHGNFIPNLSIQYV
FLNNLIFQLFNHFILPSSTLTRLVDISMFRCNNKILFVISSFFGWLIGHILLMKSIGLVLSWPW
EK--MRS-FRSNKYLVSKWRNSVSQIFSILLFITCVCYLGRMPSPIITKKLKEVEEDKSLLSLL
FDYQRWNRPLRYIENDRFSNAVRNEMSQYFFNTCASDGKQIISFTYPPSLSTFFEMIQKKM

YFRTAEKLPED--LSNWVSTTEKQRDNLRNEFINRIEAID--KLILDVVEKRARLCNDEEEQE
CLPKFYDPLLNGPYRGTIKKGYLYTKISWINKIHGIL-REFEHED-PNDSIKIEEMKKKVPRW
LYKLTDFDFEEEEGIRSRKAKRVVIYTDALIRYSQQSDFRRDIIKGSMRAQRRKTVTWEMF
QTDVHSPLFLDRIDKTPLFSFDI--SRMMNLIFRNRQEDERIAIAEAWDTIPFAQAVRSSLLVT
HSILRKYIVLPSLIIVKNIGRMLLFQFPEWYEDFKEWSREMHVKCTYNGVQLSETEFPKDW
LTDGIQIKILFPFSLKPWRRSKRKKQKNFCFLTVWGVETELPFGSPRKNPFFFEPIHKTLEKK
IRKVK-KKGFFILKILKDNIKGFLEIFKEKIRWVIKIVL--FIKS--------KDSKISNRIIHESPIRIG
SRDWANDSLTERKINDLADKTIKIRDQVEKIMKDKKK----------ITESQKYIWQISKKRSARL
IPKWNSFMKSFIERIYMGILLCITNIYKINVKFFIP--FCDLSSLSQAYVFYKLLQTQVSNKYK
SESIFQYTYPFIKDRIKDYFTRGISDSESKHKKLRNSGMNEWKNWLRNHYQYNLSRAKWS
GLAPLKWRNKVNQHRTIKNNQKLDSYE-EKDQLIHSEIKKKMKKHYRYDLLSYKYINKYI
IDLDRKYFELRIIYFYPRFFDWMGMNEQMLDRWFLPEFVPLYDRYRTNPWIIPIKLLLFDFK
SKKEAELDLLLKKYFLFQLRWGDSFNQRMTNNIKVYCLLLRLMDPSEIAISSIERGEMGLD
VMLIHKDLSLPELIKRGIFIIEPVRLSTKWDGISIMYQTIAISLTHKVQHNNYDLLVPENILSP
NHRREFRIISRFKFLWPTHRLEDLACMNRYWFDTNNGSRFSMSRIHMYQMKRHQFKSCIF
ELREILREIKNSRYFLDSWTKFDSVVSFTHIFFHQERFVKLLGPQTWSVLLS-RDS--QGSTS
NRYF---TIKGVVLLVVA-VLI----YRINNRNMVERKNLYLMGLL-----PIPMNSIGPRNDTLEES
F-WSPNINRLIVS-LLYLPKGKKISESCFMDPKESTWVLPIT----KKCIMPESNWGSRWWRN
WIGKKR--DSSCKIS---NETVAGIEISFKEKDIK-YLEFLFVSYMD--DPIRKDHGWELFDRVSP
RKKRNIINL--NSGQLFEILAKHLICYLMSAFREKRPIEVEG-FLKQQG-AEATIQSNDIEHVS
H--LFSRNKWDISLQNCAQFHVWQFHQDLFV---SWGK-NHHESDFLRN-VSRENLIWLDNM
WLVNKDR---FFS--KVRNVSSNIQYDS---TR--SIFVQVTDSSQLKGSS-DQSRENFDSIS----NE
DSE-------------YHTLINQTEIQQLKERLIL-WD--PSSLQTERTEIESD--RFPK--YPSGYS--SMS
RLFT-EREKQMNNHLFPEKIEEFL--GNPTRSIRSFFSDRW--SELH-LVSNATERFARDH----KL
LKKQQDVFSFVPSRRSE----KKEMVDIFKIITYLQN--TVSIHPISSDPGCDMVP-RDEPD-MD
SSNKISFLNKNPFCD--LFHLFHDRNKGRYTL--HHDFESE--ERFQEMADLFTLSITEP-DLVY
HKGFAFSMDSYGLDQKK--FLNEVFNSRDESKKNSL---LVLPHIFY--EENESFYRRIRKKWV
R-ISCRNGLEDPKPKIVVFASNNIMEAVNQYRLIRNLIQIQYSTYG--YIRNLSNRFSLM---NR
SDRNFEYGIQ---RDQIGNDTLNHITIMKYMINQHLSNVKK-SQKKWFD----PLISRTERSMN
RDPNAYRYKWSNGSKNFQEHLEHFVSEQKN---RFQVVFDRLRINQHSIDWSKVIDKQDLS
KSLHFFLSKS---------LLFLS-------KSL--------TFFFVSIGNIPIHRSE-IHIY-ELKGPNDQLCNQL
LESIGVQIVHLNKLKP--FLLD---DHDTS-QRSKFLINGGTISAFLFNKIP-KW-MIDSLHT--RN
NRRKFFDNTDSYFSMISHDRDNWLNPVKPFHRSSLISSFYKANLLRFLNNPHHFCFY--SNK
RFPFY----AEKTRIDNYD--LTYGGQFLHISFIRNK-IFSLCVGK--KKHVFLE-RDTISPIESQVS
DIFIPNDFPQSGDET-YNLYK---------------------------SQPLS---------TYCQPLSDMNLSDSEGK
NLHQYLS--FNSNMGLIHTPCSEKYLP--SAK--K--------RNLCLK--KCVEKGQMYRAFQRDS
AFSNLSKWNLFQTYMPWFLTSAGCKYINLILLDTFSDPL--PILSSS-HKFVSFFYDITHGSDI
SWSILQIPLW----AILPQWNL-ISEISSKCLQN--LLLPEEMIHRNNESPVPLKWTHLRSPNARE
F-LYSILFLLLVAGY-LVRTHLLFISR--VSSELQTELEKIKSLMIQSYMIELRKLLDRYPPPELN
SFWLKNLFLVALEQLGDSLEEIRSSASGGNMLLGGGP-----TYG-VKSIRSKKKDLNINLIDII
YLI-SIIPNPINPITFSRNTRHLSRTSKEIYSLIR--KNVNSDWIDDQIESWVANSDLIDDEEREF
LVQFS--TLTT--EKRIDQILLSLT-HSDHLS-----KNDSGYQMIEQPGSIYLRYLVDIH--KKYLM
NYEFNRSCLAERRIFLAHYQTITY--SQTPCGANSSHFPSHG---KPFSLRLALPPS-RGILVIGFI
GTGRSYFVKYRVTNSYVPFITVFPNKFLDDN-KGYLID-----DID-IDDSDDID----IDDSDDI---

--------------DDDLDTELLTMPNVL---TMYMT---PKIDRFEITLPLELAKAMSPCIIWIPNIHDL
YVNESNYLSLGLLVNHLP---RDCERCS-TRNILVIASTHIPQKVDPALIAPNKSNTCIKIRRLL
IPQQRKHFFILSYTRGFRLEKKMSHT--NGFGSITMGSNARDLVALTNEVLSISITQKKSIIDT
NTIRSALHRQTWDLR---SQVRSVQDHEILFYQIGRAVAQNVLLSNCSIDPISIYMKKK--SCK
EGDSYLSKWYFELGTSMKKLTILLYLLSCSAGSVAQDLWSPP--GPDEK-NWITSYGFVEND
S-DLVHG---LESALVG--YSRTE--CSQFDND--RVTLLLRSEPRN-QLDMMQNGSCSIVDQRFL
YEK--YESGFEE-GEGEGTLDLQ----QIEEDE--DLFNH--IVWAP--RLWRP---HGNLFDCIERPN
KLGFP-YWVRSFR---GKKIIYHKEDKLQENDSEFLQSGTVQYQTRDRSSKEQGFFRISQFIW
DPADQFFFLFK-DQPFVSVFSRREFFADEEMSKGLITSQTNPPTSIYKRWLI--KNTQEKHFEL
LIHRQRWLRTN-TNSSLS--NGSFRSN--TLSESYQYLSNLFLSNNGTLLDQMTKTLLRKRWL
FPDEMKHLI--HAPGERFPIPMPRSR-INGNFIDKTSSIVANILLRIIPTTSGEKEAFTYYRDG--
MSAQSEGNYAEALQNYYEAMRPEIDPYDRSYILYNIGLIHTSNGEHTKALEYYFRALERNP
FLPQASNNMAVICHY-RGEEAIRQGDSEIAEAWFDQAAEYWKQAIALTPGNYIEAQNWLKI
TGRFE--MNWRSEHIWIELITGSRKISNFCWACILFLGSLGFLLVGTSSYFGRNLISLFPSQQII
FFPQGIVMCFYGIAGLFISSYLWCTISWSVGSGYDKFDRKEGIVCIFRWGFPGRNRRIFFRF
RIRDIRSIRMEVKEGLFPRRVLYMEIGGRGDIPLTRTNEN-LTPREIEQKAAESAYFLRVPIEV
F
>Ranunculus
MEKWRFNSMLSLSKSLKCTGSIGNTNGGEDLAIN---NMIR--SNIDHLVGVSNIRSLISDDTF
LVRDSNGKNYSIYFDIENQIFEIEIEGDRSFLSELESFFSSYLNLSYPNDGSKSDNHYYDRSM
YDTQYSWNNYITSCIENYLRSEIH--IDSYISSSSDNYSKSYISNYVCSDSESFIQ---NGST-DM
IGKF-NFDISPKYSHLWVQCENCYGLNYKKFFRLKLHICEQCGYHLKMSSSERIEILIDPGT
WDAMDEDMVSVDPIEFHSEEEPYKDRIDSYQKKTGLTEAVQTGIGQLNGISIAIGVMDFQF
MGGSMGSVVGEKITRLIEYATNGFLPLIIVCASGGARMQEGSLSLMQMAKISSASYDYQS
NKKLFYVSILTSPTTGGVTASFGMLGDIIIAEPNAYIAFAGKRVIEQTLNKTVPDGSQEAEYL
FQKGLFDLIVPRNLLKGVLSELFQLHGFFPLNKNFIKMATLRADEISNIIRERIEQYNREVKI
VNTGTVLQVGDGIARIHGLDEVMAGELVEFEEKTVGIALNLESNNVGVVLMGDGLMIQE
GSSVKATGRIAQIPVSEDYLGRVINALAKPIDGRGEISASESRLIESPAPGIISRRSVYEPMQT
GLIAIDAMIPIGRGQRELIIGDRQTGKTAVATDTILNQKGQNVICVYVAIGQKASSVAQVVT
TFQEQGVMEYTIVVAETADAPATLQYLAPYTGAALAEYFMYRERHTLIIYDDLSKQAQAY
RQMSLLLRRPPGREAYPGDVFYLHSRLLERAAKLSSRLGEGSMTALPIVETQSGDVSAYIP
TNVISITDGQIFLSADLFNSGIRPAINVGISVSRVGSAAQIKAMKQVAGKLKLELAQFAELE
AFAQFASDLDKATQNQLARGQRLRELLKQSQAAPLTVEEQVVTIYTGTNGYLDSLEIGQV
KKFLVQLRTYLKSSKPQFQEIISSTKTFTEEAEVLLKEAIQEQMERFLLQEQTMRSNPTTSG
PGVPALEEKNLGRIAQIIGPVLDVSFPPGKMPNIYNALVVKGQDAVGQQINVTCEVQQLLG
NNRVRAVAMSATDGLMRGMEVIDTGAPLSVPVGGATLGRIFNVLGEPVDNLGPVDTRTTS
PIHRSAPAFIQLDTKLSIFETGIKVVDLLAPYRRGGKIGLFGGAGVGKTVLIMELINNIAKA
HGGVSVFGGVGERTREGNDLYMEMKESGVINEENIAESKVALVYGQMNEPPGARMRVGL
TALTMAEYFRDINEQDVLLFIDNIFRFVQAGSEVSALLGRMPSAVGYQPTLSTEMGSLQER
ITSTKEGSITSIQAVYVPADDLTDPAPATTFAHLDATTVLSRGLAAKGIYPAVDPLDSTSTML
QPRIVGEEHYETAQRVKQTSQRYKELQDIIAILGLDELSEEDRLTVARARKIERFLSQPFFVA
EVFTGSPGKYVGLTETIRGFQLILSGELDGLPEQAFYLVGNIDEATAKAINLDEESKSKKMT
LNLCVLTPNRIVWDSEVKEIILSTNSGQIGVLPNHAPIATAVDIGILRIRLNDQWLTVALMGG
FARIGNNEITILVNDAERGSDIDPQEAQQTLEIAQANLSSAEGKRQTIEANLALRRARTRVE

AINAI-MKKVTDSFVSLGHWPSAGSFGFNTDILATNPINLSVVLGVLIFFGKGVLSDLLDNR
KQRILSTIRNSEELRGGAIEKLEKAKARLRKVKAEADEFRTNGYSEIEREKCNLINSTYQNL
ERLENYKNETIQFEQQRAINQVRQRIFQQALQGALGTLNSCLNNELHLRTISANIGMFGAM
KEIT-MNPLISAASVIAAGLAVGLASIGPGVGQGTAAGQAVEGIARQPEAEGKIRGTLLLSL
AFMEALTIYGLVVALALLFANPFVMNVLPCSVNTLKGLYDISGVEVGQHFYWQIGGFQVH
AQVLITSWFVIAILLGSAIIAVRNPQTIPTDGQNFFEYVLEFIRDLSKTQIGEEYGPWVPFIGT
MFLFIFVSNWSGALLPWKIIQLPHGELAAPTNDINTTVALALPTSVAYFYAGLTKKGLGYF
GKYIQPTPILLPINILEDFTKPLSLSFRLFGNILADELVVVVLVSLVPSVVPIPVMFLGLFTSGI
QALIFATLAAAYIGESMEGHHMIFFTLEHVLTHISLSIILIVITIFLMNLLVYEIEGLSYSSEKG
MVASFLCITGLLVIRWIYSGHLPLSDLYESLMFLSWSFSIFYMIPYFRNKKSNLNVLTAPGTI
FTQGFATSGVLREIHQSTILVPALQSQWLMMHVSMMVLSYAALLCGSLLSVALLVILFRKS
SFTFSKYKEIQYVNEKK-----NIFLSFRNYHRYQLIQQLDCWSSRVISLGFLFLTIGILSGAVW
ANETWGSYWNWDPKETWAFITWTIFAIYLHTRTNKSLQSSNSAIVASIGFFIIWICYFGVNL
LGIGLHSYGSFAITMQKKKAFNPLLYLTSIVFLSWSISFSFKKSMESLVIHWWNAEQSEVFL
NDIQEKSALEKFIELEELLFLDEMIKESPDIHIQKLRIGIHKETIQLVKMYNEGHIHTILNFLT
NIISFAILSVFYILGNEQLVILNSWVREFIYNLSDTIKAFCILLLTDLCIGFHSPHGWELMIGS
VYKDFGFSHNNQIISGLVSTFPVILDTIFKYLIFRYLNRVSPSLVVIYHSINDMPIGVPKVPFRI
PGEEDAVWVDVY-RLHRERLLFLGQGVDSEISNQLIGLMVYLSLEDETRDLFLFINSPGGW
VVPGVAIYDTMQFVPPDVHTICMGLAASMGSFLLVGGEITKRIAFPHARVMIHQPASSFYE
AQTGEFVLEAEELLKLRETLTRVYVQRTGNPLWVVSEDMERDVFMSATEAQAHGIVDFVA
VE???MEELQ
RYLKMDRSRERDFLYSLLFQEYIYALAHDFGLQILS-KYSSLIVKRLIIRMYQQKHLIILDND
SKNKNFLGKNLYSQMISEGFAVIVEIPFALRLVSSYIEKSINLGSIHSTFPFLEDKFVHLNHVL
NILIPYPIHFELIVQNLRCWIQDASFLHLLRFFLYEYHNWNSFTTKQNSLFLKRRFFLFLYNF
HVYESESIFLFLRKKSYHLRSTSSIAFLDRTHFFGKIEHLKVVFRNDFMLWLFKDPFMHYFR
YQGKSIMSSKGTPLLMKKWKYYLVNLWECHFYFWSQPNRIHINQLSNIFLNFLGYLSSVR
PNPSVVRNQMLENAFIIDISR--NKLSTLVPIIPLIGSLAKAKFCNLSGQPISKPAWTDSLDSDI
IDRFGRICRNVSHYYSGSSKKKTLYRIKYILRLSCARTLARKHKSTVRSFLKRLGSEFLEEF
LIEEEQVLSFILPKRIWYFDIIRINDLMDL-MIIDTT-KLQ-AINSFYRSE-SLKEVYGLVWLLIP
IFILVLGIVIGVLVIVWLERQISAGVQQRIGPEYAGPLGILQALADGTKLLFKEDLLPSRGDI
YLFSIGPSIAVIAILLSYLVIPFGYHLVLADLSIGVFLWIAISSIAPIGLLMSGYGSNNKYSFLG
GLRAAAQSISYEIPLTPCVLSISLLSNSSSTVDIVEAQAKYGFWGWNLWRQPIGFTVFFISSL
AECERLPFDLPEAEEELVAGYQTEYSGIKFGLFYVASYLNLLVSSLFVTVLYLGGWNLSIPH
ILIPELFGINEMGGVFGMTIGIFITLAKAYLFLFISITTRWTLPRMRMDQLLNLGWKFLLPIS
LGNLLLTTSFQLFSLTIWHVQNENFILDSTRIFMKAFHLLLFHGSFIFPECILIFGLILLLMIDL
TSDQKDIPWLYFISSTSLVMSIAALLFRWREEPMISFSGNFQTNNFNEIFQFLILLCSTLCIPL
SVEYIECTEMAITEFLLFVLTATLGGMFLCGANDLITIFVAPECFSLCSYLLSGYTKRDVRSN
EATMKYLLMGGASSSILVHGFSWLYGSSGGEIELQEIANGLINTQMYNSPGISIALIFITVGI
GFKLSLAPSHQWTPDVYEGSPTPVVAFLSVTSKVAASALATRIFDIPFYFSSNEWHLLLEIL
AILSMILGNLIAITQTSMKRMLAYSSIGQIGYVIIGIIVGDSNDGYASMITYMLFYISMNLGT
FACIVSFGLRTGTDNIRDYAGLYTKDPFLALSLALCLLSLGGLPPLAGFFGKLHLFWCGWQ
AGLYFLVSIGLLTSVVSIYYYLKIIKLLMTGRNQEITPHVRNYRRSPLRSNNSIELSMIVCVI
ASTIPGISMNPIIAIAQDTLFMFLLHEYDIFWAFLIISSVIPILAFLISGVLAPINEGPEKLSSYE
SGIEPMGDAWLQFRIRYYMFALVFVVFDVETVFLYPWAMSFDVLGVSVFVEALIFVLILIV

GLVYAWRKGALEWSTNDFPWLTIIVGLPIFAGTLIFFFPHRGNKIIRWYTICICILELLITAYV
FCYHFQLDDPLIQLEQDYKWIHFFDFHWRLGIDGLSVGPILLTGFITTLATLAAWPVTRDSR
LFHFLMLAMYSGQIGLFSSRDLLLFFIMWEFELIPVYLLLSMWGGKKRLYSATKFILYTAG
GSIFLLMGVLGMGLYGSNEPTLNFETSANQPYPVALEILFYFGFLIGYAVKLPIIPLHTWLPD
THGEAHYSTCMLLAGILLKMGAYGLVRINMELLPHAHSIFSPWLIIVGAIQIIYAASTSLGQ
RNLKKRIAYSSVSHMGFIIIGICSITDTGLNGAILQMISHGFIGAALFFLAGTSYDRIRLVYLD
EMGGIAIPMPKIFTMFSSFSMASLALPGMSGFAAELVVFFGLITSQKYLLMPKILITFVTAIG
MILTPIYSLSMLRQMFYGYKQFKSLNSSFFDSGPRELFVSICIFLPVIGIGIYPDFVLSLSVDK
VEAILSNYFYRMMLEHVLVLSSYLLSIGIYGLITSRNMVRALMCLELILNAVNINFVTFSDL
FDNRQLKGDIFSIFVIGIAAAEAAIGLAIISAIHRNRKSTRINQSNLLNKMEHIYQDAWIIPFIP
LPIPIAIGLGLLLFPTTTKRIRRIWAFFSVLLLSIIMIFSVILSIKQIDGNPIYQYVWSWTINNDF
SLDFGNFIDPLTSIMLILITTVGIMVLIYSDNYMSHDQGYLRFFAYMSFFNASMLGLVTSSN
LIQIYIFWELVGMCSYLLIGFWFTRPLAANACQKAFVTNRVGDFSLFLGILGLYWITGSFEF
RDFFEIAKNNGYNSFFLTLCTSLLFVGAVAKSAQFPLHVWLPDAMEGPTPISALIHAATMV
AAGIFLVARLFPLFTVIPYIMNIIALVGIITLLLGATLALAQRDIKRSLAYSTMSQLGYIMLAL
GMGSYRAALFHLITHAYSKALLFLGSGSIIHSMENVVGYSPDKSQNMALMGGLTKYAPIT
KTSFLLGTLSLCGIPPLACFWSKDEILNDSWLYSPIFATIACLTAGLTAFYMFRMYLLTFEGH
LNINCKNYSAKKNNGLYSISIWGKMGSKVIQNN-YFVSTMNLKAFDNTKTFTYPYESDNT
MLFPLFVLVIFTFFIGYIGLPFHFDILSKWLIPSINFLHSDFN-SFDWYEFLINALFSVSIAYFGI
FIALFLYGPAYSYFHNFNLINFFIILWDKIINRVYNWSYNRGYIDIFYAKILIAGIRGLADFTY
FFDKKVIEGIVNGIGVISFFVAESIKYVGGG-RISSYLFFYLFYVAVFLVICLMDLPGPIHDFLL
VFLGLGFIMGGLGVVLLTNPIFSAFSLGLVLVCTSLFYTPSNSHFVAAAQLLIYVGAINVLIIF
AVMFMNGSEYYSDFHLWTVGDGVTSLVCTSIFVSLITTIPDTSWYGIVWTTRSNQIIEQDLI
NNSQQIGIHLSTDFFLPFELISIILLVALIGAISMARQMTVPTTRKDLMIVNMGPQHPSMHG
VLRLVVTLDGEDVIDCEPILGYLHRGMEKIAENRTIIQYLPYVTRWDYLATMFTEAITVNG
PEQLGNIQVPKRASYIRVIMLELSRIASHLLWLGPFMADIGAQTPFFYIFRERELIYDLFESA
TGMRMMHNYFRIGGVAADLPYGWIDKCLDFCDYFLTGVTEYQKLITRNPIFLERVEGVGII
GGEEAINWGLSGPMLRASGIQWDLRKVDHYECYDEFDWEVQWQKEGDSLARYLVRISE
MTESIKILQQALEGIPGGPYENLEVRRFDRAKDSEWNDFDYRFISKKPSPTFELSKQELYVR
VEAPKGELGIFLVGDKSVFPWRWKIRPPGFINLQILPQLVKRMKLADIMTILGSIDIIMGEV
DRMFPMVTGFMNYGQQTVRAARYIGQSLMITLSHANRLPVTIQYPYEKVITSERFRGRIH
FEFDKCIACEVCVRVCPIDLPVVDWKLETNIRKKRLLNYSIDFGICIFCGNCVEYCPTNCLS
MTEEYELSAYDRHELNYNQIALGRLPMSVIGDYTIRTITNSEIATGKRFDSKTITNYMQGRL
SAWLAKHRLVHRSLGFDYQGIETLQIKPEDWHSIAVILYVYGYNYLRSQCAYDVAPGGLL
ASVYHLTRIQYGVDQPEEVCIKVFAPRRNPRIPSVFWIWKSADFQERESYDMLGISYDNHP
RLKRILMPESWIGWPLRKDYIAPNFYEIQDAH--
----MNSIEFPLLNQTTQ-SVISTTSNDLSNWSRLSSLWPLLYGTSCCFIEFASLIGSRFDFDRYG
LVPRSSPRQADLILTAGTVTMKMAPSLVRLYEQMPEPKYVIAMGACTITGGMFSTDSYSTV
RGVDKLIPVDVYLPGCPPKPEAVIDAITKLRKKVSREIYEDRVGSQQETRCFTINHKFRVGR
STHTGNYDQGLLYQSPSISEIPNETFLKYKI-VSSHKLVNMQNRNIFSWI-EQMTRSIFVSIIIFI
ITQPSISHAYPIFAQQGYENPREATGRIVCANCHLANKPVDIEVPQAVLPDTVFEAVVRIPYD
IQVKQVLSNGKRGSLNVGAVLILPEGFELAPPNRISPEMKEKMGNLSFQSYRPTKKNILVIG
PVPGQKYSEITFPILSPDPLTNKDVYFLKYPIYVGGNRGRGQIYPDGSKSNNTVYNSTASGII
SKIVRKEKGGYEITIGDVSDGRSVVDIIPPGPELLVSEGESIKLDQPLTSNPNVGGFGQGDAE

IVLQDPSRVQGLFFFLASVILAQIFLVLKKKQFEKVQLSEMNFMSKVYDWFEERLEIQAIA
DDITSKYVPPHVNIFYCLGGITLTCFLVQVATGFAMTFYYRPTVTEAFASVQYIMTEANFG
WLIRSVHRWSASMMVLMMILHVFRVYLTGGFKKPRELTWVTGVVLGVLTASFGVTGYSL
PWDQIGYWAVKIVTGVPDAIPVIGSPLVELLRGSASVGQSTLTRFYSLHTFVLPLLTAVFML
MHFPMIRKQGISGPLMGVTKKPDLNDPVLRAKLAKGMGHNYYGEPAWPNDLLYIFPVVI
LGTIACNVGLAVLEPSMIGEPADPFATPLEILPEWYFFPVFQILRTVPNKLLGVLLMVSVPA
GLLTVPFLENVNKFQNPFRRPVATTVFLIGTAVALWLGIGATLPIDKSLTLGLFMIEVLLFGIV
LGLIPITLAGLFVTAYLQYRRGDQLDLMPTITSYFGFLLAALTITSALFIGLSKIRLIMDIVSL
AWAALMVVFTFSLSLVVWGRSGLMIIRSPESEVKILVDRDPVKTSFEEWARPGHFSRTIAK
GPDTTTWIWNLHADAHDFDSHTSDLEEISRKVFSAHFGQLSIIFLWLSGMYFHGARFSNYE
AWLSDPTHIGPSAQVVWPIVGQEILNGDVGGGFRGIQITSGFFQIWRASGITSELQLYCTAI
GALVFSALMLFAGWFHYHKAAPKLAWFQDVESMLNHHLAGLLGLGSLSWAGHQVHVSL
PINQFLDAGVDPKEIPLPHEFILNRDLLAQLYPSFAEGATPFFTLNWSKYAEFLTFRGGLDPV
TGGLWLTDIAHHHLAIAVLFLIAGHMYRTNWGIGHGLKDILEAHKGPFTGQGHKGLYEILT
TSWHAQLSLNLAMLGSLTIVVAHHMYSMPPYPYLATDYGTQLSLFTHHMWIGGFLIVGA
AAHAAIFMVRDYDPTTRYNDLLDRVLRHRDAIISHLNWACIFLGFHSFGLYIHNDTMSALG
RPQDMFSDTAIQLQPIFAQWIQNTHALAPGVTAPGATASTSLTWGGGALIAVGGKVALLPIP
LGTADFLVHHIHAFTIHVTVLILLKGVLFARSSRLIPDKANLGFRFPCDGPGRGGTCQVSA
WDHVFLGLFWMYNAISVVIFHFSWKMQSDVWGSINDQGVVTHITGGNFAQSSITINGWL
RDFLWAQASQVIQSYGSSLSAYGLFFLGAHFVWAFSLMFLFSGRGYWQELIESIVWAHNK
LKVAPAIQPRALSIVQGRAVGVTHYLLGGIATTWAFFLARIIAVGMALRFPRFSQGLAQDPT
TRRIWFGIATAHDFESHDDITEERLYQNIFASHFGQLAIIFLWTSGNLFHVAWQGNFESWVQ
DPLHVRPIAHAIWDPHFGQPAVEAFTRGGALGPVNIAYSGVYQWWYTIGLRTNEDLYTGA
LFLLFLSAISLIAGWLHLQPKWKPSVSWFKNAESRLNHHLSGLFGVSSLAWTGHLVHVAIP
ASRGEYVRWNNFLDVLPHPQGLGPLFTGQWNLYAQNPDSGSHLFGTSQGGGTAILTLLGG
FHPQTQSLWLTDIAHHHLAIAIIFLVAGHMYRTNFGIGHSIKDLLEAHIPPGGRLGRGHKGL
YDTINNSIHFQLGLALASLGVITSLVAQHMYSLPAYAFIAQDFTTQAALYTHHQYIAGFIMT
GAFAHGAIFFIRDYNPEQNEDNVLARMLDHKEAIISHLSWASLFLGFHTLGLYVHNDVML
AFGTPEKQILIEPIFAQWIQSAHGKTSYGFDVLLSSTSGPAFNAGRSIWLPGWLNAINE--NS
NSLFLTIGPGDFLVHHAIALGLHTTTLILVKGALDARGSKLMPDKKDFGYSFPCDGPGRGG
TCDISAWDAFYLAVFWMLNTIGWVTFYWHWKHITLWQGNVSQFNESSTYLMGWLRDYL
WLNSSQLINGYNPFGMNSLSVWAWMFLFGHLVWATGFMFLISWRGYWQELIETLAWAHE
RTPLANLIRWRDKPVALSIVQARLVGLAHFSVGYIFTYAAFLIASTSGKFGMSHSVKIYDTC
IGCTQCVRACPTDVLEMIPWDGCKAKQIASAPRTEDCVGCKRCESACPTDFLSVRVYLWH
ETTRSMGLAYMTTFNLPSFFVPLVGLVFPAIAMASLSLHVQKNKIVMRDLKTYLSVAPVLS
TLWFGSLAGLLIEINRLFPDALTFPFFSFMTAILERRESESLWGRFCNWVTSTENRLYIGWF
GVLMIPTLLTATSVFIIAFIAAPPVDIDGIREPVSGSLLYGNNIISGAIIPTSAAIGLHFYPIWEA
ASVDEWLYNGGPYELIVLHFLLGVACYMGREWELSFRLGMRPWIAVAYSAPVAAATAVFL
IYPIGQGSFSDGMPLGISGTFNFMIVFQAEHNILMHPFHMLGVAGVFGGSLFSAMHGSLVT
SSLIRETTENESANEGYRFGQEEETYNIVAAHGYFGRLIFQYASFNNSRSLHFFLAAWPVV
GIWFTALGISTMAFNLNGFNFNQSVVDSQGRVINTWADIINRANLGMEVMHERNAHNFPL
DLAAVEVPSINGMGLPWYRVHTVVLNDPGRLLAVHIMHTALVSGWAGSMALYELAVFDP
SDPVLDPMWRQGMFVIPFMTRLGITNSWGGWSIT-GGTITNPGIWSYEGVAGAHIVFSGLC
FLAAIWHWVYWDLQIFCDERTGKPSLDLPKIFGIHLFLSGLACFGFGAFHVTGLYGPGIWV

SDPYGLTGKVQSVNPAWGVEGFDPFVPGGIASHHIAAGTLGILAGLFHLSVRPPQRLYKGL
RMGNIETVLSSSIAAVFFAAFVVAGTMWYGSATTPIELFGPTRYQWDQGYFQQEIYRRVGA
GLAENKSLSDVWSKIPEKLAFYDYIGNNPAKGGLFRAGSMDNGDGIAVGWLGHPIFRDKE
GRELFVRRMPTFFETFPVVLVDGDGIVRADVPFRRAESKYSVEQVGVTVEFYGGELDGVS
YSDPATVKKYARRAQLGEIFELDRATLKSDGVFRSSPRGWFTFGHASFALLFFFGHIWHGA
RTLFRDVFAGIDPDLDAQVEFGAFQKLGDPTTKRQVV------------VETLFNGTLALAGRDQE
TTGFAWWAGNARLINLSGKLLGAHVAHAGLIVFWAGAMNLFEVAHFVPEKPMYEQGLIL
LPHLATLGWGIGPGGEVIDTFPYFVSGVLHLISSAVLGFGGIYHSLLGPETLEESFPFFGYV
WKDRNKMTTILGIHLILLGIGAFLLVLKALYFGGVYDTWAPGGGDVRKISNLTLSPSIIFGY
LLKSPFGGEGWIVSVDDLEDIIGGHVWLGSICIFGGIWHILTKPFAWARRALVWSGEAYLS
YSLAALSVFGFTACCFVWFNNTAYPSEFYGPTGPEASQAQAFTFLVRDQRLGANVGSAQG
PTGLGKYLMRSPTGEVIFGGETMRFWDLRAPWLEPLRGPNGLDLSRLKKDIQPWQERRS
AEYMTHAPLGSLNSVGGVATEINAVNYVSPRSWLSTSHFVLGFFLFVGHLWHAGRARAA
AAGFEKGIDRDLEPVLFMTPLNMTIALGRFTKDEKDLFDTMDDWLRRDRFVFVGWSGLL
LFPCAYFALGGWFTGTTFVTSWYTHGLASSYLEGCNFLTAAVSTPANSLAHSLLLLWGPEA
QGDFTRWCQLGGLWTFVALHGAFGLIGFMLRQFELARSVQLRPYNAIAFSGPIAVFVSVFL
IYPLGQSGWFFAPSFGVAAIFRFILFFQGFHNWTLNPFHMMGVAGVLGAALLCAIHGATVE
NTLFEDGDGANTFRAFNPTQAEETYSMVTANRFWSQIFGVAFSNKRWLHFFMLFVPVTGL
WMSALGVVGLALNLRAYDFVSQEIRAAEDPEFETFYTKNILLNEGIRAWMAAQDQPHEN
LIFPEEVLPRGNALMSGSTGERSFADIITSIRYWVIHSITIPSLFIAGWLFVSTGLAYDVFGSP
RPNEYFTENRQGIPLITGRFDSLAQLDEFSRSFMTIDRTYPIFTVRWLAVHGLAVPTVSFLGS
ISAMQFIQRMATQTIEGSSKSG-PRGTTVGSLLKPLNSEYGKVAPGWGTTPLMGVAMALFA
VFLSIILEIYNSSVLLDGISMNMLTLKLFVYTVVIFFVSLFIFGFLSNDPGRNPGREEMADTT
GRIPLWLIGTVAGILVIGLIGVFFYGSYSGLGSSLMLNIFSLICIYLNSILHSSHFLFAKLPEAY
AFFNPIVDIMPVIPLFFLLLAFVWQAAVSFRMTQSNPNEQSVELNRTSLYWGLLLIFVLAVL
FSNYFFNMEVNILAFIATALFILVPTAFLLIIYVKTVSQNDMETATLVAIFISGLLVSFTGYALY
TAFGQPSQQLRDPFEEHGDMEALVYTFLLVSTLGIIFFAIFFREPPKVPTKK--MTIAFQLAVF
ALIATSSILLISVPVVFASSDGWSSNKNFVFSGTSLWIGLVFLVAILNSLIS-------MSPQTETK
ASVGFKAGVKEYKLNYYTPEYEPKDTDTLAAFRVTPQPGVPPEEAGAAVAAESSTGTWTT
VWTDGLTSLDRYKGRCYHIEPVAGEENQYICYVAYPLDLFEEGSVTNMFTSIVGNVFGFK
ALRALRLEDLRIPVAYVKTFQGPPHGIQVERDKLNKYGRPLLGCTIKPKLGLSAKNYGRAV
YECLRGGLDFTKDDENVNSQPFMRWRDRFLFCAEAIYKAQAETGEIKGHYLNATAGTCE
DMMKRAVFARELGVPIVMHDYLTGGFTANTSLAHYCRDNGLLLHIHRAMHAVIDRQKNH
GIHFRVLAKALRMSGGDHIHSGTVVGKLEGEREITLGFVDLLRDDFIEKDRSRGIYFTQDW
VSLPGVLPVASGGIHVWHMPALTEIFGDDSVLQFGGGTLGHPWGNAPGAVANRVALEACV
QARNEGRDLAREGNEIIREACKWSLELAAACEVWKEIKFEFEAMDTL--TAIHLYKTSTPST
RNGAVGSQVKSNPRNNLIYGQRHCGKGRNARGIITVRHRGGGHKRLYRKIDFRRNEKDIS
GKIVTIEYDPNRNAYICLIHYGDGEKRYILHPRGAIIGDTIVSGTEVPISMGNALPLTDMPLG
TAIHNIEITLGKGGQLARAAGAVAKLIAKEGKSATLKLPSGEVRLISKNCSATVGQVGNVG
VNQKSLGRAGSKRWLGKRPVVRGVVMNPVDHPHGGGEGRAPIGRKKPTTPWGYPALGR
RSRKRNKYSDSLILRRRSKMIQPQTYLNVADNSGARKLMCIRIIGAS-NRRYAHIGDVIVAVI
KEAVPNMPLQRSEVIRAVIVRTCKELKRDNGMIIQYDDNAAVVIDQEGNPKGTRVFGAIAR
ELRQLNFTKIVSLAPEVLMLSPKRTRFRKQHRGRMKGISYRGNRICFGGYALQALEPAWIT
SRQIEAGRRAMTRYARRGGKIWVRIFPDKPVTVRPAETRMGSGKGSPEFWVFVVKPGRIL

YEMGGVSEIVAREAISIAASKMPIRTQFIIAGMTRIRRGYVARRRRTKIRLFASTFRGTHSRL
TRSTIQQKMRALVSAHRDRGRQKRNFRRLWISRINAAIRKM--VSYSYSRSIHDLYKRQSLL
NRKILAQIAISNTNCLYMISNKILKKV---------MIKEVYALGKHIRMSAHKARRVIDQIRGRP
YEETLMILELMPYRACYPILKLVYSAAANASHNMGFNETALIISQAEVNEGTITKKLKPRA
RGRSFAIKRPTCHITIVLQDISKDEVLWDKKMDGIKYAVFTDKSIRLLGNN-QYTSNVESGS
TRTEIKHWVELFFGVKVLAMNSHRLPGKGRRMGPIMGHTMHYRRMIITLQPGYSIPPLRK
KRTMAVPKKRTSISKKRIRKNFWKNKAYWATIKAFSLAKSLSTGNSKSFFVRQT-NMAKG
KDVRTRVILECNSCVRNDIN--KKSRGISRYITQKNRHNTPSRLELRKFCPYCYKHTIHGEIK
KMKIRASVRKICEKCRLIRRRGRIIVICSNPRHKQRQGMLRDEVAVSARTLQWKCVESRAD
SKRLYYGRFILSPLMKGQADTIGIAMRRALLGEIEGTCITRAKSE--KIPHEYSTIVGIEESVH
EILMNLKEIVLRSNLYGTRDASICVKGPGYVTAQDIISPPSVEIVDTTQHIASLTEPVDFCIGL
QIESNRGYRMKTPNNAQKGSY--PIDAVFMPVRNANHSIHSYGNGNENQEILFLEIWTNGSL
TPKEALYEASRNLIDLFIPFLHAEEQ--DQNLKDNQKNGVTLPFFTFHDGFD-KLKKNKK--I
ELKCIFIDQSELPSRTYNCLKRANIHTLLDLLNKSQEDLMKIEHFRIEDVKQILDIIQKHF-V
VIDLPKNKMLRDGN-QGMSTIPGFSQIQFEGFCRFIDQGLAEELSKFPKIEDTDQEIEFQLFV
ETYQLVEPLINERDAVYESLTYSSELYVPAGLIWKNGRDTQEQTIFIGKIPLMNSFGTFIINGI
YRIVINQILQSPGIYYRSELDHNGISVYTGTIISDWGGRSELEIDRKARIWARVSRKQKISILV
PSSAMGSNLREIIDNVCYPEIFLSFLNDKEKKKIGSKENAILEFYQQFACVGGDPVFSESLC
KELQKKFFQQRCELGRIGRRNINRRLNLDIPQNITFLLPRDIVAAADHLIRMKFGMGTLDD-
-MNHLKNKRIRSVADLLQDQFGLALVRLENVVRGTIGGAIR-HKLI-PTPHNLVTSTP----LTT
TYESFFGLHPLSQVLDRTNPLTQIVHGRKLSYLGPGGLTGRTASFRIRDIHPSHYGRICPIDT
SEGINVGLIGSLAIHARIGHWG-SLERPFY---EISEKS--KEVRVVYLSPSKDEYYMVAAGNSL
ALNRGIQEEQVVPARYRQEFLTITWEQIHLRSIFPFQYFSIGASLIPFIEHNDANRALMSSNM
QRQAVPLCRSEKCIVGTGLERQVALDSGVSAIAEHEGKIIYTDTDKIMLSGNGDTLSIPLVM
YQRSNKNTCMHQKPRVPRSKCIKKGQILADGAATVGGELALGKNVLVAYMPWEGYNFE
DAVLINERLVYRDIYTSFHIRKYEIQTHVTSQG-PERITNEIPHLEAHLLRNLDRNGIVMLGS
WVETGDILVGKLTPQIAKE-SSYAPEDRLLRAILGIQVSTAKETCLKLPIGGRGRVVDVRWI
QKKG-GSSYNPETIRVYISQKREIKVGDKVAGRHGNKGIISKILPRQDMPYLQDGTPVDMV
FNPLGVPSRMNVGQIFECSLGLAGNLLDRHYRISPFDERYEQEASRKLVFSELYQASKQTG
NPWVFEPEYPGKSRIFDGRTGDPFEQPVIIGKSYILKLIHQVDDKIHGRSSGHYALVTQQPL
RGRAKQGGQRVGEMEVWALEGFGVAHILQEMLTYKSDHIRARQEVLGTTIMGGTISNPE
DAPESFRLLVRELRSLALELNHFLVSEKNFQINRKEA-------MIDRYKHQQLQIGLVSPQQIIT
WAEKILPNGEIVGEVTKPYTFHYKTNKPEKDGLFCERIFGPIKSGICACGNYRVIGNEKEDP
KFCEQCGVEFVDSRVRRYQMGYIKLACPVTHVWYLKRLPSYIANLLDKPLKELEGLVYC
D--FSFARP-VVKNPTFLRLRGSFEYEIQSWKYSIPLFFTTQGFDTFRNREISTGASAIREQLA
DLDLRLIIDCSLLEWKELGEE-GPAGNEWEDRKMGRRKSFLVRRMELAKHFIRTNVEPER
MVLCLLPVLPPELRPIIQIDGGKLMSSDINELYRRVIYRNNTLTDLLTTSRSTPGELVMCQEK
LVQEAVDTLLDNGIRGQPTRDGHNKIYKSFSDVIEGKEGRFRETLLGKRVDYSGRSVIVVG
PSLSLHRCGLPREIAIELFHTFVIRGLIRQHIASNIGVAKSKIREKAPIVWEILQEVMQGHPVL
LNRAPTLHRLGIQAFQPILVEGRAICLHPLVCKGFNADFDGDQMAVHIPLSLEAQAEARLL
MFSHMNLLSPAIGDPISVPTQDMLMGLYVLTIGNRRGICANRYNPWNHRN---SQNEKIDDN
NHTYT--KEPYFCSSYDALGAFRQKRINLDSPLWLRWRLDQR-VIAP--REVPIEVQCESFGT
YHEIYGHYLIVRNVNKKIFCIDIRTTVGLISFYREIEEAIQGFCRACSYST-----------MAERAD
LVFHNKAIDGTAMKRLISRLIDHFGMAYTSHILDQVKTLGFQQATTTSISLGIDDLLTIPSKG

WLVQDAEQQSLILEKHHNYGNVHAVEKLRQSIEIWYATSEYLRQEMNPNFRMTDPSNPV
HIMSFSGARGNASQVHQLVGMRGLMSDPQGQMIDLPIQSNLREGLSLTEYIISCYGARKG
VVDTAVRTSDAGYLTRRLVEVVQHIVVRRTDCGTTRGISVSPRNGMIT-ERIFIQTLIGRVLA
DDLYMGSRCIAIRNQDIGVGLVNRFIT------FRSQPISIRTPFTCRSTSWICRLCYGRSPTHGD
LVELGEAVGIIAGQSIGEPGTQLTLRTFHTGGVFTGGTAEHVRAPDNGKIKFNEGLVYPTRT
RHGYPAFLCYIDLSVTIEGPDILHNVNIPPKSFILVKNDQYVESEQVIAEIRAEASTL-NFKER
VRKHIYSDSEGE-MHWSTAVYHAPEYTYGNVHLLPKTSHLWILSGVPQR--SSIVPFSLHKD
QDQINIH--SLSAKQKFISNLSLSMTNDQ-VIKKLFRSDSSV--GGWVLDY-SGPAEVLCNGHC
NLIYSA-ILDENSD--LLAKRRRNRFTIPLQSNQEKE--QKPRSGISIEIPIN--GLFRINSILAYFE
DPRYRRKSSGITKYGTIEVDSIVKKED--LIEYRR--KEFRPKYMKIDRFFFIPEEVHILPGSSSI
MIRNNSIIGVDTRITFNIRSQVGGLVRVEKKKKKIELKIFSGNIYFPGETDPISWHSGILIPPG
AGKK--NSKESK--KLKNGIYVQR--ITPTKKK--YFVLVRPVVTYERADGINLAALFPQDPLQ
ERDNVQLRVVNYILYGNGKPIRRISHTNIQLVRTCLVLNWDQDKK---GSSIEEVRASFVEIKI
NALIRDFIKMDLVKYPISYTRKRNDPVGSGLIPV-NGSDHTNLNP-FYS--KAS-----IKQSLIRH
QGTIRTLLNK---NKECQSLITLSSSNCSR--IDPFNRLKYHNN--MIKKSDILPPIRNLLGPLGI--
IPKITNFFS--FYYLIT--HNQIL-VKKNSQLDN---LKRTSPVLK---YYLIDENGR--IFNPDPSIN
MILNPFNWNWYFIHHDYCEETSTIIN-LGQFFCENVCISKSGP---HLKSGQVLIVD-VDSLVI
RSAKPYLATPGATVHGHYGEILYEGDTLVTFIYEKSRSGDITQGLPKVEQVLEVRSIDSISM
NLENRVEGWNERITRTLGIPWGFLIGAELTIAQSRISLVNKIQKVYRSQGVQIHNRHIEIIVR
QITSKVLVSE---DGMSNVFSPGELIGLLRAERTGRALEEAICYRAVLLGITRASLNTQSFISE
ASFQETARVLAKAALRGRIDWLKGLKENVVLGGMIPVGTGFKGLVHRL--S---NIS--LEIEK
KNLFKGGMR--DILFHHKELFDSCIPRNFPDTTKQSFTRFTD-------MIRRYWNINLEEMMEA
GVHFGHGTRKWNPRMAPYISAKRKGIHIINLTRTARFLSEACDLVFDAASRGKQFLIVGTK
NKAADSVASAAIRARCHYVNKKWLGGMLTNWSTTETRLHKFRDLRAEQKMGRLNHLPK
RDSAMLKRQLSHLQTYLGGIKYMTGLPDIVIIVDQQEEYTALRECITLGIPTICLIDTNCDP
DLADISIPANDDAIASIRLILTKLVSAICEGRSS-YIRNRMGQKINPLGFRLGTTQSHHSVWFA
QPKNYSGGLQEDKKIKDCIKNYVQKNRKISSG-VEGIARIKIKKRIDLIQVIIYMGFPKFLE-
GNPQ----GIEELQSNIQKEFNSVNQKLNIAITRIAKPYGQPNILAEFIAGQLKNRVSFRKAMK
KAIELTEQADTKGIQIQIAGRIDGKEIARVEWIREGRVPLQTIRAKIDYCSYTVRTIYGALGI
KIWIFAGEEMSRYRGPRFKKIRRLGVLPGLTSKRPTPGSDLRNQSRPGK-RSQYRIRLEEKQ
KLRFHYGLTERQLLKYVHIAGKAKGSTGQVLLQLLEMRLDNILFRLGMASTIPGARQLVN
HRHILVNGRIVDIPSYRCKPRDIITTRDEQKSRALIQNFMDSSPH-EELPKHLTLHPSQY--KG
SVNQIIDSKWVGLKINELLVVEYYSRQTMSRRGTAEEKTAKSDPIYRNRLVNMLVNRILKH
GKKSLAYQIIYRALKKIQQKTETNPLSVLRQAIRGVTPDIAVKARRVGGSTHQVPIEIGSTQ
GKALAIRWLLGASRKRPGRNMAFKLSSELVDAAKGSGDAIRKKEETHKMAEANRAFAHF
RMGRDTIAEIITSIRNADMDKKGTVRIASTNITENIVKILLREGFIENVRKHRVSKKYFLVST
LRHRR--NRKGPYG---MVL--KRISRPGLRIYSNYQRIPRILGGMGIVILSTSRGLMTDREARL
ERIGGEILCYIWMTKTIPRIGSRRSGRISSRKNGRRIPKGVIHVQASFNNTIVTVTDIRGRVV
SWSSAGTCGFRGTRRGTPFAAQTAAANAIRTVVDQGMQRAEVMIKGPGLGRDAALRAIR
RSGILLSFVRDVTPMPHNGCRPPKKRRVMPTIKQLIRNTRQPIKNVTKSPALRGCPQRRGT
CTRVYTITPKKPNSALRKVARVRLTSGFEITAYIPGIGHNLQEHSVVLVRGGRVKDLPGVRY
HIVRGTLDAVGVKDRQQGRSKYGVKKPKMARKSLIQREKKRQKLEQKYHLIRRSSKKEI
K---KVPSLSEKWEIHGKLQSPPRNSAPTRLHRRCFSTGRPRANYRDFGLSGHILREMVQAC
LLPGATRSSWMIKNLFVSSISQG-E-KKENRGSVEFQVFSFTNKIRRLTSHLELHRKDFLSQR

GLRKILGKRQRLLSYLSKKNKVRYKELISNLSIRESKTR--MVKLRLKRCGRKQRAIYRIVAI
DVRSRREGRDLKKVGFYDPIKNQTYSNVPAILNFLEKGAQPTGTVHDILKKAKIFK---------
--MDKSKRLFIKSKRSFRRRLPPIGSGDRIDYRNMSLISRFISEQGKILSRRVNRLTLKQQRLI
TVAIKQARILSLLPFLNNEK--QFERTESTPRTTGPRTRNKVTRSLKKNPFVANHLLIKITKLN
MRAEKDTIVTWSRASTIIPTMIGHTIAIHNGKEHLPIYITDRMVGHKLGEFAPTLNFRGHAR
NDNKSRRITLILLIKSNLVSSCMKIINSVVVVGLYYGFFTTFSIGPSYFFLLRARVLEVG---DE
KEISATTGFIAGQLMMFISIYYAPLHLALRKPHTITALVLPYLLFHFFWNNHKNFFDSVYTT
GNSMRNLNIQCIFLNSLIFQLFNHFILPSSTLARLINIYMFRYNNKMLFVASSFVGWLVGHIF
FMKWVELVLFWIRQNHLIRS-----KYLVSELKNSMSRIFSILLFIVCIYYLGRMPSPLVTKKL
KKAEEDKPLVTLLFDYKRWNRPLRYIKNDFFENAVRNEMSQYFFYTCQSDGKQKISFTYL
PSLSTFLEMIQQKMLLCITEKRSYEESYNNWVYTNDEKKKNLSNELFNRIAVLDKGFLVT
DVLEKRTRLCN-ENEQECLPKIYDPLLNGPYRGTIKELYSRT--IWINKLYDIFFKELKKK------
--INEIKKKVFQWSYKLIDLEEE--EGIRSRKAKRVVIFNDALISYSQQSDFRRDIIKGSMRAQ
RRKTVTWKLFQTRVHSPLFLDRIDKIFVLSFDI--DRILNFIFRNKKENERITIAETWDTVLFA
QTIRGCILVIQSILRKLIVLPSLIIAKNIGHMLVFKPTEWHEDFEELNKEIHVKCTYNGVQLS
ENEFPKNWLTDGIQIKILFPFCLKSRHRSKKGKKQNFYFLTVWGMEAELPFGSPRKGPSFF
ETIWKQLKKKHKKVK-VKSFVAVKIIKERTKWFGKVSNEKTKWVTKKVQ--FIKKIMKELT
KRN--IISNKMIPEFAIRIRSIDWTNYSLTEKKMKDMADRINTIRNQIEKITKDKKSRPNKTS
WDDKRLEQSKIFWQIVKRGRAKIIRKCNFFFHFCIEKIC-DIFLSIINIPSRNIQILIHFIS-DLSF
LSQAYVFYKLSQIQVSNKYNLKSLFQYTIPFIKDKIKIFFTQGIFNSESRHKNFHDFGMNEW
QSWLMGNYQYNLSQTCWSGLVPQKWRNRINKNLANSS--NFYFYEKEP--LIHYEKEEKLK
N-YKYDLLSNKYINDYDIDSDRKYLDWRTFNFCLRFFDWMGMNEEILNWWFFPEFLLLY
DAYKIQPWIIPLNLLLL-SKSNIAAELDFFLKKYFLFQLRWDNSFSEKIINNIKIYCLLLRLKN
PKEIAISSIQRDEMSMDVMLVPKATTFIKLRKRRLLLIEPARLSINWDGLFIMYQTLVILQVH
NSKYNNYDFLIPENLLSSRRRRELRIQICLRFLWPNYRLEDLACMNRYWFDTNNGSRFSM
SRIHMYPVKRHPLKSWIFELREILREIKNSHYFLDSWTKFDSVGSFTHIFFHQERFMKLFDP
RIWSILLS-RDS--QGLTSNRYFT---IKGVVLLAVA-VLIY----RINSRNMVERKNIYLMGLLP----
-IPMNSIGPRNDALEESF-GSSNINRLIVS-LLYLPKGKKISESCFMDPKESTLVLPIT----KKCI
MPESNWGSRWRRNQIGKRR--DSSCKIS---NETVAGIEISFKEKDLK-YLESPFVSYTD--DPIR
KDHDWELFDRLSPRKKRNIINL--NSGQLFEILVKHWICYLMSAFREKRPIEVEG-FFKQQG-
AGSTIQSNDIERVSH--LLSRNKWGISLQNCAQFHMWQFRQDLFV---SWGK-NPHELDFLRN
-VSRENWIWLDSVRLVNKDR---FFS--KVRNVSSNMQYDS---TR--SIFVQVTDSSQLKGSS-D
QSRDHLDSIS----HEDSE------------YHTLINQSEIQQLKERSIL-WD--PSFFQTERTEIELD--RF
PK--RLSGDS--SMSRLFT-EREKPMIIHRLPEEIEEFL--GNPTRSIRSFFSDRW--SELH-LGSNP
TEGSTRDQ----KLLKKQQDFS-FVPSRRSE----NKEMVDIFRIITYLQN--TVSIHPILSDPGCD
MVP-KDEPD-MDSSNKISFLNQNSFFY--LFHLFHDRNRGGYTL--HHDFESE--EKFQEMADL
LTLSITEP-DLVYHKGSAFSIDSYRLDQKK--FLNEVFNSRDESKKKSL---LVLPPIFY--EENES
FYQRIRKKWVQ-ISCGNALEDPKQKRVVFASNNIMKAVNQYRFIQNLIQIQYSTHG--YIRN
VSNQFFK-----RSDRNFEYGIQ---RDQIGNDTLNHRTIMKYTINQHLSNLKK-SQKKWFDP---
-LISRTERSTNRYPDAYRYKWSKGSKNLQEHLEHFVAEQKSL---FQVVFDRLR--QNSIDWS
EVIDKQYFSKSLRFFLS-----------------------KSLLFLSKSLPFFFVSIGNIPSHRSE-IHIY-ELKG
PNDQLCNQLLESIGVQIVHLNKWKS--FLLD---DHDTS-QKSKFLINGGRISPFLFNKIP-KW-
MIESFHT--RNNRRKSFDNADSYFSMISHDRDNWLNPAKPFHRSSLISSFYKANRLRFLNNP
HHFWFY--CNKRFPFYV----EKARINKYD--LTY-GQFLNILFIRKK-IFSLCVGKGKKKHVFGE

-RDTISPIESQVSNIFIPNDFPQSGDET-YNLYKS---FHFPIRPAPFVR--RALYSIADISGTPLTEG
QIVHFERTYCQPLSDMNPSDSEGKNLHQYF--NFNSNMGLIHTPYSEKYLP--SEKRKK-------
-RSLCLK--KCVEKRQMYRTFQRDSTFSNLSKLNLFQTYMPWFLTSAGCKYLNLTLLDTFS
DLL--PILSIS-QKWVSIFHDIMRGSDISWPIPQKKLW----AILPQWNL-ISEISSKCLHD--LLLS
EETIRRHNESPVPLIWAHLRSPNAREL-LYSILFFLLVAGY-LVRTHLLFVSR--ASSELQTEFE
KIKSLMIPSYMIELRKLLDGYPTSERNSFWLKNLFLVALKQFGDSLEEIRGSASGGNMLLG
GGP-----AYG-VKSIRSKKKYWNINLVDIMDLI-SIIPNPINRITFSRNTGHLSRTSKEIYSLIRK
RKNVNGDWIDDKIESWVANSDSIDDEEREFLVQFS--TLTT--EKRIDQILLSLT-HSDHLS-----
KNDSGYQMIEQPGSVYLRYLVDIH--KKNLMNYEFNRSCIAERRIFLAHSQTITY--SQTSCG
ANSFHFPSPG---KPFSLRLALSPS-RGILVIGSIGTGRSYLVKYLATNSYVPFITVFPNKFLDD
KPKGYLIDDI---DID-IDDSDDIDDS-----------------------DDIDDDLDTELLTMTNAL---TMY---
ITPKIDRFDITLPFELAKAMSPCIIWIPNIHDLYVNESNYLSLGLLVNHLS---RDCERCS-TRNI
LVIASTHIPQKVDPALIAPNKSNTCIKIRRLLIPQQRKHFFTLSYTRGFYLEKKMFHT--NGFG
SITMGSNARDLVALTNEALSISITQKKSIIDTNTIRSALHKQTWDLR---SQVRSVQDHGILFY
QIGRAIAQNVLLSNCPIDPISIYMKKK--SCKEGDSYLYKWYFELGTSMKKLTILLYLLSCSA
GSVAQDLWSPP--GPDEK-NGITSYGFVENDS-DLVHGLLEVEGVLVG--SSPTEKDCSQFYK
D--RVTLLLRSEPRN-PFDMMQNGSYSIVDQRFLYEK--YELEFEE-GGGEGALDPQ----QLEE
D----LFNH--IVWAP--RIWHP---CGNLFDCIERPNELGFP-YWARSFR---GKRIIYHK-DELEEN
DSEFLQSGTMQYQTRDRSSKEQGFFRISQFIWDPADPFLFLFK-DQPFVSVFSRREFFADQE
MPKGLLTSQTNSPTSIYKRWFI--KNTQEKHFELLIHRQRWLRT---NSSLS--NGSFRSN--TLS
ESYQYLSNLFLSN-GTLLDQMTKTLLRKRWLFPDEMKHLI--HVTGERFPIPMPRSR-INGNF
IDKTFSIVANILLRIIPTTSGEKEAFTYYRDG--MSAQSEGNYAEALQNYYEATRLEIDPYDRS
YILYNIGLIHTSNGEHTKALEYYFRALERNPFLPQAFNNMAVICHY-RGEQAIRQGDSEIAE
AWSDQAAEYWKQAIALTPGNYIEAHNWLKITRRFE--MNWRSERVWIELITGSRKASNFF
WACILFLGSTGFLLVGTSSYLGRNLISLFPSQQILFFPQGIVMSFYGIAGLFISSYLWCTISWN
VGSGYDRFDRKKGIVCIFRWGFPGRNRRIFLRFLMRDIQSIRIEVKEGIYPRRILYMEIRGQG
AVPLTRTDQN-LTPREIEQKAAELAYFLRVPIEVF
>Populus
MKKCWFHSMLSLSKSMDNLGPLENTSVSEDPILNDTEKNTYN--NVDHLVGVRDIRNLNV
DDTFLVLDNKKDGYSIYFDIENQVFGIDNNH--SFLSKLEKEFSSYWNSSYLNKGSRSDDSH
YDYSMYDNKYSWNNYINSCIDSYLR--SQIGIASSILSGSESYSESYISTYILGTGNSRLRTST
NGSDFALRENSN-LGVTQKYKHLWVQCEICYGLNYKKFFKSKMNICEQCGYHLKMSSSD
RIELSIDPGTWDPMDEEMFSLDPIDFHSEEEPYKDRIDSYQKKTGLTEAIQTGIGQLNGIPVA
IGVMDFQFMGGSMGSVVGEKITRLIEYATNQFLPLILVCASGGARMQEGSLSLMQMAKIS
SALYDYQSNKKLVYVSILTSPTTGGVTASFGMLGDIIIAEPNAYIAFAGKRVIEQTLNKTVPE
GSQAAEFLFHKGLFDPIVPRNLLKGVLSELFQLHAFFPLNHNLSRMVTIRADEISNIIRERIE
QYNREVKIVNTGTVLQVGDGIARIYGLDEVMAGELVEFEEGTIGIALNLESNNVGVVLMG
DGLMIQEGSSVKATGKIAQIPVSEAYLGRVINALAKPIDGRGAISSSESRLIESPAPGIISRRS
VYEPLQTGLIAIDSMIPIGRGQRELIIGDRQTGKTAVATDTILNQQGQNVICVYVAIGQKASS
VAQVVNALQERGAMEYTIVVAETADSPATLQYLAPYTGAALAEYFMYRERHTLIIYDDLS
KQAQAYRQMSLLLRRPPGREAYPGDVFYLHSRLLERAAKPSSSLGEGSMTALPIVETQSG
DVSAYIPTNVISITDGQIFLSADLFNAGIRPAINVGISVSRVGSAAQIKAMKQVAGKLKLELA
QFAELEAFAQFASDLDKATQNQLARGQRLRELLKQSQAAPFTVAEQIMTIYTGTNGYLDS
LEIGQVRKFLVELRTYLKTSKTQFQEIISSTKTFTEEAEALLKEAIQEQMERFLLQEQVMRI

NPTTSGPGVSALEKKNLGHIAQIIGPVLDVVFPPGKMPNIYNALVVKGRDTVSQQINVTCE
VQQLLGNNRVRAVAMSATDGLMRGMEVIDTGAPLSVPVGGATLGRIFNVLGEPVDDLGP
VDTQITSPIHRSAPAFIQLDTKLSIFETGIKVVDLLAPYRRGGKIGLFGGAGVGKTVLIMELI
NNIAKAHGGVSVFGGVGERTREGNDLYMEMKESGVINEENIAESKVALVYGQMNEPPGA
RMRVGLTALTMAEYFRDVNEQDVLLFIDNIFRFVQAGSEVSALLGRMPSAVGYQPTLSTE
MGTLQERITSTKEGSITSIQAVYVPADDLTDPAPATTFAHLDATTVLSRGLAAKGIYPAVDPL
DSTSTMLQPQIVGEEHYETAQRVKQTLQRYKELQDIIAILGLDELSEEDRLTVARARKIERF
LSQPFFVAEVFTGSPGKYVGLAETIRGFKLILSGELDSLPEQAFYLVGNIDEATAKATNLEM
ENNLKKMILSLCVLTPNRIVWDSEVKEIILSTNSGQIGVLPNHAPIATAVDIGILRIRLNDQW
LTMALMGGFARIGNNEVTVLVNDAEKGSDIDPQEAQQTLEIAEANLRKAEGKRQIIEANL
ALRRARARVEAINVISMKNITDSFVSLGHWSSAGSFGFNTDILATNPINLSVVLGVLIFFGK
GVLSDLLDNRKQRILNTIRNSEELRGGAIEQLEKARARLRKVEIEADQFRVNGYSEIEREK
LNLINSTYKTLEQLENYKNETIHFEQQRAINQVRQRVFQQALQGALGTLNSCLTNELHLRT
ISANIGMFGAMKEITNMNPLISAASVIAAGLAVGLASIGPGVGQGTAAGQAVEGIARQPEA
EGKIRGTLLLSLAFMEALTIYGLVVALALLFANPFVMNVLSYSINTLKGLYEISGVEVGQHF
YWKIGGFQVHAQVLITSWVVIVILLGSAIVTVRNPQTIPTDGQNFFEYILEFIRDVSKTQIGE
EYGPWVPFIGTLFLFIFVSNWSGALLPWKIIELPHGELAAPTNDINTTVALALLTSIAYFYAG
LSKKGLGYFGKYIQPTPILLPINILEDFTKPLSLSFRLFGNILADELVVVVLVSLVPSVVPIPV
MFLGLFTSGIQALIFATLAAAYIGESMEGHHMIFLTLEHILTHISFSVVSIVIIIHFLTLLVNEFV
GLYDSSEKGMLTTFFCLTGLLITRWIYSGHLPISDLYESLIFLSWIFSIIHMVPYFKKHKNYLS
TITAPSTFFTQGFATWGLLTDMHQSKILVPALQSQWLIMHVSMMVSGYAALLCGSLLSAAL
LVITFRKVIRIVG--KEIQYM--MESILKNTFFPSSRNYYRFQLIQQLDHWGFRILSIGFLFLTIG
ILSGAVWANEAWGSYWNWDPKETWAFITWTIFAIYFHTRTNKNLEGLNSAIVASIGFLIIWI
CYFGVNLLGIGLHSYGSFTLI-MEKKAFIPLLYLTSIVFLPWWVSFSFNKSLGSWIINWWNT
SKSETFLNDIQEKSILEKLIEFEELFLLDEMIKEYPETHLQKFRIG-HKETIQLIKMHNADRID
TILHFSTNIICFVILSGYSFLVNEELFILNSWVQEFIYNLSDTIKALSILLLTDLCIGFHSPHGW
ELMISSFYKDFGFAHNDQIISGLVSTFPVIFDTIFKYWIFRYLNRVSPSLVVIYHSMNDMPIG
VPKVPFRNPGEDSSNWIDVYNRLYRERLLFLGQDIDSEISNQLIGLMVYLSTESEIKDLYLFI
NSPGGWVIPGIAIYDTMQFVRPDVQTVCMGLAASMGSFILVGGKITKRLAFPHARVMIHQ
PFAAFYEAQIGEFVLEAEELLKLREILTRVYAQRTGKPLWVVSEDMERDVFMSAAEAQVH
GIVDLVAVA???
IEKSQRNLEIDRSRKNDFLYPLIFREYIYTFAHDRDLVGYDNKYSLLIVKRLITRMYQQNHL
IISANDSNQNTFFRKNLYFQMISEGFAVIVEIPFSLRLVSSLIVKSHNLRSIHSIFPFLEGKFPH
LNYLSEGLIPYPIHLEKLVQILRYWVKDPSSLHLLRLFLHEYWNLNSLIIKSISFFVKQRFFL
FLYNSHVYEYESVFFFLCKQSFHFRLTFYQVFLERIYFYGKIEHFVEVFTKGDSLCLLKDPFI
HYIRYQGKSIFVSKDTPLLMKKWKYYLVNLCQCHFDVCFQPQKIHINSLYKHSFALLGYLS
SVRLNLSVVRSQMLENAFLMDN--IMNKLDTTVSIIPLIGSLAKMKFCNAVGHPISKPTWAD
FSDSDIIDRFVRICRNLSHYYSGSSRKKSLYRIKYILRLSCVKTLARKHKSTVRIFLKRLGSE
LLEEFFTEEEQILFLIFPRRVWYLDIICINELSNHEMIIDTT-TVQ-AINSFSRLKS-LNEVYGII
WMLVPILTLVLGITIGILVIVWLEREISAGIQQRIGPEYAGPFGVLQALADGTKLLFKENLFP
SRGDTRLFSIGPSIAVISTLLSYSVIPFGYHFVLADLNIGVFLWIAISSIAPIGLLMSGYGSNN
KYSFLGGLRAAAQSISYEIPLTLCVLSISLLSNSSSTVDIVEAQSKYGLGGWNLWRQPIGFII
FFISSLAECERLPFDLPEAEEELVAGYQTEYSGIKFGLFYVASYLNLLVSSLFVAVLYLGGW
NISIPYISVPEFFDFEKVGRVFGTTMGILITLVKTYLFLFIPITTRWTLPRLRMDQLLNLGWK

FLLPISLGNLLLTTSSQLFSLMIWHVQNENFILDSTRIFMKAFHLLLFDGSFIFPECILIFGLIL
LLMIDSTSDQKDMPWLYFISSTSLVMSITALLFRWREEPMISFSGNFQTNNFNEIFQFLILLC
STLCIPLSVEYIECTEMAITEFLLFVLTATLGGMFLCGANDLITIFVAPECFSLCSYLLSGYTK
KDVRSNEATTKYLLMGGASSSILVHGFSWLYGSSGGEIELQEIVNGLINTQMYNSPGISIAL
IFITVGIGFKLSPAPSHQWTPDVYEGSPTPVVAFLSVTSKVAASASATRIFDIPFYFSSNEWH
LLLEILAILSMIVGNLIAITQTSMKRMLAYSSIGQIGYVIIGIIVGDSNGGYASMITYMLFYIS
MNLGTFACIVLFGLRTGTDNIRDYAGLYTKDPFLALSLALCLLSLGGLPPLAGFFGKLHLF
WCGWQAGLYFLVSIGLLTSVLSIYYYLKIIKLLMTGQNQEITPHVRNYRGSPLRSNNSIELS
MIVCVIASTIPGISMSPIIEIAQDTLFMFLLYEYDIFWAFLIISSVIPILAFLISGLLSPIRKGPEK
LSSYESGIEPMGDAWLQFRIRYYMFALVFVVFDVETVFLYPWAMSFDVLGVSVFIEALIFV
LILIVGLVYAWRKGALEWSTNSFPWLTIFVGLPISAGFLIFVFPHRGNKVIRWYTIFICVLEL
LLMTYAFSYYFQLDDPLIQLTESYKWINFFDFYWRLGIDGLSLGPILLTGFITTLATLAARPI
TRDSRLFHFLMLAMYSGQIGLFSSQNLLLFFIMWELELIPVYLLLAMWGGKQRLYSATKFI
LYTAGSSVFLLMGALGIAFYASNEPTFNFETSANQSYPVTLEILFYIGFLIAFAVKSPIIPLHT
WLPDTHGEAHYSTCMLLAGILLKMGAYGLVRINMELLSHAHSIFSSWLIIVGAIQIIYAAST
SSGQKNLKKRIAYSSVSHMGFTIIGICSISDMGLNGAILQIISHGFIGAALFFLSGTSYDRIRL
VYLDEMGGMATLMPKIFMMFSILSLASLALPGMSGFFAELVVFFGIITAKKYFLMPKILITF
VTAVGTILTPIYLLSMLRQMFYGYKLFNARNSYFFDSGPRELFVSIAILLPVISIGIYPDFVFS
LSVDKVEAILSNYFYSMMLEYVLGLSAYLFSIGIYGLITSRNMVRALMCLELILNAVNINF
VTFSDFFDSRQLKGNIFSIFVISIAAAEAAIGPAIVSSIYRNRKSIRINQLNLLNKMEHTYQYL
WIISFVTLPVPMLIGMGLLLFPVSTKKLHRIWAFPSVLLLSIVMVFSIDLFIQQINSSSIYQYV
WSWTINNDFSLEFGYLIDPLTSILLILITTVGILVLVYSDSYMSHDQGYLRFFVYMSFFNTS
MLGLVTSSNLIQIYIFWELVGMCSYLLIGFWFTRPIVSNACQKAFVTNRVGDFGLLLGILGL
YWITGSFEFQDLFEIFNNNEVHFLFVTLCAFLLFSGAIAKSAQFPLHVWLPDAMEGPTPISA
LIHAATMVAAGIFLVARLLPLFVVIPYIMKLIALIGIITVLLGATLAFAQKDIKRGLAYSTMSQ
LGYTMLALGMGSYRAALFHLITHAYSKALLFLGSGSIIHSMEVIVGYSPDKSQNMVLMGG
LTKHVPITKIAFLLGTLSLCGIPPLACFWSKDEILNDSWSYSPIFAIIAFSTAGLTAFYMFRVY
LLTFEGHLNIYFQNYSGKKNSAFYSISLWGKQGSKILKK-LLPLLTINFFWGTKR-IFSYPQE
SDNTILFPMLVLVLFTLFIGAIGNQFNFDILSKLLIPSLSLLHQNQN-SVDW-EFVTNSTFSVSI
ASFGIFIASSLYKPIYSSLQNLKFLNLVAILRDKIINVIYDWSYNRGYIDVFYAISLTEGIRRLA
ELTSFFDRRVIDGITNGVGFTSFFAGEGIKYVGGG-RISFYLLLYLFYVLIFLLISSMNLPGPIH
DFLLVFLGLGLILGGLGVVLLTNPIFSAFSLGLVLVCISLFYILSNSHFVAAAQLLIYVGAINV
LILFAVMFMNGSEYYKDFNLWTVGNGLTSLICTSLFVLLITIISNTTWYGIIWTTRANQIIEQ
DLVSNGQQIGIHLSTDFFLPFEFISIILLVALIGAIATARQMNVPATRKDFMIVNMGPHHPSM
HGVLRLILSLDGEDVIDCEPILGYLHRGMEKIAENRTIIQYLPYVTRWDYLATMFTEAITIN
GPEQLGNIQVPKRASYIRVIMLELSRIASHLLWLGPFMADIGGQTPFFYIFRERELIYDLFEA
ATGMRMMHNYFRIGGVAADLPHGWLDKCLDFCDYFLTGVAEYQKLITRNPIFLERVEGIG
IVSAEEAINWGLSGPMLRASGVRWDLRKVDHYECYDEFDWGVQWQKEGDSLARYLVRI
GEMTESVKIIQQALEGIPGGPYENLETRSFDRERNREWNDFEYRFISKKTSPAFELPKQELY
VRVEAPKGELGIFLIGDQSSFPWRWKIRPPGFINLQILPQLIKRMKLADIMTILGSIDIIMGEV
DRMFPMVTGFMNYGQQTVRAARYIGQSFMTTLSHVNRLPVTIQYPYEKLITSERFRGRIH
FEFDKCIACEVCVRVCPIDLPVVDWELETNIRKKRLLNYSIDFGICIFCGNCVEYCPTNCLS
MTEEYELSTYNRHELNYNQIALGRLPMSVIDDYTIRTILNSLIKD------------MRGPLSAWLV
KHGLVHRSLGFDYQGIETLQIKPEDWHSIAVILYVYGYNYLRSQCAYDVAPGGLLASIYHL

TRIEYGLDQPEEVCIKVFAPRKNPRIPSVFWVWKSADFQERESYDMLGISYDNHPRLKRIL
MPESWIGWPLRKDYIAPNFYEIQDAH--MNSI
EFPLLDRTTPISVISTTSNDLSNWSRLSSLWPLLYGTSCCFIEFASLIGSRFDFDRYGLVPRSSP
RQADLILTAGTVTMKMAPSLVRLYEQMPEQKYVIAMGACTITGGMFSTDSYSTVRGVDK
LIPVDVYLPGCPPKPEAIIDAITKLRKKISREIYEDRIRS--GNRCFTTNHKFHIGRTTNTGNY
DQGLLYQPP-TSKIAPEAFFKYKKSVSSPELVNMQTRKTLSWIKEEITRSISVSLMIYIITGAY
ISNAYPIFAQQGYENPREATGRIVCANCHLANKPVGIEVPQAVLPDTVFEAVVRIPYDMQL
KQVLANGKKGALNVGAVLILPEGFELAPPDRISPEMKEKIGNLSFQSYRPAKKNILVIGPVP
GQKYSEITFPILSPDPAAKKDAHFLKYPIYVGGNRGRGQIYPDGSKSNNTVYNATAAGIVS
KIIRKEKGGYEITITDAPEGRQVIDSIPPGPELLVSEGESIKLDQPLTSNPNVGGFGQGDAEIV
LQDPLRVQGLLFFLASVILAQIFLVLKKKQFEKVQLSEMNFMSKVYDWFEERLEIQTIADD
ITSKYVPPHVNIFYCLGGITLTCFLVQVATGFAMTFYYRPTVTEAFASVQYIMTEANFGWLI
RSVHRWSASMMVLMMILHVFRVYLTGGFKKPRELTWVTGVVLGVLTASFGVTGYSLPW
DQIGYWAVKIVTGVPEAIPVIGSPLVELLRGSASVGQSTLTRFYSLHTFVLPLLTAVFMLMH
FPMIRKQGISGPLMGVTKKPDLNDPVLRAKLAKGMGHNYYGEPAWPNDLLYIFPVVILGT
IACNVGLAVLEPSMIGEPADPFATPLEILPEWYFFPVFQILRTVPNKLLGVLLMVSVPAGLLT
VPFLENVNKFQNPFRRPVATTVFLIGTVVALWLGIGATLPIDKSLTLGLFMIEVFLFGIVLGLI
PITLAGLFVTAYLQYRRGDQLDLMPTLTSYFGFLLVALTITLVLFISLSKIRLIMDIVSLAWAA
LMVVFTFSLSLVVWGRSGLMIIRSPEPEVKILVDRDPIKTSFEEWARPGHFSRTIAKGPDTTT
WIWNLHADAHDFDSHTSDLEEISRKVFSAHFGQLSIIFLWLSGMYFHGARFSNYEAWLSD
PTHIGPSAQVVWPIVGQEILNGDVGGGFRGIQITSGFFQIWRASGITSELQLYCTAIGALVFA
ALMLFAGWFHYHKAAPKLAWFQDVESMLNHHLAGLLGLGSLSWAGHQVHVSLPINQFL
NAGVDPKEIPLPHEFILNRDLLAQLYPSFAEGATPFFTLNWSKYSEFLTFRGGLDPVTGGLW
LTDIAHHHLAIAILFLVAGHMYRTNWGIGHGIKDILEAHKGPFTGQGHKGLYEILTTSWHA
QLSLNLAMLGSLTIVVAHHMYAMPPYPYLATDYGTQLSLFTHHMWIGGFLIVGAAAHAAI
FMVRDYDPTTRYNDLLDRVLRHRDAIISHLNWVCIFLGFHSFGLYIHNDTMSALGRPQDM
FSDTAIQLQPVFAQWIQNTHALAPGATAPGATASTSLTWGGDDLVAVGGKVALLPIPLGTA
DFLVHHIHAFTIHVTVLILLKGVLFARSSRLIPDKANLGFRFPCDGPGRGGTCQVSAWDHV
FLGLFWMYNAISVVIFHFSWKMQSDVWGSISDQGVVTHITGGNFAQSSITINGWLRDFLW
AQASQVIQSYGSSLSAYGLFFLGAHFVWAFSLMFLFSGRGYWQELIESIVWAHNKLKVAPA
TQPRALSIIQGRAVGVTHYLLGGIATTWAFFLARIIAVGMALRFPRFSQGLAQDPTTRRIWF
GIATAHDFESHDDITEERLYQNIFASHFGQLAIIFLWTSGNLFHVAWQGNFETWVQDPLHV
RPIAHAIWDPHFGQPAVEAFTRGGALGPVNIAYSGVYQWWYTIGLRTNEDLYIGALFLLFL
SAVSLLGGWLHLQPKWKPSVSWFKNAESRLNHHLSGLFGVSSLAWTGHLVHVAIPGSRG
ESVRWNNFLDVLPHPQGLGPLFTGQWNLYAQNPDSSSHLFGTSQGAGTAILTLLGGFHPQ
TQSLWLTDMAHHHLAIAFLFLIAGHMYRTNFGIGHSIKDLLEAHIPPGGRLGRGHKGLYDT
INNSLHFQLGLALASLGVITSLVAQHMYSLPAYAFIAQDFTTQAALYTHHQYIAGFIMTGAF
AHGAIFFIRDYNPEQNEDNVLARMLDHKEAIISHLSWASLFLGFHTLGLYVHNDVMLAFG
TPEKQILIEPIFAQWIQSAHGKTSYGFDVLLSSTNSPAFNAGRSIWLPGWLNAINE--NSNSL
FLTIGPGDFLVHHAIALGLHTTTLILVKGALDARGSKLMPDKKDFGYSFPCDGPGRGGTCD
ISAWDAFYLAVFWMLNTIGWVTFYWHWKHITLWQGNVSQFNESSTYLMGWLRDYLWL
NSSQLINGYNPFGMNSLSVWAWMFLFGHLVWATGFMFLISWRGYWQELIETLAWAHERT
PLANLIRWRDKPVALSIVQARLVGLAHFSVGYIFTYAAFLIASTSGKFGMSHSVKIYDTCIG
CTQCVRACPTDVLEMIPWDGCKAKQIASAPRTEDCVGCKRCESACPTDFLSVRVYLWHE

TTRSMGLAYMTILNLPSIFVPLVGLVFPAIAMASLSLHVQKNKIFMRDLKTYLSVAPVISTL
WFGSLAGLLIEINRFFPDALTFPFFSFMTAILERRESESLWGRFCNWITSTENRLYIGWFGVL
MIPTLLTATSVFIIAFIAAPPVDIDGIREPVSGSLLYGNNIISGAIIPTSAAIGLHFYPIWEAASV
DEWLYNGGPYELIVLHFLLGVACYMGREWELSFRLGMRPWIAVAYSAPVAAATAVFLIYPI
GQGSFSDGMPLGISGTFNFMIVFQAEHNILMHPFHMLGVAGVFGGSLFSAMHGSLVTSSLI
RETTENESANEGYRFGQEEETYNIVAAHGYFGRLIFQYASFNNSRSLHFFLAAWPVVGIWF
TALGISTMAFNLNGFNFNQSVVDSQGRVINTWADIINRANLGMEVMHERNAHNFPLDLAA
VEVPSTNGMGLPWYRVHTVVLNDPGRLLAVHIMHTALVAGWAGSMALYELAVFDPSDPV
LDPMWRQGMFVIPFMTRLGITNSWGGWSIT-GGTITNPGIWSYEGVAGAHIVFSGLCFLAA
IWHWVYWDLEIFCDERTGKPSLDLPKIFGIHLFLSGVACFGFGAFHVTGLYGPGIWVSDPY
GLTGKVQSVNPAWGVEGFDPFVPGGIASHHIAAGTLGILAGLFHLSVRPPQRLYKGLRMG
NIETVLSSSIAAVFFAAFVVAGTMWYGSATTPIELFGPTRYQWDQGYFQQEIYRRVGTGLA
ENQSLSEAWSKIPEKLAFYDYIGNNPAKGGLFRAGSMDNGDGIAIGWLGHPLFRDKEGRE
LFVRRMPTFFETFPVVLVDGDGIVRADVPFRRAESKYSVEQVGVTVEFYGGELNGVSYSD
PATVKKYARRAQLGEIFELDRATLKSDGVFRSSPRGWFTFGHASFALLFFFGHIWHGSRTL
FRDVFAGIDPDLDAQVEFGAFQKLGDPTTRRQVVMKTLYSLRRFYPVETLFNGTLALAGR
DQETTGFAWWAGNARLINLSGKLLGAHVAHAGLIVFWAGAMNLFEVAHFVPEKPMYEQ
GLILLPHLATLGWGVGPGGEVIDTFPYFVSGVLHLISSAVLGFGGIYHALLGPETLEESFPFF
GYVWKDRNKMTTILGIHLILLGIGAFLLVFKALYFGGVYDTWAPGGGDVRKITNLTLSPSV
IFGYLLKSPFGGEGWIVSVDDLEDIIGGHVWLGSICILGGIWHILTKPFAWARRALVWSGEA
YLSYSLGALAVFGFIACCFVWFNNTAYPSEFYGPTGPEASQAQAFTFLVRDQRLGANVGS
AQGPTGLGKYLMRSPTGEVIFGGETMRFWDLRAPWLEPLRGPNGLDLSRLKKDIQPWQE
RRSAEYMTHAPLGSLNSVGGVATEINAVNYVSPRSWLATSHFVLGFFLFVGHLWHAGRAR
AAAAGFEKGIDRDFEPVLSMTPLNMTIALGKFTKDENDLFDIMDDWLRRDRFVFVGWSG
LLLFPCAYFALGGWFTGTTFVTSWYTHGLASSYLEGCNFLTAAVSTPANSLAHSLLLLWGP
EAQGDFTRWCQLGGLWTFVALHGAFGLIGFMLRQFELARSVQLRPYNAIAFSGPIAVFVS
VFLIYPLGQSGWFFAPSFGVAAIFRFILFFQGFHNWTLNPFHMMGVAGVLGAALLCAIHGA
TVENTLFEDGDGANTFRAFNPTQAEETYSMVTANRFWSQIFGVAFSNKRWLHFFMLFVPV
TGLWMSALGVVGLALNLRAYDFVSQEIRAAEDPEFETFYTKNILLNEGIRAWMAAQDQP
HENLIFPEEVLPRGNALMSGSTGERSFADIITSIRYWVIHSITIPSLFIAGWLFVSTGLAYDVF
GSPRPNEYFTESRQGIPLITGRFDPLEQLDEFSRSFMTIDRTYPIFTVRWLAVHGLAVPTVSF
LGSISAMQFIQRMATQSVEGSSRSG-PRRTIVGDLLKPLNSEYGKVAPGWGTTPLMGVAMA
LFAVFLSIILEIYNSSVLLDGISMNMLTLKLFVYTVVIFFVSLFIFGFLSNDPGRNPGREEMA
DTTGRIPLWIIGTVTGIPVIGLIGIFFYGSYSGLGSSLMLNILNLICICLNFALY-SSFFFTKLPE
AYAFLNPIVDVMPVIPLFFFLLAFVWQAAVSFRMTQSNPNEQNVELNRTSLYWGLLLIFVL
AVLFSNYFFNMEVNILAFIATALFILVPTAFLLIIYVKTVSQSDMETATLVAISISGLLVSFTGY
ALYTAFGQPSQQLRDPFEEHGDMEALVYTFLLVSTLGIIFFAIFFREPPKVPTKKVKMTIAF
QLAVFALIATSSILLISVPVVFSSPDGWSSNKNVVFSGTSLWIGLVFLVGILNSLIS-------MSP
QTETKAGVGFKAGVKDYKLTYYTPDYETKDTDILAAFRVTPQPGVPPEEAGAAVAAESST
GTWTTVWTDGLTSLDRYKGRCYDIEPVAGEENQFIAYVAYPLDLFEEGSVTNMFTSIVGN
VFGFKALRALRLEDLRIPPAYVKTFQGPPHGIQVERDKLNKYGRPLLGCTIKPKLGLSAKN
YGRAVYECLRGGLDFTKDDENVNSQPFMRWRDRFLFCAEALYKAQAETGEIKGHYLNAT
AGTCEEMIKRAVFARELGVPIVMHDYLTGGFTANTSLAHYCRDNGLLLHIHRAMHAVIDR
QKNHGIHFRVLAKALRMSGGDHIHSGTVVGKLEGERDITLGFVDLLRDDFVEKDRSRGIY

FTQDWVSLPGVLPVASGGIHVWHMPALTEIFGDDSVLQFGGGTLGHPWGNAPGAVANRV
ALEACVQARNEGRDLAREGNEIIREASKWSPELAAACEVWKEIKFEFQAMDTL--MAIHL
YKTSTPSTRNGAVDSQVKSNTRNNLIYGQHRCGKGRNSRGIITARHRGGGHKRLYRKIDF
RRNEKYIYGRIVTIEYDPNRNAYICLIHYGDGEKRYILHPRGAIIGDTIISGTEVPIKMGNAL
PLTDMPLGTAIHNIEITLGRGGQLARAAGAVAKLIAKEGKSATLKLPSGEVRLISKNCSATV
GQVGNAGVNQKSLGRAGSKCWLGKRPVVRGVVMNPVDHPHGGGEGRAPIGRKKPATP
WGYPALGRRSRKRNKYSDNLILRRRSKMIQSQTHLNVADNSGARELMCIRIIGTS-NRRYA
HIGDIIIAVIKEAVPNSPLERSEVIRAVIVRTSKELKRDNGMIIRYDDNAAVVIDQEGNPKGTR
IFGAIARELRQLNFTKIVSLAPEVLMLSPKRTRFRKQHRGRMKGISSRGNRICFGRYALQAL
EPAWITSRQIEAGRRAMTRNVRRGGKIWVRIFPDKPVTLRPSETRMGSGKGSPEYWVAVV
KPGRMIYEMGGVAENIARKAISIAASKMPIRTQFIILRMTRIRRGYIARRRRTKIRLFTSSFR
GAHSRLTRTMIQQKIKALFSAYRDRDRHKRNFRCLWVTRINAAIRE-NGVSYSYSTLINNL
YKRQLLLNRKILAQLAILNRNCLYLISNDMIK-----------MIKEASASGFYISMSAHKARRIID
QIRGRSYEETLMILELMPYRACYPIFKLVYSAAANASHNMGLNEASLVISKAEVNEGATM
KKFKPRARGRSYLIKRQTCHISIVLEDISYE-------MDGIKYAVVTDKSIRLLLKN-QYTSNVE
SGSTRTEIKHWVELFFGVKVIAMNSHRLPGKGRRMRPIMGHTMHYRRMIITLQPGYSIPPL
RKKRT???--????MANGKDVRIRVILECTSC-V
RK--VNKK--GISRYITQKNRHNRPSRLELRKFCPYCYKHTIHGEIKKMKIRASARKICEKCR
LIRRRGRILIICSNPRHKQRQGMVREKVRISTRTLQWKCVESRADSKRLYYGRFILSPLMK
GQADTIGIAMRRALLGEIEGTCITRAKSK--KIPHEFSTITGIQESIHEILMNLKEIVLRSNLYG
THDASICVKGPGCVTAQDIILPPSVEIIDNTQHIASLREPIDLHIGLEIERNRGYCMKPPKNF
QDGSYS--IDAVFMPVRNANHSVHSYGNGNEKQEILFLEIWTNGSLTPKEALHEASRNLIDL
FIPFLHAEEENFHLE--KNQ-HKITLPLFAFHDRLA-KLRKNQK-EIALKSIFIDQLELAPKIYN
CLKRSNIHTLWDLLKNSQEDLMKIEHFRIEDVKHIFGILKIEKHFTINLPKNKMLGDGN-GG
MSTIPGFNQIQFEGFCRFIDQGLAEELYKFPKIEDRDQEIEFQLFVETYQLVEPSIKERDAVY
ESLTYSSELYVSGGLIWKNSRDMQEQTIFIGNIPLMNSLGTSIVNGIYRIVINQILQSPGIYYR
SELNHNGISVYTGTIISDWGGRVELEIDKKARIWARVSRKQKISILVLSSAMGLNLREILEN
VCYPEIFLSFLSDKEKKKIGSRENAILEFYQQFTCVGGGPVFSESLCKELQKKFFQQRCELG
RIGRLNMNQRLNLDIPHNNTFLLPRDILAAADHLIGMKFGMGTLDD--MNHLKNKRIRSVA
DLLQDQFGLALIRLENVVRGTICGAIR-HKLI-PTPQNLVTSTP----LTTTYESFFGLHPLSQVL
DRTNPLTQIVHGRKSSYLGPGGLTGRTASFRIRDIHPSHYGRICPIDTSEGINVGLIGSLTIHA
KIGHLG-SLESPFY---EISARSK--KVRMLYLSPNRDEYYMIAAGNCLALNRGAREEQVVPA
RYRQEFLTIAWEQVRLRSFFPFQYFSIGASLIPFIEHNDANRALMSSNMQRQAVPLARSEKC
IVGTGLERQVALDSGVPAIAEHEGKIIYTDIDKIILSGNGYTVSIPLVMYQRSNKNTCMHQK
TQVQRGKCIKRGQVLADGAATVGGELALGKNILVAYMPWEGYNFEDAVLISERLVYEDV
YTSFHIRKYEIQTHVTSQG-PERITNEIPHLEAHLLRNLDKNGIVMLGSWVETGDILIGKLTP
QLAKE-SSYAPEDRLLRAILGIQVSTSKETCLKLPTGGRGRVIDVRWIQKKG-GSSYNPETIR
VYILQKREIKVGDKVAGRHGNKGIISKILPRQDMPYLQDGAPVDMVFNPLGVPSRMNVG
QIFECSLGLAGSLLDRHYRVAPFDERYEQEASRKLVFSELYEAGKQTGNPWVFEPECPGKS
RIFDGRTGDPFEQPVIIGKPYILKLIHQVADKIHGRSSGHYALVTQQPLRGRAKQGGQRVGE
MEVWALEGFGVSHILQEMLTYKSDHIRARQEVLGTTISGRTIPKPEDAPESFRLLVRELRSL
ALELKHFLISEKNFQINRKEVMNQNFSSMIDRYKHQQLRIGSVSPQQISAWTNKILPNGEIV
GEVTKPYTFHYKTNKPEKDGLFCERIFGPIKSGICACGNYRVIGDEKEEPKFCEQCGVEFV
DSRIRRYQMGYIKLACPVTHVWYLKRLPSYIANLLDKPLKQLEGLVYCDF---FARP-IAKK

PTFLRLRGSFEYEIQSWKYSIPLFFTTQGFDTFRNREISTGAGAIRELLADLDLRIILDYSSLE
WKELGEE-GPTGNEWEDRKVGRRKDFLVRRVELAKHFIRTNIEPEWMVLCLLPVLPPELR
PIIQIDGGKLMSSDINELYRRVIYRNNTLTDLLTTSRSTPGELVMCQEKLVQEAVDTLLDNGI
RGQPMRDGHNKVYKSFSDVIEGKEGRFRETMLGKRVDYSGRSVIVVGPSLSLHRCGLPRE
IAIELFQTFVIRGLIRQHLASNIGVAKSKIREKEPIVWGILQEVMRGHPILLNRAPTLHRLGIQ
AFQPILVEGRAICLHPLVRKGFNADFDGDQMAVHVPLSLEAQAEARLLMFSHMNLLSPAI
GDPISVPTQDMLMGLYVLTSGNRRGICANRYNPFNCRN---FQNEKIDANADKYIK--EPFFC
NSYDAIGAYRQKRINLESPLWLRWQLDQG-LIAS--REAPIEGHFGSLGTYHEIYGHYLIVRS
IKKKILSIYIRTTVGHIYLYREIEEAIQGFCQACSDGT-----------MAERANLFFHNKVIDGTAI
KRIISRFIDHFGMAYTSHILDQVKTLGFHQATATSISLGIDDLLTIPSKGWLVQDAEQQSLILE
KHHHYGNVHAIEKLRQSIEIWYATSEYLRQEMNPNFRMTEPFNPVHIMSFSGARGNASQV
HQLVGMRGLMSDPQGQMIDLPIQSNLREGLSLTEYIISCYGARKGVVDTAVRTSDAGYLTR
RLVEVVQHIVVRRTDCGTTRGISVSSRNGMIP-ERIFIQTLIGRVLADNIYMGLRCIATRNQD
IGIGLVNRFIT------FRTQPISIRTPFTCRSTSWICRLCYGRSPTHGDLVELGEAVGIIAGQSIGE
PGTQLTLRTFHTGGVFTGGTAEHVRAPSNGKIKFNKGLVHPTRTRHGHPAFLCSMDLYVTI
ESQDIIHNVTIPPKSFLLVQNDQYVESEQVIAEIRSGTYTL-NFTERVRKHIYSDSEGE-MHW
STDVYHASEFTYSNVHLLPKTSHLWILSGGSCR--SSIVPFSLHKDQDQINVH--SLSVER-GY
ISNP-SVNNDK-VKHKFFSSYLSSKSKSRILDY-SDLNRMICTG---FIYPT-ILHENSD--LLAKR
RKNRFIIPFQSIQE--KELMSHSDILIEIPIN--GIFRRNSIFAYFDDPQYRRKSSGITKYVAIGVH
SIVKKED--LVEYR-GVKEFQPKYMKVDRFFFIPEEVYILPESSSLMVRNNSIIGVDTQITLNT
KSRVGGLIRIERKKKKMELKIFSGDIHFPRATDKISRYSGILIPPGTVKT--NSKESKK--VKN
WIYVQR--ITPTKKK--SFVLVRPVLIYERGDGINLERLFPPDLLQEKENLKLRIVNYILYGNG
KPIQGISNTSIQLVRTCLVLNWNQDK---KSSSIEEARVYFVEVSINGLIRDFLRIHLGKSRISYI
RKRNDPSGLGLIS-DNGPDRTNINPFYSIYSKTR-----IPQSLKQNQGSISTLLNR---NMECQSL
IILSSSNCFRMDP--SNGVKSYN---VIKES-PIIPIRNLLGPLG--TALQIANF--YSFYHLL--THN
QIS-VIKYLKLDNLK-LKQTSKVL--K-YYLMDENGR--IVNHDPYSNNVLNPFKLNWYFLHH
NYCEETFTIIN-LGQFICENVCMTKNGP---RLKSGQVLIVH-ADSVILRLAKPYLATPGATVH
GHYGEILYEGDTLVTFIYEKSRSGDITQGLPKVEQVLEVRSIDSISINLEKRVENWNECITRI
VGIPWGFLIGAELTIVQSRISLVNKIQKVYRSQGVQIHNRHIEIIVRQITSKVLVSE---DGMSN
VFSPGELIGLLRAERAMRALEEAICYRTVFLGITRASLSTQSFISEASFQETARVLAKAALR
GRIDWLKGLKENVVLGGMIPVGTGFKGLAHRS--SQHKIIPFKTKKKN--LFEGEMR--DILF
HHRELFDSCISKNFYNISEQSFIGFNDS------MIRRYWNINLKEMLETGVHFGHATRKWNPK
MAPYISAKRKGIHITNLTRTARFLSEACDLVFDAASRRKQFLIVGTKNKAADPVARAAIRA
RCHYVNKKWLGGLLTNWSTTEMRLQKFRDLRMEQKTGGIHRLPKGDAARLKRQLFHLQ
TYLGGIKYMTGLPDIVIIVDQQEEYMALQECITLGIPTICLIDTNCDPDLTDISIPANDDAIAS
IRLILNKLVFAICEGRS-SYIRNPMGQKINPLGFRLGTTQDHYSLWFAQPKNFFEGLQEDQKI
RNCIKNYVQKNMKISSG-VEGIGHIEIQKRIDVIQVIIYLGFPKFLTEGK----PKRIKELQINVQ
KELNCMNRKLNISITRIENPYMHPNVLAEFIAGQLKNRVSFRKAMKKAIELTEQSNTKGIQ
VQIAGRLDGKEIARAEWVREGRVPLQTLRAKINYCSYTVRTIYGVLGIKIWIFVDEEMSRY
RGPRFKKIRRLGALPGLTSKRPRAGSDLRNQSRAGK-KSQYRIRLEEKQKLRFHYGLTERQ
LLKYVRIAAKAKGSTGQVLLQLLEMRLDNILFRLGMASTIPRARQLVNHRHILVNGRIVDI
PSYRCKPRDIITAKDEQKSRVMIQNSLDSFPQ-EELPKHLTLHPFQ--YKGLVNHIIDSKWIGL
KINELLVVEYYSRQTMSRRGTAEEKTAKSDPIYRNRLVNMLVNRILKHGKKSLAYQILYRA
MKKIQQKTETNPLSVLHQAIRGVTPDIAVKARRVGGSTHQVPIEIGSTQGKALAIRWLLGA

SRKRPGRNMVFKLSSELVDAAKGSGDAIRKKEETHRMAEANRAFAHFRMGRDTLADIITS
IRNADMDRKGTVRIPSTNITENIIKILLREGFIENVRKHQEGNFFFFALTLRHRR--NRKGRTS
-----LNLKRISRPGLRIYSNYQQIPRILGGMGIVILSTSRGIMTDREARLERIGGEILCYIWMA
KLLPRIGSRKNGRISSRKNARKIPKGVIHVQASFNNTIVTVTDVRGRVISWSSAGACGFRG
TRRGTPFAAQTAAGNAIRTVVDQGMQRAEVMIKGPGLGRDAALRAIRRSGILLSFVRDVT
PMPHNGCRPPKKRRVMPTIKQLIRNTRQPIRNVTKSPALGGCPQRRGTCTRVYTITPKKPN
SALRKVARVRLTSGFEITAYIPGIGHNSQEHSVVLVRGGRVKDLPGVRYHIVRGTLDAVGV
KDRQQGRSQYGVKKPKMARKSLIQREKKRQKLEQKYHLIRRSSKKEIS---KVPSLSDKWE
IHGKLQSPPRNSAPTRLHRRCFLTGRPRANYRDFGLSGHILRAKVHACLLPGATRSSWMV
KSSFISIISQE-D-KKENKGSVEFQIVSFTNKIRRLTSHLELHRKDYLSQRGLRKILGKRQRLL
S Y L A K K N G V R Y K E L I S Q L N I R E S K T R -
-???
MDKSKRLFLKPKRSLRRRLPPIGSGDRIDYRNMSLISRFISEQGKILSRRVNRLTLKQQRLIT
IAIKQARILSSLPFLNNER--QFEKNELVARTTGLRTRKKMTR-LKKNPFVANHLLRKINKLN
TKAEKNLIVTWSRASTIIPTMIGHTIAIHNGKEHLPIYITDRMVGHKLGEFAPTLNFRGHAK
N D N K S R
R??
???
???
???
???
???
???
???
???
???
???
???
???
?????????MKGHQFKSWIFELREILREIKNSHYFLDSWTQFNSVGSFIHIFFHQERFIKLLDSRI
WSILFS-RNS--QGSTSNRYF---TIKGVVLFVVV-VLIYR----INNRKMVERKNLYLTGLLP-----I
PMNSIGPRNDTLEESF-GSSNINRLIVS-LLYLPKGKKISESRFLDPKESTWVLPIT----KKCIM
SESNWGSRWWRNWIGKKR--DSSCKISN---ETVAGIEISFKEKDIK-YLEFLFVYYMD--DPIR
KDHDWELFDRLSPRKGRNIINL--NSGQLFEILVKDWICYLMFAFREKIPIEVEG-FFKQQG-
AGSTIQSNDIEHVSH--LFSRKKWAISLQNCAQFHMWQFRQDLFV---SWGN-NPHESDFLRN
-ISRENWIWLDNVWLVNKDR---FFS--KARNISSNIQYDS---TR--SSFVQGRDSSQLKGSS-D
QSRDHFDSIS----NEDSE------------YHTLINQREIQQLKERSIL-WD--PSFLQTERTEIESD--RF
PK--CLSGYS--SMSRLFT-EGAKEMNNHLLPEEIEEF--LGNPTRSIRSFFSDRW--SELH-LGSN
PTERSTRDQ----KLLKKEQDVS-FVPSRRSE----NKEIVNIFKTITYLQN--TVSIHPISSDPGCD
MVL-KDELD-MDSSNKISFLNKNPFFDL--FHLFHDRNGGGYTL--HHDFESE--ERFQEMAD
LFTLSITEP-DLVYHKGFAFFIDSYGLDQKQ--FLNEVFNSRDESKKKSL---LVLPPIFY--EENE
SFYRRIRKKWVR-ISCGNDLEDPKQKRVVFASNNIMEAVNQYGLIRNLIQIQYSTYG--YIRN
VLNQFFLMN---RSDRNFEYGIQ---KDQIGNDTLNHRTLMKYTINQHLSNLKQ-SQKKWFDP

--LIFLSRTERSMNWDPNSYRYKWSNGSNNFQEHLEHFISEQKSRF-LFQVVFDRLRINQYSI
DWSEVIDKKDLSKSLPFFLSKLLL---------FLS-------KFLLFLSNSLP-FFFVSFGNIPIHRSE-IH
IY-ELKGPNDQLCNQLLEPIGLQIVHLKKWKPF--LLD---DHDTS-QKSKFLINGGTISPFLFN
KIP-KW-MIDSFHTR--NNRRKSFDNTDSSFSMISHDQDNWLNPVKPFHRSSLISSFYKANRL
RFLNNLHHFCFY--CNKRFPF---Y-VEKARIKNYD--FTYG-QFLNILFIRNK-IFSLCGGK--KK
HAFLE-RDTISPIESQVSNIFIPNDFPQSGDE---SLYKS---FHFPIRPDPFVR--RAIYSIADVSGT
PLTEGQIFNFERTYCQPLSDINLSDSEGKNLHQYLN--FNSNMGLIHTPCSEKYLPSEK--RKK
--------RSLCLK--KCVEKGQMYRTFQRDSAFSTLSKWNLFQTYMPWFLTSTGYKYLNLIFL
DTFSDHLLPILSSS--QKIVSIFHDIMHGSDISWRILQKNL------WKTQWN-LISEISSKCLHN--
LLLSEEMIHRNNEP--PLISTRLRSPNVREF-LYSILFLLLVAGY-LVRTHLLFVSR--AYSELQTE
FEKVKSLMIPSYMIELRKLLDRYPTSELNSFWLKNLFLVALEQLGDLLEEIRGSASGGNML
WGGGP-----AYG-VKSIRSKKKFFNIN---LIDLI-SIIPNPINRITFSRNTRHLSHTSKEIYSLIRKR
KNVNGDWIDDKIESLVANSDSIDDKEREFLVQFS--TLTT--EKRIDQILLSLT-HSDHLS-----KN
DSGYQMIEEPGAIYLRYLVDIH--KKYLMNYEFNTPCLAERRIFLAHYQTITY--SQTSCGAN
SFHFPSHG---KPFSLRLALSPS-RGILVIGSIGTGRSYLVKYLATNSYVPFITVFLNKFLDNKP
KGFLID-----DRDDIDDSDDIDVSDDIDR---------------------DLDTELELLTRMNVL---TMDM
MP---EIDRFYITLQFELAKAMSPCIIWIPNIHDLDVNESNYLSLGLLVNYLS---RDCERCS-TR
NILVIASTHIPQKVDPALIAPNKLNTCIKIRRLLIPQQRKHVFTLSYSRGFHLEKKMFHT--KR
FGSVTMGSNVRDLVALTNEALSISITQKKSIIDTNIIRSALHRQTWDLR---SQVRSVQDHGIL
FYQIGRAVAQNVFLSNCPIDPISIYMKKK--SCNEGDSYLYKWYFELGTSMKKLTILLYLLSC
SAGSIAQDLWSLP--GPDEK-NGITYYGLVENDS-DLVHGLLEVEGALVG--SSRTEKDCSQF
DND--RVTLLLRPEPRS-PLDMMQNGSCSILDQRFLYEK--YESEFEE-GEVEGILDPQ----QIEE
D----LFNH--IVWAP--RIWSP---WGFLFDCIERPNSLGFP-YWARSFRGK---RIIYDEEDELQEN
DSEFLQSGTMQYQIRDRSSKEQGVFRISQFIWDPADPLFFLFK-DQPLVSVFSHREFFADEE
MSKGLLTSQTDPPTSIYKRWFI--KNTQEKHFELLIHRQRWLR---TKSSLS--NGFFRSN--TLS
ESYQYLSNLFLSN-GRLLDQMTKALLRKRWLFPDEMKIGF--M---------MPRSR-ITGNLIDK
TFSIVANILLRIIPTTSGEKEAFTYYRDG--MSAQSEGNYAEALQNYYEAMRLEIDPYDRSYI
LYNIGLIHTSNGEHTKALEYYFRALERNPFLPQAFNNMAVICHY-RGEQAIRQGDSEIAEAW
FDQAAEYWKQAIALTPGNYIEAQNWLKITRRFE--MSWRSEHIWIELIAGSRKISNFCWAIIL
FLGSLGFLLIGISSYLDRNLISLFPSQQILFFPQGIVMSFYGLAGLFISSYLWCTISWNVGSGY
DRFDRKEGIVCIFRWGFPGKNRRILLRLFMKDIQSIRIEVKEGFYARRVLYMEIRGQGAIPLT
RTDEN-LTPREIEQKAAELAYFLRVPIEVF
>Phaseolus
MQKWRFNSMLLFK---ESLGPIENTSLNEEPILSDIHKKINRYNSLDYLVGADNIQDFLSDKT
FLVRDNKRNSYSIYLDIEKKTLEINNNH--SFISEQENFFS-----RYLNTVIKSDYD---HYMYNT
PFSWNNNIHSCIESYFH--SQICIDSCIFGDIEKYNQ-SFYSYFFGNSSFSFITSTNDNDST--------
--------LWIECENCYQFNLKKLFQWKMNVCDYCGYLFKIDSSERIEFSIDSGTWNPMDEDM
VSTDPMKFHSKEELYKDRIDSYQIKTGLTEAVQTGTGQLNGIPVAIGIMDFNFMGGSMGSV
VGEKITRLVEYATNQLLPLILVCSSGGARMQEGCLSLMQMAKISSALYYYQTIQKLLYVSIL
TSPTTGGVTASFGMLGDIIIAEPKTYIAFAGKRVIEQTLSKAISKNSQVAEYLLHKGFVDSIV
PRHLLKGVVTELFQFHNFFSLAKNERNMVTIRADEISKIIRERIEQYNTEVKIVNTGTVLQV
GDGIARIYGLDEVMAGELVEFEEGTIGIALNLESKNVGVVLMGDGLMIQEGSSVKATGRI
AQIPVSEAYLSRVINALAKPIDGRGEILASESRLIESPAPGIISRRSVYEPLQTGLIAIDSMIPIG
RGQRELIIGDRQTGKTAVATDTILNQQGQNVICVYVAVGQKASSVAQVVTTLQERGAMEY

TIVVAETADSPATLQYLAPYTGAALAEYFMYRERHTLIIYDDLSKQAQAYRQMSLLLRRPP
GREAYPGDVFYLHSRLLERAAKLSSQLGEGSMTALPIVETQSGDVSAYIPTNVISITDGQIF
LSADLFNAGIRPAINVGISVSRVGSAAQIKAMKQVAGKLKLELAQFAELEAFAQFASDLDK
ATQNQLARGQRLRELLKQSQSAPLTVEEQIVTIYTGTNGYLDSLEIGQVRKFLVELRAYLK
TNKPQFKEIISSTNTLTGEAEALLKDAIKEQMELFLFQEQVMGINPTTSGPEVSSREKKNLG
HIVQIIGPVLDVAFPPGKMPNIYNALVVKGRDTVGQQINVTCEVQQLLGNNRVRAVAMSA
TDGLMRGMEVIDTGTPLSVPVGGATLGRIFNVLGEPIDNLGPVDTRTTSPIHRSAPTFIQLD
TKLSIFETGIKVVDLLAPYRRGGKIGLFGGAGVGKTVLIMELINNIAKAHGGVSVFGGVGE
RTREGNDLYMEMKESGVINEQNIAESKVALVYGQMNEPPGARMRVGLTALTMAEYFRDV
NEQDVLLFIDNIFRFVQAGSEVSALLGRMPSAVGYQPTLSTEMGSLQERITSTKEGSITSIQA
VYVPADDLTDPAPATTFAHLDATTVLSRGLAAKGIYPAVDPLDSTSTMLQPRIVGEEHYETA
QRVKQTLQRYKELQDIIAILGLDELSEEDRLTVARARKIERFLSQPFFVAEVFTGSAGKYVG
LVETIRGFNLILSGELDSLPEQAFYLVGNIDEATAKATNLEMESNLKKMTLNLCVLTPNRIV
WDSEVKEIILPTNSGQIGVLPNHAPIASAVDIGILRIRLKDQWLTMALMGGFARINNNEITV
LVNDAEKGSDIDPQEAQQTLEIAETNLNQAEGKRQTIEANLTLRRARTRVEAINVISMKNIT
DSFLCLGSWPSAGSFVFNTDILATNPINLSVVLGVLVFFGKGVLSDLLDNRKQKIWRTIQN
SEELQEEAIEQLEKAQARLRKVETEADRFRVNGYSEIKREKLNLIHSIYTTLEQLENYKNE
AIDFEQQRVINQVRQRVLQQALQGALGTLNSCLNNELHLRTVSANIGMFGTMKEKNNMN
PIISAASVIAAGLAVGLASIGPGVGQGTAAGQAVEGIARQPEAEGKIRGTLLLSLAFMEALTI
YGLVVALALLFANPFVMNVLLCSINTLKRLYDISAVEVGQHFYWQIGGFLVHAQVLITSW
VVIAILLVSAFLVIRNLQTIPAFGQNFFEYVLEFIRDVSKTQIGEEYGPWVPFIGTLFLFIFVS
NWSGALFPWKIIQLPHGELAAPTNDINTTVALALLTSVAYFYAGLSKKGLAYFSKYIQPTPI
LLPINILEDFTKPLSLSFRLFGNILADELVVVVLVSLVPLVVPIPVMFLGLFTSGIQALIFATLA
AAYIGESIEGHHMVFSTLEHILTHISFSVVSLLISIHLITLLLGEIIGLDNSFKKGMIITFFCITG
LLVTRWIFSGHLPFSNLYESLIFLSWTFSIFYMVLCLKKKKNYFNTIITPSILFTQGFATSGLL
TEMHQSLILVPALQSHWLMMHVSMMILGYTTLLCGSLLSVAILVITFQELIHMIGKSKEIKY
MNMNDNLLQKTPFMSYINYYRYQFIQQLDRWGYRTISLGFIFLTVGNISGAVWANEAWGS
YWNWDPKETWAFITWIIFAIYLHIRKNKKLEYLNSSIVASMGFLIIWICYLGINLLGIGLHSY
GSFTSNMKKKS--ISLLYLISIVFLPWCISFTFKKSLESWFINWWNTSQSEIFLNDIKEKSILKK
FIELKELFFLDDMLKECPKTYLQNLRTG-YKETIQLIKTHNEDRMNTILHFSTNIICFFILSGY
SILGNQELILINSLVREFIYNLSDTIKAFSILLLTDLCIGFHSTRGWELIMGFVSKDFGFAQND
QIISGLVSTFPVILDTIFKYWIFRYLNRISPSLVVIYHSMNDMPIGVPRVPFRTPGDRDASWV
DILIRLYRERLLFLGQDVDSEISNQLISLMIYLSIEKENKDLYLFINSPGGWVIPGIALYDTMQ
FVQPDVQTVCLGLAASMGSFLLAGGTITKRLAFPHA--MIHQPASSFYEAQAGEFILEAEEL
L K M R E T I T R V Y Y V Q R T G K S L W V I S E D M E R D V F M S A A E A Q A H G I V D L V A V
E??MEKYQA
YLELRRSRYQDILYPLFFRESIYGLAYGH--IDYNNKFSLLIVKRLSTRMYQQTNFILFANDS
NKKTFGGYNFDSQIILEGFGVVVEILFSLQLFISLSVKSYNLQSIHSIFPFFEDKLIYLNHKSD
IRIPYPIHLEILVQILRYWIKDVSFFHLIRFFFYYYSNWNSLFPKGISPFFSLRIFLFLYNLYVW
EYESIFLFLRNKSSQLQLKHFRVFFERIFFYEKRKHLVEISTKSYTLFFFKDTFIHYVRYQGK
SILVLKNTPLLINKWKYYFLYLWQCYFDIWAGPETIYINQLSQYSFHFLGYFLSIPQNLSVV
RSQMLKNSFLIKI--VIKKLDTIIPIIPLMRSLAKTKFCNVMGHPISKPVWANFSDFYILDRFL
RICRNFSHYYNGSAKKKSFYQIKYILRFSCIKTLARKHKSTVRTFLKKLSSKLLEEFFT-EED
LFSLIFPKRIWYLDILFRNDFVNHLMIIDLT-EIQDIH-FFFRLEF-FKEIYEILWVFVPILIFIVGI

TISVLAIVWLEREISAGIQQRIGPEYTGPFGVLQALADGTKLLFKENLIPSRGDIRLFSFGPAI
SVISIILSYSVIPFGYNFVLSDLNIGVFLWIAISSIAPIGLLMSGYGSNNKYSFLGGLRAAAQS
ISYEIPLTLCVLSISLLSNSLSTVDIVDAQSKYGFWGWNLWRQPMGFLVFLISSLAECERLPF
DLPEAEEELIAGYQTEYSGIKFGLFYVASYLNLLVSSLFVTVLYLGGSNISIPYIFVSNFFEIN
KTYGVFVTIIGIFITLVKTFLFIFVSITTRWTLPRLRIDQLLNLGWKFLLPISLGNLLLTTSSQL
FSL-----------------MKAFHLLLFDGSLIFPECILIFGLILLLMIDSTSDQKDISWFYFISSTSLVM
SITALLFRWREEPMIAFSGNLQTNNFNEIFQFLILLCSTLCIPLSVEYIECTEMAITEFLLFILT
TTLGGMFLCGANDLITIFVALECFSLCSYLLSGYTKKDVRSNEATTKYLLMGGASSSILVH
GFSWLYGSSGGEIELQEIVNGLINTQMYNSPGILIALLFITVGIGFKLSPAPSHQWTPDVYEG
SPTPVVAFLSVTSKVAASASATRIFDIPFYFSSNEWHLLLEILAILSMILGNLIAITQTSMKRM
LAYSSIGQIGYVIIGIIVGDSNGGYASMITYMLFYISMNLGTFACIVSFGLRTGTDNIRDYAG
LYTKDPYLALSLALCLLSLGGLPPLAGFFGKLHLFWCGWQAGLYFLVSIGLLTSVVSIYYY
LKIIKLLMTGRNQEITPHVRNYRRSPFRSNNSIEFSMIVCVIASTIPGISMNPIIEIAQDTLFMF
LLYEYDIFWAFLIISSLIPILAFLISGILAPISKGPEKLSSYESGIEPIGDAWLQFRIRYYMFALIF
VVFDVETVFLYPWAMSFDVLGVSVFLEAFLFVLILIVGSVYAWRKGALEWSTNDFPWLTT
VVVLPIVGGSLIVLFPHKGNKTIKWYTYCICFIDLLLIAYVFCYHFELDDPLIQLTENYKWI
HFFDFYWRFGIDGLSLGPILLTGFITTLATLSAQPVTRESKLFYFLMLAMYSGQLGTFSSRD
ILLFFIMWELELIPVYLLLSMWGGKKRLYSATKFILYTAGSSVFLLLGILGMSLYSSNEPTLN
FESLTNQSYPVALEIIFYMGFLIAFAVKSPIIPLHTWLPDTHGEAHYSTCMLLAGILLKMGAY
GLVRINMELLSRAHSIFCPWLMLLGSIQIIYAASTSLGQRNVKKRIAYSSVSHMGFLILGIGS
ISETGLNGAILQIISHGFIGAALFFLAGTSYDRLRLLYLDEMGGMAIPMPKIFTIFTTLSMAS
LALPGMSGFVAELIVLLGIITNQKYLLITKILITFVTAIGMILTPIYSLSILRQMFYGYKLFNTP
NSYFFDSGPRELFISISILIPVISIGIYPDFIFSFSADKVEAILSNFL--MIFEHALVLSAFLFSIGIY
GLITSRNMVRALMCLELILNAVNINLVTFSDFFDRRQLKGNIFSIFVIAVAAAEAAIGPAIVSS
IYRNRKSTRINQSNLLNKMEYTHQYSWIIPFIPFPVPMLIGVGLLLFPTATKKIRRIWAFPSIL
LLTIVMFFSLDLSIHQIQNSSIFQYVWSWTINNDISLEFGYLIDSLTSIMSILITTVGILVLIYSD
NYMSHDQGYLRFFAYMTLFNISMLGLVTSSNLIQIYFFWELIGMCSYLLIGFWFTRPIAAN
ACQKAFVTNRVGDFGLLLGILGIYWITGSLEFRDLFQIINN-EMNLFFVTLFALLLFCGSVA
KSAQFPLHVWLPDAMEGPTPISALIHAATMVAAGIFLVARLLPLFVLLPQIMNTIAFIGLITV
ILGATLAIAQKDIKKNLAYSTMSQLGYMMLALGMGSYRGALFHLITHAYSKALLFLGSGSI
IHSMEALVGYSPAKSQNMVFMGGLTKHVPITKFFFLVGTLSLCGIPPFACFWSKDEILNDSR
LYSPIVAIIACSAAALTAFYMFRIYLLVFEGYLNVHFLNFNGKKNSSFYSISLWGKKQVKLK
IKNLLVLLKIKFFIGMKKTACLYPNESNNTMQFSMLVLVLFTLFVGAIG--FDLDILSKLLIPFI
DLLHKDSK-FVNY-EFFTNATFSLILTFWGIFIASFFYKSVYSYLKNLNLLNLFEKFSDHFQNI
IYNWSYNHGYIDVFYEISFISSIRRLVKFNSFFDKKRIDGITNGIGITSFFLGEAIKNVGGG-RI
SSYILLYILDILIFILIYIMDLSESLHDFILVFLGSGLILGSLGVVFFTNTIFSAFSLGLVLVCVSL
FYILSNSHFVAASQLLIYVGAINVLIIFAVMFMNGSEYDQNFRVWTVGDGITLMVCTSIFIS
QINTILDTSWHGIIWTTRPNQILEQDLISTSQQIGIHLSTDFFLPFELISIILLVALIGAIFVARQ
MNISTIRKDFMIVNMGPHHPSMHGVLRLIVTLDGEDVIDCEPILGYLHRGMEKIAENRTIIQ
YLPYVTRWDYLATMFTEAITVNGPEQLGNIQVPKRASYIRVIMLELSRIASHLLWLGPFMA
DIGAQTPFFYIFREREFIYDLFEAATGMRMMHNFFRIGGVATDLPYGWVDKCSDFCDYFLT
SIAEYQKLITRNPIFLERVEGVGVVDVKEVINWGLSGPMLRASGIQWDLRKVDNYECYEE
FHWEVQWQKEGDSLARYLVRIGEMVESIKIIQQALEGLPGGPYENLEIRCFDREKEPEWN
EFEYRFISKKSSPSFELPKQELYVRIEAPKGELGIFIIGDQNGFPWRWKIHPPGFINLQILPQL

VKRMKLADIMTILGSIDIIMGEVDRMFLMVTEFINYSEQIIRAARYIGQGLMITLSHANRLP
VTIQYPYEKIISSERFRGRIHFEFDKCIACEVCVRVCPIDLPIVDWKLETDIRKKRLLNYSIDF
GICIFCGNCIEYCPTNCLSMTEEYELSTYDRHELNYNLIALGRLPVSVIDDYTIRTIQIK-------
---------MQGRLSSWLVKHGLIHRSLGFDYLGVETLQIKPEDWHSIAVILYVYGYNYLRSQCA
YDVAPGGLLASVYHLTRIEYGINQPEEVCIKIFVSRKNPRIPSIFWVWKSADFQEQESYDML
GISYDSHPRLRRILMPENWIGWPLRKDYIAPNFYEIQDAH--
-------------------MNSIEFPLLDQTTQNSVISTTLNDFSNWSRLSSLWPLLYGTSCCFIEFASLIG
SRFDFDRYGLVPRSSPRQADLILTAGTVTMKMAPSLVRLYEQMPEPKYVIAMGACTITGG
MFSTDSYSTVRGVDKLIPVDVYLPGCPPKPEAIIDAITKLRKKISREIYEDPISFQ-ENRCFTI
NHKFHVGYSTYTGNYGQEFFYQPPSTSEIAT--FF-------------MQTRNAFSCIKEGITRSISISV
MIYIIIRAPFSNAYPIFAQQGYENPREATGRIVCANCHLANKPVDIEVPQAVLPDTVFEAVVR
IPYDMQVKQVLANGKKGTLNVGAVLILPEGFELAPPDRISPEIKEKIGNLSFQNYRPTKKNI
LVVGPVPGQKYKEITFPILSPDPASKRDIHFLKYPIYVGGNRGRGQIYLDGSKSNNNVYNAT
AAGIVKKIIRKEKGGYEITIVDTLDEHEVIDIIPPGPELLVSEGESIKLDQPLTSNPNVGGFGQ
GDAEIVLQDPLRVQGLLFFLASIILAQIFLVLKKKQFEKVQLFEMNFMSKVYDWFEERLEI
QAIADDITSKYVPPHVNIFYCLGGITLTCFLVQVATGFAMTFYYRPTVTEAFASVQYIMTEA
NFGWLIRSVHRWSASMMVLMMILHVFRVYLTGGFKKPRELTWVTGVVLAVLTASFGVTG
YSLPWDQIGYWAVKIVTGVPEAIPVIGSSLVELLRGSSSVGQSTLTRFYSLHTFVLPLLTAVF
MLMHFSMIRKQGISGPLMGVTKKPDLNDPVLRAKLAKGMGHNYYGEPAWPNDLLYIFPV
VILGTIACNVGLAVLEPSMIGEPADPFATPLEILPEWYFFPVFQILRTVPNKLLGVLLMVSVP
TGLLTVPFLENVNKFQNPFRRPVATTIFLIGTVVALWLGIGATLPIEKSLTLGLFMIEVFLFGI
VLGLIPITLAGLFVTAYLQYRRGDQLDLMLTITSYFGFLLAVLIITSSLFIGLSKIQLIMDIVSI
AWAALMVVFSFSLSLVVWGRSGLMIIRSPEPEVKILVDRDPIKTSFEEWAKPGHFSRTIAKG
PDTTTWIWNLHADAHDFDSHTNDLEEISRKVFSAHFGQLSIIFLWLSGMYFHGARFSNYE
AWLSDPTHIRPSAQVVWPIVGQEILNGDVGGGFRGIQITSGFFQIWRASGITNELQLYCTAI
GALVFAALMLFAGWFHYHKAAPKLAWFQDVESMLNHHLTGLLGLGSLSWAGHQIHVSL
PINQFLNAAVDPKEIPLPHEFILNRDLLAQLYPSFAEGATPFFTLNWSKYGEFLTFRGGLDPV
TGGLWLTDIIHHHLAIAILFLIAGHMYRTNWGIGHSIKDILEAHKGPFTGQGHKGLYEILTTS
WHAQLSINLAMLGSLTIVVAHHMYSMPPYPYLATDYGTQLSLFTHHMWIGGFLIVGAAA
HAAIFMVRDYDPTIRYNDLLDRVLRHRDAIISHLNWVCIFLGFHSFGLYIHNDTMSALGRP
QDMFSDTAIQLQPIFAQWIQNTHALAPGTTAPGAATSTSLTWGGENLVAVGGKVALLPIPL
GTADFLVHHIHAFTIHVTVLILLKGVLFARSSRLIPDKANLGFRFPCDGPGRGGTCQVSAW
DHVFLGLFWMYNSISVVIFHFSWKMQSDVWGSISDQGIVTHITGGNFAQSSITINGWLRDF
LWAQASQVIQSYGSSLSAYGLFFLGAHFVWAFSLMFLFSGRGYWQELIESIVWAHNKLKV
APATQPRALSIVQGRAVGVTHYLLGGIATTWAFFLARIIAVGMALRFPSFSQGLAQDPTTRR
IWFGIATAHDFESHDDITEERLYQNIFASHFGQLAIIFLWTSGNLFHVAWQGNFETWVQDPL
HVRPIAHAIWDPHFGQPAVEAFTRGGALGPVNIAYSGVYQWWYTIGLRTNGDLYTGAIFL
LILSTISLIAGWLHLQPKWKPSVSWFKNAESRLNHHLSGLFGVSSLAWTGHLVHVAIPGSR
GEYVRWNNLLGILPHPEGLGPFFTGQWNLYAQNPDSNNHIFGTPQGAGTAILTLLGGFHPQ
TQSLWLTDIAHHHLAIAFIFLVAGHMYRTNFGIGHSIKDLLEVHTPPGGRLGRGHKGLYDTI
NNSIHFQLGLALASLGVITSLVAQHMYSLPAYAFIAQDFTTQAALYTHHQYIAGFIMTGAFA
HGAIFFIRDYNPEQNKDNVLARMLDHKEAIISHLSWASLFLGFHTLGLYVHNDVMLAFGT
PEKQILIEPIFAQWIQSAHGKTSYGFDILLSSTNSPAFNAGRSIWLPGWLNAVNE--NSNSLFL
TIGPGDFLVHHAIALGLHTTTLILVKGALDARGSKLMPDKKDFGYSFPCDGPGRGGTCDIS

AWDAFYLAVFWMLNTIGWVTFYWHWKHITLWQGNISQFNESSTYLMGWLRDYLWLNS
SQLINGYNPFGMNSLSVWAWMFLFGHLVWATGFMFLISWRGYWQELIETLAWAHERTPL
ANLIRWRDKPVALSIVQARLVGLAHFSVGYIFTYAAFLIASTSGKFGMSHSVKIYDTCIGCT
QCVRACPTDVLEMIPWGGCKAKQIASAPRTEDCVGCKRCESACPTDFLSVRVYLWHETT
RSMGLAYMI--NFPSIFVPLVGLVFPAIAMASLFLHVQKNKIFMRDLKTYLSVAPVVSTLWF
GALAGLLIEINRFFPDALIFPFFSFMTAILERRESESLWGRFCNWITSTENRLYIGWFGVLMI
PTLLTATSVFIIAFIAAPPVDIDGIREPVSGSLLYGNNIISGAIIPTSAAIGLHFYPIWEAASVDE
WLYNGGPYELIVLHFLLGVACYMGREWELSFRLGMRPWIAVAYSAPVAAATAVFLIYPIGQ
GSFSDGMPLGISGTFNFMIVFQAEHNILMHPFHMLGVAGVFGGSLFSAMHGSLVTSSLIRE
TTENESANEGYRFGQEEETYNIVAAHGYFGRLIFQYASFNNSRSLHFFLAAWPVVGIWFTA
LGISTMAFNLNGFNFNQSVVDSQGRVINTWADIINRANLGMEVMHERNAHNFPLDLAAM
EAPSVNGMGLPWYRVHTVVLNDPGRLLSVHIMHTALVAGWAGSMALYELAVFDPSDPVL
DPMWRQGMFVIPFMTRLGITNSWGGWNIT-GGAITNPGIWSYEGVAGAHIVFSGLCFLAAI
WHWVYWDLEIFCDERTGKPSLDLPKIFGIHLFLAGVACFGFGAFHVTGLYGPGIWVSDPY
GLTGRIQSVNPAWGVEGFDPFVPGGVASHHIAAGTLGILAGLFHLSVRPPQRLYKGLRMG
NIETVLSSSIAAVFFAAFVVAGTMWYGSATTPIELFGPTRYQWDQGYFQQEIYRRVGAGLA
ENQNLTEAWSKIPEKLAFYDYIGNNPAKGGLFRAGSMDNGDGIAVGWLGHPIFRDKEGHE
LFVRRMPTFFETFPVVLVDGDGIVRADVPFRRAESKYSVEQVGVTVEFYGGELNGISYSDP
TTVKKYARRAQLGEIFELDRATLKSDGVFRSSPRGWFTFGHASFALLFFFGHIWHGARTLF
RDVFAGIDPDLDAQVEFGAFQKLGDPTTRRQVVMKTLYSLRRFYHVETLFNGTLALTGRD
QETTGFAWWAGNARLINLSGKLLGAHVAHAGLIVFWAGAMNLFEVAHFVPEKPMYEQGL
ILLPHLATLGWGVGPGGEVIDTFPYFVSGVLHLISSAVLGFGGIYHALLGPETLEESFPFFGY
VWKDRNKMTTILGIHLILLGIGAFLLVFKALYFGGIYDTWAPGGGDVRKITNLTLSPSILFG
YLLKSPFGGEGWIVSVDDLEDIIGGHVWLGSICILGGIWHILTKPFAWARRALVWSGEAYL
SYSLGALSVFGFIACCFVWFNNTAYPSEFYGPTGPEASQAQAFTFLVRDQRLGANVGSAQ
GPTGLGKYLMRSPTGEVIFGGETMRFWDLRAPWLEPLRGPNGLDLSRLKKDIQPWQERR
SAEYMTHAPLGSLNSVGGVATEINAVNYVSPRSWLATSHFVLGFFLFVGHLWHAGRARA
AAAGFEKGIDRDFEPVLSMTPLNMTIALGKFTKDEKDLFDIMDDWLRRDRFVFVGWSGL
LLFPCAYFALGGWFTGTTFVTSWYTHGLASSYLEGCNFLTAAVSTPANSLSHSLLLLWGPE
AQGDFTRWCQLGGLWTFVALHGAFGLIGFMLRQFELARSVQLRPYNAIAFSGPIAVFVSV
FLIYPLGQSGWFFAPSFGVAAIFRFILFFQGFHNWTLNPFHMMGVAGVLGAALLCAIHGAT
VENTLFEDGDGANTFRAFNPTQAEETYSMVTANRFWSQIFGVAFSNKRWLHFFMLFVPVT
GLWMSALGVVGLALNLRAYDFVSQEIRAAEDPEFETFYTKNILLNEGIRAWMAAQDQPH
ENLIFPEEVLPRGNALMSGSTGERSFADIITSIRYWIIHSITIPSLFIAGWLFVSTGLAYDVFGS
PRPNEYFTENRQGIPLITGRFDPLEQLDEFSRSFMTIDRTYPIFTVRWLAVHGLAVPTVSFLG
SISAMQFIQRMATQTVEDNSRSG-PRRTVVGNLLKPLNSEYGKVAPGWGTTPLMGVAMAL
FAIFLSIILEIYNSSILLDGIYMNMLTLKLFVYTVVIFFVSLFIFGFLSNDPGRNPGREEMADT
TGRIPLWIIGTVTGITVIGLIGIFFYGSYSGLGSSLMLNIFSLVCICIHSVLYSSNFFLEKLPEAY
AFLNPIVDIMPVIPLLFFLLAFVWQAAVSFRMTQSNPNEQNVELNRTSLYWGLLLIFVLAVL
FSNYFFNMEVNILAFIATALFILVPTAFLLIIYVKTVSKSDMETATLIAISISGLLVSFTGYALY
TAFGQPSQQLRDPFEEHGDMEALVYTFLLVSTLGIIFFAIFFREPPKVPTKKVKMTIAFQLAV
FALIAISFLLLISVPVLFASPEGWSNNKNVLFSGTSLWIGLVFLVGILNSLIS-------MSPQTET
KASVGFKAGVKDYKLTYYTPDYETKDTDILAAFRVTPQPGVPPEEAGAAVAAESSTGTWT
TVWTDGLTSLDRYKGRCYHIEPVAGEENQFIAYVAYPLDLFEEGSVTNMFTSIVGNVFGFK

ALRALRLEDLRIPTAYIKTFQGPPHGIQVERDKLNKYGRPLLGCTIKPKLGLSAKNYGRAV
YECLRGGLDFTKDDENVNSQPFMRWRDRFLFCAEAIYKAQAETGEIKGHYLNATAGTCE
EMIKRAVFARELGVPIVMHDYLTGGFTANTSLAHYCRDNGLLLHIHRAMHAVIDRQKNHG
MHFRVLAKALRLSGGDHVHSGTVVGKLEGEREITLGFVDLLRDDFIEKDRSRGIYFTQDW
VSLPGVLPVASGGIHVWHMPALTEIFGDDSVLQFGGGTLGHPWGNAPGAVANRVALEACV
QARNEGRDLAREGNEIIREASKWSPELAAACEVWKEIKFEFEAMDTLD-MAIHLYKTSTPS
TRNGAVDSQVKSNPRNHLIYGQHRCGKGRNARGIITAGHRGGGHKRLYRQIDFRRNEKNI
YGRIVTIEYDPNRNASICLIHYGDGEKKYILHPRGAIIGDTIVSGTEVPIKMGNALPLTDMPL
GTAIHNIEITLGKGGQLARAAGAVAKLIAKEGKSATLKLPSGEVRLISKNCSATVGQVGNV
GVNQKNLGRAGSKCWLGKRPIVRGVVMNPVDHPHGGGEGRAPIGRKKPATPWGFPALG
RRSRKRKKYSDNLILRRRTKMIQPQTHLNVADNSGARKLMCIRILGAS-NRRYAYIGDIVVA
VIKQAVPNTNLERSEVIRAVIVRTCKQLKRSNGIIIQYDDNAAVVIDQEGNPKGTRIFCAIAR
ELRQLNFTKIVSLAPEVLMLSPQRTRFRKQHRGRMKGISSRGNRICFGRYALQALEPAWITS
RQIEAGRRAMSRNVRRGGQIWVRIFPDKPVTVRPTETRMGSGKGFPEYWVAVVKPGKILY
EMGGVPENIARKAISIASSKMPIRTQFIISGMTRIKRGYIARKRRTKIRLFVSSFRGAHSRLTR
TITQQKMKALVSAHRDRDRKKRDFRGLWISRINAVIRGNPKVSYIYSNLIHSLYTRQLLLN
R K I V A Q I A I L K E N C L F M I A N N I I K T - - - - - - - - -
-???
??????????????????????????????????MNGIKYAVFTDKSIRLLGKN-QYTFNVESGSTRTEIKH
WVELFFDVKVIAMNSHRLPVKGRRVRPIMGHTMHYRRIIITLQPGYSIPPLRKKRTMAVPK
K R T S I S K K I I R N T L W K K K G Y F T A L K A F S L A Q S L F T G N S K S F F - - K Y K
R???MKINASVRKICEK
CRLIRRRGRIIVICFNPKHKQRQGMVQEKLRVSTRTLQWKCVESRIDSKRLYYGRFILSPLM
KGQADTIGIAIRRILLGEIEGTCITCVKSE--KIPHEYSTIIGIEESVHEIFMNLKEIVLKSNMYG
IQDASISFKGPGYITAQDIILPPSVEIVDNRQHIANVTEPVHLCIELKIERNRGYRIKTLKNFQ
DGSY--DIDARFMPVRNVNYSIHSYVNGNEKQEILFLEIWTNGSLTPKEALYEASQNLIELFI
PFLHAEED--NFHLEKNQ-HKVTLPLFTFHDILVKKLRKNQK-EIALKSIFIDQLELPPRIYNC
LKRSNIHTLLELLSNSQEDLLKFEHFHVEDGKSILDILKIQKYFA-------MLGSGN-EEMSTLP
GLNQIQLEGFCRFIDRGLTEGLFKFPKIEDTDQEIEFQLFAETYQLLEPLINEKDAVYESLTY
SAELYVSTGLIWKSSRDIKKQTIFVGNIPLMNSLGTSIVNGIYRIVINQILQSPGIYYRSELDP
NGISVYTGTIISDWGGRLELEIDKKARIWARVSRKQKISILILLSAMGSNLSEILENVCYPEIF
VSFLNDKDKKKIGSKENAILEFYRQFACVGGDPVFSESLWKELQKKFFQQRCELGRIGRR
NMNQKLNLDIPQNNTFLLPRDILTAASHLIGMKFGMALLDD--INHLKNKRIRSVADLLQD
QFGLALVRLENMVRGTICGAIR-HKLI-PTPQNLVTSTP----LTTTYESFFGLHPLSQVLDQTN
PLTEIVHGRKLSYLGPGGLTGRTASFRIRDIHPSHYGRICPIDTSEGINVGLIGSLAIHARIGI
WG-AIESPFF---EISERSKR--IRMLYLSPSIDEYYMVATGNSLALTRDIQEEQIVPARYRQEFL
TIAWEQVHLRSIYPFQYFSIGASLIPFIEHNDANRALMSSNMQRQAVPLSQSEKCIVGTGLE
CQVALDSGVSAIADHEGNIVYTDTDRIFLFVNGDTLSIPLTIYQRSNKNTCMHQKPQVHRG
KFIKKGQILADGAATVGGELALGKNVLVAYMPWEGYNSEDAVLISERLVYEDIFTSFHIRK
YEIQTHMTSYG-SERITNKIPHLEANLLRNLDKNGIVILGSWVETGDVLVGKLTPQMAKE-S
SYSPEDRLLRAILGIQVSTSKETCLKLPIGGRGRVIDVRWIQKKG-GSSYNPETIRISILQKRE
IKVGDKVAGRHGNKGIVSKILSRQDMPYLQDGEPVDMVFNPLGVPSRMNVGQIFECSLGL
SGGMLDKHYRITPFDERYEQEASRKLVFSELYEASKKTSNPWIFEPEYPGKSKIFDGRTGNS
FKQPAIMGKTYILKLIHQVDDKIHGRSSGHYALVTQQPLRGRAKQGGQRVGEMEVWALE

GFGVAHILQEMLTYKSDHIKTRQEVLGTTIIGGTIPKPTDAPESFRLLVRELRSLAMELNHFL
VSEKNFRIHRKEA-------MIDQYKHQQLRIGSVSPQQMSAWAKRILPNGEIVGEVTKPYTFH
YKTNKPEKDGLFCERIFGPIKSGICACGNYRVIRDKKDDPKFCEQCGVEFIDSRIRRYQMG
YIKLACLVTHAWYLKRLPSYIANLLDKPLKELESLVYGDV--SFARP-VVKKPTFLRLRGSFE
YEIQSWKHSIPLFFTTRGFDIFRNREISSGAVAIREQLADLDLRIIMDYSLIEWKELGKE-GSP
DNEWEDRKVGRRKNFLVRRIELAKHFIRTNIEPEWMVLCLLPVLPPELRPIIQVDGGKLMS
SDINELYRRVIYRNNTLIDLLTTSRSTPGELVICQEKLVQEAVDTLLDNGIRGQPMRDGHNK
VYKSFSDIIEGKEGRFRETLLGKRVDYSGRSVIIVGPSLSLHRCGLPGEIAIELFQTFLIRGLI
RKHFASNIGIAKSKIRQKEPIVWEILQEVMQGHPVLLNRAPTLHRLGIQAFQPILVEGRAIC
LHPLVCKGFNADFDGDQMAVHVPLSLEAQAEARLLMFSHTNLLSPAIADPISVPTQDMLIG
LYILTSGNRRGIYSNRYNPRNC---RNLKNERIPNNNYKYTKK-EPFFCNSYDAIGAYQQKRI
TFDSPLWLRWRLDLR-IISS--REVPIEVHYESLGTYHEIYGHYLVVRSIKKQMRSIYIRTNVG
HISFYREIEEAVQGFCRAYSYDI------------MAERTNLMFHNKVIGGTAIKRLISRLIDHFGMA
YTSHILDQVKTLGFRQATATSISLGIDDLLTIPSKGWLVRDAEHQNLILEKQHHYGNVHAVE
KLRQSIEIWYATSEYFRQEMNPNFRMTDPFNPVHIMSFSGARGNASQVHQLVGMRGLMS
DPQGQMIDLPIQSNLREGLSLTEYIISCYGARKGVVDTAVRTSDAGYLTRRLVEVVQHIVIR
RTDCGTIRGISVNTQNEMMP-ESTWTQTLIGRVLADDIYRGSRCIAVRNQDIGIGLFNQFKT-
-----FQTQPISIRTPFTCRNTSWICRLCYGQSPTQGHLVELGEAVGIIAGQSIGEPGTQLTLRTF
HTGGVFTGGTAEQVRAPYNGKIKFNEDLVHPTRTRHGHPAFLCYIDLYVSIENGDIIHNVTI
PPKSFLLVQNNQYVKSEQVIAEILAGTYTF-NFKEKVRKHVYSDLEGE-MHWSTNVYHAS
EFKYSNVHILPKTSHLWILSGKSDR--SGSVSFSTRKDQDQLNIH--SLSTGERDICNHL--ASN
NK-IRHKLFHFNPSEKKERRISDY-SIINQIICIDHCHFTHPA-IFHDTTD--LLAKRRRNRFIIPF
QFIQERDKALMLASSISIEIPI--NGLFRRNSIFAYFDDPQYRTQSSGITKYRTIDINYILKKEDF
-VIEYP-GVKEFKTKYMKVDQFFFIPEEVYILPEFSSIMVRNNSIVEVDTPITVNIRSQVSGLV
RLEKKKKKIQLKIFSGNIYFPGEMDKISRHSAMLIPP-RTVKKNSKGSKKK--MKNWIYAQW
--ITITKKK--YFVLVRPVILYEIADRIKLIKFFSQDMLQERDNLELQVINYILSGNGKSIRGISN
TSIQLVRTCLVLNWDQDK---KVSSIEKGHASFVELSINGLVRYFLKIALVK-HISYIRKRNDP
SGSRFILD-NESDWTNINPF---FSMNS--REKVQQSLSQNH-TIHMLLN---RNDKCRSLIILSSS
NCF--QIRSFHDGKYY------KEEDPLIPIKNSLGPLGI--ALQVANLD---FYLLIT--HNQIS-INKN
GQLDK---LKETFQVF--K-YYLIDENER--IYKPDISSNILLNPFYLNWHFFHHN----TFPIIS-LG
QFICENVCIVQNAP---HLKSGQILTVQ-MDSVGIRSANPYLATPGTTVHGHYGEILSEGDILV
TFIYQKSRSGDITQGLPKVEQVLEVRSIDSISINLEKRVGTWNGRITRILGIPWGFFISAELTI
AQSRISLVNQIQKVYRSQGVHIHNRHIEIIVRQITSKVLVSE---DGMSNVFLPGELIGLLRAE
RAGRSLEESICYRVLLLGITKTSLNTQSFISEASFQETARVLSKAALRGRIDWLKGLKENVV
LGGMMPVGTGFKRIIYRSKQRQYNKIT--SETKKR---------IDMIYQS------------NLGFKNS----
---------MTKRYWNIILEEMMEAGVHFGHGTRKWNPKMSPYISANRKGIHITNLIRTARFLSE
SCDLVFHAASGGKQFLIVGTKKKAADSVARAAIRARCHYVNKKWLGGMLTNWYTTKTR
LQKFRDLRMQQKTGRFDCFPKKDAAILKRHLAQLETYLGGIKYMKGLPDIVIIIDQQEEYT
ALRECITLEIPTICLIDTNSDPDLADISIPANDDAIASIRFILNKLVFAICEGRSR-SIRNSMGQK
INPLGFRLGTTQSHDSIWFAQPTKYSENIQEDKKIRDWIKNYIQKNIRISS-GVEGIGEIKIQK
RIDLIQVIIYMGFPKLLIEGKPH----KIEEFQTNMHKKLNCVNKKLNIAIVKITNAYKHPNIL
AEFIAGQLKNRVSFRKAMKKAIELTEQAGTKGVQVQIAGRIDGKEIARVEWIREGRVPLQT
IRAKIEYCCYTVRTIYGILGIKVWIFSK--MSRYRGPCFKKIRRLGYLPGLTSKKPTVKNELR
NQLRFSK-KSQYRIRLEEKQKLRFHYGLTERQLLKYVRISGKAKGSTGQVLLQLLEMRLD

NILFRLGMAATIPQARQFINHRHVLVNGRIVDIPSYRCKPQDIITAKDEQKSKTLIQNYLDS
APR-DKLPNHLTVHPFQ--YKGLINQIIDNKWVGLKINELLVVEYYSRQTMSRRGTAEEKTA
KSDPIYRNRLVNMLVNRILKHGKKSLAYQILYRAMKNIQQKTETNPLSVLRQAIRGVTPDI
AVKARRVGGSTHQVPVEIGSAQGKALAIRWLLGASRKRPGRNMAFKLSSELVDAAKGSG
DAIRKKEETHRMAEANRAFAHFRMGKDTIANIITYIRNADINKKGMVQLPFTNITEKIVKIL
LREGFVENIRKHRENDKSFLVLTLRYRR--NRKESYKS-F--LNLKRISTPGLRIYYNYKKIPRI
LGGMGIVILSTSRGIMTDREARLEKIGGEVLCYIWMAKYIPKTGSRKNVRIGS-NHTRKIPK
GIIHVQASFNNTIVTITDVRGRVISWSSAGTCGFKGTRRGTPFAAQTAAGNAIRTVSDQGM
QRAEIMIKGPGLGRDAALRAIRRSGILLNFIRDVTPMPHNGCRSPKKRRVMPTMKQLIRNT
RQPIRNVTKSPALQGCPQRRGTCTRVYTITPKKPNSALRKVARVRLTSGFEITAYIPGIGHNL
QEHSVVLVRGGRVKDLPGVRYHIVRGTLDAVGVKDRQQGRSKYGAKKPKMARKSVIQR
EKKRQKLEQKYHLIRRSSKKEIS---KVPSLSDKWEIHGKLESLPRNSAPIRLHRRCFSTGRP
RANYRDFGLSGHILREMVHACFLPGATRSSWMVKNSFIPVLSQE-KKGK-NPGLVEFQIFQ
FTNRIRRLTSHFELHPKDYSSQTGLRKILGKRQRLLSYLSKKDGIQYKKLINQFDIRQSQIR-
-??
MEKSKRLFIKSKRSFRRRLPPIQSGDRIDYKNMGLICRFISEQGKILSRRVNRLTLKQQRLIT
IAIKQARILSSLPFLNNEK--QFEKSESTEKITTLRKK-NVTR-LKKNPFVANHLLRKINKLNT
KAEKDIILTWSRASTIIPTMIGHTIAIHNGKEHLPIYITDRMVGHKLGEFSPTLNFRGHAKND
NRSRRMIFESFILENLVFLCMKIMNSIVVVGLYYGFMTTFSIGPSYLFLLRARLVEEG---TEN
KISATTGFITGQLIIFMSIYYAPLHLALGRPHTITVIAIPYLLFQFFGNSKKNFLNYGYKNPNS
IRNFSIQRIFFQNLLFQFFNPLFLPSSIFMRFVNIYLFRCNNKVLFLT-------------KSVSILIS----
----YK-IN---

--M
KGHQFKSWIFELREILKEIKNSRYFLDSWTQFNSAGFLIHIFFHQESFIKLLDSRIWSILLS-R
NS--QGSTSNRYF---TIKYVVLFVVA-VLIYR----INNRKMVERKNPYLTRLLP-----IPMNSIGP
KNDTLEESS-ESSNINRLIVP-LLYLPKGKKISESSFLDPKESTRVLPIT----KKYIMPEFNWGS
RWWRNWIGKKS--YSSCKISN---ETIAGIEISFKEKDIK-YLEFLFVYYMD--DPIRKDHDWEF
FDRLSPRKRRNIINL--NSGQLFEILVKDWIYYLMFAFREKIPKEVEG-FFKQQG-TGSIIQSND
IEHVSH--LFLRNKRAISLQNCAQFHMWQFRQDLFV---SWGK-SPHESDFLRN-MSQENWI
WLDNVWLGNKDR---FFS--KVRNVSSNLQYDS---TR--SSFIQVTDSSQLKGSS-DQSKDSFD
SIR----NEDSK------------YHTLINQREIQQLKERSILCWD--PSFLQTERTEIESE--RFLK--NLS
GYS--SMCRLFM-EREKQMNNHLLPEEIEEF--LGNPARATRSFFSDRW--SELH-LGSNPTDRS
TRDQ----KLLKKEQKKH-LALSRRSE----KKEIVNLFKIIMYLQN--TVSIHPISSYRGCDMVP-
KDEL---DSSNKISFLNKNPFWGF--FHLFHDRNRGRYTL--HHDFESE--DLFQEMADLFTLSI
TEP-DLVYHKEFDFSIDSSGLDQKH--FLNELLNSRDESKKHSL---LVLPPLFY--EQNESFYRR
IIKKWVQ-TSCGNNLEDPKPKIVVFASNNIMEAVNQYRLIRNLIQIQYSTH--VYIRNVLN-----
----RFNCNFEYGIQ---RYQIGNDTLNHRTRMKYTINQHFSNLKK-SQKKWFDP--LILISRTER

SMNWDPNAYRYKWSNGSKNFQEHLDYFISEQNS----LQVVFDRLHINQYSIDWSEVIDKK
DLSKSLCLFLSKLLL---------FLP-------KFLLFLSNSLPSFFFVSFGGISIHRSE-IHIY-ELKGPN
DPLCNQLLESIGLQIFHLKKRKPL--LLD---DQDTS-QKSKFLINGGTISPFLFNKIP-KW-MID
SFHTR--KNRRKSFDNTDSYFSMISHDPDNWLNPVKPFHRSSLIYYFYKANQLRFLNNQYH
FCFY--CNKRFPF---Y-VEKARINNYD--FTYG-QFLKILFIRNK-IFSFCDGQ--KKHAFLK-RDT
ISPIELQVSNILIPNDFPQSGDEG-YNFYKS---FHFPIRYDPFVR--GAIYSIADISGTPLTEGQIV
HFEKTYCQPLSDMNIPDSEGKNLYQYLN--FNSNMGWIHTPCSEKYLPSEK--RKK--------RS
SCLQ--KCLEKGQMYRTFQQDSVFSTLSKWNLFQTYIPWFLTSTGYKYLNFIFLDTFSD-LL
PILSSS--QKFVSIFHDIMHGSDILWRIRQIPL------CLPQWN-LISEIPGNCFHN--LLLSEEMTH
RNNEL--LLISTHLRSLNVQEF-FYSILFLLLVAGY-LVRTHLLFVSR--VYSELQTEFEKVKSL
MIPSYMIELRKLLDRYPTSELNSFWLKNLFLVALEQLGDSLEEIRSFAFGGNMLWGGGP----
-AYG-VKSIRSKNKSWNL-----IDLI-SIIPNPINRIAFSRNTRHLSHPSKAIYSLIRKIKNVNGD
WIDDQIESWVSNTDSIDDKEKEFLVQFS--TLTT--EKRIDQILLSLT-HSDLLS-----KNNSGYQI
SEQPGAIYLRYLVDIH--KKYLMIYEFNTSCLVERHIFLANYQTITY--SQTLWGANSFHFPSH
G---KPFSLRLALPPPSRGILVIGSIGTGRSYLVKYLAKNSYVPFITVFLNKFLDNKPKGFLID--
---DSD------DIDDSDDSDD------------------IDRDLDIELELLTMMNTL---TMDMMP---EIDRFY
ITFHFELAKAMSPCIIWIPNIHDLDVNESNYLSLGLLVNYLS---RDCERCS-TRNILVIASTHIP
QKVDPALIAPNQFNTCIKIRRLLIPQQRKHFFTLSYTRGFHLEKKMSHT--NGFGSTTMGSN
VRDLVALNNEALSISIIQKKSIIDTNIISSVLHRQTWDFR---SQVRSVQDHGILFYQIGRAVSQ
NVLLSNCSIDPISIYMKKK--SCDGGDSYLYKWYFELGTSMKKLTILLYLLSCSAGSVAQDL
WSLP--GPDEK-NGITSYGLVENNS-DLVHGLLEVEGALVG--SSRTEKDCSQFDKD--RVTLL
LRSEPRN-PLNRIQNGSYSIVDQRFLYEK--YESEFEE---RGGVLDPQ----QIEED----FFNH--IV
WAP--RIWRP---WGFLFDCIERPNSLGFP-YWARSFRDK---RIIYDEEDELQENDSEFLQGGT
MQYQTRDRSSKEQGFFRISQFIWDPADPLFFLFK-DQPFVSVFSHRQFFTDEEMSRELLTSQ
TDLPTSIYKHWFI--KNTQEKHFELLIHCQRWLR---INSSSS--KGFFPSN--TLSESYQYLSNLF
LSN-EALLDQMTKTLLRKRWLFPDEIVVAI--CSNNESLVSLMPRSR-INENFIDKTFSIVANIL
LRIIPTTSGEKRAFTYYRDG--MSAQSEGNYAEALQNYYEAMRLEIDPYDRSYILYNIGLIHT
SNGEHTKALEYYFRALERNPFLPQAFNNMAVICHY-RGEQAIRQGDSEVAESWFNQAAEY
WKQAIALTPGNYIAAQNWLKITGRFE--LKKRSKEVLIYSITES-KISNIFSALIIFLGSLGLLL
VAISSYLGMDLFLFSEISNFPFIPQGATMAFYGIGGLFISFYLWWILLWNIGGGFDIFDKKNK
KVCFIRWGFPGKNRRIILKIPMNEIQSIRIIAGRGILTRTIVYMETIEQGFITLTRIEDN-LTPPEI
ANKAGELAFFLGVPLLY-
>Chloranthus
MEKWWFNSMLSLSKSMESLGPIGNTRGSEDPIINDTDKNIHSCSNVDHFFGVSDIWSFISD
ETFLVRDSNGKGYSVYFDIENRIFEIDND--SSFLSELESSFS-----SYLSNGSKNDNRYYDSY
MYDTKYSWNNHMNSCIDSYLRSEIS--IDSYISTGSDNFSDSYIYSYIFSDSESSIRTSGNDSN
FNVRERD-DFDRNQKYGRLWVQCENCYELNYRSFFRSKMNICEQCGYHLKMNSLDRIEL
SIDSGTWNPMDDDMVSMDPIEFHSMEEPYRDRIDSYQRNTGLTEAVQTGIGQLNGIPIAIG
VMDFQFMGGSMGSVVGEKITRLIEYATNESIPVIMVCASGGARMQEGSLSLMQMAKISSA
SYNYQLNKKLFYVSILTSPTTGGVTASFGMLGDIIIAEPNAYIAFAGKRVIEQTLNKTVPDG
SQVAEYSFHKGLFDPIVPRNPLKGVLSELFQLHGFFPLNQNSSGMVTMRADEISNIIRERIE
QYNREVKVVNTGTVLQVGDGIARIHGLDEVMAGELVEFEEGTIGIALNLESNNVGVVLM
GDGLMIQEGSSVKATGRIAQIPVSEAYLGRVINALAKPIDGRGEISAYESRLIESPAPGIISRR
SVYEPLQTGLIAIDSMIPIGRGQRELIIGDRQTGKTAVATDTILNQKGQNVICVYVAIGQKAS

SVAQVVTTFQERGAMEYTIVVAEMADSPATLQYLAPYTGAALAEYFMYRERHTSIIYDDP
SKQAQAYRQMSLLLRRPPGREAYPGDVFYLHSRLLERAAKLSSRLGEGSMTALPIVETQS
GDVSAYIPTNVISITDGQIFLSADLFNAGIRPAINVGISVSRVGSAAQIKAMKQVAGKSKLEL
AQFAELEAFAQFASDLDKATQNQLARGQRLRELLKQSQSAPLTVEEQIVTIYTGANGYLDS
LEIGQVKKFLVQLRTYLKTNKPQFQEIISSTRTFTEQAEALLKEIIQEQIELFLLQEQTMRINP
TTSGPGVSTLEEKILGRIAQIIGPVLDVAFPPGKMPNIYNALVVKGRDTVGQQINVTCEVQ
QLLGNNRVRAVAMSATDGLMRGMEVIDTGAPLSVPVGGATLGRIFNVLGEPVDNLGPVD
TRTTSPIHRSAPAFIQLDTKLSIFETGIKVVDLLAPYRRGGKIGLFGGAGVGKTVLIMELINN
IAKAHGGVSVFGGVGERTREGNDLYMEMKESGVINEENIAESKVALVYGQMNEPPGARM
RVGLTALTMAEYFRDVNEQDVLLFIDNIFRFVQAGSEVSALLGRMPSAVGYQPTLSTEMG
SLQERITSTKEGSITSIQAVYVPADDLTDPAPATTFAHLDATTVLSRGLAAKGIYPAVDPLDS
TSTMLQPRIVGEEHYETAQRVKQTSQRYKELQDIIAILGLDELSEEDRLTVSRARKIERFLS
QPFFVAEVFTGSPGKYVGLAETIRGFQLILSGELDGLPEQAFYLVGNIDEATAKAMNLEVE
SKLKKMTLNLCVLTPNRIIWDSEVKEIILSTNSGQIGVLPNHAPIATAVDIGILRIRLNDQWL
TMALMGGFARIGNNEITILVNDAEKGSDIDPQEAQQTLEIAEANLSRAEGKRQAIEANLAI
RRARTRVEAINVISMKNITDSFVSVGYWSSAGSFGFNTDILATNPINLSVVLGVLIFFGKGV
LSDLLDNRKQRILSTIRNSEELRGGAIEQLEKARARLRKVEMEADEFRVNGYSEIERERVN
LINATYENLERLEIYKNETIHFEQQRAINQVRQRVFQQALQGALGTLNSCLNSELHLRTISA
NIGMFGAMKEITDMNPLISAASVIAAGLAVGLASIGPGVGQGTAAGQAVEGIARQPEAEG
KIRGTLLLSLAFMEALTIYGLVVALALLFANPFVMNVLPCSINTLKVLYDISGVEVGQHFY
WQIGGFQVHAQVLITSWVVIAILLGSATIAVRNPQTIPTDGQNFFEYVLEFIRDLSKTQIGEE
YGPWVPFIGTMFLFIFVSNWSGALLPRKIIQLPHGELAAPTNDINTTVALALPTSVAYFYAG
LTKKGLGYFGKYIQPTPILLPINILEDFTKPLSLSFRLFGNILADELVVVVLVSLVPSVVPIPV
MFLGLFTSGIQALIFATLAAAYIGESMEGHHMIFATLEHILTHICFSIILIVITIHLITLLVHEIG-
LYNLSEKGMIATFFCITGLLVSRWIYSGHFPLSDLYESLIFLSWSFAIIHMVPKIRNHPNSLSTI
TAPSTIFTQGFTTSGLLTKMHQSTILVPALQSKWLMMHVSMMLLSYAALLCGSLLSIALLVI
TFRKNIDILGKSNKTQYLNEKRLHLQKASFLSFRNYHRYQLTHRLDYWSYRVISLGFTFLTI
GILSGAVWANEAWGSYWNWDPKETWAFITWTIFAIYSHTRTNKSLQGANSAIVASMGFLII
WICYFGVNLLGIGLHSYGSFTLTKEKESTPLPFPYLASIVLLPWWISLSFKKCFEPWVTHW
CNTRQSKFFLNDIQEKGTLERFIELEELFLLDKIIKYYPDTHIQRLWIRIHKETIQLVKIHNED
HNHIIFCLSTNIISFTILSGYSILGNEELFILNSWVQEFLYNLSDTIKAFSILLFTDLCIGFHSPH
GWELMIGSIYKIFGFAHNDQVISGFVSTFPVFLDTFFKYLIFRHLNRVSPSLVVIYDSINEMPI
GVPKVPFRSPGEEDAVWVDVN-RLHRERLLFLGQEVDSEISNQLVGLMVYLSIEDDTRDLY
LFINSPGGWVIPGLTIYDTMQFVPPDVHTICMGLAASMGSFILVGGEITKRLAFPHARIMIH
QPASSFYEAQAGEFILEAEELLKLRETLTRVYVQRTGNPLWIVSEDMERDVFMSATEAQAH
GIVDLVAVEMKEQKLIHEGLITESLPNGMFRVRLDNEDLILGYVSGRIRRSFIRILPGDRVKI
EVSRYDSTRGRIIYRLRNKDSNDMEELQRYLEIDRSRQQHFLYPLLFQEYIYVLAHDHGLL
GYDN-SSSLIIKRLIIRMHQQNHLMISVNHSNQNRFLGKNFDYQMISEGFAVIVEIPFSLQLV
SSLIAKFH-LRSIHSILPFLEDKFSHLNYVSDILIPYPTHLEILVQTLHRWIQDAPSLHLLRFFL
HEYTNWNSFITKSIYLFTKQRLFLFLYNSHVYECESVFIFLRKQSSYLGSKYSGAFIERTHF
YGKMEY-VVVLRNQK-LWLFKDPFMHYVRYQGKAILASKGAHLLMKKWKYHLVNFWQ
CHFYLWYQPDRIHINQLSNHSFYFLGYLSSVLLNLSAVRSQMLENSFLINTAI--KKFDTIVPI
IPLIGALAKAKFCNISGHPISKPVRVDSSDSDIIDRFGRICRNLSHYHSGSSKKQSLYRIKYIL
RFSCARTLARKHKSTVRTFLKRLGSELLEEFLTEKEQVISLIFAIRIWYLDILCINDLANHEM

IIDTT-EIK-AINSFSRSE-SLKEVYGLIWMLVPIFTPVSGITLGVLVIVWLEREISAGIQQRIGPE
YAGPLGILQALADGTKLLFKEDLLPSRGDIRLFSIGPSIAVISILLSYSVIPFYHRFVLADLSIG
VSLWIAISSIAPIGLLMSGYGSNNKYSFSGGLRAAAQSISYEIPLTPCVLSISLLSNSSSTVDI
VEAQSKYGFWGWNLWRQPIGLMVFLISSLAECERLPFDLPEAEEELVAGYQTEYSGIKSGL
FYVASYLNLLVSSLFVTVLYLGGWNFPIPYIFIPELFGINKTGGVFGTTIGILITLAKAYLFLFI
PITTRWTLPRMRMDQLLSLGWKFLLPISLGNLLLTTSSQLVSLMIWHVQNENFILDSTRIFM
KAFHLLLFHGSFIFPECILIFGLILLLMIDSTSDQKDIPWLYFISSTSLVMSITALLFRWREEPM
ISFSGNFQTNNFNEIFQFLILLCSTLCIPLSVEYIECTEMAITEFLLFVLTATLGGMFLCGAND
SITIFVAPECFSLCSYLLSGYTKRDVRSNEATTKYLLMGGASSSILVHGFSWLYGSSGGEIEL
QEIVNGLINTQMYNSPGISIALISITVGIGFKLSPAPSHQWTPDVYEGSPTPVVAFLSVTSKV
AASASATRIFDIPFYFSSNEWHLLLEILAILSMILGNLIAITQTSMKRMLAYSSIGQIGYVIIGI
IVGDSNDGYASMITYMLFYISMNLGTFARIVSFGLRTGTDNIRDYAGLYTKDPFLALSSALC
LLSLGGLPPLAGFFGKLHLFWCGWQAGLYFLVSIGLLTSVVSIYYYLKIIKLLMTGRNQEIT
PHVRNYRRSPLRSNNSIELSMIVCVIASTIPGISMNPIIAIAQDTLFMFLLHEYDIFWAFLIISS
VIPILVFLISGVLAPINEGPEKLSSYESGIEPMGNAWLQFRIRYYMFALVFVVFDVETVFLYP
WAMSFDVLGVSVFIEALIFVLIPIVGSVYAWRKGALEWSTSSFPWLTIIVVLPIFAGSSIFFLP
HRGNKIVRWYTICICLLELLLTTYTFCYHFQLDDPLIQLEEDYKWINIFDFHWRLGIDGLSI
GPILLTGFITTLATLAARPVTRDSRLFHFLMLAMYSGQIGSFSSRDLLLFFIMWELELIPVYL
LLSMWGGKKRLYSATKFILYTAGGSIFLLMGIPGMGLYGSNQPPFNFETSANQSYPVALEIL
FYFGFLIAYAVKSPIIPLHTWLPDTHGEAHYSTCMLLAGILLKMGAYGLVRINMELLPHAH
SIFSPWLMIVGTIQVIYAASTSPGQRNLKKRIAYSSVSHMAFIIIGIGSITDTGLNGAILQIISH
GFIGAALFFLAGTSYDRIRLIYLDEMGGIAIPMPKIFTMFSSFSMASLALPGMSGFVAESIVF
FGIITSPKYFFMPKILITFVMAIGMILTPIYSLSMSRQMFYGYKLFNVPNSYFVDSGPRELFV
SICIFLPVIGIGIYPDFVLSLSVDKVEAILSNYFHRMMLEHVLVLSAYLFSIGIYGLITSRNMV
RALMCLELILNAVNMNLVTFSDLFDSRQLKGDIFSIFVIAIAAAEAAIGPAIVSSIHRNRKST
RINQSNLLNKMEHTFQYAWVIPFVPLPVTMSIGLGLLLVPTATKNLRRMWAFPSVLLLSIT
MVFSSNLSIQQINGSFIYQYLWSWTINNDFSLEFGYLIDPLTSIMSILITTVGIVVLIYSDNYM
SHDQGYLRFLSYMSFSNTSMLGLVTSSNLIQIHIFWELVGMCSYLLIGFWSTRTVAANACQ
KAFVTNRIGDFGLLLGILGLYWITGSFEFRDLFEIFKNNGVHFVFATLCASLLFVGAVAKSA
QFPLHVWLPDAMEVPTPISALIHAATMVAAGIFLVARLLPIFTVIPYIMNLIALIGVITVLLGA
TLALAQRDIKRSLAYSTMSQLGYTMLALGIGSYRAALFHLITHAYSKALLFLGSGSIIHSME
PIVGYSPDKSQNMVLMGGLRKYIPITKTTFLLGTFSLCGIPPLACFWSKDEILNDSWLYSPIF
AIIAYSTAGLTAFYMFRMYLLTFEGHLHIHFQNYNSIKNGSLYSISIWGKEGPKPVKI-NFVL
STMNKVSNKE--TSVYPQESDNTMLFPLLVLVLFTLFVGSIGISGDFDILSKWLTPSINLLHK
NLSYSPDWYEFFINAIFSVSISYFGILIAFLLYGSVYSSFQNLDLINLFVILLDRIKNVIYNWS
YNRGYIDVFYATALTRGIRGLAQLTHFFDRRAIDGITNGVGVASFFVGEGIKY-VGGGRISSY
LFLYLFYIIIIIFLLIMDLPGPIHDILLVFLGSGLILGGLGVVLLTNPISSAFSLGLVLVCISLFYIP
SNSYFVAAAQLLIYVGAINVLIVFAVMFMNGSEYYNDFHLWTIGDGFTSLVCTSILFSLITTI
PDTSWYGIIWTTRSNQIIEQDLTSNVQQIGIHLSTDFYLPFELISIILLVALIGAIAMARQMTV
PATRKDLMIVNMGPHHPSMHGVLRLIVTLDGEDVIDCEPILGYLHRGMEKIAENRTIIQYL
PYVTRWDYLATMFTEAITVNAPEQLGNIQVPKRASYIRVIMLELSRIASHLLWLGPFMADI
GAQTPFFYIFRERELLYDLFEAATGMRMMHNYFRIGGVAADLPHGWVDKCLDFCDYFLT
GVAEYQKLITRNPIFLERVEGVGIIGGEEAINWGLSGPMLRASGIRWDLRKVDHYECYDEF
DWEVQWQKEGDSLARYLVRISEMTESIKIIQQALEGIPGGPYENLEVRRFDRARDAEWND

FDYRFISKKPSPTFELSKQELYARVEAPKGELGIFLIGDNSVFPWRWKIRPPGFINLQILPQLV
KRMKLADIMTILGSIDIIMGEVDRMFPMVTGFMNYGQQTVRAARYIGQSFMITLSHANRL
PVTIQYPYEKSITSERFRGRIHFELDKCIACEVCVRVCPIDLPVVDWRLETDIRKKRLLNYSI
DFGICIFCGNCVEYCPTNCLSMTEEYELSTYDRHELNYNQIALGRLPMSVIEDYTIQTILNS
QIVTDKPLNSRTITNSMQGRLSAWLVKHELVHRSLGFDYQGIETLQIKPEDWYSIAVISYVY
GYNYLRSQCAYDIAPGGLLASVYHLTRIQYGVDQPEEVCIKVFAPRRNPRIPSVFWIWKSA
DFQERESYDMLGISYDNHPRLKRILMPESWIGWPLRKDYIAPNFYEIQDAHMGNEFRCIG
RIRIYRSFNFRAYPN-------CWFSLCMAKRSIGMVLKNKKEGKDNIETIMNSIEFPLFERTAQ
NSVISTTSNDLSNWSRLSSLWPLLYGTSCCFIEFASLIGSRFDFDRYGLVPRSSPRQADLILTA
GTVTMKMAPSLVRLYEQMPEPKYVIAMGACTITGGMFSTDSYSTVRGVDKLIPVDVYLP
GCPPKPEAVIDAITKLRKRVSRETYEDRIGSQQENRCFTTNHKFHVGRSTHTGNYDQGLLY
QSPY---KPSEIFFRYKSSVSSHELVNMQNRNTFSWVKEQMTRSISVSIIIYVITRTSISNAYPIF
AQQGYENPREATGRIVCANCHLANKPVDIEVPQAVLPDTVFEAVVKIPYDMQLKQVLAN
GKKGALNVGALLILPEGFELAPPDRISPEMKEKMGNLSFQSYRPTKRNILVIGPVPGQKYS
EIVFPILSPDPATKKDVHFLKYPIYVGGNRGRGQIYPDGSKSNNTVYNARTAGIVSRIVRKE
KGGYEITIADASDGHQVVDIIPSGPELLVSEGESIKLDQPLTSNPNVGGFGQGGAEIVLQDP
LRVQGLLFFLASIILAQIFLVLKKKQFEKVQLSEMNFLNKVYDWFEERLEIQAIADDITSKY
VPPHVNIFHCLGGITLTCFLVQVATGFAMTFYYRPTVTEAFASVQYIMTEANFGWLIRSVH
RWSASMMVLMMILHVFRVYLTGGFKKPRELTWVTGVVLAVLTASFGVTGYSLPRDQIGY
WAVKIVTGVPEAIPVIGSPLVELLRGSASVGQSTLTRFYSLHTFVLPLLTAVFMLMHFPMIR
KQGISGPLMGITKKPDLTDPVLRAKLAKGMGHNYYGEPAWPNDLLYIFPVVILGTIACNV
GLAVLEPSMIGEPADPFATPLEILPEWYFFPVFQILRTVPNKLLGVLLMVSVPTGLLTVPFLE
NVNKFQNPFRRPVATTVFLIGTAVALWLGIGATLPIDKSLTLGLFMIEVFLFGIVLGLIPITLA
GLFVTAYLQYRRGDQLDLMPTITSYFGFLLAASTITSALLIGLSKIRLIMDTVSLAWAALMV
VFTFSLSLVVWGRSGLMIIRSPEPEVKIVVDRDPIKTSFEEWARPGHFSRTIAKGPDTTTWI
WNLHADAHDFDSHTSDLEEISRKVFSAHFGQLSIIFLWLSGMYFHGARFSNYEAWLSDPT
HIGPSAQVVWPIVGQEILNGDVGGGFRGIQITSGFFQIWRASGITSELQLYCTAIGALVFAAL
MLFAGWFHYHKAAPKLAWFQDVESMLNHHLAGLLGLGSLSWAGHQIHVSLPINQFLDA
GVDPKEIPLPHEFILNRDLLAQLYPSFAEGSTPFFTLNWSKYAEFLSFRGGLDPVTGGLWLT
DIAHHHLAIAILFLIAGHMYRTNWGIGHGLKDILEAHKGPFTGQGHKGLYEILTTSWHAQL
SLNLAMLGSSTIVVAHHMYSMPPYPYLAIDYGTQLSLFTHHMWIGGFLIVGAAAHAAIFM
VRDYDPTTRYNDLLDRVLRHRDAIISHLNWACIFLGFHSFGLYIHNDTMSALGRPQDMFSD
TAIQLQPIFAQWVQNTHALAPGATAPGATTSTSLTWGGAGLVAIGGKVALLPIPLGTADFLV
HHIHAFTIHVTVLILLKGVLFARSSRLIPDKANLGFRFPCDGPGRGGTCQVSAWDHVFLGL
FWMYNAISVVIFHFSWKMQSDVWGSISDQGVVTHITGGNFAQSSITINGWLRDFLWAQAS
QVIQSYGSSLSAYGLFFLGAHFVWAFSLMFLFSGRGYWQELIESIVWAHNKLKVAPATQPR
ALSIVQGRAVGVTHYLLGGIATTWAFFLARIIAVGMALRFPRFSQGLAQDPTTRRIWFGIAT
AHDFESHDDITEERLYQNIFASHFGQLAIIFLWTSGNLFHVAWQGNFQSWVQDPLHVRPIA
HAIWDPHFGQPAVEAFTRGGALGPVNIAYSGVYQWWYTIGLRTNEDLYTGALFLLFLSAIS
LIAGWLHLQPKWKPSVSWFKNAESRLNHHLSGLFGVSSLAWTGHLVHVAIPGSRGEYVR
WNNFLDVLPHPQGLGPLFTGQWNLYAQNPDSSSHLFGTSQGAGTAILTLLGGFHPQTQSL
WLTDMAHHHLAIAFIFLVAGHMYRTNFGIGHSMKDLLEAHIPPGGRLGRGHKGLYDTINN
SIHFQLGLALASLGVITSLVAQHMYSLPAYAFIAQDFTTQAALYTHHQYIAGFIMTGAFAHG
AIFFIRDYNPEQNEDNVLARMLDHKEAIKSHLSWASLFLGFHTLGLYVHNDVMLAFGTPE

KQILIEPIFAQWIQSAHGKTSYGFDVLLSSTNGPAFNAGRSIWLPGWLNAVNE--NSNSLFLT
IGPGDFLVHHAIALGLHTTTLILVKGALDARGSKLMPDKKDFGYSFPCDGPGRGGTCDISA
WDAFYLAVFWMLNTIGWVTFYWHWKHITLWQGNVSQFNESSTYLMGWLRDYLWLNSS
QLINGYNPFGTNSLSVWAWMFLFGHLVWATGFMFLISWRGYWQELIETLAWAHERTPLA
NLIRWRDKPVALSIVQARLVGLAHFSVGYIFTYAAFLIASTSGKFGMSHSVKIYDTCIGCTQ
CVRACPTDVLEMIPWDGCKAKQIAPAPRTEDCVGCKRCESACPTDFLSVRVYLSNETTRS
MGLAYMTDLNLPSIFVPFVGLVFPAIAMASLSLHVQKNKIVMRDIKTYLSVAPVLATLWFG
SLAGLLIEINRLFPDALAFPFF--MTAILERRETTSLWGRFCNWITSTENRLYIGWFGVLMIPT
LLTATSVFIIAFIAAPPVDIDGIREPVSGSLLYGNNIISGAIIPTSAAIGLHFYPIWEAASVDEW
LYNGGPYELIVLHFLLGVACYMGREWELSFRLGMRPWIAVAYSAPVAAATAVFLIYPIGQG
SFSDGMPLGISGTFNFMIVFQAEHNILMHPFHMLGVAGVFGGSLFSAMHGSLVTSSLIRET
TENESANAGYRFGQEEETYNIVAAHGYFGRLIFQYASFNNSRSLHFFLAAWPVVGIWFTAL
GISTMAFNLNGFNFNQSVVDSQGRVINTWADIINRANLGMEVMHERNAHNFPLDLAAVE
APSINGMGLPWYRVHTVVLNDPGRLLSVHIMHTALVSGWAGSMALYELAVFDPSDPVLD
PMWRQGMFVIPFMTRLGINNSWGGWSIT-GGTITNPGIWSYEGVAGAHIVFSGLCFLAAIW
HWVYWDLEIFCDERTGKPSLDLPKIFGIHLFLSGVACFGFGAFHVTGLYGPGIWVSDPYGL
TGKVQSVNPAWGAEGFDPFVPGGIASHHIAAGTLGILAGLFHLSVRPPQRLYKGLRMGNIE
TVLSSSIAAVFFAAFVVAGTMWYGSATTPIELFGPTRYQWDQGYFQQEIYRRVGTGLSENQ
SLSEAWSKIPEKLAFYDYIGNNPAKGGLFRAGPMDNGDGIAVGWLGHPIFRDKEGHELFV
RRMPTFFETFPVVLVDGDGIVRADVPFRRAESKYSVEQVGVTVEFYGGELNGVSYSDPAT
VKKYARRAQLGEIFELDRATLKSDGVFRSSPRGWFTFGHATFALLFFFGHIWHGARTLFRD
VFAGIDPDLDAQVEFGTFQKLGDPTTRRQVVMKTLYSLRRFYHVETLFNGTLALAGRDQE
TTGFAWWAGNARLINLSGKLLGAHVAHAGLIVFWAGAMNLFEVAHFVPEKPMYEQGLIL
LPHLATLGWGVGPGGEVIDTFPYFVSGVLHLISSAVLGFGGIYHALLGPETLEESFPFFGYV
WKDRNKMTTILGIHLILLGIGAFLLVLKALYFGGVYDTWAPGGGDVRKITNLTLSPSVIFG
YSLKSPFGGEGWIVSVDDLEDIIGGHVWLGSICILGGIWHILTKPFAWARRAFVWSGEAYL
SYSLGALSVFGFIACCFVWFNNTAYPSEFYGPTGPEASQAQAFTFLVRDQRLGANVGSAQ
GPTGLGKYLMRSPTGEVIFGGETMRFWDLRAPWLEPLRGPNGLDLSRLKKDIQPWQERR
SAEYMTHAPLGSLNSVGGVATEINAVNYVSPRSWLATSHFVLGFFLFVGHLWHAGRARA
AAAGFEKGIDRDFEPVLSMTPLNMTITLGRFTKEE-DLFDIMDDWLRRDRFVFVGWSGLL
LFPCAYFALGGWFTGTTFVTSWYTHGLASSYLEGCNFLTAAVSTPANSLAHSLLLLWGPEA
QGDFTRWCQLGGLWTFVALHGAFGLIGFMLRQFELARSVQLRPYNAIAFSAPIAVFVSVFL
IYPLGQSGWFFAPSFGVAAIFRFILFFQGFHNWTLNPFHMMGVAGVLGAALLCAIHGATVE
NTLFEDGDGANTFRAFNPTQAEETYSMVTANRFWSQIFGVAFSNKRWLHFFMLFVPVTGL
WMSALGVVGLALNLRAYDFVSQEIRAAEDPEFETFYTKNILLNEGIRAWMAAQDQPHEN
LIFPEEVLPRGNALMSGSTGERSFADIITSIRYWVIHSITIPSLFIAGWLFVSTGLAYDVFGSP
RPNEYFTESRQGIPLITGRFDSLEQLDEFSRSFMTIDRTYPIFTVRWLAVHGLAVPTVSFLGSI
SAMQFIQRMATQTIEDSSRSG-PRRTTTGSLLKPLNSEYGKVAPGWGTTPFMGVAMALFAI
FLSIILEIYNSSVLLDGISMNMLTLKLFVYTVVIFFVSLFIFGFLSNDPGRNPGREEMADTTG
RIPLWLIGTVTGIPVIGSIGIFFYGSYSGLGSSLMLNIFSFIFICLNSSLHSSSFFFAKLPEAYAF
FNPIVDVMPVIPVFFFLLALVWQAAVSFRMTQSNPNEQNVELNRTSLYWGLLLIFVLAVLF
SNYFFNMEVNILAFIATALFILVPTAFLLIIYVKTVSQNDMETATLVAISISGSLVSFTGYALYT
AFGQPSQQLRDPFEEHGDMEALVYTFLLVSTLGIIFFAIFFREPPKVPTKKMKMTIAFQLAV
FALIATSSILLISVPVVFASSDGWSSNKNVVFSGTSLWIGLVFLVAILNSLIS-------MSPQTET

KASVGFKAGVKEYKLNYYTPDYETKDTDILAAFRLTPQPGVPPEEAGAAVAAESSTGTWT
TVWTDGLTSLDRYKGRCYHIEPVAGEENQYIAYVAYPLDLFEEGSVTNMFTSIVGNVFGFK
ALRALRLEDLRIPPAYTKTFQGPPHGIQVERDKLNKYGRPLLGCTIKPKLGLSAKNYGRAV
YECLRGGLDFTKDDENVNSQPFMRWRDRFVFCAEALYKAQAETGEIKGHYLNATAGTCE
EMIKRAVFARELGVPIVMHDYLTGGFTANTSLAHYCRDNGLLLHIHRAMHAVIDRQKNHG
IHFRVLAKALRMSGGDHIHAGTVVGKLEGERDITLGFVDLLRDDFIEKDRSRGLYFTQDW
VSLPGVLPVASGGIHVWHMPALTEIFGDDSVLQFGGGTLGHPWGNAPGAVANRVALEACV
QARNEGRDLAREGNEVIREASKWSPELAAACEIWKEIKFEFEAMDTV--TAIHLYKTSTPST
RNGAVDSQVKSNPRNNLIYGQHHCGKGRNARGIITAGHRGGGHKRLYRKIDFRRNEKDIS
GRIVTIEYDPNRNAYICLIHYGDGEKRYILHPRGAIIGDTIVSGTEVPISMGNALPLTDMPLG
TAIHNIEITLGKGGQLARAAGAVAKLIAKEGKSATLRLPSGEVRLISKNCSATVGQVGNVG
VNQKSLGRAGSKCWLGKRPVVRGVVMNPVDHPHGGGEGRSPIGRKKPTTPWGYPALGR
RSRKRNKYSDSLILRRRK-MIQPKTHLNVADNSGARELMCIRIIGAS-NHRYAHIGDVIVAVI
KEAVPNMPLERSEVIRAVIVRTCKELKRDNGMIIRYDDNAAVVIDQEGNPRGTRVFGAIAR
ELRQLNFTKIVSLAPEVLMLSPKRTRFRKQHRGRMKGISYRGNNICFGRYALQALEPAWIT
SRQIEAGRRAMTRYARRGGKIWVRIFPDKPVTIRPTETRMGSGKGSPEYWVAVVKPGRILY
EMAGVSESIARAAISIAACKMPIRTQFVIAEMTRVRRGYIARRRRTKIRLFASTFRGAHSRL
TRTITQQKMRALVSTHRDRGRQKINFRRLWITRINAVTREN--VSYSYSRLIHDLYKRQLLL
NRKIPAQIAISNRNCLYTISSEIIK-----------MKKEVQAFARHICMSAHKMRRVIDQIRGRSYE
QTLMILELMPYRASYPIFRLIYSAAANASKTMGSKEADSLISKAEVNEGTFIKKLKPRARG
RSYPIKKPTCHIIIVLKDKSKK-------MDGIKYIVLTDKSILLLGNNQ-YTSNVESGSTRTEIKH
WIELFFGVKIIAMNSHRLPGKGRRMGPIMGHTMHYKRMIITLQPGYSIPPLIEKGTMAVPK
KRTSMSKKRIRKNIWKRKGYRAAIKAFSLAKSVSTGHSKSFLVRQ---MAKGKDVRAIVILE
CTSCVRNGVN--KESPGISRYITQRNRHNTPNRLELRKFCPYCYKHTIHGEIKKMKIRASVR
KICEKCQLIRRRGRILVICSNPRHKQRQGMVREEIAVSTRTLQWKCVESRADSKRLYYGRF
MLSPLMKGQADTIGIAMRRALLGEIEGTCITRAKLD--KVPHEYSTIVGIEESIHEIFMNLKEI
VLRSNLYGTRNASICVRGPRYVTAQDIISPPSVEIVDTTQHIASLTEPIDLCIELQIQRDRGYR
MKTPNKYQNGSY--PIDAVSMPVRNANHSIHSYGNGNEKQEILFLEIWTNGSLTPKEALHE
ASRNLIDLFIPFLHAEEEGIRL--EDNP-NRFNIPFFTFHDGLANNIRKKKK-GIALKCIFIDQSE
LPPRTYNYLKRSNINTLLDLLSNSQEDFLKIEHFRIEDVKQILDILQKH--FTIDC----MLRDG-
NEGMSTIPGFNQIQFEGFCRFIDQGLTEELHKFPKIEDTDQEIEFQLFVETYQLVEPLIKERD
AVYESLTYSSELYVPAGLIWKTSRDMQEQTVFIGNIPLMNSLGTSIVNGIYRIVINQILQSPGI
YYRSELDHNGISVYTGTIISDWGGRSELEIDRKARIWARVSRKQKISILVPSSAMGSNLREIL
DNVCYPEIFLSFPNDKEKKKFGSKENAILEFYQQFACVGGDPIFSESLCKELQKKFFQQRCE
LGRIGRRNMNRRLNLDIPQNNIFLLPRDVLAAADHLIGMKFGMGTLDD--MNHLKNKRIR
SVADLLQDQFGLALVRLENVVRGTICGAIR-HKLI-PTPHNLVTSTP----LTTTYESFFGLHPL
SQVLDRTNPLTQIVHGRKSSYLGPGGLTGRTASFRIRDIHPSHYGRICPIDTSEGINVGLIGSL
AIHARIGHWG-SIESPFY---EIYERSKE--VQMVYLSPSRDEYYMVAAGNSLALNQGIQEEQV
VPARYRQEFLTIAWEQIHLRSIFPFQYFSIGASLIPFIEHNDANRALMSSNMQRQAVPLSRSE
KCIVGTGLEHQAALDSGVSVIAEHEGKIIYTETNKIVFSGNGDTISIPLVMYQRSNKNTCM
HQNPQVQRGKCIKKGQILAGGASTVGGELALGKNILVAYMPWEGYNSEDAVLISERLVYG
DIYTSFHIRKYEIKTHVTSQG-PERITKEIPHLEANLLRNLDKNGIVMLGSWIETGDILVGKL
TPQTAKE-SSYAPEDRLLRAILGIQVSTTRETCLKLPIGGRGRVIDVRWIQKKG-GSSYNPETI
RVYISQKREIKVGDKVAGRHGNKGIISKILPRQDMPYLQDGRPVDMVFNPLGVPSRMNVG

QIFECSLGLAGDLLDRHYRIAPFDERYEQEASRKLVFPELYEASKQTANPWVFEPEYPGKS
RIFDGRTGDPFEQPVIMGKSYILKLIHQVDDKIHGRSSGHYALVTQQPLRGRAKQGGQRVG
EMEVWALEGFGVAHISQEMLTYKSDHIRARQEVLGTTIIGGTILNPEDAPESFRLLIRELRS
LALELNHFLVSEKNFQINRKEA-------MIDRYKHQQLRIGSVSPQQITAWANKILPNGEIVGE
VTKPYTFHYKTNKPEKDGLFCERIFGPIKSGICACGNYRVIGDEKEDPKFCEQCGVEFVDS
RIRRYQMGYIKLACPVTHVWYLKRLPSYIANLLDKPLKELEGLVYCDVNFSFARP-IAKKP
TFLRLRGSFEYEIQSWKYSIPLFFTTQGFDTFRNREISTGAGAIREQLADPDLRTITDHSLVE
WKELGEKGST-GNEWEDRKIGRRKDFLVRRLELAKNLIRTNVEPEWMVLRLLPVLPPELR
PIIQIDGGKPMSSDINELYRRVIYRNNTLTDPLTTSRSTPGESVMCQEKLVQEAVDTLLDNGI
RGQPMKDGHNKVYKSFSDVIEGKEGRFRETLLGKRVDYSGRSVIVVGPSLSLHRCGLPRE
IAIELFQTFVIRGLIRQHVASNIGIAKSKIREKEPIVWEILQEVMQGHPVLLNRAPTLHRLGI
QAFQPILVEGRAICLHPLVRKGFNADFDGDQMAVHVPLSLEAQAEACLLMFSHMNLLSPT
IGDPISVPTQDMLIGLYVLTIGNRRGICANRYNPCNCRNDN-DQNETVDDN-YKYTKEKEP
YFCSSYDALGAYRQKRIHLDSPLWLRWRLDQR-VIAS--REVPIEVQYESSGTHHEIYGHYLI
VRSVKKEILCIYIRTTVGHISFYREIEEAIQGFCRAYSYGT------------MAERADLVFHNKVID
GTAMKRLISRLIDHFGMAYTSHILDQVKTLGFQQATATSISLGIDDLLTIPSKGWLVQDAEQ
QSLILEKYHHYGNVHAVEKLRQSIEIWYATSEYLRQEMNPNFRMTDPSNPVHIMSFSGAR
GNASQVHQLVGMRGLMSDPQGQMIDLPIQSNLREGLSLTEYIISCYGARKGVVDTAVRTS
DAGYLTRRLVEVVQHIVVRRTDCGTIRGIFVSPQN--GMAKRIFIQTLIGRVLADDIYMGPR
CIAARNQYIGIGLVNRFIT------FRAQPISIRTPFTCRSTSWICRLCYGRSPTHGDLVELGEAV
GIIAGQSIGEPGTQLTLRTFHTGGVFTGGTAEHVRAPSNGKIKFNEDLVHPTRTRHGHPAFL
CYIDLYVTIENQDIIHNVNIPPKSFLLVQNDQYVESEQVIAEIRAGASTL--FKERVRKHIYSD
SEGE-MHWSTDVYHASEYTYGNVHLLPKTSHLWILSGSP--YRSSIVPFSLHKDQDQMNVH
--SLSVERGSISDLS--VTNDR-VRHKLFSSDLSGK-RGGILDYS-GPDRIISNGHWNFIYPA-IL
HENSD--LLAKRRRNRFIIPFQYDQEREKELMPRSGISIEIPIN--GIFRRKSILAYFDDPRYRKS
SSGITKYGTIEVDSIVKKED--LIEYRG-AKEFRPKYMKVDRFFFIPEEVHILTGSSSIMVRNN
SIIGVDTRIALNTRSRVGGLVRVEKKKKRIELKIFSGDIHFPGETDKISRHSGILIPPGTGKK--
NPKESK--KWKNWIYVQQ--ITPTKKK--YFVSVRSVVTYEIADGINLATLFPQDLLQERDNV
QLRVVNYILYGNGKPIRGISHTSIQLVRTCLVLNWDQDRNG----SIEEAHASFVEVRANALIR
DFIRIHLVKSPISYTVKRNDTASSGLIPD-NASDRPNINPFDFKAR---------VQSLTQHQGTIRT
LLNR---NKECQSLIILSSSNCSRLGP--FNGSKYHNVT---KESDPSISIRNSLGPLGI--VPKIANF
-----YYLMT--HNQILLN-KYLLLDN---LKQTFQVLQYYKYSLMDENGQ--IYNPDPYSNTILN
PFDLNWRFLHHDYCEETSTIIS-LGQFICENGCISKYGP---HIESGQVLIVH-VDSLVIRSAKP
HLATPGATVHGHYGEILYEGDTLVTFIYEKSRSGDITQGLPKVEQVLEVRSIDSISINLEKRV
EGWNERITRIIGIPWGFLIGAELTIAQSRISLVNKIQKVYRSQGVQIHNRHIEIIVRQITSKVLV
SE---DGMSNVFSPGELIGLLRAERTGRALEEAICYRAILLGITRTSLNTQSFISEASFQETARV
LAKAALRGRIDWLKGLKENVVLGGMIPVGTGFKGLVHRSR--QHNNSP--LEIKKKNLFEG
EMR--DILFHHRELFGSCIPNHFHDTTFTGFHDS---------MTRRYWNINLEEMMEAGVHFGH
GTRKWNPRMAPYISAKRKGIHITNLTRTARFLSEACDLVFDAASSGKHFLIVGTKNKAADS
VASAAIRARCHYVNKKWLGGMSTNWSTTETRLQKFRDLRAEQKMGRLNRLPKRDAAM
LKRQLSRLQTYLGGIKYMTGLPDIVIIVDQQEEYTALRECVTLGIPTICLIDTNCDPDLADIS
IPANDDAIASIRLILNKLVSAICEGRSG-YIRNRMGQKINPLGFRLG-AQGHHSLWFAQPKSY
SRGLQEDEKIKDCIKNYVQKNMRISSD-YEGIAHIEIKKRIDLIQVIIYMGSKNMLIEG--PTR-
-GIEELQINVQKEFHSANQRLNIDITEVANPYGQPNLLAEYIARQLKKRVSFRKAMKKTIEF

AEQADTKGIQVQISGRIDGKEIARVEWVREGRVPLQTIRAKIDYCSYTVRTIHGVLGIKIWV
FVDDKMSRYRGPRFKKIRRLGALPGLTSKRPRSGSDLRNQSRFGK-RSQYRIRLEEKQKLR
FHYGLTERQLLRYVRIAGKAKGSTGQVLLQLLEMRLDNTLFRLGMASTIPGARQLVNHRH
ILVNSRIVDIPSYRCKPRDIITTRDEQRSRTLIQNHIDSSPH-EELPKHLTLHSFQY--KGLVNQI
IDSKSIGLKINELLVVEYYSRQTMSRRGTAEEKTTKSDPIYRNRLVNMLVNRILKHGKKSLA
YQIIYRAVKKIQQKTETNPLSVLRQAMRGVTPDIAVKARRVGGSTHQVPLEIGSTQGKALA
IRWLLGASRKRPGRNMAFKLSSELVDAAKGSGDAIRKKEETHRMAEANRAFAHFRMGK
DTIADIITSIRNADMEKKGTVRIASTNISENIVQILLREGFIENVRKHRENNKYFLVSTLRHR
R--NRKGAYR---NIL--KRISRPGLRIYSNYQRIPRILGGMGIVILSTSRGIMTDREARLEGIGG
EILCYIWMTKPIPRIGSRKNGRISSRKNGRRIPKGVIHVQASFNNTIVTVTDVRGRVVSWSS
AGTSGFRGTRRGTPFAAQTAAGNAIRTVVDQGMQRAEVMIKGPGLGRDAALRAIRRSGL
LLSFVRDVTPMPHNGCRPPKKRRVMPTIKQLIRNTRQPIRNVTKSPALRGCPQRRGTCTRV
YTITPKKPNSALRKVARVRLTSGFEITAYIPGIGHNSQEHSVVLVRGGRVKDLPGVRYHIVR
GTLDAVGVKDRQQGRSQYGVKKPKMARKSLIQRERKRKKLEQKYHWIRRSSKTEIN---K
VPSLSDKWEIHGKLQSPPRNSAPIRLHRRCFSTGRPRANYRDFGLSGHILREMVHACLLPG
ATRSSWMVKNSFNSVIPQEE-----NKGSVEFQVFNFTNKIRRLTSHLELHRKDYLSQRGLRG
ILGKRQRLLAYLSKKNRVRYQELIGQLDIREPKTREFMVKLRLKRCGRKS--IYRIVAIDVRS
RREGRDLRKVGFYDPIKNQTYLNVPAILYFLEKGAQPTGTVHDISKRAEVFKEFKINQLK--
-MDKSKRPFRKSKRSFRKRLPPIGSGDRIDYRNMSLISRFISEQGKILSRRVNRLTLKQQRLI
TTAIKQARILSSLPFLNNEK--QLERTESIPRTTGTRIRNKVTRSLKKNPFVANHLLEKVEKLN
MREEKKIIVTWSRSSTIIPTMIGHTIAIHNGREHLPIYITDRMVGHKLGEFAPTLTFRGHARN
DNRSRRMILKSFLLGNLLSLCMKIINSVVVVGLYYGFLTTFSVGPSYLFLLRARVMEEG---T
EKEVSATTGFITGQLMMFISIYYAPLHLALGRPHTITVLVLPYLLFHFFWKNHKHFFDYGST
TRNSMRNLSIQCVFLNNLIFQLFNHFILPSSTLARLVNIYMFRCNNKMLFVTSSFVGWLIGH
ILFMKWVGLVLFWIRQNNSIRS--VLIKYLVSELRNSMARIFRILLFITCVYYLGRIPSPIVTK
KLKETEEDKPLLTLLFDYKRWNRPLRYIKNDRFENAVRNEMSQYFFHTCPSDGKQIISFTY
PPSLSTFLEMIQKKMSLCTTKPEP-EDLYNNWIYINDQKIN--
--KK---

---MKRHQFQSWIFELREILREIKNSHYFLDSWTKFDAVGSFT
QIFFHQERFMKLFDPRIWSILLS-RDS--QGSTSNRDF---TIKGVVLLVVA-VLI----YRINNRN
MVERKNLYLMGLLSIPILPIPMNSIGPRNDTLEESF-WSSNINRLIVS-LLYLPKGKKISESCF
MDPKESTWVLPIT----KKCIMPESNWGSRWWRNRIGKKR--DSSCKIS---NETVAGIEISFKE
KDIK-YLEFLFVSYTD--DPIRKGHDCELFDRLSPRKKRNIVNL--NSGQLFEILVKHLICYLM
SAFREKRPIEVGG-FFKQQG-AEATIQSNDIEHVSH--LLSRNKWGISLQNCAQFHMWQFRQ
DLFV---SWGK-NQHESDFLRN-VSRENWIWLDNVWLVNKDR---FFS--QVRNVSSNIQYDS--
-TR--SIFVQVTDSSQLKGSS-DQSRDHFDSIS----NEDSE------------YHTLINQTEIQQLKERSI
L-WD--PSFLQTERTEIESD--RFPK--SLSGYS--SMSRLFT-EREKQMNNHLLPEEIEEFL--GNP
TRSIRSFFSDRW--SELH-PGSNPTERSTRDQ----KLLKKQQDVS-FVPSRRSE----NKEMVDIF

KIITYLQN--TVSIHPISSDPGCDMVP-KDEPD-MGSSNKISFLNKNPFFDFYLFHLFHDRNKG
GYTL--HHDFESE--ERFQEMADLFTLSITEP-DLVYHKGFAFSIDSYGLDQKK--FLNEVFNSR
DESKKKSL---LVLPPIFY--EENESFYRRIRKKSVR-ISCGNDLEDPKPKIVVFASNNIMEAVN
QYRLIRNLIQIQYSTYG--YIRNVSNRFFLM---NRSDRNFEYGIQ---RDQIGNDTLNHITIMKY
TINQHLSNLKK-SQKKWFD----LLISRTERSMNRDPDAYRYKWSNGSKNFQEHLEHFVSEQ
KN---HFQVVFDRLRMNQYSIDWSEVIDKQDLSKSLRFFLSKS---------LLFLS-------KSL-LFL
SKSLPFFFVSIGNIPIHRSE-IHIY-ELKGPNDQLCNQLLESIGVQIVHLNKLKP--FLLD---DQY
TS-QRSKFLINGGTISPFLFNKTP-KW-MIDSFHT--RNNRRKSFDNTDSYFSMISHDRDNWLN
PVKPFHRSSLISSFYKANQLRFLNNPHNFWFY--CHKRFPFYV----EKTRINNYD--LTY-GQFL
NILFIRNK-RFSLCVGK--KKHVFLE-RDTISPIESQVSDIFIPNDFPQSGDET-YNLYKS---FHFP
IRSDPFVR--RAIYSIADISGTPLTEEQIVNFERTYCQPLSDMNLSDSEGKNSHQYLS--FNSN
MSLIHTPCSEKYLP--SGKRKK--------RSLCLK--KCVEKRQMYRTFQRDSAFSNL--WNLFQT
YMPWFLTSTGCKYLNFTLLDTFSDPL--PILSSS-PKFVSIFHDIMHGSDISWPIPQKKLW----V
ILPQWNL-ISEISSKCLQN--LLLSEERIRRNNESPVPLIWTHLRSPNAREF-LYSILFLLLVAGY-
LVRTHLLFVSR--ASSELQTELEKIKSLMIPSYMIELRKLLDRYPTSELNSFWLKNLFLVALE
QLGDSLEEIRGSASGDNMLLGGGP-----AYG-VKSIRSKKKYLNINLIDIIDLI-SIIPNPINRITF
SRNTRHLSRTSKEIYSLIRKRKNVNGDWIDDKIESWVANSDSIDDEEREFLIQFS--TLTTT-E
KGIDKILLSLT-HSDHLS-----KNDSGYQMIEQPGSIYLRYLVDIH--KKYLMNYEFNRSCLAE
RRIFLAHYQTITY--SQTSCGANSSHFPSHG---KPFSLRLALSPS-GGILVIGSIGTGRSYLVKY
LVTNSYLPFITVFPNKFD--KSKGYLID-----DID-IDDSDEID----IDDIDEINMDDSDEIDIDDIG
EINMDDDLDTELLTRANAL---TMYMT---PKIDRFDLTLPFELAKAMSPCIIWIPNIHDLYVN
EPNYLSLGLLVNYLS---RNCEICF-TKNILVIASTHLPQKVDPALIAPNKLNTCIKIRRLLIPQQ
RKHFLILSYTRGFHLEKKMFHT--NGFGSITMGSNARDLVALTNEALSISITQKKSIIDTNTIR
SALHRQTWDLR---SQVRSVQDHGILFYQIGRAVAQNAFLSNCPIDPISIYMKKK--SCKEGD
SYLYKWYFELGTSMKKLTILLYLLSCSAGSVAQDLWSPP--GPDEK-NWITSYGFVENDS-DL
VYG-LEVEGALVG--SSRTEKDCSQFDND--RVTLLLRSEPRN-PLDMMQNRSCSIVDQRFLY
EK--YESEFEE-GEGEGALDPQ----QIEED----LFNH--IVWAP--RIWRP---CGNLFDCIERPNEL
GFP-YWARSFR---GKRIIYHKEDELQENDSEFLQSGTMQYQTRDRSSKEQGFFRISQFIWDP
ADPFFFLFK-DPPFVSVFSRREFFADEEMSKGLITSQTNPPTSLFKRWFI--KNTQEKHFELLI
HRQRWLRT---NSSLS--NGSFRSN--TPSESYQYLSNLFLSN-GALLDQMTKTLLRKRWLFPD
EMKHLI--HVTGERFPIPMPRSR-INGNFIDKTFSIVANILLRIIPTTSGEKEAFTYYRDG--MSA
QSDGNYAEALQNYYEATRLEIDPYDRSYILYNIGLIHTSNGEHTKALEYYFRALERNPFLP
QAFNNMAVICHY-RGEQAIRQGDSEIAEAWSDQAAEYWKQAIALTPGNYIEAHNWLKITR
RFE--MNWRSERIWIELITGSRKTSNFCWACILFLGSLGFLLVGTSSYLGKNLISLLPSQQILF
FPQGIVMSFYGIAGLFISSYLWCTISWNVGSGYDRFDRKEGIVCIFRWGFPGINRRIFLRFL
MRDIQSIRMEVKEGLYSRRVLYMEIRGQGAIPLTRTDDN-LTPREIEQKAAELAYFLRVPIEL
K
>Buxus
MEKWWFNSMLSLSKSMESLGPIGNTSGGEDHIINNMDKNIH-CSNVYPLFGVRGIQGFISD
DTFLVRDSNGDSYSIYFDIENKIFEIDND--HSFLSELESSFSSYQN-----NGSNSDNPYYDRYI
YDTKYSWNNYINSCIDSYLRSEIR--IDSYILSGSDNYSDSYIYSYICS--ESSIK---NGSD-NIR
GRY-DLDLNKKYRHLWVQCENCYGLNYKKFFRSKMNICEQCGYHLKMSSSDRIELSIDTG
TWDPMDEDMVSMDPIEFHSEEELYKDRINSYQRKTGLTEAVQTGIGQLNGIPIAIGVMDFQ
FMGGSMGSVVGEKITRLIEYATNRSLPVIIVCASGGARMQEGSLSLMQMAKISSASYNYQS

NKKLFYVSILTSPTTGGVTASFGMLGDIIIAEPNAYIAFAGKRVIEQTLNKTVPDGSQTAEYL
FHKGLFDPIVPRNLLKGVLSELFQLHGFFPLNSNSIKMVTIQADEISNIIRERIEQYNREVKI
VNTGTVLQVGDGIARIHGLDEVMAGELVEFEEGTIGIALNLESNNVGVVLMGDGLMIQEG
SSVKATGRIAQIPVSEGYLGRVINALAKPIDGRGEISASESRLIESPAPGIISRRSVYEPLQTG
LIAIDSMIPIGRGQRELIIGDRQTGKTAVATDTILNQKGQNVICVYVAIGQKASSVAQVVTTF
QERGAMEYTIVVAETADSPATLQYLAPYTGAALAEYFMYRERHTSIIYDDPSKQAQAYRQ
MSLLLRRPPGREAYPGDVFYLHSRLLERAAKSSSRLGEGSMTALPIVETQSGDVSAYIPTN
VISITDGQIFLSADLFNAGIRPAINVGISVSRVGSAAQIKAMKQVAGKSKLELAQFAELEAFA
QFASDLDKATQNQLARGQRLRELLKQSQSAPLTVEEQIVTIYTGTNGYLDSLEIGQVKKFL
VQLRTYLKTNKPQFQEIISSTKTFTEEAEALLKEAIQEQMERFLLQEQTMRINPTTSGPEVS
TLEEKNLGRIAQIIGPVLDVAFPPGKMPNIYNALVVKGRDTVGQQINVTCEVQQLLGNNR
VRAVAMSATDGLTRGMEVIDTRAPLSVPVGGATLGRIFNVLGEPVDNLGPVDTRTTSPIHR
SAPAFIQLDTKLSIFETGIKVVDLLAPYRRGGKIGLFGGAGVGKTVLIMELINNIAKAHGGV
SVFGGVGERTREGNDLYMEMKESGVINEQNIAESKVALVYGQMNEPPGARMRVGLTALT
MAEYFRDVNEQDVLLFIDNIFRFVQAGSEVSALLGRMPSAVGYQPTLSTEMGSLQERITST
KEGSITSIQAVYVPADDLTDPAPATTFAHLDATTVLSRGLAAKGIYPAVDPLDSTSTMLQPRI
VGEEHYETAQRVKQTLQRYKELQDIIAILGLDELSEEDRLTVARARKIERFLSQPFFVAEVF
TGSPGKYVGLAETIRGFQLILSGELDGLPEQAFYLVGNIDEATAKAMNLEVESKLKKMTL
NLCVLTPNRIVWDSEVKEIILSTNSGQIGVLPNHAPIATAVDIGILRIRLNDQWVTMALMGG
FARIGNNEITILVNDAEKSSDIDPQEAQRTLEIAEANLRRAEGKRQTIEANLALRRARTRVE
AINVISMKNVTDSFVSLVNWPSAGSFGFNTDILATNPINLSVVLGVLIFFGKGVLSDLLDNR
KQRILSTIRNSEELRVGAIEQLEKARARLRKVEMEADEFRVNGYSEIEREKLNLINSTYKNL
ERLENYKNETIHFEQQRAINQVRQRVFQQALQGALGTLNSCLNNELHLRTISANIGMFGA
MKEITDMNPLISAASVIAAGLAVGLASIGPGVGQGTAAGQAVEGIARQPEAEGKIRGTLLL
SLAFMEALTIYGLVVALALLFANPFVMSVLPCSINTLKVLYDISGVEVGQHFYWQIGGFQV
HAQVLITSWVVIAILLGSATIAVRNPQTIPTDGQNFFEYVLEFIRDLSKTQIGEEYGPWVPFI
GTMFLFIFVSNWSGALLPWKIIQLPHGELAAPTNDINTTVALALLTSVAYFYAGLTKKGLG
YFGKYIQPTPILLPINILEDFTKPLSLSFRLFGNILADELVVVVLVSLVPSVVPIPVMFLGLFT
SGIQALIFATLAAAYIGESMEGHHMIFLTLEHILTHISFSIVSIVITIHLITLLVDEIVGLYDSLE
KGMISTFLCITGLLVTRWIYSGHLPLSNLYESLIFLSWSFSIIHMIPYFKNHKNHLSLVTAPSA
IFTQGFATSGLLTEMHQSAILVPALQSQWLMMHVSMMVLSYGALLCGSLLSVALLVITFRK
NIDIFGKRNEIQYTNERSNVLRNVSFLSLRNYRRYQLIRQLDNWSYRVISLGFIFLTIGILSGA
VWANEAWGSYWNWDPKETWAFITWTIFAIYLHTRTNKNFQGADSAIVASIGFIIIWICYFG
VNLLGIGLHSYGSFILTMAKKKAFTSLPYLASIVFLPWWISLSFNKWLESWVTNWWNTRQ
SETFLNDIQEKNILEKFIELEELFLLDEMIKEYPETHIQRR---IHKETIQLVKMHNESHIHTIL
HLSTNIICFAILSGYSILGNEGLFILNSWVQEFLYNLSDTIKAFSILLLTDLCIGFHSPHGWEL
MIGSVYKDFGFAHNDQIISGLVSTFPVILDTILKYWIFRYLNRVSPSLVVIYHSMNDMPIGVP
KVPFRNPGEEDAVWVDVYNRLHRERLLFLGQEVDSEISNQLIGLMVYLSIEDDTKDLYLFI
NSPGGWVIPGIAIYDTMQFVPPDIHTICMGLAASMGSFLLVGGEITKRLAFPHARVMIHQP
ASSFYEAQTGEFILEAEELLKLRETVTRVYVQRTGNPLWVVSEDMERDVFMSAIEAQAHG
IVDLVAVEMKEQKWIHEGLITESLPNGMFRVRLDNEDLILGYVSGKIRRSFIRILPGDRVKIE
VSRYDSSRGRIIYRLRNKDSNDMKELQGYLEIYRSRQQDFLYPLLFQEYIYVLAHDHGLEN
LG-KSSSLVVKRLIARMYQQNHLIISANNSNQNKFVGKDFYSQMILEGFAVIVEIPFSLRFVS
SLILESHNLRSIHSIFPFLEDTLSHLNYVSDILIPHPIHLEILVQSLRCWVQDAPSLHLLRFFLH

EYHNWNNFISKSIFIFSKKRFFLFLYNSYVVYECESIFVFLCKQSSHLRSTSSGALLERTHFYV
KIEDLVIVFRNDFILWLFKDPLMHYVRYQGKSILASKGTPLLMNKWKYYLVNFWQCNFYL
WSQSGRIHIKQLSNHSLDFLGYLSSVRLNPSVVKSQMLENSFIIDSAI--NKFDTIVPIISLIGS
LAKAKFCNVSGHPISKPVRADSSDSDIIDRFGQICRNLSHYHSGSSKKTSLYRIKYILRLSCA
RTLARKHKSTVRAFLKRLGLELLEEFLTEEEHVLSLIFPRRIWYLDIIRINDLANHQMIIDTT-
EVQ-AINSFSRPE-SLKEVYGLIWLLVPIFTTVSGITIGVLVIVWLEREISAGIQRRIGPEYAGP
LGILQALADGTKLLFKEDLLPSRGDTRLFSIGPSIAVISILLSYSVIPFGHHLVLADLSIGVFL
WIAISSIAPIGLLMSGYGSNNKYSFLGGLRAAAQSISYEIPLTPCVLSISLLSNSSSTVDIVEA
QSKYGFWGWNLWRQPIGFIVFIISSLAECERLPFDLPEAEEELVAGYQTEYSGIKFGLFYVA
SYLNLLISSLFVTVLYFGGWNISIPYISIPELFEINKTDGIFGTTIGIFITLAKAYLFLFISIATRW
TLPRMRMDQLLNLGWKFLLPISLGNILLTTSFQLLSLMIWHVQNENFILDSTRIFMKAFHL
LLFHGSFIFPECILIFGLILLLMIDSTSDQKDIPWLYFISSTSLVISITALLFRWREEPMISFSGN
FQTNNFNEIFQFLILLCSTLCIPLSVEYIECTEMAITEFLLFVLTATLGGMFLCGANDLITIFVA
PECFSLCSYLLSGYTKRDVRSNEATTKYLLMGGASSSILVHGFSWLYGSSGGEIELQEIVNG
LINTQMYNSPGISIALIFITVGIGFKLSPAPSHQWTPDVYEGSPTPVVAFLSVTSKVAASASA
TRIFDIPFYFSSNEWHLHLEILAILSMILGNLIAITQTSMKRMLAYSSIGQIGYVIIGIIVGDSN
DGYASMITYMLFYISMNLGTFARIVSFGLRTGTDNIRDYAGLYTKDPFLALSLALCLLSLG
GLPPLAGFFGKLHLFWCGWQAGLYFLVSIGLLTSVVSIYYYLKIIKLLMTGRNQEITPHVR
NYRRSPLRSNNSIELSMIVCVIASTIPGISMNPIIAIAQDTLFMFLLHEYDIFWAFLIISSVIPIL
AFLISGVLAPISKGPEKLSSYESGIEPMGDAWLQFRIRYYMFALVFVVFDVETVFLYPWAM
SFDVLGVSVFIEALIFVLILIVGSVYAWRKGALEWSTNYFPWLTIIVVLPISAGSLILFLPYKG
NKTIRWYTICICILELLITTYAFCYHFQLDDPLIQLEEDYKWVNFFDFHWRLGIDGLSIGPIL
LTGFITTLATLAARPVTRDSRLLHLLMLAMYSGQIGSFSSRDLLLFFIMWELELIPVYLLLS
MWGGKKRLYSATKFILYTAGGSIFLLMGVLGMDLYGSNEPTLNFETLANQSYPVALEILFY
FGFIIAFAVKSPIIPLHTWLPDTHGEAHYSTCMLLAGILLKMGAYGLIRINMELLPHAHSIFS
PWLVIVGTMQIIYAASTSPGQRNLKKRIAYSSVSHMGFIIIGIGSITDAGLNGAILQIISHGFIG
AALFFLAGTSYDRIRLIYLDEMGGIAIQMPKIFTMFSSFSMASLALPGMSGFVAELIVFFGII
TSQKYFLMPKILITFVMAIGMILTPIYSLSMSRQMFYGYKLFNTTNSFFSDSGPRELFVSISIF
LPVIGIGIYPDFVLSLSVDKVEAILSNYFYRMMLEHVLVLGAYLFSIGIYGLITSRNMVRAL
MCLELILNAVNINLVTFSDFFDSRQLKGDILSIFVIAIAAAEAAIGLAIVSSIYRNRKSTRINQ
SNLLNKMEHTYQYTLIIPFALLPVPMLIGVALIFFPTATKNVRRMWAFPSVLLLSIVMIFSFN
LSIQQINGSSIYQYVCSWTINNDFSFEFGLLIDSLTSIMLILITTVGILVLIYSDNYMSHDQGY
LRFFAYMSFSNISMLGLVTSSNLIQIHIFWELVGVCSYLLIGFWFTRPIAANACQKAFVTNR
VGDFGLLLGILGFYWITGSFEFRDLFEIFNNNQVNSLFVILCASLLFVGAVAKSAQFPLHVW
LPDAMEGPTPISALIHAATMVAAGIFLVARLLPLFTVIPYIMNIISLIGIITILLGATLALTQKDI
KRSLAYSTMSQLGYIMLALGMGSYRAALFHLITHAYSKALLFLGSGSIIHSMESIVGYSPD
KSQNMILMGGLKKYVPITKTAFFLGTLSLCGIPPLACFWSKDEIINDSWLYSPIFAIIACSTA
GLTAFYMFRTYLLTFEGHLNVNLKNYSGKKTSSFYSICLWGKEGSKTIKKN-YCLSTINFFS
FDNKETFSYPHESDNNMLFPLLVLVLFTFFVGSIGIPFDLDILSKWLTPSINLLHTNSN-SFD
WYEFVKNSIFSVSIAYFGIFISSFLYRPIYSSLQNLELINSFVSLWDKIINVIYNWSYNRGYID
FFYSIALTRSIRVISELTHFFDRRVIDGITNGVGVTSFFVGEGVRYVGSG-RISSYLFLYLFSVS
IFFLIFQMDLPGPIHDFLLVFLGSGIILGGLGVVLLTNPIFSAFSLGLVLFCISLFHIPSNSHFVA
AAQLLIYVGAINVLIIFAVMFTNGSEYYNDFHVWTVGDGVTSLVCTSIFVSLITTILDTSWY
GIIWTTRSNQIIEQDLISNGQQIGIHLSTDFFLPFELVSIILLVALIGAIAMARQMTVPSTRKDL

IIVNMGPHHPSMHGVLRLIVSLDGEDVIDCEPILGYLHRGMEKIAENRTIIQYLPYVTRWD
YLATMFTEAITINAPEQLGNIQVPKRASHIRVIMLELSRIASHLLWLGPFMADIGAQTPFFYI
FRERELIYDLFEAATGMRMMHNYFRIGGVAADLPHGWIDKCLDFCDYFLTGVVEYQKLIT
RNPVFLERVEGVGIIGGEEAINWGLSGPMLRASGIQWDLRKVDHYECYDEFDWEVQWQK
EGDSLARYLVRISEMTESIKIIQQALEGIPGGPYENLEVRRLDRTKDSEWNDFEYRFISKKPS
PTFELSKQELYVRVEAPKGELGIFLIGDQSVFPWRWKIRPPGFINLQILPQLVKRMKLADIM
TILGSIDIIMGEVDRMFPMITGFMNYGQQTVRAARYIGQSFMITLSHTNRLPVTIQYPYEKL
LTSERFRGRIHFEFDKCIACEVCVRVCPIDLPVVDWKLETYIRKKRLLNYSIDFGICIFCGNC
VEYCPTNCLSMTEEYELSTYDRHELNYNQIALGRLPMSAIGDYTIRTIMNSKTSMDKPFDS
RTITNHMQGRLSAWLVKHGLVHRSLGFDYQGIETLQIKPEDWHSIAVISYVYGYNYLRSQ
CAYDVAPGGLLASVYHLTRIQYGVDQPEEICIKVFTPRKNPRIPSVFWIWKSADFQERESY
DMLGISYDNHPRLKRILMPESWIGWPLRKDYIAPNFYEIQDAHMGDEFRCVGCICIYRSFN
FRAYPNCWFSLCMAKRSIGMVLVPD---NNKKKEEKNYIEIVMNSIEFPLVDRTTQNSVISTT
SNDLSNWSRLSSLWPLLYGTSCCFIEFASLIGSRFDFDRYGLVPRSSPRQADLILTAGTVTM
KMAPSLVRLYEQMPEPKYVIAMGACTITGGMFSTDSYSTVRGVDKLIPVDVYLPGCPPKP
EAVIDAITKLRKKVSREIYEDRILYQQENRCFTTNHKFHVGHSTLTGNYDQGLLYQS---------
----------------MQTRNTFFWIKEQMTRSISVSIIVYVITQTSISNAYPIFAQQGYENPREATGRIV
CANCHLANKPLDIEVPQAVLPDTVFEAVVRIPYDMQLKQVLANGKKGALNVGAVLILPEG
FELAPPDRISPEMKEKIGNLSFQSYRPTKKNILVIGPVPGQKYSEITFPILSPDPATKKDVHFL
KYPIYVGGNRGRGQIYPDGSKSNNTVYNATAAGIISKIIRKEKGGYEITIADASDGHQVVNI
IPPGPELLVSEGESIKLDQPLTSNPNVGGFGQGDAEIVLQDPLRVQGLLFFFASVILAQIFLVL
KKKQFEKVQLSEMNFMSKVYDWFEERLEIQAIADDITSKYVPPHVNIFYCLGGITLTCFLV
QVATGFAMTFYYRPTVTEAFASVQYIMTEANFGWLIRSVHRWSASMMVLMMILHVFRVY
LTGGFKKPRELTWVTGVVLAVLTASFGVTGYSLPRDQIGYWAVKIVTGVPEAIPVIGAPLV
ELLRGSASVGQSTLTRFYSLHTFVLPLLTAVFMLMHFPMIRKQGISGPLMGVTKKPDLNDP
VLRAKLAKGMGHNYYGEPAWPNDLLYIFPVVILGTIACNVGLAVLEPSMIGEPADPFATPL
EILPEWYFFPVFQILRTVPNKLLGVLLMVSVPAGLLIVPFLENVNKFQNPFRRPVATTVFLIG
TTVALWLGIGATLPIDKSLTLGLFMIEVFLFGIVLGLIPITLAGLFVTAYLQYRRGDQLDLMP
TITSYFGFLLAASTITPALFIGLSKIRLIMDIVSLAWAALMVVFTFSLSLVVWGRSGLMIIRSP
EPEVKILVDRDPVKTSFEEWARPGHFSRTIAKGPDTTTWIWNLHADAHDFDSHTSDLEEIS
RKVFSAHFGQLSIIFLWLSGMYFHGARFSNYEAWLSDPTHIGPSAQVVWPIVGQEILNGDV
GGGFRGIQITSGFFQIWRASGITSELQLYCTAIGALVFAALMLFAGWFHYHKAAPKLAWFQ
DVESMLNHHLAGLLGLGSLSWAGHQVHVSLPINQFLDAGVDPKEIPLPHEFILNRDLLAQ
LYPSFAEGATPFFTLNWSKYAEFLTFRGGLDPVTGGLWLTDIAHHHLAIAILFLIAGHMYRT
NWGIGHGLKDILEAHKGPFTGQGHKGLYEILTTSWHAQLSLNLAMLGSLTIVVAHHMYS
MPPYPYLATDYGTQLSLFTHHMWIGGFLIVGAAAHAAIFMVRDYDPTTRYNDLLDRVLR
HRDAIISHLNWACIFLGFHSFGLYIHNDTMSALGRPQDMFSDTAIQLQPVFAQWIQNTHAL
APSVTAPGATTSTSVTWGGGDLVAVGGKVALLPIPLGTADFLVHHIHAFTIHVTVLILLKGV
LFARSSRLIPDKANLGFRFPCDGPGRGGTCQVSAWDHVFLGLFWMYNAISVVIFHFSWKM
QSDVWGSISDQGVVTHITGGNFAQSSITINGWLRDFLWAQASQVIQSYGSSLSAYGLFFLG
AHFVWAFSLMFLFSGRGYWQELIESIVWAHNKLKVAPATQPRALSIVQGRAVGVTHYLLG
GIATTWAFFLARIIAVGMALRFPRFSQGLAQDPTTRRIWFGIATAHDFESHDDITEERLYQNI
FASHFGQLAIIFLWTSGNLFHVAWQGNFESWVQDPLHVRPIAHAIWDPHFGQPAVEAFTRG
GALGPVNIAYSGVYQWWYTIGLRTNEDLYTGALFLLFLSSISLTAGWLHLQPKWKPSVSW

FKNAESRLNHHLSGLFGVSSLAWTGHLVHVAIPGSRGEYVRWNNFLDVLPHPQGLGPLFT
GQWNLYAQNPDSSSHLFGTSQGAGTAILTLLGGFHPQTQSLWLTDIAHHHLAIAFIFLVAGH
MYRTNFGIGHSMKDLLEAHIPPGGRLGRGHKGLYDTINNSIHFQLGLALASLGVITSLVAQ
HMYSLPAYAFIAQDFTTQAALYTHHQYIAGFIMTGAFAHGAIFFIRDYNPEQNEDNVLARM
LDHKEAIISHLSWASLFLGFHTLGLYVHNDVMLAFGTPEKQILIEPIFAQWIQSAHGKTSYG
FDVLLSSTNGPAFNAGRSIWLPGWLNAINE--NSNSLFLTIGPGDFLVHHAIALGLHTTTLIL
VKGALDARGSKLMPDKKDFGYSFPCDGPGRGGTCDISAWDAFYLAVFWMLNTIGWVTF
YWHWKHITLWQGNVSQFNESSTYLMGWLRDYLWLNSSQLINGYNPFGMNSLSVWAWM
FLFGHLVWATGFMFLISWRGYWQELIETLAWAHERTPLANLIRWRDKPVALSIVQARLVGL
AHFSVGYIFTYAAFLIASTSGKFGMSHSVKIYDTCIGCTQCVRACPTDVLEMIPWDGCKAK
QIASAPRTEDCVGCKRCESACPTDFLSVRVYLWHETTRSMGLAYMN--NLPSIFVPLVGLVF
PAIAMASLSLHVQKNKIVMRDLKTYLSVAPVLSTLWFGSLAGLLIEINRLFPDALTFPFFSF
MTAILERRESESLWGRFCNWITSTENRLYIGWFGVLMIPTLLTATSVFIIAFIAAPPVDIDGIR
EPVSGSLLYGNNIISGAIIPTSAAIGLHFYPIWEAASVDEWLYNGGPYELIVLHFLLGVACY
MGREWELSFRLGMRPWIAVAYSAPVAAATAVFLIYPIGQGSFSDGMPLGISGTFNFMIVFQ
AEHNILMHPFHMLGVAGVFGGSLFSAMHGSLVTSSLIRETTENESANEGYRFGQEEETYNI
VAAHGYFGRLIFQYASFNNSRSLHFFLAAWPVIGIWFTALGISTMAFNLNGFNFNQSVVDS
QGRVINTWADIINRANLGMEVMHERNAHNFPLDLAAVEAPSTNGMGLPWYRVHTVVLN
DPGRLLAVHIMHTALVAGWAGSMALYELAVFDPSDPVLDPMWRQGMFVIPFMTRLGITNS
WGGWSIT-GGTITNPGIWSYEGVAGAHIVFSGLCFLAAIWHWVYWDLEIFCDERTGKPSLD
LPKIFGIHLFLSGVACFGFGAFHVTGLYGPGIWVSDPYGLTGKVQSVNPAWGVEGFDPFVP
GGIASHHIAAGTLGILAGLFHLSVRPPQRLYKGLRMGNIETVLSSSIAAVFFAAFVVAGTM
WYGSATTPIELFGPTRYQWDQGYFQQEIYRRVGVSLAENKSLSEAWSKIPEKLAFYDYIGN
NPAKGGLFRAGSMDNGDGIAVGWLGHPIFRDKEGRELFVRRMPTFFETFPVVLVDGDGIV
RADVPFRRAESKYSVEQVGVTVEFYGGELNGVSYSDPATVKKYARRAQLGEIFELDRATL
KSDGVFRSSPRGWFTFGHASFALLFFFGHIWHGARTLFRDVFAGIDPDLDAQVEFGAFQK
LGDPTTRRQVVMKTLYSLRRFYPVETLFNGTLALAGRDQETTGFAWWAGNARLINLSGK
LLGAHVAHAGLIVFWAGAMNLFEVAHFVPEKPMYEQGLILLPHLATLGWGVGPGGEVID
TFPYFVSGVLHLISSAVLGFGGIYHALLGPETLEESFPFFGYVWKDRNKMTTILGIHLILLGI
GAFLLVLKALYFGGVYDTWAPGGGDVRKITNLTLSPSVIFGYLLKSPFGGEGWIVSVDDLE
DIIGGHVWLGSICIVGGIWHILTKPFAWARRALVWSGEAYLSYSLGALSVFGFIACCFVWF
NNTAYPSEFYGPTGPEASQAQAFTFLVRDQRLGANVGSAQGPTGLGKYLMRSPTGEVIFG
GETMRFWDLRAPWLEPLRGPNGLDLSRLKKDIQPWQERRSAEYMTHAPLGSLNSVGGVA
TEINAVNYVSPRSWLATSHFVLGFFLFVGHLWHAGRARAAAAGFEKGIDRDFEPVLSMTP
LNMTIALGRFTKDENDLFDIMDDWLRRDRFVFVGWSGLLLFPCAYFALGGWFTGTTFVT
SWYTHGLASSYLEGCNFLTAAVSTPANSLAHSLLLLWGPEAQGDFTRWCQLGGLWTFVAL
HGAFGLIGFMLRQFELARSVQLRPYNAIAFSAPIAVFVSVFLIYPLGQSGWFFAPSFGVAAIF
RFILFFQGFHNWTLNPFHMMGVAGVLGAALLCAIHGATVENTLFEDGDGANTFRAFNPT
QAEETYSMVTANRFWSQIFGVAFSNKRWLHFFMLFVPVTGLWMSALGVVGLALNLRAY
DFVSQEIRAAEDPEFETFYTKNILLNEGIRAWMAAQDQPHENLIFPEEVLPRGNALMSGST
GERSFADIITSIRYWVIHSITIPSLFIAGWLFVSTGLAYDVFGSPRPNEYFTESRQGIPLITGRF
DSLAQLDEFSRSFMTIDRTYPIFTVRWLAVHGLAVPTVFFLGSISAMQFIQRMATQIVEGSS
RSG-PRRTTVGELLKPLNSEYGKVAPGWGTTPLMGVAMALFAVFLSIILEIYNSSVLLDGIS
MGMLTLKLFVYTVVIFFVSLFIFGFLSNDPGRNPGREEMADTTGRIPLWLIGTVTGIPVIGLI

GIFFYGSYSGLGSSLMLNIFSLICIYLNSSLHSSSFFFSKLPEAYAFLNPIVDVMPVIPVLFFLL
ALVWQAAVSFRTTQSNPNEQNVELNRTSLYWGLLLIFVLAVLFSNYFFNMEVNILAFIATA
LFILVPTAFLLIIYVKTVSQNDMETATLVAIFISGSLVSFTGYALYTAFGQPSQQLRDPFEEHG
DMEALVYTFLLVSTLGILFFAIFFREPPKVPTKKMKMTIAFQLAVFALIATSSILLISVPVVFA
SPDGWSSNKNVVFSGTSLWIVLVFLVGILNSLIS-------MSPQTETKASVGFKAGVKDYKLT
YYTPEYQTKDTDILAAFRVTPQPGVPPEEAGAAVAAESSTGTWTTVWTDGLTSLDRYKGR
CYHIEPVAGEENQFIAYVAYPLDLFEEGSVTNMFTSIVGNVFGFKALRALRLEDLRVPPAYS
KTFQGPPHGIQVERDKLNKYGRPLLGCTIKPKLGLSAKNYGRAVYECLRGGLDFTKDDEN
VNSQPFMRWRDRFVFCAEAIYKAQAETGEIKGHYLNATAGTCEEMIKRAVFARELGVPIV
MHDYLTGGFTANTSLAHYCRDNGLLLHIHRAMHAVIDRQKNHGIHFRVLAKALRMSGGD
HIHAGTVVGKLEGERDITLGFVDLLRDDFIEKDRSRGIYFTQDWVSLPGVLPVASGGIHVW
HMPALTEIFGDDSVLQFGGGTLGHPWGNAPGAVANRVALEACVQARNEGRDLAREGNEII
REASKWSPELAAACEVWKEIKFEFPAMDTL----IHLYKTSTPSTRNGAVDSQVKSNPRNNLI
YGQHRCGKGRNARGIITAGHRGGGHKRLYRKIDFRRNEKNIYGRIVTIEYDPNRNAYICLI
HYGDGEKRYILHPRGAIIGDTIVSGTEVPIKMGNALPLTDMPLGTAIHNIEITLGKGGQLAR
AAGAVAKLIAKEGKSATLKLPSGEVRLISKNCSATVGQVGNVGANQKSLGRAGSKCWLG
KRPVVRGVVMNPVDHPHGGGEGRAPIGRKKPTTPWGYPALGRRSRKRNKYSDSLILRRR
SKMIQPQTYLNVADNSGARKLMCIRIVGAS-NRRYAHIGDVIVAVIKEAVPNMPLERSEVIR
AVIVRTCKELNRGNGMIIRYDDNAAVVIDQEGNPKGTRVFGAIARELRQLNFTKIVSLAPE
VLMLSPKRTRFRKQHRGRMKGISYRGNRICFGRYALQALEPTWITSRQIEAGRRAMTRYA
RRGGKIWVRIFPDKPVTVRPTETRMGSGKGSPEYWVSVVKPGRILYEMGGVSEIVAREAIS
IAASKMPIRTQFIIAGMTRIRRGYIARRRRTKIRLFASTFRGSHSRLTRTTTQQKIRALVSAHR
DRGKQKRYFRRLWITRINAVIRDN--VFYSYSRLIHDLYKKQLLLNRKILAQIAISNRNCLYM
ISNEIIK--------------EVYALGQHICMSAHKARRVIDQIRGRSYEEALMILELMPYRACYPIFK
LVYSAAANASHNMGFNEADSVISKAEVNDGTSVKKLKPRARGRSYLIKRPTCHITIVLKDI
YLDGGVWDKKMDGIKYAVFTDKSIRLLGNN-QYTSNVESGSTRTEIKHWVELFFGVKVIAI
NSHRLPGKGRRMGPIMGHTMHYRRMIITLQPGYSIPPLRKKRTMAVPKKRISMSKKRIRK
NIWKRRGYWAAVKAFSLAKSLSTGNSKSFFVRQRQIMAKGKDVRVRVILECTSCVRNGV
N--KESLGISRYITQKNRHNTPSRLELRKFCPYCYKHTIHGEIKKMKIRASVRKICEKCRLIR
RRGRIIVICSNPRHKQRQGMVREEVRVCTRTLQWKCVESRADNKRLYYGRFILSPLMKGQ
ADTIGIAMRRALLGEIEGTCITCAKSE--KIPHEYSNIVGIEESVHEILMNLKEIVLRSNLYGT
RDASICVRGPRHVTAQDIISPPSVEIIDTTQHIASLTEPIDLCIGLQIERNRGYNMKTPKNSQD
GRY--PIDAVFMPVRNANHSIHSYGNGNEKQEILFLEIWTNGSLTPKEALHEASRNLIDLFIPF
LHANEE--NFILKDNQ-NKVALPLFTFHDRLA-KQRKNKK--ISFQCIFIDQSELPPRTYNCLKR
SNIHTLLDLLNNSQEDLMKIEHFRIEDVKQILGILQKH--FAIDLPKNKMIRDGN-EEMSTIPG
FSQIQFEGFCRFIDQGLMEELYKFPKIEDTDQEIEFQLFVETYQLAEPLIKERDAVYESLTYS
SELYVPAGLIWKTSRNMQEQTIFIGNIPLMNSLGTSIVNGIYRIVINQILQSPGIYYRSELDHN
GTSVYTGTIISDWGGRSELEIDRKARIWARVSRKQKISILVLSSAMGSNLREILDNVCYPEIF
LSFPNDKEKKKIGSKENAILEFYQQFACVGGDPVFSESLCKELQKKFFQQRCELGRIGRRN
MNRRLNLDIPQNNTFLLPRDILAAADRLIGIKFGMGTLDD--MNHLKNKRIRSVADLLQDQ
FGLALVRLENAVRGTICGAIR-HKLI-PTPQNLVTSTP----LTTTYESFFGLHPLSQVLDRTNPL
TQIVHGRKSSYLGPGGLTGRTASFRIRDIHPSHYGRICPIDTSEGINVGLIGSLAIHARIGHW
G-SLESPFY---EISERSKK--VRMLYLSPSKDEYYMVAAGNSLSLNKGIQEEQVVPARYRQEF
LTIAWEQVHLRSIFPFQYFSIGASLIPFIEHNDANRALMSSNMQRQAVPLSRSEKCIVGTGLE

RQAALDSGVSAIAEHEGKIIYTDIDKIILSGNGDTLRIPLVMYQRSNKNTCMHQKPQVQRG
KCIKKGQILADGAATVGGELALGKNVLVAYMPWEGYNSEDAVLISECLVYRDIYTSFHIRK
YEIQTHVTSQG-PERITNEIPHLEAHLLRNLDKNGIVMLGSWVETGDILVGKLTPQTSKE-SS
YAPEDRLLRAILGIQVSTSKETCLKLPIGGRGRVIDVRWIQKKG-GSNYNPETIRVYISQKRE
IKVGDKVAGRHGNKGIISKILPRQDMPYLQNGTPVDMVFNPLGVPSRMNVGQIFECSLGL
AGGLLDRHYRIAPFDERYEQEASRKLVFSELYEASKQTANPWVFEPEYPGKSRIFDGRTGD
PFEQPVIIGKSYIMKLIHQVDDKIHGRSSGHYALVTQQPLRGRAKQGGQRVGEMEVWALE
GFGVAHILQEMLTYKSDHIRARQEVLGTTIIGGTIPNPEDTPESFRLLVRELRSLALELNHFL
VSEKNFQINRKAA-------MIDRYKHQQLRIGLVSPQQISAWANKILPNGEIVGEVTKPYTFHY
KTNKPERDGLFCERIFGPIKSGICACGNYRVIGDEKEDPKFCEQCGVEFVDSRIRRYQMGYI
KLACPVTHVWYLKRIPSYIANLLDKPLKELEGLVYCD--FSFARP-VAKKPTFLRLRGSFEYE
IQSRKYSIPLFFTTQGFDTFRNREISTGAGAIREQLADPDLRIITDYSLGEWKELGEE-GPTG
NEWEDRKIGRRKDFLVRRMELAKHFIRTKVEPERMVLCLLPVLPPELRPIIQIDGGKSMSS
DINELYRRVIYRNNTLIDLLTTSRSTPRELVMCQEKLVQEAVDTLLDNGIRGQPMRDGHNK
VYKSFSDVIEGKEGRFRETLLGKRVDYSGRSVIVVGPSLSLHRCGLPREIAIELFQTFVIRGL
IRQHVASNIGVAKSKIREKEPIVWEILQEVMQGHPVLLNRAPTLHRLGIQAFQPILVEGRAI
CLHPLVCKGFNADFDGDQMAVHVPLSLEAQAEARLLMFSHMNLLSPAIGDPISVPTQDML
IGLYVLTSGNRRGICANRYNPCNRGNRVNFQNERIDDNNYKYTKEKDPYFCSSYDAIGAY
RQKRINLDSPLWLRWRLDQR-IIAS--REVPIEVHYESLGTYHEIYVNYLIVKSVKKEILCIYI
RTTVGHIYFYRKIEEAVQGFFRACSYGT--------MEVLMAERADLVFHNKVIDGTAMKRLIS
RLIDHFGMAYTSHILDQVKTLGFQQATATSISLGIDDLLTTPSKRWLVQDAEQQSFILEKHH
HYGNVHAVEKLRQSIEIWYATSEYLRQEMNTNFRMTDPSNPVHIMSFSGARGNPSQVHQL
VGMRGLMSDPQGQMIDLPIQSNLREGLSLTEYIISCYGARKGVVDTAVRTSDAGYLTRRLV
EVVQHIVVRRTDCGTIRGIYVTSRNGMMT-ERIFIQTLIGRVLADDIYMGPRCIATRNQDVG
IELVNRFIT------FRAQPISIRTPFTCRSTSWICRLCYGRSPTHGDLVELGEAVGIIAGQSIGEPG
TQLTLRTFHTGGVFTGGTAEHVRAPFNGKIKFNEDLVHPTRTRHGHPAFLCYIDLYVTIESQ
DIIHNVNIPQKSFLLVQNNQYVESEQVIAEIRAETSTF-NFKERVRKHIYSDSEGE-MHWSTD
VYHAPEYTYGNVHLLPKTSHLWVLSGGPCR--SRIVPFSLHKDQDQMNVH--SLSAERRYIS
N--LSATNDQ-VRHKLFSSDPSG--GGSILDY-SGPNRIICNGHLNFIYPT-ILHENSD--LLAKRR
RNRFILPLQSNQEREKELMPCSGISIEIPIN--GIFRRNSILAYFDDPLYRRKSSGITKYGTIEVH
SIVKKED--LIEYRG--KEFRSKYMKVDRFFFIPEEVHILPGSSYIMVRNNSIIGVDTRITLNIRS
RVGGLVRVERKKKNIELKIFSGDIHFPGETDKISRHSGILIPPGTEKK--KSKESKK-KLKNWI
YVQR--ITPTKKK--YFVLVRPVVTYEIADGINLVTLFPQDLLQERDDVQLRVVNYILYENGK
PIRGISDTSIQLVRTCLVLNCDQYKN---GSSMEEIHASFVEVRTNGLIRYFLRMDLVKSPISYI
VKRNDPSSSGLIPD-NGSDRNNINPFYSIYYKAR-----IQQSLSQHQGTIRTLLNR---NKECQS
LIILSSSNCSR--MHPFNGLKYHN---VTKESDPLIPIKNWLGPLGT--VPKIANFYS--FYHLIT--
HNQIL-VNKYLILNN---LKQIFQVLK---YYLMDENGR--IYNPDRCRNIIFNPFNFNWYFLHH
DYCEETSTIIS-PGQFICENVCISKNGP---HLKSGQVLIVH-IDSLVIRSAKPYLATPGATVHGH
YGEILYEGDTLVTFIYEKSRSGDITQGLPKVEQVLEVRSIDSISMNLEKRVEGWNERIPRILG
IPWGFLIGAELTIAQSRISLVNKIQKVYRSQGVQIHNRHIEIIVRQITSKVLVSE---EGMSNVF
SPGELIGLLRAERTGRALEEAICYRAVLLGITRASLNTQSFISEASFQETARVLAKAALRGRI
DWLKGLKENVVLGGLIPVGTGFKVLVHRS--RQHNNIP--LETKKKNIFEGEMR--DILFHHR
KLLNYCIPKNFHDTPEQSFTGFNDS------MTRRYWNINLEEMMEAGVHFGHGTRKWNPR
MAPYISAKRKGIHITNLTRTARFLSEACDLLFDAASRGKQFLIVGTKNKAADLVASAAIRA

RCHYVNKKWLGGMSTNWSTTETRLHKFRDLRAEQKTARLARLPKRDAAMLKRQLSHL
QTYLGGIKYMTRLPDIVIIVDQQEEYTAIRECVTLGIPMICLIDTNCDPDLADISIPANDDAIA
SIRLILNKLVSAICEGRSS-YIRNRMGQKINPLGFRLGTTQGHHSLWFAQPKSYSEGLQEDQ
KIRDCIKNYVQKNMRISSG-AEGIARIEIQKRIDLIQVIIYMGFPKLLIEGRPR----GVEELQIN
VQKEFNSVNRKLNIAITRIAKPYGQPNILAEFIAGQLKNRVSFRKAMKKAIELTEQADTKGI
QIQIAGRIDGKEIARVEWIREGRVPLQTIRAKIYYCSYTVRTIYGVLGIKIWIFVDEEMSRYR
GPRFKKIRRLGALPGLTSKRPRAGSDLRNQSRSGK-RSQYRIRLEEKQKLRFHYGLTERQLL
KYVRIAGKAKGSTGQVLLQLLEMRLDNILFRLGMASTIPGARQLVNHRHILVNGRIVDIPS
YRCKPRDIITAKDEQKSRALIQNYLDSSPH-EELPKHLTLHSFQY--KGLVNQIIDSKWVGLKI
NELLVVEYYSRQTMSRRGTAEEKTAKSDPIYRNRLVNMLVNRILKHGKKSLAYQIIYRAVK
KIQQKTETNPLSVLRQAIRGVTPDIAVKARRVGGSTHQVPIEIGSTQGKALAIRWLLGASR
KRPGRNMAFKLSSELVDAAKGSGDAIRKKEETHRMAEANRAFAHFRMGRDTIANIITSIR
NADMDKKGTVRIASTNITENIVKILLREGFIENVRKHRESNKYFLVSTLRHRR--SRKGPYR-
--TIL--KRISRPGLRIYSNYQRIPRILGGMGIVILSTSRGIMTDREARLEGIGGEILCYIWMAK
PIPRIGSRKNGRIGSRKSGRRIPKGVIHVQASFNNTIVTVTDVRGRVVSWSSAGTCGFRGTR
RGTPFAAQTAAGNAIRTVMDQGMQRAEVMIKGPGLGRDAALRAIRRSGLLLSFVRDVTP
MPHNGCRPPKKRRVMPTIKQLIRNTRQPIRNVTKSPALRGCPQRRGTCTRVYTITPKKPNS
ALRKVARVRLTSGFEITAYIPGIGHNSQEHSVVLVRGGRVKDLPGVRYHIVRGTLDAVGVK
DRQQGRSKYGVKKPKMARKSLIQREKKRKKLEQKYHLIRRSSKKEIS---KVPSLSDKWEI
HGKLQSPPRNSAPTRLHRRCFSTGRPRANYRDFGLSGHILREMVHACLLPGATRSSWMVK
NSFISVISQE-K-KEENKGSVEFQVFNFNNKIRILTSHLELHRKDYSSQRGLRKILGKRQRLL
SYLSKKNRVRYKELIGQLGILEQKAR--MVKLRLKRCGRKQRAIYRIVAIDIRSRREGRDLR
KVGFYDPIKNKTYSNVPSILYFLEKGAQPTVTVHDISKKAEIFKELRINQTK---MDKSKRPF
LKSKRSFRRRLPPIGSGDRIDYRNMSLISRFISEQGKILSRRVNRLTLKQQRLITIAIKQARIL
SSLPFLNNEK--QFERTEFTARTTGSRTINLVARSLKKNPFVANHLLEKIEKLNMRAKKEIIVT
WSRASTIIPTMIGHTIAIHNGKEHLPIFITDRMVGHKLGEFAPTLNFREHARNDNKSRRMIL
KSFLLGNLLSLCMKIINSVVVVGLYYGFLTTFSIGPSYLFLLRARVMEEG---TEKKVSATTG
FITGQLMMFISIYYAPLHLALGRPHTITVLALPYLLFHFFWNNHKHFFDYGSTTRNSMRNL
SIQCVFLNNLIFQLFNHFILPSSTLARLVNIYMFRCNNKMLFVTSSFVGWLIGHILFMKWVG
LVLFWIRQNHSIKS-NVLIKYLVSELRNSMARIFSILLFITCVYYLGRMPAPIVTKKLKETEE
DKPLVTLLFDYKRWNRPLRYIKNDRFENAVRNEMSQYLFYTCKNDGKQRISFTYPPSLSTF
LEMIQRKIYLCTKEKLSSEEMYNHWIYTNEQKKNNLRNEFINRIEALDKEFVSLNVLEKIT
RLCNDETEQECSPKVYDPLLNGPYRGTITNLYSSTLCLCINKIHGILYREFEHK--------VKEIS
KRVPRWSYKLITLEQEEREEIRSRKAKRVVIFNDALIRYSQQSDFRRDIIKGSMRAQRRKIV
TWKLFQANVHSPLFLDRIDKTFFFSFDI--SGMMKIIFRNREEKTRILIAETWDSILCAQVIRG
SMLVIQSIIRKYIVLPSLIIAKNISRMLLFQFPEWSEDLKDWSREIHVKCTYNGVQLSETEFP
KNWLIDGIQIKILFPFCLKPWHRSKKRKKNDFCFLTVWGMEAELPFGSPRKRLSFFEPICKE
LEKKIRKVK-KNYFIVLKILKERTKLFIKVSKEKKGWVIKRVL--FIKRIIKELNKI---IISNQMI
HESSIRIRSMDWTNSLLTEKKMKDLTDRTNTIRKKIERIKRDKKKSPNERSCDNKKLESPK
NIWQILKRRNTRLIRKWNYFIKFLIERIY-DALPYIINIPRIHGRLFTHFIS-DLSSLSQAYVFYK
LSQTQVINKYNLRSVLQYASLFPKERIRYFCTRGIFNSQSKHKRLRNFGMNEWKNWLRDH
YQYDLSQIKWSRLVLQKWRNRVNQRRTVQNKKKLGSYEKDQ--LIHYETEENLKKNYRY
DLLSYNYIHDYIIDPDRKYFDWIILNFCLRFFDWMGMNEEILNRWFFQEFVLLYNAYKIKP
WVIPIKLLLLNCTSNTEAELNFLLKRYLLFQLRWDDPFNQRMINNIKVYCLLLRLINPKEISI

SSIQSGEMSPDIIQK--DLTITELIKKGLLIIEPVRLSIKRDGQFIMYQTISISLVHKNKQNHYEL
LVPENISSPRRRRELRIRICFRFLWPNYQLEDLACMNRYWFNTNNGSRFSMSRIHMYPMKR
HQFKSWIFELREILREIKNSHSFLDSWTKFDSVGSFTHIFFHQERFMNLFDPRIWSILLS-RD
S--QGSTSNRYFT---IKGVVLFVVT-VLIY----RINNRNMVERKNLYLMGLLPI---PIPMNSIGPR
NDTLEESF-GSSNINRLIVS-LLYLPKGKKISESCFLDPKESTWVLPIT----KKCIMPESNWGSR
WWRNWIGKKR--DSSCKIS---NETVAGIEISFKEKDIK-YLEFLFVYYTD--DPIRKDHDWELF
DRLSPRKKRNIINF--NSGQLFEILVKHWICYLMSAFREKRPIEVEG-FFKQQG-AESTIQSNDI
EHVSH--LFSRNKWGISLQNCAQFHMWQFRQDLFV---SWGK-NPHESDFLRK-VSRENWIW
LDNVWLVNKDR---FFS--KVRNVSSNIQYDS---TR--SIFVQVRDSSQLKGSS-DQSRDHFDSI
R----NEDSE-------------YHTLINQREIQQLKERSIL-WD--PSFLQTERTEIESD--RFPK--CLSGY
S--SMSRLFT-EREKQMNNHLLPEEIEEFL--GNPTRSIRSFFSDRW--SELH-LGSNPTERSTRD
Q----KLLKKQQDVS-FVPSRRSE----NKEMVDIFKIITYLQN--TVSIHPISSDPGCDMVP-KDE
PD-MDSSNKISFLNKNPFLD--LFHLFHDRNRGGYTL--HHDFESE--ERFQEIADLFTLSITEP-
DLVYHKGFALSIDSYGLDQKK--FLNEVFNSRDESKNKSLS--LADPPIFY--EENESFYRRIRK
KSVR-ISCGNDLEDPKQKIVVFASNNIMEAVNQYRLIRNLIQIQYSTYG--YIRNVSNRFF---L
MNRSDRNFEYGIQ---RDQIGNDTLNHRTIMKYMINQHLSNLKK-SQKKWFDP----LISRTER
SMNRDPDAYRYKWSNGSKNFQEH---FVSEQKS---RFQVVFDRLRINQYSIDWSEVIDKKD
LSKSLRFFLSKS---------LLFLS-------KSLLFLSK-SLPFFFVSFGNIPIHRSE-IHIY-ELKGPNDQ
LCNQLLESIGVQIVHLNKLKPF--------LLDTS-QKSKFLINGGTISPFLFNKIQ-KW-MIDSFHT
--RNNRRKSFDNTDSYFSMISHDRDNWLNPVKPFHRSSLISSFYKANRLRFLNNPHHFWFY-
-CNKRFPFYV----EKARINNYD--LTY-GQFLNILFIRNK-IFSLCVGK--KKHAFLE-RDTISPIES
QVSNIFIPNDFPQGGDET-YNLYKS---FHFPIRSDPFVR--RAIYSIADISGTPLTEGQIVNFERT
YCQPLSDMNISDSEGKNFHQYLN--FNSNMGLIHTPCSEKYLP--SEKRKN--------RSLCLK--
KCVEKRQMYRTFQRDSAFSNLSKWNLFQTYMPWFLTSTGCKYLNFTLLDTFSDLL--PILS
SS-KKFVSIFNDIMHGSDISWPIPQKKW------CLPQWNL-ISEISSKCLHN--LLLSEEMIHRNN
ESPVPLIWAHLRSPNAREF-LYSILFLLLVAGY-LVRTHLLFVSR--ASSELQTEFEKIKSLMIPS
YMIELRKLLDRYPTSELNSFWLKNLFLVALEQLGDSLEEIRGSASGGNMLLGGGP-----AYG
-VKSIRSKKKYLNIN---LIDLI-SIIPNPINRITFSRNTRHLSRTSKEIYSLIRKRKNVNGDWIDD
KIESWVANSDSIDDEEREFLVQFS--TLTT--EKRIDQILLSLT-HSDHLS-----KNDSGYQMIEQP
GSIYLRYLVDIH--KKYLMNYEFNRSCLAERRIFLAHYQTITY--SQTSCGANSFHFPSHG---K
PFSLRLALSPS-RGILVIGSIGTGRSYLVKYLATNSYVPFITLFPNKFLDDKPKGYLIDD---IDI
DDIDDSDDIY------------------------DSDDIDD-LDTELLTMTNAL---TMYMT---PKIDRFDITLQ
FELAKAMSPCIIWIPNIHDLYVNESNYLSLGLLVNYLS---RDCERCS-TRNILVIASTHTPQK
VDPALIAPNKLNTCIKIRRLLIPQQRKHFFTLSYTRGFRLEKKMFHT--NGFGSITMGSNARD
LVALTNEALSISITQKKSIIDTNTIRSALHRQTWDLR---SQVRSVRDHGILFYQIGRAVAQNV
LLSNCPIDPISIYMKKK--SCKEGDSYLYKWYFELGTSMKKLTILLYLLSCSAGSVAQDLWS
LP--GPDEK-NWITSYGLVENDS-DLVHGLLEVEGALVG--SSRTEKDCSQFDND--RVTLLLR
SEPRN-PLDMMQNGSCSIVDQRFLYEK--YESEFEE-GE--GALDPQ----QIEED----LFNH--IVW
AP--RIWRP---CGNLFDCIERPNELGFP-YWARSFR---GKQIIYHKEDELQENDSEFLQSGTM
QYQTRDRSSKEQGFFRISQFIWDPADPFFFLFK-DQPFVSVFSRREFFADEEMSKGLLTSQT
NPPTSIYKRWFI--KNTQEKHFELLIHRQRWLRT---NSSLS--NGSFRSN--TLSESYQYLSNLFL
SN-GTLLDQMTKTLLRKRWLFPDEMKIGF--M---------MSRSR-INGNFIDKTFSIVANILLRIIP
TTSGEKEAFTYYRDG--MSAQSEGNYAEALQNYYEATRLEIDPYDRSYILYNIGLIHTSNGE
HTKALEYYSRALERNPFLPQAFNNMAVICHY-RGEQAIRQGDSEIAEAWSDQAAEYWKQA

IALTPGNYIEAHNWLKITGRFE--MSWRSERIWIELIMGSRKTSNFCWAFILFLGSLGFLLVG
TSSYIGRNLISLFPSQQIIFFPQGIVMSFYGIAGLFISSYLWCTISWNVGGGYDRFDRKEGRV
CIFRWGFPGINRRIFLRFLMRDIQSIRIEVKEGLYPRRVLYMEVRGQGAIPLTRTDEN-FTPRE
IEQKAAELAYFLRVPIEVF
>Illicium
MEKWWFNSMLSLSKSADSLGPIGNASGSETPIINGIDKNIH-CSNVDHLFGVRDIWSFISDD
TFLVRDSNGDSYSVYFDIENQIFEIHND--SYFLSELESAFSSYLNKSYLNSGPKSYNRNYDR
YMYDTQYSWNNHINSCIDSYLHSEIS--I------------DSDIYSYICSDSESSIRTS-NG----------DF
DINTKYGRLWVQCENCYGLNYQKFFRSKMNICERCGYYLKMSSSDRIELSIDPGTWDPM
DEDMISMDPIEFHSEEEPYRDRIDFFQRTTGLTEAVQTGIGQLNGIPIAIGVMDFQFMGGSM
GSVVGEKITRLIECATNRSLPVIIVCASGGARMQEGSLSLMQMAKISSALYNYQSNKKLFY
VSILTSPTTGGVTASFGMLGDIIIAEPNAYIAFAGKRVIEQTLKKTIPEGSQAAEYLFHKGLF
DLIVPRNPLKGVPSELFQLHGFFPLN----QMVTIRADEISNIIRERIEQYNREVKIVNTGTVLQ
VGDGIARIHGLDEVMAGELVEFEEGTIGIAPNSESNNVGVVLMGDGLMIQEGSSVKATGRI
AQIPVSEAYLGRVINALAKPIDGRGEISASESRLIESPAPGIISRRSVYEPLQTGLIAIDSMIPIG
RGQRELIIGDRQTGKTAVATDTILNQKGQNVICVYVAIGQKASSVAQVVTTFQERGAMEYT
IVVAETADSPATLQYLAPYTGAALAEYFMYRERHTLIIYDDPSKQAQAYRQMSLLLRRPPG
REAYPGDVFYLHSRLLERAAKSSSRLGEGSMTALPIVETQSGDVSAYIPTNVISITDGQIFLS
ADLFNAGIRPAINVGISVSRVGSAAQIKAMKQVAGKSKLELAQFAELEAFAQFASDLDKAT
QNQLARGQRLRELLKQSQSAPFTVEEQIVTIYTGANGYLDLLEIGQVKKFLVQLRTHLRTN
KPQFQEIICSTRTFTEQAEALLKEAIQEQIELFLLQEQAMRINPTTSGPGVSTLEERNLGRIT
QIIGPVLDVAFPPGKMPNIYNSLVVKGRDTVGQQINVTCEVQQLLGNNRIRAVAMSATDGL
MRGMEVIDTGAPLSVPVGGSTLGRIFNVLGEPVDNLGPVDTCTTSPIHRSAPAFIQLDTKFS
IFETGIKVVDLLAPYRRGGKIGLFGGAGVGKTVLIMELINNIAKAHGGVSVSGGVGERTRE
GNDLYMEMKESGVINEQNIAESKVALVYGQMNEPPGARMRVGLTALTMAEYFRDVNEQ
DVLLFIDNISRFVQAGSEVSALLGRMPSAVGYQPTLSTEMGSLQERITSTKEGSITSIQAVY
VPADDLTDPAPATTFAHLDATTVLSRGLAAKGIYPAVDPLGSTSTMLQPRIVGEKHYETAQR
VKQTSQRYKELQDIIAILGLDELSEEDRLTVARARKIERFLSQPFFVAEVFTGSPGKYVGLA
ETIRGFQLILSGELDGLPEQAFYLVGNIDEATAKAMNLEVESKLKKMTLNLCVLTPNRIIW
DSEVKEIILSTNSGQIGVLPNHSPIATAVDIGILRIRLNDQWLTMALMGGFARIGNNEITILVN
DAEKGSDIDPREAQRTLEIAEANLSRAEGKRQAIEANLALRRARTRVEAINAISMKNVNDS
FVSLGHWPFAGSFGFNTDILATNPINLSVVLGVLIFFGKGVLNDLLDNRKQRILSTIRNSEE
LRGGAIEQLEKAWARLRKVEMEADEFRVNGYSEIEREKMNLIIATNENLERLENYKNETIH
FEQQRAINQVRQRVFQQALQGALGTLKSCLNSELHLRTISDNIGTLGNMKEITDMNPLISA
ASVIAAGLAVGLASIGPGVGQGTAAGQAVEGIARQPEAEGKIRGTLLLSLAFMEALTIYGL
VVALALLFANPFVMNVLPCSINTLKGLYEISGVEVGQHFYWQIGGFQVHAQVLITSWVVI
AILLGSAAIAVRNPQTIPTDGQNFFEYVLEFIRDLSKTQIGEEYGPWVPFIGTMFLFIFVSNW
SGALLPWKIIQLPHGELAAPTNDINTTVALALPTSVAYFYAGLTKKGLGYFGKYIQPTPILLP
INILEDFTKPLSLSFRLFGNILADELVVVVLVSLVPSVVPIPVMFLGLFTSSIQALIFATLAAA
YIGESMEGHHMIFVILEHILTHISFSIIAIVITTHLMTLLVHEIVGLCDSSEKGMIATFFCITGL
LVTRWIYSGHFPLNDLYESLMFLSWSFSLILMVPYFKNHKNHFSTITAPSAICTQSFATSGLS
TEIHQSEILVPALQSHWLMMHVSMMLLSYAALLCGSLLSIALLVITFRKNIDMIGFTNEIKY
LNEKRSLLKNTSFLLFRNYHRYQLTQRLDHWSYRVIGLGFTFSTIGILSGAVWANEAWGSY
WNWDPKETWAFITWTISAIYSHTRTNKSLQGMNSAIVASMGFLIIWICYFGVNLLGIGLHS

YGSFT--VPKK-ALTPFPYLASIVFLPWGISLSFNKSLESWVINWWNTRQSETFLNDIQEKKV
LERFIELEELFLLDEMIKEYSGTHIQKLRIGIYKETIQLVRMHNQDHIHLILHFSTNIICFTILS
AYSILGNEELVILNSWVQEFLYNLSDTIKAFSILLLTDLCIGFHSPHGWELMIGLVYQNFGFA
HNEQVISGLVSTFPVIIDTILKYWIFLFLNRVSPSLVVIYHSMNEMPIGVPKVPFRNPGEEGA
VWVDVYNRLHRERLLFLGQGVDSEISNQLVGLMVYLSIEDDTRDLYLFINSPGGWVIPGIA
IYDTMQFVPPDVHTICMGLAASMGSFLLAGGEITKRLAFPHARVMIHQPASSFYEAQTGEF
ILEAEELLKLRETITRVYAQRTGNPLWVVFEDMERDVFMSATEAQAHGIVDLVAVEMKEQ
KWIHEGLITESLPNGMFRVRLDNEDLILGYVSGRIRRSFIRILPGDRVKIEVSRYDSTRGRIIY
RLRNKDSNDMEELQGYLGIDRSCQQRFLYPLLFQESIYALAHDYGLLGYDN-PSLLIVKRLI
IRMHQQNLFFILVNGSNQNRFVGRSFYSQMISEGFAGIVEIPLSMRLASSLIAKSH-LRSIHSI
FPFLEDKFSHLNHVSDILIPHPVHLEILVQTLRRWIQDAPSLHLLRFFLHGHPNWNSLITKSI
SLFSKPRFFLFLYNSYVYEYESILVFLHKQSSHLRSISSGAFLERTNFYGKTEH-LVVLRNQK
WLWLFKDPFMHYARYQGKAIMASKGTHLLMKKWKYHLVNFWQLHFYLWSQSGRIRINE
LSNHFFYFPGYLSGIRLNPSVVRGQMIENSFMIDTGI--KKFDMIVPIIPLIGSLVKAKFCNISG
YPISKSVRADSSNSDIINRFGRICRNLSHYHSGSSKKHNLYRIKYILRLSCAKTLARKHKST
VRTILKRLGSELLEEFLTEKEEILSLISPRGIWYLDIIHIHGMSNHSMIIDTT-EVQ-AINSFSRS
E-SSKEFYGLIWLLVPIFTPVSGILIGVLVIVWLEREISAGIQQRIGPEYAGPLGILQALADGT
KLLFKEDLLPSRGDIRLFSVGPSIAVISILLSYSVIPFGYRLIIADISIGVFLWIAISSIAPIGLLM
SGYGSNNKYSFSGGLRAAAQSISYEIPLTPCVLSISLLSNSSSTVDIVEAQSKYGFCGWNLW
RQPIGFIVFLISSLAECERLPFDLPEAEEELVAGYQTEYSGIKSGLFYVASYLNLLVSSLFVTV
LYLGGWNLSIPYISIPELFGINKTGGVFGSTIGILITLAKAYLFLFVPITTRWTLPRMRMDQL
LNLGWKFLLPIALGNLLLTTSSQLLSF------------------MKAFHLLLFHGSFIFPECILIFGLILLL
MIDSTSDQKDTPWLYFISSTSLVMSITALLFRWREEPMISFSGNFQTNNFNEVFQFLILLCST
LCIPLSVEYIECTEMAITEFLLFVLTATLGGMFLCGANDLITIFVAPECFSLCSYLLSGYTKR
DVRSNEATTKYLLMGGASSSILVHGFSWLYGSSGGEIELQEILNGLINTQMYNSPGISIALIS
ITVGIGFKLSPAPSHQWTPDVYEGSPTPVVAFLSVTSKVAASASATRIFDIPFYFSSNEWHLL
LEILAILSMILGNLIAITQTSMKRMLAYSSIGQIGYVIIGIIVGDSNDGYASMITYMLFYISMN
LGTFARIVSFGPRTGTDNIRDYAGLYTKDPFLALSSALCLLSLGGIPPLAGFFGKLHLFWCG
WQAGLYFLVSIGLLTSVVSIYYYLKIIKLLMTGRNKEITPHVRNYRRSPLRSNNSIELSMIVC
VIASTIPGISMNPIIAIAQDTLFMFLLHEYDIFWAFLMISSVIPILAFLISGVLAPIREGPEKLSS
YESGIEPMGDAWLQFRIRYYMFALVFVVFDVETVFLYPWAMSFDVLGVSAFIEALIFVLIPI
VGSVYAWRKGALEWSTSHFPWLTIIVVLPISAGSSIGFLPYRGNKAVRWYTICICILELLLTT
YAFCYHFQLDDPLIQLEEDYNWINLFDFNWRLGIDGLSMGPVLLTGFITTLATLAAWPVTR
DSRLFHFLMLAMYSGQIGSFSSRDLLLFFMMWELELIPIYLLLSLWGGKKRLYSATKFILYT
AGGSIFLLMGVSGMGLYSSNEPTLNFETLTNQSYPVALEIIFYIGFFIAYAAKSPIIPLHTWLP
DTHGEAHYSTCMLLAGILLKMGAYGLVRINMELLPHAHSIFSPWLMIIGAIQIIYAASTSPG
QRNLKKRIAYSSVSHMGFITIGIGSITDTGLNGSILQIISHGFIGAALFFLAGTTYDRIRLVYL
DEMGGIAILMPKMFMMFSSFSMASLALPGMSGFVAESVVFLGIITSPKYFLMPKILITFVM
AIGMILTPIYSLSMSRQIFYGYKLFNVSNSYFVDSGPRELFILICILLPVIGIGIYPDFVLSLSV
DKVEAILSSYFHRMMLEHVLVLSAYLFSIGIYGLITSRNMVRALMCLELILNAVNINLVTFS
DLFDSRQLKGDIFSIFVIAIAAAEAAIGPAIVSSIYRNRKSTRINQSNLLNKMERTYDYAWIIP
FIPLTVPMSIGLGLLLVPTATKSILRMWAFFSVSLLSIVMVFSADLSIQQINCSFIYQYLWSW
TINSDFSLELGCLIDPLTSIMLILITTVGIMVLIYSDNYMSHDQGYLRFFASMSFSNTSMLGL
VTSSNLIQIYIFWELVGMCSYLLIGFWFTRPIAANACQKAFVTNRVGDFGLLLGILGLYWIT

GSFEFRDLFKIFNNNGVNSLFVTFFASLLFLGAVAKSAQFPLHIWLPDAMEGPTPISALIHAA
TMVVAGIFLVARLLPLFTVIPYIMNLISLIGVITLLLGATFALAQRDIKKSLAYSTMSQLGYI
MLAPGIGSYRAASFHLITHAYSKALLFLGSGSIIHSMEPIVGYSPDKSQNMILMGGLTKYLP
ITKITFLLGTLSLCGIPPLACFWSKDEILNDSWLYSPIFAIIACFTTGLTAFYMFRMYLLTFEE
HLRVHFQKYSGATNSSFYSISIWGKEASQLLNS-GLLLSTMNVSFKKK--TPMYPHESDNTM
LLPLLVLVIFTLFVGFIGVRFDLDILSKWLTPSINFLHRNLNDPWDWYEFATNAIFSVSIACF
GIWIASLLYGSVYSSFQNLDLMNSFVILLEPIINVIYNWSYNRGYIDVYYATVFTRGIRGLA
KLTHSFDRRVIDGITNGFGIASFFVGEGVKY-VGGGRISSYLFLYLSYVSIFLVISYMDLPGPI
HDIFLVFLGSGLILGGLGVVLLTNPVYSAFSLGLVLVCISLFHIPSNSYFVAAAQLLIYVGAI
NVLIVFAVMFMNGSEYYNYFHLWTVGDGVTSLICTSILFSLIKTILDTSWYGIIWTTRSNQII
EQDLISNVQQIGIHLSTDFYLPFELISIILLVALVGAIAMARQMTVPATRKDLMIVNMGPHHP
SMHGVLRLIVTLDGEDVIDCEPILGYLHRGMEKIAENRTIIQYLPYVTRWDYLATMFTEAI
TVNAPEELGNIQIPKRASYIRVIMLELSRIASHLLWLGPFMADIGAQTPFFYISRERELLYDL
FEAVTGMRMMHNYFRIGGVAADLPHGWIDKCLDFCDYSLTGVAEYQKLITRNPIFLERVE
GVGIIGGEEAINWGLSGPMLRASGIQWDLRKVDHYECYDELDWEVQWQKEGDSLARYL
VRIGEMTESIKIIQQALEGIPGGPYENLEVRRFDRARDSEWNYFEYRFISKKPSPTFELSKQE
LYVRVEAPKGELGIFLIGDNSVFPWRWKIRSPGFINLQILPQLVKRMKLADIMTILGSIDIIM
GEVDRMFPMLNGFMNYGQQTVRAARYIGQSFMITLSHTNRLPVTIQYPYEKSITSERFRG
RIHFEFDKCIACEVCVRVCPIDLPVVDWRLETDIRKKRLLNYSIDFGICIFCGNCVEYCPTN
CLSMTEEYELSTYDRHELNYNQISLGRLPMSVIGDYTIQTIMNSKIAMDKSLDSRTITNYM
QGRLSAWLVKHELVHRSLGFDYQGIETLQIKPEDWHSIAVISYVVYGYNYLRSQCAYDVAP
GGLLASVYHLTRIQYGVDQPEEVCIKVFAKRRNPRIPSVFWIWKSADFQERESYDMLGISY
DNHPRLKRILMPESWIGWPLRKDYIAPNFYEIQDAH--
------------MNSMRFSLLDRTTQNSVISTTSNDLSNWSRLSSLWPLLYGTSCCFIEFASLIGSRF
DFDRYGLVPRSSPRQADIILTAGTVTMKMAPSLVRLYEQMPEPKYVIAMGACTITGGMFST
DSYSTVRGVDKLIPVDVYLPGCPPKPEAIIDAITKLRKKLSREIYEDRIGCQQENRCFTTNH
KFRVGRSTHTGNYDQG---LYQSTSEIPFESFLKYKSSVSSHELVNMTRSIS---IS---VLIISVLI
MIYVITRTSISNAYPIFAQQGYENPREATGRIVCANCHLANKPVDIEVPQAVLPNTVFEAVV
RIPYDMQLKQVLANGKRGGLNVGAVLILPEGFELAPPDRISPEMKEKMGNLSFQNYRPTK
RNILVIGPVPGQKYSEIVFPILSPDPATKKEVHFLKYPIYVGGNRGRGQIYPDGSKSNNTVY
NATGAGLVSKIVRKEKGGYEITIADASDGHQAVDIIPPGPELLVSEGEYIKLDQPLTSNPNV
GGFGQGDAEIVLQDPLRVQGLLFFLASVILAQIFLVLKKKQFEKVQLAEMNFMSKVYDWF
EERLEIQAIADDITSKYVPPHVNIFHCLGGITLTCFLVQVATGFAMTFYYRPTVTEAFASVQ
YIMTEANFGWLIRSVHRWSASMMVLMMILHVFRVYLTGGFKKPRELTWVTGVVLAVLTA
SFGVTGYSLPRDQIGYWAVKIVTGVPEAIPVIGSPLVELLRGSASVGQSTLTRFYSLHTFVLP
LLTAVFMSMHFPMIRKQGISGPLMGVTKKPDLNDPVLRAKSAKGMGHNYYGEPAWPNDL
LYIFPVVILGTIACNVGLAVLDPSMIGEPADPFATPLEILPEWYFFPVFQILRTVPNKLLGVLL
MVSVPTGLLTVPFLENVNKFQNPFRRPVATTVFLLGTAVALWLGIGATLPIDKSLTLGLFMI
EVFLFGIVLGLIPITLAGLFVTAYLQYRRGDQLDLMPTITSYFGFLLAASTITPALLIGLSKIR
LIMDIVSLAWAALMVVFTFSLSLVVWGRSGLMIIRSPEPEVKIVVDRDPVKTSFEEWARPG
HFSRTIAKGPDTTTWIWNLHADAHDFDSHTNDLEEISRKVFSAHFGQLSIIFLWLSGMYFH
GARFSNYEAWLSDPTHIGPSAQVVWPIVGQEILNGDVGGGFRGIQITSGFFQIWRASGITSE
LQLYCTAIGALVFAALMLFAGWFHYHKAAPKLAWFQDVESMLNHHLAGLLGLGSLSWA
GHQVHVSLPINQFLDAGVDPKEIPLPHEFILNRDLLAQLYPSFAEGSTPFFTLNWSKYAEFL

SFRGGLDPVTGGLWLTDIAHHHLAIAILFLIAGHMYRTNWGIGHGLKDILEAHKGPFTGKG
HKGLYEILTTSWHAQLALNLAMLGSSTIVVAHHMYSMPPYPYLAIDYGTQLSLFTHHMWI
GGFLIVGAAAHAAIFMVRDYDPTTRYNDLLDRVLRHRDAIISHLNWACIFLGFHSFGLYIH
NDTMSALGRPQDMFSDTAIQLQPIFAQWVQNTHALAPVATAPGATTSTSLTWGGGGLVAV
GGKVALLPIPLGTADFLVHHIHAFTIHVTVLILLKGVLFARSSRLIPDKANLGFRFPCDGPGR
GGTCQVSAWDHVFLGLFWMYNAISVVIFHFSWKMQSDVWGSISDQGVVTHITGGNFAQS
SITINGWLRDFLWAQASQVIQSYGSSLSAYGLFFLGAHFVWAFSLMFLFSGRGYWQELIESI
VWAHNKLKVAPATQPRALSIVQGRAVGVTHYLLGGIATTWAFFLARIIAVGMALRFPRFSQ
GLAQDPTTRRIWFGIATAHDFESHDDITEERLYQNIFASHFGQLAIIFLWTSGNLFHVAWQG
NFESWVQDPLHVRPIAHAIWDPHFGQPAVEAFTRGGALGPVNIAYSGVYQWWYTIGLRT
NEDLYTGALFLLFLSAISLIAGWLHLQPKWKPSVSWFKNAESRLNHHLSGLFGVSSLAWT
GHLVHVAIPGSRGEYVRWNNFLDVLPYPQGLGPLFTGQWNLYAQNPDSSSHLFGTSQGAG
TAILTLLGGFHPQTQSLWLTDIAHHHLAIAFVFLVAGHMYRTNFGIGHSMKDLLEAHIPPGG
RLGRGHKGLYDTINNSIHFQLGLALASLGVITSLVAQHMYSLPAYAFIAQDFTTQAALYTH
HQYIAGFIMTGAFAHGAIFFIRDYNPEQNEDNVLARMLDHKEAIKSHLSWASLFLGFHTLG
LYVHNDVMLAFGTPEKQILIEPIFAQWIQSAHGKTSYGFDVLLSSTNGPAFNAGRSIWLPG
WLNAINE--NGNSLFLTIGPGDFLVHHAIALGLHTTTLILVKGALDARGSKLMPDKKDFGYS
FPCDGPGRGGTCDISAWDAFYLAVFWMLNTIGWVTFYWHWKHITLWQGNVSQFNESST
YLMGWLRDYLWLNSSQLINGYNPFGTNSLSVWAWMFLFGHLVWATGFMFLISWRGYWQ
ELIETLAWAHERTPLANLIRWRDKPVALSIVQARLVGLAHFSVGYIFTYAAFLIASTSGKFG
MSHSVKIYDTCIGCTQCVRACPTDVLEMIPWDGCKAKQIAPAPRTEDCVGCKRCESACPT
DFLSVRVYLWYETTRSMGLAYMTDLNLPSIFVPLVGLLFPAIAMASLSLHVQKNKIVMRNI
KTYLSVAPVLATLWFGSLAGLLIEINRLFPDALAFPFFSFMTVILERRESTSLWGRFCNWITS
TENRLYIGWFGVLMIPTLLTATSVFIIAFIAAPPVDIDGIREPVSGSLLYGNNIISGAIIPTSAAI
GLHFYPIWEAASVDEWLYNGGPYELIVLHFLLGVACYMGREWELSFRLGMRPWIAVAYS
APVAAATAVFLIYPIGQGSFSDGMPLGISGTFNFMIVFQAEHNILMHPFHMLGVAGVFGGSL
FSAMHGSLVTSSLIRETTENESANAGYRFGQEEETYNIVAAHGYFGRLIFQYASFNNSRSLH
FFLAAWPVVGIWFTALGISTMAFNLNGFNFNQSVVDSQGRVINTWADIINRANLGMEVMH
ERNAHNFPLDLAAVEAPSINGMGLPWYRVHTVVLNDPGRLLSVHIMHTALVSGWAGSMA
LYELAVFDPSDPVLDPMWRQGMFVIPFMTRLGITNSWGGWSIT-GGTITNPGIWSYEGVAG
AHIVFSGLCFLAAIWHWVYWDLEIFCDERTGKPSLDLPKIFGIHLFLSGVACFGFGAFHVT
GLYGPGIWVSDPYGLTGKVQSVNPAWGAEGFDPFVPGGIASHHIAAGTLGILAGLFHLSVR
PPQRLYKGLRMGNIETVLSSSIAAVFFAAFVVAGTMWYGSATTPIELFGPTRYQWDQGYFQ
QEIYRRVGTGLAENLSLSEAWSKIPDKLAFYDYIGNNPAKGGLFRAGSMDNGDGIAVGWL
GHPIFRDKDGHELFVRRMPTFFETFPVVLVDGDGIVRADVPFRRAESKYSVEQVGVTVEF
YGGELNGVSYGDPATVKKYARRAQLGEIFELDRATLKSDGVFRSSPRGWFTFGHASFALL
FFFGHIWHGARTLFRDVFAGIDPDLDAQVEFGAFQKLGDPTTRRQVVMKTLYSLRRFYPV
ETLFNGTLALAGRDQETTGFAWWAGNARLINLSGKLLGAHVAHAGLIVFWAGAMNLFEV
AHFVPEKPMYEQGLILLPHLATLGWGVGPGGEVIDTFPYFVSGVLHLISSAVLGFGGIYHA
LLGPETLEESFPFFGYVWKDRNKMTTILGIHLILLGIGAFLLVLKALYFGGVYDTWAPGGG
DVRRITNLTLSPSVIFGYLLKSPFGGEGWIVSVDDLEDIIGGHVWVGAICILGGTWHILTKPF
AWARRALVWSGEAYLSYSLGALSVFGFIACCFVWFNNTAYPSEFYGPTGPEASQAQAFTF
LVRDQRLGANVGSAQGPTGLGKYLMRSPTGEVIFGGETMRFWDLRAPWLEPLRGPNGLD
LSRLKKDIQPWQERRSAEYMTHAPLGSLNSVGGVATEINAVNYVSPRSWLATSHFVLGFF

LFVGHLWHAGRARAAAAGFEKGIDRDFEPVLSMTPLNMTIALGRFTKEE-DLFDIMDDWL
RRDRFVFVGWSGLLLFPCAYFALGGWFTGTTFVTSWYTHGLASSYLEGCNFLTAAVSTPA
NSLAHSLLLLWGPEAQGDFTRWCQLGGLWTFVALHGAFGLIGFMLRQFELARSVQLRPY
NAIAFSAPIAVFVSVFLIYPLGQSGWFFAPSFGVAAIFRFILFFQGFHNWTLNPFHMMGVAG
VLGAALLCAIHGATVENTLFEDGDGANTFRAFNPTQAEETYSMVTANRFWSQIFGVAFSN
KRWLHFFMLFVPVTGLWMSALGVVGLALNLRAYDFVSQEIRAAEDPEFETFYTKNILLNE
GIRAWMAAQDQPHENLIFPEEVLPRGNALMSGSTGERSFADIITSIRYWVIHSITIPSLFIAG
WLFVSTGLAYDVFGSPRPNEYFTENRQGIPLITGRFDPLEQLDEFSRSFMTIDRTYPIFTVR
WLAVHGLAVPTVFFLGSISAMQFIQRMATQTIEGSSSRSGPRRTIVGDLLKPLNSEYGKVAP
GWGTTPFMGVAMALFAIFLSIILEIYNSSVLLDGISVSMLTLKLFVYTVVIFFVSLFIFGFLSN
DPGRNPGREEMADTTGRIPLWLIGTVTGIPVIGSMGIFFYGSYSGLGSSLMPNIFSLICICLN
YALHSSSFFFAKLPEAYAFFNPIVDVMPVIPVLFFLLALVWQAAVSFRTTQSNPNEQNVELN
RTSLYWGLLLIFVLAVLFSNYFFNMEVNILAFIATALFILVPTAFLLIIYVKTVSQNDMETATL
VAISISGSLVSFTGYALYTAFGQPSQQLRDPFEEHGDMEALVYTFLLVSTLGIIFFAIFFREPP
KVPNKKMKMIIAFQLAVFALIATSSILLISVPVVFASSDGWSNNKNVVFSGTSLWIGLVFLV
AILNSLIS-------MSPKTETKASVGFKAGVKDYRLTYYTPEYETKETDILAAFRVTPQPGVPP
EEAGAAVAAESSTGTWTTVWTDGLTSLDRYKGRCYHIEPVAGEENQYIAYVAYPLDLFEE
GSVTNMFTSIVGNVFGFKALRALRLEDLRIPPAYSKTFQGPPHGIQVERDKLNKYGRPLLG
CTIKPKLGLSAKNYGRAVYECLRGGLDFTKDDENVNSQPFMRWRDRFVFCAEAIYKAQA
ETGEIKGHYLNATAGTCEEMIKRAVFARELGVPIVMHDYLTGGFTANTSLAHYCRDNGLL
LHIHRAMHAVIDRQRNHGMHFRVLAKALRMSGGDHIHAGTVVGKLEGERDVTLGFVDL
LRDDFIEKDRSRGIYFTQDWVSMPGVLPVASGGIHVWHMPALTEIFGDDSVLQFGGGTLG
HPWGNAPGAVANRVALEACVQARNEGRDLAREGNEVIREASKWSPELAAACEVWREIKF
EFEAMDVL--MAIHLYKTSTPSTRNGAVDSQAKSTPQKSLIYGQHHCGKGRNARGVITTGH
RGGGHKRLYRKINFRRNEKDISGRIVTIEYDPNRNAYICLIHYGDGEKRYILHPRGARIGDT
IVSGTEVPISMGNALPLTDMSLGTAIHNIEITLGKGGQLARAAGTVAKLIAKEGKSATLRLP
SGEVRLVSKNCSATVGQVGNVGVNQKSLGRAGSKCWLGKRPVVRGVVMNPVDHPHGG
GEGRAPIGRKRPTTPWGYPALGRRSRKRNKYSDSFILRRRK-MIQPQTHLNVADNSGAREL
MCIRIIGAS-NHRYAHIGDVIVAVIKEAVPNMPLERSEVIRAVIVRTCKELKRDNGMIIRYDD
NAAVVIDQEGNPKGTRVFGAIARELRQLNFTKIVSLAPEVLMLSPKRTRFRKQHRGRMKG
VSYRGNHICFGRYALQALEPAWITSRQIEAGRRAMTRYARRGGRIWVRIFPDKPVTVRPTE
TRMGSGKGSPEYWVSVVKPGRILYEMGGVSETVARSAISIAASKMPIRTQFVIAGMIRVRR
GYIARRRRTKFFLFASTFRGAHSRLTRTTTQQKMRALVSAHRDRGRQKRDFRRLWITRINA
VTREN--G--SYSRLIHDLYKGQLLLNRKIPAQLAISNGNCLYMISNEIGSANKEIGRSSSEMI
KEVKALAQYICMSAHKARRVIDQIRGRSYEQTLMILELMPYRASYHIFKLVYSAAANASH
NKGLNEADSFISKAEVNGGVIVKKCKPRARGRSYPIKRPTCHITIVLNKRSN--------MDGIK
YAVFTEKSLRLLMVNNQYTSNVESGSTRTEIKHWVELFFGVKVIAINSHRLPRKGKRMGPI
MGHTMHYRRMIITLQPGYSIPPVIRT--MAVPKKRTSMSKKRIRRNIWRRKGYWTAVKAFS
LAKSVSTGHSKSFF------MAAGKDVRVTVILECTSCAKNG--K--KSPGVSRYITEKNRHNTP
GRLELIKFCPYCYKHTIHGEIKKMKIRASVRKICEKCRLIRRRGRIIVICPNPRHKQRQGMV
REEIAVSTRTLQWKCVESRADSKRLYYGRFVLSPLMKGQADTIGIAMRRALLGEIEGTCIT
RAKSE--KVPHEYSTIVGIEESVHEILMNLKEIVFRSHLYGTRGASICVRGPRYVTAQDIISPP
SVKIVDTTQYIASLTEPIDLCIELQIERDRGYRIKTPNNYQDGSY--PIDAVSMPVRNANHSIH
SYGNGNEKQEILFLEIWTNGSFTPKEALYEASRNLIDLFLPFLHAEEQDINV--EDNQ-NRAT

APLFTFHDGLTN-IGKRKK-GIALKRVFIDQSELPTRTYNCLKRSNIHTLLDLLSNSQEDLMR
IEHFRIEDVKQILGILQKR--FAID-------------MSTIPGSSQIQFEGFCKFIDQGLAEELHKFPK
MEDTDQEMEFQLFVETYQLVEPLIKERDAVYESLTYSSELYVSAGLIWRAGRDMRKQTVF
IGNIPLMNSLGTSLVNGIYRIVVNQILQSPGIYYRSELDHNGISVYTGTIISDWGGRSELEID
RKARIWARVSRKQKISILVPSSAMGSNLRDILDNVCYPEIFLSFPNDKEKKKIGSKENAVLE
FYQQFACVGGDPVFSESLCKELQKKFFQQRCELGRIGRRNMNQRLNLDIPQNSTFLLPRD
VLAAADHLIGMKFGMGTLDD--LNHLKNKRIRSVADLLQDQFGLALVRLENVVRGTICGA
IR-HKLI-PTPHNLVTSTP----LTTTYESFFGLHPLSQVLDRTNPLTQIVHGRKWSYLGPGGLT
GRTASFRIRDIHPSHYGRICPIDTSEGINVGLIGSLAIHARIGDWG-SIESPFY---EISERLKE--E
QMVYLSPRRDEYYMVAAGNSLALNRGIQEEQVVPARYRQEFLTIAWEQIHLRSIYPFQYFS
IGASLIPFIEHNDANRALMSSNMQRQAVPLSHSEKCIVGTGLERQAALDSGGSAIAEHEGKI
TYTDTEKIVLSGNGDTISIPLVMYQRSNKNTCMHQKPQVHRGKCVKKGQILADGAAIIGG
ELALGKNILVAYMPWEGYNSEDAVLISERLVYGDIYTSFHIRKYEIQTHVTSQG-PERITNEI
PHLEAHLLRNLDKNGIVMLGSWVETGDILVGKLTPQTAKE-SSYAPEDRLLRAILGIQVSTA
KETCLKLPIGGRGRVIDVRWIQKKR-GSSSNPETIRVYISQKREIKVGDKVAGRHGNKGIISK
ILPRQDMPYLQDGTPVDMVFNPLGIPSRMNVGQMFECSIGLAGNLLNRHYRIIPFDERYEQ
EASRKLVFPELYKASKQTANPWVFEPEYPGKSRIFDGRTGDPFEQPVIIGKSYMMKLIHQV
DDKIHGRSSGHYALVTQQPLRGRAKHGGQRVGEMEVWALEGFGVAHISQEMLTYKSDHI
RARQEVLGTTIIGGTIPNPDGAPESFRLLVRELRSLALELNHFLVSEKSFQINRKEAMNQNF
SSMIDRYKYQQLRIGSVSPQQISAWANKILPNGEIVGEVTKPYTFHYKTNKPEKDGLFCERI
SGPIKSGICACGNYRVIGDEKEGPKFCEQCGVESVDSRIRRYQMGYIKLACPVTHVWYLK
RLPSYIANLLDKPLKELEGLVYCD--FSFARP-IAKKPTFLRLRGSFEYEIQSRKYSIPLFFTTQ
GFDTFRNREISTGASAIREQLADPDLRIIMDRSLVEWKELGEEGST-GNAWEDRKIGRRKDF
LVKRMELAKHFLRTNVEPERMVLCLLPVLPPELRPIIQIDGGKPMSSDINELYRRVIYRNNT
LTDPLTTSRSTPGELVLCQEKLVQEAVDTLLDNGIRGQPMRDGHNKVYKSFSDVIEGKEGR
FRETLLGKRVDYSGRSVIVVGPSLSLHRCGLPREIAIELFQTFVIRGLIRQRVASNIGIAKSKI
REKEPIVWEILQEVMRGHPVLLNRAPTLHRLGIQAFQPIIGEGRAICLHPLVRKGFNADFD
GDQMAVHVPLSLEAQTEARLLMFSHMNLLSPAIGDPISVPTQDMLIGLYVLTIGNHRGICA
NRYNPCNYR--N-YQNETVDDN-YQYTKEKEPHFCSSYDALGAYRQKRINLYSPLWLRWRL
DQR-VIAS--REVPIEVQYESLGTYHEIYGHYQIVRSIKKEILCIYIRTTIGHISFYREIEEAVQG
FCRAYSYGT--------MEVLMAERANLVFHNKVIDGTAMKRLISRLIDHFGMAYTSHILDQVK
TLGFQQATATSISLGIDDLLTISSKGWLVQDAEQQSLLLEKHHRYGNVHAVEKLRQSIEIWY
ATSEYLRQEMNPNFRMTDPSNPVHIMSFSGARGNVSQVHQLVGMRGLMSDPQGQMIDLP
IQSNLREGLSLTEYTISCYGARKGVVDTAVRTSDAGYLTRRLVEVVQHVVVRRTDCGTIRG
ISISIRN--GMTGRILIQTLIGRVLADDVYMGLRCIAARNQDIGIGLVNRFIT------SRAQPIYIR
TPFTCRSTSWICRLCYGRSPTHGDLVELGEAVGIIAGQSIGEPGTQLTLRTFHTGGVFTGGT
AEHVRAPFNGKIKFNEDLVHPTRTRHGHPAFLCYIDLYVTIESQDILHNVNIPPKSFILVQND
QYVESEQVIAEIRARTSTF--FKEKVRKHIYSDSEGE-IHWSTDVYHVPEYTHGNVHLLPKT
THLWILSGGPGPCRRSIVPFSLHKDQDQMNLQ--SLSVEQRYISNLS--VNNDR-VRHKLFDL
DPFWK-KGGVG-YS-GPDRILSNGHWDFIYPA-ILHENSN--LLAKRRRNRFIISFQHDQEREK
KLPPRSGIKIEIPIN--GILRRNSILAYFDDPRYRRSSSGITKYGTIEVDSIVKKDD--LIEYRG-A
KELRPKYMKVDPFFFIPEEVHILPGSSPIMVRNNSIIGVDTRITLNTRSRIGGLVRVERKKKR
IELKIFSGDIHFPGETDKISRHSGILIPPETRKK--NSKESKSKQLKNWIYVQR--ITPIKKK--YF
VSVRAVVIYEIADGIHLATLFPQDLLQERDNLQLRVVNYFLYGNGKPIRGISHKSIQLVRTC

LVLNWDQDQNG----SIEEVYASFVKVRANDLIRDFIRIDLVKSPIS---KRNDTAGSGLIPV-NG
SDRTNINPFCSKAR---------IQSLSQHQGTVRTFLNR---NKEGESFLVLSSSNCSRIGP--FNGL
KYHNLTK--KESDPMIPIRNSLGPFGT--VPKIANFYSS--YQLIT--HNEILLN-KYLLLDN---PK
QTFEALKYY---FMDENGI--IYNPDPCSNIILNPFDSNWYFPHQDYCEETCTIIS-LGQFICENV
CISKHGP---RIKSGQVLIVH-VDSLVIRSAKPHLATPGATVHGHYGEILYEGNTLVTFIYEKSR
SGDITQGLPKVEQVLEVRSIDSISMNLEKRFEGWGERMTGILGIPWGFLIGAELTIAQSHFS
LVNKIQRVYRSQGVQIHNRHIEIIVRQITSKVLVSE---DGMSNVFSPGELIGLLRAERTGRAL
EEAICYRAVLLGITRASLNTQSFISEASFQETTRVLAKAALRGRIDWLKGLKENVVLGGMI
PVGTGFKGLVHHSR--EHNNIP--FEIQKKSRFEGEMR--DILFPHRELFCSCLETNFHDTSFM
GLNDS---------MTRRYWNINLEEMLEAGVHFGHGTRKWNPRMAPYISAKRKGIHITNLTRT
ARSLSEACDLVFDAASRGKHFLIVGTKDKAADSVASAAIRARCHYVNKKWLGGMLTNW
FTTETRLHKFRDLRAEQKTGRLNRLPKRDAAMLKRKLSHLQAYLGGIKYMTGLPDIVIID
QQEEYTAIRECATLGIPTICLIDTNCDPDLADIPIPANDDAIASIRLILNKLVSAICEGRSS-YIR
NHMGQKINPLGFRLGTTQSHHCLWFAQPKNYSGGLQEDEKIRDCIKNYVQKHMRVSSG-F
EGIARIGIQKKIDLIQVIIHIGFPHFLIEG--RAR--GIEELQINVQKKLNSVNRRLNIAITRVAKP
YGQPTILAEYIALQLKNRVSFRKAMKKAIELTEQADTKGIQVQISGRINGKEIARVEWIREG
RVPLQTIRAKIDHCSYAVRTIYGVLGIKIWIFLDEEMSRYRGPRFKKIRRLGSLPGLTSKRPR
SGSDLRNQSRSGK-RSQYRIRLEEKQKLRFHYGLTERQLLRYVRIAGKAKGSTGQVLLQLL
EMRLDNILFRLGMASTIPGARQLVNHRHILVNGRLVDIPSYRCKPRDIITTRDEHRSRALIQ
NSIDSSQK-EELPKHLTLHSLQYQYKGLVNQIIDSKWVGLKINELLVVEYYSRQTMSRRGT
AEEKTAKSDPIYRNRLVNMLVNRILKHGKKSLAYQIIYRAVKKIQQKTETNPLSVLRQAIR
GVTPDIAVKARRVGGSTHQVPIEIGSTQGKALAIRWLLGASRKRPGRNMAFKLSSELVDA
AKGSGDAIRKKEETHRMAEANRAFAHFRMGRDTIADIITSIRNADMGTKGMVRIASTNIT
ENIVKILLREGFIENVRKHRESNKYFLVSTLRHRR--NRKGTYR---NIL--KRISRPGLRIYSNY
QGIPRILGGMGVVILSTSRGIMTDREARLEGIGGEILCYIWMTKPIPRIGLRRNGRIGLRKN
GRRISKGVIHVQASFNNTIVTVTDVRGRVVSWSSAGTCGFRGARRGTPFAAQTAAGNAIR
TVVDQGMQRAEVMIKGPGLGRDAALRAIRRSGILLSFVRDVTPMPHNGCRPPKKRRVMP
TIKQLIRNTRQPIKNVTKSPALRGCPQRRGTCTRVYTITPKKPNSALRKVARVRLTSGFEITA
YIPGIGHNSQEHSVVLVRGGRVKDLPGVRYHIVRGTLDAVGVKDRQQGRSKYGVKKPKM
ARKSLIQRERKRDKLEQKYHLIRRSSKKQIS---KVPSLSDKWEIHGKLQSPPRNSAPIRLHR
RCFLTGRPRANYRDFGLSGHVLREMVHACLLPGATRSSWMVKKSFILVIPQEEKKG-----SV
EFQVSSFTNKIRRLTSHLELHRKDYLSQRGLRRILGKRQRLLAYLSKKNRVRYKELISQLDI
REPKTH--MVKLRLKRCGRKQRAIYRIVAIDVRSRREGRDLCKVGFYDPIKNQTYSNVPAIL
YFLERGAQPTGTVHDISKKAEVFKELRVNQTK---MDKSKRTFRKSKRSFRRRLPPIGSGDR
IDYRNMSLISRFISEQGKILSRRVNRLTLKQQRLITIAIKQARILSSLPFLNNEK--QFERTESTP
RTTGTRARKKVARSLKKNPFVANHLLGKIEKLNMREEKEIIVTWSRASTIIPTMIGHTIAIH
NGKEHLPIYITDRMVGHKLGEFAPTLTFRGHARNDNRSRRMILKSFLLGNLLSLCMKIINS
VVVVGLYYGFLTTFSIGPSYLFLLRARVMEEG---TEKEVSATTGFITGQFMMFISIYYAPLHL
ALGRPHTITVLVLPYLLFHFFWNNHKHFFDYGSTTRNSMRNLSIQCVFLNNLSFQLFNHFI
LPSSTLARLVNIYMFRCNNKMLFVTSSFVGWLIGHILFMKWVGLVLFWIRQNHSIRS-IRSN
KYLVSELRNSMARIFSILLFITCVYYLGRIPSPIVTKKLKETEEGKPLVTLLFDYLRWNRPLR
YINNDRFPNAVRNEMSQYFFDTSKRDGNKIISFTYPPSLSIFNEMIQKKIFLSTTEKLSPEGL
YNHWIYANEQKKYNLNNEFINRIEALD--KLVLDVLEQKNRLCNDENEQECLPKMYDPFL
NGPYRGRIKKYYLHVETVWINRIHDILYREFGHKD-PNNAIGLEEIGKRVPRWLYRLTDLEE

QEDEGIRSRKAKRVIIFADTLLRYSQKSDFRRNLIKGSMRAQRRKTVTWEMFQINVHSPLF
LDRIDKTFSFSFDI--SRMMNLIFRNKKEDERITIAELFDTIIFAQAIRGSMLVIQSILRKYIILPS
LIIAKNIGRMLLFQFPEWYEDWKAWSKEMHVKCTYNGVQLSETEFPKDWLTDGIQIKILF
PFCLKPWRRSKKEKKDNFCFLTIWGMEAELPFGSPRKRPSFFKPIWKEFQQKITKVK-KKR
FLFLRVFKKRTKGFLKISKEKTRWVIKIVL--FIKKKMKELTKET-SIISDKITPESSLRVGSMD
WTNYSLTEKKMKDLADRTTPIRDQIERITKDKKDSPNERSCGDKRPELQKHIWKVFKKGS
ARLIPKWNYFMKSFIQRIYIDIFLYAINMPRVNAQLFIHFISCDLSSLSQAYVFYILSQTQVLN
KYYLKSLFQHEYPFIKDKIKDYFTRGIFHSESRYKQLPNCRMNEWKNWLRGHYQYNLSH
TRWSRLVPRKWRNRIKQHHRIKKKKKWDSYEK--DQLIHYEKDKKMEKHYKYDLLSHKY
INEYIIGPDRKYLDWRIIHFCLRLFDWMGMNQEILYRWFFPEFMRLYEAYKTKPWIIPTQLL
IFHSKSNKEAELDFFLKRYFLFQLRWDDPLNQKRINNIKVYCLLLRLMDPKEIALASIQRG
EMCLAAMKIQKDLAVTELIKKGILILEPVRLSIKWDGQFIMYQTIGISLVHKSKHKNYDLL
VPENILSLRRRRELRILIRFRFIWPNYRLEDLACMNRYWFDTNNGSHFSMSRIYMYPMKR
NQLKSWIFEL----REIKNSHYFVDSWDKFDSVGSFTYIFFHQERFMKLFDPRILSILLS-HDS--
QDLTRNRYFNF-TIKGVVLLVMG-VLIFIFIYRINNRNMVERKNLYLMGLL-----PIPMNSIGL
RNDTLEESF-LSSNINRLIVS-LLYLPKEKKISESFFMDPKGRSWVLPIT----KKCIVPESNWDL
RGWRNRIVKKR--DSSCKIS---NKRVAGIEISFKDKDIK-YLEFLFVSYTD--DPIRKDRDWEL
FDRLSPRKKRNIINL--NSGQRFEILVKHLICYLMPAFHEKGSIEVEG-FFKQQG-AQATIQSN
DIEHLSH--LFSRNKWGISLQNCAQFHMWQFHQDLFV---SWGK-NKHESHFFRN-VSRENW
IWLDNVWLVNNDR---FFS--KVRNVSSNIQYDS---TR--SIFVQVTDSSQLKGSS-DQSRDPFD
SIS----NESSK-------------YHTLINQTEIQQLKERLIL-LD--PSFRQMERTETE----------------------
----REKQMDNHLFPAEVKKCL--GNPTRSIRSFFSDRW--SELP-LGSNPTERSTRDQ----KLLK
KQQDVS-FVPSRRSE----NKEMFDIFKIITYLQN--TVSIHPISSDP--DMVP-KDEPD-RDSSNKI
SFLNKNPFFD--LFHLFHDRNKEGYTF--HYDFESE--ERFPEMSDLFTLSITEP-NLVYHKGFA
FSIDSYGLDQKK--FLNEVS--WDESKKKSF---LVLPHIFY--EENESFYRRIRRKSGR-ISCRNG
LEEKKKKIVVFASNNIMGAVNQYRLIRDLIQIQYSPHG--YIRNRLNRFLLM---NRSDHNFE
YGIQ---RDQIGNDTLNHITIMKYTINQHLSNLIK-NRKKWHDSH--SLIFPTERSMNRDPDAY
RYKWSNESNNFQEHFEHFVSEQKN---HFQVVFNQFRINQYSIDWSEVIDKQDLSKSFRFFL
SNS---------LLFFS-------KSL--------PLFFLSIGNIPIHSRE-IHIY-EFKGPNHQLCNQLLESIGVQI
VHLKKLKP--FLLD---DHDIS-QRSKLLINGGTISPLFFNKIQ-GW-VIDSFHI--RKNRRKYLYN
MDSYFSMISHDRDNCMNPVKPFHRSSLISSFYKANRLPFLNNSHRFWFY--CDKRFPFYV--E
VEKARINNYD--LAY-AQFLNISFIRNK-IFSLCVGK--KKKPFLE-IDAISPIESQVSDIFIPNDFP
QSGDER-YNLYKS---FYFPTRSDLFVR--RAIYSIADIFETPLTEGQIVHFERTYCQPLSDINLS
ESEGNNLHQYLS--FNSNMGLIRIPCSEKYLP--SRKRKK--------RSLCLK--KCVEKRQMYRT
FQRNSAFSNLSKWNLFQTYMPWFFTSTGCKYINLIFLDTFADPL--PILSSS-QKFLSIFHDIM
HGSDILWPTEQKKWW----TILPQSNL-ISEISSKCLQN--LLLSEEMIHRNNESPASLIWTHLIA
PNAREF-LYSILFLLLVAGY-LVRTHLLFVSQ--TSSELQTEFEKIKSLMIPSYMVEVRKLLDRY
PPLELNFLWLKNLFLVALEQLEDFMEKIWGSASGGNILLGGGP-----TYG-VKSIRSKKKDLN
INLIDII----SIISNPINLITFSRKTRDLSRTSKEIYSWIRQRKNVNSDWIDDKIESWVANNDWI
DDEEREFLVQFS--TLTT--EKRIDQILLSLT-HSDRLS-----KNDSGYQMIEQPGSIYLRYLVDIH
--QKYLMNYEFSRFCLAERRIFLAHYQTITY--SQSSCIANSSHFRSHG---KPVSLRLVLSPS-R
GILVIGSIGTGRSYLVKYLATNSYVPFITVFPNKLLDDKPKDYFSD-----DID-IDESDDLN----Y
-------------------------DLDTELLTMTNVL---TMYMT---LKMDQFDITLQFELAEEMSPCIIWIP
NIHELYVNESNYLSLSLLVNYLS---RDCERCS-TRNILVIASTPIPQKVDPALIAPNKSNTCIKI

RRLLIPQERKHFFILSSTRGFHLEKKMFHT--NRFGSISMGSNARDLVALTSEALSISITQKKSI
IDTNTIKSALHRQTWDLR---SQVRPVQDHGILFYQIGRAVAQNALLSNCSINPISIYMKKK--
SCKKGDSYLYKWYFELGTSMKKLTILLYLLSCSAGSVAQDLWSTS--GPDEK-NGITSYGFV
ENDS-DLVHG---LEGVLVG--SSRTEKDCSQFDNAQAQVTLLLRSEPRN-QLDMMQNGSCSI
VDQRFLYEK--YESEFEE-GEREGALDPQ----QIEED----LFNH--IVWAPRTRIRRP---CGNLFD
CIERTNELGFP-YWARSFR---GKRIIYHKEDELQENDSEFLQSGTMQYQTRDRSSKEQGFFR
ISQFIWDPADPFFFLFK-DQPFVSVFSRREFFADEEMSKGLITSQTNPPTSIYKRWFI--KNTQE
KHFELLIHRQRWLRT---NSSLS--NGSFRSN--TPSESYQYLSNLFLSN-GTLLDQMTKALLRK
RWLFPDEMKHLI--HVTG------MSRSR-INGNFIDKTSSIVANILLRIIPTTSGEKEAFTYYRDG
--MSAQSEGNYAEALQNYYEATRPEIDPYDRSYILYNIGLIHTSNGEHTKALEYYFRALERN
PFLPQAFNNMAVICHY-RGEQAIRQGDSEIAEAWSDQAAEYWKQAIALTPGNYIEAQNWL
KITRRFE--MNWRSERIWIELITGSRKTSNFCWACILFLGSIGFLLVGISSYLGRNLISLFPSQQ
ILFFPQGIVMCFYGIAGLFISSYLWCTISWNVGSGYDRFDRKEGIVCIFRWGFPGINRRIFLR
FRMRDIRSIRMKVKEGLYPRRVLYMEIRGRGDIPLTRTDEN-LSPLEIEQKAAEWAYFLRVPI
EVF
>Dioscorea
MEKWWFNSILSLSKSMDSVNGIEYT---KDPILNDTEKNIL-FSTVDYLFEIRNIWSLISDDTF
LVRDSNGDSYSVYFDIENQIFEIDNN--SSFLGELESFFSS-----YLNRGSKNK---YYHYMYDT
QFSWNNHITNCIDSYLRFEIGISITSYISGDIVNYSDGYIYSFICTESGSSIRTSSNGSDFNMRE
NS-DSDINKKYRHLWVQCENCYGLNYKKFFRSKMNICEQCGYHLKMSSSDRIEVSIDPDT
WDPMDEDMVSIDPIEFHSEEEPYRDRIDSYQRKTGLTEAIQTGIGQLNGIPIAIGVMDFQFM
GGSMGSVVGEKITRLIEYATNESLPVIIVCASGGARMQEGSLSLMQMAKISSASYNYQSNK
KLFYVSILTSPTTGGVTASFGMLGDVIIAEPNAYIAFAGKRVIEQTLNKTVPDGSQSAEYLF
HKGLFDPIVPRNPLKGVLSELFQLHGFLPLNQNKKEMVTLRADEISNIIRERIEQYNIGVKI
VNTGTVLQVGDGIARIHGLDEVMAGELVEFEEGTIGIALNLESNNVGVVLMGDGLMIQEG
SSVKATGRIAQIPVSEAYLGRVINALAKPIDGRGEISASESRLIESPAPGIISRRSVYEPLQTGL
IAIDSMIPIGRGQRELIIGDRQTGKTAVATDTILNQKGQNVICVYVAIGQKASSVAQVVTTFQ
ERGAMEYTIVVAETADSPATLQYLAPYTGAALAEYFMHREQHTSIIYDDLSKQAQAYRQM
SLLLRRPPGREAYPGDVFYLHSRLLERAAKSSSRLGEGSMTALPIVETQSGDVSAYIPTNVI
SITDGQIFLSADLFNAGIRPAINVGISVSRVGSAAQIKAMKQVAGKSKLELAQFAELEAFAQ
FASDLDKATQNQLARGQRLRELLKQSQSDPLAVEEQIATIYTGANGYLDPLEIGQVKKFLV
QLRTYLKKNKSQFKEIISSTKTFTEEAEALLKGAVQEQIELFLLQEQTMRINPTTSGPMISTL
EEKNLGRIVQIIGPVLDALFPPGKMPNIYNALVVKGR--AGQQINVTCEVQQLLGNNRVRA
VAMSATDGLMRGMEVIDTGAPLSVPVGGATLGRIFNVLGEPVDNLGPVDTRTTSPIHRSAP
AFIQLDTKLSIFETGIKVVDLLAPYRRGGKIGLFGGAGVGKTVLIMELINNIAKAHGGVSV
FGGVGERTREGNDLYIEMKESGVINEKNIAESKVALVYGQMNEPPGARMRVGLTALTMAE
YFRDVNEQDVLLFIDNIFRFVQAGSEVSALLGRMPSAVGYQPTLSTEMGSLQERITSTKEG
SITSIQAVYVPADDLTDPAPATTFAHLDATTVLSRGLAAKGIYPAVDPLDSTSTMLQPGIVGE
EHYETAQKVKQTSQRYKELQDIIAILGLDELSEEDRLTVARARKIERFLSQPFFVAEVFTGSP
GKYVGLAETIRGFQLILSGELDSLPEQAFYLVGNIDEATAKAMNLEVENKLKKMTLNLCV
LTPNRIIWDSEVKEIILSTNSGQIGVLPNHAPIATAVDIGLLRIRLNDQWLTVALMGGFARIG
NNEITILGNDAEMNTDIDPQEAQQTLEIAEANLSRAEGKRQVIEANLALRRARTRIEAINVI
SMKNVTDSFVYLGHCPFAGSFAFNTDILATNPINLSIVLGVLIFFGKGVLNDLLDNRKQRIL
STIQNSEELRKKTIEQLERARDRLRKVEIEADEYRINGYSEIEREKTNLINATYDSLERLENY

KNETLHFEQQRAINKVRQRVFQEALQGALGTLNSCLNSDLHFRTISANIGILGAMEEITDM
NPLISAASVIAAGLAVGLASIGPGVGQGTAAGQAVEGIARQPEAEGKIRGTLLLSLAFMEA
LTIYGLVVALALLFANPFVMNVIPCSIKTLKGLYDISGVEVGQHLYWQIGALQIHAQVLITS
WVVIAILLGSVIIAVRNPQTIPSGGQNFFEYVLEFIRDLSKAQIGEEYGPWVPFIGTMFLFIFV
SNWSGALLPWKIIQLPHGELAAPTNDINTTVALALPTSVAYFYAGLTKKGLGYFRKYIQPT
PILLPINILEDFTKPLSLSFRLFGNILADELVVVVLVSLVPSLVPIPVMFLGLFTSGIQALIFATL
AAAYIGESMEGHHMIFVTLEHILTHISFSIISIVITIHLMTLLIHKIAGLCDSSEKGIISTFFSITG
LLITRWIYSGHFPLSNLYESLIFLSWSFSIIHMIPYLRNHKNDLSVITVPSVIFTQGFVTSCLST
EMRQSAILVPALKSQWLIMHVSMMLLSYATLLCGSLLSVALLVITFRKNMDIFCKRKETEY
LNKKRSVFKNTSFLSFRNYYKYQLTQRLDYWTYRIISLGFIFLTMGILSGAVWANEAWGSY
WNWDPKETWAFITWTIFAIYSHIRININFQGTNPALVASIGFLIIWICYFGINLLGIGLHSYGS
FTLPMKKKALI-SIPYLVSIVFLPWWVSLSFNKSLEPWITNWWNSEQSETFLNDIQEKNVLE
RFIELEELFLLDEMIKENPETHIQKFRIEIHKQTIQLIKTHNEYNLHIILHFSTNIICFVILGGYF
FLSNEELIILNSWIQELLYNLSDTIKAFSILLITDLWIGFHSTHGWELMIGSVYNDFGLAHND
QIISGLVSTFPVILDTIVKYWIFHYLNRVSPSLVIIYHSMNEMPIGVPKVPFRSPGEEDAVWV
DVN-RLHRERLLFLGQEVDNEVSNQLVGLMVYLSIEDATKDLYLFINSPGGWVIPGMAIYD
TMQFVSPDVHTICMGLAASMGSLILVGGEITKRLAFPHARVMIHQPASSFYEAQAGEFILE
AEELLKLRETLTKVYVQRTGNPLWAVSEDMERDAFMSATEAQAHGIVDLVAVEMKEQKLI
HEGVIIESLPNGMFRVRLDNEDLILGYVSGRIRRSFIRILPGDRVKIEVSRYDSTKGRIIYRLR
NKDSNDIEELQGYLEKDGSRQRHFLYPLLFQEYIYTLAHDHGLLDYDN-SSSVLVKHLITR
MYQQNYLIYSADDSNQNRIVG----SKMISEGFAVSMEIPFLLRLGSSLIPKSQNLRSIHSIFPF
LEDKSSHLNYVSDILIPHPIHLEILVQILQCWTQDVSSLHLLRFFLHEYHNSNSFITKPVYVIS
KKRFFRFLYNSYVFECEFLLVFFHKQSSYLRSRSSGVFLERTHFYEKMGN--VVCCNQKTQ
WFFKDPFLHYVRYQGKAILVSKGTHLLMRKWRSYLVHFWQYYFQFWSHPHRIHINQLSN
YSFCFLGYLSNLLINLSVVRSQMLENSFVIDILT--KKFDTRVSVIALIRSLSKAKFCTVSGHPI
SKSIWTNLSDLDIIHRFGWICRNLSHYHSGSSKKQSLYRIKYILRISCARTLARKHKSKVRA
FLQRLGSGLLQEFFREEEEILSLIFPQRIWYLDIICINDLRNLMMIIDMT-EVQ-AINSFSSSE-S
VKEVYGLISLFIPILSLILGIIGVLVIVWLEREISAAIQQRIGPEYAGPLGILQAIADGTKLLFK
EDLFPSRGNIRLFSIGPSLAVISILLSYLVMPFGYRLVLADLSIGVFLWIAISSIAPIGLLMSGY
GSNNKYSFSGGLRAAAQSISYEIPLTLCVLSISLLSNSLSTVDIVEAQSKYGFGGWNLWRQP
IGFIVFLISSLAECERLPFDLPEAEEELVAGYQTEYSGIKYGLFYVASYLNLLVSSLFVTVLYL
GGWNFFIPYIFIPELFVKKKMVVVFGMTIGIFITLVKAYLFLFISITTRWTLPRMRMDQLLNL
GWKFLLPISLGNLLLTTSFQLVSLMIWHVQNENFILDSTRIFMKAFHLLLFHGSFIFPECILIF
GLILLLMIDSTSDQKDRPWFYFISSTTLVMSITALLFRWREEPIISFSGNFQTNNFNEIFQFLIL
LCSTLCIPLSVEYIECTEMAITEFLLFVLTATLGGMFLCGANDLITLFVAPECFSLCSYLLSG
YTKRDVRSNEATMKYLLMGGASSSILVHGFSWLYGSSGGEIELQEIVNGLINTQMYNSPGI
SIALISITVGIGFKLSPAPFHQWTPDVYEGSPTPVVAFLSVTSKVAASASATRIFDIPFYFSSNE
WHLLLEILAILSMILGNLIAITQTSMKRMLAYSSIGQIGYVIIGIIVGDSNDGYASMITYMLF
YISMNLGTFACIVSFGLRTGTDNIRDYAGLYTKDPFLALSLALCLLSLGGLPPLAGFFGKLH
LFWCGWQAGLYFLVSIGLLTSVVSIYYYLKIIKLLMTGRNQEITPHVRNYRRSPLRSNNSIE
LSMTVCVIASTIPGISMNPILAIAQDTLFMFLLHEYDIFWAFLIISSIIPILAFLISGVLTPIREGS
EKLSSYESGIEPMGNAWLQFRIRYYMFALVFVVFDVETVFLYPWAMSFDVLGISVFIEAFIF
VLIPIVGLVYAWRKGALEWSTSYFPWLTIIVVLPIFAASSIFFLPHKGNKVFRWYTTCICLTE
LLLTTYVFCYHFQLDDQLIQLEEDLKCINIFDFHWRLGIDGLSIGPILLTGFITTLATLAAWP

VTRNSRLFYLLMLVMYSGQIGLFSSRDLLLFFIMWELELIPVYLLLSMWGGKKRLYSATKF
ILYTAGGSVFLLIGVLGMGLYGSNEPTLDFERLANQSYPVALEIIFYFGFLIAYAVKSPIIPLH
TWLPDTHGEAHYSTCMLLAGILLKMGAYGLIRINMELLPHAHSLFSPWLVIVGTIQIIYAAS
TSFGQRNLKKRIACSSVSHMGFIIIGIGSITDTGLNGAILQILSHGFIGAALFFLSGTSCDRIRF
VYLDEMGGISLPMPKIFTMFSSFSMASLALPGMSGFVAELVVFLGIITSPKYLLMPKILITFV
MAIGMILTPIYLLSMSRQMFYGYKLFNVPNSFFVDSGPRELFVSICIFLPVIGIGIYPDCVFSL
SVDKIEGILSNYFHRMMFEHVLVLSVYLFSIGIYGLITSRNMVRALMCLDLILNSVNMNLV
TFSDLFDSRQLKGDIFSIFVIAVAAAEAAIGLAIVSSIYRNRKSTRINQSNLLNNMEHTYQYA
YIILFLPLPVTMSIGFGLLFVPTATKNIRRMWAFVSVLLLSMVMGFSVNLAIQQINGSFIYQ
YLCSWTINNDFSLEFGYLIDPLTSIMSLLISTVGIMVLMYSDNYMSHDQGYLRFFAYMSFF
NTSMLGLVTSSNLIQIHIFWELVGMCSYLLIGFWFTRPIAASACQKAFVINRVGDFGLLLGI
LGFYWITGSLEFRDLFEIVNNNGVNFRFAILCACLLFLGAVAKSAQFPLHVWLPDAMEGPT
PISALIHAATMVAAGIFLVARLRPLFIVIPYIMNLISLIGIITLLLGATLALAQGDIKRSLAYST
VSQLGYIMLALGIGSYRAALFHLITHAYSKALLFLGSGSIIHSMEPVVGYSPDKSQNMVLM
GGLKKYVPVTRTTFLLGTLSLCGIPPLACFWSKDEILTDSWLYSPIFAIIAYFTAGLTAFYMF
RVYLLTFDGCLRVHFQNYSSTKRSLFCSVSVWGREPLKIDNQ-NLPFLILKFFSNKY--TYM
YPHESENTVLFPLLVLVLFTLVVGLIGIPFDFDILSKWLT---NPFQKNLNHSVDWYEFLTNAI
FSVSVSLFGLFIASIFYGSVYSSFQNLDLINFFVILLDQIKNVIYNWSYNRGYIDIFYTKGLT
MSIRRLSKLIQFFDRYIIDGITNGIGVASFFIGEGIRY-IGGGRISSYLFLYLSYVSIFLIFYQMD
LLGPIHDILVIFLGSVLILGGLGVVLLNNLIYSAFSLGWVLFCISLFYISSNSYFVAAAQLLIY
VGAINVLIIFAVMFISGSEYSNDSHLWTVGDGVTSVVCTSIFFSLITTIPDTSWYGIIWTTRSN
QIIEQDLISNVQQIGIHLSTNFYLPFELISIILLISLIGAITMARQMTVPDTRKGLMIVNMGPH
HPSMHGVLRLIVTLEGEDVIDCEPILGYLHRGMEKIAENRTIIQYLPYVTRWDYLATMFTE
AITVNAPEQLGNVQVPQRASYIRVIMLELSRIASHLLWLGPFMADIGAQTPFFYIFRERELL
YDLFEASTGMRMMHNYFRIGGVAADLPHGWIDKCLDFCDYFLTRVAEYEKLITRNPIFLD
RVEGVGIIGGEEAINWGLSGPMLRASGIPWDLRKVDHYECYNEFDWEVQWQKEGDSLAR
YLVRIGEMTESIKIIQQAIEGIPGGPYENLEVRRFDGATNSEWNDLEYGFISKKPSPNFELSK
QELYMRVEAPKGELGIYMIGDNSVFPWRWKIRPPGFINLQILPQLVKRMKLADIMTILGSID
IIMGEVDRMFPMVTGFINYGQQTIRAARYIGQSFIITLSHTNRLPVTIQYPYEKSIASERFRG
RIHFEFDKCIACEVCVRVCPIDLPVVDWRLEKNIKKKQLLNYSIDFGFCIFCGNCVEYCPTN
CLSMTEEYELSTYDRHELNYNQIALGRLPMSVIGDYTIQTVLNWKIDKNKLLDSRTITNY
MQSRLSDWLVKHELVHRSLGFDYQGIETLQIKTEDWDSIAVISYIYGYNYLRSQCAYDVAP
GGFLASVYHLTRIQYGIDKVEEVCLKVFVSRNNPRIPSVFWIWKSADFQERESYDMLGISY
ANHPHLKRILMPESWIGWPLRKDYITPNFYEIQDAR--
-----------MNLIEFTLLDQTTPNSVISTTSNDLSNWSRLSSLWPLLYGTSCCFIEFASLIGSRFDF
DRYGLVPRSSPRQADLILTAGTVTMKMAPSLVRLYEQMAEPKYVIAMGACTITGGMFSTD
SYSTVRGVDKLIPVDVYLPGCPPKPEAVIDAITKLRKKISREISEDRVMSQ-ENRSFTTNHKF
DIRRSTHTGNYDQGLFYQSSYTSEISR--FFKSKNSVSSHELVNMQNRNTFLGVKEQITRSIF
VSIMIYVITRASISNAYPIFAQQGYENPREATGRIVCANCHLASKPVDIEVPQAVLPDTVFEA
VVRIPYDMQLKQVLANGKKGALNVGAVLILPEGFELAPPDRISPEVKEKMGNLSFQNYRP
NKKNIIVIGPAPGQKYSEIVFPILSPDPATKKDVHFLKYPIYVGGNRGRGQIYPDGSKSNNT
VYNATSAGIVSKIVRKEKGGYEITIIDASDGHQVVDIIPRGPELLVSEGESIKLDQPLTSNPN
VGGFGQGDAEIVLQDPLRIQGLLFFLASVILAQIFLVLKKKQFEKVQLYEMNFMSKVYDW
FEERLEIQAIADDITSKYVPPHVNIFYCLGGITLTCFLVQVATGFAMTFYYRPTVTEAFSSVQ

YIMTEANFGWLIRSVHRWSASMMVLMMILHVFRVYLTGGFKKPRELTWVTGVVLAVLTA
SFGVTGYSLPRDQIGYWAVKIVTGVPEAIPVIGSPLVELLRGSASVGQSTLTRFYSLHTFVLP
LLTAVFMLMHFLMIRKQGISGPLMGITKKPDLNDPVLRAKLAKGMGHNYYGEPAWPNDL
LYIFPVVILGTIACNVGLAVLEPSMIGEPADPFATPLEILPEWYFFPVFQILRTVPNKLLGVLL
MVSVPIGLLTVPFLENVNKFQNPFRRPVATTVFLIGTAVALWLGIGATLPIDKSLTLGLFMIE
VFLFGIVLGLIPITLAGLFVTAYLQYRRGDQLDLMLTITSYFGFLLAALTITPALLISLNKIQLI
MDIVSLAWAALMVVFSFSLSLVVWGRSGLMIIRSPEPEVKIVVDRDPIKTSFEEWARPGHF
SRTIAKGPDTTTWIWNLHADAHDFDSHTSDLEEISRKVFSAHFGQLSIIFLWLSGMYFHGA
RFSNYEAWLSDPTHIGPSAQVVWPIVGQEILNGDVGGGFRGIQITSGFFQIWRASGITSELQ
LYCTAIGGLVFASLMLFAGWFHYHKAAPKLAWFQDVESMLNHHLAGLLGLGSLSWAGH
QIHVSLPINQFLDAGVDPKEIPLPHEFILNRDLLVQLYPSFAEGATPFFTLNWSKYAEFLSFR
GGLDPITGGLWLSDIAHHHLAIAILFLIAGHMYRTNWGIGHGLKDILEAHKGPFTGQGHK
GLYEILTTSWHAQLSLNLAMLGSLTIVVAHHMYSMPPYPYLAIDYGTQLSLFTHHMWIGG
FLIVGAAAHAAIFMVRDYDPTTRYNDLLDRVLRHRDAIISHLNWACIFLGFHSFGLYIHND
TMSALGRPQDMFSDTAIQLQPVFAQWVQNTHALAPGVTAPGATTSTSLTWGGDQLVAVG
GKVALLPIPLGTADFLVHHIHAFTIHVTVLILLKGVLFARSSRLIPDKANLGFRFPCDGPGRG
GTCQVSAWDHIFLGLFWMYNAISVVIFHFSWKMQSDVWGTISDQGVVTHITGGNFAQSSI
TINGWLRDFLWAQASQVIQSYGSSLSAYGLFFLGAHFVWAFSLMFLFSGRGYWQELIESII
WAHNKLKVAPATQPRALSIVQGRAVGVTHYLLGGIATTWAFFLARIIAVGMALRFPRFSQG
LAQDPTTRRIWFGIATAHDFESHDDITEERLYQNIFASHFGQLAIIFLWTSGNLFHVAWQGN
FESWIQDPLHVRPIAHAIWDPHFGQPAVEAFTRGGAIGPVNIAYSGVYQWWYTIGLRTNED
LYTGALFLLCLSAISLIAGWLHLQPKWKPSLSWFKNAESRLNHHLSGLFGVSSLAWAGHL
VHVAIPGSRGEYVRWNNFLNVLPHPQGLEPLLTGQWNLYAQNSDSSSHLFGTSKGAGTAIL
TLLGGFHPQTQSLWLTDIAHHHLAIAFLFLVAGHMYRTNFGIGHSIKDLLEAHIPPGGRLGR
GHKGLYDTINNSIHFQLGLALASLGVITSLVAQHMYSLPAYAFIAQDFTTQAALYTHHQYIA
GFIMTGAFAHGAIFFIRDYNPEQNEDNVLARMLDHKEAIISHLSWASLFLGFHTLGLYVHN
DVMLAFGTPEKQILIEPIFAQWIQSAHGKTSYGFDVLLSSTNGPAFNAGRSIWLPGWLNAI
NE--NSNSLFLTIGPGDFLVHHAIALGLHTTTLILVKGALDARGSKLMPDKKDFGYSFPCDG
PGRGGTCDISAWDAFYLAVFWMLNTIGWVTFYWHWKHITLWQGNVSQFNESSTYLMG
WLRDYLWLNSSQLINGYNPFGMNSLSVWAWMFLFGHLVWATGFMFLISWRGYWQELIET
LAWAHERTPLANLIRWRDKPVALSIVQARLVGLAHFSVGYIFTYAAFLIASTSGKFGMSHS
VKIYDTCIGCTQCVRACPTDVLEMIPWNGCKAKQIASAPRTEDCVGCKRCESACPTNFLS
VRVYLWHETTRSMGLSYMTDLNLPSIFVPLVGLVFPAIAMASLSLYVQKNKIVMRNIKTYL
STVPVLTTLWFGSLAGLLIEINRLFPDALLFPFFSFMTAILERRESTSLWGRFCNWITSTENR
LYIGWFGVLMIPTLLTATSVFIIAFIAAPPVDIDGIREPVSGSLLYGNNIISGAIIPTSAAIGLHF
YPIWEAASVDEWLYNGGPYELIVLHFLLGVACYMGREWELSFRLGMRPWIAVAYSAPVA
AATAVFLIYPIGQGSFSDGMPLGISGTFNFMIVFQAEHNILMHPFHMLGVAGVFGGSLFSA
MHGSLVTSSLIRETTENESANEGYKFGQEEETYNIVAAHGYFGRLIFQYASFNNSRSLHFLL
AAWPVVGIWFTALGISTMAFNLNGFNFNQSVVDSQGRVINTWADIINRANLGMEVMHER
NAHNFPLDLAAVEAPSTNGMGLPWYRVHTVVLNDPGRLLSVHIMHTALVSGWAGSMAL
YELAVFDPSDPVLDPMWRQGMFVIPFMTRLGITNSWGGWSIS-GGSITNPGIWSYEGVAGA
HIVFSGLCFLAAIWHWVYWDLEIFCDERTGKPSLDLPKIFGIHLFLSGLACFGFGAFHVTGL
YGPGIWVSDPYGLTGKVQSINPAWGVEGFDPFVPGGIASHHIAAGTLGILAGLFHLSVRPP
QRLYKGLRMGNIETVLSSSIAAVFFAAFVVAGTMWYGSATTPIELFGPTRYQWDQGYFQQ

EIYRRVGVGLAENLSLSEAWSKIPEKLAFYDYIGNNPAKGGLFRAGSMDNGDGIAVGWLG
HPIFRDKEGRELFVRRMPTFFETFPVVLVDVDGIVRADVPFRRAESKYSVEQVGVTVEFY
GGELNGVSYSDPTTVKKYARRAQLGEIFELDRATLKSDGVFRSSPRGWFTFGHASFALLFF
FGHIWHGARTLFRDVFAGIDPDLDAQVEFGAFQKIGDPTTRRQAVMKTLYSLRRFYPVET
LFNGTLALAGRDQETTGFAWWAGNARLINLSGKLLGAHVAHAGLIVFWAGAMNLFEVAH
FVPEKPMYEQGLILLPHLATLGWGVGPGGEVIDTFPYFVSGVLHLISSAVLGFGGIYHALL
GPETLEESFPFFGYVWKDRNKMTTILGIHLILLGIGAFLLVLKALYFGGVYDTWAPGGGDV
RKITNLTLSPSVIFGYLLKSPFGGEGWIVSVDDLEDIIGGHVWLGSICILGGIWHILTKPFAW
ARRAFVWSGEAYLSYSLGALSVFGFIACCFVWFNNTAYPSEFYGPTGPEASQAQAFTFLVR
DQRLGANVGSAQGPTGLGKYLMRSPTGEVIFGGETMRFWDLRAPWLEPLRGPNGLDLG
RLKKDIQPWQERRSAEYMTHAPLGSLNSVGGVATEINAVNYVSPRSWLATSHFVLGFFLF
VGHLWHAGRARAAAAGFEKGIDRDLEPVLSMTPLSMTIAAGRFTKEE-DLFDIMDDWLR
RDRFVFVGWSGLLLFPCAYFALGGWFTGTTFVTSWYTHGLASSYLEGCNFLTAAVSTPAN
SLAHSLLLLWGPEAQGDFTRWCQLGGLWTFVALHGAFGLIGFMLRQFELARSVQLRPYN
AIAFSAPIAVFVSVFLIYPLGQSGWFFAPSFGVAAIFRFILFFQGFHNWTLNPFHMMGVAGV
LGAALLCAIHGATVENTLFEDGDGANTFRAFNPTQAEETYSMVTANRFWSQIFGVAFSNK
RWLHFFMLFVPVTGLWMSALGVVGLALNLRAYDFVSQEIRAAEDPEFETFYTKNILLNEG
IRAWMAAQDQPHENLIFPEEVLPRGNALMSGSTGERSFADIITSIRYWVIHSITIPSLFIAGW
LFVSTGLAYDVFGSPRPNEYFTESRQGIPLITGRFDSLEQLDEFSRSFMTIDRTYPIFTVRWL
AVHGLAVPTVFFLGSISAMQFIQRMATQTVEGSS-RSGPRRTIIGDLLKPLNSEYGKVAPGW
GTTPLMGVAIALFAVFLSIILEIYNSSVLLDGISVSMLTLKLFVYTVVIFFVSLFIFGFLSNDPG
RNPGREEMADTTGRIPLWLIGTVTGIPVIGSIGIFFYGSYSGLGSSLMLNIFSLMCICLNSVF
YSSGFFFAKLPEAYALFNPIVDVMPVIPVLFFLLAFVWQAAVSFRMTQSNPNEQNVELNRT
SLYWGLLLIFVLAVLFSNYFFNMEVNILAFIATALFILVPTAFLLIIYVKTVSQNDMETATLVT
IFISGLLVSFTGYALYIAFGQPSQQLRDPFEEHGDMEALVYTFLLVSTLGIIFFAIFFREPPKVP
TKKMKMTIAFQLAVFALIATSSILLISVPVVFASSDGWSSKKNVVFSGTSLWIGLVFLVAILN
SLIS-------MSPQTETKASVGFKAGVKDYKLTYYTPDYETKDTDILAAFRVSPQPGVPPEEA
GAAVAAESSTGTWTTVWTDGLTSLDRYKGRCYHIESVVGEENQYIAYVAYPLDLFEEGSV
TNMFTSIVGNVFGFKALRALRLEDLRIPTSYSKTFQGPPHGIQVERDKLNKYGRPLLGCTI
KPKLGLSAKNYGRAVYECLRGGLDFTKDDENVNSQPFMRWRDRFLFCAEAIYKAQAETG
EIKGHYLNATAGTCEEMIKRAVFARELGAPIVMHDYLTGGFTANTSLAHYCRDNGLLLHIH
RAMHAVIDRQKNHGMHFRVLAKALRMSGGDHIHAGTVVGKLEGEREMTLGFVDLLRDD
FIEKDRSRGIFFTQDWVSMPGVLPVASGGIHVWHMPALTEIFGDDSVLQFGGGTLGHPWG
NAPGAVANRVALEACVQARNEGRDLAREGNEIIREACKWSPELAAACEVWKEIKFEFKPV
DTLDLTAIHLYKTSTPSTRNGAVDSQVKSNLRNNLISGQHRCGKGRNARGIITAGHRGGGH
KRLYRKIDFRRNEKDISGRIVTIEYDPNRNAYICLIHYGDGEKRYILHPRGAIIGDTIVSGTE
VPISMGNALPLTDMPLGTAIHNIEITLGKGGQLARAAGAVAKLIAKEGKSATLRLPSGEVRL
ISKNCLATVGQVGNVGVNQKSLGRAGSKCWLGKRPVVRGVVMNPVDHPHGGGEGRAPI
GRKKPTTPWGYPALGRRSRKRKKYSDSFILRRRK-MIQPQTLLNVADNSGARELMCIRIIG
AG-NPRYAHIGDVIVAVIKEAVPNMPLERSEVIRAVIVRTCKELKRNNGIIIRYDDNAAVVID
QEGNPKGTRVFGAIPRELRQFNFTKIVSLAPEVLMLSPKRTRFRKQHRGRMRGISCRGNHI
SFGRYALQALEPAWITARQIEAGRRAMTRYARRGGKIWVRIFPDKPVTVRPTETRMGSGK
GSPEYWVSVVKPGRILYEMSGVSETVARTAILIAASKMPIPTQFVITGMTRVRRGYTARRR
RIKIRLFASTFRGAHSRLTRTTTQQKMRALVSSHRDRGRQKRDFRRLWITRINAVTRDN--V

FHSYSKFIHNLYKRQLLLNRKILAQIAIENKNCLYMISNKISQIKEII-------------------MSVFK
ARRIINQIRGRSYEETLMILELMPYRASYPILKLVYSAAANASHNMGLNKADLFISKAEVN
GGTIVKKLRPRAQGRSYPVKKPTCHITIVLK-KSKS-------MDVIKYAVFTEKSIRLLGNNQ-Y
TSNVESGSTRTEIKHWVELFFGVKVIAMNSHRLPGKGRRMGPIMGHTMHYRRMIITLQPG
YSIPPLIEKRTMAVPKKRTSISKKRIRRNIWKKRGYLAAIKAFSLAKSVSTRHSKSFF--QKN
NMAKGKDVRVRIILECASCVRN--G--KELPGISRYITQKSRHNTPNRLELRKYCHYCRTHTI
HGEIKKMKIRASVRKICEKCRLIRRRGRIMVICSNPRHKQRQGMVREEIAESTRTLQWKCV
ESRVDSKRLYYGRFILSPLMKGQADTIGISMRRALLGEIEGTCITRAKSE--KVPHEYSTVVG
IEESVHEILFNLKEIVLRSNLYEIRDASICVRGPRYVTAQDIISPPYVEIVDTTQYIANLTEPID
LCIELEIKRDRGYRMKPITNSQDGSY--PIDAVFMPVRNANHSIHSYGNGNEKQEILFLEIWT
NGSLTPKEALYEASRNLIDLFVPFLHAEEE--DINFEENK-NKFTLPPLTFQDRLS-NLKKNKK
-GIPLKYIFIDQLELPSRTYNCLKRSNIHTLLDLLSKGQENLIKMEYFHIEDVKQILDTLQKH-
-FAVDLPKVLMIRD-GNEGMYTIPGFNQIQFEGFCRFINQGLMEELHKFPKIEDTDQEIEFQL
FVETYQLVEPLIKERDAVYESLTYSSELYVPAGLIWKTSRAMQEQTIFIGNIPLMNSLGTSII
NGIYRIVINQILQNPGVYYRSELDHNGISVYTSTIISDWGGRLELEIDRKARIWARVSRKQKI
SILVLLSAMGSNLREILDNVCYPEIFLSFPNDKEKKKIGSKENAILEFYQQFACVGGDPVFS
ESLCKELQKKFFQQRCELGRIGRRNTNQRLNLDIPQKNTFLLPRDVLAAADHLIRMKFGM
GTLDD--MNHLKNKRIRSVADLLQDQFGLALVRLENGVRGTICGAIR-HKLI-PNPKNLVTST
S----FTTTYESFFGLHPLSQVLDRTNPLTQIAHGRRLSYLGPGGLTGRTASFRIRDIHPSHYGR
ICPIDTSEGINVGLIGSLAIHVRIGHWG-SIESPFY---EISERSKK--AQMIYLSPSRDEYYMVA
AGNSLALNRGIQEEQVVPARYRQEFLTIAWEQIHLRSIFPFQYFSIGASLIPFIEHNDANRAL
MSSNMQRQAVPLSRSEKCIVGTGLERQTALDSGVSAIAEREGKIIYTDTHKIIFSSNGDTMS
IPLVMYQRSNKNTCMHQKPQVPRGKCIKKGQILADGATTVGGELALGKNVLVAHMPWE
GYNSEDAVLISERLVYEDIYTSFYIRKYEIQTHVTSQG-PERITKEIPHLEDHLLRNLDRNGI
VMLGSWIETGDILVGKLTPQTATE-SSYAPEDRLLRAILGIQVSTAKETSLKLSIGGRGRVID
VRWIQKRG-GSIYNPEMIRVYISQKREIKVGDKVAGRHGNKGIISKILPRQDMPYLQDGTPV
DMVFNPLGVPSRMNVGQIFECSLGLAGDLLKRHYRIAPFDERYEQEASRKLVFSELYSASK
QTKNPWVFEPEYPGKSRIFDGRTGDPFEQPVLIGKPYILKLIHQVDDKIHGRSSGHYALVTQ
QPLRGRAKQGGQRVGEMEVWALEGFGVAHILQEMLTYKSDHIRARQEVLGATIVGGTVP
NPEDAPESFRLLVRELRSLALELNHFLVSEKNFQINRKEA-------MIDQYKHQQLRIGPVSPQ
QIRAWANKILPNGEIIGEVTKPYTFHYKTNKPEKDGLFCERISGPIKSGICACGNYRVIGAE
KEDPKFCEQCGVEFVDSRIRRYQMGYIKLACPATHVWYLKRLPSYIANLLDKPLKELEGL
VYCD-VFSFARP-ISKKPTFLRLRGSFEYEIQSWKYSIPLFFTNQSFETFRNREISTGAGAIRE
QLADSDLQIILNNSLVEWKELGDEVIGTGNEWEDRKIRRRKDFLVKRMELAKHFIRTNVEP
KRMVLCLLPVLPPELRPIIQIDGGKLMSSDINELYRRVIYRNNTLTDLLATSRSMPGELVMC
QEKLVQEAVDTLLDNGIRGQPMKDGHNKVYKSFSDVIEGKEGRFRETLLGKRVDYSGRS
VIVVGPSLSLHQCGLPREIAIELFQTFLIHGLIKQHVASNIGIAKSKIREKEPIVWEILQEVMR
GHPILLNRAPTLHRLGIQAFQPILVEERAICLHPLVCRGFNADFDGDQMAVHVPLSLEAQA
EARLLMFSHMNLLSPAIGDPISVPTQDMLIGLYVLTIGNRRGICANRYNTYNCRNS---QNKI
LNNNKYNYIKMKATYCCSSYDALGAYRQKRIDLDSPLWLRWRLDQR-VIGS--REVPIEIQY
ESLGTYHEIYRHYLIVRSVKKKIRWIYIRTTIGHISFYREIEEAVQGFCWAYSYVI--------MGV
FMTERADLVFHNKVIDGTAMKRLISRLIDHFGMGYTSHILDQSKILGFQQATATSISLGIDD
LLTIPSKEWLVQDTEQQRFILEKHYHYANVHAVEKLRQSIEIWYATSEYLRQEMNPNFRMT
DPSNPVHLMSFSGARGNASQVHQLVGMRGLMSDPQGQMIDLPIQSNLREGLSLTEYIISCY

GARKGVVDTAVRTSDAGYLTRRLVEVVQHIIVRRTDCGTIRSISVSPRKGMTE--KIFVQTLI
GRVLADDIYMGLRCIAVRKQEIGIGLANRLIT------FRARPIYIRTPFTCRSTSWICQLCYGRS
PTHGDLVELGEAVGIIASQSIGEPGTQLTLRTFHTGGVFTGGTAEHVRAPSSGKIKFNEDLV
HRTRTRHGHPAFLCSIGLYVTLEGRDIIHNLNIPPKGLILVQNGQYVESEQLIAEIRAGAFTL-
-FKERVRKHIYSESEGE-MHWSTNVYHAPEYPYGNIHLLLKTSHLWILAGASRR--SSIVSFS
LHKDQDQMNVHSFSVEERYIYIFDLS--MTN-HRVKHKLLDTSG----DREILEYS-ISDRIISNG
HWNLISPS-ILQDN--LDFLAKRRRNRFIIPLQYDQEREKELIPCFDISIEIPIN--GILRGNSILAY
FDDPVYRRS--GITKY-TLEVDSIVK--KEDLIEYRG-TKEFN-QI-KVDRFFFIPEEVHILPGSSSI
MVRNNSLIGVHTRLTLNTRSKIGGLVRVERKKKSIELKIFSGNIHFPGETDKISRHSGILIPPG
TGKK--NFNQSKK-ELKNWIYVQR--ITPTKKK--YFVSVRPVVTYEIADGINLATLFPQDLLQ
EKDNVQLRVVNYILYGNGKSIRGIYHTS-QLVRTCLVLNWDQEQNDSIE----EVHTSFIEVR
ANDLIRDFIRIDLIKSTISYTGKRNDTGSSGLIPD---LNSTNINPFYSKSKIQS-----LTQDQETI
GM--GTLLNRNK---ECQSLIILSSSNCSQIG--PFNGSKYK---NVTKESENIIPI-DFLGSLGTIVP
-KIANFYSS-YHLIT---HNQILLK-RFLLLD---NSKQTFQVL---KYGLIDENSR--IYSPDPCSNII
LNPFRLNWCFLRHNYCEETSTIIS-LGQFFCENICLFKYGPNINIKKSGQIIIVH-VDSLVIRSA
KPYLVTLGATVHGHYGKILYEGDRLVTFIYERSRSGDITQGLPKVEQVLEVRSIDSISMNLE
KRVNSWNECISRILGRPWGFMIGSELTIAQSRISLVNRIQKVYRSQGVQIHNRHIEIIVRQVT
SRVLVSE---DGMSNVFSPGELIGLLRAERAGRALDEAICYRAILLGITRASLNTQSFISEASF
QETTRVLAKAALRGRIDWLKGLKENVVLGGIIPVGTGFQKLVHRSR--QDKNIH--LEIKKN
NLFDLEM--RDILLHHRELFCSCIQD-----SEQSFTNS---------VKRRYWNINLPEMMEAGVHF
GHGTKKWNPRMAPYISAKRKGIHITNLTITARFLSEACDLVFDAASRGKSFLIVGTKNKAA
DSVASAAIRARCHYVNKKWLGGMLTNWSTTETRLHKFRDLRAEQNMGKLNRLPKRDAA
MLKRKLSALQSYLGGIKYMTGLPDIVIIVDQEKEYMALQECAILGIPTICLIDTNCNPDLAD
ISIPANDDAIASIRLILNKLVFAICEGRYS-YIGNRMGQKINPLGFRLGTTQSHHSFWFAQPRN
FSTGLQEDEKIRNSIKNYIQKNRRISSG-FEGIARIRIKKRIDLIQVIIHIGFPNLLIEG--RTR--GI
EELQINVQKEFHFSNRRLKIAITRVEKPYEQANILAEYIALQLKNRVSFRKAMKKAIELTEQ
TDTKGIQVQIAGRIDGKEIARVEWIREGRVPLQTIRAKIDHCSYTIRTIYGVLGIKIWIFVD--
MSRYRGPRFKKIRRLGALPGLTSKRPRSGSDFKNQSRFGK-KSQYRIRLEEKQKLRFHYGL
TEQQLLRYVHIAGKAKGSTDQVLLQLLEMRLDNILFRLGMASTIPAARQLVNHRHILVNG
RIVDIPSYRCKPRDIITSKDKQRSKVLIQNSIESSPHE--LPKHLTIDS--FQYKGLVNQIIDSKLI
GLKINELLVVEYYSRQTMSRRGTAEEKTAKSDPIYRNRLVNMLVNRILKHGKKSLAYQIIY
RTVKKIQQKTETNPLSVLRQAIRGVTPDIAVKARRVGGSTHQVPIEIGSTQGKALAIRWLL
GASRKRPGRNMAFKLSSELVDAAKGSGDAIRKKEETHRMAEANRAFAHFRMGRDTIANII
TSIRNADMDKKGTVRIASTNITENIVKILLREGFIENVRKHQENQRFFLVSTLRHRR--TSKG
VYR-----TILKRISRPGLRIYSNYQRIPKILGGIGIVILSTSQGIMTDREARLKKIGGEILCYIW
MTKPIPRIGSRRNGRIGSRKNARRIPKGVIHVQASFNNTIVTVSDVRGQVISWASAGTSGFK
GTRRGTPYAAQAAAVNAIRTIIDQDMQRAEVMIKGAGFGRDAALRAIRRSGILLSFVRDVT
PMPHNGCRPPQKRRVMPTIKQLIRNTRQKIRNVTKSPALRGCPQRRGTCTRVYTITPKKPN
SALRKVARVRLTSGFEITAYIPGIGHNLQEHSVVLVRGGRVKDLPGVRYHIVRGTLDAVGV
KDRQQGRSKYGVKKPKMARKSLIHREKKRHKLEQKYQLIRRSSKKEIS---KVLPLSEKWK
IHRKLQSSPRNSAPIRLHRRCFLTGRPRANYRDFGLSGHILREMVYACLLPGAIRSSWMIKN
SFISVIPQEEKEEN--KGSVEFQVFSFTNKIRRLTSHLELHKKDFLSQRGLRKILGKRQRLLTY
L L K K N R V R Y K K L I G Q L D I R E P K I H -
-???

MDKSKQPFRKSKRSFRRRLPPIGSGDQINYRNMSLISRFISEQGKILSRRVNRLTLKQQRLIT
IAIKQARILSLLPFLNNEK--QFERAESIPRTTGPRTRNKVTRSLKKNPFVVDHLSGKIEKLN
MREEKEIIVTWSRGSTIIPAMMGHTIAIHNGKEHFPIYITDQMVGHKLGEFSPTLTFARHTR
NDNKSRRMIFKSFLLGNLLSLCIKIINSVVVVGLYYGFLTTFSIGPSYLFLLRARVMEEG---T
EKEVSATTGFITGQLMMFISIYYAPLHLALGRPHTMTVLVLPYLLFHFFWKNQKNFTSIRA
STRDSMCNLNIQCVFLNNLIFQLLNHFILPSSTLARLVNIYMFRCNNKMLFVTSSFVGWLIG
HFLFMKWLGLVLFWIPQNYSIRS----NKYLVSELRNSMGRIFSILLFITCVYYLGRMPSPIVT
KKLKETEEDKPLVNFLFDYKRWNRPLRYIKNDRFENVVRNEISQYFFYTCPSDGKKRISFT
YPLSLSTLSEMIERKIFLYTTEKLSHEGL--YWVYTNEQKKYNMSNELLSRIKVLEKEKLVI
DMLEKRTRLYNDENEQKCLHKTYDPLLNGPYRGTIKKLYSKIETFWINKIHDILSRKFEHQ
DLNDESIGIEEILKKVPRWSYKLTGLEE-EDEGLRSRKAKRVVIYTDALIRYSQQSDFRRDII
KGSMRAQRRKTVTWEMFQANMHSALFLDRIDKTFFFSSFD-ISEMRNIIFRNENKRIRIAIS
ETWDTIIFAQAIRGSMLITQSFLRKYIILPSLIIAKNIGRILLFQFPEWDEDLKEWNREMHVK
CTYNGVQLSETEFPKDWLTDGIQIKILFPFCLKPWRKPKKRKKENFCFLTIWGMEAELPFG
SPRKQSSFFEPIYKELEKKMRKVK-KKFFRVLRVFKERKKGFLKVRKEKTGWILPIVL--IRK
QIMKEFEKINDKTP-NKITRESSIQIRSMNWTNYSLIEKKMKDLADRTLTTRNQIEQITKDN
KK--------GKRLESRKKIFQILKRQYIRLLRKSDSFMKSFIEKIYINIFLCTINIFSINIQLFMNFI
SYNLSSLSQAYVFYILSQTQLFNKYHLRSVLHYTYPFLRDTIKNYCTQGIFDTKSRYKKIHK
SEINEWKNWLRDHYQYKLSQTKWFRLVPQKWGNKVNKHYIIQNKIKLDSYKKEKDQLIH
YMKQEKLKKHYRYDLLSHEYMNGYIVDMNRKYFDSRILCFPIRVFDWIGMNEERLYHWF
FPEFWLLFNAYKIKPQIIPIQLLFLNSKSNKEAELYFFLKKYLLFQLRWDDSLNQRMIDNIK
VYCLLLRLINTKEIVISSIQRGEMCLDVMLIQKDLALTELIKKGILIIEPIRLFLERGVKLIIYQ
TIGISLINK--NNHYDFFVPENILSPRRRRELRILICFRFLWPNYRLEDLACMNRYWFDTNNG
SRFSMSRIHMYPMKRHQFKSWIFELREIWREIKNSHYFLDSWIKFDSVGFFTHIFFHQERF
MKLFDPRIGSILLS-RDS--QGSTSNRYFT---IKGVVLLVVA-VLIS----RINNRKMVERKNLYL
MGLLP-----IPMNSIGPRNETLEESF-WSSNINRLIVS-LLYLPKRKKISESCFMDPQERTWVLP
IN----KKCIMPESNRGSRWWRNRIGKRR--DSSCKIS---NETVAGIEISFKEKDSK-YLEFLFLS
YT--DNPIHKDHDWELFDRLSPRKKRNIINL--NSGQLFEILGKDLICYLMSAFREKRPIEGEG
-FFKQQG-AEATIQSNDIEHVSH--LFSRNKWGISLQNCAQFHMWQFRQDLFV---SWGK-NQ
HESDFLRN-VSRENLIWLDNVWLVNKDR---FFS--KVRNVLSNIQYDS---TR--SIFVQVTDSS
QWKGFS-DQSRDHFDSIR----NVDSE------------YHTLIDQTEIQQLKERSIL-WD--PSFLQTER
TEIESD--RFPK--CLFGSS--SMSRLFT-EREKQMNNHLLPEEIEEFL--GNPTRSIRSLFSDRW--
SELH-LGSNPTERSTRDQ----KFWKKKQDVS-FVLSRRSE----NKEMVDIFKIITYLQN--TVSI
HPISSDPGCDMVP-KDEPD-MDSSNKISFLNKNPFS---------------------------EEGFQEMADLF
TLSITEP-DLVYHRGFAFSIDSYGLDEKK--FLNEVFNSRDESKKKSL---LVLPPLFY--EENESF
YRRIRKKSVR-IYCGNDLEDPKLKTAVFASNNIMEAVNQYRLIRDLIQVQYRTYG--YIRNVS
NRFFLM---NRSDRNFEYGIQ---RDQIGNDTLNHITIMKYMINQHLSNLKK-S--KWFDS----LI
SRTERSMNRDPDAYRYKWSNGSKNFQKHLEHFISEQKN---RFQVVFDRLRINQYSIDWSE
AIDKQDLSKSLRFFLSKS---------LLFLS-------KSLPFL-SKSLPFFFVSIGDIPIHRSE-IHIY-EL
KGPNDQLCNQLLESIGVQIVHLNKLKP--FLLD---DHDTS-QRPKFLINGGTILPFLFKKIQ-K
W-MIDSFHT--RKNRRKSFDNTDFYFSMISHDRDDWLNTVKPFHRSSLISSFYKANRLRFLN
DPHHFWFY--CNKRFPFYV----EKTRINNYD--LTY-GQFLNILFIRNK-IFSLCVGK--KKHILLE
-RETISPIESQVSDIFIPNDFPQSGDET-YKLYK---SFHFPIRSDPFVR--RAIYSIADISATPLTEE
QIANFERTYCQPLSDMNLFDSEGKNLHQYLS--FNSNMGLIHTPCSEKYLP--SGKRKK-------

-QSLCLK--KCVEKRRMYRTFQRDSAFSNLSKWNLFQTYMPWLLTSTGCKYLNFTLLDTFS
DPL--PIPSSS-QKFVSIFNDIMHGS--AWPIPQKKLW----AILPQWTL-ISEISSKCLQNL-LLLSE
EMIHRNNESPVLLIWTHLRSTNAREF-LYSILFLLLVAGY-LVRIHLLFVFR--ASSELQTGLEK
IKSLMIPSYMIELRKLLYRYPTSELNSFWLKNLLLVALEQLGDSLEEIRGSASGGNMLLGGG
P-----AYG-IKSIRSKKKYLNIN---LIDLI-SIIPNPINRITFSRNTRHLSRTSKEIYSLIRKRKNVN
GDWIDDKIESWVANSDSIDDEEREFLIQFS--TLTT--EKRIDQILLSLT-HSDHLS-----KNDSGY
QMIEQPGSIYLRYLVDIH--KKYLMNYEFNRSCLAERRIFLAHYQTITY--SQTSCGANSFHFP
SHG---KPFSLRLALSPS-RGILVIGSIGTGRSYLVKYLATNSYVPFITVFPNKFLDDKPKGYLI
D-----DID-IDESDD------------------------------IDDDLDTELLTMTNVL---TMYMT---PKIDQFDIT
LQFELAKAMSPCIIWIPNIHDLYVNESNYLSIGLLENYLS---RDCERCS-TRNILVIASTHIPQ
KVDPALIAPNKLNTCIKIRRLLIPQRRKHFFILSYTRGFRLEKKMFHT--NGFESITMGSNAR
DLVALINEALSSSITQKKSIIETNTIRSALHRQTWDLR---SQVRSVQDHGILFYQIGRAVAQN
VLLSNCPIDPISIYMKKK--SRKEGDSYLYEWYFELGTSMKKLTILLYLLSCSAGSVAQDLW
SSPSPGPDEK-NGITSYGFVENDS-DLVHGLLEVEGALVG--SSRTEKDCSQFDNN--RVTLLE-
--PKN-QLDMMQNGSCSIVDQRFQYEK--YESEFEE-GEGEGALDPQ----QIEED----LFNH--IV
WAP--RIWRP---CGNLFNCIERPTELGFP-YWAGSFR---GKQIIYHKEDELQENDSEFLQSGT
MQYQTRDRSSKEQGFFRISQFIWDPADPFFFLFK-DQPFVSVFSRREFFADEEMSKGLITSQ
TNPPTSIYKRWFI--KNTQEKHFELLIHRQRWLRT---NSSLS--NGSFRSN--TLSESYQYLSNLF
LSN-GTLLDQMTKTLLRKRWLFPDEMKHLI--HVTGERFPIPMPRSQ-INGNFIDKTSSIVANI
LLRIIPTTSGEKKAFTYYRDG--MSAQSEGNYAEALQNYYEATRSEIDPYDRSYILYNIGLIH
TSNGEHTKALEYYFQAIERNPFLPQAFNNMAVICHY-QGERAILRGDSEIAEAWFDQAAEY
WKQAIGLTPGNYIEAHNWLKITRRLEFEMNWRSEHIWIELITGSRKTSNFCWACILFLGSL
GFLVVGTSSYLGRNLISVFPSQQIIFFPQGIVMSFYGIAGLFISSYLWCTISWNVGSGYDRFD
RKEGIVCIFRWGFPGINRRIFLRFLMRDIQSIRMQVKEGLYPRRVLYMEIRGQGAIPLTRTDE
N-FTPREIEQKAAELAYFLRVPIEGK
>Cycas
LKEWF------ISGSIED--LIER--TSKDYIVNEMDKNKN---IKID
FN---
--NRLWVQCDNRENLLYMKYLRQNKSVCEECGYHLQMSSSDRIELSIDHGTWHPMDEDM
AAPDPIQFH--DEPHIDRITSCQIRTGLTDAVQTGIGRPNGIPIAIGVMDFQFMGGSMGSVVG
EKITRLIEYATKQFLPVIMVCASGGARMQEGSFSLMQMAKISTALYIHQLEKKLLYVSILTY
PTTGGVTASFGMLGDIIIAEPKAYIAFAGRRVIEQTSGQKVPDGLQVAEHLFDHGSFDLIVP
RSLLKGVLSEPFQLYGIPCEKERTLGMVTIRPDEISSIIRKQIKQYNQEVEVVNIGIVLQVGD
GIARIHGLDEVMAGELVEFEDGTVGIALNLESNNVGAVLMGDGLMIQEGSSVRATGKIAQ
IPVSDAYSGRVVNALAQPIDGRGKITASESRSIESPAPGIISRRSVYEPLQTGLIAIDSMIPIGR
GQRELIIGDRQTGKTAVATDTIINQKGQDVICVYVAIGQKASSVAQVVNIFQERGAMEYTIV
VAETADSPATLQYLAPYTGAALAEYFMYKEQHTLIIYDDLSKQARAYRQMSLLLRRPPGR
EAYPGDVFYLHSRLLERAAKLSSQLGEGSMTALPIVETQAGDVSAYIPTNVISITDGQIFLS
ADLFNAGIRPAINVGISVSRVGSAAQIKAMKQVAGKLKLELAQFAELEAFAQFASDLDRAT
QNQLARGQRLRELLKQSQSAPLTVEEQIATIYAGANGYLDILEIVQVRKFLVQLREYLITNK
PQFGEIIRSTRAFTEQAEALLKEAIQEHIELFLLREQKMRTNPLAL---VPTLVEKNVGHIVQII
GPVLDVVFSPGNMPNILNSLIIKGKDTAGQEINVTCEVQQLLGNNKVRAVAMSATDGLKR
GMKVIDTGAPLSVPVGGATLGRIFNVIGEPVDNLGPVDARTTSPIHRPAPAFIQLDTKLSIFE
TGIKVVDLLAPYRRGGKIGLFGGAGVGKTVLIMELINNIAKAHGGVSVFGGVGERTREGN

DLYVEMKESGVINEQDISGSKVALVYGQMNEPPGARMRVGLTALTMAEYFRDVNEQDVL
LFIDNIFRFVQAGSEVSALLGRMPSAVGYQPTLSTEMGSLQERITSTKEGSITSIQAVYVPAD
DLTDPAPATTFAHLDATTVLSRGLAAKGIYPAVDPLDSTSTMLQPWIVGEEHYETAQGVKQ
TLQRYKELQDIIAILGLDELSEEDRLTVARARKIERFLSQPFFVAEVFTGSPGKYVGLVETIR
GFKMILSGELDGLPEQAFYLVGNIDEATAKAMNLKVEN----MTLNLRVLTPNRVVWDSEV
EEIILSTNSGQIGVLPNHAPIVAALDIGITKIRLDGQWSTMALMGGFARIDNNKITILVNDAE
KGIDIDPREARETFRIAEADLARAEGKRQVIEANVALRRARTRLEAIPSVSMINGTDFSFSL
GYWPPAGGFGLNTNILGTNLINLSVVLGVLIYFGKG-LSNLLDDRKQKILSTIRDSEELYKG
ATDQLEKARARLREVEMRADEIQVNGYSQIEREKEDLINAAHENLERLEDSKNETVNFEQ
QRAIDQVRQQISRQALRRALGTLNSRLNNELHLRTIDHNISMLRAMKNTTDMNPLIPAAS
VIAAGLAVGLASIGPGVGQGTAAGQAVEGIARQPEAEGKIRGTLLLSLAFMEALTIYGLVV
ALALLFANPFVMDIVQSSIDTLNHFYEISGVEVGQHFYWQIGGFKVHAQVLITSWVVIAVL
LGSATIAVRDPQTIPTGGQNFVEYVLGFVRDLTRTQIGEEYGPWVPFIGTMFLFILVSNWSG
ALLPWKIIQLPHGELAAPTNDINTTVALALLTSVAYFYAGLAKKGLGYFGKYIQPTPVLLPI
NILEDFTKPLSLSFRLFGNILADELVVAVLVSLVPLVVPIPVMFLGLFTSAIQALIFATLAAAYI
GESMEGHHMIFITLEHILAHISFSLISVVTLAYWGT-LVHQIEGLSSSGGKGMIVTFVCTTGL
LITRWLYSGHLPLSNLYESFMFLSWSSSVIHIILEVRSQKDGLGAITAPSTMLTHGFATSGLP
KEMQQSAMLVPALQSHWLMMHVSMILLSYATLLCGSLSSIAFLIITFRRNRRILSLLSNNLY
LHEQESDLQNTFYLSFTNHRKCKLTQQLDYWSYRVIGLGFLLLTIGILSGAVWANEAWGSR
WSWDPKETWALITWIIFAIYLHTRVNKGWQDENPAIIASLGSFIVWICYLGVDLLGIGLHSY
GWLI--VAKK-ALASLRYLACLVFLPWGISISFQKGLEPWVTNWWNTGQSREFSDYLQEEN
ALERFGEIEELFLLERMVEDSSETHSQDLRIEIRKKTIQLVEMYNEDLIQIISHLLANFICFAT
PGAYFILGKEKLVVLNSWIQELFHSLSDTMKAFSILLVTDLCIGFHSPHGWELMIDSISENY
GFSHDEQRISGLVSTFPVISDTIFKYWIFRHLNRISPSLVVIYHSMNEMPIGVPKVPFRIPGEE
D------N-RLYRERLLFPGQQVDDEIANQLIGIMMYLNGEDENKDIYPYINSPGGAVIPGISIY
DAMQFVVPDVHTICMGLAASMGSSVLTGGEITKRIALPHA-VMIHQPASSYYDGQAGECI
MEAEEVLKLRDSITNVYVQRTGKPLWVISGDMERDVFMSATEAQAYGIVDLVAVEMNKQ
NLIDEGSVTESLPNGMFRVRLDNGCQVPTHISGKIRRNHVRILPGDRVKVELSPYDLTKGRI
IYRLRNKSSNDMDKFRRNGKEDTFRQRRFLYPLLFQENLYAIAYDHYLSSYNDRFSFLTVK
RLISRIRQQNGSIVSFGNQNKNKLVGRNFYSELVLEGLTVVLEVTFSIQ--SKL-NEWNSFRSI
HSIFPFMEDKFPHSNFLLDIRIPHSTHPEILVRTFRYWIQDAPSLHSLRSVLHEHRNLILSENI
LIASKEKTRLSLVLWNYYVYECESLLVPLWKRFSYSRSLSYGAFLERTTFYRKIEH-IVIFSH
DLRIWFLKDPSIHYVKDRERFLIALRGTYLLVKKWRYHLTNFWQCHFHLRSQPYRMSIDE
LSKNCFSFLGYLFSVQMKTFVVKIKMLDDSFITDPIT--KEFDPIAPTTLLIGYLAKERFCDIS
GRPTGRLAWTGLTDDNILHRFDRIWRNILHYYSGSSKKDGLYRMKYILRLPCAKTLACKH
KSAIRVVRERFGSELFTKSSPKERELISLSFS-RIWHSDILQRNPFVNSWMVINTT-DPEERVI
NLSFVLGFIKEIFGLIWIRIYILIPILGVLIGLLVIVWLERKISAGIQQRIGPEYAGPLGILQALA
DGIKLLLKEDIIPSRGDLWLFSIGPAIVVIPILSSYLVIPFGRHIVLADLSIGVFFRIAVSSIAPLG
LLMAGYGSNNKYSFSGGLRAAAQSISYEIPLACVLSISLL-SNSLGTVDIVEAQSRYGFWG
WNLWRQPIGFIAFFISSLAECERLPFDLPEAEEELVAGYQTEYSGIKFGLFYVASYLNLLASS
LFVTILYLGGWNFSIPSIPISEYFEWDGTSEVLGITMGILITLAKAYLFLFISITARWTLPRMRI
DQLLDLGWKFLLPIALGNLLLTASFQLLLL------------------MKEFHLLLFHGGSIFPECILILG
LILLLMIDLTSDQKDTPWFYFISLTSLVMSITVLLFRWREEPMISFLGNFQTSNFSKIFRFLIL
LCSTLCIPLSVEYIKCTEMAITEFLLFLLTAALGGMVLCGANDLVTIFVALECFSLCSYLLSG

YTKRDVRSNEATMKYLLMGGASSSILVYGFSWLYGLSGGEIELQEVVNGLINTQMYNSPG
ILIALISIAVGIGFKLSLVPFHQWTPDVYEGSPTPVVAFLSVTSKVAASALATRIFDLIFYFSSN
EWHLLLEILAILSMILGNLIAITQTSMKRMLAYSSMGQIGYIIIGIIAGDSKNGYASMITYML
FYIFMNLGTFACIVLFGLRTGTDNIRDYAGLYTKDPFSAFSLALCLLSLGGIPPLAGFFGKLY
LFWCGWQAGSYLLVSIGPLMSVISIYYYLKIIKLLMTERNKEITPHVQNYRRSPSSFKNSIE
LSMIVCVTASTTLGIVMNPIIAIAQDTLFMFLLFEYETFWIFLLISSLMPILAFLISRALAPISE
GPEKLTSYESGIEAMGDAWIQFRIRYYMFALVFVVFDVETVFLYPWAMSFDILGISTFIEASI
FVLILIVGSVHAWRRGALEWSMSDLPWLTIIVILPISAGSSIPLFPHRGNNIIRWYTLGICLLE
FLLMTYTFRYHFHFDDPLIQLEEDYDWIDLIDLHWRLGIDGLSIGPISLTSFVTTLATLAAW
PVTRNPRLFYFLMLAMYSGQVGLFASRDILLFFLMWELELIPVYLLISMWGGKRRLYSAT
KFILYTAGGSIFISMGASSMGLY-FDGPTLDFEILANKYYPVVLEIVFYLGFFIAHAIKLPILPL
HTWLPDTHGEAHYSTCMLLAGIPLKMGGYGLIRINMELLPHAHSLFSPWLVIVGAVQIIYA
ALTSLSQRNLKRRIAYSSVSHMGFVIVGIGSMADTSLNGAILQMISHGLIGAALFFLAGTSY
DGIRTLFLDGIGGMAIPMSKISTMFSSFSMASLALPGMSGFVAESMVLLGIITSRKYSLTFQI
VIAAIMAIGMILTPIHLLSMLRRMFYGYKFLNVLNPYLKDSGPREIFISICLFLPVIGTGTYP
DLVLSLWDKKVEAIMFRSFHE-MLEHALISGAYLFSIGIYGLITSRNMVRALMCLELILNAV
NVNLVTFSNYFDSRQVKGDIFSIFVTAIAAAEAAIGLAIALAIYRNRKSTRIDQFNLSKWME
LIYKYVWIVPFLPLSASVPIGSGSLLFPRATRGLHHIWALISISLLGIAMLLSFNLFLQQITGG
PVYQYLWSWTINKNFSPELGYLIDPLTSIMPISVTTVGIMVMIYSDNYMSHDQGYVRFFAY
LSLFNASMLGLVISPNLVQIYIFWELVGMCSYLLIGSWFTRPSAANACQKAFITNRIGDFGL
LLGILGFYQITGSFKFRYLLGRSNE-EVSSFFATLCAFLLFLGPVAKSAQFPLHVWLPDAME
GPTPISALIHAATMVAAGIFLVARLFPLFKALPFIMNLISWTGGITALLGATMALAQSDLKR
GLAYSTMSQSGYMVLALGTGSYRAALFHPITHAYSKALLFLGSGSVIHSMESLVGYCPAES
QNMALMGGLSKYMPITGTTFLLGTLSLCGIPPFACFWSKDEIISDSWLYSTILGGIARSAAG
FTAFYMSRMYFLAFEGDLRVN----LYNDPIPFYSISMWGEKESGTFTETMPQLPGNKFQFPL
DKGDPPYPKESDNTMLLPSLVLGLFTLFVGSIGVSFSSDILSQRLTL-MNHFHENHP--ENWS
EFFVDAIPSVGIAFSSTFVAFVLYGPINF-----NFSSPYLGIPDRFINVIYNWSYYRGYIDIYYN
KIFTMGIRRLAELTYLFDRWVIDGIIDRVGILGLFVGEGMKYLGGG-RTSSYLFGFLFFAVIF
LLTIFMDLPGPIRDILLVPLELGLILGGLEVVLLTNIIYSALSLGPVLVCISLLYILLNADFVAA
AQILIYVGAVNVLIVFAVMLMNNQKY-PNFPLWTVGDGITLVVCTSLFCSLITIILNTSWSEI
SMTTKSNQILEQDLTNNVQRIGAHLSTDFFLPFELLSIILLVALVGAITIARRMTVLATEKDS
VIVSMGPHHPSMHGVLRLIVTLDGEDVIDCEPILGYLHRGMEKIAENRTIVQYLPYVTRW
DYLATMFTEAVTVNAPERLGNIQVPKRASCIRVIMLELSRIAPHLLWLGPFMADIGAQTPFF
YISREREIIYDLFEAATGMRMMHNYFRIGGVAVDLPYGWIDKCLDFCDYFLSKVAEYERLI
TRNPIFLERVEGVGIIGREEAIDWGLSGPMLRASGIQWDLRKVDHYECYDEFDWEVQWQ
KEGDSLARYLVRIGEMTESIKIIRQALKIIPGGPYENLEARRLDKGKDSEWNDFEFRFISKK
PSPTFESPKQEHYVRVEAPKGELGIFLMGDDSIFPWRWKIRPPGFINLQIPPQLVKSMKLAD
IMTILGSIDIIMGEVDRMFSMVTGFIDYSQRAIQAARYIGQGFMVTLYHMNRLPMTIQYPY
DKLIPSERFRGRIHFEFDKCIAREVCVRVCPIDLPVVDWNFGRDIGKKRLENHSIDFGICIFC
GNCVEYCPTNCLSMTEEYELSTYDRHELNYDQIALGRSPISVIEDSTIRTIFSS--TMDGHLD
PRTVTSSVQGRLSAWLAKHKLAHRPLGSDYQGIETLQIKSEDRASVAVALYVYGFNYLRS
QCAYDVAPGGSLASVYHLTNIQDDADQPEEICIKVFVPRKNPRIPSIFWIWKSADFQERESY
DMLGILHESHPRLKRILMPESWIGWPLRKDYITPNFYEIQDAH------------------------------------
----------MG-------------STESPLLGQTTPNSVISTTLNDLSNWARLSSLWPLLYGTSCCFIEFAS

LIGSRFDFDRYGLVPRSSPRQADLIITAGTVTMKMAPSLVRLYEQMPGPKYVIAMGACTIT
GGMFSTDSYSTVRGVDKLIPVDVYLPGCPPKPEAIIDAIIKLRKKVAREIYEDRTKLQQGKR
YFTQNHKFRVGSTSEILSKTFESTSENSVQESRNEEYFVKSNEQEINFQKFHMRNINTYDW
MKKWMTRSISILVMIHMITRTSISNAYPIFAQQGYENPREATGRIVCANCHLAKKPVNIEVP
QSVLPDTVFEAVVQIPCDMQIKQVLANGKKGALNVGAVLILPEGFELAPPDRISPEIKEKIG
NLYFQNYRPNQKEILVIGPVPGQKYSEIVFPILSPNPATNKEVHFLKYPIYVGGNRGRGQIY
PDGSKSNNTVYNASTTGRVSKILRKEKGGYEITIDNASDGRQVVDIVPPGPELLISEGEFIK
VDQPLTNNPNVGGFGQGDAEIVLQDPLRVQGLLLLLASVILAQIFLVLKKKQFEKVQLAE
MNFMGKVYDWFEERLEIQAIADDITSKYVPPHVNIFYCLGGITLTCFLVQVATGFAMTFYY
RPTVTEAFASVQYIMTEVNFGWLIRSVHRWSASMMVLMMILHVFRVYLTGGFKKPRELT
WVTGVILAVLTVSFGVTGYSLPWDQIGYWAVKIVTGVPEAIPVIGPPLVELLRGSVSVGQS
TLTRFYSLHTFVLPLLTAVFMLMHFLMIRKQGIPGPLMGVTKKPDLNDPVLRARLAKGMG
HNYYGEPAWPNDLLYIFPVVILGTIACTIGLAVLAPSMIGEPANPLATPLEILPEWYFFPVFQI
LRTVPNKLLGVLLMASVPAGLLTVPFPENVNQFQNPFRRPVATTVFLIGTAVALWLGIGAA
LPIDESFTLGLFMIEALPSGIVLGLIPITSAGSSVTAYLQYRRGDQLDITPTITSYFGFLLASLI
FTLALFIGSSKIRLLMDIVSIAWAALMVVFTFSLSLVVWGRSGLMIIRSPEPEVKIVVERDPI
KTSFEKWAKPGHFSRTLAKGPNTTTWIWNLHADAHDFGSHTNDLEEISRKVFSAHFGQLA
IILIWLSGMYFHGARFSNYEAWLSDPTHIKPSAQVVWPIVGQEILNGDVGGGSRGIQITSGL
FQIWRASGITSELQLYCTAIGALIFAALMLFAGWFHYHKAAPKLAWFQDVESMLNHHLAG
LLGLGSLSWAGHQVHVSLPINQLLDAGVDPKEIPLPHEFISNRDLLAQLYPSFAEGLTPLFT
LNWSEYSEFLTFRGGLNPVTGGLWLTDTAHHHLAIAILFLIAGHMYRTNWGIGHSLKEILE
THKGPFTGEGHKGLYEILTTSWHAQLALNLAMLGSLTIVVAHHMYSMPPYPYLAIDYGTQ
LSLFTHHMWIGGFLIVGAAAHAAIFMVRDYDPTTQYNNLLDRVLRHRDAIVSHLNWACIF
LGFHSFGLYIHNDTMSALGRPQDMFSDTAIQLQPIFAQWVQNTHALAPGSTAPDATASTSL
TWGGGDLVAVGGKVALLPIPLGTADFLVHHIHAFTIHVTVLILLKGVLFARSSRLIPDKANL
GFRFPCDGPGRGGTCQVSAWDHVFLGLFWMYNAISVVIFHFSWKMQSDVWGSISDQGM
VTHITGGNFAQSSITINGWLRDFLWAQASQVIQSYGSSLSAYGLLFLGAHFVWAFSLMFLF
SGRGYWQELIESIVWAHNKLEVAPVIQPRALSIVQGRAVGVAHYLLGGIATTWAFFLARILA
VGMASRFPKFSQGLAQDPTTRRIWFGIATAHDFESHDDITEERLYQKIFASHFGQLAIIFLW
TSGNLFHVAWQGNFEAWVRDPLHVRPIAHAIWDLHFGQPAVEAFTRGGAPGPVNIAHSGV
YQWWYTIGLRTNEDLYTGALFLLFLSAISLIAGRFHLQPKWKPSVSWFKNAESRLDHHLS
GLFGVSSLAWTGHLVHVAIPESRGEHVRWDNFLDVLPYPRGLGPLFTGQWNLYAQNPDSS
SHLFGTSQGAGTAILTLLGGFHPQTQSLWLTDIAHHHLAIAFVFFIAGHMYRTNFGIGHSIK
DILEAHIPPGGLLGRGHKGLYNTINNSLHFQLGLALASLGVITSLVAQHMYSLPAYAFIAQD
FTTQAALYTHHQYIAGFIMTGAFAHGAIFFIRDYNPEQNRDNVLARMLEHKEAIISHLSWA
SLFLGFHTLGLYVHNDVMLAFGTPEKQILIEPIFAQWIQSAHGKALYGFDVLLSSTNSPAFN
AGQSIWLPGWLNAIND--NSNSLFLTIGPGDFLVHHAIALGLHTTTLILVKGALDARGSKLM
PDKKDFGYSFPCDGPGRGGTCDISAWDAFYLAVFWMLNTIGWVTFYWHWKHITLWQGN
VAQFNESSTYLMGWLRDYLWLNSSQLINGYNPFGMNSLSVWAWMFLFGHLVWATGFMF
LISWRGYWQELIETLAWAHERTPLANSVRWRDKPVALSIVQARLVGLAHFSVGYIFTYAAF
LIASTSGKFGMAHSVKIYDTCIGCTQCVRACPTDVLEMIPWEGCKAKQIASAPRTEDCVG
CKRCESACPTDFLSVRVYLWHETTRSMGLAYMTNPNLPSIFVPLAGLFFPAITMAFLYFYV
QKDEILMRDIKTYLSTAPVLATLWFGSLAGLLIEINRFFPDALTFPFFSFMTAILEKRESASL
WGRFCDWITSTENRLYIGWFGVLMIPTPLVATSVFIIAFIAAPPVYIDGIREPVPGSLLYGNNI

RYGAIIPTSAAIGLHLYPIWEAASVDGWHYYVGPYELIVMHLLLGVARYMGREWELSFRL
GMRPWIAVAYSAPVAAATAVFLIYPIGQGNFSDGMPLGISGTFNFMIVFQAEHNILMHPFH
MLGVAGVFGGSLFSAMHGSLVTSSLIRETTENESANAGYKFGQEGETYNIVAAHGYFGRLI
FQYASFNNSRSLHFFLAVWPVVGIWFTALGISTMAFNLNGFNFNQSVVDGQGRVINTWAD
IINRANLGMEVMHERNAHNFPLDLAVAEVTFIDGMGLPWYRVHTVVLNDPGRLLSVHIM
HTALVSGWAGSMALYELAVFDPSDPVLDPMWRQGMFVIPFMTRLGINNSWGGWSIT-GET
VTNPGLWSYEGVAGAHIVFSGLCFLAAIWHWVYWDLDIFCDERTGKPSLDLPKIFGIHLFL
SGVACFGSGAFHVTGLYGPGIWVSDPYGLTGKIQPVNPAWGAEGFDPFVPGGIASHHIAAG
ILGILAGLFHLSVRPPQRLYKGLRMGNIETVLSSSIAAVFFAAFIVAGTMWYGSATAPVELF
GPTRYQWDQGYFQQEIDRRVRAGLSENLSLSEAWSKIPEKLAFYDYIGNNPAKGGLFRAG
AMDNGDGIAVGWLGHPIFRDKEGHELFVRRMPTFFETFPVVLVDEEGIVKADVPFRRAES
KYSVEQVGVTVEFYGGELDGVSFGDPATVKKYARRAQLGEIFELDRATLKSDGVFRSSPR
GWFTFGHATFALLFFFGHIWHGARTLFRDVFAGIDPDLDAQVEFGAFQ?LGDPTTKRQIVM
KTLYSLRRFYPVETLFNGTLALAGRDQETTGFAWWAGNARLINLSGKLLGAHVAHAGLIV
FWAGAMNLFEVAHFVPEKPMYEQGLILLPHLATLGWGVGPGGEVVDTFPYFVSGVLHLIS
SAVLGFGGIYHALIGPETLEESLPFFGYVWKDRSKMTTILGIHLILLGVGAFLLVLKALYFG
GVYDTWAPGGGDVRKITNLTLSPSVIFGYLLESPFGGEGWIVSVDNLEDIIGGHVWLGSICI
FGGIWHILTKPFAWARRAFVWSGEAYLSYSLGALSVFGFIACCFVWFNNTAYPSEFYGPTG
PEASQAQAFTFLVRDQRLGANVGSAQGPTGLGKYLMRSPTGEIIFGGETMRFWDLRAPW
LEPLRGPNGLDLSRLKKDIQPWQERRSAEYMTHAPLGSLNSVGGVATEINAVNYVSPRSW
LATSHFVLGFFFFVGHLWHAGRARAAAAGFEKGIDRDFEPVLSMNPLNMTVALGKSSKEE
KNLFDIADDWLRRDRFVFVGWSGLLLFPCAYFALGGWFTGTTFVTSWYTHGLASSYLEG
CNFLTAAVSTPANSLAHSLLLLWGPEAQGDFTRWCQLGGLWTFVAFHGGFGLIGFMLRQF
ELARSVQLRPYNAIAFSAPIAVFVSVFLIYPLGQSGWFFAPSFGVAAIFRFILFFQGFHNWTL
NPFHMMGVAGVLGAALLCAIHGATVENTLFEDGDGANTFRAFNPTQAEETYSMVTANRF
WSQIFGVAFSNKRWLHFFMLFVPVTGLWMSAIGVVGLALNLRAYDFVSQEIRAAEDPEFE
TFYTKNILLNEGIRAWMAAQDQPHENLIFPEEVLPRGNALMSGNTGERSFADIITSIRYWVI
HSITIPSLFIAGWLFVSTGLAYDVFGSPRPNEYFTENRQEVPLITGRSDPLEQLNEFTRSFMT
IDRTYPIFTVRWLAVHGLAVPTVFFLGSISAMQFIQRMAAQTTDDTF-RSGPRRTVVGNLFK
PLNSEYGKVAPGWGTTPLMGAAMALFAVFLSIIPEIYNSSVLLDGIPVSMFTLKLFVYTVVI
FFVSLFIFGFLSNDPGRNPGRKEMADTTGRIPLWLIGTVTGTLVIGLIGIFFYGSYSGLGSSL
MPAM---SNICLDDA-LHDLF-LAKLPEAYAIFDPVVNVMPIIPVLFFLLAFVWQAAVSFRMT
QSNPNEQNVELNRTSLYWGLLLIFVLAVLFSNYSFNTEVNILAFIAIVLFISVPTAFLLIIYVK
TVSESNMETATLVAISISRLLVSFTGYALYTAFGQPSEQLRDPFEEHEDMEALVYTFLLVSTL
GIIFFAIFFREPPKVPDKGSKMTIAFQLAVFALIAISFLLVIGVPVVLASPDGWSSNKNVIFSG
ASLWIGLVFLVGILNSFIS-------MSPKTETKASVGFKAGVKDYRLTYYTPEYQTKDTDILAA
FRVTPQPGVPPEEAGAAVAAESSTGTWTTVWTDGLTSLDRYKGRCYDIEPVPGEENQFIAY
VAYPLDLFEEGSVTNMFTSIVGNVFGFKALRALRLEDLRVPPAYSKTFQGPPHGIQVERDK
LNKYGRPLLGCTIKPKLGLSAKNYGRAVYECLRGGLDFTKDDENVNSQPFMRWRDRFCF
CAEAIYKAQAETGEIKGHYLNATAGTCEEMIKRAVFARELGVPIVMHDYLTGGFTANTSL
AHYCRDNGLLLHIHRAMHAVIDRQRNHGMHFRVLAKALRMSGGDHIHAGTVVGKLEGE
RDVTLGFVDLLRDDFIERDRSRGIYFTQDWVSMPGVLPVASGGIHVWHMPALTEIFGDDS
VLQFGGGTLGHPWGNAPGAVANRVALEACVQARNEGRDLAREGNEVIREASKWSPELAA
ACEVWKEIKFEFEAMDVL--MAIRSYRTYTPSTRNRPISSRVRSNPQKKLTSGQHRCGKGR

NSRGIITARHRGGGHKRLYRQIDFQRNEKYIFGEIVTIEYDPNRSAYICLVHYGDGEKKYIL
HPRGVIIGDTITSGPRAPISIGNALPLTDVPLGTAIHSIEITLGKGGQLARAAGAVAELIAKEG
RSTTLRLPSGEIRLISENCSATIGQVGNVNANNGTLGKAGSKRWLGKRPRVRGVVMNPVD
HPHGGGEGRTPIGRKKPVTPWGYAALGRKSRKNNKYSDASILRRRK-MIQPQTYSNVADN
SGARKLMCIRVLKAS-NRQYAHIGDVIIAIIKEAVPNMPLKESEIVRAVVVRTCKELKRDNG
MIIRSDDNAAVIVDQEGNPRGTRVFGSVARELRQLNFAKIVSLAPEVLMLSPQKTKFRKQH
RGRMKGVSHRGNHICFGRFALQALEPAWITSGQIEAGRRAITRYARRGGKIWVRIFPDKPIT
MRPAETRMGSGKGSPEYWVSVIRPYRILYEMGGVSETVARAATEIAAYKMPIRTRFVTAS
MTRIKRGYIARKRRKKILAFASGFRGAHSKLFRTADQQKIRALVSAHRDRSKRKRDLRRL
WITRINAAARNN----VSYNKFIRYLYKRQLLSNRRILAQIAVLDKNCFSTIINNIIE-----------M
EKKIRALAKHIRMSTHRARRVIDQVRNRSYEQALMILELMPYRACYPILQLISSAAANANH
NIGLNKANLFVSRAEVNEGAILKRSQPRAQGRGYPIQKPTCHITIVLEERSRS-------MNEM
EYTILTEKKIRLLENN-QYTFDVGSRPTKTGVKNWIELFFGVKVIAMNSYRPPKKGGTVG-P
IGHPIRCRRMIIALQPGYSIPLLSEE--MAVPKKRTSKSRKRIRKNVWKGKADQAAVKAFSL
AESISTGRSKIFY--TNDKMAKGGDVRVKITLECTSCTRDS--VDKKYPGVSRYITQKNRRNT
PIRSELKKFCPYCYKHTIHGEIKKMKIRASVRKICEKCRLIRRRGRVMVICSNPRHKQRQG
MIRDEISVSTQTPRWRCIGSGADSKRLHYGRFALSPLRKGQASTIGIAMRRALLGEIEGTCI
THAKLE--KVTHEYSAIIGIEESVHDILINLKEIVLRSDPYGTREASICIVGPRNVTAQDIILPPS
VRIIDATQHIASLTKSITFDIRLWIEKDRGYRIQSPKNYQDGIF--PIDAVFMPVRNANYSIHSY
GNGNDIQEILFLEIWTNGSLAPREALYRASRNLIDLFIPFLRAEEQ--NIDGMDNQ-NGSNMP
SFSFSNISAD-ERMEEE--VAFKHIFIDQSELPPRVYNCLGRVNIHTLSDLLNYSQEDLMRIGH
FGKKSVEQVSEVLQKH--FAVDLPKNKMLLDEN-KGTSTIPEFGKIQFEGFCRFIDQGLIEEL
QNFPKIEDTDQEIESQLFGNKYELAEPLIKERNAVYQSLTYSSELYVPARLIQRNSRKIQKQT
VLIGNLPLMNSQGTFVVNGISRIVVNQILRSPGIYYSSEPDQSGITLYTSTIISDWGGRSKLEI
DGKTRIWARVSKKRKISIPILLSAMGSNLGEILDNVCYPKIFLS-ERQEQKEYLRSKKNAILE
FYKKLYCVSGDLVFSESLCKELRKKFLQQRCELGKIGRRNPNQKLNLDIPENEIFSLPQDVL
AAVDYSIRVKFGMGTLDD--MDHLKNRRIRSVADLLQNQFGLALGRLENAVRRTIRRATKR
KCL--PTPNNLVTSTP----LTTTFQDFFGSHPLSQFLDQTNPLTEIVHRRKLSYLGPGGLTGRT
ASSRIRDIHPSHYGRICPIETSEGMNAGLVASLAIRARIGHCG-SLQSPFH---KISERSEE--EH
MVYLSSGEDE--RIATGNYLALNQGIREEQVTPARYRQEFLAIAWEQIHFRSIFPFQYFSVGV
SLIPFLEHNDANRALMGSNMQRQAVPLFQPEKCIVGTGLEGQAAPDSGSAAIATQGGRITY
IDAGKITSSVDGDTVGTELVTYQRSNNNTCMHQKPRVRRGEYVKKGQILADGAATVGGE
LSLGKNILVAHMPWEGYNFEDAILISERLVYEDIYTSFHIERYGIVTCMTSQG-PERITKEIPH
LDAHLLRHLDGNGLVMLGSWVETGDVLVGKLTPQPAEE-SLRAPEGRLLQAIFGIQVSSAR
ESCLRVPIGGRGRVIDVRWIHKEE-NFGDNAEVVHVYIFQKRKIQVGDKVAGRLGNKGIIS
KILPRQDMPYLQDGTSVDMVLNPLGVLSRMNVGQIFECLPGLAGNLMNRHYRITPFDER
YEREASRKPVFPELYGASEQTANPWVFEPNHPGKNRLIDGRTGDTLEQPVTTGKAYMPKL
IHQVDDKIHARSSGPYALVTQQPLRGKSKRGGQRVGEMEVWALEGFGVAYILQEMLTLKS
DHIGARHEVLGAIITGGPIPRPGTAPESFRLLVRELRSLAPELDHAIIYENDFQIDRKEV-------
MIDRDKHQQLRIGLASPEQICAWSKRILPNGRIVGQVTKPYTLHYKTNEPEKDGSFCERIF
GPIKSGVCACGNSRVIGNEKENSKFCEQCGVEFVDSRIRRYRMGYIELACPVVHVWYSKR
LPSYIANLLAKPLKESEGPVYCD--LFIARP-IANKPTSLRSRGPFKYEIQSWRDIIPHYFSA-G
FGAFRHREIATGGDAIREQLAGLNLQILMDRSYMEWK--LGKQKSAGNGWGDRKIQRRK
DFSVRRMKLAKHFLQTDIEPEWMVLCPLPVLPPELRPIVQLGGGELITSDPNELYRRVIYR

NNTLTDLLARSRSTPGGLVICQKKLVQEAVDALLDNGIRGQPMRDSHDRPYKSFSDVIEGK
EGRSRENLLGKRVDYSGRSVIVVGPSLPLHQCGLPREIAIELFQAFVIRGLIRRHFAPNLRA
AKSIIRDKEPIVWEVLQGVMQGHPVSLNRAPTLHRLGIQAFQPILVEGRAIRLHPLVCGGF
NADSDGDQMAVHVPLSLEAQAEARLLMFSHTNLLSPAIGDPISVPTQDMLLGLYILTVGN
NQGIYGNRYHPYYS------KYKI-----FSCKK---PSFYSYDDALGAHWQKRIELDSPLWFRWG
VGLR-IITSVDREAPIEVQYESLGIFHEIYEHYRIGKNEVGEILSIYIRTTVGRIRFDREIEEAIQ
GFSREHPNKSLPAIII--RFFPMAERAKLLFH-EAMDKTAMKQLISRLINHFGMTYTSNISDQL
KTSGFQRATDAAISLGIDDLLTAPSKGWLVQDAEQQGSISEKHHHYGNVHAVEKLRQSIET
WYATSEYLRQEMNPNFRMTDPFNPVHMMSFSGSRGSTSQVHQLVGMRGLVSDPQGQIVD
LPIQSNFREGLSLTEYIISCYGARKGVVDTAVRTSDAGYLTRRLVEVVQHIVVRKTDCGTIQ
GIFVNLRERIKNRIVLQT--LIGRVLADDVYI-MRCIATRNQDIGVGLAHQLIT------LRAQPIYI
RTPSTCKSLSRICRLCYGRSPTHGNLIELGEAVGIIAGQSIGEPGTQLTLRTFHTGGIFTGDIA
EHVRAPFNGKIEFDENLVYPTRTRHGHPAFMCHNNLSITLDGQDQVHDLTISPQSLLLVQN
NQYVESEQIIAEVHARTSPS---KEKVRKHIYSNLEGE-MHWSTNVCHAPEYVQGNVHLILR
TSHLWVLSGGIHG--SSAVSFPFHKDQDQVNVQ--LPLAKHELLPDYS--VNQDR-MKHKSV
DSNFYGK-EEQISSYS-EIDRVISNEHWDSIYST-ISSDNFN--ISGKKQRNRFFVPLRYDKERG
NKGISRPNLIIKISRN--GISQRNDIFAVLDDPRYRINSSGIIKYGTIKVDSIGNKEN--YLGDQR-
ARGFRSKDMKGGRFLFIPEEVHILYESSSIMVQNNSIIRAGTQITCDIESQVGGLVRIVRIKK
KIEMRILPGDIYFPGEIHGISRHNGTLIPPGKILF-------DEFQSENWIYLQW--IIPPKEK--PFVP
VRPVVEY-IPDGPDITAPLSLDLLREGDNLQVRVGNFLLYGDGERIQVINDISIQLVRTYLVL
DWEQKD------ME-EAYAYLTEVRTNGIVRN?LQISLAKY---LYGKRKNTAGSKSMFH-NKLD
HANP--ISSNEES---------QLLSQHQGTIRALPNE---REEGGSLMVLSPSDCSRIVL--SKGSKH
YDM---VNRSDSMMQIIELSGFLGN---LSIANRSPS--SHSIT--YNKYYNIKEQPIFGN---SENT
LRVPK---WYFMDENTV--VSNFGPCRNIISDLFNSNRCFPLSKFCENPFPVVS-LGQLIRESVR
ISEDEP---LPGSGQIIAVH-EESLVIRLAELYLATRRATVHGHYGEIINEGDTLITLIYERFKSG
DIIQGLPKVEQLSEARSINSISMNLEKSFEDWNRDMTRSLGSPWGSFISSKITMEQSRIHLV
NQIQRVYRSQGVQISDKHIEIIVRQMTSKVLISG---DGMADVFLPGELIELSRAQRMDRALK
EATYYRTMLLGATRASLDTQSFISEAGFQETARVLARAALQGRIDWLKGLKENVILGGIVP
AGTGFKQSIYHSG--ERNEID--PRTGK---MTKRYWNIN
LEEMMEAGVHFGHQARKWNPRMAPYIFTERRGIHIINLTRTARFLSEACDLVFDAAGKGK
QFPIVGTKYQAADSVASAAIKARCHYVNKKWLGGMLTNWSTTGTRPRKFKDLKKEQDT
GQSNRLPKKEAAMLKRQLAQLQKYLGGIKYMTSLPDIVIIADQQEESTAIGECITLGIPTICL
VDTDCDPDLTDIPIPANDDARASIRLILNKLTSSICEGHY---SSMKMGQKMNPLGFRLGITQ
NHRSHWFAQQKNYSEDLREDEKISNCIVNYIRRHMRSSSN----IARVEIRRKIDLIQVEIHIGF
PNLLIENRG----RGSEQLRTDVRNMLNPVDGKLNIAIVKVAKPYGEPNILAEFIALQLEDRV
SLGRTVKRAIELAEQADIKGIQIQIAGRLDGNEIARVEWAREGRVPLQTIRAKIDHCYYPAQ
TIYGVLGIKIWIFGDEKMSRYRGPRLKIIRRLRTLPGLTNKRPKSRNDPTNQSSSRK-ISQYRI
RPEEKQKLRFHYGLTERQLLKYVRIAGRAKGSTGQILLQLLEMRLDNIIFRLGMALTIPGA
RQLVNHRHILVNDRVVDIPSYRCKPRDVITIIDQRRSRAMIGKNLDLSQK-DQMPNHLTLHS
SQ--YKGLVNQIIDSKWISLKINELLVVEYYSRQAMSRRSTAEKETAKSDPIYRNRLVNMLV
NRILRHGKKSLAYRILYRAMKNIQQKTEKNPLSVLRQAIRGVTPNVTVKARRVGGSTYQV
PVEIRSTQGKALAIRWLLGASRKRPGRNMAFKLSYELMDAARGNGNAIRKKEETHRMAE
ANRAFAHFRMGNDTIANIITSIRNADIVKKKTVRIIATNTTKNVVRILLQEGFIEDAREHRE
GQKSFSVLTLRYRG---RKETYIT-----TSKRTSKPGLRIYSNSQKVPKVLGGMGVVILSTSQGI

MTDREARRRRIGGEILCYVRMSKPIRKIGSRRN--------ERRIPKGVIHVQASFNNTIVTVTD
VRGQVVSWSSAGACGFKGTKRSTPFAAQTAAENAIRTLMDQGMERAEVMISGPGPGRDT
ALRAIRRSGVLLSFVRDVTPMPHNGCRPPKKRRVVPTIQQLIRNTRQPIVNGTKSPALRGC
PQRGGVCTRVYTTTPKKPNSALRKVARVRLTSGFEITAYIPGIGHNLQEHSVVLVRGGRVK
DLPGVRYHIVRGTLDAVGVKDRQQGRSLYILI---MARKSLIQREKKRQVLEQKYHSIRQSL
EREIS---EVSSLDDKWEIHRKLQSSPRNSTPTRLHRRCFLTGRSRANYRDFGLSGHVLREM
THACLLPGMKKSSWMPKNSPISLILKE------RRRSVESQIYHLTDRILRLTHHLELHRRDYSS
QRGLWQILGKRKRLLVYLSKRNRPRYEDLIGQLSIRGLKIR--MVKLRLKRCGRKQLATYRI
VAINVESRREGKALQEVGFYDPMKDQTYSNVPAILHFLEKGAQPTETVHDILEKAGIFKKF
QTNL-----MNKSKRSFR--------RLPPIGSRDQIDHKNMSSISQFISERGKILSGRVSRLTPKQQR
LMTIAIKRARIPSSSPFLNNDN--------------------MAR-LKKNPFVANHSLRKIENLNIKKEKKI
IVTWSRASTIVPAMIGHTIAVHNGKEHLPIYITDRMVGHKLGEFAPTLIPRGHAKSDNRSRR
ILRILSLPWDSISSRIEVSGPIILFGLYYGFITTLPISPSQISPMR-IFFLEG----TVDGIVTIGSITG
QLIVFLSMYYSPIYTVLGKPHAITLLVVPYMFLHEKLSNSKSLY---INSLNNSR---ILGLFMD
SLIFQLLNPIILPSPILTRLMNLFLFRYSNNISFVISSLCGWLGGHVLFINLARLVSFRIERDSPI
GY-----------------RTFSILIYYSCLLYLG-------------FDQP--WPIILFDHRRWNRPLRYIRNSPFT
NPVKTEVSQYFFDTCSSDGKQRISFTSLPSVSVFWERIRRYRNLSDTS---SEDLYYQWICTE
EQRKDNLGNEFRNRIEALSNGSPVGDVMEKGVKLSN--YQGDSFPKIHDPFLNGPFRGIIDQ
LRSPAEGSWNNQIED--EPLLTDTEIASCIGG-EKNVTSPMRWELILSHLEQKEWTKSISAFC
GKFLFNDPIVRRYSEESDFRRNLVKGSMRAQRRRTIICKMIQTGAHSPLFLFL--RMEIPTFL
KDSFYMPN-----SGERSKDRLSIAKLDFSLNQKIRGFILVTQSILRKYIILPSLIIAKNIGRMLLF
QVPEWDEDWGEWSREIHIKCTYDGIEFSETEFPDRWLRDGIQIKILFPFRLRPWHGSEKGE
KSSSSYLTTWGLETEVPFGYPK--PSFWEPIIKELKGR--LIRT-----ILSGIGRIKE------------SGP
Q--PDNRSTENLGI----DQILNKLLIGTRSANFSSEIQDYGTQTT-IEDL-DWTTTMNDQITEKY
LVNLGCSSYIESESKKRLIQIRLVQFRKRS-VRFFWKWPN---LFIERMG-DISLSVIRLIRLNIQ
FC-----TDRDMILQAYLFNEIWQIRSMNKSHSKYLLQYSYPFLKGKIKEFFIQGILDS-KEPQD
LR-----DWKEWLKCY-RYNLSSQIWSGIAPQRWRNEMSKQRTTENR---SLHSYEENRFIPYE
QQRKLNKRYRYNLFSYSYID-IIRE----------------ERDNFDITDSPRINWLFPELVGMNDTYKT
ELMIIPRKSLIREFRWAESIARELENNIICSLLSGMKDPEPYLRTGDLDLDLMSHSIK----------
--------DVSGTVKDG-------ILIVEPFRLSGVLDDQFLMYKIVSISLRFKNRFNISSSLIFEDILLP
RRRREFRILNRFAFLWPSYRLEDLACMNRYWFNTNDGSRSVMLRIRMYPMKRQK-KSWIL
KL----EEIRSFQYLFNSWTELNLVRLFTKIVSHRERLIKLFDSRILSTLLLRDLRG---SRSNQSL
T---IKGVVLLTLP-VLIYH---VNQKS-MIERKKFYSMKLFP-----IPINYAEPRNETSEEYFESFN
KNLLIFPHLFLSFPKGRKIYQSHLRNSKEDAWVLSKRKVCVMPAYNQIDSWGSRWWKNW
IIEEI--LPSWKIPQ---GSIAKIEMSLKEKDVE-HLKGFFEFYID--DLIRKDYDWEYHFDRVFM
RNKQDTID-LSSKQQVEILGNNLICYLMSAFCEKMLFEAEGPFKQQKS--KSIVESNNIKHFS
HLRLSYKEKGYLSFQNYAEF--WQFYKDSFVSWEKDQQKLELSKDILKEKFIQLNDVNND
QLFSSSQLKRSYNRSIDHFDPISENSEYGTN-MNKKDEIISENQRPITWETHSERDEKDIDSEI
DLTLNFTENEYWEPEKDLCERIFTNGYINKTEIEQLKERSILWDP--SSSIRIERTKIKSD--LSS
K--CLSEDSPIYWTKELFTEDKKSITE-LFPKERKRFI--ENFTKSIRSYFFDILSIDEPCMG-SSV
TKKPIENF----KLLKGQQDVF-FRYFRGSE----KNGIIDPWKIRTYFQ--NPSSNCAISLDPGCN
MIRKNQRDI--NKLNCILFMNRSLSRNR--FSSFCDQNKEQYIF--HNNLR--FKKRVLKITDQF
ALLITKP-NQVYDNTLAPD-QFYELNKNRFL--DQIFNHKNKLKNQSL---LILLNITD--KENE
YLDRIIEKELIQ-ISSKKGSEIGTP-----LNNYRTETSNWYK------LIRYKIHG--HLKNAFNKLY

SMN---GSSRNLKDQIR---TNWIENENLNN--VSKDTINRHPSNWRK-GQKEWFDHS----ILRT
DKCINRNLNVYKWS--NQTEYLKRCSKHLVSKQND----LKIVFDPIER--DSIGWSGSPNKKD
YSKFSLIAENT---------------------VKIFLSK-----KSISHS--AIFIPE-FFID-KLKGMNDQLFNKL
LKSIGVRIVHLKTFKPF--LLD---NHNLS-QRSKFLIDERTVAQFFYHEMPVNRFIIDLFENE--
KNCMELFDNTD--FSTISNDRDNWLNPVKLSNQSSSRASFYKANTLQFFDYSHHPRFN--YK
KRLPYYM----ERIHTKNHN--LTYG-QLFNISPIHSN-LFSLSISE--IRPVHLE-KDTISLIKSQVS
NIFLPKYLQQSGNQTFVSIYDLYKSFDLLTRLNPFVHDKIDISSIEEISTTPLTRERIANFEKTS
CQPF--LNRSDLEENNFDQYLKGGFSSNMGLIQTQSYRDDLLSEHRIRDRFVKSSSTEGSKN
RIENKAIDKRSTLFNFSKKQEMHRISKWDLFRAYTPWLLTSAWWKYLE-LLLETFPEILLN-
-SSD---QLVSIL-DIMHKSNLSWAISHQLWAL-------QCNLRTN------ILDKFFSLWNLCEMINQ-
SSVLSIWAHLRLLNAREY-EYSILILFFVLGY-FVLRYSFVVSS--AFIELQIHLERIKDLMDPSY
AIELQKLMDHPLP--GLLFSM
DIRDISIYFLDELIYSMRNRKFYSPIR--RELNRSCFSMDISGKERELLVQFL----IT--EKNISQF
GSNLT-HSHNFF-----KNEFDYQITEQPGLIYLIYLADTY--QKDLMNHEFDQSRLTERWVFLA
FCRKITS--SQIFR---GLNLTFYG---KPFSLHLGSSLS-KGILLIGPTETGRSYLVKGLAADSYV
PLVRI-LNKFLYDKGE--YSNFLDIRSMN--NVVQK
MH--------QFNLTFELAKRMSPCIIWIPNIHELNVNYLTHFFLGLLVNHLS---RDDEKDS-TRN
LLVIASTHIPKKVDPAPIAPNRLDRSINVRMLPIPQRQKEFPILLRSKGFDSEKELSCP--KEFG
SRTIGSNARDLAALANEALSISITQNKSVIGTDTIRLALYRQTWGLQSIDNQVGSGQNYEIL
PYKVGKAVIQNTLRRNSSMNPLSINNELWK----KRFSYLSKWYLEPSTTMKELTILPHILGC
LAGSAARDSWFIS--GQNRE-NWIPLDRFAEHDF-DLASG--LLESLLVE--FPWLGICRGKPD
KN--QITFAPQPKTRN-HLNVMRKGVYSMVNKMFIYKEYELK-------FQQETPGAK----QMD-
---EKLVNN--IVWAP--RIWRLSFLRSNLFDRTKRPNELGFSYQFGLFQEEQSNARQKNLQHI
SLQEFEIG-IFQFSIQYQMRSKSSNKPMFFLGRRFLWDP--GFLFQDQ-----HLVFSRREFFANE
EMLRRLYITYGSTPTFLYQRWLIENPQEKVDRFELLIHRQRWLET---NSSLS--NESFLYN--TL
SESYKYLSNLFLSN-RMLLNQMTRTLLKNRWLFPKEIEHLIHTTKDRFHISILMPRSQ-RND
NFTDKTFTIVADILLRIIPTAPGEKEAFTYYRDG--MSAQSEGEYAEALQNYYEAMRPEIDPY
DRSYILYNIGLIHMSNGEHTEALEYYFQALKRNPSLPQAFNNMAVICHY-RGEQAIRQGDP
ETAEAWSDRAAEYWKQAIALAPGNYIEAQNWLKITGRLGE-MNWRSEWLWIEPITGSRRT
SNFCRACILFFGSLGFFLVGISSYLGKNLIPVLSSQQILFVPQGIVMCFYGIAGLFISSYLWCT
ILWNVGSGYDKFDEEEGIVCLFRWGFPGRNRRTFLRFLMKDIQAIKMEVQEGLYPRRVLY
MEIKGQRDIPLARTGEN-LTLREMEQKAAELARFLRISIEVF
>Ipomoea
MEKWRIYS---LNSSLG---PIETTGED---NRKDTVKNIQSCSNVDHLFGVKDNENFISDDTFV
VFDIEN-----IFEIDND---------HSFLNEPFSS-YSDRNSSYLTNVF-SEDPYYN--MYDAQYSWN
NHINGCIDSYLQ--SQICIDTTVSGSDNSYD--IHGSVCDGGESSSRRTHTKGVDLTIRESSND
LDRTKKYRDLWLQCENCYGLNYKKFLKSKMNICEQCGYHLKMSSSDRIELLIDPGTWDP
MDEYMVSQDPIEFDSEGEQYKDRIDFYQTKTGLTEAVQTGIGQLNGIPVAIGVMDFHFLGG
SMGSVVGEKITRLIEHATNNFLPLIIVCASGGARMQEGSLSLMQMAKISSALYEYQLNKRL
FYVSILTSPTTGGVTASFGMLGDVIIVEPNAYVAFAGKRVIEQTLNQTVPEGSQEAEYLFEK
GLFDLIVPRHLLKSVLSELFEFHALFPLNQNSNQMVTIQADEISNLIRERIEQYNREVNIVN
TGTVLQVGDGIARIYGLDEVMAGELVEFEEGTIGIALNLESKNVGVVLMGDGLMIQEGSS
VKATARIAQIPVGEAYLGRVINALANPIDGRGEISASGFRLIESPAPGIISRRSVYEPLQTGLI
AIDSMIPIGRGQRELIIGDRQTGKTAVATDTILNQQGQNVICVYVAIGQKASSVAQVVNTLQ

ERGAMEYTIVVAETADSPATLQYLAPYTGAALAEYFMYRERHTLIIYDDLSKQAQAYRQM
SLLLRRPPGREAYPGDVFYLHSRLLERAAKSSSKLGEGSMTALPIVETQSGDVSAYIPTNVI
SITDGQIFLSADLFNAGIRPAINVGISVSRVGSAAQIKAMKQVAGKLKLELAQFAELEAFAQ
FASDLDKASQNQLARGQRLRELLKQSQSAPLTVAEQIITIFTGINGYLDSLELGQVKKFIVE
LRTYLKTNKPRFEEIISSTKIFTEEAEALLKEAIRDQMDRFLLQQQVMRINPTTSGSEVSAVE
KKNLGRIVKIIGPVLDVAFPPGKMPNIYNALVVQGRD--NEQTNVTCEVQQLLGNNRVRAV
AMSDTDGLMRGMEVIDTGAPISVPVGGSTLGRIFNVLGQPVDNLGPVDTNTTSPIHRSAPA
FIQLDTKLSIFETGIKVVDLLAPYRRGGKIGLFGGAGVGKTVLIMELINNIAKAHGGVSVF
GGVGERTREGNDLYLEMKESGVINEENIPESKVALVYGQMNEPPGARMRVGLTALTMAEY
FRDVNEQDVLLFIDNIFRFVQAGSEVSALLGRMPSAVGYQPTLSTEMGSLQERITSTKEGSI
TSIQAVYVPADDLTDPAPATTFAHLDATTVLSRGLAAKGIYPAVDPLDSTSTMLQPRIVGEE
HYETAQRVKQTLQRYKELQDIIAILGLDELSEEDRLTVARARKIERFLSQPFFVAEVFTGSPG
KYVGLAETIRGFQSILSGELDGLPEQAFYLVGNIDEATAKAMNLKMESDLKKMTLKLCVL
TPNRIVWDSEVKEIILSTNSGQIGILPNHAPIATAVDIGILRIRLNDQWVTMALMGGFARIGN
NEITVLVNDAEKGSDIDSLEAQQTLEIAEANFRKAEGKRQTIEANLALRRARTRVEAINAIS
MKNVTDSFLSLGPWPSAGSFGFNTDIFATNPINLSVVLGVLIFFGKGVLSDLLDNRKQRILN
TIRNSEELRGGAIEQLEKARARLRKIETEAEQFRVNGYSEIEREKLNLIQSTYKTLEQLENY
KNETIRFEQQRALNQVRQRVFQQALQRALGTLNSCLNNELHLRTISANIGMLGTMKEITD
MDPLISAASVIAAGLAVGLASIGPGVGQGTAAGQAVEGIARQPEAEGKIRGTLLLSLAFME
ALTIYGLVVALALLFANPFVMDVLSCSINTLKGLYDISGVEVGQHFYWQIGGGQVHGQVLI
TSWVVIAILLGSATIAVRNPQTIPTGGQNFFEYVLEFIRDVSKTQIGEEYGPWVPFIGTIFLFI
FVSNWSGALLPWKIIQLPHGELAAPTNDINTTVALALLTSLAYFYAGLSKKGLGYFGKYIQ
PTPILLPINILEDFTKPLSLSFRLFGNILADELVVVVLVSLVPSVVPIPVMLLGLFTSGIQALIF
ATLAAAYIGESMEGHHMIFSTLEHILTHISLSIILILMTIHLIIFLVDEIRKLYDSSEKGMLVTFF
CITGLLATHWISSEHFPLSDLYESFIFLSWSFALIHIIPY--KNTKFLSKIISPSAIFTQGFATSGLL
TEIHQSSILVPALKSEWLIMHVSMMILGYAALLCGSLLSVALLVITFRKNGKLFS--GENLFQ
KTSF----------AKNYYRTQFIQQLDFWSYRVISLGFLFLTIGILSGAVWANEAWGSYWSWDP
KETWAFITWIVFAIFLHTRTNLNLQSVNSAIVASIGFLIIWICYFGVNLVGIGLHSYGSFPSTM
AKKKAFTPLFYLSFIVFLPWWISFSFKKCLESWITNWWNTGQSEIFLNDIQEKSILEKFIELE
EFVFLDEMIKEYSETHPQEFRI-IHKETIQFIKIQNEGHIHTILHFSTNLICFVILSGYSIWGNE
NLVILNSWSREFLYNLSDTVKVFSILLLTDLCIGFHSPHGWELMIGSIYQDFGFGYNDQILS
GLVSTFPVILDTILKYWIFRYLNRVSPSLVVIYHSMNDMPIGVPKVPFRNPGDEDASWIDIY
NRLYRERFLFLVQEIDSEIANQIVGLLVYLNIEDDTKDIFLYINSPGGDVISGVAIYDMMQTV
RPGVSTICIGLAASMGSFILAGGAITRRAAFPHARVMIHQPRTSFFESHAGELLLEMKEALK
L R E R I A E I Y A Q R T G N P T S I I S R D M D R D V F F S A R Q A R I Y G I I D D I E F
S???MEEIQGY
LQ-ERSQQHNFLYPLLFQESIYALAHDRGLTGYEKKLSFQIVKRLITRIYQRIYFTTYSS--NK
SQIFGKSLYSKLLSEGFAFIVEIPFSLRFIL--ILKSQNLRSIHSIFPFLEDNFSHLNYVLDILIPYP
PHLEILVQTLHYWVKDASSLHLLRFFLHEYCNFNIFITSR-SPFSKQRFFFFLYNSYVCEYESI
FLFLRNQSLHLRSTSFGALFERNIFYGKIECFVKVFA-DFNLCLLKDASMHYFRYRGKSILA
SKGTLLLMSKWNYYFVNFWQCSFCLWFHTERIYISQLSSHSLDFMGYLSSIPLTPSTVRSQ
MLENSFLINNAI--KKFDTIVPIIPIIGSLAKAKFCNVLGNPISKPVWADLSDSDIIERFGHIYR
NFFHYYSGSSKKRSLYRIKYILRLSCARTLARKHKSTVRAFLQSLGSEFLEEFFTSEEQILSF
TFPNRIWYFDIVCINELANYQMIID-TTQIQ-AIHSFSGLESKVKEVYGIIWILAPIFTIVLGIT

MGLLVIVWLEREISAGIQQRIGPEYAGPLGILQALADGIKLLLKESLLPSRGDTRLFRIGPSIA
VISVFLSYLVIPFGYKLVLADLSIGVFFWIAASSIAPVGLLMSGYGSNNKYSFLGGLRAAAQ
SISYEIPLALCVLSISLLSNSSSTVDIVEAQSKYGFWGWHLWRQPIGFIIFIISSLAECERLPFD
LPEAEEELVAGYQTEYSGIKFGLFYVASYLNLLISSLFVTVLYLGGWNLSIPYIFVPELFDIT
KRGKVFGTIVSIVITLAKTFSFLFISIATRWTLPRLRMDQLLNLGWKFLLPISLGNLLLTTSS
QLLSLMSWHVQNENFILDSTRIFMKAFHLLLFDGSLIFPECILIFGLILLLMIDSTSDQKERP
WLYFISSTSLVMSITALLFRWREEPMISFSGNFQTNNFNEIFQFLILLCSTLCIPLSVEYIECTE
MAITEFLLFVLTATLGGMFLCGANDFITIFVAPECFSLCSYLLSGYTKKDVRSNEATMKYLL
MGGASSSILVHGFSWLYGLSGGEIELQEIVNGLINTQMYNSPGISIALIFITVGIGFKLSPAPS
HQWTPDVYEGSPTPVVAFLSVTSKVAASASATRIFDIPFYFSSNEWHLLLEILAILSMILGNL
IAITQTSMKRMLAYSSIGQIGYVIIGIIVGDSNDGYASMITYMLFYISMNLGAFACIVLFGLR
TGTDNIRDYAGLYTKDPFLALSLALCLLSLGGLPPLAGFFGKLYLFWCGWQAGLYFLVFIG
LLTSVVSIYYYLKIIKLLMTGRNQAITPHVRNYRRSP--SNNSIELSMIVCVIASTIPGISMNPII
AIAQDTLFMFLLYEYDLFWAFLIISSLIPILAFFISGVLAPISKGPEKLSTYESGIEPMGDAWL
QFRIRYYMFALVFVVFDVETVFLYPWAMSFDVLGVSVFIEAFIFVLILIGGLVYAWRKGALE
WSTNFFPWLTIIVVFPIFAGSIVFFLPHKGNRVIRWYTLCICILELLITTYTFCFHFQVDDPLIQ
LEETYKWINFFDCHWRLGIDGLSVGPILLTGFITTLATLAARPVTRDSRLFHFLMLAMYSG
QIGLFASRDLFLFFIMWELELIPVYLLLSMWGGKKRLYSATKFILYTAGGSIFLLMGVLGLT
LYGSNEPTFNFETSVNQSYPAALEIIFYIGFFIAFAVKSPILPLHTWLPDTHGEAHYSTCMLL
AGILLKMGAYGLIRINMELFPHAHSLFSPYLVIVGTIQMIYAALTSLGQRNLKRRIAYSSVS
HMGFIIIGIGSMTDTGLNGALLQIISHGFIGAALFFLAGTTYDRIRLVYLDEMGGIAIQMPKI
FTMFSSFSMASLALPGMSGFVAELLVFFGIISAKKYLFMSKILISFVMAIGIILTPIYSLSMLR
QMFYGYKLFNAPNSSFLDSGPRELFLSISILLPVIGIGMYPDFVLSLSVEKVEVILSHFFYRM
MLEYALVLSAFLFSIGIYGLITSRNMVRALMCLELILNAVNMNLVTFSYFFDNRQLKGDIFS
IFIIAIAAAEAAIGLAIVSSIYRNRKSTRIDQSNLLNNMEQTYQYAWIIPFVPLLVPMLIGAGL
LIFPTVTKNLRRMWSFQSVLLLSIVMAFSIYLSIQQINRGSIYQYVWSWIINNDFSLEFGYLI
DPLTSIMSILVTTVGILVLIYSDNYMAHDQGYLRFFTYMSFFCAAMLGLVTSSNFIQIYIFW
ELVGLCSYLLIGFWFTRPGAANACQKAFVTNRVGDFGLLLGILGFYWITGSFEFRELFQIL
NNKEVNLLFVTLCAALLFVGAIAKSAQFPLHVWLPDAMEGPTPISALIHAATLVAAGIFLV
ARLLPLFLVIPYIMYFISLIGIITVLLGATLALAQQDIKRGLAYSTMSQLGYMMVALGMGSY
RSALFHLITHAYSKALLFLAAGSVIHSMETIIGYSPEKSQNMVIMGGLTKHVPITKGAFLLG
TLSLCGIPPLACFWSKDEILNDSWLYSPIFALIAWGTVGLTAFYMFRIYLMTFEGHLNVGFK
NYSGKKNNPADSMSLWGKKGSKP----NFRFFTID---QTKISYTSDPFESENTMLFPQILLCFV
TFVIGFLGIP-NLDILTQWLHPAIYLLHHTN--STDLAEFLKHTVISGGIAYCGIFIAFLLYKPT
YSSFKSFLLINSLHVIRDKIVFVIYDWAYNRAYIDTFYKNFFVCQVRRFSQIIRSFDLRIIDQIF
NCFAFLSFIASEGIKYLGYA-RIPFYLFFYFFFVSIFIFLFYMDLPGPIHDFLLVFLGSGLILGSI
GVVLFPNPIYSAFSLGLVLVCISLFYILSNSYFVAAAQLLIYVGAINVLILFAVMFLNGSEYS
NDFPLWTLGDGITSQVCISLFISLISTILDTSWYGIIWTTRSNQIIEQDLISNSQQIGIHLSTDFF
LPFELISIILLVALIGAIVIARQMTAPATRKDLMIVNMGPHHPSMHGVLRLILTLDGEDVIDC
EPILGYLHRGMEKIAENRTIIQYLPYVTRWDYLATMFTEAITVNGPERLSNIQVPKRASYIR
VIMLELSRIASHLLWLGPFMADIGAQTPFFYIFRERELIYDLFEAATGMRMMHNFFRIGGV
AADLPHGWIDKCLDFCNYFLTGVAEYQKLITQNPIFLERVEGIGTIRAEEALNWGLSGPML
RASGIEWDLRKIDQYECYDEFAWEVQWQKEGDSLARYLVRIGEMEESIKMIQQALEGIPG
GPYENLEIRRFDRVKHPQWNDFEYRFIAKKTSPIFELSKQELYVRVEAPKGELGIFLIGDQS

VFPWRWKIRPPGLINLQILPQVVKRMKLADIMTILGSIDIIMGEVDRMFPMVTELRNYGQQ
TIRVARYIGQGFMITLSHANRLPVTIQYPYEKLITPERFRGRIHFEFDKCIACEVCVRVCPIDL
PVVDWQLETDIRKKRLLNYSIDFGLCIFCGNCVEYCPTNCLSMTEEYELSTYDRHELNFN
QIALGRLPMSVIEDYTIRKISNL----------------MQSFLSVWLVKHGIIHRSLGFDYQGIETLQI
KPEDWHSVAVILYVYGYNYLRSQCAYDVAPGGLLASVYHLTRIEYGVTQPEEVCIKVFASR
KNSRIPSVFWIWKSADFQERESYDMLGISYDNHPRLKRILMPESWIGWPLRKDYVAPHFY
EIQDAH--MNSIQFPLLDRTAKNSVISTTLNDL
SNWSRLSSLWPLLYGTSCCFIEFASLIGSRFDFDRYGLVPRSSPRQADLILTAGTVTMKMAP
SLVRLYEQMPEPKYVIAMGACTITGGMFSTDSYSTVRGVDKLIPVDVYLPGCPPKPEAIID
AITKLRKKISREIYQDRMKSQRANRCFTTNHKFRVGHSIHTGNYDQRFLSQPPSTSSIPTET
LFTYKNSVSSHELVNMQTRNTFSCIKKQITRSISVSLMLYIITRTSIASAYPIFAQQGFENPRE
ATGRIVCANCHLANKPVDIEVPQAVLPDTVFEAVVRIPYDMQLKQVLSNGKKGGLNVGA
VLILPEGFELAPPDRLSTEMKEKIGNLSFQSYRPNKKNILVVGPVPGKKYSEITFPILSPDPAT
KKDARFLKYPIYVGGNRGRGQIYPDGSKSNNTVYNATAAGIVSKIIRKEKGGYEITITDAS
DSRQVVDIIPPGPELLVSEGESIKFDQPLTSNPNVGGFGQGDAEIVLQDPLRVQGLLFFLAS
VILAQIFLVLKKKQFEKVQLAEMNFMSKVYDWFEERLEIQAIADDITSKYVPPHVNLFYCL
GGITLTCFLVQVATGFAMTFYYRPTVTEAFASVQYIMTEANFGWLIRSVHRWSASMMVLM
MILHVFRVYLTGGFKKPRELTWVTGVVLAVLTASFGVTGYSLPRDQIGYWAVKIVTGVPE
AIPVIGSPLVELLRGSASVGQSTLTRFYSLHTFVLPLLTAVFMLMHFPMIRKQGISGPLMGV
TKKPDLNDPVLRAKLAKGMGHNYYGEPAWPNDLLYIFPVVILGTIACNVGLAVLEPSMIG
EPADPFATPLEILPEWYFFPVFQILRTVPNKLLGVLLMVSVPAGLLTVPFLENVNKFQNPFR
RPVATTVFLIGTAVALWLGIGATLPIDQSLTLGLFMIEVFLFGIVLGLIPITLAGLFVTAYLQY
RRGDQLDLMLTLTSYFGFLLAALTITSALFIGLSKIRLIMDIVSLAWAALMVVFTFSLSLVV
WGRSGLMIIRSPEPEVKILVDRDPVKTSFEEWAKPGHFSRTIAKGPDTTTWIWNLHADAHD
FDSHTSDLEEISRKVFSAHFGQLSIIFLWLSGMYFHGARFSNYEAWLSDPTHIGPSAQVVW
PIVGQEILNGDVGGGFRGIQITSGFFQMWRASGITSELQLYCTAIGALVFAALMLFAGWFH
YHKAAPKLAWFQDVESMLNHHLAGLLGLGSLAWAGHQVHVSLPINQFLNAGVDPKEIPL
PHEFILNRDLMAQLYPSFAEGATPFFTLNWSKYAEFLTFRGGLDPVTGGLWLTDIAHHHLA
IAILFLIAGHMYRTNWGIGHGLKEILDAHKGPFTGQGHKGLYEILTTSWHAQLSLNLAML
GSLTIIVAHHMYSMPPYPYLATDYGTQLSLFTHHMWIGGFLIVGAAAHAAIFMVRDYDPT
TRYNDLLDRVLRHRDAIISHLNWACIFLGFHSFGLYIHNDTMSALGRPQDMFSDTAIQLQP
VFAQWIQNTHALAPGATAPGATASTSLTWGGGDFVAVGGKVALVPIPLGTADFLVHHIHAF
TIHVTVLILLKGVLFARSSRLIPDKANLGFRFPCDGPGRGGTCQVSAWDHVFLGLFWMYN
SISVVIFHFSWKMQSDVWGSISDQGVITHITGGNFAQSSITINGWLRDFLWAQASQVIQSYG
SSLSAYGLFFLGAHFVWAFSLMFLFSGRGYWQELIESIVWAHNKLKVAPATQPRALSIVQG
RAVGITHYLLGGIATTWAFFLARIIAVGMALRFPRFSQGLAQDPTTRRIWFGIATAHDFESH
DDITEERLYQNIFASHFGQLAIIFLWTSGNLFHVAWQGNFESWVQDPLHVRPIAHAIWDPH
FGQPAVEAFTRGGALGPVNIAYSGVYQWWYTIGLRTNEDLYTGALFLLFLSAISLIAGWLH
LQPKWKPSVSWFKNAESRLNHHLSGLFGVSSLAWTGHLVHVAIPASRGEYVRWNNFLEV
LPHPQGLGPLFTGQWNLYAQNPDSSSHLFGTSQGAGTAILTLLGGFHPQTQSLWLTDIAHH
HLAITFLFLVAGHMYRTNFGIGHSMKDLLDAHIPPGGRLGRGHKGLYDTINNSLHFQLGLA
LASLGVITSLVAQHMYSLPAYAFIAQDFTTQAALYTHHQYIAGFIMTGAFAHGAIFFIRDYN
PEQNEDNVLARMLDHKEAIISHLSWASLFLGFHTLGLYVHNDVMLAFGTPEKQILIEPIFA
QWIQSAHGKTSYGFDVLLSSTSGPAFNAGRSIWLPGWLNAVNE--NSNSLFLTIGPGDFLVH

HAIALGLHTTTLILVKGALDARGSKLMPDKKDFGYSFPCDGPGRGGTCDISAWDAFYLAV
FWMLNTIGWVTFYWHWKHITLWQGNVSQFNESSTYLMGWLRDYLWLNSSQLINGYNPF
GMNSLSVWAWMFLFGHLVWATGFMFLISWRGYWQELIETLAWAHERTPLANLIRWRDKP
VALSIVQARLVGLAHFSVGYIFTYAAFLIASTSGKFGMSHSVKIYDTCIGCTQCVRACPTDV
LEMIPWDGCKAKQIASAPRTEDCVGCKRCESACPTDFLSVRVYLWHETTRSMGLAY---MN
LPSIFVPLVGLVFPAIAMASLFLHVQNNKIVMRDFKTYLSVAPVLSTLWFGALAGLLIEINR
FFPDALTFPFFSFMTAILERRESQSLWGRFCNWITSTENRLYIGWFGVLMIPTLLTATSVFIIA
FIAAPPVDIDGIREPVSGSLLYGNNIISGAIIPTSAAIGLHFYPIWEAASVDEWLYNGGPYELI
VLHFLLGVACYMGREWELSFRLGMRPWIAVAYSAPVAAATAVFLIYPIGQGSFSDGMPLGI
SGTFNFMIVFQAEHNILMHPFHMLGVAGVFGGSLFSAMHGSLVTSSLIRETTENESANEGY
RFGQEEETYNIVAAHGYFGRLIFQYASFNNSRSLHFFLAAWPVVGIWFTALGISTMAFNLN
GFNFNQSVVDSQGRVINTWADIINRANLGMEVMHERNAHNFPLDLAAIEAPSTNGMGLP
WYRVHTVVLNDPGRLLSVHIMHTALVAGWAGSMALYELAVFDPSDPVLDPMWRQGMFV
IPFMTRLGITNSWGGWSIT-GGTVTNPGIWSYEGVAGAHIVFSGLCFLAAIWHWVYWDLEI
FCDERTGKPSLDLPKIFGIHLFLSGLACFGFGAFHVTGLYGPGIWVSDPYGLTGKVQPVNPA
WGVEGFDPFVPGGIASHHIAAGTLGILAGLFHLSVRPPQRLYKGLRMGNIETVLSSSIAAVF
FAAFVVAGTMWYGSATTPIELFGPTRYQWDQGYFQQEIYRRVSAGLAENQSLSEAWSKIP
EKLAFYDYIGNNPAKGGLFRAGSMDNGDGIAVGWLGHPIFRDKEGRELFVRRMPTFFETF
PVVLVDGDGIVRADVPFRRAESKYSVEQVGVTVAFYGGELNGVSYSDPTTVKKYARRAQ
LGEIFELDRATLKSDGVFRSSPRGWFTFGHASFALLFFFGHIWHGARTLFRDVFAGIDPDLD
AQVEFGAFLKLGDPTTKRQAA------------VETLFNGTLALAGRDQETTGFAWWAGNARLIN
LSGKLLGAHVAHAGLIVFWAGAMNLFEVAHFVPEKPMYEQGLILLPHLATLGWGVGPGG
EVLDTFPYFVSGVLHLISSAVLGFGGIYHALLGPETLEESFPFFGYVWKDRNKMTTILGIHL
ILLGLGAFLLVFKALYFGGVYDTWAPGGGDVRKITNLTLSPSIIFGYLLKSPFGGEGWIVSV
DDLEDIVGGHVWLGSICILGGIWHILTKPFAWARRAFVWSGEAYLSYSLGALAIFGFTACC
FVWFNNTAYPSEFYGPTGPEASQAQAFTFLVRDQRLGANVGSAQGPTGLGKYLMRSPTG
EVIFGGETMRFWDLRAPWLEPLRGPNGLDLSRLKKDIQPWQERRSAEYMTHAPLGSLNS
VGGVATEINAVNYVSPRSWLATSHFVLGFFFFVGHLWHAGRARAAAAGFEKGIDRDFEPV
LSMTPLNMTIALGQFTKDENDLFDTMDDWLRRDRFVFVGWSGLLLFPCAYFAVGGWFTG
TTFVTSWYTHGLASSYLEGCNFLTAAVSTPANSLAHSLLLLWGPEAQGDFTRWCQLGGLW
TFVALHGAFGLIGFMLRQFELARSVQLRPYNAIAFSGPIAVFVSVFLIYPLGQSGWFFAPSF
GVAAIFRFILFFQGFHNWTLNPFHMMGVAGVLGAALLCAIHGATVENTLFEDGDGANTFR
AFNPTQSEETYSMVTANRFWSQIFGVAFSNKRWLHFFMLFVPVTGLWMSALGVVGLALN
LRAYDFVSQEIRAAEDPEFETFYTKNILLNEGIRAWMAAQDQPHENLIFPEEVLPRGNALM
SGSTGERSFADIITSIRYWVIHSITIPSLFIAGWLFVSTGLAYDVFGSPRPNEYFTESRQGIPLI
TGRFDPLEQLDEFSRSFMTIDRTYPIFTVRWLAVHGLAVPTVFFLGSISAMQFIQRMATQTV
ENSSRSG-PRRTTVGKLLKPLNSEYGKVAPGWGTTPLMGVAMGLFAVFLSIILEIYNSSVLL
DGISMNMLTLKLFVYTVVIFFVSLFIFGFLSNDPGRNPGREEMADTTGRIPLWIIGTVTGLL
VIGLIGIFFYGSYSGLGSSLMLKIFSL--ICLNSALY-SSFFFGKLPEAYAFLNPIVDFMPVIPVL
FFLLAFVWQAAVSFRTTQSNPNEQNVELNRTSLYWGLLLIFVLAVLFSNYFFNMEVNILAFI
ATALFILVPTAFLLIIYVKTVSQNDMETATLVTIFLSGLLVSFTGYALYTAFGQPSQQLRDPFE
EHGDMEALVYTFLLVSTLGIIFFAIFFREPPKVPTKK--MTLAFQLAVFALIATSSILLISVPVV
FASPEGWSSNKNVVFSGTSLWIGLVFLVGILNSLIS-------MSPQTETKASVGFKAGVKDYKL
TYYTPDYQTKDTDILAAFRVTPQPGVPPEEAGAAVAAESSTGTWTTVWTDGLTSLDRYKG

RCYRIERVIGEKDQYIAYVAYPLDLFEEGSVTNMFTSIVGNVFGFKALRALRLEDLRIPTAYI
KTFQGPPHGIQVERDKLNKYGRPLLGCTIKPKLGLSAKNYGRAVYECLRGGLDFTKDDEN
VNSQPFMRWRDRFLFCAEAIYKSQAETGEIKGHYLNATAGTCEEMMKRAIFARELGVPIV
MHDYLTGGFTANTSLAHYCRDNGLLLHIHRAMHAVIDRQKNHGMHFRVLAKALRLSGG
DHIHAGTVVGKLEGEREITLGFVDLLRDDFVEQDRSRGIYFTQDWVSLPGVLPVASGGIH
VWHMPALTEIFGDDSVLQFGGGTLGHPWGNAPGAVANRVALEACVQARNEGRDLAREG
NEIIRQACKWSPELAAACEVWKEIRFEFKPVDTLDPMVIQLYKTYTPSTRNGTVNGQVKS
TRGKNLIYGQHRCGKGRNARGIITARHRGGGHKRLYRKIDFRRNEKDIYGRIVTIEYDPNR
NAYICLIHYGNGEKRYILHPRGAIIGDSIVSGTEVSIKIGNALPLTEMPLGTAIHNIEITLGKG
GQLARAAGAVAKLIAKEGKSATIKLPSGEVRLISKNCSATVGQVGNVGVNQKSLGRAGAK
RWLGKRPVVRGVVMNPVDHPHGGGEGRAPIGRKKPTTPWGYPALGRKSRKRNKYSEKFI
LRHRSKMIQPQTHLNVADNSGARELMCIRIIGAS-NRRYAHIGDIIVAVIKEAVPNMPLERSE
VVRAVIVRTCKELKRDNGMIIRYDDNAAVVIDKEGNPKGTRIFGAIARELRQFNFTKIVSL
APEVLMLSPKRTRFRKQHRGRMKGISSGGNHICFGKYALQALEPAWITSRQIEAGRRAMT
RNARRGGKIWVRIFPDKPVTVRPAETRMGSGKGSPEYWVAVVKPGRILYEMGGVTKKIAR
RAISIAASKMPIRTQFISSEMTRIKRGYIARRRRTKMHFFASSFRGAHSRLTRTMTQQEKRA
LVSAHRDRDRQKRDFRRLWITRINAVIRER--VSYSYSKFIHDLYKNQLLLNRKILAQIAISN
RKCLDMISNEIV------------VLKEVYALGQHISMSADKARRVIDQIRGRSYEETLMILELMPY
RAGYPIFKLVYSAAANASYTMASNEANLVISKAEVNEGTTVKKFKPRARGRSYPIKRTTC
HITIVLKDISLDGGLWDKKMDGIKYAVLTDKSIRLLGKN-KYTFNIESRATKIGIKLSIELFFG
VKVKDVNSHRLPAKSRR----MGQTM--RRVIITLQPGYSIQPLKKIKNMAVPKKRISKSKKRI
RKNTWKRKSFWTALKAFSLGKSLSTGNLKSFFVSQTN-MAKSKDVRVAVILECTSCVRNG
VN--KVSTGISRYITQKNRHNTPNPLELKKFCPYCYKHTIHGEIKKMKIRASVRKICEKCRLI
RRRGRIIVICSNPRHKQRQGMIREKVTVSTRTLQWKCVESRTDSKRLYYGRFILSPLMKGQ
ADTIGIAMRRALLGEIEGTCITRVKSE--KAPHEYSTIAGIQESVHEILMNLKEIVLRSNLYGT
CDASICVKGPGCVTAEDIILPPSVEIVDNTQHIAWLTEPIDFCIGLQIERNRGYILKTHH-NFD
GSY--PIDAVFMPVRNANHSIHSYGNGNEKQEILFLEIWTNGSLTPKEALHEASRNLIDLFIPF
LHMEEE--NLHLEDGE-HTIPLSPLSFHDKVA-KLRKNKK-KLALKSIFIDQLEFPPKIYNCLK
KSNISTLLDLLNKSQEDLMKIEHFHIEDVKQILGILEKH--FAIDLPKNQ-MEG---GGMTTIPG
FNQIQFEGFCRFIDQGLTEELSKFPKIEDIDQEIEFQLFVETYQLVEPLIKERDAVYDSLTYSS
ELYVSARLIWKTSRDMQEQTIFIGSIPLMNSQGTSIVNGIYRIVINQILQSPGIYYRSELDHN
GISVYTGTIISDWGGRSELEIDRKARIWARVSRKQKISILVLSSAMGSNLREILENVCYPEIFL
SFLSDKEKKKIGSKENAILEFYQQFACVDGDPIFSESLWKELQKKFFQQRCELGRIGRRNM
NRRLNLDIPQNNTFLLPRDLLAAADHLIGLKFGMGTLDD--MNHLQNKRIRSVADLLQDQF
GLALVRLENAVRGTICGAIR-HKL-IPTPQNLVTSTP----LTTTYESFFGLHPLSQVLDRTNPLT
QIVHGRKFSSLGPGGLTGRTASFRIRDIHPSHYGRICPIDTSEGINVGLIGSLAIHARIGHWG-
SLESPFY---EISERSTG--VRMLYLSPGRDEYYMVAAGNSLALNQDIQEDQVVPARYRQEFL
TIAWEQVHLRSIFPFQYFSIGASLIPFIEHNDANRALMSSNMQRQAVPLSRSEKCIVGTGVE
RQAALDSGALVIAEREGRVVYTDTDKILFSGDGETLSIPLVMYKRSNKNTCMHQKPQVQR
GKCIKKGQILADGAATVEGELALGKNVLVAYMPWEGYNSEDAVLISERLVYEDIYTSFHIK
KYEIQTHVTSQG-PEKVTNEIPHLEAHFIRNLDKNGIVKQGSWVETGDVLVGKLTPQVVKE
-SSYAPEDRLLRAILGIQVSTSKETCLKVPIGGRGRVIDVRWIQKKG-GSSYNPEMIRVYILQ
KREIKVGDKVAGRHGNKGIISKILPRQDMPYLQDGRSVDLVFNPLGVPSRMNLGQIFECSL
GLAGSLLDRHYRIAPFDERYEQEASRKVVFSELYEASKQTANPWAFEPEYPGKSRIFDGRT

GNPFEQPVLIGKPYILKLIHQVDDKIHGRSSGHYALVTQQPLRGRAKQGGQRVGEMEVWA
LEGFGVAHILQEMLTYKSDHIRARQEVLGTTIVGGTIPNPKDAPESFRLLVRELRSLALELN
HFLVSEKNFQIHRKEAMNPNFSSMIDRYKHQQLRIGSVSPQQISAWATKILPNGEIVGEVKK
PYTFLYKTNKPEKDGLFCERIFGPIKSGICACGNYRVIGDEKEEPKFCEQCGVEFIDSRIRRY
QMGYIKLACPVTHVWYLKRLPSYIANLLDKPLKELEGLVYCD--FSFARP-ITKKPTFLRLR
GLFEYEIQSWKYSIPLFFTTQGFDTFRNREISTGAGAIREQLADLDLRIIIENSLLEWKDLGE
E-EHTGNEWEDRKVGRRKDFLVRRMELAKHFLRTNIEPEWMVLCLLPVLPPELRPIIQIDG
GKLMSSDINELYRRVIYRNNTLTDLLTTSRSTPGELVMCQEKLVQEAVDTLLDNGIRGQPM
RDGHNKIYKSFSDLIEGKEGRFRETLLGKRVDYSGRSVIVVGPSLSLHRCGLPREIAIELFQ
TFVIRGLIRQHLASNIGVAKSKIREKEPIVWEILQEVMQGHPVLLNRAPTLHRLGIQAFQPV
LVEGRAICLHPLVCKGFNADFDGDQMAVHVPLSLEAQVEARLLMFSHMNLLSPAIGDPIS
VPTQDMLIGLYVLTSENRRGICLNRYTKCNR---RNSQ-TKRSDNSYKYK---EPVFCNSYDAI
GAYRQKRINLDSPLWLRWRLDQR-VITS--RETPIEVHYESLGTYYEIYGHYLIVRSLKKEIIF
IYIRTTVGHISLYREIEEAIQGFSRAGSSDT--------MEVLMAERANLVFHNKVIDGTAMKRLI
SRLINHFGMAYTSHILDQVKTLGFQQATATSISLGIDDLLTIPSKGWLVQDAEQQSLILEKH
HHYGNVHAVEKLRQSIEIWYATSEYLRQEMNPNFRMTDPLNPVHIMSFSGARGNASQVH
QLVGMRGLMSDPQGQMIDLPIQSNLREGLSLTEYIISCYGARKGVVDTAVRTSDAGYLTRR
LVEVVQHIVVRRADCGTVRGVSVSPQNGMMP-ERILIQTLIGRVLADDIYIGPRCIATRNQN
IGVGLVNRFIT------FRAQPISIRTPFTCRSTSWICRLCYGRSPTHGDLVELGEAVGIIAGQSIG
EPGTQLTLRTFHTGGVFTGGTAEHVRAPSNGKIRFHEDLVHPTRTRHGHPAFLCSIDLYVTI
ESEDILHNVNIPPKSFLLVQNDQYVESEQVIAEIRAGTST-LNLKEKVRKHIYSDSDGE-MH
WSTDVYHAPEFTYGNVHLLSKTSHLWILLGEPCR--SSLVSLSIHRDQDQKSAH--SLFVKR
RSISNLS--ETNDR-ARQKIFSAHFSGQ-EDRIADY-SDLNRIICTDHSNLVYPAPILAENSDSNL
LSKRRRNQFIIPLQSIQERKKELMPCSGISMKIP--TNGIFCGNSIIAYFDDPRYRRMSSGSTK
YGTLEMHSIVKKED--LIQYRG-VREFRPKHKKVDRFFFIPEEVHILPGSSSIMVRNNSIIGVD
TQITLNIRSRVGGLVRVEKKKKRIELKIFSGDIYFPGRADKISRHSGVLIP-PG-TRKTNEKES
KK--VKNWIYVQR--ITPSK--KKFFVLVRPVVTYEIMDGITLATLFPPDLLQERDNVQLRVVN
YILYGNGKPIRGISDTSIQLVRTCLVLNWNQDKK---SSSSEAARASVVELRTNDLIRHFLRID
FVKSPISYIGKRNDPWGLGLLAD-NGLDWTNINP------Y---SKARIQQTLNQNQGTIHTFLNR
---NKGCQSLSILSSSNCSRMDP--VNGAKYHNV---IQES-PLIPIRNSLGPLGT--SLPIANFYS--
FDRL--LTHNQIL-VTNYLQLDN---LKQPFQVLKLK-YYLIAEN--GKIYNPNPRSKILLNPLNL
NWYFLHHNYCAETS-KIMSLGQFICENVFIAKKRPP-LK--SGQVILVQ-VDSVVIRSAKPYL
ATPGATVRGLYGETFYEGDTLVTFHYEKSRSGDITQGLPKVEQVLEVRSVDSISMNLERRV
EGWNKCLTRILGIPWGFLIGAELTIAQSRISLVNKIQKVYRSQGVQIHNRHIEIIVRQITSNVL
VSE---DGMSNVFSPGELIRLLRAKRMGRALEEAICYRALLLGITKASLNTQSFISEASFQET
ARVLAKAALQGRIDWLKGLKENVVLGGVIPAGTGFRGL--VHPSKQYNNLS--LETTKKNL
FEGGVR--DILSHHRKLLDSSLSKKFHNTSHNTIHQTIIYRIYNDSMTKIYWNINLEEMMEA
GVHFGHGTRKWNPKMAPYISAKRKGIHITNLTGTARFLSEACDLVFDAASRGKQFLIVGT
KNKAADSVARASIRARCHYVNKKWLGGMLTNWSTTETRLHKFRDLRMEQKMGGLNRL
PKRDATILKRQLARLQTYLGGMKYMTGLPDIAIIVDQHKEYTALQECITLGIPTICLIDTNC
DPNLSDISIPANDDAISSIRFILNKLVFAICEGRS-SYIINPMGQKINPLGFRLGTTQGHHSLWF
SQPKNYSESLQEDQKIRNFIKNYVQKNMITSSG-VTGIARIDIQKGIDLIKVIIFMGFPKLLIE
NRPR----ATEELQMTLQKELNCVNRKLNIAITRIAKPYGNPKILAEFIAGQLKNRVSFRKAM
KKAIELAEKADTKGIQVQIAGRIDGKEIARVEWIREGRVPRQTIRAKIDYCSYPVRTIYGVL

GIKIWIFLDEAMSRYRGPRFKKIRRLGALPGLTNKRPRAESDLRNQSRSGK-KSQYCIRLEE
KQKLRFHYGLTERQLIQYVRIARKAKGSTGQVLLQLLEMRLDNILFRLGMASTIPAARQL
VNHRHILVNGRIVDIPSYRCKPRDIITSKDNQKSRALIQNSLESSPH-EELPNHLALDPSPY-Y
KGLVNQIIDTKWVGLKINEFLVIEYYSRQTMSRRGTAEKKTAKSDPIYRNRLVNMLVNRIL
KHGKKSLAYQIIYRAVKKIQQKTETNPLSVLRQAIRGVTPNITVKARRVGGSTHQVPIEIGS
TQGKALAVRWLLAASRKRPGRDMAFKLSSELVDAAKGSGDAIRKKEETLRMAEANRAFA
HFRMGRDTIAEIITSIRNADMDRKRVVRIASTNITENIVRILLREGFIENVRKHREKNKNFFV
LTLRHRR--NRKRPY---RNIFNLKRISRPGLRIYSNYQRIPKILGGMGVVILSTSRGIMTDREA
RLEGIGGEILCYIWMAKAIPRRSSRRNGRIGSRKSARRIPKGVIHVQASFNNTIVTVTDVRG
RVVSWSSAGTSGFKGTRRGTPFAAETAASNAIRTVVDRGMLRAEVMIKGPGLGRDAALR
AILQSGILLTFVRDVTPMPHNGCRPPKKRRVMPTIKQLIRNPRQPIRNVTKSPALRGCPQRR
GTCTRVYTITPKKPNSALRKVARVRLTSGFEITAYIPGIGHNLQEHSVVLVRGGRVKDLPGV
RYHIVRGTLDAVGVKDRQQGRSKYGVKKPKMARKSLIQREKKRQKLEMKYHLIRRSSKK
EIS---KVALLSDKWEFYGKLQSPPRNSAPTRLHRRCFLTGRPRANYRDFGLSGHILREMVQ
ACLLPGATRSSWMVKNLFISVISQ--EETKENRGSVEFQIFNFTNKIQRLTSHLKLHKKDYLS
QKGLRKILGKRQRLLAYLSKKNKVRY---IEKLDIRETKTH--MVKLRLKRCGRKQRAVYRIV
AIDVRSRREGKDLRKLGFYDPIRNQTYLNVPAILYFLEKGAQPTGTVQDILKRSGV--ELRP
NQAKFN-MDKSKRPFLKSKRSFRRRLPPIQSGDRIDYRNMSLISRFISEQGKILSRRVNRLTL
KQQRLITLAIKQARILSLLPF--KR--KGFQISESTARTNALKARTQVARSLKKNPFVANHLLK
KIDKLNRKAEKEIIRTWSRGSTIIPTMIGHTIAIHNGKEHLPIYITDHMVGHKLGEFASTFNF
RGHTKSDNKSRR----MRNQFPFRSVYYKIINSVLAAGLYYGFLSAFSIKTSHLLLLRVFEEEE
E---TKKRIVAKTGLIMGQILLFISIYYKPFHIALTRPRTITALGFFYLFFQIFWKSQKRVFQ-----
-NSMSN--LSCIFLNHLILQLLNTCILPSSAARRLVSIYTFRSNNKMLFVTSSFFAWFIGQILVC
EFFELAGKHIRIELAGKH-NRS-SIIRSDYFHF---FFVILFFMMSLHSFGRIPSPILLPKMSNEK
LESLISPLLFDYRRWYRPFRYIQNNRLEQAIRNEMSQYFFDTQQSDGKERISFTYPRSLSTFF
KVIKRKIN-ILLTLSN--QLDNAWVSRNKKKMNHLKNDFFNRINHLDKEKKAVEILETRTRL
CIDTTKQEYLPQMYDPFLNGPYRGKIKKELPPRETGEQNRLHDLLYENFDQNTNASKDTI
DKETKTNASKETNTVNETKPNASKKTNASKETDTVNKETVLMRYAHQPDFKHGLIKGSM
RTQRRKIVIEKLFQANAHSPLFFDRIKKKKLFSFAIPRPVQ---IFRNVKEKKRLEIGETWDTF
EITQMLRGCILIAQSSLRKNLILPSLIIGKNLGRMLLLQIPEWSEDFEEWNREKHIKCTYTGN
PVSETEFPENWLTQGIQIKIVYPFHLKPWHTS-LTSRDDVCFLTIFGRETELPFGSPRKT-SFFE
PILKELEKK---------------------IGKYEKVTAESVLKKIIIKLFKKVSKETRTNQDSRISNQIIDE
SFQKIQIPDWTNSSLIETKMQDMTDRTSTIKNQIERITEEKKK------TYKKSCRLELSIWQIV
KLK--NIICKFHYFRKFFIQRIY-DICLSTIHICQTNTQLF-----CDLSNLSQAYVFYKISQTGVV
NLSKLRSVLQHTSFFLKTQMKDSFTQGIFQS-VIHKKLQ-SITSQWKNWLRGNYQYDLSQII
WSSLMSQKWRNRVNRYCRSQKKKKWNSCGKYQ--LSHYK--DNFQKCYRYDLLSYKSIN
VYIPYLNRKYFNWENIY----FFDWMGMNEQILNRWFFPEFVRLFNVYKTKPWIIPSKLLLL
NKSLSTRELRELFMKKYFLLQLRWDQRNIRK---NVRILALPKSSINLETMTLSCIERRKLQL
NVMWKSKSNLSILKFFKGMV-VDRLGLSVKNNGQFLMYQTIGISLVHKSKNNHFDALVLE
NILSSRRRRELRILICFTFLWPNYRLEDLACMNRYWFDTNNGSRFSILRIHMYLTQKRQIKF
CIFELREIMREIKNS--FLDSWTQFNSVGSFIHIFFHQERFLKLFDPRIFSILLS-RNF--QGSTSN
RSFT---IRGVILFVVA-VLIYR----INNRNMVERKNLYLTGFLP-----IPMNSTGPRNDRLEEAV-
GSSNINRLIVS-LLYLPKGKKISESSFLNRKESTGVLSIT----KR----------------------NSSCKISN-
--ETVAEIEILFKEKDSK-SLEFLFVYYMD-DDSTHKDHDWKLADPILLER----------------LAK

DWISYLMSAFREKRPIEAG-------------IFFKQHEHVSH--LFSRNKGAISLQNCTQFHMWKFR
QDLFLLFPSWGN-NPPESYFWL-----------GNVWL-KKDR---FFS--KVRNVWSNIQYDS---TR-
-SSFVQVTDSSQLKGSS-DQSRDHLDSIS----NEDSE-------------YRTLINQREIQQLEERSIPW
DP---SFLQTERKEIESD--RFPK--NLSGYS--SMSQLFT-EREKPMINHLFPEEMEEFL--GNATR
SVRSFFSDRW--SELH-LGSNPTERSTREQ----KLLKKHLSFV-----RRSE----NKEMIHLFKIITY
LQN--TISIHSISLDPGCDMVP-KDDPD-LDSSNKISFFNKNPFFDF--FHRFHERNRGGYAL--H
HDFESE--ERLQEMADLFTLSITEP-DLVYHKGFSFSIDSYGLDQKK--FLNEVFNTRAESKKK
SL---LVLSPVLFRYEENEYFFRRIRQKRVW-ISCGNGLGDLKQ-MVVFASNNIMEAVNQYRL
IRNLIQIQYNRY----IRSVLNRFFLMN---RSDRNFEYGIQ---RDQKGKDTLSHRTLMKYMIKQ
DYA---------------------------------YIYKWSNGSKNCQEHLEHFLSEQKSCFQRFQVMFDQLRIN
QFLMNYSEVCKKFEWSRSLRFFFLFSQSLRFLGKSLHFLA-------KLLFFLSNLSFPFSCVSF
GNTPIHRSE-INIY-ELKGPNDKLCNQLLESIGFQIVHLKKLNPF--LLA------DD-GTSKFLING
GTISPFLFNKIP-KR-MIDSFHTR--TNRRKSFDNKDSYFSMIFYDQDNWLNPGKPFHRSSLIS
SFYKANRLRFLNNPHHFCFY--CNKRFPFSV----EKARNNNSY--FLYG-QFLNILFLRKK-RFS
LCVGK--KKHVFGG-RTTISPIESQVSNIFIPNDFPQSGDER-YNLDKS---FHFLSRPDPFVR--R
AIYSIVDTSGNPLTEGQIVNFERTYCQPLSDMNLSDSEGKNLHECPN--FNSNIGLIHIPCSEK
DFPSEKR--KK--------QSLCLKKCVGVQKGRMYTTFQRDSAFSILSKWNLFQTYMPSFFTST
GYKYLNSIFLDTFSDLLS--ILSSS----VSIFH-------ISWRILLWKMQFFLR----------SEISSKWL
HN---LLSKEMIHRNKKNKSSLISTHLRSPNVWEF-LYSILFLVLVAGY-LVLIHLFFVSQ--TFS
ELQTEFEKVKSLMIPSSEIELRKLLDKYPTSEPNSFWLKNLFLF------------------------------------
-----------------------------PINRIAFSINTRHLSHTSKEIYSLIRKRKNVNGDWIDDKIESWVANSD
SIHEEERKFLVQLS--ALTT--EKR---ILLSLT-HSDHLS-----KNDSGYQMIEQPGAIYLRYLVDI
H--QKHLMNYEFNISCLAERRIFLAHYQTITY--SQTSSGANSFHFPSDG---KPFSLRLALSPS-
RGILVIGSIGTGRSYLVKYLATNSYVPFITVFLNKLLDKKPK-FIAD----IDIDNIDASDDIDID
DSD--------------------DIDRDLDTELELLTWMNAL---TMDKEMM-AEINRLSITLQFELARAM
SPCIIWIPNIHDLDVNESNYLSLGLLVNHLS---RDCERCS-TRNILVIASTHIPQKVDPALIAPN
KFNTCIKLRRLLIPQQRKYFFTLSYTRGFHLEKKMFHT--NGFGSITMGPNARDLVALTNEV
LSISITQKKSIIDTNTIRSALHRQTWDLR---SQVRSVQDHGILFYQIGRAVAQNVLLSNCPIDP
ISIYLKKK--LCNEGDSYLYKWYFELGTSMKKFTILLYLLSCSAGSVAQDLWSLP--GPDEK-
NGITSYGLVENDS-DLVHGLLEVEGALVG--SSRTEKNCSQFDND--RVTLLLRPEPRN-PLD
MMQNGSCSILDQIFLYEK--YESEFEE-----GALDPNLDPQQIEED----LFNH--IVWAP--RIWRP
-----WGFLCIERPNELGFS-YWSRSFRGK---RIFYDKEDELQENDSEFLQSGTMQYKTRDRSS
KEQGFFRISQFIWDPADPLFFLFK-DRSPGSVFSRRELFADEEMSKGLLTAQTYTSRSKSTR
WFI--KNTQEKHFELLINRQRWLRT---NSSLS--NGSFRSN--TLSESYQYLSNLFLSN-GTLFD
QMTKTLLRKRWLFPDEMKIGF--M---------MPRSR-INGNFIDKTFSIVANILLRIIPTTSGEKE
AFTYYRDG--MSAQSEGNYAEALQNYYEAMRLEIDPYDRSYILYNIGLIHTSNGEHTKALE
YYFRALERNPFLPQACNNMAVICHY-RGEQAIQQGDSELAETWFDQAAEYWKQAIALTPG
NYIEAHNWLKITGRFE--MSWRSEHIWIELLPGSRKISNFCWAFILFLGSLGFLLVGISSYLG
RNLISFFPSQQIIFFPQGIVMSFYGIAGLFISSYLWCTISWNVGSGYDLFDRREGIVCIFRWGF
PGRNRRIFLRFLIKDIRSVRIEVKEGIYARRVLYMDIRGQRAIPLTRTDEN-LTPGEIEKKAAE
LAYFLRVPIEVF
>Lolium
???
???

??
??
??
???????????????????????????????MATLRVDEIHKILRERIEQYNRKVGIENIGRVVQVGDGIA
RIIGLGEIMSGELVEFAEGTRGIALNLESKNVGIVLMGDGLMIQEGSFVKATGRIAQIPVSEA
YLGRVINALAKPIDGKGEIIASESRLIESPAPSIISRRSVYEPLQTGLIAIDSMIPIGRGQRELII
GDRQTGKTAVATDTILNQKGQNVICVYVAIGQRASSVAQVVTNFHEEGAMEYTIVVAEMA
DSPATLQYLAPYTGAALAEYFMYRERHTLIIYDDLSKQAQAYRQMSLLLRRPPGREAYPG
DVFYLHSRLLERAAKLNSLLGEGSMTALPIVETQSGDVSAYIPTNVISITDGQIFLSSDLFNA
GIRPAINVGISVSRVGSAAQIKAMKQVAGKSKLELAQFAELQAFAQFASALDKTSQNQLAR
GRRLRELLKQSQANPLPVEEQIATIYTGTRGYLDSLEIEQVNKFLDDLRKHLKDTKPQFQE
ILSSSKTFTEQAEILLKEAIQEQLERFSLQQQTMRTNPTTSRPGVSTIEEKSTGRIDQIIGPVL
DVTFPPGKLPYIYNALVVQSIDTANKQINVTCEVQQLLGNNRVRAVAMSATDGLMRGME
VIDTGAPLSVPVGGATLGRIFNVLGEPVDNLGPVDSSATFPIHRSAPAFIELDTKLSIFETGIK
VVDLLAPYRRGGKIGLFGGAGVGKTVLIMELINNIAKAHGGVSVFGGVGERTREGNDLY
MEMKESGVINEKNIEESKVALVYGQMNEPPGARMRVGLTALTMAEYFRDVNKQDVLLFI
DNIFRFVQAGSEVSALLGRMPSAVGYQPTLSTEMGSLQERIASTKKGSITSIQAVYVPADDL
TDPAPATTFAHLDATTVLSRGLASKGIYPAVDPLDSTSTMLQPRIVGNEHYETAQRVKETLQ
RYKELQDIIAILGLDELSEEDRLTVARARKIERFLSQPFFVAEVFTGSAGKYVGLAETIRGFQ
LILSGELDGLPEQAFYLVGNIDEASTKAITLEEENKSKKMKLNLYVLTPKRIIWDCEVKEIIL
STNSGQIGVLPNHAPINTAVDMGPLRIRLNDQWLTAVLWSGFARVVNNEIIILGNDAELGSD
IDPEEAQEALKIAEANLSKAEGTKELVEAKLALRRARIRIEAVNWIPMKNVTHSFVFLAHW
PSAGSFGLNTNILATNLINLTVVVGVLIFFGKGVLKDLLDNRKQRILSTIRNSEELRRGTIEK
LEKARIRLQKVELEADEYRMNGYSDIEREKANLINATSISLEQLEKSKNETLYFEKQRAMN
QVRQRVFQQAVQGALGTLNSCLNTELHFRTIRANIGILGSMEWKR-MNPLIAAASVIAAGL
AVGLASIGPGVGQGTAAGQAVEGIARQPEAEGKIRGTLLLSLAFMEALTIYGLVVALALLF
ANPFVMNIIPCSIKTLKGLYDISGVEVGQHFYWQIGSFQIHAQVLITSWVVITILLGSVVIAV
RNPQTVPMDGQNFFEYVLEFIRDLSKTQIGEEYGPWVPFIGTMFLFIFVSNWSGALLPWKII
ELPHGELAAPTNDINTTVALALLTSAAYFYAGLSKKGLSYFEKYIKPTPILLPINILEDFTKPL
SLSFRLFGNILADELVVVVLVSLVPLVVPIPVMFLGLFTSGIQALIFATLAAAYIGESMEGHH
MLFATLEHILNHISFSTISIVITIHLITLLVHELGGLRDSSEKGMIVTFFSITGFLVSRWASSGH
FPLSNLYESLIFLSWALYILHTIPKIQNSKNDLSTITTPSTILTQGFATSGLLTEMHQSTILVPAL
QSQWLMMHVSMMLLSYATLLCGSLLSAAILIIRFRNNFFFFSKKKD---FYVKRSSLKSTSV
PSFPNYYKYQLTERLDSWSYRVISLGFTLLTIGILCGAVWANDAWGSYWNWDPKETWAFI
TWTIFAIYLHSRTNLNWKGTNSALVASIGFLIIWICYFGINLLGIGLHSYGSFILTMKKKKAL
PSLLYLVFIVLLPWGVSFSFNKCLELWIKNWWNTRQSETLLPYIQEKRILERFIELEELSLLD
EMIKEKLKTHAQKPPIGIHKEIIQLVKMDNEDHLHIILHFSTNIICLAILSGFFFLSKEELVILN
SWVQEFFYNLNDSIKAFFILLVTDFFVGFHSTRGWELVIRWVYNDLGWAPNELIFTIFVCSF
PVILDTCLKFWVFFCLNRLSPSLVVIYHSISEMPIGVPKVPYRIPGDEEATWVDLYNVMYRE
RTLFLGQEIRCEITNHITGLMVYLSIEDGISDIFLFINSPGGWLISGMAIFDTMQTVTPDIYTI
CLGIAASMASFILLGGEPTKRIAFPHARIMLHQPASAYYRARTPEFLLEVEELHKVREMITR
VYALRTGKPFWVVSEDMERDVFMSADEAKAYGLVDIVGDEPREAKVTFEGLVTEALPNG
MFRVRLENDTIILGYISGKIRSSSIRILMGDRVKIEVSRYDSSKGRIIYRLPHKDSKDMEKFE
GYSEKHKSRQQYFVYPLLFQEYIYAFAHDYGLFSCNNKFSSLLVKRLIIRMYQQSFWMNS

VNHPNQDRLLDSEFYSQILSEGFAIVVETPFSLRELPCPIPKFQNLHSIHSIFSFLEDKFVHLD
YLSHIEIPYPIHLEILVQLLQYRIQDVPSLHLLRFFLNNYSNWNSFITKSIFLLKKKRLFRFLY
NSYVSEYEFFLLFLRKQSSCLPLAASGTFLERIHFSKKMEH--IMYPGRKTIWFFMDPLMHY
VRYQGKALFASKGTPFLNKKWKWYLINLWQYCFSFWTQPRRIHLNQLANSCFDFMGYLS
SVPKSPFLVKNQMLDNSFLIDTLI--QKLDTIVPATALIGYLSKAQFCTGSGHPISKPIWTDLS
DWDILDRFGRICRNLFHYHSGSSKKRTLYRLKYILRLSCARTLARKHKSTVRTFMQRLGSA
FLEEFFTEEELVFSLMFTKRIWYFDIIRINDLVKPLMIIDRV-EVE-IINSFSKSE-LLKEVYGLIS
IL-PILSLLLGITIEVLVIVWLEREISASIQQRIGPEYAGPLGLLQAIADGTKLLFKEDILPSRGD
IPLFSIGPSIAVISILLSFLVIPLAYHFVLADLSIGVFLWIAISSIAPIGLLMAGYSSNNKYSFSG
GLRAAAQSISYEIPLTFCVLAISLLSNSSSTVDIVEAQSKYGFFGWNIWRQPIGFLVFLISSLA
ECERLPFDLPEAEEELVAGYQTEYSGIKYALFYLVSYLNLLVSSLFVTVLYLGGWDLSIPYIS
FFDFFKMNNAVGILEITMGIFITLTKAYLFLFISITIRWTLPRMRMDQLLNLGWKFLLPISLG
NLLLTTSFQLVSLMIWHVQNENFILDSTRIFMKAFHLLLFNGSFIFPECILIFGLILLLMIDSTS
DQKDRPWFYFISSTSLVISITALLFRWREEPIISFSGNFQTNNFNEIFQFLILLCSTLCIPLSVE
YIECTEMAITEFLLFILTATLGGMFLCGANDLITIFVAPECFSLCSYLLSGYTKRDLRSNEAT
MKYLLMGGASSSILVHGFSWLYGSSGGEIELQEIVNGLINTQMYNSPGISIALISITVGLGFK
LSPAPFHQWTPDVYEGSPTPVVAFLSVTSKVAASASATRILDIPFYFSSNEWHLLLEILAILS
MILGNLLAITQTSMKRMLAYSSIGQIGYVIIGIIVGDSNDGYASMITYMLFYISMNLGTFACI
VLFGLRTGTDNIRDYAGLYTKDPFLALSLALCLLSLGGLPPLAGFFGKLYLFWCGWQAGL
YFLVSIGLLTSVLSIYYYLKIIKLLMTGRNQEITPYVRNYRRSPLRSNNSIELSMTVCVIAST
LPGISMNPILAIAQDTLFMFLLHEYDIFWTFLIIASLIPILAFWISGLLAPVSEGPEKLSSYESG
IEPMGGAWLQFRIRYYMFALVFVVFDVETVFLYPWAMSFDVLGVSVFIEAFIFVLILVVGL
VYAWRKGALEWSMSYFPWLTILVVLPIFAGSLIFFLPHRGNKIVRWYTISICLLEFLLMTYA
FCYHFQLEDPLIQLKEDYKWIDIFDFHWRLGIDGLSLGSILLTGFITTLATLAAWPVTRNSR
LFYFLMLAMYSGQIGLFSSRDLLLFFIMWELELIPVYLLLSMWGGKRRLYSATKFILYTAG
GSIFFLIGVLGMGLYGSNELGLDLERLINQSYPATLEILLYFGFLIAYAVKLPIIPLHTWLPDT
HGEAHYSTCMLLAGILLKMGAYGLIRINMELLPHAHYLFSPWLVIIGAIQIIYAASTSLGQR
NFKKRIAYSSISHMGFIIIGIGSITNIGLNGAILQILSHGFIGATLFFLAGTASDRMRLVYLEEL
GGISIPMPKIFTMFSSFSMASLALPGMSGFVAELVVFFGLITSPKFLLMPKMLITFVMAIGMI
LTPIYLLSMLRQMFYGYKLFNVPNANFVDSGPRELFILICIFLPVIGIGIYPDFVLSLSIDRVE
ALVSNYYPKMMFELVLFLSVYLFSIGIYGLITSRNMVRALICLELILNSINLNLVTFSDLFDS
RQLKGDIFAIFVIALAAAEAAIGLSILSSIHRNRKSTRINQSNLLNNMEHTYQYAWVIPLLPL
PVIMSMGFGLFFIPTATRNLRRIWAFPSVLFLSIAMVFSIQLSIQQINGSSIYQYLWSWTVNN
DFSLEFGYLIDPLTSIMLILITTVGILVLIYSDGYMSHDEGYLRFFVYISFFTTSMLGLVTSSN
LIQIYFFWELVGMCSYLLIGFWFTRPIAASACQKAFVTNRVGDFGLLLGILGFFWITGSLEF
RDLFKIANNNGINSLLTTLCAFLLFLGAVAKSAQFPLHVWLPDAMEGPTPISALIHAATMV
AAGIFLLARLFPLFISLPLIMTLISLVGTITLFLGATLAIAQRDIKRSLAYSTMSQLGYMMLA
LGIGSYQAALFHLITHAYSKALLFLGSGSIIHSMEPLVGYSPEKSQNMVLMGGLRKYIPITR
TTFLWGTLSLCGIPPLACFWSKDEILSNSWLYSPFFGIIASFTAGLTAFYMFRIYLLTFDGYL
RVHFQNYSTTKEGYLYSISLWGKKIPKRVNR-DFILSTTKFFSAKN--TFAYPHEMGNTMLFP
LLILLLFTLFIGFIGISFELTLLSKWLTPSINLTQESSNSSINSYEFLTNAISSVSLAIFGLFIAYIL
YGSPYSFFQNLDLINSFYFFLDQVKKKIYSWSYNRGYMDIFYTRVFTLGIRRLTELTEFFDK
GIIDGITNGVGLVSFCIGEEIKY-VGGGRISSYLFFFLCYVSVFLFFFIMDLPGPIHEILMLFGG
FVLLLGGLGVVLLTNPIYSAFSLGLVLVCISLFYFLLNSYFVAVAQLLIYVGAINVLIIFAVMF

VNGSEWSKDKNYWTIGDGFTSIVCITIVFSLMTTIPDTSWYGILWTTRSNQIVEQGLINNV
QQIGIHLATDFYLPFELISIILLVSLIGAITMARQMSLPLTRKDLMIVNMGPQHPSMHGVLRL
IVTLDGEDVIDCEPILGYLHRGMEKIAENRTIIQYLPYVTRWDYLATMFTEAITVNAPEFLE
NIQIPQRASYIRVIMLELSRIASHLLWLGPFMADLGAQTPFFYIFRERELIYDLFEAATGMR
MMHNYFRIGGVAADLPYGWIDKCLDFCDYFLRGIIEYQQLIKQNPIFLERVEGVGFISGEEA
VNWGLSGPMLRASGIQWDLRKVDLYESYNQFDWKVQWQKEGDSLARYLVRISEMRESI
KIIQQAIEKIPGGPYENLEVRR-KKAKTSEWNDFEYKFLGKKPSPNFELSKQELYVRVEAPK
GELGIYLVGDDSLFPWRWKIRPPGFINLQILPQLVKKMKLADIMTILGSIDIIMGEVDRMFP
MITGFMSYGQQTIRATRYIGQSFITTLSHTNRLPITIHYPYEKSITPERFRGRIHFEFDKCIAC
EVCVRVCPIDLPLVDWRFKKDIKRKQLLNYSIDFGVCIFCGNCVEYCPTNCLSMTEEYELS
TYDRHELNYNQIALSRLPISIMEDYTIQTIRNSKIDEEKSSNSRTITDYQQGWLSNWLVKHE
VVHRSLGFDHRGIETLQIKAGDWDSIAVILYVYGYNYLRSQCAYDVAPGGSLASVYHLTRI
QYGIDNPEEVCIKVFAQKDNPRIPSVFWIWRSADFQERESYDMVGISYDNHPRLKRILMPE
SWIGWPLRKDYITPNFYEIQDAH------------------------------------MVLT-EYLD-KKKKEGKDS
IETVMNLIEFPLLDQTSSNSVISTTPNDLSNWSRLSSLWPLLYGTSCCFIEFASLIGSRFDFDR
YGLVPRSSPRQADLILTAGTVTMKMAPSLVRLYEQMPEPKYVIAMGACTITGGMFSTDSY
STVRGVDKLIPVDVYLPGCPPKPEAVIDALTKLRKKISREIVEDQTLSQ-KNRFFTTSHKLY
VRRSTHTGTYEQELLYQSPSTLDIST--FFKSKSPVPSYKLVNMENKNTFSWVKEQMTRSIS
VSIMIYVITQTSISNAYPIFAQQGYENPREATGRIVCANCHLASKPVDIEVPQAVLPDTVFEA
VLRIPYDMQLKQVLANGKKGGLNVGAVLILPEGFELAPPDRISPELKEKIGNLSFQSYRPD
KKNILVIGPVPGKKYSEIVFPILSPDPATKKDAYFLKYPIYVGGNRGRGQIYPDGSKSNNTV
YNATSTGIVKKILRKEKGGYEISIVDASDGRQVIDTIPPGPELLVSEGESIKLDQPLTSNPNV
GGFGQGDAEIVLQDPLRVQGLLFFFASVILAQVFLVLKKKQFEKVQLYEMNFMSKVYDW
FEERLEIQAIADDITSKYVPPHVNIFYCLGGITLTCFLVQVATGFAMTFYYRPTVTEAFSSVQ
YIMTEANFGWLIRSVHRWSASMMVLMMILHVFRVYLTGGFKKPRELTWVTGVVLAVLTA
SFGVTGYSLPWDQIGYWAVKIVTGVPDAIPVIGSPLVELLRGSASVGQSTLTRFYSLHTFVL
PLLTAVFMLMHFPMIRKQGISGPLMGVTKKPDLNDPVLRAKLAKGMGHNYYGEPAWPND
LLYIFPVVILGTIACNVGLAVLEPSMIGEPADPFATPLEILPEWYFFPVFQILRTVPNKLLGVL
LMVSVPTGLLTVPFLENVNKFQNPFRRPVATTVFLIGTAVALWLGIGATLPIDKSLTLGLFMI
EVFLFGIVLGLIPITLAGLFVTAYLQYRRGDQLDLMLTLTSYFGFLLAALTITPALFIGLNKIR
LIMDIVSLAWAALMVVFTFSLSLVVWGRSGLMIIRSPEPEVKIVVDRDPVKTSFEEWSRPG
HFSRTLAKGPDTTTWIWNLHADAHDFDSHTGDLEEISRKVFSAHFGQLSIIFLWLSGMYFH
GARFSNYEAWLSDPTHIGPSAQVVWPIVGQEILNGDVGGGFRGIQITSGFFQLWRASGITS
ELQLYCTAIGALIFAALMLFAGWFHYHKAAPKLAWFQDVESMLNHHLAGLLGLGSLSWA
GHQIHVSLPINQFLDAGVDPKEIPLPHEFILNRDLLAQLYPSFAEGATPFFTLNWSKYAEFLT
FRGGLDPVTGGLWLTDIAHHHLAIAILFLIAGHMYRTNWGIGHGLKDILEAHKGPFTGQG
HKGLYEILTTSWHAQLALNLAMLGSTTIVVAHHMYSMPPYPYLATDYGTQLSLFTHHMW
IGGFLIVGAAAHAAIFMVRDYDPTTRYNDLLDRVLRHRDAIISHLNWVCIFLGFHSFGLYIH
NDTMSALGRPQDMFSDTAIQLQPIFAQWVQNIHATAPGVTAPGATTSTSLTWGGGELVAVG
GKVALLPIPLGTADFLVHHIHAFTIHVTVLILLKGVLFARSSRLIPDKANLGFRFPCDGPGRG
GTCQVSAWDHVFLGLFWMYNAISVVIFHFSWKMQSDVWGTISDQGVVTHITGGNFAQSS
ITINGWLRDFLWAQASQVIQSYGSSLSAYGLFFLGAHFVWAFSLMFLFSGRGYWQELIESI
VWAHNKLKVAPATQPRALSIIQGRAVGVTHYLLGGIATTWAFFLARIIAVGMELRFPRFSQG
LAQDPTTRRIWFGIATAHDFESHDDITEERLYQNIFASHFGQLAIIFLWTSGNLFHVAWQGN

FESWIQDPLHVRPIAHAIWDPHFGQPAVEAFTRGGAAGPVNIAYSGVYQWWYTIGLRTND
DLYTGALFLLFLSTLSLIAGWLHLQPKWKPSLSWFKNAESRLNHHLSGLFGVSSLAWTGH
LVHVAIPASRGEYVRWNNFLDVLPYPQGLGPLLTGQWNLYAQNPDSSNHLFGTAQGAGTA
ILTLLGGFHPQTQSLWLTDIAHHHLAIAFIFLIAGHMYRTNFGIGHSIKDLLEAHTPPGGRLG
RGHKGLYDTINNSIHFQLGLALASLGVITSLVAQHMYSLPAYAFIAQDFTTQAALYTHHQYI
AGFIMTGAFAHGAIFFIRDYNPEQNEDNVLARMLDHKEAIISHLSWASLFLGFHTLGLYVH
NDVMLAFGTPEKQILIEPIFAQWIQSAHGKTTYGFDILLSSTNGPAFNAGRNLWLPGWLNA
VNE--NSNSLFLTIGPGDFLVHHAIALGLHTTTLILVKGALDARGSKLMPDKKDFGYSFPCD
GPGRGGTCDISAWDAFYLAVFWMLNTIGWVTFYWHWKHITLWQGNVSQFNESSTYLMG
WLRDYLWLNSSQLINGYNPFGMNSLSVWAWMFLFGHLVWATGFMFLISWRGYWQELIET
LAWAHERTPLANLIRWRDKPVALSIVQARLVGLAHFSVGYIFTYAAFLIASTSGKFGMSHS
VKIYDTCIGCTQCVRACPTDVLEMIPWDGCKAKQIASAPRTEDCVGCKRCESACPTDFLS
VRVYLGPETTRSMALSYMTDLNLPSIFVPLVGLVFPAIAMASLFLYVQKNKIVMRDIKTYL
SVAPVLSTLWFGALAGLLIEINRLFPDALSFPFF--MTAILERRESTSLWGRFCNWITSTENRL
YIGWFGVLMIPTLLTATSVFIIAFIAAPPVDIDGIREPVSGSLLYGNNIISGAIIPTSAAIGLHFY
PIWEAASVDEWLYNGGPYELIVLHFLLGVACYMGREWELSFRLGMRPWIAVAYSAPVAAA
TAVFLIYPIGQGSFSDGMPLGISGTFNFMIVFQAEHNILMHPFHMLGVAGVFGGSLFSAMH
GSLVTSSLIRETTENESANEGYKFGQEEETYNIVAAHGYFGRLIFQYASFNNSRSLHFFLAA
WPVVGIWFTALGISTMAFNLNGFNFNQSVVDSQGRVINTWADIINRANLGMEVMHERNA
HNFPLDLAALEVPSLNGMGLPWYRVHTVVLNDPGRLLAVHIMHTALVSGWAGSMALYEL
AVFDPSDPVLDPMWRQGMFVIPFMTRLGITDSWGGWSIS-GGTVTNPGIWSYEGVAGAHI
VFSGLCFLAAIWHWVYWDLAIFSDDRTGKPSLDLPKIFGIHLFLAGVACFGFGAFHVTGLY
GPGIWVSDPYGLTGKVQAVNPAWGAEGFDPFVPGGIASHHIAAGTLGILAGLFHLSVRPPQ
RLYKGLRMGNIETVLSSSIAAVFFAAFVVAGTMWYGSATTPIELFGPTRYQWDQGYFQQEI
YRRVSNGLAENLSLSEAWSKIPEKLAFYDYIGNNPAKGGLFRAGSMDNGDGIAVGWLGH
PVFRDKEGRELFVRRMPTFFETFPVVLVDEEGIVRADVPFRRAESKYSVEQVGVTVEFYG
GELNGVSYSDPATVKKYARRSQLGEIFELDRATLKSDGVFRSSPRGWFTFGHATFALLFFF
GHIWHGARTLFRDVFAGIDPDLDAQVEFGTFQKVGDPTTRKQAVMKILYSLRRFYHVETL
FNGTFVLAGRDQETTGFAWWAGNARLINLSGKLLGAHVAHAGLIVFWAGAMNLFEVAHF
VPEKPMYEQGLILLPHLATLGWGVGPGGEVLDTFPYFVSGVLHLISSAVLGFGGIYHALLG
PETLEESFPFFGYVWKDRNKMTTILGIHLILLGLGSFLLVLKALYFGGVYDTWAPGGGDVR
KITNLTLSPSVIFGYLLKSPFGGEGWIVSVDDLEDIIGGHVWLGFICVFGGIWHILTKPFAWA
RRAFVWSGEAYLSYSLAALSVFGFIACCFVWFNNTAYPSEFYGPTGPEASQAQAFTFLVRD
QRLGANVGSAQGPTGLGKYLMRSPTGEVIFGGETMRFWDLRAPWLEPLRGPNGLDLNRL
KKDIQPWQERRSAEYMTHAPLGSLNSVGGVATEINAVNYVSPRSWLSTSHFVLGFFFFVG
HLWHAGRARAAAAGFEKGIDRDLEPVLYMNPLNMTIVLGRVPKEENDLFDTMDDWLRR
DRFVFVGWSGLLLFPCAYFALGGWFTGTTFVTSWYTHGLASSYLEGCNFLTAAVSTPANS
LAHSLLLLWGPEAQGDFTRWCQLGGLWTFVALHGAFALIGFMLRQFELARSVQLRPYNAI
SFSGPIAVFVSVFLIYPLGQSGWFFAPSFGVAAIFRFILFFQGFHNWTLNPFHMMGVAGVLG
AALLCAIHGATVENTLFEDGDGANTFRAFNPTQAEETYSMVTANRFWSQIFGVAFSNKRW
LHFFMLFVPVTGLWMSAIGVVGLALNLRAYDFVSQEIRAAEDPEFETFYTKNILLNEGIRA
WMAAQDQPHENLIFPEEVLPRGNALMSGSTGERSFADIITSIRYWVIHSITIPSLFIAGWLFV
STGLAYDVFGSPRPNEYFTERRQGIPLITDRFDSLEQLDEFSRSFMTIDRTYPIFTVRWLAIH
GLAIPTVFFLGSISAMQFIQRMATQTVEDSSKPR-PKRTGAGSLLKPLNSEYGKVAPGWGTT

PFMGVAMALFAIFLAIILEIYNSSVLLDGILTNMLTLKLFVYTVVIFFVSLFIFGFLSNDPGRN
PGREEMADTTGRIPLWLIGTVTGIPVIGLVGVFFYGSYSGLGSSLMPNILSLTCIFFNSVLYP
TSFFFAKLPEAYAIFNPIVDVMPVIPLFFFLLAFVWQAAVSFRMTQSNPNEQNVELNRTSLY
WGLLLIFVLAVLFSNYFFNMEVNILAFIATALFILIPTAFLLIIYVKTVSQNDMETATLVAISIS
GLLVSFTGYALYTAFGQPSQQLRDPFEEHGDMEALVYTFLLVSTLGIIFFAIFFREPPKVPTK
RIKMTIAFQLAVFALIATSSVLVISVPLVFASPDGWSNNKNVVFSGTSLWIGLVFLVAILNSLI
S-------MSPQTETKASVGFQAGVKDYKLTYYTPEYETKDTDILAAFRVTPQPGVPPEEAGAA
VAAESSTGTWTTVWTDGLTSLDRYKGRCYHIEPVAGEDNQWICYVAYPLDLFEEGSVTN
MFTSIVGNVFGFKALRALRLEDLRIPVAYSKTFQGPPHGIQVERDKLNKYGRPLLGCTIKP
KLGLSAKNYGRACYECLRGGLDFTKDDENVNSQPFMRWRDRFVFCAEALYKAQAETGEI
KGHYLNATAGTCEEMIKRAVFARELGVPIVMHDYITGGFTANTSLAHYCRDNGLLLHIHR
AMHAVIDRQKNHGMHFRVLAKALRMSGGDHIHSGTVVGKLEGEREMTLGFVDLLRDDF
IEKDRARGIFFTQDWASMPGVIPVASGGIHVWHMPALTEIFGDDSVLQFGGGTLGHPWGN
APGAAANRVALEACVQARNEGRDLAREGNEIIRAACKWSPELAAACEVWKAIKFEFEPV
DTIDNTAKHLYKTPIPSTRKGTVDRQVKSNPRNNLIHGRHRCGKGRNSRGIITARHRGGGH
KRLYRKIDFRRNQKDISGRIVTIEYDPNRNAYICLIHYGDGEKRYILHPRGAIIGDTIVSGTK
VPISMGNALPLTDMPLGTAMHNIEITRGRGGQLARAAGAVAKLIAKEGKSATLRLPSGEVR
LVSQNCLATVGQVGNVGVNQKSLGRAGSKCWLGKRPVVRGVVMNPVDHPHGGGEGKA
PIGRKKPTTPWGYPALGRRTRKRKKYSDSFILRRRK-MIQPQTLLNVADNSGARKLMCIRVI
GAAGNQRYARIGDVIVAVIKDALPQMPLERSEVIRAVIVRTCKEFKCEDGIIIRYDDNAAVII
DQKGNPKGTRVFGAIAEELRELNFTKIVSLAPEVLMLSPKRTRFRKQHRGRMKGKSCRGN
RICFGRYALQALEPAWITARQIEAGRRAITRYARRGGKIWVRIFPDKPVTLRPTETRMGSGK
GSPEYWVAIVKPGRILYEMGGVSETIARAAMEIAASKMPIRSQFIRLEMTRVPRGYIARRRR
TKMRSFASNFRGAHLRLNRMITQQVKRAFVSSHRDRGRQKRDFRRLWITRINAATRIY--V
FDSYSKLIHNLYKKKLILNRKMLAQVAVSNPNNLYTISNKIKIIN---------RIKRIFKAQKRIN
MSVFKAQRVLDEIRWRYYEETVMILNLMPYRASYPILKLVYSASANATHYRNFDKANLFI
TKAEVSRSTIMKKFRPRARGRSFPIKKNMCHITIILN-VKK--------MDGIKYAVFTEKSLRLL
GKN-QYTFNVESGFTKTEIKHWVELFFGVKVVAVNSHRLPGKGRRIGPILGHTMHYRRMII
TLQPGYSIPLLDREKNMAVPKKRTSMSKKRIRKNIWKKKTYFSIVQAYSLAKSFSSGKPKG
FSRQQTNKMAKGKDVRIRVILECISCVRK--G--EESTGISRYSTQKNRHNTPGQLEFKKFCR
YCRKHTTHHEIKKMKIRASVRKICTKCRLIRRRGRIRVICSNPKHKQRQGMVREEVAGSTQ
TLQWKCVESRVDSKRLYYGRFILSPLRKGQADTVGIALRRALLGEIEGTCIARAKFG--SVP
HEYSTIAGIEESVQEILLNLKEIVLRSNLYGVRDASICVKGPRYITAQDIILPPSVEIVDTSQPI
ANLTEPIDFCIELQIKRDRGYQTELRKNYQDGSY--PIDAVSMPVRNVNYSIFSCGNGNEKH
EILFLEIWTNGSLTPKEALYEASRNLIDLFLPFLHAEEE--GTSFEENK-NRFTPPLFTFQKRLT
NNLKKNKK-GIPLNCIFIDQLELPSRTYNCLKRANIHTLLDLLSKTEEDLMRIDSFHMEDGK
QIWDTLEKY--LPIDLLKNKMLRNGN-EGMSTIPGFSQIQFEGFCRFINQALAEELNKFPAIK
DPDHEIAFQLFAKGYQLLEPSIKERDAVYESLTYSSELYVSARLIFGF--DVQKQTISIGNIPIM
NSLGTFIINGIYRIVINQILLSPGIYYRSELDHKGISIYTGTIISDWGGRSELAIDKKERIWARV
SRKQKISILVLSSAMGSNLREILDNVSYPEIFLSFPNAKEKKRIESKEKAILEFYQQFACVGG
DLVFSESLCEELQKKFFQQKCELGRIGRRNMNRRLNLDIPQNNTFLLPRDVLAATDHLIGM
KFGTGILDDDDMNHLKNKRIRSVADLLQDQFGLALGRLQHAVQKTIRRVFIRQSK--PTPQT
LVTPTSTSILLITTYETFFGTYPLSQVFDQTNPLTQTVHGRKVSCLGPGGLTGRTSSFRSRDI
HPSHYGRICPIDTSEGINVGLTGSLAIHARINHLWGSIESPFYEISEKAKKKKS--RQVVYLSP

NRDEYYMIAAGNSLSLNQGIQEEQVVPARYRQEFLTIAWEQIHVRSIFPFQYFSIGGSLIPFI
EHNDANRALMSSNMQRQAVPLSRSEKCIVGTGLERQTALDSGVSVIAEREGKILSTDSHKI
LLSSSGKTLSIPLVNHRRSNKNNCMHQKSRVPRGKSIKKGQILAEGAATVGGELALGKNV
LVAYMPWEGYNFEDAVLISERLVYEDIYTSFHIRKYEIQTDTTSQGSAEKITKEIPHLEAHLL
RNLDRNGVVRLGSWVETSDILVGKLTPQIASE-SSYIAEAGLLRAIFGLEVSTSKETSLKLPI
GGRGRVIDVKWIQR-------DPIMVRVYILQKREIKVGDKVAGRHGNKGIISKILPRQDMPYL
QDGTPVDMVFNPLGVPSRMNVGQIFESSLGLAGDLLKKHYRIAPFDERYEQEASRKLVFS
ELYEASKETKNPWVFEPEYPGKSRIFDGRTGDPFEQPVLIGKSYILKLIHQVDEKIHGRSTG
PYSLVTQQPVRGRAKQGGQRVGEMEVWALEGFGVAHILQEILTYKSDHLIARQEILNATIW
GKRIPNHEDPPESFRVLVRELRSLALELNHFLVSEKNFQVTREEV-------MIDQYKHQQLQIG
LVSPQQIKAWANKNLPNGEVVGEVTRPSTFHYKTDKPEKDGLFCERIFGPIKSGICACGNS
RASGAENEDERFCQKCGVEFVDSRIRRYQMGYIKLACPVTHVWYLKGLPSYIANLLDKPL
KKLEGLVYGD--FSFARP-STKKPTFLRLRGLFEEEIASCNHSISPFFSTPGFATFRNREIATGA
GAIREQLADLDLRIIIENSLVEWKELEDE-GYSGDEWEDRKRRIRKVFLIRRMQLAKHFIQT
NVEPEWMVLCLLPVLPPELRPIVYRSGDKVVTSDINELYKRVIRRNNNLAYLLKRSELAPA
DLVMCQEKLVQEAVDTLLDSGSRGQPMRDGHNKVYKSLSDVIEGKEGRFRETLLGKRVD
YSGRSVIVVGPSLSLHQCGLPLEIAIKLFQLFVIRDLITKRATSNVRIAKRKIWEKEPIVWEIL
QEVMRGHPVLLNRAPTLHRLGIQAFQPTLVEGRTISLHPLVCKGFNADFDGDQMAVHLPL
SLEAQAEARLLMFSHMNLLSPAIGDPICVPTQDMLIGLYVLTIGNRRGICANRYNSCVN-----
YPTKKVNYN----MKDKEPHFSSSYDALGAYRQKLISLDSPLWLRWKLDQR-VIGS--REVPI
EVQYESLGTYHEIYAHYLIVGNRKKEIRSIYIRTTLGHISFYRELEEAIQGFSQAYSYTI-------
-----MAERANLVFHNKEIDGTGMKRLISRLIDHFGMGYTSHILDQLKTLGFHQATTTSISLGI
EDLLTIPSKGWLVQDAEQQSFLLEKHYYYGAIHAVEKLRQSVEIWYATSEYLKQEMNSNF
RITDPSNPVYLMSFSGARGNASQVHQLVGMRGLMADPQGQMIDLPIQSNLREGLSLTEYII
SCYGARKGVVDTAVRTADAGYLTRRLVEVVQHIIVRRRDCGTIRGISVSPQNGMTE--KLF
VQTLIGRVLADDIYIGSRCIAARNQDIGIGLVNRFITAFRAQPFLAQPIYIRTPFTCRSTSWIC
QLCYGRSPTHSDLVELGEAVGIIAGQSIGEPGTQLTLRTFHTGGVFTGGTADLVRSPSNGKI
QFTENLVHPTRTRHGQPAFLCYIDLHVTIQSQDILYSVNIPSKSLILVQNDQYVKSEQVIAEI
RAGTPTL--FKERVQKHIYSESDGE-MHWSTDVYHAPEYQYGNLRRLPKTSHLWILSVSRC
R--SSIASFSLHKDQDQMNTY--------------------------------G----DREIFDYS-TLVRIMPNGHWN
LIYPS-IFKEN--SDLLAKKRRNRFVIPLQYHQEQEKELISCLGISIEIPFM--GVLHRNTIFAYF
DDPRYRKD--GIVKFRSPEEDSIFK--KEGLIEHRG-TKEFSLKY-EVDRFFFILQELHILPRSSS
LKVLDNSIIGVDTQLTKNTRSRLGGLVRVKRKKSHTELKIFSGDIHFPEEADKVLG--GCLIP
PERQKK--DSKE-SK-KSKNWVYVQRKKIIKSKEK--YFVSIRPAVAYEMDEGRNLATLFPQD
LLQEEDNLQLRLVNFISHENSKLTQRIYHTN-QFVRTCLVVNWEQEKKEKAG-------ASLVE
VRANDLIRDFLRIELIKCTISYTRKRYDRTSAGPIPH---LDHTNLNSFYSKAKIES-----LSQHQ
EAIG----TLLNRNK---EYQSLMILSASNCSQIG--LFKNPKHP---NTIKELNRRIPI-EIFGPLGVI
VP-SISNFSSS-YYLLT---HNKILLK-KYLFLD---NLKQTFQVLQGLKYSLIDENKR--ISNFD--S
NIMLDPFLLNCHFVHHDSWEETSAIIH-LGQLICENVCLFKSHI----KKSGQIFIIN-MDSFVIR
AAKPYLATTGATVNGHYGEILYKGDRLVTFIYEKSRSSDITQGLPKVEQIFEARSIDSLSPNL
ERRIEDWNERIPRILGIPWGFLIGAELTIAQSRISLVNKIQKVYRSQGVQIHNRHIEIIIRQVTS
KVRVSE---DGMSNVFSPGELIGLLRAERAGRALDESIYYRAILLGITRASLNTQSFISEASFQ
ETARVLAKAALRGRIDWLKGLKENVVLGGIIPVGTGFQKFVHRSP--QDKNLY--FEIQKQN
LFASEM--RDFLFLHTELVSSDSDITN---LEPPFTPIYTS------MTRRYWNINLKEMVEAGVHF

GHGIKKWNPKMAPYISAKRKGTHIINLARTARFLSEACDLVFDAASQGKSFLIVGTKKRAT
DLVASAAIRARCHYVNKKWFSGMLTNWSITKTRLSQFRDLRAEEKMGKFQHLPKRDVAI
LKRKLSTLQRYLGGIKYMTRLPDIVIVLDQQKEYIALRECAILGIPTISLVDTNCDPDLANIS
IPANDDTMTSIRLILNKLVFAICEGHSL-YIRNHMGQKINPLGFRLGTTQKHHSFWFAQPKN
YSEGLQEDKKIRDCIKNYIQKNRKKSSNKLESITRIEIQKEIDTIHVIIHIGFPN-LLKKKG-----
AIEELEKDLQKEIHSVNQRLNISIEKVKEPYREPNILAEYIAFQLKNRVSFRKAMKKAMELT
KKADIKGVKVKIAGRLGGKEIARAECIKKGRLPLQTIRAKIDYCCYPIRTIYGVLGVKIWIF
VDEEMSRYRGPRLKKIRRLGALPGLTRKTPKSGSNLKKKFHSGK-KEQYRIRLQEKQKLRF
HYGLTERQLLRYVHIAGKAKRSTGQVLLQLLEMRLDNILFRLGMASTIPGARQLVNHRHI
LVNGRIVNIPSFRCKPRDIITTKDNQRSKGLVQTLMASSDPK--LPKHLTIDT--LEYKGLVNKI
LDRKWVGLKINELLVVEYYSRQTMSRRGTAEKRTAKSDPIFRNRLVNMVVNRIMKDGKK
SLAYQILYRAVKKIQQKTETNPLLVLRQAIRRVTPNIGVKTRRKKGSTRKVPIEIGSKQGRA
LAIRWLLEASQKRPGRNMAFKLSSELVDAAKGSGGAIRKKEATHRMAEANRALAHFRMG
KDTIADLLTSIRNADMNKKGTVRVISTNITENIVKILLREGFIESVRKHQERNRYFLVSTLRH
QKRKTRKGIYRT---RTFLKRISRPGLRIYTNYQGIPKVLGGMGIAILSTSRGIMTDREARLNR
IGGEVLCYIWMAKAIPKIGSRKKVRIGLRKSARRITKGVIHVQASFNNTIITVTDPQGRVVF
WSSAGTCGFKSSRKASPYAGQRTAVDAIRTVG---LQRAEVMVKGAGSGRDAALRAIAKSG
VRLSCIRDVTPMPHNGCRPPKKRRLMPTVKQLIRNARQPIRNARKTAALKGCPQRRGTCA
RVYTINPKKPNSALRKVARVRLTSGFEITAYIPGIGHNLQEHSVVLVRGGRVKDLPGVRYRII
RGTLDAVAVKNRQQGRSKYGVKKPKMAKKSLIQREKKRQKLEQKYHLIRQSLKKKIRSK
VSSLSLSEKTKMREKLQSLPRNSAPTRLHRRCFLTGRPRANYRDFGLSGHVLREMVYECL
LPGATRSSWMKKRKILGFMVKEEKEEN--RGSVEFQVFSFTNKIRRLASHLELHKKDFSSE
RGLRILLGKRRRLLAYLAKKNRVRYKKLISQLNIREQ-----MVKLRLKRCGRKQQAVYRIV
AIDVRSRREGRDLRKVGFYDPIKNQTCLNVPAILYFLEKGAQPTRTVYDILRKAEFFKEKE
RTLSLLKNFRKSKQPFRKFKKPFRKRRPRIGPGDRIDYRNMSLINRFISEQGKILSRRINRLT
LKQQRLITLAIKQARILSFLPFRNYENEKQFQ-AQSISIITGSRPRKNVTRK-KMNPFVAHHL
LAKIEKVNMKEEKETIVTWSRASSILPTMVGHTIAIHNGKEHIPIYITNPMVGRKLGEFVPT
R H F T S Y A R K D T K S R
R???
??
??
??
??
??
??
??
??
??
??
??
??
??
??
??
??

??
??
??
??
??
??
??
??
??
??
??
??
??
??
??
??
??
??
??
??
??
??
??
??
??
??
??
??MPRSR-ANGNFIDKTSSIVANILLRIIPTTSGEK
KAFTYYRDG--MLAQSEGNYAEALQNYYEATRLEIDPYDRSYILYNIGLIHTSNGEHTKALE
YYFRALERNPFLPQAFNNMAVICHY-RGEQAILQGDSEIAEAWFDQAAEYWKQAIALTPG
NYIEAQNWLKITKRFEFEMNWRSEHVWVELLKGSRKRGNFFWACILFLGSLGFLSVGASS
YLGKNIISILPSQQILFFPQGVVMSFYGIAGLFISSYLWCTILWNVGSGYDRFDRKEGIVCIF
RWGFPGIKRRVFLQFLMRDIQSIRIQVKEGLSPRRILYMEIRGQGVIPLTRTDEKFFTPREME
QKAAELAYFLRVPIEVF
>Cuscuta
MERRGGNS---LNQSLDSLGPIEKTWESEG-NSKDTVKNIQSCSNVDQLFGVKDSESLLYDD
AFIVRDRNGDSYF-YLDIEN----------QTFLSEPFYS-YSSRNYSYLPQAF-SEDHNYN--VLTDE
YNFKMHINRSIDSYLQ--SQICIATTGSGSDNSY---IHGSRVYGGRSSSIRTRTKDVELTRIERP
KNLDRTKKYGYLWLQCDNCYGLNYKKVLKSKMNICEQCGYHLQMSSSDRIELSIDPGTW
DPMDEDMVSRDPIKFDSGGG-YKARIYFYQRKTGLTEAVQTGIGQLNGIPVAIGVMDFKF
MGGSMGSVVGEKITRLIEHATNKFLPLIIVSASGGARMQEGSLSLMQMAKISSALYDYQS
NKRLVYLSILTSPTAGGVTASFGMLGDIIIAEPRAYVAFAGKRVIEQTLNQTIPNDSQEAEFL
FHKGLFDLIVPRHLLKSVISELFTLHDLFPLNQNSKKMVTVRADEIRKIIRERIEQYNREVN
TLNTGTVLQVGDGIARINGLDEVMAGELVEFEEGTIGIALNLESKNVGVVLMGDGLMIKE

GSSVKATGRIAQIPVGEAYLGRVINAQANPIDGRGKISSSAFRLIESSAPGIISRRSIYEPLQT
GLIAIDSMIPIGRGQRELIIGDRQTGKTAVATDTIINQQGQNVICVYVAIGQKASSVAQVVTT
LQERGAMEYTIVVAETADSPATLQYLAPYTGAALAEYFMYSERHTLIIYDDLSKQAQAYR
QISLLLRRPPGREAYPGDVFYLHSRLLERAAKLSSKLGEGSMTALPIVETQSGDVSAYIPTN
VISITDGQIFLSADLFNAGIRPAINVGISVSRVGSAAQIKAMKKVAGKLKLELAQFAELEAF
AQFASDLDKTSQNQLARGQRLRELLKQSQSAPLTVAEQIMTIYTGINGYLDSLQLRQVRKF
LLELCTYLKTNKPRFQEIISSTKTFTEEAEDLLKEAIRDQTDRFLVKEQAMRINPTTYGSEIS
LIEKKNRGRIVQIIGPVLDVAFPPGKMPNIYNALVVKGRD--TEQMNVTCEVQQLLGNNRV
RAVAMNDTDGLMRGMEVIDMGTPITVPVGGSTLGRIFNVLGEPVDNFGPVDTNTTSTIHR
SAPAFIQLDTKLSIFETGIKVVDLLAPYRRGGKIGLFGGAGVGKTVLIMELINNIAKAHGGV
SVFGGVGERTREGNDLYMEMKESGVINEKNITESKVALVYGQMNEPPGARMRVGLTALT
MAEYFRDVNEQDVLLFIDNIFRFVQAGSEVSALLGRMPSAVGYQPTLSTEMGSLQERITST
KEGSITSIQAVYVPADDLTDPAPATTFAHLDATTVLSRGLAAKGIYPAVDPLDSTSMMLQPR
IVGEEHYETAQKVKQTLQRYKELQDIIAILGLDELSEEDRLTVARARKIERFLSQPFFVAEVF
TGSPGKYVGLAETIRGFNLILSGELDSLPEQAFYLVGNIDEATKKAMDLK-------MTLKLCV
LTPNRIVWDSEVEEIILSTNSGQIGILPTHTPIATSVDIGILRIRLNDQWLTMALMGGFARLG
NNVITVLVNDAERGSDIDPQEAQQTVEIAEANFIKAKGKRQKIEANLALRRARTRVKAISPI
LMKNVTDSFLSLGHWSSAGSFGLNTDILATNPINLSVVLGVLIFFGKGVLSDLLDNRKQRI
LKTIQNSEELGVGAVEKLEKARSRLRKVKTEAEQFLVNGYSDIEREKFNLIKSTSNTLEQL
ENDKNETLRFEQQRLIYQVRQRFFQQALQRAIGTLNSCLNNELHLRTISANIGMLGTIKEIT
DMNPLISAASVIAAGLAVGLASIGPGVGQGTAAGQAVEGIARQPEAEGKIRGTLLLSLAFM
EALTIYGLVVALALLFANPFVMDVLSCSINTLKGLYDISGVEVGQHFYWQIGDFLVHGQVL
ITSWVVIAILLGSATVAVRNPQTIPTGGQNFFEYVLEFIRDVSKTQIGEEYGPWVPFIGTMFL
FIFVSNWSGALLPWKILQLPHGELAAPTNDINTTVALALLTSAAYFYAGILKKGLGYFEKYI
KPTPILLPINILEDFTKPLSLSFRLFGNILADELVVVVLVSLVPSVVPIPVMLLGLFTSGIQALI
FATLAAAYIGESMEGHLMIVSTLEHILTHISFSIVSILITIKLRIFLADEIKKLYDSSERGMLVT
FFCITGLLATHWIYLGHFPLSDLSESLIFLSWSFALIHSIAY--KNTKLLSTITSQSTVFTQGFAT
SGILTEIQKSSILVPALQSEWLIMHVSLMILGYAALLCGSLLSVALMVITFRKDGKFFS--KEI
FYKKIFV----------YNNYYKTQLIQELDFWSYQVIGLGFIFLTIGILSGAVWANEAWGSYWSW
DPKETWAFITWIVFAIYLHTRKKRSLQGTNSAIVASIGFLIIWICYFGVNLVGLGLHSYGSFP
STMTKKKAFTPILYLSFIVFLPWWIYFSFNKCLGSWITNWWNTRESEIFLNDLQEKSILEKL
IELEEFLFVDEILKKNSETHPQEFHT-IHKEAIQFIKIQNESYIHMILRLSTNLICVVIISGFYIW
RNETLVILNSWSREFLYNLSDTVKVFSILLLTDLCIGFHSPHGWELMIGSIYQDFGFVQNDR
IISGLVSTFPVILDTILKYWIFRYLNRVSPSLVVIYHSINDMPIGVPRVRFQYRRDRSRVWIDI
YNRLYRERCLFLAHVVDSQIANQLVGLFIYLGVQDETKDIFLFINSPGGGIISGFAIYDTMQ
FVRPDIQTICVGLAASMGSFLLVGGAITKRLAFPHARVMIHQPMSAFFETQTVEAILEAEEL
L K L R E S L A E V Y V Q R T G K P G W V I A E D M E R D V F L S A T E A Q A Y G I V D V V G V
A???TEDFKID
LQLERSQQHNFIYPLLFQEYIYALVNDRDLIGYEKKFRFQIVKRLINRMYKQIHWTTYFSNS
NKRKIYGKSLYYKLLSAGFSFLFEIPFSFKFIS--ILKSQNLRSILSIFPFFEDNFSHLKYVLDLL
IPNPPHPEILVQKIRSWVQDASSLHLLQFFLQEFCSVKSLITS------KERFLFLLYNSYVCEYE
SFFLFLRNQSLHLGSISFVTLFERNIFYGKIECFVELFV-DSNLRLFKDPSMHYARYCGKSIL
ASNGGLLLMPKWQYYIVNFWQCYFYLWFDTERINLSQIASHPFYLMDYISSIAQTPSMVR
SKMLENSFLIKNDM--KIFDTFVPIIPIIGSLAKAKFCNLLGNPISKPVWSDFSDSDIIERFGRI

CRNLFHYYSGSSKKRSLYQIKYILRLSCARTLARKHKSTVRAFLQSLGPKFLEKFFTSEEQII
F L T F P N R I W Y F D I V C I N E Q A N F
H???
??
??
??
??
??
??
??
??
??
??
??
??
??
??
??
??
??
??
??
??
??
??
??
??
??
??
??
??
??
??
??
??
??
??
??
??
??
??
?????????????MQTINTFSWINQRITRSISVLLLVYIITRTSISSAYPIFAQRGYENPREATGRIVC
ANCHLANKPVDIEVPQAVLPDTVFEAVVRIPYDSQLKQVLSNGKKGGLNVGAVIILPEGFE
LAPPDRLSPQMKEKIGNLSFQNYRPNQKNIIVVGPVPGQKYSEITFPILSPDPATKKDAHFL

KYPIYVGGNRGRGQIYPDGSKSNNTFYSATAAGIVSKIIRKEKGGYEITITDASDSHQVVEII
PPGPELLVSEGEYLKFDQPLTSNPNVGGFGQGDGEIVLQDPLRVQGLLFFLASVILAQIFLV
LKKKQFEKVQLAEMNLMSKVYDWFEERLEIQAIADDITSKYVPPHVNIFYCLGGITLTCFL
VQVATGFAMTFYYRPTVTEAFASVKYIMTEANFGWLIRSVHRWSASMMVLMMILHVFRV
YLTGGFKKPRELTWVTGVVLAVLTASFGVTGYSLPRDQIGYWAVKIVTGVPEAIPVIGSPLV
ELLRGSASVGQSTLTRFYSLHTFVLPLITAVFMLMHFPMIRKQGISGPLMGVTKKPDLNDP
VLIAKLAKGMGHNYYGEPAWPNDLLYIFPVVILGTIACNVGFAVLEPSMIGEPADPFATPLE
ILPEWYFFPVFQILRTVPNKLLGVLLMGSVPAGLLTVPFFENVNKFQNPFRRPVATTFFLIGT
AVALWLGIGATLPIDQSLTLGLFMIEVFLFGIVLGIIPITLAGLFVTAYLQYRRGDRLDVMLTI
TSFFGFLVAAFTITSALFIGLSKIRLIMDIVSLAWAALMIVFTFSLSLVVWGRSGLMIIRSPEA
EGKILVDRDPVKTSFEEWARPGHFSRTIAKGPDTTTWIWNLHADAHDFDSHTSDLEEISRK
VFSAHFGQLSIIFLWLSGMYFHGARFSNYEAWLSDPTHIGPSAQVVWPIVGQEILNGDVGG
GFRGIQITSGFFQMWRASGITSELQLYCTAIGALVFAALMLFAGWFHYHKAAPKLAWFQE
VESMLNHHLAGLLGLGSLAWSGHQVHVSLPINLFLNAGVDPKEIPLPHEFILNRNLMAQL
YPSFAEGATPFFTLNWSKYAEFLTFRGGLDPVTGGLWLTDIAHHHLAVAILFLIAGHMYRT
NWGIGHGLKDILDAHKGPFTGQGHKGLYDILTTSWHAQLSLNLAMLGSLTIIVAHHMYSM
PPYPYLATDYGTQLSLFTHHMWIGGFLIVGAAAHAAIFMVRDYDPTNRYNDLLDRVLRH
RDAIISHLNWACIFLGFHSFGLYIHNDTMSALGRPQDMFSDTAIQLQPIFAQWIQNTHALAP
NATAPGATASTSLTWGG--VVAVGGKVALGPIPLGTADFLVHHIHAFTIHVTVLILLKGVLFA
RSSRLIPDKANLGFRFPCDGPGRGGTCQVSAWDHVFLGLFWMYNSISVVIFHFSWKMQSD
VWGSISDQGVINHITGGNFAQSSITINGWLRDFLWAQASQVIQSYGSSLSAYGLFFLGAHF
VWAFSLMFLFSGRGYWQELIESIVWAHNKLKVAPATQPRALSIVQGRAVGVTHYLLGGIAT
TWAFFLARIIAVGMALRFPRFSQGLAQDPTTRRIWFGIATAHDFESHDDITEERLYQNIFASH
FGQLAIIFLWTSGNLFHVAWQGNFESWVQDPLHVRPIAHTIWDPHFGQPAVEAFTRGGAL
GPVNIAYSGVYQWWYTIGLRTNEDLYTGALFLLFISAISLIAGWLHLQPKWKPSVSWFKN
AESRLNHHLSGLFGVSSLAWTGHLVHVAIPASRGEYVRWNNFLDVLPHPQGLGPLFTGQ
WNLYAQNPDSSSHLFGTSQGAGTAILTFLGGFHPQTQSLWLTDIAHHHLAITFLFLVAGHM
YRTNFGIGHSMKELLDAHIPPGGRLGRGHLGLYDTINNSLHFQLGLALASLGVITSLVAQH
MYSLPAYAFLAQDFTTQAALYTHHQYIAGFLMTGAFAHGAIFFIRDYNPEQNEDNVLARM
LDHKEAIISHLSWASLFLGFHTLGLYVHNDVMLAFGTPEKQILIEPIFAQWIQSAHGKTSYG
FDVLLSSTSGPAFNASRSIWLPGWLNAVND--SSNSLFLTIGPGDFLVHHAIALGLHTTTLILL
KGALDARGSKLMPDKKDFGYSFPCDGPGRGGTCDISAWDAFYLAVFWMLNTIGWVTFY
WHWKHITLWQGNVAQFNESSTYLMGWLRDYLWLNSSQLINGYNPFGMNSLSVWAWMF
LFGHLVWATGFMFLISWRGYWQELIETLAWAHERTPLANLIRWRDKPVALSIVQARLVGL
AHFSVGYILTYAAFLIASTSGKFGMSHFVKIYDTCIGCTQCVRACPTDVLEMIPWDRCKAK
QIASAPRTEDCVGCKRCESACPTDFLSVRVYLGRETTRSMGLAY--MSHLPSVFVPLVGLVL
PAIAMIYLFFNVQKK---MRDFKTYLSAAPVLSTLWLGALAGLLIEINRFFPDALTFPFF--MT
AILERRESENLWGRFCNWITSTENRLYIGWFGVLMIPTLLTATSVFIIAFIAAPPVDIDGIREP
VSGSLLYGNNIISGAIIPTSAAIGLHFYPIWEAASVDEWLYNGGPYELIVLHFLLGVACYMG
REWELSFRLGMRPWIAVAYSAPVAAATAVFLIYPIGQGSFSDGMPLGISGTFNFMIVFQAEH
NILMHPFHMLGVAGVFGGSLFSAMHGSLVTSSLIRETTENESANEGYRFGQEEETYNIVAA
HGYFGRLIFQYASFNNSRSLHFFLAAWPVVGIWFTALGISTMAFNLNGFNFNQSVVDSQGR
VINTWADIINRANLGMEVMHERNAHNFPLDLAAIEAPATNGMGLPWYRVHTVVLNDPGR
LLSVHIMHTALVAGWAGSMALYELAVFDPSDPVLDPMWRQGMFVIPFMTRLGITNSWGG

WGIA-GGTVTNPGLWSYEGVAGAHIAFSGLCFLAAIWHWVYWDLEVFYDERTGKPSLDL
PKIFGIHLFLSGVACFGFGAFHVTGLYGPGIWVSDPYGLTGKVQPLKPAWGAEGFDPFVPG
GIASHHIAAGTLGILAGLFHLSVRPPQRLYKGLRMGNIETVLSSSIAAVFFAAFVVAGTMW
YGSATTPIELFGPTRYQWDQGYFQQEIYRRVSAGLAENQSLSETWSKIPEKLAFYDYIGNN
PAKGGLFRAGSMDNGDGIAVGWLGHPIFRDKEGRELFVRRMPTFFETFPVLLVDGDGIVR
ADVPFRRAESKYSVEQVGVTVAFYGGELNDVSYSDPATVKKYARRAQLGEIFELDRATLK
SDGVFRSSPRGWFTFGHASFALLFFFGHIWHGARTLFRDVFAGIDPDLDAQVEFGAFQKL
GDPTTRRQAVMKTLYSLRRFYHVETLFNGTLALAGRDQETTGFAWWAGNARLINLSGKL
LGAHVAHAGLIVFWAGAMNLFEVAHFVPEKPMYEQGLILLPHLATLGWGVGPGGEVIDT
FPYFVSGVLHLISSAVLGFGGIYHALLGPETLEESFPFFGYVWKDRNKMTTILGIHLILLGIG
AFLLVLKALYFGGVYDTWAPGGGDVRKIANLTLSPSLIFGYLLKSPFGGEGWIVSVDDLED
IIGGHVWLGSICILGGIWHILTKPFAWARRAFVWSGEAYLSYSLGALSIFGFTACCFVWFNN
TAYPSEFYGPTGPEASQAQAFTFLVRDQRLGANVGSAQGPTGLGKYLMRSPTGEVIFGGE
TMRFWDLRAPWLEPLRGPNGLDLNRLKKDIQPWQERRSAEYMTHAPLGSLNSVGGVATE
INAVNYVSPRSWLATSHFVLGFFFFIGHLWHAGRARAAAAGFEKGIDRDFEPVLSMTPLN
MTIALGKLTKEEKDLFDTMDDWLRRDRFIFVGWSGLLLFPCAYFALGGWFTGTTFVTSW
YTHGLASSYLEGCNFLTAAVSTPANSLAHSLLFLWGPEAQGDFTRWCQLGGLWTFVALHG
AFGLIGFMLRQFELARSVQLRPYNAIAFSAPIAVFVSVFFIYPLGQSGWFFAPSFGVAAIFRF
IRFFQGFHNWTLNPFHMMGVAGVLGAALLCAIHGATVENTLFEDGDGANTFRAFNPTQS
EETYSMVTANRFWSQIFGVAFSNKRWLHFFMLFVPVTGLWMSALGVVGLALNLRAYDFV
SQEIRAAEDPEFETFYTKNILLNEGIRAWMAAQDQPHENLIFPEEVLPRGNALMSGNTGER
SFADIITSIRYWVIHSITIPSLFIAGWLFVSTGLAYDVFGSPRPNEYFTESRQGIPLITGRFDPL
EQLNEFSRSFMTIDRTYPIFTVRWLAVHGLAVPTVFFLGSISAMQFIQRMATQTFNKGSISG-
PRRTTVGDLLKPLNSEYGKVAPGWGTTPFMGVAMGLFAVFLSIILEIYNSSVLLDGISMNM
FTLKLFVYTVVIFFVSLFIFGFLSNDPRRNPGREEMTDTTGRIPLWIIGTVTGILVIGLIGIFFF
GSYSGLGSSLMLNFFSFIC--LNYVLYSSGFFLGKLPEAYAFLNPIINFMPVIPVLFFLLAFVW
QAAVSFRTTQSNPNEQNVELNRTSLYWGLLLIFVLAVLFSNYFFNMEVNILAFIATALLILVP
TAFLLIIYVKTAK---METATLITIFLSGLLVSFTGYALYTAFGQPSQQLRDPFEEHGDMEALV
YTFLLVSTLGIIFFAIFFREPPKVPMKKIKMTLAFQLAVFALIATSSILLISVPVVFASPEGWSN
NKNVLFSGTSLWIGLVFLVGILNSLIS-------MSPQTETKASVGFKAGVKDYKLTYYTPDYE
TKDTDILAAFRVTPQPGVPPEEAGAAVAAESSTGTWTTVWTDGLTSLDRYKGRCYRIERVI
GEKDQYIAYVAYPLDLFEEGSVTNLFTSIVGNVFGFKALRALRLEDLRIPPAYTKTFQGPPH
GIQVERDKLNKYGRPLLGCTIKPKLGLSAKNYGRAVYECLRGGLDFTKDDENVNSQPFM
RWRDRFLFCAEAIYKSQAETGEIKGHYLNATAGTCEEMLKRAFFARELGVPIIMHDYLTG
GFTANTSLAHYCRENGLLLHIHRAMHAVIDRQKNHGIHFRVLAKALRLSGGDHIHSGTVV
GKLEGEREITLGFVDLLRDDFVEQDRSRGIYFTQDWVSLPGVMPVASGGIHVWHMPALTE
IFGDDSVLQFGGGTLGHPWGNAPGAVANRVALEACVQARNEGRDLAQEGNDIIRQAGKW
SPELAAACEVWKEIRFDFKPVDTLDPMVRKLNKTYAPSTRNGTIKGQVKSARGKNLIYGK
HRCGKGRNARGIITARHRGGGHKRLYRKIDFRRNKKNIYGRIITIEYDPNRNAHICLIHYRN
GEKGYILHPRGTIIGDTIVSGTEVSIKIGNALPLTEMPLGTAIHNLEITRGKGGQLARAAGAV
AKLIAKEGKSATLKLPSGEVRLISKNCSATVGQVGNVGVNQKSLGRAGAQRWLGKRPVV
RGVVMNPVDHPHGGGEGRAPIGRKKPTTPWGYPALGRKTRKGNKYSDKFILRHRRKMIQ
PQTHLNVADNSGARELMCIRIIGAS-NRRYAHIGDIIVAVIKEAVPNMPLERSELVRAVIVRTR
KELKRDNGMIIRYDDNAAVVIDQEGNPKGTRIFGAIPRELRPLNFTKIVSLAPEVLMLSPKR

TRFRKQHRGKLKGISYRGNHICFGRYALQALEPAWLTSRQIEAGRRAMTRNARRGGKIWV
RIFPDKPVTVRPAETRMGSGKGCPEYWVAVVKPGRILYEMGGVTKKIARRAFSIAASKMPI
RTQFIISEMTRIKRGSIARRRRKKMRFFASSFRGAHSRLTRTITQQGIRALVSAHRDSDRQK
REFRRLWITRLNAVIRDM--ISYTYSKLIHDLYNNQLLLNRKILSQIAISNSKCLYMISNGILQI
----------MINEIYALGQHISMSADKARRIIDRIRGRSYEETLLILELMPYRAAYPIFKLVYSAAS
NASYTLASNEANLVIIKAEVNEGPAIKKLKPRARGRCYPIKRPTCHITIVLKDISFY------
-??
??????MAVPKKRSSKSKKRIRKNTWKIKSLWTTLKAFSLGKSLLTGNFKSFFLRQTNTMAK
NKDVRVTVILECTSCAQNDVHGNKVATGISRYITQKNRHNTPNRLEFQKFCPRCYKHTLH
GEIKNMKIRASVRQICDKCRLIRRRGRILVICYNPRHKQRQGMVREKITVSTRTLQWKCVE
SRTDSKRLYYGRFILSPLRKGQADTIGIAMRRALLAEIEGTRITRVKFA--KASHEYSTIAGIQ
ESVHEIFMNLKEIVLRSNLYGTCDASISIKGPGHVTAKDIILPPHVEIVDSTQHIAWLTEPIDL
CIGLKIERNRGYFIKTHP-NFDGSY--PIDAVFMPVRNANHSIHSYGN--EKQEILFLEIWTNGS
LTPKEALHEASRNLIDLFIPFLHMEEE--NLHLEDAD-HTISLSPFTVYDKVA-KLRKNKK-KR
SLESIFIDQLEFPPKIYNCLKKSNIFTLLDLLNNSQEDLIKIEHFRLEDVKQILGILGKH--FAL
DLPKKKMLEDG-KGGITTIPGLNQIQFEGFCRFIDQGLTEELYKFQKIEDIDQEIEFQLFAET
YQLVEPLIKERDAVYDSLTYSSELYVSAGLIRKASKDMQEQKIFIGSIPIMNSLGTSIVNGIY
RIVINQILQSPGIYYRSELDQNGISVYTGTIISDWGGRSELEIDRKARIWARVSRKQKISILVL
SSAMGSNLREIIENVCYPEILLSFLRDKEKKKIGSKENAILEFYKKFACVGGDPLFSESLCKE
LQNKFFQQRCELGRIGRRNMNRRLHLDIPQNNTFLLPRDILEATDLLIGLKFGMGTLDD--
MNHLQNKRIRSVADLLQDKFGLALVRLENAVQGTICGAIH-HKK-IPTPQNLVTSTL----LTT
TYESFFGLHPLSQVLDRTNPLTQIVHARKVSSLGPGGLTGRTASFRIRDIHPSHYGRICPIDT
SEGINVGLIGSLAIHVRIGNWG-SLESPFF---KISDRLTG--VRVLHLSPGRDEYYMVAAGNSL
ALNQDIQEDLVVPARFRQEFLTIAWEQVNLRSIFPFQYFSIGTSLIPFIEHNDANRALMSSNM
QRQAVPLAWSEKCIVGTGVERQAALDSGSLAIAEREGRVIYTDTEKILVSGDGKTISIPLVM
YQRSNKNTCMYQQPQVRRGQFIKKGQILADGAATVEGELALGKSVLVAYMPWEGYNYE
DAVLISECLVYEDIFTSFHIRKYEIQTHVTTQG-PEKVTNEIPHLEAHLIRNLDKNGIVLQGS
WVEPGDVLVGKLTPQVVKE-SSYAPEDRLLRAILGIQVSASKETCLKVPIGGRGRVIDVRWI
QKKG-GYGYNPEKIRVYILQKREIKVGDKVAGRHGNKGIISKILPRQDMPYLQDGRSVDLV
FNPLGVPSRMNVGQIFECSLGLAGSLLDRHYRIAPFDERYEQEASRKIVFSELYEASKQTA
NPWAFEPEYPGKSRIFDGRTGNTFEHPVLIGKPYILKLIHQVDDKIHGRSSGHYALVTQQPL
RGRAKQGGQRVGEMEVWALEGFGVAHILQEMLTYKSDHIRARQEVLGTTIVGGTIPNPKN
APESFRLLVRELRSLALELTHFLVSEKNFQVNKREAMNNNFSSMIDRYKHQQLQIGSVSPQ
QISAWATKILPNGEIVGEVKKPYTFLYKTNKPEKEGLFCERIFGPIKSGICACGNYRVIGDEK
EEQKFCEQCGVEFVDSRIRRYRMGCIKLACPVTHVWYLKRLPSYIANILDKPLKELEGLVY
CD--FSFARP-ITNKPTFLRLRGLLKYEIQSWKSSIPLFFTTQGFDTFRNREISTGAGAIREQLA
DLDLRILLENSLLEWKDLGEE-ENTGNEWEDRKAGRRKGFLVRRMELVKHFIRTNIEPKW
MVLCLLPVLPPELRPIIQIDGGKLMSSDINELYRRVIYRNNTLTDLLTTSRSTPGELVMCQEK
LVQEAVDTLLDNGIRGQPMRDGHNKVYKSFSDIIEGKEGRFRETLLGKRVDYSGRSVIVV
GPSLSLHQCGLPREIAIELFQPFVIRDLIKQHLASNIGVAKSKIREKEPIIWEILQDVMQGHP
VLLNRAPTLHRLGIQAFQPVLVEGRVICLHPLVCKGFNADFDGDQMAVHVPLSLEAQAEA
RLLMFSHMNLLSPAIGDPISVPTQDMLIGLYVLTSEKHRGICTNRYTNCNI---RTLQ-TKSSD
YSSKYKNPYGPFFCNSYDAIGAYRQKRINLESPLWLRWRLDRR-VITS--RETPIEVHYESRG
TYFEIYGHFLIVRSLKKKILFIYLRTTVGHISLYREIEEAIQGFSRAWSSDT--------MEVLMAE

RINLVFHNKVLDRTAMKRLISRLIDHFGMAYTSHILDQVKTLGFQQATATSISLGIDDLLTIP
SKGWLVQDAEQQSFIFEKYHHYGNVHAVEKLRQSIEIWYATSEYLRQEMNLNFRMTDPL
NPVHLMYFSGARGNASQVHQLVGMRGLMSDPQGQMIDLPIQSNLREGLSLTEYIISCYGA
RKGVVDTAVRTSDAGYLTRRLVEVVQHIVVRRTDCGTARGISVSPHNGMMP-ERMFIQTLI
GRVLADDIYIGPRCIATRNQNIGVGLVNRFLN------FRAEPILIRTPLTCRSTSWICRLCYGRS
PTHGDLVELGEAVGIIAGQSIGEPGTQLTLRTFHTGGVFTGGTAEHVRAPSNGKIRLNEDLV
HPTRTRHGYPAFFCSIYLYVTIESQDILHHVKIPPKSFILVQNDQYVESEQVIAESRAGTST-F
NYKEKVRKHIYSDSGGE-MHWSTNVYHAPEFTYGNVHLLSKTSHLWILLGEPCH--SSLVS
TSIHRDQDQMSVQ--SLSVKRRSTSKLS--ETNDE-ANQEIASADFSGK-EDRIADF-SDVNRSI
CTDHYNLVYPAPILDENSSSNCLSKRRRNQFIIPLQSIQEHKNELMPCSSISMKTPPNANGIF
WANSILAYFDDPRYRRNNSGSTKYGTLEMHSTVKKED--FIKYRG-VNEFR--QMKVERFFFI
PEEVHILPGSSSIMVRNHSLIGVDTQITLNLRSRVGGLVRVERKKKIIELKIFFGDIYFPGGA
DNISQHSGVLIP-PGTERKTNYKESKK--VKSWIDVQR--ITPSK--KKFFVLVRPVVTYEIMD
DITSATLFPPDLLQQRDNAQLRVVNYILHGTGKPIRGNYDTSIQLVRTCLVFKRNQDKK---S
YYSEAARASVVEIRTNYLIRHFLRIDFVKAPISYIGKRNDPLGLGLLAD-NGLDWTHKNP---
---Y---SEARIHQNLNQNQGTIHTFLNR---NKESQSLSILSSSNCSRMDP--ANGAKSNNV---IQ
ES-PILKISNSLGPLGT--YPPIANCDS--LNHL--LTHNQIL-VTNYFKLDN---VKPPFQVFKLK-
YYFIAEN--WKVCNYNPGSNLRLNVFDFHWNFLHHNSCAETS-KIMSLGQFICQNVCIDKT
RPP-RK--SGQVILVQ-VDSVVIRLAKPYLATPGATVHGLYGETFFGGDTVVTFNYEKSISGDI
TQGLPKVEQVLEVRSVDSISMNLEGRVEGWSKCITGILGIPWGFFIGAELTIVQSRISLVNKI
QKVYRAQGVHIHNRHIEIIVRQITSKVLVSE---DGMSNVFSPGELIGLLRAERTGRALEEPIH
YRSVFLGITKASLNTQSFISEASFQETARVLSKAALGGRIDWLKGLKENVVLGGVIPAGTG
FRGL--VDPSKQYKTIP--LKT---NLFEGGMR--DLLVHHRKLFDS-----FLNTSPS-------------M
TKRYWNINLEEMMGAGVHFGHGTRKWNPQIAPFISAKRKGIHITNLTRTARFLSEACDLVF
DAARRGKKFLIVGTNNKAAYSVARASRKARCHYVNKKWLAGMLTNWSTTETRLNKFRD
LRMEQKKGGLNHLPKRDATMFKRQLARLQTYLGGIQYMTGLPDIVIIVDQHKEYTALRE
CITLGIPTICLIDTNCDPNLSDISIPANDDAISSIRFILNKLVFAICEGRF-SYLRSPMGQKINPL
GFRLGTTQDHHSLWFSQPKNYSESLQEDKKIRDFIKNYVQNNMIKASG-AEGIARIYIQKRI
DLIQVVIFMGFPKLLIETRPQ----GIKELQRTLQKEFNFGNQKLNITITRIEKPYGNPNILAEFI
AVQLKNRVSFRKAMKKAIELAEQADTKGIQVQIAGRVDGKEIARVEWIREGRVPRQTIRA
NLDYCSYPVRTIYGVLGVKIWIFLDQAMSRYRGPRFKKIRRLGALPGLTNKRPRTVRDLR
NQSRSGK-KSQYRIRLEEKQKLRFHYGLTERQLINYVRIARKAKGSTGEILIQLLEMRLDNI
LFRLGMASTIPAARQLVNHRHIFVNGHIVDIPSYRCKPRDIITSKDKPKSGALIKNSIEAFPR-
EELPNHLTLHPAP--YKGLINQIIDTKWVGLKINELLVVEYYSRQTMSRRGTAEKKTAKYDP
IYRNRLVNMLVNRILKHGKKSLAYQIIYRAVKKIQQKTETNPLSVLRQAIRGVTPDITVKAR
RVGGSTHQVPVEIGSTQGKALAVRWLLAASRKRPGRDMAFKLSSELVDAAKGSGDAIRK
KEETHRMAEANRAFAHFRMGRDTIAEIITSIRNADMDRKRVVRIASTHITENIVKLLLREGF
FENVRKHRENNKNFFVLTLRHRR--NKKRPL---RNILKLKRISRPGLRIYYKSQRIPRILGGR
GVVIISTSRGIMTDREARLEGIGGEILFYIWMAKSIPRISSRRNGPIGSGKTVRRIPKGVIHVQ
ASFHNTIVAVTDVRGRVVSWSSAGTSGFKGTRRGTPFAAQTAATKAIRTVVDQGMLRAEV
LIKGPGLGRDAALRAIRRSGILLTFVRDVTPMPHNGCRPPKKRRVMPTIKQLIRNTRQPIKN
VTKSPALGGCPQRRGTCTRVYTITPKKPNSALRKVARVRLTSGFEITAYIPGIGHNLQEHSV
VLVRGGRVKDLPGVRYHIVRGTLDAVGVKDRQQGRSKYGVKKPKMARKSLIQRNKKRQ
KLELKYHWIRGSSKKEIH---RVPLLSDKWEIYVKLQSSPRNSAPTRLHRRCFLTGRPRANY

RDFGLSGHILREMVQACLLPGATRSSWMVKNLSIWVISQ--KENKENRGSVEFQIFNFTKKI
RRLTSHLKLHKKDFLSQKGLRKILGKRQRLLAYLSKKSKIRY---IDKLELRDTKTHQ
-??
MDKSKRPFIKSKRSFRRRLPPIKSGDRIDYRNISLISRFLSEQGKILSKRVNRFTLKQQRLITL
AIKQARILSLLPFT-KR--KGFERSELTPRTNALKARNKVARSLKKNPFVADNLFKKIDKLNI
KAEKEIIITWSRGSTIIPIMVGHTIAIHTGKEHLPIYIMDHMVGHKLGEFASTFNFRGHAKSD
NRSRR------------------------------------MEKKTNKNK-----GVAEE---TKKKVAATTGFIIGQFIRFIS
IYYAPLYVALGRPHTITILALPYLLIHLFWNTDKSFFDS--KKLNSIRNLEISSVFLNHFILQLL
NSCILPNSTLARLITIYMFRCNNKILFLTSSFFAWFIGQIFMLNSFELVLVWIRKKNYLRS-NY
L-NSIFVILFNC---LFGSLLFILSIQCLGRIPSPIPTQKLSEE-----LAALLFDYKRWTRPFRYIKN
NRLEQALRNELSQYFFDTYQSDGKNRLSFTHPISLSTFLKMMKLKIPLLLVTLYN--PLDNG
WVSRNKKQMNYRRIYFLNRVNNLDKAV-------TRTRLCIDETKQEYLPENYDPLLTGPHRG
RIKRGLLTRETIILNRLHALVSKNFNQKN---------------APRWSYKLIELEQISYYRS--RKGISV
VIFDPVVIRYPQQSDFRQGLIKDSMRTQRRKIFLQELFNANVHSPLFFDRLR----IVFSFPR----
--IFINREQKERLEIGEAWDVFPLAQIIRGLLLLTQSFIRKKIILPSLILGKNIGRILLCQIPEWSE
DLQEWNRETHIKCTYNGIPLSQKKFPENWLTEGIQIKILFPFCLKPWHAY-RTSHYDFCFLT
VWGRETEQPFGHPRKT-FFFEPVLQELDKK------------------------IVN----IKARIFSKVKINLFKR
VSKEN----DFQIRKQIMNESFE--EIPSCTNSSLIE-KMRNMSDRTRTIKKKIERMTEDKKK-----
-TYKRSYRLALSICKMVKV----LIWKFSYFQKLLNQRVY-DIFLYTIYICRMTTQLF-----CDFS
NLSQAYVVFYKISQTGVLNICKLISVLQHISFFIQTQIKDLVTEGIFKS-VIHQKLQ-PKTSQWK
NWLRLNSKYDLSPIFWFSLISQKWRNRVTKYHRPKEKNKWNSHGKYR--LSDYK--DNSK
KCYQYDLLSYKSINVDIPYLDRKYLIWKNIH----LVDWMGMNEQILNRWFFPEFVWLFNV
YKTKPWIIPSKILLLN---------ELFTKKYFLFQLRGDQ-TFFK---NIQIYCLLLRLRNRKKMTL
SCIQRRKLNLRIMPSMTNLLNVPEFLKGLV-MDPLPLSIKTDGKFLLYKIVGISLVHKSKDN
HLDVLVLENILSSRCRRELRTLICFTFIWPNYRLEDLACMNRYSFDTTNGSRFSILRFHMYL
MMRHQVKLGILELREIMREIHNYLLLLDSWTKLNSVGSLIHLFFHQERFLKLFDPRIFSILL
S-RNL--QGSPSNRYFT---IKGVILFVVG-VLIYR----INNQNMVERKNLYLTGLFP-----IPMNST
GPIKDRLEEAI-GSSNINRLSVL-LLYFPKGKNISERYFLNPKETAGVLTIT----KKGSVPESNW
GSRRWRNFIGVKRG-NFSCPIAN---ETVGGIDILFKDKFRK-NMEFTFVYYMD-D-LTHNDP
DWEFFYRLSTRKSENRINW--NSGPLFKILVKDCISYLMSAFREKRPKGEGP---------ESTIQS
NKIRHVFH--LFSIKRGTISLQNCTHFYMWQFRRDLFR---SWGN-NPPESSFLRN----VSREIS
SNAWL-NKGR---FFS--KGRNVLSNLQYDS---TG--SSLVNVTDSSQLKGSE-DQSIERLASIR--
--NEDSE-------------YHALINKREVQQLEESSMTLE---------------SD--RLPK--SLSGYC--STSQ
LFK-ERENPMINFMFPGEMEEFI--GNPTRSVRSFCSDRW--SELH-LWSNPTEKSTLDH----KF
VKNHLSFV-----RGAE----NKEIKNLRRIITYLQK--TVSIHSISSDPRWDRVP-KHDPD-MDS----
--FKKNSLFFL--FHRFHERNRGAYPL--RHDFESE--ERLQEMTELFTLLISEP-DLVYHKGFSF
YIDSYGLDKKN--LLNEVLNARAESKNKSL---WVLSPILFRYEEIEYFFKRIRQKRVWWISC
GNGLVDLKQ--------------------------NDDGY----IRNLFNILFLMN---KSDRNFEHRMQ---GDRK
GTAIFNQRTIMKCMIKQDHT---------------------------------YIYKRSNGTKNFQEHLEHFLSQQK
S---YFQVLFDQLRLIHWFEVRKKVEKKVE-SKSLRFFFLPQSLHFFFIKVLRFVT-------EVILF
LSN-SFPSLCVSFGNTPIQRSE-IYIS-ELKSSNEQLCNKLFESIGFQIVHLKKLNPF--LLE------
EF-DTSNFVINVGTRSPFLLNKIP-KR-IFDSLPTR--TNHSKYFDNKDSYFSKIFHDKDNWLN
HPKPFHRSSLISSFYKANQLRFRNHLHHFLFY--CNKRFPFSV----EKARNTNSH--FLYG-QFL
NILLFRKK-IFSLCVGQ--KKHVFWS-RATLSPIESQVSNIFIPKDFPQSGDEG-YNLDKY---FN

FLIQPDPVIR--RAISSIVETSGTPLTEAQKGDLERTYCKPLSDINLSDSEVNNFHEYPN--FNS
NMGLVHILFSGKYLSSEK-------------HSLCLKKGAGVHKERMFTTFQRDSSFSILSTWNLFQ
TYLPSFFTSTGYKYLTSIFSENFSYLLS--ILSSR----VSLFQ-------LSWRILLSKMPLFLR--------
--REISSKWLHNL--FLSKEMIRR--KTQSSLISKHLRSTKFWEF-FYSLLFLLLVAGY-LVSIHFF
FISR--AFSELQTEFKSLKSLMTPSSAIELRKLLDKYPRSEPNSFWLKNIFLVVMEQLRDSLE
EIKGFVFGLNRIGLTSG---------VKSIRFKNFKNKDFNIKGIGII-ELILRPITRIAFSLNTRHISH
TSKEIYSLIIKRKNVNGVWIDDKIESWVANSDSIHEEERKLLVQLS--ALTT--EKR---ILLSLT-
HSDHFS-----KNDSGYQMIEQPGAIYLRYLVDIH--QKHLMNYEFNTSCLAERRIFLAHSQTIT
Y--SQTSYGTNSFHFPSHG---KPFSLRLALSPS-RGILVIGSIGTGRSYLVKYLATNSYVPFITVF
PNKFLDKNPQ-FIDD----IDIDNIDASDDIDMDNSDNIDD----------------DIDRDLATELELLTW
MNAL---TMDREMK-AEIARLFITLQFELARAMSPCIIWIPNIHDLDVNESNYLSLGLLVNHL
S---RDCERCS-PINILVIASTHIPQKVDPALIAPKKLNTCIKLRRLLIPQQRKYFFTLSYTRGFH
LENKMFHT--NGFGSITMGPNARDIVALTNEVLSISITQKKSIIDTNTIRSALHRQTWDLQ---S
QVRLVQDHGILFYQIGRAVAQNVLLSNCPIDPISIYLKKK--SCNEGDSYLYKWYFELGTSM
KKLTILLYLLSCTAGSVAQDLWSLP--GPDEK-NGITSYGLVENDS-DLVHGLLEVEGALVG--
SSRTEKNCSKFEND--RVTLLLRPEPRN-PLERRQNGSCSILDQRFLSEK--DESEFEE-----GAL
AP----QQIEED----LFNH--IVWAP--TIWRP-----WGFLCIERPNELGFS-YWSRSFRGK---RILYD
EEDELEENDSEFLQSGTMQYKTRDRSSKEKGLFRISQFIWDPADPLFFLFK-DRPPGSVFSR
LELFADEEMSKGLLTSQTSLFRYKYPRWFI--NKTQEKHFEFLIHRQRCLRT---NSSLS--NRSF
RSN--TLSESYQYLSNLFLSN-GTLLDQMTKTLLRKRWLFPDEMKIGF--M---------MSRSR-I
NGNFLDKTFSIVANILLRIIPTTSGEKEAFTYYRDG--MSAQSEGNYAEALQNYYEAMRLEI
DPYDRSYILYNIGLIHTSNGEHMKALEYYFRALERNPFLPQAFNNMAVICHY-RGEKAIQQ
GDSEIAEAWFDQAAEYWKQALALTPGNYIEAHNWLKITGRFD--MSWRSEHIWIELIAGSR
KISNLCWAFILFLGSLGFFLVGTSSYLGRNLISFFPLQQIIFFPQGIVMSFYGIAGLFISSYLWC
TISWNVGSGYDLFNRKEGIVCIFRWGFPGINRRIFLRFLLKDIRSVRIEVEEGIYAGRVLYMD
IRGQRAIPLTRTDEN-LTPGEIEKKAAELAYFLRVPIEVF
>Lemna
MEKLWLNSMFSLSRSMDSLGPIRYSSGSEDPVLNAMDNKVS-FSNVEHFLDIIDIWSFISDD
TFLVRDSNGDSFSIYFDIENQIFEIDND--STFINELESSFSSYLD-SSMNSGSKKSSRYYYRYM
YDTQSSWNNHINSCIDSYLRYEI--SIDSYISGETHNYSDNYVYNFICNESRNSIRTSVNGSDF
NIRGRS-DLDINKKYRHLWVQCENCYGLNYKQFFRSRLNICEHCGYHLKMSSSERIELLID
PGTWDPLDENMVSTDPIEFHSEEEPYRDRIDSYQKKTGLTEAVQTGIGELNGIPIAIGVMDF
QFMGGSMGSVVGEKITRLIEYATNKSLPVIIVCASGGARMQEGSLSLMQMAKISSALYNY
QLNKKLFYVAILTSPTTGGVTASFGMLGDIIIAEPNAYIAFAGKRVIEQTLNKTVPDGSQAA
EYLFQKGLFDLIVPRNLLKGVLGELFQLHGFFPLT-----MVTIRADEISSIIRERIEQYTREVK
VVNTGTVLQVGDGIARVHGLDKVMAGELVEFEEGTVGIALNLESNNVGVVLMGDGLLIQ
EGSSVKATGKIAQIPVSDGYLGRVLNALAKPIDGRGEISASESRLIESPAPGIIARRSVYEPL
QTGIIAIDAMIPIGRGQRELIIGDRQTGKTAVATDTILNQKGQNVICVYVAIGQKASSVAQVV
TALQERGAMDYTIVVAETADSPATLQYLAPYTGAALAEYFMYRKQHTLIIYDDLSKQAQA
YRQMSLLLRRPPGREAYPGDVFYLHSRLLERAAKLSNELGEGSMTALPIVETQSGDVSAYI
PTNVISITDGQIFLSADLFNAGIRPAINVGISVSRVGSAAQIKAMKQVAGKLKLELAQFAEL
EAFAQFASDLDKATQNQLARGQRLRELLKQSQSAPLGVDEQIATIFTGANGYLDSLEVGQ
VRKFLVQLRTYLKKNKPQFQEIISSTKTFTPEAEALLKEAIQEHIELFLQQEQAMQINPTTS-
TAVSQLEEKNLGRVAQIIGPVLDVVFPPGKMPNIYNALVVKGQDADGQEIKVTCEVQQLL

GNNRVRAVAMSATDGLTRGMDVIDTGAPLSVPVGGATLGRIFNVLGEPVDNLGPVDTRTT
SPIHRSAPAFIQLDTKLAIFETGIKVVDLLAPYRRGGKIGLFGGAGVGKTVLIMELINNIAK
AHGGVSVFGGVGERTREGNDLYMEMKESGVINEKNITESKVALVYGQMNEPPGARMRV
GLTALTMAEYFRDVNEQDVLLFIDNIFRFVQAGSEVSALLGRMPSAVGYQPTLSTEMGSL
QERITSTKEGSITSIQAVYVPADDLTDPAPATTFAHLDATTVLSRGLAAKGIYPAVDPLDSTS
TMLQPGIVGEDHYETAQRVKETLQRYKELQDIIAILGLDELSEEDRLTVARARKIERFLSQP
FFVAEVFTGSPGKYVGLEETIRGFKLILSGELDSLPEQAFYLVGNIDEATAKAINLEVESKLK
KMTLNLCVLTPNRIVWDSEVKEIILSTNSGQIGVLPNHAPIATAVDIGLLRIRLTDQWVTVA
LMGGFARIGNNEITILGNDAEISTDIDPQEAQQALEKAEDNLRKAEGKRQAIEANLALRRA
KTRVEAINVISMQNVTDSFVSL-HWPSAGGFGFNTDILATNPINLSVVLGVVIYFGKGVLN
DLLDNRKQRILSTIRNSEELRQAAIEQLEKARARLRKVETEANDYRVNGYSEIEREKQNLI
KATSENLERLENYKNETLLFEQQRAINQVRQRVFQQALQGALGTLNSCL-SELHFRTISANI
GILGVMEE---MNPLISAASVIAAGLAVGLASIGPGVGQGTAAGQAVEGIARQPEAEGKIRG
TLLLSLAFMEALTIYGLVVALALLFANPFVMNVIPCSINTLKGLYDISGVEVGQHLYWQIGG
LQVHAQVLITSWVVIAILLGSVTLAVRNPQTIPADGQNFFEYLLEFIRDLSKTQIGEEYGPW
VPFIGTMFLFIFVSNWSGALLPWKIIQLPHGELAAPTNDINTTVALALLTSVAYFYAGLSKK
GLSYFGKYIQPTPILLPINILEDFTKPLSLSFRLFGNILADELVVVVLVSLVPLVVPIPVMFLG
LFTSGIQALIFATLAAAYIGESMEGHHMIFVTLEHILTHISFSIISVVIIIQLMTFFVHEIPALSD
SLEKGMIATFLSITGLLIIRWIYSGHFPLSNLYESLMFLSWSFAIIHMIPKIWKNKNYLSAITIP
SAIFTQAFATSGLSTEMHESGILVPALRSHWLMMHVSMMLLSYAALLVGSLFSIALLVITFR
KNGKLVVENQQMDYLNFNENVLENPSFSSFKNYYKYQLVERLDYWSSRVISIGFSFLTIGI
LSGAVWANEAWGSYWNWDPKETWAFITWTIYAIYSHTRTTKNGQGANSAIVASIGFFIIWI
CYFGVNLLGIGLHSYGSFILS---MIKE
YPETHLQKLRIGIHKETVQLVKIHNEYNLHSILHFSTNIICFAILSGYFILGNEKLVILNSWIQ
EFLYNLSDTIKAFSILLVTDLWIGFHSPHGWELMIGSVYNDFGLAHNDQIISGLVSTFPVILD
TIVKYWIFHYLNRVSPSLVVIYHSMNEMPIGVPKVPFRSPGEEDAVWVDVYNRLYRERLLF
LGQEVDSEISNQLVGLMVYLSIEDDTKDLYLFINSPGGWVIPGIAIYDTMQFVPPDVHTICM
GLAASMGSFILVGGEITKRLAFPHARVMIHQPASSFYEAQAGEFILEAEELLKLRETLTRVY
V Q R T G N P L W V V S E D M E R D V F M S A T E A Q A Y G L I D L V A V
E???MEEFKG
YLQKGGFKQQHFLYPLLFQEYIYALAHDQGLSGYGN-YSSLLVKRLITRIYQQNSFIYSVNN
SKQNRFVGKNFYYKMISEGFAIVVEIPFSLRLVSSLITKSQNLRSIHSLFPFLEDKFSHLNYV
SDILIPYPVHLEILVQILQCWIQDLPTLHLLRLIFHDYHNGSNSIPKS-SFFSKPRLYRFLYNSY
VVECESIFDFLRKSSSYLRSTSFGPLLERTHFYGKMK-G-VTCCNQKTLWLFKDPFMHYVR
YQGKCIMASKGTHLLMKKWKSYFVNLWQCHFHFWSQPSRIHINQFPHFSFYLLGYLSSVP
INPSSAKSQMLENSFLIDSFT--PKFETMISIIPMIGSLAKAKFCNLSGNPISKPAWADLSDSDII
DRFGRIYRNLSHYYSGSSKKQSLYRIKYILRLSCARTLARKHKSTVRAFLQRLGSEFFEEFF
MEQEKVLSLILPRPIWYLDIIRINDLVNHFMIIDIR-EVE-TINSFYVSE-FLKEIYELIWLFVPIF
VLILGIIIGVLVIVWLEREISAAIQQRIGPEYAGPLGILQALADGTKLLFKEDLLPSRGDRRLF
SAGPAIAVVSILLSYLVVPFGSQLVLADLSIGVFLWIAISSIAPIGLLMSGYGSNNKYSFLGGL
RAAAQSISYEIPLTLCVLSISLLSNSSSTVDIVEAQSKYGFLGWNLWRQPIGFIVFLISSLAEC
ERLPFDLPEAEEELVAGYQTEYSGIKYGLFYVASYLNLLVSSLFVTVLYLGGWNISLPYMFI
PELFGINTI---FGITIGIFITLAKAFLFLFISIATRWTLPRLRMDQLLNLGWKFLLPISLGNLLLT
TSSQLVSLMIWHVQNEVFILDSTRIFMKAFHLLLFNGSFILPECILIFGLILLLMIDSTSDQKD

RPWFYFISSTSLVMSITALLFRWREEPMISFSGNFQTNNFNEIFQFLILLCSTLCIPLSVEYIEC
TEMAITEFLLFILTATLGGMFLCGANDLITIFVALECFSLCSYLLSGYTKRDVRSNEATTKYL
LMGGASSSILVHGFSWLYGLSGGEIELQEIVNGLINTQMYNSPGISIALIFITVGIGFKLSLAP
FHQWTPDVYEGSPTPVVAFLSVTSKVAASALATRIFDIPFYFSSNEWHLLLEILAILSMILGN
LIAITQTSMKRMLAYSSIGQIGYVIIGIIVGDSNDGYASMITYMLFYIAMNLGTFARIVLFGL
RTGTDNIRDYAGLYTKDPFLALSLALCLLSLGGLPPLAGFFGKLHLFWCGWQAGLYFLVSI
GLLTSVVSIYYYLKIIKLLMTGRNQEITPHVRNYRKSPLRSNNSIELSMTVCVIASTIPGISM
NPILAIAQDTLFMFLLYEYDIFWAFLVISSVIPILAFIISGVLAPLSEGPEKLSSYESGIEPIGDA
WVQFRIRYYMFALVFVVFDVETVFLYPWAMSFDVLGVSVFIEALIFVLILIVGSVYAWRKG
ALEWSTSYFPWLTLIVVFPIFAGCFIFFLPHKGNKVVRWYTMGICLLELLIMTYVFCYHFK
LDDPLIQMEDDFQWINVFDFHWRLGIDGLSIGPILLTGFITTLATLAAWPVTRDSRLFHFLM
LAMYSGQIGLFSSRDLLLFFMMWELELIPVYLLLSMWGGKKRLYSATKFILYTAGGSIFLLI
GVLGMGLYGSNGPTLNFETVANQSYPLGLEILLYFGFLIAFAVKLPIIPLHTWLPDTHGEAH
YSTCMLLAGILLKMGAYGLIRINMEFLSHAHSIFSPWLVIVGTIQIIYAALTSLGQRNLKKRI
AYSSVSHMGFIIIGIGSLTNTGLNGAILQLLSHGFIGAALFFLGGTSCDRIRLVYLDEMGGISI
PMPKIFTMFSSFSMASLALPGMSGFVAELMVFLGIITSQKYLLMTKIIITLVMAIGIILTPIYL
LSMLRQMFYGYKLFNLSNSFFVDSGPRELFVSICIFLPVIAIGIYPDLVISLSVDKVQQILSN
YYYRMMFEHVLFLSAYLFSIGIYGLITSRSMVRALMCLELILNSVNINLVTFSDLFDSRQLK
GDIFSIFVIAIAAAEAAIGLAIVSSIYRNRKSIRINQSNLLNKMEHTYQYAWIIPLLPLPVTMSI
GFGLLLIPTATKSIRRMWAFPSVLLLSIGLIFSANLAFQQINGSFIYQYLWSWTINNDFSLEF
GYFIDPLTSVMLILITTVGIMVLIYSDNYMSHDQGYLRFFAYMSFFNASMLGLVTSSNLIQI
YIFWELVGMCSYLLIGFWFTRPSAASACQKAFITNRVGDFGLLLGILGFYWITGSFEFRDLF
ETFNK-EGNSLFPTLCAFLLFLGAIAKSAQFPLHVWLPDAMEGPTPISALIHAATMVAAGIF
LVARLLPLFTGIPYIMNIISLIGVITLLLGATLALAQRDIKRSLAYSTMSQLGYIMLALGLGS
YRAALFHLITHAYSKALLFLGSGSIIHSMEPLVGYSPDKSQNMVLMGGLTRYIPITRTTFLL
GTLSLCGIPPLACFWSKDEILSESWLYSPSFAVIAYFTAGLTAFYMFRVYLLTFEGSLHVNFQ
NYSGTQNTSAYSISLWGKEEPKSV---NSLFSTMKTKNRKN--TSMYPHESDNTMLYPLLILV
VFTLFVGFIGIPLDLNLLSKWLAPSKNLLYPNSEDSIDLYDFFINASFSVSIATGGIFLAFIFYG
SPYSPLQNLDLMNLLVVSLDKIANIVYNWSHNRGYIDIFYTNVVTQSIRELAKLTQFFDRYI
IDGITNSVGVLSFFAGEAIKYLVGGGRISFYLFFYLFFILIFVYFLFMDLTGPIHDILVVFLGL
VLILGGLGVVLFTNPIYSAFSLGLVLICVSLFYILLNSYFVAAAQLLIYVGAINVLILFAVMF
MKGSEYSNDLNLWTVGDGVTSLVCTSILFLLISTISDTSWYGIIWTTKANHIIEQDLISNVQ
QIGIHLSTDFFLPFELISIILLVALIGAISMARQMTGPTTKNDLMIVNMGPQHPSMHGVLRLI
VTLDGEDVIDCEPILGYLHRGMEKIAENRTIVQYLPYVTRWDYLATMFTEAITVNAPEQLG
NIQVPQRASYIRVIMLELSRIASHLLWLGPFMADIGAQTPFFYIFRERELIYDLFEAATGMR
MMHNYFRIGGVAADLPYGWIDKCLDFCDYFLTGIDEYEKLITRNPIFLERVEGIGIISGEEAI
NWGLSGPMLRASGISWDLRKVDQYECYNEFDWEVQWQKEGDSLARYLVRMSEMRESIK
IIQQALEGIPGGPYENLEVRR-DKTKDSEWNDFEYRFISKKPSPNFELSKQELYVRVEAPKG
ELGIFLIGDQNVFPWRWKIRPPGFINLQILPQLVKRMKLADIMTILGSIDIIMGEVDRMFPM
VTGFMSYGQQTVRAARYIGQGFIITLSHANRLPVTIQYPYEKSITSERFRGRIHFEFDKCIAC
EVCVRVCPINLPVVDWRLETNIKKKQLLNYSIDFGVCIFCGNCVEYCPTNCLSMTEEYELS
AYDRHELNYNQIALGRLPISIMGDYAIQPIMNSKIDKDKPLD-------MQSHLSAWLVKHELV
HRSLGFDYQGIETLQIKPEDWDSIAVISYVYGYNYLRSQCAYDVAPGGFLASVYHLTRIQY
GVDQPEEVCIKVFVPRENPIVPSVFWVWRSADFQERESYDMFGISYDNHPRLKRILMPES

WIGWPLRKDYITPNFYEIQDAH-----------------------------------MVLAPEYSNNK-----KESIET
VMNLIEFPSLDQTMPNSVISTTLNDLSNWSRLSSLWPLLYGTSCCFIEFAALIGSRFDFDRY
GLVPRSSPRQADLILTAGTVTMKMAPSLVRLYEQMPEPKYVIAMGACTITGGMFSTDSYST
VRGVDKLIPVDVYLPGCPPKPEAVIDAITKLRKKISREIYEDRIGSK-ENRCFTINHKFHVQR
SIHTGNYNKELLYQSPSTSEITE--FFRSKSSISSHKLVNMQNRNTFFGVKQQITRFISVSIGIY
VITQTSISNAYPIFAQQGYENPREATGRIVCANCHLASKPVDIEVPQSVLPDTVFEAVVRIPY
DTQVKQVLANGKKGGLNVGAVLILPEGFELAPPDRISPELKEKIGNISFQSYSPTKKNILVI
GPVPGQKYREIVFPILSPDPATSKDVHFLKYPIYVGGNRGRGQIYPDGSKSNNTVYNATSA
GVVSKIVRKEKKGYEIIITDPSDGREVVDIIPPGPELLVSEGESIKLDQLLTSNPNVGGFGQG
DAEIVLQDPSRVQGLLFFLASVVLAQIFLVLKKKQFEKVQLFEMNFMKKVYDWFEERLEI
QAIADDITSKYVPPHVNIFYCLGGITLTCFLVQVATGFAMTFYYRPTVTEAFSSVQYIMTEV
NFGWLIRSVHRWSASMMVLMMILHVFRVYLTGGFKKPRELTWVTGVILGVLTASFGVTG
YSLPWDQIGYWAVKIVTGVPDAIPVIGSPLVELLRGSASVGQSTLTRFYSLHTFVLPLLTAV
FMLMHFLMIRKQGISGPLMGVTKKPDLNDPVLRAKLAKGMGHNYYGEPAWPNDLLYIFP
VVILGTIACNVGLAVLEPSMIGEPADPFATPLEILPEWYFFPVFQILRTVPNKLLGVLLMVSV
PAGLLIVPFLENVNKFQNPFRRPVATTVFLIGTVVALWLGIGATLPIDKSLTLGLFMIEVLLF
GIVLGLIPITLAGLFVTAYLQYRRGDQLDLMLTITSYFGFLLAASTIASVLFIGLNKIRLIMDI
VSLAWAALMVVFTFSLSLVVWGRSGLMIISSPEPEVKILVDRNPVKTSFEQWAKPGHFSRTI
AKGPETTTWIWNLHADAHDFDSHTNDLEEISRKVFSAHFGQLSIIFLWLSGMYFHGARFS
NYEAWLSDPTHIAPSAQVVWPIVGQEILNGDVGGGFRGIQITSGFFQLWRASGITSELQLY
CTAIGALVFSALMLFAGWFHYHKAAPKLAWFQDVESMLNHHLAGLLGLGSLSWAGHQIH
VSLPINQFLNAGVDPKEIPLPHEFILNRDLLSQLYPSFAEGATPFFTLNWSKYSDFLSFRGGL
DPITGGLWLSDIAHHHLAIAILFLIAGHMYRTNWGIGHGLKDILEAHKGPFTGQGHKGLYE
ILTTSWHAQLSLNLAMLGSLTIIVAHHMYAMPPYPYLATDYGTQLSLFTHHMWIGGFLIVG
AAAHAAIFMVRDYDPTTRYNDLLDRVLRHRDAIISHLNWVCIFLGFHSFGLYIHNDTMSA
LGRPQDMFSDTAIQLQPIFAQWIQNTHALAPGTTAPGATASTSLTWGGGELVAVGGKVALL
PIPLGTADFLVHHIHAFTIHVTVLILLKGVLFARSSRLIPDKANLGFRFPCDGPGRGGTCQVS
AWDHVFLGLFWMYNSISIVIFHFSWKMQSDVWGTVSDQGVITHITGGNFAQSSTTINGWL
RDFLWAQAAQVIQSYGSSLSAYGLFFLGAHFVWAFSLMFLFSGRGYWQELIESIVWAHNK
LKVAPATQPRALSIVQGRAVGVTHYLLGGIATTWAFFLARIISVGMALRFPRFSQGLAQDPT
TRRIWFGIATAHDFESHDDITEERLYQNIFASHFGQLAIIFLWTSGNLFHVAWQGNFESWVK
DPLHVRPIAHAIWDPHFGQPAVEAFTRGGALGPVNIAYSGVYQWWYTIGLRTNEDLYTGA
LFLLFLSAISLIAGWLHLQPKWKPSVSWFKNAESRLNHHLSGLFGVSSLAWAGHLVHVAIP
GSRGEYVRWNNFLDVLPYPQGLGPLFTGQWNLYAQNPDSSSHLFGTSQGAGTAILTLLGG
FHPQTQSLWLTDMAHHHLAIAFIFLVAGHMYRTNFGIGHSIKDLLEAHNPPGGRLGRGHK
GLYDTINNSIHFQLGLALASLGVITSLVAQHMYSLPAYAFIAQDFTTQAALYTHHQYIAGFI
MTGAFAHGAIFFIRDYNPEQNEDNVLARMLAHKEAIISHLSWASLFLGFHTLGLYVHNDV
MLAFGTPEKQILIEPIFAQWIQSAHGKTSYGFDVLLSSTTGPAFNAGKSIWLPGWLNAVNE-
-SSNSLFLTIGPGDFLVHHAIALGLHTTTLILVKGALDARGSKLMPDKKDFGYSFPCDGPGR
GGTCDISAWDAFYLAVFWMLNTIGWVTFYWHWKHITLWQGNVSQFNESSTYLMGWLR
DYLWLNSSQLINGYNPFGMNSLSVWAWMFLFGHLVWATGFMFLISWRGYWQELIETLAW
AHERTPLANLIRWRDKPVALSIVQARLVGLAHFSVGYIFTYAAFLIASTSGKFGMSHSVKIY
DTCIGCTQCVRACPTDVLEMIPWDGCKAKQIASAPRTEDCVGCKRCESACPTDFLSVRVY
LGSETTRSMGLGYMTDFNLPSIFVPLVGLVFPAFAMASLFLYVQKNKIVMQDLKTYLSVAP

VLSTLWFGILAGLLIEINRLFPDALLFPFF--MTAILERRESTSLWGRFCNWVTSTENRLYIG
WFGVLMIPTLLTATSVFIIAFIAAPPVDIDGIREPVSGSLLYGNNIISGAVIPTSAAIGLHFYPI
WEAASVDEWLYNGGPYELIVLHFLLGVACYMGREWELSFRLGMRPWIAVAYSAPVAAAT
AVFLIYPIGQGSFSDGMPLGISGTFNFMIVFQAEHNILMHPFHMLGVAGVFGGSLFSAMHG
SLVTSSLIRETTENESANEGYRFGQEEETYNIVAAHGYFGRLIFQYASFNNSRSLHFFLAAW
PVVGIWFTALGISTMAFNLNGFNFNQSVVDSQGRVINTWADIINRANLGMEVMHERNAH
NFPLDLAAVEAPSTIGMGLPWYRVHTVVLNDPGRLLSVHIMHTALVAGWAGSMALYELA
VFDPSDPVLDPMWRQGMFVIPFMTRLGITNSWGGWSIT-GGTITNPGIWSYEGVAGAHIVF
SGLCFLAAIWHWVYWDLEIFCDERTGKPSLDLPKIFGIHLFLSGVACFGFGAFHVTGLYGP
GIWVSDPYGLTGKVQPVSPAWGAEGFDPFVPGGIASHHIAAGTLGILAGLFHLSVRPPQRL
YKGLRMGNIETVLSSSIAAVFFAAFVVAGTMWYGSATTPIELFGPTRYQWDQGYFQQEIY
RRISAGLAENLSLSEAWSKIPEKLAFYDYIGNNPAKGGLFRAGSMDNGDGIAIGWLGHPVF
RDKEGRELFVRRMPTFFETFPVVLVDGDGIVRADVPFRRAESKYSVEQVGVTVEFYGGEL
NGVSYSDPATVKKYARRAQLGEIFELDRATLKSDGVFRSSPRGWFTFGHASFALLFFFGHI
WHGARTLFRDVFAGIDPDLDAQVEFGAFQKLGDPTTKRQAV------------VETLFNGTLALA
GRDQETTGFAWWAGNARLINLSGKLLGAHVAHAGLIVFWAGGMNLFEVAHFVPEKPMY
EQGLILLPHLATLGWGVGPGGEVIDTFPYFVSGVLHLISSAVLGFGGIYHALLGPETLEESF
PFFGYVWKDRNKMTTILGIHLILLGLGAFLLVFKALYFGGVYDTWAPGGGDVRKITNLTL
NPSVIFGYLLKSPFGGEGWIVSVDDLEDIIGGHVWLGSICILGGIWHILTKPFAWARRAFVW
SGEAYLSYSLAALSVFGFIACCFVWFNNTAYPSEFYGPTGPEASQAQAFTFLVRDQRLGAN
VGSAQGPTGLGKYLMRSPTGEIIFGGETMRFWDLRAPWLEPLRGPNGLDLSRLKKDIQPW
QERRSAEYMTHAPLGSLNSVGGVATEINAVNYVSPRSWLSTSHFVLGFFFFVGHLWHAGR
ARAAAAGFEKGIDRDLEPVLFMTPLSMTIALGRFTKEE-DLFDIMDDWLRRDRFVFVGWS
GLLLFPCAYFALGGWFTGTTFVTSWYTHGLASSYLEGCNFLTAAVSTPANSLAHSLLLLW
GPEAQGDFTRWCQLGGLWTFVALHGAFALIGFMLRQFELARSVQLRPYNAIAFSGPIAVFV
SVFLIYPLGQSGWFFAPSFGVAAIFRFILFFQGFHNWTLNPFHMMGVAGVLGAALLCAIHG
ATVENTLFEDGDGANTFRAFNPTQAEETYSMVTANRFWSQIFGVAFSNKRWLHFFMLFVP
VTGLWMSALGVVGLALNLRAYDFVSQEIRAAEDPEFETFYTKNILLNEGIRAWMAAQDQ
PHENLIFPEEVLPRGNALMSGSTGERSFVDIITSIRYWVIHSITIPSLFIAGWLFVSTGLAYDV
FGSPRPNEYFTENRQGIPLITGRFDSLEQLDEFSRSFMTIDRTYPIFTVRWLAVHGLAVPTVS
FLGSISAMQFIQRMATQTVEGSS-KSGPRRTRVGDLLKPLNSEYGKVAPGWGTTPLMGVA
MALFAVFLCIILEIYNSSVLLDGILVSMLTLKLFVYTVVIFFVSLFIFGFLSNDPGRNPGREE
MADTTGRIPLWLIGTVVGIVVIGLLGLFFYGSYSGLGSSLMLNPFNLICICLNSALSSSG-FF
GKLPEAYAIFNPIVDIMPVIPVFFFLLAFVWQAAVSFRMTQSNPNEQNVELNRTSLYWGLL
LIFVLAVLFSNYFFNMEVNILAFIATALFILVPTAFLLIIYVKTVSQNDMETATLVAISISGLLV
SFTGYALYTAFGQPSQQLRDPFEEHGDMEALVYTFLLVSTLGIIFFAIFFREPPKVPTKK--MT
IAFQLAVFALIVTSSILLISVPVVFASSDGWSSNKNVVFSGTSLWIGLVFLVAILNSLIS-------
MSPQTETKASAGFKAGVKDYKLNYYTPEYETKDTDILAAFRVTPQPGVPPEEAGAAVAAE
SSTGTWTTVWTDGLTSLDRYKGRCYHIEPVAGEENQFIAYIAYPLDLFEEGSVTNMFTSIV
GNVFGFKALRALRLEDLRIPPAYSKTFQGPPHGIQVERDKLNKYGRPLLGCTIKPKLGLSA
KNYGRAVYECLRGGLDFTKDDENVNSQPFMRWRDRFLFCAEAIYKAQAETGEIKGHYLN
ATAGTCEEMIKRAVFARELGVPIVMHDYLTGGFTANTSLAYYCRDNGLLLHIHRAMHAVI
DRQKNHGMHFRVLAKALRMSGGDHVHSGTVVGKLEGEREMTLGFVDLLRDDFIEKDRS
RGIFFTQDWVSMPGVLPVASGGIHVWHMPALTEIFGDDSVLQFGGGTLGHPWGNAPGAV

ANRVALEACVKARNEGRDLAREGNEIIREACNWSPELAAACEVWKEIKFEYEPVDKLDVT
AIHLYKTSTPSTRNGAVDRKVKSNPRNNLIYGQHRCGKGRNARGIITAGHRGGGHKRLYR
KIDFRRNEKDIYGRIVTIEYDPNRNAYICLIHYGDGEKRYILHPRGAIIGDTIVSGTEVPISM
GNALPL-DMPLGTAIHNIEITLGKGGQLARAAGAVAKLIAKEGKSATLRLPSGEVRLISKNC
LATVGQVGNVGVNQKSLGRAGSKCWLGKRPVVRGVVMNPVDHPHGGGEGRAPIGRKR
PMTPWGYPALGKRSRKKKRYSDRFILRRRK-MIQPQTLLNVADNSGARELMCIRILGAS-N
RRYANIGDVIVAVIKEAVPNMPLERSEVIRAVIVRTRKELKRENGMRIRYDDNAAVVIDQEG
NPKGTRVFGAIARELRELNFTKIVSLAPEVLMLSPKRTRFRKQHRGRMKGTSYRGNHISFG
RYALQALEPSWITARQIEAGRRAISRYARRGGKIWVRLFPDKPVTLRPAETRMGSGKGSPE
YWVSVVKPGRILYEIGGVSENVARTAMLLAASKMPIRTQFIIEGMTRVKRGYIARRRRTKIR
LFASSFRGAHSRLTRTITQQKMRALVSAYRDRGRQKRDFRRLWITRINAVTREN--VSYSYI
RLIHNLYKRQLLLNRKILAQIAILNKNCLHIISKKIIK-----------MVTKAL-AQNIH-MSPFKAR
RVIDQIRGRSYEETLMILELMPYRAAFTILKLVYSAAANA-NNLGLNEAESFISKAEVNGGT
VLKRLRPRARGRSYRIKRPTCHITIVLK-KSK--------MDGIKYAVFTEKSIRLLGNNQ-YTSN
VESGSTRTEIKHWVELFFGVKVIAMNSHRLPRKGRKRGPIMGHTMHYRRMIITLQPGYSIP
PLIEKRTMAVPKKRTSMSKKRIRKSIWKKKAYFAALKAFSLAKSISTGQSKSFFVRQTNNM
AKGKDVRVRVILECNSCGRNENG--KEVPGVSRYVTQKNRHNTPSRLELKKFCRYCNKHTI
HVEVKKMKIRASVRKICDKCRLIRRRGRIIVICSNPRHKQRQGMVREEIAGSTRTLQWKC
VESRVDSKRLHYGRFVLSPLMKGQADTIGIAMRRALLEEMEGTCITRAKSE--KVPHEYSTI
VGIEESVHEILMNLKEIVLRSNLYGTREASICVKGPKTITAEDIILPASVEIVDTTQHIATLTEP
LDLYIGLQIQRDRGYRMRPTNNSQDGSY--PIDAVFMPVRNANHSIYSYGNGNEKQEILFLE
IWTNGSLTPKEALHEASRNLIDLFIPFLHAEEK--DINLEENK-SRFTLPLFTFHDKLV-NLTKN
KK-EIGLKCIFIDQLELPSR--------------DL---------------------------------------MLRD-GNEGMFTI
PGFSQIQFEGFCRFINQGLMEEFNKFPKIEDTDQEIEFKLFVETYQLAEPLIKERDAVYESLT
YSSELYVLAGLIWKTNRDMKEQTVFIGNIPLMNSLGTFIVNGIYRIVINQILQSPGIYYRSEL
DHNGISVYTSTIISDWGGRSELEIDRKARIWARVSRKQKISILVLLSAMGLNLREILDNVCY
PEIFLSFLNDKEKKKIGSKENAILEFYQQFACVGGDPVFSESLCKELQKKFFQQRCELGRIG
RRNINQRLNLDIPHNNTFLLPRDVLAAADHLIGMKFGMGTLDD--MNHLKNKRIRSVADL
LQDQFGLALVRLENAVRGTISGAIR-HKLI-PTPQNLVTSTS----LTTTYESFFGLHPLSQVLDR
TNPLTQIVHGRKLSYLGPGGVTGRTASFRIRDIHPSHYGRICPIDTSEGINVGLIGSLAIHVRI
GHWG-SIETPFY---EISERSKEKEARMIYLSPSRDEYYMIAAGNSLALNRGIQEEQVVPARY
RQEFLTIAWEQVHLRSIFPFQYFSIGASLIPFIEHNDANRALMSSNMQRQAVPLSRSEKCIVG
TGLERQAALDSGVSVIAEHEGKVISTDTHQIVFSGNGNTLNIPLVMYQRSNKNTCMHQKP
QVQRGKYVKKGQILADGAATVGGELALGKNVLVAYMPWEGYNFEDAVLISERLVYSDIY
TSFHIRKYEIQTHMTSQG-PERITNEIPHLEAHLLRNLDRNGIVMLGSWVETGDILVGKLTP
QIANE-SSYAPEDRLLRAILGIQVSTAKETSLKLPIGGRGRVIDVRWIQKKG-GSSYNPEIIRV
YILQKREIKVGDKVAGRHGNKGIISKILPRQDMPYLQDGTPVDMVFNPLGVPSRMNVGQI
FECSLGLAGDLLNRHYRIAPFDERYEQEASRKLVFSELYEASKQTKNPWVFEPEYPGKSKI
FDGRTGDPFEQAVLIGKSYILKLIHQVDDKIHGRSSGHYALVTQQPLRGRAKQGGQRVGE
MEVWALEGFGVAHILQEMLTYKSDHIRARQEVLGTTIIGGTIVNPEDAPESFRLLVRELRSL
ALELNHFLVSEKNFQINRKEAMNQNFYSMIDRYKHQQLRIGPVSPQQISAWANKILPNGEV
VGEVTKPYTFHYKTNKPEKDGLFCERIFGPIKSGICACGNYRVIGAEKEDPKFCEQCGVEF
VDSRVRRYQMGYIKLACPVTHVWYLKRLPSYIANLLDKPLKELEGLVYC--DFSFARP-IAK
KPTFLRLRGSFEYEIQSWKYSIPLFFTTQGFETFRNREISTGAGAIREQLADLDLRIITDNSLI

EWKELGDE-GSTGNEWEDRKIRRRKDFLVRRMELAKHFIRTNVEPEWMVLCLLPVLPPEL
RPIIQIDGGKLMSSDINELYRRVIYRNNTLTDLLTTSRSTPGELVMCQEKLVQEAVDTLLDN
GIRGQPMRDGHNKVYKSFSDVIEGKEGRFRETLLGKRVDYSGRSVIVVGPSLSLHQCGLP
REIAIELFQTFVIRGLIRQHVASNIGIAKSKIREKEPIVWEILQEVMQGHPVLLNRAPTLHRL
GIQAFQPILVEGRAICLHPLVCKGFNADFDGDQMAVHVPLSLEAQAEARLLMFSHMNLLS
PAIGDPISVPTQDMLIGLYVLTIGNPRGICANRYNFFNCKND---ENAPLDNDNYQATKEKEP
HFSSSYDALGAYRRKRIRLDSPLWLRWRLDQRVIIGS--REVPLEVQYESFGTYHEIYRHYLI
VRSVKKEICSIYIRTTVGHISFYREIEEAIQGFYRAYSYAS-----------MAERADLVFHNKVID
GAAMKRLISRLIDHFGMAYTSHILDQVKTLGFQQATATSISLGIDDLLTIPSKGWLVQDAEQ
QSLILEKHHHYGNVHAVEKLRQSIEIWYATSEYLRQEMNPNFRMTDPSNPVHLMSFSGAR
GNASQVHQLVGMRGLMSDPQGQMIDLPIQSNLREGLSLTEYIISCYGARKGVVDTAVRTS
DAGYLTRRLVEVVQHIIVRRTDCGTIRSISVSPRNGVPE--RIFIQTLIGRVLADDIYIGPRCIA
ARNQDIGIGLVNRFIS------FRAQPIHIRTPFTCRSTSWICQLCYGRSPTHGDLVELGEAVGIIA
GQSIGEPGTQLTLRTFHTGGVFTGGTAEHVRAPFNGKIKFNEDLVHPTRTRHGHPAFLCSID
LYVTIESQDIIHNVNIPPKSLILVQNDQYVESEQVIAEIRAGAATL--FKEKVRKHIYSESEGE-
MHWSTDVYHAPEYTYGNVHLLPKTSHLWILAVGTCR--YSIVSFSLHKDQDQINAHSLSLE
ER--YISDLS--VTKDLARHQLFSSDSSS---RGGTFDY--RPDGSISNGHWNLQYPF-ILHEN--S
DFLAKRRRNKFIIPLQYDQERDKELIPHFGVSIEIPIN--GILRRNSILAYFDDPRYR---RGITKY
-AVEVDSILK--KEDLIEYRG-TKELSPKY--VDRFFFIPEEVHVLPGSSLIMVRNNSIIGVDTPL
TLNIRSRVGGLVRVERKKKSIELKIFSGDIHFPGETDKISRHSGILIPPGTGKM--TSKE-SK-K
LKNWIYVQR--ITPTKKK--YFVSVRPVVTYEIADGINLATFFPKDLLQEGDNIQLRVVNYIL
YGNGKPIRGISHTS-QLVRTCLVLNWHQEEKSSIE----DSHASFVEIKTNDLTCNFIRIELGKS
PLLYTGKRNSVVSSGLINH---LDYLNSNPFYSKAKIPS-----LTKQKGTG----GTLLNGNK---E
CQSLKILLSSNYSRIG--PFNGLKYN---TVTKQSDPVTPI-NCLGLLGTVVL-KIANFYSS-CHL
IT---YNKTLLK-KYFLIE---NLSQTFQGII---FFLIDENRG--IYNLDPCSKTVFNPFDLNWRFFH
HDYCDETSTLIT-LGQIFCENVCLFKYGS---QKKSGQVIIVH-VDYLVIRSAKPYLATPGATV
HGHYGETLHEGDTLVTFIYEKSRSGDITQGLPKVEQVLEVRSIDSISTNLEKRVKAWNERIS
RILGNPWGFLISTELTIAQSRISLVNKIQKVYRSQGVQIHNRHLEIIVRQVTSKVLVSE---DG
MSNVFLPGELIGLLRAERAGRALDEAISYRAILLGITRASLNTQSFISEASFQETARVLAKA
ALRGRIDWLKGLKENVVLGGIIPVGTGFKKLVHHSR--QHKNIH--LEIKNKNLFEGKM--RD
ILFHHREFLSSCIPN---------------------MTRRYWNINLEEMLEAGVHFGHGTRKWNPRMAPF
ISAKRKGIHITNLTRTARFLSEACDLVFDAASKGKHFLIVGTKNKAADLVASAAIKARCHFV
NKKWLGGMLTNWSTTETRLQKFRDLRAEQRMGKLNSLPKRDAAMLKRQLSTLQTYLG
GIKYMTGLPDIVIIVDQQEEYTALRECVILGIPTICLIDTNCDPDIADISIPANDDAIASIRLILN
KLVSAIIDGRSS-YIRNHMGQKINPLGFRLGTTQNHYSFWFAQPKSFSMGLQEDEKIRDCIK
NYVQKNMKTSSG-FEGIARIEIQKRIDLIQVIIHIGFPNLLIEG--RRR--GIEELQTNVQKELNS
ANRRLNIAITRVEKPYGQPNILAEYIALQLKNRVSFRKAMKKAIELTEQTDTKGIQVQIAGR
IDGKEIARVEWIREGRVPLQTIRAKIDYCSYTVRTIYGVLGIKIWIFVDDKMSRYRGPRFKK
IRRLGALPGLTSKRPKSGSDLKNQLRSGK-RSQYRIRLEEKQKLRFHYGLTERQLLRYVHIA
GKAKGSTGQVLLQLLEMRLDNILFRLGMASTIPAARQLVNHRHILVNGRIVDIPSYRCKPQ
DIISTKDNQRSKALIQNNMTSSPPE--VPKHLTLDS--IQSKGLVNQIIDSKWIGLKINELLVVE
YYSRQTMSRRGTAEKKTAKSDPIYRNRLVNMLVNRILKHGKKSLAYQIIYRAVKKIQQKTE
TNPLSVLRQAIRGVTPDIAVKSRRVGGSTHQVPIEIGSTQGKALAIRWLLAASRKRPGRNM
AFKLSSELVDAAKGSGDAIRKKEETHKMAEANRAFAHFRMGRDTIAEVLTSIRNAEMNK

KGTVRIASTNITENIVKILLQEGFIENVRKHRENNKNFLVSTLR--HKRTRKIIYKT-----VLKR
ISRPGLRIYSNSQRIPRILGGMGIAILSTSQGIMTDREARLKGVGGEIWCYIWMIKPIPRISSR
RNGRIGSRKTGRRIPKGIIHVQASFNNTIVTVTDVRGRVVSWSSAGTSGFKGAKRGTPFAA
QSAAVNAIRTVVDQGMQRAEVMIKGPGPGRDAALRAIRRSGILLSFVRDITPMPHNGCRP
PKKRRVMPTIKQLIRNARQPIRNVTKSPALRGCPQRRGTCTRVYTITPKKPNSALRKVARV
RLTSGFEITAYIPGIGHNLQEHSVVLVRGGRVKDLPGVRYHIVRGTLDAVGVKDRQQGRSK
YGVKKPKMARKSLIQREKKRQKLEQKFHLIRRSLKKEIS---QIPSLSEKWEIHGKLQSLPRN
SAPTRLHRRCFLTGRPRGNYRDFGLSGHILREMVNTCLLPGATRSSWMVKNSFISIISQEEK
EEN--KGSVEFQVFSFTNKIRRLTSHLGLHKKDFSSQRGLRIILGKRQRLLAYLSKKNRVRY
KKLIGQLDIREPKTR--MVKLRLKRCGRKQRAIYRIVAIDVRSRREGRDLRRVGFYDPIKNK
TYSNVPAILDFLEKGAQPTRTVHDIFKKAELFK----------MDKSKQPFHKTKRSFRRRLPPIG
SGDRIDYRNMSLISRFISEQGKILSRRVNRLTLKQQRLITIAIKQARILSLLPFLNNEK--QFER
TESIPRATGPRTRNKVTRSIKKNPFVANHLLVKIEKLNMRQEKEIIVTWSRASTIIPTMIGHTI
AIHNGKEHLPIYITDRMVGHKLGEFAPTRSFGKHARNDIKSRRMILKFFLLGNPLSLCMKII
NSVVIVGLYYGFMTTFSIGPSYLFLLRAWLMEEG---TEKQISATTGFIMGQLIIFISIYYAPLH
LALGKPHTMTVLVLPYLLFNFFWNNNKNFLDYRSTTRNSIRNFNIQCIFLNNFIFQLFNHFI
LPSSTLARLVNIYMFRCNNKMLFVTSTFIGWLIGHIFFIKCVGLVLFWIRQNHSIQS----NKY
LLSELRNSLARIFSILLFITCVYYLGRMPLPIITKKLKETEENKPLVTFLFDCKRWNRPFRYIK
NDRFENAVRNEMSQYFFYTCPSDGKQRISFTYPPSLSTFSEMIERKISLYTTEKLSHED----W
VYTNEQKKHTLSNELINRIKTLEKKTLVLDVLEKRIRLCNDENEKECLPKMYDPFLNGPY
RGKITKLDSRIEIVWINKIHGLLKKEFEYQDYNDQFPGIEEIRKEVPRWSYKLTDLEEQEEE
EIRSRKAKRVVIYTDALIRYSQQSDFRRDLIKGSMRAQRRKTVIWEMFQANVHSPLFLDQI
DKT-FFLSFD-ISEMMNLVFRG-EENERITISETWDSIIFAQAIRGSMLVTQSILRKYIVLPSLII
AKNVGRMLLFQFPEWYEDLKDWNREMHVKCTYNGVQLSETEFPKDWLTDGIQIKILFPF
RLKPWRKSKKGKKNNFCFLTVWGREAELPFGSPRKQPSFFQPIWKELNNKIRKLEKFSLSL
VLRVSKKTRKWFLKISKEKTRWVNKIVL--VIKRIMKELEKINSIIS-NKTTDESTIRIRSTNW
TKHSLIEKKVKDLADRTTTIRNQIEQITKDKKKSPNKTNCADKNSELQKPIWQIFKRKSTR
LIRKFYYFLKYFIERIYSDILLCTINSLRTNAQLFIHFISCDLSSLSQAYVFYTLSQVQVNNKY
YLRSVFQYTHLFLKDRIKDYCTRGLFDYKPKHKKVDKSGINEWKNWLKSHYQYNLSQT
QWSRLVPQKWRNRVNQRCTIQKKIILDSYKKNQ--LIHYVKQEKLKKNYKYDLLAHKYM
NESSLYLDRKYFDCRILRFCFRLFDWMGMNKERLYRWFFPEFGLLYDAYKLKPWIIPIQFL
LLNFKSKKEAELDFFLKKYFLFQLRWDDSLNQRMINNIKVYCLLLRLINLKEIAISSIQRGE
MRLDVMLIQKDLSLTELIKRGIFIIEPVRLSIKWDEEFFIYQTLGISLVNKSKQNYYDLFVPE
NILSTRRRRELRILICFVFLWPNYRLEDLACMNRYWFDTNNGSRFSMSRIQMYPMKRHQF
KSWIFEFREILREIKNSHYFLDSWIKFDSVGSFTHIFFHQERFMKLFDPRILSILLS-RDS--QD
STSNRYFT---IKGVVLLVVA-VLIS----RINNRKMVERKNLYLMGLLPIPILPIRMNSIGHRNET
LEESF-WSSNINRLIVS-LLYLPKGKKISESCFMDPRESTWVLPIN----QKCIMPESNRGSRW
WSNRIGKKR--DSSCKIS---NETVAGIEISFKEKDSK-YLEFLFLSYT--DDRIRKDPDWELFDR
LSLRKKRNIINL--NSGQLFEILGKDLVCYLMSAFREKRPIEGEG-FFKQQG-AEATIQSNDIE
HVSHLFLFSRNKWGISLQNCAQFHMWQFRQDLFV---SWGK-NRHESDFLRN-VSRENLIW
LDNVWLVNKDR---FFS--KVRNVSSNIQYDS---TG--SIFVQVTDSSQLKGSS-DQSRDCFDSI
R----NEDSE------------YHKLIDQTEIQQLKERSIL-WD--PSFSQTERTEIESD--RFPK--CLFGS
S--SMSPLFT-EREKPINNHLLPEEIEEFL--GNPTRSIRSFFSDKW--SELH-LGSNPTERSTRDQ-
---KFLKKKQDVS-FVPSRRSE----NKEMVDIFKIITYLQN--TVSIHPISSDPGCDMVVPKDEP

D-MDSSNKISFLNKNPFLD--LFHLFYDRNNGGYTL--RHDF-ES-EERFQEMADLFTLSITEP-
DLVYHRGFAFSIDSYGLDQKK--LLNRVFNSRDESKKKSL---LVLPPLFY--EENESFYRRIRK
KSVR-IYCGNYLEDPKLKIVVFASNNIMEAVNKYRLIRNMIQIQYNTYG--YIRNVSNRFFL
M---NRSDRNYEYGIQ---KDQIGNDTLNHITIMKYTINQHLSNLKK-SQKKWFDP----LFSRTE
RSINLDPDAYRYKWSNGNNNFQEHLEHFVSEQGN---HFQVVFDRLGINQYSIDWSKAIDK
QDFSKSLRFFLSKA---------LLFFS-------KSLRFFLSKTLPFFFVSIGNIPIHRSE-IHIY-ELKGT
NDQLCNQLLESIGVQIVHLNKLKP--FLLD---DHDTS-QRPKFLINGRTILPFLFNKIP-KW-MI
DSFHT--RNNRRKSFDNTDSYFSMISHDRDNWLNPVKPFHRSSLISSFYKANRLRFLNNPHH
FWFY--CNKGFPFYV----EKTCINNYD--LTY-GQFLNILFIRNK-IFSLCVGKGKKTHIFLE-REA
ILPIDSQVSDIFISNDFPKSGDET-YNLYK---SFHFPIRPDPFVR--RAIYSIADISATPLTEEQIVH
FERTSCQPFSDMNLSDSEGKNSHQYLS--FNSNMGLIHTPCSEKYLP--SGKRKK--------QSL
CLK--KCVDKQQMYRTFQRDSALSNLSKWNLLQTYMPWFLTSTGCKYLNFTLLDAFSDSL
--PILSSS-PKFVSIFHDIMHGSDISWPIRQKKWW----AILSQWTP-ISEISSKCLQN--LLLSEETI
HRNNESPVPLIWTHLRSTNAREF-LYSILFFLLVAGY-LVRIHLLFVSR--ASSELQTELEKIKS
LMIPSYMIELRKLLYRYPTSELNSFWLKNLFRVALEQLGDSLEEIRGYASDGNMLLGRGP---
--AYG-VKSIRSKNKYLNIN---LIDLI-SIIPNPVNRITFSRNTRHLSRTSKEIYALIRKRKNVNG
DWIDDKIESWVANSDSIDDDEREFLVQFS--TLTT--EKRIDQILLSLT-HSDHLS-----KNDFGY
QMIEQPGSIYLRYLVDIH--KKSLMNYEFNRSCLAERRIFLAHYQTITY--SQTSCGANSFHFR
SHG---KPFSLRLALSPS-RGILVIGSIGTGRSYLVKYLATNSYVPFITVFPNKFLDDKPKGYLI
D-----DID-D--SNDID----LDDTDDIDIEDSD----------DIDNDLETELLTMTNVL---TMYMT---S
KIDRFDITPQFELAKAMSPCIIWIPNIHDLYVNESNYLSLGLLVNYLS---RDCERSS-TRNILVI
ASTHIPQKVDPALIAPNKSNTCIKIRRLLIPQQRKHFFILSYTRGFHLEKKMFHT--NGFGSIT
VGSNARDLVALINEALSISITQKKSIIETNTIRSALHRQTWDLR---SQVRSVQDHGILFYQIG
RAVAQNVLLSNCPIDPISIYMKKK--SCKERDSYLYKWYFELGTSIKKLTILLYLLSCSAGSV
AQDLWSPS--GPDEK-NGITSFGFVENDS-DLVHGLLEVEGALVG--FSRKEKDCSQFDNN--R
VTLLLRSEPRN-PLDMMQNGSCSIVDQRFLYEK--YESEFEE-GEG--ALDPQ----QIEED----LL
NH--IVWAP--RIWRP---CGNLFDCIERTNELGFP-YWARSFR---DKRIIFHKENELQQKDSEFL
QSGTMQYQTRDRSSKEQGFFRISQFIWDPADPFFFLFK-DQPFVSVFSRREFFADEEMSKGL
ITSQKNPPTSIYKRWFI--KNTQEKHFELLIHRQRWLRT---NSSLS--NRSFCSN--TLSESYQYL
SNLFLSN-GRLLDQMTKTLLRKRWLFPDEMKHLI--DVTGKRFPIPMPRSR-INGNFIDKTFSI
VANILLRIIPTTSGEKEAFTYYRDG--MSAQSEGNYAEALQNYYEATRPEIDPYDRSYILYNI
GLIHTSNGEHTKALEYYFRALERNPFLPQAFNNMAVICHY-RGEQAILQGDSEIAEAWSDQ
AAEYWKQAIALTPGNYIEAHNWLKITRRFE--MNWRSEHIWIEFITGSRKTSNFFWACILFL
GSLGFLVVGTSSYLGRNLISVFPSQQISFFPQGIVMSFYGIAGLFISSYLWSTILWNVGSGYD
RFDRKEGIVCIFRWGFPGKNRRIVLRFLMSDVQSIRVEVKEGLYTRRVLYMEVRGQGTIPL
TRTDEN-LTPREMEQKAAELAYFLRVPIEGF
>Carica
MEKSWFNSMLSLSKSMDS-LPIENTSISEDPVINDPDKNIYNYSNVDHLVGAKDIRNFISDD
TFLVRDSNRDSYSIYFDIENNNFEIDNDH--SFLSELESFFYSYRNSSYMNNRSKSD--HYDPY
MYDTKYSWTNHINSCIDSYLR--SQICIDSSILSGSDNYNDSYIYNYICSSENSNIRTNTNRSD
LTIKESSN-LDITQKYRHLWVQCENCYGLNYKKFLKSKMNICEQCGYYLKMSSSDRIELLV
DPGTWNPMDENMVSLDPIEFHSEEEPYKDRIDSYQRKTGLTEAVQTGTGQLNGIPIALGV
MDFQFMGGSMGSVVGEKITRLIEYATNEFLPLIIVCASGGARMQEGSLSLMQMAKISSALY
DYQSNKKLFYVSILTSPTTGGVTASFGMLGDIIIAEPNAYIAFAGKRVIEQTLNKTVPEGSQ

AAEYLFHKGLFDSIVPRNPLKGVLSELFQLHGFFPLNQNSIKMVTIRADEISNIIRERIEQYN
REVKVVTIGTVLQVGDGIARIYGLDEVMAGELVEFEEGTIGIALNLESNNVGVVLMGDGL
MIQEGSSVKATGKIAQIPVSEAYLGRVINALAKPIDGRGEISASESRLIESPAPGIISRRSVYEP
LQTGLIAIDSMIPIGRGQRELIIGDRQTGKTAVATDTILNQQGQNVICVYVAIGQKASSVAQV
VTTLQERGAMDYTIVVAETADSPATLQYLAPYTGAALAEFFMYLKQHTLIIYDDPSKQAQ
AYRQMSLLLRRPPGREAYPGDVFYLHSRLLERAAKLSSQLGEGSMTALPIVETQSGDVSA
YIPTNVISITDGQIFLSADLFNAGIRPAINVGISVSRVGSAAQIKAMKQVAGKLKLELAQFAE
LEAFAQFASDLDKATQNQLARGQRLRELLKQSQSAPLTVEEQIMTIYTGTNGYLDSLEIGQ
VRKFLVELRTYLKTNKPQFQEIISSTKTFTEEAETILKEAIQEQMERFLLQE--MRINPTASGP
GVSTLEKKNLGRIAQIIGPVLDVAFPPGKMPNIYNALVVKGRDTVGQQINVTCEVQQLLG
NNRVRAVAMSATDGLTRGMEVIDTGAPLSVPVGGATLGRIFNVLGEPVDNLGPVDTRTTS
PIHKSAPAFIQLDTKFSIFETGIKVVDLLAPYRRGGKIGLFGGAGVGKTVLIMELINNIAKA
HGGVSVFGGVGERTREGNDLYMEMKESGVINEQNLAESKVALVYGQMNEPPGARMRVG
LTALTMAEYFRDVNEQDVLLFIDNIFRFVQAGSEVSALLGRMPSAVGYQPTLSTEMGSLQE
RITSTKEGSITSIQAVYVPADDLTDPAPATTFAHLDATTVLSRGLAAKGIYPAVDPLDSTSTM
LQPRIVGEEHYETAQRVKQTLQRYKELQDIIAILGLDELSEEDRLTVARARKIERFLSQPFFV
AEVFTGSPGKYVGLAETIRGFKLILSGELDGLPEQAFYLVGNIDEATAKATQLEMESKLKK
MTLNLCVLTPNRIVWDSEVKEIILSTNSGQIGVLPNHAPIATAVDIGILRIRLNDQWLTMAL
MGGFARIGNNEITILVNDAEKSSDIDPQEAQQTLEIAEANLRKAEGKRQTIEANLALRRAR
TRVEAINAISMKNVTDSFVFLGHWPSAGSFGFNTDILATNPINLSVVLGVLIFFGKGVLSDL
LDNRKQRILNTIRNSEELRDGAIEQLEKARARLRKVEMEAEQFRVNGYSEIEREKWNLINS
TSKTLEQLENYKNETIQFEQQRAINQVRQRVFQQALQGAIGTLNSCLSNELHLRTINANIG
MFGAMKEITDMNPLISAASVIAAGLAVGLASIGPGVGQGTAAGQAVEGIARQPEAEGKIR
GTLLLSLAFMEALTIYGLVVALALLFANPFVMDVISCSSNTLK-LYDISGVEVGQHLYWQIG
GLQVHAQVLITSWVVIAILLGSAIIAVRNPQTIPTAGQNFFEYVLEFIRDVSKTQIGEEYGP
WVPFIGTMFLFIFVSNWSGALLPWKIIQLPHGELAAPTNDINTTVALALLTSVAYFYAGLTK
KGLGYFSKYIQPTPILLPINILEDFTKPLSLSFRLFGNILADELVVVVLVSLVPLVVPIPVMFL
GLFTSGIQALIFATLAAAYIGESMEGHHMIFSTLEHILIHISFSVVSIVITIYFLTLLV-EIVGLY
DSSDKGMIVTFFCITGLLAARWIYSGHFPLSNLYESLIFLSWSFSIIHMVCYFNKHKNNLNA
ITGPSAILTQGFATSGLLNKMSQSVILVPALQSQWLMMHVSMMVLGYAALLCGSLLSVALL
VITFRKVIRIFGKS-KIQYMNERSNILL--SFLSSRNYYRYQLIQQLDHWSYRIISLGFIFLTIGI
LSGAVWANEAWGSYWNWDPKETWAFITWTIFAIYLHIRTNINLGGINSAIVASMGFLIIWIC
YFGVNLLGIGLHSYGSFTLTMAKKKAFIPFLYLASIVFLPWWISLSCNKSLKTWITNSWNT
RQSETFLNDIQEKSLLEKFIQLEELFLLDEMIKEDPETHLQKLRIG-HKETIQFIQMYNEDRI
HTILHFSTNILCFVILSGYSILGNEELLIINSWVQEFLYNLSDTIKAFSILLLTDLCIGFHSPHG
WELMIGYIYKDFGFAHNDQIISGLVSTFPVILDTIFKYWIFRYLNRVSPSLVVIYHSINDMPIG
VPKVPFRSPGEEDASWVDVYNRLYRERLLFLGQEVDSEISNQLIGLMVYLSIESDTKDLYL
FINSPGGWVIPGVAIYDTMQFVQPDVQTICMGLAASMGSFILVGGEITKRLAFPHARVMIH
QPASSFYEAQVGEFILEAEELLKLRETLTRVYVQRTGKPLWVVSEDMERDVFMSATEAQA
Y G I V D L V A V
Q??MEEFQV
YLELDRFRQHDFLYPLIFREYIYALAHDHSLSANKYKYSSVIVKRFIFRMYQQNHLIISAND
SNQKQFFGNNLYYEMISAVFAVIAEIPFSLRLVSSLPAKSHNLQSIHSIFPFLEDTFSHLNYVL
DVLIPYPIHLETLVQTLRYWIKDTSSLHLLRFCLYEYCNWNWKSLK-----SIPRLFLFLYNSH

VCEYESIFFFLRNQSSHLRSTSFGVLLERILFYGKIEHLIKVFVNQDILWLVKDPFMHYIRYQ
GKSILASKDTPLLMNKWKYYFVSLWQCHFYVWSQSRRVRIKQLSKDYLDFLGYFSSLRF
NPLVVRSQMLENSFLIDN--AIKKFDTKIPIIPMIGSLAKARFCNALGHPISKPAWADSADSDI
IDRFVRICRNLSHYYSGSSKKKNLYRIKYILRLSCVKTLARKHKSTVRTFLKRVGSGFLEEF
FTEEEEVLSLIFPRHIWYLDIICINDLVNHEMIIDTI-EVQDIN-SFSRLES-LKEFYGIIWMLVPI
LALVLGITTGVLVIVWLEREISAGIQQRIGPEYAGPLGILQALADGTKLLFKENLLPSRGNT
RLFSIGPSIAVISILLSYSVIPFGNHLVLADLNIGIFLWIAISSIAPIGLLMSGYGSNNKYSFLGG
LRAAAQSISYEIPLTLCVLSISLLSNSSSTVDIVEAQSKYGFWGWNLWRQPIGFIIFLISSLAE
CERLPFDLPEAEEELVAGYQTEYSGIKFGLFYVASYLNLLVSSLFVTVLYLGGWNISIPYIFV
PELFEM---DGILGTTIGIFITLAKTYLFLFISIATRWTLPRLRMDQLLNLGWKFLLPISLGNLL
LTTSSQLLSLMIWHVQNENFILDSTRIFMKAFHLLLFDGSLIFPECILIFGLILLLMIDSTSDQ
KDIPWLYFISSTSLVMSITALLFRWREEPMISFSGNFQTNNFNEIFQFLILLCSTLCIPLSVEYI
ECTEMAITEFLLFILTATLGGMFLCGANDLITIFVAPECFSLCSYLLSGYTKKDVRSNEATTK
YLLMGGASSSILVHGFSWLYGSSGGEIELQEIVNGLINTQMYNSPGISIALIFITVGIGFKLSP
APSHQWTPDVYEGSPTPVVAFLSVTSKVAASASATRIFDIPFYFSSNEWHLLLEILAILSMIL
GNLIAITQTSMKRMLAYSSIGQIGYVIIGIIVGDSNGGYASMITYMLFYISMNLGTFACIVSF
GLRTGTDNIRDYAGLYTKDPFLALSLALCLLSLGGLPPLAGFFGKLHLFWCGWQAGLYFL
VSIGLLTSVLSIYYYLKIIKLLMTGRNQEITPHVRNYRRSPLRSNNSIELSMIVCVIASTIPGIS
MNPIIAIAQDTLFMFLLYEYDIFWAFLIISSAIPILAFLISGVLAPINKGPEKLSSYESGIEPMG
DAWLQFRIRYYMFALVFVVFDVETVFLYPWAMSFDVLGVSVFIEALIFVLILIVGSVYAWR
KGALEWSTNYFPWLTIIVVLPISAGSLIFFLPHRGNKVIRWYTICICVLELLLTTYAFCYHFQ
LDDPLVQLTEDYKWINFFDFYWRLGIDGLSIGPILLTGFITTLATLAAWPVTRDSRLFHFLM
LAMYSGQIGSFSSRDLLLFFLMWELELIPVYLLLSMWGGKKRLYSATKFILYTAGGSIFLLI
GVLGIGLYGSTEPTLNFETLTNQSYPIALEIIFYIGFLIAFAVKSPIIPLHTWLPDTHGEAHYST
CMLLAGILLKMGAYGLVRINMELLPHAHSLFSPWLVVVGTMQIIYAASTSSGQRNLKKRIA
YSSVSHMGFIIIGIGSITDPGLNGAILQIVSHGFIGAALFFLAGTSYDRIRLVYLDEMGGMAI
SMPKIFTMFSILSMASLALPGMSGFVAELIVFFGIITSQKYFLMAKILITFVMAIGMILTPIYS
LSMSRQMFYGYKLINAQNFSFFDSGPRELFVSISILLPVIGIGIYPDFLLSLSSDKVEAILSNY
FNRMLLEHVLVLSAYLFSIGIYGLITSRNMVRALMCLELILNAVNMNFVTFSDFFDKRQLK
GDIFSIFVIAIAAAEAAIGSAIVSSIYRNRKSTRINQSTLLNKMEHIYQYSWMIPFIPLPVPILL
GMGLLLFPTATKNLRRMWAFLSIFLLSIVMIFSIDLSIQQITGSSAYQYVWSWTINNEFSFEF
GNLIDPLTSIMSILITTVGILVLIYSDSYMSHDQGYLRFFAYMSFFNTSMLGLVTSSNLIQVYI
FWELVGMCSYLLIGFWFTRPIAANACQKAFVTNRVGDFGLLLGILGLYWITGSFEFQDLFE
IFNN-QVHFLFVTLCAFLLFAGPVAKSAQFPLHVWLPDAMEGPTPISALIHAATMVAAGIFL
VARLLPLFIGIPYIMYLISLIGIITVLLGATLALAQKDIKRGLAYSTMSQLGYMMLALGMGS
YRAALFHLITHAYSKALLFLGSGSIIHSMEAVVGYSPVKSQNMVLMGGLTKHVPITKTTFL
LGTLSLCGIPPLACFWSKDEILNDSWLYSPIFAVIACSTAGLTAFYMFRIYLLTFDGHLNIHF
QNYSGKKSSSFYSISIWGKEEAKPINR-LVPLLTMNFFWAVKT-TFSYPHESDNTILFPMLILV
LFTLFVGAIG--FDFDILSKLLTPSINLLHQNSN-FVDW-EFFKNATFSVSIAFFGIFIAFFLYKPA
YSSLQNLNLLNSFAILWDKIINFIYDWSYNRGYIDTFYTISLTGGIRGLAELTHFFDRRVIDGI
TNGVGITSFFIGEGIKYVGGS-RISSYLLLYLFYVLIFLFIYYMDLLGPIHDFLLVFLGSGLILG
GLGVVLFPNPIYSAFSLGFVLACISLFYILSNSHFVAAAQLLIYVGAINVLIIFAVMFMNGSE
YYRDFNLWTIGDGITSLVCTSIFLSLITTILDTSWYGVIWTTRLNQILEQDLISSSQQIGIHLS
TDFFLPFELISIILLVALIGAIAVARQMNIPATGKDFMIVNMGPHHPSMHGVLRLIVTLDGED

VIDCEPILGYLHRGMEKIAENRTIIQYLPYVTRWDYLATMFTEAITVNGPEQLGNIQVPKR
ASYIRVIMLELSRIASHLLWLGPFMADIGAQTPFFYIFRERELVYDLFEAATGMRMMHNYF
RIGGIAADLPHGWIDKCLDFCDYFLTGVAEYQKLITRNPIFLERVEGVGIIGGEEAINWGLS
GPMLRASGIEWDLRKVDHYECYDEFDWEVQWQKEGDSLARYLVRISEMTESIKIIQQALE
GIPGGPYENLEIRCFDRKKDPEWNDFDYRFISKKPSPTFELPKQELYVRVEAPKGELGIFLIG
DQSGFPWRWKIRPPGFINLQILPQLVKRMKLADIMTILGSIDIIMGEVDRMFPMVTGFMNY
GQQTIRAARYIGQGFIITLSHANRLPVTIQYPYEKLITSERFRGRIHFEFDKCIACEVCVRVC
PINLPVVDWKFETDIRKKRLLNYSIDFGICIFCGNCVEYCPTNCLSMTEEYELSTYDRHELN
YNQIALGRLPMSVIDDYTIRTILNS----------------MQGHLSVWLVKHGLVHRSLGFDYQGIE
TLQIKPEDWHSIAVILYVYGYNYLRSQCAYDVAPGGLLASVYHLTRIEYGVDQPEEVCIKV
FAPRSNPRIPSVFWVWKSADFQERESYDMLGISYDNHPRLKRILMPESWIGWPLRKDYIAP
NFYEIQDAH---MNSIEFPLLDRTTQNSVISTTL
NDLSNWSRLSSLWPLLYGTSCCFIEFASLIGSRFDFDRYGLVPRSSPRQADLILTAGTVTMK
MAPSLVRLYEQMPEPKYVIAMGACTITGGMFSTDSYSTVRGVDKLIPVDVYLPGCPPKPE
AVIDAITKLRKKISREIYEDRIRSQQGNRCFTTNHKFLFVRSTHTGNYDQELLYQPSSTSEIP-
ETFFKYKSSVSSRELVNMQTRNTLSWIKEEITRSISVSLMIYIITWASISNAYPIFAQQGYENP
REATGRIVCANCHLANKPVDIEVPQAVLPDTVFEAVVRIPYDMQLKQVLANGKKGGLNV
GAVLILPEGFELAPPDRISPEMKEKIGNLSFQNYRPTQKKILVIGPVPGQKYSEITFPILSPDP
ATNKDVHFLKYPIYVGGNRGRGQIYPDGRKSNNTVYNATAAGIVSKILRKEKGGYEITIVD
ASDGRQVVDIIPPGPELLVSEGESIKLDQPLTSNPNVGGFGQGDAEIVLQDPSRVQGLLFFL
ASVVLAQIFLVLKKKQFEKVQLSEMNFMSKVYDWFEERLEIQAIADDITSKYVPPHVNIFY
CLGGITLTCFLVQVATGFAMTFYYRPTVTEAFASVQYIMTEANFGWLIRSVHRWSASMMV
LMMILHVFRVYLTGGFKKPRELTWVTGVVLAVLTASFGVTGYSLPWDQIGYWAVKIVTGV
PEAIPVIGSPLVELLRGSASVGQSTLTRFYSLHTFVLPLLTAVFMLMHFLMIRKQGISGPLM
GVTKKPDLNDPVLRAKLAKGMGHNYYGEPAWPNDLLYIFPVVILGTIACNVGLAVLEPSM
IGEPADPFATPLEILPEWYFFPVFQILRTVPNKLLGVLLMVSVPAGLLTVPFLENVNKFQNPF
RRPVATTVFLIGTAVALWLGIGATLPIDKSLTLGLFMIEVFLFGIVLGLIPITLAGLFVTAYLQ
YRRGDQLDLMPTITSYFGFLLAALTITSALFIGLSKIRLIMDIVSLAWAALMVVFTFSLSLVV
WGRSGLMIIRSPEPEVKILVDRDPIKTSFEAWAKPGHFSRTIAKGPDTTTWIWNLHADAHD
FDSHTSDLEEISRKVFSAHFGQLSIIFLWLSGMYFHGARFSNYEAWLSDPTHIGPSAQVVW
PIVGQEILNGDVGGGFRGIQITSGFFQLWRASGITSELQLYCTAIGALIFAALMLFAGWFHY
HKAAPKLAWFQDVESMLNHHLAGLLGLGSLSWAGHQVHVSLPINQFLNAGVDPKEIPLP
HEFILNRDLLAQLYPSFAEGATPFFTLNWSKYAEFLTFRGGLDPVTGGLWLTDIAHHHLAIA
ILFLIAGHMYRTNWGIGHGLKDILEAHKGPFTGQGHKGLYEILTTSWHAQLSLNLAMLGS
LTIIVAHHMYSMPPYPYLATDYGTQLSLFTHHMWIGGFLIVGAAAHAAIFMVRDYDPTNR
YNDLLDRVLRHRDAIISHLNWVCIFLGFHSFGLYIHNDTMSALGRPQDMFSDTAIQLQPVF
AQWIQNTHALAPGATAPGETASTSLTWGGGDLVAVGGKVALLPIPLGTADFLVHHIHAFTI
HVTVLILLKGVLFARSSRLIPDKANLGFRFPCDGPGRGGTCQVSAWDHVFLGLFWMYNSI
SVVIFHFSWKMQSDVWGSINDQGVVTHITGGNFAQSSTTINGWLRDFLWAQASQVIQSYG
SSLSAYGLFFLGAHFVWAFSLMFLFSGRGYWQELIESIVWAHNKLKVAPATQPRALSIVQG
RAVGVTHYLLGGIATTWAFFLARIIAVGMALRFPRFSQGLAQDPTTRRIWFGIATAHDFESH
DDITEERLYQNIFASHFGQLAIIFLWTSGNLFHVAWQGNFEAWVQDPLHVRPIAHAIWDPH
FGQPAVEAFTRGGALGPVNIAYSGVYQWWYTIGLRTNEDLYTGALFLLFLSAISLIGGWLH
LQPKWKPSVSWFKNAESRLNHHLSGLFGVSSLAWTGHLVHVAIPGSRGEYVRWNNFLDV

LPHPQGLGPLFTGQWNLYAQNPDSSSHLFGTSQGSGTAILTLLGGFHPQTQSLWLTDIAHH
HLAIAFLFLIAGHMYRTNFGIGHSIKDLLEAHIPPGGRLGRGHKGLYDTINNSIHFQLGLAL
ASLGVITSLVAQHMYSLPAYAFIAQDFTTQAALYTHHQYIAGFIMTGAFAHGAIFFIRDYNP
EQNEDNVLARMLDHKEAIISHLSWASLFLGFHTLGLYVHNDVMLAFGTPEKQILIEPIFAQ
WIQSAHGKTSYGFDVLLSSTTGPAFNAGRSIWLPGWLNAVNE--NSNSLFLTIGPGDFLVHH
AIALGLHTTTLILVKGALDARGSKLMPDKKDFGYSFPCDGPGRGGTCDISAWDAFYLAVF
WMLNTIGWVTFYWHWKHITLWQGNVSQFNESSTYLMGWLRDYLWLNSSQLINGYNPF
GMNSLSVWAWMFLFGHLVWATGFMFLISWRGYWQELIETLAWAHERTPLANLIRWRDKP
VALSIVQARLVGLAHFSVGYIFTYAAFLIASTSGKFGMSHSVKIYDTCIGCTQCVRACPTDV
LEMIPWDGCKAKQIASAPRTEDCVGCKRCESACPTDFLSVRVYLGPETTRSMGLAYMT--N
LPSIFVPLVGLVFPAIAMASLSLHVQKNKIVMRDLKTYLSVAPVLSTLWFGSLAGLLIEINR
LFPDALTFPFFSFMTAILERRESESLWGRFCNWITSTENRLYIGWFGVLMIPTLLTATSVFIIA
FIAAPPVDIDGIREPVSGSLLYGNNIISGAIIPTSAAIGLHFYPIWEAASVDEWLYNGGPYELI
VLHFLLGVACYMGREWELSFRLGMRPWIAVAYSAPVAAATAVFLIYPIGQGSFSDGMPLGI
SGTFNFMIVFQAEHNILMHPFHMLGVAGVFGGSLFSAMHGSLVTSSLIRETTENESANEGY
RFGQEEETYNIVAAHGYFGRLIFQYASFNNSRSLHFFLAAWPVVGIWFTALGISTMAFNLN
GFNFNQSVVDSQGRVINTWADIINRANLGMEVMHERNAHNFPLDLAAVEAPSTNGMGLP
WYRVHTVVLNDPGRLLSVHIMHTALVAGWAGSMALYELAVFDPSDPVLDPMWRQGMFV
IPFMTRLGITNSWGGWSIT-GGTITNPGLWSYEGVAGAHIVFSGLCFLAAIWHWVYWDLEI
FCDERTGKPSLDLPKIFGIHLFLSGVACFGFGAFHVTGLYGPGIWVSDPYGLTGKVQPVNPA
WGVEGFDPFVPGGIASHHIAAGTLGILAGLFHLSVRPPQRLYKGLRMGNIETVLSSSIAAVF
FAAFVVAGTMWYGSATTPIELFGPTRYQWDQGYFQQEIYRRVSAGLAENQSLSEAWSKIP
EKLAFYDYIGNNPAKGGLFRAGSMDNGDGIAVGWLGHPIFRDKEGRELFVRRMPTFFETF
PVVLVDGDGIVRADVPFRRAESKYSVEQVGVTVEFYGGELNGVSYSDPATVKKYARRAQ
LGEIFELDRATLKSDGVFRSSPRGWFTFGHASFALLFFFGHIWHGARTLFRDVFAGIDPDLD
AQVEFGAFQKLGDPTTRRQVV------------VETLFNGTLALAGRDQETTGFAWWAGNARLIN
LSGKLLGAHVAHAGLIVFWAGAMNLFEVAHFVPEKPMYEQGLILLPHLATLGWGVGPGG
EVIDTFPYFVSGVLHLISSAVLGFGGIYHALLGPETLEESFPFFGYVWKDRNKMTTILGIHLI
LLGIGAFLLVFKALYFGGVYDTWAPGGGDVRKITNLTLSPSVIFGYLLKSPFGGEGWIVSV
DDLEDIIGGHVWLGSICIFGGIWHILTKPFAWARRALVWSGEAYLSYSLGALSVFGFIACCF
VWFNNTAYPSEFYGPTGPEASQAQAFTFLVRDQRLGANVGSAQGPTGLGKYLMRSPTGE
VIFGGETMRFWDLRAPWLEPLRGPNGLDLSRLKKDIQPWQERRSAEYMTHAPLGSLNSV
GGVATEINAVNYVSPRSWLATSHFVLGFFLFVGHLWHAGRARAAAAGFEKGIDRDLEPVL
FMTPLNMTIALGKFTKDENDLFDIMDDWLRRDRFVFVGWSGLLLFPCAYFAIGGWFTGT
TFVTSWYTHGLASSYLEGCNFLTAAVSTPANSLAHSLLLLWGPEAQGDFTRWCQLGGLWT
FVALHGAFGLIGFMLRQFELARSVQLRPYNAIAFSGPIAVFVSVFLIYPLGQSGWFFAPSFG
VAAIFRFILFFQGFHNWTLNPFHMMGVAGVLGAALLCAIHGATVENTLFEDGDGANTFRA
FNPTQAEETYSMVTANRFWSQIFGVAFSNKRWLHFFMLFVPVTGLWMSALGVVGLALNL
RAYDFVSQEIRAAEDPEFETFYTKNILLNEGIRAWMAAQDQPHENLIFPEEVLPRGNALMS
GSTGERSFADIITSIRYWVIHSITIPSLFIAGWLFVSTGLAYDVFGSPRPNEYFTESRQGIPLIT
GRFDSLDQLDEFSRSFMTIDRTYPIFTVRWLAVHGLAVPTVSFLGSISAMQFIQRMATQTVE
DSS-RSGPRRTVVGDLLKPLNSEYGKVAPGWGTTPLMGVAMALFAVFLSIILEIYNSSVLLD
GISMNMLTLKLFVYTVVIFFVSLFIFGFLSNDPGRNPGREEMADTTGRIPLWIIGTVTGIPVI
GLIGIFFYGSYSGLGSSLMLNIFNLICICFNSALFSSSFLFAKLPEAYAFLNPIVDVMPVIPVLF

FLLAFVWQAAVSFRMTQSNPNEQNVELNRTSLYWGLLLIFVLAVLFSNYFFNMEVNILAFI
ATALFILVPTAFLLIIYVKTVSQSDMETATLVAISISGLLVSFTGYALYTAFGQPSQQLRDPFEE
HGDMEALVYTFLLVSTLGIIFFAIFFREPPKVPTKKTKMTIAFQLAVFALIATSSILLISVPVVF
ASPDGWSSNKNVVFSGTSLWIGLVFLVGILNSLIS-------MSPQTETKASVGFKAGVKDYKL
TYYTPDYQTKDTDILAAFRVTPQPGVPPEEAGAAVAAESSTGTWTTVWTDGLTSLDRYKG
RCYGIEPVPGEESQFIAYVAYPLDLFEEGSVTNMFTSIVGNVFGFKALRALRLEDLRIPPAYI
KTFQGPPHGIQVERDKLNKYGRPLLGCTIKPKLGLSAKNYGRAVYECLRGGLDFTKDDEN
VNSQPFMRWRDRFLFCAEAIYKAQAETGEIKGHYLNATAGTCEEMIKRAVFARELGAPIIM
HDYLTGGFTANTSLAHYCRDNGLLLHIHRAMHAVIDRQKNHGMHFRVLAKALRLSGGD
HIHAGTVVGKLEGERDITLGFVDLLRDEFVEKDRSRGIFFTQDWVSLPGVLPVASGGIHV
WHMPALTEIFGDDSVLQFGGGTLGHPWGNAPGAVANRVALEACVQARNEGRDLAREGN
EIIREASKWSPELAAACEVWKEIKFEFPAVDTI--MAIHLYKTSTPSTRNRAVDSQVKSNPRN
NLIYGQHRCGKGRNARGIITARHRGGGHKRLYRKIDFRRNEKDIYGRIVTIEYDPNRNAYI
CLIHYGDGEKRYILHPRGAIIGDTIVSGTEVPIKMGNALPLTDMPLGTAIHNIEITLGKGGQL
ARAAGAVAKLIAKEGKSATLKLPSGEVRLISKNCSATVGQVGNVGVNQKSLGRAGSKCW
LGKRPVVRGVVMNPVDHPHGGGEGRAPIGRKKPATPWGYPALGRRSRKRNKYSDNLILR
RRSKMIQPQTHLNVADNSGARELMCIRIIGAS-NRRYAHIGDVIVAVIKEAVPNTPLERSEVI
RAVIVRTCKELKRNNGMIIRYDDNAAVVIDQEGNPKGTRIFGAIPRELRQLNFTKIVSLAPE
VLMLSPKRTRFRKQHRGRLKGISYRGNRICFGRYALQALEPTWITSRQIEAGRRAMTRNIR
RGGKIWVRIFPDKPVTLRPTETRMGSGKGSPEYWVAVVKPGRILYEMSGVAENIARRAISI
AASKMPIKTQFIISGMTRIKRGYIARRRRTKIRLFASSFRGAHSRLTRTITQQRIRALVSAHR
DRGRKKRDFRRLWITRINAVIRGD-GVSYSYNRFIHNLYKKQLLLNRKILAQIAISNRSCLY
TISNEIRK-----------MISKVYALGQHIRMSAHKARRVIDQIRGRSYEETLMILELMPYRACYP
IFKLVYSAAANASHNMGFNETSLVVSKAEVNEGTTVKKFKPRARGRSYPIKRPTCHITIVL
EDTTVYGGLWDKKMDGIKYAVFTDKSIRLLGKN-QYTSNVESGSTRTEIKHWVELFFGVK
VIAMNSHRLPGKSRRMGPIMGHTMHYRRMIITLQPGYSIPPLRKKRTMAVPKKRTSISKKR
IRKNLWKKKGYWAALKAFSLAKSLSTGNSKSFFVRQINNMAKGKDARVTIILECTSC-VR
N--VNKESTGISRYITQKNRHNTPSRLELRKFCPYCYKHTIHGEIKKMKIRASVRKICEKCRL
IRRRGRIIVICSNPRHKQRQGMVREKVKVSTRTLQWKCVESRTDSKRLYYGRFILSPLMKG
QADTIGIAMRRALLGEIEGTCITRAKSE--KIPHEYSTIVGIQESVHEILMNLKEIVLRSNLYG
TRDAFICVKGPGYVTAQDIVLPPSVEIVDNTQHIASLTEPIDLRIGLQIERNRGYGIKTPNNF
Q-DG-SYPIDAVFMPVQNANHSIHSYGTGNEKQEILFLEIWTNGSLTPKEALHEASRNLINLF
IPFLHAEEE--KFYLENNQ-HKVTLPFFPFHDRLA-KLRKKK-TEIAFQYIFIDQLELPPRIYNC
LKKSNIHTLLDLLNNSQEDLMKIEHFRTEDVKQILSILEKK-----------MLGDGN-EGMSTIPG
LNQIQFEGFCRFIDQGLTEELYKFPKIEDTDQEIEFQLFVETYQLVEPLIKERDAVYESLTYSS
ELYVSAGLIWKTSRNMQEQTIFIGNIPLMNSLGTSIVNGLYRIVINQILQSPGIYYRSELDHN
GISVYTGTIISDWGGRLELEIDKKARIWARVSRKQKISILVLSSAMGSNLREILENVCYPEIF
LSFLTDKEKKKIGSKENAILEFYQQFSCVGGDPIFSESLCKELQKKFFHQRCELGRIGRRNM
NRRLNLNIPQNNTFLLPRDILAAADHLIGMKFGMTLDD--MNHLKNKRIRSVADLLQDQF
GLALVRLENVVKGTICGAIR-HKLI-PTPQNLVTSTP----LTTTYESFFGLHPLSQVLDRTNPLT
QIVHGRKLSYLGPGGLTGRTANFRIRDIHPSHYGRICPIDTSEGINVGLIGSLAIHARIGDWG
-SLESPFY---KIFEKSKKA--RMLYLSPSKDEYYMVAAGNSLALNQGIQEEQVVPARYRQEFL
TIAWEEVHLRSIFPFQYFSIGASLIPFIEHNDANRALMSSNMQRQAVPLSRSEKCIVGTGLER
QVALDSGVPAIAEHEGKIVSTDTDKIILSGNGDTLNIPLVMYQRSNKNTCMHQTPQVRGG

KCIKKGQILADGAATVGGELALGKNVLVAYMPWEGYNFEDAVLISERLVYGDIYTSFHIRR
YEIQTHVTSQG-PERITNEIPHLEGHLLRNLDKNGIVRLGSWVETGDILVGKLTPQVAKE-SS
YAPEDRLLRAILGIQVSTSKETCLKLPIGGRGRVIDVRWIQKKG-GSSYNPEMIRVYISQKRE
IKVGDKVAGRHGNKGIISKILPRQDMPYLQDGRPVDMVFNPLGVPSRMNVGQIFECSLGL
AGSLLDRHYRIAPFDERYEQESSRKLVFSELYEASKQTANPWVFEPEYPGKSRIFDGRTGD
PFEQPVIIGKPYTLKLIHQVDDKIHGRSSGHYALVTQQPLRGRSKQGGQRVGEMEVWALE
GFGVAHILQEMLTYKSDHIRARQEVLGTTIIGGTIPKPEDAPESFRLLVRELRSLALELNHFL
VSEKNFQINRKEV-------MIDRYKHQQLRIGLVSPQQISAWANRILPNGEIVGEVTKPYTFHY
KTNKPEKDGLFCERIFGPIKSGICACGNYRVIGDEKEDPKFCEQCGVEFVDSRIRRYQMGYI
KLACPVTHVWYLKRLPSYIANLLDKPLKELEGLVYCD--FSFARP-ITKKPTFLRLRGSFEYE
IQSWKYSIPLFFTTQGFDTFRNREISTGAGAIREQLADLDLQIIIDYSFLEWKELGEE-GPTG
NEWEDRKIGRRKDFLVRRMELAKHFIRTNIEPEWMVLCLLPVLPPELRPIIQIDGGKLMSS
DINELYRRVIYRNNTLTDLLTTSRSTPGELVMCQEKLVQEAVDTLLDNGIRGQPMRDGHNK
VYKSFSDVIEGKEGRFRETLLGKRVDYSGRSVIVVGPSLSLHRCGLPREIAIELFQTFVIRGL
IRQHLASNMGVAKSKIREKEPIIWEILQEVMQGHPVLLNRAPTLHRLGIQAFQPILVEGRAI
CLHPLVRKGFNADFDGDQMAVHVPLSLEAQAEARLLMFSHMNLLSPAIGDPISVPTQDML
MGLYVLTSGTRRGICANRYNPCNRKN---YQNERIDDNNYKYT--KEPFFCNSYDAIGAYRQ
KRINLDSPLWLRWQLDQR-VIAS--REAPIEVHYESLGTYHEIYGHYLIVRSVKKEILCIYIRT
TVGHISLYREIEEAIQGFCRACSYDT------------MAERANLVFHNKVIDGTAIKRLISRLIDRF
GMAYTSHILDQVKTLGFQQATATSISLGIDDLLTIPSKGWLVQDAEQQSLILEKHHHYGNV
HAVEKLRQSIEIWYATSEYLRQEMNPNFRVTDPFNPVHIMSFSGARGNASQVHQLVGMRG
LMSDPQGQMIDLPIQSNLREGLSLTEYIISCYGARKGVVDTAVRTSDAGYLTRRLVEVVQH
IVVRRTDCGTIRGISVSPKNRTMP-ERIFIQTLIGRVLADDIYMGPRCIAIRNQDIGIGLVNRFI
T------FRTQSISIRTPFTCRSTSWICRLCYGRSPTHGDLVELGEAVGIIAGQSIGEPGTQLTLRT
FHTGGVFTGGTAEHVRSPSNGKIKFNEDLVHPTRTRHGHPAFLCYIDLSVVIESEDIIHNVTI
PPKSFLLVQNDQYVESEQVIAEIRAGTYTL-NYKERVRKHIYSNSEGE-MHWSTDVYHAPE
FTYSNVHLLPKTSHLWILSGGSSR--SSLVPFSLHKDQDQMNIH--SLSVEQ-ESIS--LAVNND
Q-GRHKFFSSNFSDKK-CGIPDY-SEFHRILDTGHCNLIYFA-SLHENSD--LLAKRRRNRFIIPF
QSIQEQEKELMPR-GISIEIPIN--GIFRRNSILAFFDDPRYRRKSSGILKYGTIGAHSIVKKED--
VIEYR-GVKKFKTKYMKVDRFFFIPEEVHILPESSSIMVRNNSIIGVDTRITLNIRSQVGGLV
RVERKKKSIELQIFSGDIHFPGKTDKISRHSGILIPPGRGKT--NSKESKK--LKNWIYVQR--IT
PTKKK--YFVLVRPVATYEIADGINLATLFPQDLFREKDNMQLRVVNYILYGNGKPVRGIFD
TSIQLVRTCLVLNWDQDN---KSSSVEEVRTFFVEVSTNSLIRDFLRIDLVKSHISYIRKRNDP
SGSGLISD-NGSDRIN--PFYYKER--------IQQSLSKNHGTIRTLLNR---NKECQSLIILSSSNCF
QMGP--FNHVKYHN---VIKQSDPLIPIRNSLGPVGT--AIQIANFYS--IYHLIT--HNQIS-VTKYF
QLDN---LNQIFEVI--K-YYLMDETGR--VYNPDPCSNIILNPFNLNWYFLYQNYCEETSTIIS-
LGQFICENVCLAKNGP---HLKSGQVLIVQ-VDSVVIRSAKPYLATPGATVHGHYGEILYEGD
TLVTFIYEKSRSGDITQGLPKVEQVLEVRSIDSISLNLEKRVEGWNKRITRILGIPWGFLIGA
ELTIVQSRISLVNKIQKVYRSQGVQIHNRHIEIIVRQITSKVLVSE---EGMSNVFLPGELIGLL
RAERTGRALEEAICYRAILLGITRASLNTQSFISEASFQETARVLAKAALRGRIDWLKGLKE
NVVLGGMIPAGTGFKGLVHCS--RQHTSILLETKKKNLYLFEGEMR--DIFFHHRELFDSCIS
KNLHNTSERSFIGLNDS------MAKRYWNINLEEMMEAGVHFGHGTRKWNPRMAPYISAK
RKGIHITNLTRTARFLSEACDLVFDAASRGKQFLIVGTKNKAADSVARAAIRARCHYVNKK
WLGGMLTNWSTTETRLHKFRDLRTEQKTGRLNRLPKRDAAVLKRQLSHLQTYLGGIKYM

TGLPDIVIIIDQQEEYTALRECITLGIPTISLIDTNCDPDLADISIPANDDAIASIRLILNKLVFAI
CEGHSS-YIQNSMGQKINPLGFRLGTTQSHHSLWFAQPKNYSEGLQEDKKIRDFIKNYVKK
NMRISSG-VEGIARIEIQKRIDLIQVIIYMGFPKLLIEDK----PQRIEELQRNVQKELNCVNRK
LNIAIIRIANPYGHPNILAEFIAGQLKNRVSFRKAMKKAIELTEQANTKGIQVQIAGRIDGKE
IARVEWIREGRVPLQTIEAKINYCCYRVRTIYGVLGIKIWIFVDEEMSRYRGPRFKKIRRLG
ALPGLTSQRPRAGSDLRNQSRSGK-KSQYRIRLEEKQKLRFHYGLTERQLLKYVRIAGKAK
GSTGQVLLQLLEMRLDNILFRMGMALTIPQARQLVNHRHILVNGRIVDIPSYRCKPRDTIT
VKDEQKSRALIQSFLDSSTH-EELPNHLTLHTFQ--YEGLVNQIIDRKWVGLKINELLVVEYY
SRQTMSRRGTAEEKTAKSDPIYRNRLVNMLVNRILKHGKKSLAYQIIYRALKKIQQKTETN
PLSVLRQAIRGVTPDIAVKARRVGGSTHQVPIEIGSTQGKALAIRWLLGASRKRPGRNMAF
KLSSELVDAAKGSGDAIRKKEETHRMAEANRAFAHFRMGRDTIADIITSIRNADMNRKGT
VRIASTNITENIVKILLREGFIENVRKHRENNKYFLVLTLRHRR--NRKEPYRT-I--LNLKRISR
PGLRIYSNYQQIPRILGGMGIVILSTSRGIMTDREARREGIGGEILCFIWMAKPIPRVGSRKN
VRSGSRKNARRIPKGVIHVQASFNNTIVTVTDVRGRVISWSSAGTCGFRGTRRGTPFAAQT
AAGNAIRAVVDQGMQRAEVMIKGPGLGRDAALRAIRRSGILLSFVRDVTPMPHNGCRPP
KKRRVMPTIKQLIRNTRQPIRNVTKSPALRGCPQRRGTCTRVYTITPKKPNSALRKVARVRL
TSGFEITAYIPGIGHNSQEHSVVLVRGGRVKDLPGVRYHIVRGTLDAVGVKDRQQGRSKY
GVKKPKMAKKSLIHREKKRQKLEQKYHLIRRSSKKEIS---KVPSLSDKWKIHGKLQSSPRN
SAPTRLHRRCFSTGRPRANYRDFGLSGHILREMVHACLLPGATRSSWMIKNSFIS--FQE-K-
KKENSGSVEFQVFSFTNKIRRLTSHLELHRKDYLSQRGLRKILGKRQRLLSYLSKKNRVRY
KELINQLDIRESKKISFMVKLRLKRCGRKQRAVYRIVAIDVRSRREGRDLRKVGFYDPINN
QTYLNVPAILDFLKKGAQPTETVYDISKKAGVFMELNLNQ-TKVNMDKSKRLFTKSKRSF
RRRLPPIQSGDRIDYRNMSLISRFISEQGKILSRRVNRLTLKQQRLITIAIKQARILSSLPFLNN
EK--QFERSESTTKTTGLRTRNKVTRSLKKNPFVANHLLRKIEKLNTKAEKEIIITWSRASTII
PTMIGHTIAIHNGREHLPVYITDRMVGHKLGEFAPTINFRGHAKNDNKSRRMIFKSFILGNL
VSLCMKIINSVVVVGLYYGFLTTFSVGPSYLFLLRARVMEEGEEGTEKKVSATTGFIAGQL
MMFISIYYAPLHLALGRPHTITVLALPYLLFHFFWNNHKHFFDYGSTTRNSMRNLRIQCVF
LNNLIFQLFNHFILPSSMLARLVNIYMFRCNNKMLFVTSSFVGWLIGHILFMKWVGLVLV
WIQQNNSIRS-IRSNKYLVSELRNSMARIFSILLFITCVYYLGRIPSPIFTKKLKGTEEDKPFV
TLLFDYKRWNRPFRYLKNDRFENAVRNQMSQYFFYTCQSDGKERISFTYPPSLATFLEMIQ
QKMPLFTQEKLSSNEFYNHWILTNEKKESKLKNEFLNRIKALD---LALNILEKRTRFCNNE
TKKEYLPKIYDPLLYGSYRGRIIN---FIGTVLINKIHSILYHEFEQTNPNDQG--IKEISKKVPR
WSYKLIDLEQLEGEEIRSRKAKHVVIFTDALIRYSQQSDFRRDIIKGSMRPQRRKTVTWEL
FQANAHSPLFLDRIDKPLFFFSAFDISELMKRIFRNREEKTRIEIAEAWDSLLFAQVLRGFVL
VTQSILRKYIILPSLIIVKNIARLLLFQFSEWSEDLKDWNREMHIKCTYNGVQLSETEFPKN
WLTDGIQIKILFPFCLKPWHRSK-GEKNDFCFLTVWGMETELPFGSPRKRPSFFEPLFKELK
KKIRKLKIKYFLG-LRVLKERAKIFVKVSKETKKQVSTNLL--FVKGIIKEFSKRNDSRISNQ
MIPES-VQIRSMEWTN-SLTENKIKDLTDRASTIRNQIEQITKEKKNSSNKPSYHAKILASSK
NIWQILK--NARLIRKSYYFIKFFIERIYIDIFLCIVNIPRMNTQL------CNLSSFSQAYVFYKLS
QI-VINLYKLRSVLQYTSFFLKNEIKDSFEQGIFD-QLRHKPPF-YEKNLWKNWLRSHYQYD
LPQIRWPRLVPEKWRNRVKEYCRVQNKNKWDSYEKDK--LSHYERQ-NFKKNDRYDLLSS
KSINDHIFDLDRKYLDWRILNFCLRFFDWMGMNEEILS-WFFPEFVLLYNAYKIKPWIIPSN
LLLFNSN--TEAELHFFLKRYLRFQLRWN---NERIINNIKVYCLLLRLINPREITISSIQRGELS
LDILMIQKDLTLPELMKKGILIIEPVRLSVKNDGQFIIYQTIGISLGDKNKPHYD-FLVPENIL

SPKCRREFRILICFRFLWPNFRLEDLACMNRYWFNTNNGSRFSMIRIHMYPMKGHQFKSW
IFELREILREIKNSHYFLDSWTQFNSVGSFIHIFFHQERFIKLLDPRIWSILLS-RNS--QGSTSN
RYF---TIKGVVLFVVA-VLIYR----INNRNMVERKNLYLTGLLP-----IPMNSIGPRNDTLEESF-
GSSNINRLIVS-LLYLPKGKKISESSFLDPKESTWVLPIT----KKCIMPESNWSSRWWRNWIG
KKR--DSSRKISN---ETVAGIDISFKEKDIK-YLEFLFVYYMD--DPIRKGHDWELFDRLSPSK
RRNIINL--NSGQLFEILVKDWICYLMFAFREKIPIEVEG-FFKQQG-AGSTIQSNDIEHVSH--L
FSRNKWAISLQNCAQFHMWQFRQDLFV---SWGK-NPHESDFLRN-ISRENWIWLDNVWLV
NKDR---FFS--KVRNVSSNIQYDS---TR--SSFVQVTDSSQLKGSS-DQFRDHFDSIS----NEDSE
------------YHTLINQREIQQLKERSIL-WD--PSFLQTERTEIESD--QFPK--CLSGYS--SMPRLF
T-EREKRMNNHLLPEEIEEF--LGNPTRPIRSFFSDRW--SELH-LGSNPTERSTRDQ----KLLKK
EQDVS-FVPSRRSE----NKEIVNIFKIITYLQN--TVSIHPISSDLGCDMVP-KDELD-MDSSNKI
SFLNKNPFFDL--FHLFHDRNRGGSTL--HHDFESE--ERFQEMADLFTLSITEP-DLVYHKGFA
FSIDSYGLDQKQ--FLNEVFNSRDESKKKSL---LVLPPIFY--EENESFYRRIRKKWVR-ISCGN
DLEDPKPKRVVFASNNIMEAVNQYRLIRNLIQIQ--YSTYGYIRNVLNRFFLMN---RSDRNFE
YGIQ---RDQIGNDTLNHRTIMKYTINQHLSNLKK-SQKKWFDP--LIFLSRTERSINRDPNAY
RYKWSNGSKNFQEHLEHFVSEQKSR---FQVVFDRLRINQYSIDWSEVIDKKDLSKSLRFFL
SKLLL---------FLS-------KLLLFLSNSLP-FFFVSFENIPIHRSE-IHIY-ELKGPNDQLCNQLLESI
GLQIVHLKKLKPF--LLD---DHNTS-QKSKFLINGGTISPFLFNKIP-KW-MIDSFHTR--KNRR
KSFDNTDSYFSMISHDQDNWLNPVKPFHRSSLISSFYKANRLRFLNNPHHFCFY--CNKRFP
F---Y-VEKARINNYD--FTYG-QFLNILFIRNK-IFSLCGGK--KKHAFLE-RDTISPIESQVSNIFI
SNDFPQSGDER-YNLYKS---FHFAIRSDPLVR--RAIYSIADISGTPLTEGQIVNFERTYCQPLS
DMNLSDSEEKNLHQYLN--FNSNMGLIHTPCSEKYLPSEK--RKK--------RSLCLK--KCVEK
GQMDRTFQRDSAFSTLSKWNLVQTYMPWFLTSTGYKYLNLIFLDTFSD-LLPILSSS--QKF
VSIFHDIMHGSDISWRILQKKW------CLPQWN-LISEISSKCLHN--LLLSEEMIHRNNES--PLI
STHLRSPNVREF-LYSILFLLLVAGY-LVRTHLLFVSR--AYSELQTEFEKVKSLMIPSYMIELR
KLLDRYPTSELNSFWLKNLFLVALEQLGDSLEEIRGS--GGNMLWGSDP-----AYG-VKSIRS
KKKYLNINFIDIIDLI-SIIPNPINRITFSRNTRHLSHTSKEIYSLIRKRKNVSGDWIDDKIESWV
ANSDSIDDKEREFLVQFS--TLTA--EKRIDQILLSLT-HSDHLS-----KNDSGYQMIEQPGTIYLR
YLVDIH--KKYLMNYEFNTSCLVERRIFLAHYQTITY--SQTSCGANSFHFPSHG---KPFSLRL
ALSPS-RGILVIGSIGTGRSYLVKYLATNSYVPFITVFLNKFLDNKPKGFFID-----DID--DASD
DIDASDDIDASNDID----------DSDDIDRELDTELELLTMMNAL---TMDMMS---EIDRFYITLQ
FELAKAMSPCIIWIPNIHDLDVNESNYLSLGLLVNSLS---RDCERCS-TRNILVIASTHIPKKV
DPALIAPNKLNTCIKIRRLLIPQQRKHFFTLSYTRGFHLEKKMFHT--NGFESITMGSNARDL
VALTNEALSISITQKKSIIDTNTIRSALHRQTWDLR---SQVRSVQDHGILFYQIGRAVAQNVL
LSNCPIDPISIYMKKK--SCNEGDSYLYKWYFELGTSMKKLTILLYLLSCSAGSVAQDLWSL
P--GPDEK-NGITSYGFVENDS-DLVHGLLEVEGALVG--SSRTEKDCSQFDND--RVTLLLRSE
PRN-PLYMMQNGSCSIVDQRFLYEK--YESEFEE-GEGEGVLDPQ----QIEED----LFNH--IVW
AP--RIWRP---WGFLFDCIERPNELGFP-YWARSFRGK---RIIYDEKDELQANDSEFLQSGTV
QYQTRDRSSKEQGFFRISQFIWDPADPLFFLFK-DQPFVSVFSHREFFADEEMSKGLLTSQT
DPPTSIYKRWFI--KNTQEKHFELLIHRQRWLR---TNSSLS--NGFFRSN--TPSESYQYLSNLF
LSN-GTLLDQMTKTLLRKRWLFPDEMKIGF--M---------MPRSR-INGNFIDKTFSIVANILLRII
PTTSGEKEAFTYYRDG--MSAQSEGNYAEALQNYYEAMRLEIDPYDRSYILYNIGLIHTSNG
EHTKALEYYFRALERNPFLPQAFNNMAVICHY-RGEQAIRQGDSEIAEAWFAQAAEYWKQ
AIALTPGNYIEAQNWLKITRRFE--MSWRSESIWIEFIAGSRKTSNFCWAFILFLGSLGFLLVG

TSSYLGRNLISLFPSQQIVFFPQGIVMSFYGIAGLFISSYLWSTILWNVGSGYDRFDRKEGIV
SIFRWGFPGKNRRIFLRLFMKDIQSIRIEVKEGIYARRVLYMDIRGRGAIPLTRTDEN-FTTRE
IEQKAAELAYFLRVPIEVF
>Manihot
MEKRWFNSILSLSKSMDSFSSLENTSISEDPILNDTDKNTHSYSNVDHLVGVRDIQNFGAD
ETYLVRDSNKDSYSIYFDIENKVFEIDNDH--SFLSELKSSFYSYWNSSYLNNGSRSGDSHS
DHYMYDTKYSWNNYINSCIDSYLR--SQICIDSYILSGSNNYSESYIYSYFCGSESSSLRTSTS
GSDLIIRESSNDLDITQKYKHLWVQCENCYGLNYKKFFKSRMNICEQCGYHLKMSSSDRI
ELLIDLDTWDPMDEDMVSLDPIEFHSEEEPYKDRIDSYQRKTGLSEAVQTGTGQLNGIPVA
IGVMDFRFMGGSMGSVVGEKITRLIEYATNKFLPLILVCASGGARMQEGSLSLMQMAKISS
ALYDYQSNKKLFYVSILTSPTTGGVTASFGMLGDIIIAEPNAYIAFAGKRVIEQTLNKTVPEG
SQSAEFLFHKGLFDLIVPRNLLKGVLNELLQFHDFFPLNHKSK-MVTIRADEISNIIRERIEQ
YNREVKIVNTGTVLQVGDGIARIYGLDEVMAGELVEFEEGTIGIALNLESNNVGVVLMGD
GLMIQEGSSVKATGRIAQIPVSEAYLGRVINALAKPIDGRGEISASESRLIESPAPGIISRRSV
YEPLQTGLIAIDSMIPIGRGQRELIIGDRQTGKTAVATDTILNQQGQNVICVYVAIGQKASSV
AQVVTTLQERGAMEYTIVVAETADSPATLQYLAPYTGAALAEYFMYRERHTLIIYDDLSK
QAQAYRQMSLLLRRPPGREAYPGDVFYLHSRLLERAAKLSSRLGEGSMTALPIVETQSGD
VSAYIPTNVISITDGQIFLSADLFNAGIRPAINVGISVSRVGSAAQIKAMKQVAGKLKLELAQ
FVELEAFAQFASDLDKATQNQLARGQRLRELLKQSQSAPLTVEEQIMTIYTGTNGYLDSLE
IGQVRKFLVELRTYLKTNKPQFQEIISSTKTFTEEAETLLKEAIQEQKERFVIQEQVMRINPT
TSGPGVSALEKKNLGRIAQIIGPVLDVAFPPGKMPNIYNALVVKGRDTAGQEINVTCEVQQ
LLGNNRVRAVAMSATDGLTRGMEVIDTGAPLSVPVGGATLGRIFNVLGEPVDDLGPVDTR
ATSPIHRSAPAFIQLDTKLSIFETGIKVVDLLAPYRRGGKIGLFGGAGVGKTVLIMELINNIA
KAHGGVSVFGGVGERTREGNDLYMEMKESGVINEENIAESKVALVYGQMNEPPGARMR
VGLTALTMAEYFRDVNEQDVLLFIDNIFRFVQAGSEVSALLGRMPSAVGYQPTLSTEMGS
LQERITSTKEGSITSIQAVYVPADDLTDPAPATTFAHLDATTVLSRGLAAKGIYPAVDPLDST
STMLQPQIVGEEHYETAQRVKQTLQRYKELQDIIAILGLDELSEEDRLTVARARKIERFLSQ
PFFVAEVFTGSPGKYVGLAETIRGFKLILSGELDSLPEQAFYLVGNIDEATAKATNLEMENN
LKKMTLNLCVLTPNRIVWDSEVKEIILSTNSGQIGVLPNHAPIATAVDIGILRIRLNDQWLT
MALMGGFARIGNNEITVLVNDAEKGSDIDPQEAQQTLEIAEANLRKAEGRRQIIEANLALR
RARARVEALNEISMKNITDSFVSLGHWPSAGSFGFNTDILATNLINLSVVLGVLIFFGKGVL
SDLLDNRKQRILDTIRNSEKLREGAIEQLEKARARLRKVEIEADQFRTNGYSEIEREKLNLI
NSTYKTLEQLENYKNETIHFEQQRTINQVRQRVFQQALQGALGTLNSCLTNELHLRTINAN
LGMFGAIKEITDMNPLISAASVIAAGLAVGLASIGPGVGQGTAAGQAVEGIARQPEAEGKI
RGTLLLSLAFMEALTIYGLVVALALLFANPFVMNVLSCSINTLRGLYDISGVEVGQHFYWK
IGGFQVHAQVLITSWVVIAILLGSAIVAVRNPQTIPTGGQNFFEYVLEFIRDVSKTQIGEEYG
PWVPFIGTMFLFIFVSNWAGALLPWKIIQLPHGELAAPTNDINTTVALALLTSIAYFYAGLS
KKGLGYFSKYIQPTPILLPINILEDFTKPLSLSFRLFGNILADELVVVVLVSLVPSVVPIPVMF
LGLFTSGIQALIFATLAAAYIGESMEGHHMIFSTLEHILTHISFSVVSIVITIHLITLLADEFVEL
YDSSEKGMITTFFCITGLLVTRWIFLGHLPLSDLYESLIFLSWSFSIIHMVPYFKKHKNFLSAI
TAPSTFFTQGFATSGLLTDMHQSEILVPALQSQWLMMHVSMMILGYAALLCGSLFSVAFLV
ITFRKIIRIFD--KEIQYM--AENVLRNISFLSSRNYYRFQLIQQLDDWGYRIISIGFIFLTIGILSG
AVWANEAWGSYWNWDPKETWAFITWTIFAIYFHIRTNKKLEGFHSAIVASIGFLLIWICYF
GVNLLGIGLHSYGSFTLTKWKKKTFIPLLYLTSIVFLPWWVSFLFNKSLESWIINCCNTSKS

ETFLNDIQEKSILEKFMELEDLVRLNEIIKEYPETHLQKFRIG-HKETIQLIKMHNEDRIHTIL
HFSTNIICFIILSGYSILSNEELIILNSWVQEFLYNLSDTIKAFSILLLTDLCIGFHSPHGWELMI
GSVYKDFGFAHNDQIISGLVSTFPVILDTIFKYWIFRYLNRVSPSLVVIYHSMNDMPIGVPK
VPFRNPGEDDSTWIDIN-RLYRERLLFLGQDVDSEIANQLIGLMVYLSIENATKDLYLFINSP
GGWVIPGIAIYDTMQFVRPDVQTVCMGLAASMGSFILVGGKITKRLAFPHARVMIHQPISG
YYEAQIVEFVLEAEELLKLREILTRIYAQRTGKPLWVVSEDMERDVFMSATEAQAHGIVDL
VAVA??MEE--
RYLELDRSRKNDFLYPFIFREYIYTFAHDHSLVGYDNKSSLLIVKRLITRMYQQNHLIISAN
DSNQNLFFRKNLYYQMISEGFAVIVEIPFSLRLVSSLIVKSHKLRSIHSIFPFLEDKFPHLNYV
SDILIPYPIHLEKLVQTLRYWVKDPSSLHLLRLFLHEYWNLNSLIIKFITIFIKPRFFLFLYNSH
VYEYESIFFFLRNQSFHLRSIFLRVLLERIFFYGKIEHFAEVFANQAILWLFKDPFMHYVRYQ
GKSILASKDRPFLMKKWKYYLVNLCQCHFYVWFQPEKIYINSLSKHSLNFLGYLSNVQLN
PLVVRSQMLENSFIIDKDSTMKKLDTIVPIIPLIGSLAKTKFCNAVGHPISKPIRADSADSDII
DRFVRICRNLSHYYSGSSKKKSLYRIKYILRLSCVKTLARKHKSTVRAFLKRLGSELLEEFF
TEEEQILSLIFPKRVWYLDIISINDLANHEMIIDTT-EVQ-AINSFSRLES-LNEVYGIIWVFAPI
FILVLGITTGILVIVWLEREISAGIQQRIGPEYAGPLGVLQALADGTKLLFKENLFPSRGDIRL
FSIGPSIAVISTILSYSVIPFGYHLVLTDLNIGVFLWIAISSIAPIGLLMSGYGSNNKYSFLGGL
RAAAQSISYEIPLTLCVLSISLLSNSSSTVDIVEAQSKSGFWGWNLWRQPIGFIIFFISSLAEC
ERLPFDLPEAEEELVAGYQTEYSGIKFGLFYIASYLNLLVSSLFVTVLYLGGWNISIPYIFVP
ELF--EKMSGVFGTTIGIFITLVKTYLFLFIPITTRWTLPRLRMDQLLNLGWKFLLPISLGNLL
LTTSFELLSLMIWHVQNENFILDSTRIFMKAFHLLLFDGSFIFPECILIFGLILLLMIDSTSDQ
KDIPWLYFISSTSLVMSITALLFRWREEPMISFSGNFQTNNFNEIFQFLILLCSTLCIPLSVEYI
ECTEMAITEFLLFVLTATLGGMFLCGANDLITIFVAPECFSLCSYLLSGYTKKDVRSNEATT
KYLLMGGASSSILVHAFSWLYGSSGGEIELQEIVNGLINTQMYNSPGISIALIFITVGIGFKLS
LAPSHQWTPDVYEGSPTPVVAFLSVTSKVAASASATRIFDIPFYFSSNEWHLLLEILAILSMI
VGNLIAITQTSMKRMLAYSSIGQIGYVIIGIIVGDSNGGYASMITYMLFYISMNLGTFACIVL
FGLRTGTDNIRDYAGLYTKDPFLALSLALCLLSLGGLPPLAGFFGKLHLFWCGWQAGLYF
LVLIGLLTSVVSIYYYLKIIKLLMTGRNQEITPHVRNYRRSPLRSNNSIELSMIVCVIASTIPGI
SMNPIVEIAQDTLFMFLIYEYDIFWAFLIISSVIPILAFLISGVLSPISKGPEKLSSYESGIEPIG
DAWLQFRIRYYMFALVFVVFDVETVFLYPWAMSFDVLGLSVFIEALIFVLILIVGSVYAWR
KGALEWSMNSFPWLTIFVVLPISGGSLIFLFPHRGNKVIKWYTIFICIFELLLMTYAFSYYFQ
LDDPLIQLTEDYKWIQFFDFYWRLGIDGFSLGPILLTGFITTLATLAARPITRDSRLFHFLML
AMYSGQIGLFSSQDLLLFFIMWELELIPVYLLLSMWGGKKRLYSATKFILYTAGGSVFLLM
GALGIALYGSNEPRFHFETSANQSYPVALEIFFYIGFLIAFAVKSPIIPLHTWLPDTHGEAHYS
TCMLLAGILLKMGAYGLVRINMELLPHAHSIFSPWLIIVGVMQIIYAASTSPGQRNLKKRIA
YSSVSHMGFIIIGICSISDMGLNGAILQIISHGFIGAALFFLAGTGYDRIRRVYLDEMGGMAT
SMPKIFTTFSILSLASLALPGMSGFFAELIVFFGIITGQKYLLMSKILITFVMAVGMILTPIYLL
SMLRQMFYGYKLFNAPNSYFFDSGPRELFVSISILLPVIGIGFYPDFVFSLSVDRVEAILSNY
FYRMMLEHVLVLSAYLFSIGIYGLITSRNMVRALMCLELILNAVNLNFVTFSDFFDSRQLK
GNIFSIFVIAIAAAEAAIGPAIVSAIYRNRKSIHINQSNLLNKMEHIYQYSWIIPFVTLPVPMLI
GAGLLLFPAATKKLRRMWAFPSVFLLSIVMIFSIDLSIQQINSSFIYQYIWSWTINNDFSLEF
GHLIDPLTSILSVLITTVGILVLFYSDNYMSHDQGYLRFFAYMSFFTTSMLGLVTSSNLIQIYI
FWELVGVCSYLLIGFWFTRPIASNACQKAFVTNRVGDFGLLLGILGLYWITGSFEFRDLFKI
FNN-QVNFLFVTLCAVLLFSGAIAKSAQFPLHVWLPDAMEGPTPISALIHAATMVAAGIFLV

ARLFPLFVIIPFIMNLIALIGIITVFLGATLALAQKDIKRSLAYSTMSQLGYMMLALGMGSY
RAALFHLITHAYSKALLFLGSGSIIHSMEAILGYSPDKSQNMVLMGGLTKHIPITKTAFLLG
TLSLCGIPPFACFWSKDEILNDSWLYSPIFAIIACFTAGLTAFYMFRVYLLTFDGHFNAHFQS
YSGKKNSSFYSISLWGKEGSKMLNK-LLALLTMNFFWNTKR-IFSYPHESDNTMLFPMLIL
VLFTLFIGAIGNQFDIDILSKLLTPSLNLLHQNPE-SVDW-EFVTNATFSASIAFFGIFIASFLYK
PVYSSLQNLNFFNSFAILWDKIINVIYNWSSNRGYIDAFYAISFIGGIRKLAELIHFFDKQIID
GTPNGVGVTSFFVGEGIKNVGSG-RISFYLLFYLFYALIFLLIYYMDLPGLIHDFLLVFLGLG
LILGGLGVVLLTNPIYSAFSLGLVLVCISLFYILSNSHFVAAAQLLIYVGAINVLIIFAVMFMN
GSEYYKDFNLWTVGSGVTSLVCTSIFVSLITIIPDTSWYGIIWTTKTNQIIEQDLISNGQQIGI
HLSTDFFLPFEFISIILLVALIGAIAVARQMNVPATRKDLMIVNMGPHHPSMHGVLRLIVTLD
GEDVIDCEPILGYLHRGMEKIAENRTIIQYLPYVTRWDYLATMFTEAITVNGPELLGNIQVP
KRAGYIRVIMLELSRIASHLLWLGPFMADIGAQTPFFYIFRERELVYDLFEAATGMRMMHN
FFRIGGVASDLPHGWIDKCLDFCDYFLTGVTEYQKLITRNPIFLERVEGVGIVGTEEAINWG
LSGPMLRASGVQWDLRKVDHYECYDEFDWEIQWQKEGDSLARYLVRIGEMLESIKIIQQ
ALEGIPGGPYENLETRRFDRERDSEWNDFEYRFISKKTSPTFELPKQELYVRVEAPKGELGI
FLIGDQSGFPWRWKIRPPGFINLQILPELVKRMKLADIMTILGSIDIIMGEVDRMFPMVTGF
MNYGQQTIRAARYIGQGFMITLSHANRLPVTVQYPYEKLITSERFRGRIHFEFDKCIACEV
CVRVCPIDLPVVDWKLETDIRKKRLLNYSIDFGICIFCGNCVEYCPTNCLSMTEEYELSTY
DRHELNYNQIALGRLPMSVVDDYTIRTILNSQKK------------MQGRLSAWLVKHGLVHRFL
GFDYQGIETLQIKPEDWHSIAVILYVYGYNYLRSQCAYDVAPGGLLASVYHLTRIEYGIDQ
PEEVCIKVFAPRKNPRIPSVFWVWKSADFQERESYDMLGIFYDNHPRLKRILMPESWIGWP
LRKDYIAPNFYEIQDAH--MNSIEFPLLDRTT
QISVISTTSNDLSNWSRLSSLWPLLYGTSCCFIEFASLIGSRFDFDRYGLVPRSSPRQADLILT
AGTVTMKMAPSLVRLYEQMPEPKYVIAMGACTITGGMFSTDSYSTVRGVDKLIPVDVYL
PGCPPKPEAVIDAITKLRKKISREIYEDRIRS--GKRCFTTNHKFNIERTTHTGNYDQELLYQS
PSTSKIPPETFFKYKRSVSSNELVNMQTRKTFSWIKEEITRSISVLLMIYIITWASISNAYPIFA
QQGYENPREATGRIVCANCHLANKPVDIEVPQAVLPDTVFEAVVRIPYDMQLKQVLANG
KKGALNVGAVLILPEGFELAPPDRISPEMKEKMGNLSFQSYRPTKKNILVIGPVPGQKYSEI
TFPILSPDPAAKKDVHFLKYPIYVGGNRGRGQIYPDGSKSNNTVYNATAAGIVSKIIRKEKG
GYEITITDASEGRQVIDIIPPGPELLVSEGESIKLDQPLTSNPNVGGFGQGDAEIVLQDPLRV
QGLLFFLASVILAQIFLVLKKKQFEKVQLSEMNFMSKVYDWFEERLEIQAIADDITSKYVP
PHVNIFYCLGGITLTCFLVQVATGFAMTFYYRPTVTEAFASVQYIMTEANFGWLIRSVHRW
SASMMVLMMILHVFRVYLTGGFKKPRELTWVTGVVLAVLTASFGVTGYSLPWDQIGYWA
VKIVTGVPEAIPVIGSPLVELLRGSASVGQSTLTRFYSLHTFVLPLLTAVFMLMHFPMIRKQ
GISGPLMGVTKKPDLNDPVLRAKLAKGMGHNYYGEPAWPNDLLYIFPVVILGTIACNVGL
AVLEPSMIGEPADPFATPLEILPEWYFFPVFQILRTVPNKLLGVLLMVSVPAGLLTVPFLENV
NKFQNPFRRPVATTVFLIGTAVALWLGIGATLPIDKSLTLGLFMIEVFLFGIVLGLIPITLAGLF
VTAYLQYRRGDQLDLMPTITSYFGFLLAALTITSVLFIGLSKIRLIMDIVSLAWAALMVVFT
FSLSLVVWGRSGLMIIRSPEPEVKILVDRDPIKTSFEEWARPGHFSRTIAKGPDTTTWIWNL
HADAHDFDSHTSDLEEISRKVFSAHFGQLSIIFLWLSGMYFHGARFSNYEAWLSDPTHIGP
SAQVVWPIVGQEILNGDVGGGFRGIQITSGFFQIWRASGITSELQLYCTAIGALVFAALMLF
AGWFHYHKAAPKLAWFQDVESMLNHHLAGLLGLGSLSWAGHQVHVSLPINQFLNAGVD
PKEIPLPHEFILNRDLLAQLYPSFAEGATPFFTLNWSKYSDFLTFRGGLDPVTGGLWLTDTA
HHHLAIAILFLIAGHMYRTNWGIGHGIKDILEAHKGPFTGQGHKGLYEILTTSWHAQLSLN

LAMLGSLTIVVAHHMYSMPPYPYLATDYGTQLSLFTHHMWIGGFLIVGAAAHAAIFMVR
DYDPTTRYNDLLDRVLRHRDAIISHLNWVCIFLGFHSFGLYIHNDTMSALGRPQDMFSDTA
IQLQPVFAQWIQNTHALAPGATAPGATASTSLTWGGGDLVAVGGKVALLPIPLGTADFLVH
HIHAFTIHVTVLILLKGVLFARSSRLIPDKANLGFRFPCDGPGRGGTCQVSAWDHVFLGLF
WMYNSISVVIFHFSWKMQSDVWGSISDQGVVTHITGGNFAQSSITINGWLRDFLWAQASQ
VIQSYGSSLSAYGLFFLGAHFVWAFSLMFLFSGRGYWQELIESIVWAHNKLKVAPATQPRA
LSIIQGRAVGVTHYLLGGIATTWAFFLARIIAVGMALRFPRFSQGLAQDPTTRRIWFGIATAH
DFESHDDITEERLYQNIFASHFGQLAIIFLWTSGNLFHVAWQGNFEAWVQDPLHVRPIAHAI
WDPHFGQPAVEAFTRGGAPGPVNIAYSGVYQWWYTIGLRTNEDLYIGALFLLFLSALALL
GGWLHLQPKWKPSVSWFKNAESRLNHHLSGLFGVSSLAWTGHLVHVAIPGSRGEYVRW
NNFLDVLPHPQGLGPFFTGQWNLYAQNPDSGSHLFGTSQGAGTAILTLLGGFHPQTQSLW
LTDIAHHHLAIAFIFLVAGHMYRTNFGIGHSIKDLLEAHIPPGGRLGRGHKGLYDTINNSLH
FQLGLALASLGVITSLVAQHMYSLPAYAFIAQDFTTQAALYTHHQYIAGFIMTGAFAHGAIF
FIRDYNPEQNENNVLARMLDHKEAIISHLSWASLFLGFHTLGLYVHNDVMLAFGTPEKQI
LIEPIFAQWIQSAHGKTSYGFDVLLSSTNSPAFNAGRSIWLPGWLNAINE--NSNSLFLTIGPG
DFLVHHAIALGLHTTTLILVKGALDARGSKLMPDKKDFGYSFPCDGPGRGGTCDISAWDA
FYLAVFWMLNTIGWVTFYWHWKHITLWQGNVSQFNESSTYLMGWLRDYLWLNSSQLIN
GYNPFGMNSLSVWAWMFLFGHLVWATGFMFLISWRGYWQELIETLAWAHERTPLANLIR
WRDKPVALSIVQARLVGLAHFSVGYIFTYAAFLIASTSGKFGMSHSVKIYDTCIGCTQCVR
ACPTDVLEMIPWDGCKSKQIASAPRTEDCVGCKRCESACPTDFLSVRVYLWHETTRSMGL
AYMTILNLPSIFVPLVGLVFPAIAMASLFLYVQKNKIFMRDLKTYLSVAPVISTLWFGSLAG
LLIEINRFFPDALTFPFFSFMTAILERRESESLWGRFCNWITSTENRLYIGWFGVLMIPTLLTA
TSVFIIAFIAAPPVDIDGIREPVSGSLLYGNNIISGAIIPTSAAIGLHFYPIWEAASVDEWLYNG
GPYELIVLHFLLGVACYMGREWELSFRLGMRPWIAVAYSAPVAAATAVFLIYPIGQGSFSD
GMPLGISGTFNFMIVFQAEHNILMHPFHMLGVAGVFGGSLFSAMHGSLVTSSLIRETTENE
SANEGYRFGQEEETYNIVAAHGYFGRLIFQYASFNNSRSLHFFLAAWPVVGIWFTALGIST
MAFNLNGFNFNQSVVDSQGRVINTWADIINRANLGMEVMHERNAHNFPLDLAAVEAPST
NGMGLPWYRVHTVVLNDPGRLLSVHIMHTALVAGWAGSMALYELAVFDPSDPVLDPMW
RQGMFVIPFMTRLGITNSWGGWSIT-GGTITNPGIWSYEGVAGAHIVFSGLCFLAAIWHWV
YWDLEIFCDERTGKPSLDLPKIFGIHLFLSGVACFGFGAFHVTGLYGPGIWVSDPYGLTGK
VQPVSPAWGVEGFDPFVPGGIASHHIAAGTLGILAGLFHLSVRPPQRLYKGLRMGNIETVL
SSSIAAVFFAAFVVAGTMWYGSATTPIELFGPTRYQWDQGYFQQEIYRRVSAGLAENQSLS
EAWSKIPEKLAFYDYIGNNPAKGGLFRAGSMDNGDGIAVGWLGHPIFRDKEGRELFVRR
MPTFFETFPVVLVDGDGIVRADVPFRRAESKYSVEQVGVTVEFYGGELNGVSYSDPVTVK
KYARRAQLGEIFELDRATLKSDGVFRSSPRGWFTFGHASFALLFFFGHIWHGARTLFRDVF
AGIDPDLDAQVEFGAFQKLGDPTTRRQVV------------VETLFNGTLSLAGRDQETTGFAWW
AGNARLINLSGKLLGAHVAHAGLIVFWAGAMNLFEVAHFVPEKPMYEQGLILLPHLATLG
WGVGPGGEVIDTFPYFVSGVLHLISSAVLGFGGIYHALLGPETLEESFPFFGYVWKDRNK
MTTILGIHLILLGIGAFLLVFKALYFGGVYDTWAPGGGDVRKITNLTLSPSVIFGYLLKSPFG
GEGWIVSVDDLEDIIGGHVWLGSICILGGIWHILTKPFAWARRALVWSGEAYLSYSLGALS
VFGFIACCFVWFNNTAYPSEFYGPTGPEASQAQAFTFLVRDQRLGANVGSAQGPTGLGKY
LMRSPTGEVIFGGETMRFWDLRAPWLEPLRGPNGLDLSRLKKDIQPWQERRSAEYMTHA
PLGSLNSVGGVATEINAVNYVSPRSWLATSHFVLGFFLFVGHLWHAGRARAAAAGFEKGI
DRDFEPVLSMTPLNMTIALGKFTKDENDLFDIMDDWLRRDRFVFVGWSGLLLFPCAYFAV

GGWFTGTTFVTSWYTHGLASSYLEGCNFLTAAVSTPANSLAHSLLLLWGPEAQGDFTRWC
QLGGLWTFVALHGAFGLIGFMLRQFELARSVQLRPYNAIAFSGPIAVFVSVFLIYPLGQSG
WFFAPSFGVAAIFRFILFFQGFHNWTLNPFHMMGVAGVLGAALLCAIHGATVENTLFEDG
DGANTFRAFNPTQAEETYSMVTANRFWSQIFGVAFSNKRWLHFFMLFVPVTGLWMSALG
VVGLALNLRAYDFVSQEIRAAEDPEFETFYTKNILLNEGIRAWMAAQDQPHENLIFPEEVL
PRGNALMSGSTGERSFADIITSIRYWVIHSITIPSLFIAGWLFVSTGLAYDVFGSPRPNEYFTE
SRQGIPLITGRFDPLEQLDEFSKSFMTIDRTYPIFTVRWLAVHGLAVPTVSFLGSISAMQFIQ
RMATQSVEGSSRSG-PRRTIVGDLLKPLNSEYGKVAPGWGTTPLMGVAMALFAIFLSIILEI
YNSSVLLDGISMNMLTLKLFVYTVVIFFVSLFIFGFLSNDPGRNPGREEMADTTGRIPLWIV
GTVTGILVIGLIGIFFYGSYSGLGSSLMFNFFSLICICFNSAIY-SNFFFTKLPEAYAFLNPIVDV
MPVIPVLFFLLAFVWQAAVSFRMTQSNPNEQNVELNRTSLYWGLLLIFVLAVLFSNYFFN
MEVNILAFIATALFILVPTAFLLIIYVKTVSQGDMETATLVAISISGLLVSFTGYALYTAFGQPS
QQLRDPFEEHGDMEALVYTFLLVSTLGIIFFAIFFREPPKVPTKKVKMTIAFQLAVFALIATS
SILLISVPVVFSSPDGWSSNKNVVFSGTSLWIGLVFLVGILNSLIS-------MSPQTETKASVGF
KAGVKDYKLTYYTPDYQTKDTDILAAFRVTPQPGVPPEEAGAAVAAESSTGTWTTVWTD
GLTSLDRYKGRCYGLEPVPGEENQYIAYVAYPLDLFEEGSVTNMFTSIVGNVFGFKALRAL
RLEDLRVPPAYSKTFQGPPHGIQVERDKLNKYGRPLLGCTIKPKLGLSAKNYGRAVYECLR
GGLDFTKDDENVNSQPFMRWRDRFLFCAEAIYKAQAETGEIKGHYLNATAGTCEEMIKR
AVCARELGVPIVMHDYLTGGFTANTSLAHYCRDNGLLLHIHRAMHAVIDRQKNHGMHFR
VLAKALRLSGGDHIHAGTVVGKLEGERDITLGFVDLLRDDFIEKDRSRGIYFTQDWVSLP
GVLPVASGGIHVWHMPALTEIFGDDSVLQFGGGTLGHPWGNAPGAVANRVALEACVQAR
NEGRDLAREGNDIIREASKWSPELAAACEVWKEIKFEFAAVDTLDKMAIHLYKTSTPSTRN
GAVDSQAKSNTRNNLIYGQHRCGKGRNARGIITARHRGGGHKRLYRKIDFRRNEKDIYGR
IVTIEYDPNRNAYICLIHYGDGEKRYILHPRGAIIGDTIISGTEVPIKMGNALPLTDMPLGTAI
HNIEITLGKGGQLARAAGAVAKLIAKEGKSATLKLPSGEVRLISKNCSATVGQVGNTGVN
QKSLGRAGSKCWLGKRPVVRGVVMNPVDHPHGGGEGRAPIGRKKPATPWGYPALGRRS
RKRNKYSDNLILRRRSKMIQSQTHLNVADNSGARELMCIRIIGTS-NRRYAHIGDVIVAVIKE
AAPNSPLERSEVIRAVIVRTCKELKRDNGMIIRYDDNAAVVIDQEGNPKGTRIFGAIARELR
QLNFTKIVSLAPEVLMLSPKRTRFRKQHRGRMKGIAFRGNRICFGRYALQALEPAWITSRQI
EAGRRAMTRNARRGGKIWVRIFPDKPVTLRPTETRMGSGKGSPEYWVAVVKPGRILYEM
GGVAENIARKAISIAASKMPIRTQFIISGMTRIRRGYIARRRRTKIRLFASSFRGAHSRLTRTII
QQKIRALVSAHRDRDRQKRNFRRLWVTRINAVIRE-STVSYSYSRLINNLYKRQLLLNRKIL
AQIAILNRNCLYMISNDILK-----------MIKEVYALGQHICMSPHKARRIIDQIRGRSYEETLMI
LELMPYRACYPIFKLIYSAAANASHNMGFNEANLIISKAEVNEGTTVKKLKPQARGRGYPI
KRSTCHISIVLKNISL--------MDGIKYAVFTDKSIRLLGKN-QYTFNVESGSTRTEIKHWVELF
FGVKVIAMNSHRLPGKGRRMRPIMGHTMHYRRMIITLQPGYSIPPLRKKRTMAVPKKRTSI
SKKRIRKNLWKRKGYWTVLKAFSLAKSLSTGNSKSFFVRQIKDMAKGKDIRIRVILECTTC
-TRN--VNKK--GISRYITQKNRHNTPSRLELRKFCRYCYKHTIHGEIKKMKIRASVRKICEKC
RLIRRRGRIIVICFNPRHKQRQGMVREKITISTRTLQWKCIESRTDNKRLYYGRFILSPLMK
GQADTIGIAMRRALLGEIEGTCITRAKSE--KIPHEFSTIAGIQESIHEILMNLKEIVLRSNLYG
TCDASICVKGPGYVTAQDIILPPFVEIIDNTQHIASLTKAIDLCIGLQIERNRGYRIKPTNNFQ
DGSYS--IDAVFMPVRNANHSVHSYGNGNEKQEILFLEIWTNGSLTPKEALHEASRNLIDFFI
PFLHAEEENLHLEIEKNQ-HKVTLPLFCFHDKLT-KLRKNKK-EIVLKYIFIDQSELTPKIYNC
LKRSNIHTLSDLLNKSQEDLMKIEHFRIDDVKHILGILEIEKHFTIDLPKNKMLGDGN-EGM

STIPGFNQIQFEGFCRFIDQGLTEELYKFPKIEDTDQEIEFQLFVETYQLVEPLIKEKDAVYES
LTYSSELYVSAGLIWKTSRDMQEQTIFIGNIPLMNSLGTFIINGIYRIVINQILQSPGIYYRSEL
EHNGISVYTGTIISDWGGRVELEIDRKARIWARVSRKQKISILVLSSAMGLNLKEILENVCY
PEIFLSFLNDKEKKKIGSKENAILEFYQQFTCVGGDPIFSESLCKELQKKFFQQRCELGKIG
RLNMNRKLNLDIPHNNTFLLPRDILAAADRLIGMKFGMGTLDD--MNHLKNKRIRSVADL
LQDQFGLALIRLENVVRGTICGAIR-HKLI-PTPQNLVTSTP----LTTTYESFFGLHPLSQVLDR
TNPLTQIVHGRKLSYLGPGGLTGRTASFRIRDIHPSHYGRICPIDTSEGINVGLIGSLAIHAKI
GYWG-SLESPFY---EISEGSK--KVRMFYLSPNREEYYMVAAGNSLALNRGVQEEQVAPARY
RQEFLTIAWERVHLRSIFPFQYFSIGASLIPFIEHNDANRALMSSNMQRQAVPLSRSEKCIVG
TGLERQVALDSGVPAIAEHEGKIIYTDIDKIILSGNGDTLRIPLVRYQRSNKNTCMHQKTQL
RRGKCIKKGQVLADGAATVGGELALGKNVLVAYMPWEGYNFEDAVLISERLVYEDIYTSF
HIRKYEIQTHVTSQG-PERITNEIPHLEAHLLRNLDKNGIVMLGSWVETGDILVGKLTPQMA
KE-SSYAPEDRLLRAVLGIQVSTSKETCLKLPIGGRGRVIDVRWIQKKG-GSCYNPERICVYI
LQKREIKVGDKVAGRHGNKGIISKILPRQDMPYLQDGRPVDMVFNPLGVPSRMNVGQIFE
CSLGLAGGLLDRHYRIAPFDERYEQEASRKLVFSELYEASKQTANPWVFEPEYPGKSRIFD
GRTGDPFEQPVIIGKPYILKLIHQVDDKIHGRSSGHYALVTQQPLRGRAKQGGQRVGEMEV
WALEGFGVSHILQEMLTYKSDHIRARQEVLGTTIIGGTIPKPEDAPESFRLLVRELRSLALEL
NHFLVSEKNFQITRKEA-------MIDRYKHQQLRIGSVSPQQISAWANKILPNGEIVGEVTKPY
TFHYKTNKPEKDGLFCERIFGPIKSGICACGNYRVIRNEKEDQKFCEQCGVEFVDSRIRRY
QMGYIKLACPVTHVWYLKRLPSYIANLLDKPLKELEGLVYCDF---FARP-IAKKPTFLRLR
GSFEYEIQSWKYSIPLFFTTQCFDTFRNREISTGAGAIREQLADLDLRIIIDYSSVEWKELGE
E-GPTGNEWEDRKVGRRKDFLVRRVELAKHFIRTNIEPEWMVLCLLPVLPPELRPIIQIDGG
KLMSSDINELYRRVIYRNNTLIDLLTTSRSTPGELVMCQEKLVQEAVDTLLDNGIRGQPMR
DGHNKVYKSFSDVIEGKEGRFRETMLGKRVDYSGRSVIVVGPSLSLHRCGLPREIAIELFQI
FVIRGLIRQHLASNIGVAKSKIREKEPIVWEILHEVMQGHPVLLNRAPTLHRLGIQAFQPILV
EGRAICLHPLVCKGFNADFDGDQMAVHVPLSLEAQAEARLLMFSHMNLLSPAIGDPISVPT
QDMLIGLYVLTSGNRRGICANRYNPCNRRN---YQNKRIDG--DKYTK--EPLFSNSYDALGA
YRQKRIHLDSPLWLRWQLDQR-AITS--REAPIEVHYESLGTYHEIYEHYLIVRNIKKEILCIY
IRTTVGHISLYREIEEAIQGFCQACSDGI--------MEVLMAERANLVFHNKAIDGTAIKRLISRL
IDHFGMAYTSHILDQVKTLGFQQATATSISLGIDDLLTIPSKGWLVQDAEQQSLILEKHYHY
GNVHAVEKLRQSIEIWYATSEYLRQEMNLNFRMTEPFNPVHIMSFSGARGNTSQVHQLVG
MRGLMSDPQGQMIDLPIQSNLREGLSLTEYIISCYGARKGVVDTAVRTSDAGYLTRRLVEV
VQHIVVRRTDCGTARGISVSPRNGMMP-ERIFIQTFIGRVLADNIYMGLRCIAIRNQDIGIGL
ANRFIT------FRTQTISIRTPFTCRSTSWICRLCYGRSPTHGDLVELGEAVGIIAGQSIGEPGTQ
LTLRTFHTGGVFTGGTAEHVRAPSNGKIKFNEDLVHPIRTRHGHPAFLCYIDLYVTIKSQDII
HNVTIPPKSFLLVQNDQYVESEQVIAEIRAGAYTL-NFKEKVRKHIYSDSEGE-MHWSTDV
YHAPEFTYSNVHLLPKTSHLWILSGSSCR--SSIVPFSLHKDQDQMNVH--SLSVKR-RYISSP-
SVNNDQ-VKHKFFSSDFSGK-ESGIPDY-SELNRSICTGHCNLIYST-ILYKNSD--LLAKRRRN
KFIIPFQSIQEREKELMTQSAISIEIPIN--GIFRRNSVFAYFDDPQYRKKSSGITKYGAIGVHSI
VKKED--LIEYR-GVKEFKPKYTKVDRFFFIPEEVYILPESSSLMVRNNSIIGVDTQIALNTRS
RVGGLVRVERKKKKMELKIFSGDIHFPGETDKISRHSDILIPPGTVKT--NSKESKK--VKNWI
YIQR--ITPTKKK--YFVLVRPVIIYEIANGINLETLFPQDLLQEKDNLKLRVVNYILYGTGKPI
RGISDTSIQLVRTCLVLNWDQDK---KSSSIEEARAAFVEISTNGLIRDFLRINLVKFHISYIRK
RNDPSGSEPIS-NNGSDRTNINPFYPIYSKTR-----VQQSLKQNQGTISTLLNI---NKECQSLIIL

SSSNCFQMDP--FNDVKHHN---VIKES-PIIPIRNSLGPLG--TALQIANF--YLFYHLN-ITHNQI
S-VTKYSKLYN---LKQTFQVL--K-YYLMDENGR--IVNPDPCSNSVLNPFNLNWYFLHHNY
CESFFTIIS-LGQFICENLCMAKNGP---HLKSGQVIIVH-IDSVVIRSAKPYLATPGATVHGHY
GEILYEGNTLVTFIYEKSRSGDITQGLPKVEQVLEVRSIDSISINLEKRVEGWNECITRILGIP
WGFLIGTELTIVQSRISLVNKIQKVYRSQGVQIHNRHIEIIVRQITSKVLVSE---DGMSNVFSP
GELIGLLRAERTGRALEEAICYGAILLGITRASLNTQSFISEASFQETTRVLAKAALRGRID
WLKGLKENVVLGGMIPVGTGFKGLVQGS--RQHKNIPLKTKKKN--LFEGEFRDRDILFHH
RELFDSCISKNLYDTSEQSFIGFNDS------MIRRYWNINFEEMMKAGVHFGHGTRKWNPRM
APYISAKRKGIHITNLTRTARFLSEACDLVFDAASRRKQFLIVGTKNKAADSVARAAIRARC
HYVNKKWLGGILTNWSTTETRLQKFRDLRMEQKAGRLNRLPKGDAARLKRQLAHLQTY
LGGIKYMTGLPDIVIIVDQQEEYTALRECMTLGIPTICLIDTNCDPDLADISIPTNDDAIASIR
LILNKLVFAICEGRS-SYIRNPMGQKINPLGFRLGTTQSHHSLWFAQPKNYSEGLQEDQKIR
NCIKNYVKKNAKISSG-IEGIVRIEIQKRIDVIQVIIYMGFPKLLIESR----PKRIEELQMNVQK
ELNCVNRKFNIAITRIPNPYGHPNILGEFIAGQLKNRVSFRKAMKKAIELTEQANTKGIQVQ
IAGRLDGKEIARVEWIREGRVPLQTIGAKIDYCSYTVRTIYGVLGIKIWTFPDKKMSRYRGP
RFKKIRRLGALPGLTSKRPRVGSDLRNQSRSGK-KSQYRIRLEEKQKLRFHYGVTERQLLK
YVRIAAKAKGSTGQVLLQLLEMRLDNILFRLGMASTIPRARQLVNHRHILVNGRIVDIPSY
RCKPRDIITARDEQKSRAMIQNDLDSSPQ-EELPKHLTLHPFQ--YKGLVNQIIDSKWVGLKI
NELLVVEYYSRQTMSRRGTAEEKTAKSDPIYRNRLVNMLVNRILKHGKKSLAYQIIYRAM
KKIQQKTETNPLSVLRQAIRGVTPDIAVKARRVGGSTHQVPIEIGSTQGKALAIRWLLGAS
RKRPGRNMAFKLSSELVDAAKGSGDAIRKKEETHRMAEANRAFAHFRMGRDPIADIITSI
RNADINRKGTVRIASTNITENIIKILLREGFIENVRKHQEGNKKFLVLTLRHRR--KRKGRTS-
----LNLKRISRPGLRIYSNYQKIPRILGGMGIVILSTSRGIMTDREARLERIGGEILCYIWMAK
PLPKISSRRNGRIGSRKNSRKIPKGVIHVQASFNNTIVTITDVRGRVISWSSAGTCGFRGTRR
GTPFAAQTAAGNAIRTVVDQGMQRAEVMIKGPGLGRDAALRAIRRSGILLSFVRDVTPMP
HNGCRPPKKRRVMPTIKQLIRNTRQPIRNVTKSPALGGCPQRRGTCTRVYTITPKKPNSAL
RKVARVRLTSGFEITAYIPGIGHNLQEHSVVLVRGGRVKDLPGVRYHIVRGTLDAVGVKDR
QQGRSKYGVKKPKMARKSLIQREKKRQKLEQKYHLMRRSSKKEIS---KVPSLSDKWEIH
GKLQSPPRNSAPTRLHRRCFSTGRPRANYRDFGLSGHILREMVHACLLPGATRSSWMVKN
SFISVISQE-E-KDENKGSVEFQIVSFTNKIRRLTSHFELHRKDYLSQRGLRKILGKRQRLLSY
LAKKNRVRYKELISRLDIRESKTR--MVKLRLKRCGRKQRTVYRIVAIDVRSRREGRDLRK
VGFYDPIKNQTYLNVPAILYFLEKGAQPTGTVRDILRKAGVFTELRLNQ-TQFTMDKSKRL
FLKSKRSFRRRLPPIQSGDRIDYRNMSLISRFISEQGKILSRRVNRLSLKQQRLITIAIKQARI
LSSLPFLNNEK--QFE-----------RVGRYVTR-LKKNPFVANHLLKKINKLNNKAEKEIIVTWSR
ASTIIPTMIGHTIAIHNGKEHLPIYITDRMVGHKLGEFAPTLNFRGHAKNDNKSRRMIFQSFI
PGNLVSLCMKIINSVVVVGLYYGFLTTFSMGPSYLFLLRARVIEEGEEGTEKKVSATTGFIT
GQLMMFISIYYAPLHLALGRPHTITVLALPYLLFHFFWNNHKHFFDYGSTTRNSMRNLSIQ
FVFLNNLIFQLFNHFILPSSMLVRLVNIYMFRCNNKMLFVTSSFVGWLIGHILFMKWVGLIL
VWIQQNNSIRS-FRSNKYLVSELRNSRARIFSILLFITCVYSLGRIPSPIFTKKLKETEED--LVT
ILFDSKRWNRPFRYIKNDQFENAVRKEMSQYFFYTCRSDGKERISFTYPPSLSTFLEMIQRK
ISPFTTEKLSSDELYNRWNYKNEQKKKTLNNEFINRVQALDKGYLTLNTLEKRAGLCNDK
TKKEYLPKIYDPFLSGSCRGK-IQFFSPIEMLWINKIHLILYQEFESKNPKNKSTGIKEISKQV
PRWSYKLIDLEQQE--QMRSRKTKRLEIFNNSLLYYSHQPDFRRCIIKGSVRAQRRKITIFEL
FRANVHSPLFLDRINKYLFFFFDI--VELIKKMFINIEEEEEVEIAEAWDDILFAQITRGFILVT

QSIFRKYILLPSLIIIKNIIRILFLKTPEWSEDFKDWSREIHVKCTYNGVQLSEKEFPKNWLIE
GIQIKILFPFRLKPSHKSKKAQKNDYFFLTVWGTEADLPFSASRKELSLFKPIFKKLKKKFR
KMQKNGFRV-ITILEERKKLNF--SKEKKNWIIQNIF--FRKEIINNLSKSKDSIINNNTIQKLST
PIRSMAWTNYSLTEKKMKDLSARRKIIINQIEKIKKEKEKSPNKISYNAK--KLKSSISQIVKK
RNARLAHKFHFFIKILIERIYIDIFLGIINIPRINAQLFIHFISCDISSLSQAYVFYKLSQIKIINLY
KLRSIFEY-SLFLKNEIKDFFAQGLFNSELKDKNFRNSVMNQWKNWLRSHYQYKLSQIRW
SRLTPQKWRNRINQHHMHMVQNKWNLYENEKDRLIHYKKK-NFKKHYRYNLLAYKSIN
DDIIDADRKYFDWSILSFCLRPFDWMGMNEEIKNRWFFQKFLILYNTYKIKPWAIPIKFLLF
NNKSHMEVELDFFLKRYLCFQLEWNS--NNQLIENIKFYGFLLKVTNPRKIIISSIQRQEINL
NIMMVRNSFTRTELVKRGILIIEPVRLSVKNDGQFILYQMIGILLVHKNKQNDYDLLVPENI
LSPKRRRELRIL--FIFLWPNFRLEDLACMNRYWFDTNNASRFSMVRIYIYPMKGHQLKSWI
FELREILREIK-------------SVGSFIHIFFHQERFIKLLDSRIWSILLS-RNS--QGSTSNRYF---TIK
GVVLFVVV-VLIYR----INNRKMVERKNLYLTGLLP-----IPMNSIGPRNDTLEESF-WSSNINR
LIVS-LLYLPKGKKISESSFLDPKESTWVLPIT----KKCIMSESNWGSRWWRNWIGKKR--DSS
CKISN---ETVAGIEISFKEKDIK-YLEFLFVYYMD--DPIRKDHDWELFDRLSPRKGRNIINL--
NSGQLFEILVKDWICYLMFAFREKIPIEVEG-FFKQQG-AGSTIQSNDIEHVSH--LFSRKKWA
ISLQNCAQFHMWQFRQDLFV---SWGN-NPHESDFLRN-ISRENWIWLDNVWLVNKDR---FF
S--KARNISSNIQYDS---TR--SSFVQGRNSSQLKGSS-DQSRDHFDSIS----NEDSE-------------Y
HTLINQRKIQQLKERSIL-WD--PSFLQTERTEIESD--RFPK--CLSGYS--SMSRLFT-EGEKEM
NNHLLPEEIEEF--LGNPTRSIRSFFSDRS--SELH-LGSNPTERSTRNQ----KLLKKEQDVS-FV
PSRRSE----NKEIVNIFKIITYLQN--TVSIHPISSDPGCDMVL-KDELD-MDSSNKISFLNKNPF
FDL--FHLFHDRNGGGYTL--HHDFESE--ERFQEMADLFTLSITEP-DLVYHKGFTFFIDSYGL
DQKQ--FLNEVFNSRDESKKKSL---LVLPPIFY--EENESFYRRIRKKWVR-ISCGNDLEDPKQ
KIVVFASNNIMEAVNQYGLILNLIQIQYSTYG--YIRNVLTQFFLMN---RSDRNFEYGIQ---RD
QIGNDTLNHRTIMKYTINQHLSNLKQ-SQKKWFDPLIFIFLSRTERSMNWDPNAYRYKWSN
GSKNFQEHLEHFISEQKSRF-LFQVVFDRLRINQYSIDWSEVIDKKDLSKSLRFFLSKLLL----
-----FLS-------KFLLFLSNSLP-FFFVSFGNIPIHRSE-IHIY-ELKGPNDQLCNQLVEPIGLQIVHL
KKLKPFLLLLD---DHDTS-QKSKFLINGGTISPFLFNKIP-KW-MIDSFHTR--NNRRKSFDNT
DSYFSMISHDQDNWLNPVKPFHRSSLISSFYKANRLRFLNNLHHFCFY--CNKRFPF---Y-VE
KARIKNYD--FTYG-QFLNILFIRNK-IFSLCGGK--KKHAFLE-RDTISPIESQVSNIFIPNDFPQ
SGNER-YNLYKS---FHFPIRSDPFVR--RAIYSIADISGTPLTEGQIVNFERTYCQPLSDMNLSD
SEGKNLHQYLN--FNSNMGLIHTPCSEKYLPSEK--RKK--------RSLCLK--KCVEKGQMYRT
FQRDNAFSTLSKWNLFQTYMPWFLTSTGYKYLNLIFLDTFSD-LLPILSSS--QKFLSIFHDIM
HGSDISWLIFQKRL------WKICRN-LISEISSKCLHN--LLLSEEMIHRNNEP--PLISTHLRSPN
VREF-LYSILFLLLVAGY-LVCTHLLFVSH--AYSELQTEFEKVKSLMIPSYMIELRKLLDRYPT
SELNSFWLKNLFLVALEQLGDFLEEMRGSASGGNMLWGGGP-----AYG-VKSIRSKKKFWN
IN---LIDLI-SIIPNPINRITFSRNTRHLSHTSKEIYSLIRKRKNVNGDWIDDKIESLVANSDWID
DKEREFLVQFS--TLTT--EKRIDQILLSLT-HSDHLS-----KNDSGYQMIEEPGAIYLRYLVDIH--
KKYLMNYEFNTPCLAERRIFLAYYQTTTY--SQTSCGVNSFHFPSHG---KPFSLRLALSPS-R
GILVIGSIGTGRSYLVKYLATNSYLPFVTVFLNKFLNNKPKGFLID-----DSDDIDDSDDIDDS
DDIDASDDIDVSDDIDVSD-DDIDRDFDFDTELEFLTTMDAL---TIDMMPE-IEINRFYITLQF
ELAKAMSPCIIWIPNIHDLDVNESNYLSLGLLVNYLS---RDCERCS-TRNILVIASTHIPQKV
DPALIAPNKLNTCIKIRRFLIPQQRKHFFTLSYTRGFHLENKMFHT--NGFGSITMGSNVRDL
VALTNEALSISITQKKSIIDTNIIRSALHRQTWDLR---SRVRSVQDHGIFFYQIGRAVAQNVFL

SNCPIDPISIYMKKK--SCNEGDSYLYKWYFELGTSMKKLTILLYLLSCSAGSVAQDLWSLP-
-GPDEK-NGITYYGLVENDS-DLVHGLLEVEGALVG--SSRTEKDCSQFDND--RVTLLLRPEP
RS-PLDMMQNGSCSILDQRFLYEK--YESEFEE-GEGEEVLDPQ----QIEED----LFTH--IVWAP
--RIWRP---WGFLFDCIERPNELGFP-YWARSFRGK-DEEIIYDEEDELQENDSEFLQSGTMQ
YQIRDRSSKEQGFFRISQFIWDPADPLFFLFK-DQPFVSVFSHREFFADEEMSKGLLTSQTDP
PTSIYKRWFI--KNTQEKRFELLIHRQRWLR---TNSSLS--NGFFRSN--TLSESYQYLSNLFLS
N-GTLLDQMTKALLRKRWLFPDEMKIGF--M---------MPRSR-INGNFIDKTFSVVANILLRIIP
TTSGEKEAFTYYRDG--MSAQSEGNYAEALQNYYEAMRLEIDPYDRSYILYNIGLIHTSNG
EHTKALEYYFRALERNPFLPQALNNMAVICHY-RGEQAIRQGDSEIAEAWFDQAAEYWKQ
AIALTPGNYIEAQNWLKITRRFE--MSWRSERIWIELIAGSRKTSNFCWAFILFLGSLGFLLV
GISSYLGRNLISLFPSQQIIFFPQGIVMSFYGIAGLFISSYLWCTILWNIGSGYDRFDRKEGIV
CIFRWGFPGKNRRILLRFLMKDIQSIRIEVKERIYARRVLYMEIRGRGAIPLTGTDEN-LTPRE
IEQKVAELAYFLRVPIEVF
>Trachelium
???
???
???
???
???
??????????????????????????????MVNVRADEISNIIRERIKQYNREVKIENTGSVLQVGDGIA
RIHGLDEIMAGELVEFEEGTIGIALNLESHNVGVVLMGDGLRIQEGSSVKATGRIAQIPVSE
AYLGRVINALAKPIDGRGEISSSESRLIESPAPGIISRRSVSEPLQTGLIAIDSMIPIGRGQRELI
IGDRQTGKTAVATDTILNQQGQNVICVYVAIGQKASSVAQVVTTFQEKGAMEYTIVVAEM
ANSPATLQYLAPYTGAALAEYFMYRERHTSIIYDDLSKQAQAYRQMSLLLRRPPGREAYP
GDVFYLHSRLLERAAKLSSQLGEGSMTALPIVETQSGDVSAYIPTNVISITDGQIFLSADLF
NAGIRPAINVGLSVSRVGSAAQIKAMKQVVGQLKLDLAQLTELETFAQFASDLDKGTQNQ
LARGRRLRELLKQSQSNPLAVEEQIMTIYTGINGFLDSLEIGQVNKFLDKLRKYLKEEKPK
FQEIISSTKIFTEEAEDILKKAIQKQMEL-------MRLNPTTSGSGVSTLEKNKPGRIGQIIGPVL
DVAFPPGKMPNIYNALVVKGRDTLGQPINVTCEVQQLLGNNRVRAVAMSATDGLMRGME
VIDTGTPLSVPVGGATLGRIFNVLGEPIDNLGPVDTRTTSPIHRSAPAFIDLDTKLSIFETGIK
VVDLLAPYRRGGKIGLFGGAGVGKTVLIMELINNIAKAHGGVSVFGGVGERTREGNDLY
KEMKDSGVINEQKFAESKVALVYGQMNEPPGARMRVGLTALTMAEYFRDVNKQDVLLFI
DNIFRFVQAGSEVSALLGRMPSAVGYQPTLSTEMGSLQERITSTKKGSITSIQAVYVPADDL
TDPAPATTFAHLDATTVLSRGLAAKGIYPAVDPLDSTSTMLQPRIVSDEHYQTAQSVKQTL
QRYKELQDIITILGLDELSEEDRLTVARARKIERFLSQPFFVAEVFTGSTGKYVGLVETIRGF
ELILSGKLDVLPEQAFYMVGNIDEATAKAMNLEMESNLNKMILNLCVLTPNRIIWDSEVK
EIILSTDSGQIGVLPNHAPIATAVDIGILRIRLNDQWVTVALMGGFARIGNNEITVLVNDAEK
SLDIDPQEAQQTLEIAKANLSKAEGKRQTIEANLALRRARTRVEAINEISMNNGTDSFFSLG
RWPSAGSFGFNTDILATNLINLSVVIGVLIFFGKGVLSDLLDNRKQRILNSIQDSEDLRGAA
VNQLEKARARLRKVEIEADQYRVNEYSAIKREKLKLINSIYTTLEQEENKNNETLRFEQER
AINQVQQWVLQQALQGALGTLNSCLNTELHLRTISVNIGILGAMKEITDMNPLISAASVIA
AGLAVGLASIGPGIGQGTAAGQAVEGIARQPEAEGKIRGTLLLSLAFMEALTIYGLVVALAL
LFANPFVMNILSCSTNILNGFYDISGVEVGQHFYWKIGGFQVHGQVLITSWVVIAILLGSA
ALAVRKPQTIPTGIQNFFEYVLEFIRDVSKTQIGEEYGPWVPFIGTIFLFIFVSNWSGALLPW

KIIQLPHGELAAPTNDINTTVALALLTSVAYFYAGLAKKGLGYFGKYIQPTPILLPINILEDFT
KPLSLSFRLFGNILADELVVVVLVSLVPSVVPIPVMFLGLFTSGIQALIFATLAAAYIGESME
GHHMKFSTLEHILIHLSFSICSIVITIRLINLVVTTILKPYDFSEKGIITTFLCLTGFLVTRWIYS
GHFPLSNLYESLIFLSWSFSLIHIVAY--KNKN-LSRITASSTIFTQGFATSGLLTEINKSGILVPA
LQSEWLIMHVSMMILSYAALLCGSLLSAALLVITCR--------KKKIQYRNQRIHLFGKTY---V
KNYYKIQLVQQLDYWSYRVISLGFIFLTMGILSGAVWANEAWGSYWSWDPKETWAFITWI
IFAIYLHTRTNKNFWGANSAIVAVIGFLIIWICYFGVNLLGIGLHSYGSFKGMMTKKKAFPP
RLYFAFIVFLPQCISLLFTKSLESCVTNWWNIDQSENFLNDIEETSMIEKFIELEELLLFDEIIK
EYSETHRQNLRIGIHTAIIQLSKIHNEERIHTILHLSTNIISFVILIAYSILGKKELVIINSWAQEF
LYNLNDTTKALSILFLADLCLGFHSTRGWELIISSVYKDFGFVPNDQILSCLLWILPVILDSIF
K Y W V F Y Y L N R V S P S L V V I Y H A I S
D??
??
??
????????MAEFQSALE-NRFQQHDFLYPLIFQEYIYALAHDHGLAGYDKKFSLLIVKRLITRM
YDQNYLIHSTNDCKQKPFWGNHFDSQMISEAFSVIMEIPFSLRLLSWLRVKSDNLRSIHSIF
SFLEDKISHLYYVLDILIPYPIHLEILVQALRYWLKDASSLHFVRFFLHEFHNWNSLITKVGP
SFSEQRFFFFLYNSHVCEYESIFVFLRNQSSYLRSTSYRALVERIYFYGKIEHLGEVLSRAFN
LSIFTDSFMHYVRYQGKLILASKGTSLVMNKWNYYFVNFWQSYFYLWSQPRRIHINQLSN
HSLDFLAYFSSVRRRTSTVRNQMLSKLFLSDNAI--NKFDTFVPIIPMIGSLAKSKFCNRAGH
PISKAVWLDLSDSDIIDRFGRIARNLSHYHSGSSNKKSLSRIKYILQLSCARTLAR?HKSTVR
AFLKIFGSELLEEFFTSEEQVLSLTFPRRVWYLDISCINELANHDMIIE-TTEVQ-AINSFSRLE
S-LKEIYGILWMLVPILTPVLGITIGVLVIVWLEREISAGIQQRIGPEYAGPLGILQALADGTK
LLFKENLLPSRGDSRLFSIGPPIAVISILLSYLVIPFGYHLVLGDLSIGVFLWIAISSIAPAGLLM
SGYGSNNKYSFLGGLRAAAQSISYEIPLTLCVLSISLLSNSSGTVDIVEAQSKHGFWGWNL
WRQPIGFLVFLISSLAECERLPFDLPEAEEELVAGYQTEYSGIKFGLFYVASYLNLLVSSLFV
TVLYLGGWNLSIPHISVPGLFEIKKAGRVFVTLIGIFITLAKTYLFLFIPITTRWTLPRLRMDQ
LLNLGWKFLLPISLGNLLLTTSSQLLSLMIWHVQNDNFILNFTRIFMKAFHLPLFDGSFIFPE
CILIFGLILLLMIDSTSDQKDIPWLYFISSTSLVMSIMALLFRWREEPMISFSGNFQTNNFNEI
FQVLILLCSTLCIPLSVEYIECTEMAITEFLLFVLTATLGGMFLCGANDLITIFVAPECFSFCS
YLLSGYTKKDVRSNEATMKYLLMGGASSSILVHGFSWLYGLSGGEIELQEIVNGLINTQM
YNSPGISIALIFITVGIGFKLSPAPSHQWTPDVYEGSPTPVVAFLSVTSKVAASALATRIFDIPF
YLSSNEWHLLLEILALLSMILGNLIAITQTSMKRMLAYSSIGQIGYIIIGIIVGDSNDGYASMI
TYMLFYISMNLGTFACIILFGLRTGTDNIRDYAGLYTKDPFLALSLALCLLSLGGLPPLAGF
FGKLYLFWCGWQAGLYFLVLIALVTSVLSIYYYLKIIKLLMNGRNQEITPHVGNYRRSSLR
SKNSIEFSMIVCVIASTIPGISMNPIIAIAQDSLFMLLLYEYDIFWAFLIISSLIPILAFFLSGVLA
PISKGPEKFSSYESGIEPIGDAWLQFRIRYYMFALVFVVFDVETVFLYPWSMSFDVLGVSVF
IEAFIFVLILIVGLVYAWRKGALEWSTNYFPWLTVSIFLPLSVGSVIFFLPHRGNRVIRWYSIC
ICILELLVITYLFCYHFESNDPLIQLVEDYKWINFFEFHWKLGIDGLSIGPVLLTGFITTLATL
AAWPVTRDSRLFYFLMLSMYSAQIGSFSSQDLLLFFIMWELELIPIYLLLSIWGGKKRMYS
ATKFILYTAGGSVFLLMGVLGLGLYGSNESTLDFERLINQTYPMSLEILFYIGFFIAFAVKSPI
LPLHTWLPNTHGEAHYSTCMLLAGILLKMGAYGLIRINMELLPHAHSLFSPWLLIIGTLQII
YAASTSSAQPNLKKRIAYSSVSHMGFILVGIGSITNAGLNGALLQIISHGFIGAALFFLVGTS
YDRIHLVYLNEMGGVAILMPKIFTMFSTFSMASLALPGMSGFVAELIVLWGIITSQKYLVM

PKILITFVIAIGIILTPIYSLSMARQIFYGYKLLNAPNSYVLDFGPRELFVSISIFLPVIGIGLYP
DFILSLSIDKVEVILSNVFVE-MLEHVLVLSAYLFSVGLYGLITSRNMVRALICLELIFNAVNI
NFVTFSDFFDSRHLKGSIFAIFVIAIAAAEAAIGLAILSAIYRNRKSIHINQSNLLTKMAQTFQ
YACIIPFLPLPIPILLGVGLLIFPAATKSLRRMWAFQSVLLLSLVMVFSINLSIQQINISFTYQY
VWSWIINNDFLLEFGYLMDPLTSIMSILITTVGIMVLIYSDNYMLHDRGYLRFFAYMSFFST
SMLGLVTSSNLIQIYIFWELVGICSYLLIGFWFTRPVAANACQKAFVTNRVGDFGLLLGILG
FYGLTGSFEFRDLFQIFNNNESNLFFVTLCAVLLFSGAVAKSAQFPLHVWLPDAMEGPTPIS
ALMHAATMVAAGIFLVARLLPLFRFIPHIMNFISLIGIITVFFGATLALAQKDIKRGLAYSTM
SQLGYMMLALGMGSYRSALFHLITHAYSKALLFLGSGSVIHSMQILVGYSPNKSQNMVL
MGGLRKHVPITKMSFLVGTLSLCGIPPLACFWSKDEILNDSWLYSPIFAIIAWSTAGLTGFY
MFRIYLLTFEGHLNVDLKNYSGQKKISIYSVSLWGKEREKVNKT-LLTLLTLHF-FFGNKNIF
SYPYESDNNILFPLLVLVLFTLFVGVLGIPF-LDILSKWLRPSINLFHQNSK-LADWAEFLRD
GVFSISIAYLGIILAFFFYKPLYSSLTNLDFINFFVLVYDKIINGIYDWSDNRGYIDVFYGTYF
LGGIRRVSDLTYFFDRKVVDGITNGVGLMNFIFGEGIKFFSGG-RISSYLFLYCSYFSIFLLIFS
MDLPGPIHDFILVFLGLVLILGGLAVVLLPNPIYSAFSLGLVLVCISLLYILSNSHFLAAAQLL
IYVGAINVLIIFAVMFLNGSEYYKDLNPWTVGDGVTSVVCTSLFASLITTIPDTSWYGIIWT
TKSNQIIEQDLINNSQEIGIHLSTDFLIPFELISIILLVALIGAIAVARQMTVPATRKDLMIVNM
GPHHPSMHGVLRLIVTLDGEDVIDCEPILGYLHRGMEKIAENRTIIQYLPYVTRWDYLATM
FTEAITVNAPEQLGNIQVPKRASYIRVIMLELSRIASHLLWLGPFMADIGAQTPFFYIFRERE
LIYDLFEAATGMRMMHNFFRIGGVAADLPHGWIDKCLDFCDYFLTSISEYQKLITRNPIFLE
RVEGVGIIGEEEAINWGLSGPMLRASGIQWDLRKVDHYECYDEFDWEVQWQKEGDSLAR
YLVRIGEMTESIKIIQQALEGIPGGPYENLEIRRFDRVRDPGWNDFDYRFISKKPSPTFELSK
QELYVRVEAPKGELGIFFIGDKSVFPWRWKIRPPAFINLQILPQLVKRMKLADIMTILGSIDII
MGEVDRMFPMVTEFMNYGQQTVRAARYIGQGFMITLSHANRLPVTIQYPYEKLMTSERF
RGRIHFEFDKCIACEVCVRVCPIDLPVVDWKLETDIRKKRLLNYSIDFGICIFCGNCVEYCP
TNCLSMTEEYELSTYDRHQLNYNQIALGRLPMSVIDDHTIRTILNF----------------MQGYLSA
WLVKHGLIHRSLGFDYQGIETFQIKSEDWHSIAVILYVVGYNYLRSQCAYDVAPGGLLASV
YHLTRIKYGVDQPEEVCIKVFATRRDPRIPSVFCVWKSVDFQERESYDMLGISYDNHPRLK
R I L M P D S W I G W P L R K D Y I A P N F Y E I Q D A
H???
???
???
????????????????????MQTINTFSWIKERITRSISVSLMIYILSQTSISSAYPIFAQQGFENPREAT
GRIVCANCHLANKPVDIEVPQAVLPDTVFEAVVRIPYDMQLKQVLANGKKGALNVGAVLI
LPEGFELAPPNRISPEMKEKIGNLSFQSYRPNQKNILVIGPVSGQKYSEITFPILSPDPATKKD
INFLKYPIYVGANRGRGQIYPDGSKSNNTVYNATAAGIVSKIIRKEKGGYEISIADASDGRL
VVDIIPPGPELLVSEGESIKFDQPLTSNPNVGGFGQGDAEIVLQDSVRVQGLLLFLASVILAQ
IFLVLKKKQFEKVQLSEMNFMNKVYDWFEERLEIQAIADDITSKYVPPHVNIFYCLGGITL
TCFLVQVATGFAMTFYYRPTVTDAFASVQYIMTEANFGWLIRSVHRWSASMMVLMMILH
VFRVYLTGGFKKPRELTWVTGVVLAVLTASFGVTGYSLPRDQIGYWAVKIVTGVPEAIPVI
GSPLVELLRGSTSVGQSTLTRFYSLHTFVLPLLTAVFMLMHFPMIRKQGISGPL-VITKKPDL
NDPVLRAKLAKGMGHNYYGEPAWPNDLLYIFPVVILGTIACNVGLAVLEPSMIGEPADPFA
TPLEILPEWYFFPVFQILRTVPNKLLGVLLMVSVPAGLLTVPFLENVNKFQNPFRRPVATTV
FLIGTAVALWLGIGATLPIDKSLTLGLFMIEVFLFGIVLGLIPITLAGLFVTAYLQYRRGDQLD

LMPTITSYFGFLLAALTITSALYIGLSKIRLIMDIVSLAWACF?SLYIFSFTRIWGGRWSLMIIR
SPEPEVKILVDRDPIKTSFEEWAKPGHFSRTIAKGPETTTWIWNLHADAHDFDSHTSDLEEI
SRKVFSAHFGQLSIIFLWLSGMYFHGARFSNYEAWLSDPTHIGPSAQVVWPIVGQEILNGD
VGGGFRGIQITSGFFQIWRASGITSELQLYCTAIGALIFSSLMLFAGWFHYHKAAPKLTWFQ
DVESMLNHHLAGLLGLGSLSWAGHQVHVSLPINQFLNAGVDPKEIPLPHEFLLNRDLLAQ
LFPSFAEGATPFFTLNWSKYAEFLTFRGGLDPVTGGLWLTDIAHHHLAIAILFLIAGHMYRT
NWGIGHGLKDILEAHKGPFTGQGHKGLYEILTTSWHAQLALNLAMLGSLTIVVAHHMYA
MPPYPYLATDYGTQLSLFTHHMWIGGFLIVGAAAHAAIFMVRDYDPTTRYNDLLDRVLR
HRDAIISHLNWACIFLGFHSFGLYIHNDTMSALGRPQDMFSDTAIQLQPVFAQWIQNTHAL
APGATAPGATASTSLTWGGGDLVAVGGKVALLPIPLGTADFLVHHIHAFTIHVTVLILLKGV
LFARSSRLIPDKANLGFRFPCDGPGRGGTCQVSAWDHVFLGLFWMYNAISVVIFHFSWKM
QSDVWGSISDQGVVTHITGGNFGQSSITINGWLRDFLWAQASQVIQSYGSSLSAYGLFFLG
AHFVWAFSLMFLFSGRGYWQELIESIVWAHNKLKVAPATQPRALSIVQGRAVGVTHYLLG
GIATTWAFFLARIIAVGMALRFPRFSQGLAQDPTTRRIWFGIATAHDFESHDDITEERLYQNI
FASHFGQLAIIFLWTSGNLFHVAWQGNFEFWVQDPLHVRPIAHAIWDPHFGQPAVEAFTRG
GALGPVNIAYSGVYQWWYTIGLRTNDDLYAGALFLLFLSVISLIAGWLHLQPKWKPSVSW
FKNAESRLNHHLSGLFGLSSLAWTGHLVHVAIPASRGEYVRWNNFLDVLPHPQGLGPLFT
GQWNLYAQNPDSSSHLFGTSQGAGTAILTLLGGFHPQTQSLWLTDIAHHHLAIAFLFLVAG
HMYRTNFGIGHSMKDLLDAHIPPGGRLGRGHKGLYDTINNSIHFQLGLALASLGVITSLVA
QHMYSLPAYAFIAQDFTTQAALYTHHQYIAGFIMTGAFAHGAIFFIRDYNPEQNEDNVLAR
MLDHKEAIISHLSWASLFLGFHTLGLYVHNDVMLAFGTPEKQILIEPIFAQWIQSAHGKTSY
GFDVLLSSTRGPAFNAGRSIWLPGWLNAINE--NSNSLFLKIGPGDFLVHHAIALGLHTTTLI
LVKGALDARGSKLMPDKKDFGYSFPCDGPGRGGTCDISAWDAFYLAVFWMLNTIGWVT
FYWHWKHITLWQGNVSQFNESSTYLMGWLRDYLWLNSSQLINGYNPFGMNSLSVWAW
MFLFGHLVWATGFMFLISWRGYWQELIETLAWAHERTPLANLIRWRDKPVALSIVQARLV
GLAHFSVGYIFTYAAFLIASTSGKFGMSHSVKIYDTCIGCTQCVRACPTDVLEMIPWDGCK
AKQIASAPRTEDCVGCKRCESACPTDFLSIRVYLWHETTRSMGLAYMTILSFPSLLVPLVGL
VFPAIAMVSLSLHVQKNKIVMRDLKTYLSVAPVLSTLWFGSLAGLLIEINRFFPDALTFPFF
SFMTAILERRESESLWGRFCNWITSTENRLYIGWFGVLMIPTLLTATSVFIIAFIAAPPVDIDG
IREPVSGSLLYGNNIISGAIIPTSAAIGLHFYPIWEAASVDEWLYNGGPYELIVLHFLLGVAC
YMGREWELSFRLGMRPWIAVAYSAPVAAATAVFLIYPIGQGSFSDGMPLGISGTFNFMIVF
QAEHNILMHPFHMLGVAGVFGGSLFSAMHGSLVTSSLIRETTENESANEGYRFGQEEETY
NIVAAHGYFGRLIFQYASFNNSRSLHFFLAAWPVVGIWFTALGISTMAFNLNGFNFNQSVV
DSQGRVINTWADIINRANLGMEVMHERNAHNFPLDLAAIESPSKNGMGLPWYRVHTIVL
NDPGRLLAVHIMHTALVAGWAGSMALYELAVFDPSDPVLDPMWRQGMFVIPFMTRLGIIN
SWGGWGIT-GGTITYPGIWSYEGVAGAHIVFSGLCFLAAIWHWVYWDLEIFTDERTGKPSL
DLPKIFGIHLFLAGVACFGFGAFHVTGLYGPGIWVSDPYGLTGKVQYVNPAWGVEGFDPF
VPGGIASHHIAAGTLGILAGLFHLSVRPPQRLYKGLRMGNIETVLSSSIAAVFFAAFVVAGT
MWYGSATTPIELFGPTRYQWDQGYFQQEIYRRVSTGLAKNQSLSEAWSKIPEKLAFYDYI
GNNPAKGGLFRAGSMDNGDGIAVGWLGHPIFRDKEGRELFVRRMPTFFETFPVVLVDGA
GIVRADVPFRRAESKYSVEQVGVTVEFYGGELNGVSYNDPATVKKYARRAQLGEIFELDR
ATLKSDGVFRSSPRGWFTFGHASFALLFFFGHIWHGARTLFRDVFAGIDPDLDAQVEFGAF
QKLGDPTTRRQVV------------VETLFNGTLALVGRDQESTGFAWWAGNARLINLSGKLLGA
HVAHAGLIVFWAGAMNLFEVAHFVPEKPMYEQGLILLPHLATLGWGVGPGGEVLDTFPY

FVSGVLHLISSAVLGFGGIYHALLGPETLEESFPFFGYVWKDRNKMTTILGIHLILLGIGAFL
LVFKALYFGGVYDTWAPGGGDVRKISNLTLSPSILFGYLLKSPFGGEGWIVSVDDLEDIIGG
HVWLGSICIFGGIWHILTKPFAWARRALVWSGEAYLSYSLAAISVFGFIACCFVWFNNTAYP
SEFYGPTGPEASQAQAFTFLVRDQRLGANVGSAQGPTGLGKYLMRSPTGEVIFGGETMRF
WDLRAPWLEPLRGPNGLDLSRLKKDIQPWQERRSAEYMTHAPLGSLNSVGGVATEINAV
NYVSPRSWLATSHFVLGFFFFVGHLWHAGRARAAAAGFEKGIDRDFEPVLSMTPLNMTIA
LGKFTKDENDLFDIMDDWLRRDRFVFVGWSGLLLFPCAYFALGGWFTGTTFVTSWYTHG
LASSYLEGCNFLTAAVSTPANSLSHSLLLLWGPEAQGDFTRWCQLGGLWTFVALHGAFGLI
GFMLRQFELARSVQLRPYNAIAFSGPIAVFVSVFLIYPLGQSGWFFAPSFGVAAIFRFILFFQ
GFHNWTLNPFHMMGVAGVLGAALLCAIHGATVENTLFEDGDGANTFRAFNPTQAEETYS
MVTANRFWSQIFGVAFSNKRWLHFFMLFVPVTGLWMSALGVVGLALNLRAYDFVSQEIR
AAEDPEFETFYTKNILLNEGIRAWMAAQDQPHENLIFPEEVLPRGNALMSGSTGERSFADII
TSIRYWVIHSITIPSLFIAGWLFVSTGLAYDVFGSPRPNEYFTENRQGIPVITGRFDPLEQLDE
FSRSFMTIDRTYPIFTVRWLAVHGLAVPTVSFLGSISAMQFIQRMATQTVENDFRSG-PKRT
NVGSLLKPLNSEYGKVAPGWGTTPLMGVAMALFAVFLSIILEIYNSSVLLDGISMNMLTLK
LFVYTVVIFFVSLFIFGFLSNDPSRNPGREEMAKTTGRIPLWIIGTVTGILVIGLLGIFFYGSY
SGLGSSLMLNIFSLICICLNSTLYMSSLFFAKLPEAYAFLNPIVDVMPVIPVFFFLLAFVWQA
AVSFRTTQSNPNEQNVELNRTSLYWGLLLIFVLAVLFSNYFFNMEVNILAFIATALFILVPTA
FLLIIYVKTVSQNNMEIATLVAVFISGLLVSFTGYALYTAFGQPSQQLRDPFEEHGDMEALV
YTFLLVSTLGIIFFAIFFREPPKVPTKK--MTLAFQLAVFALIATSSILLISVPVVFASPDGWSSN
KNVVFSGTSLWIGLVFLVGILNSLIS-------MSPQTETKANVGFKAGVKDYKLTYYTPDYET
KDTDILAAFRVTPQPGVPPEEAGAAVAAESSTGTWTTVWTDGLTSLDRYKGRCYHIEPVA
GEENQFIAYVAYPLDLFEEGSVTNMFTSIVGNVFGFKALRALRLEDLRIPPAYIKTFQGPPH
GIQVERDKLNKYGRPLLGCTIKPKLGLSAKNYGRAVYECLRGGLDFTKDDENVNSQPFM
RWRDRFLFCAEALYKAQAETGEIKGHYLNATAGTSEEMIKRAVFARELGVPIVMHDYITG
GFTANTSLAHYCRDNGLLLHIHRAMHAVIDRQKNHGMHFRVLAKALRMSGGDHIHSGTV
VGKLEGEREITLGFVDLLRDDFVEKDRSRGIYFTQDWVSLPGVLPVASGGIHVWHMPALT
EIFGDDSVLQFGGGTLGHPWGNAPGAVANRVALEACVQARNEGRDLAREGNEIIRKAAK
WSPELFSACEVWKEIKFEFAAMDTL--MAIHLYKTSTPSTRNGSIPR------KN-LIYGQHLCG
KGRNARGIITAGHRGGGHKRLYRKIDFQRNEKDIYGRIITIEYDPNRNAYICLIHYGDGEKR
YILHPRGAIIGDTIVSGTEVPIKMGNALPL-NMPVGTAIHNIEIALGKGGQLARAGGAVAKLI
ANEGKSATLKLPSGEVRLISKNCSATVGQVGNLGVKQKSLGRAGSKRWLGKRPIVRGVV
MNPVDHPHGGGEGRAPIGKKKPTTPWGYPALGRRTRKRKKYSENLILRRRSKMIQPQTYL
NVADNSGARELMCIRIIGAS-NRRYAHIGDVIVAVIKEAVPKMSLERSEVVRAVIVRTCKELK
RDDGMIIRYDDNAAVVIDQEGNPKGTRVFGAIAEELRQFNLTKIIALAPEVLMLSPKRTRFR
KQHRGRMKGICLRGQSICLGKYALQALEPAWITSRQIEAGRRAMTRNARRGGKVWVRIFP
DKPVTVRAAETRMGSGKGNPEYWVAVVKPGQIIYEIGGVRENIARKAIEIASSKMPIRTQFI
RQTMTRISRGYIARRRRRKTSLVVSSSG---ASLSKLITQQEIKALASSDRDRDRQKRDFRCL
WITRINSVIREG--VSYSYSKFIHDLYKRQLLLNRKILAQIAIANKNFLYLISNQILKSEVNWK
ESAGVMLQEVYALGQNISMSAHKARRVIDQIRGRSYLTTLFLLKFMPYRACSRIFKLVWS
AAANARHLLGSKNENLFISQAEVNEGSSLKRFKLQARGCSYPIKRPTCHITIVMKDRSLTG
G A W D K
K??
???????MAVPKKRTSIAKKRIRKNIWKGKAYTSALKVVTLYKQTVNSKGFGFL--RSDKMA

KGKDIRITVILECTTC-VRK--ANKT--GISRYITKKNRHNTPNRLELRKFCRYCYKHTIHGEI--
MKLRASIRKICEKCRLIRRQNRILVICSNPRHKQRQGMVREKVIVSNRTLQWKCVESRADS
KRLYYGRFILSPLLKGQADIIGIAMRRALLGELEGTCITRAKSE--KLPHEYSTLVGIQESVH
EILMNLKEIVLRSNLYGTCDAAICVKGPGYVTAQDIISPPYVEIIDNTQHIASLTEPIELCIGL
QIERNRGYLIKTAK-NFDGSY--PIDAVFMPVRNVNHSIYSYGTGNDKHEILFLEIWTNGSLT
PKEALHEAARNLIDLFIPFLHTEEE--NLDLEDNQ-HMLPFRPSILPEKFV-KIK--NK-KISLKSI
FIDQSELPPRVYNCLKRSNIYTLLDLLNNSQEDLMKIEHFRVEDLKRILDILEKN--FGIDLPQ
NKMLRDKN-EGMFTIPGFNQIQFEGFCRFIDQGLRDELSNFPKIEDPDQKMEFQLFMETYQ
LVEPLIKERDAVYESLTYSSEFYVSAGLISKIRRGMQKQTIYIGNIPLMNSLGTFIVNGIYRIV
INQIVQSPGIYFRSELDHNGIASYTGTIISDWGGRSELEIDRKARIWARVSRKQKISILVLSSA
MGSSLREILENVCYPEIFLSFLNDKEKNKIGSKENAILEFYKKFTGVGGDPIFSEYLYKELQ
KKFFQQRCELGRIGRQNMNRRLNLAIPQNNTFLLPRDILAAVDGLIEMKFGMGRLDD--M
NHLKNKRIRSVADLLQDQFGLALVRLEKVVRSTICGALR-HKLR-PRPQNLVTSPL----LTTT
YDSFFGLHPLSQVLDQTNPLTQIVHGRKLSYLGPGGLTGRTASFRIRDIHPSHYGRICPIDTS
EGINVGLIGSLTIHVRIGRWG-SLESPFY---EISKRSKG--ARMLYLSPGQDEYYMVASGNSLA
LNQGIQEEQVVPARYCQEFLTIAWEEVHLRSIFSFQYFSIGASLIPFIEHNDASRALMSSNMQ
RQAVPLSRSEKCIVGTGLEGQVALDSGALALAQHEGKIIYTDTDKIFFSGNGDTLSLPLVM
YQRSNKNTCIHQKPQVQRGKCIKKGQILASGTATVGGELALGKNVLVAYMPWEGYNFED
AVLISERLVYEDIYTSFHIRKYEIQTYMTSEG-PERVTNEIPHLEAYLLRNLDKNGIVMLGSW
VEEGDILVGKLTPQMVNE-SAYAPEDRLLRAILGIEVSTSKETCLKLPIGGRGRVIDVRWIQK
-RVGSSYNPETIRVYISQKREIKVGDKVAGRHGNKGIISKILPRQDMPYLQDGRPVDMVFNP
LGVPSRMNVGQIFESLLGLAGGLLDRHYRIGPFDERYEQEASRKLVFSELYEAGKQTSNP
WVFEPEYPGKSKIFDGRTGDPFQQPVIIGKPYILKLIHQVDDKIHGRSSGHYALVTQQPLRG
RAKQGGQRVGEMEVWALEGFGVAHILQEMLTYKSDHIKARQEVLGTTIIGGTILNPEDAP
ESFRLLVRELRSLALELNHFLISEKNFQINRKEAMNQNFSYMIDRYKHQQLRIGLVSPQQIS
SWATKILPNGELVGEVTKPSTFHYKTKKAEKDGLFCERIFGPIKSGLCACGNYREIGDKKE
DPQFCEECGVEFVDSRVRRYQMGYIKLVCPVTHVWYLKRLPSYIANLLDKPLKELEGLVY
CD--FYFARP-INKKPTFLRLRGSFEYEIESWKYSIPLFFNTQGFDTFRNREISTGAGSIREQLA
DLDLRKIRNSSLLEWKELSKE-GPTGNEWEDQKVRRRKDFLVRRMGLAKHFIRTNIEPEW
MVLCLLPVLPPELRPIIEIDGGKLMSSDINELYRRVIFRNNTLINLLTTSRFTPGELIMCQEKL
VQEAVDTLLDNGVRGQPTRDGHNKVYKSFSDVIEGKEGRFRETLLGKRVDYSGRSVIVV
GPSLSLHQCGLPREIAIELFQTFVIRGIIRKHLALSIGVAKRKIREKEPIVWEILREVMEGHPV
LLNRAPTLHRLGIQAFQPILVEGRAICLHPLVRKGFNADFDGDQMAVHVPLSFEAQAEARL
LMFSHMNLLSPAIGDPISVPTQDMLIGLYVLTSGNQRGICVNRYNPCNR---RNYQNKKIDK
KNYKYTK--EPFFSNSYDAISAYRQKGIKLDSPLWLRWRLDQR-VIAA--REAPIEIHYESLGT
YYEIYGHYLIVRSIKKDILYIYIRTTVGHISLYREIEEAIQGFCQACSYDS------------MSERAN
LVFHNKVIDGTAMKRLISRLIDHFGMAYTSHILDQVKTLGFQQATATSISLGIDDLLTLPSKR
WLVQDAEQQSFILGKHHNYGNVHAVEKLRQSIEIWYSTSEYLRQEMNPNFRMTDPFNPV
HIMSFSGARGNPSQVHQLVGMRGLMSDPQGQMIELPIQSNFREGLSLTEYIISCYGARKGV
VDTAVRTADAGYLTRRLVEVVQHIVVRRTDCGTVRGISVSPRTGMMQ-DRIFLQTLIGRVL
ADDIYIGLRCIATRNQDIGVGLVNRVIT------FRAQSISIRTPFTCRSTSWICRLCYGRSPTHG
DLVELGEAVGIIAGQSIGEPGTQLTLRTFHTGGVFTGGTAEHVRAPSNGKITFNEDLVHPTR
TRHGHPAFLCSIDVYVTIESEDIIHNVSIPSKSFLLVQNDQYVESEQVIAEIRAGTAT-LSLKE
KVRKHIYSDSEGE-MHWNTDVYHAPELKYGNVHLLPKTSHLWILLGEPWK--SSLVCLSIH

KDQDQMNAH--SRSGKRRYISNPS--VTNNE-MRPKFFSSEFSGK-GDGIPDY-SELNRIRCTG
SYNLRST-ASLDENS--DLLAKRRRNRFLIPLQSIQELENDLRPSSGISIEIP--INGIFRKNSILA
YFDDPRYRIKNSGIVEYGTIEMDPIFKKED--FIEYRG-VPELRTKH-IVDRFFFLPEEVHILPE
SSSLMVRHNSIIGVDTQITLKTRSRVGGLVQVERKKKRIELRIFSGAIHFPGKTDKISPHSSV
LIPPRT--GKTNSKEYKN--LKNWLSIQR--LRLTK--KKSFVLIRPVVTYEITGGINLDLLLPMD
LLQEKDNVQLQILNYIFYANGNRIRVISATNIQLVRTCLVLNWDQDKK---SSSSDEACASFV
EIRTNALIKDFLRIDLVKSTISYTIKRNDFSGSGLISE-PGSDRTNINPFSSIYSYS---RARIQEPL
NQNQGTIHTVLNR---NKKCQSFIILSSSNCSRMGP--FNDLKSPDV---IKES-PQIPTKKSLGPL
GT--GLQIVNFYS--FSQL--ITYNPIL-ITNYFQLDNFK---PTFQILK---YYLMDEN--GKIVNPDP
CSNIILNPFNLNWYFFHSNYCVVKASTIISLGQFICENVCIPQKGPH-LK--SGQVIIVQ-VDSV
IIRSAKPYLATPGATVHGDYGKILYKGDTLVTFIYEKSRSGDITQGLPKVEQVLEVRSIDSIS
MNLKNRVEGWNKCITRLLGIPWGFLIGAELTIVQSRISLVNKIQKVYRSQGVQIHNRHIEII
VRQITSKVLVSE---DGMSNVFSPGELVGLLRAERMGRALEEAICYRAILLGITRASLNTQSF
ISEASFQETARVLAKAALRGRIDWLKGLKENVVLAGMIPVGTGFKAKRLVHPSRQQNNIP-
-LERKEKNLFEGEMR--DLFFHHKKLLDPVLSTNFHDTSEQSFIVL----NDS--MTRRYWNITL
DEMMDASVHLGHDTKQWNPKMAPYIFPKRKGIHLTNLFTTARFLSEACDVVFNAASREN
QFLIVGTKKKAADSVLRAAIRAQCHYVNKKWLSGMLTNWSTTETRLHKFRDLRIKEKRG
RLKRLPKRDAAILKRQLSHLQRSLGGIKYMTKLPDFVIIIDQQKDYTALRECIKLGIPTISLI
DTNSDPDLASLSIPANDDSRSSIKFILNKLVFAIREGRYE-SIGNSMGQKINPLGLRLGTTQSH
HSLWFAKPKNYSKELQEDEKIRYYIKNYVEKNMT--SS-VRALIRIEIEKAVDVTTMKIYIVP
APVFNKHYRR----ESKNLQPNLQKKFASVNPKFNLTLIKIFEPYRHPNILAEFISDQLQQRIPF
RKAMKKAIELAKQANTKGIRVQIAGRIGGKEIARVEWIRDGRVPLQTIQAKIDYCCYPVRT
IYGVLGIKIWIFIEGEMSRYRGPCFKKIRRLGALPGLTNRSP---DDLRKKKSGSVKKSQYRIR
LEEKQKLRFHYGLTERQLLKYVRIAAKAKGPTGQVLLQLLEMRLDNILFRLGMAPTIPGA
RQLVNHRHILVNGRLVDIPSYRCKPRDTITAKDKQKSKALIKNSLDSLPQKLKVPPHLTLK
RRQY--KGLVNKIIDSQRFGLEINELRVVEYYSRQTMSRRGTAEKKTAKSDPIYRNRLVNML
INRILKHGKKSLAYKIFYRAVQKIRQKTEKNPLLVLREAISKVTPRLAVKARRKSGLTQQVP
IAIKSPRGKVLAIRWVLAAARKRPGRNMVFKLSSELVDASKGRGGAIRKREETHRMAEAN
QALAHFRMGKDTIADMLTSIRNADMARKRVVRIAFTNISESIVKILLREGFIENVRKHREN
NKSFLVLTLKRNR---TYKK------TFNLKRISRPSLRIYSNYQRIPKILGGMGIVILSTSRGIMT
DREARLERIGGESLCYIWMAKAILKFRSRRTRRS---RRIRRIPKGRIHVQAGFNNIIVTVTDP
QGRVLSSSSAGNCGLRGKRKGTPFAGEAVAANALSSVV---MKRAEVMIKGPGLGRDTALR
AIARSRIRITLIRDVTPLPHNGCRPPKKRRVMPTINQLIRKTRQPIRNVTKSPALKRCPQRRG
TCTRVYTINPKKPNSALRKVARVRLTSGFEITAYIPGIGHSLQEHSVVLVRGGRVKDLPGVR
YHIVRGTLDTEKVKDRQQGRSKYGVKKPKMARKGLIQREKKRQKLEQKYHLIRRSSKKE
LK---KVP---DKREVYGKLQSPPRNSAPTRLRRRCSSTGRPRATYRDFGLSGYILRQMVQAC
LLPGATRSSWMVKNSFISIISQ--EENKENRGSVEFQIVSFTNKIDRLTSHLQLHKKDYLSER
GLLKILGKRHRLLAYLSKKTRVDYKEFIDQLDKTKTRVDYKMVKLRLKRCGRKQHATYR
IVAIDVRSRREGRALRKVGFYDPINNQTYLNAPAIVYFLERGAQPTETVRDILKKSEVFKRI
SSNLPPF--MDKYKRPFRKSK-PFHKRLPPIQPGDRIDYRNISLLCRFISDQGKILSRRRNKVT
LKQQRLITIAIKQARILSSLPFINT----KTGKKLAQARKN-VKSYGKMTPA-EKKTFVANHLL
KKINKINTKTEKETIKTWSRASTIIPIMIGHTIAIHNGKEHLPIYINNEMIGHKLGEFAPTFNF
REHTKNDTKSRR--------MTLFTVFIKILNSAVVVGLYFGLLTAFSIRPSYFFLLV---------EPEK
KGAAITGLILGQLVRFLSIYNAPLHRLLCIPHAITAFFVPYLLIYFLFDNFHPSF---ISSSSAFS-

-----IFLNSFIFQLSNQIILPSSLLARLGHIYLFRCKNKLDFLTGNFVGWSIGQICLLLWIYKYR
LRSAQKQLVSKKSSKKEWFVSEVQYLIERVITIFLYITWVVYASRMPIPIITHKFESELEKET
TALSFFEPNRWDRPLRYIRNQLFVAAVQERVSQYFFDTCRSDGKERIAFTYLPALSFFKEM
MKKIRISSSTERPSFDELDKNWLENNKEKTKNFNNELKKRIEALDTGF---DILDKRTRVCT
DQTLKTCLPTKYDPFLNGPHRGRIKEVPSTSEPIPLGKTTNSVFKSLKNALFHGKSLLKEKI
RKKVPRWRFLLVKMNTLTIPEIFSYQFKELIEETKYTYEFSNRDNYRPGLIKGSMRPLRRKR
ISLRWIEPFFHSPLFYDFIDRFKIKNFIDRFKSKISKKTKRERKERKREMKQEETHIDNGLGI
RTCFLILQVNLRKFVRLPLLIILKNSLRFLLVQKPEWSEDFEDWQREVYLLATHDGGLLPE
GTWPEYWFEDGFQIKVINPFSLKAWHTPDKDDFGDLYIMPLWW-LTDYPFGDPVEEERFFS
PIFDELKPI-----------NFWIFLRRSK-IYFVVLRKCIKVLRKCIKVIIKCIKVIIKRLNDSIISNE---
---------------NSEEQIKNLIDKTNTIKNQIEIIRNEKIK-----------GESPKNILKIFKRRKVRLINKL
NYFQKCLIEKIYLDIVLCIIRIHFINKILG-----PDLSSLSQAYVFYKLSQSQ---SDKLRSVLQYT
SFFLKTEIKESFRQGIFHSELKNKKLR-FRTNPWKNWLRGH-HYTLSKWRWSRLKAKLRKR
YLKYFNLKYLKFNRHRPKKKEQNRPKKKEQNKLEKNYRYDLLAYKFISVDLVADKKIWL
EPYPSVLPFRYLNWMGMNEDLDLPWFVRQVLEPLE-YYNGPWISPSELLFRNEKMLRQR
KKQLRSKRIEEFLVYCDEVNSKLIFPSARFLSTVFKEKIKRKILFNYWKSNVATVDNMIFPK
DQVVMRQRCLGTIVFKPHALPFIQNREQIMYQAISTSLNHKMKL--FDLLVPEMILSSRRRR
ELRILNSLVFLWPNFRLEDLACMNRYWFDTSNGSRFSMLRIYMYPMNKQL-KFFIGEFREV
LRETTNS-------PQLTSVVYFIRIFFDLGRLIKLFDPLIWRIVFSYTLNPPTLRMKIRRFLNWMI
KNAIFFVVP-VLIYR----INNRNLVERKS-FLLGLFP-----IPMNYIGPKNDTLEKDV-VSYHMN
RFVLS-LLSRPKGNKISERSFLNTKE------------IQNSMPESNWLSQWWKNWIRKKR-----------
----
--NSIQFKESS-N
QSLEQFYSMM-----MQRE--------------IQQITEREIQQIKERSIL-ED--LPFLETEQAETEQAETE
QAETEQAETEQAETEQAELESNPLSLYEDEMSREEIEQFL--RNPRRYVPSFFSDGW--SELQ-
LGSNPIERSTRDQ----KLLKKEHDLA---VIRRSE----TTDMVKLFKIILYLHN--TVKIHPISSDQ
GCQAED---IFPEMADLLTLSITEP-DLVYHKGFAFSI
DSYGLDQKQ--FLNEVFKSRDEWKKKSL---LVLPPIFY--EENESFSRRIGKKWFP-ICCGNHL
AEPNPKIVVFANNKIMEAVNEYRLIRNLLQFQSSIYG--SIRNVLNRVFLLN---LSDRNFEYGI
Q---GDQIGKDTLNHRILMKYTINQHLSNLKK-SQKRWFTP--LILIAPTERSMNRDPDAYRY
KWSTWFQ------EHFVSEQKSH---FQDRFK-----------------------FSFVLSN---------------------------
----ALRFFFVSFSP--------------------------------EIVSLQNLKPF--FLD---DHEPCQKISTS-----------
---------MIDSFHTR--NNRRESFAKTHSDFSMIFDDPYNWLNPVKPFHRSSLISSFYKANRLHF
LNNSHHFCFD--CNKRFPFYV----EKAPLNNYY--FIYR-QFLNPLFIRNK-KFSLGVGQ--KTHA
FSE-RDILSAIQSEVSRIFISKDFRQSGDET-ENLYKS---FYFPSRSDPFSR--RVLYSIVDISGTPL
TEEQIVNLERTYCQPLSDINLADSEGKNLISYLN--FNSKTFVIPPSSFDKFFALERSRNKHGI
FRKEQLRSIYLR--TCYKKMRMDRNKRVDRTFSTRSKWKLFKRYLPWALTSTGFRYLTFLF
LEIVSDLWQR-IWDSRRIRVLEINLRKWRRKTMKWTMKKRKWKR----KFKKWETQFKKV
DLTFLLNRIKTAYEKFVHRETFNGSPLKSPERNRRNLREF-FIFIYLTFLVLVC-LVSTALFQILI
--DLFLIAIKFKKAQALIMDELEIELEELLAEYPPSQSPDFLVQNFFLGLLQKMLFPN-----------
------------PYG--LRVKKKRKFLNINLRPIINLI----NLISNEITFVIKTRSISHTSKDIYSFIRKRKT
VTAYWN--QIAHWVSNNYWVENEEREVLMQFV--TLRR--KRSISEILWSLTHSDEHLL-----K
DEVLPEMIEQPGEVYLRKVIDFH--KKDLLNYEFNTSSLAEKRIFLALYHTMTY--SQTSSGV
DPFPVLSRQ---KPFSLRLTLAPS-KSILVIGSIGLGRSYLVNYLAKNSYLPFITIFEDDLIDEDE

YDSDDEIEVFIESRLSEDIQAIDHRHWSEYHYWRGLKNFIKYN--IKPFDEYDADPDLEPISM
ADQTD--DMDTVLY---VATFYISLQIELAKAMSPCLIWIPNIHELDFADSDFFSLGLLAHLLS--
-WDCERSS-SSNNLVIASTHMPQKMHPSLIDTHRFNTCIKVRRPLISQQRKHFSTLSSTRGFR
LERQIFET-THGFESITLGSNAYDLAALTNEALSISLSQGKSIIDSNTIRHAVHRQTWDLR---A
ELRPINNSRIFFYQIGRVVAQYLLLRDCPIDPISIYMNKR--FCPERDPYLYRWYYQLGMSMK
KLTILIYVLNCSAGLVAQDLWSRS--ELNAN-NGIASGSLVDNNS-DLVHGLLEIEGVLEG--FS
PTEKNCSKFDKD--RVPVLRRPEPRN-PSDMIQNGFWSMIDQSLIYQK---DPSAPPIVIGGIKI
DLQK-----------MFAD-FVVWAP--RIWSP---WGFLLNW-VNANELGLP-YWSRSFFAKEDDL
KENDQEDEDQDALGEALLQSVYQTRARIRSCQEQIFFRISRFIWDPGNPLLILSD-DPAIAA
MFSSREFFPDVDEEKWFFTAEIK---SLKKSWFI--DETQEKHFEWLIQRQRWLRR--PNNSLS-
-KGSFRST--ALSESYQYLSNLFLSN-RTVVNEMQKLLVRKRWLFPDDMQNFFNEQYSPAQT
GKNMPRSR-INGNFIDKTFSIVANILLRIIPTTSGEKEAFTYYRDG--MSAQSEGNYAEALQN
YYEAMRLEIDPYDRSYILYNIGLIHTRNGEHTKALEYYFRALERNPFLPQAFNNMAVICHY-
RGEQAIRQGDSEIAEAWFDQAAEYWKQAIALTPGNYSQAYNWVKLKKPFE--MSCRSEHI
WIEPITGSRKTSNFCWTLSLFLGSLGFLLVGTSSYLGRNWISLFPSQQIVFFPQGIVMAFYGI
AGLFISCYLWCTISWNVGSGYDRFDRKEGIVCIFRWGFPGKNRRIFLRFLLKDIQSIRIEVKE
GLSARRVLSMDIRGQGALPLTRTGEN-LTPWEIEQKAAELAYFLRVPIEVF
>Guizotia
MKRWWFNSMLFLSKSTSSLGPIENAIESKDPNINDTDKNIQSYSNVDLFFGVKDIRNFISDD
TFLVKDSNGDIYSIYFDIENHIFEIDNDH--SFCSELESSFY--RNSSYLNNGSKSKNPQHDPY
MNDTQYTWNNHINSCIDSYLQ--SQICIDSYIVSGSDNSSNNYISSSICC--NSSKNADARTSD
QIIRESSTDLDVTQKYRHLWVQCENCYGLNYKKFFKSKMNLCEQCGYHLKMSSSDRIELS
IDPGTWEPMDEDMVSLDPIEFHSEEEPYKNRIDSYQRKTGLTEAVQTGIGQLDGINVAIAV
MDFQFMGGSMGSVVGEKITRLIEYATKEFLPLIIVCASGGARMQEGSLSLMQMAKISSALY
DYQSNKKLFYVPILTSPTTGGVTASFGMLGDIIIAEPNAYIAFAGKRVIEQTLNKTVPDGSQ
AAEYLFQKGLFDLIVPRNPLKSVLSELFQLHTFFPLNQN---MVTIQADEISNIIRERIEQYNR
EVKIVNTGTVLQVGDGIARIHGLDEVMAGELVEFQEGTIGIALNLESTNVGVVLMGDGLL
IQEGSSVKATGRIAQIPVSEAYLGRVINALAKPIDGRGEILSSEYRLIESPAPGIISRRSVYEPL
QTGLIAIDSMIPIGRGQRELIIGDRQTGKTAVATDTILNQQGQNVICVYVAIGQKASSVAQV
VTTFQERGAMEYTIVVAETADSPATLQYLAPYTGAALAEYFMYRERHTLIIYDDLSKQAQ
AYRQMSLLLRRPPGREAYPGDVFYLHSRLLERAAKLSSLLGEGSMTALPIVETQSGDVSAY
IPTNVISITDGQIFLSADLFNAGIRPAINVGISVSRVGSAAQIKAMKQVAGKLKLELAQFAEL
EAFAQFASDLDKATQNQLARGQRLRELLKQSQSAPLAVEEQVLTIYTGTNGYLDSLEIGQV
RKFLVELRTYLKTNKPQFQEIISSTKIFTEEAEAILKEAIQEQRERFILQEQAMRMNPTTSSS
GVATLDKKNLGRIAQIIGPVLDVAFPPGKMPNIYNALVVKGRDTVGQPINVTCEVQQLLGN
NRVRAVAMSATDGLTRGMDVVDTGAPLSVPVGGATLGRIFNVLGEPIDNLGPVDNSTTFPI
HRSAPAFIQLDTKLSIFETGIKVVDLLAPYRRGGKIGLFGGAGVGKTVLIMELINNIAKAHG
GVSVFGGVGERTREGNDLYMEMKESGVINEQNIAESKVALVYGQMNEPPGARMRVGLTA
LTMAEYFRDVNEQDVLLFVDNIFRFVQAGSEVSALLGRMPSAVGYQPTLSTEMGSLQERI
TSTKDGSITSIQAVYVPADDLTDPAPATTFAHLDATTVLSRGLAAKGIYPAVDPLDSTSTML
QPRIVGDEHYETAQQVKQTLQRYKELQDIIAILGLDELSEEDRLTVARARKIERFLSQPFFV
AEVFTGSPGKYVGLAETIRGFQLILSGELDGLPEQAFYLVGNIDEATAKAINLEMESNLKK
MTLNLCVLTPNRIVWDSEVKEIILSTNSGQIGVLPNHAPIATAVDIGILRIRLNDQWLTMAL
MGGFARIGNNEITVLVNDAEKSSDIDPQEAQQTLEIAEAALRKAEGKRQTIEANLAVRRAR

TRVEAINAISMKNVTDSFVSLGHWPSAGSFGFNTDILATNLINLSVVLGVLIFFGKGVLSDL
LDNRKQRILNTIRNSEELREGAIEQLEKARARLRKVEIEADEFRVNGYSEIEREKLNLIDST
YKTLEQLENYKNETINFEQQKASNQVRQRVFQQALQGALGTLNSCLNNELHLRTISANIGI
LAAMKQITDMNPLISAASVIAAGLAVGLASIGPGVGQGTAAGQAVEGIARQPEAEGKIRGT
LLLSLAFMEALTIYGLVVALALLFANPFVMNVLSCSINTLNGLYDLSGVEVGQHFYWKIGG
FQVHGQVLITSWVVIAILLGSATLAVRNPQTIPTGGQNFFEYVLEFIRDVSKTQIGEEYGPW
VPFIGTMFLFIFVSNWSGALLPWKIIQLPHGELAAPTNDINTTVALALLTSVAYFYAGLTKK
GLGYFGKYIQPTPILLPINILEDFTKPLSLSFRLFGKILADELVVVVLVSLVPSVVPIPVMFLG
LFTSGIQALIFATLAAAYIGESMEGHHMIFSTLEHILTHISFSIVSIVITLHLITLLGNEIIKPYD
SSEKGMIATFLCLTGLLITRWIYSGHFPLSDLYESFIFLSWSFSLIHIVPY--IRKNDLTTITASST
IFTQGFATSGLLNEIHKPTILVPALQSEWLIMHVSMMILSYAALLCGSLLSVALLVITFRN---I
FYSSKEIQYKNERNNIFQKTY---DKNYYKAQFIQQLDYWSYRVISLGFIFLTIGILSGAVWA
NEAWGSYWSWDPKETWAFITWIVFAIYLHTRTKKNLQGANSAIVATLGFLIIWICYFGVNL
LGIGLHSYGSFTLTMAKKKAFTPLLYLASIVFLPWWISLLFQKSLESWVTNWWNTRQSET
FLNDIEEKSILEKFIELEELLFLEEMIKEYSETHLQNLRI-IHKETIQLIKIHNEGRIHTILHFST
NIICFIILSGYSIFGNKELVILNSWAQEFLYNLSDTIKAFSLLLLTDLCIGFHSPHGWELMIGF
VYKDFGFVHNDQIISGLVSTFPVILDTIFKYWIFRYLNRVSPSLVVIYHSMNDMPIGVPKVP
FRSPGEEDASWVDIYNRLYRERLLFLGQEVDSEISNQLIGLMIYLSIEDDTQDLYLFINSPGG
WVIPGVALYDTMQFVQPDVHTICMGSAASMGSFILVGGEITKRLAFPHARVMIHQPAGSFS
EVATGEFILEVGELLKLRETLTRVYVQRTGKPLWVVSEDMERDVFMSATEAQAYGIVDLVA
VEMKEQKWIHEGLITESLPNGMFRVRLDNEDMILGYVSGKIRRSFIRILPGDRVKIEVSRYD
STRGRIIYRLRNKDSKDMDKFQSYLG-DRS--HYFLYPLIFQEYIYVLAHDHGLAGYDNKSS
LLIVKRLIIRMYQQNHLILSVNDSKQTPFLGKNFYSQVMSEVSSTIMEIPLSLRLISSLVVKS
DNLRSIHSIFSFLEDNFSHLNYVLDILIPYPAHMEILVQALRYWIKDASSLHLLRFFLHECHN
WDSLITKAGSSFSKHRLFFFLYTSYVCEYESGFLFLRNQSSHLRSTSSGSLIERIYFYGKIEY
LAEVFAGAFNLWLFKDSFMHYVRYQGKSILASKGTFILMNKWKYYFVNFWKSYFYLWS
QPGRIYINQLSNHSLDFLGYRSSVRLKPSMVRGQMLENTFLIDNAI--KKFDSIVPIMPLVGS
LAKSKFCNALGHPIGKAVWADLSDSDIIERFGRIYRNLSHYHSGSSKKKSLYRVKYILRLSC
ARTLARKHKSTVRAFLKRFGSRLLEEFFTEEEQVFSLTFPRRIWYLDIVCINDLANHEMIID-
TTEVQ-AINSFSRLES-LKEVYGIIWVLIPIFTPVLGITIGVLVIVWLEREISAGIQQRIGPEYAG
PLGILQALADGTKLLFKENLLPSRGDTRLFSIGPSIAVISILLSYLVIPFGYHLVLADLSIGVFL
WIAISSIAPVGLLMSGYGSNNKYSFLGGLRAAAQSISYEIPLTLCVLSISLLSNSSSTVDIVE
AQSKYGFWGWNLWRQPIGFLVFLISSLAECERLPFDLPEAEEELVAGYQTEYSGIKFGLFY
VASYLNLLVSSLFVTVLYLGGWNLSIPYIFVPELFDITKRGRVFGTIISIFITLAKTYLFLFIPIA
TRWTLPRLRMDQLLNLGWKFLLPISLGNLLLTTSSQLLSLMIWHVQNENFILDSTRIFMKA
FHLLFFDGSLIFPECILIFGLILLLMIDSTSDQKDIPWLYFISSTSLVMSITALLFRWREEPMISF
SGNFQTNNFNEIFQFLILLCSTLCIPLSVEYIECTEMAITEFLLFVLTATIGGMFLCGANDLITI
FVAPECFSLCSYLLSGYTKKDVRSNEATMKYLLMGGASSSILVHGFSWLYGSSGGEIELQE
IVNGLINTQMYNSPGISIALIFITVGIGFKLSPAPSHQWTPDVYEGSPTPVVAFLSVTSKVAA
SASATRIFDIPFYFSSNEWHLLLEILAILSMILGNLIAITQTSMKRMLAYSSIGQIGYVIIGIIV
GDSNDGYASMITYMLFYISMNLGTFACIVLFGLRTGTENIRDYAGLYTKDPFLALSLALCL
LSLGGLPPLAGFFGKLYLFWCGWQAGLYFLVLIGLLTSVVSIYYYLKIIKLLMTGRNQEITP
HVRNYRRSPLRSNNSIELSMIVCVIASTIPGISMNPIIAIAQDTLFMFLLYEYDIFWAFLIISSLI
PILVFFISGFLAPSSKGPEKLSSYESGIEPIGDAWLQFRIRYYMFALVFVVFDVETVFLYPWA

MSFDVLGVSVFVEALIFVLILIVGLVYAWRKGALEWSTNKFPWLTIIVVLPIFAGSFIFFLPH
KGNRVIRWYTICICTLELLLTTYAFCYHFRLDDPLIQLVEDYKWINFFDFRWKLGIDGLSIG
PVLLTGFITTLATLAAWPVTRDSRLFHFLMLAMYSGQIGSFSSRDLLLFFIMWELELIPVYL
LLSMWGGKKRLYSATKFILYTAGGSIFLLMGVLGVGLYGSNEPTLNFETSVNQSYPVALEII
FYIGFFIAFAVKLPILPLHTWLPDTHGEAHYSTCMLLAGILLKMGAYGLIRINMELLPHAHS
IFSPWLMIVGTIQIIYAASTSLGQRNLKKRIAYSSVSHMGFILIGIASITDTGLNGAILQIISHG
FIGAALFFLAGTSYDRIRLVYLDEMGGVAIPMPKIFTMFSSFSISSLALPGMSGFVAEVIVFL
GIITSQKYLLMPKIVITFVMAIGMILTPIYLLSMSRQMFYGYKLFNIPNSFVFDSGPRELFVSI
SIFLPVIGIGMYPDFVLSLSVDKVEGILSNYFYRMMLEHVLVLSAYLFSFVLYGLITSRNMV
RALMCLELILNAVNLNFVTFSDFFDSRQLKGAIFSIFVIAIAAAEAAIGLAIVSSIYRNRKST
RINQSNLLNKMEQTYQYAWIIPFLPLPVPMLIGLGLLLFPTATKSLRRMWAFQSVLLLSIVM
IFSMNLSIQQINSSSVYQYVWSWIINNDFSLEFGYLIDPLTSIMSILITTVGIMVLIYSDNYMS
HDHGYLRFFAYMSFFSTSMLGLVTSSNLIQIYIFWELVGMCSYLLIGFWFTRPVAAKACQK
AFVTNRVGDFGLLLGILGFYWITGSFEFRDLFQILNNNEVNFVFVTLCAILLFAGAIAKSAQ
FPLHVWLPDAMEGPTPISALIHAATMVAAGIFPVARLMPLFIVIPHIMNFISLIGIITVFFGAT
LALAQKDIKRGLAYSTMSQLGYMMLALGMGSYRSALFHLITHAYSKALLFLGSGSVIHS
METLVGYCPKKSQNMVLMGGLTKHVPITKNSFLLGTLSLCGIPPLACFWSKDEILNDSWL
YSPIFAIIAWSTAGLTAFYMCRIYLLTFEGHLNVHFQNYSGKRTTPLYSISLWGKEGSKISNK
-LVTLLKMKFFSFGNTKTYLYPYESDNTMLFPILILILFTLFVGFLGIPF-VDILSKWLTPSINL
LHQNSN-SIDWYEFCKDAVFSVSIAFFGIFIAFFLYKPVYSSFQNLDLINSFVIFSDKIKNAIY
DWSYNRGYVDAFYGTFLTAGVRKLAEFTHFFDRRIIDGIPNGVGFLSFFVAEVIKSVGGG-
RISSYLFFYFSYVSIFLLIYYMDLPGLIHDFLLVFLGLGLILGGLGVVLLANPIYSAFSLGLVF
VCISLFYILANSHFVAAAQLLIYVGAINVLIIFAVMFINGSEYSKDFHLWTVGDGVTSVVCT
SLFVSLITTIPDTSWYGIIWTTKANQIIEQDLISNSQQIGIHLSTDFFLPFEFISIILLVALIGAIAV
ARQMTVPSTRKDLMIVNMGPHHPSMHGVLRLIVTLDGEDVIDCEPILGYLHRGMEKIAEN
RTIIQYLPYVTRWDYLATMFTEAITVNAPEQLGNIQVPKRASYIRVIMLELSRIASHLLWLG
PFMADIGAQTPFFYIFRERELIYDLFEAATGMRMMHNFFRIGGVAADLPHGWIDKCLDFC
DYFLTGIAEYQKLITRNPIFLERVEGVGIIGGEEAINWGLSGPMLRASGIQWDLRKVDHYE
CYDEFDWEVQWQKEGDSLARYLVRISEMTESIKIIQQALEGIPGGPYENLEIRRFDRVKDT
VWNEFDYRFISKKPSPTFELSKQELYARVEAPKGELGIFLIGDKGVFPWRYKIRPPGFINLQI
LPQLVKRMKLADIMTILGSIDIIMGEVDRMFPTVTEFMNYGQQTVRAARYIGQGFMITLSH
ANRLPVTIQYPYEKLITSERFRGRIHFEFDKCIACEVCVRVCPIDLPVVDWKLETDIRKKRL
LNYSIDFGICIFCGNCVEYCPTNCLSMTEEYELSTFDRHELNYNQIALGRLPMSIIDDYTIRT
ILNL---------------MQGNLSAWLVKHALIHRSLGFDYQGIETLQIKPGDWHSIAVILYVYGYN
YLRSQCAYDVAPGGLLASVYHLTRIEYGVDQPEEVCIKVFAPRRDPRIPSVFWVWKSVDF
QERESYDMLGISYDNHPRLKRILMPESWIGWPLRKDYIAPNFYEIQDAH---------------------
-----------------------------------MNSIEFPLFHRTTQNSVISTTLNDLSNWSRLSSLWPLLYGTSC
CFIEFASLIGSRFDFDRYGLVPRSSPRQADLILTAGTVTMKMAPSLVRLYEQMPEPKYVIAM
GACTITGGMFSTDSYSTVRGVDKLIPVDVYLPGCPPKPEAIIDAITKLRKKISREIYPDRILS
QRENRCFTTNHKFQVGHSIHTGNYDQGFLYQPPSTSEIPPETFFKYKSSVSSHELVNMQTR
NTFYWIKEQITRSISVSLMIYIITRTSISNAYPIFAQQGYENPREATGRIVCANCHLANKPVDI
EVPQAVLPDTVFEAVVRIPYDTQLKQVLANGKKGGLNVGAVLILPEGFELAPPDRISPEIKE
KMGNLSFQSYRPNQKNILVIGPVPGQKYSEITFPILSPDPATKKDIHFLKYPIYVGGNRGRG
QIYPDGSKSNNTVYNATASGIVSKIIRKEKGGYEITIADASDGRQVVDIIPPGPELLVSEGESI

KLDQPLTSNPNVGGFGQGDAEIVLQDPLRVQGLLFFFASVILAQIFLVLKKKQFEKVQLSE
MNFMSKVYDWFEERLEIQAIADDITSKYVPPHVNIFYCLGGITLTCFLVQVATGFAMTFYY
RPTVTDAFASVQYIMTEANFGWLIRSVHRWSASMMVLMMILHVFRVYLTGGFKKPRELT
WVTGVVLAVLTASFGVTGYSLPWDQIGYWAVKIVTGVPDAIPVIGSPLVELLRGSASVGQS
TLTRFYILHTFVLPLLTAVFMLMHFPMIRKQGISGPLMGVTKKPDLNDPVLRAKLAKGMG
HNYYGEPAWPNDLLYIFPVVILGTIACNVGLAFLEPSMIGEPADPFATPLEILPEWYFFPVFQ
ILRTVPNKLLGVLLMVSVPAGLLTVPFLENVNKFQNPFRRPVATTVFLIGTAVALWLGIGAT
LPIDKSLTLGLFMIEVFLFGIVLGLIPITLAGLFVTAYLQYRRGDQLDLMPIITSYFGFLLTAL
TITSALFIGLSKIRLIMDIVSLAWAALMVVFTFSLSLVVWGRSGLMIIRSPEPEVKILVDRDHI
KTSFEEWARPGHFSRTIAKGPETTTWIWNLHADAHDFDSHTSDLEEISRKVFSAHFGQLSII
FLWLSGMYFHGARFSNYEAWLSDPTHIGPSAQVVWPIVGQEILNGDVGGGFRGIQITSGFF
QIWRASGITSELQLYCTAIGALIFAALMLFAGWFHYHKAAPKLAWFQDVESMLNHHLAGL
LGLGSLSWAGHQVHVSLPINQFLNAGVDPKEIPLPHEFILNRDLLAQLYPSFAEGATPFFTL
NWSKYADFLTFRGGLDPVTGGLWLTDTAHHHLAIAILFLIAGHMYRTNWGIGHGLKDILE
AHKGPFTGQGHKGLYEILTTSWHAQLSLNLAMLGSLTIVVAHHMYAMPPYPYLATDYGT
QLSLFTHHMWIGGFLIVGAAAHAAIFMVRDYDPTTRYNDLLDRVLRHRDAIISHLNWACI
FLGFHSFGLYIHNDTMSALGRPQDMFSDTAIQLQPVFAQWIQNTHALAPGATAPGATASTS
LTWGGGDLVAVGGKVALLPIPLGTADFLVHHIHAFTIHVTVLILLKGVLFARSSRLIPDKAN
LGFRFPCDGPGRGGTCQVSAWDHVFLGLFWMYNSISVVIFHFSWKRQSDVWGSISDQGV
VTHITGGNFAQSSITINGWLRDFLWAQASQEIQTYGSSLSAYGLFFLGAHFVWAFSLMFLFS
GRGYWQELIESIVWAHNKLKVAPATQPRALSIVQGRAVGVTHYLLGGIATTWAFFLARIIA
VGMALRFPRFSQGLAQDPTTRRIWFGIATAHDFESHDDITEERLYQNIFASHFGQLAIIFLW
TSGNLFHVALQGNFESWVKDPLHVRPIAHAIWDPHFGQPAVEAFTRGGAPGPVNIAYSGV
YQWWYTIGLRTNVDLYNGAIFLLFLSAISLIAGWLHLQPKWKPSVSWFKNAESRLNHHLS
GLFGVSSLAWTGHLVHVAIPASRGEDVRWNNFLDVLPHPQGLGPLFTGQWNLYAQNPDSS
SHLFGTSQGAGTAILTLLGGFHPQTQSLWLTDMAHHHLAIAFIFLIAGHMYRTNFGIGHSM
KDLLDAHIPPGGRLGRGHKGLYDTINNSIHFQLGLALASLGVITSLVAQHMYSLPAYAFIAQ
DFTTQAALYTHHQYIAGFIMTGAFAHGAIFFIRDYNPEQNEDNVLARMLEHKEAIISHLSW
ASLFLGFHTLGLYVHNDVMLAFGTPEKQILIEPIFAQWIQSAHGKTSYGFDILLSSTNGPAF
NAGRSIWLPGWLNAVNE--NSNSLFLTIGPGDFLVHHAIALGLHTTTLILVKGALDARGSKL
MPDKKDFGYSFPCDGPGRGGTCDISAWDAFYLAVFWMLNTIGWVTFYWHWKHITLWQG
NVSQFNESSTYLMGWLRDYLWLNSSQLINGYNPFGMNSLSVWAWMFLFGHLVWATGFM
FLISWRGYWQELIETLAWAHERTPLANLIRWRDKPVALSIVQARLVGLAHFSVGYIFTYAA
FLIASTSGKFGMSHSVKIYDTCIGCTQCVRACPTDVLEMIPWDGCKAKQIASAPRTEDCVG
CKRCESACPTDFLSVRVYLWHETTRSMGIAYMTTLNFPSVLVPLVGLVFPAIAMASLFLHV
QKNKIVMRDLKTYLSVAPVLSTLWFGALAGLLIEINRFFPDALTFPFF--MTAILERRESESL
WGRFCNWITSTENRLYIGWFGVLMIPTLLTATSVFIIAFIAAPPVDIDGIREPVSGSLLYGNNI
ISGAIIPTSAAIGLHFYPIWEAASVDEWLYNGGPYELIVLHFLLGVACYMGREWELSFRLG
MRPWIAVAYSAPVAAATAVFLIYPIGQGSFSDGMPLGISGTFNFMIVFQAEHNILMHPFHML
GVAGVFGGSLFSAMHGSLVTSSLIRENTENESANEGYRFGQEEETYNIVAAHGYFGRLIFQ
YASFNNSRSLHFFLAAWPVVGIWFTALGISTMAFNLNGFNFNQSVVDSQGRVINTWADIIN
RANLGMEVMHERNAHNFPLDLAAIEAPSTNGMGLPWYRVHTVVLNDPGRLLSVHIMHT
ALVAGWAGSMALYELAVFDPSDPVLDPMWRQGMFVIPFMTRLGITNSWGGWSIT-GGTTT
NPGIWSYEGVAGAHIMFSGLCFLAAIWHWVYWDLEIFCDERTGKPSLDLPKIFGIHLFLAG

VACFGFGAFHVTGLYGPGIWVSDPYGLTGKVQSVNPSWGVEGFDPFVPGGIASHHIAAGT
LGILAGLFHLSVRPPQRLYKGLRMGNIETVLSSSIAAVFFAAFVVAGTMWYGSATTPIELFG
PTRYQWDQGYFQQEIYRRVSAGLAENQSLSEVWSKIPEKLAFYDYIGNNPAKGGLFRAGS
MDNGDGIAVGWLGHPIFRDKEGRELFVRRMPTFFETFPVVLVDGDGIVRADVPFRRAESK
YSVEQVGVTVEFYGGELNGVSYSDPVTVKKYARRAQLGEIFELDRATLKSDGVFRSSPRG
WFTFGHASFALLFFFGHIWHGARTLFRDVFAGIDPDLDAQVEFGAFQKLGDPTTRRQAVM
KTLYSLRRFYPVETLFNGTLALAGRDQETTGFAWWAGNARLINLSGKLLGAHVAHAGLIV
FLAGAMNLFEVAHFVPEKPMYEQGLILLPHLATLGWGVGPGGEVIDTFPYFVSGVLHLISS
AVLGFGGIYHALLGPETLEESFPFFGYVWKDRNKMTTILGIHLILLGIGAFLLVFKALYFGG
VYDTWAPGGGDVRKITNLTLSPSIIFGYLLKSPFGGEGWIVSVDDLEDIIGGHVWLGSICIL
GGIWHILTKPFAWARRALVWSGEAYLSYSLAAISVFGFIACCFVWFNNTAYPSEFYGPTGP
EASQAQAFTFLVRDQRLGANVGSAQGPTGLGKYLMRSPTGEVIFGGETMRFWDLRAPWL
EPLRGPNGLDLSRLKKDIQPWQERRSAEYMTHAPLGSLNSVGGVATEINAVNYVSPRSWL
ATSHFVLGFFFFVGHLWHAGRARAAAAGFEKGIDRDFEPVLSMTPLNMTIALGKFTKDEK
DLFDIMDDWLRRDRFVFVGWSGLLLFPCAYFAVGGWFTGTTFVTSWYTHGLASSYLEGC
NFLTAAVSTPANSLAHSLLLLWGPEAQGDFTRWCQLGGLWTFVALHGAFGLIGFMLRQFE
LARSVQLRPYNAIAFSGPIAVFVSVFLIYPLGQSGWFFAPSFGVAAIFRFILFFQGFHNWTLN
PFHMMGVAGVLGAALLCAIHGATVENTLFEDGDGANTFRAFNPTQAEETYSMVTANRF
WSQIFGVAFSNKRWLHFFILFVPVTGLWMSALGVVGLALNLRAYDFVSQEIRAAEDPEFE
TFYTKNILLNEGIRAWMAAQDQPHENLIFPEEVLPRGNALMSGSTGERSFADIITSIRYWVI
HSITIPSLFIAGWLFVSTGLAYDVFGSPRPNEYFTENRQGIPLITGRFDSLEQLDEFSRSFMTI
DRTYPIFTVRWLAVHGLAVPTVSFLGSISAMQFIQRMATQTVENGSKSA-PRRTTVGSLLKP
LNSEYGKVAPGWGTTPLMGVAMALFAVFLSIILEIYNSSVLLDGISMNMLTLKLFVYTVVI
FFVSLFIFGFLSNDPGRNPGREEMADTTGRIPLWIIGTVAGILVIGLVGVFFYGSYSGLGSSL
MLNIFSLIC--LNSALYPSSLFFAKLPEAYAFLNPIVDVMPVIPLFFFLLAFVWQAAVSFRTTQ
SNPNEQNVELNRTSLYWGLLLIFVLAVLFSNYFFNMEVNILAFIATALFILVPTAFLLIIYVKT
VSQNDMETATLVAIFISGLLVSFTGYALYTAFGQPSQQLRDPFEEHGDMEALVYTFLLVSTL
GIIFFAIFFREPPKISTKK--MTLAFQLAVFALIATSSILLISVPVVFASPDGWSSNKNVVFSGTS
LWIGLVFLVGILNSLIS-------MSPQTETKASVGFKAGVKDYKLTYYTPEYETKDTDILAAFR
VTPQPGVPPEEAGAAVAAESSTGTWTTVWTDGLTSLDRYKGRCYGIEPVPGEENQFIAYVA
YPLDLFEEGSVTNMFTSIVGNVFGFKALRALRLEDLRIPTAYVKTFDGPPHGIQVERDKLN
KYGRPLLGCTIKPKLGLSAKNYGRACYECLRGGLDFTKDDENVNSQPFMRWRDRFLFCA
EAIYKAQAETGEIKGHYLNATAGTCEEMLKRAVFARELGVPIVMHDYLTGGFTANTSLSH
YCRDNGLLLHIHRAMHAVIDRQKNHGMHFRVLAKALRMSGGDHIHSGTVVGKLEGEREI
TLGFVDLLRDDFIEKDRSRGIYFTQDWVSLPGVLPVASGGIHVWHMPALTEIFGDDSVLQF
GGGTLGHPWGNAPGAVANRVALEACVQARNEGRDLATEGNEIIREATKWSPELAAACEV
WKEIKFEFQAMDTLDTMAIHLYKTSTPSTRNGTVDSQVKSNPRNNLIYGQHHCGKGRNA
RGIITAGHRGGGHKRLYRKIDFRRNEKDIYGRIVTIEYDPNRNAYICLIHYRDGEKRYILHP
RGAIIGDTIVSGTEVPIKMGNALPLTDMPLGTAIHNIEITLGKGGQLARAAGAVAKLIAKEG
KSATLKLPSGEVRLISKNCSATVGQVGNVGVNQKSLGRAGSKRWLGKRPVVRGVVMNP
VDHPHGGGEGRAPIGRKKPTTPWGYPALGKRSRKRNKYSDNLILRRRSKMIQPQTHLNVA
DNSGARELMCIRIIGAS-HRRYAHIGDVIVAVIKDAVPNMPLERSEVVRAVIVRTCKELKRD
NGMIIRYDDNAAVVIDQEGNPKGTRVFGAIARELRQFNFTKIVSLAPEVLNYNPKRTRFRK
QHRGRMKGISYRGNTICFGKYALQALEPAWITSRQIEAGRRAMTRNARRGGKIWVRIFPD

KPVTVRPAETRMGSGKGSPEYWVAVVKPGRILYEMGGVTENIARRAISIAASKMPIRAQFII
SGMTRIRRGYIARRRRTKIRLFASSFRGAHSRLTRTITQQKIRALVSAHRDRDRQKINFRRL
WITRINAAIRE-RGVCYSYSRLINDLYKRQLLLNRKILAQIAISNRNCLYMISNEIIKEVSWK
ESTG--MLNEVYALGQHISMSAHKARRVIDQIRGRSYEETLIILELMPYRACYPIFKLVYSAA
ANASSNMGSNEANLVISKAEVNKGTIMKRLKHRARGRSFAIQKPTCHITIVMKDISLDGGT
WDKKMDGIRYAVFTDKSIRLLGKN-QYTSNVESGSTRTEIKHWVELFFGVKVIAMNSHRL
RGKARRMGPIMGQTMHYRRMIITLQPGYSIPPLRKKRTMAVPKKRTSISKKRIRKNIWKRK
GYGAALKALSLGKSLSTGNSKSFFVRQTNKMAKGKDVRIPVLLECTACVRNG--VNKEST
GISRYITQKNRHNTPNRLELLKFCPYCYKHTIHGEIKKMKIRASVRKICEKCRLIRRRGRIIV
ICSNPRHKQRQGMVREKITVSTRTLQWKCVESAADSKRLLYGRFILSPLMKGQADTIGIA
MRRALLGEIEGTCITRAKSD--KISHEYSTIMGIQESVHEILMNLKEIVLRSNLYGTYEASICV
RGPGYVTAQDIILPPYVEIVDNTQHIASLTEPIELVIGLQIEKNRGYLIKAPN-TFDGSY--PIDP
VFMPVRNANHSIHSYENGN--KEILFLEIWTNGSLTPKEALHEASRNLIDLLIPFLHTKEE--N
LNLEDNQ-HIVPLPPFTFYDKLA-KLTKNKK-KMALKSIFIDQSELPPRIYNCLKRSNIYTLLD
LLNNSQEDLMKIEHFRIEDVKQILGILEKN--FVIDLPKNK---------MATIPGFNQIQFEGFCRF
IDQGLAEELSKFPKIEDTNQEIDFELFLERYQLVEPLIKERDAVYESLTYSSELYVSARLIWK
NRRYIQEQTILIGKIPLMTSLGAFIVNGIYRIVINQILQSPGIYYQSELNDNGISVYTGTIISDW
GGRLELEIDRKARIWVRVSRQQKLSILVLLSAMGLNIREILENVCYPELFLSFLNDK--KKIG
SKENAILEFYQQFACVEGDPVFSESLSKDLQKKFFQQRCELGGIGRRNMNRRLNLDIPQNN
TFLLPRDILAAADRLIRIKFGMGTLDD--MNHLQNKRIRSVADLLQEQFGLALVRLENMAR
GNIYAALK-HNWT-PTPQNLVNSTP----LTDTYKVFFRLHPLSQVLDRTNPLTQIVHGRKLSY
LGPGGLTARTATFPIRDIHPSHYGRICPIDTSEGINVGLIGSLAIHARIGRWG-SLESPFY---KIS
ERSKG--AQMLYLSPGRDEYYMVAAGNSLALNQGIQEEQVVPARYRQEFLTIAWEQVHLR
SIFAFQYFSIGASLIPFIEHNDANRALMSSNMQRQAVPLSQSEKCIVGTGLEGQAALDSGAL
AIAEHEGKVFYTDTDKILLSGNGDTLRIPLVMYQRSNKNTCMHQKPQVRRGKCIKKGQIL
AYGAATVGGELALGKNVLVAYMPWEGYNFEDAVLISERLVYEDIYTSFHIRKYEIQIN---Q
G-PERVTNEIPHLEVHLLRNLDKNGIVMLGSWVETGDILVGKLTPQMVKE-SSYAPEDRLL
RTILGMRVYTSKETCLKLPIGGRGRVIDVRWVQSSKTDETEKTESIRVYILQKREIKVGDKV
AGRHGNKGIISKILPRQDMPYLQDGSPVDMVFNPLGVPSRMNVGQIFESSLGLAGDLLDR
HYRIAPFDERYEQEASRKLVFSELYEASKQTANPWIFEPESPGKSRIFDGRTGDPFEQPVIIG
KPYILKLIHQVDDKIHGRSSGRYSRLTQQPLKGRAKKGGQRVGEMEVWALEGFGVAYILQ
EMLTYKSDHIRARQEVLGTIIFGGRIPTPEDAPESFRLFVRELRSLALELNHFLVSEKTFQLN
RKEA-------MIDRYTHQQLRIGLVSPQQISTWSKKILPNGEIVGEVTKPYTFHYKTNKPEKD
GLFCERIFGPIKSGICACGNYRVIGDEKEDPQFCEQCGVEFVDSRIRRYQMGYIKLAYPVM
HVWYLKRLPSYIVNLLDKPLNELEDLVYCG--FYFARP-IDKKPTFLRLRGLLEYEIQPWKY
RIPIFFTTRSFDTFRNREMSTGGGSIRQQLANLDLRMIIDYSLVEWKELEEE-EPTGNEWED
RKVGRRKDFLLRRMELAKHFIRTNIEPKWMVLCLLPVLPPELRPIYHIDEDKLVTSDINEIY
RRIIYRNNTLTDLLTTSIATPEELIISQEKLLQEAVDALLDNGICGQPMRDDHNRIYKSLSDVI
EGKEGRVRETLLGKRVDYSGRSVIVVGPSLSLHRCGLPREIAIELFQAFVIRDLIRKHLASNI
GVAKSQIRKKKPIVWEILQEILDDHPVLLNRAPTLHRLGIQAFLPILVEGRAICLHPLVCKGF
NADFDGDQMAVHVPLSLEAQAEARLLMFSHMNLLSPTIGDPISAPTQDMLSGLYVLTSGN
RRGICVNRYNPCNRRN---YQNED---NNYKYTKKKEPFFCNAYDAIGAYRQKRIHLGSPLW
LRWRLDQR-VIAA--REAPIEIHYESLGTYYEIYGHYLIVRSIKKEILYIYIRTTLGHIYLYREIE
EAIQGFWQCNSMLPAGIRVSPGMEVLMAERPTQVFHNKVIDGTAMKRLISRFIDHYGIGY

TSHILDQVKTLGFRQATAASISLGIDDLLTIPSKRWLVQDAEQQSFILEKHHHYGNVHAVEK
LRQSIEIWYATSEYLRQEMTPNFRMTDPFNPVHIMSFSGARGNASQVHQLVGMRGLMSDP
QGQMIDLPIQSNLREGLSLTEYIISCYGARKGVVDTAIRTSDAGYLTRRLVEVVQHIVVRRT
DCGTVRGISVSPRNGMMA-DRIFIQTLIGRVLADDIYIGSRCIATRNQDIGVGLVNRFIT------
FRAQPISIRTPFTCRSTSWICQLCYGRSPAHDDLVELGEAVGIIAGQSIGEPGTQLTLRTFHTG
GVFTGGTAEHVRAPSNGKIKFNEDLVHPTRTRHGHPAFLCSRDLYVTIESEDIIHNVCIPPKS
FLLVQNDQYVESEQVIAEIRARTST-LNLKEKVRKHIYSDSEGE-MHWNTDVYHAPEFTYG
NIHLLPKTSHLWILLGEPWR--SSLGPCSIHKDQDQMNAY--SLSVKRRYISNPS--VTNNQ-V
RHKFFSSYFSGKNGDRIPDY-SELNRMTCTGRCNLRYP-GILDGNS--DLLAKRRRNRVIIPL
DSIQEGENQLIPSSGISIEIP--RNGILRRNSILAYFDDPRYIRKSSGLTKYETRELNSIVNEED--
LIEYRG-VKVFWPKYKEVNPFFFIPVEIHILAESSSIMVRHNSIIGVDTQITLNRRSRVGGLVR
VKKKAEKIKLIIFSGDIHFPEKTNKAFR----LIPPGG--GKKNSKEYKK--LKNWLYIQR--MKL
SRYEKKYFVLVQPVVPYKKTDGINLG-LFPADLLQESDNLQLRVVNYILYYDP--ILEIWDTS
IQLVRTCLVLNWDQDKK------IEKACASFVEIRTNGLIRDFLRIDLAKSPISYTGKRNDLPGS
GLIYE-NGSDRANVNPFSSIYSSS---KARIQESLNPNQGTIHTLLNR---NKESQSLIILSSSNCS
RIGP--FNDVKSPNV---IKES-PLIPIRNSLGPLGT--GFPISNFDL--FSHL--ITHNKIL-VTNYLQL
DNLK---HIFQILK---YYLLDEN--GKIYNPYSCSNIILNPFNLNWYFLHYNYCEETS-TIVSLG
QFLCENVCIAKKGPH-LK--SGQVLILQ-VDSVVIRSAKPYLATPGATVHGHYGEILYEGDTL
VTFIYEKSRSGDITQGLPKVEQVLEVRSIDSISMNLEKRIEGWNKCITRILGIPWAFFIGAELT
IVQSRISLVNKVQKVYRSQGVQIHNRHIEIIVRQITSKVLVSE---DEMSNVFSPGELIGLLRA
ERMGRALEEAICYQAVLLGITRASMNTQSFISEASFQETARVLAKAALLGRIDWLKGLKEN
VVLGGMIPVGSGFKTP-SSEPNNIPNNIA--FELKKENLLEGEMK--DILFYHRKFFDSCLSKN
FHDTQEQSFF-----------MTRRYWNINLEEMMEAGVHFGHGTRKWNPKMAPYISAKRKGIH
ITNLTRTARFLSEACDLVFDAASRGKQFLIVGTKNKEADSVAWAAIRARCHYVNKKWLGG
MLTNWSTTETRLHKFRDLRTEQKTGGLNRLPKRDAAMLKRQLSHLQTYLGGIKYMTGLP
DIVIIVDQHEEYTPLQECITLGIPTICLIDTNCDPDLADISIPSNDYSISSIRLILNKLVFAICEGR
S-GYIRNSMGQKINPIGFRLGTTQGHHSLWFAQPKNYSEGLQEDQKIRNYIKNYVQKNMK
TSSG-VEGIARIEIQKRIDLIQIIIYMGFPKILIESRPRGI----EELQMNLQKEFHSANRKLNIAIT
RIEKPYGNPNILAEFIAGQLKNRVSFRKAMKKAIELTEQADTKGIQVQIAGRIDGKEIARVE
WIREGRVPLQTIRAKIDYCSYTVRTIYGVLGIKIWIFIEGEMSRYRGPRFKKIRRLGALPGLT
NKRPRAGSDLRNQSRSGK-KSQYRIRLEEKQKLRFHYGLTERQLLKYVRIAGKAKGSTGQ
VLLQLLEMRLDNILFRLGMAPTIPGARQLVNHRHILVNGRIVDIPSYRCKPRDTIAARDEQ
KSRALIQNSLDSSPH-EELPNHLTLQPFQ--YKGLVNQIIDSKWVGLKINELLVVEYYSRQTM
SRRGTAEEKTAKSDPIYRNRLVNMLVNRILKHGKKSLAYQIIYRAVKKIQQKTETNPLSVL
RQAIHGVTPGIAVKARRVGGSTHQVPIEIGSTQGKALAIRWLLAASRKRPGRNMAFKLSSE
LVDAAKGSGDAIRKREETHRMAEANRAFAHFRMGSDTIADIITSIRNADMYRKSVVRVAS
TNISQSIVKILLREGFIENVRKHRENNKYFLVLTLRHRR--NRKRTY---RNLLNLKRISRPGLR
IYSNYQRIPRILGGMGVVILSTSRGIMTDREARLERIGGEILCYIWMAKAIPKKGSR--GRIG
SRKSIRKIPKGVIHIQASFNNTIVTVTDVRGRVVSWSSAGTCGFRGTRRGTPFAAQTAAAN
AIRAVVDQGMQRAEVMIKGPGLGRDAALRAIRRSGILLTFVRDVTPMPHNGCRPPKKRRV
MPTIKQLIRNTRQPIRNVTKSPALRGCPQRRGTCTRVY-ITPKKPNSALRKVARVRLTSGFEI
TAYIPGIGHNSQEHSVVLVRGGRVKDLPGVRYHIVRGTLDAVGVKDRQQGRS-SAL----MA
KKSLIQREKKRQKLEQKYHLIRRSSKKEIS---KVRSLSDKWEIYGKLQSPPRNSAPTRLHRR
CFSTGRPRANYRDFGLSGHILREMVHACLLPGATRSSWMLKNSFFSIILKEQEENKENRGS

VEFQVVSFTNKIRKLTSHLELHKKDYLSQRGLRKILGKRQRLLAYLSKKNRARYKELIGQL
DIRERKTR--MVKLRLKRCGRKQ----RIVAIDVRSRREGRDLRKVGFYDPIKNQTYLNVPAIL
YFLEKGAQPTGTVQDILKKAEVFKELCSNQTKFN-MDKSKRTFLKSKRSFRKRLPPIQSGD
RIDYKNMSLISRFISEQGKILSRRVNRLTLKQQRLITIAIKQARILSLLPFLNNEK--QFERTES
TTRTPSLRARKRVTRSLKKNPFVANNLLKKINKLNTKEEKEIIITWSRASTIIPIMVGHTIAIH
NGKEHLPIYITDRMVGHKLGEFAPTLNFRGHAKSDNRSRRMILKSFLLGNLVSLCMKIINS
VVVVGLYYGFLTTFSIGPSYLFLLRAHVMEEGEEGAEKKVSATTGFITGQLIMFISIYYAPL
HLALGRPHTITVLALPYLLFHFFCNNHKHFFDYGSTTRNSMRNLSIQCVFLNNLIFQLLNH
FILPSSMLARLVNIFMFRCNSKMLFVTSSFVGWIIGHILFMKWIGLLLVWIRQNRSIR--VQS
NKYLVSELKNSMSGIFGIFLLVTCVYYLGRIPSPIFTKKLNTEEEEKPILTFFFDYNRWNRPL
RYIRKIKLKGSVRKEASQYFFYTCQSDGKQRISFTYPPSLSTFGEMIARRISLSTLEKLSPDE
QYAEWLYTNKEKKNNLNNEFINRIEALETVFLSLNILETKTRLYNTKNKNNCLVKMYDPF
LN-MYRGRMKKLFSSAETAELNKIHDILSPENEHKNPKRE---INEIGKKPPRWSYKLIELEQ
YFIKQLRTRKYEHIAFLTRGFISYLEEPDFRRAVIKGSIRIQRRKMVIWGPSQGNPHSPLFLE
K-ME-DFPFP---ISDLMKLFFN-IKKNNQED----WENIPYAQKSRSVLLLAQSIFRRYIKLPLLII
AKNIGRILLRQSPEWYEDFQDWNREIYLKCSYNGLQFSKTEFPENWLIEGFQIKILYPFHLK
PWHRSKQQDDFDSCFLTVLGMETEYPFGPPRKTPSFFEPIFKDID-----------------YKEKIRNF
RVRIALKKIKKKEAKAFLFVKQIIKELLKGNDSIISNQIIHESLSQNRS---TNYSQAEEEMKH
RIDRRNTIRNQIEITKKNK----------KNNGESPPKIWKIFK------RKNIELIIQFFIEKTYMDIFLC
IINMRRIALQLF-----FDLSYLSQKYVFLKLSQTQVINFNKLRSILQYPSFFLKNEIKGFFRQGI
FDSEVRHKKLTNYGMNPWKKWLRG-YQYDLSQITWSRLVPQKWRNGKNQCQPSQNKK
KWYSYEKDQ--FFDYKKK-NLKKNHRYDRLSYKYIHSDIIYKDRKYLGWKLN-----SFDWM
GMNEKLLIFFFFPEFVILYHKYKEKPWFIPSNLLLFN--------LKIFFKGDFPLQLRWTGRLNQ
KMMNNIQIYGLLVSLINVRKITISYIQRKEMNLDIMRRR---LNLTQLTKGILIMEPARLSVKN
DGQFFMYQIIGISLVHKSKHKNYDLLVPEKILSSRRRRELRILISFVFFWPNYRLEDLACMN
RYWFDTTNGSRFSMLRIYMYPMTGHEFKSWILELREILREIKNSHYFLDSWTQFNSVGSFI
HIFFHQERFIKLFDSRIWSILLS-HNS--QGSTSNRYFT---IKG-VILFGVAVLIYR----INNRNMV
ERKNLYLIGLLP-----IPMNSIGPRNDTLEESV-GSSNINRLIVS-LLYLPKGKKIYESSFLNPKE
STWVLPIT----KKCSMPESNWGSRWWRDWIGKKS--DSSCKISN---ETVAGIEILFKEKDLK-
YLEFVFVYYRD--DPIRKDHDWEFFDRLSLRKRQNRINL--NSGPLFEILVKHWICYLMSAFR
EKIPIEVEG-FFKQQG-AGSTIQSNDIEHVSH--LFSRNKWAISLQNCAQFHMWQFRQDLFV--
-SWGK-NPPESDFLRN-VSRENLIWLDNVWLVNKDR---FFR--KVRNVSSNIQYDS---TR--SS
FVQVRDSSQLKGSS-DQFRDHFDSIS----NEDSE------------YHTLLNQREIQQLKERSIL-WD-
-PSFLQTEGTEIESN--RFPK--CLSGSS--SMSRLFT-EREKQMINHMLPEEIEELI--GNPTRSVR
SFFSDRW--SELH-LGSNPTERSTRDQ----KLLKKQQDLS-FL--RRSE----NKEMVNLFKIITYL
QN--TVSIHPISLDSGCDMVP-KDEPD-MDSSNKISFLNKNPFFDL--FHLFHDRNRGGYTL--H
HDFESE--ERFQEMADLFTLSITEP-DLVYHKRFAFSIDSYGLDPKQ--FLNGVFNSRYEWKTT
SLLVLLVLFPIFY--EENESFYRRIRKKRVR-ISCGNDLEEPKPKIVVFASNNRMEAVNQYRLI
RNLIQIQHSTHR--YIRNVLNRFFLMN---RSDRNFEYG---IQRDQIRKDTLNHRTLMKYTINQ
HLSNLKK-SQKRWFDPL--IFFSRTERSMNRDPDAYRYKWSTGSNNFQEHLEHFVSEQKSR-
--FQVVFDRLRINPYSIDWSEVIDKKDLSKPLRFFLSK----------------------LLLFLSN-SLPFLF
VSFGNIPIHRSE-IYIY-ELKGPND---PQFLESIGLQIVHLKKLKPF--LLD---DHETC-QKSKFLI
NGGTISPFLFNKIP-KW-MIDSFHTR--KNRRKSFDNTDSYFSMIFHDQYNWLNPVKSFHRSS
LRSSFYKGNQLRFLNNPHHFCFY--CNKRFPFYV----EKARIN--NYDFTYG-QFLNILFIRNK-I

FSLCVGK--KKHAFWG-RDTISAIESQVSNIFIPKAFPQSG-DETYNLYKS---FHFPSRANPFV
R--RAIYSIAGISGTPLTEGQIVNFERTYCQPLSDMNLSDSEGKNLYQYLN--FNSNMGLIHTP
CSEKYLPSEK--RKK--------RSLCLK--KCVEKGQMYRTFQRDSAYSTLSKWNLFQTYMPWF
LTSTGYRYLKFLFLDTFSDLL--PILSS-SQKFVSIFHDIMHGSDISWRILQKKF------CLPQWN
L-ISEISSKCFHN--LLLSEEMIHRN--NESPLISTHLT--NVREF-LYAILFLLLVAAY-LVCTHLLF
VFG--ASSELQTEFEKVKSLMIPSSMIELRKILDRYPTSEPNSFWLKNLFLVALKQLGDSL----
----GGNMLLGGGPA-----YG-VKSIRSKKKYLNINLIDIIDLI-SIIPNPINRITFSRNTRHLSHTS
KEIYSLIRKRKNVNGDWIDDKIESWVANSDSIDDEKREFLVQFS--TLTT--EKRIDQILLSLT-
HSDHL-----SKNDSGYQMIEQPGAIYLRDLVDIH--KKYLMNYEFNTSSLAERRIFLAHYQTI
TY--SQTSCGANSFHFPSHG---KPFSLRLALSLS-RGILVIGSIGTGRSYLVKYLAKNSYLPFIT
VFLNKFLDNKSQGFDDI-----DID-IDAS-----------DDIDA-----------SDDI---LDMEL-ELLTSM
NALTMNMMPED--EDRLYITLQFELAKAMSPCIIWIPNIHDLDVNESNYFSLGLLVNLLSR---
DYE----TRNILVIASTHIPQKADPALIAPNKLNTCIKIRRLLIPQQRKHFFTLSYTRGFHLEKK
MFHT--NGFGSITMGSNARDLVALTNEALSISITQKKSIIDTNTIRSALHRQIWDLR---SQVRS
VQDHGILFYQIGRAVAQNVLLSNCPIDPISIYMKKK--SCNEVDYYLYNWYFELGTSMKKLT
ILLYLLSCSAGSVTQDLWSLP--GPDEK-NGITPYGLVENDS-GLVRGLLEVEGALVG--SSR---
TCSQFDKD--RVTLLLRPEPRN-PLDMMQKGSCSILDQRFLYEKD--ESEFEE-GEGEGALDR
QQ----IEED----LFNH--IVWAP--RIWRP---WGFLFDCIERPNELGFP-YWSRSFRGK---RIIYDE
EDELQENDSEFLQSGTVQYQTRDRSSKEQGLFRISQFIWDPADPLFFLFK-AQPFVSVFSHR
ELFADEEMSKGLLTPQTNPPTSLYKRWFI--KKTQEKHFELLINRQRWLR---TNRSLS--NGSF
RSN--TLSESYQYLSNLFLSN-GTLLDQMTKALLRKRWLFPDEMQIGF--MEQEKDFPFLMS
RER-INGNFIDKTFSIVANILLRIIPTTSGEKEAFTYYRDG--MSAQSEGNYAEALQNYYEAM
RLEIDPYDRSYILYNIGLIHTSNGEHTKALEYYFRALERNPFLPQAFNNMAVICHY-RGEQAI
RQGDSEIAEAWFDQAAEYWKQAIALTPGNYIEAHNWLKITRRFE--MSCRSEHIWIEPIKGA
RKISNFCWAIILFLGSLGFLLVGTSSYLGRNLISFFPSQQIVFFPQGIVMSFYGIAGLFISSYL
WCTISWNVGSGYDRFDIKDGIVCIFRWGFPGKNRRVFLRFLIKDIQSVRIEVKEGIYARRVL
YMDIRGQGAIPLTRTDEN-FTPREMEQKAAELAYFLRVPIEVF
>Fagopyrum
MEKWWFNSMLSLSKSMDSLDPIDNINSNENMILNFTEKSIHSYSNLDLLFIIGDIPNLISDET
FLIRDRKGETYSVYFDIENKIVEIDNYH----------SFSNYWNSSYG-TGSKSNDTHYDLYMYD
TKSSLNNHINGCIDSYLH--SQMRLDNYVLRDSDHYGDNYIFD---------IKTTTNG-----RD---
NLEVTKKYRNLWIQCENCYELNYKKLLKSKMRICDECGYHLKMSSSERIELSIDPGTWEP
MDEDMVSTDPIEFHSQEEPYKDRINSYQKETGLTDAVQTGIGQLNGIPVAIGVMDFQFMG
GSMGSVVGEKITRLAEYATNQNLPLIIVCASGGARMQEGSLSLMQMAKISSALFDYQSNK
KLFYVSILTSPTTGGVTASFGMLGDIIISEPSAYIAFAGKRVIEETLNTTVPEGSQEAEYLFDK
GLFDPIVPRNPLKGVLSELFRLHAFFPLNQNSIGMTTIRADEIYNIIRERIEQYNREVKIVNT
GTVLQVGDGIARIHGLDEVMAGELVKFEEGTIGIALNLESNNVGVVLMGDGLMIQEGSSV
KATGRIAQIPVSEAYLGRVINALAKPIDGRGEILASEFRLIESPAPGIISRRSVYEPLQTGLIAI
DSMIPIGRGQRELIIGDRQTGKTAVATDTILNQKGQNVICVYVAIGQKASSVAQVVTTFQER
GAMEYTIVVAETADSPAALQYLAPYTGAALAEFFMYRERHTLIIYDDLSKQAQAYRQMSL
LLRRPPGREAYPGDVFYLHSRLLERAAKLSSRLGEGSMTALPIVETQSGDVSAYIPTNVISI
TDGQIFLSADLFNAGIRPAINVGISVSRVGSAAQIKAMKQVAGKLKLELAQFTELEAFAQFA
SDLDKVTQNQLARGQRLRELLKQSQASPLGVEEQIITIYTGTNGYLDSLEIGQVSPFLDDL
RKYFKTNKPQFQEILSSTKTFTEEAEALLKEGIQEQMERFLLQ---MRINPTTSSPGVSTLEK

SNLGRVAQIIGPVLDVAFPPGKMPNIYNALVIKGRDTAGQPINVTCEVQQLLGNNRVRAVA
MSATDGLTRGMEVVDTGAPLSVPVGGATLGRIFNVLGEPVDNLGPVDTRTTSPIHRSAPAF
IQLDTKLSIFETGIKVVDLLAPYRRGGKIGLFGGAGVGKTVLIMELINNIAKAHGGVSVFG
GVGERTREGNDLYMEMKESGVINEQNISESKVALVYGQMNEPPGARMRVGLTALTMAEY
FRDVNKQDVLLFIDNIFRFVQAGSEVSALLGRMPSAVGYQPTLSTEMGSLQERITSTKQGS
ITSIQAVYVPADDLTDPAPATTFAHLDATTVLSRALAAKGIYPAVDPLDSTSTMLQPRIVGEE
HYKTAQRVKQTLQRYKELQDIIAILGLDELSEEDRLTVARARKMERFLSQPFFVAEVFTGSP
GKYVGLAETIRGFQLILSGELDGLPEQAFYLVGNIDEATAKAMNLEMENKLKKMTLNLCV
LTPNRIVLDSEVKEIILSTNSGQIGVLPNHAPIATAVDIGIVRIRLNDQWLIMALMGGFARIG
NNEITVLVNDAEKGSDIDPQEAQQTLEIAEANLRKAEGKRQTIEANLALRRARTRVEAINTI
SMKNVTDSFVSLGHWRSAGSFGFNTDIFATNPINLSVVIGVLIFFGKGVFSDLLDNRKLRIV
NTIRNSEELCGRAVEQLEKARARLRKVEMEADQFRMNGYSEIERDKLNLINSIYKTLEQLE
NYKNETIHFEQQRVINQVRLRVFQQALQGALGTLNSCLSNELHLRTINANIGMFGAMKEIT
DMNPLISAASVIAAGLAVGLASIGPGVGQGTAAGQAVEGIARQPEAEGKIRGTLLLSLAFM
EALTIYGLVVALALLFANPFVMNLLSCSINTLR-LYDISGVEVGQHFYWQIGGFQVHGQVLI
TSWVVIAILLSSAAIAVRNPQTIPTDGQNFFEYVLEFIRDVSKTQIGEEYRPWVPFIGTMFLF
IFVSNWSGALLPWKIIQLPHGELAAPTNDINTTVALALLTSVAYFYAGLTKKGLGYFSKYIQ
PTPILLPINILEDFTKPLSLSFRLFGNILADELVVVVLVSLVPLVVPIPVMFLGLFTSGIQALIF
ATLAAAYIGESMEGHHMIFFTFEHLFNHISFSIVSIVITIHLITLVVNKIVELDDSLEKGMILTF
SCITGLLVIRWIFAGHFPLSDLYESLIFLSWSFYIIHMIP---YFKNK--LITAPGAIFTQGFATSGF
FTQMKQSTILVPALQSQWLMMHVSMMILAYAALLCGSLLSVALLVITFQNNISLFGKRKKI
QYVNEKNNILQ-TYLFSLINFYRYQLIEQLDRWSSRVISLGFLFLTMGILSGAVWANEAWGS
YWNWDPKETWAFITWTIFAIYLHIKTNINLKSENSAIVASIGFLIIWICYFGVNLLGIGLHSY
GSFILKMAKKKAFIPLLFLVSIVFLPWWSFLTFQKSMEYWVTNLWNTKQSETFLSDITEKT
LLEKFIELEELLLLDDMIKEYPERHLKGLRIGIHNETIQLIKLHNEHCIHTILSFSTNIICFIILS
GYSIIGNKELIILNSWVQEFLYNLSDTIKAFSILLLTDLCIGFHSPHGWELVVGSIYKDFGFSH
NDQIVSGLVSTFPVILDTILKYWIFRHLTRVSPSLVVIYHSMNEMPIGVPKVPFRLPGDEDAT
WVDVYNRLYRSRLLFLTQEITTEISNYLIGLMVYLSIEDQTQDFYLFINCPGGWVLPGIAIF
DAMQLVLPDIYTICMGIAASMGSFILVGGEITKRFAFPHARVMIHQPASSFHEGSTGECMM
EAEELLTLRETLTKVYAQRTGQPFGVLTEDLERDVFMSAREAQAHGIIDLIAKE---------------
----MFRVRLDNEDLILGYVSGRIRRSFIRILPGDRVKVEVSRYDSTRGRIIYRLRNKDLTEME
EFQGYLE-NRSWQDDFLYPLILQESIYALVHNQDLKGYD?KYSLLVVKRLVIRMYQQNFVI
LSLNHSNKNAFFVKNLYSQRISEVFAVIAEIPVSMRVMSSLRKQYKNLQSIHSIFPFLEDKFS
RLNHVLDILIPHPVHPKILVQTIRYCVKDISCLHLLQLLLYEYCNNGITLK---NFSKKQRFLL
FLYNSYVCECESIFVFLRNQSSHLRSTSYGAFLARVYFYLKLEHLK-VFTKHCILQFFKDPFI
HYIRYQGKWILASRGTFLLMTKFKYYFVNFWQCHFYLWLQTRRIYINKSLNQPIDFIGFRL
SVRLNPSVVRSQMLENAFLIYNGI--KKFETLVPTMSLIGSLAKEKFCNVLGHPISKPAWAD
LSDSDIIRRFGRMCRNLSHYYSGSSKKGGLYRIKYILRLSCARTLARKHKSTVRAFMKRLG
SEFFEEFVFKEEKVISLILSRPIWYFDIFCIHDLASHNMIIDTTRGVQAIN-SLSILES-LKEVY
GTIWMFLPILILIFGITLGVLVIVWLERQISAGIQQRIGPEYAGPFGILQALADGTKLLFKENL
LPSRGDKGLFNVGPSIAVISILLSYSVIPFSYRLILVDLSNGVFLWIAISSIAPIGLLMSGYGSN
NKYSFLGGLRAAAQSISYEIPLTLCVLSISLISNSLSTVDIVEAQSKYGLWGWNLWRQPIGF
LVFLISSLAECERLPFDLPEAEEELVAGYQTEYSGIKFGLFYVASYLNLLVSSLFVTVLYLGG
WNFSIPYIPVPDPFELNAKYGVFVTTLGIFITLAKTFLFLFISITTRWTLPRLRMDQLLNLGW

KFLLPISLGNLLLTTSSQLLSLMIWHVQNENFILDSTRIFMKAFHLLLFDGSFIFPECILIFGLI
LLLMIDSTSDQKDIPWFYFISSTSLVMSITALLFRWREEPMISFSGNFQTNNFNEIFQFLILLC
STLCIPLSVEYIECTEMAITEFLLFVLTATLGGMFLCGANDLITIFVAPECFSLCSYLLSGYTK
KDVRSNEATTKYLLMGGASSSILVHGFSWLYGSSGGEIELQEIVNGLINTQMYNSPGISIAL
IFITVGIGFKLSPAPSHQWTPDVYEGSPTPVVAFLSVTSKVAASASATRIFDIPFYFSSNEWRL
LLEILAILSMIVGNLIAITQTSMKRMLAYSSIGQIGYVIIGIIVGDSNDGYASMITYMLFYIAM
NLGTFACIVSFGLRTGTDNIRDYAGLYTKDPPLALSLALCLLSLGGLPPLAGFFGKLHLFW
CGWQAGLYFLVSIGLLTSVVSIYYYLKIIKLLMTGRNQEITPHVRNYRRSPLRSKNSIELSMI
VCVIASTILGISMNPIIAIAQDTLFMFLLYEYDIFWAFLLISSVIPILAFLLSGILAPIRKDPEKL
SSYESGIEPIGDAWLQFRIRYYMFALVFVVFDVETVFLYPWAMSFDVLGVSVFIEALIFVLIL
IVGLVYAWRKGALEWSTNFFPWLTLIVVLPIFAGSLILFLPHRGNKVIRWYTLCICILEFLLT
TYVFCYHFQLDDPLIQLAEDYNWIHFFYFHWRLGIDGLSIGPILLTGFITTLATLAAWPVTR
DSRLFHFLMLAMYSGQIGLFSSRDLLLFFLMWELELIPVYLLLSIWGGKKRLYSATKFILYT
AAGSVFLLVGVLGIGLYGSNEPTLNLETLGNQSYPVAFEILFYIGFLIAFAVKSPIIPLHTWLP
DTHGEAHYSTCMLLAGILLKMGAYGLVRINMELLPHAHSIFSPWLMIVGTIQIIYAASASP
GQRNLKKRIAYSSVSHMGFIIIGISSITDTGFNGAILQIISHGFIGAALFFLAGTSYDRIRVVYL
DEIGGIAISMPKIFTLFSTFSMASLALPGMSGFFAELMVFFGIITSHKYFLMVKVLITFGMAI
GMILTPIYLLSMLRQMFYGYKLLNNANSFFFDSGPRELFVSLAIFLPVLGIGIYPDFVLSLSV
DKVEAILSNFFL-MMLEHVLVLSAYLFSIGIYGLITSRNLVRALMCLELILNAVNLNFVTFSD
FFDSRQLKGNIFSIFVIAIAAAEAAIGPAIVSAIYRNRKSTRINQSNLLNKMEHIYQYTWIIPF
IPLTVPLLIGAGLIIFPTTTKNLRRMWAFPSILLLSLVMLFSTKLSIQQINSHYIYQSVWSWTI
NNDFSLEFGYLVDPLTSIMSMLITTVGILVLIYSDNYMVHDQGYLRFFAYLSFFNTSMLGLV
TSSNFIQIYIFWELVGMCSYLLIGFWFTRPIAANACQKAFVTNRVGDFGLLLGILGLYWLTG
SFEFRDLFEIFNT-EVHFLVGTVCTFLLFAGAVAKSAQFPLHVWLPDAMEGPTPISALIHAAT
MVAAGIFLVARLFPLLIVTPFILNLIALVGIITLFLGATLASCSKDIKRGLAYSTMSQLGYMM
LALGMGSYRAALFHLITHAYSKALLFLGSGCVIHSMEAIVGYSPDKSQNMVFMGGLKKH
VPITKTAFLLGTLSLSGIPPLACFWSKDEIINDTWLYSPIFAIISWATVGFTAFYMFRIYLLTFE
GHLNVHFQNYNGKKSSSVYSISLWGHEGLKPINKNLFTLLPIK-FYCDNKKIVPYPHESGN
TMLFPLLVLIIFTGVIGFIGIPFDFDILSSWLTPSVNLLHLKLNNSFDWSEFLTNATLSVSIAYS
GIVLASFLYKPIYSSLQNFALINLFAFFSDKIKNVIYDWAHHRGYIDAFYTRYIIRSVRGLSQ
FINFFDRRVIDGIPNGLGVTSFFVGEGLKYVGGG-RISSYLFLYLLYASIFLLIYYMDLPGPIH
DFLLVFFGSGLILGGLGVILFTNPIFSAFSLGLVLVCISLFYIIANSQFVASAQLLIYVGAINVL
IIFAVMFINGLEYDKNLRLFTLGDGMTLVICTGIFFLLITTILNTSGYGIIWTTKLNQILEQDLI
NNSQQIGIHLSTDFFPPFELISIILLVALIGAIAVARQMTGPITRKDLMIVNMGPQHPSMHGV
LRLIVTLDGEDVIDCEPVLGYLHRGMEKIAENRTIIQYLPYVTRWDYLATMFTEAITINAPE
QLGNIQVPKRASYIRVIMLELSRIASHLLWLGPFMADIGAQTPFFYIFRERELIYDLFEAATG
MRMMHNFFRIGGIAADLPYGWIDKCLDFCEYFLPAIAEYQTLITRNPIFLERVEGVGIIGGE
EAINWGLSGPMLRASGIQWDLRKIDHYESYEEFDWEVQWQKEGDSLARYLTRIGEMAESI
KIIQQALEGIPGGPYENLEIRRCNKVRDPEWNNFEYRFISKRPSPTFELSQQELYVRVEAPK
GELGIFLIGDRSAFPWRWKIRPPGFINLQILPQLVKRMKLADIMTILGSIDIIMGEIDRMFPM
VTGFLTYGQQTVRAARYIGQGFLITLSHANRLPVTIQYPYEKLITSERFRGRIHFEFDKCIAC
EVCVRVCPIDLPVVDWKLETGIRKKRLLNYSIDFGICIFCGNCVEYCPTNCLSMTEEYELS
MYDRHELNYNQIALGRLPISIIDDYTIRTILNW----------------MQGRLSAWLVKHGLVHRSLG
FDYQGIETLQIKPEDWHSIAVILYVYGYNYLRSQCAYDVEPGGLLASVYHLTRIESGVDQP

EEVCIKVFVPRRNPRIPSVFWVWKSVDFQERESYDMLGIFYENHPRLKRILMPESWIGWPL
RKDYIAPNFYEIQDAYLGNEFRCIGCICFYRSFNFRAYPNCWFSLCMAKRSIGMVLVSKYF
NNQNKKRKNNKIERVLNSVEFLPLDRITPNSVISTTSNDLSNWSRLSSLWPLLYGTSCCFIE
FASLIGSRFDFDRYGLVPRSSPRQADLILTAGTVTMKMAPSLVRLYEQMPEPKYVIAMGAC
TITGGMFSTDSYSTVRGVDKLIPVDVYLPGCPPKPEAVIDAITKLRKKISWEIYEDRIQS--K
NRYFTIKHKFRVGRRIHTGNYNQGLLSKSPSISEIPPEKFFKYRSSVSSHEFVNMQTINTLS
WIKEQITRSISVSLIIYIITQSSISNAYPIFAQQGYENPREATGRIVCANCHLANKPVDIEVPQA
VLPDTVFEAVVRIPYDMQLKQVLANGKKGGLNVGAVLILPEGFELAPTERISPEMKEKIGN
LSFQSYRPNKKNILVIGPVPGQKYSEITFPILSPDPATKKDAHFLKYPIFVGGNRGRGQIYPD
GSKSNNTVYNATATGIVKTIIRKEKGGYEITIGDASDGRQVVDIIPPGPELLVSEGESIKLDQ
PLTSNPNVGGFGQGDTEIVLQDPLRVQGLLFFLASVILAQIFLVLKKKQFEKVQLSEMNFM
SKVYDWFEERLEIQAIADDITSKYVPPHVNIFYCLGGITLTCFLVQVATGFAMTFYYRPTVT
DAFASVQYIMTEANFGWLIRSVHRWSASMMVLMMILHVFRVYLTGGFKKPRELTWVTG
VVLAVLTASFGVTGYSLPWDQVGYWAVKIVTGVPEAIPIIGSPLVELLRGSASVGQSTLTRF
YSLHTFVLPLLTAVFMLMHFLMIRKQGISGPLMGVTKKPDLNDPVLRAKLAKGMGHNYY
GEPAWPNDLLYIFPVVILGTIACNVGLAVLEPSMIGEPADPFATPLEILPEWYFFPVFQILRTV
PNKLLGVLLMVSVPAGLLTVPFLENVNKFQNPFRRPVATTVFLVGTVVALWLGIGATLPID
KSLTLGLFMIEVLLFGIVLGLIPITLAGLFVTAYLQYRRGDQLDLMPTLTSYFGFLLAALSTT
SALFIGLSKIRLLMDIVSLAWAALMVVFTFSLSLVVWGRSGLMIIRSPEPEVKILVDRDPIKT
SFEEWARPGHFSRTIAKGPETTTWIWNLHADAHDFDSHTSDLEEISRKIFSAHFGQLSIIFL
WLSGMYFHGARFSNYEAWLSDPTHIGPSAQVVWPIVGQEILNGDVGGGFRGIQITSGFFHI
WRASGITSELQLYCTAIGALVFAALMLFAGWFHYHKAAPKLAWFQDVESMLNHHLAGLL
GLGSLSWAGHQVHVSLPINQFLNAGVDPKEIPLPHEFILNRDLLAQLYPSFAEGATPFFTLN
WSKYAEFLTFRGGLDPVTGGLWLSDIAHHHLAIAILFLIAGHMYRTNWGIGHGLKDILEAH
KGPFTGQGHKGLYEILTTSWHAQLALNLAMLGSLTIVVAHHMYSMPPYPYLATDYGTQLS
LFTHHMWIGGFLIVGAAAHAAIFMVRDYDPTTRYNDLLDRVLRHRDAIISHLNWACIFLG
FHSFGLYIHNDTMSALGRPQDMFSDTAIQLQPIFAQWIQNTHALAPSVTAPGATTSTSLTW
GGGDLVAVGGKVALLPIPLGTADFLVHHIHAFTIHVTVLILLKGVLFARSSRLIPDKANLGF
RFPCDGPGRGGTCQVSAWDHVFLGLFWMYNSISVVIFHFSWKMQSDVWGSISDQGVVT
HITGGNFAQSSITINGWLRDFLWAQASQVIQSYGSSLSAYGLFFLGAHFVWAFSLMFLFSG
RGYWQELIESIVWAHNKLKVAPATQPRALSIVQGRAVGVTHYLLGGIATTWAFFLARIIAV
GMALRFPRFSQGLAQDPTTRRIWFGIATAHDFESHDDITEERLYQNIFASHFGQLAIIFLWTS
GNLFHVAWQGNFESWVQDPLHVRPIAHAIWDPHFGQPAVEAFTRGGAPGPVNIAYSGVY
QWWYTIGLRTNEDLYTGSLFLLFLSVISLIAGWLHLQPKWKPSVSWFKNAESRLNHHLSG
LFGVSSLAWTGHLVHVAIPGSRGEYVRWNNFLDVLPHPQGLAPLFTGQWNLYAQNPDSSS
HLFGTAQGAGTAILTLLGGFHPQTQSLWLTDMAHHHLAIAFIFLVAGHMYRTNFGIGHSM
KDLLEAHIPPGGRLGRGHKGLYDTINNSIHFQLGLALASLGVITSLVAQHMYSLPAYAFIAQ
DFTTQAALYTHHQYIAGFIMTGAFAHGAIFFIRDYNPEQNEDNVLARMLEHKEAIISHLSW
ASLFLGFHTLGLYVHNDVMLAFGTPEKQILIEPIFAQWIQSAHGKTSYGFDVLLSSTNGPAF
NAGRSIWLPGWLNAVNE--KSNSLFLTIGPGDFLVHHAIALGLHTTTLILVKGALDARGSKL
MPDKKDFGYSFPCDGPGRGGTCDISAWDAFYLAVFWMLNTIGWVTFYWHWKHITLWQG
NVSQFNESSTYLMGWLRDYLWLNSSQLINGYNPFGMNSLSVWAWMFLFGHLVWATGFM
FLISWRGYWQELIETLAWAHERTPLANLIRWRDKPVALSIVQARLVGLAHFSVGYIFTYAA
FLIASTSGKFGMSHSVKIYDTCIGCTQCVRACPTDVLEMIPWDGCKAKQIASAPRTEDCVG

CKRCESACPTDFLSVRVYLWHETTRSMGLAYMTDINLPSIFVPFVGLVFPAIAMASLFLHV
QKNKIVMRNLKTYLSVAPVLSTLWFGSLAGLLIEINRFFPDALTFPFFSFMTAILERRESESL
WGRFCNWITSTENRLYIGWFGVLMIPTLLTATSVFIIAFIAAPPVDIDGIREPVSGSLLYGNNI
ISGAIIPTSAAIGLHFYPIWEAASVDEWLYNGGPYELIVLHFLLGVACYMGREWELSFRLG
MRPWIAVAYSAPVAAATAVFLIYPIGQGSFSDGMPLGISGTFNFMIVFQAEHNILMHPFHML
GVAGVFGGSLFSAMHGSLVTSSLIRETTENESANEGYRFGQEEETYNIVAAHGYFGRLIFQ
YASFNNSRSLHFFLAAWPVVGIWFTALGISTMAFNLNGFNFNQSVVDSQGRVINTWADIIN
RANLGMEVMHERNAHNFPLDLAATQAPSINGMGLPWYRVHTVVLNDPGRLISVHIMHTA
LVAGWAGSMALYELAVFDPSDPVLDPMWRQGMFVIPFMTRLGITNSWGGWSIT-GGTVTD
PGIWSYEGVAGSHIVFSGLCFLAAIWHWVYWDLEIFCDERTGKPSLDLPKIFGIHLFLSGVA
CFGFGAFHVTGLYGPGIWVSDPYGLTGKVQSVNPAWGVEGFDPFVPGGIASHHIAAGTLG
ILAGLFHLSVRPPQRLYKGLRMGNIETVLSSSIAAVFFAAFVVAGTMWYGSATTPIELFGPT
RYQWDQGYFQQEIYRRVSAGLAENQSLSEAWSKIPEKLAFYDYIGNNPAKGGLFRAGSM
DNGDGIAVGWLGHPIFRDKEGRELFVRRMPTFFETFPVVLIDGDGIVRADVPFRRAESKYS
VEQVGVTVEFYGGELNGVSYSDPATVKKYARRAQLGEIFELDRATLKSDGVFRSSPRGWF
TFGHASFALLFFFGHIWHGSRTLFRDVFAGIDPDLDSQVEFGAFQKLGDPTTRRQAVMKTL
YSLRRFYHVETLFNGTLALTSRDQETTGFAWWAGNARLINLSGKLLGAHVAHAGLIVFWA
GAMNLFEVAHFVPEKPMYEQGLILLPHLATLGWGVGPGGEVIDTFPYFVSGVLHLISSAVL
GFGGIYHALLGPETLEESFPFFGYVWKDRNKMTTILGIHLILLGLGAFLLVFKALFFGGIYD
TWAPGGGDVRKITNLTLSPSIIFGYLLKSPFGGEGWIVSVDDLEDIIGGHVWLGSICILGGI
WHILTKPFAWARRALVWSGEAYLSYSLGALSVFGFIACCFVWFNNTAYPSEFYGPTGPEAS
QAQAFTFLVRDQRLGANVGSAQGPTGLGKYLMRSPTGEVIFGGETMRFWDLRAPWLEPL
RGPNGLDLSRLKKDIQPWQERRSAEYMTHAPLGSLNSVGGVATEINAVNYVSPRSWLATS
HFVLGFFLFVGHLWHAGRARAAAAGFEKGIDRDFEPVLSMTPLNMTIALGKVTKDENDL
FDIMDDWLRRDRFVFVGWSGLLLFPCAYFAVGGWFTGTTFVTSWYTHGLASSYLEGCNF
LTAAVSTPANSLAHSLLLLWGPEAQGDFTRWCQLGGLWTFVALHGAFGLIGFMLRQFELA
RSVQLRPYNAIAFSGPIAVFVSVFLIYPLGQSGWFFAPSFGVAAIFRFILFFQGFHNWTLNPF
HMMGVAGVLGAALLCAIHGATVENTLFEDGDGANTFRAFNPTQAEETYSMVTANRFWS
QIFGVAFSNKRWLHFFMLFVPVTGLWMSALGVVGLALNLRAYDFVSQEIRAAEDPEFETF
YTKNILLNEGIRAWMAAQDQPHENLIFPEEVLPRGNALMSGSTGERSFADIITSIRYWVIHS
ITIPSLFIAGWLFVSTGLAYDVFGSPRPNEYFTESRQGIPLITGRFDSLEQLDEFSRSFMTIDR
TYPIFTVRWLAVHGLAVPTVSFLGSISAMQFIQRMATQIVESSSKSR-PRQTTVGALLKPLNS
EYGKVAPGWGTTPLMGVTMALFAVFLSIILEIYNSSVLLDGISMNMLTLKLFVYTVVIFFVS
LFIFGFLSNDPGRNPGREEMADTTGRIPLWIIGTVTGILVIGLLGIFFYGSYSGLGSSLMLNL
FSLICICLNSVLSSSIFLFAKLPEAYAFLSPIVDFMPVIPLLFFLLAFVWQAAVSFRTTQSNPNE
QNVELNRTSLYWGLLLIFVLAVLFSNYFFNMEVNILAFIATALFILVPTAFLLIIYVKTVSQN
DMETATLVAIFISGLLVSFTGYALYTAFGQPSQQLRDPFEEHGDMEALVYTFLLVSTLGIIFF
AIFFREPPKVPTKK--MTIAFQLAVFALIATSSILLISVPVVFASPDGWSSNKNFLFSGTSLWIG
LVFLVGILNSLIS-------MSPQTETKASVGFKAGVKEYKLTYYTPDYEPHDHDILAAFRVTPQ
PGVPPEEAGAAVAAESSTGTWTTVWTDGLTSLDRYKGRCYHIEPVPGEENQFIAYVAYPLD
LFEEGSVTNMFTSIVGNVFGFKALRALRLEDLRIPPAYTKTFQGPPHGIQVERDKLNKYGR
PLLGCTIKPKLGLSAKNYGRAVYECLRGGLDFTKDDENVNSQPFMRWRDRFLFCAEALF
KSQVETGEIKGHYLNATAGTCEEMIKRAVFARELGVPIVMHDYLTGGFTANTSLAHYCRD
NGLLLHIHRAMHAVIDRQKNHGIHFRVLAKALRLSGGDHIHSGTVVGKLEGERDITLGFV

DLLRDDFVEKDRSRGIYFTQPWVSLPGVIPVASGGIHVWHMPALTEIFGDDSVLQFGGGTL
GHPWGNAPGAVANRVALEACVQARNEGRDLAREGNDIIRKAAKWSPELAAACEVWKEI
KFEFQAMDTL--MAIHLYKTSTPNTGNRAVDSQVKSNPRNNLIYGQRRCGKGRNARGIITA
RHRGGGHKRLYRKIDFRRNEKDIYGRIVTIEYDPNRNAYICLIHYGDGEKRYILHPRGTLIG
DTIVSGTEVPIKMGNALPLTDMPLGTAIHNIEITLGKGGQLARAAGAVAKLIAKEGKSATL
KLPSGEVRLISKNCSATVGQVGNVGVNQKSLGRAGSKRWLGKRPVVRGVVMNPVDHPH
GGGEGRAPIGRKSPTNKWGFPALGRRSRKRNKYSDNLIIRRRSKMIQPQTYLNVADNSGA
RELMCIRIIGAS-NRRYAHIGDIIVAVIKEAIPNMPLERSEVIRAVIVRTRKELKRENGMIIRYD
DNAAVIIDQKGNPKGTRVFGAIARELRQLNFTKIVSLAPEVLMLSPKRTRFRKQHRGRMK
GISYRGNRICFGRYALQALEPAWITSRQIEAGRRAMTRNARRGGKLWVRIFPDKPVTVRPA
ETRMGSGKGSPEYWVAVVKPGRILYEISGVPEKIARKAISIAASKMPIRTQFLISEMTRVRR
GYIARRRRKKMRLFVSSFRGAHSKPIRIVAQQKIRALFSAHRDRDKKKRDFRRLWITRINA
AIRETKGIYYSYSKFIHDLYKRQLILNRKILAQIAILNRNSIYSIYNEILKKEDWKDAPEML-
MREVSALGQYIPMSVHKARRVIDQIRGRSYKETLMILELMPYRACYPIFKIIYSAAANAKH
NKGFEKEDLLVWKAEVNKGPTRKKLKPRARGRSYLIKKPTCHISVVLKDISYY------
-???
??????MAVPKKRTSISKKRIRKNIWKSKGYSAALKAFSLAKSLSTGNAKSFFIGKMSNMAK
GKDARVSVILECTSC-VRK--VNKE--GISRYITQKNRRNTPSRLELRKFCPYCYKHTIHGEIK
KMKIRASVRKICEKCRLIRRRGRIIVICSNPKHKQRQGMVEDKIRVSTRTLQWKCVESRTD
SKRLYYGRFILSPLMKGQADTIGIAMRRALLGEIEGTCITRAKSE--KIPHEYSTIVGIQESVH
EILMNLKEIVLRSTFYGTCDASICVRGPGGVTAQDIISPPYVEIVDTTQHIANLTEPIDLRIEL
QLERNRGYRIKTPNNFQDGSY--PIDVVFMPVRNVNHSIHSYSNGNEKQEILFLEIWTNGSL
TPKEALYEASRNLIDLFIPFLYAEKEN--FHLEDNQ-DRVTLPLFLFHDRLI-ELRKNKK-KIAL
KSIFIDQLELPPRIYNCLKNSNIHTLLDLLTNSQDDIMKIEHFSIEDVKQILDTLEKH--FAINL
PKKKMFRDGN-EGMSTIPGLNQIQFEGFCRFIDQGLTEELYKFPKIEDTDQEIEFQLFVETYK
LVEPLLKERDAVYESLTHSSELYVSAGLIWKTGRDMQEPTIFYWNLPLMNSLGTFIVNGIY
RIVINQILQSPGIYYRSELDHNGISVYTGTVISDWGGRSELEIDRKARIWARVSRKQKISILV
LSSAMGSNLREILENVCYPEIFLSFLTDKEKKKVGSKETAILEFYQQFACVGGDPVFSESLC
KELQKKFFQQRCELGRIGRRNMNQRLNLDIPQNNTFLLPRDILAAADRLIGMKFGMGTLD
D--MNHLKKKRIRSVADLLQDQFGLALVRLENVVRGTISGAIR-HKLI-PTPQNLVTSIP----LT
TTYESFLGLHPLSQVLDRTNPLTQIVHGRKLSYLGPGGLTGRTASFRIRDIHPSHYGRICPID
TSEGINVGLIGSLAIHARIGSWG-SLESPFY---KIFGKSK--RVQMLYLSPSRDEYYMVAAGTS
LALNRGIPEEGIVPARYRQEFLTSAWEEVHFRSIFPFQYFSIGASLIPFIEHNDANRALMSSN
MQRQAVPLSQSEKCIVGTGLERQAALDSGVLAIAEHEGKIISTETDKIILSGNGYTLNIPLV
MYQRSNKNTCMHQKARIRQGKCIKKGQILADGAATVGGELALGKNVLVAYMPWEGYNF
EDAVLISERLVYGDIYTSFHIRKYEIQTHVTSQG-PERLTNEIPHLEAHLLRNLDKNGIVVLG
AWVEAGDILVGKLTPQMAKE-SSDAPEDRLLRAILGIQVATSKETCLKLPIGSRGRVIDVRW
IQKKG-SSSSNPETIRVYISQKREIKVGDKVAGRHGNKGIISKILPRQDMPYLQDGRPVDMV
FNPLGVPSRMNVGQIFECSLGLAGGLLGRHYRIAPFDERYEQEASRKLVFSELYEASKQTA
NPWVFEPEYPGKSRIFDGRTGDPFEQPVIIGNPYILKLIHQVDDKIHGRSSGHYALVTQQPL
RGRAKQGGQRVGEMEVWALEGFGVAHILQEMLTYKSDHIRARQEVLGTTIIGGTIPNPED
APESFRLLVRELRSLALELNYFLVSEKNLQINRMEA-------MIDQYKHQQLRIGLVSPQQISA
WATKILPNGEIVGEVTKPYTFHYKTNKPEKDGLFCERIFGPIKSGICACGNFRIIGDETEEPK
FCEQCAVEFVDSRIRRYQMGYIKLACPVTHVWYLKRLPSYIANFLDKPLKELEGLVYCDF-

-AFARP-IAKKPTFLRLRGLFEYEIQSWKYSIPLFFTTQGFDTFRNREISTGASTIREQLADLD
LRTILDSSLSEWKE-----GPTGNEWEDRKVRRRKDFLVRRMELAKHFIRTNIEPEWMVLCLL
PVLPPELRPIIQIDGGKLMSSDINELYRRVIYRNNTLTDLLTTSRSTPGELVMCQEKLVQEAV
DTLLDNGIRGQPMRDGHNKVYKSFSDVIEGKEGRFRETLLGKRVDYSGRSVIVVGPSLSL
HRCGLPREIAIELFQTFVIRGLIRQDLASNIGVAKSKIRENEPIVWEILQEVMRGHPVLLNRA
PTLHRLGIQAFQPTLVEGRAICLHPLVCKGFNADFDGDQMAVHVPLSLEAQAEARLLMFS
HMNLLSPAIGDPISVPTQDMLIGLYVLTSGNRRGICANRYNPWSR---KNYKNEKWNDHNY
KYMK--EPFFCNSYDAIGAYRQKRINLDSPLWLRWQLDHR-VVAS--REAPIEVHYESLGTY
HEIYGNYLIVRSVKKEIIFIYIRTTVGHISFYREIEEAIQGFYRACSYGT--------MEVLMAKGA
NLVFHNKVIDGIAMKRLISRFIDHFGMAYTSHILDQVKTLGFQQATATSISLGIDDLLTIPSK
GWLVQDAEQQSFILEKQHHYGNIHAVEKLRQSIEIWYATSEHLRQEMNPNFRMTDPYNPV
HMMSFSGARGNASQVHQLVGMRGLMSDPQGQMIDLPIQSNLREGLSLTEYIISCYGARKG
VVDTAVRTSDAGYLTRRLVEVVQHVVVRRRDCGTTRGISVSPQTGTIP-EILFIQTLIGRVLA
DDIYKGPRCIAARNQDIGVGLVNRLIT------FRVQTISIRTPFTCRSTSWICRLCYGRSPTHGD
LVELGEAVGIIAGQSIGEPGTQLTLRTFHTGGVFTGGTAVHVRAPSNGKIKFNEDLVHPTRT
RHGHPAFLCYIDLAVTIESEDIIHNVNIPPKSFLLVQKDQYVESEQVIAEIRAGTSTL-NFKEK
VRKHIYSDSQGE-MHWSTDVDHAPEFTYGNVHLLPKTSHLWILAGRPHK--SRVATRSLHK
DQDQTNAH--FLSVEQRYISNSS--VINAY-MRHTFFSSDLSSTNGDSISDY-SERKRIVRTRHS
NLIYP----AKNSY--LLGKRRRNRFMTTFQSIQELEKELRAPSGISIDIPLN--GSFRRNSILAYF
DDPRYRRESAGITKYGTIEAHSIKKKEG--LIEYR-GVNEFRAKYMQVDRFFFIPEEVHILPR
SSAIMVRNNSIIGVDTQITLTTRSRVGGLVRVEKKKKKIGLKIFSGNIHFPGKTDKISRHRGI
LIPPGRG----QTK------LKNWSYVQR--ITPTKKR--YFVLIRPAITYEITDGISLSTLFPQDLLQE
RDKVQLRVVNYILSGNGKPIRGISDTSIQLVRTCLVLNWDHDK---KSSSIEESRASFVEVKT
NDMMRDFLRIDLVKSTISYAGKRNDPSGSGLLSD---NESDRMNPFYSIYIYSKA---RLQQPF
SQNNETVRTLLNR---TKEFQSFIILSSANCFRMGP-----FKG------LKYYDPIIPIKNSSGPFGT--
SLQIANFYS--FYRLIT--QDKIL-VTNYLQLDN---LKQTFWVMK---YYFIDDNRK--IYNPDPV
SNIVLNSFKLNWYCLHHNYYEETSARMG-LGQFLCENVCLAKNGP---RLKAGQVLIVQ-V
DSVVIRAAKPYLATPGATVHGHYGEIIYEGDTLVTFIYEKSRSGDITQGLPKVEQVLEVRSL
ESISMNLERRIDAWNERITGILGLPWGFVVGAELTIVQSRISLVNKIQKVYRSQGVQIHNRH
LEIIVHQITSKVLVSE---DGMSNVFLPGELIGLLRAERTGRALEEVISYRAVLVGITRASLNT
HSFISEASFQETARVLAKAALRGRIDWLKGLKENVVLGGKIPVGTGFKGLVHPS--SQHK--S
IPLKNKKKNLFEGEIN--DILFYYREFFNIEFLCFKNYQ----------------MTRRYWNINLEEMM
QAGVHFGHGTRKWNPRMSPYISAKRKGIHITNLTRTARFLSEACDLVFDAASKGKQFLIV
GTKTKAADSVARAAIRARCHYVNKKWLGGMLTNWSTTETRLHKFRDLRMEQKAGRLA
CLPKRDAAMLKRQLSRLQTYLGGIKYMTGLPDVVIIVDQQEEYTALRECITLGIPTICLIDT
NCDPDLADISIPANDDAIASIRLILTKLVFAICEGRS-SYIRNLMGQKINPLGFRLGTSQSHYSI
WFAQPKNYSEGLHEDQKIRDCIKNYAQQNVRASSG-VEGIARIEIQKRIDLIQVIIYMGFPK
LLIENRPR----GIEELQMNVQKKLNSVNRKLNIAITRIAKPYRHPNILAEFIAGQLKNRVSFR
KAMKKAIELTEQADTKGIQIQIAGRIDGKEIARIEWIREGRVPLQTIHAKIDYCAYTVRTIYG
VLGIKIWIFIDEEMSRYRGPRFKKIRRLGVLPGLTNKKPR---DLRNPSRSGK-RSQYRIRLEE
KQKLRFHYGITERQLLKYFRIARKAKGSTGQVLLQLLEMRLDNILFRLGMAPTIPGARQL
VNHGHVLVNGRIVDIPSYRCKPNDIVMSRDKQKSKALVQNYLDSSHS-EELPKHLTLHPFP-
-YKGVVNQIIDNKWVGLKINELLVVEYYSRQTMSRRGTAEEKTAKSDPIYRNRLVNMLVN
RILKHGKKSLAYQIIYRSVKKIQQETETNPLSVLRQAIRGVTPDIAVKARRVSGSTHQVPIEI

GSTQGKALAIRWLLGESRKRPGRNMAFKLSSELVDAAKGSGGAIRKKEETHRMAEANRA
FAHFRMSRDTIADIITSIRNADMNRKGIVRITSTKITETLVKILLREGFIENVRKHQEKNKYF
LVLTLRHRR--NRQGPY---TKFLNLKQVSSPGLRIYSNYQRLPRILGGIGILILSTSRGIMTDR
EARLEKIGGEILCYIWMAKPIPRIGSQRNRRINSRKNARKMPKGVIYVQAGFNNTIVTVTD
VRGRVVSWASAGTCGFKGAKRGTPFAAQTAAGNAIRKVVDQGMQRAEVMIKGPGLGRD
AALRAIRRSGILLSFVRDVTPMPHNGCRPPKKRRVMPTIKQLIRNTRQPIRNVTKSPALRGC
PQRRGTCTRVYTITPKKPNSALRKVARVRLTSGFEITAYIPGIGHNLQEHSVVLVRGGRVKD
LPGVRYHIVRGTLDAVGVKDRQQGRSKYGVKKPKMASQSLIQREQKRKKLEQKYHLIRR
SLKKEIS---KVTSLNEKWEIHGKLQSPPRNSAPTRLHRRCFLTGRPRANYRDFGLSGHILRE
MVHTCLLPGATRSSWMKKN---SVISQE--EK---KGSAAFQVFGFTNKIRRLSAHLELHKKD
YSSQRGLRKILGKRQRLLAYFSKKNRVRYKELINQLNIREIKR---MVKLRLKRCGRTQRAV
YRIVAIDVRSRREGRDLRKVGFYDPIKNKTYLNVPAIIYFLERGAQPTGTVYDILKKAEVF
KELRPTETKFT-MDKSKRSFLKPKRPFRRRLPPLQSGDRIDYRNMSLISRFISEQGKILSRRV
NRLTLKQQRLITIAIKQARILSLLPFLNNEK--QFERAKSAVRTTGFRTKKQVTRSLKKNPFV
ANHLLRKLDKLNTKEEKEIIVTWSRASTIIPTMIGHTIAIHNGREHLPVYITDRMIGHKLGE
FSPTLNFRGHAKNDNRSRRFFLPG-------NLCMKIVNSVVVVGLYYGFLTTFSIGPSYLVLLR
TLVMEEGEEGTEKRVAATTGFIMGQLMMFISIYYTPLHLALGRPHTITFLALPYLLFNFFWS
NH---FG--STTRNSMRNLSIQCVFLNNLIFQLFNHFVLPSSMLARLVNIYMFRCNNKMLFVT
SSFVGWLIGHILFMKCVGLVLVWIRQNFSIRSRIIRSKYSVSELKYLVSRIFSILLFISCVYSL
GRMPSPILSRKLQEEEDSKPPVSLLFDYKRWNRPLRYIKNYRFEHAVRNEMSQYFFYTCQS
DGKRRISFTYPSSLSAFFEMLRQKMYFYFFKKFVYDELHNYWIYTNEKKWRNLRNEFRD
RIETLDRGS--LDVLEKKTRLCNTKTKEEYLPKIYDPLLNGSYRGIMNFFFLPNFINKLNKV
HSILFEELYQKDPIIQRKKSIGINKKVPRWSYKLFEVEQLGKTQIRSRKAKRIVLFTQNMID
YPYEADFRRDIITGSMRVQRRKTLTGKMFPLYPYSPLFFDRLFEPFILSVIEISDLIQDIIKGSR
IERTRERISELYDILIYAHGIRAFILLIQSRLRQSIVLPSLILAKNIVRFLLRQESEWEQDIREM
NKEVYVICTYNGMPLLKPGFPKNWATEGIQLMIRFPFRLKPWHRSKPKQESPAAFLTIWGL
ETDRPFGSPLVGCYYFGPPLKKLQKTIKWSFR-----VLKSFKERTKLFLKVQKEPKKLREDS
SE------IKKDS------IINNQIIHESAIQTRPMDWTNYSLTEIKMKDLTDRTSTIRNQIERITKDK
KNSPNKTSYGVQKLAPLKKIFQILKRRNDRLIRKSHFFLKWIVERIYTDILLE--SFPYIINRLI
-----FKLSLVSQAYVVFYKLSQTQVINLYKLRSVLQYASLFLKNEIKDSFRQGIISSELKHKKLQ
NSGMNQWKNWLKSHYPYDLSERKWSKLVPQKWRNRVNQHCRVENPIKRDSSEREEKVS
LLLNKN-HFQKMYRYDLLAYQSIYDFFMYPDRKYFDTKGLS----PFDWMGMNEERLNRW
LFPQFEFFLNVYKMKPGFIPIHSLIFHSIWTWEIDLDAFMKRSFALQLKWLLSPMELFDYAL
AVLEWEKESRMTTKFGFGFIKKEELTLDPMLIR-DLNLSKILKEGIFIIEPVRIPVKHNVEFIM
YQTIRISLVHEIKQNHYDLLVPENILSSRRRRELRILSCFKFLWPNYRLEDLACMNRYWFDT
NNGSRFSILRIHMYPTKNIPLDFWVFELKEILREIKNS------------VGSLVKIFFHQERFIKLFD
PRIWSILLS---PNWRGSIIKRYFTF-TITGVVFFVVA-GLIYR----INNRNMVEKKNIYLIGLLP--
---IPMNSLGSRNYTLEESF-GSSNINRLIVS-LLYLPKGKKISESCFLDPKESTWVPPIT-------K
RFKSNVGSRWWRNWIVKKR---

------------------------------DSSQLKGSF-DQSRDRLNSIR----NADSE-------------YHTSLNQREIQ
QRKERSIP-WD--PSFLQTEETEIGSG--RFPK--CLSGYS-----RLFA-EGEKQMNNQRLPEEIEE
LL--GNPTRSIRSFFSDRW--SELH-LGSNPTERSTRDQ----KLLKKEQDFF-FCPLQAIG----KSR
NGNIFKIITYLQK--TVSIHPISSDRGCDMVP-KDEPN-MYSSNKISFLNKNPFFNL--FRLFHD

RNRGGYTL--HHDFESE--ERFQEMADLFTLSITEP-DLVYHKGFAFSIDSYGLDQKH--FLNE
VFNSRDESKKKSL---LVLPPIFY--DENESFYRRIRKKWLR-ISCGNYLKDTKEKIVVFASNNI
MEAVNQYRLIRNLIQIQYSTYG--YIINVLSRFFFILIN-RSDRNFEYGIQ---RDQIGNDTLNHR
TIMKYTINQHLSNLKK-NQKKRFDS--LILISRSERSMNRDPDAYRYKWFNGSKNFQEH---F
VSEQKSH--QVQVVFDRLRINQYWIDWSKVINKKNLSKSLSK--------------------------LILFLS
N-SLPFFFVSLGNMPIHRSE-IHIY-ELQGPNDQLCNPLLKSLGLPIVHLKKWKAD--------DHD
TS-QKSKLLINGGTISPFLFNKIP-KW-MVDS-HTR--KNRRKSFDNTDSYFSMISFDQDNWLN
PVKSFHRSSLISSFSKANRLRFLNNPHHFFFY--CNKRFPFYM----EKARIKNYD--FTYR-QFL
NILFIRNK-QFSLCVGK--KKHAFLE-RDTISPIESQVSNIFIPNYFPQRGNER-YNLYKS---FHF
PIRSDLFVR--RTIYSIADISGTPLTEEEMVNLERTYCQPLSDMNLSDSERKNLHQYLN--FNS
NMGLIHTPRSEKYLPSETSKKLR-------LWRNWLK--KGVEKGQMDRTFQRDSAFSTLSKW
NLFQTYMPWFFTSTGYKYLNFIFLDAFSDLL--PMLSSS-HKFVSILNYIMHRSKLKWRALK
RKLWG-------LWAP-ISEISWKCFRR--LLRVEEMIHRNNES--PLISTHLSSPNVREF-LYSSLLL
LLLAGC-LVQVLLFSVSLD--SSELQTEFERIKSLTIPSYTIEVYKLVDGYPKPEPNSFWLKNL
FLVAREQLEDFLTEILGFAR-----FGGGP-----AYG---VKLIQKIFFN-----LIDLI-SIIPNPINRITFS
RNTRRLSHTSKEIYSWIRKGQ---RFQTHDEIESWIETCDWFLDKERVYSFHFS--TLRP--EKG
IDQILWSLT-HIDHLV-----KSDYGYQMFEQAGAIYLRYLVDIH--QKDLMNYEFNTSCLAERR
IFLAHYQTITY--SQTSCGANSFHFPSHG-KPKPFSFRLALYPS-RGILVIGPIGTGRSYLVKSLA
TNSYLPFITVFKIVIDDLDPEDYMEALD---DVD-IDDDSDPAKEYMYALKDVDDVDDRD----
----SIYSNLDSDPELREEYTVDDEILRYIVELEIDLAP--INLQFELARTMSPCIVWIPNIHDLYV
DESE--SLGLLLNYLS---GDSERRS-TRDILVIASTHIPQKVDPALIAPNKLNTCIKIRRLLIPQQ
RKHVFTLSYTRGFHLEKKMFHT--NGFGSIALGYNARDLVAITNEALSISITQKKSIIDTNTIR
FALHRQTWELR---AHVRPVPDHGILFYQIGRAVAQNVLINCCLIDPISIYTKKQ--SCYEGDP
YLYKWFFELGTSMKKLTILLYLLGCSAGSVAQDLWSLP--GPDEK-NWITSYRLVETDS-DLV
DGLEVVEGALVG-------------------SSLLRPEPRN-PLEMMENGSRSIFDRRFLYES-----EFKEW
AEG---ADPQ----QLAED----VVDH--IVWAP--RIWQP---WGFLFDWIERPNELEFP-YWARSFR
GK---PIISDEVDELQENDWEFLQSETMEYPGRDRSSKEQVFFRKGQFIWDPGDPLFYIFN-D
ELSVFLFSHREFFADEEMSKGLLLTSQR--ETLYKRGFS--KKAKEKYFEFFINRQRRRRLR-T
IRSLY--NRSFRSN--IQSASYQYLSNLFLSN-GRLLDQMTKTLFRKRWIFPDELKIGF--M-------
--MPRSR-INGNFIDKTFSVVANILLRIIPTTSGEKEAFTYYRDG--MSAQSEGNYAEALQNYY
EAMRLEIDPYDRSYILYNIGLIHTSNGDHTKALEYYFRALERNPFLPQAFNNMAVICHY-RG
EQAIRQGDSEIAEAWFDQAAEYWKQAIALTPGNYIEAHNWLKITRRFE--MNWQSERIWIE
LIRGSRKTSNLCWAFILFLGSVGFLLVGTSSYLGRNLISLFPSQQIFFFPQGIVMSFYGIAGLF
ISSYLWATISWNVGSGYDLFDKKEGIVCIFRWGFPGKKRRIFLRFLINDIQSIRIELKEGISPR
RVLYLEIRGQGAIPLTRTDEN-LTPREMEQKAAELAYFLRVPIEVF
>Cicer
MEKWWFNSMLFLSKSIDSFGPIEKK--SEEPIVTDNDS----YSHVDYLVDVSNRQNFLSDKT
FLVRDRNSYSYSIFFAIENKILEID-------------------------------------YDSQFNWKNIINSCIENY
LR--SQICIDSDILDNSFKYNDNDVYSYICG-TSTDVITITEKESFN-----DDDDFTQKYKHLW
VQCESCYGLNYKKFFKSKMNICEHCGDHLKMSSSDRIELLIDPGTWNPRDEDMVSLDPIE
FDSEDEPYKTRLDSYQKRTGLSEAVQTGTGQINGIPVAIGIMDFQFMGGSMGSVVGEKITR
LIEYATNQLLPLIIVCASGGARMQEGSLSLMQMAKISSALYNYQINQKLFYVAILTSPTTGG
VTASFGMLGDIIIAEPNAYIAFAGKRVIEQTLNTEVPEGSQSAEFLFEKGLFDSIVPRNLLKE
VLGELFQFHGFFPLTQNGN-MVTIRADEISKIIRERIEQYNTEVKIVNTGTVLQVGDGIARIY

GLDEVMAGELVEFEEGTVGIALNLESKNVGVVLMGDGLLIQEGSSVKATGRIAQIPVSEG
YLGRVVNALAKPIDGRGEISSSESRLIESPAPGIISRRSVYEPLQTGLIAIDSMIPIGRGQRELII
GDRQTGKTAVATDTILNQQGQNVICVYVAIGQKASSVAQVVTTLQERGAMEYTIIVAETAN
SPATLQYLAPYTGAALAEYFMYRKRHTLIIYDDPSKQAQAYRQMSLLLRRPPGREAYPGD
VFYLHSRLLERAAKLSSQLGEGSMTALPIVETQSGDVSAYIPTNVISITDGQIFLSADLFNA
GIRPAINVGISVSRVGSAAQIKAMKQVAGKLKLELAQFAELEAFAQFASDLDKATQNQLAR
GQRLRELLKQSQSAPLTVEEQIITIYTGTNGYLDSLEIEQVRKFLVELRAYLKTNKPKFNEII
CSTKTFTGEAETLLKEAIQEQTELFLLQEQVMKITPTTSDTEVSALEKKNLGRVVQIIGPVL
DVVFPPGKMPNIYNALIVQGRDTVGQQINVTCEVQQLLGNNRIRAVAMSATDGLKRGME
VIDTGAPLSVPVGGATLGRIFNVLGEPIDNLGPVDTRTTSPIHRSAPAFIQLDTKLSIFETGIK
VVDLLAPYRRGGKIGLFGGAGVGKTVLIMELINNIAKAHGGVSVFGGVGERTREGNDLY
MEMKESGVINEKNIAESKVALVYGQMNEPPGARMRVGLTALTMAEYFRDVNEQDVLLFI
DNIFRFVQAGSEVSALLGRMPSAVGYQPTLGTEMGSLQERITSTKEGSITSIQAVYVPADDL
TDPAPATTFAHLDATTVLSRGLAAKGIYPAVDPLDSTSTMLQPRIVGEEHYETAQRVKETLQ
RYKELQDIIAILGLDELSEEDRLTVARARKIERFLSQPFFVAEVFTGSPGKYVALEETIRGFK
LILSGELDSLPEQAFYLVGNIDEATAKAANLT-------MTLNLCVLTPNRIVWDSEVKEIILSTN
SGQIGVLKNHAPIATAVDIGILRIRLNDQWVTMALMGGFARIGNNEITILVNDAEKGSDIDP
QEAQQTLKIAEANLNKAEGKRQTIEANLALRRARTRVEAIISI-MKNITDSFLCLSYWPSAG
SFGFDTDILATNLINLSVVLGVLVFFGKGVLTDLLDNRKQRILRTIRNSEELREGAVEQLEK
ARARLRKIEMEADRFRLNGYSEIEREKLNLINSIYTTLEQFENYKNETINFEQQKAINQVQ
QRVLQQALQGALGTLNSCLNNELHLRTIGANIGMFGAMKEKNNMNPIISAASVIAAGLAV
GLASIGPGIGQGTAAGQAVEGIARQPEAEDKIRGTLLLSLAFMEALTIYGLVVALALLFANP
FVMNVLLCSINTLNRFYDISALEVGQHFYWQIGDFQVHAQVLITSWVVIAILLISTILVVRN
PQTIPTSGQNFFEYVLEFIRDVSKTQIGEEYGPWVPFIGTLFLFIFVSNWSGALLPWKIIKLP
HGELAAPTNDINTTVALALLTSVAYFYAGISKKGLAYFGKYIQPTPILLPINILEDFTKPLSLS
FRLFGNILADELVVVVLVSLVPLVIPIPVMFLGLFTSGIQALIFATLAAAYIGESMEGHHMIFS
TLEHILTHISFSVISIVISIHLITLLVNEIVGLYDLSKKAMIITFFCVTGLLITRWFFSGHLPFSDL
YESLIFLSWGFSIFHIVPGFKKDKNLLSTIIAPSVIFTQGFATSGLLTKMHQSVILVPALQSHW
LMMHVSMMILGYAALLCGSLLSVAILVITFQEIIQILGKSKEIKYMNLNENFLRKTSFSSSIN
YYRSQFIQQLDRWSYRIISLGFLFLTIGILSGAVWANEAWGSYWNWDPKETWAFITWTIFAI
YLHTRKNKKWEGVNSSIVASIGFLIIWICYLGINLLGIGLHSYGSFTSNMAKKKAFIPLLCLT
SIVFLPWCISFTFKNSFESWIINWWNTKQSEIFVNIIQEKSILKKFIEFEELFLLDEMLKEYPE
TPLQNLRIE-YKEIIQLIKTHNQDRIHTIFHFFTNFICLLILSGYSIRGNQEIIILNSWVQEFLYN
LSDTIKAFSILLLTDLCIGFHSTHGWELMIGSVYKDFGFTQNDQIISGLLSTFPVILDTFLKY
WIFRYLNRVSPSLVVIYHSMNDMPLGVPKVPFLVPGDDEASWIDLYNRLFQERLLFLGQEV
NSEISNQLVGLMVYLSLEDKNKDLYLFINSPGGEVISGMAIFDTMQFVEAEVQTVCVGLA
ASMGSLILVGGEITKRLAFPHARVMIHQPATSFYEGQTVECMLEANELLQMRRNITNIYAQ
R T G K P S W Q I S K D M E R D L F M S A E E A Q A Y G L V D M V A D
S???MKEYQV
YLERARSRQQDFLYPLIFREYIYGLAYSQNFLGYDSKYSLLIVKRLISRMYQQNHLIISAND
SNKNPFWGKNFNSQIISEGFAIVVEIPFFLQSGSSLIIKSYNLRSIHSVFPFLEDKFTYLNYVS
DIRIPYPIHLEILVQILRYWVKDAPFFHLLRLFVYNFCNWNSFITKSIYTFSKPRLFLFLYNFY
VWEYESIFLFLRNKSSHLRLKSFSVFFERIFFYAKREHLVEVFAKPYTLKFFKDPLIHYVRY
QGKSILASRNAPILMNKWKHYFLHLWQCFFDVWSQPGTIKINQLSQHSFQLLGYFSNVRL

NRSVVRSHMLQNTFLIEI--VRKKLDIIVPIIPLIRSLAKGKFCNVLGHPISKPVWADSSDLDII
DRFLRICRNLSHYYNGSSKKKSLYQIKYILRLSCIKTLACKHKSTVRAFLKRSGSELLEEFF
TEEEEILSFIFPRRIWYLDILFSNDLVNHEMIIDTT-EVQDIN-SFSGLES-LKEVYGILWILVPIL
ILVLGITISVLAIVWLEREISAGIQQRIGPEYAGPFGVLQALADGTKLLFKENLIPSRGDIRLF
SIGPSISVISILISYSVIPFGYNFVLSDFNIGVFLWIAISSIAPIGLLMSGYGSNNKYSFLGGLRA
AAQSISYEIPLTLCVLSISLLSNSLSTVDIVDAQSKYGFWGWNLWRQPMGFIVFLISSLAEC
ERLPFDLPEAEEELVAGYQTEYSGIRFGLFYVASYLNLLISSLFVTVLYLGGSNISIPYIFVSE
FFEINKTYGVFGTTIDLFITLAKTYFFLFVSIITRWSLPRLRMDQLLNLGWKFLLPISLGNLL
LTTSSQLFSLMI*HVQNENIILESTRFFMKAFHLLLFDGSFIFPEFILIFGLILLLMIDSTSDQK
NISSFYFISSTSLVMSITALLFRWREEPMISFSGNFQTNNFNEIFQFLILLSSTLCIPLSVEYIEC
TEMAIPEFLLFILTATLGGMFLCSANDLITIFVALECFSLCSYLLSGYTKKDVRSNEATMKYL
LMGGASSSILVHGFSWLYGSSGGEIELQEIVNGLINTQMYNSPGISIALIFITVGIGFKLSLAP
SHQWTPDVYEGSPTPVVAFLSVTSKVAALASATRILDIPLYFSSNEWHLLLEILAILSMILGN
FIAITQTSMKRMLAYSSIGQIGYVIIGIIVGDSNGGYASMITYMLFYIAMNLGTFACIVSFGL
RTGTDNIRDYAGLYTKDPFLAVSLALCLLSLGGLPPLAGFFGKLYLFWCGWQAGLYFLVSI
GLLTSVVSIYYYLKIIKLVMTGRNQEITTYVRNYRRSPVRSNNSIELSMIICVIASTIPGISMN
PIIEIAQDTLFMFLLYEYDIFWTFLIISILIPILAFLISGILAPIRKGPEKLSSYESGIEPMGDAWL
QFQIRYYMFALVFVVFDVETVFLYPWAMSFDVLGVSVFIEALIFVLILIVGSVYAWRKGAL
EWSTNNFPWLTIVVVLPIAAGSLILLFPHRGNKVIRWYSICICLIDLLLTTYAFCYHFQLDDP
LIQLTENYKWINFFDFYWRLGIDGLSIGPILLTGFITTLATLAAQPVTREYPLFYFLMLAMY
SGQMGPFASRDILLFFIMWELELIPVYLLLAMWGGKKRLYSATKFILYTAGSSVFLLIGVLG
IGLYGSNEPTLNFETLTNQSYPVVLEIIFYIGFLIAFAVKSPIIPLHTWLPDTHGEAHYSTCML
LAGILLKMGAYGLVRINMEFFSHAHSIFCPWLMIVGSIQIIYAALTAFGQRNLKKRIAYSSVS
HMGFIIIGIGSISDTGLNGVILQIISHGFIGAALFFLAGTSYDRLRLLYLDEMGGMAIPMPKIF
TIFTILSMASLALPGMSGFVAELIVFFGIITSQKYLFMMKILITFVTAIGMILTPIYLLSILRQM
FYGYKFFNTPNSYFFDSGPRELFISISILIPVIGIGIYPDFIFSFSVDKVEAILSNF---MMLEHVL
VLSAYLFSIGIYGLITSRNMVRALMCLELILNAVNINLVTFSDFFDNRQLKGNIFSIFVIAIAA
AEAAIGLAIVSSIYRNRKSTRINQSNLLNKMEYTHQSSWIIPFIPLPVPILIGVGLLFFPTATK
NIRRMWVFPSIFLLTIVMIFSIDLSIHQINNSSIYQYVWSWTINNDLSLEFGYLIDSLTSIMSIL
ITTVGILVLIYSDSYMSHDQSYLRFFTYLSFFNTSMLGLVTSSNLIQVYIFWELVGMCSYLLI
GFWFTRPIAANACQKAFVTNRVGDFGLLLGILGFYWITGSLEFRDLFQIFNN-EVNIFFVTL
CALLLFCGSVAKSAQFPLHVWLPDAMEGPTPISALIHAATMVAAGIFLVARLLPLFIVIPSIM
SGIALIGIITVVLGATLAIAQKDIKKNLAYSTMSQLGYMILALGMGSYRAALFHLITHAYSK
ALLFLGSGSIIHSMEAIVGYSPDKSQNMVLMGGLTKHAPITKNAFLIGTLSLCGIPPFACFW
SKDGILNDSWLYSPIFAIIACSTAGLTAFYMFRIYLLVFEGYLNVHFQHFNGKKNSSFYSISL
WGK-EEKKKLKKLLALLTMNFFQGIKNIGFFYPQESDNTMLFSMLVLVLFTFFVGSVG--FD
LDILSKLLIPSIDLLHQNSK-SVDW-EFFTNATFSVSIAFFGILIASFFYKPVFSSLQNLNLCNL
FQIIADKIINIIYDWSYNRGYIDAFLEVSLIASVKKLAKFNYFFDRQIIDGIPNGVGISSFFMG
EAIKYVGGG-RISSYIFF---FLLIFLVICYMALPETLHDFLLVFLGSGLILGSLGVVLLTNPIFS
AFSLGLVLVCISLFYILSNSHFVAASQLLIYVGAINILIIFAVMFMNSSEYYQDFNLWTVGDG
ITLIVCTSIFVSLVTTIPDTSWYGIIWTTRPNQIIEQDLISTSQQIGIHLSTDFFLPFELISIILLVA
LIGTIVVARQMNVPTTRKDLMIVNMGPQHPSMHGVLRLIVTLDGEDVIDCEPILGYLHRG
MEKIAENRTIIQYLPYVTRWDYLATMFTEAITVNGPEQLGNIQVPKRASYIRVIMLELSRIA
SHLLWLGPFMADIGAQTPFFYIFRERELIYDLFEAATGMRMMHNFFRIGGVSADLPYGWI

DKCFDFCNYFLTRVIEYQKLITRNPIFLERVEGVGIIGREEVINWGLSGPMLRASGIQWDLR
KVDNYECYEEFDWEVQWQKEGDSLARYLVRIGEMMESIKIIQQALEGIPGGPYENLEIRSF
DREKEPEWNDFEYRFIGKKSSPTFELPKQELYVRVEAPKGELGIFLIGDQNGFPWRWKIRP
PGFINLQILPQLVKRMKLADIMTILGSIDIIMGEIDRMFLMVTGFMNYGQQTVRAARYIGQ
SFVIILSHANRLPVTIQYPYEKLITPERFRGRIHFEFDKCIACEVCVRVCPIDLPVVDWKLET
DIRKKRLLNYSIDFGICIFCGNCIEYCPTNCLSMTEEYELSTYDRHELNYNQIALGRLPMSV
IDDYTIRTIQIK----------------MQGNLSAWLVKHGIVHRSLGFDYQGIETLQIKPEDWHSIAVI
LYVVYGYNYLRSQCAYDVAPGGLLASVYHLTRIEYSLDQPEEVCIKVFAPRKNPRIPSIFWV
WKSADFQERESYDMLGISYDNHPRLKRILMPESWIGWPLRKDYIAPNFYEIQDAH----------
---MNSIEFPVLDQKTKNSVISTTLNDLSNWSRLSSLW
PLLYGTSCCFIEFASLIGSRFDFDRYGLVPRSSPRQADLILTAGTVTMKMAPSLVRLYEQMP
EPKYVIAMGACTITGGMFSTDSYSTVRGVDKLIPVDVYLPGCPPKPEAVIDAITKLRKKISR
EIYEDRISYQ-ENRCFTTNHKLDVGRSTHTGNYNQGLFYQPPSISEITT--FFKYKKVQYPNE
KVNMQTRKAFSWIKEEITRSISVLLMIYIITGAPISYAYPIFAQQGYENPREATGRIVCANCH
LANKPVDIEVPQTVLPDTVFEAVVRIPYDMQVKQVLANGKKGALNVGAVLILPEGFELAP
PDRISPEIKEKIGNLSFQTYRPTKKNIIVVGPVPGKKYSEITFPILSPDPSTKRDVHFLKYPIY
VGGNRGRGQIYPDGSKSNNNVYNATATGIVNKIIRKEKGGYEITIVDAADGREVIDIIPPGP
ELLVSEGESIKLDQPLTSNPNVGGFGQGDAEIVLQDPLRVQGLLFFFASIILAQIFLVLKKKQ
FEKVQLSEMNFMSKVYDWFEERLEIQAIADDITSKYVPPHVNIFYCLGGITLTCFLVQVAT
GFAMTFYYRPTVTEAFASVQYIMTEANFGWLIRSVHRWSASMMVLMMILHVFRVYLTGG
FKKPRELTWVTGVVLGVLTASFGVTGYSLPWDQIGYWAVKIVTGVPDAIPVIGSSVVELLR
GSASVGQSTLTRFYSLHTFVLPLLTAVFMLMHFPMIRKQGISGPLMGVTKKPDLNDPVLRA
KLAKGMGHNYYGEPAWPNDLLYIFPVVILGTIACNVGLAVLEPSMIGEPADPFATPLEILPE
WYFFPVFQILRTVPNKLLGVLLMVSVPAGLLTVPFLENVNKFQNPFRRPVATTVFLIGTAVA
LWLGIGATLPIEKSLTLGLFMIEVFLFGIVLGLIPITLAGLFVTAYLQYRRGDQLDLMPTITSY
FGFLLAVLTITAGLFISLNKIRLIMDIVSLAWAALMVVFTFSLSLVVWGRSGLMIIRSPEPKV
KILVDRDPIKTSFEQWAKPGHFSRTIAKGPDTTTWIWNLHADAHDFDSHTSDLEEISRKVF
SAHFGQLSIIFLWLSGMYFHGARFSNYEAWLNDPTHIRPSAQVVWPIVGQEILNGDVGGG
FRGIQITSGFFQIWRASGITSELQLYCTAIGALVFAALMLFAGWFHYHKAAPKLAWFQDVE
SMLNHHLAGLLGLGSLSWAGHQVHVSLPINQFLNAGVDPKEIPLPHEFILNRDLLAQLYPS
FAEGATPFFTLNWSKYADFLTFRGGLDPLTGGLWLTDIAHHHLAIAILFLIAGHMYRTNWG
IGHGIKDILEAHKGPFTGQGHKGLYEILTTSWHAQLSINLAMLGSLTIIVAHHMYAMPPYPY
LATDYGTQLSLFTHHMWIGGFLIVGAAAHAAIFMVRDYDPTTRYNDLLDRVLRHRDAIIS
HLNWVCIFLGFHSFGLYIHNDTMSALGRPQDMFSDTAIQLQPVFAQWIQNTHALAPGTTA
PGATTSTSLTWGGGDLVAVGNKVALLPIPLGTADFLVHHIHAFTIHVTVLILLKGVLFARSS
RLIPDKANLGFRFPCDGPGRGGTCQVSAWDHVFLGLFWMYNSISVVIFHFSWKMQSDVW
GSINDQGVVTHITGGNFAQSSITINGWLRDFLWAQASQVIQSYGSSLSAYGLFFLGAHFVW
AFSLMFLFSGRGYWQELIESIVWAHNKLKVAPATQPRALSIVQGRAVGVTHYLLGGIATTW
AFFLARIIAVGMALRFPRFSQGLAQDPTTRRIWFGIATAHDFESHDDITEERLYQNIFASHFG
QLAIIFLWTSGNLFHVAWQGNFEAWVQDPLHVRPIAHAIWDPHFGQPAVEAFTRGGALGP
VNIAYSGVYQWWYTIGLRTNEDLYTGALFLLFISVISLLAGWLHLQPKWKPSVSWFKNAE
SRLNHHLSGLFGVSSLAWAGHLVHVAIPGSRGEYVRWNNFLSVLPHPEGLGPLFTGQWNL
YAQNPDSSSHLFSTSQGAGTAILTLLGGFHPQTQSLWLTDIAHHHLAIAILFLIAGHMYRTN
FGIGHSIKDILEAHIPPGGRLGRGHRGLYDTINNSIHFQLGLALASLGVITSLVAQHMYSLPS

YAFIAQDFTTQAALYTHHQYIAGFIMTGAFAHGAIFFIRDYNPEQNEDNVLARMLEHKEAI
ISHLSWASLFLGFHTLGLYVHNDVMLAFGTPEKQILIEPIFAQWIQSAHGKTSYGFDVLLSS
TNSPALNAGRSIWLPGWLNAINE--NSNSLFLTIGPGDFLVHHAIALGLHTTTLILVKGALDA
RGSKLMPDKKDFGYSFPCDGPGRGGTCDISAWDAFYLAVFWMLNTIGWVTFYWHWKHI
TLWQGNVSQFNESSTYLMGWLRDYLWLNSSQLINGYNPFGMNSLSVWAWMFLFGHLVW
ATGFMFLISWRGYWQELIETLAWAHERTPLANLIRWRDKPVALSIVQARLVGLVHFSVGYI
FTYAAFLIASTSGKFGMSHSVKIYDTCIGCTQCVRACPTDVLEMIPWDGCKAKQIASAPRT
EDCVGCKRCESACPTDFLSVRVYLWHETTRSMGLAYMI--NLPSFFVPLVGLVFPAVAMAS
LFLYVQKNKIFMRDLKTYLSVAPVASTLWFVALAGLLIEINRLFPDALTFPFFSFMTAILERR
DSENLWGRFCNWITSTENRLYIGWFGVLMIPTLLTATSVFIIAFIAAPPVDIDGIREPVSGSLL
YGNNIISGAIIPTSAAIGLHFYPIWEAASVDEWLYNGGPYELIVLHFLLGVACYMGREWEL
SFRLGMRPWIAVAYSAPVAAATAVFLIYPIGQGSFSDGMPLGISGTFNFMIVFQAEHNILMH
PFHMLGVAGVFGGSLFSAMHGSLVTSSLIRETTENESANEGYRFGQEEETYNIVAAHGYFG
RLIFQYASFNNSRSLHFFLAAWPVVGIWFTALGISTMAFNLNGFNFNQSVVDSQGRVINTW
ADIINRANLGMEVMHERNAHNFPLDLAAVEAPSINGMGLPWYRVHTVVLNDPGRLLSVH
IMHTALVAGWAGSMALYELAVFDPSDPVLDPMWRQGMFVIPFMTRLGITNSWGGWNIT-G
GTITNPGIWSYEGVAGAHIVFSGLCFLAAIWHWVYWDLEIFCDERTGKPSLDLPKIFGIHLF
LAGVGCFGFGAFHVTGLFGPGIWVSDPYGLTGRVQSVNPAWGVDGFDPFVPGGIASHHIA
AGTLGILAGLFHLSVRPPQRLYKGLRMGNIETVLSSSIAAVFFAAFVVAGTMWYGSATTPIE
LFGPTRYQWDQGYFQQEIYRRVGAGLAENQSLSEAWSKIPEKLAFYDYIGNNPAKGGLFR
AGSMDNGDGIAVGWLGHPIFRDKEGRELFVRRMPTFFETFPVVLVDGDGIVRADVPFRRA
ESKYSVEQVGVTVEFYGGELNGVSYSDPATVKKYARRAQLGEIFELDRATLKSDGVFRSS
PRGWFTFGHVSFALLFFFGHIWHGARTLFRDVFAGIDPDLDAQVEFGAFQKLGDPSTKKP
VVMKTLYSLRRFYHVETLFNGTLALTGRDQETTGFAWWAGNARLINLSGKLLGAHVAHA
GLIVFWAGAMNLFEVAHFVPEKPMYEQGLILLPHLATLGWGVGPGGEVIDTFPYFVSGVL
HLISSAVLGFGGIYHALLGPETLEESFPFFGYVWKDRNKMTTILGIHLILLGIGAFLLVFKAS
YFGGIYDTWAPGGGDVRKITNLTLSPSIIFGYLLKSPFGGEGWIVSVDDLEDIIGGHVWLGS
ICILGGIWHILTKPFAWARRALVWSGEAYLSYSLGALAVFGFIACCFVWFNNTAYPSEFYGP
TGPEASQAQAFTFLVRDQRLGANVGSAQGPTGLGKYLMRSPTGEVIFGGETMRFWDLRA
PWLEPLRGPNGLDLSRLKKDIQPWQERRSAEYMTHAPLGSLNSVGGVATEINAVNYVSPR
SWLATSHFVLGFFLFVGHLWHAGRARAAAAGFEKGIDRDFEPVLSMTPLNMTIALGKFTK
DQNDLFDIMDDWLRRDRFVFVGWSGLLLFPCAYFAVGGWFTGTTFVTSWYTHGLASSYL
EGCNFLTAAVSTPANSLAHSLLLLWGPEAQGDFTRWCQLGGLWTFVALHGAFGLIGFMLR
QFELARSVQLRPYNAIAFSGPIAVFVSVFLIYPLGQSGWFFAPSFGVAAIFRFILFFQGFHNW
TLNPFHMMGVAGVLGAALLCAIHGATVENTLFEDGDGANTFRAFNPTQAEETYSMVTAN
RFWSQIFGVAFSNKRWLHFFMLFVPVTGLWMSALGVVGLALNLRAYDFVSQEIRAAEDP
EFETFYTKNILLNEGIRAWMAAQDQPHENLIFPEEVLPRGNALMSGSTGERSFADIITSIRY
WIIHSITIPSLFIAGWLFVSTGLAYDVFGSPRPNEYFTESRQGIPLITGRFDSLEQLDEFSRSF
MTIDRTYPIFTVRWLAVHGLAVPTVSFLGSISAMQFIQRMATQTVEGSSRSG-PRRTVVGDL
LKPLNSEYGKVAPGWGTTPLMAIAMALFAVFLSILLEIYNSSILLDGISMNMLTLKLFVYTI
VIFFVSLFIFGFLSNDPGRNPGREEMADTTGRIPLWIIGTVAGIAVIGLIGIFFYGSYSGLGSSL
MLNIFSSVCICIDSALYSSSFLLAKLPEAYAFLNPIVDFMPVIPLLFFLLAFVWQAAVSFRTT
QSNPNEQNVELNRTSLYWGLLLIFVLAVLFSNYFFNMEVNILAFIATALFILVPTAFLLIIYV
KTVSQSDMETATLIAISISGLIVSFTGYALYTAFGQPSQQLRDPFEEHGDMEALVYTFLLVST

LGIIFFAIFFREPPIVPTKRIKMTIAFQLAVFALIATSSILLISVPVVFASPDGWSSNKNVVFSGT
SLWIGLVFLVGILNSLIS-------MSPQTETKASVGFKAGVKDYKLTYYTPDYETKDTDILAAF
RVTPQPGVPPEEAGAAVAAESSTGTWTTVWTDGLTSLDRYKGRCYHIEPVPGEESQFIAYV
AYPLDLFEEGSVTNMFTSIVGNVFGFKALRALRLEDLRIPNAYVKTFQGPPHGIQVERDKL
NKYGRPLLGCTIKPKLGLSAKNYGRAVYECLRGGLDFTKDDENVNSQPFMRWRDRFLFC
AEAIYKSQAETGEIKGHYLNATAGTCEEMIKRAVFARELGVPIVMHDYLTGGFTANTSLSH
YCRDNGLLLHIHRAMHAVIDRQKNHGMHFRVLAKALRLSGGDHIHSGTVVGKLEGEREI
TLGFVDLLRDDFVEKDRSRGIYFTQDWVSLPGVLPVASGGIHVWHMPALTEIFGDDSVLQ
FGGGTLGHPWGNAPGAVANRVALEACVQARNEGRDLAREGNEIIRDAAKWSPELAAACE
VWKEIKFEFEAMDTL--MAIHLYKT---SSRN------------RLIYGQHHCSTGRNARGIITAKHR
GGGHKRLYRKIDFRRNEKDIYGRIVTIEYDPNRNAHICLIHYGDGEKRYILHPKGAEIGDT
VVYGTEVPIKKGNALPLTDMPLGTAIHNIEITPGRGGQLARAAGAVAKLIAKEGKSATLKL
PSGEVRLISKNCSATVGQVGNVEVNQKKLGKAGSKRWLGKRPVVRGVVMNPVDHPHGG
GEGRAPIGRKKPSTPWGYPALGRRSRKSNKYSDNLILRRRTKMIQPQTYLNVADNSGARE
LMCIRIIGAS-NRRYAYIGDIIVAVIKKAVPNTPFERSEVIRAVIVRTCKELKRSNGIIIKYDDN
AAVVIDKEGNPKGTRIFCAIARELRQLNFTKIVSLAPEVLMLSPKKTRFRKQHRGRMKGK
ACRGNKICFGKYALQALEPAWITSRQIEAGRRAMSRNVRRGGQIWIRIFPDKPVTVRPTET
RMGSGKGSPEYWVAVIKPGKILYEMGGVAENIARKAISLAASKMPIRTQFILLEMTRIKRGS
IAQKRRTKIRLFTSSFRGAHSKLTRTIAQQKIKALVSSDRDRDRKKRDFRALWISRINAIIGQ
KKNVYYIYSNLMYNLYKKQLLLNRKIVAQIAILKGNCFFMIANDIIKK---------
-??
??????????????????????????????????MNGIKYAVFTDKSIRLLGKN-QYTFNVESGSIRTEIKH
WVELFFGVKVIAINSHRLPIKGRRVR--PGHIMHYRRMIITLQPGYSIPPLRKKRTMAVPKKR
TSISKKKIRKNFWKKKAYTTALKAFSLAQSIFTGKSKSFF------MAKGKDIRITVILECTSC-D
KK--VNKE--GISRYITQKNRHNTPSRLELRKFCPFCCKHTIHAEIKKMKVAASVRKICEKCR
LIRRRGRIIVICSNPKHKQRQGMIREKVKVSTRTLQWKCVESRVDSKRLYYGRFILSPLMK
GQADTIGIAMRRILLGEIEGTCITRAKSE--KIPHEYSTIVGIQESIHEILMNLKEIVLRSNLYG
TRDASICFKGPGSVTAQDIILPPSVEIVDNTQHIANLTEPINLCMELKLERNRGYRIKTLNNI
QDESY--TIDAVFMPVRNANHSIHSYGNGNEKQEILFLEIWTNGSLTPKEALFEASRNLIDLF
IPFLHAEED--KLNLENNQ-QKVTLPLFTFHDRFVKKLRKNKK-EIALKSIFIDQLELPPRIYN
CLKKSNIHTLLELLNKSQEELMKIEHFRVEDVKNILNLLQIEKHFA-------MLGVGN-EGMS
TLPGLNQIQFEGFSRFIDRGLTEELLKFPKIEDTDQEIEFQLFVETYQLVEPAIKEKDAVYESL
TYSSELYISAGLIWKNSRDIQEQIIFIGNIPLMNSLGTSIVNGIYRIVINQILQSPGIYYRSELD
HNGISVYTATIISDWGGRLELEIDRKARIWARVSRKQKISILVLSSAMGSNLSEILENVCYPE
IFLSFLNEKEEKKIGSKENAILEFYRQFACVGGDPIFPESLCTELQKKFFQQRCELGGIGRRN
MNRRLNLDIPQNNTFLLPRDILTAADHLIGMKFGMGTLDD--MNHLKNKRIRSVADLLQDQ
FGLALVRLENIIRGTISGALR-YKLI-PTPQNLVTSTP----LTTTYESFFGLHPLSQVLDRTNPLT
QIVHGRKLSYLGPGGLTGRTASFRIRDIHPSHYGRICPIDTSEGINVGLIGSLAIHARIGHWG-
SIESPFY---EISDRSKR--IQMLYLSPSRDEYYMVATGNFLALNRGIQEEQIVPARYRQEFLTIA
WEQVHLRSIFPFQYFSIGASLIPFIEHNDANRALMSSNMQRQAVPLSRSEKCIVGTGLERQV
ALDSGVPAIAEHEGKIIYNDTDKIILYGNGNALSIPLIIYQRSNKNTCMHQKSQVQKGKCV
KKGQILADGAATVGGELALGKNVLVAYMPWEGYNSEDAVLISERLVYEDIYTSFHIRKYEI
QTHVTSHG-PERITNKIPHLEAHLLRNLDKNGIVILGSWVETGDILVGKLTPQMAKE-SSYA
PEDRLLRAILGIQVSPSKETCLKLPVGGRGRVIDVRWIHKKA-GSSYNPETIHIYISQKREIK

VGDKVAGRHGNKGIVSKILSRQDMPYLQDGRPVDMVFNPLGVPSRMNVGQIFECSLGLA
GSMLNRHYRIAPFDERYEQEASRKLVFSELYEASKQTSNPWIFEPEYPGKSGIFDGRTGNRF
EQAVIIGKPYILKLIHQVDDKIHGRSSGHYALVTQQPLKGRAKQGGQRVGEMEVWALEGF
GVAHILQEMLTYKSDHIKARQEVLGTTIIGGTIPKPVDAPESFRLLVRELRSLALELNHFLVS
EKDFQIQRKEV-------MIDQYKHQHLRIGLVSPEQISAWAKKILPNGEIVGEVTKPYTLHYKT
NKPEKDGLFCERIFGPIKSGICACGNYRVIGDQKDQPKFCEQCGVEFVDSRIRRYQMGYIK
LACPVTHVWYLKRLPSYIASLLDKPLKELEGLVYCDF--SFARP-IIKKPTFLRLRGSFEYEIQ
SWKYSIPLFFATQGFDTFRNREISSGAGAIREQLVDLDLRIIMDSSLIEWKELGEE-GSTDNE
WEDRKVGRRKNFLVRRMELAKHFIRTNIEPEWMVLCLLPVLPPELRPIIQIDGGKLMSSDI
NELYRRVIYRNNTLIDLLTTSRSTPGELVMCQEKLVQEAVDTLLDNGIRGQPMRDGHNKV
YKSFSDIIEGKEGRFRETLLGKRVDYSGRSVIVVGPSLSLHRCGLPREIAIELFQTFLIRGLIR
KNCASNIGVAKSKIREKEPIVWEILQEVMQGHPVLLNRAPTLHRLGIQAFQPILVEGRAICL
HPLVCKGFNADFDGDQMAVHVPLSLEAQAEARLLMFSHTNLLSPAIGDPISVPTQDMLIGL
YVLTSGNRRGICANRYNPFNC---RNSQNEKISDNNYKYIKK-EPFFCNSYDAIGAYRQKRIN
FDSPFWLRWRIDQC-ILSS--REVPIEVHYESLGTYYEIYGHYLVIRSIKKEIRCIYIRTTVGHIS
FYREIEEAIQGFSRAYSYGI------------MAERANLVFHNNVIGGTAIKRLISRLIDHFGMAYTS
HILDQVKTLGFRQATATSISLGIDDLLTIPSKGWLVQDAEQQSLILEKHNHYGNVHAVEKLR
QSIEIWYTTSEYLRQEMNPNFRMTDPFNPVHIMSFSGARGNASQVHQLVGMRGLMSDPQ
GQMIDLPIQSNLREGLSLTEYIISCYGARKGVVDTAVRTSDAGYLTRRLVEVVQHIVVRRT
DCGTIRGISVNTRNGMMIPERILIQTLIGRVVADDIYIGSRCIVVRNQDIGIGLINRFIT------FQ
TPTIFIRTPFTCRNTSWICRLCYGRSPIHGDLVELGEAVGIIAGQSIGEPGTQLTLRTFHTGGV
FTGGTAEYVRAPSNGKIKFNEDLVHPTRTRHGYPAFICNINLYVTIESDDIIHNMIIPSKSFLL
VQNDQYIKSEQVIAEIRAGTYTF-NLKKRFENIFILTQKGKCIGVPMCTMHPNFCIVMYIFY
QKQVIYGYYQESRAGL--IQSIFYFARIKIKLTVIL--FLLKKKR-FLTFS--QGMIQ-VKHKLFSF
ETFGTKERGITDY-SIFNQIICTDYSHLMYSA-IFNNIFY--LLAKRRRNRFVIPF--IQEQKNKL
MSPSGVSIEIPI--NGIFHRNSILAYFDDPQYRRQNSGITKYRTIGIHSLFQKED--LIEYR-GIKE
LKPKYNKVDRFFFIPEEVHILPESSSIMIRNNSLVGIGTPITLNIRSRVGGLVRLEKKKKKIEL
KIFSGNIHFPGEMDKISRHSSILIPP-GTVKKKKRKEAKK--INNWIYVQW--IATTKKK--YFV
LVRPVILYEIPDSIHFVKLFPQDLFQERDNLELRVLNYILYGNGKSIRGISDTRIQLVRTCLVL
NWDQGK---KSSSIEEAPASFVEVSTNGLIEYFIRIDLVK-HMSYIRKRNDPSGLGLIPD-KESD
RVNINPF---FSIHS--KTKIQQSLIQNH-TIRMLLN---RNKECRSWIILSSSNCF--QMRPFNNVK
YHN---GIKKDIIPINNKNSLGPLGI--ALQVANYYS--FYRLIT--HNQVS-ITNNLQLDK---LIEIC
HVM--K-YYLMDENDK--IFKPDLSKNIVLNPFHLNWYFLHHN----TLTIIS-LGQFICENICITQ
NEPH-LKLKSGQVLTVQ-MDSVIIRSANPYLATPGATIHGHYGEILDEGDILVTFIYEKSRSG
DITQGLPKVEQILEIRSIDSISMNLEKRIDAWNECIPRILGIPWGFLIGAELTIAQSRISLVNKI
QKVYRSQGVHIHNRHIEIIVRQITSKVLVSE---DGMSNVFLPGELIGLLRAERTGRALEEAIC
YRALLLGITKTSLNTQSFISEASFQETARVLAKAALRGRIDWLKGLKENVVLGGIIPVGTGF
KRIMHRSRSRQHNKIT--LKKKKK-LFEVE-I-RNLLLHHRKLFDFYHFKQFM------------------
MTKRYWNITFEEMMEAGVHFGHDTRKWNPRMAPYISAKRKGIHIINLTRTARFLSEACDL
VFDAASRGKQFLIVGTKKKAADSVTRAAIRARCHYVNKKWLGGMLTNWYTTETRLRKF
RDLRTEQKTGKPNSLPKRDAAMLKRQLSHLETYLGGIKYMTRLPDIVIIVDQQEDYTALR
ECITLGIPTICLIDTNSDPDLADIPIPANDDAIASIRLILNKLVFAICEGRSS-YIRNLMGQKIHP
LGFRLGTTQSHDSIWFAQPRNYSENLKEDKIIRDCIKNYIQKTIRISS-GVEGIGRIKIKKRID
LIQVIIYMGLPKLLLEGKPR----RIEELQINVKKKLNCVNRKINIAITRIPNAYRDPNILAEFIA

GQLKNRISFRKAIKKAIELAEQAGTKGIQVQIAGRIDGKEIARVEWIREGRVPLQTIRAKID
YCSYPVRTIYGVLGIKVWIFLNKEMSRYRGPRFKKIRRLGDLPGLTSKRPTVGSELRNQSSS
SK-KSQYRIRLEEKQKLRFHYGLTERQLLKYVRIAGKAKGSTGQVLLQLLEMRLDNILFRL
GMALTIPQARQLVNHRHVFVNGRIVDIPSYRCKPQDIITAKDDQKSITLIQNSLESSPR-EEL
PNHLTLHPSE--YKGLVNQIIDSKWVGLKINELLVVEYYSRQTMSRRGTAEKKTAKSDPIYR
NRLVNMLVNRIMKHGKKSLAYQIIYRAMKRIQQKTETNPLSVLRQAIRRVTPDIAVKARRV
SGSTHQVPTEIGSTQGKALAIRWLLGAARKRPGRNMAFKLSSELVDAAKGSGDAVRKKE
ETHRMAEANRAFAHFRGGKDTIADILTLIRNADMTRKKTVQIPLSNITENIVKILLREGFVE
NVRKHRESNKYFLVLTLR--R--NRKGSYKN-F--LHLKRISTPGLRIYSNYQKIPRVLGGMGI
VILSTSRGIMTDREARLERIGGEVLCYIWMAKSISKIGSRKNARIGS-KQTRKIPKGVIYVQ
ASFNNTIVTVTDVRGRVISWSSAGSCGFKGTRRGTPFAAQTAAANAIRTVVDQGMQRAEV
IIKGPGLGRDAALRAIRRSGILLRFIRDVTPMPHNGCRAPKKRRVMPTIKQLIRNARQPIRN
VTKSPALRGCPQRRGTCTRVYTITPKKPNSALRKVARVRLTSGFEITAYIPGIGHNLQEHSV
VLVRGGRVKDLPGVRYHIVRGTLDAVGVKDRQQGRSKYGAKKPKMAKKSLIEREKKRQ
KLEQKYHLIRRSPKKQIS---KAQSLSEKWELHGKLQSLPRNSAPTRLRRRCFSTGRPRGNC
RDFGLSGHILREMVHEGLLPGATRSSWMIKNLFIPFISQK-KKEEENPGSVEFQVVNFTNKI
RKLTSHFKLHPKDYLSQRGLRKILGKRQGLLSYLLERDKRRFEKLMSELNTRDSQIR-
-???
MDKSKRLFLKSKQSFRRSLSPIQSGDRIDYKNMSLLYRFISQQGKILSRRVNRLSLKQQRLI
TIAIKQARILSSLPFVNNGKK-KYEKRESITRTTAVRKKKKMGR-LKKNPFVANHLLRKINK
LNTKGEKEIIITWSRASTIIPTMIGHTIAIHNGREHLPIYITDRMVGHKLGEFSPTLTFRGYAK
NDTKSRRMTYRSFILKYLVPFCLKIMNSAVVTGLYYGFLTTFCIGPSYLFIIRARVRNEG---T
EMVVSATTGFITGYLIMFISIYYAPLHLALNRPHTITVLILPYIFFHFVEKNNKYFYDPRYDN
ANSIHKFNIVKEFFTNFLFQLINPLLFQSSILIRLLQICLFRSNNKLLFLTSSFVGWLIGHIFLM
KLMGFPIVWFQQKGSIKSKLIMGKYILLQLRYY--QIFVVFVFVIFLHYLGRIPAPYFYAKIAE
----KPIVTTLFDYQRWIRPLRYIKNLHFENVVRDENSQFFFHICKSDGKERISFTYPPSLSIFQ
KMMEKKMNLFTKDKISNDNDNELWSYNHNEKKKKLINEFFKRAQFLDKNYSPPDVFANS
VRLCNDDMENKYLTKIYDPFLNGRFRGEIKKNFSPRNSIFVNKIHGLLDPELEQKDEKNKC
TEINEIEKEVPRWSYKLIEVQQLTESEIRCARAERVVVFSASLVKYSREPDFNRGIIKGSMRP
QRRKTVTWKFFQSNAHSPTFLEI-IDDYPFFFGELYDDILQY-WKEDEAESRNEIEESWESIL
YGLRIRSFVLLFQSNLRKYIILPSLIITKNIIRILLLQDPEWLEDISDWRKEVHIKCTYQGVPV
SDKKLPKDWFYDGIQIRILSPFVLKPWHKSKSTKKKNLWFLTAYGIESYLDYHVPNPF-AFL
GPIFKKITKYKKDLKKRFFLV-ADVLHQGKDWFRTMLENIEKFKMESFLKSCLFGVQKSDE
----SIISTNMIGESPVIIESINLTNSSFTETRIKDLVKTTRIIKQMTEEMTEENKENSNKLTYDG
KRLELQKNLFQILKRRTVRLIRKSYSFLQIFMKRIYIDILLYIISIPRIHGQRF-----CDVPYLSQ
GYVFSKLAQIQVSNKYKFRSIFESRSIFLKDEIKNYFIRAISDSKLGHNKLRDSAMNQWTS
WLKIHYQYDLPLNIWSKLGSKKWRNKINEHRVAQNKIEYDSYGKSQ--LSLYTKQ-KTQKQ
YRYDLFSYQFLNNDIVKEDDDIVEDDIND----IRDVKIKKKTIRKRWFFSKLNILYNAYKKN
PWIVPRKSLFLQTQKDIKAERDFLLDKYLGFYLNCDTDFQESMMNNINFYCLLFRLKMKK
-FFIKSIKQGDLDIESMILVNNPTDFATTECLTFVIEPVRVSRKNLEQFFIYQTISLPLIHKSRR
NHYDLLVPENLFSTRRRRELRILISLRFLWPNYRLEDLACMNRYWFDTHNGSRFSLLRIRM
YPMQLHQFQCWVLELREVVREIKNSF--LDSLAQFNSAVSFIHIFFHQESLIKLLDFRIWNIV
LS-RNSQSSTSNRYRYFT---IKNIVLFLVA-ILIYR----IHNRKMIERKNIYLT-EFLP----IPMNSIG
RSNDTLEDSK-DSFNINRLIVP-LLYFAKRKKISEKSS--

--FIQVTDSSQSSD
EF----GDHINSIR--
----------NPTRDNPSFFSDRW--SELH-LDLNPTERSTTNQ----KLFKKEEDVS-FVLSRQSE----N
KEIANLVKIIMYLQN--TVSILPISSDSRCDMVA----KDELYSSDNISFLNKNLFLGL--FHLFHY
RNRGGYLL--HHDLKSE--ERFQEMANLFNLSITQP-DLLYHKGLSFSIDSSGLYQKQ--FLNE
VFNSKDESKKKSL---LVLPPLFY--EENESFYSRIIKKWGQ-TSYGNCLEDRKPKIVVFT--------
------KNHIIQSVNQYG--LIRNQKK---------RFDP------------------------LFYIYR----------------------
----ERSMNRDQNSYKYKWSNGSKNFQEHLAHFSSEQNSH---FQVVFDRFHINPYSIDWSKV
FDQKDLFTSLFWSKFLP------------------------FLSN-SLRFFFVSFGSIPVHRSE-IHIS-ELKRPN
DPLGNPLLESIGLQIVHLKKKKPF--FLD---DHSTS-QKSKLLIDGGTISPFLFNKIPS-----------
LHNRSKSLD----NFSTISHDQDNWLNPVKPFYRSSLISSFYKVNRLRFLNNKYNFCFD--CN
KRFSFYV----ERSCIKNYD--FMYE-QFLNTLFIRNK-IFSLCSGK--KKDVFLE-KDTISPIELEV
SKILIPNNFPQNGDEE-YNLYKS---FHFPTRSDPLVR--RAIYSIEDISGIPLTEEEIVNFERTYCE
PLSDRNLPNSEGKNLHQYLN--FNSNIGLIDIPYSEKYLPSEK--RKK--------RIRRNK--R-LAK
GYMDRAFQRKKRKRSFSKWKRFYPYIIWLLTSTGHINIKLIFRDAFSDLLRILNRNPKFLKL
VYIFNDIMYESRILR---EKVL-------LLKRNP-ISAISSKWLHNYILELCDKIYHVNNES--PFIST
HLRSLNIQEL-QFLILFFLLITGY-LILGHLIVTSR--FSSQLQAEFEQVKSFMIPSYMIELRKLL
DTYPPSELNSFGLKDIFLVALEGLGSFLKEIRASYSGGNMLSGGDP-----AYG-VKSVRSKKK
YFNII------DLISIIPNPMNRIAFLRNTRHLSHTSKMIYSVIRKRKNVNSDWLDDKIESWILNS
DFIADKEREFLVQFS--TLTT--EKRIDQILLSLT-HSDHLS-----KNNSGYQMIEQPGTIYLRNLI
DIH--KKDLMNYEFNTSCLAERRIFLAHYQTIAY--SETQPGPSCLDFPSQG---KPFSLRLDLSP
S-RGVLVIGSIGTGRSYLIKYLATNSYVPFFTIFINKFLDNYPKGFTIDP----DEE--DREGTIYS
LLPSNEGSCYNYDRNDSDD----IDR--NFDRQLKFLSMKHPIS---RDMRP---ELDLFYLTLQFE
LAKALSPCIIWIPNIHDLDRKESNDLSLGLLVNYLS---GDSETCS-TRNNLVIASTHIPQKVDP
ALIATNKLNTCIKIRRLLSPQQRKNFFTLSYTRGFHLEKKMFHT--NGFGSITLSSNLRDLVAL
TNEALTISIIQKKSIIDTNIIRSALHRQTWDFR---SKIRSIQDHGILFYQIGRAVSQNLLLSNCS
LDPISIYIKKK--SCQGGDSYLYRFYFELGTSMKKLTILLYLLSCSAGSVAQDLWSLP--GPNE
K-NGITSYRLIENDS-DLIHGLLEVESALLE-----SSPKESPFDND--RVISLLRPKPRN-PLNMIQ
NGFRSIRDERFLYEK--YESGFEE---GEGVLDPG----PLEAD----LFNH--IVWAP--RLWRP---W
DFIFDCSERSNELGFP-YWERSFRDK----MIYDEEDELQENDSEVLQDVTMQYQTRDRSSK
ERGVFRISKFIWDPVDPLFLLFQ-DDPSVSVFSHREFFADEEISRILLTSQTKYWKYLNKRW
FRKNTP--EKYFELLIDRQRWLRT---NSSLS--NRFLRSN--ILSESYQYLSNLFLSN-RTLLDQIT
KTLLKKRWLFPDEMAVTI--CSNNESSV--MTRSR-INGNFIDKTFSIVANILLRIIPTTSGEKEA
FSYYRDG--MSAQSEGNYAEALENYYEAMRLEIDPYDRSYILYNIGLIHTSNGEHTKALEY
YFRALERNPFLPQAFNNMAVICHY-RGEQAIRQGDSEIAESWFDQAAEYWKQAIALTPGN
Y I E A Q N W L K I T G R F E -
-???
??
????????
>Trifolium
??
??
??

??
??
???????????????????????????????MVTLRADEISKLIRERIEQYNTEVKIVNTGTVLQVGDGIA
RIYGLDEVMAGELVEFEDGTVGIALNLESKNVGVVLMGDGLFIQEGSSVKATGRIAQIPVS
EGYLGRVVNALAKPIDGRGEISASESRLIESPAPGIISRRSVYEPLQTGLIAIDSMIPIGRGQR
ELIIGDRQTGKTAVATDTILNQQGQNVVCVYVAIGQKASSVAQVVTTLQERGAMEYTIIVA
ETANSPATLQYLAPYTGAALAEYFMYRERHTLIIYDDLSKQAQAYRQMSLLLRRPPGREA
YPGDVFYLHSRLLERAAKLSSQLGEGSMTALPIVETQSGDVSAYIPTNVISITDGQIFLSAD
LFNAGIRPAINVGISVSRVGSAAQIKAMKQVAGKLKLELAQFVELEAFAQFSSDLDKATQN
QLARGQRLRELLKQSQSAPLTVEEQIITIYTGTNGYLDSLEVEQVRKFLVELQSYLKTNKP
QFNEIISSTKTFTGEAEALVKEAIQEHTELFLLQEQVMKVIPTTSDTEISALENKNLGRITQII
GPVLDVDFPPGKMPYIYNALRVQGRDSVGQQINVTCEVQQLLGNNRIRAVAMSATDGLK
RGLEVVDTGAALSVPVGGATLGRIFNVLGEPIDNLGPVDTRTTSPIHRSAPAFVQLDTKLSI
FETGIKVVDLLAPYRRGGKIGLFGGAGVGKTVLIMELINNIAKAHGGVSVFGGVGERTRE
GNDLYMEMKESGVINEKKIEESKVALVYGQMNEPPGARMRVGLTALTMAEYFRDVNEQD
VLLFIDNIFRFVQAGSEVSALLGRMPSAVGYQPTLGTEMGTLQERITSTKEGSITSIQAVYV
PADDLTDPAPATTFAHLDATTVLSRGLAAKGIYPAVDPLDSTSTMLQPRIVGEEHYQTAQRV
KQTLQRYKELQDIIAILGLDELSEEDRLIVARARKIERFLSQPFFVAEVFTGSSGKYVGLAET
IRGFKLILSGEFDSLPEQAFYLVGNIDEATAKAANLRN------MTLNLCVLTPNRIVWDSEVK
EIILSTNSGQIGVLKNHAPIATALDIGILRIRLNDRWLTVALMGGFARIGNNEITILANDAER
GSDIDPQEAQQTLKIAEANLNKAKGKRQTIEANLALRRARTRVEAINTISMKNITDSFLCF
VYWPSAGSFGFDTDILATNLINLSVVLGVLIFFGKGVLSDLLDNRKQRILRTIRNSEELREA
AIEQLETARARLRKVETEADRFRVNGYSEIERDKLNLINSIYTTLEQFENYKNETIHFEQQR
AINQVQQRVLQQALQGALGTLNSCLNNELHLRTISANIGMFGAMKEKKNMNPLISAASVI
AAGLAVGLASIGPGVGQGTAAGQAVEGIARQPEAEDKIRGTLLLSLAFMEALTIYGLVVAL
ALLFANPFVMNVLLCYINTLNRFFDISAVEVGQHFYWQIGDFEVHAQVLITSWVVIAILLIS
TILVVRNPQTIPTSGQNFFEYVLEFIRDVSKTQIGEEYGPWVPFIGTLFLFIFVSNWSGALLP
WKIIKLPHGELAAPTNDINTTVALALLTSVAYFYAGISKKGLAYFGKYIQPTPILLPINILEDF
TKPLSLSFRLFGNILADELVVVVLVSLVPLVVPIPVMFLGLFTSGIQALIFATLAAAYIGESM
EGHHMIFSTLEHILTHISFSVISIVISIHLITLLVNEIVGLYDLSKKAMIITFFCVTGLLITRWFF
SGHLPFSDLYESLIFLSWGFSIFHMVPCFKKEKNLLSTIIAPSVIFTQGFATSGLLTKMHQSVI
LVPALQSHWLMMHVSMMILGYAALLCGSLLSVAILVITFQEVIQTVSFTKEIKYMNMNES
VLRKTSFTSSRNYYRSQFIQQLDRWGYRIISLGFLFLTIGILSGAVWANEAWGSYWNWDPK
ETWAFITWTIFAIYLHTRKTKKIEGVNSSIVASIGFLIVWICYLGINLLGIGLHSYGSFTSNMA
KKKAFIPLLCLTSIVFLPWCISFTFKKSVESWITNWWNTKESEIFLNIIQEKDLLKKFIEFEEL
FLLDEMLKEYPETRLQNLRIE-YKETIQLIETNNQDHIHTILYFCTNIIGFLILSGYSIRSNQEL
IILNSWVKEFLYNLSDTIKAFSILLLTDFCIGFHSTHGWELMIGSVYKDFGFTQNEQIISGLVS
TFPVILDTLFKYWIFRYLNRVSPSLVVIYHSMNDMPVGVPKVPYLMPEEDEASWVDLYHL
LYEERLLFLGQEVNSEISNQLNGLMVYLSLQDKTKDLYLFINSPGGELMSGMAMFDTMQF
VKAEVQTVCVGIAVSMASLLLLGGEITKRLAFSHARVMMHEPASVFEDDKTGECMLEAD
E L L K M Y I G I I N I Y V Q R T G K P V W Q I A V D I K R D I F M S A E E A Q A Y G I V D M V A D
A???MKEYQV
YLERARSRQQDFLYPLIFREYIYGLAYSHNWGGYDNKYSLLNVKRLITRMYQQNHLIISA
NDSNKNPFLGKNFYSKIISEGFAIVVEIPLFLQLSSSLIIKSYNVRSIHSIFPFLEDKFTYLNYV

SDIRIPYPIHLEILVQILRYWVKDAPFFHLLRLFLYHFCNWNRFITKSISTFSKPRLFLFLYNF
YVCEYESIFLFLRNKSSHLRFKSFSVFLERIFFYAKREHLVEVFSKSYPLPFFKDPNIHYVRY
QGKCILASKNVPFLMNKWKHYFIHLWQCFFDVWSQPRTININQLSEHSFQLLGYFSNVRL
NRSVVRSQMLQNTFLIEI--VSKKLDIIVPIIPLIRSLAKAKFCNVLGHPISKPVWADSSDFDI
MERFLRICRNLSHYYNGSSKKKSLYRIKYILRLSCIKTLACKHKSTTRAFLKRSGSELLEEF
FTEEEEILSLIFPRRIWYLDILFRNDLVNDEMIIDTT-EVQDIN-SFSGLES-FKEVYGILWILVPI
LILVLGITISVLAIVWLEREISAGIQQRIGPEYAGPFGILQALADGTKLLFKENLIPSRGDIRLF
SIGPSISVISILISYSVIPFGYNFVLSDFNIGVFLWIAVSSIAPIGLLMSGYGSNNKYSFLGGLR
AAAQSISYEIPLTLCVLSISLLSNSLSTVDIVDAQSKYGFWGWNLWRQPMGFIVFLISSLAE
CERLPFDLPEAEEELVAGYQTEYSGIRFGLFYVASYLNLFISSLFVTVLYLGGSNISIP--YISE
FFEINKRYGVFRTTIDLFITLAKTYFFLFVSIITRWSLPRLRMDQLLNLGWKFLLPISLGNLL
L T T S S Q L F S
L???
??
??
??
??
??MFLLYEYDIFWTFLII
SIFIPILAFLISGILAPIRKGPEKLSSYESGIEPMGDAWLQFQIRYYMFALVFVVFDVETVFLY
PWAMSFDVLGVSVFIEAFIFVLILIVGSVYAWRKGALEWSTNYFPWLTIVVVFPIAAGSFIF
LFPHRGNKVIRWYSICICLIDLLLTTYAFCYHFRLDDPLIQLTENYKWIHFFDFYWRLGIDG
LSIGPILLTGFITTLATLAAQPVTREYRLFYFLMLAMYSGQIGPFSSRDILLFFIMWELELIPV
YLLLSIWGGKKRLYSATKFILYTAGASIFLLMGILGIGLYGSNEPTLNFETLTNQSYPVALEII
FYTAFLLAFAVKSPIIPFHTWLPDTHGEAHYSTCMLLAGILLKMGAYGLVRINMEFFSHAH
SIFCPWLMILGSIQIIYAASTSFGQRNLKKRIAYSSVSHMGFIIIGIGSISDTGLNGAILQIISHG
FIGAALFFLAGTSYDRLRLLYLDEMGGMAIPMPKIFTIFTILSMASLALPGMSGFVAELIVF
WGIITSQKYLFMMKILMTFGTAIGMILTPIYLLSILRQMFYGYKFFNTPNSYFFDSGPRELFI
SISILIPVIGIGIYPDFIFSFSVDKVEAILSNF---MMLEHVLVLSAYLFSIGIYGLITSRNMVRAL
MCLELILNAVNINLVTFSDFFDNRQLKGNLFSIFVIAIAAAEAAIGLAIVSSIARNRKSTRINQ
SNLLNKMEYTHQSSWIIPFIPLPVPILIGVGLLLFPTATKNLRRMWAFPSILLLMIVMIFSIDL
SIHQIENNSIYQYVWSWTINNDLSLEFGYLIDSLTSIMSILITTVGILVLIYSDSYMSHDQGYL
RFFTYLSIFNTSMLGLVTSSNLIQVYIFWELVGMCSYLLIGFWFTRPIAANACQKAFVTNRV
GDFGLLLGILGFYWITGSLEFRDLFQIFNN-EVNLFFVTLCALLLFCGSVAKSAQFPLHVWL
PDAMEGPTPISALIHAATMVAAGIFLVARLLPLFIVIPPLMSGIALIGIITVVLGATLAIAQKDI
KKNLAYSTMSQLGYMMLALGMGSYRAALFHLITHAYSKALLFLGSGSIIHSMEAIVGYSP
DKSQNMVLMGGLRKHAPITKTAFLIGTLSLCGIPPFACFWSKDEILNDSWLYSPIFAIIACST
AGLTAFYMFRIYLLVFEGYLNVHFQNFNGKKNSSFYSISLWGK-EEKKKLKKLLALLTMNF
FRGTKNIPFFYPHESDNTMLFSMLVLVLFTFFVGSVG--FDLDILSKLLIPSIDLLHQNSK-SV
DW-EFFTNATFSVNIAFFGIFIASFFYKPVFSSLQNLNLFNLFQRIADKIINILYDWSYNRGYI
DDFFEVSLIASVRKLAKFNYFFDRQVIDGIPNGVGISSFFIGESIKYVGGG-RISSYIFF---FVL
IFLVICYMDLPETLHDFLLVFLGSGLILGSLGVVLLTNPIFSAFSLGLVLVCISLLYILANSHFV
AASQLLIYVGAINILIIFAVMFVNSCEYYQDFHFWTVGDGITLIVCTSIFVSLITIIPDTSWYGI
IWTTRPNQIIEQDLISTSQQIGIHLSTDFFLPFELISIILLVALIGAIVVARQMNVPARIKDLMIV
NMGPQHPSMHGVLRLIVTLDGEDVVDCEPILGYLHRGMEKIAENRTIIQYLPYVTRWDYL

ATMFTEAITVNGPEQLGNIQVPKRASYIRVIMLELSRIASHLLWLGPFMADIGAQTPFFYIF
RERELIYDLFEAATGMRMMHNYFRIGGVAADLPYGWIDKCFDFCNYFLTRVIEYQKLITR
NPIFLERVEGVGVVGREEVLTWGLSGPMLRASGIQWDLRKVDNYECYDEFDWEVQWQK
EGDSLARYLVRIGEMMESIKIIQQALEGIPGGPYENLEIRSFDREKEPEWNDFEYRFIGKKSS
PTFELPKQELYVRVEAPKGELGIFLIGDHNGFPWRWKIRPPGFINLQILPQLVKRMKLADIM
TILGSIDIIMGEVDRMFLMVTGFMNYGQQTVRAARYIGQSFMIILSHANRLPVTIQYPYEK
LITSERFRGRIHFEFDKCIACEVCVRVCPIDLPVVDWKLETDIRKKRLLNYSIDFGICIFCGN
CIEYCPTNCLSMTEEYELSTYDRHELNYNQIALGRLPMSVIDDYTIRTILIK----------------MQ
GNLSAWLVKHGIVHRSLGFDYQGIETLQIKPEDWHSIAVILYVYGYNYLRSQCAYDVAPG
GLLASVYHLTRIEYSLDQPEEVCIKVFVPRQNPRIPSIFWVWKSADFQERESYDMLGISYD
N H P R L K R I L M P E S W I G W P L R K D Y I A P N F Y E I Q D A
H???
???
???
????????????????????MQTRNAFSWIKEEITRSISVLLMIYIITRAPISNAYPIFAQQGYENPREA
TGRIVCANCHLANKPVDIEVPQAVLPDTVFEAVVRIPYDMQVKQVLANGKKGALNVGAV
LILPEGFELAPPDRISPEIKEKIGNLSFQSYRPTKKNILVVGPVPGKKYSEITFPILSPDPATKR
DVHFLKYPIYVGGNRGRGQIYPDGSKSNNNVYNATATGIVNKIIRKEKGGYEITIVAPSDG
REVIDIIPPGPELLVSEGESIKLDQPLTSNPNVGGFGQGDTEIVLQDPLRVQGLLFFLASIILA
QIFLVLKKKQFEKVQLSEMNFMSKVYDWFEERLEIQAIADDITSKYVPPHVNIFYCLGGIT
LTCFLVQVATGFAMTFYYRPTVTEAFASVQYIMTEANFGWLIRSVHRWSASMMVLMMIL
HVFRVYLTGGFKKPRELTWVTGVVLGVLTASFGVTGYSLPWDQIGYWAVKIVTGVPDAIP
VIGSSVVELLRGSASVGQSTLTRFYSLHTFVLPLLTAVFMLMHFSMIRKQGISGPLMGITKK
PDLNDPVLRAKLAKGMGHNYYGEPAWPNDLLYIFPVVILGTIACNVGLAVLEPSMIGEPA
DPFATPLEILPEWYFFPVFQILRTVPNKLLGVLLMVSVPAGLLTVPFLENVNKFQNPFRRPV
ATTVFLIGTVVALWLGIGATLPIEKSLTLGLFMIEVFLFGIVLGLIPITLAGLFVTAYLQYRRG
DQLDIMPTITSYFGFLLAVLTITAGLFISLNKIRLIMDIVSLAWAALMVVFTFSLSLVVWGRS
GLMIIRSPEPKVKILVDRDPIKTSFEQWAKPGHFSRTIAKGPDTTTWIWNLHADAHDFDSH
TSDLEEISRKVFSAHFGQLSIIFLWLSGMYFHGARFSNYEAWLNDPTHIRPSAQVVWPIVG
QEILNGDVGGGFRGIQITSGFFQIWRASGITSELQLYCTAIGALVFAALMLFAGWFHYHKA
APKLAWFQDVESMLNHHLAGLLGLGSLSWAGHQVHVSLPINQFLNAGVDPKEIPLPHEFI
LNRDLLAQLYPSFAEGATPFFTLNWSKYADFLTFRGGLDPLTGGLWLTDIAHHHLAIAILFL
IAGHMYRTNWGIGHGIKDILEAHKGPFTGQGHKGLYEILTTSWHAQLSLNLAMLGSLTIIV
AHHMYAMPPYPYLATDYGTQLSLFTHHMWIGGFLIVGAAAHAAIFMVRDYDPTTRYNDL
LDRVLRHRDAIISHLNWVCIFLGFHSFGLYIHNDTMSALGRPQDMFSDTAIQLQPVFAQWI
QNTHALAPGTTAPGATASTSLTWGGGDLVAVGGKVALLPIPLGTADFLVHHIHAFTIHVTV
LILLKGVLFARSSRLIPDKANLGFRFPCDGPGRGGTCQVSAWDHVFLGLFWMYNSISVVIF
HFSWKMQSDVWGSINDQGVVTHITGGNFAQSSITVNGWLRDFLWAQASQVIQSYGSSLS
AYGLFFLGAHFVWAFSLMFLFSGRGYWQELIESIVWAHNKLKVAPATQPRALSIVQGRAV
GVTHYLLGGIATTWAFFLARIIAVGMALRFPRFSQGLAQDPTTRRIWFGIATAHDFESHDDI
TEERLYQNIFASHFGQLAIIFLWTSGNLFHVAWQGNFEAWVQDPLHVRPIAHAIWDPHFGQ
PAVEAFTRGGALGPVNIAYSGVYQWWYTIGLRTNEDLYTGALFLLFLSVIALLAGWLHLQ
PKWKPSVSWFKNAESRLNHHLSGLFGVSSLAWAGHLVHVAIPGSRGETVRWNNFLSVLP
HPQGLGPLFTGQWNLYAQNPDSSSHLFSTSQGAGTAILTLLGGFHPQTQSLWLTDMAHHH

LAIAILFLIAGHMYRTNFGIGHSIKDILEAHIPPGGRLGRGHKGLYDTINNSIHFQLGLALAS
LGVITSLVAQHMYSLPAYAFIAQDFTTQAALYTHHQYIAGFIMTGAFAHGAIFFIRDYNPEQ
NEDNVLARMLEHKEAIISHLSWASLFLGFHTLGLYVHNDVMLAFGTPEKQILIEPIFAQWI
QSAHGKTSYGFDVLLSSTNSPALNAGRSIWLPGWLNAINE--NSNSLFLTIGPGDFLVHHAI
ALGLHTTTLILVKGALDARGSKLMPDKKDFGYSFPCDGPGRGGTCDISAWDAFYLAVFW
MLNTIGWVTFYWHWKHITLWQGNVSQFNESSTYLMGWLRDYLWLNSSQLINGYNPFGM
NSLSVWAWMFLFGHLVWATGFMFLISWRGYWQELIETLAWAHERTPLANLIRWRDKPVA
LSIVQARLVGLVHFSVGYIFTYAAFLIASTSGKFGMSHSVKIYDTCIGCTQCVRACPTDVLE
MIPWDGCKAKQIASAPRTEDCVGCKRCESACPTDFLSVRVYLWSETTRSMGLSYMI--NLP
SLFVPLVGLVFPAVAMASLFLHVQKNKIFMRDLKTYLSVAPVASTLWFAALAGLLIEINRLF
PDALTFPFF--MTAILERRDSENLWGRFCNWITSTENRLYIGWFGVLMIPTLLTATSVFIIAFIA
APPVDIDGIREPVSGSLLYGNNIISGAIIPTSAAIGLHFYPIWEAASVDEWLYNGGPYELIVL
HFLLGVACYMGREWELSFRLGMRPWIAVAYSAPVAAATAVFLIYPIGQGSFSDGMPLGISG
TFNFMIVFQAEHNILMHPFHMLGVAGVFGGSLFSAMHGSLVTSSLIRETTENESANEGYRF
GQEEETYNIVAAHGYFGRLIFQYASFNNSRSLHFFLAAWPVVGIWFTALGISTMAFNLNGF
NFNQSVVDSQGRVINTWADIINRANLGMEVMHERNAHNFPLDLAAVEAPSINGMGLPWY
RVHTVVLNDPGRLLSVHIMHTALVAGWAGSMALYELAVFDPSDPVLDPMWRQGMFVIPF
MTRLGITNSWGGWNIT-GGTITNPGIWSYEGVAAAHIVFSGLCFLAAIWHWVYWDLEIFC
DERTGKPSLDLPKIFGIHLFLAGVGCFGFGAFHVTGLFGPGIWVSDPYGLTGRVQSVNPAW
GVDGFDPFVPGGIASHHIAAGTLGILAGLFHLSVRPPQRLYKGLRMGNIETVLSSSIAAVFF
AAFVVAGTMWYGSATTPIELFGPTRYQWDQGYFQQEIYRRVGAGLAENQNLSEAWSKIPE
KLVFYDYIGNNPAKGGLFRAGSMDNGDGIAVGWLGHPIFRDKEGRELFVRRMPTFFETFP
VVLVDGDGIVRADVPFRRAESKYSVEQVGVTVEFYGGELNGVSYSDPATVKKYARRAQL
GEIFELDRATLKSDGVFRSSPRGWFTFGHASFALLFFFGHIWHGARTLFRDVFAGIDPDLD
AQVEFGAFQKLGDPTTKKQVVMKTLYSLRRFYHVETLFNGTLALTGRDQETTGFAWWAG
NARLINLSGKLLGAHVAHAGLIVFWAGAMNLFEVAHFVPEKPMYEQGLILLPHLATLGW
GVGPGGEVIDTFPYFVSGVLHLISSAVLGFGGIYHALLGPETLEESFPFFGYVWKDRNKMT
TILGIHLILLGIGAFLLVFKAFYFGGIYDTWAPGGGDVRKITNLTLSPSIIFGYLLKSPFGGEG
WIVSVDDLEDIIGGHVWLGSICILGGIWHILTKPFAWARRALVWSGEAYLSYSLAALSVFG
FIACCFVWFNNTAYPSEFYGPTGPEASQAQAFTFLVRDQRLGANVGSAQGPTGLGKYLMR
SPTGEVIFGGETMRFWDLRAPWLEPLRGPNGLDLSRLKKDIQPWQERRSAEYMTHAPLG
SLNSVGGVATEINAVNYVSPRSWLATSHFVLGFFLFVGHLWHAGRARAAAAGFEKGIDRD
LEPVLSMTPLNMTIALGKFTKDQNDLFDIVDDWLRRDRFVFVGWSGLLLFPCAYFAVGG
WFTGTTFVTSWYTHGLASSYLEGCNFLTAAVSTPANSLAHSLLLLWGPEAQGDFTRWCQL
GGLWTFVALHGAFGLIGFMLRQFELARSVQLRPYNAIAFSGPIAVFVSVFLIYPLGQSGWFF
APSFGVAAIFRFILFFQGFHNWTLNPFHMMGVAGVLGAALLCAIHGATVENTLFEDGDGA
NTFRAFNPTQAEETYSMVTANRFWSQIFGVAFSNKRWLHFFMLFVPVTGLWMSALGVVG
LALNLRAYDFVSQEIRAAEDPEFETFYTKNILLNEGIRAWMAAQDQPHENLIFPEEVLPRG
NALMSGSTGERSFADIITSIRYWIIHSITIPSLFIAGWLFVSTGLAYDVFGSPRPNEYFTESRQ
GIPLITGRFDSLEQLDEFSRSFMTIDRTYPIFTVRWLAVHGLAIPTVSFLGSISAMQFIQRMAT
QTVEGSSRSG-PIRTVVGDLLKPLNSEYGKVAPGWGTTPLMAIAMALFAVFLSIILEIYNSSI
LLDQISMNMLTLKLFVYTIVIFFVSLFIFGFLSNDPGRNPGREEMADTTGRIPLWIIGTVTGI
VVIGLVGIFFYGSYSGLGSSLMLNIFSLVCICIHSALYSSSFFLAKLPEAYAFLNPIVDFMPVIP
LLFFLLAFVWQAAVSFRTTQSNPNEQNVELNRTSLYWGLLLIFVLAVLFSNYFFNMEVNIL

AFIATALFILVPTAFLLIIYVKTVSQSDMETATLIAISLSGLIVSFTGYALYTAFGQPSLQLRDP
FEEHGDMEALVYTFLLVSTLGIIFFAIFFREPPIVPTKKTKMTIAFQLAVFALIVTSSILLISVP
VVFASPDGWSSNKNVVFSGTSLWIGLVFLVGILNSLIS-------MAPQTETKAKVGFKAGVKD
YRLTYYTPDYETKDTDILAAFRVTPQPGVPPEEAGAAVAAESSTGTWTTVWTDGLTSLDR
YKGRCYHIEPVAGEDTQFIAYVAYPLDLFEEGSVTNMFTSIVGNVFGFKALRALRLEDLRIP
VAYVKTFQGPPHGIQVERDKLNKYGRPLLGCTIKPKLGLSAKNYGRAVYECLRGGLDFTK
DDENVNSQPFMRWRDRFLFCAEAIYKSQAETGEIKGHYLNATAGTCEEMIKRAVFARELG
VPIVMHDYLTGGFTANTTLAHYCRDNGLLLHIHRAMHAVIDRQKNHGMHFRVLAKALRL
SGGDHIHAGTVVGKLEGEREITLGFVDLLRDDYVEKDRSRGIFFTQDWVSLPGVLPVASG
GIHVWHMPALTEIFGDDSVLQFGGGTLGHPWGNAPGAVANRVALEACVQARNEGRDLAR
EGNDIIRAAGKWSPELAAACEVWKEIKFEFPAMDTL--MAIHLYKTSIPSTRNGAVDSQVKS
---NRLISGQHHCGKGRNARGIITAGHRGGGHKRLYRKIDFRRNEKDIYGRIVTIEYDPNRN
ARICLIHYGDGEKGYILHPRGSRIGDTVVYGTEVPIKMGNALPLTDMPLGTAIHNIELTLGK
GGQLARAAGAVAKLIAKEGKSATLKLPSGEVRLISKNCSATVGQVGNVGANQKSLGRAG
AKRWLGKRPVVRGSAMNPVDHPHGGGEGKAPIGRKKPSTPWGYPALGRKTRKRNKYSD
NLILRRRSKMIQPQTYLNVADNSGARELMCIRIIGAS-NRRYAYIGDIVVAVIKKAVPNSSVE
RSEVIRAVIVRTCKELKRSNGIIIKYDDNAAVLVDKEGNPKGTRIFSAIARELRQLNFTKIVS
LAPEVLNYNPKRTRFRKQHRGRMKGKAYRGNKICFGKYALQALEPAWITSRQIEAGRRA
MSRNVRRGGKIWVRIFPDKPVTVRPTETRMGSGKGSPEYWVAVVKPGKILYEMGGVPENI
ARKAISIAASKMPIRTQFILSEMNRTKRGSVARKRRTKNDSFTSGCRGARSRIYRMSSQQK
TKAFVSSDRDRDRKKRDFRSLWITRINAIIVQNEKIYNIYSNIMHNLYKSRLLLNRKIVAQM
A I L K G D C L F M I A N K I I K K - - - - - - - - -
-???
??????????????????????????????????MNEIKYAVFTDKSIRLLAKN-QYTFNVESRSTRTEIKH
WVELFFDVKVIAMNSHRLPIKGRGTRPIMGHTMHYKRMIITLQPGYSIPSLRTKR
N???--????MAKGKDIRITVILECTSC-DKK--V
NKK--GISRYITQKNRHNTPSRLELKKFCPFCRKHMIHAEIKKMKVRASVRKICEKCRLIRR
RGRIIVICSNPKHKQRQGMIREKVKVSTRTLLWKCVESRVDSKRLYYGRFILSPLMKGQAD
TIGITMRRILLGEIEGTCITRAKSE--KIPHEYSTIVGIQESIHEILMNLKQIVLRSNLYGTRDAS
ICFKGPGYVTARDIILPPSVEIVDNTQHIANLTEPINLCIELQIERARGYRKKTIDNIQDGSY--
TIDVVFMPVIHANYSIHSYLNANEKQEILFLEIWTNGSLTPKEALYEAARNLIDLFIPFLHAE
EE--NFHFENNQ-HKVTLPLFAFHDRLVKKLRKNKK-QIELKSVFIDQLELPPRIYNCLKKSDI
HTLYQLLNKSQEDLMKIEDFSVEDVKNILNILQIDQHFALI-----MHGVGN-EGMSTLPGLN
QIQFEGFCRFIDRGLTEELLKFPKVEDTDQEIEFQLFVETYQLVEPSIKEKDAVYESLTYSSE
FYISAGLIWKNSSAIQEQIIFIGNIPLMNSLGTSIVNGIYRVVINQILQSPGIYYRSELDHNGIS
VYTATIISDWGGRLELEMDRKARIWARVSRKQKISILVLSSAMGSNLSEILENVCYPEIFLSF
LNEREEEKIGSKENAILEFYRQFACVGGDPIFPESLCEELQKKFFQQRCELGGIGRRNMNR
RLNIDIPENNPFLLPRDILTAADHLIGMKFGMGTLDD--MNHLKNKRIRSVADLLQDQFGLA
LVRLENIIRGTISGAIR-YKWI-PTPQNLVTSTP----LTTTYESFFGLHPLSQVLDRTNPLTQLVH
GRKWSYLGPGGLTGRTASFRIRDIHPSHYGRICPIDTSEGINVGLIGSLAIHARIGRWG-SIES
PFY---EISERSKR--IRMLYLSPSRDEYYMVATGNYLALNRDIQEEQIVPARYRQEFLTIAWEQ
VHLRSIFPFQYFSIGASLIPFIEHNDANRALMSSNMQRQAVPLSRSEKCIVGTGLERQVALD
SGVPAIAEHEGKIVYKDTDKIVLFGNGNALSIPLIIYQRSNKNTCMHQKSQVEKGKCVKRG
QILADGAATVGGELALGKNVLVAYMPWEGYNSEDAVLISERLVYEDIFTSFHIRKYEIQTH

VTSHG-PERITNKIPHLEAHLLRHLDKNGIVILGSWVETGDILVGKLTPQMENE-SSYAPEDR
LLRAILGIQVSASKETCLKLPIGGRGRVIDVRWIHKKE-ASSDHPETIQIYISQKREIKVGDK
VAGRHGNKGIVSKILSRQDMPYLQDGRPVDMVFNPLGVPSRMNIGQLLECSLGLAGSKL
NRHYRIAPFDERYEQEASRKLVFSELYEASKETSNPWIFEPEYPGKSRLFDGRTGNPFEQPV
IIGKPYILKLIHQVDDKIHGRSTGHYALVTQQPLKGRANQGGQRVGEMEVWALEGFGVAH
ILQEMLTYKSDHINARQEVLETTINGGTIPKPVDAPESFRLLVRELRSLALELNHFLVSEKD
FQMQRKEV-------MIDQYKHQHLRIGSVSPEQISAWAKKILPNGEIVGEVTKPYTLHYKTNK
PEKDGLFCERIFGPIKSGICACGNYQVIEDKKDQPKFCEQCGVEFVDSRIRRYQMGYIKLA
CPVTHVWYLKRLPSYIASLLDKPLKELEGLVYCD---SFARS-VVKKPTFLRLRGSFEYEIQS
WKYSIPRFFG-QGYDTFRNREISSGAGAIREQLVDLDLRIIMDSSLVEWKELGEE-GSTNNE
WEDRKVGRRKNFLVRRMELAKHFILSNIEPEWMVLCLLPVLPPELRPIIQIDGGKLISSDIN
ELYRRVIGRNNTLIDLLTISRATPAEFVMCQEKLVQEAVDTLLDNGIRGQPMRDGQNKVYK
SFSDIIEGKEGRFRETLLGKRVDYSGRSVIVVGPSLSLHQCGLPREIAIELFHTFLIRGLIRKH
FASNMGVAKSKIREKEPIVWEILQEVMRGHPVLLNRAPTLHRLGIQAFQPILVEGRAICLHP
LVCKGFNADFDGDQMAVHVPLSLEAQAEAHLLMFSHTNLLSPAIGDPISVPTQDMLIGLY
VLTSGNRRGIGATRYNPFNC---RNSKNEKMRKNNSKYMKK-EPFFCNSYDAIGAYRQKRIN
FDSPFWLRWPIDEC-IMSS--REVPVEIHYESLGTYYEIYEHYLVIRGIKKEIRCIYIRTTVGHIS
FYREIEEAIQGFSRADSYAI------------MAERANLVFHNNVIGGTAIKRLISRLIDHFGMAYTS
HILDQVKTLGFRQATATSISLGIDDLLTIPSKGWLAQDAEQQSSILEKHNHYGNVHAVEKL
RQSIEIWYATSEYLRQEMNPNFRMTDPLNPVHIMSFSGARGNASQVHQLVGMRGLMSDP
QGQMIDLPIQSNLREGLSLTEYIISCYGARKGVVDTAVRTSDAGYLTRRLVEVVQHIVVRR
TDCGTIRGISVNTRNGMMA-ERILIQTLIGRVVADDIYIGSRCIVVRNQDIGIGLINRFIT------
FQTQPIFVRTPFTCRNTSWICRLCYGRSPIHGDLVELGEAVGIIAGQSIGEPGTQLTLRTFHT
GGVFTGGTAEYVRAPSNGKIKFNEDLVHPTRTRHGYPAFICNIELYVTIESHDIIHNVTVPPK
SFLLVQNDQYVKSEQVIAEIRAGTYTF-NLKERVQKHIYSDSEGE-MHWSTDVYHASEFMY
SNVHILPKTSHLWILSGKSCR--SDTIHFLLRKDQDQIHID--SLSNGKRNTSTLF--VSDDH-V
KHKFFSFKTFATKEKGISNY-SIFNQTICTDRSHFMDPA-IFHDTFN--LLAKRRRNRFLIPFPFI
QERKNELMPPSGVSVEIPI--NAIFHSNSIFAYFDDPQYRRQSSGITKYGIIGIDSVFEK----------
-----------VDQIFFIPEEVHILPESSSIMVRNNSLVGIGTPITCNIRSRVGGLVRLEKKKKKIKLKI
FSGNILFPGEGYKISRDSAILIPA-GTVKKK-RKESKK--MNNWIYVHW--ITTTKKK--YFVLV
RPVILYEIPDSIHFVKLFPQDLFQERDNLELKVVNYIRYGNEKSIRVISDTGIQLVRTCLVLN
WDQGK---KSSSIEEAPASFVEVSTNGLVEYFLRIDLVK-NTSYIRKRNDPSGLGLIAD-NELD
RININPF---FSIHS--KAKIQQSISQNH-TIRMLLN---RNKESRSWIILSSSNCF--QMRSFNNVK
YHN---GIKKDRIPINNKNSLGPLGI--ALQVANLNS--LYRFKT--YNQIS-IIKKLQLYK---LTEIF
QVI--K-YYLMDENDK--IFKPDPYGNIVLNPFHLSWYFLPNY----TFPIIS-LGQFICENICIAQN
GPH-LKLKSGQVLTVQ-MDSVIIRSANPYLATPGATIHGHYGEILYQGDILVTFLYEKSRSGD
ITQGLPKVEQILEIRSIDSISMNLEKRIDAWNECITRILGTPWGFLIGAELTIAQSRISLVNKIQ
KVYRSQGVHIHNRHIEIIVRQITSKVLVSE---DGMSNVFLPGELIGLLRAERTGRALEEAICY
RALLLGITKTSLNTQSFISEASFQETARVLAKAALRGRIDWLKGLKENVVLGGMIPVGTGF
KRIMDRSRARQEKRIT--LKKKK--LFEVE-I-RNLLFHHRKLLD---------------------------MTKR
YWNITLEEMVKARVHLGHRTRKWNPRMEPFILAKRKGTHIINLLRTARFLSEACNLVFDA
ASGGKQFLIVGTKKVAVDSVRRAAIRARCHYVNKKWLGGMLTNWYTTETRLRKFRDLRT
EQKTGKMNSLPKRDAARLKRQLSHLERYLGGIKYMTGLPDIVIILDQQEEYTALRECITLG
IPTISLIDTNSDPDLANFSIPANDDSIDSIRFILNKLVFAICEGRSS-QIRNFMGQKIHPLGFRLG

TTQNHDSIWFAKPRDYSENLKEDKIIRDCIKNYLQKT---SS-GVEGIGRIKIKKRIDLIQ--IYV
GLPKLFIEGEPR----RIEELQTNVQKKLNCVNRKINIAITRIPNVYRDPNILAEFIAGQLKNRI
PFRKAIQKAIELAEQAGTKGVQVQISGRIDGKEIARVEWFREGRVPLQTIRAKMDYCSYPV
RTIYGVLGIKVWIFLNNDMSRYRGPRFKKIRRLGALPGLTSKRPTDGSKLRNQSPPTQ-KSQ
YRIRLEEKQKLRFHYGLTERQLLKYVRIAGKAKGSTGQVLLQLLEMRLDNILFRLGMAST
IPQARQLVNHRHVLVNGRIVDIPSYRCKPQDIITAKGEQKSRTLIQNSLESSPR-GALPTHLT
LHPLQ--YKGLVNQIIDSKWVGLEMNELLVVEYYSRQTMSRRGTAEKKTAKSDPIYRNRLV
NMLVNRIMKHGKKSLAYKIIYRALKRIQQKTKTNPLYVLRQAIRRVTPNIAVKARRASGST
RQVPLEIGSTKGKALAIRWLLGASRKRPGRNMAFKLSSELVDAAKGSGDAIRKKEETRRM
AEANRAFAHFRVGKDTIADILTLIRNADMNGKRTVQIPLTNIIETIVKILLQEGFVENVRKH
RESNKDFLVLTLRYRR--NRKRSYKT-F--LNLKRISTPGLRIYSNYQQIPRVLGGMGIVILSTS
RGIMTDREARLERIGGEVLCYIWMAKSIRKIGSRKSGRIGS-KHPRKIYKGVIYVQASFNNII
VTVTDLRGRAISWSSAGSCGFKGSRRSTPFAAQTVAGNAIRTV--EGMKRADVIIKGPGPGR
DAALRAICRSGIRLRFLRDATPLPHNGCRAPKKRRIMPTIKQLIRNTRQPIRNGTKSPALRG
CPQRRGTCTRVYTITPKKPNSALRKVARVRLTSGFEITAYIPGIGHNLQEHSVVLVRGGRVK
DLPGVRYHIVRGTLDAVGVKDRQQGRSKYGAKKPKMAKKSLIEREKKRQKLEEKYHLIR
RSPKKEIR---KAQSFSEKWEIQAKLEALPRNSAPTRLRRRCFSTGRPRGNYRDFGLSGHVL
REMVHEGLLPGATRSSWMIKNSFIPVISQK-KKEEENPGSVEFQVFNFTNKIRRLTSHLELH
P K D Y L S Q R G L R I I L G K R Q R L L S Y L S K K N K I R Y K N L I N Q L G I R D S Q I R -
-???
??
???????????????MTR-QKKNPFVATHLLRKINKLNTKGEKEVIITWSRTSTIIPTMIGHTIAIHN
G K E H L P I Y I T D R M V G H K L G E F S S T Q N F K G H T K N D T R S R
R??
??
??
??
??
??
??
??
??
??
??
??
??
??
?????????KHQHPFESWVLELREILRESKNSFVDLDSWVQFNSAVACIRVFFHQERLIKLLDF
RIWSILFS-RNSQ-----RNLYFM---IKGVVLFVV----IYR----INNRKMVETKNIYLTGRLLP----IP
MNSIAPGENNLEDSK-DSFNIKRLIVP-LLYGPKGKKISERS--

---RRTDRSFFSDRW--LDLLRLGLNPAERSTRDQ----KLFKKERGIS-FVLSRRSE----KKETVN

LFKIILYLQN--TVSIHPIPSDPECDRVT----KDELLT--------------------------------VQNFESE--EIF
QEMVNLFNLSITKP-DLMYPRGFSIDLFSLRSRAKQ--SVNEVCNPRDESKKKSL---LVLPPL
FY--EENESFYPRIIQKWVQ-TSCGNDFQDPKPRIVLFTSN---RSVNQYGLIKNLIQIQYNTYG
--YIKNLWN---------QFNRNLEYGIQ---RYQIQNDIVNHRPRRKYTINRHFSNLKK------------S
RKNCLERSMNKDPNVYKYKWSNGGKNFQEYLDRFISEQNSR---FQVVFDRLYMNAYLID
WSEVIHKKDLSKSLCF------------------------------FFSK-FLPFFFVSFGNIPVHRSD-IHVS-ELK
RPNDPLGNRLLESIGLQIVHLKKRKPF--LLD---DYYTS-QKSKLLINEGTIS---------------------
-NRAS-------YFSMIFHDQDNWLNPVKPFHRSSLISSFYKVNRLRFLNNQYNFCFD--WNKRF
SFYV----ERS--WNFN--FMYG-QFLNILFIRNK-LFSLCDGK--KKHAFLD-RDTIS----QVSNILI
ANNFPQSGDDG-YNLDKS---FHFPTPSDPFVR--RAIYSIADISGTPLTEEEIVNFERTNCQPLS
DMNLPDSEGKNLHQYLN--FDSNMGLIHTPYSEKYLPSEQ--RKK--------HSSCLK--KSLEK
GQVYRNFKR--RLSTLYKLKRFRRYIIWFLTGTTHKYLNLILRETFSDLLAILRRSPKFQKFV
SIFHDIIYRRRILW---KKWS-------LSQRNP-ISKISSKCLHNFRVSMFTIFFNPNNES--ALISTR
LRWLNIEEF-LFSILLLLLVTGY-LVH-TLSFVSQ--FN-ELQKEFEQVKSFMIPSYMIELRKLLD
RYPPSELNSFGLKNIFLVAREQLKNFLEERRGSASGGNMLRGGGP-----AYG-VK---------------
---DLLSIIPNPANRIKFLINTRHFSHTSKAIYSLIRKRKYVKSDFFSDKIESWIMKTDFVAEKE
KEFLFQFS--ALTTE-EKRIDQILLSLT-NSDHLS-----KNNSGYQMMEQPGTIYLRYLMDIQ--K
KDLMTYEFNPFCLAERRIFLAHCQTITY--VENLRRTSSLGLPSRG---KPFSLRLTLSPP-RGVL
VIGSRGTGRSYLVRYLSTNSHVPFITVFLNEFLDYFPEGLELDYD--NDED--ENEGTIYSLLP
DFSDRCFNINKFRSED----LDCNFDFDRELDFLSTRDPIR---KDTIHK-WELKRFFLDFQFKL
AEAMSPCIIWIPDIHDLD---SSDVSVGLLANSLS---EESEKSS-K--NLIIASTHIPQKVDPALIA
PNKLNTCIKIRSLLIPQQRKHFFTLSYTKGFHLEKKMFFT--TGLGSITLGSTVRDLVALSNEA
LSISIIQKKSILDTNIIRSALHRKTWDFR---SQIRSVQDHGILFYQIGRAVSQNLLLSNCSIDPIS
IYMKKK--SCNGGDSFLYRWYYELGTSMKKLTILLYLLSCSAGSVAQDLWSLP--GPNEKSD
GITSYTPLENDY-DLVHGLLEVEGALVG-----SSQTGSKFDND--GVTLLLRTERRN-PLNMIQ
NGSRSILDQRFLYEK--YESGFEE---GEGVLDPQ----RLEED----FFNH--IVWAP--TIWRP---W
DFLFDCLERSNELGFP-YWEGSFRDK----EIYDEEDELQENDSEFLQNGTMQYETRYRSSQE
QGFFQISQFIWAPEDPLFLLFQ-EDPLVSMFLRREFFADEEISRIFLTCQTKYWKYLNNRWFR
KNTQKQEKNFELLIDRQRWFRT---NSSLS--NGFSRSN--TLSESYQYLSNLFLSN-GTLVAQIT
KTLLRKRWLFPDEMVLSI--WANNK-----MTRSR-INGNFIDKTFTIVANILLRIIPTTSGEKEA
FTYYRDG--MSAQSEGNYAEALQNYYEAMRLEIDPYDRSYILYNIGLIHTSNGEHTKALEY
YFRALERNPFLPQAFNNMAVICHY-RGEQAIRQGDSEIAESWFDKASEYWKQAIALTPGNY
I E A Q N W L K I T G R F E -
-??
???
????????
>Megaleranthis
MEKWWFNSMLSLSKSLEGIGPLGNTDGGEDPVIN---NMIH--NNVDHLFGVSDIRSLISDDT
FLVRDSNGENYSIYLDIENMIFEIED--DHSFLSELESFFSSYLNSSYPNDGSKSDNHYYDHS
MYDTQYNWNNHITSCIENYLRSEIN--IDSHISSSSDNYSKSYIYRYICSDSESLIQ---NGSD-D
MIGKY-DLDISQKYRHLWVQCENCYGLNYKKFFRLKLNICEQCGYHLKMSSSERIELLIDP
GTWGAMDEDMVSVDPIEFHSEEEPYKDRIDSYQKKTGLTEAVQTGIGQLNGISLAIGIMDF
QFMGGSMGSVVGEKITRLIEYATNRSLPLIIVCASGGARMQEGSLSLMQMAKISSALYNYQ
SNKKLFYVSILTSPTTGGVTASFGMLGDIIIAEPNAYIAFAGKRVIEQTLNKTVPDGSQEAEY

LFHKGLFDPIVPRNPLKGVLSELFQLHGFFPLNKNPIQMATLRADEISNIIRERIEQYNREVK
IVNTGTVLQVGDGIARIHGLDEVMAGELVEFEEGTIGIALNLESNNVGVVLMGDGLMIQE
GSSVKATGRIAQIPVSESYLGRVINALAKPIDGRGEISASESRLIESPAPGIISRRSVYEPMQT
GLIAIDAMIPIGRGQRELIIGDRQTGKTAVATDTILNQKGQNVICVYVAIGQKASSVAQVVT
TFQEQGVMEYTIVVAETADSPATLQYLAPYTGAALAEYFMYRERHTSIIYDDPSKQAQAY
RQMSLLLRRPPGREAYPGDVFYLHSRLLERAAKLSSSLGEGSMTALPIVETQSGDVSAYIP
TNVISITDGQIFLSADLFNSGIRPAINVGISVSRVGSAAQIKAMKQVAGKLKLELAQFAELE
AFAQFASDLDKATQNQLARGQRLRELLKQSQAAPLTVDEQIVTIYTGTNGYLDSLEIGQV
KKFLVQLRTYLKTNKPQFQEIISSTNTFTEEAQVLLKEAIQEQMERFILQEQTMRINPTTSG
PGVSALEEKNLGRIAQIIGPVLDVSFPLGKMPNIYNALVVKGQDTVGQQINVTCEVQQLL
GNNRVRAVAMSATDGLMRGMEVLDTGAPLSVPVGGATLGRIFNVLGEPVDNLGPVDTRT
TSPIHRSAPAFIQLDTKLSIFETGIKVVDLLAPYRRGGKIGLFGGAGVGKTVLIMELINNIAK
AHGGVSVFGGVGERTREGNDLYMEMKESGVINEKNIAESKVALVYGQMNEPPGARMRV
GLTALTMAEYFRDVNEQDVLLFIDNIFRFVQAGSEVSALLGRMPSAVGYQPTLSTEMGSL
QERITSTKEGSITSIQAVYVPADDLTDPAPATTFAHLDATTVLSRGLAAKGIYPAVDPLDSTS
TMLQPRIVGEEHYETAQRVKQTLQRYKELQDIIAILGLDELSEEDRLTVARARKIERFLSQP
FFVAEVFTGSPGKYVGLTETIRGFQLILSGELDGLPEQAFYLVGNIDEATAKAMNLDVESKL
KKMTLNLCVLTPNRIVWDSEVKEIILSTNSGQIGVLPNHAPIATAVDIGILRIRLNDQWLTIA
LMGGFARIGNNEITILVNDAERGSDIDPQEAQQTLEIAEASLSSAEGKRQTIEANLALRRAR
TRVEAINAISMKNVTDSFVSLGHWPSAGSFGFNTDILATNPINLSVVLGVLIFFGKGVLSDL
LDNRKQRILSTIRNSEELREGAIEKLEKAKARLRKVQMEADEFRVNGYSEIEREKFNLINST
YQNLERLENYKNETIQFEQQRAINQVRQRVFQQALEGALGTLNSCLNNELHLRTISANIG
MFGAMKEITDMNPLISAASVIAAGLAVGLASIGPGVGQGTAAGQAVEGIARQPEAEGKIR
GTLLLSLAFMEALTIYGLVVALALLFANPFVMNVLPCSINTLKRLYDISGVEVGQHFYWQI
GGFQVHAQVLITSWFVIAILLGSATIAVRNPQTIPTDGQNFFEYVLEFIRDLSKTQIGEEYGP
WVPFIGTMFLFIFVSNWSGALLPWKIIQLPHGELAAPTNDINTTVALALPTSVAYFYAGLTK
KGLGYFGKYIQPTPILLPINILEDFTKPLSLSFRLFGNILADELVVVVLVSLVPSVVPIPVMFL
GLFTSGIQALIFATLAAAYIGESMEGHHMIFFTLEHILTHISLSIVSIVIFIYFMNLLVHEIVGL
QNSSEKGMAATFLCITGSLVIRWIYSGHLPLSDLYESLMFLSWSFSIIHMVPYFRNHKNNLH
AITAPSAIFTQGFATSGLLTEIHQSTILVPALQSQWLMMHVSMMVLSYAALLCGSLLSVALL
VIAFRKNIDIFSKYKEIQYVNKIS-VLQNNCFISFRNYRRYRLIQQLDCWSSRVISLGFIFLTIG
ILSGAVWANEAWGSYWNWDPKETWAFITWTIFAIYLHTRTNANLQASNSAIVASMGFFFI
WICYFGVNLLGIGLHSYGSFALTMLKKKALKPLLYLTSIIFLPWSISLSFNKSMESLVTNWW
NAGQSEVFLNDIHEKNVLEKFIELEELFILDEMIKEYPDTHIQKLRIGIHKETIQLVKMYNE
GHIHTILHFSTNIISFAILSGYSILGNKELVIINSWVREFIYNLSDTIKAFSILLLTDLCIGFHSP
HGWELMIGSVYKDFGFSHNNQIISGLVSTFPVILDTILKYWIFRYLNRVSPSLVVIYHSMND
MPIGVPKVPFRSPGEEDAVWVDVY-RLHRERLLFLGQEVDSEISNQLIGLMVYLSIEDEIRD
LYLFINSPGGWVIPGVAIYDTMQFVPPDVHTICMGLAASMGSFILVGGEITKRIAFPHARVM
IHQPASSFYEAQTGEFVLEAEELLKLRETLTRVYVQRTGNPLWVVSEDMERDVFMSATEA
QAHGIVDLVAVEMKEQKWIHEGLITESLPNGMFRVRLDNEDLILGYVSGRIRRSFIRILPGD
RVKIEVSRYDSTRGRIIYRLRNKDSNDMEELQGYLKIDRSRERDFLYPLLFQEYIYALAHDH
GLENLG-KYSSLIVKRLIIRMYQQKHFFILANDSNQNKFVGKNFYSQMISEGFAVIVEIPFSL
RVVSSLIAKSFNLRSIHSIFPFLEEKLSHLNHVLDILIPYPIHLELLVQTLRCWIQDAPFLHLL
RFFLYEYHNWNSLITKINFFFSKQRLFLFLYNFYVYESESILVFLRKQSSHLRSTSSRAFLDR

THFYGKIEHFLVVFHNDFIPWLFKDPFMHYFRYQGKSIMSSKGTPLLMKKWKYYLVNLW
ECRFYFWSQPDRIHINQLSHNFLDFLGYLSNVRPNPSVVRSQMLENSFIIDIAI--NKFDTIVP
IIPLIGSLAKAKFCNLSGHPISKPAWADSSDSDIIDRFGRICRNVSHYYSGSSKKKSLYRIKYI
LRLSCARTLARKHKSTVRSFLKRLGSEFLEEFLIEEEQVLSFILPRRVWYFNIIRINDLTNHS
MIIDTT-KVQ-AINSFSRSE-SLKEIYGLMRLLVPIFTPVSGIIIGVLVIVWLERQISAGVQQRIG
PEYAGPLGILQALADGTKLLFKEDLLPSRGDISLFSIGPSIAVISILLSYSVIPFGYRLVLADLS
IGVFLWIAISSIAPIGLLMSGYGSNNKYSFLGGLRAAAQSISYEIPLTPCVLSISLLSNSSSTV
DIVEAQSKYGFWGWNLWRQPIGFAVFLISSLAECERLPFDLPEAEEELVAGYQTEYSGIKF
GLFYVASYLNLLVSSLFVTVLYLGGWNLSIPYILIPELFGI-EVGGVFGTTIGIFITLAKAYLFL
FIPITTRWTLPRMRMDQLLNLGWKFLLPISLGNLLLTTSSQLFSLMIWHVQNENFILDSTRI
FMKAFHLLLFHGSFIFPECILILGLILLLMIDLTSDQKDIPWLYFISSTSLVMSIAALLFRWRE
EPMISFSGNFQTNNFNEIFQFLILLCSTLCIPLSVEYIECTEMAITEFLLFILTATLGGMFLCGA
NDLITIFVAPECFSLCSYLLSGYTKRDVRSNEATTKYLLMGGASSSILVHGFSWLYGSSGGE
IELQEIVNGLINTQMYNSPGISIALIFITVGIGFKLSPAPSHQWTPDVYEGSPTPVVAFLSVTS
KVAASALATRILDIPFYFSSNEWHLLLEILAILSMILGNLIAITQTSMKRMLAYSSIGQIGYVI
IGIIVGDSNDGYASMITYMLFYISMNLGTFACIVSFGLRTGTDNIRDYAGLYTKDPFLALSL
ALCLLSLGGLPPLAGFFGKLHLFWCAWQAGLYFLVSIGLLTSVVSIYYYLKIIKLLMTGRN
QEITPHVRNYRRSPLRSNNSIELSMIVCVIASTIPGISMNPIIAIAQDTLFMFLLHEYDIFWAF
LIISSVIPILAFIISGVLAPINEGPEKLSSYESGIEPMGDAWLQFRIRYYMFALVFVVFDVETV
FLYPWAMSFDVLGVSVFIEALIFVLILIVGSVYAWRKGALEWSTNDFPWLTIIVVLPIFAGTF
ILFFPHRGNKVIRWYTICICLLELLLTAYAFCYYFQLDDPLIQLEQDYKWINFFDFHWRLGI
DGLSIGPILLTGFITTLATLAACPVTRDSRLFHFLMLAMYSGQIGSFSSRDLLLFFIMWELEL
IPVYLLLSMWGGKKRLYSATKFILYTAGGSIFLLMGVLGMGLYGSNEPTLNFETSANQSYP
VALEIILYFGFLIAYAVKLPIIPLHTWLPDTHGEAHYSTCMLLAGILLKMGAYGLVRINMELL
PHAHSIFSPWLMIVGAIQIIYAASTSSGQRNLKKRIAYSSVSHMGFIIIGIGSITDTGLNGAIL
QMISHGFIGAALFFLAGTSYDRIRLVYLDEMGGIAIPMPKIFTMFSSFSMASLALPGMSGFV
AELVVFFGLITSQKYLLMPKILITFVTAIGMILTPIYSLSMLRQMFYGYKQFNSQNSPFLDSG
PRELFISICIFLPVIGIGIYPDFVLSLSIDKVETIISNYFYRMMLEHVLVLSSYLLSIGIYGLITS
RNMVRALMCLELILNAVNINFVTFSDLFDSRQLKGDVFSIFVIAIAAAEAAIGLAIVSSIHRN
RKSTRINQSNLLNKMEHIYQYAWIIPFIPLPVPIAIGLGLLLVPAATKSIRRIWAFPSVLLLSIV
MLFSADLSIQQINGSPIYQYVWSWTINNDFSLDFGHFMDPLTSIMLILITTVGIMVLIYSDN
YMSHDQGYLRFFSYMSFSNASMLGLVTSSNLIQIYFFWELVGMCSYLLIGFWFTRPLAAS
ACQKAFVTNRVGDFGLFLGILGLYWITGSFEFRDLFEIAKNNGANSLFLTLCASLLFVGAV
AKSAQFPLHVWLPDAMEGPTPISALIHAATMVAAGIFLIARLLPLFTVIPYIMYIISLVGIITIL
LGATLALAQRDIKRSLAYSTMSQLGYIMLALGMGSYRAALFHLITHAYSKALLFLGSGSII
HSMETIVGYSPDKSQNMALMGGLTKYVPITKTSFLLGTLSLCGFPPLACFWSKDEILNDS
WLYSPIFAIIACFTAGLTAFYMFRMYLLTFEGHLNFNFKNYSGKKNNAFYSISIWGKKGQK
AIKKK-KNLSTMNLFSFPNKKTSTYPYESDNTMLLPLFVLVLFTLFVGSIGIPFYFDILSKWL
TPSINLLHSNSN-FFDWYEFVINAIFSVSIAYFGIFIASLLYGPAYSYFQNLNLINFFVLVWDKI
INVIYNWSYNRGYIDIFYEKTFTRCIRGLAELTQFFDRQVIDGITNGIGVTSFFVAEIFKYLG
GG-RISSYLFFFLVYVSIFVLIYYMDLPGPIHDFLVVFLGLGLIVGGLGVVLLTNPIFSAFSLG
LVLVCTSLFHIPSNSHFVAAAQLLIYVGAINVLIVFAVMFMNGSEYYNDFHLWTVGDGVTS
LVCTSIFVSLITTIPDTSWYGIIWTTRSNQILEQDLINNGQQIGIHLSTDFFLPFELISIILLVALI
GAIAMARQMTIPTTRKDLMIVNMGPQHPSMHGVLRLIVTLDGEDVIDCEPVLGYLHRGM

EKIAENRTIIQYLPYVTRWDYLATMFTEAITVNGPEQLGNIQVPKRASYLRVIMLELSRIAS
HLLWLGPFMADIGAQTPFFYILRERELIYDLFESATGMRMMHNYFRIGGVAADLPYGWID
KCLDFCDYFLTGVTDYQKLITRNPIFLERVEGVGIIGGEEAINWGLSGPMLRASGIQWDLR
KVDHYECYDEFDWEVQWQKEGDSLARYLVRINEMTESIKIIQQALEGIPGGPHENLEVRR
FDRTKDSEWNDFDYRFISKKPSPTFELSKQELYVRVEAPKGELGIFLIGDRSVFPWRWKIRP
PGFINLQILPQLVKRMKLADIMTILGSIDIIMGEVDRMFPMVTGFMNYGKQTVRAARYIGQ
SFMITLSHTNRLPVTIQYPYEKLITSERFRGRIHFEFDKCIACEVCVRVCPIDLPVVDWKLET
DIRKKRLLNYSIDFGICIFCGNCVEYCPTNCLSMTEEYELSTYDRHELNYNQIALGRLPMS
VIGDYTIRTIMNSKKSVAKPLDSRTITNFMQGRLSAWLVKHRLVHRSLGFDYQGIETLQIKP
EDWHSIAVISYVYGYNYLRSQCAYDVAPGGLLASVYHLTRIQYGVDQPEEVCIKVFAPRR
NPRIPSVFWIWKSADFQERESYDMLGISYDNHPRLKRILMPESWIGWPLRKDYIAPNFYEI
QDAH---MNYIEFPLLDRTIQNSVISTTSNDLSN
WSRLSSLWPLLYGTSCCFIEFASLIGSRFDFDRYGLVPRSSPRQADLILTAGTVTMKMAPSL
VRLYEQMPEPKYVIAMGACTITGGMFSTDSYSTVRGVDKLIPVDVYLPGCPPKPEAVIDAI
TKLRKKVSREIYEDRIGSQRETRCFTTNHKFHIGRSTHTGNYDQGLLYQSPSTSEIPNETFL
K------------MQNRNIFSWI-EQMTRSISVSIIIFIITRTSISHAYPIFAQQSYENPREATGRIVCAN
CHLANKPVDIEVPQAVLPDTVFEAVVRIPYDIQLKQVLSNGKRGSLNVGAVLILPEGFELA
PPDRISPEMKEKMGNLSFQSYRPTKKNILVIGPVPGQKYSEITFPILSPDPVTKKEVYFLKYP
IYVGGNRGRGQIYPDGSKSNNTVYNATASGTVSKIVRKEKGGYEITIADASDGHSVVDIIPP
GPELLVSEGESIKLDQPLTSNPNVGGFGQGDAEIVLQDPSRVQGLFLFLASVILAQIFLVLK
KKQFEKVQLSEMNFMSKVYDWFEERLEIQAIADDITSKYVPPHVNIFYCLGGITLTCFLVQ
VATGFAMTFYYRPTVTEAFASVQYIMTEANFGWLIRSVHRWSASMMVLMMILHVFRVYL
TGGFKKPRELTWVTGVVLAVLTASFGVTGYSLPWDQIGYWAVKIVTGVPDAIPVIGSPVVE
LLRGSASVGQSTLTRFYSLHTFVLPLLTAVFMLMHFPMIRKQGISGPLMGVTKKPDLNDPV
LRAKLAKGMGHNYYGEPAWPNDLLYIFPVVILGTIACNVGLAVLEPSMIGEPADPFATPLEI
LPEWYFFPVFQILRTVPNKLLGVLLMVSVPAGLLTVPFLENVNKFQNPFRRPVATTVFLIGT
AVALWLGIGATLPIDKSLTLGLFMIEVLLFGIVLGLIPITLAGLFVTAYLQYRRGDQLDLMPT
ITSYFGFLLAALTITSALFIGLSKIRLIMDIVSLAWAALMVVFTFSLSLVVWGRSGLMIIRSPE
SEVKILVDRDPVKTSFEEWARPGHFSRTIAKGPDTTTWIWNLHADAHDFDSHTSDLEEISR
KVFSAHFGQLSIIFLWLSGMYFHGARFSNYEAWLSDPTHIGPSAQVVWPIVGQEILNGDVG
GGFRGIQITSGFFQIWRASGITSELQLYCTAIGALVFAALMLFAGWFHYHKAAPKLAWFQD
VESMLNHHLAGLLGLGSLSWAGHQVHVSLPINQFLDAGVDPKEIPLPHEFILNRDLLAQL
YPSFAEGATPFFTLNWSKYAEFLTFRGGLDPVTGGLWLTDIAHHHLAIAILFLIAGHMYRTN
WGIGHGLKDILEAHKGPFTGQGHKGLYEILTTSWHAQLSLNLAMLGSLTIVVAHHMYSMP
PYPYLATDYGTQLSLFTHHMWIGGFLIVGAAAHAAIFMVRDYDPTTRYNDLLDRVLRHR
DAIISHLNWACIFLGFHSFGLYIHNDTMSALGRPQDMFSDTAIQLQPVFAQWIQNTHALAP
GVTAPGATTSTSLTWGGGGLVAVGGKVALLPIPLGTADFLVHHIHAFTIHVTVLILLKGVLF
ARSSRLIPDKANLGFRFPCDGPGRGGTCQVSAWDHVFLGLFWMYNAISVVIFHFSWKMQ
SDVWGSINDQGVVTHITGGNFAQSSITINGWLRDFLWAQASQVIQSYGSSLSAYGLFFLGA
HFVWAFSLMFLFSGRGYWQELIESIVWAHNKLKVAPAIQPRALSIVQGRAVGVTHYLLGGI
ATTWAFFLARIIAVGMALRFPRFSQGLAQDPTTRRIWFGIATAHDFESHDDITEERLYQNIFA
SHFGQLAIIFLWTSGNLFHVAWQGNFESWVQDPLHVRPIAHAIWDPHFGQPAVEAFTRGG
ALGPVNIAYSGVYQWWYTIGLRTNEDLYTGALFLLFLSAISLIAGWLHLQPKWKPSVSWF
KNAESRLNHHLSGLFGVSSLAWTGHLVHVAIPASRGEYVRWNNFLDVLPHPQGLGPLFTG

QWNLYAQNPDSGSHLFGTSQGGGTAILTLLGGFHPQTQSLWLTDIAHHHLAIAIIFLVAGH
MYRTNFGIGHSIKDLLEAHIPPGGRLGRGHKGLYDTINNSIHFQLGLALASLGVITSLVAQH
MYSLPAYAFIAQDFTTQAALYTHHQYIAGFIMTGAFAHGAIFFIRDYNPEQNEDNVLARML
DHKEAITSHLSWASLFLGFHTLGLYVHNDVMLAFGTPEKQILIEPIFAQWIQSAHGKTSYG
FDVLLSSTNGPAFNAGRSIWLPGWLNAINE--NSNSLFLTIGPGDFLVHHAIALGLHTTTLIL
VKGALDARGSKLMPDKKDFGYSFPCDGPGRGGTCDISAWDAFYLAVFWMLNTIGWVTF
YWHWKHITLWQGNVSQFNESSTYLMGWLRDYLWLNSSQLINGYNPFGMNSLSVWAWM
FLFGHLVWATGFMFLISWRGYWQELIETLAWAHERTPLANLIRWRDKPVALSIVQARLVGL
AHFSVGYIFTYAAFLIASTSGKFGMSHSVKIYDTCIGCTQCVRACPTDVLEMIPWDGCKAK
QIASAPRTEDCVGCKRCESACPTDFLSVRVYLWHETTRSMGLAYMTTFNLPSFFVPLVGLV
FPAIAMASLSLHVQKNKIVMRDLKTYLSVAPVLSTLWFGSLAGLLIEINRLFPDALTFPFFSF
MTAILERRESESLWGRFCNWVTSTENRLYIGWFGVLMIPTLLTATSVFIIAFIAAPPVDIDGI
REPVSGSLLYGNNIISGAIIPTSAAIGLHFYPIWEAASVDEWLYNGGPYELIVLHFLLGVACY
MGREWELSFRLGMRPWIAVAYSAPVAAATAVFLIYPIGQGSFSDGMPLGISGTFNFMIVFQ
AEHNILMHPFHMLGVAGVFGGSLFSAMHGSLVTSSLIRETTENESANEGYRFGQEEETYNI
VAAHGYFGRLIFQYASFNNSRSLHFFLAAWPVVGIWFTALGISTMAFNLNGFNFNQSVVDS
QGRVINTWADIINRANLGMEVMHERNAHNFPLDLAAVEVPSTNGMGLPWYRVHTVVLN
DPGRLLAVHIMHTALVSGWAGSMALYELAVFDPSDPVLDPMWRQGMFVIPFMTRLGITNS
WGGWSIT-GGTITNPGIWSYEGVAGAHIVFSGLCFLAAIWHWVYWDLEIFCDERTGKPSLD
LPKIFGIHLFLSGLACFGFGAFHVTGLYGPGIWVSDPYGLTGKVQSVNPAWGVEGFDPFVP
GGIASHHIAAGTLGILAGLFHLSVRPPQRLYKGLRMGNIETVLSSSIAAVFFAAFVVAGTM
WYGSATTPIELFGPTRYQWDQGYFQQEIYRRVGAGLSENQSLSEAWSKIPEKLAFYDYIGN
NPAKGGLFRAGSMDNGDGIAVGWLGHPVFRDKDGRELFVRRMPTFFETFPVVLVDGDGI
VRADVPFRRAESKYSVEQVGVTVEFYGGELDGVNYSDPATVKKYARRAQLGEIFELDRAT
LKSDGVFRSSPRGWFTFGHASFALLFFFGHIWHGARTLFRDVFAGIDPDLDAQVEFGAFQ
KLGDPTTRRQGVMKTLYSPRRFYPVETLFNGTLALAGRDQETTGFAWWAGNARLINLSG
KLLGAHVAHAGLIVFWAGAMNLFEVAHFVPEKPMYEQGLILLPHLATLGWGIGPGGEVID
TFPYFVSGVLHLISSAVLGFGGIYHSLLGPETLEESFPFFGYVWKDRNKMTTILGIHLILLGI
GAFLLVLKALYFGGVYDTWAPGGGDVRKITNLTLSPSVIFGYLLKSPFGGEGWIVSVDDLE
DIIGGHVWLGSICIFGGIWHILTKPFAWARRALVWSGEAYLSYSLAALSVFGFIACCFVWFN
NTAYPSEFYGPTGPEASQAQAFTFLVRDQRLGANVGSAQGPTGLGKYLMRSPTGEVIFGG
ETMRFWDLRAPWLEPLRGPNGLDLSRLKKDIQPWQERRSAEYMTHAPLGSLNSVGGVAT
EINAVNYVSPRSWLATSHFVLGFFLFVGHLWHAGRARAAAAGFEKGIDRDLEPVLFMTPL
NMTIALGRVTKDEKDLFDTMDDWLRRDRFVFVGWSGLLLFPCAYFALGGWFTGTTFVTS
WYTHGLASSYLEGCNFLTAAVSTPANSLAHSLLLLWGPEAQGDFTRWCQLGGLWTFVAL
HGAFGLIGFMLRQFELARSVQLRPYNAIAFSGPIAVFVSVFLIYPLGQSGWFFAPSFGVAAIF
RFILFFQGFHNWTLNPFHMMGVAGVLGAALLCAIHGATVENTLFEDGDGANTFRAFNPT
QAEETYSMVTANRFWSQIFGVAFSNKRWLHFFMLFVPVTGLWMSALGVVGLALNLRAY
DFVSQEIRAAEDPEFETFYTKNILLNEGIRAWMAAQDQPHENLIFPEEVLPRGNALMSGST
GERSFADIITSIRYWVIHSITIPSLFIAGWLFVSTGLAYDVFGSPRPNEYFTENRQGIPLITGRF
DSLAQLDEFSRSFMTIDRTYPIFTVRWLAVHGLAVPTVSFLGSISAMQFIQRMATQTVEGSS
RSG-PRRTTVGNLLKPLNSEYGKVAPGWGTTPLMGNRNGSICGIPIYYLEIYNSSVLLDGIS
MNMLTLKLFVYTVVIFFVSLFIFGFLSNDPGRNPGREEMADTTGRIPLWLIGTVAGILVIGLI
GVFFYGSYSGLGSSLMLNIFNLICIYLNSILHSSHFFFAKLPEAYAFFNPIVDVMPVIPLFFFL

LAFVWQAAVSFRMTQSNPNEQSVELNRTSLYWGLLLIFVLAVLFSNYFFNMEVNILAFIAT
ALFILVPTAFLLIIYVKTVSQNDMETATLVAIFISGLLVSFTGYALYTAFGQPSQQLRDPFEEH
GDMEALVYTFLLVSTLGIIFFAIFFREPPKVPTKK--MTIAFQLAVFALIATSSILLISVPVVFAS
SDGWSSNKNVVFSGTSLWIGLVFLVAILNSLIS-------MSPQTETKASVGFKAGVKDYKLNY
YTPEYEPKDTDTLAAFRVTPQPGVPPEEAGAAVAAESSTGTWTTVWTDGLTSLDRYKGRC
YHIEPVAGEENQYICYVAYPLDLFEEGSVTNMFTSIVGNVFGFKALRALRLEDLRIPVAYVK
TFQGPPHGIQVERDKLNKYGRPLLGCTIKPKLGLSAKNYGRAVYECLRGGLDFTKDDENV
NSQPFMRWRDRFLFCAEAIYKAQAETGEIKGHYLNATAGTCEEMMKRAVFARELGVPIV
MHDYLTGGFTANTSLAHYCRDNGLLLHIHRAMHAVIDRQKNHGMHFRVLAKALRMSGG
DHIHAGTVVGKLEGEREITLGFVDLLRDDYIEKDRSRGIYFTQDWVSLPGVLPVASGGIHV
WHMPALTEIFGDDSVLQFGGGTLGHPWGNAPGAVANRVALEACVQARNEGRDLAREGN
EIIREACKWSLELAAACEVWKEIKFEFEAMDTL--TAIHLYKTSTPSTRNGAVGSQVKSNTR
NNLIYGQRRCGKGRNARGIITARHRGGGHKRLYRKIDFRRNEKDISGRIVTIEYDPNRNAYI
CLIHYGDGEKRYILHPRGAIIGDTIVSGTEVPISMGNALPLTDMPLGTAIHNIEITLGKGGQL
ARAAGAVAKLIAKEGKLATLKLPSGEVRLISKNCSATVGQVGNVGVNQKSLGRAGSKRW
LGKRPVVRGVVMNPVDHPHGGGEGRAPIGRKKPTTPWGYPALGRRSRKRNKYSDSLILR
RRSKMIQPQTYLNVADNSGARELMCIRIIGAS-NRRYAHIGDVIVAVIKEAVPNMPLQRSEVI
RAVIVRTCKELKRDNGMIIQYDDNAAVVIDQEGNPKGTRVFGAIARELRQLNFTKIVSLAP
EVLMLSPKRTRFRKQHRGRMKGISYRGNRICFGGYALQALEPAWITSRQIEAGRRAMTRY
ARRGGKIWVRIFPDKPVTVRPTETRMGSGKGSPEYWVSVVKPGRILYEMGGVSEIVAREA
ISIAASKMPIRTQFVIAGMTRIRRGYIARRRRTKIRLFASTFRGAHSRLTRSTIQQKMRALVS
AHRDRGRQKRNFRRLWITRINAAIRES--VSHSYSRSIHDLYKRQLLLNRKILAQIAISNMNC
LYMISNKIIK-----------MIKEVYALGQHIRMSAHKARRVIDQIRGRPYEETLMILELMPYRAC
YPIFKLVYSAAANARHNMGFNETALIISQAEVNEGTIMKKLKPRARGRSYAIKKPTCHITIV
LKDISKEEVLWDKKMDGIKYAVFTDKSIRLLGNN-QYTSNVESGSTRTEIKHWVELFFGVK
VIAMNSHRLPGKGRRMGPIMGHTMHYRRMIITLQPGYSIPPLRKKRTMAVPKKRTSMSKK
RIRKNFWKNKAYWASIKAFSLAKSLSTGKSKSFFVRQTSNMAKGKDVRVRVILECNSCVR
NDIN--KESRGISRYITQKNRHNTPSRLELRKFCPHCYKHTIHGEIKKMKIRASVRKICEKCR
LIRRRGRIIVICSNPRHKQRQGMLREEVAVSTRTLQWKCVESRADSKRLYYGRFILSPLMK
GQADTIGIAMRRALLGEIEGTCITRAKSE--KIPHEYSTIVGIEESVHEILMNLKEIVLRSNLY
GTRDASICVKGPGYVTAQDIISPPSVEIVDITQHIASLTEPIDLCIELQIERNRGYRMKTPNNA
QERSY--PIDAVFMPVRNANHSIHSYGNGNEKQEILFLEIWTDGSLTPKEALYEASRNLIDLFI
PFLHAEEQ--DTKLEDNQ-SRVTLPFFTFHDGLD-KVRKNKK--IELKCIFIDQSELPSRTYNCL
KRFNIHTLLDLLNKSQEDLMKMEHFRIEDVRRILGIIKN-----IS-----MLRDGN-EGMSTIPGF
SQIQFEGFCRFIDQGLTEELYKFPKIEDTDQEIEFQLFVETYQLVEPLIKERDAVYESLTYSSE
LYVPAGFIWKTGRDTQEQTIFIGKIPLMNSLGTSIVNGIYRIVINQILQSPGIYYRSELDHNGI
SVYTGTIISDWGGRSELEIDRKARIWARVSRKQKISILVPSSAMGSNLREIIDNVCYPEIFLSF
PNDKEKKKIGSKENAILEFYQQFACVGGDPVFSESLCKELQKKFFQQRCELGRIGRRNMN
RRLNLDIPQNITFLLPRDILAAADHLIRMKFGMGTLDD--MNHLKNKRIRSVADLLQDQFG
LALVRLENAVRGTIGGAIR-HKLI-PTPHNLVTSTP----LTTTYESFFGLHPLSQVLDRTNPLTQ
IVHGRKLSYLGPGGLTGRTASFRIRDIHPSHYGRICPIDTSEGINVGLIGSLAIHARIGHWG-S
LERPFY---EISDKST-KELRVVYLSPSRDEYYMVAAGNSLALNQGIQEEQVVPARYRQEFLTI
AWEQIHLRSIFPFQYFSIGASLIPFIEHNDANRALMSSNMQRQAVPLSRSEKCIVGTGLERQ
AALDSGVSAIAEHEGKIVYTDTDKIMLSGNGDTLSIPLVMYQRSNKNTCMHQKTRVPRGK

CIKKGQILADGAATVGGELALGKNVVVAYMPWEGYNFEDAVLISERLVYGDIYTSFHIRK
YEIKTHVTSQG-PERITNEIPHLEAHLLRNLDRNGIVMLGSWVETGDILVGKLTPQMAKE-S
SYAPEDRLLRAILGIQVSTAKETCLKLPIGGRGRVIDVRWIQKKG-GSSYNPETIRVYISQKR
EIKVGDKVAGRHGNKGIISKILPRQDMPYLQDGTPVDMVFNPLGVPSRMNVGQIFECSLG
LAGDLLDRHYRISPFDERYEQEASRKLVFSELYEASKQTANPWVFEPEYPGKSRIFDGRTG
DPFEQPVIIGKSYILKLIHQVDDKIHGRSSGHYALVTQQPLRGRAKQGGQRVGEMEVWAL
EGFGVAHILQEMLTYKSDHIRARQEVLGTTIIGGTISNPEDAPESFRLLVRELRSLALELNHF
LVSEKNFQINRKEA-------MIDRYKHQQLRIGLVSPQQIIAWAKKTLPNGEMVGEVTKPYTF
HYKTNKPEKDGLFCERIFGPIKSGICACGNYRVIGDEKEDPKFCEQCGVEFVDSRVRRYQM
GYIKLACPVTHVWYLKRLPSYIANLLDKPLKELEGLVYCD--FSFARP-VVKKPTFLRLRGS
FEYEIQSWKYSIPLFFTTQGFDTFRNREISTGASAIREQLADPDLRLITDSSLLEWKELGEE-
GPTGNEWEDRKIGRRKDFLVRRMELAKHFIRTNVEPERMVLCLLPVLPPELRPIIQIDGGK
LMSSDINELYRRVIYRNNTLTDLLTTSRSTPGELVMCQEKLVQEAVDTLLDNGIRGQPMRD
GHNKVYKSFSDVIEGKEGRFRETLLGKRVDYSGRSVIVVGPSLSLHRCGLPREIAIELFHTF
VIRGLIRQHVASNIGVAKSKIREKAPIVWEILQEVMQGHPVLLNRAPTLHRLGIQAFQPILV
EGRAICLHPLVCKGFNADFDGDQMAVHIPLSLEAQAEARLLMFSHMNLLSPAIGDPISVPT
QDMLMGLYVLTIGNRRGICANRYNPWNHRN---YQNEKIDDNNHKYT--KEPYFCSSYDAL
GAYWQKRINLDSPLWLRWRLDQR-VIAP--REVPIEVQCESFGTYHEIYGHYLIVRNVKKEI
LCIDIRTTVGHISFYREIEEAIQGFCRACSYST------------MAERADLVFHNKAIDGTAMKRLI
SRLIDHFGMAYTSHILDQVKTLGFQQATSTSISLGIDDLLTIPSKGWLVQDAEQQSLILEKH
HHYGNVHAVEKLRQSIEIWYATSEYLRQEMNPNFRMTDPSNPVHIMSFSGARGNASQVH
QLVGMRGLMSDPQGQMIDLPIQSNLREGLSLTEYIISCYGARKGVVDTAVRTSDAGYLTRR
LVEVVQHIVVRRTDCGTVRGISVSPRNGMIT-ERIFIQTLIGRVLADDIYMGSRCIAIRNQDI
GIGLVNRFIT------FRAQPISIRTPFTCRSTSWICRLCYGRSPTHGDLVELGEAVGIIAGQSIGE
PGTQLTLRTFHTGGVFTGGTAEHVRAPDNGKIKFNEDLVHPTRTRHGYPAFLCYIDLYVTIE
GQDILHNVNIPPKSFILVKNDQYVESEQVIAEIRAEASTL-NFKERVRKHIYSDSEGE-MHWS
TAVYHAPEYTYGNVHLLPKTSHLWILSGVPYR--SSIVPFSLHKDQDQINIH--SLSSERKYIS
N--LSVTNDQ-VRHKLFRSDPSG--RERVLYY-SEPDRIIWNGHCNFIYPA-ILDDNSD--LLAKR
RRNRFIIPFQSNQEKE--PIPRSGISIEIPIN--GLFRRNSILAYFDDPRYRRKSSGITKYGTIEVDS
IVKKED--LIEYRR--KEFRPKYMKIDRFFFIPEEVHILPGSSSIMVRNNSIIGVATRITFNIRSRV
GGLVRVEKKKKKIELKIFSGDIYFPGETDKISWHSGILIPPGTVKK--NSKESK--TLKNWIYV
QR--VTPTKKK--YFVLVRPVVTYETADGINLATLFPQDPLQERDNVQLRVVNYILYGNGKPI
RGISHTNIQLARTCLVLNWDQDKK---GSSIEEVHASFVEVRVNDLIRDFIRIDLVKLPISYTR
KRNDLAGSGLISA-NGSDRTNLNP-FYS--KAR-----IKQLLIRHQGTIRTLLNR---NKECQSLIT
LSSSNCFR--IGLFNGLKYNNNN-MTKKSDPLLPIRNLLGPLGI--VPKITHFFS--FYYLIT--HN
QIL-VNKHSLIDN---LKQTFQVLKLK-YYLMDENGI--IYNLDPCSNMILNLFNWNWYFLHH
DYCEEISTIIS-LGQFFCENVCISKYGP---HLKSGQVLIVH-VDSLVIRSAKPYLATPGATVHG
HYGEILYEGDTLVTFIYEKSRSGDITQGLPKVEQVLEVRSIDSISMNLEKRVEGWNERITRIL
GIPWGFLIGAELTIAQSRISLVNKIQKVYRSQGVQIHNRHIEIIVRQITSKVLVSE---DGMSNV
FSPGELIGLLRAERTGRALEEAICYRAVLLGITRASLNTQSFISEASFQETARVLAKAALRGR
IDWLKGLKENVVLGGMIPVGTGFKGLVHRL--SQHSNIS--LEIEKKNLFEGGMG--DILFHH
KELFDSCTQKNFHDTAEQSFTGFTDS------MTRRYWNINLEEMMEAGVHFGHGTRKWNP
RMAPYISAKRKGIHITNLTRTARFLSEACDLVFDAASRGKQFLIVGTKNKAADSVASASIRA
RCHYVNKKWLGGMLTNWSTTETRLHKFRDLRAEQKTGRLNRLPKRDAAMLKRQLSHL

QTYLGGIKYMTGLPDIVIIVDQQEEYTALRECVTLGIPTICLIDTNCDPDLADISIPANDDAI
ASIRLILNKLVSAILEGRSS-YIRNRMGQKINPLGFRLGTTQSHHSVWFSQPKSYSEGLQED
QKIQNCIKNYVQKNMKISSG-VEGIARIKIQKRIDLIQVIIYMGFPKLLIEGSPR----GIEELQI
NVQKELNSVTRKLNIAITRVSKPYGQPNILAEFIAGQLKNRVSFRKAMKKAIELTEQADTK
GIQVQIAGRIDGKEIARVEWIREGRVPLQTIRAKIDYCSYTVRTIYGALGIKIWIFTDEEMSR
YRGPRFKKIRRLGVLPGLTSKRPTPGSDLRNQSRSGK-RSQYRIRLEEKQKLRFHYGLTERQ
LLKYVHIAGKAKGSTGQVLLQLLEMRLDNILFRLGMASTIPGARQLVNHRHILVNGRIVDI
PSYRCKPRDIITTRDEQRSRALIQNYLDSSPH-EELPKHLTLHSSQY--KGSVNQIIDSKWVGL
KINELLVVEYYSRQTMSRRGTAEEKTAKSDPIYRNRLVNMLVNRILKHGKKSLAYQIIYRA
LKKIQQKTETNPLSVLRQAIRGVTPDIAVKARRVGGSTHQVPIEIGSTQGKALAIRWLLGAS
RKRPGRNMAFKLSSELVDAAKGSGDAIRKKEETHKMAEANRAFAHFRMGRDTIAEIITSI
RNADMDKKGTVRIASTNITENIVKILLREGFIENVRKHRASKKCFLVLTLRHRR--NRKGPY
R---TVL--KRISRPGLRIYSNHQRIPRILGGMGIVILSTSRGIMTDREARLEGIGGEILCYIWM
AKTIPRIGSRRNGRIGSRKNGRRIPKGVIHVQASFNNTIVTVTDIRGRVVSWSSAGTCGFRG
TRRGTPFAAQTAAANAIRTVVDQGMQRAEVMIKGPGLGRDAALRAIRRSGILLSFVRDVT
PMPHNGCRPPKKRRVMPTIKQLIRNTRQPIKNVTKSPALRGCPQRRGTCTRVYTITPKKPN
SALRKVARVRLTSGFEITAYIPGIGHNSQEHSVVLVRGGRVKDLPGVRYHIVRGTLDAVGV
KDRQQGRSKYGVKKPKMARKSLIQRETKRQRLEQKYHLIRRSSKKEIK---KVPSLSDKWE
IHGKLQSSPRNSAPTRLHRRCFSTGRPRANYRDFGLSGHILREMVHACLLPGATRSSWMV
KNLFISVSSQEEKEENK--GSVEFQVFSFTNKIRRLTSHLELHRKDYLSQRGLRKILGKRQRL
LAYLSKKNRVRYKELISQLDIREPKTR--MVKLRLKRCGRKQRAIYRIVAIDVRSRREGRDL
RKVGFYDPIKNQTYSNVPAILYFLEKGAQPTGTVHDISKKAEIFKELRLITRNQK-MDKSKR
LFLKSKRSFRRRLPPIGSGDRIDYRNMSLISRFISEQGKILSRRVNRLTLKQQRLITIAIKQAR
ILSLLPFLNNEK--QFERTESTPRTTGPRTRNKVTRSLKKNPFVANHLLGKISKLNMRAEKDT
IVTWSRASTIIPTMIGHTIAIHNGKEHLPIYITDRMVGHKLGEFAPTLNFRGHARNDNKSRR
MILKYFLLGNLLSLCMKIINSVVVVGLYYGFLTTFSIGPSYLFLLRARVMEEG---TEKEVSA
TTGFIAGQLMMFISIYYAPLHLALGRPHTITVLVLPYLLFHFFWNNHKHFFDYGSTTRNSM
RNLSIQCVFLNNLIFQLFNHFILPSSTLARLVNIYMFRCNNKMLFVTSSFVGWLIGHILFMK
WVGLVLFWIRQNNSIRS-----KYLVSELRNSMARIFSILLFITCVYYLGRIPSPIVTKKLKETE
EDKPLLTLLFDYKRWNRPLRYIKNDQFDNTVRNEMSQYFFYTCQSDGKQRISFTYPPSLST
FLKMIQQKMFLCTIEKLSSEELYNRWVYTNEKKRNNLRNELFNRIEVLDKGSLAMDVLSK
RTRLCN--DKQECLPKMYDPLLSGPRRGTIKKLYSRT--LWINKLHDILYQEFEQKYSNDQ--L
INEINKKVPRWPYKLIDLDDE--EGIRSRKAKRMVIFNEAFIRYLEQSDFRRDIIKGAMRAQR
RKTVTWKVFQPNVHSPLFLDRIDKTFFFSFDI--SRMLNPIFRNKEENERMTIAENWESIIFS
QTVRSSLLVTQAILRKYIVLPLLIIAKNIGRMLLFQSPEWYEDWQEWSRERHVKCTFSGVQ
LSEKEFPKDWLTKGIQIKILFPFCLKPWHRSKNSKGYNFCFLTVFGMETKLPFGLPIKQPSF
FQPVFKELGKKLRKLI-KKCFLARKIIKKRTKWLLKVSKEKTRWVIKKVR--FKKRIMKELT
KEK--IIGNQMIHESSIRIRSMDWTNYSLTEKKMKDLADRTSTIRNKIEKITKDKKNSTNETS
FYDKRLESTKNFWQILKRRNARLIRKCHYFLKFFIERIY-DIFLRIINIPRITIQNLKNFIS-DLS
SLSQAYVFYKLSQTQVINKYHLKSVFQYTTPFIKDRIKYFFTQGIFHSKSRYKKLRNFEMN
EWKSWLIGNYQYDLSQNRWSRLVSKKWRNRINQSFMVQNKENFDSYEKDQ--LINYEKQ
DKLKKKYKYDLLSNKYINDYIIDPDRKYFDWRTINFCLT-------------ELFPEFLLLYNAYKI
QPWIIPINLLLFN------EELFFFLKRNFLFQFRWEPPFSQTIINNIKAYCLLLRLINPKEITISSIQ
RGEMRLDVMMIPRVTTIENLEN-MGFFIEPARLSINWDGQFIMYQTIAISRVHKSKQNHYD

FIVPENILSPRRRRELRIRNCLRFLWPNYRLEDLACMNRYWFDTNNGSRFSMSRIHMYPVK
RHPLKSWIFELREILREIKNSHYFLDSWTKFDSVGSFTHIFFHQERFMKLFDPRILSILLS-RD
S--RGSTSNRYFT---IKGVVLLAVA-VLIY----RINNRNMVERKNLYLMGLLP-----IPMNSIGPR
NDTLEESF-GSSNINRLIVS-LLYLPKGKKISESCFMDPKESTWVLPIT----KKCIMPESNWGS
RWWRNRIGKKR--DYSCKIS---NETVAGIEISFKEKDIK-YLESPFVSYMD--DPIRKDHDWEL
FDRLSPRKKRNIINW--NSGQLFEILVKHWICYLMSAFREKRPIEAEG-FFKQQG-AGSTIQSN
DIEHVSH--LLSRNKWGISLQNCAQFHMWQFRQDLFV---SWGK-NPHESDFLRN-VSRENCI
WLDNVWLVNKAR---FFS--KVRNVSSNMQYDS---TR--SIFVQVTDSSQLKGSS-DQSRDHF
DSIS----NEDSE-------------YHTLIHQREIQQLKERSIL-WD--PSFLQTERTEIESD--RFPK--CLS
GDS--SMSRLFT-ERETQMINHLLPEEIEEFL--GNPTRSIRSFFSDRW--SELH-LGSNPTEGSTR
DQ----KWLKKQQDFS-FVPSRRSE----NKEMVDIFKIITYLQN--TVSIHPISSDPGCDMVP-KD
EPD-MDSSNKISFLNQNSFFD--LFHLFHDQNRGGYTL--HHDFESE--ERFQEMADLLTLSITE
P-DLVYHKGFAFSIDSYRLD-----FLNEVFNSRDESKKKSL---LVLPPIFY--EENESFYRRIRKK
WVR-ISCGNALEDPKPKIVVFASNNIMKAVNQYRLIQNLIQIQYSTRG--YIRNVSNQFFLM--
-NRSDRNFEYGIQ---RDQIGNDTLNHRTIMKYTINQHLSNLKK-SQKKWFDP----LISRTERS
MNRDPDAYRYKWSKGSKNFQERLEHFVSEQKSR---FQVVFDQLC--QYSIDWSEVIDKQD
FSKSLRFFLS------------------------KSLLFLSKSLPFFFVSIGNIPIHRSE-IHIY-ELKGPNDQLCN
QLLESIGVQIVHLNKLKS--FLLD---DHDTS-QKSKFLINGGRISPFLFNKIP-KW-MIDSFHT--
RNNRRKSFDNTDSYFSMISHDRDNWLNPAKPFHRSSLISSFYKANRLRFLNNPHHFWFY--
CNKRFPFYV----EKARINNYD--LTY-GQFLNILFIRNK-IFSLCVGK--KKHVFGE-RDTISPIES
QVSNIFIPNDFPQSGDET-YNLYKS---FHFPIRSAPFVR--RALYSIADISGTPLTEGQIANFERT
YCQPLSDMNPSDSEGKNLHQYF--NFNSNMGLIHTPCSEKYLP--SEKRKK--------RSLCLK--
KCVEKRQMYRTFQPDSAFSNLSKWNLFQTYMPWFLTSTGCKYLNFTLLDTFSDLL--PILS
SS-KKFVSIFHDIMNGSDISRPILHNKLW----AI---WNL-ISDISSKCLHD--LLLSEEMIRRNNE
SPVPLIWAHLRSPNAREL-LYSILFLLLVAGY-LVRTHLLFVSR--ASSELQTEFEKIKSLMIPSY
MIELRKLLDGYPTSELNSFWLKNLFLVALKQLGDSLEEIRGSASGGNMLLGGGP-----AYG-
VKSIRSKKKYLNIN---LIDLI-SIIPNPINRITFSRNTRHLSRTSKEIYSLIRKRKNVNGDWIDD
KIESWVANSDSIDDEEREFLVQFS--TLTT--EKGIDQILLSLT-HSDHLS-----KNDSGYQMIEQP
GSVYLRYLIDIH--KKNLMNYEFNRSCLAERRIFLAHYQTITY--SQTSCGANSFHFPSHG---K
PFSLRLALSPS-RGILVIGSIGTGRSYLVKYLAANSYVPFITVFPNKFLDDKPKGYLIDDI---DI
D-IDDSDDIDDS------------------------DDIDDDLDTELLTMTNAL---TVYRN-MMPKIDPFDITL
PFELAKAMSPCIIWIPNIHDLYVNESNYLSLGLLVNHLS---RDCERCS-TRNILVIASTHIPKK
VDPALIAPNKSNTCIKIRRLLIPQQRKNFFTLSYTRGFHLENKMFN----GFGSITMGSNARDL
VALTNEALSISITQKKSIIDTNTIRSALHKQTWDLR---SQVRSVQDHGILFYQIGRAVAQNVL
LSNCPIDPISIYMKKK--SCKEGDSYLYKWYFELGTSMKKLTILLYLLSCSAGSVAQDLWSPP
--GPDEK-NGITSYGFVENDS-DLVHGLLEVEDVLVG--SSRTEKDCSQFDND--RVTLLLRSEP
RN-PLDMMQNGSCSIVDQRFLYEK--YESEFEE-G--EGALDPQ----QLEED----LFNH--IVWAP
--RIWHP---CGNLFDCIERPNELGFP-YWARSFR---GKRIIYHK-DELQENDSEFLQSGTMEYQ
TRDRSSKEQGFFRISQFIWDPADPFFFLFK-DQPFVSVFSRREFFADQEMSKRLLTSQTKSPT
SIYKRWFV--KNTQEKHFELLIHRQRWIRT---NSSLS--NGSFRSN--TPSESYQYLSNLFLSN-G
TLLDQMTKTLLRKRWLFPDEMKHLI--HVTGERFPIPMPRSR-INGNFIDKTFSIVANILLRIIP
TTSGEKEAFTYYRDG--MSAQSEGNYAEALQNYYEAMRLEIDPYDRSYILYNIGLIHTSNG
EHTKALEYYFRALERNPFLPQAFNNMAVICHY-RGEQAIQQGDSEIAEAWSDQAAEYWKQ
AIALTPGNYIEAHNWLKITRRFEFEMNWRSERIWIELIPGSRKTSNFCWACILFLGSLGFLLV

GTSSYLGRNLISLFPSQQILFFPQGIVMSFYGIAGLFISSYLWCTISWNVGSGYDRFDRKKGI
VCIFRWGFPGRNRRIFLRFLMRDIQSIRIEVKEGIYPRRVLYMEIRGQGSVPLTRTDEN-LTPR
EIEQKAAELSYFLRVPIEVF
>Typha
MEKWWFNSMFSLSKSMESLDSIGHTSGSEEPILNDTKKKIP-FNNVDYLFDIRDMWSFISD
DTFLVKDKNGDSYSVYFDMENLSFEIDND--SSFLSELGSFFST-----FLNRGSKKNNHYYYH
YMYDTQSRWNNHINSCIDSYLRFEV--SINSSILGGTDNSSDCYIYNFICTESGSSIRTSKNGS
KFSIRGRS-DLDINKKYRHLWVQCENCYGLNYKKFFRLKMNICEQCGYHLKMSSSDRIGV
LIDPGTWDPMDEDMVSMDPIEFHSEEEPYRDRIDFYQRKTGLTEAVQTGIGQLNGISIAIGV
MDFQFMGGSMGSVVGEKITRLIEYATNRSLPVIIVCASGGARMQEGSLSLMQMAKISSAL
YNYQSTKKLFYVSILTSPTTGGVTASFGMLGDVIIAEPNAYIAFAGKRVIEQTLNKTVPDGS
QAAEYLFHKGLFDPIVPRNPLKGVLSELFQLHGFFPLNQNSKNMVTLRADEISNIIRERIEQ
YSREVKIVNTGTVLQVGDGIARIHGLDEVMAGELVEFEEGTIGIALNLESNNVGVVLMGD
GLMIQEGSSVKATGRIAQIPVSDAYLGRVINALAKPIDGRGEISSSESRLIESPAPGIISRRSV
YEPLQTGLIAIDAMIPIGRGQRELIIGDRQTGKTAVATDTILNQKGQNVICVYVAIGQKASSV
AQVVTTFQEQGAMEYTIVVAETADSPATLQYLAPYTGAALAEHFMYRGKHTLIIYDDLSK
QAQAYRQMSLLLRRPPGREAYPGDVFYLHSRLLERAAKSSSSLGEGSMTALPIVETQSGD
VSAYIPTNVISITDGQIFLSADLFNAGIRPAINVGISVSRVGSAAQIKAMKQVAGKSKLELAQ
FAELEAFAQFASDLDKATQNQLARGQRLRELLKQSQSAPLTVEEQIATIYTGANGYLDSLEI
GQVKKFLVQLRTYLKKNKPQFQEIISSTKTFNQQAEAILKEAIQEQIELFLLQEQTMRINPT
TSSPGGSTLEEKNLGRIAQIIGPVLDAVFPPGKMPNIYNALVVKGRDTLGQQINVTCEVQQ
LLGNNRVRAVAMSATDGLMRGMEVIDTGAPLSVPVGGTTLGRIFNVLGEPVDNLGPVDTR
TTSPIHRSAPAFIQLDTKLSIFETGIKVVDLLAPYRRGGKIGLFGGAGVGKTVLIMELINNIA
KAHGGVSVFGGVGERTREGNDLYMEMKESGVINEKNLAESKVALVYGQMNEPPGARMR
VGLTALTMAEYFRDVNEQDVLLFIDNIFRFVQAGSEVSALLGRMPSAVGYQPTLSTEMGS
LQERITSTKEGSITSIQAVYVPADDLTDPAPATTFAHLDATTVLSRGLAAKGIYPAVDPLDST
STMLQPRIVGEEHYETAQRVKETLQRYKELQDIIAILGLDELSEEDRLTVARARKIERFLSQ
PFFVAEVFTGSPGKYVGLAETIRGFQLILSGELDSLPEQAFYLVGNIDEATAKAMNLEVESK
LKKMTLNLCVLTPNRIIWDSKVKEIILSTNSGQIGVLPNHAPIATAVDIGLLRIRRDDQWLTV
ALMGGFARIGNNEITILGNDAEMSTDIDPQEAQQALEIAEANLSKAEGKRQVIEANLALRR
ARTRVEAVNVISMKNVTDSFVFLGHWPSAGSFGFNTDILATNLINLSVVIGVLIFFGKGVLK
DLLDNRKQRILITIRNSEELRKGAIEQLEKARVRLRKVELEADEYRTNGYSEIEREKENLIN
ATCDSLERLENYKNETLDFEKQRAMNQVRQRVFQQALQGALGTLNSCLNTELHFRTIRA
NIGILGAMEEITDMNPLISAASVIAAGLAVGLASIGPGVGQGTAAGQAVEGIARQPEAEGKI
RGTLLLSLAFMEALTIYGLVVALALLFANPFVMNVIPCSIKTLKGLYDISGVEVGQHFYWQI
GGLQIHAQVLITSWFVIAILLGSAIIAVRNPQTIPTDGQNFFEYVLEFIRDLSKTQIGEEYGP
WVPFIGTMFLFIFVSNWSGALLPWKIIELPHGELAAPTNDINTTVALALLTSVAYFYAGLTK
KGLGYFEKYIKPTPILLPINILEDFTKPLSLSFRLFGNILADELVVVVLVSLVPSVVPIPVMFL
GLFTSGIQALIFATLAAAYIGESMEGHHMIFATLEHILSHISFSIILIVIPIHLMTLLVHEISGLR
DSSEKGMIATFLSITGLLVSRWISSGHFPLSNLYESVIFLSCGFSIIYMIPKIRNHKNDLSTITA
PSTIFTQGFATSGLLTEMHQSAILVPALQSQWLMMHVSMMLLSYAALLCGSLLSVALLVITF
RKNTTIFCKNDEIESLNEKRSVFKNTSFLSFKNYYKYQLTERLDYWSYRVISLGFTFLTIGIL
CGAVWANEAWGSYWNWDPKETWAFITWTIFAIYLHTRTNQNWKGTNSALVASIGFLIIWI
CYFGINLLGIGLHSYGSFTLTMKKKALP-SFPYLASIVFLPWWVSFSFNKCLELWVTTWWN

TRQSETLFNDIQEKNLLERFIELEELFLLDEMIKEYPETHIQKLRIGIHKETIQLVKTHNEYH
LQIILHFSTNIICFAILSGYFILGNEELVILNSWVQEFLYNLSDTIKAFSILLFTDLWIGFHSTH
GWELIIGSVYNDFGLAHNDQIISGLVSTFPVILDTIVKYWIFHYLNRVSPSLVVIYHSMNEM
PIGVPKVPFRSPGEEDAVWVDVN-RLYRERLLFLGQEVDSEISNQLVGLMVYLSIEDDTRD
LYLFINSPGGWVIPGIAIYDTMQFVPPDVHTICMGLAASMGSFILVGGEITKRLAFPHARVM
IHQPASSFYEAQAGEFILEAEELLKLRETLTKVYVQRTGNPLWVVSEDMERDVFMSATEA
QAHGIVDLVAVEMKEQKLIHEGLITESLPNGMFRVRLDNEDLILGYVSGRIRRSFIRILPGDR
VKIEVSRYDSTRGRIIYRLRNKDSNDMKQLQKYLEKVRSRKQHFLYPLLFKEYIYAFAHDY
GLLGNDN-SSSVLVKRLIIRMYQQNFLINSTNHSNQNRFIGSRFFSQMISESFAVIMEIRFSLR
LVSSSIPQFHNLRSIHSIFPFLEDKFSHLNSISDILIPHPIHLEILVQILQCRIQDVPSLHLLRFFL
HEYHNWNSLITKSIYVFSKKRLFRLLYNFYVFECEFVFVFLRKQSSYLQLTSFGTFLERIHF
YGKIEH-LVVYRNNKTLWFFTDPFMHYVRYQGKAILASKGTHLFMKKWKCYLVNFWQY
YFHFWSQPHRIHINQLSNYSFHFLGYLSSLLRNPLVVRNQMLENSYLIDTVM--TKFDTIVP
VDPLIGSLSKAKFCTLLGHPISKPIWTDLSDCDIIDRFGRICRNLSHYYSGSSKKRTLYRIKYI
LRFSCARTLARKHKSTVRTFMQRLGSVLLEEFFTEEEQVLSLIFPKRIWYLDIIRINDLVNH-
MIIDTT-EVQ-TINSFSTSE-FLKETYGLIWI-VPILTLILGITIGVLVIVWLEREISAAIQQRIGPE
YAGPLGILQALADGTKLLFKEDLLPSRGDIRLFSVGPAIAVISVLLSYLVIPFGYRLVLADLSI
GVFLWIAISSIAPIGLLMSGYGSNNKYSFSGGLRAAAQSISYEIPLTLCVLSISLLSNSSSTVD
IVEAQSKYGFWGWNLWRQPIGFIVFIISSLAECERLPFDLPEAEEELVAGYQTEYSGIKYAL
FYLASYLNLLVSSLFVTVLYLGGWNFSIPYISIPELFGINKMAGIFGMTIGIFITLTKAYLFLFI
SITTRWTLPRMRMDQLLNLGWKFLLPISLGNLLLTTSSQLVSLMIWHVQNENFILDSTRIF
MKAFHLLLFHGSFIFPECILIFGLILLLMIDSTSDQKDRPWFYFISSTSLVISITALLFRWREEP
IISFSGNFQTNNFNEIFQFLILLCSTLCIPLSVEYIECTEMAITEFLLFILTATLGGMFLCGAND
LITIFVAPECFSLCSYLLSGYTKRDVRSNEATMKYLLMGGASSSILVHGFSWLYGSSGGEIE
LQEIVNGLINTQMYNSPGISIALISITVGIGFKLSPAPFHQWTPDVYEGSPTPVVAFLSVTSKV
AASASATRIFDIPFYFSSNEWHLLLEILAILSMILGNLIAITQTSMKRMLAYSSIGQIGYVIIGI
IVGDSNDGYASMITYMLFYISMNLGTFARIVLFGLRTGTDNIRDYAGLYTKDPFLALSLAL
CLLSLGGLPPLAGFFGKLYLFWCGWQAGLYFLVSIGLLTSVVSIYYYLKIIKLLMTGRNQEI
TPYVRNYRRSPLRSNNSIELSMTVCVIASTIPGISMNPILAIAQDTLFMFLLHEYDIFWAFLII
SSVIPILAFVISGVLAPVREGPEKLSSYESGIEPMGDAWLQFRIRYYMFALVFVVFDVETVFL
YPWAMSFDVLGVSVFIEALIFVLIPIVGSVYAWRKGALEWSTSYFPWLTILVVLPIFAGSLIF
FFPHRGNKMYRWYTICICLLELLLTTYAFCYHFQLDDPLIQLKEDFKWIDVFDFHWRLGID
GLSIGPILLTGFITTLATLAAWPITRNSRLFYFLMLAMYSGQIGLFSSRDLLLFFIMWELELIP
VYLLLSMWGGKKRLYSATKFILYTAGGSIFFLIGVLGMGLYGSNEPTLDFERLINQSYPVAL
EIIFYFGFLIAYAVKLPIIPLHTWLPDTHGEAHYSTCMLLAGILLKMGAYGLIRINMELLPHA
HSLFSPWLVIIGTIQIIYAASTSLGQRNLKKRIAYSSVSHMGFIIIGIGSITDMGLNGAILQMLS
HGFIGAALFFLAGTSCDRMRLVYLEEMGGISIPMPKIFTMFSSFSMASLALPGMSGFVAEL
VIFFGLITSPKYLLMPKMLITLVMAIGMILTPIYLLSMLRQMFYGYKFFNVPNSNFLDSGPR
ELFLSICIFLPVIGIGIYPDFVLSLSVDRVQAILSNYYHRMMFEHVLFLSVYLFSIGIYGLITSR
NMVRALMCLELILNSVNINLVTFSDIFDSRQLKGDIFSIFIIAIAAAEAAIGLAIVSSIHRNRK
STRINQSNLLNNMEHTYQYAWIIPFLPLPVTMSIGVGLLLIPTATKKIRRMWAFPSVLLLSIA
MVFSANLSIQQINGSFIYQYLWSWTINNDLSLEFGYLIDPLTSIMSILITTVGIMVLIYSDNY
MSHDQGYLRFFAYMSFFNTSMLGLVTSSNLIQIYIFWELVGMCSYLLIGFWFTRPTAASAC
QKAFVTNRVGDFGLLLGILGFYWITGSFEFRDLFEISKNNGVNSLFVTLCAFLLFVGAVAK

SAQFPLHVWLPDAMEGPTPISALIHAATMVAAGIFLVARLLPLFIAIPYIMNLISLIGVITLFL
GATLALAQRDIKRSLAYSTMSQLGYIMLALGIGSYRAALFHLITHAYSKALLFLGSGSIIHS
MEPIVGYSPEKSQNMVLMGGLRRYVPITRTTFLLGTLSLCGIPPLACFWSKDEILNDSWLY
SPIFAIIAFFTTGLTAFYMFRVYLLTFDGHLRVHFQNYSSTKNSSLYSISLWGKERPQGVNR-
NLLLSTMGFFSNKK--TSMYPHESDNTMLFPLIVLVIFTLFVGSIGIHFDFDILSKWLTPSTNL
FYPTSNYSVNSYEFFTNALLSVSIASLGLFIAYILYGSSYSFFQNLDLINFFVTFLDQIKNVIY
NWSYNRGYIDIFYTRVFTIGIRGLTELTQFFDRRIIDGITNGVGVVSFFIGEGIKY-MGGGRIS
SYLFLYLFYVSLFLLIFIMDLPGPIHDILVVFLGLVLVLGGLGVVLLTNPIYSAFSLGLVLVCI
SLFYILSNAYFVAAAQLLIYVGAINVLIIFAVMFMNGSEYSKDSYLWTIGDGGTSLICTTILF
SLITTIPDTSWYGIIWTTKSNQIIEQDLINNVQQIGIHLSTDFYLPFELVSIILLVSLIGAITMAR
QMTVPVTRKDLMIVNMGPQHPSMHGVLRLIVTLDGEDVIDCEPILGYLHRGMEKIAENRT
IIQYLPYVTRWDYLATMFTEAITVNAPEQLENVQVPQRASYIRVIMLELSRIASHLLWLGPF
MADIGAQTPFFYIFRERELLYDLFEAATGMRMMHNYFRIGGVAADLPYGWIDKCLDFCDY
SLTGVDEYQKLITRNPIFLERVEGVGIIGGEEAVNWGLSGPMLRASGIPWDLRKIDFYECY
NEFNWEVQWQKEGDSLARYLVRIGEMRESIKIIQQALEGIPGGPYENLEVRR-DRAKNSE
WNDFEYRFISKKPSPNFELSKQELYVRVEAPKGELGIYLIGDNSVFPWRWKIRPPGFINLQI
LPQLVKRMKLADIMTILGSIDIIMGEVDRMFPMVTGFMNYGQQTIRAARYIGQSFIITLSHT
NRLPVTIQYPYEKSITTERFRGRIHFEFDKCIACEVCVRVCPIDLPVVDWRFEKDIKKKQLL
NYSIDFGVCIFCGNCVEYCPTNCLSMTEEYELSTYDRHELNYNQIALGRLPISIIGDYTIQT
VMNSKIDKDKPFDSRTITDYQQDRLSDWLVKHELVHRSLGFDYRGIETLQIKTEDWDSIAV
ISYVYGYNYLRSQCAYDVAPGGFLASVYHLTRIQYGIDKPEEVCIKVFAPRDNPRIPSVFWI
WRSADFQERESYDMLGISYDNHPRLKRILMPESWIGWPLRKDYITPNFYEIQDAH-----------
-------------------------MVLT-EYSDNKKKKEGKDYIETVMNLIEFPLLDETTPNSVISTTSNDLS
NWSRLSSLWPLLYGTSCCFIEFASLIGSRFDFDRYGLVPRSSPRQADLILTAGTVTMKMAPS
LVRLYEQMPEPKYVIAMGACTITGGMFSTDSYSTVRGVDKLIPVDVYLPGCPPKPEAVIDA
ITKLRKKISREIIEDRTVSQ-ENRCFTTNHKFYVRRSTHTGNYDQGLLYQSPSTSEIST--FFKS
KSSVSSYELVNMQNRNTFSWVKEQMTRSISVSIMIYVITRASISNAYPVFAQQGYENPREA
TGRIVCANCHLANKPVDIEVPQAVLPDTVFEAVVRIPYDMQLKQVLANGKKGGLNVGAV
LILPEGFELAPPDRISPELKEKIGNLSFQSYRPNKKNILVIGPVPGQKYSEIVFPILSPDPATKK
DVHFLKYPIYVGGNRGRGQIYPDGSKSNNTVYNATSAGIVSKIVRKEKGGYEISIVDASDG
RQVVDIIPRGPELLVSEGESIKLDQPLTSNPNVGGFGQGDAEIVLQDPLRVQGLLFFFASVIL
AQVFLVLKKKQFEKVQLYEMNFMSKVYDWFEERLEIQAIADDITSKYVPPHVNIFYCLGG
ITLTCFLVQVATGFAMTFYYRPTVTEAFSSVQYIMTEANFGWLIRSVHRWSASMMVLMMI
LHVFRVYLTGGFKKPRELTWVTGVVLAVLTASFGVTGYSLPRDQIGYWAVKIVTGVPEAIP
VIGSPLVELLRGSASVGQSTLTRFYSLHTFVLPLLTAVFMLMHFPMIRKQGISGPLMGITKK
PDLNDPVLRAKLAKGMGHNYYGEPAWPNDLLYIFPVVILGTIACNVGLAVLEPSMIGEPA
DPFATPLEILPEWYFFPVFQILRTVPNKLLGVLLMVSVPTGLLTVPFLENVNKFQNPFRRPV
ATTVFLIGTAVALWLGIGATLPIDKSLTLGLFMIEVFLFGIVLGLIPITLAGLFVTAYLQYRRG
DQLDLMLTITSYFGFLLAALTITLALFIGLNKIRLIMDIVSLAWAALMVVFTFSLSLVVWGR
SGLMIIRSPEPEVKIVVDRDPVKTSFEQWAKPGHFSRTIAKGPDTTTWIWNLHADAHDFDS
HTSDLEEISRKVFSAHFGQLSIIFLWLSGMYFHGARFSNYEAWLSDPTHIGPSAQVVWPIV
GQEILNGDVGGGFRGIQITSGFFQLWRASGITSELQLYCTAIGALVFASLMLFAGWFHYHK
AAPKLAWFQDVESMLNHHLAGLLGLGSLSWAGHQIHVSLPINQFLDAGVDPKEIPLPHEFI
LNRDLLAQLYPSFAEGATPFFTLNWSKYAEFLTFRGGLDPITGGLWLSDIAHHHLAIAILFLI

AGHMYRTNWGIGHGLKDILEAHKGPFTGQGHKGLYEILTTSWHAQLSLNLAMLGSLTIVV
AHHMYSMPPYPYLATDYGTQLSLFTHHMWIGGFLIVGAAAHAAIFMVRDYDPTTRYNDL
LDRVLRHRDAIISHLNWVCIFLGFHSFGLYIHNDTMSALGRPQDMFSDTAIQLQPIFAQWV
QNTHALAPGVTAPGATTSTSLTWGGGELVAVGGKVALLPIPLGTADFLVHHIHAFTIHVTV
LILLKGVLFARSSRLIPDKANLGFRFPCDGPGRGGTCQVSAWDHVFLGLFWMYNAISVVIF
HFSWKMQSDVWGTISDQGVVTHITGGNFAQSSITINGWLRDFLWAQASQVIQSYGSSLSA
YGLFFLGAHFVWAFSLMFLFSGRGYWQELIESIVWAHNKLKVAPATQPRALSIVQGRAVG
VTHYLLGGIATTWAFFLARIIAVGMALRFPRFSQGLAQDPTTRRIWFGIATAHDFESHDDIT
EERLYQNIFASHFGQLAIIFLWTSGNLFHVAWQGNFESWIQDPLHVRPIAHAIWDPHFGQPA
VEAFTRGGATGPVNIAYSGVYQWWYTIGLRTNEDLYTGALFLLFLSAISLIAGWLHLQPK
WKPSVSWFKNAESRLNHHLSGLFGVSSLAWTGHLVHVAIPASRGEYVRWNNFLDVLPYP
QGLGPLLTGQWNLYAQNPDSSSHLFGTSQGAGTAILTLLGGFHPQTQSLWLTDMAHHHLA
IAFIFLIAGHMYRTNFGIGHSIKDLLEAHTPPGGRLGRGHKGLYDTINNSIHFQLGLALASL
GVITSLVAQHMYSLPAYAFIAQDFTTQAALYTHHQYIAGFIMTGAFAHGAIFFIRDYNPEQN
EDNVLARMLDHKEAIISHLSWASLFLGFHTLGLYVHNDVMLAFGTPEKQILIEPIFAQWIQS
AHGKTSYGFDVLLSSTSGPAFNAGRSIWLPGWLNAVNE--NSNSLFLTIGPGDFLVHHAIAL
GLHTTTLILVKGALDARGSKLMPDKKDFGYSFPCDGPGRGGTCDISAWDAFYLAVFWML
NTIGWVTFYWHWKHITLWQGNVSQFNESSTYLMGWLRDYLWLNSSQLINGYNPFGMNS
LSVWAWMFLFGHLVWATGFMFLISWRGYWQELIETLAWAHERTPLANLIRWRDKPVALSI
VQARLVGLAHFSVGYIFTYAAFLIASTSGKFGMSHSVKIYDTCIGCTQCVRACPTDVLEMI
PWDGCKAKQIASAPRTEDCVGCKRCESACPTDFLSVRVYLGPETTRSMALSYMTDLNLPS
IFVPLVGLVFPAIAMASLSLHVQKNKIVMRDIKTYLSVAPVLSTLWFGSLAGLLIEINRLFPD
ALSFPFF--MTAILERRESTSLWGRFCNWITSTENRLYIGWFGVLMIPTLLTATSVFIIAFIAAP
PVDIDGIREPVSGSLLYGNNIISGAIIPTSAAIGLHFYPIWEAASVDEWLYNGGPYELIVLHF
LLGVACYMGREWELSFRLGMRPWIAVAYSAPVAAATAVFLIYPIGQGSFSDGMPLGISGTF
NFMIVFQAEHNILMHPFHMLGVAGVFGGSLFSAMHGSLVTSSLIRETTENESANEGYRFG
QEEETYNIVAAHGYFGRLIFQYASFNNSRSLHFFLAAWPVVGIWFTALGISTMAFNLNGFN
FNQSVVDSQGRVINTWADIINRANLGMEVMHERNAHNFPLDLAAVEVPSINGMGLPWYR
VHTVVLNDPGRLISVHIMHTALVAGWAGSMALYELAVFDPSDPVLDPMWRQGMFVIPFM
TRLGITNSWGGWSIS-GGTVTNPGIWSYEGVAGAHIVFSGLCFLAAIWHWVYWDLEIFTD
ERTGKPSLDLPKIFGIHLFLSGVACFGFGAFHVTGLYGPGIWVSDPYGLTGKVQSVNPAWG
AEGFDPFVPGGIASHHIAAGTLGILAGLFHLSVRPPQRLYKGLRMGNIETVLSSSIAAVFFA
AFVVAGTMWYGSATTPIELFGPTRYQWDQGYFQQEIYRRVSAGLAENLSLSEAWSKVPEK
LAFYDYIGNNPAKGGLFRAGSMDNGDGIAVGWLGHPVFKDKEGRELFVRRMPTFFETFP
VVLVDEDGIVRADVPFRRAESKYSVEQVGVTVEFYGGELNGVSYSDPATVKKYARRAQL
GEIFELDRATLKSDGVFRSSPRGWFTFGHATFALLFFFGHIWHGARTLFRDVFAGIDPDLDA
QVEFGTFQKLGDPTTKRQVVMKTLYSLRRFYHVETLFNGTLALAGRDQETTGFAWWAG
NARLINLSGKLLGAHVAHAGLIVFWAGAMNLFEVAHFVPEKPMYEQGLILLPHLATLGW
GVGPGGEVIDTFPYFVSGVLHLISSAVLGFGGIYHALLGPETLEESFPFFGYVWKDRNKMT
TILGIHLILLGIGAFLLVFKALYFGGVYDTWAPGGGDVRKITNLTLSPSVIFGYLLKSPFGGE
GWIVSVDDLEDIIGGHVWLGSICIFGGIWHILTKPFAWARRAFVWSGEAYLSYSLAALSVF
GFIACCFVWFNNTAYPSEFYGPTGPEASQAQAFTFLVRDQRLGANVGSAQGPTGLGKYL
MRSPTGEVIFGGETMRFWDLRAPWLEPLRGPNGLDLSRLKKDIQPWQERRSAEYMTHAP
LGSLNSVGGVATEINAVNYVSPRSWLATSHFVLGFFLFVGHLWHAGRARAAAAGFEKGID

RDLEPVLSMTPLNMTIALGRFTKEENDLFDIMDDWLRRDRFVFVGWSGLLLFPCAYFALG
GWFTGTTFVTSWYTHGLASSYLEGCNFLTAAVSTPANSLAHSLLLLWGPEAQGDFTRWCQ
LGGLWTFVALHGAFGLIGFMLRQFELARSVQLRPYNAIAFSGPIAVFVSVFLIYPLGQSGW
FFAPSFGVAAIFRFILFFQGFHNWTLNPFHMMGVAGVLGAALLCAIHGATVENTLFEDGDG
ANTFRAFNPTQAEETYSMVTANRFWSQIFGVAFSNKRWLHFFMLFVPVTGLWMSAIGVV
GLALNLRAYDFVSQEIRAAEDPEFETFYTKNILLNEGIRAWMAAQDQPHENLIFPEEVLPR
GNALMSGSTGERSFADIITSIRYWVIHSITIPSLFIAGWLFVSTGLAYDVFGSPRPNEYFTESR
QGIPLITGRFDSLEQLDEFSRSFMTIDRTYPIFTVRWLAVHGLAVPTVSFLGSISAMQFIQRM
ATQTVEDSSRSGAPRRTNVGSLLKPLNSEYGKVAPGWGTTPLMGVAMALFAIFLSIILEIYN
SSVLLDGIAMSMLTLKLFVYTVVIFFVSLFIFGFLSNDPGRNPGREEMADTTGRIPLWLIGT
VTGIPVIGLIGVFFYGSYSGLGSSLMLNIFSLICICLNSALYSSSFFFAKLPEAYAIFNPIVDVM
PVIPLFFFLLAFVWQAAVSFRMTQSNPNEQSVELNRTSLYWGLLLIFVLAVLFSNYFFNME
VNILAFIATALFILVPTAFLLIIYVKTVSQNDMETATLVAISISGLLVSFTGYALYTAFGQPSQQ
LRDPFEEHGDMEALVYTFLLVSTLGIIFFAIFFREPPKVPTKK--MTIAFQLAVFALIATSSVLL
ISVPVVFASPDGWSSNKNVVFSGTSLWIGLVFLVAILNSLIS-------MSPQTETKASAGFKAG
VKDYKLTYYTPEYETKDTDILAAFRVTPQPGVPPEEAGAAVAAESSTGTWTTVWTDGLTS
LDRYKGRCYHIEPVVGEENQYIAYVAYPLDLFEEGSVTNMFTSIVGNVFGFKALRALRLED
LRIPPAYSKTFQGPPHGIQVERDKLNKYGRPLLGCTIKPKLGLSAKNYGRAVYECLRGGLD
FTKDDENVNSQPFMRWRDRFLFCAEAIYKAQAETGEIKGHYLNATAGTCEEMMKRAIFA
RELGVPIVMHDYLTGGFTANTSLAHYCRDNGLLLHIHRAMHAVIDRQKNHGMHFRVLAK
ALRMSGGDHIHAGTVVGKLEGEREMTLGFVDLLRDDFIEKDRSRGIFFTQDWVSMPGVL
PVASGGIHVWHMPALTEIFGDDSVLQFGGGTLGHPWGNAPGAVANRVALEACVQARNEG
RDLAREGAEIIREACKWSPELSAACEVWKEIKFEFDPVDKLDKTAIHLYKTSTPSTRNGAV
DSQVKSNPRNNLIYGRHHCSKGRNARGIITAGHRGGGHKRLYRKIDFRRNQKDISGRIVTI
EYDPNRNAYICLIHYGDGEKRYILHPRGAIIGDTIVSGTEVPISMGNALPLTDMPLGTAIHNI
EITRGKGGQLARAAGAVAKLIAKEGKSATLRLPSGEVRLISKNCLATVGQVGNVGVNQKI
LGRAGSKCWLGKRPVVRGVVMNPVDHPHGGGEGRAPIGRKKPTTPWGYPALGRRSRKR
KKYSDCFILRRRK-MIQPQTLLNVADNSGARKLMCIRIIGAG-NHRYAHIGDVIVAVIKEAVP
NMPLERSEVIRAVIVRTCKELKRENGIIIRYDDNAAVVIDQEGNPKGTRVFGAIARELRQLN
FTKIVSLAPEVLMLSPKRTRFRKQHRGRMKGISCRGNHISFGRYALQALEPAWITARQIEAG
RRAMTRYARRGGKIWVRIFPDKPITLRPAETRMGSGKGSPEYWVSVVKPGRILYEMGGVS
ETVARAAIAIAARKMPIRTQFLISGMTRVRRGYTARRRRTKIRLFASTFRGAHSRLTRTITQQ
KMRALVSSHRDRGRQKRDFRRLWITRINAVTREN--VFYSYSRLIHNLYKKQLLLNRKILA
QIAISNKNCLYMISNKIIK-----------MIKKVL-AQHIS-MSVFKARRVIDQIRGRSYEETLMILE
LLPYRASYPILKLVYSAAANANHNMGLNEADSFISKAEVNKGTIVKKLRPRARGRSYPIK
KPRCHITIVLK-KSKS-------MDGIKYAVFTEKSIRLLGNNQ-YTSNVESGSTRTEIKHWVELF
FGVKVIAMNSHRLPGKGRRMGSIMGHTMHYRRMIITLQRGYSIPPLIEKRTMAVPKKRTS
MSKKRIRRNIWKKKGSLAAVKAFSLAKSVSTGHSKSFF--QTNTMAKGKDVRVRVILECTS
CVRN--G--KESPGISRYITQKNRHNTPSRLELRKFCRYCHKHTIHGEIKKMKIRASVRKICE
KCRLIRRRGRIIVICSNPKHKQRQGMVREEVAGSTRTIQWKCVESRVDSKRLYYGRFILSPL
MKGQADTIGIAMRRALLGEIEGTCITRAKSE--RVPHEYSTIIGIEESVHEILLNLKEIVLRSNL
YGVRDASICVRGPRYVTAQDIILPPSVEIVDPTQHIANLTEPIDLYIELQIKRDRGYRMEPTN
NSQDGSY--PIDAVSMPVRNANHSIHSYGNGNEKQEILFLEIWTNGSLTPKEALYEASRNLID
LFIPFLHAEEE--DINFEENK-NRFTLPFLTFQDRLT-NLKKNKK-GIPLNCIFIDQLELPSRTYN

CLKRSNIHTLLDLLSNGQEDLMRIEYLRMEDVKQILDTLQKN--FAIDLPKNKMLRN-GNE
GMSTIPGFSQIQFEGFCRFINQGLTAEFQKFPKIEDTDQEIEFKLFVERYQLVEPLIKERDAV
YESLTYSSELYVPAGLIWKTGRDMQEQTVFIGNIPLMNSLGTSIVNGIYRIVINQILQSPGIY
YRSELDHNGISVYTSTIISDWGGRSELEIDRKARIWARVSRKQKISILVLSSAMGSNLREILD
NVCYPEIFLSFPNDKEKKKIGSKENAILEFYQQFACVGGDPVFSESLCKELQKKFFQQRCE
LGRIGRRNMNRRLNLDIPQNNTFLLPRDVLAAADHLIGMKFGMGTLDD--MNHLKNKRIR
SVADLLQDQFGLALARLENAVRGTICGAIR-HKLI-PTPQNLVTSTS----LTTTYESFFGLHPL
SQVLDRTNPLTQIVHGRKLSYLGPGGLTGRTASFRIRDIHPSHYGRICPIDTSEGINVGLIGSL
AIHVRIGHWG-SIESPFY---EISERSKEKESQMVYLSPNRDEYYMVAAGNSLALNRGIQEEQ
VVPARYRQEFLTIAWEQIHLRSIFPFQYFSIGASLIPFIEHNDANRALMSSNMQRQAVPLSRS
EKCIVGTGLERQTALDSGVSVIAEHAGKIIYTDTHKIILSSNENTISIPLVMYQRSNKNTCM
HQKPQVWRGKSIKKGQILADGAATVGGELALGKNVLVAYMPWEGYNSEDAVLISERLVY
EDIYTSFHIRKYEIQTHVTSQG-PERITKEIPHLEAHLLRNLDRNGIVMLGSWVETGDILVG
KLTPQTANE-SSYAPEDRLLRAILGIQVSTAKETSLKLPIGGRGRVIDVRWIQKKE-GSSYNS
EMIRVYILQKREIKVGDKVAGRHGNKGIISKILPRQDMPYLQDGTPVDMVFNPLGVPSRM
NVGQIFECSLGLAGDLLKRHYRIAPFDERYEQEASRKLVFSELYEASKQTKNPWVFEPEYP
GKSRIFDGRTGDPFEQPVLIGKSYILKLIHQVDDKIHGRSSGHYALVTQQPLRGRAKQGGQ
RVGEMEVWALEGFGVAHILQEMLTYKSDHIRARQEVLGATIIGGRVTKPEDAPESFRLLVR
ELRSLALELNHFLVSEKNFQINRKEA-------MIDQYKHQQLRIGPVSPQQIKAWANKILPNGE
IVGEVTKPYTFHYKTNKPEKDGLFCERISGPIKSGICACGNYRVIGAEKEDPKFCEQCGVEF
VDSRIRRYQMGYIKLACPVTHVWYLKRLPSYIANLLDKPLKELEGLVYCDVDFSFARP-IA
KKPTFLRLRGSFEYEIQSRKYSIPLFFTTPGFETFRNREISTGAGAIREQLADSDLRIIIDNSLV
EWKELGDE-GSTGNEWEDRKIRRRKDFLVRRMELAKHFIRTNVEPERMVLCLLPVLPPEL
RPIIQIDGGKLMSSDINELYRRVIYRNNTLTDLLATSRSTPGELVMCQEKLVQEAVDTLLDN
GIRGQPMRDGHNKVYKSFSDVIEGKEGRFRETLLGKRVDYSGRSVIVVGPSLSLHQCGLP
REIAIELFQTFVIRGLIWQHGASNIGIAKSKIREKEPIVWEILQEVMQGHPVLLNRAPTLHRL
GIQAFQPILVEGRAICLHPLVCKGFNADFDGDQMAVHVPLSLEAQAEARLLMFSHMNLLS
PAIGDPISVPTQDMLIGLYVLTIGNRRGICANRYNPYNCGNY---QNKTVDNNNYRYTKEKE
PYFCSSYDALGAYRQKRISLDSPLWLRWKLDQR-VIGS--REVPIEVQYESLGTYHEIYGHY
LMVGSVKKEIRCIYIRTTLGHISFYREIEEAIQGFCRAYSYTI------------MAERADLVFHNKVI
NGTAMKRLISRLIDHFGMGYTSHILDQVKTLGFHQATATSISLGIDDLLTIPSKGWLVQDAE
QQSFILEKHHHYGNVHAVEKLRQSIEIWYATSEYLRQEMNPNFQMTDPSNPVYLMSFSGA
RGNASQIHQLVGMRGLMSDPQGQMIDLPIQSNLREGLSLTEYIISCYGARKGVVDTAVRTS
DAGYLTRRLVEVVQHIIVRRRDCGTIRGISVSPQNGMTE--KIFVQTLIGRVLADDIYIGPRCI
ATRNQDIGIGLVNRFIT------FRAQPIYIRTPFTCRSTSWICQLCYGRSPTHGDLVELGEAVGII
AGQSIGEPGTQLTLRTFHTGGVFTGGTAEHVRAPSNGKIQFNEDLVHPTRTRHGHPAFLCSI
DLYVTIESRDIIHNVNIPPKSLILVQNDQYVESEQVIAEIRAGTSTF--FKERVRKHIYSELEGE
-MHWSTDVYHAPEYTYGNVHLLPKTSHLWILAGGPCR--SSIVSFSLHKDQDQMNVDSFSV
EGR--YISDFS--MTN-DQVRHKLLYTSY----DREILDYS-RPDQIISNSHWNFIYPS-ILQEN--SD
FLAKKRRDRFIIPLQYDQEQEKELIPCFGISIEIPIN--GILRRKSILAYFDDPRYRRN--GITKY-
TIEVDSIIK--KEDLIEYRG-TKEFSPKY-KVDQFFFILEEVHILPGSSFIMVRNNSIIGVDTRLTL
NTRSRVGGLVRVERKKKYIELKIFSGDIHFPGETDKISRHSGILIPPETEKK--NSKE-SK-KLK
NWIYVQR--ITPTKKK--YFVSVRPVVTYEIADGINLATLFPQDLLQEKDNVQLRVVNYILYG
NGKSIRGIYHTS-QLVRTCLVLNWDHEQNGSRE----EVHASFVQVRANDLIRDFIRIDLVKST

ISYTGKRYDRASSGLIPD---LDRTNTNPFYSKVKIQS-----LSQHRGTVG----TLLNRTK---ECR
SLLILSSSNCSRIG--PFNSSKYN---NVTKESDPIIPI-DLLGPLGTIVP-KISNFYSS-YHLIT---HN
QILLK-KYLLLD---NFKETFQVLQVLKYFLIDENGR--IYNPDPCSNIILNPFHLDWCFLHHD
YWEEASTIIS-LGQFICENVCLFKYEP--HIKKSGQILIVN-VDSLVIRSAKPYLATPGATVHGH
YGEILYEGDTLVTFIYEKSRSGDITQGLPKVEQILEVRSIDSISMNLERRVEGWNERIPRILGI
PWGFLIGAELTIAQSRISLVNKIQKVYRSQGVQIHNRHIEIIVRQVTSKVLVSE---DGMSNVF
SPGELIGLLRAERAGRALNESICYRAILLGITRASLNTQSFISEASFQETARVLAKAALRGRI
DWLKGLKENVVLGGIIPVGTGFQKFVHRSR--QDKNIY--LEIKRKNLFELET--KDILLHHRE
LFCSYVTNNFHETSEKSFMRFNDS------MTRRYWNINLEEMMEAGVHFGHGTKKWNPRM
APYISAKRKGIHITNLARTARFLSEACDLVFDAASRGKSFLIVGTKNKAADLVASAAIRARC
HYVNKKWLGGMLTNWSTTETRLSKFRDLRAEEKMGKLNRLPKRDAAMLKRKLYTLQT
YLGGIKYMTGLPDIVIIVDQQEEYTALRECAILGIPTICLIDTNCDPDLADISIPANDDAIASIR
LILNKLVSAICEGRSS-YIRNRMGQKINPLGFRLGTTQSHHSFWFAQPKNYSAGLQEDEKIR
NCIKNYVQKNRRISSG-FEGIARIGIQKKIDLIQVIIHIGFPNLLIEG--QTR--GIEELQINVQKE
FYSVNRRLNIAITRVEKPYGQPNILSEYIALQLKNRVSFRKAMKKAIELTEQTDTKGIQVQI
AGRIDGKEIARVEWIREGRVPLQTIRAKIDHCSYTIRTIYGVLGIKIWIFVDDEMSRYRGPRF
KKIRRLGALPGLTSKRPRSGSDLKNQLRSGK-RSQYRIRLEEKQKLRFHYGLTERQLLRYV
HIAGKAKGSTGQVLLQLLEMRLDNILFRLGMAATIPGARQLVNHRHILVNGRIVDIPSYRC
KPRDIITTKDNQKSKGLIQNSIASAAHE--LPKHLTIDS--FQYKGVVNQIIDSKWIGLKINELL
VVEYYSRQTMSRRGTAEEKTAKSDPIYRNRLVNMLVNRIMKHGKKSLAYQIIYRAVKKIQ
QKTETNPLSVLRQAIRGVTPDIAVKARRVGGSTHQVPIEIGSTQGKALAIRWLLGASRKRP
GRNMAFKLSSELVDAAKGSGDAIRKKEETHRMAEANRAFAHFRMSRDTISDLITSIRNAD
MDKKRTVRIASTNITENIVKILLREGFIENVRKHQEGNKYFLVSTLRH--RKNGKKIYRT---R
TVLKRISRPGLRIYSNYQRIPKILGGMGIVILSTSRGIMTDREARLERIGGEMLFCIWMTKAI
PRIGSRRNGRISSRKNGRRIPKGVIHVQASFNNTIITVTDVRGRVVSWASAGTSGFKGTRRG
TPYAAQAAAVNAIRTVVDQGMQRAEVMIKGPGLGRDAALRAIRRSGILLSFVRDVTPMPH
NGCRPPKKRRVMPTIKQLIRNTRQPIRNVTKSPALRGCPQRRGTCTRVYTITPKKPNSALR
KVARVRLTSGFEITAYIPGIGHNLQEHSVVLVRGGRVKDLPGVRYHIVRGTLDAVGVKDRQ
QGRSKYGVKKPKMAKKSLIQRENKRQKLEQKYHLIRRSSKKKIS---KVSSLSEKWEIHGK
LQSPPRNSAPIRLHRRCFLTGRPRANYRDFGLSRHVLREMVHACLLPGATRSSWMVKNSFI
SIIPQEEKDENKNKGSVEFQVFSFTNKIRRLTSHLQLHKKDFLSQRGLRKILGKRQRLLAYL
SKKNRVRYKKLIGQLDIREPKTR--MVKLRLKRCGRKQRAIYRIVAIDVRSRREGRDLRKV
GFYDPIKNQTFLNVPAILYFLEKGAQPTGTVYDILKKAEFFKEGTSN------MDKSKQPFRKY
KRSFRRRLPPIGSGDRIDYRNMSLISRFISEQGKILSRRVNRLTLKQQRLITIAIKQARILSLLP
FLNNEK--QFERAESIPRTTGPRTRNKVTRSLKKNPFVANHLSGKIEKLNMREEKEIIVTWSR
ASTIIPTMIGHTIAIHNGKEHLPIYITDRMVGHKLGEFAPTLTFARHARNDNKSRRMILKSFL
LGNLLSLCMKIINSVVVVGLYYGFLTTFSIGPSYLFLLRARVMEEG---TEKEVSATTGFITGQ
LMMFISIYYAPLHLALGRPHTITVLVLPYLLFHFFWNNHKNFLDYGSTTRNSMRNLSIQCV
FLNNLIFQLFNHFILPSSTLARLVNIYMFRCNNKMLFVTSSFVGWLIGHILFMKWVGLVLF
WIRQNHSIRS----NKYLVSELRNSMARIFSILLFITCVYYLGRMPSPIVTKKLKETEEDKPFV
TFLFDYKRWNRPSRYIKNNRFENAIRNEMSQYFFYTCPSDGKQIISFTYPTSLSTFYEMIDK
KMSLHRTEKLSHEDQ--YWVYTNEQKKYNLNNELINRTKALEKEKIALDVLEKRSRLCND
ENEQEYLPKTYDPFLNGPCRGTIKKYYSNIEIYWINKIYGLLSRKLKDQDLNDQSNGIEEIS
KKVSRWSYKLSDLEEEEEEEIRSRKAKRVVIYTDALIRYSQQSDYRRDIIKGSMRAQRRKT

VIWEMFQANVHSPLFLDRIDKTFFFSSFD-IYEMMNRIFRG-EEKERITISETWDSIIFAQTIR
GLMLVTQSFLRKYIVLPSLIIAKNIGRMLLFQFPEWYEDLKDWNREMHVKCTYNGVQLSE
TEFPKDWLTDGIQIKILFPFCLQPWRRSKKGKKDNFCFLTVWGMEAELPFGSPRKRPSFFE
PIYKELEKKIRQVK-KKIFQLLKVS-----------KEKTKWIIKIVP--VIKRIMKEVEKVNYKIN-N
TIIGESTIQIRSMNWTNYSLIEKKMKDLSDRAITIRNQIEEITKDKKK--------GKRSESEKHIE
QVFKRKSIRLLRKSHYFLKSFIDKIYRDIFLCMINIPRKNSQLFMNFFSTDLSSLSQAYVFFK
LSQTQLLKKYHLKSLLQYTYPFIKEKIKDFCTRGIFDSKSRHKKVDKSGIIEWKNWLKGH
YQYNLSQTKWVRLVPQKWRNRINQRCTIRKKIKLDSYKKEKNQLIHYVKEEKLKKHYRY
DLLSHEYMNGYIIDLDRKYFDCRILGFCLRFFDWMGMNQESVYRWFFPEFVLLFDTYKIK
PWIIPIKLLLLNSKKSKEAELDFFLKKYFLFQLRWDDSLNQRMINNIKVYCLLLRLINPKEI
AISSIQRGEMHLDVMLIQKDLALPQLIKKGILIIEPIRLSIKRDGKFLLYQTISISLGEKNKYN
YYDFLVPEHMLSPRRRRELRILICFRFLWPNYRLEDLACMNRYWFDTNNGSRFNMLRIHM
YPMKGHQFKSWIFELREILREIKNSHYFLDSWIKFDSVGSFTHIFFHQERFMKLFDPRIWSI
LLS-RDS--QGSTSNRYFT---IKGVVLLVVA-VLIS----RINNRKMVERKNLYLMGLLP-----IPM
NSIGPRNETLEESF-WSSNINRLIVS-LLYLPKGKNISESCFMDPQEITWVLPIN----KKCIM----
RSSRWWRNQIGKKR--DFCCKIS---NETVAGIEISFKEKDSK-YLEFIFLSYM--DDPIRKDHD
WELFDRLSPRKKRNIINL--NSRQLFEILGKDLICYLMSAFREKRPIEGKG-FFKQQG-AEATI
QSNDIEHVSH--LFSRNKWGISLQNCAQFHMWQFRQDLFI---SWGK-NQHESDFLRN-VSRE
NLIWLDNVWLGNKDR---FFS--KVRNVLSNIQYDS---TR--SIFVQVTDSSQLKGSS-DQFIDH
FDSIR----NEDSE-------------YHTLIDQTEIQQLKERSIL-WD--PSFLQTEGTEIESD--RFPK--CL
FGSS--SMSRLFT-EREKQMNNHLLPEEIEEFL--GNPTRSIRSFFSDRW--SELH-LGSNPTERST
RDQ----KFLKKKQDVS-FVPSRRSE----NKEMVDTFKIITYLQK--TVSIHPISSDPGCDMVP-K
DESDMMDSSNKISFLNKNPFLD--LFHLFHDRNKGGYTL--HHDF-ES-EERFQEMADLFTLS
ITEP-DLVYHRGFSFSIDSYRLDQKK--FLNEVFNSRDESKKKSL---LVLPPLFY--EENESFYR
RIRKKSVR-IHCGNDLEDSKLKTAVFASNNIMEAVNQYRLIRNLIQIQYSTYG--YIRNVSNR
FFLM---NRSDRNFEYGIQ---RGQIGNDTLNHITIMKYTINQHLSNLKK-SQKKWFDP----LISR
TERSMNRDPDAYRYKWSNGTKNFQEHLEDFVSEQKK---RFQVVFDRLRINQYSIDWSEAI
DKQDLSKSLRFFLSKS---------LLFLS-------KSLPFL-SKSLPLFFVSIGNIPIHRSE-IHIY-ELKG
PNDQLRNQLLESIGVQIVHLNKLKP--FLLD---DHDTS-QRPKFLINGGTIFPFLFKKIP-KW-M
IDSFQT--RNNRRKSFDNTDSNFSMISHDRDNWLNPVKPFHRSSLIFSFYKANRLRFLNDPH
HFWFY--CNKRFPFYV----EKTRIHNYD--LTY-GQFLNILFIRNK-IFSLCVGK--KKHIFLE-RET
ISPIESQVSNIFIPNDFPQSGDET-YNLYK---SFHFPIRSDPFVR--RAIYSIADISATPLTEEQIVN
FERTYCQPLSDMNLSDSEGKNLHQYLS--FNSNMGLIHTPCSEKYLP--SGKRKK--------RSL
CLK--KCVEKRQMYRTFQRDSAFSNLSKWNLFQTYMPWFLTSTGCKYLNFTLLDTFSDPL-
-PILSSS-PKFVSIFHDIMHGSDISWSIPQK--------ILPQRTL-ISEISSKCLQN--LLLSEEMIHRN
NESPVPLIWTHLRSTNAREF-LYSILFLLLVAGY-LVRIHFLFVSR--ASSELQTELEKIKSLMIP
SYMIELRKLLDRYPTSELNSFWLKNLFLVALEQLGDSLEEIRGSASGGNMLLGGGP-----AY
G-VKSIRSKKKYLNSN---LIDLI-SIIPNPINRITFSRNTRHLSRTSKGIYSLIRKRKNVNGDWI
DDKIESWVANSDSIDDEEREFLVQFS--TLTT--EKKIDQILLSLT-HSDHLS-----KNDSGYQMI
EQPGSIYLRYLVDIH--KKYLMSYEFNRSCLAERRIFLAHYQTITY--SQTSCGANSFHFPSHG
---KPFSLRLALSPS-RGILVIGSIGTGRSYLVKYLATNSYVPFITVFPNKFLDDKPKGYLID-----
DID-IDDSDDID----IDDRDDIDIDDSD----------DIDDDLDTELLTMTNVL---TMYMT---PKIDR
FDITLQFELAKAMSPCIIWIPNIHDLYVNESNYLSLGLLENYLS---RDCERCS-TRNILVIAST
HIPQKVDPALIAPNKLNTCIKIRRLLIPQQRKHFFILSYTRGFHLEKKMFHT--NGFGSLTMG

SNARDLVALINEALSISITQKKSIIETNTIRSALHRQTWDLR---SQVRSVQDHGILFYQIGRAV
AQNVLLSNCPIDPISIYMKKK--SCKEGDSYLYKWYFELGTSMKKLTILLYLLSCSAGSVAQ
DLWSSP--GPDEK-NWITSYGFVENDS-DLVHGLLEVEGALVG--SSRTEKDCSQFDNN--RVT
LLLRSEPRN-QLDMMQNGSCSIVDQRFLYEK--YESEFEE-GEG--AVDPQ----QIEAD----LFN
H--IVWAP--RIWRL---CGTLFDCIERPTELGFP-YWAGSFR---GKRIIYHKEDELQENDSEFLQ
RGTMQYQTRDRSSKEQGFFRISQFIWDPADPFFFLFK-DQPFVSVFSRREFFADEEMSKGLI
TSQTNPPTSIYKRWFI--KNTQEKHFELLIHRQRWLRT---NSSLS--NGSFRSN--TLSESYQYLS
NLFLSN-GTLLDQMTKTLLRKRWLFPDEMKHLI--HVTGERFPIPMPRSR-INGNFIDKTSSIV
ANILLRIIPTTSGEKKAFTYYRDGAIMLAQSEGNYAEALQNYYEATRPEIDPYDRSYILYNI
GLIHTSNGEHTKALEYYFRALERNPFLPQAFNNMAVICHY-RGEQAILQGDSEIAEAWSDQ
AAEYWKQAIALTPGNYIEAHNWLKITKRFEFEMNWRSEHIWIELITGSRKTSNFCWACILF
LGSLGFLLVGTSSYLGRNLISVFQSQQIVFFPQGIVMSFYGIAGLFISSYLWSTILWNVGSGY
DRFDRKEGIVCIFRWGFPGINRRIFLRFLMRDIQSIRIEVKEGLSPRRVLYMEIRGQGAIPLTR
TDEN-LTPREIEQKAAELAYFLRVPIEVF
>Phoenix
MEKWWFNSMLSLNKSMDSLDAIGHTSGSEEPILNGTEKNIP-FRNVDYLFDIRDIWSLISD
DTFLVRDSNGDSFSVCFDIENQIFEIDND--SSFLSELESFFSS-----YLNSGSKRNNHYYYHYI
YDTQSSWNNHINSCIDNYLRFEV--SINSSISGGTDNYSDSYIYSFICTESGSSIRTSKNGSDFN
IRRRS-DFGRNKKYRHLWVQCENCYGLNYKKFFRSKMNICEQCGYHLKMSSSDRIELSIDP
GTWDPMDEDMVSMDPIEFHSEGEPYRDRIDSYQRKIGLTEAVQTGIGQLNGIPIAIGVMDF
KFMGGSMGSVVGEKITRLIEYATNRSLPVIIVCASGGARMQEGSLSLMQMAKISSASYNY
QSNKNLFYVSILTSPTTGGVTASFGMLGDVIVAEPNAYIAFAGKRVIEQTLKKTVPDGSQAS
EYLFHKGLFDPIVPRNPLKGVLSELFQLHGFFPLNQNSKNMVTLRADEISNIIRERIEQYNR
EVKIVNTGTVLQVGDGIARIHGLDEVMAGELVEFEEGTIGIALNLESNNVGVVLMGDGLM
IQEGSSVKATGRIAQIPVSEAYLGRVINALAKPIDGRGEISASESRLIESPAPGIISRRSVYEPL
QTGLIAIDSMIPIGRGQRELIIGDRQTGKTAVATDTILNQKGQNVICVYVAIGQKASSVAQV
VTTFQERGAMEYTIVVAETADSPATLQYLAPYTGAALAEYFMYRERHTLIIYDDLSKQAQ
AYRQMSLLLRRPPGREAYPGDVFYLHSRLLERAAKSNSRLGEGSMTALPIVETQSGDVSA
YIPTNVISITDGQIFLSADLFNAGIRPAINVGISVSRVGSAAQIKAMKQVAGKSKLELAQFAE
LEAFAQFASDLDKATQNQLARGQRLRELLKQSQSDPLTVEEQIVTIYTGANGYLDPLEIGQ
VKKFLVQLRTYLKKNKPQFQEIISSTKTFTEQAEALLREAIQEQIELFLLQEQTMRINPTPSS
PVVSTLEEKNLGRIAQIIGPVLDVVFPPGKMPNIYNALVVKSRDTVGGQQINVTCEVQQLLG
NNRVRAVAMSATDGLMRGMEVIDTGAPLSVPVGGATLGRIFNVLGEPVDNLGPVDTRTTS
PIHRSAPAFIQLDTKLSIFETGIKVVDLLAPYRRGGKIGLFGGAGVGKTVLIMELINNIAKA
HGGVSVFGGVGERTREGNDLYMEMKESGVINEKNIAESKVALVYGQMNEPPGARMRVGL
TALTMAEYFRDVNEQDVLLFIDNIFRFVQAGSEVSALLGRMPSAVGYQPTLSTEMGSLQE
RITSTKEGSITSIQAVYVPADDLTDPAPATTFAHLDATTVLSRVLAAKGIYPAVDPLDSTSTM
LQPRIVGEEHYETAQRVKQTSQRYKELQDIIAILGLDELSEEDRLTVARARKIERFLSQPFFV
AEVFTGSPGKYVGLAETIRGFQLILSGELDGLPEQAFYLVGNIDEATAKAMNLEVESKLKK
MTLNLCVLTPNRIIWDSEVKEIILSTNSGQIGVLPNHAPIATAVDIGLLRIRLNDQWLTVALM
GGFARIGNNDITILGNDAEMSTDIDPQEAQQALEIAEANLSRAEGKRQAIEANLALRRART
RVEAVNAISMKNVTDSFLSLGHWPSAGSFGFNTDILATNPINLSVVLGVLIFFGKGVLNDL
LDNRKQRILSTIRNSEELRRGAIEQLERARTRLRKVEIEADEYRMNGYSEIEREKENLINAT
CDTLERLENYKNETLHFEQQRAINQVRQRVFQQALQGALGTLNSCLNSELHFRTIRANIGI

LGAVGEITDMNPLISAASVIAAGLAVGLASIGPGVGQGTAAGQAVEGIARQPEAEGKIRGT
LLLSLAFMEALTIYGLVVALALLFANPFVMNVIPCSIKTLKGLYDISGVEVGQHFYWQIGG
LQIHAQVLITSWVVIAILLGSVIIAVRNPQTIPTDGQNFFEYVLEFIRDLSKTQIGEEYGPWV
PFIGTMFLFIFVSNWSGALLPWKIIQLPHGELAAPTNDINTTVALALPTSAAYFYAGLTKKG
LGYFGKYIQPTPILLPINILEDFTKPLSLSFRLFGNILADELVVVVLVSLVPSVVPIPVMLLGL
FTSGIQALIFATLAAAYIGESMEGHHMIFVTLEHILTNISFSIISIVITIHFMNLLVYEIVGLRDS
SEKGMIATFFSITGLLVSRWISSGHFPLSNLYESLIFLSCSFFIIHIISKKLNHKNDLSTITTPSTI
FTQGFATSGLSTEMHQSAILVPALQSQWLMMHVSMMLLSYAALLCGSLLSIALLVITFRNN
INFFCKSNEIKYLNEKRSVFKNTSFLSFRNYYKYQLTQRLDYWSYRMISLGFTFLTIGILCG
AVWANEAWGSYWSWDPKETWAFITWTIFAIYSHTRTNQSLQGTNSALIASIGFFIIWICYFG
VNLLGIGLHSYGSFTLTEKKKARV-SLPYISSIIFLPWWVSLSFNKCLEPWVTNWWNTGKS
ETFLNDIQEKNVLERFIELEELFLLDEMIKEYPETHIQKLRIGIHKETIQLVKTHNEYHLHIIL
DFSTNIICFAILSGYFILGNEELVILNSWIQEFLYNLSDTIKAFLILLVTDLWIGFHSTHGWEL
MIGSVYNDFGLVHNDQIISSLVSTFPVILDTIVKYWIFYYLNRVSPSLVVIYHSMNEMPIGVP
KVPFRSPGEEDAVWVDVYNRLYRERLLFLGQEVDSEISNQLVGLMVYLSIEDDTRDLYLFI
NSPGGWVIPGIAIYDTMQFVPPDVHTICMGLAASMGSFILVGGEITKRLAFPHARVMIHQP
ASSFYEAQAGEFILEAEELLKLRETLTKVYVQRTGNPLWVVSEDMERDVFMSATEAQVHG
IVDLVAVEMKEQKLIHEGLITESLPNGMFRVRLDNEDLILGYASGRIRRSFIRILPGDRVKIE
VSRYDSTRGRIIYRLRNKDSNDMEELQGYLEKDRSRQQHFLYPLLFKEYIYTFVHDRGLFG
YDN-SSSVLVKRSIIRMYQQNYLIYSVNDSNQNRFVGFHFYSQMILEGFAVIVEIPFLLRLVS
SLIPKSQNLRSIHSTFPFLEDKLSHLNYVSDILIPYPIHLKILVQILQFWIQDVPSLHLLRFFLH
EYHNWNSLITNSIFLFSKKRVFRFPYNSYVSECEFVLVFLRKQSSYLRLTSSGAFLERTHFY
GKIEH-IVVRRNQKTLWFFKDPFMHYVRYQGKAILVSKGTHLLMKKWKCYLVNFWQYYF
HFWSQPYRIHINQLSNCSFYFLGYLSSVLINPSAVRNQMLENSFLIDTVI--KKFDTRVPVITL
IGSLSKAKFCTVLGHPISKPIWTDLSDCDIIDRFGRICRNLSHYHSGSSKKQSLYRIKYILRFS
CARTLARKHKSTVRAFLQRLGSGLLEEFFMKEEQVVSLIFPKRIWYLDIIHINDLVNYSMII
DTT-EVQ-AINSFSTLE-SIKEIYGLIWMFVSIFTLIFGIIIGVLVIVWLEREISATIQQRIGPEYAG
PLGILQALADGTKLLFKEDLLPSRGDIRLFSVGPSIAVISILLSYLVIPFGYRLVLGDLSIGVFL
WIAISSIAPIGLLVSGYGSNNKYSFSGGLRAAAQSISYEIPLTLCVLSISLLSNSSSTVDIVEA
QSKYGFWGWNLWRQPIGFIVFLISSLAECERLPFDLPEAEEELVAGYQTEYSGIKFGLFYIA
SYLNLLLSSLFVTVLYLGGWNFSIPYISIPELFGINKMAGVFGMTIGIFITLAKAYLFLFIPITT
RWTLPRIRMDQLLNLGWKFLLPISLGNLLLTTSSQLVSLMIWHVQNENFILDSTRIFMKAF
HLLLFHGSFIFPECILIFGLILLLMIDSTSDQKDRPWFYFISSTSLVISITALLFRWREEPIISFSG
NFQTNNFNEIFQFLILLCSTLCIPLSVEYIECTEMAITEFLLFVLTATLGGMFLCGANDLITIFV
APECFSLCSYLLSGYTKRDVRSNEATTKYLLMGGASSSILVHGFSWLYGLSGGEIELQEIV
NGLINTQMYNSPGISIALISITVGIGFKLSPAPFHQWTPDIYEGSPTPVVAFLSVTSKVAASAS
ATRIFDIPFYFSSNEWHLLLEILAILSMILGNLIAITQTSMKRMLAYSSIGQIGYVIIGIIVGDS
NDGYASMITYMLFYISMNLGTFACIVSFGLRTGTDNIRDYAGLYTKDPFLALSSALCLLSLG
GLPPLAGFFGKLHLFWCGWQAGLYFLVSIGLLTSVVSIYYYLKIIKLLMTGRNQEITPYVR
NYRRSPLRSNNSIELSMTVCVIASTIPGISMNPILAIAQDTLFMFLLHEYDIFWAFLIISSVIPI
LTFVISGVLAPVSEGPEKLSSYESGIEPMGDAWLQFRIRYYMFALVFVVFDVETVFLYPWA
MSFDVLGVSVFIEAFIFVLIPIVGSVYAWRKGALEWSTSYFPWLTILVVLPIFAGSSIFFFPHR
GNKMFRWYTICICLLELLLTTYAFCYHFQLDDPLIQLEEDYKWIDIFDFHWRLGIDGLSIGP
ILLTGFITTLATLAAWPVTRNSRLFYFLMLAMYSGQIGSFSSRDLLLFFIMWELELIPVYLLL

SMWGGKKRLYSATKFILYTAGGSIFLLIGVLGMGLYGSNEPTLDFERLTNQSYPVALEIILYF
GFFIAYAVKSPIIPLHTWLPNTHGEAHYSTCMLLAGILLKMGAYGLIRINMELLPHAHSIFSP
WLVIVGTMQIIYAALISLGQRNFKKRIAYSSVSHMGFTIIGIGSITDMGLNGAILQILSHGFIG
AALFFLAGTSCDRIRLVYLDEMGGISIPMPKIFTMFSSFSMASLALPGMSGFVAESVVFFGII
TSPKYLLMPKILITFVMAIGVILTPIYLLSMSRQMFYGYKLFNVPHSNFLDSGPRELFVSICI
FLPVIGIGIYPDFVLSLSVDRVQTILSNYFYRMMFEHVLFLSVYLFSIGIYGLITSRKMVRAL
MCLELILNSVNINLVTFSDIFDNRQLKGDIFSIFVIAVAAAEAAIGLAIVSSIHRNRKSTRINQ
SNLLNNMEHTYQYAWIIPFLPLTVTMSIGFGLILIPKATKNIRRIWVFPSIFLLSIAMGFSANL
SIQQINGSFIYQYLWSWTIDIDFSLEFGYLIDPLTSIMSILITTVGVMILIYSDNYMSHDQGYL
RFFAYMSFSNTSMLGLVTSSNLIQIHIFWELVGMCSYLLIGFWFTRPIAASACQKAFVTNRV
GDFGLLLGILGLYWITGSLEFRDLFEIANNNGVSSLFATLCASLLFVGAVAKSAQFPLHVWL
PDAMEGPTPISALIHAATMVAAGIFLVARLIPLFIDIPYIMNFISLLGVITVLLGATFSLAQRDI
KRSLAYSTMSQLGYIMLALGIGSYRAALFHLITHAYSKALLFLGSGSIIHSMEPIVGYSPEK
SQNMVLMGGLTRYVPITRTTFLLGTLSLCGIPPLACFWSKDEILNDSWLYSPIFAIIPYFTIGL
TAFYMFRVYLLTFDGYLRVYFQNHSSTKNNLFYSISLWGKEAPKTFNK-NLLLSTMNFFSN
KY--TSTYPHESDNTMLFPLLVLVLFTLFVGSIGISFDFDILSKWLTPSPNLLHPNSNYSVNW
YEFFTNSIFSVSIAIFGLFIAYILYGSVYSFFQNLDLINSFVIILDQIKSVIYNWSYNRGYIDIFY
TRVLVMGIRRLTELTQFFDRHIIDGITNGVGLTSSLIGEGIRY-MGGGRISSYLFLYFFYVSIFL
LFFLMDLPGPIHDILVVFLGLVLVLGGLGVVLLTNPIFSAFSLGLVLVCISLFYILSNSYFVAA
AQLLIYVGAINVLIIFAVMFMNGSEYSNDSYFWTIGDGVTSLVCTSILFSLITIIPDTSWYGIF
WTTRSSQIIEQDLISNIQQIGIHLSTDFYLPFELISIILLVSLIGAITMARQMTIPATRKDLMIVN
MGPHHPSMHGVLRLIVTLDGEDVIDCEPILGYLHRGMEKIAENRTIIQYLPYVTRWDYLAT
MFTEAITVNAPEQLENVQVPQRASYIRVIMLELSRIASHLLWLGPFMADIGAQTPFFYIFRE
RELLYDLFEAATGMRMMHNYFRIGGVAADLPHGWIDKCLDFCDYSLTGVVEYQKLITRN
PIFLERVERVGIIGGEEAINWGLSGPMLRASGIQWDLRKVDHYECYNEFDWEVQWQKEG
DSLARYLVRIGEMGESIKIIQQALEGIPGGPYENLEVRR-DRAKNSEWNDFEYRFISKKPSP
NFELSKQELYVRVEAPKGELGIYLIGDNSIFPWRWKIRPPGFINLQILPQLVKRMKLADIMTI
LGSIDIIMGEVDRMFSMVTGFMNYSQQTIRAARYIGQSFVITLSHTNRLPITIQYPYEKSITS
ERFRGRIHFEFDKCIACEVCVRVCPIDLPVVDWRFERDIKKKQLLNYSIDFGVCIFCGNCVE
YCPTNCLSMTEEYELSTSDRHELNYNQIALGRLPMSIIGDYTIQTVMNSKIDKDKPFDSRTI
TNYQQDRLSDWLVKHELVHRSLGFDCRGIETLQIKTEDWYSIAVISYVYGYNYLRSQCAY
DVAPGGFLASVYHLTRIQYGVDKPEEVCIKVFVPKNNPRISSVFWIWKSADFQERESYDM
LGISYDNHPRLKRILMPESWIGWPLRKDYITPNFYEIQDAH------------------------------------M
VLT-EYSDNQNKKEGKDYIETVMNLIEFPLLDQTTPNSVISTTSNDLSNWSRLSSLWPLLYG
TSCCFIEFASLIGSRFDFDRYGLVPKSSPRQADLILTAGTVTMKMAPSLVRLYEQMPEPKYV
IAMGACTITGGMFSTDSYSTVRGVDKLIPVDVYLPGCPPKPEAVIDAIMKLRKKISREIFED
RTVSQ-ENRCFSTNHKFYVRRSTQTGNYDQGLLYQSPSTSEMPT--FFKSKSSISSHELVNMQ
NRNTFSWVKEQMTQSISISIMIYVITRASISNAYPIFAQQGYENPREATGRIVCANCHLANK
PVDIEVPQAVLPDTVFEAVVRIPYDMQLKQVLANGKKGALNVGAVLILPEGFELAPPDRIS
PEVREKMGNLSFQSYRPNKRNILVIGPVPGQKYSEIAFPILSPDPATRKDVHFLKYPIYVGG
NRGRGQIYPDGSKSNNTVYNATSAGIVSKIVRKEKGGYEISIVDVSDGHQVIDIIPPGPELL
VSEGESIKLDQPLTSNPNVGGFGQGDAEIVLQDPLRVQGLLFFLASVILAQVFLVLKKKQF
EKVQLYEMNFLNKVYDWFEERLEIQAIADDITSKYVPPHVNIFYCLGGITLTCFLVQVATGF
AMTFYYRPTVTEAFSSVQYIMTEANFGWLIRSVHRWSASMMVLMMILHVFRVYLTGGFK

KPRELTWVTGVVLGVLTASFGVTGYSLPRDQIGYWAVKIVTGVPEAIPVIGSPLVELLRGS
ASVGQSTLTRFYSLHTFVLPLLTAVFMLMHFPMIRKQGISGPLIPITKKPDLNDPVLRAKLA
KGMGHNYYGEPAWPNDLLYIFPVVILGTIACNVGLAVLEPSMIGEPADPFATPLEILPEWYF
FPVFQILRTVPNKLLGVLLMVSVPTGLLTVPFLENVNKFQNPFRRPVATTVFLIGTVVALWL
GIGATLPIDKSLTLGLFMIEVFLFGIVLGLIPITLAGLFVTAYLQYRRGDQLDLMPTITSYFGF
LLAALTITPALLIGLNKIRLIMDIVSLTWAALMVVFTFSLSLVVWGRSGLMIIRSPEPEVKIV
VDRDPVKTSFEEWARPGHFSRTIAKGPDTTTWIWNLHADAHDFDSHTSDLEEISRKVFSA
HFGQLSIIFLWLSGMYFHGARFSNYEAWLSDPTHIGPSAQVVWPIVGQEILNGDVGGGFR
GIQITSGFFQIWRASGITNELQLYCTAIGALVFASLMLFAGWFHYHKAAPKLAWFQDVESM
LNHHLAGLLGLGSLSWAGHQIHVSLPINQFLDAGVDPKEIPLPHEFILNRDLLAQLYPSFAE
GATPFFTLNWSKYAEFLSFRGGLDPITGGLWLSDIAHHHLAIAILFLIAGHMYRTNWGIGH
GLKDILEAHKGPFTGQGHKGLYEILTTSWHAQLSLNLAMLGSLTIVVAHHMYSMPPYPYL
AIDYGTQLSLFTHHMWIGGFLIVGAAAHAAIFMVRDYDPTTRYNDLLDRVLRHRDAIISH
LNWACIFLGFHSFGLYIHNDTMSALGRPQDMFSDTAIQLQPIFSQWVQNIHALAPGVTAPG
ATTSTSLTWGGGELVAVGGKVALLPIPLGTADFLVHHIHAFTIHVTVLILLKGVLFARSSRLI
PDKANLGFRFPCDGPGRGGTCQVSAWDHVFLGLFWMYNSISVVIFHFSWKMQSDVWGTI
SDQGVVTHITGGNFAQSSITINGWLRDFLWAQASQVIQSYGSSLSAYGLFFLGAHFIWAFSL
MFLFSGRGYWQELIESIVWAHNKLKVAPATQPRALSIVQGRAVGVTHYLLGGIATTWAFFL
ARIIAVGMALRFPRFSLGLAQDPTTRRIWFGIATAHDFESHDDITEERLYQNIFASHFGQLAII
FLWTSGNLFHVAWQGNFESWIQDPLHVRPIAHAIWDPHFGQPAVEAFTRGGAIGPVNIAYS
GVYQWWYTIGLRTNEDLYTGALFLLFLSAISLIAGWLHLQPRWKPSVSWFKNAESRLNH
HLSGLFGVSSLAWAGHLVHIAIPGSRGEYVRWNNFLDVLPYPQGLGPLFTGQWNLYAQNP
DSSSHLFGTSQGAGTAILTLLGGFHPQTQSLWLTDIAHHHLAIAFIFLVAGHMYRTNFGIGH
SIKDLLETHIPPGGRLGRGHKGLYDTINNSIHFQLGLALASLGVITSLVAQHMYSLPAYAFIA
QDFTTQAALYTHHQYIAGFIMTGAFAHGAIFFIRDYNPEQNEDNVLARMLDHKEAIKSHL
SWASLFLGFHTLGLYVHNDVMLAFGTPEKQILIEPIFAQWIQSAHGKTSYGFDVLLSSTNG
PAFNAGQSIWLPGWLNAVNE--NSNSLFLTIGPGDFLVHHAIALGLHTTTLILVKGALDARG
SKLMPDKKDFGYSFPCDGPGRGGTCDISAWDAFYLAVFWMLNTIGWVTFYWHWKHITL
WQGNVSQFNESSTYLMGWLRDYLWLNSSQLINGYNPFGMNSLSVWAWMFLFGHLVWAI
GFMFLISWRGYWQELIETLAWAHERTPLANLIRWRDKPVALSIVQARLVGLAHFSVGYIFT
YAAFLIASTSGKFGMSHSVKIYDTCIGCTQCVRACPTDVLEMIPWDGCKAKQIASAPRTED
CVGCKRCESACPTDFLSVRVYLWHETTRSMGLSYMTDLNLPSIFVPLVGLVFPAIAMASLS
LYVQKNKIVMRDIKTYLSVAPVLTTLWFGSLAGLLIEINRLFPDALSFPFFSFMTAILERRES
TSLWGRFCNWITSTENRLYIGWFGVLMIPTLLTATSVFIIAFIAAPPVDIDGIREPVSGSLLYG
NNIISGAIIPTSAAIGLHFYPIWEAASVDEWLYNGGPYELIVLHFLLGVACYMGREWELSFR
LGMRPWIAVAYSAPVAAATAVFLIYPIGQGSFSDGMPLGISGTFNFMIVFQAEHNILMHPFH
MLGVAGVFGGSLFSAMHGSLVTSSLIRETTENESANAGYRFGQEGETYNIVAAHGYFGRLI
FQYASFNNSRSLHFFLAAWPVVGIWFTALGISTMAFNLNGFNFNQSVVDSQGRVINTWADI
INRANLGMEVMHERNAHNFPLDLAAVEVPSTNGMGLPWYRVHTVVLNDPGRLLSVHIM
HTALVSGWAGSMALYELAVFDPSDPVLDPMWRQGMFVIPFMTRLGITNSWGGWSIS-GGT
ITNPGIWSYEGVAGAHIVFSGLCFLAAIWHWVYWDLEIFCDERTGKPSLDLPKIFGIHLFLS
GVACFGFGAFHVTGLYGPGIWVSDPYGLTGKVQSVNPAWGAEGFDPFVPGGIASHHIAAG
TLGILAGLFHLSVRPPQRLYKGLRMGNIETVLSSSIAAVFFAAFVVAGTMWYGSATTPIELF
GPTRYQWDQGYFQQEIYRRVSAGLAENLSLSEAWSKIPEKLAFYDYIGNNPAKGGLFRAG

SMDNGDGIAVGWLGHPVFRDKEGRELFVRRMPTFFETFPVVLVDGDGIVRADVPFRRAES
KYSVEQVGVTVEFYGGELNGVSYGDPATVKKYARRAQLGEIFELDRATLKSDGVFRSSPR
GWFTFGHATFALLFFFGHIWHGARTLFRDVFAGIDPDLDAQVEFGTFQKLGDPTTRRQVV
MKTLYSLRRFYPVETLFNGTLALAGRDQETTGFAWWAGNARLINLSGKLLGAHVAHAGL
IVFWAGAMNLFEVAHFVPEKPMYEQGLILLPHLATLGWGVGPGGEVIDTFPYFVSGVLHLI
SSAVLGFGGIYHALLGPETLEESFPFFGYVWKDRNKMTTILGIHLILLGIGAFLLVLKALYF
GGVYDTWAPGGGDVRKITNLTLSPSVIFGYLLKSPFGGEGWIVSVDDLEDIIGGHVWLGSI
CILGGIWHILTKPFAWARRAFVWSGEAYLSYSLGALSVFGFIACCFVWFNNTAYPSEFYGP
TGPEASQAQAFTFLVRDQRLGANVGSAQGPTGLGKYLMRSPTGEVIFGGETMRFWDLRA
PWLEPLRGPNGLDLSRLKKDIQPWQERRSAEYMTHAPLGSLNSVGGVATEINAVNYVSPR
SWLATSHFVLGFFLFVGHLWHAGRARAAAAGFEKGIDRDLEPVLSMTPLSMTIALGRFTK
EE-DLFDIMDDWLRRDRFVFVGWSGLLLFPCAYFALGGWFTGTTFVTSWYTHGLASSYLE
GCNFLTAAVSTPANSLAHSLLLLWGPEAQGDFTRWCQLGGLWTFVALHGAFGLIGFMLRQ
FELARSVQLRPYNAIAFSAPIAVFVSVFLIYPLGQSGWFFAPSFGVAAIFRFILFFQGFHNWT
LNPFHMMGVAGVLGAALLCAIHGATVENTLFEDGDGANTFRAFNPTQAEETYSMVTANR
FWSQIFGVAFSNKRWLHFFMLFVPVTGLWMSALGVVGLALNLRAYDFVSQEIRAAEDPEF
ETFYTKNILLNEGIRAWMAAQDQPHENLIFPEEVLPRGNALMSGSTGERSFADIITSIRYWV
IHSITIPSLFIAGWLFVSTGLAYDVFGSPRPNEYFTESRQGIPLITGRFDSLEQLDKFSRSFMT
IDRTYPIFTVRWLAVHGLAVPTVFFLGSISAMQFIQRMATQTVEGSSR-SGPRRTTVGNLLK
PLNSEYGKVAPGWGTTPLMGVAMALFAIFLSIILEIYNSSVLLDGISMSMLTLKLFVYTVVI
FFVSLFIFGFLSNDPGRNPGREEMADTTGRIPLWLIGTVTGILVIGLIGIFFYGSYSGLGSSLM
FNIFSLICICLNSVFY-SSFFFAKLPEAYAFLNPIVDVMPVIPVLFFLLAFVWQAAVSFRMTQS
NPNEQNVELNRTSLYWGLLLIFVLAVLFSNYFFNMEVNILAFIATALFILVPTAFLLIIYVKT
VSQNDMETATLVAISISGLLVSFTGYALYTAFGQPSQQLRDPFEEHGDMEALVYTFLLVSTL
GIIFFAIFFREPPKVPTKK--MTIAFQLAVFALIATSSILLISVPVVFASSDGWSSNKNVVFSGTS
LWIGLVFLVAILNSLISMSCREGLMSPQTETKASVGFKAGVKDYKLTYYTPDYETKDTDIL
AAFRVTPQPGVPPEEAGAAVAAESSTGTWTTVWTDGLTSLDRYKGRCYHIETVVGEENQ
YIAYVAYPLDLFEEGSVTNMFTSIVGNVFGFKALRALRLEDLRIPTSYSKTFQGPPHGIQVE
RDKLNKYGRPLLGCTIKPKLGLSAKNYGRAVYECLRGGLDFTKDDENVNSQPFMRWRDR
FVFCAEALYKAQAETGEIKGHYLNATAGTCEEMMKRAMCARELGVPIVMHDYLTGGFTA
NTSLAHYCRDNGLLLHIHRAMHAVIDRQKNHGMHFRVLAKALRMSGGDHIHAGTVVGK
LEGEREMTLGFVDLLRDDFIEKDRSRGIFFTQDWVSMPGVLPVASGGIHVWHMPALTEIFG
DDSVLQFGGGTLGHPWGNAPGAVANRVALEACVQARNEGRDLAREGNEIIREASKWSPE
LAAACEVWKAIKFEFEPVDKLDK--IHLYKTSTPSTRNGAVDSQVKSNPRNNLIYGQHRCG
KGRNARGIITARHRGGGHKRLYRKIDFRRNEKDISGRIVTIEYDPNRNAYICLIHYGDGEKR
YILHPRGAIIGDTIVSGTEVPISMGNALPLTDMPLGTAIHNIEITLGKGGQLARAAGAVAKLI
AKEGKSATLRLPSGEVRLISKNCLATVGQVGNVGVNQKSLGRAGSKCWLGKRPVVRGV
VMNPVDHPHGGGEGRAPIGRKKPTTPWGYPALGRRSRKRKKYSDSFILRRRK-MIQPQTL
LNVADNSGARELMCIRIIGAG-NHRYAHIGDVIVAVIKEAVPNMPLERSEVIRAVIVRTCKEL
KRDNGMIIRYDDNAAVVIDQEGNPKGTRVFGAIARELRQLNFTKIVSLAPEVLNYNPKRTR
FRKQHRGRMKGISYRGNHICFGGYALQALEPAWITARQIEAGRRAMTRYARRGGKIWVRI
FPDKPITVRPTETRMGSGKGSPEYWVSVVKPGRILYEMGGVSETIARAAIEIAACKMPIRT
QFVISRMTRVRRGYTARKRRTKIRLFASTFIGAHSRLTRTTTQQKMRALVSSHRDRGRQKR
DFRSLWITRINAVIREN--VFYNYSRLIPNLYKKQLLLNRKILAQISISNKNCLYMISNKIIK----

-------MIKKVL-AQHIC-MSVLKAQRVIDQIRGRSYEETLMILELMPYRASYPILQLVYSAAA
NANHNMGLNEADSFISKAEVNRSAIVKKLRPRARGRSYTIKKTTCHITIVLK-KSKS-------
MDGIKYAVFTEKSIRLLGNNQ-YTSNVESGSTRTEIKHWVELFFGVKVIAMNSHRLPGKGR
RMGPIMGHTMHYRRMIITLQPGYSIPPLIEKRTMAVPKKRISISKKHIRRNIWKKKGYLTAV
KAFSLAKSVSTGHSKSFF--QTNNMAKGKDVRVRVILECTSCVRN--G--KESPGISRYITQKN
RHNTSSRLELRKFCHYCHKHTIHGEIKKMKIRTSVRKICEKCRLIRRRGRIIVICSNPRHKQ
RQGMVREEVVGSTRTLRWKCVESRVDSKRLYYGRFILSPLMKGQADTIGIAMRRALLGEI
EGTCITRAKSE--KVAHEYSTIVGIEESVHEILLNLKEIILRSNLYGVIDASICVRGPRYVTAK
DIISPPYVEIVDTTQHIANLTEPIDLCIELQIKRDRGYRMKSANNSQDGSY--PIDAVSMPVRN
ANHSIHSYGNGNEKQEILFLEIWTNGSLTPKEALYEASRNLIDLFIPFLHAEEE--DINFEENK
-NRFPLPLFTFQDRLT-NLKKNKK-GIPLKCIFIDQSELPSRTYNCLKGSNIHTLSDLLNNSQE
DLMRIEFFRIEDVKQILDTLQKY--FAIDLSKNKMLRN-GNEGMYTIPAFSQIQFEGFYRFINQ
GLTEEFHKFPKIED--QEIEFKLFVETYKLVEPLIKERDAVYESLTYSSELYVPAGLIWKTGRD
MQEQTVFIGNIPLMNSLGTFIVNGIYRIVINQILQSPGIYYRSELDNNGISVYTSTIISDWGG
RSELEIDRKARIWARVSRKQKISILVLSSAMGSNLRKILDNVCYPEIFLSFPNDKEKKKIGSK
ENAILEFYQQFACVGGDPVFSDSLCKELQKKFFQQRCELGRIGRRNMNRRLNLDIPQNNT
FLLPRDVLAAADHLIGMKFGMGTLDD--MNHLKNKRIRSVADLLQDQFGLALVRLENAVR
GTICGAIR-HKLI-PTPQNLVTSTS----LTTTYESFFGLHPLSQVLDRTNPLTQIVHGRKLSYLG
PGGLTGRTASFRIRDIHPSHYGRICPIDTSEGINVGLIGSLAIHVRIGHWG-SIESPFY---EISQR
SKE--AQVVYLSPNRDEYYMVAAGNSLALNRGIQEEQVVPARYRQEFLTIAWEKIHFRSFFP
FQYFSIGASLIPFIEHNDANRALMSSNMQRQAVPLSRSEKCIVGTGLERQTALDSGVSAIAE
HEGKIIYTDPHKIILSSNGDTISIPLVMYQRSNKNTCMHQKPQVRRGKYIKKGQILADGAAT
VGGELALGKNILVAYMPWEGYNSEDAVLISERLVYEDIYTSFHIRKYEIQTHVTSQG-PERIT
KEIPHLETHLLRNLDRNGIVMLGSWVETGDILVGKLTPQTANE-SSYSPEDRLLRVILGIQVS
TAKETSLKLPIGGRGRVIDVRWIQKKG-GSSYNPEMIRVYISQKREIKVGDKVAGRHGNKGI
ISKILPRQDMPYLQDGTPVDMVFNPLGVPSRMNVGQIFECSLGLAGDLLKKHYRIAPFDE
RYEQEASRKLVFSELYEACKQTKNPWVFEPEYPGKSRIFDGRTGDPFEQPVLIGKSYILKLI
HQVDDKIHGRSSGHYALVTQQPLRGRAKQGGQRVGEMEVWALEGFGVAHILQEMLTYKS
DHIRARQEVLGATIVGGTVPNPEDAPESFRLLVRELRSLALELNHFLVSEKNFQINRKEA----
---MIDRYKHQQLRIGPVSPQQIRAWANKILPNGEIVGEVTKPYTFHYKTNKPEKDGLFCERI
FGPIKSRICACGNYRVIRAEKEDPKFCEQCGVEFVDSRIRRYQMGYIKLACPVTHVWYLK
RLPSYIANLLDKPLKELEGLVYC--DFSFARP-ISKKPTFLRLRGSFEYEIQSRKYSIPLFFTTQ
GFETFRNREISTGAGAIREQLSDSDLRIIIDNSLVEWKELGDE-GSTGNEWEDRKIRRRKDFL
VRRMELAKHFIRTNVEPEWMVLCLLPVLPPELRPIIQTDGGKLMSSDINELYRRVIYRNNT
LTDLLATSRSTPGELVMCQEKLVQEAVDTLLDNGIRGQPMRDGHNKVYKSFSDVIEGKEG
RFRETLLGKRVDYSGRSVIVVGPSLSLHQCGLPREIAIELFQTFLIRGLIRQHVASNIGIAKS
QIREKEPIVWEILQEVMRGHPVLLNRAPTLHRLGIQAFQPILVEGRAICLHPLVCKGFNADF
DGDQMAVHVPLSLEAQAEARLLMFSHINLLSPAIGDPISVPTQDMLIGLYVLTIRNRRGICA
NRHNTYNRGNY---QNNTVDNNDYKYMKEKELYFCSSYDVLGAYRQKRISLDSPLWLRW
RLDQR-VIGS--REVPIELQYESLGTYHEIYEHYLIVGNVKKEIHCIYIRTTLGHISFYREIEEAI
QGFCRAYSYAI------------MAERADLVFHNKVINGTAMKRLISRLIDHFGMAYTSHILDQVK
TLGFQQATATSISLGIDDLLTIPSKGWLVQDTEQQSFIFEKHHHYGNVHAVEKLRQSIEIWYA
TSEYLRQEMNPNFRMTDPSNPVHLMSFSGARGNASQIHQLVGMRGLMSDPQGQMIDLPI
QSNLREGLSLTEYIISCYGARKGVVDTAVRTSDAGYLTRRLVEVVQHIIVRRTDCGTIRGIS

VSPRNGMTE--KIFVQTLIGRVLADNIYMGLRCIAARNQDIGIGLVNRFIT------FRAQPIYIRT
PFTCKSASWICQLCYGRSPTHGDLVELGEAVGIIAGQSIGEPGTQLTLRTFHTGGVFTGDTA
EHVRAPSNGKIKFNEDLVYPTRTRHGHPAFLCSIDLYVTIETRDIIHNVNIPPKSLILVQNDQ
YVESEQVIAEIRAGTSTF--FKEKVRKHIYSESEGE-MHWSTDVHHASEYIHGNVHLLPKTS
HLWILAGGPRG--SSIVSFSLHKDQDQTNVHSFSFEER--YIFDLS--MTN-HRVRHKLLDTSG-
---DREILDYS-RPDRIISNGHWNFIYPS-ILQEN--SDFLAKKRRNRFIIPLQYDQERDKELMPC
FGISIEIPIN--GILRRNSILAYFDDPRYRRN--GITKY-TIEVDSIIK--KEDLIEYRG-AKEFSLKY-
KVDRFFFILEEVHILPGSSFIMVRNNSIIGVDTQLALNTRSRVGGLVRVERKKKNIELKIFSG
DIYFPGETDKISRHSGILIPPETKKK--NSKK-SK-KLKNWIYVQR--ITPTKKK--YFVSVRSVV
TYEIADGINLATLFPRDLLQEKDNVQLRVVNYILYGNGKSIRGIYHTS-QLVRTCLVLNWDQ
EKNGSIE----EVHASFVEVRTNDLIRNFIRIELVKSTISYTGKRYDRASSVLISD---LDRTNINP
FYSKAKIQS-----LTQHQGTIGI--GTLLNRNK---ECQSLIILSSSNCSRIG--PFNGSKYN---NVT
KESDPIIQI-DLPGPLGTIVP-KIANFFSS-YYLIT---HNHILLK-KYLLLD---NFKQTF---QVLKY
CLIDENRR--IYNPDPCGNIILNPFHLNWCFLHHDYCEETSTIIS-LGQFICENVCLFKYES--H
VKKSGQILIVH-VDSLVIRSAKPYLATPGATVHSHYGEILYEGDTLVTFVYEKSRSGDITQGL
PKVEQILEVRSIDSISMNLERRVEGWNERIQRILGIPWGFLIGAELTIAQSRISLVNKIQKVYR
SQGVQIHNRHIEIIVRQVTSKVLVSE---DGMSNVFLPGELIGFLRAERAGRALDEAICYRAIL
LGITRASLNTQSFISEASFQETARVLAKAALRGRIDWLKGLKENVVLGGIIPVGTGFQKLV
HPSR--QDKNIH--LEIKRKNLFELEI--RDILLHHRELFCSYVQNNFHETSEQSFTRFNDS------
MTRRYWNINLEEMMEAGVHFGHGTKKWNPRMAPYISAKRKGIHITNLTRTARFLSETCD
LVFDAASRGKNFLIVGTKNKAADSVASAAIRARCHYVNKKWLGGMLTNWSTTETRLHKF
RDLRAEQKMGKLNCLSKRDAAMLKRKLSTLQTYLGGIKYMAGLPDIVIIVDQQEEYTAL
RECVILGIPTICLIDTNCDPDLADISIPANDDAIASIRLILNKLVSAICEGRSS-YIRNRMGQKI
NPLGFRLGTTQSHRSFWFAQTKNYSVGLQEDEKIRNCIKNYVQKNRRISSG-FEGIARIGIQ
KRIDLIQVIIHIGFPNLLMEG--QTR--GIEELQMNVQKELHSVNRRLNIAITRIEKPYGQPNIL
AEYIALQLKNRVSFRKSMKKAIELTEQTDTKGIQVQIAGRIDGKEIARVEWIREGRVPLQTI
RAKIDHCSYTIRTIYGVLGIKIWIFVNEFMSRYRGPRFKKIRRLGTLPGLTSKRPKSGSDLKT
QLRSGK-RSQYRIRLEEKQKLRFHYGLTERQLVRYVHIAGKAKGSTGQVLLQLLEMRLDNI
LFRLGMASTIPGARQLVNHRHILVNGRIVDIPSYRCKPRDIITTKDKQRSKALIQNYIASPPP
E--LPKHLTIDS--FQYKGLVNQIIDSKWIGLKINELLVVEYYSRQTMSRRGTAEEKTAKSDPI
YRNRLVNMLVNRIMKHGKKSLAYQIIYRAVKKIQQKTETNPLSVLRQAIHGVTPDIAVKAR
RVGGSTHQVPIEIGSTQGKALAIRWLLGASRKRPGRNMAFKLSSELVDAAKGSGDAIRKK
EETHRMAEANRAFAHFRMGRDTIADIITSIRNADMNKKGTVRIASTNITENFVKILLREGFI
ENVRKHQENNKYFLVSTLRH--RKTRKGIYR-----TVLKCISRPGLRIYSNYQRIPKILGGMGI
VILSTSRGIMTDREARLERIGGEILCYIWMTKAIPRIGSRRNGRIGSRKNGRRIPKGVIHVQA
SFNNTIVTVTDVRGRVVSWASAGTSGFKGTRRGTPYAAQAAAVNAIRTVVDQGMQRAEV
MIKGPGLGRDAALRAIRRSGILLSLVRDVTPMPHNGCRPPKKRRVMPTIKQLIRNTRQPIR
NVTKSPALRGCPQRRGTCTRVY-ITPKKPNSALRKVARVRLTSGFEITAYIPGIGHNLQEHSV
VLVRGGRVKDLPGVRYHIVRGTLDAVGVKDRQQGRSKYGVKKPKMARKSLIQREKKRQ
KLEQKYHLIRRSSKKKIS---EVPSLSEKWEIHGKLQSPPRNSAPIRLHRRCFLTGRPRANYR
DFGLSGHILREMVHACLLPGATRSSWMIKNSFISVISQEEKEEN--KGSVEFQVLSFTSKIRR
LTSHLELHKRDFLSQRGLRIILGKRQRLLAYLSKKNRVRYKKLMDQLDIREPKTR------------
-----IYRIVAIDVRSRREGRDLRKVGFYDPVKNQTYLNVPLILYFLEKGAQPTGTVCDILRKA
ELFQE-RTSIRVN--MDKSKQPFRKYKRSFRRRLSPIGSGDRIDYRNMSLISRFISEQGKILSRR

VNKLTLKQQRLITIAIKQARILSLLPFLNNEK--QFERAESIPRTTGPRTRNKVTRSLKKNPFV
ANHLSGKIEKLNMREEKEIIVTWSRASTIIPTMIGHTIAIHNGKENLPIYITDRMVGHKLGEF
APTLTFARHARNDNKSRRMILKSFLLGNLLSLCMKIINSVVVVGLYYGFLTTFSIGPSSLFLL
RARVMEEG---TEKEVSATTGFITGQLMMFISIYYAPLHLALGRPHTITVLVLPYLLFHFFWN
NHKNFFYYGSTTRNSMRNLSIQCVFLNNLIFQLFNHFILPSSTLARLVNIYMFRCNNKMLF
VTSSFVGWLIGHILFMKWVGLVLFWIRQNHSIRS----NKYLVSELRNSMARIFSILLFITCVY
YLGRMPSPIVTKKLKEAEEDKPLVTFLFDYKRWNCPLRYIKNDRFENAVRNEMSQYFFYT
CPSNGKKKISFTYPPSLSTFSEMIERKMSLYTTKKLSHEDL--YWVYTNEQKKYNLSNELIS
RIKVLEKEKFSLDMLEKRTRFCNHENEQECLTKTYDPLLSGPCRGAIKKFDLRIKSLWINKI
HELLLREFEHQDPNNQSIGIEEISKKVPRWSYKLTVLEEQEEEEIRSRKAKRVVIYTDALIRY
SQQSDFRRDIIKGSMRTQRRKTVTWEMFQANAHSPLFLDRIDKTFFFSSFD-ISEMMNLIFR
N-EENERIAISETWDNIIFAQAIRGSMLVTQSFLRKYIVLPSLIIAKNIGRMLLFQFPEWYEDL
KEWNREMHVKCTYNGVQLSETEFPKDWLTDGIQIKILFPFSLKPWRRSTKGKKENFCFLTI
WGMEAEVPFGSPRKRPSFFEPIYKELQKIIRKIIRNNFFFVLKVF-----------KEKKRWVIKIVL
--VIKQKMKEFEKINSKIN-NKIIRESAIRIRSMNWANYSLIEKKIKDLADRTVTIRNELERITK
DNKK--------DKKSESRKHILQIFKRKKIRFIRKLKFFMKSFIEKIYIDIFLRMITIFRINAQFFM
NFISYDLSSLSQAYVFYKLSQTQLLNKYHLRSVLQYTHSFIKDKIKDYCTRGIFDSKSKQK
KIYKSGKNEWKNWLKGHYQYNLSQTKWSRLVPQKWRNRINQRSTIQNKIKFDSHKKEK
DQLIHYMKQEKFKKHYRYYLLSHKYMNEYIIALDRKYFDCRILHICLRFFDWMGMNQEK
VYRRFFPEFVPLFDAYKIKPWIIPIKLLLFNSKKKKEAELDFFLKKYFLFQLRWDDSLNQR
MINNIKVYCLLLRLINPRKIAISSIQRGEMHLDVMLIQKDLALTELIKKGVLIIEPIRLSIKRD
GKFIIYQTLGISLIDKIKYSYYDFFVPENILSPRRRRELRILICFRFLWPNYRLEDLACMNRY
WFNTNNGNRFSMSRIYMYPMKRHQFKSWIFELREILREIKNSHYFLDSWIKFDSVGSFTHI
FFHQERFMKLFDPRIWSILLS-RDS--QGSTSNRYFT---IKGVVLLVVA-VLIS----RINNRKMV
ERKNLYLMGLLPIPILPIPMNSIGPRNETLEESF-WSSNINRLIVS-LLYLPKGKKISESCFMDP
QESTWVLPIN----KKCIMPESDRSSRWWRNRIGKKR--DSSCKIS---NETVAGIEISFKEKDSK-
YLEFLFLSYT--DDPIRKDHDWELFDRLSPRKKRNIINL--NSGQLFEILGKDLICYLMSAFRE
KRPIEGEV-FFKQQG-AEATIQSNDIEHVSH--LFSRNKWGISLQNCAQFHMWQFRQDLFV---
SWGK-NQHESDFLRN-VSRENLIWLDNVWLVNKDR---FFS--KVRNVLSNIQYDS---TR--SIF
VQVRDSSQLKGSS-DQSIDHFDSIR----NEDSE-------------YHTLIDQTEIQQLKERSIL-LD--PS
FLQTERTEIESD--RFPK--CLFGSS--SMSRLFT-EREKQMNNHLLPEEIEEFL--GNPTRSIRSFF
SDRW--SELH-LGSNPTERSTRDQ----KFLKKKQDVS-FVPSRRSE----NKEMVDIFKIITYLQN
--TVSIHPISSDPGCDMVP-KDEPD-MDSSNKISFLKKNPFFD--LFHLFHDRNKGGYTL--HHD
F-ES-EERFQEMADLFTLSITEP-DLVYHRGFAFSIDSYGLDQKK--FLNEVFNSRDESKKKSL-
--LVLPPLFY--EENESFYRRIRKKSVR-IYCGNDLEDPKLKTAVFASNNIMEAVNQYRLIRNLI
QIQYSTYG--YIRNVSNRFFLM---NRSDRNFEYGIQ---RDRIGNDTLNHITIMKYTINQHLSN
LKK-SQKKWFDP----LISRTERSMNRDPDAYRYKCSNGSKNFQEHLEHFVSEQKN---RFQV
VFNRLRINQYSIDWSEAIDKQDLSKSLRFFLSKS---------LLFLS-------KSLPFL-SKSLPLFFV
SIGNIPIHRSE-IHIY-ELKGPNDQLCNQLLESIGVQIVHLNKLKPFLFLLD---DHDTS-QRPKF
LINGGTILPFLFKKIP-KW-MIDSFHT--RNNRRKSFDNTDSYFSMISQDRDNWLNPVKPFHR
SSLISSFYKANRLRFLNDPHHFWFY--CNKRFPFYV----EKTRINNYD--LTY-GQFLNILFIRNK
-IFSLCVGK--KKHIFLE-RETISPIESRVSDIFIPKDFPQSGDET-YNLYK---SFHF--RSDPFVR--
RAIYSIADISATPLTEEQIVNFERTYCQPLSDMNLSDSEGKNLYQYLS--FNSNMGLIHTPCSE
KYLP--SGKRKK--------RSLCLK--KCVEKRQMYRIFQRDSAFSNLSKWNLFQTYMPWFLTS

TGCKYLNFTLLDTFSDPL--PILSSS-QKFVSIFHDIMHGSDISWPIPQK--------ILPQWTL-ISEI
SSKCLQN--LLLSEEMIHRNNESPVPLIWTHLRSTNAREF-LYSILFLLLVAGY-LVRIHLLFVS
R--ASSELQTELEKIKSLMIPSYMIELRKLLDRYPTSELNSFWLKNLLLVALEQLGDSLEEIR
GSASGGNMLLGGGP-----AYG-VKSIRSKKKYLNIN---LIDLI-SIIPNPINRITFSRNTRHLSRT
SKEIYSLIRKRKNVNGDWIDDKIESWVANSDSIDDEEREFLVQFS--TLTT--EKRIDQILLSLT-
HSDHLS-----KNDSGYQMIEQPGSIYLRYLVDIH--KKYLMNYEFNRSCLAERRIFLAHYQTI
TY--SQTSCGANSFHFPSHG---KPFSLRLALSPS-RGILVIGSIGTGRSYLVKYLTTNSYVPFIT
VFPNKFLDDKPKGYLID-----DID-IDDSDDID----IDDSDDIDIDDSD---------DIDDDLDTELL
TMTNVL---TMYMT---PKIDRFDITLQFELAKAMSPCIIWIPNIHDLYVNESNYLSLGLLENY
LS---RDCERCS-TRNILVIASTHIPQKVDPALIAPNKLNTCIKIRRLLIPQQRKHFFILSYTRGF
HLEKKMFHT--NVFGSITMGSNARDLVVLINEALSISITQKKSIIETNTIRSALHRQTWDLR---
SQVRSVQDHGILFYQIGRAVAQNVLLSNCPMDPISIYMKKK--SCKEGDSYLYKWYFELGT
SMKKLTILLYLLSCSAGSVAQDLWSSP--GPDEK-NWITSYGFVENDS-DLVHGLLEVESALV
G--SLRTEKDCSQFDNN--RVTLLLRSEPRN-QLDMMQNGSCSIVDQRFLYEK--YESEFEE-G
EG--AVDPQ----QIEED----LFNH--IVWAP--RIWRP---CGNLFDCIERPTELGFP-YWGGSFR---
GKRIIYHKEDELQENDSEFLQSGTMQYQTRDRSSKEQGFFRISQFIWDPADPFFFLFK-DQP
FVSVFSRREFFADEEMSKGLITYQTNPPTSIYKRWFI--KNTQEKHFELLIHRQRWLRT---NSS
LS--NGSFRSN--TLSESYQYLSNLFLSN-GTLLDQMTKTLLRKRWLFPDEMKHLI--HVTGER
FPIPMPRSR-INVNFIDKTSSIVANILLRIIPTTSGEKKAFTYYRDG--MLAQSEGNYAEALQN
YYEATRPEIDPYDRSYILYNIGLIHTSNGEHTKALEYYFQALERNPFLPQAFNNMAVICHY-
RGEQAILQGDSEIAEAWFDQAAEYWKQAIALTPGNYIEAHNWLKITRRFEFEMNWRSEHI
WIEPIMGSRKTSNFFWACILFLGSLGFLVVGTSSYLGRNLISVFPSQQIIFFPQGIVMSFYGIA
GLFISSYLWCTISWNVGSGYDRFDRKEGIVCIFRWGFPGINRRIFLRLFMRDIQSIRMEVKE
GLYPRRVLYMEIRGQGAIPLTRTDEY-FTPREIEQKAAELAYFLRVPIEVF
>Oncidium
MEKCWFNSMLSLSKSMDNLDALGHTSGSEETN--DAEKKIPSFSNINYLFDSRNIWSLISDH
TFFVRNSNGDTYSVYFDIDNQIFDIDNAS--SFLSELEILF------SYLNNGSNSSN-YYY-SMYD
TQSNWNNHITSCVDSYLR--FEISLNSDIYSGIN-----YISSLICTSGNSSIKTSS--SSFNIIEKYN
DFDINTKYKHLWVQCENCYGLNYKKFFSSKMNICEHCGYHLKMSSSDRIELLIDPGTWEP
MDKDMVSMDPIEFHSEEEPYIDRISFYKRKTGLTEAVQTGVGQLNSIPIAMGVMDFQFMG
GSMGSVVGEKITRLIEYATNRSLPVIIVCASGGARMQEGSLSLMQMAKISSASYDYQSNKK
LFYVSILTSPTTGGVTASFGMLGDIIIAEPNAYIAFAGKRVIEQTLKKTVPDGSQVAEYLFHK
GLFDSIVPRNLLKGVLNELFQLHGFLPLNQD---MGTLRADEISNIIRERIEQYNREVKIVNT
GTVLQVGDGIARIHGLDEVMAGELIEFEEGTIGIALNLESNNVGVVLMGDGLMIKEGSSV
KATGRIAQIPVSEAFLGRVINALAKPIDGRGEISSSESRLIESPAPGIISRRSVYEPLQTGLIAID
SMIPIGRGQRELIIGDRQTGKTAVATDTILNQKGQNVICVYVAIGQKASSVAQVVTTFQEGG
AMEYTIVVAETADSPATLQYLAPYTGAALAEYFMYRERHTSIIYDDLSKQAQAYRQMSLL
LRRPPGREAYPGDVFYLHSRLLERAAKSSSRLGEGSMTALPIVETQSGDVSAYIPTNVISIT
DGQIFLSADLFNAGIRPAINVGISVSRVGSAAQIKAMKQVAGKSKLELAQFAELEAFAQFSS
DLDKATQNQLARGQRLRELLKQSQSDPLAVEDQIATIYTGTNGYLDALEIGQVKKFLVGL
RTYLTKKKPKFQEILSSTKIFTEEAETLLKEAIQEQIELFLLQEQTMRSNPTTSDPVVSTLEE
QNQGRIAQIIGPVLDVIFPPGKMPNIYNALVIQGRDTVGQQINVTCEVQQLLGNNRVRAVA
MSATDGLMRGMKVIDTGAPLSVPVGGATLGRIFNVLGEPVDNLGPVDTRTTSPIHRSAPAF
IQLDTNLSIFETGIKVVDLLAPYRRGGKIGLFGGAGVGKTVLIMELINNIAKAHGGVSVFG

GVGERTREGNDLYMEMKESGVINEKDIAESKVALVYGQMNEPPGARMRVGLTALTMAEY
FRDVNEQDVLLFIDNIFRFVQAGSEVSALLGRMPSAVGYQPTLSTEMGSLQERITSTKEGSI
TSIQAVYVPADDLTDPAPATTFAHLDATTVLSRGLAAKGIYPAVDPLDSTSTMLQPRIVGEE
HYETAQRVKQTSQRYKELQDIIAILGLDELSEEDRLTVARARKIERFLSQPFFVAEVFTGSPG
KYVGLAETIRGFQFILSGELDGLPEQAFYLVGNIDEATAKAINLEEESKLKKMTLNLCVLTP
NRMIWNSEVKEIILSTNSGQIGVLPNHAPIATAVDIGLLRIRLN-RWLMVALMGGFARISNN
EITILGNGAEMSTDIDPQEAQQTLEIAEANFSRAEGKRQAIEANLALRRAKTRVEAVKAIS
MKNITDSFVSVGHWPSAGSFEFNTDILATNPINLSVVLGVLIFFGKGVCASCV--RKQRILST
IRNSEELRRRAIEQLERARVRLRKVEIEADEYRTNGYFEIEREKVNLINATCNSLERLENYK
NETLFFEKQRAINQVRQQVLQQALQRALGTLNSCLNSELHFRTISANIGILRSVEEITDMNP
LISAASVIAAGLAVGLASIGPGVGQGTAAGQAVEGIARQPEAEGKIRGTLLLSLAFMEALTI
YGLVVALALLFANPFVMNVIPCSIKTLKGLYDISGVEVGQHLYWQIGGLQIHAQVLITSWV
VIAILLGSVIIAVRNPQTIPTDGQNFFEYVLEFIQDLSKTQIGEEYGPWVPFIGTMFLFIFVSN
WSGALLPWKIIKLPHGELAAPTNDINTTVALALPTSVAYFYAGISKKGLGYFGKYIQPTPIL
LPINILEDFTKPLSLSFRLFGNILADELVVVVLVSLVPSVVPIPVMFLGLFTSGIQALIFATLA
AAYIGESMEGHHMIFATLEHILTHISFSIISIVILIHLMNLLVYEIKGLRNSSEKGMIATFFSITG
FLVIRWNSSGHFPLSNLYESLIFLSWSLSIIHMIPYLGNPKNDLSAITAPSAIFTQGFATSGLST
EMHKPEILVPALQSQWLMMHVSMMLLSYAALLCGSLLSVALIMITFQKRIYSFLKKNDME
YLNEKVSVLKNTSFLSFQNFYKHQLIQRLDYWSYRVISLGFTFLTIGILSGAVWANEAWGS
YWNWDPKETWAFITWTIFAIYLHTRKNSKLQDTNSAFVASIGFLLIWICYFGINLLGIGFHS
YGSFQLIMKKKALA-SLPYLVSILFLPWWVSLSFKKCLETWFLNWWNTRQSEILLNDIQEK
NVLEKFMELEELFLLDEMIKEYSEAHMQRFRIGMHKETIQLVQRQNESHFHIILHFSTNLIC
FAILSGYFFLGNEELFILNSWIQEFLYNLSDTIKAFLILLVTDLWIGFHSTHGWELMIGSIYN
DFGLAQNDQIISGLVSTFPVILDTIVKYWIFHFLNRVSPSLVVIYHSMNEMPIGVPKVPFRSP
GEEDAAWVDVYNRLHRERLLFLGQEVDSEISNQLIGLMVYLSIEDDTRDLYLFINSPGGW
VIPGIAIYDTMQFVSPDVHTICMGLAASMGSFILVGGEITKRLAFPHARVMIHQPASSFYEA
QAGEFILEAEELLKLRETLTKVYVQRTGNPLWVISEDMERDVFMSATEAQAHGIVNLVAVE
MKEQKLIHEGLITESLPNGMFRVRLDNEDLILGYISGRIRRSFIRILPGDRVKIEMSRYDSTR
GRIIYRLRKKDSNDNGRIIFRFKKDRFWQQNFLYPLLLQEYIYSLAHYHSFIGYDN-SSLVLV
KRLITRMYQQKPLIYSVNDSNQNGFWGSHFYSQMVSEGFGVILEIPFSSRLVSSLIPKYQNL
RSIHSIFPFLEDKLLHLNYVSDLLIPYPIHLEILVQILQCWIKDVPSLHLLRLFFHESHNLNNL
ITKFIYVFSKKRFFWFLHNSYVYEFEYLFLFIRKQSSYLQLISSGVFLERTHFYGKIEY--VMC
CNQRILWFLKYTFIHYVRYQGKAILASKGTLLLMKKWKFHLVNFWQSYFHFWFQPYRIHI
KQLPNYSFSFLGYFSSVLKNPLIVRNQMLENSFLINTLT--KKLDTIVPVIYLIGSLSKAQFCT
VLGHPISKPIWTDLSDSDILDRFCRICRNICRYHSGSSKKQVLYRIKYILRLSCARTLARKHK
S T V R T F M R R L G S G F L E E F F L E E E Q S L S L I F L Q R I W Y L D I I R I N D L V D H
S??
??
??
??
??
??
??
??

??
??
??
??
??
??
??
??
???-MLEHVLFLS
VDLFSIGIYGLITSRSMVRALMCLELILNSVNLNLVTFSDIFDSRQLKGEIFSIFVIAIAAAEA
A I G L A I I S S I H R N R K S T R I N Q S N L L N
T???
??
??
??
??
??
??
??
??
??
??
??
??
??
??
??
??
??
??
??
??
????????????????????MQNRKTFSWVKEQMTRSIYVLIMIYVITRASISNAYPIFAQQGYENPRE
ATGRIVCANCHLANKPVDIEVPQAVLPDTVFEAVVRIPYDMQLKQVLANGKKGALNVGA
VLILPEGFELAPPDRISPEMKEKMGNLSFQCYRPNKRNILVIGPVPGQKYSEIVFPILSPDPAT
KKDVHFLKYPIYVGGNRGRGQIYPDGSKSNNTVYNATSAGIVSRIARKEKGGYEITIVDAS
EGRQVVDIIPPGPELLVSEGESIKLDQPLTSNPNVGGFGQGDAEIVLQDPLRVQGLLFFLAS
VILAQIFLVLKKKQFEKVQLYEMNFMSKVYDWFEERLEIQAIADDITSKYVPPHVNIFYCL
GGITLTCFLVQVATGFAMTFYYRPTVTEAFSSVQYIMTEANFGWLIRSVHRWSASMWVLM
MILHVFRVYLTGGFKKPRELTWVTGVVLAVLTASFGVTGYSLPRDQIGYWAVKIVTGVPE
AIPIIGSSLVELLRGSASVGQSTLTRFYSLHTFVLPLLTAVFMLMHFPMIRKQGISGPLMGVT
KKPDLNDPVLRAKLAKGMGHNYYGEPAWPNDLLYIFPVVILGTIACNVGLAVLEPSMIGE
PADPFATPLEILPEWYFFPVFQILRTVPNKLLGVLLMVSVPIGLLTVPFLENVNKFQNPFRRP
VATTVFLIGTAVALWLGIGATLPIDKSLTLGLFMIEVFLFGIVLGLIPITLTGLFVTAYLQYRR

GDQLDLMPILTSYFGFLLAASTITPALFIGLNKIRLIMDIVSLAWAALMVVFTFSLSLVVWG
RSGLMIIRSPEPEVKIVVDRNPIKTSFEEWARPGHFSRTIAKGPDTTTWIWNLHADAHDFDS
HTSDLEEISRKVFSAHFGQLSIIFLWLSGMYFHGARFSNYEAWLSDPTHIAPSAQVVWPIV
GQEILNGDVGGGFRGIQITSGFFQIWRASGITNELQLYCTAIGALVFAALMLFAGWFHYHK
AAPKLAWFQDVESMLNHHLAGLLGLGSLSWAGHQIHVSLPINQFLEAGVDPKEIPLPHEFI
LNRDLLAQLYPSFAEGATPFFTLNWGKYAEFLSFRGGLDPITGGLWLSDIAHHHLAIAILFLI
AGHMYRTNWAIGHGLKDILEAHKGPFTGQGHKGLYEILTTSWHAQLSLNLAMLGSLTIVV
AHHMYSMPPYPYLAIDYGTQLSLFTHHMWIGGFLIVGAAAHAAIFMVRDYDPTTRYNDL
LDRVLRHRDAIISHLNWACIFLGFHSFGLYIHNDTMSALGRPQDMFSDTAIQLQPIFAQWV
QNTHALAPSVTAPGATTSTSLTWGGGELVSVGGKVALLPIPLGTADFLVHHIHAFTIHVTVL
ILLKGVLFARSSRLIPDKANLGFRFPCDGPGRGGTCQVSAWDHVFLGLFWMYNAISVVIF
HFSWKMQSDVWGTISDQGIVTHITGGNFAQSSITINGWLRDFLWAQASQVIQSYGSSLSAY
GLFFLGAHFVWAFSLMFLFSGRGYWQELIESIVWAHNKLKVAPATQPRALSIVQGRAVGV
THYLLGGIATTWAFFLARIIAVGMTLRFPRFSQGLAQDPTTRRIWFGIATAHDFESHDDITEE
RLYQNIFASHFGQLAIIFLWTSGNLFHVAWQGNFESWIQDPLHVRPIAHAIWDPHFGQSAV
EAFTRGGAIGPVNIAYSGVYQWWYTIGLRTNGDLYTGALFLLLLSAISLIASWLHLQPKW
KPSVSWFKNAESRLNHHLSGLFGVSSLAWTGHLVHVAIPGSRGEYVRWNNFLDVLPYPQ
GLSPLFMGQWNLYAQNPDSSSHLFGTSQGAGTAILTLLGGFHPQTQSLWLTDIAHHHLAIA
FLFLVAGHMYRTNFGIGHSIKDLLEAHIPPGGRLGRGHKGLYDTINNSLHFQLGLALASLG
VITSLVAQHMYSLPAYAFIAQDFTTQAALYTHHQYIAGFIMTGAFAHGAIFFIRDYNPEQNE
DNVLARMLDHKEAIISHLSWASLFLGFHTLGLYVHNDVMLAFGTPEKQILIEPIFAQWIQS
AHGKTSYGFDVLLSSTNSPAFNAGRSIWLPGWLNAINE--NSNSLFLTIGPGDFLVHSCCAL
GLHTTTLILVKGALDARGSKLMPDKKDFGYSFPCDGPGRGGTCDISAWDAFYLAVFWML
NTIGWVTFYWHWKHITLWQGNVSQFNESSTYLMGWLRDYLWLNSSQLINGYNPFGMNS
LSVWAWMFLFGHLVWATGFMFLISWRGYWQELIETLAWAHERTPLANLIRWKDKPVALSI
VQARLVGLAHFSVGYIFTYAAFLIASTSGKFGMSHSVKIYDTCIGCTQCVRACPTDVLEMI
PWDGCKAKQIASAPRTEDCVGCKRCESACPTDFLSVRVYLWHETTRSMGLSYMIDLNFPS
IFVPLVGLVFPAIAMASLFLYVQRNKIYMRDIKTYLSTAPVLTTLWLGSLAGLLIEINRLFPD
ALSFPFFSSMTAILERRESTSLWGRFCNWITSTENRLYIGWFGVLMIPTLLTATSVFIIAFIAA
PPVDIDGIREPVSGSLLYGNNIISGAIIPTSAAIGLHFYPIWEAASVDEWLYNGGPYELIVLHF
LLGVACYMGREWELSFRLGMRPWIAVAYSAPVAAATAVFLIYPIGQGSFSDGMPLGISGTF
NFMIVFQAEHNILMHPFHMLGVAGVFGGSLFSAMHGSLVTSSLIRETTENESANEGYKFG
QEEETYNIVAAHGYFGRLIFQYASFNNSRSLHFFLAAWPVVGIWFTALGISTMAFNLNGFN
FNQSVVDSQGRVINTWADIINRANLGMEVMHERNAHNFPLDLASVEVPSINGMGLPWYR
VHTVVLNDPGRLLSVHIMHTALVSGWAGSMALYELAVFDPSDPVLDPMWRQGMFVIPFM
TRLGITNSWGGWNIS-GGTITNPGIWSYEGVAGAHILFSGLCFLAAIWHWVYWDLEIFCDE
RTGKPSLDLPKIFGIHLFLSGVACFGFGAFHVTGLYGPGIWVSDPYGLTGNVQSVNPAWGA
EGFDPFVPGGIASHHIAAGTLGILAGLFHLSVRPPQRLYKGLRMGNIETVLSSSIAAVFFAAF
VVAGTMWYGSATTPIELFGPTRYQWDQGYFQQEIYRRVGAGLAENLSLSEAWSKIPEKLA
FYDYIGNNPAKGGLFRTGSMDSGDGIAVGWLGHPIFRDKEGRELFVRRMPTFFETFPVVLV
DGDGIVRADVPFRRAESKYSVEQVGVTVEFYGGELNGVIYSDPATVKKYARRAQLGEIFE
LDRATLKSDGVFRSSPRGWFTFGHATFALLFFFGHIWHGARTLFRDVFAGIDPDLDAQVEF
GTFQKLGDPTTRRQVVMKTLYSLRRFYPVETLFNGTLALVGRDQETTGFAWWAGNARLI
NLSGKLLGAHVAHAGLIVFWAGAMNLFEVAHFVPEKPMYEQGLILLPHLATLGWGVGPG

GEVIDTFPYFVSGVLHLISSAVLGFGGIYHALLGPETLEESFPFFGYVWKDRNKMTTILGIH
LILLGIGAFLLVFKALYFGGVYDTWAPGGGDVRKITNLTLSPSVIFGYLLKSPFGGEGWIVS
VDDLEDIIGGHVWLGSICILGGIWHILTKPFAWARRAFVWSGEAYLSYSLGALSVFGFIACC
FVWFNNTAYPSEFYGPTGPEASQAQAFTFLVRDQRLGANVGSAQGPTGLGKYLMRSPTG
EVIFGGETMRFWDLRAPWLEPLRGPNGLDLSRLKKDIQPWQERRSAEYMTHAPLGSLNS
VGGVATEINAVNYVSPRSWLATSHFVLGFFLFVGHLWHAGRARAAAAGFEKGIDRDLEPV
LSMTPFSMTIAFGRFIKEENDLFDIMDDWLRRDRFVFVGWSGLLLFPCAYFALGGWFTGT
TFVTSWYTHGLASSYLEGCNFLTAAVSTPANSLAHSLLLLWGPEAQGDFTRWCQLGGLWT
FVALHGAFGLIGFMLRQFELARSVQLRPYNAIAFSAPIAVFVSVFLIYPLGQSGWFFAPSFG
VAAIFRFILFFQGFHNWTLNPFHMMGVAGVLGAALLCAIHGATVENTLFEDGDGANTFRA
FNPTQAEETYSMVTANRFWSQIFGVAFSNKRWLHFFMLFVPVTGLWMSAIGVVGLALNL
RAYDFVSQEIRAAEDPEFETFYTKNILLNEGIRAWMAAQDQPHENLIFPEEVLPRGNALMS
GSTGERSF--VITSIRYWVIHSITIPSLFIAGWLFVSTGLAYDVFGSPRPNEYFTESRQGIPLIT
GRFDSLEQLDEFSKSFMTIDRTYPIFTVRWLAVHGLAVPTVSFLGSISAMQFIQRMATKTVE
SSSRSG-TRATGIGSYLKPLNSEYGKVAPGWGTTPFMGVAMALFAIFLSIILEIYNSSVLLDG
ISINMLTLKLFVYTVVIFFVSLFIFGFLSNDPGRNPGREEMTDTTGRIPLWLIGTVTGIPVIGL
IGIFFYGSYSGLGSSLMFNIFSLICLCLNSTLYSSSFLFAKLPEAYAFFNPIIDFMPVIPLLFFLL
AFVWQAAVSFRMTQSNPNEQNVELNRTSLYWGLLLIFVLAVLFSNYFFNMEVNILALIATA
LFILVPTAFLLIIYVKTVSQNDMETATLVAISISGLLVSFTGYALYTAFGQPSQKLRDPFEEHG
DMEALVYTFLLVSTLGIIFFAIFFREPPKVPTKKMKMTIAFQLAVFALIATSLILLISVPVVFA
SSDGWSSNKNFVFSGTSLWIGLVFLVAILNSLIS-------MSPQTETKASVGFKAGVKDYKLTY
YTPDYETKDTDILAAFRVTPQPGVPPEEAGAAVAAESSTGTWTTVWTDGLTSLDRYKGRC
YHIEAVVGEENQYIAYVAYPLDLFEEGSVTNMFTSIVGNVFGFKALRALRLEDLRIPTSYSK
TFQGPPHGIQVERDKLNKYGRPLLGCTIKPKLGLSAKNYGRAVYECLRGGLDFTKDDENV
NSQPFMRWRDRFLFCAEALFKAQAETGEIKGHYLNATAGTCEEMIKRAVFARELGVPIVM
HDYLTGGFTANTSLAHYCRDNGLLLHIHRAMHAVIDRQKNHGMHFRVLAKALRMSGGD
HIHAGTVVGKLEGEREMTLGFVDLLRDDFIEKDRSRGIFFTQDWVSMPGVIPVASGGIHV
WHMPALTEIFGDDSVLQFGGGTLGHPWGNAPGAVANRVALEACVQARNEGRDLAREGN
DIIREASKWSPELAAACEIWKEITFDFDPVDKLDKTAIHLYKTSTPSTRNGAVDSQVKSNPR
KNLIYGQHHCGKGRNARGIITAGHRGGGHKRLYRKIDFRRNEKGISGRIVTIEYDPNRNAY
ICLIHYGDGEKRYILHPRGAIIGDTIVSGTEVPISMGNALPLTDMPLGTAIHNIEITLGKGGQ
LARAAGAVAKLIAKEGKSATLRLPSGEVRLISKNCLATVGQVGNVGVNQKSLGRAGSKC
WLGKRPVVRGVVMNPVDHPHGGGEGRAPIGRKKPTTPWGYPALGRRSRKRNKYSDSLIL
RRRK-MIQPQTLLNVADNSGAQELMCIRIIGTG-NHRYAHIGDVLVAVIKEAVPNMPLERSE
RIRAVIVRTCKELKRDNGMIIRYDDNAAVIIDQEGNPKGTRIFGAIARELRQLNFTKIVSLAP
EVLMLSPKRTRFRKQHRGRMKGISCRGNHICFGRYALQALEPAWITARQIEAGRRAMTRY
ARRGGKIWVRIFPDKPVTVRPTETRMGSGKGSPEYWVSVVKP---LYEMSGVSETVAKAA
MEHAKCLYELNLLFQDR-MTRVKRGYTARRRRKKNRLFASTFMGAHSRLTRTNTQQRMR
ALVSSHRDRSRKKRDFRRLWITRINAVTRED--LFYSYSLFIHNLYKRQLLLNRKILAQIALS
NKNCFSIISNKIINQK---------MIKTVL-TQHIC-MSVFKARRVIDQIRGRSYEETLMILELMPY
WASYPILKLVYSAAANASHNMGLNEADLFISKAEVNRGTIVKKLKPRARGRSYLIKKTTC
HIKISLK-KSKI-------MDGIKYAVFTEKSIRLLGNNQ-YTSNVESGSTRTEIKHWVELFFGVK
VRAMNSHRLPGKGRRMGPIMGHTMHYRRMIITLQPGYSIPPLIEKRTMAVPKKRTSMSKR
HIRRNLWKKKGSLEAVKAFSLAKSISTGKSKSFF--QKKVMAKGKDIRIIVILECTCC-VRK--

VNKE--GISRYITQKNRHNTPNRLEFRKFCRYCQKYTIHGEIKKMKIRASVRKICEKCRLIRR
RGRIIVICSNPRHKQRQGMVREEVAGSTRTLQWKCVESRVDSKRLYYGRFVLSPLMKGQA
DTIGIAMRRALLGRIEGTCITRATSE--NVLHEYSMIAGIEESVHDILINLKEIVLKSNLYGVR
DASICVRGPKYITAQDIISPPSVKIVDTTQHIANLTEPIDLCIELQIKRGRGYHMKSTNESHD
GSY--PIDIVSMPVRNANHSIHSYGNGNEKQEILFLEIWTNGSLTPKEALYEASRNLIDLLIPF
LHAEEE--DMNLEENE-KRWNLPLFSFQDRFT-NLKKNKK-GIPLTCIFIDQSELSSRTYNCLK
RSNIHTLLDLLNHSQEDLMKMEYFRIEDVKQILGTLQKH--FAINLPKKKMLQN-ENEGMS
TIPGFSQIQFEGFCRFINQGLTEEFHKFQKIEDRDQEIEFQLFVETYQLVEPLIKERDAVYESL
TYSSELYVPAVLIWKTGRNMQEQTVFLGNIPIMNSFGTSIVNGIYRIVINQILQSPGIYYRSE
LEHNGISVYTSTIISDWGGRSELEIDRKARIWARVSRKQKISILVLSSAMGSNIREILDNVCY
PEIFLSFPNDKEKKKIGSKENAILEFYLQFACVGGDPVFSESLCKELQKKFFQQRCELGKIG
RRNINRRLNLDIPQNNTFLLPRDLLAAVDHLIRMKLGMGTLDD--MNHLKNKRIRSVADLL
QDQFGLALVRLENTVRGTICGAIR-HKLI-PTPQNLVTSTL----LTTTYESFFGLHPLSQVLDR
TNPLTQIVHGRKWSYLGPGGLTGRTANFRIRDIHPSHYGRICPIDTSEGINVGLIGSLAIHVK
VGYWG-SIESPFY---ELSDRSKE--AQMVYLSPNRDEYYMVAAGNSLALNRGIQEQQVVPA
RYRQEFLTIAWEEIRLRSIFPFQYFSIGASLIPFIEHNDANRALMSSNMQRQAVPLSRSEKCIV
GTGLEGQTALDSGISAIAERKGKIIYTDTQKIIFSSNGDTLSIPLVMYQRSNKNTCMHQKTQ
VQRGKYIKKGQILAGGVATAGGELALGKNVLVAYMPWEGYNFEDAVLINERLVYEDIYTS
FHIRKYEIQTHVTSQG-PERITKDIPHLEAHLLRNLDRNGIVMLGSWIETGDILVGKLTPQTA
SE-LSYTPEDRLLRAILGIQVSTSKETSLRLPIGGRGRVIDVRWIHRKG-VSNSNPEKIRVYIS
QKREIKVGDKVAGRHGNKGIISKILPRQDMPYLQDGTPVDMVFNPLGVPSRMNVGQIFEC
SLGLAGDLLKKHYRIGPFDERYEQEASRKLVFSELYEASKKTKNPWVFEPEYPGKSRIFDG
RTGDLFEQPVLIGKSYILKLIHQVDDKIHGRSSGHYALVTQQPLRGRAKQGGQRVGEMEV
WALEGFGVAHILQEMLTYKSDHIRARQEVLGATIIGATVPKPEGAPESFRLLVRELRSLSLE
LNHFLVSEKNFQMDRKEA-------MIDQYKHQQLRIGPVSPQQIMVWAKKILPNGEIVGEVT
KPYTFHYKTNKPEKDGLFCERISGPIKSGICACGNYRGIGTEKEDPKFCEECGGEFVDSRIR
RYQMGYIKLTCPVTHVWYLKRLPSYIANLLDKSLRELEGLVYCD--FSFARP-IAKKPTFLRL
RGSFEYEIQSWKYSIPLFFTTQGFEIFRNREISTGAGAIREQLADSDLRIITDNSLVEWKELG
DE-ESTGNEWEDKKIRRRKDFLVRRIELAKNFLRTNVDPEWMVLCLLPVLPPELRPIIQIDG
GKLMSSDINELYRRVIYRNNTLTDLLATSRSTPGELIMCQEKLVQEAVDTLFDNGIRGQPM
RDGHNKVYKSFSDVIEGKEGRFRETLLGKRVDYSGRSVIVVGPLLSLHQCGLPREIAIELF
QTFVIRGLIRQDVVSNTGIAKSKIREKEPIVWEILQEVMQGHPVLLNRAPTLHRLGIQAFQP
ILVEGRAICLHPLVCKGFNADFDGDQMAVHIPLSLEAQAEARLLMFSHMNLLSPAIGDPVS
VPTQDMLIGLYVLTIGNPRGICANRYNQSNSNCR---KKETVYKNDFKYPK--DPYFSSSYDA
LGAYRQKRIHLDSPLWLRWRLDQR-VVGS--REVPIEVQYESFGNYNEIYKHYRIIGSVKREI
RCIYIRTTVGHLSFYREIEEAIQGFWRAYS-----------MEVLMAERTDLVFHNKVIDGTAMKR
LISRLIDHFGMAYTSHILDQVKALGFQRATATSISLGIDDLLTIPSKGWLVQDAEQQSFLLEK
NHHYGNVHVVEKLRQSIEIWYATSEYLRQEMNPNFRMTDPSNPVHLMSFSGARGNVSQV
HQLVGMRGLMSDPQGQMIDLPIQSNLREGLSLTEYIISCYGARKGVVDTAVRTSDAGYLTR
RLVEVVQHILVRRTDCGTIRGISVSPQNGMTE--NIFVQTLIGRVLADDIYIGLRCIATRNKDI
GIGLVNRFIT------FRQHPIYIRTPFTCRSTSWICQLCYGRSPTHGDLVELGEAVGIIAGQSIGE
PGTQLTLRTFHTGGVFTGGTAEHVRAPSNGKIKFDEDLVHPTRTRHGHPAFLCFIDLYVTIE
SRDILHNVNIPKKSLILVQNDQYVESEQVIAEIRAGTSTF--FKERVQKHIYSESEGE-MHWS
TDGYHAPEYPYSNVHLLPKTSHLWILAGGLCR--SNIVSFSLHKDQDQMNANPFSLEER--Y

IFDLS--LTN-DQVRHKLLDTFG----DREILDYS-KSYR--TKGHWNLIEPS-TYIRQ--DNSLAK
KRRNRFVIPLQYDQEQEKKLIPCFGISIKIPIN--GILRRNSILAYFDDPRYRRS--GITQY-IVEV
DSIVK--KEDLIECRG-AKEFIPKC-KVDRFFFIPEEVHILPGSSPIMVRNNSIIGVDTRLALNT
RSRVGGLVRVERKKKSMELKIFSGDIHFSGEVDKISRHSGILIPPGMVKR--NSKG-SK-KLK
NWIYVQR--ITPTKKK--YFVLVRPVVTYEIADGINLATLFSQDLLQEKDNVQLRIVNYILYG
NGKSIRGIYYTS-QLVRACLVLTWDQEKNSSIEE---EIHASFVEIRVNDLLRDFIRIELVKSTIL
YTEKRYDTAGPGLISN---LDRIHRNPFDSKAKIQS-----FPQHQETLG----TLLNRKK---ESQSF
LILSSSNCSRIG--PFNTSRYK---NATKGSDPMTPI-DLLGPLGTLVP-KIVNFCSS-YYLRT---Y
NQVVLK-KDFLLE---KFQQTFQVLQVLQYCFIDENSR--IYNPDPCSNIIWNSFHLNWCFLH
HDSCDEAWTIIS-LGQFLCENLYLLKYGS--RIKKSGQIFIVH-VDSLVIRSAKPYLVTPGATV
HGHYGETFSEGDTLITFIYEKSRSGDITQGLPKVEQILEVRSIDSISMNLERRVEGWDERIRR
ILGIPWGFFIGAELTIAQSRISLVNKIQKVYRSQGVQIHNRHIEIIVRQVTSRVLVSE---DGMS
NVFLPGEFIGLLRAERAGRVLDEAICYRAILLGITKSSLNTQSFISEASFQETARVLAKAALR
GRIDWLKGLKENVVLGGIIPVGTGFNKLVHRSK--KEKNIH--LKIKRKNLFELEM--RDILLY
HRELFCSCAPN-----SDQSFM-----------MGRRYWNINLEEMMEAGVHFGHGTNKWNPRMA
PYISAKRKGIHITNLTITARFLSEACDLVFDAASRGKKFLIVGTKKKAADLVASAAIRARCH
YVNKKCLGGMLTNWSTTETRLQKFRDLRAEQKMGKLNCLPKGDAAMLKRKLSTLQAYL
GGIKYMTGLPDIVIIVDQQEEYTVLRECAILGIPTICLIDTNCDPDLADISIPANDDAIASIRLI
LTKLVSAICEGRSS-YIKKGMGQKINPLGFRLGTTQSHHSFWFAKPKNFSMGIQEDEIIRNCI
KDYVKKNNKISSG-FEGIAHIGIQKRIDLIQIIISIGFPNFLIEG--RTR--GVEELQMNVQKEFH
SVNRRLNIAITRIEKPYGQPNILAEYIALQLKNRVSFRKAIKKAIELTEQTGTKGIQVQIAGRI
DGKEIARVEWIREGRVPLQTIRAKIDHCSHTIRTIYGVLGIKIWIFLNQEMSRYRGPRFKKIR
RLGALPGLTSKIPRSRSDLQTQLHSGK-KSQYRIRLEEKQKLRFHYGLTERQLLKYVHIAGK
AKGSTGQVLLQLLEMRLDNILFRLGMASTIPGARQLVNHRHILVNGRIVDIPSYRCKARDII
TTKDKERSKALIQNYLVSSPRE--LPNHLTIDS--LQYKGFINQIIDSKWIGLKINELLVVEYYS
RQTMSRRGTAEEKTAKSDPIYRNRLVNMLVNRILKHGKKSLAYQIIYRAVKKIQQKTETNP
LSVLRQAIRGVTPDIAVKARRVGGSTHQVPIEIGSTQGKALAIRWLLGASRKRPGRNMAFR
LSSELVDAAKGSGDAIRKKEETHRMAEANRAFAHFRMGRDIIADIITSIRNADIDQKGKVK
IVSTNITKNIVKILLREGFIENVRKHQESQKNFLVSTLRHRK--TSKG------NKINLKRISRPGL
RIYSNYQRIPKILGGMGIVILSTSRGIMTDREARLEKIGGEILCYIWMTKPIAKIGLRKNARI
GSKKNERRIPKGVIHVQASFNNTIVTVTDVRGRVVSWASAGTSGFRGTRKGTPYAAQAAA
FNAIRTLVDQGMQRAEVMIKGPGLGRDAALRSLRRNGMLLSFVRDVTPMPHNGCRPPKK
RRVMPTIKQLIRNARQPIRNVTKYPALRGCPQRRGTCTRVYTITPKKPNSALRKVARVRLTS
GFEITAYIPGIGHNSQEHSVVLVRGGRVKDLPGVRYHIVRGTLDAVGVKDRQQGRSKYGV
KKPKMARKSLIQREKKRQKLEQKYYLIRRFLKKEIS---KVPSLREQWEIHGKLQSPPRNSA
PIRLHRRCFLTGRPRANYRDFGLSGHILREMFHACLLPGATRSSWMVKNSFISVITQEEKEE
N--SGSVEFQVFHFTSKIRRLTSHLELNKRDFLSQRGLRIILGKRQRLLTYLSKKNKVRYKKL
IDQLDIREPKTR--MVKLRLKRCGRKQRVIYRIVAIDVRSRREGRDLQKVGFYDPIKNQTYS
NVPAILDFLEKGAQPTGTVYDILRKAEFFKEFRISFEKKNKMDKSKQPFRKSKRSFRRRLPP
IGSGDRIDYRNMSLISRFLSEQGKILSRRTNRLTLKQQRLITIAIKQARILSLLPFRNNEK--QL
ERVESIPRTTGPRTKNKVTRSLKKNPFVANHLSGRIEKLNRREEKEIIVTWSRASTIIPTMIG
H T I A I H N G K E H L P I Y I T D R M V G H K L G E F A P T I T F V R H A R N D N K S R
R??
??

???
???
???
???
???
???
???
???
???
???
???
???
?????????MKRHQFKSWIFELREILREIKNSHYFLDSWIKFDSVGSFTHIFFHQERFMKLFDPR
ILSILLS-RDS--QGATSNRYFT---IKGVVLLVVV-VLIS----RINNRKMVERKNLYLMGLLP-----
IPMNSIGPRKETLEESF-WSSNRNRLIVS-LLYLPKGKKISESCFMDPQESTWVIPRK----KKRI
MPESNRGSRWWRNRIGKKR--DSSCQIS---NEIVAGIEISFKEKDSK-YLEFLFLSYT--DDPIR
KDHDWELFDRLSPRKKRNIINL--NSGQLFEILGKDLICYLMSAFREKRPIQGES-FFKQQG-
AGATMQSNDIEHVSH--LFSRNKWGISLQNCAQFHMWQFRQDLFV---SWGK-NQHESYFL
RN-VSRENWIWLDNVWLVNKDR---FFS--KVRNVLSNIPYDS---TR--SIFVQVMDSSQWKG
SSSDQSRDHFDSVR----NENSE-------------YHTLIDQTEIQQLKERSII-WD--PSFLQTERTEIES
D--RFPK--CLFGSS--SMSWLFT-EPEKRMKNHLLPEEIEEFL--GNPTRSIRSFFSDRW--SELH-
LGSNPTERSTRDH----KLLNKKQDVS-FVPSRRAE----NKEMVDIFKIITYLQD--TVSIHPSEP
DPGCDMVP-KDEPD-MDSSNKISFLNKNPFFS--FFHLFHDRNKGGYAL--RHDFFES-EERFP
EMADLFTLSITEP-DLVFHRGFSFSIDSYGLDQKK--FLNEVFNSRDESKKK---------------------
---SFLIQIQYSTYG--YIRNVSNQFFLM---NRSDRNFEYGIQ---
RDQIGNDTLNHRTIMKYTINQHLSNLKK-SQKKWFDP----LISRTERSMNRDPDAYRYKWS
NGSNNFQEHLEHFVSEQKNPFQVVQVVFDQLRINQYSIDWSEAIDKEDLSKPLRFFLSKS--
-------LLFLS-------K-SLLFLSKSLPFFFVSIGNIPIHRSE-IHIY-ELKGPNDQLCNQLLESIGVQI
VHLKKLKP--FLLD---DHDTS-QRPKFLINGGTILPFLFKKIP-KR-VIDSFHT--RKNRRKSFDN
TDSYFSMISHDRDNWLNPVKPFHRSSLISSFYKANRLRFLNDPHHFWFY--CNKGFPFDV----
EKTRINNYD--RTY-GQFLNILSIRNK-IFSLCVGK--KK--------YLSPIESQVSDIFIPNDFPQSG
DET-YNLYKSYKSFHFPIRSDPFVR--GAIYSIADISATPLTEEQIVNLEKTYCQPLSDMNLSDS
EGNNLHQYLS--FNSNMGLIHTPCSEKYLP--SGKRKK--------RSLCLK--KCVEKGQMYRTF
QRD----SVSKWNLFQTYMPWFLTSTGCKYLNFTLLDTLSDPL--PILSSS-QKFVYIFHDMMH
GSDISRPFPQK--------ILPQWTL-ISEISSQCLQN--LLLSDEMIHRNNESPVPLIWAHLRSTNA
REF-LYSIFFLLLVAGY-LVRIHLLFVSR--ASSELQTELEKIKSLMIPSYMMEFRKLLDRYPTS
ELNPFWLKNLFLVVLEQLGDSLEEIRGFASGGNMLLGGGP-----AYG-VKSIRSKKKYWKIN
---LID----LIPNPINRILFSRNTRHLSRTSKEIYSLIRKRKNVNGDWIDEKIEFWVANSDLIDDE
EREFLVQFS--TLTT--EKRIDQILLSLT-HSDHLS-----KNDSGYQMIEQPGSIYLRYLVDIH--QK
DLMNYEFNRSCLAERRIFLAHYKTITY--SQTSCGANSFHSPSP--HGKPFSLRLALSPS-RGIL
VIGSIGTGRSCLVKYLATNSYVPFITVFPNKFLDDKPKGYLIDD-----ID-IDDSDDID----DS----
-----------------DDDLDIDTELLTMTNVL---TMYMT---PKIDRFDITLQFELAKAMSPCIIWIPNI
HDLHVNESNYLSLGLLENYLS---RDCERCS-TGKILVIASTHIPQKVDPALIAPNKLNTCIKIR
RLLLPQQRKHFFILSYTRGFHLEKKMFHT--NGFGSITMGSNARDLVALINEALSISITQKKSI

IEINTIRSALHRKTWDFR---SQIRSVQDHGILFYQIGRAVTQNVLLSNCPIDPISIYMKKK--SC
KGGDFSLYKWYFELGTSMKKLTILLYLLSCSAGSVAQDLWSSS--RHDEK-NWITSYGFVEN
DS-DLVHGLLEVEGALALARSSRTEKYCSQFDNN--RVTLLLRSEPRN-QLDMMQNGSCSIV
DQRFLYEK--YESEFEE-GEG--ALDPQ----QIEKD----LFNH--IVWAP--RIWRP---CGNLFDCIE
RPTELGFP-YWTGSFR---GKWIIYHKEDELQENDSEFLQSGTMQYQTRDRSSKEQGFFRPS
QFIWDLADPFFSLFK-DQPSVSVFSRREFFADEEMSKGLIASQTNPPASIYKRWFI--KNTQE
KHFELLIHRQRWFRT---NSSLS--NGSFRSN--TLSESYQYLSNLFLSN-RMLLDQMTKTLLRK
RWLFPDEMKH-------------MPRSR-INGNFIDKTSSIVANILLRIIPTTSGEKKAFTYYRDG--M
SAQSEGNYAEALQNYYEAMRPEIDPYDRSYILYNIGLIHTSNGEHTKALEYYLRAIERNPF
LPQAFNNMAVICHY-RGEQAILQGDSEIAEAWFDQAAEYWKQAIALTPGNYIEAHNWLKI
TRRFE--MNWRSENIWIEFITGSRKTSNFCWACILFLGSLGFLVVGTSSYLGKNMIFVFPSQQ
IIFFPQGIVMSFYGIAGLFISSYLWCTISWNVGSGYDRFDRKEGIMSLFRWGFPGRNRRIFLR
FFLKDIQSIRIEVREGLFPRRVLYMEIRGQGAIPLTRTDEN-LTPREIEQKAAELAYFLRVPIEV
F
>Erodium
???
???
???
???
???
???????????????????????????????MVPIRADEISNIIRERIKQYNREVNIVNMGIVLQVGDGIAR
IYGLNDVMAGELVEFEEGTTGIALNLESNNVGVVLMGGGLRIQEGSSVKGTGRIAQIPVSE
AYLGRVVNALAKPIDGRGGISASESRLIESPAPGIISRRSVYEPLQTGLIAIDSMIPIGRGQRE
LIIGDRQTGKTAVATDTILNQRGKDVICVYVAIGQKASSVAQVVTTFQETGAMKYTIVVAE
TAASPAALQYLAPYTGAALAEFFMYRERHTLIIYDDLSKQAQAYRQMSLLLRRPPGREAY
PGDVFYLHSRLLERAAKSSSNLGEGSMTALPIVETQAGDVSAYIPTNVISITDGQIFLSADL
FNSGIRPAINVGISVSRVGSAAQIKAMKQVAGKSKLDLAQFTELEAFAQFASDLDKNTQNQ
LARGRRLRELLKQSQAEPLEVEEQIMTVYTGTNGYLDSLEIGQVRKFLGNLRDYLKSNKP
QFQEIISSTKTFTEEAETLLKEAIQEQIERFTFQ---MRLNPTASSLGDSTQKP---GRITQIIGPV
LDVVFPKGKMPYIYNALVVQGRDTAGQQINVTCEVQQLLGNNRVRAVAMSATDGLSRG
MEVIDTGAPLSVPVGGSTLGRIFNVLGEPIDNLGPVDTRTTSPIHRSAPDFIELDTKFSIFET
GIKVVDLLAPYRRGGKIGLFGGAGVGKTVLIMELINNIAKAHGGVSVFGGVGERTREGND
LYKEMKESGVINEENISESKVALVYGQMNEPPGARMRVGLTALTMAEYFRDVNKQDVLL
FIDNIFRFVQAGSEVSALLGRMPSAVGYQPTLSTEMGSLQERITSTKKGSITSIQAVYVPAD
DLTDPAPATTFAHLDATTVLSRGLASKGIYPAVDPLDSTSTMLQPRIVGEEHYEIAQRVKQT
LQRYKELQDIIAILGLDELSEEDRLTVARARKIERFLSQPFFVAEVFTGSPGKYVGLAETIRG
FNLILSGELDGLPEQAFYLVGNIDEATAKATKLEKESNLKKMTLNLRVLTPKRILWDSKVK
EIILSIDDGKMGVLPNHVPLVTGLDTGILKIRLNDQWVTIALMGGFARIRNNEILVFAHDGE
KVSDIDPQEAQQTLQIAKDNLREAEGKEKTIEADQAVKRAELLVNAVNDISMKNVTDSFV
SLGRWPSAGSFGFNTDILATNLINLSVVLGVLIFFGKGVLNDLLDNRKQRILNTIRNSEELR
GGAVEELEKAHARLRKVKTDVELFRVNEYSEAERKRSNLITSAYKELEQAESLKNESIRFE
QQRAINQVRQWVVQQALEGALEILNSSLNKELHLRTINANIGLLGSMKERTYMNPLIAAA
SVIAAGLAVGLASIGPGIGQGTAAGQALEGISRQPSAEGKIRGTLLLSLAFMEALTIYGLVV
ALALLFANPFVMSVLSCSINTISGLYEVSGVEVGQHFYWQIGGFQVHGQVLITSWVVIAIL

LGSAILAVQNPQTIPTDVQNFFEYVLEFIRDVSKTQIGEEYGPWVPFIGTMFLFIFVSNWSG
ALLPWKIIQLPQGELAAPTNDINTTVALALLTSVAYFYAGLSKRGLAYFKKYIQPTPILLPINI
LEDFTKPLSLSFRLFGNILADELVVVVLLSLVPLVVPIPVMFLGLFTSGIQALIFATLAAAYIG
ESMEGHHMIFSTLEHIFNHISFSVVSIVITIHFLNLAVDETVGLYDSSEKGMLVSFFCLTGLL
MTRWACSGDFPLSDFYDSLIFLSWNLSIINMLPYLKNEKNYLSPISYLSALFTQGFATSGLL
AEMHQSKRLVPALQSQWLMMHVSMMLLSYAALLCGSLLSTALLIITFRKRKGVFGKRNN
MQYLNEKK----NTSLPSSVNYYRSQLVHQLDYWSYRIISLGFLFLTIGILSGAVWANEAWGS
YWSWDPKETWAFITWTVFAIFLHSRTNKNVEGINSAIVASIGFFIIWICYFGINLLRIGLHSY
GAFAISMKKKKVPTPFLYVASIVFLPWWISLLFTKSLEFLVTDWWNSRQSDTSWDEIEEKS
LMKKFIELEELLLLDEMLKECPKVYLENFRIGLHKETVQLIKMHNEDQIHRILHFSQTLIYF
VILSGYSILGKEQLVILNSWFQEFLYNLSDTIKALCIIAFADIFFGFHSVHTWESIISYVCKGL
GCVSTDRIPSFVRWILPSILDVAWKFWSFRYLNSLSPSLLALYSAMKDMPFGVPKVPFEFED
EVEVTWMDLYNLLFRERVLFLGRAIKPEITNHLVSLMVFLTVERDDWSPMLLINSPGGYIF
NGLSLYDTVRLSG--ARTLCVGTAVSMASVILVGGSDNKRAAFPHARVMIHQPRINKFEDE
GPEVIMEARIMLDLRHHIVEIYVLKTGQPFWVISVDLEVDTYLTPTEAKIYGLVDLILI
K???MEEFQE
YLEFDGSRQHAFLYPLISRESIYALAHDHGLANYDNKSSSLIVKRLVARMYQQSPLGICPN
DSNQNPFFGKNLYSQTISEGFTEIAEIPLFLQLVSSLISKSLNLRSIQSIFPFLEDECSHFHFVS
DVLIPHPAHLEILVEALRYWVKDAPFVHFLRFFLYECW--AFNSLGPSSFFSKARLFLFLYNS
YVCEYESMLLFLRSKSSHLRSTSSAIFLERILFYKKMESLGCIFANERILLFFKDPFLHYVRY
QGKCILILKDRPLLMNKWKDYLVKLWQCHFDVWSQPGSVHRNKLSQYSLNFMGYILSV
GSIPLVIRSRALENSFLIDN--AMNKVETIIPILSLIRSLAKENFCNPLGDPISKPTWADSSDSDI
IDRFVRMCRTLSHYHGGSSKKKSLYRLKYILRLSCVKTLARKHKSTVRSFLKKLGSALLEE
FINEEEQVLSSIFSRRIWYLNILYMNDLITQEMIIDRR-EIQDIN-SFSRFES-LQEVYGTVWGL
VPILILVVGITIGVLVIVWLEREISAGIQQRIGPEYAGPLGILQALADGTKLLFKENILPSRGDI
RLFSLGPSMAVISILLSYSVIPFSYRLVLADLNVGVFFWISIASIAPIGLLMSGYGSNNKYSFL
GGLRATAQSISYEIPLTLCVLSISLLSNSSSTVDIVEAQSKYGFWGWNFWRQPIGFIVLLISSL
AECERLPFDLPEAEEELVAGYQTEYSGIKFGLFYISSYFNLLVSSFFVTILYLGGWNLSIPGT
DFPHLFERNQAGAVFRTTIALFITLAKTFFFLFISITTRWTFPRLRMDQLLNLGWKFLLPISL
GNLLLTTSSQLFSL----------ILDSTRIFMKAFHLLLFDGSFILPECSLILGLILLLMINSTSDQK
DIPWLYFISSTSLVMSITALLFRWREEPMISFSGNFQTNNFNEIFQFLILLCSTLCIPLSVEYIE
CTEMAITEFLLFILTATLGGMLLCGANDLITIFVAAECFSLCSYLLSGYTKKDVRSNEATMK
YLLMGGASSSILIHAFSWLYGSSGGEIELQEIVNGLINTQMYNSPGISIALLFITVGIGFKLSP
APSHQWIPDVYEGSPTPVVAFLSVTSKVAASASATRIFDIPFYFSSNEWHLLLEILAILSMIL
GNLIAITQTSMKRMLAYSSIGQIGYVIIGIIVGDSNGGYASMITYMLFYIAMTLGTFACIVLF
GLRTGTDTIRDYAGLYTKDPFLALSLALCLLSLGGIPPLAGFFGKLHLFWCGWQAGLSFLV
SIGLLTSVLSIYYYIKIIKLLMTGRNQEITPHVRNYRRSPLRSTNSIEFSMIVCVIASTIPGISM
NPIIAIAQDTLFMFLLYEYDIFWVFLILSSLIPILAFLISGVLAPISEGPEKLSSYESGIEPMGDA
WLQFRIRYYMFALVFVVFDVETVFLYPWAMGFDVLGVSVFIEALVFVLILIIGSVYAWRKG
ALEWSTNYFPWLTIIVIFPISAGSLIFFLPHRGNKVTRWYTLCICSLELLLMAYAFYYHFQLD
DPLIQLVDHYKWIPFFEFYWRLGIDGLSLGPVLLTGFITTLATLAAWPVTRNPRLFYFLMLA
MYSGQIGSFSSRDLLLFFIMWELELIPVYFLLSLWGGKKRLYSATKFLLYTAGSSLFLLIGVL
GIGLFGSSAPTFHFEISAHQSYPAALEIIFYIGFLIAFAVKSPIIPFHTWLPDTHGEAHYSTCML
LAGILLKMGAYGLVRINMEFLPHAHSIFSPYLITIGAIQIVYAASTSLGQRNLKKRIAYSSVS

HMGFIIIGISSITDTGLNGAILQIISHGFIGAALFFLAGTSYDRIRLLSFDKMGGMAVAIPKVF
TMFSILSMASLALPGMSGFVAELIVFLGIITSPKLLLIPRILIPFVMAIGMILTPIYSLSMLRQM
FYGYRLLNVPNPPLFDSGARELFVSVSILLPVIAVGIYPDLILSLSVDRVEFILSHFFIEMMLE
HVLVLSAFLFSIGICGLITSRNMVRALMCLELMLNAVNMNFVTFSDFFDSRQLKGAIFSVF
VIAIGAAEAAIGPAILSSIYHNKKSTRINQSNLLRKMEYTNKYSWIMPFIPLPVPILIGMGLL
LFPTTTKHLRRIWAFPTLFLLGIVMLLSAYLSIEQLNRSYIYQYVWSWTINNDFSLEFGHLI
DPLTSTMSILITTVGLTVIIYSENYMFHDQGYLRFFYYLAFFNASMLGLVTSSNLIQIYIFWE
FVGMCSYLLIGFWFTRPLSAKACQKAFVTNRVGDFGLLLGILGLYWITGSFEFRDLFQIFK
N-EIPFFFLTLCAFLLFWGALAKSAQFPLHVWLPDAMEGPTPISALIHAATMVAAGIFLVAR
LLPLFLAIPYLLNLISLIGIITVFLGATLALAQKDIKKVLAYSTMSQLGYMMLALGMGSYRA
ALFHLITHAYSKALLFLGSGSIIHSMETIVGYSPEKSQNMVLMGGLTKHMPITKTAFLLGTL
SLCGIPPLACFWSKDEILNESWLYSPIFAIIACSTAGFTAFYMFRVYLLTFEGHLNVHFLNFS
GKRRSSLYSISLWGNE-GLKTIQPLKTSLTMNFFCGTKN-TLPYPHESENTMLFPIVVLVLFT
LFVGAIG--FDLDILSKFLTPSRNLFHQTSN-SLDW-EFITNATLSVSLSYLGIVIASFFYKPFYL
SLTKLNFMNLFVIWRDKIRNQIYDWSSNRGYIDAFYTISLIKGIIKFAEFTHFFDARVIDGIT
NGLGILSFFVGEGIKYVGGG-RVSSYLLFYLSCVLICLVISYMDLPGPIHDFLLVFLGSGLLL
GGLGVVLLTNLIYSAFALGLVFICISLFYILANSHFVAAAQLLIYVGAINILILFAVMFLNGSE
SSKDSRFGTVGDGATSLVCTTLFLSLISNILDTSWYRIIWTTRATQIIEKDLISNSQQIGIRLST
DFFLPFELISIILLVALIGAIGVARQMTIPATRKDLMIVNMGPHHPSMHGVLRLILTLDGEDVI
DCEPILGYLHRGMEKIAENRTIIQYLPYVTRWDYLATMFTEGITVNAPEKLGNIQVPKRAS
YIRTIMLELSRIASHLLWLGPFMADIGAQTPFFYIFRERELVYDLFEAATGMRMMHNYFRI
GGVAADLPHGWIDKCLDFCDYFLTGLAEYEKLITRNPIFLERVEGIGILGKEDAINWGLSGP
MLRASGIQWDLRKIDNYECYKEFDWEVQWQKEGDSLARFLVRIGEMRESIKMIQQALEG
IPGGPYENLEIRCFDRERYPEWGGFEYRFISKKPSPTFELPKQELYVRVEAPKGELGIFLIGD
QSSFPWRWKIRPPGFINLQILPQLVKRMKLADIMTILGSIDIIMGEVDRMFPMVTGFMNYG
QQTVRAARYIGQSLMITLSHANRFPVTIQYPYEKLITSERVRGRIHFEFDKCIACEVCVRVC
PIDLPVVDWKFQTDIRKKRLFNYSIDFGICIFCGNCVEYCPTNCLAMTEEYELSTYDRHEL
NYNQISLGRLPMPVIEDYTIRIILNS----------------MQGRLSAWLVKHGLIHRSLGFDYQGIET
LQIKSEDWHSIAVILYVYGYNYLRSQCAYDVAPGGLLASVYHLTRVECGVDQPEEVCIKVF
VPRNNPGIPSVFWVWKSADFQERESYDMLGISYDNHPRLKRILMPESWLGWPLRKDYITP
NFYEIQDAH---MNSIEFALLDRRTQNSVLSTT
SKDLSNWSRLSSLWPLLYGTSCCFIEFASLIGSRFDFDRYGLVPRSSPRQADLVLTAGTVTM
KMAPSLVRLYEQMPEPKYVIAMGACTITGGMFSTDSYSTVRGVDKLIPVDVYLPGCPPKP
EAVIDAITKLRKKISREINEDPIGSGSANRYFTTNHKFRGGRSIRIGNYDPGFFYQPPANSKIP
PATFFKYKSSRPSHELVNMQTRKTLSCITEEITRSISVSLMLYIITQVSVSNAYPIFAQQGYEN
PREATGRIVCANCHLANKPVDIEVPQTVLPDTVFEAVVRIPYDLQLKQVLANGKKGALNV
GAVLILPEGFELAPPDRISPEMKERIGNLPFQSYRPAKKNILVIGPVPGQKYSEIAFPILSPDPA
TNKDTHFLKYPIYVGGNRGRGQIYPDGSKSNNTVYNARAAGIVRKIIRKERGGYEITIADT
ADGREVVDIIPPGPELLVSEGESIKVDQPLTSNPNVGGFGQGDAEIVLQDSLRVQGLLCFFA
SVILAQIFLVLKKKQFEKVQLSEMNFMSKVYDWFEERLEIQAIADDITSKYVPPHVNIFYC
LGGITLTCFLVQVATGFAMTFYYRPTVSEAFSSVQYIMTEANFGWLIRSVHRWSASMMVL
MMILHVFRVYLTGGFKKPRELTWVTGVILGVLTASFGVTGYSLPRDQIGYWAVKIVTGVP
DAIPVLGAPLVELLRGSASVGQSTLTRFYSLHTFVLPLLTAVFMLMHFLMIRKQGISGPLM
GVTKKPDLNDPVLRAKLAKGMGHNYYGEPAWPNDLLYIFPVVILGTIACNVGLAVLEPSM

IGEPADPFATPLEILPEWYFFPVFQILRTVPNKLLGVLLMASVPLGLLTIPFLENVNKFQNPF
RRPVATTVFLVGTVAALWLGIGATLPIDKSLTLGLFMIEVFLFGIVLGLIPITLAGLFVTAYLQ
YRRGDQSDLMSTITSYFGFLLVALTITAALFIGLSKIRLIMDIVSLAWAALMVVFTFSLSLVV
WGRSGLMIIRSPEPEVKILVDRDPVKTSFEEWARPGHFSKTIAKGPDTTTWIWNLHADAH
DFDSHTSDLEEISRKVFSAHFGQLSIIFLWLSGMYFHGARFSNYEAWLSDPTHIGPSAQVV
WPIVGQEILNGDVGGGFRGIQITSGFFQIWRASGITSELQLYCTAIGGLVFAVLMLFAGWFH
YHKAAPKLAWFQDVESMLNHHLAGLLGLGSLSWAGHQVHVALPINEFLNAGVDPKEIPL
PHEFILNRDLLAQLYPSFAEGATPLFTLNWSKYAEFLTFRGGLDPVTGGLWLTDIAHHHLAI
AILFLIAGHMYRTNWGIGHDLKDILEAHKGPFTGQGHKGLYEILTTSWHAQLSLNLAMLG
SLTIVVAHHMYSMPPYPYLATDYGTQLSLFTHHMWIGAFLIVGAAAHAAIFMVRDYDPTT
RYNDLLDRVLRHRDAIISHLNWVCIFLGFHSFGLYIHNDTMSALGRPQDMFSDTAIQLQPV
FAQWIQNTHALAPDTTAPGATTSTSLTWGGGGLVAVGGKVALLPIPLGTADFLVHHIHAFTI
HVTVLILLKGVLFARSSRLIPDKANLGFRFPCDGPGRGGTCQVSAWDHVFLGLFWMYNAI
SVVIFHFSWKMQSDVWGSISEQGVVTHITGGNFAQSSITINGWLRDFLWAQASQVIQSYGS
SLSAYGLFFLGAHFVWAFSLMFLFSGRGYWQELIESIVWAHNKLKVAPAIQPRALSIVQGR
AVGVTHYLLGGIATTWAFFLARIIAVGMALRFPRFSRGLAQDPTTRRIWFGIATAHDFESHD
DITEERLYQNIFASHFGQLAIIFLWTSGNLFHVAWQGNFEAWVQDPLHVRPIAHAIWDPHF
GQPAVEAFTRGGALGPVNIAYSGVYQWWYTIGLRTNEDLYTGALFLLFLSAISLIGGWLHL
QPKWKPSVSWFKNAESRLNHHLSGLFGVSSLAWTGHLVHVAIPGSRGEYVRWNNFLDVL
PHPQGLGPFFTGQWNLYAQNPDSSSHLFGTSQGSGTAILTLLGGFHPQTQSLWLTDIAHHH
LAIAFLFLVAGHMYRTNFGIGHSIKDLLEAHIPPGGRLGRGHRGLYDTINNSIHFQLGLALA
SLGVITSLVAQHMYSLPAYAFIAQDFTTQAALYTHHQYIAGFIMTGAFAHGAIFFIRDYNPE
QNEDNVLARMLDHKEAIISHLSWVSLFLGFHTLGLYVHNDVMLAFGTPEKQILIEPVFAQ
WIQSAHGKTSYGFNVLLSSMNSPAFIAGRSIWLPGWLNAINE--NSNSLFLTIGPGDFLVHH
AIALGLHTTTLILVKGALDARGSKLMPDKKDFGYSFPCDGPGRGGTCDISAWDAFYLAVF
WMLNTIGWVTFYWHWKHITLWQGNVSQFNESSTYLMGWLRDYLWLNSSQLINGYNPF
GMNSLSVWAWMFLFGHLVWATGFMFLISWRGYWQELIETLAWAHERTPLANLIRWRDKP
VALSIVQARLVGLAHFSVGYIFTYAAFLIASTSGKFGMSHSVKIYDTCIGCTQCVRACPTDV
LEMIPWDGCKAKQIASAPRTEDCVGCKRCESACPTDFLSVRVYLWHETTRSMGLAYMTT
LNFPSFLVPLVGLVFPAFSIASLFLHIEKNKIFMRDLKTYLSAAPVLSTLWFGALAGLLIEIN
RFFPDALTFPFFSFMTAILARRESESLWGRFCNWITSTENRLYIGWFGVLMIPTLLTATSVFII
AFIAAPPVDIDGIREPVSGSLLYGNNIISGAIIPTSAAIGLHFYPIWEAASVDEWLYNGGPYE
LIVLHFLLGVACYMGREWELSFRLGMRPWIAVAYSAPVAAATAVFLIYPIGQGSFSDGMPL
GISGTFNFMIVFQAEHNILMHPFHMLGVAGVFGGSLFSAMHGSLVTSSLIRETTENESANE
GYRFGQEEETYNIVAAHGYFGRLIFQYASFNNSRSLHFFLAAWPVVGIWFTALGISTMAFN
LNGFNFNQSVVDSQGRVINTWADIINRANLGMEVMHERNAHNFPLDLAGVEVPSINGMG
LPWYRVHTVVLNDPGRLLSVHIMHTALVAGWAGSMALYELAVFDPSDPVLDPMWRQGM
FVIPFMTRLGITNSWGGWSIT-GGTVTNPGIWSYEGVAGAHIVFSGLCFLAAIWHWVYWD
LEIFSDERTGKPSLDLPKIFGIHLFLSGVACFGFGAFHVTGLYGPGIWVSDPYGLTGRVQSV
NPAWGVDGFDPFVPGGIASHHIAAGTLGILAGLFHLSVRPPQRLYKGLRMGNIETVLSSSIA
AVFFAAFVVAGTMWYGSATTPIELFGPTRYQWDQGYFQQEIYRRVGAGLGKNQSLSEAW
AKIPEKLAFYDYIGNNPAKGGLFRAGSMDNGDGIAVGWLGHPIFRDKEGCELFVRRMPTF
FETFPVVLVDGDGIVRADVPFRRAESKYSVEQVGVTVEFYGGQLNGVIYSDPATVKKYAR
RAQLGEIFELDRAALKSDGVFRSSPRGWFTFGHASFALLFFFGHIWHGARTLFRDVFAGID

PDLDTQVEFGAFQKLGDPTTKRQAVMKTLYSLRRFCPVETLFNGTLALAGRDQETTGFA
WWAGNARLINLSGKLLGAHVAHAGLIVFWAGAMNLFEVAHFVPEKPMYEQGFILLPHLA
TLGWGVGPGGEVIDTFPYFVSGVLHLISSAVLGFGGIYHALLGPETLEESFPFFGYVWKDR
NKMTTILGIHLILLGIGAFLLVFKALSFGGVYDTWAPGGGDVRKITNLTLSPSVLFGYLLKS
PFGGEGWIVSVDDLEDIIGGHVWLGSICILGGIWHILTKPFAWARRALVWSGEAYLSYSLA
ALSLFGFIACCFVWFNNTAYPSEFYGPTGPEASQAQAFTFLVRDQRLGANVGSAQGPTGL
GKYLMRSPTGEVIFGGETMRFWDLRAPWLEPLRGPNGLDLSRLKKDIQPWQERRSAEYM
THAPLGSLNSVGGVATEINAVNYVSPRSWLATSHFVLGFFLFVGHLWHAGRARAAAAGFE
KGIDRDFEPVLSMTPLNMTIALGKFTKDETDLFDIMDDWLRRDRFVFVGWSGLLLFPCAY
FALGGWFTGTTFVTSWYTHGLASSYLEGCNFLTAAVSTPANSLAHSLLLLWGPEAQGDFT
RWCQLGGLWTFVALHGAFGLIGFMLRQFELARSVQLRPYNAIAFSGPIAVFVSVFLIYPLG
QSGWFFAPSFGVAAIFRFILFFQGFHNWTLNPFHMMGVAGVLGAALLCAIHGATVENTLF
EDGDGANTFRAFNPTQAEETYSMVTANRFWSQIFGVAFSNKRWLHFFMLFVPVTGLWMS
ALGVVGLALNLRAYDFVSQEIRAAEDPEFETFYTKNILLNEGIRAWMAAQDQPHENLIFPE
EVLPRGNALMSGSTGERSFADIITSIRYWVIHSITIPSLFIAGWLFVSTGLAYDVFGSPRPNE
YFTESRQGIPLITGRFDPLEQLDEFSRSFMTIDRTYPIFTVRWLAVHGLAVPTVFFLGSISAM
QFIQRMDTQIVEDSSRSG-PRRTNVGSLLKPLNSEYGKVAPGWGTTPLMGVAMALFAIFLSI
LLEIYNSSVLLDGISVNMLTLKLFVYTVVIFFVSLFIFGFLSNDPGRNPGREEMADTTGRIPL
WIIGTVAGIPVIGLIGIFFYGSYSGLGSSLMINIFSLICIYLNSVLYSSTFFFGKLPEAYAFLNPI
VDFMPVIPLFFFLLAFVWQAAVSFRTTQSNPNEQNVELNRTSLYWGLLLIFVLAVLFSNYFF
NMEVNILAFIATALFILVPTAFLLIIYVKTVSQSDMETATLVAISISGLLVSFTGYALYTAFGQP
SQQLRDPFEEHGDMEALVYTFLLVSTLGIIFFSIFFREPPKVPTKK--MTIAFQLAIFALIATSV
ILLIGVPVVFASPEGWSSNKNVVFSGTSLWIGLVFLVAILNSLIS-------MSPQTETKASVGFK
AGVKDYKLTYYTPDYETKDTDILAAFRVTPQPGVPPEEAGAAVAAESSTGTWTTVWTDG
LTSLDRYKGRCYHIEPVAGEENQYIAYVAYPLDLFEEGSVTNMFTSIVGNVFGFKALRALR
LEDLRIPTAYVKTFQGPPHGIQVERDKLNKYGRPLLGCTIKPKLGLSAKNYGRAVYECLRG
GLDFTKDDENVNSQPFMRWRDRFLFCTEAIYKSQAETGEIKGHYLNATAGTCEEMMKRA
VFARELGVPIVMHDYLTGGFTANTSLAHYCRDNGLLLHIHRAMHAVIDRQKNHGIHFRVL
AKALRMSGGDHIHSGTVVGKLEGERDITLGFVDLLRDDFIEKDRSRGIYFTQDWVSLPGV
LPVASGGIHVWHMPALTEIFGDDSVLQFGGGTLGHPWGNAPGAVANRVALEACVQARNE
GKDLATEGNAIIRKACKWSPELAAACEVWKEIKFEFAAMDTL--MAIDLYKTYTPSPHNGA
VDSQVKSHARNHLIHGRHRCGKGRNARGIITAGHRGGGHKRLYRKIDFRRNEKGIYGRIV
TIEYDPNRNASICLIHYGDGEKRYILHPRGAILGDTIVSGTGGPIKMGNALPLTKIPLGTAIH
NIEIAPGKGGQLARAAGAVAKLMAKEGKSATLKLPSGEVRLISQNCSATVGQVGNVGVKR
KDLGRAGSKRWLGKRPVVRGVVMNPVDHPHGGGEGKAPIGRKKPVTPWGYPALGRRTR
KRKKYSDNLILRRRSKMIQVQTHLNVADNSGARELMCIRIIGTS-NRRYAYIGDVIVAVIKEA
VPKVSLKRSEVIRAVIVRTCKELKRDDGVIIRYDDNAAVVIDQKGNPKGTRVFGAITHELR
QKNFTKIISLAPEVLMLSPKRTKFRKEHRGRMRGVASRGSCISFGRYALQALEPTWLTSRQI
EAGRRAMTRNVRRGGKIWVRIFPDKPVTVRSAETRMGSGKGSPEYWVAVVKPGRILYEM
GGVAENIARKAISIAGSKMPIRTQFIISDMSRTRRGSTAQKRRAKMHSFASGALGAHSRLTR
VIAQEIIRALASAFRDRTRKKRDFRRLWITRLNGVIRKN--VFNSYSQLIYNLYKEQLALNR
KMLAQIAISNRNCFYTIYSYLKQKFSLQNFQ----MKEVSAFGHSISMSANKARRVIDQIRGR
SYEETLMILELMPYRACSAILKLIYSAAANGIHNMYFDERSLIISKAEVNEGRTVKKLKPR
AKGRGYPIKRSTCHITIVLRDISLH-------MDGIKYAVFTHKSRRLLGKN-QYTFNVELGSTK

TEIKYWVELFFGVKVIAMNSHRLPGKVRREG----PSLHYRRMIITLGPGYSIPPFRKKRTVA
VPKKRISSSKKRIRKNLWKGKGSGAALKAFSLAKSLSTGNSKSFDKRNQKSMAKGKDAR
VRVVLECRSCVRNG--VNNKKNGISRYITQKNRHNTSSQLELRKFCPHCFKHTIHGEIRDM
KTGASVRKICEKCRLIRRKGRLRVICSNPRHKQRQGMIREKVRVSSQRPRWKCVEFRADG
KRLYYGRFILSPLKKGQADIIGIAMRRALLGEIEGTCITYAKFE--KIPHEYTTLVGIQESVYEI
VMNLKEIVLRSNLYGTRDASICVRGPRDVTAQDIILPPSVAIVDNTQHIVTLTEPIDFCVGLQ
IERNRGYSMKIPMPTNHDDRGYPIDAVFMPVKNVNHSIHAYENGS--HEMLFFEIWTNGSL
TPKEALREASRKLINLFIPFLHAEEE--NSHLE-NNQNQHTEELSIDL--------------CGLKDIYID
QLELPPRLYNSLKGSGIHTVMDLMDKNTDALMKIPHFRHEDVEKVMNILNKN--ISQHLSQ
RRMLDDRN-EGMAVIPGLNQIQFEGFCRFVDQGLREELSKFPDIEDLDMDVCFQLFAETSK
LVEPLIKERDAVYESLTYSSELYVSAGLIWKR-YYMQKQTILIGNIPLMNSWGVFIVNGMY
RMVVNQILQSPGIYYRSELDHKGILVYTGTIISDWGGRLELEIDRKGRIWARVSRKQKISIL
VLSSAMGSNLSEILENVCYPELLLAFLYDKENATIGSKENAILEFYQQFVCVGGDPVFSDSL
CKELQNQFFRQRCELGRIGRRNINRRLNLAISQNNTFLLPRDILAATDHLIGMKFGMTLD
D--MNHLKNKRIRSVGDLLQDQLRLALIRLEKVVKRAIREAMK-GKLI-PTPQNFVTSTP----
LKTTYESFFGLHPLSQVLDETNPLTQIVHGRKWSFLGPGGLTGQTASFRRRDIHPSYYGRIC
TIDTSEGINVGLIGSLAIHARIGDFG-SLESPFY---EISEKSKR--ARILYLSSRRDEYCMIGIGN
SLALNESFQEEQIVPARYRQEFLTIAWEQVHLRSIFPFQYISIGVSLIPFIEHNDATRALMSSN
MQRQAVALSRSEKCIVGTGLEGQVALDSGVSLITEHAGKIISIHTDKILLSGNGNTLSIPLVR
YQRSNKNTCIHQQPQVRWGKCIKKGQILADGASTVGGELALGKNLLVAYMPWEGYNFED
AVLISERLVYEDILTSFHIRKYEIQIHVTNQG-PEKVTNKIPHLAPHLLRNLGKNGVVILGSW
VEAGDILVGKLRPRLAQDVS---PEDRFVDALLGTQIATSRPTCLKVPRGGRGRVIDVRWIRS
NPERESRKRERIRVYISQKREIKVGDKVAGRHGNKGIISKILPRQDMPYLQDGKPIDMVFN
PLGVPSRMNVGQIFECSLGLAGALLDRHYRVAPFDERYEQEASRKLVFSELHEASKQTGN
PWVFEPEYPGKSRIFDGRTGDPFEQPVIVGQPYILKLIHQVDDKIHGRSTGHYTPLTQQPVK
GRAKGGGP-----AGRRNGGLGSRGVWCCAYFTRDAYL--
-------------------MIDRYKHQQLRIGLVSPQQISAWANKTLPNGEIVGEVTEIEPLESETNKPKK
GGFLCERIFGPIKSGVCACGISGKLGDQKEKRTFCGDCGVEFANSQIRRYQMGYIKLTCPV
THVWYLRRLPSYIANLLDKPLKELEDLVYCD--FAFTRP-IAKNPTLLRLRGSFEYETQSWT
PSIPPFLTTQGLHTH---EIYTGAGSIREQLGDLDLRVIIDSSLAEWKEL-EE-GLTGDEWEDQK
KERRKRFLVRRMGLAKHFIQTNVKPEWMVLCLLPVLPPGLRPALHLDEFKPMGSDINQLY
LRVLSRNNALLAFLTIQPR-SEDVIMSQQKLLQEAVDTLLDNGVRGQPIRDGHNKVYKSFS
DVIEGKEGRFRKNLLGKRVDYSGRSVIVVGPSLSLHQCGLPFEIAIELFQPFVIRGLVRQHF
APNTATAKRQIREKERILWEILKEVMQGHPVLLNRAPTLHRLGIQAFQPILVEGRAICLHPL
VCKGFNADFDGDQMAVHVPLSLEAQAEARLLMFSHMNLLAPSIGDPICVPTQDMLIGLYI
LTSGNRQGLCGNRYNLSNRRN---CQTD-IDDKNSRYMKGKEPVFCNSYDAIGAYRQKRIRL
DSPFWLRWRLDQG-LIVS--RGVPVEVHYESSGTHHQIYGHFLIVRSVKKEILCIYIRTTLGHI
FLYREIEEAIQGFSQ-VSY--------------MQEPANLVFQNKVIDGDAIKRLINRLIDRFGMAYTS
HILDQVKTLGFQQATATSISLGIDDLLTIPSKGWLVQDAEQQRFLLEKHYHYGNVHAVEKL
RQSIEIWYDTSAYLRQEMNPNFRTTDPLNPVHIMSFSGARGNASQVHQLLGMRGLMSDPQ
GQMIDLPIQSNFREGLSLTEYIISSYGARKGVVDTAIRTADAGYLTRRLVEVVQHVVVRRT
DCGTTRGLSVSPRNGALP-ERFVMQTLIGRVLANDIYMSARCIAVRNQDLGISLVNQLIT----
--FQIQPISIRTPFTCRSTSWICRLCYGRSPAHGNLVELGEAVGIIAGQSIGEPGTQLTLRTFHT
GGVFTGGTAEDVRAPVNGKIQFNEDLVHPARTRHGQPAFLCSIDFYVTIEGEDIIHSVPIPAK

SFLLVQNDQYVKSEQVIAEIRAGKSTF-PLKDRVRKHIYSDSEGE-MHWSTDVYHAPEFPY
SNVHRLPKTSHLWILLGDLCR--SDPVLFSIYKDQDQMSIH--SLAAQGRYTPSPY--VKNDQ-
LRHNFFRLGLYGK-RIMIRNY-SELNESLLTARWNLPYPA-ILRDNFH--LVAKKRRNRFLIPFG
SEHGREKELRLCSDISIEMPK--NGIFRRNSILAYFDDPRYKRGGSGITKYGPIEADSIVKKKD
FHFVDYDYGVQELEPKYEEVDPLFFIPEEVHILPDSSLIMVRNNSLVGVNTQITLNKRSRAS
GLVQVDRKKKKIKVQIFSGDIHFPEGEENRSLHSGVLIPVPPRLRKTQSKESTK--WKKGIY
VQR--ITLSKK---SFVLLRPVVTYEIADAKKLTRLFPQDPVEEIDNIELEVGNYIRYGDGNLIR
GISGTSIQLVRTCLFLNWDQD---EKDSDIEEARASFVEVSTKGLIRDFLKMHLG--QISYIRK
REDPSGSGLPSE-NGPDRTNINPFSSSYSIYS--KARVRQPLSQNQ-TARILLN---RSKESQSFII
LSSSNCFQLGPVKFNDRKSHN---AKKELDPIIMIRNLSGPLGA--IPQIVNFGV--CYHLLT--K
NQIS-VMKYFQLDH---LKQTLQII--Q-YFFMDENQR--IYKPDPYSNIFLTPFNLNWYFIQHNY
WEETSTIIN-LGQFFSESVCIAKNGP---SLKSGQVLIVQQGGYRIIRSAKPYLATPGTTVHGH
YGEILSGGGTLVTFLYEKSRSADITQGLPKVERVFEVRSMDSISRNLEKRIEGWNEWIPRIL
GIPWGFLIGAELTIAQSRISLVNKIQKVYRSQGVQIHNKHIEMIVRQITSKVLVSE---DGMSN
VFLPGELIGLLRAERTGRALEEGVCYRAVFLGITRASLNTPSFLSEASFQETARVLAKAALQ
GRIDWLKGLKEHVVLGEMIPAGTGFKGLVHSS--REHKALS--LETKKKNLFEEK-A-IHFFF
YHREWFDS-------------------------MTRRYWNIGLEELLGEAVHLGHGIKKWNPRMAPYLLIS
CKDNHITNLTRTARFLAEACNLVFYAASEGKRFLIVGTTKKVSNYVVQAAIKARCHYVNK
KWLGGMLTNWSTAETMLRTFRDLRTYQNTGRLDRFPKRDAAILKRQLSHLQKYLDGVQ
YMTGLPDIVIVLDQQDGFTALLECTALGIPTIGLIDTDCDPNLVDFPIPANDESILSIRFILNKL
VLAICEGRSSSSIRNPMGQKINPLGFRLGTTQNHYSIWFKQPKKYCEGLQEDQKIRDCITN
YIDNQRDAMT-----ISRIQIEKGIDGVKVTIYMGKPQFLTEDKPPKSETRIKELKGNVEKVLH
CE-QKLKISIRKIRKPYGDPKIIAEFLANQLKSRISFRKAMQKTIELAAKEEAKGIQVQIAGRI
EGKEIARVEWLRKGRVSLQTIRTKIHFCSTTVRTIHGVLGIKIWVSK---MSRYRGPRVKKIR
RLGALPGLTSKIP---KGKKETPSPFKKKKQYHIRLEEKQKLRFHYGLTERQLLQYVRLAGK
AKGQTGQVLLQLLEMRLDNTLFRLGMASTIPQARQLVNHRHILVNGRIVDIPSYRCKPRDI
ITGREKEKSRTLIQNYLDSSSRHLTTPNHLTTQQGQQ--RGLVNKIIDRESVGLEIKELLVVE
YYSRQIMSRRGTAEKKTAKSDPIYRNRLVNMLVNRILKHGKKSLAYQILYRAMKRVQQKE
QRNPLSVLRQAIRQITPDIAVKTRRKGGSTQQVPIEIGSTQGKVLAIRWLLEAARKRSGRN
MAFKLSSELGDALKGKGAAIRKKEETRKMAEANRAFAHFRMSKDTISDILTCIRNADMA
KKETVRIRFTNIAEKIVKILLREGFIKNTREHRENNKSFLVLTLRHKS--NRRSNYRT-LLN--L
KRVSRPGRRIYSDYPKIPRILGGMGIVILSTSRGIITDREARLERVGGEILCYIWMAKPIGRY
SSRKKRRI-LRKNIRKIRKGIIHVQASFNNTIVTVTDVEGRVVTWASAGVCGFKGRRKGTPF
AAQTTTEDAIGPVVAQGMHRASVLIKGIGLGRDAALRTICRSGVRLYFIRDITPMPHNGCR
PPKKRRTMPTTQQLIRNARQPVRNATKAPALRGCPQRRGTCTRTYTINPKKPNSALRKVAR
VRLTSGFEITAFIPGIDHNLQEHSVVLVRGGRVKDLPGVKYRIVRGTLDAVGVKGRQQGRS
KYGVKKPKVATKGLIQREKKRQKLEQKYHSIRRSLKKEIR---EVRSLSEKWEIHGKLQASP
RNSAPTRLRRRCFSTGRPRANYRDFGLSRHILREMVHACLLPGATRSSWMVKTPSISVISQ
E----EKNWGSVEFQVVFFSNKIERISSHLKFHRKDYLSQRGLHKLLAKRERLLSYLSKKNF
VGYKELIIRFNIRESKTSQIMVKLRLKRYGRKQRAFYRIVAIDVRARREGRALQKVGFYDPI
NNQTHLNVRTILYFLEQGAQPTGTVHDISKKAGVFGELLLKQRTSHFLQKKKRPFLKKKR
SFRRRLPPITPEELLNYKNVSVISQFISKQGKILPRWVKGLKKKKQDLVKRAVKQARLLAL
LYFQASEK--LFHRIESSPKNTKLGEQEKLSRSFKKNPFVANHLLKKIEKLNAKGEKDSLKT
WSRASTIIPAMVGYTIYIHNGKVHFPIPITNYMVGHKLGEFVPTTSFKEDSKRDTKSR

R???
???
???
???
???
???
???
???
???
???
???
???
???
???
???
???
???
???
???
???
???
???
???
???
???
???
???
???
???
???
???
???
???
???
???
???
???
???
???
???
???
???
???
??MPRSR-INGNFIDKTFSIVANILLRIIPTTSGERE

AFSYYRDG--MSAQSEGNYAEALQNYYEAMRLEIDPYDRSYILYNIGLIHTSNGEHTKALE
YYFRALERNPFLPQAFNNMAVICHY-RGEQAIRQGDSEIAEAWFDQASEYWKQAIALTPGN
YIEAQNWLKIAKRWD--MSCQSENIWIELIAGSRKTSNFFWAVILFLGSLGFLLVGSSSYLGR
NLISFFPSQQILFFPQGVVMSFYGIAGLFISSYLWCTISWNVGSGYDQFDRKEGIVCIFRWG
FPGRNRRIFLRFLMKDIQSIRIEAKAGFSIRRILSMEIRGQGALPLIRTDEN-LTPREIEQKAAE
LAYFLRVPIEVF

>Geranium

--MVQ-
KRAQ----VKSEKRLFHGIVEPEEEE---------------IEDEPYHDRLARYKNETGLLEAIQTGKGEI
YGLPVLFGSMEFGFMGGSMGSVVGEKVARLIERATSAFMPLILVCASGGARMQEGSFSLM
QMAKISGTLYYFNENKKLFYASILASPTTGGVLASFGMLADVVLSEPNTCIAFAGPRVIEAV
LNEKVPEGSQEAEPLLEKGMLDGIIQRKQLKHCVACLFDFHGLYSFLYGNSHMVPIRADEI
SNIIRERIKEYNREVKIVNMGTVLRVGDGIARIYGLNEVMAGELVEFEEGTTGIALNLESNN
VGVVLMGGGLMIQEGSSVKATGRIAQIPVSEAYLGRVVNALAKPIDGRGEISSSESRLIESP
APGIISRRSVYEPLQTGLIAIDSMIPIGRGQRELIIGDRQTGKTAVATDTILNQQGKDVICVYV
AIGQKASSVAQVVTTFQEKGAMEYTIVVAETAASPATLQYLAPYTGAALAEFFMYRERHT
LIIYDDLSKQAQAYRQMSLLLRRPPGREAYPGDVFYLHSRLLERAAKSSSNLGEGSMTALP
IVETQSGDVSAYIPTNVISITDGQIFLSADLFNSGIRPAINVGISVSRVGSAAQIKAMKQVAG
KSKLDLAQFTELEAFAQFASDLDQNTQNQLERGRRLRELLKQSQSEPLAVEEQIMTVYTG
TNGYLDSLEIGQVRKFLGNLRDYLKSNKPQFQEIISSTKTFSEEAETLLKEAIQEQMERFRL
Q---MRLNPTASSLGDSTLKKK-RGRIAQIIGPVLDIVFPKGKMPYIYNALVVQGRDTVGQQI
YVTCEVQQLLGNNRVRAVAMSATDGLMRGMEVIDTGAPLSVPVGGATLGRIFNVLGEPID
NLGPVDTLTTSPIHRSAPDFIQLDTKLSIFETGIKVVDLLAPYRRGGKIGLFGGAGVGKTVL
IMELINNIAKAHGGVSVFGGVGERTREGNDLYKEMKESGVINDQKIAESKVALVYGQMNE
PPGARMRVGLTALTMAEYFRDINKQDVLLFIDNIFRFVQAGSEVSALLGRMPSAVGYQPTL
GTEMGSLQERITSTKKGSITSIQAVYVPADDLTDPAPATTFAHLDATTVLSRGLASKGIYPAV
DPLDSTSTMLQPRIVSAEHYEIAQRVKETLQRYKELQDIISILGLDELSEDDRLTVARARKIE
RFLSQPFFVAEVFTGSTGKYVGLAETLRGFNLILSGEFDGLPEQAFYLVGNIDEATAKATKL
ETESNLKKMTLNLCVLTPKRILWDSEVKEIILSTDSGQIGVLPNHVPIVTGLDTGILRIRLND
QWFTMVLMGGFARIANNEIIIFAHDAEKGSDIDPQEAQQTLQIAEDGFRKAEGKGQTIEVK
QALTRAQTRVNAIS---MKNVTDSFVSLGRCPFAGSFGFNTDILATNPINLSVVLGVLIFFGK
GVLNDLLDNRKQKILNTIQNSEELRGGAIKQLEKAHARLRKVKTEVDQFRVNEYSEAERK
RSNLITSTYKELERSENLKNESIRFEQQRAINQVRQRVFQQALQGALEILNSCLNKELHLRT
ISANIGLFGSMKERTYMNPLIAAASVIAAGLAVGLASIGPGIGQGTAAGRAVEGISRQPEAE
GKIRGTLLLSLAFMEALTIYGLVVALALLFANPFVMNVLACSINTLKGLYEVSSVEVGQHF
YWQIGGFQVHGQVLITSWIVIAILLGSAIIAVQNPQTIPTDVQNFFEYVLEFIRDVSKTQIGE
EYRPWVPFIGTMFLFIFVSNWSGALLPWKIIQLPQGELAAPTNDINTTVALALLTSVAYFYA
GLTKKGLGYFNKYIQPTPILLPINILEDFTKPLSLSFRLFGNILADELVVVVLLSLVPLVVPIP
VMFLGLFTSGIQALIFATLAAAYIGESMEGHHMLFLTLEHIFNHISFSVVSIVITIHFIRLVFDE
IVGLFDSSEKGMLVSFFCITGLLITRWTYSGDFPLSDLYDSLIFLSWNLSIISMLPYFKNDKN
DLSAISYPSALFTQGFATSGLLAEMHQSKRLMPALQSQWLMMHVSMMILSYAALLCGSLL
SIALLIITFQKRVFLFGKINNIQYLTERK---INTFLTSSVNYYRSQLLHQLDYWSCRIISLGFLF
LTIGILSGAVWANEAWGSYWSWDPKETCAFITWTVFAIFLHIRTNKNVEGVNSAIVASMGF

FLIWVCYFGINLLRIGLHSYGSFVISMKKKKKTIP--YLASIVFLPWWISMLFTKSLECWVT
NWWNIRQSEPFLNEIQEKSLIEKFIELEELLLLDEMVKECPEVDFQKFRIGIHKETVQLMKI
HNEDQIHTILHFSQNIIYFVILSGYSVLGKGQLVILNSWFQEFLYNLSDTIKALCLVFLSDIFL
GYHSKAIWEFIIVYVVKSFGYDYNDRIVSILGWILPSLLHIALKYMSFRYFNGLCPSLLVMY
T A M K
E???
???
???
????????MEELQEYFEFDRSWQHPFLYPLIYRESIYALAHDHGLASYDNKSSSLSVKRLIAR
MYQQNPLVICPNDSNQNPFLGNNCSSQMISEGFADIAEIPLFLQLVSSLIAKSLNLRSMQSIF
PFLEDECFHCHFVSDVLIPHPAHLEILVEALRYWVKDAPFLHFLRFFLYQDW--SCNSLSPSS
FFSKARLFLFLYNSHVCEYESILLFLRNKSSDLRSTSFRSFLERILFYKKMEVLVSIFANKRIL
LFFNDPFIHYVRYQGKYILILKDRPLLMNKWKDYLIKLWQCYFYVWSQPGRVHINKLSQH
SLNFMGYILSVQPIPLVVRSQVLENVFLIDN--PMNKVQTIIPTFYLIGSLVKAKFCNLLGQPI
SKPTWADSSDSDIIDRFVRMCRNLSHYHSGSSKKKGLYRVKYILRLSCVKTLARKHKSTV
RAFLKKSGSALLEEFINEEEQALSLIFSRRVWYLDILYINDLINQEMIIDPR-EVQYIN-SFSRF
ES-FQEVYGTVWILVPILILVLGITIGVLVIVWLERAISAGIQQRIGPEYTGPLGILQALADGT
KLFFKENLLPSRGDIRLFSLGPSMAVISILLSYSVIPFSYRLVLADLNVGVFLWIAVASIAPIG
LLMSGYGSNNKYSFLGGLRATAQSISYEIPLTLCVLSISLLSNSSSTVDIVEAQSKYGFWGW
NLWRQPIGFIVLLISSLAECERLPFDLPEAEEELVTGYQTEYSGIKFGLFYVTSYFNLLVSSL
FVTILYLGGWNLSIPWIDFPQLFEINKVGAIFATTISLFITLAKTFFFLFISITTRWTLARLRMD
QLLNLGWKFLLPISLGNLLLTTSSQLLSL----------ILDSTRIFMKAFHLLLFDGSFIFPECILIF
GLILLLMIDSTSDQIGIPWFYFISSTSLVMSITALLFRWREEPMISFSGNFQTNHFNEIFQFLIL
LCSTLAIPLSVEYIECTEMAIIEFLLFILTATLGGMLLCGANDLITIFVAPECFSLCSYLLSGYT
KKDVRSNEATMKYLLMGGASSSILVHAFSWLYGSSGGEVELQEIVNGLINTQMYNSPGISI
ALVFITVGIGFKLSPAPSHQWTPDVYEGSPTPVVAFLSVTSKVAASASATRIFDIPFYFSSNE
WHLLMEILAILSMILGNLIAITQTNMKRMLAYSSIGQIGYVIIGIIVGDSNSGYASMITYMLF
YIFMNLGTFACIVLFGLRTGTDNIRDYAGLYTKDPFLALSLALCLLSLGGLPPLAGFFGKLY
LFWCGWQAGLSLLVSIGLLTSVLSIYYYLKIIKLLMTGRNQEITSHVRNYRRSPLRSNNSIE
LSMIVCVIASTIPGISMNPIIAIAQDTLFMFLLYEYDIFWVFLIISSLIPILAFLISGVLAPISEGP
EKLSSYESGIEPMGDAWLQFRIRYYMFALVFIVFDVETVFLYPWAMSLDVLGVSIFIEAFFF
VLILIIGSVYAWRKGALEWVTNYFPWLTIIVIFPISAGSFIFFFPHRGNKVTRWYTLCICSLEL
LLTAYAFCYQFQLDDPLIQLVENYKWIHFLQFYWRLGIDGLSLGPILLTGFITTLATLAAWP
VTRNSRLFYFLMLAMYSGQIGSFSSRDLLLFFIMWEFELIPVYLLLSLWGGKKRLYSATKF
LLYTAVSSLFLLIGVLSIGLFGSNEPTFHFEISAHQSYPAALEIILYIGFLITFAVKSPIIPLHTWL
PDTHGEAHYSTCMLLAGILLKMGAYGLVRINVEFLTHAHSKFCPYLITIGAIQIVYAASTSL
GQRNLKKRIAYSSVSHMGFIIIGISSITDTGLNGAILQIISHGFIGAALFFLAGTSYDRIRLVSF
HEMGGMAVAIPKVFTMFSILSMASLALPGMSGFVAELIVFFGIITSQKFLLMPKIVITFGMAI
GMILTPIYSLSMLRQMFYGYRLLNAPTLPFFDSGPRELFVSISILLPVIGIGIYPDFLMSLSVD
RVELIVSHFFLGMILEHVLVLSAFLFSIGIGGLITSRNMVRAFMCLELMLNAVNLNFVTFSD
FFDSCQLKGAVFSIFVIAIGAAEAAIGPAILSSIYHNKKSTRINQSNLLKKMEYTNKYSWIM
PFIPLPVPMLIGVGLLLFPTTTKHIRRIWAFPTLFLLGIVILLSAYLSIQQLNRSYIYQYVWSW
TINNDFSLEFGHLIDPLTSTMSILITTVGLTVLIYSENYMFHDQGYLRFFYYMSFFNASMLG
LVTSSNLIQIYIFWEFVGMCSYLLIGFWFTRPVAAKACQKAFVTNRVGDFSFLLGILGLYWI

TGSFEFRDLFQIFNN-EIPFLFLTLCAFLLFGGALAKSAQFPLHVWLPDAMEGPTPISALIHA
ATMVAAGIFLVARLLPLFIAIPSLLNLISLIAIITVFLGATLALAQKDIKRVLAYSTMSQLGYM
MLALGVGSYRAALFHLITHAYSKALLFLGSGSIIHSMEALVGYSPEKSQNMVLMGGLTKH
MPITKTAFLLGTLSLCGIPPLACFWSKDEILNDSWLYSPIFAIIAFSTAGFTAFYMFRIYLLTF
EGHLNVHFLNYSGKRSSSLYSISLWGNT-GLKTMKNFKTSLTMNFFCGTKN-TLPYPHESET
TMLFPIVVLVLFTFFVGAIG--FDLDILSKFLTPSINLLHQKSN-SLGW-EFLTNATLSVSISYLG
IVIASFLYKPFYLSLTKLNFINLFVIWRDKIINRIYDWSYNRGYIDAFYTISLIKGIRKFAELTY
FFDARVIDAITNGLGIMSFFVGEGIKYVGGGGRVSSYLLFYLSCVLICLVISYMDLPGPIHDF
LLVFLGSGLILGGLGVVLLTNLIYSAFSLGLVFICISLFYILANSHFVAAAQLLIYVGAINILIL
FAVMFINGSEYSKDLNLGIVRDGATSLVCTTLFLSLISNILDTSWYGIIWTTRATQIVEKDLIS
NSQQIGIRLSTDFFLPFELISIILLVALIGSIAVARQMTIPTTRKDFMIVNMGPHHPSMHGVLR
LILTLDGEDVIDCEPILGYLHRGMEKIAENRTIIQYLPYVTRWDYLATMFTEGITVNAPEKL
GNIQVPKRANYIRTIMLELSRIASHLLWLGPFMADIGTQTPFFYIFRERELVYDLFEAATGM
RMMHNYFRIGGVATDLPYGWIDKCLDFCDYFLTGLAEYEKLITRNPIFLERVEGIGIIDKEE
AINWGLSGPMLRASGIQWDLRKIDNYDCYNEFDWEVQWQKEGDSLARFLVRIGEMRESI
KMIQQALEGIPGGPYENLEIRCFDRERYTEWGGFEYRFVSKKPSPTFELPKQELYMRVEAP
KGELGIFMIGDQSSFPWRWKIRPPGFINLQILPQLVKRMKLADIMTILGSIDIIMGEVDRMFP
MVTGFMNYGQQTVRAARYIGQSFMITLSHANRFPVTIQYPYEKLITSERFRGRIHFEFDKC
IACEVCVRVCPIELPVVDWKFETDIRKKRLFNYSIDFGICIFCGNCVEYCPTNCLSMTEEYE
LSTYDRHELNSNQIALGRLPMPVIEDYTIRTILNS----------------MQGRLSAWLVKHGLVHRS
LGFDYQGIETLQIKPEDWHSIAVILYVYGLNYLRYQCAYDVAPGGLLASVYHLTTVECGV
DQPEEVCIKVFVPRNNPGIPSVFWVWKSSDFQERESYDMLGISYDHHPRLKRILMPESWIG
WPLRKDYVAPNFFEIQDAH---MNFIEFAVLDR
RTQNSVLSTTPNDLSNWSRLSSLWPLLYGTSCCFIEFASLIGSRFDFDRYGLVPRSSPRQADL
VLTAGTVTMKMAPSLVRLYEQMPEPKYVIAMGACTITGGMFSTDSYSTVRGVDKLIPVDV
YLPGCPPKPEAVIDAITKLRKKISREINEDPIRS--ANRYFTTNHKFRVVRSIHIGNYDQGFFY
QPPVTSEPPPATFFKYINSRPSHELVNMQTIKTLSCITKEITRSISVSLMLYIITQASLSNAYPIF
AQQGYENPREATGRIVCANCHLANKPVDIEVPQTVLPDTVFEAVVRIPYDMQLKQVLAN
GKKGALNVGAVLILPEGFKLAPPERISPEMKERIGNPPFQSYRPAKKNIIVIGPVPGQKYSEI
TFPILSPDPATNKGVNFLKYPIYVGGNRGRGQIYPDGSKSNNTISNATATGIVSKIIRKEKGG
YEITIADTSDGRQVVDIIPPGPELLVSEGESIKVDQPLTSNPNVGGFGQGDAEIVLQDSLRVQ
GLLCFLASVILAQIFLVLKKKQFEKVQLSEMNFMSKVYDWFEERLEIQAIADDITSKYVPP
HVNIFYCLGGITLTCFLVQVATGFAMTFYYRPTVTEAFSSVQYIMTEANFGWLIRSVHRWS
ASMMVLMMILHVFRVYLTGGFKKPRELTWVTGVVLAVLTASFGVTGYSLPRDQIGYWAV
KIVTGVPDAIPVIGSPLVELLRGSASVGQSTLTRFYSLHTFVLPLLTAVFMLMHFLMIRKQGI
SGPLMGVTKKPDLNDPVLRAKLAKGMGHNYYGEPAWPNDLLYIFPVVILGTIACNVGLAI
LEPSMIGEPADPFATPLEILPEWYFFPVFQILRTVPNKLLGVLLMVSVPAGLLTVPFLENVNK
FQNPFRRPVATTVFLVGTVAALWLGIGATLPIDKSLTLGLFMIEVFLFGIVLGLIPITLAGLFV
TAYLQYRRGDQLGLMSTITSYFSFLLVALTITAALFIGLSKIRLIMDIISLAWASLMVVFTFSL
SLVVWGRSGLMIIRSPEPEVKILVDRDPVKTSFEEWARPGHFSKIIAKGPDTTTWIWNLHA
DAHDFDSHTSDLEEISRKVFSAHFGQLSIIFLWLSGMYFHGARFSNYEAWLSDPTHIGPSA
QVVWPIVGQEILNGDVGGGFRGIQITSGLFQIWRASGITSELQLYCTAIGALVFAALMLFAG
WFHYHKAAPKLAWFQDVESMLNHHLAGLLGLGSLSWAGHQVHVSLPINQFLNAGVDPK
EIPLPHEFILNRDLLAQLYPSFAEGATPLFTLNWSKYAEFLTFRGGLDPVTGGLWLTDIAHH

HLAIAILFLIAGHMYRTNWGIGHDLKDILEAHKGPFTGEGHKGLYEILTTSWHAQLSLNLA
MLGSLTIVVAHHMYSMPPYPYLATDYGTQLSLFTHHMWIGAFLIVGAAAHAAIFMVRDY
DPTIRYNDLLDRVLRHRDAIISHLNWVCIFLGFHSFGLYIHNDTMSALGRPQDMFSDTAIQL
QPVFAQWIQNTHALAPGATAPGATTSTSLTWGEGGLVAVGGKVALLPIPLGTADFLVHHIH
AFTIHVTVLILLKGVLFARSSRLIPDKANLGFRFPCDGPGRGGTCQVSAWDHVFLGLFWM
YNAISVVIFHFSWKMQSDVWGSLSEEGVVTHITGGNFAQSSITINGWLRDFLWAQASQVI
QSYGSSLSAYGLFFLGAHFVWAFSLMFLFSGRGYWQELIESIVWAHNKLKVAPATQPRALS
IVQGRAVGVTHYLLGGIATTWAFFLARIIAVGMALRFPRFSQGLAQDPTTRRIWFGIATAHD
FESHDDITEERLYQNIFASHFGQLAIIFLWTSGNLFHVAWQGNFEAWVQDPLHVRPIAHAI
WDPHFGQPAVEVFTRGGALGPVNIAYSGVYQWWYTIGLRTNEDLYTGALFLLLLSSISLIA
GWLHLQPKWKPSVSWFKNAESRLNHHLSGLFGISSLAWTGHLVHVAIPGSRGGYVRWNN
FLDVLPHPQGLGPLFTGQWNLYAQNPDSSNHFFGTSQGSGTAILTLIGGFHPQTQSLWLTDI
AHHHLAIAFLFLVAGHMYRTNFGIGHSIKDLLEAHIPPGGRLGRGHRGLYDTINNSIHFQLG
LALASLGVITSLVAQHMYSLPAYAFIAQDFTTQAALYTHHQYIAGFIMTGAFAHGAIFFIRD
YNPEQNEDNVLARMLDHKEAIISHLSWVSLFLGFHTLGLYVHNDVMLAFGTPEKQILIEPI
FAQWIQSAHGKTSYGFNVLLSSTTSPAFIAGRSIWLPGWLNAINE--NSNSLFLTIGPGDFLV
HHAIALGLHTTTLILVKGALDARGSKLMPDKKDFGYSFPCDGPGRGGTCDISAWDAFYLA
VFWMLNTIGWVTFYWHWKHITLWQGNVSQFNESSTYLMGWLRDYLWLNSSQLINGYN
PFGMNSLSVWAWMFLFGHLVWATGFMFLISWRGYWQELIETLAWAHERTPLANLIRWRD
KPVALSIVQARLVGLAHFSVGYIFTYAAFLIASTSGKFGMSHSVKIYDTCIGCTQCVRACPT
DVLEMIPWDGCKAKQIASAPRTEDCVGCKRCESACPTDFLSIRVYLWHETTRSMGLAYMT
TLNLPSILVPLVGLVFPAFSIASLFLHVQKNKIFMRDLKTYLSAAPVISTLWFGALAGLLIEI
NRFFPDALTFPFFSFMTAILERRESESLWGRFCNWITSTENRLYIGWFGVLMIPTLLTATSVFI
IAFIAAPPVDIDGIREPVSGSLLYGNNIISGAIIPTSAAIGLHFYPIWEAASVDEWLYNGGPYE
LIVLHFLLGVACYMGREWELSFRLGMRPWIAVAYSAPVAAATAVFLIYPIGQGSFSDGMPL
GISGTFNFMIVFQAEHNILMHPFHMLGVAGVFGGSLFSAMHGSLVTSSLIRETTENESANE
GYRFGQEEETYNIVAAHGYFGRLIFQYASFNNSRSLHFFLAAWPVVGIWFTALGISTMAFN
LNGFNFNQSVVDSQGRVINTWADIINRANLGMEVMHERNAHNFPLDLAAVEVPSINGMG
LPWYRVHTVVLNDPGRLLSVHIMHTALVAGWAGSMALYELAVFDPSDPVLDPMWRQGM
FVIPFMTRLGITNSWGGWSIT-GETITNPGIWSYEGVAGAHIVFSGLCFLAAIWHWVYWDL
EIFSDERTGKPSLDLPKIFGIHLFLSGVSCFGFGAFHVTGLYGPGIWVSDPYGLTGRVQFVNP
AWGVSGFDPFVPGGIASHHIAAGTLGILAGLFHLSVRPPQRLYKGLRMGNIETVLSSSIAAV
FFAAFVVAGTMWYGSATTPIELFGPTRYQWDQGYFQQEIYRRVGAGLVKNQSLSEAWSKI
PEKLSFYDYIGNNPAKGGLFRAGSMDNGDGIAVGWLGHPIFRDKEGYELFVRRMPTFFET
FPVVLVDGNGIVRADVPFRRAESKYSVEQVGVTVEFYGGELNGVIYSDPTTVKKYARRA
QLGEIFELDRAALKSDGVFRSSPRGWFTFGHASFALLFFFGHIWHGARTLFRDVFAGIDPD
LDAQVEFGAFQKLGDSTTKRQAVMKTLYSLRRFCPVETLFNGTLALAGRDQETTGFAWW
AGNARLINLSGKLLGAHVAHAGLIVFWAGAMNLFEVAHFVPEKPMYEQGFILLPHLATLG
WGIGPGGEVIDTFPYFVSGVLHLISSAVLGFGGIYHALLGPETLEESFPFFGYVWKDRNKM
TTILGIHLILLGIGAFLLVFKALYFGGVYDTWAPGGGDVRKITNLTLSPSLLFGYLLKSPFGG
EGWIVSVDNLEDIIGGHLWLGSICIFGGIWHILTKPFAWARRALVWSGEAYLSYSLGALAGF
GFIACCFVWFNNTAYPSEFYGPTGPEASQAQAFTFLVRDQRLGANVGSAQGPTGLGKYL
MRSPTGEVIFGGETMRFWDLRAPWLEPLRGPNGLDLSRLKKDIQPWQERRSAEYMTHAP
LGSLNSVGGVATEINAVNYVSPRSWLATSHFVLGFFLFVGHLWHAGRARAAAAGFEKGID

RDFEPVLSMTPLNMTIALGKFTKDENDLFDVMDDWLRRDRFVFVGWSGLLLFPCAYFAL
GGWFTGTTFVTSWYTHGLASSYLEGCNFLTAAVSTPANSLAHSLLLLWGPEAQGDLTRWC
QLGGLWTFVALHGAFGLIGFMLRQFELARSVQLRPYNAIAFSGPIAVFVSVFLIYPLGQSG
WFFAPSFGVAAIFRFILFFQGFHNWTLNPFHMMGVAGVLGAALLCAIHGATVENTLFEDG
DGANTFRAFNPTQAEETYSMVTANRFWSQIFGVAFSNKRWLHFFMLFVPVTGLWMSALG
VVGLALNLRAYDFVSQEIRAAEDPEFETFYTKNILLNEGIRAWMAAQDQPHENLIFPEEVL
PRGNALMSGSTGERSFADIITSIRYWVIHSITIPSLFIAGWLFVSTGLAYDVFGSPRPNEYFTE
SRQGIPLITGRFDPLEQLDEFSRSFMTLDRTYPIFTVRWLAVHGLAVPTVSFLGSISAMQFIQ
RMATQTVADSSRSG-PRRTNVGSLLKPLNSEYGKVAPGWGTTPLMGGAMALFAIFLSILLE
IYNSSLLLDGISMNMLTLKLFVYAVVLFFVSLFIFGFLSNDPGRNPGREEMADTTGRIPLWII
GTVAGIPVIGLIGIFFYGSYSGLGSSLMINIFSLICIYLNSVLYCSTFFFGKLPEAYAFLNPIVD
FMPVIPLFFFLLAFVWQAAVSFRTTQSNPNEQSVELNRTSLYWGLLLIFVLAVLFSNYFFNM
EVNILAFIATALFILVPTAFLLIIYVKTVSQSDMETATLVAISISGLLVSFTGYALYTAFGQPSQ
QLRDPFEEHGDMEALVYTFLLVSTLGIIFFAIFFREPPKVPTQK--MTIAFQLAVFALIATSVIL
LIGVPVVFASPEGWSSNKNVVFSGTSLWIGLVFLVGILNSLIS-------MSPQTETKASVGFKA
GVKDYKLTYYTPDYETKDTDILAAFRVTPQPGVPPEEAGAAVAAESSTGTWTTVWTDGLT
SLDRYKGRCYHIEPVAGEESQFIAYVAYPLDLFEEGSVTNMFTSIVGNVFGFKALRALRLE
DLRIPPAYVKTFQGPPHGIQVERDKLNKYGRPLLGCTIKPKLGLSAKNYGRAVYECLRGGL
DFTKDDENVNSQPFMRWRDRFLFCAEAIYKSQAETGEIKGHYLNATAGTCEEMMKRAVF
ARELGVPIVMHDYLTGGFTANTTLAHYCRDNGLLLHIHRAMHAVIDRQKNHGIHFRVLA
KALRMSGGDHIHAGTVVGKLEGERDITLGFVDLLRDNFVEKDRSRGIYFTQDWVSLPGV
LPVASGGIHVWHMPALTEIFGDDSVLQFGGGTLGHPWGNAPGAVANRVALEACVQARNE
GLDLATEGNEIIRKACKWSPELAAACEIWKEIKFEFAAMDTL--MAIHLYKTYTPSPRNGAV
DSQVKSNPRNNLIYGQHRCGKGRNARGIITAGHRGGGHKRLYRQIDFRRNVYGIYGRIVTI
EYDPNRNASICLIHYGDGEKRYILHPRGAKIGDTIVSGTRVPIKMGNALPLTRIPLGTAIHNI
ETAPGKGGQLARAAGAVAKLIAKEGKSAAFKLPSGEVRLISQNCSATVGQVGNVGVKRK
DLGRAGSKRWIGKRPVVRGVVMNPVDHPHGGGEGRSPIGRKAPTTPWGYPALGRRTRKR
KKYSENWILHRRSKMIQAQTYLNVADNSGARELMCIRIIGTN-NRRYAHIGDVIVAVIKEAV
PKRSLERSEVIRAVIVRTCKELKRDDGVIIRYDDNAAVLIDKKGNPKGTRVFGAITHELRQF
NFTKIISLAPEVLMLSPKRTKFRKEHRGRMKGIASKGSRISFGRYALQALEPAWITSRQIEA
GRRAITRNVRRGGKIWVCIFPDKPVTVRSTETRMGSGKGSPEYWVAVVKPGRILYEMGGV
TENIARKAISIAGSKMPIRTQFIIADMSRTTRGSTARKRRSKMDLFSSGARGAHSSLARVIA
QATAKALASADRDRVRQKRDFRRLWITRINAVIRKN--VFNSYNKFIFNIYKEQLVLNRKML
AQIAISNSNCLYTIYSYLKLELSLQNFQYQN---EVSAVGAYISMSANKVRRVIDQIRGRSYE
ETLMILNFMPYRACYPILKLIYSAAANGIHNMNFNKKSLIISKAEVNEGTTVKKLKPRAKG
RGYPIKRSTCQITIVLRDISLM-------MEGIQYAVFTEKSYRLLGKNN-YTSNVESGSTRT------
--MAVPKKRISSSKKRIRKNIWKGKGSEAALKALS
LAKSISTGNSKSFEFKKIKPMAKGKAARVRVVLECTSCVRNG--VNKKNKGISRYITQKNR
HNTSSQLELRKFCPHCFKHTIHGEIRDMKMGASVRKICEKCRLIRRKGRIRVICSNPRHKQ
RQGMILEKVRVTTQTPRWKCVESRADGKRLYYGRFILSPLKKGQADIIGIAMRRALLGEIE
GTCITHAKFD--KIPHEYTTLVGIQESIYEILMNLKEIVLRSHLYGTRDASICVRGPRDVTAQ
DIILPTSATIVDNTQHIVTITEPIDFCVGLQIERNRGYFIQIPMLTNHDDRGYPIDSVFMPVKN
VNHSIHSYENGI--HAMLFLEIWTNGSLTPKEALREASRKLSNLFIPFLHAEEQ--NFHFE-NN
QNEHTDELPIDF--------------CALKDIYIDQLELPARFYNCLKISGIHTVMDLLNKNTEDLMK

ITHFRQQDIEKVMHILNNT--ISQFLSRRRMLGDRN-EGMAAIPGLNQIQFEGFSRFLDQGLR
EELYKFGNIEDLDMDVCFQLFAETYKLVEPLIKERDAVYESLTYSSELYVAAGLIWKR-YS
MQKQTILIGNIPLMNSLGIFIVNGIYRMVVNQILQSPGIYYRSELDHKGFLVYTGTIISDWG
GRLELEIDRKARIWARVSRKQKISILVLSSAMGSNLRDIIENVCYPEILLAFLDDKEKGKIGS
KENAILEFYQEFVCIGGDPVFSDSLCKELQNTFFRQRCELGKIGRRNINRRLNLDISQNNKF
LLPRDILAATDHLIGIKFGMGTLDD--MNHLKNKRIRSVADLLQDQFGLALIRLEEVIKGGIR
GAIK-GELI-PTPQNFVTSTP----LKTTYESFFGLHPLSQVLDETNPLTQIVHGRKWSFLGPGG
LTGQTPSFRIRDIHPSYYGRICTIDTSEGINVGLIGSLAVHARIGHFE-SLESPFF---EISEKPKG
--ARLLYLSSRRDEYCMIGTGNSLALNEGFQEEQIVPARYRQEFLTIAWEQVHLRSIFPFQYF
SIGTSLIPFIEHNDANRALMSSNMQRQAVALSRSEKCIVGTGLEGQVALDSAVPLITEHAGK
VISIHTDKILLSGNGDTLSIPLVMYQGSNKNTCIHQKPQVRRGKCMKKGQILADGAATVG
GELALGKNLLVAYMPWEGYNFEDAILISERLVYEDIYTSFHIRKYEIKIHVTRQG-PERVTK
KIP-LRAHLLRNLDKNGVVILGSWVEAGDILVGKLTPQLAQDLS---PADKFLDALFSTQLAT
SRQTCLKLPRGRRGRVIDVRWIRSNPERE-----RIRVYISQKREIKVGDKVAGRHGNKGIISKI
LPRQDMPYLQDGKPIDMVFNPLGVPSRMNVGQIFECSLGLAGVLLDRHYRVAPFDERYEQ
EASRKLVFSELHEASKQTGNPWVFEPEYPGKSRIFDGRTGDPFEQPVIVGQPYILKLIHQVD
DKIHGRYDGPYASVTQQPVRGRAKRGGQRVGEMEVWALEGFGVAHILQEMLTYKSDHLR
TRDKIFGTTILGGEMPK--AAPESFRLLVRELRSLALELNHFLVSEKNFQMNRKEA-------MI
DRYKHQQLQIGLVSPQQISTWANKTLPNGEIVGEVTEIDHVEWETNKPKIGGFFCERIFGPI
KSGICACGYYGEFGDQKEKRTFCGDCGVEFSNCRIRRYQMGYIKLACPVTHVWYLRRLP
SYIANLLDKPLKELEDLVYCD--FAFARP-LAKKPTILRLRGSFEYETQSWKDSIPRFLTIQGL
HTFGNREISTGAGAIREQLGDLDLRVIIDSSLAEWKEL-EG-GLTGDEWEDRKIETRKRFLV
RRMGLAKHFIRTNVEPEWMVLCLLPVLPPELRPGVQIAELKLMGSDINKLYGRVLSRNND
LLHLLTIQST-PEDVVMSQERLVQEAVDTLLDNGIRGQPIRDGNNKVYKSFTDVIQGKEGRF
RETLLGKRVDYSGRSVIVVGPSLSLHQCGLPREIAIELFQTFVIRDLIRQRLAPNMGTAKSQI
REKERIVWEILQEVMQSHPVLLNRAPTLHRLGIQAFQPVLVEGRAICLHPLVCKGFNADFD
GDQMAVHVPLSLEAQAEARLLMFSHMNLLAPSIGDPICVPTQDMLIGLYILTSGNRRGLCV
NRYNLWNSIN---SQTETIDENNFRYMKGKEPLFCNSYDAIGAYRQKRIHLNSSFWFRWRLD
QR-LIVS--RGVPVEVHYESAGTHHEIYGHFLIVRSLKNEIIYIYIRTTLGHIFFYREIEEAIQGF
YQ-VSSYVT-----------MPKRAHLVFHNKVIDGDVIKRLISRLIDHFGMAYTSHILDQVKTLGF
QQATATSISLGIDDLLTIPSKGWLVQDAEQQSFLSEKHYHYGNVHAVEKLRQSIEIWYDTSE
YLRQEMNPNFRTTDPLNPVHIMSFSGARGNASQVHQLIGMRGLMSDPQGQMIELPIQSNL
REGLSLTEYIISSCGARKGVVDTAIRTADAGYLTRRLVEVVQHVVVRRTDCGTTRGLSVSP
PKGTVP-ERFFIQTLIGRILADDIYMNARCIAVRNQDLGPSLVNQLIT------FQIQPISIRNPFTC
RSTSWICRLCYGRSATHGDLVELGEAVGIIAGQSIGEPGTQLTLRTFHTGGVFTGGTAEHVR
APFNGKIKFNEDLVHPARTRHGQPAFLCYIDFYVTIESENIIHRVPIRPKSFLLVQNDQYVES
EQVIAEIRAGTSTF-ALKDRVRKYIYSDSEGE-IHWSTDVYHAPEFVYTNVHLLPKTSHLWI
LLGDLCR--SGPSLFSIYKDQDQMSIH--SLSAQGRYTPILS--VKNDQ-LRHKFLSLDLYGKRR
IPIRNY-SELNRSLLTAPCNLPYPA-ILRENFD--LVAKKRKNGFIIPFGSEHGREKKRILCSDISI
EMPI--NGILPRNSMLAYFDDPRYKRGGSGITKYGPIEADSIVKKEDFHFIDYR-EVQELQPK
YKEVDPLFFIPEEVHILPDFSFIMVRNNSIVGVNTQITLKKRSRTGGLVQVERKKEGIKLQIF
SGYIYFPEREDNISLHSDIFIPIPSRIRKTNSKESKK--WQNWIYVQR--ITLTKKR--PFVLLRPV
VTYEIADGKKLTRLFPQDPLQEMDNIQLEVANYICYGDGCLIRGISGTSIQLVRTCLILNWD
QDED-EKDSSIEEAGSSFVEVSTKGLIRDFLKINLGKSQISYIRKRDDPAG--LLSD-NGPNCT

NINPFSSSYSIYSKPKASLRQPLSQNQ-SIRTLWN---RNKECHSFIILSSSNCFQLGP--FNDGK
SHN---AKKESEPLLLIRNLSGPIGI--VSKIANFDV--CYNFLT--KNKTS-EMKYFQLEN---LKQ
TFPVI--Q-YFVMDENGI--IYKPDPYSNIFLNPFHLNWYFLQHNYCEETSTIRN-LGQFFCESV
CIAKNGP---RLKSGQVLVVQ-GGYVIIRSAKPYLATPGTTLHGHYGEILYEGGTLVTFRYEK
SRSADITQGLPKVERVFEVRSIDSISMNLENRVEGWNGYITRILGIPWGFLIGAELTIVQSRIS
LVNKIQKVYRSQGVQIHNRHIEIIVRQITAKVLVSE---DGMSNVFLPGELIGLLRAERTGRAF
EEGVFYRAVLLGITRASLNTQSFLSEASFQETARVLAKAALRGRIDWLKGLKENVVLGEMI
PVGTGLKGLVQPS--RQHKTLS--RETKKTNLFEEKKA-INSFFYHREWFDSYSCSSKNLQDT
SEESFTEFNDS----MTRRYWNIRFEELLAAGVHFGHRIKKWNPRMAPYILISSKGTHITNLIR
TAHFLAEACHLVFYAASTGKSFLIVGTKKAAANFVVRAAIKARCHYVNKKWLAGMLTN
WSTTETRLRKFRDLRAAQKTGKLGRLPKRDAAILKRQLTHLQKYLGGVQYMTGLPDIVII
VGQHNEFTALRECITLGIPTIGLIDTDCDPDLVDLPIPANDDSRRSIRFILNKLVFAICEGRSS--
IRNLMGRTINPLGFRLGTTQNHHSIWFEQPKKYCQGLQEDAKIRDRIKNYINNHRFSRTYIS
RIISRIEIEKGKGFVNVIIYMGFPRFLIEGKPSK----IKELKLDVKKVLHCG-RELTISIREIVNP
YSDPNILAEFLADQLKNRVSFRKAMKKTIELA--EKAKGIKVQIAGRIDGKEMACVKWLR
KGRVPLQTICTKIHFCSTTVRTIHGVLGIKIWVFTNREMSRYRGPRFKKICRLGSLPGLTTK
RP---PDKSSNPR-FSKKKEYQIRLKEKQKLRFHYGLTERQLLKYVRIAGKAKGPTGPVLLQ
LLEMRLDNTLFRLGMASTIPQARQLVNHRHILVNGRMVDIPSYRCKPRDIITAREKEKSRT
LIQNYLDSSP-HNKLPYHLTIHPLQY--KGLVNQIIARGWVGLKIKELLVVEYYSRQIMSRRG
TAEEKTRKYDPIYRNRLVNMLVNRILKHGKKSLAYQILYRAMKRVQQKKKMNPLSVLRE
AIRQITPRIAVKTRRKNGSARQVPIEIGSRQGKILAIRWLLEAARTRPGRNMALKLSSELMD
ALKGKGAAIRKKEETRRMAEANRAFAHFRMSKDTISDILTCIRNADMAKKETVRIGFTNI
AEKIVKILLREGFIKNTREHRENNKSFLVLTLRHRR--NRRGPYKT-LLN--LKRVSRPGLRIYS
NYPKIPRIFGGMGIVILSTSRGIITDREARLEGVGGEILCYIWMAKPIRRYSNRKNKRI-LRK
NIRKIPKGIIHVQASFSNTIVTITDVEGRVVTWASAGACGFKGRRRGTPFAAQTTTKNAIQP
LVNQGMKRANVLIKGIGRGRDAALRAIGRSGVRLNLIRDITPIPHNGCRPPKKRRTMPTTQ
QLIRNTRQPIRNATKSPALRGCPQRRGTCTRTYTINPKKPNSALRKVARVRLTSGVEITAFIP
GIDHNLQEHSVVLVRGGRVKDLPGVKYRIVRGTLDAVGVKGRQQGRSKYGVKKPKVATK
GLIQREKKRQKLEQKYHLIRRSLKKEIC---EVTSLSDKWKIHGKLQSSPRNSAPTRLRRRCF
STGRPRANYRDFGLSRHILREMVHACLLPGATRSSWMVKTPLISVISQE----EKNRGSVEFQ
VLSFTNKIERISSHLKFHRKDYLSQRGLQKLLIKRERLLSYLSKKNRVRYKELINWLDIRES
KTREIMVKLRLKRCGRKQRAVYRIVAIDVRARREGRDLQKVGFYDPINNQTHLNVPTILYF
LDQGAQPTGTVHDISKKAGVFAELLLKQRTAHFKKKKKGSFWKKKRSVRRRMSPIKLGD
QIQYRNVWLISRFISQQGKIVSRRMTRLTLKQQRLLTIAIKQARILSLLSFHANEK--LLERRE
WAAKNKKRAFRNKMSRSVKKTPFVAFHLLKKIEKLNAKAEKDSLKTWSRASTILPAMVG
Y T I F I H N G K I H F P I P I T D Y M V G H K L G E F V P T R S F K E D S K S D N K L R
R???
??
??
??
??
??
??
??

??
??
??
??
??
??
??
??
??
??
??
??
??
??
??
??
??
??
??
??
??
??
??
??
??
??
??
??
??
??
??
??
??
??
??
???MPRSR-INGNFIDKTFSIVANILLRVIPTTSGEK
EAFNYYRDG--MSAQSEGNYAEALQNYYEAMRLEIDPYDRSYILYNIGLIHTSNGEHTKAL
KYFFRALERNPFLPQAFNNMAVICHY-RGEQAIRQENSEIAEAWFDLAAEYWKQAIALTPS
NYIEAQNWLKITRRFD--MSCRSENIRIELIAGSRKTSNFLWAVILFLGSLGFLLVGASSYLGR
NWISFCPSQQILFFPQGVVMSFYGIAGLFISSYLWCTISWNVGSGYDQFDRKEGIVCIFRWG
FPGRNRRIFLRFLMKDIQSIRIEAKEGFSVHRILYMEIRGHGALPLIRTDEN-LTPREMEQKA
AELAYFLRVPIEVL
>Monsonia
??

??
??
??
??
??????????????????????????????MAPIRADEISNIIRERIKQYNREVKIVNMGTVLRVGDGIA
RVYGLNEVMAGELVEFEEGTVGIALNLESNNVGVVLMGGGFMIQEGSSVKATGRIAQIPV
SEAYLGRIVNALAKPIDGRGEISASESRLIESPAPGIISRRSVYEPLQTGLIAIDSMIPIGRGQR
ELIIGDRQTGKTAVATDTILNQRGKDVICVYVAIGQKASSVAQVVTNFQERGAMEYTIVVA
ETAASPATLQYLAPYTGAALAEFFMYRQRHTLVIYDDLSKQAQAYRQISLLLRRPPGREAY
PGDVFYLHSRLLERAAKSSSSLGEGSMTALPIVETQSGDVSAYIPTNVISITDGQIFLSADLF
NSGIRPAINVGISVSRVGSAAQIKAMKQVAGKLKLDLAQFTELEAFAQFATDLDKTTQNQL
ARGRRLREVLKQSQSEPLEVEEQIMAVYTGINGYLDSLEIGQVRKFLENLRKYLKRNKPRF
REIILSTKEFTEEAEALLKEAIQEQSVLYY-----MRLNPTTSSLGDSTLQKKKAGRIDQIIGPV
LDVVFPPGKLPYIYNALVVQGRDTVGQQIHVTCEVQQLLGNNRVRAVAMSATDGLMRG
MEVIDTGAPLSVPVGGATLGRIFNVLGEPVDNLGPVDTRTTSPIHRSAPAFIELDTKLSIFET
GIKVVDLLAPYRRGGKIGLFGGAGVGKTVLIMELINNIAKAHGGVSVFGGVGERTREGND
LYKEMKESGVINDQKLAESKVALVYGQMNEPPGARMRVGLTALTMAEYFRDVNKQDVL
LFIDNIFRFVQAGSEVSALLGRMPSAVGYQPTLSTEMGSLQERITSTKRGSITSIQAVYVPAD
DLTDPAPATTFAHLDATTVLSRGLASKGIYPAVDPLDSTSTMLQPRIVGDEHYEIAQRVKQT
LQRYKELQDIIAILGLDELTEEDRLTVARARKMERFLSQPFFVAEVFTGSTGKYVGLAETIR
GFKLILSGELDGLPEQAFYLVGNIDEATAKATTLDKESNLKKMTLNLYVLTPKRILWDSEV
KEIILSTASGQIGVLPSHAPIFTGLDIGVLKIRLNDQWFAISLMGGFARIVNNEITVLAHDGE
RSSDIDPQEAQQTLEIAQDS------------------------------MKNVTDSFVSLGRWPSAGSFGLNTD
ILATNLINLSVVLGVLIFFGKGVLKDLLDNRKQRILNTIRNAEELRGGAVEELEKAQDRLR
KVKMEVDRFRVNQYSQAEWENSEWITKTYKDLEQLENSKNESIRFEQQRAVNQVRQRVF
QRAIRGALEILNSCLNKELHLRTISANIALFRSMKEITDMNPLISAASVIAAGLAVGLASIGP
GIGQGTAAGQAVEGIARQPQAEGQIRGTLLLSLAFMEALTIYGLVVALAILFANPFVMNVL
SCSINTLKGLYDVSGVEVGQHFYWQIGGFQVHGQVLITSWVVIAILLGSAILAVQNPQTIPT
DVQNFFEYVLEFIRDVSKTQIGEEYGPWVPFIGTMFLFIFVSNWSGALFPWKIIQLPQGELA
APTNDINTTVALALLTSVAYFYAGLSKKGLGYFNKYIQPTPILLPINILEDFTKPLSLSFRLFG
NILADELVVVVLLSLVPLVVPIPVMFLGLFTSGIQALIFATLAAAYIGESVEGHHMISFTLEHI
FNHISFSVVSIVITIHFINLVVDGIVGLYDSSERGMLITFFSITGLLITRWTYSGDFPLSDLYDS
FLFLSWSLSIINMLPYFKKQKNPLSAITYPSVLFTQVFATSSLLAEMNQSKRLVPALQSQWL
MMHVSMMVLSYATLLCGSLLSVAFLVITFRKTKGVLG--NNIQYVSERK---INASLTSSVNY
YRSRLLQQLDYWSYRIISLGFLFLTIGILSGAVWADEAWGSYWSWDPKETWAFITWAIFAIF
LHIRKNKSVGSVSSAIAASTGFLIIWVCYFGINLFKTGLHSYGSFPILMAKKKTLTPLLYLAS
IVFLPSWIPLLFTKILESRFTHFWNIEQPETFLNDIEKKTLLAKFIELEELLLLDEMLNDCPK
MDLQTF---LHKETIQLVKMSNDDHIHKILHFSTSIISWVILGAWSILCKEELVILNSWVKEFL
YNLSDTLKAFCILFLIDYFLGFHSNPLWAWMLRNVYKSFGLVPTDLFFAFLNAILPAILDVS
C K Y C F F R Y F N T L S P S L I A V F G S L T
D???
??
??
????????MEEYQGYLEFYRSRQQDFLYPLISRESIYGLAHDHGLASYDNKSSSLIVKRLIARM

YQRNPLSICPNDSNQNPFIGKNLYSQMISEVFADIGEIPLFLQLVPSLITKSLNLRSMQSIFPF
LEDGCSRLNYVSDVLIPHPAHLEILVEALRYWVKDASFLHFVRFFLYEYW--TWKSLGPSSF
FSKARLFLFLYNTHVCEYESILLFLRNKSSHLRSTSYGILLERIIFYKKTEGLVAIFANERIPRF
FKDPLIHYVRYQGKYILILKDMPLLMNKWRYYLFKLWQCHFYVWSAPGGVRINKLSQDP
LNFMGYLLSLRPLPLVVRGQVLENSFLIDS--AMKKVESIIPIFALIGSLAKAKFCNPVGQPIS
KPAWADSSDSDIIDRFVRMCRNLSHYHSGSSKKKSLYRLKYILRLSCVKTLARKHKSTVRA
FLKKLGSTLLEEFLKEEQQALSLIFSRRVWYLDILYINDLANQEMIIDTR-EVQDIN-SFSRFE
SQLQEVYGTAWILVPILILVLGITIGVLVIVWLEREISAGIQQRIGPEYAGPLGILQALADGIK
LLFKENLLPSRGDIRLFSLGPSMAVISILLSYSVIPFSYRLVLADLNLGVFLWIAIASIAPVGL
LMSGYGSNNKYSFLGGLRAAAQSISYEIPLTLCVLSISLLSNSSSTVDIVEAQSKYGVWGW
NFWRQPIGFIVLLISSLAECERLPFDLPEAEEELVAGYQTEYSGIKFGLFYVTSYLNLLISSLF
VTILYLGGWNLSIPYLDFFQFFKIKKAGGILGTTMALFITLAKTFFFLFISITTRWTFPRLRM
DQLLNLGWKFLLPVSLGNLLLTTSFELLSL----------ILDSTRFFMKAFHLLLFDGSFLFPECI
LIFGLILLLMIDSTSDEKDIPWLYFISSTSLVMSITALLFRWREEPMISFSGNFQTNNFNEIFQF
LILLCSTLCIPLSVEYIECTEMAITEFLLFILTATLGGMFLCGANDLITIFVAPECFSLCSYLLS
GYTKKDVRSNEATMKYLLMGGASSSILVHAFSWLYALSGGEIELQEIVNGLINTQMYNSP
GISIALIFITVGIGFKLSPAPSHQWTPDVYEGSPTPVVAFLSVTSKVAASAPATRILDIPFYFSS
SEWHLLLEILAILSMILGNLIAITQTSMKRMLAYSSIGQIGYVIIGIIVGDSNGGYASMITYM
LFYISMNLGTFACIVLFGLRTGTDNIRDYAGLYTKDPLLALSLALCLLSLGGLPPLAGFFGK
LHLFWCGWEAGLSFLVSIGLLTSLLSIYYYLKIIKLFMTGRNQEITPHVRNYRRSPLRSNNSI
ELSMIVCVIASTIPGISMNPIIAIAQDTLFMFLLYEYDIFWVFLVVSSLIPILAFLISTVLAPTRE
GPEKLSSYESGIEPMGDAWLQFRIRYYMFALVFVVFDVETVFLYPWAMSFDVLGVSVFLE
AFLFVLILMIGSVYAWRKGALEWSTNFFPWLSIIVIFPISAGSLIFFLPHRGNKVTRWYTLGI
CSLELLLTAYAFSYHFQLDDPLIQLAEDFKWIRFFQFDWRLGIDGLSLGPVLLTGFITTLATL
AAWPLTRNSRLFYFLMLAMYSGQIGLFSSRDLLLFFIMWELELIPVYFLLSLWGGRKRLYS
ATKFLLYTAGSSVFLLIGVLGIGLYGSNEPTLNLEISAHQSYPEALEIIFYIGFLIAFAVKSPILP
LHTWLPDTHGEAHYSTCMLLAGILLKMGAYGLVRINVELLPHAHSMLSPYLIIVGAIQIVY
AASTSLGQRNLKKRIAYSSVSHMGFIIIGVASITNTGLDGAILQIISHGFIGTALFFLAGTSYD
RIRLLSFDEMGGMAIAIPKVFTMFSILSMASLALPGMSGFVAELLVFFGIITSQKFIFMSRILI
TLIMAVGMILTPIYSLSMLRQMFYGYRLLNTLTPTFFDSGPRELFVLIAILLPVIGIGIYPDFL
LSLSFDRVEFIVSNFFL-MILEHVLVLSAFLFSIGICGLITSRNMVRSLMCLELMLNAVNMNF
VTFSDFFDSRQLRGDIFSIFVIGIAAAEAAIGSAILSSIYHNKKSTHINQSNLLNKLEYINKYS
WVMPFIPLPVPMLIGAGLLLFPTTTKHLRRIWALPTIFLLGIVMLISAYLSIQQLNRGSIYQY
VWSWTIDNDFSLEFGHLIDPLTSTMSILISTVGLSVFLYSENYMFHDQGYLRFFYYLAFFNS
SMLGLVTSSNLIEIYIFWEFVGMCSYLLIGFWFTRPVAAEACQKAFVTNRVGDFALLLGILG
LYWITGSFEFRDLFQIFNN-EIPFLFLTLCAFLLFGGALAKSAQFPLHVWLPDAMEGPTPISA
LIHAATMVAAGIFLVARLLPLFIAIPYILNLISLIGILTLFLASTLALAQKDIKRVLAYSTMSQL
GYMMLALGMGSYRASLFHLITHAYSKALLFLGSGSIIHSMEALVGYSPQKSQNMVLMGG
LTKHMPITKGAFLLGTLSLCGIPPLACFWSKDEILNESWHYSPIYALIACSTAGLTAFYMFRI
YLLTFEGHLNVNFLNYSGKRSSSLYSLSLWGSLRGNEEIQSLKTSLTMNFFCGTKK-TLPYP
HESENTMLFPIVVLVLFTFFVGAIG--FGLDILSKFLTPSRNLFHQKSN-SVGW-EFLTNATLS
VSISYWGIFLASFFYKPFSFSLLKLNLMNLFVIWREKIRNRIYDWSYNRGYIDAFYRKSLM
KGIRELAEFTHFFDARVIDGITNGLGIMSFFVGEGIKYVGGG-RVSSYLLFYLSCVLICLLIS
DMDLPGPIHDFLLVFLGSGLILGGLGVLLLTNPIFSAFSLGLVFICISFFFILANCHFVAAAQL

LIYVGAINVLILFAVMFMNSSEYSKDLHLRTLGAGATSLVCTTLFLSIISNILDTSWYGIIWT
TRANQILEKDLISNSQQIGIRLSTDFYLPFELISIILLVALIGAIAAARQMTIPATRKDLMIVNM
GPHHPSMHGVLRLILTLDGEDVIDCEPVLGYLHRGMEKIAENRTIIQYLPYVTRWDYLAT
MFTEGITVNAPEKLGNTQVPKRASYIRTILLELSRIASHLLWLGPFMADIGAQTPFFYIFRER
ELVYDLFEAATGMRMMHNYFRIGGVAADLPHGWIDKCLDFCDYFLTGLAEYEKLITRNPI
FLERVEGIGIIGKEEAINWGLSGPMLRASGIQWDLRKIDNYECYNEFDWEVQWQKEGDSL
ARFLVRIGEMRESIKIIQQALEGIPGGPYENLEIRCFDRERYPEWGGFEYRFVSKKPSPTFEL
PKQELYVRVEAPKGELGIFLIGDQSAFPWRWKIRPPGFMNLQILPQLVKRMKLADIMTILG
SIDIIMGEVDRMFPMVTGFLNYGQQIVRATRYIGQSLMITLLHTNRFPITIQYPYEKFITSER
FRGRIHFEFDKCIACEVCVRVCPIDLPLVDWKFESDIRKKGLLNYSIDFGICIFCGNCVEYCP
TNCLSMTEEYELSTYDRHELNYNQIALGRLPMPVIEDYTIRTIYNSS--------------MQGPLSA
WLVKHGLVHRSLGFDYQGIETVQIKPEDWQSIAVILYVYGYNYLRSQCAYDVAPGGLLAS
VYHLTRVECGVDQPEEVCIKVFVPRNNPGIPSIFWVWKSADFQERESYDMLGISYDNHPR
LSRILMPESWIGWPLRKDYLAPNFYEIQDAH---
--MNSTEFPLLDRRTQNSVLSTTSTDLSNWSRLSSLWPLLYGTSCCFIEFASLIGSRFDFDRY
GLVPRSSPRQADLVLTAGTVTMKMAPSLVRLYEQMPEPKYVIAMGACTITGGMFSTDSYS
TVRGVDKLIPVDVYLPGCPPKPEAVIDAITKLRKKIAREIYEDPIRS--ANRYFTSNHKFRVG
RSIHIGNYDKGFLYQPPATSERHPATFLKYKSSRPSHVTNSMQTRKTLSWIKDEITGSIPVSL
MLYILMQAPLSNAYPVFAQQAYENPREATGRIVCANCHLANKPVDIEVPQTVLPDTVFEA
VVRIPYDMQLKQVLANGKRGALNVGAVLILPEGFELAPPDRISPEMKEKIGSLPFQSYRPA
KKNILVIGPVPGQKYSEITFPILSPDPATNKGVHFLKYPIYVGGNRGRGQIYPDGSKSNNTV
YNATTAGIVSKIIRKERGGYEISIA--ADGRQVVDSIPPGPELLVSEGEAIKADQPLTSNPNVG
GFGQGDAEIVLQDALRVQGLLFFLTSVILAQIFLVLKKKQFEKVQLSEMNFMSKVYDWFE
ERLEIQAIADDITSKYVPPHVNIFYCLGGITLTCFLVQVATGFAMTFYYRPTVTEAFSSVQYI
MTEANFGWLIRSVHRWSASMMVLMMILHVFRVYLTGGFKRPRELTWVTGVVLAVLTASF
GVTGYSLPRDQVGYWAVKIVTGVPEAIPVIGSPLVELLRGSASVGQSTLTRFYSLHTFVLPL
LTAVFMLMHFLMIRKQGISGPLMGVTKKPDLNDPVLRAKLAKGMGHNYYGEPAWPNDLL
YIFPVVILGTIACNVGLAVLEPSMIGEPADPFATPLEILPEWYFFPVFQILRTVPNKLLGVLL
MVSVPAGLLTVPFLENVNKFQNPFRRPVATTVFLLGTLAALWLGIGATLPIDKSLTLGLFMI
EVFLFGIVLGLVPITLAGLFVTAYLQYRRGDQLDLMSTLTSYFGFLLVALTITAALFIGLSKIR
LIMDMISLAWAALMVVFTFSLSLVVWGRSGLMIIRSPEPEVKILVDRDPIKTSFEEWARPGH
FSRTIAKGPDTTTWIWNLHADAHDFDSHTSDLEEISRKVFSAHFGQLSIIFLWLSGMYFHG
ARFSNYEAWLSDPTHIGPSAQVVWPIVGQEILNGDVGGGFRGIQITSGFFQIWRASGITNEL
QLYCTAIGALVFAALMLFAGWFHYHKAAPKLAWFQDVESMLNHHLGGLLGLGSLSWAG
HQVHVSLPINQFLNAGVDPKEIPLPHEFLLNRDLLAQLYPSFAEGATPLFTLNWSKYAEFLT
FRGGLDPVTGGLWLTDIAHHHLAIAILFLIAGHMYRTNWGIGHDLKDLLEAHKGPFTGQG
HKGLYEILTTSWHAQLSLNLAMLGSLTIVVAHHMYSMPPYPYLATDYGTQLSLFTHHMWI
GAFLIVGAAAHAAIFMVRDYDPTTRYNDLLDRVLRHRDAIISHLNWVCIFLGFHSFGLYIH
NDTMSALGRPQDMFSDTAIQLQPVFAQWIQNTHALAPGATAPGATTSSSLTWGGGGLVAV
GGKVALLPIPLGTADFLVHHIHAFTIHVTVLILLKGVLFARSSRLIPDKANLGFRFPCDGPGR
GGTCQVSAWDHVFLGLFWMYNAISVVIFHFSWKMQSDVWGSLSDQGVLTHITGGNFAQ
SSITINGWLRDFLWAQASQVIQSYGSSLSAYGLFFLGAHFVWAFSLMFLFSGRGYWQELIE
SIVWAHNKLKVAPATQPRALSIVQGRAVGVTHYLLGGIATTWAFFLARIIAVGMALRFPRFS
RGLAQDPTTRRIWFGIATAHDFESHDDITEERLYQNIFASHFGQLAVIFLWTSGNLFHVAWQ

GNFEAWVQDPLHVRPIAHAIWDPHFGQPAVEAFTRGGALGPVNIAYSGVYQWWYTIGLR
TNEDLYTGALFLLFLSAISLIAGWLHLQPKWKPSLSWFKNAESRLNHHLSGLFGVSSLAW
TGHLVHVAIPGSRGEYVRWNNLLDVLPHPQGLGPLFTGQWNLYAQNPDSSSHFFATSQGS
GTAILTLLGGFHPQTQSLWLTDIAHHHLAIAFLFLIAGHMYRTNFGIGHSIKDLLEAHIPPGG
RLGRGHRGLYDTINNSIHFQLGLALASLGVITSLVAQHMYSLPAYAFIAQDFTTQAALYTH
HQYIAGFIMTGAFAHGAIFFIRDYNPEQNEDNVLARILDHKEAIISHLSWVSLFLGFHTLGL
YVHNDVMLAFGTPEKQILIEPIFAQWIQSAHGKTSYGFDVLLSSTNSPAFIAGRSIWLPGWL
NAINE--NSNSLFLTIGPGDFLVHHAIALGLHTTTLILVKGALDARGSKLMPDKKDFGYSFP
CDGPGRGGTCDISAWDAFYLAVFWMLNTIGWVTFYWHWKHITLWQGNVSQFNESSTYL
MGWLRDYLWLNSSQLINGYNPFGMNSLSVWAWMFLFGHLVWATGFMFLISWRGYWQE
LIETLAWAHERTPLANLIRWRDKPVALSIVQARLVGLAHFSIGYIFTYAAFLIASTSGKFGMS
HSVKIYDTCIGCTQCVRACPTDVLEMIPWDGCKAKQIASAPRTEDCVGCKRCESACPTDF
LSVRVYLWHETTRSMGLAYMTTLNFPSVLVPLVGLVFPAVAIASLFLHVQKKKIFMRDLKT
YLSAAPVLSTLCFGALAGLLIEINRLFPDALTFPFFSFMTAILERRESESLWGRFCNWVTSTE
NRLYIGWFGVLMIPTLLTATSVFIIAFIAAPPVDIDGIREPVSGSLLYGNNIISGAIIPTSAAIGL
HFYPIWEAASVDEWLYNGGPYELIVLHFLLGVACYMGREWELSFRLGMRPWIAVAYSAPV
AAATAVFLIYPIGQGSFSDGMPLGISGTFNFMIVFQAEHNILMHPFHMLGVAGVFGGSLFSA
MHGSLVTSSLIRETTENESANEGYRFGQEEETYNIVAAHGYFGRLIFQYASFNNSRSLHFFL
AAWPVVGIWFTALGISTMAFNLNGFNFNQSVVDSQGRVINTWADIINRANLGMEVMHER
NAHNFPLDLAAVEAPSINGMGLPWYRVHTVVLNDPGRLLSVHIMHTALVAGWAGSMALY
ELAVFDPSDPALDPMWRQGMFVIPFMTRLGISNSWGGWSIT-GGTLTNPGIWSYEGVAGA
HIVFSGLCFLAAIWHWVYWDLEIFSDERTGKPSLDLPKIFGIHLFLSGVACFGFGAFHVTGL
YGPGIWVSDPYGLTGRVQFVNPAWGVDGFDPFVPGGIASHHIAAGTLGILAGLFHLSVRPP
QRLYKGLRMGNIETVLSSSIAAVFFAAFVVAGTMWYGSATTPIELFGPTRYQWDQGYFQQ
EIYRRVGAGLAENQSLSEAWAKIPEKLAFYDYIGNNPAKGGLFRAGSMDNGDGIALGWL
GHPIFRDKEGRELFVRRMPTFFETFPVVLVDGDGIVRADVPFRRAESKYSVEEVGVTLEFY
GGELNGVIYSDPATVKKYARRAQLGEIFELDRATLKSDGVFRSSPRGWFTFGHASFALLFF
FGHIWHGARTLFRDVFAGIDPDLDAQVEFGAFQKLGDPTTKKRAVMKILYSLRRFYPVET
LFNGTLALAGRDQETTGFAWWAGNARLINLSGKLLGAHVAHAGLIVFWAGAMNLFEVAH
FVPEKPMYEQGFILLPHLATLGWGVGPGGEVIDTFPYFVSGVLHLISSAVLGFGGIYHALL
GPETLEESFPFFGYVWKDRNKMTTILGIHLILLGIGAFLLVFKALYFGGLYDTWAPGGGDV
RKITNLTLSPSLIFAYLLKSPFGGEGWIVSVDDLEDIIGGHVWLGSICILGGIWHILTKPFAWA
RRALVWSGEAYLSYSLGALSVFGFIACCFVWFNNTAYPSEFYGPTGPEASQAQAFTFLVRD
QRLGANVGSAQGPTGLGKYLMRSPTGEVIFGGETMRFWDLRAPWLEPLRGPNGLDLSRL
KKDIQPWQERRSAEYMTHAPLGSLNSVGGVATEINAVNYVSPRSWLATSHFVLGFFLFVG
HLWHAGRARAAAAGFEKGIDRDFEPVLSMTPLNMTLALGKFTKDEKDLFDIMDDWLRR
DRFVFVGWSGLLLFPCAYFALGGWFTGTTFVTSWYTHGLASSYLEGCNFLTAAVSTPANS
LAHSLLLLWGPEAQGDFTRWCQLGGLWTFVALHGAFGLIGFMLRQFELARSVQLRPYNAI
AFSGPIAVFVSVFLIYPLGQSGWFFAPSFGVAAIFRFILFFQGFHNWTLNPFHMMGVAGVLG
AALLCAIHGATVENTLFEDGDGANTFRAFNPTQAEETYSMVTANRFWSQIFGVAFSNKRW
LHFFMLFVPVTGLWMSALGVVGLALNLRAYDFVSQEIRAAEDPEFETFYTKNILLNEGIRA
WMAAQDQPHENLIFPEEVLPRGNALMSGSTGERSFADIITSIRYWVIHSITIPSLFIAGWLFV
STGLAYDVFGSPRPNEYFTESRQGIPLITGRFDPLEQLDEFSRSFMTIDRTYPIFTVRWLAVH
GLAVPTVFFLGSISAMQFIQRMATQSVEDSSRSG-PRRTNVGSLLKPLNSEYGKVAPGWGT

APFMGVAMALFTIFLSILLEIYNSSVLLDGISMNMLTLKLFVYTVVILFVSLFIFGFLSNDPG
RNPGRAEMADTTGRIPLWIIGTVAGIPVIGLIGIFFYGSYSGLGSSLMMNTFSLICIYLNSAL
YSSTFFFGKLPEAYAFLNPIVDLMPVIPLFFFLLAFVWQAAVSFRTPQSNPNEQNVELNRTS
LYWGLLLIFVLAVLFSNYFFNMEVNILAFIATALFILVPTAFLLIIYVKTVSQSDMETATLVAI
SISGLLVSFTGYALYTAFGQPSQQLRDPFEEHGDMEALVYTFLLVSALGIIFFAIFFREPPKVP
TKK--MTIAFQLAVFALIATSLILLIGVPVVFASPEGWSSNKNVVFSGTSLWVGLVFLVGILN
SLIS-------MSPQTETKASVGFKAGVKDYKLTYYTPDYETKDTDILAAFRVTPQPGVPPEEA
GAAVAAESSTGTWTTVWTDGLTSLDRYKGRCYHIEPVAGEENQYIAYVAYPLDLFEEGSV
TNMFTSIVGNVFGFKALRALRLEDLRIPPAYVKTFQGPPHGIQVERDKLNKYGRPLLGCTI
KPKLGLSAKNYGRAVYECLRGGLDFTKDDENVNSQPFMRWRDRFLFCAEAIYKAQAETG
EIKGHYLNATAGTCEEMIKRAVFARELGVPIVMHDYLTGGFTANTSLAHYCRDNGLLLHIH
RAMHAVIDRQKNHGIHFRVLAKALRMSGGDHIHSGTVVGKLEGERDITLGFVDLLRDDF
VEKDRSRGIYFTQDWVSLPGVLPVASGGIHVWHMPALTEIFGDDSVLQFGGGTLGHPWG
NAPGAVANRVALEACVQARNEGRDLAREGNEIIRKACKWSRELAAACEVWKEIKFEFAA
MDTL--MAIHSKKTYTRRTRNGAVDSQTKANPRNGLICGQRRCDKGRNARGIITAGHRGG
GHKRLYRKIDFRRNKRGVYGRIVSIEYDPNRNACICLIHYGDGEKRYILHPRGAIIGDTVVS
GTRVPIKMGNALPLTNIPLGTTIHNIEITLGKGGQLARAAGAVARLIAKEGKSATLKLPSGE
VRLISQNCWATLGQVGNVGVNQKALGRAGSKCWLGKRPVVRGSAMNPVDHPHGGGEG
KAPIGRKRPSTPWGRPALGIKTRKRNKYSDTFILRRRSKMIQAQTYLNVADNSGAQKLMC
IRIIGTS-GRRYARIGDLIVAVIKDAAPKMSLERSEVIRAVIVRTCKELKRDDGLVIRYDDNAA
VVIDKKGNPKGTRVFGAMTYELRQKNFTKIISLAPEVIMLSPKRPKFRKEHRGRMRGRSS
RGSRISFGRYALQALEPAWLTSRQIEAGRRALTRNVRRGGKIWVRIFPDKPVTIRSTGTRMG
KGKGNPEYWVAVVKPGRILYEMGGVAENLARRAISIAGSKMPIRTQFLISDMSRTTRGPVA
HRRRIKEKSIAAGGRGSHSTLSRVISQEILRAWNSGTRDRKRKKREFRCLWMVRINGGLRG
N-VVSFTYSKLISFLHKEEIVLNRKVLAQIGTLNEANFRALFAG--S-----------MIKEVSAWGK
YIPMSANKARRVIDQIRGRSYEETLMILKFMPYRACYPILKLISSAAANGSYNMNFDKRSLI
ISKAEVNEGTSVKKMRPRARGRSDTIKRPTCHITIVLSDTSLN-------MDGIQIAGWTYKRG
RLFNQN-QYTFNVESGFTKGEVKSILEFFFKAKVIAINSHRLPGRGRGRGPIMGHRIHYKR
MILTIQPKSSIRPLLQKKRMAVPKKGISRSKKRVRKNIWKGKGSGAALKAFSLAKSFSTGK
SKGFVKKEIKKMAKGKEARVRVILECTSCVRNG--VNNK--GIFRYITQKNRHNTSSQLELR
KFCPHCFKHTIYREITKMKVGASVRKMCAKCRLIRRKNRLRVICYNPRHKQRQRMIREKV
RVSAQTPRWKCVESRADDKRLFYGRFILSPLKKGQADIIGIAMRRALLGEIEGTCITHAKF
A--KIPHEYTTIAGIKESIYEILMNLKEIVLRSNLYGTRDASICVRGPRDVTAQDIILPPSTAIV
DNTQHIFTLTEPIDFCVGLKIERGRGYSIKIPMPTDHHDSAYPIDAVFMPVRNVNHSIHSYER
GS--HEMLFLEIWTNGSLTPKEALREASRKLINLLMPFLHAEEE--NFYLVENNQNQDTEELP
IDL--------------CGLKDIYIDQLELPPRFYNCLKRSGIHTVMDLLDKNRDDLMKMTHFRQED
IEGVMNILNNN--ISQLLSRRGMLGDKN-EGMAAIPGLNQIQFKGFSRFVDQGLREELSKFP
KIEDLDKDVDFRLFAETYQLVEPLIKERDAVYESLTYCSELYVCAGFFCKS-RPMQKQTIWI
GNIPLMNSLGTFLINGIYRMVVNQILQSPGIYYRSELDHKGILVYTGTIISDWGGRLELEIDR
KARIWARVSRKQKISILVLSSAMGSNLREILENVRYPEIFLSFLADKANANIWSKENAILEF
YQQFVCVGEDPLFSDSLYKELQKKFFRQRCELGGLGRRNINRRLNLAISQSKTFLLPRDIL
AATDHLIGMKFGMGRLDD--MNHLKNKRIRSVADLLQDQFGLALIRLERVVTGAIGGALK-
HKWI-RTPQSLVTPSP----LASTYASFFGLHPLSQVLDGTNPLAQIVHGRKWSFLGPGGLTAR
TASFRIRDIHPSYYGRICTIDTSEGIKVGLMGSLAIHARIGHYG-SLESPFY---EIPEKSKR--AR

MLYLSSRRDEYSMIGTGNSLALNQGYQEEQIVPARYRQEFLTIAWEEVHLRSIFPFQYFSIG
ASLIPFIEHNDANRALMSSNMQRQAVPLSRSEKCIVGTGLEGQVALDSGVSLITEHAGKPIS
IHTDKILLSGNGDSLSIPLVMYEGSNKNTCIHQKPQVQRGKCIRRGQVLADGAATVGGEL
ALGKNVLVAYMPWEGYNFEDAVLISERLVYEGIYTSFHIRKYEIQIDETSQG-PERITKKIPH
LEAHLLRNLDKNGVVILGSWVEAGDILVGKLRPQPAQESV---PENRFVEAVFGPQIPTSRA
ACLKLPIGGRGRVIDVRWSPKGGPSNP---ERIRVYISQKREIKVGDKVAGRHGNKGVVSKIL
PRQDMPYLQDGKPVDMVFNPLGVPSRMNVGQIFECSLGLAGVLLDRHYRVAPFDERYEQ
EASRKLVFSELHEASKQTGNPWVFEPEYPGKSVLFDGRTGDPFQQPVIVGQPYILKLIHQV
DDKIHGRSSGPYAIITQQPLRGRARRGGQRVGEMEVWALEGFGVAHILQEMLTYKSDHLR
TRQKVFGTTIIGGAIPK--AAPESFRLLVRELRSLALDLNHFLLSEKNFQMNRKEA-------MID
RSKHQQLRIGLASPQQISAWANKTLPTGEIVGEVTQGFFFDFQTKKPERGGLFCERIFGPIK
SGICACGNSRPIGDEKKEREFCTECGVEFVDSRIRRYQMGYIKLACPVAHVWYLKRLPSDI
ATLLDKPLKELEAIVYCG--FSFARP-TAKKPTFLRLRGTFEYKTQSWQNGIPPFFTTYGFDT
FRNREIPTGAGGIREQLVDLDLRVIRDSSLAEWKKL-ER-GPTGNVSKDRKMETRKRFLLR
RMGFAKHLIQTNVEPEWMVLCLLPVLPPELRPMLWLDEDKVMATDINILYRQVISRNNAL
LKLLRKRRREDEDVLRSHEEMVQEAVDTLLDNGIRGQPLRDGNNRVYKSFTDVIEGKEG
RFRETLLGKRVDYSGRSVIVVGPSLSLHQCGLPREIAIELFQPFVIRGLIRQHLAPTIGAAKS
QIREEERIVWEILQEVVQGHPVLLNRAPTLHRLGIQAFQPILVEGRAIWLHPLVCKGFNADF
DGDQMAVHVPLSLEAQAEARLLMFSHMNLLAPSMGDPICVPTQDMLIGLYILTSGNRRGP
CVNRYSPWNRRN---SQTD-IYDNSARYRNGREPLFCNSYDAIGAYRQKRIHLDSPFWLRW
RPCQG-LILS--RGVPVEVHYESLGTHHEIYGHFLIVKSIKKEMRCIYIRTTLGHIFLYREIEEA
IQGFSQ-ISPS-------------MPERANLGFHNKVIDGAAIKRLISRLIDHFGMGYTSHILDQVKTL
GFQQATATSISLGIDDLLTIPSKGWLVQDAEEQSFLLEKHYHYGNVHAVEKLRQSVEIWYA
TSEYLRQEMNPNFRTTDPLNPVHVMSFSGARGSPSQVHQLIGMRGLMSDPQGQMIDLPIQ
SNLREGLSLTEYIISSYGARKGVVDTAVRTSDAGYLTRRLVEVVQHLVVRRTDCGTTRGLS
VSPPNGTVS-ERFFIQTLMGRVLADDIYLSARCIAVRNQDLGMGLVNQLIT------FQRQPISIR
TPFTCRSTSWICRLCYGRSPTHGDLVELGEAVGIIAGQSIGEPGTQLTLRTFHTGGVFTGGT
AEQVRAPINGKIKFNEDLVHPARTRHGQPALLCYIDFYVTIESEDIIHSVPIPPKSLLLVQND
QYVESEQVIAEIRAGASPP-PFQDRVRKHIYSDSEGE-MHWSTDVYHAPKKPSSNVHLLPK
TSHLWILVG---G--SGSALFSIYKDQDQMSIH--SLSAQGRYTPSLS--VKNDQ-LRQKFFSLGL
YGKNRIRIRNY-SEPNLSLATARSNPPYPA-ILRENLD--LLAKRRRKRFLIPFRSEHDREKERI
LCSDISIAIPIP-NGIFPRNGILAYFDHPRYKRGGSGITKYGTPGVDEIVKKED--CIDSR-GVQE
LQPKYGKVDPLFFIPEEVHIFPDSSSIMVRNNSLLGVNTQITLNQRSRAGGLVRVERK---VR
LQIFSGDIHFPEGADNISRHSGILIAP--RIRKPNSKESKK--LKNWTYVQR--IRPTKKKKKSF
VLLRPVVTYEIADGIRLARLFPQDLVQEMDNIQLRVVNYIRFRDGSFIRVISGTSIQLVRTCL
FLNWDQDK--GDSSDMEEARASFVEVSTKGLVRDFLRIHLG--QISDIRKRNDPSGSGLPSE-
NGPDHTNTNPFYSSY-----SKARVRQPLRQNQ-TIRTPVN---RNKECQSFLILSSSNCFQLGP--
FNDVKYHN---TKKESDPLIPIRKLSGPLGA--VPKIANFSL--CYQLIT--KNQIS-VMKYLQLD
N---LKQTFQVI--H-FFLMDENGR--IYKPDPSSTIFLNPLNLNWYFLQHHFCEETSTIIN-LGQF
LCESLCIAKNGP---RLKSGQVLIVQ-VGYVIIRSSKPYLAIPGATLHGHYGKILSAGDTLVTY
LYKKSRSSDITQGLPKVERMFEVRSTDSISMNLKERAEGWNECITRILGIPWGFLISAELTM
AQSRISLVNEIQRVYRSQGVQIQNRHIEIIVRQITSKVLVSE---DGMSNVFLPGELIGLLRAER
TGRALEEGVSYRAILLGITRASLNTQSFISEASFQETARVLAKSAIRGRLDWLKGLKENVV
LGEMIPVGTGFKGLVHPS--RQLKTLSLALETKRRSLFEGRKV-RNLFLYHRELVDS--CSSK

NFQDIA--------------MTRRYWNIKLEELLRARVHLGHRIKKWNPRMAPYVSISRKDRHITNL
TRTARFLAEACDLVFYAASKGSRFLIVGTTKNVANSVVRAAIKARCHYVNKKWLGGMLT
NWSTTEMKLRQLRDLRRAQKTGGFDRLRKRDAAISKRQLKQLQKYLGGVQYMTGLPDI
VIVVEQQPAVTALRECITLGIPTICLIDTDCDPDLVDLPIPANDDSISSIRLILNKLVFAICEGRS
S--IRNPMGQKINPLGFRLGTTQNHHSVWFEKPKNYCESLKEDKKIRDRIKNYIDNRR---SG
PMKDITRIQIEKGMDLIKVIIYMGFQELFMGDKPAKTELRVRELKVNVAKALHLLNRQLK
VVIRNISNPYGDPNILAEFLADQLKNRISFRKAMKRVIELAKEANTKGVKVQIAGRLGGRD
IARVECLLQGRVPLHTIRAKIDFCSTRVQMLYGVLGIKIWIFSDTEMSHYRGPRFKKISRLG
ALPGLTSRIPKKSKSNSKPPRSSRKKSQYRIRLEEKQRLRFHYGLTERQLLKYVRIAGKAK
GPTGQVLLQLLEMRLDNTLFRLGMASTIPRARQLVNHRHILVNGHVVDIPSYRCKPRDIIT
AREKEKSRALIQNYLDAAPR--ALPKHLTTHRRQY--KGLVNQIIDREWVGLKINEFLVVEY
YSRKIMSRRGTAEKKTEKTDPIYRNRLVNMVVNRILKHGKKAVAYQILYRALKKIEQKEK
RDPVPVLRQAIRKTTPDIGVKTRRKSGSAYQVPIEIGFKKGRVIAIRWLLEAARKRSGGNM
ALKLSSELVDATKGKGRAIRKKEATRRMAEANRALAHFRMSKDTISDLLTSIRNADMAKK
ETVRIEFTNMAENVVKILLREGFIKNARKHQENNKSFLVLTLFYRR--NRGGPNKT-IFN--LK
QVSRPGRRIYSKSHLIPRILGGVGIAILSTSRGIITDREARIEGVGGEILCYIWMAKPRRKVRI
RPLRKRRLRKKVRRLRKGIIHIQVTRNNTIVTITDRRGRVAIWASAGACGFKGRRKGTPFA
AQTTMEYAIQPLVKRGMRAANVLVNGSGRGKDAAVRTIFRNGIIPYFVQDMTPLPHNGCR
P P K K R K
L??
????????????????????????????????????VAKKALIQREKKRQKLEQKYHLIRRSSKKELH---EA
TSLSEKWKIKEKLQSLPRNSAPTRLRRRCFLTGRPRANYRDFGLSRHILREMFHACLLPGA
RRSS-MGKTPLISAISQE---KEKNRGSIEFQVFSFTNKIQRISSHLEFHRRDYLSQRGLQKLL
AKRERLLSYLSKKNGNSYKELIGRLNIRESTTSIRMVKLRLKRCGRKQRAVYRIVAIDARA
RREGRDLGKVGFYDPINNQSRLNLRTIFYFLEKGAQPTGTVHDISKKAGVFEKLRLKHSFE
HLLKKKKKYKRKSKRSFRRRLSRIPMEDKINYRNISLISQFISQEGKILSRKVNRVTLKQQR
LLTIAIKQARILSLLPFKPKRK--EFEPADKNPGFRKKEFEKKLFDPPEKNYFVANHLLKKIE
KLTDKGEKDSLKTWSRASTIIPAMVGYTIYIHNGKVHLPIPITRRMLGHKLGEFVPTRNYK
E A S K S D T K S R
R??
???
???
???
???
???
???
???
???
???
???
???
???
???
???

??
??
??
??
??
??
??
??
??
??
??
??
??
??
??
??
??
??
??
??
??
??
??
??
??
??
??
??
???MPRSR-INENFIDKTFSIVANILLRIIPTTSGERE
AFTYYRDG--MSAQSEGNYAEALQNYYEAMRLEIDPYDRSYILYNIGLIHTSNGEHTKALE
YYFRALERNPFLPQAFNNMAVICHY-RGEEAIRQGDSEISEAWFDQAAEYWKQAIALTPGN
YIEAQNWLKITKRFE--MSCRSETIWIELITGSRKTSNFFWALTLFFGSFGFLLVGTSSYLGRN
WISFYPSQQILFFPQGVVMSFYGIAGLFISSYLWCTISWNVGGGYDRFDRKEGIVCIFRWGF
PGRNRRIFLRFLMKDIQSIRIEAREGFAVRRILYMEIRGQGSLPLIRTAEN-LTPREIEQKAAEL
AYFLRVPIKWK
>Castanea
MEKGWFNSMLSLSKSMGSLGSIENTSVSEDPIRNDIDKNTLSYSNVDHLVGVRDIHNFVP
DDTFLVRD-NRDSYFICFDIENKIFEIDNAH--SFLSELESSFSNSRNFCYLNSVSNSDDPHYD
PYIYDTKYSWNKHITSCIDSYF--RSQICIDSYILSGIVNYNDNYISSYIFDTQSSSIKTSPAGSE
LTITET--DLDFTQKYRHLWVQCENCYGLNYKKFLKSKMNICEHCGHHLKMSSSDRIELLI
DPGTWVPMDEDMVSVDTIEFPLEEESYKDRIDSYQRKTGLTEAVQTGTGQLNGIPIAIGVM
DFQFMGGSMGSVVGEKITRLIEYATNRFLPLILVCASGGARMQEGSLSLMQMAKIASALY
DYQSKKKLFYVSILTSPTTGGVTASFGMLGDIIIAEPNAYIAFAGKRVIEQTLNTTVPEGSQT
AEYLFHKGQFDLIVPRNLLKDVLSELFQLHAFFSLNQNSIKMVTLRADEISNIIRERIEQYN

REVKVVNTGTVLQVGDGIARIYGLDEVMAGELVEFEEGTIGIALNLESNNVGVVLMGGG
LMIQEGSSVKATGRIAQIPVSEAYLGRVINALAKPIDGRGEISASESRLIESPAPGIISRRSVYE
PLQTGLIAIDSMIPIGRGQRELIIGDRQTGKTAVATDTILNQQGQNVICVYVAIGQKASSVAQ
VVTTLQEKGAMEYTIVVAETADSPATLQYLAPYTGAALAEYFMYRKRHTLIIYDDPSKQA
QAYRQMSLLLRRPPGREAYPGDVFYLHSRLLERAAKSGSRLGEGSMTALPIVETQSGDVS
AYIPTNVISITDGQIFLSADLFNAGIRPAINVGISVSRVGSAAQIKAMKQVAGKLKLELAQFA
ELEAFAQFASDLDKATQNQLARGQRLRELLKQSQSSPLTVEEQVMTIYTGTNGYLDSLEIG
QVKKFLVELRTYLKTNKPQFQEIISSTKIFNEEAEALLKDAIQEQMERFLLQEQVMRINPTT
SDPGVSALEKKNLGRIAQIIGPVLDVTFPPRKMPNIYNALVVKGRDTAGQQINVTCEVQQL
LGNNRVRAVAMSATDGLMRGMEVIDTGAPLSVPVGGATLGRIFNVLGEPVDNLGPVDTRT
TSPIHRSAPAFIQLDTKLSIFETGIKVVDLLAPYRRGGKIGLFGGAGVGKTVLIMELINNIAK
AHGGVSVFGGVGERTREGNDLYMEMKESGVINEQNIAESKVALVYGQMNEPPGARMRV
GLTALTMAEYFRDVNEQDVLLFIDNIFRFVQAGSEVSALLGRMPSAVGYQPTLSTEMGSL
QERITSTKEGSITSIQAVYVPADDLTDPAPATTFAHLDATTVLSRGLAAKGIYPAVDPLDSTS
TMLQPRIVGEEHYETAQRVKQTLQRYKELQDIIAILGLDELSEEDRLTVARARKIERFLSQP
FFVAEVFTGSPGKYVGLSETIRGFKLILSGELDGLPEQAFYLVGNIDEATAKATNLEMESKL
KQMTLNLCVLTPNRIVWDSEVKEIILSTNSGQIGILPNHAPIATAVDIGILRIRLTDQWLTMA
LMGGFARIGNNEITVLVNDAEKGSDIDPQEAQQTLEVAEANLSKAEGKRQTIEANLALRR
ARTRVEALNMISMKNLTDSFLSLGLWSSAGSFGFNTDILATNPINLSVVLGLLIFFGKGVLS
DLLDNRKQRILKTILNSEELRRGAIEQLEKARARLRKVEMEADQFRVNGYSDIEREKSNLI
NSTYQTLEQFEIYKNETIRFEQQRASNQVRQRVFQQALKGALGTLTSSLNNELHLRTINANI
GMFGTLTEIIDMNPLISAASVIAAGLAVGLASIGPGVGQGTAAGQAVEGIARQPEAEGKIRG
TLLLSLAFMEALTIYGLVVALALLFANPFV--MKLCYINTLKGLYDISGVEVGQHFYWRIGG
FQVHAQVIITSWVVIAILLGSAIIAVRNPQTIPTDSQNFFEYVLEFIRDVSKTQIGEEYGPWV
PFIGTLFLFIFVSNWSGALLPWKIIQLPHGELAAPTNDINTTVALALLTSVAYFYAGLSKKGL
GYFGKYIQPTPILLPINILEDFTKPLSLSFRLFGNILADELVVLVLVSLVPSVVPIPVMLLGLF
TSGIQALIFATLAAAYIGESMEGHHMISSILEHILIHISFSVVSIVITIRLLILLV-EIAGLYDSSK
KGIITTFVCITGLLVTRWIYSRHLPLSDLYESFIFLSWSLSIIHMVPYLKKHKNHLSAITAPIVI
LTQGFATSGFLTEMHQSRILVPALQSHWLMMHVSMMVLGYAALLCGSLLSVALIVITFRKII
RTFDKSNEIQYINERNNVLW--PFISSKNYYRSQLIQQLDHWSYRIISLGFIFLTMGILSGAVW
ANEAWGSYWNWDPKETWAFITWTIFAIYLHIRTNPNLEGVNSAIVASLGFLIIWICYLGVN
LLGIGLHSYGTFTLTMAKKKAFIPLLYLASIVFLPWWISLSVTKSLESWVTNWWDTRQSEI
FLNDIQEKSILKKFIELEELLFLDEMIKEYPGTHLEKLRIG-YNETIQLIKMHNEDCIHTILHF
STNMICFVILSGYSMLGNEELVILNYWVQEFLYNLSDTIKAFSILLVTDLCIGFHSPHGWEL
MIGSVYKDFGFAHNDQIISGLVSTFPVILDTILKYLIFRYLNRVSPSLVVIYHSMNDMPIGVP
KVPFRSPGEEDASWVDIN-RLYRARLLFLGQEVDSDLSNQIIGLMVYLSLEDDSKDLYLFIN
SPGGWVIPGVAIYDTMQFVQPDVQTICMGLAASIGSFLLVGGEITKRLAFPHARVMIHQPA
SSFYEAPAGEFILEAEEMMKLRETITGVYVQRTGTPLWVVSEDMERDVFMSATEAQAHGI
VDHVGVE???
MEEFQGYLELDRFRQHDFLYPLIFREYSYALAHG--LIGYDNKSSLLIVKRLITTMCQQNYLI
ISANDSKQNPFFGKNLHSKILSEGFAIIVEIPFYLRLISYLIVRFYNLRSIHSIFPFLEEKFPHLN
YSADILIPYPAHLEILVQTLRYRVKDASYLHLLRFFLHEYSNCNSLIIKYISIFSKPRFFLFLYN
SYICEYESIFLFLRNQSSHLRLTSSGVLFERLCLYRKIEHFAEVFANPVIPCFLKDPFMHYVR
YQGKSILASKDTPLLMNKWKSYLVNLWQCHFDVWSHAASIRINQLSKHSLDFLSYFSSVR

RNPAVVRNQMLENSFPLNN--APNKLDTIVPIIPLIGSLAKAKFCNAVGHPISKLTRADLSDF
EIINRFLHICRNLSHYYSGSSKKKNMYRIKYILRLSCVKTLARKHKSTARAFLKRVDSEFFQ
EFFTEEGGFISLIFPRRVWYLDIIFINGLSNHEMIIDIR-EVQ-DINSFYRVES--KWVYGIIWVL
APIFTLVLGITIGVLVIVWLEREISAGLQQRIGPEYSGPLGVLQALADGTKLLFKENLLPSRG
DTRLFSIGPSIAVIAILLSYSVIPFDYHLVLADLNIGVFLWIAVSSIAPIGLLISGYGSNNKYSF
LGGLRAAAQSISYEIPLTLCVLAISLLSNSLSTVDIVEAQSKYGFWGWNLWRQPIGFIVFLIS
SLAECERLPFDLPEAEEELVAGYQTEYSGIKFGLFYVASYLNLLISSLFVTVLYLGGWNISIP
YIFVAELFEINKAREVFGTTIGIFITLAKTYLFLFIPITTRWTLPRLRMDQLLNLGWKFLLPIS
LGNLLLTTSFQLSSLMIWHVQNENFILDSTRIFMKAFHLLLFDGSLIFPECILIFGLILLLMID
STSDQKDIPWLYFISSTSLVMSITALLFRWREEPMISFSGNFQRNNFNEIFQFLILLCSTLCIPL
SVEYIECTEMVITEFLLFVLTATLGGMFLCGANDLITIFVAPECFSLCSYLLSGYTKKDVRSN
EATMKYLLMGGASSSILVHGFSWLYGSSGGEIELQEIVSGLINTQMYNSPGISIALIFITVGI
GFKLSPAPSHQWTPDVYEGSPTPVVAFLSVTSKVAASASATRIFDIPFYLSSNEWHLLLEILA
ILSMILGNLIAITQTSMKRMLAYSSIGQIGYVIIGIIVGDSNGGYASMITYMLFYISMNLGTF
ACIVLFGLRTGTENIRDYAGLYTKDPFLALSFALCLLSLGGLPPLAGFFGKLHLFWCGWQA
GLYFLVSIGLLTSVVSIYYYLKIIKLLMTGRNQEITPHVRNYRRSPLRSNNSIELSMIICVIAS
TIPGISMNPIIEIAQDTLFMFLLYEYDIFWAFIIIASAIPILAFLISGVLAPIRKGPEKLSSYESGI
EPMGDAWLQFRIRYYMFALVFVVFDVETVFLYPWAMSFDVLGISVFIEAFIFVLILIVGSVY
AWRKGALEWSTNYFPWLTIVAVLPIFGGSLIFFLPHKGNKVIRLYTLFICIIELLLTTYAFYYY
FQLDDPLMQLTEDYKWIDFFDFYWRLGIDGLSIGPILLTGFITTLATLAAWPVTRDSRLFHF
LMLAMYSGQIGLFSSRDLLLFFIMWELELIPVYLLISVWGGKKRLYSATKFILYTAGSSVFL
LLGVLGIGLYASNEPTLNFETSANQSYPVALEIIFYIGFLIAFAVKSPIIPFHTWLPDTHGEAH
YSTCMLLAGILLKMGAYGLVRINMEFLPRAHSIFAPWLMIVGTIQIIYAASASPGQRNLKK
RIAYSSVSHMGFIIIGIGSLSDMGLNGAFLQIISHGFIGAALFFLAGTSYDRIRLVYLDEMGG
MAVPVPKIFTTFSILSMASLALPGMSGFVAELIVFFGIITSQNFFLIQKILITFVMAIGTILTPIY
SLSMLRQMFYGYKFFNAPNSYFFDSGPRELFISISIFLPVIGIGLYPDFVFSLSVDKVETLISN
FFL-MMLEHVLVVSAYLFSIGIYGLITSRNMVRALMCLELILNAVNINFVTFSDFFDSRQLK
GNIFSIFVIAIAAAEAAIGPAIVSSIYRNRKSTRINQSNLLNKMEYTYQYLWFIPFIPVLIPML
LGVGLLLFPTATRNLRRTWAFTSVLLLSIVMVFSANLSVQQINNSYIYQYVWSWTINNDFS
LEFGQLIDPLTSIMSILITTVGILVLIYSDNYMSYDQGYLRFFAYMSFFNTSMLGLVTSSNFIQ
IYIFWELVGVCSYLLIGFWFTRPIAGNACQKAFVTNRVGDFGLLLGILGFYWITGSFEFRGL
FEIVNR-EVNFLFVTLCALLLFSGAIAKSAQFPLHVWLPDAMEGPTPISALIHAATMVAAGI
FLVARLLPLFIVVPYIMNIIALIGIITLLLGATLALAQKDIKRGLAYSTMSQLGYMMLALGIG
SYRSALFHLITHAYSKALLFLGSGSIIHSMEAVVGYSPDKSQNMVLMGGLTKHVPITKTAF
LLGILSLCGIPPLACFWSKDEILNYIWLYSPIFAIIACSTAGLTAFYMFRIYLLTFEGYLNVNF
QNYNGKTNNSFYSMSLWGKE-GKNKKKKLFSLLTMNFFWGITN-TFSYPHESDSTMLFPM
FVLGIFTFFVGSIGNQFDLDILSKLLTPDINLLHQNSN-SVDW-EFVKNAGFSLSIAYLGIFIAS
FLYKPIYSSLQNFNFQNSFVIVFEKIKNVIYDWSDNRGYMDVLYVIYLTDAMRALAEFTHF
FDRRIIDGITNGVGITSFFVGEGIKYIGGG-RISSYLLLYLSYILIFLLILIMGLPGPIHDFLLVFL
GSGLILGGIGVVLLTNTIYSAFSLGLVLVCISLFYILSNSHLVAAAQLLIYVGAINVLIIFAVMF
MNGSEYYKDLNLWTVGDGVTLLICTILFCLLMATISDTSWYGIIWTTRSNQIIEQDLISNSQ
QIGIHLSTDFFLPFELISIILLAALIGAIAVARQMNIPVTRKDLMIVNMGPHHPSMHGVLRLV
VTLDGEDVIDCEPILGYLHRGMEKIAENRTIIQYLPYVTRWDYLATMFTEAITVNGPERLG
NIQVPKRASYIRVIMLELSRIASHLLWLGPFMADIGAQTPFFYIFRERELVYDLFEAATGMR

MMHNYFRIGGVAADLPYGWIDKGLDFCDYFLTGVTEYQKLITRNPIFLERVEGVGIIGRDE
VINWGLSGPMLRASGIQWDLRKIDHYECYDEFDWEVQWQKEGDSLARYLVRIGEMAESI
KIIQQALEGIPGGPYENLEIRYFDRKRYPEWHDFEYRFISKKPSPTFELPKQELYVRVEAPK
GELGIFLIGDQSGFPWRWKIRPPGFINLQILPQLVKRMKLADIMTILGSIDIIMGEVDRMFP
MVTRVMDYGQQTVRIARYIGQSFMITLSHANRLPVTIQYPYEKLITSERFRGRIHFEFDKCI
ACEVCVRVCPMDLPVVDWELETNIRKKRLLNYSIDFGICIFCGNCVEYCPTNCLSMTEEYE
LSTYDRHELNYNQIALGRLPMSVIDDYTIRTILNS-IK-------------MQGRLSAWLAKHGLVH
RSLGFDYQGIETLQIKPGDWHSIAVILYVYGYNYLRSQCAYDVAPGGLLASVYHLTRIAYGI
DQPEEVCIKVFAPRWNPRIPSVFWVWKSADFQERESYDMLGIFYDNHPRLKRILMPESWI
GWPLRKDYIAPNFYEIQDAH----------------------------------MVLALEYSDNKNKDKESKKKN
IETVMNSIEFPLLDRTAPNSVISTTSNDLSNWSRLSSLWPLLYGTSCCFIEFASLIGSRFDFDR
YGLVPRSSPRQADLILTAGTVTMKMAPSLVRLYEQMPEPKYVIAMGACTITGGMFSTDSY
TTVRGVDKLIPVDVYLPGCPPKPEAVIDAITKLRKKLSREIYEDRIRS--GSRCFTINHKFYIG
RSTHTGNYDRGLLDQPPSTSEIPLEIFFKYNKSVSSPELVNMQTINTFSWIKEEITRSISVSL
MIYIITGAPISNAYPIFAQQGYENPREATGRIVCANCHLANKPVDIEVPQAVLPDTVFEAVV
RIPYDMQLKQVLANGKKGALNVGAVLILPEGFELAPPDRISPEIKEKMGNLSFQNYRPTKK
NILVIGPVPGQKYSEITFPILSPDPTTKKDVHFLKYPIYVGGNRGRGQIYPDGSKSNNNVYN
ATAAGIVSKIIRKEKGGYEITIVDASDGRQVVDIIPPGPELLVSEGESVQLDQPLTSNPNVGG
FGQGDAEIVLQDPLRVQGLLFFLASVILAQIFLVLKKKQFEKVQLSEMNFMSKVYDWFEE
RLEIQAIADDITSKYVPPHVNIFYCLGGITLTCFLVQVATGFAMTFYYRPTVTEAFASVQYI
MTEANFGWLIRSVHRWSASMMVLMMILHVFRVYLTGGFKKPRELTWVTGVVLAVLTASF
GVTGYSLPRDQIGYWAVKIVTGVPEAIPVIGSPLVELLRGSASVGQSTLTRFYSLHTFVLPLL
TAVFMLMHFPMIRKQGISGPLMGVTKKPDLNDPVLRAKLAKGMGHNYYGEPAWPNDLL
YIFPVVILGTIACNVGLAVLEPSMIGEPADPFATPLEILPEWYFFPVFQILRTVPNKLLGVLL
MVLVPAGLLTVPFLENVNKFQNPFRRPVATTVFLIGTAVALWLGIGATLPIDKSLTLGIFMIE
VFLFGIVLGLIPITLAGLFVTAYLQYRRGDQLDLMLTITSYFGFLLAVLTITSALFIGLNKIRLI
MDIVSLAWAALMVVFTFSLSLVVWGRSGLMIIRSPEPEVKILVDRDPIKTSFEEWARPGHFS
RTIAKGPDTTTWIWNLHADAHDFDSHTSDLEEISRKVFSAHFGQLSIIFLWLSGMYFHGAR
FSNYEAWLSDPTHIGPSAQVVWPIVGQEILNGDVGGGFRGIQITSGFFQIWRASGITSELQL
YCTAIGALVFAALMLFAGWFHYHKAAPKLAWFQDVESMLNHHLAGLLGLGSLSWAGHQ
VHVSLPINQFLNAGVDPKEIPLPHEFILNRDLLAQLYPSFAEGATPFFTLNWSKYADFLTFR
GGLDPVTGGLWLTDIAHHHLAIAILFLIAGHMYRTNWGIGHGIKDILEAHKGPFTGQGHK
GLYEILTTSWHAQLSINLAMLGSLTIVVAHHMYSMPPYPYLATDYGTQLSLFTHHMWIGG
FLIVGAAAHAAIFMVRDYDPTTRYNDLLDRVLRHRDAIISHLNWVCIFLGFHSFGLYIHND
TMSALGRPQDMFSDTAIQLQPVFAQWIQNTHALAPGATAPGATTSTSLTWGGADLVSVGG
KVALLPIPLGTADFLVHHIHAFTIHVTVLILLKGVLFARSSRLIPDKANLGFRFPCDGPGRGG
TCQVSAWDHVFLGLFWMYNAISVVIFHFSWKMQSDVWGSISDQGVVTHITGGNFAQSST
TINGWLRDFLWAQASQVIQSYGSSLSAYGLFFLGAHFVWAFSLMFLFSGRGYWQELIESIV
WAHNKLKVAPAIQPRALSIVQGRAVGVAHYLLGGIATTWAFFLARIIAVGMALRFPRFSQG
LAQDPTTRRIWFGIATAHDFESHDDITEERLYQNIFASHFGQLAIIFLWTSGNLFHVAWQGN
FETWVQDPLHVRPIAHAIWDPHFGQPAVEAFTRGGALGPVNIAYSGVYQWWYTIGLRTN
GDLYTGALFLLFLSVISLIAGWLHLQPKWKPSVSWFKNAESRLNHHLSGLFGVSSLAWTG
HLVHVAIPGSRGEYVRWNNFLEVLPHPQGLGPLFTGQWNLYAQNPDSGSHLFGTSQGAGT
AILTLLGGFHPQTQSLWLTDIAHHHLAIAFIFLVAGHMYRTNFGIGHSIKDLLEAHTPPGGR

LGRGHKGLYDTINNSIHFQLGLALASLGVITSLVAQHMYSLPAYAFIAQDFTTQAALYTHH
QYIAGFIMTGAFAHGAIFFIRDYNPEQNEDNVLARMLDHKEAIISHLSWASLFLGFHTLGL
YVHNDVMLAFGTPEKQILIEPIFAQWIQSAHGKTLYGFDVLLSSTSGPAFNAGRSIWLPGW
LNAINE--NSNSLFLTIGPGDFLVHHAIALGLHTTTLILVKGALDARGSKLMPDKKDFGYSFP
CDGPGRGGTCDISAWDAFYLAVFWMLNTIGWVTFYWHWKHITLWQGNVSQFNESSTYL
MGWLRDYLWLNSSQLINGYNPFGMNSLSVWAWMFLFGHLVWATGFMFLISWRGYWQE
LIETLAWAHERTPLANLIRWRDKPVALSIVQARLVGLAHFSVGYIFTYAAFLIASTSGKFGM
SHSVKIYDTCIGCTQCVRACPTDVLEMIPWDGCKAKQIASAPRTEDCVGCKRCESACPTD
FLSVRVYLWHETTRSMGLAY--MTNLPSFFVPFVGLILPAIAMASLSLHIQKNKIFMRDLKT
YLSVAPVVSTIWFGFLAGLLIEINRLFPDALIFPFFSFMTAILERRESESLWGRFCNWITSTEN
RLYIGWFGVLMIPTLLTATSVFIIAFIAAPPVDIDGIREPVSGSLLYGNNIISGAIIPTSAAIGLH
FYPIWEAASVDEWLYNGGPYELIVLHFLLGVACYMGREWELSFRLGMRPWIAVAYSAPVA
AATAVFLIYPIGQGSFSDGMPLGISGTFNFMIVFQAEHNILMHPFHMLGVAGVFGGSLFSA
MHGSLVTSSLIRETTENESANEGYRFGQEEETYNIVAAHGYFGRLIFQYASFNNSRSLHFFL
AAWPVVGIWFTALGISTMAFNLNGFNFNQSVVDSQGRVINTWADIINRANLGMEVMHER
NAHNFPLDLASVEAPSTNGMGLPWYRVHTVVLNDPGRLISVHIMHTALVAGWAGSMALY
ELAIFDPSDPVLDPMWRQGMFVIPFMTRLGITNSWGGWSIT-GGTITNPGIWSYEGVAGAH
IVFSGLCFLAAIWHWVYWDLEIFSDERTGKPSLDLPKIFGIHLFLSGLACFGFGAFHVTGLY
GPGIWVSDPYGLTGRVQSVNPAWGVEGFDPFVPGGIASHHIAAGTLGILAGLFHLSVRPPQ
RLYKGLRMGNIETVLSSSIAAVFFAAFVVAGTMWYGSATTPIELFGPTRYQWDQGYFQQEI
YRRVSAGIAENQSLSEAWSKIPEKLAFYDYIGNNPAKGGLFRAGSMDNGDGIAVGWLGHP
IFRDKEGRELFVRRMPTFFETFPVVLVDGDGIVRADVPFRRAESKYSVEQVGVTVEFYGG
ELNGVSYSDPATVKKYARRAQLGEIFELDRATLKSDGVFRSSPRGWFTFGHASFALLFFFG
HIWHGARTLFRDVFAGIDPDLDAQVEFGTFQKLGDPTTRRQVVMKTLYSLRRFYPVETLF
NGTLALAGRDQETTGFAWWAGNARLINLSGKLLGAHVAHAGLIVFWAGAMNLFEVAHF
VPEKPMYEQGLILLPHLATLGWGVGPGGEVIDTFPYFVSGVLHLISSAVLGFGGIYHALLG
PETLEESFPFFGYVWKDRNKMTTILGIHLILLGIGAFLLVFKAIYFGGVYDTWAPGGGDVR
KITNLTLSPSVIFGYLLKSPFGGEGWIVSVDDLEDIIGGHVWLGSICILGGIWHILTKPFAWA
RRALVWSGEAYLSYSLGALAVFGFIACCFVWFNNTAYPSEFYGPTGPEASQAQAFTFLVRA
LRLGANVGSAQGPTGLGKYLMRSPTGEVIFGGETMRFWDLRAPWLEPLRGPNGLDLSRL
KKDIQPWQERRSAEYMTHAPLGSLNSVGGVATEINAVNYVSPRSWLATSHFVLGFFLFVG
HLWHAGRARAAAAGFEKGIDRDFEPVLSMTPLNMTIALGKFTKDEN-LFDIMDDWLRRD
RFVFVGWSGLLLFPCAYFALGGWFTGTTFVTSWYTHGLASSYLEGCNFLTAAVSTPANSL
AHSLLLLWGPEAQGDFTRWCQLGGLWTFVALHGAFGLIGFMLRQFELARSVQLRPYNAI
AFSAPIAVFVSVFLIYPLGQSGWFFAPSFGVAAIFRFILFFQGFHNWTLNPFHMMGVAGVLG
AALLCAIHGATVENTLFEDGDGANTFRAFNPTQAEETYSMVTANRFWSQIFGVAFSNKRW
LHFFMLFVPVTGLWMSALGVVGLALNLRAYDFVSQEIRAAEDPEFETFYTKNILLNEGIRA
WMAAQDQPHENLIFPEEVLPRGNALMSGSTGERSFADIITSIRYWVIHSITIPSLFIAGWLFV
STGLAYDVFGSPRPNEYFTESRQGIPLITGRFDPLEQLDGFSRSFMTIDRTYPIFTVRWLAVH
GLAVPTVSFLGSISAMQFIQRMATQTVENSSRSG-PRRTIVGDLLKPLNSEYGKVAPGWGTT
PLMGIAMALFAIFLSIILEIYNSSILLDGISMNMLTLKLFVYTVVVFFVSLFIFGFLSNDPGRN
PGREEMADTTGRIPLWIIGTVTGILVIGLIGIFFYGSYSGLGSSLMLNIFSLICICLNSALS-SSF
FFSKLPEAYAFLNPIVDFMPVIPLLFFLLAFVWQAAVSFRMTQSNPNEQSVELNRTSLYWG
LLLIFVLAVLFSNYFFNMEVNILAFIATALFILVPTAFLLIIYVKTVSQNDMETATLVAISISGL

LVSFTGYALYTAFGQPSQQLRDPFEEHGDMEALVYTFLLVSTLGIIFFAIFFREPPKVPTKKM
KMTIAFQLAVFALIATSAILLISVPVVFASPDGWSNNKNVVFSGTSLWIGLVFLVGILNSLIS-
------MSPQTETKASVGFKAGVKDYKLTYYTPDYQTKDTDILAAFRVTPQPGVPPEEAGAAV
AAESSTGTWTTVWTDGLTSLDRYKGRCYHIEPVAGEENQFIAYVAYPLDLFEEGSVTNMF
TSIVGNVFGFKALRALRLEDLRIPTSYSKTFQGPPHGIQVERDKLNKYGRPLLGCTIKPKLG
LSAKNYGRAVYECLRGGLDFTKDDENVNSQPFMRWRDRFLFCAEAIYKAQAETGEIKGH
YLNATAGTCEEMIKRAVFARELGAPIVMHDYLTGGFTANTSLAHYCRDNGLLLHIHRAMH
AVIDRQKNHGIHFRVLAKALRMSGGDHIHAGTVVGKLEGEREITLGFVDLLRDDYIEKDR
SRGIYFTQDWVSLPGVLPVASGGIHVWHMPALTEIFGDDSVLQFGGGTLGHPWGNAPGAV
ANRVALEACVQARNEGRDLAREGNEIIREAAKWSPELAAACEVWKEIKFEFPAMDTL--M
AIHLYKTSTPSTRNGAVDSQVKSNPRNNLIYGQHHCSKGRNARGIITAGHRGGGHKRLYR
KIDFRRNEKDIYGRIVTIEYDPNRNAYICLIHYGDGEKRYILHPRGAIIGDTIVSGTEVPIKM
GNALPLTDMPLGTAIHNIEITLGKGGQLARAAGAVAKLIAKEGKSATLKLPSGEVRLISKNC
SATVGQVGNVGVNQKSLGRAGSKCWLGKRPVVRGVVMNPVDHPHGGGEGRAPIGRKK
PATPWGYPALGRRSRKRNKYSDNLILRRRSKMIQPQTHLNVADNSGARQLMCIRIIGAS-N
RRYAHIGDVIVAVIKEAIPNTPLEKSEVIRAVIVRTCKELKRDNGMILRYDDNAAVVIDQEG
NPKGTRIFGAIARELRQLNFTKIVSLAPEVLMLSPKRARFRKQHRGRMKGISYRGNRICFG
RYALQALEPAWITSRQIEAGRRAMTRNVRRGGKIWVRIFPDKPVTVRPAETRMGSGKGSP
EYWVAIVKPGRILYEMGGVAENVARKAISIAASKMPIRTQFIISGMTRIKRGCIARRRRTKIR
LFASSFRGAHSRLTRTITQQKIRALFSAYRDRGRQKRYFRRLWITRINAAIRGN-LVYYSYSI
F I H N L Y K K Q L L L N R K I L A Q I A I L N I N C L S M I S T E I I K - - - - - - - - - - -
-???
??????????????????????????????????MNGIKYAVFTDKSIRLLGKN-QYTSNVESGSTRTEIKH
WVERFFGVKVIAMNSHRLPGKGRRMGPIMGHTMHYRRMIITLQPGYSIPPLRKKRTMAVP
KKRTSISKKRIRKNFWKREGYWVALKAFSLSKSLSTGNSKKFFVQQEINMAKGKDVRVTV
ILECTNCAVRN--ANKESKGISRYITQKNRHNTPSRLELRKFCPFCYKYTIHGEIKKMKIRAS
IRKICEKCRLIRRRGRIIVICSNPRHKQRQGMVREKVIVSTRTLQWKCVESRGDSNSLYYGR
FILSPLMKGQADTIGIAVRRALLGEIEGTCITRAKSE--KIPHEYSTIIGIQESVHEILMNLKEIV
LRSNLYGISGASICVKGPRYVTAQDIILPPSVQIVDNTQHIANLTEPINLYIELQIERDRGYRI
KTTNNSQDGSY--PIDAVFMPVRNANHSIHSYVNGNEKQEILFLEIWTNGSLTPKEALHEAS
RNLIELFIPFLHAEEE--KLHLENNQ-QKVTLPFFTCHDRLAK-LNKNST-EITLKSIFIDQSELP
PRIYNCLKGSNIHTLLDLLNNSQTDFMKMKHFRREDIKHILDILEIEKDFV-------MLGGRN-
EGMYTIPGFNQIQFEGFCRFIDQGLTEELYKFPKMEDINQEIEFQLFVETYQLVEPVIKERDA
VYESLTYSSELYVSAGLIWKNSRDMQEQTIFIGNIPLMNSLGTSIVNGIYRIVINQILQSPGIY
YLSELDNTGISVYTGTIISDWGGRLELEIDRKARIWARVSRKQKISILVLSSAMGSNLREILE
NVCYPEIFLSFLNDKEKKKIGSKENAILEFYQQFACVGGDPVFSESLCKELQKKFFQQKCE
LGRIGRRNMNQRLNLDIPQNIPFLLPRDILAAADHLIGMKFGMTLDD--MNHLKNKRIRS
VADLLQDQFGVALFRLENMIRGTIGGAIR-HKLI-PTPQNLVTSTP----LTTTYESFFGLHPLSQ
VLDRTNPLTQIVHGRKLSYLGPGGLTGRTASFRIRDIHPSHYGRICPIDTSEGINVGLIGSLAI
HARIGHWG-FLESPFY---EISERSKKV--RMLYLSPSRDEYSIVASGNSLGLNRGIQEEQVVPV
RYRQEFLTVEWERVHLRSIFTFQYFSVGASLIPFIEHNDANRALMSSNMQRQAVPLSRSEK
CIVGTGLERQVALDSGALAIAEHEGKIISTDADKILLSGKGDIISIPLIMYQRSNKNTCMHQ
KPRVPRGKCIKKGQILADGAATVGGELALGKNVLVAYMPWEGYNSEDAVLISERLVYGDI
YTSFHIRKYEIQTHVTSYG-PERITNEIPHLKAYLLRNLDKNGIVMLGSWVETGDILVGKLT

PQVAKE-SSYTPEDRLLRAILGIQVSTSKETCLKLPIGGRGRVIDVRWVQKKG-VSSYNPEKI
HVYISQKREIKVGDKVAGRHGNKGIISKILPRQDMPYLQDGRSVDMVFNPLGVPSRMNVG
QIFECSLGLAGDLLGRHYRIAPFDERYEQEASRKLVFSELYEASKQTANPWVFDPEYPGKS
RILDGRTGDPFEQPVLIGKPYILKLIHQVDDKIHGRSSGHYALVTQQPLRGRAKQGGQRVG
EMEVWALEGFGVAHILQEMLTYKSDHIRARQEILGTTIIGRTIPKPEDAPESFRLLVRELRSL
ALELNHFLVSEKNFQMNRKEA-------MIDRYKHQQLRIGSVSPQQISAWANKILPNGEIVGE
VTKPYTFHYKTNKPEKDGLFCERIFGPIKSGICACGNYRVIGDEKEDPNFCEQCGVEFVDS
RIRRYQMGYIKLACPVTHVWYLKRLPSYIANLLDKPLKELEGLVYCDY-FSFARP-IAKKPT
FLRLRGSFEYEIQSWKYSIPLFFTTQGFDAFRNREMSTGAGAIREQLADLDLRIIIDYSLVE
WKELGEE-GPPGNEWENRKVGRRKDFLVRRIEIAKHFIRTNIEPEWMVLCLLPVLPPELRPI
IQIDGGKLMSSDINELYRRVIYRNNTLIDLLATSRSTPGELVMCQEKLVQEAVDTLLDNGIR
GQPMRDGHNKVYKSFSDVIEGKEGRFRETLLGKRVDYSGRSVIVVGPSLSLHQCGLPREI
AIELFQPFVIRGLIRQHRASNMGVAKSKIREKESIVWEILQEVMQGHPILLNRAPTLHRLGI
QAFHPILVEGRAICLHPLVCKGFNADFDGDQMAVHVPLSLEAQVEARLLMFSHMNLLSPSI
GNPISLPTQDMLIGLYVLTSGNRRGICANRYNPCNRIN---YQNEKMDANSYKYTKEKEPFF
CNSYDAIGAYRQKRINLDSTLWLRWKLDQR-VIAS--REAPIEVHYESLGTYHEIYEHYLIV
RSIKKEILCTYIRTTVGPISLYREIEEAIQGLCRTCSYGT--------MEVLMAERANTVFHNKVID
GTAIKRLISRLIDHFGMAYTSHILDQVKTLGFQQATATSISLGIDDLLTIPSKGWLVQDAEQQ
SLVLEKHHYYGNVHAVEKLRQSIEIWYATSEYLRQEMNPNFRMTDPFNPVHMMSFSGAR
GNASQVHQLVGMRGLMSDPQGQMIDLPIQSNLREGLSLTEYIISCYGARKGVVDTAVRTS
DAGYLTRRLVEIVQHIVVRRTDCGTIRGISVSPQNGTIP-ERIFIQTLIGRVLADDIYMGPRCI
AVRNQDIGIGLVNRFIT------FQTQPISIRTPFTCSSTSWICQLCYGRSPAHGGLVELGEAVGII
AGQSIGEPGTQLTLRTFHTGGVFTGGTAEHVRAPSNGKIKFNEDLVHPTRTRHGHPAFICYI
YLYVTIESEDILHKVTIPPKSFLLVQNDQYVQSEQVIAEIRAGTYTF-NFKERVRKHIYSNSE
GE-MHWSTDVYHAPEFTYSNVHLLPKTSHLWIVSGGSCR--SSVASFSLQKDQDKISVH--SL
SAEG-RSISSL-SGNNDQ-VRQKFFSSDIFGK-KKQ-SEI-PDYSELNRRGHCNLIYPV-ILYENF
Y--LLAKRRRNRFIIPLQLIPEREKGLMPPSGISIEIPGN--GIFRKKSILAYFDDLQYRRKNSGI
TKYGTTGGHSILKKED--LIEYR-GVKELKPKYMKIDRFFFIPEEVHILPESSSIMVQNNSLIG
LDTRITLNTRSRAGGLVRVERKKKWIELKIFSGDIHFPGEMDKISRHNDILLPLGAAKK--N
AKESKK-KFKNWIYVQW--IPPNKKK--YFVLARPVIIYEIADGINLVTLFSQDLLHERDNME
LRIVNYILYGNGKPIRGIYGTSIQLVRTCLLLNWKQDK---KSSSVEEAHASFVEVNTNGMS
RDFLRIDLVKSHISYIRKRKDPSGSGLIF-DKRPDRTSINPFYSISSKAG-----IQQSLSQNQVTI
RMLLKR---NKECQSFIILSSSNCFQMGP--FNDVKYYNDV-IKKES-SLIPIRNSLGPLGLGRTP
QIAHF--YSFYHLIT--YNQIS-VTKYLQLD--N-FKQTFQVL--K-YYLMDENGRSIYNNPDPRG
NIVLNPFNLNWNFLHHNYCEEASRIIS-LGQFICENLCIAKNGP---HLKSGQVLIVQ-IDSVVI
RSAKAYLATPGATVHGHYGEILYEGDTLVTFIYEKSRSRDITQGLPKVEQVLEVRSIDSISM
NLEKRIEGWNKSITKIIGIPWRFLIGAELTIVQGRISLVNKIQKVYRSQGVQIHNRHIEIIVRQI
TSKVLVSE---EGMSNVFSPGELIELLRAERTGRALEEAICYRAILLGITKASLNTQSFISEASF
QETARVLAKAALRGRIDWLKGLKENVVLGGMIPVGTGFNGLVQRS--RQHTNIPLETKNN
K--LFEAEMR--DILFHHRELFYFFISRNLHDTLRQSFRGLNDS------MTRRYWNIHLEEMME
AGVHFGHGTSKWNPRMEPYISSKRKGIHIINLIRTARFLSEACDLVFDTASRGKQFLIVGTK
NKAADSVARAAIRARCHYVNKKWLGGMLTNWYTTETILHKFRDLRTEQKMGRLNRLPK
RDSAMLKRQLSHLQTYLGGIKYMTGLPDIVIIVDQQEEYMALRECMILGIPTICLIDTNCDP
DLADISIPANDDAIASIRLILNKLVFAICEGRS-SYIRNPMGQKINPLGFRLGATQGHHSLWFA

QPQNYSEGLQEDQKIRDWIKNYVQKNIQISSG-VEGIACIKIQKRIDLIQVIIYMGFPKLLLE
GKGG--PRRIEELQIDVQKEINCVNRKLNIALTRITNPYGHPNILAEFIAGQLKNRVSFRKAM
KKAIELTEQAGTKGIQVQIAGRIDGKEIARVEWIREGRVPLQTIRAKIDYCSYAVGTIYGVL
GIKIWIFVDEEMSRYRGPRFKKIRRLGALPGLTSKRPKAGSDLRNQSRSGK-KSQYRIRLEE
KQKLRFHYGLTERQLLKYVRIAGKAKGSTGRVLLQLLEMRLDNILFRLGMASTIPQARQL
VNHRHILVNGRIVDIPSYRCKPQDIITARDEQKSRALIQNCLDSSPRDEELPKHLTFHPFQ--Y
KGLVNQIIDSKWVGLKINELLVVEYYSRQTMSRRGTAEEKTAKSDPIYRNRLVNMLVNRIL
KHGKKSLAYQIIYRAMKKIQQKTETNPLSVLRQAIRGVTPDIAVKARRVGGSTHQVPIEIGS
AQGKALAIRWLLGASRKRPGRNMAFKLSSELVDAAKGSGDAIRKKEETHRMAEANRAFA
HFRMGKDTIADIITSIRNADMNRKGTVRIPSTNITENLVKILLREGFIENVRKHQENNKYFL
VLTLRHRR--NRKGPCKT-V--LNLKRISRPGLRIYSSYQRIPRILGGMGIVILSTSRGIMTDRE
ARLEGIGGEILCYIWMAKPAPRIGSRRNGRIGLR-SARRIPKGVIHVQASFNNTIVTVTDVR
GRVISWSSAGTCGFKGTRRGTPFAAQTAAGNAIRTVVDQGMQRAEVMIKGPGLGRDAAL
RAIRRSGVILSFIRDVTPMPHNGCRPPKKRRVMPTIKQLIRKTRQPIRNVTKSPALGGCPQR
RGTCTRVYTITPKKPNSALRKVARVRLTSGFEITAYIPGIGHNLQEHSVVLVRGGRVKDLPG
VRYHIVRGTLDAVGVKDRQQGRSKYGVKKPKMARKGLIQREKKRQKLEQKYHLIRRSPK
KEIG---KVPLLSEKWEIHGKLQSPPRNSAPTRLHRRCFSTGRPRANYRDFGLSGHILREMV
HACLLPGATRSSWMVQNSFISLISQE-E-KEENRGSVEFQVFSWTNKIRRLTSHLELHRKDY
SSQRGLRKILGKRQRLLSYLSKKKRVLYKYLINQLGIRESKTR--MVKLRLKRCGKKQRVV
YRIVAIDARCRREGRALRKVGFYDPLKNQTYLNVPAILYFLERGAQPTGTVHDISKKAGVF
MELCLNQQIKFNMDKSKRLYFKSKRSFRRRLPPIGSGDRIDYRNMSLISRFISEQGKILSRR
VNRLNLKQQRLITIAIKQARILSLLPFLNNEK--QFERGESAARTAGLRIKKGMTRSIKKNPF
VANHLLRKINKLNTKAEKEIIITWSRTSTIIPIMIGHTIAIHNGKEHFPIYITDRMVGHKLGEF
VPTINFRGHAKNDNRSRRMILKSFILGNPVSLCMKIINSVVVVGLYYGFLTTFSIGPSYLFLL
RARVIEEG---IEKKVSATTGFITGQLMMFISIYYAPLHLALDRPHTITVLALPYLLFHFFWNN
HKHFFDYGSTTRNSMRNLSIQCVFLNNLIFQLFNHFILPSSMLVRLVNIYMFRCNNKILFVT
SSFVGWLIGHILFMKWVGLVLVWIQQNNSIRF-IRSNKYLVSELRNSMARIFSILLFITCVYY
LGRVPSPIVTKKLKETEEDKPLVTILFDSKRWNRPFRYIKNDRFQNAVKNEMSQSFFHTCK
SDGKERISFTYPPSLSTFLKMIQRKISLFTIQKISYNELYNDWSYANEEKRNNISKKLIHRSE
TLD---LAPDVLEKRILLSNNETQKEYLTKIYDPLLNGPYRGQIKKLFLFRKKFLVNKIHGVL
NTEFDQKDPKDKHIGINEISKKVPRWSYKLIELEQQERDEIRSRKAKRVVILTDALIRYSQQ
SDFRRDIIKGSMRAQRRKTVIVELFEANVHSPLFLDRLGTPP--FFSFDIPERLKGIFLNRDEK
TRIEIAEAWDSLIVAQVIRGSLLVTQSILRKYILLPSLIIAKNTLRILLFQFPEWSEDLKDWNK
EMHVKCTYNGVPLSEKEFPKNWLTDGIQIKILFPFRLKPWHRSKKGQKNDFCFLTVWGM
EAELPFGYPRKRLSFFEPLLKELEKKNFKLKQKCFLV-INVLKERTKFFVNVAKETKKWVIK
VII--FLKKIRKEFSKINNSTIHNQMSHES-IKIRYMDWET-SLTEKKMKDLMNRTNTIINKIKT
IKKDKRRNK--ISYDEKGLESPKNIWEILK--NLRLLRKSHYFVKLFIEKLYMDILLCIINIPRI
NAQL------VDLSYLSQAYVFYKLSQT-VNNLDKLRSVFKYTSFFLKNEIREYFTQGIFDSEFR
HKKPP-YGINEWKNWLKGHYQYDLSQIKWSRLVTQKWRTRFN--HMTKNKNKCESYEYE
KTQFIHYEKA---QKQYKYDLLSYKSINDDSLDSDTKYLDWKILHFCLRFFDWMGMNEEIL
SHWFFPEFVILYNTYKIKPWVIPIKLLFFNTN--TETELELFLKRYFHFQLRWD---NQKIINNI
KVYCLLLRLRNPREIAISAIQREEMSLDILMVRKGLTFTELMKKGILIIEPVRLSVKTDGQFF
IYQTIGISLVHKSKQNYD-FIFPEKILSPRRRRELRIRICFRFLWPNYRLEDLSCMNRYWFDT
NNGSRFSLIRIQIYPMKGHQFKSWIFEFREILREIKNSHYFLDSWTQFNSVGSFIHIFFHQER

FIKLLDSRIWSILLS-RNS--QGSTSNRYF---TIKGVVLFVVV-VLIYR----INNRKMVERKNIYL
TGLLP-----IPMNFIGPRNDTLEDSF-GSSNINRLIVS-LLYLPKGKKISESLFLDPKESTWALPIT
----KKCIMPESNWGSRWWRNWIGKKR--DSSCKISNN--ETVAGIEISFKEKDIK-YLEFLFVY
YID--DPIRKAHDWELFDRLSPRKRRNRINL--NSGQLFEILVKDWIGYLIFAFREKIPIEVEG-
FFKQQV-AGSTIQSNDIEHVSY--LFSRNKWAISLQNCAQFHMWQFRQDLFF---SWGK-NPH
ESDFLRK-ISRENWIWLDNVWLVNKDR---FFS--KVRNVSSNIQYDS---TR--SSFVQVTDSSQ
LKGSS-DQSRDHFDSIS----NEDSE-------------YHTLINQREIQQLKEGSIL-WD--PSFLQTERT
EIESD--RFPK--CLSGYS--SMSRLFT-EREKQMNNHLLPEEIEEF--LGNPTRSIRSFFSDRW--S
ELH-LGSNPTERSTRDQ----KLLKKEQDVS-FVPSRRSE----NKEIVNIFKIITYLQN--TVSIHPI
SSDPGCDMVP-KDELD-MDSSNKISFLNKNPFFDL--FR---DRNRGGYTL--HHDFESE--ERF
QEMADIFTLSITEP-DLSYHKGFTFSIDSCRLDQKQ--FLNEVFNSRDESKKKYL---LVLPPIFY
--EENESFYRRIREKWVR-TSCGNDLEDPKPKIVAFASNNIIEAVNQYKLIRNLIQIQYNTCG--
YIINVLNRFFLMN---RSDRNFEYGIQ---RDQIGNDTLNHRTIMKYTINQHLSNLKK-SQKKG
FDP--RSFISRTERSMNRDPNAYRYKWSNGSKNFQEHLEHFISEQKSRF-HFQVVFDRLRINQ
YSIDWSEVIDKKDLSKSLPFFLSKLLL---------FLS-------KFLLFLSNSLP-FFFVSFGNIPIHRS
E-IHIY-ELKGPNDQLCNQLLESIGLQIVHLKKLKPF--LLD---DHDTS-QKSKFLINGGTISPFL
FNKIP-KWWMIDSFHTR--NNRRKSFENTDSYFSMISHDQDNWPNPAKPFHRSSLISSFYKA
NRLRFLNNPHHFCFD--CNKRFPF---Y-VEKARVNNYD--FTYG-QFLNILFIRNK-IFSLRGGK-
-KKHAFLE-RDTISPIELQVSNIFIPNDFPQSGDERRYNLYKS---FHFPIRSDPFVR--RAIYSIA
DIFGTPLTEGQIVNFERTYCKPPSDMNLSDSEGKNLQQYLN--FNSNMGLIRTPCSEKYLPSE
K--RKK--------RSLCLK--KCLEKGQMYRTFQRDSAFSTLSKWNLFQTYMPWFLTSTGYKYL
NLIFLDTFSD-LLPILSSS--QKFVSIFHDIMHGSDISWRILQKKL------CLPQWN-LISEISSKCL
HN--LLLSEEMIHRNNES--PLISTHLRSQNVREF-LYSILFLLLVAGY-LVRTHLLFVSR--AYSE
LQTEFEKVKSLMIPSYMIELRKLLDRYPTSELNSFWLKNLFLVALEQLGDSIEEIRGYASGG
NMLWGGGP-----AYG-VKSIRSKKKYLNINLIDLM----SIIPNPINRIAFSRNTRHLSHTSKEIY
SLIRKRKNVNGDWIDDQIESWVSNSDSIDDKEREFLVQFS--TLTT--EKRIDQILLSLT-HSDH
LS-----KNDSGYQMIEQPGAIYLRYLVDIH--KKYLMNYEFNTSCLAERRIFLAHYQTITY--S
QTSCEANSFHFPSHG---KPFSLRLALSPS-RGILVIGSIGTGQSYLVKYLAENSYVPFITVFLN
KFLDNKPKGFLID-----DSD------DID---------------------------RDLDTELELLTMTNAL---TMD
MMP---EIDRFYITLQFELAKAMSPCIIWIPNIHDLDVNESNYLSLGLLVNYLS---RDCERRS-
TRNILVIASTHIPQKVDPALIAPNKLNTCIKIRRLLIPQQRKHFFTLSYTRGFHLEKKMFHTN
ANGFGSITMGSNVRDLVALTNEALSISITQKKLIIDTNIIRSALHRQTWDLR---SQVRSVHDH
GILFYQIGRAVAQNVLLSNCSIDPISIYMKKK--SCNEGDSYLYKWYFELGTSMKKLTILLYL
LSCSAGSVAQDLWSLP--GPDEK-NGITSYGLVENDS-DLVHGLLEVEGALVG--SSRTEKDCI
QFDND--RMTLLLRPEPRN-PLDMMQTGSCSIVDQRFLYEQ--YESEFEEEGEREGVLDPQ---
-QIEED----LFNH--IVWAP--RIWRP---WGFLFDCIERPNELGFP-YWARSFRGK---RIIYDEEDE
LQENDSEFLQSGTVQYQTRDRSSKEQGFFLISQFIWDPADPLFFLFK-DPPFVSVFSHREFFA
DEEMPKGLLTYQTDLPTSIYKRWFI--KNTQEKHFELLIYRQRWLR---TNSSLS--NGFFRSN--
TPSESYQYLSNLFLSN-GTLLDQMTKTLLKKRWLFPDETKIGF--M---------MPRSR-INGNFI
DKTFSIVANILLRIIPTTSGEKEAFTYYRDG--MSAQSEGNYAEALQNYYEAMRLEIDPYDR
SYILYNIGLIHTRNGEHTKALEYYFRALERNPFLPQAFNNMAVICHY-RGEQAIRQGNSEIA
EAWFDQAAEYWKQAITLTPGNYIEAQNWLKITRRFE--MNWRSEHIWIELIAGSRKTSNFC
WAFILFLGSLGFLLVGISSYLGRNLISLFPPTQIIFFPQGIVMSFYGIAGLFISSYLWCTISWNV
GSGYDRFDRKEGIVCIFRWGFPGKNRRIFLQFFMKDVQSIRIEVKEGIYARRVLYMEIRGHG

AIPLTRTDEN-LTPRELEQKAAELAYFLRVPIEVF
>Prunus
MEKWRFHSMLFLSKSMDSLGPFE-TSVSEDPIINDMDKDILNYSNVDHLVSDRYIRNFISAD
TFFVRDSNRDGYSIYFDIENKNFEIDNDR--SFLSELKSSFYSYQTSSYINNAPKSNDTRHDR
YVYDTHYIWNNHINSCIDSYLR--SQICIDSYILSNSDNYSDSYIYSYICSSESSSIKTSTDGSD
FTIS---S---VTQKYRHLWIQCENCYGLNYKKFLKLKMNICEQCGCHLKMSSFDRIELSIDPG
TWDPMNEDMVSLDPIEFHSEEEPYKNRIDSYQKNTGLTEAVQTGTGQLNGIPVAIGVMDF
QFMGGSMGSVVGEKITRLAEYATNQFLPLIIVCASGGARMQEGSLSLMQMAKISSALYDY
QSNKKLFYVSILTSPTTGGVTASFGMLGDIIIAEPNAYIAFAGKRVIEQTLNKTVPEDSQVAE
YLFHKGLFDPIVPRNPLKGVLGELFRLHAFFPLNQN---MVTIRADEISNIIRERIEQYNREVK
VLNTGTVLQVGDGIARIYGLDEVMAGELVEFEEGTIGIALNLESNNVGVVLMGDGLLIQE
GSSVKATGRIAQIPVSEAFLGRVINALAKPIDGRGEISSSESRLIESPAPGIISRRSVYEPLQTG
LIAIDSMIPIGRGQRELIIGDRQTGKTAVATDTILNQQGQNVVCVYVAIGQKASSVAQVVTA
LQERGAMEYTIVVAETADSPATLQYLAPYTGAALAEFFMYREQHTSIIYDDPSKQAQAYR
QMSLLLRRPPGREAYPGDVFYLHSRLLERAAKSSSRLGEGSMTALPIVETQSGDVSAYIPT
NVISITDGQIFLSADLFNAGIRPAINVGISVSRVGSAAQIKAMKQVAGKSKLELAQFAELEAF
AQFASDLDKATQNQLARGQRLRELLKQSQSSPLSVEEQILTIYTGTNGYLDSLEIGQVRKFL
VELRTYLKTNKPQFQEIISSTKTFTDEAEALLKEAIQEQMERFLLQEQVMRINPTTSGPGVP
ALEKKNLGRIAQIIGPVLDVAFPPGKMPNIYNALVVRGRDAVGQQINVTCEVQQLLGNNR
VRAVAMSATDGLKRGMEVIDTGAPLSVPVGGATLGRIFNVLGEPVDNLGPVDTRTTSPIHR
SAPAFIQLDTKLSIFETGIKVVDLLAPYRRGGKIGLFGGAGVGKTVLIMELINNIAKAHGGV
SVFGGVGERTREGNDLYMEMKESGVINEQNIAESKVALVYGQMNEPPGARMRVGLTALT
MAEYFRDVNEQDVLLFIDNIFRFVQAGSEVSALLGRMPSAVGYQPTLSTEMGTLQERITST
KEGSITSIQAVYVPADDLTDPAPATTFAHLDATTVLSRGLAAKGIYPAVDPLDSTSTMLQPRI
VGEEHYETAQRVKETLQRYKELQDIIAILGLDELSEDDRLTVARARKIERFLSQPFFVAEVF
TGSPGKYVGLAETIRGFKLILSGELDGLPEQAFYLVGNIDEATAKATTLGT------MTLNLCV
LTPNRIVWDSEVKEIILSTNSGQIGVLPNHAPVATAVDIGILRIRLNDQWLTMALMGGFARI
GNNEITVLVNDAEKGSDIDPQEAQQTLEIAEANLREAEGKRQTIEANLALRRARTRVEAIN
VMSMKNVTDSFLSLGYWPSAGSFGFNTDILATNPINLSVVLGVLIFFGKGVLSDLLDNRK
QRILKTIRNSEELREGALDQLEKARARLRKVETEADQFRVNGYSEIEREKFNLINSTYKTLE
QLENYKNETIHFEQQRAINQVRQRVFQQALQGALGTLNSCLNNELHLRTISTNIGMFGTV
KEITDMNPLISAASVIAAGLAVGLASIGPGVGQGTAAGQAVEGIARQPEAEGKIRGTLLLSL
AFMEALTIYGLVVALALLFANPFVMNVLLCSIKTLEGLYDISGVEVGQHFYWQIGSFQVHA
QVLITSWVVIAILLGSAIIAVRNPQTIPTDGQNFFEYVLEFIRDVSKTQIGEEYGPWVPFIGT
MFLFIFVSNWSGALLPWKIIQLPHGELAAPTNDINTTVALALLTSVAYFYAGLTKKGLGYF
GKYIQPTPILLPINILEDFTKPLSLSFRLFGNILADELVVVVLVSLVPSVVPIPVMFLGLFTSGI
QALIFATLAAAYIGESMEGHHMIFSTLEHILTHISFSIVSIVITIHLITLLV-EIVGLYDSSEKGMI
VTFFCITGLLVTRWVCLGHLPLSDLYESLIFLSWSFSIIHMIPYFKKHKNHLSAITAPSAIFTQ
GFATSGLLTEMHQSEILVPALQSHWLMMHVSMMVLSYAALLCGSLLSLALLVITFRKFRRI
FSKGNEIQYVNERNNVLR--S-ISSQNYYRYQLIQQLDRWSYRIISLGFIFLTVGILSGAVWAN
EAWGSYWNWDPKETWAFITWTVFAIYLHIRTNKIFESINSAIVASMGFLIIWICYFGVNLLG
IGLHSYGSFTLTMAKKKVLISLLYLASIVFLPWWISLSFNKSLESWVTNWWNTRQSEIFLN
DIQEKSILKKFIDLEELLRLDEMLKEYPEAHLQKPRIE-YKETIQLIKMHNEDRTHTILHFST
NIICFVILSGYSIIGNEELVILNSWVQEFLYNLSDTIKAFSILLLTDLCIGFHSPHGWELMIGSV

YKDFGFAHNDQIISGLVSTFPVILDTIFKYWIFRYLNRVSPSLVVIYHSMNDMPIGVPRVPFR
SPGEEDALWIDVY-RLYRERLLFLGQGVDSEISNQLIGLMVYLSIEDDTKDLYLFINSPGGW
VIPGVAIYDTMQFVRPDIQTICMGLAASMGSFILVGGEITKRLAFPHA-VMIHQPAASFYEA
QTGEFILEAEELLKLRETLTRVYAQRTGKPLWVVSEDMERDVFMSAIEAQAHGIVDLVAT
E???MEEFQG
YLELDRYQQHDFLYPLIFREYIYALAHDHGLVGYDNKSSLLIIKRLISRMYKHNHFFISANA
SNQKKKLGKNLYSQKISEGFTVIVEISFSLRLVSSLTVKSYNLRSIHSIFPFLEDKFPHLNYVS
DVLIPYPIHLEILVQTLRYWVKDASSLHLLRLFLHEYYNWNSLITN--FFFSKPRLFLLLYNS
HVCEYEFILLFLRNQSSHLQLTSSGIFFERIHFYEKIKYVEEVFANPAILWFFKDPFMHYVRY
QGKSILASKDTPLLMNKWKYYLVNLWQCHSYVWSQPGRIYINKLSKHSLDFLGYFSSIRP
NLSVVRSQMLENSFITDN--AMKKLDTLVPIIPLIGSLAKVKFCNALGHPISKSTWADSSDFD
IIDRFLRICRNLSHYYSGSSRKKSLYRIKYILRLSCLKTLARKHKSTVRTFLKRLGSKLLEEF
FTEEEQILSLVFPRRIWYLDIFCINDLINYEMIIDTPE--VQDIDSFSRLEF-LKEFYGIIWVLIPI
LTLVLGITIGVLVIVWLEREISAGIQQRIGPEYAGPLGILQALADGAKLVFKENLLPSRGDTR
LFSIGPSIAVISILVSYSVAPFGYHLVLADLNIGVFLWIAISSIAPIGLLMSGYGSNNKYSFLGG
LRAAAQSISYEIPLTLCVLSISLLSNSSSTIDIVEAQSKYGFWGWNLWRQPIGFIIFIISSLAEC
ERLPFDLPEAEEELVAGYQTEYSGIKFGLFYVASYLNLLVSSLFVTVLYLGGWNISIPDIYVS
ELFEINKTCGVFGATIGIFITLAKTYLFLFIPITTRWTLPRLRMDQLLNLGWKFLLPISLGNLL
LTTSSQLFSLMIWHVQNENFILDSTRIFMKAFHLLLFDGSFIFPECILIFGLILLLMIDSTSDQ
KDIPWLYFISSTSLVMSITALLFRWREEPTISFSGNFQTNNFNEIFQFLILLCSTLCIPLSVEYIE
CTEMAITEFLLFVLTATLGGMFLCGANDLITIFVAPECFSLCSYLLSGYTKKDVRSNEATTK
YLLMGGASSSILVHGFSWLYGSSGGEIELQEIVNGLINTQMYNSPGISIALIFITVGIGFKLSP
APSHQWTPDVYEGSPTPVVAFLSVTSKVAASASATRIFDIPFYFSSNEWHLLLEILAILSMIL
GNLIAITQTSMKRMLAYSSIGQIGYVIIGIIVGDSNGGYASMITYMLFYISMNLGTFACIVLF
GLRTGTDNIRDYAGLYTKDPFLALSLALCLLSLGGLPPLAGFFGKLHLFWCGWQAGLYFL
VSIGLLTSVVSIYYYLKIIKLLMTGRNQEITPHVRNYRRSPLRSNNSIELSMIVCVIASTIPGIS
MNPIIEIAQDTLFMFLLYEYDFFWAFLIVSSVIPILAFLISGVLAPISKGPEKLSSYESGIEPMG
DAWLQFRIRYYMFALVFVVFDVETVFLYPWAMSFDVLGVSVFIEALIFVLILIVGSIYAWRK
GALEWSMNYFPWLTLVVVLPIFAGSLIFFLPHRGNKVIRWYTICICLIELLLTTYAFGYRFQL
DDPLMQLTEDYKWINFFDFYWRLGIDGISIGPILLTGFITTLATLATRPVTRDSRLFHFLMLA
MYSGQIGPFSSRDLLLFFIMWELELIPVYLLLSMWGGKKRLYSATKFILYTAGSSVFLLIGV
LGIGLYGSNEPTLNFETSANQSYPVALEIIFYIGFLIAFAVKSPIIPLHTWLPDTHGEAHYSTC
MLLAGILLKMGAYGLVRINMELLPHAHSIFSPWLMIVGTIQIIYAASTSPGQRNLKKRIAYS
SVSHMGFIIIGIGSISDTGLNGAILQIISHGFIGAALFFLAGTSYDRIRLVYLDEMGGMAIAIP
KIFTTFSILSMASLALPGMSGFVAELIIFFGILTSQKYLLMTKILITFVMAIGMILTPIYSLSML
RQMFYGYKLFNAPNSYFFDSGPRELFVSISILLPVIGIGVYPDFVSSLSVDKVEAIISNYFYR
MMLEHVLVLSAYLFSIGIYGLITSRNMVRALMCLELILNAVNINFVTFSDFFDSRQLKGNIF
SIFVIAIAAAEAAIGPAIVSSIYRNRKSTRINQSNLLNKMEYTYQYSWIIPFIPLPVPMLIGAG
LLLFPTATKNLRRMWAFPSVLLLSIVMIFSAHFSIQEINNNSVYQYVWSWTINNDFYLEFG
CLVDPLTSIMSILITTVGMMVLIYSDNYMSHDQGYLRFFAYMSFSNTSMLGLVTSSNLIQIY
IFWELVGMCSYLLIGFWFTRPSAANACQKAFVTNRVGDFGLLLGILGFYWITGSFEFRDLF
EIFNN-EVNFLFVTLCAFLLFAGAVAKSAQFPLHVWLPDAMEGPTPISALIHAATMVAAGIF
LVARLLPLFIVIPYIINLIALTGIITLLLGATLALAQKDIKKSLAYSTMSQLGYMMLALGMGS
YRVALFHLITHAYSKALLFLGSGSIIHSMETIVGYSPDKSQNMVLMGGLRKHVPITKTAFLL

GTLSLCGIPPLACFWSKDEILNDSWLYSPIFAIIACSTAGLTAFYMFRIYLLTFEGHLNVYFQ
NYSGKKNSSFYSMSLWGKE-GKKIIKTLLDFLTTKFLNGTKN-TFSYPHESDNTMLFPMLVL
VLFTLFVGAVG-LFDLDILSKFLSSSINLLHKNRN-FVDW-EFITNATFSVSIAYFGILISSFLYK
PVYSSLQNLNLFNSFVILGGKIINVIYNWSYNRGYIDFFYVISFTKGIRRLAELTHFFDRRVI
DGITNGVGIASFFVGEVIKYVGGG-RISSYLLVYVSYVLIFLFFF-MDLPGPIHDFLLVFLGSV
LILGGMGVVLFTNPIYSAFSLGLVLVCISLFYILLNSYFVAAAQLLIYVGAINVLIVFAVMFM
NGSEYYKDLNLWTVGDGVTLPVCTSIFVLLMITITDTSWYGIIWTTRSNQIIEQDLISNSQQI
GIHLSTDFFLPFEFVSIILLAALIGAIAVARQMNIPATRKDLMIVNMGPHHPSMHGVLRLIITL
DGEDVIDCEPILGYLHRGMEKIAENRTIIQYLPYVTRWDYLATMFTEAITVNGPEQLGNIQ
VPKRASYIRIIMLELSRIASHLLWLGPFMADIGAQTPFFYIFRERELVYDLFEAATGMRMM
HNFFRIGGVAADLPHGWIDKCLDFCDYFLTRVAEYQKLITRNPIFLERVEGVGIIGREEVIN
WGLSGPMLRASGIQWDLRKVDHYECYDEFDWEVQWQKEGDSLARYLVRIGEMIESIKII
QQALEGIPGGPYENLEIRYFDRERNPEWNDFEYRFISKKPSPTFELPKQELYVRVEAPKGEL
GIFLIGDQSGFPWRWKIRPPGFINLQILPQLVKRMKLADIMTILGSIDIIMGEVDRMFSMVT
GFMNYGQQTVRAARYIGQSFMITLSHANRLPVTIQYPYEKLITAERFRGRIHFEFDKCIACE
VCVRVCPIDLPVVDWKLETDIRKKRLLNYSIDFGVCIFCGNCVEYCPTNCLSMTEEYELST
YDRHELNYNQIALGRLPMSVIDDYTIRTIFNP-KKVNLLIKE------MQGRLSAWLVKHGLVH
RSLGFDYQGIETLQIKPEDWHSIAVILYVYGYNYLRSQCAYDVAPGGLLASVYHLTRIEYGI
DQPEEVCIKVFAPRRNPRIPSIFWVWKSADFQERESYDMLGIFYDNHPRLKRILMPESWIG
WPLRKDYIAPNFYEIQDAY---MNIIEFPLLDRT
TQ-SVISTTSSDLSNWSRLSSLWPLLYGTSCCFIEFASLIGSRFDFDRYGLVPRSSPRQADLILT
AGTVTMKMAPSLVRLYEQMPEPKYVIAMGACTITGGMFSTDSYSTVRGVDKLIPVDVYL
PGCPPKPEAVIDAITKLRKKISREIYEDRIRSQQENRCFTTSHKFRVGCSTHTGNYDQELLY
QPPSTSEIPPEIFFKYKS-VASHELVNMQTKNTFSWIKKEITRYISVSLMIYIITQAPLSNAYPIF
AQQGYENPREATGRIVCANCHLANKPVDIEVPQAVLPDTVFEAVVRIPYDLQLKQVLANG
KKGALNVGAVLILPEGFELAPPDRILPEIKEKIGNLSFQSYRPTKKNILVIGPVPGQKYSEITF
PILSPDPATKKDVHFLKYPIYVGGNRGRGQIYPDGSKSNNNVYNATAAGIVSKIIRKEKGG
YEITVAGASDGRQVVDIIPPGPELLVSEGESIQLDQPLTSNPNVGGFGQGDAEIVLQDPLRV
QGLLLFLASVILAQIFLVLKKKQFEKVQLSEMNFMSKIYDWFEERLEIQAIADDITSKYVPP
HVNIFYCLGGITLTCFLVQVATGFAMTFYYRPTVTEAFASVQYIMTEANFGWLIRSVHRWS
ASMMVLMMILHVFRVYLTGGFKKPRELTWVTGVVLGVLTASFGVTGYSLPRDQIGYWAV
KIVTGVPDAIPVIGSPLVELLRGSASVGQSTLTRFYSLHTFVLPLLTAVFMLMHFPMIRKQGI
SGPLMGVTKKPDLNDPVLRAKLAKGMGHNYYGEPAWPNDLLYIFPVVILGTIACNVGLA
VLEPSMIGEPADPFATPLEILPEWYFFPVFQILRTVPNKLLGVLLMVSVPAGLLTVPFLENVN
KFQNPFRRPVATTVFLIGTAVALWLGIGATLPIDKSLTLGLFMIEVFLFGIVLGLIPITLAGLFV
TAYLQYRRGDQLDLMPTITSYFGFLLAALTITSALFIGLSKIRLIMDIVNLAWAALMVVFTF
SLSLVVWGRSGLMIIRSPEPEVKILVDRDPVKTSFEEWARPGHFSRTIAKGPDTTTWIWNLH
ADAHDFDSHTSDLEEISRKVFSAHFGQLSIIFLWLSGMYFHGARFSNYEAWLSDPTHIGPS
AQVVWPIVGQEILNGDVGGGFRGIQITSGFFQIWRASGITSELQLYCTAIGALVFAALMLFA
GWFHYHKAAPKLAWFQDVESMLNHHLAGLLGLGSLSWAGHQVHVSLPINQFLNAGVDP
KEIPLPHEFILNRDLLAQLYPSFAEGATPFFTLNWSKYAEFLTFRGGLDPVTGGLWLTDIAH
HHLAIAILFLVAGHMYRTNWGIGHGIKDILEAHKGPFTGQGHKGLYEILTTSWHAQLSINL
AMLGSLTIVVAHHMYSMPPYPYLATDYGTQLSLFTHHMWIGGFLIVGAAAHAAIFMVRD
YDPTTRYNDLLDRVLRHRDAIISHLNWVCIFLGFHSFGLYIHNDTMSALGRPQDMFSDTAI

QLQPVFAQWIQNTHALAPSATAPGATTSTSLTWGGGDLVAVGGKVALLPIPLGTADFLVHH
IHAFTIHVTVLILLKGVLFARSSRLIPDKANLGFRFPCDGPGRGGTCQVSAWDHVFLGLFW
MYNAISVVIFHFSWKMQSDVWGSISDQGVVTHITGGNFAQSSITINGWLRDFLWAQASQV
IQSYGSSLSAYGLFFLGAHFVWAFSLMFLFSGRGYWQELIESIVWAHNKLKVAPATQPRAL
SIVQGRAVGVTHYLLGGIATTWAFFLARIIAVGMALRFPRFSQGLAQDPTTRRIWFGIATAH
DFESHDDITEERLYQNIFASHFGQLAIIFLWTSGNLFHVAWQGNFEAWVQDPLHVRPIAHAI
WDPHFGQPAVEAFTRGGALGPVNIAYSGVYQWWYTIGLRTNEDLYTGALFLLFLSAISLIA
GWLHLQPKWKPSVSWFKNAESRLNHHLSGLFGVSSLAWTGHLVHVAIPGSRGENVRWN
NFLDVLPHPQGLGPLFTGQWNLYAQNPDSSSHLFGTSQGAGTAILTLLGGFHPQTQSLWLT
DIAHHHLAIAFIFLIAGHMYRTNFGIGHSIKNLLEAHIPPGGRLGRGHKGLYDTINNSIHFQL
GLALASLGVITSLVAQHMYSLPAYAFIAQDFTTQAALYTHHQYIAGFIMTGAFAHGAIFFIR
DYNPEQNEDNVLARMLDHKEAIISHLSWASLFLGFHTLGLYVHNDVMLAFGTPEKQILIEP
IFAQWIQSAHGKTSYGFDVLLSSTNGPAFNAGRSIWLPGWLNAVNE--NSNSLFLTIGPGDF
LVHHAIALGLHTTTLILVKGALDARGSKLMPDKKDFGYSFPCDGPGRGGTCDISAWDAFY
LAVFWMLNTIGWVTFYWHWKHITLWQGNVSQFNESSTYLMGWLRDYLWLNSSQLINGY
NPFGMNSLSVWAWMFLFGHLVWATGFMFLISWRGYWQELIETLAWAHERTPLANLIRWR
DKPVALSIVQARLVGLAHFSVGYIFTYAAFLIASTSGKFGMSHSVKIYDTCIGCTQCVRACP
TDVLEMIPWDGCKAKQIASAPRTEDCVGCKRCESACPTDFLSVRVYLWHETTRSMGLAY
MITFSFPSIFVPLVGLVFPAIAMASLSLYVQKNKIFMRDLKTYLSVAPVVSALWFGSLAGLLI
EINRFFPDALIFPPFFSFMTAILERRESESLWGRFCNWITSTENRLYIGWFGVLMIPTLLTATSV
FIIAFIAAPPVDIDGIREPVSGSLLYGNNIISGAIIPTSAAIGLHFYPIWEAASVDEWLYNGGP
YELIVLHFLLGVACYMGREWELSFRLGMRPWIAVAYSAPVAAATAVFLIYPIGQGSFSDGM
PLGISGTFNFMIVFQAEHNILMHPFHMLGVAGVFGGSLFSAMHGSLVTSSLIRETTENESA
NEGYRFGQEEETYNIVAAHGYFGRLIFQYASFNNSRSLHFFLAAWPVVGIWFTALGISTMA
FNLNGFNFNQSVVDSQGRVINTWADIINRANLGMEVMHERNAHNFPLDLAAVEVPSING
MGLPWYRVHTVVLNDPGRLIAVHIMHTALVAGWAGSMALYELAVFDPSDPVLDPMWRQ
GMFVIPFMTRLGITNSWGGWSIT-GGTVTNPGIWSYEGVAGAHIVFSGLCFLAAIWHWVY
WDLEIFTDERTGKPSLDLPKIFGIHLFLSGVACFGFGAFHVTGLYGPGIWVSDPYGLTGRVQ
SVNPAWGVEGFDPFVPGGIASHHIAAGTLGILAGLFHLSVRPPQRLYKGLRMGNIETVLSS
SIAAVFFAAFVVAGTMWYGSATTPIELFGPTRYQWDQGYFQQEIYRRIGAGLAENQSLSEA
WSKIPEKLAFYDYIGNNPAKGGLFRAGSMDNGDGIAVGWLGHPIFRDKEGRELFVRRMPT
FFETFPVVLVDGDGIVRADVPFRRAESKYSVEQVGVTVEFYGGELNGVSYSDPATVKKYA
RRAQLGEIFELDRATLKSDGVFRSSPRGWFTFGHASFALLFFFGHIWHGARTLFRDVFAGI
DPDLDAQVEFGAFQKLGDPTTRRQVVMKTLYSLRRFYPVETLFNGTLALAGRDQETTGF
AWWAGNARLINLSGKLLGAHVAHAGLIVFWAGAMNLFEVAHFVPEKPMYEQGLILLPHL
ATLGWGVGPGGEVLDTFPYFVSGVLHLISSAVLGFGGIYHALLGPETLEESFPFFGYVWKD
RNKMTTILGIHLILLGIGAFLLVFKALYFGGVYDTWAPGGGDVRKITNLTLSPSIVFGYLLK
SPFGGEGWIVSVDDLEDIIGGHVWLGSICILGGIWHILTKPFAWARRALVWSGEAYLSYSL
AALSVFGFIACCFVWFNNTAYPSEFYGPTGPEASQAQAFTFLVRDQRLGANVGSAQGPTG
LGKYLMRSPTGEVIFGGETMRFWDLRAPWLEPLRGPNGLDLSRLKKDIQPWQERRSAEY
MTHAPLGSLNSVGGVATEINAVNYVSPRSWLATSHFVLGFFLFVGHLWHAGRARAAAAG
FEKGIDRDFEPVLSMTPLNMTIALGKFTKDENDLFDIMDDWLRRDRFVFVGWSGLLLFPC
AYFALGGWFTGTTFVTSWYTHGLASSYLEGCNFLTAAVSTPANSLAHSLLLLWGPEAQGD
FTRWCQLGGLWTFVALHGAFGLIGFMLRQFELARSVQLRPYNAIAFSGPIAVFVSVFLIYPL

GQSGWFFAPSFGVAAIFRFILFFQGFHNWTLNPFHMMGVAGVLGAALLCAIHGATVENTL
FEDGDGANTFRAFNPTQAEETYSMVTANRFWSQIFGVAFSNKRWLHFFMLFVPVTGLWM
SALGVVGLALNLRAYDFVSQEIRAAEDPEFETFYTKNILLNEGIRAWMAAQDQPHENLIFP
EEVLPRGNALMSGSTGERSFADIITSIRYWVIHSITIPSLFIAGWLFVSTGLAYDVFGSPRPN
EYFTESRQGIPLITGRFDPLEQLDEFSRSFMTIDRTYPIFTVRWLAVHGLAVPTVSFLGSISA
MQFIQRMATQTVEGSSRSGRPKRTNAGSLLKPLNSEYGKVAPGWGTTPLMGLAMALFAIF
LSIILEIYNSSILLDGISMNMLTLKLFVYTVVIFFVSLFIFGFLSNDPGRNPGREEMADTTGRI
PLWIIGTVAGILVIGLIGIFFYGSYSGLGSSLMLNIFSLIC--LNSALYSSSFFVAKLPEAYAFLN
PIVDVMPVIPLFFFLLAFVWQAAVSFRTTQSNPNEQSVELNRTSLYWGLLLIFVLAVLFSNY
FFNMEVNILAFIATALFILVPTAFLLIIYVKTVSQGDMETATLVAIFLSGLLVSFTGYALYTAF
GQPSQQLRDPFEEHGDMEALVYTFLLVSTLGIIFFAIFFREPPKVPTKKVKMTIAFQLAVFAL
IATSLILLISVPVVFASPEGWSGNKNVVFSGTSLWIGLVFLVGILNSLIS-------MSPQTETKAS
VGFKAGVKDYKLTYYTPDYETKDTDILAAFRVTPQPGVPPEEAGAAVAAESSTGTWTTV
WTDGLTSLDRYKGRCYHIEPVAGEESQFIAYVAYPLDLFEEGSVTNMFTSIVGNVFGFKAL
RALRLEDLRIPPAYVKTFQGPPHGIQVERDKLNKYGRPLLGCTIKPKLGLSAKNYGRAVYE
CLRGGLDFTKDDENVNSQPFMRWRDRFLFCAEAIYKAQAETGEIKGHYLNATAGTCEEMI
KRAVFARELGVPIVMHDYLTGGFTANTSLAHYCRDNGLLLHIHRAMHAVIDRQKNHGMH
FRVLAKALRMSGGDHIHAGTVVGKLEGEREITLGFVDLLRDDFVEKDRSRGIYFTQDWVS
LPGVLPVASGGIHVWHMPALTEIFGDDSVLQFGGGTLGHPWGNAPGAVANRVALEACVQ
ARNEGRDLAREGNEIIREASKWSPELAAACEIWKEIKFEFQAMDTL--MAIHLYKTSTPSTR
NGAVDSQ--SNPRNNLIYGQHRCGKGRNARGIITAGHRGGGHKRLYRKIDFRRNEKDIYGR
IVTIEYDPNRNAYICLIHYGDGEKRYILHPRGAIIGDTIVSGTEVPIKMGNALPLTDMPLGTA
IHNIEITLGKGGQLARAAGAVAKLIAKEGKSATLKLPSGEVRLISKNCSATVGQVGNVGVN
QKSLGKAGSKCWLGKRPVVRGVVMNPVDHPHGGGEGRAPIGRKKPATPWGYPALGRRS
RKRNKYSDNLILRRRSKMIQPQTHLNVADNSGARELMCIRIIGAS-NRRYAHIGDVIVAVIK
EAVPNTPLERSEVIRAVIVRTCKELKRDNGMIIRYDDNAAVVIDQEGNPKGTRIFGAIAREL
RQLNFTKIVSLAPEVLMLSPKRTRFRKQHRGRMKGISYRGNRICFGRYALQALEPAWITSR
QIEAGRRAMTRNARRGGKIWVRIFPDKPVTVRPTETRMGSGKGSPEYWVAVVKPGRILYE
MSGVAENIARRAISIAASKMPIRTQFILSGMTRIKRGYIARRRRTKIRLFASTFRGAHSRLTR
TITQQKIRALVSAHRDRGRQKRDFRRLWITRINAVIREN-KVYFSYSKLIHNLYKRQLLLNR
KILAQIAILNKSCLYMISNEIIK-----------MIKEVYALGQHICMSAHKARRVIDQIRGRSYEET
LMILELMPYRACYAILKLVYSAAANATHNKGFNKTSLIISKAEVNEGTTVKKLKPRARGRS
YPIKRSTCHITIVLKDISLD-------MDGIKYAVFTDKSIRLLGKN-QYTSNVESGSTRTEIKHW
FELFFGVKVIAMNSHRLPGRGRRMGPIMGHTMHYRRMIITLQPGYSIPPLRKKRTMAVPK
KRTSISKKRIRKNIWKRKGYRAALKAFSLAKSLSTGSSKSFF----VTMAKGKDVRVTVILEC
TSC-LRN--VNKESRGISRYITQKNRHNTPGRLELRKFCPCCYKHTIHGEIKKMKIRASVRKI
CEKCRLIRRRGRIIVICSNPRHKQRQGMVREKVTVSTRTLQWKCVESRADSKRLYYGRFIL
APLMKGQADTIGIAMRRALLGEIEGTCITRAKSE--KIPHEYSTIIGIQESVHEILMNLKEIVL
RSNLYGTRNASICIKGPGYVTAQDIILPPSVEIVDNTQHIANLTEPINLCIELQIERNRGYRIK
TPNNFQDGSY--PIDAVFMPVRNANHSIHSYVNGNEKQEILFLEIWTNGSLTPKEALHDASR
NLIDLFIPFLHAEEE--NLQLENNQ-HKVTLPLFTFHGRLAK-QRKNKK-EIALQYIFIDQSELF
PRVYNCLKRSNIHTLLDLLNNSQEDLMKIKHFRIEDVKHILNILEI------------MLGGGN-EAI
STIPGFNQIQFEGFCRFIEQGLTEELYKFPKIEDTDQEIEFQLFVETYQLVEPVIKERDAVYES
LTYSSELYVSAGLIWKNSRDMQEQTIFIGNIPLMNSLGTSIVNGIYRILINQILQSPGIYYRSE

LDHNGISVYTGIIISDWGGRLELEIDRKARIWARVSRKQKISILVLSSAMGSNLREILENVCY
PEIFLSFLNDKEKKKIGSKENAILEFYQQFACVGGDPVVSESLCKELQKKFFQQRCELGRV
GRRNMNRRLNLDIPQNNTFLLPRDILAAADHLIGMKFGMGTLDD--MNHLKNKRIRSVAD
LLQDQLGLALVRLENMVRGTICGAIK-HKLI-PTPQNLVTSTP----LTTTYESFFGLHPLSQVL
DRTNPLTQIVHGRKSSYLGPGGLTGRTASFRIRDIHPSHYGRICPIDTSEGINVGLIGSLAIHA
KIGYWG-SLESPFY---EISERSKK--VRMLYLSPSKDEYYMVAAGNSLALNRGIQEEQVVPTR
YRQEFLTVEWEQVHLRSIFPFQYFSIGASLIPFIEHNDANRALMSSNMQRQAVPLSRSEKCI
VGTGLERQVALDSGVPAIAEHEGKVVYTYTDKIVLSGNGDTLNIPLVIYQRSNKNTCMHQ
NPQVRRGKCIKKGQILADGAATVGGELALGKNVLVAYMPWEGYNSEDAVLISERLVYGDI
YTSFHIRKYEIQTHVTSNG-PERITNEIPHLEAHLLRNLDKNGIVRLGSWVKTGDILVGKLTP
QMAKE-SSYAPEDRLLRAILGIQVSTSKETCLKLPIGGRGRVIDVRWIQKKG-GSSYNPETIR
VYISQKREIKVGDKVAGRHGNKGIVSKILPRQDMPYLQDGRPVDMVFNPLGVPSRMNVG
QIFECSLGLAGGLLDRHYRIAPFDERYEQEASRKLVFSELYEASKQTANPWVFEPEYPGKS
RIFDGRTGDPFEQPVIIGKPYILKLIHQVDDKIHGRSSGHYALVTQQPLRGRAKQGGQRVGE
MEVWALEGFGVAHILQEMLTYKSDHIRARQEVLGTTIIGGTIPKPEDAPESFRLLVRELRSL
ALELNHFLVSEKNFQIKRKEA-------MIDQYKHQQLRIGLVSPQQISAWAQKILPNGEIVGEV
TKPYTFHYKTNKPEKDGLFCERIFGPIKGGICACGNYRVIGDEKKDPRFCEQCGVEFVDSR
IRRYQMGYIKLACPVTHVWYLKRLPSYIANLLDKPLKELEGLVYCDV-FSFARP-IAKKPTF
LRLRGSFEYEIQSWKYSIPLFFTTRGFDTFRNREISTGAGAIREQLADLDLRIIIDYSLVEWK
ELGEE-GPTGNEWEDRKVGRRKDFLVRRMELAKHFIRTNIEPEWMVLCLLPVLPPELRPII
QIDGGKLMSSDINELYRRVIYRNNTLIDLLTTSRSTPGELVMCQEKLVQEAVDTLLDNGIRG
QPMRDGHNKVYKSFSDVIEGKEGRFRETLLGKRVDYSGRSVIVVGPSLSLHRCGLPREIAI
ELFQTFVIRGLIRQHFASNIGVAKSKIREKEPVVWEILHEVMQGHPVLLNRAPTLHRLGIQA
FQPILVEGHAICLHPLVCKGFNADFDGDQMAVHVPLSLEAQAEARLLMFSHMNLLSPAIG
DPISVPTQDMLIGLYVLTSGNRRGICANRYNPCNRRN---YQNKRIDDNNYKYTKE-EPFFC
NSYDAIGAYRQKRINLDSPLWLRWRLDQR-VITS--RETPIEVHYESLGTYHEIYGHYLIVRS
IKKEIICIYVRTTVGHISLYREIEEAIQGFCRAYSYGT------------MAERANLVFHNKVIDGTAI
KRLIRRLIDHFGMAYTSHILDQVKTLGFRQATATSISLGIDDLLTIPSKGWLVQDAEQQSFIL
EKHHHYGNVHAVEKLRQSIEIWYATSEYLRQEMNPNFRMTDPFNPVHMMSFSGARGNAS
QVHQLVGMRGLMSDPQGQMIDLPIQSNLREGLSLTEYIISCYGARKGVVDTAVRTSDAGY
LTRRLVEVVQHIVVRRTDCGTIRGISVSPRNGMMP-ERIFIQTLIGRVLADDIYIGPRCIAVRN
QDIGIGLVNRFIT------LQTQPISIRTPFTCKSTSWICRLCYGRSPTHGDLVELGEAVGIIAGQSI
GEPGTQLTLRTFHTGGVFTGGTAEHVRAPSNGKIKFNEDLVHPTRTRHGHPAFLCYIDLYV
TIESENIIHNVTIPPKSLILVQNDQYVESEQVIAEIRAGAYTL-NFKERVRKHIYSDSEGE-MH
WSTDVYHAPEFTYSNVHLLPKTSHLWILSGGSCR--SSAVPFSLHKDQDQTNVH--SLSAEG-
RYIYSL-SVNNDQ-VKHK--SLNLSGK-ESTSPDY-SELNRIIYTGRCNLIFPA-ILHDNSD-FLLT
KRRRNRFIIPFQSIQEREKELMPRPSISIEIPIN--GIFRRNSILAYFDDPQYRRKSSGITKYGTV
GLHSTLKKED--LIEYR-GVKEFKPKYMKVDRFFFIPEEVHILPESSSIMVRNNSIIGRDTRIT
LNTRSRVGGLVRMERKKKRIELKIFSGDIHFPGEMDKIFRHSGILIPPGTVKK--NSKESKK--
LKNWIYVQW--ITPTKKK--YFVLVRPVIIYEITDGINLATLFPQDPLRERDNLELRVVNYIFY
GNGKPIRGISGTSIQLVRTCLMLNWDQDK---KNSSIEEAHASFVEVSANGLIQYFLRINLVK
SHTSYIRKRNDPLGSGLIS-DNRSGRT--NPFYSIYSKER-----IQQSLSQNQGTFRTLLNR---SK
ESQSLIILSSYNCFQMSP--FNDVKYYN---GIKESDPLITIRNSLGPLGT--ASQIDNF--YSFYHL
IT--HNQIS-LTKYLQLN--N-LKLTFQVL--K-YYLMDENGR--IYNSD--TNILLNPFNLNWHFL

HHDYCEETYTIIS-LGQFICENLCIAKKGS---HLKSGQVIIVQ-IDSVVIRSAKPYLATPGATV
HGHYGEILSEGDTLVTFIYEKSRSGDITQGLPKVEQVLEVRSIDSISMNLEKRVEGWNECIT
RILGIPWGFLIGAELTIVQSRISLVNKIQKVYRSQGVQIHNRHIEIIVRQITSKVLVSE---DGM
SNVFSPGELIGLLRAERTGRALEEAICYRVILLGITKASLNTQSFISEASFQETARVLAKAAL
RGRIDWLKGLKENVVLGGMIPVGTGFKGLVHRS--RQHNNIPLETKKKN--LFEGEMR--DIL
FHHRELFGFCI------------------------MTRRYWNINLEEMMEAGVHFGHGTRKWNPRMAPYIS
AKRKGIHIINLTRTARFLSEACDLVFDAASRGKQFLIVGTKNKAADSVARAAIKARCHYVN
KKWLGGMLTNWYTTETRLHKFRDLRTEQKTGRINRLPKRDAAVLKRQLFHLQTYLGGIK
YMTGLPDIVIIVDQQEEYTALRECITLGIPTICLIDTNCDPDLADISIPANDDAIASIRLILNKL
VFAICEGRS-SYIRNSMGQKINPLGFRLGGTQGHHSLWFAQPKNYSEDLQEDQKIRDCIKN
YVQKNMKISSN-VEGIARIEIQKRIDLIQVIIYMGFPKLLIEDR----TRRIEELQMNVQKELNC
VNRKLNIAIRRISNPYGHPNILAEFIAGQLKNRVSFRKAMKKAIELTEQAGTKGIQVQIAGR
IDGKEIARVEWIREGRVPLQTIRAKIDYCAYAVRTIYGVLGIKIWIFVDEEMSRYRGPRFKKI
RRLGALPGLTSKKPRAGSDLRNQSRPGK-KSQYRIRLEEKQKLRFHYGLTEQQLLKYVRIA
GKAKGSTGQVLLQLLEMRLDNILFRLGMASTIPQARQLVNHRHILVNGRIVDIPSYRCKTR
DIITVRDEQKSRALIQNYLDSPPR-EELPKHLTLHPFQ--YEGLVNQIIDSKWVGLKINELLVV
EYYSRQTMSRRGTAEEKTAKSDPIYRNRLVNMLVNRILKHGKKSLAYQIIYRAMKKIQQK
TETNPLSVLRQAIRGVTPDIAVKARRVGGSTHQVPIEIGSTQGKALAIRWLLGASRKRPGR
NMVFKLSSELVDAANGSGDAIRKKEETHRMAEANRAFAHFRMSKDTIADIITAIRNADMN
KKRTVRIPSTNTTENIVKILLQEGFIENVRKHQESNKYFLVLTLRHRR--NRKGSYRT-V--LN
LKRISRPGLRIYSNYQKIPRILGGMGIVILSTSRGIMTDRRARIEGIGGEILCYIWMAKPIPKI
GSRRNRRIGSR-NARRVPKGVIHVQASFNNTIVTVTDVRGRVISWSSAGACGFKGTRRGTP
FAAQTAAGNAIRTVVDQGMQRAEVMIKGPGLGRDAALRAIRRSGILLSFIRDVTPMPHNG
CRPPKKRRVMPTIKQLIRNTRQPIRNVTKSPALGGCPQRRGTCTRVYTITPKKPNSALRKVA
RVRLTSGFEITAYIPGIGHNLQEHSVVLVRGGRVKDLPGVRYHIVRGTLDAVGVKDRQQGR
SKYGVKKPKMARKSLIQREKKRQKLEKKYHLIRRSSKKEIS---KVPSLSEKWEIHGKLQSP
PRNSAPTRLHRRCFSTGRPRANYRDFGLSGHILREMVHACLLPGATRSSWMVKNSFISAIS
QEEKDENR--GSAEFQIVSFTNRIRRLTSHLELHKKDYLSQRGLRKILGKRQRLLSYLSKKN
RICYKELINRLDIRESKTR--MVKLRLKRCGRKQRAIYRIVAIDVRSRREGRDLRKVGFYDPI
KNQIYLNVPAILYFLEKGAQPTGTVHDISKKAGVFTELRFNQQTKFNMDKSKRILLKSKRS
FRRRLPPIQSGDRIDYRNMSLISRFISEQGKILSRRVNRLTLKQQRLITIAIKQARILSLLPFLN
N---KQFERSE-STRKKKKKIGEEVTRSLKKNPFVANHLLRKINTLNTKAEKEIIVTWSRASTI
IPTMIGHTIAIHNGKEHLPI---------------------------------MILKSFILGNLVSLCMKIINSVVVVGL
YYGFLTTFSIGPSYLFLLRARVMEEGEEGTEKKVSATTGFITGQLMMFISIYYVPLHLALGR
PHTITVLALPYLLFHFFWNNHKHFFDYGSTTRNSMRNLSIQCLFLNNLIFQLFNHFILPSSM
LVRLVNIYMFRCNNKMLFVTSSFVGWLIGHILFMKWVGLVLGWIQQNNSIRS-IRSNKYLV
SELRNSMARIFSILLFITSVYYLGRIPSPIVTKKLKETEEDKPIVTILFDYKRWNRPLRYIKNN
RFENYVRNEMSQYFFYTCQSDGKERISFTYPPSLATFFEMIKSKMSLFRKEKLPSNEFYNQ
WSYRNEHKKKKLNNKFLNRVKALDKTLIVLNVLEKRTRLCNNKTKKEYLTKIYDPFLNG
PYRGRIKKLFTPIERVCINKIHGILYLEFEQKVPNDKSIEIKEINKRVPRWSYKLIDLEQQEGE
EIRSRKAKRVVIFTDALIRYSQQPDFRRDIIKGSMRAQRRKTVTWKLFQANVHSPLFLDRI
DKS---FFFFDISARMKIIFRNRKEKARIEIAEAWDSILLAQVLRGFLLVTQSILRKYIILPSLIIA
KNIVRILLFQFPEWSEDLKDWNREMHIKCTYNGVQLSEKEFPKNWLTDGIQIKILFPFSLKP
WHRSKKDQKNDFCFLTVFGMETELPFGSPRKKLSFFEPIFKELKKKFKKLKKKCFII-LRIL

KEQKKLFLNVSKETKKRIIKSIL--FIKEIIKELSKIN-SIIGNRMIHES-IQIISQGWTN-SLTEKK
MQDLTDRTNTIINQIEKITKENKKNK--ISYDDKGSESQKNIWQILK--NVRLTRKSHYFIKYF
IERIYIDIFLCIINIPSINTQL------CDLSYFSQAYVFYKLSQT-LINLYKLKSVFQYMSLFLKNEI
KDFFTQELFNSELRHKNLR-YGMNQWTNWLRSRYQFDVSHIKWSRLVPQKWRNIFNQHC
MAQNKCDLYE----KDRLINYEKN-NFKKQYRYDILAYKSINDDIRDPDRKYFDWRIFHFCL
KLFDWMGMNEEILSCWFFPELGILYNTYKIKPWVIPIKLLVLNIN--TKGEFDFFLKRYLHFQ
LRWD---NKKISNNIKVYCLLLRLINPREITISSIQRGEMSPDILMIQKDLTLTELIKRGILIIEPI
RLSIKNDGKFIMYQTIGISLVHKSKHHYD-LFVPENILSPRRRREFRILICFRFLWPNYRLEDL
ACMNRYWFNTNNGSRFSMVRVHMYPMKGHQFKSWIFELREILREIKNSHYFLDSWTKFN
SVGSFIHIFFHQERFIKLLDSRIWSILLS-RNSHLQGSTSNRYF---TIKGVVLFVVA-VLIYR----I
NNRKMVERKNLYLTGLLP-----IPMNFIGPRNDTLEESF-GSSNINRLIVS-LLYLPKGKKISES
CFLDPKESTWVLPIT----KKCIMPESNWGSWWWRNWIGKKR--ASSCKISN---ETVAGIEISF
KEKDIK-YLEFLFVYYMD--DPIRKDHDWELFDRLSPRKRRNIINL--NSGQLFEILVKDWIC
YLMFAFREKIPVEAEG-FFKQQG-AGSTIQSNEIEHVSH--LFSRNKCAM--QNCAQFHMRQF
LQDLFF---SWGK-NPHESDFLRN-ISREN--WLDNVWLLNKDR---FFS--KIRNVSSNIQYDS---
TR--SSFVQVTDSSQLKGSS-DQSRDHFDSIS----NEDSE------------YHTLINQREIQQLKERSI
L-WD--PSFLQTERTEIESD--RFPK--CLSGYS--SMSRLFT-EREKQMNKHLLPEEIEEF--LGNP
TRSIRSFFSDRW--SELH-LGSNPTERSTRDQ----KLLKKEQDVS-FVPSRRSE----NKEIVNIFKI
ITYLQN--TVSIHPISSDPGCDMVP-KDELD-MDSSNKISFLNKNPFFDL--FHLFHDRNRGGY
TL--HHDFESE--ERFQEMADLFTLSITEP-DLVYHKGFAFSIG---LDQKQ--FLNEVFNSRDES
KKKSL---LVLPPIFY--EENESFYRRIRKKWVR-TSCGNDLEDPKPKIVVFASNNIMEAVNQY
RLIRNLIQIQYSTYG--YIRNVLNRFFLMN---RSDRNFEYGIQ---RDQIGNDTLNHRTIMKYTI
NQHLSNLKK-SQKKWFDP--LIFISRTERSVNRDPNAYRYKWSNGSKNLQEHLEHFISEQKS
RF-HFQVVFDRLRINQYSIDWSEVIDKKDLSKSLPFFLSKLLL---------FLS-------KFLLFLSNS
LP-FFFVSFGNIPIHRSE-IHIY-ELKGPNDQLCNQLLESIGLQIVHFKKLKPF--LLD---DHETS-
QKSKFLINGGTISPFLFNKIP-KW-MIDSFHTR--NNRRKSFDNMDSYFSMISHDQDNWLNPV
KPFHRSSLISSFYKANRLRFLNNPHHFCFY--CNKGFPF---Y-VEKARINNYD--FTYG-QFLNI
LFIRKK-IFSLCGGK--KKYAFLE-RDTISPIELQVSNIFIPNDFPQNGDER-YNLYKS---FHFPIR
SDPFVR--RAIYSIADISGTLLTEGQIVNFERTYCQPLSDMNLSDSEGKNLHQYLN--FNSNM
GLIHTPCSEKYLPSEK--RKK--------RGLCLK--KCLEKGQMYRTFQRDSAFSTLSKWNIFQT
YMPWFLTSTGYKYLNFIFLDTFSD-LLPILSSS--QKFVSIFHDIMHGSDISWRILQKKL------C
LPQWN-LISDISSKCLHN--LLPSEEMIHRNNES--SLISTHLRSPNVREF-LYSILFLLLVAGY-L
VRTHLLFVSR--AYSELQTEFERVKSLMIPSYMIELRKLLDRYPTSELNSFWLKNLFLVAREQ
LGDSLEEIRGSASVGNMLWGGGP-----AYG-VKSIRSKKKYLNINLIDII----SIIPNPINRIAFSR
NTRHLSHTSKEIYSLIRKRKNVNGDWIDDKIESWVSNSDSIDDKEREFLVQFS--TLTT--EKR
IDQILLSLT-HSDHLS-----KNDSGYQMIEQPGAIYLRYLVDIH--KKYLMNYEFNTSCLAERRI
FLAHYQTITY--SQTSCGANSFHFPSHG---KPFSLRLALSPS-RGILVIGSLGTGRSYLVKYLAT
NSYVPFITVFLNKFLDNKPKGFLVD-----DSD------DIDDSDDIDDS-----------------DDRDFDT
ELELLTMMNAL---TMGMMP---EIDRFYITLQFELAKAMSPCIIWIPNIHDLDVNESNYLSLG
LLVNYLS---RDCERCS-TRNILVIASTHIPKKVDPALIAPNKLNTCIKIRRLLIPQQRKHFFTLS
YTRGFHLEKKMFHT--NGFGSITMGSNVRDLVALTNEALSISITQKKSIIDTNIIRSALHRQT
WDLR---SQVRSVQDHGILFYQIGRAVAQNVLLSNCSIDPISIYMKKK--SCNEGDSYLYKWY
FELGTSMKKLTILLYLLSCSAGSVAQDLWSLP--RPDEK-NGITSYGLVENDS-DLVHGLLEV
EGAPVG--SSRTEKDCSQFDND--RVTLLLRPEPRN-PLDMMQNGSCSIVDQRFLYEQ--YESE

FEE-GEG--VLDPQ----RIEED----LFNH--IVWAP--RIWRP---WGFLFDCIERPNELGFP-YWVR
SFRGK---RIIYDEEDGLQENDSEFLQSGTMQYQTRDRSSKEQGFFRISQFIWDPADPLFFLF
K-DQPFISVFSHREFFADEEMSKGLLTSQTDLPTSIYKRWFI--KNTQERYFELLIHRQKWLR-
--TNSSLS--NGFFRSN--TPSESYQYLSKLFLSN-GTLLDQMTKTLLRKRWLFPDEMKIGF--M
---------MSRSG-INGNFIDKTFSIVANILLRIIPTTSGEKEAFTYYRDG--MSAQSEGNYAEALQ
NYYEAMRLEIDPYDRSYILYNIGLIHTSNGEHTKALEYYFRALERNPFLPQAFNNMAVICH
Y-RGEQAVQQGYSEIAEAWFDQAAEYWKQAIALTPGNYIEAQNWLKITRRFE--MSWRSER
IWIELIAGSRKTSNFCWAFILFLGSLGFLLVGTSSYFGRNFISLFPSQQIIFFPQGIVMSFYGIA
GLFISSYLWCTISWNVGSGYDRFDIKEGVVCIFRWGFPGKNRRIFLRFLMKDIQSIRIEIKEG
IYARRVLYMEIKGQGAIPLTRTDEN-LTPREIEQKAAELAYFLRVPIEVF
>Corynocarpus
MEKGWLNSMLSLSKSIDSLGPSENTSVSEGPIINDMDKNIPSYSTVDHLVGVGDIRNFISDD
TFLVRDSNRGSYSIYFDIENQIFEIDNEH--SFGSELESSFYSYHNSSYLNNVSKNDDYHYDH
YMYDTKYSWNNHINSCIDSYLR--SQICIDSYILSGSHNYSDSYIYSFICGNESSSIRTSTDVS
DLTRTE-SS---ITQKYKHLWVQCENCYGLNYKKFLKSKMNICEQCGYHLKMSSSDRIELSI
DPGTWNPLNEDMVSLDPIEFHSEEEPYKERIDSYQRKTGLTEAVQTGTGQLNGIPVAIGVM
DFQFMGGSMGSVVGEKITRLIEYATNPFLPLILVCASGGARMQEGSLSLMQMAKISSALYD
YQSNKKLFYVSILTSPTTGGVTASFGMLGDIIIAEPNAYIAFAGKRVIEQTLNKTVPEGSQA
AEYLFHKGLFDPIVPRNPLKGVLSELFQLHAFFPLNQNSIKMVTIRADEISNIIRERIEQYNR
EVKIVNTGTVLQVGDGIARIYGLDEVMAGELVEFEEGTIGIALNLESNNVGVVLMGDGLM
IQEGSSVKATGRIAQIPVSEAYLGRVINALAKPIDGRGEISASESRLIESPAPGIISRRSVYEPL
QTGLIAIDSMIPIGRGQRELIIGDRQTGKTAVATDTILNQQGQNVICVYVAIGQKASSVAQV
VTTLQERGAMEYTIVVAETADSPATLQYLAPYTGAALAEYFMYRNRHTLIIYDDPSKQAQ
AYRQMSLLLRRPPGREAYPGDVFYLHSRLLERAAKSSSSLGEGSMTALPIVETQSGDVSAY
IPTNVISITDGQIFLSADLFNAGIRPAINVGISVSRVGSAAQIKAMKQVAGKSKLELAQFAEL
EAFAQFASDLDKATQNQLARGQRLRELLKQSQSAPLTVEEQIMTIYTGTNGYLDSLEIGQV
RKFLVELRTYLKTNKPQFQEIISSTKTLTEEAEALLKEAIQEQMERFLLQEQVMRINPTTSG
PGVSTLEKKDSGRIAQIIGPVLDVAFPPGKMPNIYNALVVKGRDTVGQQINVTCEVQQLLG
NNRVRAVAMSATDGLMRGMEVIDTGAPLSVPVGGATLGRIFNVLGEPIDNLGPVDTRTTSP
IHRSAPAFIQLDTKLSIFETGIKVVDLLAPYRRGGKIGLFGGAGVGKTVLIMELINNIAKAH
GGVSVFGGVGERTREGNDLYMEMKESGVINEQNIAESKVALVYGQMNEPPGARMRVGLT
ALTMAEYFRDVNEQDVLLFIDNIFRFVQAGSEVSALLGRMPSAVGYQPTLSTEMGSLQERI
TSTKEGSITSIQAVYVPADDLTDPAPATTFAHLDATTVLSRGLAAKGIYPAVDPLDSTSTMLQ
PRIVGEEHYETAQRVKQTLQRYKELQDIIAILGLDELSEEDRLTVARARKIERFLSQPFFVAE
VFTGSPGKYVGLAETIRGFKLILSGELDGLPEQAFYLVGNIDEATAKATNLEMGSNLKKMT
LNLCVLTPNRIVWDSEVKEIILSTNSGQIGVLPNHAPIATAVDIGILRIRRDDQWLTMALMG
GFARIGNNEITVLVNDAEKGSDIDPQEAQQTLEIAEANLSKAEGKRQTIEANLALRRARTR
VEAINVISMKNVTDSFISLGYWPSAGSFGFNTDILATNPINLSVVLGVLIFFGKGVLSDLLD
NRKNRILKTIRNSEELRGGAIEQLERARSRLRKVEMEADQFRVNGYSEIEREKLNLINSTY
KTLGQLENYKNETIRFEQQRAINQVRQQVFQQALQGALGTLNNCLNNELHLRTISANLG
MFGTMKEITNMNPLISAASVIAAGLAVGLASIGPGVGQGTAAGQAVEGIARQPEAEGKIRG
TLLLSLAFMEALTIYGLVVALALLFANPFVMNVLLCSINTLKGLYDISGVEVGQHFYWQIA
GFQVHAQVLITSWVVIAILLGSAIIAVRNPQTIPTDGQNFFEYVLEFIRDVSKTQIGEEYGP
WVPFIGTMFLFIFVSNWSGALLPWKIIQLPHGELAAPTNDINTTVALALLTSVAYFYAGLTK

KGLGYFGKYIQPTPILLPINILEDFTKPLSLSFRLFGNILADELVVVVLVSLVPSVVPIPVMFL
GLFTSGIQALIFSTLAAAYIGESMEGHHMIFSTLEHILTHISFSVVSIVITIHLITLLV-EIVGLYT
SSEKGMIATFFCITGLLVTRWIYLRHLPLSDLYESLLFLSWGFSIIHMVTYFKKKKNHFNVIT
APSTIFTQGFASSGFLTEMHQSAILVPALQSQWLMMHVSMMVLGYAALLCGSLFSVALIVI
TFRKVLRIIGKNNEIQYMNERNNVLR--SFFYSKNYYKSQLIQQLDYWSYRIISLGFLFLSIGI
LSGAVWANEAWGSYWNWDPKETWAFITWTVFAIYLHTRTNKNWEGVNSAIVASMGFLII
WICYFGVNLLGIGLHSYGSFTLTMAKKKAFIPLLYLASIVFLPWWISLSFKKSLESWVTNW
WNTGQSEIFLNDLQEKSIIKKVIELEELFLLDEMLKEYPETHLQKLRIG-YKETIQLIKMHNE
DRIHTILHFSTNIICFVILSGYSILSNEELGILNSWVQEFLYNLSDTIKAFSILLLTDLCIGFHSP
HGWELMIDSVYKDFGFAHNDQIISGLVSTFPVILDTILKYWIFRYLNRVSPSLVVIYHSMND
MPIGVPKVPFRTPGDEDASWIDIYNRLYRERLLFLGQAVDSEISNQLIALMVYLSTESETKD
LYLFINSPGGWVIPGVAIYDTMQFVRPDVQTVCLGLAASMGSFILVGGAITKRLALPHARV
MIHQPSSAFYEAQTGEFILEAEELLKLRETLTMVYVQRTGKPLWVVSEDMERDVFMSATE
A Q A H G I V D L V A V
-??MKEFQGY
LELDKSRQRDFLYPLIFREYIYAFAHDHFLIGYANKSSLLIVKRLITRMYQHNHLIISTNDSN
QNPFLGKNLYAQMLSEGFAVIVEIPFSLRLVSSLIVKSYNFRSIHSIFPFLEEKFPHLNYVSDV
LIPYPLHLEILVQILRYWVKDASSLHLLRVFLHEYSNWNSLITKSISFFSKRRLFLFLYNSHV
CEYEFIFLFLRNQSSHLRLTSSRVLFERISFYGKIKHLVDVFANPAILRVRKDPFMHYVRYQG
KSILVSKDTPLLMNKWKYYLVNLWQYHFFAWSQPERIYINQLSKRSLDFLGYISSVRLNTS
VVRSQMLENSFLIDN--AMKNLDTLVPIITLLGSLAKAKFCNVLGHPISKLTWADSSDLDIID
RFVRICRNISHYYSGSSKKKSLYRIKYILRLSCVKTLARKHKSTVRAFLKRLNSEFLEEFFT
EEERVLSLIFPRQIWYLDIICINDLADQEMIIDTTE--VQEIHSFSKLEF-LKEVYGILWVLIPIF
TTVVGITIGVLVIVWLEREISAGIQQRIGPEYAGPWGVLQALADGTKLLFKENLLPSRGDT
RLFSIGPSIAVISILLSYSVIPFGYRLVLADLNIGVFLWIAISSIAPIGLLISGYGSNNKYSFLGG
LRAAAQSISYEIPLTLCVLSISLLSNSSSTVDIVEAQSKYGFWGWNLWRQPIGFIIFLISSLAE
CERLPFDLPEAEEELVAGYQTEYSGIKFGLFYVASYLNLLVSSLFVTVLYLGGWDFSIPYILV
SELFEINKTSGVFGITIGIFITLAKTYLFLFISITTRWTLPRLRMDQLLNLGWKFLLPISLGNL
LLTTSFQLFSLMIWHVQNENFILDSTRIFMKAFHLLLFDGSFIFPECILIFGLILLLMIDSTSD
QKDIPWLYFISSTSLVMSITALLFRWREEPMISFSGNFQTNNFNEIFQFLILLCSTLCIPLSVE
YIECTEMAITEFLLFVLTATLGGMFLCGANDLITIFVAPECFSLCSYLLSGYTKKDVRSNEAT
TKYLLMGGASSSILVHGFSWLYGSSGGEIELQEIVNGLLNTQMYNSPGISIALIFITVGIGFK
LSPAPSHQWTPDVYEGSPTPVVAFLSVTSKVAASASATRIFDIPFYFSSNEWHLLLEILAILS
MILGNLIAITQTSMKRMLAYSSIGQIGYVIIGIIVGDSNGGYASMITYMLFYISMNLGTFARI
VSFGLRTGTDNIRDYAGLYTKDPFLALSLALCLLSLGGLPPLAGFFGKLHLFWCGWQAGL
YFLVSIGLLTSVVSIYYYLKIIKLLMTGRNQEITPHVRNYRRSPLRSNNSIELSMIVCVIASTI
PGISMNPIIEIAQDTLFMFLLYEYDIFWVFLIISSVIPILAFLISGVLAPIRKEPEKLSSYESGIEP
MGDAWLQFRIRYYMFALVFVVFDVETVFLYPWAMSFDVLGVSVFIEALIFVLILIVGSVYA
W R K G A L E W
S??
???
???
???
???

???-MLEHVLVVSAYLFSIGIYGLIT
SRNMVRALMCLELILNAVNINFITFSDFFDSRQLKGNIFSIFVIAIAAAEAAIGPAIVSSIYRN
RKSTRINQSNLLNKMEYTYQYSWIIPFISLPVPMLIGMGLLLFPTATKNLRRMWSFPSVLLL
SIVLIFSVYLSIHQINSSYIYQYVWSWTINNDFSLDFGYLIDPLTSIMSILITTVGILVLIYSDN
YMSHDQGYLRFFAYMSFFNTAMLGLVTSSNLIQIYIFWELVGMCSYLLIGFWFTRPSAANA
CQKAFVTNRVGDFGLLLGILGLYWTTGTFEFRDLFKIFNI-EVNFLFVSLCAFLLFAGAVAK
SAQFPLHVWLPDAMEGPTPISALIHAATMVAAGIFLVARLLPLFIVIPYIMNIIALIGIITVLFG
ATLALAQKDIKRSLAYSTMSQLGYMMLALGIGSYRAALFHLITHAYSKALLFLGSGAIIHS
MESVVGYSPDKSQNMVLMGGLTKHVPIT-IFFLLGTLSLCGIPPLACFWSKDEILNDSWLYS
PIFAIIACSTAGLTAFYMFRIYLLTFEGHLNLSFKSYSPKTNNSLYSISLWGKK-GSKISQKLLS
LLTMNFFCDTKS-TFSYPYESDNTMLFPMFMLALFTLFVGVIGIPFYLDILDKWLIPSINLLH
QSSN-SVNW-EFLKNATLSVSIAFFGISLASFLYKPAYSSLQNLNFRNSVVILLEKIINVIYDW
SYNRGYIDAFYGISLIGGIRGLAELTHFFDRRVIDGITNLVGILHFFVGESIKYVGGG-RISSYI
LLYLSYVLIFILIYSMDLPGPIHDFLLVFFGSGIILGGMGVVLLTNSIYSAFSLGLVLVCIALFY
ILSNSHFVAAAQLLIYVGAINVLIIFAVMFMKGSEYSKDFPLWTVGSGVTLLVCTSIFVSLIN
TILDTSWYGIIWTTRSNQILEQDLISNSQQIGIHLSTDFFLPFELISIILLVALIGAISVARQMNE
PATRKDLMIVNMGPHHPSMHGVLRLIVTLDGEDVIDCEPILGYLHRGMEKIAENRTIIQYL
PYVTRWDYLATMFTEAITVNGPEQLGNIQVPKRASYIRVIMLELSRIASHLLWLGPFMADI
GAQTPFFYIFREREL VYDLFEAATGMRMMHNYFRIGGVAADLPYGWIDKCLDFCDYFLTG
VAEYEKLITQNPIFLERVEGVGIVGGEEGINWGLSGPMLRASGIQWDLRKVDHYECYDEF
DWEVQWQKEGDSLARYLVRLGEMTESIKIIQQALEGIPGGPYENLEIRCFDRETYPEWND
FEYRFISKKPSPTFELPKQELYVRVEAPKGELGIFLIGDQGGFPWRWKIRPPGFINLQILPQL
VKRMKLADIMTILGSIDIIMGEVDRMFPMVTGFMNYGQQTVRAARYIGQSFMITLSHANR
LPVTIQYPYEKLITSERFRGRIHFEFDKCIACEVCVRVCPIDLPVVDWKLETDIRKKRLLNY
SIDFGICIFCGNCVEYCPTNCLSMTEEYELSTYDRHELNYNQIALCRLPMSVVDDYTIRTIS
NS---QVKNK--------MQGHLSAWLVKHGLVHRSLGFDYQGIETLQIKPEDWHSIAVILYVYG
YNYLRSQCAYDVAPGGLLASVYHLTRIEYGIDQPEEVCIKVFVARRNPRIPSVFWVWKSA
DFQERESYDMLGISYDNHPRLKRILMPESWVGWPLRKDYIAPNFYEIQDAH-----------------
---MNSIEFPLLDRTTQTSVISTTSNDLSNWSRLSSLWPLLYG
TSCCFIEFASLIGSRFDFDRYGLVPRSSPRQADLILTAGTVTMKMAPSLVRLYEQMPEPKYVI
AMGACTITGGMFSTDSYSTVRGVDKLIPVDVYLPGCPPKPEAVIDAITKLRKKISREIYEDR
IKS--ENRCFTTNHKFHVGRSTHTGNYDQGLLYQPPSSEIIPPETFFKYKSSLSSYELVNMQT
RNTFSWIKKEITRSISVSLMIYIITGASVSNAYPIFAQQGYENPREATGRIVCANCHLANKPV
DIEVPQAVLPDTVFEAVVRIPYDMQLKQVLANGKKGGLNVGAVLILPEGFELAPPDRISPEI
KEKIGNLSFQSYRPTKKNILVIGPAPGQKYSEITFPILSPDPATKKDVHFLKYPIYVGGNRGR
GQIYPDGSKSNNNVYNATAAGIVSKIIRKEKGGYEITIVDASDGRQVVDIIPPGPELLVSEGE
SIKLDQPLTSNPNVGGFGQGDAEIVLQDPLRVQGLLVFLASVILAQIFLVLKKKQFEKVQLS
EMNFMSKVYDWFEERLEIQAIADDITSKYVPPHVNIFYCLGGITLTCFLVQVATGFAMTFY
YRPTVTEAFASVQYIMTEANFGWLIRSVHRWSASMMVLMMILHVFRVYLTGGFKKPREL
TWVTGVVLAVLTASFGVTGYSLPRDQIGYWAVKIVTGVPEAIPVIGSPLVELLRGSASVGQ
STLTRFYSLHTFVLPLLTAVFMLMHFPMIRKQGISGPLMGVTKKPDLNDPVLRAKLAKGM
GHNYYGEPAWPNDLLYIFPVVILGTIACNVGLAVLEPSMIGEPADPFATPLEILPEWYFFPVF
QILRTVPNKLLGVLLMVSVPAGLLTVPFLENVNKFQNPFRRPVATTVFLIGTAVALWLGIGA
TLPIDKSLTLGLFMIEVFLFGIVLGLIPITLAGLFVTAYLQYRRGDQLDLMPTITSYFGFLLAV

LTITSALFIGLSKIRLIMDIVSLAWAALMVVFTFSLSLVVWGRSGLMIIRSPEPEVKILVDRDP
IKTSFEEWARPGHFSRTIAKGPDTTTWIWNLHADAHDFDSHTSDLEEISRKVFSAHFGQLSI
IFLWLSGMYFHGARFSNYEAWLGDPTHIGPSAQVVWPIVGQEILNGDVGGGFRGIQITSGF
FQLWRASGITSELQLYCTAIGALVFAALMLFAGWFHYHKAAPKLAWFQDVESMLNHHLA
GLLGLGSLSWAGHQVHVSLPINQFLNAGVDPKEIPLPHEFILNRDLLAQLYPSFAEGATPFF
TLNWSKYAEFLTFRGGLDPVTGGLWLTDIAHHHLAIAILFLIAGHMYRTNWGIGHGIKDIL
EAHKGPFTGQGHKGLYEILTTSWHAQLSINLAMLGSLTIIVAHHMYSMPPYPYLATDYGTQ
LSLFTHHMWIGGFLIVGAAAHAAIFMVRDYDPTTRYNDLLDRVLRHRDAIISHLNWVCIF
LGFHSFGLYIHNDTMSALGRPQDMFSDTAIQLQPVFAQWIQNTHALAPGATAPGATTSTSL
TWGGGDLVAVGGKVALLPIPLGTADFLVHHIHAFTIHVTVLILLKGVLFARSSRLIPDKANL
GFRFPCDGPGRGGTCQVSAWDHVFLGLFWMYNSISVVIFHFSWKMQSDVWGSISDQGVV
THITGGNFSQSSITINGWLRDFLWAQASQVIQSYGSSLSAYGLFFLGAHFVWAFSLMFLFSG
RGYWQELIESIVWAHNKLKVAPATQPRALSIVQGRAVGVTHYLLGGIATTWAFFLARIIAV
GMALRFPRFSQGLAQDPTTRRIWFGIATAHDFESHDDITEERLYQNIFASHFGQLAIIFLWTS
GNLFHVAWQGNFEAWVQDPLHVRPIAHAIWDPHFGQPAVEAFTRGGALGPVNIAYSGVY
QWWYTIGLRTNGDLYTGALFLLFLSAISLIAGWLHLQPKWKPSVSWFKNAESRLNHHLSG
LFGVSSLAWTGHLVHVAIPGARGEYVRWNNFLDVLPHPQGLGPLFTGQWNLYAQNPDSN
NHLFGTSQGAGTAILTLLGGFHPQTQSLWLTDIAHHHLAIAFIFLVAGHMYRTNFGIGHSIK
DLLEAHIPPGGRLGRGHKGLYDTINNSIHFQLGLALASLGVITSLVAQHMYSLPAYAFIAQD
FTTQAALYTHHQYIAGFIMTGAFAHGAIFFIRDYNPEQNEDNVLARMLDHKEAIISHLSWA
SLFLGFHTLGLYVHNDVMLAFGTPEKQILIEPIFAQWIQSAHGKTSYGFDVLLSSTNGPAFN
AGRSIWLPGWLNAVNE--NSNSLFLTIGPGDFLVHHAIALGLHTTTLILVKGALDARGSKLM
PDKKDFGYSFPCDGPGRGGTCDISAWDAFYLAVFWMLNTIGWVTFYWHWKHITLWQGN
VSQFNESSTYLMGWLRDYLWLNSSQLINGYNPFGMNSLSVWAWMFLFGHLVWATGFMF
LISWRGYWQELIETLAWAHERTPLANLIRWRDKPVALSIVQARLVGLAHFSVGYIFTYAAF
LIASTSGKFGMSHSVKIYDTCIGCTQCVRACPTDVLEMIPWDGCKAKQIASAPRTEDCVG
CKRCESACPTDFLSVRVYLWHETTRSMGLAYMPTLNLPSIFVPLVGLVFPALAMASLFIHIQ
KNKIFMRDLKTYLSVAPVVSTLWFGALAGLLIEINRLFPDALIFPFFSFMTAILERRESESLW
GRFCNWITSTENRLYIGWFGVLMIPTLLTATSVFIIAFIAAPPVDIDGIREPVSGSLLYGNNIIS
GAIIPTSAAIGLHFYPIWEAASVDEWLYNGGPYELIVLHFLLGVACYMGREWELSFRLGM
RPWIAVAYSAPVAAATAVFLIYPIGQGSFSDGMPLGISGTFNFMIVFQAEHNILMHPFHMLG
VAGVFGGSLFSAMHGSLVTSSLIRETTENESANEGYRFGQEEETYNIVAAHGYFGRLIFQY
ASFNNSRSLHFFLAAWPVVGIWFTALGISTMAFNLNGFNFNQSVVDSQGRVINTWADIINR
ANLGMEVMHERNAHNFPLDLAAVEAPSLNGMGLPWYRVHTVVLNDPGRLLAVHIMHTA
LVAGWAGSMALYELAVFDPSDPVLDPMWRQGMFVIPFMTRLGIINSWGGWSIT-GGTVTN
PGIWSYEGVAGAHIVFSGLCFLAAIWHWVYWDLEIFSDERTGKPSLDLPKIFGIHLFLSGVA
CFGFGAFHVTGLYGPGIWVSDPYGLTGRVQSVNPAWGVEGFDPFVPGGIASHHIAAGTLGI
LAGLFHLSVRPPQRLYKGLRMGNIETVLSSSIAAVFFAAFVVAGTMWYGSATTPIELFGPTR
YQWDQGYFQQEIYRRVGAGLAENQSLSEAWSKIPEKLAFYDYIGNNPAKGGLFRAGSMD
NGDGIAVGWLGHPIFRDKEGRELFVRRMPTFFETFPVVLVDGDGIVRADVPFRRAESKYS
VEQVGVTVEFYGGELNGVSYSDPATVKKYARRAQLGEIFELDRATLKSDGVFRSSPRGWF
TFGHASFALLFFFGHIWHGARTLFRDVFAGIDPDLDAQVEFGAFQKLGDPTTRRQVVMKT
LYSLRRFYHVETLFNGTLALAGRDQETTGFAWWAGNARLINLSGKLLGAHVAHAGLIVF
WAGAMNLFEVAHFVPEKPMYEQGLILLPHLATLGWGVGPGGEVIDTFPYFVSGVLHLISS

AVLGFGGIYHALLGPETLEESFPFFGYVWKDRNKMTTILGIHLILLGIGAFLLVFKALYFGG
VYDTWAPGGGDVRKITNLTLSPSVIFGYLLKSPFGGEGWIVSVDDLEDIIGGHVWLGSICIF
GGIWHILTKPFAWARRALVWSGEAYLSYSLGALSVCGFIACCFVWFNNTAYPSEFYGPTGP
EASQAQAFTFLVRDQRLGANVGSAQGPTGLGKYLMRSPTGEVIFGGETMRFWDLRAPWL
EPLRGPNGLDLSRLKKDIQPWQERRSAEYMTHAPLGSLNSVGGVATEINAVNYVSPRSWL
ATSHFVLGFFLFVGHLWHAGRARAAAAGFEKGIDRDFEPVLSMTPLNMTIALGKFTKDEK
DLFDIMDDWLRRDRFVFVGWSGLLLFPCAYFALGGWFTGTTFVTSWYTHGLASSYLEGC
NFLTAAVSTPANSLAHSLLLLWGPEAQGDFTRWCQLGGLWTFVALHGAFGLIGFMLRQFE
LARSVQLRPYNAIAFSGPIAVFVSVFLIYPLGQSGWFFAPSFGVAAIFRFILFFQGFHNWTLN
PFHMMGVAGVLGAALLCAIHGATVENTLFEDGDGANTFRAFNPTQAEETYSMVTANRF
WSQIFGVAFSNKRWLHFFMLFVPVTGLWMSALGVVGLALNLRAYDFVSQEIRAAEDPEFE
TFYTKNILLNEGIRAWMAAQDQPHENLIFPEEVLPRGNALMSGSTGERSFADIITSIRYWVI
HSITIPSLFIAGWLFVSTGLAYDVFGSPRPNEYFTESRQGIPLITGRFDPLEQLDEFSRSFMTI
DKTYPIFTVRWLAVHGLAVPTVSFLGSISAMQFIQRMATQTVGDSSKSG-PRRTNVGSLLKP
LNSEYGKVAPGWGTTPLMGVAMALFAIFLSIILEIYNSSILLDGISVNMLTLKLFVYTVVIFF
VSLFIFGFLSNDPGRNPGREEMADTTGRIPLWIIGTVTGIPVIGLIGIFFYGSYSGLGSSLMLN
ILSLISICLNSVLYSSSFFFAKLPEAYAFLNPIVDVMPVIPLFFFLLAFVWQAAVSFRTTQSNP
NEQNVELNRTSLYWGLLLIFVLAVLFSNYFFNMEVNILAFIATALFILVPTAFLLIIYVKTISQ
SDMETATLVAISISGLLVSFTGYALYTAFGQPSQQLRDPFEEHGDMEALVYTFLLVSTLGIIFF
AIFFREPPKVPTKK--MTIAFQLAVFALIATSSILLISVPVVFASPDGWSSNKNVVFSGTSLWI
GLVFLVGILNSLIS-------MSPQTETKASVGFKAGVKDYKLTYYTPEYETKDTDILAAFRVTP
QPGVPPEEAGAAVAAESSTGTWTTVWTDGLTSLDRYKGRCYHIEPVAGEENQYIAYVAYP
LDLFEEGSVTNMFTSIVGNVFGFKALRALRLEDLRIPPAYVKTFQGPPHGIQVERDKLNKY
GRPLLGCTIKPKLGLSAKNYGRAVYECLRGGLDFTKDDENVNSQPFMRWRDRFLFCAEA
LFKAQAETGEIKGHYLNATAGTCEEMIKRAVFARELGVPIVMHDYLTGGFTANTSLAHYC
RDNGLLLHIHRAMHAVIDRQKNHGIHFRVLAKALRMSGGDHIHAGTVVGKLEGERDITL
GFVDLLRDDFIEKDRSRGIYFTQDWVSLPGVLPVASGGIHVWHMPALTEIFGDDSVLQFGG
GTIGHPWGNAPGAVANRVALEACVQARNEGRDLAREGNAIILEASKWSPELAAACEVWK
QIKFEFEAMDTL--MAIHLYKTSTPSTRNGAVDSQMKSNPRNNLIYGQHRCGKGRNSRGIIT
AGHRGGGHKRLYRKIDFRRNEKDIYGRIVTIEYDPNRNAYICLIHYGDGEKRYILHPRGAII
GDTIVSGTEVPIKMGNALPLTDMPLGTAIHNIEITLGKGGQLARAAGAVAKLIAKEGKSAT
LKLPSGEVRLISKNCSATVGQVGNAGVNQKSLGRAGSKCWLGKRPVVRGVVMNPVDHP
HGGGEGRAPIGRKKPATPWGYPALGRRSRKRNKYSDNLILRRRSKMIQPQSHLNVADNSG
ARELMCIRIIGAS-NRRYAHIGDVIVAVIKEAVPNTPLERSEVIRAVIVRTCKELKRENGMIIR
YDDNAAVVIDQEGNPKGTRIFGAIARELRQLNFTKIISLAPEVLMLSPKRTRFRKHHRGRM
KGISYRGNRICFGRYALQTLEPAWITSRQIEAGRRAMTRNARRGGKIWVRIFPDKPVTVRP
TETRMGSGKGSPEYWVAVVKPGRILYEMGGVAENIARKAISIAASKMPIRTQFIISGMTRIK
RGYIARRRRKKIRLFASSFRGAHSRLTRTITQQKIRALVSAYRDRGRQKRDFRRLWITRINA
VIRDN-KVYYSYSKLIHNLYKRQLLLNRKILAQIAILNRNCLYMISNEIIK-----------MIKEVY
ALGQHICMSAHKARRIIDQIRGRSYEETLMILEIMPYRACYPILKLVYSAAANASHNMGFN
EATLFISKAEVNKSTTMKKLKPRARGRSYPIKRPTCHLTIVLRDISVN-GLWDKKMDGIKYA
VFTDKSIRLLGKN-QYTSNVESGSTRTEIKHWVELFFGVKVIAMNSHRLPGKGRRMGPIKG
HTMHYRRMIITLQPGYSIPPLRKKRTMAVPKKRTSISKKRIRKNIWKRKGYWAALKAFSLA
KSLSTGNSKSFFVR--QIMAKGKDARVTVILECTSC-VRN--VNKGSMGISRYITQKNRHNTP

SRLELIKFCPRCYKHTIHGEIKKMKIRASVRKICEKCRLIRRRRRIIVICSNPRHKQRQGMV
REKIRVSTRTLKWKCVESRADSKRLYYGRFILSPLMKGQADTIGIAMRKALLGEIEGTCITR
AKSE--KIPHEYSTIVGIQESVHEIFMNLKEIVLRSNLYGTRDASICVKGPGYVTAQDIILPPS
VEIVDNTQHIANLTEPINLCIELQIERNRGYRIKTPNNFQDGSY--PIDAVFMPVRNANHSIHS
YVNGNEKQEILFLEIWTNGSLTPKEALYEASRNLIDFFIPFLHAEEE--NFHLKNNQ-HKVTL
PLFTFHDRLAK-LRKNKK-EIALKYIFIDQSELPPRIYNCLKRSNIHTLLDLLNNSPDDLMKI
KHFRIEDVKHILDILEIEKHFA-------MLGSGN-ERMSTIPGFNQIQFEGFCRFIDQGLTEELYK
FPKIEDTDQEIEFQLFVETYQLVEPLIKERDAVYESLTYSSELYVSARLIWKTSRDMQEQTIF
IGNIPLMNSLGTSIVNGIYRIVISQILQSPGIYYRSELDHKGISVYTGTIISDWGGRLELEIDR
KARIWARVSRKQKISILVLLSAMGSNLREILENVCYPEIFLSFLNDKEKKKIGSKENAILEFY
QQFSCVGGDPVFSESLCKELQKKFFQQRCELGRIGRRNLNRRLNLDIPQNNTFLLPRDILA
AADHLIGMKFGMGTLDD--MNHLKNKRIRSVADLLQDQFGLALVRLENMVRGTICGAIR-
HKLI-PTPQNLVTSTP----LTTTYESFFGLHPLSQVLDRTNPLTQIVHGRKSSYLGPGGLTGRT
ASFRIRDIHPSHYGRICPIDTSEGINVGLIGSLAIHARIGHWG-SLESPFY---EISERSKK--VRM
LYLSPSRDEYYMVATGNSLALNRGIQEEQVVPARYRQEFLTIEWEQVHLRSIFPFQYFSIGA
SLIPFIEHNDANRALMSSNMQRQAVPLSRSEKCIVGTGLERQVALDSGVPALAEHGGKIIYT
DTDKIVLSGNGYTRSIPLVMYQRSNKNTCMHQKSQVQQGKCIKKGQILADGAATVGGEL
ALGKNVVVAYMPWEGYNFEDAVLISERLVYEDIYTSFHIRKYEIQTHVTSHG-PERITNEIP
HLEAHLLRNLDKNGIVMLGSWVETGDILVGKLTPQMAKE-SSYAPEDRLLRAILGIQVSTS
KETCLKLPIGGRGRVIDVRWIQKKG-GSSYNPEMIRVYISQKREIKVGDKVAGRHGNKGIV
SKILPREDMPYLQDGRPVDMVFNPLGVPSRMNVGQIFECSLGLAGGLLDRHYRIVPFDER
YEQEASRKLVFSELYEASKQTASPWVFEPEYPGKSRIFDGRTGNPFEQPVIIGKPYILKLIHQ
VDDKIHGRSSGHYALVTQQPLRGRAKQGGQRVGEMEVWALEGFGVAHILQEMLTYKSDH
IRARQEVLGTTIIGGTIPKPEDAPESFRLLVRELRSLALELNHFLVSEKNFQINRKEAMNQKI
SSMIDRYKHQQLRIGLVSPQQISAWANKILPNGEIVGEVTKPYTFHYKTNKPEKDGLFCERI
FGPIKSGICACGNYRVIGDKKEDSKFCEQCGVEFVDSRIRRYQMGYIKLACPVTHVWYLK
RLPSYIANILDKPLKELEGLVYCD--FSFARP-IAKKPTFLRLRGSFEYEIQSWKYSIPLFFTTQ
GFDTFRNREISTGAGAIREQLADLDLRIIIDYSLVEWKELGEE-GSAGNEWEDRKVGRRKD
FLVRRMELAKHFIRTNIEPEWMVLCLLPVLPPELRPIIQIDGGKLMSSDINELYRRVIYRNNT
LIDLLTTSRSTPGELVMCQEKLVQEAVDTLLDNGIRGQPMRDGHNKVYKSFSDVIEGKEG
RFRETLLGKRVDYSGRSVIVVGPSLSLHRCGLPREIAIELFQTFVIRGLIRQHFASNIGVAKS
KIRQKEPIVWEILQEVMQGHPVLLNRAPTLHRLGIQAFQPILVEGRAICLHPLVCKGFNADF
DGDQMAVHVPLSLEAQAEARLLMFSHMNLLSPAIGDPISVPTQDMLIGLYVLTSGNRRGIC
ANRYNPCNRKN---HQNEKIDDNDYKYTKE-EPFFCNSYDAIGAYRQKRIHLDSPLWLRWR
LDQR-VIAS--REAPIEVHYESLGTHHEIYGSYLIIKSIKKEILCIYIRTTVGHISLYREIEEAIQG
FCRACSYGT--------MEVLMAERADLVFHNKVIDGTAIKRLIRRLIDHFGMAYTSHILDQIKT
LGFQQATATSISLGIDDLLTIPSKGWLVQDAEQQSLILEKHHHYGNVHAVEKLRQSIEIWYA
TSEYLRQEMNPNFRMTDPFNPVHIMSFSGARGNASQVHQLVGMRGLMSDPQGQMIDLPI
QSNLREGLSLTEYIISCYGARKGVVDTAVRTSDAGYLTRRLVEVVQHIVVRRTDCGTIRGIS
VSPRNGMMP-ERIFIQTLIGRVLADNIYMGPRCIGIRNQDIGIGLVNRFIT------FQTQPISIRTP
FTCRSTFWICRLCYGRSPTHGDLVELGEAVGIIAGQSIGEPGTQLTLRTFHTGGVFTGGTAE
HIRAPSNGKIKFNEDLVHPTRTRHGHPAFLCYIYLYVTIESEDIIHNVTIPPTSFLLVQNNQY
VESEQVIAEIRAGASTF-NFKERVRKHIYSDSEGE-MHWSTDVYHAPEFTYSNVHLLPKTSH
LWILSGGSCS--SSAVPFSLYKDQDQINVH--SLSVEG-RYISSL-SVNNDQ-VRHKFLISDPSGK

-E------------ICTGHCNLIYPA-IFHGNSD--LLATRRRNRFIIQFESLQEREKELLPLSGISIEIPIN
--GIFRRNSILAYFDDPQYKRNSSGITKYGTIGVHSILKKED--LIEYR-GVKEFKPKYMKVDR
FFFIPEEVHILPESSSIMVRNNSIIGVATRITLSIRSRVGGLVRVEKKKKRIELKIFSGDIHFPGK
MDKISRHNGILIPPERLKK--ISKKSKQ--LKNWIYVQW--ITPNKKK--YFVLVRPVIIYEIADG
INLVTLFPQDLLQERDNLELRVVNYILYGNGKPIRGISGTSIQLVRTCLVLNWDQDK---KSSS
IEEARASFVEVSTNGLVRDFLRIDLVKSHISYIRKRKDPSGSGLIS-NNESDRTNINPFYSIYS
KAR-----VEQSLSQNQGTIRTLLNS---NKERQSLIILSASNCFQMDP--FNDVKYYN---VIKES-
PIIPIRNSLGPLGA--VPQIANF--YSFYQLIT--HNQIA-VNKYLQLD--N-LKQTFHLN--K-YYL
MDENG-GIFNS-DPCSNIVLNTFNLNWHFLHHNYCEERPIIIS-LGQFICENVCIAKNRP---HL
KSGQILIVQ-VDSVVIRSAKPYLATSGATVHRHYGEILYEGDTLVTFVYEKSRSGDITQGLP
KVEQVLEVRSIDSISMSLEKRVEGWNERITKILGMPWGFLIGAELTIVQSRISLVNKIQKVY
RSQGVAIHNRHIEIIVRQITSKVLVSE---DGMSNVFSPGELIGLLRAERTGRALEEAICYRAIL
LGITKASLNTQSFISEASFQETARVLAKAALRGRIDWLKGLKENVVLGGMIPVGTGFKGL
APRS--RQHNNIPLETKKKN--FFEGEMR--DILFHHRELCDFCISKNLHDTSEQSFLGFNDS---
---MTRRYWNINLEEMMEAGVHFGHGTRKWNPRMAPYISAKRKGIHIINLTRTARFLSEAC
DLVFDAASRGKQFLIVGTKNKAADSVARAAIRARCHYVNKKWLGGMLTNWSTTETRLH
KFRDLRTEQKTGGLNRLPKRDAAMLKRQLSHLQTYLGGIKYMTGLPDIVIIGDQQEEYRA
LQECITLGIPTICLIDTNCDPDLADISIPANDDAIASIRLILNKLVFAICEGRS-SSIRNPMGQKI
NPLGFRLGANQGHHSLWFAQPQKYSEGLQEDQKIRDCIKNYVQKNMRIFSG-VEGISRIEI
QKRIDLIQVIIYMGFPKLLIEG--G--SRRIEELQMNVQKELNCVNQKLNIAITRIANPYGHPNI
LAEFIAGQLKNRVSFRKAMKKAIELTEQAGTKGIQVQIAGRLDGKEIARVEWIREGRVPLQ
TIRAKIDYCSYTVRTIYGVLGIKIWIFVDEEMSRYRGPRFKKIRRLGALPGLTSKRPRTGSDL
KNQSRSGK-KSQYRIRLEEKQKLRFHYGLTERQLLKYVRIAGKAKGSTGQVLLQLLEMRL
DNILFRLGMASTISQARQLVNHRHILVNGCIVDIPSYRCKPRDIITAKDEQRSRALIQNYLDS
SPH-EELPKDLTLHPVQ--YKGLVNQIIDSKSVGLKINELLVVEYYSRQTMSRRGTAEEKIAK
SDPIYRNRLVNMLVNRILKHGKKSLAYQILYRAMKKIQQKTETNPLSVLRQAIRGVTPDIA
VKARRVGGSTHQVPIEIGSTQGKALAIRWLLGASRKRPGRNMAFKLSSELVDAAKGSGDA
IRKKEETHRMAEANRAFAHFRMGKDTIANVITSIRNADMNRKGMVRIPSTNITENIVKILL
REGFIENVRKHRESNKDFLVLTLRHRR--NRKGTYKT-I--LNLKRISRPGLRIYSNYQRIPRIL
GGMGIVILSTSRGIMTDREARLEGIGGEIFCYIWMAKPIPRISSRRNGRIGSRTSTRRIPKGVI
HVQASFNNTIVTVTDVRGRVISWSSAGTCGFKGTRRGTPFAAQTAAGNAIRTVVDQGMQ
RAEVMIKGPGLGRDAALRAIRRSGILLSFIRDVTPMPHNGCRPPKKRRVMPTIKQLIRNTR
QPIINFTKSPALRGCPQRRGTCTRVYTITPKKPNSALRKVARVRLTSGFEITAYIPGIGHNLQE
HSVVLVRGGRVKDLPGVRYHIVRGTLDAVGVKDRQQGRSKYGVKKPKMARKSLIQREK
KRQELEQKYHLIRRSSKKEIS---KVPSLSEKWEIHGKLQSPPRNSAPTRLHRRCFLTGRPRA
NYRDFGLSGHILREMVHACLLPGATRSSWMVKNAFISVISQE-EEKKENRGSVEFQVFSFT
NKIRRLTSHLELHKKDYLSQRGLRKILGKRQRLLSYLSKKNRIRYKELINQFDIRESKTR--
MVKLRLKRCGRKQRAVYRIVAIDVRSRREGRDLRKVGFYDPIKNQTYLNVPAILYFLEKG
AQPTVTVHDISKKAGVFTELHLNQ-----MDKSKRLFLKSKRSFRRRLPPIQSGDRIDYRNMS
LISRFISEQGKILSRRVNRLTLKQQRLITIAIKQARILSLLPFLNNE--KQFERSESTARTIGLRT
RTKVTRSLKKNPFLANHLLRKIKKLNTKAEKEIIVTWSRASTIIPTMIGHTIAIHNGKEHLPI
YITDRMVGHKLGEFAPTLNFRGHAKNDNRSRRMILKSFILGNLVSLCMKIINSVVVVGLY
YGFLTTFSIGPSYLFLLRARVMEEG---TEKKVSATTGFITGQLMMFISIYYAPLHLALGRPHT
ITVLALPYLLFHFFWNNHKHFFDYGSTTRNSMRNLSIQCVFLNNLIFQLFNHFILPSSMLVR

LVNISMFRCNNKMLFVTSSFVGWLIGHILFMKWVGLVLVWIQQNNYIRS-IRSNKYLVSEL
RNSMARIFSILLFITCVSYLGRIPSPIFTKKLKETEEDKPLVTILFDYKRWNRPLRYIKKAQSL
NAVRNEMSQYFFYTCQSEGKERISFTYPPSVSTFLEMIERKISLFTSEQLSSDELDKHWRHP
NEQKRNNLNKKFLNRVEILD---LALDALEKRTRLCNDKTKKEYFPKTYDPFLNGPYRGRI
KICFSLRENTFINKIHGIFYQKMEHKDYNDKSIGIKEISKKVPRWSYKLIDLEQEHGEEIRSR
KAKRVIIYTDALIRYSQLSDFRRDIIKGSMRAQRRKTVIWKLFQANVHSPLFFDRIDKPF--F
FFFDIPGLLKTFFKNREEKIRIEIAETWDSIPFAQVIRGPLLVTQSILRKYIVLPSLIIAKNIIRM
LFFQFPEWSEDLKDWKNEMHVKCTYNGVPLSENEFPKNWLTDGIQIKILFPFHLKPWHRS
KQKKKNDFCFLTVLGLEAQRPFGSPRKRISFFEPILKEFEKKMIKLK---------------KLLRNIE
KETKKWVIQNIL--FLKEIIKELPKRNDLIISNRMIHGS-IPILSMNWANYSLREKKMKDLTDR
TNTIIKKIEKIRKDKKKNK--ISYDDKRLESPKNIWQILK--NVRLIRKSHHFLTFFVEKIYIDIL
LCMINIPRINAQL------WDLSYLSEAYVFYKISQT-FINLSKLRPVFQYTSLFLKNAIKDSFAQ
GIFHSELREKNLP-SVMNQWKNWLRGHYQYDLYTIRWSRLVPQKWRNRVSQGPMAKNK
NKCHS--YEKNLLIHYKKQ-NFKKQYIYDLLSYKSINNNIIDPDRKYFDCRILNFCLK----------
-ISRWFFPEFVILYNVYKITPWIIPIKLLLLNKN--TEVELDSFLKRYLRFQLRWD---NPRIINNI
KVYCLLLRLKNPREIAISSIQRGEMSLDILIVQKDLTLTELMQRGILIIEPVRLSVKNDGQFL
MYQTIGISLVRKNKRHYD-LLVPENILSPRGRRELRIQICFRFLWPNYRLEDLACMNRYWF
DTNNGSRFSMVRIHMYPMKGHQFKSWIFELREILREIKNSHYFLDSWTQFNSVGSFIHIFF
HQERFIKLLDSRIWSILLS-RNS--QGSTSNRYFTI-TIKGVVLFVVA-VLIYR----INNRKMVER
KNLYLTGFLP-----IPMNFIGPRNDTLEESF-GSSNINRLIVS-LLYLPKGKKISESCFLDPKEST
WVLPIT----KKCIMPESNWGSRWWRNWIGKKR--DSSCKISN---ETVAGIEISFKEKDIK-YLE
FLFVYYMD--DQIRKDHDWELFDRLSPRKRRNIINL--NSGQLFEILVKDWICYLMFAFREKI
PIEVEG-FFKQQG-AGSTIQSNDIEHVSH--LFSRNKWAISLQNCAQFHMWQFRQDLFF---SW
AK-NPHESDFLRN-ISRENWIWLDNVWLVNKDR---FFS--KVRNVLSNIQYDS---TR--SSFVQ
VTDSSQLKGSS-DQSRDHFDSIS----NEDSE-------------YHTLINQREIQQLKERAIL-WD--PSF
LQTERTEIESD--RFPK--CFSGYS--SMSRLFT-EREKQMNNHLLPEEIEEF--LGNPTRSIRSFFS
DRW--SELH-LGSNPIERSTRDQ----KLLKKEQDVS-FVPSRRSE----NKEIVNIFKIITYLQN--T
VSIHPISSDPGCDMVP-KDELD-MDSSNKISFLNKNPFFDL--FHLFHDRNRRGYAL--HHDFE
SE--ERFQEMADLFTLSITEP-DLVYHKGFALSIDSYGLDQKQ--FLNEVFNSRDESKKKYL---
LVLPPIFY--EENESFYRRIRKKWVR-TSRGNDLEDPKPKIVVFASNNIMEAVNQYRLIRNLIQ
IQYNTYG--YIRNVLNRFFLMN---RSDRNFEYGIQ---RDQIGNDTLNHRTIMKYTINQHLSNL
KK-GQKKWFDP--LIFISRTERSMNRDPNAYRYKWSNGSKNFQEHLEHFISEQKSHF-HFQV
VFDRLRINQYSIDWSEVIDKKDLSKSLPFFLSKLLL---------FLS-------KFLLFLSNSLP-FFFV
SFGNIPIHRSE-IHIY-ELKGPNDQLCNQLLESIGLQIVHLKKWKPF--LLD---DHDTS-QKSKF
LINGGTISPFLFTKIP-KW-MIDSFHTR--NNRRKSFDNTDSYFSMISHDQDNWLNPVKPFHR
SSLISSFYKANRLRFLNNPHHFCFY--CNKRFPFYVDY-VEKARINNSD--FTYG-QFLNILFIR
NK-IFSLCGGK--KKHAFLE-RDTISPIESQVSNIFIPNDFPQSGDER-YNLYKS---FHFPIRSDPF
VR--RAIYSIADISGTPLTEGQIVHFERTYCQPLSDMNLSDSEGKNLHQYLN--FNSNMGLIH
TPCSEKYLPSEK--RKK--------RSLCLK--KCLEKGQMYRTFQRDSAFSTLSKWNLFQTYMP
WFLTSTGYKYLNLIFLDTFSD-LLPILSSS--QKFVSIFHDIMHGSDISWRILQKKW------CLPQ
WN-LISEISSKCLHN--LLLSEEMIHRNNES--PLISTHLRSPNVREF-LYSILFLLLVAGY-LVRT
HLLFVSR--AYSELQTEFEKVKSLMIPSYMIELRKILDRYPTSELNSFWLKNLFLVALEQLGD
SLEEIWGYASGGNMLWGGGP-----AYG-VKSIRSKKKDWNINLINIIDLI-SIIPNPINRIAFSRN
TRHLSHTSKEIYSLIRKRKNVNGDWIDDKIESWVSNSDSIDDKEREFLVQFS--TLTT--EKRI

DQILLSLT-HSDHLS-----KNDSGYQMIEQPGAIYLRYLVDIH--KKYLMNYEFNTSCLAERRT
FLAHYQTITY--SQTSCGANSFHFPSHG---KPFSLRLALSPS-RGILVIGSIGTGRSYLVKYLAT
NSYVPFITVFLNKFLDNKPKGFLID-----DSGDIDDSEDIDDSGDIDDSDD----------------IDRD
LDTELELLTMMNAL---TMDMMP---EIDRFYITLQFELAKAMSPCIIWIPNIHDLDVNESNYL
SLGLLVNYLS---RDCERCS-TRNILVIASTHIPQKVDPALIAPNKLNTCIKIRRLLIPQQRKHFF
TLSYTRGFHLEKKMFHT--NGFGSITMGSNVRDLVALTNEALSISITQKKSIIDTNIIRSALHR
QTWDLR---SQVRSVQDHGILFYQIGRAVAQNVLLSNCSIDPISIYMKKK--SCNEGGSYLYK
WYFELGTSMKKLTILLYLLSCSAGSVAQDLWSLP--GPDEK-NGITSYGLVENDS-DLVHGV
LEVEGALVG--SSRTEKDCSRFDND--RVTLLLRPEPRN-PLDMMQNGSCSIVDQRFLYEQ--Y
ESEFEE---GEGVLDPQ----QIEED----LFNH--IVWAP--RIWRP---WGFLFDCIERPNSLGFP-YW
ARSFRGK---RIIYD---ELQENDSEFLQSGTVQYQTRDRSSKEQGFFRISQFIWDPADPLFFLF
K-DQPFVSVFSHREFFADEEMSKGLLTSQTDIPTSIYKRWFI--KNTQEKHFGLLIHRQRWLR
---TNSSLS--NGFFRSN--TPSESYQYLSNLFLSN-GTLLDQMTKTLLRKRWLFPDEMVVAI--C
YNNESLV--MPRSR-INGNFIDKTFSIVANILLRIIPTTSGEKEAFTYYRDG--MSAQSEGNYAE
ALQNYYEAMRLEIDPYDRSYILYNIGLIHTRNGEHTKALEYYFRALERNPFLPQAFNNMA
VICHY-RGEQAIRQGDSEIAEAWFNQAAEYWKQAIALTPGNYIEAQNWLKITRRFE--MSW
RSERIWIEFIRGSRKTSNFCWAFILFLGSLGFLLVGTSSYLGRDLISLFPSQQIIFFPQGIVMSF
YGIAGLFISSYLWCTISWNVGSGYDRFDRKEGIVCIFRWGFPGKNRRIFLRFLMKDIQSIRIE
VKEGIYARRVLYMEIRGQGAIPLTRTDNN-LTPREIEQKAAELAYFLRVPIEVF
>Rhizanthella
MAKYWFNLMLSLSKSMDNLNDLGHIGGNEELNENDAKKNILG---THYLFDSRNIYNLIYN
ETFLVRNSNGYHYFVYFDIENKILNINNVN-------------------YLNNVFNNNN-SYYDSMYDT
QSNWNNHIDGCIDAYLH--FEISLNSDIYDGIK-----YITDLICTSENYSIETSN--SSLRIEERSD
DFDKYAKYRHLWVQCENCYVPNYKKFLKSKMNICEYCGYHLKMSSLDRIELLIDPGTWE
SMDEDMVYEEEGSIHLEEESYIDRIDFYQRTTGLIEAVQTGIGQLNSVPIAIGVMDFQFIGGS
MGSVVGEKITRLIEYATNQLLPVILVCASGGARMQEGSLSLMQMAKISSVSYDYQSNKKL
FYVSILASPTTGGVTASFGMLGDIIIAEPNAYIAFAGKRVIEQTLNKPVPDGSQEAEYLFHK
G L F D P I V P R N L L K N V L N E L F Q L H G F L P L N K D S N
S???
??
??
??
??
??
??
??
??
??
??
??
??
??
??
??

??
??
??
??
??
??
??
??
??MPIGLPKVPFRIPGEEDAIWIDVY
NRLYRERLLFLGQEVDSEISNQLVGLMVYLSIEDNTRDLHLFINSPGGWVIPGISIYDTMQF
VPPTVHTVCIGLAASIGSLILVGGEITKRLAFPHARVMIHQPASSFYESQVGEFILEAEEFLK
LRETILKVYVQRTSKPLFVISKDIERDVFMSAKEALAYGIVDLVAV-MKEQKLIYEGLITESH
PNGMFLVRLDNQDIMMVHISGRIRQSFIRILPGDRVEIEMSRYDLTRGRIIYRLPKKNI-
-???
??
??
??
??
??
??
??
??
??
??
??
??
??
??
??
??
??
??
??
??
??
??
??
??
??
??
??
??
??
??

???
???
???
???
???
???
???
???
???
???
???
???
???
???
???
???
???
???
???
???
???
???
???
???
???
???
???
???
???
???
???
???
???
???
???
???
???
???
???
???
???
???
???
???

???
???
???
???
???
???
???
???
???
???
???
???
???
???
???
???
???
???
???
???
???
???
???
???
???
???
???
???????????????????????????????????????TATHFYNTSTSSTRNGTVDSQVESNSRNNLIYGQHR
CGKGRNARGIITAGHRGGGHKRLYRKIDFRRNEKGISGRIVTIEHDPNRNACICLIHYRDGE
KRYILHPRGARIGDTVVSGAEVPISMGNALPLTDMPLGTAIHNIEITLGKGGQLARAAGAA
AKLIAKEGQLTSLRLPSGEVRLISKNCLATVGQVGNVGANQKSLGRAGSKCWLGKRPVV
RGVAMNPIDHPHGGGEGRSPIGRKKPTTPWGYPALGIRSRKRNKYSDGLILRRRK-MIQHQ
TFLNVADNSGAKELMCIRIIGTG-NHQYAHIGDVVVAVIKEAAPDMPVERSEIIRAVIVRTCK
ELKRANGMIIRYDDNAAVIIDQEKNPKGTRIFGAIAKELRQFNLTKIVSLASEVLMLSPKRN
RFRKQHRGRMKGISCRGKSICFGKYALKALESSWITARQIEAGRRAITQHAWRREKLWVR
IFPDKPITVRPTETRMGSGKGSPEYWVSVVKPGRLIYEINGISETIAKTSMKIAACKIPVRTQ
FVIKVMVRVKRGYTARKR---LRINS----------TRTTIQQRMRALVSFHRDSNRKKIDFRCLWI
TRINAATCDV--VFHSYNIFIHNLYKNQLILNRKILMQIALSNNNYFDTICNKIMNPK--------
-??
???????????????????????????????????MNGIKYAIFTKKCLRLWGN-NQYTSNVESGSTRTEIKR
WFELFFGVKVIAMNLYLLPGKVRR----MGHTMHYRRMIITLQPGYSIPPIIEKL
T ? -
-???MKIRASIRKIC
D K C C L I R R R G Q I L V I C S N P R H K Q K Q

G??
???
???
???
???
???
???
???
???
???
???
???
???
???
???
???
???
???
???
???
???
???
???
???
???
???
???
???
???
???
???
???
???
???
???
???
???
???
???
???
???
???MKRYYWNIHLEEMMEAGVHFGH
GTKKWNPRMAPYISAKHKGIHITNLTITARFLSEACNLVFEAASHGKKFLIVGTKNKTADL
VASAAIRARCNYVNKKWPGGMLTNWSTTETRLHKLRNLIAEQKMGKLQRLTKGKAVML
NRKLSILQTYLVGIQYMARLPDIVIIVDQQKDYKVLKECAILGIPTICLIDTNCDPDCADISIP

ANDDAITSIQLILTKLVSAIYKGIKKKVVRIGMGQKINPLGFRLGITQIHYSFWFAKPKYFS
MGLQEDERIRRCIHNYVQNKKKISSG-FEGIAYMRIQKKIDLIQVMIYIGFPNLLREGLLST
W--IGEELQRNVQKKFH---RIFNIVIIKIEKPYAQPNILAEYIALQFKNRVSVRKIMKKVIELTE
QTDTKGIQVQIAGRIDGKEIARVEWIREGRVPLQTICAKIDYCSYTIRTIYGNLGIKIWIFAN
K-MSRYLGPCFKKIRRLGALPGLTNKIPRSRSDLQIQLRSIK-KSQYRLRLEEKQKFRFHYGL
TERQLLKYVRIAGKTKGSTGRVLLQLLEMRLDNILFRLGMASTIPGARQLVNHRHILVNG
DIVDIPNYRCKPLDIIAAKDKERSKAMIKNYLISSSHE--LPNHLTMDS--LQHKGFVNQIIDS
KCSGLKINELLVVEYYSRQTMSRRGTAEKKITKSDPIYRNKLINMFVNHIMKHGKKSLAY
QIIYLTVKNIKQNTETNPLSVLRRAMRRVTPNIAVKARRVGGSTHQVPIEIGSRQGKAFAIR
WLLGASQKRTGRNMAFKLSSELLDAAQGIGDAIRKKDEIHRMAEANIVFAHFHMSRDIIA
NLITSIRNADMHSKGRIQIVSTNITNNIVKILLQEGFIENIRKHKERQKNFLVSTLQHRK--TR
KR------KKIILKRISRSGLRIYSNYQQIPKILGGLGVAIISTSKGIMTDREARLQRMGGEILCYI
WMTKPSAKIGLRNNAR--------RIPKGVIYIKASFNNTIVTIADLRGRVVSWSSAGTSGFIGT
KKGTPYAAQATTLNTVRTIIDRGMRRAEIMIKGPGLGRDAALRAIRRSRISLNSVRDVTPIP
H N G C R P P K K R R
V??
????????????????????????????????????MAKMGWMQRENKRQKLEQKYAWIRRSFKKEIS---
KVTSLGEKWKIHVKLQSPPRNSAPIRLHRRCFLTGRSRANYRDFGLSGHMLLEMFHSGLL
P G A T R S S
W???
???
????????--KIKQCFRKYNRSISRRLLPIGPGDQIDYKNMSLISQFISEQGRILSRRANKLPLKQ
QRFITIAIKQARILSLLPFFRN--------------NEKELYRVDVVRSLTKNPFVAIDLLGKIERLNMR
EEKEIIVTWSRASTIIPIMIGHTIAIHNGKEHLPIYITDRMVDHKLGEFAPTLTFVRHARNDN
KSSHMIFKSFILGNPLSLYIKVINSPVVTGLYYGLTNILSIGPYYLFIIQVWFMEEEGT-KERA
SAATTGFITGQLMMFISIYYAPLHLALDRPHIITFLVLPYLLFHFFWNNQKNLLNSGSTIINS
MHNFSIQYVFLNNLLFQLFNHFILPSSTLYRLVNVNMFRCNNKIIFLISSFVGWLIGHILLMK
LVVLTLLWIRQNNFIRY-----KYLVSEFQNYGARIFSIILFITCVYYLGRMPSPIVTKKFKA----
NAFVTFFFDYTRWNRPLRYVKNDQLENALRNEMAQFFFFSCKSDGKKIISFAYPPSLSTFF
EMTRQIIFLYSIKED----LYNLWIYTNEKKKLNLNNELINRIKILEKNG---DMLEKKTILCDDP
NIKKCWPKAYDPCFNGSYRGIRKKLHSYVEIFLINKIHNML------LEYHVKSIIIEEINKKVP
RWSYKLTLAEEGEN-GIYSRRAKRVIIYNAAFMHYSQQSDFRRNLIKGSMRAQRRKTPIWG
LLQINVHSPLFLDRLDKTSLFSFFD-IKEVKNFIIRN---SEQNELMSEWDNLMFASTIRTLILI
VQSFLRKYILLPSFIIIKNSFRILLLQYPEWHQDFKKWNREIHIKCTYDGVQLSETEFPQDW
LTEGIQIKIIHPFYIKPWQKYKKYKNKNFCFLTVFGMETELPFGSPRKQPSFWGPVYKELK--
------KKILRVIPFLKK------------TKWIIKVVQ--IIKLMMEKMYKINVKIN-NGIICKSSVLIGPV
NWLNYSLIESRMKTISDKTRKIKNQIEQIAKYKQK--------NKE--------LKLKGQTIRLIRRLN
YFLKYLIEKIYIDILLCTINFFKKSVQLFN----KNSYYISQAYVFYKLSQTQLFHKYNLRHILQ
YY--LLNSKLKDYCIHRIFQFNSIGNTISMYVMNEWKNWLKGHYQYDLSEDR--KYHRLVR
LNTIHKRCMIKNKIQFYRYKKKP--FISSMENVKWKKNYRYDLLSYKYIHGYIIDLNRKYF
DCRFLKLWFIFLNYIGMDQNKLY-WVLPEFVPLMNIYKIKPWIIPIKSLFFNQGNKKELELD
SFLIRYFIFQFKWTN---SLIMENIRVYCLLLRLMKIKEIAISSIRRGEINPEQMLIQKELVFSELI
RRGIWIIEPIFLSIIRDGKFIIYQTIDIIQTSN---NLNDFLIPEHLLSPVRRREFRIQTCFKFLWPN
YRLEDLACMNRYWFNTNNSSRFSMLRIRMYSMKKHQYKSWIFEL----IETKNYNYLLDS

WSKFDSAVSFTHIFFHQERFMKLFDPRIWSIMLS-RDS--QGAIN---FT---IKGAALLVVA-VLL
S----RIHNQRMVERKNIYLMGLLP-----IPMNSIGPRNDTLEESF-WSSNTNRSIVS-LLHRKN--
------IYDLQASARVIPIK----KRCIMPEFTTGSRWWRNRIGKKR--DYGCQIV---NETIAGIEISF
KEKDSKSLKEFLFLSYTA-DDPIRKDRDWELFPSLSPR----------NSG---LLLGKYFICYLMSA
FREKIPIP----FFKQQG-TGTTMQSN---EISH--LFSRNKRSISLQTCAKFHMWQFRQ-------SW-
------SYFLRN-VSR---IWLDGVWLVNKD----------LRNVLSNIKYDPTDPTR--SISVQVTDSNQ
WKGSSSDHS------VR----IENSE-------------YQAFIDQTEIQQLKERSIL-WD--LSFLQTERTER
ESD----------LGSY--SIEKLMH-D-------HLLSEEIEEVL--GNP-----AFFSDRW--SELH-LGSNPT
ERSTIDS----KFCNKKQDVS-FVPSRQGE----KKEMVDIFKIITYLQN--TVSIHPPEPYHIPDM
G----------SSNKISFFNKKTFFS--FFHLFHDRNKGECAS--------F-LERLPKMADLFTLSITEPP
DMVYHRGFAFYIDSYGLNPNNSSEENESFYGNDLKDPK--
---------------------LKMAFASNDIME--AVNRCSTYGNVL---NRSDP--EYGIQ---RDQI--------TI
MKDRINQPLSNWKN-SPNKWVDP----MIYRTERSVNRDPDAYRYKWSNGSKYLQ---EHFV
SEQKNTFKAVQVVFDRLRIYKYLIDRSEAIDKEYLSQSLRFFLSK------------------------------S
LPFFLMSIRNIPIHKSE-IHIY-D---PNDPLCNQLLELIGVQIVHLKK-------------------------------F
LFKKIP-KR-MIDSFQF--HT-IKKSLDNTNSCFSMISHDQNNWLNPVKPFHRSSLISSFYKAN
RLRFLNDPPHFWFY--CNKRLPFDV----EKTRINNYD--LTY-GQFLNILSIHNP-IFYLCVDK--K
KDLFLE-REAISPIESQVSDIFIPKDFH---DET-YNLYK---SFHFPIRSDPFVR--RAIYSIADISTT
PITEEQ------IVNRPLSDRNLSDSEGNRLHQYLA--FNSNKGLT--PCSDKPLP--SGKRKK-------
-RSIRLK--KCVEKGQMRRTFQRDSAFYNIFKWNLVQTYMPWFLTSTGCRYLHLTLFDTLS
DPL--PILSSS-QKFVSIFHDMMHRSDVSRPIPQK--------SLPQWTLLISEISSQCLQN--LILSEE
RIHRNN---VPLIWAHLGSINVQEF-LYSIFFLLLVAGY-LVRMHLLVVFR--ASSELQTELEKIK
SLMIPSYMVEFRKLLDRYPTSELNSFWLKNLLMVVLERLGYFLEEICG-----------GP-----PY
GGVKSICSQKIYWKIN---LIHIV-SILPNPINPIIVSINTRHLSRTSKEIYSSIIK-KNGNGDRVDY
EIEFWLANSDLIDDEEREFLVQFS--ALTS--ERRIDQILWNLT-HSDHLS-----KNDSGYQMIEQ
PGSISLRYLVDIH--KKNLMNYEFNQSCLAERRIFLAHYQTIAS--SQTSCGTNSFHSPST--NG
KPFSLRLALSPY-KGILVIGSIGTGRSCLVKCLATNSYVPFITVFTNEFLDYKPKGYPIDD------
-----SD-------------------------------DIHIDMELL---------TMYMT---PKRDRFDITLQFELAKATTP
CIIWIPNIHDLHVNESNYLS-----NSLS---KDCERCS-AGKILVIASTHIPQKVDPALIAPNKLNT
CIKIRRLLIPQQRKHFFILSYTRGFRLEKRMFRA--NGFGPITMGSNAQDLITLINEALSISITQ
KKSIIETHTIRLAIHKKTWDFR---SQIRWVQDHGILFYQIGRAVAQNVLLSNCPLDPISIYMK
KK--LCQVGDSYLYKWYFELGTSMKKFTILLYLLSCSAGSVAQDLWSSY--RPNEN-KRITYY
GFVENDSSYLVHGLFEVEGAP-------AEKDCSQFDNN--LMTLLLRSEPRCGALDMTQ-----IV
DQRFLYEK--YESEVEE-G----ALDPQ----QMED-----LFNH--IVWAP--RIWRP------------------S-
YWTGCKR-------IYHKEDELQENDSELLQSGTAQYQTRDGSSKEQSFFIPSQFIWDPADSFFS
----DQPFASVFSRRAFFAYEEMS---LASQTEPPTSIYKRWFI--KNTQEKHFD---HRQRWFIN---
NR--S--NGSFRSN--ALYESYQYLSNLFLSN-GRLVDQMTRALLRKRWLFPDEMNI-------------
-???
??
??
??
????
>Hevea
MEKWRFNSILSLSKSMDSFSSLENTSISEDPILNDTDKNTYSYSNVDHLVGVRDIQNFSAD

ETFLVRDSNRDSYSIYFDIENKVFEIDNDH--SFLSELKSSFYSYWNSSYLNNGFRSGDSHYD
HYMYDTKYSWNNYINSCIDSYLR--SQICIDSYILSGSNNYSESYIYSYICGSDSSSLRTSTSG
SDLIIRENSN-LDITQKYKHLWVQCENCYGLNYKKFFKSRMNICEQCGYHLKMSSSDRIEL
LIDLGTWDPMDEDMVSLDPIEFHSEEEPYKDRIDSYQRKTGLTEAVQTGTGQLNGIPVAIG
VMDFQFMGGSMGSVVGEKITRLIEYATNKFLPLILVCASGGARMQEGSLSLMQMAKISSA
LYDYQSNKKLFYVSILTSPTTGGVTASFGMLGDIIIAEPNAYIAFAGKRVIEQTLNKTVPEGS
QSAEFLFHKGLFDLIVPRNLLKGVLNELLQLHDFFPLNHKSSRMVTIRADEISNIIRERIEQY
NREVKIVNTGTVLQVGDGIARIYGLDEVMAGELVEFEEGTIGIALNLESNNVGVVLMGDG
LMIQEGSSVKATGRIAQIPVSEAYLGRVINALAKPIDGRGEISASESRLIESPAPGIISRRSVYE
PLQTGLIAIDSMIPIGRGQRELIIGDRQTGKTAVATDTILNQQGQNVICVYVAIGQKASSVAQ
VVTTLQERGAMEYTIVVAETADSPATLQYLAPYTGAALAEYFMYRERHTLIIYDDLSKQA
QAYRQMSLLLRRPPGREAYPGDVFYLHSRLLERAAKLSSRLGEGSMTALPIVETQSGDVS
AYIPTNVISITDGQIFLSADLFNAGIRPAINVGISVSRVGSAAQIKAMKQVAGKLKLELAQFA
ELEAFAQFASDLDKATQNQLARGQRLRELLKQSQSAPLTVEEQIMTIYTGTNGYLDSLEIG
QVRKFLVELRTYLKTNKPQFQEIISSTKTFTEEAETLLKEAIQEQKERFVIQEQVMRINPTTS
GPGVSALEKKNLGRIAQIIGPVLDVAFPPGKMPNIYNALVVKGRDTAGQEINVTCEVQQLL
GNNRVRAVAMSATDGLTRGMEVIDTGAPLSVPVGGATLGRIFNVLGEPVDDLGPVDTRAT
SPIHRSAPAFIQLDTKLSIFETGIKVVDLLAPYRRGGKIGLFGGAGVGKTVLIMELINNIAKA
HGGVSVFGGVGERTREGNDLYMEMKESGVINEENIAESKVALVYGQMNEPPGARMRVGL
TALTMAEYFRDVNEQDVLLFIDNIFRFVQAGSEVSALLGRMPSAVGYQPTLSTEMGSLQE
RITSTKEGSITSIQAVYVPADDLTDPAPATTFAHLDATTVLSRGLAAKGIYPAVDPLDSTSTM
LQPQIVGEEHYETAQRVKQTLQRYKELQDIIAILGLDELSEEDRLTVARARKIERFLSQPFFV
AEVFTGSRGKYVGLAETIRGFKLILSGELDSLPEQAFYLVGNIDEATAKATNLEMENNLKK
MTLNLCVLTPNRIVWDSEVKEIILSTNSGQIGVLPNHAPIATAVDIGILRIRLNDQWLTMAL
MGGFARIGNNEITVLVNDAEKGSDIDPQEAQQTLEIAEANLRKAEGRRQIIEANLALRRAR
ARVEAINGISMKNVTDSFVSLGHWPSAGSFGFNTDILATNLINLSVVLGVLIFFGKGVLSDL
LDNRKQRILDTIRNSEKLREGAIEQLEKARARLRKVEIEADQFRTNGYSEIEREKWNLINST
YKTLEQLENYKNETIHFEQQRTINQVRQRVFQQALQGALGTLNSCLTNELHLRTINANLG
MFGAIKEITDMNPLISAASVIAAGLAVGLASIGPGVGQGTAAGQAVEGIARQPEAEGKIRG
TLLLSLAFMEALTIYGLVVALALLFANPFVMNVLSCSINTLKGLYDISGVEVGQHFYWKIG
GFQVHAQVLITSWVVIAILLGSAIVAVRNPQTIPTGGQNFFEYVLEFIRDVSKTQIGEEYGP
WVPFIGTMFLFIFVSNWAGALLPWKIIQLPHGELAAPTNDINTTVALALLTSIAYFYAGLSK
KGLGYFSKYIQPTPILLPINILEDFTKPLSLSFRLFGNILADELVVVVLVSLVPSVVPIPVMFL
GLFTSGIQALIFATLAAAYIGESMEGHHMIFSTLEHILTHISFSVVSIVITIHLITLLVDEFVELY
DSSEKGMITTFFCITGLLVIRWIFLGHLPLSDLYESLIFLSWAFSIIHMVPYFKKHKNFLSAIT
APSTFFTQGFATSGLLTDMHQSEILVPALQSQWLMMHVSMMILGYAALLCGSLFSVAFLVI
TFRKIIRIVD--KEIQYM--AENVLRNISFLSSRNYYRFQLIQQLDDWGCRIISIGFIFLTIGILSG
AVWANEAWGSYWNWDPKETWAFITWTIFAIFFHIRTNKKLEGFNSAIVASIGFLLIWICYFG
VNLLGIGLHSYGSFTLTKWKKKTFIPLLYLTSIVFLPWWISFLFNKSFESWVINWCNISKSET
FLNDIQEKSILEKFIELEELVRLDEMIKEYPETHLQKFRIG-HKETIQLIKMHNEDRIHTIFHF
STNIISFVILRGYSILSNEKLIILNSWVQEFLYNLSDTIKAFSILLLTDLCIGFHSPHGWELMIG
SVYKDFGFAHNDQIISGLVSTFPVILDTIFKYWIFRYLNRVSPSLVVIYHSMNDMPIGVPKVP
FRNPGEDDSIWIDVN-RLYRERLLFLGQDVDSEISNQLIGLMVYLSIESETKDLYLFINSPGG
WVIPGIAIYDTMQFVRPDVQTVCMGLAASMGSFILVGGKITKRLAFPHARVMIHQPIAGFY

EAQIGEFVLEAEELLKLREILTRIYAQRTGKPLWVVSEDMERDVFMSATEAQAHGIVDLVA
VA???MEE--R
YLELDRSRKNDFLYPFIFREYIYTFAHDHSLVGYDNKSSFLIVKRLITRMYQQNHLIISAND
SNQNPFFRKNLYYQMISEGFAVIVEIPFSLRLVSSLIVKSHKLRSIHSIFPLLEDKFPHLNYVS
DILIPYPIHLEKLVQTLRYWVKDPSSLHLLRLFLHEYWNLNSLIIKSIIIFIKPRFFLFLYNSHV
YEYESIFFFLRNQSFHLRSIFLRVLLERIFFYGKIEHFAEVFANQATLWLFKDPFMHYVRYQ
GKSILASKNRPFLMKKWKYYLVNLCQYHFYVWFQPEKIYINSLSKHSLNFLGYLSSVQLN
PSVVRSQMLEYSFIIDN--TIKKLDTIVPIIPLIGSLAKTKFCNAVGHPISKPIRADSSDSDILDR
FVRICRNLSHYYSGSSKKKSLYRIKYILRLSCVKTLARKHKSTVRAFLKRLGSELLEEFFTE
EEQILSLIFPKRVWYLDIISINDLANHEMIIDTT-EIQ-AINSFSRLES-LNEVYGIIWVFVPIFIL
VLGITIGILVIVWLEREISAGIQQRIGPEYAGPLGVLQALADGTKLLFKENLFPSRGDIRLFSI
GPSIAVISTLLSYSVIPFGYHLVLTDLNIGVFLWIAISSIAPIGLLMSGYGSNNKYSFLGGLRA
AAQSISYEIPLTLCVLSISLLSNSSSTVDIVEAQSKSGFWGWNLWRQPIGFIIFFISSLAECER
LPFDLPEAEEELVAGYQTEYSGIKFGLFYIASYLNLLVSSLFVTVLYLGGWNISIPYIFVPELF
--EKIGGVFGTTIGIFITLVKTYLFLFIPITTRWTLPRLRMDQLLNLGWKFLLPISLGNLLLTTS
FQLLSLMIWHVQNENFILDSTRIFMKAFHLLLFDGSFIFPECILIFGLILLLMIDSTSDQKDIP
WLYFISSTSLVMSITALLFRWREEPMISFSGNFQTNNFNEIFQFLILLCSTLCIPLSVEYIECTE
MAITEFLLFVLTATLGGMFLCGANDLITIFVAPECFSLCSYLLSGYTKKDVRSNEATTKYLL
MGGASSSILVHAFSWLYGSSGGEIELQEIVNGLINTQMYNSPGISIALIFITVGIGFKLSLAPS
HQWTPDVYEGSPTPVVAFLSVTSKVAASASATRIFDIPFYFSSNEWHLLLEILAILSMIVGNL
IAITQTSMKRMLAYSSIGQIGYVIIGIIVGDSNGGYASMITYMLFYISMNLGTFACIVLFGLR
TGTDNIRDYAGLYTKDPFLALSLALCLLSLGGLPPLAGFFGKLHLFWCGWQAGLYFLVLIG
LLTSVVSIYYYLKIIKLLMTGRNQEITPHVRNYRRSPLRSNNSIELSMIVCVIASTIPGISMNP
IVEIAQDTLFMFLIYEYDIFWAFLIISSVIPILAFLISGVLSPISKGPEKLSSYESGIEPMGDAW
LQFRIRYYMFALVFVVFDVETVFLYPWAMSFDILGLSVFIEAFIFVLILIVGSVYAWRKGAL
EWSMNSFPWLTIFVVLPISAGSLIFLFPHRGNKVIKWYTIFICAFELLLMNYVFSYYFQLDD
PLIQLTEDYKWIQFFDFYWRLGIDGFSLGPILLTGFITTLATLAARPITRDSRLFHFLMLAMY
SGQIGLFSSQDLLLFFIMWELELIPVYLLLSMWGGKKRLYSATKFILYTAGGSVFLLMGAL
GIALYGSNEPRFHFETSANQSYPVALEIFFYIGFLIAFAVKSPIIPLHTWLPDTHGEAHYSTC
MLLAGILLKMGAYGLVRINMELLSHAHSIFSPWLIIVGVMQIIYAASTSPGQRNLKKRIAYS
SVSHMGFIIIGICSISDMGLNGAILQIISHGFIGAALFFLAGTGYDRIRRVYLDEMGGMATSM
PKIFTTFSILSLASLALPGMSGFFAELIVFFGIITGQKYLLMSKILITFVMAVGMILTPIYLLSM
LRQMFYGYKLFNAPNSSFFDSGPRELFVSISILLPVIGIGIYPDFVFSLSVDRVEAILSNYFYR
MMLEHVLVLSAYLFSIGIYGLITSRNMVRALMCLELILNAVNLNFVTFSDFFDSRQLKGNIF
FHFVIAIAAAEAAIGPAIVSSIYRNRKSTRINQSNLLNKMEHIYQYSWIISFVTLPVPMLIGV
GLLLFPAATKKLRRMWAFPSVFLLSIVMIFSIDLSIQQINSSFIYQYIWSWTINNDFSLEFGHL
IDPLTSILSVLITTVGILVLFYSDNYMSHDQGYLRFFAYMSFFTTSMLGLVTSSNLIQIYIFWE
LVGVCSYLLIGFWFTRPIASNACQKAFVTNRVGDFGLLLGILGLYWITGSFEFRDLFKIFNN
-QVNFLFVTLCAFLLFSGAIAKSAQFPLHVWLPDAMEGPTPISALIHAATMVAAGIFLVARL
FPLFVIIPFIMNLIALIGIITVFLGVTLALAQKDIKRSLAYSTMSQLGYTMLALGMGSYRAAL
FHLITHAYSKALLFLGSGSIIHSMEAILGYSPDKSQNMVLMGGLTKHIPITKTAFLLGTLSLC
GIPPFACFWSKDEILNDSWLYSPIFAIIACFTAGLTAFYMFRVYLLTFDGHFNAHFKNYSGK
KNSSFYSISLWGKEGSKMLNK-LLALLTMNFFGNTKR-IFSYPHESDNTMLFPMLILVLFTL
FIGAIGNQFDIDILSKLLTPSLNLLHQNPE-SVDW-EFVTNAIFSVSIAFFGIFIASFLYKPVYSS

LLNFNFFNSFAIFWDKIINVIYNWSSNRGYIDAFYAISFIGGIRKLVELIHFFDKRIIDGITNGV
GVTSFFVGEGIKYVGSG-RISSYLLFYLFYALIFLLI--MDLPGLIHDFLLVFLGLGLILGGLGV
VLLTNPIYSAFSLGLVLVCISLFYILSNSHFVAAAQLLIYVGAINVLIIFAVMFMNGSEYYKD
FNLWTVGSGVTSLVCTSIFVSLITILPDTSWYGIIWTTRTNQIIEQDLISNGQQIGIHLSTDFFL
PFEFISIILLVALIGAIAVARQMNVPATRKDLMIVNMGPHHPSMHGVLRLIVTLDGEDVIDCE
PILGYLHRGMEKIAENRTIIQYLPYVTRWDYLATMFTEAITVNGPELLGNIQVPKRAGYIRII
MLELSRIASHLLWLGPFMADIGAQTPFFYIFREREELVYDLFEAATGMRMMHNFFRIGGVAS
DLPHGWIDKCLDFCDYFLTGVTEYQKLITRNPIFLERVEGVGIVGTEEAKNWGLSGPMLR
ASGVQWDLRKVDHYECYDEFDWEIQWQKEGDSLARYLVRIGEMMESIKIIQQALEGIPG
GPYENLEIRRFDRERDSEWNDFEYRFISKKTSPTFELPKQELYVRVEAPKGELGIFLIGDQS
GFPWRWKIRPPGFINLQILPELVKRMKLADIMTILGSIDIIMGEVDRMFPMVTGFMNYGQQ
TIRAARYIGQGFMITLSHANRLPVTIQYPYEKLITSERFRGRIHFEFDKCIACEVCVRVCPID
LPVVDWKLETDIRKKRLLNYSIDFGICIFCGNCVEYCPTNCLSMTEEYELSTYDRHELNYN
QIALGRLPMSVVDDYTIRTILNSQKK-------------MQGRLSAWLVKHGLVHRFLGFDYQGIE
TLQIKPEDWHSIAVILYVYGYNYLRSQCAYDVAPGGLLASVYHLTRIEYDIDQPEEVCIKVF
APRKNPRIPSVFWVWKSADFQERESYDMLGIFYDNHPRLKRILMPESWIGWPLRKDYIVP
NFYEIQDAH---MNSIEFPLLDRTTQISVISTTSN
DLSNWSRLSSLWPLLYGTSCCFIEFASLIGSRFDFDRYGLVPRSSPRQADLILTAGTVTMKM
APSLVRLYEQMPEPKYVIAMGACTITGGMFSTDSYSTVRGVDKLIPVDVYLPGCPPKPEAV
IDAITKLRKKISREIYEDRIRS--GKRCFTTNHKFNIERTTHTGNYDQELLYQSPSTSKIPPETF
FKYKRSVSSNELVNMQTRKTFSWIKEEITRSISVSLMIYIITWASISNAYPIFAQQGYENPRE
ATGRIVCANCHLANKPVDIEVPQAVLPDTVFEAVVRIPYNMQLKQVLANGKKGALNVGA
VLILPEGFELAPPDRISPEMKEKMGNLSFQSYRPTKKNILVIGPVPGQKYSEITFPILSPDPAA
KKDVHFLKYPIYVGGNRGRGQIYPDGSKSNNTVYNAIAAGIVSKIIRKEKGGYEITITNAS
EGRQVIDIIPPGPELLVSEGESIKLDQPLTSNPNVGGFGQGDAEIVLQDPLRVQGLLFFLASV
ILAQIFLVLKKKQFEKVQLSEMNFMSKVYDWFEERLEIQAIADDITSKYVPPHVNIFYCLG
GITLTCFLVQVATGFAMTFYYRPTVTEAFASVQYIMTEANFGWLIRSVHRWSASMMVLM
MILHVFRVYLTGGFKKPRELTWVTGVVLAVLTASFGVTGYSLPWDQIGYWAVKIVTGVPE
AIPVIGSPLVELLRGSASVGQSTLTRFYSLHTFVLPLLTAVFMLMHFSMIRKQGISGPLMGV
TKKPDLNDPVLRAKLAKGMGHNYYGEPAWPNDLLYIFPVVILGTIACNVGLAVLEPSMIG
EPADPFATPLEILPEWYFFPVFQILRTVPNKLLGVLLMVSVPAGLLTVPFLENVNKFQNPFR
RPVATTVFLIGTAVALWLGIGATLPIDKSLTLGLFMIEVFLFGIVLGLIPITLAGLFVTAYLQY
RRGDQLDLMPTITSYFGFLLAALTITSVLFIGLSKIRLIMDIVSLAWAALMVVFTFSLSLVV
WGRSGLMIIRSPEPEVKILVDRDPIKTSFEEWARPGHFSRTIAKGPDTTTWIWNLHADAHD
FDSHTSDLEEISRKVFSAHFGQLSIIFLWLSGMYFHGARFSNYEAWLSDPTHIGPSAQVVW
PIVGQEILNGDVGGGFRGIQITSGFFQIWRASGITSELQLYCTAIGALVFAALMLFAGWFHY
HKAAPKLAWFQDVESMLNHHLAGLLGLGSLSWAGHQVHVSLPINQFLNAGVDPKEIPLP
HEFILNRDLLAQLYPSFAEGATPFFTLNWSKYSDFLTFRGGLDPVTGGLWLTDTAHHHLAI
AILFLIAGHMYRTNWAIGHGIKEILEAHKGPFTGQGHKGLYEILTTSWHAQLSLNLAMLGS
LTIVVAHHMYSMPPYPYLATDYGTQLSLFTHHMWIGGFLIVGAAAHAAIFMVRDYDPTTR
YNDLLDRVLRHRDAIISHLNWVCIFLGFHSFGLYIHNDTMSALGRPQDMFSDIAIQLQPVF
AQWIQNTHALAPGATAPGATASTSLTWGGGDLVAVGGKVALLPIPLGTADFLVHHIHAFTI
HVTVLILLKGVLFARSSRLIPDKANLGFRFPCDGPGRGGTCQVSAWDHVFLGLFWMYNSI
SVVIFHFSWKMQSDVWGSISDQGVVTHITGGNFAQSSITINGWLRDFLWAQASQVIQSYG

SSLSAYGLFFLGAHFVWAFSLMFLFSGRGYWQELIESIVWAHNKLKVAPATQPRALSIIQGR
AVGVTHYLLGGIATTWAFFLARIIAVGMALRFPRFSQGLAQDPTTRRIWFGIATAHDFESHD
DITEERLYQNIFASHFGQLAIIFLWTSGNLFHVAWQGNFEAWVQDPLHVRPIAHAIWDPHF
GQPAVEAFTRGGAPGPVNIAYSGVYQWWYTIGLRTNEDLYIGALFLLFLSALALLGGWLH
LQPKWKPSVSWFKNAESRLNHHLSGLFGVSSLAWTGHLVHVAIPGSRGEYVRWNNFLDV
LPHPQGLGPLFTGQWNLYAQNPDSGSHLFGTSQGAGTAILTLLGGFHPQTQSLWLTDIAHH
HLAIAVIFLVAGHMYRTNFGIGHSIKDLLEAHIPPGGRLGRGHKGLYDTINNSLHFQLGLAL
ASLGVITSLVAQHMYSLPAYAFIAQDFTTQAALYTHHQYIAGFIMTGAFAHGAIFFIRDYNP
EQNENNVLARMLDHKEAIISHLSWASLFLGFHTLGLYVHNDVMLAFGTPEKQILIEPIFAQ
WIQSAHGKTSYGFDVLLSSTNSPAFNAGRSIWLPGWLNAINE--NSNSLFLTIGPGDFLVHH
AIALGLHTTTLILVKGALDARGSKLMPDKKDFGYSFPCDGPGRGGTCDISAWDAFYLAVF
WMLNTIGWVTFYWHWKHITLWQGNVSQFNESSTYLMGWLRDYLWLNSSQLINGYNPF
GMNSLSVWAWMFLFGHLVWATGFMFLISWRGYWQELIETLAWAHERTPLANLIRWRDKP
VALSIVQARLVGLAHFSVGYIFTYAAFLIASTSGKFGMSHSVKIYDTCIGCTQCVRACPTDV
LEMIPWDGCKSKQIASAPRTEDCVGCKRCESACPTDFLSVRVYLWHETTRSMGLAYMTIL
NSPSILVPLVGLVFPAIAMASLFLYIQKNKIFMRDLKTYLSVAPVISTLWFGSLAGLLIEINRF
FPDALTFPFFSFMTAILERRESESLWGRFCNWITSTENRLYIGWFGVLMIPTLLTATSVFIIAFI
AAPPVDIDGIREPVSGSLLYGNNIISGAIIPTSAAIGLHFYPIWEAASVDEWLYNGGPYELIV
LHFLLGVACYMGREWELSFRLGMRPWIAVAYSAPVAAATAVFLIYPIGQGSFSDGMPLGIS
GTFNFMIVFQAEHNILMHPFHMLGVAGVFGGSLFSAMHGSLVTSSLIRETTENESANEGYR
FGQEEETYNIVAAHGYFGRLIFQYASFNNSRSLHFFLAAWPVVGIWFTALGISTMAFNLNG
FNFNQSVVDSQGRVINTWADIINRANLGMEVMHERNAHNFPLDLAAVEVPSTNGMGLPW
YRVHTVVLNDPGRLLSVHIMHTALVAGWAGSMALYELAVFDPSDPVLDPMWRQGMFVIP
FMTRLGITNSWGGWSIT-GGTITNPGIWSYEGVAGAHIVFSGLCFLAAIWHWVYWDLEIFC
DERTGKPSLDLPKIFGIHLFLSGVACFGFGAFHVTGLYGPGIWVSDPYGLTGKVQPVSPAW
GVEGFDPFVPGGIASHHIAAGTLGILAGLFHLSVRPPQRLYKGLRMGNIETVLSSSIAAVFF
AAFVVAGTMWYGSATTPIELFGPTRYQWDQGYFQQEIYRRVSAGLAENQSLSEAWSKIPE
KLAFYDYIGNNPAKGGLFRAGSMDNGDGIAVGWLGHPIFRDKEGRELFVRRMPTFFETFP
VVLVDGDGIVRADVPFRRAESKYSVEQVGVTVEFYGGELNGVSYSDPATVKKYARRAQL
GEIFELDRATLKSDGVFRSSPRGWFTFGHASFALLFFFGHIWHGARTLFRDVFAGIDPDLD
AQVEFGAFQKLGDPTTRRQVV------------VETLFNGTLSLAGRDQETTGFAWWAGNARLIN
LSGKLLGAHVAHAGLIVFWAGAMNLFEVAHFVPEKPMYEQGLILLPHLATLGWGVGPGG
EVIDTFPYFVSGVLHLISSAVLGFGGIYHALLGPEILEESFPFFGYVWKDRNKMTTILGIHLI
LLGIGAFLLVFKALYFGGVYDTWAPGGGDVRKITNLTLSPSVIFGYLLKSPFGGEGWIVSV
DDLEDIIGGHVWLGSICILGGIWHILTKPFAWARRALVWSGEAYLSYSLGALSVFGFIACCF
VWFNNTAYPSEFYGPTGPEASQAQAFTFLVRDQRLGANVGSAQGPTGLGKYLMRSPTGE
VIFGGETMRFWDLRAPWLEPLRGPNGLDLSRLKKDIQPWQERRSAEYMTHAPLGSLNSV
GGVATEINAVNYVSPRSWLATSHFVLGFFLFVGHLWHAGRARAAAAGFEKGIDRDFEPVL
SMTPLNMTIALGKFTKDENDLFDIMDDWLRRDRFVFVGWSGLLLFPCAYFAVGGWFTGT
TFVTSWYTHGLASSYLEGCNFLTAAVSTPANSLAHSLLLLWGPEAQGDFTRWCQLGGLWT
FVALHGAFGLIGFMLRQFELARSVQLRPYNAIAFSAPIAVFVSVFLIYPLGQSGWFFAPSFG
VAAIFRFILFFQGFHNWTLNPFHMMGVAGVLGAALLCAIHGATVENTLFEDGDGANTFRA
FNPTQAEETYSMVTANRFWSQIFGVAFSNKRWLHFFMLFVPVTGLWMSALGVVGLALNL
RAYDFVSQEIRAAEDPEFETFYTKNILLNEGIRAWMAAQDQPHENLIFPEEVLPRGNALMS

GSTGERSFADIITSIRYWVIHSITIPSLFIAGWLFVSTGLAYDVFGSPRPNEYFTESRQGIPLIT
GRFDPLEQLDEFSKSFMTIDRTYPIFTVRWLAVHGLAVPTVSFLGSISAMQFIQRMATQSVE
GSSRSG-PRRTIVGDLLKPLNSEYGKVAPGWGTTPLMGVAMALFAIFLSIILEIYNSSVLLDG
ISMNMLTLKLFVYTVVIFFVSLFIFGFLSNDPGRNPGREEMADTTGRIPLWIVGTVTGILVIG
LIGIFFYGSYSGLGSSLMLNIFSLIFICFNSAIF-SNFFFTKLPEAYAFLNPIVDVMPVIPVLFFL
LAFVWQAAVSFRMTQSNPNEQNVELNRTSLYWGLLLIFVLAVLFSNYFFNMEVNILAFIAT
ALFILVPTAFLLIIYVKTVSQGDMETATLVAISISGLLVSFTGYALYTAFGQPSQQLRDPFEEH
GDMEALVYTFLLVSTLGIIFFAIFFREPPKVPTKKVKMTIAFQLAVFALIATSSILLISVPVVFS
SPDGWSSNKNVVFSGTSLWIGLVFLVGILNSLIS-------MSPQTETKASVGFKAGVKDYKLT
YYTPEYQTKDTDILAAFRVTPQPGVPPEEAGAAVAAESSTGTWTTVWTDGLTSLDRYKGR
CYGIEPVPGEENQYIAYVAYPLDLFEEGSVTNMFTSIVGNVFGFKALRALRLEDLRIPPAYS
KTFQGPPHGIQVERDKLNKYGRPLLGCTIKPKLGLSAKNYGRAVYECLRGGLDFTKDDEN
VNSQPFMRWRDRFLFCAEAIYKAQAETGEIKGHYLNATAGTCEEMIKRAVFARELGVPIV
MHDYLTGGFTANTSLAHYCRDNGLLLHIHRAMHAVIDRQKNHGMHFRVLAKALRLSGG
DHIHAGTVVGKLEGERDITLGFVDLLRDDFIEKDRSRGIYFTQDWVSLPGVIPVASGGIHV
WHMPALTEIFGDDSVLQFGGGTLGHPWGNAPGAVANRVALEACVQARNEGRDLAREGN
DIIREASKWSPELAAACEVWKEIKFEFEAVDTL--MAIHLYKTSTPSTRNGAVDSQAKSNTR
NNLIYGQHRCGKGRNARGIITARHRGGGHKRLYRKIDFRRNEKDIYGRIVTIEYDPNRNAY
ICLIHYGDGEKRYILHPRGAIIGDTVISGTEVPIKMGNALPLTDMPLGTAIHNIEITLGKGGQ
LARAAGAVAKLIAKEGKSATLKLPSGEVRLISKNCSATVGQVGNTGVNQKNLGRAGSKC
WLGKRPVVRGVVMNPVDHPHGGGEGRAPIGRKKPATPWGYPALGRRSRKRNKYSDNLIL
RRRSKMIQSQTHLNVADNSGARELMCIRIIGTS-NRRYAHIGDVIVAVIKEAAPNSPLERSEV
IRAVIVRTCKELKRDNGMIIRYDDNAAVVIDQEGNPKGTRIFGAIARELRQLNFTKIVSLAP
EVLMLSPKRTRFRKQHRGRMKGIAFRGNRICFGRYALQALEPAWITSRQIEAGRRAMTRN
ARRGGKIWVRIFPDKPVTLRPTETRMGSGKGSPEYWVAVVKPGRILYEMGGVAENIARKA
ISIAASKMPIRTQFIISGMTRIRRGYIARRRRTKIRLFASSFRGAHSRLTRTIIQQKIRALVSAH
RDRDRQKRNFRRLWVTRINAVIRE-SMVSYSYSRLINNLYKRQLLLNRKILAQIAILNRNCL
YMISNDILK-----------MINEVYALGQHICMSPHKARRIIDQIRGRSYEETLMILELMPYRACY
PLFKLIYSAAANASHNMGFNEVNLIISKAEVNEGTTVKKLKPQARGRGYLIKRSTCHITIIL
KNISL--------MDGIKYAVFTDKSIRLLVKN-QYTFNVESGSTRTEIKHWVELFFGVKVIAMNS
HRLPGKGRRMRPIMGHTMHYRRMIITLQPGYSIPPLRKKRTMAVPKKRTSISKKLIRKNIW
KRKGYWTVLKAFSLAKSLSTGNSKSFFVQQIKDMAKGKDIRIRVILECTTC-ARN--VNKK-
-GISRYITQKNRHNTPSRLELRKFCRYCYKHTIHGEIKKMKIRASVRKICEKCRLIRRRGRII
VICLNPRHKQRQGMVREKVTISTRTLQWKCVESRTDNKRLYYGRFILSPLMKGQADTIGI
AMRRALLGEIEGTCITRAKSE--KIPHEFSTIAGIQESIHEILMNLKEIVLRSNLYGTCNASICV
EGPGYVTAQDIILPPFVEIIDNTQHIASLTEAIDLCIGLQIERNRGYRINPTNNFQDGSYS--ID
AVFMPVRNANHSVHSYGNGNEKQEILFLEIWTNGSLTPKEALHEASRNLIDFFIPFLHAEEE
NLHLE--KNQ-HKVTLPLFTFHDRLT-KLRKNKK-EIALKYIFIDQSELTPKIYNCLKRSNIHTL
SDLLKKSQEDLMKIEHFRIDDVKHILGILEIEKHFAIDLPKNKMLGDGN-GGMSTIPGFNQI
QFEGFCRFIDQGLTEELYKFPKIEDTDQEIEFQLFVETYQLVEPLIKERDAVYESITYSSELYV
SAGLIWKTSRDMQEQTIFIGNIPLMNSLGTFIINGIYRIVINQILQSPGIYYRSELDHNGISVY
TGTIISDWGGRVELEIDRKARIWARVSRKQKISILVLSSAMGLNLREILENVCYPEIFLSFLN
DKEKKKIGSKENAILEFYQQFTCVGGDPIFSESLCKELQKKFFQQRCELGKIGRLNMNRRL
NLDIPHNNTFLLPRDILAAADRLIEMKFGMGTLDD--MNHLKNKRIRSVADLLQDQFGLAL

IRLENVVRGTICGAIR-HKLI-PTPQNLVTSTP----LTTTYESFFGLHPLSQVLDRTNPLTQIVH
GRKLSYLGPGGLTGRTASFRIRDIHPSHYGRICPIDTSEGINVGLIGSLAIHAKIGHWG-SLES
PFY---EISEGSK--KVRMFYLSPNREEYYMVAAGNYLALNRGVQEKQVAPARYRQEFLTIA
WEQVHLRSIFPFQYFSIGASLIPFIEHNDANRALMSSNMQRQAVPLSRSEKCIVGTGLERQV
ALDSGVPAIAEHEGKIIYTDIDKIILSGNGDTLRIPLVMYQRSNKNTCMHQKTQLRRGKCIK
KGQVLADGAATVGGELALGKNVLVAYMPWEGYNFEDAVLISERLVYEDIYTSFHIRKYEI
QTHVTSQG-PERITNEIPHLEAHLLRNLDKNGIVMLGSWVETGDILVGKLTPQMAKE-SSYA
PEDRLLRAILGIQVSTSKETCLKLPIGGRGRVIDVRWIQKKG-GSSYNPETIRVYILQKREIK
VGDKVAGRHGNKGIISKILPRQDMPYLQDGRPVDMVFNPLGVPSRMNVGQIFECSLGLTG
DLLDRHYRIAPFDERYEQEASRKLVFSELYEASKQTANPWVFEPEYPGKSRIFDGRTGDPF
EQPVIIGKPYILKLIHQVDDKIHGRSSGHYALVTQQPLRGRAKQGGQRVGEMEVWALEGF
GVSHILQEMLTYKSDHIRARQEVLGTTIIGGTIPKPEDAPESFRLLVRELRSLALELNHFLVS
EKNFQINRKEA-------MIDRYKHQQLRIGSVSPQQISAWANKILPNGEIVGEVTKPYTFHYKT
NKPEKDGLFCERIFGPIKSGICACGNYRVIRNEKEDQKFCEQCGVEFMDSRIRRYQMGYIK
LACPVTHVWYLKRLPSYIANLLDKPLKELEGLVYCDF---FARP-IAKKPTFLRLRGSFEYEI
QSWKYSIPLFFTTQGFDTFRNREISTGAGAIREQLADLDLRIIIDYSLVEWKELGEE-GPTGN
EWEDRKVGRRKDFLVRRVELAKHFIRTNIEPEWMVLCLLPVLPPELRPIIQIDGGKLMSSDI
NELYRRVIYRNNTLIDLLTTSRSTPGELVMCQEKLVQEAVDTLLDNGIRGQPMRDGHNKV
YKSFSDVIEGKEGRFRETMLGKRVDYSGRSVIVVGPSLSLHRCGLPREIAIELFQIFVIRGLI
RQHLASNIGVAKSKIREKEPIVWEILHEVMQGHPVLLNRAPTLHRLGIQAFQPILVEGRAIC
LHPLVCKGFNADFDGDQMAVHVPLSLEAQAEARLLMFSHMNLLSPAIGDPISVPTQDMLI
GLYVLTSGNCRGICANRYNPCNRRN---YQNKRIDG--DKYTK--EPFFSNSYDALGAYRQKR
IHLDSPLWLRWQLDQR-AITS--REAPIEVHYESLGTYHEIYEHYLIVKNIKKEILCIYIRTTVG
HISLYREIEEAIQGFCQACSDSI--------MEVLMAERANLVFHNKVIDGTAIKRLISRLIDHFG
MAYTSHILDQVKTLGFQQATATSISLGIDDLLTIPSKGWLVQDAEQQSLILEKHYHYGNVH
AIEKLRQSIEIWYATSEYLRQEMNLNFRMTEPFNPVHIMSFSGARGNTSQVHQLVGMRGL
MSDPQGQMIDLPIQSNLREGLSLTEYIISCYGARKGVVDTAVRTSDAGYLTRRLVEVVQHI
VVRRTDCGTTRGISVSPRNGMMS-ERILIQTLIGRVLADNIYMGLRCIAIRNQDIGIGLANRF
IT------FRTQTISIRTPFTCRSTSWICRLCYGRSPTHGDLVELGEAVGIIAGQSIGEPGTQLTLRT
FHTGGVFTGGTAEHVRAPSNGKIKFNEDLVHPIRTRHGHPAFLCYIDLYVTIESQDIIHNVTI
PPKSFLLVQNDQYVESEQVIAEIRARTYTL-NFKEKVRKHIYSDSEGE-MHWSTDVYHAPE
FTYSNVHLLPKTSHLWILSGSSCG--SSIVSFSLHKDQDQMNVH--SLSVKR-RYISSP-SVNN
DQ-VKHKFFSSDFSGK-ESEIPDY-SELNRIICTGHCNLIYPT-ILYENSD--LLAKRRRNKFIIPF
QSIQEREKELMTHSAISIEIPIN--GIFRRNSVFVYFDDPQYRRKSSGITKYGAIGVHSIVKKE
D--LIEYR-GVKEFKPKYTKVDCFFFIPEEVYILPESSSLMVRNNSIIGVDTQIALNTRSRVGG
LVRVERKEKKMELKIFSGDIHFPGETDKISRHSGILMPPGTVKT--NSKESKK--VKNWIYIQR
--ITPTKKK--YFVLVRPVIIYEIANGINLETLFPQDLLQEKDNLKLRVVNYILYGTGKPIRGIS
DTSIQLVRTCLVLNWDQDK---KSSSIEEARAFFVEISTNGLIRDFLRINLVKSHISYIRKRNDP
SGSELIS-NNGSDRTNINPFYPIYSKTR-----IQQSLKQNQGTISTLLNR---NKECKSLIILSSSN
CFRMDP--FNDVKHHN---GIKES-PIIPIRNSLGPLG--TALQISNF--YLFYHFNLITHNQIS-VT
KYSKLDN---LKQTFQVL--K-YYLMDENGR--IVNPDPCSNSVLNPFNLNWYFLHHNYCEER
FTIIS-LGQFICENLCMAKKGP---HLKSGQVIIVH-IDSVVIRSAKPYLATPGATVHGHYGEIL
YEGNTLVTFIYEKSRSGDITQGLPKVEQVLEVRSIDSISINLEKRVEGWNECITRILGIPWGF
LIGTELTIVQSRISLVNKIKKVYRSQGVQIHNRHIEIIVRQITSKVLVSE---DGMSNVFSPGELI

GLLRAERTGRALEEAICYGAILLGITRASLNTQSFISEASFQETTRVLAKAALRGRIDWLKG
LKENVVLGGMIPVGTGFKGLVQGS--RQHKNIPLKTKKKH--LFEGEFRDRDILFHYRELFD
SCISKNFYDTSEQPFIGFNDS------MIRRYWNINLEEMMKAGVHFGHGTRKWNPRMAPYIS
AKRKGIHITNLTRTARFLSEACDLVFDAASRRKQFLIVGTKNKAADSVARAAIRARCHYVN
KKWLGGILTNWSTTETRLQKFRDLRMEQKTGRLNRLPKGDAARLKRQLTHLQTYLGGIK
YMTGLPDIVIIVDQQEEYTALRECMTLGIPTICLIDTNCDPDLADISIPTNDDAIASIRLILNK
LVFAICEGHS-SYIRNPMGQKINPLGFRLGTTQSHHSLWFAQPKNYSEGLQEDQKIRNCIKN
YVKKNAKISSG-IEGIARIEIQKRIDVIQVIIYMGFQKLLIESR----PKRIEELQMNVQKELNCV
NRKLNITITRITNPYGHPTILAEFIAGQLKNRVSFRKAMKKAIELTEQTDTKGIQVQIAGRL
DGKEIARVEWIREGRVPLQTIGAKIDYCSYTVRTIYGVLGIKIWIFPDKKMSRYRGPRFKKI
RRLGALPGLTSKRPKVGSDLRNQSRSGK-KSQYRIRLEEKQKLRFHYGLTERQLLKYVRIA
AKAKGSTGQVLLQLLEMRLDNILFRLGMASTIPRARQLVNHRHILVNGRIVDIPSYRCKPR
DIITARAEQKSRAMIKNYLDSSPQ-EELPKHLTLHPFQ--YKGLVNQIIDSKWVGLKINELLV
VEYYSRQTMSRRGTAEEKTAKSDPIYRNRLVNMLVNRILKHGKKSLAYQIIYRAMKKIQQ
KTETNPLSVLRQAIRGVTPDIAVKARRVGGSTQQVPIEIGSTQGKALAIRWLLGASRKRPG
RNMAFKLSSELVDAAKGSGDAIRKKEETHRMAEANRAFAHFRMGRDPIADIITSIRNADM
NRKGIVRIASTNITENIIKILLREGFIENVRKHKEGNKKFLVLTLRHRR--KRKERTS-----LNL
KRISRPGLRIYSNYQKIPRILGGMGIVILSTSRGIMTDREARLERIGGEILCYIWMAKPLPKI
GSRRNGRIGSRKNSRKIPKGVIHVQASFNNTIVTVTDVRGRVISWSSAGTCGFRGTRRGTP
FAAQTAAGNAIRTVVDQGMQRAEVMIKGPGLGRDAALRAIRRSSILLSFVRDVTPMPHNG
CRPPKKRRVMPTIKQLIRNTRQPIRNVTKSPALGGCPQRRGTCTRVYTITPKKPNSALRKVA
RVRLTSGFEITAYIPGIGHNLQEHSVVLVRGGRVKDLPGVRYHIVRGTLDAVGVKDRQQGR
SKYGVKKPKMARKSLIQREKKRQKLEQKYHLMRRSSKKEIS---KVPSLSDKWEIHGKLQS
PPRNSAPTRLHRRCFSTGKPRANYRDFGLSGHILREMVHACLLPGATRSSWMVKNSFISVI
SQE-E-KDENKGSVEFQIVSFTNKIRRLTSHLELHRKDYLSQRGLRKILGKRQRLLSYLAKK
NRVRYKELIILLDIRESKTR--MVKLRLKRCGRKQRVVYRIVAIDVRSRREGRDLRKVGFYD
PIKNQTYLNVPAILYFLEKGAQPTGTVHDILRKAGVFTELRLNQ-TQFTMDKSKRLFLKSK
RSFRRRLPPIQSGDQIDYRNMSLISRFISEQGKILSRRVNRLSLKQQRLITIAIKQARILSSLPF
LNNEK--QFE-----------RVGHYVTR-LKKNPFVANHLLKKINKLNNKAEKQIIKTWSRTSTIIP
TMIGHTIAIHNGKEHLPIYITDRMVGHKLGEFSPTLNFQGHAKNDNKSHRMIFQSFILGNL
VSLCMKIINSVVVVGLYYGFLTTFSMGPSYLFLLRARVIEEGEEGTEKKVSATTGFITGQLM
MFISIYYAPLHLALGRPHTITVLALPYLLFHFFWNNHKHFFDYGSTTRNSMRNLSIQFVFL
NNLIFQLFNHFILPSSMLVRLVNIYMFRCNNKMLFVTSSFVGWLIGHILFMKWVGLILVWI
QQNNSIRS-FRSNKYLVSELRNSMARIFSILLFITCVYSLGRIPSPIFTKKLKETEED--LVTILF
DSKRWNRPFRYIKNDQFENAVRKEMSQYFFYTCRSDGKERISFTYPPSLSTFLEMIQRKISL
FTTEKLSSDELYNRWNYKNEQKKKILNNEFINRVQALDKEYLTLNTLEKRTRLCNDKTKK
KYLPKIYDPFLSGSCRGK-IQFFFSIEMLWINKIHLILYQEFESKNPKNKSTGIKEISKQVPRW
SYKLIDLEQQEGEEIRSRKAKRVVIFTDTLIHYSQQSDFRRDIIKGSVRAQRRKIAIWELFQA
NVHSPLFLDRIDKSLFFSFDI--SELIK-MFINREEKARIEIAEAWDSILFAQIIRSCVLVTQSILR
KYIILPSLIITKNIIRILFFQTPEWSEDLKDWSREMHVKCTYNGVQLSEKEFPKNWLIDGIQI
KILFPFRLKPWYKSKKVQKNDFFFLTVLGMEAELPFGSPRKRLSLFEPIFKKLEKKIRKMQ
KNGFRV-ITILEERRKLNF--LKEKKNWIIKNIF--FRKEIINNLSKSKDSIINNNTIKKLSIPIRSM
TWTNYSLTEKKMKDLSARRKIIINQIEKITKEKEKNKNKISYNAK--KLKSSISQIVKRRNVR
LARKFHFFIKILIEKIYIDIFL-IINIPRINAQLFIHFISCDISSLSQAYVFYKLSQIKIINLYKLKSI

FEY-SLFLKNEIKDFFAQGLFNSELKNKNFRNSVMNQWKNWLRSHYQYKLSQIRWSRLTP
QKWRNIINQHQHHMVQNKWNLYE--KDRLIHYKKK-NFKKHYKYNLLSYKSINDDIIDAD
RKYFDWRILNFCLRFFDWMGMNEEIKNCWFFRKFVILYNTYKIKPWAIPIQFLLFNNKSH
MEVELDFFLKRYLCFQLRWNG--NHKIINNIKVYCLLLRLTNPREIIISSIQRQEISLNILMVQ
KDLTLTELMKKGILIIEPVRLSVKNDGQFILYQMVGILLVHKNKQNDYDLLVPENILSPKRR
RELRIL--FIFLWPNFRLEDLACMNRYWFDTNNASRFSMVRIYIYPMKGHQFKSWIFELREI
LREIKNSHYFLDSWTQFNSVGSFIHIFFHQERFIKLLDSRIWSILLS-RNS--QGSTSNRYF---TI
KGVVLFVVV-VLIYR----INNRKMVERKNLYLTGLLP-----IPMNSIGPRNDTLEESF-GSSNIN
RLIVS-LLYLPKGKKISESSFLDPKESTWVLPIT----KKCIMSESNWGSRWWRNWIGKKR--D
SSCKISN---ETVAGIEISFKEKDIK-YLEFLFVYYMD--DPIRKDHDWELFDRLSPRKGRNIINL
--NSGQLFEILVKDWICYLMFAFREKIPIEVEG-FFKQQG-AGSTIQSNDIEHVSH--LFSRKKW
AISLQNCAQFHMWQFRQDLFV---SWGN-NPHESDFLSN-ISR---IWLDNVWLVNKDR---FFS
--KARNISSNIQYDS---TR--SSFVQGRNSSQLKGSS-DQSRDHFDSIS----NEDSE------------YH
TLINQRKIQQLKERSIL-WD--PSFLQTERTEIESD--RFPK--CLSGYS--SMSRLFT-EGEKEMN
NHLLPEEIEEF--LGNPTRSIRSFFSDRS--SELY-LGSNPTERSTRDQ----KLLKKEQDVS-FVPS
RRSE----NKEIVNIFKIITYLQN--TVSIHPISSDPGCDMVL-KDELD-MDSSNKISFLNKNPFFD
L--FHLFHDRNGGGYTL--HHDFESE--ERFQEMADLFTLSITEP-DLVYHKGFTFFIDSYGLD
QKQ--FLNEVFNSRDESKKKSL---LVLPPIFY--EENESFYRRIRKKWVR-ISCGNDLEDPKQKI
VVFASNNIMEAVNQYGLILNLIQIQYSTYG--YIRNVLTQFFLMN---RSDRNFEYGIQ---RDQI
GNDTLNHRTIMKYTINQHLSNLKQ-SQKKWFDP--LIFLSRTERSMNWDPNAYRYKWSNGS
KNFQEHLEHFISEQKSRF-LFQVVFDRLRINQYSIDWSEVIDKKDLSKSLRFFLSKLLL-------
--FLS-------KFLLFLSNSLP-FFFVSFGNIPIHRSE-IHIY-ELKGPNDQLCNQLVEPIGLQIVHLK
KLKPFLLLLD---DHDTS-QKSKFLINGGTISPFLFNKIT-KW-MIDSFHTR--NNRRKSFDNTDS
YFSMISHDQDNWLNPVKPFHRSSLISSFYKANRLRFLNNLHHFCFY--CNKRFPF---Y-VEKA
RIKNYD--FTYG-QFLNILFIRNK-IFSLCGGK--KKHAFLE-RDTISPIESQVSNIFIPNDFPQSG
NER-YNLYKS---FHFPIRSDPFVR--RAIYSIADISGTPLTEGQIVNFERTYCQPLSDMNLSDSE
GKNLHQYLN--FNSNMGLIHTPCSEKYLPSEK--RKK--------RSLCLK--KCVEKGQMYRTFQ
RDNAFSTLSKWNLFQTYMPWFLTSTGYKYLNLIFLDTFSD-LLPILSSS--QKFVSIFHDIMH
GSDISWRILQKKL------CLPQWN-LISEISSKCLHN--LLLSEEMIHRNNEP--PLISTHLRSPNV
REF-LYSILFLLLVAGY-LVRTHLFFVSR--AYSELQTEFEKVKSLMIPSYMIELRKLLDRYPTS
ELNSFWLKNLFLVALEQLGDFLEEMRGSASGGNMLWGGGP-----AYG-VKSIRSKKKFFNIN
---LIDLI-SIIPNPINRITFSRNTRHLSHTSKEIYSLIRKRKNVNGDWIDDKIESLVANSDSIDDK
EREFLVQFS--TLTT--EKRIDQILLSLT-HSDHLS-----KNDSGYQMIEEPGAIYLRYLVDIH--KK
YLMNYEFNTPCLVERRIFLAYYQTTTY--SQTSCGVNSFHFPSHG---KPFSLRLALSPS-RGIL
VIGSIGTGRSYLVKYLATNSYLPFITVFLNKFLDNKPKGFLID-----DSDDIDDSDDIDDSDDI
DDSDDIDVSDDIDVS--DDIDR--DFDTELEFLTRMNVL---TMDMMP---EIDRFYITLQFELAK
AMSPCIIWIPNIHDLDVNESNYLSLGLLVNYLS---RDCERCS-TRNILVIASTHIPQKVDPALI
APNKLNTCIKIRRLLIPQQRKHFFTLSYTRGFHLENKMFHT--NGFGSITMGSNVRDLVALTN
EALSISITQKKSIIDTNIIRSALHRQTWDLR---SQVRSVQDHGILFYQIGRAVAQNVFLSNCPI
DPISIYMKKK--SCNEGDSYLYKWYFELGTSMKKLTILLYLLSCSAGSVAQDLWSLP--GPDE
K-NGITYYGLVENDS-DLVHGLLEVEGALVG--SSRTEKDCSQFDND--RVTLLLRPEPRS-PL
DMMQNGSCSILDQRFLYEK--YESEFEE-GEGEEVLDPQ----QIEED----LFNH--IVWAP--RIW
RP---WGFLFDCIERPNELGFP-YWARSFRGK---RIIYDEEDELQENDSEFLQSGTMQYQIRD
RSSKEQGFFRISQFIWDPADPLFFLFK-DQPFVSVFSHREFFADEEMSKGLLTSQTDPPTSIY

KRWFI--KNMQEKHFELLIHRQRWLR---TNSSLS--NGFFRSN--TLSESYQYLSNLFLSN-GTL
LDQMTKALLRKRWLFPDEMKIGF--M---------MPRSR-ITGNFIDKTFSVVANILLRIIPTTSGE
KEAFTYYRDG--MSAQSEGNYAEALQNYYEAMRLEIDPYDRSYILYNIGLIHTSNGEHTKA
LEYYFRSLERNPFLPQALNNMAVICHY-RGEQAIRQGDSEIAEAWFDQAAEYWKQAIALTP
GNYIEAQNWLKITRRFE--MSWRSERIWIELIAGSRKTSNFCWAFILFLGSLGFLLVGISSYL
GRNLISLFPSQQIIFFPQGIVMSFYGIAGLFISSYLWCTILWNVGSGYDRFDRKEGIVCIFRW
GFPGKNRRILLRFLMKDIQSIRIEVKEGIYARRVLYMEIRGRGAIPLTRTDEN-LTPREIEQKV
AELAYFLRVPIEVF
>Jacobaea
MKRWWFNSMLFLSKSTGSLGPIENASESKDPNRNDTEKNIQSYSNVDLFFGVKDIWNFIS
DETFLVKDSNGDSYSIYFDIENHIFEIDNDH--TFSSELESSFY--RNSSYLNKGSKSKNTHHD
RYMDDTQYTWNNHINSCIDSYLQ--SHICLDTYILSGSDNYSNNYISNSICG--NSSKNENAR
R--KTIRKSYTDLDVTQKYRHLWIQCESCFGLNYKKSFQSKMNLCEQCGYHLKMSSSDRIE
LSIDPGTWEPMDEDMVSLDPIQFHSEEEPYKKRIDSYQRKTGLTEAVQTGIGQLNGINVSIA
VMDFQFMGGSMGSVVGEKITRLIEYATKEFLPLIIVCASGGARMQEGSLSLMQMAKISSAL
YDYQSNKKLFYVPILTSPTTGGVTASFGMLGDIIIAEPNAYIAFAGKRVIEQTLNKTVPDGS
QASEYLFQKGLFDLIVPRNPLKSVLSELFQLHTFFPLNQN---MVTIQADEISNIIRERIEQYN
REVKIVNTGTVLQVGDGIARIHGLDEVMAGELVEFQEGTIGIALNLESTNVGVVLMGDGL
LIQEGSSVKATGKIAQIPVSEAYLGRVINALAKPIDGRGEIISSEYRLIESAAPGIISRRSVYEP
LQTGLIAIDSMIPIGRGQRELIIGDRQTGKTAVATDTILNQQGKNVICVYVAIGQKASSVAQV
VTNFQERGAMEYTIVVAETADSPATLQYLAPYTGAALAEYFMYRERHTSIIYDDPSKQAQ
AYRQMSLLLRRPPGREAYPGDVFYLHSRLLERAAKLSSLLGEGSMTALPIVETQSGDVSAY
IPTNVISITDGQIFLSADLFNAGIRPAINVGISVSRVGSAAQIKAMKQVAGKLKLELAQFAEL
EAFAQFASDLDKATQNQLARGQRLRELLKQSQSAPLGVEEQVLTIYTGTNGYLDSLEIGQ
VRKFLVELRTYLKTNKPEFQDIISSTKTFTEEAEAILKEAIQEQRERFILQEQAMRMNPTLS
NSAVPTLDKTNLGRIAQIIGPVLDVSFPPGKMPNIYNALVVKGRDTLGEPINVTCEVQQLL
GNNRVRAVAMSATDGLTRGMDVINTGAPLSVPVGGATLGRIFNVLGEPVDNLGPVDNSTK
FPIHRSAPAFIQLDTKLSIFETGIKVVDLLAPYRRGGKIGLFGGAGVGKTVLIMELINNIAKA
HGGVSVFGGVGERTREGNDLYMEMKESGVINEKNIAESKVALVYGQMNEPPGARMRVGL
TALTMAEYFRDVNEQDVLLFIDNIFRFVQAGSEVSALLGRMPSAVGYQPTLSTEMGSLQE
RITSTKEGSITSIQAVYVPADDLTDPAPATTFAHLDATTVLSRGLAAKGIYPAVDPLDSTSTM
LQPRIVGEEHYETAQLVKQTLQRYKELQDIIAILGLDELSEEDRLTVARARKIERFLSQPFFV
AEVFTGSPGKYVGLTETIRGFQLILSGELDSLPEQAFYLVGNIDEATAKAINLEMESNVKK
MTLNLCVLTPNRIVWDSEVKEIILCTNNGQIGVLPNHAPIATAVDIGILRIRLNDQWLTMAL
MGGFARIGNNEITVLVNDAEKSGDIDPEEAQQTLEIAEDTFRKAEGKRQTIEANLALRRAR
TRVEAINAISMKNVTDSFVSLGHWPSAGSFGFNTDIFATNLINLSVVLGVLIFFGKGVLSDL
LDNRKQRILNTIRNSEELREGAIEQLEKARARLRKVEIEADQFRVNGYSEIEREKLNLIDST
YKTLEQLENYKNETINFEQQKASNQVRQRVFQQALQGALGTLNSCLNSELHLRTISANIGI
LGTMKQITDMNPLISAASVIAAGLAVGLASIGPGVGQGTAAGQAVEGIARQPEAEGKIRGT
LLLSLAFMEALTIYGLVVALALLFANPFVMNVLSCSINTLNGLYDLSGVEVGQHFYWKIGG
FQVHGQVLITSWVVIAILLGSATLAVRNPQTIPTASQNFFEYVLEFIRDVSKTQIGEEYGPW
VPFIGTMFLFIFVSNWSGALLPWKIIQLPHGELAAPTNDINTTVALALLTSVAYFYAGLSKK
GLSYFGKYIQPTPILLPINILEDFTKPLSLSFRLFGNILADELVVVVLVSLVPSVVPIPVMFLG
LFTSGIQALIFATLAAAYIGESMEGHHMIFSTLEHILTHISFSIVSIVITIHLITLLGNEIIKPYDS

SERGMIATFLCLTGLLTTRWIYSGHLPLSDLYESLIFLSWSFSLIHIVPY--IRKNYLTTITASSTI
FTQGFATSGLLNEIQKPAILVPALQSEWLIMHVSMMILSYAALLCGSLLSVALLVITFRN---IF
YSSKEIQY---RNDIFKRAS---AKNYYKAQLIQQLDYWSYRVISLGFIFLTIGILSGAVWANEA
WGSYWSWDPKETWAFITWIVFAIYLHTRTNQNLQGANSAIVATLGFLIIWICYFGVNLLGI
GLHSYGSFTLTMAKKKAFTSLLYLASIVFLPWWISLSFQKSLESWVSNWCNTRQSETFLN
DIEEKSILEKFIELEELLLLEEMIKEYSETHLQNLGI-IHKETIQLIKIHNEGHIHTILNFSTNIIC
FLILSGYSILGNKKLFILNSWAQEFLYNLSDTIKAFSLLLLTDLCIGFHSPHGWELLIGFVYK
DFGFVHNDQIISGLVSTFPVIVDTIFKYWIFRYLNRVSPSLVVIYHSMNDMPIGVPKVPFRSP
GEEDASWVDIYNRLYRERLLFLGQEVDSEISNQLIGLMIYLSIEDETKDLYLFINSPGGWVI
PGVALYDTMQFVQPDIHTICMGAAASMGSFILVGGEITKRLAFPHARVMIHQPAGSFSEVA
TGEFILEVGELLKLRETLTRVYVQRTGKPIWVVSEDMERDIFMSATEAQAYGIVDLVAVEM
KEQKWIHEGLITESLPNGMFRVRLDNEDLILGYVSGKIRRSFIRILPGDRVKIEVSRYDSSRG
RIIYRLRNKDSKDMDKSQIYLG-DRSQQHYFLYPLIFQEYIYVLAHDHGLAGYDNKCSLLI
VKRLIIRMYQQN---LFVNDSKQTPFVGENFYSQVMSEVSSIIMEIPLSLRLISSLVVKSNNLR
SIHSIFSFLEDNFSHLNYVLDILIPYPAHLEILVQALRYWIKDASSLHLLRFFLHECHNWDSLI
TKASSSFSKKRLFLFLYTSHVCEYESSFLFLRNQSSHLRSTSSGALIERIYFYGKMEHLAEVF
ARAFNLWLFKDPFMHCVRYQGKSIIASKGTFILMNKWKYYFVNFWKSHFYLWSQPGRIYI
NQLSNHSLDFLGYRSSVRLKPSMVRSQMLENAFLIDNAI--KKFDTLVPIMPLIGSLAKSKF
CNAVGRPIGKAIWADFSDSDIIERFGRIYRNLSHYHSGSSKKKSLYRVKYILRLSCARTLAR
KHKSTVRAFLKRLGSELLEEFFTEEEQVFSLTFPRRIWYLDIICINDLANHEMIID-TTEVQ-A
INSFSRLES-LKEVYGIIWMLIPILTLVLGITIGVLVIVWLEREISAGIQQRIGPEYAGPLGILQA
LADGTKLLFKENLLPSRGDTRLFSIGPSIAVISILLSYLVIPFGYHLVLADLSIGVFLWIAISSI
APVGLLMSGYGSNNKYSFLGGLRAAAQSISYEIPLTLCVLSISLLSNSSSTVDIVEAQSKYG
FWGWNLWRQPIGFLVFLISSLAECERLPFDLPEAEEELVAGYQTEYSGIKFGLFYVASYLNL
LVSSLFVTVLYLGGWNLSIPYIFVPELFEITKKGRVFGTIIGIFITLAKTYLFLFISIATRWTLP
RLRMDQLLNLGWKFLLPISLGNLLLTTSSQLLSLMIWHVQNENFILDSTRIFMKAFHLLLF
DGSLIFPECILIFGLILLLMIDSTSDQKDIPWLYFISSTSLVMSITALLFRWREEPMISFSGNFQ
TNNFNEIFQFLILLCSTLCIPLSVEYIECTEMAITEFLLFVLTATLGGMFLCGANDLITIFVAPE
CFSLCSYLLSGYTKKDVRSNEATMKYLLMGGASSSILVHGFSWLYGSSGGEIELQEIVNGL
INTQMYNSPGISIALIFITVGIGFKLSPAPSHQWTPDVYEGSPTPVVAFLSVTSKVAASASAT
RIFDIPFYFSSNEWHLLLEILAILSMILGNLIAITQTSMKRMLAYSSIGQIGYVIIGIIVGDSND
GYASMITYMLFYICMNLGTFACIVLFGLRTGTENIRDYAGLYTKDPFLALSLALCLLSLGGL
PPLAGFFGKLYLFWCGWQAGLYFLVLIGLLTSVVSIYYYLKIIKLLMTGRNQEITPHVRNY
RRSPLRSNNSIELSMIVCVIASTIPGISMNPIIAIAQDTLFMFLLYEYDIFWAFLIISSLIPILVFFI
SGFLAPISKGPEKLSSYESGIEPIGDAWLQFRIRYYMFALVFVVFDVETVFLYPWAMSFDVL
GVSVFVEALIFVLILIVGLVYAWRKGALEWSTNNFPWLTIIVVLPIFAGSLIFFLPHKGNRTIR
WYTICICMLELLLTTYAFCYHFQFDDPLIQLVEDYKWISFFDFRWKLGIDGLSIGPVLLTGFI
TTLATLAAWPVTRDSRLFHFLMLAMYSGQIGSFSSRDLLLFFIMWELELIPVYLLLCMWG
GKKRLYSATKFILYTAGGSIFLLMGVLGVGLYGSNEPTLNFETSVNQSYPVALEIIFYIGFFIA
FAVKLPILPLHTWLPDTHGEAHYSTCMLLAGILLKMGAYGLIRINMELLPHAHSIFSPWLM
IVGTIQIIYAASTSPGQRNLKKRIAYSSVSHMGFILIGIASITDTGLNGAILQIISHGFIGAALFF
LAGTSYDRIRLVYLDEMGGVAIPMPKIFTMFSSFSMASLALPGMSGFVAEVIVFLGIITSQK
YLLMPKIAITFVMAIGMILTPIYLLSMSRQMFYGYKLFNTPNSFVFDSGPRELFVSISIFLPVI
GIGLYPDFVLSLSVDKVEGILSNYFYRMMLEHVLVLSAYLFSVGLYGLITSRNMVRALMC

LELILNAVNLNFVTFSDFFDSRQLKGAIFSIFVIAIAAAEAAIGLAIVSSIYRNRKSTRINQSN
LLNKMEQIYQYAWIIPFLPLPVPMLIGLGLLLFPTATKSLRRMWAFQSILLLSIVMIFSMNLS
IQQINSSSVYQYVWSWIINNDFSLEFGYLIDPLTSIMSILITTVGIMVLIYSDNYMSHDHGYL
RFFAYMSFFSTSMLGLVTSSNLIQIYIFWELVGVCSYLLIGFWFTRPVAAKACQKAFVTNRV
GDFGLLLGILGFYWVTGSFEFRDLFQIFNNNEVNFVFVTLCAVLLFAGAIAKSAQFPLHVW
LPDAMEGPTPISALIHAATMVAAGIFLVARLMPLFIVIPHIMNFISLIGIITVFLGATLALAQK
DIKRGLAYSTMSQLGYMMLALGMGSYRSALFHLITHAYSKALLFLGSGSVIHSMETLVGY
CPKKSQNMVLMGGLTKHVPITKNSFLLGTLSLCGIPPFACFWSKDEILNDSWLYSPIFAIIA
WSTAGLTAFYMFRIYLLTFEGHLNVHFQNYSGKRNTPFYSISLWGKEGSKKSNK-LVTLLK
MKFFSFDNIKTSLYPYESDNTMLFPILILILFTLFVGFLGIPF-MDILSKWLTPSINLLHKNSN-
SIDWYEFCKDAVFSVSIASFGIFIAFFLYQPVYSSFQNLDLINSFVNFSDKIKNAIYDWSYNR
GYIDAFYGTFLIAGMRKLAEFTHFFDRRIIDGIPNGVGLMSFFVAEVIKSVGGG-RISSYLFF
YFSYVLMFLLIYYMDLPGLIHDFLLVFLGLGLILGSLGVVLLPNPIYSAFSLGLVFVCISLFY
ILSNSHFVAAAQLLIYVGAINVLIIFAVMFINGSEYSKDFQLWTVGDGVTSVVCTSIFFSLITT
IPDTSWYGIIWTTKANQIIEQDLISNSQQIGIHLSTDFFLPFEFISIILLVALIGAIAVARQMTVP
-ARKDLMIVNMGPHHPSMHGVLRLIVTLDGEDVIDCEPILGYLHRGMEKIAENRTIIQYLP
YVTRWDYLATMFTEAITVNGPEQLGNIQVPKRASYIRVIMLELSRIASHLLWLGPFMADIG
AQTPFFYIFRERELIYDLFEAATGMRMMHNFFRIGGVAADLPHGWIDKCLDFCDYFLTGIA
EYQKLITRNPIFLERVEGVGIIGGEEAINWGLSGPMLRASGIQWDLRKVDHYECYDEFDW
EIQWQKEGDSLARYLVRISEMTESIKIIQQALEGIPGGPYENLEIRRFDRVKDTVWNEFDYR
FISKKPSPTFELSKQELYTRVEAPKGELGIFLIGDKGVFPWRYKIRPPGFINLQILPQLVKGM
KLADIMTILGSIDIIMGEVDRMFPMVTEFMNYGQQTVRAARYIGQGFMITLSHANRLPVTI
QYPYEKLITSERFRGRIHFEFDKCIACEVCVRVCPIDLPVVDWKLETDIRKKRLLNYSIDFGI
CIFCGNCVEYCPTNCLSMTEEYELSTYDRHELNYNQIALGRLPMSVIDDYTIRTILNL--------
--------MQGNLSAWLVKHGLIHRSLGFDYQGIETLQIKPGDWHSIAVILYVYGYNYLRSQCA
YDVAPGGLLASVYHLTRIEYGVDQPEEVCIKVFAPRGDPRIPSVFWVWKSVDFQERESYD
MLGISYDNHPRLKRILMPESWIGWPLRKDYIAPNFYEIQDAH------------------------------------
----------------------MNSIEFPLFHRTTQNSVISTTLNDLSNWSRLSSLWPLLYGTSCCFIEFASL
IGSRFDFDRYGLVPRSSPRQADLILTAGTVTMKMAPSLVRLYEQMPEPKYVIAMGACTITG
GMFSTDSYSTVRGVDKLIPVDVYLPGCPPKPEAIIDAITKLRKKISREIYPDRILFQRENRCF
TTNHKFQVGHSIHTGNYDQGFLYQPPSTSEIPPETFFKYKSSVSSHELVNMQTRNTFSWIKE
QITRSISVSLMIYIITRTSISNAYPIFAQQGYENPREATGRIVCANCHLANKPVDIEVPQAVLP
DTVFEAVVRIPYDMQLKQVLANGKKGALNVGAVLILPEGFELAPPDRISPEIKEKMGNLSF
QSYRPNQKNILVIGPVPGQKYSEITFPILSPDPATKKDIHFLKYPIYVGGNRGRGQIYPDGSK
SNNTVYNATASGIVSKILRKEKGGYEITIADTSDGRQVVDIIPRGPELLISEGESIKLDQPLTS
NPNVGGFGQGDAEIVLQDPLRVQGLLFFFASVILAQIFLVLKKKQFEKVQLSEMNFMSKV
YDWFEERLEIQAIADDITSKYVPPHVNIFYCLGGITLTCFLVQVATGFAMTFYYRPTVTDAF
ASVQYIMTEANFGWLIRSVHRWSASMMVLMMILHVFRVYLTGGFKKPRELTWVTGVVL
AVLTASFGVTGYSLPRDQIGYWAVKIVTGVPDAIPVIGSPLVELLRGSASVGQSTLTRFYSL
HTFVLPLLTAVFMLMHFPMIRKQGISGPLMGVTKKPDLNDPVLRAKLAKGMGHNYYGEP
AWPNDLLYIFPVVILGTIACNVGLAVLEPSMIGEPADPFATPLEILPEWYFFPVFQILRTVPN
KLLGVLLMVSVPAGLLTVPFLENVNKFQNPFRRPVATTVFLIGTAVALWLGIGATLPIDKSL
TLGLFMIEVFLFGIVLGLIPITLSGLFVTAYLQYRRGDQLDLMPTITSYFGFLLTALTITSALFI
GLSKIRLIMDIVSLAWAALMVVFTFSLSLVVWGRSGLMIIRSPEPEVKILVDRDHIKTSFEE

WARPGHFSRTIAKGPETTTWIWNLHADAHDFDSHTSDLEEISRKVFSAHFGQLSIIFLWLSG
MYFHGARFSNYEAWLSDPTHIGPSAQVVWPIVGQEILNGDVGGGFRGIQITSGFFQLWRA
SGITSELQLYCTAIGALVFAALMLFAGWFHYHKAAPKLAWFQDVESMLNHHLAGLLGLG
SLSWAGHQVHVSLPINQFLNAGVDPKEIPLPHEFILNRDLLAQLYPSFAEGATPFFTLNWSK
YADFLTFRGGLDPVTGGLWLSDTAHHHLAIAILFLIAGHMYRTNWGIGHGLKDILEAHKG
PFTGQGHKGLYEILTTSWHAQLSLNLAMLGSLTIVVAHHMYAMPPYPYLATDYGTQLSLF
THHMWIGGFLIVGAAAHAAIFMVRDYDPTTRYNDLLDRVLRHRDAIISHLNWACIFLGFH
SFGLYIHNDTMSALGRPQDMFSDTAIQLQPVFAQWIQNTHALAPGATAPGATASTSLTWGG
GDLVAVGGKVALLPIPLGTADFLVHHIHAFTIHVTVLILLKGVLFARSSRLIPDKANLGFRFP
CDGPGRGGTCQVSAWDHVFLGLFWMYNAISVVIFHFSWKMQSDVWGSISDQGVVTHIT
GGNFAQSSITINGWLRDFLWAQASQVIQSYGSSLSAYGLFFLGAHFVWAFSLMFLFSGRGY
WQELIESIVWAHNKLKVAPATQPRALSIVQGRAVGVTHYLLGGIATTWAFFLARIIAVGMA
LRFPRFSQGLAQDPTTRRIWFGIATAHDFESHDDITEERLYQNIFASHFGQLAIIFLWTSGNL
FHVAWQGNFESWVQDPLHVRPIAHAIWDPHFGQPAVEAFTRGGALGPVNIAYSGVYQWW
YTIGLRTNEDLYTGALFLLFISAISLIAGWLHLQPKWKPSVSWFKNAESRLNHHLSGLFGV
SSLAWTGHLVHVAIPASRGEYVRWNNFLDVLPHPQGLSPLFTGQWNLYAQNPDSSSHLFG
TSQGAGTAILTLLGGFHPQTQSLWLTDMAHHHLAIAFVFLIAGHMYRTNFGIGHSMKDLL
DAHIPPGGRLGRGHKGLYDTINNSIHFQLGLALASLGVITSLVAQHMYSLPAYAFIAQDFTT
QAALYTHHQYIAGFIMTGAFAHGAIFFIRDYNPEQNEDNVLARMLEHKEAIISHLSWASLF
LGFHTLGLYVHNDVMLAFGTPEKQILIEPIFAQWIQSAHGKTSYGFDILLSSTNGPAFNAGR
SIWLPGWLNAINE--NSNSLFLTIGPGDFLVHHAIALGLHTTTLILVKGALDARGSKLMPDK
KDFGYSFPCDGPGRGGTCDISAWDAFYLAVFWMLNTIGWVTFYWHWKHITLWQGNVSQ
FNESSTYLMGWLRDYLWLNSSQLINGYNPFGMNSLSVWAWMFLFGHLVWATGFMFLISW
RGYWQELIETLAWAHERTPLANLIRWRDKPVALSIVQARLVGLAHFSVGYIFTYAAFLIAS
TSGKFGMSHSVKIYDTCIGCTQCVRACPTDVLEMIPWDGCKAKQIASAPRTEDCVGCKRC
ESACPTDFLSVRVYLWHETTRSMGIAYMTTLNFPSVLVPLVGLVFPAIAMASLFLHVQKNK
IVMRDLKTYLSVAPVLSTLWFASLAGLLIEINRFFPDALTFPFFSFMTAILERRESESLWGRF
CNWITSTENRLYIGWFGVLMIPTLLTATSVFIIAFIAAPPVDIDGIREPVSGSLLYGNNIISGAII
PTSAAIGLHFYPIWEAASVDEWLYNGGPYELIVLHFLLGVACYMGREWELSFRLGMRPWI
AVAYSAPVAAATAVFLIYPIGQGSFSDGMPLGISGTFNFMIVFQAEHNILMHPFHMLGVAGV
FGGSLFSAMHGSLVTSSLIRETTENESANEGYRFGQEEETYNIVAAHGYFGRLIFQYASFNN
SRSLHFFLAAWPVVGIWFTALGISTMAFNLNGFNFNQSVVDSQGRVINTWADIINRANLG
MEVMHERNAHNFPLDLAAIEAPSTNGMGLPWYRVHTVVLNDPGRLLAVHIMHTALVAG
WAGSMALYELAVFDPSDPVLDPMWRQGMFVIPFMTRLGITNSWGGWSIT-GGTITNPGIW
SYEGVAGAHIVFSGLCFLAAIWHWVYWDLEIFSDERTGKPSLDLPKIFGIHLFLAGVACFG
FGAFHVTGLYGPGIWVSDPYGLTGKVQSVNPLWGVEGFDPFVPGGIASHHIAAGTLGILA
GLFHLSVRPPQRLYKGLRMGNIETVLSSSIAAVFFAAFVVAGTMWYGSATTPIELFGPTRY
QWDQGYFQQEIYRRVSAGLAENQSLSEAWSKIPEKLAFYDYIGNNPAKGGLFRAGSMDS
GDGIAVGWLGHPIFRDKEGHELFVRRMPTFFETFPVVLVDGDGIVRADVPFRRAESKYSV
EQVGVTVEFYGGELNGVNYSDPVTVKKYARRAQLGEIFELDRATLKSDGVFRSSPRGWF
TFGHASFALLFFFGHIWHGARTLFRDVFAGIDPDLDAQVEFGAFQKLGDPTTRRQVVMKT
LYSLRRFYPVETLFNGTLALAGRDQETTGFAWWAGNARLINLSGKLLGAHVAHAGLIVFW
AGAMNLFEVAHFVPEKPMYEQGLILLPHLATLGWGVGPGGEVIDTFPYFVSGVLHLISSAV
LGFGGIYHALLGPETLEESFPFFGYVWKDRNKMTTILGIHLILLGIGAFLLVFKALYFGGVY

DTWAPGGGDVRKITNLTLSPSIIFGYLLKSPFGGEGWIVSVDDLEDIIGGHVWLGSICILGGI
WHILTKPFAWARRALVWSGEAYLSYSLAAISVFGFIACCFVWFNNTAYPSEFYGPTGPEAS
QAQAFTFLVRDQRLGANVGSAQGPTGLGKYLMRSPTGEVIFGGETMRFWDLRAPWLEPL
RGPNGLDLSRLKKDIQPWQERRSAEYMTHAPLGSLNSVGGVATEINAVNYVSPRSWLSTS
HFVLGFFFFVGHLWHAGRARAAAAGFEKGIDRDFEPVLSMTPLNMTIALGKFTKDENDLF
DIMDDWLRRDRFVFVGWSGLLLFPCAYFAVGGWFTGTTFVTSWYTHGLASSYLEGCNFL
TAAVSTPANSLAHSLLLLWGPEAQGDFTRWCQLGGLWTFVALHGAFGLIGFMLRQFELAR
SVQLRPYNAIAFSGPIAVFVSVFLIYPLGQSGWFFAPSFGVAAIFRFILFFQGFHNWTLNPFH
MMGVAGVLGAALLCAIHGATVENTLFEDGDGANTFRAFNPTQAEETYSMVTANRFWSQI
FGVAFSNKRWLHFFMLFVPVTGLWMSALGVVGLALNLRAYDFVSQEIRAAEDPEFETFYT
KNILLNEGIRAWMAAQDQPHENLIFPEEVLPRGNALMSGSTGERSFADIITSIRYWVIHSITI
PSLFIAGWLFVSTGLAYDVFGSPRPNEYFTENRQGIPLITGRFDSLEQLDEFSRSFMTIDRTY
PIFTVRWLAVHGLAVPTVSFLGSISAMQFIQRMATQTVENGSRSA-PRRTNVGSLLKPLNSE
YGKVAPGWGTTPLMGVAMALFAVFLSIILEIYNSSVLLDGISMNMLTLKLFVYTVVIFFVSL
FIFGFLSNDPGRNPGREEMADTTGRIPLWIIGTVAGILVIGLVGVFFYGSYSGLGSSLMLNIF
SLIC--LNSALYPSSLFFAKLPEAYAFLNPIVDFMPVIPLFFFLLAFVWQAAVSFRTTQSNPNE
QNVELNRTSLYWGLLLIFVLAVLFSNYFFNMEVNILAFIATALFILVPTAFLLIIYVKTVSQN
NMEIATLVAIFISGLLVSFTGYALYTAFGQPSQQLRDPFEEHGDMEALVYTFLLVSTLGIIFFA
IFFREPPKISTKK--MTLAFQLAVFALIATSSILLISVPVVFASPDGWSSNKNVVFSGTSLWIGL
VFLVGILNSLIS-------MSPQTETKASVGFKAGVKDYKLTYYTPDYETKDTDILAAFRVTPQP
GVPPEEAGAAVAAESSTGTWTTVWTDGLTSLDRYKGRCYGIEPVPGEENQFIAYVAYPLD
LFEEGSVTNMFTSIVGNVFGFKALRALRLEDLRIPKAYVKTFDGPPHGIQVERDKLNKYG
RPLLGCTIKPKLGLSAKNYGRAVYECLRGGLDFTKDDENVNSQPFMRWRDRFLFCAEAIY
KSQAETGEIKGHYLNATAGTCEDMMKRAIFARELGVPIVMHDYLTGGFTANTSLSHYCRD
NGLLLHIHRAMHAVIDRQKNHGMHFRVLAKALRMSGGDHIHSGTVVGKLEGEREITLGF
VDLLRDDFIPKDRARGIYFTQDWVSLPGVLPVASGGIHVWHMPALTEIFGDDSVLQFGGG
TLGHPWGNAPGAVANRVALEACVQARNEGRDLATEGNEIIREAAKWSPELAAACEVWKE
IKFEFQAMDTLDGMAIHLYKTSTPSTRNGTVDSQVKSNSRNNLIYGQHHCGKGRNARGIIT
AGHRGGGHKRLYRKIDFRRNEKDIYGRIVTIEYDPNRNAYICLIHYRDGEKRYILHPRGAII
GDTIVSGTEVPIKMGNALPLTDMPLGTAIHNIEITLGKGGQLARAAGAVAKLIAKEGKSAT
LKLPSGEVRLISKNCSATVGQVGNVGVNQKSLGRAGSKRWLGKRPVVRGVVMNPVDHP
HGGGEGRAPIGRKKPTTPWGYPALGKRSRKRNKYSDNLILRRRSKMIQPQTYLNVADNSG
ARQLMCIRIIGAS-NRRYAHIGDVIVAVIKDAVPNMPLERSEVVRAVIVRTCKELKRDNGMII
RYDDNAAVVIDQEGNPKGTRVFGAIARELRQFNFTKIVSLAPEVLNYNPKRTRFRKQHRG
RMKGISSRGNAICFGKYALQALEPAWITSRQIEAGRRAMTRNARRGGKIWVRIFPDKPVT
VRPAETRMGSGKGSPEYWVAVVKPGRILYEMGGVTENIARRAISIAASKMPIRAQFIISGM
TRIRRGYIARRRRTKIRLFASSFRGAHSRLTRTITQQKIRALVSAHRDRDKQKINFRRLWITR
INAAIRE-RGVCYSYSRLINDLYKRQLLLNRKILAQIAISNRNCLYMISNEIIKEVS--------ML
NEVYALGQHISMSAHKARRVIDQIRGRSYEETLMILELMPYRACYPIFKLVYSAAANASFN
MGSNEANLVISKAEVNEGSIVKRSKPRARGRSFAIQKPTCHITVVMKDISLDGGAWDKKM
DGIRYAVFTDKSIRLLGKN-QYTSNVESGSTRTEIKHWVELFFGVKVIAMNSHRLRGKARR
MGPIMGQTMHYRRMIITLQPGYSIPPLRKKRTMAVPKKRTSISKKRIRKNIWKRKGYLAAL
KALSLGKSLSTGNSKSFFVRQTNKMAKGKDVRIPVLLECTACVRNG--VNKESTGISRYITQ
KNRHNTPNRLELLKFCPYCYKHTIHGEIKKMKIRASVRKICEKCRLIRRRGRIIVICSNPRH

KQRQGMVREKITVSTRTLQWKCVESAADSKRLFYGRFILSPLMKGQADTIGIAMRRALLG
EIEGTCITCAKSE--KISHEYSTIMGIQESVHEILMNLKEIVLRSNLYGTCEASICVRGPGYVT
AQDIILPPYVEIVDNTQHIASLTEPIELVIGLQIEKNRGYLIKAPN-TFDGSY--PIDPVFMPVR
NANHSIHSYENGN--KEILFLEIWTNGSLTPKEALHEASRNLIDLLIPFLHTKEE--NLNLEDN
Q-HMVPLPPFTFYEKLA-KLTKKKK-KMALKSIFIDQSELPPRIYNCLKRSNIYTLLDLLNNS
QEDLMKMEHFRIEDVKQILGVLEKN--FVIDLPKNQ--------MSTIPGFNQIQFEGFCRFIDRG
LTEELSKFPKIEDTNQEIDFELFLERYQLVEPLIKERDAVYESLTYSSELYVSARLIWKNRRYI
QEQTILIGKIPLMTSLGAFIVNGIYRIVINQILQSPGIYYQSELNDNGISVYTGTIISDWGGRL
ELEIDRKARIWVRVSRQQKLSILVLLSAMGLNIREILENVCYPELFLSFLNDK--KKIGSKEN
AILEFYQQFACVEGDPVFSESLSKDLQKKFFQQRCELGGIGRRNMNRRLNLDIPQNNTFLL
PRDILAAADRLIRIKFGMGTLDD--MNHLQNKRIRSVADLLQEQFGLALVRLENMARGNIY
AALK-HNWT-PTPQNLVNSTP----LTDTYKVFFRLHPLSQVLDRTNPLTQIVHGRKLSYLGPG
GLTARTATFPIRDIHPSHYGRICPIDTSEGINVGLIGSLAIHARIGRWG-SLESPFY---KISERSK
G--TRMLYLSPGRDEYYMVAAGNSLALNQGIQEEQVVPARYRQEFLTIAWEQVHLRSIFSF
QYFSIGASLIPFIEHNDANRALMSSNMQRQAVPLSQSEKCIVGTGLEGQAALDSGALAIAE
HEGKIVYTDTDKILLSGNGDTLRIPLVMYQRSNKNTCMHQKPQVRRGKCIKKGQILAYGA
ATIGGELALGKNVLVAYMPWEGYNFEDAVLISERLVYEDIYTSFHIRKYEIQIN---QG-PERV
TNEIPHLEVHLLRNLDKNGIVMLGSWVETGDILVGKLTPQMVKE-SSYAPEDRLLRTILGM
RVYTSKETCLKLPIGGRGRVIDVRWVRSSKTDETEKTESIRVYILQKREIKVGDKVAGRHG
NKGIISKILPRQDMPYLQDGRPVDMVFNPLGVPSRMNVGQIFESSLGLAGDLLDRHYRIAP
FDERYEQEASRKLVFSELYEASKQTSNPWIFEPESPGKSRIFDGRTGDPFEQPVIIGKPYILK
LIHQVDDKIHGRSSGRYSRLTQQPLKGRAKKGGQRVGEMEVWALEGFGVAYILQEMLTY
KSDHIRARQEVLGTIIFGGRIPTPEDAPESFRLFVRELRSLALELNHFLVSAKTFQLNRKE
A???
??
??
??
??
??
??
???M
EVLMAERPNQVFHNKVIDGTAMKRLISRFIDHYGIGYTSHILDQVKTLGFRQATAASISLGI
DDLLTIPSKRWLVQDAEQQSFILEKHHHYGNVHAVEKLRQSIEIWYATSEYLRQEMNPNFR
MTDPFNPVHIMSFSGARGNASQVHQLVGMRGLMSDPQGQMIDLPIQSNLREGLSLTEYIIS
CYGARKGVVDTAIRTSDAGYLTRRLVEVVQHIVVRRTDCGTVRGISVSPRNGMIA-DRIFIQ
TLIGRVLADDIYIGSRCIATRNQDIGIGLVNRFIT------FRAQPISIRTPFTCRSTSWICQLCYGR
SPAHDDLVELGEAVGIIAGQSIGEPGTQLTLRTFHTGGVFTGGTAEHVRAPSNGKIKFNEDL
VHPTRTRHGHPAFLCSRDLYVTIESEDIIHNVCIPPKSFLLVQNDQYVESEQVIAEIRARTST-
LNLKEKVRKHIYSDSEGE-MHWNTDVYHAPEFTYGNIHLLPKTSHLWILLGEPWR--SSLG
PCSIHKDQDQMNAY--SLSVKRRYISNPS--VTNNQ-ARHKFFSSYFSGKNGDWIPDY-SELNR
MTCTGRCN---P-AILEGNS--DLLAKRRRNRVIIPLESLQEGENQLIPSSGISIEIP--RNGILRRN
SILAYFDDPRYIRKSSGLTKYETRELNSIVNEED--LIEYRG-VKVFWPKYKEVNPFFFIPVEV
HILAESSSIMVRHNSIIGVDTQITLNRRSRVGGLVRVKKKAEKIKLIIFSGDIHFPGKTNKAF
R----LIPPGG--GKQNYKEYKK--LKNWLYIQR--MKLSRYEKKYFVLVQPVVPYKKTDSINL

G-LFPPDLLQESDNLQLRVVNYILYYDP--ILEIWDTSIQLVRTCLVLNWDQDKK------IEKAC
ASFVEIRTNGLIRDFLRIDLAKSPISYTGKRNDLSGAGLISE-NGSDRANVNPFSSIHSYS---K
TSIQESINPNQGTIHTLLNR---NKESQSLIILSSSNCFRIGP--FNDVKSPNV---IKES-PLIPISNS
LGPLGT--GFPISNLDL--FSHL--ITHNQIL-LTNYLQLDNLK---QSFQILK---YYLLDEK--GKIY
NPYSCSNIILNPFNLNWYFIHYNYCEETS-TIVSLGQFLCENVCIAKKGPH-FK--SGQVLILQ-
VDSVVIRSAKPYLATPGATVHGHYGEILYEGDTLVTFIYEKSRSGDITQGLPKVEQVLEVRS
IDSISMNLEKRIETWNKCITRILGIPWTFLIGAELTIVQSRISLVNKVQKVYRSQGVQIHNRHI
EIIVRQITSKVLVSE---DEMSNVFLPGELIELLRAERMGRALEEAICYQAILLGITRASMNTQ
SFISEASFQETARVLAKAALLGRIDWLKGLKENVVLGGMIPVGSGFKTP-SSEPNNIPNNIA-
-FELKNKNLLEGEMK--DILFYHRKLFDSCLSKNLHDPQEQSFFRI---------MTRRYWNINLE
EMMEAGVHFGHGTRKWNPKMAPYISAKRKGIHITNLTRTARFLSEACDLVFDAASKGKQ
FLIVGTKNKEADSVAWAAIRARCHYVNKKWLGGMLTNWSTTETRLHKFRDLRTEQKTGG
LNRLPKRDAAMLKRQLSHLQTYLGGIKYMTGLPDIVIIVDQHEEYTALQECITLGIPTICLI
DTNCDPDLADISIPANDDAISSIRLILNKLVFAICEGRS-GYIRNPMGQKINPIGFRLGTSQGH
HSLWFAQPKNYSEGLQEDQKIRNYIKNYVQKNMKTSSG-VEGIARIEIQKRIDLIQIIIYMGF
PKILIESRPRGI----EELQMNLQKEIHSVNRKLNIAITRIEKPYGNPNILAEFIAGQLKNRVSFR
KAMKKAIELTEQADTKGIQVQIAGRIDGKEIARVEWIREGRVPLQTIRAKIDYCSYTVRTIY
GVLGIKIWIFIDAEMSRYRGPRFKKIRRLGALPGLTNKRPRAGSDLRNQSRSGK-KSQYRIR
LEEKQKLRFHYGLTERQLLKYVRIAGKAKGSTGQVLLQLLEMRLDNILFRLGMAPTIPGA
RQLVNHRHILVNGRIVDIPSYRCKPRDTIAARDEQKSKALIQNSLDSSPN-EELPNHLTLQAF
Q--YKGLVNQIIDSKWVGLKINELLVVEYYSRQTMSRRGTSEEKTAKSDPIYRNRLVNMLV
NRILKHGKKSLAYQIIYRAVKKIQQKTETNPLSVLRQAIHGVTPGIAVKARRVGGSTHQVP
VEIGSTQGKALAIRWLLAASRKRPGRNMAFKLSSELVDAAKGSGDAIRKREETHRMAEA
NRAFAHFRMGSDTIADIITSIRNADMYRKSVVRVASTNISQSIVKILLREGFIENVRKHREN
NKSFLVLTLRHRR--NRKRTY---RNLLNLKRISRPGLRIYSNYQRIPRILGGMGVVILSTSRGI
MTDREARLERIGGEILCYIWMAKAIPKKGSR--GRIGSRKSTRKIPKGVIHIQASFNNTIVTV
TDVRGRVVSWSSAGTCGFRGTRRGTPFAAQTAAASAIRAVVDQGMQRAEVMIKGPGLGR
DAALRAIRRSGILLTFVRDVTPMPHNGCRPPKKRRVMPTIKQLIRNTRQPIRNVTKSPALRG
CPQRRGTCTRVYTITPKKPNSALRKVARVRLTSGFEITAYIPGIGHNLQEHSVVLVRGGRVK
DLPGVRYHIVRGTLDAVGVKDRQQGRS-SAL----MAKKSLIQREKKRQKLEQKYHLIRRSS
KKEIS---KVQSLSDKWEIYGKLQSPPRNSAPTRLHRRCFSTGRPRANYRDFGLSGHILREM
VHACLLPGATRSSWMVKNLFISISLQEQEENKENRGSVEFQVVSFTNKIRRLTSHLELHKK
DYLSQRGLRKILGKRQRLLAYLSKKDRPRYKELIGQLGIRERKTP--MVKLRLKRCGRKQ--
--RIVAIDVRSRREGRDLRKVGFYDPIKNQTYLNVPAILYFLERGAQPTGTVQDILKKAEVF
KELYPNQTKYN-MDKSKRTFLKSKRSFRRRLPPIQSGDRIDYKNMSLISRFISEQGKILSRRV
NRLTLKQQRLITIAIKQARILSLLPFLNNDK--QFERTESTTRTPNLRARKRVTRSLKKNPFVA
NNLLKKINKLNTKAEKEIIITWSRASTIIPIMVGHTIAIHNGKEHLPIYITDRMVGHKLGEFA
PTLNFRGHAKSDNRSRRMILKSFLLGNLVSLCMKIINSVVVVGLYYGFLTTFSIGPSYLFLL
RAHVMEEGEEGTEKKVSATTGFITGQLIMFISIYYAPLHLALGRPHTITVLALPYLLFHFFC
NNHKHFFDYGSTTRNSMRNLSIQCVFLNNLIFQLFNHFILPSSMLARLVNIFMFRCNSKML
FVTSSFVGWIIGHILFMKWVGKI--

--MTGHEFKSWILELREILREIKNSHYFLDSWTQFNSVGSF
IHIFFQQERFIKLFDSRIWSILLS-HNS--QGSTSNRYFT---IKG-VILFGVAVLIYR----INNRNM
VERKNIYLIGLLP-----IPMNSIGPRNDTLEESV-GSSNINRLIVS-LLYLPKGKKIYESSFLNPK
ESTWVLPIT----KKCSMPESNWGSRWWRDWIGKKS--DSSCKISN---ETVAGIEILFKEKDLK
-YLEFFFVYYRD--DLMRKDHDWELFDRLSLRKRQNRINL--NSGPLFEILVKHWICYLMSA
FREKIPTESD--FLRN-VSRENLIWLDNV
WLVNKDR---FFR--KVRNVSSNIQYDS---TR--SSFVQVRDSSQLKGSS-DQSRDHFDSIS----N
EDSE-------------YHTLIHQREIQQLKERSIL-RD--PSFLQTEGTEIESN--RFPK--CLSGYS--SMS
RLFT-EREKQMINHLLTEEIEEFL--GNPTRSVRSFFSDRW--SELH-LGSNPTERSTRDQ----KL
LKKQQDLS-FL--RRSE----NKEMVNLFKIITYLQN--TVSIHPISSDSGCDMVP-KDEPD-RDSS
NKISFLTKNPFFYL--FHLFHDRNRGGCTL--NHDFELE--ERFQEMADLFTLSITEP-DLVYHK
RFAFSIDSYGLDPKQ--FLNGVFNSRYEWKTTSLLVLLVLLPIFY--EENESFYRRIRKKGVR-I
SCGNDLEEPKPKIVVFASNNIMEAVNQYRLIRNLIQIQHSTHR--YIRNVLNRFFLMN---RSD
RNFEYG---IQRDQIGKDTLNHRTLMKYTINQHLSNLKK-SQKRWFDPL--LFFSRTERSMNR
DPDAYRYKWSTGSNNFQEHLEHFVSEQKSR---FQVVFDRLRINPYSIDWSEVINKKDLSKP
LRFFLSK-------------------------LLLFLSN-SLPFLFVSFGNIPIQRSE-IYIY-ELKGPND---PQFLESI
GLQIVHLKKLKPF--LLD---DHETC-QKSKFLINGGTISPFLFNKIP-KW-MIDSFHTR--NNRR
KSFDNTDSYFSMIFHDQYNWLNPVKSFHRSSLRSSFYKANQLRFLNNPHHFCFY--CNKRF
PFYV----EKARIN--NYDFTYG-QFLNILFIRNK-IFSLCVGK--KKHAFWG-RDTISAIESQVSN
MFIPKAFPQSG-DETYNLYKY---FHFPSRSNPFVR--RAIYSIADISGTPLTEGQIVNFERTYCQ
PLSDMNLSDSEGKNLYQYLN--FNSNMGLIHTPCSEKFLPSEK--RKK--------RSLCLK--KCV
EKGQMYRTFQRDSAYSTLSKWNLFQTYMPWLLTSTGYRYLKFLFLDTFSDLL--PILSS-SQ
KFVSIFHDIMHGSDRLWRILQKKL------YLPQWNL-ISEISSKCFHN--LLLSEEMIHRN--NES
PLISTHLT--NVREF-LYAILFLLLVATY-LVCTHLLFVFG--TSSELQTEFEKVKSLMIPSSMIEL
RKLLDRYPTSELNSFWLKNLFLVALKQLGDSL--------GGNMLLRGGPA-----YG-VKSIRSKK
KYLNINLIDIIDLI-SIIPNPINRITFSRNTRHLSHTSKEIYSLIRKRKNVNGDWIDDKIESWVA
NSDSIDDEKREFLVQFS--TLTT--EKRIDQILLSLT-HSDHL-----SKNDSGYKMIEQPGAVYLR
YLVDIH--KKSLMNYEFNTSSLAERRIFLAHYQTITY--SQTSCGANSFHFPSHG---KPFSLRL
ALSLS-RGILVIGSIGTGRSYLVKYLAKNSYLPFITVFLNKFLDNKSQGFDDI-----DID-IDAN-
-----------DDIDA------------SDDI---LDMEL-ELITSMNALTMDMMPED--EDRLYIALQFELAK
AMSPCIIWIPNIHDLDVNESNYFSLGLLVNLLSR---DYE----TRNLLVIASTHIPQKVDPALIA
PNKLNTCIKIRRLLIPQQRKHFFTLSYTRGFHLEKKMFHT--NGFGSITMGSNARDLVALTNE
ALSISITQKKSIIDTNTIRFALHRQIWDLR---SQVRSVQDHGILFYQIGRAVAQNVLLSNCPID
PISIYMKKK--SCNEVDYYLYNWYFELGTSMKKLTILLYLLSCSAGSVTQDLWSLP--GPDE
K-NGITPYGLVENDS-GLVRGLLEVEGALVG--SSR---ACSQFDKD--RVTLLLRPEPRN-PLD
MMQNGSCSILDQRFLYEKD--ESEFEE-GEGEGALDRQQ----IEED----LFNH--IVWAP--RIWR
P---WGFLFDCIERPNSLGFP-YWSRSFRGK---RIIYDEEDELQENDSEFLQSGTVQYQTRDRS
SKEQGLFRISQFIWDPADPLFFLFK-AQPFVSVFSHRELFADEEMSKVLLTPQTNPPTSLYKR
WFI--KKTQEKHFELLINRQRWLR---TNRSLS--NGSFRSN--TLFESYQYLSNLFLSN-GTLLD

QMTKALLRKRWLLPDEMQIGF--MEQEKDFPFLMSRSR-INGNFIDKTFSIVANILLRIIPTTS
GEKEAFTYYRDG--MSAQSEGNYAEALQNYYEAMRLEIDPYDRSYILYNIGLIHTSNGEHT
KALEYYFRALERNPFLPQAFNNMAVICHY-RGEQAIRQGDSEIAEAWFDQAAEYWKQAIA
LTPGNYIEAHNWLKITRRFE--MSCRSEHIWIEPITGARKTGNFCWAVILFLGSLGFLLVGTS
SYLGRNLISLFPSQQIVFFPQGIVMSFYGIAGLFISSYLWCTISWNVGSGYDRFDRKEGIVCI
FRWGFPGKNRRVFLRFFIKDIQSIRIEVKEGIYARRVLYMDIRGQGAIPLTRTDEN-FTPREM
EQKAAELAYFLRVPIEVF
>Nelumbolutea
MKKWWLNSMLFLSKSMDSLGPIGNTSGSEDPVINDTDKNIH-CSNVDHLFGVRDIRSFISD
DTFLVRDSNGDSYSIYLDIENQIFEIDND--HSFLSEFENLFSSYLNSSYPINGSKSDNHYYDR
YMSDTKYSWNNHINSCIDSYLRSEIR--IDSYISSDSDNYSDSYIYTYICSDSGSSIT---TGSD-
NKRRRN-DLEKNKKSDILWVQCENCYELNYKKLLMSTMNICKQCGYHLKMSSSDRIERSI
DPGTWDPMDEDMVSTDPINFHSGEEHYKKRIDFNQRSTGLAEAVQTGIGQLNGIPVAIGV
MDFKFLGGSMGSVVGEKITRLIEYATNRSLPLIIVCASGGARMQEGSLSLMQMAKISSASY
NYQSNKKLFYVSILTSPTTGGVTASFGMLGDIIIAEPNAYIAFAGKRVVEQTLKNTIPDNSQ
AAEYSFHKGLFDPIVPRNLLKDVLSELFQLHGFFPLNQNSIKMVTIRADEISNIIRERIEQYN
REVKIVNTGTVLQVGDGIARIHGLDEVMAGELVEFEEGTIGIALNLESNNVGVVLMGDGL
MIQEGSSVKATGRIAQIPVSEAYLGRVINALAKPIDGRGEISASESRLIESPAPGIISRRSVYEP
LQTGLIAIDSMIPIGRGQRELIIGDRQTGKTAVATDTILNQKGQNVICVYVAIGQKASSVAQV
VTTFQEKGAMEYTIVVAETADSPATLQYLAPYTGAALAEYFMYREQHTSIIYDDPSKQAQ
AYRQMSLLLRRPPGREAYPGDVFYLHSRLLERAAKLSSRLGEGSMTALPIVETQSGDVSA
YIPTNVISITDGQIFLSADLFNAGIRPAINVGISVSRVGSAAQIKAMKQVAGKLKLELAQFAE
LEAFAQFASDLDKATQNQLARGQRLRELLRQSQSAPLTVDEQIVTIYTGTNGYLDSLEIGQ
VKKFLVQLRTYLKTNKPQFQEIISSTKTFTEEAEALLKEAIQEQMERFLLQEQTMRMNPTT
SGPGVSALEEKNLGRIAQIIGPVLDVAFPPGKMPNIYNALVIKGRDTAGQQINVTCEVQQL
LGNNRVRAVAMSATDGLMRGMEVIDTGAPLSVPVGGATLGRIFNVLGEPVDNLGPVDTRT
TSPIHKSAPAFIQLDTKLSIFETGIKVVDLLAPYRRGGKIGLFGGAGVGKTVLIMELINNIAK
AHGGVSVFGGVGERTREGNDLYMEMKESGVINEQNIAESKVALVYGQMNEPPGARMRV
GLTALTMAEYFRDVNEQDVLLFIDNIFRFVQAGSEVSALLGRMPSAVGYQPTLSTEMGSL
QERITSTKEGSITSIQAVYVPADDLTDPAPATTFAHLDATTVLSRGLAAKGIYPAVDPLDSTS
TMLQPRIVGEEHYETAQRVKQTLQRYKELQDIIAILGLDELSEEDRLTVARARKIERFLSQP
FFVAEVFTGSPGKYVGLTETIRGFQLILSGELDGLPEQAFYLVGNIDEATAKAMNLEVESKL
KKMTLNLCVLTPNRIVWDSEVKEIILSTNSGQIGVLPNHAPIATAVDIGILRIRLNDQWLTM
ALMGGFARIGNNEITILVNDAEKGSDIDPQEAQRTLEIAEANLSRAEGKRQTIEANLALRR
ARTRVEAINVISMKNVTDSFVSLGHWPSAGSFGFNTDILATNPINLSVVLGVLIFFGKGVLS
DLLDNRKQRILSTIRNSEELRGGAIEQLEKARARLRKVEMEADEFRVNGYSEIEREKLNLI
NSTYKNLERLENYKNETIHFEQQRAINQVRQRVFQQALQGALGTLNSCLTSELHLRTISAN
IGMFGTMKEITDMNPLISAASVIAAGLAVGLASIGPGVGQGTAAGQAVEGIARQPEAEGKI
RGTLLLSLAFMEALTIYGLVVALALLFANPFVMNVLPCSINTLKGLYDISGVEVGQHFYWQ
IGGFQVHAQVLITSWVVIAILLGSATIAVRNPQTIPTAGQNFFEYVLEFIRDLSKTQIGEEYG
PWVPFIGTMFLFIFVSNWSGALLPWKIIELPHGELAAPTNDINTTVALALPTSVAYFYAGLS
KKGLGYFAKYIQPTPILLPINILEDFTKPLSLSFRLFGNILADELVVVVLVSLVPSVVPIPVMF
LGLFTSGIQALIFATLAAAYIGESMEGHHMIFLTLEHILTHISFSIVSIVITIHLMTLLIDEIVGL
YDSSEKGMIATFFCITGFLVTRWIHSGHFPLSDLYESLMFLSWSFSIIHMVPKIGNHKNNLSE

ITTPSAIFTQGFATSGLLTDMHQSAILVPALQSQWLMMHVSMMVLSYAALLCGSLLSIALL
VITFRKNIENFSKSNEIQYVNKKSNVLRNTSFT-FRNYHRHQLIQQLDHWSYRVISLGFIFLT
IGILSGAVWANEAWGSYWNWDPKETWAFITWTIFAIYLHTRTNKKWQGANSAIVASMGFL
IIWICYFGVNLLGIGLHSYGSFTF-MPKKKAFTPLPYLASIVFLPWWISLSFNKSLESWVTN
WWNTRQSETFLNDIQEKNVLEKFIELEELFLLNEMIKEYPETHIQKLHIRIHKETIQLVKMY
NEGHIHTILHFSTNIICFAILSGYSILGSEELVILNSWVQEFLYNLSDTIKAFSILLLTDLCIGFH
SPHGWELMIGSIYKDFGFAHNEQIISGLVSTFPVILDTILKYWIFRYLNRVSPSLVVIYHSMN
DMPIGVPKVPFRSPGEEDAVWVDVN-RLHRERLLFLGQEVDSEISNQLIGLMVYLSIEDDT
KDLYLFINSPGGWVIPGIAIYDTMQFVPPDVHTICMGLAASMGSFILAGGEITKRLAFPHAR
VMIHQPASSFYEAQTGEFILEAEELLKLRETLTRVYVQRTGNPLWVVSEDMERDVFMSATE
AQAHGIVDLVAVEMKEQKWIHEGLIIESLPNGMFRVRLDNEDLILGYVSGKIRRSFIRILPG
DRVKIEVSRYDSTRGRIVYRLRNKDSNDMEELQRYLEIDRSRQQDFLYPLLFQEYIYVFAH
DHGLENLG-KSSSLIVKRVITRMYQRNHLIFLANDSNQTQCVGKNFYSQMILEGFAVIVEIP
FSLRLVSSLITKSHNLRSIHSIFSFLEDKLSRLNYVLDLLIPYPIHSEILVQTLRCWVKDTPSL
HLLRFFLHEYRNWNSLITKSSSLLKKQRFFLFLYNSHVCECESIFVFLRKQSFHLRSTSSGA
LLERTHFYGKIEHFGVVFRNDFILWLVKDPFMHYVRYQGKSILASKGTPLLMNKWKYHL
VNFWQCHFYLWSQPGRIHINQLSNHSLDFLGYLSSVRLNPSLVRSQMLENSYLIDIAS--KK
FETIVPIIPLIGSLAKAKFCNVSGHPISKPVRADSSDSDIIDRFGRICKNLSHYHSGSSKKKSL
YRIKYILRLSCARTLARKHKSTVRAFLKRLGSELLKEFFTEEEQVLSLILPRRIWYLDILRIN
DLTNPEMIIDTT-KAQ-VINSFSRSE-SLKEVYGLIWLLVPILTLISGITIGVLVIVWLEREISAGI
QQRIGPEYAGPLGILQALADGIKLLFKEDLLPSRGDARLFSIGPSIAVISILLSYSVIPFGYRLV
LADLSIGVFLWIAISSIAPIGLLMSGYGSNNKYSFSGGLRAAAQSISYEIPLTSCVLSISLLSN
SSSTVDIVEAQSKYGFWGWNLWRQPIGFVVFLISSLAECERLPFDLPEAEEELVAGYQTEY
SGIKFGLFYVASYLNLLVSSLFVTVLYLGGWNLSIPYIFIPELFGINKVGGVFETTIGIFITLA
KAYLFLFIPIATRWTLPRMRMDQLLNLGWKFLLPISLGNLLLTTSSQLLSLMIWHVQNENFI
LDSTRIFMKAFHLLLFHGSFIFPECILIFGLILLLMIDSTSDQKDIPWFYFISSTSLVMSITALL
FRWREEPMISFSGNFQTNNFNEIFQFLILLCSTLCIPLSVEYIECTEMAITEFLLFVLTATLGG
MFLCGANDLITIFVAPECFSLCSYLLSGYTKRDVRSNEATTKYLLMGGASSSILVHGFSWL
YGSSGGEIELQEIVNGLINTQMYNSPGISIALIFITVGIGFKLSPAPFHQWTPDVYEGSPTPVV
AFLSVTSKVAASASATRIFDIPFYFSSNEWHLLLEILAILSMILGNLIAITQTSMKRMLAYSSI
GQIGYVIIGIIVGDSNDGYASMITYMLFYISMNLGTFACIVSFGLRTGTDNIRDYAGLYTKDP
FLALSSALCLLSLGGLPPLAGFFGKLHLFWCGWQAGLYFLVSIGLLTSVVSIYYYLKIIKLL
MTGRNQEITPHVRNYRRSPLRSNNSIELSMIVCVIASTIPGISMNPIIAIAQDTLFMFLLHEY
DIFWAFLIISSVIPILAFLISGVLAPIREGPEKLSSYESGIEPMGDAWLQFRIRYYMFALVFVV
FDVETVFLYPWAMSFDVLGVSVFIEALIFVLILIVGSVYAWRKGALEWSMNFFPWLTIIVVL
PISAGSLIFFLPHKGNKVIRWYTISICLIELLLMTYAFCYHFKLDDPLIQLEEDYKWINIFDFH
WRLGIDGLSIGPILLTGFITTLATLAAWPVTRDSRLFHFLMLAMYSGQIGSFSSRDLLLFFIM
WELELIPVYLLLSMWGGKKRLYSATKFILYTAGGSIFLLLGVLGMGLYGSNEPTLNFETSA
NQSYPVALEIIFYFGFLIAYAVKSPIIPLHTWLPDTHGEAHYSTCMLLAGILLKMGAYGLVRI
NMEFLPHAHSIFSPWLMIVGTIQIIYAALTSPGQRNLKKRIAYSSVSHMGFTIIGIASITDTGL
NGAILQIISHGFIGAALFFLAGTSYDRIRLVYLDEMGGIAIPMPKIFTMFSSFSMASLALPGM
SGFVAELIVFFGIITSPKYLLMPKILITFVMAIGMILTPIYSLSMLRQMFYGYKLFNASNSYFF
DSGPRELFVSISIFLPVIGIGIYPDFVLSLSVDKVEAILSNYFSRMMLEHVLVLSAYLFSIGIY
GLITSRNMVRALMCLELILNAVNINFVTFSDFFDSRQLKGDIFSIFVIAIAAAEAAIGPAIVSS

IFRNRKSTRINQSNLLNKMEHTYQYAWMIPFVPLPVPMLIGVGLLFVPMATKKLRRMWAF
PSVLLLSIVMIFSANLAIQQINGSSIYQYVWSWTINNDFSLEFGYLIDPLTSIMLILITTVGVM
VLVYSDNYMSHDQGYLRFFAYMSFSNTSMLGLVTSSNLIQIYIFWELVGMCSYLLIGFWFT
RPIAANACQKAFVTNRVGDFGLLLGILGLYWITGSFEFKDLFEIVNNNGVNSLFVTLCASL
LFVGAVAKSAQFPLHVWLPDAMEGPTPISALIHAATMVAAGIFLVARLLPLFTVIPYIMNLIS
LIGIITVLLGATLALAQKDIKRSLAYSTMSQLGYMMLALGMGSYRAALFHLITHAYSKALL
FLGSGSIIHSMETIVGYSPDKSQNMVLMGGLTKYMPITKTAFLLGTLSLCGIPPLACFWSK
DEILNDSWLYSPIFAIIACSTAGLTAFYMFRIYLLTFEGPLNVHFQNYSGKKNSSFYLISIWG
KEGPKTINKN-FRLSTMNFFSFGNKEIFPYPHESDNTMLVPLLVLVFFTLFVGFIGIPFDFDIL
SKWLTPSINLLHPNWN-SVDWYAFVTNAIFSVSIAYFGIFIASLLYGPVYSSFQNLELINFFVI
LWDKIINVIYNWSYNRGYIDSFYARAFTRGIRGLAELTHFFDRRVIDGITNGVGVTSFFVGE
GIKYVGGG-RISSYLFLYLFYVSIFLLIYYMDLPGPIHDFLLVFLGSGLILGGLGVVLLTNPIY
SAFSLGLVLVCISLFYIPSNSHFVAAAQLLIYVGAINVLIIFAVMFMNGSEYYNDFHLWTIGD
GVTSLVCTSIFFSLITTILDTSWYGIIWTTRSNQIIEQDLISNGQQIGIHLSTDFFLPFELISIILL
VALIGAIAMARQMTVPATRKDLMIVNMGPHHPSMHGVLRLIVTLDGEDVIDCEPILGYLH
RGMEKIAENRTIIQYLPYVTRWDYLATMFTEAITVNAPEQLGNIQVPKRASYIRVIMLELSR
IASHLLWLGPFMADIGAQTPFFYIFRERELIYDLLEAATGMRMMHNYFRIGGVAADLPYG
WMDKCLDFCDYFLTGVAEYQKLITRNPIFLERVEGVGIIGGEEAINWGLSGPMLRASGIQW
DLRKVDHYECYDEFDWEVQWQKEGDSLARYLVRIGEMTESIKIIQQALEGIPGGPYENLEI
RRFARAKDSEWNDFEYRFISKKPSPTFELSKQELYARVEAPKGELGIYLIGDQSVFPWRWK
IRPPGFINLQILPQLVKRMKLADIMTILGSIDIIMGEVDRMLPMVTGFMNYGQQTIRAARYI
GQSFMITLSHANRLPVTIQYPYEKLITSERFRGRIHFEFDKCIACEVCVRVCPIDLPVVDWR
LETDIRKKRLLNYSIDFGICIFCGNCVEYCPTNCLSMTEEYELSTYDRHELNYNQIALGRLP
ISVIGDSTIRTIMNSKIATGKSLDSKTITNYMQGRLSAWLVKHGLVHRSLGFDYQGIETLQI
KPEDWHSIAVILYVYGYNYLRSQCAYDVAPGGLLASVYHLTRIQYGVDQPEEVCIKVFAPR
RNPRIPSVFWIWKSADFQERESYDMLGISYDNHPRLKRILMPESWIGWPLRKDYIAPNFYE
IQDAH--MNSIKFPLLDRPTQNSVISTTLNDLS
NWSRLSSLWPLLYGTSCCFIEFASLIGSRFDFDRYGLVPRSSPRQADLILTAGTVTMKMAPS
LVRLYEQMPEPKYVIAMGACTITGGMFSTDSYSTVRGVDKLIPVDVYLPGCPPKPEAVIDA
ITKLRKKVSREIYEDRIVSQQKNRCFTTNHKFHVGRSTHTGNYDQGLLYQSPSTSEIPSETF
FKYKKSVSSHELVNMQNRNTFSWIKEQMTRSISVSIMIYVITRTSISNAYPIFAQQGYENPR
EATGRIVCANCHLANKPVDIEVPQAVLPDTVFEAVVRIPYDMQLKQVLANGKKGALNVG
AVLILPEGFELAPPDRISPEMKEKIGNLSFQSYRPTKKNILVIGPVPGQKYSEITFPILSPDPAT
KKDVHFLKYPIYVGGNRGRGQIYPDGSKSNNTVYNATAAGIVSKIVRKEKGGYEITIADAS
DGHQVVDIIPPGPELLVSEGESIKLDQPLTSNPNVGGFGQGDAEIVLQDPLRVQGLLFFFAS
VILAQIFLVLKKKQFEKVQLSEMNFMSKVYDWFEERLEIQAIADDITSKYVPPHVNIFYCL
GGITLTCFLVQVATGFAMTFYYRPTVTEAFASVQYIMTEANFGWLIRSVHRWSASMMVLM
MILHVFRVYLTGGFKKPRELTWVTGVILAVLTASFGVTGYSLPWDQIGYWAVKIVTGVPEA
IPVIGSPLVELLRGSASVGQSTLTRFYSLHTFVLPLLTAVFMLMHFPMIRKQGISGPLMGVT
KKPDLNDPVLRAKLAKGMGHNYYGEPAWPNDLLYIFPVVILGTIACNVGLAVLEPSMIGE
PADPFATPLEILPEWYFFPVFQILRTVPNKLLGVLLMVSVPAGLLTVPFLENVNKFQNPFRR
PVATTVFLIGIAVALWLGIGATLPIDKSLTLGLFMIEVFLFGIVLGLIPITLAGLFVTAYLQYRR
GDQLDLMPTLTSYFGFLLAASTITSALFIGLSKIRLIMDIVSLAWAALMVVFTFSLSLVVWG
RSGLMIIRSPEPEVKILVDRDPIKTSFEEWARPGHFSRTIAKGPETTTWIWNLHADAHDFDS

HTSDLEEISRKVFSAHFGQLSIIFLWLSGMYFHGARFSNYEAWLSDPTHIGPSAQVVWPIV
GQEILNGDVGGGFRGIQITSGFFQIWRASGITSELQLYCTAIGALVFAALMLFAGWFHYHK
AAPKLAWFQDVESMLNHHLAGLLGLGSLSWAGHQVHVSLPINQFLDAGVDPKEIPLPHEF
ILNRDLLAQLYPSFAEGATPFFTLNWSKYADFLTFRGGLDPVTGGLWLTDIAHHHLAIAILF
LIAGHMYRTNWGIGHSLKDILEAHKGPFTGQGHKGLYEILTTSWHAQLSLNLAMLGSLTI
VVAHHMYSMPPYPYLATDYGTQLSLFTHHMWIGGFLIVGAAAHAAIFMVRDYDPTTRYN
DLLDRVLRHRDAIISHLNWACIFLGFHSFGLYIHNDTMSALGRPQDMFSDTAIQLQPVFAQ
WVQNTHALAPGATAPGATTSTSLTWGGGDLVAVGGKVALLPIPLGTADFLVHHIHAFTIHV
TVLILLKGVLFARSSRLIPDKANLGFRFPCDGPGRGGTCQVSAWDHVFLGLFWMYNAISV
VIFHFSWKMQSDVWGSINDQGVVTHITGGNFAQSSITINGWLRDFLWAQASQVIQSYGSS
LSAYGLFFLGAHFVWAFSLMFLFSGRGYWQELIESIVWAHNKLKVAPATQPRALSIVQGRA
VGVTHYLLGGIATTWAFFLARIIAVGMALRFPRFSQGLAQDPTTRRIWFGIATAHDFESHD
DITEERLYQNIFASHFGQLAIIFLWTSGNLFHVAWQGNFESWVQDPLHVRPIAHAIWDPHF
GQPAVEAFTRGGALGPVNIAYSGVYQWWYTIGLRTNEDLYTGSLFLLFLSALSLIAGWLH
LQPKWKPSVSWFKNAESRLNHHLSGLFGVSSLAWTGHLVHVAIPGSRGEYVRWNNFLDV
LPHPQGLGPLFTGQWNLYAQNPDSSSHLFGTSQGAGTAILTLLGGFHPQTQSLWLTDIAHH
HLAIALIFLVAGHMYRTNFGIGHSMKDLLEAHIPPGGRLGRGHKGLYDTINNSIHFQLGLA
LASLGVITSLVAQHMYSLPAYAFIAQDFTTQAALYTHHQYIAGFIMTGAFAHGAIFFIRDYN
PEQNEDNVLARMLDHKEAIISHLSWASLFLGFHTLGLYVHNDVMLAFGTPEKQILIEPIFA
QWIQSAHGKTSYGFDVLLSSTTGPAFNAGRSIWLPGWLNAINE--NSNSLFLTIGPGDFLVH
HAIALGLHTTTLILVKGALDARGSKLMPDKKDFGYSFPCDGPGRGGTCDISAWDAFYLAV
FWMLNTIGWVTFYWHWKHITLWQGNVSQFNESSTYLMGWLRDYLWLNSSQLINGYNPF
GMNSLSVWAWMFLFGHLVWATGFMFLISWRGYWQELIETLAWAHERTPLANLIRWRDKP
VALSIVQARLVGLAHFSVGYIFTYAAFLIASTSGKFGMSHSVKIYDTCIGCTQCVRACPTDV
LEMIPWDGCKAKQIASAPRTEDCVGCKRCESACPTDFLSVRVYLWHETTRSMGLAYMTT
FNLPSIFVPLVGLVFPAIAMASLFLHVQKNKIVMRDIKTYLSVAPVLSTLWFGSLAGLLIEIN
RLFPDALAFPFF--MTAILERRESESLWGRFCNWITSTENRLYIGWFGVLMIPTLLTATSVFII
AFIAAPPVDIDGIREPVSGSLLYGNNIISGAIIPTSAAIGLHFYPIWEAASVDEWLYNGGPYE
LIVLHSLLGVACYMGREWELSFRLGMRPWIAVAYSAPVAAATAVFLIYPIGQGSFSDGMPL
GISGTFNFMIVFQAEHNILMHPFHMLGVAGVFGGSLFSAMHGSLVTSSLIRETTENESANE
GYRFGQEEETYNIVAAHGYFGRLIFQYASFNNSRSLHFFLAAWPVIGIWFTALGISTMAFNL
NGFNFNQSVVDSQGRVINTWADIINRANLGMEVMHERNAHNFPLDLAAVEAPSTNGMGL
PWYRVHTVVLNDPGRLLSVHIMHTALVSGWAGSMALYELAVFDPSDPVLDPMWRQGMF
VIPFMTRLGITNSWGGWSIT-GGTITNPGLWSYEGVAGAHIVFSGLCFLAAIWHWVYWDL
EIFCDERTGKPSLDLPKIFGIHLFLSGVACFGFGAFHVTGLYGPGIWVSDPYGLTGKVQPVN
PAWGVEGFDPFVPGGIASHHIAAGTLGILAGLFHLSVRPPQRLYKGLRMGNIETVLSSSIAA
VFFAAFVVAGTMWYGSATTPIELFGPTRYQWDQGYFQQEIYRRVGAGLAENQSLSEAWS
KIPEKLAFYDYIGNNPAKGGLFRAGSMDNGDGIAVGWLGHPIFRDKEGRELFVRRMPTFF
ETFPVVLVDGDGIVRADVPFRRAESKYSVEQVGVTVEFYGGELNGVSYSDPATVKKYARR
AQLGEIFELDRATLKSDGVFRSSPRGWFTFGHASFALLFFFGHIWHGARTLFRDVFAGIDP
DLDAQVEFGTFQKLGDPTTKRQVVMKTLYSLRRFYHVETLFNGTLALAGRDQETTGFAW
WAGNARLINLSGKLLGAHVAHAGLIVFWAGAMNLFEVAHFVPEKPMYEQGLILLPHLATL
GWGVGPGGEVIDTFPYFVSGVLHLISSAVLGFGGIYHALLGPETLEESFPFFGYVWKDRNK
MTTILGIHLILLGIGAFLLVLKALYFGGVYDTWAPGGGDVRKITNLTLSPSVIFGYLLKSPFG

GEGWIVSVDDLEDIIGGHVWLGSICILGGIWHILTKPFAWARRALVWSGEAYLSYSLGALS
VFGFIACCFVWFNNTAYPSEFYGPTGPEASQAQAFTFLVRDQRLGANVGSAQGPTGLGKY
LMRSPTGEVIFGGETMRFWDLRAPWLEPLRGPNGLDLSRLKKDIQPWQERRSAEYMTHA
PLGSLNSVGGVATEINAVNYVSPRSWLATSHFVLGFFLFVGHLWHAGRARAAAAGFEKGI
DRDFEPVLSMTPLNMTIALGRFTKDENDLFDIMDDWLRRDRFVFVGWSGLLLFPCAYFAL
GGWFTGTTFVTSWYTHGLASSYLEGCNFLTAAVSTPANSLAHSLLLLWGPEAQGDFTRWC
QLGGLWTFVALHGAFGLIGFMLRQFELARSVQLRPYNAIAFSAPIAVFVSVFLIYPLGQSG
WFFAPSFGVAAIFRFILFFQGFHNWTLNPFHMMGVAGVLGAALLCAIHGATVENTLFEDG
DGANTFRAFNPTQAEETYSMVTANRFWSQIFGVAFSNKRWLHFFMLFVPVTGLWMSALG
VVGLALNLRAYDFVSQEIRAAEDPEFETFYTKNILLNEGIRAWMAAQDQPHENLIFPEEVL
PRGNALMSGSTGERSFADIITSIRYWVIHSITIPSLFIAGWLFVSTGLAYDVFGSPRPNEYFTE
SRQGIPLITDRFDPLAQLDEFSRSFMTIDRTYPIFTVRWLAVHGLAVPTVSFLGSISAMQFIQ
RMATQTVEGSSRSG-PRRTIIGDLLKPLNSEYGKVAPGWGTTPLMGVAMALFAIFLSIILEIY
NSSVLLDGISMQMLTLKLFVYTVVIFFVSLFIFGFLSNDPGRNPGREEMADTTGRIPLWLIG
TVTGIPVIGSIGIFFYGSYSGLGSSLMLNIFSLICICLNSAFHSSSFFFAKLPEAYAIFNPIVDV
MPVIPVLFFLLALVWQAAVSFRTTQSNPNEQNVELNRTSLYWGLLLIFVLAVLFSNYFFNM
EVNILAFIATALFILVPTAFLLIIYVKTVSQNDMETATLVAIFISGSLVSFTGYALYTAFGQPSQ
QLRDPFEEHGDMEALVYTFLLVSTLGIIFFAIFFREPPKVPTKKVKMTIAFQLAVFALIATSSI
LLISVPVVFASPEGWSSNKNVVFSGTTLWIGLVFLVGILNSLIS-------MSPQTETKASVGFK
AGVKDYRLTYYTPEYETKDTDILAAFRVTPQPGVPPEEAGAAVAAESSTGTWTTVWTDGL
TSLDRYKGRCYHIEPVAGEENQFIAYVAYPLDLFEEGSVTNMFTSIVGNVFGFKALRALRL
EDLRIPPAYSKTFQGPPHGIQVERDKLNKYGRPLLGCTIKPKLGLSAKNYGRAVYECLRGG
LDFTKDDENVNSQPFMRWRDRFLFCAEAIYKAQAETGEIKGHYLNATAGTCEEMIKRAVF
ARELGVPIVMHDYLTGGFTANTSLAHYCRDNGLLLHIHRAMHAVIDRQKNHGIHFRVLAK
ALRMSGGDHIHSGTVVGKLEGEREITLGFVDLLRDDFIEKDRSRGIYFTQDWVSLPGVLPV
ASGGIHVWHMPALTEIFGDDSVLQFGGGTLGHPWGNAPGAVANRVALEACVQARNEGRD
LAREGNEIIREASKWSPELAAACEVWKEIKFEFEAMDTL--TAIHLYKTSTPSTRNGAVDSQ
VKSNPRNNLIYGQHRCGKGRNARGIITAGHRGGGHKRLYRKIDFRRNEKKISGRIVTIEYD
PNRNAYICLIHYGDGEKRYILHPRGAIIGDTIVSGSEVPIKMGNALPLTEMPLGTAIHNIEITL
GKGGQLARAAGAVAKLIAKEGKSATLKLPSGEVRLISKNCSATVGQVGNVGVNQKSLGR
AGSKCWLGKRPVVRGVVMNPVDHPHGGGEGRAPIGRKKPTTPWGYPALGRRSRKRNKY
SDSLILRRRSKMIQPQTHLNVADNSGARELMCIRIIGAS-NRRYAHIGDVIVAVIKEAVPNMP
LEKSEVIRAVIVRTCKELKRDNGMIIRYDDNAAVVIDQEGNPKGTRVFGAIARELRQLNFIK
IVSLAPEVLMLSPKRTRFRKQHRGRMKGISYRGNRICFGRYALQALEPAWITSRQIEAGRR
AMTRYARRGGKIWIRIFPDKPVTIRPTETRMGSGKGSPEYWVSVVKPGRILYEMGGVSEIV
AREAISIAASKMPIRTQFVIAGMTRIKRGYIARRRRTKIRLFASTFRGAHSRLTRTITQQKMR
ALVSAHRDRGKQKRNFRRLWITRINAVIRENNGVSYSYSRLIHDLYKRQLLLNRKILAQLA
ISNRNCLYMISNEILK-----------MIKEVYALGQHIRMSAHKARRVIDQIRGRSYEETLMILEL
MPYRACYPIFKLVYSAAANASHNMGLNEADSVISKAEVNESTTMKRLKPQARGRSYPIKR
PTCHITIALKDISLDGVVWVKKMDGIKYAVFTDKSIRLLGNN-QYTSNVESGSTRTEIKHW
VELFFGVKVIAMNSHRLPGKGRRMGPIMGHTMHYRRMIITLQPGYSIPPLRKKRTMAVPK
KRTSISKKRIRKNIWKRKGYWAAVKAFSLAKSLSTGNSKSFFVR--QIMAKGKDVRVRVILE
CTNCVRNGVN--KESPGISRYITQKNRHNTPSRLELRKFCPYCFKHTIHGEIKKMKIRASVR
KICEKCRLIRRRGRIIVICSNPRHKQRQGMVREEIAVSTRTLQWKCVESRVDSKRLYYGRFI

LSPLMKGQADTIGIAMRRALLGEIEGTCITRAKSE--KIPHEYSTIVGIEESVHEILMNLKEIV
LRSNLYGTRDAFICVRGPGYVTAQDIISPPSVEIVDTTQHIASLTEPIDLCIGLQIERNRGYR
MKTPNNSQDGSY--PIDAVFMPVRNANHSIHSYGNGNEKQEILFLEIWTNGSLTPKEALHEA
SRNLINLFIPFLDAQEE--DIQLEDNQ-NKVTLPFFTFHDRLT-KLRKNKK-EIALKCIFIDQSEL
PPRTYNCLKRSNIHTLLDLLNNSQEDLMKMEHFQIEDVKQILDILQKH--FAIDLPKNKMLR
DGN-EGMSTIPGFSQIQFEGFCRFIDQGLTEELYKFPKIEDTDQEIEFQLFVETYQLVEPLINE
RDAVYESLTYSSELYVPAGLIWKTGRDMQEQTIFIGNIPLMNSLGTSIVNGIYRIVINQILQS
PGIYYRSELDHNGISVYTGTIISDWGGRSELEIDRKARIWARVSRKQKISILVPSSAMGSNLR
EILDNVCYPEIFLSFLNDKEKKKIGSKENAILEFYQQFACVGGDPVFSESLCKELQKKFFQQ
RCELGRIGRRNINRRLNLDIPQKNTFLLPRDVLAAADHLIGMKFGMGTLDD--MNHLKNK
RIRSVADLLQDQFGLALVRLENAVRGTISGAIR-HKLI-PTPQNLVTSTP----LTTTYESFFGLH
PLSQVLDRTNPLTQIVHGRKLSYLGPGGLTGRTASFRIRDIHPSHYGRICPIDTSEGINVGLIG
SLAIHARIGHWG-SLESPFY---EISERS--KEVRIVYLSPSRDEYYMVAAGNSLALNQSIQEEQ
VVPARYRQEFLTIAWEQVHLRSIFPFQYFSIGASLIPFIEHNDANRALMSSNMQRQAVPLSR
SEKCIVGTGLECQAALDSGVSAIAEHEGKIIYTDTDKIILSGNGDTLSIPLIMYQRSNKNTC
MHQKPQVRRGKCIKKGQILADGAATVGGELALGKNVLVAYMPWEGYNSEDAVLISERLV
YGDIYTSFHIRKYEIQTHVTSQG-PERITNEIPHLEAHLLRNLDRNGIVMLGSWVETGDILV
GKLTPQMAKE-SSYAPEDRLLRAILGIQVSTSKETCLKLPIGGRGRVIDVRWIQKKG-GSSY
NPETIRVYISQKREIRVGDKVAGRHGNKGIISKILPRQDMPYLQDGTPVDMVFNPLGVPSR
MNVGQIFECSLGLAGDLLDRHYRIAPFDERYEQEASRKLVFSELYEASKQTANPWVFEPE
YPGKSRIFDGRTGDPFEQPVIIGKSYILKLIHQVDDKIHGRSSGHYALVTQQPLRGRAKQGG
QRVGEMEVWALEGFGVAHILQEMLTYKSDHIRARQEVLGTTIIGGTITNPEDAPESFRLLV
RELRSLALELNHFLVSEKNFQINRKEA-------MIDRYKHQQLRIGSVSPQQIIAWAQKILPNG
EIVGEVTKPYTFHYKTNKPEKDGLFCERIFGPIKSGICACGNFRVIGDEKEDPKFCEQCGVE
FVDSRIRRYQMGYIKLACPVTHVWYLKRLPSYIANLLDKPLKELEGLVYCD--FSFARP-IA
KKPTFLRLRGLFEYEIQSWKYSIPLFFTTQGFDTFRNREISTGAGAIREQLADLDLRIIIDSSL
VEWKELGEE-GPTGNEWEDRKIGRRKDFLVRRMELAKHFIRTNVEPERMVLCLLPVLPPE
LRPIIQIDGGKLMSSDINELYRRVIYRNNTLTDLLTTSRSTPGESVMCQEKLVQEAVDTLLD
NGIRGQPMRDGHNKVYKSFSDVIEGKEGRFRETLLGKRVDYSGRSVIVVGPSLSLHRCGL
PREIAIELFQTFVIRGLIRQHVASNIGVAKSKIREKEPIVWEILQEVMQGHPVLLNRAPTLHR
LGIQAFQPILVEGRAICLHPLVRKGFNADFDGDQMAVHVPLSLEAQAEARLLMFSHRNLLS
PAIGDPISIPTQDMLIGLYVLTMGNHRGICANRYNPWNRRN---YQNERIDDNNYKYTKEKE
PYFCSSYDALGAYRQKRINLDSPLWLRWRLDQR-VIAS--REVPIEVQYESLGTYHEIYGHY
LIVRSVKKKILCIYIRTTVGHISFYREIEEAVQGFYRARSYGT--------MEVLMAERADLVFHN
KVIDGTAMKRLISRLIDHFGMAYTSHILDQVKTLGFQQATATSISLGIDDLLTIPSKGWLVR
DAEQQSLILEKHYHYGNVHAVEKLRQSIEIWYATSEYLRQEMNPNFRMTDPSNPVHIMSFS
GARGNASQVHQLVGMRGLMSDPQGQMIDLPIQSNLREGLSLTEYIISCYGARKGVVDTAV
RTSDAGYLTRRLVEVVQHIVVRRTDCGTVRGISVSPRNGMMT-ERIFIQTLMGRVLADDIY
MGPRCIAIRNQDIGIGLVNRFIT------FRAQPISIRTPFTCRSTSWICRLCYGRSPTHGDLVELG
EAVGIIAGQSIGEPGTQLTLRTFHTGGVFTGGTAEQVRAPSNGKIKFNEDLVHPTRTRHGHP
AFLCYIDLYVTIESQDIIHNVNIPPKSFLLVQNDQYVESEQVIAEIRAGTSTL-NFKERVRKHI
YSDSEGE-MHWSTDVYHAPEYTYGNVHLLPKTSHLWILSGSPCR--SNIVPFSLHKDQDQM
SVH--SLSVEGRYISN--LSVTNDQ-VRHKLFSSDPSSC-GERILDF-SGPDRIICNGHCNFIYPA-
ILHENSD--LLAKRRRNRFIIPFQSNQEREKELMPRAGISIEIPIN--GIFRRNSILAYFDDPRYR

RNSSGITKYGTIEVHSIVKKED--LIEYRR--KEFRPKYKKVDRFFFIPEEVHILPGSSSILVRN
NSIIGVDTRITLNTRSRVGGLVRVERKKKKIELKIFSGDIHFPGETDKISRHSGILIPPGAEKK-
-NSKESKT-KLKNWIYIQR--ITPTKKK--FFVLVRPVVTYEIADGINLATLFPQDLLQERDNVQ
LRVVNYILYGNGKPIRGISHTSIQLVRTCLVLNWDQDKK---GSSIEEVHASLVEVRANDLIR
DFIRMDLAKSPISYIGKRNDPAGSGLISD-NRSDRTNRNPFY---SKAR-----IQQSLSQHQGTIR
TLLNR---NKECQSLIILSSSNCSRIGIGPFNGLKYHN---VTKESDPLISIRNSFGPLGT--VPKIA
NFYSS-YYHLIT--HNQIL-INKYLLLDN---LKQTFQVLK---YYLIDENGR--IFNPDPCSNIIFNP
FNLNWYFLHHDYCEESSTIIR-LGQFLCENVCISKNGP---HLKSGQVLIVH-VDSLVIRSAKP
YLATPGATVHGHYGEILYEGDTLVTFIYEKSRSGDITQGLPKVEQVLEVRSIDSISMNLEKR
VEDWNERIARILGIPWGFLIGAELTIAQSRISLVNKIQKVYRSQGVQIHNRHIEIIVRQITSKV
VVSE---DGMSNVFLPGELIGLLRAERTGRALEEAICYRAVLLGITRASLNTQSFISEASFQET
ARVLAKAALRGRIDWLKGLKENVVLGGMIPVGTGFKGLVHRS--RQHNNIP--LEIKKKNLF
EGEMR--DILFHHKELFHFCIPKNFHDTSEQSFTGFNDS------MTRRYWNINLEEMMEAGVH
FGHGTRKWNPRMAPYISAKRKGIHITNLTRTARFLSEACDLVFDAASRGKQFLIVGTKNK
AADLVASAAIRARCHYVNKKWLGGMSTNWSTTETRLQKFRDLRAEQKAGRLNRLPKRD
AAMLKRQLFNLQTYLGGIKYMTGLPDIVIIVDQQEEYTALRECVTLGIPTICLIDTNCDPDL
ADISIPANDDAIASIRLILNKLISAICEGRSS-YIRNRMGQKINPLGFRLGTTQGHHSLWFAQP
KSYSEGLQEDQKIRDCIKNFVQKNMRISSG-FEGIARIEIQKRIDLIQVIIYMGFPKLLIESRP
R----GIEELQINVQKEFHSVNRKLNIDITRIAKPYGQPNILAEFIAGQLKNRVSFRKAMKKAI
ELTEQADTKGIQIQIAGRIDGKEIARVEWIREGRVPLQTIRAKIDYCSYPVRTIYGVLGIKIWI
FVDEEMSRYRGPRFKKIRRLGTLPGLTSKRPKAGSDFRNQARSGK-RSQYRIRLEEKQKLR
FHYGLTERQLLKYVCIARKAKGSTGQVLLQLLEMRLDNILFRLGMASTIPGARQLVNHRH
ILVNGRIVDIPSYRCKPRDIITTRDEQKSRALIQNYLDSSPH-EELPKHLTLHSFQY--KGLVN
QIIDSKWVGLKINELLVVEYYSRQTMSRRGTAEEKTAKSDPIYRNRLVNMLVNRIMKHGK
KSLAYQIIYRAVKKIQQKTETNPLSVLRQAIRGVTPDIAVKARRVGGSTHQVPIEIGSTQGK
ALAIRWLLGASRKRPGRNMAFKLSSELVDAAKGSGDAIRKKEETHRMAEANRAFAHFRM
GRDTIADIITSIRNADMDKKRTVRITSTNITENIVKILLREGFIENVRKHQENNKYFLVLTLR
HRR--NRKGPYR-----TILKRISRPGLRIYSNHQHIPKILGGMGIVILSTSRGIMTDREARREGI
GGEIVCYIWMAKPIPRIGSRRNGRIGLRKSGRRIPKGVIHVQASFNNTIVTVTDVRGRVVS
WSSAGTCGFRGTRRGTPFAAQTAAGNAIRTVVDQGMQRAEVMIKGPGLGRDAALRAIRR
SGLQLSFVRDVTPMPHNGCRPPKKRRVMPTIKQLIRNTRQPIRNVTKSPALRGCPQRRGTC
TRVYTITPKKPNSALRKVARVRLTSGFEITAYIPGIGHNSQEHSVVLVRGGRVKDLPGVRYH
IVRGTLDAVGVKDRQQGRSKYGVKKPKMAKKSLIQREKKRQKLEQKYHLIRRSSKKEIS--
-KVPSLSDKWEIHGKLQSSPRNSAPTRLRRRCFSTGRPRANYRDFGLSGHILREMVHACLL
PGATRSSWVKKNAFISVISQKEKEENENRGSVEFQVLSFTNKIRRLTSHLELHRKDYLSQR
GLRKILGKRQRLLVYLSKKNRVRYKELISQLDIREPKTR--MVKLRLKRCGRKQRAIYRIVA
IDVRSRREGRDLRKVGFYDPIKNQTYLNVPAILYFLEKGAQPTGTVHDISKKAEVFKELRV
NQTK---MDKSKRPFLKSKRSFRRRLPPIGSGDRIDYRNMSLISRFISEQGKILSRRVNRLTLK
QQRLITIAIKQARILSSLPFLNNEK--QFERTESTSRTAGPRTRNKVPRSLKKNPFVANHLLSK
IEKLNTRAEKEIIVTWSRASTIIPTMIGHTIAIHNGKEHLPIYITDRMVGHKLGEFAPTINFRG
HAKNDNRSRRMILKSFLLGNLFSLCMKIINSVVVVGLYYGFLTTFSIGPSYLFLLRARVME
EG---TEKEVSATTGFITGQLMMFISIYYAPLHLALGRPHTITVLVLPYLLFHFFWNNHKHFF
DYGSTTRNSMRNLSIQCVFLNNLIFQLFNHFILPSSTLARLVNIYMFRCNNKMLFVTSSFVG
WLIGHILFMKWVGLVLFWIRQNHSIRS-NVLIKYPVSELRNSMAQIFSILLFITCVYYLGRM

PSPIFTKKLKE----KPIVTLLFDYKRWNRPLRYIKNNRLEKAVRNEMSQYFFYTCQSDGKQR
ISFTYPPSLSTFGEMIQKKISLCTTEKLSSDELYNHWVYINEQKKDNLNNEFRNRIEALDKG
SLVLDVLEKRTRLCNDETEQKCLPKMYDPLLNGPYRGTIKNLYSRK--VWINKIHGILYREF
EQKDPNDQ--GIKEIRKKVPRWSYKLIYLEQEEVEEIRSRKCKRVIIFTEALIRYSQQSDFRR
NLIKGSMRAQRRKTIIGELFQTNAHSPLFLDRKDKTVFFSFDI--SRILKLIFRNKEENERIRIA
EAWDTFIFTQAIRGFMLITQLTLRKYIVLPSLIIAKNLCRMLLFQFPEWNEDLKELNREMHI
KCTYNGIQLSEKEFPKNWLTDGIQIKILFPFRLKPWHRSKKGKKENFCFLTVFGMEAELPF
GSPRKQPSFFEPIWKELDKKIRNVK-KKSFLVLRFLKERKKWFLKVSKEQKEWIIKKVL--FI
KRIMKEFTKVTITTIRNQIIPDLSIQIKSTGWTNYSLTEKKMKDLADRTSTIRNQIERITKEK
KNNAKETSCDNKRSESQKNIWQIFKRRSARLIRKWHFFIKFFIERLY-DTLLCIINIPRVNAQ
LFIHFIS-DLSSLSQAYVFYKLSQNQVINKYHLRSVLQYTSLFIKDRLKDYFTQGIFHSESKH
KKLRNSGMNEWKNWLKGHYQYDLSQTRWFRLVPQKWRNRVNQRRTIQNKQKLGSYEK
DQ--FIFYEKQDKFKKHYRYDLLSHKYINDYIIDLDRKYLDWRILHFCLKLFDWMGMNEEI
LNRWFFPELLLLYDTYKIKPWGIPIKLLLLNFKNNIEVELDFFLKKHLLFQLRPDYPLNKK
MINNIKVYCFLLRLKNPKEIAISSIQRREMSLDVMVIQKDITLTELIKKGIFIIDPIRLSIKWD
GQFIMYQTIGISLVHKNKHNQYDLLAPENILSPRRRRKLRIRICFKFLWPNYRLEDLACMN
RYWFDTNNGSRFNMLRIHMYPMKRHQFKSWIFELREILREIKNSHYFLDSWTKFDSVGSF
THIFFHQERFMKLFDPRIWSILLS-RDS--QGSTSNRYFT---IKGVVLLVVA-VLIY----RINNRN
MVERKNLYLMGLL-----PIPMNSIGPRNDTLEESF-GSSNINRLIVS-LLYLPKGNKISESCFMD
PKESTWVLPIT----KKCIMPESNWGSRWWRNRIGKKR--DSSCKIS---NETVAGIEISFKEKDI
K-YLEFLFVSYTD--DPIRKDHDWELFDRLSPRKKRNIINL--NSGQLFEILVKHWICYLMSAF
REKRPIEVEG-FFKQQG-AESTIQSNDIEHVSH--LFSRNKWGISLQNCAQFHMWQFRQDLF
V---SWGK-NPHESDFLRN-VSRENWIWLDNVWLVNKDR---FFS--KVRNVSSNIQYDS---TR-
-SIFVQVTDSSQLKGSS-DQSRDHFDSITIS--NEDSE-------------YHTLINQREIQQLKESSIL-W
D--PSFLQTERTEIESD--RFPK--CLSGYS--SMSRLFT-EREKQMNNHLLPEEIEEFL--GNPTRS
IRSFFSDRW--SELH-LGSNPTERSTRDQ----KLLKKQQDVS-FVPSRRSE----NKEMVDMFKII
TYLQN--TVSIHPISSDPGCNMVP-KDEPD-MDSSNKISFLNKNPFFD--LFHLFHDRNRGGYT
L--HHDFESE--ERFQEMADLFTLSITEP-DLVYHKGFAFSIDSYGLDQKK--FLNEVFNSRDES
KKKSL---LVLPPIFY--EENESFYRRIRKKSVR-ISCGNDLEDPKPKRVVFASNNIMEAVNQYR
LIRNLIQIQYRTYG--YIRNVSNRFF---LMNRSDRNFEYGIQ---RDQIGNDTLNHRTIMKYTIN
QHLSNLKK-SQKKWFDP----LISRTERSMNRDPDAYRYKWSNGSKNFQEHLEHFVSEQKS--
-RFQVVFDRLRINQYSIDWSEVIDKQDLSKSLRFFLSKS---------LLFLS-------KLLLFLSK-SL
PFFFVSFGNIPIHRSE-IHIY-ELKGPNDQLCNQLLESIGVQIVHLNKLKPF--LLD---DHDTS-Q
KSKFLINGGTISPFLFNKIP-KW-MIDSFHT--RNNRRKSFDNTDSYFSMISHDRDNWLNPVK
PFHRSSLISSFYKANRLRFLNNPHYFWFY--CNKRFPFYV----EKARINNYD--LTY-GQFLNIL
FIRNK-IFSLCVGK--KKYAFLE-RDTISPIESQVSNIFIPNDFPQSGDET-YNLYKS---FHFPIRS
DPFVR--RAIYSIADISGTPLTEGQIVNFERTYCQPLSDMNLSDSEGKNLHQYLN--FNSNMG
LIHTPCSEKYLP--SEKRKK--------RSLCLK--KCVEKRQMYRTFQRDSAFSNLSKWNLFQTY
MPWFLTSTGCKYLNFTLLDTFSDLL--PILSSS-HKFVSIFHDIMHGSDISWPIPQKKW------CL
PQWNL-ISEISSKCLHN--LLLSEEMIHRNNESPVPLIWTHLRSPNAREF-LYSILFLLLVAGY-L
VRTHLLFVSR--ASSELQTEFEKIKSLMIPSYMMELRKLLDRYPTSELNSFWLKNLFLVALE
QLGDSLEEIRGSASGGNMLLGGGP-----AYG-VKSIRSKKKYLNIN---LIDLI-SIIPNPINRITFS
RNTRHLSRTSKEIYSLIRKRKNVNGDWIDDKIESWVANSDSIDDEEREFLFQFS--TLTT--EK
RIDQILLSLT-HSDHLS-----KNNSGYQMIEQPGSIYLRYLVDIH--KKYLMNYEFNRSCLAER

RIFLAHYQTITY--SQTSCGANSFHFPSHG---KPFSLRLALSPS-RGILVIGSIGTGRSYLVKYLA
TNSYVPFITVFPNKFLDDKPKGYLNDD---IDIDDIDDSDDID----------------------DSNDIDDD
LDTELLTMTNAL---TMYMT---PKIDRFDITLQFELAKAMFPCIIWIPNIHDLYVNESNYLSLG
LLVNYLS---RDCERCS-TRNILVIASTHIPQKVDPALIAPNKLNTCIKIRRLLIPQQRKHFFTLS
YTRGFHLEKKMFHT--NGFGSITMGSNARDLVALTNEALSISITQKKSIIDTNTIRSALHRQT
WDLR---SQVRSVQDHGILFYQIGRAVAQNVLLSNCPIDPISIYMKKK--SCKEGDSYLYKWY
FELGTSMKKLTILLYLLSCSAGSVAQDLWSLP--GPDEK-NWITSYGFVENDS-DLVHGLLEV
EGALVG--SSRTEKDCSQFDND--RVTLLLRSEPRN-PLDMMQNGSCSIVDQRFLYEK--YESE
FEE-GEGEGALDPQ----QIEED----LFNH--IVWAP--RIWRP---CGNLFDCIERPNELGFP-YWA
RSFR---GKRIIYHKEDELQENDSEFLQSGTMQYQTRDRSSKEQGFFRISQFIWDPADPFFFLF
K-DQPSVSVFSRREFFADEEMSKGLLTSQTNPPTSIYKHWFI--KNTQEKHFELLIHRQRWLR
T---NSSLS--NGSFRSN--TLFESYQYLSNLFLSN-GTLLDQMTKTLLRKRWLFPDEMKHLI--H
VTGERFPIPMPRSR-INGNFIDKTSSIVANILLRIIPTTSGEKEAFTYYRDG--MSAQSEGNYAE
ALQNYYEATRLEIDPYDRSYILYNIGLIHTSNGEHTKALEYYFRALERNPFLPQAFNNMAVI
CHY-RGEQAIQQGDSEIAEAWSDQAAEYWKQAIALTPGNYIEAHNWLKITRRFE--MNWRS
ERIWIELITGSRKTSNFCWAFILFLGSLGFLLVGTSSYLGRNLISLFPSQQIIFFPQGIVMSFYG
IAGLFISSYLWCTISWNVGSGYDRFDRKEGIVCIFRWGFPGKNRRIFLRFLMRDIQSIRIEVK
EGIYPRRVLYMEIRGQGAIPLTRTDEN-LTPREIEQKAAELAYFLRVPIEVF
>Olea
MERWWFNSM--INKSMDNLGPIENTSKSED-NRK--AKNIHSYSNVDHLFGIKDIRNFISDDT
FLVRDSNGDSYS-YFDIENQIFEIDND--HSFLSELESSFYSNRNSSYLNSTS--EDPYYN--MY
DTQYSWNNHINSCIDSYLQ--SQIYMDSIVSDSS-SY----ISRCIFGSARSGIRTHTKSSDLTLRE
RSNDLDVTQKYRHLWVQCENCYGLNYKKFLKSKTNICEQCGYHLKMSSSDRIELSIDPGT
WHPMDEDMVSLDPIGFHSEEE-YKDRIDSYQRKTGLTEAVQTGIGQLNGISVAIGVMDFQF
MGGSMGSVVGEKITRLIEYATNKFLPLIIVCASGGARMQEGSLSLMQMAKISSALYDYQS
NKKLFYVSILTSPTTGGVTASFGMLGDIIIAEPNSYIAFAGKRVIEQTLNKTVPEGSQAAEYL
FQKGLFDLIVPRNPLKSVLSELFKLHAFFPLN-----MVTIRADEISNIIRERIEQYNREVKIVNT
GTVLQVGDGIARIHGLDEVMAGELVEFEEGTIGIALNLESNNVGVVLMGDGLMIQEGSSV
KATGRIAQIPVSEAYLGRVINALAKPIDGRGEISASESRLIESPAPGIISRRSVYEPLQTGLIAI
DSMIPIGRGQRELIIGDRQTGKTAVATDTILNQQGQNVICVYVAIGQKASSVAQVVTTLQER
GAMEYTIVVAETADSPATLQYLAPYTGAALAEYFMYRKQHTLIIYDDLSKQAQAYRQMSL
LLRRPPGREAYPGDVFYLHSRLLERAAKSSSSLGEGSMTALPIVETQSGDVSAYIPTNVISIT
DGQIFLSADLFNAGIRPAINVGISVSRVGSAAQIKAMKQVAGKLKLELAQFAELEAFAQFAS
DLDKATQNQLARGQRLRELVKQSQAAPLAVEEQIMTIYTGTNGYLDSLEIGQVRKFLVEL
RTYLKTNKPQFQEIISSTKTFTEEAEVLLKEAIQEQMDRFLLQEQAMRINPTTSGSGVSTLE
KQNLGRIVQIIGPVLDVAFPPGKMPNIYNALVVKGRDTVGQAINVTCEVQQLLGNNRVRA
VAMSATDGLMRGMEVIDTGAPLSVPVGGATLGRIFNVLGEPVDNLGPVDIRTTSPIHRSAP
AFIQLDTKLSIFETGIKVVDLLAPYRRGGKIGLFGGAGVGKTVLIMELINNIAKAHGGVSV
FGGVGERTREGNDLYMEMKESGVINEENIAESKVALVYGQMNEPPGARMRVGLTALTMA
EYFRDVNEQDVLLFIDNIFRFVQAGSEVSALLGRMPSAVGYQPTLSTEMGTLQERITSTKE
GSITSIQAVYVPADDLTDPAPATTFAHLDATTVLSRGLAAKGIYPAVDPLDSTSTMLQPRIVG
EEHYETAQRVKQTLQRYKELQDIIAILGLDELSEEDRLTVARARKIERFLSQPFFVAEVFTGS
PGKYVGLAETIRGFQLILSGELDGLPEQAFYLVGNIDEATAKAMNLEMESNLKKMTLNLC
VLTPNRIGWDSEVKEIILYTNSGQIGVLPNHAPIATAVDIGILRIRFKDQWLTMALMGGFARI

GNNEIIILVNDAEKGSDIDPQEAQKTLEIAEANLRKAEGKRQIIEANLALRRARTRVEAVNV
ISMKNVTDSFVSLGHWPSAGSFGFNTDILATNPINLSVVIGVLIFFGKGVLSDLLDNRKQRI
LNTIRNSEELRGGAIEQLEKARARLRKVEMEADQFRVNGYSEIEREKWNLINSTSKTLEQL
ENYKNETIQFEQQRAINQVRQRVFQQALQGALGTLNSCLNNELHLRTISANIGMLGAMKE
ITDMNPLISAASVIAAGLAVGLASIGPGVGQGTAAGQAVEGIARQPEAEGKIRGTLLLSLAF
MEALTIYGLVVALALLFANPFVMNVLSCSINTLKGLYDISAVEVGQHFYWQIGGFQVHGQ
VLITSWVVIAILLGSATIAVRNPQTIPTGGQNFFEYVLEFIRDVSKTQIGEEYGPWVPFIGTM
FLFIFVSNWSGALLPWKIIQLPHGELAAPTNDINTTVALALLTSVAYFYAGLTKKGLSYFGK
YIQPTPILLPINILEDFTKPLSLSFRLFGNILADELVVVVLVSLVPSVVPIPVMFLGLFTSGIQA
L I F A T L A A A Y I G E S M E G H
H??
??
??
??
??
??
??????MPIGVPKVPFRSPGEEDASWVDVYNRLYRERLLFLGQEVDSEISNQLIGLMVYLSIE
DDTKDLYLFINSPGGWVIPGVAIYDSMQFVRPDVHTICMGLAASMGSFILVGGEITKRLAF
PHARVMIHQPASSFYEAQTGEFILEAEELLKLRETLTRVYVQRTGKPLWVVSEDMERDVF
M S A T E A Q A Y G I V D L V A V
E??MEEIQRY
LQ-DRSQQHDFLYPLIFQEYIYGLAHDQGFPGYDNKSSLLIVKRLITQMYQQNHFLISANDS
NQNPFLGKNLYPQIISEGFAFIVEIPFSLRLILSLIVKSHNLRSIHSIFPFLEDNFSHLNFVLDILI
PHPIHLEILVQTLRYWVKDASSLHLLRFFLHEYCNWNSLITAS-SSFSKQRLFFFLYNSHVCE
YESIFVFLRKQSSHLRSTSSGALLERIYFYGKIERLVDVFV-DFNLCLFKDPFMHYVRYQGK
SILASKGTPLLMNKWKYYLVTFWQCYFSLWFHPRRIYLNQLSNHSLEFVGYLSSVRLNAS
VVRSQILENSFLINNAI--KKFDTLVPIIPLIGSLAKAKFCNVLGYPISKPVRADLSDSDIIDRF
GRICRNLSHYHSGSSKKKSLYRIKYILRLSCARTLARKHKSTVRAFLKRLGSELLEEFLMSE
EQVLSLTFRKRIWYLDIICINDLINHKMIIDNTTELQ-AIHSFSRLEF-LKEVYGIIWVLVPILTL
VLGITLGVLVIVWLEREISAGIQQRIGPEYAGPLGILQALADGVKLLFKENLFPSRGDTRLF
SIGPSIAVISILLSYSVIPFGYRLILADLSIGVFLWIAVSSLAPVGLLMSGYGSNNKYSFLGGL
RAAAQSISYEIPLTLCVLSISLLSNSSSTVDIVEAQSKYGFWGWNLWRQPIGFIVFLISSLAE
CERLPFDLPEAEEELVAGYQTEYSGIKFGLFYVASYLNLLVSSLFVTVLYLGGWNLSIPYIF
VPELFE---RGKVFGTIIGIFITLAKTYLFLFISIATRWTLPRLRMDQLLNLGWKFLLPISLGNL
LLTTSFQLLSLMIWHVQNENFILDSTRIFMKAFHLLLFDGSLIFPECILIFGLILLLMIDSTSD
QKDIPWLYFISSTSLVMSITALLFRWREEPMISFSGNFQTNNFNEIFQFLILLCSTLCIPLSVE
YIECTEMAITEFLLFVLTATLGGMFLCGANDLITIFVAPECFSLCSYLLSGYTKKDVRSNEAT
MKYLLMGGASSSILVHGFSWLYGSSGGEIELQEIVNGLINTQMYNSPGISIALIFITVGIGFK
LSPAPSHQWTPDVYEGSPTPVVAFLSVTSKVAASASATRIFDIPFYFSSNEWHLLLEILAILS
MILGNIIAITQTSMKRMLAYSSIGQIGYVIIGIIVGDSNDGYASMITYMLFYISMNLGTFACI
VLFGLRTGTDNIRDYAGLYTKDPFLALSLALCLLSLGGLPPLAGFFGKLYLFWCGWQAGL
YSLVLIGLLTSVVSIYYYLKIIKLLMTGRNQEITPHVRNYRRSPLRSNNSIELSMIVCVIASTI
PGISMNPIIAIAQDTLFMFLLYEYDIFWAFLIISSLIPILAFFISGILAPIRKGPEKLSSYESGIEP
MGDAWLQFRIRYYMFALVFVVFDVETVFLYPWAMSFDVLGVSVFIEALIFVLILIVGLVYA

WRKGALEWSTNHFPWLTIIVILPISAGSLIFFLPHRGNRVIRWYTICICILELLITTYAFCYHF
QSGDPLIQLVEDYKWIDFFDFHWRLGIDGLSIGPILLTGFITTLATLAARPVTRDSRLFHFLM
LAMYSGQIGLFSCRDLLLFFLMWELELIPVYLLLSMWGGKKRLYSATKFILYTAGGSVFLL
MGVLGIGLYGSNEPTLNLEILANQSYPVALEIIFYIGFFIAFAVKLPIIPLHTWLPDTHGEAHY
STCMLLAGILLKMGAYGLVRINMELLPHAHSIFSPWLMIVGAMQIIYAASTSFGQRNLKKR
IAYSSVSHMGFIIIGIGSITDTGLNGALLQIISHGFIGAALFFLAGTTYDRIRLVYLDEMGGIAI
PMPKIFTMFSSFSMASLALPGMSGFVAELIVFLGLITSQKYLLIPKILITFVMAIGMILTPIYS
LSMSRQMFYGYKIFNAPNSYFFDSGPRELFLSISIFLPILGIGMYPDFVLSLSVDKVEVILSN
SFYRMLLEHVLVLSAYLFSIGIYGLITSRNMVRALMCLELILNSVNINFVTFSGFFDNRQLR
GDIFSIFVIAIAAAEAAIGPAIVSSIYRNRKSTRINQSNLLNKMEQTYQYAWIIPFVPLPVPML
IGVGLILFPTATKNLRRMWAFPSILLLSIVMIFSINLSIQQINSSCIYQYVWSWTINNDFSLEF
GYLIDPLTSIMSILITTVGIMVLIYSDNYMAHDQGYLRFFAYMSFFSTSMLGLVTSSNLIQIYI
FWELVGMCSYLLIGFWFTRPIAANACQKAFVTNRVGDFGLLLGILGFYWITGSFEFRDLFE
ILNNNKVNSSFVTLCAALLFTGAVAKSAQFPLHVWLPDAMEGPTPISALIHAATMVAAGIF
LVARLLPLFIVIPYIMNLISFIGIITVLLGATLALAQKDIKRGLAYSTMSQLGYMMLALGMG
SYRSALFHLITHAYSKALLFLGSGSIIHSMETIVGYSPDKSQNMALMGGLTKHVPITRISFLL
GTISLCGIPPLACFWSKDEILNDSWLYSPIFAIIAWTTAGLTAFYMFRIYLLTFEGHLNVHFQ
NYSGKQNTPFYSISLWGKGGSKR----NFRLLKMNFFSFENKKDYSYPYESDNTMLFPLLVL
GIFTLFVGSLGIPFELDILTKWLTPSINLLHQKLN-SIDWYEFLKDAIFSVSIAYFGIFIASFLYK
PIYSSFKNFDLINLFVSLWDTILNGLYNWSYNRAYIDAFYTTSLTGAVRGLAQLTHFFDRRV
IDGITNGVGVMSFFVGEGIKYVGGG-RISSYLFFYLSCVSIFLLGLYMGLPGPIHDFLLVFLG
SGLILGSLGVVLLPNPIYSAFSLGLVLFCISLFYILSNSYFVAAAQLLIYVGAINILIIFAVMFM
SGSEYYKDFHLWTVGDGVTSIVCTSLFISLITTIPDTSWYGIIWTTKSNQIIEQDLISNSQQIG
IHLSTDFFLPFELISIILLVALIGAIAIARQMIAPTTRKDLMIVNMGPHHPSMHGVLRLIVTLD
GEDVIDCEPILGYLHRGMEKIAENRTIIQYLPYVTRWDYLATMFTEAITVNGPEQLGNIQVP
KRASYIRAIMLELSRIASHLLWLGPFMADIGAQTPFFYIFRERELIYDLFEAATGMRMMHN
YFRIGGIAADLPHGWIDKCLDFCDYFLTGVAEYQKLITRNPIFLERVEGVGIIGGEEALNWG
LSGPMLRASGIQWDLRKVDHYECYDEFDWEVQWQKEGDSLARYLVRIGEMTESIKIIQQ
ALEGIPGGPFENLEIRRFDRVKDTEWNDFEYRFISKKPSPTFELSKQELYVRVEAPKGELGIF
LIGDQSVFPWRWKIRPPGFINLQILPQLVKRMKLADIMTILGSIDIIMGEVDRMFPMVTEFM
NYGQQTIRAARYIGQGFMITLSHVNRLPVTIQYPYEKLITSERFRGRIHFEFDKCIACEVCV
RVCPIDLPVVDWKLETDIRKKRLLNYSIDFGICIFCGNCVEYCPTNCLSMTEEYELSTYNR
HELNYNQIALGRLPVSIIDDYTIRPISNS----------------MQGRLSAWLVKHGLIHRSLGFDYQ
GIETLQIKPKDWHSIAVILYVYGYNYLRSQCAYDVAPGGLLASVYHLTRIEYGVDQPEEVC
IKVFASRRNPRIPSVFWVWKSVDFQERESYDMLGISYDNHPRLKRILMPESWIGWPLRKD
Y I A P N F Y E I Q D A
H???
??
??
????????????????????MQTRNTFSWIKEQITRSISVSLMIYIITRTSISSAYPIFAQQGYENPREA
TGRIVCANCHLANKPVDIEVPQAVLPDTVFEAVVRIPYDKQLKQVLANGKKGGLNVGAV
LILPEGFELAPTDRISPEMKEKIGNLSFQSYRPNKKNILVIGPVPGQKYSEITFPILSPDPATK
KDVHFLKYPIYVGGNRGRGQIYPDGSKSNNTVYNATAAGIVSKIIRKEKGGYEISITDPSDV
RQVVDIIPPGPELLVSEGESIQFDQPLTSNPNVGGFGQGDAEIVLQDPLRVQGLLFFLASVIL

AQIFLVLKKKQFEKVQLAEMNFMSKVYDWFEERLEIQAIADDITSKYVPPHVNIFYCLGGI
TLTCFLVQVATGFAMTFYYRPTVTEAFASVQYIMTEANFGWLIRSVHRWSASMMVLMMIL
HVFRVYLTGGFKKPRELTWVTGVVLGVLTASFGVTGYSLPRDQIGYWAVKIVTGVPEAIPV
IGSPLVELLRGSASVGQSTLTRFYSLHTFVLPLLTAVFMLMHFPMIRKQGISGPLMGVTKKP
DLNDPVLRAKLAKGMGHNYYGEPAWPNDLLYIFPVVILGTIACNVGLAVLEPSMIGEPAD
PFATPLEILPEWYFFPVFQILRTVPNKLLGVLLMVSVPAGLLTVPFLENVNKFQNPFRRPVA
TTVFLIGTAVALWLGIGATLPIDKSLTLGLFMIEVFLFGIVLGLIPITLAGLFVTAYLQYRRGD
QLDLMLTITSYFGFLLAALTITSALFIGLSKIRLIMDIVSLAWAALMVVFTFSLSLVVWGRSG
LMIIRSPEPEVKILVDRDPVKTSFEEWARPGHFSRTIAKGPDTTTWIWNLHADAHDFDSHT
SDLEEISRKVFSAHFGQLSIIFLWLSGMYFHGARFSNYEAWLSDPTHIGPSAQVVWPIVGQ
EILNGDVGGGFRGIQITSGFFQIWRASGITSELQLYCTAIGALVFAALMLFAGWFHYHKAA
PKLAWFQDVESMLNHHLAGLLGLGSLSWAGHQVHVSLPINQFLNAGVDPKEIPLPHEFIL
NRELLAQLYPSFAEGATPFFTLNWSKYAEFLTFRGGLDPVTGGLWLTDIAHHHLAIAILFLI
AGHMYRTNWGIGHGLKDILEAHKGPFTGQGHKGLYEILTTSWHAQLSLNLAMLGSLTIVV
AHHMYSMPPYPYLATDYGTQLSLFTHHMWIGGFLIVGAAAHAAIFMVRDYDPTTRYNDL
LDRVLRHRDAIISHLNWACIFLGFHSFGLYIHNDTMSALGRPQDMFSDTAIQLQPVFAQWI
QNTHALAPGATAPGATASTSLTWGGGDLVAVGGKVALLPIPLGTADFLVHHIHAFTIHVTV
LILLKGVLFARSSRLIPDKANLGFRFPCDGPGRGGTCQVSAWDHVFLGLFWMYNSISVVIF
HFSWKMQSDVWGSISDQGVVTHITGGNFAQSSITINGWLRDFLWAQASQVIQSYGSSLSA
YGLFFLGAHFVWAFSLMFLFSGRGYWQELIESIVWAHNKLKVAPATQPRALSIVQGRAVG
VTHYLLGGIATTWAFFLARIIAVGMALRFPRFSQGLAQDPTTRRIWFGIATAHDFESHDDIT
EERLYQNIFASHFGQLAIIFLWTSGNLFHVAWQGNFESWVQDPLHVRPIAHAIWDPHFGQP
AVEAFTRGGALGPVNIAYSGVYQWWYTIGLRTNEDLYTGALFLLFLSAISLIAGWLHLQP
KWKPSVSWFKNAESRLNHHLSGLFGVSSLAWTGHLVHVAIPGSRGEYVRWNNFLEVLPH
PQGLGPLFTGQWNLYAQNPDSSSHLFGTSQGAGTAILTLLGGFHPQTQSLWLTDIAHHHLA
IAFIFLVAGHMYRTNFGIGHSIKDLLDAHIPPGGRLGRGHKGLYDTINNSLHFQLGLALASL
GVITSLVAQHMYSLPAYAFIAQDFTTQAALYTHHQYIAGFIMTGAFAHGAIFFIRDYNPEQN
EDNVLARMLDHKEAIISHLSWASLFLGFHTLGLYVHNDVMLAFGTPEKQILIEPIFAQWIQS
AHGKTSYGFDVLLSSTSGPAFNAGRSIWLPGWLNAVNE--NSNSLFLTIGPGDFLVHHAIAL
GLHTTTLILVKGALDARGSKLMPDKKDFGYSFPCDGPGRGGTCDISAWDAFYLAVFWML
NTIGWVTFYWHWKHITLWQGNVSQFNESSTYLMGWLRDYLWLNSSQLINGYNPFGMNS
LSVWAWMFLFGHLVWATGFMFLISWRGYWQELIETLAWAHERTPLANLIRWRDKPVALSI
VQARLVGLAHFSVGYIFTYAAFLIASTSGKFGMSHSVKIYDTCIGCTQCVRACPTDVLEMI
PWDGCKAKQIASAPRTEDCVGCKRCESACPTDFLSVRVYLWHETTRSMGLAYMTTFNFP
SIFVPLVGLVFPAIAMASLSLHVQKNKIVMRDLKTYLSVAPVLSTLWFGALAGLLIEINRFF
PDALTFPFFSFMTAILERRESESLWGRFCNWITSTENRLYIGWFGVLMIPTLLTATSVFIIAFI
AAPPVDIDGIREPVSGSLLYGNNIISGAIIPTSAAIGLHFYPIWEAASVDEWLYNGGPYELIV
LHFLLGVACYMGREWELSFRLGMRPWIAVAYSAPVAAAAAVFLIYPIGQGSFSDGMPLGIS
GTFNFMIVFQAEHNILMHPFHMLGVAGVFGGSLFSAMHGSLVTSSLIRETTENESANAGY
RFGQEEETYNIVAAHGYFGRLIFQYASFNNSRSLHFFLAAWPVVGIWFTALGISTMAFNLN
GFNFNQSVVDSQGRVINTWADIINRANLGMEVMHERNAHNFPLDLASIEVPSTNGMGLP
WYRVHTVVLNDPGRLLSVHIMHTALVAGWAGSMALYELAVFDPSDPVLDPMWRQGMFV
IPFMTRLGITNSWGGWSIT-GGTVTNPGIWSYEGVAGAHIVFSGLCFLAAIWHWVYWDLEI
FCDERTGKPSLDLPKIFGIHLFLSGVACFGFGAFHVTGLYGPGIWVSDPYGLTGKVQPVNPA

WGVEGFDPFVPGGIASHHIAAGTLGILAGLFHLSVRPPQRLYKGLRMGNIETVLSSSIAAVF
FAAFVVAGTMWYGSATTPIELFGPTRYQWDQGYFQQEIYRRVSAGLAENQSLSEAWSKIP
EKLAFYDYIGNNPAKGGLFRAGSMDNGDGIAVGWLGHPIFRDKEGRELFVRRMPTFFETF
PVVLVDGDGIVRADVPFRRAESKYSVEQVGVTVEFYGGELNGVSYSDPATVKKYARRAQ
LGEIFELDRATLKSDGVFRSSPRGWFTFGHASFALLFFFGHIWHGARTLFRDVFAGIDPDLD
AQVEFGAFQKLGDPTTRRQVVMKTLYSLRRFYHVETLFNGTLAVTGRDQETTGFAWWAG
NARLINLSGKLLGAHVAHAGLIVFWAGAMNLFEVAHFVPEKPMYEQGLILLPHLATLGW
GVGPGGEVIDTFPYFVSGVLHLISSAVLGFGGIYHALLGPETLEESFPFFGYVWKDRNKMT
TILGIHLILLGLGAFLLVFKALYFGGVYDTWAPGGGDVRKITNLTLSPSIIFGYLLKSPFGGE
GWIVSVDDLEDIIGGHVWLGSICILGGIWHILTKPFAWARRALVWSGEAYLSYSLGALSIFG
FTACCFVWFNNTAYPSEFYGPTGPEASQAQAFTFLVRDQRLGANVGSAQGPTGLGKYLM
RSPTGEVIFGGETMRFWDLRAPWLEPLRGPNGLDLSRLKKDIQPWQERRSAEYMTHAPL
GSLNSVGGVATEINAVNYVSPRSWLATSHFVLGFFFFVGHLWHAGRARAAAAGFEKGIDR
DFEPVLSMTPLNMTIALGKFTKDEN-LFDIMDDWLRRDRFVFVGWSGLLLFPCAYFALGG
WFTGTTFVTSWYTHGLASSYLEGCNFLTAAVSTPANSLAHSLLLLWGPEAQGDFTRWCQL
GGLWTFVALHGAFGLIGFMLRQFELARSVQLRPYNAIAFSAPIAVFVSVFLIYPLGQSGWFF
APSFGVAAIFRFILFFQGFHNWTLNPFHMMGVAGVLGAALLCAIHGATVENTLFEDGDGA
NTFRAFNPTQAEETYSMVTANRFWSQIFGVAFSNKRWLHFFMLFVPVTGLWMSALGVVG
LALNLRAYDFVSQEIRAAEDPEFETFYTKNILLNEGIRAWMAAQDQPHENLIFPEEVLPRG
NALMSGSTGERSFADIITSIRYWVIHSITIPSLFIAGWLFVSTGLAYDVFGSPRPNEYFTESRQ
GIPLITGRFDPLEQLDEFSRSFMTIDRTYPIFTVRWLAVHGLAVPTVFFLGSISAMQFIQRMA
TQTVENSSRSG-PRRTTVGSLLKPLNSEYGKVAPGWGTTPLMGVAMALFAVFLSIILEIYNS
SVLLDGISMNMLTLKLFVYTVVIFFVSLFIFGFLSNDPGRNPGREEMADTTGRIPLWIIGTV
TGILVIGLIGIFFYGSYSGLGSSLMLNIFSLICICLNSALY-SSFFFGKLPEAYAFLNPIVDVMP
VIPLFFFLLAFVWQAAVSFRMTQSNPNEQNVELNRTSLYWGLLLIFVLAVLFSNYFFNMEV
NILAFIATALFILVPTAFLLIIYVKTVSQNDMETATLVAIFISGLLVSFTGYALYTAFGQPSQQL
R D P F E E H G D M E A L V Y T F L L V S T L G I I F F A I F F R E P P K V P T K K M
K??-------MSPQTETKASVGF
KAGVKEYKLTYYTPQYETKDTDILAAFRVTPQPGVPPEEAGAAVAAESSTGTWTTVWTD
GLTSLDRYKGRCYHIEPVPGEADQYICYVAYPLDLFEEGSVTNMFTSIVGNVFGFKALRAL
RLEDLRIPPAYIKTFQGPPHGIQVERDKLNKYGRPLLGCTIKPKLGLSAKNYGRAVYECLR
GGLDFTKDDENVNSQPFMRWRDRFLFCAEALYKAQAETGEIKGHYLNATAGTCEEMIKR
AVFARELGVPIVMHDYLTGGFTANTSLAHYCRDNGLLLHIHRAMHAVIDRQKNHGIHFRV
LAKALRMSGGDHIHSGTVVGKLEGERDITLGFVDLLRDDFIEKDRSRGIYFTQDWVSLPG
VIPVASGGIHVWHMPALTEIFGDDSVLQFGGGTLGHPWGNAPGAVANRVALEACVKARNE
GRDLASEGNVIIREASKWSPELAAACEVWKEIKFEFQAMDTL--MAIHLYKTSTPSTRNGT
VDSQVKSNPRNNLIYGQHHCGKGRNARGIITARHRGGGHKRLYRKIDFRRNEKDIYGRIV
TIEYDPNRNAYICLIHYGDGEKRYILHPRGAIIGDTIVSGTEVPIKMGNALPLTDMPLGTAIH
NIEITLGKGGQLVRAAGAVAKLIAKEGKSATLKLPSGEVRLISKNCSATVGQVGNVGANQ
KSLGRAGSKRWLGKRPVVRGVVMNPVDHPHGGGEGRAPIGRKKPTTPWGYPALGSRSR
KRNKYSDNLILRRRSKMIQPQTHLNVADNSGARELMCIRIIGAS-NRRYAHIGDVIVAVIKEA
VPNMPLEKSEVVRAVIVRTCKELKRDSGMIIRYDDNAAVVIDQEGNPKGTRIFGAIPRELR
QFNFTKIVSLAPEVLMLSPKRTRFRKQHRGRMKGISYRGNHICFGKYALQTLEPAWITSRQI
EAGRRAMTRNARRGGKIWVRIFPDKPVTVRPAETRMGSGKGSPEYWVAVVKPGRILYEIG

GVTENIARRAISIAASKMPIRTQFIISGMTRIKRGYIARRRRTKIRLFASSFRGAHSRLTRTITQ
QKIRALVSAHRDRDKQKRNFRRLWITRINAVIRE-KGVSYSYSKLIHDLYKRQLLLNRKILA
QIAISNRNCLYMISNEIKE-VDWKESTGIIMRKEVYALGRHISLSADKARRVIDQIRGCSYEE
TLMILELMPYRACYPIFKLVYSAAANASYNMGSNEANLVISKAEVNEGTTVKKFKPRARG
RSYPIKRPTCHITIGVKDISLHGGVWDKKMDGIKYAVFTDKSIRLLGKN-QYTSNVESGSTR
TEIKHWVELFFGVKVIAMNSHRLPGKGRRMGPIMGHTMHYRRMIITLQPGYSIPPLRKKR
TMAVPKKRTSASKKRIRKNIWKRKGYGTALKAFSLGKSLSTGNSKSFFVRKK--MAKGKD
ARVTVILECTSCVRNG--VKKVSRGISRYITQKNRHNTPNRLELRKFCPYCYKHMIHGEIKK
MKIRASVRKICEKCRLIRRRGRIIVICSNPRHKQRQGMVREKVTVSTRTLQWKCVESREDS
KRLYYGRFILSPLMKGQADTIGIAMRRALLGEIEGTCITRVKSE--NVPHEYSTIAGIQESVH
EILMNLKEIVLRSNLYGTCDASICVRGPGYVTAQDIILPPYVEIVDNTQHIASLTEPIDLCIGL
QIKRNRGYLIKMPH-NFDGSY--PIDAVFMPVRNANHSIHSYGNGNEKQEILFLEIWTNGSLT
PKEALHEACRNLIDLFIPFLHKEEE--NLPLEDNQ-HTVPLSPLTFHDKLD-KLRKNKK-KIAL
KSIFIDQSEFSPRVYNCLKRSNIYTLLDLLNNSPEDLMKIEHLRLEDIKQILVILEKH--FAIDL
PKKKMLGDG-NEGMSTIPGFNQIQFEGFCRFIDQGLTEELYKFPKIEDTDQEIEFQLFVERY
QLVEPLIKERDAVYESLTYSSELYVSGGLIWKTSRDMQEQTIFIGNIPLMNSLGTSIVNGIYR
IVINQILQSPGIYYRSELDHNGISVYTGTIISDWGGRSELEIDRKARIWARVSRKQKISILVLS
SAMGSNLREILDNVCYPEIFLSFLNDKERKKIGSKENAILEFYQQFACVGGDPVFSESLCKE
LQKKFFQQRCELGRIGRRNMNRRLNLDIPQNNTFLLPRDILAAADHLIELKFGMGTLDD--
MNHLKNKRIRSVADLLQDQFGLALVRLENVVRGTICGAIR-HKL-IPTPQNLVTSTP----LTT
TYESFFGLHPLSQVLDRTNPLTQIVHGRKLSYLGPGGLTGRTASFRIRDIHPSHYGRICPIDT
SEGINVGLIGSLAIHARIGHWG-SLESPFY---EISERSTG--VRMLYLSPGRDEYYMVAAGNSL
ALNQDIQEEQVVPARYRQEFLTIAWEQVHLRSIFPFQYFSIGASLIPFIEHNDANRALMSSN
MQRQAVPLSRPEKCIVGTGLERQAALDSGALAIAEREGKIIYTDTEKILFSGNGDTLSIPLV
MYQRSNKNTCMHQKPQVQRGKCIKKGQILADGAATVGGELALGKNVLVAYMPWEGYN
SEDAVLISERLVYEDIYTSFHIRKYEIQIHVTSQG-PERITNEIPHFEAHLLRNLDKNGIVMLG
SWVETGDILVGKLTPQMVKE-SSYAPEDRLLRAILGIQVSTSKETCLKLPIGGRGRVIDVRW
IQKRG-GSSYNPETIRIYISQKREIKVGDKVAGRHGNKGIISKILPRQDMPYLQDGRPVDMV
FNPLGVPSRMNVGQIFECSLGLAGGLLDRHYRIAPFDERYEQEASRKLVFSELYEASKQTA
NPWVFEPEYPGKSRIFDGRSGNPFEQPVLIGKPYILKLIHQVDDKIHGRSSGHYALVTQQPL
RGRAKQGGQRVGEMEVWALEGFGVAHILQEMLTYKSDHIRARQEVLGTTIIGGTIPNPED
APESFRLLVRELRSLALELNHFLVSEKNFQINRKEAMNQNFSSMIDRYKHQQLRIGLVSPQ
QISAWATKILPNGEIVGEVTKPYTFHYKTNKPEKDGLFCERIFGPIKSGICACGNYRVIGDE
KEDPKFCEQCGVAFVDSRIRRYQMGYIKLACPVTHVWYLKRLPSYIANLLDKPLKELEGL
VYCD--FSFARP-ITKKPTFLRLRGLFEYEIQSWKYSIPLFFTTQGFDTFRNREISTGAGAIREQ
LADLDLRIILDNSLVEWKELGEE-GPTGNEWEDRKVGRRKDFLVRRMELAKHFIRTNIEPE
WMVLCLLPVLPPELRPIIQIDGGKLMSSDINELYRRVIYRNNTLTDLLTTSKSTPGELVMCQ
EKLVQEAVDTLLDNGIRGQPMRDGHNKVYKSFSDVIEGKEGRFRETLLGKRVDYSGRSVI
VVGPSLSLHRCGLPREIAIELFQTFVIRGLIRQHLASNIGVAKSKIREKEPIVWEILQEVMQG
HPVLLNRAPTLHRLGIQAFQPVLVEGRAICLHPLVCKGFNADFDGDQMAVHVPLSLEAQA
EARLLMFSHMNLLSPAIGDPISIPTQDMLIGLYVLTSGNRRGICVNRYNPWNR---RNYQNQ
RSNNNNYKYT---EPFFSNSYDAIGAYRQKRINLDSPLWLRWRLDQR-VIAS--RESPIEVHYE
SLGTYYEIYGHYLIVRSIKKEILFIYIRTTVGHISLYREIEEAVQGFSQACSYGTELS-----MEV
LMAERANLVFHNKVIDGTAMKRLISRLIDHFGMAYTSHILDQVKTLGFHQATATSISLGID

DLLTIPSKRWLVQDAEQQSLILEKHHHYGNVHAVEKLRQSIEIWYATSEYLRQEMNPNFR
MTDPFNPVHIMSFSGARGNASQVHQLVGMRGLMSDPQGQMIDLPIQSNLREGLSLTEYIIS
CYGARKGVVDTAVRTSDAGYLTRRLVEVVQHIVVRRTDCGTTRGISVSPRNGMMP-ERIFI
QTLMGRVLADDIYTGTRCIASRNQDIGIGLVNRFIT------FRAQPISIRTPFTCRSASWICRLC
YGRSPTHGDLVELGEAVGIIAGQSIGEPGTQLTLRTFHTGGVFTGGTAEHVRAPSNGKIKFN
EDLVHPTRTRHGHPAFLCSIDLYVTIESEDILHNVNIPSKSFLLVQNDQYVESEQVIAEIRAG
TST-LNFKEKVRKHIYSDSDGE-MHWSTDVYHPPEFTYGNVHLLPKTSHLWILLGGPCR--S
SLVSLSLHKDQDQINAH--SRSVKRRYTSNLS--ETNDP-ERQKLFSSYFYGKKEDRISDY-SD
LNRIRCNGRCNLIYP-TILHQNS--DLFSKRRRNRFIIPLQSIQERENELMPRSGISIEIP--PNGIF
RRNSILAYFDDPRYRRKSSGITKYGTIEMHSIVKKED--LIEYRG-VKAFRPKYMKVDRFFFI
PEEVHILPGSSSIMVRNNSLIGVDTQITLNIRSRVGGFVRVERKKKRIELKIFSGDIHFPGETD
KISRHSGVLIP-PG-TGKRNSKESKK--WKNWIYVQR--ITPSK--KKYFVLVRLVVTYEITDGI
NLATLFPPDLLQERDNVQLRVVNYILYGNGKPIRGISDTDIQLVRTCLVLNWDQDKKK--SS
SSEEARASFVEIRTNGLIRHFLRIDLVKSPISYIGKRNDPSGSGLLSD-NGSDCTNINPFSS--IY
---SKARIQQSLNQNQGTIHTLLNR---NTGFQSLIILSSSNCFRMGP--FNDVIKYHN--VKKES
DPRIPIKNSLGPFGT--AFPIVNFYS--FYHL--ITHNQIL-VNNYLQLDN---LKQTFQVIK---YYL
MDEN--EKIYNPEPGSNIILNPFNLNWYFLHHNYCQETS-TIISLGQFICENVCIAKNAPH-LK-
-SGQVLLVQ-VDSVVIRSAKPYLATPGATVHGHYGEILYEGDTLITFIYEKSRSGDITQGLPK
VEQVLEVRSIDSISMNLEKRVEGWNERITRILGMPWGFLIGAELTIVQSRISLVNKIQKVYR
SQGVQIHNRHIEIIVRQITSKVLVSE---DGMSNVFSPGELIGLLRAERMGRALEEAVCYRAV
LLGITRASLNTQSFISEASFQETARVLAKAALRGRIDWLKGLKENVVLGGMIPLGTGFKGL
--VPPSKQDNNSL--LETKKKNLFEGEMR--DILFHHRKLFDSCLSKNFHDTSEQSFIG------FN
DSMTRRYWNINLEEMMEAGVHFGHGARKWNPKMAPYISAKRKGIHITNLTRTARFLSEA
CDLVFDAASRGKQFLIVGTKNKAADSVAWAAIKARCHYVNKKWLGGMLTNWSTTETRL
HKFRDLRMEQKTGRLNRLPKRDAAMLKRQLSRLQTYLGGIKYMTGLPDIVIIVDQHEEYT
ALRECITLGIPTICLIDTNCDPDLADISIPANDDAISSIRLILNKLVFAICEGRS-SYIRNPMGQK
INPLGFRLGTTQGHHSLWFAQPKNYSEGLQEDQKIRDFLKNYIQKNMRISSG-VEGIARIEI
QKRIDLIQVIIFMGFPKLLIENRPR----GIQELQINLEKEFHYVNQKLNVAITRIAKPYGNPNIL
AEFIAGQLKNRVSFRKAMKKAIELTEQADTKGIQVQIAGRIDGKEIARVEWIREGRVPLQTI
RAKIDYCSYTVRTIYGVLGIKIWIFIDEEMSRYRGPRFKKIRRLGALPGLTNKRPKAGSDLR
NQSRSGK-KSQYRIRLEEKHKLRFHYGLTERQLLKYVRIAGKAKGSTGQVLLQLLEMRLD
NILFRLGMASTIPAARQLVNHRHILVNGRIVDIPSYRCKPRDIIMTKDEQKSRTLIPNSLDSS
PH-EELPNHLTLHPFQ--YKGLVNQIIDSKWVGLKINELLVVEYYSRQTMSRRGTAEEKTAK
SDPIYRNRLVNMLVNRILKHGKKSLAYQIIYRAMKKIQQKTETNPLSVLRQAIRGVTPDIAV
KARRVGGSTHQVPIEIGSTQGKALAIRWLLAASRKRPGRNMAFKLSSELVDAAKGSGDAI
RKKEETHKMAEANRAFAHFRMGRDTIAEIITSIRNADMDRKRVVRIASTNITENIVKILFRE
GFIENVRKHREKNKSFLVLNLRHRR--NRKRLY---RNFLNLKRISRPGLRIYSNSQRIPRILGG
MGIVILSTSRGIMTDREARLEGIGGEILCYIWMAKAIPRIGSRRTGRIGSRKSARRIPKGVIH
VQASFNNTIVTVTDVRGRVVSWSSAGTCGFKGTRRGTPFAAQTAAANAIRTVVDQGMQR
A E V M I K G P G L G R D A A L R A I R R S G I L L T F V R D V T P M P H N G C R P P K K R R
V??
????????????????????????????????????MAKKSLIQREKKRQKLEQKYHLIRRSSKKEIS---KVP
SLSDKWEIYGKLQSPPRNSAPTRLHRRCFSTGRPRANYRDFGLSGHILREMVHACLLPGAT
RSSWMVRNSFISVISQ--KENEENRGSVEFQVFSFTTKIRRLTSHLELHKKDYSSQRGLRKIL

GKRQRLLAYLSKKNRVRYKQLIDRLDIRETKTR--MVKLRLKRCGRKQRAVYRIVAIDVRS
RREGKDLRKVGFYDPLKNQTYLNVPAILYFLEKGAQPTGTVQDILKKAEVFKELRPNQTK
FN-MDKSKRPFLKSKRSFRRRLPPIQSGDRIDYRNMSLISRFISEQGKILSRRVNRLTLKQQR
LITIAIKQARILSLLPFLNNEK--QFERTESTARTTGLRTRNKVAGSLKKNPFVVNHLLRKIDK
LNKKAEKEIIVTWSRASTIIPTMIGHTIAIHNGKEHLPIYITDRMVGHKLGEFVPTLNFRGH
AKSDNRSRRMIFQSFLLGNLVSLCMKIINSVVVVGLYYGFLTTFSIGPSYLFLLRAQVMEE
G---TEKKVSATTGFITGQLMMFISIYYAPLHLALGRPHTITALALPYLLFHFFWNNHKHFFD
YGSTTRNSMRNFSIQCVFLNNLIFQLFNHFILPSSMLARLVNIYMFRCNNKMLFVTSSFVG
WLIGHILFMKWLGLVLVWIRQNHSIRSYIRSNKYLVSELRNSMARIFSILLFLTCVYYLGRIP
SPILTKKWKEAEEDKPLVTLLFDSNRWNRPFRYIKNNRFENAVRNEMSQYFFDTCQSAGK
ERISFTYPPSLSTFWEMIKRRISLATLEKFSSNELYNHWVYTNKEKSN-LNNEFVNRIQALD
KESLFLNILETRTRLCNDNTTNEYLPKIYDPFLNGPYRRTITKSVSPIEKVGINRIHGIIYQKI
EQKDTNVQSIRIKEISKKVPRWLYKLTELEQEA-GEIRSRKAKRVVIFTGALIRYSQQSDFRR
GIIKGSIRAQRRKIVIWELFQANVHSPLFLDRIKKSLLFSFDI--SGPIKPIFRNRKENARIEIAE
AWDTIPFAQVIRGSMLITQSLFRKYILLPSLIIAKNIGRMLLLQLPEWYEDLEEWNREMHV
KCTYNGVQLSETEFPQNWLTDGIQIKILFPFCLKPWHRSKKKEKDDFCFLTVLGMETELPF
GSPRKRPSFFEPIFKELEKKIGKFQKKYFLL-LKIFKGKIKLLRKVSKETKKWVIKSVV--FFK
KIRKELLKVNDPIISNQMIHKSFSQIASPDWTNSSLTEKKMKDLTDRTNTIRNQIERITKEKK
KSLNK----TSYNAKRLEKWQILKRRNARLISKLPFVFKFFIERIYTNIFLSIINIPRMKRKLF-E
FISYDLSYLSQAYVFYKLSQTQVINLYKLKSFLQYIPFFLKPEIKDSFTQGIIHSKLKDNRLPS
SEMNQWKNWLRGHYQYDLSQIRWSRLIPQQWRNRVYQRRMVKRKRKWNLYEKDQ--LI
DSKKEDNFQKYSKYDLLSYKSINGDILYPDRKYLDWKIINFDLRFFDWMGMNEKILKRWF
FPEFLLLYNTYKTKSWFIPSKLLLLNYKSNTEAELDLFLKRYLLFQLRWDDTLNQRMINNI
KVYCFLLRLIDPRKISISSIQRGEMSLDIMLIQKNLTLPELMK-GILIIEPIRLSVKNDGQFIMY
QTIGILLVHKSKHNHFDLLVPENIFSFRRRRKLRILICFRFLWPNYRLEDLACMNRYWFDTN
NGSRFSMLRIHLYP--
---------------------------------MNSIGPRNDTLEESV-GSSNINRLIVS-LLYLPKGKKISESCFLN
PKESTWVLPIT----KKCSMPESNWGSRWWRNWIGKKR--DSSCKISN---ETVAGIEILFKEKD
IK-YLEFLFVYYMD--DPIRKDHDWELFDRLSLRKRRNRINL--NSGPLFEILVKHWISYLMS
AFREKIPIEVEG-FFKQQG-AGSTIQSNDIEHVSH--LFSRNKWAISLQNCAQFHMWQFRQDL
FL---SWGK-NPHESDFLRN-VSRENLIWLDNVWLVNKDR---FFR--KVRNVSSNIQYDS---TR
--SSFVQVTDSSQLKGSS-DQSRDHLDSIS----NEDSE------------YHTLINQREIQQLKERSIL-
WD--PSFLQTEGTEIESD--RFPK--CLSGYS--SMSRLFT-EREKQMINHLLPEEIEEFL--GNPTR
SVRSFFSDRW--SELH-LGSNPTERSTRDQ----KLLKKQQDLS-FVPSRRSE----NKEMVNIFKII
TYLQN--TVSIHPISSDPGCDRVP-KDEPD-MDSSNKISFLNKNPFFDL--FHLFHDRNRRGYT
L--HHDFESE--ERFQEMADLFTLSITEP-DLVYHKGFAFSIDSYGLDQKQ--FLNEARDE---SK
KKS---LLVLPPIFY--EENESFSRRIRKKWVR-ISCGNDLEDPKPKIVVFASNNIMEAVNQYRLI
RNLIQIQYSTYG--YIRNVLHRFFLMN---RSDRNFEYG---IQRDQIGKDTLNHRTIMKYTINQ
HLSNLKK-SQ---FDPL--ILISRTEKSMNRDPDAYRYKWSNGSKNFQEHLEHFVSEQKSR---L
QVVFDQLRINQYSIDWSEVIDKKDLSKPLRFFLSK---------SLLFLS-------KLLFFLSN-SLPFF
CVSFGNIPIHRSE-IYIY-ELKGPNDQLCNQLLESIGLQIVHLKKWKPF--LLD---DHDTS-RKS
KFLINGGTISPFLFNKIP-KW-MIDSFHTR--NNRRKSFDNTDSYFSMIFHNQDNWLNPVKPF
HRSSLISSFYKANRLRFLNNPHHFCFY--CNTRFPFSV----EKARIY--NYDFTYG-QFLNILFIR
NK-IFSLCVGK--KKHAFGG-RDTISPIESQVSNIFIPNYFPQSG-DETYNLYKS---FHFPSRYDP

FVR--RTIYSIADISGTPLTEGQIVNFERTYCQPLSDMNLSDSEGKNLHQYLN--FNSNMGLIH
TPCSEKYLPSEK--RKK--------RSLCLK--KCVEKGQMYR---RDSAFSTLSKWNLFQTYMPW
FLTSTGYKYLNLIFLDTFSDLL--PILSS-SQKFVSIFHDIMHGSGISWRILQKKL------CLPQW
NL-ISEISSKCLHS--LLLSEEMIHRN--NESPLISTHLRSPNVREF-LYSILFLLLVAGY-LVRTHL
LFVSR--ASSELQTEFKKVKSLMIPSSMIELRKLLDRYPTSEPNSFWLKNLFLVALEQLGDSL
EEIRGSASGGNMLG---PA-----YG-VKSIRSKKKYLNIN---LIDLI----PNPINRITVSRNTRHLS
HTSKEIYSLIRKRKNVNGDWIDDKIESWVANSDSIDDEEREFLVQFS--ALTT--EKRIDQILLS
LT-HSDHL-----SKNDSGYQMIEQPGAIYLRYLVDIH--KKYLLNYEFNTSCLAERRVFLAHY
QTITY--SQTSCGTNTFHFPSHG---KPFSLRLALSPS-RGILVIGSIGTGRSYLVKYLATNSYVP
FITVFLNKFLDNKPKGFLID-----DINDIDDSDNIDASDDIDASD-----------------DIDRDLDTEL
-ELLTMMNALTMDMMPE---IDRFYITLQFELAKAMSPCIIWIPNIHDLDVNESNYLSLGLLV
NHLS------ERCS-TRNILVIASTHIPQKVDPALIAPNKLNTCIKIRRLLIPQQQKHFFTLSYTRG
FHLEKKMFHT--NGFGSITMGSNARDLVALTNEALSISITQKKSIIDTNTIRSALHRQTWDLR-
--SQVRSVQDHGILFYQIGRAVAQNVLLSNCPIDPISIYMKKR--SCNEGDSYLSKWYFELGT
SMKKLTILLYLLSCSAGSVAQDLWSLP--GPDEK-NGITSYGLVENDS-DLVHGLLEVEGALV
G--SSRTETDCSQFDND--RVTLLLRPEPRN-PLDMMQNGFCSIFDQRFLYEKY--ESEFEE-GE
GEGALDPQQ----IEED----LFNH--IVWAP--RIWRP---WGFLFDCIERPNELGFP-YWSRSFRGK
---RIIYDEEDELQENDSEFLQSGTMQYQTRDRSSKEQGLFRISQFIWDPADPLFFLFK-DQPP
GSVFSHRELFADEEMSKGLLTSQTDPPTSIYKRWFI--KNTQEKHFELLINRQRWLR---TNSS
LS--NGSFRSN--TLSESYQYLSNLFLSN-GTLLDQMTKTLLRKRWLFPDEMKIGF--M---------
MPRSR-INGNFIDKTFSIVANILLRIIPTTSGEKEAFTYYRDG--MSAQSEGNYAEALQNYYE
AMRLEIDPYDRSYILYNIGLIHTSNGEHTKALEYYFRALERNPFLPQAFNNMAVICHY-RGE
QAIRQGDSEIAEAWFDQAAEYWKQAIALTPGNYIEAHNWLKITGRFE--MSWRSEDIWIELI
TGSRKLSNLCWALILFLGSLGFLLVGTSSYLGRNLISFFPSQQIIFFPQGIVMSFYGIAGLFISS
YLWCTISWNVGSGYDRFDRKEGIVCIFRWGFPGKNRRIFLRFLIKDIQSVRIEVKEGIYARR
VLYMDIRGQGAIPLTRTDEN-LTPREIEQKAAELAYFLRVPIEVF
>Nelumbonucifera
MKKWWLNSMLFLSKSMDSLGPIGNTSGSEDPVINDTDKNIH-CSNVDHLFGVRDIRSFISD
DTFLVRDSNGDSYSIYLDIENQIFEIDND--HSFLSEFENLFSSYLNSSYPINGSKSDNHYYDR
YMSDTKYSWNNHINSCIDSYLRSEIR--IDSYISSDSDNYSDSYIYTYICSDSGSSIT---TGSD-
NKRRRN-DLEKNKKSDILWVQCENCYELNYKKLLMSTMNICKQCGYHLKMSSSDRIERSI
DPGTWDPMDEDMVSTDPINFHSGEEHYKKRIDFNQRSTGLAEAVQTGIGQLNGILVAIGV
MDFKFLGGSMGSVVGEKITRLIEYATNRSLPLIIVCASGGARMQEGSLSLMQMAKISSASY
NYQSNKKLFYVSILTSPTTGGVTASFGMLGDIIIAEPNAYIAFAGKRVVEQTLKNTIPDNSQ
AAEYSFHKGLFDPIVPRNLLKDVLSELFQLHGFFPLNQNSIKMVTIRADEISNIIRERIEQYN
REVKIVNTGTVLQVGDGIARIHGLDEVMAGELVEFEEGTIGIALNLESNNVGVVLMGDGL
MIQEGSSVKATGRIAQIPVSEAYLGRVINALAKPIDGRGEISASESRLIESPAPGIISRRSVYEP
LQTGLIAIDSMIPIGRGQRELIIGDRQTGKTAVATDTILNQKGQNVICVYVAIGQKASSVAQV
VTTFQEKGAMEYTIVVAETADSPATLQYLAPYTGAALAEYFMYREQHTSIIYDDPSKQAQ
AYRQMSLLLRRPPGREAYPGDVFYLHSRLLERAAKLSSRLGEGSMTALPIVETQSGDVSA
YIPTNVISITDGQIFLSADLFNAGIRPAINVGISVSRVGSAAQIKAMKQVAGKLKLELAQFAE
LEAFAQFASDLDKATQNQLARGQRLRELLRQSQSAPLTVDEQIVTIYTGTNGYLDSLEIGQ
VKKFLVQLRTYLKTNKPQFQEIISSTKTFTEEAEALLKEAIQEQMERFLLQEQTMRMNPTT
SGPGVSALEEKNLGRIAQIIGPVLDVAFPPGKMPNIYNALVIKGRDTAGQQINVTCEVQQL

LGNNRVRTVAMSATDGLMRGMEVIDTGAPLSVPVGGATLGRIFNVLGEPVDNLGPVDTRT
TSPIHKSAPAFIQLDTKLSIFETGIKVVDLLAPYRRGGKIGLFGGAGVGKTVLIMELINNIAK
AHGGVSVFGGVGERTREGNDLYMEMKESGVINEQNIAESKVALVYGQMNEPPGARMRV
GLTALTMAEYFRDVNEQDVLLFIDNIFRFVQAGSEVSALLGRMPSAVGYQPTLSTEMGSL
QERITSTKEGSITSIQAVYVPADDLTDPAPATTFAHLDATTVLSRGLAAKGIYPAVDPLDSTS
TMLQPRIVGEEHYETAQRVKQTLQRYKELQDIIAILGLDELSEEDRLTVARARKIERFLSQP
FFVAEVFTGSPGKYVGLTETIRGFQLILSGELDGLPEQAFYLVGNIDEATAKAMNLEVESKL
KKMTLNLCVLTPNRIVWDSEVKEIILSTNSGQIGVLPNHAPIATAVDIGILRIRLNDQWLTM
ALMGGFARIGNNEITILVNDAEKGSDIDPQEAQRTLEIAEANLSRAEGKRQTIEANLALRR
ARTRVEAINVISMKNVTDSFVSLGHWPSAGSFGFNTDILATNPINLSVVLGVLIFFGKGVLS
DLLDNRKQRILSTIRNSEELRGGAIEQLEKARARLRKVEMEADEFRVNGYSEIEREKLNLI
NSTYKNLERLENYKNETIHFEQQRAINQVRQRVFQQALQGALGTLNSCLTSELHLRTISAN
IGMFGTMKEITDMNPLISAASVIAAGLAVGLASIGPGVGQGTAAGQAVEGIARQPEAEGKI
RGTLLLSLAFMEALTIYGLVVALALLFANPFVMNVLPCSINTLKGLYDISGVEVGQHFYWQ
IGGFQVHAQVLITSWVVIAILLGSATIAVRNPQTIPTAGQNFFEYVLEFIRDLSKTQIGEEYG
PWVPFIGTMFLFIFVSNWSGALLPWKIIELPHGELAAPTNDINTTVALALPTSVAYFYAGLS
KKGLGYFAKYIQPTPILLPINILEDFTKPLSLSFRLFGNILADELVVVVLVSLVPSVVPIPVMF
LGLFTSGIQALIFATLAAAYIGESMEGHHMIFLTLEHILTHISFSIVSIVITIHLMTLLIDETVGL
YDSSEKGMIATFFCITGFLVTRWIHSGHFPLSDLYESLMFLSWSFSIIHMVPKIGNHKNHLSE
ITTPSAIFTQGFATSGLLTDMHQSAILVPALQSQWLMMHVSMMVLSYAALLCGSLLSIALL
VITFRKNIENFSKSNEIQYVNKKSNVLRNTSFT-FRNYHRHQLIQQLDHWSYRVISLGFIFLT
IGILSGAVWANEAWGSYWNWDPKETWAFITWTIFAIYLHTRTNTKWQGANSAIVASMGFL
IIWICYFGVNLLGIGLHSYGSFTF-MPKKKAFTPLPYLASIVFLPWWISLSFNKSLESWVTN
WWNTRQSETFLNDIQEKNVLEKFIELEELFLLNEMIKEYPETHIQKLHIRIHKETIQLVKMY
NEGHIHTILHFSTNIICFAILSGYSILGSEELVILNSWVQEFLYNLSDTIKAFSILLLTDLCIGFH
SPHGWELMIGSIYKDFGFAHNEQIISGLVSTFPVILDTIFKYWIFRYLNRVSPSLVVIYHSMN
DMPIGVPKVPFRSPGEEDAVWVDVINRLHRERLLFLGQEVDSEISNQLIGLMVYLSIEDDT
KDLYLFINSPGGWVIPGIAIYDTMQFVPPDVHTICMGLAASMGSFILAGGEITKRLAFPHAR
VMIHQPASSFYEAQTGEFILEAEELLKLRETLTRVYVQRTGNPLWVVSEDMERDVFMSATE
AQAHGIVDLVAVEMKEQKWIHEGLIIESLPNGMFRVRLDNEDLILGYVSGKIRRSFIRILPG
DRVKIEVSRYDSTRGRIVYRLRNKDSNDMEELQRYLEIDRSRQQDFLYPLLFQEYIYVFAH
DHGLENLG-KSSSLIVKRVITRMYQRNHLIFLANDSNQTQCVGKNFYSQMILEGFAVIVEIP
FSLRLVSSLITKSHNLRSIHSIFSFLEDKLSRLNYVLDLLIPYPIHSEILVQTLRCWVKDTPSL
HLLRFFLHEYRNWNSLITKSSSLLKKQRFFLFLYNSHVCECESIFVFLRKQSFHLRSTSSGA
LLERTHFYGKIEHFGVVFRNDFILWLVKDPFMHYVRYQGKSILASKGTPLLMNKWKYHL
VNFWQCHFYLWSQPGRIHINQLSNHSLDFLGYLSSVRLNPSLVRSQMLENSYLIDIAS--KK
FETIVPIIPLIGSLAKAKFCNVSGHPISKPVRADSSDSDIIDRFGRICKNLSHYHSGSSKKKSL
YRIKYILRLSCARTLARKHKSTVRAFLKRLGSELLKEFFTEEEQVLSLILPRRIWYLDILRIN
DLTNPEMIIDTT-KAQ-VINSFSRSE-SLKEVYGLIWLLVPILTLISGITIGVLVIVWLEREISAGI
QQRIGPEYAGPLGILQALADGIKLLFKEDLLPSRGDARLFSIGPSIAVISILLSYSVIPFGYRLV
LADLSIGVFLWIAISSIAPIGLLMSGYGSNNKYSFSGGLRAAAQSISYEIPLTSCVLSISLLSN
SSSTVDIVEAQSKYGFWGWNLWRQPIGFVVFLISSLAECERLPFDLPEAEEELVAGYQTEY
SGIKFGLFYVASYLNLLVSSLFVTVLYLGGWNLSIPYIFIPELFGINKVGGVFETTIGIFITLA
KAYLFLFIPIATRWTLPRMRMDQLLNLGWKFLLPISLGNLLLTTSSQLLSLMIWHVQNENFI

LDSTRIFMKAFHLLLFHGSFIFPECILIFGLILLLMIDSTSDQKDIPWFYFISSTSLVMSITALL
FRWREEPMISFSGNFQTNNFNEIFQFLILLCSTLCIPLSVEYIECTEMAITEFLLFVLTATLGG
MFLCGANDLITIFVAPECFSLCSYLLSGYTKRDVRSNEATTKYLLMGGASSSILVHGFSWL
YGSSGGEIELQEIVNGLINTQMYNSPGISIALIFITVGIGFKLSPAPFHQWTPDVYEGSPTPVV
AFLSVTSKVAASASATRIFDIPFYFSSNEWHLLLEILAILSMILGNLIAITQTSMKRMLAYSSI
GQIGYVIIGIIVGDSNDGYASMITYMLFYISMNLGTFACIVSFGLRTGTDNIRDYAGLYTKDP
FLALSSALCLLSLGGLPPLAGFFGKLHLFWCGWQAGLYFLVSIGLLTSVVSIYYYLKIIKLL
MTGRNQEITPHVRNYRRSPLRSNNSIELSMIVCVIASTIPGISMNPIIAIAQDTLFMFLLHEY
DIFWAFLIISSVIPILAFLISGVLAPIREGPEKLSSYESGIEPMGDAWLQFRIRYYMFALVFVV
FDVETVFLYPWAMSFDVLGVSVFIEALIFVLILIVGSVYAWRKGALEWSMNFFPWLTIIVVL
PISAGSLIFFLPHKGNKVIRWYTISICLIELLLMTYAFCYHFKLDDPLIQLEEDYKWINIFDFH
WRLGIDGLSIGPILLTGFITTLATLAAWPVTRDSRLFHFLMLAMYSGQIGSFSSRDLLLFFIM
WELELIPVYLLLSMWGGKKRLYSATKFILYTAGGSIFLLLGVLGMGLYGSNEPTLNFETSA
NQSYPVALEIIFYFGFLIAYAVKSPIIPLHTWLPDTHGEAHYSTCMLLAGILLKMGAYGLVRI
NMEFLPHAHSIFSPWLMIVGTIQIIYAALTSPGQRNLKKRIAYSSVSHMGFTIIGIASITDTGL
NGAILQIISHGFIGAALFFLAGTSYDRIRLVYLDEMGGIAIPMPKIFTMFSSFSMASLALPGM
SGFVAELIVFFGIITSPKYLLMPKILITFVMAIGMILTPIYSLSMLRQMFYGYKLFNASNSYFF
DSGPRELFVSISIFLPVIGIGIYPDFVLSLSVDKVEAILSNYFSRMMLEHVLVLSAYLFSIGIY
GLITSRNMVRALMCLELILNAVNLNFVTFSDFFDSRQLKGDIFSIFVIAIAAAEAAIGPAIVS
SIFRNRKSTRINQSNLLNKMEHTYQYAWMIPFVPLPVPMLIGVGLLFVPMATKKLRRMWA
FPSVFLLSIVMLFSANLAIQQINGSSIYQYVWSWTINNDFSLEFGYLIDPLTSIMLILITTVGV
MVLVYSDNYMSHDQGYLRFFAYMSFSNTSMLGLVTSSNLIQIYIFWELVGMCSYLLIGFW
FTRPIAANACQKAFVTNRVGDFGLLLGILGLYWITGSFEFKDLFEIVNNNGVNSLFVTLCA
SLLFVGAVAKSAQFPLHVWLPDAMEGPTPISALIHAATMVAAGIFLVARLLPLFTVIPYIMN
LISLIGIITVLLGATLALAQKDIKRSLAYSTMSQLGYMMLALGMGSYRAALFHLITHAYSK
ALLFLGSGSIIHSMETIVGYSPDKSQNMVLMGGLTKYMPITKTAFLLGTLSLCGIPPLACFW
SKDEILNDSWLYSPIFAIIACSTAGLTAFYMFRIYLLTFEGPLNVHFQNYSGKKNSSFYLISIW
GKEGPKTINKN-FRLSTMNFFSFGNKEIFPYPHESDNTMLVPLLVLVFFTLFVGFIGIPFDFDI
LSKWLTPSINLLHPNLN-SVDWYAFVTNAIFSVSIAYFGIFIASLLYGPVYSSFQNLELINFFVI
LWDKIINVIYNWSYNRGYIDSFYARAFTRGIRGLAELTHFFDRRVIDGITNGVGVTSFFVGE
GIKYVGGG-RISSYLFLYLFYVSIFLLIYYMDLPGPIHDFLLVFLGSGLILGGLGVVLLTNPIY
SAFSLGLVLVCISLFYIPSNSHFVAAAQLLIYVGAINVLIIFAVMFMNGSEYYNDFHLWTIGD
GVTSLVCTSIFFSLITTILDTSWYGIIWTTRSNQIIEQDLISNGQQIGIHLSTDFFLPFELISIILL
VALIGAIAMARQMTVPATRKDLMIVNMGPHHPSMHGVLRLIVTLDGEDVIDCEPILGYLH
RGMEKIAENRTIIQYLPYVTRWDYLATMFTEAITVNAPEQLGNIQVPKRASYIRVIMLELSR
IASHLLWLGPFMADIGAQTPFFYIFRERELIYDLLEAATGMRMMHNYFRIGGVAADLPYG
WMDKCLDFCDYFLTGVAEYQKLITRNPIFLERVEGVGIIGGEEAINWGLSGPMLRASGIQW
DLRKVDHYECYDEFDWEVQWQKEGDSLARYLVRIGEMTESIKIIQQALEGIPGGPYENLE
VRRFARAKDSEWNDFEYRFISKKPSPTFELSKQELYARVEAPKGELGIYLIGDQSVFPWRW
KIRPPGFINLQILPQLVKRMKLADIMTILGSIDIIMGEVDRMLPMVTGFMNYGQQTIRAAR
YIGQSFMITLSHANRLPVTIQYPYEKLITSERFRGRIHFEFDKCIACEVCVRVCPIDLPVVDW
RLETDIRKKRLLNYSIDFGICIFCGNCVEYCPTNCLSMTEEYELSTYDRHELNYNQIALGRL
PISVIGDSTIRTIMNSKIATGKSLDSKTITNYMQGRLSAWLVKHGLVHRSLGFDYQGIETLQI
KPEDWHSIAVILYVVGYNYLRSQCAYDVAPGGLLASVYHLTRIQYGVDQPEEVCIKVFAPR

RNPRIPSVFWIWKSADFQERESYDMLGISYDNHPRLKRILMPESWIGWPLRKDYIAPNFYE
IQDAH---MNSIKFPLLDRPTQNSVISTTLNDLS
NWSRLSSLWPLLYGTSCCFIEFASLIGSRFDFDRYGLVPRSSPRQADLILTAGTVTMKMAPS
LVRLYEQMPEPKYVIAMGACTITGGMFSTDSYSTVRGVDKLIPVDVYLPGCPPKPEAVIDA
ITKLRKKVSREIYEDRIVSQQKNRCFTTNHKFHVGRSTHTGNYDQGLLYQSPSTSEIPSETF
FKYKKSVSSHELVNMQNRNTFSWIKEQMTRSISVSIMIYVITRTSISNAYPIFAQQGYENPR
EATGRIVCANCHLANKPVDIEVPQAVLPDTVFEAVVRIPYDMQLKQVLANGKKGALNVG
AVLILPEGFELAPPDRISPEMKEKIGNLSFQSYRPTKKNILVIGPVPGQKYSEITFPILSPDPAT
KKDIHFLKYPIYVGGNRGRGQIYPDGSKSNNTVYNATAAGIVSKIVRKEKGGYEITIADAS
DGHQVVDIIPPGPELLVSEGESIKLDQPLTSNPNVGGFGQGDAEIVLQDPLRVQGLLFFFAS
VILAQIFLVLKKKQFEKVQLSEMNFMSKVYDWFEERLEIQAIADDITSKYVPPHVNIFYCL
GGITLTCFLVQVATGFAMTFYYRPTVTEAFASVQYIMTEANFGWLIRSVHRWSASMMVLM
MILHVFRVYLTGGFKKPRELTWVTGVILAVLTASFGVTGYSLPWDQIGYWAVKIVTGVPEA
IPVIGSPLVELLRGSASVGQSTLTRFYSLHTFVLPLLTAVFMLMHFPMIRKQGISGPLMGVT
KKPDLNDPVLRAKLAKGMGHNYYGEPAWPNDLLYIFPVVILGTIACNVGLAVLEPSMIGE
PADPFATPLEILPEWYFFPVFQILRTVPNKLLGVLLMVSVPAGLLTVPFLENVNKFQNPFRR
PVATTVFLIGIAVALWLGIGATLPIDKSLTLGLFMIEVFLFGIVLGLIPITLAGLFVTAYLQYRR
GDQLDLMPTLTSYFGFLLAASTITSALFIGLSKIRLIMDIVSLAWAALMVVFTFSLSLVVWG
RSGLMIIRSPEPEVKILVDRDPIKTSFEEWARPGHFSRTIAKGPETTTWIWNLHADAHDFDS
HTSDLEEISRKVFSAHFGQLSIIFLWLSGMYFHGARFSNYEAWLSDPTHIGPSAQVVWPIV
GQEILNGDVGGGFRGIQITSGFFQIWRASGITSELQLYCTAIGALVFAALMLFAGWFHYHK
AAPKLAWFQDVESMLNHHLAGLLGLGSLSWAGHQVHVSLPINQFLDAGVDPKEIPLPHEF
ILNRDLLAQLYPSFAEGATPFFTLNWSKYADFLTFRGGLDPVTGGLWLTDIAHHHLAIAILF
LIAGHMYRTNWGIGHSLKDILEAHKGPFTGQGHKGLYEILTTSWHAQLSLNLAMLGSLTI
VVAHHMYSMPPYPYLATDYGTQLSLFTHHMWIGGFLIVGAAAHAAIFMVRDYDPTTRYN
DLLDRVLRHRDAIISHLNWACIFLGFHSFGLYIHNDTMSALGRPQDMFSDTAIQLQPVFAQ
WVQNTHALAPGATAPGATTSTSLTWGGGDLVAVGGKVALLPIPLGTADFLVHHIHAFTIHV
TVLILLKGVLFARSSRLIPDKANLGFRFPCDGPGRGGTCQVSAWDHVFLGLFWMYNAISV
VIFHFSWKMQSDVWGSINDQGVVTHITGGNFAQSSITINGWLRDFLWAQASQVIQSYGSS
LSAYGLFFLGAHFVWAFSLMFLFSGRGYWQELIESIVWAHNKLKVAPATQPRALSIVQGRA
VGVTHYLLGGIATTWAFFLARIIAVGMALRFPRFSQGLAQDPTTRRIWFGIATAHDFESHD
DITEERLYQNIFASHFGQLAIIFLWTSGNLFHVAWQGNFESWVQDPLHVRPIAHAIWDPHF
GQPAVEAFTRGGALGPVNIAYSGVYQWWYTIGLRTNEDLYTGSLFLLFLSALSLIAGWLH
LQPKWKPSVSWFKNAESRLNHHLSGLFGVSSLAWTGHLVHVAIPGSRGEYVRWNNFLDV
LPHPQGLGPLFTGQWNLYAQNPDSSSHLFGTSQGAGTAILTLLGGFHPQTQSLWLTDIAHH
HLAIALIFLVAGHMYRTNFGIGHSMKDLLEAHIPPGGRLGRGHKGLYDTINNSIHFQLGLA
LASLGVITSLVAQHMYSLPAYAFIAQDFTTQAALYTHHQYIAGFIMTGAFAHGAIFFIRDYN
PEQNEDNVLARMLDHKEAIISHLSWASLFLGFHTLGLYVHNDVMLAFGTPEKQILIEPIFA
QWIQSAHGKTSYGFDVLLSSTTGPAFNAGRSIWLPGWLNAINE--NSNSLFLTIGPGDFLVH
HAIALGLHTTTLILVKGALDARGSKLMPDKKDFGYSFPCDGPGRGGTCDISAWDAFYLAV
FWMLNTIGWVTFYWHWKHITLWQGNVSQFNESSTYLMGWLRDYLWLNSSQLINGYNPF
GMNSLSVWAWMFLFGHLVWATGFMFLISWRGYWQELIETLAWAHERTPLANLIRWRDKP
VALSIVQARLVGLAHFSVGYIFTYAAFLIASTSGKFGMSHSVKIYDTCIGCTQCVRACPTDV
LEMIPWDGCKAKQIASAPRTEDCVGCKRCESACPTDFLSVRVYLWHETTRSMGLAYMTT

FNLPSIFVPLVGLVFPAIAMASLFLHVQKNKIVMRDIKTYLSVAPVLSTLWFGSLAGLLIEIN
RLFPDALAFPFF--MTAILERRESESLWGRFCNWITSTENRLYIGWFGVLMIPTLLTATSVFII
AFIAAPPVDIDGIREPVSGSLLYGNNIISGAIIPTSAAIGLHFYPIWEAASVDEWLYNGGPYE
LIVLHSLLGVACYMGREWELSFRLGMRPWIAVAYSAPVAAATAVFLIYPIGQGSFSDGMPL
GISGTFNFMIVFQAEHNILMHPFHMLGVAGVFGGSLFSAMHGSLVTSSLIRETTENESANE
GYRFGQEEETYNIVAAHGYFGRLIFQYASFNNSRSLHFFLAAWPVVGIWFTALGISTMAFN
LNGFNFNQSVVDSQGRVINTWADIINRANLGMEVMHERNAHNFPLDLAAVEAPSTNGMG
LPWYRVHTVVLNDPGRLLSVHIMHTALVSGWAGSMALYELAVFDPSDPVLDPMWRQGM
FVIPFMTRLGITNSWGGWSIT-GGTITNPGLWSYEGVAGAHIVFSGLCFLAAIWHWVYWDL
EIFCDERTGKPSLDLPKIFGIHLFLSGVACFGFGAFHVTGLYGPGIWVSDPYGLTGKVQPVN
PAWGVEGFDPFVPGGIASHHIAAGTLGILAGLFHLSVRPPQRLYKGLRMGNIETVLSSSIAA
VFFAAFVVAGTMWYGSATTPIELFGPTRYQWDQGYFQQEIYRRVGAGLAENQSLSEAWS
KIPEKLAFYDYIGNNPAKGGLFRAGSMDNGDGIAVGWLGHPIFRDKEGRELFVRRMPTFF
ETFPVVLVDGDGIVRADVPFRRAESKYSVEQVGVTVEFYGGELNGVSYSDPATVKKYARR
AQLGEIFELDRATLKSDGVFRSSPRGWFTFGHASFALLFFFGHIWHGARTLFRDVFAGIDP
DLDAQVEFGTFQKLGDPTTKRQVVMKTLYSLRRFYHVETLFNGTLALAGRDQETTGFAW
WAGNARLINLSGKLLGAHVAHAGLIVFWAGAMNLFEVAHFVPEKPMYEQGLILLPHLATL
GWGVGPGGEVIDTFPYFVSGVLHLISSAVLGFGGIYHALLGPETLEESFPFFGYVWKDRNK
MTTILGIHLILLGIGAFLLVLKALYFGGVYDTWAPGGGDVRKITNLTLSPSVIFGYLLKSPFG
GEGWIVSVDDLEDIIGGHVWLGSICILGGIWHILTKPFAWARRALVWSGEAYLSYSLGALS
VFGFIACCFVWFNNTAYPSEFYGPTGPEASQAQAFTFLVRDQRLGANVGSAQGPTGLGKY
LMRSPTGEVIFGGETMRFWDLRAPWLEPLRGPNGLDLSRLKKDIQPWQERRSAEYMTHA
PLGSLNSVGGVATEINAVNYVSPRSWLATSHFVLGFFLFVGHLWHAGRARAAAAGFEKGI
DRDFEPVLSMTPLNMTIALGRFTKDENDLFDIMDDWLRRDRFVFVGWSGLLLFPCAYFAL
GGWFTGTTFVTSWYTHGLASSYLEGCNFLTAAVSTPANSLAHSLLLLWGPEAQGDFTRWC
QLGGLWTFVALHGAFGLIGFMLRQFELARSVQLRPYNAIAFSAPIAVFVSVFLIYPLGQSG
WFFAPSFGVAAIFRFILFFQGFHNWTLNPFHMMGVAGVLGAALLCAIHGATVENTLFEDG
DGANTFRAFNPTQAEETYSMVTANRFWSQIFGVAFSNKRWLHFFMLFVPVTGLWMSALG
VVGLALNLRAYDFVSQEIRAAEDPEFETFYTKNILLNEGIRAWMAAQDQPHENLIFPEEVL
PRGNALMSGSTGERSFADIITSIRYWVIHSITIPSLFIAGWLFVSTGLAYDVFGSPRPNEYFTE
SRQGIPLITDRFDPLAQLDEFSRSFMTIDRTYPIFTVRWLAVHGLAVPTVSFLGSISAMQFIQ
RMATQTVEGSSRSG-PRRTIIGDLLKPLNSEYGKVAPGWGTTPLMGVAMALFAIFLSIILEIY
NSSVLLDGISMQMLTLKLFVYTVVIFFVSLFIFGFLSNDPGRNPGREEMADTTGRIPLWLIG
TVTGIPVIGSIGIFFYGSYSGLGSSLMLNIFSLICICLNSAFHSSSFFFAKLPEAYAIFNPIVDV
MPVIPVLFFLLALVWQAAVSFRTTQSNPNEQNVELNRTSLYWGLLLIFVLAVLFSNYFFNM
EVNILAFIATALFILVPTAFLLIIYVKTVSQNDMETATLVAIFISGSLVSFTGYALYTAFGQPSQ
QLRDPFEEHGDMEALVYTFLLVSTLGIIFFAIFFREPPKVPTKKVKMTIAFQLAVFALIATSSI
LLISVPVVFASPEGWSSNKNVVFSGTTLWIGLVFLVGILNSLIS-------MSPQTETKASVGFK
AGVKDYRLTYYTPEYETKDTDILAAFRVTPQPGVPPEEAGAAVAAESSTGTWTTVWTDGL
TSLDRYKGRCYHIEPVAGEESQFIAYVAYPLDLFEEGSVTNMFTSIVGNVFGFKALRALRLE
DLRIPPAYSKTFQGPPHGIQVERDKLNKYGRPLLGCTIKPKLGLSAKNYGRAVYECLRGGL
DFTKDDENVNSQPFMRWRDRFLFCAEAIYKAQAETGEIKGHYLNATAGTCEEMIKRAVFA
RELGVPIVMHDYLTGGFTANTSLAHYCRDNGLLLHIHRAMHAVIDRQKNHGIHFRVLAKA
LRMSGGDHIHSGTVVGKLEGEREITLGFVDLLRDDFIEKDRSRGIYFTQDWVSLPGVLPVA

SGGIHVWHMPALTEIFGDDSVLQFGGGTLGHPWGNAPGAVANRVALEACVQARNEGRDL
AREGNEIIREASKWSPELAAACEVWKEIKFEFEAMDTL--TAIHLYKTSTPSTRNGAVDSQV
KSNPRNNLIYGQHRCGKGRNARGIITAGHRGGGHKRLYRKIDFRRNEKKISGRIVTIEYDP
NRNAYICLIHYGDGEKRYILHPRGAIIGDTIVSGSEVPIKMGNALPLTEMPLGTAIHNIEITL
GKGGQLARAAGAVAKLIAKEGKSATLKLPSGEVRLISKNCSATVGQVGNVGVNQKSLGR
AGSKCWLGKRPVVRGVVMNPVDHPHGGGEGRAPIGRKKPTTPWGYPALGRRSRKRNKY
SDSLILRRRSKMIQPQTHLNVADNSGARELMCIRIIGAS-NRRYAHIGDVIVAVIKEAVPNMP
LEKSEVIRAVIVRTCKELKRDNGMIIRYDDNAAVVIDQEGNPKGTRVFGAIARELRQLNFIK
IVSLAPEVLMLSPKRTRFRKQHRGRMKGISYRGNRICFGRYALQALEPAWITSRQIEAGRR
AMTRYARRGGKIWIRIFPDKPVTIRPTETRMGSGKGSPEYWVSVVKPGRILYEMGGVSEIV
AREAISIAASKMPIRTQFVIAGMTRIKRGYIARRRRTKIRLFASTFRGAHSRLTRTITQQKMR
ALVSAHRDRGKQKRNFRRLWITRINAVIRENKGVSYSYSRLIHDLYKRQLLLNRKILAQLA
ISNRNCLYMISNEILK-----------MIKEVYALGQHIRMSAHKARRVIDQIRGRSYEETLMILEL
MPYRACYPIFKLVYSAAANASHNMGLNEADSVISKAEVNESTTMKRLKPQARGRSYPIKR
PTCHITIALKDISLDGVVWVKKMDGIKYAVFTDKSIRLLGNN-QYTSNVESGSTRTEIKHW
VELFFGVKVIAMNSHRLPGKGRRMGPIMGHTMHYRRMIITLQPGYSIPPLRKKRTMAVPK
KRTSISKKRIRKNIWKRKGYWAAVKAFSLAKSLSTGNSKSFFVR--QIMAKGKDVRVRVILE
CTNCVRNGVN--KESPGISRYITQKNRHNTPSRLELRKFCPYCFKHTIHGEIKKMKIRASVR
KICEKCRLIRRRGRIIVICSNPRHKQRQGMVREEIAVSTRTLQWKCVESRVDSKRLYYGRFI
LSPLMKGQADTIGIAMRRALLGEIEGTCITRAKSE--KIPHEYSTIVGIEESVHEILMNLKEIV
LRSNLYGTRDAFICVRGPGYVTAQDIISPPSVEIVDTTQHIASLTEPIDLCIGLQIERNRGYR
MKTPNNSQDGSY--PIDAVFMPVRNANHSIHSYGNGNEKQEILFLEIWTNGSLTPKEALHEA
SRNLIDLFIPFLDAQEE--DIQLEDNQ-NKVTLPFFTFHDRLT-KLRKNKK-EIALKCIFIDQSEL
PPRTYNCLKRSNIHTLLDLLNNSQEDLMKMFHFQIEDVKQILDILQKH--FAIDLPKNKMLR
DGN-EGMSTIPGFSQIQFEGFCRFIDQGLTEELYKFPKIEDTDQEIEFQLFVETYQLVEPLINE
RDAVYESLTYSSELYVPAGLIWKTGRDMQEQTIFIGNIPLMNSLGTSIVNGIYRIVINQILQS
PGIYYRSELDHNGISVYTGTIISDWGGRSELEIDRKARIWARVSRKQKISILVPSSAMGSNLR
EILDNVCYPEIFLSFLNDKEKKKIGSKENAILEFYQQFACVGGDPVFSESLCKELQKKFFQQ
RCELGRIGRRNINRRLNLDIPQKNTFLLPRDVLAAADHLIGMKFGMGTLDD--MNHLKNK
RIRSVADLLQDQFGLALVRLENAVRGTISGAIR-HKLI-PTPQNLVTSTP----LTTTYESFFGLH
PLSQVLDRTNPLTQIVHGRKLSYLGPGGLTGRTASFRIRDIHPSHYGRICPIDTSEGINVGLIG
SLAIHARIGHWG-SLESPFY---EISERS--KEVRIVYLSPSRDEYYMVAAGNSLALNQSIQEEQ
VVPARYRQEFLTIAWEQVHLRSIFPFQYFSIGASLIPFIEHNDANRALMSSNMQRQAVPLSR
SEKCIVGTGLECQAALDSGVSAIAEHEGKIIYTDTDKIILSGNGDTLSIPLIMYQRSNKNTC
MHQKPQVRRGKCIKKGQILADGAATVGGELALGKNVLVAYMPWEGYNSEDAVLISERLV
YGDIYTSFHIRKYEIQTHVTSQG-PERITNEIPHLEAHLLRNLDRNGIVMLGSWVETGDILV
GKLTPQMAKE-SSYAPEDRLLRAILGIQVSTSKETCLKLPIGGRGRVIDVRWIQKKG-GSSY
NPETIRVYISQKREIRVGDKVAGRHGNKGIISKILPRQDMPYLQDGTPVDMVFNPLGVPSR
MNVGQIFECSLGLAGDLLDRHYRIAPFDERYEQEASRKLVFSELYEASKQTANPWVFEPE
YPGKSRIFDGRTGDPFEQPVIIGKSYILKLIHQVDDKIHGRSSGHYALVTQQPLRGRAKQGG
QRVGEMEVWALEGFGVAHILQEMLTYKSDHIRARQEVLGTTIIGGTIINPEDAPESFRLLVR
ELRSLALELNHFLVSEKNFQINRKEA-------MIDRYKHQQLRIGSVSPQQIIAWAQKILPNGEI
VGEVTKPYTFHYKTNKPEKDGLFCERIFGPIKSGICACGNFRVIGDEKEDPKFCEQCGVEF
VDSRIRRYQMGYIKLACPVTHVWYLKRLPSYIANLLDKPLKELEGLVYCD--FSFARP-IAK

KPTFLRLRGLFEYEIQSWKYSIPLFFTTQGFDTFRNREISTGAGAIREQLADLDLRIIIDSSLV
EWKELGEE-GPTGNEWEDRKIGRRKDFLVRRMELAKHFIRTNVEPERMVLCLLPVLPPEL
RPIIQIDGGKLMSSDINELYRRVIYRNNTLTDLLTTSRSTPGESVMCQEKLVQEAVDTLLDN
GIRGQPMRDGHNKVYKSFSDVIEGKEGRFRETLLGKRVDYSGRSVIVVGPSLSLHRCGLP
REIAIELFQTFVIRGLIRQHVASNIGVAKSKIREKEPIVWEILQEVMQGHPVLLNRAPTLHRL
GIQAFQPILVEGRAICLHPLVRKGFNADFDGDQMAVHVPLSLEAQAEARLLMFSHRNLLSP
AIGDPISIPTQDMLIGLYVLTMGNHRGICANRYNPWNRRN---YKNERIDDNNYKYTKEKEP
YFCSSYDALGAYRQKRINLDSPLWLRWRLDQR-VIAS--REVPIEVQYESLGTYHEIYGHYL
IVRSVKKKILCIYIRTTVGHISFYREIEEAVQGFYRARSYGT--------MEVLMAERADLVFHN
KVIDGTAMKRLISRLIDHFGMAYTSHILDQVKTLGFQQATATSISLGIDDLLTIPSKGWLVR
DAEQQSLILEKHYHYGNVHAVEKLRQSIEIWYATSEYLRQEMNPNFRMTDPSNPVHIMSFS
GARGNASQVHQLVGMRGLMSDPQGQMIDLPIQSNLREGLSLTEYIISCYGARKGVVDTAV
RTSDAGYLTRRLVEVVQHIVVRRTDCGTVRGISVSPRNGMMT-ERIFIQTLMGRVLADDIY
MGPRCIAIRNQDIGIGLVNRFIT------FRAQPISIRTPFTCRSTSWICRLCYGRSPTHGDLVELG
EAVGIIAGQSIGEPGTQLTLRTFHTGGVFTGGTAEQVRAPSNGKIKFNEDLVHPTRTRHGHP
AFLCYIDLYVTIESQDIIHNVNIPPKSFLLVQNDQYVESEQVIAEIRAGISTL-NFKERVRKHI
YSDSEGE-MHWSTDVYHAPEYTYGNVHLLPKTSHLWILSGSPCR--SNIVPFSLHKDQDQM
SVH--SLSVEGRYISN--LSVTNDQ-VRHKLFSSDPSSC-GERILDF-SGPDRIICNGHCNFIYPA-
ILHENSD--LLAKRRRNRFIIPFQSNQEREKELMPRAGISIEIPIN--GIFRRNSILAYFDDPRYR
RNSSGITKYGTIEVHSIVKKED--LIEYRR--KEFRPKYKKVDRFFFIPEEVHILPGSSSILVRN
NSIIGVDTRITLNTRSRVGGLVRVERKKKKIELKIFSGDIHFPGETDKISRHSGILIPPGAEKK-
-NSKESKT-KLKNWIYIQR--ITPTKKK--FFVLVRPVVTYEIADGINLATLFPQDLLQERDNVQ
LRVVNYILYGNGKPIRGISHTSIQLVRTCLVLNWDQDKK---GSSIEEVHASLVEVRANDLIR
DFIRMDLAKSPISYIGKRNDPAGSGLISD-NRSDRTNRNPFY---SKAR-----IQQSLSQHQGTIR
TLLNR---NKECQSLIILSSSNCSRIGIGPFNGLKYHN---VTKESDPLISIRNSFGPLGT--VPKIA
NFYSS-YYHLIT--HNQIL-INKYLLLDN---LKQTFQVLK---YYLIDENGR--IFNPDPCSNIIFNP
FNLNWYFLHHDYCEESSTIIS-LGQFLCENVCISKNGP---HLKSGQVLIVH-VDSLVIRSAKP
YLATPGATVHGHYGEILYEGDTLVTFIYEKSRSGDITQGLPKVEQVLEVRSIDSISMNLEKR
VEDWNERIARILGIPWGFLIGAELTIAQSRISLVNKIQKVYRSQGVQIHNRHIEIIVRQITSKV
VVSE---DGMSNVFLPGELIGLLRAERTGRALEEAICYRAVLLGITRASLNTQSFISEASFQET
ARVLAKAALRGRIDWLKGLKENVVLGGIIPVGTGFKGLVHRS--RQHNNIP--LEIKKKNLF
EGEMR--DILFHHKELFHFCIPKNFHDTSEQSFTGFNDS------MTRRYWNINLEEMMEAGVH
FGHGTRKWNPRMAPYISAKRKGIHITNLTRTARFLSEACDLVFDAASRGKQFLIVGTKNK
AADLVASAAIRARCHYVNKKWLGGMSTNWSTTETRLQKFRDLRAEQKAGRLNRLPKRD
AAMLKRQLFNLQTYLGGIKYMTGLPDIVIIVDQQEEYTALRECVTLGIPTICLIDTNCDPDL
ADISIPANDDAIASIRLILNKLISAICEGRSS-YIRNRMGQKINPLGFRLGTTQGHHSLWFAQP
KSYSEGLQEDQKIRDCIKNFVQKNMRISSG-FEGIARIEIQKRIDLIQVIIYMGFPKLLIESRP
R----GIEELQINVQKEFHSVNRKLNIDITRIAKPYGQPNILAEFIAGQLKNRVSFRKAMKKAI
ELTEQADTKGIQIQIAGRIDGKEIARVEWIREGRVPLQTIRAKIDYCSYPVRTIYGVLGIKIWI
FVDEEMSRYRGPRFKKIRRLGTLPGLTSKRPKAGSDLRNQARSGK-RSQYRIRLEEKQKLR
FHYGLTERQLLKYVCIARKAKGSTGQVLLQLLEMRLDNILFRLGMASTIPGARQLVNHRH
ILVNGRIVDIPSYRCKPRDIITTRDEQKSRALIQNYLDSSPH-EELPKHLTLHSFQY--KGLVN
QIIDSKWVGLKINELLVVEYYSRQTMSRRGTAEEKTAKSDPIYRNRLVNMLVNRIMKHGK
KSLAYQIIYRAVKKIQQKTETNPLSVLRQAIRGVTPDIAVKARRVGGSTHQVPIEIGSTQGK

ALAIRWLLGASRKRPGRNMAFKLSSELVDAAKGSGDAIRKKEETHRMAEANRAFAHFRM
GRDTIADIITSIRNADMDKKRTVRITSTNITENIVKILLREGFIENVRKHQENNKYFLVLTLR
HRR--NRKGPYR-----TILKRISRPGLRIYSNHQRIPKILGGMGIVILSTSRGIMTDREARREGI
GGEIVCYIWMAKPIPRIGSRRNGRIGLRKSGRRIPKGVIHVQASFNNTIVTVTDVRGRVVS
WSSAGTCGFRGTRRGTPFAAQTAAGNAIRTVVDQGMQRAEVMIKGPGLGRDAALRAIRR
SGLQLSFVRDVTPMPHNGCRPPKKRRVMPTIKQLIRNTRQPIRNVTKSPALRGCPQRRGTC
TRVYTITPKKPNSALRKVARVRLTSGFEITAYIPGIGHNSQEHSVVLVRGGRVKDLPGVRYH
IVRGTLDAVGVKDRQQGRSKYGVKKPKMAKKSLIQREKKRQKLEQKYHLIRRSSKKEIS--
-KVPSLSDKWEIHGKLQSSPRNSAPTRLRRRCFSTGRPRANYRDFGLSGHILREMVHACLL
PGATRSSWVKKNSFISVISQKEKEENENRGSVEFQVLSFTNKIRRLTSHLELHRKDYLSQR
GLRKILGKRQRLLVYLSKKNRVRYKELISQLDIREPKTR--MVKLRLKRCGRKQRAIYRIVA
IDVRSRREGRDLRKVGFYDPIKNQTYLNVPAILYFLEKGAQPTGTVHDISKKAEVFKELRV
NQTK---MDKSKRPFLKSKRSFRRRLPPIGSGDRIDYRNMSLISRFISEQGKILSRRVNRLTLK
QQRLITIAIKQARILSSLPFLNNEK--QFERTESTSRTAGPRTRNKVPRSLKKNPFVANHLLRK
IEKLNTRAEKEIIVTWSRASTIIPTMIGHTIAIHNGKEHLPIYITDRMVGHKLGEFAPTINFRG
HAKNDNRSRRMILKSFLLGNLFSLCMKIINSVVVVGLYYGFLTTFSIGPSYLFLLRARVME
EG---TEKEVSATTGFITGQLMMFISIYYAPLHLALGRPHTITVLVLPYLLFHFFWNNHKHFF
DYGSTTRNSMRNLSIQCVFLNNLIFQLFNHFILPSSTLARLVNIYMFRCNNKMLFVTSSFVG
WLIGHILFMKWVGLVLFWIRQNHSIRS-NVLIKYPVSELRNSMAQIFSILLFITCVYYLGRM
PSPIFTKKLKE----KPIVTLLFDYKRWNRPLRYIKNNRLEKAVRNEMSQYFFYTCQSDGKQR
ISFTYPPSLSTFGEMIQKKISLCTTEKLSSDELYNHWVYINEQKKDNLNNEFRNRIEALDKG
SLVLDVLEKRTRLCNDETEQKCLPKMYDPLLNGPYRGTIKNLYSRK--VWINKIHGILYREF
EQKDPNDQ--GIKEIRKKVPRWSYKLIYLEQEEVEEIRSRKCKRVIIFTEALIRYSQQSDFRR
NLIKGSMRAQRRKTIIGELFQTNAHSPLFLDRKDKTVFFSFDI--SRILKLIFRNKEENERIRIA
EAWDTFIFTQAIRGFMLITQLTLRKYIVLPSLIIAKNLCRMLLFQFPEWNEDLKELNREMHI
KCTYNGIQLSEKEFPKNWLTDGIQIKILFPFRLKPWHRSKKGKKENFCFLTVFGMEAELPF
GSPRKQPSFFEPIWKELDKKIRNVK-KKSFLVLRFLKERKKRFLKVSKEQKEWIIKKVL--FI
KIIMKEFTKVTITTIRNNLIPDLSIQIKSTGWTNYSLTEKKTKDLADRTSTIINQIERITKEKK
NNAKETSCDNKRSESQKNIWQIFKRRSARLIRKWHFFIKFFIERLY-DTLLCIINIPRVNAQLF
IHFIS-DLSSLSQAYVFYKLSQNQVINKYHLRSVLQYTSLFIKDRLKDFFTQGIFHSESKHKK
LRNSGMNEWKNWLKGHYQYDLSQTRWFRLVPQKWRNRVNQRRTIQNKQKLGSYEKDQ
--FLFYEKQDKFKKHYRYDLLSHKYINDYIIDLDRKYFDWRILHFCLKLFDWMGMNEEILN
RWFFSELLLLYDTYKIKPWGIPIKLLLLNFKNNIEVELDFFLKKHLLFQLRPDYPLNKKMIK
NIKVYCFLLRLKNPKEIAISSIQRREMSLDVMVIQKDITLTELIKKGIFIIDPIRLSIKWDGQFI
MYQTIGISLVHKNKHNQYDLLAPENILSPRRRRKLRIRICFKFLWPNYRLEDLACMNRYW
FDTNNGSRFNMLRIHMYPMKRHQFKSWIFELREILREIKNSHYFLDSWTKFDSVGSFTHIF
FHQERFMKLFDPRIWSILLS-RDS--QGSTSNRYFT---IKGVVLLVVA-VLIY----RINNRNMVE
RKNLYLMGLL-----PIPMNSIGPRNDTLEESF-GSSNINRLIVS-LLYLPKGNKISESCFMDPKE
STWVLPIT----KKCIMPESNWGSRWWRNRIGKKR--DSSCKIS---NETVAGIEISFKEKDIK-Y
LEFLFVSYTD--DPIRKDHDWELFDRLSPRKKRNIINL--NSGQLFEILVKHWICYLMSAFRE
KRPIEVEG-FFKQQG-AESTIQSNDIEHVSH--LFSRNKWGISLQNCAQFHMWQFRQDLFV---
SWGK-NPHESDFLRN-VSRENWIWLDNVWLVNKDR---FFS--KVRNVSSNIQYDS---TR--SIF
VQVTDSSQLKGSS-DQSRDHFDSITIS--NEDSE------------YHTLINQREIQQLKESSIL-WD--P
SFLQTERTEIESD--RFPK--CLSGYS--SMSRLFT-EREKQMNNHLLPEEIEEFL--GNPTRSIRSF

FSDRW--SELH-LGSNPTERSTRDQ----KLLKKQQDVS-FVPSRRSE----NKEMVDMFKIITYL
QN--TVSIHPISSDPGCNMVP-KDEPD-MDSSNKISFLNKNPFFD--LFHLFHDRNRGGYTL--H
HDFESE--ERFQEMADLFTLSITEP-DLVYHKGFAFSIDSYGLDQKK--FLNEVFNSRDESKKK
SL---LVLPPIFY--EENESFYRRIRKKSVR-ISCGNDLEDPKPKRVVFASNNIMEAVNQYRLIRN
LIQIQYRTYG--YIRNVSNRFF---LMNRSDRNFEYGIQ---RDQIGNDTLNHRTIMKYTINQHL
SNLKK-SQKKWFDP----LISRTERSMNRDPDAYRYKWSNGSKNFQEHLEHFVSEQKS---RF
QVVFDRLRINQYSIDWSEVIDKQDLSKSLRFFLSKS---------LLFLS-------KLLLFLSK-SLPFF
FVSFGNIPIHRSE-IHIY-ELKGPNDQLCNQLLESIGVQIVHLNKLKPF--LLD---DHDTS-QKS
KFLINGGTISPFLFNKIP-KW-MIDSFHT--RNNRRKSFDNTDSYFSMISHDRDNWLNPVKPF
HRSSLISSFYKANRLRFLNNPHHFWFY--CNKRFPFYV----EKARINNYD--LTY-GQFLNILFIR
NK-IFSLCVGK--KKYAFLE-RDTISPIESQVSNIFIPNDFPQSGDET-YNLYKS---FHFPIRSDPF
VR--RAIYSIADISGTPLTEGQIVNFERTYCQPLSDMNLSDSEGKNLHQYLN--FNSNMGLIH
TPCSEKYLP--SEKRKK--------RSLCLK--KCVEKRQMYRTFQRDSAFSNLSKWNLFQTYMP
WFLTSTGCKYLNFTLLDTFSDLL--PILSSS-HKFVSIFHDIMHGSDISWPIPQKKW------CLPQ
WNL-ISEISSKCLHN--LLLSEEMIHRNNESPVPLIWTHLRSPNAREF-LYSILFLLLVAGY-LVR
THLLFVSR--ASSELQTEFEKIKSLMIPSYMMELRKLLDRYPTSELNSFWLKNLFLVALEQL
GDSLEEIRGSASGGNMLLGGGP-----AYG-VKSIRSKKKYLNIN---LIDLI-SIIPNPINRITFSRN
TRHLSRTSKEIYSLIRKRKNVNGDWIDDKIESWVANSDSIDDEEREFLFQFS--TLTT--EKRID
QILLSLT-HSDHLS-----KNNSGYQMIEQPGSIYLRYLVDIH--KKYLMNYEFNRSCLAERRIFL
AHYQTITY--SQTSCGANSFHFPSHG---KPFSLRLALSPS-RGILVIGSIGTGRSYLVKYLATNS
YVPFITVFPNKFLDDKPKGYLNDD---IDIDDIDDSDDID------------------------DSNDIDDDLDT
ELLTMTNAL---TMYMT---PKIDRFDITLQFELAKAMSPCIIWIPNIHDLYVNESNYLSLGLLV
NYLS---RDCERCS-TRNILVIASTHIPQKVDPALIAPNKSNTCIKIRRLLIPQQRKHFFTLSYTR
GFHLEKKMFHT--NGFGSITMGSNARDLVALTNEALSISITQKKSIIDTNTIRSALHRQTWDL
R---SQVRSVQDHGILFYQIGRAVAQNVLLSNCPIDPISIYMKKK--SCKEGDSYLYKWYFEL
GTSMKKLTILLYLLSCSAGSVAQDLWSLP--GPDEK-NWITSYGFVENDS-DLVHGLLEVEG
ALVG--SSRTEKDCSQFDND--RVTLLLRSEPRN-PLDMMQNGSCSIVDQRFLYEK--YESEFE
E-GEGEGALDPQ----QIEED----LFNH--IVWAP--RIWRP---CGNLFDCIERPNELGFP-YWARS
FR---GKRIIYHKEDELQENDSEFLQSGTMQYQTRDRSSKEQGFFRISQFIWDPADPFFFLFK-
DQPSVSVFSRREFFADEEMSKGLLTSQTNPPTSIYKHWFI--KNTQEKHFELLIHRQRWLRT--
-NSSLS--NGSFRSN--TLFESYQYLSNLFLSN-GTLLDQMTKTLLRKRWLFPDEMKHLI--HV
TGERFPIPMPRSR-INGNFIDKTSSIVANILLRIIPTTSGEKEAFTYYRDG--MSAQSEGNYAEA
LQNYYEATRLEIDPYDRSYILYNIGLIHTSNGEHTKALEYYFRALERNPFLPQAFNNMAVIC
HY-RGEQAIQQGDSEIAEAWSDQAAEYWKQAIALTPGNYIEAHNWLKITRRFE--MNWRSE
RIWIELITGSRKTSNFCWAFILFLGSLGFLLVGTSSYLGRNLISLFPSQQILFFPQGIVMSFYGI
AGLFISSYLWCTISWNVGSGYDRFDRKEGIVCIFRWGFPGKNRRIFLRFLMRDIQSIRIEVK
EGIYPRRVLYMEIRGQGAIPLTRTDEN-LTPREIEQKAAELAYFLRVPIEVF
>Bambusa
??
??
??
??
??
???????????????????????????????MATLRVDEIHKILRERIEQYNRKVGIENIGRVVQVGDGIA

RIIGLGEIMSGELVEFAEGTRGIALNLESKNVGIVLMGDGLMIQEGSFVKATGRIAQIPVSEA
YLGRVINALAKPIDGRGEIVASESRLIESPAPGIISRRSVYEPLQTGLIAIDSMIPIGRGQRELII
GDRQTGKTAVATDTILNQKGQDVICVYVAIGQRASSVAQVVTTFHEEGAMEYTIVVAEMA
DSPATLQYLAPYTGAALAEYFMYRERHTLIIYDDLSKQAQAYRQMSLLLRRPPGREAYPG
DVFYLHSRLLERAAKLNSLLGEGSMTALPIVETQSGDVSAYIPTNVISITDGQIFLSADLFN
AGIRPAINVGISVSRVGSAAQIKAMKQVAGKSKLELAQFAELQAFAQFASALDKTSQNQLA
RGRRLRELLKQSQSNPLPVEEQIATIYTGTRGYLDSLEIGQVKKFLDELRKHLKDTKPQFQ
EIISSSKTFTEQAEILLKEAIQEQLERFSLQEQTMRTNPTTSRPGVSTIEEKSTGRIDQIIGPVL
DITFPPGKLPYIYNALVVKSRDTAGKQINVTCEVQQLLGNNRVRAVAMSATDGLMRGMEV
IDTGAPLSVPVGGATLGRIFNVLGEPIDNLGPVDTSATFPIHRSAPAFIELDTKLSIFETGIKV
VDLLAPYRRGGKIGLFGGAGVGKTVLIMELINNIAKAHGGVSVFGGVGERTREGNDLYM
EMKESGVINEKNIEESKVALVYGQMNEPPGARMRVGLTALTMAEYFRDVNKQDVLLFIDN
IFRFVQAGSEVSALLGRMPSAVGYQPTLSTEMGSLQERITSTKKGSITSIQAVYVPADDLTD
PAPATTFAHLDATTVLSRGLASKGIYPAVDPLDSTSTMLQPRIVGNEHYETAQRVKQTLQR
YKELQDIIAILGLDELSEEDRLTVARARKIERFLSQPFFVAEVFTGSAGKYVGLAETIRGFQL
ILSGELDGLPEQAFYLVGNIDEASTKAINLEEENKLKKMKLNLYVLTPKRIIWDCEVKEIILS
TNSGQIGVLPNHAPINTAVDMGPLRIRLNDQWLTAVLWSGFARIVNNEIIILGNDAELGSDI
DPEEAQQALEIAEANLSRAEGTKELVEAKLALRRARIRVEAINWIPMKNVTHSFVFLAHW
PSAGSFGLNTDILATNLINLTVVVGVLIFFGKGVLKDLLDNRKQRILSTIRNSEELRRGTIEQ
LEKARVRLQKVELEADEYRMNRYSEIEREKANLINATSISLEQLEKSKNETLYFEKQRAMN
QVRQRVFQQAVQGALGTLNSCLNTELHFRTIRANIGILGAIESKR-MNPLIAAASVIAAGLA
VGLASIGPGVGQGTAAGQAVEGIARQPEAEGKIRGTLLLSLAFMEALTIYGLVVALALLFA
NPFVMNIIPCSIKTLKGLYDISGVEVGQHFYWQIGSFQIHAQVLITSWVVITILLGSVIIAVRN
PQTIPTDGQNFFEYVLEFIRDLSKTQIGEEYGPWVPFIGTMFLFIFVSNWSGALLPWKIIQLP
HGELAAPTNDINTTVALALLTSAAYFYAGLSKKGLSYFEKYIKPTPILLPINILEDFTKPLSLS
FRLFGNILADELVVVVLVSLVPLVVPIPVMFLGLFTSGIQALIFATLAAAYIGESMEGHHMLF
ATLEHILTHISFSTISIVITIHLITLLVRELGGLHDSSEKGMIATFLSITGFLVSRWASSGHFPLS
NLYESLIFLSWALYILHTIPKIQNSKNDLSTITTPSTILTQGFATSGLLTEMHQSTILVPALQSQ
WLMMHVSMMLLSYATLLCGSLLSAALLIIKFRKNFDFFSKKKEIEYFYAKRSALKNTSFPS
FSNYYKYQLTERLDSWSYRVISLGFTLLTIGILCGAVWANEAWGSYWNWDPKETWAFITW
TIFAIYLHSRTNPNWKGTNSALVASIGFLIIWICYFGINLLGIGLHSYGSFTLPMKKKKALPS
FLYLVFIVLLPWGVSFSFNKCLELWIKNWWNTRQSETLLTDIQEKKVLERFIELEELFLLDE
MIKEKPKTHVQKPPIGIHKEIIQLAKIDNEDHLHIILHFSTNIICLAILSGSFFLGKEELVILNS
WVQEFFYNLNDSIKAFSILLVTDFFVGFHSTRGWELLIRWVYNDLGWAPNELIFTIFVCSFP
VILDTCLKFWVFFYLNRLSPSLVVIYHSISEMPIGVPKVPYRIPGDEEATWVDLYNVMYRE
RTLFLGQEIRCEITNHITGLMVYLSIEDGISDIFLFINSPGGWLISGMAIFDTMQTVTPDIYTI
CLGIAASMASFILLGGEPTKRIAFPHARIMLHQPASAYYRARTPEFLLEVEELHKVREMITR
VYALRTGKPFWVVSEDMERDVFMSADEAKAYGLVDIVGDEPREAKVTFEGLVTEALPNG
MFRVRLENDTIILGYISGKIRSSSIRILMGDRVKIEVSRYDSSKGRIIYRLPHKDSKDMEKFE
GYSEKQKSRQQYFVYPLLFQEYIYAFAHDYGLVSCNNKFSSLLVKRLIIRMYQQNFWINSV
NHPNQDRLLDSEFYSQILSEGFAIVVEIPFSLRELSCPTTKFQNLRSIHSIFPFLEDKFLHLHY
LSHIEIPYPIHLEILVQLLQYRIQDVPSLHLLRFFLNYYSNWNSLITKSIFLLKKKRLFRFLYN
SYVSEYEFFLLFLRKQSFCLPLTSSGTFVERIHFSRKMEH--VMYPGRKTVWFFMDPLMHY
VRYQGKAILASKGTLLLKKKWKCYLVNFWQYSFSFWTQPRRIHLNQLANSCFDFLGYLS

SVPINPLLVRNQMLENSFLIDTRM--KKFDTIVPATPLIRSLSKAQFCTGSGHPISKPIWTDLS
DWDILDRFGRICRNLFHYHSGSSKKRTLYRLKYILRLSCARTLARKHKSTVRTFMQRLGSV
FLEEFFMEEEQVFSLMFTKRIWYLDIIRINDLVNPLMIIDRV-EVE-TINSFSKSE-LLKEVYGL
IWIL-PILTLLLGITIEVLVIVWLEREISASIQQRIGPEYAGPLGLLQAIADGTKLLFKEDILPSR
GDIPLFSIGPSIAVISILLSFLVIPLGYRFVLADLSIGVFLWIAISSIAPIGLLMAGYSSNNKYSF
LGGLRAAAQSISYEIPLTFCVLAISLLSNSSSTVDIVEAQSKYGFFGWNLWRQPIGFLVFLIS
SLAECERLPFDLPEAEEELVAGYQTEYSGIKYGLFYLVSYLNLLVSSLFVTVLYLGGWNLSI
PYISFFGFFQMNKMVGILEMTMGIFITLTKAYLFLFISITIRWTLPRMRMDQLLNLGWKFLL
PISLGNLLLTTSSQLVSLMIWHVQNENFILDSTRIFMKAFHLLLFHGSFIFPECILIFGLILLLM
IDSTSDQKDRPWFYFISSTSLVISITALLFRWREEPIISFSGNFQTNNFNEIFQFLILLCSTLCIP
LSVEYIECTEMAITEFLLFVLTATLGGMFLCGANDLITIFVAPECFSLCSYLLSGYTKRDLRS
NEATMKYLLMGGASSSILVHGFSWLYGSSGGEIELQEIVNGLINTQMYNSPGISIALISITVG
LGFKLSPAPFHQWTPDVYEGSPTPVVAFLSVTSKVAASASATRILDIPFYFSSNEWHLLLEIL
AILSMILGNLLAITQTSMKRMLAYSSIGQIGYVIIGIIVGDSNDGYASMITYMLFYISMNLGT
FACIVLFGLRTGTDNIRDYAGLYTKDPFLALSLALCLLSLGGLPPLAGFFGKLYLFWCGWQ
AGLYFLVSIGLLTSVLSIYYYLKIIKLLMTGRNQEITPYVRNYRRSPLRSNNSIELSMTVCVI
ASTIPGISMNPIVAIAQDTLFMFLLHEYDIFWTFLIIASLIPILAFWISGLLAPVSEGPEKLSSY
ESGIEPMGGAWLQFRIRYYMFALVFVVFDVETVFLYPWAMSFDVLGVSVFIEAFIFVLIPVV
GSVYAWRKGALEWSMSYFPWLTILVVLPIFAGSLIFFLPHRGNKIVRWYTISICLLEFLLMT
YAFCYHFQLEDPLIQLKEDSKWIDVFDFHWRLGIDGLSLGSILLTGFITTLATLAAWPVTRN
SRLFYFLMLAMYSGQIGLFSSRDLLLFFIMWELELIPVYLLLSMWGGKRRLYSATKFILYT
AGGSIFFLIGVLGMGLYGSNEPRLDLERLINRSYPATLEILFYLGFLIAYAVKLPIIPLHTWLP
DTHGEAHYSTCMLLAGILLKMGAYGLIRINMELLPHAHYLFSPWLVIIGAMQIIYAASTSL
GQRNFKKRIAYSSVSHMGFIIIGIGSITNIGLNGAILQILSHGFIGATLFFLAGTACDRMRLVY
LEELGGISIPMPKIFTMFSSFSMASLALPGMSGFVAELVVFFGLITSPKFLLMPKMLITFVMA
IGMILTPIYLLSMLRQMFYGYKLFHVPNANFVDSGPRELFLLICIFLPVIGIGIYPDFVLSLSV
DRVEALLSNYYPKMMFEHVLFLSVYLFSIGIYGLITSRNMVRALICLELILNSINLNLVTFSD
LFDSRQLKGDIFAIFVIALAAAEAAIGLSILSSIHRNRKSTRINQSNFLNNMEHTYQYAWVIP
LLPLPVIMSMGFGLILIPTATKNLRRIWAFPSVLLLSIAMVFSVHLSIQQINGSSIYQYLWSW
TVNNDFSLEFGYLIDPLTSIMLILITTVGILVLIYSDDYMSHDEGYLRFFVYISFFNTSMLGL
VTSSNLIQIYFFWELVGMCSYLLIGFWFTRPIAASACQKAFVTNRVGDFGLLLGILGFFWIT
GSLEFRDLFKIANNNGINSLLTTLCAFLLFLGAVAKSAQFPLHVWLPDAMEGPTPISALIHA
ATMVAAGVFLLARLLPLFISLPLIMSFISLVGTITLFLGATLALAQRDIKRSLAYSTMSQLGY
MMLALGIGSYQAALFHLITHAYSKALLFLGSGSVIHSMEPLVGYSPDKSQNMVLMGGLR
KYVPITRTTFLWGTLSLCGIPPLACFWSKDEILSNSWLYSPFFGIIASFTAGLTAFYMFRIYLL
TFDGYLRVHFQNYSSTKEGSLYSISLWGKRIPKGVNR-DFILSTTKFFSVKN--PFVYPHETG
NTMLFPLFILLLFTLFIGSIGIHFELTILSKWLTPSISFFQESSNSSINSYEFITNAISSVSLAMFG
LFIAYIFYGSAYSFFQNLYLKNSLVSFLDQVKKKIYSWSYNRGYIDIFYTRVFTLGIRGLTEL
TQFFDKGVIDGITNGVGLAGFCIGEEIKY-VGGGRISSYLFFFLCYVSVFLFFFLMDLPGPIH
EILVLFGEFFLLLGGIGVVLLTNPIYSAFSLGLVLICISLFYILLNSYFVAVAQLLIYVGAINVLI
IFAVMFIDGSEWSKNKNYWTIGDGFTSLVCITILFSLMTTIPDTSWYGILWTTRSNQIVEQG
LINNVQQIGIHLATDFYLPFELISIILLVSLIGAITMARQMSLPLTRKDLMIVNMGPHHPSMH
GVLRLIVTLDGEDVIDCEPILGYLHRGMEKIAENRTIIQYLPYVTRWDYLATMFTEAITVN
APEFLENIQIPQRASYIRVIMLELSRIASHLLWLGPFMADLGAQTPFFYIFRERELIYDLFEA

ATGMRMMHNYFRIGGVAADLPYGWIDKCLDFCDYFLRGVVEYQQLITQNPIFIERVEGVG
FISGEEAVNWGLSGPMLRASGIQWDLRKVDPYESYNQFDWKIQWQKEGDSLARYLVRM
GEMRESIKIIQQAVEKIPGGPYENLEVRR-KKAKNSEWNDFEYRFLGKKPSPNFELSKQELY
VRVEAPKGELGIYLVGDDSLFPWRWKIRPPGFINLQILPQLVKKMKLADIMTILGSIDIIMG
EVDRMFPMVTGFMSYGQQTIRAARYIGQSFIITLSHTNRLPITIHYPYEKSITSERFRGRIHF
EFDKCIACEVCVRVCPIDLPLVDWRFEKDIKRKQLLNYSIDFGVCIFCGNCVEYCPTSCLS
MTEEYELSTYDRHELNYNQIALSRLPISIMGDYTIQTIRNSKIDEEKSLNSRTITNYQQGWL
SNWLVKHEVVHRSLGFDHRGIETLQIKAGDWDSIAVILYVYGYNYLRSQCAYDVAPGGSL
ASVYHLTRIQYGIDNPEEICIKVFAQKDNPRIPSVFWIWKSADFQERESYDMVGISYDNHP
RLKRILMPESWIGWPLRKDYITPNFYEIQDAH------------------------------------MVLT-EYSD-K
KKKEGKDSIETVMSLIEFPLLDQTSSNSVISTTPNDLSNWSRLSSLWPLLYGTSCCFIEFASLI
GSRFDFDRYGLVPRSSPRQADLILTAGTVTMKMAPSLVRLYEQMPEPKYVIAMGACTITGG
MFSTDSYSTVRGVDKLIPVDVYLPGCPPKPEAVIDALTKLRKKISREIVEDRTLSQ-KNRCF
TTSHKLYVRRSTHTGTYEQELLYQSPSTLDIST--FFKSKSPVSSYKLVNMENRNTFSWVKE
QMIRSISVSIMIYVITRTSISNAYPIFAQQGYENPREATGRIVCANCHLANKPVDIEVPQAVLP
DTVFEAVLRIPYDMQLKQVLANGKKGGLNVGAVLILPEGFELAPPDRISPELKEKIGNLSF
QSYRPDKKNILVIGPVPGQKYSEIVFPILSPDPATKKDVHFLKYPIYVGGNRGRGQIYPDGS
KSNNTVYNATSTGIVRKILRKEKGGYEISIVDTSDGRQVIDIIPPGPELLVSEGESIKLDQPLT
SNPNVGGFGQGDAEIVLQDPLRVQGLLFFFASVILAQVFLVLKKKQFEKVQLYEMNFMSK
VYDWFEERLEIQAIADDITSKYVPPHVNIFYCLGGITLTCFLVQVATGFAMTFYYRPTVTEA
FSSVQYIMTEANFGWLIRSVHRWSASMMVLMMILHVFRVYLTGGFKKPRELTWVTGVVL
AVLTASFGVTGYSLPWDQIGYWAVKIVTGVPEAIPVIGSPLVELLRGSASVGQSTLTRFYSL
HTFVLPLLTAVFMLMHFPMIRKQGISGPLMGVTKKPDLNDPVLRAKLAKGMGHNYYGEP
AWPNDLLYIFPVVILGTIACNVGLAVLEPSMIGEPADPFATPLEILPEWYFFPVFQILRTVPN
KLLGVLLMVSVPTGLLTVPFLENVNKFQNPFRRPVATTVFLIGTAVALWLGIGATLPIEKSLT
LGLFMIEVFLFGIVLGLIPITLAGLFVTAYLQYRRGDQLDLMLTITSYFGFLLAALTITPALFI
GLNKIRLIMDIVSLAWAALMVVFTFSLSLVVWGRSGLMIIRSPEPEVKIVVDRDPVKTSFEE
WAKPGHFSRTIAKGPDTTTWIWNLHADAHDFDSHTGDLEEISRKVFSAHFGQLSIIFLWLS
GMYFHGARFSNYEAWLSDPTHIGPSAQVVWPIVGQEILNGDVGGGFRGIQITSGFFQIWR
ASGITSELQLYCTAIGALIFASLMLFAGWFHYHKAAPKLAWFQDVESMLNHHLAGLLGLG
SLSWAGHQIHVSLPINQFLDAGVDPKEIPLPHEFILNRDLLAQLYPSFAEGATPFFTLNWSK
YAEFLSFRGGLDPITGGLWLSDIAHHHLAIAILFLIAGHMYRTNWGIGHGLKDILEAHKGPF
TGQGHKGLYEILTTSWHAQLSLNLAMLGSTTIVVAHHMYSMPPYPYLATDYGTQLSLFTH
HMWIGGFLIVGAAAHAAIFMVRDYDPTTRYNDLLDRVLRHRDAIISHLNWVCIFLGFHSF
GLYIHNDTMSALGRPQDMFSDTAIQLQPIFAQWVQNIHANAPGVTAPGATTSTSLTWGGG
ELVAVGGKVALLPIPLGTADFLVHHIHAFTIHVTVLILLKGVLFARSSRLIPDKANLGFRFPC
DGPGRGGTCQVSAWDHVFLGLFWMYNAISVVIFHFSWKMQSDVWGTISDQGMVTHITG
GNFAQSSITINGWLRDFLWAQASQVIQSYGSSLSAYGLFFLGAHFVWAFSLMFLFSGRGY
WQELIESIVWAHNKLKVAPATQPRALSIIQGRAVGVTHYLLGGIATTWAFFLARIIAVGMEL
RFPRFSQGLAQDPTTRRIWFGIATAHDFESHDDITEERLYQNIFASHFGQLAIIFLWTSGNLF
HVAWQGNFESWIQDPLHVRPIAHAIWDPHFGQPAVEAFTRGGAAGPVNIAYSGVYQWWY
TIGLRTNQDLYTGALFLLFLSTLSLIAGWLHLQPKWKPSLSWFKNAESRLNHHLSGLFGVS
SLAWTGHLVHVAIPGSRGEYVRWNNFLDVLPYPQGLGPLLTGQWNLYAQNPDSSNHLFGT
AQGAGTAILTLLGGFHPQTQSLWLTDIAHHHLAIAFIFLIAGHMYRTNFGIGHSIKDLLEAH

TPPGGRLGRGHKGLYDTINNSIHFQLGLALASLGVITSLVAQHMYSLPAYAFIAQDFTTQAA
LYTHHQYIAGFIMTGAFAHGAIFFIRDYNPEQNEDNVLARMLDHKEAIISHLSWASLFLGF
HTLGLYVHNDVMLAFGTPEKQILIEPIFAQWIQSAHGKTTYGFDILLSSTSGPAFNAGRTLW
LPGWLNAVNE--NSNSLFLTIGPGDFLVHHAIALGLHTTTLILVKGALDARGSKLMPDKKDF
GYSFPCDGPGRGGTCDISAWDAFYLAVFWMLNTIGWVTFYWHWKHITLWQGNVSQFNE
SSTYLMGWLRDYLWLNSSQLINGYNPFGMNSLSVWAWMFLFGHLVWATGFMFLISWRG
YWQELIETLAWAHERTPLANLIRWRDKPVALSIVQARLVGLAHFSVGYIFTYAAFLIASTSG
KFGMSHSVKIYDTCIGCTQCVRACPTDVLEMIPWDGCKAKQIASAPRTEDCVGCKRCESA
CPTDFLSVRVYLGPETTRSMALSYMTDFNLPSIFVPLVGLVFPAIAMASLFLYVQKNKIVM
RDIKTYLSVAPVLSTLWFGALAGLLIEINRLFPDALSFPFF--MTAILERRESTSLWGRFCNWI
TSTENRLYIGWFGVLMIPTLLTATSVFIIAFIAAPPVDIDGIREPVSGSLLYGNNIISGAIIPTSA
AIGLHFYPIWEAASVDEWLYNGGPYELIVLHFLLGVACYMGREWELSFRLGMRPWIAVAY
SAPVAAAAAVFLIYPIGQGSFSDGMPLGISGTFNFMIVFQAEHNILMHPFHMLGVAGVFGG
SLFSAMHGSLVTSSLIRETTENESANEGYKFGQEEETYNIVAAHGYFGRLIFQYASFNNSRS
LHFFLAAWPVVGIWFTALGISTMAFNLNGFNFNQSVVDSQGRVINTWADIINRANLGMEV
MHERNAHNFPLDLAALEVPSLNGMGLPWYRVHTVVLNDPGRLLSVHIMHTALVSGWAG
SMALYELAVFDPSDPVLDPMWRQGMFVIPFMTRLGITNSWGGWSIS-GGTVTNPGIWSYE
GVAGAHIVFSGLCFLAAIWHWVYWDLEIFCDERTGKPSLDLPKIFGIHLFLAGVACFGFGA
FHVTGLYGPGIWVSDPYGLTGKVQAVNPAWGVEGFDPFVPGGIASHHIAAGTLGILAGLF
HLSVRPPQRLYKGLRMGNIETVLSSSIAAVFFAAFVVAGTMWYGSATTPIELFGPTRYQWD
QGYFQQEIYRRVSDGLAENLSLSEAWSKIPEKLAFYDYIGNNPAKGGLFRAGSMDNGDGI
AVGWLGHPVFRDKEGRELFVRRMPTFFETFPVVLVDEEGIVRADVPFRRAESKYSVEQVG
VTVEFYGGELNGVSYSDPATVKKYARRSQLGEIFELDRATLKSDGVFRSSPRGWFTFGHAT
FALLFFFGHIWHGARTLFRDVFAGIDPDLDAQVEFGTFQKVGDPTTRRQAVMKILYSLRRF
YHVETLFNGTFVLAGRDQETTGFAWWAGNARLINLSGKLLGAHVAHAGLIVFWAGAMN
LFEVAHFVPEKPMYEQGLILLPHLATLGWGVGPGGEVLDTFPYFVSGVLHLISSAVLGFGG
IYHALLGPETLEESFPFFGYVWKDRNKMTTILGIHLILLGIGAFLLVLKALYFGGVYDTWA
PGGGDVRKITNLTLSPSVIFGYLLKSPFGGEGWIVSVDDLEDIIGGHVWLGFICVFGGIWHI
LTKPFAWARRAFVWSGEAYLSYSLGALSVFGFIACCFVWFNNTAYPSEFYGPTGPEASQAQ
AFTFLVRDQRLGANVGSAQGPTGLGKYLMRSPTGEVIFGGETMRFWDLRAPWLEPLRGP
NGLDLSRLKKDIQPWQERRSAEYMTHAPLGSLNSVGGVATEINAVNYVSPRSWLATSHFV
LGFFFFVGHLWHAGRARAAAAGFEKGIDRDLEPVLYMNPLNMTIALGRVTKEENDLFDI
MDDWLRRDRFVFVGWSGLLLFPCAYFALGGWFTGTTFVTSWYTHGLASSYLEGCNFLTA
AVSTPANSLAHSLLLLWGPEAQGDFTRWCQLGGLWTFVALHGAFALIGFMLRQFELARSV
QLRPYNAISFSAPIAVFVSVFLIYPLGQSGWFFAPSFGVAAIFRFILFFQGFHNWTLNPFHM
MGVAGVLGAALLCAIHGATVENTLFEDGDGANTFRAFNPTQAEETYSMVTANRFWSQIF
GVAFSNKRWLHFFMLFVPVTGLWMSAIGVVGLALNLRAYDFVSQEIRAAEDPEFETFYTK
NILLNEGIRAWMAAQDQPHENLIFPEEVLPRGNALMSGSTGERSFADIITSIRYWVIHSITIP
SLFIAGWLFVSTGLAYDVFGSPRPNEYFTESRQGIPLITDRFDSLEQLDEFSRSFMTIDRTYP
IFTVRWLAVHGLAVPTVFFLGSISAMQFIQRMATQTVEDSSRPG-PRRTRAGSLLKPLNSEY
GKVAPGWGTTPFMGVAMALFAIFLSIILEIYNSSVLLDGILMNMLTLKLFVYTVVIFFVSLFI
FGFLSNDPGRNPGREEMADTTGRIPLWLIGTVTGIPVIGLIGVFFYGSYSGLGSSLMPNILSL
TCICFNSVLYPTSFFFAKLPEAYAIFNPIVDVMPVIPVLFFLLAFVWQAAVSFRMTQSNPNE
QNVELNRTSLYWGLLLIFVLAVLFSNYFFNMEVNILAFIATALFILVPTAFLLIIYVKTVSQN

DMETATLVAISISGLLVSFTGYALYTAFGQPSQQLRDPFEEHGDMEALVYTFLLVSTLGIIFF
AIFFREPPKVPTKKMKMTIAFQLAVFALIATSSVLVISVPLVFASPDGWSNNKNVVFSGTSL
WIGLVFLVAILNSLIS-------MSPQTETKASVGFKAGVKDYKLTYYTPEYETKDTDILAAFRV
TPQPGVPPEEAGAAVAAESSTGTWTTVWTDGLTSLDRYKGRCYHIEPVVGEENQYIAYVA
YPLDLFEEGSVTNMFTSIVGNVFGFKALRALRLEDLRIPTTYSKTFLGPPHGIQVERDKLN
KYGRPFLGCTIKPKLGLSAKNYGRACYECLRGGLDFTKDDENVNSQPFMRWRDRFVFCA
EALYKAQAETGEIKGHYLNATAGTCEEMIKRAVFARELGAPIVMHDYLTGGFTANTSLAH
YCRDNGLLLHIHRAMHAVIDRQKNHGMHFRVLAKALRMSGGDHIHAGTVVGKLEGERE
MTLGFVDLLRDDFIEKDRARGIFFTQDWVSMPGVIPVASGGIHVWHMPALTEIFGDDSVL
QFGGGTLGHPWGNAPGAAANRVALEACVQARNEGRDLAREGNEIIRAACKWSPELAAA
CEVWKAIKFEFEPVDKLDKTAKHLYKTPIPSTRKGTVDRQVKSNPRNNLIHGRHRCGKGR
NSRGIITARHRGGGHKRLYRKIDFRRNQKDISGRIVTIEYDPNRNAYICLIHYGDGEKRYIL
HPRGAIIGDTIVSGTKVPISMGNALPLTDMPLGTAIHNIEITRGRGGQLARAAGAVAKLIAK
EGKSATLRLPSGEVRLVSQNCLATVGQVGNVGVNQKSLGRAGSKCWLGKRPVVRGVVM
NPVDHPHGGGEGKAPIGRKKPTTPWGYPALGRRTRKRKKYSDSFILRRRK-MIQPQTLLNV
ADNSGARKLMCIRVIGAASNQRYARIGDVIVAVIKDAVPQMPLERSEVIRAVIVRTCKEFKC
EDGIIIRYDDNAAVIIDQKGNPKGTRVFGAIAEELRELNFTKIVSLAPEVLMLSPKRTRFRKQ
HRGRMKGKSCRGNRICFGRYALQALEPAWITSRQIEAGRRAMTRYARRGGKLWVRIFPDK
PVTLRPTETRMGSGKGSPEYWVAVVKPGRILYEMGGVSETVARAAISIAASKMPIRSQFLR
LEMTRVPRGYIARRRRTKMRSFASNFRGAHLRLNRMITQQVRRAFVSSHRDRGRQKRDF
RRLWITRINAATRIY--VFNSYSKLIHNLYKKELILNRKMLAQVAVSNPNNLYTISNKIKIIN---
------RIKRVFKAQKRIHMSVFKAQRVLDEIRWRYYEETVMILNLMPYRASYPILKLVYSAA
ANATHYRDFDKANLFITKAEVSRSTIMKKFRPRARGRSSPIKKTMCHITIVLN-VKKS-------
MDGIKYAVFTEKSLRLLGKN-QYTFNVESGFTKTEIKHWVELFFGVKVVAVNSHRLPRKG
RRMGPILGHTMHYRRMIITLQPGYSIPLLDREKNMAVPKKRTSMSKKRIRKNLWKKKTYF
SIVQSYSLAKSFSSGKPKGFSGQQTNNMAKGKDVRIRLILECISCVRK--G--KESTGISRYST
QKNRHNTPGQLEFKKFCRYCRKHTTHGEIKKMKIRASVRKICTKCRLIRRRGRIRVICSNP
KHKQRQGMVREEVAGSTQTLQWKCVESRVDSKRLYYGRFILSPLRKGQADTVGIALRRA
LLGEIEGTCITRAKFG--SVPHEYSTIAGIEESVQEILLNLKEIVLRSNLYGVRDASICVKGPR
YITAQDIILPPSVEIVDMAQPIANLTEPIDFCIELQIKRDRGYHTELRKNSQDGSY--PIDAVSM
PVRNVNYSIFYCGNGNEKHEILFLEIWTNGSLTPKEALYEASRNLIDLFLPFLHAEEE--GTS
FEENK-NRFTPPLLTFQKKLT-NLKKNKK-GIPLNCIFIDQLELPSRTYNCLKRANIHTLLDLL
SKTEEDLMRIDSFRMEDGKQIWDTLEKH--LPIDLPKNKMLRNGN-EGMSTIPGFSQIQFEG
FFRFINQGLAEELEKFPTIKDPDHEIAFQLFAKGYQLLEPSIKERDAVYESLTYSSELYVSAR
LIFGF--DVQKQTISIGNIPIMNSLGTFIINGIYRIVINQILLSPGIYYRSELDHKGISIYTGTIISD
WGGRSELAIDKKERIWARVSRKQKVSILVLSSAMGSNLREILDNVSYPEILLSFPNAKEKK
RIESKEKAILEFYQQFACVGGDLVFSESLCEELQKKFFQQKCELGRIGRRNMNRRLNLDIP
QNNTFLLPRDVLAATDHLIGMKFGTGILDDDDMNHLKNKRIRSVADLLQDQFGLALDRL
QHAVQKTIRRVFIRQSK--PTPQTLVTPTSTSILLITTYETFFGTYPLSQVFDQTNPLTQTVHG
RKVSCLGPGGLTGRTASFRSRDIHPSHYGRICPIDTSEGINVGLTGSLAIHARIDHWWGSIES
PFYEISEKAKEKKE--KQVVYLSPNRDEYYMIAAGNSLSLNQGIQEEQVVPARYRQEFLTIA
WEQIHVRSIFPFQYFSIGGSLIPFIEHNDANRALMSSNMQRQAVPLSRSEKCIVGTGLERQT
ALDSRVSVIAEREGKIISTDSHKILLSSSGKTISIPLVTHRRSNKNTCMHQKPRVPWGKSIKK
GQILAEGAATVGGELALGKNVLVAYMPWEGYNFEDAVLISERLVYEDIYTSFHIRKYEIQT

DTTSQGSAEKITKEIPHLEEHLLRNLDRNGVVRLGSWVETGDILVGKLTPQIASE-SSYIAE
AGLLRAIFGLEVSTSKETSLKLPIGGRGRVIDVKWIQR-------DPIMVRVYILQKREIKVGDK
VAGRHGNKGIISKILPRQDMPYLQDGTPVDMVFNPLGVPSRMNVGQIFESSLGLAGDLLK
KHYRIAPFDERYEQEASRKLVFSELYEASKQTKNPWVFEPEYPGKSRIFDGRTGDPFEQPV
LIGKSYILKLIHQVDEKIHGRSTGPYSLVTQQPVRGRAKQGGQRVGEMEVWALEGFGVAH
ILQEILTYKSDHLIARQGILNATIWGKRVPNHEDPPESFRVLVRELRSLALELNHFLVSEKNF
QVNREEV-------MIDQYKHQQLQIGLVSPQQIKAWANKNLPNGEVVGEVTRPSTFHYKTDK
PEKDGLFCERIFGPIKSRICACGNSRASVAENEDERFCQKCGVEFVDSRIRRYQMGYIKLAC
PVTHVWYLKGLPSYIANLLDKPLKKLEGLVYGD--FSFARP-SAKKPTFLRLRGLFEDEISSC
NHSISPFFSTPGFATFRNREIATGAGAIREQLADLDLRIIIENSLVEWKELEDE-GYSGDEWE
DRKRRIRKVFLIRRMQLAKHFIQTNVEPEWMVLCLLPVLPPELRPIVYRSGDKVVTSDINE
LYKRVIRRNNNLAYLLKRSELAPADLVMCQEKLVQEAVDTLLDSGSRGQPTRDGHNKVY
KSLSDVIEGKEGRFRETLLGKRVDYSGRSVIVVGPSLSLHQCGLPLEIAIKLFQLFVIRDLIT
KRATSNVRIAKRKIWEKEPIVWEILQEVMRGHPVLLNRAPTLHRLGIQAFQPTLVEGRTIC
LHPLVCKGFNADFDGDQMAVHLPLSLEAQAEARLLMFSHMNLLSPAIGDPICVPTQDMLI
GLYVLTIGNRRGICANRYNSCGN-----YPNQKVSYNN-KYTKDKEPHFSSSYDALGAYRQK
RISLDSPLWLRWKLDQR-VIGS--REVPIEVQYESLGTYHEIYAHYLIVGNRKKEIRSIYIRST
LGHISFYREIEEAIQGFSQAYSYTI------------MAERANLVFHNKEIDGTAMKRLISRLIDHFG
MGYTSHILDQIKTLGFHQATTTSISLGIEDLLTIPSKGWLVQDAEQQSFLLEKHYYYGAVHA
VEKLRQSVEIWYATSEYLKQEMNSNFRITDPSNPVYLMSFSGARGNASQVHQLVGMRGL
MADPQGQMIDLPIQSNLREGLSLTEYIISCYGARKGVVDTAVRTADAGYLTRRLVEVVQHII
VRRRDCGTIRGISVSPQNGMTE--KLFVQTLIGRVLADDIYIGSRCIAARNQDIGIGLVNRFIT
AFRAQPFRAQPIYIRTPFTCRSTSWICQLCYGRSPTHGDLVELGEAVGIIAGQSIGEPGTQLT
LRTFHTGGVFTGGTADLVRSPSNGKIQFNEDLVHPTRTRHGQPAFLCYIDLHVTIQSQDILY
SVNIPSKSLILVQNDQYVESEQVIAEIRAGTSTL--FKERVQKHIYSESDGE-MHWSTDVYHA
PEYQYGNLRRLPKTSHLWILSVSMCR--SSIASFSLHKDQDQMNTYSFSVDGR--YIFDLS--
MANDDQVRHRLLDTFG----DREILDYS-TPDRIMSNGHWNFVYPS-ILQDN--SDLLAKKRR
NRFVIPLQYHQEQEKELISCLGISIEIPFM--GVLRRNTIFAYFDDPRYRKD--GIVKFRSPEED
SILK--KEGLIEHRG-TKEFSLKY-ELDRFFFILQELHILPRSSSLKVLDNSIIGVDTQLTKNTRS
RLGGLVRVNRKKSHTELKIFSGDIHFPEEADKILG--GSLIPPEREKK--ESKE-SK-KRKNWV
YVQRKKILKSKEK--YFVSVRPAVAYEMDEGRNLATLFPQDLLQEEDNLQLRLVNFLSHEN
SKLTQRIYHTN-QFVRTCLVVNWEQEEKEEAR-------ASLVEVRANDLIRDFLRIELVKSTISY
TRRRYDRTSAGPIPN---LDRTNTNSFYSKAKIQS-----LSQHQEAIG----TLLNRNK---EYQSL
MILLASNCSRIG--LFKNSKYP---NAVKESNPRIPI-DIFGPLAAIVP-SISNFSSS-YYLLT---HNQ
ILLK-KYLFLD---NLKQTFQVLQGLKYSLIDENQR--ISNFD--SNIMLDPFHLNWHFLHHDS
WEETSAIIH-LGQFICENVCLFKSHI----KKSGQIFIVN-IDSFVIRAAKPYLATTGATVHGHYG
EILYKGDRLVTFIYEKSRSSDITQGLPKVEQIFEARSIDSLSPNLERRIEDWNERIPRILGVPW
GFLIGAELTIAQSRISLVNKIQKVYRSQGVQIHNRHIEIIIRQVTSKVRVSE---DGMSNVFSPG
ELIGLLRAERAGRALDESIYYRAILLGITRASLNTQSFISEASFQETARVLAKAALRGRIDW
LKGLKENVVLGGIIPVGTGFQKFVHRSP--QDKNLY--FEIQKKNLFASEM--RDILFLHTELVS
SDSDVTN---SEPPFTPNYTI------MTRRYWNINLKEMIEAGVHFGHGIKKWNPKMAPYISA
KRKGTHITNLARTARFLSEACDLVFDAASQGKSFLIVGTKKRAAYLVASAAIRARCHYVN
KKWFSGMLTNWSITKTRLSQFRDLRAEEKMGKFHHLPKRDVAILKRKLSTLQRYLGGIKY
MTRLPDIVIVLDQQKEYIALRECAILGIPTISLVDTNCDPDLANISIPANDDTMTSIRLILNKL

VFAICEGRSL-YIRNRMGQKINPLGFRLGTTQNHHSFWFAQPKNYSEGLQEDKKIRNCIKN
YIQKNRKKGSNKIESITHIEIQKEIDTIHVIIHIGFPN-LLKKKG-----AIEELEKDLQKEVNSVN
QRLNIAIEKVKEPYRQPSILAEYIAFQLKNRVSFRKAMKKAIELTQKADIRGVKVKIAGRL
AGKEIARAECIKKGRLPLQTIRAKIDYCCYPIRTIYGVLGVKIWIFIDEEMSRYRGPRFKKIR
RLGALPGLTRKTPKSGSNLKKKFHSGK-KEQYRIRLQEKQKLRFHYGLTERQLLRYVHIAG
KAKRSTGQVLLQLLEMRLDNILFRLGMASTIPEARQLVNHRHILVNRRIVDIPSFRCKPRDI
ITTKDNQRSKCLVQNSIASSDPK--LPKHLTIDT--LQYKGLVKKILDRKWVGLKINELLVVE
YYSRKTMSRRGTAEKRTAKSDPIFRNRLVNMVVNRIMKDGKKSLAYQILYRAVKKIQQKT
ETNPLLVLRQAIRRVTPNIGVKTRRKKGSTRKVPIEIGSKQGRALAIRWLLEASQKRPGRN
MAFKLSSELVDAAKGSGGAIRKKEATHRMAEANRALAHFRMGKDTIADLLTSIRNADMN
KKGTVRVVSTNITENIVKILLREGFIESVRKHQESNRYFLVSTLRHQKRKTRKGIYRT---RTF
LKRISRPGLRIYANYQGIPKVLGGMGIAILSTSRGIMTDREARLNRIGGEVLCYIWMTKAIP
KIGSRRKVRIGLRKSARRITKGVIHVQASFNNTIITVTDPQGRVVFWSSAGTCGFKSSRKAS
PYAGQRTAVDAIRTVG---LQRAEVMVKGAGSGRDAALRAIAKSGVRLSCIRDVTPMPHNG
CRPPKKRRLMPTVKQLIRNARQPIRNARKSAALKGCPQRRGTCARVYTINPKKPNSALRK
VARVRLTSGFEITAYIPGIGHNLQEHSVVLVRGGRVKDLPGVRYRIIRGTLDAVAVKNRQQG
RSNMGSKSQKMAKKSLIQREKKRQKLEQKYHLIRRSSKKKIRSKVSPLSLSEKTKMREKL
QSLPRNSAPTRLNRRCFLTGRPRANYRDFGLSGHILREMVYACLLPGATRSSWKKKRKIFG
FMVKEEKEEN--RGSVEFQVFSFTNKIRRLASHLELHKKDFSSERGLRRLLGKRQRLLAYL
AKKNRVRYKKLISQLDIREK-----MVKLRLKRCGRKQQAVYRIVAIDVRSRREGRDLRKVG
FYDPIKNQTCLNVPAILYFLEKGAQPTRTVYDILRKAEFFKEKEGTLS----FQKSKQTFRKSK
QTFRKRRPRIGPGDPIDYRNMSLINRFISEQGKILSRRINRLTLKQQRLITLAIKQARILSFLP
FRNYENEKQFQ-AQSISIITGPRPRKNVTRK-KTNPFVAHHLLAKIEKVNMKEEKETIVTWS
RASSILPAMVGHTIAIHNGKEHIPIYITNPMVGRKLGEFVPTRHFTSYARKDTKSR
R???
??
??
??
??
??
??
??
??
??
??
??
??
??
??
??
??
??
??
??

??
??
??
??
??
??
??
??
??
??
??
??
??
??
??
??
??
??
??
??
??
??
??
???MPRSR-INGNFIDKTSSIVANILLRIIPTTSGEK
RAFTYYRDGAIMLAQSEGNYAEALQNYYEATRLEIDPYDRSYILYNIGLIHTSNGEHTKAL
EYYFRALERNPFLPQAFNNMAVICHY-RGEQAILQGDSEIAEAWFDQAAEYWKQAIALTPG
NYIEAQNWLKITKRFEFEMNWRSEHIWIELLKGSRKRGNFFWACILFLGSLGFLSVGASSY
LGKNMISVLPSQQILFFPQGVVMSFYGIAGLFISSYLWCTILWNVGSGYDRFDRKEGIVCIF
RWGFPGIKRRVFLRFLMRDIQSIRIQVKEGLYPRRILYMEIRGQGVIPLTRTDEKFFTPREIEQ
KAAELAYFLRVPIEVF
>Spirodela
MEKWWFNSMFSLSRSMDSLGPIRYTSGSEDPVLNAMDKNVS-FSNVEHFFDIIDIWSFISD
DTFLVRDSNGDNFSIYFDIENQIFEIDNN--STFMNELESSFSSYQN-SYMNSGSKSSSRYYYP
YMYDTQSSWNNHINSCIDSYLRFEI--SIDSYISGETHNYSDNYQYNFICNESSNSIRTSVNG
SDFNIRGRS-DLDINKKYRHLWVQCENCYGLNYKKFFRSRLNICEHCGYHLKMSSSERIEL
LIDPGTWDPLDENMVSTDPIEFHSEEEPYRDRIDSYQKKTGLTEAVQTGIGQLNGIPIAIGV
MDFQFMGGSMGSVVGEKITRLIEYATKKSLPVIIVCASGGARMQEGSLSLMQMAKISSAL
YNYQLNKKLFYVAILTSPTTGGVTASFGMLGDIIIAEPNAYIAFAGKRVIEQTLNKTVPDGS
QAAEYLFQKGLFDLIVPRNLLKGVLGELFQLHGFFPLT-----MVTIRADEISNIIRERIEQYNR
EVKVVNTGTVLQVGDGIARVHGLDKVMAGELVEFQEGTVGIALNLESNNVGVVLMGDG
LLIQEGSSVKATGRIAQIPVSEAYLGRVLNALAKPIDGRGEISSSESRLIESPAPGIIARRSVYE
PLQTGIIAIDAMIPIGRGQRELIIGDRQTGKTAVATDTILNQKGQNVICVYVAIGQKASSVAQ
VVTALQERGAMEYTIVVAETADSPATLQYLAPYTGAALAEYFMYRKQHTLIIYDDLSKQA
QAYRQMSLLLRRPPGREAYPGDVFYLHSRLLERAAKLSNELGEGSMTALPIVETQSGDVS

AYIPTNVISITDGQIFLSADLFNAGIRPAINVGISVSRVGSAAQIKAMKQVAGKLKLELAQFA
ELEAFAQFASDLDKATQNQLARGQRLRELLKQSQSAPLGVEEQIATIYTGANGYLDSLEIA
QVRKFLVQLRTYLKKNKPQFKEIISSTKTFTQEAEALLKEAIQEQIELFLQQEQTMQINPTT
S-TAVSKLEEKNLGRIAQIIGPVLDVVFPPGKMPNIYNALVVKGQDAAGQQIKVTCEVQQL
LGNNRVRAVAMSATDGLTRGMDVIDTGAPLSVPVGGATLGRIFNVLGEPVDNLGPVDTTT
TSPIHRSAPAFIQLDTKLSIFETGIKVVDLLAPYRRGGKIGLFGGAGVGKTVLIMELINNIAK
AHGGVSVFGGVGERTREGNDLYMEMKESGVINEKNIAESKVALVYGQMNEPPGARMRV
GLTALTMAEFFRDVNEQDVLLFIDNIFRFVQAGSEVSALLGRMPSAVGYQPTLSTEMGSLQ
ERITSTKEGSITSIQAVYVPADDLTDPAPATTFAHLDATTVLSRGLAAKGIYPAVDPLDSTST
MLQPGIVGEEHYETAQKVKETLQRYKELQDIIAILGLDELSEDDRLTVARARKIERFLSQPF
FVAEVFTGSPGKYVGLAETIRGFKLILSGELDSLPEQAFYLVGNIDEATAKAINLEVESKLK
KMTLNLCVLTPNRIIWDSEVKEIILSTNSGQIGVLPNHAPIATAVDIGLLRIRLNDQWVTVAL
MGGFARIGNNEITILGNDAEISTDIDPQEAQQALEKAEDNLRKAEGKRQAIEANLALRRAK
TRVEAINVISMKNVTDSFVSL-HWPSAGGFGFNTDILATNPINLSVVLGVLIYFGKGVLNDL
LDNRKQRILSTIRNSEELRQAAIEQLEKARARLRKVETEANEYRVNGYSEIEREKENLINAT
SDNLERLENYKNETLLFEQQRAINQVRQRVFQQALQGALGTLNSCL-SELHFRTISANIGIL
GVMEE---MNPLISAASVIAAGLAVGLASIGPGVGQGTAAGQAVEGIARQPEAEGKIRGTLL
LSLAFMEALTIYGLVVALALLFANPFVMNVIPCSINTLTGLYDISGVEVGQHLYWQIGGLQV
HAQVLITSWVVIAILLGSVTLAVRNPQTIPADGQNFFEYLLEFIRDLSKTQIGEEYGPWVPFI
GTMFLFIFVSNWSGALLPWKIIELPHGELAAPTNDINTTVALALLTSVAYFYAGLSKKGLSY
FGKYIQPTPILLPINILEDFTKPLSLSFRLFGNILADELVVVVLVSLVPLVVPIPVMFLGLFTSG
IQALIFATLAAAYIGESMEGHHMIFVTLEHILTHISFSIISVVIIIQLLTFFVHEIPGLSDSSEKG
MIATFLSITGLLVIRWIYSGHFPLSNLYESLMFLSWSFAIIHMIPKIWKNKNYLSAITTPSAILT
QAFATSGLSTEMHQSSILVPALRSHWLMMHVSMMLLSYAALLVGSLFSIALLVITFRKNIKI
FVENQQMEYLTLNENILENTSFSSFKNYYKYQLIERLDYWSSRVISIGFTFLTIGILSGAVWA
NEAWGSYWNWDPKETWAFITWTIYAIYSHTRTTKNWQGANSAIVASIGFLIIWICYFGVNL
LGIGLHSYGSFILS---MLKEYPETHLQ
KLRIGIHKETVQLVKIHNEYNLHSILHFSTNIICFAILSGYFILGNEKLVILNSWIQEFLYNLSD
TIKAFCILLVTDLWIGFHSPHGWELMIGSVYNDFGLAHNDQIISGLVSTFPVILDTIVKYWIF
HYLNRVSPSLVVIYHSMNEMPIGVPKVPFRSPGEEDAVWVDVN-RLYRERLLFLGQEVDSE
ISNQLVGLMVYLSIEDDTKDLYLFINSPGGWVIPGIAIYDTMQFVPPDVHTICMGLAASMG
SFILVGGEITKRLAFPHARVMIHQPASSFYEAQAGEFILEAEELLKLRETLTRVYVQRTGNPL
W V V S E D M E R D V F M S A T E A Q A Y G I I D L V A V
E??MEEFKG
YLQKGGFKQQHFLYPLLFQEYIYVLAHDHGLSGYDN-SSLLLVKRLITRIYQQNSLIHSVN
DSNQNIFVGKNFYYQMISEGFAIVVEIPFSLRLVSSFIPKSQNLRSIHSIFPFLEDKFAHLNYV
SDILIPYPVHLEILVQILQCWIQDVPSLHLLRFLFHQYHNGNNCITKS-SYFSKPRLYRFLYNS
YVVEYESIFVFLRKSSSYLRSTSFGTLLERTYFYGKMK-G-VTHSNQKTLWLFKDPFMHYV
RYQGKSIMASKGTHLLMKKWKSYFVNLWQCHFHFWSQPSRIHINQFSHFSFYFLGYLSSV
PRNRSSVTTQMLENSFLIETVT--KKFETMVSIIPMIGSLSKAKFCNLSGNPISKPVWADLSD
SDIIDRFGRICRNLSHYYSGSSKKQSLYRIKYILRLSCARTLARKHKSTVRAFLQRLGSEFLE
EFFMEEEKVLSLILPRPIWYLDIIRINDLVNNLMIIDIT-EVQ-TINSFSGSE-FLKEIYELIWLFV
PIFVLILGIVIGVLVIVWLEREISAVIQQRIGPEYAGPLGILQALADGTKLLFKEDILPSRGDR
RLFGAGPAIAVVSILLSYLVIPFGSHLVLADLSIGVFLWIAISSIAPIGLLMSGYGSNNKYSFS

GGLRAAAQSISYEIPLTPCVLSISLLSNSSSTVDIVEAQSKYGFWGWNLWRQPIGFIVFLISS
LAECERLPFDLPEAEEELVAGYQTEYSGIKYGLFYVASYLNLLVSSLFVTILYLGGWNFSLP
YMFLPDLFGMNKI---FGMTIGIFITLAKAYLFLFIPIATRWTLPRMRMDQLLNLGWKFLLPIS
LGNLLLTTSSQLVSLMIWHVQNEVFILDSTRIFMKAFHLLLFNGSFIFPECILIFGLILLLMID
STSDQKDRPWFYFISSTSLVMSITALLFRWREEPMISFSGNFQTNNFNEIFQFLILLCSTLCIP
LSVEYIECTEMAITEFLLFVLTATLGGMFLCGANDSITIFVAPECFSLCSYLLSGYTKRDVRS
NEATTKYLLMGGASSSILVHGFSWLYGLSGGEIELQEIVNGLINTQMYNSPGISIALISITVGI
GFKLSPAPFHQWTPDVYEGSPTPVVAFLSVTSKVAASASATRIFDIPFYFSSNEWHLLLEILA
ILSMILGNLIAITQTSMKRMLAYSSIGQIGYVIIGIIVGDSNDGYASMITYMLFYIAMNLGTF
ARIVLFGLRTGTDNIRDYAGLYTKDPFLALSSALCLLSLGGLPPLAGFFGKLHLFWCGWQ
AGLYFLVSIGLLTSVVSIYYYLKIIKLLMTGRNQEITPHVRNYRKSPLRSNNSIELSMTVCVI
ASTIPGISMNPILAIAQDTLFMFLLHEYDIFWAFLIISSVIPILAFIISGVLAPSSEGPEKLSSYE
SGIEPMGDAWLQFRIRYYMFALVFVVFDVETVFLYPWAMSFDVLGVSVFIEALIFVLIPIVG
SVYAWRKGALEWS-----------------------------------MGICLLELLIMTYVFCYHFQLDDPLIQFE
DDFKWINIFDFHWRLGIDGLSIGPILLTGFITTLATLAAWPVTRDSRLFHFLMLAMYSGQIG
LFSSRDLLLFFMMWELELIPVYLLLSMWGGKKRLYSATKFILYTAGGSIFLLIGVLGMGLY
GSNEPTLNFETLGNQSYPLALEILLYFGFLIAFAVKSPIIPLHTWLPDTHGEAHYSTCMLLA
GILLKMGAYGLIRINMEFLSHAHSLFSPWLVIVGTIQIIYAASTSLGQRNLKKRIAYSSVSHM
GFTIIGIGSLTNTGLNGAILQLLSHGFIGAALFFLGGTSCDRIRLVYLDEMGGIAIPMPKIFTM
FSSFSMASLALPGMSGFVAESIVFLGIITSQKYLLMTKILITFVMAIGIILTPIYLLSMSRQMF
YGYKLFNLSNSFFVDSGPRELFVSICVFLPVIAIGIYPDLVISLSVDKVQAILSNYYYRMMF
EHVLFLSAYLFSIGIYGLITSRSMVRALMCLELILNSVNINLVTFSDLFDSRQLKGDIFSIFVI
AIAAAEAAIGLAIVSSIYRNRKSTRINQSNLLNKMEHTYQYAWIIPLLPLPVTMSIGFGLLLI
PTATKNIRRMWSFPSVLLLSIAMIFSANLAFQQINGSFIYQYLWSWTINNDFSLEFGYFIDPL
TSIMLILITTVGILVLIYSDNYMSHDQGYLRFFAYMSFFNASMLGLVTSSNLIQIYIFWELVG
MCSYLLIGFWFTRPSAASACQKAFVTNRVGDFGLLLGILGFYWMTGSFEFRDLFETFNK-E
GNSLFPTLCAFLLFLGAIAKSAQFPLHVWLPDAMEGPTPISALIHAATMVAAGIFLVARLLP
LFTGIPYIMNIISLIGIITLFLGATLALAQRDIKRSLAYSTMSQLGYIMLALGLGSYQAALFHL
ITHAYSKALLFLGSGSIIHSMEPLVGYSPDKSQNMVLMGGLTRYIPITRTTFLLGTLSLCGIP
PLACFWSKDEILSESWLYSPTFAVIAYFTAGLTAFYMFRVYLLTFEGNLHVQFQNYSGTQNI
SSYSISLWGKEAPKPG---NFVFSTIN--NKKN--TSMYPHESDNTMLFPLLVLVVFTLFVGFIGI
PLDLNLLSKWLAPSKNLLHPNSEDSIDLYEFFTNASFSVSIALFGIFVASIFYGSPYSSFQNL
DLINSFVLFLDKIANVIYNWSYNRGYIDLFYTNVVTQSIRELAKLTHFFDRYIIDGITNSVG
VVSFFGGEAIKYLVAGGRISSYLFFYLSFMLIFIYFSFMDLTGPIHDILVVFLGLVLILGGLGV
VLFTNPIYSAFSLGLVLVCVSLFYILLNSYFVAAAQLLIYVGAINVLIIFAVMFMKGSEYSND
FHLWTVGDGVTSLVCTSILFSLISIISDTSWYGIIWTTKSNHIIEQDLISNVQQIGIHLSTDFFL
PFELISIILLVALIGAITMARQMTGPATRNDLMIVNMGPQHPSMHGVLRLIVTLDGEDVIDC
EPILGYLHRGMEKIAENRTIVQYLPYVTRWDYLATMFTEAITVNAPEQLGNIQVPQRASYI
RVIMLELSRIASHLLWLGPFMADIGAQTPFFYIFRERELIYDLFEAATGMRMMHNYFRIGG
VAADLPYGWIDKCLDFCDYFLTVIEEYQKLITRNPIFLERVEGVGIIRGEEAINWGLSGPML
RASGISWDLRKVDQYECYNEFDWEVQWQKEGDSLARYLVRMSEMRESIKIIQQALEGIPG
GPYENLEVRR-DRTKDSEWNDFEYRFISKKPSPNFELSKQELYVRVEAPKGELGIFLIGDQN
VFPWRWKIRPPGFINLQILPQLVKRMKLADIMTILGSIDIIMGEVDRMFPMVTGFMNYGQQ
TVRAARYIGQGFMITLSHANRLPVTIQYPYEKLITSERFRGRIHFEFDKCIACEVCVRVCPIN

LPVVDWRLETNIKKKQLLNYSIDFGVCIFCGNCVEYCPTNCLSMTEEYELSAYDRHELNY
NQIALGRLPISIMGDYAIQPIMNSKIDKDKPLDSGTINNKMQSHLSAWLVKHELVHRSLGF
DYQGIETLQIKPEDWDSIAVISYVYGYNYLRSQCAYDVAPGGFLASVYHLTRIQYGVDQPE
EVCIKVFAPRQNPRVPSVFWVWRSADFQERESYDMLGISYDNHPRLKRILMPESWIGWPL
RKDYITPNFYEIQDAH--------------------------------------MVLAPEYSNNKN---KKDSIETVMNLI
EFPSLDQTTPNSVISTTLNDLSNWSRLSSLWPLLYGTSCCFIEFAALIGSRFDFDRYGLVPRS
SPRQADLILTAGTVTMKMAPSLVRLYEQMPEPKYVIAMGACTITGGMFSTDSYSTVRGVD
KLIPVDVYLPGCPPKPEAVIDAITKLRKKISREIYEDRIGSQ-ENRCFTTNHKFNVQRSIHTG
NYNQELLYQSPSTSEIPE--FFRSKSSISSHKLVNMQNRNTFLGVKEQMTRFISVSIVIYVITQ
TSISNAYPIFAQQGYENPREATGRIVCANCHLASKPVDIEVPQSVLPDTVFEAVVRIPYDTQ
VKQVLANGKKGGLNVGAVLILPEGFELAPPDRISPEMKEKIGNISFQSYGPTKRNILVVGP
VPGQKYREIVFPILSPDPATRKDVHFLKYPIYVGGNRGRGQIYPDGSKSNNTVYNATAAGV
VSKIVRKEKKGYEITITDPSDGHEVVDIIPPGPELLVSEGESIKLDQLLTSNPNVGGFGQGDA
EIVLQDPLRVQGLLFFLASVVLAQIFLVLKKKQFEKVQLFEMNFMKKVYDWFEERLEIQAI
ADDITSKYVPPHVNIFYCLGGITLTCFLVQVATGFAMTFYYRPTVTEAFASVQYIMTEANFG
WLIRSVHRWSASMMVLMMILHVFRVYLTGGFKKPRELTWVTGVVLGVLTASFGVTGYSL
PWDQIGYWAVKIVTGVPDAIPVIGSPLVELLRGSASVGQSTLTRFYSLHTFVLPLLTAVFML
MHFLMIRKQGISGPLIPITKKPDLNDPVLRAKLAKGMGHNYYGEPAWPNDLLYIFPVVILG
TIACNVGLAVLEPSMIGEPADPFATPLEILPEWYFFPVFQILRTVPNKLLGVLLMVSVPAGLL
TVPFLENVNKFQNPFRRPVATTVFLIGTVVALWLGIGATLPIDKSLTLGLFMIEVFLFGIVLG
LIPITLAGLFVTAYLQYRRGDQLDLMLTITSYFGFLLAASTITSALLIGLNKIRLIMDIVSLAW
AALMVVFTFSLSLVVWGRSGLMIISSPEPEVKILVDRNPVKTSFEQWAKPGHFSRTIAKGPE
TTTWIWNLHADAHDFDSHTNDLEEISRKVFSAHFGQLSIIFLWLSGMYFHGARFSNYEAW
LSDPTHIAPSAQVVWPIVGQEILNGDVGGGFRGIQITSGFFQLWRASGITSELQLYCTAIGAL
VFSALMLFAGWFHYHKAAPKLAWFQDVESMLNHHLAGLLGLGSLSWAGHQIHVSLPINQ
FLNAGVDPKEIPLPHEFILNRDLLAQLYPSFAEGATPFFTLNWSKYSDFLSFRGGLDPITGGL
WLSDTAHHHLAIAILFLIAGHMYRTNWGIGHGLKDILEAHKGPFTGQGHKGLYEILTTSW
HAQLSLNLAMLGSLTIIVAHHMYAMPPYPYLATDYGTQLSLFTHHMWIGGFLIVGAAAHA
AIFMVRDYDPTTRYNDLLDRVLRHRDAIISHLNWVCIFLGFHSFGLYIHNDTMSALGRPQD
MFSDTAIQLQPIFAQWIQNTHALAPGTTAPGAAASTSLTWGGGELVAVGGKVALLPIPLGT
ADFLVHHIHAFTIHVTVLILLKGVLFARSSRLIPDKANLGFRFPCDGPGRGGTCQVSAWDH
VFLGLFWMYNSISIVIFHFSWKMQSDVWGTVSDQGVVTHITGGNFAQSSTTINGWLRDFL
WAQAAQVIQSYGSSLSAYGLFFLGAHFVWAFSLMFLFSGRGYWQELIESIVWAHNKLKVA
PATQPRALSIVQGRAVGVTHYLLGGIATTWAFFLARIISVGMALRFPRFSQGLAQDPTTRRI
WFGIATAHDFESHDDITEERLYQNIFASHFGQLAIIFLWTSGNLFHVAWQGNFESWVQDPL
HVRPIAHAIWDPHFGQPAVEAFTRGGASGPVNIAYSGVYQWWYTIGLRTNEDLYTGALFL
LFLSAISLIAGWLHLQPKWKPSVSWFKNAESRLNHHLSGLFGVSSLAWAGHLVHVAIPGSR
GEYVRWNNFLDVLPYPQGLGPLFTGQWNLYAQNPDSSSHLFGTSQGAGTAILTLLGGFHP
QTQSLWLTDMAHHHLAIAFIFLIAGHMYRTNFGIGHSIKDLLEAHNPPGGRLGRGHKGLY
DTINNSIHFQLGLALASLGVVTSLVAQHMYSLPAYAFIAQDFTTQAALYTHHQYIAGFIMT
GAFAHGAIFFIRDYNPEQNEDNVLARMLAHKEAIISHLSWASLFLGFHTLGLYVHNDVML
AFGTPEKQILIEPIFAQWIQSAHGKTSYGFDVLLSSTTGPAFNAGKSIWLPGWLNAVNE--SS
NSLFLTIGPGDFLVHHAIALGLHTTTLILVKGALDARGSKLMPDKKDFGYSFPCDGPGRGG
TCDISAWDAFYLAVFWMLNTIGWVTFYWHWKHITLWQGNVSQFNESSTYLMGWLRDYL

WLNSSQLINGYNPFGMNSLSVWAWMFLFGHLVWATGFMFLISWRGYWQELIETLAWAHE
RTPLANLIRWRDKPVALSIVQARLVGLAHFSVGYIFTYAAFLIASTSAKFGMSHSVKIYDTC
IGCTQCVRACPTDVLEMIPWNGCKAKQIASAPRTEDCVGCKRCETACPTDFLSVRVYLSN
ETTRSMGLGYMTDFNLPSIFVPLVGLVFPAFAMASLFLYVQKNKIVMQDLKTYLSVAPVLS
TLWFAVLAGLLIEINRLFPDALSFPFF--MTAILERRESTSLWGRFCNWVTSTENRLYIGWFG
VLMIPTLLTATSVFIIAFIAAPPVDIDGIREPVSGSLLYGNNIISGAIIPTSAAIGLHFYPIWEAA
SVDEWLYNGGPYELIVLHFLLGVACYMGREWELSFRLGMRPWIAVAYSAPVAAATAVFLI
YPIGQGSFSDGMPLGISGTFNFMIVFQAEHNILMHPFHMLGVAGVFGGSLFSAMHGSLVTS
SLIRETTENESANEGYRFGQEEETYNIVAAHGYFGRLIFQYASFNNSRSLHFFLAAWPVVGI
WFTALGISTMAFNLNGFNFNQSVVDSQGRVINTWADIINRANLGMEVMHERNAHNFPLD
LAAVEAPSTIGMGLPWYRVHTVVLNDPGRLLSVHIMHTALVAGWAGSMALYELAVFDPS
DPVLDPMWRQGMFVIPFMTRLGITNSWGGWSIT-GGTVTNPGIWSYEGVAGAHIVFSGLC
FLAAIWHWVYWDLEIFCDERTGKPSLDLPKIFGIHLFLSGVACFGFGAFHVTGLYGPGIWV
SDPYGLTGKVQPVSPAWGAEGFDPFVPGGIASHHIAAGTLGILAGLFHLSVRPPQRLYKGL
RMGNIETVLSSSIAAVFFAAFVVAGTMWYGSATTPIELFGPTRYQWDQGYFQQEIYRRVSA
GLAENLSLSEAWAKIPEKLAFYDYIGNNPAKGGLFRAGSMDNGDGIAIGWLGHPIFRDKE
GRELFVRRMPTFFETFPVVLVDGDGIVRADVPFRRAESKYSVEQVGVTVEFYGGELNGVS
YSDPATVKKYARRAQLGEIFELDRATLKSDGVFRSSPRGWFTFGHASFALLFFFGHIWHGA
RTLFRDVFAGIDPDLDAQVEFGTFQKLGDPTTKRQAVMKILYSLRRFYHVETLFNGTLALA
GRDQETTGFAWWAGNARLINLSGKLLGAHVAHAGLIVFWAGAMNLFEVAHFVPEKPMY
EQGLILLPHLATLGWGVGPGGEVIDTFPYFVSGVLHLISSAVLGFGGIYHALLGPETLEESF
PFFGYVWKDRNKMTTILGIHLILLGLGAFLLVFKALYFGGVYDTWAPGGGDVRKITNLTL
SPSVIFGYLLKSPFGGEGWIVSVDDLEDIIGGHVWLGSICILGGIWHILTKPFAWARRAFVW
SGEAYLSYSLAALSIFGFTACCFVWFNNTAYPSEFYGPTGPEASQAQAFTFLVRDQRLGAN
VGSAQGPTGLGKYLMRSPTGEIIFGGETMRFWDLRAPWLEPLRGPNGLDLSRLKKDIQPW
QERRSAEYMTHAPLGSLNSVGGVATEINAVNYVSPRSWLATSHFVLGFFFFVGHLWHAGR
ARAAAAGFEKGIDRDLEPVLFMTPLSMTIALGRFTKEE-DLFDIMDDWLRRDRFVFVGWS
GLLLFPCAYFALGGWFTGTTFVTSWYTHGLASSYLEGCNFLTAAVSTPANSLAHSLLLLW
GPEAQGDFTRWCQLGGLWTFVALHGAFGLIGFMLRQFELARSVQLRPYNAIAFSGPIAVF
VSVFLIYPLGQSGWFFAPSFGVAAIFRFILFFQGFHNWTLNPFHMMGVAGVLGAALLCAIH
GATVENTLFEDGDGANTFRAFNPTQAEETYSMVTANRFWSQIFGVAFSNKRWLHFFMLF
VPVTGLWMSALGVVGLALNLRAYDFVSQEIRAAEDPEFETFYTKNILLNEGIRAWMAAQ
DQPHENLIFPEEVLPRGNALMSGSTGERSFADIITSIRYWVIHSITIPSLFIAGWLFVSTGLAY
DVFGSPRPNEYFTENRQGIPLITGRFDSLEQLDEFSRSFMTIDRTYPIFTVRWLAVHGLAVPT
VSFLGSISAMQFIQRMATQTVEGSS-RSGPRRTRVGDLLKPLNSEYGKVAPGWGTTPLMGV
AMALFAVFLSIILEIYNSSVLLDGILVSMLTLKLFVYTVVIFFVSLFIFGFLSNDPGRNPGREE
MADTTGRIPLWLIGTVAGILVIGSIGVFFYGSYSGLGSSLMLNILNLICICLNSVLSSSS-FFG
KLPEAYAIFNPIVDIMPVIPLFFFLLAFVWQAAVSFRMTQSNPNEQNVELNRTSLYWGLLLI
FVLAVLFSNYFFNMEVNILAFIATALFILVPTAFLLIIYVKTVSQNDMETATLVAISISGLLVSF
TGYALYTAFGQPSQQLRDPFEEHGDMEALVYTFLLVSTLGIIFFAIFFREPPKVPTKK--MTIA
FQLAVFALIATSSILLISVPVVFASSDGWSSNKNVVFSGTSLWIGLVFLVAILNSLISMSCREG
LMSPQTETKASAGFKAGVKDYKLTYYTPEYETKDTDILAAFRVTPQPGVPPEEAGAAVAA
ESSTGTWTTVWTDGLTSLDRYKGRCYHIEPVVGEENQYIAYVAYPLDLFEEGSVTNMFTSI
VGNVFGFKALRALRLEDLRIPPAYSKTFQGPPHGIQVERDKLNKYGRPLLGCTIKPKLGLS

AKNYGRAVYECLRGGLDFTKDDENVNSQPFMRWRDRFLFCAEAIYKAQAETGEIKGHYL
NATAGTCEEMIKRAVFARELGVPIVMHDYLTGGFTANTSLAHYCRDNGLLLHIHRAMHAV
IDRQKNHGMHFRVLAKALRMSGGDHIHAGTVVGKLEGEREMTLGFVDLLRDDYIEKDRS
RGIFFTQDWVSMPGVIPVASGGIHVWHMPALTEIFGDDSVLQFGGGTLGHPWGNAPGAVA
NRVALEACVQARNEGRDLAREGNEIIREACKWSPELAAACEVWKAIKFEFEPVDKLDVM
AIHLYKTSTPSTRNGAVDRKVKSNPRNNLIYGQHRCGKGRNARGIITAGHRGGGHKRLYR
KIDFRRNEKDIYGRIVTIEYDPNRNAYICLIHYGDGEKRYILHPRGAIIGDTIVSGTEVPISM
GNALPL-DMPLGTAIHNIEITLGKGGQLARAAGAVAKLIAKEGKSATLRLPSGEVRLISKNC
LATVGQVGNVGVNQKSLGRAGSKCWLGKRPVVRGVVMNPVDHPHGGGEGRAPIGRKR
PMTPWGYPALGKRSRKKKRYSDSFILRRRK-MIQPQTLLNVADNSGARELMCIRILGAS-N
RRYAHIGDVIVAVIKEAVPNMPLERSEVIRAVIVRTCKELKRENGMRIRYDDNAAVVIDQEG
NPKGTRVFGAIARELRELNFTKIVSLAPEVLNYNPKRTRFRKQHRGRMKGTSYRGNHISFG
RYALQALEPAWITARQIEAGRRAISRYARRGGKIWVRLFPDKPVTLRPTETRMGSGKGSPE
Y W V S V V K P G R I L Y E I G G V S E S V A R A A I S I A A S K M P I R T Q F I I Q
G???
???MITKAL-AQNIH-MSPFKARRVIDQIRGRSYEE
TLMILELMPYRASFSILKLVYSAAANA-NDLGLNEADSFISKAEVNGGTVLKRLRPRARGR
SYRIKRPTCHITIVLK-KSKK-------MDGIKYAVFTEKSIRLLGNNQ-YTSNVESGSTRTEIKH
WVELFFGVKVIAMNSHRLPRKGRKRGPIMGHTMQYRRMIITLQPGYSIPPLIEKRTMAVPK
KRTSMSKKRIRKSVWKKKAYLAALKAFSLAKSVSTGQSKSFFVRQTSNMAKGKDVRVRV
ILECNSCGRNENG--KEVPGVSRYVTQKNRHNTPSRLELKKFCRYCNKHTIHVEVKKMKIR
ASVRKICEKCRLIRRRGRIIVICSNPRHKQRQGMVREEIAGSTRTLQWKCVESRVDSKRLH
YGRFVLSPLMKGQADTIGIAMRRALLEEMEGTCITRAKSE--KVPHEYSTIAGIEESVHEIL
MNLKEIVLRSNLYGTRDASICVKGPRYITAQDIILPASVEIVDTTQHIATLTEPLDLCIGLQIQ
RDRGYRMRPTNDSQDGSY--PIDAVFMPVRNANHSIYSYGNGNEKQEILFLEIWTNGSLTP
KEALHEASRNLIDLFIPFLHAEEK--DINLEENK-SRFTLPLFTFHDRLA-NLTKNKK-EIALKC
IFIDQLELPSRTYNCLKRSNIHTLSDLLSNSQEDLMKIEYLHLEDIQQIVDILQKH--FTINLPK
NKMLRD-GNEGMFTIPGFSQIQFEGFCRFINQGLMEEFNKFPKIEDTDQEIEFKLFVETYQL
AEPLIKERDAVYESLTYSSELYVLAGLIWKTNRDMKEQTVFIGNIPLMNSLGTFIVNGIYRI
VINQILQSPGIYYRSELDHNGISVYTSTIISDWGGRSELEIDRKARIWARVSRKQKISILVLLS
AMGLNLREILDNVCYPEIFLSFLNDKEKKKIGSKENAILEFYQQFACVGGDPVFSESLCKE
[truncated: 451,798 more chars]
