# Supplementary material for: A precise chloroplast genome of Nelumbo nucifera (Nelumbonaceae) evaluated with Sanger, Illumina MiSeq, and PacBio RS II sequencing platforms: insight into the plastid evolution of basal eudicots
Source: BMC Plant Biol. 2014 Nov 19;14:289. doi: 10.1186/s12870-014-0289-0 (PMC4245832; doi:10.1186/s12870-014-0289-0)
Supplement: Additional file 7 — Fossil constraints used in the MCMC analyses. [file 12870_2014_289_MOESM7_ESM.pdf]

Additional file 7. Fossil constraints used in the MCMCtree analyses in this study.

Fossil constraints	Constraint Node	Min age (Mya)	Max age (Mya)	References
Root	Gymnosperms with Angiosperms	290	310	Moore *et al.* 2010
Eudicots	Crown with stem group eudicots	125		Moore *et al.* 2010
Calycanthaceae	*Calycanthus* with *Liriodendron*	113		Crepet *et al*. 2004
Platanaceae	*Platanus* with *Buxus* and core eudicots	113		Crepet *et al*. 2004
Platanaceae	*Platanus* with *Nelumbo*	110		Crane *et al*. 1993
Malpighiales	*Populus* with *Morus*	90		Crepet *et al*. 2004
Fabaceae	*Glycine* with *Morus*	60		Crepet *et al*. 2004
Monocots	Poales with other monocots	115		Linder *et al*. 2005

References:

Crane PR, Pedersen KR, Friis EM, Drinnan AN: **Early Cretaceous (Early to middle Albian) platanoid inflorescences associated with Sapindopsis leaves from the Potomac Group of eastern North America.** *Syst Bot* 1993, **18**: 328–344.

Crepet WL, Nixon KC, Gandolfo MA: **Fossil evidence and phylogeny: the age of major angiosperm clades based on mesofossil and macrofossil evidence from Cretaceous deposits**. *Am J Bot* 2004, **91**:1666–1682.

Linder HP, Rudall PJ: **Evolutionary history of Poales**. *Annu Rev Ecol Evol Syst* 2005, **36**: 107–124.

Moore MJ, Soltis PS, Bell CD, Burleigh JG, Soltis DE: **Phylogenetic analysis of 83 plastid genes further resolves the early diversification of eudicots**. *Proc Natl Acad Sci* USA 2010, **107**(10):4623-4628.
